# Supplementary material for: Investigating the Human Intestinal DNA Virome and Predicting Disease-Associated Virus–Host Interactions in Severe Myalgic Encephalomyelitis/Chronic Fatigue Syndrome (ME/CFS)
Source: Int J Mol Sci. 2023 Dec 8;24(24):17267. doi: 10.3390/ijms242417267 (PMC10744171; doi:10.3390/ijms242417267)
Supplement: Supplementary file 1 [file Table S7.pdf › Table S7.pdf]

**TableS7. VLP_viral cluster analysis**

**Table S7-1. vlp_viral_cluster_overview**

| VC | Size | Internal Weight | External Weight | Quality | P-value | Min Dist | Max Dist | Total Dist | Below Thres | Taxon Prediction Score | Avg Dist | Genera | Families | Orders | Members |
|---|---|---|---|---|---|---|---|---|---|---|---|---|---|---|---|
| VC_0_0 | 3 | 77.92734868 | 0 | 1 | 0 | 1 | 2 | 3 | 3 | 1 | 1.577350269 | 0 | 0 | 0 | AB002632,KC357596,OP297622 |
| VC_1_0 | 54 | 125467.6298 | 21828.6075 | 0.85180472 | 0 | 0 | 8.1240384 | 1431 | 1431 | 1 | 5.333219267 | 0 | 0 | 0 | AB276040,AB981169,GU911303,KP966108,MG747435,MT701591,ON170026,ON170032,ON170053,ON170059,ON170061,ON170067,ON170070,ON170073,ON170076,ON170078,ON170080,ON170081,ON170082,ON170084,ON170128,ON170130,ON170131,ON170135,ON170151,ON170173,ON170210,ON170213,ON170227,ON170230,ON170233,ON170235,ON170237,ON170239,ON170241,ON170243,ON170258,ON170261,ON170263,ON170266,ON170268,ON170270,ON170272,ON170276,ON170304,ON170305,ON170321,ON170325,ON170329,ON170344,ON170347,ON170348,ON170351,ON642070 |
| VC_10_0 | 25 | 32710.87399 | 62465.41202 | 0.34368723 | 4.59E-178 | 0 | 9.21954446 | 300 | 292 | 0.973333333 | 7.391403197 | 0 | 0 | 0 | AP011955,AP011956,AY508486,CP062430,CP062436,CP062447,DQ530361,GQ398772,KC595278,KF831354,KJ452292,KJ596420,KT809368,KT809369,KT878766,KY653123,MG029510,MG029512,MH384259,MH401416,MW924889,OP038320,OP136135,OP328423,OP493558 |
| VC_1000_0 | 2 | 219.0071484 | 717.9326858 | 0.23374729 | 0.0430783 | 2.236067977 | 2.23606798 | 1 | 1 | 1 | 2.236067977 | 0 | 0 | 0 | MG596800,OM982619 |
| VC_1001_0 | 3 | 559.7547706 | 1047.998569 | 0.34815961 | 0.0015857 | 2.828427125 | 3.87298335 | 3 | 3 | 1 | 3.339345087 | 0 | 0 | 0 | MK608336,OK499995,OL539452 |
| VC_1002_0 | 10 | 13500 | 17.25202397 | 0.9987237 | 4.34E-14 | 0 | 4.24264069 | 45 | 45 | 1 | 3.350118396 | 0 | 0 | 0 | JX126919,JX126920,JX442241,KJ094020,KJ094021,MH299806,MN114082,MN114083,MN128593,MN904502 |
| VC_1003_0 | 16 | 10140.55094 | 1168.72965 | 0.89665747 | 2.90E-29 | 0 | 3.16227766 | 120 | 120 | 1 | 2.324485898 | 0 | 0 | 0 | JQ309827,KT932700,LT630001,MK721183,MK721184,MK721185,MK721188,MK721193,MK721195,MK721196,MK721197,MK721198,MT747434,MT757399,MW623430,ON352054 |
| VC_1004_0 | 4 | 488.8968265 | 1168.72965 | 0.29493787 | 2.36E-05 | 0 | 1.41421356 | 6 | 6 | 1 | 0.707106781 | 0 | 0 | 0 | KT945995,MK360025,OL799259,ON113181 |
| VC_1005_0 | 3 | 825.8833549 | 173.7965065 | 0.82614784 | 0.0023423 | 4.69041576 | 6.244998 | 3 | 3 | 1 | 5.440192855 | 0 | 0 | 0 | JQ312117,MK637516,MT234341 |
| VC_1006_0 | 9 | 10713.71161 | 361.7935136 | 0.9673339 | 1.75E-17 | 0 | 8 | 36 | 36 | 1 | 5.505736802 | 0 | 0 | 0 | MN988470,MN988484,MN988495,MN988531,MN988537,MN988544,MN988546,MN988557,MW980069 |
| VC_1007_0 | 3 | 900 | 15722.23701 | 0.05414434 | 0.0016109 | 0 | 4.69041576 | 3 | 3 | 1 | 3.12694384 | 0 | 0 | 0 | KP280063,KY883654,KY883655 |
| VC_1007_1 | 24 | 82800 | 29636.03302 | 0.73641872 | 6.62E-124 | 1 | 9.48683298 | 276 | 245 | 0.887681159 | 6.611168308 | 0 | 0 | 0 | JQ340389,LC487410,MG649966,MG649967,MK796244,MK895508,MK907780,MN954399,MN958086,MN966730,MN966731,MN966732,MT448616,MT448617,MT459144,MT460514,MT460515,MT460516,MT460517,MT460518,MW344054,MW344055,MW344056,MW398869 |
| VC_1007_2 | 1 | 0 | 6407.39363 | 0 | 0 | 0 | 0 | 1 | 1 | 1 | 0 | 0 | 0 | 0 | KT345706 |
| VC_1008_0 | 6 | 4500 | 11255.89 | 0.28560748 | 3.92E-11 | 1 | 5.29150262 | 15 | 15 | 1 | 3.495571797 | 0 | 0 | 0 | MH645904,MH925094,MN871505,MN871510,MN871511,MT442039 |
| VC_1009_0 | 8 | 8400 | 9575.103805 | 0.46731302 | 9.07E-19 | 0 | 5.19615242 | 28 | 28 | 1 | 2.026288589 | 0 | 0 | 0 | MW824415,MW824416,MW824419,MW824422,MW824423,MW824424,MW824425,MW824426 |
| VC_101_0 | 32 | 45535.25663 | 6054.21256 | 0.88264635 | 5.38E-81 | 0 | 4.47213595 | 496 | 496 | 1 | 3.000634047 | 0 | 0 | 0 | AB647160,AB731695,JF734911,JQ617284,KX119174,KX119175,KX119176,KX119177,KX119182,KX119188,KX119189,KX119190,KX119191,KX119192,KX119193,KX119194,KX119195,KX119196,KX119197,KX119198,KX119199,KX119200,KX119201,KX119202,KX119203,KX119204,KX119205,KX119206,MW247144,MW247145,MW247147,OM515228 |
| VC_1010_0 | 2 | 194.2887561 | 0 | 1 | 0 | 2.236067977 | 2.23606798 | 1 | 1 | 1 | 2.236067977 | 0 | 0 | 0 | JQ823122,JQ823123 |
| VC_1011_0 | 2 | 300 | 9645.049985 | 0.03016576 | 0.0725063 | 4.123105626 | 4.12310563 | 1 | 1 | 1 | 4.123105626 | 0 | 0 | 0 | ON529850,ON529857 |
| VC_1011_1 | 5 | 3000 | 19944.44549 | 0.1307506 | 2.92E-07 | 4.123105626 | 9 | 10 | 9 | 0.9 | 7.135035013 | 0 | 0 | 0 | ON529851,ON529852,ON529853,ON529855,ON529860 |
| VC_1011_2 | 2 | 300 | 10674.66488 | 0.02733569 | 0.0458921 | 5.477225575 | 5.47722558 | 1 | 1 | 1 | 5.477225575 | 0 | 0 | 0 | JX100810,MH588545 |
| VC_1011_3 | 2 | 300 | 11410.68725 | 0.02561763 | 0.1159711 | 5.099019514 | 5.09901951 | 1 | 1 | 1 | 5.099019514 | 0 | 0 | 0 | JX100814,MH588544 |
| VC_1011_4 | 11 | 16500 | 33033.62055 | 0.33310709 | 1.46E-28 | 0 | 6.32455532 | 55 | 55 | 1 | 4.140975138 | 0 | 0 | 0 | JX100809,JX100811,JX100812,JX100813,JX163858,KY555142,KY555143,KY555144,KY555145,KY555146,KY555147 |
| VC_1011_5 | 1 | 0 | 5435.806853 | 0 | 0 | 0 | 0 | 1 | 1 | 1 | 0 | 0 | 0 | 0 | ON529858 |
| VC_1012_0 | 2 | 300 | 3469.412159 | 0.07958801 | 0.0607968 | 2.236067977 | 2.23606798 | 1 | 1 | 1 | 2.236067977 | 0 | 0 | 0 | KT870145,MH015255 |
| VC_1012_1 | 2 | 300 | 3552.952073 | 0.07786238 | 0.0602281 | 5.477225575 | 5.47722558 | 1 | 1 | 1 | 5.477225575 | 0 | 0 | 0 | MN602268,MT270409 |
| VC_1012_2 | 1 | 0 | 2070.376016 | 0 | 0 | 0 | 0 | 1 | 1 | 1 | 0 | 0 | 0 | 0 | OP186293 |
| VC_1013_0 | 2 | 119.1392414 | 2501.879636 | 0.04545532 | 0.0505103 | 8.717797887 | 8.71779789 | 1 | 1 | 1 | 8.717797887 | 0 | 0 | 0 | KY629563,OL963731 |
| VC_1013_1 | 1 | 0 | 1450.669934 | 0 | 0 | 0 | 0 | 1 | 1 | 1 | 0 | 0 | 0 | 0 | OL955261 |
| VC_1015_0 | 3 | 721.4154671 | 12913.59846 | 0.05290904 | 0.0014631 | 1.414213562 | 4.12310563 | 3 | 3 | 1 | 3.136767511 | 0 | 0 | 0 | JX182369,ON526970,ON526974 |
| VC_1015_1 | 26 | 62084.11352 | 26742.19063 | 0.69893838 | 2.42E-181 | 1 | 8.06225775 | 325 | 325 | 1 | 6.0655698 | 0 | 0 | 0 | JX182370,JX262376,MF766044,MF766045,MF766046,MF766047,MF766048,MG593802,MH001460,MH590601,MH651190,MH669004,MH669016,MK392363,MK392364,MK392365,MK392366,MK460245,MK524524,MN234192,MN234208,MT310863,MT701594,MT701597,MT708548,OK412919 |
| VC_1016_0 | 62 | 254833.5242 | 40187.70784 | 0.86378029 | 0 | 0 | 9.94987437 | 1891 | 1405 | 0.742993125 | 7.000905116 | 0 | 0 | 0 | JX889246,KC700556,KC700557,KC700558,KT124227,KT124228,KT124229,KT152029,KT184390,KT184391,KT186228,KT186229,KX344445,KX507343,KX507344,KX507345,KX815338,MF541403,MF541404,MF541405,MF541406,MF541407,MF541408,MF541409,MF541410,MG298964,MG757163,MH171093,MH171094,MH171095,MH171096,MH171097,MH171098,MH248947,MK433270,MK433271,MK433276,MK433278,MN096373,MN096375,MN096379,MN096381,MN204498,MN284899,MN586009,MT701598,MT711978,MT711979,MW365951,MW365953,MW435856,MZ648032,MZ648039,MZ648040,MZ820088,MZ820091,MZ820092,OL455891,OL455894,OL742563,ON208832,ON526985 |
| VC_1018_0 | 38 | 104182.0855 | 2590.558517 | 0.97573762 | 1.54E-28 | 0 | 7.34846923 | 703 | 703 | 1 | 4.553077461 | 0 | 0 | 0 | JX290549,KR604693,KT240186,KX278418,KX278419,KY953156,LC063634,LC375533,MH807811,MH807814,MH807816,MH807818,MH807819,MK095192,MK095193,MK095194,MK095195,MK095196,MK095197,MK095198,MK095199,MK095200,MK095201,MK095202,MK095203,MK095204,MK095205,MK095206,MK095207,MK095208,MK095209,MK095210,MK095211,MN270887,MN270888,MN271656,MN812690,MN812692 |
| VC_102_0 | 15 | 19089.07832 | 10144.97752 | 0.652974 | 3.42E-16 | 0 | 8.24621125 | 105 | 105 | 1 | 5.36854133 | 0 | 0 | 0 | KC330679,KC330680,KC330681,KC330682,KC330683,KC330684,KF669648,KF669651,KF669659,KU836751,MG983742,MH884513,MN727882,MT422786,MT514532 |
| VC_1020_0 | 22 | 22199.67883 | 2603.937267 | 0.89501784 | 6.66E-57 | 0 | 8.36660027 | 231 | 231 | 1 | 6.0284453 | 0 | 0 | 0 | JX483874,JX483875,JX483879,JX483880,MF403005,MF403006,MN988466,MN988488,MN988492,MN988493,MN988512,MN988513,MN988514,MN988528,MN988529,MT708544,MT708546,MW960034,MW980062,MW980065,MW980074,MZ462995 |
| VC_1021_0 | 2 | 162.7045163 | 869.6900912 | 0.15759915 | 0.0473107 | 2.645751311 | 2.64575131 | 1 | 1 | 1 | 2.645751311 | 0 | 0 | 0 | MF278336,MW043865 |
| VC_1022_0 | 2 | 107.3189051 | 789.2344642 | 0.11970164 | 0.0474878 | 3.741657387 | 3.74165739 | 1 | 1 | 1 | 3.741657387 | 0 | 0 | 0 | KY065149,MT227925 |
| VC_1023_0 | 3 | 900 | 536.9923871 | 0.62630812 | 0.0018044 | 2 | 6.92820323 | 3 | 3 | 1 | 5.187150937 | 0 | 0 | 0 | KF614509,MN988552,MT778840 |
| VC_1024_0 | 2 | 91.20895372 | 152.7901944 | 0.37380849 | 0.0858152 | 4.358898944 | 4.35889894 | 1 | 1 | 1 | 4.358898944 | 0 | 0 | 0 | KC292024,KC292028 |
| VC_1025_0 | 8 | 305.3425177 | 485.6467668 | 0.38602611 | 6.08E-16 | 0 | 1.73205081 | 28 | 28 | 1 | 0.935429417 | 0 | 0 | 0 | KP087954,MH572347,MH572348,MH572349,MH572405,MH572418,MH572455,MH572515 |
| VC_1027_0 | 1 | 0 | 27.62887329 | 0 | 0 | 0 | 0 | 1 | 1 | 1 | 0 | 0 | 0 | 0 | MK249135 |
| VC_1028_0 | 69 | 18104.10213 | 3078.874153 | 0.85465337 | 8.91E-11 | 0 | 2.64575131 | 2346 | 2346 | 1 | 1.833061586 | 0 | 0 | 0 | KC131022,KM589502,KM589511,KT264813,KT264814,KT264815,MG945202,MG945465,MG945477,MG945663,MG945838,MG945843,MH572283,MH572284,MH572285,MH572286,MH572291,MH572292,MH572293,MH572382,MH572383,MH572423,MH572424,MH572427,MH572428,MH572466,MH572495,MH572504,MH992156,MH992161,MH992184,MH992185,MH992213,MK249141,MK249147,MK249165,MK249185,MK249190,MK249197,MK249201,MK249202,MK249203,MK249211,MK249213,MK249221,MK496825,MN862333,MN862335,MN862341,MW202412,MW202414,MW202466,MW202471,MW202549,MW202587,MW202847,MW202853,MW202883,MZ364228,MZ364233,MZ364240,MZ364243,MZ364256,MZ364258,MZ364259,MZ364267,MZ364274,OL617029,OM523002 |
| VC_1029_0 | 2 | 4.161094484 | 94.55678366 | 0.04215138 | 0.4096185 | 2 | 2 | 1 | 1 | 1 | 2 | 0 | 0 | 0 | MN862339,MN862340 |
| VC_103_0 | 3 | 510.1827529 | 872.2480696 | 0.36904758 | 0.0023609 | 2.449489743 | 5.19615242 | 3 | 3 | 1 | 4.147157896 | 0 | 0 | 0 | KY565347,MH884514,OP341625 |
| VC_1030_0 | 1 | 0 | 271.0556535 | 0 | 0 | 0 | 0 | 1 | 1 | 1 | 0 | 0 | 0 | 0 | MH992169 |
| VC_1031_0 | 2 | 9.896962924 | 182.9349428 | 0.0513243 | 0.0609819 | 1.414213562 | 1.41421356 | 1 | 1 | 1 | 1.414213562 | 0 | 0 | 0 | MH572299,MH572425 |
| VC_1032_0 | 2 | 4.404128344 | 156.5666944 | 0.02735979 | 0.2601067 | 2 | 2 | 1 | 1 | 1 | 2 | 0 | 0 | 0 | MH572426,MK249189 |
| VC_1033_0 | 21 | 1783.541044 | 331.3921947 | 0.84330844 | 2.47E-08 | 0 | 2.23606798 | 210 | 210 | 1 | 1.45145382 | 0 | 0 | 0 | MG945161,MK249131,MK249144,MK249156,MK249159,MK249160,MK249167,MK249179,MK249181,MZ364226,MZ364241,MZ364244,MZ364245,MZ364246,MZ364247,MZ364249,MZ364250,MZ364260,MZ364263,MZ364266,MZ364270 |
| VC_1035_0 | 2 | 123.0256499 | 156.6416867 | 0.43989996 | 0.0597748 | 4.358898944 | 4.35889894 | 1 | 1 | 1 | 4.358898944 | 0 | 0 | 0 | S14_vs_88666,S5_vs_3501 |
| VC_1037_0 | 2 | 5.556925528 | 25.24494654 | 0.18040869 | 0.2790923 | 5.567764363 | 5.56776436 | 1 | 1 | 1 | 5.567764363 | 0 | 0 | 0 | MT152698,MZ334527 |
| VC_1038_0 | 4 | 619.9068924 | 93.2627021 | 0.86922788 | 0.0001622 | 5 | 6.78232998 | 6 | 6 | 1 | 6.016528108 | 0 | 0 | 0 | MN698240,MN698245,MN698246,MN698248 |
| VC_1039_0 | 2 | 213.8092601 | 255.9625718 | 0.45513427 | 0.0577446 | 0 | 0 | 1 | 1 | 1 | 0 | 0 | 0 | 0 | MW202462,MW202544 |
| VC_104_0 | 2 | 32.89394105 | 275.8179414 | 0.10655223 | 0.0535763 | 7.416198487 | 7.41619849 | 1 | 1 | 1 | 7.416198487 | 0 | 0 | 0 | KJ159566,KT001912 |
| VC_1040_0 | 2 | 208.8228652 | 324.0088158 | 0.3919115 | 0.0524928 | 5.477225575 | 5.47722558 | 1 | 1 | 1 | 5.477225575 | 0 | 0 | 0 | S12_vf_181024,S17_vf_174033 |
| VC_1040_1 | 1 | 0 | 363.1288949 | 0 | 0 | 0 | 0 | 1 | 1 | 1 | 0 | 0 | 0 | 0 | S8_vf_161703 |
| VC_1042_0 | 8 | 7946.549985 | 2813.770997 | 0.73850492 | 4.07E-14 | 0 | 8.88819442 | 28 | 28 | 1 | 6.788659361 | 0 | 0 | 0 | KC595511,KT070866,MG967616,MG967617,MG967618,MK288022,MW392801,MW392802 |
| VC_1043_0 | 19 | 26376.90206 | 8261.564168 | 0.76149163 | 2.72E-54 | 0 | 4 | 171 | 171 | 1 | 2.534708956 | 0 | 0 | 0 | MT074134,MT074135,MT074143,MT074144,MT074145,MT074147,MT074148,MT074149,MT074150,MT074151,MT074152,MT074153,MT074154,MT074155,MT074156,MT074157,MT074158,MT074159,MT074160 |
| VC_1044_0 | 5 | 913.6561319 | 293.2250684 | 0.757039 | 1.62E-06 | 1 | 6.63324958 | 10 | 10 | 1 | 4.893796294 | 0 | 0 | 0 | MT635598,MT806185,MT806186,S4_vf_18803,S8_vf_183045 |
| VC_1045_0 | 2 | 163.7045036 | 111.0735097 | 0.59577002 | 0.0707894 | 1.414213562 | 1.41421356 | 1 | 1 | 1 | 1.414213562 | 0 | 0 | 0 | S11_vf_104071,S3_vs_127413 |
| VC_1047_0 | 9 | 1554.693089 | 0 | 1 | 0 | 0 | 1.73205081 | 36 | 36 | 1 | 0.962250449 | 0 | 0 | 0 | KC821606,KC821623,KC821628,KC821631,MT732445,MT732446,MT732447,MT732448,MT732449 |
| VC_1048_0 | 4 | 1244.862965 | 195.4203671 | 0.86431811 | 4.61E-05 | 6.8556546 | 7.61577311 | 6 | 6 | 1 | 7.27476053 | 0 | 0 | 0 | KC821608,KC821620,KC821622,KC821633 |
| VC_1049_0 | 34 | 95078.91703 | 598.8151583 | 0.99374133 | 6.06E-59 | 0 | 7.14142843 | 561 | 561 | 1 | 5.010295196 | 0 | 0 | 0 | MN812206,MN812207,MN812208,MN812209,MN812210,MN812212,MN812213,MN812214,MN812215,MN812216,MN812217,MN812218,MN812219,MN812220,MN812221,MN812222,MN812223,MN812224,MN812225,MN812226,MN812227,MN812228,MN812229,MN812230,MN812231,MN812232,MN812233,MN812234,MN812235,MN812236,MN812237,MN812238,MN812240,MN812241 |
| VC_105_0 | 6 | 4322.879023 | 1570.296172 | 0.73353988 | 2.17E-10 | 1 | 3.46410162 | 15 | 15 | 1 | 2.598958212 | 0 | 0 | 0 | MN176222,MN176223,MN176224,MN176225,MN176226,MN176229 |
| VC_1050_0 | 2 | 257.6559444 | 7.891125769 | 0.97028351 | 0.1855467 | 2.645751311 | 2.64575131 | 1 | 1 | 1 | 2.645751311 | 0 | 0 | 0 | KR136259,KR136260 |
| VC_1051_0 | 62 | 556010.2824 | 3207.311622 | 0.99426464 | 9.85E-289 | 0 | 3.87298335 | 1891 | 1891 | 1 | 1.145404917 | 0 | 0 | 0 | MT497017,MT497065,MT497066,MT497067,MT497068,MT497069,MT497070,MT497071,MT497072,MT497073,MT497074,MT497075,MT497076,MT497077,MT497078,MT497079,MT497080,MT497081,MT497082,MT497083,MT497084,MT497085,MT497086,MT497087,MT497088,MT497089,MT497090,MT497091,MT497092,MT497093,MT497094,MT497095,MT497096,MT497097,MT497098,MT497099,MT497100,MT497101,MT497102,MT497103,MT497104,MT497105,MT497106,MT497107,MT497108,MT497109,MT497110,MT497111,MT497112,MT497113,MT497114,MT497115,MT497116,MT497117,MT497118,MT497119,MT497120,MT497121,MT497122,MT497123,MT497124,MT497125 |
| VC_1052_0 | 8 | 5476.542258 | 343.6407681 | 0.94095705 | 5.11E-16 | 0 | 0 | 28 | 28 | 1 | 0 | 0 | 0 | 0 | MT732435,MT732436,MT732437,MT732438,MT732439,MT732440,MT732441,MT732442 |
| VC_1053_0 | 5 | 3000 | 216.5316725 | 0.93268163 | 3.02E-07 | 0 | 4.89897949 | 10 | 10 | 1 | 2.939387691 | 0 | 0 | 0 | KC821609,KC821632,KT962245,KT962246,KT962247 |
| VC_1054_0 | 5 | 1793.20301 | 7672.458257 | 0.18944297 | 5.98E-06 | 3.31662479 | 8.88819442 | 10 | 10 | 1 | 7.734690381 | 0 | 0 | 0 | MT774387,MT774390,MT774392,S11_vf_104044,S13_vs_151510 |
| VC_1054_1 | 8 | 5556.142984 | 8556.040792 | 0.39371249 | 2.86E-14 | 2 | 9.59166305 | 28 | 26 | 0.928571429 | 7.667692806 | 0 | 0 | 0 | MH675552,MT074136,MT074138,MT774378,MT774388,MT774391,S3_vf_127447,S5_vf_55415 |
| VC_1054_2 | 2 | 190.1347249 | 2977.492817 | 0.06002433 | 0.0496638 | 6.164414003 | 6.164414 | 1 | 1 | 1 | 6.164414003 | 0 | 0 | 0 | KJ003983,MT774389 |
| VC_1054_3 | 1 | 0 | 1340.557066 | 0 | 0 | 0 | 0 | 1 | 1 | 1 | 0 | 0 | 0 | 0 | MT774385 |
| VC_1055_0 | 2 | 300 | 2883.48608 | 0.09423632 | 0.1489166 | 8.246211251 | 8.24621125 | 1 | 1 | 1 | 8.246211251 | 0 | 0 | 0 | S13_vs_15645,S7_vf_28429 |
| VC_1055_1 | 3 | 900 | 3434.027158 | 0.20765906 | 0.0216747 | 7.681145748 | 8.94427191 | 3 | 3 | 1 | 8.27025681 | 0 | 0 | 0 | S14_vs_15228,S3_vs_82895,S8_vs_182288 |
| VC_1055_2 | 1 | 0 | 1641.193041 | 0 | 0 | 0 | 0 | 1 | 1 | 1 | 0 | 0 | 0 | 0 | S10_vs_37379 |
| VC_1056_0 | 2 | 226.3638602 | 1256.639892 | 0.15263876 | 0.0562722 | 7 | 7 | 1 | 1 | 1 | 7 | 0 | 0 | 0 | S3_vs_80151,S4_vs_VIRSorter_13190-cat_2 |
| VC_1057_0 | 5 | 3000 | 483.694866 | 0.86115464 | 1.33E-08 | 0 | 2.44948974 | 10 | 10 | 1 | 1.469693846 | 0 | 0 | 0 | KC821612,KC821614,MT732432,MT732433,MT732434 |
| VC_1058_0 | 5 | 1862.343303 | 395.6577036 | 0.82477523 | 2.96E-07 | 0 | 4.35889894 | 10 | 10 | 1 | 3.379649917 | 0 | 0 | 0 | KC821613,KC821615,KC821617,KC821619,KC821627 |
| VC_1059_0 | 10 | 4883.715776 | 669.8730421 | 0.87938015 | 5.34E-22 | 0 | 6.8556546 | 45 | 45 | 1 | 4.220634945 | 0 | 0 | 0 | MK764442,MK764445,MK764448,MN812203,MN812204,MN812205,MW421581,MW421582,MW421583,MW421584 |
| VC_106_0 | 6 | 2554.841771 | 536.3739479 | 0.82648447 | 4.69E-10 | 2.645751311 | 7.81024968 | 15 | 15 | 1 | 6.109451441 | 0 | 0 | 0 | MH752385,MN013088,MN176219,MN176227,MN176228,X97918 |
| VC_1060_0 | 4 | 1488.546832 | 212.7056409 | 0.87497115 | 6.58E-05 | 1 | 3.60555128 | 6 | 6 | 1 | 2.469417422 | 0 | 0 | 0 | KY206887,MK017819,MN417334,OP381444 |
| VC_1061_0 | 8 | 4908.36366 | 949.5000397 | 0.83791019 | 6.60E-16 | 1 | 5.65685425 | 28 | 28 | 1 | 3.918338992 | 0 | 0 | 0 | KC959568,KU599887,KU599888,KU599889,MK764437,MK764440,MK764450,MW421576 |
| VC_1062_0 | 2 | 174.0405521 | 192.0612138 | 0.47538845 | 0.0514193 | 6.708203932 | 6.70820393 | 1 | 1 | 1 | 6.708203932 | 0 | 0 | 0 | S10_vs_14557,S8_vs_28311 |
| VC_1063_0 | 2 | 129.820729 | 181.1797453 | 0.41742936 | 0.0644425 | 5 | 5 | 1 | 1 | 1 | 5 | 0 | 0 | 0 | S16_vs_136935,S3_vs_64424 |
| VC_1064_0 | 2 | 300 | 0 | 1 | 0 | 0 | 0 | 1 | 1 | 1 | 0 | 0 | 0 | 0 | KC960489,KF981876 |
| VC_1065_0 | 4 | 1800 | 0 | 1 | 1 | 1 | 3.46410162 | 6 | 6 | 1 | 2.665279944 | 0 | 0 | 0 | KF005317,KF005318,KF005319,KF005320 |
| VC_1066_0 | 2 | 2.831332108 | 6.762113211 | 0.29513194 | 0.1855467 | 7.141428429 | 7.14142843 | 1 | 1 | 1 | 7.141428429 | 0 | 0 | 0 | KX257490,MW805362 |
| VC_1067_0 | 20 | 17617.55515 | 7165.790275 | 0.71086267 | 9.35E-59 | 0 | 7.21110255 | 190 | 190 | 1 | 5.585094879 | 0 | 0 | 0 | KF322026,KF800937,KJ502657,KY290756,KY626176,KY626177,MF481197,MG018929,MG545917,MH113812,MH113813,MH113814,MH113815,MK455769,MK455770,MK820013,MN807240,MT941682,MW451247,MW865291 |
| VC_1068_0 | 8 | 8400 | 641.2545979 | 0.9290746 | 7.68E-17 | 1 | 3.74165739 | 28 | 28 | 1 | 2.48352807 | 0 | 0 | 0 | KU245542,OK638201,OK638202,OK638203,OL343755,OL473897,OL622073,ON857939 |
| VC_1069_0 | 4 | 1800 | 209.0709601 | 0.8959365 | 4.13E-05 | 2.449489743 | 4.58257569 | 6 | 6 | 1 | 3.64296812 | 0 | 0 | 0 | MW677511,MW677527,OP009274,OP009281 |
| VC_1070_0 | 3 | 646.760985 | 174.8779999 | 0.78715956 | 0.0045758 | 6 | 7.21110255 | 3 | 3 | 1 | 6.760723454 | 0 | 0 | 0 | MG596799,MK473373,MZ605292 |
| VC_1071_0 | 7 | 3046.835091 | 35.93916961 | 0.98834194 | 4.96E-06 | 1.414213562 | 4.47213595 | 21 | 21 | 1 | 2.984375755 | 0 | 0 | 0 | KF669655,KF669657,KF669660,KM236247,KM236248,KP411017,KT001911 |
| VC_1073_0 | 2 | 147.2205052 | 1934.745737 | 0.07071224 | 0.0467364 | 8 | 8 | 1 | 1 | 1 | 8 | 0 | 0 | 0 | ON645346,OP580516 |
| VC_1073_1 | 1 | 0 | 357.924714 | 0 | 0 | 0 | 0 | 1 | 1 | 1 | 0 | 0 | 0 | 0 | KF692088 |
| VC_1074_0 | 6 | 2609.424104 | 2786.734201 | 0.48357071 | 1.23E-10 | 3.464101615 | 5.74456265 | 15 | 15 | 1 | 4.896734247 | 0 | 0 | 0 | MH834603,MH834605,MH834611,MH834614,MH834623,ON970561 |
| VC_1075_0 | 11 | 9501.726885 | 4978.021593 | 0.65620801 | 4.26E-31 | 2.449489743 | 9.48683298 | 55 | 51 | 0.927272727 | 7.77648168 | 0 | 0 | 0 | MH834598,MH834612,MH834625,MH910036,MH910037,MH910041,MW712719,ON724017,ON970598,OP434442,OP434463 |
| VC_1076_0 | 6 | 2479.439631 | 1181.479104 | 0.67727251 | 2.17E-10 | 3.605551275 | 6.08276253 | 15 | 15 | 1 | 4.992253228 | 0 | 0 | 0 | MH834621,MH834627,MK392368,MZ150784,MZ274308,ON392166 |
| VC_1078_0 | 5 | 1714.008665 | 0 | 1 | 0 | 0 | 3.60555128 | 10 | 10 | 1 | 2.682239499 | 0 | 0 | 0 | KF751793,KF751794,KF751795,KF751796,KF751797 |
| VC_1079_0 | 1 | 0 | 455.2404957 | 0 | 0 | 0 | 0 | 1 | 1 | 1 | 0 | 0 | 0 | 0 | S15_vf_75924 |
| VC_1079_1 | 1 | 0 | 487.5365533 | 0 | 0 | 0 | 0 | 1 | 1 | 1 | 0 | 0 | 0 | 0 | S5_vf_67622 |
| VC_1079_2 | 1 | 0 | 355.3488253 | 0 | 0 | 0 | 0 | 1 | 1 | 1 | 0 | 0 | 0 | 0 | S5_vf_145579 |
| VC_108_0 | 2 | 300 | 1098.612425 | 0.21449831 | 0.0462688 | 1.732050808 | 1.73205081 | 1 | 1 | 1 | 1.732050808 | 0 | 0 | 0 | ON210834,ON210835 |
| VC_108_1 | 1 | 0 | 783.2461215 | 0 | 0 | 0 | 0 | 1 | 1 | 1 | 0 | 0 | 0 | 0 | OM654379 |
| VC_1080_0 | 23 | 60947.64849 | 2518.442031 | 0.96031831 | 5.32E-41 | 0 | 1.73205081 | 253 | 253 | 1 | 0.207235319 | 0 | 0 | 0 | KT876725,KU599880,KU599881,KU599882,KU599883,KU599884,KU599885,MK764436,MK764438,MK764439,MK764443,MK764444,MK764446,MK764447,OK268422,OK268423,OK268424,OK268425,OK268426,OK268427,OK340817,OK340818,OK340819 |
| VC_1081_0 | 9 | 5588.701536 | 2979.319885 | 0.65227446 | 1.10E-21 | 0 | 5.38516481 | 36 | 36 | 1 | 3.49415694 | 0 | 0 | 0 | MG592391,MG592421,MG592436,MG592443,MG592445,MG592449,MG592661,OX241561,OX241569 |
| VC_1083_0 | 4 | 1582.746668 | 262.6631758 | 0.85766675 | 2.69E-05 | 5.196152423 | 6.55743852 | 6 | 6 | 1 | 5.843023616 | 0 | 0 | 0 | MN096357,MN096358,MT818426,ON526969 |
| VC_1084_0 | 2 | 300 | 4732.864259 | 0.0596082 | 0.0433209 | 0 | 0 | 1 | 1 | 1 | 0 | 0 | 0 | 0 | MN183282,MT771347 |
| VC_1085_0 | 2 | 289.5250765 | 4995.253623 | 0.05478471 | 0.0459564 | 5.744562647 | 5.74456265 | 1 | 1 | 1 | 5.744562647 | 0 | 0 | 0 | S12_vs_92868,S4_vs_VIRSorter_30222-cat_2 |
| VC_1085_1 | 19 | 15899.92306 | 13943.69064 | 0.53277472 | 8.05E-50 | 3 | 9.64365076 | 171 | 153 | 0.894736842 | 8.123786425 | 0 | 0 | 0 | S10_vs_26365,S11_vf_13033,S11_vs_104064,S11_vs_68151,S12_vs_163990,S12_vs_188821,S12_vs_58560,S16_vf_5270,S2_vf_21511,S3_vf_35073,S4_vs_VIRSorter_3006-cat_1,S4_vs_VIRSorter_4839-cat_2,S5_vs_85065,S7_vf_38697,S7_vs_41098,S8_vf_163760,S8_vf_41376,S9_vs_32588,S9_vs_4558 |
| VC_1085_2 | 1 | 0 | 2010.488655 | 0 | 0 | 0 | 0 | 1 | 1 | 1 | 0 | 0 | 0 | 0 | S8_vf_23955 |
| VC_1086_0 | 2 | 158.2469901 | 211.2159173 | 0.42831631 | 0.0560197 | 5.916079783 | 5.91607978 | 1 | 1 | 1 | 5.916079783 | 0 | 0 | 0 | MN988468,MN988516 |
| VC_1087_0 | 7 | 4256.29545 | 271.4932402 | 0.94003845 | 2.16E-11 | 0 | 4.12310563 | 21 | 21 | 1 | 2.712907607 | 0 | 0 | 0 | KJ510413,KM591905,MF155936,MG925349,MH001451,MK494089,MN735432 |
| VC_1088_0 | 5 | 1444.674077 | 760.1204883 | 0.65524203 | 6.75E-08 | 0 | 8.18535277 | 10 | 10 | 1 | 6.112687395 | 0 | 0 | 0 | MW584157,MW584173,MW584189,MW584206,MW584209 |
| VC_1089_0 | 3 | 283.1289559 | 0 | 1 | 0 | 1.414213562 | 1.73205081 | 3 | 3 | 1 | 1.626105059 | 0 | 0 | 0 | KJ617393,LK392619,Z47794 |
| VC_1090_0 | 8 | 5458.998334 | 3101.148069 | 0.63772254 | 3.53E-18 | 0 | 8.88819442 | 28 | 28 | 1 | 7.273862469 | 0 | 0 | 0 | KJ802832,KX987158,KY002061,MH586730,MZ726797,OL604152,ON602746,OP617744 |
| VC_1091_0 | 24 | 80315.83439 | 72549.13471 | 0.52540379 | 1.01E-131 | 0 | 6.164414 | 276 | 276 | 1 | 1.905224889 | 0 | 0 | 0 | MG592411,MG592538,MG592563,MG592564,MG592565,MG592571,MG592632,MG592633,MG592634,MG592635,MG592636,MG592637,MG592638,MG592639,MG592640,MG592642,MG592645,MG592646,MG592648,MG592649,MG592650,MG592651,MG592652,MG592653 |
| VC_1091_1 | 29 | 68834.09252 | 72909.98854 | 0.48562234 | 5.21E-178 | 0 | 9.48683298 | 406 | 389 | 0.958128079 | 7.897133867 | 0 | 0 | 0 | MG592397,MG592406,MG592407,MG592408,MG592416,MG592417,MG592428,MG592463,MG592464,MG592471,MG592475,MG592476,MG592492,MG592493,MG592528,MG592601,MG592602,MG592617,MG592618,MG592655,MH925092,MW824418,MW824430,MW824432,MW824433,MW824434,OX241427,OX241556,OX241563 |
| VC_1092_0 | 2 | 77.4797653 | 0 | 1 | 0 | 4.795831523 | 4.79583152 | 1 | 1 | 1 | 4.795831523 | 0 | 0 | 0 | KM058087,MW900438 |
| VC_1093_0 | 2 | 300 | 3613.035398 | 0.07666682 | 0.0539935 | 1 | 1 | 1 | 1 | 1 | 1 | 0 | 0 | 0 | MT234342,MT234343 |
| VC_1093_1 | 1 | 0 | 3808.57968 | 0 | 0 | 0 | 0 | 1 | 1 | 1 | 0 | 0 | 0 | 0 | KP881232 |
| VC_1093_2 | 1 | 0 | 3371.73108 | 0 | 0 | 0 | 0 | 1 | 1 | 1 | 0 | 0 | 0 | 0 | MF403007 |
| VC_1093_3 | 5 | 3000 | 13247.49433 | 0.18464386 | 0.0134358 | 4.582575695 | 7.14142843 | 10 | 10 | 1 | 6.239792949 | 0 | 0 | 0 | KM199770,MN549360,MN549361,MT778837,MT778839 |
| VC_1093_4 | 6 | 4500 | 14013.07651 | 0.24307143 | 0.0020407 | 0 | 8.66025404 | 15 | 15 | 1 | 4.744674703 | 0 | 0 | 0 | MN988486,MN988509,MN988520,MN988532,MN988534,MN988553 |
| VC_1094_0 | 1 | 0 | 63.40997071 | 0 | 0 | 0 | 0 | 1 | 1 | 1 | 0 | 0 | 0 | 0 | MG945594 |
| VC_1095_0 | 12 | 371.6639089 | 946.8868033 | 0.28187305 | 0.0016291 | 0 | 2.82842712 | 66 | 66 | 1 | 2.095388801 | 0 | 0 | 0 | KM589498,KT264826,KT264827,MG945473,MG945703,MH931003,MK496723,MN379633,MN379651,OM522999,OM523001,OM869624 |
| VC_11_0 | 19 | 26099.88132 | 8498.196227 | 0.75437374 | 1.59E-90 | 0 | 8.36660027 | 171 | 171 | 1 | 5.930331419 | 0 | 0 | 0 | AP019560,AP019561,AP019562,KX827368,KX827369,KX827370,MF428478,MF428479,MF428480,MF428481,MK075003,MK075006,MN028536,MT423823,MT423824,OM373551,OM373552,OM373553,OP168678 |
| VC_110_0 | 33 | 125262.0108 | 6762.660293 | 0.9487773 | 3.43E-84 | 0 | 7.93725393 | 528 | 528 | 1 | 6.140698321 | 0 | 0 | 0 | AB712291,HE962497,JF731128,KF192053,KJ094032,KJ127303,KT932699,KY303907,KY472224,LC547238,MH618488,MH791415,MK570225,MK721189,MK721194,MK800154,MN103542,MN871443,MN939408,MW677132,MZ333457,OL505085,OL870612,ON086985,ON113171,ON113172,ON113173,ON113174,ON113175,ON113177,ON113178,ON113179,ON113180 |

| | | | | | | | | | | | | | | |
|---|---|---|---|---|---|---|---|---|---|---|---|---|---|---|
| VC_1100_0 | 2 | 13.37055676 | 12.67709929 | 0.51331132 | 0.1855467 | 1.732050808 | 1.73205081 | 1 | 1 | 1 | 1.732050808 | 0 | 0 | 0 OM869532,OM869702 |
| VC_1101_0 | 3 | 18.99728788 | 29.48667774 | 0.3918262 | 0.0160054 | 1.414213562 | 2 | 3 | 3 | 1 | 1.804737854 | 0 | 0 | 0 MG945835,MH992170,MH992193 |
| VC_1103_0 | 2 | 21.03070523 | 8.808256688 | 0.70480687 | 0.2397501 | 0 | 0 | 1 | 1 | 1 | 0 | 0 | 0 | 0 KM589499,KM589507 |
| VC_1104_0 | 44 | 6277.579414 | 279.2492302 | 0.95741093 | 0.2718142 | 0 | 1.73205081 | 946 | 946 | 1 | 1.007584276 | 0 | 0 | 0 KM589500,KM589503,KM589504,KM589505,KM589509,KM589510,KM589512,KM589513,KP087948,MG945178,MG945463,MH572367,MH572368,MH572377,MH572378,MH572379,MH572459,MH572460,MH572461,MH572462,MH572463,MH572465,MH992180,MK249133,MK249138,MK249199,MK249207,MK249208,MK249228,MK249232,MK765579,MK765582,MK765639,MK765642,MK765643,MK765644,MK765646,MN311488,MN311490,MN311491,MZ364242,OL617032,OL617033 |
| VC_1108_0 | 52 | 16916.53921 | 5127.806859 | 0.76738676 | 1.05E-284 | 0 | 2.64575131 | 1326 | 1326 | 1 | 1.350829078 | 0 | 0 | 0 KM589508,KT264808,KT264809,KT264810,MH992165,MK249127,MK249128,MK249129,MK249139,MK249149,MK249150,MK249153,MK249154,MK249155,MK249158,MK249162,MK249163,MK249166,MK249168,MK249174,MK249176,MK249178,MK249180,MK249188,MK249192,MK249194,MK249195,MK249196,MK249198,MK249204,MK249212,MK249214,MK249219,MN862338,MZ364222,MZ364223,MZ364232,MZ364248,MZ364254,MZ364255,MZ364261,MZ364262,MZ364264,MZ364265,MZ364268,MZ364269,MZ364271,MZ364272,MZ364273,MZ364275,MZ364276,MZ364278 |
| VC_1109_0 | 2 | 8.683898524 | 718.340579 | 0.01194444 | 0.1419223 | 1.414213562 | 1.41421356 | 1 | 1 | 1 | 1.414213562 | 0 | 0 | 0 MK249132,MK249205 |
| VC_111_0 | 3 | 852.5586973 | 4183.618555 | 0.16928687 | 0.0016438 | 0 | 3.16227766 | 3 | 3 | 1 | 2.108185107 | 0 | 0 | 0 MZ398135,MZ398136,MZ399596 |
| VC_1110_0 | 2 | 4.705154151 | 98.5916735 | 0.04554984 | 0.0744573 | 1 | 1 | 1 | 1 | 1 | 1 | 0 | 0 | 0 MK249191,MN862347 |
| VC_1111_0 | 5 | 82.16170349 | 0 | 1 | 0 | 0 | 1.41421356 | 10 | 10 | 1 | 0.882842712 | 0 | 0 | 0 KM589514,MK249222,MZ364231,MZ364280,MZ364284 |
| VC_1112_0 | 4 | 65.83955943 | 0 | 1 | 0 | 0 | 1.41421356 | 6 | 6 | 1 | 1.178511302 | 0 | 0 | 0 KM589515,KM589516,MT135405,OM869497 |
| VC_1113_0 | 2 | 300 | 2061.47221 | 0.12703939 | 0.0462473 | 7.141428429 | 7.14142843 | 1 | 1 | 1 | 7.141428429 | 0 | 0 | 0 MH153807,MH153812 |
| VC_1113_1 | 1 | 0 | 1290.088917 | 0 | 0 | 0 | 0 | 1 | 1 | 1 | 0 | 0 | 0 | 0 KX774321 |
| VC_1114_0 | 20 | 57000 | 119058.9948 | 0.32375511 | 1.31E-102 | 2 | 9.05538514 | 190 | 187 | 0.984210526 | 6.337771521 | 0 | 0 | 0 MH576964,MH576965,MH576968,MH590597,MH590599,MH727564,MK359332,MK359351,MK801722,MN369750,MN369757,MN428060,MN484599,MW291014,MW507134,ON260819,ON260828,ON970579,ON970590,OP021680 |
| VC_1114_1 | 1 | 0 | 12987.97607 | 0 | 0 | 0 | 0 | 1 | 1 | 1 | 0 | 0 | 0 | 0 OL829978 |
| VC_1114_2 | 5 | 3000 | 53517.29618 | 0.0530811 | 2.64E-05 | 3.464101615 | 9.38083152 | 10 | 9 | 0.9 | 7.174740102 | 0 | 0 | 0 MF155946,MT310865,MT684590,MT897905,ON970591 |
| VC_1114_3 | 16 | 36000 | 118134.6735 | 0.23356198 | 9.11E-57 | 3.31662479 | 9.32737905 | 120 | 116 | 0.966666667 | 7.367608895 | 0 | 0 | 0 KM652554,MF347636,MF347637,MF347638,MF347639,MF358541,MF358542,MK460248,MK494112,MK977711,MN096371,MN369743,MW507136,MZ958750,OK310502,ON108650 |
| VC_1114_4 | 1 | 0 | 12111.60886 | 0 | 0 | 0 | 0 | 1 | 1 | 1 | 0 | 0 | 0 | 0 MN444876 |
| VC_1114_5 | 1 | 0 | 11926.0522 | 0 | 0 | 0 | 0 | 1 | 1 | 1 | 0 | 0 | 0 | 0 MT711976 |
| VC_1115_0 | 2 | 300 | 11152.62968 | 0.02619486 | 0.0488291 | 3.605551275 | 3.60555128 | 1 | 1 | 1 | 3.605551275 | 0 | 0 | 0 MG757153,MK620896 |
| VC_1115_1 | 2 | 300 | 11433.84881 | 0.02556706 | 0.0487196 | 4 | 4 | 1 | 1 | 1 | 4 | 0 | 0 | 0 MT024865,MT024872 |
| VC_1115_2 | 5 | 3000 | 27045.31849 | 0.09984917 | 6.85E-06 | 2.236067977 | 5.09901951 | 10 | 10 | 1 | 4.103919087 | 0 | 0 | 0 MH590589,MH651172,MW365952,MZ820095,ON456343 |
| VC_1115_3 | 6 | 4500 | 30179.19828 | 0.12976079 | 6.48E-08 | 3.605551275 | 9.11043358 | 15 | 13 | 0.866666667 | 6.980707437 | 0 | 0 | 0 MK061412,MT114162,MW435853,MZ820093,MZ820094,ON208830 |
| VC_1115_4 | 4 | 1800 | 22765.82599 | 0.07327252 | 0.0004344 | 3.741657387 | 7.93725393 | 6 | 6 | 1 | 6.887275578 | 0 | 0 | 0 MH536811,MH536814,MN369754,MT952852 |
| VC_1115_5 | 3 | 900 | 18136.15691 | 0.04727845 | 0.0100268 | 6.92820323 | 8.94427191 | 3 | 3 | 1 | 8.234745336 | 0 | 0 | 0 MT684598,MW822145,ON645339 |
| VC_1116_0 | 24 | 56854.17338 | 11027.22834 | 0.83755155 | 1.11E-125 | 1.414213562 | 7.14142843 | 276 | 276 | 1 | 4.357263141 | 0 | 0 | 0 KT365401,KU160651,KU160656,KU160665,KU160668,KU647629,MF189176,MF189177,MF189178,MF324907,MF377441,MF377442,MH825703,MK919479,MN183279,MN183283,MT024868,MT114161,MT310895,MT776811,MT889388,MW862982,OK999980,ON970601 |
| VC_1117_0 | 19 | 51246.21475 | 4594.412135 | 0.91772277 | 5.50E-89 | 0 | 5.47722558 | 171 | 171 | 1 | 3.069561112 | 0 | 0 | 0 KM879463,KU160660,KX522650,KX670786,KX670787,KX855961,KX855962,MF185718,MF185721,MF185723,MF185724,MF185725,MF668274,MH744419,MK494107,MN204499,MN204503,MN234234,MN703411 |
| VC_1118_0 | 4 | 1189.734805 | 6789.915609 | 0.14909611 | 1.57E-05 | 3.872983346 | 6.4807407 | 6 | 6 | 1 | 5.086983276 | 0 | 0 | 0 MT498055,MW601223,MZ747522,OL742560 |
| VC_1119_0 | 48 | 6865.90261 | 1192.257874 | 0.85204342 | 1.82E-44 | 0 | 2.23606798 | 1128 | 1128 | 1 | 1.407328578 | 0 | 0 | 0 KP087938,KP087942,MG883726,MG945129,MG945206,MG945670,MG945836,MG945840,MG945846,MH572362,MH572410,MH572411,MH572412,MH572486,MH572521,MH931004,MH992163,MH992164,MH992192,MH992199,MH992202,MH992206,MH992208,MH992214,MH992215,MH992216,MH992217,MH992219,MH992220,MH992222,MK765645,MN862346,MW202445,MW202609,MW202760,MW202819,MZ089752,MZ364224,MZ364225,MZ364227,MZ364277,MZ364279,MZ364283,MZ364285,MZ364286,MZ364291,OL617030,OL617031 |
| VC_112_0 | 4 | 793.3768443 | 6207.638956 | 0.1133231 | 1.27E-05 | 0 | 6 | 6 | 6 | 1 | 4.362373124 | 0 | 0 | 0 FR687252,MZ333135,OM513679,OM513680 |
| VC_1121_0 | 6 | 138.032417 | 10.41029992 | 0.92986992 | 0.1090342 | 0 | 2 | 15 | 15 | 1 | 1.013076828 | 0 | 0 | 0 MH572360,MH572361,MH572409,MH572457,MH572482,MH572483 |
| VC_1123_0 | 1 | 0 | 13.21296533 | 0 | 0 | 0 | 0 | 1 | 1 | 1 | 0 | 0 | 0 | 0 MN379632 |
| VC_1124_0 | 3 | 24.29496213 | 10.80823714 | 0.69210108 | 0.3804534 | 0 | 1.73205081 | 3 | 3 | 1 | 1.154700538 | 0 | 0 | 0 MH572366,MW202464,MW202671 |
| VC_1126_0 | 9 | 561.8254763 | 296.7257597 | 0.65438783 | 1.47E-17 | 0 | 1.41421356 | 36 | 36 | 1 | 0.791257816 | 0 | 0 | 0 MH572287,MH572288,MH572467,MH572468,MH572496,MH572497,MH572498,MH572499,MH572500 |
| VC_1127_0 | 2 | 25.55350704 | 17.84847558 | 0.58876359 | 0.0668072 | 0 | 0 | 1 | 1 | 1 | 0 | 0 | 0 | 0 KT264812,MG945475 |
| VC_1128_0 | 3 | 14.27030357 | 2.709588767 | 0.84042368 | 0.1855467 | 1 | 1.73205081 | 3 | 3 | 1 | 1.382088123 | 0 | 0 | 0 KP087943,KP087951,KP087957 |
| VC_1129_0 | 5 | 24.37967715 | 5.805742316 | 0.80766402 | 0.0124042 | 1 | 2.23606798 | 10 | 10 | 1 | 1.567486109 | 0 | 0 | 0 KP087953,MH572371,MH572373,MH572380,MK249215 |
| VC_113_0 | 91 | 464828.7657 | 20179.66462 | 0.95839317 | 0 | 0 | 7.61577311 | 4095 | 4095 | 1 | 6.033690095 | 0 | 0 | 0 AB716666,GQ413938,KF765493,KM576124,KP708985,KP708986,KT367886,KT964103,KU666550,KX211991,KX237514,KX587949,KX856662,KY000081,KY385423,KY684082,MF612071,MG972768,MH885472,MK135468,MK562503,MK598851,MK693005,MN101217,MN101219,MN101220,MN116494,MN176572,MN176573,MN380459,MN794000,MN794001,MN794002,MN794003,MT090077,MT197175,MT197176,MT360680,MT559526,MT559527,MT682065,MT701589,MT939252,MW590329,MZ208805,MZ424864,MZ571827,MZ602146,MZ634338,MZ634339,MZ634340,MZ634342,MZ634343,OK562429,OK583892,OK583893,OL362278,OL362279,OL474125,OL976437,OL979478,OL979479,OM864357,ON132389,ON146449,ON165415,ON602723,ON602724,ON602725,ON602726,ON602727,ON602728,ON602732,ON602739,ON602740,ON602743,ON602748,ON602752,ON602754,ON602756,ON602757,ON602761,ON602762,ON602767,ON809559,OP114731,OP413829,OU509533,OU509534,OX335379,OX335428 |
| VC_1130_0 | 3 | 16.61029432 | 4.860053319 | 0.77363882 | 0.1728893 | 0 | 1 | 3 | 3 | 1 | 0.666666667 | 0 | 0 | 0 KP087955,MH572364,MH572458 |
| VC_1131_0 | 2 | 3.244668459 | 2.709588767 | 0.54493253 | 0.5 | 1 | 1 | 1 | 1 | 1 | 1 | 0 | 0 | 0 MH572489,MW202618 |
| VC_1132_0 | 4 | 181.1055825 | 0 | 1 | 0 | 7.348469228 | 8.60232527 | 6 | 6 | 1 | 7.98745799 | 0 | 0 | 0 KP202971,KT001914,MF098558,ON323491 |
| VC_1133_0 | 6 | 4276.362601 | 326.5671735 | 0.92905232 | 1.82E-07 | 0 | 3.74165739 | 15 | 15 | 1 | 2.405757917 | 0 | 0 | 0 MG592536,MG592554,MG592555,MG592556,MG592591,MG592625 |
| VC_1134_0 | 2 | 152.4390534 | 0 | 1 | 0 | 3.872983346 | 3.87298335 | 1 | 1 | 1 | 3.872983346 | 0 | 0 | 0 KP836355,KP836356 |
| VC_1135_0 | 12 | 11777.59249 | 1486.146357 | 0.88795419 | 8.74E-33 | 0 | 5.29150262 | 66 | 66 | 1 | 3.716604004 | 0 | 0 | 0 MW314851,MW314853,MW314857,MW584149,MW584151,MW584184,MW584193,MW584194,MW584198,MW584200,MW584205,MW584210 |
| VC_1136_0 | 40 | 167177.2947 | 23967.44865 | 0.874611 | 2.54E-278 | 1 | 7.61577311 | 780 | 780 | 1 | 5.728244738 | 0 | 0 | 0 KU963250,MF919514,MG812487,MH153809,MH450129,MH479924,MH651166,MH651168,MH976506,MH976507,MH976508,MH976509,MH976510,MH976511,MH976512,MH976513,MH976514,MH976518,MH976519,MK937594,MK937601,MK967396,MK977702,MN329673,MN329677,MT498035,MT639640,MT723934,MT723937,MT723941,MW822146,MW924645,MZ322015,MZ681510,ON081332,ON456336,ON637761,ON724014,OP172877,OP434456 |
| VC_1138_0 | 16 | 23760.75362 | 28274.26414 | 0.45663007 | 1.40E-70 | 0 | 8.83176087 | 120 | 120 | 1 | 6.188558907 | 0 | 0 | 0 KU963253,MH669000,MK937607,MK937611,MN586026,MN586028,MN586047,MT498036,MW314847,MZ322009,MZ322020,ON392159,ON456337,ON456349,ON755186,OP068342 |
| VC_1139_0 | 5 | 2092.135305 | 353.0438723 | 0.85561636 | 0.0013002 | 2.645751311 | 8.24621125 | 10 | 10 | 1 | 6.159672454 | 0 | 0 | 0 KR063280,MK279853,MN586022,MZ820089,ON755184 |
| VC_114_0 | 8 | 3418.265927 | 10156.83342 | 0.25180412 | 1.16E-19 | 3.16227766 | 6.55743852 | 28 | 28 | 1 | 4.883686567 | 0 | 0 | 0 KF319020,KM819694,MG575418,MG575419,MK387869,MW358930,OL416096,OM234793 |
| VC_1141_0 | 4 | 1800 | 1523.267688 | 0.54163557 | 3.54E-05 | 1 | 7.81024968 | 6 | 6 | 1 | 4.672372311 | 0 | 0 | 0 MK814760,MK894436,MN484601,OP297539 |
| VC_1141_1 | 1 | 0 | 749.0408552 | 0 | 0 | 0 | 0 | 1 | 1 | 1 | 0 | 0 | 0 | 0 KR063281 |
| VC_1142_0 | 2 | 300 | 862.9571105 | 0.25796308 | 0.0532685 | 2.449489743 | 2.44948974 | 1 | 1 | 1 | 2.449489743 | 0 | 0 | 0 MN234206,MN234211 |
| VC_1142_1 | 1 | 0 | 680.924084 | 0 | 0 | 0 | 0 | 1 | 1 | 1 | 0 | 0 | 0 | 0 OK040794 |
| VC_1143_0 | 2 | 300 | 2075.161635 | 0.12630719 | 0.0532685 | 3.464101615 | 3.46410162 | 1 | 1 | 1 | 3.464101615 | 0 | 0 | 0 MN908685,MZ150783 |
| VC_1143_1 | 2 | 300 | 2194.757994 | 0.12025214 | 0.0532685 | 6.08276253 | 6.08276253 | 1 | 1 | 1 | 6.08276253 | 0 | 0 | 0 OK040793,ON637770 |
| VC_1143_2 | 1 | 0 | 968.0932973 | 0 | 0 | 0 | 0 | 1 | 1 | 1 | 0 | 0 | 0 | 0 OK040790 |
| VC_1143_3 | 1 | 0 | 1285.981472 | 0 | 0 | 0 | 0 | 1 | 1 | 1 | 0 | 0 | 0 | 0 OP068343 |
| VC_1144_0 | 2 | 14.88465382 | 0 | 1 | 0 | 0 | 0 | 1 | 1 | 1 | 0 | 0 | 0 | 0 KR704913,KR704915 |
| VC_1145_0 | 164 | 131262.2408 | 16762.04726 | 0.88676151 | 0 | 0 | 3.46410162 | 13366 | 13366 | 1 | 1.952241715 | 0 | 0 | 0 KT264749,KT264750,KT264751,KT264752,KT264753,KT264754,KT264755,KT264756,KT264757,KT264758,KT264759,KT264760,KT264761,KT264763,KT264764,KT264765,KT264766,KT264767,KT264768,KT264769,KT264771,KT264772,KT264774,KT264779,KX513865,MG025518,MG641179,MG641180,MG641186,MG883717,MG945153,MG945158,MG945163,MG945190,MG945203,MG945205,MG945207,MG945213,MG945227,MG945237,MG945238,MG945318,MG945325,MG945330,MG945333,MG945346,MG945351,MG945355,MG945361,MG945363,MG945387,MG945393,MG945398,MG945402,MG945407,MG945415,MG945424,MG945429,MG945435,MG945438,MG945444,MG945459,MG945461,MG945467,MG945476,MG945486,MG945499,MG945508,MG945517,MG945526,MG945531,MG945541,MG945548,MG945559,MG945565,MG945566,MG945570,MG945586,MG945587,MG945589,MG945592,MG945596,MG945602,MG945646,MG945671,MG945673,MG945674,MG945681,MG945691,MG945699,MG945705,MG945708,MG945710,MG945715,MG945728,MG945731,MG945744,MG945758,MG945761,MG945765,MG945768,MG945770,MG945786,MG945787,MG945793,MG945796,MG945801,MG945802,MG945820,MG945853,MG945855,MG945862,MG945864,MG945869,MK765620,MT135257,MT135263,MT135264,MT135272,MT135273,MT135278,MT135338,MZ089765,MZ089767,MZ089774,MZ089775,MZ089776,OM869524,OM869525,OM869526,OM869527,OM869528,OM869531,OM869533,OM869534,OM869535,OM869536,OM869537,OM869538,OM869543,OM869546,OM869547,OM869550,OM869606,OM869635,OM869641,OM869642,OM869643,OM869644,OM869645,OM869664,OM869697,OM869698,OM869699,OM869700,OM869701,OM869703,OM869704,OP549869,OP549872,OP549875,OP549876,S3_vf_127426,S8_vf_94361 |
| VC_1146_0 | 3 | 46.67842573 | 81.01637672 | 0.36554679 | 0.0036818 | 0 | 0 | 3 | 3 | 1 | 0 | 0 | 0 | 0 MG945359,MG945431,MG945700 |
| VC_1147_0 | 6 | 153.0090983 | 345.9918216 | 0.30663089 | 4.26E-07 | 0 | 2 | 15 | 15 | 1 | 1.396852185 | 0 | 0 | 0 KT264770,MG883711,MG945134,MG945399,MG945707,MK496726 |
| VC_1148_0 | 4 | 99.37786234 | 1875.184652 | 0.05032905 | 1.00E-05 | 0 | 2.23606798 | 6 | 6 | 1 | 1.558408522 | 0 | 0 | 0 KT264775,MG883718,MG945386,MG945775 |
| VC_1149_0 | 3 | 141.8897693 | 117.336472 | 0.54735882 | 0.0035409 | 5.916079783 | 8.42614977 | 3 | 3 | 1 | 6.940990085 | 0 | 0 | 0 S14_vs_66841,S4_vs_VIRSorter_19324-cat_2,S7_vs_49426 |
| VC_115_0 | 9 | 3185.946014 | 2981.651736 | 0.5165619 | 1.05E-22 | 2 | 8.71779789 | 36 | 36 | 1 | 6.855938508 | 0 | 0 | 0 AB720063,AP008979,AY299121,DQ777876,EU717894,MW934263,MZ701794,OK258140,OM913894 |
| VC_1150_0 | 2 | 15.66280352 | 43.93128736 | 0.26282477 | 0.1015201 | 1 | 1 | 1 | 1 | 1 | 1 | 0 | 0 | 0 MG945489,MT135282 |
| VC_1151_0 | 2 | 8.382872996 | 33.48584846 | 0.20021803 | 0.2225044 | 1.732050808 | 1.73205081 | 1 | 1 | 1 | 1.732050808 | 0 | 0 | 0 MK496719,MK496738 |
| VC_1152_0 | 26 | 2503.22939 | 1241.536504 | 0.66846085 | 8.69E-18 | 0 | 2.82842712 | 325 | 325 | 1 | 1.95874619 | 0 | 0 | 0 KT264796,KT264797,MG883722,MG945286,MG945310,MG945311,MG945312,MG945319,MG945338,MG945347,MG945385,MG945389,MG945410,MG945416,MG945437,MG945538,MG945600,MG945627,MG945635,MG945639,MG945672,MG945746,MG945767,MG945858,OM869558,OM869663 |
| VC_1153_0 | 2 | 8.683898524 | 162.7658315 | 0.05064982 | 0.0521443 | 1.414213562 | 1.41421356 | 1 | 1 | 1 | 1.414213562 | 0 | 0 | 0 MG945131,MG945742 |
| VC_1155_0 | 14 | 999.821919 | 206.6519526 | 0.82871411 | 2.15E-22 | 0 | 2.23606798 | 91 | 91 | 1 | 1.539755979 | 0 | 0 | 0 KT264776,MG883719,MG945139,MG945339,MG945394,MG945403,MG945542,MG945550,MG945645,MG945736,MG945740,MG945771,MG945819,OP209174 |
| VC_1156_0 | 5 | 173.5098916 | 216.0192195 | 0.44543498 | 2.34E-07 | 0 | 1 | 10 | 10 | 1 | 0.6 | 0 | 0 | 0 KT264781,MG945481,MG945807,MT135256,MT135266 |
| VC_1160_0 | 3 | 16.00823713 | 46.34228411 | 0.25674584 | 0.0132412 | 0 | 1 | 3 | 3 | 1 | 0.666666667 | 0 | 0 | 0 MK496751,MK496754,OM869507 |
| VC_1161_0 | 16 | 751.6964362 | 803.439401 | 0.48336384 | 4.20E-07 | 0 | 2.44948974 | 120 | 120 | 1 | 1.358146779 | 0 | 0 | 0 KT264782,KT264783,KT264833,MG945154,MG945472,MG945534,MG945826,MK496812,MK496813,MK765559,MK765603,MK765621,MK765669,MZ089756,MZ089773,S12_vs_188832 |
| VC_1162_0 | 3 | 43.56582849 | 26.84177837 | 0.61876593 | 0.0131197 | 0 | 0 | 3 | 3 | 1 | 0 | 0 | 0 | 0 MG945418,MG945549,MG945747 |
| VC_1163_0 | 1 | 0 | 81.30436937 | 0 | 0 | 0 | 0 | 1 | 1 | 1 | 0 | 0 | 0 | 0 OM869631 |
| VC_1164_0 | 5 | 158.7404882 | 32.28615214 | 0.83098613 | 0.0012095 | 0 | 1 | 10 | 10 | 1 | 0.6 | 0 | 0 | 0 MG945529,MG945568,MG945717,MG945739,MT135265 |
| VC_1165_0 | 10 | 745.3898297 | 59.27115483 | 0.92634022 | 5.60E-10 | 0 | 2.44948974 | 45 | 45 | 1 | 1.837559165 | 0 | 0 | 0 KT264785,KX513873,MG945186,MG945569,MG945689,MG945797,MG945870,MN379630,MN379640,MT135351 |
| VC_1166_0 | 2 | 25.55350704 | 55.04399297 | 0.31705086 | 0.0672973 | 0 | 0 | 1 | 1 | 1 | 0 | 0 | 0 | 0 KT264784,KX513864 |
| VC_1167_0 | 11 | 891.5340139 | 690.1519988 | 0.56366055 | 1.11E-27 | 0 | 3 | 55 | 55 | 1 | 2.122420158 | 0 | 0 | 0 KT264798,KT264799,KT264800,KT264801,MG945135,MG945136,MG945157,MG945391,MG945867,MT135258,MT135268 |
| VC_1169_0 | 8 | 397.2564869 | 973.7936722 | 0.28974614 | 4.99E-13 | 0 | 2.82842712 | 28 | 28 | 1 | 1.897876585 | 0 | 0 | 0 KT264788,KT264789,KT264790,MG945138,MG945381,MG945382,MG945780,MT135259 |
| VC_117_0 | 2 | 61.39540842 | 79.33435945 | 0.43626455 | 0.1139 | 5.196152423 | 5.19615242 | 1 | 1 | 1 | 5.196152423 | 0 | 0 | 0 MG592523,OX241425 |
| VC_1170_0 | 19 | 3796.450067 | 1267.7247 | 0.74966806 | 2.74E-68 | 0 | 2.64575131 | 171 | 171 | 1 | 1.562387072 | 0 | 0 | 0 KT264791,KT264792,KT264793,KX513868,KX513877,MG945162,MG945233,MG945334,MG945383,MG945430,MG945447,MG945514,MG945642,MG945694,MG945753,MG945781,MT135277,MZ821031,S8_vf_208119 |
| VC_1171_0 | 1 | 0 | 50.03353137 | 0 | 0 | 0 | 0 | 1 | 1 | 1 | 0 | 0 | 0 | 0 KT264794 |
| VC_1173_0 | 3 | 8.079156504 | 73.88498109 | 0.09856941 | 0.2127806 | 2 | 2.44948974 | 3 | 3 | 1 | 2.149829914 | 0 | 0 | 0 KT264795,MG945624,MG945738 |
| VC_1174_0 | 4 | 39.46660338 | 265.2533238 | 0.12951763 | 0.0233479 | 1.414213562 | 2.44948974 | 6 | 6 | 1 | 2.002227107 | 0 | 0 | 0 MG641184,MZ089778,OM869517,OM869640 |
| VC_1176_0 | 6 | 75.29178976 | 526.1737707 | 0.12518055 | 0.0364794 | 0 | 2.44948974 | 15 | 15 | 1 | 1.796640014 | 0 | 0 | 0 MK496714,MK496779,MK496792,MK496803,MZ089777,OM869545 |
| VC_1177_0 | 2 | 32.69160596 | 21.22662884 | 0.60631818 | 0.0938906 | 1.414213562 | 1.41421356 | 1 | 1 | 1 | 1.414213562 | 0 | 0 | 0 KT264803,MG945142 |
| VC_1178_0 | 3 | 64.32463117 | 9.024920896 | 0.87696011 | 0.0382613 | 1.414213562 | 1.73205081 | 3 | 3 | 1 | 1.626105059 | 0 | 0 | 0 KT264802,MG945620,MG945741 |
| VC_1179_0 | 4 | 139.0044301 | 11.42472529 | 0.92405245 | 0.005317 | 1.414213562 | 2 | 6 | 6 | 1 | 1.670763258 | 0 | 0 | 0 KT264805,MG945519,MG945693,MG945809 |
| VC_118_0 | 13 | 14359.05478 | 0 | 1 | 0 | 0 | 4.79583152 | 78 | 78 | 1 | 3.10338801 | 0 | 0 | 0 AB720064,LR743523,LR743528,LR743529,LR743531,MW802488,MW822535,MW822536,MW822537,MW822538,MW822539,OK913679,OK913680 |
| VC_1180_0 | 4 | 116.3318995 | 0 | 1 | 0 | 0 | 2.23606798 | 6 | 6 | 1 | 1.578689326 | 0 | 0 | 0 KT264806,MG945320,MG945561,MG945721 |
| VC_1181_0 | 4 | 42.90002732 | 280.87452 | 0.13249969 | 0.3444046 | 1.414213562 | 2.64575131 | 6 | 6 | 1 | 2.22279108 | 0 | 0 | 0 KT264820,KT264821,MG945644,MK765602 |
| VC_1183_0 | 2 | 34.43196865 | 47.34176251 | 0.42106393 | 0.0839909 | 0 | 0 | 1 | 1 | 1 | 0 | 0 | 0 | 0 KT264816,KT264817 |
| VC_1184_0 | 10 | 1093.190962 | 363.1525294 | 0.75064088 | 4.60E-22 | 0 | 1.41421356 | 45 | 45 | 1 | 0.690468174 | 0 | 0 | 0 KT264818,KT264819,KX513867,KX513872,KX513874,MG945573,MG945759,MG945760,MG945811,S8_vf_208146 |
| VC_1185_0 | 7 | 538.6715417 | 246.3578185 | 0.68618012 | 5.35E-13 | 0 | 1.41421356 | 21 | 21 | 1 | 0.824462309 | 0 | 0 | 0 MG883714,MG945364,MG945525,MG945719,MG945805,MT135337,MT135424 |
| VC_1186_0 | 5 | 161.2969427 | 22.00957006 | 0.87993023 | 0.0007894 | 0 | 0 | 10 | 10 | 1 | 0 | 0 | 0 | 0 OM869513,OM869516,OM869519,OM869634,OM869637 |
| VC_1187_0 | 3 | 63.0921157 | 62.53317146 | 0.50222465 | 0.002521 | 0 | 0 | 3 | 3 | 1 | 0 | 0 | 0 | 0 MG945234,MG945405,MG945827 |
| VC_1188_0 | 3 | 12.72631731 | 50.73259446 | 0.20054421 | 0.0416292 | 1.414213562 | 1.73205081 | 3 | 3 | 1 | 1.626105059 | 0 | 0 | 0 KT264822,KT264830,MK496745 |
| VC_1189_0 | 16 | 1668.574408 | 224.4361303 | 0.88143958 | 1.68E-24 | 0 | 2 | 120 | 120 | 1 | 1.099336914 | 0 | 0 | 0 KT264823,KT264824,KT264825,MG945179,MG945357,MG945413,MG945506,MG945507,MG945609,MG945676,MG945706,MG945764,MG945817,MT135267,MT135271,MT135281 |
| VC_119_0 | 9 | 2988.131862 | 6.966487696 | 0.99767404 | 0.0101136 | 1 | 6.32455532 | 36 | 36 | 1 | 4.715993154 | 0 | 0 | 0 AB757800,AB757801,JX880072,KX365747,MH059635,MK574011,MW202665,MW202744,OL959999 |
| VC_1190_0 | 5 | 196.08166 | 229.8409173 | 0.46036926 | 1.80E-07 | 0 | 2 | 10 | 10 | 1 | 1.219615242 | 0 | 0 | 0 MG945441,MG945487,MG945505,MT135255,MT135275 |
| VC_1191_0 | 9 | 593.4664997 | 101.7328885 | 0.85366373 | 1.41E-11 | 0 | 1.41421356 | 36 | 36 | 1 | 0.909108946 | 0 | 0 | 0 KT264828,MG945228,MG945426,MG945726,MG945754,MG945816,OP549870,OP549873,OP549877 |
| VC_1192_0 | 4 | 126.1842314 | 49.38445779 | 0.71871717 | 0.0002884 | 0 | 0 | 6 | 6 | 1 | 0 | 0 | 0 | 0 MG945204,MG945727,MG945785,MG945810 |
| VC_1193_0 | 2 | 3.713947622 | 47.08505212 | 0.07311064 | 0.1943736 | 2.236067977 | 2.23606798 | 1 | 1 | 1 | 2.236067977 | 0 | 0 | 0 KT264831,MN379641 |
| VC_1194_0 | 2 | 9.051870842 | 0 | 1 | 0 | 1.414213562 | 1.41421356 | 1 | 1 | 1 | 1.414213562 | 0 | 0 | 0 KT264834,MG945678 |
| VC_1195_0 | 2 | 82.17662523 | 280.7517716 | 0.22642655 | 0.0373139 | 2 | 2 | 1 | 1 | 1 | 2 | 0 | 0 | 0 S4_vf_12624,S7_vf_5843 |
| VC_1196_0 | 7 | 6300 | 3415.213038 | 0.64846751 | 1.75E-11 | 1.732050808 | 7 | 21 | 21 | 1 | 4.510617844 | 0 | 0 | 0 MN988521,MN988522,MN988523,MN988524,MN988525,MN988559,MN988561 |
| VC_1196_1 | 1 | 0 | 2203.709028 | 0 | 0 | 0 | 0 | 1 | 1 | 1 | 0 | 0 | 0 | 0 MN988560 |
| VC_1197_0 | 8 | 6535.953053 | 1466.84525 | 0.81670846 | 1.11E-14 | 0 | 6.55743852 | 28 | 28 | 1 | 4.718654796 | 0 | 0 | 0 KT755656,MH431938,MH454076,MH454080,MH460824,MH460825,MH460826,MH460827 |
| VC_1198_0 | 24 | 80164.64381 | 0 | 1 | 0 | 0 | 5 | 276 | 276 | 1 | 2.885243546 | 0 | 0 | 0 KT876724,KT876726,KU599877,KU599878,KU599879,KU599886,MK764441,MK764449,MW421577,MW421578,MW421579,MW421580,MW442825,MW442826,MW442827,MW442828,MW442829,MW442830,MW448539,MW448540,MW448541,OK340820,OK340821,OK340822 |
| VC_1199_0 | 3 | 900 | 1970.196389 | 0.31356739 | 0.0057488 | 0 | 1.73205081 | 3 | 3 | 1 | 1.154700538 | 0 | 0 | 0 MH576962,MK620894,MW291021 |
| VC_1199_1 | 2 | 300 | 1911.910669 | 0.13562935 | 0.0926982 | 3.464101615 | 3.46410162 | 1 | 1 | 1 | 3.464101615 | 0 | 0 | 0 MH744418,MT310898 |
| VC_12_0 | 21 | 20746.36293 | 70971.22892 | 0.2261983 | 2.44E-127 | 2.828427125 | 9.21954446 | 210 | 201 | 0.957142857 | 7.893140391 | 0 | 0 | 0 AP008954,AY954960,AY954961,CP062422,CP062462,CP071049,DQ530362,JQ973847,KJ140076,KJ452291,KP209285,KP893289,KT344895,MK290764,MK618716,MW238767,OM439663,OM439669,OM439672,OM439673,OM439674 |
| VC_12_1 | 2 | 188.6241017 | 9778.572207 | 0.01892449 | 0.042593 | 7.071067812 | 7.07106781 | 1 | 1 | 1 | 7.071067812 | 0 | 0 | 0 MG543995,MG564297 |
| VC_12_2 | 19 | 17946.19428 | 71370.29153 | 0.20092813 | 3.22E-104 | 0 | 8.77496439 | 171 | 171 | 1 | 7.519158643 | 0 | 0 | 0 AB370268,AP001553,AY954962,AY954963,AY954964,AY954965,AY954966,AY954967,AY954968,DQ908929,JX013863,JX094501,KC595279,KP893290,KR709302,KR709303,MK211557,MN904510,ON571632 |
| VC_120_0 | 3 | 461.4146258 | 187.7895154 | 0.71073888 | 0.0078851 | 1 | 6.55743852 | 3 | 3 | 1 | 4.679393074 | 0 | 0 | 0 AB746912,LC545443,LC545444 |
| VC_1200_0 | 73 | 357120.2681 | 6366.529218 | 0.98248484 | 0 | 0 | 8.36660027 | 2628 | 2628 | 1 | 5.945062584 | 0 | 0 | 0 KU160640,KU160643,KU160645,KU160648,KU160649,KU160652,KU160653,KU160659,KU160661,KU160662,KU160671,KU160672,KX576640,KX576641,KX621004,KX621005,KX688102,KX688103,MF140400,MF140404,MF140407,MF140424,MF140425,MF668285,MG198779,MG198781,MG198782,MG210949,MH450117,MH450118,MH450121,MH450131,MH744421,MK112526,MK112528,MK112529,MK112531,MK112535,MK112538,MK279844,MK279845,MK279847,MK279851,MK279854,MK279856,MK279860,MK279862,MK279863,MK279867,MK279868,MK279869,MK279872,MK279874,MK279875,MK279876,MK279880,MK279884,MK279886,MK279892,MK279896,MK279901,MK279907,MK660714,MK814749,MK919476,MK919484,MN586061,MN735429,MN813680,MT498045,MT498050,MW314848,OP434444 |
| VC_1201_0 | 2 | 97.49158301 | 3571.722829 | 0.02657015 | 0.0426231 | 5.744562647 | 5.74456265 | 1 | 1 | 1 | 5.744562647 | 0 | 0 | 0 KX557278,MT771339 |
| VC_1202_0 | 10 | 8273.099989 | 6293.707939 | 0.56794186 | 7.39E-30 | 0 | 5.65685425 | 45 | 45 | 1 | 3.238904209 | 0 | 0 | 0 MK016495,MK016497,MK737942,MN369765,MT310855,MT498057,MZ150782,MZ747515,ON260821,ON970583 |
| VC_1203_0 | 28 | 45793.52561 | 47575.79628 | 0.4904558 | 1.05E-220 | 0 | 8.30662386 | 378 | 378 | 1 | 6.119889509 | 0 | 0 | 0 MG839027,MG925343,MG925345,MG925347,MG944216,MG944218,MG944226,MH513982,MH590604,MH590606,MK308637,MK620901,MK620904,MK814762,MK894432,MK937602,MN096367,MN096378,MN284901,MN310543,MT553340,MT657343,MT657344,MZ005670,OK318961,ON526972,ON970571,OP068339 |
| VC_1204_0 | 25 | 70663.95727 | 25058.69185 | 0.73821565 | 4.93E-124 | 0 | 7.14142843 | 300 | 300 | 1 | 5.342705459 | 0 | 0 | 0 KU160641,KU160646,MF038790,MF038791,MF140401,MF140423,MF140432,MH399789,MH450115,MH450125,MH479926,MH825711,MN234168,MN234194,MN234221,MN284902,MN703412,MW055912,MZ209303,MZ274309,OK310503,ON456335,ON456340,ON645338,OP434462 |
| VC_1206_0 | 3 | 795.5318527 | 8226.722847 | 0.0881744 | 0.0014939 | 3.31662479 | 3.87298335 | 3 | 3 | 1 | 3.643755174 | 0 | 0 | 0 OM913586,ON970564,ON970608 |
| VC_1207_0 | 20 | 31042.24167 | 319.8125272 | 0.98980256 | 4.71E-40 | 1.732050808 | 7.54983444 | 190 | 190 | 1 | 5.406547502 | 0 | 0 | 0 KU160644,MF140397,MH834595,MH834606,MH834608,MH834609,MH834616,MH834618,MH834620,MH834624,MN234214,MN484600,MN703409,MT889373,MT952853,MW507131,ON260822,ON970604,OP297550,OP297552 |
| VC_1208_0 | 6 | 2496.129909 | 394.8512607 | 0.86341964 | 5.45E-07 | 0 | 4 | 15 | 15 | 1 | 2.59761825 | 0 | 0 | 0 KU160650,MF668266,MF668273,MF668279,MH450114,ON337195 |
| VC_1209_0 | 2 | 39.57362975 | 360.512087 | 0.09891288 | 0.064498 | 4.69041576 | 4.69041576 | 1 | 1 | 1 | 4.69041576 | 0 | 0 | 0 MW507126,MW590223 |
| VC_121_0 | 2 | 221.1034873 | 180.8230277 | 0.55010923 | 0.0815934 | 0 | 0 | 1 | 1 | 1 | 0 | 0 | 0 | 0 LC545445,LC545446 |
| VC_1210_0 | 7 | 6145.138128 | 120.4518482 | 0.98077566 | 4.04E-05 | 2.236067977 | 4.47213595 | 21 | 21 | 1 | 3.654633973 | 0 | 0 | 0 KU160654,KU160664,MF140418,MF140427,MF140434,MG099950,MN585972 |

| | | | | | | | | | | | | | | | |
|---|---|---|---|---|---|---|---|---|---|---|---|---|---|---|---|
| VC_1211_0 | 3 | 900 | 1884.509854 | 0.32321667 | 0.0016579 | 2.828427125 | 3.46410162 | 3 | 3 | 1 | 3.040318622 | 0 | 0 | 0 | MN586021,MT889394,OP434452 |
| VC_1211_1 | 3 | 840.0330961 | 1912.269992 | 0.30521097 | 0.0016355 | 2.828427125 | 5 | 3 | 3 | 1 | 4.276142375 | 0 | 0 | 0 | KU160669,KU160673,MW712735 |
| VC_1211_2 | 2 | 146.1010093 | 1763.679932 | 0.07650145 | 0.0458109 | 7.549834435 | 7.54983444 | 1 | 1 | 1 | 7.549834435 | 0 | 0 | 0 | MW862992,OM818331 |
| VC_1212_0 | 21 | 58564.40571 | 23802.48186 | 0.7110188 | 2.92E-95 | 0 | 7.41619849 | 210 | 210 | 1 | 5.406578821 | 0 | 0 | 0 | MH153803,MH153813,MN183284,MT114166,MT639641,MT657333,MW365946,MW365949,MW365950,MW712724,MW712727,MW712728,MW924638,MZ322011,MZ322018,OM203157,OP068331,OP068332,OP068336,OP068337,OP068344 |
| VC_1214_0 | 6 | 4500 | 23.55474208 | 0.99479287 | 7.04E-06 | 0 | 7.07106781 | 15 | 15 | 1 | 4.997587108 | 0 | 0 | 0 | KU230356,MK605242,MK605243,MK605244,MK605245,MK605246 |
| VC_1215_0 | 4 | 1431.373206 | 0 | 1 | 0 | 3 | 4.58257569 | 6 | 6 | 1 | 3.979988005 | 0 | 0 | 0 | KU234532,KU234533,MT426122,MZ078141 |
| VC_1216_0 | 22 | 42292.65692 | 50745.80928 | 0.45457173 | 1.02E-137 | 0 | 8.71779789 | 231 | 231 | 1 | 4.88055141 | 0 | 0 | 0 | KU963246,KU963247,MG099941,MH271295,MK279911,MK279912,MK359313,MK801734,MK875797,MN586055,MT771342,MT818424,MW055908,MW291013,MW507130,MW822147,MZ150788,MZ171371,MZ681509,OM203160,ON645343,OP297544 |
| VC_1217_0 | 41 | 139432.2045 | 6630.384057 | 0.95460587 | 0 | 0 | 9.16515139 | 820 | 818 | 0.997560976 | 6.922237236 | 0 | 0 | 0 | KU963251,KU963254,KU963259,KU998236,KU998248,KX557274,KX557275,MF919521,MH020241,MH153808,MH536818,MH576969,MK501729,MK501730,MK801721,MK801724,MK814757,MK864263,MK878896,MK919470,MK967389,MN010762,MN062704,MN096365,MN234161,MN234179,MN369740,MN585963,MN586031,MT553336,MT723935,MT723936,MT723942,MW291016,MW601225,MZ150785,MZ622164,ON724008,ON724011,ON724012,ON970567 |
| VC_1218_0 | 14 | 10850.34767 | 3039.972665 | 0.78114452 | 3.55E-53 | 2.449489743 | 9.2736185 | 91 | 90 | 0.989010989 | 6.915847147 | 0 | 0 | 0 | KU963252,KU998235,KX557279,MH479915,MK061410,MK284521,MK878901,MK937599,MK967377,MN813694,MT498059,MT522006,MT771341,MW055901 |
| VC_1219_0 | 24 | 68370.72775 | 3452.450626 | 0.95193125 | 5.36E-90 | 0 | 7.68114575 | 276 | 276 | 1 | 5.428561403 | 0 | 0 | 0 | KU998234,KX557286,MH479910,MH479917,MH669015,MK305889,MK814761,MK864264,MK864265,MK864266,MK864267,MK937603,MK967381,MN010760,MN284907,MT310872,MT521998,MT639651,MW507125,MZ005680,MZ150791,MZ622177,ON970602,ON970619 |
| VC_1221_0 | 3 | 900 | 289.4992953 | 0.75662088 | 0.0318013 | 1.732050808 | 2.64575131 | 3 | 3 | 1 | 2.275763954 | 0 | 0 | 0 | KU647626,KU647627,MN586053 |
| VC_1222_0 | 3 | 533.2385939 | 511.4283949 | 0.51043883 | 0.0018127 | 2.449489743 | 4.69041576 | 3 | 3 | 1 | 3.713301834 | 0 | 0 | 0 | MK814759,MZ209302,OM818327 |
| VC_1223_0 | 2 | 300 | 6277.201231 | 0.04561211 | 0.0487357 | 7.141428429 | 7.14142843 | 1 | 1 | 1 | 7.141428429 | 0 | 0 | 0 | KU998245,MK524501 |
| VC_1223_1 | 10 | 13500 | 9797.879709 | 0.57945187 | 2.99E-22 | 1.732050808 | 7.81024968 | 45 | 45 | 1 | 4.747876313 | 0 | 0 | 0 | KU998244,KU998246,KU998247,KX557276,MF919520,MF919537,MK376956,MK376959,MT498054,MZ388552 |
| VC_1224_0 | 4 | 1800 | 4597.756291 | 0.28134864 | 2.32E-05 | 6 | 8.30662386 | 6 | 6 | 1 | 7.551900206 | 0 | 0 | 0 | KX557272,MK977705,MN908691,MT657339 |
| VC_1224_1 | 1 | 0 | 1927.740746 | 0 | 0 | 0 | 0 | 1 | 1 | 1 | 0 | 0 | 0 | 0 | MH590603 |
| VC_1225_0 | 6 | 4398.346522 | 23.48115402 | 0.99468972 | 3.03E-05 | 0 | 6.63324958 | 15 | 15 | 1 | 3.64029784 | 0 | 0 | 0 | MH479913,MH479925,MK919478,MN586014,MN813698,MW601220 |
| VC_1226_0 | 5 | 1521.03238 | 785.5812899 | 0.65942225 | 1.79E-07 | 3.872983346 | 4.89897949 | 10 | 10 | 1 | 4.325924901 | 0 | 0 | 0 | KX557282,MK524505,MK814750,MW862986,ON724009 |
| VC_1228_0 | 4 | 855.4429069 | 992.11117 | 0.46301373 | 9.71E-05 | 3.464101615 | 5.91607978 | 6 | 6 | 1 | 5.078476877 | 0 | 0 | 0 | KX620750,KX620751,KX620753,KX620754 |
| VC_1229_0 | 5 | 95.93518044 | 1.037936793 | 0.98929665 | 0.0818672 | 3.464101615 | 5.29150262 | 10 | 10 | 1 | 4.524211323 | 0 | 0 | 0 | KX077892,KX077893,OL774866,OL774876,S9_vf_90866 |
| VC_123_0 | 4 | 1017.740682 | 4629.304057 | 0.18022536 | 1.47E-05 | 0 | 5.74456265 | 6 | 6 | 1 | 4.713584711 | 0 | 0 | 0 | JQ177061,JQ177064,KJ572844,MN379460 |
| VC_1230_0 | 2 | 83.43977665 | 175.8622872 | 0.32178601 | 0.0517377 | 5 | 5 | 1 | 1 | 1 | 5 | 0 | 0 | 0 | S12_vs_5857,S16_vs_19320 |
| VC_1231_0 | 2 | 136.6295844 | 61.9326808 | 0.68809441 | 0.0818672 | 5.291502622 | 5.29150262 | 1 | 1 | 1 | 5.291502622 | 0 | 0 | 0 | S3_vs_73361,S8_vs_176604 |
| VC_1232_0 | 3 | 554.8966051 | 62.99330938 | 0.89805092 | 0.0055862 | 0 | 1.41421356 | 3 | 3 | 1 | 0.942809042 | 0 | 0 | 0 | MT344103,MT344104,MT344105 |
| VC_1233_0 | 9 | 7461.151114 | 250.186128 | 0.96755606 | 1.20E-11 | 2.236067977 | 6.244998 | 36 | 36 | 1 | 4.696770336 | 0 | 0 | 0 | KX455876,KY290947,KY290949,KY290950,KY290952,MH179470,MH179471,MH179479,MT276994 |
| VC_1234_0 | 2 | 238.9896657 | 231.9786032 | 0.50744324 | 0.0523546 | 0 | 0 | 1 | 1 | 1 | 0 | 0 | 0 | 0 | MG592484,MG592485 |
| VC_1235_0 | 3 | 23.69291051 | 145.3618806 | 0.1401493 | 0.0102303 | 1 | 2.23606798 | 3 | 3 | 1 | 1.745355992 | 0 | 0 | 0 | KX513871,MG945160,MG945185 |
| VC_1236_0 | 3 | 28.83000093 | 42.27022988 | 0.40548393 | 0.0057267 | 1.414213562 | 1.73205081 | 3 | 3 | 1 | 1.626105059 | 0 | 0 | 0 | MT135371,MT135422,MT135432 |
| VC_1237_0 | 3 | 61.59904798 | 51.45910268 | 0.54484394 | 0.0047622 | 1 | 1.41421356 | 3 | 3 | 1 | 1.138071187 | 0 | 0 | 0 | MG945352,MG945735,MG945752 |
| VC_1238_0 | 5 | 154.847672 | 141.9896244 | 0.52165841 | 1.01E-06 | 0 | 2 | 10 | 10 | 1 | 1.312095586 | 0 | 0 | 0 | MG945210,MG945327,MG945623,MT135440,MT135446 |
| VC_124_0 | 5 | 1854.431867 | 7130.74151 | 0.20638799 | 4.49E-08 | 3.872983346 | 8.06225775 | 10 | 10 | 1 | 7.187067897 | 0 | 0 | 0 | MG592400,MG592422,MG592425,MG592448,MG592486 |
| VC_124_1 | 1 | 0 | 675.1862643 | 0 | 0 | 0 | 0 | 1 | 1 | 1 | 0 | 0 | 0 | 0 | MG592434 |
| VC_1240_0 | 3 | 61.2242334 | 121.0298436 | 0.33592792 | 0.0025602 | 1 | 1.41421356 | 3 | 3 | 1 | 1.138071187 | 0 | 0 | 0 | MG945748,MG945799,MG945800 |
| VC_1241_0 | 13 | 399.9519104 | 61.06627158 | 0.86754043 | 0.2395541 | 0 | 2 | 78 | 78 | 1 | 1.514726797 | 0 | 0 | 0 | KX513875,MG883712,MG945284,MG945502,MG945616,MG945794,MK496693,MK496774,MZ089789,OM869564,OM869565,OM869570,OM869709 |
| VC_1242_0 | 1 | 0 | 1103.053637 | 0 | 0 | 0 | 0 | 1 | 1 | 1 | 0 | 0 | 0 | 0 | S3_vs_68031 |
| VC_1243_0 | 8 | 4379.080426 | 918.5279988 | 0.82661459 | 5.25E-16 | 0 | 6.70820393 | 28 | 28 | 1 | 4.790919079 | 0 | 0 | 0 | MH727560,MH926061,MK937613,MK977706,MK977710,MK977712,MK977714,MK977715 |
| VC_1244_0 | 4 | 1261.090874 | 194.1313789 | 0.86659675 | 8.04E-07 | 0 | 0 | 6 | 6 | 1 | 0 | 0 | 0 | 0 | MF197383,MH271317,MN234233,OP172878 |
| VC_1245_0 | 2 | 61.20562286 | 7.655129122 | 0.88883175 | 0.1855467 | 2 | 2 | 1 | 1 | 1 | 2 | 0 | 0 | 0 | KY817360,OL656105 |
| VC_1246_0 | 2 | 24.65041705 | 0 | 1 | 0 | 1 | 1 | 1 | 1 | 1 | 1 | 0 | 0 | 0 | LC035386,LC210520 |
| VC_1247_0 | 2 | 300 | 41.15259355 | 0.87937189 | 0.0802903 | 1.414213562 | 1.41421356 | 1 | 1 | 1 | 1.414213562 | 0 | 0 | 0 | LC644975,LC644976 |
| VC_1248_0 | 3 | 790.6290768 | 69.25143028 | 0.91946389 | 0.0049819 | 0 | 2.82842712 | 3 | 3 | 1 | 1.885618083 | 0 | 0 | 0 | MT740726,MT740743,MT740745 |
| VC_1249_0 | 8 | 2758.177352 | 11683.64963 | 0.19098535 | 2.29E-18 | 1 | 8.88819442 | 28 | 28 | 1 | 7.514897582 | 0 | 0 | 0 | MH271298,MH271308,MH271320,MK880124,MT952845,MW862981,OK040779,ON970568 |
| VC_125_0 | 23 | 37381.25989 | 8414.756515 | 0.81625571 | 1.20E-95 | 4.123105626 | 8.71779789 | 253 | 253 | 1 | 6.763447395 | 0 | 0 | 0 | JX866719,KJ021043,KX237516,MF663761,MG197810,MG432137,MK521906,MK685667,MK798142,MN689778,MT559528,MT757392,MT894004,MZ374361,MZ398021,MZ398245,MZ504995,OK499978,ON086804,ON602758,ON807605,ON809560,OP114730 |
| VC_1250_0 | 29 | 64413.68248 | 13948.87823 | 0.82199563 | 2.00E-116 | 0 | 8.36660027 | 406 | 406 | 1 | 5.709594246 | 0 | 0 | 0 | MH834610,MH834619,MH834629,MK061413,MN703413,MN813676,MN908689,MT024869,MT024871,MT639653,MT889366,MT889375,MT889376,MT897906,MW055910,MZ005682,MZ681506,MZ747516,MZ820087,OK040777,OL455896,OL549188,OL549189,OL549191,OL549192,ON208833,ON528933,ON970616,OP434455 |
| VC_1251_0 | 11 | 16500 | 0 | 1 | 1 | 2 | 6.244998 | 55 | 55 | 1 | 4.995261868 | 0 | 0 | 0 | LR535901,LR535903,LR535904,LR535905,LR535906,LR535907,LR535908,LR535911,LR535912,LR535913,LR535917 |
| VC_1252_0 | 2 | 67.61761821 | 424.9183588 | 0.13728463 | 0.047633 | 7.937253933 | 7.93725393 | 1 | 1 | 1 | 7.937253933 | 0 | 0 | 0 | LR596902,S5_vs_120079 |
| VC_1252_1 | 1 | 0 | 178.950614 | 0 | 0 | 0 | 0 | 1 | 1 | 1 | 0 | 0 | 0 | 0 | S5_vs_122344 |
| VC_1254_0 | 2 | 217.5651607 | 1198.359601 | 0.15365588 | 0.0489136 | 6.782329983 | 6.78232998 | 1 | 1 | 1 | 6.782329983 | 0 | 0 | 0 | S3_vf_67333,S8_vf_132697 |
| VC_1254_1 | 2 | 201.1438578 | 961.1579054 | 0.17305649 | 0.0501121 | 6.708203932 | 6.70820393 | 1 | 1 | 1 | 6.708203932 | 0 | 0 | 0 | S16_vf_1037,S4_vf_31978 |
| VC_1254_2 | 2 | 80.99257333 | 1162.414956 | 0.06513759 | 0.0482694 | 8.774964387 | 8.77496439 | 1 | 1 | 1 | 8.774964387 | 0 | 0 | 0 | S15_vf_2513,S8_vf_131009 |
| VC_1254_3 | 1 | 0 | 626.3699104 | 0 | 0 | 0 | 0 | 1 | 1 | 1 | 0 | 0 | 0 | 0 | S3_vf_108959 |
| VC_1254_4 | 1 | 0 | 714.1780633 | 0 | 0 | 0 | 0 | 1 | 1 | 1 | 0 | 0 | 0 | 0 | S3_vs_16825 |
| VC_1256_0 | 3 | 345.9997551 | 93.96718652 | 0.78642217 | 0.0027785 | 5.099019514 | 6.78232998 | 3 | 3 | 1 | 6.221226493 | 0 | 0 | 0 | S11_vf_66160,S11_vs_62573,S1_vf_158961 |
| VC_1257_0 | 2 | 300 | 7890.629595 | 0.03662722 | 0.2949799 | 7.071067812 | 7.07106781 | 1 | 1 | 1 | 7.071067812 | 0 | 0 | 0 | MK250021,MK250023 |
| VC_1257_1 | 7 | 6300 | 17128.56731 | 0.26890249 | 0.0004346 | 3 | 8.48528137 | 21 | 21 | 1 | 6.216553301 | 0 | 0 | 0 | MK250020,MK250022,MK250024,MK250025,MK250026,MK250027,MK250028 |
| VC_1257_2 | 4 | 1800 | 13440.98148 | 0.11810263 | 0.0340354 | 1.414213562 | 5.09901951 | 6 | 6 | 1 | 4.043296893 | 0 | 0 | 0 | MK250015,MK250016,MK250017,MK250018 |
| VC_1257_3 | 2 | 300 | 7918.016577 | 0.03650516 | 0.2578717 | 9 | 9 | 1 | 0 | 0 | 9 | 0 | 0 | 0 | MK250019,MK250029 |
| VC_1258_0 | 24 | 3776.432019 | 4708.185078 | 0.44509163 | 1.41E-62 | 0 | 5.47722558 | 276 | 276 | 1 | 3.891717471 | 0 | 0 | 0 | S11_vs_37666,S13_vf_28309,S14_vf_114491,S14_vf_99344,S16_vf_100235,S17_vf_148303,S17_vf_174203,S17_vf_79151,S3_vf_57422,S3_vf_83536,S3_vf_84832,S4_vf_13627,S4_vf_5021,S5_vf_123990,S7_vf_19866,S7_vf_32559,S7_vf_36627,S7_vf_69348,S8_vf_196670,S8_vf_52050,S8_vs_142097,S8_vs_162053,S9_vf_70236,S9_vf_75363 |
| VC_126_0 | 2 | 91.1914171 | 153.30678 | 0.37297378 | 0.0466291 | 6.480740698 | 6.4807407 | 1 | 1 | 1 | 6.480740698 | 0 | 0 | 0 | KF977490,MW960031 |
| VC_1261_0 | 14 | 1962.570505 | 3324.439756 | 0.37120611 | 1.45E-43 | 0 | 5.19615242 | 91 | 91 | 1 | 3.556411867 | 0 | 0 | 0 | S12_vf_176437,S12_vs_183752,S13_vf_131208,S16_vf_119834,S16_vf_169895,S17_vf_67042,S2_vf_4910,S3_vf_111776,S5_vs_142450,S6_vf_45228,S7_vf_76382,S7_vs_119492,S8_vf_174559,S9_vf_34736 |
| VC_1264_0 | 1 | 0 | 136.0336636 | 0 | 0 | 0 | 0 | 1 | 1 | 1 | 0 | 0 | 0 | 0 | S11_vs_75554 |
| VC_1265_0 | 2 | 75.77784719 | 281.0129967 | 0.21238731 | 0.045523 | 1 | 1 | 1 | 1 | 1 | 1 | 0 | 0 | 0 | S4_vs_VIRSorter_4746-cat_2,S7_vs_41534 |
| VC_1267_0 | 26 | 4102.03235 | 172.3750929 | 0.95967275 | 1.84E-08 | 0 | 2.44948974 | 325 | 325 | 1 | 1.646039958 | 0 | 0 | 0 | LT986678,MG945141,MG945453,MG945455,MG945456,MG945498,MG945509,MG945564,MG945664,MG945680,MG945683,MG945713,MG945716,MG945730,MG945784,MG945790,MG945813,MG945845,MK765557,MK765655,MK765661,MK765667,OM869561,OM869610,S3_vs_127442,S3_vs_77156 |
| VC_1268_0 | 2 | 16.44095477 | 0 | 1 | 0 | 0 | 0 | 1 | 1 | 1 | 0 | 0 | 0 | 0 | MF101922,MH015251 |
| VC_1269_0 | 2 | 91.34937626 | 0 | 1 | 0 | 0 | 0 | 1 | 1 | 1 | 0 | 0 | 0 | 0 | MF443783,MF443784 |
| VC_127_0 | 17 | 13705.34969 | 1156.460682 | 0.92218575 | 1.46E-65 | 1.732050808 | 8.24621125 | 136 | 136 | 1 | 6.421434821 | 0 | 0 | 0 | AB775548,GU903191,KM360178,KX231828,KY705409,MH252365,MT012302,MT161381,MT161382,MT161383,MT161384,MT375042,MT613935,MT701586,MT708550,MZ345003,ON853583 |
| VC_1271_0 | 3 | 455.228415 | 0 | 1 | 0 | 0 | 2.23606798 | 3 | 3 | 1 | 1.490711985 | 0 | 0 | 0 | MF580955,MF580957,MF580958 |
| VC_1272_0 | 3 | 740.02469 | 171.1858724 | 0.81213357 | 0.0077456 | 0 | 1.73205081 | 3 | 3 | 1 | 1.154700538 | 0 | 0 | 0 | MF580956,MF580959,MF580960 |
| VC_1273_0 | 3 | 675.6760575 | 171.1858724 | 0.79785858 | 0.0078372 | 0 | 2 | 3 | 3 | 1 | 1.333333333 | 0 | 0 | 0 | MF580961,MF580962,MF629150 |
| VC_1274_0 | 6 | 912.509715 | 934.8553023 | 0.49395204 | 1.95E-10 | 4.69041576 | 8.24621125 | 15 | 15 | 1 | 6.584265568 | 0 | 0 | 0 | S12_vf_173208,S12_vs_188833,S3_vf_82571,S3_vs_114271,S5_vs_56083,S8_vs_125717 |
| VC_1275_0 | 4 | 306.5449029 | 103.8432319 | 0.74696337 | 0.0008689 | 0 | 1.73205081 | 6 | 6 | 1 | 0.866025404 | 0 | 0 | 0 | MF979562,MK080312,MT074429,MT074440 |
| VC_1276_0 | 3 | 15.01854406 | 0 | 1 | 1 | 0 | 1 | 3 | 3 | 1 | 0.666666667 | 0 | 0 | 0 | MG214764,MW202596,OL617037 |
| VC_1277_0 | 2 | 3.559140954 | 0 | 1 | 0 | 5 | 5 | 1 | 1 | 1 | 5 | 0 | 0 | 0 | MG269973,MW202566 |
| VC_1278_0 | 19 | 29687.78543 | 0 | 1 | 0 | 0 | 6.55743852 | 171 | 171 | 1 | 4.078194401 | 0 | 0 | 0 | MG515223,MG518519,MG518520,MG663582,MG663583,MG663584,MG663585,MG663586,MH155868,MK112550,MN119376,MN284894,MN994275,MT684596,MZ622160,OL455897,ON108647,ON970569,OP021678 |
| VC_1279_0 | 4 | 414.8892543 | 16.71342059 | 0.96127591 | 0.0062511 | 1 | 1.41421356 | 6 | 6 | 1 | 1.138071187 | 0 | 0 | 0 | MG592394,MG592402,MG592427,MG592455 |
| VC_128_0 | 2 | 82.59502521 | 419.4430667 | 0.16451944 | 0.0481843 | 5.196152423 | 5.19615242 | 1 | 1 | 1 | 5.196152423 | 0 | 0 | 0 | EF057797,EU399241 |
| VC_1280_0 | 2 | 139.5421448 | 75.76253852 | 0.64811477 | 0.1317762 | 0 | 0 | 1 | 1 | 1 | 0 | 0 | 0 | 0 | MG592533,MG592560 |
| VC_1281_0 | 2 | 280.8232941 | 4.034083846 | 0.98583823 | 0.1855467 | 2.645751311 | 2.64575131 | 1 | 1 | 1 | 2.645751311 | 0 | 0 | 0 | MK373772,OP136151 |
| VC_1282_0 | 7 | 4557.288288 | 36.52581748 | 0.99204891 | 1.33E-07 | 0 | 7.28010989 | 21 | 21 | 1 | 4.621331987 | 0 | 0 | 0 | MG592596,MW824384,MW824385,MW824386,MW824395,MW824396,MW824397 |
| VC_1283_0 | 2 | 3.91806202 | 22.56504688 | 0.1479457 | 0.2884996 | 2.236067977 | 2.23606798 | 1 | 1 | 1 | 2.236067977 | 0 | 0 | 0 | OM869552,OM869646 |
| VC_1285_0 | 3 | 24.77208602 | 8.623213379 | 0.74178362 | 0.1932381 | 0 | 1.41421356 | 3 | 3 | 1 | 0.942809042 | 0 | 0 | 0 | MG641185,MG945497,MG945667 |
| VC_1286_0 | 16 | 1252.536859 | 73.17351753 | 0.9448043 | 4.15E-13 | 0 | 2.44948974 | 120 | 120 | 1 | 1.403640257 | 0 | 0 | 0 | MG641188,MG945130,MG945256,MG945457,MG945557,MG945725,MG945757,MN379637,MN379647,MT135339,MT135348,MT135361,MT135384,MT135409,MT135435,OL617023 |
| VC_1287_0 | 44 | 151306.1092 | 0 | 1 | 0 | 0 | 8.60232527 | 946 | 946 | 1 | 5.801147403 | 0 | 0 | 0 | MG670586,MH045566,MH045567,MH045568,MH509447,MH834596,MH834599,MH834602,MH834604,MH834626,MK814753,MK937598,MK937610,MN062714,MN062717,MN183281,MN329675,MT024860,MT310853,MT310896,MT639649,MT818427,MT889365,MT889372,MT889389,MT889392,MW601221,MW601224,MW712721,MW924640,MW924647,MZ322012,MZ747513,MZ820083,OK318958,OK999979,OK999981,OL742562,OM818329,ON724018,OP172875,OP434439,OP434443,OP434445 |
| VC_1288_0 | 7 | 2040.519969 | 31.91388393 | 0.98460077 | 2.31E-05 | 2.828427125 | 6.55743852 | 21 | 21 | 1 | 5.093754399 | 0 | 0 | 0 | MG711460,S11_vs_52577,S14_vf_58919,S17_vf_97671,S5_vf_134040,S7_vf_39724,S8_vs_134805 |
| VC_1289_0 | 9 | 1901.970789 | 1988.243822 | 0.48891153 | 7.52E-16 | 3.741657387 | 10.1488916 | 36 | 26 | 0.722222222 | 7.487984906 | 0 | 0 | 0 | S11_vf_47632,S11_vs_30739,S14_vf_92240,S14_vs_117161,S3_vs_12985,S6_vs_10314,S6_vs_54606,S6_vs_56498,S7_vs_5236 |
| VC_129_0 | 1 | 0 | 155.3839691 | 0 | 0 | 0 | 0 | 1 | 1 | 1 | 0 | 0 | 0 | 0 | MF663786 |
| VC_129_1 | 1 | 0 | 134.3562715 | 0 | 0 | 0 | 0 | 1 | 1 | 1 | 0 | 0 | 0 | 0 | OL614104 |
| VC_129_2 | 1 | 0 | 168.1270506 | 0 | 0 | 0 | 0 | 1 | 1 | 1 | 0 | 0 | 0 | 0 | MT711888 |
| VC_1290_0 | 4 | 173.1669504 | 619.4515637 | 0.21847452 | 2.30E-05 | 6.403124237 | 7.28010989 | 6 | 6 | 1 | 6.956448135 | 0 | 0 | 0 | S12_vf_55175,S16_vs_75108,S17_vs_127519,S8_vs_145173 |
| VC_1291_0 | 18 | 2284.528881 | 2199.657359 | 0.50946343 | 3.88E-26 | 3.16227766 | 9.05538514 | 153 | 146 | 0.954248366 | 7.42255823 | 0 | 0 | 0 | S10_vs_18901,S13_vs_33947,S14_vs_9676,S17_vs_103009,S3_vs_18858,S3_vs_49835,S3_vs_95574,S5_vs_136679,S5_vs_145026,S5_vs_74199,S7_vs_20200,S7_vs_42563,S8_vf_72773,S8_vs_204260,S8_vs_205257,S8_vs_208113,S9_vs_4343,S9_vs_90849 |
| VC_1292_0 | 1 | 0 | 194.6578843 | 0 | 0 | 0 | 0 | 1 | 1 | 1 | 0 | 0 | 0 | 0 | S13_vf_38708 |
| VC_1294_0 | 2 | 32.290856 | 358.3449735 | 0.0826623 | 0.0460078 | 7.211102551 | 7.21110255 | 1 | 1 | 1 | 7.211102551 | 0 | 0 | 0 | S14_vs_3251,S8_vf_166403 |
| VC_1295_0 | 1 | 0 | 47.72661198 | 0 | 0 | 0 | 0 | 1 | 1 | 1 | 0 | 0 | 0 | 0 | S12_vs_123620 |
| VC_1295_1 | 1 | 0 | 74.55179773 | 0 | 0 | 0 | 0 | 1 | 1 | 1 | 0 | 0 | 0 | 0 | S13_vs_26756 |
| VC_1296_0 | 82 | 417478.7334 | 108444.577 | 0.79380154 | 0 | 0 | 8.94427191 | 3321 | 3321 | 1 | 6.48111885 | 0 | 0 | 0 | MG839015,MG839016,MG839017,MG839018,MG839020,MG839021,MG839022,MG839023,MG839026,MG839028,MG839029,MG920061,MG925352,MG944219,MG944222,MG962360,MG962367,MH045554,MH045558,MH045560,MH045562,MH045563,MH045564,MH045565,MH153802,MH153805,MH153806,MH153811,MH513981,MK620895,MK620902,MK620903,MK620905,MK757447,MK919471,MK967388,MK967391,MK967394,MK967400,MK977697,MN062702,MN062709,MN062713,MN234163,MN234177,MN234181,MN234204,MN234222,MN369737,MN617840,MN735430,MN735435,MN807246,MN892483,MT024864,MT310851,MT310856,MT310861,MT310866,MT310873,MT451985,MT498049,MT553339,MT553341,MT553343,MT771350,MT952846,MT986026,MT986027,MW055911,MW291026,MW435859,MZ005684,MZ958749,OK310506,ON260823,ON337194,ON456354,ON970581,ON970605,ON970606,OP021683 |
| VC_1299_0 | 53 | 7177.8017 | 1104.814599 | 0.86661043 | 2.27E-20 | 0 | 3.16227766 | 1378 | 1378 | 1 | 2.106428778 | 0 | 0 | 0 | MG883709,MG945260,MG945282,MG945390,MG945396,MG945452,MG945485,MG945511,MG945515,MG945618,MG945634,MG945641,MG945647,MG945743,MH572525,MH572526,MK496694,MK496775,MK765561,MK765563,MK765564,MK765568,MK765569,MK765594,MK765638,MN311493,MT135347,MT135352,MT135355,MT135366,MT135381,MT135382,MT135387,MT135444,MW202703,MW202824,MZ089786,MZ089791,MZ089795,MZ089806,OL617028,OM869549,OM869559,OM869560,OM869562,OM869569,OM869650,OP549868,OP549871,OP549874,S3_vf_54498,S3_vf_67828,S9_vf_23901 |
| VC_1300_0 | 2 | 9.227959122 | 141.8115771 | 0.06109632 | 0.1218355 | 1.732050808 | 1.73205081 | 1 | 1 | 1 | 1.732050808 | 0 | 0 | 0 | S13_vs_90967,S1_vs_100019 |
| VC_1302_0 | 2 | 14.88465382 | 0 | 1 | 0 | 0 | 0 | 1 | 1 | 1 | 0 | 0 | 0 | 0 | MG883710,MG945464 |
| VC_1303_0 | 2 | 18.73845235 | 0 | 1 | 0 | 0 | 0 | 1 | 1 | 1 | 0 | 0 | 0 | 0 | MG883713,MT135394 |
| VC_1304_0 | 7 | 331.2533324 | 33.60809704 | 0.90788805 | 1.04E-05 | 0 | 1.41421356 | 21 | 21 | 1 | 0.543533979 | 0 | 0 | 0 | MG883720,MG945384,MG945482,MG945692,MG945697,MG945698,MG945734 |
| VC_1306_0 | 4 | 80.44511239 | 17.84847558 | 0.81841668 | 0.0046941 | 0 | 1 | 6 | 6 | 1 | 0.5 | 0 | 0 | 0 | MG945724,MT135383,MT135403,MT135456 |
| VC_1307_0 | 5 | 92.10170979 | 52.50975524 | 0.63689079 | 0.0008106 | 0 | 1.73205081 | 10 | 10 | 1 | 1.258505748 | 0 | 0 | 0 | MG883724,MG945625,MG945629,MG945632,MG945633 |
| VC_1308_0 | 15 | 1509.43447 | 154.0222025 | 0.90740835 | 4.90E-21 | 0 | 2.64575131 | 105 | 105 | 1 | 2.134916383 | 0 | 0 | 0 | MG945128,MG945159,MG945188,MG945229,MG945255,MG945323,MG945332,MG945401,MG945685,MG945745,MG945774,MG945803,MG945829,MT135336,MT135441 |
| VC_1309_0 | 3 | 56.81741545 | 119.8029314 | 0.32169236 | 0.0020961 | 1.414213562 | 1.41421356 | 3 | 3 | 1 | 1.414213562 | 0 | 0 | 0 | MG945544,MG945723,MT135439 |
| VC_131_0 | 43 | 123245.9571 | 15964.82631 | 0.88531904 | 1.72E-289 | 0 | 6.63324958 | 903 | 903 | 1 | 3.916307313 | 0 | 0 | 0 | AF493143,KJ507099,KR091952,KU064779,KY798120,KY798121,KY971611,LC730319,LN889756,LN889995,LN913017,MG250485,MG775258,MN729595,MN729596,MN729597,MN729598,MN729599,MN729600,MT104465,MT104466,MT711889,MT711890,MW812345,MZ501272,MZ598487,MZ826344,MZ826345,MZ826346,MZ826347,MZ826348,MZ826349,MZ826351,MZ826352,MZ826354,OK523998,OK523999,OL581611,OM256450,OM282085,OM471789,OM630590,ON189046 |
| VC_1310_0 | 6 | 289.2836658 | 186.8980724 | 0.60750685 | 2.35E-09 | 0 | 2.23606798 | 15 | 15 | 1 | 1.52363122 | 0 | 0 | 0 | MG945414,MG945445,MG945578,MG945611,MT135367,MT135433 |
| VC_1313_0 | 4 | 44.04592753 | 31.84719472 | 0.58036784 | 0.0524826 | 1 | 2 | 6 | 6 | 1 | 1.54875479 | 0 | 0 | 0 | MG945241,MG945276,MG945448,MG945643 |
| VC_1314_0 | 6 | 162.600927 | 87.40137394 | 0.65039772 | 3.06E-07 | 0 | 1 | 15 | 15 | 1 | 0.333333333 | 0 | 0 | 0 | MG945140,MG945257,MG945449,MG945458,MG945662,MG945815 |
| VC_1315_0 | 3 | 13.32836753 | 41.68404467 | 0.24227928 | 0.1407418 | 1 | 1.41421356 | 3 | 3 | 1 | 1.138071187 | 0 | 0 | 0 | MG945580,MG945804,MG945909 |
| VC_1316_0 | 8 | 609.1191911 | 132.7773021 | 0.82102988 | 3.73E-12 | 0 | 1.41421356 | 28 | 28 | 1 | 0.63672954 | 0 | 0 | 0 | MG945166,MG945208,MG945329,MG945365,MG945512,MG945540,MG945687,MT135329 |
| VC_1317_0 | 2 | 9.896962924 | 0 | 1 | 0 | 1 | 1 | 1 | 1 | 1 | 1 | 0 | 0 | 0 | MG945165,MT135354 |
| VC_1318_0 | 5 | 154.0077848 | 47.58300488 | 0.76396241 | 7.00E-05 | 0 | 1.41421356 | 10 | 10 | 1 | 0.965685425 | 0 | 0 | 0 | MG945177,MG945349,MG945411,MG945462,MG945495 |
| VC_1319_0 | 2 | 16.44095477 | 122.029311 | 0.11873275 | 0.0438644 | 0 | 0 | 1 | 1 | 1 | 0 | 0 | 0 | 0 | MN311487,MN311492 |
| VC_132_0 | 19 | 21495.74102 | 14027.51045 | 0.6051175 | 7.61E-95 | 0 | 5.83095189 | 171 | 171 | 1 | 4.244654662 | 0 | 0 | 0 | AB775549,FR823298,GU583987,JX173487,KP025626,KX431888,KX449361,KX449362,KX449363,MH179472,MH179475,MH179478,MH268168,MK033137,MT094431,MT711887,OK094519,OM131411,ON109291 |
| VC_1320_0 | 10 | 519.4200515 | 93.2300509 | 0.84782496 | 4.60E-11 | 0 | 2.44948974 | 45 | 45 | 1 | 1.890308702 | 0 | 0 | 0 | MG945181,MG945211,MG945232,MG945340,MG945553,MG945718,MG945783,MT135261,MT135334,MZ089771 |
| VC_1321_0 | 2 | 3.91806202 | 0 | 1 | 0 | 0 | 0 | 1 | 1 | 1 | 0 | 0 | 0 | 0 | MG945183,MG945314 |
| VC_1322_0 | 6 | 52.9240673 | 22.00957286 | 0.70627914 | 0.0794428 | 0 | 2 | 15 | 15 | 1 | 1.214715465 | 0 | 0 | 0 | MG945261,MG945420,MG945470,MG945518,MK249148,MK496685 |
| VC_1324_0 | 5 | 58.72359084 | 0 | 1 | 0 | 0 | 2 | 10 | 10 | 1 | 1.292820323 | 0 | 0 | 0 | MG945189,MG945275,MG945408,MG945848,MG945852 |
| VC_1325_0 | 7 | 235.8675112 | 0 | 1 | 0 | 0 | 1.41421356 | 21 | 21 | 1 | 0.808122036 | 0 | 0 | 0 | MG945209,MG945263,MG945279,MG945404,MG945750,MG945776,MG945791 |
| VC_1326_0 | 3 | 14.74934964 | 168.742904 | 0.08038132 | 0.3137468 | 1.414213562 | 1.41421356 | 3 | 3 | 1 | 1.414213562 | 0 | 0 | 0 | MG945212,MG945582,MT135345 |
| VC_1328_0 | 3 | 55.25586821 | 26.11267818 | 0.67908142 | 0.0131197 | 1 | 1.41421356 | 3 | 3 | 1 | 1.138071187 | 0 | 0 | 0 | MG945214,MT135380,MT135423 |
| VC_1329_0 | 2 | 24.65041705 | 0 | 1 | 0 | 0 | 0 | 1 | 1 | 1 | 0 | 0 | 0 | 0 | MG945230,MT135357 |
| VC_133_0 | 17 | 40800 | 112934.6108 | 0.26539242 | 3.72E-68 | 0 | 7.28010989 | 136 | 136 | 1 | 3.751751135 | 0 | 0 | 0 | HE600015,KJ716335,KM209228,KM209270,KM209320,KY942056,KY942057,MG266157,MH807812,MH807820,MN788369,MN813048,MN813049,MN813050,MN813051,MN813052,MN813053 |
| VC_133_1 | 6 | 4500 | 75048.83872 | 0.05656902 | 7.64E-09 | 0 | 7.34846923 | 15 | 15 | 1 | 6.039441195 | 0 | 0 | 0 | MW650886,OL656110,ON184125,ON184126,ON212266,ON862890 |
| VC_133_2 | 7 | 6300 | 86434.30567 | 0.06793602 | 8.87E-12 | 6.8556546 | 9.16515139 | 21 | 19 | 0.904761905 | 7.971938225 | 0 | 0 | 0 | FJ373894,JX181829,LC373201,MK509462,MT078988,OL615011,OL615013 |
| VC_133_3 | 1 | 0 | 14252.269 | 0 | 0 | 0 | 0 | 1 | 1 | 1 | 0 | 0 | 0 | 0 | MT857002 |
| VC_1330_0 | 5 | 167.6189607 | 0 | 1 | 0 | 0 | 1 | 10 | 10 | 1 | 0.6 | 0 | 0 | 0 | MG945235,MG945392,MT135388,MT135443,MT135462 |
| VC_1331_0 | 6 | 145.5744084 | 51.80362014 | 0.7375411 | 1.96E-05 | 0 | 1.41421356 | 15 | 15 | 1 | 0.816176046 | 0 | 0 | 0 | MG945236,MG945822,MT135358,MT135377,MT135426,MT135427 |
| VC_1332_0 | 2 | 13.37055676 | 7.632009642 | 0.63661538 | 0.2701457 | 0 | 0 | 1 | 1 | 1 | 0 | 0 | 0 | 0 | MG945567,MG945631 |
| VC_1333_0 | 4 | 84.524356 | 0 | 1 | 0 | 0 | 1.73205081 | 6 | 6 | 1 | 1.215421457 | 0 | 0 | 0 | MG945252,MG945584,MN379645,OM869494 |
| VC_1334_0 | 7 | 370.0940495 | 164.8915132 | 0.69178325 | 1.30E-09 | 0 | 1 | 21 | 21 | 1 | 0.285714286 | 0 | 0 | 0 | MG945488,MG945572,MZ089802,OP549867,S10_vs_43473,S14_vf_37500,S9_vf_26805 |

| | | | | | | | | | | | | | | | | |
|---|---|---|---|---|---|---|---|---|---|---|---|---|---|---|---|---|
| VC_1335_0 | 13 | 541.5900967 | 178.7368047 | 0.7518671 | 4.07E-09 | 0 | 2.23606798 | 78 | 78 | | 1 | 1.325963793 | 0 | 0 | 0 | MG945494,MG945563,MG945585,MK765590,MN379638,MT135333,MT135379,MT135391,MT135401,MT135437,MT135445,OM869668,S7_vs_135828 |
| VC_1336_0 | 1 | 0 | 62.17687715 | 0 | 0 | 0 | 0 | 1 | 1 | | 1 | 0 | 0 | 0 | 0 | MZ089808 |
| VC_1337_0 | 3 | 56.81741545 | 87.39099022 | 0.39399517 | 0.0029471 | 1 | 1.41421356 | 3 | 3 | | 1 | 1.138071187 | 0 | 0 | 0 | MG945425,MG945789,MT135415 |
| VC_1338_0 | 4 | 33.09080068 | 21.57407236 | 0.60533939 | 0.2302791 | 1 | 2 | 6 | 6 | | 1 | 1.426746322 | 0 | 0 | 0 | MG945258,MG945434,MT135434,MT135451 |
| VC_1339_0 | 2 | 9.896962924 | 140.4211187 | 0.06584014 | 0.0501099 | 0 | 0 | 1 | 1 | | 1 | 0 | 0 | 0 | 0 | MK765572,OM869521 |
| VC_134_0 | 2 | 300 | 24133.50514 | 0.01227822 | 0.0425426 | 4.582575695 | 4.58257569 | 1 | 1 | | 1 | 4.582575695 | 0 | 0 | 0 | KP037007,MG973030 |
| VC_134_1 | 4 | 1800 | 49033.06152 | 0.03541003 | 1.22E-05 | 4.795831523 | 7.68114575 | 6 | 6 | | 1 | 6.263796994 | 0 | 0 | 0 | JX878496,KX452697,MF285619,MH929319 |
| VC_134_2 | 17 | 40800 | 197065.6999 | 0.17152536 | 1.28E-86 | 0 | 9.2736185 | 136 | 134 | 0.985294118 | | 7.342700174 | 0 | 0 | 0 | AB797215,KX147096,KY073123,MG428990,MG428991,MG770379,MK373783,MN045230,MN478483,MZ634341,ON202820,ON323462,ON399185,OX335402,OX335403,OX335405,OX335416 |
| VC_1340_0 | 7 | 339.0678367 | 29.24364528 | 0.92060078 | 4.71E-05 | 0 | 1.41421356 | 21 | 21 | | 1 | 0.908146086 | 0 | 0 | 0 | MG945278,MG945285,MG945492,MG945605,MG945751,MG945812,MG945825 |
| VC_1341_0 | 4 | 27.50182193 | 0 | 1 | 0 | 0 | 0 | 6 | 6 | | 1 | 0 | 0 | 0 | 0 | MG945280,MK765649,OM869499,OM869500 |
| VC_1343_0 | 2 | 15.66280352 | 0 | 1 | 0 | 0 | 0 | 1 | 1 | | 1 | 0 | 0 | 0 | 0 | MG945309,MG945433 |
| VC_1344_0 | 26 | 6243.461141 | 5239.029027 | 0.54373756 | 9.52E-112 | 0 | 3.31662479 | 325 | 325 | | 1 | 2.257867212 | 0 | 0 | 0 | MK765552,MK765553,MK765556,MK765560,MK765562,MK765565,MK765591,MK765592,MK765593,MK765596,MK765606,MK765607,MK765609,MK765611,MK765612,MK765613,MK765615,MK765633,MK765648,MK765651,MK765657,MK765658,MK765660,MK765666,MT135276,MT135425 |
| VC_1346_0 | 25 | 2118.163488 | 0 | 1 | 0 | 0 | 2.44948974 | 300 | 300 | | 1 | 1.560084368 | 0 | 0 | 0 | MG945316,MG945344,MG945468,MG945524,MG945556,MG945562,MG945579,MG945612,MG945640,MG945668,MG945712,MG945733,MG945806,MG945814,MK765571,MK765574,MK765576,MK765577,MK765580,MT135330,MT135406,MT135431,MT135458,OL617036,OM869623 |
| VC_1347_0 | 3 | 25.37413903 | 13.36728839 | 0.65496139 | 0.1341429 | 1 | 2 | 3 | 3 | | 1 | 1.577350269 | 0 | 0 | 0 | MG945336,MT135365,MT135448 |
| VC_1348_0 | 7 | 247.7463094 | 0 | 1 | 0 | 0 | 1.41421356 | 21 | 21 | | 1 | 0.908146086 | 0 | 0 | 0 | MG945331,MG945343,MG945490,MG945501,MG945665,MG945729,MG945782 |
| VC_1349_0 | 3 | 54.60108003 | 0 | 1 | 0 | 0 | 2.23606798 | 3 | 3 | | 1 | 1.490711985 | 0 | 0 | 0 | MG945335,MG945778,MT135385 |
| VC_135_0 | 2 | 300 | 47890.70161 | 0.00622527 | 0.0596111 | 6.164414003 | 6.164414 | 1 | 1 | | 1 | 6.164414003 | 0 | 0 | 0 | KF669653,KF669654 |
| VC_135_1 | 1 | 0 | 30989.87154 | 0 | 0 | 0 | 0 | 1 | 1 | | 1 | 0 | 0 | 0 | 0 | MG428992 |
| VC_135_10 | 10 | 13500 | 245037.2064 | 0.05221686 | 4.78E-22 | 1.732050808 | 7.61577311 | 45 | 45 | | 1 | 5.623420291 | 0 | 0 | 0 | MH709121,MN871504,MZ375357,MZ375358,OL964736,OL964737,OL964738,OL964746,OL964747,OL964753 |
| VC_135_11 | 2 | 300 | 50333.80506 | 0.0059249 | 0.0621888 | 0 | 0 | 1 | 1 | | 1 | 0 | 0 | 0 | 0 | MW006476,MW006480 |
| VC_135_12 | 2 | 300 | 59137.26825 | 0.00504734 | 0.1358917 | 1.732050808 | 1.73205081 | 1 | 1 | | 1 | 1.732050808 | 0 | 0 | 0 | MN871494,MN871495 |
| VC_135_13 | 2 | 300 | 59267.0881 | 0.00503634 | 0.1360561 | 7.348469228 | 7.34846923 | 1 | 1 | | 1 | 7.348469228 | 0 | 0 | 0 | JN126049,LR800396 |
| VC_135_14 | 3 | 900 | 87900.33796 | 0.0101351 | 0.0385373 | 5.099019514 | 6.40312424 | 3 | 3 | | 1 | 5.748902133 | 0 | 0 | 0 | KX438380,MN953776,OM681507 |
| VC_135_15 | 2 | 300 | 58364.41163 | 0.00511383 | 0.1573717 | 4.472135955 | 4.47213595 | 1 | 1 | | 1 | 4.472135955 | 0 | 0 | 0 | KX765865,MW388005 |
| VC_135_16 | 1 | 0 | 30060.61578 | 0 | 0 | 0 | 0 | 1 | 1 | | 1 | 0 | 0 | 0 | 0 | KJ174318 |
| VC_135_17 | 1 | 0 | 30469.9089 | 0 | 0 | 0 | 0 | 1 | 1 | | 1 | 0 | 0 | 0 | 0 | MH709120 |
| VC_135_18 | 1 | 0 | 29992.46851 | 0 | 0 | 0 | 0 | 1 | 1 | | 1 | 0 | 0 | 0 | 0 | KR296692 |
| VC_135_19 | 2 | 300 | 59143.86197 | 0.00504678 | 0.1332951 | 6.8556546 | 6.8556546 | 1 | 1 | | 1 | 6.8556546 | 0 | 0 | 0 | MN871500,MT074475 |
| VC_135_2 | 1 | 0 | 31147.94161 | 0 | 0 | 0 | 0 | 1 | 1 | | 1 | 0 | 0 | 0 | 0 | MT074472 |
| VC_135_20 | 1 | 0 | 29528.89115 | 0 | 0 | 0 | 0 | 1 | 1 | | 1 | 0 | 0 | 0 | 0 | OM367911 |
| VC_135_21 | 1 | 0 | 29996.18116 | 0 | 0 | 0 | 0 | 1 | 1 | | 1 | 0 | 0 | 0 | 0 | MK972690 |
| VC_135_22 | 13 | 23400 | 270917.3288 | 0.07950602 | 1.08E-46 | 1.414213562 | 10.8166538 | 78 | 64 | 0.820512821 | | 7.867173998 | 0 | 0 | 0 | FQ312032,JX128257,JX181828,KJ174317,MH427377,MH688040,MH940212,MN066127,MN342150,MW021753,MW021757,OK322699,OP610151 |
| VC_135_23 | 1 | 0 | 28600.93426 | 0 | 0 | 0 | 0 | 1 | 1 | | 1 | 0 | 0 | 0 | 0 | MT074470 |
| VC_135_24 | 1 | 0 | 30079.15063 | 0 | 0 | 0 | 0 | 1 | 1 | | 1 | 0 | 0 | 0 | 0 | MF001362 |
| VC_135_25 | 1 | 0 | 30234.56714 | 0 | 0 | 0 | 0 | 1 | 1 | | 1 | 0 | 0 | 0 | 0 | JN593240 |
| VC_135_26 | 1 | 0 | 29770.98213 | 0 | 0 | 0 | 0 | 1 | 1 | | 1 | 0 | 0 | 0 | 0 | JN673056 |
| VC_135_27 | 1 | 0 | 30810.61142 | 0 | 0 | 0 | 0 | 1 | 1 | | 1 | 0 | 0 | 0 | 0 | MG383452 |
| VC_135_28 | 3 | 900 | 84921.01462 | 0.01048694 | 0.0265049 | 4.795831523 | 9 | 3 | 2 | 0.666666667 | | 7.265277174 | 0 | 0 | 0 | LR811967,LR811969,MW355478 |
| VC_135_29 | 2 | 300 | 57909.26847 | 0.00515382 | 0.1436035 | 1 | 1 | 1 | 1 | | 1 | 1 | 0 | 0 | 0 | MN871475,MN871501 |
| VC_135_3 | 3 | 900 | 85558.7409 | 0.01040959 | 0.0093957 | 4.358898944 | 8.1240384 | 3 | 3 | | 1 | 6.677590594 | 0 | 0 | 0 | MH370368,MH370371,MT074469 |
| VC_135_30 | 2 | 300 | 57708.81565 | 0.00517163 | 0.1242281 | 0 | 0 | 1 | 1 | | 1 | 0 | 0 | 0 | 0 | KX245012,KY000003 |
| VC_135_31 | 6 | 4500 | 167558.7035 | 0.02615386 | 3.64E-06 | 1.414213562 | 8.06225775 | 15 | 15 | | 1 | 6.174372274 | 0 | 0 | 0 | HQ259103,MH370370,MT074476,MW355450,MW355475,MW355477 |
| VC_135_32 | 1 | 0 | 29895.01187 | 0 | 0 | 0 | 0 | 1 | 1 | | 1 | 0 | 0 | 0 | 0 | OM688977 |
| VC_135_33 | 1 | 0 | 28763.16319 | 0 | 0 | 0 | 0 | 1 | 1 | | 1 | 0 | 0 | 0 | 0 | MT499896 |
| VC_135_34 | 3 | 900 | 87515.16001 | 0.01017925 | 0.0276845 | 7.810249676 | 9.16515139 | 3 | 2 | 0.666666667 | | 8.304218333 | 0 | 0 | 0 | KY630163,KY787213,OK283619 |
| VC_135_35 | 1 | 0 | 30061.13231 | 0 | 0 | 0 | 0 | 1 | 1 | | 1 | 0 | 0 | 0 | 0 | MZ156570 |
| VC_135_36 | 2 | 300 | 58234.23472 | 0.00512521 | 0.1379992 | 1 | 1 | 1 | 1 | | 1 | 1 | 0 | 0 | 0 | MN871491,MN871492 |
| VC_135_37 | 1 | 0 | 29869.82357 | 0 | 0 | 0 | 0 | 1 | 1 | | 1 | 0 | 0 | 0 | 0 | MN871493 |
| VC_135_38 | 1 | 0 | 29360.27197 | 0 | 0 | 0 | 0 | 1 | 1 | | 1 | 0 | 0 | 0 | 0 | MN871502 |
| VC_135_39 | 1 | 0 | 30081.66491 | 0 | 0 | 0 | 0 | 1 | 1 | | 1 | 0 | 0 | 0 | 0 | MT074478 |
| VC_135_4 | 1 | 0 | 30186.29042 | 0 | 0 | 0 | 0 | 1 | 1 | | 1 | 0 | 0 | 0 | 0 | LR811968 |
| VC_135_40 | 1 | 0 | 30101.54336 | 0 | 0 | 0 | 0 | 1 | 1 | | 1 | 0 | 0 | 0 | 0 | MH523359 |
| VC_135_41 | 1 | 0 | 29673.01574 | 0 | 0 | 0 | 0 | 1 | 1 | | 1 | 0 | 0 | 0 | 0 | MT074474 |
| VC_135_42 | 1 | 0 | 28406.68596 | 0 | 0 | 0 | 0 | 1 | 1 | | 1 | 0 | 0 | 0 | 0 | MN445184 |
| VC_135_43 | 4 | 1800 | 119302.7685 | 0.01486341 | 6.71E-05 | 1.732050808 | 2.44948974 | 6 | 6 | | 1 | 2.015855318 | 0 | 0 | 0 | MN871496,MN871497,MN871498,MN871499 |
| VC_135_44 | 1 | 0 | 29650.12257 | 0 | 0 | 0 | 0 | 1 | 1 | | 1 | 0 | 0 | 0 | 0 | MW021754 |
| VC_135_45 | 2 | 300 | 58199.92396 | 0.00512821 | 0.0542894 | 7.549834435 | 7.54983444 | 1 | 1 | | 1 | 7.549834435 | 0 | 0 | 0 | MT074471,MT074480 |
| VC_135_46 | 1 | 0 | 29588.80914 | 0 | 0 | 0 | 0 | 1 | 1 | | 1 | 0 | 0 | 0 | 0 | MW021760 |
| VC_135_47 | 1 | 0 | 29228.39813 | 0 | 0 | 0 | 0 | 1 | 1 | | 1 | 0 | 0 | 0 | 0 | MT074479 |
| VC_135_48 | 2 | 300 | 58503.78985 | 0.00510171 | 0.0699912 | 1.732050808 | 1.73205081 | 1 | 1 | | 1 | 1.732050808 | 0 | 0 | 0 | MK972698,MK972699 |
| VC_135_49 | 1 | 0 | 30450.06526 | 0 | 0 | 0 | 0 | 1 | 1 | | 1 | 0 | 0 | 0 | 0 | ON169963 |
| VC_135_5 | 1 | 0 | 30808.96536 | 0 | 0 | 0 | 0 | 1 | 1 | | 1 | 0 | 0 | 0 | 0 | MG748547 |
| VC_135_50 | 1 | 0 | 30909.69569 | 0 | 0 | 0 | 0 | 1 | 1 | | 1 | 0 | 0 | 0 | 0 | ON169961 |
| VC_135_51 | 1 | 0 | 31516.67301 | 0 | 0 | 0 | 0 | 1 | 1 | | 1 | 0 | 0 | 0 | 0 | ON169962 |
| VC_135_6 | 1 | 0 | 30754.5663 | 0 | 0 | 0 | 0 | 1 | 1 | | 1 | 0 | 0 | 0 | 0 | MH791405 |
| VC_135_7 | 1 | 0 | 30298.74095 | 0 | 0 | 0 | 0 | 1 | 1 | | 1 | 0 | 0 | 0 | 0 | MT974436 |
| VC_135_8 | 1 | 0 | 30365.53733 | 0 | 0 | 0 | 0 | 1 | 1 | | 1 | 0 | 0 | 0 | 0 | MT074477 |
| VC_135_9 | 3 | 900 | 90552.29604 | 0.0098412 | 0.016967 | 2.236067977 | 8.54400375 | 3 | 3 | | 1 | 6.421784366 | 0 | 0 | 0 | KP797973,KX171211,MN384979 |
| VC_1350_0 | 2 | 23.09411781 | 0 | 1 | 0 | 1 | 1 | 1 | 1 | | 1 | 1 | 0 | 0 | 0 | MG945337,MT135410 |
| VC_1351_0 | 2 | 3.91806202 | 0 | 1 | 0 | 1.732050808 | 1.73205081 | 1 | 1 | | 1 | 1.732050808 | 0 | 0 | 0 | MG945345,MG945818 |
| VC_1352_0 | 3 | 17.61709367 | 0 | 1 | 0 | 0 | 1.41421356 | 3 | 3 | | 1 | 0.942809042 | 0 | 0 | 0 | MG945348,MG945702,MK496732 |
| VC_1353_0 | 4 | 78.22159039 | 21.57407236 | 0.78381754 | 0.007107 | 1 | 1.73205081 | 6 | 6 | | 1 | 1.451123717 | 0 | 0 | 0 | MG945350,MG945432,MG945755,MT135447 |
| VC_1354_0 | 6 | 125.9805341 | 64.64538573 | 0.6608783 | 7.28E-06 | 0 | 2 | 15 | 15 | | 1 | 1.284481349 | 0 | 0 | 0 | MG945354,MG945576,MG945637,MK496765,OM869571,OP209176 |
| VC_1355_0 | 3 | 76.66052112 | 0 | 1 | 1 | 0 | 0 | 3 | 3 | | 1 | 0 | 0 | 0 | 0 | MG945360,MG945478,MG945614 |
| VC_1356_0 | 4 | 33.61852726 | 5.103089971 | 0.86821083 | 0.0884832 | 0 | 1 | 6 | 6 | | 1 | 0.5 | 0 | 0 | 0 | MG945362,MK496704,MK496729,MK496782 |
| VC_1357_0 | 7 | 99.60120761 | 0 | 1 | 0 | 0 | 1.41421356 | 21 | 21 | | 1 | 0.812907991 | 0 | 0 | 0 | MG945395,MG945421,MG945480,MG945539,MG945696,MK496696,MK496711 |
| VC_1358_0 | 1 | 0 | 30.48666099 | 0 | 0 | 0 | 0 | 1 | 1 | | 1 | 0 | 0 | 0 | 0 | OM869651 |
| VC_136_0 | 3 | 900 | 8739.089174 | 0.09336982 | 0.0014146 | 2 | 3.74165739 | 3 | 3 | | 1 | 3.068586334 | 0 | 0 | 0 | MH363700,MK795384,OK428602 |
| VC_1360_0 | 2 | 19.34051075 | 0 | 1 | 0 | 0 | 0 | 1 | 1 | | 1 | 0 | 0 | 0 | 0 | MG945409,OM869563 |
| VC_1361_0 | 2 | 13.91462015 | 7.114641962 | 0.66167895 | 0.2701457 | 1 | 1 | 1 | 1 | | 1 | 1 | 0 | 0 | 0 | MG945412,MG945828 |
| VC_1362_0 | 2 | 30.01831504 | 0 | 1 | 0 | 0 | 0 | 1 | 1 | | 1 | 0 | 0 | 0 | 0 | MG945417,MG945419 |
| VC_1363_0 | 3 | 23.37613558 | 0 | 1 | 0 | 0 | 1 | 3 | 3 | | 1 | 0.666666667 | 0 | 0 | 0 | MG945427,MG945574,MT135411 |
| VC_1364_0 | 6 | 87.20025028 | 4.860053319 | 0.94720794 | 0.4563309 | 0 | 1.73205081 | 15 | 15 | | 1 | 1.26994394 | 0 | 0 | 0 | MG945439,MG945628,MK496757,MK765601,MZ089768,OM869696 |
| VC_1365_0 | 1 | 0 | 164.6656408 | 0 | 0 | 0 | 0 | 1 | 1 | | 1 | 0 | 0 | 0 | 0 | MG945749 |
| VC_1366_0 | 7 | 205.196291 | 53.91013103 | 0.79193827 | 1.99E-07 | 0 | 1.73205081 | 21 | 21 | | 1 | 1.319333969 | 0 | 0 | 0 | MZ089804,MZ089807,OM869574,OM869576,OM869577,OM869592,OM869653 |
| VC_1367_0 | 5 | 64.80701439 | 19.37349859 | 0.76985768 | 0.3301496 | 0 | 1.41421356 | 10 | 10 | | 1 | 0.882842712 | 0 | 0 | 0 | MG945469,MG945824,MT135353,MT135400,MT135402 |
| VC_1368_0 | 2 | 15.66280352 | 0 | 1 | 0 | 0 | 0 | 1 | 1 | | 1 | 0 | 0 | 0 | 0 | MG945483,MT135420 |
| VC_1369_0 | 10 | 294.1483374 | 178.5373281 | 0.62229164 | 0.0415491 | 0 | 2 | 45 | 45 | | 1 | 1.448603688 | 0 | 0 | 0 | MG945500,MG945621,MH572369,MK765583,MT135326,OM869502,OM869505,OM869587,OM869604,OM869633 |
| VC_137_0 | 2 | 300 | 9829.727356 | 0.0296158 | 0.0444253 | 0 | 0 | 1 | 1 | | 1 | 0 | 0 | 0 | 0 | MG592609,MG592621 |
| VC_137_1 | 2 | 300 | 9928.097585 | 0.02933097 | 0.0444236 | 8.544003745 | 8.54400375 | 1 | 1 | | 1 | 8.544003745 | 0 | 0 | 0 | MW824370,MW824375 |
| VC_1370_0 | 2 | 4.404128344 | 10.20617994 | 0.3014398 | 0.2397501 | 0 | 0 | 1 | 1 | | 1 | 0 | 0 | 0 | 0 | OM869510,OM869512 |
| VC_1372_0 | 2 | 4.705154151 | 10.50720854 | 0.30929805 | 0.2701457 | 0 | 0 | 1 | 1 | | 1 | 0 | 0 | 0 | 0 | MT135328,OM869626 |
| VC_1373_0 | 2 | 4.161094484 | 0 | 1 | 0 | 2.449489743 | 2.44948974 | 1 | 1 | | 1 | 2.449489743 | 0 | 0 | 0 | MG945504,MG945617 |
| VC_1374_0 | 3 | 72.26455169 | 0 | 1 | 0 | 1 | 2 | 3 | 3 | | 1 | 1.577350269 | 0 | 0 | 0 | MG945533,MT135343,MT135416 |
| VC_1375_0 | 12 | 466.0224635 | 165.6243683 | 0.7377896 | 0.1340952 | 0 | 2 | 66 | 66 | | 1 | 1.649546329 | 0 | 0 | 0 | MZ089798,MZ089799,MZ089800,MZ089801,MZ089803,MZ089805,OM869557,OM869648,OM869654,OM869655,OM869666,OM869684 |
| VC_1378_0 | 4 | 53.40147587 | 13.38635668 | 0.79956893 | 0.0112467 | 0 | 1.41421356 | 6 | 6 | | 1 | 0.971404521 | 0 | 0 | 0 | MT135349,MT135363,MT135392,MT135408 |
| VC_138_0 | 2 | 300 | 6968.322208 | 0.041275 | 0.0425908 | 3.464101615 | 3.46410162 | 1 | 1 | | 1 | 3.464101615 | 0 | 0 | 0 | MH375644,MT366762 |
| VC_1382_0 | 2 | 8.382872996 | 0 | 1 | 0 | 0 | 0 | 1 | 1 | | 1 | 0 | 0 | 0 | 0 | MG945554,MG945575 |
| VC_1383_0 | 1 | 0 | 76.07983625 | 0 | 0 | 0 | 0 | 1 | 1 | | 1 | 0 | 0 | 0 | 0 | MZ089762 |
| VC_1384_0 | 14 | 930.1547258 | 191.8777409 | 0.82899092 | 2.86E-12 | 0 | 2.64575131 | 91 | 91 | | 1 | 1.663397128 | 0 | 0 | 0 | MZ089770,MZ089772,MZ089780,OM869514,OM869529,OM869540,OM869542,OM869544,OM869548,OM869553,OM869589,OM869590,OM869638,OM869707 |
| VC_1385_0 | 4 | 25.03603156 | 109.8437294 | 0.18561741 | 0.3802584 | 1.414213562 | 1.41421356 | 6 | 6 | | 1 | 1.414213562 | 0 | 0 | 0 | MG945598,OM869551,OM869556,S1_vs_3435 |
| VC_1386_0 | 2 | 5.103089971 | 0 | 1 | 0 | 0 | 0 | 1 | 1 | | 1 | 0 | 0 | 0 | 0 | MG945615,MT135372 |
| VC_1387_0 | 6 | 121.3164044 | 81.84148276 | 0.59715331 | 0.0053961 | 0 | 1.73205081 | 15 | 15 | | 1 | 1.188513211 | 0 | 0 | 0 | MH572374,MH572375,MH572376,MH572414,MH572485,MH572522 |
| VC_1388_0 | 4 | 26.59874451 | 17.67450532 | 0.60078591 | 0.4559264 | 0 | 1 | 6 | 6 | | 1 | 0.5 | 0 | 0 | 0 | MG945619,MH572524,MH992173,MZ364234 |
| VC_1389_0 | 7 | 500.2621497 | 46.14425894 | 0.91554956 | 1.89E-07 | 0 | 2.64575131 | 21 | 21 | | 1 | 1.259881577 | 0 | 0 | 0 | MK765555,MK765567,MK765600,MK765610,MK765617,MK765659,MK765664 |
| VC_1390_0 | 2 | 3.713947622 | 0 | 1 | 0 | 1.414213562 | 1.41421356 | 1 | 1 | | 1 | 1.414213562 | 0 | 0 | 0 | MG945690,MK496692 |
| VC_1391_0 | 2 | 3.157667461 | 0 | 1 | 0 | 2 | 2 | 1 | 1 | | 1 | 2 | 0 | 0 | 0 | MG945766,MG945823 |
| VC_1392_0 | 2 | 3.713947622 | 0 | 1 | 0 | 0 | 0 | 1 | 1 | | 1 | 0 | 0 | 0 | 0 | MG945769,MG945792 |
| VC_1393_0 | 2 | 15.66280352 | 0 | 1 | 0 | 0 | 0 | 1 | 1 | | 1 | 0 | 0 | 0 | 0 | MG945788,MG945795 |
| VC_1394_0 | 4 | 86.60849513 | 31.61614953 | 0.73257564 | 0.0010054 | 0 | 1.41421356 | 6 | 6 | | 1 | 0.707106781 | 0 | 0 | 0 | MG945798,MK765653,MT135350,MT135457 |
| VC_1395_0 | 2 | 24.65041705 | 31.61614953 | 0.43810061 | 0.0802903 | 0 | 0 | 1 | 1 | | 1 | 0 | 0 | 0 | 0 | OM869639,OM869679 |
| VC_1396_0 | 2 | 19.34051075 | 0 | 1 | 0 | 0 | 0 | 1 | 1 | | 1 | 0 | 0 | 0 | 0 | MG945808,MG945849 |
| VC_1397_0 | 1 | 0 | 13.78429111 | 0 | 0 | 0 | 0 | 1 | 1 | | 1 | 0 | 0 | 0 | 0 | MG945821 |
| VC_1398_0 | 7 | 254.7302513 | 50.84499213 | 0.83360893 | 1.56E-06 | 0 | 1.73205081 | 21 | 21 | | 1 | 1.017314122 | 0 | 0 | 0 | MG945831,MT135370,MT135373,MT135418,MT135430,MT135455,MT135459 |
| VC_1399_0 | 3 | 49.41084835 | 50.84499213 | 0.49284758 | 0.0047102 | 0 | 1.41421356 | 3 | 3 | | 1 | 0.942809042 | 0 | 0 | 0 | OP549864,OP549865,OP549866 |
| VC_140_0 | 2 | 183.8207726 | 2559.407312 | 0.06700893 | 0.0447642 | 6.708203932 | 6.70820393 | 1 | 1 | | 1 | 6.708203932 | 0 | 0 | 0 | AY855346,LK985322 |
| VC_140_1 | 4 | 1096.60538 | 5113.564341 | 0.17658219 | 1.43E-05 | 3.605551275 | 8.42614977 | 6 | 6 | | 1 | 6.908124433 | 0 | 0 | 0 | DQ466086,HG531805,LN681541,LN681542 |
| VC_1400_0 | 2 | 15.11873548 | 0 | 1 | 0 | 1 | 1 | 1 | 1 | | 1 | 1 | 0 | 0 | 0 | MG945832,MT135421 |
| VC_1401_0 | 4 | 66.43830872 | 0 | 1 | 0 | 0 | 0 | 6 | 6 | | 1 | 0 | 0 | 0 | 0 | MG945839,MH572484,MZ364252,MZ364293 |
| VC_1402_0 | 7 | 213.3772691 | 19.21855242 | 0.91737361 | 0.002277 | 0 | 2 | 21 | 21 | | 1 | 1.361355108 | 0 | 0 | 0 | MG945842,MH992159,MH992175,MH992200,MK249218,MK249229,MK249233 |
| VC_1403_0 | 5 | 72.39804305 | 0 | 1 | 0 | 1.414213562 | 2 | 10 | 10 | | 1 | 1.589949494 | 0 | 0 | 0 | MG945850,MT135335,MT135340,MT135376,MT135393 |
| VC_1404_0 | 2 | 15.11873548 | 4.462118895 | 0.77211827 | 0.5 | 1 | 1 | 1 | 1 | | 1 | 1 | 0 | 0 | 0 | MT135269,MT135429 |
| VC_1405_0 | 3 | 23.57666469 | 0 | 1 | 0 | 1.414213562 | 2.23606798 | 3 | 3 | | 1 | 1.794110782 | 0 | 0 | 0 | MG945857,MG945859,MG945866 |
| VC_1406_0 | 21 | 52864.22072 | 881.774697 | 0.98359367 | 3.90E-57 | 0 | 8.24621125 | 210 | 210 | | 1 | 3.555195494 | 0 | 0 | 0 | MH271292,MH271301,MK737941,MK801731,MK801732,MN497954,MT522001,MT522003,MT639643,MT684591,MT684592,MT818420,MW055903,MW291025,MZ681513,OK040788,OK318957,ON526977,ON637767,ON755183,OP580517 |
| VC_1407_0 | 78 | 163536.6695 | 4515.368282 | 0.97313113 | 1.99E-73 | 0 | 5.47722558 | 3003 | 3003 | | 1 | 3.734033554 | 0 | 0 | 0 | MH045557,MH045561,MH371111,MH371118,MH371124,MH399783,MH576963,MH651181,MH744416,MH779512,MH825700,MK524502,MK620897,MK801730,MK878899,MK894434,MN010755,MN010759,MN284892,MN329679,MN369745,MN428062,MN428064,MN585990,MN586008,MN586010,MT310864,MT316458,MT316459,MT316462,MT498038,MT498040,MT498046,MT498047,MT498051,MT498065,MT498067,MT521990,MT639642,MT639647,MT657332,MT776807,MT952850,MW055907,MW055909,MW291020,MW321655,MW507127,MW924642,MZ005676,MZ005678,MZ028628,MZ209304,MZ274307,MZ322006,MZ322008,MZ322014,MZ322027,MZ622180,MZ648031,OK040781,OK040783,OK310504,ON081334,ON108642,ON108648,ON260812,ON260814,ON392161,ON456350,ON645341,ON645345,ON755182,ON755188,ON970573,ON970589,ON970613,OP580518 |
| VC_1408_0 | 2 | 300 | 2424.008565 | 0.11013181 | 0.0441777 | 0 | 0 | 1 | 1 | | 1 | 0 | 0 | 0 | 0 | MN703407,MN703414 |
| VC_1408_1 | 2 | 300 | 2901.198792 | 0.09371489 | 0.0437768 | 6.32455532 | 6.32455532 | 1 | 1 | | 1 | 6.32455532 | 0 | 0 | 0 | MH183162,MT639646 |
| VC_1409_0 | 10 | 8860.451032 | 2529.654581 | 0.77790772 | 1.16E-15 | 0 | 3.74165739 | 45 | 45 | | 1 | 2.51186182 | 0 | 0 | 0 | MW021755,MW021763,MW021766,OL539438,OL539439,OL539442,OL539455,OL539456,OL539464,OM135608 |
| VC_141_0 | 5 | 1500.509468 | 1805.033843 | 0.45393732 | 5.63E-08 | 4 | 7.07106781 | 10 | 10 | | 1 | 6.121615389 | 0 | 0 | 0 | OP341624,OP341626,OP341627,OP341628,OP341630 |
| VC_1411_0 | 5 | 2177.237258 | 1820.788581 | 0.54457809 | 1.54E-07 | 1 | 2.82842712 | 10 | 10 | | 1 | 2.030600053 | 0 | 0 | 0 | MH153799,MK737944,MZ456037,MZ622170,ON260825 |
| VC_1412_0 | 7 | 5217.076768 | 15896.36331 | 0.24709743 | 1.15E-14 | 2.645751311 | 8 | 21 | 21 | | 1 | 5.142584048 | 0 | 0 | 0 | MH271303,MK937591,MK937606,MK937608,MN813682,MN813684,OK040792 |
| VC_1413_0 | 2 | 195.970227 | 1013.078627 | 0.16208628 | 0.0469515 | 3.16227766 | 3.16227766 | 1 | 1 | | 1 | 3.16227766 | 0 | 0 | 0 | MT889371,ON970592 |
| VC_1414_0 | 2 | 23.57041017 | 0 | 1 | 0 | 6.8556546 | 6.8556546 | 1 | 1 | | 1 | 6.8556546 | 0 | 0 | 0 | MH155870,MK240575 |
| VC_1415_0 | 31 | 119075.9582 | 21734.63777 | 0.84564629 | 9.27E-153 | 0 | 7.21110255 | 465 | 465 | | 1 | 4.709667751 | 0 | 0 | 0 | MH155873,MH271304,MH271312,MH271321,MH590600,MK524510,MK875798,MK977698,MN062719,MN175603,MN444877,MN585980,MN585986,MN586007,MN586042,MN586059,MT657336,MT818415,MW578837,MW601217,MZ150792,MZ322026,MZ622179,MZ648035,ON081330,ON260815,ON260820,ON970563,ON970612,OP068338,OP297543 |
| VC_1416_0 | 10 | 11100.26498 | 0 | 1 | 0 | 0 | 5.09901951 | 45 | 45 | | 1 | 3.582205633 | 0 | 0 | 0 | MH229862,MH229863,MH229864,MH229865,MH536815,MH825706,MN224564,MN224565,ON456338,ON456348 |
| VC_1417_0 | 3 | 514.0898818 | 9.227103957 | 0.98236804 | 0.0404278 | 0 | 4 | 3 | 3 | | 1 | 2.666666667 | 0 | 0 | 0 | MH447526,MK064562,MK064563 |
| VC_1418_0 | 3 | 26.18558766 | 0 | 1 | 0 | 1.414213562 | 1.41421356 | 3 | 3 | | 1 | 1.414213562 | 0 | 0 | 0 | MH572274,MH572275,MH572276 |

| | | | | | | | | | | | | | | | |
|---|---|---|---|---|---|---|---|---|---|---|---|---|---|---|---|
| VC_1419_0 | 8 | 270.8878211 | 39.6309151 | 0.8723719 | 1.78E-05 | 0 | 1.41421356 | 28 | 28 | 1 | 0.353553391 | 0 | 0 | 0 | MH572353,MH572354,MH572355,MH572357,MH572358,MH572407,MH572408,MH572516 |
| VC_142_0 | 6 | 3409.993237 | 1219.593039 | 0.73656544 | 6.79E-11 | 0 | 8 | 15 | 15 | 1 | 5.275870066 | 0 | 0 | 0 | KC595514,KC595515,KC595517,KC595518,KT151956,KT151958 |
| VC_1420_0 | 6 | 142.9878794 | 0 | 1 | 0 | 0 | 1 | 15 | 15 | 1 | 0.533333333 | 0 | 0 | 0 | MH572490,MH572491,MH572517,MH572518,MH572519,MH572520 |
| VC_1421_0 | 4 | 546.2365222 | 1150.519432 | 0.32192993 | 2.32E-05 | 7.483314774 | 9.11043358 | 6 | 5 | 0.833333333 | 8.379240834 | 0 | 0 | 0 | S14_vf_109545,S15_vs_7103,S6_vf_45618,S8_vf_111790 |
| VC_1421_1 | 1 | 0 | 358.0167574 | 0 | 0 | 0 | 0 | 1 | 1 | 1 | 0 | 0 | 0 | 0 | S9_vs_10424 |
| VC_1422_0 | 5 | 2404.110025 | 0 | 1 | 0 | 0 | 1 | 10 | 10 | 1 | 0.4 | 0 | 0 | 0 | MH707430,MW354669,MW354676,MW354677,MW354678 |
| VC_1423_0 | 11 | 4658.389782 | 0 | 1 | 1 | 0 | 0 | 55 | 55 | 1 | 0 | 0 | 0 | 0 | MH730557,MZ230992,MZ230993,MZ230994,MZ230995,MZ230996,MZ230997,MZ230998,MZ230999,MZ231000,MZ231001 |
| VC_1424_0 | 31 | 59371.89837 | 7837.200777 | 0.88339078 | 7.52E-89 | 0 | 7.28010989 | 465 | 465 | 1 | 5.170556738 | 0 | 0 | 0 | MK308638,MK757449,MK878904,MK894435,MK894438,MN310546,MN369747,MT310869,MT310875,MT310887,MT310888,MT310889,MT310890,MT310891,MT498064,MT657334,MT657337,MT684587,MT771344,MW055914,MW291018,MW365947,MW365948,MW507124,MW862991,MZ388554,OM913587,ON081328,ON456346,ON970584,OP297538 |
| VC_1425_0 | 6 | 2426.014407 | 7837.200777 | 0.23637957 | 6.08E-11 | 0 | 4.35889894 | 15 | 15 | 1 | 2.423545924 | 0 | 0 | 0 | MH825698,MN062711,MT024863,ON260817,ON526980,OP068335 |
| VC_1426_0 | 2 | 138.8411673 | 33.24371058 | 0.80681794 | 0.0773645 | 6.32455532 | 6.32455532 | 1 | 1 | 1 | 6.32455532 | 0 | 0 | 0 | MK411746,MN586027 |
| VC_1427_0 | 6 | 669.1995257 | 0 | 1 | 0 | 1.732050808 | 4.69041576 | 15 | 15 | 1 | 3.580623048 | 0 | 0 | 0 | MH834607,MH834622,MK757448,MT024867,MT024870,MT889364 |
| VC_1428_0 | 2 | 190.2229751 | 44.55201117 | 0.81023527 | 0.2701457 | 0 | 0 | 1 | 1 | 1 | 0 | 0 | 0 | 0 | MH939157,MK071653 |
| VC_1429_0 | 3 | 34.9796111 | 0 | 1 | 0 | 0 | 1 | 3 | 3 | 1 | 0.666666667 | 0 | 0 | 0 | MH992154,MK249216,MK249217 |
| VC_1430_0 | 3 | 43.65724872 | 0 | 1 | 0 | 0 | 1.73205081 | 3 | 3 | 1 | 1.154700538 | 0 | 0 | 0 | MH992155,MH992196,MH992205 |
| VC_1432_0 | 2 | 15.66280352 | 0 | 1 | 0 | 0 | 0 | 1 | 1 | 1 | 0 | 0 | 0 | 0 | MH992167,MK496682 |
| VC_1433_0 | 2 | 11.07305121 | 0 | 1 | 0 | 0 | 0 | 1 | 1 | 1 | 0 | 0 | 0 | 0 | MH992171,MW202680 |
| VC_1434_0 | 1 | 0 | 276.5340135 | 0 | 0 | 0 | 0 | 1 | 1 | 1 | 0 | 0 | 0 | 0 | S16_vs_15093 |
| VC_1435_0 | 2 | 131.1442893 | 36.59400176 | 0.78183871 | 0.0737507 | 1.414213562 | 1.41421356 | 1 | 1 | 1 | 1.414213562 | 0 | 0 | 0 | ON920540,S11_vf_66522 |
| VC_1436_0 | 3 | 380.4486634 | 7.655129122 | 0.98027556 | 0.0404278 | 1 | 1.41421356 | 3 | 3 | 1 | 1.138071187 | 0 | 0 | 0 | MK305890,MK620900,OP434453 |
| VC_1437_0 | 5 | 64.60404612 | 23.43763746 | 0.73378931 | 0.4753645 | 0 | 1.73205081 | 10 | 10 | 1 | 1.17067423 | 0 | 0 | 0 | MK496679,MK496680,MK496776,OM869578,OM869711 |
| VC_1438_0 | 2 | 5.006181354 | 23.23352027 | 0.17727458 | 0.2635446 | 1.414213562 | 1.41421356 | 1 | 1 | 1 | 1.414213562 | 0 | 0 | 0 | MK496698,MK496772 |
| VC_1439_0 | 2 | 30.01831504 | 16.42996079 | 0.64627404 | 0.1317762 | 0 | 0 | 1 | 1 | 1 | 0 | 0 | 0 | 0 | MK496681,MK496760 |
| VC_144_0 | 2 | 40.43660086 | 321.3837455 | 0.11175878 | 0.0497983 | 6.708203932 | 6.70820393 | 1 | 1 | 1 | 6.708203932 | 0 | 0 | 0 | KT626446,ON699007 |
| VC_1440_0 | 2 | 21.03070523 | 0 | 1 | 0 | 0 | 0 | 1 | 1 | 1 | 0 | 0 | 0 | 0 | MK496683,MK496761 |
| VC_1441_0 | 2 | 4.404128344 | 0 | 1 | 0 | 0 | 0 | 1 | 1 | 1 | 0 | 0 | 0 | 0 | MK496686,MK496764 |
| VC_1442_0 | 3 | 36.7083078 | 0 | 1 | 0 | 0 | 1.41421356 | 3 | 3 | 1 | 0.942809042 | 0 | 0 | 0 | MK496688,MK496690,MK496766 |
| VC_1443_0 | 3 | 8.92423779 | 0 | 1 | 1 | 1 | 1.73205081 | 3 | 3 | 1 | 1.382088123 | 0 | 0 | 0 | MK496689,MK496700,MK496784 |
| VC_1444_0 | 2 | 4.705154151 | 0 | 1 | 0 | 0 | 0 | 1 | 1 | 1 | 0 | 0 | 0 | 0 | MK496697,MK496780 |
| VC_1445_0 | 2 | 6.404115778 | 0 | 1 | 0 | 0 | 0 | 1 | 1 | 1 | 0 | 0 | 0 | 0 | MK496699,MK496785 |
| VC_1446_0 | 2 | 11.07305121 | 0 | 1 | 0 | 0 | 0 | 1 | 1 | 1 | 0 | 0 | 0 | 0 | MK496701,MK496702 |
| VC_1447_0 | 3 | 8.079156504 | 0 | 1 | 0 | 1 | 1.41421356 | 3 | 3 | 1 | 1.138071187 | 0 | 0 | 0 | MK496710,MK496767,MK496768 |
| VC_1448_0 | 2 | 11.77202121 | 0 | 1 | 0 | 0 | 0 | 1 | 1 | 1 | 0 | 0 | 0 | 0 | MK496712,MK496787 |
| VC_1449_0 | 2 | 14.34058733 | 8.079156504 | 0.639641 | 0.2701457 | 0 | 0 | 1 | 1 | 1 | 0 | 0 | 0 | 0 | MK496716,MK496790 |
| VC_145_0 | 6 | 232.8513736 | 669.4525961 | 0.25806312 | 0.1374818 | 6 | 9.21954446 | 15 | 13 | 0.866666667 | 8.180215802 | 0 | 0 | 0 | GU967410,MF893271,MH844558,MW476490,S12_vs_48045,S9_vf_73311 |
| VC_145_1 | 1 | 0 | 313.8523744 | 0 | 0 | 0 | 0 | 1 | 1 | 1 | 0 | 0 | 0 | 0 | KX965989 |
| VC_1450_0 | 2 | 9.419844648 | 0 | 1 | 0 | 0 | 0 | 1 | 1 | 1 | 0 | 0 | 0 | 0 | MK496717,MK496804 |
| VC_1451_0 | 2 | 25.55350704 | 0 | 1 | 0 | 0 | 0 | 1 | 1 | 1 | 0 | 0 | 0 | 0 | MK496720,MK496796 |
| VC_1452_0 | 3 | 21.01852731 | 0 | 1 | 1 | 0 | 0 | 3 | 3 | 1 | 0 | 0 | 0 | 0 | MK496721,MK496781,MK496786 |
| VC_1453_0 | 3 | 30.43732828 | 10.93772987 | 0.73564436 | 0.0382613 | 1 | 1.41421356 | 3 | 3 | 1 | 1.138071187 | 0 | 0 | 0 | MK496725,MK496746,MK496811 |
| VC_1454_0 | 2 | 14.88465382 | 0 | 1 | 0 | 0 | 0 | 1 | 1 | 1 | 0 | 0 | 0 | 0 | MK496748,MK496815 |
| VC_1455_0 | 3 | 24.77208602 | 5.103089971 | 0.82918628 | 0.5 | 0 | 1.73205081 | 3 | 3 | 1 | 1.154700538 | 0 | 0 | 0 | MK496750,MK496816,OM869659 |
| VC_1456_0 | 2 | 16.44095477 | 0 | 1 | 0 | 0 | 0 | 1 | 1 | 1 | 0 | 0 | 0 | 0 | MK496753,MK496818 |
| VC_1457_0 | 2 | 9.896962924 | 0 | 1 | 0 | 1.414213562 | 1.41421356 | 1 | 1 | 1 | 1.414213562 | 0 | 0 | 0 | MK496756,MK496824 |
| VC_1458_0 | 2 | 14.88465382 | 0 | 1 | 0 | 0 | 0 | 1 | 1 | 1 | 0 | 0 | 0 | 0 | MK496758,MK496808 |
| VC_1459_0 | 2 | 16.44095477 | 0 | 1 | 0 | 0 | 0 | 1 | 1 | 1 | 0 | 0 | 0 | 0 | MK496770,MK496771 |
| VC_146_0 | 1 | 0 | 626.6947378 | 0 | 0 | 0 | 0 | 1 | 1 | 1 | 0 | 0 | 0 | 0 | KM983329 |
| VC_1460_0 | 2 | 3.95697869 | 0 | 1 | 0 | 0 | 0 | 1 | 1 | 1 | 0 | 0 | 0 | 0 | MK496820,OM869632 |
| VC_1461_0 | 3 | 506.7978302 | 146.4355053 | 0.77582971 | 0.0360991 | 0 | 2.23606798 | 3 | 3 | 1 | 1.490711985 | 0 | 0 | 0 | MK570053,MK570054,MK570055 |
| VC_1462_0 | 110 | 1776197.971 | 0 | 1 | 0 | 0 | 10.2469508 | 5995 | 5843 | 0.974645538 | 4.936735917 | 0 | 0 | 0 | MK719701,MK719702,MK719703,MK719704,MK719705,MK719706,MK719707,MK719708,MK719709,MK719710,MK719711,MK719712,MK719713,MK719714,MK719715,MK719716,MK719717,MK719718,MK719719,MK719720,MK719721,MK719722,MK719723,MK719724,MK719725,MK719726,MK719727,MK719728,MK719729,MK719730,MK719731,MK719732,MK719733,MK719734,MK719735,MK719736,MK719737,MK719738,MK719739,MK719740,MK719741,MK719742,MK719743,MK719744,MK719745,MK719746,MK719747,MK719748,MK719749,MK719750,MK719751,MK719752,MK719753,MK719754,MT497224,MT497225,MT497226,MT497227,MT497228,MT497229,MT497230,MT497231,MT497232,MT497233,MT497234,MT497235,MT497236,MT497237,MT497238,MT497239,MT497240,MT497241,MT497242,MT497243,MT497244,MT497245,MT497246,MT497247,MT497248,MT497249,MT497250,MT497251,MT497252,MT497253,MT497254,MT497255,MT497256,MT497257,MT497258,MT497259,MT497260,MT497261,MT497262,MT497263,MT497264,MT497265,MT497266,MT497267,MT497268,MT497269,MT497270,MT497271,MT497272,MT497273,MT497274,MT497275,MT497276,MT497277,MT497278,MT497279 |
| VC_1463_0 | 2 | 20.18560719 | 0 | 1 | 0 | 0 | 0 | 1 | 1 | 1 | 0 | 0 | 0 | 0 | MK765551,MK765650 |
| VC_1464_0 | 9 | 423.7927635 | 0 | 1 | 1 | 0 | 0 | 36 | 36 | 1 | 0 | 0 | 0 | 0 | MK765558,MK765581,MK765604,MK765622,MK765640,MK765654,MK765656,MK765663,MK765668 |
| VC_1465_0 | 2 | 9.419844648 | 0 | 1 | 0 | 0 | 0 | 1 | 1 | 1 | 0 | 0 | 0 | 0 | MK765573,MK765662 |
| VC_1466_0 | 2 | 11.07305121 | 0 | 1 | 0 | 0 | 0 | 1 | 1 | 1 | 0 | 0 | 0 | 0 | MK765575,MK765578 |
| VC_1467_0 | 14 | 419.0107822 | 2089.918329 | 0.16700782 | 0.4860965 | 0 | 2.64575131 | 91 | 91 | 1 | 1.990900852 | 0 | 0 | 0 | MK765587,MK765588,MK765598,MK765599,MK765623,MK765625,MK765628,MK765630,MK765631,MK765634,OL617020,OL617024,OL617026,OM782324 |
| VC_1468_0 | 28 | 8263.224956 | 2737.802529 | 0.75113211 | 2.07E-168 | 0 | 2.23606798 | 378 | 378 | 1 | 0.666085152 | 0 | 0 | 0 | MK765589,MN988460,MN988467,MN988469,MN988471,MN988473,MN988474,MN988475,MN988478,MN988479,MN988480,MN988481,MN988489,MN988491,MN988494,MN988498,MN988499,MN988503,MN988504,MN988511,MN988518,MW960029,MW960035,MW980067,OL617021,OL617022,OL617025,OL617027 |
| VC_1469_0 | 1 | 0 | 52.47933727 | 0 | 0 | 0 | 0 | 1 | 1 | 1 | 0 | 0 | 0 | 0 | MK765626 |
| VC_1471_0 | 2 | 10.37408269 | 0 | 1 | 0 | 0 | 0 | 1 | 1 | 1 | 0 | 0 | 0 | 0 | MK765605,MK765641 |
| VC_1472_0 | 2 | 20.72428145 | 4.933341507 | 0.80772414 | 0.1855467 | 8.306623863 | 8.30662386 | 1 | 1 | 1 | 8.306623863 | 0 | 0 | 0 | MK929790,MW202545 |
| VC_1473_0 | 2 | 13.37055676 | 0 | 1 | 0 | 0 | 0 | 1 | 1 | 1 | 0 | 0 | 0 | 0 | MN379629,MN379636 |
| VC_1474_0 | 2 | 20.18560719 | 0 | 1 | 0 | 0 | 0 | 1 | 1 | 1 | 0 | 0 | 0 | 0 | MN379639,MN379646 |
| VC_1475_0 | 2 | 14.88465382 | 0 | 1 | 0 | 0 | 0 | 1 | 1 | 1 | 0 | 0 | 0 | 0 | MN379643,MN379650 |
| VC_1477_0 | 2 | 30.38696429 | 139.0230894 | 0.17936931 | 0.0508389 | 4.795831523 | 4.79583152 | 1 | 1 | 1 | 4.795831523 | 0 | 0 | 0 | S16_vf_90328,S8_vf_13758 |
| VC_1477_1 | 1 | 0 | 39.23902606 | 0 | 0 | 0 | 0 | 1 | 1 | 1 | 0 | 0 | 0 | 0 | S12_vs_172379 |
| VC_1479_0 | 4 | 291.8550798 | 573.9252734 | 0.3371006 | 4.99E-05 | 6.08276253 | 9.16515139 | 6 | 5 | 0.833333333 | 7.687350593 | 0 | 0 | 0 | S14_vs_117165,S7_vs_87439,S8_vs_71212,S9_vs_90857 |
| VC_148_0 | 2 | 270.9469136 | 430.7355592 | 0.38613892 | 0.0516367 | 1 | 1 | 1 | 1 | 1 | 1 | 0 | 0 | 0 | MN604230,MN604698 |
| VC_1481_0 | 3 | 228.2061042 | 1905.268305 | 0.10696454 | 0.0043642 | 6.782329983 | 9 | 3 | 2 | 0.666666667 | 8.029651282 | 0 | 0 | 0 | S4_vs_VIRSorter_31196-circular-cat_2,S6_vs_15813,S7_vs_118295 |
| VC_1481_1 | 2 | 141.8246626 | 1410.103135 | 0.09138612 | 0.0450454 | 7.810249676 | 7.81024968 | 1 | 1 | 1 | 7.810249676 | 0 | 0 | 0 | S4_vs_VIRSorter_31560-circular-cat_2,S8_vf_55476 |
| VC_1481_2 | 1 | 0 | 737.5112392 | 0 | 0 | 0 | 0 | 1 | 1 | 1 | 0 | 0 | 0 | 0 | S7_vs_78507 |
| VC_1482_0 | 4 | 1800 | 0 | 1 | 1 | 0 | 1.73205081 | 6 | 6 | 1 | 1.093412989 | 0 | 0 | 0 | MN988461,MN988462,MN988463,MN988472 |
| VC_1483_0 | 2 | 15.66280352 | 0 | 1 | 0 | 0 | 0 | 1 | 1 | 1 | 0 | 0 | 0 | 0 | MN988485,MN988550 |
| VC_1484_0 | 15 | 23052.42376 | 42.95385982 | 0.99814015 | 2.09E-10 | 0 | 6.40312424 | 105 | 105 | 1 | 4.041789466 | 0 | 0 | 0 | MT732458,MT732459,MT732460,MT732461,MT732462,MT732463,MT732464,MT732465,MT732466,MT732467,MT732468,MT732469,MT732470,MT732471,MT732472 |
| VC_1485_0 | 3 | 238.1986677 | 82.96946363 | 0.74166346 | 0.0404278 | 0 | 5.65685425 | 3 | 3 | 1 | 3.771236166 | 0 | 0 | 0 | MT006234,MT006235,S16_vf_173109 |
| VC_1488_0 | 6 | 4500 | 1238.634344 | 0.78415869 | 4.40E-09 | 0 | 7.21110255 | 15 | 15 | 1 | 4.677905222 | 0 | 0 | 0 | MT074137,MT074139,MT074140,MT074141,MT074142,MT074146 |
| VC_1489_0 | 2 | 300 | 336.3070167 | 0.47147052 | 0.056629 | 2.828427125 | 2.82842712 | 1 | 1 | 1 | 2.828427125 | 0 | 0 | 0 | S1_vf_159951,S3_vf_127399 |
| VC_149_0 | 1 | 0 | 224.6635536 | 0 | 0 | 0 | 0 | 1 | 1 | 1 | 0 | 0 | 0 | 0 | HM159959 |
| VC_1490_0 | 6 | 4043.551935 | 5190.350181 | 0.43790284 | 3.28E-09 | 2.449489743 | 7.34846923 | 15 | 15 | 1 | 5.824436722 | 0 | 0 | 0 | S12_vf_188824,S13_vf_151045,S15_vf_74586,S16_vf_169985,S3_vf_18829,S4_vf_27861 |
| VC_1490_1 | 6 | 3194.201548 | 5360.767219 | 0.37337384 | 3.29E-09 | 3.31662479 | 8.42614977 | 15 | 15 | 1 | 6.213614496 | 0 | 0 | 0 | MT121964,S13_vf_56074,S15_vf_20644,S3_vf_127406,S3_vs_127445,S8_vf_147128 |
| VC_1491_0 | 4 | 60.01929299 | 0 | 1 | 0 | 0 | 1.73205081 | 6 | 6 | 1 | 1.093412989 | 0 | 0 | 0 | MT135262,MT135274,MT135389,MT135413 |
| VC_1492_0 | 8 | 718.3333485 | 147.6774406 | 0.8294739 | 2.38E-08 | 0 | 2 | 28 | 28 | 1 | 1.384139336 | 0 | 0 | 0 | MT135285,MT135289,MT135297,MT135302,MT135308,MT135314,S3_vf_127431,S9_vf_90852 |
| VC_1493_0 | 8 | 519.7870457 | 149.2543828 | 0.77691309 | 3.52E-13 | 0 | 3.60555128 | 28 | 28 | 1 | 2.567446955 | 0 | 0 | 0 | MT135288,MT135290,MT135291,MT135301,MT135303,MT135307,MT135309,MT135311 |
| VC_1494_0 | 7 | 312.2199133 | 137.2277721 | 0.69467465 | 1.45E-08 | 0 | 4 | 21 | 21 | 1 | 2.664121186 | 0 | 0 | 0 | MT135287,MT135294,MT135298,MT135299,MT135304,MT135306,MT135310 |
| VC_1495_0 | 6 | 237.2336676 | 4.136706948 | 0.98286158 | 0.0153271 | 0 | 2.44948974 | 15 | 15 | 1 | 1.53852385 | 0 | 0 | 0 | MT135292,MT135293,MT135296,MT135305,S12_vf_188838,S13_vf_150407 |
| VC_1496_0 | 2 | 5.404118571 | 0 | 1 | 0 | 1 | 1 | 1 | 1 | 1 | 1 | 0 | 0 | 0 | MT135332,MT135436 |
| VC_1497_0 | 2 | 8.081848958 | 0 | 1 | 0 | 0 | 0 | 1 | 1 | 1 | 0 | 0 | 0 | 0 | MT135346,MT135395 |
| VC_1498_0 | 8 | 282.673755 | 8.361107034 | 0.97127112 | 0.0548292 | 1 | 2.82842712 | 28 | 28 | 1 | 1.983155984 | 0 | 0 | 0 | MT135359,MT135368,MT135374,MT135378,MT135399,MT135414,MT135419,MT135463 |
| VC_1499_0 | 4 | 36.48849837 | 0 | 1 | 0 | 1.414213562 | 2 | 6 | 6 | 1 | 1.670763258 | 0 | 0 | 0 | MT135369,MT135396,MT135454,MT135460 |
| VC_15_0 | 3 | 364.882834 | 7283.041749 | 0.04771005 | 0.0014632 | 7 | 7.28010989 | 3 | 3 | 1 | 7.16373748 | 0 | 0 | 0 | KX827371,MK075002,OM373558 |
| VC_15_1 | 9 | 2801.606166 | 12617.68497 | 0.18169487 | 8.49E-23 | 4.472135955 | 9.38083152 | 36 | 33 | 0.916666667 | 7.4367008 | 0 | 0 | 0 | AY954954,MK075001,MK075004,MK450538,OM373550,OM373554,OM373555,OM373556,OM373561 |
| VC_150_0 | 3 | 490.9762647 | 1748.218122 | 0.21926469 | 0.0015441 | 2.645751311 | 4.35889894 | 3 | 3 | 1 | 3.749096981 | 0 | 0 | 0 | S11_vf_104070,S11_vf_34224,S9_vf_90871 |
| VC_1500_0 | 3 | 28.45330631 | 0 | 1 | 0 | 0 | 1.41421356 | 3 | 3 | 1 | 0.942809042 | 0 | 0 | 0 | MT135375,MT135438,MT135453 |
| VC_1501_0 | 2 | 15.66280352 | 0 | 1 | 0 | 1 | 1 | 1 | 1 | 1 | 1 | 0 | 0 | 0 | MT135397,MT135407 |
| VC_1502_0 | 4 | 46.35568681 | 0 | 1 | 0 | 0 | 2 | 6 | 6 | 1 | 1.333333333 | 0 | 0 | 0 | MT185428,MZ089750,MZ364229,MZ364288 |
| VC_1503_0 | 2 | 300 | 0 | 1 | 0 | 5.477225575 | 5.47722558 | 1 | 1 | 1 | 5.477225575 | 0 | 0 | 0 | MT234670,ON014754 |
| VC_1504_0 | 2 | 275.8296539 | 50.05679693 | 0.84639804 | 0.1056498 | 1.414213562 | 1.41421356 | 1 | 1 | 1 | 1.414213562 | 0 | 0 | 0 | ON529863,ON529867 |
| VC_1505_0 | 7 | 456.6802866 | 497.936347 | 0.47839129 | 5.41E-07 | 4.795831523 | 9.74679434 | 21 | 19 | 0.904761905 | 7.655844614 | 0 | 0 | 0 | S14_vs_32499,S17_vs_116640,S5_vf_8965,S6_vs_9784,S7_vs_557,S7_vs_944,S9_vs_28197 |
| VC_1507_0 | 2 | 9.120885143 | 43.8198354 | 0.17228487 | 0.1481349 | 6.164414003 | 6.164414 | 1 | 1 | 1 | 6.164414003 | 0 | 0 | 0 | S8_vs_148389,S8_vs_4153 |
| VC_1508_0 | 6 | 4500 | 0 | 1 | 1 | 0 | 3.60555128 | 15 | 15 | 1 | 1.860050056 | 0 | 0 | 0 | MT732450,MT732451,MT732452,MT732453,MT732454,MT732455 |
| VC_151_0 | 17 | 5958.655812 | 6295.153684 | 0.48626966 | 1.05E-68 | 4.582575695 | 9.21954446 | 136 | 133 | 0.977941176 | 7.528006294 | 0 | 0 | 0 | S11_vs_41107,S11_vs_58702,S12_vs_150812,S12_vs_6836,S12_vs_79785,S14_vf_56130,S14_vs_632,S16_vf_36146,S3_vf_100440,S3_vf_127409,S3_vf_80287,S4_vs_VIRSorter_1135-cat_2,S5_vs_145598,S7_vs_59465,S8_vf_208126,S9_vs_7881,S9_vs_90851 |
| VC_151_1 | 1 | 0 | 928.9891726 | 0 | 0 | 0 | 0 | 1 | 1 | 1 | 0 | 0 | 0 | 0 | S5_vs_77593 |
| VC_1510_0 | 4 | 668.6522594 | 0 | 1 | 0 | 0 | 1 | 6 | 6 | 1 | 0.666666667 | 0 | 0 | 0 | MT952848,MZ150790,OL455892,OP068334 |
| VC_1511_0 | 3 | 397.2517318 | 2.171746629 | 0.9945628 | 0.0744573 | 2.449489743 | 3.31662479 | 3 | 3 | 1 | 3.027579774 | 0 | 0 | 0 | S14_vf_44452,S5_vf_6032,S8_vf_71214 |
| VC_1512_0 | 2 | 25.55350704 | 0 | 1 | 0 | 0 | 0 | 1 | 1 | 1 | 0 | 0 | 0 | 0 | MT975991,OP433453 |
| VC_1513_0 | 2 | 258.6985471 | 8.820078519 | 0.96703004 | 0.1317762 | 0 | 0 | 1 | 1 | 1 | 0 | 0 | 0 | 0 | MW202613,MW202748 |
| VC_1514_0 | 2 | 195.5907949 | 31.80262472 | 0.86014272 | 0.2397501 | 0 | 0 | 1 | 1 | 1 | 0 | 0 | 0 | 0 | MW202637,MW202789 |
| VC_1515_0 | 3 | 90.05494511 | 0 | 1 | 1 | 0 | 0 | 3 | 3 | 1 | 0 | 0 | 0 | 0 | MW202486,MW202591,MW202766 |
| VC_1516_0 | 4 | 1276.387269 | 14.51568116 | 0.9887554 | 0.0066729 | 1 | 1.41421356 | 6 | 6 | 1 | 1.138071187 | 0 | 0 | 0 | MW202572,MW202631,MW202645,MW202742 |
| VC_1517_0 | 3 | 727.6743621 | 0 | 1 | 1 | 0 | 0 | 3 | 3 | 1 | 0 | 0 | 0 | 0 | MW202524,MW202718,MW202743 |
| VC_1518_0 | 2 | 246.0355269 | 0 | 1 | 0 | 0 | 0 | 1 | 1 | 1 | 0 | 0 | 0 | 0 | MW202601,MW202604 |
| VC_1519_0 | 5 | 2102.25829 | 0 | 1 | 0 | 0 | 1 | 10 | 10 | 1 | 0.6 | 0 | 0 | 0 | MW202608,MW202725,MW202812,MW202826,MW202842 |
| VC_152_0 | 10 | 2346.26882 | 1238.68899 | 0.65447599 | 1.34E-25 | 6.244997998 | 9.79795897 | 45 | 40 | 0.888888889 | 8.057338738 | 0 | 0 | 0 | S11_vs_26767,S13_vs_148294,S14_vs_117158,S3_vs_108076,S4_vs_VIRSorter_15837-cat_2,S5_vs_145601,S7_vs_48523,S7_vs_83100,S7_vs_90915,S8_vs_208118 |
| VC_1520_0 | 2 | 199.2510427 | 0 | 1 | 0 | 0 | 0 | 1 | 1 | 1 | 0 | 0 | 0 | 0 | MW202622,MW202658 |
| VC_1521_0 | 3 | 23.37613558 | 0 | 1 | 0 | 0 | 1 | 3 | 3 | 1 | 0.666666667 | 0 | 0 | 0 | MW202642,MW202843,MW202852 |
| VC_1522_0 | 2 | 267.3514695 | 0 | 1 | 0 | 0 | 0 | 1 | 1 | 1 | 0 | 0 | 0 | 0 | MW202657,MW202864 |
| VC_1523_0 | 2 | 66.87849682 | 0 | 1 | 0 | 5 | 5 | 1 | 1 | 1 | 5 | 0 | 0 | 0 | MW202753,MW202825 |
| VC_1524_0 | 7 | 1536.123581 | 148.6898226 | 0.91174701 | 0.0004866 | 0 | 5.19615242 | 21 | 21 | 1 | 3.416689082 | 0 | 0 | 0 | MW353166,MW353167,MW353168,MW353169,MW353170,MW353171,MW353172 |
| VC_1525_0 | 1 | 0 | 273.857806 | 0 | 0 | 0 | 0 | 1 | 1 | 1 | 0 | 0 | 0 | 0 | S10_vs_62776 |
| VC_1525_1 | 1 | 0 | 240.0791172 | 0 | 0 | 0 | 0 | 1 | 1 | 1 | 0 | 0 | 0 | 0 | S7_vf_37279 |
| VC_1525_2 | 1 | 0 | 154.6130728 | 0 | 0 | 0 | 0 | 1 | 1 | 1 | 0 | 0 | 0 | 0 | S10_vf_43964 |
| VC_1526_0 | 3 | 742.0719708 | 342.4439496 | 0.68424258 | 0.0030824 | 5.656854249 | 7.93725393 | 3 | 3 | 1 | 6.864702728 | 0 | 0 | 0 | MW660583,S12_vs_151292,S16_vs_127669 |
| VC_1527_0 | 2 | 227.3573366 | 397.8648883 | 0.36364244 | 0.0574875 | 7.141428429 | 7.14142843 | 1 | 1 | 1 | 7.141428429 | 0 | 0 | 0 | S10_vs_35854,S15_vf_2032 |
| VC_153_0 | 2 | 139.4959539 | 88.16163022 | 0.61274459 | 0.0952151 | 6.8556546 | 6.8556546 | 1 | 1 | 1 | 6.8556546 | 0 | 0 | 0 | S13_vs_67429,S8_vf_12212 |
| VC_1530_0 | 2 | 11.77202121 | 0 | 1 | 0 | 0 | 0 | 1 | 1 | 1 | 0 | 0 | 0 | 0 | MZ089747,MZ089748 |
| VC_1531_0 | 2 | 4.462118895 | 0 | 1 | 0 | 1 | 1 | 1 | 1 | 1 | 1 | 0 | 0 | 0 | MZ089769,OM869588 |
| VC_1532_0 | 2 | 17.65927749 | 0 | 1 | 0 | 0 | 0 | 1 | 1 | 1 | 0 | 0 | 0 | 0 | MZ089783,OM869706 |
| VC_1533_0 | 3 | 41.71718857 | 11.55007166 | 0.78316753 | 0.0382613 | 0 | 1.73205081 | 3 | 3 | 1 | 1.154700538 | 0 | 0 | 0 | MZ089787,MZ089788,OM869708 |
| VC_1534_0 | 3 | 47.76656182 | 0 | 1 | 0 | 0 | 0 | 3 | 3 | 1 | 0 | 0 | 0 | 0 | MZ089790,MZ089792,MZ089794 |
| VC_1535_0 | 4 | 73.76919516 | 0 | 1 | 0 | 0 | 1 | 6 | 6 | 1 | 0.5 | 0 | 0 | 0 | MZ089811,OM869593,OM869669,OM869712 |
| VC_1536_0 | 6 | 127.5465309 | 0 | 1 | 0 | 0 | 1 | 15 | 15 | 1 | 0.333333333 | 0 | 0 | 0 | MZ364235,MZ364236,MZ364238,MZ364239,MZ364257,MZ364289 |
| VC_1537_0 | 3 | 23.69291051 | 0 | 1 | 0 | 0 | 2 | 3 | 3 | 1 | 1.333333333 | 0 | 0 | 0 | MZ364251,MZ364282,MZ364294 |
| VC_1538_0 | 2 | 210.1818123 | 0 | 1 | 0 | 0 | 0 | 1 | 1 | 1 | 0 | 0 | 0 | 0 | MZ447863,MZ570427 |
| VC_1539_0 | 2 | 2.332437488 | 0 | 1 | 0 | 2.236067977 | 2.23606798 | 1 | 1 | 1 | 2.236067977 | 0 | 0 | 0 | MZ474488,MZ474489 |
| VC_1540_0 | 3 | 163.6625247 | 0 | 1 | 0 | 2.645751311 | 3.16227766 | 3 | 3 | 1 | 2.817926761 | 0 | 0 | 0 | OL689226,OL689227,OL689230 |
| VC_1542_0 | 2 | 21.03070523 | 0 | 1 | 0 | 0 | 0 | 1 | 1 | 1 | 0 | 0 | 0 | 0 | OM869572,OM869573 |
| VC_1543_0 | 3 | 5.391926686 | 0 | 1 | 0 | 0 | 0 | 3 | 3 | 1 | 0 | 0 | 0 | 0 | OM869583,OM869672,OM869688 |

| | | | | | | | | | | | | | | | |
|---|---|---|---|---|---|---|---|---|---|---|---|---|---|---|---|
| VC_1544_0 | 2 | 14.34058733 | 0 | 1 | 0 | 0 | 0 | 1 | 1 | 1 | 0 | 0 | 0 | 0 | OM869594,OM869713 |
| VC_1545_0 | 2 | 2.62135135 | 0 | 1 | 0 | 6.32455532 | 6.32455532 | 1 | 1 | 1 | 6.32455532 | 0 | 0 | 0 | ON014753,ON014757 |
| VC_1546_0 | 3 | 900 | 0 | 1 | 1 | 2 | 3 | 3 | 3 | 1 | 2.666666667 | 0 | 0 | 0 | ON464759,ON464763,ON464764 |
| VC_1547_0 | 2 | 300 | 0 | 1 | 0 | 1.732050808 | 1.73205081 | 1 | 1 | 1 | 1.732050808 | 0 | 0 | 0 | ON529865,ON529866 |
| VC_1548_0 | 2 | 16.44095477 | 0 | 1 | 0 | 0 | 0 | 1 | 1 | 1 | 0 | 0 | 0 | 0 | OP549878,OP549879 |
| VC_1549_0 | 5 | 346.3455804 | 192.9989846 | 0.64216014 | 1.78E-05 | 2 | 3.60555128 | 10 | 10 | 1 | 2.811721298 | 0 | 0 | 0 | S13_vs_12957,S5_vf_137260,S7_vf_20129,S8_vf_136235,S8_vf_142700 |
| VC_155_0 | 5 | 3000 | 104954.8942 | 0.02778938 | 1.35E-06 | 0 | 1.73205081 | 10 | 10 | 1 | 0.897469149 | 0 | 0 | 0 | MT080592,MT080597,MT080598,MT080599,MT080603 |
| VC_155_1 | 3 | 900 | 69846.96947 | 0.01272139 | 0.0071754 | 1 | 1.41421356 | 3 | 3 | 1 | 1.138071187 | 0 | 0 | 0 | OM850391,OM850392,ON113344 |
| VC_155_10 | 1 | 0 | 36020.69481 | 0 | 0 | 0 | 0 | 1 | 1 | 1 | 0 | 0 | 0 | 0 | KY794641 |
| VC_155_11 | 4 | 1800 | 87605.2206 | 0.02013305 | 0.0001838 | 0 | 8.94427191 | 6 | 6 | 1 | 4.906036795 | 0 | 0 | 0 | MT080582,MT080583,MT080586,MT080594 |
| VC_155_12 | 2 | 300 | 67541.00252 | 0.0044221 | 0.1705605 | 7.874007874 | 7.87400787 | 1 | 1 | 1 | 7.874007874 | 0 | 0 | 0 | MH107769,MN045228 |
| VC_155_13 | 3 | 900 | 102474.3837 | 0.00870622 | 0.0486978 | 7.348469228 | 8.88819442 | 3 | 3 | 1 | 8.220937806 | 0 | 0 | 0 | AB853330,JX878671,LR215719 |
| VC_155_14 | 1 | 0 | 35441.03665 | 0 | 0 | 0 | 0 | 1 | 1 | 1 | 0 | 0 | 0 | 0 | AB903967 |
| VC_155_15 | 16 | 36000 | 426156.0651 | 0.07789576 | 2.59E-67 | 0 | 10.7703296 | 120 | 97 | 0.808333333 | 7.02767147 | 0 | 0 | 0 | JQ686190,KM216423,KY779849,LC574321,LR215718,MF001365,MG557618,MH159197,MK250904,MN304941,MT786458,MW546073,MZ427930,MZ573923,OP235318,OP323059 |
| VC_155_16 | 1 | 0 | 34152.00357 | 0 | 0 | 0 | 0 | 1 | 1 | 1 | 0 | 0 | 0 | 0 | KJ888149 |
| VC_155_17 | 4 | 1800 | 137674.7461 | 0.01290556 | 0.0005446 | 0 | 2 | 6 | 6 | 1 | 1.333333333 | 0 | 0 | 0 | MT080585,MT080589,MT080591,MT080601 |
| VC_155_18 | 1 | 0 | 35246.5064 | 0 | 0 | 0 | 0 | 1 | 1 | 1 | 0 | 0 | 0 | 0 | MT554104 |
| VC_155_19 | 1 | 0 | 34747.59268 | 0 | 0 | 0 | 0 | 1 | 1 | 1 | 0 | 0 | 0 | 0 | OP263968 |
| VC_155_2 | 1 | 0 | 25030.15644 | 0 | 0 | 0 | 0 | 1 | 1 | 1 | 0 | 0 | 0 | 0 | LR215720 |
| VC_155_20 | 1 | 0 | 34136.03003 | 0 | 0 | 0 | 0 | 1 | 1 | 1 | 0 | 0 | 0 | 0 | KP027446 |
| VC_155_21 | 1 | 0 | 38159.85423 | 0 | 0 | 0 | 0 | 1 | 1 | 1 | 0 | 0 | 0 | 0 | MG721208 |
| VC_155_22 | 2 | 300 | 48326.59885 | 0.00616946 | 0.0708344 | 1 | 1 | 1 | 1 | 1 | 1 | 0 | 0 | 0 | MT080593,MT080596 |
| VC_155_23 | 1 | 0 | 32976.82693 | 0 | 0 | 0 | 0 | 1 | 1 | 1 | 0 | 0 | 0 | 0 | AB853331 |
| VC_155_24 | 1 | 0 | 23512.78233 | 0 | 0 | 0 | 0 | 1 | 1 | 1 | 0 | 0 | 0 | 0 | MZ417322 |
| VC_155_25 | 14 | 27300 | 262722.0301 | 0.09413078 | 1.13E-46 | 2.449489743 | 10.9544512 | 91 | 80 | 0.879120879 | 7.006047118 | 0 | 0 | 0 | MZ417315,MZ417319,MZ417320,MZ417321,MZ417327,MZ417328,MZ417332,MZ417337,MZ417340,MZ417345,MZ417346,MZ417347,MZ417350,MZ417354 |
| VC_155_26 | 1 | 0 | 23887.16614 | 0 | 0 | 0 | 0 | 1 | 1 | 1 | 0 | 0 | 0 | 0 | MZ417331 |
| VC_155_27 | 1 | 0 | 17646.42476 | 0 | 0 | 0 | 0 | 1 | 1 | 1 | 0 | 0 | 0 | 0 | KX011028 |
| VC_155_28 | 1 | 0 | 21842.96629 | 0 | 0 | 0 | 0 | 1 | 1 | 1 | 0 | 0 | 0 | 0 | MN935200 |
| VC_155_29 | 3 | 900 | 74646.08654 | 0.01191326 | 0.0058726 | 6.403124237 | 8.24621125 | 3 | 3 | 1 | 7.528863141 | 0 | 0 | 0 | KY794642,KY794643,LR215721 |
| VC_155_3 | 1 | 0 | 24038.84593 | 0 | 0 | 0 | 0 | 1 | 1 | 1 | 0 | 0 | 0 | 0 | MT787017 |
| VC_155_4 | 1 | 0 | 24449.76803 | 0 | 0 | 0 | 0 | 1 | 1 | 1 | 0 | 0 | 0 | 0 | OL741057 |
| VC_155_5 | 1 | 0 | 25706.37699 | 0 | 0 | 0 | 0 | 1 | 1 | 1 | 0 | 0 | 0 | 0 | MN091625 |
| VC_155_6 | 1 | 0 | 27149.77248 | 0 | 0 | 0 | 0 | 1 | 1 | 1 | 0 | 0 | 0 | 0 | OP297178 |
| VC_155_7 | 1 | 0 | 23677.97796 | 0 | 0 | 0 | 0 | 1 | 1 | 1 | 0 | 0 | 0 | 0 | LC648442 |
| VC_155_8 | 66 | 643500 | 774823.8211 | 0.45370457 | 0 | 0 | 10.3923048 | 2145 | 1755 | 0.818181818 | 6.463957648 | 0 | 0 | 0 | AY176327,AY954969,EU418428,FR852584,HQ163896,JX080300,JX080301,JX080302,JX080303,JX080304,JX080305,JX875065,KC012913,KF766114,KJ206559,KJ206560,KJ206561,KJ206562,KJ206563,KP687431,KP687432,KR902361,KR908644,KY581279,MF398190,MF405094,MG656408,MH136584,MH844528,MH844529,MK331930,MK331931,MK417514,MK417515,MK417516,MK584893,MK656892,MN047438,MN336261,MN336262,MN336263,MN539736,MN539737,MN539738,MT080600,MT080602,MT226657,MT338525,MW460250,MW528836,MW546064,MW546065,MW546066,MW546067,MW546068,MW546069,MW546070,MW546072,MW546074,OL362280,OL960567,OL960568,OL960569,OL960570,OM030343,OM373557 |
| VC_155_9 | 3 | 900 | 106894.1341 | 0.00834925 | 0.0275895 | 7.937253933 | 8.88819442 | 3 | 3 | 1 | 8.357219867 | 0 | 0 | 0 | AP018375,MT411892,OP263967 |
| VC_1551_0 | 2 | 30.01831504 | 0 | 1 | 0 | 0 | 0 | 1 | 1 | 1 | 0 | 0 | 0 | 0 | PF3COMN,PF3COMNY |
| VC_1552_0 | 9 | 3031.686052 | 2169.82429 | 0.58284726 | 8.43E-21 | 4 | 9.21954446 | 36 | 29 | 0.805555556 | 7.682574096 | 0 | 0 | 0 | S11_vf_84303,S11_vf_95067,S12_vf_128794,S12_vf_79666,S13_vf_109235,S1_vf_22149,S5_vf_110171,S5_vf_136076,S7_vf_76463 |
| VC_1553_0 | 2 | 29.89395945 | 97.0805033 | 0.23543285 | 0.0547993 | 8.246211251 | 8.24621125 | 1 | 1 | 1 | 8.246211251 | 0 | 0 | 0 | S12_vs_111815,S12_vs_188820 |
| VC_1554_0 | 5 | 242.1225525 | 1806.035952 | 0.11821475 | 0.0007323 | 7 | 9.38083152 | 10 | 7 | 0.7 | 8.212901039 | 0 | 0 | 0 | S1_vs_143929,S5_vs_145592,S5_vs_67958,S7_vf_29196,S8_vs_4883 |
| VC_1554_1 | 1 | 0 | 377.7046239 | 0 | 0 | 0 | 0 | 1 | 1 | 1 | 0 | 0 | 0 | 0 | S16_vf_108554 |
| VC_1554_2 | 1 | 0 | 665.4214933 | 0 | 0 | 0 | 0 | 1 | 1 | 1 | 0 | 0 | 0 | 0 | S7_vs_103530 |
| VC_1554_3 | 1 | 0 | 817.5609451 | 0 | 0 | 0 | 0 | 1 | 1 | 1 | 0 | 0 | 0 | 0 | S9_vs_50439 |
| VC_1554_4 | 1 | 0 | 1175.885075 | 0 | 0 | 0 | 0 | 1 | 1 | 1 | 0 | 0 | 0 | 0 | S9_vf_57277 |
| VC_1554_5 | 1 | 0 | 1264.169411 | 0 | 0 | 0 | 0 | 1 | 1 | 1 | 0 | 0 | 0 | 0 | S8_vf_54514 |
| VC_1557_0 | 1 | 0 | 370.719501 | 0 | 0 | 0 | 0 | 1 | 1 | 1 | 0 | 0 | 0 | 0 | S16_vf_96483 |
| VC_1558_0 | 3 | 145.8328954 | 8.826593095 | 0.94292886 | 0.0744573 | 3.16227766 | 3.87298335 | 3 | 3 | 1 | 3.450628599 | 0 | 0 | 0 | S10_vf_25241,S11_vf_28645,S3_vf_109277 |
| VC_1559_0 | 2 | 23.44010423 | 391.6638729 | 0.05646803 | 0.28163 | 8.124038405 | 8.1240384 | 1 | 1 | 1 | 8.124038405 | 0 | 0 | 0 | S11_vf_68693,S11_vs_23188 |
| VC_1559_1 | 2 | 137.0452668 | 545.830928 | 0.20068831 | 0.050872 | 8.185352772 | 8.18535277 | 1 | 1 | 1 | 8.185352772 | 0 | 0 | 0 | S15_vs_27510,S16_vs_127905 |
| VC_1559_2 | 1 | 0 | 220.9694529 | 0 | 0 | 0 | 0 | 1 | 1 | 1 | 0 | 0 | 0 | 0 | S15_vs_42043 |
| VC_156_0 | 13 | 23400 | 75008.99295 | 0.23778315 | 1.34E-51 | 0 | 9 | 78 | 77 | 0.987179487 | 6.230438546 | 0 | 0 | 0 | KF021268,KP027447,MH321490,MH321491,MH542234,MT596501,MT596502,MT596503,MT596504,MT596505,MT596506,MZ417323,MZ417325 |
| VC_1560_0 | 2 | 300 | 25.76495258 | 0.92090938 | 0.1444222 | 2.828427125 | 2.82842712 | 1 | 1 | 1 | 2.828427125 | 0 | 0 | 0 | S3_vs_45147,S9_vs_83108 |
| VC_1561_0 | 2 | 133.8780873 | 78.10597933 | 0.63154788 | 0.0737507 | 5.477225575 | 5.47722558 | 1 | 1 | 1 | 5.477225575 | 0 | 0 | 0 | S4_vf_4891,S8_vf_79769 |
| VC_1564_0 | 5 | 1458.810399 | 0 | 1 | 0 | 3 | 6.244998 | 10 | 10 | 1 | 5.139402081 | 0 | 0 | 0 | S10_vf_64371,S11_vf_27845,S14_vf_117157,S7_vf_138069,S8_vf_134943 |
| VC_1565_0 | 2 | 177.5586092 | 586.9495609 | 0.23225208 | 0.0484329 | 7 | 7 | 1 | 1 | 1 | 7 | 0 | 0 | 0 | S13_vs_83447,S9_vs_48424 |
| VC_1565_1 | 1 | 0 | 403.6701183 | 0 | 0 | 0 | 0 | 1 | 1 | 1 | 0 | 0 | 0 | 0 | S7_vs_54322 |
| VC_1566_0 | 3 | 76.11898657 | 3.028509809 | 0.96173587 | 0.1855467 | 6.08276253 | 6.63324958 | 3 | 3 | 1 | 6.424483545 | 0 | 0 | 0 | S10_vs_120489,S12_vs_188823,S8_vs_193843 |
| VC_1567_0 | 4 | 129.5327244 | 586.6931266 | 0.18085458 | 2.73E-05 | 7.211102551 | 9.32737905 | 6 | 5 | 0.833333333 | 8.208938102 | 0 | 0 | 0 | S10_vs_120541,S11_vs_104054,S7_vs_27530,S8_vs_50431 |
| VC_1568_0 | 2 | 77.14156193 | 172.9567583 | 0.30844494 | 0.0511203 | 5.567764363 | 5.56776436 | 1 | 1 | 1 | 5.567764363 | 0 | 0 | 0 | S12_vs_188828,S7_vs_134391 |
| VC_157_0 | 6 | 4500 | 57655.09693 | 0.07239953 | 1.33E-11 | 0 | 8.83176087 | 15 | 15 | 1 | 6.660043276 | 0 | 0 | 0 | KX171212,KX171213,MW430345,MZ417329,MZ417330,MZ417344 |
| VC_1570_0 | 7 | 227.202542 | 342.8365513 | 0.39857361 | 4.42E-07 | 5.916079783 | 7.48331477 | 21 | 21 | 1 | 6.729854229 | 0 | 0 | 0 | S11_vs_14882,S11_vs_79275,S3_vs_72118,S5_vs_121419,S7_vs_136164,S7_vs_138096,S8_vf_93976 |
| VC_1572_0 | 3 | 260.6178455 | 159.7205056 | 0.62001919 | 0.00577 | 5.830951895 | 6.63324958 | 3 | 3 | 1 | 6.154733825 | 0 | 0 | 0 | S3_vs_70508,S5_vs_129970,S7_vs_2599 |
| VC_1576_0 | 5 | 524.9395721 | 217.7441921 | 0.70681439 | 6.09E-07 | 2 | 8.18535277 | 10 | 10 | 1 | 7.053738084 | 0 | 0 | 0 | S10_vs_71900,S12_vf_174374,S3_vf_18080,S7_vf_117697,S8_vs_9114 |
| VC_1578_0 | 2 | 29.5578466 | 83.74268884 | 0.26088003 | 0.0574875 | 7.348469228 | 7.34846923 | 1 | 1 | 1 | 7.348469228 | 0 | 0 | 0 | S10_vs_36407,S7_vs_129271 |
| VC_1579_0 | 2 | 71.18811453 | 44.67906793 | 0.61439411 | 0.0644425 | 6.08276253 | 6.08276253 | 1 | 1 | 1 | 6.08276253 | 0 | 0 | 0 | S10_vs_36598,S16_vs_80244 |
| VC_1582_0 | 2 | 149.3535546 | 529.6211526 | 0.21996925 | 0.0876221 | 8.246211251 | 8.24621125 | 1 | 1 | 1 | 8.246211251 | 0 | 0 | 0 | S12_vs_121913,S5_vs_1338 |
| VC_1582_1 | 1 | 0 | 455.0628724 | 0 | 0 | 0 | 0 | 1 | 1 | 1 | 0 | 0 | 0 | 0 | S3_vs_39774 |
| VC_1582_2 | 1 | 0 | 556.3953796 | 0 | 0 | 0 | 0 | 1 | 1 | 1 | 0 | 0 | 0 | 0 | S14_vs_74067 |
| VC_1582_3 | 1 | 0 | 266.9464923 | 0 | 0 | 0 | 0 | 1 | 1 | 1 | 0 | 0 | 0 | 0 | S9_vf_13507 |
| VC_1583_0 | 3 | 174.8005219 | 97.15077573 | 0.64276407 | 0.008115 | 5.567764363 | 7 | 3 | 3 | 1 | 6.400337981 | 0 | 0 | 0 | S12_vs_138261,S17_vs_43307,S8_vf_16172 |
| VC_1585_0 | 3 | 196.0681785 | 3.723336358 | 0.98136389 | 0.1855467 | 1.414213562 | 2.23606798 | 3 | 3 | 1 | 1.794110782 | 0 | 0 | 0 | S11_vf_92391,S17_vf_77057,S2_vf_20910 |
| VC_1586_0 | 1 | 0 | 57.90161903 | 0 | 0 | 0 | 0 | 1 | 1 | 1 | 0 | 0 | 0 | 0 | S16_vf_173099 |
| VC_1586_1 | 1 | 0 | 52.76219137 | 0 | 0 | 0 | 0 | 1 | 1 | 1 | 0 | 0 | 0 | 0 | S3_vs_81793 |
| VC_1587_0 | 5 | 356.9016204 | 236.0065555 | 0.60195092 | 3.56E-07 | 5.830951895 | 7.21110255 | 10 | 10 | 1 | 6.446773076 | 0 | 0 | 0 | S5_vs_13090,S5_vs_139401,S5_vs_17520,S5_vs_28263,S7_vs_100640 |
| VC_159_0 | 25 | 90000 | 26899.70045 | 0.76989077 | 4.66E-143 | 0 | 8.06225775 | 300 | 300 | 1 | 5.573626113 | 0 | 0 | 0 | AP019522,JX194239,JX846612,JX846613,KP881332,KX532239,KY779848,LC492751,LC492752,MT080584,MT080587,MT080588,MT080590,MT080595,MW363798,MW546071,MW546075,MW546076,MW546077,MZ643272,ON814134,ON814135,ON814136,ON866946,OP263969 |
| VC_159_1 | 1 | 0 | 8800.494036 | 0 | 0 | 0 | 0 | 1 | 1 | 1 | 0 | 0 | 0 | 0 | LC576631 |
| VC_1590_0 | 2 | 26.66594348 | 109.2536844 | 0.19618906 | 0.0707894 | 7.483314774 | 7.48331477 | 1 | 1 | 1 | 7.483314774 | 0 | 0 | 0 | S6_vs_32886,S7_vf_134023 |
| VC_1591_0 | 2 | 6.437007657 | 42.76832858 | 0.1308193 | 0.2145977 | 6.8556546 | 6.8556546 | 1 | 1 | 1 | 6.8556546 | 0 | 0 | 0 | S16_vs_19968,S16_vs_33281 |
| VC_1594_0 | 1 | 0 | 188.5905375 | 0 | 0 | 0 | 0 | 1 | 1 | 1 | 0 | 0 | 0 | 0 | S7_vs_135679 |
| VC_1595_0 | 2 | 31.83904066 | 34.48959803 | 0.48001951 | 0.1444222 | 6.633249581 | 6.63324958 | 1 | 1 | 1 | 6.633249581 | 0 | 0 | 0 | S11_vs_6458,S5_vs_9747 |
| VC_1596_0 | 2 | 25.46117269 | 20.11600319 | 0.55863867 | 0.1444222 | 6.782329983 | 6.78232998 | 1 | 1 | 1 | 6.782329983 | 0 | 0 | 0 | S11_vs_84627,S13_vs_9438 |
| VC_1597_0 | 3 | 364.4347524 | 0 | 1 | 0 | 4.69041576 | 6.63324958 | 3 | 3 | 1 | 5.600296972 | 0 | 0 | 0 | S12_vf_10887,S12_vf_188822,S14_vf_48775 |
| VC_1598_0 | 2 | 58.30505264 | 0 | 1 | 0 | 3.741657387 | 3.74165739 | 1 | 1 | 1 | 3.741657387 | 0 | 0 | 0 | S12_vf_188830,S13_vf_7670 |
| VC_1599_0 | 2 | 16.27454365 | 37.24671099 | 0.30407627 | 0.1056498 | 5.656854249 | 5.65685425 | 1 | 1 | 1 | 5.656854249 | 0 | 0 | 0 | S15_vs_35445,S16_vs_32764 |
| VC_16_0 | 12 | 1440.711984 | 741.7982897 | 0.66011693 | 1.66E-22 | 0 | 3.60555128 | 66 | 66 | 1 | 2.414769882 | 0 | 0 | 0 | AB012573,AB012574,AB572858,AF452449,AF453500,AY242528,CP017905,CP017906,D89074,FJ904927,MN536023,MN690600 |
| VC_160_0 | 4 | 304.4031488 | 1432.716327 | 0.17523443 | 4.74E-05 | 4.358898944 | 6.63324958 | 6 | 6 | 1 | 5.845516355 | 0 | 0 | 0 | AB854109,KY117485,MN844877,OL634959 |
| VC_1601_0 | 2 | 34.35927522 | 8.781059967 | 0.7964536 | 0.1855467 | 2.828427125 | 2.82842712 | 1 | 1 | 1 | 2.828427125 | 0 | 0 | 0 | S12_vs_137069,S6_vs_56493 |
| VC_1602_0 | 4 | 314.8548616 | 80.19420294 | 0.79700192 | 0.0004423 | 5.099019514 | 6.244998 | 6 | 6 | 1 | 5.915507716 | 0 | 0 | 0 | S15_vs_18274,S16_vs_51901,S17_vs_177196,S7_vs_50194 |
| VC_1603_0 | 2 | 13.01837735 | 0 | 1 | 0 | 0 | 0 | 1 | 1 | 1 | 0 | 0 | 0 | 0 | S12_vs_183891,S9_vs_90863 |
| VC_1604_0 | 2 | 20.2668512 | 42.99884153 | 0.32034505 | 0.0707894 | 7.348469228 | 7.34846923 | 1 | 1 | 1 | 7.348469228 | 0 | 0 | 0 | S4_vs_VIRSorter_15112-cat_2,S9_vs_63485 |
| VC_1605_0 | 2 | 186.2831941 | 76.45720475 | 0.70900096 | 0.1207833 | 3.741657387 | 3.74165739 | 1 | 1 | 1 | 3.741657387 | 0 | 0 | 0 | S12_vs_26336,S5_vs_142195 |
| VC_1606_0 | 2 | 62.56069242 | 15.84252895 | 0.79793523 | 0.1444222 | 5.385164807 | 5.38516481 | 1 | 1 | 1 | 5.385164807 | 0 | 0 | 0 | S12_vs_71525,S3_vs_117894 |
| VC_1607_0 | 11 | 747.3850522 | 200.5808198 | 0.78840924 | 0.0096282 | 1 | 4.69041576 | 55 | 55 | 1 | 3.372057879 | 0 | 0 | 0 | S13_vs_87108,S16_vf_164913,S17_vf_172132,S17_vs_58025,S1_vf_125650,S2_vf_3356,S3_vf_127168,S6_vf_6314,S7_vf_27437,S8_vf_43483,S9_vs_75290 |
| VC_161_0 | 14 | 11530.37623 | 7483.601319 | 0.60641579 | 3.74E-55 | 0 | 6.78232998 | 91 | 91 | 1 | 4.202627495 | 0 | 0 | 0 | FN594518,MT104468,MT104469,MT104470,MT104471,MT104472,MT104476,MT104477,MT354570,MW627366,MZ868714,MZ868715,MZ868716,MZ868717 |
| VC_1611_0 | 4 | 384.0254139 | 288.2946208 | 0.57119436 | 0.0002752 | 4.795831523 | 6.92820323 | 6 | 6 | 1 | 5.395295822 | 0 | 0 | 0 | S16_vs_64584,S3_vs_12973,S4_vs_VIRSorter_13488-cat_2,S8_vs_5211 |
| VC_1612_0 | 2 | 274.7097096 | 817.8491531 | 0.25143699 | 0.0707894 | 6.782329983 | 6.78232998 | 1 | 1 | 1 | 6.782329983 | 0 | 0 | 0 | S14_vs_25852,S6_vs_46039 |
| VC_1612_1 | 2 | 149.0136539 | 737.5155966 | 0.16808656 | 0.0707894 | 6.708203932 | 6.70820393 | 1 | 1 | 1 | 6.708203932 | 0 | 0 | 0 | S14_vs_11510,S8_vs_179208 |
| VC_1612_2 | 1 | 0 | 497.921303 | 0 | 0 | 0 | 0 | 1 | 1 | 1 | 0 | 0 | 0 | 0 | S14_vs_70985 |
| VC_1612_3 | 1 | 0 | 497.7331821 | 0 | 0 | 0 | 0 | 1 | 1 | 1 | 0 | 0 | 0 | 0 | S5_vs_109527 |
| VC_1613_0 | 3 | 55.94678997 | 66.19054391 | 0.45806461 | 0.0164847 | 5.385164807 | 7.54983444 | 3 | 3 | 1 | 6.806104672 | 0 | 0 | 0 | S3_vs_50924,S5_vs_140946,S9_vs_58402 |
| VC_1614_0 | 3 | 45.65630351 | 0 | 1 | 0 | 2.236067977 | 3.31662479 | 3 | 3 | 1 | 2.904990143 | 0 | 0 | 0 | S14_vs_67094,S5_vs_124035,S5_vs_7444 |
| VC_1615_0 | 2 | 6.906138505 | 11.66295571 | 0.37191574 | 0.1444222 | 4.242640687 | 4.24264069 | 1 | 1 | 1 | 4.242640687 | 0 | 0 | 0 | S5_vs_145593,S7_vf_138019 |
| VC_1616_0 | 2 | 11.77805945 | 0 | 1 | 0 | 3.31662479 | 3.31662479 | 1 | 1 | 1 | 3.31662479 | 0 | 0 | 0 | S16_vs_48714,S7_vf_71527 |
| VC_1617_0 | 2 | 165.4140553 | 0 | 1 | 0 | 5.099019514 | 5.09901951 | 1 | 1 | 1 | 5.099019514 | 0 | 0 | 0 | S17_vf_177197,S4_vf_10506 |
| VC_1618_0 | 2 | 39.02111428 | 0 | 1 | 0 | 5 | 5 | 1 | 1 | 1 | 5 | 0 | 0 | 0 | S3_vf_127423,S8_vs_132359 |
| VC_1619_0 | 2 | 88.68633213 | 24.84919294 | 0.78113288 | 0.2397501 | 3.464101615 | 3.46410162 | 1 | 1 | 1 | 3.464101615 | 0 | 0 | 0 | S8_vf_208102,S8_vf_208107 |
| VC_162_0 | 12 | 10037.69586 | 1052.40236 | 0.90510432 | 8.86E-30 | 1 | 5.56776436 | 66 | 66 | 1 | 3.709839687 | 0 | 0 | 0 | FR751545,KM247287,MK629528,MN850568,MN850570,MN850583,MN850592,MN850608,MN850617,MN850624,MN850625,MW250275 |
| VC_1620_0 | 2 | 47.50351914 | 0 | 1 | 0 | 5.196152423 | 5.19615242 | 1 | 1 | 1 | 5.196152423 | 0 | 0 | 0 | S3_vs_90341,S7_vs_129858 |
| VC_163_0 | 3 | 513.9897313 | 870.4448796 | 0.37126328 | 0.001919 | 1.414213562 | 2.82842712 | 3 | 3 | 1 | 2.230710143 | 0 | 0 | 0 | MK962628,MK962638,MK962640 |
| VC_165_0 | 2 | 162.0415652 | 1067.61166 | 0.13177826 | 0.0436872 | 2.828427125 | 2.82842712 | 1 | 1 | 1 | 2.828427125 | 0 | 0 | 0 | OM913597,OM913598 |
| VC_166_0 | 2 | 155.0857491 | 165.8894832 | 0.48317046 | 0.0545565 | 3.31662479 | 3.31662479 | 1 | 1 | 1 | 3.31662479 | 0 | 0 | 0 | MF150911,MN996301 |
| VC_167_0 | 3 | 492.0479514 | 366.4827609 | 0.57312795 | 0.001956 | 1.414213562 | 3.74165739 | 3 | 3 | 1 | 2.873324188 | 0 | 0 | 0 | AB863625,MT740737,MT740741 |
| VC_168_0 | 18 | 17950.34661 | 6325.405798 | 0.73943523 | 2.99E-66 | 1.732050808 | 9.2736185 | 153 | 132 | 0.862745098 | 7.090655067 | 0 | 0 | 0 | AY349011,FJ937737,JN662425,JX104231,MH638294,MT361768,MT740727,MT740730,MT740731,MT740732,MT740733,MT740736,MT740738,MT740739,MT740740,MT740742,OM283127,OV121131 |
| VC_169_0 | 2 | 37.02207795 | 93.23131414 | 0.2842312 | 0.0599379 | 6.708203932 | 6.70820393 | 1 | 1 | 1 | 6.708203932 | 0 | 0 | 0 | MW202475,MW202867 |
| VC_17_0 | 9 | 1334.771397 | 584.7575777 | 0.69536403 | 2.73E-18 | 1.414213562 | 2.64575131 | 36 | 36 | 1 | 1.966193006 | 0 | 0 | 0 | AB043678,AB043679,MN719123,MT188662,MT188663,MT188664,MT188665,MT188666,MT193890 |
| VC_170_0 | 3 | 900 | 29330.59173 | 0.02977117 | 0.1701627 | 2 | 4 | 3 | 3 | 1 | 3.154700538 | 0 | 0 | 0 | MK268344,MK773491,MT001829 |
| VC_170_1 | 1 | 0 | 10362.14252 | 0 | 0 | 0 | 0 | 1 | 1 | 1 | 0 | 0 | 0 | 0 | MT446416 |
| VC_170_10 | 5 | 3000 | 45856.37601 | 0.06140447 | 0.0351642 | 5 | 8.36660027 | 10 | 10 | 1 | 6.756909001 | 0 | 0 | 0 | AB897757,JQ513383,MT708547,OP617745,OU509535 |
| VC_170_11 | 10 | 13500 | 76363.21553 | 0.15022832 | 2.03E-06 | 5.744562647 | 9.74679434 | 45 | 41 | 0.911111111 | 7.533132912 | 0 | 0 | 0 | KC295538,KM507819,LC494302,LT603033,MH383160,MH791411,MK327931,MK817115,MW822008,ON568341 |
| VC_170_2 | 1 | 0 | 9727.706973 | 0 | 0 | 0 | 0 | 1 | 1 | 1 | 0 | 0 | 0 | 0 | MH494197 |
| VC_170_3 | 1 | 0 | 10345.64367 | 0 | 0 | 0 | 0 | 1 | 1 | 1 | 0 | 0 | 0 | 0 | ON286972 |
| VC_170_4 | 1 | 0 | 10356.01047 | 0 | 0 | 0 | 0 | 1 | 1 | 1 | 0 | 0 | 0 | 0 | JN882285 |
| VC_170_5 | 1 | 0 | 10309.90301 | 0 | 0 | 0 | 0 | 1 | 1 | 1 | 0 | 0 | 0 | 0 | KU574722 |
| VC_170_6 | 1 | 0 | 10358.67835 | 0 | 0 | 0 | 0 | 1 | 1 | 1 | 0 | 0 | 0 | 0 | OP292654 |
| VC_170_7 | 7 | 6300 | 59857.17333 | 0.09522777 | 0.0012505 | 3 | 6.08276253 | 21 | 21 | 1 | 4.198196279 | 0 | 0 | 0 | KY630187,MT939486,MT939487,MT939488,MW879342,MW879343,MW879344 |
| VC_170_8 | 3 | 900 | 29296.90653 | 0.02980438 | 0.1484441 | 0 | 5.65685425 | 3 | 3 | 1 | 3.771236166 | 0 | 0 | 0 | LR596615,LT960551,LT960552 |
| VC_170_9 | 1 | 0 | 6471.394783 | 0 | 0 | 0 | 0 | 1 | 1 | 1 | 0 | 0 | 0 | 0 | MG592671 |
| VC_171_0 | 4 | 1800 | 6367.298849 | 0.2203911 | 1.67E-05 | 3 | 4.69041576 | 6 | 6 | 1 | 3.876563835 | 0 | 0 | 0 | LC727697,LC727698,LR588166,MK797984 |
| VC_172_0 | 26 | 78434.31536 | 20742.70131 | 0.79085173 | 7.44E-165 | 0 | 8.30662386 | 325 | 325 | 1 | 4.660748372 | 0 | 0 | 0 | AM749441,DQ163916,JX131330,KC758116,KT734862,KX198613,KX961385,KY000218,KY000219,KY000220,KY000221,MF490239,MH791403,MH791406,MN871456,MN871463,MN871469,MN871478,MN871482,MN871486,MT118302,MZ089734,MZ089741,OL763419,OM809160,ON165687 |
| VC_173_0 | 3 | 900 | 409.9391681 | 0.68705481 | 0.0017343 | 1 | 2.82842712 | 3 | 3 | 1 | 2.158059479 | 0 | 0 | 0 | AB910392,MK443266,MK803322 |
| VC_175_0 | 20 | 38188.96482 | 12705.58799 | 0.75035466 | 1.78E-110 | 1.414213562 | 7.93725393 | 190 | 190 | 1 | 5.556011134 | 0 | 0 | 0 | JQ067089,JQ067093,KU204984,KX241618,LR743532,MG018224,MT664984,OK275492,OK275493,OL674541,ON005621,ON189045,ON189047,ON331942,ON529854,ON932079,ON932080,ON932084,OP361299,OP380269 |
| VC_176_0 | 1 | 0 | 10.1787922 | 0 | 0 | 0 | 0 | 1 | 1 | 1 | 0 | 0 | 0 | 0 | KT001913 |
| VC_176_1 | 1 | 0 | 98.96459799 | 0 | 0 | 0 | 0 | 1 | 1 | 1 | 0 | 0 | 0 | 0 | KY006853 |
| VC_177_0 | 8 | 5007.584325 | 3555.472422 | 0.58478934 | 8.11E-19 | 1 | 7.14142843 | 28 | 28 | 1 | 4.075828958 | 0 | 0 | 0 | KP202969,KP202970,MH746817,MK962627,MK962633,MK962634,MT241605,MT241607 |
| VC_178_0 | 5 | 1931.295768 | 3514.102649 | 0.35466565 | 3.05E-08 | 2.236067977 | 5.83095189 | 10 | 10 | 1 | 4.580025852 | 0 | 0 | 0 | KF626665,KF626668,LR743524,MZ326855,ON189048 |
| VC_179_0 | 2 | 171.370776 | 1984.621115 | 0.07948582 | 0.0451091 | 5.385164807 | 5.38516481 | 1 | 1 | 1 | 5.385164807 | 0 | 0 | 0 | KT887558,MG707188 |
| VC_18_0 | 4 | 739.1774756 | 84.97571669 | 0.8968933 | 0.0001207 | 1 | 6.63324958 | 6 | 6 | 1 | 4.600777349 | 0 | 0 | 0 | AB016282,HM072038,OM236514,ON528935 |
| VC_180_0 | 3 | 900 | 36.78733574 | 0.96073032 | 0.0066644 | 1 | 1.73205081 | 3 | 3 | 1 | 1.382088123 | 0 | 0 | 0 | AB910393,LC102729,OK094707 |

| | | | | | | | | | | | | | | | |
|---|---|---|---|---|---|---|---|---|---|---|---|---|---|---|---|
| VC_181_0 | 4 | 878.1063183 | 1422.47706 | 0.38168854 | 1.74E-05 | 3.464101615 | 7.34846923 | 6 | 6 | 1 | 5.770075875 | 0 | 0 | 0 | MG592578,MG592660,MW865292,OX241572 |
| VC_182_0 | 2 | 211.0214733 | 4055.739796 | 0.04945706 | 0.0433539 | 6.92820323 | 6.92820323 | 1 | 1 | 1 | 6.92820323 | 0 | 0 | 0 | AF165214,HQ711985 |
| VC_182_1 | 2 | 137.4115224 | 3672.248296 | 0.03606924 | 0.0434243 | 8.306623863 | 8.30662386 | 1 | 1 | 1 | 8.306623863 | 0 | 0 | 0 | KT887557,ON857940 |
| VC_182_2 | 1 | 0 | 1982.421195 | 0 | 0 | 0 | 0 | 1 | 1 | 1 | 0 | 0 | 0 | 0 | OM141125 |
| VC_182_3 | 1 | 0 | 1529.203733 | 0 | 0 | 0 | 0 | 1 | 1 | 1 | 0 | 0 | 0 | 0 | KT968831 |
| VC_183_0 | 5 | 147.0371074 | 50.35958803 | 0.7448813 | 0.0011435 | 1.414213562 | 3.46410162 | 10 | 10 | 1 | 2.665768372 | 0 | 0 | 0 | AB910602,HG315669,KX181651,KY853667,MN335248 |
| VC_184_0 | 5 | 230.0617568 | 67.90934674 | 0.77209419 | 1.01E-05 | 1 | 2.82842712 | 10 | 10 | 1 | 2.301171671 | 0 | 0 | 0 | MH206183,MH206184,MH218848,MK512531,MN263053 |
| VC_185_0 | 53 | 219302.4631 | 20287.59008 | 0.91532374 | 0 | 0 | 6.70820393 | 1378 | 1378 | 1 | 4.275079744 | 0 | 0 | 0 | AY394005,DQ631426,DQ873690,EU272036,EU272037,HE584812,HM624080,JN808773,JN811560,JQ067085,JQ762257,JX434030,JX434031,JX434032,JX434033,KF475786,KJ477077,KM233689,KT968832,KU199707,KU199708,KU199709,LN610590,LN907801,LT608331,LT999987,MH643777,MH643778,MK144667,MK511017,MK511018,MK511019,MK511020,MK511021,MK511022,MK511023,MK511024,MK511025,MK511026,MK511027,MK511028,MK511029,MK511030,MK511031,MK511032,MK511033,MK511034,MK511035,MK511036,MK511037,MK511038,MW390886,OM541307 |
| VC_186_0 | 4 | 779.435191 | 3019.786315 | 0.20515655 | 1.38E-05 | 0 | 3.87298335 | 6 | 6 | 1 | 2.363324182 | 0 | 0 | 0 | AB916497,ON170148,ON170170,ON170354 |
| VC_188_0 | 5 | 630.1935526 | 388.1517827 | 0.61884071 | 2.16E-07 | 0 | 8.36660027 | 10 | 10 | 1 | 6.411658072 | 0 | 0 | 0 | JF974307,JF974308,KT381864,KT381865,KX077179 |
| VC_189_0 | 2 | 68.55674088 | 896.2367751 | 0.07105846 | 0.0451041 | 4.69041576 | 4.69041576 | 1 | 1 | 1 | 4.69041576 | 0 | 0 | 0 | KF302035,MZ568827 |
| VC_19_0 | 3 | 450.8740403 | 1051.039498 | 0.30019973 | 0.001599 | 0 | 7.74596669 | 3 | 3 | 1 | 5.163977795 | 0 | 0 | 0 | KM983328,KM983331,KM983334 |
| VC_190_0 | 2 | 121.1878879 | 3240.236812 | 0.03605254 | 0.0436447 | 3.31662479 | 3.31662479 | 1 | 1 | 1 | 3.31662479 | 0 | 0 | 0 | MF975721,OK490409 |
| VC_191_0 | 2 | 199.0548575 | 53.76674893 | 0.78733325 | 0.0633152 | 3.605551275 | 3.60555128 | 1 | 1 | 1 | 3.605551275 | 0 | 0 | 0 | MH727593,MZ547449 |
| VC_192_0 | 22 | 29641.82313 | 15395.86129 | 0.65815602 | 1.30E-129 | 0 | 7.61577311 | 231 | 231 | 1 | 5.190908926 | 0 | 0 | 0 | AF232233,JX495041,JX495042,JX495043,LN898172,MH719189,MH719190,MH719191,MH719193,MH719194,MH719195,MK511057,MK511058,MK511059,MK511060,MK511061,MK511063,MK511064,MK511065,MK934841,MN536027 |
| VC_193_0 | 3 | 16.37126445 | 20.50641919 | 0.44393419 | 0.0285199 | 6.708203932 | 7.14142843 | 3 | 3 | 1 | 6.925945197 | 0 | 0 | 0 | LR797314,MF959999,MK088078 |
| VC_195_0 | 4 | 1800 | 25657.37126 | 0.06555617 | 5.69E-05 | 6.403124237 | 7.68114575 | 6 | 6 | 1 | 7.012485515 | 0 | 0 | 0 | KX961630,MN150686,OK499977,OK499983 |
| VC_195_1 | 7 | 6300 | 38424.7563 | 0.14086158 | 7.05E-14 | 3.31662479 | 8.36660027 | 21 | 21 | 1 | 6.89654448 | 0 | 0 | 0 | JN797797,JN797798,KT187252,KT224359,KX987999,MH487649,MN013089 |
| VC_195_2 | 2 | 300 | 14131.01552 | 0.02078856 | 0.069205 | 6.782329983 | 6.78232998 | 1 | 1 | 1 | 6.782329983 | 0 | 0 | 0 | KJ081346,KJ676859 |
| VC_195_3 | 9 | 10800 | 44456.61062 | 0.19545173 | 2.09E-22 | 0 | 9.74679434 | 36 | 31 | 0.861111111 | 7.931741392 | 0 | 0 | 0 | JX094431,KJ451625,KT995480,KU577463,KX495186,MG602477,MH638309,MH638312,OL964058 |
| VC_195_4 | 1 | 0 | 7303.595342 | 0 | 0 | 0 | 0 | 1 | 1 | 1 | 0 | 0 | 0 | 0 | MN364664 |
| VC_196_0 | 13 | 845.7161791 | 2.322296926 | 0.99726157 | 0.001762 | 0 | 1 | 78 | 78 | 1 | 0.153846154 | 0 | 0 | 0 | AB971354,AF059242,AF059243,EF068134,FJ483840,FJ483841,FJ483842,FJ483843,FJ483844,FJ539132,FJ539133,FJ539134,FJ539135 |
| VC_197_0 | 2 | 40.6638754 | 3907.079967 | 0.01030054 | 0.0604656 | 4 | 4 | 1 | 1 | 1 | 4 | 0 | 0 | 0 | AJ604530,AJ604531 |
| VC_198_0 | 2 | 300 | 34781.94735 | 0.00855141 | 0.0423653 | 5.916079783 | 5.91607978 | 1 | 1 | 1 | 5.916079783 | 0 | 0 | 0 | KP143763,KX130863 |
| VC_198_1 | 1 | 0 | 19638.97457 | 0 | 0 | 0 | 0 | 1 | 1 | 1 | 0 | 0 | 0 | 0 | MK373792 |
| VC_198_10 | 1 | 0 | 20589.15526 | 0 | 0 | 0 | 0 | 1 | 1 | 1 | 0 | 0 | 0 | 0 | KR422353 |
| VC_198_11 | 1 | 0 | 16196.88959 | 0 | 0 | 0 | 0 | 1 | 1 | 1 | 0 | 0 | 0 | 0 | AC171169 |
| VC_198_12 | 1 | 0 | 20993.112 | 0 | 0 | 0 | 0 | 1 | 1 | 1 | 0 | 0 | 0 | 0 | LR027384 |
| VC_198_13 | 4 | 1800 | 82368.92867 | 0.02138556 | 3.29E-05 | 1.414213562 | 9 | 6 | 4 | 0.666666667 | 7.437928288 | 0 | 0 | 0 | MH370377,MH370378,MN970211,MN994502 |
| VC_198_14 | 3 | 900 | 62402.29345 | 0.01421749 | 0.0031441 | 8.717797887 | 9.16515139 | 3 | 2 | 0.666666667 | 8.866915721 | 0 | 0 | 0 | LR597655,MN022787,MN316588 |
| VC_198_15 | 3 | 900 | 61583.91703 | 0.01440371 | 0.0024502 | 8.366600265 | 9.21954446 | 3 | 2 | 0.666666667 | 8.710049489 | 0 | 0 | 0 | KJ190157,KY677846,MN994496 |
| VC_198_16 | 1 | 0 | 22033.55103 | 0 | 0 | 0 | 0 | 1 | 1 | 1 | 0 | 0 | 0 | 0 | OK272480 |
| VC_198_17 | 1 | 0 | 21271.21851 | 0 | 0 | 0 | 0 | 1 | 1 | 1 | 0 | 0 | 0 | 0 | AP019559 |
| VC_198_18 | 1 | 0 | 21877.09482 | 0 | 0 | 0 | 0 | 1 | 1 | 1 | 0 | 0 | 0 | 0 | MF402939 |
| VC_198_19 | 1 | 0 | 20637.07469 | 0 | 0 | 0 | 0 | 1 | 1 | 1 | 0 | 0 | 0 | 0 | MZ501075 |
| VC_198_2 | 2 | 300 | 41430.11593 | 0.00718905 | 0.045084 | 1.414213562 | 1.41421356 | 1 | 1 | 1 | 1.414213562 | 0 | 0 | 0 | MK373797,MK373799 |
| VC_198_20 | 4 | 1800 | 77543.28433 | 0.02268623 | 2.28E-05 | 0 | 8.54400375 | 6 | 6 | 1 | 6.794462362 | 0 | 0 | 0 | LN887948,MK972715,MK972716,MT843274 |
| VC_198_21 | 1 | 0 | 19843.1927 | 0 | 0 | 0 | 0 | 1 | 1 | 1 | 0 | 0 | 0 | 0 | KY114934 |
| VC_198_22 | 3 | 900 | 48256.93622 | 0.01830871 | 0.0014032 | 4.242640687 | 6.92820323 | 3 | 3 | 1 | 5.9843913 | 0 | 0 | 0 | KM979354,KM979355,MH203051 |
| VC_198_23 | 10 | 13360.48244 | 178219.8276 | 0.06973829 | 1.87E-27 | 5.477225575 | 9.69535971 | 45 | 38 | 0.844444444 | 8.105580876 | 0 | 0 | 0 | HQ406778,KY963424,MH102285,MN026741,MT311645,MW149273,MW149275,OK108607,OK254197,OP394141 |
| VC_198_24 | 5 | 3000 | 100345.7688 | 0.02902876 | 5.01E-07 | 4 | 9.38083152 | 10 | 7 | 0.7 | 8.224818939 | 0 | 0 | 0 | HQ665011,LR597659,MZ398246,OK143207,ON185585 |
| VC_198_25 | 1 | 0 | 19943.54715 | 0 | 0 | 0 | 0 | 1 | 1 | 1 | 0 | 0 | 0 | 0 | MN956514 |
| VC_198_26 | 1 | 0 | 19671.6445 | 0 | 0 | 0 | 0 | 1 | 1 | 1 | 0 | 0 | 0 | 0 | MG387042 |
| VC_198_27 | 1 | 0 | 19452.2304 | 0 | 0 | 0 | 0 | 1 | 1 | 1 | 0 | 0 | 0 | 0 | KR233164 |
| VC_198_28 | 5 | 3000 | 96861.16945 | 0.03004171 | 6.83E-08 | 1.414213562 | 4.24264069 | 10 | 10 | 1 | 3.028504821 | 0 | 0 | 0 | OK665835,ON185581,ON185582,ON185583,ON185584 |
| VC_198_29 | 1 | 0 | 22442.8156 | 0 | 0 | 0 | 0 | 1 | 1 | 1 | 0 | 0 | 0 | 0 | MT080103 |
| VC_198_3 | 1 | 0 | 20860.95845 | 0 | 0 | 0 | 0 | 1 | 1 | 1 | 0 | 0 | 0 | 0 | MN994504 |
| VC_198_30 | 1 | 0 | 10996.9783 | 0 | 0 | 0 | 0 | 1 | 1 | 1 | 0 | 0 | 0 | 0 | MF774688 |
| VC_198_31 | 1 | 0 | 16456.88042 | 0 | 0 | 0 | 0 | 1 | 1 | 1 | 0 | 0 | 0 | 0 | MG065692 |
| VC_198_32 | 1 | 0 | 21383.86807 | 0 | 0 | 0 | 0 | 1 | 1 | 1 | 0 | 0 | 0 | 0 | MZ501058 |
| VC_198_33 | 1 | 0 | 17015.92583 | 0 | 0 | 0 | 0 | 1 | 1 | 1 | 0 | 0 | 0 | 0 | MZ501103 |
| VC_198_34 | 1 | 0 | 19488.84179 | 0 | 0 | 0 | 0 | 1 | 1 | 1 | 0 | 0 | 0 | 0 | MN445182 |
| VC_198_35 | 1 | 0 | 18820.1029 | 0 | 0 | 0 | 0 | 1 | 1 | 1 | 0 | 0 | 0 | 0 | OK272477 |
| VC_198_36 | 1 | 0 | 20606.91157 | 0 | 0 | 0 | 0 | 1 | 1 | 1 | 0 | 0 | 0 | 0 | MT653143 |
| VC_198_37 | 1 | 0 | 20640.30201 | 0 | 0 | 0 | 0 | 1 | 1 | 1 | 0 | 0 | 0 | 0 | MT653146 |
| VC_198_38 | 3 | 900 | 51278.38713 | 0.01724852 | 0.0015492 | 1.732050808 | 3.74165739 | 3 | 3 | 1 | 3.026419823 | 0 | 0 | 0 | AY543070,AY587007,AY692264 |
| VC_198_39 | 1 | 0 | 20044.1087 | 0 | 0 | 0 | 0 | 1 | 1 | 1 | 0 | 0 | 0 | 0 | MT233524 |
| VC_198_4 | 1 | 0 | 20873.27016 | 0 | 0 | 0 | 0 | 1 | 1 | 1 | 0 | 0 | 0 | 0 | MT577844 |
| VC_198_40 | 2 | 300 | 36286.85678 | 0.00819967 | 0.044436 | 8.485281374 | 8.48528137 | 1 | 1 | 1 | 8.485281374 | 0 | 0 | 0 | MG969412,MW006479 |
| VC_198_5 | 1 | 0 | 20564.04333 | 0 | 0 | 0 | 0 | 1 | 1 | 1 | 0 | 0 | 0 | 0 | OM386657 |
| VC_198_6 | 1 | 0 | 19498.97184 | 0 | 0 | 0 | 0 | 1 | 1 | 1 | 0 | 0 | 0 | 0 | MZ501074 |
| VC_198_7 | 7 | 6300 | 127606.8677 | 0.04704762 | 1.32E-14 | 0 | 8.88819442 | 21 | 21 | 1 | 3.176010077 | 0 | 0 | 0 | MF431730,MF431731,MF431732,MF431733,MF431734,MF431735,MT074448 |
| VC_198_8 | 1 | 0 | 20510.69898 | 0 | 0 | 0 | 0 | 1 | 1 | 1 | 0 | 0 | 0 | 0 | OM386660 |
| VC_198_9 | 1 | 0 | 20152.40787 | 0 | 0 | 0 | 0 | 1 | 1 | 1 | 0 | 0 | 0 | 0 | MK373796 |
| VC_199_0 | 2 | 300 | 45320.98166 | 0.00657592 | 0.0423903 | 8.124038405 | 8.1240384 | 1 | 1 | 1 | 8.124038405 | 0 | 0 | 0 | MW006478,MW006481 |
| VC_199_1 | 1 | 0 | 27634.38416 | 0 | 0 | 0 | 0 | 1 | 1 | 1 | 0 | 0 | 0 | 0 | MK370036 |
| VC_199_10 | 1 | 0 | 26489.93696 | 0 | 0 | 0 | 0 | 1 | 1 | 1 | 0 | 0 | 0 | 0 | MN197465 |
| VC_199_11 | 1 | 0 | 25257.07357 | 0 | 0 | 0 | 0 | 1 | 1 | 1 | 0 | 0 | 0 | 0 | MF681663 |
| VC_199_12 | 1 | 0 | 27185.58552 | 0 | 0 | 0 | 0 | 1 | 1 | 1 | 0 | 0 | 0 | 0 | MT074464 |
| VC_199_13 | 5 | 3000 | 126784.3708 | 0.02311526 | 1.19E-06 | 0 | 1.73205081 | 10 | 10 | 1 | 0.897469149 | 0 | 0 | 0 | MK907267,MK907268,MK907269,MK907270,MK907271 |
| VC_199_14 | 1 | 0 | 26327.502 | 0 | 0 | 0 | 0 | 1 | 1 | 1 | 0 | 0 | 0 | 0 | LR027389 |
| VC_199_15 | 2 | 300 | 53029.93075 | 0.00562536 | 0.0672476 | 8.602325267 | 8.60232527 | 1 | 1 | 1 | 8.602325267 | 0 | 0 | 0 | OL451946,OL502173 |
| VC_199_16 | 12 | 19800 | 289793.4626 | 0.06395484 | 1.04E-25 | 1.414213562 | 9.64365076 | 66 | 62 | 0.939393939 | 7.002171715 | 0 | 0 | 0 | KY787212,MF001363,MH370369,MK393882,MK947458,MK972717,MN218190,MN218191,MN379739,MN994501,MT074450,MT074458 |
| VC_199_17 | 4 | 1800 | 107467.7315 | 0.0164733 | 0.0010673 | 6.782329983 | 9.21954446 | 6 | 5 | 0.833333333 | 8.00216241 | 0 | 0 | 0 | CP000917,MK947459,MZ501105,OL770310 |
| VC_199_18 | 2 | 300 | 52560.48126 | 0.00567532 | 0.0831288 | 9 | 9 | 1 | 0 | 0 | 9 | 0 | 0 | 0 | MT833283,MZ501072 |
| VC_199_19 | 12 | 19800 | 284523.519 | 0.06506234 | 2.14E-30 | 1 | 9.48683298 | 66 | 57 | 0.863636364 | 8.017454343 | 0 | 0 | 0 | KM236244,KU927497,KX017521,LC659915,MG065642,MH631454,MK728824,MK728825,MN187969,MW357609,OK288022,OP045498 |
| VC_199_2 | 1 | 0 | 26080.52563 | 0 | 0 | 0 | 0 | 1 | 1 | 1 | 0 | 0 | 0 | 0 | MZ501109 |
| VC_199_20 | 1 | 0 | 27453.37139 | 0 | 0 | 0 | 0 | 1 | 1 | 1 | 0 | 0 | 0 | 0 | MK005300 |
| VC_199_21 | 1 | 0 | 27262.64439 | 0 | 0 | 0 | 0 | 1 | 1 | 1 | 0 | 0 | 0 | 0 | MZ501076 |
| VC_199_22 | 1 | 0 | 26050.06305 | 0 | 0 | 0 | 0 | 1 | 1 | 1 | 0 | 0 | 0 | 0 | MW567727 |
| VC_199_23 | 1 | 0 | 27270.18001 | 0 | 0 | 0 | 0 | 1 | 1 | 1 | 0 | 0 | 0 | 0 | MH370379 |
| VC_199_24 | 1 | 0 | 26414.97538 | 0 | 0 | 0 | 0 | 1 | 1 | 1 | 0 | 0 | 0 | 0 | MZ570151 |
| VC_199_25 | 1 | 0 | 25668.8135 | 0 | 0 | 0 | 0 | 1 | 1 | 1 | 0 | 0 | 0 | 0 | MW206381 |
| VC_199_26 | 1 | 0 | 21862.30661 | 0 | 0 | 0 | 0 | 1 | 1 | 1 | 0 | 0 | 0 | 0 | HE956708 |
| VC_199_27 | 1 | 0 | 25521.95154 | 0 | 0 | 0 | 0 | 1 | 1 | 1 | 0 | 0 | 0 | 0 | MK867835 |
| VC_199_28 | 1 | 0 | 23960.33301 | 0 | 0 | 0 | 0 | 1 | 1 | 1 | 0 | 0 | 0 | 0 | MZ501108 |
| VC_199_29 | 1 | 0 | 22370.69354 | 0 | 0 | 0 | 0 | 1 | 1 | 1 | 0 | 0 | 0 | 0 | MH370375 |
| VC_199_3 | 5 | 3000 | 127731.8839 | 0.02294773 | 3.01E-06 | 4.358898944 | 9.79795897 | 10 | 8 | 0.8 | 7.829415765 | 0 | 0 | 0 | MK972704,MK972707,MK972709,MT074453,MT074457 |
| VC_199_30 | 2 | 300 | 49851.33117 | 0.0059819 | 0.0458715 | 8.660254038 | 8.66025404 | 1 | 1 | 1 | 8.660254038 | 0 | 0 | 0 | MT074447,MT074461 |
| VC_199_31 | 2 | 300 | 52977.31651 | 0.00563091 | 0.0786062 | 2 | 2 | 1 | 1 | 1 | 2 | 0 | 0 | 0 | MK770410,MK972708 |
| VC_199_32 | 12 | 19800 | 272928.0873 | 0.06763956 | 3.41E-36 | 0 | 9.74679434 | 66 | 50 | 0.757575758 | 6.759904261 | 0 | 0 | 0 | MF431736,MF431737,MF431738,MF431739,MF431740,MF431741,MK050846,MK809530,MT074449,MT074465,MT653145,ON526840 |
| VC_199_33 | 1 | 0 | 24182.90178 | 0 | 0 | 0 | 0 | 1 | 1 | 1 | 0 | 0 | 0 | 0 | MW423798 |
| VC_199_4 | 2 | 300 | 54740.47091 | 0.00545053 | 0.1176756 | 8.602325267 | 8.60232527 | 1 | 1 | 1 | 8.602325267 | 0 | 0 | 0 | MH370366,MT074455 |
| VC_199_5 | 6 | 4500 | 154527.7869 | 0.02829694 | 8.19E-08 | 5.385164807 | 8.94427191 | 15 | 15 | 1 | 7.240986422 | 0 | 0 | 0 | MH370386,MT004791,MT074452,MT074456,MT074460,MT074463 |
| VC_199_6 | 1 | 0 | 26663.22864 | 0 | 0 | 0 | 0 | 1 | 1 | 1 | 0 | 0 | 0 | 0 | MT074468 |
| VC_199_7 | 1 | 0 | 26947.28036 | 0 | 0 | 0 | 0 | 1 | 1 | 1 | 0 | 0 | 0 | 0 | MT074467 |
| VC_199_8 | 2 | 300 | 54565.30617 | 0.00546794 | 0.0867468 | 8.246211251 | 8.24621125 | 1 | 1 | 1 | 8.246211251 | 0 | 0 | 0 | MH370376,MT074454 |
| VC_199_9 | 5 | 3000 | 129744.7709 | 0.02259976 | 5.48E-06 | 1.414213562 | 9.48683298 | 10 | 7 | 0.7 | 7.691184777 | 0 | 0 | 0 | MH370367,MH370380,MT074459,MT074466,MW411578 |
| VC_2_0 | 4 | 771.6070077 | 5162.256915 | 0.1300345 | 1.31E-05 | 1.414213562 | 4.35889894 | 6 | 6 | 1 | 3.489654876 | 0 | 0 | 0 | AB008550,MK034952,MK511066,Y13918 |
| VC_20_0 | 5 | 485.3260624 | 651.726646 | 0.42682811 | 5.96E-07 | 6.32455532 | 7.61577311 | 10 | 10 | 1 | 6.905980583 | 0 | 0 | 0 | KT372714,KT852578,KX190835,KX534340,MN065183 |
| VC_200_0 | 15 | 31500 | 31443.47964 | 0.50044898 | 6.21E-68 | 2.449489743 | 5.83095189 | 105 | 105 | 1 | 4.46625316 | 0 | 0 | 0 | KX845404,MG459987,MK521907,MK630230,MN101228,MN163280,MN434094,MN434096,MT380195,MW713578,MZ571828,MZ571830,MZ634344,ON637170,OP171943 |
| VC_200_1 | 1 | 0 | 11560.65256 | 0 | 0 | 0 | 0 | 1 | 1 | 1 | 0 | 0 | 0 | 0 | MZ079855 |
| VC_200_2 | 1 | 0 | 8127.7696 | 0 | 0 | 0 | 0 | 1 | 1 | 1 | 0 | 0 | 0 | 0 | MN478377 |
| VC_201_0 | 2 | 300 | 9232.502516 | 0.03147127 | 0.0423734 | 4.795831523 | 4.79583152 | 1 | 1 | 1 | 4.795831523 | 0 | 0 | 0 | KY363465,MW057857 |
| VC_203_0 | 2 | 300 | 2263.129794 | 0.1170444 | 0.0431842 | 6.08276253 | 6.08276253 | 1 | 1 | 1 | 6.08276253 | 0 | 0 | 0 | MG676224,MG676225 |
| VC_203_1 | 1 | 0 | 1432.710093 | 0 | 0 | 0 | 0 | 1 | 1 | 1 | 0 | 0 | 0 | 0 | MK318083 |
| VC_204_0 | 2 | 300 | 13690.75309 | 0.02144273 | 0.042373 | 4.582575695 | 4.58257569 | 1 | 1 | 1 | 4.582575695 | 0 | 0 | 0 | MN095770,ON287373 |
| VC_205_0 | 5 | 43.5835107 | 285.3506821 | 0.13249918 | 0.153016 | 0 | 2.64575131 | 10 | 10 | 1 | 1.825951226 | 0 | 0 | 0 | AE002163,CMU41758,MG641183,MN862345,MW202581 |
| VC_206_0 | 28 | 2088.460802 | 6516.337756 | 0.24270885 | 0.0002883 | 0 | 2.64575131 | 378 | 378 | 1 | 2.059570554 | 0 | 0 | 0 | AJ270057,AJ550635,AY769964,MG883723,MG945324,MG945546,MG945571,MG945669,MH572277,MH572280,MH572282,MH572290,MH572330,MH572334,MH572422,MH572438,MH572444,MH572448,MH572452,MH572454,MH992157,MH992174,MH992182,MH992210,MN862343,MW202732,MW202889,OL617035 |
| VC_207_0 | 12 | 933.8259036 | 1157.567324 | 0.446509 | 9.20E-37 | 0 | 1.41421356 | 66 | 66 | 1 | 0.449977043 | 0 | 0 | 0 | KC131021,MH572341,MH572342,MH572343,MH572399,MH572400,MH572401,MH572402,MH572403,MH572453,MH572513,MH572514 |
| VC_208_0 | 1 | 0 | 38.93648301 | 0 | 0 | 0 | 0 | 1 | 1 | 1 | 0 | 0 | 0 | 0 | MH992181 |
| VC_209_0 | 3 | 19.00624542 | 171.8652553 | 0.09957613 | 0.3030924 | 1.414213562 | 2 | 3 | 3 | 1 | 1.804737854 | 0 | 0 | 0 | MH572434,MH572475,MZ286294 |
| VC_21_0 | 2 | 164.5540989 | 262.5434653 | 0.38528456 | 0.0496196 | 3.31662479 | 3.31662479 | 1 | 1 | 1 | 3.31662479 | 0 | 0 | 0 | S14_vf_117150,S3_vf_127405 |
| VC_210_0 | 105 | 244433.5382 | 355238.7144 | 0.40761189 | 0 | 0 | 10.3440804 | 5460 | 4240 | 0.776556777 | 8.438335371 | 0 | 0 | 0 | AF009630,AF011378,DQ054536,DQ227763,DQ227764,EU221285,FJ848881,FJ848882,FJ848883,FJ848884,FJ848885,GQ979703,KC182542,KC182543,KC182544,KC182545,KC182546,KC182547,KC182548,KC182549,KC182550,KC182551,KC182552,KC522412,KF676640,KM091442,KP793104,KP793105,KP793108,KP793109,KP793111,KP793113,KP793115,KP793116,KP793117,KP793118,KP793123,KP793124,KP793129,KP793134,KX346246,KX379667,KX379668,KX379670,KX379671,KY554760,KY554761,MF346933,MF448554,MF448555,MF448556,MF448557,MF448558,MF448559,MF448560,MF448561,MF448562,MF448563,MF448564,MF448565,MF448566,MF448567,MF448568,MF448569,MF448570,MF448571,MF448572,MF775703,MF775704,MF775705,MG253647,MG253648,MG253649,MG253650,MG253651,MG253652,MG253653,MG309717,MG309718,MK301437,MN552148,MN552149,MN552150,MN689514,MN689517,MN689518,MN689519,MN689520,MN689522,MN689523,MN689524,MN689525,MW032477,MW041632,MW041633,MW041634,MW041635,MW041636,MW041637,MW041638,MW041639,MW041640,S3_vf_96222,S5_vf_25423,S8_vf_45776 |
| VC_218_0 | 4 | 1077.080072 | 8742.894703 | 0.10968257 | 1.21E-05 | 0 | 4.35889894 | 6 | 6 | 1 | 2.179449472 | 0 | 0 | 0 | KY888143,KY926698,KY926699,MZ368140 |
| VC_219_0 | 2 | 156.2223151 | 517.2934447 | 0.2319505 | 0.0514456 | 8.306623863 | 8.30662386 | 1 | 1 | 1 | 8.306623863 | 0 | 0 | 0 | S10_vf_80712,S11_vf_28515 |
| VC_219_1 | 1 | 0 | 480.1410928 | 0 | 0 | 0 | 0 | 1 | 1 | 1 | 0 | 0 | 0 | 0 | S5_vs_97574 |
| VC_219_2 | 1 | 0 | 354.8550228 | 0 | 0 | 0 | 0 | 1 | 1 | 1 | 0 | 0 | 0 | 0 | S7_vs_137611 |
| VC_219_3 | 1 | 0 | 339.5022788 | 0 | 0 | 0 | 0 | 1 | 1 | 1 | 0 | 0 | 0 | 0 | S8_vs_59736 |
| VC_22_0 | 4 | 113.3758666 | 88.36668246 | 0.56198292 | 0.0874222 | 7.681145748 | 9.11043358 | 6 | 4 | 0.666666667 | 8.587254879 | 0 | 0 | 0 | JF974314,KJ746502,MF614627,MF614628 |
| VC_220_0 | 2 | 300 | 311.0481586 | 0.49095967 | 0.0471955 | 4 | 4 | 1 | 1 | 1 | 4 | 0 | 0 | 0 | KT429160,MT880872 |
| VC_221_0 | 1 | 0 | 118.559759 | 0 | 0 | 0 | 0 | 1 | 1 | 1 | 0 | 0 | 0 | 0 | S12_vf_67909 |
| VC_221_1 | 1 | 0 | 96.17799871 | 0 | 0 | 0 | 0 | 1 | 1 | 1 | 0 | 0 | 0 | 0 | S9_vf_90847 |
| VC_222_0 | 5 | 2545.518403 | 32820.87288 | 0.07197563 | 2.53E-08 | 3.741657387 | 6.40312424 | 10 | 10 | 1 | 5.340275076 | 0 | 0 | 0 | DQ398043,KF017927,KF416344,MG099947,MN585989 |
| VC_222_1 | 19 | 42977.07647 | 60196.31605 | 0.41655194 | 3.05E-107 | 0 | 6.8556546 | 171 | 171 | 1 | 3.81392702 | 0 | 0 | 0 | AF022214,AP018476,AP018480,KC787102,KC787105,KC787106,KX574455,KX758538,KX897981,LC730318,MH513985,MK494087,MN234169,MT310876,MT310883,MT310884,MT310885,MW924646,OP297553 |
| VC_222_2 | 5 | 2011.758365 | 33329.15897 | 0.05692434 | 2.56E-08 | 4.242640687 | 8.06225775 | 10 | 10 | 1 | 7.08658422 | 0 | 0 | 0 | JF946695,KT381276,MF919536,MH020237,Z18946 |
| VC_223_0 | 4 | 1632.647169 | 32492.36842 | 0.04784312 | 1.22E-05 | 2.236067977 | 4.58257569 | 6 | 6 | 1 | 3.712064485 | 0 | 0 | 0 | MF185727,MF324899,MK801735,MN369748 |
| VC_223_1 | 1 | 0 | 9521.715204 | 0 | 0 | 0 | 0 | 1 | 1 | 1 | 0 | 0 | 0 | 0 | MK814748 |
| VC_223_2 | 2 | 249.7182003 | 17837.78122 | 0.01380612 | 0.04255 | 4.242640687 | 4.24264069 | 1 | 1 | 1 | 4.242640687 | 0 | 0 | 0 | MN585968,MN586002 |
| VC_223_3 | 8 | 4962.679058 | 66055.0237 | 0.06987946 | 7.27E-20 | 3.741657387 | 9.2736185 | 28 | 24 | 0.857142857 | 7.77084591 | 0 | 0 | 0 | GU339467,JN408461,KF024721,KU761558,KU761559,KX641263,MF140403,MH825709 |
| VC_223_4 | 13 | 19452.29678 | 98480.8141 | 0.16494347 | 5.74E-51 | 0 | 8.30662386 | 78 | 78 | 1 | 4.828184227 | 0 | 0 | 0 | KJ959632,KM197169,KM677210,KU297783,KX683875,MF668269,MF919538,MH825701,MK061408,MK284522,MK967395,MZ322019,OM203159 |
| VC_223_5 | 2 | 197.4068542 | 20855.60466 | 0.00937666 | 0.0425219 | 7.141428429 | 7.14142843 | 1 | 1 | 1 | 7.141428429 | 0 | 0 | 0 | MW601216,MZ209305 |
| VC_223_6 | 42 | 164967.202 | 184770.2426 | 0.4716887 | 0 | 0 | 10 | 861 | 719 | 0.835075494 | 7.215970855 | 0 | 0 | 0 | AP018479,JN408460,JX899358,KF981601,KJ410132,KJ410134,KM463009,KM591906,KT588442,KU985093,KU985095,KX576639,KX619650,KY965065,LC730320,MF472896,MF919490,MF919500,MF919531,MH051252,MH077576,MH338234,MH697591,MH825704,MK524491,MK919472,MN234203,MN585981,MN585983,MN585998,MT310877,MT310886,MT498041,MT498060,MT498066,MT658804,MW924643,MZ355728,MZ622171,MZ622182,MZ820090,OP297537 |
| VC_223_7 | 1 | 0 | 8987.530416 | 0 | 0 | 0 | 0 | 1 | 1 | 1 | 0 | 0 | 0 | 0 | MT310858 |

| | | | | | | | | | | | | | | |
|---|---|---|---|---|---|---|---|---|---|---|---|---|---|---|
| VC_224_0 | 5 | 2310.612461 | 31399.32518 | 0.06854396 | 2.46E-08 | 3.872983346 | 6.92820323 | 10 | 10 | 1 | 5.476779411 | 0 | 0 | 0 EU744250,KU568494,KX758539,MK279857,MN703408 |
| VC_224_1 | 1 | 0 | 8026.603244 | 0 | 0 | 0 | 0 | 1 | 1 | 1 | 0 | 0 | 0 | 0 MN062708 |
| VC_225_0 | 3 | 789.1901979 | 15740.0581 | 0.04774507 | 0.0013902 | 3 | 5 | 3 | 3 | 1 | 4 | 0 | 0 | 0 KF861510,MN096376,MT639645 |
| VC_226_0 | 5 | 1610.68322 | 18703.62601 | 0.07928811 | 2.48E-08 | 2.828427125 | 8.48528137 | 10 | 10 | 1 | 6.384485748 | 0 | 0 | 0 JF957060,KC661275,KP273225,MG793454,MT553337 |
| VC_227_0 | 4 | 1467.75882 | 22621.70708 | 0.06092949 | 1.19E-05 | 5.567764363 | 6.78232998 | 6 | 6 | 1 | 6.165049233 | 0 | 0 | 0 KR080193,MN310541,MT522004,OM203161 |
| VC_228_0 | 22 | 67678.32847 | 23646.10554 | 0.74107581 | 1.30E-137 | 0 | 5.09901951 | 231 | 231 | 1 | 3.019205911 | 0 | 0 | 0 KU695581,KY471460,MF140410,MH338236,MH338237,MH536828,MK494130,MK524507,MK524511,MK524513,MK524514,MK524519,MN310547,MN586006,MN703405,MN892487,MW055904,MZ747517,MZ747521,ON108640,OP297532,OP297549 |
| VC_229_0 | 5 | 1533.315203 | 25558.14247 | 0.05659774 | 2.53E-08 | 6.244997998 | 8.42614977 | 10 | 10 | 1 | 7.638507314 | 0 | 0 | 0 KX657793,MH513984,MK494113,MK524530,OM203158 |
| VC_23_0 | 11 | 2950.297893 | 2785.025931 | 0.51440825 | 8.62E-27 | 1 | 7.93725393 | 55 | 55 | 1 | 5.464976065 | 0 | 0 | 0 AB036666,HQ906662,HQ906663,HQ906664,KX522565,MK976036,MN180248,MN180249,MN180250,MN180251,MN180252 |
| VC_230_0 | 38 | 180851.267 | 18402.29702 | 0.90764383 | 0 | 0 | 7.14142843 | 703 | 703 | 1 | 4.653610138 | 0 | 0 | 0 JF704097,JF937092,JF937094,JN049605,JN698999,JN699019,KF560332,KF560333,KF560334,KM401838,KU695582,KU985096,KX375815,KX576638,KX641261,MF668288,MG099945,MG099952,MH020240,MH051253,MH155876,MH779517,MK359352,MK359360,MK494090,MK894433,MN586039,MN728683,MT024861,MT657338,MW822143,MZ005685,MZ171376,MZ322007,MZ456034,MZ681516,OM913585,OP434447 |
| VC_231_0 | 19 | 47734.33424 | 3167.939039 | 0.93776429 | 4.68E-94 | 0 | 7.74596669 | 171 | 171 | 1 | 3.912475107 | 0 | 0 | 0 KU998249,KU998250,KU998251,KX557280,KX557283,KX557284,MF668282,MH001454,MH316566,MH513974,MK967382,MN284898,MN369756,MT522007,MZ028625,MZ622176,MZ681508,OM818328,OP021679 |
| VC_232_0 | 32 | 114206.7639 | 20621.34616 | 0.84705455 | 2.78E-254 | 0 | 9.11043358 | 496 | 494 | 0.995967742 | 5.745797411 | 0 | 0 | 0 JF704110,JN699005,KT285706,KU613353,KU716094,KU716095,KX585252,KX664447,KX685355,MG872835,MG872840,MG872842,MH371122,MK061409,MK468591,MK494108,MK524489,MK559428,MK977713,MN234167,MN234200,MN234209,MN234235,MN310542,MN369741,MN369763,MN369764,MN585974,MN585978,MN585979,MN585994,ON724016 |
| VC_234_0 | 2 | 1.161148463 | 2.322296926 | 0.33333333 | 1 | 0 | 0 | 1 | 1 | 1 | 0 | 0 | 0 | 0 AF334111,HM066936 |
| VC_235_0 | 60 | 157352.0566 | 9808.755536 | 0.94132144 | 0 | 0 | 7 | 1770 | 1770 | 1 | 4.917806789 | 0 | 0 | 0 AF063097,AY135486,AY135739,AY251033,B1U32222,HQ110084,KC139521,KC618326,KF322032,KR073660,KR073661,KT630644,KX833211,LR595869,LR595870,LR595873,LR595883,LR595885,LR595887,MF276773,MK416018,MK907236,MK907237,MK907238,MK907239,MK972710,MK972711,MN016939,MT239398,MT374855,MT374857,MT374858,MZ420230,MZ520833,OK490408,OK490461,OK490464,OK490467,OK490470,OK490473,OM046626,OM046627,OM046628,OM140653,OM475426,OM475429,OM475431,OM475433,OM475434,ON146439,ON146440,ON239131,ON470601,ON470606,ON470613,ON782130,OU784549,OU784746,OU784750,OV192280 |
| VC_236_0 | 32 | 47019.78852 | 4567.200508 | 0.91146604 | 1.94E-113 | 0 | 7.48331477 | 496 | 496 | 1 | 4.474620715 | 0 | 0 | 0 CP025874,CP025901,CP025923,CP052639,KP063541,KY271397,LR595891,MG589387,MK416015,OK490394,OK490396,OK490398,OK490400,OK490402,OK490404,OK490406,OK490412,OK490414,OK490422,OK490424,OK490426,OK490428,OK490432,OK490435,OK490437,OK490440,OK490443,OK490446,OK490449,OK490453,OK490456,OK490459 |
| VC_237_0 | 3 | 71.66456883 | 907.4755997 | 0.07319133 | 0.0064416 | 8.124038405 | 9.2736185 | 3 | 2 | 0.666666667 | 8.627646091 | 0 | 0 | 0 JQ780327,MK524177,OL964755 |
| VC_237_1 | 1 | 0 | 491.6521421 | 0 | 0 | 0 | 0 | 1 | 1 | 1 | 0 | 0 | 0 | 0 HM208537 |
| VC_238_0 | 3 | 159.3751095 | 538.1542426 | 0.22848517 | 0.0017185 | 5.656854249 | 6.92820323 | 3 | 3 | 1 | 6.355266059 | 0 | 0 | 0 MT366580,MZ496293,OP121182 |
| VC_239_0 | 2 | 126.1122463 | 10.07488542 | 0.92602175 | 0.2701457 | 1.732050808 | 1.73205081 | 1 | 1 | 1 | 1.732050808 | 0 | 0 | 0 AF065411,AF301375 |
| VC_24_0 | 3 | 518.8743476 | 707.0840841 | 0.42323976 | 0.0016238 | 3.605551275 | 4.35889894 | 3 | 3 | 1 | 3.988150073 | 0 | 0 | 0 AY133112,FN297812,JX556417 |
| VC_240_0 | 2 | 25.2828497 | 10.07488542 | 0.71505852 | 0.2701457 | 4 | 4 | 1 | 1 | 1 | 4 | 0 | 0 | 0 MH674343,MN737622 |
| VC_241_0 | 21 | 61739.13224 | 12269.71547 | 0.83421286 | 7.68E-100 | 0 | 5.09901951 | 210 | 210 | 1 | 2.748225472 | 0 | 0 | 0 AP018469,AP018470,AP018471,JN831653,KJ944841,KT361920,KX621007,KY087992,MF140402,MF140435,MH051258,MH926058,MK224497,MK524485,MK524488,MN369761,MN945904,MT310882,MW601218,ON526978,ON645342 |
| VC_243_0 | 72 | 588435.1098 | 139049.682 | 0.80886242 | 0 | 0 | 8.83176087 | 2556 | 2556 | 1 | 6.128947404 | 0 | 0 | 0 HM152764,HM152767,JF704105,JF704106,JN185608,JN643714,KJ567045,KM677211,KX585253,KX641264,KX657794,KX657795,KX657796,KY380102,MF140412,MF140428,MF140430,MF377440,MF472892,MF668267,MF919532,MG962364,MG962371,MH001453,MH051246,MH077577,MH371113,MH509444,MH513977,MH536821,MH536822,MH697578,MH727544,MK016503,MK112537,MK460247,MN234182,MN234197,MN234198,MN234205,MN234230,MN234231,MN369742,MN369746,MN908692,MW712722,MW712723,MW712730,MW712733,MW712734,MW862979,MW862984,MW862985,MW862990,MZ005674,MZ355721,MZ648037,OK040778,OK040782,OK040784,OK310505,OM818330,OM913584,ON081329,ON081331,ON260816,ON456339,ON456353,ON526984,ON687735,ON687736,ON724013 |
| VC_243_1 | 5 | 2234.793389 | 60523.75231 | 0.03560939 | 2.74E-08 | 2.236067977 | 8.30662386 | 10 | 10 | 1 | 6.288541918 | 0 | 0 | 0 MF140416,MN234165,MN234174,MN234201,MN234226 |
| VC_244_0 | 4 | 1701.278438 | 38244.20412 | 0.04259001 | 1.24E-05 | 1.732050808 | 5.83095189 | 6 | 6 | 1 | 3.978626572 | 0 | 0 | 0 KF713486,MF185722,MK310138,MK494119 |
| VC_244_1 | 13 | 15241.4621 | 71071.63731 | 0.17658342 | 1.56E-49 | 0 | 9.38083152 | 78 | 57 | 0.730769231 | 6.99515529 | 0 | 0 | 0 JF937104,JX042579,KP027199,KR080206,KX683428,KY087993,MF140398,MF140406,MF324915,MF373841,MN234199,MT723940,MW601222 |
| VC_244_2 | 11 | 12182.20407 | 74660.70876 | 0.14027862 | 2.38E-36 | 2 | 9.05538514 | 55 | 54 | 0.981818182 | 6.364072338 | 0 | 0 | 0 KY945355,MF324908,MF324909,MF324911,MF324912,MF324913,MF324914,MN234176,MN369762,MN945901,ON456351 |
| VC_244_3 | 8 | 6856.624334 | 56267.71065 | 0.10862094 | 9.24E-20 | 2.236067977 | 8.88819442 | 28 | 28 | 1 | 5.997649661 | 0 | 0 | 0 MH910038,MH910039,MH910042,MK376955,MK460246,MN284895,MT310859,MT310860 |
| VC_245_0 | 17 | 34013.10402 | 21922.69136 | 0.60807402 | 2.64E-82 | 0 | 7.07106781 | 136 | 136 | 1 | 5.01230262 | 0 | 0 | 0 JN243855,KJ567042,KM363596,KM923971,KX585251,KX636165,MF185717,MF185719,MF185720,MG962374,MH051255,MH576966,MH651171,MH697592,MW435854,MZ322016,MZ958747 |
| VC_247_0 | 4 | 1300.130412 | 382.7703357 | 0.77255323 | 1.73E-05 | 0 | 6.40312424 | 6 | 6 | 1 | 4.944050781 | 0 | 0 | 0 MW314859,MW584165,MW584169,MW584207 |
| VC_248_0 | 2 | 49.64028885 | 645.4678216 | 0.07141377 | 0.0457098 | 7.810249676 | 7.81024968 | 1 | 1 | 1 | 7.810249676 | 0 | 0 | 0 MF975720,MT121966 |
| VC_249_0 | 2 | 214.3425161 | 2357.139373 | 0.08335369 | 0.0458341 | 2.449489743 | 2.44948974 | 1 | 1 | 1 | 2.449489743 | 0 | 0 | 0 KT588074,MK340941 |
| VC_249_1 | 21 | 12049.65163 | 5734.851473 | 0.67753659 | 1.34E-82 | 0 | 10.0498756 | 210 | 178 | 0.847619048 | 7.866966729 | 0 | 0 | 0 KT630649,MK416007,MK416019,MK422455,MK433579,MK448230,MN098327,MN166823,MT135176,MT701587,MW057855,OK665842,OL791266,OM284015,OM621816,ON470579,ON470582,ON470588,ON470593,OP114732,S4_vs_VIRSorter_31980-circular-cat_2 |
| VC_25_0 | 3 | 798.3354041 | 1554.339341 | 0.33933097 | 0.0015481 | 0 | 0 | 3 | 3 | 1 | 0 | 0 | 0 | 0 JX507079,MH142219,MH142220 |
| VC_250_0 | 5 | 2461.197345 | 3351.543434 | 0.42341426 | 3.49E-08 | 0 | 0 | 10 | 10 | 1 | 0 | 0 | 0 | 0 OK490460,OK490463,OK490466,OK490469,OK490472 |
| VC_251_0 | 2 | 104.5831743 | 1547.073417 | 0.06332017 | 0.0439173 | 5.830951895 | 5.83095189 | 1 | 1 | 1 | 5.830951895 | 0 | 0 | 0 MF695815,MK416014 |
| VC_252_0 | 12 | 10745.80477 | 15627.20463 | 0.40745463 | 1.32E-41 | 0 | 7.14142843 | 66 | 66 | 1 | 1.618787636 | 0 | 0 | 0 KY271401,MK416017,OK490430,OK490433,OK490438,OK490441,OK490444,OK490447,OK490450,OK490451,OK490454,OK490458 |
| VC_253_0 | 3 | 162.3995366 | 50.76187986 | 0.76186178 | 0.0404278 | 1.732050808 | 2.64575131 | 3 | 3 | 1 | 2.12593404 | 0 | 0 | 0 AY583234,AY583235,AY583236 |
| VC_254_0 | 14 | 20749.63483 | 2006.157778 | 0.91183969 | 9.60E-24 | 0 | 9.38083152 | 91 | 88 | 0.967032967 | 6.940166829 | 0 | 0 | 0 AF234172,AF234173,AF503408,FO818745,KU760857,MH160767,MH422554,MH445380,MH445381,MZ188976,ON018986,ON470615,ON470617,OP279344 |
| VC_254_1 | 1 | 0 | 1939.492172 | 0 | 0 | 0 | 0 | 1 | 1 | 1 | 0 | 0 | 0 | 0 MF356679 |
| VC_255_0 | 11 | 9760.477132 | 4115.149547 | 0.70342604 | 3.63E-31 | 0 | 4 | 55 | 55 | 1 | 1.28942858 | 0 | 0 | 0 HQ316581,MG592412,MG592507,MG592513,MG592524,MG592527,MG592588,MG592589,MG592669,MG592670,MZ568828 |
| VC_256_0 | 2 | 63.42651701 | 1387.747848 | 0.04370703 | 0.0438898 | 6.164414003 | 6.164414 | 1 | 1 | 1 | 6.164414003 | 0 | 0 | 0 KY939598,OP380511 |
| VC_257_0 | 3 | 404.6102619 | 1523.194464 | 0.20988135 | 0.00168 | 3 | 5.19615242 | 3 | 3 | 1 | 4.295522728 | 0 | 0 | 0 AY539836,CP000622,ON170188 |
| VC_258_0 | 121 | 303923.5652 | 109062.3282 | 0.73591755 | 0 | 0 | 9 | 7260 | 7259 | 0.999862259 | 7.165646137 | 0 | 0 | 0 AF085222,AF115102,AF115103,AF145054,FJ236310,KT717083,KT717084,KT717085,KY705251,KY705256,KY705257,KY705259,KY705260,KY705261,KY705262,KY705263,KY705264,KY705265,KY705266,KY705267,KY705270,KY705271,KY705272,KY705273,KY705274,KY705275,KY705276,KY705282,KY705283,KY705284,KY705287,KY705288,KY705289,KY705290,MF161328,MF580759,MF580760,MF580761,MF580762,MF580763,MF580764,MF580765,MF580766,MF580767,MF580768,MF580769,MF580770,MF580771,MF580772,MF580773,MF580774,MF580775,MG708273,MG708274,MG708275,MH000602,MH000603,MH000604,MH375594,MH375595,MH375596,MH375597,MH375598,MH375599,MH375600,MH892351,MH892352,MH892353,MH892354,MH892356,MH892357,MH892358,MH892359,MH892360,MH892361,MH892375,MH892377,MH892378,MH892379,MH892380,MH892381,MH892382,MH937458,MH937459,MH937461,MH937462,MH937465,MH937466,MH937467,MH937468,MH937469,MH937470,MH937471,MH937472,MH937474,MH937477,MH937480,MH937481,MH937482,MH937483,MH937485,MH937486,MH937487,MH937488,MH937489,MH937490,MH937491,MH937492,MH937493,MH937495,MH937496,MH937497,MH937499,MH937500,MH937501,MH937504,MH937506,MH937508,MH973661,MH973662,MZ090946 |
| VC_26_0 | 12 | 3681.693623 | 963.1788853 | 0.7926361 | 1.57E-31 | 0 | 5.38516481 | 66 | 66 | 1 | 1.982165596 | 0 | 0 | 0 ON170017,ON170042,ON170051,ON170056,ON170060,ON170066,ON170072,ON170166,ON170169,ON170274,ON170278,ON170357 |
| VC_260_0 | 46 | 83485.62322 | 79043.5534 | 0.51366545 | 0 | 0 | 8.48528137 | 1035 | 1035 | 1 | 6.236329199 | 0 | 0 | 0 AF158600,AY699705,EF529515,FJ226752,HE861935,HG380752,HG424323,KY705258,KY705268,KY705269,KY705277,KY705278,KY705279,KY705280,KY705281,KY705285,KY705286,MH375593,MH892362,MH892363,MH892364,MH892366,MH892376,MH892383,MH892384,MH892385,MH937457,MH937460,MH937463,MH937464,MH937473,MH937475,MH937476,MH937478,MH937479,MH937484,MH937494,MH937498,MH937502,MH937503,MH937505,MH937507,MH937509,MH937510,MH937511,U88974 |
| VC_261_0 | 13 | 8253.194219 | 32521.84246 | 0.20240801 | 1.65E-51 | 1.414213562 | 6.78232998 | 78 | 78 | 1 | 5.049151107 | 0 | 0 | 0 FJ965538,KX879643,KY705252,KY705253,KY705254,KY705255,MH892355,MH892367,MH892373,MH973663,MK202159,MK202160,MK202161 |
| VC_262_0 | 14 | 11266.38368 | 36392.5916 | 0.23639584 | 2.51E-59 | 1 | 6.63324958 | 91 | 91 | 1 | 3.98042113 | 0 | 0 | 0 KU678389,KU678390,KU678391,KU678392,KX879641,KX879642,MH892350,MH892365,MH892368,MH892369,MH892370,MH892371,MH892372,MH892374 |
| VC_265_0 | 3 | 721.0382354 | 5624.008539 | 0.11363797 | 0.0014136 | 0 | 1.41421356 | 3 | 3 | 1 | 0.942809042 | 0 | 0 | 0 MK448824,MK449002,MK449003 |
| VC_266_0 | 13 | 5269.630405 | 9280.606422 | 0.36216802 | 4.05E-48 | 0 | 8.36660027 | 78 | 78 | 1 | 6.532234578 | 0 | 0 | 0 AF176025,AF323669,AF489521,AJ245616,LT985568,LT985571,LT993224,MN534316,MN534317,MN689509,MN689516,MN689521,MN689530 |
| VC_267_0 | 1 | 0 | 2174.738066 | 0 | 0 | 0 | 0 | 1 | 1 | 1 | 0 | 0 | 0 | 0 MZ308445 |
| VC_268_0 | 10 | 2590.086951 | 15682.99403 | 0.14174331 | 1.05E-24 | 1 | 9.32737905 | 45 | 42 | 0.933333333 | 7.44091888 | 0 | 0 | 0 AF242738,KX160204,KX160219,KX160220,KX456207,KX456211,KX456212,MK448706,MK448721,MN534320 |
| VC_268_1 | 20 | 8968.68847 | 38086.63233 | 0.19059882 | 1.71E-64 | 1 | 10.4403065 | 190 | 129 | 0.678947368 | 8.090092954 | 0 | 0 | 0 AY007505,FR671408,FR671409,KC348600,KC348601,KC348602,KT337360,KX077894,KY065485,KY065503,KY065504,MK448814,MK448881,MK448882,MK448896,MK448909,MK448988,MK448989,OL774869,OL774877 |
| VC_268_2 | 1 | 0 | 1830.574642 | 0 | 0 | 0 | 0 | 1 | 1 | 1 | 0 | 0 | 0 | 0 KP208803 |
| VC_268_3 | 1 | 0 | 1905.644229 | 0 | 0 | 0 | 0 | 1 | 1 | 1 | 0 | 0 | 0 | 0 MK448900 |
| VC_269_0 | 7 | 1646.255457 | 5579.529275 | 0.22783068 | 3.05E-15 | 0 | 7.68114575 | 21 | 21 | 1 | 5.263473119 | 0 | 0 | 0 AF323668,LT985569,LT985574,LT985578,LT985583,LT993229,LT996082 |
| VC_27_0 | 4 | 68.40736574 | 24.07359233 | 0.73969136 | 0.0006385 | 0 | 2.82842712 | 6 | 6 | 1 | 2.265684697 | 0 | 0 | 0 AY714348,JN848801,MN200777,MN200778 |
| VC_271_0 | 12 | 7442.587002 | 10048.16387 | 0.42551558 | 1.54E-41 | 0 | 7.21110255 | 66 | 66 | 1 | 5.276548645 | 0 | 0 | 0 DQ394807,DQ394808,DQ394809,DQ394810,KX456206,KX456208,KX456209,KX456210,KX456213,LT985575,LT985576,LT996081 |
| VC_273_0 | 2 | 126.6849879 | 1119.390031 | 0.10166722 | 0.0443946 | 6.164414003 | 6.164414 | 1 | 1 | 1 | 6.164414003 | 0 | 0 | 0 GQ452243,KT945994 |
| VC_274_0 | 7 | 3111.825832 | 6036.713233 | 0.34014456 | 4.03E-15 | 1.732050808 | 6.40312424 | 21 | 21 | 1 | 3.672350891 | 0 | 0 | 0 JX564242,JX567312,KX160206,KX160209,KX160210,KX160211,MN552144 |
| VC_275_0 | 6 | 941.1284424 | 503.3218774 | 0.65154781 | 6.32E-08 | 4.358898944 | 9.11043358 | 15 | 14 | 0.933333333 | 7.306881516 | 0 | 0 | 0 AF195902,DQ320509,HE956704,KF767351,MN830257,S14_vf_106156 |
| VC_278_0 | 11 | 1405.009807 | 1950.065525 | 0.41877146 | 1.80E-26 | 5.656854249 | 9.94987437 | 55 | 44 | 0.8 | 8.216958858 | 0 | 0 | 0 S5_vs_145590,S5_vs_145599,S5_vs_145602,S5_vs_2876,S5_vs_80498,S6_vs_56491,S7_vf_60968,S7_vs_13799,S7_vs_138109,S7_vs_48832,S9_vs_34898 |
| VC_278_1 | 1 | 0 | 341.4923085 | 0 | 0 | 0 | 0 | 1 | 1 | 1 | 0 | 0 | 0 | 0 S9_vs_90859 |
| VC_279_0 | 11 | 3337.58483 | 3560.034036 | 0.48387493 | 3.78E-30 | 1.732050808 | 9.43398113 | 55 | 48 | 0.872727273 | 7.74299066 | 0 | 0 | 0 AJ251789,AP018361,AY131267,AY605066,EU246945,KC171646,KC171647,MF999224,MH983004,MN830258,MT956952 |
| VC_280_0 | 4 | 280.3567311 | 604.4057011 | 0.31687233 | 5.08E-05 | 6.92820323 | 9.05538514 | 6 | 5 | 0.833333333 | 7.911966997 | 0 | 0 | 0 GU323708,HQ141410,MN830252,MN830253 |
| VC_281_0 | 2 | 36.01629589 | 442.1359155 | 0.07532391 | 0.0459047 | 5.656854249 | 5.65685425 | 1 | 1 | 1 | 5.656854249 | 0 | 0 | 0 AJ421943,MH220877 |
| VC_283_0 | 2 | 19.08453111 | 0 | 1 | 0 | 1 | 1 | 1 | 1 | 1 | 1 | 0 | 0 | 0 AF133242,AJ969242 |
| VC_284_0 | 3 | 130.3623164 | 0 | 1 | 0 | 1 | 3.16227766 | 3 | 3 | 1 | 2.387425887 | 0 | 0 | 0 AF155037,MG966533,MK424903 |
| VC_285_0 | 3 | 586.7723848 | 1300.592744 | 0.310895 | 0.0015177 | 0 | 0 | 3 | 3 | 1 | 0 | 0 | 0 | 0 AY029185,AY526908,AY526909 |
| VC_286_0 | 2 | 58.62628503 | 245.8166948 | 0.19256902 | 0.0484506 | 6.92820323 | 6.92820323 | 1 | 1 | 1 | 6.92820323 | 0 | 0 | 0 AY605181,MH128984 |
| VC_287_0 | 2 | 105.7896362 | 256.812209 | 0.29175151 | 0.0474412 | 3.464101615 | 3.46410162 | 1 | 1 | 1 | 3.464101615 | 0 | 0 | 0 GQ421471,MT084350 |
| VC_288_0 | 3 | 450.2366877 | 810.5042383 | 0.3571207 | 0.0017317 | 0 | 1.41421356 | 3 | 3 | 1 | 0.942809042 | 0 | 0 | 0 MK733262,MK733263,MK733264 |
| VC_289_0 | 2 | 172.7585838 | 616.0890893 | 0.2190012 | 0.0452868 | 2.645751311 | 2.64575131 | 1 | 1 | 1 | 2.645751311 | 0 | 0 | 0 MK942064,MN746332 |
| VC_29_0 | 2 | 100.2949395 | 0 | 1 | 0 | 2.828427125 | 2.82842712 | 1 | 1 | 1 | 2.828427125 | 0 | 0 | 0 AB063393,GQ403789 |
| VC_290_0 | 2 | 300 | 121348.9122 | 0.00246611 | 0.0516816 | 2.236067977 | 2.23606798 | 1 | 1 | 1 | 2.236067977 | 0 | 0 | 0 MN508614,MN508618 |
| VC_290_1 | 1 | 0 | 58480.47152 | 0 | 0 | 0 | 0 | 1 | 1 | 1 | 0 | 0 | 0 | 0 MZ502379 |
| VC_290_10 | 1 | 0 | 60009.37165 | 0 | 0 | 0 | 0 | 1 | 1 | 1 | 0 | 0 | 0 | 0 AP011113 |
| VC_290_100 | 2 | 300 | 113077.6502 | 0.00264602 | 0.0942011 | 1.414213562 | 1.41421356 | 1 | 1 | 1 | 1.414213562 | 0 | 0 | 0 MT515752,MT526905 |
| VC_290_101 | 1 | 0 | 60592.73128 | 0 | 0 | 0 | 0 | 1 | 1 | 1 | 0 | 0 | 0 | 0 LR597660 |
| VC_290_102 | 1 | 0 | 35714.12256 | 0 | 0 | 0 | 0 | 1 | 1 | 1 | 0 | 0 | 0 | 0 OK570375 |
| VC_290_103 | 1 | 0 | 58128.10642 | 0 | 0 | 0 | 0 | 1 | 1 | 1 | 0 | 0 | 0 | 0 ON528735 |
| VC_290_104 | 1 | 0 | 53847.59117 | 0 | 0 | 0 | 0 | 1 | 1 | 1 | 0 | 0 | 0 | 0 OL310488 |
| VC_290_105 | 1 | 0 | 56830.32263 | 0 | 0 | 0 | 0 | 1 | 1 | 1 | 0 | 0 | 0 | 0 MZ234013 |
| VC_290_106 | 1 | 0 | 55228.5406 | 0 | 0 | 0 | 0 | 1 | 1 | 1 | 0 | 0 | 0 | 0 MZ753803 |
| VC_290_107 | 1 | 0 | 58957.71397 | 0 | 0 | 0 | 0 | 1 | 1 | 1 | 0 | 0 | 0 | 0 ON528739 |
| VC_290_108 | 1 | 0 | 57319.32228 | 0 | 0 | 0 | 0 | 1 | 1 | 1 | 0 | 0 | 0 | 0 OK148441 |
| VC_290_109 | 2 | 300 | 113817.7378 | 0.00262886 | 0.0856325 | 1.732050808 | 1.73205081 | 1 | 1 | 1 | 1.732050808 | 0 | 0 | 0 KY608967,OK275722 |
| VC_290_11 | 1 | 0 | 61032.57068 | 0 | 0 | 0 | 0 | 1 | 1 | 1 | 0 | 0 | 0 | 0 ON528741 |
| VC_290_110 | 2 | 300 | 113036.0934 | 0.00264699 | 0.0914177 | 1.732050808 | 1.73205081 | 1 | 1 | 1 | 1.732050808 | 0 | 0 | 0 MN508616,MN508617 |
| VC_290_111 | 1 | 0 | 56977.89299 | 0 | 0 | 0 | 0 | 1 | 1 | 1 | 0 | 0 | 0 | 0 KY290975 |
| VC_290_112 | 1 | 0 | 58873.93054 | 0 | 0 | 0 | 0 | 1 | 1 | 1 | 0 | 0 | 0 | 0 OL870317 |
| VC_290_113 | 1 | 0 | 58151.8233 | 0 | 0 | 0 | 0 | 1 | 1 | 1 | 0 | 0 | 0 | 0 LR215724 |
| VC_290_114 | 1 | 0 | 60098.20794 | 0 | 0 | 0 | 0 | 1 | 1 | 1 | 0 | 0 | 0 | 0 KX130861 |
| VC_290_115 | 1 | 0 | 58911.12282 | 0 | 0 | 0 | 0 | 1 | 1 | 1 | 0 | 0 | 0 | 0 JN986846 |
| VC_290_116 | 1 | 0 | 62591.52471 | 0 | 0 | 0 | 0 | 1 | 1 | 1 | 0 | 0 | 0 | 0 MZ501089 |
| VC_290_117 | 3 | 900 | 172095.0825 | 0.00520246 | 0.0026948 | 1 | 2.23606798 | 3 | 3 | 1 | 1.745355992 | 0 | 0 | 0 MZ234031,MZ234035,MZ234038 |
| VC_290_118 | 3 | 900 | 168252.5965 | 0.00532064 | 0.009531 | 3.16227766 | 6.63324958 | 3 | 3 | 1 | 5.425422646 | 0 | 0 | 0 MZ501113,OL741432,OL870316 |
| VC_290_119 | 1 | 0 | 63015.7732 | 0 | 0 | 0 | 0 | 1 | 1 | 1 | 0 | 0 | 0 | 0 MK327939 |
| VC_290_12 | 1 | 0 | 60832.82412 | 0 | 0 | 0 | 0 | 1 | 1 | 1 | 0 | 0 | 0 | 0 MK373785 |
| VC_290_120 | 1 | 0 | 61302.59479 | 0 | 0 | 0 | 0 | 1 | 1 | 1 | 0 | 0 | 0 | 0 MK962752 |
| VC_290_121 | 2 | 300 | 116705.7537 | 0.00256398 | 0.0932659 | 1.414213562 | 1.41421356 | 1 | 1 | 1 | 1.414213562 | 0 | 0 | 0 MK327940,MK327945 |
| VC_290_122 | 1 | 0 | 57802.69066 | 0 | 0 | 0 | 0 | 1 | 1 | 1 | 0 | 0 | 0 | 0 OK274152 |
| VC_290_123 | 1 | 0 | 58212.747 | 0 | 0 | 0 | 0 | 1 | 1 | 1 | 0 | 0 | 0 | 0 OL362041 |
| VC_290_124 | 1 | 0 | 59196.70413 | 0 | 0 | 0 | 0 | 1 | 1 | 1 | 0 | 0 | 0 | 0 MK373779 |
| VC_290_125 | 1 | 0 | 60046.13409 | 0 | 0 | 0 | 0 | 1 | 1 | 1 | 0 | 0 | 0 | 0 KX130862 |
| VC_290_126 | 1 | 0 | 60127.71361 | 0 | 0 | 0 | 0 | 1 | 1 | 1 | 0 | 0 | 0 | 0 OM386666 |
| VC_290_127 | 1 | 0 | 56069.57741 | 0 | 0 | 0 | 0 | 1 | 1 | 1 | 0 | 0 | 0 | 0 MK327929 |
| VC_290_128 | 1 | 0 | 58319.09649 | 0 | 0 | 0 | 0 | 1 | 1 | 1 | 0 | 0 | 0 | 0 OK076929 |
| VC_290_129 | 1 | 0 | 59231.3252 | 0 | 0 | 0 | 0 | 1 | 1 | 1 | 0 | 0 | 0 | 0 MH992510 |
| VC_290_13 | 1 | 0 | 56634.946 | 0 | 0 | 0 | 0 | 1 | 1 | 1 | 0 | 0 | 0 | 0 ON528745 |
| VC_290_130 | 1 | 0 | 61559.00092 | 0 | 0 | 0 | 0 | 1 | 1 | 1 | 0 | 0 | 0 | 0 MN022785 |
| VC_290_131 | 1 | 0 | 57883.42301 | 0 | 0 | 0 | 0 | 1 | 1 | 1 | 0 | 0 | 0 | 0 MN716856 |
| VC_290_132 | 1 | 0 | 57947.9898 | 0 | 0 | 0 | 0 | 1 | 1 | 1 | 0 | 0 | 0 | 0 ON528740 |
| VC_290_133 | 1 | 0 | 57256.13404 | 0 | 0 | 0 | 0 | 1 | 1 | 1 | 0 | 0 | 0 | 0 MK327936 |
| VC_290_134 | 1 | 0 | 56170.13738 | 0 | 0 | 0 | 0 | 1 | 1 | 1 | 0 | 0 | 0 | 0 MK327944 |
| VC_290_135 | 1 | 0 | 59439.98868 | 0 | 0 | 0 | 0 | 1 | 1 | 1 | 0 | 0 | 0 | 0 OP168898 |
| VC_290_136 | 2 | 300 | 116612.0072 | 0.00256603 | 0.0612112 | 0 | 0 | 1 | 1 | 1 | 0 | 0 | 0 | 0 MT515753,MT515755 |
| VC_290_137 | 1 | 0 | 58947.93523 | 0 | 0 | 0 | 0 | 1 | 1 | 1 | 0 | 0 | 0 | 0 LR597657 |
| VC_290_138 | 1 | 0 | 56783.07805 | 0 | 0 | 0 | 0 | 1 | 1 | 1 | 0 | 0 | 0 | 0 MG867727 |
| VC_290_139 | 1 | 0 | 59304.31989 | 0 | 0 | 0 | 0 | 1 | 1 | 1 | 0 | 0 | 0 | 0 KX009778 |
| VC_290_14 | 1 | 0 | 60731.40111 | 0 | 0 | 0 | 0 | 1 | 1 | 1 | 0 | 0 | 0 | 0 MF001360 |
| VC_290_140 | 2 | 300 | 108387.0896 | 0.00276022 | 0.0626741 | 7.416198487 | 7.41619849 | 1 | 1 | 1 | 7.416198487 | 0 | 0 | 0 OV876900,OV877085 |
| VC_290_141 | 1 | 0 | 54956.09199 | 0 | 0 | 0 | 0 | 1 | 1 | 1 | 0 | 0 | 0 | 0 LR746311 |
| VC_290_142 | 2 | 300 | 105935.727 | 0.00282391 | 0.0579223 | 5.916079783 | 5.91607978 | 1 | 1 | 1 | 5.916079783 | 0 | 0 | 0 ON528726,ON528744 |
| VC_290_143 | 1 | 0 | 54108.14573 | 0 | 0 | 0 | 0 | 1 | 1 | 1 | 0 | 0 | 0 | 0 KX452698 |
| VC_290_144 | 1 | 0 | 53837.54752 | 0 | 0 | 0 | 0 | 1 | 1 | 1 | 0 | 0 | 0 | 0 MW341595 |
| VC_290_145 | 1 | 0 | 52176.32518 | 0 | 0 | 0 | 0 | 1 | 1 | 1 | 0 | 0 | 0 | 0 KM501444 |
| VC_290_146 | 1 | 0 | 53458.30251 | 0 | 0 | 0 | 0 | 1 | 1 | 1 | 0 | 0 | 0 | 0 ON528746 |
| VC_290_147 | 1 | 0 | 55663.35372 | 0 | 0 | 0 | 0 | 1 | 1 | 1 | 0 | 0 | 0 | 0 MZ501096 |

| | | | | | | | | | | | | | | | |
|---|---|---|---|---|---|---|---|---|---|---|---|---|---|---|---|
| VC_290_148 | 1 | 0 | 53244.28391 | 0 | 0 | 0 | 0 | 1 | 1 | 1 | 0 | 0 | 0 | 0 | JN202312 |
| VC_290_149 | 1 | 0 | 55550.44698 | 0 | 0 | 0 | 0 | 1 | 1 | 1 | 0 | 0 | 0 | 0 | ON528736 |
| VC_290_15 | 1 | 0 | 57597.28729 | 0 | 0 | 0 | 0 | 1 | 1 | 1 | 0 | 0 | 0 | 0 | LC465543 |
| VC_290_150 | 1 | 0 | 56207.26933 | 0 | 0 | 0 | 0 | 1 | 1 | 1 | 0 | 0 | 0 | 0 | MZ065353 |
| VC_290_151 | 1 | 0 | 55504.71575 | 0 | 0 | 0 | 0 | 1 | 1 | 1 | 0 | 0 | 0 | 0 | MH051915 |
| VC_290_152 | 1 | 0 | 57941.06267 | 0 | 0 | 0 | 0 | 1 | 1 | 1 | 0 | 0 | 0 | 0 | OL960580 |
| VC_290_153 | 1 | 0 | 59697.0717 | 0 | 0 | 0 | 0 | 1 | 1 | 1 | 0 | 0 | 0 | 0 | MW749000 |
| VC_290_154 | 1 | 0 | 61145.55832 | 0 | 0 | 0 | 0 | 1 | 1 | 1 | 0 | 0 | 0 | 0 | HM035025 |
| VC_290_155 | 2 | 300 | 112425.0483 | 0.00266134 | 0.0495557 | 1.414213562 | 1.41421356 | 1 | 1 | 1 | 1.414213562 | 0 | 0 | 0 | MT630408,MZ681930 |
| VC_290_156 | 1 | 0 | 56592.35848 | 0 | 0 | 0 | 0 | 1 | 1 | 1 | 0 | 0 | 0 | 0 | MK327942 |
| VC_290_157 | 1 | 0 | 55762.86319 | 0 | 0 | 0 | 0 | 1 | 1 | 1 | 0 | 0 | 0 | 0 | OL960581 |
| VC_290_158 | 1 | 0 | 59974.54316 | 0 | 0 | 0 | 0 | 1 | 1 | 1 | 0 | 0 | 0 | 0 | MF327007 |
| VC_290_159 | 1 | 0 | 55561.57192 | 0 | 0 | 0 | 0 | 1 | 1 | 1 | 0 | 0 | 0 | 0 | MH837626 |
| VC_290_16 | 1 | 0 | 61521.45936 | 0 | 0 | 0 | 0 | 1 | 1 | 1 | 0 | 0 | 0 | 0 | MK327937 |
| VC_290_160 | 1 | 0 | 54937.13253 | 0 | 0 | 0 | 0 | 1 | 1 | 1 | 0 | 0 | 0 | 0 | ON229909 |
| VC_290_161 | 1 | 0 | 53888.61195 | 0 | 0 | 0 | 0 | 1 | 1 | 1 | 0 | 0 | 0 | 0 | MT179807 |
| VC_290_162 | 1 | 0 | 49504.95961 | 0 | 0 | 0 | 0 | 1 | 1 | 1 | 0 | 0 | 0 | 0 | ON720975 |
| VC_290_163 | 2 | 300 | 104418.8889 | 0.00286481 | 0.0677207 | 2.449489743 | 2.44948974 | 1 | 1 | 1 | 2.449489743 | 0 | 0 | 0 | KF925357,KR233165 |
| VC_290_164 | 2 | 300 | 100949.6547 | 0.00296297 | 0.0656488 | 6 | 6 | 1 | 1 | 1 | 6 | 0 | 0 | 0 | MK886800,MZ502380 |
| VC_290_165 | 1 | 0 | 54454.5595 | 0 | 0 | 0 | 0 | 1 | 1 | 1 | 0 | 0 | 0 | 0 | OK018184 |
| VC_290_166 | 1 | 0 | 51393.07292 | 0 | 0 | 0 | 0 | 1 | 1 | 1 | 0 | 0 | 0 | 0 | MZ189262 |
| VC_290_167 | 1 | 0 | 51370.22161 | 0 | 0 | 0 | 0 | 1 | 1 | 1 | 0 | 0 | 0 | 0 | OM135583 |
| VC_290_168 | 1 | 0 | 53757.86124 | 0 | 0 | 0 | 0 | 1 | 1 | 1 | 0 | 0 | 0 | 0 | MW822007 |
| VC_290_169 | 1 | 0 | 62721.31134 | 0 | 0 | 0 | 0 | 1 | 1 | 1 | 0 | 0 | 0 | 0 | MK962750 |
| VC_290_17 | 1 | 0 | 61617.74625 | 0 | 0 | 0 | 0 | 1 | 1 | 1 | 0 | 0 | 0 | 0 | MK327946 |
| VC_290_170 | 1 | 0 | 53705.99124 | 0 | 0 | 0 | 0 | 1 | 1 | 1 | 0 | 0 | 0 | 0 | MH051916 |
| VC_290_171 | 1 | 0 | 54203.39425 | 0 | 0 | 0 | 0 | 1 | 1 | 1 | 0 | 0 | 0 | 0 | MT682709 |
| VC_290_172 | 1 | 0 | 53699.02219 | 0 | 0 | 0 | 0 | 1 | 1 | 1 | 0 | 0 | 0 | 0 | MW883059 |
| VC_290_173 | 1 | 0 | 55388.13865 | 0 | 0 | 0 | 0 | 1 | 1 | 1 | 0 | 0 | 0 | 0 | MT968995 |
| VC_290_174 | 1 | 0 | 57381.71241 | 0 | 0 | 0 | 0 | 1 | 1 | 1 | 0 | 0 | 0 | 0 | MZ417521 |
| VC_290_175 | 1 | 0 | 54415.61945 | 0 | 0 | 0 | 0 | 1 | 1 | 1 | 0 | 0 | 0 | 0 | MW822011 |
| VC_290_176 | 1 | 0 | 53598.51204 | 0 | 0 | 0 | 0 | 1 | 1 | 1 | 0 | 0 | 0 | 0 | MZ150757 |
| VC_290_177 | 1 | 0 | 50308.30373 | 0 | 0 | 0 | 0 | 1 | 1 | 1 | 0 | 0 | 0 | 0 | OL770073 |
| VC_290_178 | 1 | 0 | 52086.57146 | 0 | 0 | 0 | 0 | 1 | 1 | 1 | 0 | 0 | 0 | 0 | ON645936 |
| VC_290_179 | 1 | 0 | 54745.98002 | 0 | 0 | 0 | 0 | 1 | 1 | 1 | 0 | 0 | 0 | 0 | MN580668 |
| VC_290_18 | 1 | 0 | 63707.20453 | 0 | 0 | 0 | 0 | 1 | 1 | 1 | 0 | 0 | 0 | 0 | MK327928 |
| VC_290_180 | 1 | 0 | 60928.94687 | 0 | 0 | 0 | 0 | 1 | 1 | 1 | 0 | 0 | 0 | 0 | MT515756 |
| VC_290_181 | 1 | 0 | 58545.60974 | 0 | 0 | 0 | 0 | 1 | 1 | 1 | 0 | 0 | 0 | 0 | MH243439 |
| VC_290_182 | 1 | 0 | 59434.74439 | 0 | 0 | 0 | 0 | 1 | 1 | 1 | 0 | 0 | 0 | 0 | LC348379 |
| VC_290_183 | 1 | 0 | 55991.37285 | 0 | 0 | 0 | 0 | 1 | 1 | 1 | 0 | 0 | 0 | 0 | MF001359 |
| VC_290_184 | 2 | 300 | 120877.4412 | 0.00247571 | 0.070218 | 2 | 2 | 1 | 1 | 1 | 2 | 0 | 0 | 0 | MT884006,MT884007 |
| VC_290_185 | 1 | 0 | 60187.27017 | 0 | 0 | 0 | 0 | 1 | 1 | 1 | 0 | 0 | 0 | 0 | MN994497 |
| VC_290_186 | 1 | 0 | 59348.56618 | 0 | 0 | 0 | 0 | 1 | 1 | 1 | 0 | 0 | 0 | 0 | ON528742 |
| VC_290_187 | 1 | 0 | 60175.57184 | 0 | 0 | 0 | 0 | 1 | 1 | 1 | 0 | 0 | 0 | 0 | AP018932 |
| VC_290_188 | 1 | 0 | 63206.64783 | 0 | 0 | 0 | 0 | 1 | 1 | 1 | 0 | 0 | 0 | 0 | MH992121 |
| VC_290_189 | 1 | 0 | 59577.39432 | 0 | 0 | 0 | 0 | 1 | 1 | 1 | 0 | 0 | 0 | 0 | MF158046 |
| VC_290_19 | 1 | 0 | 57736.39624 | 0 | 0 | 0 | 0 | 1 | 1 | 1 | 0 | 0 | 0 | 0 | MF327008 |
| VC_290_190 | 1 | 0 | 59425.34368 | 0 | 0 | 0 | 0 | 1 | 1 | 1 | 0 | 0 | 0 | 0 | MK977694 |
| VC_290_2 | 2 | 300 | 112732.571 | 0.0026541 | 0.0615738 | 1.732050808 | 1.73205081 | 1 | 1 | 1 | 1.732050808 | 0 | 0 | 0 | HM997020,KP869105 |
| VC_290_20 | 1 | 0 | 57859.76206 | 0 | 0 | 0 | 0 | 1 | 1 | 1 | 0 | 0 | 0 | 0 | MF327009 |
| VC_290_21 | 1 | 0 | 60431.0413 | 0 | 0 | 0 | 0 | 1 | 1 | 1 | 0 | 0 | 0 | 0 | MN781580 |
| VC_290_22 | 1 | 0 | 56422.73438 | 0 | 0 | 0 | 0 | 1 | 1 | 1 | 0 | 0 | 0 | 0 | OP197928 |
| VC_290_23 | 1 | 0 | 56857.06479 | 0 | 0 | 0 | 0 | 1 | 1 | 1 | 0 | 0 | 0 | 0 | MT828552 |
| VC_290_24 | 1 | 0 | 57946.90732 | 0 | 0 | 0 | 0 | 1 | 1 | 1 | 0 | 0 | 0 | 0 | MG775043 |
| VC_290_25 | 1 | 0 | 60951.10129 | 0 | 0 | 0 | 0 | 1 | 1 | 1 | 0 | 0 | 0 | 0 | KU867876 |
| VC_290_26 | 1 | 0 | 59956.09249 | 0 | 0 | 0 | 0 | 1 | 1 | 1 | 0 | 0 | 0 | 0 | OL615012 |
| VC_290_27 | 1 | 0 | 61533.58714 | 0 | 0 | 0 | 0 | 1 | 1 | 1 | 0 | 0 | 0 | 0 | MT176424 |
| VC_290_28 | 1 | 0 | 61544.71536 | 0 | 0 | 0 | 0 | 1 | 1 | 1 | 0 | 0 | 0 | 0 | MT176426 |
| VC_290_29 | 1 | 0 | 61190.51457 | 0 | 0 | 0 | 0 | 1 | 1 | 1 | 0 | 0 | 0 | 0 | ON528727 |
| VC_290_3 | 3 | 900 | 163912.02 | 0.00546077 | 0.0032353 | 2 | 3.87298335 | 3 | 3 | 1 | 3.248655564 | 0 | 0 | 0 | FJ839693,KM607004,OK040907 |
| VC_290_30 | 1 | 0 | 59858.17069 | 0 | 0 | 0 | 0 | 1 | 1 | 1 | 0 | 0 | 0 | 0 | ON528728 |
| VC_290_31 | 1 | 0 | 59978.66688 | 0 | 0 | 0 | 0 | 1 | 1 | 1 | 0 | 0 | 0 | 0 | MN895437 |
| VC_290_32 | 1 | 0 | 61342.08984 | 0 | 0 | 0 | 0 | 1 | 1 | 1 | 0 | 0 | 0 | 0 | MZ436830 |
| VC_290_33 | 4 | 1800 | 239584.1641 | 0.00745699 | 0.0002455 | 0 | 2 | 6 | 6 | 1 | 1.276142375 | 0 | 0 | 0 | LN881726,LN881729,LN881734,LN881737 |
| VC_290_34 | 4 | 1800 | 220234.6848 | 0.00810684 | 0.0002834 | 0 | 1.73205081 | 6 | 6 | 1 | 1.093412989 | 0 | 0 | 0 | MH809535,MT515751,MT828551,MT828553 |
| VC_290_35 | 1 | 0 | 61332.03314 | 0 | 0 | 0 | 0 | 1 | 1 | 1 | 0 | 0 | 0 | 0 | MK962755 |
| VC_290_36 | 1 | 0 | 60677.76514 | 0 | 0 | 0 | 0 | 1 | 1 | 1 | 0 | 0 | 0 | 0 | MZ501065 |
| VC_290_37 | 1 | 0 | 60753.69936 | 0 | 0 | 0 | 0 | 1 | 1 | 1 | 0 | 0 | 0 | 0 | LN881732 |
| VC_290_38 | 1 | 0 | 59598.8309 | 0 | 0 | 0 | 0 | 1 | 1 | 1 | 0 | 0 | 0 | 0 | LR990704 |
| VC_290_39 | 1 | 0 | 60825.34353 | 0 | 0 | 0 | 0 | 1 | 1 | 1 | 0 | 0 | 0 | 0 | MN895434 |
| VC_290_4 | 5 | 3000 | 267889.8298 | 0.01107461 | 3.34E-07 | 0 | 1 | 10 | 10 | 1 | 0.4 | 0 | 0 | 0 | MZ291552,OP555980,OP555981,OP555982,OP572230 |
| VC_290_40 | 1 | 0 | 60058.75905 | 0 | 0 | 0 | 0 | 1 | 1 | 1 | 0 | 0 | 0 | 0 | MZ234018 |
| VC_290_41 | 1 | 0 | 60090.21314 | 0 | 0 | 0 | 0 | 1 | 1 | 1 | 0 | 0 | 0 | 0 | MZ501046 |
| VC_290_42 | 1 | 0 | 58146.97033 | 0 | 0 | 0 | 0 | 1 | 1 | 1 | 0 | 0 | 0 | 0 | MZ501050 |
| VC_290_43 | 1 | 0 | 61430.92164 | 0 | 0 | 0 | 0 | 1 | 1 | 1 | 0 | 0 | 0 | 0 | MK373786 |
| VC_290_44 | 1 | 0 | 61085.18718 | 0 | 0 | 0 | 0 | 1 | 1 | 1 | 0 | 0 | 0 | 0 | MN895438 |
| VC_290_45 | 1 | 0 | 58594.35536 | 0 | 0 | 0 | 0 | 1 | 1 | 1 | 0 | 0 | 0 | 0 | MH355584 |
| VC_290_46 | 1 | 0 | 60884.53359 | 0 | 0 | 0 | 0 | 1 | 1 | 1 | 0 | 0 | 0 | 0 | MZ501066 |
| VC_290_47 | 1 | 0 | 61746.57642 | 0 | 0 | 0 | 0 | 1 | 1 | 1 | 0 | 0 | 0 | 0 | MZ501067 |
| VC_290_48 | 1 | 0 | 61704.07677 | 0 | 0 | 0 | 0 | 1 | 1 | 1 | 0 | 0 | 0 | 0 | MN895436 |
| VC_290_49 | 1 | 0 | 58773.68941 | 0 | 0 | 0 | 0 | 1 | 1 | 1 | 0 | 0 | 0 | 0 | MT176425 |
| VC_290_5 | 1 | 0 | 55572.86922 | 0 | 0 | 0 | 0 | 1 | 1 | 1 | 0 | 0 | 0 | 0 | LC620505 |
| VC_290_50 | 1 | 0 | 60567.69095 | 0 | 0 | 0 | 0 | 1 | 1 | 1 | 0 | 0 | 0 | 0 | MZ501106 |
| VC_290_51 | 1 | 0 | 59521.03952 | 0 | 0 | 0 | 0 | 1 | 1 | 1 | 0 | 0 | 0 | 0 | MN895435 |
| VC_290_52 | 1 | 0 | 61533.61292 | 0 | 0 | 0 | 0 | 1 | 1 | 1 | 0 | 0 | 0 | 0 | MH553563 |
| VC_290_53 | 1 | 0 | 61950.31264 | 0 | 0 | 0 | 0 | 1 | 1 | 1 | 0 | 0 | 0 | 0 | MK373781 |
| VC_290_54 | 1 | 0 | 61884.47828 | 0 | 0 | 0 | 0 | 1 | 1 | 1 | 0 | 0 | 0 | 0 | MT682713 |
| VC_290_55 | 1 | 0 | 60735.99439 | 0 | 0 | 0 | 0 | 1 | 1 | 1 | 0 | 0 | 0 | 0 | ON528743 |
| VC_290_56 | 1 | 0 | 61067.45224 | 0 | 0 | 0 | 0 | 1 | 1 | 1 | 0 | 0 | 0 | 0 | MZ726795 |
| VC_290_57 | 1 | 0 | 58059.97706 | 0 | 0 | 0 | 0 | 1 | 1 | 1 | 0 | 0 | 0 | 0 | KT184311 |
| VC_290_58 | 1 | 0 | 60686.73956 | 0 | 0 | 0 | 0 | 1 | 1 | 1 | 0 | 0 | 0 | 0 | KX130864 |
| VC_290_59 | 1 | 0 | 60408.66773 | 0 | 0 | 0 | 0 | 1 | 1 | 1 | 0 | 0 | 0 | 0 | OK272473 |
| VC_290_6 | 1 | 0 | 56898.64949 | 0 | 0 | 0 | 0 | 1 | 1 | 1 | 0 | 0 | 0 | 0 | ON782582 |
| VC_290_60 | 1 | 0 | 58848.65482 | 0 | 0 | 0 | 0 | 1 | 1 | 1 | 0 | 0 | 0 | 0 | OK272476 |
| VC_290_61 | 1 | 0 | 61205.95288 | 0 | 0 | 0 | 0 | 1 | 1 | 1 | 0 | 0 | 0 | 0 | MT446387 |
| VC_290_62 | 1 | 0 | 60587.35246 | 0 | 0 | 0 | 0 | 1 | 1 | 1 | 0 | 0 | 0 | 0 | OK272484 |
| VC_290_63 | 1 | 0 | 58457.49211 | 0 | 0 | 0 | 0 | 1 | 1 | 1 | 0 | 0 | 0 | 0 | KT184312 |
| VC_290_64 | 1 | 0 | 60088.85345 | 0 | 0 | 0 | 0 | 1 | 1 | 1 | 0 | 0 | 0 | 0 | MF158045 |
| VC_290_65 | 1 | 0 | 59747.95968 | 0 | 0 | 0 | 0 | 1 | 1 | 1 | 0 | 0 | 0 | 0 | OL956808 |
| VC_290_66 | 1 | 0 | 58084.13756 | 0 | 0 | 0 | 0 | 1 | 1 | 1 | 0 | 0 | 0 | 0 | KT184308 |
| VC_290_67 | 2 | 300 | 110508.4867 | 0.00270737 | 0.09274 | 7.745966692 | 7.74596669 | 1 | 1 | 1 | 7.745966692 | 0 | 0 | 0 | LR877332,MT515754 |
| VC_290_68 | 1 | 0 | 61305.82148 | 0 | 0 | 0 | 0 | 1 | 1 | 1 | 0 | 0 | 0 | 0 | MW749001 |
| VC_290_69 | 1 | 0 | 61000.45717 | 0 | 0 | 0 | 0 | 1 | 1 | 1 | 0 | 0 | 0 | 0 | HE956711 |
| VC_290_7 | 1 | 0 | 58930.21623 | 0 | 0 | 0 | 0 | 1 | 1 | 1 | 0 | 0 | 0 | 0 | MW286157 |
| VC_290_70 | 1 | 0 | 57256.91604 | 0 | 0 | 0 | 0 | 1 | 1 | 1 | 0 | 0 | 0 | 0 | MZ501098 |
| VC_290_71 | 1 | 0 | 61070.5653 | 0 | 0 | 0 | 0 | 1 | 1 | 1 | 0 | 0 | 0 | 0 | LR215723 |
| VC_290_72 | 1 | 0 | 62284.01595 | 0 | 0 | 0 | 0 | 1 | 1 | 1 | 0 | 0 | 0 | 0 | MT682710 |
| VC_290_73 | 3 | 900 | 176064.2262 | 0.00508577 | 0.0027306 | 0 | 1 | 3 | 3 | 1 | 0.666666667 | 0 | 0 | 0 | LN881728,LN881733,LN881736 |
| VC_290_74 | 1 | 0 | 58831.96418 | 0 | 0 | 0 | 0 | 1 | 1 | 1 | 0 | 0 | 0 | 0 | MT682714 |
| VC_290_75 | 1 | 0 | 59849.27607 | 0 | 0 | 0 | 0 | 1 | 1 | 1 | 0 | 0 | 0 | 0 | MZ170040 |
| VC_290_76 | 1 | 0 | 56363.60705 | 0 | 0 | 0 | 0 | 1 | 1 | 1 | 0 | 0 | 0 | 0 | MK373787 |
| VC_290_77 | 2 | 300 | 122392.7545 | 0.00244513 | 0.0485042 | 1.732050808 | 1.73205081 | 1 | 1 | 1 | 1.732050808 | 0 | 0 | 0 | MT682712,MZ170041 |
| VC_290_78 | 13 | 23400 | 652712.5524 | 0.03460962 | 9.91E-36 | 0 | 11.1355287 | 78 | 62 | 0.794871795 | 6.932772937 | 0 | 0 | 0 | MZ234027,MZ234030,MZ234034,MZ234036,MZ234037,MZ234039,MZ234040,MZ234041,MZ234042,MZ234043,MZ234051,MZ234052,MZ234053 |
| VC_290_79 | 1 | 0 | 56225.4105 | 0 | 0 | 0 | 0 | 1 | 1 | 1 | 0 | 0 | 0 | 0 | MF001354 |
| VC_290_8 | 1 | 0 | 59222.64432 | 0 | 0 | 0 | 0 | 1 | 1 | 1 | 0 | 0 | 0 | 0 | KU925172 |
| VC_290_80 | 1 | 0 | 55370.59203 | 0 | 0 | 0 | 0 | 1 | 1 | 1 | 0 | 0 | 0 | 0 | MT764206 |
| VC_290_81 | 1 | 0 | 56069.73363 | 0 | 0 | 0 | 0 | 1 | 1 | 1 | 0 | 0 | 0 | 0 | KR269718 |
| VC_290_82 | 6 | 4500 | 347206.1865 | 0.01279477 | 2.49E-11 | 0 | 2.44948974 | 15 | 15 | 1 | 1.494635803 | 0 | 0 | 0 | KM606994,KM606995,KM606996,KM606997,KM606998,KM606999 |
| VC_290_83 | 1 | 0 | 62531.40554 | 0 | 0 | 0 | 0 | 1 | 1 | 1 | 0 | 0 | 0 | 0 | KM607000 |
| VC_290_84 | 1 | 0 | 63231.73754 | 0 | 0 | 0 | 0 | 1 | 1 | 1 | 0 | 0 | 0 | 0 | FJ839692 |
| VC_290_85 | 1 | 0 | 61052.31682 | 0 | 0 | 0 | 0 | 1 | 1 | 1 | 0 | 0 | 0 | 0 | KX828711 |
| VC_290_86 | 1 | 0 | 58792.1655 | 0 | 0 | 0 | 0 | 1 | 1 | 1 | 0 | 0 | 0 | 0 | ON528734 |
| VC_290_87 | 1 | 0 | 60985.10861 | 0 | 0 | 0 | 0 | 1 | 1 | 1 | 0 | 0 | 0 | 0 | ON528738 |
| VC_290_88 | 2 | 300 | 118485.7097 | 0.00252556 | 0.0440345 | 3.464101615 | 3.46410162 | 1 | 1 | 1 | 3.464101615 | 0 | 0 | 0 | AP018814,MH550421 |
| VC_290_89 | 2 | 300 | 111514.2575 | 0.00268302 | 0.0888744 | 2.449489743 | 2.44948974 | 1 | 1 | 1 | 2.449489743 | 0 | 0 | 0 | MG781190,MG781191 |
| VC_290_9 | 1 | 0 | 57942.28708 | 0 | 0 | 0 | 0 | 1 | 1 | 1 | 0 | 0 | 0 | 0 | MZ501052 |
| VC_290_90 | 1 | 0 | 59644.70647 | 0 | 0 | 0 | 0 | 1 | 1 | 1 | 0 | 0 | 0 | 0 | KF208315 |
| VC_290_91 | 11 | 16500 | 570398.6818 | 0.02811388 | 3.32E-35 | 0 | 7.07106781 | 55 | 55 | 1 | 3.669044122 | 0 | 0 | 0 | AF158101,HM137666,KJ477684,KJ477685,KJ477686,KM607002,KM607003,MT984581,MZ375356,OL964735,OL964745 |
| VC_290_92 | 4 | 1800 | 228335.2045 | 0.00782149 | 4.93E-05 | 1.732050808 | 3.31662479 | 6 | 6 | 1 | 2.490266304 | 0 | 0 | 0 | AP018813,LC348380,MH751506,OM339528 |
| VC_290_93 | 1 | 0 | 60342.17068 | 0 | 0 | 0 | 0 | 1 | 1 | 1 | 0 | 0 | 0 | 0 | MT932213 |
| VC_290_94 | 2 | 300 | 114808.3588 | 0.00260624 | 0.0470624 | 0 | 0 | 1 | 1 | 1 | 0 | 0 | 0 | 0 | DQ904452,KM607001 |
| VC_290_95 | 1 | 0 | 64208.06183 | 0 | 0 | 0 | 0 | 1 | 1 | 1 | 0 | 0 | 0 | 0 | JX128259 |
| VC_290_96 | 1 | 0 | 63578.72423 | 0 | 0 | 0 | 0 | 1 | 1 | 1 | 0 | 0 | 0 | 0 | ON506924 |
| VC_290_97 | 1 | 0 | 58578.95253 | 0 | 0 | 0 | 0 | 1 | 1 | 1 | 0 | 0 | 0 | 0 | LR215722 |
| VC_290_98 | 1 | 0 | 57773.31201 | 0 | 0 | 0 | 0 | 1 | 1 | 1 | 0 | 0 | 0 | 0 | ON210139 |
| VC_290_99 | 1 | 0 | 60334.78087 | 0 | 0 | 0 | 0 | 1 | 1 | 1 | 0 | 0 | 0 | 0 | KJ668714 |
| VC_291_0 | 3 | 900 | 17760.67154 | 0.04822978 | 0.0013903 | 6.32455532 | 7.48331477 | 3 | 3 | 1 | 7.097061622 | 0 | 0 | 0 | KJ025957,MN095771,OM457001 |
| VC_293_0 | 2 | 300 | 40863.71463 | 0.00728797 | 0.2241749 | 7.280109889 | 7.28010989 | 1 | 1 | 1 | 7.280109889 | 0 | 0 | 0 | MW512264,MZ501047 |
| VC_293_1 | 21 | 63000 | 308819.0903 | 0.16943724 | 5.54E-32 | 0 | 9.94987437 | 210 | 190 | 0.904761905 | 7.509750767 | 0 | 0 | 0 | KR422352,LR027383,LR027386,LR027387,LR027390,LR027391,MH243438,MK327934,MK327943,MK373778,MK373782,MK373784,MK962754,MK962756,MK962757,MN850579,MN850590,MZ234025,OL960576,OL960577,OM960734 |
| VC_293_10 | 9 | 10800 | 164869.6267 | 0.06147904 | 1.16E-06 | 7.071067812 | 9.43398113 | 36 | 24 | 0.666666667 | 8.603631143 | 0 | 0 | 0 | AY303349,KF562341,KM407600,KY703222,MH992122,MT446390,MZ501056,OM982647,ON528737 |

| | | | | | | | | | | | | | | | |
|---|---|---|---|---|---|---|---|---|---|---|---|---|---|---|---|
| VC_293_11 | 3 | 900 | 60740.29091 | 0.01460084 | 0.0653972 | 6.8556546 | 8.88819442 | 3 | 3 | 1 | 8.191869961 | 0 | 0 | 0 | MH359124,MK524178,MW882907 |
| VC_293_12 | 3 | 900 | 60627.27753 | 0.01462766 | 0.0767173 | 8.185352772 | 8.94427191 | 3 | 3 | 1 | 8.672606366 | 0 | 0 | 0 | MG589383,MZ189261,OL770074 |
| VC_293_13 | 10 | 13500 | 179972.5701 | 0.06977733 | 6.87E-07 | 1.414213562 | 9.11043358 | 45 | 43 | 0.955555556 | 7.788539478 | 0 | 0 | 0 | KF582788,MF044457,MK639187,MT782071,MW630116,MZ311865,MZ560701,MZ868638,OV754623,OV877511 |
| VC_293_14 | 1 | 0 | 21220.21874 | 0 | 0 | 0 | 0 | 1 | 1 | 1 | 0 | 0 | 0 | 0 | JX536493 |
| VC_293_15 | 2 | 300 | 40824.20482 | 0.00729497 | 0.2437369 | 7.615773106 | 7.61577311 | 1 | 1 | 1 | 7.615773106 | 0 | 0 | 0 | MK047717,ON528725 |
| VC_293_16 | 1 | 0 | 20779.52673 | 0 | 0 | 0 | 0 | 1 | 1 | 1 | 0 | 0 | 0 | 0 | KX130865 |
| VC_293_17 | 8 | 8400 | 148760.5687 | 0.05344852 | 2.12E-05 | 0 | 9.43398113 | 28 | 17 | 0.607142857 | 7.943774458 | 0 | 0 | 0 | KP869101,KP869104,KP869111,KT184309,KT184310,MK047718,MN648445,MN850622 |
| VC_293_2 | 1 | 0 | 20712.01883 | 0 | 0 | 0 | 0 | 1 | 1 | 1 | 0 | 0 | 0 | 0 | MN850651 |
| VC_293_3 | 2 | 300 | 40754.25619 | 0.0073074 | 0.251142 | 2 | 2 | 1 | 1 | 1 | 2 | 0 | 0 | 0 | MK373774,MK373775 |
| VC_293_4 | 1 | 0 | 20856.13037 | 0 | 0 | 0 | 0 | 1 | 1 | 1 | 0 | 0 | 0 | 0 | MK327933 |
| VC_293_5 | 1 | 0 | 20933.11469 | 0 | 0 | 0 | 0 | 1 | 1 | 1 | 0 | 0 | 0 | 0 | MT446423 |
| VC_293_6 | 1 | 0 | 21178.72444 | 0 | 0 | 0 | 0 | 1 | 1 | 1 | 0 | 0 | 0 | 0 | MN864145 |
| VC_293_7 | 1 | 0 | 25972.27877 | 0 | 0 | 0 | 0 | 1 | 1 | 1 | 0 | 0 | 0 | 0 | OM632671 |
| VC_293_8 | 1 | 0 | 20711.07164 | 0 | 0 | 0 | 0 | 1 | 1 | 1 | 0 | 0 | 0 | 0 | KU255730 |
| VC_293_9 | 1 | 0 | 22110.99741 | 0 | 0 | 0 | 0 | 1 | 1 | 1 | 0 | 0 | 0 | 0 | OM386662 |
| VC_294_0 | 2 | 300 | 10712.82097 | 0.02724098 | 0.0422719 | 5.567764363 | 5.56776436 | 1 | 1 | 1 | 5.567764363 | 0 | 0 | 0 | MT457552,MT457553 |
| VC_294_1 | 1 | 0 | 6382.873436 | 0 | 0 | 0 | 0 | 1 | 1 | 1 | 0 | 0 | 0 | 0 | KY971610 |
| VC_295_0 | 3 | 900 | 21022.57962 | 0.04105356 | 0.0014376 | 5.656854249 | 6.244998 | 3 | 3 | 1 | 5.882138298 | 0 | 0 | 0 | MW358927,MW385300,OK499989 |
| VC_295_1 | 1 | 0 | 7068.648191 | 0 | 0 | 0 | 0 | 1 | 1 | 1 | 0 | 0 | 0 | 0 | KX078569 |
| VC_295_2 | 2 | 300 | 13104.67937 | 0.02238024 | 0.0446001 | 2.828427125 | 2.82842712 | 1 | 1 | 1 | 2.828427125 | 0 | 0 | 0 | MG696114,MK295204 |
| VC_295_3 | 3 | 900 | 19414.92296 | 0.04430241 | 0.0015536 | 5.916079783 | 8.71779789 | 3 | 3 | 1 | 7.258435366 | 0 | 0 | 0 | KP890823,MF001355,MW367898 |
| VC_295_4 | 1 | 0 | 6774.16751 | 0 | 0 | 0 | 0 | 1 | 1 | 1 | 0 | 0 | 0 | 0 | MW057858 |
| VC_297_0 | 10 | 13500 | 125581.7888 | 0.09706519 | 3.02E-24 | 1 | 8.77496439 | 45 | 45 | 1 | 7.474032872 | 0 | 0 | 0 | MT129652,MT446396,MT446411,MT446412,MT446415,MT446421,MT611523,MW233709,MW250787,MW598459 |
| VC_297_1 | 8 | 8400 | 103715.078 | 0.07492302 | 1.64E-15 | 7.071067812 | 9.8488578 | 28 | 24 | 0.857142857 | 8.295226522 | 0 | 0 | 0 | GQ981382,KP007360,MT446420,MT446422,MT533174,MW269952,OK254198,OL451225 |
| VC_297_2 | 2 | 300 | 29413.98667 | 0.01009626 | 0.077099 | 8.124038405 | 8.1240384 | 1 | 1 | 1 | 8.124038405 | 0 | 0 | 0 | HM563683,KP007361 |
| VC_297_3 | 2 | 300 | 29312.36429 | 0.0101309 | 0.0772616 | 8.888194417 | 8.88819442 | 1 | 1 | 1 | 8.888194417 | 0 | 0 | 0 | KP007362,MT446392 |
| VC_297_4 | 2 | 300 | 32729.879 | 0.00908269 | 0.0977823 | 8.366600265 | 8.36660027 | 1 | 1 | 1 | 8.366600265 | 0 | 0 | 0 | KP007359,OM386655 |
| VC_297_5 | 14 | 27300 | 166319.1508 | 0.14099845 | 3.87E-42 | 5.567764363 | 9.48683298 | 91 | 84 | 0.923076923 | 7.823538534 | 0 | 0 | 0 | HM071924,HQ829472,KT176190,KU878968,KU878969,MH051917,MK234886,MT150133,MT682711,MW149274,MW679410,MZ561532,OK335775,OL780484 |
| VC_297_6 | 12 | 19800 | 153967.2192 | 0.11394554 | 2.07E-30 | 1 | 9.89949494 | 66 | 54 | 0.818181818 | 7.862790764 | 0 | 0 | 0 | EF469154,EU863409,MH051913,MH791409,MK310184,MN850609,MN850621,MT129654,OL770107,OP292222,OV876907,OV877765 |
| VC_297_7 | 1 | 0 | 15148.75096 | 0 | 0 | 0 | 0 | 1 | 1 | 1 | 0 | 0 | 0 | 0 | MK295203 |
| VC_299_0 | 4 | 684.8229087 | 3263.017434 | 0.17346773 | 1.33E-05 | 0 | 8.66025404 | 6 | 6 | 1 | 6.62821126 | 0 | 0 | 0 | MK448704,MK448707,MK448709,MK448710 |
| VC_3_0 | 17 | 13970.56805 | 10910.60523 | 0.56149153 | 7.19E-80 | 0 | 6.55743852 | 136 | 136 | 1 | 4.898300906 | 0 | 0 | 0 | CP000623,CP000624,CP008753,DQ087285,GU911304,HM461982,JX681814,LC528882,LC667452,MW072790,MZ375754,OM304382,OM638608,OM638609,OM638610,OM638611,ON135435 |
| VC_30_0 | 2 | 234.349383 | 7411.857485 | 0.0306491 | 0.0427903 | 1.732050808 | 1.73205081 | 1 | 1 | 1 | 1.732050808 | 0 | 0 | 0 | MW722521,MW790496 |
| VC_30_1 | 53 | 135303.1883 | 53910.09258 | 0.71508294 | 0 | 0 | 9.74679434 | 1378 | 1285 | 0.932510885 | 7.38035055 | 0 | 0 | 0 | AB102868,AB362338,AB426868,AF217253,AF527608,AY052766,BK000583,CP051272,CP051278,CP051279,CP051285,CP051288,DQ003260,EU570103,FJ000341,GQ422450,GQ422451,GU573886,JF900176,JQ806763,KR296686,KR296687,KR296688,KU878967,KU927491,KU927492,KU927494,KU927495,KU927496,KU927498,KX776161,LC730322,LT714109,MF188997,MG407615,MG969415,MH370387,MK770414,MK770415,MK972685,MK972686,MK972687,MK972692,MK972693,MK972694,MN161573,MT774487,MT932212,MW013502,MW013503,MW355480,OP265889,OP377721 |
| VC_300_0 | 1 | 0 | 1293.335714 | 0 | 0 | 0 | 0 | 1 | 1 | 1 | 0 | 0 | 0 | 0 | MK448750 |
| VC_300_1 | 1 | 0 | 1578.149098 | 0 | 0 | 0 | 0 | 1 | 1 | 1 | 0 | 0 | 0 | 0 | MK448995 |
| VC_300_2 | 2 | 111.6740249 | 3240.458308 | 0.03331432 | 0.0429953 | 7.071067812 | 7.07106781 | 1 | 1 | 1 | 7.071067812 | 0 | 0 | 0 | MK448740,MK448917 |
| VC_300_3 | 19 | 10847.58458 | 30377.279 | 0.26313209 | 1.21E-92 | 1 | 9.8488578 | 171 | 140 | 0.81871345 | 8.190149882 | 0 | 0 | 0 | MK448675,MK448676,MK448688,MK448690,MK448694,MK448772,MK448778,MK448782,MK448850,MK448859,MK448877,MK448931,MK448955,MK448962,MK448966,MK448973,MK448975,MK449004,OL774870 |
| VC_301_0 | 2 | 238.9896657 | 2570.904803 | 0.0850529 | 0.0426452 | 0 | 0 | 1 | 1 | 1 | 0 | 0 | 0 | 0 | MK448832,MK449008 |
| VC_302_0 | 2 | 53.69674747 | 1565.311117 | 0.03316645 | 0.0551198 | 8.717797887 | 8.71779789 | 1 | 1 | 1 | 8.717797887 | 0 | 0 | 0 | MK819239,ON857931 |
| VC_303_0 | 6 | 1848.552226 | 1612.749244 | 0.53406276 | 4.02E-11 | 0 | 5.91607978 | 15 | 15 | 1 | 4.272578936 | 0 | 0 | 0 | MG592403,MG592404,MG592405,MG592551,MN592897,MW824406 |
| VC_304_0 | 5 | 1451.593554 | 12335.71343 | 0.10528478 | 7.48E-08 | 4.898979486 | 9.43398113 | 10 | 9 | 0.9 | 7.68797595 | 0 | 0 | 0 | KM389210,KU199710,MW748993,MZ089728,ON778007 |
| VC_304_1 | 20 | 27800.87168 | 21558.41362 | 0.56323489 | 3.18E-93 | 0 | 9.64365076 | 190 | 146 | 0.768421053 | 6.309387252 | 0 | 0 | 0 | HQ711984,JX403939,KU310943,MK511001,MK511002,MK511003,MK511004,MK511005,MK511006,MK511007,MK511008,MK511009,MK511010,MK511011,MK511012,MK511013,MK511014,MK511015,MK511016,MZ773939 |
| VC_305_0 | 1 | 0 | 380.7340416 | 0 | 0 | 0 | 0 | 1 | 1 | 1 | 0 | 0 | 0 | 0 | KU708004 |
| VC_305_1 | 1 | 0 | 488.5999779 | 0 | 0 | 0 | 0 | 1 | 1 | 1 | 0 | 0 | 0 | 0 | MW862109 |
| VC_306_0 | 24 | 78273.9992 | 771.496357 | 0.99023984 | 9.83E-68 | 0 | 4.47213595 | 276 | 276 | 1 | 1.837628284 | 0 | 0 | 0 | KX581090,KX581091,KX581092,KX581093,KX581094,KX581095,KX581096,KX581097,KX581098,KX581099,KX581100,KY658673,KY658674,KY658675,KY658676,KY658677,KY658678,KY658679,KY658680,MK672799,MK672800,MK672801,MK672803,MK672804 |
| VC_307_0 | 2 | 76.77043144 | 529.1590387 | 0.12669863 | 0.0455437 | 6 | 6 | 1 | 1 | 1 | 6 | 0 | 0 | 0 | HQ141411,OP650937 |
| VC_307_1 | 1 | 0 | 455.8132318 | 0 | 0 | 0 | 0 | 1 | 1 | 1 | 0 | 0 | 0 | 0 | MG788324 |
| VC_308_0 | 2 | 22.57124069 | 154.1402182 | 0.12772936 | 0.050698 | 1.732050808 | 1.73205081 | 1 | 1 | 1 | 1.732050808 | 0 | 0 | 0 | LT985589,LT993247 |
| VC_309_0 | 2 | 64.50787151 | 291.294691 | 0.18130244 | 0.0495975 | 7 | 7 | 1 | 1 | 1 | 7 | 0 | 0 | 0 | MH837542,MH837543 |
| VC_31_0 | 10 | 5451.7115 | 5176.770636 | 0.51293415 | 4.93E-30 | 0 | 3 | 45 | 45 | 1 | 2.237413959 | 0 | 0 | 0 | AF157835,EU794049,LR794146,LR794147,LR794148,LR794149,LR794150,LR794151,LR794152,LR797923 |
| VC_310_0 | 155 | 172051.4321 | 363.0474498 | 0.99789433 | 4.34E-56 | 0 | 2.64575131 | 11935 | 11935 | 1 | 1.325213926 | 0 | 0 | 0 | AF176027,AF176028,AF176029,AF176030,AF176031,AF176032,AF176033,AF176034,AF274751,AF299300,AF299301,AF299302,AF299303,AF299304,AF299305,AF299306,AF299307,AF299308,AF299309,AF299310,AF299311,AF299312,AF299313,AF299314,CP004084,DQ079880,DQ079881,DQ079882,DQ079883,DQ079884,DQ079885,DQ079886,DQ079887,DQ079888,DQ079889,DQ079890,DQ079891,DQ079892,DQ079893,DQ079894,DQ079895,EF380009,EF380010,EF380011,EF380012,EF380013,EF380014,EF380015,EF380016,EF380017,EF380018,EF380019,EF380020,EF380021,EF380022,EF380023,EF380024,EF380025,EF380026,EF380027,EF380028,EF380029,EF380030,EF380031,EF380032,FJ849058,GQ153912,GQ153913,GQ153914,GQ153915,GU385905,HM753658,HM753659,HM753660,HM753661,HM753662,HM753663,HM753664,HM753665,HM753666,HM753667,HM753668,HM753669,HM753670,HM753671,HM753672,HM753673,HM753674,HM753675,HM753676,HM753677,HM753678,HM753679,HM753680,HM753681,HM753682,HM753683,HM753684,HM753685,HM753686,HM753687,HM753688,HM753689,HM753690,HM753691,HM753692,HM753693,HM753694,HM753695,HM753696,HM753697,HM753698,HM753699,HM753700,HM753701,HM753702,HM753703,HM753704,HM753705,HM753706,HM753707,HM753708,HM753709,HM753710,HM753711,HM753712,HM753713,HM753714,HM753715,HM753716,HM753717,HM753718,HM775306,HM775307,HM775308,HM775309,HM775310,HM775311,JF719726,JF719727,JF719728,JQ764986,JQ764987,JQ764988,JQ764989,JQ764990,KJ997912,KY653237,MH378441,MH378442,MH378443,MN385565,MT947439,PX1CG,S13CG |
| VC_311_0 | 11 | 6337.398315 | 1734.499521 | 0.78511875 | 7.13E-25 | 1 | 7.34846923 | 55 | 55 | 1 | 4.61864606 | 0 | 0 | 0 | AF189021,FJ867910,FJ867911,FJ867912,FJ867913,FJ867914,JQ809650,MF431615,MF431616,MF431617,MK613346 |
| VC_312_0 | 12 | 4002.509104 | 2996.595234 | 0.57186019 | 2.92E-33 | 4.358898944 | 9.53939201 | 66 | 56 | 0.848484848 | 8.138585731 | 0 | 0 | 0 | GU557055,MK613343,MK613344,MK613345,MW514246,MW514247,MZ892987,MZ892988,MZ892989,MZ892990,MZ892991,MZ892992 |
| VC_313_0 | 2 | 60.18621931 | 0 | 1 | 0 | 0 | 0 | 1 | 1 | 1 | 0 | 0 | 0 | 0 | AF191797,MW557853 |
| VC_315_0 | 4 | 112.8252449 | 435.520522 | 0.20575566 | 4.18E-05 | 7.211102551 | 8.36660027 | 6 | 6 | 1 | 7.801164047 | 0 | 0 | 0 | MF041990,MN830256,S14_vf_72953,X98106 |
| VC_317_0 | 33 | 158399.8148 | 22506.14213 | 0.87559203 | 2.56E-181 | 0 | 6.32455532 | 528 | 528 | 1 | 4.613391631 | 0 | 0 | 0 | AF222060,AY190604,KC292020,KC292022,MN901520,MN901521,MW450817,MZ334492,MZ334493,MZ334494,MZ334495,MZ334496,MZ334497,MZ334498,MZ334499,MZ334500,MZ334501,MZ334502,MZ334503,MZ334504,MZ334505,MZ334506,MZ334507,MZ334508,MZ334509,MZ334510,MZ334511,MZ334512,MZ334513,MZ334514,MZ334515,MZ334516,OK649958 |
| VC_318_0 | 6 | 4488.476701 | 18172.63288 | 0.19806959 | 4.85E-11 | 0 | 4.58257569 | 15 | 15 | 1 | 3.552051462 | 0 | 0 | 0 | KC117376,KC292021,MZ334517,MZ334518,MZ334519,MZ334520 |
| VC_319_0 | 2 | 14.18051779 | 451.7657029 | 0.03043381 | 0.0456357 | 7.615773106 | 7.61577311 | 1 | 1 | 1 | 7.615773106 | 0 | 0 | 0 | KC292026,MZ334525 |
| VC_32_0 | 2 | 173.9122675 | 4537.154463 | 0.03691569 | 0.0426536 | 5.477225575 | 5.47722558 | 1 | 1 | 1 | 5.477225575 | 0 | 0 | 0 | JQ182733,JQ182734 |
| VC_32_1 | 16 | 7850.205097 | 32157.02206 | 0.19621967 | 8.79E-73 | 1.414213562 | 9.69535971 | 120 | 100 | 0.833333333 | 8.266120525 | 0 | 0 | 0 | AF335538,AF547987,CP000711,JF974339,JN600960,KJ832078,MH717096,MN340231,MT813197,MT833387,MZ043897,OM022251,ON470589,ON470594,ON470605,OV696608 |
| VC_320_0 | 2 | 300 | 1459.943968 | 0.17045997 | 0.092556 | 2.236067977 | 2.23606798 | 1 | 1 | 1 | 2.236067977 | 0 | 0 | 0 | KC292029,MZ334524 |
| VC_320_1 | 3 | 900 | 1700.976563 | 0.34602388 | 0.0018738 | 0 | 6.78232998 | 3 | 3 | 1 | 4.521553322 | 0 | 0 | 0 | KC117377,KC292027,MZ334523 |
| VC_321_0 | 2 | 7.00617577 | 32.51215697 | 0.17728925 | 1.68E-07 | 0 | 0 | 1 | 1 | 1 | 0 | 0 | 0 | 0 | DQ836063,JX625144 |
| VC_322_0 | 12 | 462.4076008 | 55.73512623 | 0.89243287 | 1.12E-26 | 0 | 0 | 66 | 66 | 1 | 0 | 0 | 0 | 0 | AF227250,EF108464,EF108465,EF204939,EF204940,FJ483837,FJ483838,FJ483839,JQ966307,LT898437,MK213795,MT830620 |
| VC_323_0 | 3 | 687.3883073 | 233.1055047 | 0.74676038 | 0.0017834 | 1.732050808 | 2 | 3 | 3 | 1 | 1.821367205 | 0 | 0 | 0 | MZ326866,MZ611865,ON711490 |
| VC_324_0 | 2 | 137.8567761 | 3011.717368 | 0.04376997 | 0.0437641 | 4.472135955 | 4.47213595 | 1 | 1 | 1 | 4.472135955 | 0 | 0 | 0 | KY065466,KY065468 |
| VC_33_0 | 9 | 3366.094567 | 6508.810979 | 0.3408736 | 3.88E-24 | 0 | 9.69535971 | 36 | 25 | 0.694444444 | 7.898677711 | 0 | 0 | 0 | KY271395,KY271396,MK448231,MK448233,MK448235,MK714353,OK490410,OK490415,OK490416 |
| VC_33_1 | 1 | 0 | 1152.599679 | 0 | 0 | 0 | 0 | 1 | 1 | 1 | 0 | 0 | 0 | 0 | OK499971 |
| VC_33_2 | 1 | 0 | 1262.898834 | 0 | 0 | 0 | 0 | 1 | 1 | 1 | 0 | 0 | 0 | 0 | HQ201308 |
| VC_331_0 | 2 | 71.71045775 | 2412.042205 | 0.02887182 | 0.0433832 | 7.810249676 | 7.81024968 | 1 | 1 | 1 | 7.810249676 | 0 | 0 | 0 | MK448821,MK448822 |
| VC_331_1 | 5 | 2123.711906 | 11879.49794 | 0.15165894 | 2.81E-08 | 0 | 6.8556546 | 10 | 10 | 1 | 3.983210017 | 0 | 0 | 0 | MK448757,MK448758,MK448759,MK448939,OL774872 |
| VC_331_2 | 7 | 1637.320664 | 8901.397489 | 0.15536241 | 1.95E-13 | 4.582575695 | 9.32737905 | 21 | 20 | 0.952380952 | 7.631160262 | 0 | 0 | 0 | MK448735,MK448743,MK448893,MK448903,MK448915,MK448919,MK448921 |
| VC_332_0 | 3 | 210.1887551 | 937.3890373 | 0.18315861 | 0.0019809 | 6.782329983 | 8.18535277 | 3 | 3 | 1 | 7.592644144 | 0 | 0 | 0 | S5_vs_84946,S7_vs_86377,S9_vs_1966 |
| VC_335_0 | 2 | 118.7565425 | 28839.09512 | 0.00410101 | 0.0442556 | 8.831760866 | 8.83176087 | 1 | 1 | 1 | 8.831760866 | 0 | 0 | 0 | MH513971,ON970586 |
| VC_335_1 | 2 | 139.9168139 | 32659.79519 | 0.00426579 | 0.0433665 | 8.602325267 | 8.60232527 | 1 | 1 | 1 | 8.602325267 | 0 | 0 | 0 | JN398369,MN617845 |
| VC_335_10 | 3 | 594.4341685 | 48883.00237 | 0.01201425 | 0.0014083 | 4.898979486 | 8.06225775 | 3 | 3 | 1 | 6.653745745 | 0 | 0 | 0 | MH744414,MK359331,MN119379 |
| VC_335_11 | 1 | 0 | 14591.56213 | 0 | 0 | 0 | 0 | 1 | 1 | 1 | 0 | 0 | 0 | 0 | MH001458 |
| VC_335_12 | 1 | 0 | 17281.68132 | 0 | 0 | 0 | 0 | 1 | 1 | 1 | 0 | 0 | 0 | 0 | MN813700 |
| VC_335_13 | 2 | 277.6923879 | 33491.08398 | 0.00822335 | 0.0424856 | 4.242640687 | 4.24264069 | 1 | 1 | 1 | 4.242640687 | 0 | 0 | 0 | EU744249,EU744252 |
| VC_335_14 | 3 | 462.5106076 | 48812.75548 | 0.00938626 | 0.0014083 | 8.185352772 | 8.71779789 | 3 | 3 | 1 | 8.521134899 | 0 | 0 | 0 | GU247132,KT818595,MT889384 |
| VC_335_15 | 1 | 0 | 14389.43669 | 0 | 0 | 0 | 0 | 1 | 1 | 1 | 0 | 0 | 0 | 0 | KJ174156 |
| VC_335_16 | 2 | 300 | 31538.7708 | 0.00942247 | 0.0424861 | 2.449489743 | 2.44948974 | 1 | 1 | 1 | 2.449489743 | 0 | 0 | 0 | MK310141,MK524523 |
| VC_335_17 | 1 | 0 | 17786.40802 | 0 | 0 | 0 | 0 | 1 | 1 | 1 | 0 | 0 | 0 | 0 | KC701493 |
| VC_335_18 | 1 | 0 | 15599.09017 | 0 | 0 | 0 | 0 | 1 | 1 | 1 | 0 | 0 | 0 | 0 | OL549190 |
| VC_335_19 | 3 | 497.8021766 | 45883.9703 | 0.01073271 | 0.0014078 | 6 | 8.60232527 | 3 | 3 | 1 | 7.69586888 | 0 | 0 | 0 | JN020140,MH338238,MK967387 |
| VC_335_2 | 1 | 0 | 16784.60785 | 0 | 0 | 0 | 0 | 1 | 1 | 1 | 0 | 0 | 0 | 0 | JF937099 |
| VC_335_20 | 1 | 0 | 15267.63081 | 0 | 0 | 0 | 0 | 1 | 1 | 1 | 0 | 0 | 0 | 0 | MN585970 |
| VC_335_21 | 5 | 1696.12178 | 79750.53685 | 0.02082494 | 2.44E-07 | 0 | 9.53939201 | 10 | 8 | 0.8 | 7.160281335 | 0 | 0 | 0 | EU744248,KX522649,KX522943,KX574454,MK524497 |
| VC_335_22 | 1 | 0 | 15112.39919 | 0 | 0 | 0 | 0 | 1 | 1 | 1 | 0 | 0 | 0 | 0 | MK524499 |
| VC_335_23 | 17 | 19310.66073 | 250988.4321 | 0.07144183 | 1.15E-74 | 4.358898944 | 10.0498756 | 136 | 87 | 0.639705882 | 8.460710332 | 0 | 0 | 0 | JP937103,KC661273,KT259047,MG920060,MG925351,MH020244,MH450113,MH450130,MH576959,MH576971,MH576974,MK359299,MK820638,MT639654,MW507123,MW712732,ON970627 |
| VC_335_24 | 3 | 481.5117795 | 53246.00272 | 0.00896211 | 0.0014605 | 7.681145748 | 8.94427191 | 3 | 3 | 1 | 8.350522477 | 0 | 0 | 0 | JP957057,MH230876,MN585976 |
| VC_335_25 | 1 | 0 | 17060.07373 | 0 | 0 | 0 | 0 | 1 | 1 | 1 | 0 | 0 | 0 | 0 | MZ648034 |
| VC_335_26 | 1 | 0 | 16588.58012 | 0 | 0 | 0 | 0 | 1 | 1 | 1 | 0 | 0 | 0 | 0 | MG944220 |
| VC_335_27 | 6 | 2503.313062 | 91569.33372 | 0.02661042 | 2.77E-10 | 2.828427125 | 9.69535971 | 15 | 12 | 0.8 | 7.395816482 | 0 | 0 | 0 | KX683876,KX702320,MG099951,MH000606,MH651180,MT952847 |
| VC_335_28 | 2 | 137.4417648 | 31211.71757 | 0.00438422 | 0.0424905 | 8.306623863 | 8.30662386 | 1 | 1 | 1 | 8.306623863 | 0 | 0 | 0 | MH536816,MH632118 |
| VC_335_29 | 1 | 0 | 16364.05333 | 0 | 0 | 0 | 0 | 1 | 1 | 1 | 0 | 0 | 0 | 0 | KX369584 |
| VC_335_3 | 1 | 0 | 16053.5579 | 0 | 0 | 0 | 0 | 1 | 1 | 1 | 0 | 0 | 0 | 0 | KF493880 |
| VC_335_30 | 3 | 545.2391504 | 46850.54635 | 0.01150396 | 0.0014256 | 4.795831523 | 8.60232527 | 3 | 3 | 1 | 7.153471513 | 0 | 0 | 0 | KP027196,KP027203,MW862983 |
| VC_335_31 | 2 | 117.8376882 | 32435.78953 | 0.0036198 | 0.053952 | 9 | 9 | 1 | 0 | 0 | 9 | 0 | 0 | 0 | MF919508,MN585997 |
| VC_335_32 | 1 | 0 | 17430.71364 | 0 | 0 | 0 | 0 | 1 | 1 | 1 | 0 | 0 | 0 | 0 | ON208829 |
| VC_335_33 | 4 | 823.2808754 | 54652.61921 | 0.01484033 | 1.44E-05 | 7.549834435 | 8.83176087 | 6 | 6 | 1 | 8.344148423 | 0 | 0 | 0 | KF560331,MK112532,MK359316,OM203162 |
| VC_335_34 | 2 | 138.0666865 | 30884.12157 | 0.00445058 | 0.0424911 | 8.366600265 | 8.36660027 | 1 | 1 | 1 | 8.366600265 | 0 | 0 | 0 | AF271693,MZ355726 |
| VC_335_35 | 2 | 127.7319994 | 32026.80059 | 0.00397244 | 0.0442566 | 8.485281374 | 8.48528137 | 1 | 1 | 1 | 8.485281374 | 0 | 0 | 0 | KP027205,MH697581 |
| VC_335_36 | 2 | 119.0627658 | 33305.51811 | 0.00356213 | 0.0592887 | 8.774964387 | 8.77496439 | 1 | 1 | 1 | 8.774964387 | 0 | 0 | 0 | JF937100,MK112530 |
| VC_335_37 | 4 | 704.8720331 | 62196.32744 | 0.01120602 | 0.0001956 | 7.416198487 | 9.16515139 | 6 | 4 | 0.666666667 | 8.552617406 | 0 | 0 | 0 | JN699016,JX307705,KT363731,MN119377 |
| VC_335_38 | 1 | 0 | 16195.82879 | 0 | 0 | 0 | 0 | 1 | 1 | 1 | 0 | 0 | 0 | 0 | KX712238 |
| VC_335_39 | 1 | 0 | 17035.83902 | 0 | 0 | 0 | 0 | 1 | 1 | 1 | 0 | 0 | 0 | 0 | MK524525 |
| VC_335_4 | 1 | 0 | 15927.97959 | 0 | 0 | 0 | 0 | 1 | 1 | 1 | 0 | 0 | 0 | 0 | KX369586 |
| VC_335_40 | 2 | 137.0177258 | 33801.85862 | 0.00403719 | 0.0424838 | 8.485281374 | 8.48528137 | 1 | 1 | 1 | 8.485281374 | 0 | 0 | 0 | MH576975,MK967401 |
| VC_335_41 | 2 | 128.4460394 | 28490.43123 | 0.00448816 | 0.0424908 | 8.366600265 | 8.36660027 | 1 | 1 | 1 | 8.366600265 | 0 | 0 | 0 | JN699000,MG962370 |
| VC_335_42 | 2 | 118.5148326 | 30040.01816 | 0.00392973 | 0.0499106 | 8.426149773 | 8.42614977 | 1 | 1 | 1 | 8.426149773 | 0 | 0 | 0 | KY213952,MK112540 |
| VC_335_43 | 4 | 895.3756757 | 63073.20318 | 0.01399712 | 4.04E-05 | 0 | 8.94427191 | 6 | 6 | 1 | 7.263079288 | 0 | 0 | 0 | AP018477,AP018478,KF416340,MH632117 |
| VC_335_44 | 3 | 728.5135043 | 45683.39145 | 0.01569669 | 0.0014083 | 3.741657387 | 5.91607978 | 3 | 3 | 1 | 5.014300659 | 0 | 0 | 0 | EU744251,JN660814,MH669011 |
| VC_335_45 | 4 | 1015.550326 | 69842.60173 | 0.01433216 | 1.38E-05 | 6.08276253 | 9.05538514 | 6 | 5 | 0.833333333 | 7.832372876 | 0 | 0 | 0 | KJ409696,KT326768,MK494116,OK040791 |
| VC_335_46 | 1 | 0 | 17889.22225 | 0 | 0 | 0 | 0 | 1 | 1 | 1 | 0 | 0 | 0 | 0 | MK814754 |
| VC_335_47 | 1 | 0 | 15449.53934 | 0 | 0 | 0 | 0 | 1 | 1 | 1 | 0 | 0 | 0 | 0 | MW435858 |
| VC_335_48 | 1 | 0 | 17446.91417 | 0 | 0 | 0 | 0 | 1 | 1 | 1 | 0 | 0 | 0 | 0 | ON677304 |
| VC_335_49 | 17 | 20398.85129 | 258693.1243 | 0.07309007 | 2.06E-71 | 3.16227766 | 10.6301458 | 136 | 105 | 0.772058824 | 8.22596587 | 0 | 0 | 0 | EU826470,JN572689,JQ698665,JQ809703,KF024724,KF279415,KF279416,KM597531,KR080201,MF668283,MK359312,MK524506,MK524531,MN428054,MN586000,MT657342,MZ322004 |
| VC_335_5 | 11 | 7786.25639 | 158239.1448 | 0.04689798 | 3.11E-34 | 6.403124237 | 9.94987437 | 55 | 42 | 0.763636364 | 8.509909372 | 0 | 0 | 0 | AY500152,JF937108,JF937110,KJ194585,KT626047,MG872833,MG925344,MG962372,MK359354,MN585999,MT310897 |
| VC_335_50 | 2 | 149.3823731 | 34610.4858 | 0.00429755 | 0.0424888 | 8.544003745 | 8.54400375 | 1 | 1 | 1 | 8.544003745 | 0 | 0 | 0 | MH371110,MN369760 |
| VC_335_51 | 3 | 424.3415132 | 52379.41132 | 0.0080362 | 0.0036506 | 7.937253933 | 9.05538514 | 3 | 2 | 0.666666667 | 8.512214272 | 0 | 0 | 0 | MK061415,MN585985,MT776812 |
| VC_335_52 | 4 | 1255.675531 | 68137.70084 | 0.01809503 | 1.22E-05 | 0 | 8.54400375 | 6 | 6 | 1 | 5.736322004 | 0 | 0 | 0 | MG812486,MG812489,MG812495,MG872836 |
| VC_335_53 | 2 | 131.0946035 | 33725.8174 | 0.00387202 | 0.0433617 | 8.426149773 | 8.42614977 | 1 | 1 | 1 | 8.426149773 | 0 | 0 | 0 | KJ690250,MN585991 |
| VC_335_54 | 5 | 1199.926317 | 80256.93151 | 0.01473082 | 9.22E-06 | 7.211102551 | 9.32737905 | 10 | 7 | 0.7 | 8.63969743 | 0 | 0 | 0 | JN153085,MH230878,MH697575,MN062710,MN234212 |
| VC_335_55 | 2 | 183.1718388 | 30153.37957 | 0.00603799 | 0.04249 | 7 | 7 | 1 | 1 | 1 | 7 | 0 | 0 | 0 | MK305893,MZ355722 |
| VC_335_56 | 7 | 2945.684083 | 118764.7768 | 0.02420239 | 1.13E-12 | 5.916079783 | 9.53939201 | 21 | 19 | 0.904761905 | 8.248639306 | 0 | 0 | 0 | KT388014,KX550443,MH020236,MH399782,MH590595,MK359300,MK359304 |
| VC_335_57 | 1 | 0 | 17213.11613 | 0 | 0 | 0 | 0 | 1 | 1 | 1 | 0 | 0 | 0 | 0 | KY224001 |
| VC_335_58 | 1 | 0 | 16102.02583 | 0 | 0 | 0 | 0 | 1 | 1 | 1 | 0 | 0 | 0 | 0 | ON970624 |
| VC_335_59 | 1 | 0 | 16083.1322 | 0 | 0 | 0 | 0 | 1 | 1 | 1 | 0 | 0 | 0 | 0 | MT771340 |
| VC_335_6 | 1 | 0 | 16295.85221 | 0 | 0 | 0 | 0 | 1 | 1 | 1 | 0 | 0 | 0 | 0 | MN444869 |
| VC_335_60 | 2 | 126.3771395 | 33058.98049 | 0.00380822 | 0.042487 | 8.660254038 | 8.66025404 | 1 | 1 | 1 | 8.660254038 | 0 | 0 | 0 | JN687951,MH338239 |
| VC_335_61 | 8 | 5042.334163 | 122887.2202 | 0.03941493 | 1.66E-19 | 0 | 9.69535971 | 28 | 22 | 0.785714286 | 7.441945008 | 0 | 0 | 0 | JN153086,JN698998,MG925340,MG925342,MH450133,MH479919,MK310142,MT639644 |

| | | | | | | | | | | | | | | | |
|---|---|---|---|---|---|---|---|---|---|---|---|---|---|---|---|
| VC_335_62 | 3 | 892.7794182 | 46862.20521 | 0.018695 | 0.0014081 | 1.732050808 | 3.16227766 | 3 | 3 | 1 | 2.631442823 | 0 | 0 | 0 | OL455898,OM105884,OP021677 |
| VC_335_7 | 3 | 415.4140613 | 48387.28346 | 0.00851211 | 0.0026022 | 8 | 9.21954446 | 3 | 2 | 0.666666667 | 8.664836282 | 0 | 0 | 0 | KR997929,KR997934,MG944221 |
| VC_335_8 | 2 | 300 | 32469.98746 | 0.00915472 | 0.0424873 | 2 | 2 | 1 | 1 | 1 | 2 | 0 | 0 | 0 | KF024726,KP027204 |
| VC_335_9 | 2 | 183.8436572 | 33098.63271 | 0.00552374 | 0.0424907 | 7.348469228 | 7.34846923 | 1 | 1 | 1 | 7.348469228 | 0 | 0 | 0 | AY500153,KT438500 |
| VC_336_0 | 1 | 0 | 205.9822211 | 0 | 0 | 0 | 0 | 1 | 1 | 1 | 0 | 0 | 0 | 0 | KJ608189 |
| VC_337_0 | 1 | 0 | 16.6648012 | 0 | 0 | 0 | 0 | 1 | 1 | 1 | 0 | 0 | 0 | 0 | MW202895 |
| VC_338_0 | 12 | 814.9216114 | 98.81067295 | 0.89186037 | 4.46E-12 | 0 | 2 | 66 | 66 | 1 | 1.238268715 | 0 | 0 | 0 | AF306496,MH992158,MH992160,MH992177,MH992178,MH992189,MH992191,MH992194,MH992197,MH992207,MH992218,MW202535 |
| VC_339_0 | 11 | 16500 | 19005.55032 | 0.46471608 | 7.57E-37 | 0 | 3 | 55 | 55 | 1 | 2.233767648 | 0 | 0 | 0 | EU710883,FQ482083,HQ728263,MG589384,MH426725,MK759884,MK770412,MN604407,OK483342,OM522317,ON706965 |
| VC_34_0 | 1 | 0 | 654.6683599 | 0 | 0 | 0 | 0 | 1 | 1 | 1 | 0 | 0 | 0 | 0 | MH443100 |
| VC_340_0 | 2 | 266.167885 | 52302.43931 | 0.00506325 | 0.0453542 | 8.185352772 | 8.18535277 | 1 | 1 | 1 | 8.185352772 | 0 | 0 | 0 | MG646672,MH586731 |
| VC_340_1 | 17 | 34441.96615 | 364448.0486 | 0.08634452 | 1.39E-57 | 1.732050808 | 10.2469508 | 136 | 112 | 0.823529412 | 7.472644634 | 0 | 0 | 0 | AF320576,JF461087,JX181822,KP143762,KY626162,MF370225,MG251391,MG251392,MG969405,MG969406,MK965969,MK965970,MW006474,MW006475,MW006482,MZ327261,MZ327262 |
| VC_340_10 | 1 | 0 | 24586.21305 | 0 | 0 | 0 | 0 | 1 | 1 | 1 | 0 | 0 | 0 | 0 | MN994498 |
| VC_340_11 | 15 | 27218.24462 | 317292.6203 | 0.07900548 | 1.03E-62 | 0 | 9.2736185 | 105 | 98 | 0.933333333 | 7.981978837 | 0 | 0 | 0 | KP010413,MG646669,MG646670,MG646671,MN227145,MN481367,MN655999,MW311372,MW321605,MW362867,MZ150758,OL474141,OL800606,OL825705,ON239128 |
| VC_340_12 | 2 | 188.9278998 | 46098.77586 | 0.0040816 | 0.2739453 | 8.888194417 | 8.88819442 | 1 | 1 | 1 | 8.888194417 | 0 | 0 | 0 | MG065664,OK272472 |
| VC_340_13 | 1 | 0 | 26096.29341 | 0 | 0 | 0 | 0 | 1 | 1 | 1 | 0 | 0 | 0 | 0 | KM657822 |
| VC_340_14 | 1 | 0 | 24881.26597 | 0 | 0 | 0 | 0 | 1 | 1 | 1 | 0 | 0 | 0 | 0 | MG969404 |
| VC_340_15 | 1 | 0 | 35050.78939 | 0 | 0 | 0 | 0 | 1 | 1 | 1 | 0 | 0 | 0 | 0 | GU169904 |
| VC_340_16 | 4 | 1648.637927 | 103274.3292 | 0.01571284 | 1.93E-05 | 6.782329983 | 8.88819442 | 6 | 6 | 1 | 8.307422475 | 0 | 0 | 0 | KF055347,KX405003,MT409176,MW423797 |
| VC_340_17 | 4 | 1617.776406 | 98566.46695 | 0.01614801 | 3.75E-05 | 2 | 8.88819442 | 6 | 6 | 1 | 6.821291528 | 0 | 0 | 0 | KM092515,MT322327,OK040806,OK040807 |
| VC_340_18 | 7 | 5668.095333 | 168018.0414 | 0.03263413 | 4.95E-13 | 5.830951895 | 9.48683298 | 21 | 17 | 0.80952381 | 8.100208534 | 0 | 0 | 0 | JX560968,MK524176,MN882542,MT833282,MT887289,MW296865,ON540300 |
| VC_340_19 | 1 | 0 | 25846.65273 | 0 | 0 | 0 | 0 | 1 | 1 | 1 | 0 | 0 | 0 | 0 | ON868915 |
| VC_340_2 | 2 | 257.7316128 | 52472.81105 | 0.00488771 | 0.0443991 | 8.366600265 | 8.36660027 | 1 | 1 | 1 | 8.366600265 | 0 | 0 | 0 | MN850631,MN850637 |
| VC_340_20 | 4 | 1746.203902 | 99294.26725 | 0.01728222 | 1.22E-05 | 0 | 7.61577311 | 6 | 6 | 1 | 4.279291074 | 0 | 0 | 0 | LC739530,LC739531,LC739532,ON210138 |
| VC_340_21 | 1 | 0 | 23517.35712 | 0 | 0 | 0 | 0 | 1 | 1 | 1 | 0 | 0 | 0 | 0 | MH051914 |
| VC_340_22 | 5 | 2774.602895 | 124055.7133 | 0.0218765 | 6.49E-08 | 4.582575695 | 8.94427191 | 10 | 10 | 1 | 6.70791858 | 0 | 0 | 0 | MZ311863,OM386661,OM386663,OM386664,OM386665 |
| VC_340_3 | 24 | 69756.83172 | 509072.1195 | 0.12051372 | 4.00E-140 | 6.557438524 | 10.0498756 | 276 | 201 | 0.72826087 | 8.604064902 | 0 | 0 | 0 | KX377933,LR880803,MK962758,MN850564,MN850566,MN850577,MN850580,MN850585,MN850603,MN850605,MN850610,MN850611,MN850614,MN850619,MN850633,MN850635,MN850636,MN850639,MN850644,MN850647,MZ501086,OK272490,OM630585,OX016465 |
| VC_340_4 | 2 | 276.4694128 | 52996.96662 | 0.00518963 | 0.0425355 | 8.366600265 | 8.36660027 | 1 | 1 | 1 | 8.366600265 | 0 | 0 | 0 | JN225449,KR014248 |
| VC_340_5 | 1 | 0 | 25855.66598 | 0 | 0 | 0 | 0 | 1 | 1 | 1 | 0 | 0 | 0 | 0 | MN994499 |
| VC_340_6 | 3 | 844.3903312 | 78540.09041 | 0.01063672 | 0.0016653 | 5 | 8.48528137 | 3 | 3 | 1 | 7.182513041 | 0 | 0 | 0 | LR597658,MZ726791,MZ726792 |
| VC_340_7 | 18 | 41321.94869 | 400267.8276 | 0.09357542 | 7.04E-90 | 1 | 10.0498756 | 153 | 119 | 0.777777778 | 7.869979305 | 0 | 0 | 0 | EU877232,KP869100,KP869106,KP869109,KP869110,KP869113,KT184313,KT184314,KT184315,KT184316,LC519489,LR699048,MH571750,MK482688,MK482689,MK482690,MT446388,MW481326 |
| VC_340_8 | 2 | 244.6808458 | 50292.72667 | 0.00484158 | 0.0434599 | 8.485281374 | 8.48528137 | 1 | 1 | 1 | 8.485281374 | 0 | 0 | 0 | LC473039,MN994503 |
| VC_340_9 | 1 | 0 | 25651.70062 | 0 | 0 | 0 | 0 | 1 | 1 | 1 | 0 | 0 | 0 | 0 | LR597663 |
| VC_341_0 | 27 | 102888.8963 | 100531.3543 | 0.50579476 | 3.60E-212 | 0 | 9.59166305 | 351 | 304 | 0.866096866 | 6.121019687 | 0 | 0 | 0 | KM236239,KT001916,KT363872,KT454805,KT454806,KY654690,MF158040,MF158041,MF158043,MF158044,MF327003,MF327004,MF327005,MH791400,MH791413,MH920362,MK562502,MK562504,MK562505,MN342151,MN850587,OK539913,OM953433,ON528729,ON528730,ON528731,ON528732 |
| VC_342_0 | 4 | 1800 | 18759.78271 | 0.08754956 | 1.23E-05 | 0 | 4.79583152 | 6 | 6 | 1 | 3.925845044 | 0 | 0 | 0 | MK290737,MK290738,MN270891,MN270892 |
| VC_343_0 | 3 | 900 | 21356.23837 | 0.0404381 | 0.0014125 | 3.605551275 | 4.79583152 | 3 | 3 | 1 | 4.214674495 | 0 | 0 | 0 | MW749004,MW749010,OK283618 |
| VC_344_0 | 30 | 35687.84727 | 32544.84505 | 0.5230315 | 3.01E-208 | 0 | 9 | 435 | 433 | 0.995402299 | 6.867338216 | 0 | 0 | 0 | FN391954,KT337342,KY065453,KY065454,KY065458,KY065459,KY065460,KY065461,KY065462,KY065463,KY065469,KY065470,KY065471,KY065494,KY065495,KY065499,MK448725,MK448732,MK448739,MK448830,MK448842,MK448894,MK448901,MK448912,MK448914,MK448918,MK448920,MT900487,OL774868,S9_vf_15012 |
| VC_346_0 | 1 | 0 | 811.6816354 | 0 | 0 | 0 | 0 | 1 | 1 | 1 | 0 | 0 | 0 | 0 | MK449000 |
| VC_347_0 | 6 | 2316.986668 | 3867.987871 | 0.37461539 | 2.50E-11 | 0 | 5.65685425 | 15 | 15 | 1 | 3.141487628 | 0 | 0 | 0 | MK448804,MK448805,MK448807,MK448808,MK448815,MK448991 |
| VC_348_0 | 6 | 1715.517016 | 1157.675942 | 0.59707686 | 9.30E-11 | 3.741657387 | 7.28010989 | 15 | 15 | 1 | 6.334641411 | 0 | 0 | 0 | MT980836,MT980837,MT980838,MT980839,MT980840,MT980841 |
| VC_349_0 | 5 | 59.93079461 | 253.8167416 | 0.19101598 | 0.0290066 | 6.244997998 | 8.77496439 | 10 | 10 | 1 | 7.623771931 | 0 | 0 | 0 | HQ332138,KU517658,MF595878,S11_vs_8456,S5_vs_20268 |
| VC_35_0 | 35 | 146593.0922 | 193498.3805 | 0.43104019 | 0 | 0 | 8.77496439 | 595 | 595 | 1 | 4.202204325 | 0 | 0 | 0 | AF125520,AP000363,AP004422,AP004402,AP005153,AP005154,AP012529,AP012531,AP012532,AP012535,EU311208,HM208303,KJ909655,KP682372,KP682373,KP682375,KP682376,KP682377,KP682378,KP682379,KP682380,KP682382,KP682383,KP682384,KP682385,KP682386,KP682387,KP682388,KP682389,KP682390,KP682391,KP682392,KU238068,KU238069,KU238070 |
| VC_350_0 | 2 | 30.46620581 | 27.95567117 | 0.52148625 | 0.1444222 | 8.831760866 | 8.83176087 | 1 | 1 | 1 | 8.831760866 | 0 | 0 | 0 | MW462221,MZ047271 |
| VC_351_0 | 9 | 991.3497573 | 64.24177642 | 0.93914144 | 8.73E-07 | 0 | 5 | 36 | 36 | 1 | 3.751616527 | 0 | 0 | 0 | AF323671,LT985572,LT985581,LT985584,LT985586,LT985587,LT985593,LT993227,LT996080 |
| VC_352_0 | 31 | 26592.64572 | 1260.533198 | 0.95474365 | 4.59E-55 | 0 | 8.83176087 | 465 | 465 | 1 | 7.15087471 | 0 | 0 | 0 | AF338467,AP013538,AP013539,AY939843,EF372997,GU071093,GU071100,GU071102,GU071104,GU071107,GU943054,HM559717,HQ316584,HQ317388,HQ317389,HQ332137,HQ332140,HQ337022,HQ633062,HQ634152,HQ634188,KC310802,KC310803,KC310804,KC310805,KC310806,MH540083,MK016662,MT424636,MW015080,MZ803112 |
| VC_353_0 | 3 | 30.11687098 | 104.2911885 | 0.22407042 | 0.1872072 | 6.164414003 | 8.30662386 | 3 | 3 | 1 | 7.204155431 | 0 | 0 | 0 | AF396866,MW202489,OK085710 |
| VC_354_0 | 2 | 55.32185474 | 250.3489367 | 0.18098509 | 0.0658859 | 8 | 8 | 1 | 1 | 1 | 8 | 0 | 0 | 0 | MN988526,MN988556 |
| VC_356_0 | 21 | 63000 | 53479.84947 | 0.54086608 | 5.93E-76 | 0 | 7.34846923 | 210 | 210 | 1 | 4.629051515 | 0 | 0 | 0 | AF399011,AP019418,JX233784,KU521356,LC727695,LC727696,LC727699,LC727701,MF805716,MN563784,MN871480,MN871489,MT133560,MT995926,MT995927,MT995928,MW057919,MZ444140,ON631220,OP310983,OP310984 |
| VC_356_1 | 3 | 900 | 24196.71532 | 0.03586127 | 0.0630094 | 2.828427125 | 3.46410162 | 3 | 3 | 1 | 3.040318622 | 0 | 0 | 0 | MK599315,MN103543,OU343167 |
| VC_356_2 | 1 | 0 | 7349.528853 | 0 | 0 | 0 | 0 | 1 | 1 | 1 | 0 | 0 | 0 | 0 | MF063068 |
| VC_356_3 | 1 | 0 | 8267.777134 | 0 | 0 | 0 | 0 | 1 | 1 | 1 | 0 | 0 | 0 | 0 | MK552327 |
| VC_356_4 | 1 | 0 | 8179.065526 | 0 | 0 | 0 | 0 | 1 | 1 | 1 | 0 | 0 | 0 | 0 | EU197055 |
| VC_356_5 | 1 | 0 | 8425.045688 | 0 | 0 | 0 | 0 | 1 | 1 | 1 | 0 | 0 | 0 | 0 | MF042360 |
| VC_356_6 | 1 | 0 | 8652.274817 | 0 | 0 | 0 | 0 | 1 | 1 | 1 | 0 | 0 | 0 | 0 | HQ630627 |
| VC_357_0 | 2 | 300 | 4148.55846 | 0.06743758 | 0.0434584 | 3.605551275 | 3.60555128 | 1 | 1 | 1 | 3.605551275 | 0 | 0 | 0 | AP017924,AP017925 |
| VC_357_1 | 1 | 0 | 2149.402275 | 0 | 0 | 0 | 0 | 1 | 1 | 1 | 0 | 0 | 0 | 0 | LC667451 |
| VC_358_0 | 4 | 1800 | 2173.082867 | 0.45304869 | 2.03E-05 | 0 | 3.46410162 | 6 | 6 | 1 | 1.732050808 | 0 | 0 | 0 | MK308674,MK308675,MK308676,MK308677 |
| VC_358_1 | 2 | 300 | 2181.091441 | 0.12091453 | 0.0463912 | 2.828427125 | 2.82842712 | 1 | 1 | 1 | 2.828427125 | 0 | 0 | 0 | MT811961,OM319461 |
| VC_358_2 | 1 | 0 | 1361.279932 | 0 | 0 | 0 | 0 | 1 | 1 | 1 | 0 | 0 | 0 | 0 | OV032902 |
| VC_358_3 | 1 | 0 | 1449.147948 | 0 | 0 | 0 | 0 | 1 | 1 | 1 | 0 | 0 | 0 | 0 | MH595538 |
| VC_359_0 | 3 | 900 | 5062.42659 | 0.15094525 | 0.0015527 | 6.403124237 | 6.78232998 | 3 | 3 | 1 | 6.580964248 | 0 | 0 | 0 | LR881104,MN871444,MN871445 |
| VC_36_0 | 4 | 373.4917457 | 5880.367023 | 0.05972181 | 5.43E-05 | 6.633249581 | 7.48331477 | 6 | 6 | 1 | 6.991144846 | 0 | 0 | 0 | HQ201307,JQ182729,MH178096,MN688132 |
| VC_360_0 | 22 | 20084.14881 | 836.8617066 | 0.95999898 | 2.47E-21 | 1 | 7.81024968 | 231 | 231 | 1 | 5.56966756 | 0 | 0 | 0 | AJ344259,AJ414696,AJ748296,AJ875026,FM164764,KP282675,KX712143,KY744228,KY744229,KY744230,KY744231,KY744232,KY744233,KY744234,KY744235,MK064565,MN876841,MN876842,MN876843,OM401712,OM401713,OM401714 |
| VC_361_0 | 7 | 2595.304798 | 179.5863192 | 0.93528167 | 2.32E-08 | 0 | 8.66025404 | 21 | 21 | 1 | 6.029744064 | 0 | 0 | 0 | AF440571,AM087120,AM087121,AM087122,AM087123,EU545650,KX467643 |
| VC_362_0 | 2 | 79.25472564 | 167.1313469 | 0.32166885 | 0.0613455 | 3.605551275 | 3.60555128 | 1 | 1 | 1 | 3.605551275 | 0 | 0 | 0 | AJ854042,MK064564 |
| VC_363_0 | 3 | 123.888636 | 15.84948891 | 0.8865772 | 0.0184442 | 5.567764363 | 7.21110255 | 3 | 3 | 1 | 6.115543759 | 0 | 0 | 0 | AM419438,HQ332141,MW344765 |
| VC_364_0 | 3 | 675.8388333 | 251.27303 | 0.72897226 | 0.0113272 | 1 | 6.55743852 | 3 | 3 | 1 | 4.679393074 | 0 | 0 | 0 | AF440695,MK002701,MK450543 |
| VC_365_0 | 6 | 2158.565364 | 1842.196832 | 0.53953853 | 2.23E-11 | 0 | 7.87400787 | 15 | 15 | 1 | 6.214943619 | 0 | 0 | 0 | AF447491,AY453853,CP000625,OK095358,OM892879,OP293079 |
| VC_366_0 | 4 | 654.29507 | 3125.567429 | 0.17310023 | 1.32E-05 | 7.549834435 | 9.05538514 | 6 | 5 | 0.833333333 | 8.230053652 | 0 | 0 | 0 | MN512538,MN812722,MT227924,OP125534 |
| VC_367_0 | 2 | 14.51064128 | 271.1896292 | 0.05078974 | 0.0658161 | 7.280109889 | 7.28010989 | 1 | 1 | 1 | 7.280109889 | 0 | 0 | 0 | MT121967,MW651859 |
| VC_368_0 | 5 | 1083.11497 | 749.1640935 | 0.59112992 | 2.06E-07 | 0 | 3.60555128 | 10 | 10 | 1 | 1.44222051 | 0 | 0 | 0 | ON170062,ON170209,ON170231,ON170302,ON170328 |
| VC_369_0 | 2 | 53.13010178 | 214.6116818 | 0.19843784 | 0.0678891 | 6.8556546 | 6.8556546 | 1 | 1 | 1 | 6.8556546 | 0 | 0 | 0 | HQ634187,MK105819 |
| VC_37_0 | 4 | 1023.941755 | 1343.54254 | 0.43250203 | 1.94E-05 | 1.732050808 | 4.35889894 | 6 | 6 | 1 | 3.203663739 | 0 | 0 | 0 | AY374448,CP038007,CP072507,KY271402 |
| VC_370_0 | 53 | 126907.3161 | 14537.02497 | 0.89722441 | 0 | 0 | 6.63324958 | 1378 | 1378 | 1 | 2.523892394 | 0 | 0 | 0 | MT740734,ON170024,ON170027,ON170031,ON170052,ON170057,ON170058,ON170068,ON170071,ON170075,ON170077,ON170079,ON170129,ON170133,ON170134,ON170136,ON170150,ON170172,ON170175,ON170179,ON170181,ON170182,ON170185,ON170187,ON170208,ON170214,ON170228,ON170229,ON170232,ON170234,ON170236,ON170238,ON170240,ON170242,ON170254,ON170257,ON170262,ON170264,ON170265,ON170267,ON170275,ON170279,ON170286,ON170287,ON170303,ON170317,ON170323,ON170324,ON170326,ON170327,ON170343,ON170355,ON170359 |
| VC_371_0 | 3 | 148.1302887 | 139.7700552 | 0.51451932 | 0.0234271 | 0 | 0 | 3 | 3 | 1 | 0 | 0 | 0 | 0 | ON170149,ON170171,ON170322 |
| VC_372_0 | 7 | 2567.845159 | 4389.807694 | 0.36906773 | 4.92E-14 | 0 | 8.77496439 | 21 | 21 | 1 | 6.84867358 | 0 | 0 | 0 | JN585957,MF612073,MH426726,MK618715,MT251347,MT251348,MW722080 |
| VC_373_0 | 358 | 942236.0133 | 37799.15346 | 0.96143082 | 0 | 0 | 2.44948974 | 63903 | 63903 | 1 | 1.242094692 | 0 | 0 | 0 | AF454431,AY751298,DQ079896,DQ079897,DQ079899,DQ079900,DQ079901,DQ079902,DQ079903,DQ079904,DQ079906,DQ079908,DQ079909,GQ153920,GQ153922,GQ153923,JF719729,JF719730,JF719731,KX266300,KX266301,KX266302,KX266303,KX266304,KX266305,KX266306,KX266307,KX266308,KX266309,KX266310,KX266311,KX266312,KX266313,KX266314,KX266315,KX266316,KX266317,KX266318,KX266319,KX266320,KX266321,KX266322,KX266323,KX266324,KX266325,KX266326,KX266327,KX266328,KX266329,KX266330,KX266331,KX266332,KX266333,KX266334,KX266335,KX266336,KX266337,KX266338,KX266339,KX266340,KX266341,KX266342,KX266343,KX266344,KX266345,KX266346,KX266347,KX266348,KX266349,KX266350,KX266351,KX266352,KX266353,KX266354,KX266355,KX266356,KX266357,KX266358,KX266359,KX266360,KX266361,KX266362,KX266363,KX266364,KX266365,KX266366,KX266367,KX266368,KX266369,KX266370,KX266371,KX266372,KX266373,KX266374,KX266375,KX266376,KX266377,KX266378,KX266379,KX266380,KX266381,KX266382,KX266383,KX266384,KX266385,KX266386,KX266387,KX266388,KX266389,KX266390,KX266391,KX266392,KX266393,KX266394,KX266395,KX266396,KX266397,KX266398,KX266399,KX266400,KX266401,KX266402,KX266403,KX266404,KX266405,KX266406,KX266407,KX266408,KX266409,KX266410,KX266411,KX266412,KX266413,KX266414,KX266415,KX266416,KX266417,KX266418,KX266419,KX266420,KX266421,KX266422,KX266423,KX266424,KX266425,KX266426,KX266427,KX266428,KX266429,KX266430,KX266431,KX266432,KX266433,KX266434,KX266435,KX266436,KX266437,KX266438,KX266439,KX266440,KX266441,KX266442,KX266443,KX266444,KX266445,KX266446,KX266447,KX266448,KX266449,KX266450,KX266451,KX266452,KX266453,KX266454,KX266455,KX266456,KX266457,KX266458,KX266459,KX266460,KX266461,KX266462,KX266463,KX266464,KX266465,KX266466,KX266467,KX266468,KX266469,KX266470,KX266471,KX266472,KX266473,KX266474,KX266475,KX266476,KX266477,KX266478,KX266479,KX266480,KX266481,KX266482,KX266483,KX266484,KX266485,KX266486,KX266487,KX266488,KX266489,KX266490,KX266491,KX266492,KX266493,KX266494,KX266495,KX266496,KX266497,KX266498,KX266499,KX266500,KX266501,KX266502,KX266503,KX266504,KX266505,KX266506,KX266507,KX266508,KX266509,KX266510,KX266511,KX266512,KX266513,KX266514,KX266515,KX266516,KX266517,KX266518,KX266519,KX266520,KX266521,KX266522,KX266523,KX266524,KX266525,KX266526,KX266527,KX266528,KX266529,KX266530,KX266531,KX266532,KX266533,KX266534,KX266535,KX266536,KX266537,KX266538,KX266539,KX266540,KX266541,KX266542,KX266543,KX266544,KX266545,KX266546,KX266547,KX266548,KX266549,KX266550,KX266551,KX266552,KX266553,KX266554,KX266555,KX266556,KX266557,KX266558,KX266559,KX266560,KX266561,KX266562,KX266563,KX266564,KX266565,KX266566,KX266567,KX266568,KX266569,KX266570,KX266571,KX266572,KX266573,KX266574,KX266575,KX266576,KX266577,KX266578,KX266579,KX266580,KX266581,KX266582,KX266583,KX266584,KX266585,KX266586,KX266587,KX266588,KX266589,KX266590,KX266591,KX266592,KX266593,KX266594,KX266595,KX266596,KX266597,KX266598,KX266599,KX266600,KX266601,KX266602,KX266603,KX266604,KX266605,KX266606,KX266607,KX266608,KX266609,KX266610,KX266611,KX266612,KX266613,KX266614,KX266615,KX266616,KX266617,KX266618,KX266619,KX266620,KX266621,KX266622,KX266623,KX266624,KX266625,KX266626,KX266627,KX266628,KX266629,KX266630,KX266631,KX266632,KX266633,KX266634,KX266635,KX266636,KX266637,MG563770 |
| VC_375_0 | 2 | 9.419844648 | 4696.628712 | 0.00200165 | 0.1669227 | 2 | 2 | 1 | 1 | 1 | 2 | 0 | 0 | 0 | GQ153921,KC237308 |
| VC_376_0 | 2 | 29.06407253 | 7.25180957 | 0.800313 | 0.2701457 | 0 | 0 | 1 | 1 | 1 | 0 | 0 | 0 | 0 | OK338687,OM055665 |
| VC_377_0 | 16 | 23263.53648 | 12952.4886 | 0.64235477 | 2.27E-62 | 0 | 7.81024968 | 120 | 120 | 1 | 3.926788423 | 0 | 0 | 0 | AP008986,KY210139,MG944227,MG944228,MG944229,MG944230,MG944231,MG944232,MG944233,MG944234,MG944235,MG944236,MN461279,OK275494,ON996340,OP067662 |
| VC_38_0 | 4 | 688.0551279 | 3252.757929 | 0.17459725 | 1.66E-05 | 5.830951895 | 8.06225775 | 6 | 6 | 1 | 7.249280792 | 0 | 0 | 0 | MK416013,MK416022,MK448234,MK448237 |
| VC_38_1 | 1 | 0 | 791.3029139 | 0 | 0 | 0 | 0 | 1 | 1 | 1 | 0 | 0 | 0 | 0 | OL617042 |
| VC_380_0 | 6 | 2667.020192 | 2562.921998 | 0.50995214 | 3.66E-11 | 0 | 7.93725393 | 15 | 15 | 1 | 5.425412902 | 0 | 0 | 0 | JQ177063,KY290953,KY290954,MH179474,MK804891,MK804892 |
| VC_383_0 | 3 | 178.3914844 | 851.305076 | 0.17324665 | 0.0016142 | 6.164414003 | 8.48528137 | 3 | 3 | 1 | 7.46522069 | 0 | 0 | 0 | S12_vs_93357,S14_vs_2870,S16_vs_120622 |
| VC_384_0 | 2 | 180.3249487 | 56746.5249 | 0.00316766 | 0.0526541 | 8.717797887 | 8.71779789 | 1 | 1 | 1 | 8.717797887 | 0 | 0 | 0 | MT897903,ON456342 |
| VC_384_1 | 11 | 10836.54074 | 275563.1917 | 0.03783712 | 3.19E-34 | 0 | 10.0498756 | 55 | 47 | 0.854545455 | 7.96321926 | 0 | 0 | 0 | KJ595576,KM347890,KR997967,KY006474,MH926060,MK279910,MK524512,MK524526,MN585967,MN586058,MW534377 |
| VC_384_10 | 1 | 0 | 26560.08364 | 0 | 0 | 0 | 0 | 1 | 1 | 1 | 0 | 0 | 0 | 0 | MH590594 |
| VC_384_11 | 31 | 83195.54182 | 815461.0775 | 0.09257768 | 1.88E-148 | 0 | 10.6301458 | 465 | 326 | 0.701075269 | 8.527191855 | 0 | 0 | 0 | KX592589,MF155947,MF668276,MF919528,MF919539,MG757159,MH230874,MH513980,MH590588,MK112539,MK112546,MK112551,MK279843,MK279865,MK279870,MK279873,MK279878,MK279894,MK279895,MK279897,MN369744,MT310868,MT316460,MT316463,MW534381,MW712720,MW862980,MW862993,MZ648038,ON637769,OP434459 |
| VC_384_12 | 4 | 1272.67191 | 100445.886 | 0.0125117 | 1.51E-05 | 4.242640687 | 9.05538514 | 6 | 5 | 0.833333333 | 7.528073504 | 0 | 0 | 0 | MK112544,MT310867,MT952849,ON456332 |
| VC_384_13 | 33 | 107715.6383 | 495812.3845 | 0.17847661 | 0 | 0 | 10.0995049 | 528 | 463 | 0.876893939 | 7.685665692 | 0 | 0 | 0 | JN192463,KM363597,KR029086,KR029087,KX576645,KX576646,KX576647,KX702319,KY385380,KY385382,KY385383,MF919511,MG757164,MG757165,MG944223,MG962363,MH051251,MH155874,MH316568,MH371107,MH479916,MH479918,MH513973,MH576958,MK279850,MK279858,MK279864,MK279883,MK279890,MK279891,MN585965,MN945903,ON456330 |
| VC_384_14 | 1 | 0 | 26777.80929 | 0 | 0 | 0 | 0 | 1 | 1 | 1 | 0 | 0 | 0 | 0 | MN369738 |
| VC_384_15 | 44 | 168137.8312 | 1041816.418 | 0.13896214 | 0 | 4.123105626 | 10.198039 | 946 | 707 | 0.747357294 | 8.47829338 | 0 | 0 | 0 | GQ303264,GQ303266,JF704103,JF704109,JF937097,JF957056,JN006063,JN638752,JN638753,JN699008,JN699010,KM408320,KP027208,KT599441,KU867907,KX578071,KX670813,KX683292,KX683293,KY676783,MG770212,MG925346,MG925350,MG944225,MH399775,MH399786,MH576970,MH651184,MH825702,MH825705,MK112527,MK112536,MK112555,MK279859,MK279861,MK279866,MK279879,MK279881,MK279882,MK279885,MK279887,MK279888,MW712726 |
| VC_384_16 | 42 | 155699.1516 | 1006197.322 | 0.13400432 | 1.00E-279 | 0 | 10.198039 | 861 | 627 | 0.728222997 | 8.472045595 | 0 | 0 | 0 | AF547430,DQ398046,FJ174694,GU247133,GU247134,JF704102,JN699009,JX649096,JX649097,JX649100,KJ194580,KJ194583,KX369585,KX620786,MG757158,MG925348,MG962365,MH371114,MH371117,MH371125,MH399773,MH399780,MH450116,MH450122,MH479914,MH479921,MH651175,MH651186,MH727555,MH744417,MH779516,MK112542,MK112553,MK279846,MK279852,MK279871,MK494104,MN945899,MT897909,MW534379,MW534380,MZ622173 |
| VC_384_17 | 3 | 672.1153247 | 88615.43094 | 0.00752754 | 0.0018797 | 0 | 8.36660027 | 3 | 3 | 1 | 5.57773351 | 0 | 0 | 0 | JF937095,JN698990,JN699017 |
| VC_384_18 | 10 | 9002.065369 | 289826.9112 | 0.03012447 | 7.05E-23 | 0 | 9.43398113 | 45 | 36 | 0.8 | 7.681119688 | 0 | 0 | 0 | JF704091,JF937109,JN698989,KJ194579,KP027197,KP027209,KR816508,KX576643,KY965066,MT897901 |
| VC_384_19 | 1 | 0 | 29086.75858 | 0 | 0 | 0 | 0 | 1 | 1 | 1 | 0 | 0 | 0 | 0 | MZ622172 |
| VC_384_2 | 8 | 5363.049335 | 226003.4025 | 0.02317989 | 8.68E-17 | 6.32455532 | 9.48683298 | 28 | 24 | 0.857142857 | 8.052410028 | 0 | 0 | 0 | KC576784,KJ567044,MG925356,MG962375,MH077582,MK112552,MK279889,MN096366 |
| VC_384_20 | 1 | 0 | 29395.42304 | 0 | 0 | 0 | 0 | 1 | 1 | 1 | 0 | 0 | 0 | 0 | MN703415 |
| VC_384_21 | 2 | 169.6541079 | 51352.45323 | 0.00329284 | 0.0422899 | 8.888194417 | 8.88819442 | 1 | 1 | 1 | 8.888194417 | 0 | 0 | 0 | JN006064,KR781349 |
| VC_384_3 | 22 | 43738.36964 | 512968.3377 | 0.07856627 | 4.68E-136 | 5.477225575 | 9.69535971 | 231 | 209 | 0.904761905 | 8.015235961 | 0 | 0 | 0 | GQ303259,JF704099,JX649098,JX649099,KC661274,KC691258,KF713485,KJ174157,KJ538723,KM236502,MF919503,MF919507,MF919523,MF919530,MG925339,MG962362,MH230875,MH779500,MK279877,MK279908,MN444868,MT897902 |
| VC_384_4 | 5 | 1897.195931 | 139250.4343 | 0.01344122 | 2.85E-07 | 0 | 8.88819442 | 10 | 10 | 1 | 7.618358723 | 0 | 0 | 0 | KT364588,MH576954,MH576967,MH651177,MK279904 |
| VC_384_5 | 2 | 181.1565145 | 52086.12306 | 0.00346596 | 0.0422893 | 8.602325267 | 8.60232527 | 1 | 1 | 1 | 8.602325267 | 0 | 0 | 0 | MH051264,MK310139 |
| VC_384_6 | 1 | 0 | 20771.60316 | 0 | 0 | 0 | 0 | 1 | 1 | 1 | 0 | 0 | 0 | 0 | MF919519 |
| VC_384_7 | 1 | 0 | 26021.02413 | 0 | 0 | 0 | 0 | 1 | 1 | 1 | 0 | 0 | 0 | 0 | ON970594 |
| VC_384_8 | 1 | 0 | 24339.98383 | 0 | 0 | 0 | 0 | 1 | 1 | 1 | 0 | 0 | 0 | 0 | MZ648041 |
| VC_384_9 | 4 | 1106.384103 | 100251.5305 | 0.01091562 | 2.33E-05 | 6.244997998 | 9.38083152 | 6 | 4 | 0.666666667 | 8.31236511 | 0 | 0 | 0 | MT776813,MT889369,MZ681520,ON970577 |
| VC_385_0 | 24 | 80159.16828 | 65169.47804 | 0.5515717 | 6.82E-144 | 0 | 6.244998 | 276 | 276 | 1 | 3.627152774 | 0 | 0 | 0 | DQ398044,EU770221,JF704094,JF704104,JN699011,KF279411,KF279412,KF279413,KF493881,KR080194,KR080200,KT270441,KX589269,MH513970,MK524486,MN234171,MN369759,MN428050,MT310870,MT639648,MT818419,MT897910,MW435857,ON970575 |
| VC_385_1 | 9 | 9203.670853 | 38522.85755 | 0.19284183 | 2.20E-24 | 0 | 8.24621125 | 36 | 36 | 1 | 5.080135532 | 0 | 0 | 0 | JN699007,KF024727,KF416341,KR080199,KY224000,MH727561,MK524490,MN284893,MT889381 |
| VC_385_2 | 5 | 1797.698185 | 23283.07184 | 0.07167636 | 3.09E-08 | 5.656854249 | 9.05538514 | 10 | 9 | 0.9 | 7.571257399 | 0 | 0 | 0 | MH744423,MK059749,MN617843,MW055906,OL455893 |
| VC_385_3 | 5 | 2976.914932 | 23660.46826 | 0.11175703 | 3.12E-08 | 0 | 4.47213595 | 10 | 10 | 1 | 3.779188896 | 0 | 0 | 0 | KF024729,KJ433973,KJ433974,KJ433975,KJ433976 |
| VC_385_4 | 3 | 528.6531951 | 15537.62715 | 0.03290452 | 0.0014674 | 6.633249581 | 8.83176087 | 3 | 3 | 1 | 7.983430607 | 0 | 0 | 0 | MN813693,MZ150786,OP297541 |
| VC_386_0 | 10 | 5817.772196 | 1987.125453 | 0.74540019 | 1.69E-21 | 2 | 6.164414 | 45 | 45 | 1 | 4.78686672 | 0 | 0 | 0 | AJ006589,AJ550940,JX182372,MK433266,MK450421,MK450426,MK450431,MK450433,MK686068,MK686069 |
| VC_387_0 | 2 | 99.83354675 | 699.7820192 | 0.12485193 | 0.0442374 | 4 | 4 | 1 | 1 | 1 | 4 | 0 | 0 | 0 | GQ379227,MK937595 |
| VC_388_0 | 3 | 334.5841102 | 1015.952226 | 0.24774166 | 0.0019271 | 0 | 6.70820393 | 3 | 3 | 1 | 4.472135955 | 0 | 0 | 0 | MG593800,MG593803,MH001457 |
| VC_389_0 | 3 | 389.5594933 | 1246.552045 | 0.23810082 | 0.0021008 | 4 | 4.35889894 | 3 | 3 | 1 | 4.16066819 | 0 | 0 | 0 | MT701593,MT701595,MT701596 |
| VC_390_0 | 2 | 155.1562446 | 188.377673 | 0.45164753 | 0.0547993 | 4.582575695 | 4.58257569 | 1 | 1 | 1 | 4.582575695 | 0 | 0 | 0 | EU409559,S14_vf_78139 |
| VC_392_0 | 9 | 4880.683936 | 1603.14449 | 0.75274724 | 1.45E-17 | 1.732050808 | 7.93725393 | 36 | 36 | 1 | 6.518984517 | 0 | 0 | 0 | AJ242593,DQ003637,KJ094022,KP399678,MH341452,MZ361825,MZ361826,MZ361827,ON456151 |
| VC_393_0 | 5 | 907.642719 | 1022.206178 | 0.47031802 | 7.52E-08 | 4.242640687 | 9 | 10 | 9 | 0.9 | 7.458931357 | 0 | 0 | 0 | AJ312240,DQ003639,JX120799,KJ094023,MT500540 |
| VC_394_0 | 3 | 236.710404 | 1259.129367 | 0.15824583 | 0.004542 | 7.071067812 | 8.06225775 | 3 | 3 | 1 | 7.647858412 | 0 | 0 | 0 | DQ003642,KP399677,MH341451 |
| VC_396_0 | 3 | 97.02769626 | 156.4997869 | 0.38271076 | 0.002359 | 6.164414003 | 7.54983444 | 3 | 3 | 1 | 6.998119443 | 0 | 0 | 0 | KR905068,KU848187,LC625742 |

| | | | | | | | | | | | | | | |
|---|---|---|---|---|---|---|---|---|---|---|---|---|---|---|
| VC_398_0 | 51 | 163126.5205 | 23505.82726 | 0.87405277 | 0 | 0 | 6.164414 | 1275 | 1275 | 1 | 4.163273787 | 0 | 0 | 0 | AJ251805,AJ318471,EU547803,JX421753,KC960671,KM253764,KP313531,KP791805,KP791806,KP791807,KT008108,KU687347,KU687348,KX130726,KX452696,KX778611,LC730324,MF285616,MF285617,MF285620,MH717709,MK608335,MN013074,MN149904,MT419366,MT813142,MW296877,MW600722,MZ359670,MZ428229,OK149215,OL362281,OL362282,OL362283,OL691920,OL744209,OL744212,OL744217,OL800605,ON528720,ON528721,ON528722,ON528723,ON528724,ON950090,OP617747,OX335419,OX335420,OX335431,OX335434,OX335437 |
| VC_399_0 | 49 | 121850.8107 | 31872.64859 | 0.79266243 | 0 | 0 | 7 | 1176 | 1176 | 1 | 5.000250071 | 0 | 0 | 0 | AM183667,EU734170,EU734171,FJ194439,GQ468526,HQ333270,JQ965702,KF700371,KM366096,KP090453,KP090454,KY084243,LT960606,LT961836,LT961837,LT961838,LT961840,LT961841,LT961842,LT961843,LT961844,LT961845,LT961846,LT962379,LT962380,LT962475,LT962906,LT962907,MG471392,MH031343,MH809534,MK685668,MK907285,MN080711,MN252582,MT374859,MT515757,MT542512,MT625440,MW147603,MW367418,MW767996,MZ501055,MZ501064,OK663600,OL800603,OL856139,ON754979,ON922920 |
| VC_4_0 | 7 | 3688.377234 | 1026.637652 | 0.78226205 | 4.13E-15 | 0 | 3.74165739 | 21 | 21 | 1 | 2.14880429 | 0 | 0 | 0 | DQ426904,DQ426905,JN255163,KP137432,KP137434,KP137436,KP137438 |
| VC_40_0 | 6 | 1961.292795 | 1493.386414 | 0.56772067 | 6.04E-10 | 1.414213562 | 9.05538514 | 15 | 13 | 0.866666667 | 6.714387411 | 0 | 0 | 0 | KU052037,OM046621,OM046622,OM046623,OM046625,OM046629 |
| VC_40_1 | 2 | 52.96117204 | 1208.815885 | 0.04197348 | 0.1124915 | 8.94427191 | 8.94427191 | 1 | 1 | 1 | 8.94427191 | 0 | 0 | 0 | MN013086,OL396571 |
| VC_400_0 | 3 | 370.2313009 | 8472.409704 | 0.04186886 | 0.0013837 | 3.16227766 | 4.89897949 | 3 | 3 | 1 | 4.1777977 | 0 | 0 | 0 | HQ728265,MN450150,ON715521 |
| VC_401_0 | 4 | 602.4697697 | 16971.52444 | 0.03428189 | 1.19E-05 | 1 | 7 | 6 | 6 | 1 | 5.268252116 | 0 | 0 | 0 | KT321314,ON602733,OX335378,OX335441 |
| VC_402_0 | 3 | 544.1790599 | 10118.30596 | 0.05103679 | 0.0013844 | 1 | 1.73205081 | 3 | 3 | 1 | 1.382088123 | 0 | 0 | 0 | MF979561,MW001769,ON995367 |
| VC_403_0 | 4 | 699.5937517 | 14441.03022 | 0.0462064 | 1.18E-05 | 3.872983346 | 5.74456265 | 6 | 6 | 1 | 4.973149053 | 0 | 0 | 0 | EU652770,KX078568,MW358931,OK499982 |
| VC_405_0 | 126 | 900700.8954 | 36123.80106 | 0.96144017 | 0 | 0 | 7.28010989 | 7875 | 7875 | 1 | 4.589371852 | 0 | 0 | 0 | AY247822,AY264774,AY264775,AY264776,AY264777,AY264778,EU734174,FR667955,GU071091,JQ957925,JQ965700,JQ965701,JQ965703,JX000007,JX872508,KM242061,KU927499,LC730325,LR745706,LR745707,LR745708,LR745709,LR745710,MG488277,MG832642,MG833025,MG966531,MH382198,MH717098,MH717099,MH779619,MK310182,MK542821,MN026740,MN098329,MN481365,MN481366,MT350293,MT543041,MT681669,MW074885,MW883060,MZ220766,MZ318361,MZ318362,MZ318363,MZ375233,MZ375234,MZ375235,MZ375236,MZ375237,MZ375238,MZ375239,MZ375240,MZ375241,MZ375242,MZ375243,MZ375244,MZ375245,MZ375246,MZ375247,MZ375248,MZ375249,MZ375250,MZ375251,MZ375252,MZ375253,MZ375254,MZ375255,MZ375256,MZ375257,MZ375258,MZ375259,MZ375260,MZ375261,MZ375262,MZ375263,MZ375264,MZ375265,MZ375266,MZ375267,MZ375268,MZ375269,MZ375270,MZ375271,MZ375272,MZ375273,MZ375274,MZ375275,MZ375276,MZ375277,MZ375278,MZ375279,MZ375280,MZ375281,MZ375282,MZ375283,MZ375284,MZ375285,MZ375286,MZ375287,MZ375288,MZ375289,MZ375290,MZ375291,MZ375292,MZ375293,MZ375294,MZ501078,MZ501081,MZ833439,OK042080,OL964739,OL964740,OL964741,OL964748,OL964749,OL964750,OM630586,ON528715,ON528716,ON528717,ON528719,ON568193,OP331230,V01146 |
| VC_406_0 | 7 | 3213.220276 | 15039.25412 | 0.176043 | 2.84E-15 | 1.414213562 | 4 | 21 | 21 | 1 | 3.290631022 | 0 | 0 | 0 | MH059639,MH460459,MN013082,MN013085,MN812693,MT822288,ON287378 |
| VC_407_0 | 2 | 163.9433305 | 7695.979639 | 0.02085813 | 0.0420683 | 2.236067977 | 2.23606798 | 1 | 1 | 1 | 2.236067977 | 0 | 0 | 0 | MH370477,MT465573 |
| VC_408_0 | 5 | 1833.338457 | 16440.8332 | 0.10032403 | 2.40E-08 | 1 | 2.44948974 | 10 | 10 | 1 | 1.903600885 | 0 | 0 | 0 | MN184885,MT822284,MT822285,MT822286,MT822287 |
| VC_409_0 | 2 | 112.0423157 | 1496.240964 | 0.06966578 | 0.0423923 | 4.123105626 | 4.12310563 | 1 | 1 | 1 | 4.123105626 | 0 | 0 | 0 | MW057854,MW358928 |
| VC_41_0 | 3 | 900 | 0 | 1 | 1 | 3.741657387 | 4.89897949 | 3 | 3 | 1 | 4.443684211 | 0 | 0 | 0 | AB231700,KF356199,OP394178 |
| VC_411_0 | 48 | 7725.515267 | 8342.8854 | 0.48078931 | 4.49E-81 | 0 | 2.44948974 | 1128 | 1128 | 1 | 1.48586544 | 0 | 0 | 0 | KP087939,KP087945,KT264811,MG945164,MG945682,MG945837,MH572269,MH572314,MH572315,MH572316,MH572317,MH572318,MH572319,MH572320,MH572321,MH572325,MH572326,MH572328,MH572329,MH572336,MH572351,MH572394,MH572395,MH572396,MH572397,MH572404,MH572419,MH572431,MH572439,MH572441,MH572443,MH572445,MH572446,MH572451,MH572473,MH572487,MH572492,MH572493,MH572494,MH572503,MH572510,MH572511,MH992195,MH992211,MK249143,MK249151,MK249223,MK249231 |
| VC_412_0 | 25 | 2086.823902 | 1611.926078 | 0.56419707 | 1.16E-22 | 0 | 2.44948974 | 300 | 300 | 1 | 1.795576552 | 0 | 0 | 0 | MH572279,MH572300,MH572301,MH572302,MH572303,MH572305,MH572306,MH572307,MH572308,MH572309,MH572310,MH572311,MH572312,MH572313,MH572345,MH572392,MH572393,MH572406,MH572435,MH572436,MH572437,MH572469,MH572480,MH572508,MH572509 |
| VC_414_0 | 1 | 0 | 137.6516988 | 0 | 0 | 0 | 0 | 1 | 1 | 1 | 0 | 0 | 0 | 0 | MH572340 |
| VC_415_0 | 1 | 0 | 23.5195531 | 0 | 0 | 0 | 0 | 1 | 1 | 1 | 0 | 0 | 0 | 0 | MH572479 |
| VC_416_0 | 1 | 0 | 26.63766397 | 0 | 0 | 0 | 0 | 1 | 1 | 1 | 0 | 0 | 0 | 0 | MH572512 |
| VC_417_0 | 20 | 3065.329545 | 1592.292767 | 0.65813184 | 3.21E-84 | 0 | 2.23606798 | 190 | 190 | 1 | 1.198881836 | 0 | 0 | 0 | KC131023,KC131025,MH572294,MH572295,MH572296,MH572384,MH572385,MH572386,MH572387,MH572388,MH572389,MH572390,MH572391,MH572430,MH572470,MH572471,MH572472,MH572505,MH572506,MH572507 |
| VC_419_0 | 11 | 9793.10035 | 553.3450067 | 0.94651835 | 4.59E-27 | 0 | 6.70820393 | 55 | 55 | 1 | 5.293483378 | 0 | 0 | 0 | KP790011,KU998237,KU998238,KY471268,KY471269,MH651189,MH697580,MN010758,MT553344,MW132713,ON260826 |
| VC_42_0 | 5 | 1484.2683 | 16106.08413 | 0.08437968 | 5.35E-08 | 4.123105626 | 5.47722558 | 10 | 10 | 1 | 4.961042449 | 0 | 0 | 0 | LC567830,LC567838,LC567842,LC616079,ON416862 |
| VC_420_0 | 3 | 355.1338582 | 723.1531553 | 0.32935003 | 0.0017473 | 3 | 5.47722558 | 3 | 3 | 1 | 4.620796794 | 0 | 0 | 0 | AJ278322,MG324353,MG324354 |
| VC_421_0 | 9 | 6630.628612 | 1124.639728 | 0.85498378 | 1.57E-21 | 0 | 0 | 36 | 36 | 1 | 0 | 0 | 0 | 0 | KY566218,KY613597,KY624610,KY624611,KY624612,KY624613,KY624614,KY624615,KY624616 |
| VC_422_0 | 3 | 69.88629408 | 421.6842959 | 0.1421694 | 0.0183859 | 7.071067812 | 8.42614977 | 3 | 3 | 1 | 7.546215338 | 0 | 0 | 0 | KY203335,KY250482,MW582634 |
| VC_423_0 | 7 | 2488.581221 | 671.9715129 | 0.78738798 | 3.41E-13 | 2.449489743 | 7.41619849 | 21 | 21 | 1 | 6.268348397 | 0 | 0 | 0 | MG198783,MG198784,MH271305,MH271318,MT521997,MZ681507,ON456352 |
| VC_424_0 | 3 | 68.17234799 | 470.9567442 | 0.12644902 | 0.0336712 | 7.211102551 | 8.88819442 | 3 | 3 | 1 | 7.926814239 | 0 | 0 | 0 | LC644972,LC644974,MT843275 |
| VC_425_0 | 40 | 55047.75821 | 10493.45414 | 0.83989533 | 0 | 0 | 9.05538514 | 780 | 778 | 0.997435897 | 7.264226413 | 0 | 0 | 0 | AJ302074,DQ113772,HF563658,JX879087,KC413988,KT337358,KT337364,KT388093,KX550065,KY065455,KY065479,KY065487,KY065490,KY065491,KY065492,KY065493,KY065501,KY065502,KY349816,MK448671,MK448708,MK448714,MK448831,MK448834,MK448861,MK448874,MK448875,MK448886,MK448887,MK448897,MK448898,MK448905,MK448925,MK448927,MK449006,MK449009,MT900488,MW495853,OL774873,S9_vf_4115 |
| VC_426_0 | 3 | 184.3274769 | 4313.003969 | 0.04098597 | 0.001593 | 7.141428429 | 8.1240384 | 3 | 3 | 1 | 7.775908194 | 0 | 0 | 0 | KY065465,MK448733,MK448904 |
| VC_426_1 | 1 | 0 | 1171.260533 | 0 | 0 | 0 | 0 | 1 | 1 | 1 | 0 | 0 | 0 | 0 | MK448820 |
| VC_427_0 | 37 | 71368.79146 | 53215.60298 | 0.57285499 | 0 | 1.414213562 | 9.53939201 | 666 | 631 | 0.947447447 | 7.27462438 | 0 | 0 | 0 | FR671405,FR671406,FR671407,FR671410,FR671411,HG799490,HG799496,HG799497,KJ417497,KT337339,KT337340,KT337341,KT337352,KT337354,KT337355,KT337356,KT337359,KT337362,KY065450,KY065451,KY065452,KY065456,KY065472,KY065473,KY065474,KY065475,KY065476,KY065477,KY065478,KY065480,KY065486,KY065488,KY065496,KY065497,KY065500,KY065505,OK173051 |
| VC_428_0 | 1 | 0 | 2052.847789 | 0 | 0 | 0 | 0 | 1 | 1 | 1 | 0 | 0 | 0 | 0 | KY065449 |
| VC_429_0 | 14 | 8389.998617 | 24106.30979 | 0.25818313 | 1.78E-58 | 0 | 9 | 91 | 90 | 0.989010989 | 7.181532971 | 0 | 0 | 0 | MK448686,MK448762,MK448788,MK448791,MK448793,MK448862,MK448949,MK448951,MK448954,MK448958,MK448959,MK448960,MK448965,MK448977 |
| VC_43_0 | 16 | 22254.41953 | 88074.08341 | 0.20171052 | 1.96E-55 | 0 | 11 | 120 | 116 | 0.966666667 | 4.866937692 | 0 | 0 | 0 | AJ556162,JQ347801,LC727891,LC727892,LC727893,LC727894,LC727895,LC727896,LC727897,LC727898,LC727899,LC727900,LC727901,LC727902,ON470597,ON470625 |
| VC_43_1 | 10 | 7344.968052 | 55272.9526 | 0.11729818 | 2.72E-30 | 2.236067977 | 10 | 45 | 37 | 0.822222222 | 7.293530987 | 0 | 0 | 0 | AP012530,AP012538,AP012539,AP012540,FJ184280,FJ188381,FM180578,LC645408,LC645425,LC645426 |
| VC_430_0 | 2 | 19.63536058 | 72.05283716 | 0.21415363 | 0.1228694 | 7 | 7 | 1 | 1 | 1 | 7 | 0 | 0 | 0 | S3_vf_106401,S5_vf_28061 |
| VC_431_0 | 5 | 1160.329382 | 3439.645597 | 0.25224689 | 3.43E-08 | 1.414213562 | 6.78232998 | 10 | 10 | 1 | 5.776864618 | 0 | 0 | 0 | MK448680,MK448684,MK448698,MK448769,MK448895 |
| VC_433_0 | 5 | 1521.543883 | 4501.95136 | 0.25260149 | 2.50E-08 | 0 | 3 | 10 | 10 | 1 | 2.383623342 | 0 | 0 | 0 | MF034659,MF166859,MT902335,MZ995503,MZ995506 |
| VC_434_0 | 45 | 173561.7685 | 9535.054217 | 0.94792343 | 4.05E-274 | 0 | 4.69041576 | 990 | 990 | 1 | 2.82465251 | 0 | 0 | 0 | AJ505558,AM265638,AM910651,EU056923,EU236438,FN263372,JQ307386,JX501340,JX997978,KC969441,KM067278,KR054031,KU743887,KX711710,KY618819,LC727700,LN610574,LN610580,MH107770,MH979674,MN553585,MN553587,MN553588,MN553589,MN602045,MN615698,MN615699,MN692672,MN871455,MN871483,MN901924,MW117144,MW406975,MW406976,MZ553931,MZ687409,OK539824,OL754589,OL802210,ON857926,ON857928,ON857933,ON857935,OP292288,OP556577 |
| VC_435_0 | 7 | 1143.90233 | 121.3119826 | 0.90411744 | 0.0001857 | 0 | 5 | 21 | 21 | 1 | 3.309097027 | 0 | 0 | 0 | AJ536073,AY257527,AY701338,CP013282,EU408779,MZ089978,MZ089979 |
| VC_437_0 | 3 | 115.4682582 | 963.8774671 | 0.10697986 | 0.0019603 | 8 | 8.88819442 | 3 | 3 | 1 | 8.43811473 | 0 | 0 | 0 | CP013974,MG575421,MZ424863 |
| VC_439_0 | 3 | 92.09655327 | 14.98078915 | 0.86009375 | 0.0184442 | 6.08276253 | 7.34846923 | 3 | 3 | 1 | 6.762295453 | 0 | 0 | 0 | S12_vf_48278,S13_vs_98591,S8_vs_208114 |
| VC_44_0 | 49 | 175047.3863 | 213401.7986 | 0.45063136 | 0 | 0 | 9.64365076 | 1176 | 1012 | 0.860544218 | 6.787187114 | 0 | 0 | 0 | AP012534,HE664024,HG792102,HG792103,HG792104,HG792105,HG803182,HG803183,HG803184,HG803185,HQ424691,JQ011318,KF971864,KP682371,KP682374,KR781488,KU298437,KY914478,LC645409,LC645410,LC645411,LC645412,LC645413,LC645414,LC645415,LC645416,LC645417,LC645418,LC645419,LC645420,LC645421,LC645422,LC645423,LC645424,LC645427,LC645438,LC645442,LC645443,LC645444,LC645445,LC645446,LC645447,LC645448,LC645449,LC645450,LC645451,LC727903,MG710528,MN067333 |
| VC_440_0 | 33 | 124429.309 | 1000.686215 | 0.99202195 | 3.33E-84 | 0 | 6 | 528 | 528 | 1 | 3.166296407 | 0 | 0 | 0 | KM366099,KX826077,KY608966,MF740800,MH181876,MK568062,MK673511,MK770411,MK770413,MN379740,MT012730,MT074434,MT074435,MT074436,MT074437,MT074438,MT074439,MT074441,MT074442,MT074443,MT074444,MT074445,MT074446,MT663719,MT764207,MT768059,MW413353,MW508890,MW602648,MZ127825,OM313460,OM313461,OP132241 |
| VC_441_0 | 2 | 271.5373983 | 1405.900692 | 0.16187626 | 0.0542632 | 2.645751311 | 2.64575131 | 1 | 1 | 1 | 2.645751311 | 0 | 0 | 0 | KT588075,MZ514874 |
| VC_441_1 | 8 | 2979.801122 | 2494.753021 | 0.54430024 | 6.31E-13 | 2.828427125 | 9.38083152 | 28 | 27 | 0.964285714 | 7.501091613 | 0 | 0 | 0 | CAKLQA020000001,JX403940,KP861230,KT588073,MH115576,MT361972,MZ675741,ON391949 |
| VC_442_0 | 3 | 251.1528639 | 309.1034666 | 0.44828206 | 0.0022273 | 7.483314774 | 8.66025404 | 3 | 3 | 1 | 7.897801082 | 0 | 0 | 0 | S16_vs_116956,S16_vs_173079,S7_vs_48708 |
| VC_445_0 | 9 | 2679.31945 | 13330.1673 | 0.16735824 | 1.03E-21 | 0 | 8.71779789 | 36 | 36 | 1 | 6.974198472 | 0 | 0 | 0 | MK448692,MK448702,MK448711,MK448716,MK448724,MK448730,MK448744,MK448745,MK448923 |
| VC_446_0 | 2 | 300 | 8174.313823 | 0.0354011 | 0.0431804 | 0 | 0 | 1 | 1 | 1 | 0 | 0 | 0 | 0 | MN695334,MN695335 |
| VC_446_1 | 1 | 0 | 8444.425424 | 0 | 0 | 0 | 0 | 1 | 1 | 1 | 0 | 0 | 0 | 0 | MH412654 |
| VC_446_10 | 2 | 300 | 13892.91075 | 0.02113731 | 0.0422707 | 1.732050808 | 1.73205081 | 1 | 1 | 1 | 1.732050808 | 0 | 0 | 0 | GU071098,HQ317391 |
| VC_446_11 | 1 | 0 | 9974.219889 | 0 | 0 | 0 | 0 | 1 | 1 | 1 | 0 | 0 | 0 | 0 | HQ633071 |
| VC_446_12 | 1 | 0 | 7192.089038 | 0 | 0 | 0 | 0 | 1 | 1 | 1 | 0 | 0 | 0 | 0 | MW015081 |
| VC_446_13 | 1 | 0 | 8799.136686 | 0 | 0 | 0 | 0 | 1 | 1 | 1 | 0 | 0 | 0 | 0 | MK799832 |
| VC_446_14 | 1 | 0 | 8335.430008 | 0 | 0 | 0 | 0 | 1 | 1 | 1 | 0 | 0 | 0 | 0 | MN018232 |
| VC_446_15 | 3 | 900 | 21083.38687 | 0.04094001 | 0.0014432 | 1.414213562 | 2.64575131 | 3 | 3 | 1 | 2.098677617 | 0 | 0 | 0 | KU686204,KU686205,KU686206 |
| VC_446_16 | 1 | 0 | 7059.211077 | 0 | 0 | 0 | 0 | 1 | 1 | 1 | 0 | 0 | 0 | 0 | MH629685 |
| VC_446_2 | 1 | 0 | 10236.21164 | 0 | 0 | 0 | 0 | 1 | 1 | 1 | 0 | 0 | 0 | 0 | GU071095 |
| VC_446_3 | 1 | 0 | 9045.42156 | 0 | 0 | 0 | 0 | 1 | 1 | 1 | 0 | 0 | 0 | 0 | MF351863 |
| VC_446_4 | 1 | 0 | 8916.436835 | 0 | 0 | 0 | 0 | 1 | 1 | 1 | 0 | 0 | 0 | 0 | MT408532 |
| VC_446_5 | 1 | 0 | 10910.51199 | 0 | 0 | 0 | 0 | 1 | 1 | 1 | 0 | 0 | 0 | 0 | MK867354 |
| VC_446_6 | 1 | 0 | 5310.404254 | 0 | 0 | 0 | 0 | 1 | 1 | 1 | 0 | 0 | 0 | 0 | MH319749 |
| VC_446_7 | 1 | 0 | 7444.630478 | 0 | 0 | 0 | 0 | 1 | 1 | 1 | 0 | 0 | 0 | 0 | MH319754 |
| VC_446_8 | 1 | 0 | 7133.857162 | 0 | 0 | 0 | 0 | 1 | 1 | 1 | 0 | 0 | 0 | 0 | KM359505 |
| VC_446_9 | 3 | 900 | 23762.55119 | 0.03649258 | 0.0015183 | 2.236067977 | 4.12310563 | 3 | 3 | 1 | 3.366943663 | 0 | 0 | 0 | AY939844,GU071092,HQ632825 |
| VC_447_0 | 6 | 4500 | 53591.53852 | 0.07746395 | 2.45E-11 | 1.414213562 | 2.44948974 | 15 | 15 | 1 | 1.939724305 | 0 | 0 | 0 | HQ634177,KU686192,KU686193,KU686194,KU686195,KU686196 |
| VC_447_1 | 9 | 10800 | 74295.93743 | 0.12691558 | 1.53E-24 | 1 | 9.79795897 | 36 | 31 | 0.861111111 | 6.785481915 | 0 | 0 | 0 | KJ019054,KJ019069,KJ019082,KJ019089,KJ019094,KJ019156,KU686200,KU686201,KU686202 |
| VC_447_10 | 1 | 0 | 10550.32869 | 0 | 0 | 0 | 0 | 1 | 1 | 1 | 0 | 0 | 0 | 0 | KU686211 |
| VC_447_2 | 1 | 0 | 10358.98572 | 0 | 0 | 0 | 0 | 1 | 1 | 1 | 0 | 0 | 0 | 0 | KF156338 |
| VC_447_3 | 3 | 900 | 30315.24785 | 0.02883206 | 0.0018719 | 1.414213562 | 2.64575131 | 3 | 3 | 1 | 2.098677617 | 0 | 0 | 0 | KU686197,KU686198,KU686199 |
| VC_447_4 | 9 | 10800 | 74269.33664 | 0.12695526 | 9.71E-24 | 0 | 6.78232998 | 36 | 36 | 1 | 4.631535894 | 0 | 0 | 0 | GU071105,KU594607,KX349291,KX349292,KX349293,KX349294,KX349295,KX349296,KX349297 |
| VC_447_5 | 2 | 300 | 17178.89297 | 0.01716356 | 0.0635784 | 4.69041576 | 4.69041576 | 1 | 1 | 1 | 4.69041576 | 0 | 0 | 0 | AJ630128,LN828717 |
| VC_447_6 | 1 | 0 | 11354.88836 | 0 | 0 | 0 | 0 | 1 | 1 | 1 | 0 | 0 | 0 | 0 | KU594606 |
| VC_447_7 | 1 | 0 | 10978.44488 | 0 | 0 | 0 | 0 | 1 | 1 | 1 | 0 | 0 | 0 | 0 | MW147367 |
| VC_447_8 | 1 | 0 | 10441.58882 | 0 | 0 | 0 | 0 | 1 | 1 | 1 | 0 | 0 | 0 | 0 | FM207411 |
| VC_447_9 | 1 | 0 | 10906.14064 | 0 | 0 | 0 | 0 | 1 | 1 | 1 | 0 | 0 | 0 | 0 | MW117966 |
| VC_449_0 | 2 | 300 | 6958.053018 | 0.0413334 | 0.0432801 | 7.348469228 | 7.34846923 | 1 | 1 | 1 | 7.348469228 | 0 | 0 | 0 | MT162466,MT162467 |
| VC_449_1 | 1 | 0 | 3899.411129 | 0 | 0 | 0 | 0 | 1 | 1 | 1 | 0 | 0 | 0 | 0 | MK016664 |
| VC_45_0 | 38 | 83305.49623 | 60695.9294 | 0.57850466 | 0 | 0 | 9.79795897 | 703 | 679 | 0.965860597 | 6.64068718 | 0 | 0 | 0 | EU078592,JQ086376,JQ182735,KT232076,LAMCG,LC730321,LR595850,LR595859,LR595860,LR595861,LR595863,LR595864,LR595865,LR595866,LR597635,LR597636,LR597639,LR597643,LR597644,LR597648,LR597650,LR597651,LR597652,LR597653,MF374379,MH547045,OL657226,OL657227,OL657228,OM418625,OM418626,OM418627,OM475427,OM475428,OM475430,OM475432,OM475435,OV696612 |
| VC_450_0 | 4 | 1800 | 3774.475973 | 0.32290031 | 1.28E-05 | 0 | 2.82842712 | 6 | 6 | 1 | 1.794110782 | 0 | 0 | 0 | MZ577095,MZ577096,MZ577097,MZ577098 |
| VC_451_0 | 4 | 1800 | 1456.160563 | 0.55279829 | 1.83E-05 | 2 | 8.18535277 | 6 | 6 | 1 | 6.341155883 | 0 | 0 | 0 | KF381361,KR052480,KR052481,KR052482 |
| VC_452_0 | 6 | 520.4592804 | 5205.600524 | 0.09089309 | 5.87E-05 | 4.123105626 | 8.88819442 | 15 | 15 | 1 | 7.117642466 | 0 | 0 | 0 | MH319720,MH319733,MH319742,MH319753,MH319758,MH327487 |
| VC_453_0 | 2 | 29.07868857 | 978.8124229 | 0.02885102 | 0.0936118 | 8.544003745 | 8.54400375 | 1 | 1 | 1 | 8.544003745 | 0 | 0 | 0 | MH319750,MH319759 |
| VC_454_0 | 4 | 183.5113307 | 0 | 1 | 0 | 1.732050808 | 3.87298335 | 6 | 6 | 1 | 2.974977029 | 0 | 0 | 0 | AJ635161,AY722806,MN876845,MT047590 |
| VC_456_0 | 4 | 1800 | 19834.45141 | 0.08320063 | 5.77E-05 | 1.732050808 | 3 | 6 | 6 | 1 | 2.461117863 | 0 | 0 | 0 | MK838116,MN102098,MN131137,MN159079 |
| VC_456_1 | 3 | 900 | 16074.73829 | 0.05301996 | 0.0058045 | 2.645751311 | 5.19615242 | 3 | 3 | 1 | 4.313641082 | 0 | 0 | 0 | MN642089,ON287370,ON287375 |
| VC_456_2 | 4 | 1800 | 19805.63041 | 0.08331162 | 0.0001187 | 7.416198487 | 9.48683298 | 6 | 5 | 0.833333333 | 8.568747127 | 0 | 0 | 0 | JN627160,MK689364,MZ348422,MZ501267 |
| VC_456_3 | 3 | 900 | 16315.73949 | 0.05227774 | 0.0032482 | 3 | 5.65685425 | 3 | 3 | 1 | 4.617668891 | 0 | 0 | 0 | MN032614,MN453779,OL964756 |
| VC_456_4 | 2 | 300 | 11621.92382 | 0.02516372 | 0.0943647 | 6.480740698 | 6.4807407 | 1 | 1 | 1 | 6.480740698 | 0 | 0 | 0 | MK774614,OM810292 |
| VC_457_0 | 7 | 6300 | 18418.53262 | 0.2548695 | 5.10E-14 | 2.645751311 | 10.198039 | 21 | 15 | 0.714285714 | 7.47035602 | 0 | 0 | 0 | AY266303,GU396103,HM452126,MG250483,MH791401,MH791407,MN871440 |
| VC_457_1 | 1 | 0 | 4834.182146 | 0 | 0 | 0 | 0 | 1 | 1 | 1 | 0 | 0 | 0 | 0 | MT161459 |
| VC_457_2 | 2 | 300 | 10108.82022 | 0.02882171 | 0.1229216 | 5.385164807 | 5.38516481 | 1 | 1 | 1 | 5.385164807 | 0 | 0 | 0 | MH791414,MW272540 |
| VC_457_3 | 2 | 300 | 10200.0456 | 0.0285713 | 0.1065262 | 3.605551275 | 3.60555128 | 1 | 1 | 1 | 3.605551275 | 0 | 0 | 0 | GU459069,KY290955 |
| VC_457_4 | 10 | 13500 | 27207.56178 | 0.33163372 | 3.76E-23 | 5 | 9.94987437 | 45 | 42 | 0.933333333 | 7.360303405 | 0 | 0 | 0 | JX123262,MF448340,MF498773,MF498775,MH791398,MH791404,MN871441,MN871442,MT713136,OM339718 |
| VC_458_0 | 2 | 191.0754653 | 44.11143276 | 0.81244094 | 0.2701457 | 1.732050808 | 1.73205081 | 1 | 1 | 1 | 1.732050808 | 0 | 0 | 0 | AJ783769,JQ287645 |
| VC_459_0 | 2 | 300 | 460.3268866 | 0.39456713 | 0.0773645 | 2.236067977 | 2.23606798 | 1 | 1 | 1 | 2.236067977 | 0 | 0 | 0 | MT732443,MT732444 |
| VC_459_1 | 2 | 95.77647854 | 651.827689 | 0.12811122 | 0.0677329 | 8.774964387 | 8.77496439 | 1 | 1 | 1 | 8.774964387 | 0 | 0 | 0 | KC821607,KC821618 |
| VC_460_0 | 4 | 699.0226741 | 230.66755 | 0.75188773 | 0.0001223 | 4.472135955 | 6 | 6 | 6 | 1 | 5.165250435 | 0 | 0 | 0 | HG322870,KP238129,KP282677,KP282678 |
| VC_461_0 | 3 | 590.5548578 | 125.494149 | 0.82474084 | 0.0049007 | 0 | 3.16227766 | 3 | 3 | 1 | 2.108185107 | 0 | 0 | 0 | AJ888457,KX607101,KX607102 |
| VC_462_0 | 4 | 1800 | 4138.549846 | 0.3031043 | 0.0002115 | 0 | 6.32455532 | 6 | 6 | 1 | 4.094198404 | 0 | 0 | 0 | KU878088,LC680884,MF360957,MW749003 |
| VC_462_1 | 1 | 0 | 1921.329896 | 0 | 0 | 0 | 0 | 1 | 1 | 1 | 0 | 0 | 0 | 0 | KT895374 |
| VC_463_0 | 7 | 6300 | 4250.614184 | 0.59712164 | 1.21E-11 | 3 | 8.88819442 | 21 | 21 | 1 | 7.026236964 | 0 | 0 | 0 | LC680885,MN091626,MW218148,MW248466,MW349128,MW349129,MZ779063 |
| VC_464_0 | 16 | 36000 | 1663.806594 | 0.95582479 | 1.75E-34 | 0 | 2.23606798 | 120 | 120 | 1 | 0.385698076 | 0 | 0 | 0 | AM076770,MN617773,MT366931,MT366932,MT366933,MT366934,MT366935,MT366936,MT366937,MT366938,MT366939,MT366940,MT366941,MT366942,MT366943,MT366944 |
| VC_465_0 | 80 | 339070.2464 | 403.5509498 | 0.99881125 | 2.64E-127 | 0 | 7.41619849 | 3160 | 3160 | 1 | 5.572048164 | 0 | 0 | 0 | AM084414,DQ111067,EU734172,HG813241,HG818823,HG818824,KC109329,KJ748011,KT990215,KU687349,KU687350,KU687351,KX669227,KX689784,KY295894,KY962008,LT594300,MF630921,MG845865,MH400309,MH464253,MH491167,MK033136,MK301532,MK610268,MK903277,MK903279,MK903281,MN067430,MN218775,MN414250,MN510331,MN518893,MN518894,MN715150,MN812686,MT350292,MT446393,MT446394,MT496969,MT682707,MT808983,MT862763,MW258709,MW345254,MW416012,MW748991,MW876471,MZ223858,MZ234009,MZ234011,MZ234021,MZ234022,MZ234023,MZ234024,MZ234044,MZ234045,MZ234046,MZ234047,MZ311861,MZ311862,MZ398247,MZ648215,MZ726794,MZ923531,OK210074,OL362275,OL362276,OL539729,OL773676,OL773677,OM256482,OM355495,OM457002,OM867676,OM908436,OM937123,ON528733,ON548433,ON637250 |
| VC_466_0 | 66 | 231264.0663 | 18871.76783 | 0.92455392 | 1.85E-253 | 0 | 8.24621125 | 2145 | 2145 | 1 | 5.917967067 | 0 | 0 | 0 | AM084415,AY288927,AY370673,AY370674,GU595417,JN986844,JQ837901,KM366097,KM370384,KM819695,KM819696,KP890822,KU946962,KX279892,KY295891,KY295892,KY295893,KY295897,KY295899,KY435490,KY744566,LC730323,MG004687,MG251390,MG775042,MH729818,MK373771,MK373773,MK380014,MN384978,MT496971,MT680615,MW394389,MZ031013,MZ234012,MZ234019,MZ234020,MZ234032,MZ234049,MZ234050,MZ398244,MZ573924,MZ747518,MZ997839,MZ997840,MZ997841,OK625527,OL362272,OL362273,OL744215,OL744218,ON148527,ON185580,ON210144,ON210145,ON461912,ON526839,ON602763,ON602764,ON602765,OP018999,OP038547,OP079918,OP413828,OP413830,OX335382 |
| VC_467_0 | 6 | 2405.587015 | 18769.9686 | 0.11360207 | 2.24E-11 | 0 | 4 | 15 | 15 | 1 | 2.657579136 | 0 | 0 | 0 | EF160123,FQ482084,FQ482086,MG736918,OK570184,OM486945 |
| VC_469_0 | 18 | 28023.86613 | 33124.15146 | 0.45829558 | 6.73E-92 | 0 | 7.54983444 | 153 | 153 | 1 | 5.464752943 | 0 | 0 | 0 | DQ121662,JF770475,JQ182736,KC333879,KC579452,KF771236,KF771237,KF771238,KF771239,KJ668713,KT825490,MF158039,MF158042,MW286156,OL539447,OL539451,OL539453,OL539467 |
| VC_47_0 | 12 | 5535.177707 | 9966.507234 | 0.35706942 | 1.07E-39 | 0 | 8.77496439 | 66 | 66 | 1 | 6.994701136 | 0 | 0 | 0 | AJ298298,AY055382,KC736978,KC814930,KU927493,MK733259,MK733260,MK733261,MZ501270,ON470584,ON470603,SFU82619 |
| VC_470_0 | 62 | 365605.0307 | 39724.57182 | 0.9019944 | 0 | 0 | 8 | 1891 | 1891 | 1 | 3.83484609 | 0 | 0 | 0 | AM156909,JN986845,KX130960,KY398841,MG050172,MG065688,MK372342,MK373793,MK907226,MK907227,MK907228,MK907229,MK907230,MK907231,MK907232,MK907233,MK907234,MK907235,MK907240,MK907241,MK907242,MK907243,MK907244,MK907245,MK907246,MK907247,MK907248,MK907249,MK907250,MK907251,MK907252,MK907253,MK907254,MK907255,MK907256,MK907257,MK907258,MK907259,MK907260,MK907261,MK907262,MK907263,MK907264,MK907265,MK907266,MK907272,MK907273,MK907274,MK907275,MK907276,MN164484,MN840485,MT511058,MT682706,MW057918,MZ501051,MZ501080,MZ501087,OM258170,ON081052,OP087522,OP251154 |
| VC_472_0 | 5 | 1664.037575 | 7053.15695 | 0.19089141 | 3.03E-08 | 0 | 3.31662479 | 10 | 10 | 1 | 2.520266907 | 0 | 0 | 0 | MH059632,MH807810,MH807813,MH807815,MH807817 |
| VC_473_0 | 3 | 233.0540112 | 40.64762977 | 0.85148927 | 0.0094518 | 4.123105626 | 5.56776436 | 3 | 3 | 1 | 5.056031854 | 0 | 0 | 0 | KC292023,MG550112,MZ334526 |
| VC_474_0 | 19 | 26872.8709 | 45190.48872 | 0.37290616 | 2.87E-105 | 0 | 8.54400375 | 171 | 171 | 1 | 7.153407194 | 0 | 0 | 0 | JF974294,JF974312,MK327140,MT682064,MW119258,MW722082,MZ318367,MZ440881,MZ836210,OL362277,OL617041,ON602730,ON602737,ON602738,ON602744,ON602759,ON602760,OP373729,OP481875 |
| VC_475_0 | 96 | 669729.599 | 50164.43318 | 0.93031692 | 0 | 0 | 9.32737905 | 4560 | 4366 | 0.95745614 | 5.931787047 | 0 | 0 | 0 | AM491472,KX017520,KX523699,KY860935,MF158038,MH023293,MH321493,MH424446,MH717097,MH791402,MH791410,MH791412,MK112901,MK599416,MK759854,MK947460,MN692673,MN737551,MN871490,MN871503,MT768060,MW427215,MW477799,MZ375232,MZ375295,MZ375296,MZ375297,MZ375298,MZ375299,MZ375300,MZ375301,MZ375302,MZ375303,MZ375304,MZ375305,MZ375306,MZ375307,MZ375308,MZ375309,MZ375310,MZ375311,MZ375312,MZ375313,MZ375314,MZ375315,MZ375316,MZ375317,MZ375318,MZ375319,MZ375320,MZ375321,MZ375322,MZ375323,MZ375324,MZ375325,MZ375326,MZ375327,MZ375328,MZ375329,MZ375330,MZ375331,MZ375332,MZ375333,MZ375334,MZ375335,MZ375336,MZ375337,MZ375338,MZ375339,MZ375340,MZ375341,MZ375342,MZ375343,MZ375344,MZ375345,MZ375346,MZ375347,MZ375348,MZ375349,MZ375350,MZ375351,MZ375352,MZ375353,MZ375354,MZ375355,MZ489634,MZ726796,OL362274,OL964734,OL964742,OL964743,OL964744,OL964751,OL964752,ON239132,ON637251 |
| VC_477_0 | 4 | 789.566061 | 219.4795113 | 0.78248801 | 2.71E-05 | 1 | 2.82842712 | 6 | 6 | 1 | 2.232634022 | 0 | 0 | 0 | AM749121,DQ386162,KR153145,MT430910 |
| VC_478_0 | 8 | 5886.392394 | 16592.05024 | 0.26186834 | 1.58E-19 | 1.732050808 | 8.24621125 | 28 | 28 | 1 | 5.438312341 | 0 | 0 | 0 | JQ067087,KU356689,LN610577,LN610584,LN610585,MT108725,MT108731,MZ926748 |
| VC_479_0 | 7 | 5059.614315 | 10302.0933 | 0.32936536 | 2.27E-15 | 2.236067977 | 4.12310563 | 21 | 21 | 1 | 3.173498152 | 0 | 0 | 0 | JQ067084,KX129925,KX898399,KX898400,MN504636,MW835180,ON911540 |
| VC_48_0 | 2 | 204.5078301 | 11165.82279 | 0.01798609 | 0.0423614 | 5.477225575 | 5.47722558 | 1 | 1 | 1 | 5.477225575 | 0 | 0 | 0 | KF766125,KJ603229 |

| | | | | | | | | | | | | | | | | |
|---|---|---|---|---|---|---|---|---|---|---|---|---|---|---|---|---|
| VC_480_0 | 3 | 84.65335182 | 188.8149475 | 0.30955453 | 0.0025003 | 4.898979486 | 7.81024968 | 3 | 3 | | 1 | 6.31807572 | 0 | 0 | 0 | MW091529,MW202777,MW462191 |
| VC_481_0 | 5 | 3000 | 1422.566272 | 0.67833918 | 3.77E-08 | 4 | 6 | 10 | 10 | | 1 | 4.996699561 | 0 | 0 | 0 | MN862068,ON529856,ON529859,ON529862,ON529864 |
| VC_482_0 | 4 | 1059.485372 | 6382.625513 | 0.14236356 | 1.52E-05 | 2.449489743 | 6.55743852 | 6 | 6 | | 1 | 5.085990191 | 0 | 0 | 0 | MN988465,MN988541,MN988554,ON014755 |
| VC_483_0 | 2 | 214.4777622 | 1242.929967 | 0.14716387 | 0.0434186 | 1.732050808 | 1.73205081 | 1 | 1 | | 1 | 1.732050808 | 0 | 0 | 0 | MT536174,OL960029 |
| VC_484_0 | 3 | 289.5763709 | 243.0931 | 0.54363238 | 0.001761 | 5.477225575 | 7 | 3 | 3 | | 1 | 6.395143169 | 0 | 0 | 0 | MN698239,MN698241,MW574966 |
| VC_485_0 | 2 | 232.0019479 | 2682.562189 | 0.07960091 | 0.0433428 | 3.464101615 | 3.46410162 | 1 | 1 | | 1 | 3.464101615 | 0 | 0 | 0 | MW677525,OP009285 |
| VC_486_0 | 47 | 214384.627 | 23231.50389 | 0.90223095 | 5.66E-148 | 0 | 7.48331477 | 1081 | 1081 | | 1 | 4.439782171 | 0 | 0 | 0 | AM910650,AY078382,HE983844,HF543949,HG518155,JN254801,JX863101,KC294142,KF856712,KP233880,KR054029,LC648443,LN610578,MF768469,MG432151,MG589385,MG589386,MG845684,MH973725,MK837011,MK837012,MN508619,MN553586,MN562749,MN871476,MN871484,MT108723,MT108724,MT118288,MT119362,MT119371,MT119373,MW329986,MW512831,MW526260,MZ089732,MZ089733,MZ089738,MZ089739,MZ089740,MZ089742,OL441337,ON156559,ON376263,ON857927,ON857936,OP342787 |
| VC_487_0 | 1 | 0 | 1083.368301 | 0 | 0 | 0 | 0 | 1 | 1 | | 1 | 0 | 0 | 0 | 0 | CP067352 |
| VC_487_1 | 1 | 0 | 1208.671975 | 0 | 0 | 0 | 0 | 1 | 1 | | 1 | 0 | 0 | 0 | 0 | CP069348 |
| VC_487_2 | 2 | 300 | 2629.095659 | 0.10242069 | 0.0544882 | 0 | 0 | 1 | 1 | | 1 | 0 | 0 | 0 | 0 | CP011970,LN681537 |
| VC_487_3 | 2 | 300 | 2500.244485 | 0.1071335 | 0.0593376 | 5.656854249 | 5.65685425 | 1 | 1 | | 1 | 5.656854249 | 0 | 0 | 0 | MF547662,MF547663 |
| VC_487_4 | 1 | 0 | 1440.240635 | 0 | 0 | 0 | 0 | 1 | 1 | | 1 | 0 | 0 | 0 | 0 | CP067347 |
| VC_488_0 | 14 | 27300 | 4758.192073 | 0.85157641 | 2.15E-49 | 0 | 8.71779789 | 91 | 91 | | 1 | 5.938817616 | 0 | 0 | 0 | HM029250,KF926093,KM677185,KY554768,KY554769,KY554770,KY554771,KY554772,KY554773,KY554774,KY554775,KY554776,KY554777,MN552145 |
| VC_489_0 | 3 | 900 | 10615.79543 | 0.07815352 | 0.0019868 | 5 | 7.48331477 | 3 | 3 | | 1 | 6.587808221 | 0 | 0 | 0 | OM236516,OM634653,ON133270 |
| VC_489_1 | 3 | 900 | 14574.87281 | 0.0581588 | 0.0111698 | 6.480740698 | 7.74596669 | 3 | 3 | | 1 | 7.003012458 | 0 | 0 | 0 | OK499972,OK499987,OK499994 |
| VC_489_10 | 2 | 300 | 10220.09991 | 0.02851684 | 0.125647 | 8.602325267 | 8.60232527 | 1 | 1 | | 1 | 8.602325267 | 0 | 0 | 0 | OK499992,ON548418 |
| VC_489_2 | 3 | 900 | 14498.76514 | 0.05844624 | 0.0130225 | 7.874007874 | 8.30662386 | 3 | 3 | | 1 | 8.039295223 | 0 | 0 | 0 | KC481682,MT254578,ON548420 |
| VC_489_3 | 1 | 0 | 4252.072427 | 0 | 0 | 0 | 0 | 1 | 1 | | 1 | 0 | 0 | 0 | 0 | ON881243 |
| VC_489_4 | 1 | 0 | 4333.571402 | 0 | 0 | 0 | 0 | 1 | 1 | | 1 | 0 | 0 | 0 | 0 | MH884508 |
| VC_489_5 | 2 | 300 | 9880.630807 | 0.02946772 | 0.061969 | 7.745966692 | 7.74596669 | 1 | 1 | | 1 | 7.745966692 | 0 | 0 | 0 | ON548417,ON548419 |
| VC_489_6 | 1 | 0 | 5272.646328 | 0 | 0 | 0 | 0 | 1 | 1 | | 1 | 0 | 0 | 0 | 0 | KT070867 |
| VC_489_7 | 1 | 0 | 5330.693594 | 0 | 0 | 0 | 0 | 1 | 1 | | 1 | 0 | 0 | 0 | 0 | MW084976 |
| VC_489_8 | 2 | 300 | 10012.50146 | 0.0290909 | 0.0991199 | 8.94427191 | 8.94427191 | 1 | 1 | | 1 | 8.94427191 | 0 | 0 | 0 | MK288021,OK499991 |
| VC_489_9 | 1 | 0 | 5348.410882 | 0 | 0 | 0 | 0 | 1 | 1 | | 1 | 0 | 0 | 0 | 0 | ON548421 |
| VC_49_0 | 48 | 335673.2083 | 97576.08485 | 0.77478074 | 0 | 0 | 5.65685425 | 1128 | 1128 | | 1 | 1.061405149 | 0 | 0 | 0 | LC616031,LC616032,LC616033,LC616034,LC616035,LC616036,LC616037,LC616038,LC616039,LC616040,LC616041,LC616042,LC616043,LC616044,LC616045,LC616046,LC616047,LC616048,LC616049,LC616050,LC616051,LC616052,LC616053,LC616054,LC616055,LC616056,LC616057,LC616058,LC616059,LC616060,LC616061,LC616062,LC616063,LC616064,LC616065,LC616066,LC616067,LC616068,LC616069,LC616070,LC616071,LC616072,LC616073,LC616074,LC616075,LC616076,LC616077,LC616078 |
| VC_490_0 | 1 | 0 | 52.49307917 | 0 | 0 | 0 | 0 | 1 | 1 | | 1 | 0 | 0 | 0 | 0 | S11_vf_3790 |
| VC_490_1 | 1 | 0 | 84.98969025 | 0 | 0 | 0 | 0 | 1 | 1 | | 1 | 0 | 0 | 0 | 0 | S8_vf_173225 |
| VC_490_2 | 1 | 0 | 95.55272641 | 0 | 0 | 0 | 0 | 1 | 1 | | 1 | 0 | 0 | 0 | 0 | S4_vf_31973 |
| VC_490_3 | 1 | 0 | 70.73016617 | 0 | 0 | 0 | 0 | 1 | 1 | | 1 | 0 | 0 | 0 | 0 | S12_vf_188819 |
| VC_491_0 | 2 | 300 | 1865.024673 | 0.13856655 | 0.0554641 | 8.366600265 | 8.36660027 | 1 | 1 | | 1 | 8.366600265 | 0 | 0 | 0 | S12_vf_169796,S8_vf_208110 |
| VC_491_1 | 1 | 0 | 939.5761905 | 0 | 0 | 0 | 0 | 1 | 1 | | 1 | 0 | 0 | 0 | 0 | S13_vf_4901 |
| VC_491_2 | 1 | 0 | 1127.606307 | 0 | 0 | 0 | 0 | 1 | 1 | | 1 | 0 | 0 | 0 | 0 | S8_vf_11794 |
| VC_491_3 | 1 | 0 | 825.1361986 | 0 | 0 | 0 | 0 | 1 | 1 | | 1 | 0 | 0 | 0 | 0 | S10_vf_120738 |
| VC_492_0 | 20 | 19795.61393 | 5303.12675 | 0.78870945 | 1.30E-94 | 0 | 8.18535277 | 190 | 190 | | 1 | 5.613356449 | 0 | 0 | 0 | AY150271,DQ126339,JN391180,JQ011317,JQ691610,KC911856,KC911857,KP869107,KP869108,KX231829,MK416010,MK422448,MN187550,MT261384,MT580116,MT580117,MW205203,MW722083,OK490417,S5_vf_74661 |
| VC_493_0 | 4 | 1800 | 23.4635754 | 0.98713241 | 0.0025103 | 0 | 3.74165739 | 6 | 6 | | 1 | 2.641065979 | 0 | 0 | 0 | AP011616,AP011617,DQ997624,MK257744 |
| VC_494_0 | 2 | 300 | 567.1774106 | 0.34594997 | 0.0527127 | 5.567764363 | 5.56776436 | 1 | 1 | | 1 | 5.567764363 | 0 | 0 | 0 | MG592415,OX241562 |
| VC_494_1 | 1 | 0 | 358.8078541 | 0 | 0 | 0 | 0 | 1 | 1 | | 1 | 0 | 0 | 0 | 0 | MW073017 |
| VC_495_0 | 17 | 20009.28534 | 1991.388214 | 0.90948513 | 1.98E-55 | 0 | 2 | 136 | 136 | | 1 | 1.137372402 | 0 | 0 | 0 | OM416763,OM416764,OM416765,OM416766,OM416767,OM416768,OM416769,OM416770,OM416771,OM416772,OM416773,OM416774,OM416775,OM416776,OM416777,OM416778,OM416779 |
| VC_496_0 | 22 | 22073.13163 | 14105.14672 | 0.61012112 | 2.72E-120 | 0 | 7.93725393 | 231 | 231 | | 1 | 6.081473424 | 0 | 0 | 0 | AP013540,AP013541,AP013542,AP013545,KC465901,MH579717,MH598798,MH598800,MH598801,MH598802,MH598803,MH598804,MH598805,MH598806,MH598807,MT375520,MT375521,MT375526,MT375527,MT375528,MT375530,MT375531 |
| VC_497_0 | 4 | 458.7096697 | 4310.326253 | 0.09618499 | 0.000475 | 2.828427125 | 6.92820323 | 6 | 6 | | 1 | 5.087307028 | 0 | 0 | 0 | JX483873,KT997847,MW023914,MW980070 |
| VC_498_0 | 4 | 292.0963479 | 712.7778659 | 0.29067951 | 1.56E-05 | 4 | 5.38516481 | 6 | 6 | | 1 | 4.941346208 | 0 | 0 | 0 | AP013546,AP013547,AP013548,MW822601 |
| VC_5_0 | 27 | 20972.99216 | 875.7983245 | 0.95991548 | 3.95E-88 | 0 | 7.14142843 | 351 | 351 | | 1 | 5.691127293 | 0 | 0 | 0 | AB374228,AF125163,AY027935,BHU24159,DQ114220,DQ674738,HG803181,HQ110083,KF361475,KT887559,KU504502,KX058879,KX774374,KY045851,LR597637,LR597638,LR597640,LR597649,MG592573,MK972712,MK972713,MK972714,MN563793,MT662115,OM095401,OP484843,S11_vs_5077 |
| VC_50_0 | 10 | 12388.40846 | 59438.06838 | 0.17247691 | 6.24E-31 | 0 | 2.82842712 | 45 | 45 | | 1 | 1.87260851 | 0 | 0 | 0 | LC645428,LC645429,LC645430,LC645431,LC645432,LC645433,LC645434,LC645435,LC645436,LC645437 |
| VC_500_0 | 3 | 533.3699765 | 1163.579739 | 0.31431101 | 0.0015636 | 1.414213562 | 1.73205081 | 3 | 3 | | 1 | 1.626105059 | 0 | 0 | 0 | KJ183191,KJ183192,KJ183193 |
| VC_501_0 | 3 | 413.275649 | 1344.622359 | 0.23509649 | 0.0015564 | 2 | 8.24621125 | 3 | 3 | | 1 | 6.164140834 | 0 | 0 | 0 | MN698242,MN698243,MN698247 |
| VC_502_0 | 18 | 19413.5654 | 2626.302631 | 0.88083855 | 1.19E-72 | 0 | 8 | 153 | 153 | | 1 | 5.592536978 | 0 | 0 | 0 | KC465898,MT375525,MT647605,MW273920,MW273922,MW273923,MW273924,MW273925,MW273926,OP131293,OP131294,OP131295,OP131296,OP131297,OP131299,OP131300,OP131301,OP131302 |
| VC_503_0 | 2 | 82.10374986 | 240.4246458 | 0.25456286 | 0.0510242 | 5.099019514 | 5.09901951 | 1 | 1 | | 1 | 5.099019514 | 0 | 0 | 0 | MT375519,MT375529 |
| VC_504_0 | 4 | 1800 | 5247.483973 | 0.2554103 | 1.25E-05 | 1.732050808 | 6.92820323 | 6 | 6 | | 1 | 5.173031417 | 0 | 0 | 0 | JQ245707,OP053360,OP053361,OP053362 |
| VC_506_0 | 8 | 8400 | 20561.67836 | 0.29003844 | 2.69E-18 | 0 | 7.34846923 | 28 | 28 | | 1 | 5.287198181 | 0 | 0 | 0 | MN871446,MN871447,MN871448,MN871449,MN871451,MN871452,MN871453,MW394391 |
| VC_506_1 | 8 | 8400 | 20636.89231 | 0.28928716 | 3.62E-18 | 2 | 6.92820323 | 28 | 28 | | 1 | 4.525775496 | 0 | 0 | 0 | MF285618,MK994515,MW021758,OK040170,OL539468,ON287372,ON287376,ON375847 |
| VC_506_2 | 2 | 254.9794341 | 7610.718829 | 0.03241663 | 0.0448892 | 8.717797887 | 8.71779789 | 1 | 1 | | 1 | 8.717797887 | 0 | 0 | 0 | KF623294,MN334766 |
| VC_507_0 | 3 | 900 | 12418.8948 | 0.06757317 | 0.0174084 | 0 | 8.94427191 | 3 | 3 | | 1 | 5.96284794 | 0 | 0 | 0 | MN445183,MW845760,MW879340 |
| VC_507_1 | 11 | 16500 | 19278.06023 | 0.46117648 | 1.66E-24 | 1.732050808 | 6.70820393 | 55 | 55 | | 1 | 3.302973778 | 0 | 0 | 0 | KF806589,KU886222,KU886223,KU886224,KU886225,MG655267,MG655268,MG655269,MG655270,MK514281,MZ501268 |
| VC_507_2 | 1 | 0 | 4756.059191 | 0 | 0 | 0 | 0 | 1 | 1 | | 1 | 0 | 0 | 0 | 0 | MT661596 |
| VC_508_0 | 2 | 300 | 29634.81181 | 0.01002178 | 0.0465514 | 4.123105626 | 4.12310563 | 1 | 1 | | 1 | 4.123105626 | 0 | 0 | 0 | AP014714,AP014715 |
| VC_508_1 | 5 | 3000 | 65320.89984 | 0.04391043 | 3.83E-08 | 5.567764363 | 8.42614977 | 10 | 10 | | 1 | 7.704845158 | 0 | 0 | 0 | MN087708,MN882610,MZ348423,OL989991,ON630910 |
| VC_508_2 | 1 | 0 | 14848.28081 | 0 | 0 | 0 | 0 | 1 | 1 | | 1 | 0 | 0 | 0 | 0 | KF835987 |
| VC_508_3 | 2 | 300 | 22427.0339 | 0.01320014 | 0.0429685 | 7.937253933 | 7.93725393 | 1 | 1 | | 1 | 7.937253933 | 0 | 0 | 0 | HE956709,KY593455 |
| VC_508_4 | 1 | 0 | 10764.28114 | 0 | 0 | 0 | 0 | 1 | 1 | | 1 | 0 | 0 | 0 | 0 | KP202158 |
| VC_508_5 | 1 | 0 | 10851.41235 | 0 | 0 | 0 | 0 | 1 | 1 | | 1 | 0 | 0 | 0 | 0 | MH059636 |
| VC_508_6 | 1 | 0 | 11101.33177 | 0 | 0 | 0 | 0 | 1 | 1 | | 1 | 0 | 0 | 0 | 0 | MT920315 |
| VC_509_0 | 36 | 189000 | 168179.5192 | 0.52914568 | 0 | 2.236067977 | 11.1803399 | 630 | 612 | 0.971428571 | 1 | 5.983972297 | 0 | 0 | 0 | CP062992,KR269720,KT239446,KX258185,KY000080,LR877331,MG751100,MH333064,MH729874,MK421971,MN101226,MN101229,MN101230,MN434095,MT580897,MT701588,MW021752,MW239157,MZ560702,MZ602148,MZ890186,OK499980,OK499981,OK499996,OK499997,OK631811,OL539458,OL870318,OL870319,OM337876,OM337877,ON630219,ON550260,OP331213,OP617748,OU509536 |
| VC_509_1 | 1 | 0 | 13612.10127 | 0 | 0 | 0 | 0 | 1 | 1 | | 1 | 0 | 0 | 0 | 0 | MN101225 |
| VC_51_0 | 36 | 136425.6073 | 87730.27682 | 0.60861934 | 0 | 0 | 6.244998 | 630 | 630 | | 1 | 3.370415202 | 0 | 0 | 0 | LC727840,LC727841,LC727842,LC727843,LC727844,LC727845,LC727846,LC727847,LC727848,LC727849,LC727850,LC727851,LC727852,LC727853,LC727854,LC727855,LC727856,LC727857,LC727858,LC727859,LC727860,LC727861,LC727862,LC727863,LC727864,LC727865,LC727866,LC727867,LC727868,LC727869,LC727870,LC727871,LC727872,LC727873,LC727874,LC727875 |
| VC_510_0 | 2 | 300 | 18727.41469 | 0.01576672 | 0.3293445 | 3.16227766 | 3.16227766 | 1 | 1 | | 1 | 3.16227766 | 0 | 0 | 0 | HQ698922,MK240351 |
| VC_510_1 | 20 | 57000 | 79624.49477 | 0.41720191 | 3.80E-17 | 0 | 5.09901951 | 190 | 190 | | 1 | 4.047077882 | 0 | 0 | 0 | CAKLQC020000001,CAKLQD020000001,MH165274,MH460829,MH713599,MN648195,MN662249,MN709128,MN723850,MN732883,MN782535,MT385367,MT409116,MT741943,OL763420,OL770258,OL770259,OL770261,OL770262,OP585103 |
| VC_510_2 | 8 | 8400 | 60454.60655 | 0.12199619 | 0.0121343 | 1 | 5 | 28 | 28 | | 1 | 3.852043461 | 0 | 0 | 0 | MK278860,MN864865,MT949699,MW388001,MW388002,MW388003,MW388004,OL770260 |
| VC_510_3 | 1 | 0 | 9821.375805 | 0 | 0 | 0 | 0 | 1 | 1 | | 1 | 0 | 0 | 0 | 0 | GU911519 |
| VC_510_4 | 1 | 0 | 9702.648412 | 0 | 0 | 0 | 0 | 1 | 1 | | 1 | 0 | 0 | 0 | 0 | HM004124 |
| VC_510_5 | 1 | 0 | 7647.332589 | 0 | 0 | 0 | 0 | 1 | 1 | | 1 | 0 | 0 | 0 | 0 | HM032710 |
| VC_510_6 | 1 | 0 | 7345.696533 | 0 | 0 | 0 | 0 | 1 | 1 | | 1 | 0 | 0 | 0 | 0 | HM114315 |
| VC_511_0 | 1 | 0 | 2946.396096 | 0 | 0 | 0 | 0 | 1 | 1 | | 1 | 0 | 0 | 0 | 0 | KT932418 |
| VC_511_1 | 1 | 0 | 5539.127686 | 0 | 0 | 0 | 0 | 1 | 1 | | 1 | 0 | 0 | 0 | 0 | MG592562 |
| VC_511_10 | 1 | 0 | 4185.182067 | 0 | 0 | 0 | 0 | 1 | 1 | | 1 | 0 | 0 | 0 | 0 | HQ634156 |
| VC_511_11 | 1 | 0 | 2934.581116 | 0 | 0 | 0 | 0 | 1 | 1 | | 1 | 0 | 0 | 0 | 0 | KX507046 |
| VC_511_12 | 2 | 300 | 9025.215533 | 0.03217084 | 0.0444208 | 2.645751311 | 2.64575131 | 1 | 1 | | 1 | 2.645751311 | 0 | 0 | 0 | MW824371,MW824372 |
| VC_511_13 | 1 | 0 | 4880.862817 | 0 | 0 | 0 | 0 | 1 | 1 | | 1 | 0 | 0 | 0 | 0 | MW824376 |
| VC_511_14 | 1 | 0 | 5060.879309 | 0 | 0 | 0 | 0 | 1 | 1 | | 1 | 0 | 0 | 0 | 0 | HQ316579 |
| VC_511_15 | 1 | 0 | 5062.80114 | 0 | 0 | 0 | 0 | 1 | 1 | | 1 | 0 | 0 | 0 | 0 | HQ634195 |
| VC_511_16 | 1 | 0 | 4124.375212 | 0 | 0 | 0 | 0 | 1 | 1 | | 1 | 0 | 0 | 0 | 0 | MG592553 |
| VC_511_2 | 1 | 0 | 4449.633424 | 0 | 0 | 0 | 0 | 1 | 1 | | 1 | 0 | 0 | 0 | 0 | MG592529 |
| VC_511_3 | 1 | 0 | 3814.890469 | 0 | 0 | 0 | 0 | 1 | 1 | | 1 | 0 | 0 | 0 | 0 | OX241437 |
| VC_511_4 | 1 | 0 | 4261.147308 | 0 | 0 | 0 | 0 | 1 | 1 | | 1 | 0 | 0 | 0 | 0 | OX241455 |
| VC_511_5 | 1 | 0 | 2787.484237 | 0 | 0 | 0 | 0 | 1 | 1 | | 1 | 0 | 0 | 0 | 0 | AP014858 |
| VC_511_6 | 1 | 0 | 3392.334265 | 0 | 0 | 0 | 0 | 1 | 1 | | 1 | 0 | 0 | 0 | 0 | MG592495 |
| VC_511_7 | 1 | 0 | 3888.863673 | 0 | 0 | 0 | 0 | 1 | 1 | | 1 | 0 | 0 | 0 | 0 | HQ634157 |
| VC_511_8 | 1 | 0 | 3291.470512 | 0 | 0 | 0 | 0 | 1 | 1 | | 1 | 0 | 0 | 0 | 0 | MG592441 |
| VC_511_9 | 1 | 0 | 3284.726228 | 0 | 0 | 0 | 0 | 1 | 1 | | 1 | 0 | 0 | 0 | 0 | ON922990 |
| VC_513_0 | 39 | 222300 | 499986.4488 | 0.30777263 | 2.92E-97 | 0 | 7.68114575 | 741 | 741 | | 1 | 3.952205067 | 0 | 0 | 0 | KY883638,KY883639,KY883640,MH310933,MH310935,MN419153,MW794150,MW794151,MW794152,MW794153,MW794154,MW794155,MW794156,MW794157,MW794158,MW794159,MW794160,MW794161,MW794162,MW794163,MW794164,MW794165,MW794166,MW794167,MW794168,MW794169,MW794170,MW794171,MW794172,MW794173,MW794176,MW794177,MW794179,MW794180,MW794182,MW794183,MW794188,MW794190,MZ292654 |
| VC_513_1 | 41 | 246000 | 504819.3603 | 0.32764206 | 2.55E-115 | 0 | 10.4403065 | 820 | 747 | 0.91097561 | 1 | 6.478268626 | 0 | 0 | 0 | HQ641347,HQ641348,HQ641349,HQ641350,HQ641351,HQ641352,HQ641353,HQ641354,KY065147,KY883634,KY883635,KY883636,KY883637,MH310934,MH310936,MN402506,MW794140,MW794141,MW794142,MW794143,MW794144,MW794145,MW794146,MW794147,MW794148,MW794149,MW794174,MW794175,MW794178,MW794181,MW794184,MW794185,MW794186,MW794187,MW794189,MW794191,MW794192,MW911612,MW911613,MW911614,MW911615 |
| VC_514_0 | 1 | 0 | 1153.995706 | 0 | 0 | 0 | 0 | 1 | 1 | | 1 | 0 | 0 | 0 | 0 | KF302034 |
| VC_514_1 | 1 | 0 | 809.262212 | 0 | 0 | 0 | 0 | 1 | 1 | | 1 | 0 | 0 | 0 | 0 | MF370964 |
| VC_514_2 | 1 | 0 | 855.6521158 | 0 | 0 | 0 | 0 | 1 | 1 | | 1 | 0 | 0 | 0 | 0 | KR534323 |
| VC_514_3 | 1 | 0 | 1168.582793 | 0 | 0 | 0 | 0 | 1 | 1 | | 1 | 0 | 0 | 0 | 0 | KX822733 |
| VC_515_0 | 8 | 2808.550416 | 4259.713163 | 0.39734659 | 2.32E-18 | 1 | 7.68114575 | 28 | 28 | | 1 | 5.824701134 | 0 | 0 | 0 | AP014887,AY328852,AY328853,KF854250,KX196154,KY622015,MG592395,OL658619 |
| VC_516_0 | 24 | 30861.8392 | 5208.433061 | 0.85560317 | 3.39E-104 | 0 | 9.38083152 | 276 | 262 | 0.949275362 | 1 | 7.694473556 | 0 | 0 | 0 | KT160311,MG592392,MG592401,MG592414,MG592451,MG592461,MG592462,MG592467,MG592468,MG592472,MG592499,MG592526,MG592531,MG592541,MG592542,MG592547,MG592561,MG592572,MG592590,MG592592,MG592611,MW824380,MW824381,MW824382 |
| VC_517_0 | 3 | 553.4178436 | 117.7651084 | 0.82454097 | 0.0030824 | 0 | 2.82842712 | 3 | 3 | | 1 | 1.885618083 | 0 | 0 | 0 | OM287554,OM732336,OM732337 |
| VC_518_0 | 16 | 15604.49824 | 26.38026333 | 0.9983123 | 1.50E-10 | 0 | 4.58257569 | 120 | 120 | | 1 | 3.609799117 | 0 | 0 | 0 | AP014888,MG592509,MG592546,MG592620,MG592623,OX241426,OX241428,OX241430,OX241431,OX241432,OX241441,OX241448,OX241449,OX241494,OX241567,OX241568 |
| VC_519_0 | 2 | 300 | 5742.36837 | 0.04964941 | 0.0461462 | 5.656854249 | 5.65685425 | 1 | 1 | | 1 | 5.656854249 | 0 | 0 | 0 | S11_vf_104035,S13_vf_11246 |
| VC_519_1 | 1 | 0 | 2952.956608 | 0 | 0 | 0 | 0 | 1 | 1 | | 1 | 0 | 0 | 0 | 0 | MT774405 |
| VC_519_10 | 2 | 200.6866863 | 4391.566682 | 0.04370114 | 0.0461448 | 3.741657387 | 3.74165739 | 1 | 1 | | 1 | 3.741657387 | 0 | 0 | 0 | MT774379,S4_vf_31981 |
| VC_519_11 | 7 | 3360.88393 | 12615.3362 | 0.2103679 | 2.21E-14 | 3.605551275 | 9.74679434 | 21 | 16 | 0.761904762 | 1 | 7.883482526 | 0 | 0 | 0 | MT774375,MT774376,MT774380,MT774382,S13_vf_143422,S17_vf_81232,S4_vf_4838 |
| VC_519_12 | 1 | 0 | 3377.659755 | 0 | 0 | 0 | 0 | 1 | 1 | | 1 | 0 | 0 | 0 | 0 | MT774396 |
| VC_519_2 | 2 | 238.6989355 | 5155.53955 | 0.04425072 | 0.051063 | 8.062257748 | 8.06225775 | 1 | 1 | | 1 | 8.062257748 | 0 | 0 | 0 | MT774407,S3_vf_115304 |
| VC_519_3 | 1 | 0 | 2549.837781 | 0 | 0 | 0 | 0 | 1 | 1 | | 1 | 0 | 0 | 0 | 0 | MT774404 |
| VC_519_4 | 1 | 0 | 3075.451842 | 0 | 0 | 0 | 0 | 1 | 1 | | 1 | 0 | 0 | 0 | 0 | MT774400 |
| VC_519_5 | 2 | 300 | 6007.045481 | 0.04756585 | 0.0460179 | 5.916079783 | 5.91607978 | 1 | 1 | | 1 | 5.916079783 | 0 | 0 | 0 | MT774397,S8_vf_131802 |
| VC_519_6 | 2 | 199.3406096 | 6221.847677 | 0.03104419 | 0.0618107 | 8.602325267 | 8.60232527 | 1 | 1 | | 1 | 8.602325267 | 0 | 0 | 0 | MT774395,S9_vf_76769 |
| VC_519_7 | 2 | 300 | 6302.1246 | 0.04543992 | 0.0485535 | 6.164414003 | 6.164414 | 1 | 1 | | 1 | 6.164414003 | 0 | 0 | 0 | S10_vf_3644,S3_vf_25163 |
| VC_519_8 | 2 | 300 | 6522.124076 | 0.04397457 | 0.0485535 | 5.099019514 | 5.09901951 | 1 | 1 | | 1 | 5.099019514 | 0 | 0 | 0 | MT774399,MT774403 |
| VC_519_9 | 1 | 0 | 3447.285043 | 0 | 0 | 0 | 0 | 1 | 1 | | 1 | 0 | 0 | 0 | 0 | MT774398 |
| VC_52_0 | 9 | 6227.405632 | 4920.291097 | 0.55862711 | 7.19E-23 | 0 | 4.69041576 | 36 | 36 | | 1 | 3.401792912 | 0 | 0 | 0 | AF083977,GQ357916,KP010268,LR595867,MT374852,MT374853,MT374854,MT374856,MT591577 |
| VC_520_0 | 2 | 188.4159497 | 2869.662096 | 0.06161254 | 0.0461448 | 4.898979486 | 4.89897949 | 1 | 1 | | 1 | 4.898979486 | 0 | 0 | 0 | MT774408,MT774409 |
| VC_520_1 | 2 | 233.1874828 | 2686.311825 | 0.07987242 | 0.0461393 | 4.898979486 | 4.89897949 | 1 | 1 | | 1 | 4.898979486 | 0 | 0 | 0 | MT774410,MT774411 |
| VC_523_0 | 3 | 900 | 131.2367964 | 0.87273845 | 0.0318013 | 0 | 1.73205081 | 3 | 3 | | 1 | 1.154700538 | 0 | 0 | 0 | MW353175,MW353176,MW353177 |
| VC_524_0 | 8 | 8400 | 3005.342739 | 0.73649694 | 7.13E-17 | 1.414213562 | 7.14142843 | 28 | 28 | | 1 | 4.73885141 | 0 | 0 | 0 | KR560069,MK580972,MN095772,MN098328,MZ042490,MZ326868,MZ398248,OL702939 |
| VC_524_1 | 1 | 0 | 1774.629052 | 0 | 0 | 0 | 0 | 1 | 1 | | 1 | 0 | 0 | 0 | 0 | ON758385 |
| VC_525_0 | 8 | 8084.459827 | 10165.70312 | 0.44298014 | 1.45E-19 | 0 | 4 | 28 | 28 | | 1 | 2.950966002 | 0 | 0 | 0 | AP018462,AP018463,KY130461,MH001446,MH001450,MK494114,MK494131,MK494132 |
| VC_526_0 | 32 | 148773.0948 | 15849.08335 | 0.90372449 | 9.12E-241 | 0 | 3.31662479 | 496 | 496 | | 1 | 1.053959571 | 0 | 0 | 0 | AY129334,DQ398048,JN618996,JN698991,JN699004,KF024722,KM101117,KR997932,KT365402,KT880194,KX443696,MG757162,MG812490,MG812491,MH001452,MH051249,MH590591,MH697584,MH727546,MK061411,MK620898,MK814751,MN234225,MN428049,MN586045,MN586046,MN813678,MT639652,MW712731,MZ456036,ON970587,ON970593 |
| VC_527_0 | 37 | 199783.0331 | 6620.715671 | 0.96792347 | 2.18E-283 | 0 | 4.35889894 | 666 | 666 | | 1 | 2.494687492 | 0 | 0 | 0 | DQ398049,EU816589,FJ641182,JF704095,JN698992,JN699003,JN699006,JN699018,KF493879,KJ194581,KJ194584,KP027207,KR080203,KR080205,MG839014,MG920059,MG925338,MG925353,MH051265,MH316565,MH513976,MH576977,MK061414,MK359357,MN096359,MN096360,MN234223,MN444872,MN444873,MT310871,MT310892,MT952851,MW924644,MZ171375,MZ171381,OK318960,OP434441 |
| VC_528_0 | 12 | 11077.09382 | 17162.59462 | 0.39225269 | 1.30E-43 | 0 | 9 | 66 | 64 | 0.96969697 | 1 | 7.037173591 | 0 | 0 | 0 | MH153810,MK801725,MN234196,MN369758,MN908693,MT657335,OK216879,OM105886,ON081333,ON108644,ON260813,OP434461 |
| VC_529_0 | 3 | 604.6353644 | 4940.671536 | 0.10903551 | 0.0014127 | 6.32455532 | 7.54983444 | 3 | 3 | | 1 | 6.810609427 | 0 | 0 | 0 | JN698995,OP172873,OP172874 |
| VC_53_0 | 10 | 2863.160107 | 13559.90482 | 0.17433775 | 1.48E-25 | 1.414213562 | 8.54400375 | 45 | 45 | | 1 | 6.624197138 | 0 | 0 | 0 | AB285204,JQ182728,LR595868,LR597641,MF807953,MW057859,ON470578,ON470598,ON470618,OV696610 |
| VC_531_0 | 32 | 107478.7504 | 49223.76971 | 0.68587761 | 1.81E-283 | 0 | 7.07106781 | 496 | 496 | | 1 | 4.711232911 | 0 | 0 | 0 | AP018485,JF704111,JF957058,KC172839,KJ510415,KM592966,KT326767,KX640831,KX670828,KX683423,KY464936,LC730317,MF373840,MG812492,MH020248,MH047632,MH047633,MH051259,MH316563,MH976517,MK284519,MK494127,MK524496,MN062706,MN062712,MN586063,MT310878,MT310879,MT310880,MT310881,MW712718,ON970623 |
| VC_532_0 | 75 | 621708.0507 | 40203.15046 | 0.93926202 | 0 | 0 | 8.48528137 | 2775 | 2775 | | 1 | 5.184703418 | 0 | 0 | 0 | AY129332,HM755814,JF704098,JF704101,JF704114,KC661272,KC661281,KF279418,KM101119,KM233454,KM233455,KP017310,KP017311,KP027202,KT359365,KT381277,KU255188,KU578077,KU984914,KU985091,KX458237,KX507362,KX523125,KX664448,KX670788,KX808129,KY965063,MF185729,MF185732,MF185733,MG757161,MG925341,MG925355,MH020238,MH077583,MH155864,MH338240,MH371121,MH399776,MH450119,MH450124,MH450126,MH450128,MH513975,MH536813,MH536825,MH576949,MH651179,MH727554,MK112543,MK279903,MK279913,MK284518,MK310140,MK359341,MK433273,MK494096,MK494128,MK875800,MK967399,MN062718,MN096368,MN284900,MN497953,MN585969,MN585984,MN586048,MN813699,MT657331,MT897907,MW507128,MZ681518,MZ747514,OK318959,OK999978 |
| VC_533_0 | 16 | 25427.27521 | 42108.65418 | 0.37649997 | 5.71E-78 | 0 | 6.8556546 | 120 | 120 | | 1 | 4.47315232 | 0 | 0 | 0 | JQ512844,JX307704,JX411619,KC661279,KJ156985,KM101120,MF773750,MH001459,MK494124,MN586038,MN807247,MT522002,MZ322017,MZ355723,MZ622159,ON337196 |
| VC_534_0 | 3 | 804.9531318 | 973.3198557 | 0.45266005 | 0.0014309 | 0 | 4.79583152 | 3 | 3 | | 1 | 3.197221016 | 0 | 0 | 0 | MW314854,MW584159,MW584197 |
| VC_535_0 | 18 | 35438.96635 | 186707.6093 | 0.15952965 | 2.46E-95 | 0 | 8.48528137 | 153 | 153 | | 1 | 4.577889885 | 0 | 0 | 0 | JF704093,JN243857,KX550442,KX579975,KX641260,KY083059,MF668278,MK279898,MK359310,MK359322,MK494115,MK494117,MK494129,MK875799,MN284905,MN813697,MZ322023,ON970614 |

| | | | | | | | | | | | | | | | |
|---|---|---|---|---|---|---|---|---|---|---|---|---|---|---|---|
| VC_535_1 | 108 | 1133274.326 | 203327.5699 | 0.84787724 | 0 | 0 | 9.43398113 | 5778 | 5752 | 0.995500173 | 6.241694953 | 0 | 0 | 0 | GQ303263,HM152766,JF792674,JN243856,JN561150,JN699015,JQ809701,JQ896627,JX307702,JX307703,KC661271,KF024733,KF841475,KF841476,KF954506,KF959563,KJ510414,KP027198,KP057620,KT365397,KT716493,KU055616,KU867906,KU985092,KU985094,KX458236,KX550441,KX557234,KX619651,KX648373,KX808130,KX817174,KX817175,KY006473,KY083058,KY083060,KY083061,KY083062,KY083063,KY204244,KY204245,KY204246,KY204247,KY204248,KY204249,KY204250,KY204251,KY471266,KY549155,MF919496,MF919509,MG099942,MG944217,MG944224,MG962373,MH020249,MH051247,MH077581,MH155875,MH155879,MH230877,MH399777,MH399785,MH479909,MH479911,MH509443,MH536817,MH651170,MH697588,MH727563,MH825710,MK279902,MK310145,MK359301,MK359323,MK359325,MK359340,MK359356,MK494088,MK494093,MK494094,MK494097,MK494101,MK494125,MK494126,MK878895,MK878905,MN728684,MN813696,MT114158,MT490373,MT498056,MT553338,MT553346,MT723939,MT818417,MT889382,MT889386,MT889390,MW291019,MZ005673,MZ171380,MZ322025,MZ355725,MZ747509,ON637771,ON970565,OP297529 |
| VC_536_0 | 6 | 2413.5198 | 16298.40073 | 0.12898301 | 1.99E-11 | 2.236067977 | 7.61577311 | 15 | 15 | 1 | 6.25639766 | 0 | 0 | 0 | AP018487,FJ168659,HM152765,JN699001,MN234195,MN234236 |
| VC_536_1 | 2 | 146.0462777 | 3767.430557 | 0.0373188 | 0.0428124 | 7.615773106 | 7.61577311 | 1 | 1 | 1 | 7.615773106 | 0 | 0 | 0 | AY129333,KP027201 |
| VC_537_0 | 39 | 162984.0645 | 37054.15823 | 0.81476461 | 0.00E+00 | 0 | 8.66025404 | 741 | 741 | 1 | 4.793252917 | 0 | 0 | 0 | JN412591,JN572061,KC691256,KF841477,KP027200,KR080195,KT347313,KU985090,KX641262,MF133446,MF140399,MF281061,MF373843,MF472894,MF919492,MG198785,MK016499,MK494122,MK524508,MK814758,MK820639,MK919480,MK937604,MN010757,MN096356,MN585977,MN807248,MN807249,MN807250,MN892484,MN892486,MN908683,MT024866,MT498061,MW055902,ON637760,ON970574,OP172867,OP434454 |
| VC_538_0 | 1 | 0 | 11972.17609 | 0 | 0 | 0 | 0 | 1 | 1 | 1 | 0 | 0 | 0 | 0 | KT309034 |
| VC_538_1 | 1 | 0 | 10340.24428 | 0 | 0 | 0 | 0 | 1 | 1 | 1 | 0 | 0 | 0 | 0 | MT889374 |
| VC_538_10 | 1 | 0 | 10723.36581 | 0 | 0 | 0 | 0 | 1 | 1 | 1 | 0 | 0 | 0 | 0 | MN369752 |
| VC_538_11 | 1 | 0 | 11159.05552 | 0 | 0 | 0 | 0 | 1 | 1 | 1 | 0 | 0 | 0 | 0 | MN585995 |
| VC_538_12 | 1 | 0 | 10709.91001 | 0 | 0 | 0 | 0 | 1 | 1 | 1 | 0 | 0 | 0 | 0 | MN735431 |
| VC_538_13 | 1 | 0 | 10739.59055 | 0 | 0 | 0 | 0 | 1 | 1 | 1 | 0 | 0 | 0 | 0 | KT281793 |
| VC_538_14 | 1 | 0 | 11454.52657 | 0 | 0 | 0 | 0 | 1 | 1 | 1 | 0 | 0 | 0 | 0 | MT684595 |
| VC_538_15 | 2 | 175.1668896 | 22281.03871 | 0.00780038 | 0.042396 | 9 | 9 | 1 | 0 | 0 | 9 | 0 | 0 | 0 | MF668270,MT684593 |
| VC_538_16 | 1 | 0 | 10440.30511 | 0 | 0 | 0 | 0 | 1 | 1 | 1 | 0 | 0 | 0 | 0 | KX808131 |
| VC_538_17 | 1 | 0 | 8561.523618 | 0 | 0 | 0 | 0 | 1 | 1 | 1 | 0 | 0 | 0 | 0 | MN369739 |
| VC_538_18 | 1 | 0 | 10833.63493 | 0 | 0 | 0 | 0 | 1 | 1 | 1 | 0 | 0 | 0 | 0 | MN586001 |
| VC_538_19 | 2 | 222.3054585 | 22592.57019 | 0.00974388 | 0.0423845 | 7.348469228 | 7.34846923 | 1 | 1 | 1 | 7.348469228 | 0 | 0 | 0 | KM652553,MN369749 |
| VC_538_2 | 1 | 0 | 10634.83604 | 0 | 0 | 0 | 0 | 1 | 1 | 1 | 0 | 0 | 0 | 0 | KR824843 |
| VC_538_20 | 1 | 0 | 11333.52524 | 0 | 0 | 0 | 0 | 1 | 1 | 1 | 0 | 0 | 0 | 0 | JN699012 |
| VC_538_21 | 1 | 0 | 10768.51707 | 0 | 0 | 0 | 0 | 1 | 1 | 1 | 0 | 0 | 0 | 0 | MH669001 |
| VC_538_22 | 1 | 0 | 10254.86879 | 0 | 0 | 0 | 0 | 1 | 1 | 1 | 0 | 0 | 0 | 0 | OP068340 |
| VC_538_23 | 2 | 180.3249487 | 23268.81084 | 0.00769005 | 0.0423659 | 8.717797887 | 8.71779789 | 1 | 1 | 1 | 8.717797887 | 0 | 0 | 0 | JN859129,KF024723 |
| VC_538_24 | 1 | 0 | 11193.52866 | 0 | 0 | 0 | 0 | 1 | 1 | 1 | 0 | 0 | 0 | 0 | EF536069 |
| VC_538_25 | 1 | 0 | 9812.13733 | 0 | 0 | 0 | 0 | 1 | 1 | 1 | 0 | 0 | 0 | 0 | MH590598 |
| VC_538_26 | 3 | 864.0355576 | 32617.6608 | 0.02580621 | 0.001401 | 5.099019514 | 6.244998 | 3 | 3 | 1 | 5.696193386 | 0 | 0 | 0 | HQ728524,JN698996,JX121091 |
| VC_538_27 | 2 | 300 | 20956.21901 | 0.01411352 | 0.0423837 | 0 | 0 | 1 | 1 | 1 | 0 | 0 | 0 | 0 | KT281792,KY471267 |
| VC_538_28 | 2 | 300 | 21964.76939 | 0.0134742 | 0.0423847 | 3.605551275 | 3.60555128 | 1 | 1 | 1 | 3.605551275 | 0 | 0 | 0 | JN020141,MT114167 |
| VC_538_29 | 1 | 0 | 9194.317177 | 0 | 0 | 0 | 0 | 1 | 1 | 1 | 0 | 0 | 0 | 0 | KM597530 |
| VC_538_3 | 1 | 0 | 9658.509955 | 0 | 0 | 0 | 0 | 1 | 1 | 1 | 0 | 0 | 0 | 0 | MF668287 |
| VC_538_30 | 2 | 163.4223636 | 22421.90007 | 0.00723578 | 0.045531 | 8.831760866 | 8.83176087 | 1 | 1 | 1 | 8.831760866 | 0 | 0 | 0 | MH825707,ON526976 |
| VC_538_31 | 11 | 9896.272929 | 100824.8227 | 0.08938019 | 2.17E-36 | 0 | 9.8488578 | 55 | 33 | 0.6 | 8.51832669 | 0 | 0 | 0 | FJ174690,GU060500,JF937093,KT281795,MH020235,MH077580,MK016494,MN585973,MW924649,MZ681519,OP297536 |
| VC_538_32 | 1 | 0 | 12085.36495 | 0 | 0 | 0 | 0 | 1 | 1 | 1 | 0 | 0 | 0 | 0 | MH651178 |
| VC_538_33 | 2 | 193.9474751 | 22194.16428 | 0.00866297 | 0.0423825 | 8.185352772 | 8.18535277 | 1 | 1 | 1 | 8.185352772 | 0 | 0 | 0 | MH371123,MN369755 |
| VC_538_34 | 1 | 0 | 11982.20605 | 0 | 0 | 0 | 0 | 1 | 1 | 1 | 0 | 0 | 0 | 0 | MH077578 |
| VC_538_35 | 3 | 853.4061587 | 33396.13013 | 0.02491731 | 0.0014014 | 3 | 6.4807407 | 3 | 3 | 1 | 5.075101115 | 0 | 0 | 0 | KR997930,KR997931,KR997968 |
| VC_538_36 | 1 | 0 | 9918.872749 | 0 | 0 | 0 | 0 | 1 | 1 | 1 | 0 | 0 | 0 | 0 | MH744415 |
| VC_538_37 | 1 | 0 | 10527.89244 | 0 | 0 | 0 | 0 | 1 | 1 | 1 | 0 | 0 | 0 | 0 | JF937102 |
| VC_538_38 | 6 | 3201.978573 | 58727.31119 | 0.05170378 | 1.75E-11 | 3.31662479 | 10.0498756 | 15 | 13 | 0.866666667 | 7.79849748 | 0 | 0 | 0 | KJ025956,MH155865,MH155871,MH590602,MK524517,MT310893 |
| VC_538_39 | 1 | 0 | 11891.89705 | 0 | 0 | 0 | 0 | 1 | 1 | 1 | 0 | 0 | 0 | 0 | MH371120 |
| VC_538_4 | 1 | 0 | 10555.97303 | 0 | 0 | 0 | 0 | 1 | 1 | 1 | 0 | 0 | 0 | 0 | MH155866 |
| VC_538_40 | 4 | 1200.35027 | 29226.73209 | 0.03945006 | 1.22E-05 | 5.567764363 | 7.81024968 | 6 | 6 | 1 | 7.136524503 | 0 | 0 | 0 | KU865302,KY965064,MH371115,MT553345 |
| VC_538_41 | 2 | 300 | 21501.3631 | 0.01376061 | 0.0423854 | 3.741657387 | 3.74165739 | 1 | 1 | 1 | 3.741657387 | 0 | 0 | 0 | MH651183,MN586011 |
| VC_538_42 | 1 | 0 | 7641.929537 | 0 | 0 | 0 | 0 | 1 | 1 | 1 | 0 | 0 | 0 | 0 | MH399771 |
| VC_538_43 | 3 | 616.7152864 | 31383.19043 | 0.01927241 | 0.0016296 | 6.92820323 | 8.71779789 | 3 | 3 | 1 | 8.082775461 | 0 | 0 | 0 | JN020143,ON456356,ON970562 |
| VC_538_44 | 1 | 0 | 11798.52291 | 0 | 0 | 0 | 0 | 1 | 1 | 1 | 0 | 0 | 0 | 0 | KY348865 |
| VC_538_45 | 7 | 4154.217036 | 61557.25212 | 0.06321906 | 5.05E-15 | 5.744562647 | 9.11043358 | 21 | 17 | 0.80952381 | 7.861683874 | 0 | 0 | 0 | KT895281,MG872841,MH316564,MH399784,MH651169,MH834617,MK494110 |
| VC_538_46 | 1 | 0 | 9970.002544 | 0 | 0 | 0 | 0 | 1 | 1 | 1 | 0 | 0 | 0 | 0 | MZ274306 |
| VC_538_47 | 1 | 0 | 10163.47604 | 0 | 0 | 0 | 0 | 1 | 1 | 1 | 0 | 0 | 0 | 0 | MT684586 |
| VC_538_48 | 1 | 0 | 9556.780925 | 0 | 0 | 0 | 0 | 1 | 1 | 1 | 0 | 0 | 0 | 0 | MZ171378 |
| VC_538_49 | 1 | 0 | 10746.74527 | 0 | 0 | 0 | 0 | 1 | 1 | 1 | 0 | 0 | 0 | 0 | KM101122 |
| VC_538_5 | 1 | 0 | 10872.90987 | 0 | 0 | 0 | 0 | 1 | 1 | 1 | 0 | 0 | 0 | 0 | MH727548 |
| VC_538_50 | 1 | 0 | 10900.28018 | 0 | 0 | 0 | 0 | 1 | 1 | 1 | 0 | 0 | 0 | 0 | MT897904 |
| VC_538_51 | 1 | 0 | 10612.63742 | 0 | 0 | 0 | 0 | 1 | 1 | 1 | 0 | 0 | 0 | 0 | MH051257 |
| VC_538_52 | 1 | 0 | 8358.612819 | 0 | 0 | 0 | 0 | 1 | 1 | 1 | 0 | 0 | 0 | 0 | KC736071 |
| VC_538_53 | 1 | 0 | 9940.884731 | 0 | 0 | 0 | 0 | 1 | 1 | 1 | 0 | 0 | 0 | 0 | KY385384 |
| VC_538_54 | 1 | 0 | 9517.968329 | 0 | 0 | 0 | 0 | 1 | 1 | 1 | 0 | 0 | 0 | 0 | OM105885 |
| VC_538_55 | 1 | 0 | 10696.66186 | 0 | 0 | 0 | 0 | 1 | 1 | 1 | 0 | 0 | 0 | 0 | OP297542 |
| VC_538_56 | 1 | 0 | 9120.488892 | 0 | 0 | 0 | 0 | 1 | 1 | 1 | 0 | 0 | 0 | 0 | KM279937 |
| VC_538_57 | 1 | 0 | 9703.862867 | 0 | 0 | 0 | 0 | 1 | 1 | 1 | 0 | 0 | 0 | 0 | MK359343 |
| VC_538_58 | 3 | 718.7619879 | 33802.76899 | 0.02082069 | 0.0014007 | 0 | 8.18535277 | 3 | 3 | 1 | 5.456901848 | 0 | 0 | 0 | DQ398050,EU816590,ON970595 |
| VC_538_59 | 5 | 2718.434617 | 56922.15648 | 0.04558028 | 2.56E-08 | 1.732050808 | 7.21110255 | 10 | 10 | 1 | 5.04516783 | 0 | 0 | 0 | JN542517,MH001448,MN585987,MN586005,MZ681512 |
| VC_538_6 | 3 | 900 | 32099.75453 | 0.02727293 | 0.0014013 | 0 | 1.41421356 | 3 | 3 | 1 | 0.942809042 | 0 | 0 | 0 | MN586004,MT684597,MT684601 |
| VC_538_60 | 2 | 229.5593844 | 24997.59452 | 0.00909969 | 0.0423877 | 6.8556546 | 6.8556546 | 1 | 1 | 1 | 6.8556546 | 0 | 0 | 0 | DQ398045,KT279576 |
| VC_538_61 | 1 | 0 | 11299.11173 | 0 | 0 | 0 | 0 | 1 | 1 | 1 | 0 | 0 | 0 | 0 | MW055915 |
| VC_538_62 | 1 | 0 | 11150.03096 | 0 | 0 | 0 | 0 | 1 | 1 | 1 | 0 | 0 | 0 | 0 | ON970566 |
| VC_538_63 | 1 | 0 | 11777.36436 | 0 | 0 | 0 | 0 | 1 | 1 | 1 | 0 | 0 | 0 | 0 | MF919501 |
| VC_538_64 | 1 | 0 | 11241.04427 | 0 | 0 | 0 | 0 | 1 | 1 | 1 | 0 | 0 | 0 | 0 | MG962361 |
| VC_538_65 | 1 | 0 | 11343.66189 | 0 | 0 | 0 | 0 | 1 | 1 | 1 | 0 | 0 | 0 | 0 | MZ747519 |
| VC_538_66 | 1 | 0 | 10818.74084 | 0 | 0 | 0 | 0 | 1 | 1 | 1 | 0 | 0 | 0 | 0 | MN586012 |
| VC_538_67 | 1 | 0 | 10362.65265 | 0 | 0 | 0 | 0 | 1 | 1 | 1 | 0 | 0 | 0 | 0 | ON970610 |
| VC_538_68 | 1 | 0 | 11334.62994 | 0 | 0 | 0 | 0 | 1 | 1 | 1 | 0 | 0 | 0 | 0 | KM347889 |
| VC_538_69 | 1 | 0 | 11532.19562 | 0 | 0 | 0 | 0 | 1 | 1 | 1 | 0 | 0 | 0 | 0 | MH669014 |
| VC_538_7 | 1 | 0 | 11168.75001 | 0 | 0 | 0 | 0 | 1 | 1 | 1 | 0 | 0 | 0 | 0 | MN310548 |
| VC_538_70 | 1 | 0 | 11173.35564 | 0 | 0 | 0 | 0 | 1 | 1 | 1 | 0 | 0 | 0 | 0 | KC661280 |
| VC_538_71 | 1 | 0 | 11069.22166 | 0 | 0 | 0 | 0 | 1 | 1 | 1 | 0 | 0 | 0 | 0 | FJ174693 |
| VC_538_72 | 1 | 0 | 11240.30496 | 0 | 0 | 0 | 0 | 1 | 1 | 1 | 0 | 0 | 0 | 0 | MK494118 |
| VC_538_73 | 2 | 253.5515828 | 23487.08254 | 0.01068007 | 0.0423726 | 6.708203932 | 6.70820393 | 1 | 1 | 1 | 6.708203932 | 0 | 0 | 0 | KR935214,MH669012 |
| VC_538_74 | 2 | 172.0252577 | 22844.60324 | 0.00747396 | 0.0423941 | 8.888194417 | 8.88819442 | 1 | 1 | 1 | 8.888194417 | 0 | 0 | 0 | JF704117,MK112556 |
| VC_538_75 | 1 | 0 | 11864.78552 | 0 | 0 | 0 | 0 | 1 | 1 | 1 | 0 | 0 | 0 | 0 | MN310544 |
| VC_538_76 | 2 | 196.2785356 | 21315.57084 | 0.00912421 | 0.0423862 | 7.745966692 | 7.74596669 | 1 | 1 | 1 | 7.745966692 | 0 | 0 | 0 | JN398368,MT114164 |
| VC_538_77 | 3 | 701.8055356 | 31749.06643 | 0.02162671 | 0.0014009 | 1.414213562 | 8.24621125 | 3 | 3 | 1 | 5.928154406 | 0 | 0 | 0 | JQ300538,KX576644,MK016496 |
| VC_538_78 | 2 | 188.295776 | 23491.23667 | 0.00795184 | 0.0423848 | 8.062257748 | 8.06225775 | 1 | 1 | 1 | 8.062257748 | 0 | 0 | 0 | MH590596,OP297528 |
| VC_538_79 | 1 | 0 | 10751.73271 | 0 | 0 | 0 | 0 | 1 | 1 | 1 | 0 | 0 | 0 | 0 | KC661278 |
| VC_538_8 | 1 | 0 | 10740.4758 | 0 | 0 | 0 | 0 | 1 | 1 | 1 | 0 | 0 | 0 | 0 | MZ958744 |
| VC_538_80 | 3 | 900 | 30005.8785 | 0.02912067 | 0.001401 | 0 | 2.64575131 | 3 | 3 | 1 | 1.763834207 | 0 | 0 | 0 | MH651188,MH669003,MK359315 |
| VC_538_81 | 1 | 0 | 11937.20855 | 0 | 0 | 0 | 0 | 1 | 1 | 1 | 0 | 0 | 0 | 0 | MF919526 |
| VC_538_82 | 1 | 0 | 9740.520911 | 0 | 0 | 0 | 0 | 1 | 1 | 1 | 0 | 0 | 0 | 0 | MN310545 |
| VC_538_83 | 1 | 0 | 9622.994603 | 0 | 0 | 0 | 0 | 1 | 1 | 1 | 0 | 0 | 0 | 0 | KX610764 |
| VC_538_84 | 1 | 0 | 11183.37417 | 0 | 0 | 0 | 0 | 1 | 1 | 1 | 0 | 0 | 0 | 0 | ON970585 |
| VC_538_85 | 2 | 300 | 20214.08373 | 0.0146241 | 0.042376 | 0 | 0 | 1 | 1 | 1 | 0 | 0 | 0 | 0 | KT895280,MN369751 |
| VC_538_86 | 1 | 0 | 8959.104563 | 0 | 0 | 0 | 0 | 1 | 1 | 1 | 0 | 0 | 0 | 0 | MZ747512 |
| VC_538_87 | 1 | 0 | 10917.67952 | 0 | 0 | 0 | 0 | 1 | 1 | 1 | 0 | 0 | 0 | 0 | MK524503 |
| VC_538_88 | 1 | 0 | 10103.48689 | 0 | 0 | 0 | 0 | 1 | 1 | 1 | 0 | 0 | 0 | 0 | JN020142 |
| VC_538_89 | 1 | 0 | 11016.54697 | 0 | 0 | 0 | 0 | 1 | 1 | 1 | 0 | 0 | 0 | 0 | MN585975 |
| VC_538_9 | 1 | 0 | 9300.428011 | 0 | 0 | 0 | 0 | 1 | 1 | 1 | 0 | 0 | 0 | 0 | KF114874 |
| VC_538_90 | 1 | 0 | 10192.93905 | 0 | 0 | 0 | 0 | 1 | 1 | 1 | 0 | 0 | 0 | 0 | MK016504 |
| VC_538_91 | 1 | 0 | 11185.75414 | 0 | 0 | 0 | 0 | 1 | 1 | 1 | 0 | 0 | 0 | 0 | MZ209299 |
| VC_538_92 | 5 | 3000 | 42911.12673 | 0.06534364 | 2.55E-08 | 0 | 3.16227766 | 10 | 10 | 1 | 1.516227766 | 0 | 0 | 0 | KM402757,MK359307,MN428051,MT889396,MT889397 |
| VC_538_93 | 2 | 202.0254052 | 19381.84801 | 0.01031591 | 0.0423792 | 8.366600265 | 8.36660027 | 1 | 1 | 1 | 8.366600265 | 0 | 0 | 0 | JF937098,MH632119 |
| VC_538_94 | 1 | 0 | 9682.5854 | 0 | 0 | 0 | 0 | 1 | 1 | 1 | 0 | 0 | 0 | 0 | MK967397 |
| VC_538_95 | 1 | 0 | 10590.25706 | 0 | 0 | 0 | 0 | 1 | 1 | 1 | 0 | 0 | 0 | 0 | MK224592 |
| VC_538_96 | 1 | 0 | 10640.52348 | 0 | 0 | 0 | 0 | 1 | 1 | 1 | 0 | 0 | 0 | 0 | AY129330 |
| VC_538_97 | 1 | 0 | 9410.128505 | 0 | 0 | 0 | 0 | 1 | 1 | 1 | 0 | 0 | 0 | 0 | MG925354 |
| VC_538_98 | 1 | 0 | 7189.968716 | 0 | 0 | 0 | 0 | 1 | 1 | 1 | 0 | 0 | 0 | 0 | MG812488 |
| VC_539_0 | 37 | 102932.8256 | 10061.74882 | 0.9109537 | 3.31E-267 | 0 | 8.30662386 | 666 | 666 | 1 | 5.617991731 | 0 | 0 | 0 | JN256079,JN624851,KC576783,KF986246,KM588359,KU935726,KU935727,KU935728,KU935729,KU935730,KU935731,KX756439,MG099936,MG099948,MH316570,MH399787,MH697576,MH697585,MH697593,MH926055,MK392367,MK524500,MK524509,MK524515,MK524518,MK524520,MK524522,MK524528,MK977708,MT498042,MT498044,MT498048,MT684588,MT723932,MZ622162,OL742561,OP297540 |
| VC_54_0 | 87 | 236241.9717 | 3414.032225 | 0.98575445 | 2.35E-136 | 0 | 6.164414 | 3741 | 3741 | 1 | 1.064977125 | 0 | 0 | 0 | AB259123,AB434711,AB937974,AB981170,KX179905,MF716957,ON170020,ON170023,ON170029,ON170030,ON170034,ON170037,ON170041,ON170050,ON170055,ON170087,ON170089,ON170091,ON170093,ON170095,ON170097,ON170101,ON170103,ON170105,ON170107,ON170109,ON170111,ON170113,ON170115,ON170117,ON170119,ON170121,ON170125,ON170127,ON170132,ON170143,ON170145,ON170147,ON170153,ON170155,ON170157,ON170159,ON170161,ON170163,ON170165,ON170168,ON170193,ON170195,ON170197,ON170199,ON170201,ON170203,ON170205,ON170207,ON170212,ON170216,ON170218,ON170220,ON170223,ON170226,ON170245,ON170247,ON170249,ON170251,ON170253,ON170273,ON170277,ON170281,ON170283,ON170285,ON170290,ON170292,ON170294,ON170296,ON170299,ON170301,ON170308,ON170312,ON170314,ON170316,ON170331,ON170333,ON170335,ON170337,ON170339,ON170341,ON170353 |
| VC_540_0 | 3 | 399.5460687 | 4412.600882 | 0.08302865 | 0.0014734 | 5.744562647 | 7.41619849 | 3 | 3 | 1 | 6.790621228 | 0 | 0 | 0 | KU963256,MN175604,ON645344 |
| VC_540_1 | 5 | 1058.129444 | 9402.517658 | 0.10115334 | 3.27E-08 | 5.744562647 | 9.38083152 | 10 | 8 | 0.8 | 7.787670622 | 0 | 0 | 0 | MN428053,MN585966,MT521995,OP297548,OP434457 |
| VC_541_0 | 1 | 0 | 5121.700345 | 0 | 0 | 0 | 0 | 1 | 1 | 1 | 0 | 0 | 0 | 0 | MK977709 |
| VC_542_0 | 2 | 300 | 2396.98709 | 0.11123524 | 0.0450696 | 1.414213562 | 1.41421356 | 1 | 1 | 1 | 1.414213562 | 0 | 0 | 0 | MW584176,MW584199 |
| VC_542_1 | 1 | 0 | 1418.782728 | 0 | 0 | 0 | 0 | 1 | 1 | 1 | 0 | 0 | 0 | 0 | MW584188 |
| VC_543_0 | 25 | 56314.40122 | 66547.53619 | 0.45835515 | 3.07E-174 | 0 | 8.60232527 | 300 | 300 | 1 | 5.879004775 | 0 | 0 | 0 | KU963255,KU963257,KX557273,KX557287,MH479912,MH479927,MK016492,MK112547,MK801727,MK814755,MK820642,MK875793,MK875796,MK878898,MK919483,MT723938,MT889398,MT952844,MW712717,MW924648,MW924650,MZ171374,MZ274304,OP297546,OP434440 |
| VC_543_1 | 5 | 1200.425778 | 23600.98797 | 0.04840151 | 5.28E-07 | 7.549834435 | 9 | 10 | 9 | 0.9 | 8.386121703 | 0 | 0 | 0 | KU963249,KU963263,MK919469,MK967384,MT639650 |
| VC_544_0 | 6 | 4500 | 5854.356519 | 0.43459968 | 5.13E-10 | 1.732050808 | 7.54983444 | 15 | 15 | 1 | 6.450145554 | 0 | 0 | 0 | MG557979,MG765277,MG765279,MH809529,MH809530,MH809531 |
| VC_544_1 | 1 | 0 | 2351.808838 | 0 | 0 | 0 | 0 | 1 | 1 | 1 | 0 | 0 | 0 | 0 | MF787246 |
| VC_544_2 | 1 | 0 | 1809.626434 | 0 | 0 | 0 | 0 | 1 | 1 | 1 | 0 | 0 | 0 | 0 | AY682195 |
| VC_544_3 | 1 | 0 | 1380.436912 | 0 | 0 | 0 | 0 | 1 | 1 | 1 | 0 | 0 | 0 | 0 | MK504444 |
| VC_545_0 | 3 | 193.5546419 | 635.0202979 | 0.23359944 | 0.0016011 | 3.872983346 | 5.19615242 | 3 | 3 | 1 | 4.586517176 | 0 | 0 | 0 | MW202473,MW202478,MW202738 |
| VC_546_0 | 3 | 168.0605724 | 810.0911684 | 0.17181442 | 0.0016147 | 6 | 6.4807407 | 3 | 3 | 1 | 6.215051567 | 0 | 0 | 0 | MW202640,MW202722,OL791268 |
| VC_547_0 | 2 | 192.9725515 | 181.5460717 | 0.51525489 | 0.050121 | 1.414213562 | 1.41421356 | 1 | 1 | 1 | 1.414213562 | 0 | 0 | 0 | MW824391,MW824401 |
| VC_548_0 | 9 | 6131.241473 | 1515.305607 | 0.80183139 | 7.48E-13 | 2.645751311 | 7.07106781 | 36 | 36 | 1 | 4.525014673 | 0 | 0 | 0 | AP019417,KJ936628,MF431726,MG592626,MK483107,MN062185,MW217515,MW451248,MW748992 |
| VC_549_0 | 9 | 10800 | 30794.1289 | 0.25965203 | 1.30E-09 | 2.645751311 | 6.244998 | 36 | 36 | 1 | 4.539354477 | 0 | 0 | 0 | KC131129,KP671755,KT919972,KT919973,MK568540,MT612988,MT612989,ON000910,ON190025 |
| VC_549_1 | 6 | 4500 | 25933.14439 | 0.1478651 | 0.0001681 | 2.828427125 | 8.83176087 | 15 | 15 | 1 | 5.808049561 | 0 | 0 | 0 | AY283928,JN849462,MN794232,MT135026,OK905446,ON190024 |
| VC_549_2 | 1 | 0 | 5815.520386 | 0 | 0 | 0 | 0 | 1 | 1 | 1 | 0 | 0 | 0 | 0 | HQ317393 |
| VC_549_3 | 1 | 0 | 5814.354947 | 0 | 0 | 0 | 0 | 1 | 1 | 1 | 0 | 0 | 0 | 0 | MW824377 |
| VC_549_4 | 1 | 0 | 5808.248989 | 0 | 0 | 0 | 0 | 1 | 1 | 1 | 0 | 0 | 0 | 0 | MG592456 |
| VC_549_5 | 1 | 0 | 8182.08452 | 0 | 0 | 0 | 0 | 1 | 1 | 1 | 0 | 0 | 0 | 0 | MT135025 |
| VC_549_6 | 1 | 0 | 5783.76994 | 0 | 0 | 0 | 0 | 1 | 1 | 1 | 0 | 0 | 0 | 0 | MT135024 |
| VC_55_0 | 5 | 343.1530764 | 348.2146689 | 0.49633944 | 1.92E-06 | 0 | 4 | 10 | 10 | 1 | 2.422693876 | 0 | 0 | 0 | HM064452,ON170025,ON170069,ON170139,ON170320 |
| VC_550_0 | 2 | 300 | 449.9458128 | 0.4000289 | 0.1056498 | 1.414213562 | 1.41421356 | 1 | 1 | 1 | 1.414213562 | 0 | 0 | 0 | AP019524,AP019525 |
| VC_550_1 | 1 | 0 | 375.908071 | 0 | 0 | 0 | 0 | 1 | 1 | 1 | 0 | 0 | 0 | 0 | LC168164 |
| VC_551_0 | 2 | 300 | 859.7133863 | 0.25868461 | 0.0546959 | 0 | 0 | 1 | 1 | 1 | 0 | 0 | 0 | 0 | LR756501,LR756503 |
| VC_551_1 | 1 | 0 | 910.9819274 | 0 | 0 | 0 | 0 | 1 | 1 | 1 | 0 | 0 | 0 | 0 | LR745206 |

| | | | | | | | | | | | | | | |
|---|---|---|---|---|---|---|---|---|---|---|---|---|---|---|
| VC_551_2 | 1 | 0 | 915.6123884 | 0 | 0 | 0 | 0 | 1 | 1 | 1 | 0 | 0 | 0 | 0 | LR756502 |
| VC_551_3 | 1 | 0 | 722.3420264 | 0 | 0 | 0 | 0 | 1 | 1 | 1 | 0 | 0 | 0 | 0 | LR756504 |
| VC_552_0 | 9 | 10206.52736 | 0 | 1 | 0 | 0 | 4.24264069 | 36 | 36 | 1 | 2.756800211 | 0 | 0 | 0 | AP019527,FJ429185,KY554762,KY554763,KY554764,KY554765,KY554766,KY554767,MN528768 |
| VC_553_0 | 3 | 545.6186476 | 5710.013206 | 0.08722039 | 0.0014633 | 5.656854249 | 7.14142843 | 3 | 3 | 1 | 6.599427559 | 0 | 0 | 0 | EF153632,MT708549,MW460246 |
| VC_558_0 | 2 | 25.73744265 | 941.6483408 | 0.02660515 | 0.0526787 | 8.246211251 | 8.24621125 | 1 | 1 | 1 | 8.246211251 | 0 | 0 | 0 | S11_vs_76784,S7_vs_111831 |
| VC_559_0 | 3 | 170.0127634 | 0 | 1 | 0 | 3 | 3.74165739 | 3 | 3 | 1 | 3.352760726 | 0 | 0 | 0 | AY048850,BK013337,BK013338 |
| VC_56_0 | 6 | 493.3854267 | 241.5578092 | 0.67132454 | 1.01E-07 | 0 | 2.23606798 | 15 | 15 | 1 | 1.454741697 | 0 | 0 | 0 | AB259124,AB828698,AB931172,JQ408219,LC066596,MT495635 |
| VC_560_0 | 15 | 12605.86473 | 31693.45673 | 0.28456112 | 4.21E-68 | 1 | 8.36660027 | 105 | 105 | 1 | 6.644672412 | 0 | 0 | 0 | KC139518,KT287080,MF158036,MF158037,MH424444,MK214385,MK479295,MN070121,MN393079,MT012729,MW725301,MZ004649,MZ501269,ON720976,S17_vf_177199 |
| VC_561_0 | 3 | 128.937636 | 249.0402016 | 0.34112486 | 0.0020745 | 6.08276253 | 7.34846923 | 3 | 3 | 1 | 6.810410586 | 0 | 0 | 0 | AY082070,JQ660954,MW478290 |
| VC_563_0 | 6 | 3513.564294 | 556.5872024 | 0.86325148 | 5.19E-07 | 0 | 3.16227766 | 15 | 15 | 1 | 1.913044717 | 0 | 0 | 0 | MG592399,MG592418,MG592419,MG592510,MG592511,MG592579 |
| VC_564_0 | 2 | 246.0355269 | 456.2571201 | 0.35033191 | 0.0594094 | 0 | 0 | 1 | 1 | 1 | 0 | 0 | 0 | 0 | MG592447,MG592453 |
| VC_566_0 | 2 | 300 | 21768.88922 | 0.0135938 | 0.362514 | 8.660254038 | 8.66025404 | 1 | 1 | 1 | 8.660254038 | 0 | 0 | 0 | JN201525,MH399772 |
| VC_566_1 | 2 | 300 | 21835.36214 | 0.01355297 | 0.3111998 | 7.937253933 | 7.93725393 | 1 | 1 | 1 | 7.937253933 | 0 | 0 | 0 | MN586029,MW314846 |
| VC_566_10 | 1 | 0 | 11476.21745 | 0 | 0 | 0 | 0 | 1 | 1 | 1 | 0 | 0 | 0 | 0 | AY129338 |
| VC_566_11 | 1 | 0 | 11338.57976 | 0 | 0 | 0 | 0 | 1 | 1 | 1 | 0 | 0 | 0 | 0 | JF937101 |
| VC_566_2 | 3 | 900 | 31855.69884 | 0.02747613 | 0.1419306 | 4.69041576 | 5.65685425 | 3 | 3 | 1 | 5.148763174 | 0 | 0 | 0 | JN698997,KM400683,MF919534 |
| VC_566_3 | 4 | 1800 | 41294.97791 | 0.04176821 | 0.072186 | 4.358898944 | 8.48528137 | 6 | 6 | 1 | 6.592672932 | 0 | 0 | 0 | KM925136,MF072690,MF133445,MF668284 |
| VC_566_4 | 16 | 36000 | 107052.7776 | 0.25165537 | 1.97E-07 | 4.472135955 | 9.59166305 | 120 | 113 | 0.941666667 | 7.461739702 | 0 | 0 | 0 | KF114875,MH077579,MH669017,MH697582,MH697583,MH727551,MK524504,MK524521,MK524527,MK524529,MK967379,MK967402,MN062701,MN183286,MZ622169,MZ958748 |
| VC_566_5 | 1 | 0 | 11223.23744 | 0 | 0 | 0 | 0 | 1 | 1 | 1 | 0 | 0 | 0 | 0 | MF919504 |
| VC_566_6 | 2 | 300 | 21972.25061 | 0.01346968 | 0.2189071 | 6.32455532 | 6.32455532 | 1 | 1 | 1 | 6.32455532 | 0 | 0 | 0 | KF006818,MF919512 |
| VC_566_7 | 4 | 1800 | 42177.13952 | 0.04093036 | 0.003384 | 6.403124237 | 8.94427191 | 6 | 6 | 1 | 7.722585294 | 0 | 0 | 0 | JF937090,JF957059,KM101123,MK279849 |
| VC_566_8 | 1 | 0 | 11191.51565 | 0 | 0 | 0 | 0 | 1 | 1 | 1 | 0 | 0 | 0 | 0 | MW132714 |
| VC_566_9 | 1 | 0 | 11194.68168 | 0 | 0 | 0 | 0 | 1 | 1 | 1 | 0 | 0 | 0 | 0 | MK524516 |
| VC_57_0 | 3 | 415.7639771 | 1089.984299 | 0.27611785 | 0.0014927 | 1.732050808 | 2.82842712 | 3 | 3 | 1 | 2.265515303 | 0 | 0 | 0 | CP006891,CP023680,CP023686 |
| VC_570_0 | 12 | 18777.97644 | 4694.261354 | 0.80000793 | 1.80E-35 | 0 | 8.30662386 | 66 | 66 | 1 | 5.696831437 | 0 | 0 | 0 | MW314855,MW314856,MW314858,MW584152,MW584158,MW584161,MW584164,MW584171,MW584179,MW584180,MW584196,MW584204 |
| VC_571_0 | 16 | 34952.33356 | 349210.9573 | 0.09098301 | 4.35E-58 | 0 | 10.3440804 | 120 | 80 | 0.666666667 | 8.305420408 | 0 | 0 | 0 | JF937091,JF937106,MG872831,MG872832,MG872843,MH399774,MH399778,MH536820,MK016502,MK359320,MK801726,MK937593,MN096361,MN586013,MT723943,MW291023 |
| VC_571_1 | 4 | 1745.019177 | 108610.3764 | 0.01581272 | 0.0003361 | 6.782329983 | 9.32737905 | 6 | 4 | 0.666666667 | 8.397948149 | 0 | 0 | 0 | MF919541,MH576956,MN586043,MN586044 |
| VC_571_2 | 17 | 40703.38768 | 381222.4758 | 0.09647047 | 1.26E-75 | 0 | 9.2736185 | 136 | 133 | 0.977941176 | 7.37256991 | 0 | 0 | 0 | EU816591,JF937096,JN006061,JN006062,KF188414,KR080204,KT222941,KT222942,KU865303,KX817173,KY319168,MH513972,MH513978,MN586032,MN586051,MT114165,MZ274305 |
| VC_571_3 | 1 | 0 | 28103.30722 | 0 | 0 | 0 | 0 | 1 | 1 | 1 | 0 | 0 | 0 | 0 | KF306380 |
| VC_571_4 | 2 | 300 | 49887.98835 | 0.00597753 | 0.0430414 | 3.741657387 | 3.74165739 | 1 | 1 | 1 | 3.741657387 | 0 | 0 | 0 | JN412590,KF416343 |
| VC_571_5 | 2 | 281.0022461 | 51458.81489 | 0.00543106 | 0.0445194 | 8.888194417 | 8.88819442 | 1 | 1 | 1 | 8.888194417 | 0 | 0 | 0 | MK016491,MK016498 |
| VC_571_6 | 22 | 67972.70197 | 495361.902 | 0.12066133 | 2.92E-91 | 0 | 10.2956301 | 231 | 187 | 0.80952381 | 7.78923934 | 0 | 0 | 0 | EU816588,KX611831,KX834009,MG757160,MG872837,MH371112,MH513983,MH536829,MH651174,MH669002,MH727557,MH779503,MK559429,MN428059,MN586034,MN586037,MT952854,MZ622157,MZ681505,OL742559,ON970597,ON970600 |
| VC_571_7 | 28 | 111666.4178 | 564676.658 | 0.16510322 | 1.51E-155 | 0 | 10.3440804 | 378 | 322 | 0.851851852 | 7.846339488 | 0 | 0 | 0 | AY129331,DQ398041,GQ303265,JN382248,JN391441,KC661277,KC691255,KC748971,KF279417,KT020852,KT373978,KY549152,MF668277,MH000607,MH536827,MH576953,MK359309,MK620893,MK757445,MN586017,MN586019,MN586035,MN586041,MN586050,MZ322024,MZ622158,MZ622163,ON456345 |
| VC_571_8 | 19 | 50522.52344 | 439368.4838 | 0.10313013 | 1.96E-77 | 0 | 9.89949494 | 171 | 149 | 0.871345029 | 7.47701522 | 0 | 0 | 0 | KC748969,KF024732,KF493883,KF562099,KF562100,KF562101,KF562102,MF919506,MF919524,MF919525,MF919529,MF919535,MF919540,MG099953,MH020247,MH590587,MK433262,MN096364,MT522005 |
| VC_574_0 | 2 | 78.62221093 | 276.211882 | 0.22157457 | 0.0474591 | 6.557438524 | 6.55743852 | 1 | 1 | 1 | 6.557438524 | 0 | 0 | 0 | JN698994,MPU46938 |
| VC_575_0 | 13 | 13934.71723 | 4666.210947 | 0.74914096 | 1.09E-45 | 0 | 7.21110255 | 78 | 78 | 1 | 5.125806796 | 0 | 0 | 0 | MW314852,MW584148,MW584150,MW584154,MW584156,MW584162,MW584163,MW584181,MW584182,MW584192,MW584203,MW584208,MW584212 |
| VC_577_0 | 21 | 63000 | 583.0682672 | 0.99082982 | 2.90E-67 | 0 | 5.09901951 | 210 | 210 | 1 | 3.642219586 | 0 | 0 | 0 | AY129335,JN698993,KF017002,KF024730,KJ829260,KT281796,MG099943,MG872838,MH020246,MH590592,MH926062,MN428052,MN428058,MN585964,MT818418,MT818425,MW314845,MW712737,MZ209306,MZ747511,ON260829 |
| VC_578_0 | 3 | 464.9823391 | 5297.937863 | 0.08068519 | 0.0014625 | 8.717797887 | 9.16515139 | 3 | 2 | 0.666666667 | 8.904903381 | 0 | 0 | 0 | MK937597,MN096370,OP434458 |
| VC_578_1 | 11 | 10099.48545 | 10432.12438 | 0.49189935 | 6.16E-35 | 2 | 9.53939201 | 55 | 43 | 0.781818182 | 8.297248607 | 0 | 0 | 0 | MG099939,MH651176,MH651185,MH779504,MK279906,MK376961,MK814752,MZ388553,MZ747510,ON456334,ON526981 |
| VC_578_2 | 1 | 0 | 2288.63679 | 0 | 0 | 0 | 0 | 1 | 1 | 1 | 0 | 0 | 0 | 0 | MH536812 |
| VC_578_3 | 1 | 0 | 2439.457894 | 0 | 0 | 0 | 0 | 1 | 1 | 1 | 0 | 0 | 0 | 0 | MW534375 |
| VC_579_0 | 2 | 300 | 92697.08439 | 0.00322591 | 0.2520392 | 8.426149773 | 8.42614977 | 1 | 1 | 1 | 8.426149773 | 0 | 0 | 0 | JN204348,JN412592 |
| VC_579_1 | 2 | 300 | 93200.75987 | 0.00320853 | 0.2401055 | 9 | 9 | 1 | 0 | 0 | 9 | 0 | 0 | 0 | GQ303260,MT553335 |
| VC_579_10 | 1 | 0 | 47415.80885 | 0 | 0 | 0 | 0 | 1 | 1 | 1 | 0 | 0 | 0 | 0 | MK112534 |
| VC_579_11 | 2 | 300 | 93375.73507 | 0.00320254 | 0.23233 | 4.358898944 | 4.35889894 | 1 | 1 | 1 | 4.358898944 | 0 | 0 | 0 | KT281790,MT889395 |
| VC_579_12 | 3 | 900 | 139214.1473 | 0.00642333 | 0.1315873 | 7.348469228 | 7.68114575 | 3 | 3 | 1 | 7.481937821 | 0 | 0 | 0 | KC748970,MH825697,MK359348 |
| VC_579_13 | 1 | 0 | 47273.79781 | 0 | 0 | 0 | 0 | 1 | 1 | 1 | 0 | 0 | 0 | 0 | MK359321 |
| VC_579_14 | 8 | 8400 | 361018.7683 | 0.02273842 | 0.0009588 | 2.449489743 | 9.79795897 | 28 | 23 | 0.821428571 | 7.955531127 | 0 | 0 | 0 | MG872839,MH316561,MK359303,MK359311,MK359358,MK450427,ON526982,ON637768 |
| VC_579_15 | 1 | 0 | 47365.78334 | 0 | 0 | 0 | 0 | 1 | 1 | 1 | 0 | 0 | 0 | 0 | MK359342 |
| VC_579_16 | 1 | 0 | 47441.21734 | 0 | 0 | 0 | 0 | 1 | 1 | 1 | 0 | 0 | 0 | 0 | ON755189 |
| VC_579_17 | 1 | 0 | 47494.53829 | 0 | 0 | 0 | 0 | 1 | 1 | 1 | 0 | 0 | 0 | 0 | MK359328 |
| VC_579_18 | 1 | 0 | 47474.99736 | 0 | 0 | 0 | 0 | 1 | 1 | 1 | 0 | 0 | 0 | 0 | MN183285 |
| VC_579_19 | 1 | 0 | 47494.43568 | 0 | 0 | 0 | 0 | 1 | 1 | 1 | 0 | 0 | 0 | 0 | ON637764 |
| VC_579_2 | 2 | 300 | 94415.60467 | 0.00316738 | 0.3625686 | 8.366600265 | 8.36660027 | 1 | 1 | 1 | 8.366600265 | 0 | 0 | 0 | EU826471,MH727543 |
| VC_579_20 | 17 | 40800 | 717290.9354 | 0.0538194 | 1.12E-15 | 0 | 9.21954446 | 136 | 135 | 0.992647059 | 5.130990877 | 0 | 0 | 0 | JF704096,KR080196,MF919493,MH697586,MH697590,MK359326,MK359335,MK359338,MK359339,MK359347,MK359362,MK450432,MN428066,MZ028626,MZ681511,ON081335,ON260824 |
| VC_579_21 | 1 | 0 | 47009.34342 | 0 | 0 | 0 | 0 | 1 | 1 | 1 | 0 | 0 | 0 | 0 | MT723945 |
| VC_579_22 | 4 | 1800 | 179167.6482 | 0.00994653 | 0.0097955 | 7.141428429 | 9.38083152 | 6 | 5 | 0.833333333 | 8.031610371 | 0 | 0 | 0 | JF704092,MH590593,MK450423,MZ958745 |
| VC_579_23 | 1 | 0 | 47073.57067 | 0 | 0 | 0 | 0 | 1 | 1 | 1 | 0 | 0 | 0 | 0 | KX369583 |
| VC_579_24 | 1 | 0 | 47223.61186 | 0 | 0 | 0 | 0 | 1 | 1 | 1 | 0 | 0 | 0 | 0 | OP021681 |
| VC_579_25 | 1 | 0 | 47653.70245 | 0 | 0 | 0 | 0 | 1 | 1 | 1 | 0 | 0 | 0 | 0 | EU826468 |
| VC_579_26 | 1 | 0 | 47613.62104 | 0 | 0 | 0 | 0 | 1 | 1 | 1 | 0 | 0 | 0 | 0 | EU826469 |
| VC_579_27 | 2 | 300 | 94587.1988 | 0.00316165 | 0.3625686 | 6.708203932 | 6.70820393 | 1 | 1 | 1 | 6.708203932 | 0 | 0 | 0 | MK359361,MW712729 |
| VC_579_28 | 1 | 0 | 47412.99286 | 0 | 0 | 0 | 0 | 1 | 1 | 1 | 0 | 0 | 0 | 0 | MK359333 |
| VC_579_29 | 1 | 0 | 47654.6156 | 0 | 0 | 0 | 0 | 1 | 1 | 1 | 0 | 0 | 0 | 0 | MT818416 |
| VC_579_3 | 1 | 0 | 47241.72056 | 0 | 0 | 0 | 0 | 1 | 1 | 1 | 0 | 0 | 0 | 0 | MK359355 |
| VC_579_30 | 3 | 900 | 140710.3299 | 0.00635547 | 0.2520363 | 6.782329983 | 8.94427191 | 3 | 3 | 1 | 7.950213433 | 0 | 0 | 0 | EU826467,JN699625,MK359344 |
| VC_579_31 | 1 | 0 | 47689.85358 | 0 | 0 | 0 | 0 | 1 | 1 | 1 | 0 | 0 | 0 | 0 | DQ398053 |
| VC_579_32 | 1 | 0 | 47645.10457 | 0 | 0 | 0 | 0 | 1 | 1 | 1 | 0 | 0 | 0 | 0 | MK310143 |
| VC_579_33 | 1 | 0 | 47497.66085 | 0 | 0 | 0 | 0 | 1 | 1 | 1 | 0 | 0 | 0 | 0 | MH910040 |
| VC_579_34 | 1 | 0 | 47663.07484 | 0 | 0 | 0 | 0 | 1 | 1 | 1 | 0 | 0 | 0 | 0 | MK112549 |
| VC_579_35 | 1 | 0 | 47536.5044 | 0 | 0 | 0 | 0 | 1 | 1 | 1 | 0 | 0 | 0 | 0 | MT818423 |
| VC_579_36 | 4 | 1800 | 186297.924 | 0.00956948 | 0.1544064 | 0 | 8.60232527 | 6 | 6 | 1 | 6.722553718 | 0 | 0 | 0 | MK359336,MK359363,MK359366,MN234193 |
| VC_579_37 | 1 | 0 | 47448.66095 | 0 | 0 | 0 | 0 | 1 | 1 | 1 | 0 | 0 | 0 | 0 | KT365400 |
| VC_579_38 | 2 | 300 | 94504.65845 | 0.0031644 | 0.3645618 | 7.615773106 | 7.61577311 | 1 | 1 | 1 | 7.615773106 | 0 | 0 | 0 | MK878897,MN428065 |
| VC_579_39 | 3 | 900 | 141067.7617 | 0.00633947 | 0.2714162 | 7.810249676 | 9.32737905 | 3 | 2 | 0.666666667 | 8.420555711 | 0 | 0 | 0 | MK359364,MT723944,OP297534 |
| VC_579_4 | 1 | 0 | 47558.2647 | 0 | 0 | 0 | 0 | 1 | 1 | 1 | 0 | 0 | 0 | 0 | MK359353 |
| VC_579_40 | 1 | 0 | 47586.55275 | 0 | 0 | 0 | 0 | 1 | 1 | 1 | 0 | 0 | 0 | 0 | MN276192 |
| VC_579_41 | 1 | 0 | 47707.64209 | 0 | 0 | 0 | 0 | 1 | 1 | 1 | 0 | 0 | 0 | 0 | MK450424 |
| VC_579_42 | 1 | 0 | 47532.79144 | 0 | 0 | 0 | 0 | 1 | 1 | 1 | 0 | 0 | 0 | 0 | MZ856343 |
| VC_579_43 | 1 | 0 | 47540.76024 | 0 | 0 | 0 | 0 | 1 | 1 | 1 | 0 | 0 | 0 | 0 | MK359337 |
| VC_579_44 | 1 | 0 | 47044.1606 | 0 | 0 | 0 | 0 | 1 | 1 | 1 | 0 | 0 | 0 | 0 | MK359345 |
| VC_579_45 | 1 | 0 | 47500.70047 | 0 | 0 | 0 | 0 | 1 | 1 | 1 | 0 | 0 | 0 | 0 | MK359327 |
| VC_579_46 | 1 | 0 | 47104.26486 | 0 | 0 | 0 | 0 | 1 | 1 | 1 | 0 | 0 | 0 | 0 | MK359349 |
| VC_579_47 | 3 | 900 | 140636.7861 | 0.00635877 | 0.2194023 | 6.92820323 | 8.71779789 | 3 | 3 | 1 | 7.709771964 | 0 | 0 | 0 | MK112541,MK112545,MK112548 |
| VC_579_48 | 2 | 300 | 93957.09973 | 0.00318278 | 0.3196949 | 7.810249676 | 7.8102468 | 1 | 1 | 1 | 7.810249676 | 0 | 0 | 0 | KF024734,KX721256 |
| VC_579_49 | 7 | 6300 | 319141.7596 | 0.0193583 | 0.0108557 | 6.32455532 | 9.94987437 | 21 | 18 | 0.857142857 | 8.265139531 | 0 | 0 | 0 | JN624850,KC748968,KX721255,MH779498,MK112554,MK359334,OP068341 |
| VC_579_5 | 1 | 0 | 47547.9479 | 0 | 0 | 0 | 0 | 1 | 1 | 1 | 0 | 0 | 0 | 0 | KC691257 |
| VC_579_50 | 15 | 31500 | 643413.6077 | 0.04667264 | 1.32E-11 | 4.123105626 | 9.94987437 | 105 | 97 | 0.923809524 | 7.416660423 | 0 | 0 | 0 | JQ911768,KJ595575,KM881426,KT321476,MF919499,MH051266,MH316567,MH669008,MH825713,MK359314,MK359365,MK450429,MK450434,OK040789,OP021684 |
| VC_579_51 | 1 | 0 | 47327.60558 | 0 | 0 | 0 | 0 | 1 | 1 | 1 | 0 | 0 | 0 | 0 | MK112533 |
| VC_579_52 | 1 | 0 | 47347.65405 | 0 | 0 | 0 | 0 | 1 | 1 | 1 | 0 | 0 | 0 | 0 | MH744422 |
| VC_579_53 | 1 | 0 | 47606.32233 | 0 | 0 | 0 | 0 | 1 | 1 | 1 | 0 | 0 | 0 | 0 | MK359306 |
| VC_579_54 | 1 | 0 | 47279.47223 | 0 | 0 | 0 | 0 | 1 | 1 | 1 | 0 | 0 | 0 | 0 | MH779502 |
| VC_579_55 | 1 | 0 | 47248.37401 | 0 | 0 | 0 | 0 | 1 | 1 | 1 | 0 | 0 | 0 | 0 | MF919494 |
| VC_579_56 | 1 | 0 | 47260.46056 | 0 | 0 | 0 | 0 | 1 | 1 | 1 | 0 | 0 | 0 | 0 | MH727558 |
| VC_579_57 | 1 | 0 | 47132.29183 | 0 | 0 | 0 | 0 | 1 | 1 | 1 | 0 | 0 | 0 | 0 | MK450425 |
| VC_579_58 | 1 | 0 | 47680.618 | 0 | 0 | 0 | 0 | 1 | 1 | 1 | 0 | 0 | 0 | 0 | MZ388551 |
| VC_579_59 | 1 | 0 | 47524.24078 | 0 | 0 | 0 | 0 | 1 | 1 | 1 | 0 | 0 | 0 | 0 | MK359317 |
| VC_579_6 | 2 | 300 | 93556.10768 | 0.00319638 | 0.2770066 | 8.888194417 | 8.88819442 | 1 | 1 | 1 | 8.888194417 | 0 | 0 | 0 | JF704116,KT365398 |
| VC_579_60 | 1 | 0 | 47640.19019 | 0 | 0 | 0 | 0 | 1 | 1 | 1 | 0 | 0 | 0 | 0 | MN183280 |
| VC_579_61 | 2 | 300 | 94551.39197 | 0.00316284 | 0.3548019 | 8.888194417 | 8.88819442 | 1 | 1 | 1 | 8.888194417 | 0 | 0 | 0 | MH051260,MK359318 |
| VC_579_62 | 1 | 0 | 47486.55464 | 0 | 0 | 0 | 0 | 1 | 1 | 1 | 0 | 0 | 0 | 0 | MK359305 |
| VC_579_63 | 1 | 0 | 47683.03741 | 0 | 0 | 0 | 0 | 1 | 1 | 1 | 0 | 0 | 0 | 0 | JN699626 |
| VC_579_64 | 1 | 0 | 47408.69841 | 0 | 0 | 0 | 0 | 1 | 1 | 1 | 0 | 0 | 0 | 0 | MK359350 |
| VC_579_65 | 2 | 300 | 94819.91347 | 0.00315391 | 0.3906136 | 7.874007874 | 7.87400787 | 1 | 1 | 1 | 7.874007874 | 0 | 0 | 0 | MK450422,MW291012 |
| VC_579_66 | 1 | 0 | 47677.61531 | 0 | 0 | 0 | 0 | 1 | 1 | 1 | 0 | 0 | 0 | 0 | MK359330 |
| VC_579_67 | 1 | 0 | 47681.3949 | 0 | 0 | 0 | 0 | 1 | 1 | 1 | 0 | 0 | 0 | 0 | OP021682 |
| VC_579_68 | 1 | 0 | 47613.58438 | 0 | 0 | 0 | 0 | 1 | 1 | 1 | 0 | 0 | 0 | 0 | MG812494 |
| VC_579_69 | 1 | 0 | 47491.91331 | 0 | 0 | 0 | 0 | 1 | 1 | 1 | 0 | 0 | 0 | 0 | MK359329 |
| VC_579_7 | 1 | 0 | 47451.05621 | 0 | 0 | 0 | 0 | 1 | 1 | 1 | 0 | 0 | 0 | 0 | MH051261 |
| VC_579_70 | 1 | 0 | 47632.31419 | 0 | 0 | 0 | 0 | 1 | 1 | 1 | 0 | 0 | 0 | 0 | ON456344 |
| VC_579_71 | 1 | 0 | 47076.49827 | 0 | 0 | 0 | 0 | 1 | 1 | 1 | 0 | 0 | 0 | 0 | MK359346 |
| VC_579_72 | 1 | 0 | 47582.61778 | 0 | 0 | 0 | 0 | 1 | 1 | 1 | 0 | 0 | 0 | 0 | MG812493 |
| VC_579_73 | 2 | 300 | 91644.73 | 0.00326283 | 0.1736488 | 6.708203932 | 6.70820393 | 1 | 1 | 1 | 6.708203932 | 0 | 0 | 0 | MH697579,MK359359 |
| VC_579_74 | 1 | 0 | 46907.31388 | 0 | 0 | 0 | 0 | 1 | 1 | 1 | 0 | 0 | 0 | 0 | MF919495 |
| VC_579_75 | 1 | 0 | 47549.85734 | 0 | 0 | 0 | 0 | 1 | 1 | 1 | 0 | 0 | 0 | 0 | ON551388 |
| VC_579_76 | 1 | 0 | 47095.19332 | 0 | 0 | 0 | 0 | 1 | 1 | 1 | 0 | 0 | 0 | 0 | MW822148 |
| VC_579_77 | 3 | 900 | 138458.9152 | 0.00645814 | 0.1082494 | 7.745966692 | 9.48683298 | 3 | 2 | 0.666666667 | 8.368935849 | 0 | 0 | 0 | MF919513,MK359308,MN062707 |
| VC_579_78 | 1 | 0 | 47330.8901 | 0 | 0 | 0 | 0 | 1 | 1 | 1 | 0 | 0 | 0 | 0 | JN699627 |
| VC_579_79 | 1 | 0 | 47411.96643 | 0 | 0 | 0 | 0 | 1 | 1 | 1 | 0 | 0 | 0 | 0 | MN735434 |
| VC_579_8 | 2 | 300 | 94175.98366 | 0.00317541 | 0.3348483 | 5.744562647 | 5.74456265 | 1 | 1 | 1 | 5.744562647 | 0 | 0 | 0 | AY129337,JN699013 |
| VC_579_80 | 1 | 0 | 47578.30683 | 0 | 0 | 0 | 0 | 1 | 1 | 1 | 0 | 0 | 0 | 0 | JN412588 |
| VC_579_81 | 1 | 0 | 46568.45616 | 0 | 0 | 0 | 0 | 1 | 1 | 1 | 0 | 0 | 0 | 0 | KX752698 |
| VC_579_82 | 3 | 900 | 139149.2096 | 0.00642631 | 0.1326333 | 8 | 9.43398113 | 3 | 2 | 0.666666667 | 8.580201665 | 0 | 0 | 0 | GQ303262,KX831080,MF919527 |
| VC_579_9 | 3 | 900 | 139259.1103 | 0.00642127 | 0.188183 | 7.211102551 | 9.64365076 | 3 | 2 | 0.666666667 | 8.264002415 | 0 | 0 | 0 | KF006817,KX781992,MK359324 |
| VC_580_0 | 2 | 300 | 26999.33656 | 0.01098928 | 0.0434872 | 7.549834435 | 7.54983444 | 1 | 1 | 1 | 7.549834435 | 0 | 0 | 0 | EU826466,MF668280 |
| VC_580_1 | 1 | 0 | 15021.85707 | 0 | 0 | 0 | 0 | 1 | 1 | 1 | 0 | 0 | 0 | 0 | MZ820085 |
| VC_583_0 | 2 | 300 | 3908.389405 | 0.07128618 | 0.043574 | 4.358898944 | 4.35889894 | 1 | 1 | 1 | 4.358898944 | 0 | 0 | 0 | KJ567043,MK016501 |
| VC_584_0 | 3 | 806.2163935 | 1589.374117 | 0.33654182 | 0.0014966 | 1 | 2.64575131 | 3 | 3 | 1 | 2.031747018 | 0 | 0 | 0 | MW584155,MW584175,MW584201 |
| VC_585_0 | 20 | 51333.19391 | 9955.855264 | 0.83755899 | 1.23E-107 | 0 | 5.91607978 | 190 | 190 | 1 | 2.340816186 | 0 | 0 | 0 | DQ398047,DQ398051,EU676000,FJ168660,FJ168661,FJ168662,JF937107,JN699014,KJ194582,MH025888,MH316562,MH399788,MH834615,MK494102,MK524498,MK559427,MN119378,MN234227,MN284908,MT889385 |
| VC_586_0 | 2 | 280.5965723 | 2583.042512 | 0.09798601 | 0.0437602 | 3.16227766 | 3.16227766 | 1 | 1 | 1 | 3.16227766 | 0 | 0 | 0 | AY129339,MK279909 |
| VC_587_0 | 8 | 7406.262265 | 7688.222864 | 0.49066015 | 2.33E-19 | 2 | 6.4807407 | 28 | 28 | 1 | 3.692026831 | 0 | 0 | 0 | EU770222,FJ174691,KF986247,KJ567046,MG962376,MH509445,MN010756,MT521996 |
| VC_588_0 | 8 | 8321.877036 | 6291.620643 | 0.56946511 | 2.33E-19 | 1 | 3.74165739 | 28 | 28 | 1 | 2.697423168 | 0 | 0 | 0 | JF704112,KF416342,KT591076,KY969628,MH001447,MH926059,MK757446,MT684602 |
| VC_589_0 | 3 | 900 | 3442.591943 | 0.2072495 | 0.0015006 | 1.732050808 | 2.44948974 | 3 | 3 | 1 | 2.139202843 | 0 | 0 | 0 | JN412589,KR997933,ON108649 |
| VC_59_0 | 4 | 557.1363132 | 814.2755328 | 0.40625018 | 1.32E-05 | 0 | 3 | 6 | 6 | 1 | 1.776142375 | 0 | 0 | 0 | ON470596,ON470604,ON470624,ON470627 |
| VC_590_0 | 9 | 8833.764836 | 9006.007465 | 0.49517251 | 3.61E-24 | 0 | 6.63324958 | 36 | 36 | 1 | 3.7813655 | 0 | 0 | 0 | KX557285,MH025890,MH271313,MH576972,MT639639,MT771345,MT771348,MZ171372,ON970620 |
| VC_591_0 | 4 | 1168.361777 | 4989.655122 | 0.1897302 | 1.41E-05 | 2.828427125 | 8.36660027 | 6 | 6 | 1 | 6.460381676 | 0 | 0 | 0 | MG757156,MH834613,MN585993,MT776806 |
| VC_592_0 | 16 | 31854.01997 | 12025.67253 | 0.72593991 | 2.12E-71 | 0 | 6.63324958 | 120 | 120 | 1 | 4.221284356 | 0 | 0 | 0 | MN234162,MN234190,MN234207,MN234220,MN234232,MN586003,MZ005671,OL455887,OL455890,OL455895,ON755185,ON755187,ON970611,OP172868,OP172870,OP172876 |
| VC_593_0 | 2 | 300 | 2446.181402 | 0.1092426 | 0.0436081 | 0 | 0 | 1 | 1 | 1 | 0 | 0 | 0 | 0 | MN813687,ON108643 |
| VC_594_0 | 7 | 3336.285683 | 8905.150509 | 0.27254038 | 8.34E-15 | 1.732050808 | 7.68114575 | 21 | 21 | 1 | 5.262939248 | 0 | 0 | 0 | JX901189,KF024728,MK279840,MW460248,MZ152914,OM793623,ON108645 |

| | | | | | | | | | | | | | | | |
|---|---|---|---|---|---|---|---|---|---|---|---|---|---|---|---|
| VC_595_0 | 35 | 137483.8063 | 18590.78245 | 0.88088527 | 5.26E-211 | 0 | 6 | 595 | 595 | 1 | 4.089582088 | 0 | 0 | 0 | AY216660,HM035024,JX912252,KP085586,KU194205,KU194206,KX828710,KY985004,LC516895,MF093736,MF468274,MG049919,MH051911,MH051912,MH285980,MH917278,MK213796,MK335533,MK972831,MN153797,MN296515,MN337573,MN342247,MN393473,MT427400,MT682715,MZ501092,OL770108,OL800706,OL960578,OL960579,OL960582,ON185577,OP094641,OP455115 |
| VC_596_0 | 4 | 1174.701444 | 18289.69427 | 0.06035129 | 1.23E-05 | 2.645751311 | 8.48528137 | 6 | 6 | 1 | 6.122655419 | 0 | 0 | 0 | MF996376,MH051918,MT023084,MZ501053 |
| VC_597_0 | 6 | 1562.995419 | 131.8229648 | 0.92222001 | 7.01E-08 | 1 | 8.24621125 | 15 | 15 | 1 | 5.974610183 | 0 | 0 | 0 | AY236756,JN051154,JX486087,MG765275,MG765278,MH809528 |
| VC_598_0 | 2 | 25.73386029 | 109.522559 | 0.19025981 | 0.0612488 | 7.483314774 | 7.48331477 | 1 | 1 | 1 | 7.483314774 | 0 | 0 | 0 | MG020111,MK504442 |
| VC_599_0 | 2 | 300 | 4621.860148 | 0.06095256 | 0.0423669 | 2.645751311 | 2.64575131 | 1 | 1 | 1 | 2.645751311 | 0 | 0 | 0 | KC954775,OM638103 |
| VC_6_0 | 44 | 107578.2199 | 11052.13476 | 0.90683552 | 0 | 0 | 6.8556546 | 946 | 946 | 1 | 4.202784737 | 0 | 0 | 0 | AY319521,CP019275,CP025900,HM770079,KT630647,KY271398,MK416009,MK416011,MK416012,MK416020,MK422449,MK422453,MK422454,MK433577,MK433578,MK433580,MK433581,MK433582,OK490395,OK490397,OK490399,OK490401,OK490403,OK490405,OK490407,OK490411,OK490413,OK490418,OK490423,OK490425,OK490427,OK490429,OK490431,OK490434,OK490436,OK490439,OK490442,OK490445,OK490448,OK490452,OK490455,OK490457,ON470620,OV696611 |
| VC_600_0 | 1 | 0 | 617.5331418 | 0 | 0 | 0 | 0 | 1 | 1 | 1 | 0 | 0 | 0 | 0 | MW805361 |
| VC_600_1 | 1 | 0 | 722.7907488 | 0 | 0 | 0 | 0 | 1 | 1 | 1 | 0 | 0 | 0 | 0 | OM982621 |
| VC_601_0 | 4 | 1800 | 29157.81005 | 0.05814365 | 3.92E-05 | 2 | 8.18535277 | 6 | 6 | 1 | 5.644110395 | 0 | 0 | 0 | MN184887,MN732867,OP480062,OP480063 |
| VC_601_1 | 3 | 900 | 13763.87312 | 0.06137533 | 0.0019803 | 0 | 1 | 3 | 3 | 1 | 0.666666667 | 0 | 0 | 0 | OK136180,OK181858,OK181861 |
| VC_601_2 | 1 | 0 | 8828.624257 | 0 | 0 | 0 | 0 | 1 | 1 | 1 | 0 | 0 | 0 | 0 | KM190144 |
| VC_601_3 | 12 | 19800 | 64852.64105 | 0.23389701 | 9.40E-33 | 4.795831523 | 9.89949494 | 66 | 57 | 0.863636364 | 7.513816288 | 0 | 0 | 0 | KT934943,KX452695,KY652726,MG250486,MK024806,MT178275,MT438396,MT939253,MZ602147,ON602729,ON602736,OP296941 |
| VC_601_4 | 3 | 900 | 25082.76809 | 0.03463834 | 0.0194703 | 7.348469228 | 8.06225775 | 3 | 3 | 1 | 7.740325551 | 0 | 0 | 0 | JN882284,KF550303,MZ311864 |
| VC_601_5 | 4 | 1800 | 32483.13632 | 0.05250395 | 0.0006906 | 5.477225575 | 9.48683298 | 6 | 5 | 0.833333333 | 7.52142506 | 0 | 0 | 0 | GU070616,JX181824,MW006477,MZ326168 |
| VC_601_6 | 1 | 0 | 7606.723145 | 0 | 0 | 0 | 0 | 1 | 1 | 1 | 0 | 0 | 0 | 0 | MG969411 |
| VC_603_0 | 28 | 83590.31444 | 25078.32194 | 0.76922208 | 1.07E-184 | 0 | 8 | 378 | 378 | 1 | 5.084829332 | 0 | 0 | 0 | AY308796,KM236241,KX015770,KX015771,KY657202,KY694971,LR597656,MG241338,MH102284,MH491968,MH729819,MN994500,MT478992,MW021748,MW021750,MW598258,MZ501059,OK499973,OK499984,OL539443,OL539444,OL539461,OL539462,OL539465,OL539466,ON843697,OP292673,OP292674 |
| VC_604_0 | 4 | 1292.097532 | 23058.09676 | 0.05306313 | 1.25E-05 | 4.242640687 | 8.1240384 | 6 | 6 | 1 | 6.341460459 | 0 | 0 | 0 | MN019128,MW032452,MZ501077,MZ501085 |
| VC_604_1 | 3 | 523.9505147 | 16307.70113 | 0.03112888 | 0.0014233 | 4.795831523 | 7.87400787 | 3 | 3 | 1 | 6.695345961 | 0 | 0 | 0 | MK778457,MN090155,MZ501099 |
| VC_604_2 | 1 | 0 | 4231.596456 | 0 | 0 | 0 | 0 | 1 | 1 | 1 | 0 | 0 | 0 | 0 | MW689258 |
| VC_604_3 | 1 | 0 | 5493.715152 | 0 | 0 | 0 | 0 | 1 | 1 | 1 | 0 | 0 | 0 | 0 | OM317910 |
| VC_605_0 | 3 | 685.4025328 | 13248.04302 | 0.04919117 | 0.0014007 | 4.472135955 | 5.38516481 | 3 | 3 | 1 | 4.884377428 | 0 | 0 | 0 | MN101222,OK019720,ON045001 |
| VC_606_0 | 2 | 75.61692717 | 169.4239783 | 0.308589 | 0.0880711 | 7.810249676 | 7.81024968 | 1 | 1 | 1 | 7.810249676 | 0 | 0 | 0 | AY320035,KT221034 |
| VC_607_0 | 2 | 10.14755269 | 120.5939043 | 0.07761542 | 0.0533837 | 7.416198487 | 7.41619849 | 1 | 1 | 1 | 7.416198487 | 0 | 0 | 0 | KU958700,MH744420 |
| VC_608_0 | 2 | 36.92500421 | 0 | 1 | 0 | 2 | 2 | 1 | 1 | 1 | 2 | 0 | 0 | 0 | AY324828,MN710383 |
| VC_609_0 | 27 | 105300 | 602.0001547 | 0.9943155 | 1.48E-107 | 2.449489743 | 7.21110255 | 351 | 351 | 1 | 5.276747218 | 0 | 0 | 0 | AY343333,EF437941,EU863408,HE978309,KY683735,MF327006,MH560358,MK327932,MK327935,MK327941,MK327947,MK573636,MK962753,MN655998,MN709127,MN850574,MN850615,MN882349,MN894885,MT478991,MT764208,MW748999,MZ504876,MZ504877,MZ504878,ON286974,OX001802 |
| VC_610_0 | 14 | 19290.70096 | 18098.8283 | 0.51593859 | 2.94E-57 | 0 | 4.58257569 | 91 | 91 | 1 | 2.11587904 | 0 | 0 | 0 | CP059058,KP063118,MG030346,MN062188,MN604053,MN840486,MW145139,OK499974,OK499975,OK499986,OK499998,OK500000,OL741431,OM962992 |
| VC_611_0 | 50 | 263731.5019 | 97266.11115 | 0.73056301 | 0 | 0 | 7.41619849 | 1225 | 1225 | 1 | 4.071210299 | 0 | 0 | 0 | JN871591,JQ340774,JX094499,JX296113,KC139512,KC139514,KC139515,KC139519,KC677662,KM366098,KM458633,KR296689,KR296691,KU927500,KX452699,KX452700,KX765862,KX765863,KX765864,KY014601,KY620117,LC553736,LR026998,MG280946,MH631453,MH791395,MH791396,MK249308,MK286578,MN871450,MT499897,MT499898,MT675124,MT675125,MT675126,MW057860,MW149272,MW355449,MW355460,MW355467,MW355470,MW355474,MW355476,MW355479,OL606627,OL699877,OM025231,OP453108,OP535471,OP535472 |
| VC_612_0 | 5 | 2744.194642 | 3312.195479 | 0.45310731 | 5.09E-08 | 0 | 4.58257569 | 10 | 10 | 1 | 1.833030278 | 0 | 0 | 0 | KM581061,MH015249,MH015252,MH015254,MH015258 |
| VC_613_0 | 2 | 209.3736196 | 2668.068258 | 0.0727638 | 0.0431414 | 3.872983346 | 3.87298335 | 1 | 1 | 1 | 3.872983346 | 0 | 0 | 0 | KT321316,OK041469 |
| VC_614_0 | 4 | 192.9831286 | 3089.068136 | 0.05879955 | 0.0072582 | 6.403124237 | 8.54400375 | 6 | 6 | 1 | 7.964181499 | 0 | 0 | 0 | HQ632859,KY249644,MF476925,MW269554 |
| VC_617_0 | 6 | 3147.468162 | 14420.1317 | 0.17916324 | 1.89E-11 | 2 | 6.78232998 | 15 | 15 | 1 | 3.924864783 | 0 | 0 | 0 | MK838113,MN317029,MT952005,MW978786,OL840901,OL964754 |
| VC_618_0 | 50 | 50276.74679 | 56.45387248 | 0.9988784 | 2.66E-10 | 0 | 6.92820323 | 1225 | 1225 | 1 | 4.878632408 | 0 | 0 | 0 | AY370762,AY388628,AY423772,EU030938,EU030939,FJ870915,FJ870916,FJ870917,KC618393,KY563228,KY579375,MK054204,MK054205,MK054206,MK054207,MK054208,MK054209,MK054210,MK054211,MK054212,MK054213,MK054214,MK054215,MK054216,MK054217,MK054218,MK054219,MK054220,MK054221,MK054222,MK054223,MK054224,MK054225,MK054226,MK054227,MK054228,MK054229,MK054230,MK054231,MK054232,MK054233,MK054234,MK054235,MK054236,MK054237,MN496305,MN496306,MN496307,MN496308,X07234 |
| VC_619_0 | 3 | 900 | 15756.79941 | 0.05403199 | 0.0270529 | 2.449489743 | 5.83095189 | 3 | 3 | 1 | 4.645765296 | 0 | 0 | 0 | MK577502,MN871507,ON042478 |
| VC_619_1 | 7 | 6300 | 28044.97215 | 0.18343296 | 3.05E-08 | 0 | 3.16227766 | 21 | 21 | 1 | 2.540839297 | 0 | 0 | 0 | AY375531,AY962392,KY290948,KY290951,KY290956,KY290957,KY290958 |
| VC_619_2 | 10 | 13500 | 30995.75613 | 0.30339972 | 5.36E-17 | 3.872983346 | 7.28010989 | 45 | 45 | 1 | 5.629866963 | 0 | 0 | 0 | DQ529280,HM452125,JN377894,JN377895,JX306041,MF479730,MH179476,MH179477,MW584871,OL840900 |
| VC_62_0 | 12 | 1608.102594 | 501.1602551 | 0.76240028 | 6.80E-23 | 0 | 1.41421356 | 66 | 66 | 1 | 0.54913092 | 0 | 0 | 0 | AB334720,AB334721,F1CCG,GQ153916,GQ153917,GQ153918,GQ153919,JF719732,JF719733,JF719734,JX412914,PFDCG |
| VC_621_0 | 2 | 188.2745059 | 637.9216123 | 0.22788113 | 0.0443952 | 1.732050808 | 1.73205081 | 1 | 1 | 1 | 1.732050808 | 0 | 0 | 0 | JN190960,MW677528 |
| VC_622_0 | 9 | 3399.343526 | 26.55458064 | 0.99224887 | 5.86E-08 | 0 | 1.41421356 | 36 | 36 | 1 | 0.314269681 | 0 | 0 | 0 | AY441783,AY848684,AY848685,AY848686,AY848687,AY848688,AY848689,OK041468,OM630588 |
| VC_623_0 | 22 | 20949.2457 | 5770.220761 | 0.78404431 | 1.71E-81 | 0 | 8.1240384 | 231 | 231 | 1 | 5.755723423 | 0 | 0 | 0 | KR093634,KR093635,KR093636,KR093637,KR093639,KR093640,KR093641,KR093642,KR093643,KR093644,KR093645,KR093646,KR093647,KR093648,KR093649,KR093650,KR093651,KR093652,KR093653,KR093654,KR093655,KR093656 |
| VC_625_0 | 2 | 72.56088058 | 269.5093123 | 0.21212278 | 0.0477797 | 5.656854249 | 5.65685425 | 1 | 1 | 1 | 5.656854249 | 0 | 0 | 0 | EF455602,KF188409 |
| VC_627_0 | 16 | 18643.74513 | 1691.973298 | 0.91679796 | 8.89E-52 | 0 | 5.47722558 | 120 | 120 | 1 | 2.700670241 | 0 | 0 | 0 | AY505112,AY510084,JF712866,KM612259,KM612260,KM612261,KM612262,KM612263,KM612264,KM612265,KY883642,KY883647,KY883656,MH105773,MW824387,MW824404 |
| VC_628_0 | 5 | 1581.316987 | 2098.937882 | 0.42967594 | 4.03E-08 | 1.414213562 | 4.79583152 | 10 | 10 | 1 | 3.76301008 | 0 | 0 | 0 | MG674163,MT992243,MW176032,MW176034,MW590698 |
| VC_629_0 | 4 | 171.5010366 | 181.6532898 | 0.48562632 | 7.58E-05 | 6.244997998 | 7.48331477 | 6 | 6 | 1 | 6.962169599 | 0 | 0 | 0 | MF074189,MW202496,MW202711,MW202879 |
| VC_630_0 | 4 | 710.8294337 | 91.21251294 | 0.88627464 | 0.0001226 | 6.164414003 | 9.21954446 | 6 | 4 | 0.666666667 | 8.146450401 | 0 | 0 | 0 | S15_vs_71968,S16_vs_142561,S3_vs_17168,S5_vs_20500 |
| VC_631_0 | 2 | 94.73065177 | 0 | 1 | 0 | 1 | 1 | 1 | 1 | 1 | 1 | 0 | 0 | 0 | AY569307,GU080336 |
| VC_632_0 | 9 | 6978.468063 | 348.6023041 | 0.95242269 | 7.27E-12 | 0 | 1.41421356 | 36 | 36 | 1 | 0.971404521 | 0 | 0 | 0 | AY575774,DQ163914,KU884561,KU884562,KU884563,KU884564,KU884565,MK514283,ON857941 |
| VC_633_0 | 2 | 12.42738916 | 338.1867282 | 0.03544463 | 0.1056498 | 4 | 4 | 1 | 1 | 1 | 4 | 0 | 0 | 0 | JF412296,OM519319 |
| VC_634_0 | 2 | 68.84894869 | 196.2998211 | 0.25966158 | 0.0559417 | 6 | 6 | 1 | 1 | 1 | 6 | 0 | 0 | 0 | CAKLQA020000003,MZ326864 |
| VC_636_0 | 13 | 21435.62101 | 13008.89569 | 0.62232318 | 1.45E-40 | 2.828427125 | 8.18535277 | 78 | 78 | 1 | 4.432820219 | 0 | 0 | 0 | JX128258,MH051335,MH816966,MK373776,MK373777,MK903282,MN038177,MN176217,MT446405,MT446408,OL856099,ON210137,OP352608 |
| VC_637_0 | 27 | 92540.66891 | 12541.47234 | 0.88065077 | 3.61E-154 | 0 | 6.63324958 | 351 | 351 | 1 | 2.174825626 | 0 | 0 | 0 | KT361654,KT361655,KT361656,KT361657,OP494865,OP503543,OP503968,OP503969,OP503970,OP503971,OP503972,OP503973,OP503974,OP503975,OP503976,OP503977,OP503978,OP503979,OP503980,OP503981,OP503982,OP503983,OP503984,OP503985,OP503986,OP503987,OP503988 |
| VC_638_0 | 2 | 6.131678344 | 83.26455719 | 0.06858989 | 0.4371835 | 7.681145748 | 7.68114575 | 1 | 1 | 1 | 7.681145748 | 0 | 0 | 0 | MH884512,S5_vf_145585 |
| VC_64_0 | 2 | 187.8213234 | 1478.290812 | 0.1127303 | 0.0430903 | 0 | 0 | 1 | 1 | 1 | 0 | 0 | 0 | 0 | AP009146,DQ785801 |
| VC_640_0 | 1 | 0 | 474.5289431 | 0 | 0 | 0 | 0 | 1 | 1 | 1 | 0 | 0 | 0 | 0 | S9_vs_90846 |
| VC_641_0 | 2 | 179.2638278 | 570.8176699 | 0.23899247 | 0.0455601 | 5.477225575 | 5.47722558 | 1 | 1 | 1 | 5.477225575 | 0 | 0 | 0 | S3_vs_127391,S4_vs_VIRSorter_31976-circular-cat_2 |
| VC_642_0 | 3 | 28.22551428 | 196.0795598 | 0.12583538 | 0.2762225 | 6.403124237 | 8 | 3 | 3 | 1 | 7.227744709 | 0 | 0 | 0 | EF579802,KX911187,S7_vs_73280 |
| VC_643_0 | 2 | 9.024707333 | 726.3290314 | 0.01227261 | 0.3829237 | 8.124038405 | 8.1240384 | 1 | 1 | 1 | 8.124038405 | 0 | 0 | 0 | S11_vs_104047,S3_vs_29077 |
| VC_645_0 | 20 | 37922.01907 | 6680.022242 | 0.85023057 | 1.10E-70 | 0 | 6.164414 | 190 | 190 | 1 | 3.328174658 | 0 | 0 | 0 | AY625898,KC262634,KC900378,LT603684,MF490237,MK511039,MK511040,MK511041,MK511042,MK511043,MK511044,MK511045,MK511046,MK511047,MK511048,MK511049,MK511050,MK511051,MT118301,MZ285879 |
| VC_646_0 | 3 | 598.0368738 | 4293.043933 | 0.1222709 | 0.001808 | 1.414213562 | 5.29150262 | 3 | 3 | 1 | 3.999072936 | 0 | 0 | 0 | MT152150,MT491207,MT491208 |
| VC_647_0 | 37 | 81345.56499 | 57768.45877 | 0.58474022 | 4.08E-296 | 0 | 10.2956301 | 666 | 615 | 0.923423423 | 6.290653613 | 0 | 0 | 0 | FM864213,FN997652,KC348603,KC581799,KT336320,KT336321,KX077896,MK359990,MK448674,MK448741,MK448752,MK448811,MK448819,MK448828,MK448983,MN270258,MN270259,MN270260,MN270261,MN270262,MN270263,MN270264,MN270265,MN270266,MN270267,MN270268,MN270269,MN270270,MN270271,MN270272,MN270273,MN270274,MN270275,MN270276,MN270277,MN270278,MN270279 |
| VC_649_0 | 2 | 300 | 1735.093927 | 0.14741334 | 0.0467009 | 7.348469228 | 7.34846923 | 1 | 1 | 1 | 7.348469228 | 0 | 0 | 0 | S14_vs_66139,S5_vs_40848 |
| VC_649_1 | 14 | 6295.667286 | 5751.195648 | 0.52259807 | 1.40E-43 | 0 | 9.8488578 | 91 | 76 | 0.835164835 | 7.091518818 | 0 | 0 | 0 | MF172979,MK448667,MK448700,MK448720,MK448839,MK448841,MK448843,MK448934,MK448997,MW387018,S11_vs_42927,S13_vs_23314,S14_vs_96111,S5_vs_8847 |
| VC_649_2 | 2 | 181.9950425 | 2193.843944 | 0.07660243 | 0.0453418 | 5.099019514 | 5.09901951 | 1 | 1 | 1 | 5.099019514 | 0 | 0 | 0 | S13_vs_30167,S3_vs_119605 |
| VC_649_3 | 2 | 57.49904909 | 1826.778332 | 0.03051517 | 0.0817229 | 8.717797887 | 8.71779789 | 1 | 1 | 1 | 8.717797887 | 0 | 0 | 0 | S13_vs_30101,S8_vs_149700 |
| VC_649_4 | 1 | 0 | 994.013441 | 0 | 0 | 0 | 0 | 1 | 1 | 1 | 0 | 0 | 0 | 0 | S8_vs_115958 |
| VC_649_5 | 1 | 0 | 1032.096372 | 0 | 0 | 0 | 0 | 1 | 1 | 1 | 0 | 0 | 0 | 0 | S9_vs_90727 |
| VC_65_0 | 3 | 764.9506684 | 596.045227 | 0.56205215 | 0.0020879 | 2.828427125 | 3.74165739 | 3 | 3 | 1 | 3.344728709 | 0 | 0 | 0 | JN022534,MK798143,MK798144 |
| VC_650_0 | 3 | 900 | 3582.429799 | 0.20078396 | 0.001617 | 4 | 5.38516481 | 3 | 3 | 1 | 4.726998777 | 0 | 0 | 0 | KU052488,MG252693,MG765276 |
| VC_650_1 | 6 | 3921.880819 | 5447.772702 | 0.41857266 | 4.24E-11 | 2.828427125 | 8 | 15 | 15 | 1 | 6.33927 | 0 | 0 | 0 | JX486088,KX223815,KY381600,MG744354,MG765274,ON117106 |
| VC_653_0 | 9 | 3625.178345 | 12031.07236 | 0.23154831 | 2.31E-24 | 0 | 9.11043358 | 36 | 35 | 0.972222222 | 7.079862765 | 0 | 0 | 0 | KM289195,MK448681,MK448683,MK448783,MK448790,MK448798,MK448833,MK448967,MK449010 |
| VC_654_0 | 115 | 521226.9647 | 192465.1332 | 0.7303247 | 0 | 0 | 9.64365076 | 6555 | 5745 | 0.876430206 | 7.896581085 | 0 | 0 | 0 | AY730274,EF177456,HE775250,HG934469,HG934470,JQ007353,JX202565,KC139511,KR270151,KT881477,KT962832,KU595434,KU951146,KU951147,KX245013,KX405002,KX649889,KY379853,KY626163,LC507823,MF001356,MF001366,MF431252,MG646668,MG969408,MG969409,MG969410,MH358359,MH370358,MH370359,MH370360,MH370361,MH370362,MH370363,MH370365,MH370372,MH370373,MH370374,MH370381,MH370384,MH370385,MH424443,MH424445,MH992509,MK249126,MK761195,MK761196,MK761197,MK761198,MK761199,MK770409,MK972691,MK972700,MK972701,MK972702,MK972703,MK972705,MK972706,MN336264,MN336265,MN336266,MN393078,MN722429,MN850616,MT074431,MT074432,MT074451,MT074462,MT074473,MT074481,MT074482,MT074483,MT074484,MT588083,MT632017,MT632018,MT632019,MT632020,MT653137,MT653144,MT677934,MT740291,MW201959,MW355463,MW355464,MW355465,MW715619,MW790495,MZ156567,MZ156568,MZ156569,MZ274225,MZ955866,OK108608,OK108609,OK143508,OL436139,OL519843,OL652658,OL770276,OM210019,OM291373,OM339548,ON167532,ON381768,ON409568,ON409569,ON409570,ON454038,ON526838,ON540301,ON720977,ON996339,OP484850,OP494211 |
| VC_654_1 | 27 | 55284.36439 | 159745.2969 | 0.25710111 | 6.29E-210 | 0 | 9.69535971 | 351 | 346 | 0.985754986 | 5.519142779 | 0 | 0 | 0 | JX297445,KF562864,KF562865,KY176369,MK578530,MK759883,MK931446,MT074430,MT677933,MW355451,MW355452,MW355453,MW355454,MW355455,MW355456,MW355457,MW355458,MW355459,MW355461,MW355462,MW355466,MW355468,MW355469,MW355471,MW355472,MW355473,OK169616 |
| VC_654_2 | 2 | 62.56375318 | 16402.47473 | 0.00379979 | 0.0715507 | 8.717797887 | 8.71779789 | 1 | 1 | 1 | 8.717797887 | 0 | 0 | 0 | KF148055,KM272358 |
| VC_655_0 | 45 | 136123.0735 | 40166.10024 | 0.77215787 | 0 | 0 | 8.77496439 | 990 | 990 | 1 | 7.066828327 | 0 | 0 | 0 | GU196277,GU196278,GU196279,GU196280,GU196281,KY295895,KY295896,KY295898,MF153391,MG099933,MG252615,MG852086,MH051334,MK542015,MK737937,MK931438,MK931439,MK931440,MK931441,MN927225,MN927226,MT711523,MT711524,MT711525,MT711526,MT711527,MT711528,MW331437,MW722821,MZ234010,MZ234048,MZ576217,MZ576218,MZ832314,MZ997838,OK539620,OL539727,OM272052,OM632667,OM632668,OM810255,ON001686,ON391668,ON548432,ON556632 |
| VC_656_0 | 2 | 148.4586164 | 395.140854 | 0.27310295 | 0.0447308 | 5.744562647 | 5.74456265 | 1 | 1 | 1 | 5.744562647 | 0 | 0 | 0 | MK813940,OM033133 |
| VC_657_0 | 7 | 3116.64442 | 552.1332649 | 0.84950485 | 9.11E-12 | 2.449489743 | 3.87298335 | 21 | 21 | 1 | 3.311013351 | 0 | 0 | 0 | AY739900,EU340421,KF188410,KJ564036,KJ564037,KJ564038,MG913376 |
| VC_658_0 | 3 | 42.82335186 | 281.9561733 | 0.13185361 | 0.0099355 | 8.426149773 | 9.16515139 | 3 | 2 | 0.666666667 | 8.788755183 | 0 | 0 | 0 | S10_vs_2538,S14_vs_117160,S5_vs_145581 |
| VC_66_0 | 2 | 300 | 1376.660432 | 0.17892711 | 0.044462 | 7.141428429 | 7.14142843 | 1 | 1 | 1 | 7.141428429 | 0 | 0 | 0 | MT366760,MT366761 |
| VC_660_0 | 2 | 33.32578518 | 871.37175 | 0.03683638 | 0.043753 | 8.185352772 | 8.18535277 | 1 | 1 | 1 | 8.185352772 | 0 | 0 | 0 | S5_vs_138980,S7_vs_69630 |
| VC_661_0 | 7 | 2442.791722 | 13842.2947 | 0.15000177 | 4.22E-15 | 0 | 9.32737905 | 21 | 16 | 0.761904762 | 7.939462238 | 0 | 0 | 0 | HQ632860,MG592506,MG592545,MG592567,MG592568,MG592600,MG592630 |
| VC_661_1 | 1 | 0 | 2745.932731 | 0 | 0 | 0 | 0 | 1 | 1 | 1 | 0 | 0 | 0 | 0 | OX241574 |
| VC_661_2 | 6 | 1403.099899 | 6980.882929 | 0.16735482 | 3.78E-11 | 5.744562647 | 8.1240384 | 15 | 15 | 1 | 7.216197275 | 0 | 0 | 0 | AY772740,JF974313,MG676223,MK097141,ON714422,OP375635 |
| VC_661_3 | 9 | 4079.958855 | 15130.21552 | 0.21238531 | 9.91E-19 | 0 | 9.32737905 | 36 | 25 | 0.694444444 | 7.723742783 | 0 | 0 | 0 | KX507045,MG592494,MG592501,MG592508,MG592514,MG592583,MG592587,MG592595,MG592644 |
| VC_661_4 | 2 | 288.7405972 | 5305.848863 | 0.05161069 | 0.0439552 | 3.605551275 | 3.60555128 | 1 | 1 | 1 | 3.605551275 | 0 | 0 | 0 | MW824412,MW824413 |
| VC_661_5 | 4 | 851.4402042 | 8668.671458 | 0.08943595 | 3.34E-05 | 0 | 8.60232527 | 6 | 6 | 1 | 6.748278696 | 0 | 0 | 0 | MG592544,MG592664,MG592665,OX241573 |
| VC_662_0 | 2 | 49.22595042 | 405.7437394 | 0.10819611 | 0.0491465 | 7.681145748 | 7.68114575 | 1 | 1 | 1 | 7.681145748 | 0 | 0 | 0 | JF974287,MK249872 |
| VC_663_0 | 5 | 650.0261435 | 328.1492943 | 0.6645292 | 2.58E-07 | 7.280109889 | 8.54400375 | 10 | 10 | 1 | 7.837972392 | 0 | 0 | 0 | KC542353,KP994596,KU747973,MK387309,MN313256 |
| VC_664_0 | 8 | 3978.968696 | 8759.530348 | 0.31235773 | 1.07E-18 | 4.472135955 | 7.34846923 | 28 | 28 | 1 | 6.587604139 | 0 | 0 | 0 | MG592390,MG592420,MG592423,MG592439,MG592444,MG592454,MG592465,MG592466 |
| VC_665_0 | 29 | 66926.07733 | 6446.753367 | 0.91213705 | 5.45E-161 | 0 | 8.1240384 | 406 | 406 | 1 | 6.928512056 | 0 | 0 | 0 | MG592393,MG592477,MG592478,MG592518,MG592540,MG592552,MG592569,MG592570,MG592608,MG592614,MG592622,MG592627,MG592628,MG592641,MG592647,MG592656,MW824369,MW824373,MW824374,MW824378,MW824379,MW824383,OX241429,OX241438,OX241498,OX241557,OX241558,OX241575,OX241591 |
| VC_666_0 | 11 | 6960.3842 | 4995.378033 | 0.58217821 | 3.97E-34 | 1.732050808 | 5.74456265 | 55 | 55 | 1 | 4.321025167 | 0 | 0 | 0 | CP069347,HG796225,HG798901,JX145342,LK985321,LN681538,LN681540,MK473382,MT193276,MW512571,MW512572 |
| VC_667_0 | 12 | 602.00655 | 1468.991595 | 0.29068425 | 1.27E-08 | 5.744562647 | 8.60232527 | 66 | 66 | 1 | 7.477396533 | 0 | 0 | 0 | KX190832,S11_vs_104038,S11_vs_81775,S16_vs_124703,S16_vs_4662,S4_vf_13759,S6_vs_33711,S6_vs_38250,S7_vs_56529,S8_vs_175235,S8_vs_176720,S9_vs_45812 |
| VC_669_0 | 2 | 23.80949342 | 160.4982803 | 0.12918334 | 0.0478577 | 5.291502622 | 5.29150262 | 1 | 1 | 1 | 5.291502622 | 0 | 0 | 0 | S11_vs_104041,S8_vs_182331 |
| VC_67_0 | 2 | 300 | 1246.506409 | 0.19398562 | 0.0451258 | 3.16227766 | 3.16227766 | 1 | 1 | 1 | 3.16227766 | 0 | 0 | 0 | MK552140,MK552141 |
| VC_67_1 | 1 | 0 | 906.7504629 | 0 | 0 | 0 | 0 | 1 | 1 | 1 | 0 | 0 | 0 | 0 | MN094788 |
| VC_67_2 | 1 | 0 | 1091.181279 | 0 | 0 | 0 | 0 | 1 | 1 | 1 | 0 | 0 | 0 | 0 | MW460245 |
| VC_67_3 | 1 | 0 | 1059.493516 | 0 | 0 | 0 | 0 | 1 | 1 | 1 | 0 | 0 | 0 | 0 | AB366653 |
| VC_670_0 | 4 | 602.6663804 | 851.2733437 | 0.41450575 | 1.71E-05 | 5.196152423 | 8.06225775 | 6 | 6 | 1 | 6.435744992 | 0 | 0 | 0 | S16_vs_89511,S4_vs_VIRSorter_4959-cat_2,S6_vs_12945,S6_vs_39025 |
| VC_671_0 | 4 | 1174.407639 | 1294.799018 | 0.47562145 | 1.92E-05 | 1 | 7.14142843 | 6 | 6 | 1 | 5.583143613 | 0 | 0 | 0 | EU874396,GU229986,KX227758,KX227759 |
| VC_673_0 | 4 | 824.5882564 | 2027.581374 | 0.28910912 | 1.74E-05 | 1 | 7.14142843 | 6 | 6 | 1 | 5.506636027 | 0 | 0 | 0 | KT725776,KX227757,KX227760,MW302330 |
| VC_674_0 | 2 | 300 | 27158.12321 | 0.01092573 | 0.042955 | 2 | 2 | 1 | 1 | 1 | 2 | 0 | 0 | 0 | GU071099,MH512890 |
| VC_674_1 | 1 | 0 | 13907.09136 | 0 | 0 | 0 | 0 | 1 | 1 | 1 | 0 | 0 | 0 | 0 | GU071103 |
| VC_674_2 | 3 | 900 | 38715.47024 | 0.0227184 | 0.0013924 | 2 | 7.54983444 | 3 | 3 | 1 | 5.609981442 | 0 | 0 | 0 | AY940168,HQ337021,HQ634176 |
| VC_674_3 | 1 | 0 | 15076.34762 | 0 | 0 | 0 | 0 | 1 | 1 | 1 | 0 | 0 | 0 | 0 | HQ634175 |
| VC_674_4 | 1 | 0 | 15024.85331 | 0 | 0 | 0 | 0 | 1 | 1 | 1 | 0 | 0 | 0 | 0 | JN371769 |
| VC_674_5 | 3 | 900 | 54314.76793 | 0.01629999 | 0.0013937 | 1 | 2.44948974 | 3 | 3 | 1 | 1.895185907 | 0 | 0 | 0 | KU686207,KU686208,KU686209 |
| VC_674_6 | 2 | 300 | 38627.22178 | 0.00770669 | 0.0422471 | 2 | 2 | 1 | 1 | 1 | 2 | 0 | 0 | 0 | GU071106,HQ634190 |
| VC_674_7 | 3 | 900 | 54209.71036 | 0.01633106 | 0.0014306 | 5.385164807 | 7 | 3 | 3 | 1 | 5.954130127 | 0 | 0 | 0 | HQ634189,MH920639,MH920640 |
| VC_674_8 | 1 | 0 | 19215.54707 | 0 | 0 | 0 | 0 | 1 | 1 | 1 | 0 | 0 | 0 | 0 | GU071094 |
| VC_674_9 | 1 | 0 | 17271.0697 | 0 | 0 | 0 | 0 | 1 | 1 | 1 | 0 | 0 | 0 | 0 | GU071097 |
| VC_675_0 | 2 | 300 | 33625.42304 | 0.00884293 | 0.0773488 | 1.414213562 | 1.41421356 | 1 | 1 | 1 | 1.414213562 | 0 | 0 | 0 | HQ316583,HQ316603 |
| VC_675_1 | 46 | 310500 | 55097.43514 | 0.8492948 | 0 | 0 | 6.40312424 | 1035 | 1035 | 1 | 1.322924465 | 0 | 0 | 0 | KJ019028,KJ019029,KJ019031,KJ019032,KJ019034,KJ019035,KJ019036,KJ019046,KJ019047,KJ019048,KJ019050,KJ019056,KJ019057,KJ019062,KJ019070,KJ019072,KJ019073,KJ019074,KJ019075,KJ019077,KJ019078,KJ019079,KJ019080,KJ019083,KJ019112,KJ019113,KJ019115,KJ019117,KJ019118,KJ019119,KJ019120,KJ019121,KJ019124,KJ019125,KJ019126,KJ019127,KJ019129,KJ019130,KJ019131,KJ019136,KJ019139,KJ019140,KJ019160,KJ019162,KJ019164,KJ019165 |
| VC_676_0 | 3 | 900 | 40585.27048 | 0.02169445 | 0.0754085 | 3.741657387 | 5.09901951 | 3 | 3 | 1 | 4.646565471 | 0 | 0 | 0 | KU686212,KU686213,MT586120 |
| VC_676_1 | 41 | 246000 | 83569.29779 | 0.74642875 | 3.640412223 | 0 | 7.21110255 | 820 | 820 | 1 | 4.601362915 | 0 | 0 | 0 | KJ019037,KJ019045,KJ019052,KJ019053,KJ019058,KJ019059,KJ019066,KJ019085,KJ019086,KJ019090,KJ019091,KJ019092,KJ019093,KJ019095,KJ019096,KJ019097,KJ019098,KJ019099,KJ019100,KJ019101,KJ019102,KJ019103,KJ019105,KJ019106,KJ019107,KJ019111,KJ019123,KJ019141,KJ019142,KJ019143,KJ019144,KJ019145,KJ019146,KJ019147,KJ019148,KJ019149,KJ019150,KJ019151,KJ019152,KJ019155,KJ019159 |
| VC_678_0 | 2 | 5.208108209 | 203.641529 | 0.02493712 | 0.4142553 | 5.196152423 | 5.19615242 | 1 | 1 | 1 | 5.196152423 | 0 | 0 | 0 | MH319737,MH319746 |
| VC_68_0 | 2 | 300 | 21207.40808 | 0.01394868 | 0.3064239 | 6.782329983 | 6.78232998 | 1 | 1 | 1 | 6.782329983 | 0 | 0 | 0 | KY448244,MW366843 |
| VC_68_1 | 8 | 8400 | 70445.12975 | 0.10653797 | 0.0010675 | 0 | 3.74165739 | 28 | 28 | 1 | 2.939946756 | 0 | 0 | 0 | MZ475891,MZ475892,MZ475893,MZ475894,MZ475895,MZ475896,MZ475897,MZ475898 |
| VC_68_2 | 2 | 300 | 21217.53335 | 0.01394212 | 0.3064239 | 2.645751311 | 2.64575131 | 1 | 1 | 1 | 2.645751311 | 0 | 0 | 0 | KY984068,MZ443769 |
| VC_68_3 | 20 | 57000 | 104148.5747 | 0.35371085 | 2.11E-23 | 0 | 8.48528137 | 190 | 190 | 1 | 6.942078762 | 0 | 0 | 0 | MH248138,MH460463,MZ443770,MZ443771,MZ443772,MZ443773,MZ443774,MZ443776,MZ443777,MZ443778,MZ443779,MZ443780,MZ443782,MZ443783,MZ443784,MZ443785,MZ443786,MZ443787,MZ443788,MZ443789 |
| VC_68_4 | 4 | 1800 | 39959.15962 | 0.04310432 | 0.0905697 | 0 | 4.58257569 | 6 | 6 | 1 | 3.159537972 | 0 | 0 | 0 | MH389777,MH460460,MH460461,MH460462 |
| VC_68_5 | 1 | 0 | 6293.462585 | 0 | 0 | 0 | 0 | 1 | 1 | 1 | 0 | 0 | 0 | 0 | LT598654 |
| VC_681_0 | 62 | 567300 | 251264.3564 | 0.69304264 | 0 | 2.236067977 | 7.61577311 | 1891 | 1891 | 1 | 4.887167278 | 0 | 0 | 0 | HQ317290,HQ317291,HQ317292,KX349226,KX349227,KX349228,KX349229,KX349230,KX349231,KX349232,KX349233,KX349234,KX349235,KX349236,KX349237,KX349238,KX349239,KX349240,KX349241,KX349242,KX349243,KX349244,KX349245,KX349246,KX349247,KX349248,KX349249,KX349250,KX349251,KX349252,KX349253,KX349254,KX349255,KX349256,KX349257,KX349258,KX349259,KX349260,KX349261,KX349262,KX349263,KX349264,KX349265,KX349266,KX349267,KX349268,KX349269,KX349270,KX349271,KX349272,KX349273,KX349274,KX349275,KX349276,KX349277,KX349278,KX349279,KX349280,KX349281,KX349282,KX349283,KX349284 |
| VC_684_0 | 4 | 701.277157 | 0 | 1 | 0 | 2.449489743 | 3.74165739 | 6 | 6 | 1 | 3.327831774 | 0 | 0 | 0 | AY950802,JN968479,KC252997,KT809302 |
| VC_685_0 | 7 | 6300 | 9273.083331 | 0.40454417 | 5.45E-15 | 0 | 7.21110255 | 21 | 21 | 1 | 5.564690762 | 0 | 0 | 0 | KF929199,KJ804259,MT596499,MZ417336,MZ417339,MZ417343,MZ417348 |
| VC_685_1 | 1 | 0 | 2039.846747 | 0 | 0 | 0 | 0 | 1 | 1 | 1 | 0 | 0 | 0 | 0 | MH356730 |

| | | | | | | | | | | | | | | | |
|---|---|---|---|---|---|---|---|---|---|---|---|---|---|---|---|
| VC_685_2 | 1 | 0 | 1649.726702 | 0 | 0 | 0 | 0 | 1 | 1 | 1 | 0 | 0 | 0 | 0 | MK893987 |
| VC_686_0 | 25 | 90000 | 73593.58743 | 0.55014381 | 8.51E-141 | 3.31662479 | 9.59166305 | 300 | 288 | 0.96 | 6.259798712 | 0 | 0 | 0 | GU295964,HQ918180,KT001918,KT001919,KT321315,KX130727,LR746310,LT607758,MN101223,MT157285,MW448170,MZ322895,MZ612130,MZ707156,MZ707157,OK631812,OK631813,OM103621,OM971648,OM971649,ON515458,OP045497,OP684128,OX335396,OX335407 |
| VC_686_1 | 1 | 0 | 10438.99939 | 0 | 0 | 0 | 0 | 1 | 1 | 1 | 0 | 0 | 0 | 0 | OP617746 |
| VC_686_2 | 1 | 0 | 9334.121113 | 0 | 0 | 0 | 0 | 1 | 1 | 1 | 0 | 0 | 0 | 0 | MN101221 |
| VC_686_3 | 1 | 0 | 6512.688244 | 0 | 0 | 0 | 0 | 1 | 1 | 1 | 0 | 0 | 0 | 0 | MN101224 |
| VC_687_0 | 21 | 63000 | 111627.9901 | 0.36076691 | 1.34E-66 | 3.741657387 | 7.93725393 | 210 | 210 | 1 | 6.051823405 | 0 | 0 | 0 | JN882287,KT381880,KX431559,KX431560,KX443552,LC589952,MH823906,MN508621,MN508622,MN508623,MN508624,MT341500,MW021751,MW021756,MW250785,MW629017,MW767161,OL539470,OL539471,OL551674,ON157416 |
| VC_687_1 | 10 | 13500 | 93408.27315 | 0.12627648 | 2.95E-12 | 2.236067977 | 9.05538514 | 45 | 44 | 0.977777778 | 7.066805594 | 0 | 0 | 0 | AY967407,HE858210,HE981739,HM134276,KC801932,KM236237,KR869820,KX245890,MT334653,MW021749 |
| VC_687_2 | 1 | 0 | 13708.87637 | 0 | 0 | 0 | 0 | 1 | 1 | 1 | 0 | 0 | 0 | 0 | MN508817 |
| VC_687_3 | 1 | 0 | 13160.77362 | 0 | 0 | 0 | 0 | 1 | 1 | 1 | 0 | 0 | 0 | 0 | MN038175 |
| VC_687_4 | 1 | 0 | 6658.140017 | 0 | 0 | 0 | 0 | 1 | 1 | 1 | 0 | 0 | 0 | 0 | OK210075 |
| VC_687_5 | 1 | 0 | 10614.92048 | 0 | 0 | 0 | 0 | 1 | 1 | 1 | 0 | 0 | 0 | 0 | MT560058 |
| VC_688_0 | 2 | 186.8651157 | 85.31065237 | 0.68656044 | 0.2701457 | 2.645751311 | 2.64575131 | 1 | 1 | 1 | 2.645751311 | 0 | 0 | 0 | CAKLQA020000002,MW176033 |
| VC_689_0 | 5 | 2204.690277 | 2038.878423 | 0.51953684 | 4.06E-08 | 1.414213562 | 8.24621125 | 10 | 10 | 1 | 5.836921642 | 0 | 0 | 0 | MN937349,MW393850,MZ326859,MZ398240,MZ398241 |
| VC_69_0 | 1 | 0 | 895.7330037 | 0 | 0 | 0 | 0 | 1 | 1 | 1 | 0 | 0 | 0 | 0 | AP018399 |
| VC_69_1 | 1 | 0 | 918.8795066 | 0 | 0 | 0 | 0 | 1 | 1 | 1 | 0 | 0 | 0 | 0 | MN047793 |
| VC_690_0 | 12 | 1019.718333 | 0 | 1 | 0 | 0 | 1.41421356 | 66 | 66 | 1 | 0.54913092 | 0 | 0 | 0 | BCP1,MG883715,MG883725,MG945215,MG945341,MG945520,MG945551,MG945686,MG945756,MT135344,MT135404,MT135412 |
| VC_691_0 | 18 | 25441.20234 | 11620.03011 | 0.68646401 | 3.57E-60 | 0 | 9.48683298 | 153 | 145 | 0.947712418 | 6.529641915 | 0 | 0 | 0 | BK010471,MH616963,MK069403,MK238400,MT006214,MT774381,MT774383,MT774386,MW067000,MW067001,MW067002,MW067003,S11_vf_104059,S11_vf_43997,S12_vs_14927,S3_vf_17748,S8_vf_158225,S8_vf_87755 |
| VC_691_1 | 5 | 1387.85059 | 7259.486917 | 0.16049456 | 6.97E-08 | 4.69041576 | 10.198039 | 10 | 8 | 0.8 | 7.776033353 | 0 | 0 | 0 | MH552500,MT774384,MT774394,S8_vf_166677,S9_vf_51656 |
| VC_693_0 | 4 | 659.9297707 | 1539.711348 | 0.30001702 | 2.01E-05 | 0 | 8.30662386 | 6 | 6 | 1 | 6.311270784 | 0 | 0 | 0 | MT774393,S10_vf_36880,S16_vf_151450,S4_vf_26417 |
| VC_694_0 | 2 | 67.91582835 | 302.0418302 | 0.1835773 | 0.0505939 | 5 | 5 | 1 | 1 | 1 | 5 | 0 | 0 | 0 | S14_vs_78164,S5_vs_93839 |
| VC_695_0 | 17 | 4732.181786 | 1851.268995 | 0.7187996 | 2.00E-23 | 2.828427125 | 10 | 136 | 123 | 0.904411765 | 7.13595425 | 0 | 0 | 0 | BK010646,S10_vs_58014,S11_vs_3277,S11_vs_58930,S11_vs_84848,S11_vs_98193,S12_vs_141337,S12_vs_23796,S17_vs_2079,S2_vs_10289,S3_vs_119660,S5_vs_123256,S5_vs_76500,S7_vs_11081,S8_vf_117796,S8_vs_99362,S9_vs_21850 |
| VC_695_1 | 1 | 0 | 787.958138 | 0 | 0 | 0 | 0 | 1 | 1 | 1 | 0 | 0 | 0 | 0 | S5_vs_131510 |
| VC_696_0 | 3 | 345.6545889 | 596.0343897 | 0.36705812 | 0.0018328 | 4.472135955 | 6.32455532 | 3 | 3 | 1 | 5.653701759 | 0 | 0 | 0 | S5_vs_69083,S5_vs_79992,S7_vs_119679 |
| VC_697_0 | 3 | 76.90503073 | 5.681602547 | 0.93120433 | 0.1855467 | 3.741657387 | 4.47213595 | 3 | 3 | 1 | 4.228643099 | 0 | 0 | 0 | BK013345,BK013346,BK013347 |
| VC_698_0 | 2 | 92.75330077 | 0 | 1 | 0 | 5.744562647 | 5.74456265 | 1 | 1 | 1 | 5.744562647 | 0 | 0 | 0 | BK059895,OM373202 |
| VC_699_0 | 5 | 2503.981629 | 301.4591705 | 0.89254481 | 2.25E-07 | 3.31662479 | 6.08276253 | 10 | 10 | 1 | 4.994404776 | 0 | 0 | 0 | BK061308,BK061309,BK061310,BK061311,BK061312 |
| VC_7_0 | 28 | 35841.19451 | 56303.32606 | 0.38896718 | 3.08E-213 | 0 | 9.21954446 | 378 | 376 | 0.994708995 | 7.32541991 | 0 | 0 | 0 | AB009866,AB044554,AB243556,AF424783,CP062426,CP062435,CP062445,CP062457,EF462197,EF462199,FJ713816,GQ915271,KC342645,KX232515,KY389063,KY389064,KY653125,KY695153,MH384260,MH401414,MH401415,MW147366,MW192778,MW627293,MW650841,OM439664,OP136133,OP493557 |
| VC_70_0 | 5 | 3000 | 18.38274407 | 0.99390974 | 4.20E-05 | 0 | 6.4807407 | 10 | 10 | 1 | 4.659164571 | 0 | 0 | 0 | MN988515,MN988535,MN988540,MW980063,MW980064 |
| VC_700_0 | 5 | 2475.580068 | 3875.101549 | 0.38981329 | 5.15E-08 | 0 | 5.29150262 | 10 | 10 | 1 | 3.353378039 | 0 | 0 | 0 | MW353181,MW584168,MW584172,MW584178,MW584190 |
| VC_701_0 | 16 | 15863.87602 | 37779.29756 | 0.29572963 | 3.61E-76 | 0 | 9.43398113 | 120 | 110 | 0.916666667 | 7.675232537 | 0 | 0 | 0 | MG872834,MH513979,MH651167,MK284520,MK433274,MK433275,MK820641,MN062715,MT521999,MW534378,MW601219,MZ322028,MZ622161,MZ622165,ON108651,OP434460 |
| VC_702_0 | 2 | 300 | 1112.964736 | 0.21231952 | 0.0460806 | 2.449489743 | 2.44948974 | 1 | 1 | 1 | 2.449489743 | 0 | 0 | 0 | MK494099,ON260818 |
| VC_702_1 | 2 | 300 | 1072.993194 | 0.21850072 | 0.0867209 | 2.645751311 | 2.64575131 | 1 | 1 | 1 | 2.645751311 | 0 | 0 | 0 | KU728633,MK977701 |
| VC_703_0 | 3 | 900 | 640.8367318 | 0.58409822 | 0.0074733 | 0 | 1.41421356 | 3 | 3 | 1 | 0.942809042 | 0 | 0 | 0 | KF114879,KF114880,KX656785 |
| VC_703_1 | 2 | 167.2766476 | 584.2589732 | 0.2225798 | 0.0858152 | 4 | 4 | 1 | 1 | 1 | 4 | 0 | 0 | 0 | KF114876,KF114877 |
| VC_704_0 | 2 | 273.8448341 | 1.607552593 | 0.99416395 | 0.5 | 1.414213562 | 1.41421356 | 1 | 1 | 1 | 1.414213562 | 0 | 0 | 0 | MF974396,MF974397 |
| VC_705_0 | 2 | 69.1251122 | 97.30727313 | 0.4153345 | 0.0439379 | 4.123105626 | 4.12310563 | 1 | 1 | 1 | 4.123105626 | 0 | 0 | 0 | DQ490056,MW791312 |
| VC_706_0 | 52 | 152403.3476 | 3315.009715 | 0.9787115 | 2.58E-93 | 0 | 4.79583152 | 1326 | 1326 | 1 | 2.951281886 | 0 | 0 | 0 | C2PVCG,KX373685,KX373686,KX373687,KX373688,KX373689,KX373690,KX373691,KX373692,L67CG,MF443119,MF443120,MF443121,MF443122,MF443123,MF443124,MF443125,MF443126,MH779518,MH779519,MH779520,MH779521,MH779522,MH779523,MH779524,MH779525,MH779526,MH779527,MK301442,MK301443,MN337887,MN689503,MN689504,MN689505,MN689506,MN689507,MN689508,MN689510,MN689511,MN689512,MN689513,MN689515,MN689526,MN689527,MN689528,MN689529,MN689531,MN689532,MN689533,MZ955869,S6_vf_3435,S7_vf_89197 |
| VC_707_0 | 2 | 300 | 2603.476599 | 0.10332441 | 0.0464169 | 1 | 1 | 1 | 1 | 1 | 1 | 0 | 0 | 0 | MT939241,ON506926 |
| VC_707_1 | 5 | 3000 | 3986.171287 | 0.42941976 | 6.31E-08 | 2.828427125 | 4.24264069 | 10 | 10 | 1 | 3.514585849 | 0 | 0 | 0 | CAJCJZ010000002,LT546029,LT615366,MT119360,MT119361 |
| VC_708_0 | 2 | 300 | 420.0480612 | 0.41663886 | 0.0463109 | 1 | 1 | 1 | 1 | 1 | 1 | 0 | 0 | 0 | MF001358,MF001361 |
| VC_71_0 | 27 | 19805.93447 | 13405.24547 | 0.59636347 | 2.31E-107 | 0 | 9.05538514 | 351 | 349 | 0.994301994 | 6.73295832 | 0 | 0 | 0 | AB920995,AB983711,GU393987,HG793132,HG796219,HG796220,HG796221,KC853746,KT381879,KX660669,KY316062,KY464836,KY981272,MF621978,MH684921,MN191861,MT740728,MZ326863,OK319016,OK335783,ON170174,ON170178,ON170184,ON170190,ON170318,ON170358,ON529868 |
| VC_710_0 | 1 | 0 | 3175.778298 | 0 | 0 | 0 | 0 | 1 | 1 | 1 | 0 | 0 | 0 | 0 | MW672041 |
| VC_711_0 | 48 | 171888.3766 | 37291.60484 | 0.82172479 | 6.73E-251 | 0 | 7.87400787 | 1128 | 1128 | 1 | 5.82346555 | 0 | 0 | 0 | CAJDJX010000002,CAJDKF010000002,JX193904,KF728385,KF733017,KJ127304,KR131750,KT932701,KX284704,LC623721,MF678788,MF678789,MF678790,MG264739,MG708276,MH193369,MH203383,MH203384,MH355583,MH375074,MK125140,MK360024,MK721186,MK721187,MK721190,MK721191,MK721199,MK721200,MK982307,MT119359,MT520979,MT627482,MT661597,MT661598,MT829326,MT857001,MZ182246,MZ272341,MZ326865,MZ333461,OL505084,OL539449,OL799256,OL799257,ON113176,ON113334,OP254195,OP559177 |
| VC_712_0 | 5 | 2189.688678 | 454.9907898 | 0.82795995 | 2.89E-07 | 2 | 5.56776436 | 10 | 10 | 1 | 4.37314319 | 0 | 0 | 0 | KJ507100,MT104473,MT104475,MZ348425,MZ868718 |
| VC_713_0 | 26 | 56948.95767 | 3908.579213 | 0.93577494 | 2.07E-82 | 1 | 8.36660027 | 325 | 325 | 1 | 6.986859523 | 0 | 0 | 0 | HE806280,HM368260,JX976549,KJ817802,KP861229,KP861231,KU510289,KY670595,KY829116,MF346584,MH853786,MH853787,MH853788,MN166083,MN516421,MN516422,MZ593728,MZ618622,MZ712044,OL743187,OM471864,ON036882,ON036883,ON237674,ON480525,OP585104 |
| VC_714_0 | 71 | 299990.8907 | 20866.6401 | 0.93496603 | 0 | 0 | 6.4807407 | 2485 | 2485 | 1 | 4.870621324 | 0 | 0 | 0 | CAKLQB020000001,CAKLQG020000001,HQ186308,JX658790,KC311669,KR149290,KT339321,KT388102,KT388103,KT804908,KY082667,KY268295,KY268296,KY888680,MF033347,MF033348,MF033349,MF033350,MF033351,MG459218,MG599035,MH042230,MH763831,MH800200,MK089780,MK257719,MK257720,MK257721,MK257722,MK257723,MK278859,MN061582,MN212906,MN294712,MN395291,MN433707,MN604238,MN604239,MN614471,MN651570,MN807295,MT263719,MT633129,MT741944,MW056501,MW056502,MW056503,MW345241,MW366783,MW366784,MW459163,MW926912,MZ099557,MZ593174,MZ868724,MZ868725,MZ868726,MZ936314,MZ936315,MZ936316,MZ967493,OK546191,OL770263,OL964948,OM925528,ON210142,ON513429,OP019135,OP171942,OP291336,OP508218 |
| VC_715_0 | 10 | 13500 | 3281.535979 | 0.80445556 | 7.57E-25 | 4.123105626 | 7.28010989 | 45 | 45 | 1 | 5.907179569 | 0 | 0 | 0 | KJ628499,MH746814,MH800198,MH800199,MK170160,MT188223,MT623546,MW349133,OL960030,OP585105 |
| VC_715_1 | 1 | 0 | 2089.118867 | 0 | 0 | 0 | 0 | 1 | 1 | 1 | 0 | 0 | 0 | 0 | KJ473422 |
| VC_716_0 | 2 | 300 | 1738.393905 | 0.14717469 | 0.0475008 | 8.717797887 | 8.71779789 | 1 | 1 | 1 | 8.717797887 | 0 | 0 | 0 | MN276049,MZ477002 |
| VC_716_1 | 2 | 300 | 2024.64376 | 0.12905203 | 0.0488584 | 8.306623863 | 8.30662386 | 1 | 1 | 1 | 8.306623863 | 0 | 0 | 0 | MH133207,MZ043613 |
| VC_716_2 | 1 | 0 | 1244.939227 | 0 | 0 | 0 | 0 | 1 | 1 | 1 | 0 | 0 | 0 | 0 | CAKLQE020000001 |
| VC_717_0 | 5 | 3000 | 867.2483568 | 0.77574537 | 1.21E-07 | 5.916079783 | 7.61577311 | 10 | 10 | 1 | 6.736913996 | 0 | 0 | 0 | KM672662,KY000079,MW316731,MW316732,MW316733 |
| VC_719_0 | 16 | 16688.52182 | 6233.785103 | 0.72804722 | 1.88E-65 | 0 | 8.24621125 | 120 | 120 | 1 | 4.798018361 | 0 | 0 | 0 | DQ150593,DQ222851,DQ222853,DQ222855,DQ289555,DQ289556,JQ062992,KY963369,KY963370,KY963371,MK085976,MT745954,MT745955,MT745956,OK499979,OK499999 |
| VC_720_0 | 6 | 363.0964404 | 308.584674 | 0.54057861 | 2.24E-07 | 5.744562647 | 8.18535277 | 15 | 15 | 1 | 6.871900126 | 0 | 0 | 0 | S14_vf_72335,S14_vs_59347,S3_vs_127387,S7_vf_114476,S7_vs_13041,S7_vs_47050 |
| VC_721_0 | 11 | 6157.336092 | 803.4970456 | 0.88456884 | 1.16E-25 | 2 | 8.06225775 | 55 | 55 | 1 | 6.190584445 | 0 | 0 | 0 | KF591601,MF614100,MH363708,MK105855,MK125141,MK651787,MN850581,MN850588,MN850623,MW258710,MW258711 |
| VC_722_0 | 2 | 153.6820465 | 0 | 1 | 0 | 2.449489743 | 2.44948974 | 1 | 1 | 1 | 2.449489743 | 0 | 0 | 0 | CP002495,CP091911 |
| VC_723_0 | 20 | 16845.98975 | 6092.00382 | 0.73441427 | 2.08E-96 | 3.31662479 | 10.198039 | 190 | 133 | 0.7 | 7.623343818 | 0 | 0 | 0 | MG711462,S10_vf_24791,S11_vs_10172,S11_vs_19892,S11_vs_23357,S11_vs_40945,S16_vf_97279,S3_vf_8058,S3_vs_1143,S3_vs_54303,S4_vs_VIRSorter_7386-circular-cat_2,S5_vs_38949,S6_vs_56496,S7_vf_5439,S7_vs_4956,S7_vs_6626,S8_vf_151384,S8_vs_47265,S9_vs_27813,S9_vs_56354 |
| VC_724_0 | 6 | 298.4608676 | 1266.763043 | 0.19068254 | 1.23E-09 | 6 | 7.74596669 | 15 | 15 | 1 | 7.030450127 | 0 | 0 | 0 | S5_vs_107105,S5_vs_145597,S8_vs_76852,S9_vs_5676,S9_vs_84046,S9_vs_90860 |
| VC_725_0 | 47 | 156724.6241 | 24480.29042 | 0.86490272 | 1.10E-270 | 0 | 7.81024968 | 1081 | 1081 | 1 | 5.723794409 | 0 | 0 | 0 | KF010834,KP202972,KP296791,KP296793,KP296794,KP296795,KP296796,KT361649,KT361650,KT361652,MG727695,MG727696,MG727698,MG727699,MG727700,MG727701,MG727702,MH431931,MH431932,MH431933,MH431934,MH431935,MH431936,MH431937,MH454077,MH454078,MH454079,MH454082,MH454083,MH454084,MW927511,MW927513,MW927514,MW927515,MW927516,MW927517,MW927519,MW927520,MW927521,MW927522,MW927523,MW927524,MW927525,OP503992,OP503993,OP503994,OP503995 |
| VC_727_0 | 8 | 3228.871082 | 2059.242592 | 0.61059033 | 3.09E-18 | 1.414213562 | 9.16515139 | 28 | 26 | 0.928571429 | 6.886054916 | 0 | 0 | 0 | LR596903,MG711461,S13_vs_35816,S16_vs_86116,S3_vf_99734,S4_vs_VIRSorter_8622-cat_2,S8_vs_206526,S9_vf_64844 |
| VC_728_0 | 7 | 1657.445196 | 5412.544657 | 0.23443389 | 1.43E-14 | 6.08276253 | 9.74679434 | 21 | 17 | 0.80952381 | 7.9905072 | 0 | 0 | 0 | MG711466,S16_vf_6102,S16_vs_18440,S3_vs_42569,S7_vs_134583,S8_vf_101283,S8_vf_170878 |
| VC_728_1 | 1 | 0 | 1007.063075 | 0 | 0 | 0 | 0 | 1 | 1 | 1 | 0 | 0 | 0 | 0 | S5_vs_124677 |
| VC_73_0 | 2 | 156.3387935 | 1873.80323 | 0.0770088 | 0.0438199 | 3.16227766 | 3.16227766 | 1 | 1 | 1 | 3.16227766 | 0 | 0 | 0 | JN882298,MG201401 |
| VC_731_0 | 3 | 781.9535829 | 567.7310344 | 0.57936022 | 0.0020001 | 0 | 1.73205081 | 3 | 3 | 1 | 1.154700538 | 0 | 0 | 0 | ON464760,ON464761,ON464762 |
| VC_732_0 | 3 | 377.935552 | 1703.638025 | 0.18156243 | 0.0015642 | 5.385164807 | 6.63324958 | 3 | 3 | 1 | 6.191950971 | 0 | 0 | 0 | S11_vs_11994,S5_vf_122568,S5_vs_130958 |
| VC_734_0 | 4 | 872.648033 | 499.5469496 | 0.63595046 | 3.32E-05 | 3.741657387 | 9.59166305 | 6 | 5 | 0.833333333 | 6.917246021 | 0 | 0 | 0 | KP137433,KP137435,KP137437,KP137439 |
| VC_735_0 | 2 | 300 | 235.8783159 | 0.55982859 | 0.1056498 | 1 | 1 | 1 | 1 | 1 | 1 | 0 | 0 | 0 | KT151955,KT151957 |
| VC_735_1 | 1 | 0 | 217.93969 | 0 | 0 | 0 | 0 | 1 | 1 | 1 | 0 | 0 | 0 | 0 | CP027117 |
| VC_736_0 | 9 | 1122.59087 | 0 | 1 | 0 | 0 | 1 | 36 | 36 | 1 | 0.5 | 0 | 0 | 0 | CP045566,MN890136,MN890137,MN890138,MN890139,MN890140,MN890141,MN890142,MN890143 |
| VC_737_0 | 13 | 18835.66419 | 18219.66409 | 0.5083119 | 2.35E-47 | 2.236067977 | 5.38516481 | 78 | 78 | 1 | 4.284242638 | 0 | 0 | 0 | KJ419279,KP719132,KP719133,KP719134,KY926791,MK165087,MK373794,MK373795,MT682705,MZ501057,MZ501068,OM386652,ON922918 |
| VC_738_0 | 9 | 9455.510035 | 3833.008359 | 0.71155487 | 6.13E-20 | 0 | 8.71779789 | 36 | 36 | 1 | 7.246134483 | 0 | 0 | 0 | CP053388,CP054387,CP058330,CP101890,CP101896,JQ965645,MK422451,MZ779062,OV696614 |
| VC_739_0 | 3 | 900 | 4842.067684 | 0.15673796 | 0.0018484 | 4.358898944 | 5.29150262 | 3 | 3 | 1 | 4.81541103 | 0 | 0 | 0 | MT770738,MT778838,MW960030 |
| VC_739_1 | 6 | 4500 | 5867.494096 | 0.43404896 | 1.46E-10 | 0 | 8.83176087 | 15 | 15 | 1 | 6.228076692 | 0 | 0 | 0 | JX483881,MN988505,MN988506,MN988543,MN988547,MN988548 |
| VC_740_0 | 4 | 1800 | 2462.719303 | 0.42226566 | 2.64E-05 | 2.449489743 | 7.61577311 | 6 | 6 | 1 | 5.976817439 | 0 | 0 | 0 | MG018927,MT354569,OM140839,OM223115 |
| VC_741_0 | 3 | 31.31521383 | 792.6514924 | 0.03800544 | 0.11349 | 8.185352772 | 8.88819442 | 3 | 3 | 1 | 8.597115025 | 0 | 0 | 0 | JN116824,KR053195,MH153804 |
| VC_742_0 | 11 | 6211.738094 | 6099.235984 | 0.50456918 | 9.35E-29 | 0 | 7.41619849 | 55 | 55 | 1 | 5.515265293 | 0 | 0 | 0 | JX182371,KY092479,KY092480,KY092481,KY092482,KY092483,KY092484,KY676784,MH825699,MK305888,MT711975 |
| VC_743_0 | 3 | 899.7241921 | 1374.967293 | 0.3955368 | 0.0014974 | 2.828427125 | 3.60555128 | 3 | 3 | 1 | 3.346509892 | 0 | 0 | 0 | MK279841,MT316461,MT723933 |
| VC_745_0 | 9 | 3357.755479 | 15.10940652 | 0.9955203 | 2.29E-06 | 0 | 5.29150262 | 36 | 36 | 1 | 3.682573764 | 0 | 0 | 0 | CP060690,HQ377372,HQ377373,JF773396,KX879601,KX879602,KY661963,MT899443,MT899444 |
| VC_746_0 | 2 | 102.4013791 | 176.8160653 | 0.3667442 | 0.0499745 | 6.164414003 | 6.164414 | 1 | 1 | 1 | 6.164414003 | 0 | 0 | 0 | KX190833,MK759918 |
| VC_747_0 | 2 | 30.34843415 | 23.07561483 | 0.5680669 | 0.0938906 | 5.830951895 | 5.83095189 | 1 | 1 | 1 | 5.830951895 | 0 | 0 | 0 | S3_vs_98675,S5_vs_137154 |
| VC_748_0 | 2 | 70.47855895 | 174.704334 | 0.287453 | 0.0524928 | 8.888194417 | 8.88819442 | 1 | 1 | 1 | 8.888194417 | 0 | 0 | 0 | S7_vf_84974,S8_vf_187742 |
| VC_749_0 | 5 | 1742.623852 | 1961.870053 | 0.47040808 | 4.52E-08 | 0 | 3.74165739 | 10 | 10 | 1 | 2.503506579 | 0 | 0 | 0 | HM568888,KU057941,LN681535,LN681536,LN881738 |
| VC_75_0 | 15 | 12699.2035 | 11816.66039 | 0.51799943 | 1.04E-60 | 2 | 7.07106781 | 105 | 105 | 1 | 4.887694655 | 0 | 0 | 0 | KF626667,KU197013,KU595432,KU595433,LR778216,MF375456,MK903278,MN478375,OK258138,OK275495,ON911539,ON932078,ON932081,ON932083,ON960072 |
| VC_750_0 | 2 | 46.61464704 | 1.336657025 | 0.9721247 | 0.5 | 2.449489743 | 2.44948974 | 1 | 1 | 1 | 2.449489743 | 0 | 0 | 0 | S11_vf_104048,S6_vf_25710 |
| VC_751_0 | 2 | 90.02770364 | 43.00097857 | 0.67675408 | 0.1207833 | 3.31662479 | 3.31662479 | 1 | 1 | 1 | 3.31662479 | 0 | 0 | 0 | CP084202,S16_vs_173096 |
| VC_752_0 | 2 | 77.22330741 | 41.66432155 | 0.64954872 | 0.1444222 | 4.358898944 | 4.35889894 | 1 | 1 | 1 | 4.358898944 | 0 | 0 | 0 | S12_vs_86928,S5_vs_86562 |
| VC_753_0 | 26 | 97500 | 4470.530377 | 0.9561586 | 6.90E-122 | 0 | 7.68114575 | 325 | 325 | 1 | 4.322761581 | 0 | 0 | 0 | DQ003638,DQ004855,JQ797329,JX126918,KJ094029,KJ094030,KJ094031,KJ094033,KJ535721,KJ535722,KJ586794,KJ586795,KJ591604,KJ591605,KJ668715,KM373208,MK792752,MN128594,MN172529,MN939539,MN939540,MT178766,MT178767,MT438761,MT668623,MT668624 |
| VC_754_0 | 2 | 284.8741477 | 110.0686174 | 0.72130489 | 0.0691431 | 0 | 0 | 1 | 1 | 1 | 0 | 0 | 0 | 0 | MN830254,MN830255 |
| VC_755_0 | 2 | 76.56267498 | 246.87831 | 0.23671297 | 0.0500261 | 3.605551275 | 3.60555128 | 1 | 1 | 1 | 3.605551275 | 0 | 0 | 0 | DQ003641,EU855793 |
| VC_757_0 | 5 | 514.3486768 | 4775.716552 | 0.09722917 | 3.65E-06 | 2.828427125 | 9.43398113 | 10 | 6 | 0.6 | 7.805861818 | 0 | 0 | 0 | KY065457,KY065464,MK448672,MK448849,MK448889 |
| VC_759_0 | 2 | 64.86329226 | 330.6904961 | 0.16398097 | 0.0494264 | 6.480740698 | 6.4807407 | 1 | 1 | 1 | 6.480740698 | 0 | 0 | 0 | S11_vf_104052,S11_vf_10907 |
| VC_76_0 | 2 | 164.5657953 | 2784.491229 | 0.05580285 | 0.0436907 | 1.732050808 | 1.73205081 | 1 | 1 | 1 | 1.732050808 | 0 | 0 | 0 | MZ666965,MZ666966 |
| VC_760_0 | 15 | 2938.380937 | 0 | 1 | 0 | 0 | 2 | 105 | 105 | 1 | 1.057342236 | 0 | 0 | 0 | DQ079870,DQ079871,DQ079872,DQ079873,DQ079874,DQ079875,DQ079876,DQ079878,DQ079879,DQ085810,GQ149088,MG945254,MK629527,X60322,X60323 |
| VC_762_0 | 4 | 495.73251 | 613.653757 | 0.44685294 | 3.52E-05 | 0 | 1.73205081 | 6 | 6 | 1 | 1.093412989 | 0 | 0 | 0 | MT006233,MT006238,MT006239,MT006240 |
| VC_764_0 | 23 | 42211.81892 | 9302.527108 | 0.81941871 | 3.54E-98 | 0 | 8.18535277 | 253 | 253 | 1 | 4.279196873 | 0 | 0 | 0 | DQ163912,KJ959591,KY707339,MK510962,MK510963,MK510964,MK510965,MK510968,MK510969,MK510970,MK510971,MK510974,MK510975,MK510976,MK510978,MK510984,MK510985,MK510986,MK510987,MK510988,MK510990,MK510992,MK510993 |
| VC_765_0 | 12 | 14692.99908 | 6721.744727 | 0.68611603 | 1.44E-38 | 0 | 2.44948974 | 66 | 66 | 1 | 0.527426811 | 0 | 0 | 0 | MK510966,MK510967,MK510972,MK510973,MK510977,MK510979,MK510980,MK510981,MK510982,MK510983,MK510989,MK510991 |
| VC_766_0 | 40 | 89908.49051 | 29800.37793 | 0.75105956 | 1.58E-208 | 0 | 8.71779789 | 780 | 780 | 1 | 5.701184377 | 0 | 0 | 0 | DQ163913,HG962376,JF939047,JQ067092,JQ307387,KM434185,KR537871,KR537872,KR935213,KR935215,KU197014,LC552830,LR743530,MG897800,MK165657,MK165658,MK574078,MN062187,MT118304,MT118305,MW512832,MW677514,MW677526,MZ501266,MZ927745,MZ927746,OK094665,OK330455,OK330456,OK539825,OL802211,OM234791,ON189042,ON189043,ON189044,ON911538,ON932082,ON932085,OP009282,OP422637 |
| VC_767_0 | 1 | 0 | 2747.874411 | 0 | 0 | 0 | 0 | 1 | 1 | 1 | 0 | 0 | 0 | 0 | MG366115 |
| VC_768_0 | 4 | 1294.838811 | 6087.291964 | 0.17540177 | 1.47E-05 | 1.414213562 | 2.64575131 | 6 | 6 | 1 | 1.9463942 | 0 | 0 | 0 | KP313532,MK962635,MK962636,MK962637 |
| VC_769_0 | 3 | 514.2038969 | 6036.589603 | 0.0784949 | 0.0015398 | 2 | 3.87298335 | 3 | 3 | 1 | 3.159511541 | 0 | 0 | 0 | MN734437,MN734438,MN734439 |
| VC_77_0 | 37 | 199800 | 12926.88178 | 0.9392325 | 1.99E-249 | 0 | 8.36660027 | 666 | 666 | 1 | 4.593315182 | 0 | 0 | 0 | GU988610,HE983845,HQ832595,KC862297,KC862298,KC862300,KR052143,KT736033,KT804923,KU297675,KU497559,KU761955,KY000083,KY073228,LN610572,LN610575,LN610586,LN610587,MF974178,MH370478,MH725810,MH791399,MH791408,MH791416,MK138526,MK224498,MK340761,MK443264,MN013356,MN563785,MN871470,MN871481,MN871488,MW526259,OK539826,ON169972,ON361135 |
| VC_77_1 | 1 | 0 | 8854.234065 | 0 | 0 | 0 | 0 | 1 | 1 | 1 | 0 | 0 | 0 | 0 | MK443265 |
| VC_771_0 | 8 | 6681.159634 | 2237.679916 | 0.74910638 | 1.29E-18 | 0 | 3.60555128 | 28 | 28 | 1 | 2.356362535 | 0 | 0 | 0 | HQ641345,HQ641346,KM224878,KM224879,KY883657,KY883658,MT066160,MT767883 |
| VC_772_0 | 9 | 5195.327603 | 6433.155595 | 0.44677603 | 2.11E-23 | 2.828427125 | 7.48331477 | 36 | 36 | 1 | 5.715751918 | 0 | 0 | 0 | DQ163915,KU535860,LT986460,MF788075,MG676466,MW929175,MZ089731,MZ727333,ON857932 |
| VC_773_0 | 2 | 152.8085503 | 789.782834 | 0.16211537 | 0.0457299 | 2.828427125 | 2.82842712 | 1 | 1 | 1 | 2.828427125 | 0 | 0 | 0 | KC751414,KF302037 |
| VC_774_0 | 2 | 221.1034873 | 2837.726684 | 0.07228368 | 0.0446702 | 0 | 0 | 1 | 1 | 1 | 0 | 0 | 0 | 0 | MG592599,MG592619 |
| VC_775_0 | 18 | 24897.93919 | 8914.12343 | 0.73636263 | 1.05E-80 | 0 | 6.70820393 | 153 | 153 | 1 | 4.340089816 | 0 | 0 | 0 | MG592629,MW824407,MW824408,MW824409,MW865297,MW865299,MW865302,MW865307,OL676770,OX241435,OX241436,OX241445,OX241446,OX241447,OX241501,OX241504,OX241559,OX241560 |
| VC_776_0 | 3 | 880.0563877 | 1000.735949 | 0.46791789 | 0.0018064 | 3 | 5.38516481 | 3 | 3 | 1 | 4.428048098 | 0 | 0 | 0 | MN988507,MN988510,MN988527 |
| VC_777_0 | 3 | 44.71677739 | 19.90030938 | 0.69202713 | 0.046645 | 7 | 7.21110255 | 3 | 3 | 1 | 7.117510326 | 0 | 0 | 0 | MW202785,MW202796,OK172329 |
| VC_778_0 | 13 | 1656.570163 | 4761.40578 | 0.25811411 | 1.94E-31 | 3.872983346 | 9.79795897 | 78 | 73 | 0.935897436 | 7.443657994 | 0 | 0 | 0 | S11_vs_11267,S12_vs_99092,S14_vs_62178,S16_vs_26078,S17_vs_121041,S1_vs_62409,S5_vf_11450,S5_vs_49480,S6_vs_56490,S7_vs_55409,S8_vs_143608,S9_vs_73764,S9_vs_75050 |
| VC_779_0 | 19 | 3886.739758 | 3727.009785 | 0.51048957 | 5.98E-78 | 4.582575695 | 9.43398113 | 171 | 158 | 0.923976608 | 7.520187006 | 0 | 0 | 0 | S11_vs_34137,S12_vs_106329,S13_vf_22175,S14_vs_117166,S14_vs_61530,S16_vs_103726,S16_vs_92150,S17_vs_41845,S17_vs_73830,S3_vs_28402,S4_vs_VIRSorter_12831-circular-cat_2,S5_vs_58859,S7_vs_122151,S7_vs_38113,S7_vs_38834,S7_vs_82140,S8_vs_154231,S9_vf_79919,S9_vs_46498 |
| VC_78_0 | 20 | 57000 | 2133.997662 | 0.96391251 | 9.64E-94 | 0 | 6.70820393 | 190 | 190 | 1 | 4.229773102 | 0 | 0 | 0 | AB472900,KC862295,KC862296,KC862299,KC862301,KM434186,KY994101,LN610573,LN610576,LN610581,LN610582,LN610583,MK799650,MT108728,MT108730,MT118294,MT118295,MT118300,OM421595,ON653033 |
| VC_780_0 | 1 | 0 | 174.5362059 | 0 | 0 | 0 | 0 | 1 | 1 | 1 | 0 | 0 | 0 | 0 | S16_vs_34751 |
| VC_782_0 | 5 | 305.8410081 | 151.8597086 | 0.66821177 | 1.17E-05 | 5.291502622 | 8.54400375 | 10 | 10 | 1 | 6.72927369 | 0 | 0 | 0 | S14_vs_102698,S14_vs_110290,S1_vs_91270,S5_vs_3183,S9_vs_26179 |
| VC_783_0 | 67 | 466773.7556 | 55080.58653 | 0.89445218 | 0 | 0 | 5.47722558 | 2211 | 2211 | 1 | 2.185786347 | 0 | 0 | 0 | DQ398042,EU568876,FJ973624,GQ303261,JN412593,JN699002,KC787103,KC787104,KC787107,KC787108,KC787109,KC787110,KC787111,KC787112,KJ725374,KM923970,KT347314,KT355472,KT355474,KT365399,KX443326,KX534004,KX588251,KX641265,KX641266,KX664455,MF668268,MF668272,MF919497,MG099946,MH001455,MH001456,MH045569,MH077584,MH371116,MH371119,MH450127,MH479920,MH590605,MH779505,MH779507,MH779509,MH779513,MH779514,MH779515,MK305884,MK310146,MK433277,MK433279,MK524493,MK524494,MK919475,MN444875,MT818422,MW119570,MW291028,MZ171377,MZ747508,ON392160,ON392162,ON637759,ON637765,ON637766,ON645337,ON970618,OP172872,OP294982 |
| VC_784_0 | 9 | 5326.063214 | 54892.58647 | 0.08844541 | 3.00E-24 | 3 | 6.8556546 | 36 | 36 | 1 | 5.625723684 | 0 | 0 | 0 | KJ410133,KR080197,KR080198,KR080202,MF141540,MK494105,MN234183,MN234185,MN234219 |
| VC_785_0 | 2 | 300 | 4679.871978 | 0.06024251 | 0.0490479 | 5.656854249 | 5.65685425 | 1 | 1 | 1 | 5.656854249 | 0 | 0 | 0 | MW584160,MW584202 |
| VC_785_1 | 9 | 10800 | 18555.28953 | 0.36790644 | 1.56E-20 | 0 | 6.92820323 | 36 | 36 | 1 | 3.504694552 | 0 | 0 | 0 | JN699628,KC900379,KT591491,MH697587,MK937612,MN586024,MT658803,MW713383,OP297551 |
| VC_785_2 | 7 | 6155.959761 | 17568.08249 | 0.25948191 | 1.81E-13 | 2.645751311 | 8.71779789 | 21 | 21 | 1 | 6.239868842 | 0 | 0 | 0 | JF937105,KY223999,KY783914,MF319184,MT657341,MT658802,MW578836 |
| VC_786_0 | 4 | 1800 | 3315.341539 | 0.35188266 | 2.53E-05 | 0 | 3.74165739 | 6 | 6 | 1 | 2.687325274 | 0 | 0 | 0 | DQ398052,KP027195,KY385381,MN585992 |
| VC_787_0 | 132 | 647077.6221 | 0 | 1 | 0 | 0 | 7.54983444 | 8646 | 8646 | 1 | 5.802307653 | 0 | 0 | 0 | DQ431235,FJ706171,FJ706172,JX262215,JX262216,JX262217,JX262218,JX262219,JX262220,JX262221,JX262222,JX262223,JX262224,JX262225,JX570702,JX570703,JX570704,JX570705,JX570706,JX570707,JX570708,JX570709,JX570710,JX570711,JX570712,JX570713,JX570714,KJ578758,KJ578759,KJ578760,KJ578761,KJ578762,KJ578763,KJ578764,KJ578765,KJ578766,KJ578767,KJ578768,KJ578769,KJ578770,KJ578771,KJ578772,KJ578773,KJ578774,KJ578775,KJ578776,KJ578777,KJ578778,KJ578779,KJ578780,KJ578781,KJ578782,KJ578783,KJ578784,KJ578785,KJ578786,KJ578787,KJ578788,KJ578789,KJ578790,KJ578791,KJ578792,KJ722067,KR337643,KR337644,KR337645,KR337646,KR337647,KR337648,KR337649,KR337650,KR337651,KR337652,KR337653,KR337654,KR347352,KR347353,KR347354,KR347355,KR902978,KR902979,KR902980,KR902981,KR902982,KR902983,KR902984,KR902985,KR902986,KR902987,KT934381,KY926792,KY926793,MF919491,MF919505,MF919515,MF919516,MF919522,MF919533,MG820632,MG820633,MG820634,MG820635,MG820636,MG820637,MG820638,MG820639,MG820640,MG820641,MG820642,MG820643,MG820644,MG820645,MN329678,MN813675,MN813677,MN813679,MN813683,MN813688,MN813689,MN813690,MN813691,MN813692,MT647607,MW161461,MW161462,MW161463,MW161464,MW161465,MW161466,MW161467,MW161468,MW505928 |
| VC_788_0 | 2 | 136.1993734 | 473.1705948 | 0.22350851 | 0.0494469 | 4 | 4 | 1 | 1 | 1 | 4 | 0 | 0 | 0 | LN681534,MN718463 |
| VC_789_0 | 34 | 117933.6645 | 10720.50171 | 0.91667194 | 6.00E-160 | 0 | 8.30662386 | 561 | 561 | 1 | 6.534063008 | 0 | 0 | 0 | JF923796,JF923797,KR053201,MF919510,MF919542,MG757157,MG757166,MH536819,MH536824,MH576960,MH669007,MH669013,MH727547,MH727552,MH744424,MH779499,MH779501,MH779508,MH779510,MK376954,MK376960,MK376962,MK433272,MK977699,MN369753,MN369766,MN428063,MT889393,MT986025,MW507129,MZ322010,MZ322022,MZ622178,ON456333 |
| VC_79_0 | 2 | 300 | 9638.824043 | 0.03018466 | 0.0438261 | 6.403124237 | 6.40312424 | 1 | 1 | 1 | 6.403124237 | 0 | 0 | 0 | MG018930,MZ605293 |
| VC_79_1 | 6 | 4500 | 28881.55179 | 0.134805 | 1.09E-10 | 0 | 4.47213595 | 15 | 15 | 1 | 3.313315198 | 0 | 0 | 0 | KU130126,KU130127,KU130128,KU130129,KU130130,KU130131 |

| | | | | | | | | | | | | | | | |
|---|---|---|---|---|---|---|---|---|---|---|---|---|---|---|---|
| VC_79_2 | 1 | 0 | 7237.579711 | 0 | 0 | 0 | 0 | 1 | 1 | 1 | 0 | 0 | 0 | 0 | OK040171 |
| VC_79_3 | 11 | 16500 | 47920.40777 | 0.25613001 | 4.16E-14 | 1.732050808 | 7.48331477 | 55 | 55 | 1 | 5.422404343 | 0 | 0 | 0 | KJ409772,MN905160,MT670417,MT670418,MT670419,MT670420,MT670421,MT670422,MW175491,MZ348426,MZ868713 |
| VC_79_4 | 1 | 0 | 7318.654826 | 0 | 0 | 0 | 0 | 1 | 1 | 1 | 0 | 0 | 0 | 0 | MN812694 |
| VC_79_5 | 6 | 4500 | 37059.40087 | 0.10827875 | 0.0002501 | 0 | 7.34846923 | 15 | 15 | 1 | 5.279130029 | 0 | 0 | 0 | LN887844,MZ826350,MZ826353,OK258139,OK275491,OM982620 |
| VC_79_6 | 1 | 0 | 7537.00374 | 0 | 0 | 0 | 0 | 1 | 1 | 1 | 0 | 0 | 0 | 0 | MF893340 |
| VC_790_0 | 8 | 7740.15034 | 3347.840112 | 0.69806611 | 9.30E-19 | 0 | 3.74165739 | 28 | 28 | 1 | 2.767178385 | 0 | 0 | 0 | MG198776,MG198777,MG198778,MG198780,MH926056,MH926057,MH976516,MK977700 |
| VC_791_0 | 2 | 300 | 5982.639321 | 0.04775063 | 0.044857 | 0 | 0 | 1 | 1 | 1 | 0 | 0 | 0 | 0 | MW452562,MW582531 |
| VC_792_0 | 5 | 1175.725738 | 238.5844639 | 0.83130684 | 5.95E-06 | 6.633249581 | 8.83176087 | 10 | 10 | 1 | 8.256962053 | 0 | 0 | 0 | DQ500118,MN988496,MW960032,OP484857,OP484858 |
| VC_793_0 | 2 | 224.6858398 | 71.29840367 | 0.75911419 | 0.0839909 | 0 | 0 | 1 | 1 | 1 | 0 | 0 | 0 | 0 | MN988490,MN988502 |
| VC_794_0 | 6 | 4500 | 143235.1449 | 0.03045992 | 0.1898717 | 1 | 8.48528137 | 15 | 15 | 1 | 5.120721746 | 0 | 0 | 0 | MG065644,MG065662,MG065672,MG065674,MG065677,MG065690 |
| VC_794_1 | 2 | 300 | 50075.09438 | 0.00595532 | 0.4231764 | 8.717797887 | 8.71779789 | 1 | 1 | 1 | 8.717797887 | 0 | 0 | 0 | MK962749,ON286975 |
| VC_794_10 | 8 | 8400 | 185472.6467 | 0.04332741 | 0.0911817 | 0 | 9.79795897 | 28 | 26 | 0.928571429 | 6.615135979 | 0 | 0 | 0 | MN850630,MT884008,MT884009,MT884010,MT884011,MZ501082,MZ501083,MZ501102 |
| VC_794_11 | 6 | 4500 | 143026.5183 | 0.03050299 | 0.1616922 | 1.414213562 | 9.48683298 | 15 | 12 | 0.8 | 7.896730496 | 0 | 0 | 0 | LC739541,LC739542,MH491969,MH752840,MZ501048,MZ501094 |
| VC_794_12 | 1 | 0 | 24005.05789 | 0 | 0 | 0 | 0 | 1 | 1 | 1 | 0 | 0 | 0 | 0 | MG065659 |
| VC_794_13 | 1 | 0 | 24022.39242 | 0 | 0 | 0 | 0 | 1 | 1 | 1 | 0 | 0 | 0 | 0 | MG065653 |
| VC_794_14 | 3 | 900 | 74312.98577 | 0.01196602 | 0.3602049 | 1 | 8.06225775 | 3 | 3 | 1 | 5.687419249 | 0 | 0 | 0 | MG065654,MG065655,MG065666 |
| VC_794_15 | 5 | 3000 | 120517.718 | 0.02428801 | 0.2385514 | 1.414213562 | 9.16515139 | 10 | 9 | 0.9 | 7.618194443 | 0 | 0 | 0 | DQ832317,KC690136,KP869102,KP869103,KP869112 |
| VC_794_16 | 10 | 13500 | 226357.0792 | 0.05628352 | 0.0588681 | 1.414213562 | 9.89949494 | 45 | 39 | 0.866666667 | 7.991383739 | 0 | 0 | 0 | KJ190158,LC739538,LC739540,MG065646,MG065647,MG065658,MG065661,MG065682,MG963916,MN850642 |
| VC_794_17 | 2 | 300 | 50132.12793 | 0.00594859 | 0.4297409 | 7.874007874 | 7.87400787 | 1 | 1 | 1 | 7.874007874 | 0 | 0 | 0 | MW748998,MZ501073 |
| VC_794_18 | 1 | 0 | 25215.3912 | 0 | 0 | 0 | 0 | 1 | 1 | 1 | 0 | 0 | 0 | 0 | MZ501061 |
| VC_794_19 | 16 | 36000 | 333239.6899 | 0.09749764 | 0.0038337 | 5.477225575 | 9.59166305 | 120 | 97 | 0.808333333 | 8.220898076 | 0 | 0 | 0 | KT001917,LR699804,MG065635,MK327930,MK373780,MN850576,MN850578,MN850595,MN850597,MN850612,MN850626,MN850627,MZ501054,MZ501062,OK272482,OV101294 |
| VC_794_2 | 1 | 0 | 25326.66415 | 0 | 0 | 0 | 0 | 1 | 1 | 1 | 0 | 0 | 0 | 0 | MZ501060 |
| VC_794_20 | 2 | 300 | 49968.93318 | 0.0059679 | 0.4113141 | 8.831760866 | 8.83176087 | 1 | 1 | 1 | 8.831760866 | 0 | 0 | 0 | MN850593,MT682716 |
| VC_794_3 | 1 | 0 | 25301.01446 | 0 | 0 | 0 | 0 | 1 | 1 | 1 | 0 | 0 | 0 | 0 | MZ501093 |
| VC_794_4 | 4 | 1800 | 97491.56102 | 0.01812843 | 0.269817 | 5.196152423 | 8.06225775 | 6 | 6 | 1 | 6.948725067 | 0 | 0 | 0 | JQ031132,MK883717,MK883718,MW883061 |
| VC_794_5 | 1 | 0 | 25335.94468 | 0 | 0 | 0 | 0 | 1 | 1 | 1 | 0 | 0 | 0 | 0 | MT740314 |
| VC_794_6 | 3 | 900 | 74236.17678 | 0.01197825 | 0.3602049 | 7.874007874 | 8.18535277 | 3 | 3 | 1 | 7.998871526 | 0 | 0 | 0 | MN850646,MN850649,MZ501111 |
| VC_794_7 | 2 | 300 | 49992.4834 | 0.00596511 | 0.4113129 | 1.414213562 | 1.41421356 | 1 | 1 | 1 | 1.414213562 | 0 | 0 | 0 | LR694610,LR694611 |
| VC_794_8 | 2 | 300 | 50027.102 | 0.005961 | 0.4170632 | 8.426149773 | 8.42614977 | 1 | 1 | 1 | 8.426149773 | 0 | 0 | 0 | KR698074,ON286973 |
| VC_794_9 | 7 | 6300 | 164494.4644 | 0.03688644 | 0.1266963 | 0 | 8.60232527 | 21 | 21 | 1 | 7.15404982 | 0 | 0 | 0 | KY683736,LN881727,LN881735,LR597647,LR694165,MG022439,MG022440 |
| VC_795_0 | 23 | 33427.64361 | 27378.12057 | 0.54974465 | 4.13E-147 | 0 | 6.63324958 | 253 | 253 | 1 | 4.681915739 | 0 | 0 | 0 | DQ864624,MK448678,MK448689,MK448693,MK448696,MK448697,MK448699,MK448766,MK448779,MK448784,MK448789,MK448797,MK448852,MK448866,MK448867,MK448868,MK448871,MK448947,MK448952,MK448961,MK448968,MK448974,MK448976 |
| VC_796_0 | 5 | 1873.385832 | 3864.926593 | 0.32646982 | 4.45E-08 | 0 | 5 | 10 | 10 | 1 | 3.357615421 | 0 | 0 | 0 | GQ478081,GQ478082,GQ478083,GQ478084,GQ478085 |
| VC_797_0 | 3 | 247.5010248 | 6625.602244 | 0.03601008 | 0.0014977 | 8.485281374 | 8.94427191 | 3 | 3 | 1 | 8.67729285 | 0 | 0 | 0 | MK448854,MK448933,MK448950 |
| VC_797_1 | 14 | 7385.381842 | 19132.16515 | 0.27850924 | 1.10E-56 | 0 | 10.1488916 | 91 | 69 | 0.758241758 | 8.127152968 | 0 | 0 | 0 | MK448673,MK448765,MK448775,MK448794,MK448795,MK448858,MK448864,MK448941,MK448944,MK448945,MK448946,MK448953,MK448957,MK448970 |
| VC_799_0 | 3 | 210.6505254 | 233.2381108 | 0.47455715 | 0.0038188 | 2.236067977 | 5 | 3 | 3 | 1 | 4.045015821 | 0 | 0 | 0 | EF537008,KF598865,MT457475 |
| VC_8_0 | 58 | 215473.9919 | 44598.46415 | 0.82851523 | 0 | 0 | 9.2736185 | 1653 | 1644 | 0.994555354 | 6.674113308 | 0 | 0 | 0 | AB045978,AF424782,AP009363,AP012341,AY954955,AY954956,AY954957,CP062434,CP062442,CP062444,CP062461,EF462198,EU861005,JQ779023,JQ957932,JX174275,KF598856,KT186243,KY056620,LC541428,MF580410,MG029509,MG029511,MG029513,MG029514,MG029515,MG029516,MG029517,MG029518,MG770897,MH384261,MK348510,MK801680,MK801681,MK940809,MT366568,MT899504,MW924497,OL690335,OL840290,OM304856,OM439665,OM439667,ON856257,ON856258,ON989481,OP038318,OP038319,OP136131,OP136132,OP136134,OP328420,OP328422,OP374154,OP374155,OP374156,OP584903,OP584904 |
| VC_80_0 | 64 | 343985.5148 | 478874.4279 | 0.41803653 | 0 | 0 | 10.198039 | 2016 | 1542 | 0.764880952 | 8.236851446 | 0 | 0 | 0 | AB560486,AP018815,FM201282,JN254800,KP340287,KP340288,KR869157,KX171208,LC102730,LC105987,LC105988,LC105989,LC105990,LC472883,LC472884,LN610579,LN610588,MF490240,MG897799,MN871458,MN871459,MN871461,MN871462,MN871464,MN871465,MN871466,MN871473,MN871479,MN871485,MN871487,MN871508,MN871509,MT118291,MT118297,MT118298,MT118299,MT119363,MT119365,MT119368,MT119376,MT133561,MT133562,MT349888,MT413450,MT933737,MW512833,MW595221,MZ553930,OL362268,OL362269,OL754588,OM953790,ON324181,ON375838,ON375839,ON375840,ON529291,ON857930,ON857934,ON857942,OP310975,OP310977,OP314870,OP314871 |
| VC_80_1 | 2 | 171.3642905 | 28441.64492 | 0.00598903 | 0.0425126 | 8.124038405 | 8.1240384 | 1 | 1 | 1 | 8.124038405 | 0 | 0 | 0 | LC472881,MW406974 |
| VC_80_10 | 1 | 0 | 15079.77714 | 0 | 0 | 0 | 0 | 1 | 1 | 1 | 0 | 0 | 0 | 0 | AP019535 |
| VC_80_11 | 1 | 0 | 14432.90034 | 0 | 0 | 0 | 0 | 1 | 1 | 1 | 0 | 0 | 0 | 0 | KY000082 |
| VC_80_2 | 1 | 0 | 13867.10236 | 0 | 0 | 0 | 0 | 1 | 1 | 1 | 0 | 0 | 0 | 0 | FM201281 |
| VC_80_3 | 1 | 0 | 14681.84012 | 0 | 0 | 0 | 0 | 1 | 1 | 1 | 0 | 0 | 0 | 0 | OP310979 |
| VC_80_4 | 3 | 528.3460319 | 49549.02077 | 0.0105506 | 0.0036791 | 7.615773106 | 9.21954446 | 3 | 2 | 0.666666667 | 8.172154437 | 0 | 0 | 0 | MF623055,MT118289,MT118292 |
| VC_80_5 | 2 | 161.3364419 | 34921.0745 | 0.00459878 | 0.0721803 | 8.717797887 | 8.71779789 | 1 | 1 | 1 | 8.717797887 | 0 | 0 | 0 | MK837009,MW595220 |
| VC_80_6 | 11 | 9351.317832 | 167982.9174 | 0.05273273 | 3.60E-19 | 2.236067977 | 9.53939201 | 55 | 41 | 0.745454545 | 7.971332033 | 0 | 0 | 0 | MK837010,MN871457,MN871460,MN871467,MT108729,MT491204,MT491205,MT491206,MW512830,MW512834,OP310974 |
| VC_80_7 | 33 | 95574.61529 | 347762.0739 | 0.21558021 | 1.86E-286 | 0 | 10.1488916 | 528 | 444 | 0.840909091 | 7.875683632 | 0 | 0 | 0 | FM887021,FM897211,GU815091,KR054033,KU198331,MF490241,MF768470,MK318076,MN131141,MN131142,MN131143,MN615700,MN615701,MN615702,MT108727,MT118290,MT119366,MT119369,MZ285878,ON857925,ON857929,ON857937,ON857938,OP310970,OP310971,OP310972,OP310973,OP310976,OP310978,OP310980,OP310981,OP310982,OP314872 |
| VC_80_8 | 1 | 0 | 14890.72651 | 0 | 0 | 0 | 0 | 1 | 1 | 1 | 0 | 0 | 0 | 0 | KM434184 |
| VC_80_9 | 2 | 150.2219865 | 24448.97901 | 0.00610678 | 0.0514 | 7.416198487 | 7.41619849 | 1 | 1 | 1 | 7.416198487 | 0 | 0 | 0 | MK340760,MN871468 |
| VC_800_0 | 4 | 743.858183 | 174.758538 | 0.80975903 | 0.0001457 | 2 | 4.58257569 | 6 | 6 | 1 | 3.686969088 | 0 | 0 | 0 | DQ875742,OM897575,ON677538,OP056300 |
| VC_801_0 | 37 | 119463.7351 | 91166.07226 | 0.56717393 | 0 | 0 | 8.54400375 | 666 | 666 | 1 | 6.240049647 | 0 | 0 | 0 | EF056009,HQ259105,JX415535,JX880034,KC206276,KF192075,KF562340,KF620435,KJ135004,LR999871,MG208881,MG770228,MG922974,MH358458,MH669274,MH733496,MH992123,MK931297,MT682708,MW175414,MW630115,MZ358387,MZ394712,MZ501049,OM632664,OM632665,OM632666,OM632669,OM632670,OM632672,OM982646,ON602749,ON602751,ON922919,OP312987,OV049961,OW991345 |
| VC_801_1 | 11 | 9883.9761 | 52554.40224 | 0.15829969 | 2.91E-36 | 0 | 8.66025404 | 55 | 55 | 1 | 6.569343817 | 0 | 0 | 0 | KF787094,KF787095,KT321317,MG018928,MK962626,MK962629,MK962630,MK962631,MK962632,MK962639,MK962641 |
| VC_801_2 | 5 | 2429.677211 | 25506.33861 | 0.08697293 | 3.00E-08 | 2 | 7 | 10 | 10 | 1 | 4.613887505 | 0 | 0 | 0 | KF806588,KX098389,KX098390,KX098391,MH160392 |
| VC_801_3 | 7 | 2552.62277 | 30870.26853 | 0.07637349 | 2.71E-11 | 2.645751311 | 9.69535971 | 21 | 15 | 0.714285714 | 7.456925631 | 0 | 0 | 0 | MG983743,MK863032,MN062186,MZ326856,MZ326857,MZ326861,OM687511 |
| VC_802_0 | 8 | 6349.308589 | 6000.247632 | 0.51413253 | 1.05E-19 | 3.605551275 | 7.48331477 | 28 | 28 | 1 | 5.888299469 | 0 | 0 | 0 | KC139517,KC139520,KM236242,MH899585,MN163281,MW544066,OM858838,OM908766 |
| VC_803_0 | 2 | 268.2023214 | 6930.691777 | 0.03725604 | 0.0430744 | 2.236067977 | 2.23606798 | 1 | 1 | 1 | 2.236067977 | 0 | 0 | 0 | KU356690,KU356691 |
| VC_804_0 | 3 | 877.4742561 | 2162.009636 | 0.28869186 | 0.0014331 | 0 | 3.46410162 | 3 | 3 | 1 | 2.309401077 | 0 | 0 | 0 | MG592603,MG592604,MG592610 |
| VC_805_0 | 3 | 604.2474847 | 8964.169568 | 0.0631502 | 0.0014513 | 2.236067977 | 7.74596669 | 3 | 3 | 1 | 5.887726806 | 0 | 0 | 0 | MN228696,MW960036,MW960038 |
| VC_806_0 | 6 | 3100.703117 | 2570.038516 | 0.5467897 | 6.28E-11 | 2.236067977 | 8.54400375 | 15 | 15 | 1 | 6.155311269 | 0 | 0 | 0 | KC438282,KC438283,KP280062,KY065148,KY612839,OP313112 |
| VC_808_0 | 3 | 900 | 10310.93296 | 0.08027878 | 0.0014557 | 0 | 0 | 3 | 3 | 1 | 0 | 0 | 0 | 0 | MT234338,MT234339,MT234340 |
| VC_809_0 | 7 | 5747.172096 | 5405.698093 | 0.51530879 | 8.33E-15 | 0 | 6.55743852 | 21 | 21 | 1 | 3.647790413 | 0 | 0 | 0 | KX889068,MG592409,MG592410,MG592470,MG592519,MG592521,MG592668 |
| VC_809_1 | 3 | 713.759999 | 3556.048212 | 0.16716442 | 0.0015712 | 0 | 7.68114575 | 3 | 3 | 1 | 5.120763832 | 0 | 0 | 0 | HQ634194,HQ634196,JF974296 |
| VC_81_0 | 41 | 204820.7896 | 147406.5602 | 0.58150166 | 0 | 0 | 10.5830052 | 820 | 804 | 0.980487805 | 5.065439501 | 0 | 0 | 0 | DQ163917,EU716414,KP981875,KR054028,KR054030,KT254130,KT254131,KT254132,KT254133,KT372690,KT372691,KT372692,KT372693,KT372694,KT372695,KT372696,KT372697,KT372698,LC472882,LN610589,MH536736,MK317959,MN508620,MN563783,MN564818,MN871454,MT108726,MT118293,MT118303,MT119364,MT119367,MT119370,MT119372,MT119374,MT119375,MT119377,MT119378,MT133563,MW557846,MZ089729,ON361134 |
| VC_814_0 | 1 | 0 | 719.8200382 | 0 | 0 | 0 | 0 | 1 | 1 | 1 | 0 | 0 | 0 | 0 | MK448818 |
| VC_815_0 | 3 | 241.501453 | 0 | 1 | 0 | 2.236067977 | 4.69041576 | 3 | 3 | 1 | 3.683196454 | 0 | 0 | 0 | EF432053,KP282673,KP282674 |
| VC_816_0 | 35 | 105323.2765 | 1890.536984 | 0.98236667 | 8.52E-163 | 0 | 8.54400375 | 595 | 595 | 1 | 5.68601442 | 0 | 0 | 0 | EF460875,HQ728264,KP534715,KY087898,MF044458,MH191398,MN045229,MN098326,MN117721,MN122072,MN136198,MN812688,MN850594,MT230534,MT446397,MT446398,MT446399,MT446400,MT446402,MT446403,MT446406,MT446409,MT446419,MW147598,MW147599,MW147600,MW147601,MW147602,MW349138,OK073976,OK129343,OK148439,OL741059,OL960574,ON778463 |
| VC_817_0 | 12 | 10686.62009 | 1890.536984 | 0.84968487 | 2.49E-39 | 0 | 7.07106781 | 66 | 66 | 1 | 4.738283606 | 0 | 0 | 0 | KJ002054,KJ858521,KY709296,LR813619,MT623578,MW380983,MW380985,MZ336020,OL770365,OM810291,OP056089 |
| VC_818_0 | 3 | 60.94400742 | 0 | 1 | 0 | 1.414213562 | 2.23606798 | 3 | 3 | 1 | 1.794110782 | 0 | 0 | 0 | EF506570,SVU28974,X51344 |
| VC_819_0 | 19 | 46069.0188 | 21334.4613 | 0.68348131 | 1.75E-94 | 0 | 6.40312424 | 171 | 171 | 1 | 4.524674234 | 0 | 0 | 0 | KM236243,KP064094,KX534337,LN881725,LR025195,LR025196,LR025197,LR025198,LT907986,MH230177,MK672798,MZ501071,MZ501091,MZ501104,MZ501110,MZ501112,OL539454,OL960573,OL960575 |
| VC_820_0 | 5 | 2930.284056 | 5896.824536 | 0.3319642 | 3.19E-08 | 0 | 2 | 10 | 10 | 1 | 1.4 | 0 | 0 | 0 | MK816297,MN029011,MN871471,MN871472,MN871474 |
| VC_821_0 | 5 | 2421.48247 | 7733.425667 | 0.23845439 | 3.05E-08 | 0 | 6 | 10 | 10 | 1 | 4.548244464 | 0 | 0 | 0 | KX198614,MT795651,MW114771,ON457558,ON457559 |
| VC_823_0 | 4 | 1664.142952 | 1283.911288 | 0.56448858 | 1.47E-05 | 1.414213562 | 2.23606798 | 6 | 6 | 1 | 1.710107921 | 0 | 0 | 0 | KJ489010,KJ489011,KJ489012,KJ489013 |
| VC_824_0 | 3 | 714.5997429 | 705.0152536 | 0.50337574 | 0.0015712 | 1.414213562 | 3.31662479 | 3 | 3 | 1 | 2.576946118 | 0 | 0 | 0 | EU081845,MK779875,MN552143 |
| VC_825_0 | 17 | 37736.77519 | 7379.695332 | 0.83643013 | 2.40E-75 | 0 | 5.29150262 | 136 | 136 | 1 | 4.317211283 | 0 | 0 | 0 | KU160642,KU647628,MF189170,MF189171,MF189172,MF189173,MF189174,MF189175,MF324906,MH450120,MK919473,MK967398,MN284897,MZ209300,MZ209307,MZ747520,OP434451 |
| VC_826_0 | 7 | 349.2177991 | 6513.650497 | 0.05088511 | 0.0009007 | 4.898979486 | 8.83176087 | 21 | 21 | 1 | 7.079512639 | 0 | 0 | 0 | KY053532,MT006236,S13_vs_113593,S13_vs_152223,S16_vf_100378,S3_vf_44405,S5_vf_77701 |
| VC_829_0 | 4 | 1440.150857 | 148.746326 | 0.90638392 | 0.0023859 | 2 | 7.14142843 | 6 | 6 | 1 | 4.764998774 | 0 | 0 | 0 | EU100883,EU100884,KX987127,MH992131 |
| VC_83_0 | 24 | 47633.41721 | 17219.59504 | 0.73448273 | 1.26E-109 | 0 | 3.60555128 | 276 | 276 | 1 | 1.712322506 | 0 | 0 | 0 | DQ029335,FJ409640,HQ641340,HQ641341,HQ641342,HQ641343,HQ641344,JQ780163,KY883641,KY883643,KY883644,KY883645,KY883646,KY883648,KY883649,KY883650,KY883651,KY883652,KY883653,MF574151,MG957431,MT215167,ON464735,ON464736 |
| VC_830_0 | 3 | 428.3401815 | 386.6702529 | 0.52556405 | 0.0017478 | 0 | 4.12310563 | 3 | 3 | 1 | 2.748737084 | 0 | 0 | 0 | KP792623,MW202557,MW202655 |
| VC_831_0 | 17 | 14698.66635 | 1530.783287 | 0.90567867 | 3.04E-58 | 0 | 6.244998 | 136 | 136 | 1 | 3.68989543 | 0 | 0 | 0 | EU124666,MG592549,MG592550,MG592581,MG592582,MK796797,MW824388,MW824389,MW824392,MW824393,MW824394,MW824398,MW824399,MW824402,MW824403,MW824405,OL762410 |
| VC_832_0 | 2 | 60.86213409 | 105.0685258 | 0.36679258 | 0.0507352 | 5.656854249 | 5.65685425 | 1 | 1 | 1 | 5.656854249 | 0 | 0 | 0 | MW202520,MW202674 |
| VC_833_0 | 2 | 282.0130799 | 1296.57908 | 0.17864847 | 0.0370009 | 1.732050808 | 1.73205081 | 1 | 1 | 1 | 1.732050808 | 0 | 0 | 0 | KJ028219,MG770216 |
| VC_834_0 | 18 | 44973.62704 | 4362.206063 | 0.91158138 | 4.34E-52 | 0 | 2.44948974 | 153 | 153 | 1 | 0.797589782 | 0 | 0 | 0 | EU203571,KJ538722,KT222940,KT246485,KT454972,MF919517,MH697577,MH727549,MK494095,MK919482,MT114159,MT657330,MT684594,MT818421,MW924639,MZ005672,MZ622181,OP297533 |
| VC_836_0 | 3 | 900 | 6876.745824 | 0.11572964 | 0.0015762 | 3.16227766 | 4.69041576 | 3 | 3 | 1 | 4.108276458 | 0 | 0 | 0 | HM997019,MG873442,MW296032 |
| VC_836_1 | 4 | 1800 | 6250.726693 | 0.2235823 | 1.57E-05 | 3.16227766 | 4.58257569 | 6 | 6 | 1 | 3.828890753 | 0 | 0 | 0 | LC519601,MT028297,MT664722,MT740244 |
| VC_836_2 | 2 | 300 | 4251.650271 | 0.06591016 | 0.0448186 | 1.732050808 | 1.73205081 | 1 | 1 | 1 | 1.732050808 | 0 | 0 | 0 | JN882286,MW343794 |
| VC_836_3 | 1 | 0 | 2223.073817 | 0 | 0 | 0 | 0 | 1 | 1 | 1 | 0 | 0 | 0 | 0 | OL799327 |
| VC_837_0 | 12 | 19800 | 65290.07023 | 0.2326946 | 4.84E-40 | 0 | 6.32455532 | 66 | 66 | 1 | 4.721547599 | 0 | 0 | 0 | JX415536,LC553734,MK310183,MT446407,MT446413,MT446414,MZ234015,MZ234017,MZ342906,MZ398243,OK148440,OP293233 |
| VC_837_1 | 24 | 79803.2004 | 69977.70856 | 0.53279955 | 1.80E-126 | 3.605551275 | 8.24621125 | 276 | 276 | 1 | 6.138831822 | 0 | 0 | 0 | EU330206,JX867715,KP981730,KM044272,KP308307,LC553735,MG673519,MG748548,MG976803,MG983840,MK373770,MN045231,MN148435,MN508615,MN715356,MT127620,MT129653,MT129655,MT446386,MT446410,MW741821,MW929182,MZ234028,MZ648214 |
| VC_838_0 | 3 | 230.7988385 | 0 | 1 | 0 | 0 | 4.24264069 | 3 | 3 | 1 | 2.828427125 | 0 | 0 | 0 | EU350361,KY296500,KY296501 |
| VC_839_0 | 8 | 5442.095294 | 60.87244613 | 0.98893825 | 3.30E-11 | 0 | 4.89897949 | 28 | 28 | 1 | 3.728409574 | 0 | 0 | 0 | EU588980,GQ443085,JF767208,JF767209,JF767210,MH999280,MZ401006,MZ995505 |
| VC_84_0 | 6 | 2482.969301 | 1418.816055 | 0.63636748 | 4.88E-11 | 1 | 4.24264069 | 15 | 15 | 1 | 3.221323242 | 0 | 0 | 0 | AB597179,KP743639,OK274245,OK318991,OP313111,OP617560 |
| VC_842_0 | 14 | 6493.935579 | 3373.949806 | 0.65808786 | 8.40E-42 | 0 | 4.47213595 | 91 | 91 | 1 | 2.504760607 | 0 | 0 | 0 | EU622808,EU771092,KU831549,MF407276,MT459794,MW354668,MW354670,MW477480,MZ570263,OL744111,OM249958,ON042750,PZACG,X99260 |
| VC_843_0 | 20 | 19000.1848 | 748.5837607 | 0.96209466 | 8.62E-37 | 0 | 5.65685425 | 190 | 190 | 1 | 4.129435499 | 0 | 0 | 0 | KC685370,KX349899,KX349900,KX349901,KX961631,KY947509,MF156577,MF156578,MF156580,MH817022,MK084630,MK284526,MK284527,MK284528,MN082624,MT777452,MW012634,MW348917,MZ384014,OL744112 |
| VC_844_0 | 3 | 175.6179293 | 1639.918415 | 0.09673061 | 0.0015985 | 1.414213562 | 4.69041576 | 3 | 3 | 1 | 3.598348361 | 0 | 0 | 0 | KT780304,KY821088,KY921761 |
| VC_847_0 | 2 | 37.37993913 | 335.7027239 | 0.10019211 | 0.0454835 | 1.414213562 | 1.41421356 | 1 | 1 | 1 | 1.414213562 | 0 | 0 | 0 | S13_vs_68102,S8_vf_203044 |
| VC_848_0 | 124 | 598166.387 | 32518.72313 | 0.94843905 | 0 | 0 | 8.48528137 | 7626 | 7626 | 1 | 6.663940833 | 0 | 0 | 0 | EU734173,GQ413937,HG994092,HM480846,KR149291,KT367885,KU183006,KX591654,KX712070,KX712071,KY389315,KY389316,KY450753,KY652723,KY652724,KY652725,LC413193,LC413194,LC413195,LN866626,MF285615,MG835568,MH172261,MH172262,MH172263,MH172264,MH587638,MK134560,MK203841,MK380015,MK380016,MK521905,MK618658,MK795385,MK903728,MN052874,MN101216,MN101218,MN101227,MN149903,MN176574,MN434093,MN585130,MT425185,MT446417,MT446418,MT542697,MT813140,MT813141,MT966872,MT966873,MW042787,MW042788,MW042789,MW042790,MW042791,MW042792,MW042793,MW042794,MW042795,MW042796,MW042797,MW042798,MW042799,MW042800,MW042801,MW042803,MW042804,MW042805,MW042806,MW042807,MW042808,MW042809,MW042810,MW042811,MW042812,MZ424865,MZ571832,OK138556,OL409034,OL799328,OM194188,OM194189,OM194190,ON148528,ON148529,ON571554,ON602742,ON602755,ON880501,OP013026,OP045496,OP103917,OP103918,OP103919,OP103920,OP103921,OP103922,OP103923,OP256047,OU342754,OX335359,OX335360,OX335375,OX335376,OX335377,OX335389,OX335390,OX335392,OX335394,OX335398,OX335401,OX335408,OX335409,OX335413,OX335414,OX335418,OX335426,OX335427,OX335433,OX335438,OX335440,OX335443 |
| VC_849_0 | 2 | 206.7010888 | 280.4992976 | 0.42426298 | 0.0524992 | 1.414213562 | 1.41421356 | 1 | 1 | 1 | 1.414213562 | 0 | 0 | 0 | KP063902,KP063903 |
| VC_850_0 | 91 | 653962.1358 | 4332.023954 | 0.99341932 | 1.80E-170 | 0 | 4.79583152 | 4095 | 4095 | 1 | 0.906742905 | 0 | 0 | 0 | ON170019,ON170022,ON170028,ON170033,ON170036,ON170038,ON170040,ON170049,ON170054,ON170074,ON170086,ON170088,ON170090,ON170092,ON170094,ON170096,ON170098,ON170100,ON170102,ON170104,ON170106,ON170108,ON170110,ON170112,ON170114,ON170116,ON170118,ON170120,ON170122,ON170124,ON170126,ON170142,ON170144,ON170146,ON170152,ON170154,ON170156,ON170158,ON170160,ON170162,ON170164,ON170167,ON170176,ON170180,ON170186,ON170189,ON170192,ON170194,ON170196,ON170198,ON170200,ON170202,ON170204,ON170206,ON170211,ON170215,ON170217,ON170219,ON170221,ON170222,ON170225,ON170244,ON170246,ON170248,ON170250,ON170252,ON170271,ON170280,ON170282,ON170289,ON170291,ON170293,ON170295,ON170297,ON170298,ON170300,ON170306,ON170307,ON170311,ON170313,ON170315,ON170330,ON170332,ON170334,ON170336,ON170338,ON170340,ON170352,ON170356,ON170360 |
| VC_851_0 | 6 | 2208.287035 | 747.5864436 | 0.74708442 | 3.97E-10 | 0 | 6.40312424 | 15 | 15 | 1 | 4.839038974 | 0 | 0 | 0 | KJ018213,MG592431,MG592432,MG592435,MG592458,MG592666 |
| VC_852_0 | 3 | 486.4399631 | 509.1687837 | 0.48858547 | 0.0015642 | 2 | 4 | 3 | 3 | 1 | 3.247219129 | 0 | 0 | 0 | JQ619704,MG784342,MH598512 |
| VC_853_0 | 5 | 1675.840598 | 242.7056908 | 0.873495 | 1.68E-06 | 1 | 4.47213595 | 10 | 10 | 1 | 3.302470595 | 0 | 0 | 0 | EU887664,JX887877,KF296717,KX190834,MT136606 |
| VC_854_0 | 6 | 3004.634679 | 2840.536862 | 0.51403704 | 1.71E-10 | 2.645751311 | 4.35889894 | 15 | 15 | 1 | 3.653147576 | 0 | 0 | 0 | FJ008913,HE608841,MN078104,MT630433,MW314138,MW916539 |
| VC_855_0 | 12 | 6500.119196 | 4211.599211 | 0.6068232 | 2.85E-32 | 3.872983346 | 7.34846923 | 66 | 66 | 1 | 5.724387455 | 0 | 0 | 0 | JQ867099,MN929097,OK632025,S10_vf_18671,S13_vf_87873,S16_vs_173102,S2_vs_7306,S3_vs_127404,S3_vs_89898,S4_vs_VIRSorter_6022-circular-cat_1,S4_vs_VIRSorter_9913-cat_1,S5_vs_1572 |
| VC_857_0 | 16 | 21597.36479 | 1583.961708 | 0.93167079 | 4.09E-66 | 0 | 7.61577311 | 120 | 120 | 1 | 6.071992627 | 0 | 0 | 0 | FJ591093,FJ591094,FR682616,FR719956,HQ317387,KJ621082,KJ803031,KU885988,KU885989,KU885990,MH015250,MH015253,MH015256,MH015257,MH015259,MZ773648 |
| VC_858_0 | 7 | 259.8274179 | 0 | 1 | 0 | 0 | 3.31662479 | 21 | 21 | 1 | 2.450549183 | 0 | 0 | 0 | FJ685651,GU321093,JN882264,JN882266,KF056323,MW344764,MW344766 |
| VC_859_0 | 67 | 333714.2663 | 13123.90219 | 0.96216131 | 0 | 0 | 6.40312424 | 2211 | 2211 | 1 | 4.99848368 | 0 | 0 | 0 | FJ750948,GQ502199,JN984867,JQ086375,JX865427,KM233151,KM896878,KX534335,KX534336,KX534338,KX534339,KX534341,LN881730,LN881731,LR597646,LR990702,LR999870,LT960609,MG065648,MG581355,MK138527,MK373789,MK373790,MK373791,MK728541,MN850575,MN850589,MN850628,MN850629,MN850650,MT331608,MT446389,MT446391,MT478993,MT884012,MT884013,MT884014,MT884015,MT951623,MW749005,MW822006,MW862804,MZ234008,MZ234014,MZ234016,MZ234026,MZ234029,MZ501070,MZ501088,MZ501095,MZ501097,MZ501107,OK272474,OK272488,OK310512,OK310513,OK310514,OK499985,OK499988,OK499993,OL615010,OL770278,OM262540,OM262541,ON184124,ON210136,OP046317 |
| VC_86_0 | 2 | 54.33140152 | 233.4753848 | 0.18877735 | 0.0443912 | 5.385164807 | 5.38516481 | 1 | 1 | 1 | 5.385164807 | 0 | 0 | 0 | S13_vf_151755,S8_vf_106946 |
| VC_860_0 | 9 | 5926.128729 | 1746.794251 | 0.77234305 | 3.12E-17 | 0 | 6.164414 | 36 | 36 | 1 | 4.392069024 | 0 | 0 | 0 | KT367887,MN656993,MT075871,MW419910,OL539440,OM687892,ON602734,ON602747,ON602766 |
| VC_861_0 | 29 | 70857.09397 | 329.2875197 | 0.99537429 | 5.79E-49 | 0 | 3.74165739 | 406 | 406 | 1 | 1.765107108 | 0 | 0 | 0 | FJ896200,LR794124,MF754111,MF754112,MK474470,MN974282,MT178448,MT501516,MW250641,MW331544,MZ020222,MZ182247,MZ516827,MZ592921,OK349506,OK585158,OK585159,OK585160,OM362522,OM799543,ON080940,ON080941,ON457554,ON457555,ON457556,ON457557,ON864052,ON872379,OP117450 |
| VC_862_0 | 29 | 120350.8566 | 19690.61107 | 0.85939443 | 1.89E-138 | 0 | 5.29150262 | 406 | 406 | 1 | 2.260645968 | 0 | 0 | 0 | FN422399,HG962375,JX194238,KM411958,KM411959,KM411960,KM974184,KP994390,KR052142,KR054032,KU948710,KX171209,KX171210,MF490236,MF490238,MH356729,MN553583,MN553584,MN906996,MT080102,MZ955867,OK665488,OL519844,OM234792,OM937766,ON815901,ON815902,ON815903,ON857943 |
| VC_863_0 | 5 | 3000 | 15376.63024 | 0.16325082 | 5.27E-08 | 1.414213562 | 4.12310563 | 10 | 10 | 1 | 3.168442053 | 0 | 0 | 0 | FN422398,LC064302,MT416090,OL519842,OM782452 |
| VC_865_0 | 10 | 12157.286 | 8188.970062 | 0.59751956 | 9.87E-28 | 0 | 6.08276253 | 45 | 45 | 1 | 4.819756981 | 0 | 0 | 0 | KY083726,KY514264,KY514265,KY549659,KY769270,MH059638,MN812685,MN812687,MN812691,MZ348421 |
| VC_866_0 | 2 | 283.1339543 | 4717.811939 | 0.05661608 | 0.0443988 | 3.464101615 | 3.46410162 | 1 | 1 | 1 | 3.464101615 | 0 | 0 | 0 | MG775260,MH179480 |
| VC_867_0 | 5 | 3000 | 7690.659519 | 0.2806188 | 5.62E-08 | 0 | 7.21110255 | 10 | 10 | 1 | 5.86727123 | 0 | 0 | 0 | FN667788,FN667789,HE815464,MK408758,MT932329 |
| VC_868_0 | 27 | 105300 | 7051.137139 | 0.93724018 | 1.26E-123 | 0 | 5.74456265 | 351 | 351 | 1 | 3.567060959 | 0 | 0 | 0 | FR823450,GU296433,JN132397,JX569801,KF148616,KX229736,KX236333,KX879627,MH107028,MT863714,MT863715,MT863716,MT863717,MT863718,MT863719,MT863720,MT863721,MT863722,MT863723,MT863724,MT863725,MT863726,MT863727,MT863728,MT863729,MT863730,MT863731 |
| VC_869_0 | 3 | 900 | 1838.462251 | 0.3286516 | 0.001685 | 4.123105626 | 6.40312424 | 3 | 3 | 1 | 5.563547955 | 0 | 0 | 0 | MK387337,MW451250,OM249648 |
| VC_87_0 | 3 | 900 | 20735.99453 | 0.04159735 | 0.0017652 | 1.414213562 | 7.74596669 | 3 | 3 | 1 | 5.591984454 | 0 | 0 | 0 | KJ010547,KJ010548,KM051843 |
| VC_87_1 | 4 | 1800 | 34163.85672 | 0.05005025 | 3.31E-05 | 2 | 6.244998 | 6 | 6 | 1 | 5.074713749 | 0 | 0 | 0 | MW419084,MW419085,MW419086,MW419087 |
| VC_87_10 | 1 | 0 | 8705.13931 | 0 | 0 | 0 | 0 | 1 | 1 | 1 | 0 | 0 | 0 | 0 | MN043729 |
| VC_87_11 | 1 | 0 | 8184.51753 | 0 | 0 | 0 | 0 | 1 | 1 | 1 | 0 | 0 | 0 | 0 | MN940411 |
| VC_87_12 | 1 | 0 | 9024.288428 | 0 | 0 | 0 | 0 | 1 | 1 | 1 | 0 | 0 | 0 | 0 | MT151604 |
| VC_87_13 | 1 | 0 | 6426.752002 | 0 | 0 | 0 | 0 | 1 | 1 | 1 | 0 | 0 | 0 | 0 | KU640380 |

| | | | | | | | | | | | | | | |
|---|---|---|---|---|---|---|---|---|---|---|---|---|---|---|
| VC_87_2 | 3 | 900 | 26349.28449 | 0.03302839 | 0.0026702 | 6 | 7.34846923 | 3 | 3 | 1 | 6.660572936 | 0 | 0 | 0 KC699836,MW749002,MW749007 |
| VC_87_3 | 19 | 51300 | 89429.87071 | 0.36452815 | 1.44E-73 | 0 | 10.198039 | 171 | 138 | 0.807017544 | 7.83877131 | 0 | 0 | 0 AB930182,AP013029,KF669652,LT960608,MF422185,MG000860,MW354667,MZ501260,MZ501261,MZ501262,MZ501263,MZ501265,OM982668,OM982669,OM982670,OM982671,OM982672,OM982673,OM982674 |
| VC_87_4 | 3 | 900 | 29931.91093 | 0.02919054 | 0.0187474 | 4.358898944 | 6.63324958 | 3 | 3 | 1 | 5.745715508 | 0 | 0 | 0 KY368640,MN043730,OK349510 |
| VC_87_5 | 1 | 0 | 10735.01474 | 0 | 0 | 0 | 0 | 1 | 1 | 1 | 0 | 0 | 0 | 0 MH606185 |
| VC_87_6 | 1 | 0 | 7377.040835 | 0 | 0 | 0 | 0 | 1 | 1 | 1 | 0 | 0 | 0 | 0 JX238501 |
| VC_87_7 | 1 | 0 | 7308.588352 | 0 | 0 | 0 | 0 | 1 | 1 | 1 | 0 | 0 | 0 | 0 KM236246 |
| VC_87_8 | 1 | 0 | 7266.130398 | 0 | 0 | 0 | 0 | 1 | 1 | 1 | 0 | 0 | 0 | 0 KU253712 |
| VC_87_9 | 1 | 0 | 8143.749723 | 0 | 0 | 0 | 0 | 1 | 1 | 1 | 0 | 0 | 0 | 0 KM236245 |
| VC_870_0 | 2 | 300 | 200.7580289 | 0.59909174 | 0.0764249 | 2.645751311 | 2.64575131 | 1 | 1 | 1 | 2.645751311 | 0 | 0 | 0 MK388689,OK570186 |
| VC_871_0 | 5 | 3000 | 45444.94628 | 0.06192596 | 0.0007806 | 0 | 6.32455532 | 10 | 10 | 1 | 4.197077977 | 0 | 0 | 0 LC739533,LC739534,LC739535,LC739537,LC739539 |
| VC_871_1 | 26 | 97500 | 57189.73093 | 0.63029394 | 7.85E-128 | 2.645751311 | 9.16515139 | 325 | 319 | 0.981538462 | 6.137217131 | 0 | 0 | 0 JX561091,KX552041,KX664695,LR597654,LR746137,MG065650,MH051333,MH160766,MH252123,MK373788,MN850565,MN850571,MN850598,MN850618,MN850648,MT496970,MT944117,MW175890,MZ726793,OL505078,OL739525,OM362897,OM386656,OM386659,OM386667,OP562383 |
| VC_872_0 | 28 | 113400 | 30375.42942 | 0.78873004 | 4.59E-191 | 0 | 6.92820323 | 378 | 378 | 1 | 5.016619389 | 0 | 0 | 0 FR775895,KU522583,LC739536,LR865361,MF805809,MG065639,MG065645,MG065656,MG065689,MH675927,MH816848,MN850573,MN850584,MN850601,MN850632,MN850645,MT932211,MZ501063,MZ501084,MZ501100,OK210077,OK272470,OM386653,OM386654,OM386658,OM401812,OP177727,OX 090893 |
| VC_872_1 | 1 | 0 | 10171.88337 | 0 | 0 | 0 | 0 | 1 | 1 | 1 | 0 | 0 | 0 | 0 OP617743 |
| VC_873_0 | 3 | 900 | 12363.93521 | 0.06785317 | 0.0015206 | 5.830951895 | 7.68114575 | 3 | 3 | 1 | 7.020644026 | 0 | 0 | 0 MH533020,ON287369,ON287374 |
| VC_873_1 | 1 | 0 | 6614.186247 | 0 | 0 | 0 | 0 | 1 | 1 | 1 | 0 | 0 | 0 | 0 MZ520832 |
| VC_873_2 | 2 | 300 | 11940.77646 | 0.02450825 | 0.1297745 | 8.246211251 | 8.24621125 | 1 | 1 | 1 | 8.246211251 | 0 | 0 | 0 JQ015307,KY514263 |
| VC_873_3 | 2 | 300 | 12054.19861 | 0.02428324 | 0.1481177 | 2 | 2 | 1 | 1 | 1 | 2 | 0 | 0 | 0 MF979560,MF979563 |
| VC_873_4 | 2 | 300 | 12316.23315 | 0.02377889 | 0.1339287 | 4.242640687 | 4.24264069 | 1 | 1 | 1 | 4.242640687 | 0 | 0 | 0 MT552976,MT560059 |
| VC_873_5 | 3 | 900 | 17439.91792 | 0.04907328 | 0.0238918 | 4.582575695 | 6.63324958 | 3 | 3 | 1 | 5.594529879 | 0 | 0 | 0 JQ691612,KC954774,KT353109 |
| VC_873_6 | 7 | 6300 | 32227.14608 | 0.16352107 | 1.40E-08 | 0 | 9.11043358 | 21 | 20 | 0.952380952 | 4.916218409 | 0 | 0 | 0 JQ691611,MN013077,OL744211,OL744214,OL744216,OL744219,OL744220 |
| VC_873_7 | 1 | 0 | 6567.5418 | 0 | 0 | 0 | 0 | 1 | 1 | 1 | 0 | 0 | 0 | 0 ON712643 |
| VC_873_8 | 1 | 0 | 6420.166128 | 0 | 0 | 0 | 0 | 1 | 1 | 1 | 0 | 0 | 0 | 0 MW367417 |
| VC_873_9 | 1 | 0 | 6645.748652 | 0 | 0 | 0 | 0 | 1 | 1 | 1 | 0 | 0 | 0 | 0 MW250786 |
| VC_874_0 | 3 | 900 | 9273.748331 | 0.08846297 | 0.0015211 | 0 | 1 | 3 | 3 | 1 | 0.666666667 | 0 | 0 | 0 OK136181,OK181860,OK181862 |
| VC_875_0 | 2 | 235.3909864 | 383.4784774 | 0.38035644 | 0.0558704 | 0 | 0 | 1 | 1 | 1 | 0 | 0 | 0 | 0 MH444512,MK932884 |
| VC_876_0 | 2 | 150.4049412 | 179.4924601 | 0.4559143 | 0.0551081 | 3.464101615 | 3.46410162 | 1 | 1 | 1 | 3.464101615 | 0 | 0 | 0 GQ141189,S4_vs_VIRSorter_16493-cat_2 |
| VC_877_0 | 2 | 2.739105882 | 7.343481123 | 0.27166697 | 0.2701457 | 7.483314774 | 7.48331477 | 1 | 1 | 1 | 7.483314774 | 0 | 0 | 0 MG941013,MW073100 |
| VC_878_0 | 2 | 166.1084651 | 471.7057618 | 0.26043393 | 0.05822 | 3.464101615 | 3.46410162 | 1 | 1 | 1 | 3.464101615 | 0 | 0 | 0 S16_vs_78361,S3_vs_123834 |
| VC_879_0 | 5 | 217.6950112 | 385.1074224 | 0.36113824 | 0.0002312 | 5.567764363 | 8.18535277 | 10 | 10 | 1 | 7.328395648 | 0 | 0 | 0 MT006237,S3_vf_70746,S4_vf_28601,S5_vs_63924,S8_vf_197061 |
| VC_880_0 | 4 | 77.94449651 | 994.7013274 | 0.07266564 | 0.1119989 | 5.477225575 | 7.54983444 | 6 | 6 | 1 | 6.727246263 | 0 | 0 | 0 GQ241246,KF669650,S3_vf_54353,S8_vf_170430 |
| VC_881_0 | 7 | 6300 | 11969.79742 | 0.34483141 | 9.21E-14 | 2.449489743 | 4.12310563 | 21 | 21 | 1 | 3.448152834 | 0 | 0 | 0 MH371108,MH371109,MH536823,MZ355724,MZ456035,ON260827,ON970603 |
| VC_881_1 | 13 | 17229.36607 | 12177.49894 | 0.58589605 | 4.36E-37 | 0 | 8.94427191 | 78 | 78 | 1 | 6.791594401 | 0 | 0 | 0 KJ173786,MH271300,MH271307,MH271310,MH271316,MH399779,MK376963,MK919474,MK937596,MN096374,MN908690,MZ622166,OP068333 |
| VC_882_0 | 11 | 13670.56499 | 826.5439362 | 0.9429856 | 2.38E-27 | 0 | 4.58257569 | 55 | 55 | 1 | 2.131513 | 0 | 0 | 0 KU160638,KU160639,KU160647,KU160663,KU160666,MF185731,MN234164,MN234217,MN234224,MT310874,MT986028 |
| VC_883_0 | 5 | 205.6536409 | 379.6173115 | 0.35138194 | 3.94E-05 | 6.633249581 | 8.94427191 | 10 | 10 | 1 | 7.960161717 | 0 | 0 | 0 MF668275,MN234178,MZ923504,MZ955870,OV696619 |
| VC_885_0 | 9 | 9226.915596 | 585.4614959 | 0.94033439 | 3.36E-21 | 1 | 6.4807407 | 36 | 36 | 1 | 4.651904773 | 0 | 0 | 0 MH019215,MH019216,MK305891,MT498037,MT498053,MT897908,MT936332,MZ648033,MZ648036 |
| VC_886_0 | 16 | 22183.67151 | 134.0746412 | 0.99399246 | 2.69E-37 | 0 | 0 | 120 | 120 | 1 | 0 | 0 | 0 | 0 MN023176,MN023177,MN023178,MN023179,MN023180,MN023181,MN023182,MN023183,MN023184,MN023185,MN023186,MN023187,MN023188,MN023189,MN023190,MN023191 |
| VC_888_0 | 9 | 5152.280964 | 4660.295433 | 0.52506913 | 6.62E-20 | 0 | 2.44948974 | 36 | 36 | 1 | 1.779981331 | 0 | 0 | 0 JQ659259,KC013021,KC013022,KC013023,KC013024,KC013025,KX555527,MN552146,OV696621 |
| VC_889_0 | 13 | 10105.82046 | 4660.295433 | 0.6843926 | 1.56E-32 | 0 | 4.89897949 | 78 | 78 | 1 | 3.298946323 | 0 | 0 | 0 GQ451696,KC013026,KC013027,KC013028,KC013029,KM262191,KM262192,KX578042,KX578043,KX578044,MN552147,S7_vf_63894,S8_vf_129423 |
| VC_89_0 | 8 | 8400 | 6853.845454 | 0.55068081 | 2.62E-15 | 3.605551275 | 6.4807407 | 28 | 28 | 1 | 5.121328049 | 0 | 0 | 0 FJ230960,KF669649,KY368639,MT601270,MT601271,MW001214,MZ751040,OM236513 |
| VC_89_1 | 3 | 900 | 4670.762423 | 0.16155778 | 0.0022208 | 2.645751311 | 4.89897949 | 3 | 3 | 1 | 4.113520773 | 0 | 0 | 0 KC595512,KC595513,KF554508 |
| VC_89_2 | 1 | 0 | 1816.704757 | 0 | 0 | 0 | 0 | 1 | 1 | 1 | 0 | 0 | 0 | 0 MH884511 |
| VC_891_0 | 11 | 11665.43363 | 407.0935915 | 0.96627934 | 1.96E-12 | 0 | 6.164414 | 55 | 55 | 1 | 3.295094572 | 0 | 0 | 0 KP876465,KP876466,KT989433,KU189325,KU530220,KY365739,MF975638,MH178381,MH178382,MK686071,ON526979 |
| VC_892_0 | 2 | 300 | 3650.347579 | 0.07594268 | 0.0636853 | 7.615773106 | 7.61577311 | 1 | 1 | 1 | 7.615773106 | 0 | 0 | 0 MK804893,MK813939 |
| VC_892_1 | 1 | 0 | 2232.273034 | 0 | 0 | 0 | 0 | 1 | 1 | 1 | 0 | 0 | 0 | 0 MK813938 |
| VC_892_2 | 1 | 0 | 2299.05273 | 0 | 0 | 0 | 0 | 1 | 1 | 1 | 0 | 0 | 0 | 0 MK813941 |
| VC_892_3 | 2 | 300 | 3740.985573 | 0.07423931 | 0.0653094 | 2 | 2 | 1 | 1 | 1 | 2 | 0 | 0 | 0 OM033134,OM033135 |
| VC_892_4 | 1 | 0 | 2075.718924 | 0 | 0 | 0 | 0 | 1 | 1 | 1 | 0 | 0 | 0 | 0 OM033136 |
| VC_893_0 | 4 | 1799.163576 | 21065.79372 | 0.0786865 | 1.41E-05 | 2 | 3.16227766 | 6 | 6 | 1 | 2.682841463 | 0 | 0 | 0 JN035618,KR063278,MW534374,ON526973 |
| VC_893_1 | 20 | 56467.86587 | 61367.51375 | 0.47920978 | 1.07E-107 | 0 | 3.74165739 | 190 | 190 | 1 | 2.388197052 | 0 | 0 | 0 KU998240,MG099935,MH025889,MK450428,MK450430,MK450435,MK450436,MK494106,MK494109,MK494121,MK878903,MK919477,MK919481,ON724015,ON970582,ON970588,ON970596,ON970607,OP297547,OP434450 |
| VC_893_2 | 3 | 863.0660217 | 17995.41081 | 0.04576542 | 0.0014959 | 0 | 4.89897949 | 3 | 3 | 1 | 3.265986324 | 0 | 0 | 0 KR011062,KR011063,KR011064 |
| VC_894_0 | 6 | 2410.33113 | 5190.336548 | 0.31712097 | 2.36E-11 | 1.414213562 | 8.54400375 | 15 | 15 | 1 | 6.668070979 | 0 | 0 | 0 KP790008,KP790009,KP790010,KR063279,KX557281,ON526975 |
| VC_895_0 | 25 | 87508.81782 | 64218.49037 | 0.57675061 | 1.15E-159 | 0 | 6.55743852 | 300 | 300 | 1 | 4.585911655 | 0 | 0 | 0 KU963245,KU998241,MG099940,MG757152,MG757155,MG770211,MG770214,MK359302,MK433261,MK433263,MK433265,MK433267,MK801728,MK801733,MK967390,MN444874,MN945905,MT114160,MT889368,MT889370,MT889379,MT986029,MW507132,MW507133,ON456331 |
| VC_896_0 | 9 | 10688.35276 | 30358.63916 | 0.26039308 | 3.38E-24 | 0 | 4.12310563 | 36 | 36 | 1 | 2.674366436 | 0 | 0 | 0 KU963258,KU963262,KU998242,KU998243,MH576976,MK494098,MN234166,OM203156,OP434448 |
| VC_897_0 | 5 | 3000 | 4171.710833 | 0.41831023 | 3.48E-08 | 0 | 3.87298335 | 10 | 10 | 1 | 2.589970548 | 0 | 0 | 0 KU998252,KU998253,KU998254,MH479923,MN096363 |
| VC_898_0 | 2 | 300 | 7762.445068 | 0.03720956 | 0.045271 | 2.828427125 | 2.82842712 | 1 | 1 | 1 | 2.828427125 | 0 | 0 | 0 GU580941,GU580942 |
| VC_898_1 | 27 | 81102.55051 | 10080.36195 | 0.889449 | 8.93E-139 | 0 | 7.68114575 | 351 | 351 | 1 | 5.444768938 | 0 | 0 | 0 MG962368,MG962369,MK376953,MK376957,MN010761,MN204501,MN284896,MN284904,MN585971,MN585982,MN585988,MN585996,MT310850,MT498039,MT498052,MT521994,MW291022,MW862987,MZ005675,MZ005683,MZ171370,MZ209301,MZ388556,MZ622168,ON526971,ON970572,ON970622 |
| VC_899_0 | 2 | 198.6249078 | 3970.799102 | 0.04763845 | 0.042744 | 3.464101615 | 3.46410162 | 1 | 1 | 1 | 3.464101615 | 0 | 0 | 0 MH153800,MK814756 |
| VC_899_1 | 1 | 0 | 2016.50187 | 0 | 0 | 0 | 0 | 1 | 1 | 1 | 0 | 0 | 0 | 0 MH153801 |
| VC_9_0 | 38 | 73145.77432 | 77389.06789 | 0.48590594 | 0 | 0 | 8.83176087 | 703 | 703 | 1 | 7.566579362 | 0 | 0 | 0 AB370205,AF424781,AP008953,AY954951,AY954952,AY954953,CP062427,CP062451,CP062454,DQ517338,DQ530359,DQ530360,EU861004,GU477322,HQ127381,JQ779024,JX274646,JX274647,KC677663,KJ716334,MG557619,MH321492,MK658834,MK801682,MK801683,MT013111,MW554488,OM439662 ,OM439666,OM439668,OM439670,OM439671,OM439675,ON911714,ON911715,ON911716,ON911717,ON911718 |
| VC_90_0 | 1 | 0 | 113.5200897 | 0 | 0 | 0 | 0 | 1 | 1 | 1 | 0 | 0 | 0 | 0 KT624200 |
| VC_90_1 | 1 | 0 | 175.5655197 | 0 | 0 | 0 | 0 | 1 | 1 | 1 | 0 | 0 | 0 | 0 MF418016 |
| VC_901_0 | 31 | 93082.94252 | 19949.48029 | 0.82350657 | 7.77E-155 | 0 | 9.48683298 | 465 | 381 | 0.819354839 | 5.544227595 | 0 | 0 | 0 KX670789,KX670790,MF467948,MF467949,MG757154,MH155877,MH155880,MH669006,MK433259,MK433260,MK433268,MK433269,MK967386,MN204492,MN204494,MN204495,MN204496,MN204497,MN204500,MN204504,MN586054,MN586056,MT310852,MT310854,MT657340,MT684589,MT684600, MW055905,MW145539,MW712736,ON108646 |
| VC_902_0 | 3 | 172.8890474 | 19.44432883 | 0.89890299 | 0.0103188 | 4.898979486 | 6.164414 | 3 | 3 | 1 | 5.631448461 | 0 | 0 | 0 GU936715,HM480106,MG271909 |
| VC_903_0 | 3 | 514.7052895 | 313.1846198 | 0.62170741 | 0.001851 | 0 | 1 | 3 | 3 | 1 | 0.666666667 | 0 | 0 | 0 KR935216,KR935217,KT253150 |
| VC_904_0 | 10 | 882.2321641 | 10.92351692 | 0.98776975 | 0.0005157 | 0 | 3.16227766 | 45 | 45 | 1 | 1.949558002 | 0 | 0 | 0 GU942563,HQ224500,KJ619459,KP972568,KP972569,KR063267,KR063268,KT728930,KT728931,MF155889 |
| VC_905_0 | 4 | 258.0070621 | 667.191715 | 0.27886663 | 5.89E-05 | 5.291502622 | 7.48331477 | 6 | 6 | 1 | 6.145142646 | 0 | 0 | 0 S13_vs_121307,S1_vf_50591,S3_vs_100785,S5_vs_116235 |
| VC_907_0 | 32 | 41172.69733 | 149.7179355 | 0.99637683 | 3.12E-22 | 0 | 7.41619849 | 496 | 496 | 1 | 5.854186207 | 0 | 0 | 0 HE956707,HE956710,JN651747,KC107834,KT184661,KT949345,KX756572,KY318515,LN878149,MF683623,MG878892,MH179473,MH992513,MK053931,MK330684,MK838107,MK838108,MK838109,MK838110,MK838111,MN509793,MT682386,MT682387,MT682388,MT682389,MT682390,MT682391,MW 250276,MZ592920,OL362270,OM654404,ON287377 |
| VC_908_0 | 36 | 131407.4793 | 752.9320983 | 0.99430289 | 1.45E-62 | 0 | 3 | 630 | 630 | 1 | 1.282631085 | 0 | 0 | 0 HF569088,HF569089,HF569090,HF569091,HF569092,HG428758,JN939331,JN939332,KC556893,KC556894,KC556895,KC556896,KC556897,KC556898,KJ133688,KJ133689,KJ133690,KJ133691,KJ133692,KJ133693,KJ133694,KJ133695,KJ133696,KJ133697,KJ133698,KJ133699,KJ133700,KJ133701,KJ133702,KJ 133703,KJ133704,KJ133705,KJ133706,KJ133707,KY056619,MT002975 |
| VC_909_0 | 2 | 6.595576295 | 34.42639697 | 0.16078155 | 0.1654493 | 2.828427125 | 2.82842712 | 1 | 1 | 1 | 2.828427125 | 0 | 0 | 0 HG007973,HM150760 |
| VC_91_0 | 2 | 300 | 6393.970929 | 0.04481645 | 0.074949 | 5.656854249 | 5.65685425 | 1 | 1 | 1 | 5.656854249 | 0 | 0 | 0 MT601273,MT601274 |
| VC_91_1 | 2 | 300 | 6459.572925 | 0.0443815 | 0.0535311 | 6.633249581 | 6.63324958 | 1 | 1 | 1 | 6.633249581 | 0 | 0 | 0 MT601272,OL580764 |
| VC_91_2 | 5 | 3000 | 11817.44192 | 0.2024641 | 2.74E-06 | 1.414213562 | 6.92820323 | 10 | 10 | 1 | 4.80147403 | 0 | 0 | 0 KY030782,MT366945,MT366946,MT366947,MT366948 |
| VC_91_3 | 1 | 0 | 3630.953735 | 0 | 0 | 0 | 0 | 1 | 1 | 1 | 0 | 0 | 0 | 0 AF020713 |
| VC_91_4 | 1 | 0 | 3446.770864 | 0 | 0 | 0 | 0 | 1 | 1 | 1 | 0 | 0 | 0 | 0 ON107264 |
| VC_91_5 | 1 | 0 | 3349.272028 | 0 | 0 | 0 | 0 | 1 | 1 | 1 | 0 | 0 | 0 | 0 MZ969646 |
| VC_91_6 | 1 | 0 | 2960.988033 | 0 | 0 | 0 | 0 | 1 | 1 | 1 | 0 | 0 | 0 | 0 OM236515 |
| VC_910_0 | 2 | 117.4479429 | 56.6968783 | 0.67442685 | 0.0818672 | 4.795831523 | 4.79583152 | 1 | 1 | 1 | 4.795831523 | 0 | 0 | 0 KP282679,KP282680 |
| VC_911_0 | 20 | 57000 | 486.3474713 | 0.99153977 | 1.68E-47 | 0 | 5 | 190 | 190 | 1 | 3.471682614 | 0 | 0 | 0 HG992588,JQ692107,JX556418,MF754113,MF754114,MF754115,MF754116,MG602476,MG893203,MH599087,MH925090,MN215888,MW451246,MW451251,MZ592922,OK483201,OK625546,OL314400,OP547477,OP595601 |
| VC_912_0 | 10 | 13500 | 54187.43638 | 0.19944617 | 6.62E-30 | 2 | 8.36660027 | 45 | 45 | 1 | 6.953825071 | 0 | 0 | 0 JF966203,KJ489397,KJ489398,KJ489400,KU737346,MF288921,MF288922,MF498901,MH638310,MK215646 |
| VC_912_1 | 12 | 19800 | 61906.03817 | 0.24233215 | 1.58E-29 | 0 | 8.88819442 | 66 | 66 | 1 | 7.231172083 | 0 | 0 | 0 KF208639,KF669647,KJ489402,KT307976,KU737345,KU737347,MF765814,MN038178,MW281503,OK499976,OK499990,OM350011 |
| VC_912_2 | 2 | 300 | 16343.61795 | 0.01802493 | 0.1190515 | 1 | 1 | 1 | 1 | 1 | 1 | 0 | 0 | 0 JN790865,JN797796 |
| VC_912_3 | 2 | 300 | 16276.35699 | 0.01809807 | 0.1209976 | 6.08276253 | 6.08276253 | 1 | 1 | 1 | 6.08276253 | 0 | 0 | 0 KF669662,KY888882 |
| VC_912_4 | 1 | 0 | 8380.573283 | 0 | 0 | 0 | 0 | 1 | 1 | 1 | 0 | 0 | 0 | 0 MN153503 |
| VC_912_5 | 1 | 0 | 8374.371149 | 0 | 0 | 0 | 0 | 1 | 1 | 1 | 0 | 0 | 0 | 0 MW787012 |
| VC_912_6 | 1 | 0 | 8460.560659 | 0 | 0 | 0 | 0 | 1 | 1 | 1 | 0 | 0 | 0 | 0 OM743306 |
| VC_913_0 | 28 | 113400 | 69324.68186 | 0.62060581 | 5.79E-66 | 0 | 9.2736185 | 378 | 372 | 0.984126984 | 5.8583255 | 0 | 0 | 0 KJ489399,KJ489401,KT207918,KU737344,KU737348,KU737349,KU737350,KU737351,KU737352,KX147229,KX156152,KX349902,KX349903,KX961629,KX961632,MF288917,MF288918,MF288919,MF288920,MG763894,MH538193,MH638311,MN038176,MN038179,MT584805,OK500001,OM350012,OM350 013 |
| VC_913_1 | 8 | 8400 | 67848.99259 | 0.11016539 | 0.0010677 | 2.236067977 | 9.79795897 | 28 | 21 | 0.75 | 6.416757534 | 0 | 0 | 0 HM144387,JN654439,KC430106,KU847400,KX011169,MW392803,MW584228,MW596414 |
| VC_914_0 | 11 | 12320.67143 | 390.2525065 | 0.96929786 | 8.90E-27 | 1.414213562 | 4.12310563 | 55 | 55 | 1 | 2.609763728 | 0 | 0 | 0 MW677509,MW677510,MW677512,MW677515,MW677517,MW677520,OP009272,OP009275,OP009278,OP009280,OP009286 |
| VC_915_0 | 2 | 257.8432128 | 1630.787862 | 0.13652386 | 0.0464766 | 5.567764363 | 5.56776436 | 1 | 1 | 1 | 5.567764363 | 0 | 0 | 0 MN988459,MN988558 |
| VC_915_1 | 1 | 0 | 970.1615999 | 0 | 0 | 0 | 0 | 1 | 1 | 1 | 0 | 0 | 0 | 0 MW677529 |
| VC_917_0 | 28 | 113381.8121 | 94068.36818 | 0.5465496 | 1.94E-189 | 0 | 6.70820393 | 378 | 378 | 1 | 3.953489101 | 0 | 0 | 0 JF744988,JN680858,KC661276,KC691254,KF024731,KT591489,KU234099,KU997639,KX507361,KX580961,KX580962,MF140422,MF185728,MF185730,MF324910,MH779511,MH834600,MK937600,MN096380,MN234228,MN586030,MN586052,MN703406,MN703410,MT758688,MW578835,MZ171373,OL45 5899 |
| VC_918_0 | 16 | 35901.63155 | 85988.18897 | 0.29454167 | 2.49E-71 | 0 | 6.40312424 | 120 | 120 | 1 | 4.331197159 | 0 | 0 | 0 KF024725,KT281791,KT281794,KT372003,MF140405,MF140413,MF140414,MF140421,MH727559,MK878900,MN096377,MT889383,MT889391,MW291024,MZ622183,ON755181 |
| VC_919_0 | 3 | 894.2670062 | 1671.285908 | 0.34856697 | 0.001687 | 4.472135955 | 5.74456265 | 3 | 3 | 1 | 5.137617008 | 0 | 0 | 0 MH651182,MK967378,MT658805 |
| VC_919_1 | 4 | 1800 | 2187.023946 | 0.45146456 | 1.54E-05 | 2 | 6 | 6 | 6 | 1 | 4.260305182 | 0 | 0 | 0 MH976515,MN444870,MT684599,OK040786 |
| VC_92_0 | 1 | 0 | 213.0808089 | 0 | 0 | 0 | 0 | 1 | 1 | 1 | 0 | 0 | 0 | 0 LC597490 |
| VC_92_1 | 1 | 0 | 309.8711893 | 0 | 0 | 0 | 0 | 1 | 1 | 1 | 0 | 0 | 0 | 0 OM634661 |
| VC_92_2 | 1 | 0 | 115.9611787 | 0 | 0 | 0 | 0 | 1 | 1 | 1 | 0 | 0 | 0 | 0 EF583821 |
| VC_920_0 | 2 | 16.04352999 | 131.8961513 | 0.10844643 | 0.138861 | 6.782329983 | 6.78232998 | 1 | 1 | 1 | 6.782329983 | 0 | 0 | 0 S14_vs_73301,S5_vs_83886 |
| VC_921_0 | 4 | 266.9754035 | 165.6477639 | 0.61710843 | 5.36E-05 | 5.477225575 | 8.77496439 | 6 | 6 | 1 | 7.59520964 | 0 | 0 | 0 S2_vf_21630,S5_vf_145588,S7_vf_68546,S8_vs_136922 |
| VC_922_0 | 1 | 0 | 140.8281426 | 0 | 0 | 0 | 0 | 1 | 1 | 1 | 0 | 0 | 0 | 0 S10_vs_77004 |
| VC_923_0 | 7 | 187.2782079 | 1364.379977 | 0.12069553 | 0.0004856 | 6 | 9.11043358 | 21 | 20 | 0.952380952 | 7.852574405 | 0 | 0 | 0 S14_vs_14737,S14_vs_39797,S4_vs_VIRSorter_31171-circular-cat_2,S4_vs_VIRSorter_31977-circular-cat_2,S5_vf_6506,S5_vs_58766,S7_vs_28733 |
| VC_923_1 | 1 | 0 | 203.037655 | 0 | 0 | 0 | 0 | 1 | 1 | 1 | 0 | 0 | 0 | 0 S3_vf_61860 |
| VC_923_2 | 1 | 0 | 230.1288622 | 0 | 0 | 0 | 0 | 1 | 1 | 1 | 0 | 0 | 0 | 0 S6_vs_29152 |
| VC_925_0 | 3 | 485.4681358 | 281.0452069 | 0.63334597 | 0.0030785 | 2 | 5.09901951 | 3 | 3 | 1 | 3.999333 | 0 | 0 | 0 HM588722,KF302032,KF302033 |
| VC_926_0 | 2 | 95.49765638 | 249.3248668 | 0.27694727 | 0.0574875 | 6.32455532 | 6.32455532 | 1 | 1 | 1 | 6.32455532 | 0 | 0 | 0 KF302036,KX365748 |
| VC_927_0 | 5 | 822.9030687 | 826.7010486 | 0.49884882 | 1.94E-07 | 4.69041576 | 7 | 10 | 10 | 1 | 6.402193855 | 0 | 0 | 0 KR060090,KX912252,KY379511,MF370965,MT002875 |
| VC_928_0 | 6 | 897.6496051 | 1310.570218 | 0.40650373 | 3.17E-10 | 1 | 3.46410162 | 15 | 15 | 1 | 2.258077258 | 0 | 0 | 0 HM640230,JN800508,KY996523,MH999279,MW815121,OP022426 |
| VC_929_0 | 10 | 3887.671124 | 1522.850113 | 0.71853911 | 8.42E-23 | 0 | 3.46410162 | 45 | 45 | 1 | 2.214779374 | 0 | 0 | 0 JQ729990,JQ729991,JQ729992,MH248069,MW057920,MZ401005,MZ401007,MZ401008,MZ401009,OL547738 |
| VC_93_0 | 22 | 69300 | 61831.6174 | 0.52847667 | 3.97E-111 | 0 | 8.42614977 | 231 | 231 | 1 | 6.254413275 | 0 | 0 | 0 AB609718,AP009390,AP018714,AP018715,CAJDJY010000002,CAJDJZ010000002,KJ801817,KR049063,LC596377,LC596378,LC596379,LR031359,MK268686,MK693030,MK721192,MN854830,MW633168,MZ420150,ON113167,ON113168,ON113169,ON286976 |
| VC_93_1 | 1 | 0 | 7249.641933 | 0 | 0 | 0 | 0 | 1 | 1 | 1 | 0 | 0 | 0 | 0 OP559178 |
| VC_930_0 | 4 | 352.2016572 | 1019.050926 | 0.25684667 | 2.75E-05 | 0 | 4.12310563 | 6 | 6 | 1 | 2.697074804 | 0 | 0 | 0 MH393889,MK017820,OM638104,OP256049 |
| VC_932_0 | 5 | 2283.509912 | 184.6514295 | 0.92518665 | 5.03E-06 | 0 | 6.92820323 | 10 | 10 | 1 | 5.202732593 | 0 | 0 | 0 KF623293,MW824400,MW824417,MW824421,MW824431 |
| VC_933_0 | 3 | 900 | 3.238952501 | 0.99641407 | 0.0532916 | 0 | 7 | 3 | 3 | 1 | 4.666666667 | 0 | 0 | 0 HQ316582,MG592662,MG592672 |
| VC_934_0 | 7 | 3648.031491 | 1309.55715 | 0.73584796 | 6.66E-13 | 0 | 5.47722558 | 21 | 21 | 1 | 3.175463184 | 0 | 0 | 0 MW824414,MW824420,MW824427,MW824428,MW824429,MW865319,OX241434 |
| VC_935_0 | 6 | 3984.421751 | 13211.78254 | 0.23170356 | 1.50E-10 | 0 | 8.1240384 | 15 | 15 | 1 | 6.254827105 | 0 | 0 | 0 JF713456,MG592585,MG592586,MG720309,MK559459,MT735629 |
| VC_935_1 | 4 | 1800 | 10438.93219 | 0.14707165 | 2.28E-05 | 0 | 4.58257569 | 6 | 6 | 1 | 3.177976178 | 0 | 0 | 0 HQ316604,KX198615,MT451873,ON015836 |
| VC_935_2 | 7 | 5584.809796 | 14101.44272 | 0.28369085 | 3.35E-13 | 0 | 7.28010989 | 21 | 21 | 1 | 3.702864892 | 0 | 0 | 0 KY022433,MH925091,MN871506,MW411580,OK483199,OP547478,OV032860 |
| VC_936_0 | 3 | 900 | 1764.312389 | 0.33779823 | 0.0017622 | 3 | 3.60555128 | 3 | 3 | 1 | 3.255942979 | 0 | 0 | 0 MG189906,MT110073,MZ326862 |
| VC_938_0 | 3 | 667.4312446 | 340.3016453 | 0.66230968 | 0.0036689 | 3 | 5 | 3 | 3 | 1 | 4 | 0 | 0 | 0 HQ317384,HQ332142,KM233261 |
| VC_939_0 | 3 | 623.9904153 | 610.452646 | 0.50548335 | 0.0027547 | 0 | 6.63324958 | 3 | 3 | 1 | 4.422166387 | 0 | 0 | 0 MN988533,MN988538,MW980068 |
| VC_94_0 | 22 | 69300 | 54396.93961 | 0.56024021 | 7.06E-119 | 2.645751311 | 8.71779789 | 231 | 231 | 1 | 6.794186059 | 0 | 0 | 0 CAJDKA010000002,KP339049,KY549443,LR990835,MH791397,MH880817,MN027503,MN241318,MT856905,MT909815,MW004544,MW004545,MZ147816,MZ333462,OL799258,OL799260,OL870611,OM966901,ON113170,ON125307,ON506927,ON506928 |
| VC_940_0 | 2 | 25.93656681 | 25.97725206 | 0.49960815 | 0.0737507 | 6.164414003 | 6.164414 | 1 | 1 | 1 | 6.164414003 | 0 | 0 | 0 JQ177062,S11_vs_104032 |
| VC_941_0 | 5 | 1558.094165 | 543.970021 | 0.74122102 | 7.24E-06 | 3.872983346 | 4.79583152 | 10 | 10 | 1 | 4.326114384 | 0 | 0 | 0 HQ317386,MG592522,MG640035,MT720689,OM837731 |
| VC_942_0 | 2 | 94.25884099 | 1.209484116 | 0.98733104 | 0.5 | 8.366600265 | 8.36660027 | 1 | 1 | 1 | 8.366600265 | 0 | 0 | 0 HQ317390,MZ568826 |
| VC_943_0 | 6 | 3732.829742 | 9743.605362 | 0.27698941 | 1.51E-11 | 0 | 4.35889894 | 15 | 15 | 1 | 1.829714663 | 0 | 0 | 0 HQ317392,KC821610,KC821616,KC821629,KC821630,KC821634 |
| VC_944_0 | 76 | 714931.4885 | 15429.04086 | 0.97887476 | 2.10E-297 | 0 | 6.08276253 | 2850 | 2850 | 1 | 3.006768721 | 0 | 0 | 0 KM873719,KY951963,KY951964,KY979235,KY979236,KY979237,KY979238,KY979239,KY979240,KY979241,KY979242,KY979243,KY979244,KY979245,KY979246,KY979247,KY992519,KY992520,MK756083,MK756084,MK756085,MK756086,MK756087,MK756088,MK756089,MK756090,MK756091,MK7 56092,MK756093,MK756094,MK756095,MK756096,MT431535,MT585273,MT585274,MT585275,MT585276,MT585277,MT585278,MT585279,MT585280,MT585281,MT585282,MT585283,MT585284,MT585285,MT585286,MT585287,MT585288,MT585289,MT585290,MT585291,MT585292,MT585293,MT585 294,MT585295,MT585296,MT585297,MT585298,MT585299,MT585300,MT585301,MT585302,MT585303,MT585304,MT585305,MT585306,MT585307,MT585308,MT585309,MT585310,MT585311,MT585312,MT585313,MT585314,MT585315 |
| VC_945_0 | 2 | 3.61565195 | 32.77687898 | 0.09935148 | 0.165703 | 6.244997998 | 6.244998 | 1 | 1 | 1 | 6.244997998 | 0 | 0 | 0 HQ332136,MW452941 |
| VC_946_0 | 7 | 385.760515 | 78.62545245 | 0.83068943 | 4.67E-07 | 1.732050808 | 4.69041576 | 21 | 21 | 1 | 3.935898613 | 0 | 0 | 0 S11_vs_104053,S14_vf_117164,S15_vf_55639,S15_vs_1407,S4_vf_2524,S5_vs_145605,S7_vf_135778 |
| VC_947_0 | 4 | 1490.074184 | 295.7165254 | 0.83440583 | 7.59E-05 | 0 | 5 | 6 | 6 | 1 | 2.816496581 | 0 | 0 | 0 HQ396194,MZ995504,ON922917,ON933637 |
| VC_948_0 | 3 | 184.7161216 | 470.8121484 | 0.28178208 | 0.002469 | 6.08276253 | 8.83176087 | 3 | 3 | 1 | 7.858259145 | 0 | 0 | 0 S17_vs_160502,S17_vs_39515,S3_vf_73597 |
| VC_948_1 | 1 | 0 | 449.3548514 | 0 | 0 | 0 | 0 | 1 | 1 | 1 | 0 | 0 | 0 | 0 S4_vs_VIRSorter_31975-circular-cat_1 |

| | | | | | | | | | | | | | | | |
|---|---|---|---|---|---|---|---|---|---|---|---|---|---|---|---|
| VC_949_0 | 2 | 168.5544177 | 288.7586163 | 0.36857558 | 0.0583387 | 5.830951895 | 5.83095189 | 1 | 1 | 1 | 5.830951895 | 0 | 0 | 0 | S17_vf_35138,S4_vf_12614 |
| VC_95_0 | 2 | 300 | 2617.963162 | 0.10281144 | 0.0432886 | 4.69041576 | 4.69041576 | 1 | 1 | 1 | 4.69041576 | 0 | 0 | 0 | FJ822135,MT758689 |
| VC_950_0 | 5 | 860.0684318 | 629.8868468 | 0.57724446 | 2.12E-07 | 4.242640687 | 8 | 10 | 10 | 1 | 6.827952039 | 0 | 0 | 0 | S10_vs_67471,S12_vf_60224,S12_vf_69136,S14_vs_66208,S7_vs_138173 |
| VC_951_0 | 2 | 157.0344535 | 577.4547442 | 0.2138009 | 0.0498857 | 5.916079783 | 5.91607978 | 1 | 1 | 1 | 5.916079783 | 0 | 0 | 0 | S11_vs_1937,S8_vs_9987 |
| VC_951_1 | 2 | 74.86609501 | 566.2586212 | 0.11677306 | 0.0490775 | 6.92820323 | 6.92820323 | 1 | 1 | 1 | 6.92820323 | 0 | 0 | 0 | S5_vf_18461,S8_vf_208120 |
| VC_951_2 | 1 | 0 | 429.5897703 | 0 | 0 | 0 | 0 | 1 | 1 | 1 | 0 | 0 | 0 | 0 | S11_vf_70015 |
| VC_952_0 | 39 | 101261.7366 | 10456.59954 | 0.90640212 | 7.09E-169 | 0 | 8.30662386 | 741 | 741 | 1 | 6.305444185 | 0 | 0 | 0 | HQ403646,KU963260,KU998239,MG770215,MH271302,MH450132,MK279900,MK875791,MK875795,MK967383,MK967385,MN062716,MN234215,MN284906,MN310549,MN444871,MN444878,MN586015,MN586036,MN586049,MN586057,MT776808,MT776809,MT776810,MT889367,MT889378,MW507135,MW862988,MZ150793,MZ322013,MZ622167,OK040785,OL455888,OL829977,ON970576,ON970599,ON970609,ON970621,OP297531 |
| VC_953_0 | 2 | 300 | 1527.104834 | 0.16419419 | 0.044437 | 2.828427125 | 2.82842712 | 1 | 1 | 1 | 2.828427125 | 0 | 0 | 0 | MN204493,MW822144 |
| VC_954_0 | 2 | 205.715538 | 11.52102448 | 0.94696554 | 0.2701457 | 0 | 0 | 1 | 1 | 1 | 0 | 0 | 0 | 0 | HQ632857,HQ632858 |
| VC_955_0 | 2 | 12.76576405 | 33.63029077 | 0.27514762 | 0.1056498 | 8.246211251 | 8.24621125 | 1 | 1 | 1 | 8.246211251 | 0 | 0 | 0 | KR072689,MK291442 |
| VC_956_0 | 23 | 73517.34793 | 4361.797183 | 0.94399274 | 5.31E-93 | 1 | 6.40312424 | 253 | 253 | 1 | 3.997941658 | 0 | 0 | 0 | MW677513,MW677516,MW677518,MW677519,MW677521,MW677522,MW677523,MW677524,OP009265,OP009266,OP009267,OP009268,OP009269,OP009270,OP009271,OP009273,OP009276,OP009277,OP009279,OP009283,OP009284,OP009287,OP009288 |
| VC_957_0 | 34 | 54265.49906 | 6472.606952 | 0.89343417 | 5.31E-162 | 0 | 9.16515139 | 561 | 548 | 0.976827094 | 7.587825603 | 0 | 0 | 0 | MG592442,MG592450,MG592457,MG592460,MG592482,MG592483,MG592488,MG592489,MG592490,MG592502,MG592503,MG592504,MG592505,MG592516,MG592517,MG592525,MG592530,MG592532,MG592534,MG592535,MG592539,MG592543,MG592557,MG592558,MG592559,MG592576,MG592593,MG592594,MG592597,MG592598,MG592605,MG592606,MG592607,MG592631 |
| VC_958_0 | 3 | 136.5432247 | 359.6641055 | 0.27517374 | 0.0486127 | 6.08276253 | 7.54983444 | 3 | 3 | 1 | 6.599003656 | 0 | 0 | 0 | KJ847052,KX982260,MN592896 |
| VC_959_0 | 3 | 529.3422963 | 32.4488227 | 0.94224041 | 0.0056961 | 0 | 5.38516481 | 3 | 3 | 1 | 3.590109871 | 0 | 0 | 0 | MG592520,OX241566,OX241570 |
| VC_96_0 | 2 | 300 | 2085.876179 | 0.12573997 | 0.0427482 | 5.099019514 | 5.09901951 | 1 | 1 | 1 | 5.099019514 | 0 | 0 | 0 | HM242243,OM135992 |
| VC_960_0 | 2 | 2.091212004 | 22.23675584 | 0.08595917 | 0.2431172 | 8.660254038 | 8.66025404 | 1 | 1 | 1 | 8.660254038 | 0 | 0 | 0 | HQ698895,KJ410740 |
| VC_961_0 | 3 | 706.4135829 | 195.7998594 | 0.78297834 | 0.0055862 | 0 | 1 | 3 | 3 | 1 | 0.666666667 | 0 | 0 | 0 | JF974286,JF974321,MT490303 |
| VC_962_0 | 4 | 1800 | 536.7853088 | 0.77028899 | 9.80E-05 | 0 | 3.16227766 | 6 | 6 | 1 | 1.58113883 | 0 | 0 | 0 | HQ634192,KC821604,KC821621,KC821625 |
| VC_963_0 | 4 | 549.6392981 | 1380.37858 | 0.28478456 | 5.86E-05 | 7 | 9 | 6 | 5 | 0.833333333 | 8.015016047 | 0 | 0 | 0 | S16_vf_87190,S6_vf_56486,S7_vs_137915,S8_vs_208103 |
| VC_963_1 | 1 | 0 | 639.2934125 | 0 | 0 | 0 | 0 | 1 | 1 | 1 | 0 | 0 | 0 | 0 | S11_vf_104033 |
| VC_965_0 | 2 | 15.86929696 | 23.55474208 | 0.40252844 | 0.1056498 | 8.185352772 | 8.18535277 | 1 | 1 | 1 | 8.185352772 | 0 | 0 | 0 | HQ683709,MG209611 |
| VC_966_0 | 8 | 2561.255634 | 4480.200262 | 0.3637395 | 5.85E-18 | 0 | 7 | 28 | 28 | 1 | 5.71239497 | 0 | 0 | 0 | KR093625,KR093626,KR093627,KR093628,KR093629,KR093630,KR093631,KR093633 |
| VC_967_0 | 5 | 584.4059273 | 4.715095495 | 0.99199639 | 0.020629 | 2.449489743 | 6.244998 | 10 | 10 | 1 | 5.162006681 | 0 | 0 | 0 | JF344709,S11_vs_93101,S15_vs_76390,S17_vs_138036,S8_vs_208129 |
| VC_968_0 | 2 | 260.2998497 | 43.07286908 | 0.85801997 | 0.0750078 | 0 | 0 | 1 | 1 | 1 | 0 | 0 | 0 | 0 | MT119766,MT210154 |
| VC_969_0 | 2 | 16.96170363 | 7.174547233 | 0.70274807 | 0.2701457 | 6.708203932 | 6.70820393 | 1 | 1 | 1 | 6.708203932 | 0 | 0 | 0 | MN988536,OP028995 |
| VC_97_0 | 3 | 900 | 3531.274302 | 0.20310185 | 0.0014708 | 5 | 7.74596669 | 3 | 3 | 1 | 6.765267043 | 0 | 0 | 0 | MK504443,MK504446,OM293948 |
| VC_970_0 | 2 | 300 | 23.77956793 | 0.9265563 | 0.1855467 | 1.732050808 | 1.73205081 | 1 | 1 | 1 | 1.732050808 | 0 | 0 | 0 | MN602266,MN602267 |
| VC_971_0 | 40 | 142955.8868 | 67497.55691 | 0.67927559 | 0 | 0 | 10.198039 | 780 | 730 | 0.935897436 | 6.445293508 | 0 | 0 | 0 | JN672684,KR269719,KX237515,KY780482,MN013075,MN013076,MN013078,MN013079,MN013080,MN013083,MN013087,MT701592,MW021762,MW021764,MW021765,MW042802,MW417503,MW722081,MZ428221,MZ428224,MZ598515,MZ634348,MZ634350,MZ634351,OL539450,OL539472,OL865411,OM065837,OM065838,OM065839,OM065841,OM328157,OM735690,OP611406,OV877339,OV877387,OV877766,OX335361,OX335395,OX335410 |
| VC_972_0 | 99 | 761106.0591 | 74117.04318 | 0.91126078 | 0 | 0 | 9.74679434 | 4851 | 4816 | 0.992784993 | 6.942443969 | 0 | 0 | 0 | JF501022,KP658157,KR262148,KT001920,KY575286,LR757892,MF415412,MF612072,MG552615,MG894052,MH445453,MH633484,MH633485,MH633486,MH633487,MH688453,MH844531,MK170446,MK278861,MK521904,MK618657,MK931442,MK931443,MK931444,MK931445,MN379832,MN395285,MN395286,MN689779,MN783016,MT894005,MW655991,MW672037,MZ172460,MZ221764,MZ398242,MZ428222,MZ428223,MZ428225,MZ428226,MZ428227,MZ428228,MZ571831,MZ571833,MZ571834,MZ634345,MZ634346,OK138555,OK655936,OL539448,OL539460,OL674249,OL702938,OL744210,OL744213,OM032871,OM065840,OM065842,OM065843,OM687893,OM938991,OM938992,OM964875,ON489264,ON602735,ON602741,ON602750,ON881905,OP267563,OP413832,OP617742,OU342755,OW251746,OX335369,OX335370,OX335371,OX335380,OX335381,OX335383,OX335384,OX335385,OX335386,OX335387,OX335388,OX335391,OX335393,OX335400,OX335404,OX335411,OX335412,OX335417,OX335421,OX335422,OX335423,OX335429,OX335430,OX335432,OX335435,OX335439 |
| VC_973_0 | 15 | 31500 | 0 | 1 | 1 | 0 | 4 | 105 | 105 | 1 | 2.82037923 | 0 | 0 | 0 | JF704100,KJ538721,KX159477,MG757167,MH727553,MH727562,MK061407,MK279842,MK305892,MK494123,MN329674,MN329676,MN428056,MN428061,ON637772 |
| VC_974_0 | 34 | 125547.9217 | 32309.9996 | 0.79532228 | 4.34E-174 | 0 | 6.70820393 | 561 | 561 | 1 | 4.801789614 | 0 | 0 | 0 | JF704107,JN083852,JN083853,JN408459,JQ684677,JX042578,KF017001,KF493882,KF560330,KM101118,KT004677,KT246486,KT438501,MG099934,MG099937,MG099938,MG099944,MG099949,MH020239,MH051250,MH051254,MH051256,MH338235,MH338241,MK305887,MK494091,MN096372,MN586016,MN586060,MN586062,MN617844,OL455889,ON970625,ON970626 |
| VC_975_0 | 40 | 136073.5429 | 61491.03969 | 0.68875474 | 0 | 0 | 8.36660027 | 780 | 780 | 1 | 6.24999193 | 0 | 0 | 0 | KC710998,MG065636,MG065640,MG065641,MG065643,MG065649,MG065657,MG065663,MG065665,MG065667,MG065669,MG065670,MG065671,MG065673,MG065675,MG065679,MG065680,MG065681,MG065687,MK373798,MN055691,MN432485,MN850567,MN850569,MN850572,MN850591,MN850600,MN850602,MN850607,MN850640,MT074433,MT127619,MT360681,MZ501079,MZ501090,OK272479,OK272487,OL539459,ON185589,ON528718 |
| VC_976_0 | 36 | 151270.5624 | 41843.35266 | 0.78332295 | 0 | 0 | 7.81024968 | 630 | 630 | 1 | 4.77516521 | 0 | 0 | 0 | LR027385,LR027388,LR596614,LT841304,LT961732,MF564201,MK962751,MN781674,MN850582,MN850586,MN850596,MN850604,MN850606,MN850613,MN850620,MN850634,MN850638,MN850641,MN850643,MZ234033,MZ501069,MZ501101,OK272471,OK272478,OK272481,OK272483,OK272485,OK272486,OK272489,OL539445,OL539446,ON185586,ON185587,ON185588,ON711395,OU734268 |
| VC_977_0 | 2 | 118.8020985 | 0 | 1 | 0 | 0 | 0 | 1 | 1 | 1 | 0 | 0 | 0 | 0 | JF917095,JQ010983 |
| VC_978_0 | 6 | 2067.702852 | 465.2801931 | 0.81631137 | 3.24E-10 | 0 | 8.06225775 | 15 | 15 | 1 | 6.453563135 | 0 | 0 | 0 | MG592398,MG592496,MG592497,MG592584,MG592612,MG592613 |
| VC_98_0 | 30 | 27963.79356 | 11009.27474 | 0.71751583 | 1.98E-151 | 0 | 2.64575131 | 435 | 435 | 1 | 1.647346546 | 0 | 0 | 0 | AB626962,AB626963,AF513032,AF513033,AY954949,EU136189,HF937074,KJ210330,KM366100,KU992911,KY000084,KY000085,LC709172,MG766218,MG766219,MH107118,MH972260,MK764384,MK903033,MK922546,MK922547,MK922548,MK936475,MK936476,MN098325,MN150710,MT926124,MW286254,MW363799,MZ359091 |
| VC_980_0 | 11 | 14740.65195 | 5361.59887 | 0.73328365 | 5.62E-32 | 0 | 5.29150262 | 55 | 55 | 1 | 3.218661496 | 0 | 0 | 0 | JX483876,JX483877,JX483878,MN988482,MN988500,MN988501,MN988508,MN988530,MN988545,MN988549,MW980073 |
| VC_981_0 | 9 | 4651.457632 | 3933.020355 | 0.54184513 | 6.34E-23 | 2.828427125 | 8.24621125 | 36 | 36 | 1 | 6.813621841 | 0 | 0 | 0 | MG592429,MG592438,MG592487,MG592566,MG592654,MG592657,MG592658,MG592659,MZ369152 |
| VC_982_0 | 3 | 58.85544189 | 47.43177153 | 0.55373963 | 0.0341237 | 6.480740698 | 7.61577311 | 3 | 3 | 1 | 7.102538785 | 0 | 0 | 0 | MK291441,MK291443,MK291444 |
| VC_984_0 | 5 | 362.6681182 | 1760.95523 | 0.17077799 | 1.58E-07 | 2.236067977 | 4.24264069 | 10 | 10 | 1 | 3.499764209 | 0 | 0 | 0 | JN116822,MG812496,MH271296,MK660712,MN234213 |
| VC_985_0 | 38 | 61656.86555 | 4396.813133 | 0.93343576 | 1.86E-189 | 0 | 3.31662479 | 703 | 703 | 1 | 0.700224354 | 0 | 0 | 0 | KT355471,KT355473,KT355475,KT783672,KU160655,KU160657,KU160658,KU160667,KU160670,KU160674,KX443695,KX576642,KX610765,KY434670,MF140409,MF140415,MF140417,MF140419,MF140420,MF140426,MF140429,MF140431,MF140433,MH576948,MH576950,MH576951,MH576952,MH576955,MH576957,MH576961,MH779506,MK494103,MK494111,MK737943,MK820640,MN617842,MN813695,ON970580 |
| VC_986_0 | 3 | 172.2476174 | 1496.2096 | 0.10323766 | 0.0008244 | 0 | 2.64575131 | 3 | 3 | 1 | 1.763834207 | 0 | 0 | 0 | MH834594,MH834597,MH834628 |
| VC_987_0 | 2 | 219.0398067 | 18.39076508 | 0.92254256 | 0.092556 | 3.31662479 | 3.31662479 | 1 | 1 | 1 | 3.31662479 | 0 | 0 | 0 | JN116828,MW654182 |
| VC_988_0 | 2 | 37.90715363 | 146.5732983 | 0.2054806 | 0.0511826 | 8.246211251 | 8.24621125 | 1 | 1 | 1 | 8.246211251 | 0 | 0 | 0 | JN116823,MK967393 |
| VC_989_0 | 13 | 19930.7004 | 3185.171689 | 0.86220846 | 5.85E-34 | 0 | 3.16227766 | 78 | 78 | 1 | 2.102148219 | 0 | 0 | 0 | KU963261,MH399781,MH509446,MK279893,MK305885,MK359319,MK376958,MK967392,MW291015,MW534376,MZ005677,ON637762,ON637763 |
| VC_99_0 | 5 | 113.2170662 | 1517.180545 | 0.06944138 | 0.0188617 | 2.645751311 | 4.12310563 | 10 | 10 | 1 | 3.445671806 | 0 | 0 | 0 | AY212251,KJ528544,LC628082,LC644557,ON637248 |
| VC_990_0 | 3 | 900 | 266.6306927 | 0.77145236 | 0.0086538 | 3.31662479 | 3.74165739 | 3 | 3 | 1 | 3.458302322 | 0 | 0 | 0 | MZ322021,ON970578,OP297535 |
| VC_991_0 | 39 | 162449.3144 | 0 | 1 | 0 | 0 | 4.89897949 | 741 | 741 | 1 | 2.348671665 | 0 | 0 | 0 | JN116826,JN116827,KT372002,KT375356,KT959213,KT990218,KX550082,KX611788,KX712236,KX712237,KY549153,KY549154,MF189179,MF324898,MF324900,MF324901,MF324902,MF324903,MF324904,MF324905,MF373842,MF537628,MH271291,MH271293,MH271294,MH271297,MH271299,MH271306,MH271309,MH271311,MH271314,MH271315,MH271319,MH316569,MK524487,MK524495,MN945902,MW291027,MZ402608 |
| VC_992_0 | 1 | 0 | 50.1051464 | 0 | 0 | 0 | 0 | 1 | 1 | 1 | 0 | 0 | 0 | 0 | JN175269 |
| VC_992_1 | 1 | 0 | 254.2358947 | 0 | 0 | 0 | 0 | 1 | 1 | 1 | 0 | 0 | 0 | 0 | KU862660 |
| VC_993_0 | 3 | 477.3439596 | 2861.036315 | 0.14298669 | 0.0015199 | 4.582575695 | 5.47722558 | 3 | 3 | 1 | 5.085317898 | 0 | 0 | 0 | MW570842,MW584170,MW584191 |
| VC_994_0 | 5 | 3000 | 57663.85561 | 0.04945284 | 0.0001619 | 1.732050808 | 6 | 10 | 10 | 1 | 4.673294096 | 0 | 0 | 0 | JX094500,KX397367,KX397372,MK514282,MW845759 |
| VC_994_1 | 8 | 8400 | 87407.23582 | 0.08767605 | 0.0786335 | 0 | 5.65685425 | 28 | 28 | 1 | 3.902897941 | 0 | 0 | 0 | KX397368,KX397370,KX397371,MT710307,MW384823,MW812340,MW812342,MW812343 |
| VC_994_2 | 5 | 3000 | 59078.96532 | 0.04832555 | 0.1162949 | 1.414213562 | 7.54983444 | 10 | 10 | 1 | 6.37000768 | 0 | 0 | 0 | KX397365,KX397366,MW760841,MW812338,MW812341 |
| VC_994_3 | 3 | 900 | 37182.75386 | 0.02363274 | 0.2478436 | 3.31662479 | 5.91607978 | 3 | 3 | 1 | 4.963186274 | 0 | 0 | 0 | KX397369,MH426724,MW845758 |
| VC_994_4 | 3 | 900 | 37186.41569 | 0.02363047 | 0.2418559 | 3 | 5.83095189 | 3 | 3 | 1 | 4.738705567 | 0 | 0 | 0 | JX316028,KX397364,KX397373 |
| VC_994_5 | 19 | 51300 | 144381.1165 | 0.26216122 | 1.56E-11 | 0 | 8.18535277 | 171 | 171 | 1 | 4.285256603 | 0 | 0 | 0 | JN641803,KU726251,LR535902,LR535909,LR535910,LR535914,LR535915,LR535916,LR535918,LR535919,LR535920,LR535921,MH221129,MN445185,MT679221,MT799840,MW812339,OK428535,OM456492 |
| VC_994_6 | 1 | 0 | 12558.22855 | 0 | 0 | 0 | 0 | 1 | 1 | 1 | 0 | 0 | 0 | 0 | MH221128 |
| VC_995_0 | 2 | 49.35099876 | 29.35430136 | 0.62703527 | 0.1444222 | 7.745966692 | 7.74596669 | 1 | 1 | 1 | 7.745966692 | 0 | 0 | 0 | JN712910,S4_vf_22219 |
| VC_996_0 | 3 | 107.4241856 | 49.35475684 | 0.68519524 | 0.0140929 | 2.236067977 | 3.31662479 | 3 | 3 | 1 | 2.904990143 | 0 | 0 | 0 | KX344510,KY264020,MW344767 |
| VC_997_0 | 4 | 230.1187532 | 40.88295494 | 0.84914134 | 0.0012073 | 1 | 3.74165739 | 6 | 6 | 1 | 2.468349637 | 0 | 0 | 0 | KY965934,MG550110,MG550111,MG550113 |
| VC_998_0 | 2 | 23.99231729 | 40.88295494 | 0.36982222 | 0.0876221 | 2 | 2 | 1 | 1 | 1 | 2 | 0 | 0 | 0 | JN882267,OM621814 |

**Table S7-2. VLP_genome_by_genome_overview**

| Genome | Order | Family | Genus | VC | VC Status | Size | VC Subcluster | VC Subcluster Size | Quality | Adj P-value | Topology Confidence Score | Genera in VC | Families in VC | Orders in VC | Genus Confidence Score | Reference |
|---|---|---|---|---|---|---|---|---|---|---|---|---|---|---|---|---|
| AB002632 | Unassigned | Unassigned | Unassigned | 0_0 | Clustered | 3 | VC_0_0 | 3 | 1 | 1 | 1 | 0 | 0 | 0 | 1 | Ref |
| AB008550 | Unassigned | Unassigned | Unassigned | 2_0 | Clustered | 4 | VC_2_0 | 4 | 0.13 | 0.99998694 | 0.13 | 0 | 0 | 0 | 1 | Ref |
| AB009866 | Unassigned | Unassigned | Unassigned | 7_0 | Clustered | 28 | VC_7_0 | 28 | 0.389 | 1 | 0.389 | 0 | 0 | 0 | 0.9947 | Ref |
| AB012573 | Unassigned | Unassigned | Unassigned | 16_0 | Clustered | 12 | VC_16_0 | 12 | 0.6601 | 1 | 0.6601 | 0 | 0 | 0 | 1 | Ref |
| AB012574 | Unassigned | Unassigned | Unassigned | 16_0 | Clustered | 12 | VC_16_0 | 12 | 0.6601 | 1 | 0.6601 | 0 | 0 | 0 | 1 | Ref |
| AB016282 | Unassigned | Unassigned | Unassigned | 18_0 | Clustered | 4 | VC_18_0 | 4 | 0.8969 | 0.99987935 | 0.8968 | 0 | 0 | 0 | 1 | Ref |
| AB036666 | Unassigned | Unassigned | Unassigned | 23_0 | Clustered | 11 | VC_23_0 | 11 | 0.5144 | 1 | 0.5144 | 0 | 0 | 0 | 1 | Ref |
| AB043678 | Unassigned | Unassigned | Unassigned | 17_0 | Clustered | 9 | VC_17_0 | 9 | 0.6954 | 1 | 0.6954 | 0 | 0 | 0 | 1 | Ref |
| AB043679 | Unassigned | Unassigned | Unassigned | 17_0 | Clustered | 9 | VC_17_0 | 9 | 0.6954 | 1 | 0.6954 | 0 | 0 | 0 | 1 | Ref |
| AB044554 | Unassigned | Unassigned | Unassigned | 7_0 | Clustered | 28 | VC_7_0 | 28 | 0.389 | 1 | 0.389 | 0 | 0 | 0 | 0.9947 | Ref |
| AB045978 | Unassigned | Unassigned | Unassigned | 8_0 | Clustered | 58 | VC_8_0 | 58 | 0.8285 | 1 | 0.8285 | 0 | 0 | 0 | 0.9946 | Ref |
| AB063393 | Unassigned | Unassigned | Unassigned | 29_0 | Clustered | 2 | VC_29_0 | 2 | 1 | 1 | 1 | 0 | 0 | 0 | 1 | Ref |
| AB102868 | Unassigned | Unassigned | Unassigned | 30_1 | Clustered | 53 | VC_30_1 | 53 | 0.7151 | 1 | 0.7151 | 0 | 0 | 0 | 0.9325 | Ref |
| AB231700 | Unassigned | Unassigned | Unassigned | 41_0 | Clustered | 3 | VC_41_0 | 3 | 1 | 0 | 0 | 0 | 0 | 0 | 1 | Ref |
| AB243556 | Unassigned | Unassigned | Unassigned | 7_0 | Clustered | 28 | VC_7_0 | 28 | 0.389 | 1 | 0.389 | 0 | 0 | 0 | 0.9947 | Ref |
| AB259123 | Unassigned | Unassigned | Unassigned | 54_0 | Clustered | 87 | VC_54_0 | 87 | 0.9858 | 1 | 0.9858 | 0 | 0 | 0 | 1 | Ref |
| AB259124 | Unassigned | Unassigned | Unassigned | 56_0 | Clustered | 6 | VC_56_0 | 6 | 0.6713 | 0.9999999 | 0.6713 | 0 | 0 | 0 | 1 | Ref |
| AB276040 | Unassigned | Unassigned | Unassigned | 1_0 | Clustered | 54 | VC_1_0 | 54 | 0.8518 | 1 | 0.8518 | 0 | 0 | 0 | 1 | Ref |
| AB285204 | Unassigned | Unassigned | Unassigned | 53_0 | Clustered | 10 | VC_53_0 | 10 | 0.1743 | 1 | 0.1743 | 0 | 0 | 0 | 1 | Ref |
| AB334720 | Unassigned | Unassigned | Unassigned | 62_0 | Clustered | 12 | VC_62_0 | 12 | 0.7624 | 1 | 0.7624 | 0 | 0 | 0 | 1 | Ref |
| AB334721 | Unassigned | Unassigned | Unassigned | 62_0 | Clustered | 12 | VC_62_0 | 12 | 0.7624 | 1 | 0.7624 | 0 | 0 | 0 | 1 | Ref |
| AB362338 | Unassigned | Unassigned | Unassigned | 30_1 | Clustered | 53 | VC_30_1 | 53 | 0.7151 | 1 | 0.7151 | 0 | 0 | 0 | 0.9325 | Ref |
| AB366653 | Unassigned | Unassigned | Unassigned | 67_3 | Clustered/Singleton | 1 | VC_67_3 | 1 | 0 | 1 | 0 | 0 | 0 | 0 | 1 | Ref |
| AB370205 | Unassigned | Unassigned | Unassigned | 9_0 | Clustered | 38 | VC_9_0 | 38 | 0.4859 | 1 | 0.4859 | 0 | 0 | 0 | 1 | Ref |
| AB370268 | Unassigned | Unassigned | Unassigned | 12_2 | Clustered | 19 | VC_12_2 | 19 | 0.2009 | 1 | 0.2009 | 0 | 0 | 0 | 1 | Ref |
| AB374228 | Unassigned | Unassigned | Unassigned | 5_0 | Clustered | 27 | VC_5_0 | 27 | 0.9599 | 1 | 0.9599 | 0 | 0 | 0 | 1 | Ref |
| AB426868 | Unassigned | Unassigned | Unassigned | 30_1 | Clustered | 53 | VC_30_1 | 53 | 0.7151 | 1 | 0.7151 | 0 | 0 | 0 | 0.9325 | Ref |
| AB434711 | Unassigned | Unassigned | Unassigned | 54_0 | Clustered | 87 | VC_54_0 | 87 | 0.9858 | 1 | 0.9858 | 0 | 0 | 0 | 1 | Ref |
| AB472900 | Unassigned | Unassigned | Unassigned | 78_0 | Clustered | 20 | VC_78_0 | 20 | 0.9639 | 1 | 0.9639 | 0 | 0 | 0 | 1 | Ref |
| AB560486 | Unassigned | Unassigned | Unassigned | 80_0 | Clustered | 64 | VC_80_0 | 64 | 0.418 | 1 | 0.418 | 0 | 0 | 0 | 0.7649 | Ref |
| AB572858 | Unassigned | Unassigned | Unassigned | 16_0 | Clustered | 12 | VC_16_0 | 12 | 0.6601 | 1 | 0.6601 | 0 | 0 | 0 | 1 | Ref |
| AB597179 | Unassigned | Unassigned | Unassigned | 84_0 | Clustered | 6 | VC_84_0 | 6 | 0.6364 | 1 | 0.6364 | 0 | 0 | 0 | 1 | Ref |
| AB609718 | Unassigned | Unassigned | Unassigned | 93_0 | Clustered | 22 | VC_93_0 | 22 | 0.5285 | 1 | 0.5285 | 0 | 0 | 0 | 1 | Ref |
| AB626962 | Unassigned | Unassigned | Unassigned | 98_0 | Clustered | 30 | VC_98_0 | 30 | 0.7175 | 1 | 0.7175 | 0 | 0 | 0 | 1 | Ref |
| AB626963 | Unassigned | Unassigned | Unassigned | 98_0 | Clustered | 30 | VC_98_0 | 30 | 0.7175 | 1 | 0.7175 | 0 | 0 | 0 | 1 | Ref |
| AB647160 | Unassigned | Unassigned | Unassigned | 101_0 | Clustered | 32 | VC_101_0 | 32 | 0.8826 | 1 | 0.8826 | 0 | 0 | 0 | 1 | Ref |
| AB712291 | Unassigned | Unassigned | Unassigned | 110_0 | Clustered | 33 | VC_110_0 | 33 | 0.9488 | 1 | 0.9488 | 0 | 0 | 0 | 1 | Ref |
| AB716666 | Unassigned | Unassigned | Unassigned | 113_0 | Clustered | 91 | VC_113_0 | 91 | 0.9584 | 1 | 0.9584 | 0 | 0 | 0 | 1 | Ref |
| AB720063 | Unassigned | Unassigned | Unassigned | 115_0 | Clustered | 9 | VC_115_0 | 9 | 0.5166 | 1 | 0.5166 | 0 | 0 | 0 | 1 | Ref |
| AB720064 | Unassigned | Unassigned | Unassigned | 118_0 | Clustered | 13 | VC_118_0 | 13 | 1 | 1 | 1 | 0 | 0 | 0 | 1 | Ref |
| AB731695 | Unassigned | Unassigned | Unassigned | 101_0 | Clustered | 32 | VC_101_0 | 32 | 0.8826 | 1 | 0.8826 | 0 | 0 | 0 | 1 | Ref |
| AB746912 | Unassigned | Unassigned | Unassigned | 120_0 | Clustered | 3 | VC_120_0 | 3 | 0.7107 | 0.99211494 | 0.7051 | 0 | 0 | 0 | 1 | Ref |
| AB757800 | Unassigned | Unassigned | Unassigned | 119_0 | Clustered | 9 | VC_119_0 | 9 | 0.9977 | 0.98988637 | 0.9876 | 0 | 0 | 0 | 1 | Ref |
| AB757801 | Unassigned | Unassigned | Unassigned | 119_0 | Clustered | 9 | VC_119_0 | 9 | 0.9977 | 0.98988637 | 0.9876 | 0 | 0 | 0 | 1 | Ref |
| AB775548 | Unassigned | Unassigned | Unassigned | 127_0 | Clustered | 17 | VC_127_0 | 17 | 0.9222 | 1 | 0.9222 | 0 | 0 | 0 | 1 | Ref |
| AB775549 | Unassigned | Unassigned | Unassigned | 132_0 | Clustered | 19 | VC_132_0 | 19 | 0.6051 | 1 | 0.6051 | 0 | 0 | 0 | 1 | Ref |
| AB797215 | Unassigned | Unassigned | Unassigned | 134_2 | Clustered | 17 | VC_134_2 | 17 | 0.1715 | 1 | 0.1715 | 0 | 0 | 0 | 0.9853 | Ref |
| AB828698 | Unassigned | Unassigned | Unassigned | 56_0 | Clustered | 6 | VC_56_0 | 6 | 0.6713 | 0.9999999 | 0.6713 | 0 | 0 | 0 | 1 | Ref |
| AB853330 | Unassigned | Unassigned | Unassigned | 155_13 | Clustered | 3 | VC_155_13 | 3 | 0.0087 | 0.95130224 | 0.0083 | 0 | 0 | 0 | 1 | Ref |
| AB853331 | Unassigned | Unassigned | Unassigned | 155_23 | Clustered/Singleton | 1 | VC_155_23 | 1 | 0 | 1 | 0 | 0 | 0 | 0 | 1 | Ref |
| AB854109 | Unassigned | Unassigned | Unassigned | 160_0 | Clustered | 4 | VC_160_0 | 4 | 0.1752 | 0.9999526 | 0.1752 | 0 | 0 | 0 | 1 | Ref |
| AB863625 | Unassigned | Unassigned | Unassigned | 167_0 | Clustered | 3 | VC_167_0 | 3 | 0.5731 | 0.99804396 | 0.572 | 0 | 0 | 0 | 1 | Ref |
| AB897757 | Unassigned | Unassigned | Unassigned | 170_10 | Clustered | 5 | VC_170_10 | 5 | 0.0614 | 0.96483582 | 0.0592 | 0 | 0 | 0 | 1 | Ref |
| AB903967 | Unassigned | Unassigned | Unassigned | 155_14 | Clustered/Singleton | 1 | VC_155_14 | 1 | 0 | 1 | 0 | 0 | 0 | 0 | 1 | Ref |
| AB910392 | Unassigned | Unassigned | Unassigned | 173_0 | Clustered | 3 | VC_173_0 | 3 | 0.6871 | 0.99826575 | 0.6859 | 0 | 0 | 0 | 1 | Ref |
| AB910393 | Unassigned | Unassigned | Unassigned | 180_0 | Clustered | 3 | VC_180_0 | 3 | 0.9607 | 0.99333557 | 0.9543 | 0 | 0 | 0 | 1 | Ref |
| AB910602 | Unassigned | Unassigned | Unassigned | 183_0 | Clustered | 5 | VC_183_0 | 5 | 0.7449 | 0.99885646 | 0.744 | 0 | 0 | 0 | 1 | Ref |
| AB916497 | Unassigned | Unassigned | Unassigned | 186_0 | Clustered | 4 | VC_186_0 | 4 | 0.2052 | 0.99998618 | 0.2052 | 0 | 0 | 0 | 1 | Ref |
| AB920995 | Unassigned | Unassigned | Unassigned | 71_0 | Clustered | 27 | VC_71_0 | 27 | 0.5964 | 1 | 0.5964 | 0 | 0 | 0 | 0.9943 | Ref |
| AB930182 | Unassigned | Unassigned | Unassigned | 87_3 | Clustered | 19 | VC_87_3 | 19 | 0.3645 | 1 | 0.3645 | 0 | 0 | 0 | 0.807 | Ref |
| AB931172 | Unassigned | Unassigned | Unassigned | 56_0 | Clustered | 6 | VC_56_0 | 6 | 0.6713 | 0.9999999 | 0.6713 | 0 | 0 | 0 | 1 | Ref |
| AB937974 | Unassigned | Unassigned | Unassigned | 54_0 | Clustered | 87 | VC_54_0 | 87 | 0.9858 | 1 | 0.9858 | 0 | 0 | 0 | 1 | Ref |
| AB971354 | Unassigned | Unassigned | Unassigned | 196_0 | Clustered | 13 | VC_196_0 | 13 | 0.9973 | 0.99823802 | 0.9955 | 0 | 0 | 0 | 1 | Ref |
| AB981169 | Unassigned | Unassigned | Unassigned | 1_0 | Clustered | 54 | VC_1_0 | 54 | 0.8518 | 1 | 0.8518 | 0 | 0 | 0 | 1 | Ref |
| AB981170 | Unassigned | Unassigned | Unassigned | 54_0 | Clustered | 87 | VC_54_0 | 87 | 0.9858 | 1 | 0.9858 | 0 | 0 | 0 | 1 | Ref |
| AB983711 | Unassigned | Unassigned | Unassigned | 71_0 | Clustered | 27 | VC_71_0 | 27 | 0.5964 | 1 | 0.5964 | 0 | 0 | 0 | 0.9943 | Ref |
| AC171169 | Unassigned | Unassigned | Unassigned | 198_11 | Clustered/Singleton | 1 | VC_198_11 | 1 | 0 | 1 | 0 | 0 | 0 | 0 | 1 | Ref |
| AE002163 | Unassigned | Unassigned | Unassigned | 205_0 | Clustered | 5 | VC_205_0 | 5 | 0.1325 | 0.84698401 | 0.1122 | 0 | 0 | 0 | 1 | Ref |
| AF009630 | Unassigned | Unassigned | Unassigned | 210_0 | Clustered | 105 | VC_210_0 | 105 | 0.4076 | 1 | 0.4076 | 0 | 0 | 0 | 0.7766 | Ref |
| AF011378 | Unassigned | Unassigned | Unassigned | 210_0 | Clustered | 105 | VC_210_0 | 105 | 0.4076 | 1 | 0.4076 | 0 | 0 | 0 | 0.7766 | Ref |
| AF020713 | Unassigned | Unassigned | Unassigned | 91_3 | Clustered/Singleton | 1 | VC_91_3 | 1 | 0 | 1 | 0 | 0 | 0 | 0 | 1 | Ref |
| AF022214 | Unassigned | Unassigned | Unassigned | 222_1 | Clustered | 19 | VC_222_1 | 19 | 0.4166 | 1 | 0.4166 | 0 | 0 | 0 | 1 | Ref |
| AF059242 | Unassigned | Unassigned | Unassigned | 196_0 | Clustered | 13 | VC_196_0 | 13 | 0.9973 | 0.99823802 | 0.9955 | 0 | 0 | 0 | 1 | Ref |
| AF059243 | Unassigned | Unassigned | Unassigned | 196_0 | Clustered | 13 | VC_196_0 | 13 | 0.9973 | 0.99823802 | 0.9955 | 0 | 0 | 0 | 1 | Ref |
| AF063097 | Unassigned | Unassigned | Unassigned | 235_0 | Clustered | 60 | VC_235_0 | 60 | 0.9413 | 1 | 0.9413 | 0 | 0 | 0 | 1 | Ref |
| AF065411 | Unassigned | Unassigned | Unassigned | 239_0 | Clustered | 2 | VC_239_0 | 2 | 0.926 | 0.72985431 | 0.6759 | 0 | 0 | 0 | 1 | Ref |
| AF083977 | Unassigned | Unassigned | Unassigned | 52_0 | Clustered | 9 | VC_52_0 | 9 | 0.5586 | 1 | 0.5586 | 0 | 0 | 0 | 1 | Ref |
| AF085222 | Unassigned | Unassigned | Unassigned | 258_0 | Clustered | 121 | VC_258_0 | 121 | 0.7359 | 1 | 0.7359 | 0 | 0 | 0 | 0.9999 | Ref |

| | | | | | | | | | | | | | | | | |
|---|---|---|---|---|---|---|---|---|---|---|---|---|---|---|---|---|
| AF115102 | Unassigned | Unassigned | Unassigned | 258_0 | Clustered | 121 | VC_258_0 | 121 | 0.7359 | 1 | 0.7359 | 0 | 0 | 0 | 0.9999 | Ref |
| AF115103 | Unassigned | Unassigned | Unassigned | 258_0 | Clustered | 121 | VC_258_0 | 121 | 0.7359 | 1 | 0.7359 | 0 | 0 | 0 | 0.9999 | Ref |
| AF125163 | Unassigned | Unassigned | Unassigned | 5_0 | Clustered | 27 | VC_5_0 | 27 | 0.9599 | 1 | 0.9599 | 0 | 0 | 0 | 1 | Ref |
| AF125520 | Unassigned | Unassigned | Unassigned | 35_0 | Clustered | 35 | VC_35_0 | 35 | 0.431 | 1 | 0.431 | 0 | 0 | 0 | 1 | Ref |
| AF133242 | Unassigned | Unassigned | Unassigned | 283_0 | Clustered | 2 | VC_283_0 | 2 | 1 | 1 | 1 | 0 | 0 | 0 | 1 | Ref |
| AF145054 | Unassigned | Unassigned | Unassigned | 258_0 | Clustered | 121 | VC_258_0 | 121 | 0.7359 | 1 | 0.7359 | 0 | 0 | 0 | 0.9999 | Ref |
| AF155037 | Unassigned | Unassigned | Unassigned | 284_0 | Clustered | 3 | VC_284_0 | 3 | 1 | 1 | 1 | 0 | 0 | 0 | 1 | Ref |
| AF157835 | Unassigned | Unassigned | Unassigned | 31_0 | Clustered | 10 | VC_31_0 | 10 | 0.5129 | 1 | 0.5129 | 0 | 0 | 0 | 1 | Ref |
| AF158101 | Unassigned | Unassigned | Unassigned | 290_91 | Clustered | 11 | VC_290_91 | 11 | 0.0281 | 1 | 0.0281 | 0 | 0 | 0 | 1 | Ref |
| AF158600 | Unassigned | Unassigned | Unassigned | 260_0 | Clustered | 46 | VC_260_0 | 46 | 0.5137 | 1 | 0.5137 | 0 | 0 | 0 | 1 | Ref |
| AF165214 | Unassigned | Unassigned | Unassigned | 182_0 | Clustered | 2 | VC_182_0 | 2 | 0.0495 | 0.95664605 | 0.0473 | 0 | 0 | 0 | 1 | Ref |
| AF176025 | Unassigned | Unassigned | Unassigned | 266_0 | Clustered | 13 | VC_266_0 | 13 | 0.3622 | 1 | 0.3622 | 0 | 0 | 0 | 1 | Ref |
| AF176027 | Unassigned | Unassigned | Unassigned | 310_0 | Clustered | 155 | VC_310_0 | 155 | 0.9979 | 1 | 0.9979 | 0 | 0 | 0 | 1 | Ref |
| AF176028 | Unassigned | Unassigned | Unassigned | 310_0 | Clustered | 155 | VC_310_0 | 155 | 0.9979 | 1 | 0.9979 | 0 | 0 | 0 | 1 | Ref |
| AF176029 | Unassigned | Unassigned | Unassigned | 310_0 | Clustered | 155 | VC_310_0 | 155 | 0.9979 | 1 | 0.9979 | 0 | 0 | 0 | 1 | Ref |
| AF176030 | Unassigned | Unassigned | Unassigned | 310_0 | Clustered | 155 | VC_310_0 | 155 | 0.9979 | 1 | 0.9979 | 0 | 0 | 0 | 1 | Ref |
| AF176031 | Unassigned | Unassigned | Unassigned | 310_0 | Clustered | 155 | VC_310_0 | 155 | 0.9979 | 1 | 0.9979 | 0 | 0 | 0 | 1 | Ref |
| AF176032 | Unassigned | Unassigned | Unassigned | 310_0 | Clustered | 155 | VC_310_0 | 155 | 0.9979 | 1 | 0.9979 | 0 | 0 | 0 | 1 | Ref |
| AF176033 | Unassigned | Unassigned | Unassigned | 310_0 | Clustered | 155 | VC_310_0 | 155 | 0.9979 | 1 | 0.9979 | 0 | 0 | 0 | 1 | Ref |
| AF176034 | Unassigned | Unassigned | Unassigned | 310_0 | Clustered | 155 | VC_310_0 | 155 | 0.9979 | 1 | 0.9979 | 0 | 0 | 0 | 1 | Ref |
| AF189021 | Unassigned | Unassigned | Unassigned | 311_0 | Clustered | 11 | VC_311_0 | 11 | 0.7851 | 1 | 0.7851 | 0 | 0 | 0 | 1 | Ref |
| AF191797 | Unassigned | Unassigned | Unassigned | 313_0 | Clustered | 2 | VC_313_0 | 2 | 1 | 1 | 1 | 0 | 0 | 0 | 1 | Ref |
| AF195902 | Unassigned | Unassigned | Unassigned | 275_0 | Clustered | 6 | VC_275_0 | 6 | 0.6515 | 0.99999994 | 0.6515 | 0 | 0 | 0 | 0.9333 | Ref |
| AF217253 | Unassigned | Unassigned | Unassigned | 30_1 | Clustered | 53 | VC_30_1 | 53 | 0.7151 | 1 | 0.7151 | 0 | 0 | 0 | 0.9325 | Ref |
| AF222060 | Unassigned | Unassigned | Unassigned | 317_0 | Clustered | 33 | VC_317_0 | 33 | 0.8756 | 1 | 0.8756 | 0 | 0 | 0 | 1 | Ref |
| AF227250 | Unassigned | Unassigned | Unassigned | 322_0 | Clustered | 12 | VC_322_0 | 12 | 0.8924 | 1 | 0.8924 | 0 | 0 | 0 | 1 | Ref |
| AF232233 | Unassigned | Unassigned | Unassigned | 192_0 | Clustered | 22 | VC_192_0 | 22 | 0.6582 | 1 | 0.6582 | 0 | 0 | 0 | 1 | Ref |
| AF234172 | Unassigned | Unassigned | Unassigned | 254_0 | Clustered | 14 | VC_254_0 | 14 | 0.9118 | 1 | 0.9118 | 0 | 0 | 0 | 0.967 | Ref |
| AF234173 | Unassigned | Unassigned | Unassigned | 254_0 | Clustered | 14 | VC_254_0 | 14 | 0.9118 | 1 | 0.9118 | 0 | 0 | 0 | 0.967 | Ref |
| AF242738 | Unassigned | Unassigned | Unassigned | 268_0 | Clustered | 10 | VC_268_0 | 10 | 0.1417 | 1 | 0.1417 | 0 | 0 | 0 | 0.9333 | Ref |
| AF271693 | Unassigned | Unassigned | Unassigned | 335_34 | Clustered | 2 | VC_335_34 | 2 | 0.0045 | 0.95750889 | 0.0043 | 0 | 0 | 0 | 1 | Ref |
| AF274751 | Unassigned | Unassigned | Unassigned | 310_0 | Clustered | 155 | VC_310_0 | 155 | 0.9979 | 1 | 0.9979 | 0 | 0 | 0 | 1 | Ref |
| AF299300 | Unassigned | Unassigned | Unassigned | 310_0 | Clustered | 155 | VC_310_0 | 155 | 0.9979 | 1 | 0.9979 | 0 | 0 | 0 | 1 | Ref |
| AF299301 | Unassigned | Unassigned | Unassigned | 310_0 | Clustered | 155 | VC_310_0 | 155 | 0.9979 | 1 | 0.9979 | 0 | 0 | 0 | 1 | Ref |
| AF299302 | Unassigned | Unassigned | Unassigned | 310_0 | Clustered | 155 | VC_310_0 | 155 | 0.9979 | 1 | 0.9979 | 0 | 0 | 0 | 1 | Ref |
| AF299303 | Unassigned | Unassigned | Unassigned | 310_0 | Clustered | 155 | VC_310_0 | 155 | 0.9979 | 1 | 0.9979 | 0 | 0 | 0 | 1 | Ref |
| AF299304 | Unassigned | Unassigned | Unassigned | 310_0 | Clustered | 155 | VC_310_0 | 155 | 0.9979 | 1 | 0.9979 | 0 | 0 | 0 | 1 | Ref |
| AF299305 | Unassigned | Unassigned | Unassigned | 310_0 | Clustered | 155 | VC_310_0 | 155 | 0.9979 | 1 | 0.9979 | 0 | 0 | 0 | 1 | Ref |
| AF299306 | Unassigned | Unassigned | Unassigned | 310_0 | Clustered | 155 | VC_310_0 | 155 | 0.9979 | 1 | 0.9979 | 0 | 0 | 0 | 1 | Ref |
| AF299307 | Unassigned | Unassigned | Unassigned | 310_0 | Clustered | 155 | VC_310_0 | 155 | 0.9979 | 1 | 0.9979 | 0 | 0 | 0 | 1 | Ref |
| AF299308 | Unassigned | Unassigned | Unassigned | 310_0 | Clustered | 155 | VC_310_0 | 155 | 0.9979 | 1 | 0.9979 | 0 | 0 | 0 | 1 | Ref |
| AF299309 | Unassigned | Unassigned | Unassigned | 310_0 | Clustered | 155 | VC_310_0 | 155 | 0.9979 | 1 | 0.9979 | 0 | 0 | 0 | 1 | Ref |
| AF299310 | Unassigned | Unassigned | Unassigned | 310_0 | Clustered | 155 | VC_310_0 | 155 | 0.9979 | 1 | 0.9979 | 0 | 0 | 0 | 1 | Ref |
| AF299311 | Unassigned | Unassigned | Unassigned | 310_0 | Clustered | 155 | VC_310_0 | 155 | 0.9979 | 1 | 0.9979 | 0 | 0 | 0 | 1 | Ref |
| AF299312 | Unassigned | Unassigned | Unassigned | 310_0 | Clustered | 155 | VC_310_0 | 155 | 0.9979 | 1 | 0.9979 | 0 | 0 | 0 | 1 | Ref |
| AF299313 | Unassigned | Unassigned | Unassigned | 310_0 | Clustered | 155 | VC_310_0 | 155 | 0.9979 | 1 | 0.9979 | 0 | 0 | 0 | 1 | Ref |
| AF299314 | Unassigned | Unassigned | Unassigned | 310_0 | Clustered | 155 | VC_310_0 | 155 | 0.9979 | 1 | 0.9979 | 0 | 0 | 0 | 1 | Ref |
| AF301375 | Unassigned | Unassigned | Unassigned | 239_0 | Clustered | 2 | VC_239_0 | 2 | 0.926 | 0.72985431 | 0.6759 | 0 | 0 | 0 | 1 | Ref |
| AF306496 | Unassigned | Unassigned | Unassigned | 338_0 | Clustered | 12 | VC_338_0 | 12 | 0.8919 | 1 | 0.8919 | 0 | 0 | 0 | 1 | Ref |
| AF320576 | Unassigned | Unassigned | Unassigned | 340_1 | Clustered | 17 | VC_340_1 | 17 | 0.0863 | 1 | 0.0863 | 0 | 0 | 0 | 0.8235 | Ref |
| AF323668 | Unassigned | Unassigned | Unassigned | 269_0 | Clustered | 7 | VC_269_0 | 7 | 0.2278 | 1 | 0.2278 | 0 | 0 | 0 | 1 | Ref |
| AF323669 | Unassigned | Unassigned | Unassigned | 266_0 | Clustered | 13 | VC_266_0 | 13 | 0.3622 | 1 | 0.3622 | 0 | 0 | 0 | 1 | Ref |
| AF323671 | Unassigned | Unassigned | Unassigned | 351_0 | Clustered | 9 | VC_351_0 | 9 | 0.9391 | 0.99999913 | 0.9391 | 0 | 0 | 0 | 1 | Ref |
| AF334111 | Unassigned | Unassigned | Unassigned | 234_0 | Clustered | 2 | VC_234_0 | 2 | 0.3333 | 0 | 0 | 0 | 0 | 0 | 1 | Ref |
| AF335538 | Unassigned | Unassigned | Unassigned | 32_1 | Clustered | 16 | VC_32_1 | 16 | 0.1962 | 1 | 0.1962 | 0 | 0 | 0 | 0.8333 | Ref |
| AF338467 | Unassigned | Unassigned | Unassigned | 352_0 | Clustered | 31 | VC_352_0 | 31 | 0.9547 | 1 | 0.9547 | 0 | 0 | 0 | 1 | Ref |
| AF396866 | Unassigned | Unassigned | Unassigned | 353_0 | Clustered | 3 | VC_353_0 | 3 | 0.2241 | 0.81279277 | 0.1821 | 0 | 0 | 0 | 1 | Ref |
| AF399011 | Unassigned | Unassigned | Unassigned | 356_0 | Clustered | 21 | VC_356_0 | 21 | 0.5409 | 1 | 0.5409 | 0 | 0 | 0 | 1 | Ref |
| AF424781 | Unassigned | Unassigned | Unassigned | 9_0 | Clustered | 38 | VC_9_0 | 38 | 0.4859 | 1 | 0.4859 | 0 | 0 | 0 | 1 | Ref |
| AF424782 | Unassigned | Unassigned | Unassigned | 8_0 | Clustered | 58 | VC_8_0 | 58 | 0.8285 | 1 | 0.8285 | 0 | 0 | 0 | 0.9946 | Ref |
| AF424783 | Unassigned | Unassigned | Unassigned | 7_0 | Clustered | 28 | VC_7_0 | 28 | 0.389 | 1 | 0.389 | 0 | 0 | 0 | 0.9947 | Ref |
| AF440571 | Unassigned | Unassigned | Unassigned | 361_0 | Clustered | 7 | VC_361_0 | 7 | 0.9353 | 0.99999998 | 0.9353 | 0 | 0 | 0 | 1 | Ref |
| AF440695 | Unassigned | Unassigned | Unassigned | 364_0 | Clustered | 3 | VC_364_0 | 3 | 0.729 | 0.98867275 | 0.7207 | 0 | 0 | 0 | 1 | Ref |
| AF447491 | Unassigned | Unassigned | Unassigned | 365_0 | Clustered | 6 | VC_365_0 | 6 | 0.5395 | 1 | 0.5395 | 0 | 0 | 0 | 1 | Ref |
| AF452449 | Unassigned | Unassigned | Unassigned | 16_0 | Clustered | 12 | VC_16_0 | 12 | 0.6601 | 1 | 0.6601 | 0 | 0 | 0 | 1 | Ref |
| AF453500 | Unassigned | Unassigned | Unassigned | 16_0 | Clustered | 12 | VC_16_0 | 12 | 0.6601 | 1 | 0.6601 | 0 | 0 | 0 | 1 | Ref |
| AF454431 | Unassigned | Unassigned | Unassigned | 373_0 | Clustered | 358 | VC_373_0 | 358 | 0.9614 | 1 | 0.9614 | 0 | 0 | 0 | 1 | Ref |
| AF489521 | Unassigned | Unassigned | Unassigned | 266_0 | Clustered | 13 | VC_266_0 | 13 | 0.3622 | 1 | 0.3622 | 0 | 0 | 0 | 1 | Ref |
| AF493143 | Unassigned | Unassigned | Unassigned | 131_0 | Clustered | 43 | VC_131_0 | 43 | 0.8853 | 1 | 0.8853 | 0 | 0 | 0 | 1 | Ref |
| AF503408 | Unassigned | Unassigned | Unassigned | 254_0 | Clustered | 14 | VC_254_0 | 14 | 0.9118 | 1 | 0.9118 | 0 | 0 | 0 | 0.967 | Ref |
| AF513032 | Unassigned | Unassigned | Unassigned | 98_0 | Clustered | 30 | VC_98_0 | 30 | 0.7175 | 1 | 0.7175 | 0 | 0 | 0 | 1 | Ref |
| AF513033 | Unassigned | Unassigned | Unassigned | 98_0 | Clustered | 30 | VC_98_0 | 30 | 0.7175 | 1 | 0.7175 | 0 | 0 | 0 | 1 | Ref |
| AF527608 | Unassigned | Unassigned | Unassigned | 30_1 | Clustered | 53 | VC_30_1 | 53 | 0.7151 | 1 | 0.7151 | 0 | 0 | 0 | 0.9325 | Ref |
| AF547430 | Unassigned | Unassigned | Unassigned | 384_16 | Clustered | 42 | VC_384_16 | 42 | 0.134 | 1 | 0.134 | 0 | 0 | 0 | 0.7282 | Ref |
| AF547987 | Unassigned | Unassigned | Unassigned | 32_1 | Clustered | 16 | VC_32_1 | 16 | 0.1962 | 1 | 0.1962 | 0 | 0 | 0 | 0.8333 | Ref |
| AJ006589 | Unassigned | Unassigned | Unassigned | 386_0 | Clustered | 10 | VC_386_0 | 10 | 0.7454 | 1 | 0.7454 | 0 | 0 | 0 | 1 | Ref |
| AJ242593 | Unassigned | Unassigned | Unassigned | 392_0 | Clustered | 9 | VC_392_0 | 9 | 0.7527 | 1 | 0.7527 | 0 | 0 | 0 | 1 | Ref |
| AJ245616 | Unassigned | Unassigned | Unassigned | 266_0 | Clustered | 13 | VC_266_0 | 13 | 0.3622 | 1 | 0.3622 | 0 | 0 | 0 | 1 | Ref |
| AJ251789 | Unassigned | Unassigned | Unassigned | 279_0 | Clustered | 11 | VC_279_0 | 11 | 0.4839 | 1 | 0.4839 | 0 | 0 | 0 | 0.8727 | Ref |
| AJ251805 | Unassigned | Unassigned | Unassigned | 398_0 | Clustered | 51 | VC_398_0 | 51 | 0.8741 | 1 | 0.8741 | 0 | 0 | 0 | 1 | Ref |
| AJ270057 | Unassigned | Unassigned | Unassigned | 206_0 | Clustered | 28 | VC_206_0 | 28 | 0.2427 | 0.99971175 | 0.2426 | 0 | 0 | 0 | 1 | Ref |

| | | | | | | | | | | | | | | | | |
|---|---|---|---|---|---|---|---|---|---|---|---|---|---|---|---|---|
| AJ278322 | Unassigned | Unassigned | Unassigned | 420_0 | Clustered | 3 | VC_420_0 | 3 | 0.3294 | 0.99825267 | 0.3288 | 0 | 0 | 0 | 1 | Ref |
| AJ298298 | Unassigned | Unassigned | Unassigned | 47_0 | Clustered | 12 | VC_47_0 | 12 | 0.3571 | 1 | 0.3571 | 0 | 0 | 0 | 1 | Ref |
| AJ302074 | Unassigned | Unassigned | Unassigned | 425_0 | Clustered | 40 | VC_425_0 | 40 | 0.8399 | 1 | 0.8399 | 0 | 0 | 0 | 0.9974 | Ref |
| AJ312240 | Unassigned | Unassigned | Unassigned | 393_0 | Clustered | 5 | VC_393_0 | 5 | 0.4703 | 0.99999992 | 0.4703 | 0 | 0 | 0 | 0.9 | Ref |
| AJ318471 | Unassigned | Unassigned | Unassigned | 398_0 | Clustered | 51 | VC_398_0 | 51 | 0.8741 | 1 | 0.8741 | 0 | 0 | 0 | 1 | Ref |
| AJ344259 | Unassigned | Unassigned | Unassigned | 360_0 | Clustered | 22 | VC_360_0 | 22 | 0.96 | 1 | 0.96 | 0 | 0 | 0 | 1 | Ref |
| AJ414696 | Unassigned | Unassigned | Unassigned | 360_0 | Clustered | 22 | VC_360_0 | 22 | 0.96 | 1 | 0.96 | 0 | 0 | 0 | 1 | Ref |
| AJ421943 | Unassigned | Unassigned | Unassigned | 281_0 | Clustered | 2 | VC_281_0 | 2 | 0.0753 | 0.9540953 | 0.0719 | 0 | 0 | 0 | 1 | Ref |
| AJ505558 | Unassigned | Unassigned | Unassigned | 434_0 | Clustered | 45 | VC_434_0 | 45 | 0.9479 | 1 | 0.9479 | 0 | 0 | 0 | 1 | Ref |
| AJ536073 | Unassigned | Unassigned | Unassigned | 435_0 | Clustered | 7 | VC_435_0 | 7 | 0.9041 | 0.9998143 | 0.9039 | 0 | 0 | 0 | 1 | Ref |
| AJ550635 | Unassigned | Unassigned | Unassigned | 206_0 | Clustered | 28 | VC_206_0 | 28 | 0.2427 | 0.99971175 | 0.2426 | 0 | 0 | 0 | 1 | Ref |
| AJ550940 | Unassigned | Unassigned | Unassigned | 386_0 | Clustered | 10 | VC_386_0 | 10 | 0.7454 | 1 | 0.7454 | 0 | 0 | 0 | 1 | Ref |
| AJ556162 | Unassigned | Unassigned | Unassigned | 43_0 | Clustered | 16 | VC_43_0 | 16 | 0.2017 | 1 | 0.2017 | 0 | 0 | 0 | 0.9667 | Ref |
| AJ604530 | Unassigned | Unassigned | Unassigned | 197_0 | Clustered | 2 | VC_197_0 | 2 | 0.0103 | 0.93953444 | 0.0097 | 0 | 0 | 0 | 1 | Ref |
| AJ604531 | Unassigned | Unassigned | Unassigned | 197_0 | Clustered | 2 | VC_197_0 | 2 | 0.0103 | 0.93953444 | 0.0097 | 0 | 0 | 0 | 1 | Ref |
| AJ630128 | Unassigned | Unassigned | Unassigned | 447_5 | Clustered | 2 | VC_447_5 | 2 | 0.0172 | 0.93642158 | 0.0161 | 0 | 0 | 0 | 1 | Ref |
| AJ635161 | Unassigned | Unassigned | Unassigned | 454_0 | Clustered | 4 | VC_454_0 | 4 | 1 | 1 | 1 | 0 | 0 | 0 | 1 | Ref |
| AJ748296 | Unassigned | Unassigned | Unassigned | 360_0 | Clustered | 22 | VC_360_0 | 22 | 0.96 | 1 | 0.96 | 0 | 0 | 0 | 1 | Ref |
| AJ783769 | Unassigned | Unassigned | Unassigned | 458_0 | Clustered | 2 | VC_458_0 | 2 | 0.8124 | 0.72985431 | 0.593 | 0 | 0 | 0 | 1 | Ref |
| AJ854042 | Unassigned | Unassigned | Unassigned | 362_0 | Clustered | 2 | VC_362_0 | 2 | 0.3217 | 0.93865445 | 0.3019 | 0 | 0 | 0 | 1 | Ref |
| AJ875026 | Unassigned | Unassigned | Unassigned | 360_0 | Clustered | 22 | VC_360_0 | 22 | 0.96 | 1 | 0.96 | 0 | 0 | 0 | 1 | Ref |
| AJ888457 | Unassigned | Unassigned | Unassigned | 461_0 | Clustered | 3 | VC_461_0 | 3 | 0.8247 | 0.99509929 | 0.8207 | 0 | 0 | 0 | 1 | Ref |
| AJ969242 | Unassigned | Unassigned | Unassigned | 283_0 | Clustered | 2 | VC_283_0 | 2 | 1 | 1 | 1 | 0 | 0 | 0 | 1 | Ref |
| AM076770 | Unassigned | Unassigned | Unassigned | 464_0 | Clustered | 16 | VC_464_0 | 16 | 0.9558 | 1 | 0.9558 | 0 | 0 | 0 | 1 | Ref |
| AM084414 | Unassigned | Unassigned | Unassigned | 465_0 | Clustered | 80 | VC_465_0 | 80 | 0.9988 | 1 | 0.9988 | 0 | 0 | 0 | 1 | Ref |
| AM084415 | Unassigned | Unassigned | Unassigned | 466_0 | Clustered | 66 | VC_466_0 | 66 | 0.9246 | 1 | 0.9246 | 0 | 0 | 0 | 1 | Ref |
| AM087120 | Unassigned | Unassigned | Unassigned | 361_0 | Clustered | 7 | VC_361_0 | 7 | 0.9353 | 0.99999998 | 0.9353 | 0 | 0 | 0 | 1 | Ref |
| AM087121 | Unassigned | Unassigned | Unassigned | 361_0 | Clustered | 7 | VC_361_0 | 7 | 0.9353 | 0.99999998 | 0.9353 | 0 | 0 | 0 | 1 | Ref |
| AM087122 | Unassigned | Unassigned | Unassigned | 361_0 | Clustered | 7 | VC_361_0 | 7 | 0.9353 | 0.99999998 | 0.9353 | 0 | 0 | 0 | 1 | Ref |
| AM087123 | Unassigned | Unassigned | Unassigned | 361_0 | Clustered | 7 | VC_361_0 | 7 | 0.9353 | 0.99999998 | 0.9353 | 0 | 0 | 0 | 1 | Ref |
| AM156909 | Unassigned | Unassigned | Unassigned | 470_0 | Clustered | 62 | VC_470_0 | 62 | 0.902 | 1 | 0.902 | 0 | 0 | 0 | 1 | Ref |
| AM183667 | Unassigned | Unassigned | Unassigned | 399_0 | Clustered | 49 | VC_399_0 | 49 | 0.7927 | 1 | 0.7927 | 0 | 0 | 0 | 1 | Ref |
| AM265638 | Unassigned | Unassigned | Unassigned | 434_0 | Clustered | 45 | VC_434_0 | 45 | 0.9479 | 1 | 0.9479 | 0 | 0 | 0 | 1 | Ref |
| AM419438 | Unassigned | Unassigned | Unassigned | 363_0 | Clustered | 3 | VC_363_0 | 3 | 0.8866 | 0.98155579 | 0.8702 | 0 | 0 | 0 | 1 | Ref |
| AM491472 | Unassigned | Unassigned | Unassigned | 475_0 | Clustered | 96 | VC_475_0 | 96 | 0.9303 | 1 | 0.9303 | 0 | 0 | 0 | 0.9575 | Ref |
| AM749121 | Unassigned | Unassigned | Unassigned | 477_0 | Clustered | 4 | VC_477_0 | 4 | 0.7825 | 0.99997292 | 0.7825 | 0 | 0 | 0 | 1 | Ref |
| AM749441 | Unassigned | Unassigned | Unassigned | 172_0 | Clustered | 26 | VC_172_0 | 26 | 0.7909 | 1 | 0.7909 | 0 | 0 | 0 | 1 | Ref |
| AM910650 | Unassigned | Unassigned | Unassigned | 486_0 | Clustered | 47 | VC_486_0 | 47 | 0.9022 | 1 | 0.9022 | 0 | 0 | 0 | 1 | Ref |
| AM910651 | Unassigned | Unassigned | Unassigned | 434_0 | Clustered | 45 | VC_434_0 | 45 | 0.9479 | 1 | 0.9479 | 0 | 0 | 0 | 1 | Ref |
| AP000363 | Unassigned | Unassigned | Unassigned | 35_0 | Clustered | 35 | VC_35_0 | 35 | 0.431 | 1 | 0.431 | 0 | 0 | 0 | 1 | Ref |
| AP000422 | Unassigned | Unassigned | Unassigned | 35_0 | Clustered | 35 | VC_35_0 | 35 | 0.431 | 1 | 0.431 | 0 | 0 | 0 | 1 | Ref |
| AP001553 | Unassigned | Unassigned | Unassigned | 12_2 | Clustered | 19 | VC_12_2 | 19 | 0.2009 | 1 | 0.2009 | 0 | 0 | 0 | 1 | Ref |
| AP004402 | Unassigned | Unassigned | Unassigned | 35_0 | Clustered | 35 | VC_35_0 | 35 | 0.431 | 1 | 0.431 | 0 | 0 | 0 | 1 | Ref |
| AP005153 | Unassigned | Unassigned | Unassigned | 35_0 | Clustered | 35 | VC_35_0 | 35 | 0.431 | 1 | 0.431 | 0 | 0 | 0 | 1 | Ref |
| AP005154 | Unassigned | Unassigned | Unassigned | 35_0 | Clustered | 35 | VC_35_0 | 35 | 0.431 | 1 | 0.431 | 0 | 0 | 0 | 1 | Ref |
| AP008953 | Unassigned | Unassigned | Unassigned | 9_0 | Clustered | 38 | VC_9_0 | 38 | 0.4859 | 1 | 0.4859 | 0 | 0 | 0 | 1 | Ref |
| AP008954 | Unassigned | Unassigned | Unassigned | 12_0 | Clustered | 21 | VC_12_0 | 21 | 0.2262 | 1 | 0.2262 | 0 | 0 | 0 | 0.9571 | Ref |
| AP008979 | Unassigned | Unassigned | Unassigned | 115_0 | Clustered | 9 | VC_115_0 | 9 | 0.5166 | 1 | 0.5166 | 0 | 0 | 0 | 1 | Ref |
| AP008986 | Unassigned | Unassigned | Unassigned | 377_0 | Clustered | 16 | VC_377_0 | 16 | 0.6424 | 1 | 0.6424 | 0 | 0 | 0 | 1 | Ref |
| AP009146 | Unassigned | Unassigned | Unassigned | 64_0 | Clustered | 2 | VC_64_0 | 2 | 0.1127 | 0.95690971 | 0.1079 | 0 | 0 | 0 | 1 | Ref |
| AP009363 | Unassigned | Unassigned | Unassigned | 8_0 | Clustered | 58 | VC_8_0 | 58 | 0.8285 | 1 | 0.8285 | 0 | 0 | 0 | 0.9946 | Ref |
| AP009390 | Unassigned | Unassigned | Unassigned | 93_0 | Clustered | 22 | VC_93_0 | 22 | 0.5285 | 1 | 0.5285 | 0 | 0 | 0 | 1 | Ref |
| AP011113 | Unassigned | Unassigned | Unassigned | 290_10 | Clustered/Singleton | 1 | VC_290_10 | 1 | 0 | 1 | 0 | 0 | 0 | 0 | 1 | Ref |
| AP011616 | Unassigned | Unassigned | Unassigned | 493_0 | Clustered | 4 | VC_493_0 | 4 | 0.9871 | 0.99748973 | 0.9847 | 0 | 0 | 0 | 1 | Ref |
| AP011617 | Unassigned | Unassigned | Unassigned | 493_0 | Clustered | 4 | VC_493_0 | 4 | 0.9871 | 0.99748973 | 0.9847 | 0 | 0 | 0 | 1 | Ref |
| AP011955 | Unassigned | Unassigned | Unassigned | 10_0 | Clustered | 25 | VC_10_0 | 25 | 0.3437 | 1 | 0.3437 | 0 | 0 | 0 | 0.9733 | Ref |
| AP011956 | Unassigned | Unassigned | Unassigned | 10_0 | Clustered | 25 | VC_10_0 | 25 | 0.3437 | 1 | 0.3437 | 0 | 0 | 0 | 0.9733 | Ref |
| AP012341 | Unassigned | Unassigned | Unassigned | 8_0 | Clustered | 58 | VC_8_0 | 58 | 0.8285 | 1 | 0.8285 | 0 | 0 | 0 | 0.9946 | Ref |
| AP012529 | Unassigned | Unassigned | Unassigned | 35_0 | Clustered | 35 | VC_35_0 | 35 | 0.431 | 1 | 0.431 | 0 | 0 | 0 | 1 | Ref |
| AP012530 | Unassigned | Unassigned | Unassigned | 43_1 | Clustered | 10 | VC_43_1 | 10 | 0.1173 | 1 | 0.1173 | 0 | 0 | 0 | 0.8222 | Ref |
| AP012531 | Unassigned | Unassigned | Unassigned | 35_0 | Clustered | 35 | VC_35_0 | 35 | 0.431 | 1 | 0.431 | 0 | 0 | 0 | 1 | Ref |
| AP012532 | Unassigned | Unassigned | Unassigned | 35_0 | Clustered | 35 | VC_35_0 | 35 | 0.431 | 1 | 0.431 | 0 | 0 | 0 | 1 | Ref |
| AP012534 | Unassigned | Unassigned | Unassigned | 44_0 | Clustered | 49 | VC_44_0 | 49 | 0.4506 | 1 | 0.4506 | 0 | 0 | 0 | 0.8605 | Ref |
| AP012535 | Unassigned | Unassigned | Unassigned | 35_0 | Clustered | 35 | VC_35_0 | 35 | 0.431 | 1 | 0.431 | 0 | 0 | 0 | 1 | Ref |
| AP012538 | Unassigned | Unassigned | Unassigned | 43_1 | Clustered | 10 | VC_43_1 | 10 | 0.1173 | 1 | 0.1173 | 0 | 0 | 0 | 0.8222 | Ref |
| AP012539 | Unassigned | Unassigned | Unassigned | 43_1 | Clustered | 10 | VC_43_1 | 10 | 0.1173 | 1 | 0.1173 | 0 | 0 | 0 | 0.8222 | Ref |
| AP012540 | Unassigned | Unassigned | Unassigned | 43_1 | Clustered | 10 | VC_43_1 | 10 | 0.1173 | 1 | 0.1173 | 0 | 0 | 0 | 0.8222 | Ref |
| AP013029 | Unassigned | Unassigned | Unassigned | 87_3 | Clustered | 19 | VC_87_3 | 19 | 0.3645 | 1 | 0.3645 | 0 | 0 | 0 | 0.807 | Ref |
| AP013538 | Unassigned | Unassigned | Unassigned | 352_0 | Clustered | 31 | VC_352_0 | 31 | 0.9547 | 1 | 0.9547 | 0 | 0 | 0 | 1 | Ref |
| AP013539 | Unassigned | Unassigned | Unassigned | 352_0 | Clustered | 31 | VC_352_0 | 31 | 0.9547 | 1 | 0.9547 | 0 | 0 | 0 | 1 | Ref |
| AP013540 | Unassigned | Unassigned | Unassigned | 496_0 | Clustered | 22 | VC_496_0 | 22 | 0.6101 | 1 | 0.6101 | 0 | 0 | 0 | 1 | Ref |
| AP013541 | Unassigned | Unassigned | Unassigned | 496_0 | Clustered | 22 | VC_496_0 | 22 | 0.6101 | 1 | 0.6101 | 0 | 0 | 0 | 1 | Ref |
| AP013542 | Unassigned | Unassigned | Unassigned | 496_0 | Clustered | 22 | VC_496_0 | 22 | 0.6101 | 1 | 0.6101 | 0 | 0 | 0 | 1 | Ref |
| AP013545 | Unassigned | Unassigned | Unassigned | 496_0 | Clustered | 22 | VC_496_0 | 22 | 0.6101 | 1 | 0.6101 | 0 | 0 | 0 | 1 | Ref |
| AP013546 | Unassigned | Unassigned | Unassigned | 498_0 | Clustered | 4 | VC_498_0 | 4 | 0.2907 | 0.99998435 | 0.2907 | 0 | 0 | 0 | 1 | Ref |
| AP013547 | Unassigned | Unassigned | Unassigned | 498_0 | Clustered | 4 | VC_498_0 | 4 | 0.2907 | 0.99998435 | 0.2907 | 0 | 0 | 0 | 1 | Ref |
| AP013548 | Unassigned | Unassigned | Unassigned | 498_0 | Clustered | 4 | VC_498_0 | 4 | 0.2907 | 0.99998435 | 0.2907 | 0 | 0 | 0 | 1 | Ref |
| AP014714 | Unassigned | Unassigned | Unassigned | 508_0 | Clustered | 2 | VC_508_0 | 2 | 0.01 | 0.95344863 | 0.0096 | 0 | 0 | 0 | 1 | Ref |
| AP014715 | Unassigned | Unassigned | Unassigned | 508_0 | Clustered | 2 | VC_508_0 | 2 | 0.01 | 0.95344863 | 0.0096 | 0 | 0 | 0 | 1 | Ref |
| AP014858 | Unassigned | Unassigned | Unassigned | 511_5 | Clustered/Singleton | 1 | VC_511_5 | 1 | 0 | 1 | 0 | 0 | 0 | 0 | 1 | Ref |
| AP014887 | Unassigned | Unassigned | Unassigned | 515_0 | Clustered | 8 | VC_515_0 | 8 | 0.3973 | 1 | 0.3973 | 0 | 0 | 0 | 1 | Ref |

| | | | | | | | | | | | | | | | | |
|---|---|---|---|---|---|---|---|---|---|---|---|---|---|---|---|---|
| AP014888 | Unassigned | Unassigned | Unassigned | 518_0 | Clustered | 16 | VC_518_0 | 16 | 0.9983 | 1 | 0.9983 | 0 | 0 | 0 | 1 | Ref |
| AP017924 | Unassigned | Unassigned | Unassigned | 357_0 | Clustered | 2 | VC_357_0 | 2 | 0.0674 | 0.95654162 | 0.0645 | 0 | 0 | 0 | 1 | Ref |
| AP017925 | Unassigned | Unassigned | Unassigned | 357_0 | Clustered | 2 | VC_357_0 | 2 | 0.0674 | 0.95654162 | 0.0645 | 0 | 0 | 0 | 1 | Ref |
| AP018361 | Unassigned | Unassigned | Unassigned | 279_0 | Clustered | 11 | VC_279_0 | 11 | 0.4839 | 1 | 0.4839 | 0 | 0 | 0 | 0.8727 | Ref |
| AP018375 | Unassigned | Unassigned | Unassigned | 155_9 | Clustered | 3 | VC_155_9 | 3 | 0.0083 | 0.97241045 | 0.0081 | 0 | 0 | 0 | 1 | Ref |
| AP018399 | Unassigned | Unassigned | Unassigned | 69_0 | Clustered/Singleton | 1 | VC_69_0 | 1 | 0 | 1 | 0 | 0 | 0 | 0 | 1 | Ref |
| AP018462 | Unassigned | Unassigned | Unassigned | 525_0 | Clustered | 8 | VC_525_0 | 8 | 0.443 | 1 | 0.443 | 0 | 0 | 0 | 1 | Ref |
| AP018463 | Unassigned | Unassigned | Unassigned | 525_0 | Clustered | 8 | VC_525_0 | 8 | 0.443 | 1 | 0.443 | 0 | 0 | 0 | 1 | Ref |
| AP018469 | Unassigned | Unassigned | Unassigned | 241_0 | Clustered | 21 | VC_241_0 | 21 | 0.8342 | 1 | 0.8342 | 0 | 0 | 0 | 1 | Ref |
| AP018470 | Unassigned | Unassigned | Unassigned | 241_0 | Clustered | 21 | VC_241_0 | 21 | 0.8342 | 1 | 0.8342 | 0 | 0 | 0 | 1 | Ref |
| AP018471 | Unassigned | Unassigned | Unassigned | 241_0 | Clustered | 21 | VC_241_0 | 21 | 0.8342 | 1 | 0.8342 | 0 | 0 | 0 | 1 | Ref |
| AP018476 | Unassigned | Unassigned | Unassigned | 222_1 | Clustered | 19 | VC_222_1 | 19 | 0.4166 | 1 | 0.4166 | 0 | 0 | 0 | 1 | Ref |
| AP018477 | Unassigned | Unassigned | Unassigned | 335_43 | Clustered | 4 | VC_335_43 | 4 | 0.014 | 0.99995965 | 0.014 | 0 | 0 | 0 | 1 | Ref |
| AP018478 | Unassigned | Unassigned | Unassigned | 335_43 | Clustered | 4 | VC_335_43 | 4 | 0.014 | 0.99995965 | 0.014 | 0 | 0 | 0 | 1 | Ref |
| AP018479 | Unassigned | Unassigned | Unassigned | 223_6 | Clustered | 42 | VC_223_6 | 42 | 0.4717 | 1 | 0.4717 | 0 | 0 | 0 | 0.8351 | Ref |
| AP018480 | Unassigned | Unassigned | Unassigned | 222_1 | Clustered | 19 | VC_222_1 | 19 | 0.4166 | 1 | 0.4166 | 0 | 0 | 0 | 1 | Ref |
| AP018485 | Unassigned | Unassigned | Unassigned | 531_0 | Clustered | 32 | VC_531_0 | 32 | 0.6859 | 1 | 0.6859 | 0 | 0 | 0 | 1 | Ref |
| AP018487 | Unassigned | Unassigned | Unassigned | 536_0 | Clustered | 6 | VC_536_0 | 6 | 0.129 | 1 | 0.129 | 0 | 0 | 0 | 1 | Ref |
| AP018714 | Unassigned | Unassigned | Unassigned | 93_0 | Clustered | 22 | VC_93_0 | 22 | 0.5285 | 1 | 0.5285 | 0 | 0 | 0 | 1 | Ref |
| AP018715 | Unassigned | Unassigned | Unassigned | 93_0 | Clustered | 22 | VC_93_0 | 22 | 0.5285 | 1 | 0.5285 | 0 | 0 | 0 | 1 | Ref |
| AP018813 | Unassigned | Unassigned | Unassigned | 290_92 | Clustered | 4 | VC_290_92 | 4 | 0.0078 | 0.99995066 | 0.0078 | 0 | 0 | 0 | 1 | Ref |
| AP018814 | Unassigned | Unassigned | Unassigned | 290_88 | Clustered | 2 | VC_290_88 | 2 | 0.0025 | 0.95596547 | 0.0024 | 0 | 0 | 0 | 1 | Ref |
| AP018815 | Unassigned | Unassigned | Unassigned | 80_0 | Clustered | 64 | VC_80_0 | 64 | 0.418 | 1 | 0.418 | 0 | 0 | 0 | 0.7649 | Ref |
| AP018932 | Unassigned | Unassigned | Unassigned | 290_187 | Clustered/Singleton | 1 | VC_290_187 | 1 | 0 | 1 | 0 | 0 | 0 | 0 | 1 | Ref |
| AP019417 | Unassigned | Unassigned | Unassigned | 548_0 | Clustered | 9 | VC_548_0 | 9 | 0.8018 | 1 | 0.8018 | 0 | 0 | 0 | 1 | Ref |
| AP019418 | Unassigned | Unassigned | Unassigned | 356_0 | Clustered | 21 | VC_356_0 | 21 | 0.5409 | 1 | 0.5409 | 0 | 0 | 0 | 1 | Ref |
| AP019522 | Unassigned | Unassigned | Unassigned | 159_0 | Clustered | 25 | VC_159_0 | 25 | 0.7699 | 1 | 0.7699 | 0 | 0 | 0 | 1 | Ref |
| AP019524 | Unassigned | Unassigned | Unassigned | 550_0 | Clustered | 2 | VC_550_0 | 2 | 0.4 | 0.89435023 | 0.3578 | 0 | 0 | 0 | 1 | Ref |
| AP019525 | Unassigned | Unassigned | Unassigned | 550_0 | Clustered | 2 | VC_550_0 | 2 | 0.4 | 0.89435023 | 0.3578 | 0 | 0 | 0 | 1 | Ref |
| AP019527 | Unassigned | Unassigned | Unassigned | 552_0 | Clustered | 9 | VC_552_0 | 9 | 1 | 1 | 1 | 0 | 0 | 0 | 1 | Ref |
| AP019535 | Unassigned | Unassigned | Unassigned | 80_10 | Clustered/Singleton | 1 | VC_80_10 | 1 | 0 | 1 | 0 | 0 | 0 | 0 | 1 | Ref |
| AP019559 | Unassigned | Unassigned | Unassigned | 198_17 | Clustered/Singleton | 1 | VC_198_17 | 1 | 0 | 1 | 0 | 0 | 0 | 0 | 1 | Ref |
| AP019560 | Unassigned | Unassigned | Unassigned | 11_0 | Clustered | 19 | VC_11_0 | 19 | 0.7544 | 1 | 0.7544 | 0 | 0 | 0 | 1 | Ref |
| AP019561 | Unassigned | Unassigned | Unassigned | 11_0 | Clustered | 19 | VC_11_0 | 19 | 0.7544 | 1 | 0.7544 | 0 | 0 | 0 | 1 | Ref |
| AP019562 | Unassigned | Unassigned | Unassigned | 11_0 | Clustered | 19 | VC_11_0 | 19 | 0.7544 | 1 | 0.7544 | 0 | 0 | 0 | 1 | Ref |
| AY007505 | Unassigned | Unassigned | Unassigned | 268_1 | Clustered | 20 | VC_268_1 | 20 | 0.1906 | 1 | 0.1906 | 0 | 0 | 0 | 0.6789 | Ref |
| AY027935 | Unassigned | Unassigned | Unassigned | 5_0 | Clustered | 27 | VC_5_0 | 27 | 0.9599 | 1 | 0.9599 | 0 | 0 | 0 | 1 | Ref |
| AY029185 | Unassigned | Unassigned | Unassigned | 285_0 | Clustered | 3 | VC_285_0 | 3 | 0.3109 | 0.99848226 | 0.3104 | 0 | 0 | 0 | 1 | Ref |
| AY048850 | Unassigned | Unassigned | Unassigned | 559_0 | Clustered | 3 | VC_559_0 | 3 | 1 | 1 | 1 | 0 | 0 | 0 | 1 | Ref |
| AY052766 | Unassigned | Unassigned | Unassigned | 30_1 | Clustered | 53 | VC_30_1 | 53 | 0.7151 | 1 | 0.7151 | 0 | 0 | 0 | 0.9325 | Ref |
| AY055382 | Unassigned | Unassigned | Unassigned | 47_0 | Clustered | 12 | VC_47_0 | 12 | 0.3571 | 1 | 0.3571 | 0 | 0 | 0 | 1 | Ref |
| AY078382 | Unassigned | Unassigned | Unassigned | 486_0 | Clustered | 47 | VC_486_0 | 47 | 0.9022 | 1 | 0.9022 | 0 | 0 | 0 | 1 | Ref |
| AY082070 | Unassigned | Unassigned | Unassigned | 561_0 | Clustered | 3 | VC_561_0 | 3 | 0.3411 | 0.99792547 | 0.3404 | 0 | 0 | 0 | 1 | Ref |
| AY129330 | Unassigned | Unassigned | Unassigned | 538_96 | Clustered/Singleton | 1 | VC_538_96 | 1 | 0 | 1 | 0 | 0 | 0 | 0 | 1 | Ref |
| AY129331 | Unassigned | Unassigned | Unassigned | 571_7 | Clustered | 28 | VC_571_7 | 28 | 0.1651 | 1 | 0.1651 | 0 | 0 | 0 | 0.8519 | Ref |
| AY129332 | Unassigned | Unassigned | Unassigned | 532_0 | Clustered | 75 | VC_532_0 | 75 | 0.9393 | 1 | 0.9393 | 0 | 0 | 0 | 1 | Ref |
| AY129333 | Unassigned | Unassigned | Unassigned | 536_1 | Clustered | 2 | VC_536_1 | 2 | 0.0373 | 0.95718761 | 0.0357 | 0 | 0 | 0 | 1 | Ref |
| AY129334 | Unassigned | Unassigned | Unassigned | 526_0 | Clustered | 32 | VC_526_0 | 32 | 0.9037 | 1 | 0.9037 | 0 | 0 | 0 | 1 | Ref |
| AY129335 | Unassigned | Unassigned | Unassigned | 577_0 | Clustered | 21 | VC_577_0 | 21 | 0.9908 | 1 | 0.9908 | 0 | 0 | 0 | 1 | Ref |
| AY129337 | Unassigned | Unassigned | Unassigned | 579_8 | Clustered | 2 | VC_579_8 | 2 | 0.0032 | 0.66515174 | 0.0021 | 0 | 0 | 0 | 1 | Ref |
| AY129338 | Unassigned | Unassigned | Unassigned | 566_10 | Clustered/Singleton | 1 | VC_566_10 | 1 | 0 | 1 | 0 | 0 | 0 | 0 | 1 | Ref |
| AY129339 | Unassigned | Unassigned | Unassigned | 586_0 | Clustered | 2 | VC_586_0 | 2 | 0.098 | 0.95623977 | 0.0937 | 0 | 0 | 0 | 1 | Ref |
| AY131267 | Unassigned | Unassigned | Unassigned | 279_0 | Clustered | 11 | VC_279_0 | 11 | 0.4839 | 1 | 0.4839 | 0 | 0 | 0 | 0.8727 | Ref |
| AY133112 | Unassigned | Unassigned | Unassigned | 24_0 | Clustered | 3 | VC_24_0 | 3 | 0.4232 | 0.9983762 | 0.4226 | 0 | 0 | 0 | 1 | Ref |
| AY135486 | Unassigned | Unassigned | Unassigned | 235_0 | Clustered | 60 | VC_235_0 | 60 | 0.9413 | 1 | 0.9413 | 0 | 0 | 0 | 1 | Ref |
| AY135739 | Unassigned | Unassigned | Unassigned | 235_0 | Clustered | 60 | VC_235_0 | 60 | 0.9413 | 1 | 0.9413 | 0 | 0 | 0 | 1 | Ref |
| AY150271 | Unassigned | Unassigned | Unassigned | 492_0 | Clustered | 20 | VC_492_0 | 20 | 0.7887 | 1 | 0.7887 | 0 | 0 | 0 | 1 | Ref |
| AY176327 | Unassigned | Unassigned | Unassigned | 155_8 | Clustered | 66 | VC_155_8 | 66 | 0.4537 | 1 | 0.4537 | 0 | 0 | 0 | 0.8182 | Ref |
| AY190604 | Unassigned | Unassigned | Unassigned | 317_0 | Clustered | 33 | VC_317_0 | 33 | 0.8756 | 1 | 0.8756 | 0 | 0 | 0 | 1 | Ref |
| AY212251 | Unassigned | Unassigned | Unassigned | 99_0 | Clustered | 5 | VC_99_0 | 5 | 0.0694 | 0.98113835 | 0.0681 | 0 | 0 | 0 | 1 | Ref |
| AY216660 | Unassigned | Unassigned | Unassigned | 595_0 | Clustered | 35 | VC_595_0 | 35 | 0.8809 | 1 | 0.8809 | 0 | 0 | 0 | 1 | Ref |
| AY236756 | Unassigned | Unassigned | Unassigned | 597_0 | Clustered | 6 | VC_597_0 | 6 | 0.9222 | 0.99999993 | 0.9222 | 0 | 0 | 0 | 1 | Ref |
| AY242528 | Unassigned | Unassigned | Unassigned | 16_0 | Clustered | 12 | VC_16_0 | 12 | 0.6601 | 1 | 0.6601 | 0 | 0 | 0 | 1 | Ref |
| AY247822 | Unassigned | Unassigned | Unassigned | 405_0 | Clustered | 126 | VC_405_0 | 126 | 0.9614 | 1 | 0.9614 | 0 | 0 | 0 | 1 | Ref |
| AY251033 | Unassigned | Unassigned | Unassigned | 235_0 | Clustered | 60 | VC_235_0 | 60 | 0.9413 | 1 | 0.9413 | 0 | 0 | 0 | 1 | Ref |
| AY257527 | Unassigned | Unassigned | Unassigned | 435_0 | Clustered | 7 | VC_435_0 | 7 | 0.9041 | 0.9998143 | 0.9039 | 0 | 0 | 0 | 1 | Ref |
| AY264774 | Unassigned | Unassigned | Unassigned | 405_0 | Clustered | 126 | VC_405_0 | 126 | 0.9614 | 1 | 0.9614 | 0 | 0 | 0 | 1 | Ref |
| AY264775 | Unassigned | Unassigned | Unassigned | 405_0 | Clustered | 126 | VC_405_0 | 126 | 0.9614 | 1 | 0.9614 | 0 | 0 | 0 | 1 | Ref |
| AY264776 | Unassigned | Unassigned | Unassigned | 405_0 | Clustered | 126 | VC_405_0 | 126 | 0.9614 | 1 | 0.9614 | 0 | 0 | 0 | 1 | Ref |
| AY264777 | Unassigned | Unassigned | Unassigned | 405_0 | Clustered | 126 | VC_405_0 | 126 | 0.9614 | 1 | 0.9614 | 0 | 0 | 0 | 1 | Ref |
| AY264778 | Unassigned | Unassigned | Unassigned | 405_0 | Clustered | 126 | VC_405_0 | 126 | 0.9614 | 1 | 0.9614 | 0 | 0 | 0 | 1 | Ref |
| AY266303 | Unassigned | Unassigned | Unassigned | 457_0 | Clustered | 7 | VC_457_0 | 7 | 0.2549 | 1 | 0.2549 | 0 | 0 | 0 | 0.7143 | Ref |
| AY283928 | Unassigned | Unassigned | Unassigned | 549_1 | Clustered | 6 | VC_549_1 | 6 | 0.1479 | 0.9998319 | 0.1478 | 0 | 0 | 0 | 1 | Ref |
| AY288927 | Unassigned | Unassigned | Unassigned | 466_0 | Clustered | 66 | VC_466_0 | 66 | 0.9246 | 1 | 0.9246 | 0 | 0 | 0 | 1 | Ref |
| AY299121 | Unassigned | Unassigned | Unassigned | 115_0 | Clustered | 9 | VC_115_0 | 9 | 0.5166 | 1 | 0.5166 | 0 | 0 | 0 | 1 | Ref |
| AY303349 | Unassigned | Unassigned | Unassigned | 293_10 | Clustered | 9 | VC_293_10 | 9 | 0.0615 | 0.99999884 | 0.0615 | 0 | 0 | 0 | 0.6667 | Ref |
| AY308796 | Unassigned | Unassigned | Unassigned | 603_0 | Clustered | 28 | VC_603_0 | 28 | 0.7692 | 1 | 0.7692 | 0 | 0 | 0 | 1 | Ref |
| AY319521 | Unassigned | Unassigned | Unassigned | 6_0 | Clustered | 44 | VC_6_0 | 44 | 0.9068 | 1 | 0.9068 | 0 | 0 | 0 | 1 | Ref |
| AY320035 | Unassigned | Unassigned | Unassigned | 606_0 | Clustered | 2 | VC_606_0 | 2 | 0.3086 | 0.91192888 | 0.2814 | 0 | 0 | 0 | 1 | Ref |
| AY324828 | Unassigned | Unassigned | Unassigned | 608_0 | Clustered | 2 | VC_608_0 | 2 | 1 | 1 | 1 | 0 | 0 | 0 | 1 | Ref |
| AY328852 | Unassigned | Unassigned | Unassigned | 515_0 | Clustered | 8 | VC_515_0 | 8 | 0.3973 | 1 | 0.3973 | 0 | 0 | 0 | 1 | Ref |

| | | | | | | | | | | | | | | | | |
|---|---|---|---|---|---|---|---|---|---|---|---|---|---|---|---|---|
| AY328853 | Unassigned | Unassigned | Unassigned | 515_0 | Clustered | 8 | VC_515_0 | 8 | 0.3973 | 1 | 0.3973 | 0 | 0 | 0 | 1 | Ref |
| AY343333 | Unassigned | Unassigned | Unassigned | 609_0 | Clustered | 27 | VC_609_0 | 27 | 0.9943 | 1 | 0.9943 | 0 | 0 | 0 | 1 | Ref |
| AY349011 | Unassigned | Unassigned | Unassigned | 168_0 | Clustered | 18 | VC_168_0 | 18 | 0.7394 | 1 | 0.7394 | 0 | 0 | 0 | 0.8627 | Ref |
| AY370673 | Unassigned | Unassigned | Unassigned | 466_0 | Clustered | 66 | VC_466_0 | 66 | 0.9246 | 1 | 0.9246 | 0 | 0 | 0 | 1 | Ref |
| AY370674 | Unassigned | Unassigned | Unassigned | 466_0 | Clustered | 66 | VC_466_0 | 66 | 0.9246 | 1 | 0.9246 | 0 | 0 | 0 | 1 | Ref |
| AY370762 | Unassigned | Unassigned | Unassigned | 618_0 | Clustered | 50 | VC_618_0 | 50 | 0.9989 | 1 | 0.9989 | 0 | 0 | 0 | 1 | Ref |
| AY374448 | Unassigned | Unassigned | Unassigned | 37_0 | Clustered | 4 | VC_37_0 | 4 | 0.4325 | 0.99998062 | 0.4325 | 0 | 0 | 0 | 1 | Ref |
| AY375531 | Unassigned | Unassigned | Unassigned | 619_1 | Clustered | 7 | VC_619_1 | 7 | 0.1834 | 0.99999997 | 0.1834 | 0 | 0 | 0 | 1 | Ref |
| AY388628 | Unassigned | Unassigned | Unassigned | 618_0 | Clustered | 50 | VC_618_0 | 50 | 0.9989 | 1 | 0.9989 | 0 | 0 | 0 | 1 | Ref |
| AY394005 | Unassigned | Unassigned | Unassigned | 185_0 | Clustered | 53 | VC_185_0 | 53 | 0.9153 | 1 | 0.9153 | 0 | 0 | 0 | 1 | Ref |
| AY423772 | Unassigned | Unassigned | Unassigned | 618_0 | Clustered | 50 | VC_618_0 | 50 | 0.9989 | 1 | 0.9989 | 0 | 0 | 0 | 1 | Ref |
| AY441783 | Unassigned | Unassigned | Unassigned | 622_0 | Clustered | 9 | VC_622_0 | 9 | 0.9922 | 0.99999994 | 0.9922 | 0 | 0 | 0 | 1 | Ref |
| AY453853 | Unassigned | Unassigned | Unassigned | 365_0 | Clustered | 6 | VC_365_0 | 6 | 0.5395 | 1 | 0.5395 | 0 | 0 | 0 | 1 | Ref |
| AY500152 | Unassigned | Unassigned | Unassigned | 335_5 | Clustered | 11 | VC_335_5 | 11 | 0.0469 | 1 | 0.0469 | 0 | 0 | 0 | 0.7636 | Ref |
| AY500153 | Unassigned | Unassigned | Unassigned | 335_9 | Clustered | 2 | VC_335_9 | 2 | 0.0055 | 0.95750934 | 0.0053 | 0 | 0 | 0 | 1 | Ref |
| AY505112 | Unassigned | Unassigned | Unassigned | 627_0 | Clustered | 16 | VC_627_0 | 16 | 0.9168 | 1 | 0.9168 | 0 | 0 | 0 | 1 | Ref |
| AY508486 | Unassigned | Unassigned | Unassigned | 10_0 | Clustered | 25 | VC_10_0 | 25 | 0.3437 | 1 | 0.3437 | 0 | 0 | 0 | 0.9733 | Ref |
| AY510084 | Unassigned | Unassigned | Unassigned | 627_0 | Clustered | 16 | VC_627_0 | 16 | 0.9168 | 1 | 0.9168 | 0 | 0 | 0 | 1 | Ref |
| AY526908 | Unassigned | Unassigned | Unassigned | 285_0 | Clustered | 3 | VC_285_0 | 3 | 0.3109 | 0.99848226 | 0.3104 | 0 | 0 | 0 | 1 | Ref |
| AY526909 | Unassigned | Unassigned | Unassigned | 285_0 | Clustered | 3 | VC_285_0 | 3 | 0.3109 | 0.99848226 | 0.3104 | 0 | 0 | 0 | 1 | Ref |
| AY539836 | Unassigned | Unassigned | Unassigned | 257_0 | Clustered | 3 | VC_257_0 | 3 | 0.2099 | 0.99831999 | 0.2095 | 0 | 0 | 0 | 1 | Ref |
| AY543070 | Unassigned | Unassigned | Unassigned | 198_38 | Clustered | 3 | VC_198_38 | 3 | 0.0172 | 0.99845083 | 0.0172 | 0 | 0 | 0 | 1 | Ref |
| AY569307 | Unassigned | Unassigned | Unassigned | 631_0 | Clustered | 2 | VC_631_0 | 2 | 1 | 1 | 1 | 0 | 0 | 0 | 1 | Ref |
| AY575774 | Unassigned | Unassigned | Unassigned | 632_0 | Clustered | 9 | VC_632_0 | 9 | 0.9524 | 1 | 0.9524 | 0 | 0 | 0 | 1 | Ref |
| AY583234 | Unassigned | Unassigned | Unassigned | 253_0 | Clustered | 3 | VC_253_0 | 3 | 0.7619 | 0.9595722 | 0.7311 | 0 | 0 | 0 | 1 | Ref |
| AY583235 | Unassigned | Unassigned | Unassigned | 253_0 | Clustered | 3 | VC_253_0 | 3 | 0.7619 | 0.9595722 | 0.7311 | 0 | 0 | 0 | 1 | Ref |
| AY583236 | Unassigned | Unassigned | Unassigned | 253_0 | Clustered | 3 | VC_253_0 | 3 | 0.7619 | 0.9595722 | 0.7311 | 0 | 0 | 0 | 1 | Ref |
| AY587007 | Unassigned | Unassigned | Unassigned | 198_38 | Clustered | 3 | VC_198_38 | 3 | 0.0172 | 0.99845083 | 0.0172 | 0 | 0 | 0 | 1 | Ref |
| AY605066 | Unassigned | Unassigned | Unassigned | 279_0 | Clustered | 11 | VC_279_0 | 11 | 0.4839 | 1 | 0.4839 | 0 | 0 | 0 | 0.8727 | Ref |
| AY605181 | Unassigned | Unassigned | Unassigned | 286_0 | Clustered | 2 | VC_286_0 | 2 | 0.1926 | 0.95154944 | 0.1832 | 0 | 0 | 0 | 1 | Ref |
| AY625898 | Unassigned | Unassigned | Unassigned | 645_0 | Clustered | 20 | VC_645_0 | 20 | 0.8502 | 1 | 0.8502 | 0 | 0 | 0 | 1 | Ref |
| AY682195 | Unassigned | Unassigned | Unassigned | 544_2 | Clustered/Singleton | 1 | VC_544_2 | 1 | 0 | 1 | 0 | 0 | 0 | 0 | 1 | Ref |
| AY692264 | Unassigned | Unassigned | Unassigned | 198_38 | Clustered | 3 | VC_198_38 | 3 | 0.0172 | 0.99845083 | 0.0172 | 0 | 0 | 0 | 1 | Ref |
| AY699705 | Unassigned | Unassigned | Unassigned | 260_0 | Clustered | 46 | VC_260_0 | 46 | 0.5137 | 1 | 0.5137 | 0 | 0 | 0 | 1 | Ref |
| AY701338 | Unassigned | Unassigned | Unassigned | 435_0 | Clustered | 7 | VC_435_0 | 7 | 0.9041 | 0.9998143 | 0.9039 | 0 | 0 | 0 | 1 | Ref |
| AY714348 | Unassigned | Unassigned | Unassigned | 27_0 | Clustered | 4 | VC_27_0 | 4 | 0.7397 | 0.99936153 | 0.7392 | 0 | 0 | 0 | 1 | Ref |
| AY722806 | Unassigned | Unassigned | Unassigned | 454_0 | Clustered | 4 | VC_454_0 | 4 | 1 | 1 | 1 | 0 | 0 | 0 | 1 | Ref |
| AY730274 | Unassigned | Unassigned | Unassigned | 654_0 | Clustered | 115 | VC_654_0 | 115 | 0.7303 | 1 | 0.7303 | 0 | 0 | 0 | 0.8764 | Ref |
| AY739900 | Unassigned | Unassigned | Unassigned | 657_0 | Clustered | 7 | VC_657_0 | 7 | 0.8495 | 1 | 0.8495 | 0 | 0 | 0 | 1 | Ref |
| AY751298 | Unassigned | Unassigned | Unassigned | 373_0 | Clustered | 358 | VC_373_0 | 358 | 0.9614 | 1 | 0.9614 | 0 | 0 | 0 | 1 | Ref |
| AY769964 | Unassigned | Unassigned | Unassigned | 206_0 | Clustered | 28 | VC_206_0 | 28 | 0.2427 | 0.99971175 | 0.2426 | 0 | 0 | 0 | 1 | Ref |
| AY772740 | Unassigned | Unassigned | Unassigned | 661_2 | Clustered | 6 | VC_661_2 | 6 | 0.1674 | 1 | 0.1674 | 0 | 0 | 0 | 1 | Ref |
| AY848684 | Unassigned | Unassigned | Unassigned | 622_0 | Clustered | 9 | VC_622_0 | 9 | 0.9922 | 0.99999994 | 0.9922 | 0 | 0 | 0 | 1 | Ref |
| AY848685 | Unassigned | Unassigned | Unassigned | 622_0 | Clustered | 9 | VC_622_0 | 9 | 0.9922 | 0.99999994 | 0.9922 | 0 | 0 | 0 | 1 | Ref |
| AY848686 | Unassigned | Unassigned | Unassigned | 622_0 | Clustered | 9 | VC_622_0 | 9 | 0.9922 | 0.99999994 | 0.9922 | 0 | 0 | 0 | 1 | Ref |
| AY848687 | Unassigned | Unassigned | Unassigned | 622_0 | Clustered | 9 | VC_622_0 | 9 | 0.9922 | 0.99999994 | 0.9922 | 0 | 0 | 0 | 1 | Ref |
| AY848688 | Unassigned | Unassigned | Unassigned | 622_0 | Clustered | 9 | VC_622_0 | 9 | 0.9922 | 0.99999994 | 0.9922 | 0 | 0 | 0 | 1 | Ref |
| AY848689 | Unassigned | Unassigned | Unassigned | 622_0 | Clustered | 9 | VC_622_0 | 9 | 0.9922 | 0.99999994 | 0.9922 | 0 | 0 | 0 | 1 | Ref |
| AY855346 | Unassigned | Unassigned | Unassigned | 140_0 | Clustered | 2 | VC_140_0 | 2 | 0.067 | 0.95523577 | 0.064 | 0 | 0 | 0 | 1 | Ref |
| AY939843 | Unassigned | Unassigned | Unassigned | 352_0 | Clustered | 31 | VC_352_0 | 31 | 0.9547 | 1 | 0.9547 | 0 | 0 | 0 | 1 | Ref |
| AY939844 | Unassigned | Unassigned | Unassigned | 446_9 | Clustered | 3 | VC_446_9 | 3 | 0.0365 | 0.99848173 | 0.0364 | 0 | 0 | 0 | 1 | Ref |
| AY940168 | Unassigned | Unassigned | Unassigned | 674_2 | Clustered | 3 | VC_674_2 | 3 | 0.0227 | 0.99860756 | 0.0227 | 0 | 0 | 0 | 1 | Ref |
| AY950802 | Unassigned | Unassigned | Unassigned | 684_0 | Clustered | 4 | VC_684_0 | 4 | 1 | 1 | 1 | 0 | 0 | 0 | 1 | Ref |
| AY954949 | Unassigned | Unassigned | Unassigned | 98_0 | Clustered | 30 | VC_98_0 | 30 | 0.7175 | 1 | 0.7175 | 0 | 0 | 0 | 1 | Ref |
| AY954951 | Unassigned | Unassigned | Unassigned | 9_0 | Clustered | 38 | VC_9_0 | 38 | 0.4859 | 1 | 0.4859 | 0 | 0 | 0 | 1 | Ref |
| AY954952 | Unassigned | Unassigned | Unassigned | 9_0 | Clustered | 38 | VC_9_0 | 38 | 0.4859 | 1 | 0.4859 | 0 | 0 | 0 | 1 | Ref |
| AY954953 | Unassigned | Unassigned | Unassigned | 9_0 | Clustered | 38 | VC_9_0 | 38 | 0.4859 | 1 | 0.4859 | 0 | 0 | 0 | 1 | Ref |
| AY954954 | Unassigned | Unassigned | Unassigned | 15_1 | Clustered | 9 | VC_15_1 | 9 | 0.1817 | 1 | 0.1817 | 0 | 0 | 0 | 0.9167 | Ref |
| AY954955 | Unassigned | Unassigned | Unassigned | 8_0 | Clustered | 58 | VC_8_0 | 58 | 0.8285 | 1 | 0.8285 | 0 | 0 | 0 | 0.9946 | Ref |
| AY954956 | Unassigned | Unassigned | Unassigned | 8_0 | Clustered | 58 | VC_8_0 | 58 | 0.8285 | 1 | 0.8285 | 0 | 0 | 0 | 0.9946 | Ref |
| AY954957 | Unassigned | Unassigned | Unassigned | 8_0 | Clustered | 58 | VC_8_0 | 58 | 0.8285 | 1 | 0.8285 | 0 | 0 | 0 | 0.9946 | Ref |
| AY954960 | Unassigned | Unassigned | Unassigned | 12_0 | Clustered | 21 | VC_12_0 | 21 | 0.2262 | 1 | 0.2262 | 0 | 0 | 0 | 0.9571 | Ref |
| AY954961 | Unassigned | Unassigned | Unassigned | 12_0 | Clustered | 21 | VC_12_0 | 21 | 0.2262 | 1 | 0.2262 | 0 | 0 | 0 | 0.9571 | Ref |
| AY954962 | Unassigned | Unassigned | Unassigned | 12_2 | Clustered | 19 | VC_12_2 | 19 | 0.2009 | 1 | 0.2009 | 0 | 0 | 0 | 1 | Ref |
| AY954963 | Unassigned | Unassigned | Unassigned | 12_2 | Clustered | 19 | VC_12_2 | 19 | 0.2009 | 1 | 0.2009 | 0 | 0 | 0 | 1 | Ref |
| AY954964 | Unassigned | Unassigned | Unassigned | 12_2 | Clustered | 19 | VC_12_2 | 19 | 0.2009 | 1 | 0.2009 | 0 | 0 | 0 | 1 | Ref |
| AY954965 | Unassigned | Unassigned | Unassigned | 12_2 | Clustered | 19 | VC_12_2 | 19 | 0.2009 | 1 | 0.2009 | 0 | 0 | 0 | 1 | Ref |
| AY954966 | Unassigned | Unassigned | Unassigned | 12_2 | Clustered | 19 | VC_12_2 | 19 | 0.2009 | 1 | 0.2009 | 0 | 0 | 0 | 1 | Ref |
| AY954967 | Unassigned | Unassigned | Unassigned | 12_2 | Clustered | 19 | VC_12_2 | 19 | 0.2009 | 1 | 0.2009 | 0 | 0 | 0 | 1 | Ref |
| AY954968 | Unassigned | Unassigned | Unassigned | 12_2 | Clustered | 19 | VC_12_2 | 19 | 0.2009 | 1 | 0.2009 | 0 | 0 | 0 | 1 | Ref |
| AY954969 | Unassigned | Unassigned | Unassigned | 155_8 | Clustered | 66 | VC_155_8 | 66 | 0.4537 | 1 | 0.4537 | 0 | 0 | 0 | 0.8182 | Ref |
| AY962392 | Unassigned | Unassigned | Unassigned | 619_1 | Clustered | 7 | VC_619_1 | 7 | 0.1834 | 0.99999997 | 0.1834 | 0 | 0 | 0 | 1 | Ref |
| AY967407 | Unassigned | Unassigned | Unassigned | 687_1 | Clustered | 10 | VC_687_1 | 10 | 0.1263 | 1 | 0.1263 | 0 | 0 | 0 | 0.9778 | Ref |
| B1U32222 | Unassigned | Unassigned | Unassigned | 235_0 | Clustered | 60 | VC_235_0 | 60 | 0.9413 | 1 | 0.9413 | 0 | 0 | 0 | 1 | Ref |
| BCP1 | Unassigned | Unassigned | Unassigned | 690_0 | Clustered | 12 | VC_690_0 | 12 | 1 | 1 | 1 | 0 | 0 | 0 | 1 | Ref |
| BHU24159 | Unassigned | Unassigned | Unassigned | 5_0 | Clustered | 27 | VC_5_0 | 27 | 0.9599 | 1 | 0.9599 | 0 | 0 | 0 | 1 | Ref |
| BK000583 | Unassigned | Unassigned | Unassigned | 30_1 | Clustered | 53 | VC_30_1 | 53 | 0.7151 | 1 | 0.7151 | 0 | 0 | 0 | 0.9325 | Ref |
| BK010471 | Unassigned | Unassigned | Unassigned | 691_0 | Clustered | 18 | VC_691_0 | 18 | 0.6865 | 1 | 0.6865 | 0 | 0 | 0 | 0.9477 | Ref |
| BK010646 | Unassigned | Unassigned | Unassigned | 695_0 | Clustered | 17 | VC_695_0 | 17 | 0.7188 | 1 | 0.7188 | 0 | 0 | 0 | 0.9044 | Ref |
| BK013337 | Unassigned | Unassigned | Unassigned | 559_0 | Clustered | 3 | VC_559_0 | 3 | 1 | 1 | 1 | 0 | 0 | 0 | 1 | Ref |
| BK013338 | Unassigned | Unassigned | Unassigned | 559_0 | Clustered | 3 | VC_559_0 | 3 | 1 | 1 | 1 | 0 | 0 | 0 | 1 | Ref |

| | | | | | | | | | | | | | | | | |
|---|---|---|---|---|---|---|---|---|---|---|---|---|---|---|---|---|
| BK013345 | Unassigned | Unassigned | Unassigned | 697_0 | Clustered | 3 | VC_697_0 | 3 | 0.9312 | 0.81445332 | 0.7584 | 0 | 0 | 0 | 1 | Ref |
| BK013346 | Unassigned | Unassigned | Unassigned | 697_0 | Clustered | 3 | VC_697_0 | 3 | 0.9312 | 0.81445332 | 0.7584 | 0 | 0 | 0 | 1 | Ref |
| BK013347 | Unassigned | Unassigned | Unassigned | 697_0 | Clustered | 3 | VC_697_0 | 3 | 0.9312 | 0.81445332 | 0.7584 | 0 | 0 | 0 | 1 | Ref |
| BK059895 | Unassigned | Unassigned | Unassigned | 698_0 | Clustered | 2 | VC_698_0 | 2 | 1 | 1 | 1 | 0 | 0 | 0 | 1 | Ref |
| BK061308 | Unassigned | Unassigned | Unassigned | 699_0 | Clustered | 5 | VC_699_0 | 5 | 0.8925 | 0.99999977 | 0.8925 | 0 | 0 | 0 | 1 | Ref |
| BK061309 | Unassigned | Unassigned | Unassigned | 699_0 | Clustered | 5 | VC_699_0 | 5 | 0.8925 | 0.99999977 | 0.8925 | 0 | 0 | 0 | 1 | Ref |
| BK061310 | Unassigned | Unassigned | Unassigned | 699_0 | Clustered | 5 | VC_699_0 | 5 | 0.8925 | 0.99999977 | 0.8925 | 0 | 0 | 0 | 1 | Ref |
| BK061311 | Unassigned | Unassigned | Unassigned | 699_0 | Clustered | 5 | VC_699_0 | 5 | 0.8925 | 0.99999977 | 0.8925 | 0 | 0 | 0 | 1 | Ref |
| BK061312 | Unassigned | Unassigned | Unassigned | 699_0 | Clustered | 5 | VC_699_0 | 5 | 0.8925 | 0.99999977 | 0.8925 | 0 | 0 | 0 | 1 | Ref |
| C2PVCG | Unassigned | Unassigned | Unassigned | 706_0 | Clustered | 52 | VC_706_0 | 52 | 0.9787 | 1 | 0.9787 | 0 | 0 | 0 | 1 | Ref |
| CAJCJZ010000002 | Unassigned | Unassigned | Unassigned | 707_1 | Clustered | 5 | VC_707_1 | 5 | 0.4294 | 0.99999994 | 0.4294 | 0 | 0 | 0 | 1 | Ref |
| CAJDJX010000002 | Unassigned | Unassigned | Unassigned | 711_0 | Clustered | 48 | VC_711_0 | 48 | 0.8217 | 1 | 0.8217 | 0 | 0 | 0 | 1 | Ref |
| CAJDJY010000002 | Unassigned | Unassigned | Unassigned | 93_0 | Clustered | 22 | VC_93_0 | 22 | 0.5285 | 1 | 0.5285 | 0 | 0 | 0 | 1 | Ref |
| CAJDJZ010000002 | Unassigned | Unassigned | Unassigned | 93_0 | Clustered | 22 | VC_93_0 | 22 | 0.5285 | 1 | 0.5285 | 0 | 0 | 0 | 1 | Ref |
| CAJDKA010000002 | Unassigned | Unassigned | Unassigned | 94_0 | Clustered | 22 | VC_94_0 | 22 | 0.5602 | 1 | 0.5602 | 0 | 0 | 0 | 1 | Ref |
| CAJDKF010000002 | Unassigned | Unassigned | Unassigned | 711_0 | Clustered | 48 | VC_711_0 | 48 | 0.8217 | 1 | 0.8217 | 0 | 0 | 0 | 1 | Ref |
| CAKLQA020000001 | Unassigned | Unassigned | Unassigned | 441_1 | Clustered | 8 | VC_441_1 | 8 | 0.5443 | 1 | 0.5443 | 0 | 0 | 0 | 0.9643 | Ref |
| CAKLQA020000002 | Unassigned | Unassigned | Unassigned | 688_0 | Clustered | 2 | VC_688_0 | 2 | 0.6866 | 0.72985431 | 0.5011 | 0 | 0 | 0 | 1 | Ref |
| CAKLQA020000003 | Unassigned | Unassigned | Unassigned | 634_0 | Clustered | 2 | VC_634_0 | 2 | 0.2597 | 0.94405832 | 0.2451 | 0 | 0 | 0 | 1 | Ref |
| CAKLQB020000001 | Unassigned | Unassigned | Unassigned | 714_0 | Clustered | 71 | VC_714_0 | 71 | 0.935 | 1 | 0.935 | 0 | 0 | 0 | 1 | Ref |
| CAKLQC020000001 | Unassigned | Unassigned | Unassigned | 510_1 | Clustered | 20 | VC_510_1 | 20 | 0.4172 | 1 | 0.4172 | 0 | 0 | 0 | 1 | Ref |
| CAKLQD020000001 | Unassigned | Unassigned | Unassigned | 510_1 | Clustered | 20 | VC_510_1 | 20 | 0.4172 | 1 | 0.4172 | 0 | 0 | 0 | 1 | Ref |
| CAKLQE020000001 | Unassigned | Unassigned | Unassigned | 716_2 | Clustered/Singleton | 1 | VC_716_2 | 1 | 0 | 1 | 0 | 0 | 0 | 0 | 1 | Ref |
| CAKLQG020000001 | Unassigned | Unassigned | Unassigned | 714_0 | Clustered | 71 | VC_714_0 | 71 | 0.935 | 1 | 0.935 | 0 | 0 | 0 | 1 | Ref |
| CMU41758 | Unassigned | Unassigned | Unassigned | 205_0 | Clustered | 5 | VC_205_0 | 5 | 0.1325 | 0.84698401 | 0.1122 | 0 | 0 | 0 | 1 | Ref |
| CP000622 | Unassigned | Unassigned | Unassigned | 257_0 | Clustered | 3 | VC_257_0 | 3 | 0.2099 | 0.99831999 | 0.2095 | 0 | 0 | 0 | 1 | Ref |
| CP000623 | Unassigned | Unassigned | Unassigned | 3_0 | Clustered | 17 | VC_3_0 | 17 | 0.5615 | 1 | 0.5615 | 0 | 0 | 0 | 1 | Ref |
| CP000624 | Unassigned | Unassigned | Unassigned | 3_0 | Clustered | 17 | VC_3_0 | 17 | 0.5615 | 1 | 0.5615 | 0 | 0 | 0 | 1 | Ref |
| CP000625 | Unassigned | Unassigned | Unassigned | 365_0 | Clustered | 6 | VC_365_0 | 6 | 0.5395 | 1 | 0.5395 | 0 | 0 | 0 | 1 | Ref |
| CP000711 | Unassigned | Unassigned | Unassigned | 32_1 | Clustered | 16 | VC_32_1 | 16 | 0.1962 | 1 | 0.1962 | 0 | 0 | 0 | 0.8333 | Ref |
| CP000917 | Unassigned | Unassigned | Unassigned | 199_17 | Clustered | 4 | VC_199_17 | 4 | 0.0165 | 0.99893269 | 0.0165 | 0 | 0 | 0 | 0.8333 | Ref |
| CP002495 | Unassigned | Unassigned | Unassigned | 722_0 | Clustered | 2 | VC_722_0 | 2 | 1 | 1 | 1 | 0 | 0 | 0 | 1 | Ref |
| CP004084 | Unassigned | Unassigned | Unassigned | 310_0 | Clustered | 155 | VC_310_0 | 155 | 0.9979 | 1 | 0.9979 | 0 | 0 | 0 | 1 | Ref |
| CP006891 | Unassigned | Unassigned | Unassigned | 57_0 | Clustered | 3 | VC_57_0 | 3 | 0.2761 | 0.99850726 | 0.2757 | 0 | 0 | 0 | 1 | Ref |
| CP008753 | Unassigned | Unassigned | Unassigned | 3_0 | Clustered | 17 | VC_3_0 | 17 | 0.5615 | 1 | 0.5615 | 0 | 0 | 0 | 1 | Ref |
| CP011970 | Unassigned | Unassigned | Unassigned | 487_2 | Clustered | 2 | VC_487_2 | 2 | 0.1024 | 0.94551181 | 0.0968 | 0 | 0 | 0 | 1 | Ref |
| CP013282 | Unassigned | Unassigned | Unassigned | 435_0 | Clustered | 7 | VC_435_0 | 7 | 0.9041 | 0.9998143 | 0.9039 | 0 | 0 | 0 | 1 | Ref |
| CP013974 | Unassigned | Unassigned | Unassigned | 437_0 | Clustered | 3 | VC_437_0 | 3 | 0.107 | 0.99803965 | 0.1068 | 0 | 0 | 0 | 1 | Ref |
| CP017905 | Unassigned | Unassigned | Unassigned | 16_0 | Clustered | 12 | VC_16_0 | 12 | 0.6601 | 1 | 0.6601 | 0 | 0 | 0 | 1 | Ref |
| CP017906 | Unassigned | Unassigned | Unassigned | 16_0 | Clustered | 12 | VC_16_0 | 12 | 0.6601 | 1 | 0.6601 | 0 | 0 | 0 | 1 | Ref |
| CP019275 | Unassigned | Unassigned | Unassigned | 6_0 | Clustered | 44 | VC_6_0 | 44 | 0.9068 | 1 | 0.9068 | 0 | 0 | 0 | 1 | Ref |
| CP023680 | Unassigned | Unassigned | Unassigned | 57_0 | Clustered | 3 | VC_57_0 | 3 | 0.2761 | 0.99850726 | 0.2757 | 0 | 0 | 0 | 1 | Ref |
| CP023686 | Unassigned | Unassigned | Unassigned | 57_0 | Clustered | 3 | VC_57_0 | 3 | 0.2761 | 0.99850726 | 0.2757 | 0 | 0 | 0 | 1 | Ref |
| CP025874 | Unassigned | Unassigned | Unassigned | 236_0 | Clustered | 32 | VC_236_0 | 32 | 0.9115 | 1 | 0.9115 | 0 | 0 | 0 | 1 | Ref |
| CP025900 | Unassigned | Unassigned | Unassigned | 6_0 | Clustered | 44 | VC_6_0 | 44 | 0.9068 | 1 | 0.9068 | 0 | 0 | 0 | 1 | Ref |
| CP025901 | Unassigned | Unassigned | Unassigned | 236_0 | Clustered | 32 | VC_236_0 | 32 | 0.9115 | 1 | 0.9115 | 0 | 0 | 0 | 1 | Ref |
| CP025923 | Unassigned | Unassigned | Unassigned | 236_0 | Clustered | 32 | VC_236_0 | 32 | 0.9115 | 1 | 0.9115 | 0 | 0 | 0 | 1 | Ref |
| CP027117 | Unassigned | Unassigned | Unassigned | 735_1 | Clustered/Singleton | 1 | VC_735_1 | 1 | 0 | 1 | 0 | 0 | 0 | 0 | 1 | Ref |
| CP038007 | Unassigned | Unassigned | Unassigned | 37_0 | Clustered | 4 | VC_37_0 | 4 | 0.4325 | 0.99998062 | 0.4325 | 0 | 0 | 0 | 1 | Ref |
| CP045566 | Unassigned | Unassigned | Unassigned | 736_0 | Clustered | 9 | VC_736_0 | 9 | 1 | 1 | 1 | 0 | 0 | 0 | 1 | Ref |
| CP051272 | Unassigned | Unassigned | Unassigned | 30_1 | Clustered | 53 | VC_30_1 | 53 | 0.7151 | 1 | 0.7151 | 0 | 0 | 0 | 0.9325 | Ref |
| CP051278 | Unassigned | Unassigned | Unassigned | 30_1 | Clustered | 53 | VC_30_1 | 53 | 0.7151 | 1 | 0.7151 | 0 | 0 | 0 | 0.9325 | Ref |
| CP051279 | Unassigned | Unassigned | Unassigned | 30_1 | Clustered | 53 | VC_30_1 | 53 | 0.7151 | 1 | 0.7151 | 0 | 0 | 0 | 0.9325 | Ref |
| CP051285 | Unassigned | Unassigned | Unassigned | 30_1 | Clustered | 53 | VC_30_1 | 53 | 0.7151 | 1 | 0.7151 | 0 | 0 | 0 | 0.9325 | Ref |
| CP051288 | Unassigned | Unassigned | Unassigned | 30_1 | Clustered | 53 | VC_30_1 | 53 | 0.7151 | 1 | 0.7151 | 0 | 0 | 0 | 0.9325 | Ref |
| CP052639 | Unassigned | Unassigned | Unassigned | 236_0 | Clustered | 32 | VC_236_0 | 32 | 0.9115 | 1 | 0.9115 | 0 | 0 | 0 | 1 | Ref |
| CP053388 | Unassigned | Unassigned | Unassigned | 738_0 | Clustered | 9 | VC_738_0 | 9 | 0.7116 | 1 | 0.7116 | 0 | 0 | 0 | 1 | Ref |
| CP054387 | Unassigned | Unassigned | Unassigned | 738_0 | Clustered | 9 | VC_738_0 | 9 | 0.7116 | 1 | 0.7116 | 0 | 0 | 0 | 1 | Ref |
| CP058330 | Unassigned | Unassigned | Unassigned | 738_0 | Clustered | 9 | VC_738_0 | 9 | 0.7116 | 1 | 0.7116 | 0 | 0 | 0 | 1 | Ref |
| CP059058 | Unassigned | Unassigned | Unassigned | 610_0 | Clustered | 14 | VC_610_0 | 14 | 0.5159 | 1 | 0.5159 | 0 | 0 | 0 | 1 | Ref |
| CP060690 | Unassigned | Unassigned | Unassigned | 745_0 | Clustered | 9 | VC_745_0 | 9 | 0.9955 | 0.99999771 | 0.9955 | 0 | 0 | 0 | 1 | Ref |
| CP062422 | Unassigned | Unassigned | Unassigned | 12_0 | Clustered | 21 | VC_12_0 | 21 | 0.2262 | 1 | 0.2262 | 0 | 0 | 0 | 0.9571 | Ref |
| CP062426 | Unassigned | Unassigned | Unassigned | 7_0 | Clustered | 28 | VC_7_0 | 28 | 0.389 | 1 | 0.389 | 0 | 0 | 0 | 0.9947 | Ref |
| CP062427 | Unassigned | Unassigned | Unassigned | 9_0 | Clustered | 38 | VC_9_0 | 38 | 0.4859 | 1 | 0.4859 | 0 | 0 | 0 | 1 | Ref |
| CP062430 | Unassigned | Unassigned | Unassigned | 10_0 | Clustered | 25 | VC_10_0 | 25 | 0.3437 | 1 | 0.3437 | 0 | 0 | 0 | 0.9733 | Ref |
| CP062434 | Unassigned | Unassigned | Unassigned | 8_0 | Clustered | 58 | VC_8_0 | 58 | 0.8285 | 1 | 0.8285 | 0 | 0 | 0 | 0.9946 | Ref |
| CP062435 | Unassigned | Unassigned | Unassigned | 7_0 | Clustered | 28 | VC_7_0 | 28 | 0.389 | 1 | 0.389 | 0 | 0 | 0 | 0.9947 | Ref |
| CP062436 | Unassigned | Unassigned | Unassigned | 10_0 | Clustered | 25 | VC_10_0 | 25 | 0.3437 | 1 | 0.3437 | 0 | 0 | 0 | 0.9733 | Ref |
| CP062442 | Unassigned | Unassigned | Unassigned | 8_0 | Clustered | 58 | VC_8_0 | 58 | 0.8285 | 1 | 0.8285 | 0 | 0 | 0 | 0.9946 | Ref |
| CP062444 | Unassigned | Unassigned | Unassigned | 8_0 | Clustered | 58 | VC_8_0 | 58 | 0.8285 | 1 | 0.8285 | 0 | 0 | 0 | 0.9946 | Ref |
| CP062445 | Unassigned | Unassigned | Unassigned | 7_0 | Clustered | 28 | VC_7_0 | 28 | 0.389 | 1 | 0.389 | 0 | 0 | 0 | 0.9947 | Ref |
| CP062447 | Unassigned | Unassigned | Unassigned | 10_0 | Clustered | 25 | VC_10_0 | 25 | 0.3437 | 1 | 0.3437 | 0 | 0 | 0 | 0.9733 | Ref |
| CP062451 | Unassigned | Unassigned | Unassigned | 9_0 | Clustered | 38 | VC_9_0 | 38 | 0.4859 | 1 | 0.4859 | 0 | 0 | 0 | 1 | Ref |
| CP062454 | Unassigned | Unassigned | Unassigned | 9_0 | Clustered | 38 | VC_9_0 | 38 | 0.4859 | 1 | 0.4859 | 0 | 0 | 0 | 1 | Ref |
| CP062457 | Unassigned | Unassigned | Unassigned | 7_0 | Clustered | 28 | VC_7_0 | 28 | 0.389 | 1 | 0.389 | 0 | 0 | 0 | 0.9947 | Ref |
| CP062461 | Unassigned | Unassigned | Unassigned | 8_0 | Clustered | 58 | VC_8_0 | 58 | 0.8285 | 1 | 0.8285 | 0 | 0 | 0 | 0.9946 | Ref |
| CP062462 | Unassigned | Unassigned | Unassigned | 12_0 | Clustered | 21 | VC_12_0 | 21 | 0.2262 | 1 | 0.2262 | 0 | 0 | 0 | 0.9571 | Ref |
| CP062992 | Unassigned | Unassigned | Unassigned | 509_0 | Clustered | 36 | VC_509_0 | 36 | 0.5291 | 1 | 0.5291 | 0 | 0 | 0 | 0.9714 | Ref |
| CP067347 | Unassigned | Unassigned | Unassigned | 487_4 | Clustered/Singleton | 1 | VC_487_4 | 1 | 0 | 1 | 0 | 0 | 0 | 0 | 1 | Ref |
| CP067352 | Unassigned | Unassigned | Unassigned | 487_0 | Clustered/Singleton | 1 | VC_487_0 | 1 | 0 | 1 | 0 | 0 | 0 | 0 | 1 | Ref |
| CP069347 | Unassigned | Unassigned | Unassigned | 666_0 | Clustered | 11 | VC_666_0 | 11 | 0.5822 | 1 | 0.5822 | 0 | 0 | 0 | 1 | Ref |

| | | | | | | | | | | | | | | | | |
|---|---|---|---|---|---|---|---|---|---|---|---|---|---|---|---|---|
| CP069348 | Unassigned | Unassigned | Unassigned | 487_1 | Clustered/Singleton | 1 | VC_487_1 | 1 | 0 | 1 | 0 | 0 | 0 | 0 | 1 | Ref |
| CP071049 | Unassigned | Unassigned | Unassigned | 12_0 | Clustered | 21 | VC_12_0 | 21 | 0.2262 | 1 | 0.2262 | 0 | 0 | 0 | 0.9571 | Ref |
| CP072507 | Unassigned | Unassigned | Unassigned | 37_0 | Clustered | 4 | VC_37_0 | 4 | 0.4325 | 0.99998062 | 0.4325 | 0 | 0 | 0 | 1 | Ref |
| CP084202 | Unassigned | Unassigned | Unassigned | 751_0 | Clustered | 2 | VC_751_0 | 2 | 0.6768 | 0.87921671 | 0.595 | 0 | 0 | 0 | 1 | Ref |
| CP091911 | Unassigned | Unassigned | Unassigned | 722_0 | Clustered | 2 | VC_722_0 | 2 | 1 | 1 | 1 | 0 | 0 | 0 | 1 | Ref |
| CP101890 | Unassigned | Unassigned | Unassigned | 738_0 | Clustered | 9 | VC_738_0 | 9 | 0.7116 | 1 | 0.7116 | 0 | 0 | 0 | 1 | Ref |
| CP101896 | Unassigned | Unassigned | Unassigned | 738_0 | Clustered | 9 | VC_738_0 | 9 | 0.7116 | 1 | 0.7116 | 0 | 0 | 0 | 1 | Ref |
| D89074 | Unassigned | Unassigned | Unassigned | 16_0 | Clustered | 12 | VC_16_0 | 12 | 0.6601 | 1 | 0.6601 | 0 | 0 | 0 | 1 | Ref |
| DQ003260 | Unassigned | Unassigned | Unassigned | 30_1 | Clustered | 53 | VC_30_1 | 53 | 0.7151 | 1 | 0.7151 | 0 | 0 | 0 | 0.9325 | Ref |
| DQ003637 | Unassigned | Unassigned | Unassigned | 392_0 | Clustered | 9 | VC_392_0 | 9 | 0.7527 | 1 | 0.7527 | 0 | 0 | 0 | 1 | Ref |
| DQ003638 | Unassigned | Unassigned | Unassigned | 753_0 | Clustered | 26 | VC_753_0 | 26 | 0.9562 | 1 | 0.9562 | 0 | 0 | 0 | 1 | Ref |
| DQ003639 | Unassigned | Unassigned | Unassigned | 393_0 | Clustered | 5 | VC_393_0 | 5 | 0.4703 | 0.99999992 | 0.4703 | 0 | 0 | 0 | 0.9 | Ref |
| DQ003641 | Unassigned | Unassigned | Unassigned | 755_0 | Clustered | 2 | VC_755_0 | 2 | 0.2367 | 0.9499739 | 0.2249 | 0 | 0 | 0 | 1 | Ref |
| DQ003642 | Unassigned | Unassigned | Unassigned | 394_0 | Clustered | 3 | VC_394_0 | 3 | 0.1582 | 0.99545803 | 0.1575 | 0 | 0 | 0 | 1 | Ref |
| DQ004855 | Unassigned | Unassigned | Unassigned | 753_0 | Clustered | 26 | VC_753_0 | 26 | 0.9562 | 1 | 0.9562 | 0 | 0 | 0 | 1 | Ref |
| DQ029335 | Unassigned | Unassigned | Unassigned | 83_0 | Clustered | 24 | VC_83_0 | 24 | 0.7345 | 1 | 0.7345 | 0 | 0 | 0 | 1 | Ref |
| DQ054536 | Unassigned | Unassigned | Unassigned | 210_0 | Clustered | 105 | VC_210_0 | 105 | 0.4076 | 1 | 0.4076 | 0 | 0 | 0 | 0.7766 | Ref |
| DQ079870 | Unassigned | Unassigned | Unassigned | 760_0 | Clustered | 15 | VC_760_0 | 15 | 1 | 1 | 1 | 0 | 0 | 0 | 1 | Ref |
| DQ079871 | Unassigned | Unassigned | Unassigned | 760_0 | Clustered | 15 | VC_760_0 | 15 | 1 | 1 | 1 | 0 | 0 | 0 | 1 | Ref |
| DQ079872 | Unassigned | Unassigned | Unassigned | 760_0 | Clustered | 15 | VC_760_0 | 15 | 1 | 1 | 1 | 0 | 0 | 0 | 1 | Ref |
| DQ079873 | Unassigned | Unassigned | Unassigned | 760_0 | Clustered | 15 | VC_760_0 | 15 | 1 | 1 | 1 | 0 | 0 | 0 | 1 | Ref |
| DQ079874 | Unassigned | Unassigned | Unassigned | 760_0 | Clustered | 15 | VC_760_0 | 15 | 1 | 1 | 1 | 0 | 0 | 0 | 1 | Ref |
| DQ079875 | Unassigned | Unassigned | Unassigned | 760_0 | Clustered | 15 | VC_760_0 | 15 | 1 | 1 | 1 | 0 | 0 | 0 | 1 | Ref |
| DQ079876 | Unassigned | Unassigned | Unassigned | 760_0 | Clustered | 15 | VC_760_0 | 15 | 1 | 1 | 1 | 0 | 0 | 0 | 1 | Ref |
| DQ079878 | Unassigned | Unassigned | Unassigned | 760_0 | Clustered | 15 | VC_760_0 | 15 | 1 | 1 | 1 | 0 | 0 | 0 | 1 | Ref |
| DQ079879 | Unassigned | Unassigned | Unassigned | 760_0 | Clustered | 15 | VC_760_0 | 15 | 1 | 1 | 1 | 0 | 0 | 0 | 1 | Ref |
| DQ079880 | Unassigned | Unassigned | Unassigned | 310_0 | Clustered | 155 | VC_310_0 | 155 | 0.9979 | 1 | 0.9979 | 0 | 0 | 0 | 1 | Ref |
| DQ079881 | Unassigned | Unassigned | Unassigned | 310_0 | Clustered | 155 | VC_310_0 | 155 | 0.9979 | 1 | 0.9979 | 0 | 0 | 0 | 1 | Ref |
| DQ079882 | Unassigned | Unassigned | Unassigned | 310_0 | Clustered | 155 | VC_310_0 | 155 | 0.9979 | 1 | 0.9979 | 0 | 0 | 0 | 1 | Ref |
| DQ079883 | Unassigned | Unassigned | Unassigned | 310_0 | Clustered | 155 | VC_310_0 | 155 | 0.9979 | 1 | 0.9979 | 0 | 0 | 0 | 1 | Ref |
| DQ079884 | Unassigned | Unassigned | Unassigned | 310_0 | Clustered | 155 | VC_310_0 | 155 | 0.9979 | 1 | 0.9979 | 0 | 0 | 0 | 1 | Ref |
| DQ079885 | Unassigned | Unassigned | Unassigned | 310_0 | Clustered | 155 | VC_310_0 | 155 | 0.9979 | 1 | 0.9979 | 0 | 0 | 0 | 1 | Ref |
| DQ079886 | Unassigned | Unassigned | Unassigned | 310_0 | Clustered | 155 | VC_310_0 | 155 | 0.9979 | 1 | 0.9979 | 0 | 0 | 0 | 1 | Ref |
| DQ079887 | Unassigned | Unassigned | Unassigned | 310_0 | Clustered | 155 | VC_310_0 | 155 | 0.9979 | 1 | 0.9979 | 0 | 0 | 0 | 1 | Ref |
| DQ079888 | Unassigned | Unassigned | Unassigned | 310_0 | Clustered | 155 | VC_310_0 | 155 | 0.9979 | 1 | 0.9979 | 0 | 0 | 0 | 1 | Ref |
| DQ079889 | Unassigned | Unassigned | Unassigned | 310_0 | Clustered | 155 | VC_310_0 | 155 | 0.9979 | 1 | 0.9979 | 0 | 0 | 0 | 1 | Ref |
| DQ079890 | Unassigned | Unassigned | Unassigned | 310_0 | Clustered | 155 | VC_310_0 | 155 | 0.9979 | 1 | 0.9979 | 0 | 0 | 0 | 1 | Ref |
| DQ079891 | Unassigned | Unassigned | Unassigned | 310_0 | Clustered | 155 | VC_310_0 | 155 | 0.9979 | 1 | 0.9979 | 0 | 0 | 0 | 1 | Ref |
| DQ079892 | Unassigned | Unassigned | Unassigned | 310_0 | Clustered | 155 | VC_310_0 | 155 | 0.9979 | 1 | 0.9979 | 0 | 0 | 0 | 1 | Ref |
| DQ079893 | Unassigned | Unassigned | Unassigned | 310_0 | Clustered | 155 | VC_310_0 | 155 | 0.9979 | 1 | 0.9979 | 0 | 0 | 0 | 1 | Ref |
| DQ079894 | Unassigned | Unassigned | Unassigned | 310_0 | Clustered | 155 | VC_310_0 | 155 | 0.9979 | 1 | 0.9979 | 0 | 0 | 0 | 1 | Ref |
| DQ079895 | Unassigned | Unassigned | Unassigned | 310_0 | Clustered | 155 | VC_310_0 | 155 | 0.9979 | 1 | 0.9979 | 0 | 0 | 0 | 1 | Ref |
| DQ079896 | Unassigned | Unassigned | Unassigned | 373_0 | Clustered | 358 | VC_373_0 | 358 | 0.9614 | 1 | 0.9614 | 0 | 0 | 0 | 1 | Ref |
| DQ079897 | Unassigned | Unassigned | Unassigned | 373_0 | Clustered | 358 | VC_373_0 | 358 | 0.9614 | 1 | 0.9614 | 0 | 0 | 0 | 1 | Ref |
| DQ079899 | Unassigned | Unassigned | Unassigned | 373_0 | Clustered | 358 | VC_373_0 | 358 | 0.9614 | 1 | 0.9614 | 0 | 0 | 0 | 1 | Ref |
| DQ079900 | Unassigned | Unassigned | Unassigned | 373_0 | Clustered | 358 | VC_373_0 | 358 | 0.9614 | 1 | 0.9614 | 0 | 0 | 0 | 1 | Ref |
| DQ079901 | Unassigned | Unassigned | Unassigned | 373_0 | Clustered | 358 | VC_373_0 | 358 | 0.9614 | 1 | 0.9614 | 0 | 0 | 0 | 1 | Ref |
| DQ079902 | Unassigned | Unassigned | Unassigned | 373_0 | Clustered | 358 | VC_373_0 | 358 | 0.9614 | 1 | 0.9614 | 0 | 0 | 0 | 1 | Ref |
| DQ079903 | Unassigned | Unassigned | Unassigned | 373_0 | Clustered | 358 | VC_373_0 | 358 | 0.9614 | 1 | 0.9614 | 0 | 0 | 0 | 1 | Ref |
| DQ079904 | Unassigned | Unassigned | Unassigned | 373_0 | Clustered | 358 | VC_373_0 | 358 | 0.9614 | 1 | 0.9614 | 0 | 0 | 0 | 1 | Ref |
| DQ079906 | Unassigned | Unassigned | Unassigned | 373_0 | Clustered | 358 | VC_373_0 | 358 | 0.9614 | 1 | 0.9614 | 0 | 0 | 0 | 1 | Ref |
| DQ079908 | Unassigned | Unassigned | Unassigned | 373_0 | Clustered | 358 | VC_373_0 | 358 | 0.9614 | 1 | 0.9614 | 0 | 0 | 0 | 1 | Ref |
| DQ079909 | Unassigned | Unassigned | Unassigned | 373_0 | Clustered | 358 | VC_373_0 | 358 | 0.9614 | 1 | 0.9614 | 0 | 0 | 0 | 1 | Ref |
| DQ085810 | Unassigned | Unassigned | Unassigned | 760_0 | Clustered | 15 | VC_760_0 | 15 | 1 | 1 | 1 | 0 | 0 | 0 | 1 | Ref |
| DQ087285 | Unassigned | Unassigned | Unassigned | 3_0 | Clustered | 17 | VC_3_0 | 17 | 0.5615 | 1 | 0.5615 | 0 | 0 | 0 | 1 | Ref |
| DQ111067 | Unassigned | Unassigned | Unassigned | 465_0 | Clustered | 80 | VC_465_0 | 80 | 0.9988 | 1 | 0.9988 | 0 | 0 | 0 | 1 | Ref |
| DQ113772 | Unassigned | Unassigned | Unassigned | 425_0 | Clustered | 40 | VC_425_0 | 40 | 0.8399 | 1 | 0.8399 | 0 | 0 | 0 | 0.9974 | Ref |
| DQ114220 | Unassigned | Unassigned | Unassigned | 5_0 | Clustered | 27 | VC_5_0 | 27 | 0.9599 | 1 | 0.9599 | 0 | 0 | 0 | 1 | Ref |
| DQ121662 | Unassigned | Unassigned | Unassigned | 469_0 | Clustered | 18 | VC_469_0 | 18 | 0.4583 | 1 | 0.4583 | 0 | 0 | 0 | 1 | Ref |
| DQ126339 | Unassigned | Unassigned | Unassigned | 492_0 | Clustered | 20 | VC_492_0 | 20 | 0.7887 | 1 | 0.7887 | 0 | 0 | 0 | 1 | Ref |
| DQ150593 | Unassigned | Unassigned | Unassigned | 719_0 | Clustered | 16 | VC_719_0 | 16 | 0.728 | 1 | 0.728 | 0 | 0 | 0 | 1 | Ref |
| DQ163912 | Unassigned | Unassigned | Unassigned | 764_0 | Clustered | 23 | VC_764_0 | 23 | 0.8194 | 1 | 0.8194 | 0 | 0 | 0 | 1 | Ref |
| DQ163913 | Unassigned | Unassigned | Unassigned | 766_0 | Clustered | 40 | VC_766_0 | 40 | 0.7511 | 1 | 0.7511 | 0 | 0 | 0 | 1 | Ref |
| DQ163914 | Unassigned | Unassigned | Unassigned | 632_0 | Clustered | 9 | VC_632_0 | 9 | 0.9524 | 1 | 0.9524 | 0 | 0 | 0 | 1 | Ref |
| DQ163915 | Unassigned | Unassigned | Unassigned | 772_0 | Clustered | 9 | VC_772_0 | 9 | 0.4468 | 1 | 0.4468 | 0 | 0 | 0 | 1 | Ref |
| DQ163916 | Unassigned | Unassigned | Unassigned | 172_0 | Clustered | 26 | VC_172_0 | 26 | 0.7909 | 1 | 0.7909 | 0 | 0 | 0 | 1 | Ref |
| DQ163917 | Unassigned | Unassigned | Unassigned | 81_0 | Clustered | 41 | VC_81_0 | 41 | 0.5815 | 1 | 0.5815 | 0 | 0 | 0 | 0.9805 | Ref |
| DQ222851 | Unassigned | Unassigned | Unassigned | 719_0 | Clustered | 16 | VC_719_0 | 16 | 0.728 | 1 | 0.728 | 0 | 0 | 0 | 1 | Ref |
| DQ222853 | Unassigned | Unassigned | Unassigned | 719_0 | Clustered | 16 | VC_719_0 | 16 | 0.728 | 1 | 0.728 | 0 | 0 | 0 | 1 | Ref |
| DQ222855 | Unassigned | Unassigned | Unassigned | 719_0 | Clustered | 16 | VC_719_0 | 16 | 0.728 | 1 | 0.728 | 0 | 0 | 0 | 1 | Ref |
| DQ227763 | Unassigned | Unassigned | Unassigned | 210_0 | Clustered | 105 | VC_210_0 | 105 | 0.4076 | 1 | 0.4076 | 0 | 0 | 0 | 0.7766 | Ref |
| DQ227764 | Unassigned | Unassigned | Unassigned | 210_0 | Clustered | 105 | VC_210_0 | 105 | 0.4076 | 1 | 0.4076 | 0 | 0 | 0 | 0.7766 | Ref |
| DQ289555 | Unassigned | Unassigned | Unassigned | 719_0 | Clustered | 16 | VC_719_0 | 16 | 0.728 | 1 | 0.728 | 0 | 0 | 0 | 1 | Ref |
| DQ289556 | Unassigned | Unassigned | Unassigned | 719_0 | Clustered | 16 | VC_719_0 | 16 | 0.728 | 1 | 0.728 | 0 | 0 | 0 | 1 | Ref |
| DQ320509 | Unassigned | Unassigned | Unassigned | 275_0 | Clustered | 6 | VC_275_0 | 6 | 0.6515 | 0.99999994 | 0.6515 | 0 | 0 | 0 | 0.9333 | Ref |
| DQ386162 | Unassigned | Unassigned | Unassigned | 477_0 | Clustered | 4 | VC_477_0 | 4 | 0.7825 | 0.99997292 | 0.7825 | 0 | 0 | 0 | 1 | Ref |
| DQ394807 | Unassigned | Unassigned | Unassigned | 271_0 | Clustered | 12 | VC_271_0 | 12 | 0.4255 | 1 | 0.4255 | 0 | 0 | 0 | 1 | Ref |
| DQ394808 | Unassigned | Unassigned | Unassigned | 271_0 | Clustered | 12 | VC_271_0 | 12 | 0.4255 | 1 | 0.4255 | 0 | 0 | 0 | 1 | Ref |
| DQ394809 | Unassigned | Unassigned | Unassigned | 271_0 | Clustered | 12 | VC_271_0 | 12 | 0.4255 | 1 | 0.4255 | 0 | 0 | 0 | 1 | Ref |
| DQ394810 | Unassigned | Unassigned | Unassigned | 271_0 | Clustered | 12 | VC_271_0 | 12 | 0.4255 | 1 | 0.4255 | 0 | 0 | 0 | 1 | Ref |
| DQ398041 | Unassigned | Unassigned | Unassigned | 571_7 | Clustered | 28 | VC_571_7 | 28 | 0.1651 | 1 | 0.1651 | 0 | 0 | 0 | 0.8519 | Ref |

| | | | | | | | | | | | | | | | | |
|---|---|---|---|---|---|---|---|---|---|---|---|---|---|---|---|---|
| DQ398042 | Unassigned | Unassigned | Unassigned | 783_0 | Clustered | 67 | VC_783_0 | 67 | 0.8945 | 1 | 0.8945 | 0 | 0 | 0 | 1 | Ref |
| DQ398043 | Unassigned | Unassigned | Unassigned | 222_0 | Clustered | 5 | VC_222_0 | 5 | 0.072 | 0.99999997 | 0.072 | 0 | 0 | 0 | 1 | Ref |
| DQ398044 | Unassigned | Unassigned | Unassigned | 385_0 | Clustered | 24 | VC_385_0 | 24 | 0.5516 | 1 | 0.5516 | 0 | 0 | 0 | 1 | Ref |
| DQ398045 | Unassigned | Unassigned | Unassigned | 538_60 | Clustered | 2 | VC_538_60 | 2 | 0.0091 | 0.95761233 | 0.0087 | 0 | 0 | 0 | 1 | Ref |
| DQ398046 | Unassigned | Unassigned | Unassigned | 384_16 | Clustered | 42 | VC_384_16 | 42 | 0.134 | 1 | 0.134 | 0 | 0 | 0 | 0.7282 | Ref |
| DQ398047 | Unassigned | Unassigned | Unassigned | 585_0 | Clustered | 20 | VC_585_0 | 20 | 0.8376 | 1 | 0.8376 | 0 | 0 | 0 | 1 | Ref |
| DQ398048 | Unassigned | Unassigned | Unassigned | 526_0 | Clustered | 32 | VC_526_0 | 32 | 0.9037 | 1 | 0.9037 | 0 | 0 | 0 | 1 | Ref |
| DQ398049 | Unassigned | Unassigned | Unassigned | 527_0 | Clustered | 37 | VC_527_0 | 37 | 0.9679 | 1 | 0.9679 | 0 | 0 | 0 | 1 | Ref |
| DQ398050 | Unassigned | Unassigned | Unassigned | 538_58 | Clustered | 3 | VC_538_58 | 3 | 0.0208 | 0.9985993 | 0.0208 | 0 | 0 | 0 | 1 | Ref |
| DQ398051 | Unassigned | Unassigned | Unassigned | 585_0 | Clustered | 20 | VC_585_0 | 20 | 0.8376 | 1 | 0.8376 | 0 | 0 | 0 | 1 | Ref |
| DQ398052 | Unassigned | Unassigned | Unassigned | 786_0 | Clustered | 4 | VC_786_0 | 4 | 0.3519 | 0.99997469 | 0.3519 | 0 | 0 | 0 | 1 | Ref |
| DQ398053 | Unassigned | Unassigned | Unassigned | 579_31 | Clustered/Singleton | 1 | VC_579_31 | 1 | 0 | 1 | 0 | 0 | 0 | 0 | 1 | Ref |
| DQ426904 | Unassigned | Unassigned | Unassigned | 4_0 | Clustered | 7 | VC_4_0 | 7 | 0.7823 | 1 | 0.7823 | 0 | 0 | 0 | 1 | Ref |
| DQ426905 | Unassigned | Unassigned | Unassigned | 4_0 | Clustered | 7 | VC_4_0 | 7 | 0.7823 | 1 | 0.7823 | 0 | 0 | 0 | 1 | Ref |
| DQ431235 | Unassigned | Unassigned | Unassigned | 787_0 | Clustered | 132 | VC_787_0 | 132 | 1 | 1 | 1 | 0 | 0 | 0 | 1 | Ref |
| DQ466086 | Unassigned | Unassigned | Unassigned | 140_1 | Clustered | 4 | VC_140_1 | 4 | 0.1766 | 0.99998565 | 0.1766 | 0 | 0 | 0 | 1 | Ref |
| DQ490056 | Unassigned | Unassigned | Unassigned | 705_0 | Clustered | 2 | VC_705_0 | 2 | 0.4153 | 0.95606213 | 0.3971 | 0 | 0 | 0 | 1 | Ref |
| DQ500118 | Unassigned | Unassigned | Unassigned | 792_0 | Clustered | 5 | VC_792_0 | 5 | 0.8313 | 0.99999405 | 0.8313 | 0 | 0 | 0 | 1 | Ref |
| DQ517338 | Unassigned | Unassigned | Unassigned | 9_0 | Clustered | 38 | VC_9_0 | 38 | 0.4859 | 1 | 0.4859 | 0 | 0 | 0 | 1 | Ref |
| DQ529280 | Unassigned | Unassigned | Unassigned | 619_2 | Clustered | 10 | VC_619_2 | 10 | 0.3034 | 1 | 0.3034 | 0 | 0 | 0 | 1 | Ref |
| DQ530359 | Unassigned | Unassigned | Unassigned | 9_0 | Clustered | 38 | VC_9_0 | 38 | 0.4859 | 1 | 0.4859 | 0 | 0 | 0 | 1 | Ref |
| DQ530360 | Unassigned | Unassigned | Unassigned | 9_0 | Clustered | 38 | VC_9_0 | 38 | 0.4859 | 1 | 0.4859 | 0 | 0 | 0 | 1 | Ref |
| DQ530361 | Unassigned | Unassigned | Unassigned | 10_0 | Clustered | 25 | VC_10_0 | 25 | 0.3437 | 1 | 0.3437 | 0 | 0 | 0 | 0.9733 | Ref |
| DQ530362 | Unassigned | Unassigned | Unassigned | 12_0 | Clustered | 21 | VC_12_0 | 21 | 0.2262 | 1 | 0.2262 | 0 | 0 | 0 | 0.9571 | Ref |
| DQ631426 | Unassigned | Unassigned | Unassigned | 185_0 | Clustered | 53 | VC_185_0 | 53 | 0.9153 | 1 | 0.9153 | 0 | 0 | 0 | 1 | Ref |
| DQ674738 | Unassigned | Unassigned | Unassigned | 5_0 | Clustered | 27 | VC_5_0 | 27 | 0.9599 | 1 | 0.9599 | 0 | 0 | 0 | 1 | Ref |
| DQ777876 | Unassigned | Unassigned | Unassigned | 115_0 | Clustered | 9 | VC_115_0 | 9 | 0.5166 | 1 | 0.5166 | 0 | 0 | 0 | 1 | Ref |
| DQ785801 | Unassigned | Unassigned | Unassigned | 64_0 | Clustered | 2 | VC_64_0 | 2 | 0.1127 | 0.95690971 | 0.1079 | 0 | 0 | 0 | 1 | Ref |
| DQ832317 | Unassigned | Unassigned | Unassigned | 794_15 | Clustered | 5 | VC_794_15 | 5 | 0.0243 | 0.76144861 | 0.0185 | 0 | 0 | 0 | 0.9 | Ref |
| DQ836063 | Unassigned | Unassigned | Unassigned | 321_0 | Clustered | 2 | VC_321_0 | 2 | 0.1773 | 0.99999983 | 0.1773 | 0 | 0 | 0 | 1 | Ref |
| DQ864624 | Unassigned | Unassigned | Unassigned | 795_0 | Clustered | 23 | VC_795_0 | 23 | 0.5497 | 1 | 0.5497 | 0 | 0 | 0 | 1 | Ref |
| DQ873690 | Unassigned | Unassigned | Unassigned | 185_0 | Clustered | 53 | VC_185_0 | 53 | 0.9153 | 1 | 0.9153 | 0 | 0 | 0 | 1 | Ref |
| DQ875742 | Unassigned | Unassigned | Unassigned | 800_0 | Clustered | 4 | VC_800_0 | 4 | 0.8098 | 0.99985435 | 0.8096 | 0 | 0 | 0 | 1 | Ref |
| DQ904452 | Unassigned | Unassigned | Unassigned | 290_94 | Clustered | 2 | VC_290_94 | 2 | 0.0026 | 0.95293762 | 0.0025 | 0 | 0 | 0 | 1 | Ref |
| DQ908929 | Unassigned | Unassigned | Unassigned | 12_2 | Clustered | 19 | VC_12_2 | 19 | 0.2009 | 1 | 0.2009 | 0 | 0 | 0 | 1 | Ref |
| DQ997624 | Unassigned | Unassigned | Unassigned | 493_0 | Clustered | 4 | VC_493_0 | 4 | 0.9871 | 0.99748973 | 0.9847 | 0 | 0 | 0 | 1 | Ref |
| EF056009 | Unassigned | Unassigned | Unassigned | 801_0 | Clustered | 37 | VC_801_0 | 37 | 0.5672 | 1 | 0.5672 | 0 | 0 | 0 | 1 | Ref |
| EF057797 | Unassigned | Unassigned | Unassigned | 128_0 | Clustered | 2 | VC_128_0 | 2 | 0.1645 | 0.95181568 | 0.1566 | 0 | 0 | 0 | 1 | Ref |
| EF068134 | Unassigned | Unassigned | Unassigned | 196_0 | Clustered | 13 | VC_196_0 | 13 | 0.9973 | 0.99823802 | 0.9955 | 0 | 0 | 0 | 1 | Ref |
| EF108464 | Unassigned | Unassigned | Unassigned | 322_0 | Clustered | 12 | VC_322_0 | 12 | 0.8924 | 1 | 0.8924 | 0 | 0 | 0 | 1 | Ref |
| EF108465 | Unassigned | Unassigned | Unassigned | 322_0 | Clustered | 12 | VC_322_0 | 12 | 0.8924 | 1 | 0.8924 | 0 | 0 | 0 | 1 | Ref |
| EF153632 | Unassigned | Unassigned | Unassigned | 553_0 | Clustered | 3 | VC_553_0 | 3 | 0.0872 | 0.99853671 | 0.0871 | 0 | 0 | 0 | 1 | Ref |
| EF160123 | Unassigned | Unassigned | Unassigned | 467_0 | Clustered | 6 | VC_467_0 | 6 | 0.1136 | 1 | 0.1136 | 0 | 0 | 0 | 1 | Ref |
| EF177456 | Unassigned | Unassigned | Unassigned | 654_0 | Clustered | 115 | VC_654_0 | 115 | 0.7303 | 1 | 0.7303 | 0 | 0 | 0 | 0.8764 | Ref |
| EF204939 | Unassigned | Unassigned | Unassigned | 322_0 | Clustered | 12 | VC_322_0 | 12 | 0.8924 | 1 | 0.8924 | 0 | 0 | 0 | 1 | Ref |
| EF204940 | Unassigned | Unassigned | Unassigned | 322_0 | Clustered | 12 | VC_322_0 | 12 | 0.8924 | 1 | 0.8924 | 0 | 0 | 0 | 1 | Ref |
| EF372997 | Unassigned | Unassigned | Unassigned | 352_0 | Clustered | 31 | VC_352_0 | 31 | 0.9547 | 1 | 0.9547 | 0 | 0 | 0 | 1 | Ref |
| EF380009 | Unassigned | Unassigned | Unassigned | 310_0 | Clustered | 155 | VC_310_0 | 155 | 0.9979 | 1 | 0.9979 | 0 | 0 | 0 | 1 | Ref |
| EF380010 | Unassigned | Unassigned | Unassigned | 310_0 | Clustered | 155 | VC_310_0 | 155 | 0.9979 | 1 | 0.9979 | 0 | 0 | 0 | 1 | Ref |
| EF380011 | Unassigned | Unassigned | Unassigned | 310_0 | Clustered | 155 | VC_310_0 | 155 | 0.9979 | 1 | 0.9979 | 0 | 0 | 0 | 1 | Ref |
| EF380012 | Unassigned | Unassigned | Unassigned | 310_0 | Clustered | 155 | VC_310_0 | 155 | 0.9979 | 1 | 0.9979 | 0 | 0 | 0 | 1 | Ref |
| EF380013 | Unassigned | Unassigned | Unassigned | 310_0 | Clustered | 155 | VC_310_0 | 155 | 0.9979 | 1 | 0.9979 | 0 | 0 | 0 | 1 | Ref |
| EF380014 | Unassigned | Unassigned | Unassigned | 310_0 | Clustered | 155 | VC_310_0 | 155 | 0.9979 | 1 | 0.9979 | 0 | 0 | 0 | 1 | Ref |
| EF380015 | Unassigned | Unassigned | Unassigned | 310_0 | Clustered | 155 | VC_310_0 | 155 | 0.9979 | 1 | 0.9979 | 0 | 0 | 0 | 1 | Ref |
| EF380016 | Unassigned | Unassigned | Unassigned | 310_0 | Clustered | 155 | VC_310_0 | 155 | 0.9979 | 1 | 0.9979 | 0 | 0 | 0 | 1 | Ref |
| EF380017 | Unassigned | Unassigned | Unassigned | 310_0 | Clustered | 155 | VC_310_0 | 155 | 0.9979 | 1 | 0.9979 | 0 | 0 | 0 | 1 | Ref |
| EF380018 | Unassigned | Unassigned | Unassigned | 310_0 | Clustered | 155 | VC_310_0 | 155 | 0.9979 | 1 | 0.9979 | 0 | 0 | 0 | 1 | Ref |
| EF380019 | Unassigned | Unassigned | Unassigned | 310_0 | Clustered | 155 | VC_310_0 | 155 | 0.9979 | 1 | 0.9979 | 0 | 0 | 0 | 1 | Ref |
| EF380020 | Unassigned | Unassigned | Unassigned | 310_0 | Clustered | 155 | VC_310_0 | 155 | 0.9979 | 1 | 0.9979 | 0 | 0 | 0 | 1 | Ref |
| EF380021 | Unassigned | Unassigned | Unassigned | 310_0 | Clustered | 155 | VC_310_0 | 155 | 0.9979 | 1 | 0.9979 | 0 | 0 | 0 | 1 | Ref |
| EF380022 | Unassigned | Unassigned | Unassigned | 310_0 | Clustered | 155 | VC_310_0 | 155 | 0.9979 | 1 | 0.9979 | 0 | 0 | 0 | 1 | Ref |
| EF380023 | Unassigned | Unassigned | Unassigned | 310_0 | Clustered | 155 | VC_310_0 | 155 | 0.9979 | 1 | 0.9979 | 0 | 0 | 0 | 1 | Ref |
| EF380024 | Unassigned | Unassigned | Unassigned | 310_0 | Clustered | 155 | VC_310_0 | 155 | 0.9979 | 1 | 0.9979 | 0 | 0 | 0 | 1 | Ref |
| EF380025 | Unassigned | Unassigned | Unassigned | 310_0 | Clustered | 155 | VC_310_0 | 155 | 0.9979 | 1 | 0.9979 | 0 | 0 | 0 | 1 | Ref |
| EF380026 | Unassigned | Unassigned | Unassigned | 310_0 | Clustered | 155 | VC_310_0 | 155 | 0.9979 | 1 | 0.9979 | 0 | 0 | 0 | 1 | Ref |
| EF380027 | Unassigned | Unassigned | Unassigned | 310_0 | Clustered | 155 | VC_310_0 | 155 | 0.9979 | 1 | 0.9979 | 0 | 0 | 0 | 1 | Ref |
| EF380028 | Unassigned | Unassigned | Unassigned | 310_0 | Clustered | 155 | VC_310_0 | 155 | 0.9979 | 1 | 0.9979 | 0 | 0 | 0 | 1 | Ref |
| EF380029 | Unassigned | Unassigned | Unassigned | 310_0 | Clustered | 155 | VC_310_0 | 155 | 0.9979 | 1 | 0.9979 | 0 | 0 | 0 | 1 | Ref |
| EF380030 | Unassigned | Unassigned | Unassigned | 310_0 | Clustered | 155 | VC_310_0 | 155 | 0.9979 | 1 | 0.9979 | 0 | 0 | 0 | 1 | Ref |
| EF380031 | Unassigned | Unassigned | Unassigned | 310_0 | Clustered | 155 | VC_310_0 | 155 | 0.9979 | 1 | 0.9979 | 0 | 0 | 0 | 1 | Ref |
| EF380032 | Unassigned | Unassigned | Unassigned | 310_0 | Clustered | 155 | VC_310_0 | 155 | 0.9979 | 1 | 0.9979 | 0 | 0 | 0 | 1 | Ref |
| EF432053 | Unassigned | Unassigned | Unassigned | 815_0 | Clustered | 3 | VC_815_0 | 3 | 1 | 1 | 1 | 0 | 0 | 0 | 1 | Ref |
| EF437941 | Unassigned | Unassigned | Unassigned | 609_0 | Clustered | 27 | VC_609_0 | 27 | 0.9943 | 1 | 0.9943 | 0 | 0 | 0 | 1 | Ref |
| EF455602 | Unassigned | Unassigned | Unassigned | 625_0 | Clustered | 2 | VC_625_0 | 2 | 0.2121 | 0.95222028 | 0.202 | 0 | 0 | 0 | 1 | Ref |
| EF460875 | Unassigned | Unassigned | Unassigned | 816_0 | Clustered | 35 | VC_816_0 | 35 | 0.9824 | 1 | 0.9824 | 0 | 0 | 0 | 1 | Ref |
| EF462197 | Unassigned | Unassigned | Unassigned | 7_0 | Clustered | 28 | VC_7_0 | 28 | 0.389 | 1 | 0.389 | 0 | 0 | 0 | 0.9947 | Ref |
| EF462198 | Unassigned | Unassigned | Unassigned | 8_0 | Clustered | 58 | VC_8_0 | 58 | 0.8285 | 1 | 0.8285 | 0 | 0 | 0 | 0.9946 | Ref |
| EF462199 | Unassigned | Unassigned | Unassigned | 7_0 | Clustered | 28 | VC_7_0 | 28 | 0.389 | 1 | 0.389 | 0 | 0 | 0 | 0.9947 | Ref |
| EF469154 | Unassigned | Unassigned | Unassigned | 297_6 | Clustered | 12 | VC_297_6 | 12 | 0.1139 | 1 | 0.1139 | 0 | 0 | 0 | 0.8182 | Ref |
| EF506570 | Unassigned | Unassigned | Unassigned | 818_0 | Clustered | 3 | VC_818_0 | 3 | 1 | 1 | 1 | 0 | 0 | 0 | 1 | Ref |
| EF529515 | Unassigned | Unassigned | Unassigned | 260_0 | Clustered | 46 | VC_260_0 | 46 | 0.5137 | 1 | 0.5137 | 0 | 0 | 0 | 1 | Ref |

| | | | | | | | | | | | | | | | | |
|---|---|---|---|---|---|---|---|---|---|---|---|---|---|---|---|---|
| EF536069 | Unassigned | Unassigned | Unassigned | 538_24 | Clustered/Singleton | 1 | VC_538_24 | 1 | 0 | 1 | 0 | 0 | 0 | 0 | 1 | Ref |
| EF537008 | Unassigned | Unassigned | Unassigned | 799_0 | Clustered | 3 | VC_799_0 | 3 | 0.4746 | 0.9961812 | 0.4727 | 0 | 0 | 0 | 1 | Ref |
| EF579802 | Unassigned | Unassigned | Unassigned | 642_0 | Clustered | 3 | VC_642_0 | 3 | 0.1258 | 0.72377751 | 0.0911 | 0 | 0 | 0 | 1 | Ref |
| EF583821 | Unassigned | Unassigned | Unassigned | 92_2 | Clustered/Singleton | 1 | VC_92_2 | 1 | 0 | 1 | 0 | 0 | 0 | 0 | 1 | Ref |
| EU030938 | Unassigned | Unassigned | Unassigned | 618_0 | Clustered | 50 | VC_618_0 | 50 | 0.9989 | 1 | 0.9989 | 0 | 0 | 0 | 1 | Ref |
| EU030939 | Unassigned | Unassigned | Unassigned | 618_0 | Clustered | 50 | VC_618_0 | 50 | 0.9989 | 1 | 0.9989 | 0 | 0 | 0 | 1 | Ref |
| EU056923 | Unassigned | Unassigned | Unassigned | 434_0 | Clustered | 45 | VC_434_0 | 45 | 0.9479 | 1 | 0.9479 | 0 | 0 | 0 | 1 | Ref |
| EU078592 | Unassigned | Unassigned | Unassigned | 45_0 | Clustered | 38 | VC_45_0 | 38 | 0.5785 | 1 | 0.5785 | 0 | 0 | 0 | 0.9659 | Ref |
| EU081845 | Unassigned | Unassigned | Unassigned | 824_0 | Clustered | 3 | VC_824_0 | 3 | 0.5034 | 0.9984288 | 0.5026 | 0 | 0 | 0 | 1 | Ref |
| EU100883 | Unassigned | Unassigned | Unassigned | 829_0 | Clustered | 4 | VC_829_0 | 4 | 0.9064 | 0.99761409 | 0.9042 | 0 | 0 | 0 | 1 | Ref |
| EU100884 | Unassigned | Unassigned | Unassigned | 829_0 | Clustered | 4 | VC_829_0 | 4 | 0.9064 | 0.99761409 | 0.9042 | 0 | 0 | 0 | 1 | Ref |
| EU124666 | Unassigned | Unassigned | Unassigned | 831_0 | Clustered | 17 | VC_831_0 | 17 | 0.9057 | 1 | 0.9057 | 0 | 0 | 0 | 1 | Ref |
| EU136189 | Unassigned | Unassigned | Unassigned | 98_0 | Clustered | 30 | VC_98_0 | 30 | 0.7175 | 1 | 0.7175 | 0 | 0 | 0 | 1 | Ref |
| EU197055 | Unassigned | Unassigned | Unassigned | 356_4 | Clustered/Singleton | 1 | VC_356_4 | 1 | 0 | 1 | 0 | 0 | 0 | 0 | 1 | Ref |
| EU203571 | Unassigned | Unassigned | Unassigned | 834_0 | Clustered | 18 | VC_834_0 | 18 | 0.9116 | 1 | 0.9116 | 0 | 0 | 0 | 1 | Ref |
| EU221285 | Unassigned | Unassigned | Unassigned | 210_0 | Clustered | 105 | VC_210_0 | 105 | 0.4076 | 1 | 0.4076 | 0 | 0 | 0 | 0.7766 | Ref |
| EU236438 | Unassigned | Unassigned | Unassigned | 434_0 | Clustered | 45 | VC_434_0 | 45 | 0.9479 | 1 | 0.9479 | 0 | 0 | 0 | 1 | Ref |
| EU246945 | Unassigned | Unassigned | Unassigned | 279_0 | Clustered | 11 | VC_279_0 | 11 | 0.4839 | 1 | 0.4839 | 0 | 0 | 0 | 0.8727 | Ref |
| EU272036 | Unassigned | Unassigned | Unassigned | 185_0 | Clustered | 53 | VC_185_0 | 53 | 0.9153 | 1 | 0.9153 | 0 | 0 | 0 | 1 | Ref |
| EU272037 | Unassigned | Unassigned | Unassigned | 185_0 | Clustered | 53 | VC_185_0 | 53 | 0.9153 | 1 | 0.9153 | 0 | 0 | 0 | 1 | Ref |
| EU311208 | Unassigned | Unassigned | Unassigned | 35_0 | Clustered | 35 | VC_35_0 | 35 | 0.431 | 1 | 0.431 | 0 | 0 | 0 | 1 | Ref |
| EU330206 | Unassigned | Unassigned | Unassigned | 837_1 | Clustered | 24 | VC_837_1 | 24 | 0.5328 | 1 | 0.5328 | 0 | 0 | 0 | 1 | Ref |
| EU340421 | Unassigned | Unassigned | Unassigned | 657_0 | Clustered | 7 | VC_657_0 | 7 | 0.8495 | 1 | 0.8495 | 0 | 0 | 0 | 1 | Ref |
| EU350361 | Unassigned | Unassigned | Unassigned | 838_0 | Clustered | 3 | VC_838_0 | 3 | 1 | 1 | 1 | 0 | 0 | 0 | 1 | Ref |
| EU399241 | Unassigned | Unassigned | Unassigned | 128_0 | Clustered | 2 | VC_128_0 | 2 | 0.1645 | 0.95181568 | 0.1566 | 0 | 0 | 0 | 1 | Ref |
| EU408779 | Unassigned | Unassigned | Unassigned | 435_0 | Clustered | 7 | VC_435_0 | 7 | 0.9041 | 0.9998143 | 0.9039 | 0 | 0 | 0 | 1 | Ref |
| EU409559 | Unassigned | Unassigned | Unassigned | 390_0 | Clustered | 2 | VC_390_0 | 2 | 0.4516 | 0.94520071 | 0.4269 | 0 | 0 | 0 | 1 | Ref |
| EU418428 | Unassigned | Unassigned | Unassigned | 155_8 | Clustered | 66 | VC_155_8 | 66 | 0.4537 | 1 | 0.4537 | 0 | 0 | 0 | 0.8182 | Ref |
| EU545650 | Unassigned | Unassigned | Unassigned | 361_0 | Clustered | 7 | VC_361_0 | 7 | 0.9353 | 0.99999998 | 0.9353 | 0 | 0 | 0 | 1 | Ref |
| EU547803 | Unassigned | Unassigned | Unassigned | 398_0 | Clustered | 51 | VC_398_0 | 51 | 0.8741 | 1 | 0.8741 | 0 | 0 | 0 | 1 | Ref |
| EU568876 | Unassigned | Unassigned | Unassigned | 783_0 | Clustered | 67 | VC_783_0 | 67 | 0.8945 | 1 | 0.8945 | 0 | 0 | 0 | 1 | Ref |
| EU570103 | Unassigned | Unassigned | Unassigned | 30_1 | Clustered | 53 | VC_30_1 | 53 | 0.7151 | 1 | 0.7151 | 0 | 0 | 0 | 0.9325 | Ref |
| EU588980 | Unassigned | Unassigned | Unassigned | 839_0 | Clustered | 8 | VC_839_0 | 8 | 0.9889 | 1 | 0.9889 | 0 | 0 | 0 | 1 | Ref |
| EU622808 | Unassigned | Unassigned | Unassigned | 842_0 | Clustered | 14 | VC_842_0 | 14 | 0.6581 | 1 | 0.6581 | 0 | 0 | 0 | 1 | Ref |
| EU652770 | Unassigned | Unassigned | Unassigned | 403_0 | Clustered | 4 | VC_403_0 | 4 | 0.0462 | 0.99998824 | 0.0462 | 0 | 0 | 0 | 1 | Ref |
| EU676000 | Unassigned | Unassigned | Unassigned | 585_0 | Clustered | 20 | VC_585_0 | 20 | 0.8376 | 1 | 0.8376 | 0 | 0 | 0 | 1 | Ref |
| EU710883 | Unassigned | Unassigned | Unassigned | 339_0 | Clustered | 11 | VC_339_0 | 11 | 0.4647 | 1 | 0.4647 | 0 | 0 | 0 | 1 | Ref |
| EU716414 | Unassigned | Unassigned | Unassigned | 81_0 | Clustered | 41 | VC_81_0 | 41 | 0.5815 | 1 | 0.5815 | 0 | 0 | 0 | 0.9805 | Ref |
| EU717894 | Unassigned | Unassigned | Unassigned | 115_0 | Clustered | 9 | VC_115_0 | 9 | 0.5166 | 1 | 0.5166 | 0 | 0 | 0 | 1 | Ref |
| EU734170 | Unassigned | Unassigned | Unassigned | 399_0 | Clustered | 49 | VC_399_0 | 49 | 0.7927 | 1 | 0.7927 | 0 | 0 | 0 | 1 | Ref |
| EU734171 | Unassigned | Unassigned | Unassigned | 399_0 | Clustered | 49 | VC_399_0 | 49 | 0.7927 | 1 | 0.7927 | 0 | 0 | 0 | 1 | Ref |
| EU734172 | Unassigned | Unassigned | Unassigned | 465_0 | Clustered | 80 | VC_465_0 | 80 | 0.9988 | 1 | 0.9988 | 0 | 0 | 0 | 1 | Ref |
| EU734173 | Unassigned | Unassigned | Unassigned | 848_0 | Clustered | 124 | VC_848_0 | 124 | 0.9484 | 1 | 0.9484 | 0 | 0 | 0 | 1 | Ref |
| EU734174 | Unassigned | Unassigned | Unassigned | 405_0 | Clustered | 126 | VC_405_0 | 126 | 0.9614 | 1 | 0.9614 | 0 | 0 | 0 | 1 | Ref |
| EU744248 | Unassigned | Unassigned | Unassigned | 335_21 | Clustered | 5 | VC_335_21 | 5 | 0.0208 | 0.99999976 | 0.0208 | 0 | 0 | 0 | 0.8 | Ref |
| EU744249 | Unassigned | Unassigned | Unassigned | 335_13 | Clustered | 2 | VC_335_13 | 2 | 0.0082 | 0.95751436 | 0.0079 | 0 | 0 | 0 | 1 | Ref |
| EU744250 | Unassigned | Unassigned | Unassigned | 224_0 | Clustered | 5 | VC_224_0 | 5 | 0.0685 | 0.99999998 | 0.0685 | 0 | 0 | 0 | 1 | Ref |
| EU744251 | Unassigned | Unassigned | Unassigned | 335_44 | Clustered | 3 | VC_335_44 | 3 | 0.0157 | 0.99859171 | 0.0157 | 0 | 0 | 0 | 1 | Ref |
| EU744252 | Unassigned | Unassigned | Unassigned | 335_13 | Clustered | 2 | VC_335_13 | 2 | 0.0082 | 0.95751436 | 0.0079 | 0 | 0 | 0 | 1 | Ref |
| EU770221 | Unassigned | Unassigned | Unassigned | 385_0 | Clustered | 24 | VC_385_0 | 24 | 0.5516 | 1 | 0.5516 | 0 | 0 | 0 | 1 | Ref |
| EU770222 | Unassigned | Unassigned | Unassigned | 587_0 | Clustered | 8 | VC_587_0 | 8 | 0.4907 | 1 | 0.4907 | 0 | 0 | 0 | 1 | Ref |
| EU771092 | Unassigned | Unassigned | Unassigned | 842_0 | Clustered | 14 | VC_842_0 | 14 | 0.6581 | 1 | 0.6581 | 0 | 0 | 0 | 1 | Ref |
| EU794049 | Unassigned | Unassigned | Unassigned | 31_0 | Clustered | 10 | VC_31_0 | 10 | 0.5129 | 1 | 0.5129 | 0 | 0 | 0 | 1 | Ref |
| EU816588 | Unassigned | Unassigned | Unassigned | 571_6 | Clustered | 22 | VC_571_6 | 22 | 0.1207 | 1 | 0.1207 | 0 | 0 | 0 | 0.8095 | Ref |
| EU816589 | Unassigned | Unassigned | Unassigned | 527_0 | Clustered | 37 | VC_527_0 | 37 | 0.9679 | 1 | 0.9679 | 0 | 0 | 0 | 1 | Ref |
| EU816590 | Unassigned | Unassigned | Unassigned | 538_58 | Clustered | 3 | VC_538_58 | 3 | 0.0208 | 0.9985993 | 0.0208 | 0 | 0 | 0 | 1 | Ref |
| EU816591 | Unassigned | Unassigned | Unassigned | 571_2 | Clustered | 17 | VC_571_2 | 17 | 0.0965 | 1 | 0.0965 | 0 | 0 | 0 | 0.9779 | Ref |
| EU826466 | Unassigned | Unassigned | Unassigned | 580_0 | Clustered | 2 | VC_580_0 | 2 | 0.011 | 0.95651281 | 0.0105 | 0 | 0 | 0 | 1 | Ref |
| EU826467 | Unassigned | Unassigned | Unassigned | 579_30 | Clustered | 3 | VC_579_30 | 3 | 0.0064 | 0.74796372 | 0.0048 | 0 | 0 | 0 | 1 | Ref |
| EU826468 | Unassigned | Unassigned | Unassigned | 579_25 | Clustered/Singleton | 1 | VC_579_25 | 1 | 0 | 1 | 0 | 0 | 0 | 0 | 1 | Ref |
| EU826469 | Unassigned | Unassigned | Unassigned | 579_26 | Clustered/Singleton | 1 | VC_579_26 | 1 | 0 | 1 | 0 | 0 | 0 | 0 | 1 | Ref |
| EU826470 | Unassigned | Unassigned | Unassigned | 335_49 | Clustered | 17 | VC_335_49 | 17 | 0.0731 | 1 | 0.0731 | 0 | 0 | 0 | 0.7721 | Ref |
| EU826471 | Unassigned | Unassigned | Unassigned | 579_2 | Clustered | 2 | VC_579_2 | 2 | 0.0032 | 0.63743142 | 0.002 | 0 | 0 | 0 | 1 | Ref |
| EU855793 | Unassigned | Unassigned | Unassigned | 755_0 | Clustered | 2 | VC_755_0 | 2 | 0.2367 | 0.9499739 | 0.2249 | 0 | 0 | 0 | 1 | Ref |
| EU861004 | Unassigned | Unassigned | Unassigned | 9_0 | Clustered | 38 | VC_9_0 | 38 | 0.4859 | 1 | 0.4859 | 0 | 0 | 0 | 1 | Ref |
| EU861005 | Unassigned | Unassigned | Unassigned | 8_0 | Clustered | 58 | VC_8_0 | 58 | 0.8285 | 1 | 0.8285 | 0 | 0 | 0 | 0.9946 | Ref |
| EU863408 | Unassigned | Unassigned | Unassigned | 609_0 | Clustered | 27 | VC_609_0 | 27 | 0.9943 | 1 | 0.9943 | 0 | 0 | 0 | 1 | Ref |
| EU863409 | Unassigned | Unassigned | Unassigned | 297_6 | Clustered | 12 | VC_297_6 | 12 | 0.1139 | 1 | 0.1139 | 0 | 0 | 0 | 0.8182 | Ref |
| EU874396 | Unassigned | Unassigned | Unassigned | 671_0 | Clustered | 4 | VC_671_0 | 4 | 0.4756 | 0.99998076 | 0.4756 | 0 | 0 | 0 | 1 | Ref |
| EU877232 | Unassigned | Unassigned | Unassigned | 340_7 | Clustered | 18 | VC_340_7 | 18 | 0.0936 | 1 | 0.0936 | 0 | 0 | 0 | 0.7778 | Ref |
| EU887664 | Unassigned | Unassigned | Unassigned | 853_0 | Clustered | 5 | VC_853_0 | 5 | 0.8735 | 0.99999832 | 0.8735 | 0 | 0 | 0 | 1 | Ref |
| F1CCG | Unassigned | Unassigned | Unassigned | 62_0 | Clustered | 12 | VC_62_0 | 12 | 0.7624 | 1 | 0.7624 | 0 | 0 | 0 | 1 | Ref |
| FJ000341 | Unassigned | Unassigned | Unassigned | 30_1 | Clustered | 53 | VC_30_1 | 53 | 0.7151 | 1 | 0.7151 | 0 | 0 | 0 | 0.9325 | Ref |
| FJ008913 | Unassigned | Unassigned | Unassigned | 854_0 | Clustered | 6 | VC_854_0 | 6 | 0.514 | 1 | 0.514 | 0 | 0 | 0 | 1 | Ref |
| FJ168659 | Unassigned | Unassigned | Unassigned | 536_0 | Clustered | 6 | VC_536_0 | 6 | 0.129 | 1 | 0.129 | 0 | 0 | 0 | 1 | Ref |
| FJ168660 | Unassigned | Unassigned | Unassigned | 585_0 | Clustered | 20 | VC_585_0 | 20 | 0.8376 | 1 | 0.8376 | 0 | 0 | 0 | 1 | Ref |
| FJ168661 | Unassigned | Unassigned | Unassigned | 585_0 | Clustered | 20 | VC_585_0 | 20 | 0.8376 | 1 | 0.8376 | 0 | 0 | 0 | 1 | Ref |
| FJ168662 | Unassigned | Unassigned | Unassigned | 585_0 | Clustered | 20 | VC_585_0 | 20 | 0.8376 | 1 | 0.8376 | 0 | 0 | 0 | 1 | Ref |
| FJ174690 | Unassigned | Unassigned | Unassigned | 538_31 | Clustered | 11 | VC_538_31 | 11 | 0.0894 | 1 | 0.0894 | 0 | 0 | 0 | 0.6 | Ref |
| FJ174691 | Unassigned | Unassigned | Unassigned | 587_0 | Clustered | 8 | VC_587_0 | 8 | 0.4907 | 1 | 0.4907 | 0 | 0 | 0 | 1 | Ref |
| FJ174693 | Unassigned | Unassigned | Unassigned | 538_71 | Clustered/Singleton | 1 | VC_538_71 | 1 | 0 | 1 | 0 | 0 | 0 | 0 | 1 | Ref |

| | | | | | | | | | | | | | | | | |
|---|---|---|---|---|---|---|---|---|---|---|---|---|---|---|---|---|
| FJ174694 | Unassigned | Unassigned | Unassigned | 384_16 | Clustered | 42 | VC_384_16 | 42 | 0.134 | 1 | 0.134 | 0 | 0 | 0 | 0.7282 | Ref |
| FJ184280 | Unassigned | Unassigned | Unassigned | 43_1 | Clustered | 10 | VC_43_1 | 10 | 0.1173 | 1 | 0.1173 | 0 | 0 | 0 | 0.8222 | Ref |
| FJ188381 | Unassigned | Unassigned | Unassigned | 43_1 | Clustered | 10 | VC_43_1 | 10 | 0.1173 | 1 | 0.1173 | 0 | 0 | 0 | 0.8222 | Ref |
| FJ194439 | Unassigned | Unassigned | Unassigned | 399_0 | Clustered | 49 | VC_399_0 | 49 | 0.7927 | 1 | 0.7927 | 0 | 0 | 0 | 1 | Ref |
| FJ226752 | Unassigned | Unassigned | Unassigned | 260_0 | Clustered | 46 | VC_260_0 | 46 | 0.5137 | 1 | 0.5137 | 0 | 0 | 0 | 1 | Ref |
| FJ230960 | Unassigned | Unassigned | Unassigned | 89_0 | Clustered | 8 | VC_89_0 | 8 | 0.5507 | 1 | 0.5507 | 0 | 0 | 0 | 1 | Ref |
| FJ236310 | Unassigned | Unassigned | Unassigned | 258_0 | Clustered | 121 | VC_258_0 | 121 | 0.7359 | 1 | 0.7359 | 0 | 0 | 0 | 0.9999 | Ref |
| FJ373894 | Unassigned | Unassigned | Unassigned | 133_2 | Clustered | 7 | VC_133_2 | 7 | 0.0679 | 1 | 0.0679 | 0 | 0 | 0 | 0.9048 | Ref |
| FJ409640 | Unassigned | Unassigned | Unassigned | 83_0 | Clustered | 24 | VC_83_0 | 24 | 0.7345 | 1 | 0.7345 | 0 | 0 | 0 | 1 | Ref |
| FJ429185 | Unassigned | Unassigned | Unassigned | 552_0 | Clustered | 9 | VC_552_0 | 9 | 1 | 1 | 1 | 0 | 0 | 0 | 1 | Ref |
| FJ483837 | Unassigned | Unassigned | Unassigned | 322_0 | Clustered | 12 | VC_322_0 | 12 | 0.8924 | 1 | 0.8924 | 0 | 0 | 0 | 1 | Ref |
| FJ483838 | Unassigned | Unassigned | Unassigned | 322_0 | Clustered | 12 | VC_322_0 | 12 | 0.8924 | 1 | 0.8924 | 0 | 0 | 0 | 1 | Ref |
| FJ483839 | Unassigned | Unassigned | Unassigned | 322_0 | Clustered | 12 | VC_322_0 | 12 | 0.8924 | 1 | 0.8924 | 0 | 0 | 0 | 1 | Ref |
| FJ483840 | Unassigned | Unassigned | Unassigned | 196_0 | Clustered | 13 | VC_196_0 | 13 | 0.9973 | 0.99823802 | 0.9955 | 0 | 0 | 0 | 1 | Ref |
| FJ483841 | Unassigned | Unassigned | Unassigned | 196_0 | Clustered | 13 | VC_196_0 | 13 | 0.9973 | 0.99823802 | 0.9955 | 0 | 0 | 0 | 1 | Ref |
| FJ483842 | Unassigned | Unassigned | Unassigned | 196_0 | Clustered | 13 | VC_196_0 | 13 | 0.9973 | 0.99823802 | 0.9955 | 0 | 0 | 0 | 1 | Ref |
| FJ483843 | Unassigned | Unassigned | Unassigned | 196_0 | Clustered | 13 | VC_196_0 | 13 | 0.9973 | 0.99823802 | 0.9955 | 0 | 0 | 0 | 1 | Ref |
| FJ483844 | Unassigned | Unassigned | Unassigned | 196_0 | Clustered | 13 | VC_196_0 | 13 | 0.9973 | 0.99823802 | 0.9955 | 0 | 0 | 0 | 1 | Ref |
| FJ539132 | Unassigned | Unassigned | Unassigned | 196_0 | Clustered | 13 | VC_196_0 | 13 | 0.9973 | 0.99823802 | 0.9955 | 0 | 0 | 0 | 1 | Ref |
| FJ539133 | Unassigned | Unassigned | Unassigned | 196_0 | Clustered | 13 | VC_196_0 | 13 | 0.9973 | 0.99823802 | 0.9955 | 0 | 0 | 0 | 1 | Ref |
| FJ539134 | Unassigned | Unassigned | Unassigned | 196_0 | Clustered | 13 | VC_196_0 | 13 | 0.9973 | 0.99823802 | 0.9955 | 0 | 0 | 0 | 1 | Ref |
| FJ539135 | Unassigned | Unassigned | Unassigned | 196_0 | Clustered | 13 | VC_196_0 | 13 | 0.9973 | 0.99823802 | 0.9955 | 0 | 0 | 0 | 1 | Ref |
| FJ591093 | Unassigned | Unassigned | Unassigned | 857_0 | Clustered | 16 | VC_857_0 | 16 | 0.9317 | 1 | 0.9317 | 0 | 0 | 0 | 1 | Ref |
| FJ591094 | Unassigned | Unassigned | Unassigned | 857_0 | Clustered | 16 | VC_857_0 | 16 | 0.9317 | 1 | 0.9317 | 0 | 0 | 0 | 1 | Ref |
| FJ641182 | Unassigned | Unassigned | Unassigned | 527_0 | Clustered | 37 | VC_527_0 | 37 | 0.9679 | 1 | 0.9679 | 0 | 0 | 0 | 1 | Ref |
| FJ685651 | Unassigned | Unassigned | Unassigned | 858_0 | Clustered | 7 | VC_858_0 | 7 | 1 | 1 | 1 | 0 | 0 | 0 | 1 | Ref |
| FJ706171 | Unassigned | Unassigned | Unassigned | 787_0 | Clustered | 132 | VC_787_0 | 132 | 1 | 1 | 1 | 0 | 0 | 0 | 1 | Ref |
| FJ706172 | Unassigned | Unassigned | Unassigned | 787_0 | Clustered | 132 | VC_787_0 | 132 | 1 | 1 | 1 | 0 | 0 | 0 | 1 | Ref |
| FJ713816 | Unassigned | Unassigned | Unassigned | 7_0 | Clustered | 28 | VC_7_0 | 28 | 0.389 | 1 | 0.389 | 0 | 0 | 0 | 0.9947 | Ref |
| FJ750948 | Unassigned | Unassigned | Unassigned | 859_0 | Clustered | 67 | VC_859_0 | 67 | 0.9622 | 1 | 0.9622 | 0 | 0 | 0 | 1 | Ref |
| FJ822135 | Unassigned | Unassigned | Unassigned | 95_0 | Clustered | 2 | VC_95_0 | 2 | 0.1028 | 0.95671136 | 0.0984 | 0 | 0 | 0 | 1 | Ref |
| FJ839692 | Unassigned | Unassigned | Unassigned | 290_84 | Clustered/Singleton | 1 | VC_290_84 | 1 | 0 | 1 | 0 | 0 | 0 | 0 | 1 | Ref |
| FJ839693 | Unassigned | Unassigned | Unassigned | 290_3 | Clustered | 3 | VC_290_3 | 3 | 0.0055 | 0.99676466 | 0.0054 | 0 | 0 | 0 | 1 | Ref |
| FJ848881 | Unassigned | Unassigned | Unassigned | 210_0 | Clustered | 105 | VC_210_0 | 105 | 0.4076 | 1 | 0.4076 | 0 | 0 | 0 | 0.7766 | Ref |
| FJ848882 | Unassigned | Unassigned | Unassigned | 210_0 | Clustered | 105 | VC_210_0 | 105 | 0.4076 | 1 | 0.4076 | 0 | 0 | 0 | 0.7766 | Ref |
| FJ848883 | Unassigned | Unassigned | Unassigned | 210_0 | Clustered | 105 | VC_210_0 | 105 | 0.4076 | 1 | 0.4076 | 0 | 0 | 0 | 0.7766 | Ref |
| FJ848884 | Unassigned | Unassigned | Unassigned | 210_0 | Clustered | 105 | VC_210_0 | 105 | 0.4076 | 1 | 0.4076 | 0 | 0 | 0 | 0.7766 | Ref |
| FJ848885 | Unassigned | Unassigned | Unassigned | 210_0 | Clustered | 105 | VC_210_0 | 105 | 0.4076 | 1 | 0.4076 | 0 | 0 | 0 | 0.7766 | Ref |
| FJ849058 | Unassigned | Unassigned | Unassigned | 310_0 | Clustered | 155 | VC_310_0 | 155 | 0.9979 | 1 | 0.9979 | 0 | 0 | 0 | 1 | Ref |
| FJ867910 | Unassigned | Unassigned | Unassigned | 311_0 | Clustered | 11 | VC_311_0 | 11 | 0.7851 | 1 | 0.7851 | 0 | 0 | 0 | 1 | Ref |
| FJ867911 | Unassigned | Unassigned | Unassigned | 311_0 | Clustered | 11 | VC_311_0 | 11 | 0.7851 | 1 | 0.7851 | 0 | 0 | 0 | 1 | Ref |
| FJ867912 | Unassigned | Unassigned | Unassigned | 311_0 | Clustered | 11 | VC_311_0 | 11 | 0.7851 | 1 | 0.7851 | 0 | 0 | 0 | 1 | Ref |
| FJ867913 | Unassigned | Unassigned | Unassigned | 311_0 | Clustered | 11 | VC_311_0 | 11 | 0.7851 | 1 | 0.7851 | 0 | 0 | 0 | 1 | Ref |
| FJ867914 | Unassigned | Unassigned | Unassigned | 311_0 | Clustered | 11 | VC_311_0 | 11 | 0.7851 | 1 | 0.7851 | 0 | 0 | 0 | 1 | Ref |
| FJ870915 | Unassigned | Unassigned | Unassigned | 618_0 | Clustered | 50 | VC_618_0 | 50 | 0.9989 | 1 | 0.9989 | 0 | 0 | 0 | 1 | Ref |
| FJ870916 | Unassigned | Unassigned | Unassigned | 618_0 | Clustered | 50 | VC_618_0 | 50 | 0.9989 | 1 | 0.9989 | 0 | 0 | 0 | 1 | Ref |
| FJ870917 | Unassigned | Unassigned | Unassigned | 618_0 | Clustered | 50 | VC_618_0 | 50 | 0.9989 | 1 | 0.9989 | 0 | 0 | 0 | 1 | Ref |
| FJ896200 | Unassigned | Unassigned | Unassigned | 861_0 | Clustered | 29 | VC_861_0 | 29 | 0.9954 | 1 | 0.9954 | 0 | 0 | 0 | 1 | Ref |
| FJ904927 | Unassigned | Unassigned | Unassigned | 16_0 | Clustered | 12 | VC_16_0 | 12 | 0.6601 | 1 | 0.6601 | 0 | 0 | 0 | 1 | Ref |
| FJ937737 | Unassigned | Unassigned | Unassigned | 168_0 | Clustered | 18 | VC_168_0 | 18 | 0.7394 | 1 | 0.7394 | 0 | 0 | 0 | 0.8627 | Ref |
| FJ965538 | Unassigned | Unassigned | Unassigned | 261_0 | Clustered | 13 | VC_261_0 | 13 | 0.2024 | 1 | 0.2024 | 0 | 0 | 0 | 1 | Ref |
| FJ973624 | Unassigned | Unassigned | Unassigned | 783_0 | Clustered | 67 | VC_783_0 | 67 | 0.8945 | 1 | 0.8945 | 0 | 0 | 0 | 1 | Ref |
| FM164764 | Unassigned | Unassigned | Unassigned | 360_0 | Clustered | 22 | VC_360_0 | 22 | 0.96 | 1 | 0.96 | 0 | 0 | 0 | 1 | Ref |
| FM180578 | Unassigned | Unassigned | Unassigned | 43_1 | Clustered | 10 | VC_43_1 | 10 | 0.1173 | 1 | 0.1173 | 0 | 0 | 0 | 0.8222 | Ref |
| FM201281 | Unassigned | Unassigned | Unassigned | 80_2 | Clustered/Singleton | 1 | VC_80_2 | 1 | 0 | 1 | 0 | 0 | 0 | 0 | 1 | Ref |
| FM201282 | Unassigned | Unassigned | Unassigned | 80_0 | Clustered | 64 | VC_80_0 | 64 | 0.418 | 1 | 0.418 | 0 | 0 | 0 | 0.7649 | Ref |
| FM207411 | Unassigned | Unassigned | Unassigned | 447_8 | Clustered/Singleton | 1 | VC_447_8 | 1 | 0 | 1 | 0 | 0 | 0 | 0 | 1 | Ref |
| FM864213 | Unassigned | Unassigned | Unassigned | 647_0 | Clustered | 37 | VC_647_0 | 37 | 0.5847 | 1 | 0.5847 | 0 | 0 | 0 | 0.9234 | Ref |
| FM887021 | Unassigned | Unassigned | Unassigned | 80_7 | Clustered | 33 | VC_80_7 | 33 | 0.2156 | 1 | 0.2156 | 0 | 0 | 0 | 0.8409 | Ref |
| FM897211 | Unassigned | Unassigned | Unassigned | 80_7 | Clustered | 33 | VC_80_7 | 33 | 0.2156 | 1 | 0.2156 | 0 | 0 | 0 | 0.8409 | Ref |
| FN263372 | Unassigned | Unassigned | Unassigned | 434_0 | Clustered | 45 | VC_434_0 | 45 | 0.9479 | 1 | 0.9479 | 0 | 0 | 0 | 1 | Ref |
| FN297812 | Unassigned | Unassigned | Unassigned | 24_0 | Clustered | 3 | VC_24_0 | 3 | 0.4232 | 0.9983762 | 0.4226 | 0 | 0 | 0 | 1 | Ref |
| FN391954 | Unassigned | Unassigned | Unassigned | 344_0 | Clustered | 30 | VC_344_0 | 30 | 0.523 | 1 | 0.523 | 0 | 0 | 0 | 0.9954 | Ref |
| FN422398 | Unassigned | Unassigned | Unassigned | 863_0 | Clustered | 5 | VC_863_0 | 5 | 0.1633 | 0.99999995 | 0.1633 | 0 | 0 | 0 | 1 | Ref |
| FN422399 | Unassigned | Unassigned | Unassigned | 862_0 | Clustered | 29 | VC_862_0 | 29 | 0.8594 | 1 | 0.8594 | 0 | 0 | 0 | 1 | Ref |
| FN594518 | Unassigned | Unassigned | Unassigned | 161_0 | Clustered | 14 | VC_161_0 | 14 | 0.6064 | 1 | 0.6064 | 0 | 0 | 0 | 1 | Ref |
| FN667788 | Unassigned | Unassigned | Unassigned | 867_0 | Clustered | 5 | VC_867_0 | 5 | 0.2806 | 0.99999994 | 0.2806 | 0 | 0 | 0 | 1 | Ref |
| FN667789 | Unassigned | Unassigned | Unassigned | 867_0 | Clustered | 5 | VC_867_0 | 5 | 0.2806 | 0.99999994 | 0.2806 | 0 | 0 | 0 | 1 | Ref |
| FN997652 | Unassigned | Unassigned | Unassigned | 647_0 | Clustered | 37 | VC_647_0 | 37 | 0.5847 | 1 | 0.5847 | 0 | 0 | 0 | 0.9234 | Ref |
| FO818745 | Unassigned | Unassigned | Unassigned | 254_0 | Clustered | 14 | VC_254_0 | 14 | 0.9118 | 1 | 0.9118 | 0 | 0 | 0 | 0.967 | Ref |
| FQ312032 | Unassigned | Unassigned | Unassigned | 135_22 | Clustered | 13 | VC_135_22 | 13 | 0.0795 | 1 | 0.0795 | 0 | 0 | 0 | 0.8205 | Ref |
| FQ482083 | Unassigned | Unassigned | Unassigned | 339_0 | Clustered | 11 | VC_339_0 | 11 | 0.4647 | 1 | 0.4647 | 0 | 0 | 0 | 1 | Ref |
| FQ482084 | Unassigned | Unassigned | Unassigned | 467_0 | Clustered | 6 | VC_467_0 | 6 | 0.1136 | 1 | 0.1136 | 0 | 0 | 0 | 1 | Ref |
| FQ482086 | Unassigned | Unassigned | Unassigned | 467_0 | Clustered | 6 | VC_467_0 | 6 | 0.1136 | 1 | 0.1136 | 0 | 0 | 0 | 1 | Ref |
| FR667955 | Unassigned | Unassigned | Unassigned | 405_0 | Clustered | 126 | VC_405_0 | 126 | 0.9614 | 1 | 0.9614 | 0 | 0 | 0 | 1 | Ref |
| FR671405 | Unassigned | Unassigned | Unassigned | 427_0 | Clustered | 37 | VC_427_0 | 37 | 0.5729 | 1 | 0.5729 | 0 | 0 | 0 | 0.9474 | Ref |
| FR671406 | Unassigned | Unassigned | Unassigned | 427_0 | Clustered | 37 | VC_427_0 | 37 | 0.5729 | 1 | 0.5729 | 0 | 0 | 0 | 0.9474 | Ref |
| FR671407 | Unassigned | Unassigned | Unassigned | 427_0 | Clustered | 37 | VC_427_0 | 37 | 0.5729 | 1 | 0.5729 | 0 | 0 | 0 | 0.9474 | Ref |
| FR671408 | Unassigned | Unassigned | Unassigned | 268_1 | Clustered | 20 | VC_268_1 | 20 | 0.1906 | 1 | 0.1906 | 0 | 0 | 0 | 0.6789 | Ref |
| FR671409 | Unassigned | Unassigned | Unassigned | 268_1 | Clustered | 20 | VC_268_1 | 20 | 0.1906 | 1 | 0.1906 | 0 | 0 | 0 | 0.6789 | Ref |
| FR671410 | Unassigned | Unassigned | Unassigned | 427_0 | Clustered | 37 | VC_427_0 | 37 | 0.5729 | 1 | 0.5729 | 0 | 0 | 0 | 0.9474 | Ref |

| | | | | | | | | | | | | | | | | |
|---|---|---|---|---|---|---|---|---|---|---|---|---|---|---|---|---|
| FR671411 | Unassigned | Unassigned | Unassigned | 427_0 | Clustered | 37 | VC_427_0 | 37 | 0.5729 | 1 | 0.5729 | 0 | 0 | 0 | 0.9474 | Ref |
| FR682616 | Unassigned | Unassigned | Unassigned | 857_0 | Clustered | 16 | VC_857_0 | 16 | 0.9317 | 1 | 0.9317 | 0 | 0 | 0 | 1 | Ref |
| FR687252 | Unassigned | Unassigned | Unassigned | 112_0 | Clustered | 4 | VC_112_0 | 4 | 0.1133 | 0.99998733 | 0.1133 | 0 | 0 | 0 | 1 | Ref |
| FR719956 | Unassigned | Unassigned | Unassigned | 857_0 | Clustered | 16 | VC_857_0 | 16 | 0.9317 | 1 | 0.9317 | 0 | 0 | 0 | 1 | Ref |
| FR751545 | Unassigned | Unassigned | Unassigned | 162_0 | Clustered | 12 | VC_162_0 | 12 | 0.9051 | 1 | 0.9051 | 0 | 0 | 0 | 1 | Ref |
| FR775895 | Unassigned | Unassigned | Unassigned | 872_0 | Clustered | 28 | VC_872_0 | 28 | 0.7887 | 1 | 0.7887 | 0 | 0 | 0 | 1 | Ref |
| FR823298 | Unassigned | Unassigned | Unassigned | 132_0 | Clustered | 19 | VC_132_0 | 19 | 0.6051 | 1 | 0.6051 | 0 | 0 | 0 | 1 | Ref |
| FR823450 | Unassigned | Unassigned | Unassigned | 868_0 | Clustered | 27 | VC_868_0 | 27 | 0.9372 | 1 | 0.9372 | 0 | 0 | 0 | 1 | Ref |
| FR852584 | Unassigned | Unassigned | Unassigned | 155_8 | Clustered | 66 | VC_155_8 | 66 | 0.4537 | 1 | 0.4537 | 0 | 0 | 0 | 0.8182 | Ref |
| GQ141189 | Unassigned | Unassigned | Unassigned | 876_0 | Clustered | 2 | VC_876_0 | 2 | 0.4559 | 0.94489191 | 0.4308 | 0 | 0 | 0 | 1 | Ref |
| GQ149088 | Unassigned | Unassigned | Unassigned | 760_0 | Clustered | 15 | VC_760_0 | 15 | 1 | 1 | 1 | 0 | 0 | 0 | 1 | Ref |
| GQ153912 | Unassigned | Unassigned | Unassigned | 310_0 | Clustered | 155 | VC_310_0 | 155 | 0.9979 | 1 | 0.9979 | 0 | 0 | 0 | 1 | Ref |
| GQ153913 | Unassigned | Unassigned | Unassigned | 310_0 | Clustered | 155 | VC_310_0 | 155 | 0.9979 | 1 | 0.9979 | 0 | 0 | 0 | 1 | Ref |
| GQ153914 | Unassigned | Unassigned | Unassigned | 310_0 | Clustered | 155 | VC_310_0 | 155 | 0.9979 | 1 | 0.9979 | 0 | 0 | 0 | 1 | Ref |
| GQ153915 | Unassigned | Unassigned | Unassigned | 310_0 | Clustered | 155 | VC_310_0 | 155 | 0.9979 | 1 | 0.9979 | 0 | 0 | 0 | 1 | Ref |
| GQ153916 | Unassigned | Unassigned | Unassigned | 62_0 | Clustered | 12 | VC_62_0 | 12 | 0.7624 | 1 | 0.7624 | 0 | 0 | 0 | 1 | Ref |
| GQ153917 | Unassigned | Unassigned | Unassigned | 62_0 | Clustered | 12 | VC_62_0 | 12 | 0.7624 | 1 | 0.7624 | 0 | 0 | 0 | 1 | Ref |
| GQ153918 | Unassigned | Unassigned | Unassigned | 62_0 | Clustered | 12 | VC_62_0 | 12 | 0.7624 | 1 | 0.7624 | 0 | 0 | 0 | 1 | Ref |
| GQ153919 | Unassigned | Unassigned | Unassigned | 62_0 | Clustered | 12 | VC_62_0 | 12 | 0.7624 | 1 | 0.7624 | 0 | 0 | 0 | 1 | Ref |
| GQ153920 | Unassigned | Unassigned | Unassigned | 373_0 | Clustered | 358 | VC_373_0 | 358 | 0.9614 | 1 | 0.9614 | 0 | 0 | 0 | 1 | Ref |
| GQ153921 | Unassigned | Unassigned | Unassigned | 375_0 | Clustered | 2 | VC_375_0 | 2 | 0.002 | 0.83307735 | 0.0017 | 0 | 0 | 0 | 1 | Ref |
| GQ153922 | Unassigned | Unassigned | Unassigned | 373_0 | Clustered | 358 | VC_373_0 | 358 | 0.9614 | 1 | 0.9614 | 0 | 0 | 0 | 1 | Ref |
| GQ153923 | Unassigned | Unassigned | Unassigned | 373_0 | Clustered | 358 | VC_373_0 | 358 | 0.9614 | 1 | 0.9614 | 0 | 0 | 0 | 1 | Ref |
| GQ241246 | Unassigned | Unassigned | Unassigned | 880_0 | Clustered | 4 | VC_880_0 | 4 | 0.0727 | 0.88800114 | 0.0645 | 0 | 0 | 0 | 1 | Ref |
| GQ303259 | Unassigned | Unassigned | Unassigned | 384_3 | Clustered | 22 | VC_384_3 | 22 | 0.0786 | 1 | 0.0786 | 0 | 0 | 0 | 0.9048 | Ref |
| GQ303260 | Unassigned | Unassigned | Unassigned | 579_1 | Clustered | 2 | VC_579_1 | 2 | 0.0032 | 0.75989446 | 0.0024 | 0 | 0 | 0 | 0 | Ref |
| GQ303261 | Unassigned | Unassigned | Unassigned | 783_0 | Clustered | 67 | VC_783_0 | 67 | 0.8945 | 1 | 0.8945 | 0 | 0 | 0 | 1 | Ref |
| GQ303262 | Unassigned | Unassigned | Unassigned | 579_82 | Clustered | 3 | VC_579_82 | 3 | 0.0064 | 0.86736674 | 0.0056 | 0 | 0 | 0 | 0.6667 | Ref |
| GQ303263 | Unassigned | Unassigned | Unassigned | 535_1 | Clustered | 108 | VC_535_1 | 108 | 0.8479 | 1 | 0.8479 | 0 | 0 | 0 | 0.9955 | Ref |
| GQ303264 | Unassigned | Unassigned | Unassigned | 384_15 | Clustered | 44 | VC_384_15 | 44 | 0.139 | 1 | 0.139 | 0 | 0 | 0 | 0.7474 | Ref |
| GQ303265 | Unassigned | Unassigned | Unassigned | 571_7 | Clustered | 28 | VC_571_7 | 28 | 0.1651 | 1 | 0.1651 | 0 | 0 | 0 | 0.8519 | Ref |
| GQ303266 | Unassigned | Unassigned | Unassigned | 384_15 | Clustered | 44 | VC_384_15 | 44 | 0.139 | 1 | 0.139 | 0 | 0 | 0 | 0.7474 | Ref |
| GQ357916 | Unassigned | Unassigned | Unassigned | 52_0 | Clustered | 9 | VC_52_0 | 9 | 0.5586 | 1 | 0.5586 | 0 | 0 | 0 | 1 | Ref |
| GQ379227 | Unassigned | Unassigned | Unassigned | 387_0 | Clustered | 2 | VC_387_0 | 2 | 0.1249 | 0.95576264 | 0.1193 | 0 | 0 | 0 | 1 | Ref |
| GQ398772 | Unassigned | Unassigned | Unassigned | 10_0 | Clustered | 25 | VC_10_0 | 25 | 0.3437 | 1 | 0.3437 | 0 | 0 | 0 | 0.9733 | Ref |
| GQ403789 | Unassigned | Unassigned | Unassigned | 29_0 | Clustered | 2 | VC_29_0 | 2 | 1 | 1 | 1 | 0 | 0 | 0 | 1 | Ref |
| GQ413937 | Unassigned | Unassigned | Unassigned | 848_0 | Clustered | 124 | VC_848_0 | 124 | 0.9484 | 1 | 0.9484 | 0 | 0 | 0 | 1 | Ref |
| GQ413938 | Unassigned | Unassigned | Unassigned | 113_0 | Clustered | 91 | VC_113_0 | 91 | 0.9584 | 1 | 0.9584 | 0 | 0 | 0 | 1 | Ref |
| GQ421471 | Unassigned | Unassigned | Unassigned | 287_0 | Clustered | 2 | VC_287_0 | 2 | 0.2918 | 0.9525588 | 0.2779 | 0 | 0 | 0 | 1 | Ref |
| GQ422450 | Unassigned | Unassigned | Unassigned | 30_1 | Clustered | 53 | VC_30_1 | 53 | 0.7151 | 1 | 0.7151 | 0 | 0 | 0 | 0.9325 | Ref |
| GQ422451 | Unassigned | Unassigned | Unassigned | 30_1 | Clustered | 53 | VC_30_1 | 53 | 0.7151 | 1 | 0.7151 | 0 | 0 | 0 | 0.9325 | Ref |
| GQ443085 | Unassigned | Unassigned | Unassigned | 839_0 | Clustered | 8 | VC_839_0 | 8 | 0.9889 | 1 | 0.9889 | 0 | 0 | 0 | 1 | Ref |
| GQ451696 | Unassigned | Unassigned | Unassigned | 889_0 | Clustered | 13 | VC_889_0 | 13 | 0.6844 | 1 | 0.6844 | 0 | 0 | 0 | 1 | Ref |
| GQ452243 | Unassigned | Unassigned | Unassigned | 273_0 | Clustered | 2 | VC_273_0 | 2 | 0.1017 | 0.95560536 | 0.0972 | 0 | 0 | 0 | 1 | Ref |
| GQ468526 | Unassigned | Unassigned | Unassigned | 399_0 | Clustered | 49 | VC_399_0 | 49 | 0.7927 | 1 | 0.7927 | 0 | 0 | 0 | 1 | Ref |
| GQ478081 | Unassigned | Unassigned | Unassigned | 796_0 | Clustered | 5 | VC_796_0 | 5 | 0.3265 | 0.99999996 | 0.3265 | 0 | 0 | 0 | 1 | Ref |
| GQ478082 | Unassigned | Unassigned | Unassigned | 796_0 | Clustered | 5 | VC_796_0 | 5 | 0.3265 | 0.99999996 | 0.3265 | 0 | 0 | 0 | 1 | Ref |
| GQ478083 | Unassigned | Unassigned | Unassigned | 796_0 | Clustered | 5 | VC_796_0 | 5 | 0.3265 | 0.99999996 | 0.3265 | 0 | 0 | 0 | 1 | Ref |
| GQ478084 | Unassigned | Unassigned | Unassigned | 796_0 | Clustered | 5 | VC_796_0 | 5 | 0.3265 | 0.99999996 | 0.3265 | 0 | 0 | 0 | 1 | Ref |
| GQ478085 | Unassigned | Unassigned | Unassigned | 796_0 | Clustered | 5 | VC_796_0 | 5 | 0.3265 | 0.99999996 | 0.3265 | 0 | 0 | 0 | 1 | Ref |
| GQ502199 | Unassigned | Unassigned | Unassigned | 859_0 | Clustered | 67 | VC_859_0 | 67 | 0.9622 | 1 | 0.9622 | 0 | 0 | 0 | 1 | Ref |
| GQ915271 | Unassigned | Unassigned | Unassigned | 7_0 | Clustered | 28 | VC_7_0 | 28 | 0.389 | 1 | 0.389 | 0 | 0 | 0 | 0.9947 | Ref |
| GQ979703 | Unassigned | Unassigned | Unassigned | 210_0 | Clustered | 105 | VC_210_0 | 105 | 0.4076 | 1 | 0.4076 | 0 | 0 | 0 | 0.7766 | Ref |
| GQ981382 | Unassigned | Unassigned | Unassigned | 297_1 | Clustered | 8 | VC_297_1 | 8 | 0.0749 | 1 | 0.0749 | 0 | 0 | 0 | 0.8571 | Ref |
| GU060500 | Unassigned | Unassigned | Unassigned | 538_31 | Clustered | 11 | VC_538_31 | 11 | 0.0894 | 1 | 0.0894 | 0 | 0 | 0 | 0.6 | Ref |
| GU070616 | Unassigned | Unassigned | Unassigned | 601_5 | Clustered | 4 | VC_601_5 | 4 | 0.0525 | 0.99930943 | 0.0525 | 0 | 0 | 0 | 0.8333 | Ref |
| GU071091 | Unassigned | Unassigned | Unassigned | 405_0 | Clustered | 126 | VC_405_0 | 126 | 0.9614 | 1 | 0.9614 | 0 | 0 | 0 | 1 | Ref |
| GU071092 | Unassigned | Unassigned | Unassigned | 446_9 | Clustered | 3 | VC_446_9 | 3 | 0.0365 | 0.99848173 | 0.0364 | 0 | 0 | 0 | 1 | Ref |
| GU071093 | Unassigned | Unassigned | Unassigned | 352_0 | Clustered | 31 | VC_352_0 | 31 | 0.9547 | 1 | 0.9547 | 0 | 0 | 0 | 1 | Ref |
| GU071094 | Unassigned | Unassigned | Unassigned | 674_8 | Clustered/Singleton | 1 | VC_674_8 | 1 | 0 | 1 | 0 | 0 | 0 | 0 | 1 | Ref |
| GU071095 | Unassigned | Unassigned | Unassigned | 446_2 | Clustered/Singleton | 1 | VC_446_2 | 1 | 0 | 1 | 0 | 0 | 0 | 0 | 1 | Ref |
| GU071097 | Unassigned | Unassigned | Unassigned | 674_9 | Clustered/Singleton | 1 | VC_674_9 | 1 | 0 | 1 | 0 | 0 | 0 | 0 | 1 | Ref |
| GU071098 | Unassigned | Unassigned | Unassigned | 446_10 | Clustered | 2 | VC_446_10 | 2 | 0.0211 | 0.95772926 | 0.0202 | 0 | 0 | 0 | 1 | Ref |
| GU071099 | Unassigned | Unassigned | Unassigned | 674_0 | Clustered | 2 | VC_674_0 | 2 | 0.0109 | 0.95704495 | 0.0105 | 0 | 0 | 0 | 1 | Ref |
| GU071100 | Unassigned | Unassigned | Unassigned | 352_0 | Clustered | 31 | VC_352_0 | 31 | 0.9547 | 1 | 0.9547 | 0 | 0 | 0 | 1 | Ref |
| GU071102 | Unassigned | Unassigned | Unassigned | 352_0 | Clustered | 31 | VC_352_0 | 31 | 0.9547 | 1 | 0.9547 | 0 | 0 | 0 | 1 | Ref |
| GU071103 | Unassigned | Unassigned | Unassigned | 674_1 | Clustered/Singleton | 1 | VC_674_1 | 1 | 0 | 1 | 0 | 0 | 0 | 0 | 1 | Ref |
| GU071104 | Unassigned | Unassigned | Unassigned | 352_0 | Clustered | 31 | VC_352_0 | 31 | 0.9547 | 1 | 0.9547 | 0 | 0 | 0 | 1 | Ref |
| GU071105 | Unassigned | Unassigned | Unassigned | 447_4 | Clustered | 9 | VC_447_4 | 9 | 0.127 | 1 | 0.127 | 0 | 0 | 0 | 1 | Ref |
| GU071106 | Unassigned | Unassigned | Unassigned | 674_6 | Clustered | 2 | VC_674_6 | 2 | 0.0077 | 0.9577529 | 0.0074 | 0 | 0 | 0 | 1 | Ref |
| GU071107 | Unassigned | Unassigned | Unassigned | 352_0 | Clustered | 31 | VC_352_0 | 31 | 0.9547 | 1 | 0.9547 | 0 | 0 | 0 | 1 | Ref |
| GU080336 | Unassigned | Unassigned | Unassigned | 631_0 | Clustered | 2 | VC_631_0 | 2 | 1 | 1 | 1 | 0 | 0 | 0 | 1 | Ref |
| GU169904 | Unassigned | Unassigned | Unassigned | 340_15 | Clustered/Singleton | 1 | VC_340_15 | 1 | 0 | 1 | 0 | 0 | 0 | 0 | 1 | Ref |
| GU196277 | Unassigned | Unassigned | Unassigned | 655_0 | Clustered | 45 | VC_655_0 | 45 | 0.7722 | 1 | 0.7722 | 0 | 0 | 0 | 1 | Ref |
| GU196278 | Unassigned | Unassigned | Unassigned | 655_0 | Clustered | 45 | VC_655_0 | 45 | 0.7722 | 1 | 0.7722 | 0 | 0 | 0 | 1 | Ref |
| GU196279 | Unassigned | Unassigned | Unassigned | 655_0 | Clustered | 45 | VC_655_0 | 45 | 0.7722 | 1 | 0.7722 | 0 | 0 | 0 | 1 | Ref |
| GU196280 | Unassigned | Unassigned | Unassigned | 655_0 | Clustered | 45 | VC_655_0 | 45 | 0.7722 | 1 | 0.7722 | 0 | 0 | 0 | 1 | Ref |
| GU196281 | Unassigned | Unassigned | Unassigned | 655_0 | Clustered | 45 | VC_655_0 | 45 | 0.7722 | 1 | 0.7722 | 0 | 0 | 0 | 1 | Ref |
| GU229986 | Unassigned | Unassigned | Unassigned | 671_0 | Clustered | 4 | VC_671_0 | 4 | 0.4756 | 0.99998076 | 0.4756 | 0 | 0 | 0 | 1 | Ref |
| GU247132 | Unassigned | Unassigned | Unassigned | 335_14 | Clustered | 3 | VC_335_14 | 3 | 0.0094 | 0.9985917 | 0.0094 | 0 | 0 | 0 | 1 | Ref |
| GU247133 | Unassigned | Unassigned | Unassigned | 384_16 | Clustered | 42 | VC_384_16 | 42 | 0.134 | 1 | 0.134 | 0 | 0 | 0 | 0.7282 | Ref |

| | | | | | | | | | | | | | | | | |
|---|---|---|---|---|---|---|---|---|---|---|---|---|---|---|---|---|
| GU247134 | Unassigned | Unassigned | Unassigned | 384_16 | Clustered | 42 | VC_384_16 | 42 | 0.134 | 1 | 0.134 | 0 | 0 | 0 | 0.7282 | Ref |
| GU295964 | Unassigned | Unassigned | Unassigned | 686_0 | Clustered | 25 | VC_686_0 | 25 | 0.5501 | 1 | 0.5501 | 0 | 0 | 0 | 0.96 | Ref |
| GU296433 | Unassigned | Unassigned | Unassigned | 868_0 | Clustered | 27 | VC_868_0 | 27 | 0.9372 | 1 | 0.9372 | 0 | 0 | 0 | 1 | Ref |
| GU321093 | Unassigned | Unassigned | Unassigned | 858_0 | Clustered | 7 | VC_858_0 | 7 | 1 | 1 | 1 | 0 | 0 | 0 | 1 | Ref |
| GU323708 | Unassigned | Unassigned | Unassigned | 280_0 | Clustered | 4 | VC_280_0 | 4 | 0.3169 | 0.99994916 | 0.3169 | 0 | 0 | 0 | 0.8333 | Ref |
| GU339467 | Unassigned | Unassigned | Unassigned | 223_3 | Clustered | 8 | VC_223_3 | 8 | 0.0699 | 1 | 0.0699 | 0 | 0 | 0 | 0.8571 | Ref |
| GU385905 | Unassigned | Unassigned | Unassigned | 310_0 | Clustered | 155 | VC_310_0 | 155 | 0.9979 | 1 | 0.9979 | 0 | 0 | 0 | 1 | Ref |
| GU393987 | Unassigned | Unassigned | Unassigned | 71_0 | Clustered | 27 | VC_71_0 | 27 | 0.5964 | 1 | 0.5964 | 0 | 0 | 0 | 0.9943 | Ref |
| GU396103 | Unassigned | Unassigned | Unassigned | 457_0 | Clustered | 7 | VC_457_0 | 7 | 0.2549 | 1 | 0.2549 | 0 | 0 | 0 | 0.7143 | Ref |
| GU459069 | Unassigned | Unassigned | Unassigned | 457_3 | Clustered | 2 | VC_457_3 | 2 | 0.0286 | 0.89347378 | 0.0255 | 0 | 0 | 0 | 1 | Ref |
| GU477322 | Unassigned | Unassigned | Unassigned | 9_0 | Clustered | 38 | VC_9_0 | 38 | 0.4859 | 1 | 0.4859 | 0 | 0 | 0 | 1 | Ref |
| GU557055 | Unassigned | Unassigned | Unassigned | 312_0 | Clustered | 12 | VC_312_0 | 12 | 0.5719 | 1 | 0.5719 | 0 | 0 | 0 | 0.8485 | Ref |
| GU573886 | Unassigned | Unassigned | Unassigned | 30_1 | Clustered | 53 | VC_30_1 | 53 | 0.7151 | 1 | 0.7151 | 0 | 0 | 0 | 0.9325 | Ref |
| GU580941 | Unassigned | Unassigned | Unassigned | 898_0 | Clustered | 2 | VC_898_0 | 2 | 0.0372 | 0.95472903 | 0.0355 | 0 | 0 | 0 | 1 | Ref |
| GU580942 | Unassigned | Unassigned | Unassigned | 898_0 | Clustered | 2 | VC_898_0 | 2 | 0.0372 | 0.95472903 | 0.0355 | 0 | 0 | 0 | 1 | Ref |
| GU583987 | Unassigned | Unassigned | Unassigned | 132_0 | Clustered | 19 | VC_132_0 | 19 | 0.6051 | 1 | 0.6051 | 0 | 0 | 0 | 1 | Ref |
| GU595417 | Unassigned | Unassigned | Unassigned | 466_0 | Clustered | 66 | VC_466_0 | 66 | 0.9246 | 1 | 0.9246 | 0 | 0 | 0 | 1 | Ref |
| GU815091 | Unassigned | Unassigned | Unassigned | 80_7 | Clustered | 33 | VC_80_7 | 33 | 0.2156 | 1 | 0.2156 | 0 | 0 | 0 | 0.8409 | Ref |
| GU903191 | Unassigned | Unassigned | Unassigned | 127_0 | Clustered | 17 | VC_127_0 | 17 | 0.9222 | 1 | 0.9222 | 0 | 0 | 0 | 1 | Ref |
| GU911303 | Unassigned | Unassigned | Unassigned | 1_0 | Clustered | 54 | VC_1_0 | 54 | 0.8518 | 1 | 0.8518 | 0 | 0 | 0 | 1 | Ref |
| GU911304 | Unassigned | Unassigned | Unassigned | 3_0 | Clustered | 17 | VC_3_0 | 17 | 0.5615 | 1 | 0.5615 | 0 | 0 | 0 | 1 | Ref |
| GU911519 | Unassigned | Unassigned | Unassigned | 510_3 | Clustered/Singleton | 1 | VC_510_3 | 1 | 0 | 1 | 0 | 0 | 0 | 0 | 1 | Ref |
| GU936715 | Unassigned | Unassigned | Unassigned | 902_0 | Clustered | 3 | VC_902_0 | 3 | 0.8989 | 0.98968122 | 0.8896 | 0 | 0 | 0 | 1 | Ref |
| GU942563 | Unassigned | Unassigned | Unassigned | 904_0 | Clustered | 10 | VC_904_0 | 10 | 0.9878 | 0.99948433 | 0.9873 | 0 | 0 | 0 | 1 | Ref |
| GU943054 | Unassigned | Unassigned | Unassigned | 352_0 | Clustered | 31 | VC_352_0 | 31 | 0.9547 | 1 | 0.9547 | 0 | 0 | 0 | 1 | Ref |
| GU967410 | Unassigned | Unassigned | Unassigned | 145_0 | Clustered | 6 | VC_145_0 | 6 | 0.2581 | 0.86251824 | 0.2226 | 0 | 0 | 0 | 0.8667 | Ref |
| GU988610 | Unassigned | Unassigned | Unassigned | 77_0 | Clustered | 37 | VC_77_0 | 37 | 0.9392 | 1 | 0.9392 | 0 | 0 | 0 | 1 | Ref |
| HE584812 | Unassigned | Unassigned | Unassigned | 185_0 | Clustered | 53 | VC_185_0 | 53 | 0.9153 | 1 | 0.9153 | 0 | 0 | 0 | 1 | Ref |
| HE600015 | Unassigned | Unassigned | Unassigned | 133_0 | Clustered | 17 | VC_133_0 | 17 | 0.2654 | 1 | 0.2654 | 0 | 0 | 0 | 1 | Ref |
| HE608841 | Unassigned | Unassigned | Unassigned | 854_0 | Clustered | 6 | VC_854_0 | 6 | 0.514 | 1 | 0.514 | 0 | 0 | 0 | 1 | Ref |
| HE664024 | Unassigned | Unassigned | Unassigned | 44_0 | Clustered | 49 | VC_44_0 | 49 | 0.4506 | 1 | 0.4506 | 0 | 0 | 0 | 0.8605 | Ref |
| HE775250 | Unassigned | Unassigned | Unassigned | 654_0 | Clustered | 115 | VC_654_0 | 115 | 0.7303 | 1 | 0.7303 | 0 | 0 | 0 | 0.8764 | Ref |
| HE806280 | Unassigned | Unassigned | Unassigned | 713_0 | Clustered | 26 | VC_713_0 | 26 | 0.9358 | 1 | 0.9358 | 0 | 0 | 0 | 1 | Ref |
| HE815464 | Unassigned | Unassigned | Unassigned | 867_0 | Clustered | 5 | VC_867_0 | 5 | 0.2806 | 0.99999994 | 0.2806 | 0 | 0 | 0 | 1 | Ref |
| HE858210 | Unassigned | Unassigned | Unassigned | 687_1 | Clustered | 10 | VC_687_1 | 10 | 0.1263 | 1 | 0.1263 | 0 | 0 | 0 | 0.9778 | Ref |
| HE861935 | Unassigned | Unassigned | Unassigned | 260_0 | Clustered | 46 | VC_260_0 | 46 | 0.5137 | 1 | 0.5137 | 0 | 0 | 0 | 1 | Ref |
| HE956704 | Unassigned | Unassigned | Unassigned | 275_0 | Clustered | 6 | VC_275_0 | 6 | 0.6515 | 0.99999994 | 0.6515 | 0 | 0 | 0 | 0.9333 | Ref |
| HE956707 | Unassigned | Unassigned | Unassigned | 907_0 | Clustered | 32 | VC_907_0 | 32 | 0.9964 | 1 | 0.9964 | 0 | 0 | 0 | 1 | Ref |
| HE956708 | Unassigned | Unassigned | Unassigned | 199_26 | Clustered/Singleton | 1 | VC_199_26 | 1 | 0 | 1 | 0 | 0 | 0 | 0 | 1 | Ref |
| HE956709 | Unassigned | Unassigned | Unassigned | 508_3 | Clustered | 2 | VC_508_3 | 2 | 0.0132 | 0.95703149 | 0.0126 | 0 | 0 | 0 | 1 | Ref |
| HE956710 | Unassigned | Unassigned | Unassigned | 907_0 | Clustered | 32 | VC_907_0 | 32 | 0.9964 | 1 | 0.9964 | 0 | 0 | 0 | 1 | Ref |
| HE956711 | Unassigned | Unassigned | Unassigned | 290_69 | Clustered/Singleton | 1 | VC_290_69 | 1 | 0 | 1 | 0 | 0 | 0 | 0 | 1 | Ref |
| HE962497 | Unassigned | Unassigned | Unassigned | 110_0 | Clustered | 33 | VC_110_0 | 33 | 0.9488 | 1 | 0.9488 | 0 | 0 | 0 | 1 | Ref |
| HE978309 | Unassigned | Unassigned | Unassigned | 609_0 | Clustered | 27 | VC_609_0 | 27 | 0.9943 | 1 | 0.9943 | 0 | 0 | 0 | 1 | Ref |
| HE981739 | Unassigned | Unassigned | Unassigned | 687_1 | Clustered | 10 | VC_687_1 | 10 | 0.1263 | 1 | 0.1263 | 0 | 0 | 0 | 0.9778 | Ref |
| HE983844 | Unassigned | Unassigned | Unassigned | 486_0 | Clustered | 47 | VC_486_0 | 47 | 0.9022 | 1 | 0.9022 | 0 | 0 | 0 | 1 | Ref |
| HE983845 | Unassigned | Unassigned | Unassigned | 77_0 | Clustered | 37 | VC_77_0 | 37 | 0.9392 | 1 | 0.9392 | 0 | 0 | 0 | 1 | Ref |
| HF543949 | Unassigned | Unassigned | Unassigned | 486_0 | Clustered | 47 | VC_486_0 | 47 | 0.9022 | 1 | 0.9022 | 0 | 0 | 0 | 1 | Ref |
| HF563658 | Unassigned | Unassigned | Unassigned | 425_0 | Clustered | 40 | VC_425_0 | 40 | 0.8399 | 1 | 0.8399 | 0 | 0 | 0 | 0.9974 | Ref |
| HF569088 | Unassigned | Unassigned | Unassigned | 908_0 | Clustered | 36 | VC_908_0 | 36 | 0.9943 | 1 | 0.9943 | 0 | 0 | 0 | 1 | Ref |
| HF569089 | Unassigned | Unassigned | Unassigned | 908_0 | Clustered | 36 | VC_908_0 | 36 | 0.9943 | 1 | 0.9943 | 0 | 0 | 0 | 1 | Ref |
| HF569090 | Unassigned | Unassigned | Unassigned | 908_0 | Clustered | 36 | VC_908_0 | 36 | 0.9943 | 1 | 0.9943 | 0 | 0 | 0 | 1 | Ref |
| HF569091 | Unassigned | Unassigned | Unassigned | 908_0 | Clustered | 36 | VC_908_0 | 36 | 0.9943 | 1 | 0.9943 | 0 | 0 | 0 | 1 | Ref |
| HF569092 | Unassigned | Unassigned | Unassigned | 908_0 | Clustered | 36 | VC_908_0 | 36 | 0.9943 | 1 | 0.9943 | 0 | 0 | 0 | 1 | Ref |
| HF937074 | Unassigned | Unassigned | Unassigned | 98_0 | Clustered | 30 | VC_98_0 | 30 | 0.7175 | 1 | 0.7175 | 0 | 0 | 0 | 1 | Ref |
| HG007973 | Unassigned | Unassigned | Unassigned | 909_0 | Clustered | 2 | VC_909_0 | 2 | 0.1608 | 0.83455073 | 0.1342 | 0 | 0 | 0 | 1 | Ref |
| HG315669 | Unassigned | Unassigned | Unassigned | 183_0 | Clustered | 5 | VC_183_0 | 5 | 0.7449 | 0.99885646 | 0.744 | 0 | 0 | 0 | 1 | Ref |
| HG322870 | Unassigned | Unassigned | Unassigned | 460_0 | Clustered | 4 | VC_460_0 | 4 | 0.7519 | 0.99987768 | 0.7518 | 0 | 0 | 0 | 1 | Ref |
| HG380752 | Unassigned | Unassigned | Unassigned | 260_0 | Clustered | 46 | VC_260_0 | 46 | 0.5137 | 1 | 0.5137 | 0 | 0 | 0 | 1 | Ref |
| HG424323 | Unassigned | Unassigned | Unassigned | 260_0 | Clustered | 46 | VC_260_0 | 46 | 0.5137 | 1 | 0.5137 | 0 | 0 | 0 | 1 | Ref |
| HG428758 | Unassigned | Unassigned | Unassigned | 908_0 | Clustered | 36 | VC_908_0 | 36 | 0.9943 | 1 | 0.9943 | 0 | 0 | 0 | 1 | Ref |
| HG518155 | Unassigned | Unassigned | Unassigned | 486_0 | Clustered | 47 | VC_486_0 | 47 | 0.9022 | 1 | 0.9022 | 0 | 0 | 0 | 1 | Ref |
| HG531805 | Unassigned | Unassigned | Unassigned | 140_1 | Clustered | 4 | VC_140_1 | 4 | 0.1766 | 0.99998565 | 0.1766 | 0 | 0 | 0 | 1 | Ref |
| HG792102 | Unassigned | Unassigned | Unassigned | 44_0 | Clustered | 49 | VC_44_0 | 49 | 0.4506 | 1 | 0.4506 | 0 | 0 | 0 | 0.8605 | Ref |
| HG792103 | Unassigned | Unassigned | Unassigned | 44_0 | Clustered | 49 | VC_44_0 | 49 | 0.4506 | 1 | 0.4506 | 0 | 0 | 0 | 0.8605 | Ref |
| HG792104 | Unassigned | Unassigned | Unassigned | 44_0 | Clustered | 49 | VC_44_0 | 49 | 0.4506 | 1 | 0.4506 | 0 | 0 | 0 | 0.8605 | Ref |
| HG792105 | Unassigned | Unassigned | Unassigned | 44_0 | Clustered | 49 | VC_44_0 | 49 | 0.4506 | 1 | 0.4506 | 0 | 0 | 0 | 0.8605 | Ref |
| HG793132 | Unassigned | Unassigned | Unassigned | 71_0 | Clustered | 27 | VC_71_0 | 27 | 0.5964 | 1 | 0.5964 | 0 | 0 | 0 | 0.9943 | Ref |
| HG796219 | Unassigned | Unassigned | Unassigned | 71_0 | Clustered | 27 | VC_71_0 | 27 | 0.5964 | 1 | 0.5964 | 0 | 0 | 0 | 0.9943 | Ref |
| HG796220 | Unassigned | Unassigned | Unassigned | 71_0 | Clustered | 27 | VC_71_0 | 27 | 0.5964 | 1 | 0.5964 | 0 | 0 | 0 | 0.9943 | Ref |
| HG796221 | Unassigned | Unassigned | Unassigned | 71_0 | Clustered | 27 | VC_71_0 | 27 | 0.5964 | 1 | 0.5964 | 0 | 0 | 0 | 0.9943 | Ref |
| HG796225 | Unassigned | Unassigned | Unassigned | 666_0 | Clustered | 11 | VC_666_0 | 11 | 0.5822 | 1 | 0.5822 | 0 | 0 | 0 | 1 | Ref |
| HG798901 | Unassigned | Unassigned | Unassigned | 666_0 | Clustered | 11 | VC_666_0 | 11 | 0.5822 | 1 | 0.5822 | 0 | 0 | 0 | 1 | Ref |
| HG799490 | Unassigned | Unassigned | Unassigned | 427_0 | Clustered | 37 | VC_427_0 | 37 | 0.5729 | 1 | 0.5729 | 0 | 0 | 0 | 0.9474 | Ref |
| HG799496 | Unassigned | Unassigned | Unassigned | 427_0 | Clustered | 37 | VC_427_0 | 37 | 0.5729 | 1 | 0.5729 | 0 | 0 | 0 | 0.9474 | Ref |
| HG799497 | Unassigned | Unassigned | Unassigned | 427_0 | Clustered | 37 | VC_427_0 | 37 | 0.5729 | 1 | 0.5729 | 0 | 0 | 0 | 0.9474 | Ref |
| HG803181 | Unassigned | Unassigned | Unassigned | 5_0 | Clustered | 27 | VC_5_0 | 27 | 0.9599 | 1 | 0.9599 | 0 | 0 | 0 | 1 | Ref |
| HG803182 | Unassigned | Unassigned | Unassigned | 44_0 | Clustered | 49 | VC_44_0 | 49 | 0.4506 | 1 | 0.4506 | 0 | 0 | 0 | 0.8605 | Ref |
| HG803183 | Unassigned | Unassigned | Unassigned | 44_0 | Clustered | 49 | VC_44_0 | 49 | 0.4506 | 1 | 0.4506 | 0 | 0 | 0 | 0.8605 | Ref |
| HG803184 | Unassigned | Unassigned | Unassigned | 44_0 | Clustered | 49 | VC_44_0 | 49 | 0.4506 | 1 | 0.4506 | 0 | 0 | 0 | 0.8605 | Ref |
| HG803185 | Unassigned | Unassigned | Unassigned | 44_0 | Clustered | 49 | VC_44_0 | 49 | 0.4506 | 1 | 0.4506 | 0 | 0 | 0 | 0.8605 | Ref |

| | | | | | | | | | | | | | | | | |
|---|---|---|---|---|---|---|---|---|---|---|---|---|---|---|---|---|
| HG813241 | Unassigned | Unassigned | Unassigned | 465_0 | Clustered | 80 | VC_465_0 | 80 | 0.9988 | 1 | 0.9988 | 0 | 0 | 0 | 1 | Ref |
| HG818823 | Unassigned | Unassigned | Unassigned | 465_0 | Clustered | 80 | VC_465_0 | 80 | 0.9988 | 1 | 0.9988 | 0 | 0 | 0 | 1 | Ref |
| HG818824 | Unassigned | Unassigned | Unassigned | 465_0 | Clustered | 80 | VC_465_0 | 80 | 0.9988 | 1 | 0.9988 | 0 | 0 | 0 | 1 | Ref |
| HG934469 | Unassigned | Unassigned | Unassigned | 654_0 | Clustered | 115 | VC_654_0 | 115 | 0.7303 | 1 | 0.7303 | 0 | 0 | 0 | 0.8764 | Ref |
| HG934470 | Unassigned | Unassigned | Unassigned | 654_0 | Clustered | 115 | VC_654_0 | 115 | 0.7303 | 1 | 0.7303 | 0 | 0 | 0 | 0.8764 | Ref |
| HG962375 | Unassigned | Unassigned | Unassigned | 862_0 | Clustered | 29 | VC_862_0 | 29 | 0.8594 | 1 | 0.8594 | 0 | 0 | 0 | 1 | Ref |
| HG962376 | Unassigned | Unassigned | Unassigned | 766_0 | Clustered | 40 | VC_766_0 | 40 | 0.7511 | 1 | 0.7511 | 0 | 0 | 0 | 1 | Ref |
| HG992588 | Unassigned | Unassigned | Unassigned | 911_0 | Clustered | 20 | VC_911_0 | 20 | 0.9915 | 1 | 0.9915 | 0 | 0 | 0 | 1 | Ref |
| HG994092 | Unassigned | Unassigned | Unassigned | 848_0 | Clustered | 124 | VC_848_0 | 124 | 0.9484 | 1 | 0.9484 | 0 | 0 | 0 | 1 | Ref |
| HM004124 | Unassigned | Unassigned | Unassigned | 510_4 | Clustered/Singleton | 1 | VC_510_4 | 1 | 0 | 1 | 0 | 0 | 0 | 0 | 1 | Ref |
| HM029250 | Unassigned | Unassigned | Unassigned | 488_0 | Clustered | 14 | VC_488_0 | 14 | 0.8516 | 1 | 0.8516 | 0 | 0 | 0 | 1 | Ref |
| HM032710 | Unassigned | Unassigned | Unassigned | 510_5 | Clustered/Singleton | 1 | VC_510_5 | 1 | 0 | 1 | 0 | 0 | 0 | 0 | 1 | Ref |
| HM035024 | Unassigned | Unassigned | Unassigned | 595_0 | Clustered | 35 | VC_595_0 | 35 | 0.8809 | 1 | 0.8809 | 0 | 0 | 0 | 1 | Ref |
| HM035025 | Unassigned | Unassigned | Unassigned | 290_154 | Clustered/Singleton | 1 | VC_290_154 | 1 | 0 | 1 | 0 | 0 | 0 | 0 | 1 | Ref |
| HM064452 | Unassigned | Unassigned | Unassigned | 55_0 | Clustered | 5 | VC_55_0 | 5 | 0.4963 | 0.99999808 | 0.4963 | 0 | 0 | 0 | 1 | Ref |
| HM066936 | Unassigned | Unassigned | Unassigned | 234_0 | Clustered | 2 | VC_234_0 | 2 | 0.3333 | 0 | 0 | 0 | 0 | 0 | 1 | Ref |
| HM071924 | Unassigned | Unassigned | Unassigned | 297_5 | Clustered | 14 | VC_297_5 | 14 | 0.141 | 1 | 0.141 | 0 | 0 | 0 | 0.9231 | Ref |
| HM072038 | Unassigned | Unassigned | Unassigned | 18_0 | Clustered | 4 | VC_18_0 | 4 | 0.8969 | 0.99987935 | 0.8968 | 0 | 0 | 0 | 1 | Ref |
| HM114315 | Unassigned | Unassigned | Unassigned | 510_6 | Clustered/Singleton | 1 | VC_510_6 | 1 | 0 | 1 | 0 | 0 | 0 | 0 | 1 | Ref |
| HM134276 | Unassigned | Unassigned | Unassigned | 687_1 | Clustered | 10 | VC_687_1 | 10 | 0.1263 | 1 | 0.1263 | 0 | 0 | 0 | 0.9778 | Ref |
| HM137666 | Unassigned | Unassigned | Unassigned | 290_91 | Clustered | 11 | VC_290_91 | 11 | 0.0281 | 1 | 0.0281 | 0 | 0 | 0 | 1 | Ref |
| HM144387 | Unassigned | Unassigned | Unassigned | 913_1 | Clustered | 8 | VC_913_1 | 8 | 0.1102 | 0.9989323 | 0.11 | 0 | 0 | 0 | 0.75 | Ref |
| HM150760 | Unassigned | Unassigned | Unassigned | 909_0 | Clustered | 2 | VC_909_0 | 2 | 0.1608 | 0.83455073 | 0.1342 | 0 | 0 | 0 | 1 | Ref |
| HM152764 | Unassigned | Unassigned | Unassigned | 243_0 | Clustered | 72 | VC_243_0 | 72 | 0.8089 | 1 | 0.8089 | 0 | 0 | 0 | 1 | Ref |
| HM152765 | Unassigned | Unassigned | Unassigned | 536_0 | Clustered | 6 | VC_536_0 | 6 | 0.129 | 1 | 0.129 | 0 | 0 | 0 | 1 | Ref |
| HM152766 | Unassigned | Unassigned | Unassigned | 535_1 | Clustered | 108 | VC_535_1 | 108 | 0.8479 | 1 | 0.8479 | 0 | 0 | 0 | 0.9955 | Ref |
| HM152767 | Unassigned | Unassigned | Unassigned | 243_0 | Clustered | 72 | VC_243_0 | 72 | 0.8089 | 1 | 0.8089 | 0 | 0 | 0 | 1 | Ref |
| HM159959 | Unassigned | Unassigned | Unassigned | 149_0 | Clustered/Singleton | 1 | VC_149_0 | 1 | 0 | 1 | 0 | 0 | 0 | 0 | 1 | Ref |
| HM208303 | Unassigned | Unassigned | Unassigned | 35_0 | Clustered | 35 | VC_35_0 | 35 | 0.431 | 1 | 0.431 | 0 | 0 | 0 | 1 | Ref |
| HM208537 | Unassigned | Unassigned | Unassigned | 237_1 | Clustered/Singleton | 1 | VC_237_1 | 1 | 0 | 1 | 0 | 0 | 0 | 0 | 1 | Ref |
| HM242243 | Unassigned | Unassigned | Unassigned | 96_0 | Clustered | 2 | VC_96_0 | 2 | 0.1257 | 0.95725182 | 0.1204 | 0 | 0 | 0 | 1 | Ref |
| HM368260 | Unassigned | Unassigned | Unassigned | 713_0 | Clustered | 26 | VC_713_0 | 26 | 0.9358 | 1 | 0.9358 | 0 | 0 | 0 | 1 | Ref |
| HM452125 | Unassigned | Unassigned | Unassigned | 619_2 | Clustered | 10 | VC_619_2 | 10 | 0.3034 | 1 | 0.3034 | 0 | 0 | 0 | 1 | Ref |
| HM452126 | Unassigned | Unassigned | Unassigned | 457_0 | Clustered | 7 | VC_457_0 | 7 | 0.2549 | 1 | 0.2549 | 0 | 0 | 0 | 0.7143 | Ref |
| HM461982 | Unassigned | Unassigned | Unassigned | 3_0 | Clustered | 17 | VC_3_0 | 17 | 0.5615 | 1 | 0.5615 | 0 | 0 | 0 | 1 | Ref |
| HM480106 | Unassigned | Unassigned | Unassigned | 902_0 | Clustered | 3 | VC_902_0 | 3 | 0.8989 | 0.98968122 | 0.8896 | 0 | 0 | 0 | 1 | Ref |
| HM480846 | Unassigned | Unassigned | Unassigned | 848_0 | Clustered | 124 | VC_848_0 | 124 | 0.9484 | 1 | 0.9484 | 0 | 0 | 0 | 1 | Ref |
| HM559717 | Unassigned | Unassigned | Unassigned | 352_0 | Clustered | 31 | VC_352_0 | 31 | 0.9547 | 1 | 0.9547 | 0 | 0 | 0 | 1 | Ref |
| HM563683 | Unassigned | Unassigned | Unassigned | 297_2 | Clustered | 2 | VC_297_2 | 2 | 0.0101 | 0.92290095 | 0.0093 | 0 | 0 | 0 | 1 | Ref |
| HM568888 | Unassigned | Unassigned | Unassigned | 749_0 | Clustered | 5 | VC_749_0 | 5 | 0.4704 | 0.99999995 | 0.4704 | 0 | 0 | 0 | 1 | Ref |
| HM588722 | Unassigned | Unassigned | Unassigned | 925_0 | Clustered | 3 | VC_925_0 | 3 | 0.6333 | 0.99692152 | 0.6314 | 0 | 0 | 0 | 1 | Ref |
| HM624080 | Unassigned | Unassigned | Unassigned | 185_0 | Clustered | 53 | VC_185_0 | 53 | 0.9153 | 1 | 0.9153 | 0 | 0 | 0 | 1 | Ref |
| HM640230 | Unassigned | Unassigned | Unassigned | 928_0 | Clustered | 6 | VC_928_0 | 6 | 0.4065 | 1 | 0.4065 | 0 | 0 | 0 | 1 | Ref |
| HM753658 | Unassigned | Unassigned | Unassigned | 310_0 | Clustered | 155 | VC_310_0 | 155 | 0.9979 | 1 | 0.9979 | 0 | 0 | 0 | 1 | Ref |
| HM753659 | Unassigned | Unassigned | Unassigned | 310_0 | Clustered | 155 | VC_310_0 | 155 | 0.9979 | 1 | 0.9979 | 0 | 0 | 0 | 1 | Ref |
| HM753660 | Unassigned | Unassigned | Unassigned | 310_0 | Clustered | 155 | VC_310_0 | 155 | 0.9979 | 1 | 0.9979 | 0 | 0 | 0 | 1 | Ref |
| HM753661 | Unassigned | Unassigned | Unassigned | 310_0 | Clustered | 155 | VC_310_0 | 155 | 0.9979 | 1 | 0.9979 | 0 | 0 | 0 | 1 | Ref |
| HM753662 | Unassigned | Unassigned | Unassigned | 310_0 | Clustered | 155 | VC_310_0 | 155 | 0.9979 | 1 | 0.9979 | 0 | 0 | 0 | 1 | Ref |
| HM753663 | Unassigned | Unassigned | Unassigned | 310_0 | Clustered | 155 | VC_310_0 | 155 | 0.9979 | 1 | 0.9979 | 0 | 0 | 0 | 1 | Ref |
| HM753664 | Unassigned | Unassigned | Unassigned | 310_0 | Clustered | 155 | VC_310_0 | 155 | 0.9979 | 1 | 0.9979 | 0 | 0 | 0 | 1 | Ref |
| HM753665 | Unassigned | Unassigned | Unassigned | 310_0 | Clustered | 155 | VC_310_0 | 155 | 0.9979 | 1 | 0.9979 | 0 | 0 | 0 | 1 | Ref |
| HM753666 | Unassigned | Unassigned | Unassigned | 310_0 | Clustered | 155 | VC_310_0 | 155 | 0.9979 | 1 | 0.9979 | 0 | 0 | 0 | 1 | Ref |
| HM753667 | Unassigned | Unassigned | Unassigned | 310_0 | Clustered | 155 | VC_310_0 | 155 | 0.9979 | 1 | 0.9979 | 0 | 0 | 0 | 1 | Ref |
| HM753668 | Unassigned | Unassigned | Unassigned | 310_0 | Clustered | 155 | VC_310_0 | 155 | 0.9979 | 1 | 0.9979 | 0 | 0 | 0 | 1 | Ref |
| HM753669 | Unassigned | Unassigned | Unassigned | 310_0 | Clustered | 155 | VC_310_0 | 155 | 0.9979 | 1 | 0.9979 | 0 | 0 | 0 | 1 | Ref |
| HM753670 | Unassigned | Unassigned | Unassigned | 310_0 | Clustered | 155 | VC_310_0 | 155 | 0.9979 | 1 | 0.9979 | 0 | 0 | 0 | 1 | Ref |
| HM753671 | Unassigned | Unassigned | Unassigned | 310_0 | Clustered | 155 | VC_310_0 | 155 | 0.9979 | 1 | 0.9979 | 0 | 0 | 0 | 1 | Ref |
| HM753672 | Unassigned | Unassigned | Unassigned | 310_0 | Clustered | 155 | VC_310_0 | 155 | 0.9979 | 1 | 0.9979 | 0 | 0 | 0 | 1 | Ref |
| HM753673 | Unassigned | Unassigned | Unassigned | 310_0 | Clustered | 155 | VC_310_0 | 155 | 0.9979 | 1 | 0.9979 | 0 | 0 | 0 | 1 | Ref |
| HM753674 | Unassigned | Unassigned | Unassigned | 310_0 | Clustered | 155 | VC_310_0 | 155 | 0.9979 | 1 | 0.9979 | 0 | 0 | 0 | 1 | Ref |
| HM753675 | Unassigned | Unassigned | Unassigned | 310_0 | Clustered | 155 | VC_310_0 | 155 | 0.9979 | 1 | 0.9979 | 0 | 0 | 0 | 1 | Ref |
| HM753676 | Unassigned | Unassigned | Unassigned | 310_0 | Clustered | 155 | VC_310_0 | 155 | 0.9979 | 1 | 0.9979 | 0 | 0 | 0 | 1 | Ref |
| HM753677 | Unassigned | Unassigned | Unassigned | 310_0 | Clustered | 155 | VC_310_0 | 155 | 0.9979 | 1 | 0.9979 | 0 | 0 | 0 | 1 | Ref |
| HM753678 | Unassigned | Unassigned | Unassigned | 310_0 | Clustered | 155 | VC_310_0 | 155 | 0.9979 | 1 | 0.9979 | 0 | 0 | 0 | 1 | Ref |
| HM753679 | Unassigned | Unassigned | Unassigned | 310_0 | Clustered | 155 | VC_310_0 | 155 | 0.9979 | 1 | 0.9979 | 0 | 0 | 0 | 1 | Ref |
| HM753680 | Unassigned | Unassigned | Unassigned | 310_0 | Clustered | 155 | VC_310_0 | 155 | 0.9979 | 1 | 0.9979 | 0 | 0 | 0 | 1 | Ref |
| HM753681 | Unassigned | Unassigned | Unassigned | 310_0 | Clustered | 155 | VC_310_0 | 155 | 0.9979 | 1 | 0.9979 | 0 | 0 | 0 | 1 | Ref |
| HM753682 | Unassigned | Unassigned | Unassigned | 310_0 | Clustered | 155 | VC_310_0 | 155 | 0.9979 | 1 | 0.9979 | 0 | 0 | 0 | 1 | Ref |
| HM753683 | Unassigned | Unassigned | Unassigned | 310_0 | Clustered | 155 | VC_310_0 | 155 | 0.9979 | 1 | 0.9979 | 0 | 0 | 0 | 1 | Ref |
| HM753684 | Unassigned | Unassigned | Unassigned | 310_0 | Clustered | 155 | VC_310_0 | 155 | 0.9979 | 1 | 0.9979 | 0 | 0 | 0 | 1 | Ref |
| HM753685 | Unassigned | Unassigned | Unassigned | 310_0 | Clustered | 155 | VC_310_0 | 155 | 0.9979 | 1 | 0.9979 | 0 | 0 | 0 | 1 | Ref |
| HM753686 | Unassigned | Unassigned | Unassigned | 310_0 | Clustered | 155 | VC_310_0 | 155 | 0.9979 | 1 | 0.9979 | 0 | 0 | 0 | 1 | Ref |
| HM753687 | Unassigned | Unassigned | Unassigned | 310_0 | Clustered | 155 | VC_310_0 | 155 | 0.9979 | 1 | 0.9979 | 0 | 0 | 0 | 1 | Ref |
| HM753688 | Unassigned | Unassigned | Unassigned | 310_0 | Clustered | 155 | VC_310_0 | 155 | 0.9979 | 1 | 0.9979 | 0 | 0 | 0 | 1 | Ref |
| HM753689 | Unassigned | Unassigned | Unassigned | 310_0 | Clustered | 155 | VC_310_0 | 155 | 0.9979 | 1 | 0.9979 | 0 | 0 | 0 | 1 | Ref |
| HM753690 | Unassigned | Unassigned | Unassigned | 310_0 | Clustered | 155 | VC_310_0 | 155 | 0.9979 | 1 | 0.9979 | 0 | 0 | 0 | 1 | Ref |
| HM753691 | Unassigned | Unassigned | Unassigned | 310_0 | Clustered | 155 | VC_310_0 | 155 | 0.9979 | 1 | 0.9979 | 0 | 0 | 0 | 1 | Ref |
| HM753692 | Unassigned | Unassigned | Unassigned | 310_0 | Clustered | 155 | VC_310_0 | 155 | 0.9979 | 1 | 0.9979 | 0 | 0 | 0 | 1 | Ref |
| HM753693 | Unassigned | Unassigned | Unassigned | 310_0 | Clustered | 155 | VC_310_0 | 155 | 0.9979 | 1 | 0.9979 | 0 | 0 | 0 | 1 | Ref |
| HM753694 | Unassigned | Unassigned | Unassigned | 310_0 | Clustered | 155 | VC_310_0 | 155 | 0.9979 | 1 | 0.9979 | 0 | 0 | 0 | 1 | Ref |
| HM753695 | Unassigned | Unassigned | Unassigned | 310_0 | Clustered | 155 | VC_310_0 | 155 | 0.9979 | 1 | 0.9979 | 0 | 0 | 0 | 1 | Ref |

| | | | | | | | | | | | | | | | | |
|---|---|---|---|---|---|---|---|---|---|---|---|---|---|---|---|---|
| HM753696 | Unassigned | Unassigned | Unassigned | 310_0 | Clustered | 155 | VC_310_0 | 155 | 0.9979 | 1 | 0.9979 | 0 | 0 | 0 | 1 | Ref |
| HM753697 | Unassigned | Unassigned | Unassigned | 310_0 | Clustered | 155 | VC_310_0 | 155 | 0.9979 | 1 | 0.9979 | 0 | 0 | 0 | 1 | Ref |
| HM753698 | Unassigned | Unassigned | Unassigned | 310_0 | Clustered | 155 | VC_310_0 | 155 | 0.9979 | 1 | 0.9979 | 0 | 0 | 0 | 1 | Ref |
| HM753699 | Unassigned | Unassigned | Unassigned | 310_0 | Clustered | 155 | VC_310_0 | 155 | 0.9979 | 1 | 0.9979 | 0 | 0 | 0 | 1 | Ref |
| HM753700 | Unassigned | Unassigned | Unassigned | 310_0 | Clustered | 155 | VC_310_0 | 155 | 0.9979 | 1 | 0.9979 | 0 | 0 | 0 | 1 | Ref |
| HM753701 | Unassigned | Unassigned | Unassigned | 310_0 | Clustered | 155 | VC_310_0 | 155 | 0.9979 | 1 | 0.9979 | 0 | 0 | 0 | 1 | Ref |
| HM753702 | Unassigned | Unassigned | Unassigned | 310_0 | Clustered | 155 | VC_310_0 | 155 | 0.9979 | 1 | 0.9979 | 0 | 0 | 0 | 1 | Ref |
| HM753703 | Unassigned | Unassigned | Unassigned | 310_0 | Clustered | 155 | VC_310_0 | 155 | 0.9979 | 1 | 0.9979 | 0 | 0 | 0 | 1 | Ref |
| HM753704 | Unassigned | Unassigned | Unassigned | 310_0 | Clustered | 155 | VC_310_0 | 155 | 0.9979 | 1 | 0.9979 | 0 | 0 | 0 | 1 | Ref |
| HM753705 | Unassigned | Unassigned | Unassigned | 310_0 | Clustered | 155 | VC_310_0 | 155 | 0.9979 | 1 | 0.9979 | 0 | 0 | 0 | 1 | Ref |
| HM753706 | Unassigned | Unassigned | Unassigned | 310_0 | Clustered | 155 | VC_310_0 | 155 | 0.9979 | 1 | 0.9979 | 0 | 0 | 0 | 1 | Ref |
| HM753707 | Unassigned | Unassigned | Unassigned | 310_0 | Clustered | 155 | VC_310_0 | 155 | 0.9979 | 1 | 0.9979 | 0 | 0 | 0 | 1 | Ref |
| HM753708 | Unassigned | Unassigned | Unassigned | 310_0 | Clustered | 155 | VC_310_0 | 155 | 0.9979 | 1 | 0.9979 | 0 | 0 | 0 | 1 | Ref |
| HM753709 | Unassigned | Unassigned | Unassigned | 310_0 | Clustered | 155 | VC_310_0 | 155 | 0.9979 | 1 | 0.9979 | 0 | 0 | 0 | 1 | Ref |
| HM753710 | Unassigned | Unassigned | Unassigned | 310_0 | Clustered | 155 | VC_310_0 | 155 | 0.9979 | 1 | 0.9979 | 0 | 0 | 0 | 1 | Ref |
| HM753711 | Unassigned | Unassigned | Unassigned | 310_0 | Clustered | 155 | VC_310_0 | 155 | 0.9979 | 1 | 0.9979 | 0 | 0 | 0 | 1 | Ref |
| HM753712 | Unassigned | Unassigned | Unassigned | 310_0 | Clustered | 155 | VC_310_0 | 155 | 0.9979 | 1 | 0.9979 | 0 | 0 | 0 | 1 | Ref |
| HM753713 | Unassigned | Unassigned | Unassigned | 310_0 | Clustered | 155 | VC_310_0 | 155 | 0.9979 | 1 | 0.9979 | 0 | 0 | 0 | 1 | Ref |
| HM753714 | Unassigned | Unassigned | Unassigned | 310_0 | Clustered | 155 | VC_310_0 | 155 | 0.9979 | 1 | 0.9979 | 0 | 0 | 0 | 1 | Ref |
| HM753715 | Unassigned | Unassigned | Unassigned | 310_0 | Clustered | 155 | VC_310_0 | 155 | 0.9979 | 1 | 0.9979 | 0 | 0 | 0 | 1 | Ref |
| HM753716 | Unassigned | Unassigned | Unassigned | 310_0 | Clustered | 155 | VC_310_0 | 155 | 0.9979 | 1 | 0.9979 | 0 | 0 | 0 | 1 | Ref |
| HM753717 | Unassigned | Unassigned | Unassigned | 310_0 | Clustered | 155 | VC_310_0 | 155 | 0.9979 | 1 | 0.9979 | 0 | 0 | 0 | 1 | Ref |
| HM753718 | Unassigned | Unassigned | Unassigned | 310_0 | Clustered | 155 | VC_310_0 | 155 | 0.9979 | 1 | 0.9979 | 0 | 0 | 0 | 1 | Ref |
| HM755814 | Unassigned | Unassigned | Unassigned | 532_0 | Clustered | 75 | VC_532_0 | 75 | 0.9393 | 1 | 0.9393 | 0 | 0 | 0 | 1 | Ref |
| HM770079 | Unassigned | Unassigned | Unassigned | 6_0 | Clustered | 44 | VC_6_0 | 44 | 0.9068 | 1 | 0.9068 | 0 | 0 | 0 | 1 | Ref |
| HM775306 | Unassigned | Unassigned | Unassigned | 310_0 | Clustered | 155 | VC_310_0 | 155 | 0.9979 | 1 | 0.9979 | 0 | 0 | 0 | 1 | Ref |
| HM775307 | Unassigned | Unassigned | Unassigned | 310_0 | Clustered | 155 | VC_310_0 | 155 | 0.9979 | 1 | 0.9979 | 0 | 0 | 0 | 1 | Ref |
| HM775308 | Unassigned | Unassigned | Unassigned | 310_0 | Clustered | 155 | VC_310_0 | 155 | 0.9979 | 1 | 0.9979 | 0 | 0 | 0 | 1 | Ref |
| HM775309 | Unassigned | Unassigned | Unassigned | 310_0 | Clustered | 155 | VC_310_0 | 155 | 0.9979 | 1 | 0.9979 | 0 | 0 | 0 | 1 | Ref |
| HM775310 | Unassigned | Unassigned | Unassigned | 310_0 | Clustered | 155 | VC_310_0 | 155 | 0.9979 | 1 | 0.9979 | 0 | 0 | 0 | 1 | Ref |
| HM775311 | Unassigned | Unassigned | Unassigned | 310_0 | Clustered | 155 | VC_310_0 | 155 | 0.9979 | 1 | 0.9979 | 0 | 0 | 0 | 1 | Ref |
| HM997019 | Unassigned | Unassigned | Unassigned | 836_0 | Clustered | 3 | VC_836_0 | 3 | 0.1157 | 0.99842379 | 0.1155 | 0 | 0 | 0 | 1 | Ref |
| HM997020 | Unassigned | Unassigned | Unassigned | 290_2 | Clustered | 2 | VC_290_2 | 2 | 0.0027 | 0.93842619 | 0.0025 | 0 | 0 | 0 | 1 | Ref |
| HQ110083 | Unassigned | Unassigned | Unassigned | 5_0 | Clustered | 27 | VC_5_0 | 27 | 0.9599 | 1 | 0.9599 | 0 | 0 | 0 | 1 | Ref |
| HQ110084 | Unassigned | Unassigned | Unassigned | 235_0 | Clustered | 60 | VC_235_0 | 60 | 0.9413 | 1 | 0.9413 | 0 | 0 | 0 | 1 | Ref |
| HQ127381 | Unassigned | Unassigned | Unassigned | 9_0 | Clustered | 38 | VC_9_0 | 38 | 0.4859 | 1 | 0.4859 | 0 | 0 | 0 | 1 | Ref |
| HQ141410 | Unassigned | Unassigned | Unassigned | 280_0 | Clustered | 4 | VC_280_0 | 4 | 0.3169 | 0.99994916 | 0.3169 | 0 | 0 | 0 | 0.8333 | Ref |
| HQ141411 | Unassigned | Unassigned | Unassigned | 307_0 | Clustered | 2 | VC_307_0 | 2 | 0.1267 | 0.95445632 | 0.1209 | 0 | 0 | 0 | 1 | Ref |
| HQ163896 | Unassigned | Unassigned | Unassigned | 155_8 | Clustered | 66 | VC_155_8 | 66 | 0.4537 | 1 | 0.4537 | 0 | 0 | 0 | 0.8182 | Ref |
| HQ186308 | Unassigned | Unassigned | Unassigned | 714_0 | Clustered | 71 | VC_714_0 | 71 | 0.935 | 1 | 0.935 | 0 | 0 | 0 | 1 | Ref |
| HQ201307 | Unassigned | Unassigned | Unassigned | 36_0 | Clustered | 4 | VC_36_0 | 4 | 0.0597 | 0.99994573 | 0.0597 | 0 | 0 | 0 | 1 | Ref |
| HQ201308 | Unassigned | Unassigned | Unassigned | 33_2 | Clustered/Singleton | 1 | VC_33_2 | 1 | 0 | 1 | 0 | 0 | 0 | 0 | 1 | Ref |
| HQ224500 | Unassigned | Unassigned | Unassigned | 904_0 | Clustered | 10 | VC_904_0 | 10 | 0.9878 | 0.99948433 | 0.9873 | 0 | 0 | 0 | 1 | Ref |
| HQ259103 | Unassigned | Unassigned | Unassigned | 135_31 | Clustered | 6 | VC_135_31 | 6 | 0.0262 | 0.99999636 | 0.0262 | 0 | 0 | 0 | 1 | Ref |
| HQ259105 | Unassigned | Unassigned | Unassigned | 801_0 | Clustered | 37 | VC_801_0 | 37 | 0.5672 | 1 | 0.5672 | 0 | 0 | 0 | 1 | Ref |
| HQ316579 | Unassigned | Unassigned | Unassigned | 511_14 | Clustered/Singleton | 1 | VC_511_14 | 1 | 0 | 1 | 0 | 0 | 0 | 0 | 1 | Ref |
| HQ316581 | Unassigned | Unassigned | Unassigned | 255_0 | Clustered | 11 | VC_255_0 | 11 | 0.7034 | 1 | 0.7034 | 0 | 0 | 0 | 1 | Ref |
| HQ316582 | Unassigned | Unassigned | Unassigned | 933_0 | Clustered | 3 | VC_933_0 | 3 | 0.9964 | 0.94670842 | 0.9433 | 0 | 0 | 0 | 1 | Ref |
| HQ316583 | Unassigned | Unassigned | Unassigned | 675_0 | Clustered | 2 | VC_675_0 | 2 | 0.0088 | 0.92265122 | 0.0082 | 0 | 0 | 0 | 1 | Ref |
| HQ316584 | Unassigned | Unassigned | Unassigned | 352_0 | Clustered | 31 | VC_352_0 | 31 | 0.9547 | 1 | 0.9547 | 0 | 0 | 0 | 1 | Ref |
| HQ316603 | Unassigned | Unassigned | Unassigned | 675_0 | Clustered | 2 | VC_675_0 | 2 | 0.0088 | 0.92265122 | 0.0082 | 0 | 0 | 0 | 1 | Ref |
| HQ316604 | Unassigned | Unassigned | Unassigned | 935_1 | Clustered | 4 | VC_935_1 | 4 | 0.1471 | 0.99997723 | 0.1471 | 0 | 0 | 0 | 1 | Ref |
| HQ317290 | Unassigned | Unassigned | Unassigned | 681_0 | Clustered | 62 | VC_681_0 | 62 | 0.693 | 1 | 0.693 | 0 | 0 | 0 | 1 | Ref |
| HQ317291 | Unassigned | Unassigned | Unassigned | 681_0 | Clustered | 62 | VC_681_0 | 62 | 0.693 | 1 | 0.693 | 0 | 0 | 0 | 1 | Ref |
| HQ317292 | Unassigned | Unassigned | Unassigned | 681_0 | Clustered | 62 | VC_681_0 | 62 | 0.693 | 1 | 0.693 | 0 | 0 | 0 | 1 | Ref |
| HQ317384 | Unassigned | Unassigned | Unassigned | 938_0 | Clustered | 3 | VC_938_0 | 3 | 0.6623 | 0.99633114 | 0.6599 | 0 | 0 | 0 | 1 | Ref |
| HQ317386 | Unassigned | Unassigned | Unassigned | 941_0 | Clustered | 5 | VC_941_0 | 5 | 0.7412 | 0.99999276 | 0.7412 | 0 | 0 | 0 | 1 | Ref |
| HQ317387 | Unassigned | Unassigned | Unassigned | 857_0 | Clustered | 16 | VC_857_0 | 16 | 0.9317 | 1 | 0.9317 | 0 | 0 | 0 | 1 | Ref |
| HQ317388 | Unassigned | Unassigned | Unassigned | 352_0 | Clustered | 31 | VC_352_0 | 31 | 0.9547 | 1 | 0.9547 | 0 | 0 | 0 | 1 | Ref |
| HQ317389 | Unassigned | Unassigned | Unassigned | 352_0 | Clustered | 31 | VC_352_0 | 31 | 0.9547 | 1 | 0.9547 | 0 | 0 | 0 | 1 | Ref |
| HQ317390 | Unassigned | Unassigned | Unassigned | 942_0 | Clustered | 2 | VC_942_0 | 2 | 0.9873 | 0.5 | 0.4937 | 0 | 0 | 0 | 1 | Ref |
| HQ317391 | Unassigned | Unassigned | Unassigned | 446_10 | Clustered | 2 | VC_446_10 | 2 | 0.0211 | 0.95772926 | 0.0202 | 0 | 0 | 0 | 1 | Ref |
| HQ317392 | Unassigned | Unassigned | Unassigned | 943_0 | Clustered | 6 | VC_943_0 | 6 | 0.277 | 1 | 0.277 | 0 | 0 | 0 | 1 | Ref |
| HQ317393 | Unassigned | Unassigned | Unassigned | 549_2 | Clustered/Singleton | 1 | VC_549_2 | 1 | 0 | 1 | 0 | 0 | 0 | 0 | 1 | Ref |
| HQ332136 | Unassigned | Unassigned | Unassigned | 945_0 | Clustered | 2 | VC_945_0 | 2 | 0.0994 | 0.83429703 | 0.0829 | 0 | 0 | 0 | 1 | Ref |
| HQ332137 | Unassigned | Unassigned | Unassigned | 352_0 | Clustered | 31 | VC_352_0 | 31 | 0.9547 | 1 | 0.9547 | 0 | 0 | 0 | 1 | Ref |
| HQ332138 | Unassigned | Unassigned | Unassigned | 349_0 | Clustered | 5 | VC_349_0 | 5 | 0.191 | 0.97099341 | 0.1855 | 0 | 0 | 0 | 1 | Ref |
| HQ332140 | Unassigned | Unassigned | Unassigned | 352_0 | Clustered | 31 | VC_352_0 | 31 | 0.9547 | 1 | 0.9547 | 0 | 0 | 0 | 1 | Ref |
| HQ332141 | Unassigned | Unassigned | Unassigned | 363_0 | Clustered | 3 | VC_363_0 | 3 | 0.8866 | 0.98155579 | 0.8702 | 0 | 0 | 0 | 1 | Ref |
| HQ332142 | Unassigned | Unassigned | Unassigned | 938_0 | Clustered | 3 | VC_938_0 | 3 | 0.6623 | 0.99633114 | 0.6599 | 0 | 0 | 0 | 1 | Ref |
| HQ333270 | Unassigned | Unassigned | Unassigned | 399_0 | Clustered | 49 | VC_399_0 | 49 | 0.7927 | 1 | 0.7927 | 0 | 0 | 0 | 1 | Ref |
| HQ337021 | Unassigned | Unassigned | Unassigned | 674_2 | Clustered | 3 | VC_674_2 | 3 | 0.0227 | 0.99860756 | 0.0227 | 0 | 0 | 0 | 1 | Ref |
| HQ337022 | Unassigned | Unassigned | Unassigned | 352_0 | Clustered | 31 | VC_352_0 | 31 | 0.9547 | 1 | 0.9547 | 0 | 0 | 0 | 1 | Ref |
| HQ377372 | Unassigned | Unassigned | Unassigned | 745_0 | Clustered | 9 | VC_745_0 | 9 | 0.9955 | 0.99999771 | 0.9955 | 0 | 0 | 0 | 1 | Ref |
| HQ377373 | Unassigned | Unassigned | Unassigned | 745_0 | Clustered | 9 | VC_745_0 | 9 | 0.9955 | 0.99999771 | 0.9955 | 0 | 0 | 0 | 1 | Ref |
| HQ396194 | Unassigned | Unassigned | Unassigned | 947_0 | Clustered | 4 | VC_947_0 | 4 | 0.8344 | 0.99992411 | 0.8343 | 0 | 0 | 0 | 1 | Ref |
| HQ403646 | Unassigned | Unassigned | Unassigned | 952_0 | Clustered | 39 | VC_952_0 | 39 | 0.9064 | 1 | 0.9064 | 0 | 0 | 0 | 1 | Ref |
| HQ406778 | Unassigned | Unassigned | Unassigned | 198_23 | Clustered | 10 | VC_198_23 | 10 | 0.0697 | 1 | 0.0697 | 0 | 0 | 0 | 0.8444 | Ref |
| HQ424691 | Unassigned | Unassigned | Unassigned | 44_0 | Clustered | 49 | VC_44_0 | 49 | 0.4506 | 1 | 0.4506 | 0 | 0 | 0 | 0.8605 | Ref |
| HQ630627 | Unassigned | Unassigned | Unassigned | 356_6 | Clustered/Singleton | 1 | VC_356_6 | 1 | 0 | 1 | 0 | 0 | 0 | 0 | 1 | Ref |
| HQ632825 | Unassigned | Unassigned | Unassigned | 446_9 | Clustered | 3 | VC_446_9 | 3 | 0.0365 | 0.99848173 | 0.0364 | 0 | 0 | 0 | 1 | Ref |

| | | | | | | | | | | | | | | | | |
|---|---|---|---|---|---|---|---|---|---|---|---|---|---|---|---|---|
| HQ632857 | Unassigned | Unassigned | Unassigned | 954_0 | Clustered | 2 | VC_954_0 | 2 | 0.947 | 0.72985431 | 0.6911 | 0 | 0 | 0 | 1 | Ref |
| HQ632858 | Unassigned | Unassigned | Unassigned | 954_0 | Clustered | 2 | VC_954_0 | 2 | 0.947 | 0.72985431 | 0.6911 | 0 | 0 | 0 | 1 | Ref |
| HQ632859 | Unassigned | Unassigned | Unassigned | 614_0 | Clustered | 4 | VC_614_0 | 4 | 0.0588 | 0.99274182 | 0.0584 | 0 | 0 | 0 | 1 | Ref |
| HQ632860 | Unassigned | Unassigned | Unassigned | 661_0 | Clustered | 7 | VC_661_0 | 7 | 0.15 | 1 | 0.15 | 0 | 0 | 0 | 0.7619 | Ref |
| HQ633062 | Unassigned | Unassigned | Unassigned | 352_0 | Clustered | 31 | VC_352_0 | 31 | 0.9547 | 1 | 0.9547 | 0 | 0 | 0 | 1 | Ref |
| HQ633071 | Unassigned | Unassigned | Unassigned | 446_11 | Clustered/Singleton | 1 | VC_446_11 | 1 | 0 | 1 | 0 | 0 | 0 | 0 | 1 | Ref |
| HQ634152 | Unassigned | Unassigned | Unassigned | 352_0 | Clustered | 31 | VC_352_0 | 31 | 0.9547 | 1 | 0.9547 | 0 | 0 | 0 | 1 | Ref |
| HQ634156 | Unassigned | Unassigned | Unassigned | 511_10 | Clustered/Singleton | 1 | VC_511_10 | 1 | 0 | 1 | 0 | 0 | 0 | 0 | 1 | Ref |
| HQ634157 | Unassigned | Unassigned | Unassigned | 511_7 | Clustered/Singleton | 1 | VC_511_7 | 1 | 0 | 1 | 0 | 0 | 0 | 0 | 1 | Ref |
| HQ634175 | Unassigned | Unassigned | Unassigned | 674_3 | Clustered/Singleton | 1 | VC_674_3 | 1 | 0 | 1 | 0 | 0 | 0 | 0 | 1 | Ref |
| HQ634176 | Unassigned | Unassigned | Unassigned | 674_2 | Clustered | 3 | VC_674_2 | 3 | 0.0227 | 0.99860756 | 0.0227 | 0 | 0 | 0 | 1 | Ref |
| HQ634177 | Unassigned | Unassigned | Unassigned | 447_0 | Clustered | 6 | VC_447_0 | 6 | 0.0775 | 1 | 0.0775 | 0 | 0 | 0 | 1 | Ref |
| HQ634187 | Unassigned | Unassigned | Unassigned | 369_0 | Clustered | 2 | VC_369_0 | 2 | 0.1984 | 0.93211095 | 0.185 | 0 | 0 | 0 | 1 | Ref |
| HQ634188 | Unassigned | Unassigned | Unassigned | 352_0 | Clustered | 31 | VC_352_0 | 31 | 0.9547 | 1 | 0.9547 | 0 | 0 | 0 | 1 | Ref |
| HQ634189 | Unassigned | Unassigned | Unassigned | 674_7 | Clustered | 3 | VC_674_7 | 3 | 0.0163 | 0.99856944 | 0.0163 | 0 | 0 | 0 | 1 | Ref |
| HQ634190 | Unassigned | Unassigned | Unassigned | 674_6 | Clustered | 2 | VC_674_6 | 2 | 0.0077 | 0.9577529 | 0.0074 | 0 | 0 | 0 | 1 | Ref |
| HQ634192 | Unassigned | Unassigned | Unassigned | 962_0 | Clustered | 4 | VC_962_0 | 4 | 0.7703 | 0.999902 | 0.7702 | 0 | 0 | 0 | 1 | Ref |
| HQ634194 | Unassigned | Unassigned | Unassigned | 809_1 | Clustered | 3 | VC_809_1 | 3 | 0.1672 | 0.99842878 | 0.1669 | 0 | 0 | 0 | 1 | Ref |
| HQ634195 | Unassigned | Unassigned | Unassigned | 511_15 | Clustered/Singleton | 1 | VC_511_15 | 1 | 0 | 1 | 0 | 0 | 0 | 0 | 1 | Ref |
| HQ634196 | Unassigned | Unassigned | Unassigned | 809_1 | Clustered | 3 | VC_809_1 | 3 | 0.1672 | 0.99842878 | 0.1669 | 0 | 0 | 0 | 1 | Ref |
| HQ641340 | Unassigned | Unassigned | Unassigned | 83_0 | Clustered | 24 | VC_83_0 | 24 | 0.7345 | 1 | 0.7345 | 0 | 0 | 0 | 1 | Ref |
| HQ641341 | Unassigned | Unassigned | Unassigned | 83_0 | Clustered | 24 | VC_83_0 | 24 | 0.7345 | 1 | 0.7345 | 0 | 0 | 0 | 1 | Ref |
| HQ641342 | Unassigned | Unassigned | Unassigned | 83_0 | Clustered | 24 | VC_83_0 | 24 | 0.7345 | 1 | 0.7345 | 0 | 0 | 0 | 1 | Ref |
| HQ641343 | Unassigned | Unassigned | Unassigned | 83_0 | Clustered | 24 | VC_83_0 | 24 | 0.7345 | 1 | 0.7345 | 0 | 0 | 0 | 1 | Ref |
| HQ641344 | Unassigned | Unassigned | Unassigned | 83_0 | Clustered | 24 | VC_83_0 | 24 | 0.7345 | 1 | 0.7345 | 0 | 0 | 0 | 1 | Ref |
| HQ641345 | Unassigned | Unassigned | Unassigned | 771_0 | Clustered | 8 | VC_771_0 | 8 | 0.7491 | 1 | 0.7491 | 0 | 0 | 0 | 1 | Ref |
| HQ641346 | Unassigned | Unassigned | Unassigned | 771_0 | Clustered | 8 | VC_771_0 | 8 | 0.7491 | 1 | 0.7491 | 0 | 0 | 0 | 1 | Ref |
| HQ641347 | Unassigned | Unassigned | Unassigned | 513_1 | Clustered | 41 | VC_513_1 | 41 | 0.3276 | 1 | 0.3276 | 0 | 0 | 0 | 0.911 | Ref |
| HQ641348 | Unassigned | Unassigned | Unassigned | 513_1 | Clustered | 41 | VC_513_1 | 41 | 0.3276 | 1 | 0.3276 | 0 | 0 | 0 | 0.911 | Ref |
| HQ641349 | Unassigned | Unassigned | Unassigned | 513_1 | Clustered | 41 | VC_513_1 | 41 | 0.3276 | 1 | 0.3276 | 0 | 0 | 0 | 0.911 | Ref |
| HQ641350 | Unassigned | Unassigned | Unassigned | 513_1 | Clustered | 41 | VC_513_1 | 41 | 0.3276 | 1 | 0.3276 | 0 | 0 | 0 | 0.911 | Ref |
| HQ641351 | Unassigned | Unassigned | Unassigned | 513_1 | Clustered | 41 | VC_513_1 | 41 | 0.3276 | 1 | 0.3276 | 0 | 0 | 0 | 0.911 | Ref |
| HQ641352 | Unassigned | Unassigned | Unassigned | 513_1 | Clustered | 41 | VC_513_1 | 41 | 0.3276 | 1 | 0.3276 | 0 | 0 | 0 | 0.911 | Ref |
| HQ641353 | Unassigned | Unassigned | Unassigned | 513_1 | Clustered | 41 | VC_513_1 | 41 | 0.3276 | 1 | 0.3276 | 0 | 0 | 0 | 0.911 | Ref |
| HQ641354 | Unassigned | Unassigned | Unassigned | 513_1 | Clustered | 41 | VC_513_1 | 41 | 0.3276 | 1 | 0.3276 | 0 | 0 | 0 | 0.911 | Ref |
| HQ665011 | Unassigned | Unassigned | Unassigned | 198_24 | Clustered | 5 | VC_198_24 | 5 | 0.029 | 0.9999995 | 0.029 | 0 | 0 | 0 | 0.7 | Ref |
| HQ683709 | Unassigned | Unassigned | Unassigned | 965_0 | Clustered | 2 | VC_965_0 | 2 | 0.4025 | 0.89435023 | 0.36 | 0 | 0 | 0 | 1 | Ref |
| HQ698895 | Unassigned | Unassigned | Unassigned | 960_0 | Clustered | 2 | VC_960_0 | 2 | 0.086 | 0.75688284 | 0.0651 | 0 | 0 | 0 | 1 | Ref |
| HQ698922 | Unassigned | Unassigned | Unassigned | 510_0 | Clustered | 2 | VC_510_0 | 2 | 0.0158 | 0.67065552 | 0.0106 | 0 | 0 | 0 | 1 | Ref |
| HQ711984 | Unassigned | Unassigned | Unassigned | 304_1 | Clustered | 20 | VC_304_1 | 20 | 0.5632 | 1 | 0.5632 | 0 | 0 | 0 | 0.7684 | Ref |
| HQ711985 | Unassigned | Unassigned | Unassigned | 182_0 | Clustered | 2 | VC_182_0 | 2 | 0.0495 | 0.95664605 | 0.0473 | 0 | 0 | 0 | 1 | Ref |
| HQ728263 | Unassigned | Unassigned | Unassigned | 339_0 | Clustered | 11 | VC_339_0 | 11 | 0.4647 | 1 | 0.4647 | 0 | 0 | 0 | 1 | Ref |
| HQ728264 | Unassigned | Unassigned | Unassigned | 816_0 | Clustered | 35 | VC_816_0 | 35 | 0.9824 | 1 | 0.9824 | 0 | 0 | 0 | 1 | Ref |
| HQ728265 | Unassigned | Unassigned | Unassigned | 400_0 | Clustered | 3 | VC_400_0 | 3 | 0.0419 | 0.99861633 | 0.0418 | 0 | 0 | 0 | 1 | Ref |
| HQ728524 | Unassigned | Unassigned | Unassigned | 538_26 | Clustered | 3 | VC_538_26 | 3 | 0.0258 | 0.99859898 | 0.0258 | 0 | 0 | 0 | 1 | Ref |
| HQ829472 | Unassigned | Unassigned | Unassigned | 297_5 | Clustered | 14 | VC_297_5 | 14 | 0.141 | 1 | 0.141 | 0 | 0 | 0 | 0.9231 | Ref |
| HQ832595 | Unassigned | Unassigned | Unassigned | 77_0 | Clustered | 37 | VC_77_0 | 37 | 0.9392 | 1 | 0.9392 | 0 | 0 | 0 | 1 | Ref |
| HQ906662 | Unassigned | Unassigned | Unassigned | 23_0 | Clustered | 11 | VC_23_0 | 11 | 0.5144 | 1 | 0.5144 | 0 | 0 | 0 | 1 | Ref |
| HQ906663 | Unassigned | Unassigned | Unassigned | 23_0 | Clustered | 11 | VC_23_0 | 11 | 0.5144 | 1 | 0.5144 | 0 | 0 | 0 | 1 | Ref |
| HQ906664 | Unassigned | Unassigned | Unassigned | 23_0 | Clustered | 11 | VC_23_0 | 11 | 0.5144 | 1 | 0.5144 | 0 | 0 | 0 | 1 | Ref |
| HQ918180 | Unassigned | Unassigned | Unassigned | 686_0 | Clustered | 25 | VC_686_0 | 25 | 0.5501 | 1 | 0.5501 | 0 | 0 | 0 | 0.96 | Ref |
| JF344709 | Unassigned | Unassigned | Unassigned | 967_0 | Clustered | 5 | VC_967_0 | 5 | 0.992 | 0.97937098 | 0.9715 | 0 | 0 | 0 | 1 | Ref |
| JF412296 | Unassigned | Unassigned | Unassigned | 633_0 | Clustered | 2 | VC_633_0 | 2 | 0.0354 | 0.89435023 | 0.0317 | 0 | 0 | 0 | 1 | Ref |
| JF461087 | Unassigned | Unassigned | Unassigned | 340_1 | Clustered | 17 | VC_340_1 | 17 | 0.0863 | 1 | 0.0863 | 0 | 0 | 0 | 0.8235 | Ref |
| JF501022 | Unassigned | Unassigned | Unassigned | 972_0 | Clustered | 99 | VC_972_0 | 99 | 0.9113 | 1 | 0.9113 | 0 | 0 | 0 | 0.9928 | Ref |
| JF704091 | Unassigned | Unassigned | Unassigned | 384_18 | Clustered | 10 | VC_384_18 | 10 | 0.0301 | 1 | 0.0301 | 0 | 0 | 0 | 0.8 | Ref |
| JF704092 | Unassigned | Unassigned | Unassigned | 579_22 | Clustered | 4 | VC_579_22 | 4 | 0.0099 | 0.99020449 | 0.0098 | 0 | 0 | 0 | 0.8333 | Ref |
| JF704093 | Unassigned | Unassigned | Unassigned | 535_0 | Clustered | 18 | VC_535_0 | 18 | 0.1595 | 1 | 0.1595 | 0 | 0 | 0 | 1 | Ref |
| JF704094 | Unassigned | Unassigned | Unassigned | 385_0 | Clustered | 24 | VC_385_0 | 24 | 0.5516 | 1 | 0.5516 | 0 | 0 | 0 | 1 | Ref |
| JF704095 | Unassigned | Unassigned | Unassigned | 527_0 | Clustered | 37 | VC_527_0 | 37 | 0.9679 | 1 | 0.9679 | 0 | 0 | 0 | 1 | Ref |
| JF704096 | Unassigned | Unassigned | Unassigned | 579_20 | Clustered | 17 | VC_579_20 | 17 | 0.0538 | 1 | 0.0538 | 0 | 0 | 0 | 0.9926 | Ref |
| JF704097 | Unassigned | Unassigned | Unassigned | 230_0 | Clustered | 38 | VC_230_0 | 38 | 0.9076 | 1 | 0.9076 | 0 | 0 | 0 | 1 | Ref |
| JF704098 | Unassigned | Unassigned | Unassigned | 532_0 | Clustered | 75 | VC_532_0 | 75 | 0.9393 | 1 | 0.9393 | 0 | 0 | 0 | 1 | Ref |
| JF704099 | Unassigned | Unassigned | Unassigned | 384_3 | Clustered | 22 | VC_384_3 | 22 | 0.0786 | 1 | 0.0786 | 0 | 0 | 0 | 0.9048 | Ref |
| JF704100 | Unassigned | Unassigned | Unassigned | 973_0 | Clustered | 15 | VC_973_0 | 15 | 1 | 0 | 0 | 0 | 0 | 0 | 1 | Ref |
| JF704101 | Unassigned | Unassigned | Unassigned | 532_0 | Clustered | 75 | VC_532_0 | 75 | 0.9393 | 1 | 0.9393 | 0 | 0 | 0 | 1 | Ref |
| JF704102 | Unassigned | Unassigned | Unassigned | 384_16 | Clustered | 42 | VC_384_16 | 42 | 0.134 | 1 | 0.134 | 0 | 0 | 0 | 0.7282 | Ref |
| JF704103 | Unassigned | Unassigned | Unassigned | 384_15 | Clustered | 44 | VC_384_15 | 44 | 0.139 | 1 | 0.139 | 0 | 0 | 0 | 0.7474 | Ref |
| JF704104 | Unassigned | Unassigned | Unassigned | 385_0 | Clustered | 24 | VC_385_0 | 24 | 0.5516 | 1 | 0.5516 | 0 | 0 | 0 | 1 | Ref |
| JF704105 | Unassigned | Unassigned | Unassigned | 243_0 | Clustered | 72 | VC_243_0 | 72 | 0.8089 | 1 | 0.8089 | 0 | 0 | 0 | 1 | Ref |
| JF704106 | Unassigned | Unassigned | Unassigned | 243_0 | Clustered | 72 | VC_243_0 | 72 | 0.8089 | 1 | 0.8089 | 0 | 0 | 0 | 1 | Ref |
| JF704107 | Unassigned | Unassigned | Unassigned | 974_0 | Clustered | 34 | VC_974_0 | 34 | 0.7953 | 1 | 0.7953 | 0 | 0 | 0 | 1 | Ref |
| JF704109 | Unassigned | Unassigned | Unassigned | 384_15 | Clustered | 44 | VC_384_15 | 44 | 0.139 | 1 | 0.139 | 0 | 0 | 0 | 0.7474 | Ref |
| JF704110 | Unassigned | Unassigned | Unassigned | 232_0 | Clustered | 32 | VC_232_0 | 32 | 0.8471 | 1 | 0.8471 | 0 | 0 | 0 | 0.996 | Ref |
| JF704111 | Unassigned | Unassigned | Unassigned | 531_0 | Clustered | 32 | VC_531_0 | 32 | 0.6859 | 1 | 0.6859 | 0 | 0 | 0 | 1 | Ref |
| JF704112 | Unassigned | Unassigned | Unassigned | 588_0 | Clustered | 8 | VC_588_0 | 8 | 0.5695 | 1 | 0.5695 | 0 | 0 | 0 | 1 | Ref |
| JF704114 | Unassigned | Unassigned | Unassigned | 532_0 | Clustered | 75 | VC_532_0 | 75 | 0.9393 | 1 | 0.9393 | 0 | 0 | 0 | 1 | Ref |
| JF704116 | Unassigned | Unassigned | Unassigned | 579_6 | Clustered | 2 | VC_579_6 | 2 | 0.0032 | 0.72299343 | 0.0023 | 0 | 0 | 0 | 1 | Ref |
| JF704117 | Unassigned | Unassigned | Unassigned | 538_74 | Clustered | 2 | VC_538_74 | 2 | 0.0075 | 0.95760586 | 0.0072 | 0 | 0 | 0 | 1 | Ref |
| JF712866 | Unassigned | Unassigned | Unassigned | 627_0 | Clustered | 16 | VC_627_0 | 16 | 0.9168 | 1 | 0.9168 | 0 | 0 | 0 | 1 | Ref |
| JF713456 | Unassigned | Unassigned | Unassigned | 935_0 | Clustered | 6 | VC_935_0 | 6 | 0.2317 | 1 | 0.2317 | 0 | 0 | 0 | 1 | Ref |

| | | | | | | | | | | | | | | | | |
|---|---|---|---|---|---|---|---|---|---|---|---|---|---|---|---|---|
| JF719726 | Unassigned | Unassigned | Unassigned | 310_0 | Clustered | 155 | VC_310_0 | 155 | 0.9979 | 1 | 0.9979 | 0 | 0 | 0 | 1 | Ref |
| JF719727 | Unassigned | Unassigned | Unassigned | 310_0 | Clustered | 155 | VC_310_0 | 155 | 0.9979 | 1 | 0.9979 | 0 | 0 | 0 | 1 | Ref |
| JF719728 | Unassigned | Unassigned | Unassigned | 310_0 | Clustered | 155 | VC_310_0 | 155 | 0.9979 | 1 | 0.9979 | 0 | 0 | 0 | 1 | Ref |
| JF719729 | Unassigned | Unassigned | Unassigned | 373_0 | Clustered | 358 | VC_373_0 | 358 | 0.9614 | 1 | 0.9614 | 0 | 0 | 0 | 1 | Ref |
| JF719730 | Unassigned | Unassigned | Unassigned | 373_0 | Clustered | 358 | VC_373_0 | 358 | 0.9614 | 1 | 0.9614 | 0 | 0 | 0 | 1 | Ref |
| JF719731 | Unassigned | Unassigned | Unassigned | 373_0 | Clustered | 358 | VC_373_0 | 358 | 0.9614 | 1 | 0.9614 | 0 | 0 | 0 | 1 | Ref |
| JF719732 | Unassigned | Unassigned | Unassigned | 62_0 | Clustered | 12 | VC_62_0 | 12 | 0.7624 | 1 | 0.7624 | 0 | 0 | 0 | 1 | Ref |
| JF719733 | Unassigned | Unassigned | Unassigned | 62_0 | Clustered | 12 | VC_62_0 | 12 | 0.7624 | 1 | 0.7624 | 0 | 0 | 0 | 1 | Ref |
| JF719734 | Unassigned | Unassigned | Unassigned | 62_0 | Clustered | 12 | VC_62_0 | 12 | 0.7624 | 1 | 0.7624 | 0 | 0 | 0 | 1 | Ref |
| JF731128 | Unassigned | Unassigned | Unassigned | 110_0 | Clustered | 33 | VC_110_0 | 33 | 0.9488 | 1 | 0.9488 | 0 | 0 | 0 | 1 | Ref |
| JF734911 | Unassigned | Unassigned | Unassigned | 101_0 | Clustered | 32 | VC_101_0 | 32 | 0.8826 | 1 | 0.8826 | 0 | 0 | 0 | 1 | Ref |
| JF744988 | Unassigned | Unassigned | Unassigned | 917_0 | Clustered | 28 | VC_917_0 | 28 | 0.5465 | 1 | 0.5465 | 0 | 0 | 0 | 1 | Ref |
| JF767208 | Unassigned | Unassigned | Unassigned | 839_0 | Clustered | 8 | VC_839_0 | 8 | 0.9889 | 1 | 0.9889 | 0 | 0 | 0 | 1 | Ref |
| JF767209 | Unassigned | Unassigned | Unassigned | 839_0 | Clustered | 8 | VC_839_0 | 8 | 0.9889 | 1 | 0.9889 | 0 | 0 | 0 | 1 | Ref |
| JF767210 | Unassigned | Unassigned | Unassigned | 839_0 | Clustered | 8 | VC_839_0 | 8 | 0.9889 | 1 | 0.9889 | 0 | 0 | 0 | 1 | Ref |
| JF770475 | Unassigned | Unassigned | Unassigned | 469_0 | Clustered | 18 | VC_469_0 | 18 | 0.4583 | 1 | 0.4583 | 0 | 0 | 0 | 1 | Ref |
| JF773396 | Unassigned | Unassigned | Unassigned | 745_0 | Clustered | 9 | VC_745_0 | 9 | 0.9955 | 0.99999771 | 0.9955 | 0 | 0 | 0 | 1 | Ref |
| JF792674 | Unassigned | Unassigned | Unassigned | 535_1 | Clustered | 108 | VC_535_1 | 108 | 0.8479 | 1 | 0.8479 | 0 | 0 | 0 | 0.9955 | Ref |
| JF900176 | Unassigned | Unassigned | Unassigned | 30_1 | Clustered | 53 | VC_30_1 | 53 | 0.7151 | 1 | 0.7151 | 0 | 0 | 0 | 0.9325 | Ref |
| JF917095 | Unassigned | Unassigned | Unassigned | 977_0 | Clustered | 2 | VC_977_0 | 2 | 1 | 1 | 1 | 0 | 0 | 0 | 1 | Ref |
| JF923796 | Unassigned | Unassigned | Unassigned | 789_0 | Clustered | 34 | VC_789_0 | 34 | 0.9167 | 1 | 0.9167 | 0 | 0 | 0 | 1 | Ref |
| JF923797 | Unassigned | Unassigned | Unassigned | 789_0 | Clustered | 34 | VC_789_0 | 34 | 0.9167 | 1 | 0.9167 | 0 | 0 | 0 | 1 | Ref |
| JF937090 | Unassigned | Unassigned | Unassigned | 566_7 | Clustered | 4 | VC_566_7 | 4 | 0.0409 | 0.99661604 | 0.0408 | 0 | 0 | 0 | 1 | Ref |
| JF937091 | Unassigned | Unassigned | Unassigned | 571_0 | Clustered | 16 | VC_571_0 | 16 | 0.091 | 1 | 0.091 | 0 | 0 | 0 | 0.6667 | Ref |
| JF937092 | Unassigned | Unassigned | Unassigned | 230_0 | Clustered | 38 | VC_230_0 | 38 | 0.9076 | 1 | 0.9076 | 0 | 0 | 0 | 1 | Ref |
| JF937093 | Unassigned | Unassigned | Unassigned | 538_31 | Clustered | 11 | VC_538_31 | 11 | 0.0894 | 1 | 0.0894 | 0 | 0 | 0 | 0.6 | Ref |
| JF937094 | Unassigned | Unassigned | Unassigned | 230_0 | Clustered | 38 | VC_230_0 | 38 | 0.9076 | 1 | 0.9076 | 0 | 0 | 0 | 1 | Ref |
| JF937095 | Unassigned | Unassigned | Unassigned | 384_17 | Clustered | 3 | VC_384_17 | 3 | 0.0075 | 0.99812032 | 0.0075 | 0 | 0 | 0 | 1 | Ref |
| JF937096 | Unassigned | Unassigned | Unassigned | 571_2 | Clustered | 17 | VC_571_2 | 17 | 0.0965 | 1 | 0.0965 | 0 | 0 | 0 | 0.9779 | Ref |
| JF937097 | Unassigned | Unassigned | Unassigned | 384_15 | Clustered | 44 | VC_384_15 | 44 | 0.139 | 1 | 0.139 | 0 | 0 | 0 | 0.7474 | Ref |
| JF937098 | Unassigned | Unassigned | Unassigned | 538_93 | Clustered | 2 | VC_538_93 | 2 | 0.0103 | 0.95762079 | 0.0099 | 0 | 0 | 0 | 1 | Ref |
| JF937099 | Unassigned | Unassigned | Unassigned | 335_2 | Clustered/Singleton | 1 | VC_335_2 | 1 | 0 | 1 | 0 | 0 | 0 | 0 | 1 | Ref |
| JF937100 | Unassigned | Unassigned | Unassigned | 335_36 | Clustered | 2 | VC_335_36 | 2 | 0.0036 | 0.94071129 | 0.0034 | 0 | 0 | 0 | 1 | Ref |
| JF937101 | Unassigned | Unassigned | Unassigned | 566_11 | Clustered/Singleton | 1 | VC_566_11 | 1 | 0 | 1 | 0 | 0 | 0 | 0 | 1 | Ref |
| JF937102 | Unassigned | Unassigned | Unassigned | 538_37 | Clustered/Singleton | 1 | VC_538_37 | 1 | 0 | 1 | 0 | 0 | 0 | 0 | 1 | Ref |
| JF937103 | Unassigned | Unassigned | Unassigned | 335_23 | Clustered | 17 | VC_335_23 | 17 | 0.0714 | 1 | 0.0714 | 0 | 0 | 0 | 0.6397 | Ref |
| JF937104 | Unassigned | Unassigned | Unassigned | 244_1 | Clustered | 13 | VC_244_1 | 13 | 0.1766 | 1 | 0.1766 | 0 | 0 | 0 | 0.7308 | Ref |
| JF937105 | Unassigned | Unassigned | Unassigned | 785_2 | Clustered | 7 | VC_785_2 | 7 | 0.2595 | 1 | 0.2595 | 0 | 0 | 0 | 1 | Ref |
| JF937106 | Unassigned | Unassigned | Unassigned | 571_0 | Clustered | 16 | VC_571_0 | 16 | 0.091 | 1 | 0.091 | 0 | 0 | 0 | 0.6667 | Ref |
| JF937107 | Unassigned | Unassigned | Unassigned | 585_0 | Clustered | 20 | VC_585_0 | 20 | 0.8376 | 1 | 0.8376 | 0 | 0 | 0 | 1 | Ref |
| JF937108 | Unassigned | Unassigned | Unassigned | 335_5 | Clustered | 11 | VC_335_5 | 11 | 0.0469 | 1 | 0.0469 | 0 | 0 | 0 | 0.7636 | Ref |
| JF937109 | Unassigned | Unassigned | Unassigned | 384_18 | Clustered | 10 | VC_384_18 | 10 | 0.0301 | 1 | 0.0301 | 0 | 0 | 0 | 0.8 | Ref |
| JF937110 | Unassigned | Unassigned | Unassigned | 335_5 | Clustered | 11 | VC_335_5 | 11 | 0.0469 | 1 | 0.0469 | 0 | 0 | 0 | 0.7636 | Ref |
| JF939047 | Unassigned | Unassigned | Unassigned | 766_0 | Clustered | 40 | VC_766_0 | 40 | 0.7511 | 1 | 0.7511 | 0 | 0 | 0 | 1 | Ref |
| JF946695 | Unassigned | Unassigned | Unassigned | 222_2 | Clustered | 5 | VC_222_2 | 5 | 0.0569 | 0.99999997 | 0.0569 | 0 | 0 | 0 | 1 | Ref |
| JF957056 | Unassigned | Unassigned | Unassigned | 384_15 | Clustered | 44 | VC_384_15 | 44 | 0.139 | 1 | 0.139 | 0 | 0 | 0 | 0.7474 | Ref |
| JF957057 | Unassigned | Unassigned | Unassigned | 335_24 | Clustered | 3 | VC_335_24 | 3 | 0.009 | 0.99853949 | 0.0089 | 0 | 0 | 0 | 1 | Ref |
| JF957058 | Unassigned | Unassigned | Unassigned | 531_0 | Clustered | 32 | VC_531_0 | 32 | 0.6859 | 1 | 0.6859 | 0 | 0 | 0 | 1 | Ref |
| JF957059 | Unassigned | Unassigned | Unassigned | 566_7 | Clustered | 4 | VC_566_7 | 4 | 0.0409 | 0.99661604 | 0.0408 | 0 | 0 | 0 | 1 | Ref |
| JF957060 | Unassigned | Unassigned | Unassigned | 226_0 | Clustered | 5 | VC_226_0 | 5 | 0.0793 | 0.99999998 | 0.0793 | 0 | 0 | 0 | 1 | Ref |
| JF966203 | Unassigned | Unassigned | Unassigned | 912_0 | Clustered | 10 | VC_912_0 | 10 | 0.1994 | 1 | 0.1994 | 0 | 0 | 0 | 1 | Ref |
| JF974286 | Unassigned | Unassigned | Unassigned | 961_0 | Clustered | 3 | VC_961_0 | 3 | 0.783 | 0.9944138 | 0.7786 | 0 | 0 | 0 | 1 | Ref |
| JF974287 | Unassigned | Unassigned | Unassigned | 662_0 | Clustered | 2 | VC_662_0 | 2 | 0.1082 | 0.95085347 | 0.1029 | 0 | 0 | 0 | 1 | Ref |
| JF974294 | Unassigned | Unassigned | Unassigned | 474_0 | Clustered | 19 | VC_474_0 | 19 | 0.3729 | 1 | 0.3729 | 0 | 0 | 0 | 1 | Ref |
| JF974296 | Unassigned | Unassigned | Unassigned | 809_1 | Clustered | 3 | VC_809_1 | 3 | 0.1672 | 0.99842878 | 0.1669 | 0 | 0 | 0 | 1 | Ref |
| JF974307 | Unassigned | Unassigned | Unassigned | 188_0 | Clustered | 5 | VC_188_0 | 5 | 0.6188 | 0.99999978 | 0.6188 | 0 | 0 | 0 | 1 | Ref |
| JF974308 | Unassigned | Unassigned | Unassigned | 188_0 | Clustered | 5 | VC_188_0 | 5 | 0.6188 | 0.99999978 | 0.6188 | 0 | 0 | 0 | 1 | Ref |
| JF974312 | Unassigned | Unassigned | Unassigned | 474_0 | Clustered | 19 | VC_474_0 | 19 | 0.3729 | 1 | 0.3729 | 0 | 0 | 0 | 1 | Ref |
| JF974313 | Unassigned | Unassigned | Unassigned | 661_2 | Clustered | 6 | VC_661_2 | 6 | 0.1674 | 1 | 0.1674 | 0 | 0 | 0 | 1 | Ref |
| JF974314 | Unassigned | Unassigned | Unassigned | 22_0 | Clustered | 4 | VC_22_0 | 4 | 0.562 | 0.91257778 | 0.5129 | 0 | 0 | 0 | 0.6667 | Ref |
| JF974321 | Unassigned | Unassigned | Unassigned | 961_0 | Clustered | 3 | VC_961_0 | 3 | 0.783 | 0.9944138 | 0.7786 | 0 | 0 | 0 | 1 | Ref |
| JF974339 | Unassigned | Unassigned | Unassigned | 32_1 | Clustered | 16 | VC_32_1 | 16 | 0.1962 | 1 | 0.1962 | 0 | 0 | 0 | 0.8333 | Ref |
| JN006061 | Unassigned | Unassigned | Unassigned | 571_2 | Clustered | 17 | VC_571_2 | 17 | 0.0965 | 1 | 0.0965 | 0 | 0 | 0 | 0.9779 | Ref |
| JN006062 | Unassigned | Unassigned | Unassigned | 571_2 | Clustered | 17 | VC_571_2 | 17 | 0.0965 | 1 | 0.0965 | 0 | 0 | 0 | 0.9779 | Ref |
| JN006063 | Unassigned | Unassigned | Unassigned | 384_15 | Clustered | 44 | VC_384_15 | 44 | 0.139 | 1 | 0.139 | 0 | 0 | 0 | 0.7474 | Ref |
| JN006064 | Unassigned | Unassigned | Unassigned | 384_21 | Clustered | 2 | VC_384_21 | 2 | 0.0033 | 0.95771013 | 0.0032 | 0 | 0 | 0 | 1 | Ref |
| JN020140 | Unassigned | Unassigned | Unassigned | 335_19 | Clustered | 3 | VC_335_19 | 3 | 0.0107 | 0.99859221 | 0.0107 | 0 | 0 | 0 | 1 | Ref |
| JN020141 | Unassigned | Unassigned | Unassigned | 538_28 | Clustered | 2 | VC_538_28 | 2 | 0.0135 | 0.95761531 | 0.0129 | 0 | 0 | 0 | 1 | Ref |
| JN020142 | Unassigned | Unassigned | Unassigned | 538_88 | Clustered/Singleton | 1 | VC_538_88 | 1 | 0 | 1 | 0 | 0 | 0 | 0 | 1 | Ref |
| JN020143 | Unassigned | Unassigned | Unassigned | 538_43 | Clustered | 3 | VC_538_43 | 3 | 0.0193 | 0.99837043 | 0.0192 | 0 | 0 | 0 | 1 | Ref |
| JN022534 | Unassigned | Unassigned | Unassigned | 65_0 | Clustered | 3 | VC_65_0 | 3 | 0.5621 | 0.99791205 | 0.5609 | 0 | 0 | 0 | 1 | Ref |
| JN035618 | Unassigned | Unassigned | Unassigned | 893_0 | Clustered | 4 | VC_893_0 | 4 | 0.0787 | 0.99998595 | 0.0787 | 0 | 0 | 0 | 1 | Ref |
| JN049605 | Unassigned | Unassigned | Unassigned | 230_0 | Clustered | 38 | VC_230_0 | 38 | 0.9076 | 1 | 0.9076 | 0 | 0 | 0 | 1 | Ref |
| JN051154 | Unassigned | Unassigned | Unassigned | 597_0 | Clustered | 6 | VC_597_0 | 6 | 0.9222 | 0.99999993 | 0.9222 | 0 | 0 | 0 | 1 | Ref |
| JN083852 | Unassigned | Unassigned | Unassigned | 974_0 | Clustered | 34 | VC_974_0 | 34 | 0.7953 | 1 | 0.7953 | 0 | 0 | 0 | 1 | Ref |
| JN083853 | Unassigned | Unassigned | Unassigned | 974_0 | Clustered | 34 | VC_974_0 | 34 | 0.7953 | 1 | 0.7953 | 0 | 0 | 0 | 1 | Ref |
| JN116822 | Unassigned | Unassigned | Unassigned | 984_0 | Clustered | 5 | VC_984_0 | 5 | 0.1708 | 0.99999984 | 0.1708 | 0 | 0 | 0 | 1 | Ref |
| JN116823 | Unassigned | Unassigned | Unassigned | 988_0 | Clustered | 2 | VC_988_0 | 2 | 0.2055 | 0.9488174 | 0.195 | 0 | 0 | 0 | 1 | Ref |
| JN116824 | Unassigned | Unassigned | Unassigned | 741_0 | Clustered | 3 | VC_741_0 | 3 | 0.038 | 0.88651 | 0.0337 | 0 | 0 | 0 | 1 | Ref |
| JN116826 | Unassigned | Unassigned | Unassigned | 991_0 | Clustered | 39 | VC_991_0 | 39 | 1 | 1 | 1 | 0 | 0 | 0 | 1 | Ref |
| JN116827 | Unassigned | Unassigned | Unassigned | 991_0 | Clustered | 39 | VC_991_0 | 39 | 1 | 1 | 1 | 0 | 0 | 0 | 1 | Ref |

| | | | | | | | | | | | | | | | | |
|---|---|---|---|---|---|---|---|---|---|---|---|---|---|---|---|---|
| JN116828 | Unassigned | Unassigned | Unassigned | 987_0 | Clustered | 2 | VC_987_0 | 2 | 0.9225 | 0.90744399 | 0.8372 | 0 | 0 | 0 | 1 | Ref |
| JN126049 | Unassigned | Unassigned | Unassigned | 135_13 | Clustered | 2 | VC_135_13 | 2 | 0.005 | 0.86394392 | 0.0044 | 0 | 0 | 0 | 1 | Ref |
| JN132397 | Unassigned | Unassigned | Unassigned | 868_0 | Clustered | 27 | VC_868_0 | 27 | 0.9372 | 1 | 0.9372 | 0 | 0 | 0 | 1 | Ref |
| JN153085 | Unassigned | Unassigned | Unassigned | 335_54 | Clustered | 5 | VC_335_54 | 5 | 0.0147 | 0.99999078 | 0.0147 | 0 | 0 | 0 | 0.7 | Ref |
| JN153086 | Unassigned | Unassigned | Unassigned | 335_61 | Clustered | 8 | VC_335_61 | 8 | 0.0394 | 1 | 0.0394 | 0 | 0 | 0 | 0.7857 | Ref |
| JN175269 | Unassigned | Unassigned | Unassigned | 992_0 | Clustered/Singleton | 1 | VC_992_0 | 1 | 0 | 1 | 0 | 0 | 0 | 0 | 1 | Ref |
| JN185608 | Unassigned | Unassigned | Unassigned | 243_0 | Clustered | 72 | VC_243_0 | 72 | 0.8089 | 1 | 0.8089 | 0 | 0 | 0 | 1 | Ref |
| JN190960 | Unassigned | Unassigned | Unassigned | 621_0 | Clustered | 2 | VC_621_0 | 2 | 0.2279 | 0.95560476 | 0.2178 | 0 | 0 | 0 | 1 | Ref |
| JN192463 | Unassigned | Unassigned | Unassigned | 384_13 | Clustered | 33 | VC_384_13 | 33 | 0.1785 | 1 | 0.1785 | 0 | 0 | 0 | 0.8769 | Ref |
| JN201525 | Unassigned | Unassigned | Unassigned | 566_0 | Clustered | 2 | VC_566_0 | 2 | 0.0136 | 0.63748598 | 0.0087 | 0 | 0 | 0 | 1 | Ref |
| JN202312 | Unassigned | Unassigned | Unassigned | 290_148 | Clustered/Singleton | 1 | VC_290_148 | 1 | 0 | 1 | 0 | 0 | 0 | 0 | 1 | Ref |
| JN204348 | Unassigned | Unassigned | Unassigned | 579_0 | Clustered | 2 | VC_579_0 | 2 | 0.0032 | 0.74796081 | 0.0024 | 0 | 0 | 0 | 1 | Ref |
| JN225449 | Unassigned | Unassigned | Unassigned | 340_4 | Clustered | 2 | VC_340_4 | 2 | 0.0052 | 0.95746455 | 0.005 | 0 | 0 | 0 | 1 | Ref |
| JN243855 | Unassigned | Unassigned | Unassigned | 245_0 | Clustered | 17 | VC_245_0 | 17 | 0.6081 | 1 | 0.6081 | 0 | 0 | 0 | 1 | Ref |
| JN243856 | Unassigned | Unassigned | Unassigned | 535_1 | Clustered | 108 | VC_535_1 | 108 | 0.8479 | 1 | 0.8479 | 0 | 0 | 0 | 0.9955 | Ref |
| JN243857 | Unassigned | Unassigned | Unassigned | 535_0 | Clustered | 18 | VC_535_0 | 18 | 0.1595 | 1 | 0.1595 | 0 | 0 | 0 | 1 | Ref |
| JN254800 | Unassigned | Unassigned | Unassigned | 80_0 | Clustered | 64 | VC_80_0 | 64 | 0.418 | 1 | 0.418 | 0 | 0 | 0 | 0.7649 | Ref |
| JN254801 | Unassigned | Unassigned | Unassigned | 486_0 | Clustered | 47 | VC_486_0 | 47 | 0.9022 | 1 | 0.9022 | 0 | 0 | 0 | 1 | Ref |
| JN255163 | Unassigned | Unassigned | Unassigned | 4_0 | Clustered | 7 | VC_4_0 | 7 | 0.7823 | 1 | 0.7823 | 0 | 0 | 0 | 1 | Ref |
| JN256079 | Unassigned | Unassigned | Unassigned | 539_0 | Clustered | 37 | VC_539_0 | 37 | 0.911 | 1 | 0.911 | 0 | 0 | 0 | 1 | Ref |
| JN371769 | Unassigned | Unassigned | Unassigned | 674_4 | Clustered/Singleton | 1 | VC_674_4 | 1 | 0 | 1 | 0 | 0 | 0 | 0 | 1 | Ref |
| JN377894 | Unassigned | Unassigned | Unassigned | 619_2 | Clustered | 10 | VC_619_2 | 10 | 0.3034 | 1 | 0.3034 | 0 | 0 | 0 | 1 | Ref |
| JN377895 | Unassigned | Unassigned | Unassigned | 619_2 | Clustered | 10 | VC_619_2 | 10 | 0.3034 | 1 | 0.3034 | 0 | 0 | 0 | 1 | Ref |
| JN382248 | Unassigned | Unassigned | Unassigned | 571_7 | Clustered | 28 | VC_571_7 | 28 | 0.1651 | 1 | 0.1651 | 0 | 0 | 0 | 0.8519 | Ref |
| JN391180 | Unassigned | Unassigned | Unassigned | 492_0 | Clustered | 20 | VC_492_0 | 20 | 0.7887 | 1 | 0.7887 | 0 | 0 | 0 | 1 | Ref |
| JN391441 | Unassigned | Unassigned | Unassigned | 571_7 | Clustered | 28 | VC_571_7 | 28 | 0.1651 | 1 | 0.1651 | 0 | 0 | 0 | 0.8519 | Ref |
| JN398368 | Unassigned | Unassigned | Unassigned | 538_76 | Clustered | 2 | VC_538_76 | 2 | 0.0091 | 0.95761376 | 0.0087 | 0 | 0 | 0 | 1 | Ref |
| JN398369 | Unassigned | Unassigned | Unassigned | 335_1 | Clustered | 2 | VC_335_1 | 2 | 0.0043 | 0.95663348 | 0.0041 | 0 | 0 | 0 | 1 | Ref |
| JN408459 | Unassigned | Unassigned | Unassigned | 974_0 | Clustered | 34 | VC_974_0 | 34 | 0.7953 | 1 | 0.7953 | 0 | 0 | 0 | 1 | Ref |
| JN408460 | Unassigned | Unassigned | Unassigned | 223_6 | Clustered | 42 | VC_223_6 | 42 | 0.4717 | 1 | 0.4717 | 0 | 0 | 0 | 0.8351 | Ref |
| JN408461 | Unassigned | Unassigned | Unassigned | 223_3 | Clustered | 8 | VC_223_3 | 8 | 0.0699 | 1 | 0.0699 | 0 | 0 | 0 | 0.8571 | Ref |
| JN412588 | Unassigned | Unassigned | Unassigned | 579_80 | Clustered/Singleton | 1 | VC_579_80 | 1 | 0 | 1 | 0 | 0 | 0 | 0 | 1 | Ref |
| JN412589 | Unassigned | Unassigned | Unassigned | 589_0 | Clustered | 3 | VC_589_0 | 3 | 0.2072 | 0.99849937 | 0.2069 | 0 | 0 | 0 | 1 | Ref |
| JN412590 | Unassigned | Unassigned | Unassigned | 571_4 | Clustered | 2 | VC_571_4 | 2 | 0.006 | 0.95695857 | 0.0057 | 0 | 0 | 0 | 1 | Ref |
| JN412591 | Unassigned | Unassigned | Unassigned | 537_0 | Clustered | 39 | VC_537_0 | 39 | 0.8148 | 1 | 0.8148 | 0 | 0 | 0 | 1 | Ref |
| JN412592 | Unassigned | Unassigned | Unassigned | 579_0 | Clustered | 2 | VC_579_0 | 2 | 0.0032 | 0.74796081 | 0.0024 | 0 | 0 | 0 | 1 | Ref |
| JN412593 | Unassigned | Unassigned | Unassigned | 783_0 | Clustered | 67 | VC_783_0 | 67 | 0.8945 | 1 | 0.8945 | 0 | 0 | 0 | 1 | Ref |
| JN542517 | Unassigned | Unassigned | Unassigned | 538_59 | Clustered | 5 | VC_538_59 | 5 | 0.0456 | 0.99999997 | 0.0456 | 0 | 0 | 0 | 1 | Ref |
| JN561150 | Unassigned | Unassigned | Unassigned | 535_1 | Clustered | 108 | VC_535_1 | 108 | 0.8479 | 1 | 0.8479 | 0 | 0 | 0 | 0.9955 | Ref |
| JN572061 | Unassigned | Unassigned | Unassigned | 537_0 | Clustered | 39 | VC_537_0 | 39 | 0.8148 | 1 | 0.8148 | 0 | 0 | 0 | 1 | Ref |
| JN572689 | Unassigned | Unassigned | Unassigned | 335_49 | Clustered | 17 | VC_335_49 | 17 | 0.0731 | 1 | 0.0731 | 0 | 0 | 0 | 0.7721 | Ref |
| JN585957 | Unassigned | Unassigned | Unassigned | 372_0 | Clustered | 7 | VC_372_0 | 7 | 0.3691 | 1 | 0.3691 | 0 | 0 | 0 | 1 | Ref |
| JN593240 | Unassigned | Unassigned | Unassigned | 135_25 | Clustered/Singleton | 1 | VC_135_25 | 1 | 0 | 1 | 0 | 0 | 0 | 0 | 1 | Ref |
| JN600960 | Unassigned | Unassigned | Unassigned | 32_1 | Clustered | 16 | VC_32_1 | 16 | 0.1962 | 1 | 0.1962 | 0 | 0 | 0 | 0.8333 | Ref |
| JN618996 | Unassigned | Unassigned | Unassigned | 526_0 | Clustered | 32 | VC_526_0 | 32 | 0.9037 | 1 | 0.9037 | 0 | 0 | 0 | 1 | Ref |
| JN624850 | Unassigned | Unassigned | Unassigned | 579_49 | Clustered | 7 | VC_579_49 | 7 | 0.0194 | 0.98914431 | 0.0191 | 0 | 0 | 0 | 0.8571 | Ref |
| JN624851 | Unassigned | Unassigned | Unassigned | 539_0 | Clustered | 37 | VC_539_0 | 37 | 0.911 | 1 | 0.911 | 0 | 0 | 0 | 1 | Ref |
| JN627160 | Unassigned | Unassigned | Unassigned | 456_2 | Clustered | 4 | VC_456_2 | 4 | 0.0833 | 0.99988129 | 0.0833 | 0 | 0 | 0 | 0.8333 | Ref |
| JN638752 | Unassigned | Unassigned | Unassigned | 384_15 | Clustered | 44 | VC_384_15 | 44 | 0.139 | 1 | 0.139 | 0 | 0 | 0 | 0.7474 | Ref |
| JN638753 | Unassigned | Unassigned | Unassigned | 384_15 | Clustered | 44 | VC_384_15 | 44 | 0.139 | 1 | 0.139 | 0 | 0 | 0 | 0.7474 | Ref |
| JN641803 | Unassigned | Unassigned | Unassigned | 994_5 | Clustered | 19 | VC_994_5 | 19 | 0.2622 | 1 | 0.2622 | 0 | 0 | 0 | 1 | Ref |
| JN643714 | Unassigned | Unassigned | Unassigned | 243_0 | Clustered | 72 | VC_243_0 | 72 | 0.8089 | 1 | 0.8089 | 0 | 0 | 0 | 1 | Ref |
| JN651747 | Unassigned | Unassigned | Unassigned | 907_0 | Clustered | 32 | VC_907_0 | 32 | 0.9964 | 1 | 0.9964 | 0 | 0 | 0 | 1 | Ref |
| JN654439 | Unassigned | Unassigned | Unassigned | 913_1 | Clustered | 8 | VC_913_1 | 8 | 0.1102 | 0.9989323 | 0.11 | 0 | 0 | 0 | 0.75 | Ref |
| JN660814 | Unassigned | Unassigned | Unassigned | 335_44 | Clustered | 3 | VC_335_44 | 3 | 0.0157 | 0.99859171 | 0.0157 | 0 | 0 | 0 | 1 | Ref |
| JN662425 | Unassigned | Unassigned | Unassigned | 168_0 | Clustered | 18 | VC_168_0 | 18 | 0.7394 | 1 | 0.7394 | 0 | 0 | 0 | 0.8627 | Ref |
| JN672684 | Unassigned | Unassigned | Unassigned | 971_0 | Clustered | 40 | VC_971_0 | 40 | 0.6793 | 1 | 0.6793 | 0 | 0 | 0 | 0.9359 | Ref |
| JN673056 | Unassigned | Unassigned | Unassigned | 135_26 | Clustered/Singleton | 1 | VC_135_26 | 1 | 0 | 1 | 0 | 0 | 0 | 0 | 1 | Ref |
| JN680858 | Unassigned | Unassigned | Unassigned | 917_0 | Clustered | 28 | VC_917_0 | 28 | 0.5465 | 1 | 0.5465 | 0 | 0 | 0 | 1 | Ref |
| JN687951 | Unassigned | Unassigned | Unassigned | 335_60 | Clustered | 2 | VC_335_60 | 2 | 0.0038 | 0.957513 | 0.0036 | 0 | 0 | 0 | 1 | Ref |
| JN698989 | Unassigned | Unassigned | Unassigned | 384_18 | Clustered | 10 | VC_384_18 | 10 | 0.0301 | 1 | 0.0301 | 0 | 0 | 0 | 0.8 | Ref |
| JN698990 | Unassigned | Unassigned | Unassigned | 384_17 | Clustered | 3 | VC_384_17 | 3 | 0.0075 | 0.99812032 | 0.0075 | 0 | 0 | 0 | 1 | Ref |
| JN698991 | Unassigned | Unassigned | Unassigned | 526_0 | Clustered | 32 | VC_526_0 | 32 | 0.9037 | 1 | 0.9037 | 0 | 0 | 0 | 1 | Ref |
| JN698992 | Unassigned | Unassigned | Unassigned | 527_0 | Clustered | 37 | VC_527_0 | 37 | 0.9679 | 1 | 0.9679 | 0 | 0 | 0 | 1 | Ref |
| JN698993 | Unassigned | Unassigned | Unassigned | 577_0 | Clustered | 21 | VC_577_0 | 21 | 0.9908 | 1 | 0.9908 | 0 | 0 | 0 | 1 | Ref |
| JN698994 | Unassigned | Unassigned | Unassigned | 574_0 | Clustered | 2 | VC_574_0 | 2 | 0.2216 | 0.9525409 | 0.2111 | 0 | 0 | 0 | 1 | Ref |
| JN698995 | Unassigned | Unassigned | Unassigned | 529_0 | Clustered | 3 | VC_529_0 | 3 | 0.109 | 0.99858726 | 0.1089 | 0 | 0 | 0 | 1 | Ref |
| JN698996 | Unassigned | Unassigned | Unassigned | 538_26 | Clustered | 3 | VC_538_26 | 3 | 0.0258 | 0.99859898 | 0.0258 | 0 | 0 | 0 | 1 | Ref |
| JN698997 | Unassigned | Unassigned | Unassigned | 566_2 | Clustered | 3 | VC_566_2 | 3 | 0.0275 | 0.85806939 | 0.0236 | 0 | 0 | 0 | 1 | Ref |
| JN698998 | Unassigned | Unassigned | Unassigned | 335_61 | Clustered | 8 | VC_335_61 | 8 | 0.0394 | 1 | 0.0394 | 0 | 0 | 0 | 0.7857 | Ref |
| JN698999 | Unassigned | Unassigned | Unassigned | 230_0 | Clustered | 38 | VC_230_0 | 38 | 0.9076 | 1 | 0.9076 | 0 | 0 | 0 | 1 | Ref |
| JN699000 | Unassigned | Unassigned | Unassigned | 335_41 | Clustered | 2 | VC_335_41 | 2 | 0.0045 | 0.9575092 | 0.0043 | 0 | 0 | 0 | 1 | Ref |
| JN699001 | Unassigned | Unassigned | Unassigned | 536_0 | Clustered | 6 | VC_536_0 | 6 | 0.129 | 1 | 0.129 | 0 | 0 | 0 | 1 | Ref |
| JN699002 | Unassigned | Unassigned | Unassigned | 783_0 | Clustered | 67 | VC_783_0 | 67 | 0.8945 | 1 | 0.8945 | 0 | 0 | 0 | 1 | Ref |
| JN699003 | Unassigned | Unassigned | Unassigned | 527_0 | Clustered | 37 | VC_527_0 | 37 | 0.9679 | 1 | 0.9679 | 0 | 0 | 0 | 1 | Ref |
| JN699004 | Unassigned | Unassigned | Unassigned | 526_0 | Clustered | 32 | VC_526_0 | 32 | 0.9037 | 1 | 0.9037 | 0 | 0 | 0 | 1 | Ref |
| JN699005 | Unassigned | Unassigned | Unassigned | 232_0 | Clustered | 32 | VC_232_0 | 32 | 0.8471 | 1 | 0.8471 | 0 | 0 | 0 | 0.996 | Ref |
| JN699006 | Unassigned | Unassigned | Unassigned | 527_0 | Clustered | 37 | VC_527_0 | 37 | 0.9679 | 1 | 0.9679 | 0 | 0 | 0 | 1 | Ref |
| JN699007 | Unassigned | Unassigned | Unassigned | 385_1 | Clustered | 9 | VC_385_1 | 9 | 0.1928 | 1 | 0.1928 | 0 | 0 | 0 | 1 | Ref |
| JN699008 | Unassigned | Unassigned | Unassigned | 384_15 | Clustered | 44 | VC_384_15 | 44 | 0.139 | 1 | 0.139 | 0 | 0 | 0 | 0.7474 | Ref |
| JN699009 | Unassigned | Unassigned | Unassigned | 384_16 | Clustered | 42 | VC_384_16 | 42 | 0.134 | 1 | 0.134 | 0 | 0 | 0 | 0.7282 | Ref |

| | | | | | | | | | | | | | | | | |
|---|---|---|---|---|---|---|---|---|---|---|---|---|---|---|---|---|
| JN699010 | Unassigned | Unassigned | Unassigned | 384_15 | Clustered | 44 | VC_384_15 | 44 | 0.139 | 1 | 0.139 | 0 | 0 | 0 | 0.7474 | Ref |
| JN699011 | Unassigned | Unassigned | Unassigned | 385_0 | Clustered | 24 | VC_385_0 | 24 | 0.5516 | 1 | 0.5516 | 0 | 0 | 0 | 1 | Ref |
| JN699012 | Unassigned | Unassigned | Unassigned | 538_20 | Clustered/Singleton | 1 | VC_538_20 | 1 | 0 | 1 | 0 | 0 | 0 | 0 | 1 | Ref |
| JN699013 | Unassigned | Unassigned | Unassigned | 579_8 | Clustered | 2 | VC_579_8 | 2 | 0.0032 | 0.66515174 | 0.0021 | 0 | 0 | 0 | 1 | Ref |
| JN699014 | Unassigned | Unassigned | Unassigned | 585_0 | Clustered | 20 | VC_585_0 | 20 | 0.8376 | 1 | 0.8376 | 0 | 0 | 0 | 1 | Ref |
| JN699015 | Unassigned | Unassigned | Unassigned | 535_1 | Clustered | 108 | VC_535_1 | 108 | 0.8479 | 1 | 0.8479 | 0 | 0 | 0 | 0.9955 | Ref |
| JN699016 | Unassigned | Unassigned | Unassigned | 335_37 | Clustered | 4 | VC_335_37 | 4 | 0.0112 | 0.99980442 | 0.0112 | 0 | 0 | 0 | 0.6667 | Ref |
| JN699017 | Unassigned | Unassigned | Unassigned | 384_17 | Clustered | 3 | VC_384_17 | 3 | 0.0075 | 0.99812032 | 0.0075 | 0 | 0 | 0 | 1 | Ref |
| JN699018 | Unassigned | Unassigned | Unassigned | 527_0 | Clustered | 37 | VC_527_0 | 37 | 0.9679 | 1 | 0.9679 | 0 | 0 | 0 | 1 | Ref |
| JN699019 | Unassigned | Unassigned | Unassigned | 230_0 | Clustered | 38 | VC_230_0 | 38 | 0.9076 | 1 | 0.9076 | 0 | 0 | 0 | 1 | Ref |
| JN699625 | Unassigned | Unassigned | Unassigned | 579_30 | Clustered | 3 | VC_579_30 | 3 | 0.0064 | 0.74796372 | 0.0048 | 0 | 0 | 0 | 1 | Ref |
| JN699626 | Unassigned | Unassigned | Unassigned | 579_63 | Clustered/Singleton | 1 | VC_579_63 | 1 | 0 | 1 | 0 | 0 | 0 | 0 | 1 | Ref |
| JN699627 | Unassigned | Unassigned | Unassigned | 579_78 | Clustered/Singleton | 1 | VC_579_78 | 1 | 0 | 1 | 0 | 0 | 0 | 0 | 1 | Ref |
| JN699628 | Unassigned | Unassigned | Unassigned | 785_1 | Clustered | 9 | VC_785_1 | 9 | 0.3679 | 1 | 0.3679 | 0 | 0 | 0 | 1 | Ref |
| JN712910 | Unassigned | Unassigned | Unassigned | 995_0 | Clustered | 2 | VC_995_0 | 2 | 0.627 | 0.85557782 | 0.5365 | 0 | 0 | 0 | 1 | Ref |
| JN790865 | Unassigned | Unassigned | Unassigned | 912_2 | Clustered | 2 | VC_912_2 | 2 | 0.018 | 0.88094852 | 0.0159 | 0 | 0 | 0 | 1 | Ref |
| JN797796 | Unassigned | Unassigned | Unassigned | 912_2 | Clustered | 2 | VC_912_2 | 2 | 0.018 | 0.88094852 | 0.0159 | 0 | 0 | 0 | 1 | Ref |
| JN797797 | Unassigned | Unassigned | Unassigned | 195_1 | Clustered | 7 | VC_195_1 | 7 | 0.1409 | 1 | 0.1409 | 0 | 0 | 0 | 1 | Ref |
| JN797798 | Unassigned | Unassigned | Unassigned | 195_1 | Clustered | 7 | VC_195_1 | 7 | 0.1409 | 1 | 0.1409 | 0 | 0 | 0 | 1 | Ref |
| JN800508 | Unassigned | Unassigned | Unassigned | 928_0 | Clustered | 6 | VC_928_0 | 6 | 0.4065 | 1 | 0.4065 | 0 | 0 | 0 | 1 | Ref |
| JN808773 | Unassigned | Unassigned | Unassigned | 185_0 | Clustered | 53 | VC_185_0 | 53 | 0.9153 | 1 | 0.9153 | 0 | 0 | 0 | 1 | Ref |
| JN811560 | Unassigned | Unassigned | Unassigned | 185_0 | Clustered | 53 | VC_185_0 | 53 | 0.9153 | 1 | 0.9153 | 0 | 0 | 0 | 1 | Ref |
| JN831653 | Unassigned | Unassigned | Unassigned | 241_0 | Clustered | 21 | VC_241_0 | 21 | 0.8342 | 1 | 0.8342 | 0 | 0 | 0 | 1 | Ref |
| JN848801 | Unassigned | Unassigned | Unassigned | 27_0 | Clustered | 4 | VC_27_0 | 4 | 0.7397 | 0.99936153 | 0.7392 | 0 | 0 | 0 | 1 | Ref |
| JN849462 | Unassigned | Unassigned | Unassigned | 549_1 | Clustered | 6 | VC_549_1 | 6 | 0.1479 | 0.9998319 | 0.1478 | 0 | 0 | 0 | 1 | Ref |
| JN859129 | Unassigned | Unassigned | Unassigned | 538_23 | Clustered | 2 | VC_538_23 | 2 | 0.0077 | 0.95763408 | 0.0074 | 0 | 0 | 0 | 1 | Ref |
| JN871591 | Unassigned | Unassigned | Unassigned | 611_0 | Clustered | 50 | VC_611_0 | 50 | 0.7306 | 1 | 0.7306 | 0 | 0 | 0 | 1 | Ref |
| JN882264 | Unassigned | Unassigned | Unassigned | 858_0 | Clustered | 7 | VC_858_0 | 7 | 1 | 1 | 1 | 0 | 0 | 0 | 1 | Ref |
| JN882266 | Unassigned | Unassigned | Unassigned | 858_0 | Clustered | 7 | VC_858_0 | 7 | 1 | 1 | 1 | 0 | 0 | 0 | 1 | Ref |
| JN882267 | Unassigned | Unassigned | Unassigned | 998_0 | Clustered | 2 | VC_998_0 | 2 | 0.3698 | 0.91237788 | 0.3374 | 0 | 0 | 0 | 1 | Ref |
| JN882284 | Unassigned | Unassigned | Unassigned | 601_4 | Clustered | 3 | VC_601_4 | 3 | 0.0346 | 0.98052968 | 0.034 | 0 | 0 | 0 | 1 | Ref |
| JN882285 | Unassigned | Unassigned | Unassigned | 170_4 | Clustered/Singleton | 1 | VC_170_4 | 1 | 0 | 1 | 0 | 0 | 0 | 0 | 1 | Ref |
| JN882286 | Unassigned | Unassigned | Unassigned | 836_2 | Clustered | 2 | VC_836_2 | 2 | 0.0659 | 0.95518138 | 0.063 | 0 | 0 | 0 | 1 | Ref |
| JN882287 | Unassigned | Unassigned | Unassigned | 687_0 | Clustered | 21 | VC_687_0 | 21 | 0.3608 | 1 | 0.3608 | 0 | 0 | 0 | 1 | Ref |
| JN882298 | Unassigned | Unassigned | Unassigned | 73_0 | Clustered | 2 | VC_73_0 | 2 | 0.077 | 0.95618005 | 0.0736 | 0 | 0 | 0 | 1 | Ref |
| JN939331 | Unassigned | Unassigned | Unassigned | 908_0 | Clustered | 36 | VC_908_0 | 36 | 0.9943 | 1 | 0.9943 | 0 | 0 | 0 | 1 | Ref |
| JN939332 | Unassigned | Unassigned | Unassigned | 908_0 | Clustered | 36 | VC_908_0 | 36 | 0.9943 | 1 | 0.9943 | 0 | 0 | 0 | 1 | Ref |
| JN968479 | Unassigned | Unassigned | Unassigned | 684_0 | Clustered | 4 | VC_684_0 | 4 | 1 | 1 | 1 | 0 | 0 | 0 | 1 | Ref |
| JN984867 | Unassigned | Unassigned | Unassigned | 859_0 | Clustered | 67 | VC_859_0 | 67 | 0.9622 | 1 | 0.9622 | 0 | 0 | 0 | 1 | Ref |
| JN986844 | Unassigned | Unassigned | Unassigned | 466_0 | Clustered | 66 | VC_466_0 | 66 | 0.9246 | 1 | 0.9246 | 0 | 0 | 0 | 1 | Ref |
| JN986845 | Unassigned | Unassigned | Unassigned | 470_0 | Clustered | 62 | VC_470_0 | 62 | 0.902 | 1 | 0.902 | 0 | 0 | 0 | 1 | Ref |
| JN986846 | Unassigned | Unassigned | Unassigned | 290_115 | Clustered/Singleton | 1 | VC_290_115 | 1 | 0 | 1 | 0 | 0 | 0 | 0 | 1 | Ref |
| JQ007353 | Unassigned | Unassigned | Unassigned | 654_0 | Clustered | 115 | VC_654_0 | 115 | 0.7303 | 1 | 0.7303 | 0 | 0 | 0 | 0.8764 | Ref |
| JQ010983 | Unassigned | Unassigned | Unassigned | 977_0 | Clustered | 2 | VC_977_0 | 2 | 1 | 1 | 1 | 0 | 0 | 0 | 1 | Ref |
| JQ011317 | Unassigned | Unassigned | Unassigned | 492_0 | Clustered | 20 | VC_492_0 | 20 | 0.7887 | 1 | 0.7887 | 0 | 0 | 0 | 1 | Ref |
| JQ011318 | Unassigned | Unassigned | Unassigned | 44_0 | Clustered | 49 | VC_44_0 | 49 | 0.4506 | 1 | 0.4506 | 0 | 0 | 0 | 0.8605 | Ref |
| JQ015307 | Unassigned | Unassigned | Unassigned | 873_2 | Clustered | 2 | VC_873_2 | 2 | 0.0245 | 0.87022547 | 0.0213 | 0 | 0 | 0 | 1 | Ref |
| JQ031132 | Unassigned | Unassigned | Unassigned | 794_4 | Clustered | 4 | VC_794_4 | 4 | 0.0181 | 0.73018303 | 0.0132 | 0 | 0 | 0 | 1 | Ref |
| JQ062992 | Unassigned | Unassigned | Unassigned | 719_0 | Clustered | 16 | VC_719_0 | 16 | 0.728 | 1 | 0.728 | 0 | 0 | 0 | 1 | Ref |
| JQ067084 | Unassigned | Unassigned | Unassigned | 479_0 | Clustered | 7 | VC_479_0 | 7 | 0.3294 | 1 | 0.3294 | 0 | 0 | 0 | 1 | Ref |
| JQ067085 | Unassigned | Unassigned | Unassigned | 185_0 | Clustered | 53 | VC_185_0 | 53 | 0.9153 | 1 | 0.9153 | 0 | 0 | 0 | 1 | Ref |
| JQ067087 | Unassigned | Unassigned | Unassigned | 478_0 | Clustered | 8 | VC_478_0 | 8 | 0.2619 | 1 | 0.2619 | 0 | 0 | 0 | 1 | Ref |
| JQ067089 | Unassigned | Unassigned | Unassigned | 175_0 | Clustered | 20 | VC_175_0 | 20 | 0.7504 | 1 | 0.7504 | 0 | 0 | 0 | 1 | Ref |
| JQ067092 | Unassigned | Unassigned | Unassigned | 766_0 | Clustered | 40 | VC_766_0 | 40 | 0.7511 | 1 | 0.7511 | 0 | 0 | 0 | 1 | Ref |
| JQ067093 | Unassigned | Unassigned | Unassigned | 175_0 | Clustered | 20 | VC_175_0 | 20 | 0.7504 | 1 | 0.7504 | 0 | 0 | 0 | 1 | Ref |
| JQ086375 | Unassigned | Unassigned | Unassigned | 859_0 | Clustered | 67 | VC_859_0 | 67 | 0.9622 | 1 | 0.9622 | 0 | 0 | 0 | 1 | Ref |
| JQ086376 | Unassigned | Unassigned | Unassigned | 45_0 | Clustered | 38 | VC_45_0 | 38 | 0.5785 | 1 | 0.5785 | 0 | 0 | 0 | 0.9659 | Ref |
| JQ177061 | Unassigned | Unassigned | Unassigned | 123_0 | Clustered | 4 | VC_123_0 | 4 | 0.1802 | 0.99998531 | 0.1802 | 0 | 0 | 0 | 1 | Ref |
| JQ177062 | Unassigned | Unassigned | Unassigned | 940_0 | Clustered | 2 | VC_940_0 | 2 | 0.4996 | 0.9262493 | 0.4628 | 0 | 0 | 0 | 1 | Ref |
| JQ177063 | Unassigned | Unassigned | Unassigned | 380_0 | Clustered | 6 | VC_380_0 | 6 | 0.51 | 1 | 0.51 | 0 | 0 | 0 | 1 | Ref |
| JQ177064 | Unassigned | Unassigned | Unassigned | 123_0 | Clustered | 4 | VC_123_0 | 4 | 0.1802 | 0.99998531 | 0.1802 | 0 | 0 | 0 | 1 | Ref |
| JQ182728 | Unassigned | Unassigned | Unassigned | 53_0 | Clustered | 10 | VC_53_0 | 10 | 0.1743 | 1 | 0.1743 | 0 | 0 | 0 | 1 | Ref |
| JQ182729 | Unassigned | Unassigned | Unassigned | 36_0 | Clustered | 4 | VC_36_0 | 4 | 0.0597 | 0.99994573 | 0.0597 | 0 | 0 | 0 | 1 | Ref |
| JQ182733 | Unassigned | Unassigned | Unassigned | 32_0 | Clustered | 2 | VC_32_0 | 2 | 0.0369 | 0.95734637 | 0.0353 | 0 | 0 | 0 | 1 | Ref |
| JQ182734 | Unassigned | Unassigned | Unassigned | 32_0 | Clustered | 2 | VC_32_0 | 2 | 0.0369 | 0.95734637 | 0.0353 | 0 | 0 | 0 | 1 | Ref |
| JQ182735 | Unassigned | Unassigned | Unassigned | 45_0 | Clustered | 38 | VC_45_0 | 38 | 0.5785 | 1 | 0.5785 | 0 | 0 | 0 | 0.9659 | Ref |
| JQ182736 | Unassigned | Unassigned | Unassigned | 469_0 | Clustered | 18 | VC_469_0 | 18 | 0.4583 | 1 | 0.4583 | 0 | 0 | 0 | 1 | Ref |
| JQ245707 | Unassigned | Unassigned | Unassigned | 504_0 | Clustered | 4 | VC_504_0 | 4 | 0.2554 | 0.99998753 | 0.2554 | 0 | 0 | 0 | 1 | Ref |
| JQ287645 | Unassigned | Unassigned | Unassigned | 458_0 | Clustered | 2 | VC_458_0 | 2 | 0.8124 | 0.72985431 | 0.593 | 0 | 0 | 0 | 1 | Ref |
| JQ300538 | Unassigned | Unassigned | Unassigned | 538_77 | Clustered | 3 | VC_538_77 | 3 | 0.0216 | 0.99859911 | 0.0216 | 0 | 0 | 0 | 1 | Ref |
| JQ307386 | Unassigned | Unassigned | Unassigned | 434_0 | Clustered | 45 | VC_434_0 | 45 | 0.9479 | 1 | 0.9479 | 0 | 0 | 0 | 1 | Ref |
| JQ307387 | Unassigned | Unassigned | Unassigned | 766_0 | Clustered | 40 | VC_766_0 | 40 | 0.7511 | 1 | 0.7511 | 0 | 0 | 0 | 1 | Ref |
| JQ309827 | Unassigned | Unassigned | Unassigned | 1003_0 | Clustered | 16 | VC_1003_0 | 16 | 0.8967 | 1 | 0.8967 | 0 | 0 | 0 | 1 | Ref |
| JQ312117 | Unassigned | Unassigned | Unassigned | 1005_0 | Clustered | 3 | VC_1005_0 | 3 | 0.8261 | 0.99765766 | 0.8242 | 0 | 0 | 0 | 1 | Ref |
| JQ340389 | Unassigned | Unassigned | Unassigned | 1007_1 | Clustered | 24 | VC_1007_1 | 24 | 0.7364 | 1 | 0.7364 | 0 | 0 | 0 | 0.8877 | Ref |
| JQ340774 | Unassigned | Unassigned | Unassigned | 611_0 | Clustered | 50 | VC_611_0 | 50 | 0.7306 | 1 | 0.7306 | 0 | 0 | 0 | 1 | Ref |
| JQ347801 | Unassigned | Unassigned | Unassigned | 43_0 | Clustered | 16 | VC_43_0 | 16 | 0.2017 | 1 | 0.2017 | 0 | 0 | 0 | 0.9667 | Ref |
| JQ408219 | Unassigned | Unassigned | Unassigned | 56_0 | Clustered | 6 | VC_56_0 | 6 | 0.6713 | 0.9999999 | 0.6713 | 0 | 0 | 0 | 1 | Ref |
| JQ512844 | Unassigned | Unassigned | Unassigned | 533_0 | Clustered | 16 | VC_533_0 | 16 | 0.3765 | 1 | 0.3765 | 0 | 0 | 0 | 1 | Ref |
| JQ513383 | Unassigned | Unassigned | Unassigned | 170_10 | Clustered | 5 | VC_170_10 | 5 | 0.0614 | 0.96483582 | 0.0592 | 0 | 0 | 0 | 1 | Ref |
| JQ617284 | Unassigned | Unassigned | Unassigned | 101_0 | Clustered | 32 | VC_101_0 | 32 | 0.8826 | 1 | 0.8826 | 0 | 0 | 0 | 1 | Ref |

| | | | | | | | | | | | | | | | | |
|---|---|---|---|---|---|---|---|---|---|---|---|---|---|---|---|---|
| JQ619704 | Unassigned | Unassigned | Unassigned | 852_0 | Clustered | 3 | VC_852_0 | 3 | 0.4886 | 0.99843584 | 0.4878 | 0 | 0 | 0 | 1 | Ref |
| JQ659259 | Unassigned | Unassigned | Unassigned | 888_0 | Clustered | 9 | VC_888_0 | 9 | 0.5251 | 1 | 0.5251 | 0 | 0 | 0 | 1 | Ref |
| JQ660954 | Unassigned | Unassigned | Unassigned | 561_0 | Clustered | 3 | VC_561_0 | 3 | 0.3411 | 0.99792547 | 0.3404 | 0 | 0 | 0 | 1 | Ref |
| JQ684677 | Unassigned | Unassigned | Unassigned | 974_0 | Clustered | 34 | VC_974_0 | 34 | 0.7953 | 1 | 0.7953 | 0 | 0 | 0 | 1 | Ref |
| JQ686190 | Unassigned | Unassigned | Unassigned | 155_15 | Clustered | 16 | VC_155_15 | 16 | 0.0779 | 1 | 0.0779 | 0 | 0 | 0 | 0.8083 | Ref |
| JQ691610 | Unassigned | Unassigned | Unassigned | 492_0 | Clustered | 20 | VC_492_0 | 20 | 0.7887 | 1 | 0.7887 | 0 | 0 | 0 | 1 | Ref |
| JQ691611 | Unassigned | Unassigned | Unassigned | 873_6 | Clustered | 7 | VC_873_6 | 7 | 0.1635 | 0.99999999 | 0.1635 | 0 | 0 | 0 | 0.9524 | Ref |
| JQ691612 | Unassigned | Unassigned | Unassigned | 873_5 | Clustered | 3 | VC_873_5 | 3 | 0.0491 | 0.9761082 | 0.0479 | 0 | 0 | 0 | 1 | Ref |
| JQ692107 | Unassigned | Unassigned | Unassigned | 911_0 | Clustered | 20 | VC_911_0 | 20 | 0.9915 | 1 | 0.9915 | 0 | 0 | 0 | 1 | Ref |
| JQ698665 | Unassigned | Unassigned | Unassigned | 335_49 | Clustered | 17 | VC_335_49 | 17 | 0.0731 | 1 | 0.0731 | 0 | 0 | 0 | 0.7721 | Ref |
| JQ729990 | Unassigned | Unassigned | Unassigned | 929_0 | Clustered | 10 | VC_929_0 | 10 | 0.7185 | 1 | 0.7185 | 0 | 0 | 0 | 1 | Ref |
| JQ729991 | Unassigned | Unassigned | Unassigned | 929_0 | Clustered | 10 | VC_929_0 | 10 | 0.7185 | 1 | 0.7185 | 0 | 0 | 0 | 1 | Ref |
| JQ729992 | Unassigned | Unassigned | Unassigned | 929_0 | Clustered | 10 | VC_929_0 | 10 | 0.7185 | 1 | 0.7185 | 0 | 0 | 0 | 1 | Ref |
| JQ762257 | Unassigned | Unassigned | Unassigned | 185_0 | Clustered | 53 | VC_185_0 | 53 | 0.9153 | 1 | 0.9153 | 0 | 0 | 0 | 1 | Ref |
| JQ764986 | Unassigned | Unassigned | Unassigned | 310_0 | Clustered | 155 | VC_310_0 | 155 | 0.9979 | 1 | 0.9979 | 0 | 0 | 0 | 1 | Ref |
| JQ764987 | Unassigned | Unassigned | Unassigned | 310_0 | Clustered | 155 | VC_310_0 | 155 | 0.9979 | 1 | 0.9979 | 0 | 0 | 0 | 1 | Ref |
| JQ764988 | Unassigned | Unassigned | Unassigned | 310_0 | Clustered | 155 | VC_310_0 | 155 | 0.9979 | 1 | 0.9979 | 0 | 0 | 0 | 1 | Ref |
| JQ764989 | Unassigned | Unassigned | Unassigned | 310_0 | Clustered | 155 | VC_310_0 | 155 | 0.9979 | 1 | 0.9979 | 0 | 0 | 0 | 1 | Ref |
| JQ764990 | Unassigned | Unassigned | Unassigned | 310_0 | Clustered | 155 | VC_310_0 | 155 | 0.9979 | 1 | 0.9979 | 0 | 0 | 0 | 1 | Ref |
| JQ779023 | Unassigned | Unassigned | Unassigned | 8_0 | Clustered | 58 | VC_8_0 | 58 | 0.8285 | 1 | 0.8285 | 0 | 0 | 0 | 0.9946 | Ref |
| JQ779024 | Unassigned | Unassigned | Unassigned | 9_0 | Clustered | 38 | VC_9_0 | 38 | 0.4859 | 1 | 0.4859 | 0 | 0 | 0 | 1 | Ref |
| JQ780163 | Unassigned | Unassigned | Unassigned | 83_0 | Clustered | 24 | VC_83_0 | 24 | 0.7345 | 1 | 0.7345 | 0 | 0 | 0 | 1 | Ref |
| JQ780327 | Unassigned | Unassigned | Unassigned | 237_0 | Clustered | 3 | VC_237_0 | 3 | 0.0732 | 0.99355836 | 0.0727 | 0 | 0 | 0 | 0.6667 | Ref |
| JQ797329 | Unassigned | Unassigned | Unassigned | 753_0 | Clustered | 26 | VC_753_0 | 26 | 0.9562 | 1 | 0.9562 | 0 | 0 | 0 | 1 | Ref |
| JQ806763 | Unassigned | Unassigned | Unassigned | 30_1 | Clustered | 53 | VC_30_1 | 53 | 0.7151 | 1 | 0.7151 | 0 | 0 | 0 | 0.9325 | Ref |
| JQ809650 | Unassigned | Unassigned | Unassigned | 311_0 | Clustered | 11 | VC_311_0 | 11 | 0.7851 | 1 | 0.7851 | 0 | 0 | 0 | 1 | Ref |
| JQ809701 | Unassigned | Unassigned | Unassigned | 535_1 | Clustered | 108 | VC_535_1 | 108 | 0.8479 | 1 | 0.8479 | 0 | 0 | 0 | 0.9955 | Ref |
| JQ809703 | Unassigned | Unassigned | Unassigned | 335_49 | Clustered | 17 | VC_335_49 | 17 | 0.0731 | 1 | 0.0731 | 0 | 0 | 0 | 0.7721 | Ref |
| JQ823122 | Unassigned | Unassigned | Unassigned | 1010_0 | Clustered | 2 | VC_1010_0 | 2 | 1 | 1 | 1 | 0 | 0 | 0 | 1 | Ref |
| JQ823123 | Unassigned | Unassigned | Unassigned | 1010_0 | Clustered | 2 | VC_1010_0 | 2 | 1 | 1 | 1 | 0 | 0 | 0 | 1 | Ref |
| JQ837901 | Unassigned | Unassigned | Unassigned | 466_0 | Clustered | 66 | VC_466_0 | 66 | 0.9246 | 1 | 0.9246 | 0 | 0 | 0 | 1 | Ref |
| JQ867099 | Unassigned | Unassigned | Unassigned | 855_0 | Clustered | 12 | VC_855_0 | 12 | 0.6068 | 1 | 0.6068 | 0 | 0 | 0 | 1 | Ref |
| JQ896627 | Unassigned | Unassigned | Unassigned | 535_1 | Clustered | 108 | VC_535_1 | 108 | 0.8479 | 1 | 0.8479 | 0 | 0 | 0 | 0.9955 | Ref |
| JQ911768 | Unassigned | Unassigned | Unassigned | 579_50 | Clustered | 15 | VC_579_50 | 15 | 0.0467 | 1 | 0.0467 | 0 | 0 | 0 | 0.9238 | Ref |
| JQ957925 | Unassigned | Unassigned | Unassigned | 405_0 | Clustered | 126 | VC_405_0 | 126 | 0.9614 | 1 | 0.9614 | 0 | 0 | 0 | 1 | Ref |
| JQ957932 | Unassigned | Unassigned | Unassigned | 8_0 | Clustered | 58 | VC_8_0 | 58 | 0.8285 | 1 | 0.8285 | 0 | 0 | 0 | 0.9946 | Ref |
| JQ965645 | Unassigned | Unassigned | Unassigned | 738_0 | Clustered | 9 | VC_738_0 | 9 | 0.7116 | 1 | 0.7116 | 0 | 0 | 0 | 1 | Ref |
| JQ965700 | Unassigned | Unassigned | Unassigned | 405_0 | Clustered | 126 | VC_405_0 | 126 | 0.9614 | 1 | 0.9614 | 0 | 0 | 0 | 1 | Ref |
| JQ965701 | Unassigned | Unassigned | Unassigned | 405_0 | Clustered | 126 | VC_405_0 | 126 | 0.9614 | 1 | 0.9614 | 0 | 0 | 0 | 1 | Ref |
| JQ965702 | Unassigned | Unassigned | Unassigned | 399_0 | Clustered | 49 | VC_399_0 | 49 | 0.7927 | 1 | 0.7927 | 0 | 0 | 0 | 1 | Ref |
| JQ965703 | Unassigned | Unassigned | Unassigned | 405_0 | Clustered | 126 | VC_405_0 | 126 | 0.9614 | 1 | 0.9614 | 0 | 0 | 0 | 1 | Ref |
| JQ966307 | Unassigned | Unassigned | Unassigned | 322_0 | Clustered | 12 | VC_322_0 | 12 | 0.8924 | 1 | 0.8924 | 0 | 0 | 0 | 1 | Ref |
| JQ973847 | Unassigned | Unassigned | Unassigned | 12_0 | Clustered | 21 | VC_12_0 | 21 | 0.2262 | 1 | 0.2262 | 0 | 0 | 0 | 0.9571 | Ref |
| JX000007 | Unassigned | Unassigned | Unassigned | 405_0 | Clustered | 126 | VC_405_0 | 126 | 0.9614 | 1 | 0.9614 | 0 | 0 | 0 | 1 | Ref |
| JX013863 | Unassigned | Unassigned | Unassigned | 12_2 | Clustered | 19 | VC_12_2 | 19 | 0.2009 | 1 | 0.2009 | 0 | 0 | 0 | 1 | Ref |
| JX042578 | Unassigned | Unassigned | Unassigned | 974_0 | Clustered | 34 | VC_974_0 | 34 | 0.7953 | 1 | 0.7953 | 0 | 0 | 0 | 1 | Ref |
| JX042579 | Unassigned | Unassigned | Unassigned | 244_1 | Clustered | 13 | VC_244_1 | 13 | 0.1766 | 1 | 0.1766 | 0 | 0 | 0 | 0.7308 | Ref |
| JX080300 | Unassigned | Unassigned | Unassigned | 155_8 | Clustered | 66 | VC_155_8 | 66 | 0.4537 | 1 | 0.4537 | 0 | 0 | 0 | 0.8182 | Ref |
| JX080301 | Unassigned | Unassigned | Unassigned | 155_8 | Clustered | 66 | VC_155_8 | 66 | 0.4537 | 1 | 0.4537 | 0 | 0 | 0 | 0.8182 | Ref |
| JX080302 | Unassigned | Unassigned | Unassigned | 155_8 | Clustered | 66 | VC_155_8 | 66 | 0.4537 | 1 | 0.4537 | 0 | 0 | 0 | 0.8182 | Ref |
| JX080303 | Unassigned | Unassigned | Unassigned | 155_8 | Clustered | 66 | VC_155_8 | 66 | 0.4537 | 1 | 0.4537 | 0 | 0 | 0 | 0.8182 | Ref |
| JX080304 | Unassigned | Unassigned | Unassigned | 155_8 | Clustered | 66 | VC_155_8 | 66 | 0.4537 | 1 | 0.4537 | 0 | 0 | 0 | 0.8182 | Ref |
| JX080305 | Unassigned | Unassigned | Unassigned | 155_8 | Clustered | 66 | VC_155_8 | 66 | 0.4537 | 1 | 0.4537 | 0 | 0 | 0 | 0.8182 | Ref |
| JX094431 | Unassigned | Unassigned | Unassigned | 195_3 | Clustered | 9 | VC_195_3 | 9 | 0.1955 | 1 | 0.1955 | 0 | 0 | 0 | 0.8611 | Ref |
| JX094499 | Unassigned | Unassigned | Unassigned | 611_0 | Clustered | 50 | VC_611_0 | 50 | 0.7306 | 1 | 0.7306 | 0 | 0 | 0 | 1 | Ref |
| JX094500 | Unassigned | Unassigned | Unassigned | 994_0 | Clustered | 5 | VC_994_0 | 5 | 0.0495 | 0.99983811 | 0.0494 | 0 | 0 | 0 | 1 | Ref |
| JX094501 | Unassigned | Unassigned | Unassigned | 12_2 | Clustered | 19 | VC_12_2 | 19 | 0.2009 | 1 | 0.2009 | 0 | 0 | 0 | 1 | Ref |
| JX100809 | Unassigned | Unassigned | Unassigned | 1011_4 | Clustered | 11 | VC_1011_4 | 11 | 0.3331 | 1 | 0.3331 | 0 | 0 | 0 | 1 | Ref |
| JX100810 | Unassigned | Unassigned | Unassigned | 1011_2 | Clustered | 2 | VC_1011_2 | 2 | 0.0273 | 0.95410794 | 0.0261 | 0 | 0 | 0 | 1 | Ref |
| JX100811 | Unassigned | Unassigned | Unassigned | 1011_4 | Clustered | 11 | VC_1011_4 | 11 | 0.3331 | 1 | 0.3331 | 0 | 0 | 0 | 1 | Ref |
| JX100812 | Unassigned | Unassigned | Unassigned | 1011_4 | Clustered | 11 | VC_1011_4 | 11 | 0.3331 | 1 | 0.3331 | 0 | 0 | 0 | 1 | Ref |
| JX100813 | Unassigned | Unassigned | Unassigned | 1011_4 | Clustered | 11 | VC_1011_4 | 11 | 0.3331 | 1 | 0.3331 | 0 | 0 | 0 | 1 | Ref |
| JX100814 | Unassigned | Unassigned | Unassigned | 1011_3 | Clustered | 2 | VC_1011_3 | 2 | 0.0256 | 0.88402892 | 0.0226 | 0 | 0 | 0 | 1 | Ref |
| JX104231 | Unassigned | Unassigned | Unassigned | 168_0 | Clustered | 18 | VC_168_0 | 18 | 0.7394 | 1 | 0.7394 | 0 | 0 | 0 | 0.8627 | Ref |
| JX120799 | Unassigned | Unassigned | Unassigned | 393_0 | Clustered | 5 | VC_393_0 | 5 | 0.4703 | 0.99999992 | 0.4703 | 0 | 0 | 0 | 0.9 | Ref |
| JX121091 | Unassigned | Unassigned | Unassigned | 538_26 | Clustered | 3 | VC_538_26 | 3 | 0.0258 | 0.99859898 | 0.0258 | 0 | 0 | 0 | 1 | Ref |
| JX123262 | Unassigned | Unassigned | Unassigned | 457_4 | Clustered | 10 | VC_457_4 | 10 | 0.3316 | 1 | 0.3316 | 0 | 0 | 0 | 0.9333 | Ref |
| JX126918 | Unassigned | Unassigned | Unassigned | 753_0 | Clustered | 26 | VC_753_0 | 26 | 0.9562 | 1 | 0.9562 | 0 | 0 | 0 | 1 | Ref |
| JX126919 | Unassigned | Unassigned | Unassigned | 1002_0 | Clustered | 10 | VC_1002_0 | 10 | 0.9987 | 1 | 0.9987 | 0 | 0 | 0 | 1 | Ref |
| JX126920 | Unassigned | Unassigned | Unassigned | 1002_0 | Clustered | 10 | VC_1002_0 | 10 | 0.9987 | 1 | 0.9987 | 0 | 0 | 0 | 1 | Ref |
| JX128257 | Unassigned | Unassigned | Unassigned | 135_22 | Clustered | 13 | VC_135_22 | 13 | 0.0795 | 1 | 0.0795 | 0 | 0 | 0 | 0.8205 | Ref |
| JX128258 | Unassigned | Unassigned | Unassigned | 636_0 | Clustered | 13 | VC_636_0 | 13 | 0.6223 | 1 | 0.6223 | 0 | 0 | 0 | 1 | Ref |
| JX128259 | Unassigned | Unassigned | Unassigned | 290_95 | Clustered/Singleton | 1 | VC_290_95 | 1 | 0 | 1 | 0 | 0 | 0 | 0 | 1 | Ref |
| JX131330 | Unassigned | Unassigned | Unassigned | 172_0 | Clustered | 26 | VC_172_0 | 26 | 0.7909 | 1 | 0.7909 | 0 | 0 | 0 | 1 | Ref |
| JX145342 | Unassigned | Unassigned | Unassigned | 666_0 | Clustered | 11 | VC_666_0 | 11 | 0.5822 | 1 | 0.5822 | 0 | 0 | 0 | 1 | Ref |
| JX163858 | Unassigned | Unassigned | Unassigned | 1011_4 | Clustered | 11 | VC_1011_4 | 11 | 0.3331 | 1 | 0.3331 | 0 | 0 | 0 | 1 | Ref |
| JX173487 | Unassigned | Unassigned | Unassigned | 132_0 | Clustered | 19 | VC_132_0 | 19 | 0.6051 | 1 | 0.6051 | 0 | 0 | 0 | 1 | Ref |
| JX174275 | Unassigned | Unassigned | Unassigned | 8_0 | Clustered | 58 | VC_8_0 | 58 | 0.8285 | 1 | 0.8285 | 0 | 0 | 0 | 0.9946 | Ref |
| JX181822 | Unassigned | Unassigned | Unassigned | 340_1 | Clustered | 17 | VC_340_1 | 17 | 0.0863 | 1 | 0.0863 | 0 | 0 | 0 | 0.8235 | Ref |
| JX181824 | Unassigned | Unassigned | Unassigned | 601_5 | Clustered | 4 | VC_601_5 | 4 | 0.0525 | 0.99930943 | 0.0525 | 0 | 0 | 0 | 0.8333 | Ref |
| JX181828 | Unassigned | Unassigned | Unassigned | 135_22 | Clustered | 13 | VC_135_22 | 13 | 0.0795 | 1 | 0.0795 | 0 | 0 | 0 | 0.8205 | Ref |

| | | | | | | | | | | | | | | | | |
|---|---|---|---|---|---|---|---|---|---|---|---|---|---|---|---|---|
| JX181829 | Unassigned | Unassigned | Unassigned | 133_2 | Clustered | 7 | VC_133_2 | 7 | 0.0679 | 1 | 0.0679 | 0 | 0 | 0 | 0.9048 | Ref |
| JX182369 | Unassigned | Unassigned | Unassigned | 1015_0 | Clustered | 3 | VC_1015_0 | 3 | 0.0529 | 0.99853685 | 0.0528 | 0 | 0 | 0 | 1 | Ref |
| JX182370 | Unassigned | Unassigned | Unassigned | 1015_1 | Clustered | 26 | VC_1015_1 | 26 | 0.6989 | 1 | 0.6989 | 0 | 0 | 0 | 1 | Ref |
| JX182371 | Unassigned | Unassigned | Unassigned | 742_0 | Clustered | 11 | VC_742_0 | 11 | 0.5046 | 1 | 0.5046 | 0 | 0 | 0 | 1 | Ref |
| JX182372 | Unassigned | Unassigned | Unassigned | 386_0 | Clustered | 10 | VC_386_0 | 10 | 0.7454 | 1 | 0.7454 | 0 | 0 | 0 | 1 | Ref |
| JX193904 | Unassigned | Unassigned | Unassigned | 711_0 | Clustered | 48 | VC_711_0 | 48 | 0.8217 | 1 | 0.8217 | 0 | 0 | 0 | 1 | Ref |
| JX194238 | Unassigned | Unassigned | Unassigned | 862_0 | Clustered | 29 | VC_862_0 | 29 | 0.8594 | 1 | 0.8594 | 0 | 0 | 0 | 1 | Ref |
| JX194239 | Unassigned | Unassigned | Unassigned | 159_0 | Clustered | 25 | VC_159_0 | 25 | 0.7699 | 1 | 0.7699 | 0 | 0 | 0 | 1 | Ref |
| JX202565 | Unassigned | Unassigned | Unassigned | 654_0 | Clustered | 115 | VC_654_0 | 115 | 0.7303 | 1 | 0.7303 | 0 | 0 | 0 | 0.8764 | Ref |
| JX233784 | Unassigned | Unassigned | Unassigned | 356_0 | Clustered | 21 | VC_356_0 | 21 | 0.5409 | 1 | 0.5409 | 0 | 0 | 0 | 1 | Ref |
| JX238501 | Unassigned | Unassigned | Unassigned | 87_6 | Clustered/Singleton | 1 | VC_87_6 | 1 | 0 | 1 | 0 | 0 | 0 | 0 | 1 | Ref |
| JX262215 | Unassigned | Unassigned | Unassigned | 787_0 | Clustered | 132 | VC_787_0 | 132 | 1 | 1 | 1 | 0 | 0 | 0 | 1 | Ref |
| JX262216 | Unassigned | Unassigned | Unassigned | 787_0 | Clustered | 132 | VC_787_0 | 132 | 1 | 1 | 1 | 0 | 0 | 0 | 1 | Ref |
| JX262217 | Unassigned | Unassigned | Unassigned | 787_0 | Clustered | 132 | VC_787_0 | 132 | 1 | 1 | 1 | 0 | 0 | 0 | 1 | Ref |
| JX262218 | Unassigned | Unassigned | Unassigned | 787_0 | Clustered | 132 | VC_787_0 | 132 | 1 | 1 | 1 | 0 | 0 | 0 | 1 | Ref |
| JX262219 | Unassigned | Unassigned | Unassigned | 787_0 | Clustered | 132 | VC_787_0 | 132 | 1 | 1 | 1 | 0 | 0 | 0 | 1 | Ref |
| JX262220 | Unassigned | Unassigned | Unassigned | 787_0 | Clustered | 132 | VC_787_0 | 132 | 1 | 1 | 1 | 0 | 0 | 0 | 1 | Ref |
| JX262221 | Unassigned | Unassigned | Unassigned | 787_0 | Clustered | 132 | VC_787_0 | 132 | 1 | 1 | 1 | 0 | 0 | 0 | 1 | Ref |
| JX262222 | Unassigned | Unassigned | Unassigned | 787_0 | Clustered | 132 | VC_787_0 | 132 | 1 | 1 | 1 | 0 | 0 | 0 | 1 | Ref |
| JX262223 | Unassigned | Unassigned | Unassigned | 787_0 | Clustered | 132 | VC_787_0 | 132 | 1 | 1 | 1 | 0 | 0 | 0 | 1 | Ref |
| JX262224 | Unassigned | Unassigned | Unassigned | 787_0 | Clustered | 132 | VC_787_0 | 132 | 1 | 1 | 1 | 0 | 0 | 0 | 1 | Ref |
| JX262225 | Unassigned | Unassigned | Unassigned | 787_0 | Clustered | 132 | VC_787_0 | 132 | 1 | 1 | 1 | 0 | 0 | 0 | 1 | Ref |
| JX262376 | Unassigned | Unassigned | Unassigned | 1015_1 | Clustered | 26 | VC_1015_1 | 26 | 0.6989 | 1 | 0.6989 | 0 | 0 | 0 | 1 | Ref |
| JX274646 | Unassigned | Unassigned | Unassigned | 9_0 | Clustered | 38 | VC_9_0 | 38 | 0.4859 | 1 | 0.4859 | 0 | 0 | 0 | 1 | Ref |
| JX274647 | Unassigned | Unassigned | Unassigned | 9_0 | Clustered | 38 | VC_9_0 | 38 | 0.4859 | 1 | 0.4859 | 0 | 0 | 0 | 1 | Ref |
| JX290549 | Unassigned | Unassigned | Unassigned | 1018_0 | Clustered | 38 | VC_1018_0 | 38 | 0.9757 | 1 | 0.9757 | 0 | 0 | 0 | 1 | Ref |
| JX296113 | Unassigned | Unassigned | Unassigned | 611_0 | Clustered | 50 | VC_611_0 | 50 | 0.7306 | 1 | 0.7306 | 0 | 0 | 0 | 1 | Ref |
| JX297445 | Unassigned | Unassigned | Unassigned | 654_1 | Clustered | 27 | VC_654_1 | 27 | 0.2571 | 1 | 0.2571 | 0 | 0 | 0 | 0.9858 | Ref |
| JX306041 | Unassigned | Unassigned | Unassigned | 619_2 | Clustered | 10 | VC_619_2 | 10 | 0.3034 | 1 | 0.3034 | 0 | 0 | 0 | 1 | Ref |
| JX307702 | Unassigned | Unassigned | Unassigned | 535_1 | Clustered | 108 | VC_535_1 | 108 | 0.8479 | 1 | 0.8479 | 0 | 0 | 0 | 0.9955 | Ref |
| JX307703 | Unassigned | Unassigned | Unassigned | 535_1 | Clustered | 108 | VC_535_1 | 108 | 0.8479 | 1 | 0.8479 | 0 | 0 | 0 | 0.9955 | Ref |
| JX307704 | Unassigned | Unassigned | Unassigned | 533_0 | Clustered | 16 | VC_533_0 | 16 | 0.3765 | 1 | 0.3765 | 0 | 0 | 0 | 1 | Ref |
| JX307705 | Unassigned | Unassigned | Unassigned | 335_37 | Clustered | 4 | VC_335_37 | 4 | 0.0112 | 0.99980442 | 0.0112 | 0 | 0 | 0 | 0.6667 | Ref |
| JX316028 | Unassigned | Unassigned | Unassigned | 994_4 | Clustered | 3 | VC_994_4 | 3 | 0.0236 | 0.75814415 | 0.0179 | 0 | 0 | 0 | 1 | Ref |
| JX403939 | Unassigned | Unassigned | Unassigned | 304_1 | Clustered | 20 | VC_304_1 | 20 | 0.5632 | 1 | 0.5632 | 0 | 0 | 0 | 0.7684 | Ref |
| JX403940 | Unassigned | Unassigned | Unassigned | 441_1 | Clustered | 8 | VC_441_1 | 8 | 0.5443 | 1 | 0.5443 | 0 | 0 | 0 | 0.9643 | Ref |
| JX411619 | Unassigned | Unassigned | Unassigned | 533_0 | Clustered | 16 | VC_533_0 | 16 | 0.3765 | 1 | 0.3765 | 0 | 0 | 0 | 1 | Ref |
| JX412914 | Unassigned | Unassigned | Unassigned | 62_0 | Clustered | 12 | VC_62_0 | 12 | 0.7624 | 1 | 0.7624 | 0 | 0 | 0 | 1 | Ref |
| JX415535 | Unassigned | Unassigned | Unassigned | 801_0 | Clustered | 37 | VC_801_0 | 37 | 0.5672 | 1 | 0.5672 | 0 | 0 | 0 | 1 | Ref |
| JX415536 | Unassigned | Unassigned | Unassigned | 837_0 | Clustered | 12 | VC_837_0 | 12 | 0.2327 | 1 | 0.2327 | 0 | 0 | 0 | 1 | Ref |
| JX421753 | Unassigned | Unassigned | Unassigned | 398_0 | Clustered | 51 | VC_398_0 | 51 | 0.8741 | 1 | 0.8741 | 0 | 0 | 0 | 1 | Ref |
| JX434030 | Unassigned | Unassigned | Unassigned | 185_0 | Clustered | 53 | VC_185_0 | 53 | 0.9153 | 1 | 0.9153 | 0 | 0 | 0 | 1 | Ref |
| JX434031 | Unassigned | Unassigned | Unassigned | 185_0 | Clustered | 53 | VC_185_0 | 53 | 0.9153 | 1 | 0.9153 | 0 | 0 | 0 | 1 | Ref |
| JX434032 | Unassigned | Unassigned | Unassigned | 185_0 | Clustered | 53 | VC_185_0 | 53 | 0.9153 | 1 | 0.9153 | 0 | 0 | 0 | 1 | Ref |
| JX434033 | Unassigned | Unassigned | Unassigned | 185_0 | Clustered | 53 | VC_185_0 | 53 | 0.9153 | 1 | 0.9153 | 0 | 0 | 0 | 1 | Ref |
| JX442241 | Unassigned | Unassigned | Unassigned | 1002_0 | Clustered | 10 | VC_1002_0 | 10 | 0.9987 | 1 | 0.9987 | 0 | 0 | 0 | 1 | Ref |
| JX483873 | Unassigned | Unassigned | Unassigned | 497_0 | Clustered | 4 | VC_497_0 | 4 | 0.0962 | 0.999525 | 0.0961 | 0 | 0 | 0 | 1 | Ref |
| JX483874 | Unassigned | Unassigned | Unassigned | 1020_0 | Clustered | 22 | VC_1020_0 | 22 | 0.895 | 1 | 0.895 | 0 | 0 | 0 | 1 | Ref |
| JX483875 | Unassigned | Unassigned | Unassigned | 1020_0 | Clustered | 22 | VC_1020_0 | 22 | 0.895 | 1 | 0.895 | 0 | 0 | 0 | 1 | Ref |
| JX483876 | Unassigned | Unassigned | Unassigned | 980_0 | Clustered | 11 | VC_980_0 | 11 | 0.7333 | 1 | 0.7333 | 0 | 0 | 0 | 1 | Ref |
| JX483877 | Unassigned | Unassigned | Unassigned | 980_0 | Clustered | 11 | VC_980_0 | 11 | 0.7333 | 1 | 0.7333 | 0 | 0 | 0 | 1 | Ref |
| JX483878 | Unassigned | Unassigned | Unassigned | 980_0 | Clustered | 11 | VC_980_0 | 11 | 0.7333 | 1 | 0.7333 | 0 | 0 | 0 | 1 | Ref |
| JX483879 | Unassigned | Unassigned | Unassigned | 1020_0 | Clustered | 22 | VC_1020_0 | 22 | 0.895 | 1 | 0.895 | 0 | 0 | 0 | 1 | Ref |
| JX483880 | Unassigned | Unassigned | Unassigned | 1020_0 | Clustered | 22 | VC_1020_0 | 22 | 0.895 | 1 | 0.895 | 0 | 0 | 0 | 1 | Ref |
| JX483881 | Unassigned | Unassigned | Unassigned | 739_1 | Clustered | 6 | VC_739_1 | 6 | 0.434 | 1 | 0.434 | 0 | 0 | 0 | 1 | Ref |
| JX486087 | Unassigned | Unassigned | Unassigned | 597_0 | Clustered | 6 | VC_597_0 | 6 | 0.9222 | 0.99999993 | 0.9222 | 0 | 0 | 0 | 1 | Ref |
| JX486088 | Unassigned | Unassigned | Unassigned | 650_1 | Clustered | 6 | VC_650_1 | 6 | 0.4186 | 1 | 0.4186 | 0 | 0 | 0 | 1 | Ref |
| JX495041 | Unassigned | Unassigned | Unassigned | 192_0 | Clustered | 22 | VC_192_0 | 22 | 0.6582 | 1 | 0.6582 | 0 | 0 | 0 | 1 | Ref |
| JX495042 | Unassigned | Unassigned | Unassigned | 192_0 | Clustered | 22 | VC_192_0 | 22 | 0.6582 | 1 | 0.6582 | 0 | 0 | 0 | 1 | Ref |
| JX495043 | Unassigned | Unassigned | Unassigned | 192_0 | Clustered | 22 | VC_192_0 | 22 | 0.6582 | 1 | 0.6582 | 0 | 0 | 0 | 1 | Ref |
| JX501340 | Unassigned | Unassigned | Unassigned | 434_0 | Clustered | 45 | VC_434_0 | 45 | 0.9479 | 1 | 0.9479 | 0 | 0 | 0 | 1 | Ref |
| JX507079 | Unassigned | Unassigned | Unassigned | 25_0 | Clustered | 3 | VC_25_0 | 3 | 0.3393 | 0.99845192 | 0.3388 | 0 | 0 | 0 | 1 | Ref |
| JX536493 | Unassigned | Unassigned | Unassigned | 293_14 | Clustered/Singleton | 1 | VC_293_14 | 1 | 0 | 1 | 0 | 0 | 0 | 0 | 1 | Ref |
| JX556417 | Unassigned | Unassigned | Unassigned | 24_0 | Clustered | 3 | VC_24_0 | 3 | 0.4232 | 0.9983762 | 0.4226 | 0 | 0 | 0 | 1 | Ref |
| JX556418 | Unassigned | Unassigned | Unassigned | 911_0 | Clustered | 20 | VC_911_0 | 20 | 0.9915 | 1 | 0.9915 | 0 | 0 | 0 | 1 | Ref |
| JX560968 | Unassigned | Unassigned | Unassigned | 340_18 | Clustered | 7 | VC_340_18 | 7 | 0.0326 | 1 | 0.0326 | 0 | 0 | 0 | 0.8095 | Ref |
| JX561091 | Unassigned | Unassigned | Unassigned | 871_1 | Clustered | 26 | VC_871_1 | 26 | 0.6303 | 1 | 0.6303 | 0 | 0 | 0 | 0.9815 | Ref |
| JX564242 | Unassigned | Unassigned | Unassigned | 274_0 | Clustered | 7 | VC_274_0 | 7 | 0.3401 | 1 | 0.3401 | 0 | 0 | 0 | 1 | Ref |
| JX567312 | Unassigned | Unassigned | Unassigned | 274_0 | Clustered | 7 | VC_274_0 | 7 | 0.3401 | 1 | 0.3401 | 0 | 0 | 0 | 1 | Ref |
| JX569801 | Unassigned | Unassigned | Unassigned | 868_0 | Clustered | 27 | VC_868_0 | 27 | 0.9372 | 1 | 0.9372 | 0 | 0 | 0 | 1 | Ref |
| JX570702 | Unassigned | Unassigned | Unassigned | 787_0 | Clustered | 132 | VC_787_0 | 132 | 1 | 1 | 1 | 0 | 0 | 0 | 1 | Ref |
| JX570703 | Unassigned | Unassigned | Unassigned | 787_0 | Clustered | 132 | VC_787_0 | 132 | 1 | 1 | 1 | 0 | 0 | 0 | 1 | Ref |
| JX570704 | Unassigned | Unassigned | Unassigned | 787_0 | Clustered | 132 | VC_787_0 | 132 | 1 | 1 | 1 | 0 | 0 | 0 | 1 | Ref |
| JX570705 | Unassigned | Unassigned | Unassigned | 787_0 | Clustered | 132 | VC_787_0 | 132 | 1 | 1 | 1 | 0 | 0 | 0 | 1 | Ref |
| JX570706 | Unassigned | Unassigned | Unassigned | 787_0 | Clustered | 132 | VC_787_0 | 132 | 1 | 1 | 1 | 0 | 0 | 0 | 1 | Ref |
| JX570707 | Unassigned | Unassigned | Unassigned | 787_0 | Clustered | 132 | VC_787_0 | 132 | 1 | 1 | 1 | 0 | 0 | 0 | 1 | Ref |
| JX570708 | Unassigned | Unassigned | Unassigned | 787_0 | Clustered | 132 | VC_787_0 | 132 | 1 | 1 | 1 | 0 | 0 | 0 | 1 | Ref |
| JX570709 | Unassigned | Unassigned | Unassigned | 787_0 | Clustered | 132 | VC_787_0 | 132 | 1 | 1 | 1 | 0 | 0 | 0 | 1 | Ref |
| JX570710 | Unassigned | Unassigned | Unassigned | 787_0 | Clustered | 132 | VC_787_0 | 132 | 1 | 1 | 1 | 0 | 0 | 0 | 1 | Ref |
| JX570711 | Unassigned | Unassigned | Unassigned | 787_0 | Clustered | 132 | VC_787_0 | 132 | 1 | 1 | 1 | 0 | 0 | 0 | 1 | Ref |
| JX570712 | Unassigned | Unassigned | Unassigned | 787_0 | Clustered | 132 | VC_787_0 | 132 | 1 | 1 | 1 | 0 | 0 | 0 | 1 | Ref |

| | | | | | | | | | | | | | | | | |
|---|---|---|---|---|---|---|---|---|---|---|---|---|---|---|---|---|
| JX570713 | Unassigned | Unassigned | Unassigned | 787_0 | Clustered | 132 | VC_787_0 | 132 | 1 | 1 | 1 | 0 | 0 | 0 | 1 | Ref |
| JX570714 | Unassigned | Unassigned | Unassigned | 787_0 | Clustered | 132 | VC_787_0 | 132 | 1 | 1 | 1 | 0 | 0 | 0 | 1 | Ref |
| JX625144 | Unassigned | Unassigned | Unassigned | 321_0 | Clustered | 2 | VC_321_0 | 2 | 0.1773 | 0.99999983 | 0.1773 | 0 | 0 | 0 | 1 | Ref |
| JX649096 | Unassigned | Unassigned | Unassigned | 384_16 | Clustered | 42 | VC_384_16 | 42 | 0.134 | 1 | 0.134 | 0 | 0 | 0 | 0.7282 | Ref |
| JX649097 | Unassigned | Unassigned | Unassigned | 384_16 | Clustered | 42 | VC_384_16 | 42 | 0.134 | 1 | 0.134 | 0 | 0 | 0 | 0.7282 | Ref |
| JX649098 | Unassigned | Unassigned | Unassigned | 384_3 | Clustered | 22 | VC_384_3 | 22 | 0.0786 | 1 | 0.0786 | 0 | 0 | 0 | 0.9048 | Ref |
| JX649099 | Unassigned | Unassigned | Unassigned | 384_3 | Clustered | 22 | VC_384_3 | 22 | 0.0786 | 1 | 0.0786 | 0 | 0 | 0 | 0.9048 | Ref |
| JX649100 | Unassigned | Unassigned | Unassigned | 384_16 | Clustered | 42 | VC_384_16 | 42 | 0.134 | 1 | 0.134 | 0 | 0 | 0 | 0.7282 | Ref |
| JX658790 | Unassigned | Unassigned | Unassigned | 714_0 | Clustered | 71 | VC_714_0 | 71 | 0.935 | 1 | 0.935 | 0 | 0 | 0 | 1 | Ref |
| JX681814 | Unassigned | Unassigned | Unassigned | 3_0 | Clustered | 17 | VC_3_0 | 17 | 0.5615 | 1 | 0.5615 | 0 | 0 | 0 | 1 | Ref |
| JX846612 | Unassigned | Unassigned | Unassigned | 159_0 | Clustered | 25 | VC_159_0 | 25 | 0.7699 | 1 | 0.7699 | 0 | 0 | 0 | 1 | Ref |
| JX846613 | Unassigned | Unassigned | Unassigned | 159_0 | Clustered | 25 | VC_159_0 | 25 | 0.7699 | 1 | 0.7699 | 0 | 0 | 0 | 1 | Ref |
| JX863101 | Unassigned | Unassigned | Unassigned | 486_0 | Clustered | 47 | VC_486_0 | 47 | 0.9022 | 1 | 0.9022 | 0 | 0 | 0 | 1 | Ref |
| JX865427 | Unassigned | Unassigned | Unassigned | 859_0 | Clustered | 67 | VC_859_0 | 67 | 0.9622 | 1 | 0.9622 | 0 | 0 | 0 | 1 | Ref |
| JX866719 | Unassigned | Unassigned | Unassigned | 125_0 | Clustered | 23 | VC_125_0 | 23 | 0.8163 | 1 | 0.8163 | 0 | 0 | 0 | 1 | Ref |
| JX867715 | Unassigned | Unassigned | Unassigned | 837_1 | Clustered | 24 | VC_837_1 | 24 | 0.5328 | 1 | 0.5328 | 0 | 0 | 0 | 1 | Ref |
| JX872508 | Unassigned | Unassigned | Unassigned | 405_0 | Clustered | 126 | VC_405_0 | 126 | 0.9614 | 1 | 0.9614 | 0 | 0 | 0 | 1 | Ref |
| JX875065 | Unassigned | Unassigned | Unassigned | 155_8 | Clustered | 66 | VC_155_8 | 66 | 0.4537 | 1 | 0.4537 | 0 | 0 | 0 | 0.8182 | Ref |
| JX878496 | Unassigned | Unassigned | Unassigned | 134_1 | Clustered | 4 | VC_134_1 | 4 | 0.0354 | 0.99998777 | 0.0354 | 0 | 0 | 0 | 1 | Ref |
| JX878671 | Unassigned | Unassigned | Unassigned | 155_13 | Clustered | 3 | VC_155_13 | 3 | 0.0087 | 0.95130224 | 0.0083 | 0 | 0 | 0 | 1 | Ref |
| JX879087 | Unassigned | Unassigned | Unassigned | 425_0 | Clustered | 40 | VC_425_0 | 40 | 0.8399 | 1 | 0.8399 | 0 | 0 | 0 | 0.9974 | Ref |
| JX880034 | Unassigned | Unassigned | Unassigned | 801_0 | Clustered | 37 | VC_801_0 | 37 | 0.5672 | 1 | 0.5672 | 0 | 0 | 0 | 1 | Ref |
| JX880072 | Unassigned | Unassigned | Unassigned | 119_0 | Clustered | 9 | VC_119_0 | 9 | 0.9977 | 0.98988637 | 0.9876 | 0 | 0 | 0 | 1 | Ref |
| JX887877 | Unassigned | Unassigned | Unassigned | 853_0 | Clustered | 5 | VC_853_0 | 5 | 0.8735 | 0.99999832 | 0.8735 | 0 | 0 | 0 | 1 | Ref |
| JX889246 | Unassigned | Unassigned | Unassigned | 1016_0 | Clustered | 62 | VC_1016_0 | 62 | 0.8638 | 1 | 0.8638 | 0 | 0 | 0 | 0.743 | Ref |
| JX899358 | Unassigned | Unassigned | Unassigned | 223_6 | Clustered | 42 | VC_223_6 | 42 | 0.4717 | 1 | 0.4717 | 0 | 0 | 0 | 0.8351 | Ref |
| JX901189 | Unassigned | Unassigned | Unassigned | 594_0 | Clustered | 7 | VC_594_0 | 7 | 0.2725 | 1 | 0.2725 | 0 | 0 | 0 | 1 | Ref |
| JX912252 | Unassigned | Unassigned | Unassigned | 595_0 | Clustered | 35 | VC_595_0 | 35 | 0.8809 | 1 | 0.8809 | 0 | 0 | 0 | 1 | Ref |
| JX976549 | Unassigned | Unassigned | Unassigned | 713_0 | Clustered | 26 | VC_713_0 | 26 | 0.9358 | 1 | 0.9358 | 0 | 0 | 0 | 1 | Ref |
| JX997978 | Unassigned | Unassigned | Unassigned | 434_0 | Clustered | 45 | VC_434_0 | 45 | 0.9479 | 1 | 0.9479 | 0 | 0 | 0 | 1 | Ref |
| KC012913 | Unassigned | Unassigned | Unassigned | 155_8 | Clustered | 66 | VC_155_8 | 66 | 0.4537 | 1 | 0.4537 | 0 | 0 | 0 | 0.8182 | Ref |
| KC013021 | Unassigned | Unassigned | Unassigned | 888_0 | Clustered | 9 | VC_888_0 | 9 | 0.5251 | 1 | 0.5251 | 0 | 0 | 0 | 1 | Ref |
| KC013022 | Unassigned | Unassigned | Unassigned | 888_0 | Clustered | 9 | VC_888_0 | 9 | 0.5251 | 1 | 0.5251 | 0 | 0 | 0 | 1 | Ref |
| KC013023 | Unassigned | Unassigned | Unassigned | 888_0 | Clustered | 9 | VC_888_0 | 9 | 0.5251 | 1 | 0.5251 | 0 | 0 | 0 | 1 | Ref |
| KC013024 | Unassigned | Unassigned | Unassigned | 888_0 | Clustered | 9 | VC_888_0 | 9 | 0.5251 | 1 | 0.5251 | 0 | 0 | 0 | 1 | Ref |
| KC013025 | Unassigned | Unassigned | Unassigned | 888_0 | Clustered | 9 | VC_888_0 | 9 | 0.5251 | 1 | 0.5251 | 0 | 0 | 0 | 1 | Ref |
| KC013026 | Unassigned | Unassigned | Unassigned | 889_0 | Clustered | 13 | VC_889_0 | 13 | 0.6844 | 1 | 0.6844 | 0 | 0 | 0 | 1 | Ref |
| KC013027 | Unassigned | Unassigned | Unassigned | 889_0 | Clustered | 13 | VC_889_0 | 13 | 0.6844 | 1 | 0.6844 | 0 | 0 | 0 | 1 | Ref |
| KC013028 | Unassigned | Unassigned | Unassigned | 889_0 | Clustered | 13 | VC_889_0 | 13 | 0.6844 | 1 | 0.6844 | 0 | 0 | 0 | 1 | Ref |
| KC013029 | Unassigned | Unassigned | Unassigned | 889_0 | Clustered | 13 | VC_889_0 | 13 | 0.6844 | 1 | 0.6844 | 0 | 0 | 0 | 1 | Ref |
| KC107834 | Unassigned | Unassigned | Unassigned | 907_0 | Clustered | 32 | VC_907_0 | 32 | 0.9964 | 1 | 0.9964 | 0 | 0 | 0 | 1 | Ref |
| KC109329 | Unassigned | Unassigned | Unassigned | 465_0 | Clustered | 80 | VC_465_0 | 80 | 0.9988 | 1 | 0.9988 | 0 | 0 | 0 | 1 | Ref |
| KC117376 | Unassigned | Unassigned | Unassigned | 318_0 | Clustered | 6 | VC_318_0 | 6 | 0.1981 | 1 | 0.1981 | 0 | 0 | 0 | 1 | Ref |
| KC117377 | Unassigned | Unassigned | Unassigned | 320_1 | Clustered | 3 | VC_320_1 | 3 | 0.346 | 0.99812624 | 0.3454 | 0 | 0 | 0 | 1 | Ref |
| KC131021 | Unassigned | Unassigned | Unassigned | 207_0 | Clustered | 12 | VC_207_0 | 12 | 0.4465 | 1 | 0.4465 | 0 | 0 | 0 | 1 | Ref |
| KC131022 | Unassigned | Unassigned | Unassigned | 1028_0 | Clustered | 69 | VC_1028_0 | 69 | 0.8547 | 1 | 0.8547 | 0 | 0 | 0 | 1 | Ref |
| KC131023 | Unassigned | Unassigned | Unassigned | 417_0 | Clustered | 20 | VC_417_0 | 20 | 0.6581 | 1 | 0.6581 | 0 | 0 | 0 | 1 | Ref |
| KC131025 | Unassigned | Unassigned | Unassigned | 417_0 | Clustered | 20 | VC_417_0 | 20 | 0.6581 | 1 | 0.6581 | 0 | 0 | 0 | 1 | Ref |
| KC131129 | Unassigned | Unassigned | Unassigned | 549_0 | Clustered | 9 | VC_549_0 | 9 | 0.2597 | 1 | 0.2597 | 0 | 0 | 0 | 1 | Ref |
| KC139511 | Unassigned | Unassigned | Unassigned | 654_0 | Clustered | 115 | VC_654_0 | 115 | 0.7303 | 1 | 0.7303 | 0 | 0 | 0 | 0.8764 | Ref |
| KC139512 | Unassigned | Unassigned | Unassigned | 611_0 | Clustered | 50 | VC_611_0 | 50 | 0.7306 | 1 | 0.7306 | 0 | 0 | 0 | 1 | Ref |
| KC139514 | Unassigned | Unassigned | Unassigned | 611_0 | Clustered | 50 | VC_611_0 | 50 | 0.7306 | 1 | 0.7306 | 0 | 0 | 0 | 1 | Ref |
| KC139515 | Unassigned | Unassigned | Unassigned | 611_0 | Clustered | 50 | VC_611_0 | 50 | 0.7306 | 1 | 0.7306 | 0 | 0 | 0 | 1 | Ref |
| KC139517 | Unassigned | Unassigned | Unassigned | 802_0 | Clustered | 8 | VC_802_0 | 8 | 0.5141 | 1 | 0.5141 | 0 | 0 | 0 | 1 | Ref |
| KC139518 | Unassigned | Unassigned | Unassigned | 560_0 | Clustered | 15 | VC_560_0 | 15 | 0.2846 | 1 | 0.2846 | 0 | 0 | 0 | 1 | Ref |
| KC139519 | Unassigned | Unassigned | Unassigned | 611_0 | Clustered | 50 | VC_611_0 | 50 | 0.7306 | 1 | 0.7306 | 0 | 0 | 0 | 1 | Ref |
| KC139520 | Unassigned | Unassigned | Unassigned | 802_0 | Clustered | 8 | VC_802_0 | 8 | 0.5141 | 1 | 0.5141 | 0 | 0 | 0 | 1 | Ref |
| KC139521 | Unassigned | Unassigned | Unassigned | 235_0 | Clustered | 60 | VC_235_0 | 60 | 0.9413 | 1 | 0.9413 | 0 | 0 | 0 | 1 | Ref |
| KC171646 | Unassigned | Unassigned | Unassigned | 279_0 | Clustered | 11 | VC_279_0 | 11 | 0.4839 | 1 | 0.4839 | 0 | 0 | 0 | 0.8727 | Ref |
| KC171647 | Unassigned | Unassigned | Unassigned | 279_0 | Clustered | 11 | VC_279_0 | 11 | 0.4839 | 1 | 0.4839 | 0 | 0 | 0 | 0.8727 | Ref |
| KC172839 | Unassigned | Unassigned | Unassigned | 531_0 | Clustered | 32 | VC_531_0 | 32 | 0.6859 | 1 | 0.6859 | 0 | 0 | 0 | 1 | Ref |
| KC182542 | Unassigned | Unassigned | Unassigned | 210_0 | Clustered | 105 | VC_210_0 | 105 | 0.4076 | 1 | 0.4076 | 0 | 0 | 0 | 0.7766 | Ref |
| KC182543 | Unassigned | Unassigned | Unassigned | 210_0 | Clustered | 105 | VC_210_0 | 105 | 0.4076 | 1 | 0.4076 | 0 | 0 | 0 | 0.7766 | Ref |
| KC182544 | Unassigned | Unassigned | Unassigned | 210_0 | Clustered | 105 | VC_210_0 | 105 | 0.4076 | 1 | 0.4076 | 0 | 0 | 0 | 0.7766 | Ref |
| KC182545 | Unassigned | Unassigned | Unassigned | 210_0 | Clustered | 105 | VC_210_0 | 105 | 0.4076 | 1 | 0.4076 | 0 | 0 | 0 | 0.7766 | Ref |
| KC182546 | Unassigned | Unassigned | Unassigned | 210_0 | Clustered | 105 | VC_210_0 | 105 | 0.4076 | 1 | 0.4076 | 0 | 0 | 0 | 0.7766 | Ref |
| KC182547 | Unassigned | Unassigned | Unassigned | 210_0 | Clustered | 105 | VC_210_0 | 105 | 0.4076 | 1 | 0.4076 | 0 | 0 | 0 | 0.7766 | Ref |
| KC182548 | Unassigned | Unassigned | Unassigned | 210_0 | Clustered | 105 | VC_210_0 | 105 | 0.4076 | 1 | 0.4076 | 0 | 0 | 0 | 0.7766 | Ref |
| KC182549 | Unassigned | Unassigned | Unassigned | 210_0 | Clustered | 105 | VC_210_0 | 105 | 0.4076 | 1 | 0.4076 | 0 | 0 | 0 | 0.7766 | Ref |
| KC182550 | Unassigned | Unassigned | Unassigned | 210_0 | Clustered | 105 | VC_210_0 | 105 | 0.4076 | 1 | 0.4076 | 0 | 0 | 0 | 0.7766 | Ref |
| KC182551 | Unassigned | Unassigned | Unassigned | 210_0 | Clustered | 105 | VC_210_0 | 105 | 0.4076 | 1 | 0.4076 | 0 | 0 | 0 | 0.7766 | Ref |
| KC182552 | Unassigned | Unassigned | Unassigned | 210_0 | Clustered | 105 | VC_210_0 | 105 | 0.4076 | 1 | 0.4076 | 0 | 0 | 0 | 0.7766 | Ref |
| KC206276 | Unassigned | Unassigned | Unassigned | 801_0 | Clustered | 37 | VC_801_0 | 37 | 0.5672 | 1 | 0.5672 | 0 | 0 | 0 | 1 | Ref |
| KC237308 | Unassigned | Unassigned | Unassigned | 375_0 | Clustered | 2 | VC_375_0 | 2 | 0.002 | 0.83307735 | 0.0017 | 0 | 0 | 0 | 1 | Ref |
| KC252997 | Unassigned | Unassigned | Unassigned | 684_0 | Clustered | 4 | VC_684_0 | 4 | 1 | 1 | 1 | 0 | 0 | 0 | 1 | Ref |
| KC262634 | Unassigned | Unassigned | Unassigned | 645_0 | Clustered | 20 | VC_645_0 | 20 | 0.8502 | 1 | 0.8502 | 0 | 0 | 0 | 1 | Ref |
| KC292020 | Unassigned | Unassigned | Unassigned | 317_0 | Clustered | 33 | VC_317_0 | 33 | 0.8756 | 1 | 0.8756 | 0 | 0 | 0 | 1 | Ref |
| KC292021 | Unassigned | Unassigned | Unassigned | 318_0 | Clustered | 6 | VC_318_0 | 6 | 0.1981 | 1 | 0.1981 | 0 | 0 | 0 | 1 | Ref |
| KC292022 | Unassigned | Unassigned | Unassigned | 317_0 | Clustered | 33 | VC_317_0 | 33 | 0.8756 | 1 | 0.8756 | 0 | 0 | 0 | 1 | Ref |
| KC292023 | Unassigned | Unassigned | Unassigned | 473_0 | Clustered | 3 | VC_473_0 | 3 | 0.8515 | 0.99054823 | 0.8434 | 0 | 0 | 0 | 1 | Ref |
| KC292024 | Unassigned | Unassigned | Unassigned | 1024_0 | Clustered | 2 | VC_1024_0 | 2 | 0.3738 | 0.91418477 | 0.3417 | 0 | 0 | 0 | 1 | Ref |

| | | | | | | | | | | | | | | | | |
|---|---|---|---|---|---|---|---|---|---|---|---|---|---|---|---|---|
| KC292026 | Unassigned | Unassigned | Unassigned | 319_0 | Clustered | 2 | VC_319_0 | 2 | 0.0304 | 0.95436427 | 0.029 | 0 | 0 | 0 | 1 | Ref |
| KC292027 | Unassigned | Unassigned | Unassigned | 320_1 | Clustered | 3 | VC_320_1 | 3 | 0.346 | 0.99812624 | 0.3454 | 0 | 0 | 0 | 1 | Ref |
| KC292028 | Unassigned | Unassigned | Unassigned | 1024_0 | Clustered | 2 | VC_1024_0 | 2 | 0.3738 | 0.91418477 | 0.3417 | 0 | 0 | 0 | 1 | Ref |
| KC292029 | Unassigned | Unassigned | Unassigned | 320_0 | Clustered | 2 | VC_320_0 | 2 | 0.1705 | 0.90744399 | 0.1547 | 0 | 0 | 0 | 1 | Ref |
| KC294142 | Unassigned | Unassigned | Unassigned | 486_0 | Clustered | 47 | VC_486_0 | 47 | 0.9022 | 1 | 0.9022 | 0 | 0 | 0 | 1 | Ref |
| KC295538 | Unassigned | Unassigned | Unassigned | 170_11 | Clustered | 10 | VC_170_11 | 10 | 0.1502 | 0.99999797 | 0.1502 | 0 | 0 | 0 | 0.9111 | Ref |
| KC310802 | Unassigned | Unassigned | Unassigned | 352_0 | Clustered | 31 | VC_352_0 | 31 | 0.9547 | 1 | 0.9547 | 0 | 0 | 0 | 1 | Ref |
| KC310803 | Unassigned | Unassigned | Unassigned | 352_0 | Clustered | 31 | VC_352_0 | 31 | 0.9547 | 1 | 0.9547 | 0 | 0 | 0 | 1 | Ref |
| KC310804 | Unassigned | Unassigned | Unassigned | 352_0 | Clustered | 31 | VC_352_0 | 31 | 0.9547 | 1 | 0.9547 | 0 | 0 | 0 | 1 | Ref |
| KC310805 | Unassigned | Unassigned | Unassigned | 352_0 | Clustered | 31 | VC_352_0 | 31 | 0.9547 | 1 | 0.9547 | 0 | 0 | 0 | 1 | Ref |
| KC310806 | Unassigned | Unassigned | Unassigned | 352_0 | Clustered | 31 | VC_352_0 | 31 | 0.9547 | 1 | 0.9547 | 0 | 0 | 0 | 1 | Ref |
| KC311669 | Unassigned | Unassigned | Unassigned | 714_0 | Clustered | 71 | VC_714_0 | 71 | 0.935 | 1 | 0.935 | 0 | 0 | 0 | 1 | Ref |
| KC330679 | Unassigned | Unassigned | Unassigned | 102_0 | Clustered | 15 | VC_102_0 | 15 | 0.653 | 1 | 0.653 | 0 | 0 | 0 | 1 | Ref |
| KC330680 | Unassigned | Unassigned | Unassigned | 102_0 | Clustered | 15 | VC_102_0 | 15 | 0.653 | 1 | 0.653 | 0 | 0 | 0 | 1 | Ref |
| KC330681 | Unassigned | Unassigned | Unassigned | 102_0 | Clustered | 15 | VC_102_0 | 15 | 0.653 | 1 | 0.653 | 0 | 0 | 0 | 1 | Ref |
| KC330682 | Unassigned | Unassigned | Unassigned | 102_0 | Clustered | 15 | VC_102_0 | 15 | 0.653 | 1 | 0.653 | 0 | 0 | 0 | 1 | Ref |
| KC330683 | Unassigned | Unassigned | Unassigned | 102_0 | Clustered | 15 | VC_102_0 | 15 | 0.653 | 1 | 0.653 | 0 | 0 | 0 | 1 | Ref |
| KC330684 | Unassigned | Unassigned | Unassigned | 102_0 | Clustered | 15 | VC_102_0 | 15 | 0.653 | 1 | 0.653 | 0 | 0 | 0 | 1 | Ref |
| KC333879 | Unassigned | Unassigned | Unassigned | 469_0 | Clustered | 18 | VC_469_0 | 18 | 0.4583 | 1 | 0.4583 | 0 | 0 | 0 | 1 | Ref |
| KC342645 | Unassigned | Unassigned | Unassigned | 7_0 | Clustered | 28 | VC_7_0 | 28 | 0.389 | 1 | 0.389 | 0 | 0 | 0 | 0.9947 | Ref |
| KC348600 | Unassigned | Unassigned | Unassigned | 268_1 | Clustered | 20 | VC_268_1 | 20 | 0.1906 | 1 | 0.1906 | 0 | 0 | 0 | 0.6789 | Ref |
| KC348601 | Unassigned | Unassigned | Unassigned | 268_1 | Clustered | 20 | VC_268_1 | 20 | 0.1906 | 1 | 0.1906 | 0 | 0 | 0 | 0.6789 | Ref |
| KC348602 | Unassigned | Unassigned | Unassigned | 268_1 | Clustered | 20 | VC_268_1 | 20 | 0.1906 | 1 | 0.1906 | 0 | 0 | 0 | 0.6789 | Ref |
| KC348603 | Unassigned | Unassigned | Unassigned | 647_0 | Clustered | 37 | VC_647_0 | 37 | 0.5847 | 1 | 0.5847 | 0 | 0 | 0 | 0.9234 | Ref |
| KC357596 | Unassigned | Unassigned | Unassigned | 0_0 | Clustered | 3 | VC_0_0 | 3 | 1 | 1 | 1 | 0 | 0 | 0 | 1 | Ref |
| KC413988 | Unassigned | Unassigned | Unassigned | 425_0 | Clustered | 40 | VC_425_0 | 40 | 0.8399 | 1 | 0.8399 | 0 | 0 | 0 | 0.9974 | Ref |
| KC430106 | Unassigned | Unassigned | Unassigned | 913_1 | Clustered | 8 | VC_913_1 | 8 | 0.1102 | 0.9989323 | 0.11 | 0 | 0 | 0 | 0.75 | Ref |
| KC438282 | Unassigned | Unassigned | Unassigned | 806_0 | Clustered | 6 | VC_806_0 | 6 | 0.5468 | 1 | 0.5468 | 0 | 0 | 0 | 1 | Ref |
| KC438283 | Unassigned | Unassigned | Unassigned | 806_0 | Clustered | 6 | VC_806_0 | 6 | 0.5468 | 1 | 0.5468 | 0 | 0 | 0 | 1 | Ref |
| KC465898 | Unassigned | Unassigned | Unassigned | 502_0 | Clustered | 18 | VC_502_0 | 18 | 0.8808 | 1 | 0.8808 | 0 | 0 | 0 | 1 | Ref |
| KC465901 | Unassigned | Unassigned | Unassigned | 496_0 | Clustered | 22 | VC_496_0 | 22 | 0.6101 | 1 | 0.6101 | 0 | 0 | 0 | 1 | Ref |
| KC481682 | Unassigned | Unassigned | Unassigned | 489_2 | Clustered | 3 | VC_489_2 | 3 | 0.0584 | 0.98697745 | 0.0577 | 0 | 0 | 0 | 1 | Ref |
| KC522412 | Unassigned | Unassigned | Unassigned | 210_0 | Clustered | 105 | VC_210_0 | 105 | 0.4076 | 1 | 0.4076 | 0 | 0 | 0 | 0.7766 | Ref |
| KC542353 | Unassigned | Unassigned | Unassigned | 663_0 | Clustered | 5 | VC_663_0 | 5 | 0.6645 | 0.99999974 | 0.6645 | 0 | 0 | 0 | 1 | Ref |
| KC556893 | Unassigned | Unassigned | Unassigned | 908_0 | Clustered | 36 | VC_908_0 | 36 | 0.9943 | 1 | 0.9943 | 0 | 0 | 0 | 1 | Ref |
| KC556894 | Unassigned | Unassigned | Unassigned | 908_0 | Clustered | 36 | VC_908_0 | 36 | 0.9943 | 1 | 0.9943 | 0 | 0 | 0 | 1 | Ref |
| KC556895 | Unassigned | Unassigned | Unassigned | 908_0 | Clustered | 36 | VC_908_0 | 36 | 0.9943 | 1 | 0.9943 | 0 | 0 | 0 | 1 | Ref |
| KC556896 | Unassigned | Unassigned | Unassigned | 908_0 | Clustered | 36 | VC_908_0 | 36 | 0.9943 | 1 | 0.9943 | 0 | 0 | 0 | 1 | Ref |
| KC556897 | Unassigned | Unassigned | Unassigned | 908_0 | Clustered | 36 | VC_908_0 | 36 | 0.9943 | 1 | 0.9943 | 0 | 0 | 0 | 1 | Ref |
| KC556898 | Unassigned | Unassigned | Unassigned | 908_0 | Clustered | 36 | VC_908_0 | 36 | 0.9943 | 1 | 0.9943 | 0 | 0 | 0 | 1 | Ref |
| KC576783 | Unassigned | Unassigned | Unassigned | 539_0 | Clustered | 37 | VC_539_0 | 37 | 0.911 | 1 | 0.911 | 0 | 0 | 0 | 1 | Ref |
| KC576784 | Unassigned | Unassigned | Unassigned | 384_2 | Clustered | 8 | VC_384_2 | 8 | 0.0232 | 1 | 0.0232 | 0 | 0 | 0 | 0.8571 | Ref |
| KC579452 | Unassigned | Unassigned | Unassigned | 469_0 | Clustered | 18 | VC_469_0 | 18 | 0.4583 | 1 | 0.4583 | 0 | 0 | 0 | 1 | Ref |
| KC581799 | Unassigned | Unassigned | Unassigned | 647_0 | Clustered | 37 | VC_647_0 | 37 | 0.5847 | 1 | 0.5847 | 0 | 0 | 0 | 0.9234 | Ref |
| KC595278 | Unassigned | Unassigned | Unassigned | 10_0 | Clustered | 25 | VC_10_0 | 25 | 0.3437 | 1 | 0.3437 | 0 | 0 | 0 | 0.9733 | Ref |
| KC595279 | Unassigned | Unassigned | Unassigned | 12_2 | Clustered | 19 | VC_12_2 | 19 | 0.2009 | 1 | 0.2009 | 0 | 0 | 0 | 1 | Ref |
| KC595511 | Unassigned | Unassigned | Unassigned | 1042_0 | Clustered | 8 | VC_1042_0 | 8 | 0.7385 | 1 | 0.7385 | 0 | 0 | 0 | 1 | Ref |
| KC595512 | Unassigned | Unassigned | Unassigned | 89_1 | Clustered | 3 | VC_89_1 | 3 | 0.1616 | 0.99777924 | 0.1612 | 0 | 0 | 0 | 1 | Ref |
| KC595513 | Unassigned | Unassigned | Unassigned | 89_1 | Clustered | 3 | VC_89_1 | 3 | 0.1616 | 0.99777924 | 0.1612 | 0 | 0 | 0 | 1 | Ref |
| KC595514 | Unassigned | Unassigned | Unassigned | 142_0 | Clustered | 6 | VC_142_0 | 6 | 0.7366 | 1 | 0.7366 | 0 | 0 | 0 | 1 | Ref |
| KC595515 | Unassigned | Unassigned | Unassigned | 142_0 | Clustered | 6 | VC_142_0 | 6 | 0.7366 | 1 | 0.7366 | 0 | 0 | 0 | 1 | Ref |
| KC595517 | Unassigned | Unassigned | Unassigned | 142_0 | Clustered | 6 | VC_142_0 | 6 | 0.7366 | 1 | 0.7366 | 0 | 0 | 0 | 1 | Ref |
| KC595518 | Unassigned | Unassigned | Unassigned | 142_0 | Clustered | 6 | VC_142_0 | 6 | 0.7366 | 1 | 0.7366 | 0 | 0 | 0 | 1 | Ref |
| KC618326 | Unassigned | Unassigned | Unassigned | 235_0 | Clustered | 60 | VC_235_0 | 60 | 0.9413 | 1 | 0.9413 | 0 | 0 | 0 | 1 | Ref |
| KC618393 | Unassigned | Unassigned | Unassigned | 618_0 | Clustered | 50 | VC_618_0 | 50 | 0.9989 | 1 | 0.9989 | 0 | 0 | 0 | 1 | Ref |
| KC661271 | Unassigned | Unassigned | Unassigned | 535_1 | Clustered | 108 | VC_535_1 | 108 | 0.8479 | 1 | 0.8479 | 0 | 0 | 0 | 0.9955 | Ref |
| KC661272 | Unassigned | Unassigned | Unassigned | 532_0 | Clustered | 75 | VC_532_0 | 75 | 0.9393 | 1 | 0.9393 | 0 | 0 | 0 | 1 | Ref |
| KC661273 | Unassigned | Unassigned | Unassigned | 335_23 | Clustered | 17 | VC_335_23 | 17 | 0.0714 | 1 | 0.0714 | 0 | 0 | 0 | 0.6397 | Ref |
| KC661274 | Unassigned | Unassigned | Unassigned | 384_3 | Clustered | 22 | VC_384_3 | 22 | 0.0786 | 1 | 0.0786 | 0 | 0 | 0 | 0.9048 | Ref |
| KC661275 | Unassigned | Unassigned | Unassigned | 226_0 | Clustered | 5 | VC_226_0 | 5 | 0.0793 | 0.99999998 | 0.0793 | 0 | 0 | 0 | 1 | Ref |
| KC661276 | Unassigned | Unassigned | Unassigned | 917_0 | Clustered | 28 | VC_917_0 | 28 | 0.5465 | 1 | 0.5465 | 0 | 0 | 0 | 1 | Ref |
| KC661277 | Unassigned | Unassigned | Unassigned | 571_7 | Clustered | 28 | VC_571_7 | 28 | 0.1651 | 1 | 0.1651 | 0 | 0 | 0 | 0.8519 | Ref |
| KC661278 | Unassigned | Unassigned | Unassigned | 538_79 | Clustered/Singleton | 1 | VC_538_79 | 1 | 0 | 1 | 0 | 0 | 0 | 0 | 1 | Ref |
| KC661279 | Unassigned | Unassigned | Unassigned | 533_0 | Clustered | 16 | VC_533_0 | 16 | 0.3765 | 1 | 0.3765 | 0 | 0 | 0 | 1 | Ref |
| KC661280 | Unassigned | Unassigned | Unassigned | 538_70 | Clustered/Singleton | 1 | VC_538_70 | 1 | 0 | 1 | 0 | 0 | 0 | 0 | 1 | Ref |
| KC661281 | Unassigned | Unassigned | Unassigned | 532_0 | Clustered | 75 | VC_532_0 | 75 | 0.9393 | 1 | 0.9393 | 0 | 0 | 0 | 1 | Ref |
| KC677662 | Unassigned | Unassigned | Unassigned | 611_0 | Clustered | 50 | VC_611_0 | 50 | 0.7306 | 1 | 0.7306 | 0 | 0 | 0 | 1 | Ref |
| KC677663 | Unassigned | Unassigned | Unassigned | 9_0 | Clustered | 38 | VC_9_0 | 38 | 0.4859 | 1 | 0.4859 | 0 | 0 | 0 | 1 | Ref |
| KC685370 | Unassigned | Unassigned | Unassigned | 843_0 | Clustered | 20 | VC_843_0 | 20 | 0.9621 | 1 | 0.9621 | 0 | 0 | 0 | 1 | Ref |
| KC690136 | Unassigned | Unassigned | Unassigned | 794_15 | Clustered | 5 | VC_794_15 | 5 | 0.0243 | 0.76144861 | 0.0185 | 0 | 0 | 0 | 0.9 | Ref |
| KC691254 | Unassigned | Unassigned | Unassigned | 917_0 | Clustered | 28 | VC_917_0 | 28 | 0.5465 | 1 | 0.5465 | 0 | 0 | 0 | 1 | Ref |
| KC691255 | Unassigned | Unassigned | Unassigned | 571_7 | Clustered | 28 | VC_571_7 | 28 | 0.1651 | 1 | 0.1651 | 0 | 0 | 0 | 0.8519 | Ref |
| KC691256 | Unassigned | Unassigned | Unassigned | 537_0 | Clustered | 39 | VC_537_0 | 39 | 0.8148 | 1 | 0.8148 | 0 | 0 | 0 | 1 | Ref |
| KC691257 | Unassigned | Unassigned | Unassigned | 579_5 | Clustered/Singleton | 1 | VC_579_5 | 1 | 0 | 1 | 0 | 0 | 0 | 0 | 1 | Ref |
| KC691258 | Unassigned | Unassigned | Unassigned | 384_3 | Clustered | 22 | VC_384_3 | 22 | 0.0786 | 1 | 0.0786 | 0 | 0 | 0 | 0.9048 | Ref |
| KC699836 | Unassigned | Unassigned | Unassigned | 87_2 | Clustered | 3 | VC_87_2 | 3 | 0.033 | 0.99732979 | 0.0329 | 0 | 0 | 0 | 1 | Ref |
| KC700556 | Unassigned | Unassigned | Unassigned | 1016_0 | Clustered | 62 | VC_1016_0 | 62 | 0.8638 | 1 | 0.8638 | 0 | 0 | 0 | 0.743 | Ref |
| KC700557 | Unassigned | Unassigned | Unassigned | 1016_0 | Clustered | 62 | VC_1016_0 | 62 | 0.8638 | 1 | 0.8638 | 0 | 0 | 0 | 0.743 | Ref |
| KC700558 | Unassigned | Unassigned | Unassigned | 1016_0 | Clustered | 62 | VC_1016_0 | 62 | 0.8638 | 1 | 0.8638 | 0 | 0 | 0 | 0.743 | Ref |
| KC701493 | Unassigned | Unassigned | Unassigned | 335_17 | Clustered/Singleton | 1 | VC_335_17 | 1 | 0 | 1 | 0 | 0 | 0 | 0 | 1 | Ref |
| KC710998 | Unassigned | Unassigned | Unassigned | 975_0 | Clustered | 40 | VC_975_0 | 40 | 0.6888 | 1 | 0.6888 | 0 | 0 | 0 | 1 | Ref |

| | | | | | | | | | | | | | | | | |
|---|---|---|---|---|---|---|---|---|---|---|---|---|---|---|---|---|
| KC736071 | Unassigned | Unassigned | Unassigned | 538_52 | Clustered/Singleton | 1 | VC_538_52 | 1 | 0 | 1 | 0 | 0 | 0 | 0 | 1 | Ref |
| KC736978 | Unassigned | Unassigned | Unassigned | 47_0 | Clustered | 12 | VC_47_0 | 12 | 0.3571 | 1 | 0.3571 | 0 | 0 | 0 | 1 | Ref |
| KC748968 | Unassigned | Unassigned | Unassigned | 579_49 | Clustered | 7 | VC_579_49 | 7 | 0.0194 | 0.98914431 | 0.0191 | 0 | 0 | 0 | 0.8571 | Ref |
| KC748969 | Unassigned | Unassigned | Unassigned | 571_8 | Clustered | 19 | VC_571_8 | 19 | 0.1031 | 1 | 0.1031 | 0 | 0 | 0 | 0.8713 | Ref |
| KC748970 | Unassigned | Unassigned | Unassigned | 579_12 | Clustered | 3 | VC_579_12 | 3 | 0.0064 | 0.86841267 | 0.0056 | 0 | 0 | 0 | 1 | Ref |
| KC748971 | Unassigned | Unassigned | Unassigned | 571_7 | Clustered | 28 | VC_571_7 | 28 | 0.1651 | 1 | 0.1651 | 0 | 0 | 0 | 0.8519 | Ref |
| KC751414 | Unassigned | Unassigned | Unassigned | 773_0 | Clustered | 2 | VC_773_0 | 2 | 0.1621 | 0.95427014 | 0.1547 | 0 | 0 | 0 | 1 | Ref |
| KC758116 | Unassigned | Unassigned | Unassigned | 172_0 | Clustered | 26 | VC_172_0 | 26 | 0.7909 | 1 | 0.7909 | 0 | 0 | 0 | 1 | Ref |
| KC787102 | Unassigned | Unassigned | Unassigned | 222_1 | Clustered | 19 | VC_222_1 | 19 | 0.4166 | 1 | 0.4166 | 0 | 0 | 0 | 1 | Ref |
| KC787103 | Unassigned | Unassigned | Unassigned | 783_0 | Clustered | 67 | VC_783_0 | 67 | 0.8945 | 1 | 0.8945 | 0 | 0 | 0 | 1 | Ref |
| KC787104 | Unassigned | Unassigned | Unassigned | 783_0 | Clustered | 67 | VC_783_0 | 67 | 0.8945 | 1 | 0.8945 | 0 | 0 | 0 | 1 | Ref |
| KC787105 | Unassigned | Unassigned | Unassigned | 222_1 | Clustered | 19 | VC_222_1 | 19 | 0.4166 | 1 | 0.4166 | 0 | 0 | 0 | 1 | Ref |
| KC787106 | Unassigned | Unassigned | Unassigned | 222_1 | Clustered | 19 | VC_222_1 | 19 | 0.4166 | 1 | 0.4166 | 0 | 0 | 0 | 1 | Ref |
| KC787107 | Unassigned | Unassigned | Unassigned | 783_0 | Clustered | 67 | VC_783_0 | 67 | 0.8945 | 1 | 0.8945 | 0 | 0 | 0 | 1 | Ref |
| KC787108 | Unassigned | Unassigned | Unassigned | 783_0 | Clustered | 67 | VC_783_0 | 67 | 0.8945 | 1 | 0.8945 | 0 | 0 | 0 | 1 | Ref |
| KC787109 | Unassigned | Unassigned | Unassigned | 783_0 | Clustered | 67 | VC_783_0 | 67 | 0.8945 | 1 | 0.8945 | 0 | 0 | 0 | 1 | Ref |
| KC787110 | Unassigned | Unassigned | Unassigned | 783_0 | Clustered | 67 | VC_783_0 | 67 | 0.8945 | 1 | 0.8945 | 0 | 0 | 0 | 1 | Ref |
| KC787111 | Unassigned | Unassigned | Unassigned | 783_0 | Clustered | 67 | VC_783_0 | 67 | 0.8945 | 1 | 0.8945 | 0 | 0 | 0 | 1 | Ref |
| KC787112 | Unassigned | Unassigned | Unassigned | 783_0 | Clustered | 67 | VC_783_0 | 67 | 0.8945 | 1 | 0.8945 | 0 | 0 | 0 | 1 | Ref |
| KC801932 | Unassigned | Unassigned | Unassigned | 687_1 | Clustered | 10 | VC_687_1 | 10 | 0.1263 | 1 | 0.1263 | 0 | 0 | 0 | 0.9778 | Ref |
| KC814930 | Unassigned | Unassigned | Unassigned | 47_0 | Clustered | 12 | VC_47_0 | 12 | 0.3571 | 1 | 0.3571 | 0 | 0 | 0 | 1 | Ref |
| KC821604 | Unassigned | Unassigned | Unassigned | 962_0 | Clustered | 4 | VC_962_0 | 4 | 0.7703 | 0.999902 | 0.7702 | 0 | 0 | 0 | 1 | Ref |
| KC821606 | Unassigned | Unassigned | Unassigned | 1047_0 | Clustered | 9 | VC_1047_0 | 9 | 1 | 1 | 1 | 0 | 0 | 0 | 1 | Ref |
| KC821607 | Unassigned | Unassigned | Unassigned | 459_1 | Clustered | 2 | VC_459_1 | 2 | 0.1281 | 0.93226714 | 0.1194 | 0 | 0 | 0 | 1 | Ref |
| KC821608 | Unassigned | Unassigned | Unassigned | 1048_0 | Clustered | 4 | VC_1048_0 | 4 | 0.8643 | 0.99995392 | 0.8643 | 0 | 0 | 0 | 1 | Ref |
| KC821609 | Unassigned | Unassigned | Unassigned | 1053_0 | Clustered | 5 | VC_1053_0 | 5 | 0.9327 | 0.9999997 | 0.9327 | 0 | 0 | 0 | 1 | Ref |
| KC821610 | Unassigned | Unassigned | Unassigned | 943_0 | Clustered | 6 | VC_943_0 | 6 | 0.277 | 1 | 0.277 | 0 | 0 | 0 | 1 | Ref |
| KC821612 | Unassigned | Unassigned | Unassigned | 1057_0 | Clustered | 5 | VC_1057_0 | 5 | 0.8612 | 0.99999999 | 0.8612 | 0 | 0 | 0 | 1 | Ref |
| KC821613 | Unassigned | Unassigned | Unassigned | 1058_0 | Clustered | 5 | VC_1058_0 | 5 | 0.8248 | 0.9999997 | 0.8248 | 0 | 0 | 0 | 1 | Ref |
| KC821614 | Unassigned | Unassigned | Unassigned | 1057_0 | Clustered | 5 | VC_1057_0 | 5 | 0.8612 | 0.99999999 | 0.8612 | 0 | 0 | 0 | 1 | Ref |
| KC821615 | Unassigned | Unassigned | Unassigned | 1058_0 | Clustered | 5 | VC_1058_0 | 5 | 0.8248 | 0.9999997 | 0.8248 | 0 | 0 | 0 | 1 | Ref |
| KC821616 | Unassigned | Unassigned | Unassigned | 943_0 | Clustered | 6 | VC_943_0 | 6 | 0.277 | 1 | 0.277 | 0 | 0 | 0 | 1 | Ref |
| KC821617 | Unassigned | Unassigned | Unassigned | 1058_0 | Clustered | 5 | VC_1058_0 | 5 | 0.8248 | 0.9999997 | 0.8248 | 0 | 0 | 0 | 1 | Ref |
| KC821618 | Unassigned | Unassigned | Unassigned | 459_1 | Clustered | 2 | VC_459_1 | 2 | 0.1281 | 0.93226714 | 0.1194 | 0 | 0 | 0 | 1 | Ref |
| KC821619 | Unassigned | Unassigned | Unassigned | 1058_0 | Clustered | 5 | VC_1058_0 | 5 | 0.8248 | 0.9999997 | 0.8248 | 0 | 0 | 0 | 1 | Ref |
| KC821620 | Unassigned | Unassigned | Unassigned | 1048_0 | Clustered | 4 | VC_1048_0 | 4 | 0.8643 | 0.99995392 | 0.8643 | 0 | 0 | 0 | 1 | Ref |
| KC821621 | Unassigned | Unassigned | Unassigned | 962_0 | Clustered | 4 | VC_962_0 | 4 | 0.7703 | 0.999902 | 0.7702 | 0 | 0 | 0 | 1 | Ref |
| KC821622 | Unassigned | Unassigned | Unassigned | 1048_0 | Clustered | 4 | VC_1048_0 | 4 | 0.8643 | 0.99995392 | 0.8643 | 0 | 0 | 0 | 1 | Ref |
| KC821623 | Unassigned | Unassigned | Unassigned | 1047_0 | Clustered | 9 | VC_1047_0 | 9 | 1 | 1 | 1 | 0 | 0 | 0 | 1 | Ref |
| KC821625 | Unassigned | Unassigned | Unassigned | 962_0 | Clustered | 4 | VC_962_0 | 4 | 0.7703 | 0.999902 | 0.7702 | 0 | 0 | 0 | 1 | Ref |
| KC821627 | Unassigned | Unassigned | Unassigned | 1058_0 | Clustered | 5 | VC_1058_0 | 5 | 0.8248 | 0.9999997 | 0.8248 | 0 | 0 | 0 | 1 | Ref |
| KC821628 | Unassigned | Unassigned | Unassigned | 1047_0 | Clustered | 9 | VC_1047_0 | 9 | 1 | 1 | 1 | 0 | 0 | 0 | 1 | Ref |
| KC821629 | Unassigned | Unassigned | Unassigned | 943_0 | Clustered | 6 | VC_943_0 | 6 | 0.277 | 1 | 0.277 | 0 | 0 | 0 | 1 | Ref |
| KC821630 | Unassigned | Unassigned | Unassigned | 943_0 | Clustered | 6 | VC_943_0 | 6 | 0.277 | 1 | 0.277 | 0 | 0 | 0 | 1 | Ref |
| KC821631 | Unassigned | Unassigned | Unassigned | 1047_0 | Clustered | 9 | VC_1047_0 | 9 | 1 | 1 | 1 | 0 | 0 | 0 | 1 | Ref |
| KC821632 | Unassigned | Unassigned | Unassigned | 1053_0 | Clustered | 5 | VC_1053_0 | 5 | 0.9327 | 0.9999997 | 0.9327 | 0 | 0 | 0 | 1 | Ref |
| KC821633 | Unassigned | Unassigned | Unassigned | 1048_0 | Clustered | 4 | VC_1048_0 | 4 | 0.8643 | 0.99995392 | 0.8643 | 0 | 0 | 0 | 1 | Ref |
| KC821634 | Unassigned | Unassigned | Unassigned | 943_0 | Clustered | 6 | VC_943_0 | 6 | 0.277 | 1 | 0.277 | 0 | 0 | 0 | 1 | Ref |
| KC853746 | Unassigned | Unassigned | Unassigned | 71_0 | Clustered | 27 | VC_71_0 | 27 | 0.5964 | 1 | 0.5964 | 0 | 0 | 0 | 0.9943 | Ref |
| KC862295 | Unassigned | Unassigned | Unassigned | 78_0 | Clustered | 20 | VC_78_0 | 20 | 0.9639 | 1 | 0.9639 | 0 | 0 | 0 | 1 | Ref |
| KC862296 | Unassigned | Unassigned | Unassigned | 78_0 | Clustered | 20 | VC_78_0 | 20 | 0.9639 | 1 | 0.9639 | 0 | 0 | 0 | 1 | Ref |
| KC862297 | Unassigned | Unassigned | Unassigned | 77_0 | Clustered | 37 | VC_77_0 | 37 | 0.9392 | 1 | 0.9392 | 0 | 0 | 0 | 1 | Ref |
| KC862298 | Unassigned | Unassigned | Unassigned | 77_0 | Clustered | 37 | VC_77_0 | 37 | 0.9392 | 1 | 0.9392 | 0 | 0 | 0 | 1 | Ref |
| KC862299 | Unassigned | Unassigned | Unassigned | 78_0 | Clustered | 20 | VC_78_0 | 20 | 0.9639 | 1 | 0.9639 | 0 | 0 | 0 | 1 | Ref |
| KC862300 | Unassigned | Unassigned | Unassigned | 77_0 | Clustered | 37 | VC_77_0 | 37 | 0.9392 | 1 | 0.9392 | 0 | 0 | 0 | 1 | Ref |
| KC862301 | Unassigned | Unassigned | Unassigned | 78_0 | Clustered | 20 | VC_78_0 | 20 | 0.9639 | 1 | 0.9639 | 0 | 0 | 0 | 1 | Ref |
| KC900378 | Unassigned | Unassigned | Unassigned | 645_0 | Clustered | 20 | VC_645_0 | 20 | 0.8502 | 1 | 0.8502 | 0 | 0 | 0 | 1 | Ref |
| KC900379 | Unassigned | Unassigned | Unassigned | 785_1 | Clustered | 9 | VC_785_1 | 9 | 0.3679 | 1 | 0.3679 | 0 | 0 | 0 | 1 | Ref |
| KC911856 | Unassigned | Unassigned | Unassigned | 492_0 | Clustered | 20 | VC_492_0 | 20 | 0.7887 | 1 | 0.7887 | 0 | 0 | 0 | 1 | Ref |
| KC911857 | Unassigned | Unassigned | Unassigned | 492_0 | Clustered | 20 | VC_492_0 | 20 | 0.7887 | 1 | 0.7887 | 0 | 0 | 0 | 1 | Ref |
| KC954774 | Unassigned | Unassigned | Unassigned | 873_5 | Clustered | 3 | VC_873_5 | 3 | 0.0491 | 0.9761082 | 0.0479 | 0 | 0 | 0 | 1 | Ref |
| KC954775 | Unassigned | Unassigned | Unassigned | 599_0 | Clustered | 2 | VC_599_0 | 2 | 0.061 | 0.95763305 | 0.0584 | 0 | 0 | 0 | 1 | Ref |
| KC959568 | Unassigned | Unassigned | Unassigned | 1061_0 | Clustered | 8 | VC_1061_0 | 8 | 0.8379 | 1 | 0.8379 | 0 | 0 | 0 | 1 | Ref |
| KC960489 | Unassigned | Unassigned | Unassigned | 1064_0 | Clustered | 2 | VC_1064_0 | 2 | 1 | 1 | 1 | 0 | 0 | 0 | 1 | Ref |
| KC960671 | Unassigned | Unassigned | Unassigned | 398_0 | Clustered | 51 | VC_398_0 | 51 | 0.8741 | 1 | 0.8741 | 0 | 0 | 0 | 1 | Ref |
| KC969441 | Unassigned | Unassigned | Unassigned | 434_0 | Clustered | 45 | VC_434_0 | 45 | 0.9479 | 1 | 0.9479 | 0 | 0 | 0 | 1 | Ref |
| KF005317 | Unassigned | Unassigned | Unassigned | 1065_0 | Clustered | 4 | VC_1065_0 | 4 | 1 | 0 | 0 | 0 | 0 | 0 | 1 | Ref |
| KF005318 | Unassigned | Unassigned | Unassigned | 1065_0 | Clustered | 4 | VC_1065_0 | 4 | 1 | 0 | 0 | 0 | 0 | 0 | 1 | Ref |
| KF005319 | Unassigned | Unassigned | Unassigned | 1065_0 | Clustered | 4 | VC_1065_0 | 4 | 1 | 0 | 0 | 0 | 0 | 0 | 1 | Ref |
| KF005320 | Unassigned | Unassigned | Unassigned | 1065_0 | Clustered | 4 | VC_1065_0 | 4 | 1 | 0 | 0 | 0 | 0 | 0 | 1 | Ref |
| KF006817 | Unassigned | Unassigned | Unassigned | 579_9 | Clustered | 3 | VC_579_9 | 3 | 0.0064 | 0.81181703 | 0.0052 | 0 | 0 | 0 | 0.6667 | Ref |
| KF006818 | Unassigned | Unassigned | Unassigned | 566_6 | Clustered | 2 | VC_566_6 | 2 | 0.0135 | 0.7810929 | 0.0105 | 0 | 0 | 0 | 1 | Ref |
| KF010834 | Unassigned | Unassigned | Unassigned | 725_0 | Clustered | 47 | VC_725_0 | 47 | 0.8649 | 1 | 0.8649 | 0 | 0 | 0 | 1 | Ref |
| KF017001 | Unassigned | Unassigned | Unassigned | 974_0 | Clustered | 34 | VC_974_0 | 34 | 0.7953 | 1 | 0.7953 | 0 | 0 | 0 | 1 | Ref |
| KF017002 | Unassigned | Unassigned | Unassigned | 577_0 | Clustered | 21 | VC_577_0 | 21 | 0.9908 | 1 | 0.9908 | 0 | 0 | 0 | 1 | Ref |
| KF017927 | Unassigned | Unassigned | Unassigned | 222_0 | Clustered | 5 | VC_222_0 | 5 | 0.072 | 0.99999997 | 0.072 | 0 | 0 | 0 | 1 | Ref |
| KF021268 | Unassigned | Unassigned | Unassigned | 156_0 | Clustered | 13 | VC_156_0 | 13 | 0.2378 | 1 | 0.2378 | 0 | 0 | 0 | 0.9872 | Ref |
| KF024721 | Unassigned | Unassigned | Unassigned | 223_3 | Clustered | 8 | VC_223_3 | 8 | 0.0699 | 1 | 0.0699 | 0 | 0 | 0 | 0.8571 | Ref |
| KF024722 | Unassigned | Unassigned | Unassigned | 526_0 | Clustered | 32 | VC_526_0 | 32 | 0.9037 | 1 | 0.9037 | 0 | 0 | 0 | 1 | Ref |
| KF024723 | Unassigned | Unassigned | Unassigned | 538_23 | Clustered | 2 | VC_538_23 | 2 | 0.0077 | 0.95763408 | 0.0074 | 0 | 0 | 0 | 1 | Ref |
| KF024724 | Unassigned | Unassigned | Unassigned | 335_49 | Clustered | 17 | VC_335_49 | 17 | 0.0731 | 1 | 0.0731 | 0 | 0 | 0 | 0.7721 | Ref |

| | | | | | | | | | | | | | | | | |
|---|---|---|---|---|---|---|---|---|---|---|---|---|---|---|---|---|
| KF024725 | Unassigned | Unassigned | Unassigned | 918_0 | Clustered | 16 | VC_918_0 | 16 | 0.2945 | 1 | 0.2945 | 0 | 0 | 0 | 1 | Ref |
| KF024726 | Unassigned | Unassigned | Unassigned | 335_8 | Clustered | 2 | VC_335_8 | 2 | 0.0092 | 0.95751268 | 0.0088 | 0 | 0 | 0 | 1 | Ref |
| KF024727 | Unassigned | Unassigned | Unassigned | 385_1 | Clustered | 9 | VC_385_1 | 9 | 0.1928 | 1 | 0.1928 | 0 | 0 | 0 | 1 | Ref |
| KF024728 | Unassigned | Unassigned | Unassigned | 594_0 | Clustered | 7 | VC_594_0 | 7 | 0.2725 | 1 | 0.2725 | 0 | 0 | 0 | 1 | Ref |
| KF024729 | Unassigned | Unassigned | Unassigned | 385_3 | Clustered | 5 | VC_385_3 | 5 | 0.1118 | 0.99999997 | 0.1118 | 0 | 0 | 0 | 1 | Ref |
| KF024730 | Unassigned | Unassigned | Unassigned | 577_0 | Clustered | 21 | VC_577_0 | 21 | 0.9908 | 1 | 0.9908 | 0 | 0 | 0 | 1 | Ref |
| KF024731 | Unassigned | Unassigned | Unassigned | 917_0 | Clustered | 28 | VC_917_0 | 28 | 0.5465 | 1 | 0.5465 | 0 | 0 | 0 | 1 | Ref |
| KF024732 | Unassigned | Unassigned | Unassigned | 571_8 | Clustered | 19 | VC_571_8 | 19 | 0.1031 | 1 | 0.1031 | 0 | 0 | 0 | 0.8713 | Ref |
| KF024733 | Unassigned | Unassigned | Unassigned | 535_1 | Clustered | 108 | VC_535_1 | 108 | 0.8479 | 1 | 0.8479 | 0 | 0 | 0 | 0.9955 | Ref |
| KF024734 | Unassigned | Unassigned | Unassigned | 579_48 | Clustered | 2 | VC_579_48 | 2 | 0.0032 | 0.68030515 | 0.0022 | 0 | 0 | 0 | 1 | Ref |
| KF055347 | Unassigned | Unassigned | Unassigned | 340_16 | Clustered | 4 | VC_340_16 | 4 | 0.0157 | 0.9999807 | 0.0157 | 0 | 0 | 0 | 1 | Ref |
| KF056323 | Unassigned | Unassigned | Unassigned | 858_0 | Clustered | 7 | VC_858_0 | 7 | 1 | 1 | 1 | 0 | 0 | 0 | 1 | Ref |
| KF114874 | Unassigned | Unassigned | Unassigned | 538_9 | Clustered/Singleton | 1 | VC_538_9 | 1 | 0 | 1 | 0 | 0 | 0 | 0 | 1 | Ref |
| KF114875 | Unassigned | Unassigned | Unassigned | 566_4 | Clustered | 16 | VC_566_4 | 16 | 0.2517 | 0.9999998 | 0.2517 | 0 | 0 | 0 | 0.9417 | Ref |
| KF114876 | Unassigned | Unassigned | Unassigned | 703_1 | Clustered | 2 | VC_703_1 | 2 | 0.2226 | 0.91418477 | 0.2035 | 0 | 0 | 0 | 1 | Ref |
| KF114877 | Unassigned | Unassigned | Unassigned | 703_1 | Clustered | 2 | VC_703_1 | 2 | 0.2226 | 0.91418477 | 0.2035 | 0 | 0 | 0 | 1 | Ref |
| KF114879 | Unassigned | Unassigned | Unassigned | 703_0 | Clustered | 3 | VC_703_0 | 3 | 0.5841 | 0.99252665 | 0.5797 | 0 | 0 | 0 | 1 | Ref |
| KF114880 | Unassigned | Unassigned | Unassigned | 703_0 | Clustered | 3 | VC_703_0 | 3 | 0.5841 | 0.99252665 | 0.5797 | 0 | 0 | 0 | 1 | Ref |
| KF148055 | Unassigned | Unassigned | Unassigned | 654_2 | Clustered | 2 | VC_654_2 | 2 | 0.0038 | 0.92844927 | 0.0035 | 0 | 0 | 0 | 1 | Ref |
| KF148616 | Unassigned | Unassigned | Unassigned | 868_0 | Clustered | 27 | VC_868_0 | 27 | 0.9372 | 1 | 0.9372 | 0 | 0 | 0 | 1 | Ref |
| KF156338 | Unassigned | Unassigned | Unassigned | 447_2 | Clustered/Singleton | 1 | VC_447_2 | 1 | 0 | 1 | 0 | 0 | 0 | 0 | 1 | Ref |
| KF188409 | Unassigned | Unassigned | Unassigned | 625_0 | Clustered | 2 | VC_625_0 | 2 | 0.2121 | 0.95222028 | 0.202 | 0 | 0 | 0 | 1 | Ref |
| KF188410 | Unassigned | Unassigned | Unassigned | 657_0 | Clustered | 7 | VC_657_0 | 7 | 0.8495 | 1 | 0.8495 | 0 | 0 | 0 | 1 | Ref |
| KF188414 | Unassigned | Unassigned | Unassigned | 571_2 | Clustered | 17 | VC_571_2 | 17 | 0.0965 | 1 | 0.0965 | 0 | 0 | 0 | 0.9779 | Ref |
| KF192053 | Unassigned | Unassigned | Unassigned | 110_0 | Clustered | 33 | VC_110_0 | 33 | 0.9488 | 1 | 0.9488 | 0 | 0 | 0 | 1 | Ref |
| KF192075 | Unassigned | Unassigned | Unassigned | 801_0 | Clustered | 37 | VC_801_0 | 37 | 0.5672 | 1 | 0.5672 | 0 | 0 | 0 | 1 | Ref |
| KF208315 | Unassigned | Unassigned | Unassigned | 290_90 | Clustered/Singleton | 1 | VC_290_90 | 1 | 0 | 1 | 0 | 0 | 0 | 0 | 1 | Ref |
| KF208639 | Unassigned | Unassigned | Unassigned | 912_1 | Clustered | 12 | VC_912_1 | 12 | 0.2423 | 1 | 0.2423 | 0 | 0 | 0 | 1 | Ref |
| KF279411 | Unassigned | Unassigned | Unassigned | 385_0 | Clustered | 24 | VC_385_0 | 24 | 0.5516 | 1 | 0.5516 | 0 | 0 | 0 | 1 | Ref |
| KF279412 | Unassigned | Unassigned | Unassigned | 385_0 | Clustered | 24 | VC_385_0 | 24 | 0.5516 | 1 | 0.5516 | 0 | 0 | 0 | 1 | Ref |
| KF279413 | Unassigned | Unassigned | Unassigned | 385_0 | Clustered | 24 | VC_385_0 | 24 | 0.5516 | 1 | 0.5516 | 0 | 0 | 0 | 1 | Ref |
| KF279415 | Unassigned | Unassigned | Unassigned | 335_49 | Clustered | 17 | VC_335_49 | 17 | 0.0731 | 1 | 0.0731 | 0 | 0 | 0 | 0.7721 | Ref |
| KF279416 | Unassigned | Unassigned | Unassigned | 335_49 | Clustered | 17 | VC_335_49 | 17 | 0.0731 | 1 | 0.0731 | 0 | 0 | 0 | 0.7721 | Ref |
| KF279417 | Unassigned | Unassigned | Unassigned | 571_7 | Clustered | 28 | VC_571_7 | 28 | 0.1651 | 1 | 0.1651 | 0 | 0 | 0 | 0.8519 | Ref |
| KF279418 | Unassigned | Unassigned | Unassigned | 532_0 | Clustered | 75 | VC_532_0 | 75 | 0.9393 | 1 | 0.9393 | 0 | 0 | 0 | 1 | Ref |
| KF296717 | Unassigned | Unassigned | Unassigned | 853_0 | Clustered | 5 | VC_853_0 | 5 | 0.8735 | 0.99999832 | 0.8735 | 0 | 0 | 0 | 1 | Ref |
| KF302032 | Unassigned | Unassigned | Unassigned | 925_0 | Clustered | 3 | VC_925_0 | 3 | 0.6333 | 0.99692152 | 0.6314 | 0 | 0 | 0 | 1 | Ref |
| KF302033 | Unassigned | Unassigned | Unassigned | 925_0 | Clustered | 3 | VC_925_0 | 3 | 0.6333 | 0.99692152 | 0.6314 | 0 | 0 | 0 | 1 | Ref |
| KF302034 | Unassigned | Unassigned | Unassigned | 514_0 | Clustered/Singleton | 1 | VC_514_0 | 1 | 0 | 1 | 0 | 0 | 0 | 0 | 1 | Ref |
| KF302035 | Unassigned | Unassigned | Unassigned | 189_0 | Clustered | 2 | VC_189_0 | 2 | 0.0711 | 0.95489589 | 0.0679 | 0 | 0 | 0 | 1 | Ref |
| KF302036 | Unassigned | Unassigned | Unassigned | 926_0 | Clustered | 2 | VC_926_0 | 2 | 0.2769 | 0.94251254 | 0.261 | 0 | 0 | 0 | 1 | Ref |
| KF302037 | Unassigned | Unassigned | Unassigned | 773_0 | Clustered | 2 | VC_773_0 | 2 | 0.1621 | 0.95427014 | 0.1547 | 0 | 0 | 0 | 1 | Ref |
| KF306380 | Unassigned | Unassigned | Unassigned | 571_3 | Clustered/Singleton | 1 | VC_571_3 | 1 | 0 | 1 | 0 | 0 | 0 | 0 | 1 | Ref |
| KF319020 | Unassigned | Unassigned | Unassigned | 114_0 | Clustered | 8 | VC_114_0 | 8 | 0.2518 | 1 | 0.2518 | 0 | 0 | 0 | 1 | Ref |
| KF322026 | Unassigned | Unassigned | Unassigned | 1067_0 | Clustered | 20 | VC_1067_0 | 20 | 0.7109 | 1 | 0.7109 | 0 | 0 | 0 | 1 | Ref |
| KF322032 | Unassigned | Unassigned | Unassigned | 235_0 | Clustered | 60 | VC_235_0 | 60 | 0.9413 | 1 | 0.9413 | 0 | 0 | 0 | 1 | Ref |
| KF356199 | Unassigned | Unassigned | Unassigned | 41_0 | Clustered | 3 | VC_41_0 | 3 | 1 | 0 | 0 | 0 | 0 | 0 | 1 | Ref |
| KF361475 | Unassigned | Unassigned | Unassigned | 5_0 | Clustered | 27 | VC_5_0 | 27 | 0.9599 | 1 | 0.9599 | 0 | 0 | 0 | 1 | Ref |
| KF381361 | Unassigned | Unassigned | Unassigned | 451_0 | Clustered | 4 | VC_451_0 | 4 | 0.5528 | 0.99998171 | 0.5528 | 0 | 0 | 0 | 1 | Ref |
| KF416340 | Unassigned | Unassigned | Unassigned | 335_43 | Clustered | 4 | VC_335_43 | 4 | 0.014 | 0.99995965 | 0.014 | 0 | 0 | 0 | 1 | Ref |
| KF416341 | Unassigned | Unassigned | Unassigned | 385_1 | Clustered | 9 | VC_385_1 | 9 | 0.1928 | 1 | 0.1928 | 0 | 0 | 0 | 1 | Ref |
| KF416342 | Unassigned | Unassigned | Unassigned | 588_0 | Clustered | 8 | VC_588_0 | 8 | 0.5695 | 1 | 0.5695 | 0 | 0 | 0 | 1 | Ref |
| KF416343 | Unassigned | Unassigned | Unassigned | 571_4 | Clustered | 2 | VC_571_4 | 2 | 0.006 | 0.95695857 | 0.0057 | 0 | 0 | 0 | 1 | Ref |
| KF416344 | Unassigned | Unassigned | Unassigned | 222_0 | Clustered | 5 | VC_222_0 | 5 | 0.072 | 0.99999997 | 0.072 | 0 | 0 | 0 | 1 | Ref |
| KF475786 | Unassigned | Unassigned | Unassigned | 185_0 | Clustered | 53 | VC_185_0 | 53 | 0.9153 | 1 | 0.9153 | 0 | 0 | 0 | 1 | Ref |
| KF493879 | Unassigned | Unassigned | Unassigned | 527_0 | Clustered | 37 | VC_527_0 | 37 | 0.9679 | 1 | 0.9679 | 0 | 0 | 0 | 1 | Ref |
| KF493880 | Unassigned | Unassigned | Unassigned | 335_3 | Clustered/Singleton | 1 | VC_335_3 | 1 | 0 | 1 | 0 | 0 | 0 | 0 | 1 | Ref |
| KF493881 | Unassigned | Unassigned | Unassigned | 385_0 | Clustered | 24 | VC_385_0 | 24 | 0.5516 | 1 | 0.5516 | 0 | 0 | 0 | 1 | Ref |
| KF493882 | Unassigned | Unassigned | Unassigned | 974_0 | Clustered | 34 | VC_974_0 | 34 | 0.7953 | 1 | 0.7953 | 0 | 0 | 0 | 1 | Ref |
| KF493883 | Unassigned | Unassigned | Unassigned | 571_8 | Clustered | 19 | VC_571_8 | 19 | 0.1031 | 1 | 0.1031 | 0 | 0 | 0 | 0.8713 | Ref |
| KF534715 | Unassigned | Unassigned | Unassigned | 816_0 | Clustered | 35 | VC_816_0 | 35 | 0.9824 | 1 | 0.9824 | 0 | 0 | 0 | 1 | Ref |
| KF550303 | Unassigned | Unassigned | Unassigned | 601_4 | Clustered | 3 | VC_601_4 | 3 | 0.0346 | 0.98052968 | 0.034 | 0 | 0 | 0 | 1 | Ref |
| KF554508 | Unassigned | Unassigned | Unassigned | 89_1 | Clustered | 3 | VC_89_1 | 3 | 0.1616 | 0.99777924 | 0.1612 | 0 | 0 | 0 | 1 | Ref |
| KF560330 | Unassigned | Unassigned | Unassigned | 974_0 | Clustered | 34 | VC_974_0 | 34 | 0.7953 | 1 | 0.7953 | 0 | 0 | 0 | 1 | Ref |
| KF560331 | Unassigned | Unassigned | Unassigned | 335_33 | Clustered | 4 | VC_335_33 | 4 | 0.0148 | 0.99998565 | 0.0148 | 0 | 0 | 0 | 1 | Ref |
| KF560332 | Unassigned | Unassigned | Unassigned | 230_0 | Clustered | 38 | VC_230_0 | 38 | 0.9076 | 1 | 0.9076 | 0 | 0 | 0 | 1 | Ref |
| KF560333 | Unassigned | Unassigned | Unassigned | 230_0 | Clustered | 38 | VC_230_0 | 38 | 0.9076 | 1 | 0.9076 | 0 | 0 | 0 | 1 | Ref |
| KF560334 | Unassigned | Unassigned | Unassigned | 230_0 | Clustered | 38 | VC_230_0 | 38 | 0.9076 | 1 | 0.9076 | 0 | 0 | 0 | 1 | Ref |
| KF562099 | Unassigned | Unassigned | Unassigned | 571_8 | Clustered | 19 | VC_571_8 | 19 | 0.1031 | 1 | 0.1031 | 0 | 0 | 0 | 0.8713 | Ref |
| KF562100 | Unassigned | Unassigned | Unassigned | 571_8 | Clustered | 19 | VC_571_8 | 19 | 0.1031 | 1 | 0.1031 | 0 | 0 | 0 | 0.8713 | Ref |
| KF562101 | Unassigned | Unassigned | Unassigned | 571_8 | Clustered | 19 | VC_571_8 | 19 | 0.1031 | 1 | 0.1031 | 0 | 0 | 0 | 0.8713 | Ref |
| KF562102 | Unassigned | Unassigned | Unassigned | 571_8 | Clustered | 19 | VC_571_8 | 19 | 0.1031 | 1 | 0.1031 | 0 | 0 | 0 | 0.8713 | Ref |
| KF562340 | Unassigned | Unassigned | Unassigned | 801_0 | Clustered | 37 | VC_801_0 | 37 | 0.5672 | 1 | 0.5672 | 0 | 0 | 0 | 1 | Ref |
| KF562341 | Unassigned | Unassigned | Unassigned | 293_10 | Clustered | 9 | VC_293_10 | 9 | 0.0615 | 0.99999884 | 0.0615 | 0 | 0 | 0 | 0.6667 | Ref |
| KF562864 | Unassigned | Unassigned | Unassigned | 654_1 | Clustered | 27 | VC_654_1 | 27 | 0.2571 | 1 | 0.2571 | 0 | 0 | 0 | 0.9858 | Ref |
| KF562865 | Unassigned | Unassigned | Unassigned | 654_1 | Clustered | 27 | VC_654_1 | 27 | 0.2571 | 1 | 0.2571 | 0 | 0 | 0 | 0.9858 | Ref |
| KF582788 | Unassigned | Unassigned | Unassigned | 293_13 | Clustered | 10 | VC_293_13 | 10 | 0.0698 | 0.99999931 | 0.0698 | 0 | 0 | 0 | 0.9556 | Ref |
| KF591601 | Unassigned | Unassigned | Unassigned | 721_0 | Clustered | 11 | VC_721_0 | 11 | 0.8846 | 1 | 0.8846 | 0 | 0 | 0 | 1 | Ref |
| KF598856 | Unassigned | Unassigned | Unassigned | 8_0 | Clustered | 58 | VC_8_0 | 58 | 0.8285 | 1 | 0.8285 | 0 | 0 | 0 | 0.9946 | Ref |
| KF598865 | Unassigned | Unassigned | Unassigned | 799_0 | Clustered | 3 | VC_799_0 | 3 | 0.4746 | 0.9961812 | 0.4727 | 0 | 0 | 0 | 1 | Ref |
| KF614509 | Unassigned | Unassigned | Unassigned | 1023_0 | Clustered | 3 | VC_1023_0 | 3 | 0.6263 | 0.99819562 | 0.6252 | 0 | 0 | 0 | 1 | Ref |

| | | | | | | | | | | | | | | | | |
|---|---|---|---|---|---|---|---|---|---|---|---|---|---|---|---|---|
| KF620435 | Unassigned | Unassigned | Unassigned | 801_0 | Clustered | 37 | VC_801_0 | 37 | 0.5672 | 1 | 0.5672 | 0 | 0 | 0 | 1 | Ref |
| KF623293 | Unassigned | Unassigned | Unassigned | 932_0 | Clustered | 5 | VC_932_0 | 5 | 0.9252 | 0.99999497 | 0.9252 | 0 | 0 | 0 | 1 | Ref |
| KF623294 | Unassigned | Unassigned | Unassigned | 506_2 | Clustered | 2 | VC_506_2 | 2 | 0.0324 | 0.95511082 | 0.031 | 0 | 0 | 0 | 1 | Ref |
| KF626665 | Unassigned | Unassigned | Unassigned | 178_0 | Clustered | 5 | VC_178_0 | 5 | 0.3547 | 0.99999997 | 0.3547 | 0 | 0 | 0 | 1 | Ref |
| KF626667 | Unassigned | Unassigned | Unassigned | 75_0 | Clustered | 15 | VC_75_0 | 15 | 0.518 | 1 | 0.518 | 0 | 0 | 0 | 1 | Ref |
| KF626668 | Unassigned | Unassigned | Unassigned | 178_0 | Clustered | 5 | VC_178_0 | 5 | 0.3547 | 0.99999997 | 0.3547 | 0 | 0 | 0 | 1 | Ref |
| KF669647 | Unassigned | Unassigned | Unassigned | 912_1 | Clustered | 12 | VC_912_1 | 12 | 0.2423 | 1 | 0.2423 | 0 | 0 | 0 | 1 | Ref |
| KF669648 | Unassigned | Unassigned | Unassigned | 102_0 | Clustered | 15 | VC_102_0 | 15 | 0.653 | 1 | 0.653 | 0 | 0 | 0 | 1 | Ref |
| KF669649 | Unassigned | Unassigned | Unassigned | 89_0 | Clustered | 8 | VC_89_0 | 8 | 0.5507 | 1 | 0.5507 | 0 | 0 | 0 | 1 | Ref |
| KF669650 | Unassigned | Unassigned | Unassigned | 880_0 | Clustered | 4 | VC_880_0 | 4 | 0.0727 | 0.88800114 | 0.0645 | 0 | 0 | 0 | 1 | Ref |
| KF669651 | Unassigned | Unassigned | Unassigned | 102_0 | Clustered | 15 | VC_102_0 | 15 | 0.653 | 1 | 0.653 | 0 | 0 | 0 | 1 | Ref |
| KF669652 | Unassigned | Unassigned | Unassigned | 87_3 | Clustered | 19 | VC_87_3 | 19 | 0.3645 | 1 | 0.3645 | 0 | 0 | 0 | 0.807 | Ref |
| KF669653 | Unassigned | Unassigned | Unassigned | 135_0 | Clustered | 2 | VC_135_0 | 2 | 0.0062 | 0.94038885 | 0.0059 | 0 | 0 | 0 | 1 | Ref |
| KF669654 | Unassigned | Unassigned | Unassigned | 135_0 | Clustered | 2 | VC_135_0 | 2 | 0.0062 | 0.94038885 | 0.0059 | 0 | 0 | 0 | 1 | Ref |
| KF669655 | Unassigned | Unassigned | Unassigned | 1071_0 | Clustered | 7 | VC_1071_0 | 7 | 0.9883 | 0.99999504 | 0.9883 | 0 | 0 | 0 | 1 | Ref |
| KF669657 | Unassigned | Unassigned | Unassigned | 1071_0 | Clustered | 7 | VC_1071_0 | 7 | 0.9883 | 0.99999504 | 0.9883 | 0 | 0 | 0 | 1 | Ref |
| KF669659 | Unassigned | Unassigned | Unassigned | 102_0 | Clustered | 15 | VC_102_0 | 15 | 0.653 | 1 | 0.653 | 0 | 0 | 0 | 1 | Ref |
| KF669660 | Unassigned | Unassigned | Unassigned | 1071_0 | Clustered | 7 | VC_1071_0 | 7 | 0.9883 | 0.99999504 | 0.9883 | 0 | 0 | 0 | 1 | Ref |
| KF669662 | Unassigned | Unassigned | Unassigned | 912_3 | Clustered | 2 | VC_912_3 | 2 | 0.0181 | 0.87900236 | 0.0159 | 0 | 0 | 0 | 1 | Ref |
| KF676640 | Unassigned | Unassigned | Unassigned | 210_0 | Clustered | 105 | VC_210_0 | 105 | 0.4076 | 1 | 0.4076 | 0 | 0 | 0 | 0.7766 | Ref |
| KF692088 | Unassigned | Unassigned | Unassigned | 1073_1 | Clustered/Singleton | 1 | VC_1073_1 | 1 | 0 | 1 | 0 | 0 | 0 | 0 | 1 | Ref |
| KF700371 | Unassigned | Unassigned | Unassigned | 399_0 | Clustered | 49 | VC_399_0 | 49 | 0.7927 | 1 | 0.7927 | 0 | 0 | 0 | 1 | Ref |
| KF713485 | Unassigned | Unassigned | Unassigned | 384_3 | Clustered | 22 | VC_384_3 | 22 | 0.0786 | 1 | 0.0786 | 0 | 0 | 0 | 0.9048 | Ref |
| KF713486 | Unassigned | Unassigned | Unassigned | 244_0 | Clustered | 4 | VC_244_0 | 4 | 0.0426 | 0.99998759 | 0.0426 | 0 | 0 | 0 | 1 | Ref |
| KF728385 | Unassigned | Unassigned | Unassigned | 711_0 | Clustered | 48 | VC_711_0 | 48 | 0.8217 | 1 | 0.8217 | 0 | 0 | 0 | 1 | Ref |
| KF733017 | Unassigned | Unassigned | Unassigned | 711_0 | Clustered | 48 | VC_711_0 | 48 | 0.8217 | 1 | 0.8217 | 0 | 0 | 0 | 1 | Ref |
| KF751793 | Unassigned | Unassigned | Unassigned | 1078_0 | Clustered | 5 | VC_1078_0 | 5 | 1 | 1 | 1 | 0 | 0 | 0 | 1 | Ref |
| KF751794 | Unassigned | Unassigned | Unassigned | 1078_0 | Clustered | 5 | VC_1078_0 | 5 | 1 | 1 | 1 | 0 | 0 | 0 | 1 | Ref |
| KF751795 | Unassigned | Unassigned | Unassigned | 1078_0 | Clustered | 5 | VC_1078_0 | 5 | 1 | 1 | 1 | 0 | 0 | 0 | 1 | Ref |
| KF751796 | Unassigned | Unassigned | Unassigned | 1078_0 | Clustered | 5 | VC_1078_0 | 5 | 1 | 1 | 1 | 0 | 0 | 0 | 1 | Ref |
| KF751797 | Unassigned | Unassigned | Unassigned | 1078_0 | Clustered | 5 | VC_1078_0 | 5 | 1 | 1 | 1 | 0 | 0 | 0 | 1 | Ref |
| KF765493 | Unassigned | Unassigned | Unassigned | 113_0 | Clustered | 91 | VC_113_0 | 91 | 0.9584 | 1 | 0.9584 | 0 | 0 | 0 | 1 | Ref |
| KF766114 | Unassigned | Unassigned | Unassigned | 155_8 | Clustered | 66 | VC_155_8 | 66 | 0.4537 | 1 | 0.4537 | 0 | 0 | 0 | 0.8182 | Ref |
| KF766125 | Unassigned | Unassigned | Unassigned | 48_0 | Clustered | 2 | VC_48_0 | 2 | 0.018 | 0.95763863 | 0.0172 | 0 | 0 | 0 | 1 | Ref |
| KF767351 | Unassigned | Unassigned | Unassigned | 275_0 | Clustered | 6 | VC_275_0 | 6 | 0.6515 | 0.99999994 | 0.6515 | 0 | 0 | 0 | 0.9333 | Ref |
| KF771236 | Unassigned | Unassigned | Unassigned | 469_0 | Clustered | 18 | VC_469_0 | 18 | 0.4583 | 1 | 0.4583 | 0 | 0 | 0 | 1 | Ref |
| KF771237 | Unassigned | Unassigned | Unassigned | 469_0 | Clustered | 18 | VC_469_0 | 18 | 0.4583 | 1 | 0.4583 | 0 | 0 | 0 | 1 | Ref |
| KF771238 | Unassigned | Unassigned | Unassigned | 469_0 | Clustered | 18 | VC_469_0 | 18 | 0.4583 | 1 | 0.4583 | 0 | 0 | 0 | 1 | Ref |
| KF771239 | Unassigned | Unassigned | Unassigned | 469_0 | Clustered | 18 | VC_469_0 | 18 | 0.4583 | 1 | 0.4583 | 0 | 0 | 0 | 1 | Ref |
| KF787094 | Unassigned | Unassigned | Unassigned | 801_1 | Clustered | 11 | VC_801_1 | 11 | 0.1583 | 1 | 0.1583 | 0 | 0 | 0 | 1 | Ref |
| KF787095 | Unassigned | Unassigned | Unassigned | 801_1 | Clustered | 11 | VC_801_1 | 11 | 0.1583 | 1 | 0.1583 | 0 | 0 | 0 | 1 | Ref |
| KF800937 | Unassigned | Unassigned | Unassigned | 1067_0 | Clustered | 20 | VC_1067_0 | 20 | 0.7109 | 1 | 0.7109 | 0 | 0 | 0 | 1 | Ref |
| KF806588 | Unassigned | Unassigned | Unassigned | 801_2 | Clustered | 5 | VC_801_2 | 5 | 0.087 | 0.99999997 | 0.087 | 0 | 0 | 0 | 1 | Ref |
| KF806589 | Unassigned | Unassigned | Unassigned | 507_1 | Clustered | 11 | VC_507_1 | 11 | 0.4612 | 1 | 0.4612 | 0 | 0 | 0 | 1 | Ref |
| KF831354 | Unassigned | Unassigned | Unassigned | 10_0 | Clustered | 25 | VC_10_0 | 25 | 0.3437 | 1 | 0.3437 | 0 | 0 | 0 | 0.9733 | Ref |
| KF835987 | Unassigned | Unassigned | Unassigned | 508_2 | Clustered/Singleton | 1 | VC_508_2 | 1 | 0 | 1 | 0 | 0 | 0 | 0 | 1 | Ref |
| KF841475 | Unassigned | Unassigned | Unassigned | 535_1 | Clustered | 108 | VC_535_1 | 108 | 0.8479 | 1 | 0.8479 | 0 | 0 | 0 | 0.9955 | Ref |
| KF841476 | Unassigned | Unassigned | Unassigned | 535_1 | Clustered | 108 | VC_535_1 | 108 | 0.8479 | 1 | 0.8479 | 0 | 0 | 0 | 0.9955 | Ref |
| KF841477 | Unassigned | Unassigned | Unassigned | 537_0 | Clustered | 39 | VC_537_0 | 39 | 0.8148 | 1 | 0.8148 | 0 | 0 | 0 | 1 | Ref |
| KF854250 | Unassigned | Unassigned | Unassigned | 515_0 | Clustered | 8 | VC_515_0 | 8 | 0.3973 | 1 | 0.3973 | 0 | 0 | 0 | 1 | Ref |
| KF856712 | Unassigned | Unassigned | Unassigned | 486_0 | Clustered | 47 | VC_486_0 | 47 | 0.9022 | 1 | 0.9022 | 0 | 0 | 0 | 1 | Ref |
| KF861510 | Unassigned | Unassigned | Unassigned | 225_0 | Clustered | 3 | VC_225_0 | 3 | 0.0477 | 0.99860979 | 0.0477 | 0 | 0 | 0 | 1 | Ref |
| KF925357 | Unassigned | Unassigned | Unassigned | 290_163 | Clustered | 2 | VC_290_163 | 2 | 0.0029 | 0.93227927 | 0.0027 | 0 | 0 | 0 | 1 | Ref |
| KF926093 | Unassigned | Unassigned | Unassigned | 488_0 | Clustered | 14 | VC_488_0 | 14 | 0.8516 | 1 | 0.8516 | 0 | 0 | 0 | 1 | Ref |
| KF929199 | Unassigned | Unassigned | Unassigned | 685_0 | Clustered | 7 | VC_685_0 | 7 | 0.4045 | 1 | 0.4045 | 0 | 0 | 0 | 1 | Ref |
| KF954506 | Unassigned | Unassigned | Unassigned | 535_1 | Clustered | 108 | VC_535_1 | 108 | 0.8479 | 1 | 0.8479 | 0 | 0 | 0 | 0.9955 | Ref |
| KF959563 | Unassigned | Unassigned | Unassigned | 535_1 | Clustered | 108 | VC_535_1 | 108 | 0.8479 | 1 | 0.8479 | 0 | 0 | 0 | 0.9955 | Ref |
| KF971864 | Unassigned | Unassigned | Unassigned | 44_0 | Clustered | 49 | VC_44_0 | 49 | 0.4506 | 1 | 0.4506 | 0 | 0 | 0 | 0.8605 | Ref |
| KF977490 | Unassigned | Unassigned | Unassigned | 126_0 | Clustered | 2 | VC_126_0 | 2 | 0.373 | 0.95337092 | 0.3556 | 0 | 0 | 0 | 1 | Ref |
| KF981601 | Unassigned | Unassigned | Unassigned | 223_6 | Clustered | 42 | VC_223_6 | 42 | 0.4717 | 1 | 0.4717 | 0 | 0 | 0 | 0.8351 | Ref |
| KF981730 | Unassigned | Unassigned | Unassigned | 837_1 | Clustered | 24 | VC_837_1 | 24 | 0.5328 | 1 | 0.5328 | 0 | 0 | 0 | 1 | Ref |
| KF981875 | Unassigned | Unassigned | Unassigned | 81_0 | Clustered | 41 | VC_81_0 | 41 | 0.5815 | 1 | 0.5815 | 0 | 0 | 0 | 0.9805 | Ref |
| KF981876 | Unassigned | Unassigned | Unassigned | 1064_0 | Clustered | 2 | VC_1064_0 | 2 | 1 | 1 | 1 | 0 | 0 | 0 | 1 | Ref |
| KF986246 | Unassigned | Unassigned | Unassigned | 539_0 | Clustered | 37 | VC_539_0 | 37 | 0.911 | 1 | 0.911 | 0 | 0 | 0 | 1 | Ref |
| KF986247 | Unassigned | Unassigned | Unassigned | 587_0 | Clustered | 8 | VC_587_0 | 8 | 0.4907 | 1 | 0.4907 | 0 | 0 | 0 | 1 | Ref |
| KJ002054 | Unassigned | Unassigned | Unassigned | 817_0 | Clustered | 12 | VC_817_0 | 12 | 0.8497 | 1 | 0.8497 | 0 | 0 | 0 | 1 | Ref |
| KJ003983 | Unassigned | Unassigned | Unassigned | 1054_2 | Clustered | 2 | VC_1054_2 | 2 | 0.06 | 0.95033624 | 0.057 | 0 | 0 | 0 | 1 | Ref |
| KJ010547 | Unassigned | Unassigned | Unassigned | 87_0 | Clustered | 3 | VC_87_0 | 3 | 0.0416 | 0.99823485 | 0.0415 | 0 | 0 | 0 | 1 | Ref |
| KJ010548 | Unassigned | Unassigned | Unassigned | 87_0 | Clustered | 3 | VC_87_0 | 3 | 0.0416 | 0.99823485 | 0.0415 | 0 | 0 | 0 | 1 | Ref |
| KJ018213 | Unassigned | Unassigned | Unassigned | 851_0 | Clustered | 6 | VC_851_0 | 6 | 0.7471 | 1 | 0.7471 | 0 | 0 | 0 | 1 | Ref |
| KJ019028 | Unassigned | Unassigned | Unassigned | 675_1 | Clustered | 46 | VC_675_1 | 46 | 0.8493 | 1 | 0.8493 | 0 | 0 | 0 | 1 | Ref |
| KJ019029 | Unassigned | Unassigned | Unassigned | 675_1 | Clustered | 46 | VC_675_1 | 46 | 0.8493 | 1 | 0.8493 | 0 | 0 | 0 | 1 | Ref |
| KJ019031 | Unassigned | Unassigned | Unassigned | 675_1 | Clustered | 46 | VC_675_1 | 46 | 0.8493 | 1 | 0.8493 | 0 | 0 | 0 | 1 | Ref |
| KJ019032 | Unassigned | Unassigned | Unassigned | 675_1 | Clustered | 46 | VC_675_1 | 46 | 0.8493 | 1 | 0.8493 | 0 | 0 | 0 | 1 | Ref |
| KJ019034 | Unassigned | Unassigned | Unassigned | 675_1 | Clustered | 46 | VC_675_1 | 46 | 0.8493 | 1 | 0.8493 | 0 | 0 | 0 | 1 | Ref |
| KJ019035 | Unassigned | Unassigned | Unassigned | 675_1 | Clustered | 46 | VC_675_1 | 46 | 0.8493 | 1 | 0.8493 | 0 | 0 | 0 | 1 | Ref |
| KJ019036 | Unassigned | Unassigned | Unassigned | 675_1 | Clustered | 46 | VC_675_1 | 46 | 0.8493 | 1 | 0.8493 | 0 | 0 | 0 | 1 | Ref |
| KJ019037 | Unassigned | Unassigned | Unassigned | 676_1 | Clustered | 41 | VC_676_1 | 41 | 0.7464 | 1 | 0.7464 | 0 | 0 | 0 | 1 | Ref |
| KJ019045 | Unassigned | Unassigned | Unassigned | 676_1 | Clustered | 41 | VC_676_1 | 41 | 0.7464 | 1 | 0.7464 | 0 | 0 | 0 | 1 | Ref |
| KJ019046 | Unassigned | Unassigned | Unassigned | 675_1 | Clustered | 46 | VC_675_1 | 46 | 0.8493 | 1 | 0.8493 | 0 | 0 | 0 | 1 | Ref |
| KJ019047 | Unassigned | Unassigned | Unassigned | 675_1 | Clustered | 46 | VC_675_1 | 46 | 0.8493 | 1 | 0.8493 | 0 | 0 | 0 | 1 | Ref |

| | | | | | | | | | | | | | | | | |
|---|---|---|---|---|---|---|---|---|---|---|---|---|---|---|---|---|
| KJ019048 | Unassigned | Unassigned | Unassigned | 675_1 | Clustered | 46 | VC_675_1 | 46 | 0.8493 | 1 | 0.8493 | 0 | 0 | 0 | 1 | Ref |
| KJ019050 | Unassigned | Unassigned | Unassigned | 675_1 | Clustered | 46 | VC_675_1 | 46 | 0.8493 | 1 | 0.8493 | 0 | 0 | 0 | 1 | Ref |
| KJ019052 | Unassigned | Unassigned | Unassigned | 676_1 | Clustered | 41 | VC_676_1 | 41 | 0.7464 | 1 | 0.7464 | 0 | 0 | 0 | 1 | Ref |
| KJ019053 | Unassigned | Unassigned | Unassigned | 676_1 | Clustered | 41 | VC_676_1 | 41 | 0.7464 | 1 | 0.7464 | 0 | 0 | 0 | 1 | Ref |
| KJ019054 | Unassigned | Unassigned | Unassigned | 447_1 | Clustered | 9 | VC_447_1 | 9 | 0.1269 | 1 | 0.1269 | 0 | 0 | 0 | 0.8611 | Ref |
| KJ019056 | Unassigned | Unassigned | Unassigned | 675_1 | Clustered | 46 | VC_675_1 | 46 | 0.8493 | 1 | 0.8493 | 0 | 0 | 0 | 1 | Ref |
| KJ019057 | Unassigned | Unassigned | Unassigned | 675_1 | Clustered | 46 | VC_675_1 | 46 | 0.8493 | 1 | 0.8493 | 0 | 0 | 0 | 1 | Ref |
| KJ019058 | Unassigned | Unassigned | Unassigned | 676_1 | Clustered | 41 | VC_676_1 | 41 | 0.7464 | 1 | 0.7464 | 0 | 0 | 0 | 1 | Ref |
| KJ019059 | Unassigned | Unassigned | Unassigned | 676_1 | Clustered | 41 | VC_676_1 | 41 | 0.7464 | 1 | 0.7464 | 0 | 0 | 0 | 1 | Ref |
| KJ019062 | Unassigned | Unassigned | Unassigned | 675_1 | Clustered | 46 | VC_675_1 | 46 | 0.8493 | 1 | 0.8493 | 0 | 0 | 0 | 1 | Ref |
| KJ019066 | Unassigned | Unassigned | Unassigned | 676_1 | Clustered | 41 | VC_676_1 | 41 | 0.7464 | 1 | 0.7464 | 0 | 0 | 0 | 1 | Ref |
| KJ019069 | Unassigned | Unassigned | Unassigned | 447_1 | Clustered | 9 | VC_447_1 | 9 | 0.1269 | 1 | 0.1269 | 0 | 0 | 0 | 0.8611 | Ref |
| KJ019070 | Unassigned | Unassigned | Unassigned | 675_1 | Clustered | 46 | VC_675_1 | 46 | 0.8493 | 1 | 0.8493 | 0 | 0 | 0 | 1 | Ref |
| KJ019072 | Unassigned | Unassigned | Unassigned | 675_1 | Clustered | 46 | VC_675_1 | 46 | 0.8493 | 1 | 0.8493 | 0 | 0 | 0 | 1 | Ref |
| KJ019073 | Unassigned | Unassigned | Unassigned | 675_1 | Clustered | 46 | VC_675_1 | 46 | 0.8493 | 1 | 0.8493 | 0 | 0 | 0 | 1 | Ref |
| KJ019074 | Unassigned | Unassigned | Unassigned | 675_1 | Clustered | 46 | VC_675_1 | 46 | 0.8493 | 1 | 0.8493 | 0 | 0 | 0 | 1 | Ref |
| KJ019075 | Unassigned | Unassigned | Unassigned | 675_1 | Clustered | 46 | VC_675_1 | 46 | 0.8493 | 1 | 0.8493 | 0 | 0 | 0 | 1 | Ref |
| KJ019077 | Unassigned | Unassigned | Unassigned | 675_1 | Clustered | 46 | VC_675_1 | 46 | 0.8493 | 1 | 0.8493 | 0 | 0 | 0 | 1 | Ref |
| KJ019078 | Unassigned | Unassigned | Unassigned | 675_1 | Clustered | 46 | VC_675_1 | 46 | 0.8493 | 1 | 0.8493 | 0 | 0 | 0 | 1 | Ref |
| KJ019079 | Unassigned | Unassigned | Unassigned | 675_1 | Clustered | 46 | VC_675_1 | 46 | 0.8493 | 1 | 0.8493 | 0 | 0 | 0 | 1 | Ref |
| KJ019080 | Unassigned | Unassigned | Unassigned | 675_1 | Clustered | 46 | VC_675_1 | 46 | 0.8493 | 1 | 0.8493 | 0 | 0 | 0 | 1 | Ref |
| KJ019082 | Unassigned | Unassigned | Unassigned | 447_1 | Clustered | 9 | VC_447_1 | 9 | 0.1269 | 1 | 0.1269 | 0 | 0 | 0 | 0.8611 | Ref |
| KJ019083 | Unassigned | Unassigned | Unassigned | 675_1 | Clustered | 46 | VC_675_1 | 46 | 0.8493 | 1 | 0.8493 | 0 | 0 | 0 | 1 | Ref |
| KJ019085 | Unassigned | Unassigned | Unassigned | 676_1 | Clustered | 41 | VC_676_1 | 41 | 0.7464 | 1 | 0.7464 | 0 | 0 | 0 | 1 | Ref |
| KJ019086 | Unassigned | Unassigned | Unassigned | 676_1 | Clustered | 41 | VC_676_1 | 41 | 0.7464 | 1 | 0.7464 | 0 | 0 | 0 | 1 | Ref |
| KJ019089 | Unassigned | Unassigned | Unassigned | 447_1 | Clustered | 9 | VC_447_1 | 9 | 0.1269 | 1 | 0.1269 | 0 | 0 | 0 | 0.8611 | Ref |
| KJ019090 | Unassigned | Unassigned | Unassigned | 676_1 | Clustered | 41 | VC_676_1 | 41 | 0.7464 | 1 | 0.7464 | 0 | 0 | 0 | 1 | Ref |
| KJ019091 | Unassigned | Unassigned | Unassigned | 676_1 | Clustered | 41 | VC_676_1 | 41 | 0.7464 | 1 | 0.7464 | 0 | 0 | 0 | 1 | Ref |
| KJ019092 | Unassigned | Unassigned | Unassigned | 676_1 | Clustered | 41 | VC_676_1 | 41 | 0.7464 | 1 | 0.7464 | 0 | 0 | 0 | 1 | Ref |
| KJ019093 | Unassigned | Unassigned | Unassigned | 676_1 | Clustered | 41 | VC_676_1 | 41 | 0.7464 | 1 | 0.7464 | 0 | 0 | 0 | 1 | Ref |
| KJ019094 | Unassigned | Unassigned | Unassigned | 447_1 | Clustered | 9 | VC_447_1 | 9 | 0.1269 | 1 | 0.1269 | 0 | 0 | 0 | 0.8611 | Ref |
| KJ019095 | Unassigned | Unassigned | Unassigned | 676_1 | Clustered | 41 | VC_676_1 | 41 | 0.7464 | 1 | 0.7464 | 0 | 0 | 0 | 1 | Ref |
| KJ019096 | Unassigned | Unassigned | Unassigned | 676_1 | Clustered | 41 | VC_676_1 | 41 | 0.7464 | 1 | 0.7464 | 0 | 0 | 0 | 1 | Ref |
| KJ019097 | Unassigned | Unassigned | Unassigned | 676_1 | Clustered | 41 | VC_676_1 | 41 | 0.7464 | 1 | 0.7464 | 0 | 0 | 0 | 1 | Ref |
| KJ019098 | Unassigned | Unassigned | Unassigned | 676_1 | Clustered | 41 | VC_676_1 | 41 | 0.7464 | 1 | 0.7464 | 0 | 0 | 0 | 1 | Ref |
| KJ019099 | Unassigned | Unassigned | Unassigned | 676_1 | Clustered | 41 | VC_676_1 | 41 | 0.7464 | 1 | 0.7464 | 0 | 0 | 0 | 1 | Ref |
| KJ019100 | Unassigned | Unassigned | Unassigned | 676_1 | Clustered | 41 | VC_676_1 | 41 | 0.7464 | 1 | 0.7464 | 0 | 0 | 0 | 1 | Ref |
| KJ019101 | Unassigned | Unassigned | Unassigned | 676_1 | Clustered | 41 | VC_676_1 | 41 | 0.7464 | 1 | 0.7464 | 0 | 0 | 0 | 1 | Ref |
| KJ019102 | Unassigned | Unassigned | Unassigned | 676_1 | Clustered | 41 | VC_676_1 | 41 | 0.7464 | 1 | 0.7464 | 0 | 0 | 0 | 1 | Ref |
| KJ019103 | Unassigned | Unassigned | Unassigned | 676_1 | Clustered | 41 | VC_676_1 | 41 | 0.7464 | 1 | 0.7464 | 0 | 0 | 0 | 1 | Ref |
| KJ019105 | Unassigned | Unassigned | Unassigned | 676_1 | Clustered | 41 | VC_676_1 | 41 | 0.7464 | 1 | 0.7464 | 0 | 0 | 0 | 1 | Ref |
| KJ019106 | Unassigned | Unassigned | Unassigned | 676_1 | Clustered | 41 | VC_676_1 | 41 | 0.7464 | 1 | 0.7464 | 0 | 0 | 0 | 1 | Ref |
| KJ019107 | Unassigned | Unassigned | Unassigned | 676_1 | Clustered | 41 | VC_676_1 | 41 | 0.7464 | 1 | 0.7464 | 0 | 0 | 0 | 1 | Ref |
| KJ019111 | Unassigned | Unassigned | Unassigned | 676_1 | Clustered | 41 | VC_676_1 | 41 | 0.7464 | 1 | 0.7464 | 0 | 0 | 0 | 1 | Ref |
| KJ019112 | Unassigned | Unassigned | Unassigned | 675_1 | Clustered | 46 | VC_675_1 | 46 | 0.8493 | 1 | 0.8493 | 0 | 0 | 0 | 1 | Ref |
| KJ019113 | Unassigned | Unassigned | Unassigned | 675_1 | Clustered | 46 | VC_675_1 | 46 | 0.8493 | 1 | 0.8493 | 0 | 0 | 0 | 1 | Ref |
| KJ019115 | Unassigned | Unassigned | Unassigned | 675_1 | Clustered | 46 | VC_675_1 | 46 | 0.8493 | 1 | 0.8493 | 0 | 0 | 0 | 1 | Ref |
| KJ019117 | Unassigned | Unassigned | Unassigned | 675_1 | Clustered | 46 | VC_675_1 | 46 | 0.8493 | 1 | 0.8493 | 0 | 0 | 0 | 1 | Ref |
| KJ019118 | Unassigned | Unassigned | Unassigned | 675_1 | Clustered | 46 | VC_675_1 | 46 | 0.8493 | 1 | 0.8493 | 0 | 0 | 0 | 1 | Ref |
| KJ019119 | Unassigned | Unassigned | Unassigned | 675_1 | Clustered | 46 | VC_675_1 | 46 | 0.8493 | 1 | 0.8493 | 0 | 0 | 0 | 1 | Ref |
| KJ019120 | Unassigned | Unassigned | Unassigned | 675_1 | Clustered | 46 | VC_675_1 | 46 | 0.8493 | 1 | 0.8493 | 0 | 0 | 0 | 1 | Ref |
| KJ019121 | Unassigned | Unassigned | Unassigned | 675_1 | Clustered | 46 | VC_675_1 | 46 | 0.8493 | 1 | 0.8493 | 0 | 0 | 0 | 1 | Ref |
| KJ019123 | Unassigned | Unassigned | Unassigned | 676_1 | Clustered | 41 | VC_676_1 | 41 | 0.7464 | 1 | 0.7464 | 0 | 0 | 0 | 1 | Ref |
| KJ019124 | Unassigned | Unassigned | Unassigned | 675_1 | Clustered | 46 | VC_675_1 | 46 | 0.8493 | 1 | 0.8493 | 0 | 0 | 0 | 1 | Ref |
| KJ019125 | Unassigned | Unassigned | Unassigned | 675_1 | Clustered | 46 | VC_675_1 | 46 | 0.8493 | 1 | 0.8493 | 0 | 0 | 0 | 1 | Ref |
| KJ019126 | Unassigned | Unassigned | Unassigned | 675_1 | Clustered | 46 | VC_675_1 | 46 | 0.8493 | 1 | 0.8493 | 0 | 0 | 0 | 1 | Ref |
| KJ019127 | Unassigned | Unassigned | Unassigned | 675_1 | Clustered | 46 | VC_675_1 | 46 | 0.8493 | 1 | 0.8493 | 0 | 0 | 0 | 1 | Ref |
| KJ019129 | Unassigned | Unassigned | Unassigned | 675_1 | Clustered | 46 | VC_675_1 | 46 | 0.8493 | 1 | 0.8493 | 0 | 0 | 0 | 1 | Ref |
| KJ019130 | Unassigned | Unassigned | Unassigned | 675_1 | Clustered | 46 | VC_675_1 | 46 | 0.8493 | 1 | 0.8493 | 0 | 0 | 0 | 1 | Ref |
| KJ019131 | Unassigned | Unassigned | Unassigned | 675_1 | Clustered | 46 | VC_675_1 | 46 | 0.8493 | 1 | 0.8493 | 0 | 0 | 0 | 1 | Ref |
| KJ019136 | Unassigned | Unassigned | Unassigned | 675_1 | Clustered | 46 | VC_675_1 | 46 | 0.8493 | 1 | 0.8493 | 0 | 0 | 0 | 1 | Ref |
| KJ019139 | Unassigned | Unassigned | Unassigned | 675_1 | Clustered | 46 | VC_675_1 | 46 | 0.8493 | 1 | 0.8493 | 0 | 0 | 0 | 1 | Ref |
| KJ019140 | Unassigned | Unassigned | Unassigned | 675_1 | Clustered | 46 | VC_675_1 | 46 | 0.8493 | 1 | 0.8493 | 0 | 0 | 0 | 1 | Ref |
| KJ019141 | Unassigned | Unassigned | Unassigned | 676_1 | Clustered | 41 | VC_676_1 | 41 | 0.7464 | 1 | 0.7464 | 0 | 0 | 0 | 1 | Ref |
| KJ019142 | Unassigned | Unassigned | Unassigned | 676_1 | Clustered | 41 | VC_676_1 | 41 | 0.7464 | 1 | 0.7464 | 0 | 0 | 0 | 1 | Ref |
| KJ019143 | Unassigned | Unassigned | Unassigned | 676_1 | Clustered | 41 | VC_676_1 | 41 | 0.7464 | 1 | 0.7464 | 0 | 0 | 0 | 1 | Ref |
| KJ019144 | Unassigned | Unassigned | Unassigned | 676_1 | Clustered | 41 | VC_676_1 | 41 | 0.7464 | 1 | 0.7464 | 0 | 0 | 0 | 1 | Ref |
| KJ019145 | Unassigned | Unassigned | Unassigned | 676_1 | Clustered | 41 | VC_676_1 | 41 | 0.7464 | 1 | 0.7464 | 0 | 0 | 0 | 1 | Ref |
| KJ019146 | Unassigned | Unassigned | Unassigned | 676_1 | Clustered | 41 | VC_676_1 | 41 | 0.7464 | 1 | 0.7464 | 0 | 0 | 0 | 1 | Ref |
| KJ019147 | Unassigned | Unassigned | Unassigned | 676_1 | Clustered | 41 | VC_676_1 | 41 | 0.7464 | 1 | 0.7464 | 0 | 0 | 0 | 1 | Ref |
| KJ019148 | Unassigned | Unassigned | Unassigned | 676_1 | Clustered | 41 | VC_676_1 | 41 | 0.7464 | 1 | 0.7464 | 0 | 0 | 0 | 1 | Ref |
| KJ019149 | Unassigned | Unassigned | Unassigned | 676_1 | Clustered | 41 | VC_676_1 | 41 | 0.7464 | 1 | 0.7464 | 0 | 0 | 0 | 1 | Ref |
| KJ019150 | Unassigned | Unassigned | Unassigned | 676_1 | Clustered | 41 | VC_676_1 | 41 | 0.7464 | 1 | 0.7464 | 0 | 0 | 0 | 1 | Ref |
| KJ019151 | Unassigned | Unassigned | Unassigned | 676_1 | Clustered | 41 | VC_676_1 | 41 | 0.7464 | 1 | 0.7464 | 0 | 0 | 0 | 1 | Ref |
| KJ019152 | Unassigned | Unassigned | Unassigned | 676_1 | Clustered | 41 | VC_676_1 | 41 | 0.7464 | 1 | 0.7464 | 0 | 0 | 0 | 1 | Ref |
| KJ019155 | Unassigned | Unassigned | Unassigned | 676_1 | Clustered | 41 | VC_676_1 | 41 | 0.7464 | 1 | 0.7464 | 0 | 0 | 0 | 1 | Ref |
| KJ019156 | Unassigned | Unassigned | Unassigned | 447_1 | Clustered | 9 | VC_447_1 | 9 | 0.1269 | 1 | 0.1269 | 0 | 0 | 0 | 0.8611 | Ref |
| KJ019159 | Unassigned | Unassigned | Unassigned | 676_1 | Clustered | 41 | VC_676_1 | 41 | 0.7464 | 1 | 0.7464 | 0 | 0 | 0 | 1 | Ref |
| KJ019160 | Unassigned | Unassigned | Unassigned | 675_1 | Clustered | 46 | VC_675_1 | 46 | 0.8493 | 1 | 0.8493 | 0 | 0 | 0 | 1 | Ref |
| KJ019162 | Unassigned | Unassigned | Unassigned | 675_1 | Clustered | 46 | VC_675_1 | 46 | 0.8493 | 1 | 0.8493 | 0 | 0 | 0 | 1 | Ref |
| KJ019164 | Unassigned | Unassigned | Unassigned | 675_1 | Clustered | 46 | VC_675_1 | 46 | 0.8493 | 1 | 0.8493 | 0 | 0 | 0 | 1 | Ref |

| | | | | | | | | | | | | | | | | |
|---|---|---|---|---|---|---|---|---|---|---|---|---|---|---|---|---|
| KJ019165 | Unassigned | Unassigned | Unassigned | 675_1 | Clustered | 46 | VC_675_1 | 46 | 0.8493 | 1 | 0.8493 | 0 | 0 | 0 | 1 | Ref |
| KJ021043 | Unassigned | Unassigned | Unassigned | 125_0 | Clustered | 23 | VC_125_0 | 23 | 0.8163 | 1 | 0.8163 | 0 | 0 | 0 | 1 | Ref |
| KJ025956 | Unassigned | Unassigned | Unassigned | 538_38 | Clustered | 6 | VC_538_38 | 6 | 0.0517 | 1 | 0.0517 | 0 | 0 | 0 | 0.8667 | Ref |
| KJ025957 | Unassigned | Unassigned | Unassigned | 291_0 | Clustered | 3 | VC_291_0 | 3 | 0.0482 | 0.99860971 | 0.0482 | 0 | 0 | 0 | 1 | Ref |
| KJ028219 | Unassigned | Unassigned | Unassigned | 833_0 | Clustered | 2 | VC_833_0 | 2 | 0.1786 | 0.96299906 | 0.172 | 0 | 0 | 0 | 1 | Ref |
| KJ081346 | Unassigned | Unassigned | Unassigned | 195_2 | Clustered | 2 | VC_195_2 | 2 | 0.0208 | 0.93079499 | 0.0193 | 0 | 0 | 0 | 1 | Ref |
| KJ094020 | Unassigned | Unassigned | Unassigned | 1002_0 | Clustered | 10 | VC_1002_0 | 10 | 0.9987 | 1 | 0.9987 | 0 | 0 | 0 | 1 | Ref |
| KJ094021 | Unassigned | Unassigned | Unassigned | 1002_0 | Clustered | 10 | VC_1002_0 | 10 | 0.9987 | 1 | 0.9987 | 0 | 0 | 0 | 1 | Ref |
| KJ094022 | Unassigned | Unassigned | Unassigned | 392_0 | Clustered | 9 | VC_392_0 | 9 | 0.7527 | 1 | 0.7527 | 0 | 0 | 0 | 1 | Ref |
| KJ094023 | Unassigned | Unassigned | Unassigned | 393_0 | Clustered | 5 | VC_393_0 | 5 | 0.4703 | 0.99999992 | 0.4703 | 0 | 0 | 0 | 0.9 | Ref |
| KJ094029 | Unassigned | Unassigned | Unassigned | 753_0 | Clustered | 26 | VC_753_0 | 26 | 0.9562 | 1 | 0.9562 | 0 | 0 | 0 | 1 | Ref |
| KJ094030 | Unassigned | Unassigned | Unassigned | 753_0 | Clustered | 26 | VC_753_0 | 26 | 0.9562 | 1 | 0.9562 | 0 | 0 | 0 | 1 | Ref |
| KJ094031 | Unassigned | Unassigned | Unassigned | 753_0 | Clustered | 26 | VC_753_0 | 26 | 0.9562 | 1 | 0.9562 | 0 | 0 | 0 | 1 | Ref |
| KJ094032 | Unassigned | Unassigned | Unassigned | 110_0 | Clustered | 33 | VC_110_0 | 33 | 0.9488 | 1 | 0.9488 | 0 | 0 | 0 | 1 | Ref |
| KJ094033 | Unassigned | Unassigned | Unassigned | 753_0 | Clustered | 26 | VC_753_0 | 26 | 0.9562 | 1 | 0.9562 | 0 | 0 | 0 | 1 | Ref |
| KJ127303 | Unassigned | Unassigned | Unassigned | 110_0 | Clustered | 33 | VC_110_0 | 33 | 0.9488 | 1 | 0.9488 | 0 | 0 | 0 | 1 | Ref |
| KJ127304 | Unassigned | Unassigned | Unassigned | 711_0 | Clustered | 48 | VC_711_0 | 48 | 0.8217 | 1 | 0.8217 | 0 | 0 | 0 | 1 | Ref |
| KJ133688 | Unassigned | Unassigned | Unassigned | 908_0 | Clustered | 36 | VC_908_0 | 36 | 0.9943 | 1 | 0.9943 | 0 | 0 | 0 | 1 | Ref |
| KJ133689 | Unassigned | Unassigned | Unassigned | 908_0 | Clustered | 36 | VC_908_0 | 36 | 0.9943 | 1 | 0.9943 | 0 | 0 | 0 | 1 | Ref |
| KJ133690 | Unassigned | Unassigned | Unassigned | 908_0 | Clustered | 36 | VC_908_0 | 36 | 0.9943 | 1 | 0.9943 | 0 | 0 | 0 | 1 | Ref |
| KJ133691 | Unassigned | Unassigned | Unassigned | 908_0 | Clustered | 36 | VC_908_0 | 36 | 0.9943 | 1 | 0.9943 | 0 | 0 | 0 | 1 | Ref |
| KJ133692 | Unassigned | Unassigned | Unassigned | 908_0 | Clustered | 36 | VC_908_0 | 36 | 0.9943 | 1 | 0.9943 | 0 | 0 | 0 | 1 | Ref |
| KJ133693 | Unassigned | Unassigned | Unassigned | 908_0 | Clustered | 36 | VC_908_0 | 36 | 0.9943 | 1 | 0.9943 | 0 | 0 | 0 | 1 | Ref |
| KJ133694 | Unassigned | Unassigned | Unassigned | 908_0 | Clustered | 36 | VC_908_0 | 36 | 0.9943 | 1 | 0.9943 | 0 | 0 | 0 | 1 | Ref |
| KJ133695 | Unassigned | Unassigned | Unassigned | 908_0 | Clustered | 36 | VC_908_0 | 36 | 0.9943 | 1 | 0.9943 | 0 | 0 | 0 | 1 | Ref |
| KJ133696 | Unassigned | Unassigned | Unassigned | 908_0 | Clustered | 36 | VC_908_0 | 36 | 0.9943 | 1 | 0.9943 | 0 | 0 | 0 | 1 | Ref |
| KJ133697 | Unassigned | Unassigned | Unassigned | 908_0 | Clustered | 36 | VC_908_0 | 36 | 0.9943 | 1 | 0.9943 | 0 | 0 | 0 | 1 | Ref |
| KJ133698 | Unassigned | Unassigned | Unassigned | 908_0 | Clustered | 36 | VC_908_0 | 36 | 0.9943 | 1 | 0.9943 | 0 | 0 | 0 | 1 | Ref |
| KJ133699 | Unassigned | Unassigned | Unassigned | 908_0 | Clustered | 36 | VC_908_0 | 36 | 0.9943 | 1 | 0.9943 | 0 | 0 | 0 | 1 | Ref |
| KJ133700 | Unassigned | Unassigned | Unassigned | 908_0 | Clustered | 36 | VC_908_0 | 36 | 0.9943 | 1 | 0.9943 | 0 | 0 | 0 | 1 | Ref |
| KJ133701 | Unassigned | Unassigned | Unassigned | 908_0 | Clustered | 36 | VC_908_0 | 36 | 0.9943 | 1 | 0.9943 | 0 | 0 | 0 | 1 | Ref |
| KJ133702 | Unassigned | Unassigned | Unassigned | 908_0 | Clustered | 36 | VC_908_0 | 36 | 0.9943 | 1 | 0.9943 | 0 | 0 | 0 | 1 | Ref |
| KJ133703 | Unassigned | Unassigned | Unassigned | 908_0 | Clustered | 36 | VC_908_0 | 36 | 0.9943 | 1 | 0.9943 | 0 | 0 | 0 | 1 | Ref |
| KJ133704 | Unassigned | Unassigned | Unassigned | 908_0 | Clustered | 36 | VC_908_0 | 36 | 0.9943 | 1 | 0.9943 | 0 | 0 | 0 | 1 | Ref |
| KJ133705 | Unassigned | Unassigned | Unassigned | 908_0 | Clustered | 36 | VC_908_0 | 36 | 0.9943 | 1 | 0.9943 | 0 | 0 | 0 | 1 | Ref |
| KJ133706 | Unassigned | Unassigned | Unassigned | 908_0 | Clustered | 36 | VC_908_0 | 36 | 0.9943 | 1 | 0.9943 | 0 | 0 | 0 | 1 | Ref |
| KJ133707 | Unassigned | Unassigned | Unassigned | 908_0 | Clustered | 36 | VC_908_0 | 36 | 0.9943 | 1 | 0.9943 | 0 | 0 | 0 | 1 | Ref |
| KJ135004 | Unassigned | Unassigned | Unassigned | 801_0 | Clustered | 37 | VC_801_0 | 37 | 0.5672 | 1 | 0.5672 | 0 | 0 | 0 | 1 | Ref |
| KJ140076 | Unassigned | Unassigned | Unassigned | 12_0 | Clustered | 21 | VC_12_0 | 21 | 0.2262 | 1 | 0.2262 | 0 | 0 | 0 | 0.9571 | Ref |
| KJ156985 | Unassigned | Unassigned | Unassigned | 533_0 | Clustered | 16 | VC_533_0 | 16 | 0.3765 | 1 | 0.3765 | 0 | 0 | 0 | 1 | Ref |
| KJ159566 | Unassigned | Unassigned | Unassigned | 104_0 | Clustered | 2 | VC_104_0 | 2 | 0.1066 | 0.94642368 | 0.1008 | 0 | 0 | 0 | 1 | Ref |
| KJ173786 | Unassigned | Unassigned | Unassigned | 881_1 | Clustered | 13 | VC_881_1 | 13 | 0.5859 | 1 | 0.5859 | 0 | 0 | 0 | 1 | Ref |
| KJ174156 | Unassigned | Unassigned | Unassigned | 335_15 | Clustered/Singleton | 1 | VC_335_15 | 1 | 0 | 1 | 0 | 0 | 0 | 0 | 1 | Ref |
| KJ174157 | Unassigned | Unassigned | Unassigned | 384_3 | Clustered | 22 | VC_384_3 | 22 | 0.0786 | 1 | 0.0786 | 0 | 0 | 0 | 0.9048 | Ref |
| KJ174317 | Unassigned | Unassigned | Unassigned | 135_22 | Clustered | 13 | VC_135_22 | 13 | 0.0795 | 1 | 0.0795 | 0 | 0 | 0 | 0.8205 | Ref |
| KJ174318 | Unassigned | Unassigned | Unassigned | 135_16 | Clustered/Singleton | 1 | VC_135_16 | 1 | 0 | 1 | 0 | 0 | 0 | 0 | 1 | Ref |
| KJ183191 | Unassigned | Unassigned | Unassigned | 500_0 | Clustered | 3 | VC_500_0 | 3 | 0.3143 | 0.99843638 | 0.3138 | 0 | 0 | 0 | 1 | Ref |
| KJ183192 | Unassigned | Unassigned | Unassigned | 500_0 | Clustered | 3 | VC_500_0 | 3 | 0.3143 | 0.99843638 | 0.3138 | 0 | 0 | 0 | 1 | Ref |
| KJ183193 | Unassigned | Unassigned | Unassigned | 500_0 | Clustered | 3 | VC_500_0 | 3 | 0.3143 | 0.99843638 | 0.3138 | 0 | 0 | 0 | 1 | Ref |
| KJ190157 | Unassigned | Unassigned | Unassigned | 198_15 | Clustered | 3 | VC_198_15 | 3 | 0.0144 | 0.99754982 | 0.0144 | 0 | 0 | 0 | 0.6667 | Ref |
| KJ190158 | Unassigned | Unassigned | Unassigned | 794_16 | Clustered | 10 | VC_794_16 | 10 | 0.0563 | 0.9411319 | 0.053 | 0 | 0 | 0 | 0.8667 | Ref |
| KJ194579 | Unassigned | Unassigned | Unassigned | 384_18 | Clustered | 10 | VC_384_18 | 10 | 0.0301 | 1 | 0.0301 | 0 | 0 | 0 | 0.8 | Ref |
| KJ194580 | Unassigned | Unassigned | Unassigned | 384_16 | Clustered | 42 | VC_384_16 | 42 | 0.134 | 1 | 0.134 | 0 | 0 | 0 | 0.7282 | Ref |
| KJ194581 | Unassigned | Unassigned | Unassigned | 527_0 | Clustered | 37 | VC_527_0 | 37 | 0.9679 | 1 | 0.9679 | 0 | 0 | 0 | 1 | Ref |
| KJ194582 | Unassigned | Unassigned | Unassigned | 585_0 | Clustered | 20 | VC_585_0 | 20 | 0.8376 | 1 | 0.8376 | 0 | 0 | 0 | 1 | Ref |
| KJ194583 | Unassigned | Unassigned | Unassigned | 384_16 | Clustered | 42 | VC_384_16 | 42 | 0.134 | 1 | 0.134 | 0 | 0 | 0 | 0.7282 | Ref |
| KJ194584 | Unassigned | Unassigned | Unassigned | 527_0 | Clustered | 37 | VC_527_0 | 37 | 0.9679 | 1 | 0.9679 | 0 | 0 | 0 | 1 | Ref |
| KJ194585 | Unassigned | Unassigned | Unassigned | 335_5 | Clustered | 11 | VC_335_5 | 11 | 0.0469 | 1 | 0.0469 | 0 | 0 | 0 | 0.7636 | Ref |
| KJ206559 | Unassigned | Unassigned | Unassigned | 155_8 | Clustered | 66 | VC_155_8 | 66 | 0.4537 | 1 | 0.4537 | 0 | 0 | 0 | 0.8182 | Ref |
| KJ206560 | Unassigned | Unassigned | Unassigned | 155_8 | Clustered | 66 | VC_155_8 | 66 | 0.4537 | 1 | 0.4537 | 0 | 0 | 0 | 0.8182 | Ref |
| KJ206561 | Unassigned | Unassigned | Unassigned | 155_8 | Clustered | 66 | VC_155_8 | 66 | 0.4537 | 1 | 0.4537 | 0 | 0 | 0 | 0.8182 | Ref |
| KJ206562 | Unassigned | Unassigned | Unassigned | 155_8 | Clustered | 66 | VC_155_8 | 66 | 0.4537 | 1 | 0.4537 | 0 | 0 | 0 | 0.8182 | Ref |
| KJ206563 | Unassigned | Unassigned | Unassigned | 155_8 | Clustered | 66 | VC_155_8 | 66 | 0.4537 | 1 | 0.4537 | 0 | 0 | 0 | 0.8182 | Ref |
| KJ210330 | Unassigned | Unassigned | Unassigned | 98_0 | Clustered | 30 | VC_98_0 | 30 | 0.7175 | 1 | 0.7175 | 0 | 0 | 0 | 1 | Ref |
| KJ409696 | Unassigned | Unassigned | Unassigned | 335_45 | Clustered | 4 | VC_335_45 | 4 | 0.0143 | 0.99998616 | 0.0143 | 0 | 0 | 0 | 0.8333 | Ref |
| KJ409772 | Unassigned | Unassigned | Unassigned | 79_3 | Clustered | 11 | VC_79_3 | 11 | 0.2561 | 1 | 0.2561 | 0 | 0 | 0 | 1 | Ref |
| KJ410132 | Unassigned | Unassigned | Unassigned | 223_6 | Clustered | 42 | VC_223_6 | 42 | 0.4717 | 1 | 0.4717 | 0 | 0 | 0 | 0.8351 | Ref |
| KJ410133 | Unassigned | Unassigned | Unassigned | 784_0 | Clustered | 9 | VC_784_0 | 9 | 0.0884 | 1 | 0.0884 | 0 | 0 | 0 | 1 | Ref |
| KJ410134 | Unassigned | Unassigned | Unassigned | 223_6 | Clustered | 42 | VC_223_6 | 42 | 0.4717 | 1 | 0.4717 | 0 | 0 | 0 | 0.8351 | Ref |
| KJ410740 | Unassigned | Unassigned | Unassigned | 960_0 | Clustered | 2 | VC_960_0 | 2 | 0.086 | 0.75688284 | 0.0651 | 0 | 0 | 0 | 1 | Ref |
| KJ417497 | Unassigned | Unassigned | Unassigned | 427_0 | Clustered | 37 | VC_427_0 | 37 | 0.5729 | 1 | 0.5729 | 0 | 0 | 0 | 0.9474 | Ref |
| KJ419279 | Unassigned | Unassigned | Unassigned | 737_0 | Clustered | 13 | VC_737_0 | 13 | 0.5083 | 1 | 0.5083 | 0 | 0 | 0 | 1 | Ref |
| KJ433973 | Unassigned | Unassigned | Unassigned | 385_3 | Clustered | 5 | VC_385_3 | 5 | 0.1118 | 0.99999997 | 0.1118 | 0 | 0 | 0 | 1 | Ref |
| KJ433974 | Unassigned | Unassigned | Unassigned | 385_3 | Clustered | 5 | VC_385_3 | 5 | 0.1118 | 0.99999997 | 0.1118 | 0 | 0 | 0 | 1 | Ref |
| KJ433975 | Unassigned | Unassigned | Unassigned | 385_3 | Clustered | 5 | VC_385_3 | 5 | 0.1118 | 0.99999997 | 0.1118 | 0 | 0 | 0 | 1 | Ref |
| KJ433976 | Unassigned | Unassigned | Unassigned | 385_3 | Clustered | 5 | VC_385_3 | 5 | 0.1118 | 0.99999997 | 0.1118 | 0 | 0 | 0 | 1 | Ref |
| KJ451625 | Unassigned | Unassigned | Unassigned | 195_3 | Clustered | 9 | VC_195_3 | 9 | 0.1955 | 1 | 0.1955 | 0 | 0 | 0 | 0.8611 | Ref |
| KJ452291 | Unassigned | Unassigned | Unassigned | 12_0 | Clustered | 21 | VC_12_0 | 21 | 0.2262 | 1 | 0.2262 | 0 | 0 | 0 | 0.9571 | Ref |
| KJ452292 | Unassigned | Unassigned | Unassigned | 10_0 | Clustered | 25 | VC_10_0 | 25 | 0.3437 | 1 | 0.3437 | 0 | 0 | 0 | 0.9733 | Ref |
| KJ473422 | Unassigned | Unassigned | Unassigned | 715_1 | Clustered/Singleton | 1 | VC_715_1 | 1 | 0 | 1 | 0 | 0 | 0 | 0 | 1 | Ref |
| KJ477077 | Unassigned | Unassigned | Unassigned | 185_0 | Clustered | 53 | VC_185_0 | 53 | 0.9153 | 1 | 0.9153 | 0 | 0 | 0 | 1 | Ref |

| | | | | | | | | | | | | | | | | |
|---|---|---|---|---|---|---|---|---|---|---|---|---|---|---|---|---|
| KJ477684 | Unassigned | Unassigned | Unassigned | 290_91 | Clustered | 11 | VC_290_91 | 11 | 0.0281 | 1 | 0.0281 | 0 | 0 | 0 | 1 | Ref |
| KJ477685 | Unassigned | Unassigned | Unassigned | 290_91 | Clustered | 11 | VC_290_91 | 11 | 0.0281 | 1 | 0.0281 | 0 | 0 | 0 | 1 | Ref |
| KJ477686 | Unassigned | Unassigned | Unassigned | 290_91 | Clustered | 11 | VC_290_91 | 11 | 0.0281 | 1 | 0.0281 | 0 | 0 | 0 | 1 | Ref |
| KJ489010 | Unassigned | Unassigned | Unassigned | 823_0 | Clustered | 4 | VC_823_0 | 4 | 0.5645 | 0.99998533 | 0.5645 | 0 | 0 | 0 | 1 | Ref |
| KJ489011 | Unassigned | Unassigned | Unassigned | 823_0 | Clustered | 4 | VC_823_0 | 4 | 0.5645 | 0.99998533 | 0.5645 | 0 | 0 | 0 | 1 | Ref |
| KJ489012 | Unassigned | Unassigned | Unassigned | 823_0 | Clustered | 4 | VC_823_0 | 4 | 0.5645 | 0.99998533 | 0.5645 | 0 | 0 | 0 | 1 | Ref |
| KJ489013 | Unassigned | Unassigned | Unassigned | 823_0 | Clustered | 4 | VC_823_0 | 4 | 0.5645 | 0.99998533 | 0.5645 | 0 | 0 | 0 | 1 | Ref |
| KJ489397 | Unassigned | Unassigned | Unassigned | 912_0 | Clustered | 10 | VC_912_0 | 10 | 0.1994 | 1 | 0.1994 | 0 | 0 | 0 | 1 | Ref |
| KJ489398 | Unassigned | Unassigned | Unassigned | 912_0 | Clustered | 10 | VC_912_0 | 10 | 0.1994 | 1 | 0.1994 | 0 | 0 | 0 | 1 | Ref |
| KJ489399 | Unassigned | Unassigned | Unassigned | 913_0 | Clustered | 28 | VC_913_0 | 28 | 0.6206 | 1 | 0.6206 | 0 | 0 | 0 | 0.9841 | Ref |
| KJ489400 | Unassigned | Unassigned | Unassigned | 912_0 | Clustered | 10 | VC_912_0 | 10 | 0.1994 | 1 | 0.1994 | 0 | 0 | 0 | 1 | Ref |
| KJ489401 | Unassigned | Unassigned | Unassigned | 913_0 | Clustered | 28 | VC_913_0 | 28 | 0.6206 | 1 | 0.6206 | 0 | 0 | 0 | 0.9841 | Ref |
| KJ489402 | Unassigned | Unassigned | Unassigned | 912_1 | Clustered | 12 | VC_912_1 | 12 | 0.2423 | 1 | 0.2423 | 0 | 0 | 0 | 1 | Ref |
| KJ502657 | Unassigned | Unassigned | Unassigned | 1067_0 | Clustered | 20 | VC_1067_0 | 20 | 0.7109 | 1 | 0.7109 | 0 | 0 | 0 | 1 | Ref |
| KJ507099 | Unassigned | Unassigned | Unassigned | 131_0 | Clustered | 43 | VC_131_0 | 43 | 0.8853 | 1 | 0.8853 | 0 | 0 | 0 | 1 | Ref |
| KJ507100 | Unassigned | Unassigned | Unassigned | 712_0 | Clustered | 5 | VC_712_0 | 5 | 0.828 | 0.99999971 | 0.828 | 0 | 0 | 0 | 1 | Ref |
| KJ510413 | Unassigned | Unassigned | Unassigned | 1087_0 | Clustered | 7 | VC_1087_0 | 7 | 0.94 | 1 | 0.94 | 0 | 0 | 0 | 1 | Ref |
| KJ510414 | Unassigned | Unassigned | Unassigned | 535_1 | Clustered | 108 | VC_535_1 | 108 | 0.8479 | 1 | 0.8479 | 0 | 0 | 0 | 0.9955 | Ref |
| KJ510415 | Unassigned | Unassigned | Unassigned | 531_0 | Clustered | 32 | VC_531_0 | 32 | 0.6859 | 1 | 0.6859 | 0 | 0 | 0 | 1 | Ref |
| KJ528544 | Unassigned | Unassigned | Unassigned | 99_0 | Clustered | 5 | VC_99_0 | 5 | 0.0694 | 0.98113835 | 0.0681 | 0 | 0 | 0 | 1 | Ref |
| KJ535721 | Unassigned | Unassigned | Unassigned | 753_0 | Clustered | 26 | VC_753_0 | 26 | 0.9562 | 1 | 0.9562 | 0 | 0 | 0 | 1 | Ref |
| KJ535722 | Unassigned | Unassigned | Unassigned | 753_0 | Clustered | 26 | VC_753_0 | 26 | 0.9562 | 1 | 0.9562 | 0 | 0 | 0 | 1 | Ref |
| KJ538721 | Unassigned | Unassigned | Unassigned | 973_0 | Clustered | 15 | VC_973_0 | 15 | 1 | 0 | 0 | 0 | 0 | 0 | 1 | Ref |
| KJ538722 | Unassigned | Unassigned | Unassigned | 834_0 | Clustered | 18 | VC_834_0 | 18 | 0.9116 | 1 | 0.9116 | 0 | 0 | 0 | 1 | Ref |
| KJ538723 | Unassigned | Unassigned | Unassigned | 384_3 | Clustered | 22 | VC_384_3 | 22 | 0.0786 | 1 | 0.0786 | 0 | 0 | 0 | 0.9048 | Ref |
| KJ564036 | Unassigned | Unassigned | Unassigned | 657_0 | Clustered | 7 | VC_657_0 | 7 | 0.8495 | 1 | 0.8495 | 0 | 0 | 0 | 1 | Ref |
| KJ564037 | Unassigned | Unassigned | Unassigned | 657_0 | Clustered | 7 | VC_657_0 | 7 | 0.8495 | 1 | 0.8495 | 0 | 0 | 0 | 1 | Ref |
| KJ564038 | Unassigned | Unassigned | Unassigned | 657_0 | Clustered | 7 | VC_657_0 | 7 | 0.8495 | 1 | 0.8495 | 0 | 0 | 0 | 1 | Ref |
| KJ567042 | Unassigned | Unassigned | Unassigned | 245_0 | Clustered | 17 | VC_245_0 | 17 | 0.6081 | 1 | 0.6081 | 0 | 0 | 0 | 1 | Ref |
| KJ567043 | Unassigned | Unassigned | Unassigned | 583_0 | Clustered | 2 | VC_583_0 | 2 | 0.0713 | 0.956426 | 0.0682 | 0 | 0 | 0 | 1 | Ref |
| KJ567044 | Unassigned | Unassigned | Unassigned | 384_2 | Clustered | 8 | VC_384_2 | 8 | 0.0232 | 1 | 0.0232 | 0 | 0 | 0 | 0.8571 | Ref |
| KJ567045 | Unassigned | Unassigned | Unassigned | 243_0 | Clustered | 72 | VC_243_0 | 72 | 0.8089 | 1 | 0.8089 | 0 | 0 | 0 | 1 | Ref |
| KJ567046 | Unassigned | Unassigned | Unassigned | 587_0 | Clustered | 8 | VC_587_0 | 8 | 0.4907 | 1 | 0.4907 | 0 | 0 | 0 | 1 | Ref |
| KJ572844 | Unassigned | Unassigned | Unassigned | 123_0 | Clustered | 4 | VC_123_0 | 4 | 0.1802 | 0.99998531 | 0.1802 | 0 | 0 | 0 | 1 | Ref |
| KJ578758 | Unassigned | Unassigned | Unassigned | 787_0 | Clustered | 132 | VC_787_0 | 132 | 1 | 1 | 1 | 0 | 0 | 0 | 1 | Ref |
| KJ578759 | Unassigned | Unassigned | Unassigned | 787_0 | Clustered | 132 | VC_787_0 | 132 | 1 | 1 | 1 | 0 | 0 | 0 | 1 | Ref |
| KJ578760 | Unassigned | Unassigned | Unassigned | 787_0 | Clustered | 132 | VC_787_0 | 132 | 1 | 1 | 1 | 0 | 0 | 0 | 1 | Ref |
| KJ578761 | Unassigned | Unassigned | Unassigned | 787_0 | Clustered | 132 | VC_787_0 | 132 | 1 | 1 | 1 | 0 | 0 | 0 | 1 | Ref |
| KJ578762 | Unassigned | Unassigned | Unassigned | 787_0 | Clustered | 132 | VC_787_0 | 132 | 1 | 1 | 1 | 0 | 0 | 0 | 1 | Ref |
| KJ578763 | Unassigned | Unassigned | Unassigned | 787_0 | Clustered | 132 | VC_787_0 | 132 | 1 | 1 | 1 | 0 | 0 | 0 | 1 | Ref |
| KJ578764 | Unassigned | Unassigned | Unassigned | 787_0 | Clustered | 132 | VC_787_0 | 132 | 1 | 1 | 1 | 0 | 0 | 0 | 1 | Ref |
| KJ578765 | Unassigned | Unassigned | Unassigned | 787_0 | Clustered | 132 | VC_787_0 | 132 | 1 | 1 | 1 | 0 | 0 | 0 | 1 | Ref |
| KJ578766 | Unassigned | Unassigned | Unassigned | 787_0 | Clustered | 132 | VC_787_0 | 132 | 1 | 1 | 1 | 0 | 0 | 0 | 1 | Ref |
| KJ578767 | Unassigned | Unassigned | Unassigned | 787_0 | Clustered | 132 | VC_787_0 | 132 | 1 | 1 | 1 | 0 | 0 | 0 | 1 | Ref |
| KJ578768 | Unassigned | Unassigned | Unassigned | 787_0 | Clustered | 132 | VC_787_0 | 132 | 1 | 1 | 1 | 0 | 0 | 0 | 1 | Ref |
| KJ578769 | Unassigned | Unassigned | Unassigned | 787_0 | Clustered | 132 | VC_787_0 | 132 | 1 | 1 | 1 | 0 | 0 | 0 | 1 | Ref |
| KJ578770 | Unassigned | Unassigned | Unassigned | 787_0 | Clustered | 132 | VC_787_0 | 132 | 1 | 1 | 1 | 0 | 0 | 0 | 1 | Ref |
| KJ578771 | Unassigned | Unassigned | Unassigned | 787_0 | Clustered | 132 | VC_787_0 | 132 | 1 | 1 | 1 | 0 | 0 | 0 | 1 | Ref |
| KJ578772 | Unassigned | Unassigned | Unassigned | 787_0 | Clustered | 132 | VC_787_0 | 132 | 1 | 1 | 1 | 0 | 0 | 0 | 1 | Ref |
| KJ578773 | Unassigned | Unassigned | Unassigned | 787_0 | Clustered | 132 | VC_787_0 | 132 | 1 | 1 | 1 | 0 | 0 | 0 | 1 | Ref |
| KJ578774 | Unassigned | Unassigned | Unassigned | 787_0 | Clustered | 132 | VC_787_0 | 132 | 1 | 1 | 1 | 0 | 0 | 0 | 1 | Ref |
| KJ578775 | Unassigned | Unassigned | Unassigned | 787_0 | Clustered | 132 | VC_787_0 | 132 | 1 | 1 | 1 | 0 | 0 | 0 | 1 | Ref |
| KJ578776 | Unassigned | Unassigned | Unassigned | 787_0 | Clustered | 132 | VC_787_0 | 132 | 1 | 1 | 1 | 0 | 0 | 0 | 1 | Ref |
| KJ578777 | Unassigned | Unassigned | Unassigned | 787_0 | Clustered | 132 | VC_787_0 | 132 | 1 | 1 | 1 | 0 | 0 | 0 | 1 | Ref |
| KJ578778 | Unassigned | Unassigned | Unassigned | 787_0 | Clustered | 132 | VC_787_0 | 132 | 1 | 1 | 1 | 0 | 0 | 0 | 1 | Ref |
| KJ578779 | Unassigned | Unassigned | Unassigned | 787_0 | Clustered | 132 | VC_787_0 | 132 | 1 | 1 | 1 | 0 | 0 | 0 | 1 | Ref |
| KJ578780 | Unassigned | Unassigned | Unassigned | 787_0 | Clustered | 132 | VC_787_0 | 132 | 1 | 1 | 1 | 0 | 0 | 0 | 1 | Ref |
| KJ578781 | Unassigned | Unassigned | Unassigned | 787_0 | Clustered | 132 | VC_787_0 | 132 | 1 | 1 | 1 | 0 | 0 | 0 | 1 | Ref |
| KJ578782 | Unassigned | Unassigned | Unassigned | 787_0 | Clustered | 132 | VC_787_0 | 132 | 1 | 1 | 1 | 0 | 0 | 0 | 1 | Ref |
| KJ578783 | Unassigned | Unassigned | Unassigned | 787_0 | Clustered | 132 | VC_787_0 | 132 | 1 | 1 | 1 | 0 | 0 | 0 | 1 | Ref |
| KJ578784 | Unassigned | Unassigned | Unassigned | 787_0 | Clustered | 132 | VC_787_0 | 132 | 1 | 1 | 1 | 0 | 0 | 0 | 1 | Ref |
| KJ578785 | Unassigned | Unassigned | Unassigned | 787_0 | Clustered | 132 | VC_787_0 | 132 | 1 | 1 | 1 | 0 | 0 | 0 | 1 | Ref |
| KJ578786 | Unassigned | Unassigned | Unassigned | 787_0 | Clustered | 132 | VC_787_0 | 132 | 1 | 1 | 1 | 0 | 0 | 0 | 1 | Ref |
| KJ578787 | Unassigned | Unassigned | Unassigned | 787_0 | Clustered | 132 | VC_787_0 | 132 | 1 | 1 | 1 | 0 | 0 | 0 | 1 | Ref |
| KJ578788 | Unassigned | Unassigned | Unassigned | 787_0 | Clustered | 132 | VC_787_0 | 132 | 1 | 1 | 1 | 0 | 0 | 0 | 1 | Ref |
| KJ578789 | Unassigned | Unassigned | Unassigned | 787_0 | Clustered | 132 | VC_787_0 | 132 | 1 | 1 | 1 | 0 | 0 | 0 | 1 | Ref |
| KJ578790 | Unassigned | Unassigned | Unassigned | 787_0 | Clustered | 132 | VC_787_0 | 132 | 1 | 1 | 1 | 0 | 0 | 0 | 1 | Ref |
| KJ578791 | Unassigned | Unassigned | Unassigned | 787_0 | Clustered | 132 | VC_787_0 | 132 | 1 | 1 | 1 | 0 | 0 | 0 | 1 | Ref |
| KJ578792 | Unassigned | Unassigned | Unassigned | 787_0 | Clustered | 132 | VC_787_0 | 132 | 1 | 1 | 1 | 0 | 0 | 0 | 1 | Ref |
| KJ586794 | Unassigned | Unassigned | Unassigned | 753_0 | Clustered | 26 | VC_753_0 | 26 | 0.9562 | 1 | 0.9562 | 0 | 0 | 0 | 1 | Ref |
| KJ586795 | Unassigned | Unassigned | Unassigned | 753_0 | Clustered | 26 | VC_753_0 | 26 | 0.9562 | 1 | 0.9562 | 0 | 0 | 0 | 1 | Ref |
| KJ591604 | Unassigned | Unassigned | Unassigned | 753_0 | Clustered | 26 | VC_753_0 | 26 | 0.9562 | 1 | 0.9562 | 0 | 0 | 0 | 1 | Ref |
| KJ591605 | Unassigned | Unassigned | Unassigned | 753_0 | Clustered | 26 | VC_753_0 | 26 | 0.9562 | 1 | 0.9562 | 0 | 0 | 0 | 1 | Ref |
| KJ595575 | Unassigned | Unassigned | Unassigned | 579_50 | Clustered | 15 | VC_579_50 | 15 | 0.0467 | 1 | 0.0467 | 0 | 0 | 0 | 0.9238 | Ref |
| KJ595576 | Unassigned | Unassigned | Unassigned | 384_1 | Clustered | 11 | VC_384_1 | 11 | 0.0378 | 1 | 0.0378 | 0 | 0 | 0 | 0.8545 | Ref |
| KJ596420 | Unassigned | Unassigned | Unassigned | 10_0 | Clustered | 25 | VC_10_0 | 25 | 0.3437 | 1 | 0.3437 | 0 | 0 | 0 | 0.9733 | Ref |
| KJ603229 | Unassigned | Unassigned | Unassigned | 48_0 | Clustered | 2 | VC_48_0 | 2 | 0.018 | 0.95763863 | 0.0172 | 0 | 0 | 0 | 1 | Ref |
| KJ608189 | Unassigned | Unassigned | Unassigned | 336_0 | Clustered/Singleton | 1 | VC_336_0 | 1 | 0 | 1 | 0 | 0 | 0 | 0 | 1 | Ref |
| KJ617393 | Unassigned | Unassigned | Unassigned | 1089_0 | Clustered | 3 | VC_1089_0 | 3 | 1 | 1 | 1 | 0 | 0 | 0 | 1 | Ref |
| KJ619459 | Unassigned | Unassigned | Unassigned | 904_0 | Clustered | 10 | VC_904_0 | 10 | 0.9878 | 0.99948433 | 0.9873 | 0 | 0 | 0 | 1 | Ref |
| KJ621082 | Unassigned | Unassigned | Unassigned | 857_0 | Clustered | 16 | VC_857_0 | 16 | 0.9317 | 1 | 0.9317 | 0 | 0 | 0 | 1 | Ref |

| | | | | | | | | | | | | | | | |
|---|---|---|---|---|---|---|---|---|---|---|---|---|---|---|---|
| KJ628499 | Unassigned | Unassigned | Unassigned | 715_0 | Clustered | 10 | VC_715_0 | 10 | 0.8045 | 1 | 0.8045 | 0 | 0 | 0 | 1 Ref |
| KJ668713 | Unassigned | Unassigned | Unassigned | 469_0 | Clustered | 18 | VC_469_0 | 18 | 0.4583 | 1 | 0.4583 | 0 | 0 | 0 | 1 Ref |
| KJ668714 | Unassigned | Unassigned | Unassigned | 290_99 | Clustered/Singleton | 1 | VC_290_99 | 1 | 0 | 1 | 0 | 0 | 0 | 0 | 1 Ref |
| KJ668715 | Unassigned | Unassigned | Unassigned | 753_0 | Clustered | 26 | VC_753_0 | 26 | 0.9562 | 1 | 0.9562 | 0 | 0 | 0 | 1 Ref |
| KJ676859 | Unassigned | Unassigned | Unassigned | 195_2 | Clustered | 2 | VC_195_2 | 2 | 0.0208 | 0.93079499 | 0.0193 | 0 | 0 | 0 | 1 Ref |
| KJ690250 | Unassigned | Unassigned | Unassigned | 335_53 | Clustered | 2 | VC_335_53 | 2 | 0.0039 | 0.95663826 | 0.0037 | 0 | 0 | 0 | 1 Ref |
| KJ716334 | Unassigned | Unassigned | Unassigned | 9_0 | Clustered | 38 | VC_9_0 | 38 | 0.4859 | 1 | 0.4859 | 0 | 0 | 0 | 1 Ref |
| KJ716335 | Unassigned | Unassigned | Unassigned | 133_0 | Clustered | 17 | VC_133_0 | 17 | 0.2654 | 1 | 0.2654 | 0 | 0 | 0 | 1 Ref |
| KJ722067 | Unassigned | Unassigned | Unassigned | 787_0 | Clustered | 132 | VC_787_0 | 132 | 1 | 1 | 1 | 0 | 0 | 0 | 1 Ref |
| KJ725374 | Unassigned | Unassigned | Unassigned | 783_0 | Clustered | 67 | VC_783_0 | 67 | 0.8945 | 1 | 0.8945 | 0 | 0 | 0 | 1 Ref |
| KJ746502 | Unassigned | Unassigned | Unassigned | 22_0 | Clustered | 4 | VC_22_0 | 4 | 0.562 | 0.91257778 | 0.5129 | 0 | 0 | 0 | 0.6667 Ref |
| KJ748011 | Unassigned | Unassigned | Unassigned | 465_0 | Clustered | 80 | VC_465_0 | 80 | 0.9988 | 1 | 0.9988 | 0 | 0 | 0 | 1 Ref |
| KJ801817 | Unassigned | Unassigned | Unassigned | 93_0 | Clustered | 22 | VC_93_0 | 22 | 0.5285 | 1 | 0.5285 | 0 | 0 | 0 | 1 Ref |
| KJ802832 | Unassigned | Unassigned | Unassigned | 1090_0 | Clustered | 8 | VC_1090_0 | 8 | 0.6377 | 1 | 0.6377 | 0 | 0 | 0 | 1 Ref |
| KJ803031 | Unassigned | Unassigned | Unassigned | 857_0 | Clustered | 16 | VC_857_0 | 16 | 0.9317 | 1 | 0.9317 | 0 | 0 | 0 | 1 Ref |
| KJ804259 | Unassigned | Unassigned | Unassigned | 685_0 | Clustered | 7 | VC_685_0 | 7 | 0.4045 | 1 | 0.4045 | 0 | 0 | 0 | 1 Ref |
| KJ817802 | Unassigned | Unassigned | Unassigned | 713_0 | Clustered | 26 | VC_713_0 | 26 | 0.9358 | 1 | 0.9358 | 0 | 0 | 0 | 1 Ref |
| KJ829260 | Unassigned | Unassigned | Unassigned | 577_0 | Clustered | 21 | VC_577_0 | 21 | 0.9908 | 1 | 0.9908 | 0 | 0 | 0 | 1 Ref |
| KJ832078 | Unassigned | Unassigned | Unassigned | 32_1 | Clustered | 16 | VC_32_1 | 16 | 0.1962 | 1 | 0.1962 | 0 | 0 | 0 | 0.8333 Ref |
| KJ847052 | Unassigned | Unassigned | Unassigned | 958_0 | Clustered | 3 | VC_958_0 | 3 | 0.2752 | 0.95138727 | 0.2618 | 0 | 0 | 0 | 1 Ref |
| KJ858521 | Unassigned | Unassigned | Unassigned | 817_0 | Clustered | 12 | VC_817_0 | 12 | 0.8497 | 1 | 0.8497 | 0 | 0 | 0 | 1 Ref |
| KJ888149 | Unassigned | Unassigned | Unassigned | 155_16 | Clustered/Singleton | 1 | VC_155_16 | 1 | 0 | 1 | 0 | 0 | 0 | 0 | 1 Ref |
| KJ909655 | Unassigned | Unassigned | Unassigned | 35_0 | Clustered | 35 | VC_35_0 | 35 | 0.431 | 1 | 0.431 | 0 | 0 | 0 | 1 Ref |
| KJ936628 | Unassigned | Unassigned | Unassigned | 548_0 | Clustered | 9 | VC_548_0 | 9 | 0.8018 | 1 | 0.8018 | 0 | 0 | 0 | 1 Ref |
| KJ944841 | Unassigned | Unassigned | Unassigned | 241_0 | Clustered | 21 | VC_241_0 | 21 | 0.8342 | 1 | 0.8342 | 0 | 0 | 0 | 1 Ref |
| KJ959591 | Unassigned | Unassigned | Unassigned | 764_0 | Clustered | 23 | VC_764_0 | 23 | 0.8194 | 1 | 0.8194 | 0 | 0 | 0 | 1 Ref |
| KJ959632 | Unassigned | Unassigned | Unassigned | 223_4 | Clustered | 13 | VC_223_4 | 13 | 0.1649 | 1 | 0.1649 | 0 | 0 | 0 | 1 Ref |
| KJ997912 | Unassigned | Unassigned | Unassigned | 310_0 | Clustered | 155 | VC_310_0 | 155 | 0.9979 | 1 | 0.9979 | 0 | 0 | 0 | 1 Ref |
| KM044272 | Unassigned | Unassigned | Unassigned | 837_1 | Clustered | 24 | VC_837_1 | 24 | 0.5328 | 1 | 0.5328 | 0 | 0 | 0 | 1 Ref |
| KM051843 | Unassigned | Unassigned | Unassigned | 87_0 | Clustered | 3 | VC_87_0 | 3 | 0.0416 | 0.99823485 | 0.0415 | 0 | 0 | 0 | 1 Ref |
| KM058087 | Unassigned | Unassigned | Unassigned | 1092_0 | Clustered | 2 | VC_1092_0 | 2 | 1 | 1 | 1 | 0 | 0 | 0 | 1 Ref |
| KM067278 | Unassigned | Unassigned | Unassigned | 434_0 | Clustered | 45 | VC_434_0 | 45 | 0.9479 | 1 | 0.9479 | 0 | 0 | 0 | 1 Ref |
| KM091442 | Unassigned | Unassigned | Unassigned | 210_0 | Clustered | 105 | VC_210_0 | 105 | 0.4076 | 1 | 0.4076 | 0 | 0 | 0 | 0.7766 Ref |
| KM092515 | Unassigned | Unassigned | Unassigned | 340_17 | Clustered | 4 | VC_340_17 | 4 | 0.0161 | 0.99996247 | 0.0161 | 0 | 0 | 0 | 1 Ref |
| KM101117 | Unassigned | Unassigned | Unassigned | 526_0 | Clustered | 32 | VC_526_0 | 32 | 0.9037 | 1 | 0.9037 | 0 | 0 | 0 | 1 Ref |
| KM101118 | Unassigned | Unassigned | Unassigned | 974_0 | Clustered | 34 | VC_974_0 | 34 | 0.7953 | 1 | 0.7953 | 0 | 0 | 0 | 1 Ref |
| KM101119 | Unassigned | Unassigned | Unassigned | 532_0 | Clustered | 75 | VC_532_0 | 75 | 0.9393 | 1 | 0.9393 | 0 | 0 | 0 | 1 Ref |
| KM101120 | Unassigned | Unassigned | Unassigned | 533_0 | Clustered | 16 | VC_533_0 | 16 | 0.3765 | 1 | 0.3765 | 0 | 0 | 0 | 1 Ref |
| KM101122 | Unassigned | Unassigned | Unassigned | 538_49 | Clustered/Singleton | 1 | VC_538_49 | 1 | 0 | 1 | 0 | 0 | 0 | 0 | 1 Ref |
| KM101123 | Unassigned | Unassigned | Unassigned | 566_7 | Clustered | 4 | VC_566_7 | 4 | 0.0409 | 0.99661604 | 0.0408 | 0 | 0 | 0 | 1 Ref |
| KM190144 | Unassigned | Unassigned | Unassigned | 601_2 | Clustered/Singleton | 1 | VC_601_2 | 1 | 0 | 1 | 0 | 0 | 0 | 0 | 1 Ref |
| KM197169 | Unassigned | Unassigned | Unassigned | 223_4 | Clustered | 13 | VC_223_4 | 13 | 0.1649 | 1 | 0.1649 | 0 | 0 | 0 | 1 Ref |
| KM199770 | Unassigned | Unassigned | Unassigned | 1093_3 | Clustered | 5 | VC_1093_3 | 5 | 0.1846 | 0.98656422 | 0.1822 | 0 | 0 | 0 | 1 Ref |
| KM209228 | Unassigned | Unassigned | Unassigned | 133_0 | Clustered | 17 | VC_133_0 | 17 | 0.2654 | 1 | 0.2654 | 0 | 0 | 0 | 1 Ref |
| KM209270 | Unassigned | Unassigned | Unassigned | 133_0 | Clustered | 17 | VC_133_0 | 17 | 0.2654 | 1 | 0.2654 | 0 | 0 | 0 | 1 Ref |
| KM209320 | Unassigned | Unassigned | Unassigned | 133_0 | Clustered | 17 | VC_133_0 | 17 | 0.2654 | 1 | 0.2654 | 0 | 0 | 0 | 1 Ref |
| KM216423 | Unassigned | Unassigned | Unassigned | 155_15 | Clustered | 16 | VC_155_15 | 16 | 0.0779 | 1 | 0.0779 | 0 | 0 | 0 | 0.8083 Ref |
| KM224878 | Unassigned | Unassigned | Unassigned | 771_0 | Clustered | 8 | VC_771_0 | 8 | 0.7491 | 1 | 0.7491 | 0 | 0 | 0 | 1 Ref |
| KM224879 | Unassigned | Unassigned | Unassigned | 771_0 | Clustered | 8 | VC_771_0 | 8 | 0.7491 | 1 | 0.7491 | 0 | 0 | 0 | 1 Ref |
| KM233151 | Unassigned | Unassigned | Unassigned | 859_0 | Clustered | 67 | VC_859_0 | 67 | 0.9622 | 1 | 0.9622 | 0 | 0 | 0 | 1 Ref |
| KM233261 | Unassigned | Unassigned | Unassigned | 938_0 | Clustered | 3 | VC_938_0 | 3 | 0.6623 | 0.99633114 | 0.6599 | 0 | 0 | 0 | 1 Ref |
| KM233454 | Unassigned | Unassigned | Unassigned | 532_0 | Clustered | 75 | VC_532_0 | 75 | 0.9393 | 1 | 0.9393 | 0 | 0 | 0 | 1 Ref |
| KM233455 | Unassigned | Unassigned | Unassigned | 532_0 | Clustered | 75 | VC_532_0 | 75 | 0.9393 | 1 | 0.9393 | 0 | 0 | 0 | 1 Ref |
| KM233689 | Unassigned | Unassigned | Unassigned | 185_0 | Clustered | 53 | VC_185_0 | 53 | 0.9153 | 1 | 0.9153 | 0 | 0 | 0 | 1 Ref |
| KM236237 | Unassigned | Unassigned | Unassigned | 687_1 | Clustered | 10 | VC_687_1 | 10 | 0.1263 | 1 | 0.1263 | 0 | 0 | 0 | 0.9778 Ref |
| KM236239 | Unassigned | Unassigned | Unassigned | 341_0 | Clustered | 27 | VC_341_0 | 27 | 0.5058 | 1 | 0.5058 | 0 | 0 | 0 | 0.8661 Ref |
| KM236241 | Unassigned | Unassigned | Unassigned | 603_0 | Clustered | 28 | VC_603_0 | 28 | 0.7692 | 1 | 0.7692 | 0 | 0 | 0 | 1 Ref |
| KM236242 | Unassigned | Unassigned | Unassigned | 802_0 | Clustered | 8 | VC_802_0 | 8 | 0.5141 | 1 | 0.5141 | 0 | 0 | 0 | 1 Ref |
| KM236243 | Unassigned | Unassigned | Unassigned | 819_0 | Clustered | 19 | VC_819_0 | 19 | 0.6835 | 1 | 0.6835 | 0 | 0 | 0 | 1 Ref |
| KM236244 | Unassigned | Unassigned | Unassigned | 199_19 | Clustered | 12 | VC_199_19 | 12 | 0.0651 | 1 | 0.0651 | 0 | 0 | 0 | 0.8636 Ref |
| KM236245 | Unassigned | Unassigned | Unassigned | 87_9 | Clustered/Singleton | 1 | VC_87_9 | 1 | 0 | 1 | 0 | 0 | 0 | 0 | 1 Ref |
| KM236246 | Unassigned | Unassigned | Unassigned | 87_7 | Clustered/Singleton | 1 | VC_87_7 | 1 | 0 | 1 | 0 | 0 | 0 | 0 | 1 Ref |
| KM236247 | Unassigned | Unassigned | Unassigned | 1071_0 | Clustered | 7 | VC_1071_0 | 7 | 0.9883 | 0.99999504 | 0.9883 | 0 | 0 | 0 | 1 Ref |
| KM236248 | Unassigned | Unassigned | Unassigned | 1071_0 | Clustered | 7 | VC_1071_0 | 7 | 0.9883 | 0.99999504 | 0.9883 | 0 | 0 | 0 | 1 Ref |
| KM236502 | Unassigned | Unassigned | Unassigned | 384_3 | Clustered | 22 | VC_384_3 | 22 | 0.0786 | 1 | 0.0786 | 0 | 0 | 0 | 0.9048 Ref |
| KM242061 | Unassigned | Unassigned | Unassigned | 405_0 | Clustered | 126 | VC_405_0 | 126 | 0.9614 | 1 | 0.9614 | 0 | 0 | 0 | 1 Ref |
| KM247287 | Unassigned | Unassigned | Unassigned | 162_0 | Clustered | 12 | VC_162_0 | 12 | 0.9051 | 1 | 0.9051 | 0 | 0 | 0 | 1 Ref |
| KM253764 | Unassigned | Unassigned | Unassigned | 398_0 | Clustered | 51 | VC_398_0 | 51 | 0.8741 | 1 | 0.8741 | 0 | 0 | 0 | 1 Ref |
| KM262191 | Unassigned | Unassigned | Unassigned | 889_0 | Clustered | 13 | VC_889_0 | 13 | 0.6844 | 1 | 0.6844 | 0 | 0 | 0 | 1 Ref |
| KM262192 | Unassigned | Unassigned | Unassigned | 889_0 | Clustered | 13 | VC_889_0 | 13 | 0.6844 | 1 | 0.6844 | 0 | 0 | 0 | 1 Ref |
| KM272358 | Unassigned | Unassigned | Unassigned | 654_2 | Clustered | 2 | VC_654_2 | 2 | 0.0038 | 0.92844927 | 0.0035 | 0 | 0 | 0 | 1 Ref |
| KM279937 | Unassigned | Unassigned | Unassigned | 538_56 | Clustered/Singleton | 1 | VC_538_56 | 1 | 0 | 1 | 0 | 0 | 0 | 0 | 1 Ref |
| KM289195 | Unassigned | Unassigned | Unassigned | 653_0 | Clustered | 9 | VC_653_0 | 9 | 0.2315 | 1 | 0.2315 | 0 | 0 | 0 | 0.9722 Ref |
| KM347889 | Unassigned | Unassigned | Unassigned | 538_68 | Clustered/Singleton | 1 | VC_538_68 | 1 | 0 | 1 | 0 | 0 | 0 | 0 | 1 Ref |
| KM347890 | Unassigned | Unassigned | Unassigned | 384_1 | Clustered | 11 | VC_384_1 | 11 | 0.0378 | 1 | 0.0378 | 0 | 0 | 0 | 0.8545 Ref |
| KM359505 | Unassigned | Unassigned | Unassigned | 446_8 | Clustered/Singleton | 1 | VC_446_8 | 1 | 0 | 1 | 0 | 0 | 0 | 0 | 1 Ref |
| KM360178 | Unassigned | Unassigned | Unassigned | 127_0 | Clustered | 17 | VC_127_0 | 17 | 0.9222 | 1 | 0.9222 | 0 | 0 | 0 | 1 Ref |
| KM363596 | Unassigned | Unassigned | Unassigned | 245_0 | Clustered | 17 | VC_245_0 | 17 | 0.6081 | 1 | 0.6081 | 0 | 0 | 0 | 1 Ref |
| KM363597 | Unassigned | Unassigned | Unassigned | 384_13 | Clustered | 33 | VC_384_13 | 33 | 0.1785 | 1 | 0.1785 | 0 | 0 | 0 | 0.8769 Ref |
| KM366096 | Unassigned | Unassigned | Unassigned | 399_0 | Clustered | 49 | VC_399_0 | 49 | 0.7927 | 1 | 0.7927 | 0 | 0 | 0 | 1 Ref |
| KM366097 | Unassigned | Unassigned | Unassigned | 466_0 | Clustered | 66 | VC_466_0 | 66 | 0.9246 | 1 | 0.9246 | 0 | 0 | 0 | 1 Ref |

| | | | | | | | | | | | | | | | | |
|---|---|---|---|---|---|---|---|---|---|---|---|---|---|---|---|---|
| KM366098 | Unassigned | Unassigned | Unassigned | 611_0 | Clustered | 50 | VC_611_0 | 50 | 0.7306 | 1 | 0.7306 | 0 | 0 | 0 | 1 | Ref |
| KM366099 | Unassigned | Unassigned | Unassigned | 440_0 | Clustered | 33 | VC_440_0 | 33 | 0.992 | 1 | 0.992 | 0 | 0 | 0 | 1 | Ref |
| KM366100 | Unassigned | Unassigned | Unassigned | 98_0 | Clustered | 30 | VC_98_0 | 30 | 0.7175 | 1 | 0.7175 | 0 | 0 | 0 | 1 | Ref |
| KM370384 | Unassigned | Unassigned | Unassigned | 466_0 | Clustered | 66 | VC_466_0 | 66 | 0.9246 | 1 | 0.9246 | 0 | 0 | 0 | 1 | Ref |
| KM373208 | Unassigned | Unassigned | Unassigned | 753_0 | Clustered | 26 | VC_753_0 | 26 | 0.9562 | 1 | 0.9562 | 0 | 0 | 0 | 1 | Ref |
| KM389210 | Unassigned | Unassigned | Unassigned | 304_0 | Clustered | 5 | VC_304_0 | 5 | 0.1053 | 0.99999993 | 0.1053 | 0 | 0 | 0 | 0.9 | Ref |
| KM400683 | Unassigned | Unassigned | Unassigned | 566_2 | Clustered | 3 | VC_566_2 | 3 | 0.0275 | 0.85806939 | 0.0236 | 0 | 0 | 0 | 1 | Ref |
| KM401838 | Unassigned | Unassigned | Unassigned | 230_0 | Clustered | 38 | VC_230_0 | 38 | 0.9076 | 1 | 0.9076 | 0 | 0 | 0 | 1 | Ref |
| KM402757 | Unassigned | Unassigned | Unassigned | 538_92 | Clustered | 5 | VC_538_92 | 5 | 0.0653 | 0.99999997 | 0.0653 | 0 | 0 | 0 | 1 | Ref |
| KM407600 | Unassigned | Unassigned | Unassigned | 293_10 | Clustered | 9 | VC_293_10 | 9 | 0.0615 | 0.99999884 | 0.0615 | 0 | 0 | 0 | 0.6667 | Ref |
| KM408320 | Unassigned | Unassigned | Unassigned | 384_15 | Clustered | 44 | VC_384_15 | 44 | 0.139 | 1 | 0.139 | 0 | 0 | 0 | 0.7474 | Ref |
| KM411958 | Unassigned | Unassigned | Unassigned | 862_0 | Clustered | 29 | VC_862_0 | 29 | 0.8594 | 1 | 0.8594 | 0 | 0 | 0 | 1 | Ref |
| KM411959 | Unassigned | Unassigned | Unassigned | 862_0 | Clustered | 29 | VC_862_0 | 29 | 0.8594 | 1 | 0.8594 | 0 | 0 | 0 | 1 | Ref |
| KM411960 | Unassigned | Unassigned | Unassigned | 862_0 | Clustered | 29 | VC_862_0 | 29 | 0.8594 | 1 | 0.8594 | 0 | 0 | 0 | 1 | Ref |
| KM434184 | Unassigned | Unassigned | Unassigned | 80_8 | Clustered/Singleton | 1 | VC_80_8 | 1 | 0 | 1 | 0 | 0 | 0 | 0 | 1 | Ref |
| KM434185 | Unassigned | Unassigned | Unassigned | 766_0 | Clustered | 40 | VC_766_0 | 40 | 0.7511 | 1 | 0.7511 | 0 | 0 | 0 | 1 | Ref |
| KM434186 | Unassigned | Unassigned | Unassigned | 78_0 | Clustered | 20 | VC_78_0 | 20 | 0.9639 | 1 | 0.9639 | 0 | 0 | 0 | 1 | Ref |
| KM458633 | Unassigned | Unassigned | Unassigned | 611_0 | Clustered | 50 | VC_611_0 | 50 | 0.7306 | 1 | 0.7306 | 0 | 0 | 0 | 1 | Ref |
| KM463009 | Unassigned | Unassigned | Unassigned | 223_6 | Clustered | 42 | VC_223_6 | 42 | 0.4717 | 1 | 0.4717 | 0 | 0 | 0 | 0.8351 | Ref |
| KM501444 | Unassigned | Unassigned | Unassigned | 290_145 | Clustered/Singleton | 1 | VC_290_145 | 1 | 0 | 1 | 0 | 0 | 0 | 0 | 1 | Ref |
| KM507819 | Unassigned | Unassigned | Unassigned | 170_11 | Clustered | 10 | VC_170_11 | 10 | 0.1502 | 0.99999797 | 0.1502 | 0 | 0 | 0 | 0.9111 | Ref |
| KM576124 | Unassigned | Unassigned | Unassigned | 113_0 | Clustered | 91 | VC_113_0 | 91 | 0.9584 | 1 | 0.9584 | 0 | 0 | 0 | 1 | Ref |
| KM581061 | Unassigned | Unassigned | Unassigned | 612_0 | Clustered | 5 | VC_612_0 | 5 | 0.4531 | 0.99999995 | 0.4531 | 0 | 0 | 0 | 1 | Ref |
| KM588359 | Unassigned | Unassigned | Unassigned | 539_0 | Clustered | 37 | VC_539_0 | 37 | 0.911 | 1 | 0.911 | 0 | 0 | 0 | 1 | Ref |
| KM589498 | Unassigned | Unassigned | Unassigned | 1095_0 | Clustered | 12 | VC_1095_0 | 12 | 0.2819 | 0.99837092 | 0.2814 | 0 | 0 | 0 | 1 | Ref |
| KM589499 | Unassigned | Unassigned | Unassigned | 1103_0 | Clustered | 2 | VC_1103_0 | 2 | 0.7048 | 0.76024994 | 0.5358 | 0 | 0 | 0 | 1 | Ref |
| KM589500 | Unassigned | Unassigned | Unassigned | 1104_0 | Clustered | 44 | VC_1104_0 | 44 | 0.9574 | 0.72818579 | 0.6972 | 0 | 0 | 0 | 1 | Ref |
| KM589502 | Unassigned | Unassigned | Unassigned | 1028_0 | Clustered | 69 | VC_1028_0 | 69 | 0.8547 | 1 | 0.8547 | 0 | 0 | 0 | 1 | Ref |
| KM589503 | Unassigned | Unassigned | Unassigned | 1104_0 | Clustered | 44 | VC_1104_0 | 44 | 0.9574 | 0.72818579 | 0.6972 | 0 | 0 | 0 | 1 | Ref |
| KM589504 | Unassigned | Unassigned | Unassigned | 1104_0 | Clustered | 44 | VC_1104_0 | 44 | 0.9574 | 0.72818579 | 0.6972 | 0 | 0 | 0 | 1 | Ref |
| KM589505 | Unassigned | Unassigned | Unassigned | 1104_0 | Clustered | 44 | VC_1104_0 | 44 | 0.9574 | 0.72818579 | 0.6972 | 0 | 0 | 0 | 1 | Ref |
| KM589507 | Unassigned | Unassigned | Unassigned | 1103_0 | Clustered | 2 | VC_1103_0 | 2 | 0.7048 | 0.76024994 | 0.5358 | 0 | 0 | 0 | 1 | Ref |
| KM589508 | Unassigned | Unassigned | Unassigned | 1108_0 | Clustered | 52 | VC_1108_0 | 52 | 0.7674 | 1 | 0.7674 | 0 | 0 | 0 | 1 | Ref |
| KM589509 | Unassigned | Unassigned | Unassigned | 1104_0 | Clustered | 44 | VC_1104_0 | 44 | 0.9574 | 0.72818579 | 0.6972 | 0 | 0 | 0 | 1 | Ref |
| KM589510 | Unassigned | Unassigned | Unassigned | 1104_0 | Clustered | 44 | VC_1104_0 | 44 | 0.9574 | 0.72818579 | 0.6972 | 0 | 0 | 0 | 1 | Ref |
| KM589511 | Unassigned | Unassigned | Unassigned | 1028_0 | Clustered | 69 | VC_1028_0 | 69 | 0.8547 | 1 | 0.8547 | 0 | 0 | 0 | 1 | Ref |
| KM589512 | Unassigned | Unassigned | Unassigned | 1104_0 | Clustered | 44 | VC_1104_0 | 44 | 0.9574 | 0.72818579 | 0.6972 | 0 | 0 | 0 | 1 | Ref |
| KM589513 | Unassigned | Unassigned | Unassigned | 1104_0 | Clustered | 44 | VC_1104_0 | 44 | 0.9574 | 0.72818579 | 0.6972 | 0 | 0 | 0 | 1 | Ref |
| KM589514 | Unassigned | Unassigned | Unassigned | 1111_0 | Clustered | 5 | VC_1111_0 | 5 | 1 | 1 | 1 | 0 | 0 | 0 | 1 | Ref |
| KM589515 | Unassigned | Unassigned | Unassigned | 1112_0 | Clustered | 4 | VC_1112_0 | 4 | 1 | 1 | 1 | 0 | 0 | 0 | 1 | Ref |
| KM589516 | Unassigned | Unassigned | Unassigned | 1112_0 | Clustered | 4 | VC_1112_0 | 4 | 1 | 1 | 1 | 0 | 0 | 0 | 1 | Ref |
| KM591905 | Unassigned | Unassigned | Unassigned | 1087_0 | Clustered | 7 | VC_1087_0 | 7 | 0.94 | 1 | 0.94 | 0 | 0 | 0 | 1 | Ref |
| KM591906 | Unassigned | Unassigned | Unassigned | 223_6 | Clustered | 42 | VC_223_6 | 42 | 0.4717 | 1 | 0.4717 | 0 | 0 | 0 | 0.8351 | Ref |
| KM592966 | Unassigned | Unassigned | Unassigned | 531_0 | Clustered | 32 | VC_531_0 | 32 | 0.6859 | 1 | 0.6859 | 0 | 0 | 0 | 1 | Ref |
| KM597530 | Unassigned | Unassigned | Unassigned | 538_29 | Clustered/Singleton | 1 | VC_538_29 | 1 | 0 | 1 | 0 | 0 | 0 | 0 | 1 | Ref |
| KM597531 | Unassigned | Unassigned | Unassigned | 335_49 | Clustered | 17 | VC_335_49 | 17 | 0.0731 | 1 | 0.0731 | 0 | 0 | 0 | 0.7721 | Ref |
| KM606994 | Unassigned | Unassigned | Unassigned | 290_82 | Clustered | 6 | VC_290_82 | 6 | 0.0128 | 1 | 0.0128 | 0 | 0 | 0 | 1 | Ref |
| KM606995 | Unassigned | Unassigned | Unassigned | 290_82 | Clustered | 6 | VC_290_82 | 6 | 0.0128 | 1 | 0.0128 | 0 | 0 | 0 | 1 | Ref |
| KM606996 | Unassigned | Unassigned | Unassigned | 290_82 | Clustered | 6 | VC_290_82 | 6 | 0.0128 | 1 | 0.0128 | 0 | 0 | 0 | 1 | Ref |
| KM606997 | Unassigned | Unassigned | Unassigned | 290_82 | Clustered | 6 | VC_290_82 | 6 | 0.0128 | 1 | 0.0128 | 0 | 0 | 0 | 1 | Ref |
| KM606998 | Unassigned | Unassigned | Unassigned | 290_82 | Clustered | 6 | VC_290_82 | 6 | 0.0128 | 1 | 0.0128 | 0 | 0 | 0 | 1 | Ref |
| KM606999 | Unassigned | Unassigned | Unassigned | 290_82 | Clustered | 6 | VC_290_82 | 6 | 0.0128 | 1 | 0.0128 | 0 | 0 | 0 | 1 | Ref |
| KM607000 | Unassigned | Unassigned | Unassigned | 290_83 | Clustered/Singleton | 1 | VC_290_83 | 1 | 0 | 1 | 0 | 0 | 0 | 0 | 1 | Ref |
| KM607001 | Unassigned | Unassigned | Unassigned | 290_94 | Clustered | 2 | VC_290_94 | 2 | 0.0026 | 0.95293762 | 0.0025 | 0 | 0 | 0 | 1 | Ref |
| KM607002 | Unassigned | Unassigned | Unassigned | 290_91 | Clustered | 11 | VC_290_91 | 11 | 0.0281 | 1 | 0.0281 | 0 | 0 | 0 | 1 | Ref |
| KM607003 | Unassigned | Unassigned | Unassigned | 290_91 | Clustered | 11 | VC_290_91 | 11 | 0.0281 | 1 | 0.0281 | 0 | 0 | 0 | 1 | Ref |
| KM607004 | Unassigned | Unassigned | Unassigned | 290_3 | Clustered | 3 | VC_290_3 | 3 | 0.0055 | 0.99676466 | 0.0054 | 0 | 0 | 0 | 1 | Ref |
| KM612259 | Unassigned | Unassigned | Unassigned | 627_0 | Clustered | 16 | VC_627_0 | 16 | 0.9168 | 1 | 0.9168 | 0 | 0 | 0 | 1 | Ref |
| KM612260 | Unassigned | Unassigned | Unassigned | 627_0 | Clustered | 16 | VC_627_0 | 16 | 0.9168 | 1 | 0.9168 | 0 | 0 | 0 | 1 | Ref |
| KM612261 | Unassigned | Unassigned | Unassigned | 627_0 | Clustered | 16 | VC_627_0 | 16 | 0.9168 | 1 | 0.9168 | 0 | 0 | 0 | 1 | Ref |
| KM612262 | Unassigned | Unassigned | Unassigned | 627_0 | Clustered | 16 | VC_627_0 | 16 | 0.9168 | 1 | 0.9168 | 0 | 0 | 0 | 1 | Ref |
| KM612263 | Unassigned | Unassigned | Unassigned | 627_0 | Clustered | 16 | VC_627_0 | 16 | 0.9168 | 1 | 0.9168 | 0 | 0 | 0 | 1 | Ref |
| KM612264 | Unassigned | Unassigned | Unassigned | 627_0 | Clustered | 16 | VC_627_0 | 16 | 0.9168 | 1 | 0.9168 | 0 | 0 | 0 | 1 | Ref |
| KM612265 | Unassigned | Unassigned | Unassigned | 627_0 | Clustered | 16 | VC_627_0 | 16 | 0.9168 | 1 | 0.9168 | 0 | 0 | 0 | 1 | Ref |
| KM652553 | Unassigned | Unassigned | Unassigned | 538_19 | Clustered | 2 | VC_538_19 | 2 | 0.0097 | 0.95761552 | 0.0093 | 0 | 0 | 0 | 1 | Ref |
| KM652554 | Unassigned | Unassigned | Unassigned | 1114_3 | Clustered | 16 | VC_1114_3 | 16 | 0.2336 | 1 | 0.2336 | 0 | 0 | 0 | 0.9667 | Ref |
| KM657822 | Unassigned | Unassigned | Unassigned | 340_13 | Clustered/Singleton | 1 | VC_340_13 | 1 | 0 | 1 | 0 | 0 | 0 | 0 | 1 | Ref |
| KM672662 | Unassigned | Unassigned | Unassigned | 717_0 | Clustered | 5 | VC_717_0 | 5 | 0.7757 | 0.99999988 | 0.7757 | 0 | 0 | 0 | 1 | Ref |
| KM677185 | Unassigned | Unassigned | Unassigned | 488_0 | Clustered | 14 | VC_488_0 | 14 | 0.8516 | 1 | 0.8516 | 0 | 0 | 0 | 1 | Ref |
| KM677210 | Unassigned | Unassigned | Unassigned | 223_4 | Clustered | 13 | VC_223_4 | 13 | 0.1649 | 1 | 0.1649 | 0 | 0 | 0 | 1 | Ref |
| KM677211 | Unassigned | Unassigned | Unassigned | 243_0 | Clustered | 72 | VC_243_0 | 72 | 0.8089 | 1 | 0.8089 | 0 | 0 | 0 | 1 | Ref |
| KM819694 | Unassigned | Unassigned | Unassigned | 114_0 | Clustered | 8 | VC_114_0 | 8 | 0.2518 | 1 | 0.2518 | 0 | 0 | 0 | 1 | Ref |
| KM819695 | Unassigned | Unassigned | Unassigned | 466_0 | Clustered | 66 | VC_466_0 | 66 | 0.9246 | 1 | 0.9246 | 0 | 0 | 0 | 1 | Ref |
| KM819696 | Unassigned | Unassigned | Unassigned | 466_0 | Clustered | 66 | VC_466_0 | 66 | 0.9246 | 1 | 0.9246 | 0 | 0 | 0 | 1 | Ref |
| KM873719 | Unassigned | Unassigned | Unassigned | 944_0 | Clustered | 76 | VC_944_0 | 76 | 0.9789 | 1 | 0.9789 | 0 | 0 | 0 | 1 | Ref |
| KM879463 | Unassigned | Unassigned | Unassigned | 1117_0 | Clustered | 19 | VC_1117_0 | 19 | 0.9177 | 1 | 0.9177 | 0 | 0 | 0 | 1 | Ref |
| KM881426 | Unassigned | Unassigned | Unassigned | 579_50 | Clustered | 15 | VC_579_50 | 15 | 0.0467 | 1 | 0.0467 | 0 | 0 | 0 | 0.9238 | Ref |
| KM896878 | Unassigned | Unassigned | Unassigned | 859_0 | Clustered | 67 | VC_859_0 | 67 | 0.9622 | 1 | 0.9622 | 0 | 0 | 0 | 1 | Ref |
| KM923970 | Unassigned | Unassigned | Unassigned | 783_0 | Clustered | 67 | VC_783_0 | 67 | 0.8945 | 1 | 0.8945 | 0 | 0 | 0 | 1 | Ref |
| KM923971 | Unassigned | Unassigned | Unassigned | 245_0 | Clustered | 17 | VC_245_0 | 17 | 0.6081 | 1 | 0.6081 | 0 | 0 | 0 | 1 | Ref |
| KM925136 | Unassigned | Unassigned | Unassigned | 566_3 | Clustered | 4 | VC_566_3 | 4 | 0.0418 | 0.92781401 | 0.0388 | 0 | 0 | 0 | 1 | Ref |

| | | | | | | | | | | | | | | | | |
|---|---|---|---|---|---|---|---|---|---|---|---|---|---|---|---|---|
| KM974184 | Unassigned | Unassigned | Unassigned | 862_0 | Clustered | 29 | VC_862_0 | 29 | 0.8594 | 1 | 0.8594 | 0 | 0 | 0 | 1 | Ref |
| KM979354 | Unassigned | Unassigned | Unassigned | 198_22 | Clustered | 3 | VC_198_22 | 3 | 0.0183 | 0.99859679 | 0.0183 | 0 | 0 | 0 | 1 | Ref |
| KM979355 | Unassigned | Unassigned | Unassigned | 198_22 | Clustered | 3 | VC_198_22 | 3 | 0.0183 | 0.99859679 | 0.0183 | 0 | 0 | 0 | 1 | Ref |
| KM983328 | Unassigned | Unassigned | Unassigned | 19_0 | Clustered | 3 | VC_19_0 | 3 | 0.3002 | 0.99840096 | 0.2997 | 0 | 0 | 0 | 1 | Ref |
| KM983329 | Unassigned | Unassigned | Unassigned | 146_0 | Clustered/Singleton | 1 | VC_146_0 | 1 | 0 | 1 | 0 | 0 | 0 | 0 | 1 | Ref |
| KM983331 | Unassigned | Unassigned | Unassigned | 19_0 | Clustered | 3 | VC_19_0 | 3 | 0.3002 | 0.99840096 | 0.2997 | 0 | 0 | 0 | 1 | Ref |
| KM983334 | Unassigned | Unassigned | Unassigned | 19_0 | Clustered | 3 | VC_19_0 | 3 | 0.3002 | 0.99840096 | 0.2997 | 0 | 0 | 0 | 1 | Ref |
| KP007359 | Unassigned | Unassigned | Unassigned | 297_4 | Clustered | 2 | VC_297_4 | 2 | 0.0091 | 0.90221774 | 0.0082 | 0 | 0 | 0 | 1 | Ref |
| KP007360 | Unassigned | Unassigned | Unassigned | 297_1 | Clustered | 8 | VC_297_1 | 8 | 0.0749 | 1 | 0.0749 | 0 | 0 | 0 | 0.8571 | Ref |
| KP007361 | Unassigned | Unassigned | Unassigned | 297_2 | Clustered | 2 | VC_297_2 | 2 | 0.0101 | 0.92290095 | 0.0093 | 0 | 0 | 0 | 1 | Ref |
| KP007362 | Unassigned | Unassigned | Unassigned | 297_3 | Clustered | 2 | VC_297_3 | 2 | 0.0101 | 0.92273843 | 0.0093 | 0 | 0 | 0 | 1 | Ref |
| KP010268 | Unassigned | Unassigned | Unassigned | 52_0 | Clustered | 9 | VC_52_0 | 9 | 0.5586 | 1 | 0.5586 | 0 | 0 | 0 | 1 | Ref |
| KP010413 | Unassigned | Unassigned | Unassigned | 340_11 | Clustered | 15 | VC_340_11 | 15 | 0.079 | 1 | 0.079 | 0 | 0 | 0 | 0.9333 | Ref |
| KP017310 | Unassigned | Unassigned | Unassigned | 532_0 | Clustered | 75 | VC_532_0 | 75 | 0.9393 | 1 | 0.9393 | 0 | 0 | 0 | 1 | Ref |
| KP017311 | Unassigned | Unassigned | Unassigned | 532_0 | Clustered | 75 | VC_532_0 | 75 | 0.9393 | 1 | 0.9393 | 0 | 0 | 0 | 1 | Ref |
| KP025626 | Unassigned | Unassigned | Unassigned | 132_0 | Clustered | 19 | VC_132_0 | 19 | 0.6051 | 1 | 0.6051 | 0 | 0 | 0 | 1 | Ref |
| KP027195 | Unassigned | Unassigned | Unassigned | 786_0 | Clustered | 4 | VC_786_0 | 4 | 0.3519 | 0.99997469 | 0.3519 | 0 | 0 | 0 | 1 | Ref |
| KP027196 | Unassigned | Unassigned | Unassigned | 335_30 | Clustered | 3 | VC_335_30 | 3 | 0.0115 | 0.99857442 | 0.0115 | 0 | 0 | 0 | 1 | Ref |
| KP027197 | Unassigned | Unassigned | Unassigned | 384_18 | Clustered | 10 | VC_384_18 | 10 | 0.0301 | 1 | 0.0301 | 0 | 0 | 0 | 0.8 | Ref |
| KP027198 | Unassigned | Unassigned | Unassigned | 535_1 | Clustered | 108 | VC_535_1 | 108 | 0.8479 | 1 | 0.8479 | 0 | 0 | 0 | 0.9955 | Ref |
| KP027199 | Unassigned | Unassigned | Unassigned | 244_1 | Clustered | 13 | VC_244_1 | 13 | 0.1766 | 1 | 0.1766 | 0 | 0 | 0 | 0.7308 | Ref |
| KP027200 | Unassigned | Unassigned | Unassigned | 537_0 | Clustered | 39 | VC_537_0 | 39 | 0.8148 | 1 | 0.8148 | 0 | 0 | 0 | 1 | Ref |
| KP027201 | Unassigned | Unassigned | Unassigned | 536_1 | Clustered | 2 | VC_536_1 | 2 | 0.0373 | 0.95718761 | 0.0357 | 0 | 0 | 0 | 1 | Ref |
| KP027202 | Unassigned | Unassigned | Unassigned | 532_0 | Clustered | 75 | VC_532_0 | 75 | 0.9393 | 1 | 0.9393 | 0 | 0 | 0 | 1 | Ref |
| KP027203 | Unassigned | Unassigned | Unassigned | 335_30 | Clustered | 3 | VC_335_30 | 3 | 0.0115 | 0.99857442 | 0.0115 | 0 | 0 | 0 | 1 | Ref |
| KP027204 | Unassigned | Unassigned | Unassigned | 335_8 | Clustered | 2 | VC_335_8 | 2 | 0.0092 | 0.95751268 | 0.0088 | 0 | 0 | 0 | 1 | Ref |
| KP027205 | Unassigned | Unassigned | Unassigned | 335_35 | Clustered | 2 | VC_335_35 | 2 | 0.004 | 0.95574341 | 0.0038 | 0 | 0 | 0 | 1 | Ref |
| KP027207 | Unassigned | Unassigned | Unassigned | 527_0 | Clustered | 37 | VC_527_0 | 37 | 0.9679 | 1 | 0.9679 | 0 | 0 | 0 | 1 | Ref |
| KP027208 | Unassigned | Unassigned | Unassigned | 384_15 | Clustered | 44 | VC_384_15 | 44 | 0.139 | 1 | 0.139 | 0 | 0 | 0 | 0.7474 | Ref |
| KP027209 | Unassigned | Unassigned | Unassigned | 384_18 | Clustered | 10 | VC_384_18 | 10 | 0.0301 | 1 | 0.0301 | 0 | 0 | 0 | 0.8 | Ref |
| KP027446 | Unassigned | Unassigned | Unassigned | 155_20 | Clustered/Singleton | 1 | VC_155_20 | 1 | 0 | 1 | 0 | 0 | 0 | 0 | 1 | Ref |
| KP027447 | Unassigned | Unassigned | Unassigned | 156_0 | Clustered | 13 | VC_156_0 | 13 | 0.2378 | 1 | 0.2378 | 0 | 0 | 0 | 0.9872 | Ref |
| KP037007 | Unassigned | Unassigned | Unassigned | 134_0 | Clustered | 2 | VC_134_0 | 2 | 0.0123 | 0.95745739 | 0.0118 | 0 | 0 | 0 | 1 | Ref |
| KP057620 | Unassigned | Unassigned | Unassigned | 535_1 | Clustered | 108 | VC_535_1 | 108 | 0.8479 | 1 | 0.8479 | 0 | 0 | 0 | 0.9955 | Ref |
| KP063118 | Unassigned | Unassigned | Unassigned | 610_0 | Clustered | 14 | VC_610_0 | 14 | 0.5159 | 1 | 0.5159 | 0 | 0 | 0 | 1 | Ref |
| KP063541 | Unassigned | Unassigned | Unassigned | 236_0 | Clustered | 32 | VC_236_0 | 32 | 0.9115 | 1 | 0.9115 | 0 | 0 | 0 | 1 | Ref |
| KP063902 | Unassigned | Unassigned | Unassigned | 849_0 | Clustered | 2 | VC_849_0 | 2 | 0.4243 | 0.94750084 | 0.402 | 0 | 0 | 0 | 1 | Ref |
| KP063903 | Unassigned | Unassigned | Unassigned | 849_0 | Clustered | 2 | VC_849_0 | 2 | 0.4243 | 0.94750084 | 0.402 | 0 | 0 | 0 | 1 | Ref |
| KP064094 | Unassigned | Unassigned | Unassigned | 819_0 | Clustered | 19 | VC_819_0 | 19 | 0.6835 | 1 | 0.6835 | 0 | 0 | 0 | 1 | Ref |
| KP085586 | Unassigned | Unassigned | Unassigned | 595_0 | Clustered | 35 | VC_595_0 | 35 | 0.8809 | 1 | 0.8809 | 0 | 0 | 0 | 1 | Ref |
| KP087938 | Unassigned | Unassigned | Unassigned | 1119_0 | Clustered | 48 | VC_1119_0 | 48 | 0.852 | 1 | 0.852 | 0 | 0 | 0 | 1 | Ref |
| KP087939 | Unassigned | Unassigned | Unassigned | 411_0 | Clustered | 48 | VC_411_0 | 48 | 0.4808 | 1 | 0.4808 | 0 | 0 | 0 | 1 | Ref |
| KP087942 | Unassigned | Unassigned | Unassigned | 1119_0 | Clustered | 48 | VC_1119_0 | 48 | 0.852 | 1 | 0.852 | 0 | 0 | 0 | 1 | Ref |
| KP087943 | Unassigned | Unassigned | Unassigned | 1128_0 | Clustered | 3 | VC_1128_0 | 3 | 0.8404 | 0.81445332 | 0.6845 | 0 | 0 | 0 | 1 | Ref |
| KP087945 | Unassigned | Unassigned | Unassigned | 411_0 | Clustered | 48 | VC_411_0 | 48 | 0.4808 | 1 | 0.4808 | 0 | 0 | 0 | 1 | Ref |
| KP087948 | Unassigned | Unassigned | Unassigned | 1104_0 | Clustered | 44 | VC_1104_0 | 44 | 0.9574 | 0.72818579 | 0.6972 | 0 | 0 | 0 | 1 | Ref |
| KP087951 | Unassigned | Unassigned | Unassigned | 1128_0 | Clustered | 3 | VC_1128_0 | 3 | 0.8404 | 0.81445332 | 0.6845 | 0 | 0 | 0 | 1 | Ref |
| KP087953 | Unassigned | Unassigned | Unassigned | 1129_0 | Clustered | 5 | VC_1129_0 | 5 | 0.8077 | 0.98759579 | 0.7976 | 0 | 0 | 0 | 1 | Ref |
| KP087954 | Unassigned | Unassigned | Unassigned | 1025_0 | Clustered | 8 | VC_1025_0 | 8 | 0.386 | 1 | 0.386 | 0 | 0 | 0 | 1 | Ref |
| KP087955 | Unassigned | Unassigned | Unassigned | 1130_0 | Clustered | 3 | VC_1130_0 | 3 | 0.7736 | 0.82711071 | 0.6399 | 0 | 0 | 0 | 1 | Ref |
| KP087957 | Unassigned | Unassigned | Unassigned | 1128_0 | Clustered | 3 | VC_1128_0 | 3 | 0.8404 | 0.81445332 | 0.6845 | 0 | 0 | 0 | 1 | Ref |
| KP090453 | Unassigned | Unassigned | Unassigned | 399_0 | Clustered | 49 | VC_399_0 | 49 | 0.7927 | 1 | 0.7927 | 0 | 0 | 0 | 1 | Ref |
| KP090454 | Unassigned | Unassigned | Unassigned | 399_0 | Clustered | 49 | VC_399_0 | 49 | 0.7927 | 1 | 0.7927 | 0 | 0 | 0 | 1 | Ref |
| KP137432 | Unassigned | Unassigned | Unassigned | 4_0 | Clustered | 7 | VC_4_0 | 7 | 0.7823 | 1 | 0.7823 | 0 | 0 | 0 | 1 | Ref |
| KP137433 | Unassigned | Unassigned | Unassigned | 734_0 | Clustered | 4 | VC_734_0 | 4 | 0.636 | 0.99996676 | 0.6359 | 0 | 0 | 0 | 0.8333 | Ref |
| KP137434 | Unassigned | Unassigned | Unassigned | 4_0 | Clustered | 7 | VC_4_0 | 7 | 0.7823 | 1 | 0.7823 | 0 | 0 | 0 | 1 | Ref |
| KP137435 | Unassigned | Unassigned | Unassigned | 734_0 | Clustered | 4 | VC_734_0 | 4 | 0.636 | 0.99996676 | 0.6359 | 0 | 0 | 0 | 0.8333 | Ref |
| KP137436 | Unassigned | Unassigned | Unassigned | 4_0 | Clustered | 7 | VC_4_0 | 7 | 0.7823 | 1 | 0.7823 | 0 | 0 | 0 | 1 | Ref |
| KP137437 | Unassigned | Unassigned | Unassigned | 734_0 | Clustered | 4 | VC_734_0 | 4 | 0.636 | 0.99996676 | 0.6359 | 0 | 0 | 0 | 0.8333 | Ref |
| KP137438 | Unassigned | Unassigned | Unassigned | 4_0 | Clustered | 7 | VC_4_0 | 7 | 0.7823 | 1 | 0.7823 | 0 | 0 | 0 | 1 | Ref |
| KP137439 | Unassigned | Unassigned | Unassigned | 734_0 | Clustered | 4 | VC_734_0 | 4 | 0.636 | 0.99996676 | 0.6359 | 0 | 0 | 0 | 0.8333 | Ref |
| KP143762 | Unassigned | Unassigned | Unassigned | 340_1 | Clustered | 17 | VC_340_1 | 17 | 0.0863 | 1 | 0.0863 | 0 | 0 | 0 | 0.8235 | Ref |
| KP143763 | Unassigned | Unassigned | Unassigned | 198_0 | Clustered | 2 | VC_198_0 | 2 | 0.0086 | 0.95763473 | 0.0082 | 0 | 0 | 0 | 1 | Ref |
| KP202158 | Unassigned | Unassigned | Unassigned | 508_4 | Clustered/Singleton | 1 | VC_508_4 | 1 | 0 | 1 | 0 | 0 | 0 | 0 | 1 | Ref |
| KP202969 | Unassigned | Unassigned | Unassigned | 177_0 | Clustered | 8 | VC_177_0 | 8 | 0.5848 | 1 | 0.5848 | 0 | 0 | 0 | 1 | Ref |
| KP202970 | Unassigned | Unassigned | Unassigned | 177_0 | Clustered | 8 | VC_177_0 | 8 | 0.5848 | 1 | 0.5848 | 0 | 0 | 0 | 1 | Ref |
| KP202971 | Unassigned | Unassigned | Unassigned | 1132_0 | Clustered | 4 | VC_1132_0 | 4 | 1 | 1 | 1 | 0 | 0 | 0 | 1 | Ref |
| KP202972 | Unassigned | Unassigned | Unassigned | 725_0 | Clustered | 47 | VC_725_0 | 47 | 0.8649 | 1 | 0.8649 | 0 | 0 | 0 | 1 | Ref |
| KP208803 | Unassigned | Unassigned | Unassigned | 268_2 | Clustered/Singleton | 1 | VC_268_2 | 1 | 0 | 1 | 0 | 0 | 0 | 0 | 1 | Ref |
| KP209285 | Unassigned | Unassigned | Unassigned | 12_0 | Clustered | 21 | VC_12_0 | 21 | 0.2262 | 1 | 0.2262 | 0 | 0 | 0 | 0.9571 | Ref |
| KP233880 | Unassigned | Unassigned | Unassigned | 486_0 | Clustered | 47 | VC_486_0 | 47 | 0.9022 | 1 | 0.9022 | 0 | 0 | 0 | 1 | Ref |
| KP238129 | Unassigned | Unassigned | Unassigned | 460_0 | Clustered | 4 | VC_460_0 | 4 | 0.7519 | 0.99987768 | 0.7518 | 0 | 0 | 0 | 1 | Ref |
| KP273225 | Unassigned | Unassigned | Unassigned | 226_0 | Clustered | 5 | VC_226_0 | 5 | 0.0793 | 0.99999998 | 0.0793 | 0 | 0 | 0 | 1 | Ref |
| KP280062 | Unassigned | Unassigned | Unassigned | 806_0 | Clustered | 6 | VC_806_0 | 6 | 0.5468 | 1 | 0.5468 | 0 | 0 | 0 | 1 | Ref |
| KP280063 | Unassigned | Unassigned | Unassigned | 1007_0 | Clustered | 3 | VC_1007_0 | 3 | 0.0541 | 0.99838914 | 0.0541 | 0 | 0 | 0 | 1 | Ref |
| KP282673 | Unassigned | Unassigned | Unassigned | 815_0 | Clustered | 3 | VC_815_0 | 3 | 1 | 1 | 1 | 0 | 0 | 0 | 1 | Ref |
| KP282674 | Unassigned | Unassigned | Unassigned | 815_0 | Clustered | 3 | VC_815_0 | 3 | 1 | 1 | 1 | 0 | 0 | 0 | 1 | Ref |
| KP282675 | Unassigned | Unassigned | Unassigned | 360_0 | Clustered | 22 | VC_360_0 | 22 | 0.96 | 1 | 0.96 | 0 | 0 | 0 | 1 | Ref |
| KP282677 | Unassigned | Unassigned | Unassigned | 460_0 | Clustered | 4 | VC_460_0 | 4 | 0.7519 | 0.99987768 | 0.7518 | 0 | 0 | 0 | 1 | Ref |
| KP282678 | Unassigned | Unassigned | Unassigned | 460_0 | Clustered | 4 | VC_460_0 | 4 | 0.7519 | 0.99987768 | 0.7518 | 0 | 0 | 0 | 1 | Ref |
| KP282679 | Unassigned | Unassigned | Unassigned | 910_0 | Clustered | 2 | VC_910_0 | 2 | 0.6744 | 0.91813282 | 0.6192 | 0 | 0 | 0 | 1 | Ref |

| | | | | | | | | | | | | | | | | |
|---|---|---|---|---|---|---|---|---|---|---|---|---|---|---|---|---|
| KP282680 | Unassigned | Unassigned | Unassigned | 910_0 | Clustered | 2 | VC_910_0 | 2 | 0.6744 | 0.91813282 | 0.6192 | 0 | 0 | 0 | 1 | Ref |
| KP296791 | Unassigned | Unassigned | Unassigned | 725_0 | Clustered | 47 | VC_725_0 | 47 | 0.8649 | 1 | 0.8649 | 0 | 0 | 0 | 1 | Ref |
| KP296793 | Unassigned | Unassigned | Unassigned | 725_0 | Clustered | 47 | VC_725_0 | 47 | 0.8649 | 1 | 0.8649 | 0 | 0 | 0 | 1 | Ref |
| KP296794 | Unassigned | Unassigned | Unassigned | 725_0 | Clustered | 47 | VC_725_0 | 47 | 0.8649 | 1 | 0.8649 | 0 | 0 | 0 | 1 | Ref |
| KP296795 | Unassigned | Unassigned | Unassigned | 725_0 | Clustered | 47 | VC_725_0 | 47 | 0.8649 | 1 | 0.8649 | 0 | 0 | 0 | 1 | Ref |
| KP296796 | Unassigned | Unassigned | Unassigned | 725_0 | Clustered | 47 | VC_725_0 | 47 | 0.8649 | 1 | 0.8649 | 0 | 0 | 0 | 1 | Ref |
| KP308307 | Unassigned | Unassigned | Unassigned | 837_1 | Clustered | 24 | VC_837_1 | 24 | 0.5328 | 1 | 0.5328 | 0 | 0 | 0 | 1 | Ref |
| KP313531 | Unassigned | Unassigned | Unassigned | 398_0 | Clustered | 51 | VC_398_0 | 51 | 0.8741 | 1 | 0.8741 | 0 | 0 | 0 | 1 | Ref |
| KP313532 | Unassigned | Unassigned | Unassigned | 768_0 | Clustered | 4 | VC_768_0 | 4 | 0.1754 | 0.99998526 | 0.1754 | 0 | 0 | 0 | 1 | Ref |
| KP339049 | Unassigned | Unassigned | Unassigned | 94_0 | Clustered | 22 | VC_94_0 | 22 | 0.5602 | 1 | 0.5602 | 0 | 0 | 0 | 1 | Ref |
| KP340287 | Unassigned | Unassigned | Unassigned | 80_0 | Clustered | 64 | VC_80_0 | 64 | 0.418 | 1 | 0.418 | 0 | 0 | 0 | 0.7649 | Ref |
| KP340288 | Unassigned | Unassigned | Unassigned | 80_0 | Clustered | 64 | VC_80_0 | 64 | 0.418 | 1 | 0.418 | 0 | 0 | 0 | 0.7649 | Ref |
| KP343639 | Unassigned | Unassigned | Unassigned | 84_0 | Clustered | 6 | VC_84_0 | 6 | 0.6364 | 1 | 0.6364 | 0 | 0 | 0 | 1 | Ref |
| KP399677 | Unassigned | Unassigned | Unassigned | 394_0 | Clustered | 3 | VC_394_0 | 3 | 0.1582 | 0.99545803 | 0.1575 | 0 | 0 | 0 | 1 | Ref |
| KP399678 | Unassigned | Unassigned | Unassigned | 392_0 | Clustered | 9 | VC_392_0 | 9 | 0.7527 | 1 | 0.7527 | 0 | 0 | 0 | 1 | Ref |
| KP411017 | Unassigned | Unassigned | Unassigned | 1071_0 | Clustered | 7 | VC_1071_0 | 7 | 0.9883 | 0.99999504 | 0.9883 | 0 | 0 | 0 | 1 | Ref |
| KP658157 | Unassigned | Unassigned | Unassigned | 972_0 | Clustered | 99 | VC_972_0 | 99 | 0.9113 | 1 | 0.9113 | 0 | 0 | 0 | 0.9928 | Ref |
| KP671755 | Unassigned | Unassigned | Unassigned | 549_0 | Clustered | 9 | VC_549_0 | 9 | 0.2597 | 1 | 0.2597 | 0 | 0 | 0 | 1 | Ref |
| KP682371 | Unassigned | Unassigned | Unassigned | 44_0 | Clustered | 49 | VC_44_0 | 49 | 0.4506 | 1 | 0.4506 | 0 | 0 | 0 | 0.8605 | Ref |
| KP682372 | Unassigned | Unassigned | Unassigned | 35_0 | Clustered | 35 | VC_35_0 | 35 | 0.431 | 1 | 0.431 | 0 | 0 | 0 | 1 | Ref |
| KP682373 | Unassigned | Unassigned | Unassigned | 35_0 | Clustered | 35 | VC_35_0 | 35 | 0.431 | 1 | 0.431 | 0 | 0 | 0 | 1 | Ref |
| KP682374 | Unassigned | Unassigned | Unassigned | 44_0 | Clustered | 49 | VC_44_0 | 49 | 0.4506 | 1 | 0.4506 | 0 | 0 | 0 | 0.8605 | Ref |
| KP682375 | Unassigned | Unassigned | Unassigned | 35_0 | Clustered | 35 | VC_35_0 | 35 | 0.431 | 1 | 0.431 | 0 | 0 | 0 | 1 | Ref |
| KP682376 | Unassigned | Unassigned | Unassigned | 35_0 | Clustered | 35 | VC_35_0 | 35 | 0.431 | 1 | 0.431 | 0 | 0 | 0 | 1 | Ref |
| KP682377 | Unassigned | Unassigned | Unassigned | 35_0 | Clustered | 35 | VC_35_0 | 35 | 0.431 | 1 | 0.431 | 0 | 0 | 0 | 1 | Ref |
| KP682378 | Unassigned | Unassigned | Unassigned | 35_0 | Clustered | 35 | VC_35_0 | 35 | 0.431 | 1 | 0.431 | 0 | 0 | 0 | 1 | Ref |
| KP682379 | Unassigned | Unassigned | Unassigned | 35_0 | Clustered | 35 | VC_35_0 | 35 | 0.431 | 1 | 0.431 | 0 | 0 | 0 | 1 | Ref |
| KP682380 | Unassigned | Unassigned | Unassigned | 35_0 | Clustered | 35 | VC_35_0 | 35 | 0.431 | 1 | 0.431 | 0 | 0 | 0 | 1 | Ref |
| KP682382 | Unassigned | Unassigned | Unassigned | 35_0 | Clustered | 35 | VC_35_0 | 35 | 0.431 | 1 | 0.431 | 0 | 0 | 0 | 1 | Ref |
| KP682383 | Unassigned | Unassigned | Unassigned | 35_0 | Clustered | 35 | VC_35_0 | 35 | 0.431 | 1 | 0.431 | 0 | 0 | 0 | 1 | Ref |
| KP682384 | Unassigned | Unassigned | Unassigned | 35_0 | Clustered | 35 | VC_35_0 | 35 | 0.431 | 1 | 0.431 | 0 | 0 | 0 | 1 | Ref |
| KP682385 | Unassigned | Unassigned | Unassigned | 35_0 | Clustered | 35 | VC_35_0 | 35 | 0.431 | 1 | 0.431 | 0 | 0 | 0 | 1 | Ref |
| KP682386 | Unassigned | Unassigned | Unassigned | 35_0 | Clustered | 35 | VC_35_0 | 35 | 0.431 | 1 | 0.431 | 0 | 0 | 0 | 1 | Ref |
| KP682387 | Unassigned | Unassigned | Unassigned | 35_0 | Clustered | 35 | VC_35_0 | 35 | 0.431 | 1 | 0.431 | 0 | 0 | 0 | 1 | Ref |
| KP682388 | Unassigned | Unassigned | Unassigned | 35_0 | Clustered | 35 | VC_35_0 | 35 | 0.431 | 1 | 0.431 | 0 | 0 | 0 | 1 | Ref |
| KP682389 | Unassigned | Unassigned | Unassigned | 35_0 | Clustered | 35 | VC_35_0 | 35 | 0.431 | 1 | 0.431 | 0 | 0 | 0 | 1 | Ref |
| KP682390 | Unassigned | Unassigned | Unassigned | 35_0 | Clustered | 35 | VC_35_0 | 35 | 0.431 | 1 | 0.431 | 0 | 0 | 0 | 1 | Ref |
| KP682391 | Unassigned | Unassigned | Unassigned | 35_0 | Clustered | 35 | VC_35_0 | 35 | 0.431 | 1 | 0.431 | 0 | 0 | 0 | 1 | Ref |
| KP682392 | Unassigned | Unassigned | Unassigned | 35_0 | Clustered | 35 | VC_35_0 | 35 | 0.431 | 1 | 0.431 | 0 | 0 | 0 | 1 | Ref |
| KP687431 | Unassigned | Unassigned | Unassigned | 155_8 | Clustered | 66 | VC_155_8 | 66 | 0.4537 | 1 | 0.4537 | 0 | 0 | 0 | 0.8182 | Ref |
| KP687432 | Unassigned | Unassigned | Unassigned | 155_8 | Clustered | 66 | VC_155_8 | 66 | 0.4537 | 1 | 0.4537 | 0 | 0 | 0 | 0.8182 | Ref |
| KP708985 | Unassigned | Unassigned | Unassigned | 113_0 | Clustered | 91 | VC_113_0 | 91 | 0.9584 | 1 | 0.9584 | 0 | 0 | 0 | 1 | Ref |
| KP708986 | Unassigned | Unassigned | Unassigned | 113_0 | Clustered | 91 | VC_113_0 | 91 | 0.9584 | 1 | 0.9584 | 0 | 0 | 0 | 1 | Ref |
| KP719132 | Unassigned | Unassigned | Unassigned | 737_0 | Clustered | 13 | VC_737_0 | 13 | 0.5083 | 1 | 0.5083 | 0 | 0 | 0 | 1 | Ref |
| KP719133 | Unassigned | Unassigned | Unassigned | 737_0 | Clustered | 13 | VC_737_0 | 13 | 0.5083 | 1 | 0.5083 | 0 | 0 | 0 | 1 | Ref |
| KP719134 | Unassigned | Unassigned | Unassigned | 737_0 | Clustered | 13 | VC_737_0 | 13 | 0.5083 | 1 | 0.5083 | 0 | 0 | 0 | 1 | Ref |
| KP790008 | Unassigned | Unassigned | Unassigned | 894_0 | Clustered | 6 | VC_894_0 | 6 | 0.3171 | 1 | 0.3171 | 0 | 0 | 0 | 1 | Ref |
| KP790009 | Unassigned | Unassigned | Unassigned | 894_0 | Clustered | 6 | VC_894_0 | 6 | 0.3171 | 1 | 0.3171 | 0 | 0 | 0 | 1 | Ref |
| KP790010 | Unassigned | Unassigned | Unassigned | 894_0 | Clustered | 6 | VC_894_0 | 6 | 0.3171 | 1 | 0.3171 | 0 | 0 | 0 | 1 | Ref |
| KP790011 | Unassigned | Unassigned | Unassigned | 419_0 | Clustered | 11 | VC_419_0 | 11 | 0.9465 | 1 | 0.9465 | 0 | 0 | 0 | 1 | Ref |
| KP791805 | Unassigned | Unassigned | Unassigned | 398_0 | Clustered | 51 | VC_398_0 | 51 | 0.8741 | 1 | 0.8741 | 0 | 0 | 0 | 1 | Ref |
| KP791806 | Unassigned | Unassigned | Unassigned | 398_0 | Clustered | 51 | VC_398_0 | 51 | 0.8741 | 1 | 0.8741 | 0 | 0 | 0 | 1 | Ref |
| KP791807 | Unassigned | Unassigned | Unassigned | 398_0 | Clustered | 51 | VC_398_0 | 51 | 0.8741 | 1 | 0.8741 | 0 | 0 | 0 | 1 | Ref |
| KP792623 | Unassigned | Unassigned | Unassigned | 830_0 | Clustered | 3 | VC_830_0 | 3 | 0.5256 | 0.99825218 | 0.5246 | 0 | 0 | 0 | 1 | Ref |
| KP793104 | Unassigned | Unassigned | Unassigned | 210_0 | Clustered | 105 | VC_210_0 | 105 | 0.4076 | 1 | 0.4076 | 0 | 0 | 0 | 0.7766 | Ref |
| KP793105 | Unassigned | Unassigned | Unassigned | 210_0 | Clustered | 105 | VC_210_0 | 105 | 0.4076 | 1 | 0.4076 | 0 | 0 | 0 | 0.7766 | Ref |
| KP793108 | Unassigned | Unassigned | Unassigned | 210_0 | Clustered | 105 | VC_210_0 | 105 | 0.4076 | 1 | 0.4076 | 0 | 0 | 0 | 0.7766 | Ref |
| KP793109 | Unassigned | Unassigned | Unassigned | 210_0 | Clustered | 105 | VC_210_0 | 105 | 0.4076 | 1 | 0.4076 | 0 | 0 | 0 | 0.7766 | Ref |
| KP793111 | Unassigned | Unassigned | Unassigned | 210_0 | Clustered | 105 | VC_210_0 | 105 | 0.4076 | 1 | 0.4076 | 0 | 0 | 0 | 0.7766 | Ref |
| KP793113 | Unassigned | Unassigned | Unassigned | 210_0 | Clustered | 105 | VC_210_0 | 105 | 0.4076 | 1 | 0.4076 | 0 | 0 | 0 | 0.7766 | Ref |
| KP793115 | Unassigned | Unassigned | Unassigned | 210_0 | Clustered | 105 | VC_210_0 | 105 | 0.4076 | 1 | 0.4076 | 0 | 0 | 0 | 0.7766 | Ref |
| KP793116 | Unassigned | Unassigned | Unassigned | 210_0 | Clustered | 105 | VC_210_0 | 105 | 0.4076 | 1 | 0.4076 | 0 | 0 | 0 | 0.7766 | Ref |
| KP793117 | Unassigned | Unassigned | Unassigned | 210_0 | Clustered | 105 | VC_210_0 | 105 | 0.4076 | 1 | 0.4076 | 0 | 0 | 0 | 0.7766 | Ref |
| KP793118 | Unassigned | Unassigned | Unassigned | 210_0 | Clustered | 105 | VC_210_0 | 105 | 0.4076 | 1 | 0.4076 | 0 | 0 | 0 | 0.7766 | Ref |
| KP793123 | Unassigned | Unassigned | Unassigned | 210_0 | Clustered | 105 | VC_210_0 | 105 | 0.4076 | 1 | 0.4076 | 0 | 0 | 0 | 0.7766 | Ref |
| KP793124 | Unassigned | Unassigned | Unassigned | 210_0 | Clustered | 105 | VC_210_0 | 105 | 0.4076 | 1 | 0.4076 | 0 | 0 | 0 | 0.7766 | Ref |
| KP793129 | Unassigned | Unassigned | Unassigned | 210_0 | Clustered | 105 | VC_210_0 | 105 | 0.4076 | 1 | 0.4076 | 0 | 0 | 0 | 0.7766 | Ref |
| KP793134 | Unassigned | Unassigned | Unassigned | 210_0 | Clustered | 105 | VC_210_0 | 105 | 0.4076 | 1 | 0.4076 | 0 | 0 | 0 | 0.7766 | Ref |
| KP797973 | Unassigned | Unassigned | Unassigned | 135_9 | Clustered | 3 | VC_135_9 | 3 | 0.0098 | 0.98303301 | 0.0097 | 0 | 0 | 0 | 1 | Ref |
| KP836355 | Unassigned | Unassigned | Unassigned | 1134_0 | Clustered | 2 | VC_1134_0 | 2 | 1 | 1 | 1 | 0 | 0 | 0 | 1 | Ref |
| KP836356 | Unassigned | Unassigned | Unassigned | 1134_0 | Clustered | 2 | VC_1134_0 | 2 | 1 | 1 | 1 | 0 | 0 | 0 | 1 | Ref |
| KP861229 | Unassigned | Unassigned | Unassigned | 713_0 | Clustered | 26 | VC_713_0 | 26 | 0.9358 | 1 | 0.9358 | 0 | 0 | 0 | 1 | Ref |
| KP861230 | Unassigned | Unassigned | Unassigned | 441_1 | Clustered | 8 | VC_441_1 | 8 | 0.5443 | 1 | 0.5443 | 0 | 0 | 0 | 0.9643 | Ref |
| KP861231 | Unassigned | Unassigned | Unassigned | 713_0 | Clustered | 26 | VC_713_0 | 26 | 0.9358 | 1 | 0.9358 | 0 | 0 | 0 | 1 | Ref |
| KP869100 | Unassigned | Unassigned | Unassigned | 340_7 | Clustered | 18 | VC_340_7 | 18 | 0.0936 | 1 | 0.0936 | 0 | 0 | 0 | 0.7778 | Ref |
| KP869101 | Unassigned | Unassigned | Unassigned | 293_17 | Clustered | 8 | VC_293_17 | 8 | 0.0534 | 0.99997879 | 0.0534 | 0 | 0 | 0 | 0.6071 | Ref |
| KP869102 | Unassigned | Unassigned | Unassigned | 794_15 | Clustered | 5 | VC_794_15 | 5 | 0.0243 | 0.76144861 | 0.0185 | 0 | 0 | 0 | 0.9 | Ref |
| KP869103 | Unassigned | Unassigned | Unassigned | 794_15 | Clustered | 5 | VC_794_15 | 5 | 0.0243 | 0.76144861 | 0.0185 | 0 | 0 | 0 | 0.9 | Ref |
| KP869104 | Unassigned | Unassigned | Unassigned | 293_17 | Clustered | 8 | VC_293_17 | 8 | 0.0534 | 0.99997879 | 0.0534 | 0 | 0 | 0 | 0.6071 | Ref |
| KP869105 | Unassigned | Unassigned | Unassigned | 290_2 | Clustered | 2 | VC_290_2 | 2 | 0.0027 | 0.93842619 | 0.0025 | 0 | 0 | 0 | 1 | Ref |
| KP869106 | Unassigned | Unassigned | Unassigned | 340_7 | Clustered | 18 | VC_340_7 | 18 | 0.0936 | 1 | 0.0936 | 0 | 0 | 0 | 0.7778 | Ref |

| | | | | | | | | | | | | | | | | |
|---|---|---|---|---|---|---|---|---|---|---|---|---|---|---|---|---|
| KP869107 | Unassigned | Unassigned | Unassigned | 492_0 | Clustered | 20 | VC_492_0 | 20 | 0.7887 | 1 | 0.7887 | 0 | 0 | 0 | 1 | Ref |
| KP869108 | Unassigned | Unassigned | Unassigned | 492_0 | Clustered | 20 | VC_492_0 | 20 | 0.7887 | 1 | 0.7887 | 0 | 0 | 0 | 1 | Ref |
| KP869109 | Unassigned | Unassigned | Unassigned | 340_7 | Clustered | 18 | VC_340_7 | 18 | 0.0936 | 1 | 0.0936 | 0 | 0 | 0 | 0.7778 | Ref |
| KP869110 | Unassigned | Unassigned | Unassigned | 340_7 | Clustered | 18 | VC_340_7 | 18 | 0.0936 | 1 | 0.0936 | 0 | 0 | 0 | 0.7778 | Ref |
| KP869111 | Unassigned | Unassigned | Unassigned | 293_17 | Clustered | 8 | VC_293_17 | 8 | 0.0534 | 0.99997879 | 0.0534 | 0 | 0 | 0 | 0.6071 | Ref |
| KP869112 | Unassigned | Unassigned | Unassigned | 794_15 | Clustered | 5 | VC_794_15 | 5 | 0.0243 | 0.76144861 | 0.0185 | 0 | 0 | 0 | 0.9 | Ref |
| KP869113 | Unassigned | Unassigned | Unassigned | 340_7 | Clustered | 18 | VC_340_7 | 18 | 0.0936 | 1 | 0.0936 | 0 | 0 | 0 | 0.7778 | Ref |
| KP876465 | Unassigned | Unassigned | Unassigned | 891_0 | Clustered | 11 | VC_891_0 | 11 | 0.9663 | 1 | 0.9663 | 0 | 0 | 0 | 1 | Ref |
| KP876466 | Unassigned | Unassigned | Unassigned | 891_0 | Clustered | 11 | VC_891_0 | 11 | 0.9663 | 1 | 0.9663 | 0 | 0 | 0 | 1 | Ref |
| KP881232 | Unassigned | Unassigned | Unassigned | 1093_1 | Clustered/Singleton | 1 | VC_1093_1 | 1 | 0 | 1 | 0 | 0 | 0 | 0 | 1 | Ref |
| KP881332 | Unassigned | Unassigned | Unassigned | 159_0 | Clustered | 25 | VC_159_0 | 25 | 0.7699 | 1 | 0.7699 | 0 | 0 | 0 | 1 | Ref |
| KP890822 | Unassigned | Unassigned | Unassigned | 466_0 | Clustered | 66 | VC_466_0 | 66 | 0.9246 | 1 | 0.9246 | 0 | 0 | 0 | 1 | Ref |
| KP890823 | Unassigned | Unassigned | Unassigned | 295_3 | Clustered | 3 | VC_295_3 | 3 | 0.0443 | 0.99844642 | 0.0442 | 0 | 0 | 0 | 1 | Ref |
| KP893289 | Unassigned | Unassigned | Unassigned | 12_0 | Clustered | 21 | VC_12_0 | 21 | 0.2262 | 1 | 0.2262 | 0 | 0 | 0 | 0.9571 | Ref |
| KP893290 | Unassigned | Unassigned | Unassigned | 12_2 | Clustered | 19 | VC_12_2 | 19 | 0.2009 | 1 | 0.2009 | 0 | 0 | 0 | 1 | Ref |
| KP966108 | Unassigned | Unassigned | Unassigned | 1_0 | Clustered | 54 | VC_1_0 | 54 | 0.8518 | 1 | 0.8518 | 0 | 0 | 0 | 1 | Ref |
| KP972568 | Unassigned | Unassigned | Unassigned | 904_0 | Clustered | 10 | VC_904_0 | 10 | 0.9878 | 0.99948433 | 0.9873 | 0 | 0 | 0 | 1 | Ref |
| KP972569 | Unassigned | Unassigned | Unassigned | 904_0 | Clustered | 10 | VC_904_0 | 10 | 0.9878 | 0.99948433 | 0.9873 | 0 | 0 | 0 | 1 | Ref |
| KP994390 | Unassigned | Unassigned | Unassigned | 862_0 | Clustered | 29 | VC_862_0 | 29 | 0.8594 | 1 | 0.8594 | 0 | 0 | 0 | 1 | Ref |
| KP994596 | Unassigned | Unassigned | Unassigned | 663_0 | Clustered | 5 | VC_663_0 | 5 | 0.6645 | 0.99999974 | 0.6645 | 0 | 0 | 0 | 1 | Ref |
| KR011062 | Unassigned | Unassigned | Unassigned | 893_2 | Clustered | 3 | VC_893_2 | 3 | 0.0458 | 0.99850411 | 0.0457 | 0 | 0 | 0 | 1 | Ref |
| KR011063 | Unassigned | Unassigned | Unassigned | 893_2 | Clustered | 3 | VC_893_2 | 3 | 0.0458 | 0.99850411 | 0.0457 | 0 | 0 | 0 | 1 | Ref |
| KR011064 | Unassigned | Unassigned | Unassigned | 893_2 | Clustered | 3 | VC_893_2 | 3 | 0.0458 | 0.99850411 | 0.0457 | 0 | 0 | 0 | 1 | Ref |
| KR014248 | Unassigned | Unassigned | Unassigned | 340_4 | Clustered | 2 | VC_340_4 | 2 | 0.0052 | 0.95746455 | 0.005 | 0 | 0 | 0 | 1 | Ref |
| KR029086 | Unassigned | Unassigned | Unassigned | 384_13 | Clustered | 33 | VC_384_13 | 33 | 0.1785 | 1 | 0.1785 | 0 | 0 | 0 | 0.8769 | Ref |
| KR029087 | Unassigned | Unassigned | Unassigned | 384_13 | Clustered | 33 | VC_384_13 | 33 | 0.1785 | 1 | 0.1785 | 0 | 0 | 0 | 0.8769 | Ref |
| KR049063 | Unassigned | Unassigned | Unassigned | 93_0 | Clustered | 22 | VC_93_0 | 22 | 0.5285 | 1 | 0.5285 | 0 | 0 | 0 | 1 | Ref |
| KR052142 | Unassigned | Unassigned | Unassigned | 862_0 | Clustered | 29 | VC_862_0 | 29 | 0.8594 | 1 | 0.8594 | 0 | 0 | 0 | 1 | Ref |
| KR052143 | Unassigned | Unassigned | Unassigned | 77_0 | Clustered | 37 | VC_77_0 | 37 | 0.9392 | 1 | 0.9392 | 0 | 0 | 0 | 1 | Ref |
| KR052480 | Unassigned | Unassigned | Unassigned | 451_0 | Clustered | 4 | VC_451_0 | 4 | 0.5528 | 0.99998171 | 0.5528 | 0 | 0 | 0 | 1 | Ref |
| KR052481 | Unassigned | Unassigned | Unassigned | 451_0 | Clustered | 4 | VC_451_0 | 4 | 0.5528 | 0.99998171 | 0.5528 | 0 | 0 | 0 | 1 | Ref |
| KR052482 | Unassigned | Unassigned | Unassigned | 451_0 | Clustered | 4 | VC_451_0 | 4 | 0.5528 | 0.99998171 | 0.5528 | 0 | 0 | 0 | 1 | Ref |
| KR053195 | Unassigned | Unassigned | Unassigned | 741_0 | Clustered | 3 | VC_741_0 | 3 | 0.038 | 0.88651 | 0.0337 | 0 | 0 | 0 | 1 | Ref |
| KR053201 | Unassigned | Unassigned | Unassigned | 789_0 | Clustered | 34 | VC_789_0 | 34 | 0.9167 | 1 | 0.9167 | 0 | 0 | 0 | 1 | Ref |
| KR054028 | Unassigned | Unassigned | Unassigned | 81_0 | Clustered | 41 | VC_81_0 | 41 | 0.5815 | 1 | 0.5815 | 0 | 0 | 0 | 0.9805 | Ref |
| KR054029 | Unassigned | Unassigned | Unassigned | 486_0 | Clustered | 47 | VC_486_0 | 47 | 0.9022 | 1 | 0.9022 | 0 | 0 | 0 | 1 | Ref |
| KR054030 | Unassigned | Unassigned | Unassigned | 81_0 | Clustered | 41 | VC_81_0 | 41 | 0.5815 | 1 | 0.5815 | 0 | 0 | 0 | 0.9805 | Ref |
| KR054031 | Unassigned | Unassigned | Unassigned | 434_0 | Clustered | 45 | VC_434_0 | 45 | 0.9479 | 1 | 0.9479 | 0 | 0 | 0 | 1 | Ref |
| KR054032 | Unassigned | Unassigned | Unassigned | 862_0 | Clustered | 29 | VC_862_0 | 29 | 0.8594 | 1 | 0.8594 | 0 | 0 | 0 | 1 | Ref |
| KR054033 | Unassigned | Unassigned | Unassigned | 80_7 | Clustered | 33 | VC_80_7 | 33 | 0.2156 | 1 | 0.2156 | 0 | 0 | 0 | 0.8409 | Ref |
| KR060090 | Unassigned | Unassigned | Unassigned | 927_0 | Clustered | 5 | VC_927_0 | 5 | 0.4988 | 0.99999981 | 0.4988 | 0 | 0 | 0 | 1 | Ref |
| KR063267 | Unassigned | Unassigned | Unassigned | 904_0 | Clustered | 10 | VC_904_0 | 10 | 0.9878 | 0.99948433 | 0.9873 | 0 | 0 | 0 | 1 | Ref |
| KR063268 | Unassigned | Unassigned | Unassigned | 904_0 | Clustered | 10 | VC_904_0 | 10 | 0.9878 | 0.99948433 | 0.9873 | 0 | 0 | 0 | 1 | Ref |
| KR063278 | Unassigned | Unassigned | Unassigned | 893_0 | Clustered | 4 | VC_893_0 | 4 | 0.0787 | 0.99998595 | 0.0787 | 0 | 0 | 0 | 1 | Ref |
| KR063279 | Unassigned | Unassigned | Unassigned | 894_0 | Clustered | 6 | VC_894_0 | 6 | 0.3171 | 1 | 0.3171 | 0 | 0 | 0 | 1 | Ref |
| KR063280 | Unassigned | Unassigned | Unassigned | 1139_0 | Clustered | 5 | VC_1139_0 | 5 | 0.8556 | 0.99869977 | 0.8545 | 0 | 0 | 0 | 1 | Ref |
| KR063281 | Unassigned | Unassigned | Unassigned | 1141_1 | Clustered/Singleton | 1 | VC_1141_1 | 1 | 0 | 1 | 0 | 0 | 0 | 0 | 1 | Ref |
| KR072689 | Unassigned | Unassigned | Unassigned | 955_0 | Clustered | 2 | VC_955_0 | 2 | 0.2751 | 0.89435023 | 0.2461 | 0 | 0 | 0 | 1 | Ref |
| KR073660 | Unassigned | Unassigned | Unassigned | 235_0 | Clustered | 60 | VC_235_0 | 60 | 0.9413 | 1 | 0.9413 | 0 | 0 | 0 | 1 | Ref |
| KR073661 | Unassigned | Unassigned | Unassigned | 235_0 | Clustered | 60 | VC_235_0 | 60 | 0.9413 | 1 | 0.9413 | 0 | 0 | 0 | 1 | Ref |
| KR080193 | Unassigned | Unassigned | Unassigned | 227_0 | Clustered | 4 | VC_227_0 | 4 | 0.0609 | 0.99998808 | 0.0609 | 0 | 0 | 0 | 1 | Ref |
| KR080194 | Unassigned | Unassigned | Unassigned | 385_0 | Clustered | 24 | VC_385_0 | 24 | 0.5516 | 1 | 0.5516 | 0 | 0 | 0 | 1 | Ref |
| KR080195 | Unassigned | Unassigned | Unassigned | 537_0 | Clustered | 39 | VC_537_0 | 39 | 0.8148 | 1 | 0.8148 | 0 | 0 | 0 | 1 | Ref |
| KR080196 | Unassigned | Unassigned | Unassigned | 579_20 | Clustered | 17 | VC_579_20 | 17 | 0.0538 | 1 | 0.0538 | 0 | 0 | 0 | 0.9926 | Ref |
| KR080197 | Unassigned | Unassigned | Unassigned | 784_0 | Clustered | 9 | VC_784_0 | 9 | 0.0884 | 1 | 0.0884 | 0 | 0 | 0 | 1 | Ref |
| KR080198 | Unassigned | Unassigned | Unassigned | 784_0 | Clustered | 9 | VC_784_0 | 9 | 0.0884 | 1 | 0.0884 | 0 | 0 | 0 | 1 | Ref |
| KR080199 | Unassigned | Unassigned | Unassigned | 385_1 | Clustered | 9 | VC_385_1 | 9 | 0.1928 | 1 | 0.1928 | 0 | 0 | 0 | 1 | Ref |
| KR080200 | Unassigned | Unassigned | Unassigned | 385_0 | Clustered | 24 | VC_385_0 | 24 | 0.5516 | 1 | 0.5516 | 0 | 0 | 0 | 1 | Ref |
| KR080201 | Unassigned | Unassigned | Unassigned | 335_49 | Clustered | 17 | VC_335_49 | 17 | 0.0731 | 1 | 0.0731 | 0 | 0 | 0 | 0.7721 | Ref |
| KR080202 | Unassigned | Unassigned | Unassigned | 784_0 | Clustered | 9 | VC_784_0 | 9 | 0.0884 | 1 | 0.0884 | 0 | 0 | 0 | 1 | Ref |
| KR080203 | Unassigned | Unassigned | Unassigned | 527_0 | Clustered | 37 | VC_527_0 | 37 | 0.9679 | 1 | 0.9679 | 0 | 0 | 0 | 1 | Ref |
| KR080204 | Unassigned | Unassigned | Unassigned | 571_2 | Clustered | 17 | VC_571_2 | 17 | 0.0965 | 1 | 0.0965 | 0 | 0 | 0 | 0.9779 | Ref |
| KR080205 | Unassigned | Unassigned | Unassigned | 527_0 | Clustered | 37 | VC_527_0 | 37 | 0.9679 | 1 | 0.9679 | 0 | 0 | 0 | 1 | Ref |
| KR080206 | Unassigned | Unassigned | Unassigned | 244_1 | Clustered | 13 | VC_244_1 | 13 | 0.1766 | 1 | 0.1766 | 0 | 0 | 0 | 0.7308 | Ref |
| KR091952 | Unassigned | Unassigned | Unassigned | 131_0 | Clustered | 43 | VC_131_0 | 43 | 0.8853 | 1 | 0.8853 | 0 | 0 | 0 | 1 | Ref |
| KR093625 | Unassigned | Unassigned | Unassigned | 966_0 | Clustered | 8 | VC_966_0 | 8 | 0.3637 | 1 | 0.3637 | 0 | 0 | 0 | 1 | Ref |
| KR093626 | Unassigned | Unassigned | Unassigned | 966_0 | Clustered | 8 | VC_966_0 | 8 | 0.3637 | 1 | 0.3637 | 0 | 0 | 0 | 1 | Ref |
| KR093627 | Unassigned | Unassigned | Unassigned | 966_0 | Clustered | 8 | VC_966_0 | 8 | 0.3637 | 1 | 0.3637 | 0 | 0 | 0 | 1 | Ref |
| KR093628 | Unassigned | Unassigned | Unassigned | 966_0 | Clustered | 8 | VC_966_0 | 8 | 0.3637 | 1 | 0.3637 | 0 | 0 | 0 | 1 | Ref |
| KR093629 | Unassigned | Unassigned | Unassigned | 966_0 | Clustered | 8 | VC_966_0 | 8 | 0.3637 | 1 | 0.3637 | 0 | 0 | 0 | 1 | Ref |
| KR093630 | Unassigned | Unassigned | Unassigned | 966_0 | Clustered | 8 | VC_966_0 | 8 | 0.3637 | 1 | 0.3637 | 0 | 0 | 0 | 1 | Ref |
| KR093631 | Unassigned | Unassigned | Unassigned | 966_0 | Clustered | 8 | VC_966_0 | 8 | 0.3637 | 1 | 0.3637 | 0 | 0 | 0 | 1 | Ref |
| KR093633 | Unassigned | Unassigned | Unassigned | 966_0 | Clustered | 8 | VC_966_0 | 8 | 0.3637 | 1 | 0.3637 | 0 | 0 | 0 | 1 | Ref |
| KR093634 | Unassigned | Unassigned | Unassigned | 623_0 | Clustered | 22 | VC_623_0 | 22 | 0.784 | 1 | 0.784 | 0 | 0 | 0 | 1 | Ref |
| KR093635 | Unassigned | Unassigned | Unassigned | 623_0 | Clustered | 22 | VC_623_0 | 22 | 0.784 | 1 | 0.784 | 0 | 0 | 0 | 1 | Ref |
| KR093636 | Unassigned | Unassigned | Unassigned | 623_0 | Clustered | 22 | VC_623_0 | 22 | 0.784 | 1 | 0.784 | 0 | 0 | 0 | 1 | Ref |
| KR093637 | Unassigned | Unassigned | Unassigned | 623_0 | Clustered | 22 | VC_623_0 | 22 | 0.784 | 1 | 0.784 | 0 | 0 | 0 | 1 | Ref |
| KR093639 | Unassigned | Unassigned | Unassigned | 623_0 | Clustered | 22 | VC_623_0 | 22 | 0.784 | 1 | 0.784 | 0 | 0 | 0 | 1 | Ref |
| KR093640 | Unassigned | Unassigned | Unassigned | 623_0 | Clustered | 22 | VC_623_0 | 22 | 0.784 | 1 | 0.784 | 0 | 0 | 0 | 1 | Ref |
| KR093641 | Unassigned | Unassigned | Unassigned | 623_0 | Clustered | 22 | VC_623_0 | 22 | 0.784 | 1 | 0.784 | 0 | 0 | 0 | 1 | Ref |
| KR093642 | Unassigned | Unassigned | Unassigned | 623_0 | Clustered | 22 | VC_623_0 | 22 | 0.784 | 1 | 0.784 | 0 | 0 | 0 | 1 | Ref |

| | | | | | | | | | | | | | | | | |
|---|---|---|---|---|---|---|---|---|---|---|---|---|---|---|---|---|
| KR093643 | Unassigned | Unassigned | Unassigned | 623_0 | Clustered | 22 | VC_623_0 | 22 | 0.784 | 1 | 0.784 | 0 | 0 | 0 | 1 | Ref |
| KR093644 | Unassigned | Unassigned | Unassigned | 623_0 | Clustered | 22 | VC_623_0 | 22 | 0.784 | 1 | 0.784 | 0 | 0 | 0 | 1 | Ref |
| KR093645 | Unassigned | Unassigned | Unassigned | 623_0 | Clustered | 22 | VC_623_0 | 22 | 0.784 | 1 | 0.784 | 0 | 0 | 0 | 1 | Ref |
| KR093646 | Unassigned | Unassigned | Unassigned | 623_0 | Clustered | 22 | VC_623_0 | 22 | 0.784 | 1 | 0.784 | 0 | 0 | 0 | 1 | Ref |
| KR093647 | Unassigned | Unassigned | Unassigned | 623_0 | Clustered | 22 | VC_623_0 | 22 | 0.784 | 1 | 0.784 | 0 | 0 | 0 | 1 | Ref |
| KR093648 | Unassigned | Unassigned | Unassigned | 623_0 | Clustered | 22 | VC_623_0 | 22 | 0.784 | 1 | 0.784 | 0 | 0 | 0 | 1 | Ref |
| KR093649 | Unassigned | Unassigned | Unassigned | 623_0 | Clustered | 22 | VC_623_0 | 22 | 0.784 | 1 | 0.784 | 0 | 0 | 0 | 1 | Ref |
| KR093650 | Unassigned | Unassigned | Unassigned | 623_0 | Clustered | 22 | VC_623_0 | 22 | 0.784 | 1 | 0.784 | 0 | 0 | 0 | 1 | Ref |
| KR093651 | Unassigned | Unassigned | Unassigned | 623_0 | Clustered | 22 | VC_623_0 | 22 | 0.784 | 1 | 0.784 | 0 | 0 | 0 | 1 | Ref |
| KR093652 | Unassigned | Unassigned | Unassigned | 623_0 | Clustered | 22 | VC_623_0 | 22 | 0.784 | 1 | 0.784 | 0 | 0 | 0 | 1 | Ref |
| KR093653 | Unassigned | Unassigned | Unassigned | 623_0 | Clustered | 22 | VC_623_0 | 22 | 0.784 | 1 | 0.784 | 0 | 0 | 0 | 1 | Ref |
| KR093654 | Unassigned | Unassigned | Unassigned | 623_0 | Clustered | 22 | VC_623_0 | 22 | 0.784 | 1 | 0.784 | 0 | 0 | 0 | 1 | Ref |
| KR093655 | Unassigned | Unassigned | Unassigned | 623_0 | Clustered | 22 | VC_623_0 | 22 | 0.784 | 1 | 0.784 | 0 | 0 | 0 | 1 | Ref |
| KR093656 | Unassigned | Unassigned | Unassigned | 623_0 | Clustered | 22 | VC_623_0 | 22 | 0.784 | 1 | 0.784 | 0 | 0 | 0 | 1 | Ref |
| KR131750 | Unassigned | Unassigned | Unassigned | 711_0 | Clustered | 48 | VC_711_0 | 48 | 0.8217 | 1 | 0.8217 | 0 | 0 | 0 | 1 | Ref |
| KR136259 | Unassigned | Unassigned | Unassigned | 1050_0 | Clustered | 2 | VC_1050_0 | 2 | 0.9703 | 0.81445332 | 0.7903 | 0 | 0 | 0 | 1 | Ref |
| KR136260 | Unassigned | Unassigned | Unassigned | 1050_0 | Clustered | 2 | VC_1050_0 | 2 | 0.9703 | 0.81445332 | 0.7903 | 0 | 0 | 0 | 1 | Ref |
| KR149290 | Unassigned | Unassigned | Unassigned | 714_0 | Clustered | 71 | VC_714_0 | 71 | 0.935 | 1 | 0.935 | 0 | 0 | 0 | 1 | Ref |
| KR149291 | Unassigned | Unassigned | Unassigned | 848_0 | Clustered | 124 | VC_848_0 | 124 | 0.9484 | 1 | 0.9484 | 0 | 0 | 0 | 1 | Ref |
| KR153145 | Unassigned | Unassigned | Unassigned | 477_0 | Clustered | 4 | VC_477_0 | 4 | 0.7825 | 0.99997292 | 0.7825 | 0 | 0 | 0 | 1 | Ref |
| KR233164 | Unassigned | Unassigned | Unassigned | 198_27 | Clustered/Singleton | 1 | VC_198_27 | 1 | 0 | 1 | 0 | 0 | 0 | 0 | 1 | Ref |
| KR233165 | Unassigned | Unassigned | Unassigned | 290_163 | Clustered | 2 | VC_290_163 | 2 | 0.0029 | 0.93227927 | 0.0027 | 0 | 0 | 0 | 1 | Ref |
| KR262148 | Unassigned | Unassigned | Unassigned | 972_0 | Clustered | 99 | VC_972_0 | 99 | 0.9113 | 1 | 0.9113 | 0 | 0 | 0 | 0.9928 | Ref |
| KR269718 | Unassigned | Unassigned | Unassigned | 290_81 | Clustered/Singleton | 1 | VC_290_81 | 1 | 0 | 1 | 0 | 0 | 0 | 0 | 1 | Ref |
| KR269719 | Unassigned | Unassigned | Unassigned | 971_0 | Clustered | 40 | VC_971_0 | 40 | 0.6793 | 1 | 0.6793 | 0 | 0 | 0 | 0.9359 | Ref |
| KR269720 | Unassigned | Unassigned | Unassigned | 509_0 | Clustered | 36 | VC_509_0 | 36 | 0.5291 | 1 | 0.5291 | 0 | 0 | 0 | 0.9714 | Ref |
| KR270151 | Unassigned | Unassigned | Unassigned | 654_0 | Clustered | 115 | VC_654_0 | 115 | 0.7303 | 1 | 0.7303 | 0 | 0 | 0 | 0.8764 | Ref |
| KR296686 | Unassigned | Unassigned | Unassigned | 30_1 | Clustered | 53 | VC_30_1 | 53 | 0.7151 | 1 | 0.7151 | 0 | 0 | 0 | 0.9325 | Ref |
| KR296687 | Unassigned | Unassigned | Unassigned | 30_1 | Clustered | 53 | VC_30_1 | 53 | 0.7151 | 1 | 0.7151 | 0 | 0 | 0 | 0.9325 | Ref |
| KR296688 | Unassigned | Unassigned | Unassigned | 30_1 | Clustered | 53 | VC_30_1 | 53 | 0.7151 | 1 | 0.7151 | 0 | 0 | 0 | 0.9325 | Ref |
| KR296689 | Unassigned | Unassigned | Unassigned | 611_0 | Clustered | 50 | VC_611_0 | 50 | 0.7306 | 1 | 0.7306 | 0 | 0 | 0 | 1 | Ref |
| KR296691 | Unassigned | Unassigned | Unassigned | 611_0 | Clustered | 50 | VC_611_0 | 50 | 0.7306 | 1 | 0.7306 | 0 | 0 | 0 | 1 | Ref |
| KR296692 | Unassigned | Unassigned | Unassigned | 135_18 | Clustered/Singleton | 1 | VC_135_18 | 1 | 0 | 1 | 0 | 0 | 0 | 0 | 1 | Ref |
| KR337643 | Unassigned | Unassigned | Unassigned | 787_0 | Clustered | 132 | VC_787_0 | 132 | 1 | 1 | 1 | 0 | 0 | 0 | 1 | Ref |
| KR337644 | Unassigned | Unassigned | Unassigned | 787_0 | Clustered | 132 | VC_787_0 | 132 | 1 | 1 | 1 | 0 | 0 | 0 | 1 | Ref |
| KR337645 | Unassigned | Unassigned | Unassigned | 787_0 | Clustered | 132 | VC_787_0 | 132 | 1 | 1 | 1 | 0 | 0 | 0 | 1 | Ref |
| KR337646 | Unassigned | Unassigned | Unassigned | 787_0 | Clustered | 132 | VC_787_0 | 132 | 1 | 1 | 1 | 0 | 0 | 0 | 1 | Ref |
| KR337647 | Unassigned | Unassigned | Unassigned | 787_0 | Clustered | 132 | VC_787_0 | 132 | 1 | 1 | 1 | 0 | 0 | 0 | 1 | Ref |
| KR337648 | Unassigned | Unassigned | Unassigned | 787_0 | Clustered | 132 | VC_787_0 | 132 | 1 | 1 | 1 | 0 | 0 | 0 | 1 | Ref |
| KR337649 | Unassigned | Unassigned | Unassigned | 787_0 | Clustered | 132 | VC_787_0 | 132 | 1 | 1 | 1 | 0 | 0 | 0 | 1 | Ref |
| KR337650 | Unassigned | Unassigned | Unassigned | 787_0 | Clustered | 132 | VC_787_0 | 132 | 1 | 1 | 1 | 0 | 0 | 0 | 1 | Ref |
| KR337651 | Unassigned | Unassigned | Unassigned | 787_0 | Clustered | 132 | VC_787_0 | 132 | 1 | 1 | 1 | 0 | 0 | 0 | 1 | Ref |
| KR337652 | Unassigned | Unassigned | Unassigned | 787_0 | Clustered | 132 | VC_787_0 | 132 | 1 | 1 | 1 | 0 | 0 | 0 | 1 | Ref |
| KR337653 | Unassigned | Unassigned | Unassigned | 787_0 | Clustered | 132 | VC_787_0 | 132 | 1 | 1 | 1 | 0 | 0 | 0 | 1 | Ref |
| KR337654 | Unassigned | Unassigned | Unassigned | 787_0 | Clustered | 132 | VC_787_0 | 132 | 1 | 1 | 1 | 0 | 0 | 0 | 1 | Ref |
| KR347352 | Unassigned | Unassigned | Unassigned | 787_0 | Clustered | 132 | VC_787_0 | 132 | 1 | 1 | 1 | 0 | 0 | 0 | 1 | Ref |
| KR347353 | Unassigned | Unassigned | Unassigned | 787_0 | Clustered | 132 | VC_787_0 | 132 | 1 | 1 | 1 | 0 | 0 | 0 | 1 | Ref |
| KR347354 | Unassigned | Unassigned | Unassigned | 787_0 | Clustered | 132 | VC_787_0 | 132 | 1 | 1 | 1 | 0 | 0 | 0 | 1 | Ref |
| KR347355 | Unassigned | Unassigned | Unassigned | 787_0 | Clustered | 132 | VC_787_0 | 132 | 1 | 1 | 1 | 0 | 0 | 0 | 1 | Ref |
| KR422352 | Unassigned | Unassigned | Unassigned | 293_1 | Clustered | 21 | VC_293_1 | 21 | 0.1694 | 1 | 0.1694 | 0 | 0 | 0 | 0.9048 | Ref |
| KR422353 | Unassigned | Unassigned | Unassigned | 198_10 | Clustered/Singleton | 1 | VC_198_10 | 1 | 0 | 1 | 0 | 0 | 0 | 0 | 1 | Ref |
| KR534323 | Unassigned | Unassigned | Unassigned | 514_2 | Clustered/Singleton | 1 | VC_514_2 | 1 | 0 | 1 | 0 | 0 | 0 | 0 | 1 | Ref |
| KR537871 | Unassigned | Unassigned | Unassigned | 766_0 | Clustered | 40 | VC_766_0 | 40 | 0.7511 | 1 | 0.7511 | 0 | 0 | 0 | 1 | Ref |
| KR537872 | Unassigned | Unassigned | Unassigned | 766_0 | Clustered | 40 | VC_766_0 | 40 | 0.7511 | 1 | 0.7511 | 0 | 0 | 0 | 1 | Ref |
| KR560069 | Unassigned | Unassigned | Unassigned | 524_0 | Clustered | 8 | VC_524_0 | 8 | 0.7365 | 1 | 0.7365 | 0 | 0 | 0 | 1 | Ref |
| KR604693 | Unassigned | Unassigned | Unassigned | 1018_0 | Clustered | 38 | VC_1018_0 | 38 | 0.9757 | 1 | 0.9757 | 0 | 0 | 0 | 1 | Ref |
| KR698074 | Unassigned | Unassigned | Unassigned | 794_8 | Clustered | 2 | VC_794_8 | 2 | 0.006 | 0.58293682 | 0.0035 | 0 | 0 | 0 | 1 | Ref |
| KR704913 | Unassigned | Unassigned | Unassigned | 1144_0 | Clustered | 2 | VC_1144_0 | 2 | 1 | 1 | 1 | 0 | 0 | 0 | 1 | Ref |
| KR704915 | Unassigned | Unassigned | Unassigned | 1144_0 | Clustered | 2 | VC_1144_0 | 2 | 1 | 1 | 1 | 0 | 0 | 0 | 1 | Ref |
| KR709302 | Unassigned | Unassigned | Unassigned | 12_2 | Clustered | 19 | VC_12_2 | 19 | 0.2009 | 1 | 0.2009 | 0 | 0 | 0 | 1 | Ref |
| KR709303 | Unassigned | Unassigned | Unassigned | 12_2 | Clustered | 19 | VC_12_2 | 19 | 0.2009 | 1 | 0.2009 | 0 | 0 | 0 | 1 | Ref |
| KR781349 | Unassigned | Unassigned | Unassigned | 384_21 | Clustered | 2 | VC_384_21 | 2 | 0.0033 | 0.95771013 | 0.0032 | 0 | 0 | 0 | 1 | Ref |
| KR781488 | Unassigned | Unassigned | Unassigned | 44_0 | Clustered | 49 | VC_44_0 | 49 | 0.4506 | 1 | 0.4506 | 0 | 0 | 0 | 0.8605 | Ref |
| KR816508 | Unassigned | Unassigned | Unassigned | 384_18 | Clustered | 10 | VC_384_18 | 10 | 0.0301 | 1 | 0.0301 | 0 | 0 | 0 | 0.8 | Ref |
| KR824843 | Unassigned | Unassigned | Unassigned | 538_2 | Clustered/Singleton | 1 | VC_538_2 | 1 | 0 | 1 | 0 | 0 | 0 | 0 | 1 | Ref |
| KR869157 | Unassigned | Unassigned | Unassigned | 80_0 | Clustered | 64 | VC_80_0 | 64 | 0.418 | 1 | 0.418 | 0 | 0 | 0 | 0.7649 | Ref |
| KR869820 | Unassigned | Unassigned | Unassigned | 687_1 | Clustered | 10 | VC_687_1 | 10 | 0.1263 | 1 | 0.1263 | 0 | 0 | 0 | 0.9778 | Ref |
| KR902361 | Unassigned | Unassigned | Unassigned | 155_8 | Clustered | 66 | VC_155_8 | 66 | 0.4537 | 1 | 0.4537 | 0 | 0 | 0 | 0.8182 | Ref |
| KR902978 | Unassigned | Unassigned | Unassigned | 787_0 | Clustered | 132 | VC_787_0 | 132 | 1 | 1 | 1 | 0 | 0 | 0 | 1 | Ref |
| KR902979 | Unassigned | Unassigned | Unassigned | 787_0 | Clustered | 132 | VC_787_0 | 132 | 1 | 1 | 1 | 0 | 0 | 0 | 1 | Ref |
| KR902980 | Unassigned | Unassigned | Unassigned | 787_0 | Clustered | 132 | VC_787_0 | 132 | 1 | 1 | 1 | 0 | 0 | 0 | 1 | Ref |
| KR902981 | Unassigned | Unassigned | Unassigned | 787_0 | Clustered | 132 | VC_787_0 | 132 | 1 | 1 | 1 | 0 | 0 | 0 | 1 | Ref |
| KR902982 | Unassigned | Unassigned | Unassigned | 787_0 | Clustered | 132 | VC_787_0 | 132 | 1 | 1 | 1 | 0 | 0 | 0 | 1 | Ref |
| KR902983 | Unassigned | Unassigned | Unassigned | 787_0 | Clustered | 132 | VC_787_0 | 132 | 1 | 1 | 1 | 0 | 0 | 0 | 1 | Ref |
| KR902984 | Unassigned | Unassigned | Unassigned | 787_0 | Clustered | 132 | VC_787_0 | 132 | 1 | 1 | 1 | 0 | 0 | 0 | 1 | Ref |
| KR902985 | Unassigned | Unassigned | Unassigned | 787_0 | Clustered | 132 | VC_787_0 | 132 | 1 | 1 | 1 | 0 | 0 | 0 | 1 | Ref |
| KR902986 | Unassigned | Unassigned | Unassigned | 787_0 | Clustered | 132 | VC_787_0 | 132 | 1 | 1 | 1 | 0 | 0 | 0 | 1 | Ref |
| KR902987 | Unassigned | Unassigned | Unassigned | 787_0 | Clustered | 132 | VC_787_0 | 132 | 1 | 1 | 1 | 0 | 0 | 0 | 1 | Ref |
| KR905068 | Unassigned | Unassigned | Unassigned | 396_0 | Clustered | 3 | VC_396_0 | 3 | 0.3827 | 0.99764103 | 0.3818 | 0 | 0 | 0 | 1 | Ref |
| KR908644 | Unassigned | Unassigned | Unassigned | 155_8 | Clustered | 66 | VC_155_8 | 66 | 0.4537 | 1 | 0.4537 | 0 | 0 | 0 | 0.8182 | Ref |
| KR935213 | Unassigned | Unassigned | Unassigned | 766_0 | Clustered | 40 | VC_766_0 | 40 | 0.7511 | 1 | 0.7511 | 0 | 0 | 0 | 1 | Ref |

| | | | | | | | | | | | | | | | | |
|---|---|---|---|---|---|---|---|---|---|---|---|---|---|---|---|---|
| KR935214 | Unassigned | Unassigned | Unassigned | 538_73 | Clustered | 2 | VC_538_73 | 2 | 0.0107 | 0.95762743 | 0.0102 | 0 | 0 | 0 | 1 | Ref |
| KR935215 | Unassigned | Unassigned | Unassigned | 766_0 | Clustered | 40 | VC_766_0 | 40 | 0.7511 | 1 | 0.7511 | 0 | 0 | 0 | 1 | Ref |
| KR935216 | Unassigned | Unassigned | Unassigned | 903_0 | Clustered | 3 | VC_903_0 | 3 | 0.6217 | 0.99814904 | 0.6206 | 0 | 0 | 0 | 1 | Ref |
| KR935217 | Unassigned | Unassigned | Unassigned | 903_0 | Clustered | 3 | VC_903_0 | 3 | 0.6217 | 0.99814904 | 0.6206 | 0 | 0 | 0 | 1 | Ref |
| KR997929 | Unassigned | Unassigned | Unassigned | 335_7 | Clustered | 3 | VC_335_7 | 3 | 0.0085 | 0.99739782 | 0.0085 | 0 | 0 | 0 | 0.6667 | Ref |
| KR997930 | Unassigned | Unassigned | Unassigned | 538_35 | Clustered | 3 | VC_538_35 | 3 | 0.0249 | 0.99859857 | 0.0249 | 0 | 0 | 0 | 1 | Ref |
| KR997931 | Unassigned | Unassigned | Unassigned | 538_35 | Clustered | 3 | VC_538_35 | 3 | 0.0249 | 0.99859857 | 0.0249 | 0 | 0 | 0 | 1 | Ref |
| KR997932 | Unassigned | Unassigned | Unassigned | 526_0 | Clustered | 32 | VC_526_0 | 32 | 0.9037 | 1 | 0.9037 | 0 | 0 | 0 | 1 | Ref |
| KR997933 | Unassigned | Unassigned | Unassigned | 589_0 | Clustered | 3 | VC_589_0 | 3 | 0.2072 | 0.99849937 | 0.2069 | 0 | 0 | 0 | 1 | Ref |
| KR997934 | Unassigned | Unassigned | Unassigned | 335_7 | Clustered | 3 | VC_335_7 | 3 | 0.0085 | 0.99739782 | 0.0085 | 0 | 0 | 0 | 0.6667 | Ref |
| KR997967 | Unassigned | Unassigned | Unassigned | 384_1 | Clustered | 11 | VC_384_1 | 11 | 0.0378 | 1 | 0.0378 | 0 | 0 | 0 | 0.8545 | Ref |
| KR997968 | Unassigned | Unassigned | Unassigned | 538_35 | Clustered | 3 | VC_538_35 | 3 | 0.0249 | 0.99859857 | 0.0249 | 0 | 0 | 0 | 1 | Ref |
| KT001911 | Unassigned | Unassigned | Unassigned | 1071_0 | Clustered | 7 | VC_1071_0 | 7 | 0.9883 | 0.99999504 | 0.9883 | 0 | 0 | 0 | 1 | Ref |
| KT001912 | Unassigned | Unassigned | Unassigned | 104_0 | Clustered | 2 | VC_104_0 | 2 | 0.1066 | 0.94642368 | 0.1008 | 0 | 0 | 0 | 1 | Ref |
| KT001913 | Unassigned | Unassigned | Unassigned | 176_0 | Clustered/Singleton | 1 | VC_176_0 | 1 | 0 | 1 | 0 | 0 | 0 | 0 | 1 | Ref |
| KT001914 | Unassigned | Unassigned | Unassigned | 1132_0 | Clustered | 4 | VC_1132_0 | 4 | 1 | 1 | 1 | 0 | 0 | 0 | 1 | Ref |
| KT001916 | Unassigned | Unassigned | Unassigned | 341_0 | Clustered | 27 | VC_341_0 | 27 | 0.5058 | 1 | 0.5058 | 0 | 0 | 0 | 0.8661 | Ref |
| KT001917 | Unassigned | Unassigned | Unassigned | 794_19 | Clustered | 16 | VC_794_19 | 16 | 0.0975 | 0.99616626 | 0.0971 | 0 | 0 | 0 | 0.8083 | Ref |
| KT001918 | Unassigned | Unassigned | Unassigned | 686_0 | Clustered | 25 | VC_686_0 | 25 | 0.5501 | 1 | 0.5501 | 0 | 0 | 0 | 0.96 | Ref |
| KT001919 | Unassigned | Unassigned | Unassigned | 686_0 | Clustered | 25 | VC_686_0 | 25 | 0.5501 | 1 | 0.5501 | 0 | 0 | 0 | 0.96 | Ref |
| KT001920 | Unassigned | Unassigned | Unassigned | 972_0 | Clustered | 99 | VC_972_0 | 99 | 0.9113 | 1 | 0.9113 | 0 | 0 | 0 | 0.9928 | Ref |
| KT004677 | Unassigned | Unassigned | Unassigned | 974_0 | Clustered | 34 | VC_974_0 | 34 | 0.7953 | 1 | 0.7953 | 0 | 0 | 0 | 1 | Ref |
| KT008108 | Unassigned | Unassigned | Unassigned | 398_0 | Clustered | 51 | VC_398_0 | 51 | 0.8741 | 1 | 0.8741 | 0 | 0 | 0 | 1 | Ref |
| KT020852 | Unassigned | Unassigned | Unassigned | 571_7 | Clustered | 28 | VC_571_7 | 28 | 0.1651 | 1 | 0.1651 | 0 | 0 | 0 | 0.8519 | Ref |
| KT070866 | Unassigned | Unassigned | Unassigned | 1042_0 | Clustered | 8 | VC_1042_0 | 8 | 0.7385 | 1 | 0.7385 | 0 | 0 | 0 | 1 | Ref |
| KT070867 | Unassigned | Unassigned | Unassigned | 489_6 | Clustered/Singleton | 1 | VC_489_6 | 1 | 0 | 1 | 0 | 0 | 0 | 0 | 1 | Ref |
| KT124227 | Unassigned | Unassigned | Unassigned | 1016_0 | Clustered | 62 | VC_1016_0 | 62 | 0.8638 | 1 | 0.8638 | 0 | 0 | 0 | 0.743 | Ref |
| KT124228 | Unassigned | Unassigned | Unassigned | 1016_0 | Clustered | 62 | VC_1016_0 | 62 | 0.8638 | 1 | 0.8638 | 0 | 0 | 0 | 0.743 | Ref |
| KT124229 | Unassigned | Unassigned | Unassigned | 1016_0 | Clustered | 62 | VC_1016_0 | 62 | 0.8638 | 1 | 0.8638 | 0 | 0 | 0 | 0.743 | Ref |
| KT151955 | Unassigned | Unassigned | Unassigned | 735_0 | Clustered | 2 | VC_735_0 | 2 | 0.5598 | 0.89435023 | 0.5007 | 0 | 0 | 0 | 1 | Ref |
| KT151956 | Unassigned | Unassigned | Unassigned | 142_0 | Clustered | 6 | VC_142_0 | 6 | 0.7366 | 1 | 0.7366 | 0 | 0 | 0 | 1 | Ref |
| KT151957 | Unassigned | Unassigned | Unassigned | 735_0 | Clustered | 2 | VC_735_0 | 2 | 0.5598 | 0.89435023 | 0.5007 | 0 | 0 | 0 | 1 | Ref |
| KT151958 | Unassigned | Unassigned | Unassigned | 142_0 | Clustered | 6 | VC_142_0 | 6 | 0.7366 | 1 | 0.7366 | 0 | 0 | 0 | 1 | Ref |
| KT152029 | Unassigned | Unassigned | Unassigned | 1016_0 | Clustered | 62 | VC_1016_0 | 62 | 0.8638 | 1 | 0.8638 | 0 | 0 | 0 | 0.743 | Ref |
| KT160311 | Unassigned | Unassigned | Unassigned | 516_0 | Clustered | 24 | VC_516_0 | 24 | 0.8556 | 1 | 0.8556 | 0 | 0 | 0 | 0.9493 | Ref |
| KT176190 | Unassigned | Unassigned | Unassigned | 297_5 | Clustered | 14 | VC_297_5 | 14 | 0.141 | 1 | 0.141 | 0 | 0 | 0 | 0.9231 | Ref |
| KT184308 | Unassigned | Unassigned | Unassigned | 290_66 | Clustered/Singleton | 1 | VC_290_66 | 1 | 0 | 1 | 0 | 0 | 0 | 0 | 1 | Ref |
| KT184309 | Unassigned | Unassigned | Unassigned | 293_17 | Clustered | 8 | VC_293_17 | 8 | 0.0534 | 0.99997879 | 0.0534 | 0 | 0 | 0 | 0.6071 | Ref |
| KT184310 | Unassigned | Unassigned | Unassigned | 293_17 | Clustered | 8 | VC_293_17 | 8 | 0.0534 | 0.99997879 | 0.0534 | 0 | 0 | 0 | 0.6071 | Ref |
| KT184311 | Unassigned | Unassigned | Unassigned | 290_57 | Clustered/Singleton | 1 | VC_290_57 | 1 | 0 | 1 | 0 | 0 | 0 | 0 | 1 | Ref |
| KT184312 | Unassigned | Unassigned | Unassigned | 290_63 | Clustered/Singleton | 1 | VC_290_63 | 1 | 0 | 1 | 0 | 0 | 0 | 0 | 1 | Ref |
| KT184313 | Unassigned | Unassigned | Unassigned | 340_7 | Clustered | 18 | VC_340_7 | 18 | 0.0936 | 1 | 0.0936 | 0 | 0 | 0 | 0.7778 | Ref |
| KT184314 | Unassigned | Unassigned | Unassigned | 340_7 | Clustered | 18 | VC_340_7 | 18 | 0.0936 | 1 | 0.0936 | 0 | 0 | 0 | 0.7778 | Ref |
| KT184315 | Unassigned | Unassigned | Unassigned | 340_7 | Clustered | 18 | VC_340_7 | 18 | 0.0936 | 1 | 0.0936 | 0 | 0 | 0 | 0.7778 | Ref |
| KT184316 | Unassigned | Unassigned | Unassigned | 340_7 | Clustered | 18 | VC_340_7 | 18 | 0.0936 | 1 | 0.0936 | 0 | 0 | 0 | 0.7778 | Ref |
| KT184390 | Unassigned | Unassigned | Unassigned | 1016_0 | Clustered | 62 | VC_1016_0 | 62 | 0.8638 | 1 | 0.8638 | 0 | 0 | 0 | 0.743 | Ref |
| KT184391 | Unassigned | Unassigned | Unassigned | 1016_0 | Clustered | 62 | VC_1016_0 | 62 | 0.8638 | 1 | 0.8638 | 0 | 0 | 0 | 0.743 | Ref |
| KT184661 | Unassigned | Unassigned | Unassigned | 907_0 | Clustered | 32 | VC_907_0 | 32 | 0.9964 | 1 | 0.9964 | 0 | 0 | 0 | 1 | Ref |
| KT186228 | Unassigned | Unassigned | Unassigned | 1016_0 | Clustered | 62 | VC_1016_0 | 62 | 0.8638 | 1 | 0.8638 | 0 | 0 | 0 | 0.743 | Ref |
| KT186229 | Unassigned | Unassigned | Unassigned | 1016_0 | Clustered | 62 | VC_1016_0 | 62 | 0.8638 | 1 | 0.8638 | 0 | 0 | 0 | 0.743 | Ref |
| KT186243 | Unassigned | Unassigned | Unassigned | 8_0 | Clustered | 58 | VC_8_0 | 58 | 0.8285 | 1 | 0.8285 | 0 | 0 | 0 | 0.9946 | Ref |
| KT187252 | Unassigned | Unassigned | Unassigned | 195_1 | Clustered | 7 | VC_195_1 | 7 | 0.1409 | 1 | 0.1409 | 0 | 0 | 0 | 1 | Ref |
| KT207918 | Unassigned | Unassigned | Unassigned | 913_0 | Clustered | 28 | VC_913_0 | 28 | 0.6206 | 1 | 0.6206 | 0 | 0 | 0 | 0.9841 | Ref |
| KT221034 | Unassigned | Unassigned | Unassigned | 606_0 | Clustered | 2 | VC_606_0 | 2 | 0.3086 | 0.91192888 | 0.2814 | 0 | 0 | 0 | 1 | Ref |
| KT222940 | Unassigned | Unassigned | Unassigned | 834_0 | Clustered | 18 | VC_834_0 | 18 | 0.9116 | 1 | 0.9116 | 0 | 0 | 0 | 1 | Ref |
| KT222941 | Unassigned | Unassigned | Unassigned | 571_2 | Clustered | 17 | VC_571_2 | 17 | 0.0965 | 1 | 0.0965 | 0 | 0 | 0 | 0.9779 | Ref |
| KT222942 | Unassigned | Unassigned | Unassigned | 571_2 | Clustered | 17 | VC_571_2 | 17 | 0.0965 | 1 | 0.0965 | 0 | 0 | 0 | 0.9779 | Ref |
| KT224359 | Unassigned | Unassigned | Unassigned | 195_1 | Clustered | 7 | VC_195_1 | 7 | 0.1409 | 1 | 0.1409 | 0 | 0 | 0 | 1 | Ref |
| KT232076 | Unassigned | Unassigned | Unassigned | 45_0 | Clustered | 38 | VC_45_0 | 38 | 0.5785 | 1 | 0.5785 | 0 | 0 | 0 | 0.9659 | Ref |
| KT239446 | Unassigned | Unassigned | Unassigned | 509_0 | Clustered | 36 | VC_509_0 | 36 | 0.5291 | 1 | 0.5291 | 0 | 0 | 0 | 0.9714 | Ref |
| KT240186 | Unassigned | Unassigned | Unassigned | 1018_0 | Clustered | 38 | VC_1018_0 | 38 | 0.9757 | 1 | 0.9757 | 0 | 0 | 0 | 1 | Ref |
| KT246485 | Unassigned | Unassigned | Unassigned | 834_0 | Clustered | 18 | VC_834_0 | 18 | 0.9116 | 1 | 0.9116 | 0 | 0 | 0 | 1 | Ref |
| KT246486 | Unassigned | Unassigned | Unassigned | 974_0 | Clustered | 34 | VC_974_0 | 34 | 0.7953 | 1 | 0.7953 | 0 | 0 | 0 | 1 | Ref |
| KT253150 | Unassigned | Unassigned | Unassigned | 903_0 | Clustered | 3 | VC_903_0 | 3 | 0.6217 | 0.99814904 | 0.6206 | 0 | 0 | 0 | 1 | Ref |
| KT254130 | Unassigned | Unassigned | Unassigned | 81_0 | Clustered | 41 | VC_81_0 | 41 | 0.5815 | 1 | 0.5815 | 0 | 0 | 0 | 0.9805 | Ref |
| KT254131 | Unassigned | Unassigned | Unassigned | 81_0 | Clustered | 41 | VC_81_0 | 41 | 0.5815 | 1 | 0.5815 | 0 | 0 | 0 | 0.9805 | Ref |
| KT254132 | Unassigned | Unassigned | Unassigned | 81_0 | Clustered | 41 | VC_81_0 | 41 | 0.5815 | 1 | 0.5815 | 0 | 0 | 0 | 0.9805 | Ref |
| KT254133 | Unassigned | Unassigned | Unassigned | 81_0 | Clustered | 41 | VC_81_0 | 41 | 0.5815 | 1 | 0.5815 | 0 | 0 | 0 | 0.9805 | Ref |
| KT259047 | Unassigned | Unassigned | Unassigned | 335_23 | Clustered | 17 | VC_335_23 | 17 | 0.0714 | 1 | 0.0714 | 0 | 0 | 0 | 0.6397 | Ref |
| KT264749 | Unassigned | Unassigned | Unassigned | 1145_0 | Clustered | 164 | VC_1145_0 | 164 | 0.8868 | 1 | 0.8868 | 0 | 0 | 0 | 1 | Ref |
| KT264750 | Unassigned | Unassigned | Unassigned | 1145_0 | Clustered | 164 | VC_1145_0 | 164 | 0.8868 | 1 | 0.8868 | 0 | 0 | 0 | 1 | Ref |
| KT264751 | Unassigned | Unassigned | Unassigned | 1145_0 | Clustered | 164 | VC_1145_0 | 164 | 0.8868 | 1 | 0.8868 | 0 | 0 | 0 | 1 | Ref |
| KT264752 | Unassigned | Unassigned | Unassigned | 1145_0 | Clustered | 164 | VC_1145_0 | 164 | 0.8868 | 1 | 0.8868 | 0 | 0 | 0 | 1 | Ref |
| KT264753 | Unassigned | Unassigned | Unassigned | 1145_0 | Clustered | 164 | VC_1145_0 | 164 | 0.8868 | 1 | 0.8868 | 0 | 0 | 0 | 1 | Ref |
| KT264754 | Unassigned | Unassigned | Unassigned | 1145_0 | Clustered | 164 | VC_1145_0 | 164 | 0.8868 | 1 | 0.8868 | 0 | 0 | 0 | 1 | Ref |
| KT264755 | Unassigned | Unassigned | Unassigned | 1145_0 | Clustered | 164 | VC_1145_0 | 164 | 0.8868 | 1 | 0.8868 | 0 | 0 | 0 | 1 | Ref |
| KT264756 | Unassigned | Unassigned | Unassigned | 1145_0 | Clustered | 164 | VC_1145_0 | 164 | 0.8868 | 1 | 0.8868 | 0 | 0 | 0 | 1 | Ref |
| KT264757 | Unassigned | Unassigned | Unassigned | 1145_0 | Clustered | 164 | VC_1145_0 | 164 | 0.8868 | 1 | 0.8868 | 0 | 0 | 0 | 1 | Ref |
| KT264758 | Unassigned | Unassigned | Unassigned | 1145_0 | Clustered | 164 | VC_1145_0 | 164 | 0.8868 | 1 | 0.8868 | 0 | 0 | 0 | 1 | Ref |
| KT264759 | Unassigned | Unassigned | Unassigned | 1145_0 | Clustered | 164 | VC_1145_0 | 164 | 0.8868 | 1 | 0.8868 | 0 | 0 | 0 | 1 | Ref |
| KT264760 | Unassigned | Unassigned | Unassigned | 1145_0 | Clustered | 164 | VC_1145_0 | 164 | 0.8868 | 1 | 0.8868 | 0 | 0 | 0 | 1 | Ref |

| | | | | | | | | | | | | | | | | |
|---|---|---|---|---|---|---|---|---|---|---|---|---|---|---|---|---|
| KT264761 | Unassigned | Unassigned | Unassigned | 1145_0 | Clustered | 164 | VC_1145_0 | 164 | 0.8868 | 1 | 0.8868 | 0 | 0 | 0 | 1 | Ref |
| KT264763 | Unassigned | Unassigned | Unassigned | 1145_0 | Clustered | 164 | VC_1145_0 | 164 | 0.8868 | 1 | 0.8868 | 0 | 0 | 0 | 1 | Ref |
| KT264764 | Unassigned | Unassigned | Unassigned | 1145_0 | Clustered | 164 | VC_1145_0 | 164 | 0.8868 | 1 | 0.8868 | 0 | 0 | 0 | 1 | Ref |
| KT264765 | Unassigned | Unassigned | Unassigned | 1145_0 | Clustered | 164 | VC_1145_0 | 164 | 0.8868 | 1 | 0.8868 | 0 | 0 | 0 | 1 | Ref |
| KT264766 | Unassigned | Unassigned | Unassigned | 1145_0 | Clustered | 164 | VC_1145_0 | 164 | 0.8868 | 1 | 0.8868 | 0 | 0 | 0 | 1 | Ref |
| KT264767 | Unassigned | Unassigned | Unassigned | 1145_0 | Clustered | 164 | VC_1145_0 | 164 | 0.8868 | 1 | 0.8868 | 0 | 0 | 0 | 1 | Ref |
| KT264768 | Unassigned | Unassigned | Unassigned | 1145_0 | Clustered | 164 | VC_1145_0 | 164 | 0.8868 | 1 | 0.8868 | 0 | 0 | 0 | 1 | Ref |
| KT264769 | Unassigned | Unassigned | Unassigned | 1145_0 | Clustered | 164 | VC_1145_0 | 164 | 0.8868 | 1 | 0.8868 | 0 | 0 | 0 | 1 | Ref |
| KT264770 | Unassigned | Unassigned | Unassigned | 1147_0 | Clustered | 6 | VC_1147_0 | 6 | 0.3066 | 0.99999957 | 0.3066 | 0 | 0 | 0 | 1 | Ref |
| KT264771 | Unassigned | Unassigned | Unassigned | 1145_0 | Clustered | 164 | VC_1145_0 | 164 | 0.8868 | 1 | 0.8868 | 0 | 0 | 0 | 1 | Ref |
| KT264772 | Unassigned | Unassigned | Unassigned | 1145_0 | Clustered | 164 | VC_1145_0 | 164 | 0.8868 | 1 | 0.8868 | 0 | 0 | 0 | 1 | Ref |
| KT264774 | Unassigned | Unassigned | Unassigned | 1145_0 | Clustered | 164 | VC_1145_0 | 164 | 0.8868 | 1 | 0.8868 | 0 | 0 | 0 | 1 | Ref |
| KT264775 | Unassigned | Unassigned | Unassigned | 1148_0 | Clustered | 4 | VC_1148_0 | 4 | 0.0503 | 0.99998999 | 0.0503 | 0 | 0 | 0 | 1 | Ref |
| KT264776 | Unassigned | Unassigned | Unassigned | 1155_0 | Clustered | 14 | VC_1155_0 | 14 | 0.8287 | 1 | 0.8287 | 0 | 0 | 0 | 1 | Ref |
| KT264779 | Unassigned | Unassigned | Unassigned | 1145_0 | Clustered | 164 | VC_1145_0 | 164 | 0.8868 | 1 | 0.8868 | 0 | 0 | 0 | 1 | Ref |
| KT264781 | Unassigned | Unassigned | Unassigned | 1156_0 | Clustered | 5 | VC_1156_0 | 5 | 0.4454 | 0.99999977 | 0.4454 | 0 | 0 | 0 | 1 | Ref |
| KT264782 | Unassigned | Unassigned | Unassigned | 1161_0 | Clustered | 16 | VC_1161_0 | 16 | 0.4834 | 0.99999958 | 0.4834 | 0 | 0 | 0 | 1 | Ref |
| KT264783 | Unassigned | Unassigned | Unassigned | 1161_0 | Clustered | 16 | VC_1161_0 | 16 | 0.4834 | 0.99999958 | 0.4834 | 0 | 0 | 0 | 1 | Ref |
| KT264784 | Unassigned | Unassigned | Unassigned | 1166_0 | Clustered | 2 | VC_1166_0 | 2 | 0.3171 | 0.93270267 | 0.2957 | 0 | 0 | 0 | 1 | Ref |
| KT264785 | Unassigned | Unassigned | Unassigned | 1165_0 | Clustered | 10 | VC_1165_0 | 10 | 0.9263 | 1 | 0.9263 | 0 | 0 | 0 | 1 | Ref |
| KT264788 | Unassigned | Unassigned | Unassigned | 1169_0 | Clustered | 8 | VC_1169_0 | 8 | 0.2897 | 1 | 0.2897 | 0 | 0 | 0 | 1 | Ref |
| KT264789 | Unassigned | Unassigned | Unassigned | 1169_0 | Clustered | 8 | VC_1169_0 | 8 | 0.2897 | 1 | 0.2897 | 0 | 0 | 0 | 1 | Ref |
| KT264790 | Unassigned | Unassigned | Unassigned | 1169_0 | Clustered | 8 | VC_1169_0 | 8 | 0.2897 | 1 | 0.2897 | 0 | 0 | 0 | 1 | Ref |
| KT264791 | Unassigned | Unassigned | Unassigned | 1170_0 | Clustered | 19 | VC_1170_0 | 19 | 0.7497 | 1 | 0.7497 | 0 | 0 | 0 | 1 | Ref |
| KT264792 | Unassigned | Unassigned | Unassigned | 1170_0 | Clustered | 19 | VC_1170_0 | 19 | 0.7497 | 1 | 0.7497 | 0 | 0 | 0 | 1 | Ref |
| KT264793 | Unassigned | Unassigned | Unassigned | 1170_0 | Clustered | 19 | VC_1170_0 | 19 | 0.7497 | 1 | 0.7497 | 0 | 0 | 0 | 1 | Ref |
| KT264794 | Unassigned | Unassigned | Unassigned | 1171_0 | Clustered/Singleton | 1 | VC_1171_0 | 1 | 0 | 1 | 0 | 0 | 0 | 0 | 1 | Ref |
| KT264795 | Unassigned | Unassigned | Unassigned | 1173_0 | Clustered | 3 | VC_1173_0 | 3 | 0.0986 | 0.78721935 | 0.0776 | 0 | 0 | 0 | 1 | Ref |
| KT264796 | Unassigned | Unassigned | Unassigned | 1152_0 | Clustered | 26 | VC_1152_0 | 26 | 0.6685 | 1 | 0.6685 | 0 | 0 | 0 | 1 | Ref |
| KT264797 | Unassigned | Unassigned | Unassigned | 1152_0 | Clustered | 26 | VC_1152_0 | 26 | 0.6685 | 1 | 0.6685 | 0 | 0 | 0 | 1 | Ref |
| KT264798 | Unassigned | Unassigned | Unassigned | 1167_0 | Clustered | 11 | VC_1167_0 | 11 | 0.5637 | 1 | 0.5637 | 0 | 0 | 0 | 1 | Ref |
| KT264799 | Unassigned | Unassigned | Unassigned | 1167_0 | Clustered | 11 | VC_1167_0 | 11 | 0.5637 | 1 | 0.5637 | 0 | 0 | 0 | 1 | Ref |
| KT264800 | Unassigned | Unassigned | Unassigned | 1167_0 | Clustered | 11 | VC_1167_0 | 11 | 0.5637 | 1 | 0.5637 | 0 | 0 | 0 | 1 | Ref |
| KT264801 | Unassigned | Unassigned | Unassigned | 1167_0 | Clustered | 11 | VC_1167_0 | 11 | 0.5637 | 1 | 0.5637 | 0 | 0 | 0 | 1 | Ref |
| KT264802 | Unassigned | Unassigned | Unassigned | 1178_0 | Clustered | 3 | VC_1178_0 | 3 | 0.877 | 0.96173875 | 0.8434 | 0 | 0 | 0 | 1 | Ref |
| KT264803 | Unassigned | Unassigned | Unassigned | 1177_0 | Clustered | 2 | VC_1177_0 | 2 | 0.6063 | 0.90610936 | 0.5494 | 0 | 0 | 0 | 1 | Ref |
| KT264805 | Unassigned | Unassigned | Unassigned | 1179_0 | Clustered | 4 | VC_1179_0 | 4 | 0.9241 | 0.99468305 | 0.9191 | 0 | 0 | 0 | 1 | Ref |
| KT264806 | Unassigned | Unassigned | Unassigned | 1180_0 | Clustered | 4 | VC_1180_0 | 4 | 1 | 1 | 1 | 0 | 0 | 0 | 1 | Ref |
| KT264808 | Unassigned | Unassigned | Unassigned | 1108_0 | Clustered | 52 | VC_1108_0 | 52 | 0.7674 | 1 | 0.7674 | 0 | 0 | 0 | 1 | Ref |
| KT264809 | Unassigned | Unassigned | Unassigned | 1108_0 | Clustered | 52 | VC_1108_0 | 52 | 0.7674 | 1 | 0.7674 | 0 | 0 | 0 | 1 | Ref |
| KT264810 | Unassigned | Unassigned | Unassigned | 1108_0 | Clustered | 52 | VC_1108_0 | 52 | 0.7674 | 1 | 0.7674 | 0 | 0 | 0 | 1 | Ref |
| KT264811 | Unassigned | Unassigned | Unassigned | 411_0 | Clustered | 48 | VC_411_0 | 48 | 0.4808 | 1 | 0.4808 | 0 | 0 | 0 | 1 | Ref |
| KT264812 | Unassigned | Unassigned | Unassigned | 1127_0 | Clustered | 2 | VC_1127_0 | 2 | 0.5888 | 0.9331928 | 0.5494 | 0 | 0 | 0 | 1 | Ref |
| KT264813 | Unassigned | Unassigned | Unassigned | 1028_0 | Clustered | 69 | VC_1028_0 | 69 | 0.8547 | 1 | 0.8547 | 0 | 0 | 0 | 1 | Ref |
| KT264814 | Unassigned | Unassigned | Unassigned | 1028_0 | Clustered | 69 | VC_1028_0 | 69 | 0.8547 | 1 | 0.8547 | 0 | 0 | 0 | 1 | Ref |
| KT264815 | Unassigned | Unassigned | Unassigned | 1028_0 | Clustered | 69 | VC_1028_0 | 69 | 0.8547 | 1 | 0.8547 | 0 | 0 | 0 | 1 | Ref |
| KT264816 | Unassigned | Unassigned | Unassigned | 1183_0 | Clustered | 2 | VC_1183_0 | 2 | 0.4211 | 0.91600907 | 0.3857 | 0 | 0 | 0 | 1 | Ref |
| KT264817 | Unassigned | Unassigned | Unassigned | 1183_0 | Clustered | 2 | VC_1183_0 | 2 | 0.4211 | 0.91600907 | 0.3857 | 0 | 0 | 0 | 1 | Ref |
| KT264818 | Unassigned | Unassigned | Unassigned | 1184_0 | Clustered | 10 | VC_1184_0 | 10 | 0.7506 | 1 | 0.7506 | 0 | 0 | 0 | 1 | Ref |
| KT264819 | Unassigned | Unassigned | Unassigned | 1184_0 | Clustered | 10 | VC_1184_0 | 10 | 0.7506 | 1 | 0.7506 | 0 | 0 | 0 | 1 | Ref |
| KT264820 | Unassigned | Unassigned | Unassigned | 1181_0 | Clustered | 4 | VC_1181_0 | 4 | 0.1325 | 0.65559538 | 0.0869 | 0 | 0 | 0 | 1 | Ref |
| KT264821 | Unassigned | Unassigned | Unassigned | 1181_0 | Clustered | 4 | VC_1181_0 | 4 | 0.1325 | 0.65559538 | 0.0869 | 0 | 0 | 0 | 1 | Ref |
| KT264822 | Unassigned | Unassigned | Unassigned | 1188_0 | Clustered | 3 | VC_1188_0 | 3 | 0.2005 | 0.95837083 | 0.1922 | 0 | 0 | 0 | 1 | Ref |
| KT264823 | Unassigned | Unassigned | Unassigned | 1189_0 | Clustered | 16 | VC_1189_0 | 16 | 0.8814 | 1 | 0.8814 | 0 | 0 | 0 | 1 | Ref |
| KT264824 | Unassigned | Unassigned | Unassigned | 1189_0 | Clustered | 16 | VC_1189_0 | 16 | 0.8814 | 1 | 0.8814 | 0 | 0 | 0 | 1 | Ref |
| KT264825 | Unassigned | Unassigned | Unassigned | 1189_0 | Clustered | 16 | VC_1189_0 | 16 | 0.8814 | 1 | 0.8814 | 0 | 0 | 0 | 1 | Ref |
| KT264826 | Unassigned | Unassigned | Unassigned | 1095_0 | Clustered | 12 | VC_1095_0 | 12 | 0.2819 | 0.99837092 | 0.2814 | 0 | 0 | 0 | 1 | Ref |
| KT264827 | Unassigned | Unassigned | Unassigned | 1095_0 | Clustered | 12 | VC_1095_0 | 12 | 0.2819 | 0.99837092 | 0.2814 | 0 | 0 | 0 | 1 | Ref |
| KT264828 | Unassigned | Unassigned | Unassigned | 1191_0 | Clustered | 9 | VC_1191_0 | 9 | 0.8537 | 1 | 0.8537 | 0 | 0 | 0 | 1 | Ref |
| KT264830 | Unassigned | Unassigned | Unassigned | 1188_0 | Clustered | 3 | VC_1188_0 | 3 | 0.2005 | 0.95837083 | 0.1922 | 0 | 0 | 0 | 1 | Ref |
| KT264831 | Unassigned | Unassigned | Unassigned | 1193_0 | Clustered | 2 | VC_1193_0 | 2 | 0.0731 | 0.80562642 | 0.0589 | 0 | 0 | 0 | 1 | Ref |
| KT264833 | Unassigned | Unassigned | Unassigned | 1161_0 | Clustered | 16 | VC_1161_0 | 16 | 0.4834 | 0.99999958 | 0.4834 | 0 | 0 | 0 | 1 | Ref |
| KT264834 | Unassigned | Unassigned | Unassigned | 1194_0 | Clustered | 2 | VC_1194_0 | 2 | 1 | 1 | 1 | 0 | 0 | 0 | 1 | Ref |
| KT270441 | Unassigned | Unassigned | Unassigned | 385_0 | Clustered | 24 | VC_385_0 | 24 | 0.5516 | 1 | 0.5516 | 0 | 0 | 0 | 1 | Ref |
| KT279576 | Unassigned | Unassigned | Unassigned | 538_60 | Clustered | 2 | VC_538_60 | 2 | 0.0091 | 0.95761233 | 0.0087 | 0 | 0 | 0 | 1 | Ref |
| KT281790 | Unassigned | Unassigned | Unassigned | 579_11 | Clustered | 2 | VC_579_11 | 2 | 0.0032 | 0.76766997 | 0.0025 | 0 | 0 | 0 | 1 | Ref |
| KT281791 | Unassigned | Unassigned | Unassigned | 918_0 | Clustered | 16 | VC_918_0 | 16 | 0.2945 | 1 | 0.2945 | 0 | 0 | 0 | 1 | Ref |
| KT281792 | Unassigned | Unassigned | Unassigned | 538_27 | Clustered | 2 | VC_538_27 | 2 | 0.0141 | 0.95761635 | 0.0135 | 0 | 0 | 0 | 1 | Ref |
| KT281793 | Unassigned | Unassigned | Unassigned | 538_13 | Clustered/Singleton | 1 | VC_538_13 | 1 | 0 | 1 | 0 | 0 | 0 | 0 | 1 | Ref |
| KT281794 | Unassigned | Unassigned | Unassigned | 918_0 | Clustered | 16 | VC_918_0 | 16 | 0.2945 | 1 | 0.2945 | 0 | 0 | 0 | 1 | Ref |
| KT281795 | Unassigned | Unassigned | Unassigned | 538_31 | Clustered | 11 | VC_538_31 | 11 | 0.0894 | 1 | 0.0894 | 0 | 0 | 0 | 0.6 | Ref |
| KT281796 | Unassigned | Unassigned | Unassigned | 577_0 | Clustered | 21 | VC_577_0 | 21 | 0.9908 | 1 | 0.9908 | 0 | 0 | 0 | 1 | Ref |
| KT285706 | Unassigned | Unassigned | Unassigned | 232_0 | Clustered | 32 | VC_232_0 | 32 | 0.8471 | 1 | 0.8471 | 0 | 0 | 0 | 0.996 | Ref |
| KT287080 | Unassigned | Unassigned | Unassigned | 560_0 | Clustered | 15 | VC_560_0 | 15 | 0.2846 | 1 | 0.2846 | 0 | 0 | 0 | 1 | Ref |
| KT307976 | Unassigned | Unassigned | Unassigned | 912_1 | Clustered | 12 | VC_912_1 | 12 | 0.2423 | 1 | 0.2423 | 0 | 0 | 0 | 1 | Ref |
| KT309034 | Unassigned | Unassigned | Unassigned | 538_0 | Clustered/Singleton | 1 | VC_538_0 | 1 | 0 | 1 | 0 | 0 | 0 | 0 | 1 | Ref |
| KT321314 | Unassigned | Unassigned | Unassigned | 401_0 | Clustered | 4 | VC_401_0 | 4 | 0.0343 | 0.99998813 | 0.0343 | 0 | 0 | 0 | 1 | Ref |
| KT321315 | Unassigned | Unassigned | Unassigned | 686_0 | Clustered | 25 | VC_686_0 | 25 | 0.5501 | 1 | 0.5501 | 0 | 0 | 0 | 0.96 | Ref |
| KT321316 | Unassigned | Unassigned | Unassigned | 613_0 | Clustered | 2 | VC_613_0 | 2 | 0.0728 | 0.95685859 | 0.0696 | 0 | 0 | 0 | 1 | Ref |
| KT321317 | Unassigned | Unassigned | Unassigned | 801_1 | Clustered | 11 | VC_801_1 | 11 | 0.1583 | 1 | 0.1583 | 0 | 0 | 0 | 1 | Ref |
| KT321476 | Unassigned | Unassigned | Unassigned | 579_50 | Clustered | 15 | VC_579_50 | 15 | 0.0467 | 1 | 0.0467 | 0 | 0 | 0 | 0.9238 | Ref |

| | | | | | | | | | | | | | | | | |
|---|---|---|---|---|---|---|---|---|---|---|---|---|---|---|---|---|
| KT326767 | Unassigned | Unassigned | Unassigned | 531_0 | Clustered | 32 | VC_531_0 | 32 | 0.6859 | 1 | 0.6859 | 0 | 0 | 0 | 1 | Ref |
| KT326768 | Unassigned | Unassigned | Unassigned | 335_45 | Clustered | 4 | VC_335_45 | 4 | 0.0143 | 0.99998616 | 0.0143 | 0 | 0 | 0 | 0.8333 | Ref |
| KT336320 | Unassigned | Unassigned | Unassigned | 647_0 | Clustered | 37 | VC_647_0 | 37 | 0.5847 | 1 | 0.5847 | 0 | 0 | 0 | 0.9234 | Ref |
| KT336321 | Unassigned | Unassigned | Unassigned | 647_0 | Clustered | 37 | VC_647_0 | 37 | 0.5847 | 1 | 0.5847 | 0 | 0 | 0 | 0.9234 | Ref |
| KT337339 | Unassigned | Unassigned | Unassigned | 427_0 | Clustered | 37 | VC_427_0 | 37 | 0.5729 | 1 | 0.5729 | 0 | 0 | 0 | 0.9474 | Ref |
| KT337340 | Unassigned | Unassigned | Unassigned | 427_0 | Clustered | 37 | VC_427_0 | 37 | 0.5729 | 1 | 0.5729 | 0 | 0 | 0 | 0.9474 | Ref |
| KT337341 | Unassigned | Unassigned | Unassigned | 427_0 | Clustered | 37 | VC_427_0 | 37 | 0.5729 | 1 | 0.5729 | 0 | 0 | 0 | 0.9474 | Ref |
| KT337342 | Unassigned | Unassigned | Unassigned | 344_0 | Clustered | 30 | VC_344_0 | 30 | 0.523 | 1 | 0.523 | 0 | 0 | 0 | 0.9954 | Ref |
| KT337352 | Unassigned | Unassigned | Unassigned | 427_0 | Clustered | 37 | VC_427_0 | 37 | 0.5729 | 1 | 0.5729 | 0 | 0 | 0 | 0.9474 | Ref |
| KT337354 | Unassigned | Unassigned | Unassigned | 427_0 | Clustered | 37 | VC_427_0 | 37 | 0.5729 | 1 | 0.5729 | 0 | 0 | 0 | 0.9474 | Ref |
| KT337355 | Unassigned | Unassigned | Unassigned | 427_0 | Clustered | 37 | VC_427_0 | 37 | 0.5729 | 1 | 0.5729 | 0 | 0 | 0 | 0.9474 | Ref |
| KT337356 | Unassigned | Unassigned | Unassigned | 427_0 | Clustered | 37 | VC_427_0 | 37 | 0.5729 | 1 | 0.5729 | 0 | 0 | 0 | 0.9474 | Ref |
| KT337358 | Unassigned | Unassigned | Unassigned | 425_0 | Clustered | 40 | VC_425_0 | 40 | 0.8399 | 1 | 0.8399 | 0 | 0 | 0 | 0.9974 | Ref |
| KT337359 | Unassigned | Unassigned | Unassigned | 427_0 | Clustered | 37 | VC_427_0 | 37 | 0.5729 | 1 | 0.5729 | 0 | 0 | 0 | 0.9474 | Ref |
| KT337360 | Unassigned | Unassigned | Unassigned | 268_1 | Clustered | 20 | VC_268_1 | 20 | 0.1906 | 1 | 0.1906 | 0 | 0 | 0 | 0.6789 | Ref |
| KT337362 | Unassigned | Unassigned | Unassigned | 427_0 | Clustered | 37 | VC_427_0 | 37 | 0.5729 | 1 | 0.5729 | 0 | 0 | 0 | 0.9474 | Ref |
| KT337364 | Unassigned | Unassigned | Unassigned | 425_0 | Clustered | 40 | VC_425_0 | 40 | 0.8399 | 1 | 0.8399 | 0 | 0 | 0 | 0.9974 | Ref |
| KT339321 | Unassigned | Unassigned | Unassigned | 714_0 | Clustered | 71 | VC_714_0 | 71 | 0.935 | 1 | 0.935 | 0 | 0 | 0 | 1 | Ref |
| KT344895 | Unassigned | Unassigned | Unassigned | 12_0 | Clustered | 21 | VC_12_0 | 21 | 0.2262 | 1 | 0.2262 | 0 | 0 | 0 | 0.9571 | Ref |
| KT345706 | Unassigned | Unassigned | Unassigned | 1007_2 | Clustered/Singleton | 1 | VC_1007_2 | 1 | 0 | 1 | 0 | 0 | 0 | 0 | 1 | Ref |
| KT347313 | Unassigned | Unassigned | Unassigned | 537_0 | Clustered | 39 | VC_537_0 | 39 | 0.8148 | 1 | 0.8148 | 0 | 0 | 0 | 1 | Ref |
| KT347314 | Unassigned | Unassigned | Unassigned | 783_0 | Clustered | 67 | VC_783_0 | 67 | 0.8945 | 1 | 0.8945 | 0 | 0 | 0 | 1 | Ref |
| KT353109 | Unassigned | Unassigned | Unassigned | 873_5 | Clustered | 3 | VC_873_5 | 3 | 0.0491 | 0.9761082 | 0.0479 | 0 | 0 | 0 | 1 | Ref |
| KT355471 | Unassigned | Unassigned | Unassigned | 985_0 | Clustered | 38 | VC_985_0 | 38 | 0.9334 | 1 | 0.9334 | 0 | 0 | 0 | 1 | Ref |
| KT355472 | Unassigned | Unassigned | Unassigned | 783_0 | Clustered | 67 | VC_783_0 | 67 | 0.8945 | 1 | 0.8945 | 0 | 0 | 0 | 1 | Ref |
| KT355473 | Unassigned | Unassigned | Unassigned | 985_0 | Clustered | 38 | VC_985_0 | 38 | 0.9334 | 1 | 0.9334 | 0 | 0 | 0 | 1 | Ref |
| KT355474 | Unassigned | Unassigned | Unassigned | 783_0 | Clustered | 67 | VC_783_0 | 67 | 0.8945 | 1 | 0.8945 | 0 | 0 | 0 | 1 | Ref |
| KT355475 | Unassigned | Unassigned | Unassigned | 985_0 | Clustered | 38 | VC_985_0 | 38 | 0.9334 | 1 | 0.9334 | 0 | 0 | 0 | 1 | Ref |
| KT359365 | Unassigned | Unassigned | Unassigned | 532_0 | Clustered | 75 | VC_532_0 | 75 | 0.9393 | 1 | 0.9393 | 0 | 0 | 0 | 1 | Ref |
| KT361649 | Unassigned | Unassigned | Unassigned | 725_0 | Clustered | 47 | VC_725_0 | 47 | 0.8649 | 1 | 0.8649 | 0 | 0 | 0 | 1 | Ref |
| KT361650 | Unassigned | Unassigned | Unassigned | 725_0 | Clustered | 47 | VC_725_0 | 47 | 0.8649 | 1 | 0.8649 | 0 | 0 | 0 | 1 | Ref |
| KT361652 | Unassigned | Unassigned | Unassigned | 725_0 | Clustered | 47 | VC_725_0 | 47 | 0.8649 | 1 | 0.8649 | 0 | 0 | 0 | 1 | Ref |
| KT361654 | Unassigned | Unassigned | Unassigned | 637_0 | Clustered | 27 | VC_637_0 | 27 | 0.8807 | 1 | 0.8807 | 0 | 0 | 0 | 1 | Ref |
| KT361655 | Unassigned | Unassigned | Unassigned | 637_0 | Clustered | 27 | VC_637_0 | 27 | 0.8807 | 1 | 0.8807 | 0 | 0 | 0 | 1 | Ref |
| KT361656 | Unassigned | Unassigned | Unassigned | 637_0 | Clustered | 27 | VC_637_0 | 27 | 0.8807 | 1 | 0.8807 | 0 | 0 | 0 | 1 | Ref |
| KT361657 | Unassigned | Unassigned | Unassigned | 637_0 | Clustered | 27 | VC_637_0 | 27 | 0.8807 | 1 | 0.8807 | 0 | 0 | 0 | 1 | Ref |
| KT361920 | Unassigned | Unassigned | Unassigned | 241_0 | Clustered | 21 | VC_241_0 | 21 | 0.8342 | 1 | 0.8342 | 0 | 0 | 0 | 1 | Ref |
| KT363731 | Unassigned | Unassigned | Unassigned | 335_37 | Clustered | 4 | VC_335_37 | 4 | 0.0112 | 0.99980442 | 0.0112 | 0 | 0 | 0 | 0.6667 | Ref |
| KT363872 | Unassigned | Unassigned | Unassigned | 341_0 | Clustered | 27 | VC_341_0 | 27 | 0.5058 | 1 | 0.5058 | 0 | 0 | 0 | 0.8661 | Ref |
| KT364588 | Unassigned | Unassigned | Unassigned | 384_4 | Clustered | 5 | VC_384_4 | 5 | 0.0134 | 0.99999971 | 0.0134 | 0 | 0 | 0 | 1 | Ref |
| KT365397 | Unassigned | Unassigned | Unassigned | 535_1 | Clustered | 108 | VC_535_1 | 108 | 0.8479 | 1 | 0.8479 | 0 | 0 | 0 | 0.9955 | Ref |
| KT365398 | Unassigned | Unassigned | Unassigned | 579_6 | Clustered | 2 | VC_579_6 | 2 | 0.0032 | 0.72299343 | 0.0023 | 0 | 0 | 0 | 1 | Ref |
| KT365399 | Unassigned | Unassigned | Unassigned | 783_0 | Clustered | 67 | VC_783_0 | 67 | 0.8945 | 1 | 0.8945 | 0 | 0 | 0 | 1 | Ref |
| KT365400 | Unassigned | Unassigned | Unassigned | 579_37 | Clustered/Singleton | 1 | VC_579_37 | 1 | 0 | 1 | 0 | 0 | 0 | 0 | 1 | Ref |
| KT365401 | Unassigned | Unassigned | Unassigned | 1116_0 | Clustered | 24 | VC_1116_0 | 24 | 0.8376 | 1 | 0.8376 | 0 | 0 | 0 | 1 | Ref |
| KT365402 | Unassigned | Unassigned | Unassigned | 526_0 | Clustered | 32 | VC_526_0 | 32 | 0.9037 | 1 | 0.9037 | 0 | 0 | 0 | 1 | Ref |
| KT367885 | Unassigned | Unassigned | Unassigned | 848_0 | Clustered | 124 | VC_848_0 | 124 | 0.9484 | 1 | 0.9484 | 0 | 0 | 0 | 1 | Ref |
| KT367886 | Unassigned | Unassigned | Unassigned | 113_0 | Clustered | 91 | VC_113_0 | 91 | 0.9584 | 1 | 0.9584 | 0 | 0 | 0 | 1 | Ref |
| KT367887 | Unassigned | Unassigned | Unassigned | 860_0 | Clustered | 9 | VC_860_0 | 9 | 0.7723 | 1 | 0.7723 | 0 | 0 | 0 | 1 | Ref |
| KT372002 | Unassigned | Unassigned | Unassigned | 991_0 | Clustered | 39 | VC_991_0 | 39 | 1 | 1 | 1 | 0 | 0 | 0 | 1 | Ref |
| KT372003 | Unassigned | Unassigned | Unassigned | 918_0 | Clustered | 16 | VC_918_0 | 16 | 0.2945 | 1 | 0.2945 | 0 | 0 | 0 | 1 | Ref |
| KT372690 | Unassigned | Unassigned | Unassigned | 81_0 | Clustered | 41 | VC_81_0 | 41 | 0.5815 | 1 | 0.5815 | 0 | 0 | 0 | 0.9805 | Ref |
| KT372691 | Unassigned | Unassigned | Unassigned | 81_0 | Clustered | 41 | VC_81_0 | 41 | 0.5815 | 1 | 0.5815 | 0 | 0 | 0 | 0.9805 | Ref |
| KT372692 | Unassigned | Unassigned | Unassigned | 81_0 | Clustered | 41 | VC_81_0 | 41 | 0.5815 | 1 | 0.5815 | 0 | 0 | 0 | 0.9805 | Ref |
| KT372693 | Unassigned | Unassigned | Unassigned | 81_0 | Clustered | 41 | VC_81_0 | 41 | 0.5815 | 1 | 0.5815 | 0 | 0 | 0 | 0.9805 | Ref |
| KT372694 | Unassigned | Unassigned | Unassigned | 81_0 | Clustered | 41 | VC_81_0 | 41 | 0.5815 | 1 | 0.5815 | 0 | 0 | 0 | 0.9805 | Ref |
| KT372695 | Unassigned | Unassigned | Unassigned | 81_0 | Clustered | 41 | VC_81_0 | 41 | 0.5815 | 1 | 0.5815 | 0 | 0 | 0 | 0.9805 | Ref |
| KT372696 | Unassigned | Unassigned | Unassigned | 81_0 | Clustered | 41 | VC_81_0 | 41 | 0.5815 | 1 | 0.5815 | 0 | 0 | 0 | 0.9805 | Ref |
| KT372697 | Unassigned | Unassigned | Unassigned | 81_0 | Clustered | 41 | VC_81_0 | 41 | 0.5815 | 1 | 0.5815 | 0 | 0 | 0 | 0.9805 | Ref |
| KT372698 | Unassigned | Unassigned | Unassigned | 81_0 | Clustered | 41 | VC_81_0 | 41 | 0.5815 | 1 | 0.5815 | 0 | 0 | 0 | 0.9805 | Ref |
| KT372714 | Unassigned | Unassigned | Unassigned | 20_0 | Clustered | 5 | VC_20_0 | 5 | 0.4268 | 0.9999994 | 0.4268 | 0 | 0 | 0 | 1 | Ref |
| KT373978 | Unassigned | Unassigned | Unassigned | 571_7 | Clustered | 28 | VC_571_7 | 28 | 0.1651 | 1 | 0.1651 | 0 | 0 | 0 | 0.8519 | Ref |
| KT375356 | Unassigned | Unassigned | Unassigned | 991_0 | Clustered | 39 | VC_991_0 | 39 | 1 | 1 | 1 | 0 | 0 | 0 | 1 | Ref |
| KT381276 | Unassigned | Unassigned | Unassigned | 222_2 | Clustered | 5 | VC_222_2 | 5 | 0.0569 | 0.99999997 | 0.0569 | 0 | 0 | 0 | 1 | Ref |
| KT381277 | Unassigned | Unassigned | Unassigned | 532_0 | Clustered | 75 | VC_532_0 | 75 | 0.9393 | 1 | 0.9393 | 0 | 0 | 0 | 1 | Ref |
| KT381864 | Unassigned | Unassigned | Unassigned | 188_0 | Clustered | 5 | VC_188_0 | 5 | 0.6188 | 0.99999978 | 0.6188 | 0 | 0 | 0 | 1 | Ref |
| KT381865 | Unassigned | Unassigned | Unassigned | 188_0 | Clustered | 5 | VC_188_0 | 5 | 0.6188 | 0.99999978 | 0.6188 | 0 | 0 | 0 | 1 | Ref |
| KT381879 | Unassigned | Unassigned | Unassigned | 71_0 | Clustered | 27 | VC_71_0 | 27 | 0.5964 | 1 | 0.5964 | 0 | 0 | 0 | 0.9943 | Ref |
| KT381880 | Unassigned | Unassigned | Unassigned | 687_0 | Clustered | 21 | VC_687_0 | 21 | 0.3608 | 1 | 0.3608 | 0 | 0 | 0 | 1 | Ref |
| KT388014 | Unassigned | Unassigned | Unassigned | 335_56 | Clustered | 7 | VC_335_56 | 7 | 0.0242 | 1 | 0.0242 | 0 | 0 | 0 | 0.9048 | Ref |
| KT388093 | Unassigned | Unassigned | Unassigned | 425_0 | Clustered | 40 | VC_425_0 | 40 | 0.8399 | 1 | 0.8399 | 0 | 0 | 0 | 0.9974 | Ref |
| KT388102 | Unassigned | Unassigned | Unassigned | 714_0 | Clustered | 71 | VC_714_0 | 71 | 0.935 | 1 | 0.935 | 0 | 0 | 0 | 1 | Ref |
| KT388103 | Unassigned | Unassigned | Unassigned | 714_0 | Clustered | 71 | VC_714_0 | 71 | 0.935 | 1 | 0.935 | 0 | 0 | 0 | 1 | Ref |
| KT429160 | Unassigned | Unassigned | Unassigned | 220_0 | Clustered | 2 | VC_220_0 | 2 | 0.491 | 0.9528045 | 0.4678 | 0 | 0 | 0 | 1 | Ref |
| KT438500 | Unassigned | Unassigned | Unassigned | 335_9 | Clustered | 2 | VC_335_9 | 2 | 0.0055 | 0.95750934 | 0.0053 | 0 | 0 | 0 | 1 | Ref |
| KT438501 | Unassigned | Unassigned | Unassigned | 974_0 | Clustered | 34 | VC_974_0 | 34 | 0.7953 | 1 | 0.7953 | 0 | 0 | 0 | 1 | Ref |
| KT454805 | Unassigned | Unassigned | Unassigned | 341_0 | Clustered | 27 | VC_341_0 | 27 | 0.5058 | 1 | 0.5058 | 0 | 0 | 0 | 0.8661 | Ref |
| KT454806 | Unassigned | Unassigned | Unassigned | 341_0 | Clustered | 27 | VC_341_0 | 27 | 0.5058 | 1 | 0.5058 | 0 | 0 | 0 | 0.8661 | Ref |
| KT454972 | Unassigned | Unassigned | Unassigned | 834_0 | Clustered | 18 | VC_834_0 | 18 | 0.9116 | 1 | 0.9116 | 0 | 0 | 0 | 1 | Ref |
| KT588073 | Unassigned | Unassigned | Unassigned | 441_1 | Clustered | 8 | VC_441_1 | 8 | 0.5443 | 1 | 0.5443 | 0 | 0 | 0 | 0.9643 | Ref |
| KT588074 | Unassigned | Unassigned | Unassigned | 249_0 | Clustered | 2 | VC_249_0 | 2 | 0.0834 | 0.95416588 | 0.0795 | 0 | 0 | 0 | 1 | Ref |

| | | | | | | | | | | | | | | | | |
|---|---|---|---|---|---|---|---|---|---|---|---|---|---|---|---|---|
| KT588075 | Unassigned | Unassigned | Unassigned | 441_0 | Clustered | 2 | VC_441_0 | 2 | 0.1619 | 0.94573679 | 0.1531 | 0 | 0 | 0 | 1 | Ref |
| KT588442 | Unassigned | Unassigned | Unassigned | 223_6 | Clustered | 42 | VC_223_6 | 42 | 0.4717 | 1 | 0.4717 | 0 | 0 | 0 | 0.8351 | Ref |
| KT591076 | Unassigned | Unassigned | Unassigned | 588_0 | Clustered | 8 | VC_588_0 | 8 | 0.5695 | 1 | 0.5695 | 0 | 0 | 0 | 1 | Ref |
| KT591489 | Unassigned | Unassigned | Unassigned | 917_0 | Clustered | 28 | VC_917_0 | 28 | 0.5465 | 1 | 0.5465 | 0 | 0 | 0 | 1 | Ref |
| KT591491 | Unassigned | Unassigned | Unassigned | 785_1 | Clustered | 9 | VC_785_1 | 9 | 0.3679 | 1 | 0.3679 | 0 | 0 | 0 | 1 | Ref |
| KT599441 | Unassigned | Unassigned | Unassigned | 384_15 | Clustered | 44 | VC_384_15 | 44 | 0.139 | 1 | 0.139 | 0 | 0 | 0 | 0.7474 | Ref |
| KT624200 | Unassigned | Unassigned | Unassigned | 90_0 | Clustered/Singleton | 1 | VC_90_0 | 1 | 0 | 1 | 0 | 0 | 0 | 0 | 1 | Ref |
| KT626047 | Unassigned | Unassigned | Unassigned | 335_5 | Clustered | 11 | VC_335_5 | 11 | 0.0469 | 1 | 0.0469 | 0 | 0 | 0 | 0.7636 | Ref |
| KT626446 | Unassigned | Unassigned | Unassigned | 144_0 | Clustered | 2 | VC_144_0 | 2 | 0.1118 | 0.95020171 | 0.1062 | 0 | 0 | 0 | 1 | Ref |
| KT630644 | Unassigned | Unassigned | Unassigned | 235_0 | Clustered | 60 | VC_235_0 | 60 | 0.9413 | 1 | 0.9413 | 0 | 0 | 0 | 1 | Ref |
| KT630647 | Unassigned | Unassigned | Unassigned | 6_0 | Clustered | 44 | VC_6_0 | 44 | 0.9068 | 1 | 0.9068 | 0 | 0 | 0 | 1 | Ref |
| KT630649 | Unassigned | Unassigned | Unassigned | 249_1 | Clustered | 21 | VC_249_1 | 21 | 0.6775 | 1 | 0.6775 | 0 | 0 | 0 | 0.8476 | Ref |
| KT716493 | Unassigned | Unassigned | Unassigned | 535_1 | Clustered | 108 | VC_535_1 | 108 | 0.8479 | 1 | 0.8479 | 0 | 0 | 0 | 0.9955 | Ref |
| KT717083 | Unassigned | Unassigned | Unassigned | 258_0 | Clustered | 121 | VC_258_0 | 121 | 0.7359 | 1 | 0.7359 | 0 | 0 | 0 | 0.9999 | Ref |
| KT717084 | Unassigned | Unassigned | Unassigned | 258_0 | Clustered | 121 | VC_258_0 | 121 | 0.7359 | 1 | 0.7359 | 0 | 0 | 0 | 0.9999 | Ref |
| KT717085 | Unassigned | Unassigned | Unassigned | 258_0 | Clustered | 121 | VC_258_0 | 121 | 0.7359 | 1 | 0.7359 | 0 | 0 | 0 | 0.9999 | Ref |
| KT725776 | Unassigned | Unassigned | Unassigned | 673_0 | Clustered | 4 | VC_673_0 | 4 | 0.2891 | 0.99998263 | 0.2891 | 0 | 0 | 0 | 1 | Ref |
| KT728930 | Unassigned | Unassigned | Unassigned | 904_0 | Clustered | 10 | VC_904_0 | 10 | 0.9878 | 0.99948433 | 0.9873 | 0 | 0 | 0 | 1 | Ref |
| KT728931 | Unassigned | Unassigned | Unassigned | 904_0 | Clustered | 10 | VC_904_0 | 10 | 0.9878 | 0.99948433 | 0.9873 | 0 | 0 | 0 | 1 | Ref |
| KT734862 | Unassigned | Unassigned | Unassigned | 172_0 | Clustered | 26 | VC_172_0 | 26 | 0.7909 | 1 | 0.7909 | 0 | 0 | 0 | 1 | Ref |
| KT736033 | Unassigned | Unassigned | Unassigned | 77_0 | Clustered | 37 | VC_77_0 | 37 | 0.9392 | 1 | 0.9392 | 0 | 0 | 0 | 1 | Ref |
| KT755656 | Unassigned | Unassigned | Unassigned | 1197_0 | Clustered | 8 | VC_1197_0 | 8 | 0.8167 | 1 | 0.8167 | 0 | 0 | 0 | 1 | Ref |
| KT780304 | Unassigned | Unassigned | Unassigned | 844_0 | Clustered | 3 | VC_844_0 | 3 | 0.0967 | 0.99840149 | 0.0966 | 0 | 0 | 0 | 1 | Ref |
| KT783672 | Unassigned | Unassigned | Unassigned | 985_0 | Clustered | 38 | VC_985_0 | 38 | 0.9334 | 1 | 0.9334 | 0 | 0 | 0 | 1 | Ref |
| KT804908 | Unassigned | Unassigned | Unassigned | 714_0 | Clustered | 71 | VC_714_0 | 71 | 0.935 | 1 | 0.935 | 0 | 0 | 0 | 1 | Ref |
| KT804923 | Unassigned | Unassigned | Unassigned | 77_0 | Clustered | 37 | VC_77_0 | 37 | 0.9392 | 1 | 0.9392 | 0 | 0 | 0 | 1 | Ref |
| KT809302 | Unassigned | Unassigned | Unassigned | 684_0 | Clustered | 4 | VC_684_0 | 4 | 1 | 1 | 1 | 0 | 0 | 0 | 1 | Ref |
| KT809368 | Unassigned | Unassigned | Unassigned | 10_0 | Clustered | 25 | VC_10_0 | 25 | 0.3437 | 1 | 0.3437 | 0 | 0 | 0 | 0.9733 | Ref |
| KT809369 | Unassigned | Unassigned | Unassigned | 10_0 | Clustered | 25 | VC_10_0 | 25 | 0.3437 | 1 | 0.3437 | 0 | 0 | 0 | 0.9733 | Ref |
| KT818595 | Unassigned | Unassigned | Unassigned | 335_14 | Clustered | 3 | VC_335_14 | 3 | 0.0094 | 0.9985917 | 0.0094 | 0 | 0 | 0 | 1 | Ref |
| KT825490 | Unassigned | Unassigned | Unassigned | 469_0 | Clustered | 18 | VC_469_0 | 18 | 0.4583 | 1 | 0.4583 | 0 | 0 | 0 | 1 | Ref |
| KT852578 | Unassigned | Unassigned | Unassigned | 20_0 | Clustered | 5 | VC_20_0 | 5 | 0.4268 | 0.9999994 | 0.4268 | 0 | 0 | 0 | 1 | Ref |
| KT870145 | Unassigned | Unassigned | Unassigned | 1012_0 | Clustered | 2 | VC_1012_0 | 2 | 0.0796 | 0.93920319 | 0.0747 | 0 | 0 | 0 | 1 | Ref |
| KT876724 | Unassigned | Unassigned | Unassigned | 1198_0 | Clustered | 24 | VC_1198_0 | 24 | 1 | 1 | 1 | 0 | 0 | 0 | 1 | Ref |
| KT876725 | Unassigned | Unassigned | Unassigned | 1080_0 | Clustered | 23 | VC_1080_0 | 23 | 0.9603 | 1 | 0.9603 | 0 | 0 | 0 | 1 | Ref |
| KT876726 | Unassigned | Unassigned | Unassigned | 1198_0 | Clustered | 24 | VC_1198_0 | 24 | 1 | 1 | 1 | 0 | 0 | 0 | 1 | Ref |
| KT878766 | Unassigned | Unassigned | Unassigned | 10_0 | Clustered | 25 | VC_10_0 | 25 | 0.3437 | 1 | 0.3437 | 0 | 0 | 0 | 0.9733 | Ref |
| KT880194 | Unassigned | Unassigned | Unassigned | 526_0 | Clustered | 32 | VC_526_0 | 32 | 0.9037 | 1 | 0.9037 | 0 | 0 | 0 | 1 | Ref |
| KT881477 | Unassigned | Unassigned | Unassigned | 654_0 | Clustered | 115 | VC_654_0 | 115 | 0.7303 | 1 | 0.7303 | 0 | 0 | 0 | 0.8764 | Ref |
| KT887557 | Unassigned | Unassigned | Unassigned | 182_1 | Clustered | 2 | VC_182_1 | 2 | 0.0361 | 0.95657568 | 0.0345 | 0 | 0 | 0 | 1 | Ref |
| KT887558 | Unassigned | Unassigned | Unassigned | 179_0 | Clustered | 2 | VC_179_0 | 2 | 0.0795 | 0.95489091 | 0.0759 | 0 | 0 | 0 | 1 | Ref |
| KT887559 | Unassigned | Unassigned | Unassigned | 5_0 | Clustered | 27 | VC_5_0 | 27 | 0.9599 | 1 | 0.9599 | 0 | 0 | 0 | 1 | Ref |
| KT895280 | Unassigned | Unassigned | Unassigned | 538_85 | Clustered | 2 | VC_538_85 | 2 | 0.0146 | 0.95762405 | 0.014 | 0 | 0 | 0 | 1 | Ref |
| KT895281 | Unassigned | Unassigned | Unassigned | 538_45 | Clustered | 7 | VC_538_45 | 7 | 0.0632 | 1 | 0.0632 | 0 | 0 | 0 | 0.8095 | Ref |
| KT895374 | Unassigned | Unassigned | Unassigned | 462_1 | Clustered/Singleton | 1 | VC_462_1 | 1 | 0 | 1 | 0 | 0 | 0 | 0 | 1 | Ref |
| KT919972 | Unassigned | Unassigned | Unassigned | 549_0 | Clustered | 9 | VC_549_0 | 9 | 0.2597 | 1 | 0.2597 | 0 | 0 | 0 | 1 | Ref |
| KT919973 | Unassigned | Unassigned | Unassigned | 549_0 | Clustered | 9 | VC_549_0 | 9 | 0.2597 | 1 | 0.2597 | 0 | 0 | 0 | 1 | Ref |
| KT932418 | Unassigned | Unassigned | Unassigned | 511_0 | Clustered/Singleton | 1 | VC_511_0 | 1 | 0 | 1 | 0 | 0 | 0 | 0 | 1 | Ref |
| KT932699 | Unassigned | Unassigned | Unassigned | 110_0 | Clustered | 33 | VC_110_0 | 33 | 0.9488 | 1 | 0.9488 | 0 | 0 | 0 | 1 | Ref |
| KT932700 | Unassigned | Unassigned | Unassigned | 1003_0 | Clustered | 16 | VC_1003_0 | 16 | 0.8967 | 1 | 0.8967 | 0 | 0 | 0 | 1 | Ref |
| KT932701 | Unassigned | Unassigned | Unassigned | 711_0 | Clustered | 48 | VC_711_0 | 48 | 0.8217 | 1 | 0.8217 | 0 | 0 | 0 | 1 | Ref |
| KT934381 | Unassigned | Unassigned | Unassigned | 787_0 | Clustered | 132 | VC_787_0 | 132 | 1 | 1 | 1 | 0 | 0 | 0 | 1 | Ref |
| KT934943 | Unassigned | Unassigned | Unassigned | 601_3 | Clustered | 12 | VC_601_3 | 12 | 0.2339 | 1 | 0.2339 | 0 | 0 | 0 | 0.8636 | Ref |
| KT945994 | Unassigned | Unassigned | Unassigned | 273_0 | Clustered | 2 | VC_273_0 | 2 | 0.1017 | 0.95560536 | 0.0972 | 0 | 0 | 0 | 1 | Ref |
| KT945995 | Unassigned | Unassigned | Unassigned | 1004_0 | Clustered | 4 | VC_1004_0 | 4 | 0.2949 | 0.99997642 | 0.2949 | 0 | 0 | 0 | 1 | Ref |
| KT949345 | Unassigned | Unassigned | Unassigned | 907_0 | Clustered | 32 | VC_907_0 | 32 | 0.9964 | 1 | 0.9964 | 0 | 0 | 0 | 1 | Ref |
| KT959213 | Unassigned | Unassigned | Unassigned | 991_0 | Clustered | 39 | VC_991_0 | 39 | 1 | 1 | 1 | 0 | 0 | 0 | 1 | Ref |
| KT962245 | Unassigned | Unassigned | Unassigned | 1053_0 | Clustered | 5 | VC_1053_0 | 5 | 0.9327 | 0.9999997 | 0.9327 | 0 | 0 | 0 | 1 | Ref |
| KT962246 | Unassigned | Unassigned | Unassigned | 1053_0 | Clustered | 5 | VC_1053_0 | 5 | 0.9327 | 0.9999997 | 0.9327 | 0 | 0 | 0 | 1 | Ref |
| KT962247 | Unassigned | Unassigned | Unassigned | 1053_0 | Clustered | 5 | VC_1053_0 | 5 | 0.9327 | 0.9999997 | 0.9327 | 0 | 0 | 0 | 1 | Ref |
| KT962832 | Unassigned | Unassigned | Unassigned | 654_0 | Clustered | 115 | VC_654_0 | 115 | 0.7303 | 1 | 0.7303 | 0 | 0 | 0 | 0.8764 | Ref |
| KT964103 | Unassigned | Unassigned | Unassigned | 113_0 | Clustered | 91 | VC_113_0 | 91 | 0.9584 | 1 | 0.9584 | 0 | 0 | 0 | 1 | Ref |
| KT968831 | Unassigned | Unassigned | Unassigned | 182_3 | Clustered/Singleton | 1 | VC_182_3 | 1 | 0 | 1 | 0 | 0 | 0 | 0 | 1 | Ref |
| KT968832 | Unassigned | Unassigned | Unassigned | 185_0 | Clustered | 53 | VC_185_0 | 53 | 0.9153 | 1 | 0.9153 | 0 | 0 | 0 | 1 | Ref |
| KT989433 | Unassigned | Unassigned | Unassigned | 891_0 | Clustered | 11 | VC_891_0 | 11 | 0.9663 | 1 | 0.9663 | 0 | 0 | 0 | 1 | Ref |
| KT990215 | Unassigned | Unassigned | Unassigned | 465_0 | Clustered | 80 | VC_465_0 | 80 | 0.9988 | 1 | 0.9988 | 0 | 0 | 0 | 1 | Ref |
| KT990218 | Unassigned | Unassigned | Unassigned | 991_0 | Clustered | 39 | VC_991_0 | 39 | 1 | 1 | 1 | 0 | 0 | 0 | 1 | Ref |
| KT995480 | Unassigned | Unassigned | Unassigned | 195_3 | Clustered | 9 | VC_195_3 | 9 | 0.1955 | 1 | 0.1955 | 0 | 0 | 0 | 0.8611 | Ref |
| KT997847 | Unassigned | Unassigned | Unassigned | 497_0 | Clustered | 4 | VC_497_0 | 4 | 0.0962 | 0.999525 | 0.0961 | 0 | 0 | 0 | 1 | Ref |
| KU052037 | Unassigned | Unassigned | Unassigned | 40_0 | Clustered | 6 | VC_40_0 | 6 | 0.5677 | 1 | 0.5677 | 0 | 0 | 0 | 0.8667 | Ref |
| KU052488 | Unassigned | Unassigned | Unassigned | 650_0 | Clustered | 3 | VC_650_0 | 3 | 0.2008 | 0.99838296 | 0.2005 | 0 | 0 | 0 | 1 | Ref |
| KU055616 | Unassigned | Unassigned | Unassigned | 535_1 | Clustered | 108 | VC_535_1 | 108 | 0.8479 | 1 | 0.8479 | 0 | 0 | 0 | 0.9955 | Ref |
| KU057941 | Unassigned | Unassigned | Unassigned | 749_0 | Clustered | 5 | VC_749_0 | 5 | 0.4704 | 0.99999995 | 0.4704 | 0 | 0 | 0 | 1 | Ref |
| KU064779 | Unassigned | Unassigned | Unassigned | 131_0 | Clustered | 43 | VC_131_0 | 43 | 0.8853 | 1 | 0.8853 | 0 | 0 | 0 | 1 | Ref |
| KU130126 | Unassigned | Unassigned | Unassigned | 79_1 | Clustered | 6 | VC_79_1 | 6 | 0.1348 | 1 | 0.1348 | 0 | 0 | 0 | 1 | Ref |
| KU130127 | Unassigned | Unassigned | Unassigned | 79_1 | Clustered | 6 | VC_79_1 | 6 | 0.1348 | 1 | 0.1348 | 0 | 0 | 0 | 1 | Ref |
| KU130128 | Unassigned | Unassigned | Unassigned | 79_1 | Clustered | 6 | VC_79_1 | 6 | 0.1348 | 1 | 0.1348 | 0 | 0 | 0 | 1 | Ref |
| KU130129 | Unassigned | Unassigned | Unassigned | 79_1 | Clustered | 6 | VC_79_1 | 6 | 0.1348 | 1 | 0.1348 | 0 | 0 | 0 | 1 | Ref |
| KU130130 | Unassigned | Unassigned | Unassigned | 79_1 | Clustered | 6 | VC_79_1 | 6 | 0.1348 | 1 | 0.1348 | 0 | 0 | 0 | 1 | Ref |
| KU130131 | Unassigned | Unassigned | Unassigned | 79_1 | Clustered | 6 | VC_79_1 | 6 | 0.1348 | 1 | 0.1348 | 0 | 0 | 0 | 1 | Ref |
| KU160638 | Unassigned | Unassigned | Unassigned | 882_0 | Clustered | 11 | VC_882_0 | 11 | 0.943 | 1 | 0.943 | 0 | 0 | 0 | 1 | Ref |

| | | | | | | | | | | | | | | | | |
|---|---|---|---|---|---|---|---|---|---|---|---|---|---|---|---|---|
| KU160639 | Unassigned | Unassigned | Unassigned | 882_0 | Clustered | 11 | VC_882_0 | 11 | 0.943 | 1 | 0.943 | 0 | 0 | 0 | 1 | Ref |
| KU160640 | Unassigned | Unassigned | Unassigned | 1200_0 | Clustered | 73 | VC_1200_0 | 73 | 0.9825 | 1 | 0.9825 | 0 | 0 | 0 | 1 | Ref |
| KU160641 | Unassigned | Unassigned | Unassigned | 1204_0 | Clustered | 25 | VC_1204_0 | 25 | 0.7382 | 1 | 0.7382 | 0 | 0 | 0 | 1 | Ref |
| KU160642 | Unassigned | Unassigned | Unassigned | 825_0 | Clustered | 17 | VC_825_0 | 17 | 0.8364 | 1 | 0.8364 | 0 | 0 | 0 | 1 | Ref |
| KU160643 | Unassigned | Unassigned | Unassigned | 1200_0 | Clustered | 73 | VC_1200_0 | 73 | 0.9825 | 1 | 0.9825 | 0 | 0 | 0 | 1 | Ref |
| KU160644 | Unassigned | Unassigned | Unassigned | 1207_0 | Clustered | 20 | VC_1207_0 | 20 | 0.9898 | 1 | 0.9898 | 0 | 0 | 0 | 1 | Ref |
| KU160645 | Unassigned | Unassigned | Unassigned | 1200_0 | Clustered | 73 | VC_1200_0 | 73 | 0.9825 | 1 | 0.9825 | 0 | 0 | 0 | 1 | Ref |
| KU160646 | Unassigned | Unassigned | Unassigned | 1204_0 | Clustered | 25 | VC_1204_0 | 25 | 0.7382 | 1 | 0.7382 | 0 | 0 | 0 | 1 | Ref |
| KU160647 | Unassigned | Unassigned | Unassigned | 882_0 | Clustered | 11 | VC_882_0 | 11 | 0.943 | 1 | 0.943 | 0 | 0 | 0 | 1 | Ref |
| KU160648 | Unassigned | Unassigned | Unassigned | 1200_0 | Clustered | 73 | VC_1200_0 | 73 | 0.9825 | 1 | 0.9825 | 0 | 0 | 0 | 1 | Ref |
| KU160649 | Unassigned | Unassigned | Unassigned | 1200_0 | Clustered | 73 | VC_1200_0 | 73 | 0.9825 | 1 | 0.9825 | 0 | 0 | 0 | 1 | Ref |
| KU160650 | Unassigned | Unassigned | Unassigned | 1208_0 | Clustered | 6 | VC_1208_0 | 6 | 0.8634 | 0.99999946 | 0.8634 | 0 | 0 | 0 | 1 | Ref |
| KU160651 | Unassigned | Unassigned | Unassigned | 1116_0 | Clustered | 24 | VC_1116_0 | 24 | 0.8376 | 1 | 0.8376 | 0 | 0 | 0 | 1 | Ref |
| KU160652 | Unassigned | Unassigned | Unassigned | 1200_0 | Clustered | 73 | VC_1200_0 | 73 | 0.9825 | 1 | 0.9825 | 0 | 0 | 0 | 1 | Ref |
| KU160653 | Unassigned | Unassigned | Unassigned | 1200_0 | Clustered | 73 | VC_1200_0 | 73 | 0.9825 | 1 | 0.9825 | 0 | 0 | 0 | 1 | Ref |
| KU160654 | Unassigned | Unassigned | Unassigned | 1210_0 | Clustered | 7 | VC_1210_0 | 7 | 0.9808 | 0.99995963 | 0.9807 | 0 | 0 | 0 | 1 | Ref |
| KU160655 | Unassigned | Unassigned | Unassigned | 985_0 | Clustered | 38 | VC_985_0 | 38 | 0.9334 | 1 | 0.9334 | 0 | 0 | 0 | 1 | Ref |
| KU160656 | Unassigned | Unassigned | Unassigned | 1116_0 | Clustered | 24 | VC_1116_0 | 24 | 0.8376 | 1 | 0.8376 | 0 | 0 | 0 | 1 | Ref |
| KU160657 | Unassigned | Unassigned | Unassigned | 985_0 | Clustered | 38 | VC_985_0 | 38 | 0.9334 | 1 | 0.9334 | 0 | 0 | 0 | 1 | Ref |
| KU160658 | Unassigned | Unassigned | Unassigned | 985_0 | Clustered | 38 | VC_985_0 | 38 | 0.9334 | 1 | 0.9334 | 0 | 0 | 0 | 1 | Ref |
| KU160659 | Unassigned | Unassigned | Unassigned | 1200_0 | Clustered | 73 | VC_1200_0 | 73 | 0.9825 | 1 | 0.9825 | 0 | 0 | 0 | 1 | Ref |
| KU160660 | Unassigned | Unassigned | Unassigned | 1117_0 | Clustered | 19 | VC_1117_0 | 19 | 0.9177 | 1 | 0.9177 | 0 | 0 | 0 | 1 | Ref |
| KU160661 | Unassigned | Unassigned | Unassigned | 1200_0 | Clustered | 73 | VC_1200_0 | 73 | 0.9825 | 1 | 0.9825 | 0 | 0 | 0 | 1 | Ref |
| KU160662 | Unassigned | Unassigned | Unassigned | 1200_0 | Clustered | 73 | VC_1200_0 | 73 | 0.9825 | 1 | 0.9825 | 0 | 0 | 0 | 1 | Ref |
| KU160663 | Unassigned | Unassigned | Unassigned | 882_0 | Clustered | 11 | VC_882_0 | 11 | 0.943 | 1 | 0.943 | 0 | 0 | 0 | 1 | Ref |
| KU160664 | Unassigned | Unassigned | Unassigned | 1210_0 | Clustered | 7 | VC_1210_0 | 7 | 0.9808 | 0.99995963 | 0.9807 | 0 | 0 | 0 | 1 | Ref |
| KU160665 | Unassigned | Unassigned | Unassigned | 1116_0 | Clustered | 24 | VC_1116_0 | 24 | 0.8376 | 1 | 0.8376 | 0 | 0 | 0 | 1 | Ref |
| KU160666 | Unassigned | Unassigned | Unassigned | 882_0 | Clustered | 11 | VC_882_0 | 11 | 0.943 | 1 | 0.943 | 0 | 0 | 0 | 1 | Ref |
| KU160667 | Unassigned | Unassigned | Unassigned | 985_0 | Clustered | 38 | VC_985_0 | 38 | 0.9334 | 1 | 0.9334 | 0 | 0 | 0 | 1 | Ref |
| KU160668 | Unassigned | Unassigned | Unassigned | 1116_0 | Clustered | 24 | VC_1116_0 | 24 | 0.8376 | 1 | 0.8376 | 0 | 0 | 0 | 1 | Ref |
| KU160669 | Unassigned | Unassigned | Unassigned | 1211_1 | Clustered | 3 | VC_1211_1 | 3 | 0.3052 | 0.99836447 | 0.3047 | 0 | 0 | 0 | 1 | Ref |
| KU160670 | Unassigned | Unassigned | Unassigned | 985_0 | Clustered | 38 | VC_985_0 | 38 | 0.9334 | 1 | 0.9334 | 0 | 0 | 0 | 1 | Ref |
| KU160671 | Unassigned | Unassigned | Unassigned | 1200_0 | Clustered | 73 | VC_1200_0 | 73 | 0.9825 | 1 | 0.9825 | 0 | 0 | 0 | 1 | Ref |
| KU160672 | Unassigned | Unassigned | Unassigned | 1200_0 | Clustered | 73 | VC_1200_0 | 73 | 0.9825 | 1 | 0.9825 | 0 | 0 | 0 | 1 | Ref |
| KU160673 | Unassigned | Unassigned | Unassigned | 1211_1 | Clustered | 3 | VC_1211_1 | 3 | 0.3052 | 0.99836447 | 0.3047 | 0 | 0 | 0 | 1 | Ref |
| KU160674 | Unassigned | Unassigned | Unassigned | 985_0 | Clustered | 38 | VC_985_0 | 38 | 0.9334 | 1 | 0.9334 | 0 | 0 | 0 | 1 | Ref |
| KU183006 | Unassigned | Unassigned | Unassigned | 848_0 | Clustered | 124 | VC_848_0 | 124 | 0.9484 | 1 | 0.9484 | 0 | 0 | 0 | 1 | Ref |
| KU189325 | Unassigned | Unassigned | Unassigned | 891_0 | Clustered | 11 | VC_891_0 | 11 | 0.9663 | 1 | 0.9663 | 0 | 0 | 0 | 1 | Ref |
| KU194205 | Unassigned | Unassigned | Unassigned | 595_0 | Clustered | 35 | VC_595_0 | 35 | 0.8809 | 1 | 0.8809 | 0 | 0 | 0 | 1 | Ref |
| KU194206 | Unassigned | Unassigned | Unassigned | 595_0 | Clustered | 35 | VC_595_0 | 35 | 0.8809 | 1 | 0.8809 | 0 | 0 | 0 | 1 | Ref |
| KU197013 | Unassigned | Unassigned | Unassigned | 75_0 | Clustered | 15 | VC_75_0 | 15 | 0.518 | 1 | 0.518 | 0 | 0 | 0 | 1 | Ref |
| KU197014 | Unassigned | Unassigned | Unassigned | 766_0 | Clustered | 40 | VC_766_0 | 40 | 0.7511 | 1 | 0.7511 | 0 | 0 | 0 | 1 | Ref |
| KU198331 | Unassigned | Unassigned | Unassigned | 80_7 | Clustered | 33 | VC_80_7 | 33 | 0.2156 | 1 | 0.2156 | 0 | 0 | 0 | 0.8409 | Ref |
| KU199707 | Unassigned | Unassigned | Unassigned | 185_0 | Clustered | 53 | VC_185_0 | 53 | 0.9153 | 1 | 0.9153 | 0 | 0 | 0 | 1 | Ref |
| KU199708 | Unassigned | Unassigned | Unassigned | 185_0 | Clustered | 53 | VC_185_0 | 53 | 0.9153 | 1 | 0.9153 | 0 | 0 | 0 | 1 | Ref |
| KU199709 | Unassigned | Unassigned | Unassigned | 185_0 | Clustered | 53 | VC_185_0 | 53 | 0.9153 | 1 | 0.9153 | 0 | 0 | 0 | 1 | Ref |
| KU199710 | Unassigned | Unassigned | Unassigned | 304_0 | Clustered | 5 | VC_304_0 | 5 | 0.1053 | 0.99999993 | 0.1053 | 0 | 0 | 0 | 0.9 | Ref |
| KU204984 | Unassigned | Unassigned | Unassigned | 175_0 | Clustered | 20 | VC_175_0 | 20 | 0.7504 | 1 | 0.7504 | 0 | 0 | 0 | 1 | Ref |
| KU230356 | Unassigned | Unassigned | Unassigned | 1214_0 | Clustered | 6 | VC_1214_0 | 6 | 0.9948 | 0.99999296 | 0.9948 | 0 | 0 | 0 | 1 | Ref |
| KU234099 | Unassigned | Unassigned | Unassigned | 917_0 | Clustered | 28 | VC_917_0 | 28 | 0.5465 | 1 | 0.5465 | 0 | 0 | 0 | 1 | Ref |
| KU234532 | Unassigned | Unassigned | Unassigned | 1215_0 | Clustered | 4 | VC_1215_0 | 4 | 1 | 1 | 1 | 0 | 0 | 0 | 1 | Ref |
| KU234533 | Unassigned | Unassigned | Unassigned | 1215_0 | Clustered | 4 | VC_1215_0 | 4 | 1 | 1 | 1 | 0 | 0 | 0 | 1 | Ref |
| KU238068 | Unassigned | Unassigned | Unassigned | 35_0 | Clustered | 35 | VC_35_0 | 35 | 0.431 | 1 | 0.431 | 0 | 0 | 0 | 1 | Ref |
| KU238069 | Unassigned | Unassigned | Unassigned | 35_0 | Clustered | 35 | VC_35_0 | 35 | 0.431 | 1 | 0.431 | 0 | 0 | 0 | 1 | Ref |
| KU238070 | Unassigned | Unassigned | Unassigned | 35_0 | Clustered | 35 | VC_35_0 | 35 | 0.431 | 1 | 0.431 | 0 | 0 | 0 | 1 | Ref |
| KU245542 | Unassigned | Unassigned | Unassigned | 1068_0 | Clustered | 8 | VC_1068_0 | 8 | 0.9291 | 1 | 0.9291 | 0 | 0 | 0 | 1 | Ref |
| KU253712 | Unassigned | Unassigned | Unassigned | 87_8 | Clustered/Singleton | 1 | VC_87_8 | 1 | 0 | 1 | 0 | 0 | 0 | 0 | 1 | Ref |
| KU255188 | Unassigned | Unassigned | Unassigned | 532_0 | Clustered | 75 | VC_532_0 | 75 | 0.9393 | 1 | 0.9393 | 0 | 0 | 0 | 1 | Ref |
| KU255730 | Unassigned | Unassigned | Unassigned | 293_8 | Clustered/Singleton | 1 | VC_293_8 | 1 | 0 | 1 | 0 | 0 | 0 | 0 | 1 | Ref |
| KU297675 | Unassigned | Unassigned | Unassigned | 77_0 | Clustered | 37 | VC_77_0 | 37 | 0.9392 | 1 | 0.9392 | 0 | 0 | 0 | 1 | Ref |
| KU297783 | Unassigned | Unassigned | Unassigned | 223_4 | Clustered | 13 | VC_223_4 | 13 | 0.1649 | 1 | 0.1649 | 0 | 0 | 0 | 1 | Ref |
| KU298437 | Unassigned | Unassigned | Unassigned | 44_0 | Clustered | 49 | VC_44_0 | 49 | 0.4506 | 1 | 0.4506 | 0 | 0 | 0 | 0.8605 | Ref |
| KU310943 | Unassigned | Unassigned | Unassigned | 304_1 | Clustered | 20 | VC_304_1 | 20 | 0.5632 | 1 | 0.5632 | 0 | 0 | 0 | 0.7684 | Ref |
| KU356689 | Unassigned | Unassigned | Unassigned | 478_0 | Clustered | 8 | VC_478_0 | 8 | 0.2619 | 1 | 0.2619 | 0 | 0 | 0 | 1 | Ref |
| KU356690 | Unassigned | Unassigned | Unassigned | 803_0 | Clustered | 2 | VC_803_0 | 2 | 0.0373 | 0.95692559 | 0.0357 | 0 | 0 | 0 | 1 | Ref |
| KU356691 | Unassigned | Unassigned | Unassigned | 803_0 | Clustered | 2 | VC_803_0 | 2 | 0.0373 | 0.95692559 | 0.0357 | 0 | 0 | 0 | 1 | Ref |
| KU497559 | Unassigned | Unassigned | Unassigned | 77_0 | Clustered | 37 | VC_77_0 | 37 | 0.9392 | 1 | 0.9392 | 0 | 0 | 0 | 1 | Ref |
| KU504502 | Unassigned | Unassigned | Unassigned | 5_0 | Clustered | 27 | VC_5_0 | 27 | 0.9599 | 1 | 0.9599 | 0 | 0 | 0 | 1 | Ref |
| KU510289 | Unassigned | Unassigned | Unassigned | 713_0 | Clustered | 26 | VC_713_0 | 26 | 0.9358 | 1 | 0.9358 | 0 | 0 | 0 | 1 | Ref |
| KU517658 | Unassigned | Unassigned | Unassigned | 349_0 | Clustered | 5 | VC_349_0 | 5 | 0.191 | 0.97099341 | 0.1855 | 0 | 0 | 0 | 1 | Ref |
| KU521356 | Unassigned | Unassigned | Unassigned | 356_0 | Clustered | 21 | VC_356_0 | 21 | 0.5409 | 1 | 0.5409 | 0 | 0 | 0 | 1 | Ref |
| KU522583 | Unassigned | Unassigned | Unassigned | 872_0 | Clustered | 28 | VC_872_0 | 28 | 0.7887 | 1 | 0.7887 | 0 | 0 | 0 | 1 | Ref |
| KU530220 | Unassigned | Unassigned | Unassigned | 891_0 | Clustered | 11 | VC_891_0 | 11 | 0.9663 | 1 | 0.9663 | 0 | 0 | 0 | 1 | Ref |
| KU535860 | Unassigned | Unassigned | Unassigned | 772_0 | Clustered | 9 | VC_772_0 | 9 | 0.4468 | 1 | 0.4468 | 0 | 0 | 0 | 1 | Ref |
| KU568494 | Unassigned | Unassigned | Unassigned | 224_0 | Clustered | 5 | VC_224_0 | 5 | 0.0685 | 0.99999998 | 0.0685 | 0 | 0 | 0 | 1 | Ref |
| KU574722 | Unassigned | Unassigned | Unassigned | 170_5 | Clustered/Singleton | 1 | VC_170_5 | 1 | 0 | 1 | 0 | 0 | 0 | 0 | 1 | Ref |
| KU577463 | Unassigned | Unassigned | Unassigned | 195_3 | Clustered | 9 | VC_195_3 | 9 | 0.1955 | 1 | 0.1955 | 0 | 0 | 0 | 0.8611 | Ref |
| KU578077 | Unassigned | Unassigned | Unassigned | 532_0 | Clustered | 75 | VC_532_0 | 75 | 0.9393 | 1 | 0.9393 | 0 | 0 | 0 | 1 | Ref |
| KU594606 | Unassigned | Unassigned | Unassigned | 447_6 | Clustered/Singleton | 1 | VC_447_6 | 1 | 0 | 1 | 0 | 0 | 0 | 0 | 1 | Ref |
| KU594607 | Unassigned | Unassigned | Unassigned | 447_4 | Clustered | 9 | VC_447_4 | 9 | 0.127 | 1 | 0.127 | 0 | 0 | 0 | 1 | Ref |
| KU595432 | Unassigned | Unassigned | Unassigned | 75_0 | Clustered | 15 | VC_75_0 | 15 | 0.518 | 1 | 0.518 | 0 | 0 | 0 | 1 | Ref |

| | | | | | | | | | | | | | | | | |
|---|---|---|---|---|---|---|---|---|---|---|---|---|---|---|---|---|
| KU595433 | Unassigned | Unassigned | Unassigned | 75_0 | Clustered | 15 | VC_75_0 | 15 | 0.518 | 1 | 0.518 | 0 | 0 | 0 | 1 | Ref |
| KU595434 | Unassigned | Unassigned | Unassigned | 654_0 | Clustered | 115 | VC_654_0 | 115 | 0.7303 | 1 | 0.7303 | 0 | 0 | 0 | 0.8764 | Ref |
| KU599877 | Unassigned | Unassigned | Unassigned | 1198_0 | Clustered | 24 | VC_1198_0 | 24 | 1 | 1 | 1 | 0 | 0 | 0 | 1 | Ref |
| KU599878 | Unassigned | Unassigned | Unassigned | 1198_0 | Clustered | 24 | VC_1198_0 | 24 | 1 | 1 | 1 | 0 | 0 | 0 | 1 | Ref |
| KU599879 | Unassigned | Unassigned | Unassigned | 1198_0 | Clustered | 24 | VC_1198_0 | 24 | 1 | 1 | 1 | 0 | 0 | 0 | 1 | Ref |
| KU599880 | Unassigned | Unassigned | Unassigned | 1080_0 | Clustered | 23 | VC_1080_0 | 23 | 0.9603 | 1 | 0.9603 | 0 | 0 | 0 | 1 | Ref |
| KU599881 | Unassigned | Unassigned | Unassigned | 1080_0 | Clustered | 23 | VC_1080_0 | 23 | 0.9603 | 1 | 0.9603 | 0 | 0 | 0 | 1 | Ref |
| KU599882 | Unassigned | Unassigned | Unassigned | 1080_0 | Clustered | 23 | VC_1080_0 | 23 | 0.9603 | 1 | 0.9603 | 0 | 0 | 0 | 1 | Ref |
| KU599883 | Unassigned | Unassigned | Unassigned | 1080_0 | Clustered | 23 | VC_1080_0 | 23 | 0.9603 | 1 | 0.9603 | 0 | 0 | 0 | 1 | Ref |
| KU599884 | Unassigned | Unassigned | Unassigned | 1080_0 | Clustered | 23 | VC_1080_0 | 23 | 0.9603 | 1 | 0.9603 | 0 | 0 | 0 | 1 | Ref |
| KU599885 | Unassigned | Unassigned | Unassigned | 1080_0 | Clustered | 23 | VC_1080_0 | 23 | 0.9603 | 1 | 0.9603 | 0 | 0 | 0 | 1 | Ref |
| KU599886 | Unassigned | Unassigned | Unassigned | 1198_0 | Clustered | 24 | VC_1198_0 | 24 | 1 | 1 | 1 | 0 | 0 | 0 | 1 | Ref |
| KU599887 | Unassigned | Unassigned | Unassigned | 1061_0 | Clustered | 8 | VC_1061_0 | 8 | 0.8379 | 1 | 0.8379 | 0 | 0 | 0 | 1 | Ref |
| KU599888 | Unassigned | Unassigned | Unassigned | 1061_0 | Clustered | 8 | VC_1061_0 | 8 | 0.8379 | 1 | 0.8379 | 0 | 0 | 0 | 1 | Ref |
| KU599889 | Unassigned | Unassigned | Unassigned | 1061_0 | Clustered | 8 | VC_1061_0 | 8 | 0.8379 | 1 | 0.8379 | 0 | 0 | 0 | 1 | Ref |
| KU613353 | Unassigned | Unassigned | Unassigned | 232_0 | Clustered | 32 | VC_232_0 | 32 | 0.8471 | 1 | 0.8471 | 0 | 0 | 0 | 0.996 | Ref |
| KU640380 | Unassigned | Unassigned | Unassigned | 87_13 | Clustered/Singleton | 1 | VC_87_13 | 1 | 0 | 1 | 0 | 0 | 0 | 0 | 1 | Ref |
| KU647626 | Unassigned | Unassigned | Unassigned | 1221_0 | Clustered | 3 | VC_1221_0 | 3 | 0.7566 | 0.96819872 | 0.7326 | 0 | 0 | 0 | 1 | Ref |
| KU647627 | Unassigned | Unassigned | Unassigned | 1221_0 | Clustered | 3 | VC_1221_0 | 3 | 0.7566 | 0.96819872 | 0.7326 | 0 | 0 | 0 | 1 | Ref |
| KU647628 | Unassigned | Unassigned | Unassigned | 825_0 | Clustered | 17 | VC_825_0 | 17 | 0.8364 | 1 | 0.8364 | 0 | 0 | 0 | 1 | Ref |
| KU647629 | Unassigned | Unassigned | Unassigned | 1116_0 | Clustered | 24 | VC_1116_0 | 24 | 0.8376 | 1 | 0.8376 | 0 | 0 | 0 | 1 | Ref |
| KU666550 | Unassigned | Unassigned | Unassigned | 113_0 | Clustered | 91 | VC_113_0 | 91 | 0.9584 | 1 | 0.9584 | 0 | 0 | 0 | 1 | Ref |
| KU678389 | Unassigned | Unassigned | Unassigned | 262_0 | Clustered | 14 | VC_262_0 | 14 | 0.2364 | 1 | 0.2364 | 0 | 0 | 0 | 1 | Ref |
| KU678390 | Unassigned | Unassigned | Unassigned | 262_0 | Clustered | 14 | VC_262_0 | 14 | 0.2364 | 1 | 0.2364 | 0 | 0 | 0 | 1 | Ref |
| KU678391 | Unassigned | Unassigned | Unassigned | 262_0 | Clustered | 14 | VC_262_0 | 14 | 0.2364 | 1 | 0.2364 | 0 | 0 | 0 | 1 | Ref |
| KU678392 | Unassigned | Unassigned | Unassigned | 262_0 | Clustered | 14 | VC_262_0 | 14 | 0.2364 | 1 | 0.2364 | 0 | 0 | 0 | 1 | Ref |
| KU686192 | Unassigned | Unassigned | Unassigned | 447_0 | Clustered | 6 | VC_447_0 | 6 | 0.0775 | 1 | 0.0775 | 0 | 0 | 0 | 1 | Ref |
| KU686193 | Unassigned | Unassigned | Unassigned | 447_0 | Clustered | 6 | VC_447_0 | 6 | 0.0775 | 1 | 0.0775 | 0 | 0 | 0 | 1 | Ref |
| KU686194 | Unassigned | Unassigned | Unassigned | 447_0 | Clustered | 6 | VC_447_0 | 6 | 0.0775 | 1 | 0.0775 | 0 | 0 | 0 | 1 | Ref |
| KU686195 | Unassigned | Unassigned | Unassigned | 447_0 | Clustered | 6 | VC_447_0 | 6 | 0.0775 | 1 | 0.0775 | 0 | 0 | 0 | 1 | Ref |
| KU686196 | Unassigned | Unassigned | Unassigned | 447_0 | Clustered | 6 | VC_447_0 | 6 | 0.0775 | 1 | 0.0775 | 0 | 0 | 0 | 1 | Ref |
| KU686197 | Unassigned | Unassigned | Unassigned | 447_3 | Clustered | 3 | VC_447_3 | 3 | 0.0288 | 0.9981281 | 0.0288 | 0 | 0 | 0 | 1 | Ref |
| KU686198 | Unassigned | Unassigned | Unassigned | 447_3 | Clustered | 3 | VC_447_3 | 3 | 0.0288 | 0.9981281 | 0.0288 | 0 | 0 | 0 | 1 | Ref |
| KU686199 | Unassigned | Unassigned | Unassigned | 447_3 | Clustered | 3 | VC_447_3 | 3 | 0.0288 | 0.9981281 | 0.0288 | 0 | 0 | 0 | 1 | Ref |
| KU686200 | Unassigned | Unassigned | Unassigned | 447_1 | Clustered | 9 | VC_447_1 | 9 | 0.1269 | 1 | 0.1269 | 0 | 0 | 0 | 0.8611 | Ref |
| KU686201 | Unassigned | Unassigned | Unassigned | 447_1 | Clustered | 9 | VC_447_1 | 9 | 0.1269 | 1 | 0.1269 | 0 | 0 | 0 | 0.8611 | Ref |
| KU686202 | Unassigned | Unassigned | Unassigned | 447_1 | Clustered | 9 | VC_447_1 | 9 | 0.1269 | 1 | 0.1269 | 0 | 0 | 0 | 0.8611 | Ref |
| KU686204 | Unassigned | Unassigned | Unassigned | 446_15 | Clustered | 3 | VC_446_15 | 3 | 0.0409 | 0.99855682 | 0.0409 | 0 | 0 | 0 | 1 | Ref |
| KU686205 | Unassigned | Unassigned | Unassigned | 446_15 | Clustered | 3 | VC_446_15 | 3 | 0.0409 | 0.99855682 | 0.0409 | 0 | 0 | 0 | 1 | Ref |
| KU686206 | Unassigned | Unassigned | Unassigned | 446_15 | Clustered | 3 | VC_446_15 | 3 | 0.0409 | 0.99855682 | 0.0409 | 0 | 0 | 0 | 1 | Ref |
| KU686207 | Unassigned | Unassigned | Unassigned | 674_5 | Clustered | 3 | VC_674_5 | 3 | 0.0163 | 0.9986063 | 0.0163 | 0 | 0 | 0 | 1 | Ref |
| KU686208 | Unassigned | Unassigned | Unassigned | 674_5 | Clustered | 3 | VC_674_5 | 3 | 0.0163 | 0.9986063 | 0.0163 | 0 | 0 | 0 | 1 | Ref |
| KU686209 | Unassigned | Unassigned | Unassigned | 674_5 | Clustered | 3 | VC_674_5 | 3 | 0.0163 | 0.9986063 | 0.0163 | 0 | 0 | 0 | 1 | Ref |
| KU686211 | Unassigned | Unassigned | Unassigned | 447_10 | Clustered/Singleton | 1 | VC_447_10 | 1 | 0 | 1 | 0 | 0 | 0 | 0 | 1 | Ref |
| KU686212 | Unassigned | Unassigned | Unassigned | 676_0 | Clustered | 3 | VC_676_0 | 3 | 0.0217 | 0.92459149 | 0.0201 | 0 | 0 | 0 | 1 | Ref |
| KU686213 | Unassigned | Unassigned | Unassigned | 676_0 | Clustered | 3 | VC_676_0 | 3 | 0.0217 | 0.92459149 | 0.0201 | 0 | 0 | 0 | 1 | Ref |
| KU687347 | Unassigned | Unassigned | Unassigned | 398_0 | Clustered | 51 | VC_398_0 | 51 | 0.8741 | 1 | 0.8741 | 0 | 0 | 0 | 1 | Ref |
| KU687348 | Unassigned | Unassigned | Unassigned | 398_0 | Clustered | 51 | VC_398_0 | 51 | 0.8741 | 1 | 0.8741 | 0 | 0 | 0 | 1 | Ref |
| KU687349 | Unassigned | Unassigned | Unassigned | 465_0 | Clustered | 80 | VC_465_0 | 80 | 0.9988 | 1 | 0.9988 | 0 | 0 | 0 | 1 | Ref |
| KU687350 | Unassigned | Unassigned | Unassigned | 465_0 | Clustered | 80 | VC_465_0 | 80 | 0.9988 | 1 | 0.9988 | 0 | 0 | 0 | 1 | Ref |
| KU687351 | Unassigned | Unassigned | Unassigned | 465_0 | Clustered | 80 | VC_465_0 | 80 | 0.9988 | 1 | 0.9988 | 0 | 0 | 0 | 1 | Ref |
| KU695581 | Unassigned | Unassigned | Unassigned | 228_0 | Clustered | 22 | VC_228_0 | 22 | 0.7411 | 1 | 0.7411 | 0 | 0 | 0 | 1 | Ref |
| KU695582 | Unassigned | Unassigned | Unassigned | 230_0 | Clustered | 38 | VC_230_0 | 38 | 0.9076 | 1 | 0.9076 | 0 | 0 | 0 | 1 | Ref |
| KU708004 | Unassigned | Unassigned | Unassigned | 305_0 | Clustered/Singleton | 1 | VC_305_0 | 1 | 0 | 1 | 0 | 0 | 0 | 0 | 1 | Ref |
| KU716094 | Unassigned | Unassigned | Unassigned | 232_0 | Clustered | 32 | VC_232_0 | 32 | 0.8471 | 1 | 0.8471 | 0 | 0 | 0 | 0.996 | Ref |
| KU716095 | Unassigned | Unassigned | Unassigned | 232_0 | Clustered | 32 | VC_232_0 | 32 | 0.8471 | 1 | 0.8471 | 0 | 0 | 0 | 0.996 | Ref |
| KU726251 | Unassigned | Unassigned | Unassigned | 994_5 | Clustered | 19 | VC_994_5 | 19 | 0.2622 | 1 | 0.2622 | 0 | 0 | 0 | 1 | Ref |
| KU728633 | Unassigned | Unassigned | Unassigned | 702_1 | Clustered | 2 | VC_702_1 | 2 | 0.2185 | 0.91327915 | 0.1996 | 0 | 0 | 0 | 1 | Ref |
| KU737344 | Unassigned | Unassigned | Unassigned | 913_0 | Clustered | 28 | VC_913_0 | 28 | 0.6206 | 1 | 0.6206 | 0 | 0 | 0 | 0.9841 | Ref |
| KU737345 | Unassigned | Unassigned | Unassigned | 912_1 | Clustered | 12 | VC_912_1 | 12 | 0.2423 | 1 | 0.2423 | 0 | 0 | 0 | 1 | Ref |
| KU737346 | Unassigned | Unassigned | Unassigned | 912_0 | Clustered | 10 | VC_912_0 | 10 | 0.1994 | 1 | 0.1994 | 0 | 0 | 0 | 1 | Ref |
| KU737347 | Unassigned | Unassigned | Unassigned | 912_1 | Clustered | 12 | VC_912_1 | 12 | 0.2423 | 1 | 0.2423 | 0 | 0 | 0 | 1 | Ref |
| KU737348 | Unassigned | Unassigned | Unassigned | 913_0 | Clustered | 28 | VC_913_0 | 28 | 0.6206 | 1 | 0.6206 | 0 | 0 | 0 | 0.9841 | Ref |
| KU737349 | Unassigned | Unassigned | Unassigned | 913_0 | Clustered | 28 | VC_913_0 | 28 | 0.6206 | 1 | 0.6206 | 0 | 0 | 0 | 0.9841 | Ref |
| KU737350 | Unassigned | Unassigned | Unassigned | 913_0 | Clustered | 28 | VC_913_0 | 28 | 0.6206 | 1 | 0.6206 | 0 | 0 | 0 | 0.9841 | Ref |
| KU737351 | Unassigned | Unassigned | Unassigned | 913_0 | Clustered | 28 | VC_913_0 | 28 | 0.6206 | 1 | 0.6206 | 0 | 0 | 0 | 0.9841 | Ref |
| KU737352 | Unassigned | Unassigned | Unassigned | 913_0 | Clustered | 28 | VC_913_0 | 28 | 0.6206 | 1 | 0.6206 | 0 | 0 | 0 | 0.9841 | Ref |
| KU743887 | Unassigned | Unassigned | Unassigned | 434_0 | Clustered | 45 | VC_434_0 | 45 | 0.9479 | 1 | 0.9479 | 0 | 0 | 0 | 1 | Ref |
| KU747973 | Unassigned | Unassigned | Unassigned | 663_0 | Clustered | 5 | VC_663_0 | 5 | 0.6645 | 0.99999974 | 0.6645 | 0 | 0 | 0 | 1 | Ref |
| KU760857 | Unassigned | Unassigned | Unassigned | 254_0 | Clustered | 14 | VC_254_0 | 14 | 0.9118 | 1 | 0.9118 | 0 | 0 | 0 | 0.967 | Ref |
| KU761558 | Unassigned | Unassigned | Unassigned | 223_3 | Clustered | 8 | VC_223_3 | 8 | 0.0699 | 1 | 0.0699 | 0 | 0 | 0 | 0.8571 | Ref |
| KU761559 | Unassigned | Unassigned | Unassigned | 223_3 | Clustered | 8 | VC_223_3 | 8 | 0.0699 | 1 | 0.0699 | 0 | 0 | 0 | 0.8571 | Ref |
| KU761955 | Unassigned | Unassigned | Unassigned | 77_0 | Clustered | 37 | VC_77_0 | 37 | 0.9392 | 1 | 0.9392 | 0 | 0 | 0 | 1 | Ref |
| KU831549 | Unassigned | Unassigned | Unassigned | 842_0 | Clustered | 14 | VC_842_0 | 14 | 0.6581 | 1 | 0.6581 | 0 | 0 | 0 | 1 | Ref |
| KU836751 | Unassigned | Unassigned | Unassigned | 102_0 | Clustered | 15 | VC_102_0 | 15 | 0.653 | 1 | 0.653 | 0 | 0 | 0 | 1 | Ref |
| KU847400 | Unassigned | Unassigned | Unassigned | 913_1 | Clustered | 8 | VC_913_1 | 8 | 0.1102 | 0.9989323 | 0.11 | 0 | 0 | 0 | 0.75 | Ref |
| KU848187 | Unassigned | Unassigned | Unassigned | 396_0 | Clustered | 3 | VC_396_0 | 3 | 0.3827 | 0.99764103 | 0.3818 | 0 | 0 | 0 | 1 | Ref |
| KU862660 | Unassigned | Unassigned | Unassigned | 992_1 | Clustered/Singleton | 1 | VC_992_1 | 1 | 0 | 1 | 0 | 0 | 0 | 0 | 1 | Ref |
| KU865302 | Unassigned | Unassigned | Unassigned | 538_40 | Clustered | 4 | VC_538_40 | 4 | 0.0395 | 0.99998776 | 0.0394 | 0 | 0 | 0 | 1 | Ref |
| KU865303 | Unassigned | Unassigned | Unassigned | 571_2 | Clustered | 17 | VC_571_2 | 17 | 0.0965 | 1 | 0.0965 | 0 | 0 | 0 | 0.9779 | Ref |
| KU867876 | Unassigned | Unassigned | Unassigned | 290_25 | Clustered/Singleton | 1 | VC_290_25 | 1 | 0 | 1 | 0 | 0 | 0 | 0 | 1 | Ref |

| | | | | | | | | | | | | | | | | |
|---|---|---|---|---|---|---|---|---|---|---|---|---|---|---|---|---|
| KU867906 | Unassigned | Unassigned | Unassigned | 535_1 | Clustered | 108 | VC_535_1 | 108 | 0.8479 | 1 | 0.8479 | 0 | 0 | 0 | 0.9955 | Ref |
| KU867907 | Unassigned | Unassigned | Unassigned | 384_15 | Clustered | 44 | VC_384_15 | 44 | 0.139 | 1 | 0.139 | 0 | 0 | 0 | 0.7474 | Ref |
| KU878088 | Unassigned | Unassigned | Unassigned | 462_0 | Clustered | 4 | VC_462_0 | 4 | 0.3031 | 0.99978853 | 0.303 | 0 | 0 | 0 | 1 | Ref |
| KU878967 | Unassigned | Unassigned | Unassigned | 30_1 | Clustered | 53 | VC_30_1 | 53 | 0.7151 | 1 | 0.7151 | 0 | 0 | 0 | 0.9325 | Ref |
| KU878968 | Unassigned | Unassigned | Unassigned | 297_5 | Clustered | 14 | VC_297_5 | 14 | 0.141 | 1 | 0.141 | 0 | 0 | 0 | 0.9231 | Ref |
| KU878969 | Unassigned | Unassigned | Unassigned | 297_5 | Clustered | 14 | VC_297_5 | 14 | 0.141 | 1 | 0.141 | 0 | 0 | 0 | 0.9231 | Ref |
| KU884561 | Unassigned | Unassigned | Unassigned | 632_0 | Clustered | 9 | VC_632_0 | 9 | 0.9524 | 1 | 0.9524 | 0 | 0 | 0 | 1 | Ref |
| KU884562 | Unassigned | Unassigned | Unassigned | 632_0 | Clustered | 9 | VC_632_0 | 9 | 0.9524 | 1 | 0.9524 | 0 | 0 | 0 | 1 | Ref |
| KU884563 | Unassigned | Unassigned | Unassigned | 632_0 | Clustered | 9 | VC_632_0 | 9 | 0.9524 | 1 | 0.9524 | 0 | 0 | 0 | 1 | Ref |
| KU884564 | Unassigned | Unassigned | Unassigned | 632_0 | Clustered | 9 | VC_632_0 | 9 | 0.9524 | 1 | 0.9524 | 0 | 0 | 0 | 1 | Ref |
| KU884565 | Unassigned | Unassigned | Unassigned | 632_0 | Clustered | 9 | VC_632_0 | 9 | 0.9524 | 1 | 0.9524 | 0 | 0 | 0 | 1 | Ref |
| KU885988 | Unassigned | Unassigned | Unassigned | 857_0 | Clustered | 16 | VC_857_0 | 16 | 0.9317 | 1 | 0.9317 | 0 | 0 | 0 | 1 | Ref |
| KU885989 | Unassigned | Unassigned | Unassigned | 857_0 | Clustered | 16 | VC_857_0 | 16 | 0.9317 | 1 | 0.9317 | 0 | 0 | 0 | 1 | Ref |
| KU885990 | Unassigned | Unassigned | Unassigned | 857_0 | Clustered | 16 | VC_857_0 | 16 | 0.9317 | 1 | 0.9317 | 0 | 0 | 0 | 1 | Ref |
| KU886222 | Unassigned | Unassigned | Unassigned | 507_1 | Clustered | 11 | VC_507_1 | 11 | 0.4612 | 1 | 0.4612 | 0 | 0 | 0 | 1 | Ref |
| KU886223 | Unassigned | Unassigned | Unassigned | 507_1 | Clustered | 11 | VC_507_1 | 11 | 0.4612 | 1 | 0.4612 | 0 | 0 | 0 | 1 | Ref |
| KU886224 | Unassigned | Unassigned | Unassigned | 507_1 | Clustered | 11 | VC_507_1 | 11 | 0.4612 | 1 | 0.4612 | 0 | 0 | 0 | 1 | Ref |
| KU886225 | Unassigned | Unassigned | Unassigned | 507_1 | Clustered | 11 | VC_507_1 | 11 | 0.4612 | 1 | 0.4612 | 0 | 0 | 0 | 1 | Ref |
| KU925172 | Unassigned | Unassigned | Unassigned | 290_8 | Clustered/Singleton | 1 | VC_290_8 | 1 | 0 | 1 | 0 | 0 | 0 | 0 | 1 | Ref |
| KU927491 | Unassigned | Unassigned | Unassigned | 30_1 | Clustered | 53 | VC_30_1 | 53 | 0.7151 | 1 | 0.7151 | 0 | 0 | 0 | 0.9325 | Ref |
| KU927492 | Unassigned | Unassigned | Unassigned | 30_1 | Clustered | 53 | VC_30_1 | 53 | 0.7151 | 1 | 0.7151 | 0 | 0 | 0 | 0.9325 | Ref |
| KU927493 | Unassigned | Unassigned | Unassigned | 47_0 | Clustered | 12 | VC_47_0 | 12 | 0.3571 | 1 | 0.3571 | 0 | 0 | 0 | 1 | Ref |
| KU927494 | Unassigned | Unassigned | Unassigned | 30_1 | Clustered | 53 | VC_30_1 | 53 | 0.7151 | 1 | 0.7151 | 0 | 0 | 0 | 0.9325 | Ref |
| KU927495 | Unassigned | Unassigned | Unassigned | 30_1 | Clustered | 53 | VC_30_1 | 53 | 0.7151 | 1 | 0.7151 | 0 | 0 | 0 | 0.9325 | Ref |
| KU927496 | Unassigned | Unassigned | Unassigned | 30_1 | Clustered | 53 | VC_30_1 | 53 | 0.7151 | 1 | 0.7151 | 0 | 0 | 0 | 0.9325 | Ref |
| KU927497 | Unassigned | Unassigned | Unassigned | 199_19 | Clustered | 12 | VC_199_19 | 12 | 0.0651 | 1 | 0.0651 | 0 | 0 | 0 | 0.8636 | Ref |
| KU927498 | Unassigned | Unassigned | Unassigned | 30_1 | Clustered | 53 | VC_30_1 | 53 | 0.7151 | 1 | 0.7151 | 0 | 0 | 0 | 0.9325 | Ref |
| KU927499 | Unassigned | Unassigned | Unassigned | 405_0 | Clustered | 126 | VC_405_0 | 126 | 0.9614 | 1 | 0.9614 | 0 | 0 | 0 | 1 | Ref |
| KU927500 | Unassigned | Unassigned | Unassigned | 611_0 | Clustered | 50 | VC_611_0 | 50 | 0.7306 | 1 | 0.7306 | 0 | 0 | 0 | 1 | Ref |
| KU935726 | Unassigned | Unassigned | Unassigned | 539_0 | Clustered | 37 | VC_539_0 | 37 | 0.911 | 1 | 0.911 | 0 | 0 | 0 | 1 | Ref |
| KU935727 | Unassigned | Unassigned | Unassigned | 539_0 | Clustered | 37 | VC_539_0 | 37 | 0.911 | 1 | 0.911 | 0 | 0 | 0 | 1 | Ref |
| KU935728 | Unassigned | Unassigned | Unassigned | 539_0 | Clustered | 37 | VC_539_0 | 37 | 0.911 | 1 | 0.911 | 0 | 0 | 0 | 1 | Ref |
| KU935729 | Unassigned | Unassigned | Unassigned | 539_0 | Clustered | 37 | VC_539_0 | 37 | 0.911 | 1 | 0.911 | 0 | 0 | 0 | 1 | Ref |
| KU935730 | Unassigned | Unassigned | Unassigned | 539_0 | Clustered | 37 | VC_539_0 | 37 | 0.911 | 1 | 0.911 | 0 | 0 | 0 | 1 | Ref |
| KU935731 | Unassigned | Unassigned | Unassigned | 539_0 | Clustered | 37 | VC_539_0 | 37 | 0.911 | 1 | 0.911 | 0 | 0 | 0 | 1 | Ref |
| KU946962 | Unassigned | Unassigned | Unassigned | 466_0 | Clustered | 66 | VC_466_0 | 66 | 0.9246 | 1 | 0.9246 | 0 | 0 | 0 | 1 | Ref |
| KU948710 | Unassigned | Unassigned | Unassigned | 862_0 | Clustered | 29 | VC_862_0 | 29 | 0.8594 | 1 | 0.8594 | 0 | 0 | 0 | 1 | Ref |
| KU951146 | Unassigned | Unassigned | Unassigned | 654_0 | Clustered | 115 | VC_654_0 | 115 | 0.7303 | 1 | 0.7303 | 0 | 0 | 0 | 0.8764 | Ref |
| KU951147 | Unassigned | Unassigned | Unassigned | 654_0 | Clustered | 115 | VC_654_0 | 115 | 0.7303 | 1 | 0.7303 | 0 | 0 | 0 | 0.8764 | Ref |
| KU958700 | Unassigned | Unassigned | Unassigned | 607_0 | Clustered | 2 | VC_607_0 | 2 | 0.0776 | 0.94661631 | 0.0735 | 0 | 0 | 0 | 1 | Ref |
| KU963245 | Unassigned | Unassigned | Unassigned | 895_0 | Clustered | 25 | VC_895_0 | 25 | 0.5768 | 1 | 0.5768 | 0 | 0 | 0 | 1 | Ref |
| KU963246 | Unassigned | Unassigned | Unassigned | 1216_0 | Clustered | 22 | VC_1216_0 | 22 | 0.4546 | 1 | 0.4546 | 0 | 0 | 0 | 1 | Ref |
| KU963247 | Unassigned | Unassigned | Unassigned | 1216_0 | Clustered | 22 | VC_1216_0 | 22 | 0.4546 | 1 | 0.4546 | 0 | 0 | 0 | 1 | Ref |
| KU963249 | Unassigned | Unassigned | Unassigned | 543_1 | Clustered | 5 | VC_543_1 | 5 | 0.0484 | 0.99999947 | 0.0484 | 0 | 0 | 0 | 0.9 | Ref |
| KU963250 | Unassigned | Unassigned | Unassigned | 1136_0 | Clustered | 40 | VC_1136_0 | 40 | 0.8746 | 1 | 0.8746 | 0 | 0 | 0 | 1 | Ref |
| KU963251 | Unassigned | Unassigned | Unassigned | 1217_0 | Clustered | 41 | VC_1217_0 | 41 | 0.9546 | 1 | 0.9546 | 0 | 0 | 0 | 0.9976 | Ref |
| KU963252 | Unassigned | Unassigned | Unassigned | 1218_0 | Clustered | 14 | VC_1218_0 | 14 | 0.7811 | 1 | 0.7811 | 0 | 0 | 0 | 0.989 | Ref |
| KU963253 | Unassigned | Unassigned | Unassigned | 1138_0 | Clustered | 16 | VC_1138_0 | 16 | 0.4566 | 1 | 0.4566 | 0 | 0 | 0 | 1 | Ref |
| KU963254 | Unassigned | Unassigned | Unassigned | 1217_0 | Clustered | 41 | VC_1217_0 | 41 | 0.9546 | 1 | 0.9546 | 0 | 0 | 0 | 0.9976 | Ref |
| KU963255 | Unassigned | Unassigned | Unassigned | 543_0 | Clustered | 25 | VC_543_0 | 25 | 0.4584 | 1 | 0.4584 | 0 | 0 | 0 | 1 | Ref |
| KU963256 | Unassigned | Unassigned | Unassigned | 540_0 | Clustered | 3 | VC_540_0 | 3 | 0.083 | 0.9985266 | 0.0829 | 0 | 0 | 0 | 1 | Ref |
| KU963257 | Unassigned | Unassigned | Unassigned | 543_0 | Clustered | 25 | VC_543_0 | 25 | 0.4584 | 1 | 0.4584 | 0 | 0 | 0 | 1 | Ref |
| KU963258 | Unassigned | Unassigned | Unassigned | 896_0 | Clustered | 9 | VC_896_0 | 9 | 0.2604 | 1 | 0.2604 | 0 | 0 | 0 | 1 | Ref |
| KU963259 | Unassigned | Unassigned | Unassigned | 1217_0 | Clustered | 41 | VC_1217_0 | 41 | 0.9546 | 1 | 0.9546 | 0 | 0 | 0 | 0.9976 | Ref |
| KU963260 | Unassigned | Unassigned | Unassigned | 952_0 | Clustered | 39 | VC_952_0 | 39 | 0.9064 | 1 | 0.9064 | 0 | 0 | 0 | 1 | Ref |
| KU963261 | Unassigned | Unassigned | Unassigned | 989_0 | Clustered | 13 | VC_989_0 | 13 | 0.8622 | 1 | 0.8622 | 0 | 0 | 0 | 1 | Ref |
| KU963262 | Unassigned | Unassigned | Unassigned | 896_0 | Clustered | 9 | VC_896_0 | 9 | 0.2604 | 1 | 0.2604 | 0 | 0 | 0 | 1 | Ref |
| KU963263 | Unassigned | Unassigned | Unassigned | 543_1 | Clustered | 5 | VC_543_1 | 5 | 0.0484 | 0.99999947 | 0.0484 | 0 | 0 | 0 | 0.9 | Ref |
| KU984914 | Unassigned | Unassigned | Unassigned | 532_0 | Clustered | 75 | VC_532_0 | 75 | 0.9393 | 1 | 0.9393 | 0 | 0 | 0 | 1 | Ref |
| KU985090 | Unassigned | Unassigned | Unassigned | 537_0 | Clustered | 39 | VC_537_0 | 39 | 0.8148 | 1 | 0.8148 | 0 | 0 | 0 | 1 | Ref |
| KU985091 | Unassigned | Unassigned | Unassigned | 532_0 | Clustered | 75 | VC_532_0 | 75 | 0.9393 | 1 | 0.9393 | 0 | 0 | 0 | 1 | Ref |
| KU985092 | Unassigned | Unassigned | Unassigned | 535_1 | Clustered | 108 | VC_535_1 | 108 | 0.8479 | 1 | 0.8479 | 0 | 0 | 0 | 0.9955 | Ref |
| KU985093 | Unassigned | Unassigned | Unassigned | 223_6 | Clustered | 42 | VC_223_6 | 42 | 0.4717 | 1 | 0.4717 | 0 | 0 | 0 | 0.8351 | Ref |
| KU985094 | Unassigned | Unassigned | Unassigned | 535_1 | Clustered | 108 | VC_535_1 | 108 | 0.8479 | 1 | 0.8479 | 0 | 0 | 0 | 0.9955 | Ref |
| KU985095 | Unassigned | Unassigned | Unassigned | 223_6 | Clustered | 42 | VC_223_6 | 42 | 0.4717 | 1 | 0.4717 | 0 | 0 | 0 | 0.8351 | Ref |
| KU985096 | Unassigned | Unassigned | Unassigned | 230_0 | Clustered | 38 | VC_230_0 | 38 | 0.9076 | 1 | 0.9076 | 0 | 0 | 0 | 1 | Ref |
| KU992911 | Unassigned | Unassigned | Unassigned | 98_0 | Clustered | 30 | VC_98_0 | 30 | 0.7175 | 1 | 0.7175 | 0 | 0 | 0 | 1 | Ref |
| KU997639 | Unassigned | Unassigned | Unassigned | 917_0 | Clustered | 28 | VC_917_0 | 28 | 0.5465 | 1 | 0.5465 | 0 | 0 | 0 | 1 | Ref |
| KU998234 | Unassigned | Unassigned | Unassigned | 1219_0 | Clustered | 24 | VC_1219_0 | 24 | 0.9519 | 1 | 0.9519 | 0 | 0 | 0 | 1 | Ref |
| KU998235 | Unassigned | Unassigned | Unassigned | 1218_0 | Clustered | 14 | VC_1218_0 | 14 | 0.7811 | 1 | 0.7811 | 0 | 0 | 0 | 0.989 | Ref |
| KU998236 | Unassigned | Unassigned | Unassigned | 1217_0 | Clustered | 41 | VC_1217_0 | 41 | 0.9546 | 1 | 0.9546 | 0 | 0 | 0 | 0.9976 | Ref |
| KU998237 | Unassigned | Unassigned | Unassigned | 419_0 | Clustered | 11 | VC_419_0 | 11 | 0.9465 | 1 | 0.9465 | 0 | 0 | 0 | 1 | Ref |
| KU998238 | Unassigned | Unassigned | Unassigned | 419_0 | Clustered | 11 | VC_419_0 | 11 | 0.9465 | 1 | 0.9465 | 0 | 0 | 0 | 1 | Ref |
| KU998239 | Unassigned | Unassigned | Unassigned | 952_0 | Clustered | 39 | VC_952_0 | 39 | 0.9064 | 1 | 0.9064 | 0 | 0 | 0 | 1 | Ref |
| KU998240 | Unassigned | Unassigned | Unassigned | 893_1 | Clustered | 20 | VC_893_1 | 20 | 0.4792 | 1 | 0.4792 | 0 | 0 | 0 | 1 | Ref |
| KU998241 | Unassigned | Unassigned | Unassigned | 895_0 | Clustered | 25 | VC_895_0 | 25 | 0.5768 | 1 | 0.5768 | 0 | 0 | 0 | 1 | Ref |
| KU998242 | Unassigned | Unassigned | Unassigned | 896_0 | Clustered | 9 | VC_896_0 | 9 | 0.2604 | 1 | 0.2604 | 0 | 0 | 0 | 1 | Ref |
| KU998243 | Unassigned | Unassigned | Unassigned | 896_0 | Clustered | 9 | VC_896_0 | 9 | 0.2604 | 1 | 0.2604 | 0 | 0 | 0 | 1 | Ref |
| KU998244 | Unassigned | Unassigned | Unassigned | 1223_1 | Clustered | 10 | VC_1223_1 | 10 | 0.5795 | 1 | 0.5795 | 0 | 0 | 0 | 1 | Ref |
| KU998245 | Unassigned | Unassigned | Unassigned | 1223_0 | Clustered | 2 | VC_1223_0 | 2 | 0.0456 | 0.95126432 | 0.0434 | 0 | 0 | 0 | 1 | Ref |
| KU998246 | Unassigned | Unassigned | Unassigned | 1223_1 | Clustered | 10 | VC_1223_1 | 10 | 0.5795 | 1 | 0.5795 | 0 | 0 | 0 | 1 | Ref |

| | | | | | | | | | | | | | | | | |
|---|---|---|---|---|---|---|---|---|---|---|---|---|---|---|---|---|
| KU998247 | Unassigned | Unassigned | Unassigned | 1223_1 | Clustered | 10 | VC_1223_1 | 10 | 0.5795 | 1 | 0.5795 | 0 | 0 | 0 | 1 | Ref |
| KU998248 | Unassigned | Unassigned | Unassigned | 1217_0 | Clustered | 41 | VC_1217_0 | 41 | 0.9546 | 1 | 0.9546 | 0 | 0 | 0 | 0.9976 | Ref |
| KU998249 | Unassigned | Unassigned | Unassigned | 231_0 | Clustered | 19 | VC_231_0 | 19 | 0.9378 | 1 | 0.9378 | 0 | 0 | 0 | 1 | Ref |
| KU998250 | Unassigned | Unassigned | Unassigned | 231_0 | Clustered | 19 | VC_231_0 | 19 | 0.9378 | 1 | 0.9378 | 0 | 0 | 0 | 1 | Ref |
| KU998251 | Unassigned | Unassigned | Unassigned | 231_0 | Clustered | 19 | VC_231_0 | 19 | 0.9378 | 1 | 0.9378 | 0 | 0 | 0 | 1 | Ref |
| KU998252 | Unassigned | Unassigned | Unassigned | 897_0 | Clustered | 5 | VC_897_0 | 5 | 0.4183 | 0.99999997 | 0.4183 | 0 | 0 | 0 | 1 | Ref |
| KU998253 | Unassigned | Unassigned | Unassigned | 897_0 | Clustered | 5 | VC_897_0 | 5 | 0.4183 | 0.99999997 | 0.4183 | 0 | 0 | 0 | 1 | Ref |
| KU998254 | Unassigned | Unassigned | Unassigned | 897_0 | Clustered | 5 | VC_897_0 | 5 | 0.4183 | 0.99999997 | 0.4183 | 0 | 0 | 0 | 1 | Ref |
| KX009778 | Unassigned | Unassigned | Unassigned | 290_139 | Clustered/Singleton | 1 | VC_290_139 | 1 | 0 | 1 | 0 | 0 | 0 | 0 | 1 | Ref |
| KX011028 | Unassigned | Unassigned | Unassigned | 155_27 | Clustered/Singleton | 1 | VC_155_27 | 1 | 0 | 1 | 0 | 0 | 0 | 0 | 1 | Ref |
| KX011169 | Unassigned | Unassigned | Unassigned | 913_1 | Clustered | 8 | VC_913_1 | 8 | 0.1102 | 0.9989323 | 0.11 | 0 | 0 | 0 | 0.75 | Ref |
| KX015770 | Unassigned | Unassigned | Unassigned | 603_0 | Clustered | 28 | VC_603_0 | 28 | 0.7692 | 1 | 0.7692 | 0 | 0 | 0 | 1 | Ref |
| KX015771 | Unassigned | Unassigned | Unassigned | 603_0 | Clustered | 28 | VC_603_0 | 28 | 0.7692 | 1 | 0.7692 | 0 | 0 | 0 | 1 | Ref |
| KX017520 | Unassigned | Unassigned | Unassigned | 475_0 | Clustered | 96 | VC_475_0 | 96 | 0.9303 | 1 | 0.9303 | 0 | 0 | 0 | 0.9575 | Ref |
| KX017521 | Unassigned | Unassigned | Unassigned | 199_19 | Clustered | 12 | VC_199_19 | 12 | 0.0651 | 1 | 0.0651 | 0 | 0 | 0 | 0.8636 | Ref |
| KX058879 | Unassigned | Unassigned | Unassigned | 5_0 | Clustered | 27 | VC_5_0 | 27 | 0.9599 | 1 | 0.9599 | 0 | 0 | 0 | 1 | Ref |
| KX077179 | Unassigned | Unassigned | Unassigned | 188_0 | Clustered | 5 | VC_188_0 | 5 | 0.6188 | 0.99999978 | 0.6188 | 0 | 0 | 0 | 1 | Ref |
| KX077892 | Unassigned | Unassigned | Unassigned | 1229_0 | Clustered | 5 | VC_1229_0 | 5 | 0.9893 | 0.91813282 | 0.9083 | 0 | 0 | 0 | 1 | Ref |
| KX077893 | Unassigned | Unassigned | Unassigned | 1229_0 | Clustered | 5 | VC_1229_0 | 5 | 0.9893 | 0.91813282 | 0.9083 | 0 | 0 | 0 | 1 | Ref |
| KX077894 | Unassigned | Unassigned | Unassigned | 268_1 | Clustered | 20 | VC_268_1 | 20 | 0.1906 | 1 | 0.1906 | 0 | 0 | 0 | 0.6789 | Ref |
| KX077896 | Unassigned | Unassigned | Unassigned | 647_0 | Clustered | 37 | VC_647_0 | 37 | 0.5847 | 1 | 0.5847 | 0 | 0 | 0 | 0.9234 | Ref |
| KX078568 | Unassigned | Unassigned | Unassigned | 403_0 | Clustered | 4 | VC_403_0 | 4 | 0.0462 | 0.99998824 | 0.0462 | 0 | 0 | 0 | 1 | Ref |
| KX078569 | Unassigned | Unassigned | Unassigned | 295_1 | Clustered/Singleton | 1 | VC_295_1 | 1 | 0 | 1 | 0 | 0 | 0 | 0 | 1 | Ref |
| KX098389 | Unassigned | Unassigned | Unassigned | 801_2 | Clustered | 5 | VC_801_2 | 5 | 0.087 | 0.99999997 | 0.087 | 0 | 0 | 0 | 1 | Ref |
| KX098390 | Unassigned | Unassigned | Unassigned | 801_2 | Clustered | 5 | VC_801_2 | 5 | 0.087 | 0.99999997 | 0.087 | 0 | 0 | 0 | 1 | Ref |
| KX098391 | Unassigned | Unassigned | Unassigned | 801_2 | Clustered | 5 | VC_801_2 | 5 | 0.087 | 0.99999997 | 0.087 | 0 | 0 | 0 | 1 | Ref |
| KX119174 | Unassigned | Unassigned | Unassigned | 101_0 | Clustered | 32 | VC_101_0 | 32 | 0.8826 | 1 | 0.8826 | 0 | 0 | 0 | 1 | Ref |
| KX119175 | Unassigned | Unassigned | Unassigned | 101_0 | Clustered | 32 | VC_101_0 | 32 | 0.8826 | 1 | 0.8826 | 0 | 0 | 0 | 1 | Ref |
| KX119176 | Unassigned | Unassigned | Unassigned | 101_0 | Clustered | 32 | VC_101_0 | 32 | 0.8826 | 1 | 0.8826 | 0 | 0 | 0 | 1 | Ref |
| KX119177 | Unassigned | Unassigned | Unassigned | 101_0 | Clustered | 32 | VC_101_0 | 32 | 0.8826 | 1 | 0.8826 | 0 | 0 | 0 | 1 | Ref |
| KX119182 | Unassigned | Unassigned | Unassigned | 101_0 | Clustered | 32 | VC_101_0 | 32 | 0.8826 | 1 | 0.8826 | 0 | 0 | 0 | 1 | Ref |
| KX119188 | Unassigned | Unassigned | Unassigned | 101_0 | Clustered | 32 | VC_101_0 | 32 | 0.8826 | 1 | 0.8826 | 0 | 0 | 0 | 1 | Ref |
| KX119189 | Unassigned | Unassigned | Unassigned | 101_0 | Clustered | 32 | VC_101_0 | 32 | 0.8826 | 1 | 0.8826 | 0 | 0 | 0 | 1 | Ref |
| KX119190 | Unassigned | Unassigned | Unassigned | 101_0 | Clustered | 32 | VC_101_0 | 32 | 0.8826 | 1 | 0.8826 | 0 | 0 | 0 | 1 | Ref |
| KX119191 | Unassigned | Unassigned | Unassigned | 101_0 | Clustered | 32 | VC_101_0 | 32 | 0.8826 | 1 | 0.8826 | 0 | 0 | 0 | 1 | Ref |
| KX119192 | Unassigned | Unassigned | Unassigned | 101_0 | Clustered | 32 | VC_101_0 | 32 | 0.8826 | 1 | 0.8826 | 0 | 0 | 0 | 1 | Ref |
| KX119193 | Unassigned | Unassigned | Unassigned | 101_0 | Clustered | 32 | VC_101_0 | 32 | 0.8826 | 1 | 0.8826 | 0 | 0 | 0 | 1 | Ref |
| KX119194 | Unassigned | Unassigned | Unassigned | 101_0 | Clustered | 32 | VC_101_0 | 32 | 0.8826 | 1 | 0.8826 | 0 | 0 | 0 | 1 | Ref |
| KX119195 | Unassigned | Unassigned | Unassigned | 101_0 | Clustered | 32 | VC_101_0 | 32 | 0.8826 | 1 | 0.8826 | 0 | 0 | 0 | 1 | Ref |
| KX119196 | Unassigned | Unassigned | Unassigned | 101_0 | Clustered | 32 | VC_101_0 | 32 | 0.8826 | 1 | 0.8826 | 0 | 0 | 0 | 1 | Ref |
| KX119197 | Unassigned | Unassigned | Unassigned | 101_0 | Clustered | 32 | VC_101_0 | 32 | 0.8826 | 1 | 0.8826 | 0 | 0 | 0 | 1 | Ref |
| KX119198 | Unassigned | Unassigned | Unassigned | 101_0 | Clustered | 32 | VC_101_0 | 32 | 0.8826 | 1 | 0.8826 | 0 | 0 | 0 | 1 | Ref |
| KX119199 | Unassigned | Unassigned | Unassigned | 101_0 | Clustered | 32 | VC_101_0 | 32 | 0.8826 | 1 | 0.8826 | 0 | 0 | 0 | 1 | Ref |
| KX119200 | Unassigned | Unassigned | Unassigned | 101_0 | Clustered | 32 | VC_101_0 | 32 | 0.8826 | 1 | 0.8826 | 0 | 0 | 0 | 1 | Ref |
| KX119201 | Unassigned | Unassigned | Unassigned | 101_0 | Clustered | 32 | VC_101_0 | 32 | 0.8826 | 1 | 0.8826 | 0 | 0 | 0 | 1 | Ref |
| KX119202 | Unassigned | Unassigned | Unassigned | 101_0 | Clustered | 32 | VC_101_0 | 32 | 0.8826 | 1 | 0.8826 | 0 | 0 | 0 | 1 | Ref |
| KX119203 | Unassigned | Unassigned | Unassigned | 101_0 | Clustered | 32 | VC_101_0 | 32 | 0.8826 | 1 | 0.8826 | 0 | 0 | 0 | 1 | Ref |
| KX119204 | Unassigned | Unassigned | Unassigned | 101_0 | Clustered | 32 | VC_101_0 | 32 | 0.8826 | 1 | 0.8826 | 0 | 0 | 0 | 1 | Ref |
| KX119205 | Unassigned | Unassigned | Unassigned | 101_0 | Clustered | 32 | VC_101_0 | 32 | 0.8826 | 1 | 0.8826 | 0 | 0 | 0 | 1 | Ref |
| KX119206 | Unassigned | Unassigned | Unassigned | 101_0 | Clustered | 32 | VC_101_0 | 32 | 0.8826 | 1 | 0.8826 | 0 | 0 | 0 | 1 | Ref |
| KX129925 | Unassigned | Unassigned | Unassigned | 479_0 | Clustered | 7 | VC_479_0 | 7 | 0.3294 | 1 | 0.3294 | 0 | 0 | 0 | 1 | Ref |
| KX130726 | Unassigned | Unassigned | Unassigned | 398_0 | Clustered | 51 | VC_398_0 | 51 | 0.8741 | 1 | 0.8741 | 0 | 0 | 0 | 1 | Ref |
| KX130727 | Unassigned | Unassigned | Unassigned | 686_0 | Clustered | 25 | VC_686_0 | 25 | 0.5501 | 1 | 0.5501 | 0 | 0 | 0 | 0.96 | Ref |
| KX130861 | Unassigned | Unassigned | Unassigned | 290_114 | Clustered/Singleton | 1 | VC_290_114 | 1 | 0 | 1 | 0 | 0 | 0 | 0 | 1 | Ref |
| KX130862 | Unassigned | Unassigned | Unassigned | 290_125 | Clustered/Singleton | 1 | VC_290_125 | 1 | 0 | 1 | 0 | 0 | 0 | 0 | 1 | Ref |
| KX130863 | Unassigned | Unassigned | Unassigned | 198_0 | Clustered | 2 | VC_198_0 | 2 | 0.0086 | 0.95763473 | 0.0082 | 0 | 0 | 0 | 1 | Ref |
| KX130864 | Unassigned | Unassigned | Unassigned | 290_58 | Clustered/Singleton | 1 | VC_290_58 | 1 | 0 | 1 | 0 | 0 | 0 | 0 | 1 | Ref |
| KX130865 | Unassigned | Unassigned | Unassigned | 293_16 | Clustered/Singleton | 1 | VC_293_16 | 1 | 0 | 1 | 0 | 0 | 0 | 0 | 1 | Ref |
| KX130960 | Unassigned | Unassigned | Unassigned | 470_0 | Clustered | 62 | VC_470_0 | 62 | 0.902 | 1 | 0.902 | 0 | 0 | 0 | 1 | Ref |
| KX147096 | Unassigned | Unassigned | Unassigned | 134_2 | Clustered | 17 | VC_134_2 | 17 | 0.1715 | 1 | 0.1715 | 0 | 0 | 0 | 0.9853 | Ref |
| KX147229 | Unassigned | Unassigned | Unassigned | 913_0 | Clustered | 28 | VC_913_0 | 28 | 0.6206 | 1 | 0.6206 | 0 | 0 | 0 | 0.9841 | Ref |
| KX156152 | Unassigned | Unassigned | Unassigned | 913_0 | Clustered | 28 | VC_913_0 | 28 | 0.6206 | 1 | 0.6206 | 0 | 0 | 0 | 0.9841 | Ref |
| KX159477 | Unassigned | Unassigned | Unassigned | 973_0 | Clustered | 15 | VC_973_0 | 15 | 1 | 0 | 0 | 0 | 0 | 0 | 1 | Ref |
| KX160204 | Unassigned | Unassigned | Unassigned | 268_0 | Clustered | 10 | VC_268_0 | 10 | 0.1417 | 1 | 0.1417 | 0 | 0 | 0 | 0.9333 | Ref |
| KX160206 | Unassigned | Unassigned | Unassigned | 274_0 | Clustered | 7 | VC_274_0 | 7 | 0.3401 | 1 | 0.3401 | 0 | 0 | 0 | 1 | Ref |
| KX160209 | Unassigned | Unassigned | Unassigned | 274_0 | Clustered | 7 | VC_274_0 | 7 | 0.3401 | 1 | 0.3401 | 0 | 0 | 0 | 1 | Ref |
| KX160210 | Unassigned | Unassigned | Unassigned | 274_0 | Clustered | 7 | VC_274_0 | 7 | 0.3401 | 1 | 0.3401 | 0 | 0 | 0 | 1 | Ref |
| KX160211 | Unassigned | Unassigned | Unassigned | 274_0 | Clustered | 7 | VC_274_0 | 7 | 0.3401 | 1 | 0.3401 | 0 | 0 | 0 | 1 | Ref |
| KX160219 | Unassigned | Unassigned | Unassigned | 268_0 | Clustered | 10 | VC_268_0 | 10 | 0.1417 | 1 | 0.1417 | 0 | 0 | 0 | 0.9333 | Ref |
| KX160220 | Unassigned | Unassigned | Unassigned | 268_0 | Clustered | 10 | VC_268_0 | 10 | 0.1417 | 1 | 0.1417 | 0 | 0 | 0 | 0.9333 | Ref |
| KX171208 | Unassigned | Unassigned | Unassigned | 80_0 | Clustered | 64 | VC_80_0 | 64 | 0.418 | 1 | 0.418 | 0 | 0 | 0 | 0.7649 | Ref |
| KX171209 | Unassigned | Unassigned | Unassigned | 862_0 | Clustered | 29 | VC_862_0 | 29 | 0.8594 | 1 | 0.8594 | 0 | 0 | 0 | 1 | Ref |
| KX171210 | Unassigned | Unassigned | Unassigned | 862_0 | Clustered | 29 | VC_862_0 | 29 | 0.8594 | 1 | 0.8594 | 0 | 0 | 0 | 1 | Ref |
| KX171211 | Unassigned | Unassigned | Unassigned | 135_9 | Clustered | 3 | VC_135_9 | 3 | 0.0098 | 0.98303301 | 0.0097 | 0 | 0 | 0 | 1 | Ref |
| KX171212 | Unassigned | Unassigned | Unassigned | 157_0 | Clustered | 6 | VC_157_0 | 6 | 0.0724 | 1 | 0.0724 | 0 | 0 | 0 | 1 | Ref |
| KX171213 | Unassigned | Unassigned | Unassigned | 157_0 | Clustered | 6 | VC_157_0 | 6 | 0.0724 | 1 | 0.0724 | 0 | 0 | 0 | 1 | Ref |
| KX179905 | Unassigned | Unassigned | Unassigned | 54_0 | Clustered | 87 | VC_54_0 | 87 | 0.9858 | 1 | 0.9858 | 0 | 0 | 0 | 1 | Ref |
| KX181651 | Unassigned | Unassigned | Unassigned | 183_0 | Clustered | 5 | VC_183_0 | 5 | 0.7449 | 0.99885646 | 0.744 | 0 | 0 | 0 | 1 | Ref |
| KX190832 | Unassigned | Unassigned | Unassigned | 667_0 | Clustered | 12 | VC_667_0 | 12 | 0.2907 | 0.99999999 | 0.2907 | 0 | 0 | 0 | 1 | Ref |
| KX190833 | Unassigned | Unassigned | Unassigned | 746_0 | Clustered | 2 | VC_746_0 | 2 | 0.3667 | 0.95002548 | 0.3484 | 0 | 0 | 0 | 1 | Ref |
| KX190834 | Unassigned | Unassigned | Unassigned | 853_0 | Clustered | 5 | VC_853_0 | 5 | 0.8735 | 0.99999832 | 0.8735 | 0 | 0 | 0 | 1 | Ref |

| | | | | | | | | | | | | | | | | |
|---|---|---|---|---|---|---|---|---|---|---|---|---|---|---|---|---|
| KX190835 | Unassigned | Unassigned | Unassigned | 20_0 | Clustered | 5 | VC_20_0 | 5 | 0.4268 | 0.9999994 | 0.4268 | 0 | 0 | 0 | 1 | Ref |
| KX196154 | Unassigned | Unassigned | Unassigned | 515_0 | Clustered | 8 | VC_515_0 | 8 | 0.3973 | 1 | 0.3973 | 0 | 0 | 0 | 1 | Ref |
| KX198613 | Unassigned | Unassigned | Unassigned | 172_0 | Clustered | 26 | VC_172_0 | 26 | 0.7909 | 1 | 0.7909 | 0 | 0 | 0 | 1 | Ref |
| KX198614 | Unassigned | Unassigned | Unassigned | 821_0 | Clustered | 5 | VC_821_0 | 5 | 0.2385 | 0.99999997 | 0.2385 | 0 | 0 | 0 | 1 | Ref |
| KX198615 | Unassigned | Unassigned | Unassigned | 935_1 | Clustered | 4 | VC_935_1 | 4 | 0.1471 | 0.99997723 | 0.1471 | 0 | 0 | 0 | 1 | Ref |
| KX211991 | Unassigned | Unassigned | Unassigned | 113_0 | Clustered | 91 | VC_113_0 | 91 | 0.9584 | 1 | 0.9584 | 0 | 0 | 0 | 1 | Ref |
| KX223815 | Unassigned | Unassigned | Unassigned | 650_1 | Clustered | 6 | VC_650_1 | 6 | 0.4186 | 1 | 0.4186 | 0 | 0 | 0 | 1 | Ref |
| KX227757 | Unassigned | Unassigned | Unassigned | 673_0 | Clustered | 4 | VC_673_0 | 4 | 0.2891 | 0.99998263 | 0.2891 | 0 | 0 | 0 | 1 | Ref |
| KX227758 | Unassigned | Unassigned | Unassigned | 671_0 | Clustered | 4 | VC_671_0 | 4 | 0.4756 | 0.99998076 | 0.4756 | 0 | 0 | 0 | 1 | Ref |
| KX227759 | Unassigned | Unassigned | Unassigned | 671_0 | Clustered | 4 | VC_671_0 | 4 | 0.4756 | 0.99998076 | 0.4756 | 0 | 0 | 0 | 1 | Ref |
| KX227760 | Unassigned | Unassigned | Unassigned | 673_0 | Clustered | 4 | VC_673_0 | 4 | 0.2891 | 0.99998263 | 0.2891 | 0 | 0 | 0 | 1 | Ref |
| KX229736 | Unassigned | Unassigned | Unassigned | 868_0 | Clustered | 27 | VC_868_0 | 27 | 0.9372 | 1 | 0.9372 | 0 | 0 | 0 | 1 | Ref |
| KX231828 | Unassigned | Unassigned | Unassigned | 127_0 | Clustered | 17 | VC_127_0 | 17 | 0.9222 | 1 | 0.9222 | 0 | 0 | 0 | 1 | Ref |
| KX231829 | Unassigned | Unassigned | Unassigned | 492_0 | Clustered | 20 | VC_492_0 | 20 | 0.7887 | 1 | 0.7887 | 0 | 0 | 0 | 1 | Ref |
| KX232515 | Unassigned | Unassigned | Unassigned | 7_0 | Clustered | 28 | VC_7_0 | 28 | 0.389 | 1 | 0.389 | 0 | 0 | 0 | 0.9947 | Ref |
| KX236333 | Unassigned | Unassigned | Unassigned | 868_0 | Clustered | 27 | VC_868_0 | 27 | 0.9372 | 1 | 0.9372 | 0 | 0 | 0 | 1 | Ref |
| KX237514 | Unassigned | Unassigned | Unassigned | 113_0 | Clustered | 91 | VC_113_0 | 91 | 0.9584 | 1 | 0.9584 | 0 | 0 | 0 | 1 | Ref |
| KX237515 | Unassigned | Unassigned | Unassigned | 971_0 | Clustered | 40 | VC_971_0 | 40 | 0.6793 | 1 | 0.6793 | 0 | 0 | 0 | 0.9359 | Ref |
| KX237516 | Unassigned | Unassigned | Unassigned | 125_0 | Clustered | 23 | VC_125_0 | 23 | 0.8163 | 1 | 0.8163 | 0 | 0 | 0 | 1 | Ref |
| KX241618 | Unassigned | Unassigned | Unassigned | 175_0 | Clustered | 20 | VC_175_0 | 20 | 0.7504 | 1 | 0.7504 | 0 | 0 | 0 | 1 | Ref |
| KX245012 | Unassigned | Unassigned | Unassigned | 135_30 | Clustered | 2 | VC_135_30 | 2 | 0.0052 | 0.87577187 | 0.0045 | 0 | 0 | 0 | 1 | Ref |
| KX245013 | Unassigned | Unassigned | Unassigned | 654_0 | Clustered | 115 | VC_654_0 | 115 | 0.7303 | 1 | 0.7303 | 0 | 0 | 0 | 0.8764 | Ref |
| KX245890 | Unassigned | Unassigned | Unassigned | 687_1 | Clustered | 10 | VC_687_1 | 10 | 0.1263 | 1 | 0.1263 | 0 | 0 | 0 | 0.9778 | Ref |
| KX257490 | Unassigned | Unassigned | Unassigned | 1066_0 | Clustered | 2 | VC_1066_0 | 2 | 0.2951 | 0.81445332 | 0.2404 | 0 | 0 | 0 | 1 | Ref |
| KX258185 | Unassigned | Unassigned | Unassigned | 509_0 | Clustered | 36 | VC_509_0 | 36 | 0.5291 | 1 | 0.5291 | 0 | 0 | 0 | 0.9714 | Ref |
| KX266300 | Unassigned | Unassigned | Unassigned | 373_0 | Clustered | 358 | VC_373_0 | 358 | 0.9614 | 1 | 0.9614 | 0 | 0 | 0 | 1 | Ref |
| KX266301 | Unassigned | Unassigned | Unassigned | 373_0 | Clustered | 358 | VC_373_0 | 358 | 0.9614 | 1 | 0.9614 | 0 | 0 | 0 | 1 | Ref |
| KX266302 | Unassigned | Unassigned | Unassigned | 373_0 | Clustered | 358 | VC_373_0 | 358 | 0.9614 | 1 | 0.9614 | 0 | 0 | 0 | 1 | Ref |
| KX266303 | Unassigned | Unassigned | Unassigned | 373_0 | Clustered | 358 | VC_373_0 | 358 | 0.9614 | 1 | 0.9614 | 0 | 0 | 0 | 1 | Ref |
| KX266304 | Unassigned | Unassigned | Unassigned | 373_0 | Clustered | 358 | VC_373_0 | 358 | 0.9614 | 1 | 0.9614 | 0 | 0 | 0 | 1 | Ref |
| KX266305 | Unassigned | Unassigned | Unassigned | 373_0 | Clustered | 358 | VC_373_0 | 358 | 0.9614 | 1 | 0.9614 | 0 | 0 | 0 | 1 | Ref |
| KX266306 | Unassigned | Unassigned | Unassigned | 373_0 | Clustered | 358 | VC_373_0 | 358 | 0.9614 | 1 | 0.9614 | 0 | 0 | 0 | 1 | Ref |
| KX266307 | Unassigned | Unassigned | Unassigned | 373_0 | Clustered | 358 | VC_373_0 | 358 | 0.9614 | 1 | 0.9614 | 0 | 0 | 0 | 1 | Ref |
| KX266308 | Unassigned | Unassigned | Unassigned | 373_0 | Clustered | 358 | VC_373_0 | 358 | 0.9614 | 1 | 0.9614 | 0 | 0 | 0 | 1 | Ref |
| KX266309 | Unassigned | Unassigned | Unassigned | 373_0 | Clustered | 358 | VC_373_0 | 358 | 0.9614 | 1 | 0.9614 | 0 | 0 | 0 | 1 | Ref |
| KX266310 | Unassigned | Unassigned | Unassigned | 373_0 | Clustered | 358 | VC_373_0 | 358 | 0.9614 | 1 | 0.9614 | 0 | 0 | 0 | 1 | Ref |
| KX266311 | Unassigned | Unassigned | Unassigned | 373_0 | Clustered | 358 | VC_373_0 | 358 | 0.9614 | 1 | 0.9614 | 0 | 0 | 0 | 1 | Ref |
| KX266312 | Unassigned | Unassigned | Unassigned | 373_0 | Clustered | 358 | VC_373_0 | 358 | 0.9614 | 1 | 0.9614 | 0 | 0 | 0 | 1 | Ref |
| KX266313 | Unassigned | Unassigned | Unassigned | 373_0 | Clustered | 358 | VC_373_0 | 358 | 0.9614 | 1 | 0.9614 | 0 | 0 | 0 | 1 | Ref |
| KX266314 | Unassigned | Unassigned | Unassigned | 373_0 | Clustered | 358 | VC_373_0 | 358 | 0.9614 | 1 | 0.9614 | 0 | 0 | 0 | 1 | Ref |
| KX266315 | Unassigned | Unassigned | Unassigned | 373_0 | Clustered | 358 | VC_373_0 | 358 | 0.9614 | 1 | 0.9614 | 0 | 0 | 0 | 1 | Ref |
| KX266316 | Unassigned | Unassigned | Unassigned | 373_0 | Clustered | 358 | VC_373_0 | 358 | 0.9614 | 1 | 0.9614 | 0 | 0 | 0 | 1 | Ref |
| KX266317 | Unassigned | Unassigned | Unassigned | 373_0 | Clustered | 358 | VC_373_0 | 358 | 0.9614 | 1 | 0.9614 | 0 | 0 | 0 | 1 | Ref |
| KX266318 | Unassigned | Unassigned | Unassigned | 373_0 | Clustered | 358 | VC_373_0 | 358 | 0.9614 | 1 | 0.9614 | 0 | 0 | 0 | 1 | Ref |
| KX266319 | Unassigned | Unassigned | Unassigned | 373_0 | Clustered | 358 | VC_373_0 | 358 | 0.9614 | 1 | 0.9614 | 0 | 0 | 0 | 1 | Ref |
| KX266320 | Unassigned | Unassigned | Unassigned | 373_0 | Clustered | 358 | VC_373_0 | 358 | 0.9614 | 1 | 0.9614 | 0 | 0 | 0 | 1 | Ref |
| KX266321 | Unassigned | Unassigned | Unassigned | 373_0 | Clustered | 358 | VC_373_0 | 358 | 0.9614 | 1 | 0.9614 | 0 | 0 | 0 | 1 | Ref |
| KX266322 | Unassigned | Unassigned | Unassigned | 373_0 | Clustered | 358 | VC_373_0 | 358 | 0.9614 | 1 | 0.9614 | 0 | 0 | 0 | 1 | Ref |
| KX266323 | Unassigned | Unassigned | Unassigned | 373_0 | Clustered | 358 | VC_373_0 | 358 | 0.9614 | 1 | 0.9614 | 0 | 0 | 0 | 1 | Ref |
| KX266324 | Unassigned | Unassigned | Unassigned | 373_0 | Clustered | 358 | VC_373_0 | 358 | 0.9614 | 1 | 0.9614 | 0 | 0 | 0 | 1 | Ref |
| KX266325 | Unassigned | Unassigned | Unassigned | 373_0 | Clustered | 358 | VC_373_0 | 358 | 0.9614 | 1 | 0.9614 | 0 | 0 | 0 | 1 | Ref |
| KX266326 | Unassigned | Unassigned | Unassigned | 373_0 | Clustered | 358 | VC_373_0 | 358 | 0.9614 | 1 | 0.9614 | 0 | 0 | 0 | 1 | Ref |
| KX266327 | Unassigned | Unassigned | Unassigned | 373_0 | Clustered | 358 | VC_373_0 | 358 | 0.9614 | 1 | 0.9614 | 0 | 0 | 0 | 1 | Ref |
| KX266328 | Unassigned | Unassigned | Unassigned | 373_0 | Clustered | 358 | VC_373_0 | 358 | 0.9614 | 1 | 0.9614 | 0 | 0 | 0 | 1 | Ref |
| KX266329 | Unassigned | Unassigned | Unassigned | 373_0 | Clustered | 358 | VC_373_0 | 358 | 0.9614 | 1 | 0.9614 | 0 | 0 | 0 | 1 | Ref |
| KX266330 | Unassigned | Unassigned | Unassigned | 373_0 | Clustered | 358 | VC_373_0 | 358 | 0.9614 | 1 | 0.9614 | 0 | 0 | 0 | 1 | Ref |
| KX266331 | Unassigned | Unassigned | Unassigned | 373_0 | Clustered | 358 | VC_373_0 | 358 | 0.9614 | 1 | 0.9614 | 0 | 0 | 0 | 1 | Ref |
| KX266332 | Unassigned | Unassigned | Unassigned | 373_0 | Clustered | 358 | VC_373_0 | 358 | 0.9614 | 1 | 0.9614 | 0 | 0 | 0 | 1 | Ref |
| KX266333 | Unassigned | Unassigned | Unassigned | 373_0 | Clustered | 358 | VC_373_0 | 358 | 0.9614 | 1 | 0.9614 | 0 | 0 | 0 | 1 | Ref |
| KX266334 | Unassigned | Unassigned | Unassigned | 373_0 | Clustered | 358 | VC_373_0 | 358 | 0.9614 | 1 | 0.9614 | 0 | 0 | 0 | 1 | Ref |
| KX266335 | Unassigned | Unassigned | Unassigned | 373_0 | Clustered | 358 | VC_373_0 | 358 | 0.9614 | 1 | 0.9614 | 0 | 0 | 0 | 1 | Ref |
| KX266336 | Unassigned | Unassigned | Unassigned | 373_0 | Clustered | 358 | VC_373_0 | 358 | 0.9614 | 1 | 0.9614 | 0 | 0 | 0 | 1 | Ref |
| KX266337 | Unassigned | Unassigned | Unassigned | 373_0 | Clustered | 358 | VC_373_0 | 358 | 0.9614 | 1 | 0.9614 | 0 | 0 | 0 | 1 | Ref |
| KX266338 | Unassigned | Unassigned | Unassigned | 373_0 | Clustered | 358 | VC_373_0 | 358 | 0.9614 | 1 | 0.9614 | 0 | 0 | 0 | 1 | Ref |
| KX266339 | Unassigned | Unassigned | Unassigned | 373_0 | Clustered | 358 | VC_373_0 | 358 | 0.9614 | 1 | 0.9614 | 0 | 0 | 0 | 1 | Ref |
| KX266340 | Unassigned | Unassigned | Unassigned | 373_0 | Clustered | 358 | VC_373_0 | 358 | 0.9614 | 1 | 0.9614 | 0 | 0 | 0 | 1 | Ref |
| KX266341 | Unassigned | Unassigned | Unassigned | 373_0 | Clustered | 358 | VC_373_0 | 358 | 0.9614 | 1 | 0.9614 | 0 | 0 | 0 | 1 | Ref |
| KX266342 | Unassigned | Unassigned | Unassigned | 373_0 | Clustered | 358 | VC_373_0 | 358 | 0.9614 | 1 | 0.9614 | 0 | 0 | 0 | 1 | Ref |
| KX266343 | Unassigned | Unassigned | Unassigned | 373_0 | Clustered | 358 | VC_373_0 | 358 | 0.9614 | 1 | 0.9614 | 0 | 0 | 0 | 1 | Ref |
| KX266344 | Unassigned | Unassigned | Unassigned | 373_0 | Clustered | 358 | VC_373_0 | 358 | 0.9614 | 1 | 0.9614 | 0 | 0 | 0 | 1 | Ref |
| KX266345 | Unassigned | Unassigned | Unassigned | 373_0 | Clustered | 358 | VC_373_0 | 358 | 0.9614 | 1 | 0.9614 | 0 | 0 | 0 | 1 | Ref |
| KX266346 | Unassigned | Unassigned | Unassigned | 373_0 | Clustered | 358 | VC_373_0 | 358 | 0.9614 | 1 | 0.9614 | 0 | 0 | 0 | 1 | Ref |
| KX266347 | Unassigned | Unassigned | Unassigned | 373_0 | Clustered | 358 | VC_373_0 | 358 | 0.9614 | 1 | 0.9614 | 0 | 0 | 0 | 1 | Ref |
| KX266348 | Unassigned | Unassigned | Unassigned | 373_0 | Clustered | 358 | VC_373_0 | 358 | 0.9614 | 1 | 0.9614 | 0 | 0 | 0 | 1 | Ref |
| KX266349 | Unassigned | Unassigned | Unassigned | 373_0 | Clustered | 358 | VC_373_0 | 358 | 0.9614 | 1 | 0.9614 | 0 | 0 | 0 | 1 | Ref |
| KX266350 | Unassigned | Unassigned | Unassigned | 373_0 | Clustered | 358 | VC_373_0 | 358 | 0.9614 | 1 | 0.9614 | 0 | 0 | 0 | 1 | Ref |
| KX266351 | Unassigned | Unassigned | Unassigned | 373_0 | Clustered | 358 | VC_373_0 | 358 | 0.9614 | 1 | 0.9614 | 0 | 0 | 0 | 1 | Ref |
| KX266352 | Unassigned | Unassigned | Unassigned | 373_0 | Clustered | 358 | VC_373_0 | 358 | 0.9614 | 1 | 0.9614 | 0 | 0 | 0 | 1 | Ref |
| KX266353 | Unassigned | Unassigned | Unassigned | 373_0 | Clustered | 358 | VC_373_0 | 358 | 0.9614 | 1 | 0.9614 | 0 | 0 | 0 | 1 | Ref |
| KX266354 | Unassigned | Unassigned | Unassigned | 373_0 | Clustered | 358 | VC_373_0 | 358 | 0.9614 | 1 | 0.9614 | 0 | 0 | 0 | 1 | Ref |
| KX266355 | Unassigned | Unassigned | Unassigned | 373_0 | Clustered | 358 | VC_373_0 | 358 | 0.9614 | 1 | 0.9614 | 0 | 0 | 0 | 1 | Ref |

| | | | | | | | | | | | | | | | | |
|---|---|---|---|---|---|---|---|---|---|---|---|---|---|---|---|---|
| KX266356 | Unassigned | Unassigned | Unassigned | 373_0 | Clustered | 358 | VC_373_0 | 358 | 0.9614 | 1 | 0.9614 | 0 | 0 | 0 | 1 | Ref |
| KX266357 | Unassigned | Unassigned | Unassigned | 373_0 | Clustered | 358 | VC_373_0 | 358 | 0.9614 | 1 | 0.9614 | 0 | 0 | 0 | 1 | Ref |
| KX266358 | Unassigned | Unassigned | Unassigned | 373_0 | Clustered | 358 | VC_373_0 | 358 | 0.9614 | 1 | 0.9614 | 0 | 0 | 0 | 1 | Ref |
| KX266359 | Unassigned | Unassigned | Unassigned | 373_0 | Clustered | 358 | VC_373_0 | 358 | 0.9614 | 1 | 0.9614 | 0 | 0 | 0 | 1 | Ref |
| KX266360 | Unassigned | Unassigned | Unassigned | 373_0 | Clustered | 358 | VC_373_0 | 358 | 0.9614 | 1 | 0.9614 | 0 | 0 | 0 | 1 | Ref |
| KX266361 | Unassigned | Unassigned | Unassigned | 373_0 | Clustered | 358 | VC_373_0 | 358 | 0.9614 | 1 | 0.9614 | 0 | 0 | 0 | 1 | Ref |
| KX266362 | Unassigned | Unassigned | Unassigned | 373_0 | Clustered | 358 | VC_373_0 | 358 | 0.9614 | 1 | 0.9614 | 0 | 0 | 0 | 1 | Ref |
| KX266363 | Unassigned | Unassigned | Unassigned | 373_0 | Clustered | 358 | VC_373_0 | 358 | 0.9614 | 1 | 0.9614 | 0 | 0 | 0 | 1 | Ref |
| KX266364 | Unassigned | Unassigned | Unassigned | 373_0 | Clustered | 358 | VC_373_0 | 358 | 0.9614 | 1 | 0.9614 | 0 | 0 | 0 | 1 | Ref |
| KX266365 | Unassigned | Unassigned | Unassigned | 373_0 | Clustered | 358 | VC_373_0 | 358 | 0.9614 | 1 | 0.9614 | 0 | 0 | 0 | 1 | Ref |
| KX266366 | Unassigned | Unassigned | Unassigned | 373_0 | Clustered | 358 | VC_373_0 | 358 | 0.9614 | 1 | 0.9614 | 0 | 0 | 0 | 1 | Ref |
| KX266367 | Unassigned | Unassigned | Unassigned | 373_0 | Clustered | 358 | VC_373_0 | 358 | 0.9614 | 1 | 0.9614 | 0 | 0 | 0 | 1 | Ref |
| KX266368 | Unassigned | Unassigned | Unassigned | 373_0 | Clustered | 358 | VC_373_0 | 358 | 0.9614 | 1 | 0.9614 | 0 | 0 | 0 | 1 | Ref |
| KX266369 | Unassigned | Unassigned | Unassigned | 373_0 | Clustered | 358 | VC_373_0 | 358 | 0.9614 | 1 | 0.9614 | 0 | 0 | 0 | 1 | Ref |
| KX266370 | Unassigned | Unassigned | Unassigned | 373_0 | Clustered | 358 | VC_373_0 | 358 | 0.9614 | 1 | 0.9614 | 0 | 0 | 0 | 1 | Ref |
| KX266371 | Unassigned | Unassigned | Unassigned | 373_0 | Clustered | 358 | VC_373_0 | 358 | 0.9614 | 1 | 0.9614 | 0 | 0 | 0 | 1 | Ref |
| KX266372 | Unassigned | Unassigned | Unassigned | 373_0 | Clustered | 358 | VC_373_0 | 358 | 0.9614 | 1 | 0.9614 | 0 | 0 | 0 | 1 | Ref |
| KX266373 | Unassigned | Unassigned | Unassigned | 373_0 | Clustered | 358 | VC_373_0 | 358 | 0.9614 | 1 | 0.9614 | 0 | 0 | 0 | 1 | Ref |
| KX266374 | Unassigned | Unassigned | Unassigned | 373_0 | Clustered | 358 | VC_373_0 | 358 | 0.9614 | 1 | 0.9614 | 0 | 0 | 0 | 1 | Ref |
| KX266375 | Unassigned | Unassigned | Unassigned | 373_0 | Clustered | 358 | VC_373_0 | 358 | 0.9614 | 1 | 0.9614 | 0 | 0 | 0 | 1 | Ref |
| KX266376 | Unassigned | Unassigned | Unassigned | 373_0 | Clustered | 358 | VC_373_0 | 358 | 0.9614 | 1 | 0.9614 | 0 | 0 | 0 | 1 | Ref |
| KX266377 | Unassigned | Unassigned | Unassigned | 373_0 | Clustered | 358 | VC_373_0 | 358 | 0.9614 | 1 | 0.9614 | 0 | 0 | 0 | 1 | Ref |
| KX266378 | Unassigned | Unassigned | Unassigned | 373_0 | Clustered | 358 | VC_373_0 | 358 | 0.9614 | 1 | 0.9614 | 0 | 0 | 0 | 1 | Ref |
| KX266379 | Unassigned | Unassigned | Unassigned | 373_0 | Clustered | 358 | VC_373_0 | 358 | 0.9614 | 1 | 0.9614 | 0 | 0 | 0 | 1 | Ref |
| KX266380 | Unassigned | Unassigned | Unassigned | 373_0 | Clustered | 358 | VC_373_0 | 358 | 0.9614 | 1 | 0.9614 | 0 | 0 | 0 | 1 | Ref |
| KX266381 | Unassigned | Unassigned | Unassigned | 373_0 | Clustered | 358 | VC_373_0 | 358 | 0.9614 | 1 | 0.9614 | 0 | 0 | 0 | 1 | Ref |
| KX266382 | Unassigned | Unassigned | Unassigned | 373_0 | Clustered | 358 | VC_373_0 | 358 | 0.9614 | 1 | 0.9614 | 0 | 0 | 0 | 1 | Ref |
| KX266383 | Unassigned | Unassigned | Unassigned | 373_0 | Clustered | 358 | VC_373_0 | 358 | 0.9614 | 1 | 0.9614 | 0 | 0 | 0 | 1 | Ref |
| KX266384 | Unassigned | Unassigned | Unassigned | 373_0 | Clustered | 358 | VC_373_0 | 358 | 0.9614 | 1 | 0.9614 | 0 | 0 | 0 | 1 | Ref |
| KX266385 | Unassigned | Unassigned | Unassigned | 373_0 | Clustered | 358 | VC_373_0 | 358 | 0.9614 | 1 | 0.9614 | 0 | 0 | 0 | 1 | Ref |
| KX266386 | Unassigned | Unassigned | Unassigned | 373_0 | Clustered | 358 | VC_373_0 | 358 | 0.9614 | 1 | 0.9614 | 0 | 0 | 0 | 1 | Ref |
| KX266387 | Unassigned | Unassigned | Unassigned | 373_0 | Clustered | 358 | VC_373_0 | 358 | 0.9614 | 1 | 0.9614 | 0 | 0 | 0 | 1 | Ref |
| KX266388 | Unassigned | Unassigned | Unassigned | 373_0 | Clustered | 358 | VC_373_0 | 358 | 0.9614 | 1 | 0.9614 | 0 | 0 | 0 | 1 | Ref |
| KX266389 | Unassigned | Unassigned | Unassigned | 373_0 | Clustered | 358 | VC_373_0 | 358 | 0.9614 | 1 | 0.9614 | 0 | 0 | 0 | 1 | Ref |
| KX266390 | Unassigned | Unassigned | Unassigned | 373_0 | Clustered | 358 | VC_373_0 | 358 | 0.9614 | 1 | 0.9614 | 0 | 0 | 0 | 1 | Ref |
| KX266391 | Unassigned | Unassigned | Unassigned | 373_0 | Clustered | 358 | VC_373_0 | 358 | 0.9614 | 1 | 0.9614 | 0 | 0 | 0 | 1 | Ref |
| KX266392 | Unassigned | Unassigned | Unassigned | 373_0 | Clustered | 358 | VC_373_0 | 358 | 0.9614 | 1 | 0.9614 | 0 | 0 | 0 | 1 | Ref |
| KX266393 | Unassigned | Unassigned | Unassigned | 373_0 | Clustered | 358 | VC_373_0 | 358 | 0.9614 | 1 | 0.9614 | 0 | 0 | 0 | 1 | Ref |
| KX266394 | Unassigned | Unassigned | Unassigned | 373_0 | Clustered | 358 | VC_373_0 | 358 | 0.9614 | 1 | 0.9614 | 0 | 0 | 0 | 1 | Ref |
| KX266395 | Unassigned | Unassigned | Unassigned | 373_0 | Clustered | 358 | VC_373_0 | 358 | 0.9614 | 1 | 0.9614 | 0 | 0 | 0 | 1 | Ref |
| KX266396 | Unassigned | Unassigned | Unassigned | 373_0 | Clustered | 358 | VC_373_0 | 358 | 0.9614 | 1 | 0.9614 | 0 | 0 | 0 | 1 | Ref |
| KX266397 | Unassigned | Unassigned | Unassigned | 373_0 | Clustered | 358 | VC_373_0 | 358 | 0.9614 | 1 | 0.9614 | 0 | 0 | 0 | 1 | Ref |
| KX266398 | Unassigned | Unassigned | Unassigned | 373_0 | Clustered | 358 | VC_373_0 | 358 | 0.9614 | 1 | 0.9614 | 0 | 0 | 0 | 1 | Ref |
| KX266399 | Unassigned | Unassigned | Unassigned | 373_0 | Clustered | 358 | VC_373_0 | 358 | 0.9614 | 1 | 0.9614 | 0 | 0 | 0 | 1 | Ref |
| KX266400 | Unassigned | Unassigned | Unassigned | 373_0 | Clustered | 358 | VC_373_0 | 358 | 0.9614 | 1 | 0.9614 | 0 | 0 | 0 | 1 | Ref |
| KX266401 | Unassigned | Unassigned | Unassigned | 373_0 | Clustered | 358 | VC_373_0 | 358 | 0.9614 | 1 | 0.9614 | 0 | 0 | 0 | 1 | Ref |
| KX266402 | Unassigned | Unassigned | Unassigned | 373_0 | Clustered | 358 | VC_373_0 | 358 | 0.9614 | 1 | 0.9614 | 0 | 0 | 0 | 1 | Ref |
| KX266403 | Unassigned | Unassigned | Unassigned | 373_0 | Clustered | 358 | VC_373_0 | 358 | 0.9614 | 1 | 0.9614 | 0 | 0 | 0 | 1 | Ref |
| KX266404 | Unassigned | Unassigned | Unassigned | 373_0 | Clustered | 358 | VC_373_0 | 358 | 0.9614 | 1 | 0.9614 | 0 | 0 | 0 | 1 | Ref |
| KX266405 | Unassigned | Unassigned | Unassigned | 373_0 | Clustered | 358 | VC_373_0 | 358 | 0.9614 | 1 | 0.9614 | 0 | 0 | 0 | 1 | Ref |
| KX266406 | Unassigned | Unassigned | Unassigned | 373_0 | Clustered | 358 | VC_373_0 | 358 | 0.9614 | 1 | 0.9614 | 0 | 0 | 0 | 1 | Ref |
| KX266407 | Unassigned | Unassigned | Unassigned | 373_0 | Clustered | 358 | VC_373_0 | 358 | 0.9614 | 1 | 0.9614 | 0 | 0 | 0 | 1 | Ref |
| KX266408 | Unassigned | Unassigned | Unassigned | 373_0 | Clustered | 358 | VC_373_0 | 358 | 0.9614 | 1 | 0.9614 | 0 | 0 | 0 | 1 | Ref |
| KX266409 | Unassigned | Unassigned | Unassigned | 373_0 | Clustered | 358 | VC_373_0 | 358 | 0.9614 | 1 | 0.9614 | 0 | 0 | 0 | 1 | Ref |
| KX266410 | Unassigned | Unassigned | Unassigned | 373_0 | Clustered | 358 | VC_373_0 | 358 | 0.9614 | 1 | 0.9614 | 0 | 0 | 0 | 1 | Ref |
| KX266411 | Unassigned | Unassigned | Unassigned | 373_0 | Clustered | 358 | VC_373_0 | 358 | 0.9614 | 1 | 0.9614 | 0 | 0 | 0 | 1 | Ref |
| KX266412 | Unassigned | Unassigned | Unassigned | 373_0 | Clustered | 358 | VC_373_0 | 358 | 0.9614 | 1 | 0.9614 | 0 | 0 | 0 | 1 | Ref |
| KX266413 | Unassigned | Unassigned | Unassigned | 373_0 | Clustered | 358 | VC_373_0 | 358 | 0.9614 | 1 | 0.9614 | 0 | 0 | 0 | 1 | Ref |
| KX266414 | Unassigned | Unassigned | Unassigned | 373_0 | Clustered | 358 | VC_373_0 | 358 | 0.9614 | 1 | 0.9614 | 0 | 0 | 0 | 1 | Ref |
| KX266415 | Unassigned | Unassigned | Unassigned | 373_0 | Clustered | 358 | VC_373_0 | 358 | 0.9614 | 1 | 0.9614 | 0 | 0 | 0 | 1 | Ref |
| KX266416 | Unassigned | Unassigned | Unassigned | 373_0 | Clustered | 358 | VC_373_0 | 358 | 0.9614 | 1 | 0.9614 | 0 | 0 | 0 | 1 | Ref |
| KX266417 | Unassigned | Unassigned | Unassigned | 373_0 | Clustered | 358 | VC_373_0 | 358 | 0.9614 | 1 | 0.9614 | 0 | 0 | 0 | 1 | Ref |
| KX266418 | Unassigned | Unassigned | Unassigned | 373_0 | Clustered | 358 | VC_373_0 | 358 | 0.9614 | 1 | 0.9614 | 0 | 0 | 0 | 1 | Ref |
| KX266419 | Unassigned | Unassigned | Unassigned | 373_0 | Clustered | 358 | VC_373_0 | 358 | 0.9614 | 1 | 0.9614 | 0 | 0 | 0 | 1 | Ref |
| KX266420 | Unassigned | Unassigned | Unassigned | 373_0 | Clustered | 358 | VC_373_0 | 358 | 0.9614 | 1 | 0.9614 | 0 | 0 | 0 | 1 | Ref |
| KX266421 | Unassigned | Unassigned | Unassigned | 373_0 | Clustered | 358 | VC_373_0 | 358 | 0.9614 | 1 | 0.9614 | 0 | 0 | 0 | 1 | Ref |
| KX266422 | Unassigned | Unassigned | Unassigned | 373_0 | Clustered | 358 | VC_373_0 | 358 | 0.9614 | 1 | 0.9614 | 0 | 0 | 0 | 1 | Ref |
| KX266423 | Unassigned | Unassigned | Unassigned | 373_0 | Clustered | 358 | VC_373_0 | 358 | 0.9614 | 1 | 0.9614 | 0 | 0 | 0 | 1 | Ref |
| KX266424 | Unassigned | Unassigned | Unassigned | 373_0 | Clustered | 358 | VC_373_0 | 358 | 0.9614 | 1 | 0.9614 | 0 | 0 | 0 | 1 | Ref |
| KX266425 | Unassigned | Unassigned | Unassigned | 373_0 | Clustered | 358 | VC_373_0 | 358 | 0.9614 | 1 | 0.9614 | 0 | 0 | 0 | 1 | Ref |
| KX266426 | Unassigned | Unassigned | Unassigned | 373_0 | Clustered | 358 | VC_373_0 | 358 | 0.9614 | 1 | 0.9614 | 0 | 0 | 0 | 1 | Ref |
| KX266427 | Unassigned | Unassigned | Unassigned | 373_0 | Clustered | 358 | VC_373_0 | 358 | 0.9614 | 1 | 0.9614 | 0 | 0 | 0 | 1 | Ref |
| KX266428 | Unassigned | Unassigned | Unassigned | 373_0 | Clustered | 358 | VC_373_0 | 358 | 0.9614 | 1 | 0.9614 | 0 | 0 | 0 | 1 | Ref |
| KX266429 | Unassigned | Unassigned | Unassigned | 373_0 | Clustered | 358 | VC_373_0 | 358 | 0.9614 | 1 | 0.9614 | 0 | 0 | 0 | 1 | Ref |
| KX266430 | Unassigned | Unassigned | Unassigned | 373_0 | Clustered | 358 | VC_373_0 | 358 | 0.9614 | 1 | 0.9614 | 0 | 0 | 0 | 1 | Ref |
| KX266431 | Unassigned | Unassigned | Unassigned | 373_0 | Clustered | 358 | VC_373_0 | 358 | 0.9614 | 1 | 0.9614 | 0 | 0 | 0 | 1 | Ref |
| KX266432 | Unassigned | Unassigned | Unassigned | 373_0 | Clustered | 358 | VC_373_0 | 358 | 0.9614 | 1 | 0.9614 | 0 | 0 | 0 | 1 | Ref |
| KX266433 | Unassigned | Unassigned | Unassigned | 373_0 | Clustered | 358 | VC_373_0 | 358 | 0.9614 | 1 | 0.9614 | 0 | 0 | 0 | 1 | Ref |
| KX266434 | Unassigned | Unassigned | Unassigned | 373_0 | Clustered | 358 | VC_373_0 | 358 | 0.9614 | 1 | 0.9614 | 0 | 0 | 0 | 1 | Ref |
| KX266435 | Unassigned | Unassigned | Unassigned | 373_0 | Clustered | 358 | VC_373_0 | 358 | 0.9614 | 1 | 0.9614 | 0 | 0 | 0 | 1 | Ref |
| KX266436 | Unassigned | Unassigned | Unassigned | 373_0 | Clustered | 358 | VC_373_0 | 358 | 0.9614 | 1 | 0.9614 | 0 | 0 | 0 | 1 | Ref |

| | | | | | | | | | | | | | | | | |
|---|---|---|---|---|---|---|---|---|---|---|---|---|---|---|---|---|
| KX266437 | Unassigned | Unassigned | Unassigned | 373_0 | Clustered | 358 | VC_373_0 | 358 | 0.9614 | 1 | 0.9614 | 0 | 0 | 0 | 1 | Ref |
| KX266438 | Unassigned | Unassigned | Unassigned | 373_0 | Clustered | 358 | VC_373_0 | 358 | 0.9614 | 1 | 0.9614 | 0 | 0 | 0 | 1 | Ref |
| KX266439 | Unassigned | Unassigned | Unassigned | 373_0 | Clustered | 358 | VC_373_0 | 358 | 0.9614 | 1 | 0.9614 | 0 | 0 | 0 | 1 | Ref |
| KX266440 | Unassigned | Unassigned | Unassigned | 373_0 | Clustered | 358 | VC_373_0 | 358 | 0.9614 | 1 | 0.9614 | 0 | 0 | 0 | 1 | Ref |
| KX266441 | Unassigned | Unassigned | Unassigned | 373_0 | Clustered | 358 | VC_373_0 | 358 | 0.9614 | 1 | 0.9614 | 0 | 0 | 0 | 1 | Ref |
| KX266442 | Unassigned | Unassigned | Unassigned | 373_0 | Clustered | 358 | VC_373_0 | 358 | 0.9614 | 1 | 0.9614 | 0 | 0 | 0 | 1 | Ref |
| KX266443 | Unassigned | Unassigned | Unassigned | 373_0 | Clustered | 358 | VC_373_0 | 358 | 0.9614 | 1 | 0.9614 | 0 | 0 | 0 | 1 | Ref |
| KX266444 | Unassigned | Unassigned | Unassigned | 373_0 | Clustered | 358 | VC_373_0 | 358 | 0.9614 | 1 | 0.9614 | 0 | 0 | 0 | 1 | Ref |
| KX266445 | Unassigned | Unassigned | Unassigned | 373_0 | Clustered | 358 | VC_373_0 | 358 | 0.9614 | 1 | 0.9614 | 0 | 0 | 0 | 1 | Ref |
| KX266446 | Unassigned | Unassigned | Unassigned | 373_0 | Clustered | 358 | VC_373_0 | 358 | 0.9614 | 1 | 0.9614 | 0 | 0 | 0 | 1 | Ref |
| KX266447 | Unassigned | Unassigned | Unassigned | 373_0 | Clustered | 358 | VC_373_0 | 358 | 0.9614 | 1 | 0.9614 | 0 | 0 | 0 | 1 | Ref |
| KX266448 | Unassigned | Unassigned | Unassigned | 373_0 | Clustered | 358 | VC_373_0 | 358 | 0.9614 | 1 | 0.9614 | 0 | 0 | 0 | 1 | Ref |
| KX266449 | Unassigned | Unassigned | Unassigned | 373_0 | Clustered | 358 | VC_373_0 | 358 | 0.9614 | 1 | 0.9614 | 0 | 0 | 0 | 1 | Ref |
| KX266450 | Unassigned | Unassigned | Unassigned | 373_0 | Clustered | 358 | VC_373_0 | 358 | 0.9614 | 1 | 0.9614 | 0 | 0 | 0 | 1 | Ref |
| KX266451 | Unassigned | Unassigned | Unassigned | 373_0 | Clustered | 358 | VC_373_0 | 358 | 0.9614 | 1 | 0.9614 | 0 | 0 | 0 | 1 | Ref |
| KX266452 | Unassigned | Unassigned | Unassigned | 373_0 | Clustered | 358 | VC_373_0 | 358 | 0.9614 | 1 | 0.9614 | 0 | 0 | 0 | 1 | Ref |
| KX266453 | Unassigned | Unassigned | Unassigned | 373_0 | Clustered | 358 | VC_373_0 | 358 | 0.9614 | 1 | 0.9614 | 0 | 0 | 0 | 1 | Ref |
| KX266454 | Unassigned | Unassigned | Unassigned | 373_0 | Clustered | 358 | VC_373_0 | 358 | 0.9614 | 1 | 0.9614 | 0 | 0 | 0 | 1 | Ref |
| KX266455 | Unassigned | Unassigned | Unassigned | 373_0 | Clustered | 358 | VC_373_0 | 358 | 0.9614 | 1 | 0.9614 | 0 | 0 | 0 | 1 | Ref |
| KX266456 | Unassigned | Unassigned | Unassigned | 373_0 | Clustered | 358 | VC_373_0 | 358 | 0.9614 | 1 | 0.9614 | 0 | 0 | 0 | 1 | Ref |
| KX266457 | Unassigned | Unassigned | Unassigned | 373_0 | Clustered | 358 | VC_373_0 | 358 | 0.9614 | 1 | 0.9614 | 0 | 0 | 0 | 1 | Ref |
| KX266458 | Unassigned | Unassigned | Unassigned | 373_0 | Clustered | 358 | VC_373_0 | 358 | 0.9614 | 1 | 0.9614 | 0 | 0 | 0 | 1 | Ref |
| KX266459 | Unassigned | Unassigned | Unassigned | 373_0 | Clustered | 358 | VC_373_0 | 358 | 0.9614 | 1 | 0.9614 | 0 | 0 | 0 | 1 | Ref |
| KX266460 | Unassigned | Unassigned | Unassigned | 373_0 | Clustered | 358 | VC_373_0 | 358 | 0.9614 | 1 | 0.9614 | 0 | 0 | 0 | 1 | Ref |
| KX266461 | Unassigned | Unassigned | Unassigned | 373_0 | Clustered | 358 | VC_373_0 | 358 | 0.9614 | 1 | 0.9614 | 0 | 0 | 0 | 1 | Ref |
| KX266462 | Unassigned | Unassigned | Unassigned | 373_0 | Clustered | 358 | VC_373_0 | 358 | 0.9614 | 1 | 0.9614 | 0 | 0 | 0 | 1 | Ref |
| KX266463 | Unassigned | Unassigned | Unassigned | 373_0 | Clustered | 358 | VC_373_0 | 358 | 0.9614 | 1 | 0.9614 | 0 | 0 | 0 | 1 | Ref |
| KX266464 | Unassigned | Unassigned | Unassigned | 373_0 | Clustered | 358 | VC_373_0 | 358 | 0.9614 | 1 | 0.9614 | 0 | 0 | 0 | 1 | Ref |
| KX266465 | Unassigned | Unassigned | Unassigned | 373_0 | Clustered | 358 | VC_373_0 | 358 | 0.9614 | 1 | 0.9614 | 0 | 0 | 0 | 1 | Ref |
| KX266466 | Unassigned | Unassigned | Unassigned | 373_0 | Clustered | 358 | VC_373_0 | 358 | 0.9614 | 1 | 0.9614 | 0 | 0 | 0 | 1 | Ref |
| KX266467 | Unassigned | Unassigned | Unassigned | 373_0 | Clustered | 358 | VC_373_0 | 358 | 0.9614 | 1 | 0.9614 | 0 | 0 | 0 | 1 | Ref |
| KX266468 | Unassigned | Unassigned | Unassigned | 373_0 | Clustered | 358 | VC_373_0 | 358 | 0.9614 | 1 | 0.9614 | 0 | 0 | 0 | 1 | Ref |
| KX266469 | Unassigned | Unassigned | Unassigned | 373_0 | Clustered | 358 | VC_373_0 | 358 | 0.9614 | 1 | 0.9614 | 0 | 0 | 0 | 1 | Ref |
| KX266470 | Unassigned | Unassigned | Unassigned | 373_0 | Clustered | 358 | VC_373_0 | 358 | 0.9614 | 1 | 0.9614 | 0 | 0 | 0 | 1 | Ref |
| KX266471 | Unassigned | Unassigned | Unassigned | 373_0 | Clustered | 358 | VC_373_0 | 358 | 0.9614 | 1 | 0.9614 | 0 | 0 | 0 | 1 | Ref |
| KX266472 | Unassigned | Unassigned | Unassigned | 373_0 | Clustered | 358 | VC_373_0 | 358 | 0.9614 | 1 | 0.9614 | 0 | 0 | 0 | 1 | Ref |
| KX266473 | Unassigned | Unassigned | Unassigned | 373_0 | Clustered | 358 | VC_373_0 | 358 | 0.9614 | 1 | 0.9614 | 0 | 0 | 0 | 1 | Ref |
| KX266474 | Unassigned | Unassigned | Unassigned | 373_0 | Clustered | 358 | VC_373_0 | 358 | 0.9614 | 1 | 0.9614 | 0 | 0 | 0 | 1 | Ref |
| KX266475 | Unassigned | Unassigned | Unassigned | 373_0 | Clustered | 358 | VC_373_0 | 358 | 0.9614 | 1 | 0.9614 | 0 | 0 | 0 | 1 | Ref |
| KX266476 | Unassigned | Unassigned | Unassigned | 373_0 | Clustered | 358 | VC_373_0 | 358 | 0.9614 | 1 | 0.9614 | 0 | 0 | 0 | 1 | Ref |
| KX266477 | Unassigned | Unassigned | Unassigned | 373_0 | Clustered | 358 | VC_373_0 | 358 | 0.9614 | 1 | 0.9614 | 0 | 0 | 0 | 1 | Ref |
| KX266478 | Unassigned | Unassigned | Unassigned | 373_0 | Clustered | 358 | VC_373_0 | 358 | 0.9614 | 1 | 0.9614 | 0 | 0 | 0 | 1 | Ref |
| KX266479 | Unassigned | Unassigned | Unassigned | 373_0 | Clustered | 358 | VC_373_0 | 358 | 0.9614 | 1 | 0.9614 | 0 | 0 | 0 | 1 | Ref |
| KX266480 | Unassigned | Unassigned | Unassigned | 373_0 | Clustered | 358 | VC_373_0 | 358 | 0.9614 | 1 | 0.9614 | 0 | 0 | 0 | 1 | Ref |
| KX266481 | Unassigned | Unassigned | Unassigned | 373_0 | Clustered | 358 | VC_373_0 | 358 | 0.9614 | 1 | 0.9614 | 0 | 0 | 0 | 1 | Ref |
| KX266482 | Unassigned | Unassigned | Unassigned | 373_0 | Clustered | 358 | VC_373_0 | 358 | 0.9614 | 1 | 0.9614 | 0 | 0 | 0 | 1 | Ref |
| KX266483 | Unassigned | Unassigned | Unassigned | 373_0 | Clustered | 358 | VC_373_0 | 358 | 0.9614 | 1 | 0.9614 | 0 | 0 | 0 | 1 | Ref |
| KX266484 | Unassigned | Unassigned | Unassigned | 373_0 | Clustered | 358 | VC_373_0 | 358 | 0.9614 | 1 | 0.9614 | 0 | 0 | 0 | 1 | Ref |
| KX266485 | Unassigned | Unassigned | Unassigned | 373_0 | Clustered | 358 | VC_373_0 | 358 | 0.9614 | 1 | 0.9614 | 0 | 0 | 0 | 1 | Ref |
| KX266486 | Unassigned | Unassigned | Unassigned | 373_0 | Clustered | 358 | VC_373_0 | 358 | 0.9614 | 1 | 0.9614 | 0 | 0 | 0 | 1 | Ref |
| KX266487 | Unassigned | Unassigned | Unassigned | 373_0 | Clustered | 358 | VC_373_0 | 358 | 0.9614 | 1 | 0.9614 | 0 | 0 | 0 | 1 | Ref |
| KX266488 | Unassigned | Unassigned | Unassigned | 373_0 | Clustered | 358 | VC_373_0 | 358 | 0.9614 | 1 | 0.9614 | 0 | 0 | 0 | 1 | Ref |
| KX266489 | Unassigned | Unassigned | Unassigned | 373_0 | Clustered | 358 | VC_373_0 | 358 | 0.9614 | 1 | 0.9614 | 0 | 0 | 0 | 1 | Ref |
| KX266490 | Unassigned | Unassigned | Unassigned | 373_0 | Clustered | 358 | VC_373_0 | 358 | 0.9614 | 1 | 0.9614 | 0 | 0 | 0 | 1 | Ref |
| KX266491 | Unassigned | Unassigned | Unassigned | 373_0 | Clustered | 358 | VC_373_0 | 358 | 0.9614 | 1 | 0.9614 | 0 | 0 | 0 | 1 | Ref |
| KX266492 | Unassigned | Unassigned | Unassigned | 373_0 | Clustered | 358 | VC_373_0 | 358 | 0.9614 | 1 | 0.9614 | 0 | 0 | 0 | 1 | Ref |
| KX266493 | Unassigned | Unassigned | Unassigned | 373_0 | Clustered | 358 | VC_373_0 | 358 | 0.9614 | 1 | 0.9614 | 0 | 0 | 0 | 1 | Ref |
| KX266494 | Unassigned | Unassigned | Unassigned | 373_0 | Clustered | 358 | VC_373_0 | 358 | 0.9614 | 1 | 0.9614 | 0 | 0 | 0 | 1 | Ref |
| KX266495 | Unassigned | Unassigned | Unassigned | 373_0 | Clustered | 358 | VC_373_0 | 358 | 0.9614 | 1 | 0.9614 | 0 | 0 | 0 | 1 | Ref |
| KX266496 | Unassigned | Unassigned | Unassigned | 373_0 | Clustered | 358 | VC_373_0 | 358 | 0.9614 | 1 | 0.9614 | 0 | 0 | 0 | 1 | Ref |
| KX266497 | Unassigned | Unassigned | Unassigned | 373_0 | Clustered | 358 | VC_373_0 | 358 | 0.9614 | 1 | 0.9614 | 0 | 0 | 0 | 1 | Ref |
| KX266498 | Unassigned | Unassigned | Unassigned | 373_0 | Clustered | 358 | VC_373_0 | 358 | 0.9614 | 1 | 0.9614 | 0 | 0 | 0 | 1 | Ref |
| KX266499 | Unassigned | Unassigned | Unassigned | 373_0 | Clustered | 358 | VC_373_0 | 358 | 0.9614 | 1 | 0.9614 | 0 | 0 | 0 | 1 | Ref |
| KX266500 | Unassigned | Unassigned | Unassigned | 373_0 | Clustered | 358 | VC_373_0 | 358 | 0.9614 | 1 | 0.9614 | 0 | 0 | 0 | 1 | Ref |
| KX266501 | Unassigned | Unassigned | Unassigned | 373_0 | Clustered | 358 | VC_373_0 | 358 | 0.9614 | 1 | 0.9614 | 0 | 0 | 0 | 1 | Ref |
| KX266502 | Unassigned | Unassigned | Unassigned | 373_0 | Clustered | 358 | VC_373_0 | 358 | 0.9614 | 1 | 0.9614 | 0 | 0 | 0 | 1 | Ref |
| KX266503 | Unassigned | Unassigned | Unassigned | 373_0 | Clustered | 358 | VC_373_0 | 358 | 0.9614 | 1 | 0.9614 | 0 | 0 | 0 | 1 | Ref |
| KX266504 | Unassigned | Unassigned | Unassigned | 373_0 | Clustered | 358 | VC_373_0 | 358 | 0.9614 | 1 | 0.9614 | 0 | 0 | 0 | 1 | Ref |
| KX266505 | Unassigned | Unassigned | Unassigned | 373_0 | Clustered | 358 | VC_373_0 | 358 | 0.9614 | 1 | 0.9614 | 0 | 0 | 0 | 1 | Ref |
| KX266506 | Unassigned | Unassigned | Unassigned | 373_0 | Clustered | 358 | VC_373_0 | 358 | 0.9614 | 1 | 0.9614 | 0 | 0 | 0 | 1 | Ref |
| KX266507 | Unassigned | Unassigned | Unassigned | 373_0 | Clustered | 358 | VC_373_0 | 358 | 0.9614 | 1 | 0.9614 | 0 | 0 | 0 | 1 | Ref |
| KX266508 | Unassigned | Unassigned | Unassigned | 373_0 | Clustered | 358 | VC_373_0 | 358 | 0.9614 | 1 | 0.9614 | 0 | 0 | 0 | 1 | Ref |
| KX266509 | Unassigned | Unassigned | Unassigned | 373_0 | Clustered | 358 | VC_373_0 | 358 | 0.9614 | 1 | 0.9614 | 0 | 0 | 0 | 1 | Ref |
| KX266510 | Unassigned | Unassigned | Unassigned | 373_0 | Clustered | 358 | VC_373_0 | 358 | 0.9614 | 1 | 0.9614 | 0 | 0 | 0 | 1 | Ref |
| KX266511 | Unassigned | Unassigned | Unassigned | 373_0 | Clustered | 358 | VC_373_0 | 358 | 0.9614 | 1 | 0.9614 | 0 | 0 | 0 | 1 | Ref |
| KX266512 | Unassigned | Unassigned | Unassigned | 373_0 | Clustered | 358 | VC_373_0 | 358 | 0.9614 | 1 | 0.9614 | 0 | 0 | 0 | 1 | Ref |
| KX266513 | Unassigned | Unassigned | Unassigned | 373_0 | Clustered | 358 | VC_373_0 | 358 | 0.9614 | 1 | 0.9614 | 0 | 0 | 0 | 1 | Ref |
| KX266514 | Unassigned | Unassigned | Unassigned | 373_0 | Clustered | 358 | VC_373_0 | 358 | 0.9614 | 1 | 0.9614 | 0 | 0 | 0 | 1 | Ref |
| KX266515 | Unassigned | Unassigned | Unassigned | 373_0 | Clustered | 358 | VC_373_0 | 358 | 0.9614 | 1 | 0.9614 | 0 | 0 | 0 | 1 | Ref |
| KX266516 | Unassigned | Unassigned | Unassigned | 373_0 | Clustered | 358 | VC_373_0 | 358 | 0.9614 | 1 | 0.9614 | 0 | 0 | 0 | 1 | Ref |
| KX266517 | Unassigned | Unassigned | Unassigned | 373_0 | Clustered | 358 | VC_373_0 | 358 | 0.9614 | 1 | 0.9614 | 0 | 0 | 0 | 1 | Ref |

| | | | | | | | | | | | | | | | | |
|---|---|---|---|---|---|---|---|---|---|---|---|---|---|---|---|---|
| KX266518 | Unassigned | Unassigned | Unassigned | 373_0 | Clustered | 358 | VC_373_0 | 358 | 0.9614 | 1 | 0.9614 | 0 | 0 | 0 | 1 | Ref |
| KX266519 | Unassigned | Unassigned | Unassigned | 373_0 | Clustered | 358 | VC_373_0 | 358 | 0.9614 | 1 | 0.9614 | 0 | 0 | 0 | 1 | Ref |
| KX266520 | Unassigned | Unassigned | Unassigned | 373_0 | Clustered | 358 | VC_373_0 | 358 | 0.9614 | 1 | 0.9614 | 0 | 0 | 0 | 1 | Ref |
| KX266521 | Unassigned | Unassigned | Unassigned | 373_0 | Clustered | 358 | VC_373_0 | 358 | 0.9614 | 1 | 0.9614 | 0 | 0 | 0 | 1 | Ref |
| KX266522 | Unassigned | Unassigned | Unassigned | 373_0 | Clustered | 358 | VC_373_0 | 358 | 0.9614 | 1 | 0.9614 | 0 | 0 | 0 | 1 | Ref |
| KX266523 | Unassigned | Unassigned | Unassigned | 373_0 | Clustered | 358 | VC_373_0 | 358 | 0.9614 | 1 | 0.9614 | 0 | 0 | 0 | 1 | Ref |
| KX266524 | Unassigned | Unassigned | Unassigned | 373_0 | Clustered | 358 | VC_373_0 | 358 | 0.9614 | 1 | 0.9614 | 0 | 0 | 0 | 1 | Ref |
| KX266525 | Unassigned | Unassigned | Unassigned | 373_0 | Clustered | 358 | VC_373_0 | 358 | 0.9614 | 1 | 0.9614 | 0 | 0 | 0 | 1 | Ref |
| KX266526 | Unassigned | Unassigned | Unassigned | 373_0 | Clustered | 358 | VC_373_0 | 358 | 0.9614 | 1 | 0.9614 | 0 | 0 | 0 | 1 | Ref |
| KX266527 | Unassigned | Unassigned | Unassigned | 373_0 | Clustered | 358 | VC_373_0 | 358 | 0.9614 | 1 | 0.9614 | 0 | 0 | 0 | 1 | Ref |
| KX266528 | Unassigned | Unassigned | Unassigned | 373_0 | Clustered | 358 | VC_373_0 | 358 | 0.9614 | 1 | 0.9614 | 0 | 0 | 0 | 1 | Ref |
| KX266529 | Unassigned | Unassigned | Unassigned | 373_0 | Clustered | 358 | VC_373_0 | 358 | 0.9614 | 1 | 0.9614 | 0 | 0 | 0 | 1 | Ref |
| KX266530 | Unassigned | Unassigned | Unassigned | 373_0 | Clustered | 358 | VC_373_0 | 358 | 0.9614 | 1 | 0.9614 | 0 | 0 | 0 | 1 | Ref |
| KX266531 | Unassigned | Unassigned | Unassigned | 373_0 | Clustered | 358 | VC_373_0 | 358 | 0.9614 | 1 | 0.9614 | 0 | 0 | 0 | 1 | Ref |
| KX266532 | Unassigned | Unassigned | Unassigned | 373_0 | Clustered | 358 | VC_373_0 | 358 | 0.9614 | 1 | 0.9614 | 0 | 0 | 0 | 1 | Ref |
| KX266533 | Unassigned | Unassigned | Unassigned | 373_0 | Clustered | 358 | VC_373_0 | 358 | 0.9614 | 1 | 0.9614 | 0 | 0 | 0 | 1 | Ref |
| KX266534 | Unassigned | Unassigned | Unassigned | 373_0 | Clustered | 358 | VC_373_0 | 358 | 0.9614 | 1 | 0.9614 | 0 | 0 | 0 | 1 | Ref |
| KX266535 | Unassigned | Unassigned | Unassigned | 373_0 | Clustered | 358 | VC_373_0 | 358 | 0.9614 | 1 | 0.9614 | 0 | 0 | 0 | 1 | Ref |
| KX266536 | Unassigned | Unassigned | Unassigned | 373_0 | Clustered | 358 | VC_373_0 | 358 | 0.9614 | 1 | 0.9614 | 0 | 0 | 0 | 1 | Ref |
| KX266537 | Unassigned | Unassigned | Unassigned | 373_0 | Clustered | 358 | VC_373_0 | 358 | 0.9614 | 1 | 0.9614 | 0 | 0 | 0 | 1 | Ref |
| KX266538 | Unassigned | Unassigned | Unassigned | 373_0 | Clustered | 358 | VC_373_0 | 358 | 0.9614 | 1 | 0.9614 | 0 | 0 | 0 | 1 | Ref |
| KX266539 | Unassigned | Unassigned | Unassigned | 373_0 | Clustered | 358 | VC_373_0 | 358 | 0.9614 | 1 | 0.9614 | 0 | 0 | 0 | 1 | Ref |
| KX266540 | Unassigned | Unassigned | Unassigned | 373_0 | Clustered | 358 | VC_373_0 | 358 | 0.9614 | 1 | 0.9614 | 0 | 0 | 0 | 1 | Ref |
| KX266541 | Unassigned | Unassigned | Unassigned | 373_0 | Clustered | 358 | VC_373_0 | 358 | 0.9614 | 1 | 0.9614 | 0 | 0 | 0 | 1 | Ref |
| KX266542 | Unassigned | Unassigned | Unassigned | 373_0 | Clustered | 358 | VC_373_0 | 358 | 0.9614 | 1 | 0.9614 | 0 | 0 | 0 | 1 | Ref |
| KX266543 | Unassigned | Unassigned | Unassigned | 373_0 | Clustered | 358 | VC_373_0 | 358 | 0.9614 | 1 | 0.9614 | 0 | 0 | 0 | 1 | Ref |
| KX266544 | Unassigned | Unassigned | Unassigned | 373_0 | Clustered | 358 | VC_373_0 | 358 | 0.9614 | 1 | 0.9614 | 0 | 0 | 0 | 1 | Ref |
| KX266545 | Unassigned | Unassigned | Unassigned | 373_0 | Clustered | 358 | VC_373_0 | 358 | 0.9614 | 1 | 0.9614 | 0 | 0 | 0 | 1 | Ref |
| KX266546 | Unassigned | Unassigned | Unassigned | 373_0 | Clustered | 358 | VC_373_0 | 358 | 0.9614 | 1 | 0.9614 | 0 | 0 | 0 | 1 | Ref |
| KX266547 | Unassigned | Unassigned | Unassigned | 373_0 | Clustered | 358 | VC_373_0 | 358 | 0.9614 | 1 | 0.9614 | 0 | 0 | 0 | 1 | Ref |
| KX266548 | Unassigned | Unassigned | Unassigned | 373_0 | Clustered | 358 | VC_373_0 | 358 | 0.9614 | 1 | 0.9614 | 0 | 0 | 0 | 1 | Ref |
| KX266549 | Unassigned | Unassigned | Unassigned | 373_0 | Clustered | 358 | VC_373_0 | 358 | 0.9614 | 1 | 0.9614 | 0 | 0 | 0 | 1 | Ref |
| KX266550 | Unassigned | Unassigned | Unassigned | 373_0 | Clustered | 358 | VC_373_0 | 358 | 0.9614 | 1 | 0.9614 | 0 | 0 | 0 | 1 | Ref |
| KX266551 | Unassigned | Unassigned | Unassigned | 373_0 | Clustered | 358 | VC_373_0 | 358 | 0.9614 | 1 | 0.9614 | 0 | 0 | 0 | 1 | Ref |
| KX266552 | Unassigned | Unassigned | Unassigned | 373_0 | Clustered | 358 | VC_373_0 | 358 | 0.9614 | 1 | 0.9614 | 0 | 0 | 0 | 1 | Ref |
| KX266553 | Unassigned | Unassigned | Unassigned | 373_0 | Clustered | 358 | VC_373_0 | 358 | 0.9614 | 1 | 0.9614 | 0 | 0 | 0 | 1 | Ref |
| KX266554 | Unassigned | Unassigned | Unassigned | 373_0 | Clustered | 358 | VC_373_0 | 358 | 0.9614 | 1 | 0.9614 | 0 | 0 | 0 | 1 | Ref |
| KX266555 | Unassigned | Unassigned | Unassigned | 373_0 | Clustered | 358 | VC_373_0 | 358 | 0.9614 | 1 | 0.9614 | 0 | 0 | 0 | 1 | Ref |
| KX266556 | Unassigned | Unassigned | Unassigned | 373_0 | Clustered | 358 | VC_373_0 | 358 | 0.9614 | 1 | 0.9614 | 0 | 0 | 0 | 1 | Ref |
| KX266557 | Unassigned | Unassigned | Unassigned | 373_0 | Clustered | 358 | VC_373_0 | 358 | 0.9614 | 1 | 0.9614 | 0 | 0 | 0 | 1 | Ref |
| KX266558 | Unassigned | Unassigned | Unassigned | 373_0 | Clustered | 358 | VC_373_0 | 358 | 0.9614 | 1 | 0.9614 | 0 | 0 | 0 | 1 | Ref |
| KX266559 | Unassigned | Unassigned | Unassigned | 373_0 | Clustered | 358 | VC_373_0 | 358 | 0.9614 | 1 | 0.9614 | 0 | 0 | 0 | 1 | Ref |
| KX266560 | Unassigned | Unassigned | Unassigned | 373_0 | Clustered | 358 | VC_373_0 | 358 | 0.9614 | 1 | 0.9614 | 0 | 0 | 0 | 1 | Ref |
| KX266561 | Unassigned | Unassigned | Unassigned | 373_0 | Clustered | 358 | VC_373_0 | 358 | 0.9614 | 1 | 0.9614 | 0 | 0 | 0 | 1 | Ref |
| KX266562 | Unassigned | Unassigned | Unassigned | 373_0 | Clustered | 358 | VC_373_0 | 358 | 0.9614 | 1 | 0.9614 | 0 | 0 | 0 | 1 | Ref |
| KX266563 | Unassigned | Unassigned | Unassigned | 373_0 | Clustered | 358 | VC_373_0 | 358 | 0.9614 | 1 | 0.9614 | 0 | 0 | 0 | 1 | Ref |
| KX266564 | Unassigned | Unassigned | Unassigned | 373_0 | Clustered | 358 | VC_373_0 | 358 | 0.9614 | 1 | 0.9614 | 0 | 0 | 0 | 1 | Ref |
| KX266565 | Unassigned | Unassigned | Unassigned | 373_0 | Clustered | 358 | VC_373_0 | 358 | 0.9614 | 1 | 0.9614 | 0 | 0 | 0 | 1 | Ref |
| KX266566 | Unassigned | Unassigned | Unassigned | 373_0 | Clustered | 358 | VC_373_0 | 358 | 0.9614 | 1 | 0.9614 | 0 | 0 | 0 | 1 | Ref |
| KX266567 | Unassigned | Unassigned | Unassigned | 373_0 | Clustered | 358 | VC_373_0 | 358 | 0.9614 | 1 | 0.9614 | 0 | 0 | 0 | 1 | Ref |
| KX266568 | Unassigned | Unassigned | Unassigned | 373_0 | Clustered | 358 | VC_373_0 | 358 | 0.9614 | 1 | 0.9614 | 0 | 0 | 0 | 1 | Ref |
| KX266569 | Unassigned | Unassigned | Unassigned | 373_0 | Clustered | 358 | VC_373_0 | 358 | 0.9614 | 1 | 0.9614 | 0 | 0 | 0 | 1 | Ref |
| KX266570 | Unassigned | Unassigned | Unassigned | 373_0 | Clustered | 358 | VC_373_0 | 358 | 0.9614 | 1 | 0.9614 | 0 | 0 | 0 | 1 | Ref |
| KX266571 | Unassigned | Unassigned | Unassigned | 373_0 | Clustered | 358 | VC_373_0 | 358 | 0.9614 | 1 | 0.9614 | 0 | 0 | 0 | 1 | Ref |
| KX266572 | Unassigned | Unassigned | Unassigned | 373_0 | Clustered | 358 | VC_373_0 | 358 | 0.9614 | 1 | 0.9614 | 0 | 0 | 0 | 1 | Ref |
| KX266573 | Unassigned | Unassigned | Unassigned | 373_0 | Clustered | 358 | VC_373_0 | 358 | 0.9614 | 1 | 0.9614 | 0 | 0 | 0 | 1 | Ref |
| KX266574 | Unassigned | Unassigned | Unassigned | 373_0 | Clustered | 358 | VC_373_0 | 358 | 0.9614 | 1 | 0.9614 | 0 | 0 | 0 | 1 | Ref |
| KX266575 | Unassigned | Unassigned | Unassigned | 373_0 | Clustered | 358 | VC_373_0 | 358 | 0.9614 | 1 | 0.9614 | 0 | 0 | 0 | 1 | Ref |
| KX266576 | Unassigned | Unassigned | Unassigned | 373_0 | Clustered | 358 | VC_373_0 | 358 | 0.9614 | 1 | 0.9614 | 0 | 0 | 0 | 1 | Ref |
| KX266577 | Unassigned | Unassigned | Unassigned | 373_0 | Clustered | 358 | VC_373_0 | 358 | 0.9614 | 1 | 0.9614 | 0 | 0 | 0 | 1 | Ref |
| KX266578 | Unassigned | Unassigned | Unassigned | 373_0 | Clustered | 358 | VC_373_0 | 358 | 0.9614 | 1 | 0.9614 | 0 | 0 | 0 | 1 | Ref |
| KX266579 | Unassigned | Unassigned | Unassigned | 373_0 | Clustered | 358 | VC_373_0 | 358 | 0.9614 | 1 | 0.9614 | 0 | 0 | 0 | 1 | Ref |
| KX266580 | Unassigned | Unassigned | Unassigned | 373_0 | Clustered | 358 | VC_373_0 | 358 | 0.9614 | 1 | 0.9614 | 0 | 0 | 0 | 1 | Ref |
| KX266581 | Unassigned | Unassigned | Unassigned | 373_0 | Clustered | 358 | VC_373_0 | 358 | 0.9614 | 1 | 0.9614 | 0 | 0 | 0 | 1 | Ref |
| KX266582 | Unassigned | Unassigned | Unassigned | 373_0 | Clustered | 358 | VC_373_0 | 358 | 0.9614 | 1 | 0.9614 | 0 | 0 | 0 | 1 | Ref |
| KX266583 | Unassigned | Unassigned | Unassigned | 373_0 | Clustered | 358 | VC_373_0 | 358 | 0.9614 | 1 | 0.9614 | 0 | 0 | 0 | 1 | Ref |
| KX266584 | Unassigned | Unassigned | Unassigned | 373_0 | Clustered | 358 | VC_373_0 | 358 | 0.9614 | 1 | 0.9614 | 0 | 0 | 0 | 1 | Ref |
| KX266585 | Unassigned | Unassigned | Unassigned | 373_0 | Clustered | 358 | VC_373_0 | 358 | 0.9614 | 1 | 0.9614 | 0 | 0 | 0 | 1 | Ref |
| KX266586 | Unassigned | Unassigned | Unassigned | 373_0 | Clustered | 358 | VC_373_0 | 358 | 0.9614 | 1 | 0.9614 | 0 | 0 | 0 | 1 | Ref |
| KX266587 | Unassigned | Unassigned | Unassigned | 373_0 | Clustered | 358 | VC_373_0 | 358 | 0.9614 | 1 | 0.9614 | 0 | 0 | 0 | 1 | Ref |
| KX266588 | Unassigned | Unassigned | Unassigned | 373_0 | Clustered | 358 | VC_373_0 | 358 | 0.9614 | 1 | 0.9614 | 0 | 0 | 0 | 1 | Ref |
| KX266589 | Unassigned | Unassigned | Unassigned | 373_0 | Clustered | 358 | VC_373_0 | 358 | 0.9614 | 1 | 0.9614 | 0 | 0 | 0 | 1 | Ref |
| KX266590 | Unassigned | Unassigned | Unassigned | 373_0 | Clustered | 358 | VC_373_0 | 358 | 0.9614 | 1 | 0.9614 | 0 | 0 | 0 | 1 | Ref |
| KX266591 | Unassigned | Unassigned | Unassigned | 373_0 | Clustered | 358 | VC_373_0 | 358 | 0.9614 | 1 | 0.9614 | 0 | 0 | 0 | 1 | Ref |
| KX266592 | Unassigned | Unassigned | Unassigned | 373_0 | Clustered | 358 | VC_373_0 | 358 | 0.9614 | 1 | 0.9614 | 0 | 0 | 0 | 1 | Ref |
| KX266593 | Unassigned | Unassigned | Unassigned | 373_0 | Clustered | 358 | VC_373_0 | 358 | 0.9614 | 1 | 0.9614 | 0 | 0 | 0 | 1 | Ref |
| KX266594 | Unassigned | Unassigned | Unassigned | 373_0 | Clustered | 358 | VC_373_0 | 358 | 0.9614 | 1 | 0.9614 | 0 | 0 | 0 | 1 | Ref |
| KX266595 | Unassigned | Unassigned | Unassigned | 373_0 | Clustered | 358 | VC_373_0 | 358 | 0.9614 | 1 | 0.9614 | 0 | 0 | 0 | 1 | Ref |
| KX266596 | Unassigned | Unassigned | Unassigned | 373_0 | Clustered | 358 | VC_373_0 | 358 | 0.9614 | 1 | 0.9614 | 0 | 0 | 0 | 1 | Ref |
| KX266597 | Unassigned | Unassigned | Unassigned | 373_0 | Clustered | 358 | VC_373_0 | 358 | 0.9614 | 1 | 0.9614 | 0 | 0 | 0 | 1 | Ref |
| KX266598 | Unassigned | Unassigned | Unassigned | 373_0 | Clustered | 358 | VC_373_0 | 358 | 0.9614 | 1 | 0.9614 | 0 | 0 | 0 | 1 | Ref |

| | | | | | | | | | | | | | | | | |
|---|---|---|---|---|---|---|---|---|---|---|---|---|---|---|---|---|
| KX266599 | Unassigned | Unassigned | Unassigned | 373_0 | Clustered | 358 | VC_373_0 | 358 | 0.9614 | 1 | 0.9614 | 0 | 0 | 0 | 1 | Ref |
| KX266600 | Unassigned | Unassigned | Unassigned | 373_0 | Clustered | 358 | VC_373_0 | 358 | 0.9614 | 1 | 0.9614 | 0 | 0 | 0 | 1 | Ref |
| KX266601 | Unassigned | Unassigned | Unassigned | 373_0 | Clustered | 358 | VC_373_0 | 358 | 0.9614 | 1 | 0.9614 | 0 | 0 | 0 | 1 | Ref |
| KX266602 | Unassigned | Unassigned | Unassigned | 373_0 | Clustered | 358 | VC_373_0 | 358 | 0.9614 | 1 | 0.9614 | 0 | 0 | 0 | 1 | Ref |
| KX266603 | Unassigned | Unassigned | Unassigned | 373_0 | Clustered | 358 | VC_373_0 | 358 | 0.9614 | 1 | 0.9614 | 0 | 0 | 0 | 1 | Ref |
| KX266604 | Unassigned | Unassigned | Unassigned | 373_0 | Clustered | 358 | VC_373_0 | 358 | 0.9614 | 1 | 0.9614 | 0 | 0 | 0 | 1 | Ref |
| KX266605 | Unassigned | Unassigned | Unassigned | 373_0 | Clustered | 358 | VC_373_0 | 358 | 0.9614 | 1 | 0.9614 | 0 | 0 | 0 | 1 | Ref |
| KX266606 | Unassigned | Unassigned | Unassigned | 373_0 | Clustered | 358 | VC_373_0 | 358 | 0.9614 | 1 | 0.9614 | 0 | 0 | 0 | 1 | Ref |
| KX266607 | Unassigned | Unassigned | Unassigned | 373_0 | Clustered | 358 | VC_373_0 | 358 | 0.9614 | 1 | 0.9614 | 0 | 0 | 0 | 1 | Ref |
| KX266608 | Unassigned | Unassigned | Unassigned | 373_0 | Clustered | 358 | VC_373_0 | 358 | 0.9614 | 1 | 0.9614 | 0 | 0 | 0 | 1 | Ref |
| KX266609 | Unassigned | Unassigned | Unassigned | 373_0 | Clustered | 358 | VC_373_0 | 358 | 0.9614 | 1 | 0.9614 | 0 | 0 | 0 | 1 | Ref |
| KX266610 | Unassigned | Unassigned | Unassigned | 373_0 | Clustered | 358 | VC_373_0 | 358 | 0.9614 | 1 | 0.9614 | 0 | 0 | 0 | 1 | Ref |
| KX266611 | Unassigned | Unassigned | Unassigned | 373_0 | Clustered | 358 | VC_373_0 | 358 | 0.9614 | 1 | 0.9614 | 0 | 0 | 0 | 1 | Ref |
| KX266612 | Unassigned | Unassigned | Unassigned | 373_0 | Clustered | 358 | VC_373_0 | 358 | 0.9614 | 1 | 0.9614 | 0 | 0 | 0 | 1 | Ref |
| KX266613 | Unassigned | Unassigned | Unassigned | 373_0 | Clustered | 358 | VC_373_0 | 358 | 0.9614 | 1 | 0.9614 | 0 | 0 | 0 | 1 | Ref |
| KX266614 | Unassigned | Unassigned | Unassigned | 373_0 | Clustered | 358 | VC_373_0 | 358 | 0.9614 | 1 | 0.9614 | 0 | 0 | 0 | 1 | Ref |
| KX266615 | Unassigned | Unassigned | Unassigned | 373_0 | Clustered | 358 | VC_373_0 | 358 | 0.9614 | 1 | 0.9614 | 0 | 0 | 0 | 1 | Ref |
| KX266616 | Unassigned | Unassigned | Unassigned | 373_0 | Clustered | 358 | VC_373_0 | 358 | 0.9614 | 1 | 0.9614 | 0 | 0 | 0 | 1 | Ref |
| KX266617 | Unassigned | Unassigned | Unassigned | 373_0 | Clustered | 358 | VC_373_0 | 358 | 0.9614 | 1 | 0.9614 | 0 | 0 | 0 | 1 | Ref |
| KX266618 | Unassigned | Unassigned | Unassigned | 373_0 | Clustered | 358 | VC_373_0 | 358 | 0.9614 | 1 | 0.9614 | 0 | 0 | 0 | 1 | Ref |
| KX266619 | Unassigned | Unassigned | Unassigned | 373_0 | Clustered | 358 | VC_373_0 | 358 | 0.9614 | 1 | 0.9614 | 0 | 0 | 0 | 1 | Ref |
| KX266620 | Unassigned | Unassigned | Unassigned | 373_0 | Clustered | 358 | VC_373_0 | 358 | 0.9614 | 1 | 0.9614 | 0 | 0 | 0 | 1 | Ref |
| KX266621 | Unassigned | Unassigned | Unassigned | 373_0 | Clustered | 358 | VC_373_0 | 358 | 0.9614 | 1 | 0.9614 | 0 | 0 | 0 | 1 | Ref |
| KX266622 | Unassigned | Unassigned | Unassigned | 373_0 | Clustered | 358 | VC_373_0 | 358 | 0.9614 | 1 | 0.9614 | 0 | 0 | 0 | 1 | Ref |
| KX266623 | Unassigned | Unassigned | Unassigned | 373_0 | Clustered | 358 | VC_373_0 | 358 | 0.9614 | 1 | 0.9614 | 0 | 0 | 0 | 1 | Ref |
| KX266624 | Unassigned | Unassigned | Unassigned | 373_0 | Clustered | 358 | VC_373_0 | 358 | 0.9614 | 1 | 0.9614 | 0 | 0 | 0 | 1 | Ref |
| KX266625 | Unassigned | Unassigned | Unassigned | 373_0 | Clustered | 358 | VC_373_0 | 358 | 0.9614 | 1 | 0.9614 | 0 | 0 | 0 | 1 | Ref |
| KX266626 | Unassigned | Unassigned | Unassigned | 373_0 | Clustered | 358 | VC_373_0 | 358 | 0.9614 | 1 | 0.9614 | 0 | 0 | 0 | 1 | Ref |
| KX266627 | Unassigned | Unassigned | Unassigned | 373_0 | Clustered | 358 | VC_373_0 | 358 | 0.9614 | 1 | 0.9614 | 0 | 0 | 0 | 1 | Ref |
| KX266628 | Unassigned | Unassigned | Unassigned | 373_0 | Clustered | 358 | VC_373_0 | 358 | 0.9614 | 1 | 0.9614 | 0 | 0 | 0 | 1 | Ref |
| KX266629 | Unassigned | Unassigned | Unassigned | 373_0 | Clustered | 358 | VC_373_0 | 358 | 0.9614 | 1 | 0.9614 | 0 | 0 | 0 | 1 | Ref |
| KX266630 | Unassigned | Unassigned | Unassigned | 373_0 | Clustered | 358 | VC_373_0 | 358 | 0.9614 | 1 | 0.9614 | 0 | 0 | 0 | 1 | Ref |
| KX266631 | Unassigned | Unassigned | Unassigned | 373_0 | Clustered | 358 | VC_373_0 | 358 | 0.9614 | 1 | 0.9614 | 0 | 0 | 0 | 1 | Ref |
| KX266632 | Unassigned | Unassigned | Unassigned | 373_0 | Clustered | 358 | VC_373_0 | 358 | 0.9614 | 1 | 0.9614 | 0 | 0 | 0 | 1 | Ref |
| KX266633 | Unassigned | Unassigned | Unassigned | 373_0 | Clustered | 358 | VC_373_0 | 358 | 0.9614 | 1 | 0.9614 | 0 | 0 | 0 | 1 | Ref |
| KX266634 | Unassigned | Unassigned | Unassigned | 373_0 | Clustered | 358 | VC_373_0 | 358 | 0.9614 | 1 | 0.9614 | 0 | 0 | 0 | 1 | Ref |
| KX266635 | Unassigned | Unassigned | Unassigned | 373_0 | Clustered | 358 | VC_373_0 | 358 | 0.9614 | 1 | 0.9614 | 0 | 0 | 0 | 1 | Ref |
| KX266636 | Unassigned | Unassigned | Unassigned | 373_0 | Clustered | 358 | VC_373_0 | 358 | 0.9614 | 1 | 0.9614 | 0 | 0 | 0 | 1 | Ref |
| KX266637 | Unassigned | Unassigned | Unassigned | 373_0 | Clustered | 358 | VC_373_0 | 358 | 0.9614 | 1 | 0.9614 | 0 | 0 | 0 | 1 | Ref |
| KX278418 | Unassigned | Unassigned | Unassigned | 1018_0 | Clustered | 38 | VC_1018_0 | 38 | 0.9757 | 1 | 0.9757 | 0 | 0 | 0 | 1 | Ref |
| KX278419 | Unassigned | Unassigned | Unassigned | 1018_0 | Clustered | 38 | VC_1018_0 | 38 | 0.9757 | 1 | 0.9757 | 0 | 0 | 0 | 1 | Ref |
| KX279892 | Unassigned | Unassigned | Unassigned | 466_0 | Clustered | 66 | VC_466_0 | 66 | 0.9246 | 1 | 0.9246 | 0 | 0 | 0 | 1 | Ref |
| KX284704 | Unassigned | Unassigned | Unassigned | 711_0 | Clustered | 48 | VC_711_0 | 48 | 0.8217 | 1 | 0.8217 | 0 | 0 | 0 | 1 | Ref |
| KX344445 | Unassigned | Unassigned | Unassigned | 1016_0 | Clustered | 62 | VC_1016_0 | 62 | 0.8638 | 1 | 0.8638 | 0 | 0 | 0 | 0.743 | Ref |
| KX344510 | Unassigned | Unassigned | Unassigned | 996_0 | Clustered | 3 | VC_996_0 | 3 | 0.6852 | 0.9859071 | 0.6755 | 0 | 0 | 0 | 1 | Ref |
| KX346246 | Unassigned | Unassigned | Unassigned | 210_0 | Clustered | 105 | VC_210_0 | 105 | 0.4076 | 1 | 0.4076 | 0 | 0 | 0 | 0.7766 | Ref |
| KX349226 | Unassigned | Unassigned | Unassigned | 681_0 | Clustered | 62 | VC_681_0 | 62 | 0.693 | 1 | 0.693 | 0 | 0 | 0 | 1 | Ref |
| KX349227 | Unassigned | Unassigned | Unassigned | 681_0 | Clustered | 62 | VC_681_0 | 62 | 0.693 | 1 | 0.693 | 0 | 0 | 0 | 1 | Ref |
| KX349228 | Unassigned | Unassigned | Unassigned | 681_0 | Clustered | 62 | VC_681_0 | 62 | 0.693 | 1 | 0.693 | 0 | 0 | 0 | 1 | Ref |
| KX349229 | Unassigned | Unassigned | Unassigned | 681_0 | Clustered | 62 | VC_681_0 | 62 | 0.693 | 1 | 0.693 | 0 | 0 | 0 | 1 | Ref |
| KX349230 | Unassigned | Unassigned | Unassigned | 681_0 | Clustered | 62 | VC_681_0 | 62 | 0.693 | 1 | 0.693 | 0 | 0 | 0 | 1 | Ref |
| KX349231 | Unassigned | Unassigned | Unassigned | 681_0 | Clustered | 62 | VC_681_0 | 62 | 0.693 | 1 | 0.693 | 0 | 0 | 0 | 1 | Ref |
| KX349232 | Unassigned | Unassigned | Unassigned | 681_0 | Clustered | 62 | VC_681_0 | 62 | 0.693 | 1 | 0.693 | 0 | 0 | 0 | 1 | Ref |
| KX349233 | Unassigned | Unassigned | Unassigned | 681_0 | Clustered | 62 | VC_681_0 | 62 | 0.693 | 1 | 0.693 | 0 | 0 | 0 | 1 | Ref |
| KX349234 | Unassigned | Unassigned | Unassigned | 681_0 | Clustered | 62 | VC_681_0 | 62 | 0.693 | 1 | 0.693 | 0 | 0 | 0 | 1 | Ref |
| KX349235 | Unassigned | Unassigned | Unassigned | 681_0 | Clustered | 62 | VC_681_0 | 62 | 0.693 | 1 | 0.693 | 0 | 0 | 0 | 1 | Ref |
| KX349236 | Unassigned | Unassigned | Unassigned | 681_0 | Clustered | 62 | VC_681_0 | 62 | 0.693 | 1 | 0.693 | 0 | 0 | 0 | 1 | Ref |
| KX349237 | Unassigned | Unassigned | Unassigned | 681_0 | Clustered | 62 | VC_681_0 | 62 | 0.693 | 1 | 0.693 | 0 | 0 | 0 | 1 | Ref |
| KX349238 | Unassigned | Unassigned | Unassigned | 681_0 | Clustered | 62 | VC_681_0 | 62 | 0.693 | 1 | 0.693 | 0 | 0 | 0 | 1 | Ref |
| KX349239 | Unassigned | Unassigned | Unassigned | 681_0 | Clustered | 62 | VC_681_0 | 62 | 0.693 | 1 | 0.693 | 0 | 0 | 0 | 1 | Ref |
| KX349240 | Unassigned | Unassigned | Unassigned | 681_0 | Clustered | 62 | VC_681_0 | 62 | 0.693 | 1 | 0.693 | 0 | 0 | 0 | 1 | Ref |
| KX349241 | Unassigned | Unassigned | Unassigned | 681_0 | Clustered | 62 | VC_681_0 | 62 | 0.693 | 1 | 0.693 | 0 | 0 | 0 | 1 | Ref |
| KX349242 | Unassigned | Unassigned | Unassigned | 681_0 | Clustered | 62 | VC_681_0 | 62 | 0.693 | 1 | 0.693 | 0 | 0 | 0 | 1 | Ref |
| KX349243 | Unassigned | Unassigned | Unassigned | 681_0 | Clustered | 62 | VC_681_0 | 62 | 0.693 | 1 | 0.693 | 0 | 0 | 0 | 1 | Ref |
| KX349244 | Unassigned | Unassigned | Unassigned | 681_0 | Clustered | 62 | VC_681_0 | 62 | 0.693 | 1 | 0.693 | 0 | 0 | 0 | 1 | Ref |
| KX349245 | Unassigned | Unassigned | Unassigned | 681_0 | Clustered | 62 | VC_681_0 | 62 | 0.693 | 1 | 0.693 | 0 | 0 | 0 | 1 | Ref |
| KX349246 | Unassigned | Unassigned | Unassigned | 681_0 | Clustered | 62 | VC_681_0 | 62 | 0.693 | 1 | 0.693 | 0 | 0 | 0 | 1 | Ref |
| KX349247 | Unassigned | Unassigned | Unassigned | 681_0 | Clustered | 62 | VC_681_0 | 62 | 0.693 | 1 | 0.693 | 0 | 0 | 0 | 1 | Ref |
| KX349248 | Unassigned | Unassigned | Unassigned | 681_0 | Clustered | 62 | VC_681_0 | 62 | 0.693 | 1 | 0.693 | 0 | 0 | 0 | 1 | Ref |
| KX349249 | Unassigned | Unassigned | Unassigned | 681_0 | Clustered | 62 | VC_681_0 | 62 | 0.693 | 1 | 0.693 | 0 | 0 | 0 | 1 | Ref |
| KX349250 | Unassigned | Unassigned | Unassigned | 681_0 | Clustered | 62 | VC_681_0 | 62 | 0.693 | 1 | 0.693 | 0 | 0 | 0 | 1 | Ref |
| KX349251 | Unassigned | Unassigned | Unassigned | 681_0 | Clustered | 62 | VC_681_0 | 62 | 0.693 | 1 | 0.693 | 0 | 0 | 0 | 1 | Ref |
| KX349252 | Unassigned | Unassigned | Unassigned | 681_0 | Clustered | 62 | VC_681_0 | 62 | 0.693 | 1 | 0.693 | 0 | 0 | 0 | 1 | Ref |
| KX349253 | Unassigned | Unassigned | Unassigned | 681_0 | Clustered | 62 | VC_681_0 | 62 | 0.693 | 1 | 0.693 | 0 | 0 | 0 | 1 | Ref |
| KX349254 | Unassigned | Unassigned | Unassigned | 681_0 | Clustered | 62 | VC_681_0 | 62 | 0.693 | 1 | 0.693 | 0 | 0 | 0 | 1 | Ref |
| KX349255 | Unassigned | Unassigned | Unassigned | 681_0 | Clustered | 62 | VC_681_0 | 62 | 0.693 | 1 | 0.693 | 0 | 0 | 0 | 1 | Ref |
| KX349256 | Unassigned | Unassigned | Unassigned | 681_0 | Clustered | 62 | VC_681_0 | 62 | 0.693 | 1 | 0.693 | 0 | 0 | 0 | 1 | Ref |
| KX349257 | Unassigned | Unassigned | Unassigned | 681_0 | Clustered | 62 | VC_681_0 | 62 | 0.693 | 1 | 0.693 | 0 | 0 | 0 | 1 | Ref |
| KX349258 | Unassigned | Unassigned | Unassigned | 681_0 | Clustered | 62 | VC_681_0 | 62 | 0.693 | 1 | 0.693 | 0 | 0 | 0 | 1 | Ref |
| KX349259 | Unassigned | Unassigned | Unassigned | 681_0 | Clustered | 62 | VC_681_0 | 62 | 0.693 | 1 | 0.693 | 0 | 0 | 0 | 1 | Ref |
| KX349260 | Unassigned | Unassigned | Unassigned | 681_0 | Clustered | 62 | VC_681_0 | 62 | 0.693 | 1 | 0.693 | 0 | 0 | 0 | 1 | Ref |

| | | | | | | | | | | | | | | | | |
|---|---|---|---|---|---|---|---|---|---|---|---|---|---|---|---|---|
| KX349261 | Unassigned | Unassigned | Unassigned | 681_0 | Clustered | 62 | VC_681_0 | 62 | 0.693 | 1 | 0.693 | 0 | 0 | 0 | 1 | Ref |
| KX349262 | Unassigned | Unassigned | Unassigned | 681_0 | Clustered | 62 | VC_681_0 | 62 | 0.693 | 1 | 0.693 | 0 | 0 | 0 | 1 | Ref |
| KX349263 | Unassigned | Unassigned | Unassigned | 681_0 | Clustered | 62 | VC_681_0 | 62 | 0.693 | 1 | 0.693 | 0 | 0 | 0 | 1 | Ref |
| KX349264 | Unassigned | Unassigned | Unassigned | 681_0 | Clustered | 62 | VC_681_0 | 62 | 0.693 | 1 | 0.693 | 0 | 0 | 0 | 1 | Ref |
| KX349265 | Unassigned | Unassigned | Unassigned | 681_0 | Clustered | 62 | VC_681_0 | 62 | 0.693 | 1 | 0.693 | 0 | 0 | 0 | 1 | Ref |
| KX349266 | Unassigned | Unassigned | Unassigned | 681_0 | Clustered | 62 | VC_681_0 | 62 | 0.693 | 1 | 0.693 | 0 | 0 | 0 | 1 | Ref |
| KX349267 | Unassigned | Unassigned | Unassigned | 681_0 | Clustered | 62 | VC_681_0 | 62 | 0.693 | 1 | 0.693 | 0 | 0 | 0 | 1 | Ref |
| KX349268 | Unassigned | Unassigned | Unassigned | 681_0 | Clustered | 62 | VC_681_0 | 62 | 0.693 | 1 | 0.693 | 0 | 0 | 0 | 1 | Ref |
| KX349269 | Unassigned | Unassigned | Unassigned | 681_0 | Clustered | 62 | VC_681_0 | 62 | 0.693 | 1 | 0.693 | 0 | 0 | 0 | 1 | Ref |
| KX349270 | Unassigned | Unassigned | Unassigned | 681_0 | Clustered | 62 | VC_681_0 | 62 | 0.693 | 1 | 0.693 | 0 | 0 | 0 | 1 | Ref |
| KX349271 | Unassigned | Unassigned | Unassigned | 681_0 | Clustered | 62 | VC_681_0 | 62 | 0.693 | 1 | 0.693 | 0 | 0 | 0 | 1 | Ref |
| KX349272 | Unassigned | Unassigned | Unassigned | 681_0 | Clustered | 62 | VC_681_0 | 62 | 0.693 | 1 | 0.693 | 0 | 0 | 0 | 1 | Ref |
| KX349273 | Unassigned | Unassigned | Unassigned | 681_0 | Clustered | 62 | VC_681_0 | 62 | 0.693 | 1 | 0.693 | 0 | 0 | 0 | 1 | Ref |
| KX349274 | Unassigned | Unassigned | Unassigned | 681_0 | Clustered | 62 | VC_681_0 | 62 | 0.693 | 1 | 0.693 | 0 | 0 | 0 | 1 | Ref |
| KX349275 | Unassigned | Unassigned | Unassigned | 681_0 | Clustered | 62 | VC_681_0 | 62 | 0.693 | 1 | 0.693 | 0 | 0 | 0 | 1 | Ref |
| KX349276 | Unassigned | Unassigned | Unassigned | 681_0 | Clustered | 62 | VC_681_0 | 62 | 0.693 | 1 | 0.693 | 0 | 0 | 0 | 1 | Ref |
| KX349277 | Unassigned | Unassigned | Unassigned | 681_0 | Clustered | 62 | VC_681_0 | 62 | 0.693 | 1 | 0.693 | 0 | 0 | 0 | 1 | Ref |
| KX349278 | Unassigned | Unassigned | Unassigned | 681_0 | Clustered | 62 | VC_681_0 | 62 | 0.693 | 1 | 0.693 | 0 | 0 | 0 | 1 | Ref |
| KX349279 | Unassigned | Unassigned | Unassigned | 681_0 | Clustered | 62 | VC_681_0 | 62 | 0.693 | 1 | 0.693 | 0 | 0 | 0 | 1 | Ref |
| KX349280 | Unassigned | Unassigned | Unassigned | 681_0 | Clustered | 62 | VC_681_0 | 62 | 0.693 | 1 | 0.693 | 0 | 0 | 0 | 1 | Ref |
| KX349281 | Unassigned | Unassigned | Unassigned | 681_0 | Clustered | 62 | VC_681_0 | 62 | 0.693 | 1 | 0.693 | 0 | 0 | 0 | 1 | Ref |
| KX349282 | Unassigned | Unassigned | Unassigned | 681_0 | Clustered | 62 | VC_681_0 | 62 | 0.693 | 1 | 0.693 | 0 | 0 | 0 | 1 | Ref |
| KX349283 | Unassigned | Unassigned | Unassigned | 681_0 | Clustered | 62 | VC_681_0 | 62 | 0.693 | 1 | 0.693 | 0 | 0 | 0 | 1 | Ref |
| KX349284 | Unassigned | Unassigned | Unassigned | 681_0 | Clustered | 62 | VC_681_0 | 62 | 0.693 | 1 | 0.693 | 0 | 0 | 0 | 1 | Ref |
| KX349291 | Unassigned | Unassigned | Unassigned | 447_4 | Clustered | 9 | VC_447_4 | 9 | 0.127 | 1 | 0.127 | 0 | 0 | 0 | 1 | Ref |
| KX349292 | Unassigned | Unassigned | Unassigned | 447_4 | Clustered | 9 | VC_447_4 | 9 | 0.127 | 1 | 0.127 | 0 | 0 | 0 | 1 | Ref |
| KX349293 | Unassigned | Unassigned | Unassigned | 447_4 | Clustered | 9 | VC_447_4 | 9 | 0.127 | 1 | 0.127 | 0 | 0 | 0 | 1 | Ref |
| KX349294 | Unassigned | Unassigned | Unassigned | 447_4 | Clustered | 9 | VC_447_4 | 9 | 0.127 | 1 | 0.127 | 0 | 0 | 0 | 1 | Ref |
| KX349295 | Unassigned | Unassigned | Unassigned | 447_4 | Clustered | 9 | VC_447_4 | 9 | 0.127 | 1 | 0.127 | 0 | 0 | 0 | 1 | Ref |
| KX349296 | Unassigned | Unassigned | Unassigned | 447_4 | Clustered | 9 | VC_447_4 | 9 | 0.127 | 1 | 0.127 | 0 | 0 | 0 | 1 | Ref |
| KX349297 | Unassigned | Unassigned | Unassigned | 447_4 | Clustered | 9 | VC_447_4 | 9 | 0.127 | 1 | 0.127 | 0 | 0 | 0 | 1 | Ref |
| KX349899 | Unassigned | Unassigned | Unassigned | 843_0 | Clustered | 20 | VC_843_0 | 20 | 0.9621 | 1 | 0.9621 | 0 | 0 | 0 | 1 | Ref |
| KX349900 | Unassigned | Unassigned | Unassigned | 843_0 | Clustered | 20 | VC_843_0 | 20 | 0.9621 | 1 | 0.9621 | 0 | 0 | 0 | 1 | Ref |
| KX349901 | Unassigned | Unassigned | Unassigned | 843_0 | Clustered | 20 | VC_843_0 | 20 | 0.9621 | 1 | 0.9621 | 0 | 0 | 0 | 1 | Ref |
| KX349902 | Unassigned | Unassigned | Unassigned | 913_0 | Clustered | 28 | VC_913_0 | 28 | 0.6206 | 1 | 0.6206 | 0 | 0 | 0 | 0.9841 | Ref |
| KX349903 | Unassigned | Unassigned | Unassigned | 913_0 | Clustered | 28 | VC_913_0 | 28 | 0.6206 | 1 | 0.6206 | 0 | 0 | 0 | 0.9841 | Ref |
| KX365747 | Unassigned | Unassigned | Unassigned | 119_0 | Clustered | 9 | VC_119_0 | 9 | 0.9977 | 0.98988637 | 0.9876 | 0 | 0 | 0 | 1 | Ref |
| KX365748 | Unassigned | Unassigned | Unassigned | 926_0 | Clustered | 2 | VC_926_0 | 2 | 0.2769 | 0.94251254 | 0.261 | 0 | 0 | 0 | 1 | Ref |
| KX369583 | Unassigned | Unassigned | Unassigned | 579_23 | Clustered/Singleton | 1 | VC_579_23 | 1 | 0 | 1 | 0 | 0 | 0 | 0 | 1 | Ref |
| KX369584 | Unassigned | Unassigned | Unassigned | 335_29 | Clustered/Singleton | 1 | VC_335_29 | 1 | 0 | 1 | 0 | 0 | 0 | 0 | 1 | Ref |
| KX369585 | Unassigned | Unassigned | Unassigned | 384_16 | Clustered | 42 | VC_384_16 | 42 | 0.134 | 1 | 0.134 | 0 | 0 | 0 | 0.7282 | Ref |
| KX369586 | Unassigned | Unassigned | Unassigned | 335_4 | Clustered/Singleton | 1 | VC_335_4 | 1 | 0 | 1 | 0 | 0 | 0 | 0 | 1 | Ref |
| KX373685 | Unassigned | Unassigned | Unassigned | 706_0 | Clustered | 52 | VC_706_0 | 52 | 0.9787 | 1 | 0.9787 | 0 | 0 | 0 | 1 | Ref |
| KX373686 | Unassigned | Unassigned | Unassigned | 706_0 | Clustered | 52 | VC_706_0 | 52 | 0.9787 | 1 | 0.9787 | 0 | 0 | 0 | 1 | Ref |
| KX373687 | Unassigned | Unassigned | Unassigned | 706_0 | Clustered | 52 | VC_706_0 | 52 | 0.9787 | 1 | 0.9787 | 0 | 0 | 0 | 1 | Ref |
| KX373688 | Unassigned | Unassigned | Unassigned | 706_0 | Clustered | 52 | VC_706_0 | 52 | 0.9787 | 1 | 0.9787 | 0 | 0 | 0 | 1 | Ref |
| KX373689 | Unassigned | Unassigned | Unassigned | 706_0 | Clustered | 52 | VC_706_0 | 52 | 0.9787 | 1 | 0.9787 | 0 | 0 | 0 | 1 | Ref |
| KX373690 | Unassigned | Unassigned | Unassigned | 706_0 | Clustered | 52 | VC_706_0 | 52 | 0.9787 | 1 | 0.9787 | 0 | 0 | 0 | 1 | Ref |
| KX373691 | Unassigned | Unassigned | Unassigned | 706_0 | Clustered | 52 | VC_706_0 | 52 | 0.9787 | 1 | 0.9787 | 0 | 0 | 0 | 1 | Ref |
| KX373692 | Unassigned | Unassigned | Unassigned | 706_0 | Clustered | 52 | VC_706_0 | 52 | 0.9787 | 1 | 0.9787 | 0 | 0 | 0 | 1 | Ref |
| KX375815 | Unassigned | Unassigned | Unassigned | 230_0 | Clustered | 38 | VC_230_0 | 38 | 0.9076 | 1 | 0.9076 | 0 | 0 | 0 | 1 | Ref |
| KX377933 | Unassigned | Unassigned | Unassigned | 340_3 | Clustered | 24 | VC_340_3 | 24 | 0.1205 | 1 | 0.1205 | 0 | 0 | 0 | 0.7283 | Ref |
| KX379667 | Unassigned | Unassigned | Unassigned | 210_0 | Clustered | 105 | VC_210_0 | 105 | 0.4076 | 1 | 0.4076 | 0 | 0 | 0 | 0.7766 | Ref |
| KX379668 | Unassigned | Unassigned | Unassigned | 210_0 | Clustered | 105 | VC_210_0 | 105 | 0.4076 | 1 | 0.4076 | 0 | 0 | 0 | 0.7766 | Ref |
| KX379670 | Unassigned | Unassigned | Unassigned | 210_0 | Clustered | 105 | VC_210_0 | 105 | 0.4076 | 1 | 0.4076 | 0 | 0 | 0 | 0.7766 | Ref |
| KX379671 | Unassigned | Unassigned | Unassigned | 210_0 | Clustered | 105 | VC_210_0 | 105 | 0.4076 | 1 | 0.4076 | 0 | 0 | 0 | 0.7766 | Ref |
| KX397364 | Unassigned | Unassigned | Unassigned | 994_4 | Clustered | 3 | VC_994_4 | 3 | 0.0236 | 0.75814415 | 0.0179 | 0 | 0 | 0 | 1 | Ref |
| KX397365 | Unassigned | Unassigned | Unassigned | 994_2 | Clustered | 5 | VC_994_2 | 5 | 0.0483 | 0.88370509 | 0.0427 | 0 | 0 | 0 | 1 | Ref |
| KX397366 | Unassigned | Unassigned | Unassigned | 994_2 | Clustered | 5 | VC_994_2 | 5 | 0.0483 | 0.88370509 | 0.0427 | 0 | 0 | 0 | 1 | Ref |
| KX397367 | Unassigned | Unassigned | Unassigned | 994_0 | Clustered | 5 | VC_994_0 | 5 | 0.0495 | 0.99983811 | 0.0494 | 0 | 0 | 0 | 1 | Ref |
| KX397368 | Unassigned | Unassigned | Unassigned | 994_1 | Clustered | 8 | VC_994_1 | 8 | 0.0877 | 0.92136649 | 0.0808 | 0 | 0 | 0 | 1 | Ref |
| KX397369 | Unassigned | Unassigned | Unassigned | 994_3 | Clustered | 3 | VC_994_3 | 3 | 0.0236 | 0.75215638 | 0.0178 | 0 | 0 | 0 | 1 | Ref |
| KX397370 | Unassigned | Unassigned | Unassigned | 994_1 | Clustered | 8 | VC_994_1 | 8 | 0.0877 | 0.92136649 | 0.0808 | 0 | 0 | 0 | 1 | Ref |
| KX397371 | Unassigned | Unassigned | Unassigned | 994_1 | Clustered | 8 | VC_994_1 | 8 | 0.0877 | 0.92136649 | 0.0808 | 0 | 0 | 0 | 1 | Ref |
| KX397372 | Unassigned | Unassigned | Unassigned | 994_0 | Clustered | 5 | VC_994_0 | 5 | 0.0495 | 0.99983811 | 0.0494 | 0 | 0 | 0 | 1 | Ref |
| KX397373 | Unassigned | Unassigned | Unassigned | 994_4 | Clustered | 3 | VC_994_4 | 3 | 0.0236 | 0.75814415 | 0.0179 | 0 | 0 | 0 | 1 | Ref |
| KX405002 | Unassigned | Unassigned | Unassigned | 654_0 | Clustered | 115 | VC_654_0 | 115 | 0.7303 | 1 | 0.7303 | 0 | 0 | 0 | 0.8764 | Ref |
| KX405003 | Unassigned | Unassigned | Unassigned | 340_16 | Clustered | 4 | VC_340_16 | 4 | 0.0157 | 0.9999807 | 0.0157 | 0 | 0 | 0 | 1 | Ref |
| KX431559 | Unassigned | Unassigned | Unassigned | 687_0 | Clustered | 21 | VC_687_0 | 21 | 0.3608 | 1 | 0.3608 | 0 | 0 | 0 | 1 | Ref |
| KX431560 | Unassigned | Unassigned | Unassigned | 687_0 | Clustered | 21 | VC_687_0 | 21 | 0.3608 | 1 | 0.3608 | 0 | 0 | 0 | 1 | Ref |
| KX431888 | Unassigned | Unassigned | Unassigned | 132_0 | Clustered | 19 | VC_132_0 | 19 | 0.6051 | 1 | 0.6051 | 0 | 0 | 0 | 1 | Ref |
| KX438380 | Unassigned | Unassigned | Unassigned | 135_14 | Clustered | 3 | VC_135_14 | 3 | 0.0101 | 0.96146265 | 0.0097 | 0 | 0 | 0 | 1 | Ref |
| KX443326 | Unassigned | Unassigned | Unassigned | 783_0 | Clustered | 67 | VC_783_0 | 67 | 0.8945 | 1 | 0.8945 | 0 | 0 | 0 | 1 | Ref |
| KX443552 | Unassigned | Unassigned | Unassigned | 687_0 | Clustered | 21 | VC_687_0 | 21 | 0.3608 | 1 | 0.3608 | 0 | 0 | 0 | 1 | Ref |
| KX443695 | Unassigned | Unassigned | Unassigned | 985_0 | Clustered | 38 | VC_985_0 | 38 | 0.9334 | 1 | 0.9334 | 0 | 0 | 0 | 1 | Ref |
| KX443696 | Unassigned | Unassigned | Unassigned | 526_0 | Clustered | 32 | VC_526_0 | 32 | 0.9037 | 1 | 0.9037 | 0 | 0 | 0 | 1 | Ref |
| KX449361 | Unassigned | Unassigned | Unassigned | 132_0 | Clustered | 19 | VC_132_0 | 19 | 0.6051 | 1 | 0.6051 | 0 | 0 | 0 | 1 | Ref |
| KX449362 | Unassigned | Unassigned | Unassigned | 132_0 | Clustered | 19 | VC_132_0 | 19 | 0.6051 | 1 | 0.6051 | 0 | 0 | 0 | 1 | Ref |
| KX449363 | Unassigned | Unassigned | Unassigned | 132_0 | Clustered | 19 | VC_132_0 | 19 | 0.6051 | 1 | 0.6051 | 0 | 0 | 0 | 1 | Ref |
| KX452695 | Unassigned | Unassigned | Unassigned | 601_3 | Clustered | 12 | VC_601_3 | 12 | 0.2339 | 1 | 0.2339 | 0 | 0 | 0 | 0.8636 | Ref |
| KX452696 | Unassigned | Unassigned | Unassigned | 398_0 | Clustered | 51 | VC_398_0 | 51 | 0.8741 | 1 | 0.8741 | 0 | 0 | 0 | 1 | Ref |

| | | | | | | | | | | | | | | | | |
|---|---|---|---|---|---|---|---|---|---|---|---|---|---|---|---|---|
| KX452697 | Unassigned | Unassigned | Unassigned | 134_1 | Clustered | 4 | VC_134_1 | 4 | 0.0354 | 0.99998777 | 0.0354 | 0 | 0 | 0 | 1 | Ref |
| KX452698 | Unassigned | Unassigned | Unassigned | 290_143 | Clustered/Singleton | 1 | VC_290_143 | 1 | 0 | 1 | 0 | 0 | 0 | 0 | 1 | Ref |
| KX452699 | Unassigned | Unassigned | Unassigned | 611_0 | Clustered | 50 | VC_611_0 | 50 | 0.7306 | 1 | 0.7306 | 0 | 0 | 0 | 1 | Ref |
| KX452700 | Unassigned | Unassigned | Unassigned | 611_0 | Clustered | 50 | VC_611_0 | 50 | 0.7306 | 1 | 0.7306 | 0 | 0 | 0 | 1 | Ref |
| KX455876 | Unassigned | Unassigned | Unassigned | 1233_0 | Clustered | 9 | VC_1233_0 | 9 | 0.9676 | 1 | 0.9676 | 0 | 0 | 0 | 1 | Ref |
| KX456206 | Unassigned | Unassigned | Unassigned | 271_0 | Clustered | 12 | VC_271_0 | 12 | 0.4255 | 1 | 0.4255 | 0 | 0 | 0 | 1 | Ref |
| KX456207 | Unassigned | Unassigned | Unassigned | 268_0 | Clustered | 10 | VC_268_0 | 10 | 0.1417 | 1 | 0.1417 | 0 | 0 | 0 | 0.9333 | Ref |
| KX456208 | Unassigned | Unassigned | Unassigned | 271_0 | Clustered | 12 | VC_271_0 | 12 | 0.4255 | 1 | 0.4255 | 0 | 0 | 0 | 1 | Ref |
| KX456209 | Unassigned | Unassigned | Unassigned | 271_0 | Clustered | 12 | VC_271_0 | 12 | 0.4255 | 1 | 0.4255 | 0 | 0 | 0 | 1 | Ref |
| KX456210 | Unassigned | Unassigned | Unassigned | 271_0 | Clustered | 12 | VC_271_0 | 12 | 0.4255 | 1 | 0.4255 | 0 | 0 | 0 | 1 | Ref |
| KX456211 | Unassigned | Unassigned | Unassigned | 268_0 | Clustered | 10 | VC_268_0 | 10 | 0.1417 | 1 | 0.1417 | 0 | 0 | 0 | 0.9333 | Ref |
| KX456212 | Unassigned | Unassigned | Unassigned | 268_0 | Clustered | 10 | VC_268_0 | 10 | 0.1417 | 1 | 0.1417 | 0 | 0 | 0 | 0.9333 | Ref |
| KX456213 | Unassigned | Unassigned | Unassigned | 271_0 | Clustered | 12 | VC_271_0 | 12 | 0.4255 | 1 | 0.4255 | 0 | 0 | 0 | 1 | Ref |
| KX458236 | Unassigned | Unassigned | Unassigned | 535_1 | Clustered | 108 | VC_535_1 | 108 | 0.8479 | 1 | 0.8479 | 0 | 0 | 0 | 0.9955 | Ref |
| KX458237 | Unassigned | Unassigned | Unassigned | 532_0 | Clustered | 75 | VC_532_0 | 75 | 0.9393 | 1 | 0.9393 | 0 | 0 | 0 | 1 | Ref |
| KX467643 | Unassigned | Unassigned | Unassigned | 361_0 | Clustered | 7 | VC_361_0 | 7 | 0.9353 | 0.99999998 | 0.9353 | 0 | 0 | 0 | 1 | Ref |
| KX495186 | Unassigned | Unassigned | Unassigned | 195_3 | Clustered | 9 | VC_195_3 | 9 | 0.1955 | 1 | 0.1955 | 0 | 0 | 0 | 0.8611 | Ref |
| KX507045 | Unassigned | Unassigned | Unassigned | 661_3 | Clustered | 9 | VC_661_3 | 9 | 0.2124 | 1 | 0.2124 | 0 | 0 | 0 | 0.6944 | Ref |
| KX507046 | Unassigned | Unassigned | Unassigned | 511_11 | Clustered/Singleton | 1 | VC_511_11 | 1 | 0 | 1 | 0 | 0 | 0 | 0 | 1 | Ref |
| KX507343 | Unassigned | Unassigned | Unassigned | 1016_0 | Clustered | 62 | VC_1016_0 | 62 | 0.8638 | 1 | 0.8638 | 0 | 0 | 0 | 0.743 | Ref |
| KX507344 | Unassigned | Unassigned | Unassigned | 1016_0 | Clustered | 62 | VC_1016_0 | 62 | 0.8638 | 1 | 0.8638 | 0 | 0 | 0 | 0.743 | Ref |
| KX507345 | Unassigned | Unassigned | Unassigned | 1016_0 | Clustered | 62 | VC_1016_0 | 62 | 0.8638 | 1 | 0.8638 | 0 | 0 | 0 | 0.743 | Ref |
| KX507361 | Unassigned | Unassigned | Unassigned | 917_0 | Clustered | 28 | VC_917_0 | 28 | 0.5465 | 1 | 0.5465 | 0 | 0 | 0 | 1 | Ref |
| KX507362 | Unassigned | Unassigned | Unassigned | 532_0 | Clustered | 75 | VC_532_0 | 75 | 0.9393 | 1 | 0.9393 | 0 | 0 | 0 | 1 | Ref |
| KX513864 | Unassigned | Unassigned | Unassigned | 1166_0 | Clustered | 2 | VC_1166_0 | 2 | 0.3171 | 0.93270267 | 0.2957 | 0 | 0 | 0 | 1 | Ref |
| KX513865 | Unassigned | Unassigned | Unassigned | 1145_0 | Clustered | 164 | VC_1145_0 | 164 | 0.8868 | 1 | 0.8868 | 0 | 0 | 0 | 1 | Ref |
| KX513867 | Unassigned | Unassigned | Unassigned | 1184_0 | Clustered | 10 | VC_1184_0 | 10 | 0.7506 | 1 | 0.7506 | 0 | 0 | 0 | 1 | Ref |
| KX513868 | Unassigned | Unassigned | Unassigned | 1170_0 | Clustered | 19 | VC_1170_0 | 19 | 0.7497 | 1 | 0.7497 | 0 | 0 | 0 | 1 | Ref |
| KX513871 | Unassigned | Unassigned | Unassigned | 1235_0 | Clustered | 3 | VC_1235_0 | 3 | 0.1401 | 0.98976974 | 0.1387 | 0 | 0 | 0 | 1 | Ref |
| KX513872 | Unassigned | Unassigned | Unassigned | 1184_0 | Clustered | 10 | VC_1184_0 | 10 | 0.7506 | 1 | 0.7506 | 0 | 0 | 0 | 1 | Ref |
| KX513873 | Unassigned | Unassigned | Unassigned | 1165_0 | Clustered | 10 | VC_1165_0 | 10 | 0.9263 | 1 | 0.9263 | 0 | 0 | 0 | 1 | Ref |
| KX513874 | Unassigned | Unassigned | Unassigned | 1184_0 | Clustered | 10 | VC_1184_0 | 10 | 0.7506 | 1 | 0.7506 | 0 | 0 | 0 | 1 | Ref |
| KX513875 | Unassigned | Unassigned | Unassigned | 1241_0 | Clustered | 13 | VC_1241_0 | 13 | 0.8675 | 0.76044592 | 0.6597 | 0 | 0 | 0 | 1 | Ref |
| KX513877 | Unassigned | Unassigned | Unassigned | 1170_0 | Clustered | 19 | VC_1170_0 | 19 | 0.7497 | 1 | 0.7497 | 0 | 0 | 0 | 1 | Ref |
| KX522565 | Unassigned | Unassigned | Unassigned | 23_0 | Clustered | 11 | VC_23_0 | 11 | 0.5144 | 1 | 0.5144 | 0 | 0 | 0 | 1 | Ref |
| KX522649 | Unassigned | Unassigned | Unassigned | 335_21 | Clustered | 5 | VC_335_21 | 5 | 0.0208 | 0.99999976 | 0.0208 | 0 | 0 | 0 | 0.8 | Ref |
| KX522650 | Unassigned | Unassigned | Unassigned | 1117_0 | Clustered | 19 | VC_1117_0 | 19 | 0.9177 | 1 | 0.9177 | 0 | 0 | 0 | 1 | Ref |
| KX522943 | Unassigned | Unassigned | Unassigned | 335_21 | Clustered | 5 | VC_335_21 | 5 | 0.0208 | 0.99999976 | 0.0208 | 0 | 0 | 0 | 0.8 | Ref |
| KX523125 | Unassigned | Unassigned | Unassigned | 532_0 | Clustered | 75 | VC_532_0 | 75 | 0.9393 | 1 | 0.9393 | 0 | 0 | 0 | 1 | Ref |
| KX523699 | Unassigned | Unassigned | Unassigned | 475_0 | Clustered | 96 | VC_475_0 | 96 | 0.9303 | 1 | 0.9303 | 0 | 0 | 0 | 0.9575 | Ref |
| KX532239 | Unassigned | Unassigned | Unassigned | 159_0 | Clustered | 25 | VC_159_0 | 25 | 0.7699 | 1 | 0.7699 | 0 | 0 | 0 | 1 | Ref |
| KX534004 | Unassigned | Unassigned | Unassigned | 783_0 | Clustered | 67 | VC_783_0 | 67 | 0.8945 | 1 | 0.8945 | 0 | 0 | 0 | 1 | Ref |
| KX534335 | Unassigned | Unassigned | Unassigned | 859_0 | Clustered | 67 | VC_859_0 | 67 | 0.9622 | 1 | 0.9622 | 0 | 0 | 0 | 1 | Ref |
| KX534336 | Unassigned | Unassigned | Unassigned | 859_0 | Clustered | 67 | VC_859_0 | 67 | 0.9622 | 1 | 0.9622 | 0 | 0 | 0 | 1 | Ref |
| KX534337 | Unassigned | Unassigned | Unassigned | 819_0 | Clustered | 19 | VC_819_0 | 19 | 0.6835 | 1 | 0.6835 | 0 | 0 | 0 | 1 | Ref |
| KX534338 | Unassigned | Unassigned | Unassigned | 859_0 | Clustered | 67 | VC_859_0 | 67 | 0.9622 | 1 | 0.9622 | 0 | 0 | 0 | 1 | Ref |
| KX534339 | Unassigned | Unassigned | Unassigned | 859_0 | Clustered | 67 | VC_859_0 | 67 | 0.9622 | 1 | 0.9622 | 0 | 0 | 0 | 1 | Ref |
| KX534340 | Unassigned | Unassigned | Unassigned | 20_0 | Clustered | 5 | VC_20_0 | 5 | 0.4268 | 0.9999994 | 0.4268 | 0 | 0 | 0 | 1 | Ref |
| KX534341 | Unassigned | Unassigned | Unassigned | 859_0 | Clustered | 67 | VC_859_0 | 67 | 0.9622 | 1 | 0.9622 | 0 | 0 | 0 | 1 | Ref |
| KX550065 | Unassigned | Unassigned | Unassigned | 425_0 | Clustered | 40 | VC_425_0 | 40 | 0.8399 | 1 | 0.8399 | 0 | 0 | 0 | 0.9974 | Ref |
| KX550082 | Unassigned | Unassigned | Unassigned | 991_0 | Clustered | 39 | VC_991_0 | 39 | 1 | 1 | 1 | 0 | 0 | 0 | 1 | Ref |
| KX550441 | Unassigned | Unassigned | Unassigned | 535_1 | Clustered | 108 | VC_535_1 | 108 | 0.8479 | 1 | 0.8479 | 0 | 0 | 0 | 0.9955 | Ref |
| KX550442 | Unassigned | Unassigned | Unassigned | 535_0 | Clustered | 18 | VC_535_0 | 18 | 0.1595 | 1 | 0.1595 | 0 | 0 | 0 | 1 | Ref |
| KX550443 | Unassigned | Unassigned | Unassigned | 335_56 | Clustered | 7 | VC_335_56 | 7 | 0.0242 | 1 | 0.0242 | 0 | 0 | 0 | 0.9048 | Ref |
| KX552041 | Unassigned | Unassigned | Unassigned | 871_1 | Clustered | 26 | VC_871_1 | 26 | 0.6303 | 1 | 0.6303 | 0 | 0 | 0 | 0.9815 | Ref |
| KX555527 | Unassigned | Unassigned | Unassigned | 888_0 | Clustered | 9 | VC_888_0 | 9 | 0.5251 | 1 | 0.5251 | 0 | 0 | 0 | 1 | Ref |
| KX557234 | Unassigned | Unassigned | Unassigned | 535_1 | Clustered | 108 | VC_535_1 | 108 | 0.8479 | 1 | 0.8479 | 0 | 0 | 0 | 0.9955 | Ref |
| KX557272 | Unassigned | Unassigned | Unassigned | 1224_0 | Clustered | 4 | VC_1224_0 | 4 | 0.2813 | 0.99997676 | 0.2813 | 0 | 0 | 0 | 1 | Ref |
| KX557273 | Unassigned | Unassigned | Unassigned | 543_0 | Clustered | 25 | VC_543_0 | 25 | 0.4584 | 1 | 0.4584 | 0 | 0 | 0 | 1 | Ref |
| KX557274 | Unassigned | Unassigned | Unassigned | 1217_0 | Clustered | 41 | VC_1217_0 | 41 | 0.9546 | 1 | 0.9546 | 0 | 0 | 0 | 0.9976 | Ref |
| KX557275 | Unassigned | Unassigned | Unassigned | 1217_0 | Clustered | 41 | VC_1217_0 | 41 | 0.9546 | 1 | 0.9546 | 0 | 0 | 0 | 0.9976 | Ref |
| KX557276 | Unassigned | Unassigned | Unassigned | 1223_1 | Clustered | 10 | VC_1223_1 | 10 | 0.5795 | 1 | 0.5795 | 0 | 0 | 0 | 1 | Ref |
| KX557278 | Unassigned | Unassigned | Unassigned | 1201_0 | Clustered | 2 | VC_1201_0 | 2 | 0.0266 | 0.95737687 | 0.0254 | 0 | 0 | 0 | 1 | Ref |
| KX557279 | Unassigned | Unassigned | Unassigned | 1218_0 | Clustered | 14 | VC_1218_0 | 14 | 0.7811 | 1 | 0.7811 | 0 | 0 | 0 | 0.989 | Ref |
| KX557280 | Unassigned | Unassigned | Unassigned | 231_0 | Clustered | 19 | VC_231_0 | 19 | 0.9378 | 1 | 0.9378 | 0 | 0 | 0 | 1 | Ref |
| KX557281 | Unassigned | Unassigned | Unassigned | 894_0 | Clustered | 6 | VC_894_0 | 6 | 0.3171 | 1 | 0.3171 | 0 | 0 | 0 | 1 | Ref |
| KX557282 | Unassigned | Unassigned | Unassigned | 1226_0 | Clustered | 5 | VC_1226_0 | 5 | 0.6594 | 0.99999982 | 0.6594 | 0 | 0 | 0 | 1 | Ref |
| KX557283 | Unassigned | Unassigned | Unassigned | 231_0 | Clustered | 19 | VC_231_0 | 19 | 0.9378 | 1 | 0.9378 | 0 | 0 | 0 | 1 | Ref |
| KX557284 | Unassigned | Unassigned | Unassigned | 231_0 | Clustered | 19 | VC_231_0 | 19 | 0.9378 | 1 | 0.9378 | 0 | 0 | 0 | 1 | Ref |
| KX557285 | Unassigned | Unassigned | Unassigned | 590_0 | Clustered | 9 | VC_590_0 | 9 | 0.4952 | 1 | 0.4952 | 0 | 0 | 0 | 1 | Ref |
| KX557286 | Unassigned | Unassigned | Unassigned | 1219_0 | Clustered | 24 | VC_1219_0 | 24 | 0.9519 | 1 | 0.9519 | 0 | 0 | 0 | 1 | Ref |
| KX557287 | Unassigned | Unassigned | Unassigned | 543_0 | Clustered | 25 | VC_543_0 | 25 | 0.4584 | 1 | 0.4584 | 0 | 0 | 0 | 1 | Ref |
| KX574454 | Unassigned | Unassigned | Unassigned | 335_21 | Clustered | 5 | VC_335_21 | 5 | 0.0208 | 0.99999976 | 0.0208 | 0 | 0 | 0 | 0.8 | Ref |
| KX574455 | Unassigned | Unassigned | Unassigned | 222_1 | Clustered | 19 | VC_222_1 | 19 | 0.4166 | 1 | 0.4166 | 0 | 0 | 0 | 1 | Ref |
| KX576638 | Unassigned | Unassigned | Unassigned | 230_0 | Clustered | 38 | VC_230_0 | 38 | 0.9076 | 1 | 0.9076 | 0 | 0 | 0 | 1 | Ref |
| KX576639 | Unassigned | Unassigned | Unassigned | 223_6 | Clustered | 42 | VC_223_6 | 42 | 0.4717 | 1 | 0.4717 | 0 | 0 | 0 | 0.8351 | Ref |
| KX576640 | Unassigned | Unassigned | Unassigned | 1200_0 | Clustered | 73 | VC_1200_0 | 73 | 0.9825 | 1 | 0.9825 | 0 | 0 | 0 | 1 | Ref |
| KX576641 | Unassigned | Unassigned | Unassigned | 1200_0 | Clustered | 73 | VC_1200_0 | 73 | 0.9825 | 1 | 0.9825 | 0 | 0 | 0 | 1 | Ref |
| KX576642 | Unassigned | Unassigned | Unassigned | 985_0 | Clustered | 38 | VC_985_0 | 38 | 0.9334 | 1 | 0.9334 | 0 | 0 | 0 | 1 | Ref |
| KX576643 | Unassigned | Unassigned | Unassigned | 384_18 | Clustered | 10 | VC_384_18 | 10 | 0.0301 | 1 | 0.0301 | 0 | 0 | 0 | 0.8 | Ref |
| KX576644 | Unassigned | Unassigned | Unassigned | 538_77 | Clustered | 3 | VC_538_77 | 3 | 0.0216 | 0.99859911 | 0.0216 | 0 | 0 | 0 | 1 | Ref |

| | | | | | | | | | | | | | | | | |
|---|---|---|---|---|---|---|---|---|---|---|---|---|---|---|---|---|
| KX576645 | Unassigned | Unassigned | Unassigned | 384_13 | Clustered | 33 | VC_384_13 | 33 | 0.1785 | 1 | 0.1785 | 0 | 0 | 0 | 0.8769 | Ref |
| KX576646 | Unassigned | Unassigned | Unassigned | 384_13 | Clustered | 33 | VC_384_13 | 33 | 0.1785 | 1 | 0.1785 | 0 | 0 | 0 | 0.8769 | Ref |
| KX576647 | Unassigned | Unassigned | Unassigned | 384_13 | Clustered | 33 | VC_384_13 | 33 | 0.1785 | 1 | 0.1785 | 0 | 0 | 0 | 0.8769 | Ref |
| KX578042 | Unassigned | Unassigned | Unassigned | 889_0 | Clustered | 13 | VC_889_0 | 13 | 0.6844 | 1 | 0.6844 | 0 | 0 | 0 | 1 | Ref |
| KX578043 | Unassigned | Unassigned | Unassigned | 889_0 | Clustered | 13 | VC_889_0 | 13 | 0.6844 | 1 | 0.6844 | 0 | 0 | 0 | 1 | Ref |
| KX578044 | Unassigned | Unassigned | Unassigned | 889_0 | Clustered | 13 | VC_889_0 | 13 | 0.6844 | 1 | 0.6844 | 0 | 0 | 0 | 1 | Ref |
| KX578071 | Unassigned | Unassigned | Unassigned | 384_15 | Clustered | 44 | VC_384_15 | 44 | 0.139 | 1 | 0.139 | 0 | 0 | 0 | 0.7474 | Ref |
| KX579975 | Unassigned | Unassigned | Unassigned | 535_0 | Clustered | 18 | VC_535_0 | 18 | 0.1595 | 1 | 0.1595 | 0 | 0 | 0 | 1 | Ref |
| KX580961 | Unassigned | Unassigned | Unassigned | 917_0 | Clustered | 28 | VC_917_0 | 28 | 0.5465 | 1 | 0.5465 | 0 | 0 | 0 | 1 | Ref |
| KX580962 | Unassigned | Unassigned | Unassigned | 917_0 | Clustered | 28 | VC_917_0 | 28 | 0.5465 | 1 | 0.5465 | 0 | 0 | 0 | 1 | Ref |
| KX581090 | Unassigned | Unassigned | Unassigned | 306_0 | Clustered | 24 | VC_306_0 | 24 | 0.9902 | 1 | 0.9902 | 0 | 0 | 0 | 1 | Ref |
| KX581091 | Unassigned | Unassigned | Unassigned | 306_0 | Clustered | 24 | VC_306_0 | 24 | 0.9902 | 1 | 0.9902 | 0 | 0 | 0 | 1 | Ref |
| KX581092 | Unassigned | Unassigned | Unassigned | 306_0 | Clustered | 24 | VC_306_0 | 24 | 0.9902 | 1 | 0.9902 | 0 | 0 | 0 | 1 | Ref |
| KX581093 | Unassigned | Unassigned | Unassigned | 306_0 | Clustered | 24 | VC_306_0 | 24 | 0.9902 | 1 | 0.9902 | 0 | 0 | 0 | 1 | Ref |
| KX581094 | Unassigned | Unassigned | Unassigned | 306_0 | Clustered | 24 | VC_306_0 | 24 | 0.9902 | 1 | 0.9902 | 0 | 0 | 0 | 1 | Ref |
| KX581095 | Unassigned | Unassigned | Unassigned | 306_0 | Clustered | 24 | VC_306_0 | 24 | 0.9902 | 1 | 0.9902 | 0 | 0 | 0 | 1 | Ref |
| KX581096 | Unassigned | Unassigned | Unassigned | 306_0 | Clustered | 24 | VC_306_0 | 24 | 0.9902 | 1 | 0.9902 | 0 | 0 | 0 | 1 | Ref |
| KX581097 | Unassigned | Unassigned | Unassigned | 306_0 | Clustered | 24 | VC_306_0 | 24 | 0.9902 | 1 | 0.9902 | 0 | 0 | 0 | 1 | Ref |
| KX581098 | Unassigned | Unassigned | Unassigned | 306_0 | Clustered | 24 | VC_306_0 | 24 | 0.9902 | 1 | 0.9902 | 0 | 0 | 0 | 1 | Ref |
| KX581099 | Unassigned | Unassigned | Unassigned | 306_0 | Clustered | 24 | VC_306_0 | 24 | 0.9902 | 1 | 0.9902 | 0 | 0 | 0 | 1 | Ref |
| KX581100 | Unassigned | Unassigned | Unassigned | 306_0 | Clustered | 24 | VC_306_0 | 24 | 0.9902 | 1 | 0.9902 | 0 | 0 | 0 | 1 | Ref |
| KX585251 | Unassigned | Unassigned | Unassigned | 245_0 | Clustered | 17 | VC_245_0 | 17 | 0.6081 | 1 | 0.6081 | 0 | 0 | 0 | 1 | Ref |
| KX585252 | Unassigned | Unassigned | Unassigned | 232_0 | Clustered | 32 | VC_232_0 | 32 | 0.8471 | 1 | 0.8471 | 0 | 0 | 0 | 0.996 | Ref |
| KX585253 | Unassigned | Unassigned | Unassigned | 243_0 | Clustered | 72 | VC_243_0 | 72 | 0.8089 | 1 | 0.8089 | 0 | 0 | 0 | 1 | Ref |
| KX587949 | Unassigned | Unassigned | Unassigned | 113_0 | Clustered | 91 | VC_113_0 | 91 | 0.9584 | 1 | 0.9584 | 0 | 0 | 0 | 1 | Ref |
| KX588251 | Unassigned | Unassigned | Unassigned | 783_0 | Clustered | 67 | VC_783_0 | 67 | 0.8945 | 1 | 0.8945 | 0 | 0 | 0 | 1 | Ref |
| KX589269 | Unassigned | Unassigned | Unassigned | 385_0 | Clustered | 24 | VC_385_0 | 24 | 0.5516 | 1 | 0.5516 | 0 | 0 | 0 | 1 | Ref |
| KX591654 | Unassigned | Unassigned | Unassigned | 848_0 | Clustered | 124 | VC_848_0 | 124 | 0.9484 | 1 | 0.9484 | 0 | 0 | 0 | 1 | Ref |
| KX592589 | Unassigned | Unassigned | Unassigned | 384_11 | Clustered | 31 | VC_384_11 | 31 | 0.0926 | 1 | 0.0926 | 0 | 0 | 0 | 0.7011 | Ref |
| KX607101 | Unassigned | Unassigned | Unassigned | 461_0 | Clustered | 3 | VC_461_0 | 3 | 0.8247 | 0.99509929 | 0.8207 | 0 | 0 | 0 | 1 | Ref |
| KX607102 | Unassigned | Unassigned | Unassigned | 461_0 | Clustered | 3 | VC_461_0 | 3 | 0.8247 | 0.99509929 | 0.8207 | 0 | 0 | 0 | 1 | Ref |
| KX610764 | Unassigned | Unassigned | Unassigned | 538_83 | Clustered/Singleton | 1 | VC_538_83 | 1 | 0 | 1 | 0 | 0 | 0 | 0 | 1 | Ref |
| KX610765 | Unassigned | Unassigned | Unassigned | 985_0 | Clustered | 38 | VC_985_0 | 38 | 0.9334 | 1 | 0.9334 | 0 | 0 | 0 | 1 | Ref |
| KX611788 | Unassigned | Unassigned | Unassigned | 991_0 | Clustered | 39 | VC_991_0 | 39 | 1 | 1 | 1 | 0 | 0 | 0 | 1 | Ref |
| KX611831 | Unassigned | Unassigned | Unassigned | 571_6 | Clustered | 22 | VC_571_6 | 22 | 0.1207 | 1 | 0.1207 | 0 | 0 | 0 | 0.8095 | Ref |
| KX619650 | Unassigned | Unassigned | Unassigned | 223_6 | Clustered | 42 | VC_223_6 | 42 | 0.4717 | 1 | 0.4717 | 0 | 0 | 0 | 0.8351 | Ref |
| KX619651 | Unassigned | Unassigned | Unassigned | 535_1 | Clustered | 108 | VC_535_1 | 108 | 0.8479 | 1 | 0.8479 | 0 | 0 | 0 | 0.9955 | Ref |
| KX620750 | Unassigned | Unassigned | Unassigned | 1228_0 | Clustered | 4 | VC_1228_0 | 4 | 0.463 | 0.99990292 | 0.463 | 0 | 0 | 0 | 1 | Ref |
| KX620751 | Unassigned | Unassigned | Unassigned | 1228_0 | Clustered | 4 | VC_1228_0 | 4 | 0.463 | 0.99990292 | 0.463 | 0 | 0 | 0 | 1 | Ref |
| KX620753 | Unassigned | Unassigned | Unassigned | 1228_0 | Clustered | 4 | VC_1228_0 | 4 | 0.463 | 0.99990292 | 0.463 | 0 | 0 | 0 | 1 | Ref |
| KX620754 | Unassigned | Unassigned | Unassigned | 1228_0 | Clustered | 4 | VC_1228_0 | 4 | 0.463 | 0.99990292 | 0.463 | 0 | 0 | 0 | 1 | Ref |
| KX620786 | Unassigned | Unassigned | Unassigned | 384_16 | Clustered | 42 | VC_384_16 | 42 | 0.134 | 1 | 0.134 | 0 | 0 | 0 | 0.7282 | Ref |
| KX621004 | Unassigned | Unassigned | Unassigned | 1200_0 | Clustered | 73 | VC_1200_0 | 73 | 0.9825 | 1 | 0.9825 | 0 | 0 | 0 | 1 | Ref |
| KX621005 | Unassigned | Unassigned | Unassigned | 1200_0 | Clustered | 73 | VC_1200_0 | 73 | 0.9825 | 1 | 0.9825 | 0 | 0 | 0 | 1 | Ref |
| KX621007 | Unassigned | Unassigned | Unassigned | 241_0 | Clustered | 21 | VC_241_0 | 21 | 0.8342 | 1 | 0.8342 | 0 | 0 | 0 | 1 | Ref |
| KX636165 | Unassigned | Unassigned | Unassigned | 245_0 | Clustered | 17 | VC_245_0 | 17 | 0.6081 | 1 | 0.6081 | 0 | 0 | 0 | 1 | Ref |
| KX640831 | Unassigned | Unassigned | Unassigned | 531_0 | Clustered | 32 | VC_531_0 | 32 | 0.6859 | 1 | 0.6859 | 0 | 0 | 0 | 1 | Ref |
| KX641260 | Unassigned | Unassigned | Unassigned | 535_0 | Clustered | 18 | VC_535_0 | 18 | 0.1595 | 1 | 0.1595 | 0 | 0 | 0 | 1 | Ref |
| KX641261 | Unassigned | Unassigned | Unassigned | 230_0 | Clustered | 38 | VC_230_0 | 38 | 0.9076 | 1 | 0.9076 | 0 | 0 | 0 | 1 | Ref |
| KX641262 | Unassigned | Unassigned | Unassigned | 537_0 | Clustered | 39 | VC_537_0 | 39 | 0.8148 | 1 | 0.8148 | 0 | 0 | 0 | 1 | Ref |
| KX641263 | Unassigned | Unassigned | Unassigned | 223_3 | Clustered | 8 | VC_223_3 | 8 | 0.0699 | 1 | 0.0699 | 0 | 0 | 0 | 0.8571 | Ref |
| KX641264 | Unassigned | Unassigned | Unassigned | 243_0 | Clustered | 72 | VC_243_0 | 72 | 0.8089 | 1 | 0.8089 | 0 | 0 | 0 | 1 | Ref |
| KX641265 | Unassigned | Unassigned | Unassigned | 783_0 | Clustered | 67 | VC_783_0 | 67 | 0.8945 | 1 | 0.8945 | 0 | 0 | 0 | 1 | Ref |
| KX641266 | Unassigned | Unassigned | Unassigned | 783_0 | Clustered | 67 | VC_783_0 | 67 | 0.8945 | 1 | 0.8945 | 0 | 0 | 0 | 1 | Ref |
| KX648373 | Unassigned | Unassigned | Unassigned | 535_1 | Clustered | 108 | VC_535_1 | 108 | 0.8479 | 1 | 0.8479 | 0 | 0 | 0 | 0.9955 | Ref |
| KX649889 | Unassigned | Unassigned | Unassigned | 654_0 | Clustered | 115 | VC_654_0 | 115 | 0.7303 | 1 | 0.7303 | 0 | 0 | 0 | 0.8764 | Ref |
| KX656785 | Unassigned | Unassigned | Unassigned | 703_0 | Clustered | 3 | VC_703_0 | 3 | 0.5841 | 0.99252665 | 0.5797 | 0 | 0 | 0 | 1 | Ref |
| KX657793 | Unassigned | Unassigned | Unassigned | 229_0 | Clustered | 5 | VC_229_0 | 5 | 0.0566 | 0.99999997 | 0.0566 | 0 | 0 | 0 | 1 | Ref |
| KX657794 | Unassigned | Unassigned | Unassigned | 243_0 | Clustered | 72 | VC_243_0 | 72 | 0.8089 | 1 | 0.8089 | 0 | 0 | 0 | 1 | Ref |
| KX657795 | Unassigned | Unassigned | Unassigned | 243_0 | Clustered | 72 | VC_243_0 | 72 | 0.8089 | 1 | 0.8089 | 0 | 0 | 0 | 1 | Ref |
| KX657796 | Unassigned | Unassigned | Unassigned | 243_0 | Clustered | 72 | VC_243_0 | 72 | 0.8089 | 1 | 0.8089 | 0 | 0 | 0 | 1 | Ref |
| KX660669 | Unassigned | Unassigned | Unassigned | 71_0 | Clustered | 27 | VC_71_0 | 27 | 0.5964 | 1 | 0.5964 | 0 | 0 | 0 | 0.9943 | Ref |
| KX664447 | Unassigned | Unassigned | Unassigned | 232_0 | Clustered | 32 | VC_232_0 | 32 | 0.8471 | 1 | 0.8471 | 0 | 0 | 0 | 0.996 | Ref |
| KX664448 | Unassigned | Unassigned | Unassigned | 532_0 | Clustered | 75 | VC_532_0 | 75 | 0.9393 | 1 | 0.9393 | 0 | 0 | 0 | 1 | Ref |
| KX664455 | Unassigned | Unassigned | Unassigned | 783_0 | Clustered | 67 | VC_783_0 | 67 | 0.8945 | 1 | 0.8945 | 0 | 0 | 0 | 1 | Ref |
| KX664695 | Unassigned | Unassigned | Unassigned | 871_1 | Clustered | 26 | VC_871_1 | 26 | 0.6303 | 1 | 0.6303 | 0 | 0 | 0 | 0.9815 | Ref |
| KX669227 | Unassigned | Unassigned | Unassigned | 465_0 | Clustered | 80 | VC_465_0 | 80 | 0.9988 | 1 | 0.9988 | 0 | 0 | 0 | 1 | Ref |
| KX670786 | Unassigned | Unassigned | Unassigned | 1117_0 | Clustered | 19 | VC_1117_0 | 19 | 0.9177 | 1 | 0.9177 | 0 | 0 | 0 | 1 | Ref |
| KX670787 | Unassigned | Unassigned | Unassigned | 1117_0 | Clustered | 19 | VC_1117_0 | 19 | 0.9177 | 1 | 0.9177 | 0 | 0 | 0 | 1 | Ref |
| KX670788 | Unassigned | Unassigned | Unassigned | 532_0 | Clustered | 75 | VC_532_0 | 75 | 0.9393 | 1 | 0.9393 | 0 | 0 | 0 | 1 | Ref |
| KX670789 | Unassigned | Unassigned | Unassigned | 901_0 | Clustered | 31 | VC_901_0 | 31 | 0.8235 | 1 | 0.8235 | 0 | 0 | 0 | 0.8194 | Ref |
| KX670790 | Unassigned | Unassigned | Unassigned | 901_0 | Clustered | 31 | VC_901_0 | 31 | 0.8235 | 1 | 0.8235 | 0 | 0 | 0 | 0.8194 | Ref |
| KX670813 | Unassigned | Unassigned | Unassigned | 384_15 | Clustered | 44 | VC_384_15 | 44 | 0.139 | 1 | 0.139 | 0 | 0 | 0 | 0.7474 | Ref |
| KX670828 | Unassigned | Unassigned | Unassigned | 531_0 | Clustered | 32 | VC_531_0 | 32 | 0.6859 | 1 | 0.6859 | 0 | 0 | 0 | 1 | Ref |
| KX683292 | Unassigned | Unassigned | Unassigned | 384_15 | Clustered | 44 | VC_384_15 | 44 | 0.139 | 1 | 0.139 | 0 | 0 | 0 | 0.7474 | Ref |
| KX683293 | Unassigned | Unassigned | Unassigned | 384_15 | Clustered | 44 | VC_384_15 | 44 | 0.139 | 1 | 0.139 | 0 | 0 | 0 | 0.7474 | Ref |
| KX683423 | Unassigned | Unassigned | Unassigned | 531_0 | Clustered | 32 | VC_531_0 | 32 | 0.6859 | 1 | 0.6859 | 0 | 0 | 0 | 1 | Ref |
| KX683428 | Unassigned | Unassigned | Unassigned | 244_1 | Clustered | 13 | VC_244_1 | 13 | 0.1766 | 1 | 0.1766 | 0 | 0 | 0 | 0.7308 | Ref |
| KX683875 | Unassigned | Unassigned | Unassigned | 223_4 | Clustered | 13 | VC_223_4 | 13 | 0.1649 | 1 | 0.1649 | 0 | 0 | 0 | 1 | Ref |
| KX683876 | Unassigned | Unassigned | Unassigned | 335_27 | Clustered | 6 | VC_335_27 | 6 | 0.0266 | 1 | 0.0266 | 0 | 0 | 0 | 0.8 | Ref |
| KX685355 | Unassigned | Unassigned | Unassigned | 232_0 | Clustered | 32 | VC_232_0 | 32 | 0.8471 | 1 | 0.8471 | 0 | 0 | 0 | 0.996 | Ref |

| | | | | | | | | | | | | | | | | |
|---|---|---|---|---|---|---|---|---|---|---|---|---|---|---|---|---|
| KX688102 | Unassigned | Unassigned | Unassigned | 1200_0 | Clustered | 73 | VC_1200_0 | 73 | 0.9825 | 1 | 0.9825 | 0 | 0 | 0 | 1 | Ref |
| KX688103 | Unassigned | Unassigned | Unassigned | 1200_0 | Clustered | 73 | VC_1200_0 | 73 | 0.9825 | 1 | 0.9825 | 0 | 0 | 0 | 1 | Ref |
| KX689784 | Unassigned | Unassigned | Unassigned | 465_0 | Clustered | 80 | VC_465_0 | 80 | 0.9988 | 1 | 0.9988 | 0 | 0 | 0 | 1 | Ref |
| KX702319 | Unassigned | Unassigned | Unassigned | 384_13 | Clustered | 33 | VC_384_13 | 33 | 0.1785 | 1 | 0.1785 | 0 | 0 | 0 | 0.8769 | Ref |
| KX702320 | Unassigned | Unassigned | Unassigned | 335_27 | Clustered | 6 | VC_335_27 | 6 | 0.0266 | 1 | 0.0266 | 0 | 0 | 0 | 0.8 | Ref |
| KX711710 | Unassigned | Unassigned | Unassigned | 434_0 | Clustered | 45 | VC_434_0 | 45 | 0.9479 | 1 | 0.9479 | 0 | 0 | 0 | 1 | Ref |
| KX712070 | Unassigned | Unassigned | Unassigned | 848_0 | Clustered | 124 | VC_848_0 | 124 | 0.9484 | 1 | 0.9484 | 0 | 0 | 0 | 1 | Ref |
| KX712071 | Unassigned | Unassigned | Unassigned | 848_0 | Clustered | 124 | VC_848_0 | 124 | 0.9484 | 1 | 0.9484 | 0 | 0 | 0 | 1 | Ref |
| KX712143 | Unassigned | Unassigned | Unassigned | 360_0 | Clustered | 22 | VC_360_0 | 22 | 0.96 | 1 | 0.96 | 0 | 0 | 0 | 1 | Ref |
| KX712236 | Unassigned | Unassigned | Unassigned | 991_0 | Clustered | 39 | VC_991_0 | 39 | 1 | 1 | 1 | 0 | 0 | 0 | 1 | Ref |
| KX712237 | Unassigned | Unassigned | Unassigned | 991_0 | Clustered | 39 | VC_991_0 | 39 | 1 | 1 | 1 | 0 | 0 | 0 | 1 | Ref |
| KX712238 | Unassigned | Unassigned | Unassigned | 335_38 | Clustered/Singleton | 1 | VC_335_38 | 1 | 0 | 1 | 0 | 0 | 0 | 0 | 1 | Ref |
| KX721255 | Unassigned | Unassigned | Unassigned | 579_49 | Clustered | 7 | VC_579_49 | 7 | 0.0194 | 0.98914431 | 0.0191 | 0 | 0 | 0 | 0.8571 | Ref |
| KX721256 | Unassigned | Unassigned | Unassigned | 579_48 | Clustered | 2 | VC_579_48 | 2 | 0.0032 | 0.68030515 | 0.0022 | 0 | 0 | 0 | 1 | Ref |
| KX752698 | Unassigned | Unassigned | Unassigned | 579_81 | Clustered/Singleton | 1 | VC_579_81 | 1 | 0 | 1 | 0 | 0 | 0 | 0 | 1 | Ref |
| KX756439 | Unassigned | Unassigned | Unassigned | 539_0 | Clustered | 37 | VC_539_0 | 37 | 0.911 | 1 | 0.911 | 0 | 0 | 0 | 1 | Ref |
| KX756572 | Unassigned | Unassigned | Unassigned | 907_0 | Clustered | 32 | VC_907_0 | 32 | 0.9964 | 1 | 0.9964 | 0 | 0 | 0 | 1 | Ref |
| KX758538 | Unassigned | Unassigned | Unassigned | 222_1 | Clustered | 19 | VC_222_1 | 19 | 0.4166 | 1 | 0.4166 | 0 | 0 | 0 | 1 | Ref |
| KX758539 | Unassigned | Unassigned | Unassigned | 224_0 | Clustered | 5 | VC_224_0 | 5 | 0.0685 | 0.99999998 | 0.0685 | 0 | 0 | 0 | 1 | Ref |
| KX765862 | Unassigned | Unassigned | Unassigned | 611_0 | Clustered | 50 | VC_611_0 | 50 | 0.7306 | 1 | 0.7306 | 0 | 0 | 0 | 1 | Ref |
| KX765863 | Unassigned | Unassigned | Unassigned | 611_0 | Clustered | 50 | VC_611_0 | 50 | 0.7306 | 1 | 0.7306 | 0 | 0 | 0 | 1 | Ref |
| KX765864 | Unassigned | Unassigned | Unassigned | 611_0 | Clustered | 50 | VC_611_0 | 50 | 0.7306 | 1 | 0.7306 | 0 | 0 | 0 | 1 | Ref |
| KX765865 | Unassigned | Unassigned | Unassigned | 135_15 | Clustered | 2 | VC_135_15 | 2 | 0.0051 | 0.84262835 | 0.0043 | 0 | 0 | 0 | 1 | Ref |
| KX774321 | Unassigned | Unassigned | Unassigned | 1113_1 | Clustered/Singleton | 1 | VC_1113_1 | 1 | 0 | 1 | 0 | 0 | 0 | 0 | 1 | Ref |
| KX774374 | Unassigned | Unassigned | Unassigned | 5_0 | Clustered | 27 | VC_5_0 | 27 | 0.9599 | 1 | 0.9599 | 0 | 0 | 0 | 1 | Ref |
| KX776161 | Unassigned | Unassigned | Unassigned | 30_1 | Clustered | 53 | VC_30_1 | 53 | 0.7151 | 1 | 0.7151 | 0 | 0 | 0 | 0.9325 | Ref |
| KX778611 | Unassigned | Unassigned | Unassigned | 398_0 | Clustered | 51 | VC_398_0 | 51 | 0.8741 | 1 | 0.8741 | 0 | 0 | 0 | 1 | Ref |
| KX781992 | Unassigned | Unassigned | Unassigned | 579_9 | Clustered | 3 | VC_579_9 | 3 | 0.0064 | 0.81181703 | 0.0052 | 0 | 0 | 0 | 0.6667 | Ref |
| KX808129 | Unassigned | Unassigned | Unassigned | 532_0 | Clustered | 75 | VC_532_0 | 75 | 0.9393 | 1 | 0.9393 | 0 | 0 | 0 | 1 | Ref |
| KX808130 | Unassigned | Unassigned | Unassigned | 535_1 | Clustered | 108 | VC_535_1 | 108 | 0.8479 | 1 | 0.8479 | 0 | 0 | 0 | 0.9955 | Ref |
| KX808131 | Unassigned | Unassigned | Unassigned | 538_16 | Clustered/Singleton | 1 | VC_538_16 | 1 | 0 | 1 | 0 | 0 | 0 | 0 | 1 | Ref |
| KX815338 | Unassigned | Unassigned | Unassigned | 1016_0 | Clustered | 62 | VC_1016_0 | 62 | 0.8638 | 1 | 0.8638 | 0 | 0 | 0 | 0.743 | Ref |
| KX817173 | Unassigned | Unassigned | Unassigned | 571_2 | Clustered | 17 | VC_571_2 | 17 | 0.0965 | 1 | 0.0965 | 0 | 0 | 0 | 0.9779 | Ref |
| KX817174 | Unassigned | Unassigned | Unassigned | 535_1 | Clustered | 108 | VC_535_1 | 108 | 0.8479 | 1 | 0.8479 | 0 | 0 | 0 | 0.9955 | Ref |
| KX817175 | Unassigned | Unassigned | Unassigned | 535_1 | Clustered | 108 | VC_535_1 | 108 | 0.8479 | 1 | 0.8479 | 0 | 0 | 0 | 0.9955 | Ref |
| KX822733 | Unassigned | Unassigned | Unassigned | 514_3 | Clustered/Singleton | 1 | VC_514_3 | 1 | 0 | 1 | 0 | 0 | 0 | 0 | 1 | Ref |
| KX826077 | Unassigned | Unassigned | Unassigned | 440_0 | Clustered | 33 | VC_440_0 | 33 | 0.992 | 1 | 0.992 | 0 | 0 | 0 | 1 | Ref |
| KX827368 | Unassigned | Unassigned | Unassigned | 11_0 | Clustered | 19 | VC_11_0 | 19 | 0.7544 | 1 | 0.7544 | 0 | 0 | 0 | 1 | Ref |
| KX827369 | Unassigned | Unassigned | Unassigned | 11_0 | Clustered | 19 | VC_11_0 | 19 | 0.7544 | 1 | 0.7544 | 0 | 0 | 0 | 1 | Ref |
| KX827370 | Unassigned | Unassigned | Unassigned | 11_0 | Clustered | 19 | VC_11_0 | 19 | 0.7544 | 1 | 0.7544 | 0 | 0 | 0 | 1 | Ref |
| KX827371 | Unassigned | Unassigned | Unassigned | 15_0 | Clustered | 3 | VC_15_0 | 3 | 0.0477 | 0.99853676 | 0.0476 | 0 | 0 | 0 | 1 | Ref |
| KX828710 | Unassigned | Unassigned | Unassigned | 595_0 | Clustered | 35 | VC_595_0 | 35 | 0.8809 | 1 | 0.8809 | 0 | 0 | 0 | 1 | Ref |
| KX828711 | Unassigned | Unassigned | Unassigned | 290_85 | Clustered/Singleton | 1 | VC_290_85 | 1 | 0 | 1 | 0 | 0 | 0 | 0 | 1 | Ref |
| KX831080 | Unassigned | Unassigned | Unassigned | 579_82 | Clustered | 3 | VC_579_82 | 3 | 0.0064 | 0.86736674 | 0.0056 | 0 | 0 | 0 | 0.6667 | Ref |
| KX833211 | Unassigned | Unassigned | Unassigned | 235_0 | Clustered | 60 | VC_235_0 | 60 | 0.9413 | 1 | 0.9413 | 0 | 0 | 0 | 1 | Ref |
| KX834009 | Unassigned | Unassigned | Unassigned | 571_6 | Clustered | 22 | VC_571_6 | 22 | 0.1207 | 1 | 0.1207 | 0 | 0 | 0 | 0.8095 | Ref |
| KX845404 | Unassigned | Unassigned | Unassigned | 200_0 | Clustered | 15 | VC_200_0 | 15 | 0.5004 | 1 | 0.5004 | 0 | 0 | 0 | 1 | Ref |
| KX855961 | Unassigned | Unassigned | Unassigned | 1117_0 | Clustered | 19 | VC_1117_0 | 19 | 0.9177 | 1 | 0.9177 | 0 | 0 | 0 | 1 | Ref |
| KX855962 | Unassigned | Unassigned | Unassigned | 1117_0 | Clustered | 19 | VC_1117_0 | 19 | 0.9177 | 1 | 0.9177 | 0 | 0 | 0 | 1 | Ref |
| KX856662 | Unassigned | Unassigned | Unassigned | 113_0 | Clustered | 91 | VC_113_0 | 91 | 0.9584 | 1 | 0.9584 | 0 | 0 | 0 | 1 | Ref |
| KX879601 | Unassigned | Unassigned | Unassigned | 745_0 | Clustered | 9 | VC_745_0 | 9 | 0.9955 | 0.99999771 | 0.9955 | 0 | 0 | 0 | 1 | Ref |
| KX879602 | Unassigned | Unassigned | Unassigned | 745_0 | Clustered | 9 | VC_745_0 | 9 | 0.9955 | 0.99999771 | 0.9955 | 0 | 0 | 0 | 1 | Ref |
| KX879627 | Unassigned | Unassigned | Unassigned | 868_0 | Clustered | 27 | VC_868_0 | 27 | 0.9372 | 1 | 0.9372 | 0 | 0 | 0 | 1 | Ref |
| KX879641 | Unassigned | Unassigned | Unassigned | 262_0 | Clustered | 14 | VC_262_0 | 14 | 0.2364 | 1 | 0.2364 | 0 | 0 | 0 | 1 | Ref |
| KX879642 | Unassigned | Unassigned | Unassigned | 262_0 | Clustered | 14 | VC_262_0 | 14 | 0.2364 | 1 | 0.2364 | 0 | 0 | 0 | 1 | Ref |
| KX879643 | Unassigned | Unassigned | Unassigned | 261_0 | Clustered | 13 | VC_261_0 | 13 | 0.2024 | 1 | 0.2024 | 0 | 0 | 0 | 1 | Ref |
| KX889068 | Unassigned | Unassigned | Unassigned | 809_0 | Clustered | 7 | VC_809_0 | 7 | 0.5153 | 1 | 0.5153 | 0 | 0 | 0 | 1 | Ref |
| KX897981 | Unassigned | Unassigned | Unassigned | 222_1 | Clustered | 19 | VC_222_1 | 19 | 0.4166 | 1 | 0.4166 | 0 | 0 | 0 | 1 | Ref |
| KX898399 | Unassigned | Unassigned | Unassigned | 479_0 | Clustered | 7 | VC_479_0 | 7 | 0.3294 | 1 | 0.3294 | 0 | 0 | 0 | 1 | Ref |
| KX898400 | Unassigned | Unassigned | Unassigned | 479_0 | Clustered | 7 | VC_479_0 | 7 | 0.3294 | 1 | 0.3294 | 0 | 0 | 0 | 1 | Ref |
| KX911187 | Unassigned | Unassigned | Unassigned | 642_0 | Clustered | 3 | VC_642_0 | 3 | 0.1258 | 0.72377751 | 0.0911 | 0 | 0 | 0 | 1 | Ref |
| KX912252 | Unassigned | Unassigned | Unassigned | 927_0 | Clustered | 5 | VC_927_0 | 5 | 0.4988 | 0.99999981 | 0.4988 | 0 | 0 | 0 | 1 | Ref |
| KX961385 | Unassigned | Unassigned | Unassigned | 172_0 | Clustered | 26 | VC_172_0 | 26 | 0.7909 | 1 | 0.7909 | 0 | 0 | 0 | 1 | Ref |
| KX961629 | Unassigned | Unassigned | Unassigned | 913_0 | Clustered | 28 | VC_913_0 | 28 | 0.6206 | 1 | 0.6206 | 0 | 0 | 0 | 0.9841 | Ref |
| KX961630 | Unassigned | Unassigned | Unassigned | 195_0 | Clustered | 4 | VC_195_0 | 4 | 0.0656 | 0.9999431 | 0.0656 | 0 | 0 | 0 | 1 | Ref |
| KX961631 | Unassigned | Unassigned | Unassigned | 843_0 | Clustered | 20 | VC_843_0 | 20 | 0.9621 | 1 | 0.9621 | 0 | 0 | 0 | 1 | Ref |
| KX961632 | Unassigned | Unassigned | Unassigned | 913_0 | Clustered | 28 | VC_913_0 | 28 | 0.6206 | 1 | 0.6206 | 0 | 0 | 0 | 0.9841 | Ref |
| KX965989 | Unassigned | Unassigned | Unassigned | 145_1 | Clustered/Singleton | 1 | VC_145_1 | 1 | 0 | 1 | 0 | 0 | 0 | 0 | 1 | Ref |
| KX982260 | Unassigned | Unassigned | Unassigned | 958_0 | Clustered | 3 | VC_958_0 | 3 | 0.2752 | 0.95138727 | 0.2618 | 0 | 0 | 0 | 1 | Ref |
| KX987127 | Unassigned | Unassigned | Unassigned | 829_0 | Clustered | 4 | VC_829_0 | 4 | 0.9064 | 0.99761409 | 0.9042 | 0 | 0 | 0 | 1 | Ref |
| KX987158 | Unassigned | Unassigned | Unassigned | 1090_0 | Clustered | 8 | VC_1090_0 | 8 | 0.6377 | 1 | 0.6377 | 0 | 0 | 0 | 1 | Ref |
| KX987999 | Unassigned | Unassigned | Unassigned | 195_1 | Clustered | 7 | VC_195_1 | 7 | 0.1409 | 1 | 0.1409 | 0 | 0 | 0 | 1 | Ref |
| KY000003 | Unassigned | Unassigned | Unassigned | 135_30 | Clustered | 2 | VC_135_30 | 2 | 0.0052 | 0.87577187 | 0.0045 | 0 | 0 | 0 | 1 | Ref |
| KY000079 | Unassigned | Unassigned | Unassigned | 717_0 | Clustered | 5 | VC_717_0 | 5 | 0.7757 | 0.99999988 | 0.7757 | 0 | 0 | 0 | 1 | Ref |
| KY000080 | Unassigned | Unassigned | Unassigned | 509_0 | Clustered | 36 | VC_509_0 | 36 | 0.5291 | 1 | 0.5291 | 0 | 0 | 0 | 0.9714 | Ref |
| KY000081 | Unassigned | Unassigned | Unassigned | 113_0 | Clustered | 91 | VC_113_0 | 91 | 0.9584 | 1 | 0.9584 | 0 | 0 | 0 | 1 | Ref |
| KY000082 | Unassigned | Unassigned | Unassigned | 80_11 | Clustered/Singleton | 1 | VC_80_11 | 1 | 0 | 1 | 0 | 0 | 0 | 0 | 1 | Ref |
| KY000083 | Unassigned | Unassigned | Unassigned | 77_0 | Clustered | 37 | VC_77_0 | 37 | 0.9392 | 1 | 0.9392 | 0 | 0 | 0 | 1 | Ref |
| KY000084 | Unassigned | Unassigned | Unassigned | 98_0 | Clustered | 30 | VC_98_0 | 30 | 0.7175 | 1 | 0.7175 | 0 | 0 | 0 | 1 | Ref |
| KY000085 | Unassigned | Unassigned | Unassigned | 98_0 | Clustered | 30 | VC_98_0 | 30 | 0.7175 | 1 | 0.7175 | 0 | 0 | 0 | 1 | Ref |
| KY000218 | Unassigned | Unassigned | Unassigned | 172_0 | Clustered | 26 | VC_172_0 | 26 | 0.7909 | 1 | 0.7909 | 0 | 0 | 0 | 1 | Ref |

| | | | | | | | | | | | | | | | | |
|---|---|---|---|---|---|---|---|---|---|---|---|---|---|---|---|---|
| KY000219 | Unassigned | Unassigned | Unassigned | 172_0 | Clustered | 26 | VC_172_0 | 26 | 0.7909 | 1 | 0.7909 | 0 | 0 | 0 | 1 | Ref |
| KY000220 | Unassigned | Unassigned | Unassigned | 172_0 | Clustered | 26 | VC_172_0 | 26 | 0.7909 | 1 | 0.7909 | 0 | 0 | 0 | 1 | Ref |
| KY000221 | Unassigned | Unassigned | Unassigned | 172_0 | Clustered | 26 | VC_172_0 | 26 | 0.7909 | 1 | 0.7909 | 0 | 0 | 0 | 1 | Ref |
| KY002061 | Unassigned | Unassigned | Unassigned | 1090_0 | Clustered | 8 | VC_1090_0 | 8 | 0.6377 | 1 | 0.6377 | 0 | 0 | 0 | 1 | Ref |
| KY006473 | Unassigned | Unassigned | Unassigned | 535_1 | Clustered | 108 | VC_535_1 | 108 | 0.8479 | 1 | 0.8479 | 0 | 0 | 0 | 0.9955 | Ref |
| KY006474 | Unassigned | Unassigned | Unassigned | 384_1 | Clustered | 11 | VC_384_1 | 11 | 0.0378 | 1 | 0.0378 | 0 | 0 | 0 | 0.8545 | Ref |
| KY006853 | Unassigned | Unassigned | Unassigned | 176_1 | Clustered/Singleton | 1 | VC_176_1 | 1 | 0 | 1 | 0 | 0 | 0 | 0 | 1 | Ref |
| KY014601 | Unassigned | Unassigned | Unassigned | 611_0 | Clustered | 50 | VC_611_0 | 50 | 0.7306 | 1 | 0.7306 | 0 | 0 | 0 | 1 | Ref |
| KY022433 | Unassigned | Unassigned | Unassigned | 935_2 | Clustered | 7 | VC_935_2 | 7 | 0.2837 | 1 | 0.2837 | 0 | 0 | 0 | 1 | Ref |
| KY030782 | Unassigned | Unassigned | Unassigned | 91_2 | Clustered | 5 | VC_91_2 | 5 | 0.2025 | 0.99999726 | 0.2025 | 0 | 0 | 0 | 1 | Ref |
| KY045851 | Unassigned | Unassigned | Unassigned | 5_0 | Clustered | 27 | VC_5_0 | 27 | 0.9599 | 1 | 0.9599 | 0 | 0 | 0 | 1 | Ref |
| KY053532 | Unassigned | Unassigned | Unassigned | 826_0 | Clustered | 7 | VC_826_0 | 7 | 0.0509 | 0.99909926 | 0.0508 | 0 | 0 | 0 | 1 | Ref |
| KY056619 | Unassigned | Unassigned | Unassigned | 908_0 | Clustered | 36 | VC_908_0 | 36 | 0.9943 | 1 | 0.9943 | 0 | 0 | 0 | 1 | Ref |
| KY056620 | Unassigned | Unassigned | Unassigned | 8_0 | Clustered | 58 | VC_8_0 | 58 | 0.8285 | 1 | 0.8285 | 0 | 0 | 0 | 0.9946 | Ref |
| KY065147 | Unassigned | Unassigned | Unassigned | 513_1 | Clustered | 41 | VC_513_1 | 41 | 0.3276 | 1 | 0.3276 | 0 | 0 | 0 | 0.911 | Ref |
| KY065148 | Unassigned | Unassigned | Unassigned | 806_0 | Clustered | 6 | VC_806_0 | 6 | 0.5468 | 1 | 0.5468 | 0 | 0 | 0 | 1 | Ref |
| KY065149 | Unassigned | Unassigned | Unassigned | 1022_0 | Clustered | 2 | VC_1022_0 | 2 | 0.1197 | 0.95251219 | 0.114 | 0 | 0 | 0 | 1 | Ref |
| KY065449 | Unassigned | Unassigned | Unassigned | 428_0 | Clustered/Singleton | 1 | VC_428_0 | 1 | 0 | 1 | 0 | 0 | 0 | 0 | 1 | Ref |
| KY065450 | Unassigned | Unassigned | Unassigned | 427_0 | Clustered | 37 | VC_427_0 | 37 | 0.5729 | 1 | 0.5729 | 0 | 0 | 0 | 0.9474 | Ref |
| KY065451 | Unassigned | Unassigned | Unassigned | 427_0 | Clustered | 37 | VC_427_0 | 37 | 0.5729 | 1 | 0.5729 | 0 | 0 | 0 | 0.9474 | Ref |
| KY065452 | Unassigned | Unassigned | Unassigned | 427_0 | Clustered | 37 | VC_427_0 | 37 | 0.5729 | 1 | 0.5729 | 0 | 0 | 0 | 0.9474 | Ref |
| KY065453 | Unassigned | Unassigned | Unassigned | 344_0 | Clustered | 30 | VC_344_0 | 30 | 0.523 | 1 | 0.523 | 0 | 0 | 0 | 0.9954 | Ref |
| KY065454 | Unassigned | Unassigned | Unassigned | 344_0 | Clustered | 30 | VC_344_0 | 30 | 0.523 | 1 | 0.523 | 0 | 0 | 0 | 0.9954 | Ref |
| KY065455 | Unassigned | Unassigned | Unassigned | 425_0 | Clustered | 40 | VC_425_0 | 40 | 0.8399 | 1 | 0.8399 | 0 | 0 | 0 | 0.9974 | Ref |
| KY065456 | Unassigned | Unassigned | Unassigned | 427_0 | Clustered | 37 | VC_427_0 | 37 | 0.5729 | 1 | 0.5729 | 0 | 0 | 0 | 0.9474 | Ref |
| KY065457 | Unassigned | Unassigned | Unassigned | 757_0 | Clustered | 5 | VC_757_0 | 5 | 0.0972 | 0.99999635 | 0.0972 | 0 | 0 | 0 | 0.6 | Ref |
| KY065458 | Unassigned | Unassigned | Unassigned | 344_0 | Clustered | 30 | VC_344_0 | 30 | 0.523 | 1 | 0.523 | 0 | 0 | 0 | 0.9954 | Ref |
| KY065459 | Unassigned | Unassigned | Unassigned | 344_0 | Clustered | 30 | VC_344_0 | 30 | 0.523 | 1 | 0.523 | 0 | 0 | 0 | 0.9954 | Ref |
| KY065460 | Unassigned | Unassigned | Unassigned | 344_0 | Clustered | 30 | VC_344_0 | 30 | 0.523 | 1 | 0.523 | 0 | 0 | 0 | 0.9954 | Ref |
| KY065461 | Unassigned | Unassigned | Unassigned | 344_0 | Clustered | 30 | VC_344_0 | 30 | 0.523 | 1 | 0.523 | 0 | 0 | 0 | 0.9954 | Ref |
| KY065462 | Unassigned | Unassigned | Unassigned | 344_0 | Clustered | 30 | VC_344_0 | 30 | 0.523 | 1 | 0.523 | 0 | 0 | 0 | 0.9954 | Ref |
| KY065463 | Unassigned | Unassigned | Unassigned | 344_0 | Clustered | 30 | VC_344_0 | 30 | 0.523 | 1 | 0.523 | 0 | 0 | 0 | 0.9954 | Ref |
| KY065464 | Unassigned | Unassigned | Unassigned | 757_0 | Clustered | 5 | VC_757_0 | 5 | 0.0972 | 0.99999635 | 0.0972 | 0 | 0 | 0 | 0.6 | Ref |
| KY065465 | Unassigned | Unassigned | Unassigned | 426_0 | Clustered | 3 | VC_426_0 | 3 | 0.041 | 0.99840705 | 0.0409 | 0 | 0 | 0 | 1 | Ref |
| KY065466 | Unassigned | Unassigned | Unassigned | 324_0 | Clustered | 2 | VC_324_0 | 2 | 0.0438 | 0.9562359 | 0.0419 | 0 | 0 | 0 | 1 | Ref |
| KY065468 | Unassigned | Unassigned | Unassigned | 324_0 | Clustered | 2 | VC_324_0 | 2 | 0.0438 | 0.9562359 | 0.0419 | 0 | 0 | 0 | 1 | Ref |
| KY065469 | Unassigned | Unassigned | Unassigned | 344_0 | Clustered | 30 | VC_344_0 | 30 | 0.523 | 1 | 0.523 | 0 | 0 | 0 | 0.9954 | Ref |
| KY065470 | Unassigned | Unassigned | Unassigned | 344_0 | Clustered | 30 | VC_344_0 | 30 | 0.523 | 1 | 0.523 | 0 | 0 | 0 | 0.9954 | Ref |
| KY065471 | Unassigned | Unassigned | Unassigned | 344_0 | Clustered | 30 | VC_344_0 | 30 | 0.523 | 1 | 0.523 | 0 | 0 | 0 | 0.9954 | Ref |
| KY065472 | Unassigned | Unassigned | Unassigned | 427_0 | Clustered | 37 | VC_427_0 | 37 | 0.5729 | 1 | 0.5729 | 0 | 0 | 0 | 0.9474 | Ref |
| KY065473 | Unassigned | Unassigned | Unassigned | 427_0 | Clustered | 37 | VC_427_0 | 37 | 0.5729 | 1 | 0.5729 | 0 | 0 | 0 | 0.9474 | Ref |
| KY065474 | Unassigned | Unassigned | Unassigned | 427_0 | Clustered | 37 | VC_427_0 | 37 | 0.5729 | 1 | 0.5729 | 0 | 0 | 0 | 0.9474 | Ref |
| KY065475 | Unassigned | Unassigned | Unassigned | 427_0 | Clustered | 37 | VC_427_0 | 37 | 0.5729 | 1 | 0.5729 | 0 | 0 | 0 | 0.9474 | Ref |
| KY065476 | Unassigned | Unassigned | Unassigned | 427_0 | Clustered | 37 | VC_427_0 | 37 | 0.5729 | 1 | 0.5729 | 0 | 0 | 0 | 0.9474 | Ref |
| KY065477 | Unassigned | Unassigned | Unassigned | 427_0 | Clustered | 37 | VC_427_0 | 37 | 0.5729 | 1 | 0.5729 | 0 | 0 | 0 | 0.9474 | Ref |
| KY065478 | Unassigned | Unassigned | Unassigned | 427_0 | Clustered | 37 | VC_427_0 | 37 | 0.5729 | 1 | 0.5729 | 0 | 0 | 0 | 0.9474 | Ref |
| KY065479 | Unassigned | Unassigned | Unassigned | 425_0 | Clustered | 40 | VC_425_0 | 40 | 0.8399 | 1 | 0.8399 | 0 | 0 | 0 | 0.9974 | Ref |
| KY065480 | Unassigned | Unassigned | Unassigned | 427_0 | Clustered | 37 | VC_427_0 | 37 | 0.5729 | 1 | 0.5729 | 0 | 0 | 0 | 0.9474 | Ref |
| KY065485 | Unassigned | Unassigned | Unassigned | 268_1 | Clustered | 20 | VC_268_1 | 20 | 0.1906 | 1 | 0.1906 | 0 | 0 | 0 | 0.6789 | Ref |
| KY065486 | Unassigned | Unassigned | Unassigned | 427_0 | Clustered | 37 | VC_427_0 | 37 | 0.5729 | 1 | 0.5729 | 0 | 0 | 0 | 0.9474 | Ref |
| KY065487 | Unassigned | Unassigned | Unassigned | 425_0 | Clustered | 40 | VC_425_0 | 40 | 0.8399 | 1 | 0.8399 | 0 | 0 | 0 | 0.9974 | Ref |
| KY065488 | Unassigned | Unassigned | Unassigned | 427_0 | Clustered | 37 | VC_427_0 | 37 | 0.5729 | 1 | 0.5729 | 0 | 0 | 0 | 0.9474 | Ref |
| KY065490 | Unassigned | Unassigned | Unassigned | 425_0 | Clustered | 40 | VC_425_0 | 40 | 0.8399 | 1 | 0.8399 | 0 | 0 | 0 | 0.9974 | Ref |
| KY065491 | Unassigned | Unassigned | Unassigned | 425_0 | Clustered | 40 | VC_425_0 | 40 | 0.8399 | 1 | 0.8399 | 0 | 0 | 0 | 0.9974 | Ref |
| KY065492 | Unassigned | Unassigned | Unassigned | 425_0 | Clustered | 40 | VC_425_0 | 40 | 0.8399 | 1 | 0.8399 | 0 | 0 | 0 | 0.9974 | Ref |
| KY065493 | Unassigned | Unassigned | Unassigned | 425_0 | Clustered | 40 | VC_425_0 | 40 | 0.8399 | 1 | 0.8399 | 0 | 0 | 0 | 0.9974 | Ref |
| KY065494 | Unassigned | Unassigned | Unassigned | 344_0 | Clustered | 30 | VC_344_0 | 30 | 0.523 | 1 | 0.523 | 0 | 0 | 0 | 0.9954 | Ref |
| KY065495 | Unassigned | Unassigned | Unassigned | 344_0 | Clustered | 30 | VC_344_0 | 30 | 0.523 | 1 | 0.523 | 0 | 0 | 0 | 0.9954 | Ref |
| KY065496 | Unassigned | Unassigned | Unassigned | 427_0 | Clustered | 37 | VC_427_0 | 37 | 0.5729 | 1 | 0.5729 | 0 | 0 | 0 | 0.9474 | Ref |
| KY065497 | Unassigned | Unassigned | Unassigned | 427_0 | Clustered | 37 | VC_427_0 | 37 | 0.5729 | 1 | 0.5729 | 0 | 0 | 0 | 0.9474 | Ref |
| KY065499 | Unassigned | Unassigned | Unassigned | 344_0 | Clustered | 30 | VC_344_0 | 30 | 0.523 | 1 | 0.523 | 0 | 0 | 0 | 0.9954 | Ref |
| KY065500 | Unassigned | Unassigned | Unassigned | 427_0 | Clustered | 37 | VC_427_0 | 37 | 0.5729 | 1 | 0.5729 | 0 | 0 | 0 | 0.9474 | Ref |
| KY065501 | Unassigned | Unassigned | Unassigned | 425_0 | Clustered | 40 | VC_425_0 | 40 | 0.8399 | 1 | 0.8399 | 0 | 0 | 0 | 0.9974 | Ref |
| KY065502 | Unassigned | Unassigned | Unassigned | 425_0 | Clustered | 40 | VC_425_0 | 40 | 0.8399 | 1 | 0.8399 | 0 | 0 | 0 | 0.9974 | Ref |
| KY065503 | Unassigned | Unassigned | Unassigned | 268_1 | Clustered | 20 | VC_268_1 | 20 | 0.1906 | 1 | 0.1906 | 0 | 0 | 0 | 0.6789 | Ref |
| KY065504 | Unassigned | Unassigned | Unassigned | 268_1 | Clustered | 20 | VC_268_1 | 20 | 0.1906 | 1 | 0.1906 | 0 | 0 | 0 | 0.6789 | Ref |
| KY065505 | Unassigned | Unassigned | Unassigned | 427_0 | Clustered | 37 | VC_427_0 | 37 | 0.5729 | 1 | 0.5729 | 0 | 0 | 0 | 0.9474 | Ref |
| KY073123 | Unassigned | Unassigned | Unassigned | 134_2 | Clustered | 17 | VC_134_2 | 17 | 0.1715 | 1 | 0.1715 | 0 | 0 | 0 | 0.9853 | Ref |
| KY073228 | Unassigned | Unassigned | Unassigned | 77_0 | Clustered | 37 | VC_77_0 | 37 | 0.9392 | 1 | 0.9392 | 0 | 0 | 0 | 1 | Ref |
| KY082667 | Unassigned | Unassigned | Unassigned | 714_0 | Clustered | 71 | VC_714_0 | 71 | 0.935 | 1 | 0.935 | 0 | 0 | 0 | 1 | Ref |
| KY083058 | Unassigned | Unassigned | Unassigned | 535_1 | Clustered | 108 | VC_535_1 | 108 | 0.8479 | 1 | 0.8479 | 0 | 0 | 0 | 0.9955 | Ref |
| KY083059 | Unassigned | Unassigned | Unassigned | 535_0 | Clustered | 18 | VC_535_0 | 18 | 0.1595 | 1 | 0.1595 | 0 | 0 | 0 | 1 | Ref |
| KY083060 | Unassigned | Unassigned | Unassigned | 535_1 | Clustered | 108 | VC_535_1 | 108 | 0.8479 | 1 | 0.8479 | 0 | 0 | 0 | 0.9955 | Ref |
| KY083061 | Unassigned | Unassigned | Unassigned | 535_1 | Clustered | 108 | VC_535_1 | 108 | 0.8479 | 1 | 0.8479 | 0 | 0 | 0 | 0.9955 | Ref |
| KY083062 | Unassigned | Unassigned | Unassigned | 535_1 | Clustered | 108 | VC_535_1 | 108 | 0.8479 | 1 | 0.8479 | 0 | 0 | 0 | 0.9955 | Ref |
| KY083063 | Unassigned | Unassigned | Unassigned | 535_1 | Clustered | 108 | VC_535_1 | 108 | 0.8479 | 1 | 0.8479 | 0 | 0 | 0 | 0.9955 | Ref |
| KY083726 | Unassigned | Unassigned | Unassigned | 865_0 | Clustered | 10 | VC_865_0 | 10 | 0.5975 | 1 | 0.5975 | 0 | 0 | 0 | 1 | Ref |
| KY084243 | Unassigned | Unassigned | Unassigned | 399_0 | Clustered | 49 | VC_399_0 | 49 | 0.7927 | 1 | 0.7927 | 0 | 0 | 0 | 1 | Ref |
| KY087898 | Unassigned | Unassigned | Unassigned | 816_0 | Clustered | 35 | VC_816_0 | 35 | 0.9824 | 1 | 0.9824 | 0 | 0 | 0 | 1 | Ref |
| KY087992 | Unassigned | Unassigned | Unassigned | 241_0 | Clustered | 21 | VC_241_0 | 21 | 0.8342 | 1 | 0.8342 | 0 | 0 | 0 | 1 | Ref |
| KY087993 | Unassigned | Unassigned | Unassigned | 244_1 | Clustered | 13 | VC_244_1 | 13 | 0.1766 | 1 | 0.1766 | 0 | 0 | 0 | 0.7308 | Ref |

| | | | | | | | | | | | | | | | | |
|---|---|---|---|---|---|---|---|---|---|---|---|---|---|---|---|---|
| KY092479 | Unassigned | Unassigned | Unassigned | 742_0 | Clustered | 11 | VC_742_0 | 11 | 0.5046 | 1 | 0.5046 | 0 | 0 | 0 | 1 | Ref |
| KY092480 | Unassigned | Unassigned | Unassigned | 742_0 | Clustered | 11 | VC_742_0 | 11 | 0.5046 | 1 | 0.5046 | 0 | 0 | 0 | 1 | Ref |
| KY092481 | Unassigned | Unassigned | Unassigned | 742_0 | Clustered | 11 | VC_742_0 | 11 | 0.5046 | 1 | 0.5046 | 0 | 0 | 0 | 1 | Ref |
| KY092482 | Unassigned | Unassigned | Unassigned | 742_0 | Clustered | 11 | VC_742_0 | 11 | 0.5046 | 1 | 0.5046 | 0 | 0 | 0 | 1 | Ref |
| KY092483 | Unassigned | Unassigned | Unassigned | 742_0 | Clustered | 11 | VC_742_0 | 11 | 0.5046 | 1 | 0.5046 | 0 | 0 | 0 | 1 | Ref |
| KY092484 | Unassigned | Unassigned | Unassigned | 742_0 | Clustered | 11 | VC_742_0 | 11 | 0.5046 | 1 | 0.5046 | 0 | 0 | 0 | 1 | Ref |
| KY114934 | Unassigned | Unassigned | Unassigned | 198_21 | Clustered/Singleton | 1 | VC_198_21 | 1 | 0 | 1 | 0 | 0 | 0 | 0 | 1 | Ref |
| KY117485 | Unassigned | Unassigned | Unassigned | 160_0 | Clustered | 4 | VC_160_0 | 4 | 0.1752 | 0.9999526 | 0.1752 | 0 | 0 | 0 | 1 | Ref |
| KY130461 | Unassigned | Unassigned | Unassigned | 525_0 | Clustered | 8 | VC_525_0 | 8 | 0.443 | 1 | 0.443 | 0 | 0 | 0 | 1 | Ref |
| KY176369 | Unassigned | Unassigned | Unassigned | 654_1 | Clustered | 27 | VC_654_1 | 27 | 0.2571 | 1 | 0.2571 | 0 | 0 | 0 | 0.9858 | Ref |
| KY203335 | Unassigned | Unassigned | Unassigned | 422_0 | Clustered | 3 | VC_422_0 | 3 | 0.1422 | 0.98161408 | 0.1396 | 0 | 0 | 0 | 1 | Ref |
| KY204244 | Unassigned | Unassigned | Unassigned | 535_1 | Clustered | 108 | VC_535_1 | 108 | 0.8479 | 1 | 0.8479 | 0 | 0 | 0 | 0.9955 | Ref |
| KY204245 | Unassigned | Unassigned | Unassigned | 535_1 | Clustered | 108 | VC_535_1 | 108 | 0.8479 | 1 | 0.8479 | 0 | 0 | 0 | 0.9955 | Ref |
| KY204246 | Unassigned | Unassigned | Unassigned | 535_1 | Clustered | 108 | VC_535_1 | 108 | 0.8479 | 1 | 0.8479 | 0 | 0 | 0 | 0.9955 | Ref |
| KY204247 | Unassigned | Unassigned | Unassigned | 535_1 | Clustered | 108 | VC_535_1 | 108 | 0.8479 | 1 | 0.8479 | 0 | 0 | 0 | 0.9955 | Ref |
| KY204248 | Unassigned | Unassigned | Unassigned | 535_1 | Clustered | 108 | VC_535_1 | 108 | 0.8479 | 1 | 0.8479 | 0 | 0 | 0 | 0.9955 | Ref |
| KY204249 | Unassigned | Unassigned | Unassigned | 535_1 | Clustered | 108 | VC_535_1 | 108 | 0.8479 | 1 | 0.8479 | 0 | 0 | 0 | 0.9955 | Ref |
| KY204250 | Unassigned | Unassigned | Unassigned | 535_1 | Clustered | 108 | VC_535_1 | 108 | 0.8479 | 1 | 0.8479 | 0 | 0 | 0 | 0.9955 | Ref |
| KY204251 | Unassigned | Unassigned | Unassigned | 535_1 | Clustered | 108 | VC_535_1 | 108 | 0.8479 | 1 | 0.8479 | 0 | 0 | 0 | 0.9955 | Ref |
| KY206887 | Unassigned | Unassigned | Unassigned | 1060_0 | Clustered | 4 | VC_1060_0 | 4 | 0.875 | 0.99993415 | 0.8749 | 0 | 0 | 0 | 1 | Ref |
| KY210139 | Unassigned | Unassigned | Unassigned | 377_0 | Clustered | 16 | VC_377_0 | 16 | 0.6424 | 1 | 0.6424 | 0 | 0 | 0 | 1 | Ref |
| KY213952 | Unassigned | Unassigned | Unassigned | 335_42 | Clustered | 2 | VC_335_42 | 2 | 0.0039 | 0.95008941 | 0.0037 | 0 | 0 | 0 | 1 | Ref |
| KY223999 | Unassigned | Unassigned | Unassigned | 785_2 | Clustered | 7 | VC_785_2 | 7 | 0.2595 | 1 | 0.2595 | 0 | 0 | 0 | 1 | Ref |
| KY224000 | Unassigned | Unassigned | Unassigned | 385_1 | Clustered | 9 | VC_385_1 | 9 | 0.1928 | 1 | 0.1928 | 0 | 0 | 0 | 1 | Ref |
| KY224001 | Unassigned | Unassigned | Unassigned | 335_57 | Clustered/Singleton | 1 | VC_335_57 | 1 | 0 | 1 | 0 | 0 | 0 | 0 | 1 | Ref |
| KY249644 | Unassigned | Unassigned | Unassigned | 614_0 | Clustered | 4 | VC_614_0 | 4 | 0.0588 | 0.99274182 | 0.0584 | 0 | 0 | 0 | 1 | Ref |
| KY250482 | Unassigned | Unassigned | Unassigned | 422_0 | Clustered | 3 | VC_422_0 | 3 | 0.1422 | 0.98161408 | 0.1396 | 0 | 0 | 0 | 1 | Ref |
| KY264020 | Unassigned | Unassigned | Unassigned | 996_0 | Clustered | 3 | VC_996_0 | 3 | 0.6852 | 0.9859071 | 0.6755 | 0 | 0 | 0 | 1 | Ref |
| KY268295 | Unassigned | Unassigned | Unassigned | 714_0 | Clustered | 71 | VC_714_0 | 71 | 0.935 | 1 | 0.935 | 0 | 0 | 0 | 1 | Ref |
| KY268296 | Unassigned | Unassigned | Unassigned | 714_0 | Clustered | 71 | VC_714_0 | 71 | 0.935 | 1 | 0.935 | 0 | 0 | 0 | 1 | Ref |
| KY271395 | Unassigned | Unassigned | Unassigned | 33_0 | Clustered | 9 | VC_33_0 | 9 | 0.3409 | 1 | 0.3409 | 0 | 0 | 0 | 0.6944 | Ref |
| KY271396 | Unassigned | Unassigned | Unassigned | 33_0 | Clustered | 9 | VC_33_0 | 9 | 0.3409 | 1 | 0.3409 | 0 | 0 | 0 | 0.6944 | Ref |
| KY271397 | Unassigned | Unassigned | Unassigned | 236_0 | Clustered | 32 | VC_236_0 | 32 | 0.9115 | 1 | 0.9115 | 0 | 0 | 0 | 1 | Ref |
| KY271398 | Unassigned | Unassigned | Unassigned | 6_0 | Clustered | 44 | VC_6_0 | 44 | 0.9068 | 1 | 0.9068 | 0 | 0 | 0 | 1 | Ref |
| KY271401 | Unassigned | Unassigned | Unassigned | 252_0 | Clustered | 12 | VC_252_0 | 12 | 0.4075 | 1 | 0.4075 | 0 | 0 | 0 | 1 | Ref |
| KY271402 | Unassigned | Unassigned | Unassigned | 37_0 | Clustered | 4 | VC_37_0 | 4 | 0.4325 | 0.99998062 | 0.4325 | 0 | 0 | 0 | 1 | Ref |
| KY290756 | Unassigned | Unassigned | Unassigned | 1067_0 | Clustered | 20 | VC_1067_0 | 20 | 0.7109 | 1 | 0.7109 | 0 | 0 | 0 | 1 | Ref |
| KY290947 | Unassigned | Unassigned | Unassigned | 1233_0 | Clustered | 9 | VC_1233_0 | 9 | 0.9676 | 1 | 0.9676 | 0 | 0 | 0 | 1 | Ref |
| KY290948 | Unassigned | Unassigned | Unassigned | 619_1 | Clustered | 7 | VC_619_1 | 7 | 0.1834 | 0.99999997 | 0.1834 | 0 | 0 | 0 | 1 | Ref |
| KY290949 | Unassigned | Unassigned | Unassigned | 1233_0 | Clustered | 9 | VC_1233_0 | 9 | 0.9676 | 1 | 0.9676 | 0 | 0 | 0 | 1 | Ref |
| KY290950 | Unassigned | Unassigned | Unassigned | 1233_0 | Clustered | 9 | VC_1233_0 | 9 | 0.9676 | 1 | 0.9676 | 0 | 0 | 0 | 1 | Ref |
| KY290951 | Unassigned | Unassigned | Unassigned | 619_1 | Clustered | 7 | VC_619_1 | 7 | 0.1834 | 0.99999997 | 0.1834 | 0 | 0 | 0 | 1 | Ref |
| KY290952 | Unassigned | Unassigned | Unassigned | 1233_0 | Clustered | 9 | VC_1233_0 | 9 | 0.9676 | 1 | 0.9676 | 0 | 0 | 0 | 1 | Ref |
| KY290953 | Unassigned | Unassigned | Unassigned | 380_0 | Clustered | 6 | VC_380_0 | 6 | 0.51 | 1 | 0.51 | 0 | 0 | 0 | 1 | Ref |
| KY290954 | Unassigned | Unassigned | Unassigned | 380_0 | Clustered | 6 | VC_380_0 | 6 | 0.51 | 1 | 0.51 | 0 | 0 | 0 | 1 | Ref |
| KY290955 | Unassigned | Unassigned | Unassigned | 457_3 | Clustered | 2 | VC_457_3 | 2 | 0.0286 | 0.89347378 | 0.0255 | 0 | 0 | 0 | 1 | Ref |
| KY290956 | Unassigned | Unassigned | Unassigned | 619_1 | Clustered | 7 | VC_619_1 | 7 | 0.1834 | 0.99999997 | 0.1834 | 0 | 0 | 0 | 1 | Ref |
| KY290957 | Unassigned | Unassigned | Unassigned | 619_1 | Clustered | 7 | VC_619_1 | 7 | 0.1834 | 0.99999997 | 0.1834 | 0 | 0 | 0 | 1 | Ref |
| KY290958 | Unassigned | Unassigned | Unassigned | 619_1 | Clustered | 7 | VC_619_1 | 7 | 0.1834 | 0.99999997 | 0.1834 | 0 | 0 | 0 | 1 | Ref |
| KY290975 | Unassigned | Unassigned | Unassigned | 290_111 | Clustered/Singleton | 1 | VC_290_111 | 1 | 0 | 1 | 0 | 0 | 0 | 0 | 1 | Ref |
| KY295891 | Unassigned | Unassigned | Unassigned | 466_0 | Clustered | 66 | VC_466_0 | 66 | 0.9246 | 1 | 0.9246 | 0 | 0 | 0 | 1 | Ref |
| KY295892 | Unassigned | Unassigned | Unassigned | 466_0 | Clustered | 66 | VC_466_0 | 66 | 0.9246 | 1 | 0.9246 | 0 | 0 | 0 | 1 | Ref |
| KY295893 | Unassigned | Unassigned | Unassigned | 466_0 | Clustered | 66 | VC_466_0 | 66 | 0.9246 | 1 | 0.9246 | 0 | 0 | 0 | 1 | Ref |
| KY295894 | Unassigned | Unassigned | Unassigned | 465_0 | Clustered | 80 | VC_465_0 | 80 | 0.9988 | 1 | 0.9988 | 0 | 0 | 0 | 1 | Ref |
| KY295895 | Unassigned | Unassigned | Unassigned | 655_0 | Clustered | 45 | VC_655_0 | 45 | 0.7722 | 1 | 0.7722 | 0 | 0 | 0 | 1 | Ref |
| KY295896 | Unassigned | Unassigned | Unassigned | 655_0 | Clustered | 45 | VC_655_0 | 45 | 0.7722 | 1 | 0.7722 | 0 | 0 | 0 | 1 | Ref |
| KY295897 | Unassigned | Unassigned | Unassigned | 466_0 | Clustered | 66 | VC_466_0 | 66 | 0.9246 | 1 | 0.9246 | 0 | 0 | 0 | 1 | Ref |
| KY295898 | Unassigned | Unassigned | Unassigned | 655_0 | Clustered | 45 | VC_655_0 | 45 | 0.7722 | 1 | 0.7722 | 0 | 0 | 0 | 1 | Ref |
| KY295899 | Unassigned | Unassigned | Unassigned | 466_0 | Clustered | 66 | VC_466_0 | 66 | 0.9246 | 1 | 0.9246 | 0 | 0 | 0 | 1 | Ref |
| KY296500 | Unassigned | Unassigned | Unassigned | 838_0 | Clustered | 3 | VC_838_0 | 3 | 1 | 1 | 1 | 0 | 0 | 0 | 1 | Ref |
| KY296501 | Unassigned | Unassigned | Unassigned | 838_0 | Clustered | 3 | VC_838_0 | 3 | 1 | 1 | 1 | 0 | 0 | 0 | 1 | Ref |
| KY303907 | Unassigned | Unassigned | Unassigned | 110_0 | Clustered | 33 | VC_110_0 | 33 | 0.9488 | 1 | 0.9488 | 0 | 0 | 0 | 1 | Ref |
| KY316062 | Unassigned | Unassigned | Unassigned | 71_0 | Clustered | 27 | VC_71_0 | 27 | 0.5964 | 1 | 0.5964 | 0 | 0 | 0 | 0.9943 | Ref |
| KY318515 | Unassigned | Unassigned | Unassigned | 907_0 | Clustered | 32 | VC_907_0 | 32 | 0.9964 | 1 | 0.9964 | 0 | 0 | 0 | 1 | Ref |
| KY319168 | Unassigned | Unassigned | Unassigned | 571_2 | Clustered | 17 | VC_571_2 | 17 | 0.0965 | 1 | 0.0965 | 0 | 0 | 0 | 0.9779 | Ref |
| KY348865 | Unassigned | Unassigned | Unassigned | 538_44 | Clustered/Singleton | 1 | VC_538_44 | 1 | 0 | 1 | 0 | 0 | 0 | 0 | 1 | Ref |
| KY349816 | Unassigned | Unassigned | Unassigned | 425_0 | Clustered | 40 | VC_425_0 | 40 | 0.8399 | 1 | 0.8399 | 0 | 0 | 0 | 0.9974 | Ref |
| KY363465 | Unassigned | Unassigned | Unassigned | 201_0 | Clustered | 2 | VC_201_0 | 2 | 0.0315 | 0.95762656 | 0.0301 | 0 | 0 | 0 | 1 | Ref |
| KY365739 | Unassigned | Unassigned | Unassigned | 891_0 | Clustered | 11 | VC_891_0 | 11 | 0.9663 | 1 | 0.9663 | 0 | 0 | 0 | 1 | Ref |
| KY368639 | Unassigned | Unassigned | Unassigned | 89_0 | Clustered | 8 | VC_89_0 | 8 | 0.5507 | 1 | 0.5507 | 0 | 0 | 0 | 1 | Ref |
| KY368640 | Unassigned | Unassigned | Unassigned | 87_4 | Clustered | 3 | VC_87_4 | 3 | 0.0292 | 0.98125255 | 0.0286 | 0 | 0 | 0 | 1 | Ref |
| KY379511 | Unassigned | Unassigned | Unassigned | 927_0 | Clustered | 5 | VC_927_0 | 5 | 0.4988 | 0.99999981 | 0.4988 | 0 | 0 | 0 | 1 | Ref |
| KY379853 | Unassigned | Unassigned | Unassigned | 654_0 | Clustered | 115 | VC_654_0 | 115 | 0.7303 | 1 | 0.7303 | 0 | 0 | 0 | 0.8764 | Ref |
| KY380102 | Unassigned | Unassigned | Unassigned | 243_0 | Clustered | 72 | VC_243_0 | 72 | 0.8089 | 1 | 0.8089 | 0 | 0 | 0 | 1 | Ref |
| KY381600 | Unassigned | Unassigned | Unassigned | 650_1 | Clustered | 6 | VC_650_1 | 6 | 0.4186 | 1 | 0.4186 | 0 | 0 | 0 | 1 | Ref |
| KY385380 | Unassigned | Unassigned | Unassigned | 384_13 | Clustered | 33 | VC_384_13 | 33 | 0.1785 | 1 | 0.1785 | 0 | 0 | 0 | 0.8769 | Ref |
| KY385381 | Unassigned | Unassigned | Unassigned | 786_0 | Clustered | 4 | VC_786_0 | 4 | 0.3519 | 0.99997469 | 0.3519 | 0 | 0 | 0 | 1 | Ref |
| KY385382 | Unassigned | Unassigned | Unassigned | 384_13 | Clustered | 33 | VC_384_13 | 33 | 0.1785 | 1 | 0.1785 | 0 | 0 | 0 | 0.8769 | Ref |
| KY385383 | Unassigned | Unassigned | Unassigned | 384_13 | Clustered | 33 | VC_384_13 | 33 | 0.1785 | 1 | 0.1785 | 0 | 0 | 0 | 0.8769 | Ref |
| KY385384 | Unassigned | Unassigned | Unassigned | 538_53 | Clustered/Singleton | 1 | VC_538_53 | 1 | 0 | 1 | 0 | 0 | 0 | 0 | 1 | Ref |
| KY385423 | Unassigned | Unassigned | Unassigned | 113_0 | Clustered | 91 | VC_113_0 | 91 | 0.9584 | 1 | 0.9584 | 0 | 0 | 0 | 1 | Ref |

| | | | | | | | | | | | | | | | | |
|---|---|---|---|---|---|---|---|---|---|---|---|---|---|---|---|---|
| KY389063 | Unassigned | Unassigned | Unassigned | 7_0 | Clustered | 28 | VC_7_0 | 28 | 0.389 | 1 | 0.389 | 0 | 0 | 0 | 0.9947 | Ref |
| KY389064 | Unassigned | Unassigned | Unassigned | 7_0 | Clustered | 28 | VC_7_0 | 28 | 0.389 | 1 | 0.389 | 0 | 0 | 0 | 0.9947 | Ref |
| KY389315 | Unassigned | Unassigned | Unassigned | 848_0 | Clustered | 124 | VC_848_0 | 124 | 0.9484 | 1 | 0.9484 | 0 | 0 | 0 | 1 | Ref |
| KY389316 | Unassigned | Unassigned | Unassigned | 848_0 | Clustered | 124 | VC_848_0 | 124 | 0.9484 | 1 | 0.9484 | 0 | 0 | 0 | 1 | Ref |
| KY398841 | Unassigned | Unassigned | Unassigned | 470_0 | Clustered | 62 | VC_470_0 | 62 | 0.902 | 1 | 0.902 | 0 | 0 | 0 | 1 | Ref |
| KY434670 | Unassigned | Unassigned | Unassigned | 985_0 | Clustered | 38 | VC_985_0 | 38 | 0.9334 | 1 | 0.9334 | 0 | 0 | 0 | 1 | Ref |
| KY435490 | Unassigned | Unassigned | Unassigned | 466_0 | Clustered | 66 | VC_466_0 | 66 | 0.9246 | 1 | 0.9246 | 0 | 0 | 0 | 1 | Ref |
| KY448244 | Unassigned | Unassigned | Unassigned | 68_0 | Clustered | 2 | VC_68_0 | 2 | 0.0139 | 0.69357611 | 0.0097 | 0 | 0 | 0 | 1 | Ref |
| KY450753 | Unassigned | Unassigned | Unassigned | 848_0 | Clustered | 124 | VC_848_0 | 124 | 0.9484 | 1 | 0.9484 | 0 | 0 | 0 | 1 | Ref |
| KY464836 | Unassigned | Unassigned | Unassigned | 71_0 | Clustered | 27 | VC_71_0 | 27 | 0.5964 | 1 | 0.5964 | 0 | 0 | 0 | 0.9943 | Ref |
| KY464936 | Unassigned | Unassigned | Unassigned | 531_0 | Clustered | 32 | VC_531_0 | 32 | 0.6859 | 1 | 0.6859 | 0 | 0 | 0 | 1 | Ref |
| KY471266 | Unassigned | Unassigned | Unassigned | 535_1 | Clustered | 108 | VC_535_1 | 108 | 0.8479 | 1 | 0.8479 | 0 | 0 | 0 | 0.9955 | Ref |
| KY471267 | Unassigned | Unassigned | Unassigned | 538_27 | Clustered | 2 | VC_538_27 | 2 | 0.0141 | 0.95761635 | 0.0135 | 0 | 0 | 0 | 1 | Ref |
| KY471268 | Unassigned | Unassigned | Unassigned | 419_0 | Clustered | 11 | VC_419_0 | 11 | 0.9465 | 1 | 0.9465 | 0 | 0 | 0 | 1 | Ref |
| KY471269 | Unassigned | Unassigned | Unassigned | 419_0 | Clustered | 11 | VC_419_0 | 11 | 0.9465 | 1 | 0.9465 | 0 | 0 | 0 | 1 | Ref |
| KY471460 | Unassigned | Unassigned | Unassigned | 228_0 | Clustered | 22 | VC_228_0 | 22 | 0.7411 | 1 | 0.7411 | 0 | 0 | 0 | 1 | Ref |
| KY472224 | Unassigned | Unassigned | Unassigned | 110_0 | Clustered | 33 | VC_110_0 | 33 | 0.9488 | 1 | 0.9488 | 0 | 0 | 0 | 1 | Ref |
| KY514263 | Unassigned | Unassigned | Unassigned | 873_2 | Clustered | 2 | VC_873_2 | 2 | 0.0245 | 0.87022547 | 0.0213 | 0 | 0 | 0 | 1 | Ref |
| KY514264 | Unassigned | Unassigned | Unassigned | 865_0 | Clustered | 10 | VC_865_0 | 10 | 0.5975 | 1 | 0.5975 | 0 | 0 | 0 | 1 | Ref |
| KY514265 | Unassigned | Unassigned | Unassigned | 865_0 | Clustered | 10 | VC_865_0 | 10 | 0.5975 | 1 | 0.5975 | 0 | 0 | 0 | 1 | Ref |
| KY549152 | Unassigned | Unassigned | Unassigned | 571_7 | Clustered | 28 | VC_571_7 | 28 | 0.1651 | 1 | 0.1651 | 0 | 0 | 0 | 0.8519 | Ref |
| KY549153 | Unassigned | Unassigned | Unassigned | 991_0 | Clustered | 39 | VC_991_0 | 39 | 1 | 1 | 1 | 0 | 0 | 0 | 1 | Ref |
| KY549154 | Unassigned | Unassigned | Unassigned | 991_0 | Clustered | 39 | VC_991_0 | 39 | 1 | 1 | 1 | 0 | 0 | 0 | 1 | Ref |
| KY549155 | Unassigned | Unassigned | Unassigned | 535_1 | Clustered | 108 | VC_535_1 | 108 | 0.8479 | 1 | 0.8479 | 0 | 0 | 0 | 0.9955 | Ref |
| KY549443 | Unassigned | Unassigned | Unassigned | 94_0 | Clustered | 22 | VC_94_0 | 22 | 0.5602 | 1 | 0.5602 | 0 | 0 | 0 | 1 | Ref |
| KY549659 | Unassigned | Unassigned | Unassigned | 865_0 | Clustered | 10 | VC_865_0 | 10 | 0.5975 | 1 | 0.5975 | 0 | 0 | 0 | 1 | Ref |
| KY554760 | Unassigned | Unassigned | Unassigned | 210_0 | Clustered | 105 | VC_210_0 | 105 | 0.4076 | 1 | 0.4076 | 0 | 0 | 0 | 0.7766 | Ref |
| KY554761 | Unassigned | Unassigned | Unassigned | 210_0 | Clustered | 105 | VC_210_0 | 105 | 0.4076 | 1 | 0.4076 | 0 | 0 | 0 | 0.7766 | Ref |
| KY554762 | Unassigned | Unassigned | Unassigned | 552_0 | Clustered | 9 | VC_552_0 | 9 | 1 | 1 | 1 | 0 | 0 | 0 | 1 | Ref |
| KY554763 | Unassigned | Unassigned | Unassigned | 552_0 | Clustered | 9 | VC_552_0 | 9 | 1 | 1 | 1 | 0 | 0 | 0 | 1 | Ref |
| KY554764 | Unassigned | Unassigned | Unassigned | 552_0 | Clustered | 9 | VC_552_0 | 9 | 1 | 1 | 1 | 0 | 0 | 0 | 1 | Ref |
| KY554765 | Unassigned | Unassigned | Unassigned | 552_0 | Clustered | 9 | VC_552_0 | 9 | 1 | 1 | 1 | 0 | 0 | 0 | 1 | Ref |
| KY554766 | Unassigned | Unassigned | Unassigned | 552_0 | Clustered | 9 | VC_552_0 | 9 | 1 | 1 | 1 | 0 | 0 | 0 | 1 | Ref |
| KY554767 | Unassigned | Unassigned | Unassigned | 552_0 | Clustered | 9 | VC_552_0 | 9 | 1 | 1 | 1 | 0 | 0 | 0 | 1 | Ref |
| KY554768 | Unassigned | Unassigned | Unassigned | 488_0 | Clustered | 14 | VC_488_0 | 14 | 0.8516 | 1 | 0.8516 | 0 | 0 | 0 | 1 | Ref |
| KY554769 | Unassigned | Unassigned | Unassigned | 488_0 | Clustered | 14 | VC_488_0 | 14 | 0.8516 | 1 | 0.8516 | 0 | 0 | 0 | 1 | Ref |
| KY554770 | Unassigned | Unassigned | Unassigned | 488_0 | Clustered | 14 | VC_488_0 | 14 | 0.8516 | 1 | 0.8516 | 0 | 0 | 0 | 1 | Ref |
| KY554771 | Unassigned | Unassigned | Unassigned | 488_0 | Clustered | 14 | VC_488_0 | 14 | 0.8516 | 1 | 0.8516 | 0 | 0 | 0 | 1 | Ref |
| KY554772 | Unassigned | Unassigned | Unassigned | 488_0 | Clustered | 14 | VC_488_0 | 14 | 0.8516 | 1 | 0.8516 | 0 | 0 | 0 | 1 | Ref |
| KY554773 | Unassigned | Unassigned | Unassigned | 488_0 | Clustered | 14 | VC_488_0 | 14 | 0.8516 | 1 | 0.8516 | 0 | 0 | 0 | 1 | Ref |
| KY554774 | Unassigned | Unassigned | Unassigned | 488_0 | Clustered | 14 | VC_488_0 | 14 | 0.8516 | 1 | 0.8516 | 0 | 0 | 0 | 1 | Ref |
| KY554775 | Unassigned | Unassigned | Unassigned | 488_0 | Clustered | 14 | VC_488_0 | 14 | 0.8516 | 1 | 0.8516 | 0 | 0 | 0 | 1 | Ref |
| KY554776 | Unassigned | Unassigned | Unassigned | 488_0 | Clustered | 14 | VC_488_0 | 14 | 0.8516 | 1 | 0.8516 | 0 | 0 | 0 | 1 | Ref |
| KY554777 | Unassigned | Unassigned | Unassigned | 488_0 | Clustered | 14 | VC_488_0 | 14 | 0.8516 | 1 | 0.8516 | 0 | 0 | 0 | 1 | Ref |
| KY555142 | Unassigned | Unassigned | Unassigned | 1011_4 | Clustered | 11 | VC_1011_4 | 11 | 0.3331 | 1 | 0.3331 | 0 | 0 | 0 | 1 | Ref |
| KY555143 | Unassigned | Unassigned | Unassigned | 1011_4 | Clustered | 11 | VC_1011_4 | 11 | 0.3331 | 1 | 0.3331 | 0 | 0 | 0 | 1 | Ref |
| KY555144 | Unassigned | Unassigned | Unassigned | 1011_4 | Clustered | 11 | VC_1011_4 | 11 | 0.3331 | 1 | 0.3331 | 0 | 0 | 0 | 1 | Ref |
| KY555145 | Unassigned | Unassigned | Unassigned | 1011_4 | Clustered | 11 | VC_1011_4 | 11 | 0.3331 | 1 | 0.3331 | 0 | 0 | 0 | 1 | Ref |
| KY555146 | Unassigned | Unassigned | Unassigned | 1011_4 | Clustered | 11 | VC_1011_4 | 11 | 0.3331 | 1 | 0.3331 | 0 | 0 | 0 | 1 | Ref |
| KY555147 | Unassigned | Unassigned | Unassigned | 1011_4 | Clustered | 11 | VC_1011_4 | 11 | 0.3331 | 1 | 0.3331 | 0 | 0 | 0 | 1 | Ref |
| KY563228 | Unassigned | Unassigned | Unassigned | 618_0 | Clustered | 50 | VC_618_0 | 50 | 0.9989 | 1 | 0.9989 | 0 | 0 | 0 | 1 | Ref |
| KY565347 | Unassigned | Unassigned | Unassigned | 103_0 | Clustered | 3 | VC_103_0 | 3 | 0.369 | 0.99763908 | 0.3682 | 0 | 0 | 0 | 1 | Ref |
| KY566218 | Unassigned | Unassigned | Unassigned | 421_0 | Clustered | 9 | VC_421_0 | 9 | 0.855 | 1 | 0.855 | 0 | 0 | 0 | 1 | Ref |
| KY575286 | Unassigned | Unassigned | Unassigned | 972_0 | Clustered | 99 | VC_972_0 | 99 | 0.9113 | 1 | 0.9113 | 0 | 0 | 0 | 0.9928 | Ref |
| KY579375 | Unassigned | Unassigned | Unassigned | 618_0 | Clustered | 50 | VC_618_0 | 50 | 0.9989 | 1 | 0.9989 | 0 | 0 | 0 | 1 | Ref |
| KY581279 | Unassigned | Unassigned | Unassigned | 155_8 | Clustered | 66 | VC_155_8 | 66 | 0.4537 | 1 | 0.4537 | 0 | 0 | 0 | 0.8182 | Ref |
| KY593455 | Unassigned | Unassigned | Unassigned | 508_3 | Clustered | 2 | VC_508_3 | 2 | 0.0132 | 0.95703149 | 0.0126 | 0 | 0 | 0 | 1 | Ref |
| KY608966 | Unassigned | Unassigned | Unassigned | 440_0 | Clustered | 33 | VC_440_0 | 33 | 0.992 | 1 | 0.992 | 0 | 0 | 0 | 1 | Ref |
| KY608967 | Unassigned | Unassigned | Unassigned | 290_109 | Clustered | 2 | VC_290_109 | 2 | 0.0026 | 0.91436748 | 0.0024 | 0 | 0 | 0 | 1 | Ref |
| KY612839 | Unassigned | Unassigned | Unassigned | 806_0 | Clustered | 6 | VC_806_0 | 6 | 0.5468 | 1 | 0.5468 | 0 | 0 | 0 | 1 | Ref |
| KY613597 | Unassigned | Unassigned | Unassigned | 421_0 | Clustered | 9 | VC_421_0 | 9 | 0.855 | 1 | 0.855 | 0 | 0 | 0 | 1 | Ref |
| KY618819 | Unassigned | Unassigned | Unassigned | 434_0 | Clustered | 45 | VC_434_0 | 45 | 0.9479 | 1 | 0.9479 | 0 | 0 | 0 | 1 | Ref |
| KY620117 | Unassigned | Unassigned | Unassigned | 611_0 | Clustered | 50 | VC_611_0 | 50 | 0.7306 | 1 | 0.7306 | 0 | 0 | 0 | 1 | Ref |
| KY622015 | Unassigned | Unassigned | Unassigned | 515_0 | Clustered | 8 | VC_515_0 | 8 | 0.3973 | 1 | 0.3973 | 0 | 0 | 0 | 1 | Ref |
| KY624610 | Unassigned | Unassigned | Unassigned | 421_0 | Clustered | 9 | VC_421_0 | 9 | 0.855 | 1 | 0.855 | 0 | 0 | 0 | 1 | Ref |
| KY624611 | Unassigned | Unassigned | Unassigned | 421_0 | Clustered | 9 | VC_421_0 | 9 | 0.855 | 1 | 0.855 | 0 | 0 | 0 | 1 | Ref |
| KY624612 | Unassigned | Unassigned | Unassigned | 421_0 | Clustered | 9 | VC_421_0 | 9 | 0.855 | 1 | 0.855 | 0 | 0 | 0 | 1 | Ref |
| KY624613 | Unassigned | Unassigned | Unassigned | 421_0 | Clustered | 9 | VC_421_0 | 9 | 0.855 | 1 | 0.855 | 0 | 0 | 0 | 1 | Ref |
| KY624614 | Unassigned | Unassigned | Unassigned | 421_0 | Clustered | 9 | VC_421_0 | 9 | 0.855 | 1 | 0.855 | 0 | 0 | 0 | 1 | Ref |
| KY624615 | Unassigned | Unassigned | Unassigned | 421_0 | Clustered | 9 | VC_421_0 | 9 | 0.855 | 1 | 0.855 | 0 | 0 | 0 | 1 | Ref |
| KY624616 | Unassigned | Unassigned | Unassigned | 421_0 | Clustered | 9 | VC_421_0 | 9 | 0.855 | 1 | 0.855 | 0 | 0 | 0 | 1 | Ref |
| KY626162 | Unassigned | Unassigned | Unassigned | 340_1 | Clustered | 17 | VC_340_1 | 17 | 0.0863 | 1 | 0.0863 | 0 | 0 | 0 | 0.8235 | Ref |
| KY626163 | Unassigned | Unassigned | Unassigned | 654_0 | Clustered | 115 | VC_654_0 | 115 | 0.7303 | 1 | 0.7303 | 0 | 0 | 0 | 0.8764 | Ref |
| KY626176 | Unassigned | Unassigned | Unassigned | 1067_0 | Clustered | 20 | VC_1067_0 | 20 | 0.7109 | 1 | 0.7109 | 0 | 0 | 0 | 1 | Ref |
| KY626177 | Unassigned | Unassigned | Unassigned | 1067_0 | Clustered | 20 | VC_1067_0 | 20 | 0.7109 | 1 | 0.7109 | 0 | 0 | 0 | 1 | Ref |
| KY629563 | Unassigned | Unassigned | Unassigned | 1013_0 | Clustered | 2 | VC_1013_0 | 2 | 0.0455 | 0.94948967 | 0.0432 | 0 | 0 | 0 | 1 | Ref |
| KY630163 | Unassigned | Unassigned | Unassigned | 135_34 | Clustered | 3 | VC_135_34 | 3 | 0.0102 | 0.97231548 | 0.0099 | 0 | 0 | 0 | 0.6667 | Ref |
| KY630187 | Unassigned | Unassigned | Unassigned | 170_7 | Clustered | 7 | VC_170_7 | 7 | 0.0952 | 0.99874946 | 0.0951 | 0 | 0 | 0 | 1 | Ref |
| KY652723 | Unassigned | Unassigned | Unassigned | 848_0 | Clustered | 124 | VC_848_0 | 124 | 0.9484 | 1 | 0.9484 | 0 | 0 | 0 | 1 | Ref |
| KY652724 | Unassigned | Unassigned | Unassigned | 848_0 | Clustered | 124 | VC_848_0 | 124 | 0.9484 | 1 | 0.9484 | 0 | 0 | 0 | 1 | Ref |
| KY652725 | Unassigned | Unassigned | Unassigned | 848_0 | Clustered | 124 | VC_848_0 | 124 | 0.9484 | 1 | 0.9484 | 0 | 0 | 0 | 1 | Ref |

| | | | | | | | | | | | | | | | |
|---|---|---|---|---|---|---|---|---|---|---|---|---|---|---|---|
| KY652726 | Unassigned | Unassigned | Unassigned | 601_3 | Clustered | 12 | VC_601_3 | 12 | 0.2339 | 1 | 0.2339 | 0 | 0 | 0 | 0.8636 Ref |
| KY653123 | Unassigned | Unassigned | Unassigned | 10_0 | Clustered | 25 | VC_10_0 | 25 | 0.3437 | 1 | 0.3437 | 0 | 0 | 0 | 0.9733 Ref |
| KY653125 | Unassigned | Unassigned | Unassigned | 7_0 | Clustered | 28 | VC_7_0 | 28 | 0.389 | 1 | 0.389 | 0 | 0 | 0 | 0.9947 Ref |
| KY653237 | Unassigned | Unassigned | Unassigned | 310_0 | Clustered | 155 | VC_310_0 | 155 | 0.9979 | 1 | 0.9979 | 0 | 0 | 0 | 1 Ref |
| KY654690 | Unassigned | Unassigned | Unassigned | 341_0 | Clustered | 27 | VC_341_0 | 27 | 0.5058 | 1 | 0.5058 | 0 | 0 | 0 | 0.8661 Ref |
| KY657202 | Unassigned | Unassigned | Unassigned | 603_0 | Clustered | 28 | VC_603_0 | 28 | 0.7692 | 1 | 0.7692 | 0 | 0 | 0 | 1 Ref |
| KY658673 | Unassigned | Unassigned | Unassigned | 306_0 | Clustered | 24 | VC_306_0 | 24 | 0.9902 | 1 | 0.9902 | 0 | 0 | 0 | 1 Ref |
| KY658674 | Unassigned | Unassigned | Unassigned | 306_0 | Clustered | 24 | VC_306_0 | 24 | 0.9902 | 1 | 0.9902 | 0 | 0 | 0 | 1 Ref |
| KY658675 | Unassigned | Unassigned | Unassigned | 306_0 | Clustered | 24 | VC_306_0 | 24 | 0.9902 | 1 | 0.9902 | 0 | 0 | 0 | 1 Ref |
| KY658676 | Unassigned | Unassigned | Unassigned | 306_0 | Clustered | 24 | VC_306_0 | 24 | 0.9902 | 1 | 0.9902 | 0 | 0 | 0 | 1 Ref |
| KY658677 | Unassigned | Unassigned | Unassigned | 306_0 | Clustered | 24 | VC_306_0 | 24 | 0.9902 | 1 | 0.9902 | 0 | 0 | 0 | 1 Ref |
| KY658678 | Unassigned | Unassigned | Unassigned | 306_0 | Clustered | 24 | VC_306_0 | 24 | 0.9902 | 1 | 0.9902 | 0 | 0 | 0 | 1 Ref |
| KY658679 | Unassigned | Unassigned | Unassigned | 306_0 | Clustered | 24 | VC_306_0 | 24 | 0.9902 | 1 | 0.9902 | 0 | 0 | 0 | 1 Ref |
| KY658680 | Unassigned | Unassigned | Unassigned | 306_0 | Clustered | 24 | VC_306_0 | 24 | 0.9902 | 1 | 0.9902 | 0 | 0 | 0 | 1 Ref |
| KY661963 | Unassigned | Unassigned | Unassigned | 745_0 | Clustered | 9 | VC_745_0 | 9 | 0.9955 | 0.99999771 | 0.9955 | 0 | 0 | 0 | 1 Ref |
| KY670595 | Unassigned | Unassigned | Unassigned | 713_0 | Clustered | 26 | VC_713_0 | 26 | 0.9358 | 1 | 0.9358 | 0 | 0 | 0 | 1 Ref |
| KY676783 | Unassigned | Unassigned | Unassigned | 384_15 | Clustered | 44 | VC_384_15 | 44 | 0.139 | 1 | 0.139 | 0 | 0 | 0 | 0.7474 Ref |
| KY676784 | Unassigned | Unassigned | Unassigned | 742_0 | Clustered | 11 | VC_742_0 | 11 | 0.5046 | 1 | 0.5046 | 0 | 0 | 0 | 1 Ref |
| KY677846 | Unassigned | Unassigned | Unassigned | 198_15 | Clustered | 3 | VC_198_15 | 3 | 0.0144 | 0.99754982 | 0.0144 | 0 | 0 | 0 | 0.6667 Ref |
| KY683735 | Unassigned | Unassigned | Unassigned | 609_0 | Clustered | 27 | VC_609_0 | 27 | 0.9943 | 1 | 0.9943 | 0 | 0 | 0 | 1 Ref |
| KY683736 | Unassigned | Unassigned | Unassigned | 794_9 | Clustered | 7 | VC_794_9 | 7 | 0.0369 | 0.87330366 | 0.0322 | 0 | 0 | 0 | 1 Ref |
| KY684082 | Unassigned | Unassigned | Unassigned | 113_0 | Clustered | 91 | VC_113_0 | 91 | 0.9584 | 1 | 0.9584 | 0 | 0 | 0 | 1 Ref |
| KY694971 | Unassigned | Unassigned | Unassigned | 603_0 | Clustered | 28 | VC_603_0 | 28 | 0.7692 | 1 | 0.7692 | 0 | 0 | 0 | 1 Ref |
| KY695153 | Unassigned | Unassigned | Unassigned | 7_0 | Clustered | 28 | VC_7_0 | 28 | 0.389 | 1 | 0.389 | 0 | 0 | 0 | 0.9947 Ref |
| KY703222 | Unassigned | Unassigned | Unassigned | 293_10 | Clustered | 9 | VC_293_10 | 9 | 0.0615 | 0.99999884 | 0.0615 | 0 | 0 | 0 | 0.6667 Ref |
| KY705251 | Unassigned | Unassigned | Unassigned | 258_0 | Clustered | 121 | VC_258_0 | 121 | 0.7359 | 1 | 0.7359 | 0 | 0 | 0 | 0.9999 Ref |
| KY705252 | Unassigned | Unassigned | Unassigned | 261_0 | Clustered | 13 | VC_261_0 | 13 | 0.2024 | 1 | 0.2024 | 0 | 0 | 0 | 1 Ref |
| KY705253 | Unassigned | Unassigned | Unassigned | 261_0 | Clustered | 13 | VC_261_0 | 13 | 0.2024 | 1 | 0.2024 | 0 | 0 | 0 | 1 Ref |
| KY705254 | Unassigned | Unassigned | Unassigned | 261_0 | Clustered | 13 | VC_261_0 | 13 | 0.2024 | 1 | 0.2024 | 0 | 0 | 0 | 1 Ref |
| KY705255 | Unassigned | Unassigned | Unassigned | 261_0 | Clustered | 13 | VC_261_0 | 13 | 0.2024 | 1 | 0.2024 | 0 | 0 | 0 | 1 Ref |
| KY705256 | Unassigned | Unassigned | Unassigned | 258_0 | Clustered | 121 | VC_258_0 | 121 | 0.7359 | 1 | 0.7359 | 0 | 0 | 0 | 0.9999 Ref |
| KY705257 | Unassigned | Unassigned | Unassigned | 258_0 | Clustered | 121 | VC_258_0 | 121 | 0.7359 | 1 | 0.7359 | 0 | 0 | 0 | 0.9999 Ref |
| KY705258 | Unassigned | Unassigned | Unassigned | 260_0 | Clustered | 46 | VC_260_0 | 46 | 0.5137 | 1 | 0.5137 | 0 | 0 | 0 | 1 Ref |
| KY705259 | Unassigned | Unassigned | Unassigned | 258_0 | Clustered | 121 | VC_258_0 | 121 | 0.7359 | 1 | 0.7359 | 0 | 0 | 0 | 0.9999 Ref |
| KY705260 | Unassigned | Unassigned | Unassigned | 258_0 | Clustered | 121 | VC_258_0 | 121 | 0.7359 | 1 | 0.7359 | 0 | 0 | 0 | 0.9999 Ref |
| KY705261 | Unassigned | Unassigned | Unassigned | 258_0 | Clustered | 121 | VC_258_0 | 121 | 0.7359 | 1 | 0.7359 | 0 | 0 | 0 | 0.9999 Ref |
| KY705262 | Unassigned | Unassigned | Unassigned | 258_0 | Clustered | 121 | VC_258_0 | 121 | 0.7359 | 1 | 0.7359 | 0 | 0 | 0 | 0.9999 Ref |
| KY705263 | Unassigned | Unassigned | Unassigned | 258_0 | Clustered | 121 | VC_258_0 | 121 | 0.7359 | 1 | 0.7359 | 0 | 0 | 0 | 0.9999 Ref |
| KY705264 | Unassigned | Unassigned | Unassigned | 258_0 | Clustered | 121 | VC_258_0 | 121 | 0.7359 | 1 | 0.7359 | 0 | 0 | 0 | 0.9999 Ref |
| KY705265 | Unassigned | Unassigned | Unassigned | 258_0 | Clustered | 121 | VC_258_0 | 121 | 0.7359 | 1 | 0.7359 | 0 | 0 | 0 | 0.9999 Ref |
| KY705266 | Unassigned | Unassigned | Unassigned | 258_0 | Clustered | 121 | VC_258_0 | 121 | 0.7359 | 1 | 0.7359 | 0 | 0 | 0 | 0.9999 Ref |
| KY705267 | Unassigned | Unassigned | Unassigned | 258_0 | Clustered | 121 | VC_258_0 | 121 | 0.7359 | 1 | 0.7359 | 0 | 0 | 0 | 0.9999 Ref |
| KY705268 | Unassigned | Unassigned | Unassigned | 260_0 | Clustered | 46 | VC_260_0 | 46 | 0.5137 | 1 | 0.5137 | 0 | 0 | 0 | 1 Ref |
| KY705269 | Unassigned | Unassigned | Unassigned | 260_0 | Clustered | 46 | VC_260_0 | 46 | 0.5137 | 1 | 0.5137 | 0 | 0 | 0 | 1 Ref |
| KY705270 | Unassigned | Unassigned | Unassigned | 258_0 | Clustered | 121 | VC_258_0 | 121 | 0.7359 | 1 | 0.7359 | 0 | 0 | 0 | 0.9999 Ref |
| KY705271 | Unassigned | Unassigned | Unassigned | 258_0 | Clustered | 121 | VC_258_0 | 121 | 0.7359 | 1 | 0.7359 | 0 | 0 | 0 | 0.9999 Ref |
| KY705272 | Unassigned | Unassigned | Unassigned | 258_0 | Clustered | 121 | VC_258_0 | 121 | 0.7359 | 1 | 0.7359 | 0 | 0 | 0 | 0.9999 Ref |
| KY705273 | Unassigned | Unassigned | Unassigned | 258_0 | Clustered | 121 | VC_258_0 | 121 | 0.7359 | 1 | 0.7359 | 0 | 0 | 0 | 0.9999 Ref |
| KY705274 | Unassigned | Unassigned | Unassigned | 258_0 | Clustered | 121 | VC_258_0 | 121 | 0.7359 | 1 | 0.7359 | 0 | 0 | 0 | 0.9999 Ref |
| KY705275 | Unassigned | Unassigned | Unassigned | 258_0 | Clustered | 121 | VC_258_0 | 121 | 0.7359 | 1 | 0.7359 | 0 | 0 | 0 | 0.9999 Ref |
| KY705276 | Unassigned | Unassigned | Unassigned | 258_0 | Clustered | 121 | VC_258_0 | 121 | 0.7359 | 1 | 0.7359 | 0 | 0 | 0 | 0.9999 Ref |
| KY705277 | Unassigned | Unassigned | Unassigned | 260_0 | Clustered | 46 | VC_260_0 | 46 | 0.5137 | 1 | 0.5137 | 0 | 0 | 0 | 1 Ref |
| KY705278 | Unassigned | Unassigned | Unassigned | 260_0 | Clustered | 46 | VC_260_0 | 46 | 0.5137 | 1 | 0.5137 | 0 | 0 | 0 | 1 Ref |
| KY705279 | Unassigned | Unassigned | Unassigned | 260_0 | Clustered | 46 | VC_260_0 | 46 | 0.5137 | 1 | 0.5137 | 0 | 0 | 0 | 1 Ref |
| KY705280 | Unassigned | Unassigned | Unassigned | 260_0 | Clustered | 46 | VC_260_0 | 46 | 0.5137 | 1 | 0.5137 | 0 | 0 | 0 | 1 Ref |
| KY705281 | Unassigned | Unassigned | Unassigned | 260_0 | Clustered | 46 | VC_260_0 | 46 | 0.5137 | 1 | 0.5137 | 0 | 0 | 0 | 1 Ref |
| KY705282 | Unassigned | Unassigned | Unassigned | 258_0 | Clustered | 121 | VC_258_0 | 121 | 0.7359 | 1 | 0.7359 | 0 | 0 | 0 | 0.9999 Ref |
| KY705283 | Unassigned | Unassigned | Unassigned | 258_0 | Clustered | 121 | VC_258_0 | 121 | 0.7359 | 1 | 0.7359 | 0 | 0 | 0 | 0.9999 Ref |
| KY705284 | Unassigned | Unassigned | Unassigned | 258_0 | Clustered | 121 | VC_258_0 | 121 | 0.7359 | 1 | 0.7359 | 0 | 0 | 0 | 0.9999 Ref |
| KY705285 | Unassigned | Unassigned | Unassigned | 260_0 | Clustered | 46 | VC_260_0 | 46 | 0.5137 | 1 | 0.5137 | 0 | 0 | 0 | 1 Ref |
| KY705286 | Unassigned | Unassigned | Unassigned | 260_0 | Clustered | 46 | VC_260_0 | 46 | 0.5137 | 1 | 0.5137 | 0 | 0 | 0 | 1 Ref |
| KY705287 | Unassigned | Unassigned | Unassigned | 258_0 | Clustered | 121 | VC_258_0 | 121 | 0.7359 | 1 | 0.7359 | 0 | 0 | 0 | 0.9999 Ref |
| KY705288 | Unassigned | Unassigned | Unassigned | 258_0 | Clustered | 121 | VC_258_0 | 121 | 0.7359 | 1 | 0.7359 | 0 | 0 | 0 | 0.9999 Ref |
| KY705289 | Unassigned | Unassigned | Unassigned | 258_0 | Clustered | 121 | VC_258_0 | 121 | 0.7359 | 1 | 0.7359 | 0 | 0 | 0 | 0.9999 Ref |
| KY705290 | Unassigned | Unassigned | Unassigned | 258_0 | Clustered | 121 | VC_258_0 | 121 | 0.7359 | 1 | 0.7359 | 0 | 0 | 0 | 0.9999 Ref |
| KY705409 | Unassigned | Unassigned | Unassigned | 127_0 | Clustered | 17 | VC_127_0 | 17 | 0.9222 | 1 | 0.9222 | 0 | 0 | 0 | 1 Ref |
| KY707339 | Unassigned | Unassigned | Unassigned | 764_0 | Clustered | 23 | VC_764_0 | 23 | 0.8194 | 1 | 0.8194 | 0 | 0 | 0 | 1 Ref |
| KY709296 | Unassigned | Unassigned | Unassigned | 817_0 | Clustered | 12 | VC_817_0 | 12 | 0.8497 | 1 | 0.8497 | 0 | 0 | 0 | 1 Ref |
| KY744228 | Unassigned | Unassigned | Unassigned | 360_0 | Clustered | 22 | VC_360_0 | 22 | 0.96 | 1 | 0.96 | 0 | 0 | 0 | 1 Ref |
| KY744229 | Unassigned | Unassigned | Unassigned | 360_0 | Clustered | 22 | VC_360_0 | 22 | 0.96 | 1 | 0.96 | 0 | 0 | 0 | 1 Ref |
| KY744230 | Unassigned | Unassigned | Unassigned | 360_0 | Clustered | 22 | VC_360_0 | 22 | 0.96 | 1 | 0.96 | 0 | 0 | 0 | 1 Ref |
| KY744231 | Unassigned | Unassigned | Unassigned | 360_0 | Clustered | 22 | VC_360_0 | 22 | 0.96 | 1 | 0.96 | 0 | 0 | 0 | 1 Ref |
| KY744232 | Unassigned | Unassigned | Unassigned | 360_0 | Clustered | 22 | VC_360_0 | 22 | 0.96 | 1 | 0.96 | 0 | 0 | 0 | 1 Ref |
| KY744233 | Unassigned | Unassigned | Unassigned | 360_0 | Clustered | 22 | VC_360_0 | 22 | 0.96 | 1 | 0.96 | 0 | 0 | 0 | 1 Ref |
| KY744234 | Unassigned | Unassigned | Unassigned | 360_0 | Clustered | 22 | VC_360_0 | 22 | 0.96 | 1 | 0.96 | 0 | 0 | 0 | 1 Ref |
| KY744235 | Unassigned | Unassigned | Unassigned | 360_0 | Clustered | 22 | VC_360_0 | 22 | 0.96 | 1 | 0.96 | 0 | 0 | 0 | 1 Ref |
| KY744566 | Unassigned | Unassigned | Unassigned | 466_0 | Clustered | 66 | VC_466_0 | 66 | 0.9246 | 1 | 0.9246 | 0 | 0 | 0 | 1 Ref |
| KY769270 | Unassigned | Unassigned | Unassigned | 865_0 | Clustered | 10 | VC_865_0 | 10 | 0.5975 | 1 | 0.5975 | 0 | 0 | 0 | 1 Ref |
| KY779848 | Unassigned | Unassigned | Unassigned | 159_0 | Clustered | 25 | VC_159_0 | 25 | 0.7699 | 1 | 0.7699 | 0 | 0 | 0 | 1 Ref |
| KY779849 | Unassigned | Unassigned | Unassigned | 155_15 | Clustered | 16 | VC_155_15 | 16 | 0.0779 | 1 | 0.0779 | 0 | 0 | 0 | 0.8083 Ref |
| KY780482 | Unassigned | Unassigned | Unassigned | 971_0 | Clustered | 40 | VC_971_0 | 40 | 0.6793 | 1 | 0.6793 | 0 | 0 | 0 | 0.9359 Ref |

| | | | | | | | | | | | | | | | | |
|---|---|---|---|---|---|---|---|---|---|---|---|---|---|---|---|---|
| KY783914 | Unassigned | Unassigned | Unassigned | 785_2 | Clustered | 7 | VC_785_2 | 7 | 0.2595 | 1 | 0.2595 | 0 | 0 | 0 | 1 | Ref |
| KY787212 | Unassigned | Unassigned | Unassigned | 199_16 | Clustered | 12 | VC_199_16 | 12 | 0.064 | 1 | 0.064 | 0 | 0 | 0 | 0.9394 | Ref |
| KY787213 | Unassigned | Unassigned | Unassigned | 135_34 | Clustered | 3 | VC_135_34 | 3 | 0.0102 | 0.97231548 | 0.0099 | 0 | 0 | 0 | 0.6667 | Ref |
| KY794641 | Unassigned | Unassigned | Unassigned | 155_10 | Clustered/Singleton | 1 | VC_155_10 | 1 | 0 | 1 | 0 | 0 | 0 | 0 | 1 | Ref |
| KY794642 | Unassigned | Unassigned | Unassigned | 155_29 | Clustered | 3 | VC_155_29 | 3 | 0.0119 | 0.99412742 | 0.0118 | 0 | 0 | 0 | 1 | Ref |
| KY794643 | Unassigned | Unassigned | Unassigned | 155_29 | Clustered | 3 | VC_155_29 | 3 | 0.0119 | 0.99412742 | 0.0118 | 0 | 0 | 0 | 1 | Ref |
| KY798120 | Unassigned | Unassigned | Unassigned | 131_0 | Clustered | 43 | VC_131_0 | 43 | 0.8853 | 1 | 0.8853 | 0 | 0 | 0 | 1 | Ref |
| KY798121 | Unassigned | Unassigned | Unassigned | 131_0 | Clustered | 43 | VC_131_0 | 43 | 0.8853 | 1 | 0.8853 | 0 | 0 | 0 | 1 | Ref |
| KY817360 | Unassigned | Unassigned | Unassigned | 1245_0 | Clustered | 2 | VC_1245_0 | 2 | 0.8888 | 0.81445332 | 0.7239 | 0 | 0 | 0 | 1 | Ref |
| KY821088 | Unassigned | Unassigned | Unassigned | 844_0 | Clustered | 3 | VC_844_0 | 3 | 0.0967 | 0.99840149 | 0.0966 | 0 | 0 | 0 | 1 | Ref |
| KY829116 | Unassigned | Unassigned | Unassigned | 713_0 | Clustered | 26 | VC_713_0 | 26 | 0.9358 | 1 | 0.9358 | 0 | 0 | 0 | 1 | Ref |
| KY853667 | Unassigned | Unassigned | Unassigned | 183_0 | Clustered | 5 | VC_183_0 | 5 | 0.7449 | 0.99885646 | 0.744 | 0 | 0 | 0 | 1 | Ref |
| KY860935 | Unassigned | Unassigned | Unassigned | 475_0 | Clustered | 96 | VC_475_0 | 96 | 0.9303 | 1 | 0.9303 | 0 | 0 | 0 | 0.9575 | Ref |
| KY883634 | Unassigned | Unassigned | Unassigned | 513_1 | Clustered | 41 | VC_513_1 | 41 | 0.3276 | 1 | 0.3276 | 0 | 0 | 0 | 0.911 | Ref |
| KY883635 | Unassigned | Unassigned | Unassigned | 513_1 | Clustered | 41 | VC_513_1 | 41 | 0.3276 | 1 | 0.3276 | 0 | 0 | 0 | 0.911 | Ref |
| KY883636 | Unassigned | Unassigned | Unassigned | 513_1 | Clustered | 41 | VC_513_1 | 41 | 0.3276 | 1 | 0.3276 | 0 | 0 | 0 | 0.911 | Ref |
| KY883637 | Unassigned | Unassigned | Unassigned | 513_1 | Clustered | 41 | VC_513_1 | 41 | 0.3276 | 1 | 0.3276 | 0 | 0 | 0 | 0.911 | Ref |
| KY883638 | Unassigned | Unassigned | Unassigned | 513_0 | Clustered | 39 | VC_513_0 | 39 | 0.3078 | 1 | 0.3078 | 0 | 0 | 0 | 1 | Ref |
| KY883639 | Unassigned | Unassigned | Unassigned | 513_0 | Clustered | 39 | VC_513_0 | 39 | 0.3078 | 1 | 0.3078 | 0 | 0 | 0 | 1 | Ref |
| KY883640 | Unassigned | Unassigned | Unassigned | 513_0 | Clustered | 39 | VC_513_0 | 39 | 0.3078 | 1 | 0.3078 | 0 | 0 | 0 | 1 | Ref |
| KY883641 | Unassigned | Unassigned | Unassigned | 83_0 | Clustered | 24 | VC_83_0 | 24 | 0.7345 | 1 | 0.7345 | 0 | 0 | 0 | 1 | Ref |
| KY883642 | Unassigned | Unassigned | Unassigned | 627_0 | Clustered | 16 | VC_627_0 | 16 | 0.9168 | 1 | 0.9168 | 0 | 0 | 0 | 1 | Ref |
| KY883643 | Unassigned | Unassigned | Unassigned | 83_0 | Clustered | 24 | VC_83_0 | 24 | 0.7345 | 1 | 0.7345 | 0 | 0 | 0 | 1 | Ref |
| KY883644 | Unassigned | Unassigned | Unassigned | 83_0 | Clustered | 24 | VC_83_0 | 24 | 0.7345 | 1 | 0.7345 | 0 | 0 | 0 | 1 | Ref |
| KY883645 | Unassigned | Unassigned | Unassigned | 83_0 | Clustered | 24 | VC_83_0 | 24 | 0.7345 | 1 | 0.7345 | 0 | 0 | 0 | 1 | Ref |
| KY883646 | Unassigned | Unassigned | Unassigned | 83_0 | Clustered | 24 | VC_83_0 | 24 | 0.7345 | 1 | 0.7345 | 0 | 0 | 0 | 1 | Ref |
| KY883647 | Unassigned | Unassigned | Unassigned | 627_0 | Clustered | 16 | VC_627_0 | 16 | 0.9168 | 1 | 0.9168 | 0 | 0 | 0 | 1 | Ref |
| KY883648 | Unassigned | Unassigned | Unassigned | 83_0 | Clustered | 24 | VC_83_0 | 24 | 0.7345 | 1 | 0.7345 | 0 | 0 | 0 | 1 | Ref |
| KY883649 | Unassigned | Unassigned | Unassigned | 83_0 | Clustered | 24 | VC_83_0 | 24 | 0.7345 | 1 | 0.7345 | 0 | 0 | 0 | 1 | Ref |
| KY883650 | Unassigned | Unassigned | Unassigned | 83_0 | Clustered | 24 | VC_83_0 | 24 | 0.7345 | 1 | 0.7345 | 0 | 0 | 0 | 1 | Ref |
| KY883651 | Unassigned | Unassigned | Unassigned | 83_0 | Clustered | 24 | VC_83_0 | 24 | 0.7345 | 1 | 0.7345 | 0 | 0 | 0 | 1 | Ref |
| KY883652 | Unassigned | Unassigned | Unassigned | 83_0 | Clustered | 24 | VC_83_0 | 24 | 0.7345 | 1 | 0.7345 | 0 | 0 | 0 | 1 | Ref |
| KY883653 | Unassigned | Unassigned | Unassigned | 83_0 | Clustered | 24 | VC_83_0 | 24 | 0.7345 | 1 | 0.7345 | 0 | 0 | 0 | 1 | Ref |
| KY883654 | Unassigned | Unassigned | Unassigned | 1007_0 | Clustered | 3 | VC_1007_0 | 3 | 0.0541 | 0.99838914 | 0.0541 | 0 | 0 | 0 | 1 | Ref |
| KY883655 | Unassigned | Unassigned | Unassigned | 1007_0 | Clustered | 3 | VC_1007_0 | 3 | 0.0541 | 0.99838914 | 0.0541 | 0 | 0 | 0 | 1 | Ref |
| KY883656 | Unassigned | Unassigned | Unassigned | 627_0 | Clustered | 16 | VC_627_0 | 16 | 0.9168 | 1 | 0.9168 | 0 | 0 | 0 | 1 | Ref |
| KY883657 | Unassigned | Unassigned | Unassigned | 771_0 | Clustered | 8 | VC_771_0 | 8 | 0.7491 | 1 | 0.7491 | 0 | 0 | 0 | 1 | Ref |
| KY883658 | Unassigned | Unassigned | Unassigned | 771_0 | Clustered | 8 | VC_771_0 | 8 | 0.7491 | 1 | 0.7491 | 0 | 0 | 0 | 1 | Ref |
| KY888143 | Unassigned | Unassigned | Unassigned | 218_0 | Clustered | 4 | VC_218_0 | 4 | 0.1097 | 0.99998788 | 0.1097 | 0 | 0 | 0 | 1 | Ref |
| KY888680 | Unassigned | Unassigned | Unassigned | 714_0 | Clustered | 71 | VC_714_0 | 71 | 0.935 | 1 | 0.935 | 0 | 0 | 0 | 1 | Ref |
| KY888882 | Unassigned | Unassigned | Unassigned | 912_3 | Clustered | 2 | VC_912_3 | 2 | 0.0181 | 0.87900236 | 0.0159 | 0 | 0 | 0 | 1 | Ref |
| KY914478 | Unassigned | Unassigned | Unassigned | 44_0 | Clustered | 49 | VC_44_0 | 49 | 0.4506 | 1 | 0.4506 | 0 | 0 | 0 | 0.8605 | Ref |
| KY921761 | Unassigned | Unassigned | Unassigned | 844_0 | Clustered | 3 | VC_844_0 | 3 | 0.0967 | 0.99840149 | 0.0966 | 0 | 0 | 0 | 1 | Ref |
| KY926698 | Unassigned | Unassigned | Unassigned | 218_0 | Clustered | 4 | VC_218_0 | 4 | 0.1097 | 0.99998788 | 0.1097 | 0 | 0 | 0 | 1 | Ref |
| KY926699 | Unassigned | Unassigned | Unassigned | 218_0 | Clustered | 4 | VC_218_0 | 4 | 0.1097 | 0.99998788 | 0.1097 | 0 | 0 | 0 | 1 | Ref |
| KY926791 | Unassigned | Unassigned | Unassigned | 737_0 | Clustered | 13 | VC_737_0 | 13 | 0.5083 | 1 | 0.5083 | 0 | 0 | 0 | 1 | Ref |
| KY926792 | Unassigned | Unassigned | Unassigned | 787_0 | Clustered | 132 | VC_787_0 | 132 | 1 | 1 | 1 | 0 | 0 | 0 | 1 | Ref |
| KY926793 | Unassigned | Unassigned | Unassigned | 787_0 | Clustered | 132 | VC_787_0 | 132 | 1 | 1 | 1 | 0 | 0 | 0 | 1 | Ref |
| KY939598 | Unassigned | Unassigned | Unassigned | 256_0 | Clustered | 2 | VC_256_0 | 2 | 0.0437 | 0.95611024 | 0.0418 | 0 | 0 | 0 | 1 | Ref |
| KY942056 | Unassigned | Unassigned | Unassigned | 133_0 | Clustered | 17 | VC_133_0 | 17 | 0.2654 | 1 | 0.2654 | 0 | 0 | 0 | 1 | Ref |
| KY942057 | Unassigned | Unassigned | Unassigned | 133_0 | Clustered | 17 | VC_133_0 | 17 | 0.2654 | 1 | 0.2654 | 0 | 0 | 0 | 1 | Ref |
| KY945355 | Unassigned | Unassigned | Unassigned | 244_2 | Clustered | 11 | VC_244_2 | 11 | 0.1403 | 1 | 0.1403 | 0 | 0 | 0 | 0.9818 | Ref |
| KY947509 | Unassigned | Unassigned | Unassigned | 843_0 | Clustered | 20 | VC_843_0 | 20 | 0.9621 | 1 | 0.9621 | 0 | 0 | 0 | 1 | Ref |
| KY951963 | Unassigned | Unassigned | Unassigned | 944_0 | Clustered | 76 | VC_944_0 | 76 | 0.9789 | 1 | 0.9789 | 0 | 0 | 0 | 1 | Ref |
| KY951964 | Unassigned | Unassigned | Unassigned | 944_0 | Clustered | 76 | VC_944_0 | 76 | 0.9789 | 1 | 0.9789 | 0 | 0 | 0 | 1 | Ref |
| KY953156 | Unassigned | Unassigned | Unassigned | 1018_0 | Clustered | 38 | VC_1018_0 | 38 | 0.9757 | 1 | 0.9757 | 0 | 0 | 0 | 1 | Ref |
| KY962008 | Unassigned | Unassigned | Unassigned | 465_0 | Clustered | 80 | VC_465_0 | 80 | 0.9988 | 1 | 0.9988 | 0 | 0 | 0 | 1 | Ref |
| KY963369 | Unassigned | Unassigned | Unassigned | 719_0 | Clustered | 16 | VC_719_0 | 16 | 0.728 | 1 | 0.728 | 0 | 0 | 0 | 1 | Ref |
| KY963370 | Unassigned | Unassigned | Unassigned | 719_0 | Clustered | 16 | VC_719_0 | 16 | 0.728 | 1 | 0.728 | 0 | 0 | 0 | 1 | Ref |
| KY963371 | Unassigned | Unassigned | Unassigned | 719_0 | Clustered | 16 | VC_719_0 | 16 | 0.728 | 1 | 0.728 | 0 | 0 | 0 | 1 | Ref |
| KY963424 | Unassigned | Unassigned | Unassigned | 198_23 | Clustered | 10 | VC_198_23 | 10 | 0.0697 | 1 | 0.0697 | 0 | 0 | 0 | 0.8444 | Ref |
| KY965063 | Unassigned | Unassigned | Unassigned | 532_0 | Clustered | 75 | VC_532_0 | 75 | 0.9393 | 1 | 0.9393 | 0 | 0 | 0 | 1 | Ref |
| KY965064 | Unassigned | Unassigned | Unassigned | 538_40 | Clustered | 4 | VC_538_40 | 4 | 0.0395 | 0.99998776 | 0.0394 | 0 | 0 | 0 | 1 | Ref |
| KY965065 | Unassigned | Unassigned | Unassigned | 223_6 | Clustered | 42 | VC_223_6 | 42 | 0.4717 | 1 | 0.4717 | 0 | 0 | 0 | 0.8351 | Ref |
| KY965066 | Unassigned | Unassigned | Unassigned | 384_18 | Clustered | 10 | VC_384_18 | 10 | 0.0301 | 1 | 0.0301 | 0 | 0 | 0 | 0.8 | Ref |
| KY965934 | Unassigned | Unassigned | Unassigned | 997_0 | Clustered | 4 | VC_997_0 | 4 | 0.8491 | 0.99879268 | 0.8481 | 0 | 0 | 0 | 1 | Ref |
| KY969628 | Unassigned | Unassigned | Unassigned | 588_0 | Clustered | 8 | VC_588_0 | 8 | 0.5695 | 1 | 0.5695 | 0 | 0 | 0 | 1 | Ref |
| KY971610 | Unassigned | Unassigned | Unassigned | 294_1 | Clustered/Singleton | 1 | VC_294_1 | 1 | 0 | 1 | 0 | 0 | 0 | 0 | 1 | Ref |
| KY971611 | Unassigned | Unassigned | Unassigned | 131_0 | Clustered | 43 | VC_131_0 | 43 | 0.8853 | 1 | 0.8853 | 0 | 0 | 0 | 1 | Ref |
| KY979235 | Unassigned | Unassigned | Unassigned | 944_0 | Clustered | 76 | VC_944_0 | 76 | 0.9789 | 1 | 0.9789 | 0 | 0 | 0 | 1 | Ref |
| KY979236 | Unassigned | Unassigned | Unassigned | 944_0 | Clustered | 76 | VC_944_0 | 76 | 0.9789 | 1 | 0.9789 | 0 | 0 | 0 | 1 | Ref |
| KY979237 | Unassigned | Unassigned | Unassigned | 944_0 | Clustered | 76 | VC_944_0 | 76 | 0.9789 | 1 | 0.9789 | 0 | 0 | 0 | 1 | Ref |
| KY979238 | Unassigned | Unassigned | Unassigned | 944_0 | Clustered | 76 | VC_944_0 | 76 | 0.9789 | 1 | 0.9789 | 0 | 0 | 0 | 1 | Ref |
| KY979239 | Unassigned | Unassigned | Unassigned | 944_0 | Clustered | 76 | VC_944_0 | 76 | 0.9789 | 1 | 0.9789 | 0 | 0 | 0 | 1 | Ref |
| KY979240 | Unassigned | Unassigned | Unassigned | 944_0 | Clustered | 76 | VC_944_0 | 76 | 0.9789 | 1 | 0.9789 | 0 | 0 | 0 | 1 | Ref |
| KY979241 | Unassigned | Unassigned | Unassigned | 944_0 | Clustered | 76 | VC_944_0 | 76 | 0.9789 | 1 | 0.9789 | 0 | 0 | 0 | 1 | Ref |
| KY979242 | Unassigned | Unassigned | Unassigned | 944_0 | Clustered | 76 | VC_944_0 | 76 | 0.9789 | 1 | 0.9789 | 0 | 0 | 0 | 1 | Ref |
| KY979243 | Unassigned | Unassigned | Unassigned | 944_0 | Clustered | 76 | VC_944_0 | 76 | 0.9789 | 1 | 0.9789 | 0 | 0 | 0 | 1 | Ref |
| KY979244 | Unassigned | Unassigned | Unassigned | 944_0 | Clustered | 76 | VC_944_0 | 76 | 0.9789 | 1 | 0.9789 | 0 | 0 | 0 | 1 | Ref |
| KY979245 | Unassigned | Unassigned | Unassigned | 944_0 | Clustered | 76 | VC_944_0 | 76 | 0.9789 | 1 | 0.9789 | 0 | 0 | 0 | 1 | Ref |
| KY979246 | Unassigned | Unassigned | Unassigned | 944_0 | Clustered | 76 | VC_944_0 | 76 | 0.9789 | 1 | 0.9789 | 0 | 0 | 0 | 1 | Ref |

| | | | | | | | | | | | | | | | | |
|---|---|---|---|---|---|---|---|---|---|---|---|---|---|---|---|---|
| KY979247 | Unassigned | Unassigned | Unassigned | 944_0 | Clustered | 76 | VC_944_0 | 76 | 0.9789 | 1 | 0.9789 | 0 | 0 | 0 | 1 | Ref |
| KY981272 | Unassigned | Unassigned | Unassigned | 71_0 | Clustered | 27 | VC_71_0 | 27 | 0.5964 | 1 | 0.5964 | 0 | 0 | 0 | 0.9943 | Ref |
| KY984068 | Unassigned | Unassigned | Unassigned | 68_2 | Clustered | 2 | VC_68_2 | 2 | 0.0139 | 0.69357611 | 0.0097 | 0 | 0 | 0 | 1 | Ref |
| KY985004 | Unassigned | Unassigned | Unassigned | 595_0 | Clustered | 35 | VC_595_0 | 35 | 0.8809 | 1 | 0.8809 | 0 | 0 | 0 | 1 | Ref |
| KY992519 | Unassigned | Unassigned | Unassigned | 944_0 | Clustered | 76 | VC_944_0 | 76 | 0.9789 | 1 | 0.9789 | 0 | 0 | 0 | 1 | Ref |
| KY992520 | Unassigned | Unassigned | Unassigned | 944_0 | Clustered | 76 | VC_944_0 | 76 | 0.9789 | 1 | 0.9789 | 0 | 0 | 0 | 1 | Ref |
| KY994101 | Unassigned | Unassigned | Unassigned | 78_0 | Clustered | 20 | VC_78_0 | 20 | 0.9639 | 1 | 0.9639 | 0 | 0 | 0 | 1 | Ref |
| KY996523 | Unassigned | Unassigned | Unassigned | 928_0 | Clustered | 6 | VC_928_0 | 6 | 0.4065 | 1 | 0.4065 | 0 | 0 | 0 | 1 | Ref |
| L67CG | Unassigned | Unassigned | Unassigned | 706_0 | Clustered | 52 | VC_706_0 | 52 | 0.9787 | 1 | 0.9787 | 0 | 0 | 0 | 1 | Ref |
| LAMCG | Unassigned | Unassigned | Unassigned | 45_0 | Clustered | 38 | VC_45_0 | 38 | 0.5785 | 1 | 0.5785 | 0 | 0 | 0 | 0.9659 | Ref |
| LC035386 | Unassigned | Unassigned | Unassigned | 1246_0 | Clustered | 2 | VC_1246_0 | 2 | 1 | 1 | 1 | 0 | 0 | 0 | 1 | Ref |
| LC063634 | Unassigned | Unassigned | Unassigned | 1018_0 | Clustered | 38 | VC_1018_0 | 38 | 0.9757 | 1 | 0.9757 | 0 | 0 | 0 | 1 | Ref |
| LC064302 | Unassigned | Unassigned | Unassigned | 863_0 | Clustered | 5 | VC_863_0 | 5 | 0.1633 | 0.99999995 | 0.1633 | 0 | 0 | 0 | 1 | Ref |
| LC066596 | Unassigned | Unassigned | Unassigned | 56_0 | Clustered | 6 | VC_56_0 | 6 | 0.6713 | 0.9999999 | 0.6713 | 0 | 0 | 0 | 1 | Ref |
| LC102729 | Unassigned | Unassigned | Unassigned | 180_0 | Clustered | 3 | VC_180_0 | 3 | 0.9607 | 0.99333557 | 0.9543 | 0 | 0 | 0 | 1 | Ref |
| LC102730 | Unassigned | Unassigned | Unassigned | 80_0 | Clustered | 64 | VC_80_0 | 64 | 0.418 | 1 | 0.418 | 0 | 0 | 0 | 0.7649 | Ref |
| LC105987 | Unassigned | Unassigned | Unassigned | 80_0 | Clustered | 64 | VC_80_0 | 64 | 0.418 | 1 | 0.418 | 0 | 0 | 0 | 0.7649 | Ref |
| LC105988 | Unassigned | Unassigned | Unassigned | 80_0 | Clustered | 64 | VC_80_0 | 64 | 0.418 | 1 | 0.418 | 0 | 0 | 0 | 0.7649 | Ref |
| LC105989 | Unassigned | Unassigned | Unassigned | 80_0 | Clustered | 64 | VC_80_0 | 64 | 0.418 | 1 | 0.418 | 0 | 0 | 0 | 0.7649 | Ref |
| LC105990 | Unassigned | Unassigned | Unassigned | 80_0 | Clustered | 64 | VC_80_0 | 64 | 0.418 | 1 | 0.418 | 0 | 0 | 0 | 0.7649 | Ref |
| LC168164 | Unassigned | Unassigned | Unassigned | 550_1 | Clustered/Singleton | 1 | VC_550_1 | 1 | 0 | 1 | 0 | 0 | 0 | 0 | 1 | Ref |
| LC210520 | Unassigned | Unassigned | Unassigned | 1246_0 | Clustered | 2 | VC_1246_0 | 2 | 1 | 1 | 1 | 0 | 0 | 0 | 1 | Ref |
| LC348379 | Unassigned | Unassigned | Unassigned | 290_182 | Clustered/Singleton | 1 | VC_290_182 | 1 | 0 | 1 | 0 | 0 | 0 | 0 | 1 | Ref |
| LC348380 | Unassigned | Unassigned | Unassigned | 290_92 | Clustered | 4 | VC_290_92 | 4 | 0.0078 | 0.99995066 | 0.0078 | 0 | 0 | 0 | 1 | Ref |
| LC373201 | Unassigned | Unassigned | Unassigned | 133_2 | Clustered | 7 | VC_133_2 | 7 | 0.0679 | 1 | 0.0679 | 0 | 0 | 0 | 0.9048 | Ref |
| LC375533 | Unassigned | Unassigned | Unassigned | 1018_0 | Clustered | 38 | VC_1018_0 | 38 | 0.9757 | 1 | 0.9757 | 0 | 0 | 0 | 1 | Ref |
| LC413193 | Unassigned | Unassigned | Unassigned | 848_0 | Clustered | 124 | VC_848_0 | 124 | 0.9484 | 1 | 0.9484 | 0 | 0 | 0 | 1 | Ref |
| LC413194 | Unassigned | Unassigned | Unassigned | 848_0 | Clustered | 124 | VC_848_0 | 124 | 0.9484 | 1 | 0.9484 | 0 | 0 | 0 | 1 | Ref |
| LC413195 | Unassigned | Unassigned | Unassigned | 848_0 | Clustered | 124 | VC_848_0 | 124 | 0.9484 | 1 | 0.9484 | 0 | 0 | 0 | 1 | Ref |
| LC465543 | Unassigned | Unassigned | Unassigned | 290_15 | Clustered/Singleton | 1 | VC_290_15 | 1 | 0 | 1 | 0 | 0 | 0 | 0 | 1 | Ref |
| LC472881 | Unassigned | Unassigned | Unassigned | 80_1 | Clustered | 2 | VC_80_1 | 2 | 0.006 | 0.95748743 | 0.0057 | 0 | 0 | 0 | 1 | Ref |
| LC472882 | Unassigned | Unassigned | Unassigned | 81_0 | Clustered | 41 | VC_81_0 | 41 | 0.5815 | 1 | 0.5815 | 0 | 0 | 0 | 0.9805 | Ref |
| LC472883 | Unassigned | Unassigned | Unassigned | 80_0 | Clustered | 64 | VC_80_0 | 64 | 0.418 | 1 | 0.418 | 0 | 0 | 0 | 0.7649 | Ref |
| LC472884 | Unassigned | Unassigned | Unassigned | 80_0 | Clustered | 64 | VC_80_0 | 64 | 0.418 | 1 | 0.418 | 0 | 0 | 0 | 0.7649 | Ref |
| LC473039 | Unassigned | Unassigned | Unassigned | 340_8 | Clustered | 2 | VC_340_8 | 2 | 0.0048 | 0.95654014 | 0.0046 | 0 | 0 | 0 | 1 | Ref |
| LC487410 | Unassigned | Unassigned | Unassigned | 1007_1 | Clustered | 24 | VC_1007_1 | 24 | 0.7364 | 1 | 0.7364 | 0 | 0 | 0 | 0.8877 | Ref |
| LC492751 | Unassigned | Unassigned | Unassigned | 159_0 | Clustered | 25 | VC_159_0 | 25 | 0.7699 | 1 | 0.7699 | 0 | 0 | 0 | 1 | Ref |
| LC492752 | Unassigned | Unassigned | Unassigned | 159_0 | Clustered | 25 | VC_159_0 | 25 | 0.7699 | 1 | 0.7699 | 0 | 0 | 0 | 1 | Ref |
| LC494302 | Unassigned | Unassigned | Unassigned | 170_11 | Clustered | 10 | VC_170_11 | 10 | 0.1502 | 0.99999797 | 0.1502 | 0 | 0 | 0 | 0.9111 | Ref |
| LC507823 | Unassigned | Unassigned | Unassigned | 654_0 | Clustered | 115 | VC_654_0 | 115 | 0.7303 | 1 | 0.7303 | 0 | 0 | 0 | 0.8764 | Ref |
| LC516895 | Unassigned | Unassigned | Unassigned | 595_0 | Clustered | 35 | VC_595_0 | 35 | 0.8809 | 1 | 0.8809 | 0 | 0 | 0 | 1 | Ref |
| LC519489 | Unassigned | Unassigned | Unassigned | 340_7 | Clustered | 18 | VC_340_7 | 18 | 0.0936 | 1 | 0.0936 | 0 | 0 | 0 | 0.7778 | Ref |
| LC519601 | Unassigned | Unassigned | Unassigned | 836_1 | Clustered | 4 | VC_836_1 | 4 | 0.2236 | 0.99998426 | 0.2236 | 0 | 0 | 0 | 1 | Ref |
| LC528882 | Unassigned | Unassigned | Unassigned | 3_0 | Clustered | 17 | VC_3_0 | 17 | 0.5615 | 1 | 0.5615 | 0 | 0 | 0 | 1 | Ref |
| LC541428 | Unassigned | Unassigned | Unassigned | 8_0 | Clustered | 58 | VC_8_0 | 58 | 0.8285 | 1 | 0.8285 | 0 | 0 | 0 | 0.9946 | Ref |
| LC545443 | Unassigned | Unassigned | Unassigned | 120_0 | Clustered | 3 | VC_120_0 | 3 | 0.7107 | 0.99211494 | 0.7051 | 0 | 0 | 0 | 1 | Ref |
| LC545444 | Unassigned | Unassigned | Unassigned | 120_0 | Clustered | 3 | VC_120_0 | 3 | 0.7107 | 0.99211494 | 0.7051 | 0 | 0 | 0 | 1 | Ref |
| LC545445 | Unassigned | Unassigned | Unassigned | 121_0 | Clustered | 2 | VC_121_0 | 2 | 0.5501 | 0.91840661 | 0.5052 | 0 | 0 | 0 | 1 | Ref |
| LC545446 | Unassigned | Unassigned | Unassigned | 121_0 | Clustered | 2 | VC_121_0 | 2 | 0.5501 | 0.91840661 | 0.5052 | 0 | 0 | 0 | 1 | Ref |
| LC547238 | Unassigned | Unassigned | Unassigned | 110_0 | Clustered | 33 | VC_110_0 | 33 | 0.9488 | 1 | 0.9488 | 0 | 0 | 0 | 1 | Ref |
| LC552830 | Unassigned | Unassigned | Unassigned | 766_0 | Clustered | 40 | VC_766_0 | 40 | 0.7511 | 1 | 0.7511 | 0 | 0 | 0 | 1 | Ref |
| LC553734 | Unassigned | Unassigned | Unassigned | 837_0 | Clustered | 12 | VC_837_0 | 12 | 0.2327 | 1 | 0.2327 | 0 | 0 | 0 | 1 | Ref |
| LC553735 | Unassigned | Unassigned | Unassigned | 837_1 | Clustered | 24 | VC_837_1 | 24 | 0.5328 | 1 | 0.5328 | 0 | 0 | 0 | 1 | Ref |
| LC553736 | Unassigned | Unassigned | Unassigned | 611_0 | Clustered | 50 | VC_611_0 | 50 | 0.7306 | 1 | 0.7306 | 0 | 0 | 0 | 1 | Ref |
| LC567830 | Unassigned | Unassigned | Unassigned | 42_0 | Clustered | 5 | VC_42_0 | 5 | 0.0844 | 0.99999995 | 0.0844 | 0 | 0 | 0 | 1 | Ref |
| LC567838 | Unassigned | Unassigned | Unassigned | 42_0 | Clustered | 5 | VC_42_0 | 5 | 0.0844 | 0.99999995 | 0.0844 | 0 | 0 | 0 | 1 | Ref |
| LC567842 | Unassigned | Unassigned | Unassigned | 42_0 | Clustered | 5 | VC_42_0 | 5 | 0.0844 | 0.99999995 | 0.0844 | 0 | 0 | 0 | 1 | Ref |
| LC574321 | Unassigned | Unassigned | Unassigned | 155_15 | Clustered | 16 | VC_155_15 | 16 | 0.0779 | 1 | 0.0779 | 0 | 0 | 0 | 0.8083 | Ref |
| LC576631 | Unassigned | Unassigned | Unassigned | 159_1 | Clustered/Singleton | 1 | VC_159_1 | 1 | 0 | 1 | 0 | 0 | 0 | 0 | 1 | Ref |
| LC589952 | Unassigned | Unassigned | Unassigned | 687_0 | Clustered | 21 | VC_687_0 | 21 | 0.3608 | 1 | 0.3608 | 0 | 0 | 0 | 1 | Ref |
| LC596377 | Unassigned | Unassigned | Unassigned | 93_0 | Clustered | 22 | VC_93_0 | 22 | 0.5285 | 1 | 0.5285 | 0 | 0 | 0 | 1 | Ref |
| LC596378 | Unassigned | Unassigned | Unassigned | 93_0 | Clustered | 22 | VC_93_0 | 22 | 0.5285 | 1 | 0.5285 | 0 | 0 | 0 | 1 | Ref |
| LC596379 | Unassigned | Unassigned | Unassigned | 93_0 | Clustered | 22 | VC_93_0 | 22 | 0.5285 | 1 | 0.5285 | 0 | 0 | 0 | 1 | Ref |
| LC597490 | Unassigned | Unassigned | Unassigned | 92_0 | Clustered/Singleton | 1 | VC_92_0 | 1 | 0 | 1 | 0 | 0 | 0 | 0 | 1 | Ref |
| LC616031 | Unassigned | Unassigned | Unassigned | 49_0 | Clustered | 48 | VC_49_0 | 48 | 0.7748 | 1 | 0.7748 | 0 | 0 | 0 | 1 | Ref |
| LC616032 | Unassigned | Unassigned | Unassigned | 49_0 | Clustered | 48 | VC_49_0 | 48 | 0.7748 | 1 | 0.7748 | 0 | 0 | 0 | 1 | Ref |
| LC616033 | Unassigned | Unassigned | Unassigned | 49_0 | Clustered | 48 | VC_49_0 | 48 | 0.7748 | 1 | 0.7748 | 0 | 0 | 0 | 1 | Ref |
| LC616034 | Unassigned | Unassigned | Unassigned | 49_0 | Clustered | 48 | VC_49_0 | 48 | 0.7748 | 1 | 0.7748 | 0 | 0 | 0 | 1 | Ref |
| LC616035 | Unassigned | Unassigned | Unassigned | 49_0 | Clustered | 48 | VC_49_0 | 48 | 0.7748 | 1 | 0.7748 | 0 | 0 | 0 | 1 | Ref |
| LC616036 | Unassigned | Unassigned | Unassigned | 49_0 | Clustered | 48 | VC_49_0 | 48 | 0.7748 | 1 | 0.7748 | 0 | 0 | 0 | 1 | Ref |
| LC616037 | Unassigned | Unassigned | Unassigned | 49_0 | Clustered | 48 | VC_49_0 | 48 | 0.7748 | 1 | 0.7748 | 0 | 0 | 0 | 1 | Ref |
| LC616038 | Unassigned | Unassigned | Unassigned | 49_0 | Clustered | 48 | VC_49_0 | 48 | 0.7748 | 1 | 0.7748 | 0 | 0 | 0 | 1 | Ref |
| LC616039 | Unassigned | Unassigned | Unassigned | 49_0 | Clustered | 48 | VC_49_0 | 48 | 0.7748 | 1 | 0.7748 | 0 | 0 | 0 | 1 | Ref |
| LC616040 | Unassigned | Unassigned | Unassigned | 49_0 | Clustered | 48 | VC_49_0 | 48 | 0.7748 | 1 | 0.7748 | 0 | 0 | 0 | 1 | Ref |
| LC616041 | Unassigned | Unassigned | Unassigned | 49_0 | Clustered | 48 | VC_49_0 | 48 | 0.7748 | 1 | 0.7748 | 0 | 0 | 0 | 1 | Ref |
| LC616042 | Unassigned | Unassigned | Unassigned | 49_0 | Clustered | 48 | VC_49_0 | 48 | 0.7748 | 1 | 0.7748 | 0 | 0 | 0 | 1 | Ref |
| LC616043 | Unassigned | Unassigned | Unassigned | 49_0 | Clustered | 48 | VC_49_0 | 48 | 0.7748 | 1 | 0.7748 | 0 | 0 | 0 | 1 | Ref |
| LC616044 | Unassigned | Unassigned | Unassigned | 49_0 | Clustered | 48 | VC_49_0 | 48 | 0.7748 | 1 | 0.7748 | 0 | 0 | 0 | 1 | Ref |
| LC616045 | Unassigned | Unassigned | Unassigned | 49_0 | Clustered | 48 | VC_49_0 | 48 | 0.7748 | 1 | 0.7748 | 0 | 0 | 0 | 1 | Ref |
| LC616046 | Unassigned | Unassigned | Unassigned | 49_0 | Clustered | 48 | VC_49_0 | 48 | 0.7748 | 1 | 0.7748 | 0 | 0 | 0 | 1 | Ref |
| LC616047 | Unassigned | Unassigned | Unassigned | 49_0 | Clustered | 48 | VC_49_0 | 48 | 0.7748 | 1 | 0.7748 | 0 | 0 | 0 | 1 | Ref |

| | | | | | | | | | | | | | | | | |
|---|---|---|---|---|---|---|---|---|---|---|---|---|---|---|---|---|
| LC616048 | Unassigned | Unassigned | Unassigned | 49_0 | Clustered | 48 | VC_49_0 | 48 | 0.7748 | 1 | 0.7748 | 0 | 0 | 0 | 1 | Ref |
| LC616049 | Unassigned | Unassigned | Unassigned | 49_0 | Clustered | 48 | VC_49_0 | 48 | 0.7748 | 1 | 0.7748 | 0 | 0 | 0 | 1 | Ref |
| LC616050 | Unassigned | Unassigned | Unassigned | 49_0 | Clustered | 48 | VC_49_0 | 48 | 0.7748 | 1 | 0.7748 | 0 | 0 | 0 | 1 | Ref |
| LC616051 | Unassigned | Unassigned | Unassigned | 49_0 | Clustered | 48 | VC_49_0 | 48 | 0.7748 | 1 | 0.7748 | 0 | 0 | 0 | 1 | Ref |
| LC616052 | Unassigned | Unassigned | Unassigned | 49_0 | Clustered | 48 | VC_49_0 | 48 | 0.7748 | 1 | 0.7748 | 0 | 0 | 0 | 1 | Ref |
| LC616053 | Unassigned | Unassigned | Unassigned | 49_0 | Clustered | 48 | VC_49_0 | 48 | 0.7748 | 1 | 0.7748 | 0 | 0 | 0 | 1 | Ref |
| LC616054 | Unassigned | Unassigned | Unassigned | 49_0 | Clustered | 48 | VC_49_0 | 48 | 0.7748 | 1 | 0.7748 | 0 | 0 | 0 | 1 | Ref |
| LC616055 | Unassigned | Unassigned | Unassigned | 49_0 | Clustered | 48 | VC_49_0 | 48 | 0.7748 | 1 | 0.7748 | 0 | 0 | 0 | 1 | Ref |
| LC616056 | Unassigned | Unassigned | Unassigned | 49_0 | Clustered | 48 | VC_49_0 | 48 | 0.7748 | 1 | 0.7748 | 0 | 0 | 0 | 1 | Ref |
| LC616057 | Unassigned | Unassigned | Unassigned | 49_0 | Clustered | 48 | VC_49_0 | 48 | 0.7748 | 1 | 0.7748 | 0 | 0 | 0 | 1 | Ref |
| LC616058 | Unassigned | Unassigned | Unassigned | 49_0 | Clustered | 48 | VC_49_0 | 48 | 0.7748 | 1 | 0.7748 | 0 | 0 | 0 | 1 | Ref |
| LC616059 | Unassigned | Unassigned | Unassigned | 49_0 | Clustered | 48 | VC_49_0 | 48 | 0.7748 | 1 | 0.7748 | 0 | 0 | 0 | 1 | Ref |
| LC616060 | Unassigned | Unassigned | Unassigned | 49_0 | Clustered | 48 | VC_49_0 | 48 | 0.7748 | 1 | 0.7748 | 0 | 0 | 0 | 1 | Ref |
| LC616061 | Unassigned | Unassigned | Unassigned | 49_0 | Clustered | 48 | VC_49_0 | 48 | 0.7748 | 1 | 0.7748 | 0 | 0 | 0 | 1 | Ref |
| LC616062 | Unassigned | Unassigned | Unassigned | 49_0 | Clustered | 48 | VC_49_0 | 48 | 0.7748 | 1 | 0.7748 | 0 | 0 | 0 | 1 | Ref |
| LC616063 | Unassigned | Unassigned | Unassigned | 49_0 | Clustered | 48 | VC_49_0 | 48 | 0.7748 | 1 | 0.7748 | 0 | 0 | 0 | 1 | Ref |
| LC616064 | Unassigned | Unassigned | Unassigned | 49_0 | Clustered | 48 | VC_49_0 | 48 | 0.7748 | 1 | 0.7748 | 0 | 0 | 0 | 1 | Ref |
| LC616065 | Unassigned | Unassigned | Unassigned | 49_0 | Clustered | 48 | VC_49_0 | 48 | 0.7748 | 1 | 0.7748 | 0 | 0 | 0 | 1 | Ref |
| LC616066 | Unassigned | Unassigned | Unassigned | 49_0 | Clustered | 48 | VC_49_0 | 48 | 0.7748 | 1 | 0.7748 | 0 | 0 | 0 | 1 | Ref |
| LC616067 | Unassigned | Unassigned | Unassigned | 49_0 | Clustered | 48 | VC_49_0 | 48 | 0.7748 | 1 | 0.7748 | 0 | 0 | 0 | 1 | Ref |
| LC616068 | Unassigned | Unassigned | Unassigned | 49_0 | Clustered | 48 | VC_49_0 | 48 | 0.7748 | 1 | 0.7748 | 0 | 0 | 0 | 1 | Ref |
| LC616069 | Unassigned | Unassigned | Unassigned | 49_0 | Clustered | 48 | VC_49_0 | 48 | 0.7748 | 1 | 0.7748 | 0 | 0 | 0 | 1 | Ref |
| LC616070 | Unassigned | Unassigned | Unassigned | 49_0 | Clustered | 48 | VC_49_0 | 48 | 0.7748 | 1 | 0.7748 | 0 | 0 | 0 | 1 | Ref |
| LC616071 | Unassigned | Unassigned | Unassigned | 49_0 | Clustered | 48 | VC_49_0 | 48 | 0.7748 | 1 | 0.7748 | 0 | 0 | 0 | 1 | Ref |
| LC616072 | Unassigned | Unassigned | Unassigned | 49_0 | Clustered | 48 | VC_49_0 | 48 | 0.7748 | 1 | 0.7748 | 0 | 0 | 0 | 1 | Ref |
| LC616073 | Unassigned | Unassigned | Unassigned | 49_0 | Clustered | 48 | VC_49_0 | 48 | 0.7748 | 1 | 0.7748 | 0 | 0 | 0 | 1 | Ref |
| LC616074 | Unassigned | Unassigned | Unassigned | 49_0 | Clustered | 48 | VC_49_0 | 48 | 0.7748 | 1 | 0.7748 | 0 | 0 | 0 | 1 | Ref |
| LC616075 | Unassigned | Unassigned | Unassigned | 49_0 | Clustered | 48 | VC_49_0 | 48 | 0.7748 | 1 | 0.7748 | 0 | 0 | 0 | 1 | Ref |
| LC616076 | Unassigned | Unassigned | Unassigned | 49_0 | Clustered | 48 | VC_49_0 | 48 | 0.7748 | 1 | 0.7748 | 0 | 0 | 0 | 1 | Ref |
| LC616077 | Unassigned | Unassigned | Unassigned | 49_0 | Clustered | 48 | VC_49_0 | 48 | 0.7748 | 1 | 0.7748 | 0 | 0 | 0 | 1 | Ref |
| LC616078 | Unassigned | Unassigned | Unassigned | 49_0 | Clustered | 48 | VC_49_0 | 48 | 0.7748 | 1 | 0.7748 | 0 | 0 | 0 | 1 | Ref |
| LC616079 | Unassigned | Unassigned | Unassigned | 42_0 | Clustered | 5 | VC_42_0 | 5 | 0.0844 | 0.99999995 | 0.0844 | 0 | 0 | 0 | 1 | Ref |
| LC620505 | Unassigned | Unassigned | Unassigned | 290_5 | Clustered/Singleton | 1 | VC_290_5 | 1 | 0 | 1 | 0 | 0 | 0 | 0 | 1 | Ref |
| LC623721 | Unassigned | Unassigned | Unassigned | 711_0 | Clustered | 48 | VC_711_0 | 48 | 0.8217 | 1 | 0.8217 | 0 | 0 | 0 | 1 | Ref |
| LC625742 | Unassigned | Unassigned | Unassigned | 396_0 | Clustered | 3 | VC_396_0 | 3 | 0.3827 | 0.99764103 | 0.3818 | 0 | 0 | 0 | 1 | Ref |
| LC628082 | Unassigned | Unassigned | Unassigned | 99_0 | Clustered | 5 | VC_99_0 | 5 | 0.0694 | 0.98113835 | 0.0681 | 0 | 0 | 0 | 1 | Ref |
| LC644557 | Unassigned | Unassigned | Unassigned | 99_0 | Clustered | 5 | VC_99_0 | 5 | 0.0694 | 0.98113835 | 0.0681 | 0 | 0 | 0 | 1 | Ref |
| LC644972 | Unassigned | Unassigned | Unassigned | 424_0 | Clustered | 3 | VC_424_0 | 3 | 0.1264 | 0.96632884 | 0.1222 | 0 | 0 | 0 | 1 | Ref |
| LC644974 | Unassigned | Unassigned | Unassigned | 424_0 | Clustered | 3 | VC_424_0 | 3 | 0.1264 | 0.96632884 | 0.1222 | 0 | 0 | 0 | 1 | Ref |
| LC644975 | Unassigned | Unassigned | Unassigned | 1247_0 | Clustered | 2 | VC_1247_0 | 2 | 0.8794 | 0.9197097 | 0.8088 | 0 | 0 | 0 | 1 | Ref |
| LC644976 | Unassigned | Unassigned | Unassigned | 1247_0 | Clustered | 2 | VC_1247_0 | 2 | 0.8794 | 0.9197097 | 0.8088 | 0 | 0 | 0 | 1 | Ref |
| LC645408 | Unassigned | Unassigned | Unassigned | 43_1 | Clustered | 10 | VC_43_1 | 10 | 0.1173 | 1 | 0.1173 | 0 | 0 | 0 | 0.8222 | Ref |
| LC645409 | Unassigned | Unassigned | Unassigned | 44_0 | Clustered | 49 | VC_44_0 | 49 | 0.4506 | 1 | 0.4506 | 0 | 0 | 0 | 0.8605 | Ref |
| LC645410 | Unassigned | Unassigned | Unassigned | 44_0 | Clustered | 49 | VC_44_0 | 49 | 0.4506 | 1 | 0.4506 | 0 | 0 | 0 | 0.8605 | Ref |
| LC645411 | Unassigned | Unassigned | Unassigned | 44_0 | Clustered | 49 | VC_44_0 | 49 | 0.4506 | 1 | 0.4506 | 0 | 0 | 0 | 0.8605 | Ref |
| LC645412 | Unassigned | Unassigned | Unassigned | 44_0 | Clustered | 49 | VC_44_0 | 49 | 0.4506 | 1 | 0.4506 | 0 | 0 | 0 | 0.8605 | Ref |
| LC645413 | Unassigned | Unassigned | Unassigned | 44_0 | Clustered | 49 | VC_44_0 | 49 | 0.4506 | 1 | 0.4506 | 0 | 0 | 0 | 0.8605 | Ref |
| LC645414 | Unassigned | Unassigned | Unassigned | 44_0 | Clustered | 49 | VC_44_0 | 49 | 0.4506 | 1 | 0.4506 | 0 | 0 | 0 | 0.8605 | Ref |
| LC645415 | Unassigned | Unassigned | Unassigned | 44_0 | Clustered | 49 | VC_44_0 | 49 | 0.4506 | 1 | 0.4506 | 0 | 0 | 0 | 0.8605 | Ref |
| LC645416 | Unassigned | Unassigned | Unassigned | 44_0 | Clustered | 49 | VC_44_0 | 49 | 0.4506 | 1 | 0.4506 | 0 | 0 | 0 | 0.8605 | Ref |
| LC645417 | Unassigned | Unassigned | Unassigned | 44_0 | Clustered | 49 | VC_44_0 | 49 | 0.4506 | 1 | 0.4506 | 0 | 0 | 0 | 0.8605 | Ref |
| LC645418 | Unassigned | Unassigned | Unassigned | 44_0 | Clustered | 49 | VC_44_0 | 49 | 0.4506 | 1 | 0.4506 | 0 | 0 | 0 | 0.8605 | Ref |
| LC645419 | Unassigned | Unassigned | Unassigned | 44_0 | Clustered | 49 | VC_44_0 | 49 | 0.4506 | 1 | 0.4506 | 0 | 0 | 0 | 0.8605 | Ref |
| LC645420 | Unassigned | Unassigned | Unassigned | 44_0 | Clustered | 49 | VC_44_0 | 49 | 0.4506 | 1 | 0.4506 | 0 | 0 | 0 | 0.8605 | Ref |
| LC645421 | Unassigned | Unassigned | Unassigned | 44_0 | Clustered | 49 | VC_44_0 | 49 | 0.4506 | 1 | 0.4506 | 0 | 0 | 0 | 0.8605 | Ref |
| LC645422 | Unassigned | Unassigned | Unassigned | 44_0 | Clustered | 49 | VC_44_0 | 49 | 0.4506 | 1 | 0.4506 | 0 | 0 | 0 | 0.8605 | Ref |
| LC645423 | Unassigned | Unassigned | Unassigned | 44_0 | Clustered | 49 | VC_44_0 | 49 | 0.4506 | 1 | 0.4506 | 0 | 0 | 0 | 0.8605 | Ref |
| LC645424 | Unassigned | Unassigned | Unassigned | 44_0 | Clustered | 49 | VC_44_0 | 49 | 0.4506 | 1 | 0.4506 | 0 | 0 | 0 | 0.8605 | Ref |
| LC645425 | Unassigned | Unassigned | Unassigned | 43_1 | Clustered | 10 | VC_43_1 | 10 | 0.1173 | 1 | 0.1173 | 0 | 0 | 0 | 0.8222 | Ref |
| LC645426 | Unassigned | Unassigned | Unassigned | 43_1 | Clustered | 10 | VC_43_1 | 10 | 0.1173 | 1 | 0.1173 | 0 | 0 | 0 | 0.8222 | Ref |
| LC645427 | Unassigned | Unassigned | Unassigned | 44_0 | Clustered | 49 | VC_44_0 | 49 | 0.4506 | 1 | 0.4506 | 0 | 0 | 0 | 0.8605 | Ref |
| LC645428 | Unassigned | Unassigned | Unassigned | 50_0 | Clustered | 10 | VC_50_0 | 10 | 0.1725 | 1 | 0.1725 | 0 | 0 | 0 | 1 | Ref |
| LC645429 | Unassigned | Unassigned | Unassigned | 50_0 | Clustered | 10 | VC_50_0 | 10 | 0.1725 | 1 | 0.1725 | 0 | 0 | 0 | 1 | Ref |
| LC645430 | Unassigned | Unassigned | Unassigned | 50_0 | Clustered | 10 | VC_50_0 | 10 | 0.1725 | 1 | 0.1725 | 0 | 0 | 0 | 1 | Ref |
| LC645431 | Unassigned | Unassigned | Unassigned | 50_0 | Clustered | 10 | VC_50_0 | 10 | 0.1725 | 1 | 0.1725 | 0 | 0 | 0 | 1 | Ref |
| LC645432 | Unassigned | Unassigned | Unassigned | 50_0 | Clustered | 10 | VC_50_0 | 10 | 0.1725 | 1 | 0.1725 | 0 | 0 | 0 | 1 | Ref |
| LC645433 | Unassigned | Unassigned | Unassigned | 50_0 | Clustered | 10 | VC_50_0 | 10 | 0.1725 | 1 | 0.1725 | 0 | 0 | 0 | 1 | Ref |
| LC645434 | Unassigned | Unassigned | Unassigned | 50_0 | Clustered | 10 | VC_50_0 | 10 | 0.1725 | 1 | 0.1725 | 0 | 0 | 0 | 1 | Ref |
| LC645435 | Unassigned | Unassigned | Unassigned | 50_0 | Clustered | 10 | VC_50_0 | 10 | 0.1725 | 1 | 0.1725 | 0 | 0 | 0 | 1 | Ref |
| LC645436 | Unassigned | Unassigned | Unassigned | 50_0 | Clustered | 10 | VC_50_0 | 10 | 0.1725 | 1 | 0.1725 | 0 | 0 | 0 | 1 | Ref |
| LC645437 | Unassigned | Unassigned | Unassigned | 50_0 | Clustered | 10 | VC_50_0 | 10 | 0.1725 | 1 | 0.1725 | 0 | 0 | 0 | 1 | Ref |
| LC645438 | Unassigned | Unassigned | Unassigned | 44_0 | Clustered | 49 | VC_44_0 | 49 | 0.4506 | 1 | 0.4506 | 0 | 0 | 0 | 0.8605 | Ref |
| LC645442 | Unassigned | Unassigned | Unassigned | 44_0 | Clustered | 49 | VC_44_0 | 49 | 0.4506 | 1 | 0.4506 | 0 | 0 | 0 | 0.8605 | Ref |
| LC645443 | Unassigned | Unassigned | Unassigned | 44_0 | Clustered | 49 | VC_44_0 | 49 | 0.4506 | 1 | 0.4506 | 0 | 0 | 0 | 0.8605 | Ref |
| LC645444 | Unassigned | Unassigned | Unassigned | 44_0 | Clustered | 49 | VC_44_0 | 49 | 0.4506 | 1 | 0.4506 | 0 | 0 | 0 | 0.8605 | Ref |
| LC645445 | Unassigned | Unassigned | Unassigned | 44_0 | Clustered | 49 | VC_44_0 | 49 | 0.4506 | 1 | 0.4506 | 0 | 0 | 0 | 0.8605 | Ref |
| LC645446 | Unassigned | Unassigned | Unassigned | 44_0 | Clustered | 49 | VC_44_0 | 49 | 0.4506 | 1 | 0.4506 | 0 | 0 | 0 | 0.8605 | Ref |
| LC645447 | Unassigned | Unassigned | Unassigned | 44_0 | Clustered | 49 | VC_44_0 | 49 | 0.4506 | 1 | 0.4506 | 0 | 0 | 0 | 0.8605 | Ref |
| LC645448 | Unassigned | Unassigned | Unassigned | 44_0 | Clustered | 49 | VC_44_0 | 49 | 0.4506 | 1 | 0.4506 | 0 | 0 | 0 | 0.8605 | Ref |
| LC645449 | Unassigned | Unassigned | Unassigned | 44_0 | Clustered | 49 | VC_44_0 | 49 | 0.4506 | 1 | 0.4506 | 0 | 0 | 0 | 0.8605 | Ref |
| LC645450 | Unassigned | Unassigned | Unassigned | 44_0 | Clustered | 49 | VC_44_0 | 49 | 0.4506 | 1 | 0.4506 | 0 | 0 | 0 | 0.8605 | Ref |

| | | | | | | | | | | | | | | | | |
|---|---|---|---|---|---|---|---|---|---|---|---|---|---|---|---|---|
| LC645451 | Unassigned | Unassigned | Unassigned | 44_0 | Clustered | 49 | VC_44_0 | 49 | 0.4506 | 1 | 0.4506 | 0 | 0 | 0 | 0.8605 | Ref |
| LC648442 | Unassigned | Unassigned | Unassigned | 155_7 | Clustered/Singleton | 1 | VC_155_7 | 1 | 0 | 1 | 0 | 0 | 0 | 0 | 1 | Ref |
| LC648443 | Unassigned | Unassigned | Unassigned | 486_0 | Clustered | 47 | VC_486_0 | 47 | 0.9022 | 1 | 0.9022 | 0 | 0 | 0 | 1 | Ref |
| LC659915 | Unassigned | Unassigned | Unassigned | 199_19 | Clustered | 12 | VC_199_19 | 12 | 0.0651 | 1 | 0.0651 | 0 | 0 | 0 | 0.8636 | Ref |
| LC667451 | Unassigned | Unassigned | Unassigned | 357_1 | Clustered/Singleton | 1 | VC_357_1 | 1 | 0 | 1 | 0 | 0 | 0 | 0 | 1 | Ref |
| LC667452 | Unassigned | Unassigned | Unassigned | 3_0 | Clustered | 17 | VC_3_0 | 17 | 0.5615 | 1 | 0.5615 | 0 | 0 | 0 | 1 | Ref |
| LC680884 | Unassigned | Unassigned | Unassigned | 462_0 | Clustered | 4 | VC_462_0 | 4 | 0.3031 | 0.99978853 | 0.303 | 0 | 0 | 0 | 1 | Ref |
| LC680885 | Unassigned | Unassigned | Unassigned | 463_0 | Clustered | 7 | VC_463_0 | 7 | 0.5971 | 1 | 0.5971 | 0 | 0 | 0 | 1 | Ref |
| LC709172 | Unassigned | Unassigned | Unassigned | 98_0 | Clustered | 30 | VC_98_0 | 30 | 0.7175 | 1 | 0.7175 | 0 | 0 | 0 | 1 | Ref |
| LC727695 | Unassigned | Unassigned | Unassigned | 356_0 | Clustered | 21 | VC_356_0 | 21 | 0.5409 | 1 | 0.5409 | 0 | 0 | 0 | 1 | Ref |
| LC727696 | Unassigned | Unassigned | Unassigned | 356_0 | Clustered | 21 | VC_356_0 | 21 | 0.5409 | 1 | 0.5409 | 0 | 0 | 0 | 1 | Ref |
| LC727697 | Unassigned | Unassigned | Unassigned | 171_0 | Clustered | 4 | VC_171_0 | 4 | 0.2204 | 0.99998328 | 0.2204 | 0 | 0 | 0 | 1 | Ref |
| LC727698 | Unassigned | Unassigned | Unassigned | 171_0 | Clustered | 4 | VC_171_0 | 4 | 0.2204 | 0.99998328 | 0.2204 | 0 | 0 | 0 | 1 | Ref |
| LC727699 | Unassigned | Unassigned | Unassigned | 356_0 | Clustered | 21 | VC_356_0 | 21 | 0.5409 | 1 | 0.5409 | 0 | 0 | 0 | 1 | Ref |
| LC727700 | Unassigned | Unassigned | Unassigned | 434_0 | Clustered | 45 | VC_434_0 | 45 | 0.9479 | 1 | 0.9479 | 0 | 0 | 0 | 1 | Ref |
| LC727701 | Unassigned | Unassigned | Unassigned | 356_0 | Clustered | 21 | VC_356_0 | 21 | 0.5409 | 1 | 0.5409 | 0 | 0 | 0 | 1 | Ref |
| LC727840 | Unassigned | Unassigned | Unassigned | 51_0 | Clustered | 36 | VC_51_0 | 36 | 0.6086 | 1 | 0.6086 | 0 | 0 | 0 | 1 | Ref |
| LC727841 | Unassigned | Unassigned | Unassigned | 51_0 | Clustered | 36 | VC_51_0 | 36 | 0.6086 | 1 | 0.6086 | 0 | 0 | 0 | 1 | Ref |
| LC727842 | Unassigned | Unassigned | Unassigned | 51_0 | Clustered | 36 | VC_51_0 | 36 | 0.6086 | 1 | 0.6086 | 0 | 0 | 0 | 1 | Ref |
| LC727843 | Unassigned | Unassigned | Unassigned | 51_0 | Clustered | 36 | VC_51_0 | 36 | 0.6086 | 1 | 0.6086 | 0 | 0 | 0 | 1 | Ref |
| LC727844 | Unassigned | Unassigned | Unassigned | 51_0 | Clustered | 36 | VC_51_0 | 36 | 0.6086 | 1 | 0.6086 | 0 | 0 | 0 | 1 | Ref |
| LC727845 | Unassigned | Unassigned | Unassigned | 51_0 | Clustered | 36 | VC_51_0 | 36 | 0.6086 | 1 | 0.6086 | 0 | 0 | 0 | 1 | Ref |
| LC727846 | Unassigned | Unassigned | Unassigned | 51_0 | Clustered | 36 | VC_51_0 | 36 | 0.6086 | 1 | 0.6086 | 0 | 0 | 0 | 1 | Ref |
| LC727847 | Unassigned | Unassigned | Unassigned | 51_0 | Clustered | 36 | VC_51_0 | 36 | 0.6086 | 1 | 0.6086 | 0 | 0 | 0 | 1 | Ref |
| LC727848 | Unassigned | Unassigned | Unassigned | 51_0 | Clustered | 36 | VC_51_0 | 36 | 0.6086 | 1 | 0.6086 | 0 | 0 | 0 | 1 | Ref |
| LC727849 | Unassigned | Unassigned | Unassigned | 51_0 | Clustered | 36 | VC_51_0 | 36 | 0.6086 | 1 | 0.6086 | 0 | 0 | 0 | 1 | Ref |
| LC727850 | Unassigned | Unassigned | Unassigned | 51_0 | Clustered | 36 | VC_51_0 | 36 | 0.6086 | 1 | 0.6086 | 0 | 0 | 0 | 1 | Ref |
| LC727851 | Unassigned | Unassigned | Unassigned | 51_0 | Clustered | 36 | VC_51_0 | 36 | 0.6086 | 1 | 0.6086 | 0 | 0 | 0 | 1 | Ref |
| LC727852 | Unassigned | Unassigned | Unassigned | 51_0 | Clustered | 36 | VC_51_0 | 36 | 0.6086 | 1 | 0.6086 | 0 | 0 | 0 | 1 | Ref |
| LC727853 | Unassigned | Unassigned | Unassigned | 51_0 | Clustered | 36 | VC_51_0 | 36 | 0.6086 | 1 | 0.6086 | 0 | 0 | 0 | 1 | Ref |
| LC727854 | Unassigned | Unassigned | Unassigned | 51_0 | Clustered | 36 | VC_51_0 | 36 | 0.6086 | 1 | 0.6086 | 0 | 0 | 0 | 1 | Ref |
| LC727855 | Unassigned | Unassigned | Unassigned | 51_0 | Clustered | 36 | VC_51_0 | 36 | 0.6086 | 1 | 0.6086 | 0 | 0 | 0 | 1 | Ref |
| LC727856 | Unassigned | Unassigned | Unassigned | 51_0 | Clustered | 36 | VC_51_0 | 36 | 0.6086 | 1 | 0.6086 | 0 | 0 | 0 | 1 | Ref |
| LC727857 | Unassigned | Unassigned | Unassigned | 51_0 | Clustered | 36 | VC_51_0 | 36 | 0.6086 | 1 | 0.6086 | 0 | 0 | 0 | 1 | Ref |
| LC727858 | Unassigned | Unassigned | Unassigned | 51_0 | Clustered | 36 | VC_51_0 | 36 | 0.6086 | 1 | 0.6086 | 0 | 0 | 0 | 1 | Ref |
| LC727859 | Unassigned | Unassigned | Unassigned | 51_0 | Clustered | 36 | VC_51_0 | 36 | 0.6086 | 1 | 0.6086 | 0 | 0 | 0 | 1 | Ref |
| LC727860 | Unassigned | Unassigned | Unassigned | 51_0 | Clustered | 36 | VC_51_0 | 36 | 0.6086 | 1 | 0.6086 | 0 | 0 | 0 | 1 | Ref |
| LC727861 | Unassigned | Unassigned | Unassigned | 51_0 | Clustered | 36 | VC_51_0 | 36 | 0.6086 | 1 | 0.6086 | 0 | 0 | 0 | 1 | Ref |
| LC727862 | Unassigned | Unassigned | Unassigned | 51_0 | Clustered | 36 | VC_51_0 | 36 | 0.6086 | 1 | 0.6086 | 0 | 0 | 0 | 1 | Ref |
| LC727863 | Unassigned | Unassigned | Unassigned | 51_0 | Clustered | 36 | VC_51_0 | 36 | 0.6086 | 1 | 0.6086 | 0 | 0 | 0 | 1 | Ref |
| LC727864 | Unassigned | Unassigned | Unassigned | 51_0 | Clustered | 36 | VC_51_0 | 36 | 0.6086 | 1 | 0.6086 | 0 | 0 | 0 | 1 | Ref |
| LC727865 | Unassigned | Unassigned | Unassigned | 51_0 | Clustered | 36 | VC_51_0 | 36 | 0.6086 | 1 | 0.6086 | 0 | 0 | 0 | 1 | Ref |
| LC727866 | Unassigned | Unassigned | Unassigned | 51_0 | Clustered | 36 | VC_51_0 | 36 | 0.6086 | 1 | 0.6086 | 0 | 0 | 0 | 1 | Ref |
| LC727867 | Unassigned | Unassigned | Unassigned | 51_0 | Clustered | 36 | VC_51_0 | 36 | 0.6086 | 1 | 0.6086 | 0 | 0 | 0 | 1 | Ref |
| LC727868 | Unassigned | Unassigned | Unassigned | 51_0 | Clustered | 36 | VC_51_0 | 36 | 0.6086 | 1 | 0.6086 | 0 | 0 | 0 | 1 | Ref |
| LC727869 | Unassigned | Unassigned | Unassigned | 51_0 | Clustered | 36 | VC_51_0 | 36 | 0.6086 | 1 | 0.6086 | 0 | 0 | 0 | 1 | Ref |
| LC727870 | Unassigned | Unassigned | Unassigned | 51_0 | Clustered | 36 | VC_51_0 | 36 | 0.6086 | 1 | 0.6086 | 0 | 0 | 0 | 1 | Ref |
| LC727871 | Unassigned | Unassigned | Unassigned | 51_0 | Clustered | 36 | VC_51_0 | 36 | 0.6086 | 1 | 0.6086 | 0 | 0 | 0 | 1 | Ref |
| LC727872 | Unassigned | Unassigned | Unassigned | 51_0 | Clustered | 36 | VC_51_0 | 36 | 0.6086 | 1 | 0.6086 | 0 | 0 | 0 | 1 | Ref |
| LC727873 | Unassigned | Unassigned | Unassigned | 51_0 | Clustered | 36 | VC_51_0 | 36 | 0.6086 | 1 | 0.6086 | 0 | 0 | 0 | 1 | Ref |
| LC727874 | Unassigned | Unassigned | Unassigned | 51_0 | Clustered | 36 | VC_51_0 | 36 | 0.6086 | 1 | 0.6086 | 0 | 0 | 0 | 1 | Ref |
| LC727875 | Unassigned | Unassigned | Unassigned | 51_0 | Clustered | 36 | VC_51_0 | 36 | 0.6086 | 1 | 0.6086 | 0 | 0 | 0 | 1 | Ref |
| LC727891 | Unassigned | Unassigned | Unassigned | 43_0 | Clustered | 16 | VC_43_0 | 16 | 0.2017 | 1 | 0.2017 | 0 | 0 | 0 | 0.9667 | Ref |
| LC727892 | Unassigned | Unassigned | Unassigned | 43_0 | Clustered | 16 | VC_43_0 | 16 | 0.2017 | 1 | 0.2017 | 0 | 0 | 0 | 0.9667 | Ref |
| LC727893 | Unassigned | Unassigned | Unassigned | 43_0 | Clustered | 16 | VC_43_0 | 16 | 0.2017 | 1 | 0.2017 | 0 | 0 | 0 | 0.9667 | Ref |
| LC727894 | Unassigned | Unassigned | Unassigned | 43_0 | Clustered | 16 | VC_43_0 | 16 | 0.2017 | 1 | 0.2017 | 0 | 0 | 0 | 0.9667 | Ref |
| LC727895 | Unassigned | Unassigned | Unassigned | 43_0 | Clustered | 16 | VC_43_0 | 16 | 0.2017 | 1 | 0.2017 | 0 | 0 | 0 | 0.9667 | Ref |
| LC727896 | Unassigned | Unassigned | Unassigned | 43_0 | Clustered | 16 | VC_43_0 | 16 | 0.2017 | 1 | 0.2017 | 0 | 0 | 0 | 0.9667 | Ref |
| LC727897 | Unassigned | Unassigned | Unassigned | 43_0 | Clustered | 16 | VC_43_0 | 16 | 0.2017 | 1 | 0.2017 | 0 | 0 | 0 | 0.9667 | Ref |
| LC727898 | Unassigned | Unassigned | Unassigned | 43_0 | Clustered | 16 | VC_43_0 | 16 | 0.2017 | 1 | 0.2017 | 0 | 0 | 0 | 0.9667 | Ref |
| LC727899 | Unassigned | Unassigned | Unassigned | 43_0 | Clustered | 16 | VC_43_0 | 16 | 0.2017 | 1 | 0.2017 | 0 | 0 | 0 | 0.9667 | Ref |
| LC727900 | Unassigned | Unassigned | Unassigned | 43_0 | Clustered | 16 | VC_43_0 | 16 | 0.2017 | 1 | 0.2017 | 0 | 0 | 0 | 0.9667 | Ref |
| LC727901 | Unassigned | Unassigned | Unassigned | 43_0 | Clustered | 16 | VC_43_0 | 16 | 0.2017 | 1 | 0.2017 | 0 | 0 | 0 | 0.9667 | Ref |
| LC727902 | Unassigned | Unassigned | Unassigned | 43_0 | Clustered | 16 | VC_43_0 | 16 | 0.2017 | 1 | 0.2017 | 0 | 0 | 0 | 0.9667 | Ref |
| LC727903 | Unassigned | Unassigned | Unassigned | 44_0 | Clustered | 49 | VC_44_0 | 49 | 0.4506 | 1 | 0.4506 | 0 | 0 | 0 | 0.8605 | Ref |
| LC730317 | Unassigned | Unassigned | Unassigned | 531_0 | Clustered | 32 | VC_531_0 | 32 | 0.6859 | 1 | 0.6859 | 0 | 0 | 0 | 1 | Ref |
| LC730318 | Unassigned | Unassigned | Unassigned | 222_1 | Clustered | 19 | VC_222_1 | 19 | 0.4166 | 1 | 0.4166 | 0 | 0 | 0 | 1 | Ref |
| LC730319 | Unassigned | Unassigned | Unassigned | 131_0 | Clustered | 43 | VC_131_0 | 43 | 0.8853 | 1 | 0.8853 | 0 | 0 | 0 | 1 | Ref |
| LC730320 | Unassigned | Unassigned | Unassigned | 223_6 | Clustered | 42 | VC_223_6 | 42 | 0.4717 | 1 | 0.4717 | 0 | 0 | 0 | 0.8351 | Ref |
| LC730321 | Unassigned | Unassigned | Unassigned | 45_0 | Clustered | 38 | VC_45_0 | 38 | 0.5785 | 1 | 0.5785 | 0 | 0 | 0 | 0.9659 | Ref |
| LC730322 | Unassigned | Unassigned | Unassigned | 30_1 | Clustered | 53 | VC_30_1 | 53 | 0.7151 | 1 | 0.7151 | 0 | 0 | 0 | 0.9325 | Ref |
| LC730323 | Unassigned | Unassigned | Unassigned | 466_0 | Clustered | 66 | VC_466_0 | 66 | 0.9246 | 1 | 0.9246 | 0 | 0 | 0 | 1 | Ref |
| LC730324 | Unassigned | Unassigned | Unassigned | 398_0 | Clustered | 51 | VC_398_0 | 51 | 0.8741 | 1 | 0.8741 | 0 | 0 | 0 | 1 | Ref |
| LC730325 | Unassigned | Unassigned | Unassigned | 405_0 | Clustered | 126 | VC_405_0 | 126 | 0.9614 | 1 | 0.9614 | 0 | 0 | 0 | 1 | Ref |
| LC739530 | Unassigned | Unassigned | Unassigned | 340_20 | Clustered | 4 | VC_340_20 | 4 | 0.0173 | 0.99998777 | 0.0173 | 0 | 0 | 0 | 1 | Ref |
| LC739531 | Unassigned | Unassigned | Unassigned | 340_20 | Clustered | 4 | VC_340_20 | 4 | 0.0173 | 0.99998777 | 0.0173 | 0 | 0 | 0 | 1 | Ref |
| LC739532 | Unassigned | Unassigned | Unassigned | 340_20 | Clustered | 4 | VC_340_20 | 4 | 0.0173 | 0.99998777 | 0.0173 | 0 | 0 | 0 | 1 | Ref |
| LC739533 | Unassigned | Unassigned | Unassigned | 871_0 | Clustered | 5 | VC_871_0 | 5 | 0.0619 | 0.99921937 | 0.0619 | 0 | 0 | 0 | 1 | Ref |
| LC739534 | Unassigned | Unassigned | Unassigned | 871_0 | Clustered | 5 | VC_871_0 | 5 | 0.0619 | 0.99921937 | 0.0619 | 0 | 0 | 0 | 1 | Ref |
| LC739535 | Unassigned | Unassigned | Unassigned | 871_0 | Clustered | 5 | VC_871_0 | 5 | 0.0619 | 0.99921937 | 0.0619 | 0 | 0 | 0 | 1 | Ref |
| LC739536 | Unassigned | Unassigned | Unassigned | 872_0 | Clustered | 28 | VC_872_0 | 28 | 0.7887 | 1 | 0.7887 | 0 | 0 | 0 | 1 | Ref |

| | | | | | | | | | | | | | | | | |
|---|---|---|---|---|---|---|---|---|---|---|---|---|---|---|---|---|
| LC739537 | Unassigned | Unassigned | Unassigned | 871_0 | Clustered | 5 | VC_871_0 | 5 | 0.0619 | 0.99921937 | 0.0619 | 0 | 0 | 0 | 1 | Ref |
| LC739538 | Unassigned | Unassigned | Unassigned | 794_16 | Clustered | 10 | VC_794_16 | 10 | 0.0563 | 0.9411319 | 0.053 | 0 | 0 | 0 | 0.8667 | Ref |
| LC739539 | Unassigned | Unassigned | Unassigned | 871_0 | Clustered | 5 | VC_871_0 | 5 | 0.0619 | 0.99921937 | 0.0619 | 0 | 0 | 0 | 1 | Ref |
| LC739540 | Unassigned | Unassigned | Unassigned | 794_16 | Clustered | 10 | VC_794_16 | 10 | 0.0563 | 0.9411319 | 0.053 | 0 | 0 | 0 | 0.8667 | Ref |
| LC739541 | Unassigned | Unassigned | Unassigned | 794_11 | Clustered | 6 | VC_794_11 | 6 | 0.0305 | 0.83830784 | 0.0256 | 0 | 0 | 0 | 0.8 | Ref |
| LC739542 | Unassigned | Unassigned | Unassigned | 794_11 | Clustered | 6 | VC_794_11 | 6 | 0.0305 | 0.83830784 | 0.0256 | 0 | 0 | 0 | 0.8 | Ref |
| LK392619 | Unassigned | Unassigned | Unassigned | 1089_0 | Clustered | 3 | VC_1089_0 | 3 | 1 | 1 | 1 | 0 | 0 | 0 | 1 | Ref |
| LK985321 | Unassigned | Unassigned | Unassigned | 666_0 | Clustered | 11 | VC_666_0 | 11 | 0.5822 | 1 | 0.5822 | 0 | 0 | 0 | 1 | Ref |
| LK985322 | Unassigned | Unassigned | Unassigned | 140_0 | Clustered | 2 | VC_140_0 | 2 | 0.067 | 0.95523577 | 0.064 | 0 | 0 | 0 | 1 | Ref |
| LN610572 | Unassigned | Unassigned | Unassigned | 77_0 | Clustered | 37 | VC_77_0 | 37 | 0.9392 | 1 | 0.9392 | 0 | 0 | 0 | 1 | Ref |
| LN610573 | Unassigned | Unassigned | Unassigned | 78_0 | Clustered | 20 | VC_78_0 | 20 | 0.9639 | 1 | 0.9639 | 0 | 0 | 0 | 1 | Ref |
| LN610574 | Unassigned | Unassigned | Unassigned | 434_0 | Clustered | 45 | VC_434_0 | 45 | 0.9479 | 1 | 0.9479 | 0 | 0 | 0 | 1 | Ref |
| LN610575 | Unassigned | Unassigned | Unassigned | 77_0 | Clustered | 37 | VC_77_0 | 37 | 0.9392 | 1 | 0.9392 | 0 | 0 | 0 | 1 | Ref |
| LN610576 | Unassigned | Unassigned | Unassigned | 78_0 | Clustered | 20 | VC_78_0 | 20 | 0.9639 | 1 | 0.9639 | 0 | 0 | 0 | 1 | Ref |
| LN610577 | Unassigned | Unassigned | Unassigned | 478_0 | Clustered | 8 | VC_478_0 | 8 | 0.2619 | 1 | 0.2619 | 0 | 0 | 0 | 1 | Ref |
| LN610578 | Unassigned | Unassigned | Unassigned | 486_0 | Clustered | 47 | VC_486_0 | 47 | 0.9022 | 1 | 0.9022 | 0 | 0 | 0 | 1 | Ref |
| LN610579 | Unassigned | Unassigned | Unassigned | 80_0 | Clustered | 64 | VC_80_0 | 64 | 0.418 | 1 | 0.418 | 0 | 0 | 0 | 0.7649 | Ref |
| LN610580 | Unassigned | Unassigned | Unassigned | 434_0 | Clustered | 45 | VC_434_0 | 45 | 0.9479 | 1 | 0.9479 | 0 | 0 | 0 | 1 | Ref |
| LN610581 | Unassigned | Unassigned | Unassigned | 78_0 | Clustered | 20 | VC_78_0 | 20 | 0.9639 | 1 | 0.9639 | 0 | 0 | 0 | 1 | Ref |
| LN610582 | Unassigned | Unassigned | Unassigned | 78_0 | Clustered | 20 | VC_78_0 | 20 | 0.9639 | 1 | 0.9639 | 0 | 0 | 0 | 1 | Ref |
| LN610583 | Unassigned | Unassigned | Unassigned | 78_0 | Clustered | 20 | VC_78_0 | 20 | 0.9639 | 1 | 0.9639 | 0 | 0 | 0 | 1 | Ref |
| LN610584 | Unassigned | Unassigned | Unassigned | 478_0 | Clustered | 8 | VC_478_0 | 8 | 0.2619 | 1 | 0.2619 | 0 | 0 | 0 | 1 | Ref |
| LN610585 | Unassigned | Unassigned | Unassigned | 478_0 | Clustered | 8 | VC_478_0 | 8 | 0.2619 | 1 | 0.2619 | 0 | 0 | 0 | 1 | Ref |
| LN610586 | Unassigned | Unassigned | Unassigned | 77_0 | Clustered | 37 | VC_77_0 | 37 | 0.9392 | 1 | 0.9392 | 0 | 0 | 0 | 1 | Ref |
| LN610587 | Unassigned | Unassigned | Unassigned | 77_0 | Clustered | 37 | VC_77_0 | 37 | 0.9392 | 1 | 0.9392 | 0 | 0 | 0 | 1 | Ref |
| LN610588 | Unassigned | Unassigned | Unassigned | 80_0 | Clustered | 64 | VC_80_0 | 64 | 0.418 | 1 | 0.418 | 0 | 0 | 0 | 0.7649 | Ref |
| LN610589 | Unassigned | Unassigned | Unassigned | 81_0 | Clustered | 41 | VC_81_0 | 41 | 0.5815 | 1 | 0.5815 | 0 | 0 | 0 | 0.9805 | Ref |
| LN610590 | Unassigned | Unassigned | Unassigned | 185_0 | Clustered | 53 | VC_185_0 | 53 | 0.9153 | 1 | 0.9153 | 0 | 0 | 0 | 1 | Ref |
| LN681534 | Unassigned | Unassigned | Unassigned | 788_0 | Clustered | 2 | VC_788_0 | 2 | 0.2235 | 0.95055313 | 0.2125 | 0 | 0 | 0 | 1 | Ref |
| LN681535 | Unassigned | Unassigned | Unassigned | 749_0 | Clustered | 5 | VC_749_0 | 5 | 0.4704 | 0.99999995 | 0.4704 | 0 | 0 | 0 | 1 | Ref |
| LN681536 | Unassigned | Unassigned | Unassigned | 749_0 | Clustered | 5 | VC_749_0 | 5 | 0.4704 | 0.99999995 | 0.4704 | 0 | 0 | 0 | 1 | Ref |
| LN681537 | Unassigned | Unassigned | Unassigned | 487_2 | Clustered | 2 | VC_487_2 | 2 | 0.1024 | 0.94551181 | 0.0968 | 0 | 0 | 0 | 1 | Ref |
| LN681538 | Unassigned | Unassigned | Unassigned | 666_0 | Clustered | 11 | VC_666_0 | 11 | 0.5822 | 1 | 0.5822 | 0 | 0 | 0 | 1 | Ref |
| LN681540 | Unassigned | Unassigned | Unassigned | 666_0 | Clustered | 11 | VC_666_0 | 11 | 0.5822 | 1 | 0.5822 | 0 | 0 | 0 | 1 | Ref |
| LN681541 | Unassigned | Unassigned | Unassigned | 140_1 | Clustered | 4 | VC_140_1 | 4 | 0.1766 | 0.99998565 | 0.1766 | 0 | 0 | 0 | 1 | Ref |
| LN681542 | Unassigned | Unassigned | Unassigned | 140_1 | Clustered | 4 | VC_140_1 | 4 | 0.1766 | 0.99998565 | 0.1766 | 0 | 0 | 0 | 1 | Ref |
| LN828717 | Unassigned | Unassigned | Unassigned | 447_5 | Clustered | 2 | VC_447_5 | 2 | 0.0172 | 0.93642158 | 0.0161 | 0 | 0 | 0 | 1 | Ref |
| LN866626 | Unassigned | Unassigned | Unassigned | 848_0 | Clustered | 124 | VC_848_0 | 124 | 0.9484 | 1 | 0.9484 | 0 | 0 | 0 | 1 | Ref |
| LN878149 | Unassigned | Unassigned | Unassigned | 907_0 | Clustered | 32 | VC_907_0 | 32 | 0.9964 | 1 | 0.9964 | 0 | 0 | 0 | 1 | Ref |
| LN881725 | Unassigned | Unassigned | Unassigned | 819_0 | Clustered | 19 | VC_819_0 | 19 | 0.6835 | 1 | 0.6835 | 0 | 0 | 0 | 1 | Ref |
| LN881726 | Unassigned | Unassigned | Unassigned | 290_33 | Clustered | 4 | VC_290_33 | 4 | 0.0075 | 0.99975449 | 0.0075 | 0 | 0 | 0 | 1 | Ref |
| LN881727 | Unassigned | Unassigned | Unassigned | 794_9 | Clustered | 7 | VC_794_9 | 7 | 0.0369 | 0.87330366 | 0.0322 | 0 | 0 | 0 | 1 | Ref |
| LN881728 | Unassigned | Unassigned | Unassigned | 290_73 | Clustered | 3 | VC_290_73 | 3 | 0.0051 | 0.99726944 | 0.0051 | 0 | 0 | 0 | 1 | Ref |
| LN881729 | Unassigned | Unassigned | Unassigned | 290_33 | Clustered | 4 | VC_290_33 | 4 | 0.0075 | 0.99975449 | 0.0075 | 0 | 0 | 0 | 1 | Ref |
| LN881730 | Unassigned | Unassigned | Unassigned | 859_0 | Clustered | 67 | VC_859_0 | 67 | 0.9622 | 1 | 0.9622 | 0 | 0 | 0 | 1 | Ref |
| LN881731 | Unassigned | Unassigned | Unassigned | 859_0 | Clustered | 67 | VC_859_0 | 67 | 0.9622 | 1 | 0.9622 | 0 | 0 | 0 | 1 | Ref |
| LN881732 | Unassigned | Unassigned | Unassigned | 290_37 | Clustered/Singleton | 1 | VC_290_37 | 1 | 0 | 1 | 0 | 0 | 0 | 0 | 1 | Ref |
| LN881733 | Unassigned | Unassigned | Unassigned | 290_73 | Clustered | 3 | VC_290_73 | 3 | 0.0051 | 0.99726944 | 0.0051 | 0 | 0 | 0 | 1 | Ref |
| LN881734 | Unassigned | Unassigned | Unassigned | 290_33 | Clustered | 4 | VC_290_33 | 4 | 0.0075 | 0.99975449 | 0.0075 | 0 | 0 | 0 | 1 | Ref |
| LN881735 | Unassigned | Unassigned | Unassigned | 794_9 | Clustered | 7 | VC_794_9 | 7 | 0.0369 | 0.87330366 | 0.0322 | 0 | 0 | 0 | 1 | Ref |
| LN881736 | Unassigned | Unassigned | Unassigned | 290_73 | Clustered | 3 | VC_290_73 | 3 | 0.0051 | 0.99726944 | 0.0051 | 0 | 0 | 0 | 1 | Ref |
| LN881737 | Unassigned | Unassigned | Unassigned | 290_33 | Clustered | 4 | VC_290_33 | 4 | 0.0075 | 0.99975449 | 0.0075 | 0 | 0 | 0 | 1 | Ref |
| LN881738 | Unassigned | Unassigned | Unassigned | 749_0 | Clustered | 5 | VC_749_0 | 5 | 0.4704 | 0.99999995 | 0.4704 | 0 | 0 | 0 | 1 | Ref |
| LN887844 | Unassigned | Unassigned | Unassigned | 79_5 | Clustered | 6 | VC_79_5 | 6 | 0.1083 | 0.99974986 | 0.1083 | 0 | 0 | 0 | 1 | Ref |
| LN887948 | Unassigned | Unassigned | Unassigned | 198_20 | Clustered | 4 | VC_198_20 | 4 | 0.0227 | 0.99997722 | 0.0227 | 0 | 0 | 0 | 1 | Ref |
| LN889756 | Unassigned | Unassigned | Unassigned | 131_0 | Clustered | 43 | VC_131_0 | 43 | 0.8853 | 1 | 0.8853 | 0 | 0 | 0 | 1 | Ref |
| LN889995 | Unassigned | Unassigned | Unassigned | 131_0 | Clustered | 43 | VC_131_0 | 43 | 0.8853 | 1 | 0.8853 | 0 | 0 | 0 | 1 | Ref |
| LN898172 | Unassigned | Unassigned | Unassigned | 192_0 | Clustered | 22 | VC_192_0 | 22 | 0.6582 | 1 | 0.6582 | 0 | 0 | 0 | 1 | Ref |
| LN907801 | Unassigned | Unassigned | Unassigned | 185_0 | Clustered | 53 | VC_185_0 | 53 | 0.9153 | 1 | 0.9153 | 0 | 0 | 0 | 1 | Ref |
| LN913017 | Unassigned | Unassigned | Unassigned | 131_0 | Clustered | 43 | VC_131_0 | 43 | 0.8853 | 1 | 0.8853 | 0 | 0 | 0 | 1 | Ref |
| LR025195 | Unassigned | Unassigned | Unassigned | 819_0 | Clustered | 19 | VC_819_0 | 19 | 0.6835 | 1 | 0.6835 | 0 | 0 | 0 | 1 | Ref |
| LR025196 | Unassigned | Unassigned | Unassigned | 819_0 | Clustered | 19 | VC_819_0 | 19 | 0.6835 | 1 | 0.6835 | 0 | 0 | 0 | 1 | Ref |
| LR025197 | Unassigned | Unassigned | Unassigned | 819_0 | Clustered | 19 | VC_819_0 | 19 | 0.6835 | 1 | 0.6835 | 0 | 0 | 0 | 1 | Ref |
| LR025198 | Unassigned | Unassigned | Unassigned | 819_0 | Clustered | 19 | VC_819_0 | 19 | 0.6835 | 1 | 0.6835 | 0 | 0 | 0 | 1 | Ref |
| LR026998 | Unassigned | Unassigned | Unassigned | 611_0 | Clustered | 50 | VC_611_0 | 50 | 0.7306 | 1 | 0.7306 | 0 | 0 | 0 | 1 | Ref |
| LR027383 | Unassigned | Unassigned | Unassigned | 293_1 | Clustered | 21 | VC_293_1 | 21 | 0.1694 | 1 | 0.1694 | 0 | 0 | 0 | 0.9048 | Ref |
| LR027384 | Unassigned | Unassigned | Unassigned | 198_12 | Clustered/Singleton | 1 | VC_198_12 | 1 | 0 | 1 | 0 | 0 | 0 | 0 | 1 | Ref |
| LR027385 | Unassigned | Unassigned | Unassigned | 976_0 | Clustered | 36 | VC_976_0 | 36 | 0.7833 | 1 | 0.7833 | 0 | 0 | 0 | 1 | Ref |
| LR027386 | Unassigned | Unassigned | Unassigned | 293_1 | Clustered | 21 | VC_293_1 | 21 | 0.1694 | 1 | 0.1694 | 0 | 0 | 0 | 0.9048 | Ref |
| LR027387 | Unassigned | Unassigned | Unassigned | 293_1 | Clustered | 21 | VC_293_1 | 21 | 0.1694 | 1 | 0.1694 | 0 | 0 | 0 | 0.9048 | Ref |
| LR027388 | Unassigned | Unassigned | Unassigned | 976_0 | Clustered | 36 | VC_976_0 | 36 | 0.7833 | 1 | 0.7833 | 0 | 0 | 0 | 1 | Ref |
| LR027389 | Unassigned | Unassigned | Unassigned | 199_14 | Clustered/Singleton | 1 | VC_199_14 | 1 | 0 | 1 | 0 | 0 | 0 | 0 | 1 | Ref |
| LR027390 | Unassigned | Unassigned | Unassigned | 293_1 | Clustered | 21 | VC_293_1 | 21 | 0.1694 | 1 | 0.1694 | 0 | 0 | 0 | 0.9048 | Ref |
| LR027391 | Unassigned | Unassigned | Unassigned | 293_1 | Clustered | 21 | VC_293_1 | 21 | 0.1694 | 1 | 0.1694 | 0 | 0 | 0 | 0.9048 | Ref |
| LR031359 | Unassigned | Unassigned | Unassigned | 93_0 | Clustered | 22 | VC_93_0 | 22 | 0.5285 | 1 | 0.5285 | 0 | 0 | 0 | 1 | Ref |
| LR215718 | Unassigned | Unassigned | Unassigned | 155_15 | Clustered | 16 | VC_155_15 | 16 | 0.0779 | 1 | 0.0779 | 0 | 0 | 0 | 0.8083 | Ref |
| LR215719 | Unassigned | Unassigned | Unassigned | 155_13 | Clustered | 3 | VC_155_13 | 3 | 0.0087 | 0.95130224 | 0.0083 | 0 | 0 | 0 | 1 | Ref |
| LR215720 | Unassigned | Unassigned | Unassigned | 155_2 | Clustered/Singleton | 1 | VC_155_2 | 1 | 0 | 1 | 0 | 0 | 0 | 0 | 1 | Ref |
| LR215721 | Unassigned | Unassigned | Unassigned | 155_29 | Clustered | 3 | VC_155_29 | 3 | 0.0119 | 0.99412742 | 0.0118 | 0 | 0 | 0 | 1 | Ref |
| LR215722 | Unassigned | Unassigned | Unassigned | 290_97 | Clustered/Singleton | 1 | VC_290_97 | 1 | 0 | 1 | 0 | 0 | 0 | 0 | 1 | Ref |
| LR215723 | Unassigned | Unassigned | Unassigned | 290_71 | Clustered/Singleton | 1 | VC_290_71 | 1 | 0 | 1 | 0 | 0 | 0 | 0 | 1 | Ref |

| | | | | | | | | | | | | | | | | |
|---|---|---|---|---|---|---|---|---|---|---|---|---|---|---|---|---|
| LR215724 | Unassigned | Unassigned | Unassigned | 290_113 | Clustered/Singleton | 1 | VC_290_113 | 1 | 0 | 1 | 0 | 0 | 0 | 0 | 1 | Ref |
| LR535901 | Unassigned | Unassigned | Unassigned | 1251_0 | Clustered | 11 | VC_1251_0 | 11 | 1 | 0 | 0 | 0 | 0 | 0 | 1 | Ref |
| LR535902 | Unassigned | Unassigned | Unassigned | 994_5 | Clustered | 19 | VC_994_5 | 19 | 0.2622 | 1 | 0.2622 | 0 | 0 | 0 | 1 | Ref |
| LR535903 | Unassigned | Unassigned | Unassigned | 1251_0 | Clustered | 11 | VC_1251_0 | 11 | 1 | 0 | 0 | 0 | 0 | 0 | 1 | Ref |
| LR535904 | Unassigned | Unassigned | Unassigned | 1251_0 | Clustered | 11 | VC_1251_0 | 11 | 1 | 0 | 0 | 0 | 0 | 0 | 1 | Ref |
| LR535905 | Unassigned | Unassigned | Unassigned | 1251_0 | Clustered | 11 | VC_1251_0 | 11 | 1 | 0 | 0 | 0 | 0 | 0 | 1 | Ref |
| LR535906 | Unassigned | Unassigned | Unassigned | 1251_0 | Clustered | 11 | VC_1251_0 | 11 | 1 | 0 | 0 | 0 | 0 | 0 | 1 | Ref |
| LR535907 | Unassigned | Unassigned | Unassigned | 1251_0 | Clustered | 11 | VC_1251_0 | 11 | 1 | 0 | 0 | 0 | 0 | 0 | 1 | Ref |
| LR535908 | Unassigned | Unassigned | Unassigned | 1251_0 | Clustered | 11 | VC_1251_0 | 11 | 1 | 0 | 0 | 0 | 0 | 0 | 1 | Ref |
| LR535909 | Unassigned | Unassigned | Unassigned | 994_5 | Clustered | 19 | VC_994_5 | 19 | 0.2622 | 1 | 0.2622 | 0 | 0 | 0 | 1 | Ref |
| LR535910 | Unassigned | Unassigned | Unassigned | 994_5 | Clustered | 19 | VC_994_5 | 19 | 0.2622 | 1 | 0.2622 | 0 | 0 | 0 | 1 | Ref |
| LR535911 | Unassigned | Unassigned | Unassigned | 1251_0 | Clustered | 11 | VC_1251_0 | 11 | 1 | 0 | 0 | 0 | 0 | 0 | 1 | Ref |
| LR535912 | Unassigned | Unassigned | Unassigned | 1251_0 | Clustered | 11 | VC_1251_0 | 11 | 1 | 0 | 0 | 0 | 0 | 0 | 1 | Ref |
| LR535913 | Unassigned | Unassigned | Unassigned | 1251_0 | Clustered | 11 | VC_1251_0 | 11 | 1 | 0 | 0 | 0 | 0 | 0 | 1 | Ref |
| LR535914 | Unassigned | Unassigned | Unassigned | 994_5 | Clustered | 19 | VC_994_5 | 19 | 0.2622 | 1 | 0.2622 | 0 | 0 | 0 | 1 | Ref |
| LR535915 | Unassigned | Unassigned | Unassigned | 994_5 | Clustered | 19 | VC_994_5 | 19 | 0.2622 | 1 | 0.2622 | 0 | 0 | 0 | 1 | Ref |
| LR535916 | Unassigned | Unassigned | Unassigned | 994_5 | Clustered | 19 | VC_994_5 | 19 | 0.2622 | 1 | 0.2622 | 0 | 0 | 0 | 1 | Ref |
| LR535917 | Unassigned | Unassigned | Unassigned | 1251_0 | Clustered | 11 | VC_1251_0 | 11 | 1 | 0 | 0 | 0 | 0 | 0 | 1 | Ref |
| LR535918 | Unassigned | Unassigned | Unassigned | 994_5 | Clustered | 19 | VC_994_5 | 19 | 0.2622 | 1 | 0.2622 | 0 | 0 | 0 | 1 | Ref |
| LR535919 | Unassigned | Unassigned | Unassigned | 994_5 | Clustered | 19 | VC_994_5 | 19 | 0.2622 | 1 | 0.2622 | 0 | 0 | 0 | 1 | Ref |
| LR535920 | Unassigned | Unassigned | Unassigned | 994_5 | Clustered | 19 | VC_994_5 | 19 | 0.2622 | 1 | 0.2622 | 0 | 0 | 0 | 1 | Ref |
| LR535921 | Unassigned | Unassigned | Unassigned | 994_5 | Clustered | 19 | VC_994_5 | 19 | 0.2622 | 1 | 0.2622 | 0 | 0 | 0 | 1 | Ref |
| LR588166 | Unassigned | Unassigned | Unassigned | 171_0 | Clustered | 4 | VC_171_0 | 4 | 0.2204 | 0.99998328 | 0.2204 | 0 | 0 | 0 | 1 | Ref |
| LR595850 | Unassigned | Unassigned | Unassigned | 45_0 | Clustered | 38 | VC_45_0 | 38 | 0.5785 | 1 | 0.5785 | 0 | 0 | 0 | 0.9659 | Ref |
| LR595859 | Unassigned | Unassigned | Unassigned | 45_0 | Clustered | 38 | VC_45_0 | 38 | 0.5785 | 1 | 0.5785 | 0 | 0 | 0 | 0.9659 | Ref |
| LR595860 | Unassigned | Unassigned | Unassigned | 45_0 | Clustered | 38 | VC_45_0 | 38 | 0.5785 | 1 | 0.5785 | 0 | 0 | 0 | 0.9659 | Ref |
| LR595861 | Unassigned | Unassigned | Unassigned | 45_0 | Clustered | 38 | VC_45_0 | 38 | 0.5785 | 1 | 0.5785 | 0 | 0 | 0 | 0.9659 | Ref |
| LR595863 | Unassigned | Unassigned | Unassigned | 45_0 | Clustered | 38 | VC_45_0 | 38 | 0.5785 | 1 | 0.5785 | 0 | 0 | 0 | 0.9659 | Ref |
| LR595864 | Unassigned | Unassigned | Unassigned | 45_0 | Clustered | 38 | VC_45_0 | 38 | 0.5785 | 1 | 0.5785 | 0 | 0 | 0 | 0.9659 | Ref |
| LR595865 | Unassigned | Unassigned | Unassigned | 45_0 | Clustered | 38 | VC_45_0 | 38 | 0.5785 | 1 | 0.5785 | 0 | 0 | 0 | 0.9659 | Ref |
| LR595866 | Unassigned | Unassigned | Unassigned | 45_0 | Clustered | 38 | VC_45_0 | 38 | 0.5785 | 1 | 0.5785 | 0 | 0 | 0 | 0.9659 | Ref |
| LR595867 | Unassigned | Unassigned | Unassigned | 52_0 | Clustered | 9 | VC_52_0 | 9 | 0.5586 | 1 | 0.5586 | 0 | 0 | 0 | 1 | Ref |
| LR595868 | Unassigned | Unassigned | Unassigned | 53_0 | Clustered | 10 | VC_53_0 | 10 | 0.1743 | 1 | 0.1743 | 0 | 0 | 0 | 1 | Ref |
| LR595869 | Unassigned | Unassigned | Unassigned | 235_0 | Clustered | 60 | VC_235_0 | 60 | 0.9413 | 1 | 0.9413 | 0 | 0 | 0 | 1 | Ref |
| LR595870 | Unassigned | Unassigned | Unassigned | 235_0 | Clustered | 60 | VC_235_0 | 60 | 0.9413 | 1 | 0.9413 | 0 | 0 | 0 | 1 | Ref |
| LR595873 | Unassigned | Unassigned | Unassigned | 235_0 | Clustered | 60 | VC_235_0 | 60 | 0.9413 | 1 | 0.9413 | 0 | 0 | 0 | 1 | Ref |
| LR595883 | Unassigned | Unassigned | Unassigned | 235_0 | Clustered | 60 | VC_235_0 | 60 | 0.9413 | 1 | 0.9413 | 0 | 0 | 0 | 1 | Ref |
| LR595885 | Unassigned | Unassigned | Unassigned | 235_0 | Clustered | 60 | VC_235_0 | 60 | 0.9413 | 1 | 0.9413 | 0 | 0 | 0 | 1 | Ref |
| LR595887 | Unassigned | Unassigned | Unassigned | 235_0 | Clustered | 60 | VC_235_0 | 60 | 0.9413 | 1 | 0.9413 | 0 | 0 | 0 | 1 | Ref |
| LR595891 | Unassigned | Unassigned | Unassigned | 236_0 | Clustered | 32 | VC_236_0 | 32 | 0.9115 | 1 | 0.9115 | 0 | 0 | 0 | 1 | Ref |
| LR596614 | Unassigned | Unassigned | Unassigned | 976_0 | Clustered | 36 | VC_976_0 | 36 | 0.7833 | 1 | 0.7833 | 0 | 0 | 0 | 1 | Ref |
| LR596615 | Unassigned | Unassigned | Unassigned | 170_8 | Clustered | 3 | VC_170_8 | 3 | 0.0298 | 0.85155589 | 0.0254 | 0 | 0 | 0 | 1 | Ref |
| LR596902 | Unassigned | Unassigned | Unassigned | 1252_0 | Clustered | 2 | VC_1252_0 | 2 | 0.1373 | 0.95236699 | 0.1307 | 0 | 0 | 0 | 1 | Ref |
| LR596903 | Unassigned | Unassigned | Unassigned | 727_0 | Clustered | 8 | VC_727_0 | 8 | 0.6106 | 1 | 0.6106 | 0 | 0 | 0 | 0.9286 | Ref |
| LR597635 | Unassigned | Unassigned | Unassigned | 45_0 | Clustered | 38 | VC_45_0 | 38 | 0.5785 | 1 | 0.5785 | 0 | 0 | 0 | 0.9659 | Ref |
| LR597636 | Unassigned | Unassigned | Unassigned | 45_0 | Clustered | 38 | VC_45_0 | 38 | 0.5785 | 1 | 0.5785 | 0 | 0 | 0 | 0.9659 | Ref |
| LR597637 | Unassigned | Unassigned | Unassigned | 5_0 | Clustered | 27 | VC_5_0 | 27 | 0.9599 | 1 | 0.9599 | 0 | 0 | 0 | 1 | Ref |
| LR597638 | Unassigned | Unassigned | Unassigned | 5_0 | Clustered | 27 | VC_5_0 | 27 | 0.9599 | 1 | 0.9599 | 0 | 0 | 0 | 1 | Ref |
| LR597639 | Unassigned | Unassigned | Unassigned | 45_0 | Clustered | 38 | VC_45_0 | 38 | 0.5785 | 1 | 0.5785 | 0 | 0 | 0 | 0.9659 | Ref |
| LR597640 | Unassigned | Unassigned | Unassigned | 5_0 | Clustered | 27 | VC_5_0 | 27 | 0.9599 | 1 | 0.9599 | 0 | 0 | 0 | 1 | Ref |
| LR597641 | Unassigned | Unassigned | Unassigned | 53_0 | Clustered | 10 | VC_53_0 | 10 | 0.1743 | 1 | 0.1743 | 0 | 0 | 0 | 1 | Ref |
| LR597643 | Unassigned | Unassigned | Unassigned | 45_0 | Clustered | 38 | VC_45_0 | 38 | 0.5785 | 1 | 0.5785 | 0 | 0 | 0 | 0.9659 | Ref |
| LR597644 | Unassigned | Unassigned | Unassigned | 45_0 | Clustered | 38 | VC_45_0 | 38 | 0.5785 | 1 | 0.5785 | 0 | 0 | 0 | 0.9659 | Ref |
| LR597646 | Unassigned | Unassigned | Unassigned | 859_0 | Clustered | 67 | VC_859_0 | 67 | 0.9622 | 1 | 0.9622 | 0 | 0 | 0 | 1 | Ref |
| LR597647 | Unassigned | Unassigned | Unassigned | 794_9 | Clustered | 7 | VC_794_9 | 7 | 0.0369 | 0.87330366 | 0.0322 | 0 | 0 | 0 | 1 | Ref |
| LR597648 | Unassigned | Unassigned | Unassigned | 45_0 | Clustered | 38 | VC_45_0 | 38 | 0.5785 | 1 | 0.5785 | 0 | 0 | 0 | 0.9659 | Ref |
| LR597649 | Unassigned | Unassigned | Unassigned | 5_0 | Clustered | 27 | VC_5_0 | 27 | 0.9599 | 1 | 0.9599 | 0 | 0 | 0 | 1 | Ref |
| LR597650 | Unassigned | Unassigned | Unassigned | 45_0 | Clustered | 38 | VC_45_0 | 38 | 0.5785 | 1 | 0.5785 | 0 | 0 | 0 | 0.9659 | Ref |
| LR597651 | Unassigned | Unassigned | Unassigned | 45_0 | Clustered | 38 | VC_45_0 | 38 | 0.5785 | 1 | 0.5785 | 0 | 0 | 0 | 0.9659 | Ref |
| LR597652 | Unassigned | Unassigned | Unassigned | 45_0 | Clustered | 38 | VC_45_0 | 38 | 0.5785 | 1 | 0.5785 | 0 | 0 | 0 | 0.9659 | Ref |
| LR597653 | Unassigned | Unassigned | Unassigned | 45_0 | Clustered | 38 | VC_45_0 | 38 | 0.5785 | 1 | 0.5785 | 0 | 0 | 0 | 0.9659 | Ref |
| LR597654 | Unassigned | Unassigned | Unassigned | 871_1 | Clustered | 26 | VC_871_1 | 26 | 0.6303 | 1 | 0.6303 | 0 | 0 | 0 | 0.9815 | Ref |
| LR597655 | Unassigned | Unassigned | Unassigned | 198_14 | Clustered | 3 | VC_198_14 | 3 | 0.0142 | 0.99685589 | 0.0142 | 0 | 0 | 0 | 0.6667 | Ref |
| LR597656 | Unassigned | Unassigned | Unassigned | 603_0 | Clustered | 28 | VC_603_0 | 28 | 0.7692 | 1 | 0.7692 | 0 | 0 | 0 | 1 | Ref |
| LR597657 | Unassigned | Unassigned | Unassigned | 290_137 | Clustered/Singleton | 1 | VC_290_137 | 1 | 0 | 1 | 0 | 0 | 0 | 0 | 1 | Ref |
| LR597658 | Unassigned | Unassigned | Unassigned | 340_6 | Clustered | 3 | VC_340_6 | 3 | 0.0106 | 0.99833469 | 0.0106 | 0 | 0 | 0 | 1 | Ref |
| LR597659 | Unassigned | Unassigned | Unassigned | 198_24 | Clustered | 5 | VC_198_24 | 5 | 0.029 | 0.9999995 | 0.029 | 0 | 0 | 0 | 0.7 | Ref |
| LR597660 | Unassigned | Unassigned | Unassigned | 290_101 | Clustered/Singleton | 1 | VC_290_101 | 1 | 0 | 1 | 0 | 0 | 0 | 0 | 1 | Ref |
| LR597663 | Unassigned | Unassigned | Unassigned | 340_9 | Clustered/Singleton | 1 | VC_340_9 | 1 | 0 | 1 | 0 | 0 | 0 | 0 | 1 | Ref |
| LR694165 | Unassigned | Unassigned | Unassigned | 794_9 | Clustered | 7 | VC_794_9 | 7 | 0.0369 | 0.87330366 | 0.0322 | 0 | 0 | 0 | 1 | Ref |
| LR694610 | Unassigned | Unassigned | Unassigned | 794_7 | Clustered | 2 | VC_794_7 | 2 | 0.006 | 0.58868714 | 0.0035 | 0 | 0 | 0 | 1 | Ref |
| LR694611 | Unassigned | Unassigned | Unassigned | 794_7 | Clustered | 2 | VC_794_7 | 2 | 0.006 | 0.58868714 | 0.0035 | 0 | 0 | 0 | 1 | Ref |
| LR699048 | Unassigned | Unassigned | Unassigned | 340_7 | Clustered | 18 | VC_340_7 | 18 | 0.0936 | 1 | 0.0936 | 0 | 0 | 0 | 0.7778 | Ref |
| LR699804 | Unassigned | Unassigned | Unassigned | 794_19 | Clustered | 16 | VC_794_19 | 16 | 0.0975 | 0.99616626 | 0.0971 | 0 | 0 | 0 | 0.8083 | Ref |
| LR743523 | Unassigned | Unassigned | Unassigned | 118_0 | Clustered | 13 | VC_118_0 | 13 | 1 | 1 | 1 | 0 | 0 | 0 | 1 | Ref |
| LR743524 | Unassigned | Unassigned | Unassigned | 178_0 | Clustered | 5 | VC_178_0 | 5 | 0.3547 | 0.99999997 | 0.3547 | 0 | 0 | 0 | 1 | Ref |
| LR743528 | Unassigned | Unassigned | Unassigned | 118_0 | Clustered | 13 | VC_118_0 | 13 | 1 | 1 | 1 | 0 | 0 | 0 | 1 | Ref |
| LR743529 | Unassigned | Unassigned | Unassigned | 118_0 | Clustered | 13 | VC_118_0 | 13 | 1 | 1 | 1 | 0 | 0 | 0 | 1 | Ref |
| LR743530 | Unassigned | Unassigned | Unassigned | 766_0 | Clustered | 40 | VC_766_0 | 40 | 0.7511 | 1 | 0.7511 | 0 | 0 | 0 | 1 | Ref |
| LR743531 | Unassigned | Unassigned | Unassigned | 118_0 | Clustered | 13 | VC_118_0 | 13 | 1 | 1 | 1 | 0 | 0 | 0 | 1 | Ref |
| LR743532 | Unassigned | Unassigned | Unassigned | 175_0 | Clustered | 20 | VC_175_0 | 20 | 0.7504 | 1 | 0.7504 | 0 | 0 | 0 | 1 | Ref |

| | | | | | | | | | | | | | | | | |
|---|---|---|---|---|---|---|---|---|---|---|---|---|---|---|---|---|
| LR745206 | Unassigned | Unassigned | Unassigned | 551_1 | Clustered/Singleton | 1 | VC_551_1 | 1 | 0 | 1 | 0 | 0 | 0 | 0 | 1 | Ref |
| LR745706 | Unassigned | Unassigned | Unassigned | 405_0 | Clustered | 126 | VC_405_0 | 126 | 0.9614 | 1 | 0.9614 | 0 | 0 | 0 | 1 | Ref |
| LR745707 | Unassigned | Unassigned | Unassigned | 405_0 | Clustered | 126 | VC_405_0 | 126 | 0.9614 | 1 | 0.9614 | 0 | 0 | 0 | 1 | Ref |
| LR745708 | Unassigned | Unassigned | Unassigned | 405_0 | Clustered | 126 | VC_405_0 | 126 | 0.9614 | 1 | 0.9614 | 0 | 0 | 0 | 1 | Ref |
| LR745709 | Unassigned | Unassigned | Unassigned | 405_0 | Clustered | 126 | VC_405_0 | 126 | 0.9614 | 1 | 0.9614 | 0 | 0 | 0 | 1 | Ref |
| LR745710 | Unassigned | Unassigned | Unassigned | 405_0 | Clustered | 126 | VC_405_0 | 126 | 0.9614 | 1 | 0.9614 | 0 | 0 | 0 | 1 | Ref |
| LR746137 | Unassigned | Unassigned | Unassigned | 871_1 | Clustered | 26 | VC_871_1 | 26 | 0.6303 | 1 | 0.6303 | 0 | 0 | 0 | 0.9815 | Ref |
| LR746310 | Unassigned | Unassigned | Unassigned | 686_0 | Clustered | 25 | VC_686_0 | 25 | 0.5501 | 1 | 0.5501 | 0 | 0 | 0 | 0.96 | Ref |
| LR746311 | Unassigned | Unassigned | Unassigned | 290_141 | Clustered/Singleton | 1 | VC_290_141 | 1 | 0 | 1 | 0 | 0 | 0 | 0 | 1 | Ref |
| LR756501 | Unassigned | Unassigned | Unassigned | 551_0 | Clustered | 2 | VC_551_0 | 2 | 0.2587 | 0.94530405 | 0.2445 | 0 | 0 | 0 | 1 | Ref |
| LR756502 | Unassigned | Unassigned | Unassigned | 551_2 | Clustered/Singleton | 1 | VC_551_2 | 1 | 0 | 1 | 0 | 0 | 0 | 0 | 1 | Ref |
| LR756503 | Unassigned | Unassigned | Unassigned | 551_0 | Clustered | 2 | VC_551_0 | 2 | 0.2587 | 0.94530405 | 0.2445 | 0 | 0 | 0 | 1 | Ref |
| LR756504 | Unassigned | Unassigned | Unassigned | 551_3 | Clustered/Singleton | 1 | VC_551_3 | 1 | 0 | 1 | 0 | 0 | 0 | 0 | 1 | Ref |
| LR757892 | Unassigned | Unassigned | Unassigned | 972_0 | Clustered | 99 | VC_972_0 | 99 | 0.9113 | 1 | 0.9113 | 0 | 0 | 0 | 0.9928 | Ref |
| LR778216 | Unassigned | Unassigned | Unassigned | 75_0 | Clustered | 15 | VC_75_0 | 15 | 0.518 | 1 | 0.518 | 0 | 0 | 0 | 1 | Ref |
| LR794124 | Unassigned | Unassigned | Unassigned | 861_0 | Clustered | 29 | VC_861_0 | 29 | 0.9954 | 1 | 0.9954 | 0 | 0 | 0 | 1 | Ref |
| LR794146 | Unassigned | Unassigned | Unassigned | 31_0 | Clustered | 10 | VC_31_0 | 10 | 0.5129 | 1 | 0.5129 | 0 | 0 | 0 | 1 | Ref |
| LR794147 | Unassigned | Unassigned | Unassigned | 31_0 | Clustered | 10 | VC_31_0 | 10 | 0.5129 | 1 | 0.5129 | 0 | 0 | 0 | 1 | Ref |
| LR794148 | Unassigned | Unassigned | Unassigned | 31_0 | Clustered | 10 | VC_31_0 | 10 | 0.5129 | 1 | 0.5129 | 0 | 0 | 0 | 1 | Ref |
| LR794149 | Unassigned | Unassigned | Unassigned | 31_0 | Clustered | 10 | VC_31_0 | 10 | 0.5129 | 1 | 0.5129 | 0 | 0 | 0 | 1 | Ref |
| LR794150 | Unassigned | Unassigned | Unassigned | 31_0 | Clustered | 10 | VC_31_0 | 10 | 0.5129 | 1 | 0.5129 | 0 | 0 | 0 | 1 | Ref |
| LR794151 | Unassigned | Unassigned | Unassigned | 31_0 | Clustered | 10 | VC_31_0 | 10 | 0.5129 | 1 | 0.5129 | 0 | 0 | 0 | 1 | Ref |
| LR794152 | Unassigned | Unassigned | Unassigned | 31_0 | Clustered | 10 | VC_31_0 | 10 | 0.5129 | 1 | 0.5129 | 0 | 0 | 0 | 1 | Ref |
| LR797314 | Unassigned | Unassigned | Unassigned | 193_0 | Clustered | 3 | VC_193_0 | 3 | 0.4439 | 0.97148006 | 0.4313 | 0 | 0 | 0 | 1 | Ref |
| LR797923 | Unassigned | Unassigned | Unassigned | 31_0 | Clustered | 10 | VC_31_0 | 10 | 0.5129 | 1 | 0.5129 | 0 | 0 | 0 | 1 | Ref |
| LR800396 | Unassigned | Unassigned | Unassigned | 135_13 | Clustered | 2 | VC_135_13 | 2 | 0.005 | 0.86394392 | 0.0044 | 0 | 0 | 0 | 1 | Ref |
| LR811967 | Unassigned | Unassigned | Unassigned | 135_28 | Clustered | 3 | VC_135_28 | 3 | 0.0105 | 0.97349514 | 0.0102 | 0 | 0 | 0 | 0.6667 | Ref |
| LR811968 | Unassigned | Unassigned | Unassigned | 135_4 | Clustered/Singleton | 1 | VC_135_4 | 1 | 0 | 1 | 0 | 0 | 0 | 0 | 1 | Ref |
| LR811969 | Unassigned | Unassigned | Unassigned | 135_28 | Clustered | 3 | VC_135_28 | 3 | 0.0105 | 0.97349514 | 0.0102 | 0 | 0 | 0 | 0.6667 | Ref |
| LR813619 | Unassigned | Unassigned | Unassigned | 817_0 | Clustered | 12 | VC_817_0 | 12 | 0.8497 | 1 | 0.8497 | 0 | 0 | 0 | 1 | Ref |
| LR865361 | Unassigned | Unassigned | Unassigned | 872_0 | Clustered | 28 | VC_872_0 | 28 | 0.7887 | 1 | 0.7887 | 0 | 0 | 0 | 1 | Ref |
| LR877331 | Unassigned | Unassigned | Unassigned | 509_0 | Clustered | 36 | VC_509_0 | 36 | 0.5291 | 1 | 0.5291 | 0 | 0 | 0 | 0.9714 | Ref |
| LR877332 | Unassigned | Unassigned | Unassigned | 290_67 | Clustered | 2 | VC_290_67 | 2 | 0.0027 | 0.90726003 | 0.0025 | 0 | 0 | 0 | 1 | Ref |
| LR880803 | Unassigned | Unassigned | Unassigned | 340_3 | Clustered | 24 | VC_340_3 | 24 | 0.1205 | 1 | 0.1205 | 0 | 0 | 0 | 0.7283 | Ref |
| LR881104 | Unassigned | Unassigned | Unassigned | 359_0 | Clustered | 3 | VC_359_0 | 3 | 0.1509 | 0.99844728 | 0.1507 | 0 | 0 | 0 | 1 | Ref |
| LR990702 | Unassigned | Unassigned | Unassigned | 859_0 | Clustered | 67 | VC_859_0 | 67 | 0.9622 | 1 | 0.9622 | 0 | 0 | 0 | 1 | Ref |
| LR990704 | Unassigned | Unassigned | Unassigned | 290_38 | Clustered/Singleton | 1 | VC_290_38 | 1 | 0 | 1 | 0 | 0 | 0 | 0 | 1 | Ref |
| LR990835 | Unassigned | Unassigned | Unassigned | 94_0 | Clustered | 22 | VC_94_0 | 22 | 0.5602 | 1 | 0.5602 | 0 | 0 | 0 | 1 | Ref |
| LR999870 | Unassigned | Unassigned | Unassigned | 859_0 | Clustered | 67 | VC_859_0 | 67 | 0.9622 | 1 | 0.9622 | 0 | 0 | 0 | 1 | Ref |
| LR999871 | Unassigned | Unassigned | Unassigned | 801_0 | Clustered | 37 | VC_801_0 | 37 | 0.5672 | 1 | 0.5672 | 0 | 0 | 0 | 1 | Ref |
| LT546029 | Unassigned | Unassigned | Unassigned | 707_1 | Clustered | 5 | VC_707_1 | 5 | 0.4294 | 0.99999994 | 0.4294 | 0 | 0 | 0 | 1 | Ref |
| LT594300 | Unassigned | Unassigned | Unassigned | 465_0 | Clustered | 80 | VC_465_0 | 80 | 0.9988 | 1 | 0.9988 | 0 | 0 | 0 | 1 | Ref |
| LT598654 | Unassigned | Unassigned | Unassigned | 68_5 | Clustered/Singleton | 1 | VC_68_5 | 1 | 0 | 1 | 0 | 0 | 0 | 0 | 1 | Ref |
| LT603033 | Unassigned | Unassigned | Unassigned | 170_11 | Clustered | 10 | VC_170_11 | 10 | 0.1502 | 0.99999797 | 0.1502 | 0 | 0 | 0 | 0.9111 | Ref |
| LT603684 | Unassigned | Unassigned | Unassigned | 645_0 | Clustered | 20 | VC_645_0 | 20 | 0.8502 | 1 | 0.8502 | 0 | 0 | 0 | 1 | Ref |
| LT607758 | Unassigned | Unassigned | Unassigned | 686_0 | Clustered | 25 | VC_686_0 | 25 | 0.5501 | 1 | 0.5501 | 0 | 0 | 0 | 0.96 | Ref |
| LT608331 | Unassigned | Unassigned | Unassigned | 185_0 | Clustered | 53 | VC_185_0 | 53 | 0.9153 | 1 | 0.9153 | 0 | 0 | 0 | 1 | Ref |
| LT615366 | Unassigned | Unassigned | Unassigned | 707_1 | Clustered | 5 | VC_707_1 | 5 | 0.4294 | 0.99999994 | 0.4294 | 0 | 0 | 0 | 1 | Ref |
| LT630001 | Unassigned | Unassigned | Unassigned | 1003_0 | Clustered | 16 | VC_1003_0 | 16 | 0.8967 | 1 | 0.8967 | 0 | 0 | 0 | 1 | Ref |
| LT714109 | Unassigned | Unassigned | Unassigned | 30_1 | Clustered | 53 | VC_30_1 | 53 | 0.7151 | 1 | 0.7151 | 0 | 0 | 0 | 0.9325 | Ref |
| LT841304 | Unassigned | Unassigned | Unassigned | 976_0 | Clustered | 36 | VC_976_0 | 36 | 0.7833 | 1 | 0.7833 | 0 | 0 | 0 | 1 | Ref |
| LT898437 | Unassigned | Unassigned | Unassigned | 322_0 | Clustered | 12 | VC_322_0 | 12 | 0.8924 | 1 | 0.8924 | 0 | 0 | 0 | 1 | Ref |
| LT907986 | Unassigned | Unassigned | Unassigned | 819_0 | Clustered | 19 | VC_819_0 | 19 | 0.6835 | 1 | 0.6835 | 0 | 0 | 0 | 1 | Ref |
| LT960551 | Unassigned | Unassigned | Unassigned | 170_8 | Clustered | 3 | VC_170_8 | 3 | 0.0298 | 0.85155589 | 0.0254 | 0 | 0 | 0 | 1 | Ref |
| LT960552 | Unassigned | Unassigned | Unassigned | 170_8 | Clustered | 3 | VC_170_8 | 3 | 0.0298 | 0.85155589 | 0.0254 | 0 | 0 | 0 | 1 | Ref |
| LT960606 | Unassigned | Unassigned | Unassigned | 399_0 | Clustered | 49 | VC_399_0 | 49 | 0.7927 | 1 | 0.7927 | 0 | 0 | 0 | 1 | Ref |
| LT960608 | Unassigned | Unassigned | Unassigned | 87_3 | Clustered | 19 | VC_87_3 | 19 | 0.3645 | 1 | 0.3645 | 0 | 0 | 0 | 0.807 | Ref |
| LT960609 | Unassigned | Unassigned | Unassigned | 859_0 | Clustered | 67 | VC_859_0 | 67 | 0.9622 | 1 | 0.9622 | 0 | 0 | 0 | 1 | Ref |
| LT961732 | Unassigned | Unassigned | Unassigned | 976_0 | Clustered | 36 | VC_976_0 | 36 | 0.7833 | 1 | 0.7833 | 0 | 0 | 0 | 1 | Ref |
| LT961836 | Unassigned | Unassigned | Unassigned | 399_0 | Clustered | 49 | VC_399_0 | 49 | 0.7927 | 1 | 0.7927 | 0 | 0 | 0 | 1 | Ref |
| LT961837 | Unassigned | Unassigned | Unassigned | 399_0 | Clustered | 49 | VC_399_0 | 49 | 0.7927 | 1 | 0.7927 | 0 | 0 | 0 | 1 | Ref |
| LT961838 | Unassigned | Unassigned | Unassigned | 399_0 | Clustered | 49 | VC_399_0 | 49 | 0.7927 | 1 | 0.7927 | 0 | 0 | 0 | 1 | Ref |
| LT961840 | Unassigned | Unassigned | Unassigned | 399_0 | Clustered | 49 | VC_399_0 | 49 | 0.7927 | 1 | 0.7927 | 0 | 0 | 0 | 1 | Ref |
| LT961841 | Unassigned | Unassigned | Unassigned | 399_0 | Clustered | 49 | VC_399_0 | 49 | 0.7927 | 1 | 0.7927 | 0 | 0 | 0 | 1 | Ref |
| LT961842 | Unassigned | Unassigned | Unassigned | 399_0 | Clustered | 49 | VC_399_0 | 49 | 0.7927 | 1 | 0.7927 | 0 | 0 | 0 | 1 | Ref |
| LT961843 | Unassigned | Unassigned | Unassigned | 399_0 | Clustered | 49 | VC_399_0 | 49 | 0.7927 | 1 | 0.7927 | 0 | 0 | 0 | 1 | Ref |
| LT961844 | Unassigned | Unassigned | Unassigned | 399_0 | Clustered | 49 | VC_399_0 | 49 | 0.7927 | 1 | 0.7927 | 0 | 0 | 0 | 1 | Ref |
| LT961845 | Unassigned | Unassigned | Unassigned | 399_0 | Clustered | 49 | VC_399_0 | 49 | 0.7927 | 1 | 0.7927 | 0 | 0 | 0 | 1 | Ref |
| LT961846 | Unassigned | Unassigned | Unassigned | 399_0 | Clustered | 49 | VC_399_0 | 49 | 0.7927 | 1 | 0.7927 | 0 | 0 | 0 | 1 | Ref |
| LT962379 | Unassigned | Unassigned | Unassigned | 399_0 | Clustered | 49 | VC_399_0 | 49 | 0.7927 | 1 | 0.7927 | 0 | 0 | 0 | 1 | Ref |
| LT962380 | Unassigned | Unassigned | Unassigned | 399_0 | Clustered | 49 | VC_399_0 | 49 | 0.7927 | 1 | 0.7927 | 0 | 0 | 0 | 1 | Ref |
| LT962475 | Unassigned | Unassigned | Unassigned | 399_0 | Clustered | 49 | VC_399_0 | 49 | 0.7927 | 1 | 0.7927 | 0 | 0 | 0 | 1 | Ref |
| LT962906 | Unassigned | Unassigned | Unassigned | 399_0 | Clustered | 49 | VC_399_0 | 49 | 0.7927 | 1 | 0.7927 | 0 | 0 | 0 | 1 | Ref |
| LT962907 | Unassigned | Unassigned | Unassigned | 399_0 | Clustered | 49 | VC_399_0 | 49 | 0.7927 | 1 | 0.7927 | 0 | 0 | 0 | 1 | Ref |
| LT985568 | Unassigned | Unassigned | Unassigned | 266_0 | Clustered | 13 | VC_266_0 | 13 | 0.3622 | 1 | 0.3622 | 0 | 0 | 0 | 1 | Ref |
| LT985569 | Unassigned | Unassigned | Unassigned | 269_0 | Clustered | 7 | VC_269_0 | 7 | 0.2278 | 1 | 0.2278 | 0 | 0 | 0 | 1 | Ref |
| LT985571 | Unassigned | Unassigned | Unassigned | 266_0 | Clustered | 13 | VC_266_0 | 13 | 0.3622 | 1 | 0.3622 | 0 | 0 | 0 | 1 | Ref |
| LT985572 | Unassigned | Unassigned | Unassigned | 351_0 | Clustered | 9 | VC_351_0 | 9 | 0.9391 | 0.99999913 | 0.9391 | 0 | 0 | 0 | 1 | Ref |
| LT985574 | Unassigned | Unassigned | Unassigned | 269_0 | Clustered | 7 | VC_269_0 | 7 | 0.2278 | 1 | 0.2278 | 0 | 0 | 0 | 1 | Ref |
| LT985575 | Unassigned | Unassigned | Unassigned | 271_0 | Clustered | 12 | VC_271_0 | 12 | 0.4255 | 1 | 0.4255 | 0 | 0 | 0 | 1 | Ref |
| LT985576 | Unassigned | Unassigned | Unassigned | 271_0 | Clustered | 12 | VC_271_0 | 12 | 0.4255 | 1 | 0.4255 | 0 | 0 | 0 | 1 | Ref |

| | | | | | | | | | | | | | | | | |
|---|---|---|---|---|---|---|---|---|---|---|---|---|---|---|---|---|
| LT985578 | Unassigned | Unassigned | Unassigned | 269_0 | Clustered | 7 | VC_269_0 | 7 | 0.2278 | 1 | 0.2278 | 0 | 0 | 0 | 1 | Ref |
| LT985581 | Unassigned | Unassigned | Unassigned | 351_0 | Clustered | 9 | VC_351_0 | 9 | 0.9391 | 0.99999913 | 0.9391 | 0 | 0 | 0 | 1 | Ref |
| LT985583 | Unassigned | Unassigned | Unassigned | 269_0 | Clustered | 7 | VC_269_0 | 7 | 0.2278 | 1 | 0.2278 | 0 | 0 | 0 | 1 | Ref |
| LT985584 | Unassigned | Unassigned | Unassigned | 351_0 | Clustered | 9 | VC_351_0 | 9 | 0.9391 | 0.99999913 | 0.9391 | 0 | 0 | 0 | 1 | Ref |
| LT985586 | Unassigned | Unassigned | Unassigned | 351_0 | Clustered | 9 | VC_351_0 | 9 | 0.9391 | 0.99999913 | 0.9391 | 0 | 0 | 0 | 1 | Ref |
| LT985587 | Unassigned | Unassigned | Unassigned | 351_0 | Clustered | 9 | VC_351_0 | 9 | 0.9391 | 0.99999913 | 0.9391 | 0 | 0 | 0 | 1 | Ref |
| LT985589 | Unassigned | Unassigned | Unassigned | 308_0 | Clustered | 2 | VC_308_0 | 2 | 0.1277 | 0.94930205 | 0.1213 | 0 | 0 | 0 | 1 | Ref |
| LT985593 | Unassigned | Unassigned | Unassigned | 351_0 | Clustered | 9 | VC_351_0 | 9 | 0.9391 | 0.99999913 | 0.9391 | 0 | 0 | 0 | 1 | Ref |
| LT986460 | Unassigned | Unassigned | Unassigned | 772_0 | Clustered | 9 | VC_772_0 | 9 | 0.4468 | 1 | 0.4468 | 0 | 0 | 0 | 1 | Ref |
| LT986678 | Unassigned | Unassigned | Unassigned | 1267_0 | Clustered | 26 | VC_1267_0 | 26 | 0.9597 | 0.99999998 | 0.9597 | 0 | 0 | 0 | 1 | Ref |
| LT993224 | Unassigned | Unassigned | Unassigned | 266_0 | Clustered | 13 | VC_266_0 | 13 | 0.3622 | 1 | 0.3622 | 0 | 0 | 0 | 1 | Ref |
| LT993227 | Unassigned | Unassigned | Unassigned | 351_0 | Clustered | 9 | VC_351_0 | 9 | 0.9391 | 0.99999913 | 0.9391 | 0 | 0 | 0 | 1 | Ref |
| LT993229 | Unassigned | Unassigned | Unassigned | 269_0 | Clustered | 7 | VC_269_0 | 7 | 0.2278 | 1 | 0.2278 | 0 | 0 | 0 | 1 | Ref |
| LT993247 | Unassigned | Unassigned | Unassigned | 308_0 | Clustered | 2 | VC_308_0 | 2 | 0.1277 | 0.94930205 | 0.1213 | 0 | 0 | 0 | 1 | Ref |
| LT996080 | Unassigned | Unassigned | Unassigned | 351_0 | Clustered | 9 | VC_351_0 | 9 | 0.9391 | 0.99999913 | 0.9391 | 0 | 0 | 0 | 1 | Ref |
| LT996081 | Unassigned | Unassigned | Unassigned | 271_0 | Clustered | 12 | VC_271_0 | 12 | 0.4255 | 1 | 0.4255 | 0 | 0 | 0 | 1 | Ref |
| LT996082 | Unassigned | Unassigned | Unassigned | 269_0 | Clustered | 7 | VC_269_0 | 7 | 0.2278 | 1 | 0.2278 | 0 | 0 | 0 | 1 | Ref |
| LT999987 | Unassigned | Unassigned | Unassigned | 185_0 | Clustered | 53 | VC_185_0 | 53 | 0.9153 | 1 | 0.9153 | 0 | 0 | 0 | 1 | Ref |
| MF001354 | Unassigned | Unassigned | Unassigned | 290_79 | Clustered/Singleton | 1 | VC_290_79 | 1 | 0 | 1 | 0 | 0 | 0 | 0 | 1 | Ref |
| MF001355 | Unassigned | Unassigned | Unassigned | 295_3 | Clustered | 3 | VC_295_3 | 3 | 0.0443 | 0.99844642 | 0.0442 | 0 | 0 | 0 | 1 | Ref |
| MF001356 | Unassigned | Unassigned | Unassigned | 654_0 | Clustered | 115 | VC_654_0 | 115 | 0.7303 | 1 | 0.7303 | 0 | 0 | 0 | 0.8764 | Ref |
| MF001358 | Unassigned | Unassigned | Unassigned | 708_0 | Clustered | 2 | VC_708_0 | 2 | 0.4166 | 0.95368907 | 0.3973 | 0 | 0 | 0 | 1 | Ref |
| MF001359 | Unassigned | Unassigned | Unassigned | 290_183 | Clustered/Singleton | 1 | VC_290_183 | 1 | 0 | 1 | 0 | 0 | 0 | 0 | 1 | Ref |
| MF001360 | Unassigned | Unassigned | Unassigned | 290_14 | Clustered/Singleton | 1 | VC_290_14 | 1 | 0 | 1 | 0 | 0 | 0 | 0 | 1 | Ref |
| MF001361 | Unassigned | Unassigned | Unassigned | 708_0 | Clustered | 2 | VC_708_0 | 2 | 0.4166 | 0.95368907 | 0.3973 | 0 | 0 | 0 | 1 | Ref |
| MF001362 | Unassigned | Unassigned | Unassigned | 135_24 | Clustered/Singleton | 1 | VC_135_24 | 1 | 0 | 1 | 0 | 0 | 0 | 0 | 1 | Ref |
| MF001363 | Unassigned | Unassigned | Unassigned | 199_16 | Clustered | 12 | VC_199_16 | 12 | 0.064 | 1 | 0.064 | 0 | 0 | 0 | 0.9394 | Ref |
| MF001365 | Unassigned | Unassigned | Unassigned | 155_15 | Clustered | 16 | VC_155_15 | 16 | 0.0779 | 1 | 0.0779 | 0 | 0 | 0 | 0.8083 | Ref |
| MF001366 | Unassigned | Unassigned | Unassigned | 654_0 | Clustered | 115 | VC_654_0 | 115 | 0.7303 | 1 | 0.7303 | 0 | 0 | 0 | 0.8764 | Ref |
| MF033347 | Unassigned | Unassigned | Unassigned | 714_0 | Clustered | 71 | VC_714_0 | 71 | 0.935 | 1 | 0.935 | 0 | 0 | 0 | 1 | Ref |
| MF033348 | Unassigned | Unassigned | Unassigned | 714_0 | Clustered | 71 | VC_714_0 | 71 | 0.935 | 1 | 0.935 | 0 | 0 | 0 | 1 | Ref |
| MF033349 | Unassigned | Unassigned | Unassigned | 714_0 | Clustered | 71 | VC_714_0 | 71 | 0.935 | 1 | 0.935 | 0 | 0 | 0 | 1 | Ref |
| MF033350 | Unassigned | Unassigned | Unassigned | 714_0 | Clustered | 71 | VC_714_0 | 71 | 0.935 | 1 | 0.935 | 0 | 0 | 0 | 1 | Ref |
| MF033351 | Unassigned | Unassigned | Unassigned | 714_0 | Clustered | 71 | VC_714_0 | 71 | 0.935 | 1 | 0.935 | 0 | 0 | 0 | 1 | Ref |
| MF034659 | Unassigned | Unassigned | Unassigned | 433_0 | Clustered | 5 | VC_433_0 | 5 | 0.2526 | 0.99999998 | 0.2526 | 0 | 0 | 0 | 1 | Ref |
| MF038790 | Unassigned | Unassigned | Unassigned | 1204_0 | Clustered | 25 | VC_1204_0 | 25 | 0.7382 | 1 | 0.7382 | 0 | 0 | 0 | 1 | Ref |
| MF038791 | Unassigned | Unassigned | Unassigned | 1204_0 | Clustered | 25 | VC_1204_0 | 25 | 0.7382 | 1 | 0.7382 | 0 | 0 | 0 | 1 | Ref |
| MF041990 | Unassigned | Unassigned | Unassigned | 315_0 | Clustered | 4 | VC_315_0 | 4 | 0.2058 | 0.99995817 | 0.2057 | 0 | 0 | 0 | 1 | Ref |
| MF042360 | Unassigned | Unassigned | Unassigned | 356_5 | Clustered/Singleton | 1 | VC_356_5 | 1 | 0 | 1 | 0 | 0 | 0 | 0 | 1 | Ref |
| MF044457 | Unassigned | Unassigned | Unassigned | 293_13 | Clustered | 10 | VC_293_13 | 10 | 0.0698 | 0.99999931 | 0.0698 | 0 | 0 | 0 | 0.9556 | Ref |
| MF044458 | Unassigned | Unassigned | Unassigned | 816_0 | Clustered | 35 | VC_816_0 | 35 | 0.9824 | 1 | 0.9824 | 0 | 0 | 0 | 1 | Ref |
| MF063068 | Unassigned | Unassigned | Unassigned | 356_2 | Clustered/Singleton | 1 | VC_356_2 | 1 | 0 | 1 | 0 | 0 | 0 | 0 | 1 | Ref |
| MF072690 | Unassigned | Unassigned | Unassigned | 566_3 | Clustered | 4 | VC_566_3 | 4 | 0.0418 | 0.92781401 | 0.0388 | 0 | 0 | 0 | 1 | Ref |
| MF074189 | Unassigned | Unassigned | Unassigned | 629_0 | Clustered | 4 | VC_629_0 | 4 | 0.4856 | 0.99992419 | 0.4856 | 0 | 0 | 0 | 1 | Ref |
| MF093736 | Unassigned | Unassigned | Unassigned | 595_0 | Clustered | 35 | VC_595_0 | 35 | 0.8809 | 1 | 0.8809 | 0 | 0 | 0 | 1 | Ref |
| MF098558 | Unassigned | Unassigned | Unassigned | 1132_0 | Clustered | 4 | VC_1132_0 | 4 | 1 | 1 | 1 | 0 | 0 | 0 | 1 | Ref |
| MF101922 | Unassigned | Unassigned | Unassigned | 1268_0 | Clustered | 2 | VC_1268_0 | 2 | 1 | 1 | 1 | 0 | 0 | 0 | 1 | Ref |
| MF133445 | Unassigned | Unassigned | Unassigned | 566_3 | Clustered | 4 | VC_566_3 | 4 | 0.0418 | 0.92781401 | 0.0388 | 0 | 0 | 0 | 1 | Ref |
| MF133446 | Unassigned | Unassigned | Unassigned | 537_0 | Clustered | 39 | VC_537_0 | 39 | 0.8148 | 1 | 0.8148 | 0 | 0 | 0 | 1 | Ref |
| MF140397 | Unassigned | Unassigned | Unassigned | 1207_0 | Clustered | 20 | VC_1207_0 | 20 | 0.9898 | 1 | 0.9898 | 0 | 0 | 0 | 1 | Ref |
| MF140398 | Unassigned | Unassigned | Unassigned | 244_1 | Clustered | 13 | VC_244_1 | 13 | 0.1766 | 1 | 0.1766 | 0 | 0 | 0 | 0.7308 | Ref |
| MF140399 | Unassigned | Unassigned | Unassigned | 537_0 | Clustered | 39 | VC_537_0 | 39 | 0.8148 | 1 | 0.8148 | 0 | 0 | 0 | 1 | Ref |
| MF140400 | Unassigned | Unassigned | Unassigned | 1200_0 | Clustered | 73 | VC_1200_0 | 73 | 0.9825 | 1 | 0.9825 | 0 | 0 | 0 | 1 | Ref |
| MF140401 | Unassigned | Unassigned | Unassigned | 1204_0 | Clustered | 25 | VC_1204_0 | 25 | 0.7382 | 1 | 0.7382 | 0 | 0 | 0 | 1 | Ref |
| MF140402 | Unassigned | Unassigned | Unassigned | 241_0 | Clustered | 21 | VC_241_0 | 21 | 0.8342 | 1 | 0.8342 | 0 | 0 | 0 | 1 | Ref |
| MF140403 | Unassigned | Unassigned | Unassigned | 223_3 | Clustered | 8 | VC_223_3 | 8 | 0.0699 | 1 | 0.0699 | 0 | 0 | 0 | 0.8571 | Ref |
| MF140404 | Unassigned | Unassigned | Unassigned | 1200_0 | Clustered | 73 | VC_1200_0 | 73 | 0.9825 | 1 | 0.9825 | 0 | 0 | 0 | 1 | Ref |
| MF140405 | Unassigned | Unassigned | Unassigned | 918_0 | Clustered | 16 | VC_918_0 | 16 | 0.2945 | 1 | 0.2945 | 0 | 0 | 0 | 1 | Ref |
| MF140406 | Unassigned | Unassigned | Unassigned | 244_1 | Clustered | 13 | VC_244_1 | 13 | 0.1766 | 1 | 0.1766 | 0 | 0 | 0 | 0.7308 | Ref |
| MF140407 | Unassigned | Unassigned | Unassigned | 1200_0 | Clustered | 73 | VC_1200_0 | 73 | 0.9825 | 1 | 0.9825 | 0 | 0 | 0 | 1 | Ref |
| MF140409 | Unassigned | Unassigned | Unassigned | 985_0 | Clustered | 38 | VC_985_0 | 38 | 0.9334 | 1 | 0.9334 | 0 | 0 | 0 | 1 | Ref |
| MF140410 | Unassigned | Unassigned | Unassigned | 228_0 | Clustered | 22 | VC_228_0 | 22 | 0.7411 | 1 | 0.7411 | 0 | 0 | 0 | 1 | Ref |
| MF140412 | Unassigned | Unassigned | Unassigned | 243_0 | Clustered | 72 | VC_243_0 | 72 | 0.8089 | 1 | 0.8089 | 0 | 0 | 0 | 1 | Ref |
| MF140413 | Unassigned | Unassigned | Unassigned | 918_0 | Clustered | 16 | VC_918_0 | 16 | 0.2945 | 1 | 0.2945 | 0 | 0 | 0 | 1 | Ref |
| MF140414 | Unassigned | Unassigned | Unassigned | 918_0 | Clustered | 16 | VC_918_0 | 16 | 0.2945 | 1 | 0.2945 | 0 | 0 | 0 | 1 | Ref |
| MF140415 | Unassigned | Unassigned | Unassigned | 985_0 | Clustered | 38 | VC_985_0 | 38 | 0.9334 | 1 | 0.9334 | 0 | 0 | 0 | 1 | Ref |
| MF140416 | Unassigned | Unassigned | Unassigned | 243_1 | Clustered | 5 | VC_243_1 | 5 | 0.0356 | 0.99999997 | 0.0356 | 0 | 0 | 0 | 1 | Ref |
| MF140417 | Unassigned | Unassigned | Unassigned | 985_0 | Clustered | 38 | VC_985_0 | 38 | 0.9334 | 1 | 0.9334 | 0 | 0 | 0 | 1 | Ref |
| MF140418 | Unassigned | Unassigned | Unassigned | 1210_0 | Clustered | 7 | VC_1210_0 | 7 | 0.9808 | 0.99995963 | 0.9807 | 0 | 0 | 0 | 1 | Ref |
| MF140419 | Unassigned | Unassigned | Unassigned | 985_0 | Clustered | 38 | VC_985_0 | 38 | 0.9334 | 1 | 0.9334 | 0 | 0 | 0 | 1 | Ref |
| MF140420 | Unassigned | Unassigned | Unassigned | 985_0 | Clustered | 38 | VC_985_0 | 38 | 0.9334 | 1 | 0.9334 | 0 | 0 | 0 | 1 | Ref |
| MF140421 | Unassigned | Unassigned | Unassigned | 918_0 | Clustered | 16 | VC_918_0 | 16 | 0.2945 | 1 | 0.2945 | 0 | 0 | 0 | 1 | Ref |
| MF140422 | Unassigned | Unassigned | Unassigned | 917_0 | Clustered | 28 | VC_917_0 | 28 | 0.5465 | 1 | 0.5465 | 0 | 0 | 0 | 1 | Ref |
| MF140423 | Unassigned | Unassigned | Unassigned | 1204_0 | Clustered | 25 | VC_1204_0 | 25 | 0.7382 | 1 | 0.7382 | 0 | 0 | 0 | 1 | Ref |
| MF140424 | Unassigned | Unassigned | Unassigned | 1200_0 | Clustered | 73 | VC_1200_0 | 73 | 0.9825 | 1 | 0.9825 | 0 | 0 | 0 | 1 | Ref |
| MF140425 | Unassigned | Unassigned | Unassigned | 1200_0 | Clustered | 73 | VC_1200_0 | 73 | 0.9825 | 1 | 0.9825 | 0 | 0 | 0 | 1 | Ref |
| MF140426 | Unassigned | Unassigned | Unassigned | 985_0 | Clustered | 38 | VC_985_0 | 38 | 0.9334 | 1 | 0.9334 | 0 | 0 | 0 | 1 | Ref |
| MF140427 | Unassigned | Unassigned | Unassigned | 1210_0 | Clustered | 7 | VC_1210_0 | 7 | 0.9808 | 0.99995963 | 0.9807 | 0 | 0 | 0 | 1 | Ref |
| MF140428 | Unassigned | Unassigned | Unassigned | 243_0 | Clustered | 72 | VC_243_0 | 72 | 0.8089 | 1 | 0.8089 | 0 | 0 | 0 | 1 | Ref |
| MF140429 | Unassigned | Unassigned | Unassigned | 985_0 | Clustered | 38 | VC_985_0 | 38 | 0.9334 | 1 | 0.9334 | 0 | 0 | 0 | 1 | Ref |
| MF140430 | Unassigned | Unassigned | Unassigned | 243_0 | Clustered | 72 | VC_243_0 | 72 | 0.8089 | 1 | 0.8089 | 0 | 0 | 0 | 1 | Ref |

| | | | | | | | | | | | | | | | | |
|---|---|---|---|---|---|---|---|---|---|---|---|---|---|---|---|---|
| MF140431 | Unassigned | Unassigned | Unassigned | 985_0 | Clustered | 38 | VC_985_0 | 38 | 0.9334 | 1 | 0.9334 | 0 | 0 | 0 | 1 | Ref |
| MF140432 | Unassigned | Unassigned | Unassigned | 1204_0 | Clustered | 25 | VC_1204_0 | 25 | 0.7382 | 1 | 0.7382 | 0 | 0 | 0 | 1 | Ref |
| MF140433 | Unassigned | Unassigned | Unassigned | 985_0 | Clustered | 38 | VC_985_0 | 38 | 0.9334 | 1 | 0.9334 | 0 | 0 | 0 | 1 | Ref |
| MF140434 | Unassigned | Unassigned | Unassigned | 1210_0 | Clustered | 7 | VC_1210_0 | 7 | 0.9808 | 0.99995963 | 0.9807 | 0 | 0 | 0 | 1 | Ref |
| MF140435 | Unassigned | Unassigned | Unassigned | 241_0 | Clustered | 21 | VC_241_0 | 21 | 0.8342 | 1 | 0.8342 | 0 | 0 | 0 | 1 | Ref |
| MF141540 | Unassigned | Unassigned | Unassigned | 784_0 | Clustered | 9 | VC_784_0 | 9 | 0.0884 | 1 | 0.0884 | 0 | 0 | 0 | 1 | Ref |
| MF150911 | Unassigned | Unassigned | Unassigned | 166_0 | Clustered | 2 | VC_166_0 | 2 | 0.4832 | 0.94544348 | 0.4568 | 0 | 0 | 0 | 1 | Ref |
| MF153391 | Unassigned | Unassigned | Unassigned | 655_0 | Clustered | 45 | VC_655_0 | 45 | 0.7722 | 1 | 0.7722 | 0 | 0 | 0 | 1 | Ref |
| MF155889 | Unassigned | Unassigned | Unassigned | 904_0 | Clustered | 10 | VC_904_0 | 10 | 0.9878 | 0.99948433 | 0.9873 | 0 | 0 | 0 | 1 | Ref |
| MF155936 | Unassigned | Unassigned | Unassigned | 1087_0 | Clustered | 7 | VC_1087_0 | 7 | 0.94 | 1 | 0.94 | 0 | 0 | 0 | 1 | Ref |
| MF155946 | Unassigned | Unassigned | Unassigned | 1114_2 | Clustered | 5 | VC_1114_2 | 5 | 0.0531 | 0.99997362 | 0.0531 | 0 | 0 | 0 | 0.9 | Ref |
| MF155947 | Unassigned | Unassigned | Unassigned | 384_11 | Clustered | 31 | VC_384_11 | 31 | 0.0926 | 1 | 0.0926 | 0 | 0 | 0 | 0.7011 | Ref |
| MF156577 | Unassigned | Unassigned | Unassigned | 843_0 | Clustered | 20 | VC_843_0 | 20 | 0.9621 | 1 | 0.9621 | 0 | 0 | 0 | 1 | Ref |
| MF156578 | Unassigned | Unassigned | Unassigned | 843_0 | Clustered | 20 | VC_843_0 | 20 | 0.9621 | 1 | 0.9621 | 0 | 0 | 0 | 1 | Ref |
| MF156580 | Unassigned | Unassigned | Unassigned | 843_0 | Clustered | 20 | VC_843_0 | 20 | 0.9621 | 1 | 0.9621 | 0 | 0 | 0 | 1 | Ref |
| MF158036 | Unassigned | Unassigned | Unassigned | 560_0 | Clustered | 15 | VC_560_0 | 15 | 0.2846 | 1 | 0.2846 | 0 | 0 | 0 | 1 | Ref |
| MF158037 | Unassigned | Unassigned | Unassigned | 560_0 | Clustered | 15 | VC_560_0 | 15 | 0.2846 | 1 | 0.2846 | 0 | 0 | 0 | 1 | Ref |
| MF158038 | Unassigned | Unassigned | Unassigned | 475_0 | Clustered | 96 | VC_475_0 | 96 | 0.9303 | 1 | 0.9303 | 0 | 0 | 0 | 0.9575 | Ref |
| MF158039 | Unassigned | Unassigned | Unassigned | 469_0 | Clustered | 18 | VC_469_0 | 18 | 0.4583 | 1 | 0.4583 | 0 | 0 | 0 | 1 | Ref |
| MF158040 | Unassigned | Unassigned | Unassigned | 341_0 | Clustered | 27 | VC_341_0 | 27 | 0.5058 | 1 | 0.5058 | 0 | 0 | 0 | 0.8661 | Ref |
| MF158041 | Unassigned | Unassigned | Unassigned | 341_0 | Clustered | 27 | VC_341_0 | 27 | 0.5058 | 1 | 0.5058 | 0 | 0 | 0 | 0.8661 | Ref |
| MF158042 | Unassigned | Unassigned | Unassigned | 469_0 | Clustered | 18 | VC_469_0 | 18 | 0.4583 | 1 | 0.4583 | 0 | 0 | 0 | 1 | Ref |
| MF158043 | Unassigned | Unassigned | Unassigned | 341_0 | Clustered | 27 | VC_341_0 | 27 | 0.5058 | 1 | 0.5058 | 0 | 0 | 0 | 0.8661 | Ref |
| MF158044 | Unassigned | Unassigned | Unassigned | 341_0 | Clustered | 27 | VC_341_0 | 27 | 0.5058 | 1 | 0.5058 | 0 | 0 | 0 | 0.8661 | Ref |
| MF158045 | Unassigned | Unassigned | Unassigned | 290_64 | Clustered/Singleton | 1 | VC_290_64 | 1 | 0 | 1 | 0 | 0 | 0 | 0 | 1 | Ref |
| MF158046 | Unassigned | Unassigned | Unassigned | 290_189 | Clustered/Singleton | 1 | VC_290_189 | 1 | 0 | 1 | 0 | 0 | 0 | 0 | 1 | Ref |
| MF161328 | Unassigned | Unassigned | Unassigned | 258_0 | Clustered | 121 | VC_258_0 | 121 | 0.7359 | 1 | 0.7359 | 0 | 0 | 0 | 0.9999 | Ref |
| MF166859 | Unassigned | Unassigned | Unassigned | 433_0 | Clustered | 5 | VC_433_0 | 5 | 0.2526 | 0.99999998 | 0.2526 | 0 | 0 | 0 | 1 | Ref |
| MF172979 | Unassigned | Unassigned | Unassigned | 649_1 | Clustered | 14 | VC_649_1 | 14 | 0.5226 | 1 | 0.5226 | 0 | 0 | 0 | 0.8352 | Ref |
| MF185717 | Unassigned | Unassigned | Unassigned | 245_0 | Clustered | 17 | VC_245_0 | 17 | 0.6081 | 1 | 0.6081 | 0 | 0 | 0 | 1 | Ref |
| MF185718 | Unassigned | Unassigned | Unassigned | 1117_0 | Clustered | 19 | VC_1117_0 | 19 | 0.9177 | 1 | 0.9177 | 0 | 0 | 0 | 1 | Ref |
| MF185719 | Unassigned | Unassigned | Unassigned | 245_0 | Clustered | 17 | VC_245_0 | 17 | 0.6081 | 1 | 0.6081 | 0 | 0 | 0 | 1 | Ref |
| MF185720 | Unassigned | Unassigned | Unassigned | 245_0 | Clustered | 17 | VC_245_0 | 17 | 0.6081 | 1 | 0.6081 | 0 | 0 | 0 | 1 | Ref |
| MF185721 | Unassigned | Unassigned | Unassigned | 1117_0 | Clustered | 19 | VC_1117_0 | 19 | 0.9177 | 1 | 0.9177 | 0 | 0 | 0 | 1 | Ref |
| MF185722 | Unassigned | Unassigned | Unassigned | 244_0 | Clustered | 4 | VC_244_0 | 4 | 0.0426 | 0.99998759 | 0.0426 | 0 | 0 | 0 | 1 | Ref |
| MF185723 | Unassigned | Unassigned | Unassigned | 1117_0 | Clustered | 19 | VC_1117_0 | 19 | 0.9177 | 1 | 0.9177 | 0 | 0 | 0 | 1 | Ref |
| MF185724 | Unassigned | Unassigned | Unassigned | 1117_0 | Clustered | 19 | VC_1117_0 | 19 | 0.9177 | 1 | 0.9177 | 0 | 0 | 0 | 1 | Ref |
| MF185725 | Unassigned | Unassigned | Unassigned | 1117_0 | Clustered | 19 | VC_1117_0 | 19 | 0.9177 | 1 | 0.9177 | 0 | 0 | 0 | 1 | Ref |
| MF185727 | Unassigned | Unassigned | Unassigned | 223_0 | Clustered | 4 | VC_223_0 | 4 | 0.0478 | 0.9999878 | 0.0478 | 0 | 0 | 0 | 1 | Ref |
| MF185728 | Unassigned | Unassigned | Unassigned | 917_0 | Clustered | 28 | VC_917_0 | 28 | 0.5465 | 1 | 0.5465 | 0 | 0 | 0 | 1 | Ref |
| MF185729 | Unassigned | Unassigned | Unassigned | 532_0 | Clustered | 75 | VC_532_0 | 75 | 0.9393 | 1 | 0.9393 | 0 | 0 | 0 | 1 | Ref |
| MF185730 | Unassigned | Unassigned | Unassigned | 917_0 | Clustered | 28 | VC_917_0 | 28 | 0.5465 | 1 | 0.5465 | 0 | 0 | 0 | 1 | Ref |
| MF185731 | Unassigned | Unassigned | Unassigned | 882_0 | Clustered | 11 | VC_882_0 | 11 | 0.943 | 1 | 0.943 | 0 | 0 | 0 | 1 | Ref |
| MF185732 | Unassigned | Unassigned | Unassigned | 532_0 | Clustered | 75 | VC_532_0 | 75 | 0.9393 | 1 | 0.9393 | 0 | 0 | 0 | 1 | Ref |
| MF185733 | Unassigned | Unassigned | Unassigned | 532_0 | Clustered | 75 | VC_532_0 | 75 | 0.9393 | 1 | 0.9393 | 0 | 0 | 0 | 1 | Ref |
| MF188997 | Unassigned | Unassigned | Unassigned | 30_1 | Clustered | 53 | VC_30_1 | 53 | 0.7151 | 1 | 0.7151 | 0 | 0 | 0 | 0.9325 | Ref |
| MF189170 | Unassigned | Unassigned | Unassigned | 825_0 | Clustered | 17 | VC_825_0 | 17 | 0.8364 | 1 | 0.8364 | 0 | 0 | 0 | 1 | Ref |
| MF189171 | Unassigned | Unassigned | Unassigned | 825_0 | Clustered | 17 | VC_825_0 | 17 | 0.8364 | 1 | 0.8364 | 0 | 0 | 0 | 1 | Ref |
| MF189172 | Unassigned | Unassigned | Unassigned | 825_0 | Clustered | 17 | VC_825_0 | 17 | 0.8364 | 1 | 0.8364 | 0 | 0 | 0 | 1 | Ref |
| MF189173 | Unassigned | Unassigned | Unassigned | 825_0 | Clustered | 17 | VC_825_0 | 17 | 0.8364 | 1 | 0.8364 | 0 | 0 | 0 | 1 | Ref |
| MF189174 | Unassigned | Unassigned | Unassigned | 825_0 | Clustered | 17 | VC_825_0 | 17 | 0.8364 | 1 | 0.8364 | 0 | 0 | 0 | 1 | Ref |
| MF189175 | Unassigned | Unassigned | Unassigned | 825_0 | Clustered | 17 | VC_825_0 | 17 | 0.8364 | 1 | 0.8364 | 0 | 0 | 0 | 1 | Ref |
| MF189176 | Unassigned | Unassigned | Unassigned | 1116_0 | Clustered | 24 | VC_1116_0 | 24 | 0.8376 | 1 | 0.8376 | 0 | 0 | 0 | 1 | Ref |
| MF189177 | Unassigned | Unassigned | Unassigned | 1116_0 | Clustered | 24 | VC_1116_0 | 24 | 0.8376 | 1 | 0.8376 | 0 | 0 | 0 | 1 | Ref |
| MF189178 | Unassigned | Unassigned | Unassigned | 1116_0 | Clustered | 24 | VC_1116_0 | 24 | 0.8376 | 1 | 0.8376 | 0 | 0 | 0 | 1 | Ref |
| MF189179 | Unassigned | Unassigned | Unassigned | 991_0 | Clustered | 39 | VC_991_0 | 39 | 1 | 1 | 1 | 0 | 0 | 0 | 1 | Ref |
| MF197383 | Unassigned | Unassigned | Unassigned | 1244_0 | Clustered | 4 | VC_1244_0 | 4 | 0.8666 | 0.9999992 | 0.8666 | 0 | 0 | 0 | 1 | Ref |
| MF276773 | Unassigned | Unassigned | Unassigned | 235_0 | Clustered | 60 | VC_235_0 | 60 | 0.9413 | 1 | 0.9413 | 0 | 0 | 0 | 1 | Ref |
| MF278336 | Unassigned | Unassigned | Unassigned | 1021_0 | Clustered | 2 | VC_1021_0 | 2 | 0.1576 | 0.95268934 | 0.1501 | 0 | 0 | 0 | 1 | Ref |
| MF281061 | Unassigned | Unassigned | Unassigned | 537_0 | Clustered | 39 | VC_537_0 | 39 | 0.8148 | 1 | 0.8148 | 0 | 0 | 0 | 1 | Ref |
| MF285615 | Unassigned | Unassigned | Unassigned | 848_0 | Clustered | 124 | VC_848_0 | 124 | 0.9484 | 1 | 0.9484 | 0 | 0 | 0 | 1 | Ref |
| MF285616 | Unassigned | Unassigned | Unassigned | 398_0 | Clustered | 51 | VC_398_0 | 51 | 0.8741 | 1 | 0.8741 | 0 | 0 | 0 | 1 | Ref |
| MF285617 | Unassigned | Unassigned | Unassigned | 398_0 | Clustered | 51 | VC_398_0 | 51 | 0.8741 | 1 | 0.8741 | 0 | 0 | 0 | 1 | Ref |
| MF285618 | Unassigned | Unassigned | Unassigned | 506_1 | Clustered | 8 | VC_506_1 | 8 | 0.2893 | 1 | 0.2893 | 0 | 0 | 0 | 1 | Ref |
| MF285619 | Unassigned | Unassigned | Unassigned | 134_1 | Clustered | 4 | VC_134_1 | 4 | 0.0354 | 0.99998777 | 0.0354 | 0 | 0 | 0 | 1 | Ref |
| MF285620 | Unassigned | Unassigned | Unassigned | 398_0 | Clustered | 51 | VC_398_0 | 51 | 0.8741 | 1 | 0.8741 | 0 | 0 | 0 | 1 | Ref |
| MF288917 | Unassigned | Unassigned | Unassigned | 913_0 | Clustered | 28 | VC_913_0 | 28 | 0.6206 | 1 | 0.6206 | 0 | 0 | 0 | 0.9841 | Ref |
| MF288918 | Unassigned | Unassigned | Unassigned | 913_0 | Clustered | 28 | VC_913_0 | 28 | 0.6206 | 1 | 0.6206 | 0 | 0 | 0 | 0.9841 | Ref |
| MF288919 | Unassigned | Unassigned | Unassigned | 913_0 | Clustered | 28 | VC_913_0 | 28 | 0.6206 | 1 | 0.6206 | 0 | 0 | 0 | 0.9841 | Ref |
| MF288920 | Unassigned | Unassigned | Unassigned | 913_0 | Clustered | 28 | VC_913_0 | 28 | 0.6206 | 1 | 0.6206 | 0 | 0 | 0 | 0.9841 | Ref |
| MF288921 | Unassigned | Unassigned | Unassigned | 912_0 | Clustered | 10 | VC_912_0 | 10 | 0.1994 | 1 | 0.1994 | 0 | 0 | 0 | 1 | Ref |
| MF288922 | Unassigned | Unassigned | Unassigned | 912_0 | Clustered | 10 | VC_912_0 | 10 | 0.1994 | 1 | 0.1994 | 0 | 0 | 0 | 1 | Ref |
| MF319184 | Unassigned | Unassigned | Unassigned | 785_2 | Clustered | 7 | VC_785_2 | 7 | 0.2595 | 1 | 0.2595 | 0 | 0 | 0 | 1 | Ref |
| MF324898 | Unassigned | Unassigned | Unassigned | 991_0 | Clustered | 39 | VC_991_0 | 39 | 1 | 1 | 1 | 0 | 0 | 0 | 1 | Ref |
| MF324899 | Unassigned | Unassigned | Unassigned | 223_0 | Clustered | 4 | VC_223_0 | 4 | 0.0478 | 0.9999878 | 0.0478 | 0 | 0 | 0 | 1 | Ref |
| MF324900 | Unassigned | Unassigned | Unassigned | 991_0 | Clustered | 39 | VC_991_0 | 39 | 1 | 1 | 1 | 0 | 0 | 0 | 1 | Ref |
| MF324901 | Unassigned | Unassigned | Unassigned | 991_0 | Clustered | 39 | VC_991_0 | 39 | 1 | 1 | 1 | 0 | 0 | 0 | 1 | Ref |
| MF324902 | Unassigned | Unassigned | Unassigned | 991_0 | Clustered | 39 | VC_991_0 | 39 | 1 | 1 | 1 | 0 | 0 | 0 | 1 | Ref |
| MF324903 | Unassigned | Unassigned | Unassigned | 991_0 | Clustered | 39 | VC_991_0 | 39 | 1 | 1 | 1 | 0 | 0 | 0 | 1 | Ref |
| MF324904 | Unassigned | Unassigned | Unassigned | 991_0 | Clustered | 39 | VC_991_0 | 39 | 1 | 1 | 1 | 0 | 0 | 0 | 1 | Ref |
| MF324905 | Unassigned | Unassigned | Unassigned | 991_0 | Clustered | 39 | VC_991_0 | 39 | 1 | 1 | 1 | 0 | 0 | 0 | 1 | Ref |

| | | | | | | | | | | | | | | | | |
|---|---|---|---|---|---|---|---|---|---|---|---|---|---|---|---|---|
| MF324906 | Unassigned | Unassigned | Unassigned | 825_0 | Clustered | 17 | VC_825_0 | 17 | 0.8364 | 1 | 0.8364 | 0 | 0 | 0 | 1 | Ref |
| MF324907 | Unassigned | Unassigned | Unassigned | 1116_0 | Clustered | 24 | VC_1116_0 | 24 | 0.8376 | 1 | 0.8376 | 0 | 0 | 0 | 1 | Ref |
| MF324908 | Unassigned | Unassigned | Unassigned | 244_2 | Clustered | 11 | VC_244_2 | 11 | 0.1403 | 1 | 0.1403 | 0 | 0 | 0 | 0.9818 | Ref |
| MF324909 | Unassigned | Unassigned | Unassigned | 244_2 | Clustered | 11 | VC_244_2 | 11 | 0.1403 | 1 | 0.1403 | 0 | 0 | 0 | 0.9818 | Ref |
| MF324910 | Unassigned | Unassigned | Unassigned | 917_0 | Clustered | 28 | VC_917_0 | 28 | 0.5465 | 1 | 0.5465 | 0 | 0 | 0 | 1 | Ref |
| MF324911 | Unassigned | Unassigned | Unassigned | 244_2 | Clustered | 11 | VC_244_2 | 11 | 0.1403 | 1 | 0.1403 | 0 | 0 | 0 | 0.9818 | Ref |
| MF324912 | Unassigned | Unassigned | Unassigned | 244_2 | Clustered | 11 | VC_244_2 | 11 | 0.1403 | 1 | 0.1403 | 0 | 0 | 0 | 0.9818 | Ref |
| MF324913 | Unassigned | Unassigned | Unassigned | 244_2 | Clustered | 11 | VC_244_2 | 11 | 0.1403 | 1 | 0.1403 | 0 | 0 | 0 | 0.9818 | Ref |
| MF324914 | Unassigned | Unassigned | Unassigned | 244_2 | Clustered | 11 | VC_244_2 | 11 | 0.1403 | 1 | 0.1403 | 0 | 0 | 0 | 0.9818 | Ref |
| MF324915 | Unassigned | Unassigned | Unassigned | 244_1 | Clustered | 13 | VC_244_1 | 13 | 0.1766 | 1 | 0.1766 | 0 | 0 | 0 | 0.7308 | Ref |
| MF327003 | Unassigned | Unassigned | Unassigned | 341_0 | Clustered | 27 | VC_341_0 | 27 | 0.5058 | 1 | 0.5058 | 0 | 0 | 0 | 0.8661 | Ref |
| MF327004 | Unassigned | Unassigned | Unassigned | 341_0 | Clustered | 27 | VC_341_0 | 27 | 0.5058 | 1 | 0.5058 | 0 | 0 | 0 | 0.8661 | Ref |
| MF327005 | Unassigned | Unassigned | Unassigned | 341_0 | Clustered | 27 | VC_341_0 | 27 | 0.5058 | 1 | 0.5058 | 0 | 0 | 0 | 0.8661 | Ref |
| MF327006 | Unassigned | Unassigned | Unassigned | 609_0 | Clustered | 27 | VC_609_0 | 27 | 0.9943 | 1 | 0.9943 | 0 | 0 | 0 | 1 | Ref |
| MF327007 | Unassigned | Unassigned | Unassigned | 290_158 | Clustered/Singleton | 1 | VC_290_158 | 1 | 0 | 1 | 0 | 0 | 0 | 0 | 1 | Ref |
| MF327008 | Unassigned | Unassigned | Unassigned | 290_19 | Clustered/Singleton | 1 | VC_290_19 | 1 | 0 | 1 | 0 | 0 | 0 | 0 | 1 | Ref |
| MF327009 | Unassigned | Unassigned | Unassigned | 290_20 | Clustered/Singleton | 1 | VC_290_20 | 1 | 0 | 1 | 0 | 0 | 0 | 0 | 1 | Ref |
| MF346584 | Unassigned | Unassigned | Unassigned | 713_0 | Clustered | 26 | VC_713_0 | 26 | 0.9358 | 1 | 0.9358 | 0 | 0 | 0 | 1 | Ref |
| MF346933 | Unassigned | Unassigned | Unassigned | 210_0 | Clustered | 105 | VC_210_0 | 105 | 0.4076 | 1 | 0.4076 | 0 | 0 | 0 | 0.7766 | Ref |
| MF347636 | Unassigned | Unassigned | Unassigned | 1114_3 | Clustered | 16 | VC_1114_3 | 16 | 0.2336 | 1 | 0.2336 | 0 | 0 | 0 | 0.9667 | Ref |
| MF347637 | Unassigned | Unassigned | Unassigned | 1114_3 | Clustered | 16 | VC_1114_3 | 16 | 0.2336 | 1 | 0.2336 | 0 | 0 | 0 | 0.9667 | Ref |
| MF347638 | Unassigned | Unassigned | Unassigned | 1114_3 | Clustered | 16 | VC_1114_3 | 16 | 0.2336 | 1 | 0.2336 | 0 | 0 | 0 | 0.9667 | Ref |
| MF347639 | Unassigned | Unassigned | Unassigned | 1114_3 | Clustered | 16 | VC_1114_3 | 16 | 0.2336 | 1 | 0.2336 | 0 | 0 | 0 | 0.9667 | Ref |
| MF351863 | Unassigned | Unassigned | Unassigned | 446_3 | Clustered/Singleton | 1 | VC_446_3 | 1 | 0 | 1 | 0 | 0 | 0 | 0 | 1 | Ref |
| MF356679 | Unassigned | Unassigned | Unassigned | 254_1 | Clustered/Singleton | 1 | VC_254_1 | 1 | 0 | 1 | 0 | 0 | 0 | 0 | 1 | Ref |
| MF358541 | Unassigned | Unassigned | Unassigned | 1114_3 | Clustered | 16 | VC_1114_3 | 16 | 0.2336 | 1 | 0.2336 | 0 | 0 | 0 | 0.9667 | Ref |
| MF358542 | Unassigned | Unassigned | Unassigned | 1114_3 | Clustered | 16 | VC_1114_3 | 16 | 0.2336 | 1 | 0.2336 | 0 | 0 | 0 | 0.9667 | Ref |
| MF360957 | Unassigned | Unassigned | Unassigned | 462_0 | Clustered | 4 | VC_462_0 | 4 | 0.3031 | 0.99978853 | 0.303 | 0 | 0 | 0 | 1 | Ref |
| MF370225 | Unassigned | Unassigned | Unassigned | 340_1 | Clustered | 17 | VC_340_1 | 17 | 0.0863 | 1 | 0.0863 | 0 | 0 | 0 | 0.8235 | Ref |
| MF370964 | Unassigned | Unassigned | Unassigned | 514_1 | Clustered/Singleton | 1 | VC_514_1 | 1 | 0 | 1 | 0 | 0 | 0 | 0 | 1 | Ref |
| MF370965 | Unassigned | Unassigned | Unassigned | 927_0 | Clustered | 5 | VC_927_0 | 5 | 0.4988 | 0.99999981 | 0.4988 | 0 | 0 | 0 | 1 | Ref |
| MF373840 | Unassigned | Unassigned | Unassigned | 531_0 | Clustered | 32 | VC_531_0 | 32 | 0.6859 | 1 | 0.6859 | 0 | 0 | 0 | 1 | Ref |
| MF373841 | Unassigned | Unassigned | Unassigned | 244_1 | Clustered | 13 | VC_244_1 | 13 | 0.1766 | 1 | 0.1766 | 0 | 0 | 0 | 0.7308 | Ref |
| MF373842 | Unassigned | Unassigned | Unassigned | 991_0 | Clustered | 39 | VC_991_0 | 39 | 1 | 1 | 1 | 0 | 0 | 0 | 1 | Ref |
| MF373843 | Unassigned | Unassigned | Unassigned | 537_0 | Clustered | 39 | VC_537_0 | 39 | 0.8148 | 1 | 0.8148 | 0 | 0 | 0 | 1 | Ref |
| MF374379 | Unassigned | Unassigned | Unassigned | 45_0 | Clustered | 38 | VC_45_0 | 38 | 0.5785 | 1 | 0.5785 | 0 | 0 | 0 | 0.9659 | Ref |
| MF375456 | Unassigned | Unassigned | Unassigned | 75_0 | Clustered | 15 | VC_75_0 | 15 | 0.518 | 1 | 0.518 | 0 | 0 | 0 | 1 | Ref |
| MF377440 | Unassigned | Unassigned | Unassigned | 243_0 | Clustered | 72 | VC_243_0 | 72 | 0.8089 | 1 | 0.8089 | 0 | 0 | 0 | 1 | Ref |
| MF377441 | Unassigned | Unassigned | Unassigned | 1116_0 | Clustered | 24 | VC_1116_0 | 24 | 0.8376 | 1 | 0.8376 | 0 | 0 | 0 | 1 | Ref |
| MF377442 | Unassigned | Unassigned | Unassigned | 1116_0 | Clustered | 24 | VC_1116_0 | 24 | 0.8376 | 1 | 0.8376 | 0 | 0 | 0 | 1 | Ref |
| MF398190 | Unassigned | Unassigned | Unassigned | 155_8 | Clustered | 66 | VC_155_8 | 66 | 0.4537 | 1 | 0.4537 | 0 | 0 | 0 | 0.8182 | Ref |
| MF402939 | Unassigned | Unassigned | Unassigned | 198_18 | Clustered/Singleton | 1 | VC_198_18 | 1 | 0 | 1 | 0 | 0 | 0 | 0 | 1 | Ref |
| MF403005 | Unassigned | Unassigned | Unassigned | 1020_0 | Clustered | 22 | VC_1020_0 | 22 | 0.895 | 1 | 0.895 | 0 | 0 | 0 | 1 | Ref |
| MF403006 | Unassigned | Unassigned | Unassigned | 1020_0 | Clustered | 22 | VC_1020_0 | 22 | 0.895 | 1 | 0.895 | 0 | 0 | 0 | 1 | Ref |
| MF403007 | Unassigned | Unassigned | Unassigned | 1093_2 | Clustered/Singleton | 1 | VC_1093_2 | 1 | 0 | 1 | 0 | 0 | 0 | 0 | 1 | Ref |
| MF405094 | Unassigned | Unassigned | Unassigned | 155_8 | Clustered | 66 | VC_155_8 | 66 | 0.4537 | 1 | 0.4537 | 0 | 0 | 0 | 0.8182 | Ref |
| MF407276 | Unassigned | Unassigned | Unassigned | 842_0 | Clustered | 14 | VC_842_0 | 14 | 0.6581 | 1 | 0.6581 | 0 | 0 | 0 | 1 | Ref |
| MF415412 | Unassigned | Unassigned | Unassigned | 972_0 | Clustered | 99 | VC_972_0 | 99 | 0.9113 | 1 | 0.9113 | 0 | 0 | 0 | 0.9928 | Ref |
| MF418016 | Unassigned | Unassigned | Unassigned | 90_1 | Clustered/Singleton | 1 | VC_90_1 | 1 | 0 | 1 | 0 | 0 | 0 | 0 | 1 | Ref |
| MF422185 | Unassigned | Unassigned | Unassigned | 87_3 | Clustered | 19 | VC_87_3 | 19 | 0.3645 | 1 | 0.3645 | 0 | 0 | 0 | 0.807 | Ref |
| MF428478 | Unassigned | Unassigned | Unassigned | 11_0 | Clustered | 19 | VC_11_0 | 19 | 0.7544 | 1 | 0.7544 | 0 | 0 | 0 | 1 | Ref |
| MF428479 | Unassigned | Unassigned | Unassigned | 11_0 | Clustered | 19 | VC_11_0 | 19 | 0.7544 | 1 | 0.7544 | 0 | 0 | 0 | 1 | Ref |
| MF428480 | Unassigned | Unassigned | Unassigned | 11_0 | Clustered | 19 | VC_11_0 | 19 | 0.7544 | 1 | 0.7544 | 0 | 0 | 0 | 1 | Ref |
| MF428481 | Unassigned | Unassigned | Unassigned | 11_0 | Clustered | 19 | VC_11_0 | 19 | 0.7544 | 1 | 0.7544 | 0 | 0 | 0 | 1 | Ref |
| MF431252 | Unassigned | Unassigned | Unassigned | 654_0 | Clustered | 115 | VC_654_0 | 115 | 0.7303 | 1 | 0.7303 | 0 | 0 | 0 | 0.8764 | Ref |
| MF431615 | Unassigned | Unassigned | Unassigned | 311_0 | Clustered | 11 | VC_311_0 | 11 | 0.7851 | 1 | 0.7851 | 0 | 0 | 0 | 1 | Ref |
| MF431616 | Unassigned | Unassigned | Unassigned | 311_0 | Clustered | 11 | VC_311_0 | 11 | 0.7851 | 1 | 0.7851 | 0 | 0 | 0 | 1 | Ref |
| MF431617 | Unassigned | Unassigned | Unassigned | 311_0 | Clustered | 11 | VC_311_0 | 11 | 0.7851 | 1 | 0.7851 | 0 | 0 | 0 | 1 | Ref |
| MF431726 | Unassigned | Unassigned | Unassigned | 548_0 | Clustered | 9 | VC_548_0 | 9 | 0.8018 | 1 | 0.8018 | 0 | 0 | 0 | 1 | Ref |
| MF431730 | Unassigned | Unassigned | Unassigned | 198_7 | Clustered | 7 | VC_198_7 | 7 | 0.047 | 1 | 0.047 | 0 | 0 | 0 | 1 | Ref |
| MF431731 | Unassigned | Unassigned | Unassigned | 198_7 | Clustered | 7 | VC_198_7 | 7 | 0.047 | 1 | 0.047 | 0 | 0 | 0 | 1 | Ref |
| MF431732 | Unassigned | Unassigned | Unassigned | 198_7 | Clustered | 7 | VC_198_7 | 7 | 0.047 | 1 | 0.047 | 0 | 0 | 0 | 1 | Ref |
| MF431733 | Unassigned | Unassigned | Unassigned | 198_7 | Clustered | 7 | VC_198_7 | 7 | 0.047 | 1 | 0.047 | 0 | 0 | 0 | 1 | Ref |
| MF431734 | Unassigned | Unassigned | Unassigned | 198_7 | Clustered | 7 | VC_198_7 | 7 | 0.047 | 1 | 0.047 | 0 | 0 | 0 | 1 | Ref |
| MF431735 | Unassigned | Unassigned | Unassigned | 198_7 | Clustered | 7 | VC_198_7 | 7 | 0.047 | 1 | 0.047 | 0 | 0 | 0 | 1 | Ref |
| MF431736 | Unassigned | Unassigned | Unassigned | 199_32 | Clustered | 12 | VC_199_32 | 12 | 0.0676 | 1 | 0.0676 | 0 | 0 | 0 | 0.7576 | Ref |
| MF431737 | Unassigned | Unassigned | Unassigned | 199_32 | Clustered | 12 | VC_199_32 | 12 | 0.0676 | 1 | 0.0676 | 0 | 0 | 0 | 0.7576 | Ref |
| MF431738 | Unassigned | Unassigned | Unassigned | 199_32 | Clustered | 12 | VC_199_32 | 12 | 0.0676 | 1 | 0.0676 | 0 | 0 | 0 | 0.7576 | Ref |
| MF431739 | Unassigned | Unassigned | Unassigned | 199_32 | Clustered | 12 | VC_199_32 | 12 | 0.0676 | 1 | 0.0676 | 0 | 0 | 0 | 0.7576 | Ref |
| MF431740 | Unassigned | Unassigned | Unassigned | 199_32 | Clustered | 12 | VC_199_32 | 12 | 0.0676 | 1 | 0.0676 | 0 | 0 | 0 | 0.7576 | Ref |
| MF431741 | Unassigned | Unassigned | Unassigned | 199_32 | Clustered | 12 | VC_199_32 | 12 | 0.0676 | 1 | 0.0676 | 0 | 0 | 0 | 0.7576 | Ref |
| MF443119 | Unassigned | Unassigned | Unassigned | 706_0 | Clustered | 52 | VC_706_0 | 52 | 0.9787 | 1 | 0.9787 | 0 | 0 | 0 | 1 | Ref |
| MF443120 | Unassigned | Unassigned | Unassigned | 706_0 | Clustered | 52 | VC_706_0 | 52 | 0.9787 | 1 | 0.9787 | 0 | 0 | 0 | 1 | Ref |
| MF443121 | Unassigned | Unassigned | Unassigned | 706_0 | Clustered | 52 | VC_706_0 | 52 | 0.9787 | 1 | 0.9787 | 0 | 0 | 0 | 1 | Ref |
| MF443122 | Unassigned | Unassigned | Unassigned | 706_0 | Clustered | 52 | VC_706_0 | 52 | 0.9787 | 1 | 0.9787 | 0 | 0 | 0 | 1 | Ref |
| MF443123 | Unassigned | Unassigned | Unassigned | 706_0 | Clustered | 52 | VC_706_0 | 52 | 0.9787 | 1 | 0.9787 | 0 | 0 | 0 | 1 | Ref |
| MF443124 | Unassigned | Unassigned | Unassigned | 706_0 | Clustered | 52 | VC_706_0 | 52 | 0.9787 | 1 | 0.9787 | 0 | 0 | 0 | 1 | Ref |
| MF443125 | Unassigned | Unassigned | Unassigned | 706_0 | Clustered | 52 | VC_706_0 | 52 | 0.9787 | 1 | 0.9787 | 0 | 0 | 0 | 1 | Ref |
| MF443126 | Unassigned | Unassigned | Unassigned | 706_0 | Clustered | 52 | VC_706_0 | 52 | 0.9787 | 1 | 0.9787 | 0 | 0 | 0 | 1 | Ref |
| MF443783 | Unassigned | Unassigned | Unassigned | 1269_0 | Clustered | 2 | VC_1269_0 | 2 | 1 | 1 | 1 | 0 | 0 | 0 | 1 | Ref |
| MF443784 | Unassigned | Unassigned | Unassigned | 1269_0 | Clustered | 2 | VC_1269_0 | 2 | 1 | 1 | 1 | 0 | 0 | 0 | 1 | Ref |

| | | | | | | | | | | | | | | | | |
|---|---|---|---|---|---|---|---|---|---|---|---|---|---|---|---|---|
| MF448340 | Unassigned | Unassigned | Unassigned | 457_4 | Clustered | 10 | VC_457_4 | 10 | 0.3316 | 1 | 0.3316 | 0 | 0 | 0 | 0.9333 | Ref |
| MF448554 | Unassigned | Unassigned | Unassigned | 210_0 | Clustered | 105 | VC_210_0 | 105 | 0.4076 | 1 | 0.4076 | 0 | 0 | 0 | 0.7766 | Ref |
| MF448555 | Unassigned | Unassigned | Unassigned | 210_0 | Clustered | 105 | VC_210_0 | 105 | 0.4076 | 1 | 0.4076 | 0 | 0 | 0 | 0.7766 | Ref |
| MF448556 | Unassigned | Unassigned | Unassigned | 210_0 | Clustered | 105 | VC_210_0 | 105 | 0.4076 | 1 | 0.4076 | 0 | 0 | 0 | 0.7766 | Ref |
| MF448557 | Unassigned | Unassigned | Unassigned | 210_0 | Clustered | 105 | VC_210_0 | 105 | 0.4076 | 1 | 0.4076 | 0 | 0 | 0 | 0.7766 | Ref |
| MF448558 | Unassigned | Unassigned | Unassigned | 210_0 | Clustered | 105 | VC_210_0 | 105 | 0.4076 | 1 | 0.4076 | 0 | 0 | 0 | 0.7766 | Ref |
| MF448559 | Unassigned | Unassigned | Unassigned | 210_0 | Clustered | 105 | VC_210_0 | 105 | 0.4076 | 1 | 0.4076 | 0 | 0 | 0 | 0.7766 | Ref |
| MF448560 | Unassigned | Unassigned | Unassigned | 210_0 | Clustered | 105 | VC_210_0 | 105 | 0.4076 | 1 | 0.4076 | 0 | 0 | 0 | 0.7766 | Ref |
| MF448561 | Unassigned | Unassigned | Unassigned | 210_0 | Clustered | 105 | VC_210_0 | 105 | 0.4076 | 1 | 0.4076 | 0 | 0 | 0 | 0.7766 | Ref |
| MF448562 | Unassigned | Unassigned | Unassigned | 210_0 | Clustered | 105 | VC_210_0 | 105 | 0.4076 | 1 | 0.4076 | 0 | 0 | 0 | 0.7766 | Ref |
| MF448563 | Unassigned | Unassigned | Unassigned | 210_0 | Clustered | 105 | VC_210_0 | 105 | 0.4076 | 1 | 0.4076 | 0 | 0 | 0 | 0.7766 | Ref |
| MF448564 | Unassigned | Unassigned | Unassigned | 210_0 | Clustered | 105 | VC_210_0 | 105 | 0.4076 | 1 | 0.4076 | 0 | 0 | 0 | 0.7766 | Ref |
| MF448565 | Unassigned | Unassigned | Unassigned | 210_0 | Clustered | 105 | VC_210_0 | 105 | 0.4076 | 1 | 0.4076 | 0 | 0 | 0 | 0.7766 | Ref |
| MF448566 | Unassigned | Unassigned | Unassigned | 210_0 | Clustered | 105 | VC_210_0 | 105 | 0.4076 | 1 | 0.4076 | 0 | 0 | 0 | 0.7766 | Ref |
| MF448567 | Unassigned | Unassigned | Unassigned | 210_0 | Clustered | 105 | VC_210_0 | 105 | 0.4076 | 1 | 0.4076 | 0 | 0 | 0 | 0.7766 | Ref |
| MF448568 | Unassigned | Unassigned | Unassigned | 210_0 | Clustered | 105 | VC_210_0 | 105 | 0.4076 | 1 | 0.4076 | 0 | 0 | 0 | 0.7766 | Ref |
| MF448569 | Unassigned | Unassigned | Unassigned | 210_0 | Clustered | 105 | VC_210_0 | 105 | 0.4076 | 1 | 0.4076 | 0 | 0 | 0 | 0.7766 | Ref |
| MF448570 | Unassigned | Unassigned | Unassigned | 210_0 | Clustered | 105 | VC_210_0 | 105 | 0.4076 | 1 | 0.4076 | 0 | 0 | 0 | 0.7766 | Ref |
| MF448571 | Unassigned | Unassigned | Unassigned | 210_0 | Clustered | 105 | VC_210_0 | 105 | 0.4076 | 1 | 0.4076 | 0 | 0 | 0 | 0.7766 | Ref |
| MF448572 | Unassigned | Unassigned | Unassigned | 210_0 | Clustered | 105 | VC_210_0 | 105 | 0.4076 | 1 | 0.4076 | 0 | 0 | 0 | 0.7766 | Ref |
| MF467948 | Unassigned | Unassigned | Unassigned | 901_0 | Clustered | 31 | VC_901_0 | 31 | 0.8235 | 1 | 0.8235 | 0 | 0 | 0 | 0.8194 | Ref |
| MF467949 | Unassigned | Unassigned | Unassigned | 901_0 | Clustered | 31 | VC_901_0 | 31 | 0.8235 | 1 | 0.8235 | 0 | 0 | 0 | 0.8194 | Ref |
| MF468274 | Unassigned | Unassigned | Unassigned | 595_0 | Clustered | 35 | VC_595_0 | 35 | 0.8809 | 1 | 0.8809 | 0 | 0 | 0 | 1 | Ref |
| MF472892 | Unassigned | Unassigned | Unassigned | 243_0 | Clustered | 72 | VC_243_0 | 72 | 0.8089 | 1 | 0.8089 | 0 | 0 | 0 | 1 | Ref |
| MF472894 | Unassigned | Unassigned | Unassigned | 537_0 | Clustered | 39 | VC_537_0 | 39 | 0.8148 | 1 | 0.8148 | 0 | 0 | 0 | 1 | Ref |
| MF472896 | Unassigned | Unassigned | Unassigned | 223_6 | Clustered | 42 | VC_223_6 | 42 | 0.4717 | 1 | 0.4717 | 0 | 0 | 0 | 0.8351 | Ref |
| MF476925 | Unassigned | Unassigned | Unassigned | 614_0 | Clustered | 4 | VC_614_0 | 4 | 0.0588 | 0.99274182 | 0.0584 | 0 | 0 | 0 | 1 | Ref |
| MF479730 | Unassigned | Unassigned | Unassigned | 619_2 | Clustered | 10 | VC_619_2 | 10 | 0.3034 | 1 | 0.3034 | 0 | 0 | 0 | 1 | Ref |
| MF481197 | Unassigned | Unassigned | Unassigned | 1067_0 | Clustered | 20 | VC_1067_0 | 20 | 0.7109 | 1 | 0.7109 | 0 | 0 | 0 | 1 | Ref |
| MF490236 | Unassigned | Unassigned | Unassigned | 862_0 | Clustered | 29 | VC_862_0 | 29 | 0.8594 | 1 | 0.8594 | 0 | 0 | 0 | 1 | Ref |
| MF490237 | Unassigned | Unassigned | Unassigned | 645_0 | Clustered | 20 | VC_645_0 | 20 | 0.8502 | 1 | 0.8502 | 0 | 0 | 0 | 1 | Ref |
| MF490238 | Unassigned | Unassigned | Unassigned | 862_0 | Clustered | 29 | VC_862_0 | 29 | 0.8594 | 1 | 0.8594 | 0 | 0 | 0 | 1 | Ref |
| MF490239 | Unassigned | Unassigned | Unassigned | 172_0 | Clustered | 26 | VC_172_0 | 26 | 0.7909 | 1 | 0.7909 | 0 | 0 | 0 | 1 | Ref |
| MF490240 | Unassigned | Unassigned | Unassigned | 80_0 | Clustered | 64 | VC_80_0 | 64 | 0.418 | 1 | 0.418 | 0 | 0 | 0 | 0.7649 | Ref |
| MF490241 | Unassigned | Unassigned | Unassigned | 80_7 | Clustered | 33 | VC_80_7 | 33 | 0.2156 | 1 | 0.2156 | 0 | 0 | 0 | 0.8409 | Ref |
| MF498773 | Unassigned | Unassigned | Unassigned | 457_4 | Clustered | 10 | VC_457_4 | 10 | 0.3316 | 1 | 0.3316 | 0 | 0 | 0 | 0.9333 | Ref |
| MF498775 | Unassigned | Unassigned | Unassigned | 457_4 | Clustered | 10 | VC_457_4 | 10 | 0.3316 | 1 | 0.3316 | 0 | 0 | 0 | 0.9333 | Ref |
| MF498901 | Unassigned | Unassigned | Unassigned | 912_0 | Clustered | 10 | VC_912_0 | 10 | 0.1994 | 1 | 0.1994 | 0 | 0 | 0 | 1 | Ref |
| MF537628 | Unassigned | Unassigned | Unassigned | 991_0 | Clustered | 39 | VC_991_0 | 39 | 1 | 1 | 1 | 0 | 0 | 0 | 1 | Ref |
| MF541403 | Unassigned | Unassigned | Unassigned | 1016_0 | Clustered | 62 | VC_1016_0 | 62 | 0.8638 | 1 | 0.8638 | 0 | 0 | 0 | 0.743 | Ref |
| MF541404 | Unassigned | Unassigned | Unassigned | 1016_0 | Clustered | 62 | VC_1016_0 | 62 | 0.8638 | 1 | 0.8638 | 0 | 0 | 0 | 0.743 | Ref |
| MF541405 | Unassigned | Unassigned | Unassigned | 1016_0 | Clustered | 62 | VC_1016_0 | 62 | 0.8638 | 1 | 0.8638 | 0 | 0 | 0 | 0.743 | Ref |
| MF541406 | Unassigned | Unassigned | Unassigned | 1016_0 | Clustered | 62 | VC_1016_0 | 62 | 0.8638 | 1 | 0.8638 | 0 | 0 | 0 | 0.743 | Ref |
| MF541407 | Unassigned | Unassigned | Unassigned | 1016_0 | Clustered | 62 | VC_1016_0 | 62 | 0.8638 | 1 | 0.8638 | 0 | 0 | 0 | 0.743 | Ref |
| MF541408 | Unassigned | Unassigned | Unassigned | 1016_0 | Clustered | 62 | VC_1016_0 | 62 | 0.8638 | 1 | 0.8638 | 0 | 0 | 0 | 0.743 | Ref |
| MF541409 | Unassigned | Unassigned | Unassigned | 1016_0 | Clustered | 62 | VC_1016_0 | 62 | 0.8638 | 1 | 0.8638 | 0 | 0 | 0 | 0.743 | Ref |
| MF541410 | Unassigned | Unassigned | Unassigned | 1016_0 | Clustered | 62 | VC_1016_0 | 62 | 0.8638 | 1 | 0.8638 | 0 | 0 | 0 | 0.743 | Ref |
| MF547662 | Unassigned | Unassigned | Unassigned | 487_3 | Clustered | 2 | VC_487_3 | 2 | 0.1071 | 0.94066235 | 0.1008 | 0 | 0 | 0 | 1 | Ref |
| MF547663 | Unassigned | Unassigned | Unassigned | 487_3 | Clustered | 2 | VC_487_3 | 2 | 0.1071 | 0.94066235 | 0.1008 | 0 | 0 | 0 | 1 | Ref |
| MF564201 | Unassigned | Unassigned | Unassigned | 976_0 | Clustered | 36 | VC_976_0 | 36 | 0.7833 | 1 | 0.7833 | 0 | 0 | 0 | 1 | Ref |
| MF574151 | Unassigned | Unassigned | Unassigned | 83_0 | Clustered | 24 | VC_83_0 | 24 | 0.7345 | 1 | 0.7345 | 0 | 0 | 0 | 1 | Ref |
| MF580410 | Unassigned | Unassigned | Unassigned | 8_0 | Clustered | 58 | VC_8_0 | 58 | 0.8285 | 1 | 0.8285 | 0 | 0 | 0 | 0.9946 | Ref |
| MF580759 | Unassigned | Unassigned | Unassigned | 258_0 | Clustered | 121 | VC_258_0 | 121 | 0.7359 | 1 | 0.7359 | 0 | 0 | 0 | 0.9999 | Ref |
| MF580760 | Unassigned | Unassigned | Unassigned | 258_0 | Clustered | 121 | VC_258_0 | 121 | 0.7359 | 1 | 0.7359 | 0 | 0 | 0 | 0.9999 | Ref |
| MF580761 | Unassigned | Unassigned | Unassigned | 258_0 | Clustered | 121 | VC_258_0 | 121 | 0.7359 | 1 | 0.7359 | 0 | 0 | 0 | 0.9999 | Ref |
| MF580762 | Unassigned | Unassigned | Unassigned | 258_0 | Clustered | 121 | VC_258_0 | 121 | 0.7359 | 1 | 0.7359 | 0 | 0 | 0 | 0.9999 | Ref |
| MF580763 | Unassigned | Unassigned | Unassigned | 258_0 | Clustered | 121 | VC_258_0 | 121 | 0.7359 | 1 | 0.7359 | 0 | 0 | 0 | 0.9999 | Ref |
| MF580764 | Unassigned | Unassigned | Unassigned | 258_0 | Clustered | 121 | VC_258_0 | 121 | 0.7359 | 1 | 0.7359 | 0 | 0 | 0 | 0.9999 | Ref |
| MF580765 | Unassigned | Unassigned | Unassigned | 258_0 | Clustered | 121 | VC_258_0 | 121 | 0.7359 | 1 | 0.7359 | 0 | 0 | 0 | 0.9999 | Ref |
| MF580766 | Unassigned | Unassigned | Unassigned | 258_0 | Clustered | 121 | VC_258_0 | 121 | 0.7359 | 1 | 0.7359 | 0 | 0 | 0 | 0.9999 | Ref |
| MF580767 | Unassigned | Unassigned | Unassigned | 258_0 | Clustered | 121 | VC_258_0 | 121 | 0.7359 | 1 | 0.7359 | 0 | 0 | 0 | 0.9999 | Ref |
| MF580768 | Unassigned | Unassigned | Unassigned | 258_0 | Clustered | 121 | VC_258_0 | 121 | 0.7359 | 1 | 0.7359 | 0 | 0 | 0 | 0.9999 | Ref |
| MF580769 | Unassigned | Unassigned | Unassigned | 258_0 | Clustered | 121 | VC_258_0 | 121 | 0.7359 | 1 | 0.7359 | 0 | 0 | 0 | 0.9999 | Ref |
| MF580770 | Unassigned | Unassigned | Unassigned | 258_0 | Clustered | 121 | VC_258_0 | 121 | 0.7359 | 1 | 0.7359 | 0 | 0 | 0 | 0.9999 | Ref |
| MF580771 | Unassigned | Unassigned | Unassigned | 258_0 | Clustered | 121 | VC_258_0 | 121 | 0.7359 | 1 | 0.7359 | 0 | 0 | 0 | 0.9999 | Ref |
| MF580772 | Unassigned | Unassigned | Unassigned | 258_0 | Clustered | 121 | VC_258_0 | 121 | 0.7359 | 1 | 0.7359 | 0 | 0 | 0 | 0.9999 | Ref |
| MF580773 | Unassigned | Unassigned | Unassigned | 258_0 | Clustered | 121 | VC_258_0 | 121 | 0.7359 | 1 | 0.7359 | 0 | 0 | 0 | 0.9999 | Ref |
| MF580774 | Unassigned | Unassigned | Unassigned | 258_0 | Clustered | 121 | VC_258_0 | 121 | 0.7359 | 1 | 0.7359 | 0 | 0 | 0 | 0.9999 | Ref |
| MF580775 | Unassigned | Unassigned | Unassigned | 258_0 | Clustered | 121 | VC_258_0 | 121 | 0.7359 | 1 | 0.7359 | 0 | 0 | 0 | 0.9999 | Ref |
| MF580955 | Unassigned | Unassigned | Unassigned | 1271_0 | Clustered | 3 | VC_1271_0 | 3 | 1 | 1 | 1 | 0 | 0 | 0 | 1 | Ref |
| MF580956 | Unassigned | Unassigned | Unassigned | 1272_0 | Clustered | 3 | VC_1272_0 | 3 | 0.8121 | 0.99225442 | 0.8058 | 0 | 0 | 0 | 1 | Ref |
| MF580957 | Unassigned | Unassigned | Unassigned | 1271_0 | Clustered | 3 | VC_1271_0 | 3 | 1 | 1 | 1 | 0 | 0 | 0 | 1 | Ref |
| MF580958 | Unassigned | Unassigned | Unassigned | 1271_0 | Clustered | 3 | VC_1271_0 | 3 | 1 | 1 | 1 | 0 | 0 | 0 | 1 | Ref |
| MF580959 | Unassigned | Unassigned | Unassigned | 1272_0 | Clustered | 3 | VC_1272_0 | 3 | 0.8121 | 0.99225442 | 0.8058 | 0 | 0 | 0 | 1 | Ref |
| MF580960 | Unassigned | Unassigned | Unassigned | 1272_0 | Clustered | 3 | VC_1272_0 | 3 | 0.8121 | 0.99225442 | 0.8058 | 0 | 0 | 0 | 1 | Ref |
| MF580961 | Unassigned | Unassigned | Unassigned | 1273_0 | Clustered | 3 | VC_1273_0 | 3 | 0.7979 | 0.99216275 | 0.7916 | 0 | 0 | 0 | 1 | Ref |
| MF580962 | Unassigned | Unassigned | Unassigned | 1273_0 | Clustered | 3 | VC_1273_0 | 3 | 0.7979 | 0.99216275 | 0.7916 | 0 | 0 | 0 | 1 | Ref |
| MF595878 | Unassigned | Unassigned | Unassigned | 349_0 | Clustered | 5 | VC_349_0 | 5 | 0.191 | 0.97099341 | 0.1855 | 0 | 0 | 0 | 1 | Ref |
| MF612071 | Unassigned | Unassigned | Unassigned | 113_0 | Clustered | 91 | VC_113_0 | 91 | 0.9584 | 1 | 0.9584 | 0 | 0 | 0 | 1 | Ref |
| MF612072 | Unassigned | Unassigned | Unassigned | 972_0 | Clustered | 99 | VC_972_0 | 99 | 0.9113 | 1 | 0.9113 | 0 | 0 | 0 | 0.9928 | Ref |
| MF612073 | Unassigned | Unassigned | Unassigned | 372_0 | Clustered | 7 | VC_372_0 | 7 | 0.3691 | 1 | 0.3691 | 0 | 0 | 0 | 1 | Ref |

| | | | | | | | | | | | | | | | | |
|---|---|---|---|---|---|---|---|---|---|---|---|---|---|---|---|---|
| MF614100 | Unassigned | Unassigned | Unassigned | 721_0 | Clustered | 11 | VC_721_0 | 11 | 0.8846 | 1 | 0.8846 | 0 | 0 | 0 | 1 | Ref |
| MF614627 | Unassigned | Unassigned | Unassigned | 22_0 | Clustered | 4 | VC_22_0 | 4 | 0.562 | 0.91257778 | 0.5129 | 0 | 0 | 0 | 0.6667 | Ref |
| MF614628 | Unassigned | Unassigned | Unassigned | 22_0 | Clustered | 4 | VC_22_0 | 4 | 0.562 | 0.91257778 | 0.5129 | 0 | 0 | 0 | 0.6667 | Ref |
| MF621978 | Unassigned | Unassigned | Unassigned | 71_0 | Clustered | 27 | VC_71_0 | 27 | 0.5964 | 1 | 0.5964 | 0 | 0 | 0 | 0.9943 | Ref |
| MF623055 | Unassigned | Unassigned | Unassigned | 80_4 | Clustered | 3 | VC_80_4 | 3 | 0.0106 | 0.99632092 | 0.0105 | 0 | 0 | 0 | 0.6667 | Ref |
| MF629150 | Unassigned | Unassigned | Unassigned | 1273_0 | Clustered | 3 | VC_1273_0 | 3 | 0.7979 | 0.99216275 | 0.7916 | 0 | 0 | 0 | 1 | Ref |
| MF630921 | Unassigned | Unassigned | Unassigned | 465_0 | Clustered | 80 | VC_465_0 | 80 | 0.9988 | 1 | 0.9988 | 0 | 0 | 0 | 1 | Ref |
| MF663761 | Unassigned | Unassigned | Unassigned | 125_0 | Clustered | 23 | VC_125_0 | 23 | 0.8163 | 1 | 0.8163 | 0 | 0 | 0 | 1 | Ref |
| MF663786 | Unassigned | Unassigned | Unassigned | 129_0 | Clustered/Singleton | 1 | VC_129_0 | 1 | 0 | 1 | 0 | 0 | 0 | 0 | 1 | Ref |
| MF668266 | Unassigned | Unassigned | Unassigned | 1208_0 | Clustered | 6 | VC_1208_0 | 6 | 0.8634 | 0.99999946 | 0.8634 | 0 | 0 | 0 | 1 | Ref |
| MF668267 | Unassigned | Unassigned | Unassigned | 243_0 | Clustered | 72 | VC_243_0 | 72 | 0.8089 | 1 | 0.8089 | 0 | 0 | 0 | 1 | Ref |
| MF668268 | Unassigned | Unassigned | Unassigned | 783_0 | Clustered | 67 | VC_783_0 | 67 | 0.8945 | 1 | 0.8945 | 0 | 0 | 0 | 1 | Ref |
| MF668269 | Unassigned | Unassigned | Unassigned | 223_4 | Clustered | 13 | VC_223_4 | 13 | 0.1649 | 1 | 0.1649 | 0 | 0 | 0 | 1 | Ref |
| MF668270 | Unassigned | Unassigned | Unassigned | 538_15 | Clustered | 2 | VC_538_15 | 2 | 0.0078 | 0.957604 | 0.0075 | 0 | 0 | 0 | 0 | Ref |
| MF668272 | Unassigned | Unassigned | Unassigned | 783_0 | Clustered | 67 | VC_783_0 | 67 | 0.8945 | 1 | 0.8945 | 0 | 0 | 0 | 1 | Ref |
| MF668273 | Unassigned | Unassigned | Unassigned | 1208_0 | Clustered | 6 | VC_1208_0 | 6 | 0.8634 | 0.99999946 | 0.8634 | 0 | 0 | 0 | 1 | Ref |
| MF668274 | Unassigned | Unassigned | Unassigned | 1117_0 | Clustered | 19 | VC_1117_0 | 19 | 0.9177 | 1 | 0.9177 | 0 | 0 | 0 | 1 | Ref |
| MF668275 | Unassigned | Unassigned | Unassigned | 883_0 | Clustered | 5 | VC_883_0 | 5 | 0.3514 | 0.99996062 | 0.3514 | 0 | 0 | 0 | 1 | Ref |
| MF668276 | Unassigned | Unassigned | Unassigned | 384_11 | Clustered | 31 | VC_384_11 | 31 | 0.0926 | 1 | 0.0926 | 0 | 0 | 0 | 0.7011 | Ref |
| MF668277 | Unassigned | Unassigned | Unassigned | 571_7 | Clustered | 28 | VC_571_7 | 28 | 0.1651 | 1 | 0.1651 | 0 | 0 | 0 | 0.8519 | Ref |
| MF668278 | Unassigned | Unassigned | Unassigned | 535_0 | Clustered | 18 | VC_535_0 | 18 | 0.1595 | 1 | 0.1595 | 0 | 0 | 0 | 1 | Ref |
| MF668279 | Unassigned | Unassigned | Unassigned | 1208_0 | Clustered | 6 | VC_1208_0 | 6 | 0.8634 | 0.99999946 | 0.8634 | 0 | 0 | 0 | 1 | Ref |
| MF668280 | Unassigned | Unassigned | Unassigned | 580_0 | Clustered | 2 | VC_580_0 | 2 | 0.011 | 0.95651281 | 0.0105 | 0 | 0 | 0 | 1 | Ref |
| MF668282 | Unassigned | Unassigned | Unassigned | 231_0 | Clustered | 19 | VC_231_0 | 19 | 0.9378 | 1 | 0.9378 | 0 | 0 | 0 | 1 | Ref |
| MF668283 | Unassigned | Unassigned | Unassigned | 335_49 | Clustered | 17 | VC_335_49 | 17 | 0.0731 | 1 | 0.0731 | 0 | 0 | 0 | 0.7721 | Ref |
| MF668284 | Unassigned | Unassigned | Unassigned | 566_3 | Clustered | 4 | VC_566_3 | 4 | 0.0418 | 0.92781401 | 0.0388 | 0 | 0 | 0 | 1 | Ref |
| MF668285 | Unassigned | Unassigned | Unassigned | 1200_0 | Clustered | 73 | VC_1200_0 | 73 | 0.9825 | 1 | 0.9825 | 0 | 0 | 0 | 1 | Ref |
| MF668287 | Unassigned | Unassigned | Unassigned | 538_3 | Clustered/Singleton | 1 | VC_538_3 | 1 | 0 | 1 | 0 | 0 | 0 | 0 | 1 | Ref |
| MF668288 | Unassigned | Unassigned | Unassigned | 230_0 | Clustered | 38 | VC_230_0 | 38 | 0.9076 | 1 | 0.9076 | 0 | 0 | 0 | 1 | Ref |
| MF678788 | Unassigned | Unassigned | Unassigned | 711_0 | Clustered | 48 | VC_711_0 | 48 | 0.8217 | 1 | 0.8217 | 0 | 0 | 0 | 1 | Ref |
| MF678789 | Unassigned | Unassigned | Unassigned | 711_0 | Clustered | 48 | VC_711_0 | 48 | 0.8217 | 1 | 0.8217 | 0 | 0 | 0 | 1 | Ref |
| MF678790 | Unassigned | Unassigned | Unassigned | 711_0 | Clustered | 48 | VC_711_0 | 48 | 0.8217 | 1 | 0.8217 | 0 | 0 | 0 | 1 | Ref |
| MF681663 | Unassigned | Unassigned | Unassigned | 199_11 | Clustered/Singleton | 1 | VC_199_11 | 1 | 0 | 1 | 0 | 0 | 0 | 0 | 1 | Ref |
| MF683623 | Unassigned | Unassigned | Unassigned | 907_0 | Clustered | 32 | VC_907_0 | 32 | 0.9964 | 1 | 0.9964 | 0 | 0 | 0 | 1 | Ref |
| MF695815 | Unassigned | Unassigned | Unassigned | 251_0 | Clustered | 2 | VC_251_0 | 2 | 0.0633 | 0.95608273 | 0.0605 | 0 | 0 | 0 | 1 | Ref |
| MF716957 | Unassigned | Unassigned | Unassigned | 54_0 | Clustered | 87 | VC_54_0 | 87 | 0.9858 | 1 | 0.9858 | 0 | 0 | 0 | 1 | Ref |
| MF740800 | Unassigned | Unassigned | Unassigned | 440_0 | Clustered | 33 | VC_440_0 | 33 | 0.992 | 1 | 0.992 | 0 | 0 | 0 | 1 | Ref |
| MF754111 | Unassigned | Unassigned | Unassigned | 861_0 | Clustered | 29 | VC_861_0 | 29 | 0.9954 | 1 | 0.9954 | 0 | 0 | 0 | 1 | Ref |
| MF754112 | Unassigned | Unassigned | Unassigned | 861_0 | Clustered | 29 | VC_861_0 | 29 | 0.9954 | 1 | 0.9954 | 0 | 0 | 0 | 1 | Ref |
| MF754113 | Unassigned | Unassigned | Unassigned | 911_0 | Clustered | 20 | VC_911_0 | 20 | 0.9915 | 1 | 0.9915 | 0 | 0 | 0 | 1 | Ref |
| MF754114 | Unassigned | Unassigned | Unassigned | 911_0 | Clustered | 20 | VC_911_0 | 20 | 0.9915 | 1 | 0.9915 | 0 | 0 | 0 | 1 | Ref |
| MF754115 | Unassigned | Unassigned | Unassigned | 911_0 | Clustered | 20 | VC_911_0 | 20 | 0.9915 | 1 | 0.9915 | 0 | 0 | 0 | 1 | Ref |
| MF754116 | Unassigned | Unassigned | Unassigned | 911_0 | Clustered | 20 | VC_911_0 | 20 | 0.9915 | 1 | 0.9915 | 0 | 0 | 0 | 1 | Ref |
| MF765814 | Unassigned | Unassigned | Unassigned | 912_1 | Clustered | 12 | VC_912_1 | 12 | 0.2423 | 1 | 0.2423 | 0 | 0 | 0 | 1 | Ref |
| MF766044 | Unassigned | Unassigned | Unassigned | 1015_1 | Clustered | 26 | VC_1015_1 | 26 | 0.6989 | 1 | 0.6989 | 0 | 0 | 0 | 1 | Ref |
| MF766045 | Unassigned | Unassigned | Unassigned | 1015_1 | Clustered | 26 | VC_1015_1 | 26 | 0.6989 | 1 | 0.6989 | 0 | 0 | 0 | 1 | Ref |
| MF766046 | Unassigned | Unassigned | Unassigned | 1015_1 | Clustered | 26 | VC_1015_1 | 26 | 0.6989 | 1 | 0.6989 | 0 | 0 | 0 | 1 | Ref |
| MF766047 | Unassigned | Unassigned | Unassigned | 1015_1 | Clustered | 26 | VC_1015_1 | 26 | 0.6989 | 1 | 0.6989 | 0 | 0 | 0 | 1 | Ref |
| MF766048 | Unassigned | Unassigned | Unassigned | 1015_1 | Clustered | 26 | VC_1015_1 | 26 | 0.6989 | 1 | 0.6989 | 0 | 0 | 0 | 1 | Ref |
| MF768469 | Unassigned | Unassigned | Unassigned | 486_0 | Clustered | 47 | VC_486_0 | 47 | 0.9022 | 1 | 0.9022 | 0 | 0 | 0 | 1 | Ref |
| MF768470 | Unassigned | Unassigned | Unassigned | 80_7 | Clustered | 33 | VC_80_7 | 33 | 0.2156 | 1 | 0.2156 | 0 | 0 | 0 | 0.8409 | Ref |
| MF773750 | Unassigned | Unassigned | Unassigned | 533_0 | Clustered | 16 | VC_533_0 | 16 | 0.3765 | 1 | 0.3765 | 0 | 0 | 0 | 1 | Ref |
| MF774688 | Unassigned | Unassigned | Unassigned | 198_30 | Clustered/Singleton | 1 | VC_198_30 | 1 | 0 | 1 | 0 | 0 | 0 | 0 | 1 | Ref |
| MF775703 | Unassigned | Unassigned | Unassigned | 210_0 | Clustered | 105 | VC_210_0 | 105 | 0.4076 | 1 | 0.4076 | 0 | 0 | 0 | 0.7766 | Ref |
| MF775704 | Unassigned | Unassigned | Unassigned | 210_0 | Clustered | 105 | VC_210_0 | 105 | 0.4076 | 1 | 0.4076 | 0 | 0 | 0 | 0.7766 | Ref |
| MF775705 | Unassigned | Unassigned | Unassigned | 210_0 | Clustered | 105 | VC_210_0 | 105 | 0.4076 | 1 | 0.4076 | 0 | 0 | 0 | 0.7766 | Ref |
| MF787246 | Unassigned | Unassigned | Unassigned | 544_1 | Clustered/Singleton | 1 | VC_544_1 | 1 | 0 | 1 | 0 | 0 | 0 | 0 | 1 | Ref |
| MF788075 | Unassigned | Unassigned | Unassigned | 772_0 | Clustered | 9 | VC_772_0 | 9 | 0.4468 | 1 | 0.4468 | 0 | 0 | 0 | 1 | Ref |
| MF805716 | Unassigned | Unassigned | Unassigned | 356_0 | Clustered | 21 | VC_356_0 | 21 | 0.5409 | 1 | 0.5409 | 0 | 0 | 0 | 1 | Ref |
| MF805809 | Unassigned | Unassigned | Unassigned | 872_0 | Clustered | 28 | VC_872_0 | 28 | 0.7887 | 1 | 0.7887 | 0 | 0 | 0 | 1 | Ref |
| MF807953 | Unassigned | Unassigned | Unassigned | 53_0 | Clustered | 10 | VC_53_0 | 10 | 0.1743 | 1 | 0.1743 | 0 | 0 | 0 | 1 | Ref |
| MF893271 | Unassigned | Unassigned | Unassigned | 145_0 | Clustered | 6 | VC_145_0 | 6 | 0.2581 | 0.86251824 | 0.2226 | 0 | 0 | 0 | 0.8667 | Ref |
| MF893340 | Unassigned | Unassigned | Unassigned | 79_6 | Clustered/Singleton | 1 | VC_79_6 | 1 | 0 | 1 | 0 | 0 | 0 | 0 | 1 | Ref |
| MF919490 | Unassigned | Unassigned | Unassigned | 223_6 | Clustered | 42 | VC_223_6 | 42 | 0.4717 | 1 | 0.4717 | 0 | 0 | 0 | 0.8351 | Ref |
| MF919491 | Unassigned | Unassigned | Unassigned | 787_0 | Clustered | 132 | VC_787_0 | 132 | 1 | 1 | 1 | 0 | 0 | 0 | 1 | Ref |
| MF919492 | Unassigned | Unassigned | Unassigned | 537_0 | Clustered | 39 | VC_537_0 | 39 | 0.8148 | 1 | 0.8148 | 0 | 0 | 0 | 1 | Ref |
| MF919493 | Unassigned | Unassigned | Unassigned | 579_20 | Clustered | 17 | VC_579_20 | 17 | 0.0538 | 1 | 0.0538 | 0 | 0 | 0 | 0.9926 | Ref |
| MF919494 | Unassigned | Unassigned | Unassigned | 579_55 | Clustered/Singleton | 1 | VC_579_55 | 1 | 0 | 1 | 0 | 0 | 0 | 0 | 1 | Ref |
| MF919495 | Unassigned | Unassigned | Unassigned | 579_74 | Clustered/Singleton | 1 | VC_579_74 | 1 | 0 | 1 | 0 | 0 | 0 | 0 | 1 | Ref |
| MF919496 | Unassigned | Unassigned | Unassigned | 535_1 | Clustered | 108 | VC_535_1 | 108 | 0.8479 | 1 | 0.8479 | 0 | 0 | 0 | 0.9955 | Ref |
| MF919497 | Unassigned | Unassigned | Unassigned | 783_0 | Clustered | 67 | VC_783_0 | 67 | 0.8945 | 1 | 0.8945 | 0 | 0 | 0 | 1 | Ref |
| MF919499 | Unassigned | Unassigned | Unassigned | 579_50 | Clustered | 15 | VC_579_50 | 15 | 0.0467 | 1 | 0.0467 | 0 | 0 | 0 | 0.9238 | Ref |
| MF919500 | Unassigned | Unassigned | Unassigned | 223_6 | Clustered | 42 | VC_223_6 | 42 | 0.4717 | 1 | 0.4717 | 0 | 0 | 0 | 0.8351 | Ref |
| MF919501 | Unassigned | Unassigned | Unassigned | 538_63 | Clustered/Singleton | 1 | VC_538_63 | 1 | 0 | 1 | 0 | 0 | 0 | 0 | 1 | Ref |
| MF919503 | Unassigned | Unassigned | Unassigned | 384_3 | Clustered | 22 | VC_384_3 | 22 | 0.0786 | 1 | 0.0786 | 0 | 0 | 0 | 0.9048 | Ref |
| MF919504 | Unassigned | Unassigned | Unassigned | 566_5 | Clustered/Singleton | 1 | VC_566_5 | 1 | 0 | 1 | 0 | 0 | 0 | 0 | 1 | Ref |
| MF919505 | Unassigned | Unassigned | Unassigned | 787_0 | Clustered | 132 | VC_787_0 | 132 | 1 | 1 | 1 | 0 | 0 | 0 | 1 | Ref |
| MF919506 | Unassigned | Unassigned | Unassigned | 571_8 | Clustered | 19 | VC_571_8 | 19 | 0.1031 | 1 | 0.1031 | 0 | 0 | 0 | 0.8713 | Ref |
| MF919507 | Unassigned | Unassigned | Unassigned | 384_3 | Clustered | 22 | VC_384_3 | 22 | 0.0786 | 1 | 0.0786 | 0 | 0 | 0 | 0.9048 | Ref |
| MF919508 | Unassigned | Unassigned | Unassigned | 335_31 | Clustered | 2 | VC_335_31 | 2 | 0.0036 | 0.94604798 | 0.0034 | 0 | 0 | 0 | 0 | Ref |
| MF919509 | Unassigned | Unassigned | Unassigned | 535_1 | Clustered | 108 | VC_535_1 | 108 | 0.8479 | 1 | 0.8479 | 0 | 0 | 0 | 0.9955 | Ref |

| | | | | | | | | | | | | | | | | |
|---|---|---|---|---|---|---|---|---|---|---|---|---|---|---|---|---|
| MF919510 | Unassigned | Unassigned | Unassigned | 789_0 | Clustered | 34 | VC_789_0 | 34 | 0.9167 | 1 | 0.9167 | 0 | 0 | 0 | 1 | Ref |
| MF919511 | Unassigned | Unassigned | Unassigned | 384_13 | Clustered | 33 | VC_384_13 | 33 | 0.1785 | 1 | 0.1785 | 0 | 0 | 0 | 0.8769 | Ref |
| MF919512 | Unassigned | Unassigned | Unassigned | 566_6 | Clustered | 2 | VC_566_6 | 2 | 0.0135 | 0.7810929 | 0.0105 | 0 | 0 | 0 | 1 | Ref |
| MF919513 | Unassigned | Unassigned | Unassigned | 579_77 | Clustered | 3 | VC_579_77 | 3 | 0.0065 | 0.89175063 | 0.0058 | 0 | 0 | 0 | 0.6667 | Ref |
| MF919514 | Unassigned | Unassigned | Unassigned | 1136_0 | Clustered | 40 | VC_1136_0 | 40 | 0.8746 | 1 | 0.8746 | 0 | 0 | 0 | 1 | Ref |
| MF919515 | Unassigned | Unassigned | Unassigned | 787_0 | Clustered | 132 | VC_787_0 | 132 | 1 | 1 | 1 | 0 | 0 | 0 | 1 | Ref |
| MF919516 | Unassigned | Unassigned | Unassigned | 787_0 | Clustered | 132 | VC_787_0 | 132 | 1 | 1 | 1 | 0 | 0 | 0 | 1 | Ref |
| MF919517 | Unassigned | Unassigned | Unassigned | 834_0 | Clustered | 18 | VC_834_0 | 18 | 0.9116 | 1 | 0.9116 | 0 | 0 | 0 | 1 | Ref |
| MF919519 | Unassigned | Unassigned | Unassigned | 384_6 | Clustered/Singleton | 1 | VC_384_6 | 1 | 0 | 1 | 0 | 0 | 0 | 0 | 1 | Ref |
| MF919520 | Unassigned | Unassigned | Unassigned | 1223_1 | Clustered | 10 | VC_1223_1 | 10 | 0.5795 | 1 | 0.5795 | 0 | 0 | 0 | 1 | Ref |
| MF919521 | Unassigned | Unassigned | Unassigned | 1217_0 | Clustered | 41 | VC_1217_0 | 41 | 0.9546 | 1 | 0.9546 | 0 | 0 | 0 | 0.9976 | Ref |
| MF919522 | Unassigned | Unassigned | Unassigned | 787_0 | Clustered | 132 | VC_787_0 | 132 | 1 | 1 | 1 | 0 | 0 | 0 | 1 | Ref |
| MF919523 | Unassigned | Unassigned | Unassigned | 384_3 | Clustered | 22 | VC_384_3 | 22 | 0.0786 | 1 | 0.0786 | 0 | 0 | 0 | 0.9048 | Ref |
| MF919524 | Unassigned | Unassigned | Unassigned | 571_8 | Clustered | 19 | VC_571_8 | 19 | 0.1031 | 1 | 0.1031 | 0 | 0 | 0 | 0.8713 | Ref |
| MF919525 | Unassigned | Unassigned | Unassigned | 571_8 | Clustered | 19 | VC_571_8 | 19 | 0.1031 | 1 | 0.1031 | 0 | 0 | 0 | 0.8713 | Ref |
| MF919526 | Unassigned | Unassigned | Unassigned | 538_81 | Clustered/Singleton | 1 | VC_538_81 | 1 | 0 | 1 | 0 | 0 | 0 | 0 | 1 | Ref |
| MF919527 | Unassigned | Unassigned | Unassigned | 579_82 | Clustered | 3 | VC_579_82 | 3 | 0.0064 | 0.86736674 | 0.0056 | 0 | 0 | 0 | 0.6667 | Ref |
| MF919528 | Unassigned | Unassigned | Unassigned | 384_11 | Clustered | 31 | VC_384_11 | 31 | 0.0926 | 1 | 0.0926 | 0 | 0 | 0 | 0.7011 | Ref |
| MF919529 | Unassigned | Unassigned | Unassigned | 571_8 | Clustered | 19 | VC_571_8 | 19 | 0.1031 | 1 | 0.1031 | 0 | 0 | 0 | 0.8713 | Ref |
| MF919530 | Unassigned | Unassigned | Unassigned | 384_3 | Clustered | 22 | VC_384_3 | 22 | 0.0786 | 1 | 0.0786 | 0 | 0 | 0 | 0.9048 | Ref |
| MF919531 | Unassigned | Unassigned | Unassigned | 223_6 | Clustered | 42 | VC_223_6 | 42 | 0.4717 | 1 | 0.4717 | 0 | 0 | 0 | 0.8351 | Ref |
| MF919532 | Unassigned | Unassigned | Unassigned | 243_0 | Clustered | 72 | VC_243_0 | 72 | 0.8089 | 1 | 0.8089 | 0 | 0 | 0 | 1 | Ref |
| MF919533 | Unassigned | Unassigned | Unassigned | 787_0 | Clustered | 132 | VC_787_0 | 132 | 1 | 1 | 1 | 0 | 0 | 0 | 1 | Ref |
| MF919534 | Unassigned | Unassigned | Unassigned | 566_2 | Clustered | 3 | VC_566_2 | 3 | 0.0275 | 0.85806939 | 0.0236 | 0 | 0 | 0 | 1 | Ref |
| MF919535 | Unassigned | Unassigned | Unassigned | 571_8 | Clustered | 19 | VC_571_8 | 19 | 0.1031 | 1 | 0.1031 | 0 | 0 | 0 | 0.8713 | Ref |
| MF919536 | Unassigned | Unassigned | Unassigned | 222_2 | Clustered | 5 | VC_222_2 | 5 | 0.0569 | 0.99999997 | 0.0569 | 0 | 0 | 0 | 1 | Ref |
| MF919537 | Unassigned | Unassigned | Unassigned | 1223_1 | Clustered | 10 | VC_1223_1 | 10 | 0.5795 | 1 | 0.5795 | 0 | 0 | 0 | 1 | Ref |
| MF919538 | Unassigned | Unassigned | Unassigned | 223_4 | Clustered | 13 | VC_223_4 | 13 | 0.1649 | 1 | 0.1649 | 0 | 0 | 0 | 1 | Ref |
| MF919539 | Unassigned | Unassigned | Unassigned | 384_11 | Clustered | 31 | VC_384_11 | 31 | 0.0926 | 1 | 0.0926 | 0 | 0 | 0 | 0.7011 | Ref |
| MF919540 | Unassigned | Unassigned | Unassigned | 571_8 | Clustered | 19 | VC_571_8 | 19 | 0.1031 | 1 | 0.1031 | 0 | 0 | 0 | 0.8713 | Ref |
| MF919541 | Unassigned | Unassigned | Unassigned | 571_1 | Clustered | 4 | VC_571_1 | 4 | 0.0158 | 0.99966394 | 0.0158 | 0 | 0 | 0 | 0.6667 | Ref |
| MF919542 | Unassigned | Unassigned | Unassigned | 789_0 | Clustered | 34 | VC_789_0 | 34 | 0.9167 | 1 | 0.9167 | 0 | 0 | 0 | 1 | Ref |
| MF959999 | Unassigned | Unassigned | Unassigned | 193_0 | Clustered | 3 | VC_193_0 | 3 | 0.4439 | 0.97148006 | 0.4313 | 0 | 0 | 0 | 1 | Ref |
| MF974178 | Unassigned | Unassigned | Unassigned | 77_0 | Clustered | 37 | VC_77_0 | 37 | 0.9392 | 1 | 0.9392 | 0 | 0 | 0 | 1 | Ref |
| MF974396 | Unassigned | Unassigned | Unassigned | 704_0 | Clustered | 2 | VC_704_0 | 2 | 0.9942 | 0.5 | 0.4971 | 0 | 0 | 0 | 1 | Ref |
| MF974397 | Unassigned | Unassigned | Unassigned | 704_0 | Clustered | 2 | VC_704_0 | 2 | 0.9942 | 0.5 | 0.4971 | 0 | 0 | 0 | 1 | Ref |
| MF975638 | Unassigned | Unassigned | Unassigned | 891_0 | Clustered | 11 | VC_891_0 | 11 | 0.9663 | 1 | 0.9663 | 0 | 0 | 0 | 1 | Ref |
| MF975720 | Unassigned | Unassigned | Unassigned | 248_0 | Clustered | 2 | VC_248_0 | 2 | 0.0714 | 0.95429016 | 0.0681 | 0 | 0 | 0 | 1 | Ref |
| MF975721 | Unassigned | Unassigned | Unassigned | 190_0 | Clustered | 2 | VC_190_0 | 2 | 0.0361 | 0.95635532 | 0.0345 | 0 | 0 | 0 | 1 | Ref |
| MF979560 | Unassigned | Unassigned | Unassigned | 873_3 | Clustered | 2 | VC_873_3 | 2 | 0.0243 | 0.85188229 | 0.0207 | 0 | 0 | 0 | 1 | Ref |
| MF979561 | Unassigned | Unassigned | Unassigned | 402_0 | Clustered | 3 | VC_402_0 | 3 | 0.051 | 0.99861562 | 0.051 | 0 | 0 | 0 | 1 | Ref |
| MF979562 | Unassigned | Unassigned | Unassigned | 1275_0 | Clustered | 4 | VC_1275_0 | 4 | 0.747 | 0.9991311 | 0.7463 | 0 | 0 | 0 | 1 | Ref |
| MF979563 | Unassigned | Unassigned | Unassigned | 873_3 | Clustered | 2 | VC_873_3 | 2 | 0.0243 | 0.85188229 | 0.0207 | 0 | 0 | 0 | 1 | Ref |
| MF996376 | Unassigned | Unassigned | Unassigned | 596_0 | Clustered | 4 | VC_596_0 | 4 | 0.0604 | 0.99998766 | 0.0604 | 0 | 0 | 0 | 1 | Ref |
| MF999224 | Unassigned | Unassigned | Unassigned | 279_0 | Clustered | 11 | VC_279_0 | 11 | 0.4839 | 1 | 0.4839 | 0 | 0 | 0 | 0.8727 | Ref |
| MG000860 | Unassigned | Unassigned | Unassigned | 87_3 | Clustered | 19 | VC_87_3 | 19 | 0.3645 | 1 | 0.3645 | 0 | 0 | 0 | 0.807 | Ref |
| MG004687 | Unassigned | Unassigned | Unassigned | 466_0 | Clustered | 66 | VC_466_0 | 66 | 0.9246 | 1 | 0.9246 | 0 | 0 | 0 | 1 | Ref |
| MG018224 | Unassigned | Unassigned | Unassigned | 175_0 | Clustered | 20 | VC_175_0 | 20 | 0.7504 | 1 | 0.7504 | 0 | 0 | 0 | 1 | Ref |
| MG018927 | Unassigned | Unassigned | Unassigned | 740_0 | Clustered | 4 | VC_740_0 | 4 | 0.4223 | 0.99997355 | 0.4223 | 0 | 0 | 0 | 1 | Ref |
| MG018928 | Unassigned | Unassigned | Unassigned | 801_1 | Clustered | 11 | VC_801_1 | 11 | 0.1583 | 1 | 0.1583 | 0 | 0 | 0 | 1 | Ref |
| MG018929 | Unassigned | Unassigned | Unassigned | 1067_0 | Clustered | 20 | VC_1067_0 | 20 | 0.7109 | 1 | 0.7109 | 0 | 0 | 0 | 1 | Ref |
| MG018930 | Unassigned | Unassigned | Unassigned | 79_0 | Clustered | 2 | VC_79_0 | 2 | 0.0302 | 0.95617387 | 0.0289 | 0 | 0 | 0 | 1 | Ref |
| MG020111 | Unassigned | Unassigned | Unassigned | 598_0 | Clustered | 2 | VC_598_0 | 2 | 0.1903 | 0.93875121 | 0.1786 | 0 | 0 | 0 | 1 | Ref |
| MG022439 | Unassigned | Unassigned | Unassigned | 794_9 | Clustered | 7 | VC_794_9 | 7 | 0.0369 | 0.87330366 | 0.0322 | 0 | 0 | 0 | 1 | Ref |
| MG022440 | Unassigned | Unassigned | Unassigned | 794_9 | Clustered | 7 | VC_794_9 | 7 | 0.0369 | 0.87330366 | 0.0322 | 0 | 0 | 0 | 1 | Ref |
| MG025518 | Unassigned | Unassigned | Unassigned | 1145_0 | Clustered | 164 | VC_1145_0 | 164 | 0.8868 | 1 | 0.8868 | 0 | 0 | 0 | 1 | Ref |
| MG029509 | Unassigned | Unassigned | Unassigned | 8_0 | Clustered | 58 | VC_8_0 | 58 | 0.8285 | 1 | 0.8285 | 0 | 0 | 0 | 0.9946 | Ref |
| MG029510 | Unassigned | Unassigned | Unassigned | 10_0 | Clustered | 25 | VC_10_0 | 25 | 0.3437 | 1 | 0.3437 | 0 | 0 | 0 | 0.9733 | Ref |
| MG029511 | Unassigned | Unassigned | Unassigned | 8_0 | Clustered | 58 | VC_8_0 | 58 | 0.8285 | 1 | 0.8285 | 0 | 0 | 0 | 0.9946 | Ref |
| MG029512 | Unassigned | Unassigned | Unassigned | 10_0 | Clustered | 25 | VC_10_0 | 25 | 0.3437 | 1 | 0.3437 | 0 | 0 | 0 | 0.9733 | Ref |
| MG029513 | Unassigned | Unassigned | Unassigned | 8_0 | Clustered | 58 | VC_8_0 | 58 | 0.8285 | 1 | 0.8285 | 0 | 0 | 0 | 0.9946 | Ref |
| MG029514 | Unassigned | Unassigned | Unassigned | 8_0 | Clustered | 58 | VC_8_0 | 58 | 0.8285 | 1 | 0.8285 | 0 | 0 | 0 | 0.9946 | Ref |
| MG029515 | Unassigned | Unassigned | Unassigned | 8_0 | Clustered | 58 | VC_8_0 | 58 | 0.8285 | 1 | 0.8285 | 0 | 0 | 0 | 0.9946 | Ref |
| MG029516 | Unassigned | Unassigned | Unassigned | 8_0 | Clustered | 58 | VC_8_0 | 58 | 0.8285 | 1 | 0.8285 | 0 | 0 | 0 | 0.9946 | Ref |
| MG029517 | Unassigned | Unassigned | Unassigned | 8_0 | Clustered | 58 | VC_8_0 | 58 | 0.8285 | 1 | 0.8285 | 0 | 0 | 0 | 0.9946 | Ref |
| MG029518 | Unassigned | Unassigned | Unassigned | 8_0 | Clustered | 58 | VC_8_0 | 58 | 0.8285 | 1 | 0.8285 | 0 | 0 | 0 | 0.9946 | Ref |
| MG030346 | Unassigned | Unassigned | Unassigned | 610_0 | Clustered | 14 | VC_610_0 | 14 | 0.5159 | 1 | 0.5159 | 0 | 0 | 0 | 1 | Ref |
| MG049919 | Unassigned | Unassigned | Unassigned | 595_0 | Clustered | 35 | VC_595_0 | 35 | 0.8809 | 1 | 0.8809 | 0 | 0 | 0 | 1 | Ref |
| MG050172 | Unassigned | Unassigned | Unassigned | 470_0 | Clustered | 62 | VC_470_0 | 62 | 0.902 | 1 | 0.902 | 0 | 0 | 0 | 1 | Ref |
| MG065635 | Unassigned | Unassigned | Unassigned | 794_19 | Clustered | 16 | VC_794_19 | 16 | 0.0975 | 0.99616626 | 0.0971 | 0 | 0 | 0 | 0.8083 | Ref |
| MG065636 | Unassigned | Unassigned | Unassigned | 975_0 | Clustered | 40 | VC_975_0 | 40 | 0.6888 | 1 | 0.6888 | 0 | 0 | 0 | 1 | Ref |
| MG065639 | Unassigned | Unassigned | Unassigned | 872_0 | Clustered | 28 | VC_872_0 | 28 | 0.7887 | 1 | 0.7887 | 0 | 0 | 0 | 1 | Ref |
| MG065640 | Unassigned | Unassigned | Unassigned | 975_0 | Clustered | 40 | VC_975_0 | 40 | 0.6888 | 1 | 0.6888 | 0 | 0 | 0 | 1 | Ref |
| MG065641 | Unassigned | Unassigned | Unassigned | 975_0 | Clustered | 40 | VC_975_0 | 40 | 0.6888 | 1 | 0.6888 | 0 | 0 | 0 | 1 | Ref |
| MG065642 | Unassigned | Unassigned | Unassigned | 199_19 | Clustered | 12 | VC_199_19 | 12 | 0.0651 | 1 | 0.0651 | 0 | 0 | 0 | 0.8636 | Ref |
| MG065643 | Unassigned | Unassigned | Unassigned | 975_0 | Clustered | 40 | VC_975_0 | 40 | 0.6888 | 1 | 0.6888 | 0 | 0 | 0 | 1 | Ref |
| MG065644 | Unassigned | Unassigned | Unassigned | 794_0 | Clustered | 6 | VC_794_0 | 6 | 0.0305 | 0.8101283 | 0.0247 | 0 | 0 | 0 | 1 | Ref |
| MG065645 | Unassigned | Unassigned | Unassigned | 872_0 | Clustered | 28 | VC_872_0 | 28 | 0.7887 | 1 | 0.7887 | 0 | 0 | 0 | 1 | Ref |
| MG065646 | Unassigned | Unassigned | Unassigned | 794_16 | Clustered | 10 | VC_794_16 | 10 | 0.0563 | 0.9411319 | 0.053 | 0 | 0 | 0 | 0.8667 | Ref |
| MG065647 | Unassigned | Unassigned | Unassigned | 794_16 | Clustered | 10 | VC_794_16 | 10 | 0.0563 | 0.9411319 | 0.053 | 0 | 0 | 0 | 0.8667 | Ref |
| MG065648 | Unassigned | Unassigned | Unassigned | 859_0 | Clustered | 67 | VC_859_0 | 67 | 0.9622 | 1 | 0.9622 | 0 | 0 | 0 | 1 | Ref |

| | | | | | | | | | | | | | | | | |
|---|---|---|---|---|---|---|---|---|---|---|---|---|---|---|---|---|
| MG065649 | Unassigned | Unassigned | Unassigned | 975_0 | Clustered | 40 | VC_975_0 | 40 | 0.6888 | 1 | 0.6888 | 0 | 0 | 0 | 1 | Ref |
| MG065650 | Unassigned | Unassigned | Unassigned | 871_1 | Clustered | 26 | VC_871_1 | 26 | 0.6303 | 1 | 0.6303 | 0 | 0 | 0 | 0.9815 | Ref |
| MG065653 | Unassigned | Unassigned | Unassigned | 794_13 | Clustered/Singleton | 1 | VC_794_13 | 1 | 0 | 1 | 0 | 0 | 0 | 0 | 1 | Ref |
| MG065654 | Unassigned | Unassigned | Unassigned | 794_14 | Clustered | 3 | VC_794_14 | 3 | 0.012 | 0.63979507 | 0.0077 | 0 | 0 | 0 | 1 | Ref |
| MG065655 | Unassigned | Unassigned | Unassigned | 794_14 | Clustered | 3 | VC_794_14 | 3 | 0.012 | 0.63979507 | 0.0077 | 0 | 0 | 0 | 1 | Ref |
| MG065656 | Unassigned | Unassigned | Unassigned | 872_0 | Clustered | 28 | VC_872_0 | 28 | 0.7887 | 1 | 0.7887 | 0 | 0 | 0 | 1 | Ref |
| MG065657 | Unassigned | Unassigned | Unassigned | 975_0 | Clustered | 40 | VC_975_0 | 40 | 0.6888 | 1 | 0.6888 | 0 | 0 | 0 | 1 | Ref |
| MG065658 | Unassigned | Unassigned | Unassigned | 794_16 | Clustered | 10 | VC_794_16 | 10 | 0.0563 | 0.9411319 | 0.053 | 0 | 0 | 0 | 0.8667 | Ref |
| MG065659 | Unassigned | Unassigned | Unassigned | 794_12 | Clustered/Singleton | 1 | VC_794_12 | 1 | 0 | 1 | 0 | 0 | 0 | 0 | 1 | Ref |
| MG065661 | Unassigned | Unassigned | Unassigned | 794_16 | Clustered | 10 | VC_794_16 | 10 | 0.0563 | 0.9411319 | 0.053 | 0 | 0 | 0 | 0.8667 | Ref |
| MG065662 | Unassigned | Unassigned | Unassigned | 794_0 | Clustered | 6 | VC_794_0 | 6 | 0.0305 | 0.8101283 | 0.0247 | 0 | 0 | 0 | 1 | Ref |
| MG065663 | Unassigned | Unassigned | Unassigned | 975_0 | Clustered | 40 | VC_975_0 | 40 | 0.6888 | 1 | 0.6888 | 0 | 0 | 0 | 1 | Ref |
| MG065664 | Unassigned | Unassigned | Unassigned | 340_12 | Clustered | 2 | VC_340_12 | 2 | 0.0041 | 0.72605466 | 0.003 | 0 | 0 | 0 | 1 | Ref |
| MG065665 | Unassigned | Unassigned | Unassigned | 975_0 | Clustered | 40 | VC_975_0 | 40 | 0.6888 | 1 | 0.6888 | 0 | 0 | 0 | 1 | Ref |
| MG065666 | Unassigned | Unassigned | Unassigned | 794_14 | Clustered | 3 | VC_794_14 | 3 | 0.012 | 0.63979507 | 0.0077 | 0 | 0 | 0 | 1 | Ref |
| MG065667 | Unassigned | Unassigned | Unassigned | 975_0 | Clustered | 40 | VC_975_0 | 40 | 0.6888 | 1 | 0.6888 | 0 | 0 | 0 | 1 | Ref |
| MG065669 | Unassigned | Unassigned | Unassigned | 975_0 | Clustered | 40 | VC_975_0 | 40 | 0.6888 | 1 | 0.6888 | 0 | 0 | 0 | 1 | Ref |
| MG065670 | Unassigned | Unassigned | Unassigned | 975_0 | Clustered | 40 | VC_975_0 | 40 | 0.6888 | 1 | 0.6888 | 0 | 0 | 0 | 1 | Ref |
| MG065671 | Unassigned | Unassigned | Unassigned | 975_0 | Clustered | 40 | VC_975_0 | 40 | 0.6888 | 1 | 0.6888 | 0 | 0 | 0 | 1 | Ref |
| MG065672 | Unassigned | Unassigned | Unassigned | 794_0 | Clustered | 6 | VC_794_0 | 6 | 0.0305 | 0.8101283 | 0.0247 | 0 | 0 | 0 | 1 | Ref |
| MG065673 | Unassigned | Unassigned | Unassigned | 975_0 | Clustered | 40 | VC_975_0 | 40 | 0.6888 | 1 | 0.6888 | 0 | 0 | 0 | 1 | Ref |
| MG065674 | Unassigned | Unassigned | Unassigned | 794_0 | Clustered | 6 | VC_794_0 | 6 | 0.0305 | 0.8101283 | 0.0247 | 0 | 0 | 0 | 1 | Ref |
| MG065675 | Unassigned | Unassigned | Unassigned | 975_0 | Clustered | 40 | VC_975_0 | 40 | 0.6888 | 1 | 0.6888 | 0 | 0 | 0 | 1 | Ref |
| MG065677 | Unassigned | Unassigned | Unassigned | 794_0 | Clustered | 6 | VC_794_0 | 6 | 0.0305 | 0.8101283 | 0.0247 | 0 | 0 | 0 | 1 | Ref |
| MG065679 | Unassigned | Unassigned | Unassigned | 975_0 | Clustered | 40 | VC_975_0 | 40 | 0.6888 | 1 | 0.6888 | 0 | 0 | 0 | 1 | Ref |
| MG065680 | Unassigned | Unassigned | Unassigned | 975_0 | Clustered | 40 | VC_975_0 | 40 | 0.6888 | 1 | 0.6888 | 0 | 0 | 0 | 1 | Ref |
| MG065681 | Unassigned | Unassigned | Unassigned | 975_0 | Clustered | 40 | VC_975_0 | 40 | 0.6888 | 1 | 0.6888 | 0 | 0 | 0 | 1 | Ref |
| MG065682 | Unassigned | Unassigned | Unassigned | 794_16 | Clustered | 10 | VC_794_16 | 10 | 0.0563 | 0.9411319 | 0.053 | 0 | 0 | 0 | 0.8667 | Ref |
| MG065687 | Unassigned | Unassigned | Unassigned | 975_0 | Clustered | 40 | VC_975_0 | 40 | 0.6888 | 1 | 0.6888 | 0 | 0 | 0 | 1 | Ref |
| MG065688 | Unassigned | Unassigned | Unassigned | 470_0 | Clustered | 62 | VC_470_0 | 62 | 0.902 | 1 | 0.902 | 0 | 0 | 0 | 1 | Ref |
| MG065689 | Unassigned | Unassigned | Unassigned | 872_0 | Clustered | 28 | VC_872_0 | 28 | 0.7887 | 1 | 0.7887 | 0 | 0 | 0 | 1 | Ref |
| MG065690 | Unassigned | Unassigned | Unassigned | 794_0 | Clustered | 6 | VC_794_0 | 6 | 0.0305 | 0.8101283 | 0.0247 | 0 | 0 | 0 | 1 | Ref |
| MG065692 | Unassigned | Unassigned | Unassigned | 198_31 | Clustered/Singleton | 1 | VC_198_31 | 1 | 0 | 1 | 0 | 0 | 0 | 0 | 1 | Ref |
| MG099933 | Unassigned | Unassigned | Unassigned | 655_0 | Clustered | 45 | VC_655_0 | 45 | 0.7722 | 1 | 0.7722 | 0 | 0 | 0 | 1 | Ref |
| MG099934 | Unassigned | Unassigned | Unassigned | 974_0 | Clustered | 34 | VC_974_0 | 34 | 0.7953 | 1 | 0.7953 | 0 | 0 | 0 | 1 | Ref |
| MG099935 | Unassigned | Unassigned | Unassigned | 893_1 | Clustered | 20 | VC_893_1 | 20 | 0.4792 | 1 | 0.4792 | 0 | 0 | 0 | 1 | Ref |
| MG099936 | Unassigned | Unassigned | Unassigned | 539_0 | Clustered | 37 | VC_539_0 | 37 | 0.911 | 1 | 0.911 | 0 | 0 | 0 | 1 | Ref |
| MG099937 | Unassigned | Unassigned | Unassigned | 974_0 | Clustered | 34 | VC_974_0 | 34 | 0.7953 | 1 | 0.7953 | 0 | 0 | 0 | 1 | Ref |
| MG099938 | Unassigned | Unassigned | Unassigned | 974_0 | Clustered | 34 | VC_974_0 | 34 | 0.7953 | 1 | 0.7953 | 0 | 0 | 0 | 1 | Ref |
| MG099939 | Unassigned | Unassigned | Unassigned | 578_1 | Clustered | 11 | VC_578_1 | 11 | 0.4919 | 1 | 0.4919 | 0 | 0 | 0 | 0.7818 | Ref |
| MG099940 | Unassigned | Unassigned | Unassigned | 895_0 | Clustered | 25 | VC_895_0 | 25 | 0.5768 | 1 | 0.5768 | 0 | 0 | 0 | 1 | Ref |
| MG099941 | Unassigned | Unassigned | Unassigned | 1216_0 | Clustered | 22 | VC_1216_0 | 22 | 0.4546 | 1 | 0.4546 | 0 | 0 | 0 | 1 | Ref |
| MG099942 | Unassigned | Unassigned | Unassigned | 535_1 | Clustered | 108 | VC_535_1 | 108 | 0.8479 | 1 | 0.8479 | 0 | 0 | 0 | 0.9955 | Ref |
| MG099943 | Unassigned | Unassigned | Unassigned | 577_0 | Clustered | 21 | VC_577_0 | 21 | 0.9908 | 1 | 0.9908 | 0 | 0 | 0 | 1 | Ref |
| MG099944 | Unassigned | Unassigned | Unassigned | 974_0 | Clustered | 34 | VC_974_0 | 34 | 0.7953 | 1 | 0.7953 | 0 | 0 | 0 | 1 | Ref |
| MG099945 | Unassigned | Unassigned | Unassigned | 230_0 | Clustered | 38 | VC_230_0 | 38 | 0.9076 | 1 | 0.9076 | 0 | 0 | 0 | 1 | Ref |
| MG099946 | Unassigned | Unassigned | Unassigned | 783_0 | Clustered | 67 | VC_783_0 | 67 | 0.8945 | 1 | 0.8945 | 0 | 0 | 0 | 1 | Ref |
| MG099947 | Unassigned | Unassigned | Unassigned | 222_0 | Clustered | 5 | VC_222_0 | 5 | 0.072 | 0.99999997 | 0.072 | 0 | 0 | 0 | 1 | Ref |
| MG099948 | Unassigned | Unassigned | Unassigned | 539_0 | Clustered | 37 | VC_539_0 | 37 | 0.911 | 1 | 0.911 | 0 | 0 | 0 | 1 | Ref |
| MG099949 | Unassigned | Unassigned | Unassigned | 974_0 | Clustered | 34 | VC_974_0 | 34 | 0.7953 | 1 | 0.7953 | 0 | 0 | 0 | 1 | Ref |
| MG099950 | Unassigned | Unassigned | Unassigned | 1210_0 | Clustered | 7 | VC_1210_0 | 7 | 0.9808 | 0.99995963 | 0.9807 | 0 | 0 | 0 | 1 | Ref |
| MG099951 | Unassigned | Unassigned | Unassigned | 335_27 | Clustered | 6 | VC_335_27 | 6 | 0.0266 | 1 | 0.0266 | 0 | 0 | 0 | 0.8 | Ref |
| MG099952 | Unassigned | Unassigned | Unassigned | 230_0 | Clustered | 38 | VC_230_0 | 38 | 0.9076 | 1 | 0.9076 | 0 | 0 | 0 | 1 | Ref |
| MG099953 | Unassigned | Unassigned | Unassigned | 571_8 | Clustered | 19 | VC_571_8 | 19 | 0.1031 | 1 | 0.1031 | 0 | 0 | 0 | 0.8713 | Ref |
| MG189906 | Unassigned | Unassigned | Unassigned | 936_0 | Clustered | 3 | VC_936_0 | 3 | 0.3378 | 0.99823775 | 0.3372 | 0 | 0 | 0 | 1 | Ref |
| MG197810 | Unassigned | Unassigned | Unassigned | 125_0 | Clustered | 23 | VC_125_0 | 23 | 0.8163 | 1 | 0.8163 | 0 | 0 | 0 | 1 | Ref |
| MG198776 | Unassigned | Unassigned | Unassigned | 790_0 | Clustered | 8 | VC_790_0 | 8 | 0.6981 | 1 | 0.6981 | 0 | 0 | 0 | 1 | Ref |
| MG198777 | Unassigned | Unassigned | Unassigned | 790_0 | Clustered | 8 | VC_790_0 | 8 | 0.6981 | 1 | 0.6981 | 0 | 0 | 0 | 1 | Ref |
| MG198778 | Unassigned | Unassigned | Unassigned | 790_0 | Clustered | 8 | VC_790_0 | 8 | 0.6981 | 1 | 0.6981 | 0 | 0 | 0 | 1 | Ref |
| MG198779 | Unassigned | Unassigned | Unassigned | 1200_0 | Clustered | 73 | VC_1200_0 | 73 | 0.9825 | 1 | 0.9825 | 0 | 0 | 0 | 1 | Ref |
| MG198780 | Unassigned | Unassigned | Unassigned | 790_0 | Clustered | 8 | VC_790_0 | 8 | 0.6981 | 1 | 0.6981 | 0 | 0 | 0 | 1 | Ref |
| MG198781 | Unassigned | Unassigned | Unassigned | 1200_0 | Clustered | 73 | VC_1200_0 | 73 | 0.9825 | 1 | 0.9825 | 0 | 0 | 0 | 1 | Ref |
| MG198782 | Unassigned | Unassigned | Unassigned | 1200_0 | Clustered | 73 | VC_1200_0 | 73 | 0.9825 | 1 | 0.9825 | 0 | 0 | 0 | 1 | Ref |
| MG198783 | Unassigned | Unassigned | Unassigned | 423_0 | Clustered | 7 | VC_423_0 | 7 | 0.7874 | 1 | 0.7874 | 0 | 0 | 0 | 1 | Ref |
| MG198784 | Unassigned | Unassigned | Unassigned | 423_0 | Clustered | 7 | VC_423_0 | 7 | 0.7874 | 1 | 0.7874 | 0 | 0 | 0 | 1 | Ref |
| MG198785 | Unassigned | Unassigned | Unassigned | 537_0 | Clustered | 39 | VC_537_0 | 39 | 0.8148 | 1 | 0.8148 | 0 | 0 | 0 | 1 | Ref |
| MG201401 | Unassigned | Unassigned | Unassigned | 73_0 | Clustered | 2 | VC_73_0 | 2 | 0.077 | 0.95618005 | 0.0736 | 0 | 0 | 0 | 1 | Ref |
| MG208881 | Unassigned | Unassigned | Unassigned | 801_0 | Clustered | 37 | VC_801_0 | 37 | 0.5672 | 1 | 0.5672 | 0 | 0 | 0 | 1 | Ref |
| MG209611 | Unassigned | Unassigned | Unassigned | 965_0 | Clustered | 2 | VC_965_0 | 2 | 0.4025 | 0.89435023 | 0.36 | 0 | 0 | 0 | 1 | Ref |
| MG210949 | Unassigned | Unassigned | Unassigned | 1200_0 | Clustered | 73 | VC_1200_0 | 73 | 0.9825 | 1 | 0.9825 | 0 | 0 | 0 | 1 | Ref |
| MG214764 | Unassigned | Unassigned | Unassigned | 1276_0 | Clustered | 3 | VC_1276_0 | 3 | 1 | 0 | 0 | 0 | 0 | 0 | 1 | Ref |
| MG241338 | Unassigned | Unassigned | Unassigned | 603_0 | Clustered | 28 | VC_603_0 | 28 | 0.7692 | 1 | 0.7692 | 0 | 0 | 0 | 1 | Ref |
| MG250483 | Unassigned | Unassigned | Unassigned | 457_0 | Clustered | 7 | VC_457_0 | 7 | 0.2549 | 1 | 0.2549 | 0 | 0 | 0 | 0.7143 | Ref |
| MG250485 | Unassigned | Unassigned | Unassigned | 131_0 | Clustered | 43 | VC_131_0 | 43 | 0.8853 | 1 | 0.8853 | 0 | 0 | 0 | 1 | Ref |
| MG250486 | Unassigned | Unassigned | Unassigned | 601_3 | Clustered | 12 | VC_601_3 | 12 | 0.2339 | 1 | 0.2339 | 0 | 0 | 0 | 0.8636 | Ref |
| MG251390 | Unassigned | Unassigned | Unassigned | 466_0 | Clustered | 66 | VC_466_0 | 66 | 0.9246 | 1 | 0.9246 | 0 | 0 | 0 | 1 | Ref |
| MG251391 | Unassigned | Unassigned | Unassigned | 340_1 | Clustered | 17 | VC_340_1 | 17 | 0.0863 | 1 | 0.0863 | 0 | 0 | 0 | 0.8235 | Ref |
| MG251392 | Unassigned | Unassigned | Unassigned | 340_1 | Clustered | 17 | VC_340_1 | 17 | 0.0863 | 1 | 0.0863 | 0 | 0 | 0 | 0.8235 | Ref |
| MG252615 | Unassigned | Unassigned | Unassigned | 655_0 | Clustered | 45 | VC_655_0 | 45 | 0.7722 | 1 | 0.7722 | 0 | 0 | 0 | 1 | Ref |
| MG252693 | Unassigned | Unassigned | Unassigned | 650_0 | Clustered | 3 | VC_650_0 | 3 | 0.2008 | 0.99838296 | 0.2005 | 0 | 0 | 0 | 1 | Ref |
| MG253647 | Unassigned | Unassigned | Unassigned | 210_0 | Clustered | 105 | VC_210_0 | 105 | 0.4076 | 1 | 0.4076 | 0 | 0 | 0 | 0.7766 | Ref |

| | | | | | | | | | | | | | | | | |
|---|---|---|---|---|---|---|---|---|---|---|---|---|---|---|---|---|
| MG253648 | Unassigned | Unassigned | Unassigned | 210_0 | Clustered | 105 | VC_210_0 | 105 | 0.4076 | 1 | 0.4076 | 0 | 0 | 0 | 0.7766 | Ref |
| MG253649 | Unassigned | Unassigned | Unassigned | 210_0 | Clustered | 105 | VC_210_0 | 105 | 0.4076 | 1 | 0.4076 | 0 | 0 | 0 | 0.7766 | Ref |
| MG253650 | Unassigned | Unassigned | Unassigned | 210_0 | Clustered | 105 | VC_210_0 | 105 | 0.4076 | 1 | 0.4076 | 0 | 0 | 0 | 0.7766 | Ref |
| MG253651 | Unassigned | Unassigned | Unassigned | 210_0 | Clustered | 105 | VC_210_0 | 105 | 0.4076 | 1 | 0.4076 | 0 | 0 | 0 | 0.7766 | Ref |
| MG253652 | Unassigned | Unassigned | Unassigned | 210_0 | Clustered | 105 | VC_210_0 | 105 | 0.4076 | 1 | 0.4076 | 0 | 0 | 0 | 0.7766 | Ref |
| MG253653 | Unassigned | Unassigned | Unassigned | 210_0 | Clustered | 105 | VC_210_0 | 105 | 0.4076 | 1 | 0.4076 | 0 | 0 | 0 | 0.7766 | Ref |
| MG264739 | Unassigned | Unassigned | Unassigned | 711_0 | Clustered | 48 | VC_711_0 | 48 | 0.8217 | 1 | 0.8217 | 0 | 0 | 0 | 1 | Ref |
| MG266157 | Unassigned | Unassigned | Unassigned | 133_0 | Clustered | 17 | VC_133_0 | 17 | 0.2654 | 1 | 0.2654 | 0 | 0 | 0 | 1 | Ref |
| MG269973 | Unassigned | Unassigned | Unassigned | 1277_0 | Clustered | 2 | VC_1277_0 | 2 | 1 | 1 | 1 | 0 | 0 | 0 | 1 | Ref |
| MG271909 | Unassigned | Unassigned | Unassigned | 902_0 | Clustered | 3 | VC_902_0 | 3 | 0.8989 | 0.98968122 | 0.8896 | 0 | 0 | 0 | 1 | Ref |
| MG280946 | Unassigned | Unassigned | Unassigned | 611_0 | Clustered | 50 | VC_611_0 | 50 | 0.7306 | 1 | 0.7306 | 0 | 0 | 0 | 1 | Ref |
| MG298964 | Unassigned | Unassigned | Unassigned | 1016_0 | Clustered | 62 | VC_1016_0 | 62 | 0.8638 | 1 | 0.8638 | 0 | 0 | 0 | 0.743 | Ref |
| MG309717 | Unassigned | Unassigned | Unassigned | 210_0 | Clustered | 105 | VC_210_0 | 105 | 0.4076 | 1 | 0.4076 | 0 | 0 | 0 | 0.7766 | Ref |
| MG309718 | Unassigned | Unassigned | Unassigned | 210_0 | Clustered | 105 | VC_210_0 | 105 | 0.4076 | 1 | 0.4076 | 0 | 0 | 0 | 0.7766 | Ref |
| MG324353 | Unassigned | Unassigned | Unassigned | 420_0 | Clustered | 3 | VC_420_0 | 3 | 0.3294 | 0.99825267 | 0.3288 | 0 | 0 | 0 | 1 | Ref |
| MG324354 | Unassigned | Unassigned | Unassigned | 420_0 | Clustered | 3 | VC_420_0 | 3 | 0.3294 | 0.99825267 | 0.3288 | 0 | 0 | 0 | 1 | Ref |
| MG366115 | Unassigned | Unassigned | Unassigned | 767_0 | Clustered/Singleton | 1 | VC_767_0 | 1 | 0 | 1 | 0 | 0 | 0 | 0 | 1 | Ref |
| MG383452 | Unassigned | Unassigned | Unassigned | 135_27 | Clustered/Singleton | 1 | VC_135_27 | 1 | 0 | 1 | 0 | 0 | 0 | 0 | 1 | Ref |
| MG387042 | Unassigned | Unassigned | Unassigned | 198_26 | Clustered/Singleton | 1 | VC_198_26 | 1 | 0 | 1 | 0 | 0 | 0 | 0 | 1 | Ref |
| MG407615 | Unassigned | Unassigned | Unassigned | 30_1 | Clustered | 53 | VC_30_1 | 53 | 0.7151 | 1 | 0.7151 | 0 | 0 | 0 | 0.9325 | Ref |
| MG428990 | Unassigned | Unassigned | Unassigned | 134_2 | Clustered | 17 | VC_134_2 | 17 | 0.1715 | 1 | 0.1715 | 0 | 0 | 0 | 0.9853 | Ref |
| MG428991 | Unassigned | Unassigned | Unassigned | 134_2 | Clustered | 17 | VC_134_2 | 17 | 0.1715 | 1 | 0.1715 | 0 | 0 | 0 | 0.9853 | Ref |
| MG428992 | Unassigned | Unassigned | Unassigned | 135_1 | Clustered/Singleton | 1 | VC_135_1 | 1 | 0 | 1 | 0 | 0 | 0 | 0 | 1 | Ref |
| MG432137 | Unassigned | Unassigned | Unassigned | 125_0 | Clustered | 23 | VC_125_0 | 23 | 0.8163 | 1 | 0.8163 | 0 | 0 | 0 | 1 | Ref |
| MG432151 | Unassigned | Unassigned | Unassigned | 486_0 | Clustered | 47 | VC_486_0 | 47 | 0.9022 | 1 | 0.9022 | 0 | 0 | 0 | 1 | Ref |
| MG459218 | Unassigned | Unassigned | Unassigned | 714_0 | Clustered | 71 | VC_714_0 | 71 | 0.935 | 1 | 0.935 | 0 | 0 | 0 | 1 | Ref |
| MG459987 | Unassigned | Unassigned | Unassigned | 200_0 | Clustered | 15 | VC_200_0 | 15 | 0.5004 | 1 | 0.5004 | 0 | 0 | 0 | 1 | Ref |
| MG471392 | Unassigned | Unassigned | Unassigned | 399_0 | Clustered | 49 | VC_399_0 | 49 | 0.7927 | 1 | 0.7927 | 0 | 0 | 0 | 1 | Ref |
| MG488277 | Unassigned | Unassigned | Unassigned | 405_0 | Clustered | 126 | VC_405_0 | 126 | 0.9614 | 1 | 0.9614 | 0 | 0 | 0 | 1 | Ref |
| MG515223 | Unassigned | Unassigned | Unassigned | 1278_0 | Clustered | 19 | VC_1278_0 | 19 | 1 | 1 | 1 | 0 | 0 | 0 | 1 | Ref |
| MG518519 | Unassigned | Unassigned | Unassigned | 1278_0 | Clustered | 19 | VC_1278_0 | 19 | 1 | 1 | 1 | 0 | 0 | 0 | 1 | Ref |
| MG518520 | Unassigned | Unassigned | Unassigned | 1278_0 | Clustered | 19 | VC_1278_0 | 19 | 1 | 1 | 1 | 0 | 0 | 0 | 1 | Ref |
| MG543995 | Unassigned | Unassigned | Unassigned | 12_1 | Clustered | 2 | VC_12_1 | 2 | 0.0189 | 0.95740699 | 0.0181 | 0 | 0 | 0 | 1 | Ref |
| MG545917 | Unassigned | Unassigned | Unassigned | 1067_0 | Clustered | 20 | VC_1067_0 | 20 | 0.7109 | 1 | 0.7109 | 0 | 0 | 0 | 1 | Ref |
| MG550110 | Unassigned | Unassigned | Unassigned | 997_0 | Clustered | 4 | VC_997_0 | 4 | 0.8491 | 0.99879268 | 0.8481 | 0 | 0 | 0 | 1 | Ref |
| MG550111 | Unassigned | Unassigned | Unassigned | 997_0 | Clustered | 4 | VC_997_0 | 4 | 0.8491 | 0.99879268 | 0.8481 | 0 | 0 | 0 | 1 | Ref |
| MG550112 | Unassigned | Unassigned | Unassigned | 473_0 | Clustered | 3 | VC_473_0 | 3 | 0.8515 | 0.99054823 | 0.8434 | 0 | 0 | 0 | 1 | Ref |
| MG550113 | Unassigned | Unassigned | Unassigned | 997_0 | Clustered | 4 | VC_997_0 | 4 | 0.8491 | 0.99879268 | 0.8481 | 0 | 0 | 0 | 1 | Ref |
| MG552615 | Unassigned | Unassigned | Unassigned | 972_0 | Clustered | 99 | VC_972_0 | 99 | 0.9113 | 1 | 0.9113 | 0 | 0 | 0 | 0.9928 | Ref |
| MG557618 | Unassigned | Unassigned | Unassigned | 155_15 | Clustered | 16 | VC_155_15 | 16 | 0.0779 | 1 | 0.0779 | 0 | 0 | 0 | 0.8083 | Ref |
| MG557619 | Unassigned | Unassigned | Unassigned | 9_0 | Clustered | 38 | VC_9_0 | 38 | 0.4859 | 1 | 0.4859 | 0 | 0 | 0 | 1 | Ref |
| MG557979 | Unassigned | Unassigned | Unassigned | 544_0 | Clustered | 6 | VC_544_0 | 6 | 0.4346 | 1 | 0.4346 | 0 | 0 | 0 | 1 | Ref |
| MG563770 | Unassigned | Unassigned | Unassigned | 373_0 | Clustered | 358 | VC_373_0 | 358 | 0.9614 | 1 | 0.9614 | 0 | 0 | 0 | 1 | Ref |
| MG564297 | Unassigned | Unassigned | Unassigned | 12_1 | Clustered | 2 | VC_12_1 | 2 | 0.0189 | 0.95740699 | 0.0181 | 0 | 0 | 0 | 1 | Ref |
| MG575418 | Unassigned | Unassigned | Unassigned | 114_0 | Clustered | 8 | VC_114_0 | 8 | 0.2518 | 1 | 0.2518 | 0 | 0 | 0 | 1 | Ref |
| MG575419 | Unassigned | Unassigned | Unassigned | 114_0 | Clustered | 8 | VC_114_0 | 8 | 0.2518 | 1 | 0.2518 | 0 | 0 | 0 | 1 | Ref |
| MG575421 | Unassigned | Unassigned | Unassigned | 437_0 | Clustered | 3 | VC_437_0 | 3 | 0.107 | 0.99803965 | 0.1068 | 0 | 0 | 0 | 1 | Ref |
| MG581355 | Unassigned | Unassigned | Unassigned | 859_0 | Clustered | 67 | VC_859_0 | 67 | 0.9622 | 1 | 0.9622 | 0 | 0 | 0 | 1 | Ref |
| MG589383 | Unassigned | Unassigned | Unassigned | 293_12 | Clustered | 3 | VC_293_12 | 3 | 0.0146 | 0.92328265 | 0.0135 | 0 | 0 | 0 | 1 | Ref |
| MG589384 | Unassigned | Unassigned | Unassigned | 339_0 | Clustered | 11 | VC_339_0 | 11 | 0.4647 | 1 | 0.4647 | 0 | 0 | 0 | 1 | Ref |
| MG589385 | Unassigned | Unassigned | Unassigned | 486_0 | Clustered | 47 | VC_486_0 | 47 | 0.9022 | 1 | 0.9022 | 0 | 0 | 0 | 1 | Ref |
| MG589386 | Unassigned | Unassigned | Unassigned | 486_0 | Clustered | 47 | VC_486_0 | 47 | 0.9022 | 1 | 0.9022 | 0 | 0 | 0 | 1 | Ref |
| MG589387 | Unassigned | Unassigned | Unassigned | 236_0 | Clustered | 32 | VC_236_0 | 32 | 0.9115 | 1 | 0.9115 | 0 | 0 | 0 | 1 | Ref |
| MG592390 | Unassigned | Unassigned | Unassigned | 664_0 | Clustered | 8 | VC_664_0 | 8 | 0.3124 | 1 | 0.3124 | 0 | 0 | 0 | 1 | Ref |
| MG592391 | Unassigned | Unassigned | Unassigned | 1081_0 | Clustered | 9 | VC_1081_0 | 9 | 0.6523 | 1 | 0.6523 | 0 | 0 | 0 | 1 | Ref |
| MG592392 | Unassigned | Unassigned | Unassigned | 516_0 | Clustered | 24 | VC_516_0 | 24 | 0.8556 | 1 | 0.8556 | 0 | 0 | 0 | 0.9493 | Ref |
| MG592393 | Unassigned | Unassigned | Unassigned | 665_0 | Clustered | 29 | VC_665_0 | 29 | 0.9121 | 1 | 0.9121 | 0 | 0 | 0 | 1 | Ref |
| MG592394 | Unassigned | Unassigned | Unassigned | 1279_0 | Clustered | 4 | VC_1279_0 | 4 | 0.9613 | 0.99374885 | 0.9553 | 0 | 0 | 0 | 1 | Ref |
| MG592395 | Unassigned | Unassigned | Unassigned | 515_0 | Clustered | 8 | VC_515_0 | 8 | 0.3973 | 1 | 0.3973 | 0 | 0 | 0 | 1 | Ref |
| MG592397 | Unassigned | Unassigned | Unassigned | 1091_1 | Clustered | 29 | VC_1091_1 | 29 | 0.4856 | 1 | 0.4856 | 0 | 0 | 0 | 0.9581 | Ref |
| MG592398 | Unassigned | Unassigned | Unassigned | 978_0 | Clustered | 6 | VC_978_0 | 6 | 0.8163 | 1 | 0.8163 | 0 | 0 | 0 | 1 | Ref |
| MG592399 | Unassigned | Unassigned | Unassigned | 563_0 | Clustered | 6 | VC_563_0 | 6 | 0.8633 | 0.99999948 | 0.8633 | 0 | 0 | 0 | 1 | Ref |
| MG592400 | Unassigned | Unassigned | Unassigned | 124_0 | Clustered | 5 | VC_124_0 | 5 | 0.2064 | 0.99999996 | 0.2064 | 0 | 0 | 0 | 1 | Ref |
| MG592401 | Unassigned | Unassigned | Unassigned | 516_0 | Clustered | 24 | VC_516_0 | 24 | 0.8556 | 1 | 0.8556 | 0 | 0 | 0 | 0.9493 | Ref |
| MG592402 | Unassigned | Unassigned | Unassigned | 1279_0 | Clustered | 4 | VC_1279_0 | 4 | 0.9613 | 0.99374885 | 0.9553 | 0 | 0 | 0 | 1 | Ref |
| MG592403 | Unassigned | Unassigned | Unassigned | 303_0 | Clustered | 6 | VC_303_0 | 6 | 0.5341 | 1 | 0.5341 | 0 | 0 | 0 | 1 | Ref |
| MG592404 | Unassigned | Unassigned | Unassigned | 303_0 | Clustered | 6 | VC_303_0 | 6 | 0.5341 | 1 | 0.5341 | 0 | 0 | 0 | 1 | Ref |
| MG592405 | Unassigned | Unassigned | Unassigned | 303_0 | Clustered | 6 | VC_303_0 | 6 | 0.5341 | 1 | 0.5341 | 0 | 0 | 0 | 1 | Ref |
| MG592406 | Unassigned | Unassigned | Unassigned | 1091_1 | Clustered | 29 | VC_1091_1 | 29 | 0.4856 | 1 | 0.4856 | 0 | 0 | 0 | 0.9581 | Ref |
| MG592407 | Unassigned | Unassigned | Unassigned | 1091_1 | Clustered | 29 | VC_1091_1 | 29 | 0.4856 | 1 | 0.4856 | 0 | 0 | 0 | 0.9581 | Ref |
| MG592408 | Unassigned | Unassigned | Unassigned | 1091_1 | Clustered | 29 | VC_1091_1 | 29 | 0.4856 | 1 | 0.4856 | 0 | 0 | 0 | 0.9581 | Ref |
| MG592409 | Unassigned | Unassigned | Unassigned | 809_0 | Clustered | 7 | VC_809_0 | 7 | 0.5153 | 1 | 0.5153 | 0 | 0 | 0 | 1 | Ref |
| MG592410 | Unassigned | Unassigned | Unassigned | 809_0 | Clustered | 7 | VC_809_0 | 7 | 0.5153 | 1 | 0.5153 | 0 | 0 | 0 | 1 | Ref |
| MG592411 | Unassigned | Unassigned | Unassigned | 1091_0 | Clustered | 24 | VC_1091_0 | 24 | 0.5254 | 1 | 0.5254 | 0 | 0 | 0 | 1 | Ref |
| MG592412 | Unassigned | Unassigned | Unassigned | 255_0 | Clustered | 11 | VC_255_0 | 11 | 0.7034 | 1 | 0.7034 | 0 | 0 | 0 | 1 | Ref |
| MG592414 | Unassigned | Unassigned | Unassigned | 516_0 | Clustered | 24 | VC_516_0 | 24 | 0.8556 | 1 | 0.8556 | 0 | 0 | 0 | 0.9493 | Ref |
| MG592415 | Unassigned | Unassigned | Unassigned | 494_0 | Clustered | 2 | VC_494_0 | 2 | 0.3459 | 0.94728727 | 0.3277 | 0 | 0 | 0 | 1 | Ref |
| MG592416 | Unassigned | Unassigned | Unassigned | 1091_1 | Clustered | 29 | VC_1091_1 | 29 | 0.4856 | 1 | 0.4856 | 0 | 0 | 0 | 0.9581 | Ref |
| MG592417 | Unassigned | Unassigned | Unassigned | 1091_1 | Clustered | 29 | VC_1091_1 | 29 | 0.4856 | 1 | 0.4856 | 0 | 0 | 0 | 0.9581 | Ref |
| MG592418 | Unassigned | Unassigned | Unassigned | 563_0 | Clustered | 6 | VC_563_0 | 6 | 0.8633 | 0.99999948 | 0.8633 | 0 | 0 | 0 | 1 | Ref |
| MG592419 | Unassigned | Unassigned | Unassigned | 563_0 | Clustered | 6 | VC_563_0 | 6 | 0.8633 | 0.99999948 | 0.8633 | 0 | 0 | 0 | 1 | Ref |

| | | | | | | | | | | | | | | | | |
|---|---|---|---|---|---|---|---|---|---|---|---|---|---|---|---|---|
| MG592420 | Unassigned | Unassigned | Unassigned | 664_0 | Clustered | 8 | VC_664_0 | 8 | 0.3124 | 1 | 0.3124 | 0 | 0 | 0 | 1 | Ref |
| MG592421 | Unassigned | Unassigned | Unassigned | 1081_0 | Clustered | 9 | VC_1081_0 | 9 | 0.6523 | 1 | 0.6523 | 0 | 0 | 0 | 1 | Ref |
| MG592422 | Unassigned | Unassigned | Unassigned | 124_0 | Clustered | 5 | VC_124_0 | 5 | 0.2064 | 0.99999996 | 0.2064 | 0 | 0 | 0 | 1 | Ref |
| MG592423 | Unassigned | Unassigned | Unassigned | 664_0 | Clustered | 8 | VC_664_0 | 8 | 0.3124 | 1 | 0.3124 | 0 | 0 | 0 | 1 | Ref |
| MG592425 | Unassigned | Unassigned | Unassigned | 124_0 | Clustered | 5 | VC_124_0 | 5 | 0.2064 | 0.99999996 | 0.2064 | 0 | 0 | 0 | 1 | Ref |
| MG592427 | Unassigned | Unassigned | Unassigned | 1279_0 | Clustered | 4 | VC_1279_0 | 4 | 0.9613 | 0.99374885 | 0.9553 | 0 | 0 | 0 | 1 | Ref |
| MG592428 | Unassigned | Unassigned | Unassigned | 1091_1 | Clustered | 29 | VC_1091_1 | 29 | 0.4856 | 1 | 0.4856 | 0 | 0 | 0 | 0.9581 | Ref |
| MG592429 | Unassigned | Unassigned | Unassigned | 981_0 | Clustered | 9 | VC_981_0 | 9 | 0.5418 | 1 | 0.5418 | 0 | 0 | 0 | 1 | Ref |
| MG592431 | Unassigned | Unassigned | Unassigned | 851_0 | Clustered | 6 | VC_851_0 | 6 | 0.7471 | 1 | 0.7471 | 0 | 0 | 0 | 1 | Ref |
| MG592432 | Unassigned | Unassigned | Unassigned | 851_0 | Clustered | 6 | VC_851_0 | 6 | 0.7471 | 1 | 0.7471 | 0 | 0 | 0 | 1 | Ref |
| MG592434 | Unassigned | Unassigned | Unassigned | 124_1 | Clustered/Singleton | 1 | VC_124_1 | 1 | 0 | 1 | 0 | 0 | 0 | 0 | 1 | Ref |
| MG592435 | Unassigned | Unassigned | Unassigned | 851_0 | Clustered | 6 | VC_851_0 | 6 | 0.7471 | 1 | 0.7471 | 0 | 0 | 0 | 1 | Ref |
| MG592436 | Unassigned | Unassigned | Unassigned | 1081_0 | Clustered | 9 | VC_1081_0 | 9 | 0.6523 | 1 | 0.6523 | 0 | 0 | 0 | 1 | Ref |
| MG592438 | Unassigned | Unassigned | Unassigned | 981_0 | Clustered | 9 | VC_981_0 | 9 | 0.5418 | 1 | 0.5418 | 0 | 0 | 0 | 1 | Ref |
| MG592439 | Unassigned | Unassigned | Unassigned | 664_0 | Clustered | 8 | VC_664_0 | 8 | 0.3124 | 1 | 0.3124 | 0 | 0 | 0 | 1 | Ref |
| MG592441 | Unassigned | Unassigned | Unassigned | 511_8 | Clustered/Singleton | 1 | VC_511_8 | 1 | 0 | 1 | 0 | 0 | 0 | 0 | 1 | Ref |
| MG592442 | Unassigned | Unassigned | Unassigned | 957_0 | Clustered | 34 | VC_957_0 | 34 | 0.8934 | 1 | 0.8934 | 0 | 0 | 0 | 0.9768 | Ref |
| MG592443 | Unassigned | Unassigned | Unassigned | 1081_0 | Clustered | 9 | VC_1081_0 | 9 | 0.6523 | 1 | 0.6523 | 0 | 0 | 0 | 1 | Ref |
| MG592444 | Unassigned | Unassigned | Unassigned | 664_0 | Clustered | 8 | VC_664_0 | 8 | 0.3124 | 1 | 0.3124 | 0 | 0 | 0 | 1 | Ref |
| MG592445 | Unassigned | Unassigned | Unassigned | 1081_0 | Clustered | 9 | VC_1081_0 | 9 | 0.6523 | 1 | 0.6523 | 0 | 0 | 0 | 1 | Ref |
| MG592447 | Unassigned | Unassigned | Unassigned | 564_0 | Clustered | 2 | VC_564_0 | 2 | 0.3503 | 0.94059057 | 0.3295 | 0 | 0 | 0 | 1 | Ref |
| MG592448 | Unassigned | Unassigned | Unassigned | 124_0 | Clustered | 5 | VC_124_0 | 5 | 0.2064 | 0.99999996 | 0.2064 | 0 | 0 | 0 | 1 | Ref |
| MG592449 | Unassigned | Unassigned | Unassigned | 1081_0 | Clustered | 9 | VC_1081_0 | 9 | 0.6523 | 1 | 0.6523 | 0 | 0 | 0 | 1 | Ref |
| MG592450 | Unassigned | Unassigned | Unassigned | 957_0 | Clustered | 34 | VC_957_0 | 34 | 0.8934 | 1 | 0.8934 | 0 | 0 | 0 | 0.9768 | Ref |
| MG592451 | Unassigned | Unassigned | Unassigned | 516_0 | Clustered | 24 | VC_516_0 | 24 | 0.8556 | 1 | 0.8556 | 0 | 0 | 0 | 0.9493 | Ref |
| MG592453 | Unassigned | Unassigned | Unassigned | 564_0 | Clustered | 2 | VC_564_0 | 2 | 0.3503 | 0.94059057 | 0.3295 | 0 | 0 | 0 | 1 | Ref |
| MG592454 | Unassigned | Unassigned | Unassigned | 664_0 | Clustered | 8 | VC_664_0 | 8 | 0.3124 | 1 | 0.3124 | 0 | 0 | 0 | 1 | Ref |
| MG592455 | Unassigned | Unassigned | Unassigned | 1279_0 | Clustered | 4 | VC_1279_0 | 4 | 0.9613 | 0.99374885 | 0.9553 | 0 | 0 | 0 | 1 | Ref |
| MG592456 | Unassigned | Unassigned | Unassigned | 549_4 | Clustered/Singleton | 1 | VC_549_4 | 1 | 0 | 1 | 0 | 0 | 0 | 0 | 1 | Ref |
| MG592457 | Unassigned | Unassigned | Unassigned | 957_0 | Clustered | 34 | VC_957_0 | 34 | 0.8934 | 1 | 0.8934 | 0 | 0 | 0 | 0.9768 | Ref |
| MG592458 | Unassigned | Unassigned | Unassigned | 851_0 | Clustered | 6 | VC_851_0 | 6 | 0.7471 | 1 | 0.7471 | 0 | 0 | 0 | 1 | Ref |
| MG592460 | Unassigned | Unassigned | Unassigned | 957_0 | Clustered | 34 | VC_957_0 | 34 | 0.8934 | 1 | 0.8934 | 0 | 0 | 0 | 0.9768 | Ref |
| MG592461 | Unassigned | Unassigned | Unassigned | 516_0 | Clustered | 24 | VC_516_0 | 24 | 0.8556 | 1 | 0.8556 | 0 | 0 | 0 | 0.9493 | Ref |
| MG592462 | Unassigned | Unassigned | Unassigned | 516_0 | Clustered | 24 | VC_516_0 | 24 | 0.8556 | 1 | 0.8556 | 0 | 0 | 0 | 0.9493 | Ref |
| MG592463 | Unassigned | Unassigned | Unassigned | 1091_1 | Clustered | 29 | VC_1091_1 | 29 | 0.4856 | 1 | 0.4856 | 0 | 0 | 0 | 0.9581 | Ref |
| MG592464 | Unassigned | Unassigned | Unassigned | 1091_1 | Clustered | 29 | VC_1091_1 | 29 | 0.4856 | 1 | 0.4856 | 0 | 0 | 0 | 0.9581 | Ref |
| MG592465 | Unassigned | Unassigned | Unassigned | 664_0 | Clustered | 8 | VC_664_0 | 8 | 0.3124 | 1 | 0.3124 | 0 | 0 | 0 | 1 | Ref |
| MG592466 | Unassigned | Unassigned | Unassigned | 664_0 | Clustered | 8 | VC_664_0 | 8 | 0.3124 | 1 | 0.3124 | 0 | 0 | 0 | 1 | Ref |
| MG592467 | Unassigned | Unassigned | Unassigned | 516_0 | Clustered | 24 | VC_516_0 | 24 | 0.8556 | 1 | 0.8556 | 0 | 0 | 0 | 0.9493 | Ref |
| MG592468 | Unassigned | Unassigned | Unassigned | 516_0 | Clustered | 24 | VC_516_0 | 24 | 0.8556 | 1 | 0.8556 | 0 | 0 | 0 | 0.9493 | Ref |
| MG592470 | Unassigned | Unassigned | Unassigned | 809_0 | Clustered | 7 | VC_809_0 | 7 | 0.5153 | 1 | 0.5153 | 0 | 0 | 0 | 1 | Ref |
| MG592471 | Unassigned | Unassigned | Unassigned | 1091_1 | Clustered | 29 | VC_1091_1 | 29 | 0.4856 | 1 | 0.4856 | 0 | 0 | 0 | 0.9581 | Ref |
| MG592472 | Unassigned | Unassigned | Unassigned | 516_0 | Clustered | 24 | VC_516_0 | 24 | 0.8556 | 1 | 0.8556 | 0 | 0 | 0 | 0.9493 | Ref |
| MG592475 | Unassigned | Unassigned | Unassigned | 1091_1 | Clustered | 29 | VC_1091_1 | 29 | 0.4856 | 1 | 0.4856 | 0 | 0 | 0 | 0.9581 | Ref |
| MG592476 | Unassigned | Unassigned | Unassigned | 1091_1 | Clustered | 29 | VC_1091_1 | 29 | 0.4856 | 1 | 0.4856 | 0 | 0 | 0 | 0.9581 | Ref |
| MG592477 | Unassigned | Unassigned | Unassigned | 665_0 | Clustered | 29 | VC_665_0 | 29 | 0.9121 | 1 | 0.9121 | 0 | 0 | 0 | 1 | Ref |
| MG592478 | Unassigned | Unassigned | Unassigned | 665_0 | Clustered | 29 | VC_665_0 | 29 | 0.9121 | 1 | 0.9121 | 0 | 0 | 0 | 1 | Ref |
| MG592482 | Unassigned | Unassigned | Unassigned | 957_0 | Clustered | 34 | VC_957_0 | 34 | 0.8934 | 1 | 0.8934 | 0 | 0 | 0 | 0.9768 | Ref |
| MG592483 | Unassigned | Unassigned | Unassigned | 957_0 | Clustered | 34 | VC_957_0 | 34 | 0.8934 | 1 | 0.8934 | 0 | 0 | 0 | 0.9768 | Ref |
| MG592484 | Unassigned | Unassigned | Unassigned | 1234_0 | Clustered | 2 | VC_1234_0 | 2 | 0.5074 | 0.94764538 | 0.4809 | 0 | 0 | 0 | 1 | Ref |
| MG592485 | Unassigned | Unassigned | Unassigned | 1234_0 | Clustered | 2 | VC_1234_0 | 2 | 0.5074 | 0.94764538 | 0.4809 | 0 | 0 | 0 | 1 | Ref |
| MG592486 | Unassigned | Unassigned | Unassigned | 124_0 | Clustered | 5 | VC_124_0 | 5 | 0.2064 | 0.99999996 | 0.2064 | 0 | 0 | 0 | 1 | Ref |
| MG592487 | Unassigned | Unassigned | Unassigned | 981_0 | Clustered | 9 | VC_981_0 | 9 | 0.5418 | 1 | 0.5418 | 0 | 0 | 0 | 1 | Ref |
| MG592488 | Unassigned | Unassigned | Unassigned | 957_0 | Clustered | 34 | VC_957_0 | 34 | 0.8934 | 1 | 0.8934 | 0 | 0 | 0 | 0.9768 | Ref |
| MG592489 | Unassigned | Unassigned | Unassigned | 957_0 | Clustered | 34 | VC_957_0 | 34 | 0.8934 | 1 | 0.8934 | 0 | 0 | 0 | 0.9768 | Ref |
| MG592490 | Unassigned | Unassigned | Unassigned | 957_0 | Clustered | 34 | VC_957_0 | 34 | 0.8934 | 1 | 0.8934 | 0 | 0 | 0 | 0.9768 | Ref |
| MG592492 | Unassigned | Unassigned | Unassigned | 1091_1 | Clustered | 29 | VC_1091_1 | 29 | 0.4856 | 1 | 0.4856 | 0 | 0 | 0 | 0.9581 | Ref |
| MG592493 | Unassigned | Unassigned | Unassigned | 1091_1 | Clustered | 29 | VC_1091_1 | 29 | 0.4856 | 1 | 0.4856 | 0 | 0 | 0 | 0.9581 | Ref |
| MG592494 | Unassigned | Unassigned | Unassigned | 661_3 | Clustered | 9 | VC_661_3 | 9 | 0.2124 | 1 | 0.2124 | 0 | 0 | 0 | 0.6944 | Ref |
| MG592495 | Unassigned | Unassigned | Unassigned | 511_6 | Clustered/Singleton | 1 | VC_511_6 | 1 | 0 | 1 | 0 | 0 | 0 | 0 | 1 | Ref |
| MG592496 | Unassigned | Unassigned | Unassigned | 978_0 | Clustered | 6 | VC_978_0 | 6 | 0.8163 | 1 | 0.8163 | 0 | 0 | 0 | 1 | Ref |
| MG592497 | Unassigned | Unassigned | Unassigned | 978_0 | Clustered | 6 | VC_978_0 | 6 | 0.8163 | 1 | 0.8163 | 0 | 0 | 0 | 1 | Ref |
| MG592499 | Unassigned | Unassigned | Unassigned | 516_0 | Clustered | 24 | VC_516_0 | 24 | 0.8556 | 1 | 0.8556 | 0 | 0 | 0 | 0.9493 | Ref |
| MG592501 | Unassigned | Unassigned | Unassigned | 661_3 | Clustered | 9 | VC_661_3 | 9 | 0.2124 | 1 | 0.2124 | 0 | 0 | 0 | 0.6944 | Ref |
| MG592502 | Unassigned | Unassigned | Unassigned | 957_0 | Clustered | 34 | VC_957_0 | 34 | 0.8934 | 1 | 0.8934 | 0 | 0 | 0 | 0.9768 | Ref |
| MG592503 | Unassigned | Unassigned | Unassigned | 957_0 | Clustered | 34 | VC_957_0 | 34 | 0.8934 | 1 | 0.8934 | 0 | 0 | 0 | 0.9768 | Ref |
| MG592504 | Unassigned | Unassigned | Unassigned | 957_0 | Clustered | 34 | VC_957_0 | 34 | 0.8934 | 1 | 0.8934 | 0 | 0 | 0 | 0.9768 | Ref |
| MG592505 | Unassigned | Unassigned | Unassigned | 957_0 | Clustered | 34 | VC_957_0 | 34 | 0.8934 | 1 | 0.8934 | 0 | 0 | 0 | 0.9768 | Ref |
| MG592506 | Unassigned | Unassigned | Unassigned | 661_0 | Clustered | 7 | VC_661_0 | 7 | 0.15 | 1 | 0.15 | 0 | 0 | 0 | 0.7619 | Ref |
| MG592507 | Unassigned | Unassigned | Unassigned | 255_0 | Clustered | 11 | VC_255_0 | 11 | 0.7034 | 1 | 0.7034 | 0 | 0 | 0 | 1 | Ref |
| MG592508 | Unassigned | Unassigned | Unassigned | 661_3 | Clustered | 9 | VC_661_3 | 9 | 0.2124 | 1 | 0.2124 | 0 | 0 | 0 | 0.6944 | Ref |
| MG592509 | Unassigned | Unassigned | Unassigned | 518_0 | Clustered | 16 | VC_518_0 | 16 | 0.9983 | 1 | 0.9983 | 0 | 0 | 0 | 1 | Ref |
| MG592510 | Unassigned | Unassigned | Unassigned | 563_0 | Clustered | 6 | VC_563_0 | 6 | 0.8633 | 0.99999948 | 0.8633 | 0 | 0 | 0 | 1 | Ref |
| MG592511 | Unassigned | Unassigned | Unassigned | 563_0 | Clustered | 6 | VC_563_0 | 6 | 0.8633 | 0.99999948 | 0.8633 | 0 | 0 | 0 | 1 | Ref |
| MG592513 | Unassigned | Unassigned | Unassigned | 255_0 | Clustered | 11 | VC_255_0 | 11 | 0.7034 | 1 | 0.7034 | 0 | 0 | 0 | 1 | Ref |
| MG592514 | Unassigned | Unassigned | Unassigned | 661_3 | Clustered | 9 | VC_661_3 | 9 | 0.2124 | 1 | 0.2124 | 0 | 0 | 0 | 0.6944 | Ref |
| MG592516 | Unassigned | Unassigned | Unassigned | 957_0 | Clustered | 34 | VC_957_0 | 34 | 0.8934 | 1 | 0.8934 | 0 | 0 | 0 | 0.9768 | Ref |
| MG592517 | Unassigned | Unassigned | Unassigned | 957_0 | Clustered | 34 | VC_957_0 | 34 | 0.8934 | 1 | 0.8934 | 0 | 0 | 0 | 0.9768 | Ref |
| MG592518 | Unassigned | Unassigned | Unassigned | 665_0 | Clustered | 29 | VC_665_0 | 29 | 0.9121 | 1 | 0.9121 | 0 | 0 | 0 | 1 | Ref |
| MG592519 | Unassigned | Unassigned | Unassigned | 809_0 | Clustered | 7 | VC_809_0 | 7 | 0.5153 | 1 | 0.5153 | 0 | 0 | 0 | 1 | Ref |
| MG592520 | Unassigned | Unassigned | Unassigned | 959_0 | Clustered | 3 | VC_959_0 | 3 | 0.9422 | 0.99430386 | 0.9369 | 0 | 0 | 0 | 1 | Ref |

| | | | | | | | | | | | | | | | | |
|---|---|---|---|---|---|---|---|---|---|---|---|---|---|---|---|---|
| MG592521 | Unassigned | Unassigned | Unassigned | 809_0 | Clustered | 7 | VC_809_0 | 7 | 0.5153 | 1 | 0.5153 | 0 | 0 | 0 | 1 | Ref |
| MG592522 | Unassigned | Unassigned | Unassigned | 941_0 | Clustered | 5 | VC_941_0 | 5 | 0.7412 | 0.99999276 | 0.7412 | 0 | 0 | 0 | 1 | Ref |
| MG592523 | Unassigned | Unassigned | Unassigned | 117_0 | Clustered | 2 | VC_117_0 | 2 | 0.4363 | 0.8861 | 0.3866 | 0 | 0 | 0 | 1 | Ref |
| MG592524 | Unassigned | Unassigned | Unassigned | 255_0 | Clustered | 11 | VC_255_0 | 11 | 0.7034 | 1 | 0.7034 | 0 | 0 | 0 | 1 | Ref |
| MG592525 | Unassigned | Unassigned | Unassigned | 957_0 | Clustered | 34 | VC_957_0 | 34 | 0.8934 | 1 | 0.8934 | 0 | 0 | 0 | 0.9768 | Ref |
| MG592526 | Unassigned | Unassigned | Unassigned | 516_0 | Clustered | 24 | VC_516_0 | 24 | 0.8556 | 1 | 0.8556 | 0 | 0 | 0 | 0.9493 | Ref |
| MG592527 | Unassigned | Unassigned | Unassigned | 255_0 | Clustered | 11 | VC_255_0 | 11 | 0.7034 | 1 | 0.7034 | 0 | 0 | 0 | 1 | Ref |
| MG592528 | Unassigned | Unassigned | Unassigned | 1091_1 | Clustered | 29 | VC_1091_1 | 29 | 0.4856 | 1 | 0.4856 | 0 | 0 | 0 | 0.9581 | Ref |
| MG592529 | Unassigned | Unassigned | Unassigned | 511_2 | Clustered/Singleton | 1 | VC_511_2 | 1 | 0 | 1 | 0 | 0 | 0 | 0 | 1 | Ref |
| MG592530 | Unassigned | Unassigned | Unassigned | 957_0 | Clustered | 34 | VC_957_0 | 34 | 0.8934 | 1 | 0.8934 | 0 | 0 | 0 | 0.9768 | Ref |
| MG592531 | Unassigned | Unassigned | Unassigned | 516_0 | Clustered | 24 | VC_516_0 | 24 | 0.8556 | 1 | 0.8556 | 0 | 0 | 0 | 0.9493 | Ref |
| MG592532 | Unassigned | Unassigned | Unassigned | 957_0 | Clustered | 34 | VC_957_0 | 34 | 0.8934 | 1 | 0.8934 | 0 | 0 | 0 | 0.9768 | Ref |
| MG592533 | Unassigned | Unassigned | Unassigned | 1280_0 | Clustered | 2 | VC_1280_0 | 2 | 0.6481 | 0.86822376 | 0.5627 | 0 | 0 | 0 | 1 | Ref |
| MG592534 | Unassigned | Unassigned | Unassigned | 957_0 | Clustered | 34 | VC_957_0 | 34 | 0.8934 | 1 | 0.8934 | 0 | 0 | 0 | 0.9768 | Ref |
| MG592535 | Unassigned | Unassigned | Unassigned | 957_0 | Clustered | 34 | VC_957_0 | 34 | 0.8934 | 1 | 0.8934 | 0 | 0 | 0 | 0.9768 | Ref |
| MG592536 | Unassigned | Unassigned | Unassigned | 1133_0 | Clustered | 6 | VC_1133_0 | 6 | 0.9291 | 0.99999982 | 0.9291 | 0 | 0 | 0 | 1 | Ref |
| MG592538 | Unassigned | Unassigned | Unassigned | 1091_0 | Clustered | 24 | VC_1091_0 | 24 | 0.5254 | 1 | 0.5254 | 0 | 0 | 0 | 1 | Ref |
| MG592539 | Unassigned | Unassigned | Unassigned | 957_0 | Clustered | 34 | VC_957_0 | 34 | 0.8934 | 1 | 0.8934 | 0 | 0 | 0 | 0.9768 | Ref |
| MG592540 | Unassigned | Unassigned | Unassigned | 665_0 | Clustered | 29 | VC_665_0 | 29 | 0.9121 | 1 | 0.9121 | 0 | 0 | 0 | 1 | Ref |
| MG592541 | Unassigned | Unassigned | Unassigned | 516_0 | Clustered | 24 | VC_516_0 | 24 | 0.8556 | 1 | 0.8556 | 0 | 0 | 0 | 0.9493 | Ref |
| MG592542 | Unassigned | Unassigned | Unassigned | 516_0 | Clustered | 24 | VC_516_0 | 24 | 0.8556 | 1 | 0.8556 | 0 | 0 | 0 | 0.9493 | Ref |
| MG592543 | Unassigned | Unassigned | Unassigned | 957_0 | Clustered | 34 | VC_957_0 | 34 | 0.8934 | 1 | 0.8934 | 0 | 0 | 0 | 0.9768 | Ref |
| MG592544 | Unassigned | Unassigned | Unassigned | 661_5 | Clustered | 4 | VC_661_5 | 4 | 0.0894 | 0.99996664 | 0.0894 | 0 | 0 | 0 | 1 | Ref |
| MG592545 | Unassigned | Unassigned | Unassigned | 661_0 | Clustered | 7 | VC_661_0 | 7 | 0.15 | 1 | 0.15 | 0 | 0 | 0 | 0.7619 | Ref |
| MG592546 | Unassigned | Unassigned | Unassigned | 518_0 | Clustered | 16 | VC_518_0 | 16 | 0.9983 | 1 | 0.9983 | 0 | 0 | 0 | 1 | Ref |
| MG592547 | Unassigned | Unassigned | Unassigned | 516_0 | Clustered | 24 | VC_516_0 | 24 | 0.8556 | 1 | 0.8556 | 0 | 0 | 0 | 0.9493 | Ref |
| MG592549 | Unassigned | Unassigned | Unassigned | 831_0 | Clustered | 17 | VC_831_0 | 17 | 0.9057 | 1 | 0.9057 | 0 | 0 | 0 | 1 | Ref |
| MG592550 | Unassigned | Unassigned | Unassigned | 831_0 | Clustered | 17 | VC_831_0 | 17 | 0.9057 | 1 | 0.9057 | 0 | 0 | 0 | 1 | Ref |
| MG592551 | Unassigned | Unassigned | Unassigned | 303_0 | Clustered | 6 | VC_303_0 | 6 | 0.5341 | 1 | 0.5341 | 0 | 0 | 0 | 1 | Ref |
| MG592552 | Unassigned | Unassigned | Unassigned | 665_0 | Clustered | 29 | VC_665_0 | 29 | 0.9121 | 1 | 0.9121 | 0 | 0 | 0 | 1 | Ref |
| MG592553 | Unassigned | Unassigned | Unassigned | 511_16 | Clustered/Singleton | 1 | VC_511_16 | 1 | 0 | 1 | 0 | 0 | 0 | 0 | 1 | Ref |
| MG592554 | Unassigned | Unassigned | Unassigned | 1133_0 | Clustered | 6 | VC_1133_0 | 6 | 0.9291 | 0.99999982 | 0.9291 | 0 | 0 | 0 | 1 | Ref |
| MG592555 | Unassigned | Unassigned | Unassigned | 1133_0 | Clustered | 6 | VC_1133_0 | 6 | 0.9291 | 0.99999982 | 0.9291 | 0 | 0 | 0 | 1 | Ref |
| MG592556 | Unassigned | Unassigned | Unassigned | 1133_0 | Clustered | 6 | VC_1133_0 | 6 | 0.9291 | 0.99999982 | 0.9291 | 0 | 0 | 0 | 1 | Ref |
| MG592557 | Unassigned | Unassigned | Unassigned | 957_0 | Clustered | 34 | VC_957_0 | 34 | 0.8934 | 1 | 0.8934 | 0 | 0 | 0 | 0.9768 | Ref |
| MG592558 | Unassigned | Unassigned | Unassigned | 957_0 | Clustered | 34 | VC_957_0 | 34 | 0.8934 | 1 | 0.8934 | 0 | 0 | 0 | 0.9768 | Ref |
| MG592559 | Unassigned | Unassigned | Unassigned | 957_0 | Clustered | 34 | VC_957_0 | 34 | 0.8934 | 1 | 0.8934 | 0 | 0 | 0 | 0.9768 | Ref |
| MG592560 | Unassigned | Unassigned | Unassigned | 1280_0 | Clustered | 2 | VC_1280_0 | 2 | 0.6481 | 0.86822376 | 0.5627 | 0 | 0 | 0 | 1 | Ref |
| MG592561 | Unassigned | Unassigned | Unassigned | 516_0 | Clustered | 24 | VC_516_0 | 24 | 0.8556 | 1 | 0.8556 | 0 | 0 | 0 | 0.9493 | Ref |
| MG592562 | Unassigned | Unassigned | Unassigned | 511_1 | Clustered/Singleton | 1 | VC_511_1 | 1 | 0 | 1 | 0 | 0 | 0 | 0 | 1 | Ref |
| MG592563 | Unassigned | Unassigned | Unassigned | 1091_0 | Clustered | 24 | VC_1091_0 | 24 | 0.5254 | 1 | 0.5254 | 0 | 0 | 0 | 1 | Ref |
| MG592564 | Unassigned | Unassigned | Unassigned | 1091_0 | Clustered | 24 | VC_1091_0 | 24 | 0.5254 | 1 | 0.5254 | 0 | 0 | 0 | 1 | Ref |
| MG592565 | Unassigned | Unassigned | Unassigned | 1091_0 | Clustered | 24 | VC_1091_0 | 24 | 0.5254 | 1 | 0.5254 | 0 | 0 | 0 | 1 | Ref |
| MG592566 | Unassigned | Unassigned | Unassigned | 981_0 | Clustered | 9 | VC_981_0 | 9 | 0.5418 | 1 | 0.5418 | 0 | 0 | 0 | 1 | Ref |
| MG592567 | Unassigned | Unassigned | Unassigned | 661_0 | Clustered | 7 | VC_661_0 | 7 | 0.15 | 1 | 0.15 | 0 | 0 | 0 | 0.7619 | Ref |
| MG592568 | Unassigned | Unassigned | Unassigned | 661_0 | Clustered | 7 | VC_661_0 | 7 | 0.15 | 1 | 0.15 | 0 | 0 | 0 | 0.7619 | Ref |
| MG592569 | Unassigned | Unassigned | Unassigned | 665_0 | Clustered | 29 | VC_665_0 | 29 | 0.9121 | 1 | 0.9121 | 0 | 0 | 0 | 1 | Ref |
| MG592570 | Unassigned | Unassigned | Unassigned | 665_0 | Clustered | 29 | VC_665_0 | 29 | 0.9121 | 1 | 0.9121 | 0 | 0 | 0 | 1 | Ref |
| MG592571 | Unassigned | Unassigned | Unassigned | 1091_0 | Clustered | 24 | VC_1091_0 | 24 | 0.5254 | 1 | 0.5254 | 0 | 0 | 0 | 1 | Ref |
| MG592572 | Unassigned | Unassigned | Unassigned | 516_0 | Clustered | 24 | VC_516_0 | 24 | 0.8556 | 1 | 0.8556 | 0 | 0 | 0 | 0.9493 | Ref |
| MG592573 | Unassigned | Unassigned | Unassigned | 5_0 | Clustered | 27 | VC_5_0 | 27 | 0.9599 | 1 | 0.9599 | 0 | 0 | 0 | 1 | Ref |
| MG592576 | Unassigned | Unassigned | Unassigned | 957_0 | Clustered | 34 | VC_957_0 | 34 | 0.8934 | 1 | 0.8934 | 0 | 0 | 0 | 0.9768 | Ref |
| MG592578 | Unassigned | Unassigned | Unassigned | 181_0 | Clustered | 4 | VC_181_0 | 4 | 0.3817 | 0.99998259 | 0.3817 | 0 | 0 | 0 | 1 | Ref |
| MG592579 | Unassigned | Unassigned | Unassigned | 563_0 | Clustered | 6 | VC_563_0 | 6 | 0.8633 | 0.99999948 | 0.8633 | 0 | 0 | 0 | 1 | Ref |
| MG592581 | Unassigned | Unassigned | Unassigned | 831_0 | Clustered | 17 | VC_831_0 | 17 | 0.9057 | 1 | 0.9057 | 0 | 0 | 0 | 1 | Ref |
| MG592582 | Unassigned | Unassigned | Unassigned | 831_0 | Clustered | 17 | VC_831_0 | 17 | 0.9057 | 1 | 0.9057 | 0 | 0 | 0 | 1 | Ref |
| MG592583 | Unassigned | Unassigned | Unassigned | 661_3 | Clustered | 9 | VC_661_3 | 9 | 0.2124 | 1 | 0.2124 | 0 | 0 | 0 | 0.6944 | Ref |
| MG592584 | Unassigned | Unassigned | Unassigned | 978_0 | Clustered | 6 | VC_978_0 | 6 | 0.8163 | 1 | 0.8163 | 0 | 0 | 0 | 1 | Ref |
| MG592585 | Unassigned | Unassigned | Unassigned | 935_0 | Clustered | 6 | VC_935_0 | 6 | 0.2317 | 1 | 0.2317 | 0 | 0 | 0 | 1 | Ref |
| MG592586 | Unassigned | Unassigned | Unassigned | 935_0 | Clustered | 6 | VC_935_0 | 6 | 0.2317 | 1 | 0.2317 | 0 | 0 | 0 | 1 | Ref |
| MG592587 | Unassigned | Unassigned | Unassigned | 661_3 | Clustered | 9 | VC_661_3 | 9 | 0.2124 | 1 | 0.2124 | 0 | 0 | 0 | 0.6944 | Ref |
| MG592588 | Unassigned | Unassigned | Unassigned | 255_0 | Clustered | 11 | VC_255_0 | 11 | 0.7034 | 1 | 0.7034 | 0 | 0 | 0 | 1 | Ref |
| MG592589 | Unassigned | Unassigned | Unassigned | 255_0 | Clustered | 11 | VC_255_0 | 11 | 0.7034 | 1 | 0.7034 | 0 | 0 | 0 | 1 | Ref |
| MG592590 | Unassigned | Unassigned | Unassigned | 516_0 | Clustered | 24 | VC_516_0 | 24 | 0.8556 | 1 | 0.8556 | 0 | 0 | 0 | 0.9493 | Ref |
| MG592591 | Unassigned | Unassigned | Unassigned | 1133_0 | Clustered | 6 | VC_1133_0 | 6 | 0.9291 | 0.99999982 | 0.9291 | 0 | 0 | 0 | 1 | Ref |
| MG592592 | Unassigned | Unassigned | Unassigned | 516_0 | Clustered | 24 | VC_516_0 | 24 | 0.8556 | 1 | 0.8556 | 0 | 0 | 0 | 0.9493 | Ref |
| MG592593 | Unassigned | Unassigned | Unassigned | 957_0 | Clustered | 34 | VC_957_0 | 34 | 0.8934 | 1 | 0.8934 | 0 | 0 | 0 | 0.9768 | Ref |
| MG592594 | Unassigned | Unassigned | Unassigned | 957_0 | Clustered | 34 | VC_957_0 | 34 | 0.8934 | 1 | 0.8934 | 0 | 0 | 0 | 0.9768 | Ref |
| MG592595 | Unassigned | Unassigned | Unassigned | 661_3 | Clustered | 9 | VC_661_3 | 9 | 0.2124 | 1 | 0.2124 | 0 | 0 | 0 | 0.6944 | Ref |
| MG592596 | Unassigned | Unassigned | Unassigned | 1282_0 | Clustered | 7 | VC_1282_0 | 7 | 0.992 | 0.99999987 | 0.992 | 0 | 0 | 0 | 1 | Ref |
| MG592597 | Unassigned | Unassigned | Unassigned | 957_0 | Clustered | 34 | VC_957_0 | 34 | 0.8934 | 1 | 0.8934 | 0 | 0 | 0 | 0.9768 | Ref |
| MG592598 | Unassigned | Unassigned | Unassigned | 957_0 | Clustered | 34 | VC_957_0 | 34 | 0.8934 | 1 | 0.8934 | 0 | 0 | 0 | 0.9768 | Ref |
| MG592599 | Unassigned | Unassigned | Unassigned | 774_0 | Clustered | 2 | VC_774_0 | 2 | 0.0723 | 0.95532983 | 0.0691 | 0 | 0 | 0 | 1 | Ref |
| MG592600 | Unassigned | Unassigned | Unassigned | 661_0 | Clustered | 7 | VC_661_0 | 7 | 0.15 | 1 | 0.15 | 0 | 0 | 0 | 0.7619 | Ref |
| MG592601 | Unassigned | Unassigned | Unassigned | 1091_1 | Clustered | 29 | VC_1091_1 | 29 | 0.4856 | 1 | 0.4856 | 0 | 0 | 0 | 0.9581 | Ref |
| MG592602 | Unassigned | Unassigned | Unassigned | 1091_1 | Clustered | 29 | VC_1091_1 | 29 | 0.4856 | 1 | 0.4856 | 0 | 0 | 0 | 0.9581 | Ref |
| MG592603 | Unassigned | Unassigned | Unassigned | 804_0 | Clustered | 3 | VC_804_0 | 3 | 0.2887 | 0.99856687 | 0.2883 | 0 | 0 | 0 | 1 | Ref |
| MG592604 | Unassigned | Unassigned | Unassigned | 804_0 | Clustered | 3 | VC_804_0 | 3 | 0.2887 | 0.99856687 | 0.2883 | 0 | 0 | 0 | 1 | Ref |
| MG592605 | Unassigned | Unassigned | Unassigned | 957_0 | Clustered | 34 | VC_957_0 | 34 | 0.8934 | 1 | 0.8934 | 0 | 0 | 0 | 0.9768 | Ref |
| MG592606 | Unassigned | Unassigned | Unassigned | 957_0 | Clustered | 34 | VC_957_0 | 34 | 0.8934 | 1 | 0.8934 | 0 | 0 | 0 | 0.9768 | Ref |
| MG592607 | Unassigned | Unassigned | Unassigned | 957_0 | Clustered | 34 | VC_957_0 | 34 | 0.8934 | 1 | 0.8934 | 0 | 0 | 0 | 0.9768 | Ref |

| | | | | | | | | | | | | | | | | |
|---|---|---|---|---|---|---|---|---|---|---|---|---|---|---|---|---|
| MG592608 | Unassigned | Unassigned | Unassigned | 665_0 | Clustered | 29 | VC_665_0 | 29 | 0.9121 | 1 | 0.9121 | 0 | 0 | 0 | 1 | Ref |
| MG592609 | Unassigned | Unassigned | Unassigned | 137_0 | Clustered | 2 | VC_137_0 | 2 | 0.0296 | 0.95557467 | 0.0283 | 0 | 0 | 0 | 1 | Ref |
| MG592610 | Unassigned | Unassigned | Unassigned | 804_0 | Clustered | 3 | VC_804_0 | 3 | 0.2887 | 0.99856687 | 0.2883 | 0 | 0 | 0 | 1 | Ref |
| MG592611 | Unassigned | Unassigned | Unassigned | 516_0 | Clustered | 24 | VC_516_0 | 24 | 0.8556 | 1 | 0.8556 | 0 | 0 | 0 | 0.9493 | Ref |
| MG592612 | Unassigned | Unassigned | Unassigned | 978_0 | Clustered | 6 | VC_978_0 | 6 | 0.8163 | 1 | 0.8163 | 0 | 0 | 0 | 1 | Ref |
| MG592613 | Unassigned | Unassigned | Unassigned | 978_0 | Clustered | 6 | VC_978_0 | 6 | 0.8163 | 1 | 0.8163 | 0 | 0 | 0 | 1 | Ref |
| MG592614 | Unassigned | Unassigned | Unassigned | 665_0 | Clustered | 29 | VC_665_0 | 29 | 0.9121 | 1 | 0.9121 | 0 | 0 | 0 | 1 | Ref |
| MG592617 | Unassigned | Unassigned | Unassigned | 1091_1 | Clustered | 29 | VC_1091_1 | 29 | 0.4856 | 1 | 0.4856 | 0 | 0 | 0 | 0.9581 | Ref |
| MG592618 | Unassigned | Unassigned | Unassigned | 1091_1 | Clustered | 29 | VC_1091_1 | 29 | 0.4856 | 1 | 0.4856 | 0 | 0 | 0 | 0.9581 | Ref |
| MG592619 | Unassigned | Unassigned | Unassigned | 774_0 | Clustered | 2 | VC_774_0 | 2 | 0.0723 | 0.95532983 | 0.0691 | 0 | 0 | 0 | 1 | Ref |
| MG592620 | Unassigned | Unassigned | Unassigned | 518_0 | Clustered | 16 | VC_518_0 | 16 | 0.9983 | 1 | 0.9983 | 0 | 0 | 0 | 1 | Ref |
| MG592621 | Unassigned | Unassigned | Unassigned | 137_0 | Clustered | 2 | VC_137_0 | 2 | 0.0296 | 0.95557467 | 0.0283 | 0 | 0 | 0 | 1 | Ref |
| MG592622 | Unassigned | Unassigned | Unassigned | 665_0 | Clustered | 29 | VC_665_0 | 29 | 0.9121 | 1 | 0.9121 | 0 | 0 | 0 | 1 | Ref |
| MG592623 | Unassigned | Unassigned | Unassigned | 518_0 | Clustered | 16 | VC_518_0 | 16 | 0.9983 | 1 | 0.9983 | 0 | 0 | 0 | 1 | Ref |
| MG592625 | Unassigned | Unassigned | Unassigned | 1133_0 | Clustered | 6 | VC_1133_0 | 6 | 0.9291 | 0.99999982 | 0.9291 | 0 | 0 | 0 | 1 | Ref |
| MG592626 | Unassigned | Unassigned | Unassigned | 548_0 | Clustered | 9 | VC_548_0 | 9 | 0.8018 | 1 | 0.8018 | 0 | 0 | 0 | 1 | Ref |
| MG592627 | Unassigned | Unassigned | Unassigned | 665_0 | Clustered | 29 | VC_665_0 | 29 | 0.9121 | 1 | 0.9121 | 0 | 0 | 0 | 1 | Ref |
| MG592628 | Unassigned | Unassigned | Unassigned | 665_0 | Clustered | 29 | VC_665_0 | 29 | 0.9121 | 1 | 0.9121 | 0 | 0 | 0 | 1 | Ref |
| MG592629 | Unassigned | Unassigned | Unassigned | 775_0 | Clustered | 18 | VC_775_0 | 18 | 0.7364 | 1 | 0.7364 | 0 | 0 | 0 | 1 | Ref |
| MG592630 | Unassigned | Unassigned | Unassigned | 661_0 | Clustered | 7 | VC_661_0 | 7 | 0.15 | 1 | 0.15 | 0 | 0 | 0 | 0.7619 | Ref |
| MG592631 | Unassigned | Unassigned | Unassigned | 957_0 | Clustered | 34 | VC_957_0 | 34 | 0.8934 | 1 | 0.8934 | 0 | 0 | 0 | 0.9768 | Ref |
| MG592632 | Unassigned | Unassigned | Unassigned | 1091_0 | Clustered | 24 | VC_1091_0 | 24 | 0.5254 | 1 | 0.5254 | 0 | 0 | 0 | 1 | Ref |
| MG592633 | Unassigned | Unassigned | Unassigned | 1091_0 | Clustered | 24 | VC_1091_0 | 24 | 0.5254 | 1 | 0.5254 | 0 | 0 | 0 | 1 | Ref |
| MG592634 | Unassigned | Unassigned | Unassigned | 1091_0 | Clustered | 24 | VC_1091_0 | 24 | 0.5254 | 1 | 0.5254 | 0 | 0 | 0 | 1 | Ref |
| MG592635 | Unassigned | Unassigned | Unassigned | 1091_0 | Clustered | 24 | VC_1091_0 | 24 | 0.5254 | 1 | 0.5254 | 0 | 0 | 0 | 1 | Ref |
| MG592636 | Unassigned | Unassigned | Unassigned | 1091_0 | Clustered | 24 | VC_1091_0 | 24 | 0.5254 | 1 | 0.5254 | 0 | 0 | 0 | 1 | Ref |
| MG592637 | Unassigned | Unassigned | Unassigned | 1091_0 | Clustered | 24 | VC_1091_0 | 24 | 0.5254 | 1 | 0.5254 | 0 | 0 | 0 | 1 | Ref |
| MG592638 | Unassigned | Unassigned | Unassigned | 1091_0 | Clustered | 24 | VC_1091_0 | 24 | 0.5254 | 1 | 0.5254 | 0 | 0 | 0 | 1 | Ref |
| MG592639 | Unassigned | Unassigned | Unassigned | 1091_0 | Clustered | 24 | VC_1091_0 | 24 | 0.5254 | 1 | 0.5254 | 0 | 0 | 0 | 1 | Ref |
| MG592640 | Unassigned | Unassigned | Unassigned | 1091_0 | Clustered | 24 | VC_1091_0 | 24 | 0.5254 | 1 | 0.5254 | 0 | 0 | 0 | 1 | Ref |
| MG592641 | Unassigned | Unassigned | Unassigned | 665_0 | Clustered | 29 | VC_665_0 | 29 | 0.9121 | 1 | 0.9121 | 0 | 0 | 0 | 1 | Ref |
| MG592642 | Unassigned | Unassigned | Unassigned | 1091_0 | Clustered | 24 | VC_1091_0 | 24 | 0.5254 | 1 | 0.5254 | 0 | 0 | 0 | 1 | Ref |
| MG592644 | Unassigned | Unassigned | Unassigned | 661_3 | Clustered | 9 | VC_661_3 | 9 | 0.2124 | 1 | 0.2124 | 0 | 0 | 0 | 0.6944 | Ref |
| MG592645 | Unassigned | Unassigned | Unassigned | 1091_0 | Clustered | 24 | VC_1091_0 | 24 | 0.5254 | 1 | 0.5254 | 0 | 0 | 0 | 1 | Ref |
| MG592646 | Unassigned | Unassigned | Unassigned | 1091_0 | Clustered | 24 | VC_1091_0 | 24 | 0.5254 | 1 | 0.5254 | 0 | 0 | 0 | 1 | Ref |
| MG592647 | Unassigned | Unassigned | Unassigned | 665_0 | Clustered | 29 | VC_665_0 | 29 | 0.9121 | 1 | 0.9121 | 0 | 0 | 0 | 1 | Ref |
| MG592648 | Unassigned | Unassigned | Unassigned | 1091_0 | Clustered | 24 | VC_1091_0 | 24 | 0.5254 | 1 | 0.5254 | 0 | 0 | 0 | 1 | Ref |
| MG592649 | Unassigned | Unassigned | Unassigned | 1091_0 | Clustered | 24 | VC_1091_0 | 24 | 0.5254 | 1 | 0.5254 | 0 | 0 | 0 | 1 | Ref |
| MG592650 | Unassigned | Unassigned | Unassigned | 1091_0 | Clustered | 24 | VC_1091_0 | 24 | 0.5254 | 1 | 0.5254 | 0 | 0 | 0 | 1 | Ref |
| MG592651 | Unassigned | Unassigned | Unassigned | 1091_0 | Clustered | 24 | VC_1091_0 | 24 | 0.5254 | 1 | 0.5254 | 0 | 0 | 0 | 1 | Ref |
| MG592652 | Unassigned | Unassigned | Unassigned | 1091_0 | Clustered | 24 | VC_1091_0 | 24 | 0.5254 | 1 | 0.5254 | 0 | 0 | 0 | 1 | Ref |
| MG592653 | Unassigned | Unassigned | Unassigned | 1091_0 | Clustered | 24 | VC_1091_0 | 24 | 0.5254 | 1 | 0.5254 | 0 | 0 | 0 | 1 | Ref |
| MG592654 | Unassigned | Unassigned | Unassigned | 981_0 | Clustered | 9 | VC_981_0 | 9 | 0.5418 | 1 | 0.5418 | 0 | 0 | 0 | 1 | Ref |
| MG592655 | Unassigned | Unassigned | Unassigned | 1091_1 | Clustered | 29 | VC_1091_1 | 29 | 0.4856 | 1 | 0.4856 | 0 | 0 | 0 | 0.9581 | Ref |
| MG592656 | Unassigned | Unassigned | Unassigned | 665_0 | Clustered | 29 | VC_665_0 | 29 | 0.9121 | 1 | 0.9121 | 0 | 0 | 0 | 1 | Ref |
| MG592657 | Unassigned | Unassigned | Unassigned | 981_0 | Clustered | 9 | VC_981_0 | 9 | 0.5418 | 1 | 0.5418 | 0 | 0 | 0 | 1 | Ref |
| MG592658 | Unassigned | Unassigned | Unassigned | 981_0 | Clustered | 9 | VC_981_0 | 9 | 0.5418 | 1 | 0.5418 | 0 | 0 | 0 | 1 | Ref |
| MG592659 | Unassigned | Unassigned | Unassigned | 981_0 | Clustered | 9 | VC_981_0 | 9 | 0.5418 | 1 | 0.5418 | 0 | 0 | 0 | 1 | Ref |
| MG592660 | Unassigned | Unassigned | Unassigned | 181_0 | Clustered | 4 | VC_181_0 | 4 | 0.3817 | 0.99998259 | 0.3817 | 0 | 0 | 0 | 1 | Ref |
| MG592661 | Unassigned | Unassigned | Unassigned | 1081_0 | Clustered | 9 | VC_1081_0 | 9 | 0.6523 | 1 | 0.6523 | 0 | 0 | 0 | 1 | Ref |
| MG592662 | Unassigned | Unassigned | Unassigned | 933_0 | Clustered | 3 | VC_933_0 | 3 | 0.9964 | 0.94670842 | 0.9433 | 0 | 0 | 0 | 1 | Ref |
| MG592664 | Unassigned | Unassigned | Unassigned | 661_5 | Clustered | 4 | VC_661_5 | 4 | 0.0894 | 0.99996664 | 0.0894 | 0 | 0 | 0 | 1 | Ref |
| MG592665 | Unassigned | Unassigned | Unassigned | 661_5 | Clustered | 4 | VC_661_5 | 4 | 0.0894 | 0.99996664 | 0.0894 | 0 | 0 | 0 | 1 | Ref |
| MG592666 | Unassigned | Unassigned | Unassigned | 851_0 | Clustered | 6 | VC_851_0 | 6 | 0.7471 | 1 | 0.7471 | 0 | 0 | 0 | 1 | Ref |
| MG592668 | Unassigned | Unassigned | Unassigned | 809_0 | Clustered | 7 | VC_809_0 | 7 | 0.5153 | 1 | 0.5153 | 0 | 0 | 0 | 1 | Ref |
| MG592669 | Unassigned | Unassigned | Unassigned | 255_0 | Clustered | 11 | VC_255_0 | 11 | 0.7034 | 1 | 0.7034 | 0 | 0 | 0 | 1 | Ref |
| MG592670 | Unassigned | Unassigned | Unassigned | 255_0 | Clustered | 11 | VC_255_0 | 11 | 0.7034 | 1 | 0.7034 | 0 | 0 | 0 | 1 | Ref |
| MG592671 | Unassigned | Unassigned | Unassigned | 170_9 | Clustered/Singleton | 1 | VC_170_9 | 1 | 0 | 1 | 0 | 0 | 0 | 0 | 1 | Ref |
| MG592672 | Unassigned | Unassigned | Unassigned | 933_0 | Clustered | 3 | VC_933_0 | 3 | 0.9964 | 0.94670842 | 0.9433 | 0 | 0 | 0 | 1 | Ref |
| MG593800 | Unassigned | Unassigned | Unassigned | 388_0 | Clustered | 3 | VC_388_0 | 3 | 0.2477 | 0.99807286 | 0.2473 | 0 | 0 | 0 | 1 | Ref |
| MG593802 | Unassigned | Unassigned | Unassigned | 1015_1 | Clustered | 26 | VC_1015_1 | 26 | 0.6989 | 1 | 0.6989 | 0 | 0 | 0 | 1 | Ref |
| MG593803 | Unassigned | Unassigned | Unassigned | 388_0 | Clustered | 3 | VC_388_0 | 3 | 0.2477 | 0.99807286 | 0.2473 | 0 | 0 | 0 | 1 | Ref |
| MG596799 | Unassigned | Unassigned | Unassigned | 1070_0 | Clustered | 3 | VC_1070_0 | 3 | 0.7872 | 0.99542416 | 0.7836 | 0 | 0 | 0 | 1 | Ref |
| MG596800 | Unassigned | Unassigned | Unassigned | 1000_0 | Clustered | 2 | VC_1000_0 | 2 | 0.2337 | 0.95692166 | 0.2237 | 0 | 0 | 0 | 1 | Ref |
| MG599035 | Unassigned | Unassigned | Unassigned | 714_0 | Clustered | 71 | VC_714_0 | 71 | 0.935 | 1 | 0.935 | 0 | 0 | 0 | 1 | Ref |
| MG602476 | Unassigned | Unassigned | Unassigned | 911_0 | Clustered | 20 | VC_911_0 | 20 | 0.9915 | 1 | 0.9915 | 0 | 0 | 0 | 1 | Ref |
| MG602477 | Unassigned | Unassigned | Unassigned | 195_3 | Clustered | 9 | VC_195_3 | 9 | 0.1955 | 1 | 0.1955 | 0 | 0 | 0 | 0.8611 | Ref |
| MG640035 | Unassigned | Unassigned | Unassigned | 941_0 | Clustered | 5 | VC_941_0 | 5 | 0.7412 | 0.99999276 | 0.7412 | 0 | 0 | 0 | 1 | Ref |
| MG641179 | Unassigned | Unassigned | Unassigned | 1145_0 | Clustered | 164 | VC_1145_0 | 164 | 0.8868 | 1 | 0.8868 | 0 | 0 | 0 | 1 | Ref |
| MG641180 | Unassigned | Unassigned | Unassigned | 1145_0 | Clustered | 164 | VC_1145_0 | 164 | 0.8868 | 1 | 0.8868 | 0 | 0 | 0 | 1 | Ref |
| MG641183 | Unassigned | Unassigned | Unassigned | 205_0 | Clustered | 5 | VC_205_0 | 5 | 0.1325 | 0.84698401 | 0.1122 | 0 | 0 | 0 | 1 | Ref |
| MG641184 | Unassigned | Unassigned | Unassigned | 1174_0 | Clustered | 4 | VC_1174_0 | 4 | 0.1295 | 0.97665214 | 0.1265 | 0 | 0 | 0 | 1 | Ref |
| MG641185 | Unassigned | Unassigned | Unassigned | 1285_0 | Clustered | 3 | VC_1285_0 | 3 | 0.7418 | 0.80676188 | 0.5984 | 0 | 0 | 0 | 1 | Ref |
| MG641186 | Unassigned | Unassigned | Unassigned | 1145_0 | Clustered | 164 | VC_1145_0 | 164 | 0.8868 | 1 | 0.8868 | 0 | 0 | 0 | 1 | Ref |
| MG641188 | Unassigned | Unassigned | Unassigned | 1286_0 | Clustered | 16 | VC_1286_0 | 16 | 0.9448 | 1 | 0.9448 | 0 | 0 | 0 | 1 | Ref |
| MG646668 | Unassigned | Unassigned | Unassigned | 654_0 | Clustered | 115 | VC_654_0 | 115 | 0.7303 | 1 | 0.7303 | 0 | 0 | 0 | 0.8764 | Ref |
| MG646669 | Unassigned | Unassigned | Unassigned | 340_11 | Clustered | 15 | VC_340_11 | 15 | 0.079 | 1 | 0.079 | 0 | 0 | 0 | 0.9333 | Ref |
| MG646670 | Unassigned | Unassigned | Unassigned | 340_11 | Clustered | 15 | VC_340_11 | 15 | 0.079 | 1 | 0.079 | 0 | 0 | 0 | 0.9333 | Ref |
| MG646671 | Unassigned | Unassigned | Unassigned | 340_11 | Clustered | 15 | VC_340_11 | 15 | 0.079 | 1 | 0.079 | 0 | 0 | 0 | 0.9333 | Ref |
| MG646672 | Unassigned | Unassigned | Unassigned | 340_0 | Clustered | 2 | VC_340_0 | 2 | 0.0051 | 0.9546458 | 0.0048 | 0 | 0 | 0 | 1 | Ref |
| MG649966 | Unassigned | Unassigned | Unassigned | 1007_1 | Clustered | 24 | VC_1007_1 | 24 | 0.7364 | 1 | 0.7364 | 0 | 0 | 0 | 0.8877 | Ref |

| | | | | | | | | | | | | | | | | |
|---|---|---|---|---|---|---|---|---|---|---|---|---|---|---|---|---|
| MG649967 | Unassigned | Unassigned | Unassigned | 1007_1 | Clustered | 24 | VC_1007_1 | 24 | 0.7364 | 1 | 0.7364 | 0 | 0 | 0 | 0.8877 | Ref |
| MG655267 | Unassigned | Unassigned | Unassigned | 507_1 | Clustered | 11 | VC_507_1 | 11 | 0.4612 | 1 | 0.4612 | 0 | 0 | 0 | 1 | Ref |
| MG655268 | Unassigned | Unassigned | Unassigned | 507_1 | Clustered | 11 | VC_507_1 | 11 | 0.4612 | 1 | 0.4612 | 0 | 0 | 0 | 1 | Ref |
| MG655269 | Unassigned | Unassigned | Unassigned | 507_1 | Clustered | 11 | VC_507_1 | 11 | 0.4612 | 1 | 0.4612 | 0 | 0 | 0 | 1 | Ref |
| MG655270 | Unassigned | Unassigned | Unassigned | 507_1 | Clustered | 11 | VC_507_1 | 11 | 0.4612 | 1 | 0.4612 | 0 | 0 | 0 | 1 | Ref |
| MG656408 | Unassigned | Unassigned | Unassigned | 155_8 | Clustered | 66 | VC_155_8 | 66 | 0.4537 | 1 | 0.4537 | 0 | 0 | 0 | 0.8182 | Ref |
| MG663582 | Unassigned | Unassigned | Unassigned | 1278_0 | Clustered | 19 | VC_1278_0 | 19 | 1 | 1 | 1 | 0 | 0 | 0 | 1 | Ref |
| MG663583 | Unassigned | Unassigned | Unassigned | 1278_0 | Clustered | 19 | VC_1278_0 | 19 | 1 | 1 | 1 | 0 | 0 | 0 | 1 | Ref |
| MG663584 | Unassigned | Unassigned | Unassigned | 1278_0 | Clustered | 19 | VC_1278_0 | 19 | 1 | 1 | 1 | 0 | 0 | 0 | 1 | Ref |
| MG663585 | Unassigned | Unassigned | Unassigned | 1278_0 | Clustered | 19 | VC_1278_0 | 19 | 1 | 1 | 1 | 0 | 0 | 0 | 1 | Ref |
| MG663586 | Unassigned | Unassigned | Unassigned | 1278_0 | Clustered | 19 | VC_1278_0 | 19 | 1 | 1 | 1 | 0 | 0 | 0 | 1 | Ref |
| MG670586 | Unassigned | Unassigned | Unassigned | 1287_0 | Clustered | 44 | VC_1287_0 | 44 | 1 | 1 | 1 | 0 | 0 | 0 | 1 | Ref |
| MG673519 | Unassigned | Unassigned | Unassigned | 837_1 | Clustered | 24 | VC_837_1 | 24 | 0.5328 | 1 | 0.5328 | 0 | 0 | 0 | 1 | Ref |
| MG674163 | Unassigned | Unassigned | Unassigned | 628_0 | Clustered | 5 | VC_628_0 | 5 | 0.4297 | 0.99999996 | 0.4297 | 0 | 0 | 0 | 1 | Ref |
| MG676223 | Unassigned | Unassigned | Unassigned | 661_2 | Clustered | 6 | VC_661_2 | 6 | 0.1674 | 1 | 0.1674 | 0 | 0 | 0 | 1 | Ref |
| MG676224 | Unassigned | Unassigned | Unassigned | 203_0 | Clustered | 2 | VC_203_0 | 2 | 0.117 | 0.95681582 | 0.112 | 0 | 0 | 0 | 1 | Ref |
| MG676225 | Unassigned | Unassigned | Unassigned | 203_0 | Clustered | 2 | VC_203_0 | 2 | 0.117 | 0.95681582 | 0.112 | 0 | 0 | 0 | 1 | Ref |
| MG676466 | Unassigned | Unassigned | Unassigned | 772_0 | Clustered | 9 | VC_772_0 | 9 | 0.4468 | 1 | 0.4468 | 0 | 0 | 0 | 1 | Ref |
| MG696114 | Unassigned | Unassigned | Unassigned | 295_2 | Clustered | 2 | VC_295_2 | 2 | 0.0224 | 0.95539992 | 0.0214 | 0 | 0 | 0 | 1 | Ref |
| MG707188 | Unassigned | Unassigned | Unassigned | 179_0 | Clustered | 2 | VC_179_0 | 2 | 0.0795 | 0.95489091 | 0.0759 | 0 | 0 | 0 | 1 | Ref |
| MG708273 | Unassigned | Unassigned | Unassigned | 258_0 | Clustered | 121 | VC_258_0 | 121 | 0.7359 | 1 | 0.7359 | 0 | 0 | 0 | 0.9999 | Ref |
| MG708274 | Unassigned | Unassigned | Unassigned | 258_0 | Clustered | 121 | VC_258_0 | 121 | 0.7359 | 1 | 0.7359 | 0 | 0 | 0 | 0.9999 | Ref |
| MG708275 | Unassigned | Unassigned | Unassigned | 258_0 | Clustered | 121 | VC_258_0 | 121 | 0.7359 | 1 | 0.7359 | 0 | 0 | 0 | 0.9999 | Ref |
| MG708276 | Unassigned | Unassigned | Unassigned | 711_0 | Clustered | 48 | VC_711_0 | 48 | 0.8217 | 1 | 0.8217 | 0 | 0 | 0 | 1 | Ref |
| MG710528 | Unassigned | Unassigned | Unassigned | 44_0 | Clustered | 49 | VC_44_0 | 49 | 0.4506 | 1 | 0.4506 | 0 | 0 | 0 | 0.8605 | Ref |
| MG711460 | Unassigned | Unassigned | Unassigned | 1288_0 | Clustered | 7 | VC_1288_0 | 7 | 0.9846 | 0.99997693 | 0.9846 | 0 | 0 | 0 | 1 | Ref |
| MG711461 | Unassigned | Unassigned | Unassigned | 727_0 | Clustered | 8 | VC_727_0 | 8 | 0.6106 | 1 | 0.6106 | 0 | 0 | 0 | 0.9286 | Ref |
| MG711462 | Unassigned | Unassigned | Unassigned | 723_0 | Clustered | 20 | VC_723_0 | 20 | 0.7344 | 1 | 0.7344 | 0 | 0 | 0 | 0.7 | Ref |
| MG711466 | Unassigned | Unassigned | Unassigned | 728_0 | Clustered | 7 | VC_728_0 | 7 | 0.2344 | 1 | 0.2344 | 0 | 0 | 0 | 0.8095 | Ref |
| MG720309 | Unassigned | Unassigned | Unassigned | 935_0 | Clustered | 6 | VC_935_0 | 6 | 0.2317 | 1 | 0.2317 | 0 | 0 | 0 | 1 | Ref |
| MG721208 | Unassigned | Unassigned | Unassigned | 155_21 | Clustered/Singleton | 1 | VC_155_21 | 1 | 0 | 1 | 0 | 0 | 0 | 0 | 1 | Ref |
| MG727695 | Unassigned | Unassigned | Unassigned | 725_0 | Clustered | 47 | VC_725_0 | 47 | 0.8649 | 1 | 0.8649 | 0 | 0 | 0 | 1 | Ref |
| MG727696 | Unassigned | Unassigned | Unassigned | 725_0 | Clustered | 47 | VC_725_0 | 47 | 0.8649 | 1 | 0.8649 | 0 | 0 | 0 | 1 | Ref |
| MG727698 | Unassigned | Unassigned | Unassigned | 725_0 | Clustered | 47 | VC_725_0 | 47 | 0.8649 | 1 | 0.8649 | 0 | 0 | 0 | 1 | Ref |
| MG727699 | Unassigned | Unassigned | Unassigned | 725_0 | Clustered | 47 | VC_725_0 | 47 | 0.8649 | 1 | 0.8649 | 0 | 0 | 0 | 1 | Ref |
| MG727700 | Unassigned | Unassigned | Unassigned | 725_0 | Clustered | 47 | VC_725_0 | 47 | 0.8649 | 1 | 0.8649 | 0 | 0 | 0 | 1 | Ref |
| MG727701 | Unassigned | Unassigned | Unassigned | 725_0 | Clustered | 47 | VC_725_0 | 47 | 0.8649 | 1 | 0.8649 | 0 | 0 | 0 | 1 | Ref |
| MG727702 | Unassigned | Unassigned | Unassigned | 725_0 | Clustered | 47 | VC_725_0 | 47 | 0.8649 | 1 | 0.8649 | 0 | 0 | 0 | 1 | Ref |
| MG736918 | Unassigned | Unassigned | Unassigned | 467_0 | Clustered | 6 | VC_467_0 | 6 | 0.1136 | 1 | 0.1136 | 0 | 0 | 0 | 1 | Ref |
| MG744354 | Unassigned | Unassigned | Unassigned | 650_1 | Clustered | 6 | VC_650_1 | 6 | 0.4186 | 1 | 0.4186 | 0 | 0 | 0 | 1 | Ref |
| MG747435 | Unassigned | Unassigned | Unassigned | 1_0 | Clustered | 54 | VC_1_0 | 54 | 0.8518 | 1 | 0.8518 | 0 | 0 | 0 | 1 | Ref |
| MG748547 | Unassigned | Unassigned | Unassigned | 135_5 | Clustered/Singleton | 1 | VC_135_5 | 1 | 0 | 1 | 0 | 0 | 0 | 0 | 1 | Ref |
| MG748548 | Unassigned | Unassigned | Unassigned | 837_1 | Clustered | 24 | VC_837_1 | 24 | 0.5328 | 1 | 0.5328 | 0 | 0 | 0 | 1 | Ref |
| MG751100 | Unassigned | Unassigned | Unassigned | 509_0 | Clustered | 36 | VC_509_0 | 36 | 0.5291 | 1 | 0.5291 | 0 | 0 | 0 | 0.9714 | Ref |
| MG757152 | Unassigned | Unassigned | Unassigned | 895_0 | Clustered | 25 | VC_895_0 | 25 | 0.5768 | 1 | 0.5768 | 0 | 0 | 0 | 1 | Ref |
| MG757153 | Unassigned | Unassigned | Unassigned | 1115_0 | Clustered | 2 | VC_1115_0 | 2 | 0.0262 | 0.95117088 | 0.0249 | 0 | 0 | 0 | 1 | Ref |
| MG757154 | Unassigned | Unassigned | Unassigned | 901_0 | Clustered | 31 | VC_901_0 | 31 | 0.8235 | 1 | 0.8235 | 0 | 0 | 0 | 0.8194 | Ref |
| MG757155 | Unassigned | Unassigned | Unassigned | 895_0 | Clustered | 25 | VC_895_0 | 25 | 0.5768 | 1 | 0.5768 | 0 | 0 | 0 | 1 | Ref |
| MG757156 | Unassigned | Unassigned | Unassigned | 591_0 | Clustered | 4 | VC_591_0 | 4 | 0.1897 | 0.9999859 | 0.1897 | 0 | 0 | 0 | 1 | Ref |
| MG757157 | Unassigned | Unassigned | Unassigned | 789_0 | Clustered | 34 | VC_789_0 | 34 | 0.9167 | 1 | 0.9167 | 0 | 0 | 0 | 1 | Ref |
| MG757158 | Unassigned | Unassigned | Unassigned | 384_16 | Clustered | 42 | VC_384_16 | 42 | 0.134 | 1 | 0.134 | 0 | 0 | 0 | 0.7282 | Ref |
| MG757159 | Unassigned | Unassigned | Unassigned | 384_11 | Clustered | 31 | VC_384_11 | 31 | 0.0926 | 1 | 0.0926 | 0 | 0 | 0 | 0.7011 | Ref |
| MG757160 | Unassigned | Unassigned | Unassigned | 571_6 | Clustered | 22 | VC_571_6 | 22 | 0.1207 | 1 | 0.1207 | 0 | 0 | 0 | 0.8095 | Ref |
| MG757161 | Unassigned | Unassigned | Unassigned | 532_0 | Clustered | 75 | VC_532_0 | 75 | 0.9393 | 1 | 0.9393 | 0 | 0 | 0 | 1 | Ref |
| MG757162 | Unassigned | Unassigned | Unassigned | 526_0 | Clustered | 32 | VC_526_0 | 32 | 0.9037 | 1 | 0.9037 | 0 | 0 | 0 | 1 | Ref |
| MG757163 | Unassigned | Unassigned | Unassigned | 1016_0 | Clustered | 62 | VC_1016_0 | 62 | 0.8638 | 1 | 0.8638 | 0 | 0 | 0 | 0.743 | Ref |
| MG757164 | Unassigned | Unassigned | Unassigned | 384_13 | Clustered | 33 | VC_384_13 | 33 | 0.1785 | 1 | 0.1785 | 0 | 0 | 0 | 0.8769 | Ref |
| MG757165 | Unassigned | Unassigned | Unassigned | 384_13 | Clustered | 33 | VC_384_13 | 33 | 0.1785 | 1 | 0.1785 | 0 | 0 | 0 | 0.8769 | Ref |
| MG757166 | Unassigned | Unassigned | Unassigned | 789_0 | Clustered | 34 | VC_789_0 | 34 | 0.9167 | 1 | 0.9167 | 0 | 0 | 0 | 1 | Ref |
| MG757167 | Unassigned | Unassigned | Unassigned | 973_0 | Clustered | 15 | VC_973_0 | 15 | 1 | 0 | 0 | 0 | 0 | 0 | 1 | Ref |
| MG763894 | Unassigned | Unassigned | Unassigned | 913_0 | Clustered | 28 | VC_913_0 | 28 | 0.6206 | 1 | 0.6206 | 0 | 0 | 0 | 0.9841 | Ref |
| MG765274 | Unassigned | Unassigned | Unassigned | 650_1 | Clustered | 6 | VC_650_1 | 6 | 0.4186 | 1 | 0.4186 | 0 | 0 | 0 | 1 | Ref |
| MG765275 | Unassigned | Unassigned | Unassigned | 597_0 | Clustered | 6 | VC_597_0 | 6 | 0.9222 | 0.99999993 | 0.9222 | 0 | 0 | 0 | 1 | Ref |
| MG765276 | Unassigned | Unassigned | Unassigned | 650_0 | Clustered | 3 | VC_650_0 | 3 | 0.2008 | 0.99838296 | 0.2005 | 0 | 0 | 0 | 1 | Ref |
| MG765277 | Unassigned | Unassigned | Unassigned | 544_0 | Clustered | 6 | VC_544_0 | 6 | 0.4346 | 1 | 0.4346 | 0 | 0 | 0 | 1 | Ref |
| MG765278 | Unassigned | Unassigned | Unassigned | 597_0 | Clustered | 6 | VC_597_0 | 6 | 0.9222 | 0.99999993 | 0.9222 | 0 | 0 | 0 | 1 | Ref |
| MG765279 | Unassigned | Unassigned | Unassigned | 544_0 | Clustered | 6 | VC_544_0 | 6 | 0.4346 | 1 | 0.4346 | 0 | 0 | 0 | 1 | Ref |
| MG766218 | Unassigned | Unassigned | Unassigned | 98_0 | Clustered | 30 | VC_98_0 | 30 | 0.7175 | 1 | 0.7175 | 0 | 0 | 0 | 1 | Ref |
| MG766219 | Unassigned | Unassigned | Unassigned | 98_0 | Clustered | 30 | VC_98_0 | 30 | 0.7175 | 1 | 0.7175 | 0 | 0 | 0 | 1 | Ref |
| MG770211 | Unassigned | Unassigned | Unassigned | 895_0 | Clustered | 25 | VC_895_0 | 25 | 0.5768 | 1 | 0.5768 | 0 | 0 | 0 | 1 | Ref |
| MG770212 | Unassigned | Unassigned | Unassigned | 384_15 | Clustered | 44 | VC_384_15 | 44 | 0.139 | 1 | 0.139 | 0 | 0 | 0 | 0.7474 | Ref |
| MG770214 | Unassigned | Unassigned | Unassigned | 895_0 | Clustered | 25 | VC_895_0 | 25 | 0.5768 | 1 | 0.5768 | 0 | 0 | 0 | 1 | Ref |
| MG770215 | Unassigned | Unassigned | Unassigned | 952_0 | Clustered | 39 | VC_952_0 | 39 | 0.9064 | 1 | 0.9064 | 0 | 0 | 0 | 1 | Ref |
| MG770216 | Unassigned | Unassigned | Unassigned | 833_0 | Clustered | 2 | VC_833_0 | 2 | 0.1786 | 0.96299906 | 0.172 | 0 | 0 | 0 | 1 | Ref |
| MG770228 | Unassigned | Unassigned | Unassigned | 801_0 | Clustered | 37 | VC_801_0 | 37 | 0.5672 | 1 | 0.5672 | 0 | 0 | 0 | 1 | Ref |
| MG770379 | Unassigned | Unassigned | Unassigned | 134_2 | Clustered | 17 | VC_134_2 | 17 | 0.1715 | 1 | 0.1715 | 0 | 0 | 0 | 0.9853 | Ref |
| MG770897 | Unassigned | Unassigned | Unassigned | 8_0 | Clustered | 58 | VC_8_0 | 58 | 0.8285 | 1 | 0.8285 | 0 | 0 | 0 | 0.9946 | Ref |
| MG775042 | Unassigned | Unassigned | Unassigned | 466_0 | Clustered | 66 | VC_466_0 | 66 | 0.9246 | 1 | 0.9246 | 0 | 0 | 0 | 1 | Ref |
| MG775043 | Unassigned | Unassigned | Unassigned | 290_24 | Clustered/Singleton | 1 | VC_290_24 | 1 | 0 | 1 | 0 | 0 | 0 | 0 | 1 | Ref |
| MG775258 | Unassigned | Unassigned | Unassigned | 131_0 | Clustered | 43 | VC_131_0 | 43 | 0.8853 | 1 | 0.8853 | 0 | 0 | 0 | 1 | Ref |
| MG775260 | Unassigned | Unassigned | Unassigned | 866_0 | Clustered | 2 | VC_866_0 | 2 | 0.0566 | 0.95560116 | 0.0541 | 0 | 0 | 0 | 1 | Ref |

| | | | | | | | | | | | | | | | | |
|---|---|---|---|---|---|---|---|---|---|---|---|---|---|---|---|---|
| MG781190 | Unassigned | Unassigned | Unassigned | 290_89 | Clustered | 2 | VC_290_89 | 2 | 0.0027 | 0.91112556 | 0.0024 | 0 | 0 | 0 | 1 | Ref |
| MG781191 | Unassigned | Unassigned | Unassigned | 290_89 | Clustered | 2 | VC_290_89 | 2 | 0.0027 | 0.91112556 | 0.0024 | 0 | 0 | 0 | 1 | Ref |
| MG784342 | Unassigned | Unassigned | Unassigned | 852_0 | Clustered | 3 | VC_852_0 | 3 | 0.4886 | 0.99843584 | 0.4878 | 0 | 0 | 0 | 1 | Ref |
| MG788324 | Unassigned | Unassigned | Unassigned | 307_1 | Clustered/Singleton | 1 | VC_307_1 | 1 | 0 | 1 | 0 | 0 | 0 | 0 | 1 | Ref |
| MG793454 | Unassigned | Unassigned | Unassigned | 226_0 | Clustered | 5 | VC_226_0 | 5 | 0.0793 | 0.99999998 | 0.0793 | 0 | 0 | 0 | 1 | Ref |
| MG812486 | Unassigned | Unassigned | Unassigned | 335_52 | Clustered | 4 | VC_335_52 | 4 | 0.0181 | 0.99998785 | 0.0181 | 0 | 0 | 0 | 1 | Ref |
| MG812487 | Unassigned | Unassigned | Unassigned | 1136_0 | Clustered | 40 | VC_1136_0 | 40 | 0.8746 | 1 | 0.8746 | 0 | 0 | 0 | 1 | Ref |
| MG812488 | Unassigned | Unassigned | Unassigned | 538_98 | Clustered/Singleton | 1 | VC_538_98 | 1 | 0 | 1 | 0 | 0 | 0 | 0 | 1 | Ref |
| MG812489 | Unassigned | Unassigned | Unassigned | 335_52 | Clustered | 4 | VC_335_52 | 4 | 0.0181 | 0.99998785 | 0.0181 | 0 | 0 | 0 | 1 | Ref |
| MG812490 | Unassigned | Unassigned | Unassigned | 526_0 | Clustered | 32 | VC_526_0 | 32 | 0.9037 | 1 | 0.9037 | 0 | 0 | 0 | 1 | Ref |
| MG812491 | Unassigned | Unassigned | Unassigned | 526_0 | Clustered | 32 | VC_526_0 | 32 | 0.9037 | 1 | 0.9037 | 0 | 0 | 0 | 1 | Ref |
| MG812492 | Unassigned | Unassigned | Unassigned | 531_0 | Clustered | 32 | VC_531_0 | 32 | 0.6859 | 1 | 0.6859 | 0 | 0 | 0 | 1 | Ref |
| MG812493 | Unassigned | Unassigned | Unassigned | 579_72 | Clustered/Singleton | 1 | VC_579_72 | 1 | 0 | 1 | 0 | 0 | 0 | 0 | 1 | Ref |
| MG812494 | Unassigned | Unassigned | Unassigned | 579_68 | Clustered/Singleton | 1 | VC_579_68 | 1 | 0 | 1 | 0 | 0 | 0 | 0 | 1 | Ref |
| MG812495 | Unassigned | Unassigned | Unassigned | 335_52 | Clustered | 4 | VC_335_52 | 4 | 0.0181 | 0.99998785 | 0.0181 | 0 | 0 | 0 | 1 | Ref |
| MG812496 | Unassigned | Unassigned | Unassigned | 984_0 | Clustered | 5 | VC_984_0 | 5 | 0.1708 | 0.99999984 | 0.1708 | 0 | 0 | 0 | 1 | Ref |
| MG820632 | Unassigned | Unassigned | Unassigned | 787_0 | Clustered | 132 | VC_787_0 | 132 | 1 | 1 | 1 | 0 | 0 | 0 | 1 | Ref |
| MG820633 | Unassigned | Unassigned | Unassigned | 787_0 | Clustered | 132 | VC_787_0 | 132 | 1 | 1 | 1 | 0 | 0 | 0 | 1 | Ref |
| MG820634 | Unassigned | Unassigned | Unassigned | 787_0 | Clustered | 132 | VC_787_0 | 132 | 1 | 1 | 1 | 0 | 0 | 0 | 1 | Ref |
| MG820635 | Unassigned | Unassigned | Unassigned | 787_0 | Clustered | 132 | VC_787_0 | 132 | 1 | 1 | 1 | 0 | 0 | 0 | 1 | Ref |
| MG820636 | Unassigned | Unassigned | Unassigned | 787_0 | Clustered | 132 | VC_787_0 | 132 | 1 | 1 | 1 | 0 | 0 | 0 | 1 | Ref |
| MG820637 | Unassigned | Unassigned | Unassigned | 787_0 | Clustered | 132 | VC_787_0 | 132 | 1 | 1 | 1 | 0 | 0 | 0 | 1 | Ref |
| MG820638 | Unassigned | Unassigned | Unassigned | 787_0 | Clustered | 132 | VC_787_0 | 132 | 1 | 1 | 1 | 0 | 0 | 0 | 1 | Ref |
| MG820639 | Unassigned | Unassigned | Unassigned | 787_0 | Clustered | 132 | VC_787_0 | 132 | 1 | 1 | 1 | 0 | 0 | 0 | 1 | Ref |
| MG820640 | Unassigned | Unassigned | Unassigned | 787_0 | Clustered | 132 | VC_787_0 | 132 | 1 | 1 | 1 | 0 | 0 | 0 | 1 | Ref |
| MG820641 | Unassigned | Unassigned | Unassigned | 787_0 | Clustered | 132 | VC_787_0 | 132 | 1 | 1 | 1 | 0 | 0 | 0 | 1 | Ref |
| MG820642 | Unassigned | Unassigned | Unassigned | 787_0 | Clustered | 132 | VC_787_0 | 132 | 1 | 1 | 1 | 0 | 0 | 0 | 1 | Ref |
| MG820643 | Unassigned | Unassigned | Unassigned | 787_0 | Clustered | 132 | VC_787_0 | 132 | 1 | 1 | 1 | 0 | 0 | 0 | 1 | Ref |
| MG820644 | Unassigned | Unassigned | Unassigned | 787_0 | Clustered | 132 | VC_787_0 | 132 | 1 | 1 | 1 | 0 | 0 | 0 | 1 | Ref |
| MG820645 | Unassigned | Unassigned | Unassigned | 787_0 | Clustered | 132 | VC_787_0 | 132 | 1 | 1 | 1 | 0 | 0 | 0 | 1 | Ref |
| MG832642 | Unassigned | Unassigned | Unassigned | 405_0 | Clustered | 126 | VC_405_0 | 126 | 0.9614 | 1 | 0.9614 | 0 | 0 | 0 | 1 | Ref |
| MG833025 | Unassigned | Unassigned | Unassigned | 405_0 | Clustered | 126 | VC_405_0 | 126 | 0.9614 | 1 | 0.9614 | 0 | 0 | 0 | 1 | Ref |
| MG835568 | Unassigned | Unassigned | Unassigned | 848_0 | Clustered | 124 | VC_848_0 | 124 | 0.9484 | 1 | 0.9484 | 0 | 0 | 0 | 1 | Ref |
| MG839014 | Unassigned | Unassigned | Unassigned | 527_0 | Clustered | 37 | VC_527_0 | 37 | 0.9679 | 1 | 0.9679 | 0 | 0 | 0 | 1 | Ref |
| MG839015 | Unassigned | Unassigned | Unassigned | 1296_0 | Clustered | 82 | VC_1296_0 | 82 | 0.7938 | 1 | 0.7938 | 0 | 0 | 0 | 1 | Ref |
| MG839016 | Unassigned | Unassigned | Unassigned | 1296_0 | Clustered | 82 | VC_1296_0 | 82 | 0.7938 | 1 | 0.7938 | 0 | 0 | 0 | 1 | Ref |
| MG839017 | Unassigned | Unassigned | Unassigned | 1296_0 | Clustered | 82 | VC_1296_0 | 82 | 0.7938 | 1 | 0.7938 | 0 | 0 | 0 | 1 | Ref |
| MG839018 | Unassigned | Unassigned | Unassigned | 1296_0 | Clustered | 82 | VC_1296_0 | 82 | 0.7938 | 1 | 0.7938 | 0 | 0 | 0 | 1 | Ref |
| MG839020 | Unassigned | Unassigned | Unassigned | 1296_0 | Clustered | 82 | VC_1296_0 | 82 | 0.7938 | 1 | 0.7938 | 0 | 0 | 0 | 1 | Ref |
| MG839021 | Unassigned | Unassigned | Unassigned | 1296_0 | Clustered | 82 | VC_1296_0 | 82 | 0.7938 | 1 | 0.7938 | 0 | 0 | 0 | 1 | Ref |
| MG839022 | Unassigned | Unassigned | Unassigned | 1296_0 | Clustered | 82 | VC_1296_0 | 82 | 0.7938 | 1 | 0.7938 | 0 | 0 | 0 | 1 | Ref |
| MG839023 | Unassigned | Unassigned | Unassigned | 1296_0 | Clustered | 82 | VC_1296_0 | 82 | 0.7938 | 1 | 0.7938 | 0 | 0 | 0 | 1 | Ref |
| MG839026 | Unassigned | Unassigned | Unassigned | 1296_0 | Clustered | 82 | VC_1296_0 | 82 | 0.7938 | 1 | 0.7938 | 0 | 0 | 0 | 1 | Ref |
| MG839027 | Unassigned | Unassigned | Unassigned | 1203_0 | Clustered | 28 | VC_1203_0 | 28 | 0.4905 | 1 | 0.4905 | 0 | 0 | 0 | 1 | Ref |
| MG839028 | Unassigned | Unassigned | Unassigned | 1296_0 | Clustered | 82 | VC_1296_0 | 82 | 0.7938 | 1 | 0.7938 | 0 | 0 | 0 | 1 | Ref |
| MG839029 | Unassigned | Unassigned | Unassigned | 1296_0 | Clustered | 82 | VC_1296_0 | 82 | 0.7938 | 1 | 0.7938 | 0 | 0 | 0 | 1 | Ref |
| MG845684 | Unassigned | Unassigned | Unassigned | 486_0 | Clustered | 47 | VC_486_0 | 47 | 0.9022 | 1 | 0.9022 | 0 | 0 | 0 | 1 | Ref |
| MG845865 | Unassigned | Unassigned | Unassigned | 465_0 | Clustered | 80 | VC_465_0 | 80 | 0.9988 | 1 | 0.9988 | 0 | 0 | 0 | 1 | Ref |
| MG852086 | Unassigned | Unassigned | Unassigned | 655_0 | Clustered | 45 | VC_655_0 | 45 | 0.7722 | 1 | 0.7722 | 0 | 0 | 0 | 1 | Ref |
| MG867727 | Unassigned | Unassigned | Unassigned | 290_138 | Clustered/Singleton | 1 | VC_290_138 | 1 | 0 | 1 | 0 | 0 | 0 | 0 | 1 | Ref |
| MG872831 | Unassigned | Unassigned | Unassigned | 571_0 | Clustered | 16 | VC_571_0 | 16 | 0.091 | 1 | 0.091 | 0 | 0 | 0 | 0.6667 | Ref |
| MG872832 | Unassigned | Unassigned | Unassigned | 571_0 | Clustered | 16 | VC_571_0 | 16 | 0.091 | 1 | 0.091 | 0 | 0 | 0 | 0.6667 | Ref |
| MG872833 | Unassigned | Unassigned | Unassigned | 335_5 | Clustered | 11 | VC_335_5 | 11 | 0.0469 | 1 | 0.0469 | 0 | 0 | 0 | 0.7636 | Ref |
| MG872834 | Unassigned | Unassigned | Unassigned | 701_0 | Clustered | 16 | VC_701_0 | 16 | 0.2957 | 1 | 0.2957 | 0 | 0 | 0 | 0.9167 | Ref |
| MG872835 | Unassigned | Unassigned | Unassigned | 232_0 | Clustered | 32 | VC_232_0 | 32 | 0.8471 | 1 | 0.8471 | 0 | 0 | 0 | 0.996 | Ref |
| MG872836 | Unassigned | Unassigned | Unassigned | 335_52 | Clustered | 4 | VC_335_52 | 4 | 0.0181 | 0.99998785 | 0.0181 | 0 | 0 | 0 | 1 | Ref |
| MG872837 | Unassigned | Unassigned | Unassigned | 571_6 | Clustered | 22 | VC_571_6 | 22 | 0.1207 | 1 | 0.1207 | 0 | 0 | 0 | 0.8095 | Ref |
| MG872838 | Unassigned | Unassigned | Unassigned | 577_0 | Clustered | 21 | VC_577_0 | 21 | 0.9908 | 1 | 0.9908 | 0 | 0 | 0 | 1 | Ref |
| MG872839 | Unassigned | Unassigned | Unassigned | 579_14 | Clustered | 8 | VC_579_14 | 8 | 0.0227 | 0.99904117 | 0.0227 | 0 | 0 | 0 | 0.8214 | Ref |
| MG872840 | Unassigned | Unassigned | Unassigned | 232_0 | Clustered | 32 | VC_232_0 | 32 | 0.8471 | 1 | 0.8471 | 0 | 0 | 0 | 0.996 | Ref |
| MG872841 | Unassigned | Unassigned | Unassigned | 538_45 | Clustered | 7 | VC_538_45 | 7 | 0.0632 | 1 | 0.0632 | 0 | 0 | 0 | 0.8095 | Ref |
| MG872842 | Unassigned | Unassigned | Unassigned | 232_0 | Clustered | 32 | VC_232_0 | 32 | 0.8471 | 1 | 0.8471 | 0 | 0 | 0 | 0.996 | Ref |
| MG872843 | Unassigned | Unassigned | Unassigned | 571_0 | Clustered | 16 | VC_571_0 | 16 | 0.091 | 1 | 0.091 | 0 | 0 | 0 | 0.6667 | Ref |
| MG873442 | Unassigned | Unassigned | Unassigned | 836_0 | Clustered | 3 | VC_836_0 | 3 | 0.1157 | 0.99842379 | 0.1155 | 0 | 0 | 0 | 1 | Ref |
| MG878892 | Unassigned | Unassigned | Unassigned | 907_0 | Clustered | 32 | VC_907_0 | 32 | 0.9964 | 1 | 0.9964 | 0 | 0 | 0 | 1 | Ref |
| MG883709 | Unassigned | Unassigned | Unassigned | 1299_0 | Clustered | 53 | VC_1299_0 | 53 | 0.8666 | 1 | 0.8666 | 0 | 0 | 0 | 1 | Ref |
| MG883710 | Unassigned | Unassigned | Unassigned | 1302_0 | Clustered | 2 | VC_1302_0 | 2 | 1 | 1 | 1 | 0 | 0 | 0 | 1 | Ref |
| MG883711 | Unassigned | Unassigned | Unassigned | 1147_0 | Clustered | 6 | VC_1147_0 | 6 | 0.3066 | 0.99999957 | 0.3066 | 0 | 0 | 0 | 1 | Ref |
| MG883712 | Unassigned | Unassigned | Unassigned | 1241_0 | Clustered | 13 | VC_1241_0 | 13 | 0.8675 | 0.76044592 | 0.6597 | 0 | 0 | 0 | 1 | Ref |
| MG883713 | Unassigned | Unassigned | Unassigned | 1303_0 | Clustered | 2 | VC_1303_0 | 2 | 1 | 1 | 1 | 0 | 0 | 0 | 1 | Ref |
| MG883714 | Unassigned | Unassigned | Unassigned | 1185_0 | Clustered | 7 | VC_1185_0 | 7 | 0.6862 | 1 | 0.6862 | 0 | 0 | 0 | 1 | Ref |
| MG883715 | Unassigned | Unassigned | Unassigned | 690_0 | Clustered | 12 | VC_690_0 | 12 | 1 | 1 | 1 | 0 | 0 | 0 | 1 | Ref |
| MG883717 | Unassigned | Unassigned | Unassigned | 1145_0 | Clustered | 164 | VC_1145_0 | 164 | 0.8868 | 1 | 0.8868 | 0 | 0 | 0 | 1 | Ref |
| MG883718 | Unassigned | Unassigned | Unassigned | 1148_0 | Clustered | 4 | VC_1148_0 | 4 | 0.0503 | 0.99998999 | 0.0503 | 0 | 0 | 0 | 1 | Ref |
| MG883719 | Unassigned | Unassigned | Unassigned | 1155_0 | Clustered | 14 | VC_1155_0 | 14 | 0.8287 | 1 | 0.8287 | 0 | 0 | 0 | 1 | Ref |
| MG883720 | Unassigned | Unassigned | Unassigned | 1304_0 | Clustered | 7 | VC_1304_0 | 7 | 0.9079 | 0.99998959 | 0.9079 | 0 | 0 | 0 | 1 | Ref |
| MG883722 | Unassigned | Unassigned | Unassigned | 1152_0 | Clustered | 26 | VC_1152_0 | 26 | 0.6685 | 1 | 0.6685 | 0 | 0 | 0 | 1 | Ref |
| MG883723 | Unassigned | Unassigned | Unassigned | 206_0 | Clustered | 28 | VC_206_0 | 28 | 0.2427 | 0.99971175 | 0.2426 | 0 | 0 | 0 | 1 | Ref |
| MG883724 | Unassigned | Unassigned | Unassigned | 1307_0 | Clustered | 5 | VC_1307_0 | 5 | 0.6369 | 0.99918945 | 0.6364 | 0 | 0 | 0 | 1 | Ref |
| MG883725 | Unassigned | Unassigned | Unassigned | 690_0 | Clustered | 12 | VC_690_0 | 12 | 1 | 1 | 1 | 0 | 0 | 0 | 1 | Ref |
| MG883726 | Unassigned | Unassigned | Unassigned | 1119_0 | Clustered | 48 | VC_1119_0 | 48 | 0.852 | 1 | 0.852 | 0 | 0 | 0 | 1 | Ref |

| | | | | | | | | | | | | | | | | |
|---|---|---|---|---|---|---|---|---|---|---|---|---|---|---|---|---|
| MG893203 | Unassigned | Unassigned | Unassigned | 911_0 | Clustered | 20 | VC_911_0 | 20 | 0.9915 | 1 | 0.9915 | 0 | 0 | 0 | 1 | Ref |
| MG894052 | Unassigned | Unassigned | Unassigned | 972_0 | Clustered | 99 | VC_972_0 | 99 | 0.9113 | 1 | 0.9113 | 0 | 0 | 0 | 0.9928 | Ref |
| MG897799 | Unassigned | Unassigned | Unassigned | 80_0 | Clustered | 64 | VC_80_0 | 64 | 0.418 | 1 | 0.418 | 0 | 0 | 0 | 0.7649 | Ref |
| MG897800 | Unassigned | Unassigned | Unassigned | 766_0 | Clustered | 40 | VC_766_0 | 40 | 0.7511 | 1 | 0.7511 | 0 | 0 | 0 | 1 | Ref |
| MG913376 | Unassigned | Unassigned | Unassigned | 657_0 | Clustered | 7 | VC_657_0 | 7 | 0.8495 | 1 | 0.8495 | 0 | 0 | 0 | 1 | Ref |
| MG920059 | Unassigned | Unassigned | Unassigned | 527_0 | Clustered | 37 | VC_527_0 | 37 | 0.9679 | 1 | 0.9679 | 0 | 0 | 0 | 1 | Ref |
| MG920060 | Unassigned | Unassigned | Unassigned | 335_23 | Clustered | 17 | VC_335_23 | 17 | 0.0714 | 1 | 0.0714 | 0 | 0 | 0 | 0.6397 | Ref |
| MG920061 | Unassigned | Unassigned | Unassigned | 1296_0 | Clustered | 82 | VC_1296_0 | 82 | 0.7938 | 1 | 0.7938 | 0 | 0 | 0 | 1 | Ref |
| MG922974 | Unassigned | Unassigned | Unassigned | 801_0 | Clustered | 37 | VC_801_0 | 37 | 0.5672 | 1 | 0.5672 | 0 | 0 | 0 | 1 | Ref |
| MG925338 | Unassigned | Unassigned | Unassigned | 527_0 | Clustered | 37 | VC_527_0 | 37 | 0.9679 | 1 | 0.9679 | 0 | 0 | 0 | 1 | Ref |
| MG925339 | Unassigned | Unassigned | Unassigned | 384_3 | Clustered | 22 | VC_384_3 | 22 | 0.0786 | 1 | 0.0786 | 0 | 0 | 0 | 0.9048 | Ref |
| MG925340 | Unassigned | Unassigned | Unassigned | 335_61 | Clustered | 8 | VC_335_61 | 8 | 0.0394 | 1 | 0.0394 | 0 | 0 | 0 | 0.7857 | Ref |
| MG925341 | Unassigned | Unassigned | Unassigned | 532_0 | Clustered | 75 | VC_532_0 | 75 | 0.9393 | 1 | 0.9393 | 0 | 0 | 0 | 1 | Ref |
| MG925342 | Unassigned | Unassigned | Unassigned | 335_61 | Clustered | 8 | VC_335_61 | 8 | 0.0394 | 1 | 0.0394 | 0 | 0 | 0 | 0.7857 | Ref |
| MG925343 | Unassigned | Unassigned | Unassigned | 1203_0 | Clustered | 28 | VC_1203_0 | 28 | 0.4905 | 1 | 0.4905 | 0 | 0 | 0 | 1 | Ref |
| MG925344 | Unassigned | Unassigned | Unassigned | 335_5 | Clustered | 11 | VC_335_5 | 11 | 0.0469 | 1 | 0.0469 | 0 | 0 | 0 | 0.7636 | Ref |
| MG925345 | Unassigned | Unassigned | Unassigned | 1203_0 | Clustered | 28 | VC_1203_0 | 28 | 0.4905 | 1 | 0.4905 | 0 | 0 | 0 | 1 | Ref |
| MG925346 | Unassigned | Unassigned | Unassigned | 384_15 | Clustered | 44 | VC_384_15 | 44 | 0.139 | 1 | 0.139 | 0 | 0 | 0 | 0.7474 | Ref |
| MG925347 | Unassigned | Unassigned | Unassigned | 1203_0 | Clustered | 28 | VC_1203_0 | 28 | 0.4905 | 1 | 0.4905 | 0 | 0 | 0 | 1 | Ref |
| MG925348 | Unassigned | Unassigned | Unassigned | 384_16 | Clustered | 42 | VC_384_16 | 42 | 0.134 | 1 | 0.134 | 0 | 0 | 0 | 0.7282 | Ref |
| MG925349 | Unassigned | Unassigned | Unassigned | 1087_0 | Clustered | 7 | VC_1087_0 | 7 | 0.94 | 1 | 0.94 | 0 | 0 | 0 | 1 | Ref |
| MG925350 | Unassigned | Unassigned | Unassigned | 384_15 | Clustered | 44 | VC_384_15 | 44 | 0.139 | 1 | 0.139 | 0 | 0 | 0 | 0.7474 | Ref |
| MG925351 | Unassigned | Unassigned | Unassigned | 335_23 | Clustered | 17 | VC_335_23 | 17 | 0.0714 | 1 | 0.0714 | 0 | 0 | 0 | 0.6397 | Ref |
| MG925352 | Unassigned | Unassigned | Unassigned | 1296_0 | Clustered | 82 | VC_1296_0 | 82 | 0.7938 | 1 | 0.7938 | 0 | 0 | 0 | 1 | Ref |
| MG925353 | Unassigned | Unassigned | Unassigned | 527_0 | Clustered | 37 | VC_527_0 | 37 | 0.9679 | 1 | 0.9679 | 0 | 0 | 0 | 1 | Ref |
| MG925354 | Unassigned | Unassigned | Unassigned | 538_97 | Clustered/Singleton | 1 | VC_538_97 | 1 | 0 | 1 | 0 | 0 | 0 | 0 | 1 | Ref |
| MG925355 | Unassigned | Unassigned | Unassigned | 532_0 | Clustered | 75 | VC_532_0 | 75 | 0.9393 | 1 | 0.9393 | 0 | 0 | 0 | 1 | Ref |
| MG925356 | Unassigned | Unassigned | Unassigned | 384_2 | Clustered | 8 | VC_384_2 | 8 | 0.0232 | 1 | 0.0232 | 0 | 0 | 0 | 0.8571 | Ref |
| MG941013 | Unassigned | Unassigned | Unassigned | 877_0 | Clustered | 2 | VC_877_0 | 2 | 0.2717 | 0.72985431 | 0.1983 | 0 | 0 | 0 | 1 | Ref |
| MG944216 | Unassigned | Unassigned | Unassigned | 1203_0 | Clustered | 28 | VC_1203_0 | 28 | 0.4905 | 1 | 0.4905 | 0 | 0 | 0 | 1 | Ref |
| MG944217 | Unassigned | Unassigned | Unassigned | 535_1 | Clustered | 108 | VC_535_1 | 108 | 0.8479 | 1 | 0.8479 | 0 | 0 | 0 | 0.9955 | Ref |
| MG944218 | Unassigned | Unassigned | Unassigned | 1203_0 | Clustered | 28 | VC_1203_0 | 28 | 0.4905 | 1 | 0.4905 | 0 | 0 | 0 | 1 | Ref |
| MG944219 | Unassigned | Unassigned | Unassigned | 1296_0 | Clustered | 82 | VC_1296_0 | 82 | 0.7938 | 1 | 0.7938 | 0 | 0 | 0 | 1 | Ref |
| MG944220 | Unassigned | Unassigned | Unassigned | 335_26 | Clustered/Singleton | 1 | VC_335_26 | 1 | 0 | 1 | 0 | 0 | 0 | 0 | 1 | Ref |
| MG944221 | Unassigned | Unassigned | Unassigned | 335_7 | Clustered | 3 | VC_335_7 | 3 | 0.0085 | 0.99739782 | 0.0085 | 0 | 0 | 0 | 0.6667 | Ref |
| MG944222 | Unassigned | Unassigned | Unassigned | 1296_0 | Clustered | 82 | VC_1296_0 | 82 | 0.7938 | 1 | 0.7938 | 0 | 0 | 0 | 1 | Ref |
| MG944223 | Unassigned | Unassigned | Unassigned | 384_13 | Clustered | 33 | VC_384_13 | 33 | 0.1785 | 1 | 0.1785 | 0 | 0 | 0 | 0.8769 | Ref |
| MG944224 | Unassigned | Unassigned | Unassigned | 535_1 | Clustered | 108 | VC_535_1 | 108 | 0.8479 | 1 | 0.8479 | 0 | 0 | 0 | 0.9955 | Ref |
| MG944225 | Unassigned | Unassigned | Unassigned | 384_15 | Clustered | 44 | VC_384_15 | 44 | 0.139 | 1 | 0.139 | 0 | 0 | 0 | 0.7474 | Ref |
| MG944226 | Unassigned | Unassigned | Unassigned | 1203_0 | Clustered | 28 | VC_1203_0 | 28 | 0.4905 | 1 | 0.4905 | 0 | 0 | 0 | 1 | Ref |
| MG944227 | Unassigned | Unassigned | Unassigned | 377_0 | Clustered | 16 | VC_377_0 | 16 | 0.6424 | 1 | 0.6424 | 0 | 0 | 0 | 1 | Ref |
| MG944228 | Unassigned | Unassigned | Unassigned | 377_0 | Clustered | 16 | VC_377_0 | 16 | 0.6424 | 1 | 0.6424 | 0 | 0 | 0 | 1 | Ref |
| MG944229 | Unassigned | Unassigned | Unassigned | 377_0 | Clustered | 16 | VC_377_0 | 16 | 0.6424 | 1 | 0.6424 | 0 | 0 | 0 | 1 | Ref |
| MG944230 | Unassigned | Unassigned | Unassigned | 377_0 | Clustered | 16 | VC_377_0 | 16 | 0.6424 | 1 | 0.6424 | 0 | 0 | 0 | 1 | Ref |
| MG944231 | Unassigned | Unassigned | Unassigned | 377_0 | Clustered | 16 | VC_377_0 | 16 | 0.6424 | 1 | 0.6424 | 0 | 0 | 0 | 1 | Ref |
| MG944232 | Unassigned | Unassigned | Unassigned | 377_0 | Clustered | 16 | VC_377_0 | 16 | 0.6424 | 1 | 0.6424 | 0 | 0 | 0 | 1 | Ref |
| MG944233 | Unassigned | Unassigned | Unassigned | 377_0 | Clustered | 16 | VC_377_0 | 16 | 0.6424 | 1 | 0.6424 | 0 | 0 | 0 | 1 | Ref |
| MG944234 | Unassigned | Unassigned | Unassigned | 377_0 | Clustered | 16 | VC_377_0 | 16 | 0.6424 | 1 | 0.6424 | 0 | 0 | 0 | 1 | Ref |
| MG944235 | Unassigned | Unassigned | Unassigned | 377_0 | Clustered | 16 | VC_377_0 | 16 | 0.6424 | 1 | 0.6424 | 0 | 0 | 0 | 1 | Ref |
| MG944236 | Unassigned | Unassigned | Unassigned | 377_0 | Clustered | 16 | VC_377_0 | 16 | 0.6424 | 1 | 0.6424 | 0 | 0 | 0 | 1 | Ref |
| MG945128 | Unassigned | Unassigned | Unassigned | 1308_0 | Clustered | 15 | VC_1308_0 | 15 | 0.9074 | 1 | 0.9074 | 0 | 0 | 0 | 1 | Ref |
| MG945129 | Unassigned | Unassigned | Unassigned | 1119_0 | Clustered | 48 | VC_1119_0 | 48 | 0.852 | 1 | 0.852 | 0 | 0 | 0 | 1 | Ref |
| MG945130 | Unassigned | Unassigned | Unassigned | 1286_0 | Clustered | 16 | VC_1286_0 | 16 | 0.9448 | 1 | 0.9448 | 0 | 0 | 0 | 1 | Ref |
| MG945131 | Unassigned | Unassigned | Unassigned | 1153_0 | Clustered | 2 | VC_1153_0 | 2 | 0.0506 | 0.94785574 | 0.048 | 0 | 0 | 0 | 1 | Ref |
| MG945134 | Unassigned | Unassigned | Unassigned | 1147_0 | Clustered | 6 | VC_1147_0 | 6 | 0.3066 | 0.99999957 | 0.3066 | 0 | 0 | 0 | 1 | Ref |
| MG945135 | Unassigned | Unassigned | Unassigned | 1167_0 | Clustered | 11 | VC_1167_0 | 11 | 0.5637 | 1 | 0.5637 | 0 | 0 | 0 | 1 | Ref |
| MG945136 | Unassigned | Unassigned | Unassigned | 1167_0 | Clustered | 11 | VC_1167_0 | 11 | 0.5637 | 1 | 0.5637 | 0 | 0 | 0 | 1 | Ref |
| MG945138 | Unassigned | Unassigned | Unassigned | 1169_0 | Clustered | 8 | VC_1169_0 | 8 | 0.2897 | 1 | 0.2897 | 0 | 0 | 0 | 1 | Ref |
| MG945139 | Unassigned | Unassigned | Unassigned | 1155_0 | Clustered | 14 | VC_1155_0 | 14 | 0.8287 | 1 | 0.8287 | 0 | 0 | 0 | 1 | Ref |
| MG945140 | Unassigned | Unassigned | Unassigned | 1314_0 | Clustered | 6 | VC_1314_0 | 6 | 0.6504 | 0.99999969 | 0.6504 | 0 | 0 | 0 | 1 | Ref |
| MG945141 | Unassigned | Unassigned | Unassigned | 1267_0 | Clustered | 26 | VC_1267_0 | 26 | 0.9597 | 0.99999998 | 0.9597 | 0 | 0 | 0 | 1 | Ref |
| MG945142 | Unassigned | Unassigned | Unassigned | 1177_0 | Clustered | 2 | VC_1177_0 | 2 | 0.6063 | 0.90610936 | 0.5494 | 0 | 0 | 0 | 1 | Ref |
| MG945153 | Unassigned | Unassigned | Unassigned | 1145_0 | Clustered | 164 | VC_1145_0 | 164 | 0.8868 | 1 | 0.8868 | 0 | 0 | 0 | 1 | Ref |
| MG945154 | Unassigned | Unassigned | Unassigned | 1161_0 | Clustered | 16 | VC_1161_0 | 16 | 0.4834 | 0.99999958 | 0.4834 | 0 | 0 | 0 | 1 | Ref |
| MG945157 | Unassigned | Unassigned | Unassigned | 1167_0 | Clustered | 11 | VC_1167_0 | 11 | 0.5637 | 1 | 0.5637 | 0 | 0 | 0 | 1 | Ref |
| MG945158 | Unassigned | Unassigned | Unassigned | 1145_0 | Clustered | 164 | VC_1145_0 | 164 | 0.8868 | 1 | 0.8868 | 0 | 0 | 0 | 1 | Ref |
| MG945159 | Unassigned | Unassigned | Unassigned | 1308_0 | Clustered | 15 | VC_1308_0 | 15 | 0.9074 | 1 | 0.9074 | 0 | 0 | 0 | 1 | Ref |
| MG945160 | Unassigned | Unassigned | Unassigned | 1235_0 | Clustered | 3 | VC_1235_0 | 3 | 0.1401 | 0.98976974 | 0.1387 | 0 | 0 | 0 | 1 | Ref |
| MG945161 | Unassigned | Unassigned | Unassigned | 1033_0 | Clustered | 21 | VC_1033_0 | 21 | 0.8433 | 0.99999998 | 0.8433 | 0 | 0 | 0 | 1 | Ref |
| MG945162 | Unassigned | Unassigned | Unassigned | 1170_0 | Clustered | 19 | VC_1170_0 | 19 | 0.7497 | 1 | 0.7497 | 0 | 0 | 0 | 1 | Ref |
| MG945163 | Unassigned | Unassigned | Unassigned | 1145_0 | Clustered | 164 | VC_1145_0 | 164 | 0.8868 | 1 | 0.8868 | 0 | 0 | 0 | 1 | Ref |
| MG945164 | Unassigned | Unassigned | Unassigned | 411_0 | Clustered | 48 | VC_411_0 | 48 | 0.4808 | 1 | 0.4808 | 0 | 0 | 0 | 1 | Ref |
| MG945165 | Unassigned | Unassigned | Unassigned | 1317_0 | Clustered | 2 | VC_1317_0 | 2 | 1 | 1 | 1 | 0 | 0 | 0 | 1 | Ref |
| MG945166 | Unassigned | Unassigned | Unassigned | 1316_0 | Clustered | 8 | VC_1316_0 | 8 | 0.821 | 1 | 0.821 | 0 | 0 | 0 | 1 | Ref |
| MG945177 | Unassigned | Unassigned | Unassigned | 1318_0 | Clustered | 5 | VC_1318_0 | 5 | 0.764 | 0.99993003 | 0.7639 | 0 | 0 | 0 | 1 | Ref |
| MG945178 | Unassigned | Unassigned | Unassigned | 1104_0 | Clustered | 44 | VC_1104_0 | 44 | 0.9574 | 0.72818579 | 0.6972 | 0 | 0 | 0 | 1 | Ref |
| MG945179 | Unassigned | Unassigned | Unassigned | 1189_0 | Clustered | 16 | VC_1189_0 | 16 | 0.8814 | 1 | 0.8814 | 0 | 0 | 0 | 1 | Ref |
| MG945181 | Unassigned | Unassigned | Unassigned | 1320_0 | Clustered | 10 | VC_1320_0 | 10 | 0.8478 | 1 | 0.8478 | 0 | 0 | 0 | 1 | Ref |
| MG945183 | Unassigned | Unassigned | Unassigned | 1321_0 | Clustered | 2 | VC_1321_0 | 2 | 1 | 1 | 1 | 0 | 0 | 0 | 1 | Ref |
| MG945185 | Unassigned | Unassigned | Unassigned | 1235_0 | Clustered | 3 | VC_1235_0 | 3 | 0.1401 | 0.98976974 | 0.1387 | 0 | 0 | 0 | 1 | Ref |
| MG945186 | Unassigned | Unassigned | Unassigned | 1165_0 | Clustered | 10 | VC_1165_0 | 10 | 0.9263 | 1 | 0.9263 | 0 | 0 | 0 | 1 | Ref |

| | | | | | | | | | | | | | | | | |
|---|---|---|---|---|---|---|---|---|---|---|---|---|---|---|---|---|
| MG945188 | Unassigned | Unassigned | Unassigned | 1308_0 | Clustered | 15 | VC_1308_0 | 15 | 0.9074 | 1 | 0.9074 | 0 | 0 | 0 | 1 | Ref |
| MG945189 | Unassigned | Unassigned | Unassigned | 1324_0 | Clustered | 5 | VC_1324_0 | 5 | 1 | 1 | 1 | 0 | 0 | 0 | 1 | Ref |
| MG945190 | Unassigned | Unassigned | Unassigned | 1145_0 | Clustered | 164 | VC_1145_0 | 164 | 0.8868 | 1 | 0.8868 | 0 | 0 | 0 | 1 | Ref |
| MG945202 | Unassigned | Unassigned | Unassigned | 1028_0 | Clustered | 69 | VC_1028_0 | 69 | 0.8547 | 1 | 0.8547 | 0 | 0 | 0 | 1 | Ref |
| MG945203 | Unassigned | Unassigned | Unassigned | 1145_0 | Clustered | 164 | VC_1145_0 | 164 | 0.8868 | 1 | 0.8868 | 0 | 0 | 0 | 1 | Ref |
| MG945204 | Unassigned | Unassigned | Unassigned | 1192_0 | Clustered | 4 | VC_1192_0 | 4 | 0.7187 | 0.99971165 | 0.7185 | 0 | 0 | 0 | 1 | Ref |
| MG945205 | Unassigned | Unassigned | Unassigned | 1145_0 | Clustered | 164 | VC_1145_0 | 164 | 0.8868 | 1 | 0.8868 | 0 | 0 | 0 | 1 | Ref |
| MG945206 | Unassigned | Unassigned | Unassigned | 1119_0 | Clustered | 48 | VC_1119_0 | 48 | 0.852 | 1 | 0.852 | 0 | 0 | 0 | 1 | Ref |
| MG945207 | Unassigned | Unassigned | Unassigned | 1145_0 | Clustered | 164 | VC_1145_0 | 164 | 0.8868 | 1 | 0.8868 | 0 | 0 | 0 | 1 | Ref |
| MG945208 | Unassigned | Unassigned | Unassigned | 1316_0 | Clustered | 8 | VC_1316_0 | 8 | 0.821 | 1 | 0.821 | 0 | 0 | 0 | 1 | Ref |
| MG945209 | Unassigned | Unassigned | Unassigned | 1325_0 | Clustered | 7 | VC_1325_0 | 7 | 1 | 1 | 1 | 0 | 0 | 0 | 1 | Ref |
| MG945210 | Unassigned | Unassigned | Unassigned | 1238_0 | Clustered | 5 | VC_1238_0 | 5 | 0.5217 | 0.99999899 | 0.5217 | 0 | 0 | 0 | 1 | Ref |
| MG945211 | Unassigned | Unassigned | Unassigned | 1320_0 | Clustered | 10 | VC_1320_0 | 10 | 0.8478 | 1 | 0.8478 | 0 | 0 | 0 | 1 | Ref |
| MG945212 | Unassigned | Unassigned | Unassigned | 1326_0 | Clustered | 3 | VC_1326_0 | 3 | 0.0804 | 0.6862532 | 0.0552 | 0 | 0 | 0 | 1 | Ref |
| MG945213 | Unassigned | Unassigned | Unassigned | 1145_0 | Clustered | 164 | VC_1145_0 | 164 | 0.8868 | 1 | 0.8868 | 0 | 0 | 0 | 1 | Ref |
| MG945214 | Unassigned | Unassigned | Unassigned | 1328_0 | Clustered | 3 | VC_1328_0 | 3 | 0.6791 | 0.98688028 | 0.6702 | 0 | 0 | 0 | 1 | Ref |
| MG945215 | Unassigned | Unassigned | Unassigned | 690_0 | Clustered | 12 | VC_690_0 | 12 | 1 | 1 | 1 | 0 | 0 | 0 | 1 | Ref |
| MG945227 | Unassigned | Unassigned | Unassigned | 1145_0 | Clustered | 164 | VC_1145_0 | 164 | 0.8868 | 1 | 0.8868 | 0 | 0 | 0 | 1 | Ref |
| MG945228 | Unassigned | Unassigned | Unassigned | 1191_0 | Clustered | 9 | VC_1191_0 | 9 | 0.8537 | 1 | 0.8537 | 0 | 0 | 0 | 1 | Ref |
| MG945229 | Unassigned | Unassigned | Unassigned | 1308_0 | Clustered | 15 | VC_1308_0 | 15 | 0.9074 | 1 | 0.9074 | 0 | 0 | 0 | 1 | Ref |
| MG945230 | Unassigned | Unassigned | Unassigned | 1329_0 | Clustered | 2 | VC_1329_0 | 2 | 1 | 1 | 1 | 0 | 0 | 0 | 1 | Ref |
| MG945232 | Unassigned | Unassigned | Unassigned | 1320_0 | Clustered | 10 | VC_1320_0 | 10 | 0.8478 | 1 | 0.8478 | 0 | 0 | 0 | 1 | Ref |
| MG945233 | Unassigned | Unassigned | Unassigned | 1170_0 | Clustered | 19 | VC_1170_0 | 19 | 0.7497 | 1 | 0.7497 | 0 | 0 | 0 | 1 | Ref |
| MG945234 | Unassigned | Unassigned | Unassigned | 1187_0 | Clustered | 3 | VC_1187_0 | 3 | 0.5022 | 0.99747895 | 0.501 | 0 | 0 | 0 | 1 | Ref |
| MG945235 | Unassigned | Unassigned | Unassigned | 1330_0 | Clustered | 5 | VC_1330_0 | 5 | 1 | 1 | 1 | 0 | 0 | 0 | 1 | Ref |
| MG945236 | Unassigned | Unassigned | Unassigned | 1331_0 | Clustered | 6 | VC_1331_0 | 6 | 0.7375 | 0.9999804 | 0.7375 | 0 | 0 | 0 | 1 | Ref |
| MG945237 | Unassigned | Unassigned | Unassigned | 1145_0 | Clustered | 164 | VC_1145_0 | 164 | 0.8868 | 1 | 0.8868 | 0 | 0 | 0 | 1 | Ref |
| MG945238 | Unassigned | Unassigned | Unassigned | 1145_0 | Clustered | 164 | VC_1145_0 | 164 | 0.8868 | 1 | 0.8868 | 0 | 0 | 0 | 1 | Ref |
| MG945241 | Unassigned | Unassigned | Unassigned | 1313_0 | Clustered | 4 | VC_1313_0 | 4 | 0.5804 | 0.94751743 | 0.5499 | 0 | 0 | 0 | 1 | Ref |
| MG945252 | Unassigned | Unassigned | Unassigned | 1333_0 | Clustered | 4 | VC_1333_0 | 4 | 1 | 1 | 1 | 0 | 0 | 0 | 1 | Ref |
| MG945254 | Unassigned | Unassigned | Unassigned | 760_0 | Clustered | 15 | VC_760_0 | 15 | 1 | 1 | 1 | 0 | 0 | 0 | 1 | Ref |
| MG945255 | Unassigned | Unassigned | Unassigned | 1308_0 | Clustered | 15 | VC_1308_0 | 15 | 0.9074 | 1 | 0.9074 | 0 | 0 | 0 | 1 | Ref |
| MG945256 | Unassigned | Unassigned | Unassigned | 1286_0 | Clustered | 16 | VC_1286_0 | 16 | 0.9448 | 1 | 0.9448 | 0 | 0 | 0 | 1 | Ref |
| MG945257 | Unassigned | Unassigned | Unassigned | 1314_0 | Clustered | 6 | VC_1314_0 | 6 | 0.6504 | 0.99999969 | 0.6504 | 0 | 0 | 0 | 1 | Ref |
| MG945258 | Unassigned | Unassigned | Unassigned | 1338_0 | Clustered | 4 | VC_1338_0 | 4 | 0.6053 | 0.76972092 | 0.4659 | 0 | 0 | 0 | 1 | Ref |
| MG945260 | Unassigned | Unassigned | Unassigned | 1299_0 | Clustered | 53 | VC_1299_0 | 53 | 0.8666 | 1 | 0.8666 | 0 | 0 | 0 | 1 | Ref |
| MG945261 | Unassigned | Unassigned | Unassigned | 1322_0 | Clustered | 6 | VC_1322_0 | 6 | 0.7063 | 0.92055725 | 0.6502 | 0 | 0 | 0 | 1 | Ref |
| MG945263 | Unassigned | Unassigned | Unassigned | 1325_0 | Clustered | 7 | VC_1325_0 | 7 | 1 | 1 | 1 | 0 | 0 | 0 | 1 | Ref |
| MG945275 | Unassigned | Unassigned | Unassigned | 1324_0 | Clustered | 5 | VC_1324_0 | 5 | 1 | 1 | 1 | 0 | 0 | 0 | 1 | Ref |
| MG945276 | Unassigned | Unassigned | Unassigned | 1313_0 | Clustered | 4 | VC_1313_0 | 4 | 0.5804 | 0.94751743 | 0.5499 | 0 | 0 | 0 | 1 | Ref |
| MG945278 | Unassigned | Unassigned | Unassigned | 1340_0 | Clustered | 7 | VC_1340_0 | 7 | 0.9206 | 0.99995294 | 0.9206 | 0 | 0 | 0 | 1 | Ref |
| MG945279 | Unassigned | Unassigned | Unassigned | 1325_0 | Clustered | 7 | VC_1325_0 | 7 | 1 | 1 | 1 | 0 | 0 | 0 | 1 | Ref |
| MG945280 | Unassigned | Unassigned | Unassigned | 1341_0 | Clustered | 4 | VC_1341_0 | 4 | 1 | 1 | 1 | 0 | 0 | 0 | 1 | Ref |
| MG945282 | Unassigned | Unassigned | Unassigned | 1299_0 | Clustered | 53 | VC_1299_0 | 53 | 0.8666 | 1 | 0.8666 | 0 | 0 | 0 | 1 | Ref |
| MG945284 | Unassigned | Unassigned | Unassigned | 1241_0 | Clustered | 13 | VC_1241_0 | 13 | 0.8675 | 0.76044592 | 0.6597 | 0 | 0 | 0 | 1 | Ref |
| MG945285 | Unassigned | Unassigned | Unassigned | 1340_0 | Clustered | 7 | VC_1340_0 | 7 | 0.9206 | 0.99995294 | 0.9206 | 0 | 0 | 0 | 1 | Ref |
| MG945286 | Unassigned | Unassigned | Unassigned | 1152_0 | Clustered | 26 | VC_1152_0 | 26 | 0.6685 | 1 | 0.6685 | 0 | 0 | 0 | 1 | Ref |
| MG945309 | Unassigned | Unassigned | Unassigned | 1343_0 | Clustered | 2 | VC_1343_0 | 2 | 1 | 1 | 1 | 0 | 0 | 0 | 1 | Ref |
| MG945310 | Unassigned | Unassigned | Unassigned | 1152_0 | Clustered | 26 | VC_1152_0 | 26 | 0.6685 | 1 | 0.6685 | 0 | 0 | 0 | 1 | Ref |
| MG945311 | Unassigned | Unassigned | Unassigned | 1152_0 | Clustered | 26 | VC_1152_0 | 26 | 0.6685 | 1 | 0.6685 | 0 | 0 | 0 | 1 | Ref |
| MG945312 | Unassigned | Unassigned | Unassigned | 1152_0 | Clustered | 26 | VC_1152_0 | 26 | 0.6685 | 1 | 0.6685 | 0 | 0 | 0 | 1 | Ref |
| MG945314 | Unassigned | Unassigned | Unassigned | 1321_0 | Clustered | 2 | VC_1321_0 | 2 | 1 | 1 | 1 | 0 | 0 | 0 | 1 | Ref |
| MG945316 | Unassigned | Unassigned | Unassigned | 1346_0 | Clustered | 25 | VC_1346_0 | 25 | 1 | 1 | 1 | 0 | 0 | 0 | 1 | Ref |
| MG945318 | Unassigned | Unassigned | Unassigned | 1145_0 | Clustered | 164 | VC_1145_0 | 164 | 0.8868 | 1 | 0.8868 | 0 | 0 | 0 | 1 | Ref |
| MG945319 | Unassigned | Unassigned | Unassigned | 1152_0 | Clustered | 26 | VC_1152_0 | 26 | 0.6685 | 1 | 0.6685 | 0 | 0 | 0 | 1 | Ref |
| MG945320 | Unassigned | Unassigned | Unassigned | 1180_0 | Clustered | 4 | VC_1180_0 | 4 | 1 | 1 | 1 | 0 | 0 | 0 | 1 | Ref |
| MG945323 | Unassigned | Unassigned | Unassigned | 1308_0 | Clustered | 15 | VC_1308_0 | 15 | 0.9074 | 1 | 0.9074 | 0 | 0 | 0 | 1 | Ref |
| MG945324 | Unassigned | Unassigned | Unassigned | 206_0 | Clustered | 28 | VC_206_0 | 28 | 0.2427 | 0.99971175 | 0.2426 | 0 | 0 | 0 | 1 | Ref |
| MG945325 | Unassigned | Unassigned | Unassigned | 1145_0 | Clustered | 164 | VC_1145_0 | 164 | 0.8868 | 1 | 0.8868 | 0 | 0 | 0 | 1 | Ref |
| MG945327 | Unassigned | Unassigned | Unassigned | 1238_0 | Clustered | 5 | VC_1238_0 | 5 | 0.5217 | 0.99999899 | 0.5217 | 0 | 0 | 0 | 1 | Ref |
| MG945329 | Unassigned | Unassigned | Unassigned | 1316_0 | Clustered | 8 | VC_1316_0 | 8 | 0.821 | 1 | 0.821 | 0 | 0 | 0 | 1 | Ref |
| MG945330 | Unassigned | Unassigned | Unassigned | 1145_0 | Clustered | 164 | VC_1145_0 | 164 | 0.8868 | 1 | 0.8868 | 0 | 0 | 0 | 1 | Ref |
| MG945331 | Unassigned | Unassigned | Unassigned | 1348_0 | Clustered | 7 | VC_1348_0 | 7 | 1 | 1 | 1 | 0 | 0 | 0 | 1 | Ref |
| MG945332 | Unassigned | Unassigned | Unassigned | 1308_0 | Clustered | 15 | VC_1308_0 | 15 | 0.9074 | 1 | 0.9074 | 0 | 0 | 0 | 1 | Ref |
| MG945333 | Unassigned | Unassigned | Unassigned | 1145_0 | Clustered | 164 | VC_1145_0 | 164 | 0.8868 | 1 | 0.8868 | 0 | 0 | 0 | 1 | Ref |
| MG945334 | Unassigned | Unassigned | Unassigned | 1170_0 | Clustered | 19 | VC_1170_0 | 19 | 0.7497 | 1 | 0.7497 | 0 | 0 | 0 | 1 | Ref |
| MG945335 | Unassigned | Unassigned | Unassigned | 1349_0 | Clustered | 3 | VC_1349_0 | 3 | 1 | 1 | 1 | 0 | 0 | 0 | 1 | Ref |
| MG945336 | Unassigned | Unassigned | Unassigned | 1347_0 | Clustered | 3 | VC_1347_0 | 3 | 0.655 | 0.86585706 | 0.5671 | 0 | 0 | 0 | 1 | Ref |
| MG945337 | Unassigned | Unassigned | Unassigned | 1350_0 | Clustered | 2 | VC_1350_0 | 2 | 1 | 1 | 1 | 0 | 0 | 0 | 1 | Ref |
| MG945338 | Unassigned | Unassigned | Unassigned | 1152_0 | Clustered | 26 | VC_1152_0 | 26 | 0.6685 | 1 | 0.6685 | 0 | 0 | 0 | 1 | Ref |
| MG945339 | Unassigned | Unassigned | Unassigned | 1155_0 | Clustered | 14 | VC_1155_0 | 14 | 0.8287 | 1 | 0.8287 | 0 | 0 | 0 | 1 | Ref |
| MG945340 | Unassigned | Unassigned | Unassigned | 1320_0 | Clustered | 10 | VC_1320_0 | 10 | 0.8478 | 1 | 0.8478 | 0 | 0 | 0 | 1 | Ref |
| MG945341 | Unassigned | Unassigned | Unassigned | 690_0 | Clustered | 12 | VC_690_0 | 12 | 1 | 1 | 1 | 0 | 0 | 0 | 1 | Ref |
| MG945343 | Unassigned | Unassigned | Unassigned | 1348_0 | Clustered | 7 | VC_1348_0 | 7 | 1 | 1 | 1 | 0 | 0 | 0 | 1 | Ref |
| MG945344 | Unassigned | Unassigned | Unassigned | 1346_0 | Clustered | 25 | VC_1346_0 | 25 | 1 | 1 | 1 | 0 | 0 | 0 | 1 | Ref |
| MG945345 | Unassigned | Unassigned | Unassigned | 1351_0 | Clustered | 2 | VC_1351_0 | 2 | 1 | 1 | 1 | 0 | 0 | 0 | 1 | Ref |
| MG945346 | Unassigned | Unassigned | Unassigned | 1145_0 | Clustered | 164 | VC_1145_0 | 164 | 0.8868 | 1 | 0.8868 | 0 | 0 | 0 | 1 | Ref |
| MG945347 | Unassigned | Unassigned | Unassigned | 1152_0 | Clustered | 26 | VC_1152_0 | 26 | 0.6685 | 1 | 0.6685 | 0 | 0 | 0 | 1 | Ref |
| MG945348 | Unassigned | Unassigned | Unassigned | 1352_0 | Clustered | 3 | VC_1352_0 | 3 | 1 | 1 | 1 | 0 | 0 | 0 | 1 | Ref |
| MG945349 | Unassigned | Unassigned | Unassigned | 1318_0 | Clustered | 5 | VC_1318_0 | 5 | 0.764 | 0.99993003 | 0.7639 | 0 | 0 | 0 | 1 | Ref |
| MG945350 | Unassigned | Unassigned | Unassigned | 1353_0 | Clustered | 4 | VC_1353_0 | 4 | 0.7838 | 0.99289298 | 0.7782 | 0 | 0 | 0 | 1 | Ref |

| | | | | | | | | | | | | | | | | |
|---|---|---|---|---|---|---|---|---|---|---|---|---|---|---|---|---|
| MG945351 | Unassigned | Unassigned | Unassigned | 1145_0 | Clustered | 164 | VC_1145_0 | 164 | 0.8868 | 1 | 0.8868 | 0 | 0 | 0 | 1 | Ref |
| MG945352 | Unassigned | Unassigned | Unassigned | 1237_0 | Clustered | 3 | VC_1237_0 | 3 | 0.5448 | 0.99523779 | 0.5422 | 0 | 0 | 0 | 1 | Ref |
| MG945354 | Unassigned | Unassigned | Unassigned | 1354_0 | Clustered | 6 | VC_1354_0 | 6 | 0.6609 | 0.99999272 | 0.6609 | 0 | 0 | 0 | 1 | Ref |
| MG945355 | Unassigned | Unassigned | Unassigned | 1145_0 | Clustered | 164 | VC_1145_0 | 164 | 0.8868 | 1 | 0.8868 | 0 | 0 | 0 | 1 | Ref |
| MG945357 | Unassigned | Unassigned | Unassigned | 1189_0 | Clustered | 16 | VC_1189_0 | 16 | 0.8814 | 1 | 0.8814 | 0 | 0 | 0 | 1 | Ref |
| MG945359 | Unassigned | Unassigned | Unassigned | 1146_0 | Clustered | 3 | VC_1146_0 | 3 | 0.3655 | 0.99631816 | 0.3642 | 0 | 0 | 0 | 1 | Ref |
| MG945360 | Unassigned | Unassigned | Unassigned | 1355_0 | Clustered | 3 | VC_1355_0 | 3 | 1 | 0 | 0 | 0 | 0 | 0 | 1 | Ref |
| MG945361 | Unassigned | Unassigned | Unassigned | 1145_0 | Clustered | 164 | VC_1145_0 | 164 | 0.8868 | 1 | 0.8868 | 0 | 0 | 0 | 1 | Ref |
| MG945362 | Unassigned | Unassigned | Unassigned | 1356_0 | Clustered | 4 | VC_1356_0 | 4 | 0.8682 | 0.91151676 | 0.7914 | 0 | 0 | 0 | 1 | Ref |
| MG945363 | Unassigned | Unassigned | Unassigned | 1145_0 | Clustered | 164 | VC_1145_0 | 164 | 0.8868 | 1 | 0.8868 | 0 | 0 | 0 | 1 | Ref |
| MG945364 | Unassigned | Unassigned | Unassigned | 1185_0 | Clustered | 7 | VC_1185_0 | 7 | 0.6862 | 1 | 0.6862 | 0 | 0 | 0 | 1 | Ref |
| MG945365 | Unassigned | Unassigned | Unassigned | 1316_0 | Clustered | 8 | VC_1316_0 | 8 | 0.821 | 1 | 0.821 | 0 | 0 | 0 | 1 | Ref |
| MG945381 | Unassigned | Unassigned | Unassigned | 1169_0 | Clustered | 8 | VC_1169_0 | 8 | 0.2897 | 1 | 0.2897 | 0 | 0 | 0 | 1 | Ref |
| MG945382 | Unassigned | Unassigned | Unassigned | 1169_0 | Clustered | 8 | VC_1169_0 | 8 | 0.2897 | 1 | 0.2897 | 0 | 0 | 0 | 1 | Ref |
| MG945383 | Unassigned | Unassigned | Unassigned | 1170_0 | Clustered | 19 | VC_1170_0 | 19 | 0.7497 | 1 | 0.7497 | 0 | 0 | 0 | 1 | Ref |
| MG945384 | Unassigned | Unassigned | Unassigned | 1304_0 | Clustered | 7 | VC_1304_0 | 7 | 0.9079 | 0.99998959 | 0.9079 | 0 | 0 | 0 | 1 | Ref |
| MG945385 | Unassigned | Unassigned | Unassigned | 1152_0 | Clustered | 26 | VC_1152_0 | 26 | 0.6685 | 1 | 0.6685 | 0 | 0 | 0 | 1 | Ref |
| MG945386 | Unassigned | Unassigned | Unassigned | 1148_0 | Clustered | 4 | VC_1148_0 | 4 | 0.0503 | 0.99998999 | 0.0503 | 0 | 0 | 0 | 1 | Ref |
| MG945387 | Unassigned | Unassigned | Unassigned | 1145_0 | Clustered | 164 | VC_1145_0 | 164 | 0.8868 | 1 | 0.8868 | 0 | 0 | 0 | 1 | Ref |
| MG945389 | Unassigned | Unassigned | Unassigned | 1152_0 | Clustered | 26 | VC_1152_0 | 26 | 0.6685 | 1 | 0.6685 | 0 | 0 | 0 | 1 | Ref |
| MG945390 | Unassigned | Unassigned | Unassigned | 1299_0 | Clustered | 53 | VC_1299_0 | 53 | 0.8666 | 1 | 0.8666 | 0 | 0 | 0 | 1 | Ref |
| MG945391 | Unassigned | Unassigned | Unassigned | 1167_0 | Clustered | 11 | VC_1167_0 | 11 | 0.5637 | 1 | 0.5637 | 0 | 0 | 0 | 1 | Ref |
| MG945392 | Unassigned | Unassigned | Unassigned | 1330_0 | Clustered | 5 | VC_1330_0 | 5 | 1 | 1 | 1 | 0 | 0 | 0 | 1 | Ref |
| MG945393 | Unassigned | Unassigned | Unassigned | 1145_0 | Clustered | 164 | VC_1145_0 | 164 | 0.8868 | 1 | 0.8868 | 0 | 0 | 0 | 1 | Ref |
| MG945394 | Unassigned | Unassigned | Unassigned | 1155_0 | Clustered | 14 | VC_1155_0 | 14 | 0.8287 | 1 | 0.8287 | 0 | 0 | 0 | 1 | Ref |
| MG945395 | Unassigned | Unassigned | Unassigned | 1357_0 | Clustered | 7 | VC_1357_0 | 7 | 1 | 1 | 1 | 0 | 0 | 0 | 1 | Ref |
| MG945396 | Unassigned | Unassigned | Unassigned | 1299_0 | Clustered | 53 | VC_1299_0 | 53 | 0.8666 | 1 | 0.8666 | 0 | 0 | 0 | 1 | Ref |
| MG945398 | Unassigned | Unassigned | Unassigned | 1145_0 | Clustered | 164 | VC_1145_0 | 164 | 0.8868 | 1 | 0.8868 | 0 | 0 | 0 | 1 | Ref |
| MG945399 | Unassigned | Unassigned | Unassigned | 1147_0 | Clustered | 6 | VC_1147_0 | 6 | 0.3066 | 0.99999957 | 0.3066 | 0 | 0 | 0 | 1 | Ref |
| MG945401 | Unassigned | Unassigned | Unassigned | 1308_0 | Clustered | 15 | VC_1308_0 | 15 | 0.9074 | 1 | 0.9074 | 0 | 0 | 0 | 1 | Ref |
| MG945402 | Unassigned | Unassigned | Unassigned | 1145_0 | Clustered | 164 | VC_1145_0 | 164 | 0.8868 | 1 | 0.8868 | 0 | 0 | 0 | 1 | Ref |
| MG945403 | Unassigned | Unassigned | Unassigned | 1155_0 | Clustered | 14 | VC_1155_0 | 14 | 0.8287 | 1 | 0.8287 | 0 | 0 | 0 | 1 | Ref |
| MG945404 | Unassigned | Unassigned | Unassigned | 1325_0 | Clustered | 7 | VC_1325_0 | 7 | 1 | 1 | 1 | 0 | 0 | 0 | 1 | Ref |
| MG945405 | Unassigned | Unassigned | Unassigned | 1187_0 | Clustered | 3 | VC_1187_0 | 3 | 0.5022 | 0.99747895 | 0.501 | 0 | 0 | 0 | 1 | Ref |
| MG945407 | Unassigned | Unassigned | Unassigned | 1145_0 | Clustered | 164 | VC_1145_0 | 164 | 0.8868 | 1 | 0.8868 | 0 | 0 | 0 | 1 | Ref |
| MG945408 | Unassigned | Unassigned | Unassigned | 1324_0 | Clustered | 5 | VC_1324_0 | 5 | 1 | 1 | 1 | 0 | 0 | 0 | 1 | Ref |
| MG945409 | Unassigned | Unassigned | Unassigned | 1360_0 | Clustered | 2 | VC_1360_0 | 2 | 1 | 1 | 1 | 0 | 0 | 0 | 1 | Ref |
| MG945410 | Unassigned | Unassigned | Unassigned | 1152_0 | Clustered | 26 | VC_1152_0 | 26 | 0.6685 | 1 | 0.6685 | 0 | 0 | 0 | 1 | Ref |
| MG945411 | Unassigned | Unassigned | Unassigned | 1318_0 | Clustered | 5 | VC_1318_0 | 5 | 0.764 | 0.99993003 | 0.7639 | 0 | 0 | 0 | 1 | Ref |
| MG945412 | Unassigned | Unassigned | Unassigned | 1361_0 | Clustered | 2 | VC_1361_0 | 2 | 0.6617 | 0.72985431 | 0.4829 | 0 | 0 | 0 | 1 | Ref |
| MG945413 | Unassigned | Unassigned | Unassigned | 1189_0 | Clustered | 16 | VC_1189_0 | 16 | 0.8814 | 1 | 0.8814 | 0 | 0 | 0 | 1 | Ref |
| MG945414 | Unassigned | Unassigned | Unassigned | 1310_0 | Clustered | 6 | VC_1310_0 | 6 | 0.6075 | 1 | 0.6075 | 0 | 0 | 0 | 1 | Ref |
| MG945415 | Unassigned | Unassigned | Unassigned | 1145_0 | Clustered | 164 | VC_1145_0 | 164 | 0.8868 | 1 | 0.8868 | 0 | 0 | 0 | 1 | Ref |
| MG945416 | Unassigned | Unassigned | Unassigned | 1152_0 | Clustered | 26 | VC_1152_0 | 26 | 0.6685 | 1 | 0.6685 | 0 | 0 | 0 | 1 | Ref |
| MG945417 | Unassigned | Unassigned | Unassigned | 1362_0 | Clustered | 2 | VC_1362_0 | 2 | 1 | 1 | 1 | 0 | 0 | 0 | 1 | Ref |
| MG945418 | Unassigned | Unassigned | Unassigned | 1162_0 | Clustered | 3 | VC_1162_0 | 3 | 0.6188 | 0.98688028 | 0.6106 | 0 | 0 | 0 | 1 | Ref |
| MG945419 | Unassigned | Unassigned | Unassigned | 1362_0 | Clustered | 2 | VC_1362_0 | 2 | 1 | 1 | 1 | 0 | 0 | 0 | 1 | Ref |
| MG945420 | Unassigned | Unassigned | Unassigned | 1322_0 | Clustered | 6 | VC_1322_0 | 6 | 0.7063 | 0.92055725 | 0.6502 | 0 | 0 | 0 | 1 | Ref |
| MG945421 | Unassigned | Unassigned | Unassigned | 1357_0 | Clustered | 7 | VC_1357_0 | 7 | 1 | 1 | 1 | 0 | 0 | 0 | 1 | Ref |
| MG945424 | Unassigned | Unassigned | Unassigned | 1145_0 | Clustered | 164 | VC_1145_0 | 164 | 0.8868 | 1 | 0.8868 | 0 | 0 | 0 | 1 | Ref |
| MG945425 | Unassigned | Unassigned | Unassigned | 1337_0 | Clustered | 3 | VC_1337_0 | 3 | 0.394 | 0.99705286 | 0.3928 | 0 | 0 | 0 | 1 | Ref |
| MG945426 | Unassigned | Unassigned | Unassigned | 1191_0 | Clustered | 9 | VC_1191_0 | 9 | 0.8537 | 1 | 0.8537 | 0 | 0 | 0 | 1 | Ref |
| MG945427 | Unassigned | Unassigned | Unassigned | 1363_0 | Clustered | 3 | VC_1363_0 | 3 | 1 | 1 | 1 | 0 | 0 | 0 | 1 | Ref |
| MG945429 | Unassigned | Unassigned | Unassigned | 1145_0 | Clustered | 164 | VC_1145_0 | 164 | 0.8868 | 1 | 0.8868 | 0 | 0 | 0 | 1 | Ref |
| MG945430 | Unassigned | Unassigned | Unassigned | 1170_0 | Clustered | 19 | VC_1170_0 | 19 | 0.7497 | 1 | 0.7497 | 0 | 0 | 0 | 1 | Ref |
| MG945431 | Unassigned | Unassigned | Unassigned | 1146_0 | Clustered | 3 | VC_1146_0 | 3 | 0.3655 | 0.99631816 | 0.3642 | 0 | 0 | 0 | 1 | Ref |
| MG945432 | Unassigned | Unassigned | Unassigned | 1353_0 | Clustered | 4 | VC_1353_0 | 4 | 0.7838 | 0.99289298 | 0.7782 | 0 | 0 | 0 | 1 | Ref |
| MG945433 | Unassigned | Unassigned | Unassigned | 1343_0 | Clustered | 2 | VC_1343_0 | 2 | 1 | 1 | 1 | 0 | 0 | 0 | 1 | Ref |
| MG945434 | Unassigned | Unassigned | Unassigned | 1338_0 | Clustered | 4 | VC_1338_0 | 4 | 0.6053 | 0.76972092 | 0.4659 | 0 | 0 | 0 | 1 | Ref |
| MG945435 | Unassigned | Unassigned | Unassigned | 1145_0 | Clustered | 164 | VC_1145_0 | 164 | 0.8868 | 1 | 0.8868 | 0 | 0 | 0 | 1 | Ref |
| MG945437 | Unassigned | Unassigned | Unassigned | 1152_0 | Clustered | 26 | VC_1152_0 | 26 | 0.6685 | 1 | 0.6685 | 0 | 0 | 0 | 1 | Ref |
| MG945438 | Unassigned | Unassigned | Unassigned | 1145_0 | Clustered | 164 | VC_1145_0 | 164 | 0.8868 | 1 | 0.8868 | 0 | 0 | 0 | 1 | Ref |
| MG945439 | Unassigned | Unassigned | Unassigned | 1364_0 | Clustered | 6 | VC_1364_0 | 6 | 0.9472 | 0.54366909 | 0.515 | 0 | 0 | 0 | 1 | Ref |
| MG945441 | Unassigned | Unassigned | Unassigned | 1190_0 | Clustered | 5 | VC_1190_0 | 5 | 0.4604 | 0.99999982 | 0.4604 | 0 | 0 | 0 | 1 | Ref |
| MG945444 | Unassigned | Unassigned | Unassigned | 1145_0 | Clustered | 164 | VC_1145_0 | 164 | 0.8868 | 1 | 0.8868 | 0 | 0 | 0 | 1 | Ref |
| MG945445 | Unassigned | Unassigned | Unassigned | 1310_0 | Clustered | 6 | VC_1310_0 | 6 | 0.6075 | 1 | 0.6075 | 0 | 0 | 0 | 1 | Ref |
| MG945447 | Unassigned | Unassigned | Unassigned | 1170_0 | Clustered | 19 | VC_1170_0 | 19 | 0.7497 | 1 | 0.7497 | 0 | 0 | 0 | 1 | Ref |
| MG945448 | Unassigned | Unassigned | Unassigned | 1313_0 | Clustered | 4 | VC_1313_0 | 4 | 0.5804 | 0.94751743 | 0.5499 | 0 | 0 | 0 | 1 | Ref |
| MG945449 | Unassigned | Unassigned | Unassigned | 1314_0 | Clustered | 6 | VC_1314_0 | 6 | 0.6504 | 0.99999969 | 0.6504 | 0 | 0 | 0 | 1 | Ref |
| MG945452 | Unassigned | Unassigned | Unassigned | 1299_0 | Clustered | 53 | VC_1299_0 | 53 | 0.8666 | 1 | 0.8666 | 0 | 0 | 0 | 1 | Ref |
| MG945453 | Unassigned | Unassigned | Unassigned | 1267_0 | Clustered | 26 | VC_1267_0 | 26 | 0.9597 | 0.99999998 | 0.9597 | 0 | 0 | 0 | 1 | Ref |
| MG945455 | Unassigned | Unassigned | Unassigned | 1267_0 | Clustered | 26 | VC_1267_0 | 26 | 0.9597 | 0.99999998 | 0.9597 | 0 | 0 | 0 | 1 | Ref |
| MG945456 | Unassigned | Unassigned | Unassigned | 1267_0 | Clustered | 26 | VC_1267_0 | 26 | 0.9597 | 0.99999998 | 0.9597 | 0 | 0 | 0 | 1 | Ref |
| MG945457 | Unassigned | Unassigned | Unassigned | 1286_0 | Clustered | 16 | VC_1286_0 | 16 | 0.9448 | 1 | 0.9448 | 0 | 0 | 0 | 1 | Ref |
| MG945458 | Unassigned | Unassigned | Unassigned | 1314_0 | Clustered | 6 | VC_1314_0 | 6 | 0.6504 | 0.99999969 | 0.6504 | 0 | 0 | 0 | 1 | Ref |
| MG945459 | Unassigned | Unassigned | Unassigned | 1145_0 | Clustered | 164 | VC_1145_0 | 164 | 0.8868 | 1 | 0.8868 | 0 | 0 | 0 | 1 | Ref |
| MG945461 | Unassigned | Unassigned | Unassigned | 1145_0 | Clustered | 164 | VC_1145_0 | 164 | 0.8868 | 1 | 0.8868 | 0 | 0 | 0 | 1 | Ref |
| MG945462 | Unassigned | Unassigned | Unassigned | 1318_0 | Clustered | 5 | VC_1318_0 | 5 | 0.764 | 0.99993003 | 0.7639 | 0 | 0 | 0 | 1 | Ref |
| MG945463 | Unassigned | Unassigned | Unassigned | 1104_0 | Clustered | 44 | VC_1104_0 | 44 | 0.9574 | 0.72818579 | 0.6972 | 0 | 0 | 0 | 1 | Ref |
| MG945464 | Unassigned | Unassigned | Unassigned | 1302_0 | Clustered | 2 | VC_1302_0 | 2 | 1 | 1 | 1 | 0 | 0 | 0 | 1 | Ref |
| MG945465 | Unassigned | Unassigned | Unassigned | 1028_0 | Clustered | 69 | VC_1028_0 | 69 | 0.8547 | 1 | 0.8547 | 0 | 0 | 0 | 1 | Ref |

| | | | | | | | | | | | | | | | | |
|---|---|---|---|---|---|---|---|---|---|---|---|---|---|---|---|---|
| MG945467 | Unassigned | Unassigned | Unassigned | 1145_0 | Clustered | 164 | VC_1145_0 | 164 | 0.8868 | 1 | 0.8868 | 0 | 0 | 0 | 1 | Ref |
| MG945468 | Unassigned | Unassigned | Unassigned | 1346_0 | Clustered | 25 | VC_1346_0 | 25 | 1 | 1 | 1 | 0 | 0 | 0 | 1 | Ref |
| MG945469 | Unassigned | Unassigned | Unassigned | 1367_0 | Clustered | 5 | VC_1367_0 | 5 | 0.7699 | 0.66985041 | 0.5157 | 0 | 0 | 0 | 1 | Ref |
| MG945470 | Unassigned | Unassigned | Unassigned | 1322_0 | Clustered | 6 | VC_1322_0 | 6 | 0.7063 | 0.92055725 | 0.6502 | 0 | 0 | 0 | 1 | Ref |
| MG945472 | Unassigned | Unassigned | Unassigned | 1161_0 | Clustered | 16 | VC_1161_0 | 16 | 0.4834 | 0.99999958 | 0.4834 | 0 | 0 | 0 | 1 | Ref |
| MG945473 | Unassigned | Unassigned | Unassigned | 1095_0 | Clustered | 12 | VC_1095_0 | 12 | 0.2819 | 0.99837092 | 0.2814 | 0 | 0 | 0 | 1 | Ref |
| MG945475 | Unassigned | Unassigned | Unassigned | 1127_0 | Clustered | 2 | VC_1127_0 | 2 | 0.5888 | 0.9331928 | 0.5494 | 0 | 0 | 0 | 1 | Ref |
| MG945476 | Unassigned | Unassigned | Unassigned | 1145_0 | Clustered | 164 | VC_1145_0 | 164 | 0.8868 | 1 | 0.8868 | 0 | 0 | 0 | 1 | Ref |
| MG945477 | Unassigned | Unassigned | Unassigned | 1028_0 | Clustered | 69 | VC_1028_0 | 69 | 0.8547 | 1 | 0.8547 | 0 | 0 | 0 | 1 | Ref |
| MG945478 | Unassigned | Unassigned | Unassigned | 1355_0 | Clustered | 3 | VC_1355_0 | 3 | 1 | 0 | 0 | 0 | 0 | 0 | 1 | Ref |
| MG945480 | Unassigned | Unassigned | Unassigned | 1357_0 | Clustered | 7 | VC_1357_0 | 7 | 1 | 1 | 1 | 0 | 0 | 0 | 1 | Ref |
| MG945481 | Unassigned | Unassigned | Unassigned | 1156_0 | Clustered | 5 | VC_1156_0 | 5 | 0.4454 | 0.99999977 | 0.4454 | 0 | 0 | 0 | 1 | Ref |
| MG945482 | Unassigned | Unassigned | Unassigned | 1304_0 | Clustered | 7 | VC_1304_0 | 7 | 0.9079 | 0.99998959 | 0.9079 | 0 | 0 | 0 | 1 | Ref |
| MG945483 | Unassigned | Unassigned | Unassigned | 1368_0 | Clustered | 2 | VC_1368_0 | 2 | 1 | 1 | 1 | 0 | 0 | 0 | 1 | Ref |
| MG945485 | Unassigned | Unassigned | Unassigned | 1299_0 | Clustered | 53 | VC_1299_0 | 53 | 0.8666 | 1 | 0.8666 | 0 | 0 | 0 | 1 | Ref |
| MG945486 | Unassigned | Unassigned | Unassigned | 1145_0 | Clustered | 164 | VC_1145_0 | 164 | 0.8868 | 1 | 0.8868 | 0 | 0 | 0 | 1 | Ref |
| MG945487 | Unassigned | Unassigned | Unassigned | 1190_0 | Clustered | 5 | VC_1190_0 | 5 | 0.4604 | 0.99999982 | 0.4604 | 0 | 0 | 0 | 1 | Ref |
| MG945488 | Unassigned | Unassigned | Unassigned | 1334_0 | Clustered | 7 | VC_1334_0 | 7 | 0.6918 | 1 | 0.6918 | 0 | 0 | 0 | 1 | Ref |
| MG945489 | Unassigned | Unassigned | Unassigned | 1150_0 | Clustered | 2 | VC_1150_0 | 2 | 0.2628 | 0.89847992 | 0.2361 | 0 | 0 | 0 | 1 | Ref |
| MG945490 | Unassigned | Unassigned | Unassigned | 1348_0 | Clustered | 7 | VC_1348_0 | 7 | 1 | 1 | 1 | 0 | 0 | 0 | 1 | Ref |
| MG945492 | Unassigned | Unassigned | Unassigned | 1340_0 | Clustered | 7 | VC_1340_0 | 7 | 0.9206 | 0.99995294 | 0.9206 | 0 | 0 | 0 | 1 | Ref |
| MG945494 | Unassigned | Unassigned | Unassigned | 1335_0 | Clustered | 13 | VC_1335_0 | 13 | 0.7519 | 1 | 0.7519 | 0 | 0 | 0 | 1 | Ref |
| MG945495 | Unassigned | Unassigned | Unassigned | 1318_0 | Clustered | 5 | VC_1318_0 | 5 | 0.764 | 0.99993003 | 0.7639 | 0 | 0 | 0 | 1 | Ref |
| MG945497 | Unassigned | Unassigned | Unassigned | 1285_0 | Clustered | 3 | VC_1285_0 | 3 | 0.7418 | 0.80676188 | 0.5984 | 0 | 0 | 0 | 1 | Ref |
| MG945498 | Unassigned | Unassigned | Unassigned | 1267_0 | Clustered | 26 | VC_1267_0 | 26 | 0.9597 | 0.99999998 | 0.9597 | 0 | 0 | 0 | 1 | Ref |
| MG945499 | Unassigned | Unassigned | Unassigned | 1145_0 | Clustered | 164 | VC_1145_0 | 164 | 0.8868 | 1 | 0.8868 | 0 | 0 | 0 | 1 | Ref |
| MG945500 | Unassigned | Unassigned | Unassigned | 1369_0 | Clustered | 10 | VC_1369_0 | 10 | 0.6223 | 0.95845085 | 0.5964 | 0 | 0 | 0 | 1 | Ref |
| MG945501 | Unassigned | Unassigned | Unassigned | 1348_0 | Clustered | 7 | VC_1348_0 | 7 | 1 | 1 | 1 | 0 | 0 | 0 | 1 | Ref |
| MG945502 | Unassigned | Unassigned | Unassigned | 1241_0 | Clustered | 13 | VC_1241_0 | 13 | 0.8675 | 0.76044592 | 0.6597 | 0 | 0 | 0 | 1 | Ref |
| MG945504 | Unassigned | Unassigned | Unassigned | 1373_0 | Clustered | 2 | VC_1373_0 | 2 | 1 | 1 | 1 | 0 | 0 | 0 | 1 | Ref |
| MG945505 | Unassigned | Unassigned | Unassigned | 1190_0 | Clustered | 5 | VC_1190_0 | 5 | 0.4604 | 0.99999982 | 0.4604 | 0 | 0 | 0 | 1 | Ref |
| MG945506 | Unassigned | Unassigned | Unassigned | 1189_0 | Clustered | 16 | VC_1189_0 | 16 | 0.8814 | 1 | 0.8814 | 0 | 0 | 0 | 1 | Ref |
| MG945507 | Unassigned | Unassigned | Unassigned | 1189_0 | Clustered | 16 | VC_1189_0 | 16 | 0.8814 | 1 | 0.8814 | 0 | 0 | 0 | 1 | Ref |
| MG945508 | Unassigned | Unassigned | Unassigned | 1145_0 | Clustered | 164 | VC_1145_0 | 164 | 0.8868 | 1 | 0.8868 | 0 | 0 | 0 | 1 | Ref |
| MG945509 | Unassigned | Unassigned | Unassigned | 1267_0 | Clustered | 26 | VC_1267_0 | 26 | 0.9597 | 0.99999998 | 0.9597 | 0 | 0 | 0 | 1 | Ref |
| MG945511 | Unassigned | Unassigned | Unassigned | 1299_0 | Clustered | 53 | VC_1299_0 | 53 | 0.8666 | 1 | 0.8666 | 0 | 0 | 0 | 1 | Ref |
| MG945512 | Unassigned | Unassigned | Unassigned | 1316_0 | Clustered | 8 | VC_1316_0 | 8 | 0.821 | 1 | 0.821 | 0 | 0 | 0 | 1 | Ref |
| MG945514 | Unassigned | Unassigned | Unassigned | 1170_0 | Clustered | 19 | VC_1170_0 | 19 | 0.7497 | 1 | 0.7497 | 0 | 0 | 0 | 1 | Ref |
| MG945515 | Unassigned | Unassigned | Unassigned | 1299_0 | Clustered | 53 | VC_1299_0 | 53 | 0.8666 | 1 | 0.8666 | 0 | 0 | 0 | 1 | Ref |
| MG945517 | Unassigned | Unassigned | Unassigned | 1145_0 | Clustered | 164 | VC_1145_0 | 164 | 0.8868 | 1 | 0.8868 | 0 | 0 | 0 | 1 | Ref |
| MG945518 | Unassigned | Unassigned | Unassigned | 1322_0 | Clustered | 6 | VC_1322_0 | 6 | 0.7063 | 0.92055725 | 0.6502 | 0 | 0 | 0 | 1 | Ref |
| MG945519 | Unassigned | Unassigned | Unassigned | 1179_0 | Clustered | 4 | VC_1179_0 | 4 | 0.9241 | 0.99468305 | 0.9191 | 0 | 0 | 0 | 1 | Ref |
| MG945520 | Unassigned | Unassigned | Unassigned | 690_0 | Clustered | 12 | VC_690_0 | 12 | 1 | 1 | 1 | 0 | 0 | 0 | 1 | Ref |
| MG945524 | Unassigned | Unassigned | Unassigned | 1346_0 | Clustered | 25 | VC_1346_0 | 25 | 1 | 1 | 1 | 0 | 0 | 0 | 1 | Ref |
| MG945525 | Unassigned | Unassigned | Unassigned | 1185_0 | Clustered | 7 | VC_1185_0 | 7 | 0.6862 | 1 | 0.6862 | 0 | 0 | 0 | 1 | Ref |
| MG945526 | Unassigned | Unassigned | Unassigned | 1145_0 | Clustered | 164 | VC_1145_0 | 164 | 0.8868 | 1 | 0.8868 | 0 | 0 | 0 | 1 | Ref |
| MG945529 | Unassigned | Unassigned | Unassigned | 1164_0 | Clustered | 5 | VC_1164_0 | 5 | 0.831 | 0.99879053 | 0.83 | 0 | 0 | 0 | 1 | Ref |
| MG945531 | Unassigned | Unassigned | Unassigned | 1145_0 | Clustered | 164 | VC_1145_0 | 164 | 0.8868 | 1 | 0.8868 | 0 | 0 | 0 | 1 | Ref |
| MG945533 | Unassigned | Unassigned | Unassigned | 1374_0 | Clustered | 3 | VC_1374_0 | 3 | 1 | 1 | 1 | 0 | 0 | 0 | 1 | Ref |
| MG945534 | Unassigned | Unassigned | Unassigned | 1161_0 | Clustered | 16 | VC_1161_0 | 16 | 0.4834 | 0.99999958 | 0.4834 | 0 | 0 | 0 | 1 | Ref |
| MG945538 | Unassigned | Unassigned | Unassigned | 1152_0 | Clustered | 26 | VC_1152_0 | 26 | 0.6685 | 1 | 0.6685 | 0 | 0 | 0 | 1 | Ref |
| MG945539 | Unassigned | Unassigned | Unassigned | 1357_0 | Clustered | 7 | VC_1357_0 | 7 | 1 | 1 | 1 | 0 | 0 | 0 | 1 | Ref |
| MG945540 | Unassigned | Unassigned | Unassigned | 1316_0 | Clustered | 8 | VC_1316_0 | 8 | 0.821 | 1 | 0.821 | 0 | 0 | 0 | 1 | Ref |
| MG945541 | Unassigned | Unassigned | Unassigned | 1145_0 | Clustered | 164 | VC_1145_0 | 164 | 0.8868 | 1 | 0.8868 | 0 | 0 | 0 | 1 | Ref |
| MG945542 | Unassigned | Unassigned | Unassigned | 1155_0 | Clustered | 14 | VC_1155_0 | 14 | 0.8287 | 1 | 0.8287 | 0 | 0 | 0 | 1 | Ref |
| MG945544 | Unassigned | Unassigned | Unassigned | 1309_0 | Clustered | 3 | VC_1309_0 | 3 | 0.3217 | 0.99790393 | 0.321 | 0 | 0 | 0 | 1 | Ref |
| MG945546 | Unassigned | Unassigned | Unassigned | 206_0 | Clustered | 28 | VC_206_0 | 28 | 0.2427 | 0.99971175 | 0.2426 | 0 | 0 | 0 | 1 | Ref |
| MG945548 | Unassigned | Unassigned | Unassigned | 1145_0 | Clustered | 164 | VC_1145_0 | 164 | 0.8868 | 1 | 0.8868 | 0 | 0 | 0 | 1 | Ref |
| MG945549 | Unassigned | Unassigned | Unassigned | 1162_0 | Clustered | 3 | VC_1162_0 | 3 | 0.6188 | 0.98688028 | 0.6106 | 0 | 0 | 0 | 1 | Ref |
| MG945550 | Unassigned | Unassigned | Unassigned | 1155_0 | Clustered | 14 | VC_1155_0 | 14 | 0.8287 | 1 | 0.8287 | 0 | 0 | 0 | 1 | Ref |
| MG945551 | Unassigned | Unassigned | Unassigned | 690_0 | Clustered | 12 | VC_690_0 | 12 | 1 | 1 | 1 | 0 | 0 | 0 | 1 | Ref |
| MG945553 | Unassigned | Unassigned | Unassigned | 1320_0 | Clustered | 10 | VC_1320_0 | 10 | 0.8478 | 1 | 0.8478 | 0 | 0 | 0 | 1 | Ref |
| MG945554 | Unassigned | Unassigned | Unassigned | 1382_0 | Clustered | 2 | VC_1382_0 | 2 | 1 | 1 | 1 | 0 | 0 | 0 | 1 | Ref |
| MG945556 | Unassigned | Unassigned | Unassigned | 1346_0 | Clustered | 25 | VC_1346_0 | 25 | 1 | 1 | 1 | 0 | 0 | 0 | 1 | Ref |
| MG945557 | Unassigned | Unassigned | Unassigned | 1286_0 | Clustered | 16 | VC_1286_0 | 16 | 0.9448 | 1 | 0.9448 | 0 | 0 | 0 | 1 | Ref |
| MG945559 | Unassigned | Unassigned | Unassigned | 1145_0 | Clustered | 164 | VC_1145_0 | 164 | 0.8868 | 1 | 0.8868 | 0 | 0 | 0 | 1 | Ref |
| MG945561 | Unassigned | Unassigned | Unassigned | 1180_0 | Clustered | 4 | VC_1180_0 | 4 | 1 | 1 | 1 | 0 | 0 | 0 | 1 | Ref |
| MG945562 | Unassigned | Unassigned | Unassigned | 1346_0 | Clustered | 25 | VC_1346_0 | 25 | 1 | 1 | 1 | 0 | 0 | 0 | 1 | Ref |
| MG945563 | Unassigned | Unassigned | Unassigned | 1335_0 | Clustered | 13 | VC_1335_0 | 13 | 0.7519 | 1 | 0.7519 | 0 | 0 | 0 | 1 | Ref |
| MG945564 | Unassigned | Unassigned | Unassigned | 1267_0 | Clustered | 26 | VC_1267_0 | 26 | 0.9597 | 0.99999998 | 0.9597 | 0 | 0 | 0 | 1 | Ref |
| MG945565 | Unassigned | Unassigned | Unassigned | 1145_0 | Clustered | 164 | VC_1145_0 | 164 | 0.8868 | 1 | 0.8868 | 0 | 0 | 0 | 1 | Ref |
| MG945566 | Unassigned | Unassigned | Unassigned | 1145_0 | Clustered | 164 | VC_1145_0 | 164 | 0.8868 | 1 | 0.8868 | 0 | 0 | 0 | 1 | Ref |
| MG945567 | Unassigned | Unassigned | Unassigned | 1332_0 | Clustered | 2 | VC_1332_0 | 2 | 0.6366 | 0.72985431 | 0.4646 | 0 | 0 | 0 | 1 | Ref |
| MG945568 | Unassigned | Unassigned | Unassigned | 1164_0 | Clustered | 5 | VC_1164_0 | 5 | 0.831 | 0.99879053 | 0.83 | 0 | 0 | 0 | 1 | Ref |
| MG945569 | Unassigned | Unassigned | Unassigned | 1165_0 | Clustered | 10 | VC_1165_0 | 10 | 0.9263 | 1 | 0.9263 | 0 | 0 | 0 | 1 | Ref |
| MG945570 | Unassigned | Unassigned | Unassigned | 1145_0 | Clustered | 164 | VC_1145_0 | 164 | 0.8868 | 1 | 0.8868 | 0 | 0 | 0 | 1 | Ref |
| MG945571 | Unassigned | Unassigned | Unassigned | 206_0 | Clustered | 28 | VC_206_0 | 28 | 0.2427 | 0.99971175 | 0.2426 | 0 | 0 | 0 | 1 | Ref |
| MG945572 | Unassigned | Unassigned | Unassigned | 1334_0 | Clustered | 7 | VC_1334_0 | 7 | 0.6918 | 1 | 0.6918 | 0 | 0 | 0 | 1 | Ref |
| MG945573 | Unassigned | Unassigned | Unassigned | 1184_0 | Clustered | 10 | VC_1184_0 | 10 | 0.7506 | 1 | 0.7506 | 0 | 0 | 0 | 1 | Ref |
| MG945574 | Unassigned | Unassigned | Unassigned | 1363_0 | Clustered | 3 | VC_1363_0 | 3 | 1 | 1 | 1 | 0 | 0 | 0 | 1 | Ref |
| MG945575 | Unassigned | Unassigned | Unassigned | 1382_0 | Clustered | 2 | VC_1382_0 | 2 | 1 | 1 | 1 | 0 | 0 | 0 | 1 | Ref |

| | | | | | | | | | | | | | | | | |
|---|---|---|---|---|---|---|---|---|---|---|---|---|---|---|---|---|
| MG945576 | Unassigned | Unassigned | Unassigned | 1354_0 | Clustered | 6 | VC_1354_0 | 6 | 0.6609 | 0.99999272 | 0.6609 | 0 | 0 | 0 | 1 | Ref |
| MG945578 | Unassigned | Unassigned | Unassigned | 1310_0 | Clustered | 6 | VC_1310_0 | 6 | 0.6075 | 1 | 0.6075 | 0 | 0 | 0 | 1 | Ref |
| MG945579 | Unassigned | Unassigned | Unassigned | 1346_0 | Clustered | 25 | VC_1346_0 | 25 | 1 | 1 | 1 | 0 | 0 | 0 | 1 | Ref |
| MG945580 | Unassigned | Unassigned | Unassigned | 1315_0 | Clustered | 3 | VC_1315_0 | 3 | 0.2423 | 0.85925819 | 0.2082 | 0 | 0 | 0 | 1 | Ref |
| MG945582 | Unassigned | Unassigned | Unassigned | 1326_0 | Clustered | 3 | VC_1326_0 | 3 | 0.0804 | 0.6862532 | 0.0552 | 0 | 0 | 0 | 1 | Ref |
| MG945584 | Unassigned | Unassigned | Unassigned | 1333_0 | Clustered | 4 | VC_1333_0 | 4 | 1 | 1 | 1 | 0 | 0 | 0 | 1 | Ref |
| MG945585 | Unassigned | Unassigned | Unassigned | 1335_0 | Clustered | 13 | VC_1335_0 | 13 | 0.7519 | 1 | 0.7519 | 0 | 0 | 0 | 1 | Ref |
| MG945586 | Unassigned | Unassigned | Unassigned | 1145_0 | Clustered | 164 | VC_1145_0 | 164 | 0.8868 | 1 | 0.8868 | 0 | 0 | 0 | 1 | Ref |
| MG945587 | Unassigned | Unassigned | Unassigned | 1145_0 | Clustered | 164 | VC_1145_0 | 164 | 0.8868 | 1 | 0.8868 | 0 | 0 | 0 | 1 | Ref |
| MG945589 | Unassigned | Unassigned | Unassigned | 1145_0 | Clustered | 164 | VC_1145_0 | 164 | 0.8868 | 1 | 0.8868 | 0 | 0 | 0 | 1 | Ref |
| MG945592 | Unassigned | Unassigned | Unassigned | 1145_0 | Clustered | 164 | VC_1145_0 | 164 | 0.8868 | 1 | 0.8868 | 0 | 0 | 0 | 1 | Ref |
| MG945594 | Unassigned | Unassigned | Unassigned | 1094_0 | Clustered/Singleton | 1 | VC_1094_0 | 1 | 0 | 1 | 0 | 0 | 0 | 0 | 1 | Ref |
| MG945596 | Unassigned | Unassigned | Unassigned | 1145_0 | Clustered | 164 | VC_1145_0 | 164 | 0.8868 | 1 | 0.8868 | 0 | 0 | 0 | 1 | Ref |
| MG945598 | Unassigned | Unassigned | Unassigned | 1385_0 | Clustered | 4 | VC_1385_0 | 4 | 0.1856 | 0.61974159 | 0.115 | 0 | 0 | 0 | 1 | Ref |
| MG945600 | Unassigned | Unassigned | Unassigned | 1152_0 | Clustered | 26 | VC_1152_0 | 26 | 0.6685 | 1 | 0.6685 | 0 | 0 | 0 | 1 | Ref |
| MG945602 | Unassigned | Unassigned | Unassigned | 1145_0 | Clustered | 164 | VC_1145_0 | 164 | 0.8868 | 1 | 0.8868 | 0 | 0 | 0 | 1 | Ref |
| MG945605 | Unassigned | Unassigned | Unassigned | 1340_0 | Clustered | 7 | VC_1340_0 | 7 | 0.9206 | 0.99995294 | 0.9206 | 0 | 0 | 0 | 1 | Ref |
| MG945609 | Unassigned | Unassigned | Unassigned | 1189_0 | Clustered | 16 | VC_1189_0 | 16 | 0.8814 | 1 | 0.8814 | 0 | 0 | 0 | 1 | Ref |
| MG945611 | Unassigned | Unassigned | Unassigned | 1310_0 | Clustered | 6 | VC_1310_0 | 6 | 0.6075 | 1 | 0.6075 | 0 | 0 | 0 | 1 | Ref |
| MG945612 | Unassigned | Unassigned | Unassigned | 1346_0 | Clustered | 25 | VC_1346_0 | 25 | 1 | 1 | 1 | 0 | 0 | 0 | 1 | Ref |
| MG945614 | Unassigned | Unassigned | Unassigned | 1355_0 | Clustered | 3 | VC_1355_0 | 3 | 1 | 0 | 0 | 0 | 0 | 0 | 1 | Ref |
| MG945615 | Unassigned | Unassigned | Unassigned | 1386_0 | Clustered | 2 | VC_1386_0 | 2 | 1 | 1 | 1 | 0 | 0 | 0 | 1 | Ref |
| MG945616 | Unassigned | Unassigned | Unassigned | 1241_0 | Clustered | 13 | VC_1241_0 | 13 | 0.8675 | 0.76044592 | 0.6597 | 0 | 0 | 0 | 1 | Ref |
| MG945617 | Unassigned | Unassigned | Unassigned | 1373_0 | Clustered | 2 | VC_1373_0 | 2 | 1 | 1 | 1 | 0 | 0 | 0 | 1 | Ref |
| MG945618 | Unassigned | Unassigned | Unassigned | 1299_0 | Clustered | 53 | VC_1299_0 | 53 | 0.8666 | 1 | 0.8666 | 0 | 0 | 0 | 1 | Ref |
| MG945619 | Unassigned | Unassigned | Unassigned | 1388_0 | Clustered | 4 | VC_1388_0 | 4 | 0.6008 | 0.54407361 | 0.3269 | 0 | 0 | 0 | 1 | Ref |
| MG945620 | Unassigned | Unassigned | Unassigned | 1178_0 | Clustered | 3 | VC_1178_0 | 3 | 0.877 | 0.96173875 | 0.8434 | 0 | 0 | 0 | 1 | Ref |
| MG945621 | Unassigned | Unassigned | Unassigned | 1369_0 | Clustered | 10 | VC_1369_0 | 10 | 0.6223 | 0.95845085 | 0.5964 | 0 | 0 | 0 | 1 | Ref |
| MG945623 | Unassigned | Unassigned | Unassigned | 1238_0 | Clustered | 5 | VC_1238_0 | 5 | 0.5217 | 0.99999899 | 0.5217 | 0 | 0 | 0 | 1 | Ref |
| MG945624 | Unassigned | Unassigned | Unassigned | 1173_0 | Clustered | 3 | VC_1173_0 | 3 | 0.0986 | 0.78721935 | 0.0776 | 0 | 0 | 0 | 1 | Ref |
| MG945625 | Unassigned | Unassigned | Unassigned | 1307_0 | Clustered | 5 | VC_1307_0 | 5 | 0.6369 | 0.99918945 | 0.6364 | 0 | 0 | 0 | 1 | Ref |
| MG945627 | Unassigned | Unassigned | Unassigned | 1152_0 | Clustered | 26 | VC_1152_0 | 26 | 0.6685 | 1 | 0.6685 | 0 | 0 | 0 | 1 | Ref |
| MG945628 | Unassigned | Unassigned | Unassigned | 1364_0 | Clustered | 6 | VC_1364_0 | 6 | 0.9472 | 0.54366909 | 0.515 | 0 | 0 | 0 | 1 | Ref |
| MG945629 | Unassigned | Unassigned | Unassigned | 1307_0 | Clustered | 5 | VC_1307_0 | 5 | 0.6369 | 0.99918945 | 0.6364 | 0 | 0 | 0 | 1 | Ref |
| MG945631 | Unassigned | Unassigned | Unassigned | 1332_0 | Clustered | 2 | VC_1332_0 | 2 | 0.6366 | 0.72985431 | 0.4646 | 0 | 0 | 0 | 1 | Ref |
| MG945632 | Unassigned | Unassigned | Unassigned | 1307_0 | Clustered | 5 | VC_1307_0 | 5 | 0.6369 | 0.99918945 | 0.6364 | 0 | 0 | 0 | 1 | Ref |
| MG945633 | Unassigned | Unassigned | Unassigned | 1307_0 | Clustered | 5 | VC_1307_0 | 5 | 0.6369 | 0.99918945 | 0.6364 | 0 | 0 | 0 | 1 | Ref |
| MG945634 | Unassigned | Unassigned | Unassigned | 1299_0 | Clustered | 53 | VC_1299_0 | 53 | 0.8666 | 1 | 0.8666 | 0 | 0 | 0 | 1 | Ref |
| MG945635 | Unassigned | Unassigned | Unassigned | 1152_0 | Clustered | 26 | VC_1152_0 | 26 | 0.6685 | 1 | 0.6685 | 0 | 0 | 0 | 1 | Ref |
| MG945637 | Unassigned | Unassigned | Unassigned | 1354_0 | Clustered | 6 | VC_1354_0 | 6 | 0.6609 | 0.99999272 | 0.6609 | 0 | 0 | 0 | 1 | Ref |
| MG945639 | Unassigned | Unassigned | Unassigned | 1152_0 | Clustered | 26 | VC_1152_0 | 26 | 0.6685 | 1 | 0.6685 | 0 | 0 | 0 | 1 | Ref |
| MG945640 | Unassigned | Unassigned | Unassigned | 1346_0 | Clustered | 25 | VC_1346_0 | 25 | 1 | 1 | 1 | 0 | 0 | 0 | 1 | Ref |
| MG945641 | Unassigned | Unassigned | Unassigned | 1299_0 | Clustered | 53 | VC_1299_0 | 53 | 0.8666 | 1 | 0.8666 | 0 | 0 | 0 | 1 | Ref |
| MG945642 | Unassigned | Unassigned | Unassigned | 1170_0 | Clustered | 19 | VC_1170_0 | 19 | 0.7497 | 1 | 0.7497 | 0 | 0 | 0 | 1 | Ref |
| MG945643 | Unassigned | Unassigned | Unassigned | 1313_0 | Clustered | 4 | VC_1313_0 | 4 | 0.5804 | 0.94751743 | 0.5499 | 0 | 0 | 0 | 1 | Ref |
| MG945644 | Unassigned | Unassigned | Unassigned | 1181_0 | Clustered | 4 | VC_1181_0 | 4 | 0.1325 | 0.65559538 | 0.0869 | 0 | 0 | 0 | 1 | Ref |
| MG945645 | Unassigned | Unassigned | Unassigned | 1155_0 | Clustered | 14 | VC_1155_0 | 14 | 0.8287 | 1 | 0.8287 | 0 | 0 | 0 | 1 | Ref |
| MG945646 | Unassigned | Unassigned | Unassigned | 1145_0 | Clustered | 164 | VC_1145_0 | 164 | 0.8868 | 1 | 0.8868 | 0 | 0 | 0 | 1 | Ref |
| MG945647 | Unassigned | Unassigned | Unassigned | 1299_0 | Clustered | 53 | VC_1299_0 | 53 | 0.8666 | 1 | 0.8666 | 0 | 0 | 0 | 1 | Ref |
| MG945662 | Unassigned | Unassigned | Unassigned | 1314_0 | Clustered | 6 | VC_1314_0 | 6 | 0.6504 | 0.99999969 | 0.6504 | 0 | 0 | 0 | 1 | Ref |
| MG945663 | Unassigned | Unassigned | Unassigned | 1028_0 | Clustered | 69 | VC_1028_0 | 69 | 0.8547 | 1 | 0.8547 | 0 | 0 | 0 | 1 | Ref |
| MG945664 | Unassigned | Unassigned | Unassigned | 1267_0 | Clustered | 26 | VC_1267_0 | 26 | 0.9597 | 0.99999998 | 0.9597 | 0 | 0 | 0 | 1 | Ref |
| MG945665 | Unassigned | Unassigned | Unassigned | 1348_0 | Clustered | 7 | VC_1348_0 | 7 | 1 | 1 | 1 | 0 | 0 | 0 | 1 | Ref |
| MG945667 | Unassigned | Unassigned | Unassigned | 1285_0 | Clustered | 3 | VC_1285_0 | 3 | 0.7418 | 0.80676188 | 0.5984 | 0 | 0 | 0 | 1 | Ref |
| MG945668 | Unassigned | Unassigned | Unassigned | 1346_0 | Clustered | 25 | VC_1346_0 | 25 | 1 | 1 | 1 | 0 | 0 | 0 | 1 | Ref |
| MG945669 | Unassigned | Unassigned | Unassigned | 206_0 | Clustered | 28 | VC_206_0 | 28 | 0.2427 | 0.99971175 | 0.2426 | 0 | 0 | 0 | 1 | Ref |
| MG945670 | Unassigned | Unassigned | Unassigned | 1119_0 | Clustered | 48 | VC_1119_0 | 48 | 0.852 | 1 | 0.852 | 0 | 0 | 0 | 1 | Ref |
| MG945671 | Unassigned | Unassigned | Unassigned | 1145_0 | Clustered | 164 | VC_1145_0 | 164 | 0.8868 | 1 | 0.8868 | 0 | 0 | 0 | 1 | Ref |
| MG945672 | Unassigned | Unassigned | Unassigned | 1152_0 | Clustered | 26 | VC_1152_0 | 26 | 0.6685 | 1 | 0.6685 | 0 | 0 | 0 | 1 | Ref |
| MG945673 | Unassigned | Unassigned | Unassigned | 1145_0 | Clustered | 164 | VC_1145_0 | 164 | 0.8868 | 1 | 0.8868 | 0 | 0 | 0 | 1 | Ref |
| MG945674 | Unassigned | Unassigned | Unassigned | 1145_0 | Clustered | 164 | VC_1145_0 | 164 | 0.8868 | 1 | 0.8868 | 0 | 0 | 0 | 1 | Ref |
| MG945676 | Unassigned | Unassigned | Unassigned | 1189_0 | Clustered | 16 | VC_1189_0 | 16 | 0.8814 | 1 | 0.8814 | 0 | 0 | 0 | 1 | Ref |
| MG945678 | Unassigned | Unassigned | Unassigned | 1194_0 | Clustered | 2 | VC_1194_0 | 2 | 1 | 1 | 1 | 0 | 0 | 0 | 1 | Ref |
| MG945680 | Unassigned | Unassigned | Unassigned | 1267_0 | Clustered | 26 | VC_1267_0 | 26 | 0.9597 | 0.99999998 | 0.9597 | 0 | 0 | 0 | 1 | Ref |
| MG945681 | Unassigned | Unassigned | Unassigned | 1145_0 | Clustered | 164 | VC_1145_0 | 164 | 0.8868 | 1 | 0.8868 | 0 | 0 | 0 | 1 | Ref |
| MG945682 | Unassigned | Unassigned | Unassigned | 411_0 | Clustered | 48 | VC_411_0 | 48 | 0.4808 | 1 | 0.4808 | 0 | 0 | 0 | 1 | Ref |
| MG945683 | Unassigned | Unassigned | Unassigned | 1267_0 | Clustered | 26 | VC_1267_0 | 26 | 0.9597 | 0.99999998 | 0.9597 | 0 | 0 | 0 | 1 | Ref |
| MG945685 | Unassigned | Unassigned | Unassigned | 1308_0 | Clustered | 15 | VC_1308_0 | 15 | 0.9074 | 1 | 0.9074 | 0 | 0 | 0 | 1 | Ref |
| MG945686 | Unassigned | Unassigned | Unassigned | 690_0 | Clustered | 12 | VC_690_0 | 12 | 1 | 1 | 1 | 0 | 0 | 0 | 1 | Ref |
| MG945687 | Unassigned | Unassigned | Unassigned | 1316_0 | Clustered | 8 | VC_1316_0 | 8 | 0.821 | 1 | 0.821 | 0 | 0 | 0 | 1 | Ref |
| MG945689 | Unassigned | Unassigned | Unassigned | 1165_0 | Clustered | 10 | VC_1165_0 | 10 | 0.9263 | 1 | 0.9263 | 0 | 0 | 0 | 1 | Ref |
| MG945690 | Unassigned | Unassigned | Unassigned | 1390_0 | Clustered | 2 | VC_1390_0 | 2 | 1 | 1 | 1 | 0 | 0 | 0 | 1 | Ref |
| MG945691 | Unassigned | Unassigned | Unassigned | 1145_0 | Clustered | 164 | VC_1145_0 | 164 | 0.8868 | 1 | 0.8868 | 0 | 0 | 0 | 1 | Ref |
| MG945692 | Unassigned | Unassigned | Unassigned | 1304_0 | Clustered | 7 | VC_1304_0 | 7 | 0.9079 | 0.99998959 | 0.9079 | 0 | 0 | 0 | 1 | Ref |
| MG945693 | Unassigned | Unassigned | Unassigned | 1179_0 | Clustered | 4 | VC_1179_0 | 4 | 0.9241 | 0.99468305 | 0.9191 | 0 | 0 | 0 | 1 | Ref |
| MG945694 | Unassigned | Unassigned | Unassigned | 1170_0 | Clustered | 19 | VC_1170_0 | 19 | 0.7497 | 1 | 0.7497 | 0 | 0 | 0 | 1 | Ref |
| MG945696 | Unassigned | Unassigned | Unassigned | 1357_0 | Clustered | 7 | VC_1357_0 | 7 | 1 | 1 | 1 | 0 | 0 | 0 | 1 | Ref |
| MG945697 | Unassigned | Unassigned | Unassigned | 1304_0 | Clustered | 7 | VC_1304_0 | 7 | 0.9079 | 0.99998959 | 0.9079 | 0 | 0 | 0 | 1 | Ref |
| MG945698 | Unassigned | Unassigned | Unassigned | 1304_0 | Clustered | 7 | VC_1304_0 | 7 | 0.9079 | 0.99998959 | 0.9079 | 0 | 0 | 0 | 1 | Ref |
| MG945699 | Unassigned | Unassigned | Unassigned | 1145_0 | Clustered | 164 | VC_1145_0 | 164 | 0.8868 | 1 | 0.8868 | 0 | 0 | 0 | 1 | Ref |
| MG945700 | Unassigned | Unassigned | Unassigned | 1146_0 | Clustered | 3 | VC_1146_0 | 3 | 0.3655 | 0.99631816 | 0.3642 | 0 | 0 | 0 | 1 | Ref |

| | | | | | | | | | | | | | | | | |
|---|---|---|---|---|---|---|---|---|---|---|---|---|---|---|---|---|
| MG945702 | Unassigned | Unassigned | Unassigned | 1352_0 | Clustered | 3 | VC_1352_0 | 3 | 1 | 1 | 1 | 0 | 0 | 0 | 1 | Ref |
| MG945703 | Unassigned | Unassigned | Unassigned | 1095_0 | Clustered | 12 | VC_1095_0 | 12 | 0.2819 | 0.99837092 | 0.2814 | 0 | 0 | 0 | 1 | Ref |
| MG945705 | Unassigned | Unassigned | Unassigned | 1145_0 | Clustered | 164 | VC_1145_0 | 164 | 0.8868 | 1 | 0.8868 | 0 | 0 | 0 | 1 | Ref |
| MG945706 | Unassigned | Unassigned | Unassigned | 1189_0 | Clustered | 16 | VC_1189_0 | 16 | 0.8814 | 1 | 0.8814 | 0 | 0 | 0 | 1 | Ref |
| MG945707 | Unassigned | Unassigned | Unassigned | 1147_0 | Clustered | 6 | VC_1147_0 | 6 | 0.3066 | 0.99999957 | 0.3066 | 0 | 0 | 0 | 1 | Ref |
| MG945708 | Unassigned | Unassigned | Unassigned | 1145_0 | Clustered | 164 | VC_1145_0 | 164 | 0.8868 | 1 | 0.8868 | 0 | 0 | 0 | 1 | Ref |
| MG945710 | Unassigned | Unassigned | Unassigned | 1145_0 | Clustered | 164 | VC_1145_0 | 164 | 0.8868 | 1 | 0.8868 | 0 | 0 | 0 | 1 | Ref |
| MG945712 | Unassigned | Unassigned | Unassigned | 1346_0 | Clustered | 25 | VC_1346_0 | 25 | 1 | 1 | 1 | 0 | 0 | 0 | 1 | Ref |
| MG945713 | Unassigned | Unassigned | Unassigned | 1267_0 | Clustered | 26 | VC_1267_0 | 26 | 0.9597 | 0.99999998 | 0.9597 | 0 | 0 | 0 | 1 | Ref |
| MG945715 | Unassigned | Unassigned | Unassigned | 1145_0 | Clustered | 164 | VC_1145_0 | 164 | 0.8868 | 1 | 0.8868 | 0 | 0 | 0 | 1 | Ref |
| MG945716 | Unassigned | Unassigned | Unassigned | 1267_0 | Clustered | 26 | VC_1267_0 | 26 | 0.9597 | 0.99999998 | 0.9597 | 0 | 0 | 0 | 1 | Ref |
| MG945717 | Unassigned | Unassigned | Unassigned | 1164_0 | Clustered | 5 | VC_1164_0 | 5 | 0.831 | 0.99879053 | 0.83 | 0 | 0 | 0 | 1 | Ref |
| MG945718 | Unassigned | Unassigned | Unassigned | 1320_0 | Clustered | 10 | VC_1320_0 | 10 | 0.8478 | 1 | 0.8478 | 0 | 0 | 0 | 1 | Ref |
| MG945719 | Unassigned | Unassigned | Unassigned | 1185_0 | Clustered | 7 | VC_1185_0 | 7 | 0.6862 | 1 | 0.6862 | 0 | 0 | 0 | 1 | Ref |
| MG945721 | Unassigned | Unassigned | Unassigned | 1180_0 | Clustered | 4 | VC_1180_0 | 4 | 1 | 1 | 1 | 0 | 0 | 0 | 1 | Ref |
| MG945723 | Unassigned | Unassigned | Unassigned | 1309_0 | Clustered | 3 | VC_1309_0 | 3 | 0.3217 | 0.99790393 | 0.321 | 0 | 0 | 0 | 1 | Ref |
| MG945724 | Unassigned | Unassigned | Unassigned | 1306_0 | Clustered | 4 | VC_1306_0 | 4 | 0.8184 | 0.99530591 | 0.8146 | 0 | 0 | 0 | 1 | Ref |
| MG945725 | Unassigned | Unassigned | Unassigned | 1286_0 | Clustered | 16 | VC_1286_0 | 16 | 0.9448 | 1 | 0.9448 | 0 | 0 | 0 | 1 | Ref |
| MG945726 | Unassigned | Unassigned | Unassigned | 1191_0 | Clustered | 9 | VC_1191_0 | 9 | 0.8537 | 1 | 0.8537 | 0 | 0 | 0 | 1 | Ref |
| MG945727 | Unassigned | Unassigned | Unassigned | 1192_0 | Clustered | 4 | VC_1192_0 | 4 | 0.7187 | 0.99971165 | 0.7185 | 0 | 0 | 0 | 1 | Ref |
| MG945728 | Unassigned | Unassigned | Unassigned | 1145_0 | Clustered | 164 | VC_1145_0 | 164 | 0.8868 | 1 | 0.8868 | 0 | 0 | 0 | 1 | Ref |
| MG945729 | Unassigned | Unassigned | Unassigned | 1348_0 | Clustered | 7 | VC_1348_0 | 7 | 1 | 1 | 1 | 0 | 0 | 0 | 1 | Ref |
| MG945730 | Unassigned | Unassigned | Unassigned | 1267_0 | Clustered | 26 | VC_1267_0 | 26 | 0.9597 | 0.99999998 | 0.9597 | 0 | 0 | 0 | 1 | Ref |
| MG945731 | Unassigned | Unassigned | Unassigned | 1145_0 | Clustered | 164 | VC_1145_0 | 164 | 0.8868 | 1 | 0.8868 | 0 | 0 | 0 | 1 | Ref |
| MG945733 | Unassigned | Unassigned | Unassigned | 1346_0 | Clustered | 25 | VC_1346_0 | 25 | 1 | 1 | 1 | 0 | 0 | 0 | 1 | Ref |
| MG945734 | Unassigned | Unassigned | Unassigned | 1304_0 | Clustered | 7 | VC_1304_0 | 7 | 0.9079 | 0.99998959 | 0.9079 | 0 | 0 | 0 | 1 | Ref |
| MG945735 | Unassigned | Unassigned | Unassigned | 1237_0 | Clustered | 3 | VC_1237_0 | 3 | 0.5448 | 0.99523779 | 0.5422 | 0 | 0 | 0 | 1 | Ref |
| MG945736 | Unassigned | Unassigned | Unassigned | 1155_0 | Clustered | 14 | VC_1155_0 | 14 | 0.8287 | 1 | 0.8287 | 0 | 0 | 0 | 1 | Ref |
| MG945738 | Unassigned | Unassigned | Unassigned | 1173_0 | Clustered | 3 | VC_1173_0 | 3 | 0.0986 | 0.78721935 | 0.0776 | 0 | 0 | 0 | 1 | Ref |
| MG945739 | Unassigned | Unassigned | Unassigned | 1164_0 | Clustered | 5 | VC_1164_0 | 5 | 0.831 | 0.99879053 | 0.83 | 0 | 0 | 0 | 1 | Ref |
| MG945740 | Unassigned | Unassigned | Unassigned | 1155_0 | Clustered | 14 | VC_1155_0 | 14 | 0.8287 | 1 | 0.8287 | 0 | 0 | 0 | 1 | Ref |
| MG945741 | Unassigned | Unassigned | Unassigned | 1178_0 | Clustered | 3 | VC_1178_0 | 3 | 0.877 | 0.96173875 | 0.8434 | 0 | 0 | 0 | 1 | Ref |
| MG945742 | Unassigned | Unassigned | Unassigned | 1153_0 | Clustered | 2 | VC_1153_0 | 2 | 0.0506 | 0.94785574 | 0.048 | 0 | 0 | 0 | 1 | Ref |
| MG945743 | Unassigned | Unassigned | Unassigned | 1299_0 | Clustered | 53 | VC_1299_0 | 53 | 0.8666 | 1 | 0.8666 | 0 | 0 | 0 | 1 | Ref |
| MG945744 | Unassigned | Unassigned | Unassigned | 1145_0 | Clustered | 164 | VC_1145_0 | 164 | 0.8868 | 1 | 0.8868 | 0 | 0 | 0 | 1 | Ref |
| MG945745 | Unassigned | Unassigned | Unassigned | 1308_0 | Clustered | 15 | VC_1308_0 | 15 | 0.9074 | 1 | 0.9074 | 0 | 0 | 0 | 1 | Ref |
| MG945746 | Unassigned | Unassigned | Unassigned | 1152_0 | Clustered | 26 | VC_1152_0 | 26 | 0.6685 | 1 | 0.6685 | 0 | 0 | 0 | 1 | Ref |
| MG945747 | Unassigned | Unassigned | Unassigned | 1162_0 | Clustered | 3 | VC_1162_0 | 3 | 0.6188 | 0.98688028 | 0.6106 | 0 | 0 | 0 | 1 | Ref |
| MG945748 | Unassigned | Unassigned | Unassigned | 1240_0 | Clustered | 3 | VC_1240_0 | 3 | 0.3359 | 0.99743975 | 0.3351 | 0 | 0 | 0 | 1 | Ref |
| MG945749 | Unassigned | Unassigned | Unassigned | 1365_0 | Clustered/Singleton | 1 | VC_1365_0 | 1 | 0 | 1 | 0 | 0 | 0 | 0 | 1 | Ref |
| MG945750 | Unassigned | Unassigned | Unassigned | 1325_0 | Clustered | 7 | VC_1325_0 | 7 | 1 | 1 | 1 | 0 | 0 | 0 | 1 | Ref |
| MG945751 | Unassigned | Unassigned | Unassigned | 1340_0 | Clustered | 7 | VC_1340_0 | 7 | 0.9206 | 0.99995294 | 0.9206 | 0 | 0 | 0 | 1 | Ref |
| MG945752 | Unassigned | Unassigned | Unassigned | 1237_0 | Clustered | 3 | VC_1237_0 | 3 | 0.5448 | 0.99523779 | 0.5422 | 0 | 0 | 0 | 1 | Ref |
| MG945753 | Unassigned | Unassigned | Unassigned | 1170_0 | Clustered | 19 | VC_1170_0 | 19 | 0.7497 | 1 | 0.7497 | 0 | 0 | 0 | 1 | Ref |
| MG945754 | Unassigned | Unassigned | Unassigned | 1191_0 | Clustered | 9 | VC_1191_0 | 9 | 0.8537 | 1 | 0.8537 | 0 | 0 | 0 | 1 | Ref |
| MG945755 | Unassigned | Unassigned | Unassigned | 1353_0 | Clustered | 4 | VC_1353_0 | 4 | 0.7838 | 0.99289298 | 0.7782 | 0 | 0 | 0 | 1 | Ref |
| MG945756 | Unassigned | Unassigned | Unassigned | 690_0 | Clustered | 12 | VC_690_0 | 12 | 1 | 1 | 1 | 0 | 0 | 0 | 1 | Ref |
| MG945757 | Unassigned | Unassigned | Unassigned | 1286_0 | Clustered | 16 | VC_1286_0 | 16 | 0.9448 | 1 | 0.9448 | 0 | 0 | 0 | 1 | Ref |
| MG945758 | Unassigned | Unassigned | Unassigned | 1145_0 | Clustered | 164 | VC_1145_0 | 164 | 0.8868 | 1 | 0.8868 | 0 | 0 | 0 | 1 | Ref |
| MG945759 | Unassigned | Unassigned | Unassigned | 1184_0 | Clustered | 10 | VC_1184_0 | 10 | 0.7506 | 1 | 0.7506 | 0 | 0 | 0 | 1 | Ref |
| MG945760 | Unassigned | Unassigned | Unassigned | 1184_0 | Clustered | 10 | VC_1184_0 | 10 | 0.7506 | 1 | 0.7506 | 0 | 0 | 0 | 1 | Ref |
| MG945761 | Unassigned | Unassigned | Unassigned | 1145_0 | Clustered | 164 | VC_1145_0 | 164 | 0.8868 | 1 | 0.8868 | 0 | 0 | 0 | 1 | Ref |
| MG945764 | Unassigned | Unassigned | Unassigned | 1189_0 | Clustered | 16 | VC_1189_0 | 16 | 0.8814 | 1 | 0.8814 | 0 | 0 | 0 | 1 | Ref |
| MG945765 | Unassigned | Unassigned | Unassigned | 1145_0 | Clustered | 164 | VC_1145_0 | 164 | 0.8868 | 1 | 0.8868 | 0 | 0 | 0 | 1 | Ref |
| MG945766 | Unassigned | Unassigned | Unassigned | 1391_0 | Clustered | 2 | VC_1391_0 | 2 | 1 | 1 | 1 | 0 | 0 | 0 | 1 | Ref |
| MG945767 | Unassigned | Unassigned | Unassigned | 1152_0 | Clustered | 26 | VC_1152_0 | 26 | 0.6685 | 1 | 0.6685 | 0 | 0 | 0 | 1 | Ref |
| MG945768 | Unassigned | Unassigned | Unassigned | 1145_0 | Clustered | 164 | VC_1145_0 | 164 | 0.8868 | 1 | 0.8868 | 0 | 0 | 0 | 1 | Ref |
| MG945769 | Unassigned | Unassigned | Unassigned | 1392_0 | Clustered | 2 | VC_1392_0 | 2 | 1 | 1 | 1 | 0 | 0 | 0 | 1 | Ref |
| MG945770 | Unassigned | Unassigned | Unassigned | 1145_0 | Clustered | 164 | VC_1145_0 | 164 | 0.8868 | 1 | 0.8868 | 0 | 0 | 0 | 1 | Ref |
| MG945771 | Unassigned | Unassigned | Unassigned | 1155_0 | Clustered | 14 | VC_1155_0 | 14 | 0.8287 | 1 | 0.8287 | 0 | 0 | 0 | 1 | Ref |
| MG945774 | Unassigned | Unassigned | Unassigned | 1308_0 | Clustered | 15 | VC_1308_0 | 15 | 0.9074 | 1 | 0.9074 | 0 | 0 | 0 | 1 | Ref |
| MG945775 | Unassigned | Unassigned | Unassigned | 1148_0 | Clustered | 4 | VC_1148_0 | 4 | 0.0503 | 0.99998999 | 0.0503 | 0 | 0 | 0 | 1 | Ref |
| MG945776 | Unassigned | Unassigned | Unassigned | 1325_0 | Clustered | 7 | VC_1325_0 | 7 | 1 | 1 | 1 | 0 | 0 | 0 | 1 | Ref |
| MG945778 | Unassigned | Unassigned | Unassigned | 1349_0 | Clustered | 3 | VC_1349_0 | 3 | 1 | 1 | 1 | 0 | 0 | 0 | 1 | Ref |
| MG945780 | Unassigned | Unassigned | Unassigned | 1169_0 | Clustered | 8 | VC_1169_0 | 8 | 0.2897 | 1 | 0.2897 | 0 | 0 | 0 | 1 | Ref |
| MG945781 | Unassigned | Unassigned | Unassigned | 1170_0 | Clustered | 19 | VC_1170_0 | 19 | 0.7497 | 1 | 0.7497 | 0 | 0 | 0 | 1 | Ref |
| MG945782 | Unassigned | Unassigned | Unassigned | 1348_0 | Clustered | 7 | VC_1348_0 | 7 | 1 | 1 | 1 | 0 | 0 | 0 | 1 | Ref |
| MG945783 | Unassigned | Unassigned | Unassigned | 1320_0 | Clustered | 10 | VC_1320_0 | 10 | 0.8478 | 1 | 0.8478 | 0 | 0 | 0 | 1 | Ref |
| MG945784 | Unassigned | Unassigned | Unassigned | 1267_0 | Clustered | 26 | VC_1267_0 | 26 | 0.9597 | 0.99999998 | 0.9597 | 0 | 0 | 0 | 1 | Ref |
| MG945785 | Unassigned | Unassigned | Unassigned | 1192_0 | Clustered | 4 | VC_1192_0 | 4 | 0.7187 | 0.99971165 | 0.7185 | 0 | 0 | 0 | 1 | Ref |
| MG945786 | Unassigned | Unassigned | Unassigned | 1145_0 | Clustered | 164 | VC_1145_0 | 164 | 0.8868 | 1 | 0.8868 | 0 | 0 | 0 | 1 | Ref |
| MG945787 | Unassigned | Unassigned | Unassigned | 1145_0 | Clustered | 164 | VC_1145_0 | 164 | 0.8868 | 1 | 0.8868 | 0 | 0 | 0 | 1 | Ref |
| MG945788 | Unassigned | Unassigned | Unassigned | 1393_0 | Clustered | 2 | VC_1393_0 | 2 | 1 | 1 | 1 | 0 | 0 | 0 | 1 | Ref |
| MG945789 | Unassigned | Unassigned | Unassigned | 1337_0 | Clustered | 3 | VC_1337_0 | 3 | 0.394 | 0.99705286 | 0.3928 | 0 | 0 | 0 | 1 | Ref |
| MG945790 | Unassigned | Unassigned | Unassigned | 1267_0 | Clustered | 26 | VC_1267_0 | 26 | 0.9597 | 0.99999998 | 0.9597 | 0 | 0 | 0 | 1 | Ref |
| MG945791 | Unassigned | Unassigned | Unassigned | 1325_0 | Clustered | 7 | VC_1325_0 | 7 | 1 | 1 | 1 | 0 | 0 | 0 | 1 | Ref |
| MG945792 | Unassigned | Unassigned | Unassigned | 1392_0 | Clustered | 2 | VC_1392_0 | 2 | 1 | 1 | 1 | 0 | 0 | 0 | 1 | Ref |
| MG945793 | Unassigned | Unassigned | Unassigned | 1145_0 | Clustered | 164 | VC_1145_0 | 164 | 0.8868 | 1 | 0.8868 | 0 | 0 | 0 | 1 | Ref |
| MG945794 | Unassigned | Unassigned | Unassigned | 1241_0 | Clustered | 13 | VC_1241_0 | 13 | 0.8675 | 0.76044592 | 0.6597 | 0 | 0 | 0 | 1 | Ref |
| MG945795 | Unassigned | Unassigned | Unassigned | 1393_0 | Clustered | 2 | VC_1393_0 | 2 | 1 | 1 | 1 | 0 | 0 | 0 | 1 | Ref |
| MG945796 | Unassigned | Unassigned | Unassigned | 1145_0 | Clustered | 164 | VC_1145_0 | 164 | 0.8868 | 1 | 0.8868 | 0 | 0 | 0 | 1 | Ref |

| | | | | | | | | | | | | | | | | |
|---|---|---|---|---|---|---|---|---|---|---|---|---|---|---|---|---|
| MG945797 | Unassigned | Unassigned | Unassigned | 1165_0 | Clustered | 10 | VC_1165_0 | 10 | 0.9263 | 1 | 0.9263 | 0 | 0 | 0 | 1 | Ref |
| MG945798 | Unassigned | Unassigned | Unassigned | 1394_0 | Clustered | 4 | VC_1394_0 | 4 | 0.7326 | 0.99899459 | 0.7318 | 0 | 0 | 0 | 1 | Ref |
| MG945799 | Unassigned | Unassigned | Unassigned | 1240_0 | Clustered | 3 | VC_1240_0 | 3 | 0.3359 | 0.99743975 | 0.3351 | 0 | 0 | 0 | 1 | Ref |
| MG945800 | Unassigned | Unassigned | Unassigned | 1240_0 | Clustered | 3 | VC_1240_0 | 3 | 0.3359 | 0.99743975 | 0.3351 | 0 | 0 | 0 | 1 | Ref |
| MG945801 | Unassigned | Unassigned | Unassigned | 1145_0 | Clustered | 164 | VC_1145_0 | 164 | 0.8868 | 1 | 0.8868 | 0 | 0 | 0 | 1 | Ref |
| MG945802 | Unassigned | Unassigned | Unassigned | 1145_0 | Clustered | 164 | VC_1145_0 | 164 | 0.8868 | 1 | 0.8868 | 0 | 0 | 0 | 1 | Ref |
| MG945803 | Unassigned | Unassigned | Unassigned | 1308_0 | Clustered | 15 | VC_1308_0 | 15 | 0.9074 | 1 | 0.9074 | 0 | 0 | 0 | 1 | Ref |
| MG945804 | Unassigned | Unassigned | Unassigned | 1315_0 | Clustered | 3 | VC_1315_0 | 3 | 0.2423 | 0.85925819 | 0.2082 | 0 | 0 | 0 | 1 | Ref |
| MG945805 | Unassigned | Unassigned | Unassigned | 1185_0 | Clustered | 7 | VC_1185_0 | 7 | 0.6862 | 1 | 0.6862 | 0 | 0 | 0 | 1 | Ref |
| MG945806 | Unassigned | Unassigned | Unassigned | 1346_0 | Clustered | 25 | VC_1346_0 | 25 | 1 | 1 | 1 | 0 | 0 | 0 | 1 | Ref |
| MG945807 | Unassigned | Unassigned | Unassigned | 1156_0 | Clustered | 5 | VC_1156_0 | 5 | 0.4454 | 0.99999977 | 0.4454 | 0 | 0 | 0 | 1 | Ref |
| MG945808 | Unassigned | Unassigned | Unassigned | 1396_0 | Clustered | 2 | VC_1396_0 | 2 | 1 | 1 | 1 | 0 | 0 | 0 | 1 | Ref |
| MG945809 | Unassigned | Unassigned | Unassigned | 1179_0 | Clustered | 4 | VC_1179_0 | 4 | 0.9241 | 0.99468305 | 0.9191 | 0 | 0 | 0 | 1 | Ref |
| MG945810 | Unassigned | Unassigned | Unassigned | 1192_0 | Clustered | 4 | VC_1192_0 | 4 | 0.7187 | 0.99971165 | 0.7185 | 0 | 0 | 0 | 1 | Ref |
| MG945811 | Unassigned | Unassigned | Unassigned | 1184_0 | Clustered | 10 | VC_1184_0 | 10 | 0.7506 | 1 | 0.7506 | 0 | 0 | 0 | 1 | Ref |
| MG945812 | Unassigned | Unassigned | Unassigned | 1340_0 | Clustered | 7 | VC_1340_0 | 7 | 0.9206 | 0.99995294 | 0.9206 | 0 | 0 | 0 | 1 | Ref |
| MG945813 | Unassigned | Unassigned | Unassigned | 1267_0 | Clustered | 26 | VC_1267_0 | 26 | 0.9597 | 0.99999998 | 0.9597 | 0 | 0 | 0 | 1 | Ref |
| MG945814 | Unassigned | Unassigned | Unassigned | 1346_0 | Clustered | 25 | VC_1346_0 | 25 | 1 | 1 | 1 | 0 | 0 | 0 | 1 | Ref |
| MG945815 | Unassigned | Unassigned | Unassigned | 1314_0 | Clustered | 6 | VC_1314_0 | 6 | 0.6504 | 0.99999969 | 0.6504 | 0 | 0 | 0 | 1 | Ref |
| MG945816 | Unassigned | Unassigned | Unassigned | 1191_0 | Clustered | 9 | VC_1191_0 | 9 | 0.8537 | 1 | 0.8537 | 0 | 0 | 0 | 1 | Ref |
| MG945817 | Unassigned | Unassigned | Unassigned | 1189_0 | Clustered | 16 | VC_1189_0 | 16 | 0.8814 | 1 | 0.8814 | 0 | 0 | 0 | 1 | Ref |
| MG945818 | Unassigned | Unassigned | Unassigned | 1351_0 | Clustered | 2 | VC_1351_0 | 2 | 1 | 1 | 1 | 0 | 0 | 0 | 1 | Ref |
| MG945819 | Unassigned | Unassigned | Unassigned | 1155_0 | Clustered | 14 | VC_1155_0 | 14 | 0.8287 | 1 | 0.8287 | 0 | 0 | 0 | 1 | Ref |
| MG945820 | Unassigned | Unassigned | Unassigned | 1145_0 | Clustered | 164 | VC_1145_0 | 164 | 0.8868 | 1 | 0.8868 | 0 | 0 | 0 | 1 | Ref |
| MG945821 | Unassigned | Unassigned | Unassigned | 1397_0 | Clustered/Singleton | 1 | VC_1397_0 | 1 | 0 | 1 | 0 | 0 | 0 | 0 | 1 | Ref |
| MG945822 | Unassigned | Unassigned | Unassigned | 1331_0 | Clustered | 6 | VC_1331_0 | 6 | 0.7375 | 0.9999804 | 0.7375 | 0 | 0 | 0 | 1 | Ref |
| MG945823 | Unassigned | Unassigned | Unassigned | 1391_0 | Clustered | 2 | VC_1391_0 | 2 | 1 | 1 | 1 | 0 | 0 | 0 | 1 | Ref |
| MG945824 | Unassigned | Unassigned | Unassigned | 1367_0 | Clustered | 5 | VC_1367_0 | 5 | 0.7699 | 0.66985041 | 0.5157 | 0 | 0 | 0 | 1 | Ref |
| MG945825 | Unassigned | Unassigned | Unassigned | 1340_0 | Clustered | 7 | VC_1340_0 | 7 | 0.9206 | 0.99995294 | 0.9206 | 0 | 0 | 0 | 1 | Ref |
| MG945826 | Unassigned | Unassigned | Unassigned | 1161_0 | Clustered | 16 | VC_1161_0 | 16 | 0.4834 | 0.99999958 | 0.4834 | 0 | 0 | 0 | 1 | Ref |
| MG945827 | Unassigned | Unassigned | Unassigned | 1187_0 | Clustered | 3 | VC_1187_0 | 3 | 0.5022 | 0.99747895 | 0.501 | 0 | 0 | 0 | 1 | Ref |
| MG945828 | Unassigned | Unassigned | Unassigned | 1361_0 | Clustered | 2 | VC_1361_0 | 2 | 0.6617 | 0.72985431 | 0.4829 | 0 | 0 | 0 | 1 | Ref |
| MG945829 | Unassigned | Unassigned | Unassigned | 1308_0 | Clustered | 15 | VC_1308_0 | 15 | 0.9074 | 1 | 0.9074 | 0 | 0 | 0 | 1 | Ref |
| MG945831 | Unassigned | Unassigned | Unassigned | 1398_0 | Clustered | 7 | VC_1398_0 | 7 | 0.8336 | 0.99999844 | 0.8336 | 0 | 0 | 0 | 1 | Ref |
| MG945832 | Unassigned | Unassigned | Unassigned | 1400_0 | Clustered | 2 | VC_1400_0 | 2 | 1 | 1 | 1 | 0 | 0 | 0 | 1 | Ref |
| MG945835 | Unassigned | Unassigned | Unassigned | 1101_0 | Clustered | 3 | VC_1101_0 | 3 | 0.3918 | 0.9839946 | 0.3856 | 0 | 0 | 0 | 1 | Ref |
| MG945836 | Unassigned | Unassigned | Unassigned | 1119_0 | Clustered | 48 | VC_1119_0 | 48 | 0.852 | 1 | 0.852 | 0 | 0 | 0 | 1 | Ref |
| MG945837 | Unassigned | Unassigned | Unassigned | 411_0 | Clustered | 48 | VC_411_0 | 48 | 0.4808 | 1 | 0.4808 | 0 | 0 | 0 | 1 | Ref |
| MG945838 | Unassigned | Unassigned | Unassigned | 1028_0 | Clustered | 69 | VC_1028_0 | 69 | 0.8547 | 1 | 0.8547 | 0 | 0 | 0 | 1 | Ref |
| MG945839 | Unassigned | Unassigned | Unassigned | 1401_0 | Clustered | 4 | VC_1401_0 | 4 | 1 | 1 | 1 | 0 | 0 | 0 | 1 | Ref |
| MG945840 | Unassigned | Unassigned | Unassigned | 1119_0 | Clustered | 48 | VC_1119_0 | 48 | 0.852 | 1 | 0.852 | 0 | 0 | 0 | 1 | Ref |
| MG945842 | Unassigned | Unassigned | Unassigned | 1402_0 | Clustered | 7 | VC_1402_0 | 7 | 0.9174 | 0.99772305 | 0.9153 | 0 | 0 | 0 | 1 | Ref |
| MG945843 | Unassigned | Unassigned | Unassigned | 1028_0 | Clustered | 69 | VC_1028_0 | 69 | 0.8547 | 1 | 0.8547 | 0 | 0 | 0 | 1 | Ref |
| MG945845 | Unassigned | Unassigned | Unassigned | 1267_0 | Clustered | 26 | VC_1267_0 | 26 | 0.9597 | 0.99999998 | 0.9597 | 0 | 0 | 0 | 1 | Ref |
| MG945846 | Unassigned | Unassigned | Unassigned | 1119_0 | Clustered | 48 | VC_1119_0 | 48 | 0.852 | 1 | 0.852 | 0 | 0 | 0 | 1 | Ref |
| MG945848 | Unassigned | Unassigned | Unassigned | 1324_0 | Clustered | 5 | VC_1324_0 | 5 | 1 | 1 | 1 | 0 | 0 | 0 | 1 | Ref |
| MG945849 | Unassigned | Unassigned | Unassigned | 1396_0 | Clustered | 2 | VC_1396_0 | 2 | 1 | 1 | 1 | 0 | 0 | 0 | 1 | Ref |
| MG945850 | Unassigned | Unassigned | Unassigned | 1403_0 | Clustered | 5 | VC_1403_0 | 5 | 1 | 1 | 1 | 0 | 0 | 0 | 1 | Ref |
| MG945852 | Unassigned | Unassigned | Unassigned | 1324_0 | Clustered | 5 | VC_1324_0 | 5 | 1 | 1 | 1 | 0 | 0 | 0 | 1 | Ref |
| MG945853 | Unassigned | Unassigned | Unassigned | 1145_0 | Clustered | 164 | VC_1145_0 | 164 | 0.8868 | 1 | 0.8868 | 0 | 0 | 0 | 1 | Ref |
| MG945855 | Unassigned | Unassigned | Unassigned | 1145_0 | Clustered | 164 | VC_1145_0 | 164 | 0.8868 | 1 | 0.8868 | 0 | 0 | 0 | 1 | Ref |
| MG945857 | Unassigned | Unassigned | Unassigned | 1405_0 | Clustered | 3 | VC_1405_0 | 3 | 1 | 1 | 1 | 0 | 0 | 0 | 1 | Ref |
| MG945858 | Unassigned | Unassigned | Unassigned | 1152_0 | Clustered | 26 | VC_1152_0 | 26 | 0.6685 | 1 | 0.6685 | 0 | 0 | 0 | 1 | Ref |
| MG945859 | Unassigned | Unassigned | Unassigned | 1405_0 | Clustered | 3 | VC_1405_0 | 3 | 1 | 1 | 1 | 0 | 0 | 0 | 1 | Ref |
| MG945862 | Unassigned | Unassigned | Unassigned | 1145_0 | Clustered | 164 | VC_1145_0 | 164 | 0.8868 | 1 | 0.8868 | 0 | 0 | 0 | 1 | Ref |
| MG945864 | Unassigned | Unassigned | Unassigned | 1145_0 | Clustered | 164 | VC_1145_0 | 164 | 0.8868 | 1 | 0.8868 | 0 | 0 | 0 | 1 | Ref |
| MG945866 | Unassigned | Unassigned | Unassigned | 1405_0 | Clustered | 3 | VC_1405_0 | 3 | 1 | 1 | 1 | 0 | 0 | 0 | 1 | Ref |
| MG945867 | Unassigned | Unassigned | Unassigned | 1167_0 | Clustered | 11 | VC_1167_0 | 11 | 0.5637 | 1 | 0.5637 | 0 | 0 | 0 | 1 | Ref |
| MG945869 | Unassigned | Unassigned | Unassigned | 1145_0 | Clustered | 164 | VC_1145_0 | 164 | 0.8868 | 1 | 0.8868 | 0 | 0 | 0 | 1 | Ref |
| MG945870 | Unassigned | Unassigned | Unassigned | 1165_0 | Clustered | 10 | VC_1165_0 | 10 | 0.9263 | 1 | 0.9263 | 0 | 0 | 0 | 1 | Ref |
| MG945909 | Unassigned | Unassigned | Unassigned | 1315_0 | Clustered | 3 | VC_1315_0 | 3 | 0.2423 | 0.85925819 | 0.2082 | 0 | 0 | 0 | 1 | Ref |
| MG957431 | Unassigned | Unassigned | Unassigned | 83_0 | Clustered | 24 | VC_83_0 | 24 | 0.7345 | 1 | 0.7345 | 0 | 0 | 0 | 1 | Ref |
| MG962360 | Unassigned | Unassigned | Unassigned | 1296_0 | Clustered | 82 | VC_1296_0 | 82 | 0.7938 | 1 | 0.7938 | 0 | 0 | 0 | 1 | Ref |
| MG962361 | Unassigned | Unassigned | Unassigned | 538_64 | Clustered/Singleton | 1 | VC_538_64 | 1 | 0 | 1 | 0 | 0 | 0 | 0 | 1 | Ref |
| MG962362 | Unassigned | Unassigned | Unassigned | 384_3 | Clustered | 22 | VC_384_3 | 22 | 0.0786 | 1 | 0.0786 | 0 | 0 | 0 | 0.9048 | Ref |
| MG962363 | Unassigned | Unassigned | Unassigned | 384_13 | Clustered | 33 | VC_384_13 | 33 | 0.1785 | 1 | 0.1785 | 0 | 0 | 0 | 0.8769 | Ref |
| MG962364 | Unassigned | Unassigned | Unassigned | 243_0 | Clustered | 72 | VC_243_0 | 72 | 0.8089 | 1 | 0.8089 | 0 | 0 | 0 | 1 | Ref |
| MG962365 | Unassigned | Unassigned | Unassigned | 384_16 | Clustered | 42 | VC_384_16 | 42 | 0.134 | 1 | 0.134 | 0 | 0 | 0 | 0.7282 | Ref |
| MG962367 | Unassigned | Unassigned | Unassigned | 1296_0 | Clustered | 82 | VC_1296_0 | 82 | 0.7938 | 1 | 0.7938 | 0 | 0 | 0 | 1 | Ref |
| MG962368 | Unassigned | Unassigned | Unassigned | 898_1 | Clustered | 27 | VC_898_1 | 27 | 0.8894 | 1 | 0.8894 | 0 | 0 | 0 | 1 | Ref |
| MG962369 | Unassigned | Unassigned | Unassigned | 898_1 | Clustered | 27 | VC_898_1 | 27 | 0.8894 | 1 | 0.8894 | 0 | 0 | 0 | 1 | Ref |
| MG962370 | Unassigned | Unassigned | Unassigned | 335_41 | Clustered | 2 | VC_335_41 | 2 | 0.0045 | 0.9575092 | 0.0043 | 0 | 0 | 0 | 1 | Ref |
| MG962371 | Unassigned | Unassigned | Unassigned | 243_0 | Clustered | 72 | VC_243_0 | 72 | 0.8089 | 1 | 0.8089 | 0 | 0 | 0 | 1 | Ref |
| MG962372 | Unassigned | Unassigned | Unassigned | 335_5 | Clustered | 11 | VC_335_5 | 11 | 0.0469 | 1 | 0.0469 | 0 | 0 | 0 | 0.7636 | Ref |
| MG962373 | Unassigned | Unassigned | Unassigned | 535_1 | Clustered | 108 | VC_535_1 | 108 | 0.8479 | 1 | 0.8479 | 0 | 0 | 0 | 0.9955 | Ref |
| MG962374 | Unassigned | Unassigned | Unassigned | 245_0 | Clustered | 17 | VC_245_0 | 17 | 0.6081 | 1 | 0.6081 | 0 | 0 | 0 | 1 | Ref |
| MG962375 | Unassigned | Unassigned | Unassigned | 384_2 | Clustered | 8 | VC_384_2 | 8 | 0.0232 | 1 | 0.0232 | 0 | 0 | 0 | 0.8571 | Ref |
| MG962376 | Unassigned | Unassigned | Unassigned | 587_0 | Clustered | 8 | VC_587_0 | 8 | 0.4907 | 1 | 0.4907 | 0 | 0 | 0 | 1 | Ref |
| MG963916 | Unassigned | Unassigned | Unassigned | 794_16 | Clustered | 10 | VC_794_16 | 10 | 0.0563 | 0.9411319 | 0.053 | 0 | 0 | 0 | 0.8667 | Ref |
| MG966531 | Unassigned | Unassigned | Unassigned | 405_0 | Clustered | 126 | VC_405_0 | 126 | 0.9614 | 1 | 0.9614 | 0 | 0 | 0 | 1 | Ref |
| MG966533 | Unassigned | Unassigned | Unassigned | 284_0 | Clustered | 3 | VC_284_0 | 3 | 1 | 1 | 1 | 0 | 0 | 0 | 1 | Ref |

| | | | | | | | | | | | | | | | | |
|---|---|---|---|---|---|---|---|---|---|---|---|---|---|---|---|---|
| MG967616 | Unassigned | Unassigned | Unassigned | 1042_0 | Clustered | 8 | VC_1042_0 | 8 | 0.7385 | 1 | 0.7385 | 0 | 0 | 0 | 1 | Ref |
| MG967617 | Unassigned | Unassigned | Unassigned | 1042_0 | Clustered | 8 | VC_1042_0 | 8 | 0.7385 | 1 | 0.7385 | 0 | 0 | 0 | 1 | Ref |
| MG967618 | Unassigned | Unassigned | Unassigned | 1042_0 | Clustered | 8 | VC_1042_0 | 8 | 0.7385 | 1 | 0.7385 | 0 | 0 | 0 | 1 | Ref |
| MG969404 | Unassigned | Unassigned | Unassigned | 340_14 | Clustered/Singleton | 1 | VC_340_14 | 1 | 0 | 1 | 0 | 0 | 0 | 0 | 1 | Ref |
| MG969405 | Unassigned | Unassigned | Unassigned | 340_1 | Clustered | 17 | VC_340_1 | 17 | 0.0863 | 1 | 0.0863 | 0 | 0 | 0 | 0.8235 | Ref |
| MG969406 | Unassigned | Unassigned | Unassigned | 340_1 | Clustered | 17 | VC_340_1 | 17 | 0.0863 | 1 | 0.0863 | 0 | 0 | 0 | 0.8235 | Ref |
| MG969408 | Unassigned | Unassigned | Unassigned | 654_0 | Clustered | 115 | VC_654_0 | 115 | 0.7303 | 1 | 0.7303 | 0 | 0 | 0 | 0.8764 | Ref |
| MG969409 | Unassigned | Unassigned | Unassigned | 654_0 | Clustered | 115 | VC_654_0 | 115 | 0.7303 | 1 | 0.7303 | 0 | 0 | 0 | 0.8764 | Ref |
| MG969410 | Unassigned | Unassigned | Unassigned | 654_0 | Clustered | 115 | VC_654_0 | 115 | 0.7303 | 1 | 0.7303 | 0 | 0 | 0 | 0.8764 | Ref |
| MG969411 | Unassigned | Unassigned | Unassigned | 601_6 | Clustered/Singleton | 1 | VC_601_6 | 1 | 0 | 1 | 0 | 0 | 0 | 0 | 1 | Ref |
| MG969412 | Unassigned | Unassigned | Unassigned | 198_40 | Clustered | 2 | VC_198_40 | 2 | 0.0082 | 0.95556395 | 0.0078 | 0 | 0 | 0 | 1 | Ref |
| MG969415 | Unassigned | Unassigned | Unassigned | 30_1 | Clustered | 53 | VC_30_1 | 53 | 0.7151 | 1 | 0.7151 | 0 | 0 | 0 | 0.9325 | Ref |
| MG972768 | Unassigned | Unassigned | Unassigned | 113_0 | Clustered | 91 | VC_113_0 | 91 | 0.9584 | 1 | 0.9584 | 0 | 0 | 0 | 1 | Ref |
| MG973030 | Unassigned | Unassigned | Unassigned | 134_0 | Clustered | 2 | VC_134_0 | 2 | 0.0123 | 0.95745739 | 0.0118 | 0 | 0 | 0 | 1 | Ref |
| MG976803 | Unassigned | Unassigned | Unassigned | 837_1 | Clustered | 24 | VC_837_1 | 24 | 0.5328 | 1 | 0.5328 | 0 | 0 | 0 | 1 | Ref |
| MG983742 | Unassigned | Unassigned | Unassigned | 102_0 | Clustered | 15 | VC_102_0 | 15 | 0.653 | 1 | 0.653 | 0 | 0 | 0 | 1 | Ref |
| MG983743 | Unassigned | Unassigned | Unassigned | 801_3 | Clustered | 7 | VC_801_3 | 7 | 0.0764 | 1 | 0.0764 | 0 | 0 | 0 | 0.7143 | Ref |
| MG983840 | Unassigned | Unassigned | Unassigned | 837_1 | Clustered | 24 | VC_837_1 | 24 | 0.5328 | 1 | 0.5328 | 0 | 0 | 0 | 1 | Ref |
| MH000602 | Unassigned | Unassigned | Unassigned | 258_0 | Clustered | 121 | VC_258_0 | 121 | 0.7359 | 1 | 0.7359 | 0 | 0 | 0 | 0.9999 | Ref |
| MH000603 | Unassigned | Unassigned | Unassigned | 258_0 | Clustered | 121 | VC_258_0 | 121 | 0.7359 | 1 | 0.7359 | 0 | 0 | 0 | 0.9999 | Ref |
| MH000604 | Unassigned | Unassigned | Unassigned | 258_0 | Clustered | 121 | VC_258_0 | 121 | 0.7359 | 1 | 0.7359 | 0 | 0 | 0 | 0.9999 | Ref |
| MH000606 | Unassigned | Unassigned | Unassigned | 335_27 | Clustered | 6 | VC_335_27 | 6 | 0.0266 | 1 | 0.0266 | 0 | 0 | 0 | 0.8 | Ref |
| MH000607 | Unassigned | Unassigned | Unassigned | 571_7 | Clustered | 28 | VC_571_7 | 28 | 0.1651 | 1 | 0.1651 | 0 | 0 | 0 | 0.8519 | Ref |
| MH001446 | Unassigned | Unassigned | Unassigned | 525_0 | Clustered | 8 | VC_525_0 | 8 | 0.443 | 1 | 0.443 | 0 | 0 | 0 | 1 | Ref |
| MH001447 | Unassigned | Unassigned | Unassigned | 588_0 | Clustered | 8 | VC_588_0 | 8 | 0.5695 | 1 | 0.5695 | 0 | 0 | 0 | 1 | Ref |
| MH001448 | Unassigned | Unassigned | Unassigned | 538_59 | Clustered | 5 | VC_538_59 | 5 | 0.0456 | 0.99999997 | 0.0456 | 0 | 0 | 0 | 1 | Ref |
| MH001450 | Unassigned | Unassigned | Unassigned | 525_0 | Clustered | 8 | VC_525_0 | 8 | 0.443 | 1 | 0.443 | 0 | 0 | 0 | 1 | Ref |
| MH001451 | Unassigned | Unassigned | Unassigned | 1087_0 | Clustered | 7 | VC_1087_0 | 7 | 0.94 | 1 | 0.94 | 0 | 0 | 0 | 1 | Ref |
| MH001452 | Unassigned | Unassigned | Unassigned | 526_0 | Clustered | 32 | VC_526_0 | 32 | 0.9037 | 1 | 0.9037 | 0 | 0 | 0 | 1 | Ref |
| MH001453 | Unassigned | Unassigned | Unassigned | 243_0 | Clustered | 72 | VC_243_0 | 72 | 0.8089 | 1 | 0.8089 | 0 | 0 | 0 | 1 | Ref |
| MH001454 | Unassigned | Unassigned | Unassigned | 231_0 | Clustered | 19 | VC_231_0 | 19 | 0.9378 | 1 | 0.9378 | 0 | 0 | 0 | 1 | Ref |
| MH001455 | Unassigned | Unassigned | Unassigned | 783_0 | Clustered | 67 | VC_783_0 | 67 | 0.8945 | 1 | 0.8945 | 0 | 0 | 0 | 1 | Ref |
| MH001456 | Unassigned | Unassigned | Unassigned | 783_0 | Clustered | 67 | VC_783_0 | 67 | 0.8945 | 1 | 0.8945 | 0 | 0 | 0 | 1 | Ref |
| MH001457 | Unassigned | Unassigned | Unassigned | 388_0 | Clustered | 3 | VC_388_0 | 3 | 0.2477 | 0.99807286 | 0.2473 | 0 | 0 | 0 | 1 | Ref |
| MH001458 | Unassigned | Unassigned | Unassigned | 335_11 | Clustered/Singleton | 1 | VC_335_11 | 1 | 0 | 1 | 0 | 0 | 0 | 0 | 1 | Ref |
| MH001459 | Unassigned | Unassigned | Unassigned | 533_0 | Clustered | 16 | VC_533_0 | 16 | 0.3765 | 1 | 0.3765 | 0 | 0 | 0 | 1 | Ref |
| MH001460 | Unassigned | Unassigned | Unassigned | 1015_1 | Clustered | 26 | VC_1015_1 | 26 | 0.6989 | 1 | 0.6989 | 0 | 0 | 0 | 1 | Ref |
| MH015249 | Unassigned | Unassigned | Unassigned | 612_0 | Clustered | 5 | VC_612_0 | 5 | 0.4531 | 0.99999995 | 0.4531 | 0 | 0 | 0 | 1 | Ref |
| MH015250 | Unassigned | Unassigned | Unassigned | 857_0 | Clustered | 16 | VC_857_0 | 16 | 0.9317 | 1 | 0.9317 | 0 | 0 | 0 | 1 | Ref |
| MH015251 | Unassigned | Unassigned | Unassigned | 1268_0 | Clustered | 2 | VC_1268_0 | 2 | 1 | 1 | 1 | 0 | 0 | 0 | 1 | Ref |
| MH015252 | Unassigned | Unassigned | Unassigned | 612_0 | Clustered | 5 | VC_612_0 | 5 | 0.4531 | 0.99999995 | 0.4531 | 0 | 0 | 0 | 1 | Ref |
| MH015253 | Unassigned | Unassigned | Unassigned | 857_0 | Clustered | 16 | VC_857_0 | 16 | 0.9317 | 1 | 0.9317 | 0 | 0 | 0 | 1 | Ref |
| MH015254 | Unassigned | Unassigned | Unassigned | 612_0 | Clustered | 5 | VC_612_0 | 5 | 0.4531 | 0.99999995 | 0.4531 | 0 | 0 | 0 | 1 | Ref |
| MH015255 | Unassigned | Unassigned | Unassigned | 1012_0 | Clustered | 2 | VC_1012_0 | 2 | 0.0796 | 0.93920319 | 0.0747 | 0 | 0 | 0 | 1 | Ref |
| MH015256 | Unassigned | Unassigned | Unassigned | 857_0 | Clustered | 16 | VC_857_0 | 16 | 0.9317 | 1 | 0.9317 | 0 | 0 | 0 | 1 | Ref |
| MH015257 | Unassigned | Unassigned | Unassigned | 857_0 | Clustered | 16 | VC_857_0 | 16 | 0.9317 | 1 | 0.9317 | 0 | 0 | 0 | 1 | Ref |
| MH015258 | Unassigned | Unassigned | Unassigned | 612_0 | Clustered | 5 | VC_612_0 | 5 | 0.4531 | 0.99999995 | 0.4531 | 0 | 0 | 0 | 1 | Ref |
| MH015259 | Unassigned | Unassigned | Unassigned | 857_0 | Clustered | 16 | VC_857_0 | 16 | 0.9317 | 1 | 0.9317 | 0 | 0 | 0 | 1 | Ref |
| MH019215 | Unassigned | Unassigned | Unassigned | 885_0 | Clustered | 9 | VC_885_0 | 9 | 0.9403 | 1 | 0.9403 | 0 | 0 | 0 | 1 | Ref |
| MH019216 | Unassigned | Unassigned | Unassigned | 885_0 | Clustered | 9 | VC_885_0 | 9 | 0.9403 | 1 | 0.9403 | 0 | 0 | 0 | 1 | Ref |
| MH020235 | Unassigned | Unassigned | Unassigned | 538_31 | Clustered | 11 | VC_538_31 | 11 | 0.0894 | 1 | 0.0894 | 0 | 0 | 0 | 0.6 | Ref |
| MH020236 | Unassigned | Unassigned | Unassigned | 335_56 | Clustered | 7 | VC_335_56 | 7 | 0.0242 | 1 | 0.0242 | 0 | 0 | 0 | 0.9048 | Ref |
| MH020237 | Unassigned | Unassigned | Unassigned | 222_2 | Clustered | 5 | VC_222_2 | 5 | 0.0569 | 0.99999997 | 0.0569 | 0 | 0 | 0 | 1 | Ref |
| MH020238 | Unassigned | Unassigned | Unassigned | 532_0 | Clustered | 75 | VC_532_0 | 75 | 0.9393 | 1 | 0.9393 | 0 | 0 | 0 | 1 | Ref |
| MH020239 | Unassigned | Unassigned | Unassigned | 974_0 | Clustered | 34 | VC_974_0 | 34 | 0.7953 | 1 | 0.7953 | 0 | 0 | 0 | 1 | Ref |
| MH020240 | Unassigned | Unassigned | Unassigned | 230_0 | Clustered | 38 | VC_230_0 | 38 | 0.9076 | 1 | 0.9076 | 0 | 0 | 0 | 1 | Ref |
| MH020241 | Unassigned | Unassigned | Unassigned | 1217_0 | Clustered | 41 | VC_1217_0 | 41 | 0.9546 | 1 | 0.9546 | 0 | 0 | 0 | 0.9976 | Ref |
| MH020244 | Unassigned | Unassigned | Unassigned | 335_23 | Clustered | 17 | VC_335_23 | 17 | 0.0714 | 1 | 0.0714 | 0 | 0 | 0 | 0.6397 | Ref |
| MH020246 | Unassigned | Unassigned | Unassigned | 577_0 | Clustered | 21 | VC_577_0 | 21 | 0.9908 | 1 | 0.9908 | 0 | 0 | 0 | 1 | Ref |
| MH020247 | Unassigned | Unassigned | Unassigned | 571_8 | Clustered | 19 | VC_571_8 | 19 | 0.1031 | 1 | 0.1031 | 0 | 0 | 0 | 0.8713 | Ref |
| MH020248 | Unassigned | Unassigned | Unassigned | 531_0 | Clustered | 32 | VC_531_0 | 32 | 0.6859 | 1 | 0.6859 | 0 | 0 | 0 | 1 | Ref |
| MH020249 | Unassigned | Unassigned | Unassigned | 535_1 | Clustered | 108 | VC_535_1 | 108 | 0.8479 | 1 | 0.8479 | 0 | 0 | 0 | 0.9955 | Ref |
| MH023293 | Unassigned | Unassigned | Unassigned | 475_0 | Clustered | 96 | VC_475_0 | 96 | 0.9303 | 1 | 0.9303 | 0 | 0 | 0 | 0.9575 | Ref |
| MH025888 | Unassigned | Unassigned | Unassigned | 585_0 | Clustered | 20 | VC_585_0 | 20 | 0.8376 | 1 | 0.8376 | 0 | 0 | 0 | 1 | Ref |
| MH025889 | Unassigned | Unassigned | Unassigned | 893_1 | Clustered | 20 | VC_893_1 | 20 | 0.4792 | 1 | 0.4792 | 0 | 0 | 0 | 1 | Ref |
| MH025890 | Unassigned | Unassigned | Unassigned | 590_0 | Clustered | 9 | VC_590_0 | 9 | 0.4952 | 1 | 0.4952 | 0 | 0 | 0 | 1 | Ref |
| MH031343 | Unassigned | Unassigned | Unassigned | 399_0 | Clustered | 49 | VC_399_0 | 49 | 0.7927 | 1 | 0.7927 | 0 | 0 | 0 | 1 | Ref |
| MH042230 | Unassigned | Unassigned | Unassigned | 714_0 | Clustered | 71 | VC_714_0 | 71 | 0.935 | 1 | 0.935 | 0 | 0 | 0 | 1 | Ref |
| MH045554 | Unassigned | Unassigned | Unassigned | 1296_0 | Clustered | 82 | VC_1296_0 | 82 | 0.7938 | 1 | 0.7938 | 0 | 0 | 0 | 1 | Ref |
| MH045557 | Unassigned | Unassigned | Unassigned | 1407_0 | Clustered | 78 | VC_1407_0 | 78 | 0.9731 | 1 | 0.9731 | 0 | 0 | 0 | 1 | Ref |
| MH045558 | Unassigned | Unassigned | Unassigned | 1296_0 | Clustered | 82 | VC_1296_0 | 82 | 0.7938 | 1 | 0.7938 | 0 | 0 | 0 | 1 | Ref |
| MH045560 | Unassigned | Unassigned | Unassigned | 1296_0 | Clustered | 82 | VC_1296_0 | 82 | 0.7938 | 1 | 0.7938 | 0 | 0 | 0 | 1 | Ref |
| MH045561 | Unassigned | Unassigned | Unassigned | 1407_0 | Clustered | 78 | VC_1407_0 | 78 | 0.9731 | 1 | 0.9731 | 0 | 0 | 0 | 1 | Ref |
| MH045562 | Unassigned | Unassigned | Unassigned | 1296_0 | Clustered | 82 | VC_1296_0 | 82 | 0.7938 | 1 | 0.7938 | 0 | 0 | 0 | 1 | Ref |
| MH045563 | Unassigned | Unassigned | Unassigned | 1296_0 | Clustered | 82 | VC_1296_0 | 82 | 0.7938 | 1 | 0.7938 | 0 | 0 | 0 | 1 | Ref |
| MH045564 | Unassigned | Unassigned | Unassigned | 1296_0 | Clustered | 82 | VC_1296_0 | 82 | 0.7938 | 1 | 0.7938 | 0 | 0 | 0 | 1 | Ref |
| MH045565 | Unassigned | Unassigned | Unassigned | 1296_0 | Clustered | 82 | VC_1296_0 | 82 | 0.7938 | 1 | 0.7938 | 0 | 0 | 0 | 1 | Ref |
| MH045566 | Unassigned | Unassigned | Unassigned | 1287_0 | Clustered | 44 | VC_1287_0 | 44 | 1 | 1 | 1 | 0 | 0 | 0 | 1 | Ref |
| MH045567 | Unassigned | Unassigned | Unassigned | 1287_0 | Clustered | 44 | VC_1287_0 | 44 | 1 | 1 | 1 | 0 | 0 | 0 | 1 | Ref |
| MH045568 | Unassigned | Unassigned | Unassigned | 1287_0 | Clustered | 44 | VC_1287_0 | 44 | 1 | 1 | 1 | 0 | 0 | 0 | 1 | Ref |
| MH045569 | Unassigned | Unassigned | Unassigned | 783_0 | Clustered | 67 | VC_783_0 | 67 | 0.8945 | 1 | 0.8945 | 0 | 0 | 0 | 1 | Ref |

| | | | | | | | | | | | | | | | | |
|---|---|---|---|---|---|---|---|---|---|---|---|---|---|---|---|---|
| MH047632 | Unassigned | Unassigned | Unassigned | 531_0 | Clustered | 32 | VC_531_0 | 32 | 0.6859 | 1 | 0.6859 | 0 | 0 | 0 | 1 | Ref |
| MH047633 | Unassigned | Unassigned | Unassigned | 531_0 | Clustered | 32 | VC_531_0 | 32 | 0.6859 | 1 | 0.6859 | 0 | 0 | 0 | 1 | Ref |
| MH051246 | Unassigned | Unassigned | Unassigned | 243_0 | Clustered | 72 | VC_243_0 | 72 | 0.8089 | 1 | 0.8089 | 0 | 0 | 0 | 1 | Ref |
| MH051247 | Unassigned | Unassigned | Unassigned | 535_1 | Clustered | 108 | VC_535_1 | 108 | 0.8479 | 1 | 0.8479 | 0 | 0 | 0 | 0.9955 | Ref |
| MH051249 | Unassigned | Unassigned | Unassigned | 526_0 | Clustered | 32 | VC_526_0 | 32 | 0.9037 | 1 | 0.9037 | 0 | 0 | 0 | 1 | Ref |
| MH051250 | Unassigned | Unassigned | Unassigned | 974_0 | Clustered | 34 | VC_974_0 | 34 | 0.7953 | 1 | 0.7953 | 0 | 0 | 0 | 1 | Ref |
| MH051251 | Unassigned | Unassigned | Unassigned | 384_13 | Clustered | 33 | VC_384_13 | 33 | 0.1785 | 1 | 0.1785 | 0 | 0 | 0 | 0.8769 | Ref |
| MH051252 | Unassigned | Unassigned | Unassigned | 223_6 | Clustered | 42 | VC_223_6 | 42 | 0.4717 | 1 | 0.4717 | 0 | 0 | 0 | 0.8351 | Ref |
| MH051253 | Unassigned | Unassigned | Unassigned | 230_0 | Clustered | 38 | VC_230_0 | 38 | 0.9076 | 1 | 0.9076 | 0 | 0 | 0 | 1 | Ref |
| MH051254 | Unassigned | Unassigned | Unassigned | 974_0 | Clustered | 34 | VC_974_0 | 34 | 0.7953 | 1 | 0.7953 | 0 | 0 | 0 | 1 | Ref |
| MH051255 | Unassigned | Unassigned | Unassigned | 245_0 | Clustered | 17 | VC_245_0 | 17 | 0.6081 | 1 | 0.6081 | 0 | 0 | 0 | 1 | Ref |
| MH051256 | Unassigned | Unassigned | Unassigned | 974_0 | Clustered | 34 | VC_974_0 | 34 | 0.7953 | 1 | 0.7953 | 0 | 0 | 0 | 1 | Ref |
| MH051257 | Unassigned | Unassigned | Unassigned | 538_51 | Clustered/Singleton | 1 | VC_538_51 | 1 | 0 | 1 | 0 | 0 | 0 | 0 | 1 | Ref |
| MH051258 | Unassigned | Unassigned | Unassigned | 241_0 | Clustered | 21 | VC_241_0 | 21 | 0.8342 | 1 | 0.8342 | 0 | 0 | 0 | 1 | Ref |
| MH051259 | Unassigned | Unassigned | Unassigned | 531_0 | Clustered | 32 | VC_531_0 | 32 | 0.6859 | 1 | 0.6859 | 0 | 0 | 0 | 1 | Ref |
| MH051260 | Unassigned | Unassigned | Unassigned | 579_61 | Clustered | 2 | VC_579_61 | 2 | 0.0032 | 0.64519808 | 0.002 | 0 | 0 | 0 | 1 | Ref |
| MH051261 | Unassigned | Unassigned | Unassigned | 579_7 | Clustered/Singleton | 1 | VC_579_7 | 1 | 0 | 1 | 0 | 0 | 0 | 0 | 1 | Ref |
| MH051264 | Unassigned | Unassigned | Unassigned | 384_5 | Clustered | 2 | VC_384_5 | 2 | 0.0035 | 0.95771071 | 0.0033 | 0 | 0 | 0 | 1 | Ref |
| MH051265 | Unassigned | Unassigned | Unassigned | 527_0 | Clustered | 37 | VC_527_0 | 37 | 0.9679 | 1 | 0.9679 | 0 | 0 | 0 | 1 | Ref |
| MH051266 | Unassigned | Unassigned | Unassigned | 579_50 | Clustered | 15 | VC_579_50 | 15 | 0.0467 | 1 | 0.0467 | 0 | 0 | 0 | 0.9238 | Ref |
| MH051333 | Unassigned | Unassigned | Unassigned | 871_1 | Clustered | 26 | VC_871_1 | 26 | 0.6303 | 1 | 0.6303 | 0 | 0 | 0 | 0.9815 | Ref |
| MH051334 | Unassigned | Unassigned | Unassigned | 655_0 | Clustered | 45 | VC_655_0 | 45 | 0.7722 | 1 | 0.7722 | 0 | 0 | 0 | 1 | Ref |
| MH051335 | Unassigned | Unassigned | Unassigned | 636_0 | Clustered | 13 | VC_636_0 | 13 | 0.6223 | 1 | 0.6223 | 0 | 0 | 0 | 1 | Ref |
| MH051911 | Unassigned | Unassigned | Unassigned | 595_0 | Clustered | 35 | VC_595_0 | 35 | 0.8809 | 1 | 0.8809 | 0 | 0 | 0 | 1 | Ref |
| MH051912 | Unassigned | Unassigned | Unassigned | 595_0 | Clustered | 35 | VC_595_0 | 35 | 0.8809 | 1 | 0.8809 | 0 | 0 | 0 | 1 | Ref |
| MH051913 | Unassigned | Unassigned | Unassigned | 297_6 | Clustered | 12 | VC_297_6 | 12 | 0.1139 | 1 | 0.1139 | 0 | 0 | 0 | 0.8182 | Ref |
| MH051914 | Unassigned | Unassigned | Unassigned | 340_21 | Clustered/Singleton | 1 | VC_340_21 | 1 | 0 | 1 | 0 | 0 | 0 | 0 | 1 | Ref |
| MH051915 | Unassigned | Unassigned | Unassigned | 290_151 | Clustered/Singleton | 1 | VC_290_151 | 1 | 0 | 1 | 0 | 0 | 0 | 0 | 1 | Ref |
| MH051916 | Unassigned | Unassigned | Unassigned | 290_170 | Clustered/Singleton | 1 | VC_290_170 | 1 | 0 | 1 | 0 | 0 | 0 | 0 | 1 | Ref |
| MH051917 | Unassigned | Unassigned | Unassigned | 297_5 | Clustered | 14 | VC_297_5 | 14 | 0.141 | 1 | 0.141 | 0 | 0 | 0 | 0.9231 | Ref |
| MH051918 | Unassigned | Unassigned | Unassigned | 596_0 | Clustered | 4 | VC_596_0 | 4 | 0.0604 | 0.99998766 | 0.0604 | 0 | 0 | 0 | 1 | Ref |
| MH059632 | Unassigned | Unassigned | Unassigned | 472_0 | Clustered | 5 | VC_472_0 | 5 | 0.1909 | 0.99999997 | 0.1909 | 0 | 0 | 0 | 1 | Ref |
| MH059635 | Unassigned | Unassigned | Unassigned | 119_0 | Clustered | 9 | VC_119_0 | 9 | 0.9977 | 0.98988637 | 0.9876 | 0 | 0 | 0 | 1 | Ref |
| MH059636 | Unassigned | Unassigned | Unassigned | 508_5 | Clustered/Singleton | 1 | VC_508_5 | 1 | 0 | 1 | 0 | 0 | 0 | 0 | 1 | Ref |
| MH059638 | Unassigned | Unassigned | Unassigned | 865_0 | Clustered | 10 | VC_865_0 | 10 | 0.5975 | 1 | 0.5975 | 0 | 0 | 0 | 1 | Ref |
| MH059639 | Unassigned | Unassigned | Unassigned | 406_0 | Clustered | 7 | VC_406_0 | 7 | 0.176 | 1 | 0.176 | 0 | 0 | 0 | 1 | Ref |
| MH077576 | Unassigned | Unassigned | Unassigned | 223_6 | Clustered | 42 | VC_223_6 | 42 | 0.4717 | 1 | 0.4717 | 0 | 0 | 0 | 0.8351 | Ref |
| MH077577 | Unassigned | Unassigned | Unassigned | 243_0 | Clustered | 72 | VC_243_0 | 72 | 0.8089 | 1 | 0.8089 | 0 | 0 | 0 | 1 | Ref |
| MH077578 | Unassigned | Unassigned | Unassigned | 538_34 | Clustered/Singleton | 1 | VC_538_34 | 1 | 0 | 1 | 0 | 0 | 0 | 0 | 1 | Ref |
| MH077579 | Unassigned | Unassigned | Unassigned | 566_4 | Clustered | 16 | VC_566_4 | 16 | 0.2517 | 0.9999998 | 0.2517 | 0 | 0 | 0 | 0.9417 | Ref |
| MH077580 | Unassigned | Unassigned | Unassigned | 538_31 | Clustered | 11 | VC_538_31 | 11 | 0.0894 | 1 | 0.0894 | 0 | 0 | 0 | 0.6 | Ref |
| MH077581 | Unassigned | Unassigned | Unassigned | 535_1 | Clustered | 108 | VC_535_1 | 108 | 0.8479 | 1 | 0.8479 | 0 | 0 | 0 | 0.9955 | Ref |
| MH077582 | Unassigned | Unassigned | Unassigned | 384_2 | Clustered | 8 | VC_384_2 | 8 | 0.0232 | 1 | 0.0232 | 0 | 0 | 0 | 0.8571 | Ref |
| MH077583 | Unassigned | Unassigned | Unassigned | 532_0 | Clustered | 75 | VC_532_0 | 75 | 0.9393 | 1 | 0.9393 | 0 | 0 | 0 | 1 | Ref |
| MH077584 | Unassigned | Unassigned | Unassigned | 783_0 | Clustered | 67 | VC_783_0 | 67 | 0.8945 | 1 | 0.8945 | 0 | 0 | 0 | 1 | Ref |
| MH102284 | Unassigned | Unassigned | Unassigned | 603_0 | Clustered | 28 | VC_603_0 | 28 | 0.7692 | 1 | 0.7692 | 0 | 0 | 0 | 1 | Ref |
| MH102285 | Unassigned | Unassigned | Unassigned | 198_23 | Clustered | 10 | VC_198_23 | 10 | 0.0697 | 1 | 0.0697 | 0 | 0 | 0 | 0.8444 | Ref |
| MH105773 | Unassigned | Unassigned | Unassigned | 627_0 | Clustered | 16 | VC_627_0 | 16 | 0.9168 | 1 | 0.9168 | 0 | 0 | 0 | 1 | Ref |
| MH107028 | Unassigned | Unassigned | Unassigned | 868_0 | Clustered | 27 | VC_868_0 | 27 | 0.9372 | 1 | 0.9372 | 0 | 0 | 0 | 1 | Ref |
| MH107118 | Unassigned | Unassigned | Unassigned | 98_0 | Clustered | 30 | VC_98_0 | 30 | 0.7175 | 1 | 0.7175 | 0 | 0 | 0 | 1 | Ref |
| MH107769 | Unassigned | Unassigned | Unassigned | 155_12 | Clustered | 2 | VC_155_12 | 2 | 0.0044 | 0.82943952 | 0.0037 | 0 | 0 | 0 | 1 | Ref |
| MH107770 | Unassigned | Unassigned | Unassigned | 434_0 | Clustered | 45 | VC_434_0 | 45 | 0.9479 | 1 | 0.9479 | 0 | 0 | 0 | 1 | Ref |
| MH113812 | Unassigned | Unassigned | Unassigned | 1067_0 | Clustered | 20 | VC_1067_0 | 20 | 0.7109 | 1 | 0.7109 | 0 | 0 | 0 | 1 | Ref |
| MH113813 | Unassigned | Unassigned | Unassigned | 1067_0 | Clustered | 20 | VC_1067_0 | 20 | 0.7109 | 1 | 0.7109 | 0 | 0 | 0 | 1 | Ref |
| MH113814 | Unassigned | Unassigned | Unassigned | 1067_0 | Clustered | 20 | VC_1067_0 | 20 | 0.7109 | 1 | 0.7109 | 0 | 0 | 0 | 1 | Ref |
| MH113815 | Unassigned | Unassigned | Unassigned | 1067_0 | Clustered | 20 | VC_1067_0 | 20 | 0.7109 | 1 | 0.7109 | 0 | 0 | 0 | 1 | Ref |
| MH115576 | Unassigned | Unassigned | Unassigned | 441_1 | Clustered | 8 | VC_441_1 | 8 | 0.5443 | 1 | 0.5443 | 0 | 0 | 0 | 0.9643 | Ref |
| MH128984 | Unassigned | Unassigned | Unassigned | 286_0 | Clustered | 2 | VC_286_0 | 2 | 0.1926 | 0.95154944 | 0.1832 | 0 | 0 | 0 | 1 | Ref |
| MH133207 | Unassigned | Unassigned | Unassigned | 716_1 | Clustered | 2 | VC_716_1 | 2 | 0.1291 | 0.95114164 | 0.1227 | 0 | 0 | 0 | 1 | Ref |
| MH136584 | Unassigned | Unassigned | Unassigned | 155_8 | Clustered | 66 | VC_155_8 | 66 | 0.4537 | 1 | 0.4537 | 0 | 0 | 0 | 0.8182 | Ref |
| MH142219 | Unassigned | Unassigned | Unassigned | 25_0 | Clustered | 3 | VC_25_0 | 3 | 0.3393 | 0.99845192 | 0.3388 | 0 | 0 | 0 | 1 | Ref |
| MH142220 | Unassigned | Unassigned | Unassigned | 25_0 | Clustered | 3 | VC_25_0 | 3 | 0.3393 | 0.99845192 | 0.3388 | 0 | 0 | 0 | 1 | Ref |
| MH153799 | Unassigned | Unassigned | Unassigned | 1411_0 | Clustered | 5 | VC_1411_0 | 5 | 0.5446 | 0.99999985 | 0.5446 | 0 | 0 | 0 | 1 | Ref |
| MH153800 | Unassigned | Unassigned | Unassigned | 899_0 | Clustered | 2 | VC_899_0 | 2 | 0.0476 | 0.95725601 | 0.0456 | 0 | 0 | 0 | 1 | Ref |
| MH153801 | Unassigned | Unassigned | Unassigned | 899_1 | Clustered/Singleton | 1 | VC_899_1 | 1 | 0 | 1 | 0 | 0 | 0 | 0 | 1 | Ref |
| MH153802 | Unassigned | Unassigned | Unassigned | 1296_0 | Clustered | 82 | VC_1296_0 | 82 | 0.7938 | 1 | 0.7938 | 0 | 0 | 0 | 1 | Ref |
| MH153803 | Unassigned | Unassigned | Unassigned | 1212_0 | Clustered | 21 | VC_1212_0 | 21 | 0.711 | 1 | 0.711 | 0 | 0 | 0 | 1 | Ref |
| MH153804 | Unassigned | Unassigned | Unassigned | 741_0 | Clustered | 3 | VC_741_0 | 3 | 0.038 | 0.88651 | 0.0337 | 0 | 0 | 0 | 1 | Ref |
| MH153805 | Unassigned | Unassigned | Unassigned | 1296_0 | Clustered | 82 | VC_1296_0 | 82 | 0.7938 | 1 | 0.7938 | 0 | 0 | 0 | 1 | Ref |
| MH153806 | Unassigned | Unassigned | Unassigned | 1296_0 | Clustered | 82 | VC_1296_0 | 82 | 0.7938 | 1 | 0.7938 | 0 | 0 | 0 | 1 | Ref |
| MH153807 | Unassigned | Unassigned | Unassigned | 1113_0 | Clustered | 2 | VC_1113_0 | 2 | 0.127 | 0.95375267 | 0.1212 | 0 | 0 | 0 | 1 | Ref |
| MH153808 | Unassigned | Unassigned | Unassigned | 1217_0 | Clustered | 41 | VC_1217_0 | 41 | 0.9546 | 1 | 0.9546 | 0 | 0 | 0 | 0.9976 | Ref |
| MH153809 | Unassigned | Unassigned | Unassigned | 1136_0 | Clustered | 40 | VC_1136_0 | 40 | 0.8746 | 1 | 0.8746 | 0 | 0 | 0 | 1 | Ref |
| MH153810 | Unassigned | Unassigned | Unassigned | 528_0 | Clustered | 12 | VC_528_0 | 12 | 0.3923 | 1 | 0.3923 | 0 | 0 | 0 | 0.9697 | Ref |
| MH153811 | Unassigned | Unassigned | Unassigned | 1296_0 | Clustered | 82 | VC_1296_0 | 82 | 0.7938 | 1 | 0.7938 | 0 | 0 | 0 | 1 | Ref |
| MH153812 | Unassigned | Unassigned | Unassigned | 1113_0 | Clustered | 2 | VC_1113_0 | 2 | 0.127 | 0.95375267 | 0.1212 | 0 | 0 | 0 | 1 | Ref |
| MH153813 | Unassigned | Unassigned | Unassigned | 1212_0 | Clustered | 21 | VC_1212_0 | 21 | 0.711 | 1 | 0.711 | 0 | 0 | 0 | 1 | Ref |
| MH155864 | Unassigned | Unassigned | Unassigned | 532_0 | Clustered | 75 | VC_532_0 | 75 | 0.9393 | 1 | 0.9393 | 0 | 0 | 0 | 1 | Ref |
| MH155865 | Unassigned | Unassigned | Unassigned | 538_38 | Clustered | 6 | VC_538_38 | 6 | 0.0517 | 1 | 0.0517 | 0 | 0 | 0 | 0.8667 | Ref |
| MH155866 | Unassigned | Unassigned | Unassigned | 538_4 | Clustered/Singleton | 1 | VC_538_4 | 1 | 0 | 1 | 0 | 0 | 0 | 0 | 1 | Ref |
| MH155868 | Unassigned | Unassigned | Unassigned | 1278_0 | Clustered | 19 | VC_1278_0 | 19 | 1 | 1 | 1 | 0 | 0 | 0 | 1 | Ref |

| | | | | | | | | | | | | | | | | |
|---|---|---|---|---|---|---|---|---|---|---|---|---|---|---|---|---|
| MH155870 | Unassigned | Unassigned | Unassigned | 1414_0 | Clustered | 2 | VC_1414_0 | 2 | 1 | 1 | 1 | 0 | 0 | 0 | 1 | Ref |
| MH155871 | Unassigned | Unassigned | Unassigned | 538_38 | Clustered | 6 | VC_538_38 | 6 | 0.0517 | 1 | 0.0517 | 0 | 0 | 0 | 0.8667 | Ref |
| MH155873 | Unassigned | Unassigned | Unassigned | 1415_0 | Clustered | 31 | VC_1415_0 | 31 | 0.8456 | 1 | 0.8456 | 0 | 0 | 0 | 1 | Ref |
| MH155874 | Unassigned | Unassigned | Unassigned | 384_13 | Clustered | 33 | VC_384_13 | 33 | 0.1785 | 1 | 0.1785 | 0 | 0 | 0 | 0.8769 | Ref |
| MH155875 | Unassigned | Unassigned | Unassigned | 535_1 | Clustered | 108 | VC_535_1 | 108 | 0.8479 | 1 | 0.8479 | 0 | 0 | 0 | 0.9955 | Ref |
| MH155876 | Unassigned | Unassigned | Unassigned | 230_0 | Clustered | 38 | VC_230_0 | 38 | 0.9076 | 1 | 0.9076 | 0 | 0 | 0 | 1 | Ref |
| MH155877 | Unassigned | Unassigned | Unassigned | 901_0 | Clustered | 31 | VC_901_0 | 31 | 0.8235 | 1 | 0.8235 | 0 | 0 | 0 | 0.8194 | Ref |
| MH155879 | Unassigned | Unassigned | Unassigned | 535_1 | Clustered | 108 | VC_535_1 | 108 | 0.8479 | 1 | 0.8479 | 0 | 0 | 0 | 0.9955 | Ref |
| MH155880 | Unassigned | Unassigned | Unassigned | 901_0 | Clustered | 31 | VC_901_0 | 31 | 0.8235 | 1 | 0.8235 | 0 | 0 | 0 | 0.8194 | Ref |
| MH159197 | Unassigned | Unassigned | Unassigned | 155_15 | Clustered | 16 | VC_155_15 | 16 | 0.0779 | 1 | 0.0779 | 0 | 0 | 0 | 0.8083 | Ref |
| MH160392 | Unassigned | Unassigned | Unassigned | 801_2 | Clustered | 5 | VC_801_2 | 5 | 0.087 | 0.99999997 | 0.087 | 0 | 0 | 0 | 1 | Ref |
| MH160766 | Unassigned | Unassigned | Unassigned | 871_1 | Clustered | 26 | VC_871_1 | 26 | 0.6303 | 1 | 0.6303 | 0 | 0 | 0 | 0.9815 | Ref |
| MH160767 | Unassigned | Unassigned | Unassigned | 254_0 | Clustered | 14 | VC_254_0 | 14 | 0.9118 | 1 | 0.9118 | 0 | 0 | 0 | 0.967 | Ref |
| MH165274 | Unassigned | Unassigned | Unassigned | 510_1 | Clustered | 20 | VC_510_1 | 20 | 0.4172 | 1 | 0.4172 | 0 | 0 | 0 | 1 | Ref |
| MH171093 | Unassigned | Unassigned | Unassigned | 1016_0 | Clustered | 62 | VC_1016_0 | 62 | 0.8638 | 1 | 0.8638 | 0 | 0 | 0 | 0.743 | Ref |
| MH171094 | Unassigned | Unassigned | Unassigned | 1016_0 | Clustered | 62 | VC_1016_0 | 62 | 0.8638 | 1 | 0.8638 | 0 | 0 | 0 | 0.743 | Ref |
| MH171095 | Unassigned | Unassigned | Unassigned | 1016_0 | Clustered | 62 | VC_1016_0 | 62 | 0.8638 | 1 | 0.8638 | 0 | 0 | 0 | 0.743 | Ref |
| MH171096 | Unassigned | Unassigned | Unassigned | 1016_0 | Clustered | 62 | VC_1016_0 | 62 | 0.8638 | 1 | 0.8638 | 0 | 0 | 0 | 0.743 | Ref |
| MH171097 | Unassigned | Unassigned | Unassigned | 1016_0 | Clustered | 62 | VC_1016_0 | 62 | 0.8638 | 1 | 0.8638 | 0 | 0 | 0 | 0.743 | Ref |
| MH171098 | Unassigned | Unassigned | Unassigned | 1016_0 | Clustered | 62 | VC_1016_0 | 62 | 0.8638 | 1 | 0.8638 | 0 | 0 | 0 | 0.743 | Ref |
| MH172261 | Unassigned | Unassigned | Unassigned | 848_0 | Clustered | 124 | VC_848_0 | 124 | 0.9484 | 1 | 0.9484 | 0 | 0 | 0 | 1 | Ref |
| MH172262 | Unassigned | Unassigned | Unassigned | 848_0 | Clustered | 124 | VC_848_0 | 124 | 0.9484 | 1 | 0.9484 | 0 | 0 | 0 | 1 | Ref |
| MH172263 | Unassigned | Unassigned | Unassigned | 848_0 | Clustered | 124 | VC_848_0 | 124 | 0.9484 | 1 | 0.9484 | 0 | 0 | 0 | 1 | Ref |
| MH172264 | Unassigned | Unassigned | Unassigned | 848_0 | Clustered | 124 | VC_848_0 | 124 | 0.9484 | 1 | 0.9484 | 0 | 0 | 0 | 1 | Ref |
| MH178096 | Unassigned | Unassigned | Unassigned | 36_0 | Clustered | 4 | VC_36_0 | 4 | 0.0597 | 0.99994573 | 0.0597 | 0 | 0 | 0 | 1 | Ref |
| MH178381 | Unassigned | Unassigned | Unassigned | 891_0 | Clustered | 11 | VC_891_0 | 11 | 0.9663 | 1 | 0.9663 | 0 | 0 | 0 | 1 | Ref |
| MH178382 | Unassigned | Unassigned | Unassigned | 891_0 | Clustered | 11 | VC_891_0 | 11 | 0.9663 | 1 | 0.9663 | 0 | 0 | 0 | 1 | Ref |
| MH179470 | Unassigned | Unassigned | Unassigned | 1233_0 | Clustered | 9 | VC_1233_0 | 9 | 0.9676 | 1 | 0.9676 | 0 | 0 | 0 | 1 | Ref |
| MH179471 | Unassigned | Unassigned | Unassigned | 1233_0 | Clustered | 9 | VC_1233_0 | 9 | 0.9676 | 1 | 0.9676 | 0 | 0 | 0 | 1 | Ref |
| MH179472 | Unassigned | Unassigned | Unassigned | 132_0 | Clustered | 19 | VC_132_0 | 19 | 0.6051 | 1 | 0.6051 | 0 | 0 | 0 | 1 | Ref |
| MH179473 | Unassigned | Unassigned | Unassigned | 907_0 | Clustered | 32 | VC_907_0 | 32 | 0.9964 | 1 | 0.9964 | 0 | 0 | 0 | 1 | Ref |
| MH179474 | Unassigned | Unassigned | Unassigned | 380_0 | Clustered | 6 | VC_380_0 | 6 | 0.51 | 1 | 0.51 | 0 | 0 | 0 | 1 | Ref |
| MH179475 | Unassigned | Unassigned | Unassigned | 132_0 | Clustered | 19 | VC_132_0 | 19 | 0.6051 | 1 | 0.6051 | 0 | 0 | 0 | 1 | Ref |
| MH179476 | Unassigned | Unassigned | Unassigned | 619_2 | Clustered | 10 | VC_619_2 | 10 | 0.3034 | 1 | 0.3034 | 0 | 0 | 0 | 1 | Ref |
| MH179477 | Unassigned | Unassigned | Unassigned | 619_2 | Clustered | 10 | VC_619_2 | 10 | 0.3034 | 1 | 0.3034 | 0 | 0 | 0 | 1 | Ref |
| MH179478 | Unassigned | Unassigned | Unassigned | 132_0 | Clustered | 19 | VC_132_0 | 19 | 0.6051 | 1 | 0.6051 | 0 | 0 | 0 | 1 | Ref |
| MH179479 | Unassigned | Unassigned | Unassigned | 1233_0 | Clustered | 9 | VC_1233_0 | 9 | 0.9676 | 1 | 0.9676 | 0 | 0 | 0 | 1 | Ref |
| MH179480 | Unassigned | Unassigned | Unassigned | 866_0 | Clustered | 2 | VC_866_0 | 2 | 0.0566 | 0.95560116 | 0.0541 | 0 | 0 | 0 | 1 | Ref |
| MH181876 | Unassigned | Unassigned | Unassigned | 440_0 | Clustered | 33 | VC_440_0 | 33 | 0.992 | 1 | 0.992 | 0 | 0 | 0 | 1 | Ref |
| MH183162 | Unassigned | Unassigned | Unassigned | 1408_1 | Clustered | 2 | VC_1408_1 | 2 | 0.0937 | 0.95622325 | 0.0896 | 0 | 0 | 0 | 1 | Ref |
| MH191398 | Unassigned | Unassigned | Unassigned | 816_0 | Clustered | 35 | VC_816_0 | 35 | 0.9824 | 1 | 0.9824 | 0 | 0 | 0 | 1 | Ref |
| MH193369 | Unassigned | Unassigned | Unassigned | 711_0 | Clustered | 48 | VC_711_0 | 48 | 0.8217 | 1 | 0.8217 | 0 | 0 | 0 | 1 | Ref |
| MH203051 | Unassigned | Unassigned | Unassigned | 198_22 | Clustered | 3 | VC_198_22 | 3 | 0.0183 | 0.99859679 | 0.0183 | 0 | 0 | 0 | 1 | Ref |
| MH203383 | Unassigned | Unassigned | Unassigned | 711_0 | Clustered | 48 | VC_711_0 | 48 | 0.8217 | 1 | 0.8217 | 0 | 0 | 0 | 1 | Ref |
| MH203384 | Unassigned | Unassigned | Unassigned | 711_0 | Clustered | 48 | VC_711_0 | 48 | 0.8217 | 1 | 0.8217 | 0 | 0 | 0 | 1 | Ref |
| MH206183 | Unassigned | Unassigned | Unassigned | 184_0 | Clustered | 5 | VC_184_0 | 5 | 0.7721 | 0.99998991 | 0.7721 | 0 | 0 | 0 | 1 | Ref |
| MH206184 | Unassigned | Unassigned | Unassigned | 184_0 | Clustered | 5 | VC_184_0 | 5 | 0.7721 | 0.99998991 | 0.7721 | 0 | 0 | 0 | 1 | Ref |
| MH218848 | Unassigned | Unassigned | Unassigned | 184_0 | Clustered | 5 | VC_184_0 | 5 | 0.7721 | 0.99998991 | 0.7721 | 0 | 0 | 0 | 1 | Ref |
| MH220877 | Unassigned | Unassigned | Unassigned | 281_0 | Clustered | 2 | VC_281_0 | 2 | 0.0753 | 0.9540953 | 0.0719 | 0 | 0 | 0 | 1 | Ref |
| MH221128 | Unassigned | Unassigned | Unassigned | 994_6 | Clustered/Singleton | 1 | VC_994_6 | 1 | 0 | 1 | 0 | 0 | 0 | 0 | 1 | Ref |
| MH221129 | Unassigned | Unassigned | Unassigned | 994_5 | Clustered | 19 | VC_994_5 | 19 | 0.2622 | 1 | 0.2622 | 0 | 0 | 0 | 1 | Ref |
| MH229862 | Unassigned | Unassigned | Unassigned | 1416_0 | Clustered | 10 | VC_1416_0 | 10 | 1 | 1 | 1 | 0 | 0 | 0 | 1 | Ref |
| MH229863 | Unassigned | Unassigned | Unassigned | 1416_0 | Clustered | 10 | VC_1416_0 | 10 | 1 | 1 | 1 | 0 | 0 | 0 | 1 | Ref |
| MH229864 | Unassigned | Unassigned | Unassigned | 1416_0 | Clustered | 10 | VC_1416_0 | 10 | 1 | 1 | 1 | 0 | 0 | 0 | 1 | Ref |
| MH229865 | Unassigned | Unassigned | Unassigned | 1416_0 | Clustered | 10 | VC_1416_0 | 10 | 1 | 1 | 1 | 0 | 0 | 0 | 1 | Ref |
| MH230177 | Unassigned | Unassigned | Unassigned | 819_0 | Clustered | 19 | VC_819_0 | 19 | 0.6835 | 1 | 0.6835 | 0 | 0 | 0 | 1 | Ref |
| MH230874 | Unassigned | Unassigned | Unassigned | 384_11 | Clustered | 31 | VC_384_11 | 31 | 0.0926 | 1 | 0.0926 | 0 | 0 | 0 | 0.7011 | Ref |
| MH230875 | Unassigned | Unassigned | Unassigned | 384_3 | Clustered | 22 | VC_384_3 | 22 | 0.0786 | 1 | 0.0786 | 0 | 0 | 0 | 0.9048 | Ref |
| MH230876 | Unassigned | Unassigned | Unassigned | 335_24 | Clustered | 3 | VC_335_24 | 3 | 0.009 | 0.99853949 | 0.0089 | 0 | 0 | 0 | 1 | Ref |
| MH230877 | Unassigned | Unassigned | Unassigned | 535_1 | Clustered | 108 | VC_535_1 | 108 | 0.8479 | 1 | 0.8479 | 0 | 0 | 0 | 0.9955 | Ref |
| MH230878 | Unassigned | Unassigned | Unassigned | 335_54 | Clustered | 5 | VC_335_54 | 5 | 0.0147 | 0.99999078 | 0.0147 | 0 | 0 | 0 | 0.7 | Ref |
| MH243438 | Unassigned | Unassigned | Unassigned | 293_1 | Clustered | 21 | VC_293_1 | 21 | 0.1694 | 1 | 0.1694 | 0 | 0 | 0 | 0.9048 | Ref |
| MH243439 | Unassigned | Unassigned | Unassigned | 290_181 | Clustered/Singleton | 1 | VC_290_181 | 1 | 0 | 1 | 0 | 0 | 0 | 0 | 1 | Ref |
| MH248069 | Unassigned | Unassigned | Unassigned | 929_0 | Clustered | 10 | VC_929_0 | 10 | 0.7185 | 1 | 0.7185 | 0 | 0 | 0 | 1 | Ref |
| MH248138 | Unassigned | Unassigned | Unassigned | 68_3 | Clustered | 20 | VC_68_3 | 20 | 0.3537 | 1 | 0.3537 | 0 | 0 | 0 | 1 | Ref |
| MH248947 | Unassigned | Unassigned | Unassigned | 1016_0 | Clustered | 62 | VC_1016_0 | 62 | 0.8638 | 1 | 0.8638 | 0 | 0 | 0 | 0.743 | Ref |
| MH252123 | Unassigned | Unassigned | Unassigned | 871_1 | Clustered | 26 | VC_871_1 | 26 | 0.6303 | 1 | 0.6303 | 0 | 0 | 0 | 0.9815 | Ref |
| MH252365 | Unassigned | Unassigned | Unassigned | 127_0 | Clustered | 17 | VC_127_0 | 17 | 0.9222 | 1 | 0.9222 | 0 | 0 | 0 | 1 | Ref |
| MH268168 | Unassigned | Unassigned | Unassigned | 132_0 | Clustered | 19 | VC_132_0 | 19 | 0.6051 | 1 | 0.6051 | 0 | 0 | 0 | 1 | Ref |
| MH271291 | Unassigned | Unassigned | Unassigned | 991_0 | Clustered | 39 | VC_991_0 | 39 | 1 | 1 | 1 | 0 | 0 | 0 | 1 | Ref |
| MH271292 | Unassigned | Unassigned | Unassigned | 1406_0 | Clustered | 21 | VC_1406_0 | 21 | 0.9836 | 1 | 0.9836 | 0 | 0 | 0 | 1 | Ref |
| MH271293 | Unassigned | Unassigned | Unassigned | 991_0 | Clustered | 39 | VC_991_0 | 39 | 1 | 1 | 1 | 0 | 0 | 0 | 1 | Ref |
| MH271294 | Unassigned | Unassigned | Unassigned | 991_0 | Clustered | 39 | VC_991_0 | 39 | 1 | 1 | 1 | 0 | 0 | 0 | 1 | Ref |
| MH271295 | Unassigned | Unassigned | Unassigned | 1216_0 | Clustered | 22 | VC_1216_0 | 22 | 0.4546 | 1 | 0.4546 | 0 | 0 | 0 | 1 | Ref |
| MH271296 | Unassigned | Unassigned | Unassigned | 984_0 | Clustered | 5 | VC_984_0 | 5 | 0.1708 | 0.99999984 | 0.1708 | 0 | 0 | 0 | 1 | Ref |
| MH271297 | Unassigned | Unassigned | Unassigned | 991_0 | Clustered | 39 | VC_991_0 | 39 | 1 | 1 | 1 | 0 | 0 | 0 | 1 | Ref |
| MH271298 | Unassigned | Unassigned | Unassigned | 1249_0 | Clustered | 8 | VC_1249_0 | 8 | 0.191 | 1 | 0.191 | 0 | 0 | 0 | 1 | Ref |
| MH271299 | Unassigned | Unassigned | Unassigned | 991_0 | Clustered | 39 | VC_991_0 | 39 | 1 | 1 | 1 | 0 | 0 | 0 | 1 | Ref |
| MH271300 | Unassigned | Unassigned | Unassigned | 881_1 | Clustered | 13 | VC_881_1 | 13 | 0.5859 | 1 | 0.5859 | 0 | 0 | 0 | 1 | Ref |
| MH271301 | Unassigned | Unassigned | Unassigned | 1406_0 | Clustered | 21 | VC_1406_0 | 21 | 0.9836 | 1 | 0.9836 | 0 | 0 | 0 | 1 | Ref |
| MH271302 | Unassigned | Unassigned | Unassigned | 952_0 | Clustered | 39 | VC_952_0 | 39 | 0.9064 | 1 | 0.9064 | 0 | 0 | 0 | 1 | Ref |

| | | | | | | | | | | | | | | | |
|---|---|---|---|---|---|---|---|---|---|---|---|---|---|---|---|
| MH271303 | Unassigned | Unassigned | Unassigned | 1412_0 | Clustered | 7 | VC_1412_0 | 7 | 0.2471 | 1 | 0.2471 | 0 | 0 | 0 | 1 Ref |
| MH271304 | Unassigned | Unassigned | Unassigned | 1415_0 | Clustered | 31 | VC_1415_0 | 31 | 0.8456 | 1 | 0.8456 | 0 | 0 | 0 | 1 Ref |
| MH271305 | Unassigned | Unassigned | Unassigned | 423_0 | Clustered | 7 | VC_423_0 | 7 | 0.7874 | 1 | 0.7874 | 0 | 0 | 0 | 1 Ref |
| MH271306 | Unassigned | Unassigned | Unassigned | 991_0 | Clustered | 39 | VC_991_0 | 39 | 1 | 1 | 1 | 0 | 0 | 0 | 1 Ref |
| MH271307 | Unassigned | Unassigned | Unassigned | 881_1 | Clustered | 13 | VC_881_1 | 13 | 0.5859 | 1 | 0.5859 | 0 | 0 | 0 | 1 Ref |
| MH271308 | Unassigned | Unassigned | Unassigned | 1249_0 | Clustered | 8 | VC_1249_0 | 8 | 0.191 | 1 | 0.191 | 0 | 0 | 0 | 1 Ref |
| MH271309 | Unassigned | Unassigned | Unassigned | 991_0 | Clustered | 39 | VC_991_0 | 39 | 1 | 1 | 1 | 0 | 0 | 0 | 1 Ref |
| MH271310 | Unassigned | Unassigned | Unassigned | 881_1 | Clustered | 13 | VC_881_1 | 13 | 0.5859 | 1 | 0.5859 | 0 | 0 | 0 | 1 Ref |
| MH271311 | Unassigned | Unassigned | Unassigned | 991_0 | Clustered | 39 | VC_991_0 | 39 | 1 | 1 | 1 | 0 | 0 | 0 | 1 Ref |
| MH271312 | Unassigned | Unassigned | Unassigned | 1415_0 | Clustered | 31 | VC_1415_0 | 31 | 0.8456 | 1 | 0.8456 | 0 | 0 | 0 | 1 Ref |
| MH271313 | Unassigned | Unassigned | Unassigned | 590_0 | Clustered | 9 | VC_590_0 | 9 | 0.4952 | 1 | 0.4952 | 0 | 0 | 0 | 1 Ref |
| MH271314 | Unassigned | Unassigned | Unassigned | 991_0 | Clustered | 39 | VC_991_0 | 39 | 1 | 1 | 1 | 0 | 0 | 0 | 1 Ref |
| MH271315 | Unassigned | Unassigned | Unassigned | 991_0 | Clustered | 39 | VC_991_0 | 39 | 1 | 1 | 1 | 0 | 0 | 0 | 1 Ref |
| MH271316 | Unassigned | Unassigned | Unassigned | 881_1 | Clustered | 13 | VC_881_1 | 13 | 0.5859 | 1 | 0.5859 | 0 | 0 | 0 | 1 Ref |
| MH271317 | Unassigned | Unassigned | Unassigned | 1244_0 | Clustered | 4 | VC_1244_0 | 4 | 0.8666 | 0.9999992 | 0.8666 | 0 | 0 | 0 | 1 Ref |
| MH271318 | Unassigned | Unassigned | Unassigned | 423_0 | Clustered | 7 | VC_423_0 | 7 | 0.7874 | 1 | 0.7874 | 0 | 0 | 0 | 1 Ref |
| MH271319 | Unassigned | Unassigned | Unassigned | 991_0 | Clustered | 39 | VC_991_0 | 39 | 1 | 1 | 1 | 0 | 0 | 0 | 1 Ref |
| MH271320 | Unassigned | Unassigned | Unassigned | 1249_0 | Clustered | 8 | VC_1249_0 | 8 | 0.191 | 1 | 0.191 | 0 | 0 | 0 | 1 Ref |
| MH271321 | Unassigned | Unassigned | Unassigned | 1415_0 | Clustered | 31 | VC_1415_0 | 31 | 0.8456 | 1 | 0.8456 | 0 | 0 | 0 | 1 Ref |
| MH285980 | Unassigned | Unassigned | Unassigned | 595_0 | Clustered | 35 | VC_595_0 | 35 | 0.8809 | 1 | 0.8809 | 0 | 0 | 0 | 1 Ref |
| MH299806 | Unassigned | Unassigned | Unassigned | 1002_0 | Clustered | 10 | VC_1002_0 | 10 | 0.9987 | 1 | 0.9987 | 0 | 0 | 0 | 1 Ref |
| MH310933 | Unassigned | Unassigned | Unassigned | 513_0 | Clustered | 39 | VC_513_0 | 39 | 0.3078 | 1 | 0.3078 | 0 | 0 | 0 | 1 Ref |
| MH310934 | Unassigned | Unassigned | Unassigned | 513_1 | Clustered | 41 | VC_513_1 | 41 | 0.3276 | 1 | 0.3276 | 0 | 0 | 0 | 0.911 Ref |
| MH310935 | Unassigned | Unassigned | Unassigned | 513_0 | Clustered | 39 | VC_513_0 | 39 | 0.3078 | 1 | 0.3078 | 0 | 0 | 0 | 1 Ref |
| MH310936 | Unassigned | Unassigned | Unassigned | 513_1 | Clustered | 41 | VC_513_1 | 41 | 0.3276 | 1 | 0.3276 | 0 | 0 | 0 | 0.911 Ref |
| MH316561 | Unassigned | Unassigned | Unassigned | 579_14 | Clustered | 8 | VC_579_14 | 8 | 0.0227 | 0.99904117 | 0.0227 | 0 | 0 | 0 | 0.8214 Ref |
| MH316562 | Unassigned | Unassigned | Unassigned | 585_0 | Clustered | 20 | VC_585_0 | 20 | 0.8376 | 1 | 0.8376 | 0 | 0 | 0 | 1 Ref |
| MH316563 | Unassigned | Unassigned | Unassigned | 531_0 | Clustered | 32 | VC_531_0 | 32 | 0.6859 | 1 | 0.6859 | 0 | 0 | 0 | 1 Ref |
| MH316564 | Unassigned | Unassigned | Unassigned | 538_45 | Clustered | 7 | VC_538_45 | 7 | 0.0632 | 1 | 0.0632 | 0 | 0 | 0 | 0.8095 Ref |
| MH316565 | Unassigned | Unassigned | Unassigned | 527_0 | Clustered | 37 | VC_527_0 | 37 | 0.9679 | 1 | 0.9679 | 0 | 0 | 0 | 1 Ref |
| MH316566 | Unassigned | Unassigned | Unassigned | 231_0 | Clustered | 19 | VC_231_0 | 19 | 0.9378 | 1 | 0.9378 | 0 | 0 | 0 | 1 Ref |
| MH316567 | Unassigned | Unassigned | Unassigned | 579_50 | Clustered | 15 | VC_579_50 | 15 | 0.0467 | 1 | 0.0467 | 0 | 0 | 0 | 0.9238 Ref |
| MH316568 | Unassigned | Unassigned | Unassigned | 384_13 | Clustered | 33 | VC_384_13 | 33 | 0.1785 | 1 | 0.1785 | 0 | 0 | 0 | 0.8769 Ref |
| MH316569 | Unassigned | Unassigned | Unassigned | 991_0 | Clustered | 39 | VC_991_0 | 39 | 1 | 1 | 1 | 0 | 0 | 0 | 1 Ref |
| MH316570 | Unassigned | Unassigned | Unassigned | 539_0 | Clustered | 37 | VC_539_0 | 37 | 0.911 | 1 | 0.911 | 0 | 0 | 0 | 1 Ref |
| MH319720 | Unassigned | Unassigned | Unassigned | 452_0 | Clustered | 6 | VC_452_0 | 6 | 0.0909 | 0.9999413 | 0.0909 | 0 | 0 | 0 | 1 Ref |
| MH319733 | Unassigned | Unassigned | Unassigned | 452_0 | Clustered | 6 | VC_452_0 | 6 | 0.0909 | 0.9999413 | 0.0909 | 0 | 0 | 0 | 1 Ref |
| MH319737 | Unassigned | Unassigned | Unassigned | 678_0 | Clustered | 2 | VC_678_0 | 2 | 0.0249 | 0.58574469 | 0.0146 | 0 | 0 | 0 | 1 Ref |
| MH319742 | Unassigned | Unassigned | Unassigned | 452_0 | Clustered | 6 | VC_452_0 | 6 | 0.0909 | 0.9999413 | 0.0909 | 0 | 0 | 0 | 1 Ref |
| MH319746 | Unassigned | Unassigned | Unassigned | 678_0 | Clustered | 2 | VC_678_0 | 2 | 0.0249 | 0.58574469 | 0.0146 | 0 | 0 | 0 | 1 Ref |
| MH319749 | Unassigned | Unassigned | Unassigned | 446_6 | Clustered/Singleton | 1 | VC_446_6 | 1 | 0 | 1 | 0 | 0 | 0 | 0 | 1 Ref |
| MH319750 | Unassigned | Unassigned | Unassigned | 453_0 | Clustered | 2 | VC_453_0 | 2 | 0.0289 | 0.90638816 | 0.0262 | 0 | 0 | 0 | 1 Ref |
| MH319753 | Unassigned | Unassigned | Unassigned | 452_0 | Clustered | 6 | VC_452_0 | 6 | 0.0909 | 0.9999413 | 0.0909 | 0 | 0 | 0 | 1 Ref |
| MH319754 | Unassigned | Unassigned | Unassigned | 446_7 | Clustered/Singleton | 1 | VC_446_7 | 1 | 0 | 1 | 0 | 0 | 0 | 0 | 1 Ref |
| MH319758 | Unassigned | Unassigned | Unassigned | 452_0 | Clustered | 6 | VC_452_0 | 6 | 0.0909 | 0.9999413 | 0.0909 | 0 | 0 | 0 | 1 Ref |
| MH319759 | Unassigned | Unassigned | Unassigned | 453_0 | Clustered | 2 | VC_453_0 | 2 | 0.0289 | 0.90638816 | 0.0262 | 0 | 0 | 0 | 1 Ref |
| MH321490 | Unassigned | Unassigned | Unassigned | 156_0 | Clustered | 13 | VC_156_0 | 13 | 0.2378 | 1 | 0.2378 | 0 | 0 | 0 | 0.9872 Ref |
| MH321491 | Unassigned | Unassigned | Unassigned | 156_0 | Clustered | 13 | VC_156_0 | 13 | 0.2378 | 1 | 0.2378 | 0 | 0 | 0 | 0.9872 Ref |
| MH321492 | Unassigned | Unassigned | Unassigned | 9_0 | Clustered | 38 | VC_9_0 | 38 | 0.4859 | 1 | 0.4859 | 0 | 0 | 0 | 1 Ref |
| MH321493 | Unassigned | Unassigned | Unassigned | 475_0 | Clustered | 96 | VC_475_0 | 96 | 0.9303 | 1 | 0.9303 | 0 | 0 | 0 | 0.9575 Ref |
| MH327487 | Unassigned | Unassigned | Unassigned | 452_0 | Clustered | 6 | VC_452_0 | 6 | 0.0909 | 0.9999413 | 0.0909 | 0 | 0 | 0 | 1 Ref |
| MH333064 | Unassigned | Unassigned | Unassigned | 509_0 | Clustered | 36 | VC_509_0 | 36 | 0.5291 | 1 | 0.5291 | 0 | 0 | 0 | 0.9714 Ref |
| MH338234 | Unassigned | Unassigned | Unassigned | 223_6 | Clustered | 42 | VC_223_6 | 42 | 0.4717 | 1 | 0.4717 | 0 | 0 | 0 | 0.8351 Ref |
| MH338235 | Unassigned | Unassigned | Unassigned | 974_0 | Clustered | 34 | VC_974_0 | 34 | 0.7953 | 1 | 0.7953 | 0 | 0 | 0 | 1 Ref |
| MH338236 | Unassigned | Unassigned | Unassigned | 228_0 | Clustered | 22 | VC_228_0 | 22 | 0.7411 | 1 | 0.7411 | 0 | 0 | 0 | 1 Ref |
| MH338237 | Unassigned | Unassigned | Unassigned | 228_0 | Clustered | 22 | VC_228_0 | 22 | 0.7411 | 1 | 0.7411 | 0 | 0 | 0 | 1 Ref |
| MH338238 | Unassigned | Unassigned | Unassigned | 335_19 | Clustered | 3 | VC_335_19 | 3 | 0.0107 | 0.99859221 | 0.0107 | 0 | 0 | 0 | 1 Ref |
| MH338239 | Unassigned | Unassigned | Unassigned | 335_60 | Clustered | 2 | VC_335_60 | 2 | 0.0038 | 0.957513 | 0.0036 | 0 | 0 | 0 | 1 Ref |
| MH338240 | Unassigned | Unassigned | Unassigned | 532_0 | Clustered | 75 | VC_532_0 | 75 | 0.9393 | 1 | 0.9393 | 0 | 0 | 0 | 1 Ref |
| MH338241 | Unassigned | Unassigned | Unassigned | 974_0 | Clustered | 34 | VC_974_0 | 34 | 0.7953 | 1 | 0.7953 | 0 | 0 | 0 | 1 Ref |
| MH341451 | Unassigned | Unassigned | Unassigned | 394_0 | Clustered | 3 | VC_394_0 | 3 | 0.1582 | 0.99545803 | 0.1575 | 0 | 0 | 0 | 1 Ref |
| MH341452 | Unassigned | Unassigned | Unassigned | 392_0 | Clustered | 9 | VC_392_0 | 9 | 0.7527 | 1 | 0.7527 | 0 | 0 | 0 | 1 Ref |
| MH355583 | Unassigned | Unassigned | Unassigned | 711_0 | Clustered | 48 | VC_711_0 | 48 | 0.8217 | 1 | 0.8217 | 0 | 0 | 0 | 1 Ref |
| MH355584 | Unassigned | Unassigned | Unassigned | 290_45 | Clustered/Singleton | 1 | VC_290_45 | 1 | 0 | 1 | 0 | 0 | 0 | 0 | 1 Ref |
| MH356729 | Unassigned | Unassigned | Unassigned | 862_0 | Clustered | 29 | VC_862_0 | 29 | 0.8594 | 1 | 0.8594 | 0 | 0 | 0 | 1 Ref |
| MH356730 | Unassigned | Unassigned | Unassigned | 685_1 | Clustered/Singleton | 1 | VC_685_1 | 1 | 0 | 1 | 0 | 0 | 0 | 0 | 1 Ref |
| MH358359 | Unassigned | Unassigned | Unassigned | 654_0 | Clustered | 115 | VC_654_0 | 115 | 0.7303 | 1 | 0.7303 | 0 | 0 | 0 | 0.8764 Ref |
| MH358458 | Unassigned | Unassigned | Unassigned | 801_0 | Clustered | 37 | VC_801_0 | 37 | 0.5672 | 1 | 0.5672 | 0 | 0 | 0 | 1 Ref |
| MH359124 | Unassigned | Unassigned | Unassigned | 293_11 | Clustered | 3 | VC_293_11 | 3 | 0.0146 | 0.93460284 | 0.0136 | 0 | 0 | 0 | 1 Ref |
| MH363700 | Unassigned | Unassigned | Unassigned | 136_0 | Clustered | 3 | VC_136_0 | 3 | 0.0934 | 0.99858535 | 0.0932 | 0 | 0 | 0 | 1 Ref |
| MH363708 | Unassigned | Unassigned | Unassigned | 721_0 | Clustered | 11 | VC_721_0 | 11 | 0.8846 | 1 | 0.8846 | 0 | 0 | 0 | 1 Ref |
| MH370358 | Unassigned | Unassigned | Unassigned | 654_0 | Clustered | 115 | VC_654_0 | 115 | 0.7303 | 1 | 0.7303 | 0 | 0 | 0 | 0.8764 Ref |
| MH370359 | Unassigned | Unassigned | Unassigned | 654_0 | Clustered | 115 | VC_654_0 | 115 | 0.7303 | 1 | 0.7303 | 0 | 0 | 0 | 0.8764 Ref |
| MH370360 | Unassigned | Unassigned | Unassigned | 654_0 | Clustered | 115 | VC_654_0 | 115 | 0.7303 | 1 | 0.7303 | 0 | 0 | 0 | 0.8764 Ref |
| MH370361 | Unassigned | Unassigned | Unassigned | 654_0 | Clustered | 115 | VC_654_0 | 115 | 0.7303 | 1 | 0.7303 | 0 | 0 | 0 | 0.8764 Ref |
| MH370362 | Unassigned | Unassigned | Unassigned | 654_0 | Clustered | 115 | VC_654_0 | 115 | 0.7303 | 1 | 0.7303 | 0 | 0 | 0 | 0.8764 Ref |
| MH370363 | Unassigned | Unassigned | Unassigned | 654_0 | Clustered | 115 | VC_654_0 | 115 | 0.7303 | 1 | 0.7303 | 0 | 0 | 0 | 0.8764 Ref |
| MH370365 | Unassigned | Unassigned | Unassigned | 654_0 | Clustered | 115 | VC_654_0 | 115 | 0.7303 | 1 | 0.7303 | 0 | 0 | 0 | 0.8764 Ref |
| MH370366 | Unassigned | Unassigned | Unassigned | 199_4 | Clustered | 2 | VC_199_4 | 2 | 0.0055 | 0.88232441 | 0.0048 | 0 | 0 | 0 | 1 Ref |
| MH370367 | Unassigned | Unassigned | Unassigned | 199_9 | Clustered | 5 | VC_199_9 | 5 | 0.0226 | 0.99999452 | 0.0226 | 0 | 0 | 0 | 0.7 Ref |
| MH370368 | Unassigned | Unassigned | Unassigned | 135_3 | Clustered | 3 | VC_135_3 | 3 | 0.0104 | 0.99060433 | 0.0103 | 0 | 0 | 0 | 1 Ref |

| | | | | | | | | | | | | | | | | |
|---|---|---|---|---|---|---|---|---|---|---|---|---|---|---|---|---|
| MH370369 | Unassigned | Unassigned | Unassigned | 199_16 | Clustered | 12 | VC_199_16 | 12 | 0.064 | 1 | 0.064 | 0 | 0 | 0 | 0.9394 | Ref |
| MH370370 | Unassigned | Unassigned | Unassigned | 135_31 | Clustered | 6 | VC_135_31 | 6 | 0.0262 | 0.99999636 | 0.0262 | 0 | 0 | 0 | 1 | Ref |
| MH370371 | Unassigned | Unassigned | Unassigned | 135_3 | Clustered | 3 | VC_135_3 | 3 | 0.0104 | 0.99060433 | 0.0103 | 0 | 0 | 0 | 1 | Ref |
| MH370372 | Unassigned | Unassigned | Unassigned | 654_0 | Clustered | 115 | VC_654_0 | 115 | 0.7303 | 1 | 0.7303 | 0 | 0 | 0 | 0.8764 | Ref |
| MH370373 | Unassigned | Unassigned | Unassigned | 654_0 | Clustered | 115 | VC_654_0 | 115 | 0.7303 | 1 | 0.7303 | 0 | 0 | 0 | 0.8764 | Ref |
| MH370374 | Unassigned | Unassigned | Unassigned | 654_0 | Clustered | 115 | VC_654_0 | 115 | 0.7303 | 1 | 0.7303 | 0 | 0 | 0 | 0.8764 | Ref |
| MH370375 | Unassigned | Unassigned | Unassigned | 199_29 | Clustered/Singleton | 1 | VC_199_29 | 1 | 0 | 1 | 0 | 0 | 0 | 0 | 1 | Ref |
| MH370376 | Unassigned | Unassigned | Unassigned | 199_8 | Clustered | 2 | VC_199_8 | 2 | 0.0055 | 0.91325319 | 0.005 | 0 | 0 | 0 | 1 | Ref |
| MH370377 | Unassigned | Unassigned | Unassigned | 198_13 | Clustered | 4 | VC_198_13 | 4 | 0.0214 | 0.99996706 | 0.0214 | 0 | 0 | 0 | 0.6667 | Ref |
| MH370378 | Unassigned | Unassigned | Unassigned | 198_13 | Clustered | 4 | VC_198_13 | 4 | 0.0214 | 0.99996706 | 0.0214 | 0 | 0 | 0 | 0.6667 | Ref |
| MH370379 | Unassigned | Unassigned | Unassigned | 199_23 | Clustered/Singleton | 1 | VC_199_23 | 1 | 0 | 1 | 0 | 0 | 0 | 0 | 1 | Ref |
| MH370380 | Unassigned | Unassigned | Unassigned | 199_9 | Clustered | 5 | VC_199_9 | 5 | 0.0226 | 0.99999452 | 0.0226 | 0 | 0 | 0 | 0.7 | Ref |
| MH370381 | Unassigned | Unassigned | Unassigned | 654_0 | Clustered | 115 | VC_654_0 | 115 | 0.7303 | 1 | 0.7303 | 0 | 0 | 0 | 0.8764 | Ref |
| MH370384 | Unassigned | Unassigned | Unassigned | 654_0 | Clustered | 115 | VC_654_0 | 115 | 0.7303 | 1 | 0.7303 | 0 | 0 | 0 | 0.8764 | Ref |
| MH370385 | Unassigned | Unassigned | Unassigned | 654_0 | Clustered | 115 | VC_654_0 | 115 | 0.7303 | 1 | 0.7303 | 0 | 0 | 0 | 0.8764 | Ref |
| MH370386 | Unassigned | Unassigned | Unassigned | 199_5 | Clustered | 6 | VC_199_5 | 6 | 0.0283 | 0.99999992 | 0.0283 | 0 | 0 | 0 | 1 | Ref |
| MH370387 | Unassigned | Unassigned | Unassigned | 30_1 | Clustered | 53 | VC_30_1 | 53 | 0.7151 | 1 | 0.7151 | 0 | 0 | 0 | 0.9325 | Ref |
| MH370477 | Unassigned | Unassigned | Unassigned | 407_0 | Clustered | 2 | VC_407_0 | 2 | 0.0209 | 0.95793174 | 0.02 | 0 | 0 | 0 | 1 | Ref |
| MH370478 | Unassigned | Unassigned | Unassigned | 77_0 | Clustered | 37 | VC_77_0 | 37 | 0.9392 | 1 | 0.9392 | 0 | 0 | 0 | 1 | Ref |
| MH371107 | Unassigned | Unassigned | Unassigned | 384_13 | Clustered | 33 | VC_384_13 | 33 | 0.1785 | 1 | 0.1785 | 0 | 0 | 0 | 0.8769 | Ref |
| MH371108 | Unassigned | Unassigned | Unassigned | 881_0 | Clustered | 7 | VC_881_0 | 7 | 0.3448 | 1 | 0.3448 | 0 | 0 | 0 | 1 | Ref |
| MH371109 | Unassigned | Unassigned | Unassigned | 881_0 | Clustered | 7 | VC_881_0 | 7 | 0.3448 | 1 | 0.3448 | 0 | 0 | 0 | 1 | Ref |
| MH371110 | Unassigned | Unassigned | Unassigned | 335_50 | Clustered | 2 | VC_335_50 | 2 | 0.0043 | 0.95751115 | 0.0041 | 0 | 0 | 0 | 1 | Ref |
| MH371111 | Unassigned | Unassigned | Unassigned | 1407_0 | Clustered | 78 | VC_1407_0 | 78 | 0.9731 | 1 | 0.9731 | 0 | 0 | 0 | 1 | Ref |
| MH371112 | Unassigned | Unassigned | Unassigned | 571_6 | Clustered | 22 | VC_571_6 | 22 | 0.1207 | 1 | 0.1207 | 0 | 0 | 0 | 0.8095 | Ref |
| MH371113 | Unassigned | Unassigned | Unassigned | 243_0 | Clustered | 72 | VC_243_0 | 72 | 0.8089 | 1 | 0.8089 | 0 | 0 | 0 | 1 | Ref |
| MH371114 | Unassigned | Unassigned | Unassigned | 384_16 | Clustered | 42 | VC_384_16 | 42 | 0.134 | 1 | 0.134 | 0 | 0 | 0 | 0.7282 | Ref |
| MH371115 | Unassigned | Unassigned | Unassigned | 538_40 | Clustered | 4 | VC_538_40 | 4 | 0.0395 | 0.99998776 | 0.0394 | 0 | 0 | 0 | 1 | Ref |
| MH371116 | Unassigned | Unassigned | Unassigned | 783_0 | Clustered | 67 | VC_783_0 | 67 | 0.8945 | 1 | 0.8945 | 0 | 0 | 0 | 1 | Ref |
| MH371117 | Unassigned | Unassigned | Unassigned | 384_16 | Clustered | 42 | VC_384_16 | 42 | 0.134 | 1 | 0.134 | 0 | 0 | 0 | 0.7282 | Ref |
| MH371118 | Unassigned | Unassigned | Unassigned | 1407_0 | Clustered | 78 | VC_1407_0 | 78 | 0.9731 | 1 | 0.9731 | 0 | 0 | 0 | 1 | Ref |
| MH371119 | Unassigned | Unassigned | Unassigned | 783_0 | Clustered | 67 | VC_783_0 | 67 | 0.8945 | 1 | 0.8945 | 0 | 0 | 0 | 1 | Ref |
| MH371120 | Unassigned | Unassigned | Unassigned | 538_39 | Clustered/Singleton | 1 | VC_538_39 | 1 | 0 | 1 | 0 | 0 | 0 | 0 | 1 | Ref |
| MH371121 | Unassigned | Unassigned | Unassigned | 532_0 | Clustered | 75 | VC_532_0 | 75 | 0.9393 | 1 | 0.9393 | 0 | 0 | 0 | 1 | Ref |
| MH371122 | Unassigned | Unassigned | Unassigned | 232_0 | Clustered | 32 | VC_232_0 | 32 | 0.8471 | 1 | 0.8471 | 0 | 0 | 0 | 0.996 | Ref |
| MH371123 | Unassigned | Unassigned | Unassigned | 538_33 | Clustered | 2 | VC_538_33 | 2 | 0.0087 | 0.95761754 | 0.0083 | 0 | 0 | 0 | 1 | Ref |
| MH371124 | Unassigned | Unassigned | Unassigned | 1407_0 | Clustered | 78 | VC_1407_0 | 78 | 0.9731 | 1 | 0.9731 | 0 | 0 | 0 | 1 | Ref |
| MH371125 | Unassigned | Unassigned | Unassigned | 384_16 | Clustered | 42 | VC_384_16 | 42 | 0.134 | 1 | 0.134 | 0 | 0 | 0 | 0.7282 | Ref |
| MH375074 | Unassigned | Unassigned | Unassigned | 711_0 | Clustered | 48 | VC_711_0 | 48 | 0.8217 | 1 | 0.8217 | 0 | 0 | 0 | 1 | Ref |
| MH375593 | Unassigned | Unassigned | Unassigned | 260_0 | Clustered | 46 | VC_260_0 | 46 | 0.5137 | 1 | 0.5137 | 0 | 0 | 0 | 1 | Ref |
| MH375594 | Unassigned | Unassigned | Unassigned | 258_0 | Clustered | 121 | VC_258_0 | 121 | 0.7359 | 1 | 0.7359 | 0 | 0 | 0 | 0.9999 | Ref |
| MH375595 | Unassigned | Unassigned | Unassigned | 258_0 | Clustered | 121 | VC_258_0 | 121 | 0.7359 | 1 | 0.7359 | 0 | 0 | 0 | 0.9999 | Ref |
| MH375596 | Unassigned | Unassigned | Unassigned | 258_0 | Clustered | 121 | VC_258_0 | 121 | 0.7359 | 1 | 0.7359 | 0 | 0 | 0 | 0.9999 | Ref |
| MH375597 | Unassigned | Unassigned | Unassigned | 258_0 | Clustered | 121 | VC_258_0 | 121 | 0.7359 | 1 | 0.7359 | 0 | 0 | 0 | 0.9999 | Ref |
| MH375598 | Unassigned | Unassigned | Unassigned | 258_0 | Clustered | 121 | VC_258_0 | 121 | 0.7359 | 1 | 0.7359 | 0 | 0 | 0 | 0.9999 | Ref |
| MH375599 | Unassigned | Unassigned | Unassigned | 258_0 | Clustered | 121 | VC_258_0 | 121 | 0.7359 | 1 | 0.7359 | 0 | 0 | 0 | 0.9999 | Ref |
| MH375600 | Unassigned | Unassigned | Unassigned | 258_0 | Clustered | 121 | VC_258_0 | 121 | 0.7359 | 1 | 0.7359 | 0 | 0 | 0 | 0.9999 | Ref |
| MH375644 | Unassigned | Unassigned | Unassigned | 138_0 | Clustered | 2 | VC_138_0 | 2 | 0.0413 | 0.95740923 | 0.0395 | 0 | 0 | 0 | 1 | Ref |
| MH378441 | Unassigned | Unassigned | Unassigned | 310_0 | Clustered | 155 | VC_310_0 | 155 | 0.9979 | 1 | 0.9979 | 0 | 0 | 0 | 1 | Ref |
| MH378442 | Unassigned | Unassigned | Unassigned | 310_0 | Clustered | 155 | VC_310_0 | 155 | 0.9979 | 1 | 0.9979 | 0 | 0 | 0 | 1 | Ref |
| MH378443 | Unassigned | Unassigned | Unassigned | 310_0 | Clustered | 155 | VC_310_0 | 155 | 0.9979 | 1 | 0.9979 | 0 | 0 | 0 | 1 | Ref |
| MH382198 | Unassigned | Unassigned | Unassigned | 405_0 | Clustered | 126 | VC_405_0 | 126 | 0.9614 | 1 | 0.9614 | 0 | 0 | 0 | 1 | Ref |
| MH383160 | Unassigned | Unassigned | Unassigned | 170_11 | Clustered | 10 | VC_170_11 | 10 | 0.1502 | 0.99999797 | 0.1502 | 0 | 0 | 0 | 0.9111 | Ref |
| MH384259 | Unassigned | Unassigned | Unassigned | 10_0 | Clustered | 25 | VC_10_0 | 25 | 0.3437 | 1 | 0.3437 | 0 | 0 | 0 | 0.9733 | Ref |
| MH384260 | Unassigned | Unassigned | Unassigned | 7_0 | Clustered | 28 | VC_7_0 | 28 | 0.389 | 1 | 0.389 | 0 | 0 | 0 | 0.9947 | Ref |
| MH384261 | Unassigned | Unassigned | Unassigned | 8_0 | Clustered | 58 | VC_8_0 | 58 | 0.8285 | 1 | 0.8285 | 0 | 0 | 0 | 0.9946 | Ref |
| MH389777 | Unassigned | Unassigned | Unassigned | 68_4 | Clustered | 4 | VC_68_4 | 4 | 0.0431 | 0.90943035 | 0.0392 | 0 | 0 | 0 | 1 | Ref |
| MH393889 | Unassigned | Unassigned | Unassigned | 930_0 | Clustered | 4 | VC_930_0 | 4 | 0.2568 | 0.99997246 | 0.2568 | 0 | 0 | 0 | 1 | Ref |
| MH399771 | Unassigned | Unassigned | Unassigned | 538_42 | Clustered/Singleton | 1 | VC_538_42 | 1 | 0 | 1 | 0 | 0 | 0 | 0 | 1 | Ref |
| MH399772 | Unassigned | Unassigned | Unassigned | 566_0 | Clustered | 2 | VC_566_0 | 2 | 0.0136 | 0.63748598 | 0.0087 | 0 | 0 | 0 | 1 | Ref |
| MH399773 | Unassigned | Unassigned | Unassigned | 384_16 | Clustered | 42 | VC_384_16 | 42 | 0.134 | 1 | 0.134 | 0 | 0 | 0 | 0.7282 | Ref |
| MH399774 | Unassigned | Unassigned | Unassigned | 571_0 | Clustered | 16 | VC_571_0 | 16 | 0.091 | 1 | 0.091 | 0 | 0 | 0 | 0.6667 | Ref |
| MH399775 | Unassigned | Unassigned | Unassigned | 384_15 | Clustered | 44 | VC_384_15 | 44 | 0.139 | 1 | 0.139 | 0 | 0 | 0 | 0.7474 | Ref |
| MH399776 | Unassigned | Unassigned | Unassigned | 532_0 | Clustered | 75 | VC_532_0 | 75 | 0.9393 | 1 | 0.9393 | 0 | 0 | 0 | 1 | Ref |
| MH399777 | Unassigned | Unassigned | Unassigned | 535_1 | Clustered | 108 | VC_535_1 | 108 | 0.8479 | 1 | 0.8479 | 0 | 0 | 0 | 0.9955 | Ref |
| MH399778 | Unassigned | Unassigned | Unassigned | 571_0 | Clustered | 16 | VC_571_0 | 16 | 0.091 | 1 | 0.091 | 0 | 0 | 0 | 0.6667 | Ref |
| MH399779 | Unassigned | Unassigned | Unassigned | 881_1 | Clustered | 13 | VC_881_1 | 13 | 0.5859 | 1 | 0.5859 | 0 | 0 | 0 | 1 | Ref |
| MH399780 | Unassigned | Unassigned | Unassigned | 384_16 | Clustered | 42 | VC_384_16 | 42 | 0.134 | 1 | 0.134 | 0 | 0 | 0 | 0.7282 | Ref |
| MH399781 | Unassigned | Unassigned | Unassigned | 989_0 | Clustered | 13 | VC_989_0 | 13 | 0.8622 | 1 | 0.8622 | 0 | 0 | 0 | 1 | Ref |
| MH399782 | Unassigned | Unassigned | Unassigned | 335_56 | Clustered | 7 | VC_335_56 | 7 | 0.0242 | 1 | 0.0242 | 0 | 0 | 0 | 0.9048 | Ref |
| MH399783 | Unassigned | Unassigned | Unassigned | 1407_0 | Clustered | 78 | VC_1407_0 | 78 | 0.9731 | 1 | 0.9731 | 0 | 0 | 0 | 1 | Ref |
| MH399784 | Unassigned | Unassigned | Unassigned | 538_45 | Clustered | 7 | VC_538_45 | 7 | 0.0632 | 1 | 0.0632 | 0 | 0 | 0 | 0.8095 | Ref |
| MH399785 | Unassigned | Unassigned | Unassigned | 535_1 | Clustered | 108 | VC_535_1 | 108 | 0.8479 | 1 | 0.8479 | 0 | 0 | 0 | 0.9955 | Ref |
| MH399786 | Unassigned | Unassigned | Unassigned | 384_15 | Clustered | 44 | VC_384_15 | 44 | 0.139 | 1 | 0.139 | 0 | 0 | 0 | 0.7474 | Ref |
| MH399787 | Unassigned | Unassigned | Unassigned | 539_0 | Clustered | 37 | VC_539_0 | 37 | 0.911 | 1 | 0.911 | 0 | 0 | 0 | 1 | Ref |
| MH399788 | Unassigned | Unassigned | Unassigned | 585_0 | Clustered | 20 | VC_585_0 | 20 | 0.8376 | 1 | 0.8376 | 0 | 0 | 0 | 1 | Ref |
| MH399789 | Unassigned | Unassigned | Unassigned | 1204_0 | Clustered | 25 | VC_1204_0 | 25 | 0.7382 | 1 | 0.7382 | 0 | 0 | 0 | 1 | Ref |
| MH400309 | Unassigned | Unassigned | Unassigned | 465_0 | Clustered | 80 | VC_465_0 | 80 | 0.9988 | 1 | 0.9988 | 0 | 0 | 0 | 1 | Ref |
| MH401414 | Unassigned | Unassigned | Unassigned | 7_0 | Clustered | 28 | VC_7_0 | 28 | 0.389 | 1 | 0.389 | 0 | 0 | 0 | 0.9947 | Ref |
| MH401415 | Unassigned | Unassigned | Unassigned | 7_0 | Clustered | 28 | VC_7_0 | 28 | 0.389 | 1 | 0.389 | 0 | 0 | 0 | 0.9947 | Ref |
| MH401416 | Unassigned | Unassigned | Unassigned | 10_0 | Clustered | 25 | VC_10_0 | 25 | 0.3437 | 1 | 0.3437 | 0 | 0 | 0 | 0.9733 | Ref |

| | | | | | | | | | | | | | | | | |
|---|---|---|---|---|---|---|---|---|---|---|---|---|---|---|---|---|
| MH412654 | Unassigned | Unassigned | Unassigned | 446_1 | Clustered/Singleton | 1 | VC_446_1 | 1 | 0 | 1 | 0 | 0 | 0 | 0 | 1 | Ref |
| MH422554 | Unassigned | Unassigned | Unassigned | 254_0 | Clustered | 14 | VC_254_0 | 14 | 0.9118 | 1 | 0.9118 | 0 | 0 | 0 | 0.967 | Ref |
| MH424443 | Unassigned | Unassigned | Unassigned | 654_0 | Clustered | 115 | VC_654_0 | 115 | 0.7303 | 1 | 0.7303 | 0 | 0 | 0 | 0.8764 | Ref |
| MH424444 | Unassigned | Unassigned | Unassigned | 560_0 | Clustered | 15 | VC_560_0 | 15 | 0.2846 | 1 | 0.2846 | 0 | 0 | 0 | 1 | Ref |
| MH424445 | Unassigned | Unassigned | Unassigned | 654_0 | Clustered | 115 | VC_654_0 | 115 | 0.7303 | 1 | 0.7303 | 0 | 0 | 0 | 0.8764 | Ref |
| MH424446 | Unassigned | Unassigned | Unassigned | 475_0 | Clustered | 96 | VC_475_0 | 96 | 0.9303 | 1 | 0.9303 | 0 | 0 | 0 | 0.9575 | Ref |
| MH426724 | Unassigned | Unassigned | Unassigned | 994_3 | Clustered | 3 | VC_994_3 | 3 | 0.0236 | 0.75215638 | 0.0178 | 0 | 0 | 0 | 1 | Ref |
| MH426725 | Unassigned | Unassigned | Unassigned | 339_0 | Clustered | 11 | VC_339_0 | 11 | 0.4647 | 1 | 0.4647 | 0 | 0 | 0 | 1 | Ref |
| MH426726 | Unassigned | Unassigned | Unassigned | 372_0 | Clustered | 7 | VC_372_0 | 7 | 0.3691 | 1 | 0.3691 | 0 | 0 | 0 | 1 | Ref |
| MH427377 | Unassigned | Unassigned | Unassigned | 135_22 | Clustered | 13 | VC_135_22 | 13 | 0.0795 | 1 | 0.0795 | 0 | 0 | 0 | 0.8205 | Ref |
| MH431931 | Unassigned | Unassigned | Unassigned | 725_0 | Clustered | 47 | VC_725_0 | 47 | 0.8649 | 1 | 0.8649 | 0 | 0 | 0 | 1 | Ref |
| MH431932 | Unassigned | Unassigned | Unassigned | 725_0 | Clustered | 47 | VC_725_0 | 47 | 0.8649 | 1 | 0.8649 | 0 | 0 | 0 | 1 | Ref |
| MH431933 | Unassigned | Unassigned | Unassigned | 725_0 | Clustered | 47 | VC_725_0 | 47 | 0.8649 | 1 | 0.8649 | 0 | 0 | 0 | 1 | Ref |
| MH431934 | Unassigned | Unassigned | Unassigned | 725_0 | Clustered | 47 | VC_725_0 | 47 | 0.8649 | 1 | 0.8649 | 0 | 0 | 0 | 1 | Ref |
| MH431935 | Unassigned | Unassigned | Unassigned | 725_0 | Clustered | 47 | VC_725_0 | 47 | 0.8649 | 1 | 0.8649 | 0 | 0 | 0 | 1 | Ref |
| MH431936 | Unassigned | Unassigned | Unassigned | 725_0 | Clustered | 47 | VC_725_0 | 47 | 0.8649 | 1 | 0.8649 | 0 | 0 | 0 | 1 | Ref |
| MH431937 | Unassigned | Unassigned | Unassigned | 725_0 | Clustered | 47 | VC_725_0 | 47 | 0.8649 | 1 | 0.8649 | 0 | 0 | 0 | 1 | Ref |
| MH431938 | Unassigned | Unassigned | Unassigned | 1197_0 | Clustered | 8 | VC_1197_0 | 8 | 0.8167 | 1 | 0.8167 | 0 | 0 | 0 | 1 | Ref |
| MH443100 | Unassigned | Unassigned | Unassigned | 34_0 | Clustered/Singleton | 1 | VC_34_0 | 1 | 0 | 1 | 0 | 0 | 0 | 0 | 1 | Ref |
| MH444512 | Unassigned | Unassigned | Unassigned | 875_0 | Clustered | 2 | VC_875_0 | 2 | 0.3804 | 0.94412959 | 0.3591 | 0 | 0 | 0 | 1 | Ref |
| MH445380 | Unassigned | Unassigned | Unassigned | 254_0 | Clustered | 14 | VC_254_0 | 14 | 0.9118 | 1 | 0.9118 | 0 | 0 | 0 | 0.967 | Ref |
| MH445381 | Unassigned | Unassigned | Unassigned | 254_0 | Clustered | 14 | VC_254_0 | 14 | 0.9118 | 1 | 0.9118 | 0 | 0 | 0 | 0.967 | Ref |
| MH445453 | Unassigned | Unassigned | Unassigned | 972_0 | Clustered | 99 | VC_972_0 | 99 | 0.9113 | 1 | 0.9113 | 0 | 0 | 0 | 0.9928 | Ref |
| MH447526 | Unassigned | Unassigned | Unassigned | 1417_0 | Clustered | 3 | VC_1417_0 | 3 | 0.9824 | 0.9595722 | 0.9427 | 0 | 0 | 0 | 1 | Ref |
| MH450113 | Unassigned | Unassigned | Unassigned | 335_23 | Clustered | 17 | VC_335_23 | 17 | 0.0714 | 1 | 0.0714 | 0 | 0 | 0 | 0.6397 | Ref |
| MH450114 | Unassigned | Unassigned | Unassigned | 1208_0 | Clustered | 6 | VC_1208_0 | 6 | 0.8634 | 0.99999946 | 0.8634 | 0 | 0 | 0 | 1 | Ref |
| MH450115 | Unassigned | Unassigned | Unassigned | 1204_0 | Clustered | 25 | VC_1204_0 | 25 | 0.7382 | 1 | 0.7382 | 0 | 0 | 0 | 1 | Ref |
| MH450116 | Unassigned | Unassigned | Unassigned | 384_16 | Clustered | 42 | VC_384_16 | 42 | 0.134 | 1 | 0.134 | 0 | 0 | 0 | 0.7282 | Ref |
| MH450117 | Unassigned | Unassigned | Unassigned | 1200_0 | Clustered | 73 | VC_1200_0 | 73 | 0.9825 | 1 | 0.9825 | 0 | 0 | 0 | 1 | Ref |
| MH450118 | Unassigned | Unassigned | Unassigned | 1200_0 | Clustered | 73 | VC_1200_0 | 73 | 0.9825 | 1 | 0.9825 | 0 | 0 | 0 | 1 | Ref |
| MH450119 | Unassigned | Unassigned | Unassigned | 532_0 | Clustered | 75 | VC_532_0 | 75 | 0.9393 | 1 | 0.9393 | 0 | 0 | 0 | 1 | Ref |
| MH450120 | Unassigned | Unassigned | Unassigned | 825_0 | Clustered | 17 | VC_825_0 | 17 | 0.8364 | 1 | 0.8364 | 0 | 0 | 0 | 1 | Ref |
| MH450121 | Unassigned | Unassigned | Unassigned | 1200_0 | Clustered | 73 | VC_1200_0 | 73 | 0.9825 | 1 | 0.9825 | 0 | 0 | 0 | 1 | Ref |
| MH450122 | Unassigned | Unassigned | Unassigned | 384_16 | Clustered | 42 | VC_384_16 | 42 | 0.134 | 1 | 0.134 | 0 | 0 | 0 | 0.7282 | Ref |
| MH450124 | Unassigned | Unassigned | Unassigned | 532_0 | Clustered | 75 | VC_532_0 | 75 | 0.9393 | 1 | 0.9393 | 0 | 0 | 0 | 1 | Ref |
| MH450125 | Unassigned | Unassigned | Unassigned | 1204_0 | Clustered | 25 | VC_1204_0 | 25 | 0.7382 | 1 | 0.7382 | 0 | 0 | 0 | 1 | Ref |
| MH450126 | Unassigned | Unassigned | Unassigned | 532_0 | Clustered | 75 | VC_532_0 | 75 | 0.9393 | 1 | 0.9393 | 0 | 0 | 0 | 1 | Ref |
| MH450127 | Unassigned | Unassigned | Unassigned | 783_0 | Clustered | 67 | VC_783_0 | 67 | 0.8945 | 1 | 0.8945 | 0 | 0 | 0 | 1 | Ref |
| MH450128 | Unassigned | Unassigned | Unassigned | 532_0 | Clustered | 75 | VC_532_0 | 75 | 0.9393 | 1 | 0.9393 | 0 | 0 | 0 | 1 | Ref |
| MH450129 | Unassigned | Unassigned | Unassigned | 1136_0 | Clustered | 40 | VC_1136_0 | 40 | 0.8746 | 1 | 0.8746 | 0 | 0 | 0 | 1 | Ref |
| MH450130 | Unassigned | Unassigned | Unassigned | 335_23 | Clustered | 17 | VC_335_23 | 17 | 0.0714 | 1 | 0.0714 | 0 | 0 | 0 | 0.6397 | Ref |
| MH450131 | Unassigned | Unassigned | Unassigned | 1200_0 | Clustered | 73 | VC_1200_0 | 73 | 0.9825 | 1 | 0.9825 | 0 | 0 | 0 | 1 | Ref |
| MH450132 | Unassigned | Unassigned | Unassigned | 952_0 | Clustered | 39 | VC_952_0 | 39 | 0.9064 | 1 | 0.9064 | 0 | 0 | 0 | 1 | Ref |
| MH450133 | Unassigned | Unassigned | Unassigned | 335_61 | Clustered | 8 | VC_335_61 | 8 | 0.0394 | 1 | 0.0394 | 0 | 0 | 0 | 0.7857 | Ref |
| MH454076 | Unassigned | Unassigned | Unassigned | 1197_0 | Clustered | 8 | VC_1197_0 | 8 | 0.8167 | 1 | 0.8167 | 0 | 0 | 0 | 1 | Ref |
| MH454077 | Unassigned | Unassigned | Unassigned | 725_0 | Clustered | 47 | VC_725_0 | 47 | 0.8649 | 1 | 0.8649 | 0 | 0 | 0 | 1 | Ref |
| MH454078 | Unassigned | Unassigned | Unassigned | 725_0 | Clustered | 47 | VC_725_0 | 47 | 0.8649 | 1 | 0.8649 | 0 | 0 | 0 | 1 | Ref |
| MH454079 | Unassigned | Unassigned | Unassigned | 725_0 | Clustered | 47 | VC_725_0 | 47 | 0.8649 | 1 | 0.8649 | 0 | 0 | 0 | 1 | Ref |
| MH454080 | Unassigned | Unassigned | Unassigned | 1197_0 | Clustered | 8 | VC_1197_0 | 8 | 0.8167 | 1 | 0.8167 | 0 | 0 | 0 | 1 | Ref |
| MH454082 | Unassigned | Unassigned | Unassigned | 725_0 | Clustered | 47 | VC_725_0 | 47 | 0.8649 | 1 | 0.8649 | 0 | 0 | 0 | 1 | Ref |
| MH454083 | Unassigned | Unassigned | Unassigned | 725_0 | Clustered | 47 | VC_725_0 | 47 | 0.8649 | 1 | 0.8649 | 0 | 0 | 0 | 1 | Ref |
| MH454084 | Unassigned | Unassigned | Unassigned | 725_0 | Clustered | 47 | VC_725_0 | 47 | 0.8649 | 1 | 0.8649 | 0 | 0 | 0 | 1 | Ref |
| MH460459 | Unassigned | Unassigned | Unassigned | 406_0 | Clustered | 7 | VC_406_0 | 7 | 0.176 | 1 | 0.176 | 0 | 0 | 0 | 1 | Ref |
| MH460460 | Unassigned | Unassigned | Unassigned | 68_4 | Clustered | 4 | VC_68_4 | 4 | 0.0431 | 0.90943035 | 0.0392 | 0 | 0 | 0 | 1 | Ref |
| MH460461 | Unassigned | Unassigned | Unassigned | 68_4 | Clustered | 4 | VC_68_4 | 4 | 0.0431 | 0.90943035 | 0.0392 | 0 | 0 | 0 | 1 | Ref |
| MH460462 | Unassigned | Unassigned | Unassigned | 68_4 | Clustered | 4 | VC_68_4 | 4 | 0.0431 | 0.90943035 | 0.0392 | 0 | 0 | 0 | 1 | Ref |
| MH460463 | Unassigned | Unassigned | Unassigned | 68_3 | Clustered | 20 | VC_68_3 | 20 | 0.3537 | 1 | 0.3537 | 0 | 0 | 0 | 1 | Ref |
| MH460824 | Unassigned | Unassigned | Unassigned | 1197_0 | Clustered | 8 | VC_1197_0 | 8 | 0.8167 | 1 | 0.8167 | 0 | 0 | 0 | 1 | Ref |
| MH460825 | Unassigned | Unassigned | Unassigned | 1197_0 | Clustered | 8 | VC_1197_0 | 8 | 0.8167 | 1 | 0.8167 | 0 | 0 | 0 | 1 | Ref |
| MH460826 | Unassigned | Unassigned | Unassigned | 1197_0 | Clustered | 8 | VC_1197_0 | 8 | 0.8167 | 1 | 0.8167 | 0 | 0 | 0 | 1 | Ref |
| MH460827 | Unassigned | Unassigned | Unassigned | 1197_0 | Clustered | 8 | VC_1197_0 | 8 | 0.8167 | 1 | 0.8167 | 0 | 0 | 0 | 1 | Ref |
| MH460829 | Unassigned | Unassigned | Unassigned | 510_1 | Clustered | 20 | VC_510_1 | 20 | 0.4172 | 1 | 0.4172 | 0 | 0 | 0 | 1 | Ref |
| MH464253 | Unassigned | Unassigned | Unassigned | 465_0 | Clustered | 80 | VC_465_0 | 80 | 0.9988 | 1 | 0.9988 | 0 | 0 | 0 | 1 | Ref |
| MH479909 | Unassigned | Unassigned | Unassigned | 535_1 | Clustered | 108 | VC_535_1 | 108 | 0.8479 | 1 | 0.8479 | 0 | 0 | 0 | 0.9955 | Ref |
| MH479910 | Unassigned | Unassigned | Unassigned | 1219_0 | Clustered | 24 | VC_1219_0 | 24 | 0.9519 | 1 | 0.9519 | 0 | 0 | 0 | 1 | Ref |
| MH479911 | Unassigned | Unassigned | Unassigned | 535_1 | Clustered | 108 | VC_535_1 | 108 | 0.8479 | 1 | 0.8479 | 0 | 0 | 0 | 0.9955 | Ref |
| MH479912 | Unassigned | Unassigned | Unassigned | 543_0 | Clustered | 25 | VC_543_0 | 25 | 0.4584 | 1 | 0.4584 | 0 | 0 | 0 | 1 | Ref |
| MH479913 | Unassigned | Unassigned | Unassigned | 1225_0 | Clustered | 6 | VC_1225_0 | 6 | 0.9947 | 0.99996974 | 0.9947 | 0 | 0 | 0 | 1 | Ref |
| MH479914 | Unassigned | Unassigned | Unassigned | 384_16 | Clustered | 42 | VC_384_16 | 42 | 0.134 | 1 | 0.134 | 0 | 0 | 0 | 0.7282 | Ref |
| MH479915 | Unassigned | Unassigned | Unassigned | 1218_0 | Clustered | 14 | VC_1218_0 | 14 | 0.7811 | 1 | 0.7811 | 0 | 0 | 0 | 0.989 | Ref |
| MH479916 | Unassigned | Unassigned | Unassigned | 384_13 | Clustered | 33 | VC_384_13 | 33 | 0.1785 | 1 | 0.1785 | 0 | 0 | 0 | 0.8769 | Ref |
| MH479917 | Unassigned | Unassigned | Unassigned | 1219_0 | Clustered | 24 | VC_1219_0 | 24 | 0.9519 | 1 | 0.9519 | 0 | 0 | 0 | 1 | Ref |
| MH479918 | Unassigned | Unassigned | Unassigned | 384_13 | Clustered | 33 | VC_384_13 | 33 | 0.1785 | 1 | 0.1785 | 0 | 0 | 0 | 0.8769 | Ref |
| MH479919 | Unassigned | Unassigned | Unassigned | 335_61 | Clustered | 8 | VC_335_61 | 8 | 0.0394 | 1 | 0.0394 | 0 | 0 | 0 | 0.7857 | Ref |
| MH479920 | Unassigned | Unassigned | Unassigned | 783_0 | Clustered | 67 | VC_783_0 | 67 | 0.8945 | 1 | 0.8945 | 0 | 0 | 0 | 1 | Ref |
| MH479921 | Unassigned | Unassigned | Unassigned | 384_16 | Clustered | 42 | VC_384_16 | 42 | 0.134 | 1 | 0.134 | 0 | 0 | 0 | 0.7282 | Ref |
| MH479923 | Unassigned | Unassigned | Unassigned | 897_0 | Clustered | 5 | VC_897_0 | 5 | 0.4183 | 0.99999997 | 0.4183 | 0 | 0 | 0 | 1 | Ref |
| MH479924 | Unassigned | Unassigned | Unassigned | 1136_0 | Clustered | 40 | VC_1136_0 | 40 | 0.8746 | 1 | 0.8746 | 0 | 0 | 0 | 1 | Ref |
| MH479925 | Unassigned | Unassigned | Unassigned | 1225_0 | Clustered | 6 | VC_1225_0 | 6 | 0.9947 | 0.99996974 | 0.9947 | 0 | 0 | 0 | 1 | Ref |
| MH479926 | Unassigned | Unassigned | Unassigned | 1204_0 | Clustered | 25 | VC_1204_0 | 25 | 0.7382 | 1 | 0.7382 | 0 | 0 | 0 | 1 | Ref |
| MH479927 | Unassigned | Unassigned | Unassigned | 543_0 | Clustered | 25 | VC_543_0 | 25 | 0.4584 | 1 | 0.4584 | 0 | 0 | 0 | 1 | Ref |

| | | | | | | | | | | | | | | | | |
|---|---|---|---|---|---|---|---|---|---|---|---|---|---|---|---|---|
| MH487649 | Unassigned | Unassigned | Unassigned | 195_1 | Clustered | 7 | VC_195_1 | 7 | 0.1409 | 1 | 0.1409 | 0 | 0 | 0 | 1 | Ref |
| MH491167 | Unassigned | Unassigned | Unassigned | 465_0 | Clustered | 80 | VC_465_0 | 80 | 0.9988 | 1 | 0.9988 | 0 | 0 | 0 | 1 | Ref |
| MH491968 | Unassigned | Unassigned | Unassigned | 603_0 | Clustered | 28 | VC_603_0 | 28 | 0.7692 | 1 | 0.7692 | 0 | 0 | 0 | 1 | Ref |
| MH491969 | Unassigned | Unassigned | Unassigned | 794_11 | Clustered | 6 | VC_794_11 | 6 | 0.0305 | 0.83830784 | 0.0256 | 0 | 0 | 0 | 0.8 | Ref |
| MH494197 | Unassigned | Unassigned | Unassigned | 170_2 | Clustered/Singleton | 1 | VC_170_2 | 1 | 0 | 1 | 0 | 0 | 0 | 0 | 1 | Ref |
| MH509443 | Unassigned | Unassigned | Unassigned | 535_1 | Clustered | 108 | VC_535_1 | 108 | 0.8479 | 1 | 0.8479 | 0 | 0 | 0 | 0.9955 | Ref |
| MH509444 | Unassigned | Unassigned | Unassigned | 243_0 | Clustered | 72 | VC_243_0 | 72 | 0.8089 | 1 | 0.8089 | 0 | 0 | 0 | 1 | Ref |
| MH509445 | Unassigned | Unassigned | Unassigned | 587_0 | Clustered | 8 | VC_587_0 | 8 | 0.4907 | 1 | 0.4907 | 0 | 0 | 0 | 1 | Ref |
| MH509446 | Unassigned | Unassigned | Unassigned | 989_0 | Clustered | 13 | VC_989_0 | 13 | 0.8622 | 1 | 0.8622 | 0 | 0 | 0 | 1 | Ref |
| MH509447 | Unassigned | Unassigned | Unassigned | 1287_0 | Clustered | 44 | VC_1287_0 | 44 | 1 | 1 | 1 | 0 | 0 | 0 | 1 | Ref |
| MH512890 | Unassigned | Unassigned | Unassigned | 674_0 | Clustered | 2 | VC_674_0 | 2 | 0.0109 | 0.95704495 | 0.0105 | 0 | 0 | 0 | 1 | Ref |
| MH513970 | Unassigned | Unassigned | Unassigned | 385_0 | Clustered | 24 | VC_385_0 | 24 | 0.5516 | 1 | 0.5516 | 0 | 0 | 0 | 1 | Ref |
| MH513971 | Unassigned | Unassigned | Unassigned | 335_0 | Clustered | 2 | VC_335_0 | 2 | 0.0041 | 0.95574439 | 0.0039 | 0 | 0 | 0 | 1 | Ref |
| MH513972 | Unassigned | Unassigned | Unassigned | 571_2 | Clustered | 17 | VC_571_2 | 17 | 0.0965 | 1 | 0.0965 | 0 | 0 | 0 | 0.9779 | Ref |
| MH513973 | Unassigned | Unassigned | Unassigned | 384_13 | Clustered | 33 | VC_384_13 | 33 | 0.1785 | 1 | 0.1785 | 0 | 0 | 0 | 0.8769 | Ref |
| MH513974 | Unassigned | Unassigned | Unassigned | 231_0 | Clustered | 19 | VC_231_0 | 19 | 0.9378 | 1 | 0.9378 | 0 | 0 | 0 | 1 | Ref |
| MH513975 | Unassigned | Unassigned | Unassigned | 532_0 | Clustered | 75 | VC_532_0 | 75 | 0.9393 | 1 | 0.9393 | 0 | 0 | 0 | 1 | Ref |
| MH513976 | Unassigned | Unassigned | Unassigned | 527_0 | Clustered | 37 | VC_527_0 | 37 | 0.9679 | 1 | 0.9679 | 0 | 0 | 0 | 1 | Ref |
| MH513977 | Unassigned | Unassigned | Unassigned | 243_0 | Clustered | 72 | VC_243_0 | 72 | 0.8089 | 1 | 0.8089 | 0 | 0 | 0 | 1 | Ref |
| MH513978 | Unassigned | Unassigned | Unassigned | 571_2 | Clustered | 17 | VC_571_2 | 17 | 0.0965 | 1 | 0.0965 | 0 | 0 | 0 | 0.9779 | Ref |
| MH513979 | Unassigned | Unassigned | Unassigned | 701_0 | Clustered | 16 | VC_701_0 | 16 | 0.2957 | 1 | 0.2957 | 0 | 0 | 0 | 0.9167 | Ref |
| MH513980 | Unassigned | Unassigned | Unassigned | 384_11 | Clustered | 31 | VC_384_11 | 31 | 0.0926 | 1 | 0.0926 | 0 | 0 | 0 | 0.7011 | Ref |
| MH513981 | Unassigned | Unassigned | Unassigned | 1296_0 | Clustered | 82 | VC_1296_0 | 82 | 0.7938 | 1 | 0.7938 | 0 | 0 | 0 | 1 | Ref |
| MH513982 | Unassigned | Unassigned | Unassigned | 1203_0 | Clustered | 28 | VC_1203_0 | 28 | 0.4905 | 1 | 0.4905 | 0 | 0 | 0 | 1 | Ref |
| MH513983 | Unassigned | Unassigned | Unassigned | 571_6 | Clustered | 22 | VC_571_6 | 22 | 0.1207 | 1 | 0.1207 | 0 | 0 | 0 | 0.8095 | Ref |
| MH513984 | Unassigned | Unassigned | Unassigned | 229_0 | Clustered | 5 | VC_229_0 | 5 | 0.0566 | 0.99999997 | 0.0566 | 0 | 0 | 0 | 1 | Ref |
| MH513985 | Unassigned | Unassigned | Unassigned | 222_1 | Clustered | 19 | VC_222_1 | 19 | 0.4166 | 1 | 0.4166 | 0 | 0 | 0 | 1 | Ref |
| MH523359 | Unassigned | Unassigned | Unassigned | 135_40 | Clustered/Singleton | 1 | VC_135_40 | 1 | 0 | 1 | 0 | 0 | 0 | 0 | 1 | Ref |
| MH533020 | Unassigned | Unassigned | Unassigned | 873_0 | Clustered | 3 | VC_873_0 | 3 | 0.0679 | 0.9984794 | 0.0677 | 0 | 0 | 0 | 1 | Ref |
| MH536736 | Unassigned | Unassigned | Unassigned | 81_0 | Clustered | 41 | VC_81_0 | 41 | 0.5815 | 1 | 0.5815 | 0 | 0 | 0 | 0.9805 | Ref |
| MH536811 | Unassigned | Unassigned | Unassigned | 1115_4 | Clustered | 4 | VC_1115_4 | 4 | 0.0733 | 0.99956559 | 0.0732 | 0 | 0 | 0 | 1 | Ref |
| MH536812 | Unassigned | Unassigned | Unassigned | 578_2 | Clustered/Singleton | 1 | VC_578_2 | 1 | 0 | 1 | 0 | 0 | 0 | 0 | 1 | Ref |
| MH536813 | Unassigned | Unassigned | Unassigned | 532_0 | Clustered | 75 | VC_532_0 | 75 | 0.9393 | 1 | 0.9393 | 0 | 0 | 0 | 1 | Ref |
| MH536814 | Unassigned | Unassigned | Unassigned | 1115_4 | Clustered | 4 | VC_1115_4 | 4 | 0.0733 | 0.99956559 | 0.0732 | 0 | 0 | 0 | 1 | Ref |
| MH536815 | Unassigned | Unassigned | Unassigned | 1416_0 | Clustered | 10 | VC_1416_0 | 10 | 1 | 1 | 1 | 0 | 0 | 0 | 1 | Ref |
| MH536816 | Unassigned | Unassigned | Unassigned | 335_28 | Clustered | 2 | VC_335_28 | 2 | 0.0044 | 0.95750955 | 0.0042 | 0 | 0 | 0 | 1 | Ref |
| MH536817 | Unassigned | Unassigned | Unassigned | 535_1 | Clustered | 108 | VC_535_1 | 108 | 0.8479 | 1 | 0.8479 | 0 | 0 | 0 | 0.9955 | Ref |
| MH536818 | Unassigned | Unassigned | Unassigned | 1217_0 | Clustered | 41 | VC_1217_0 | 41 | 0.9546 | 1 | 0.9546 | 0 | 0 | 0 | 0.9976 | Ref |
| MH536819 | Unassigned | Unassigned | Unassigned | 789_0 | Clustered | 34 | VC_789_0 | 34 | 0.9167 | 1 | 0.9167 | 0 | 0 | 0 | 1 | Ref |
| MH536820 | Unassigned | Unassigned | Unassigned | 571_0 | Clustered | 16 | VC_571_0 | 16 | 0.091 | 1 | 0.091 | 0 | 0 | 0 | 0.6667 | Ref |
| MH536821 | Unassigned | Unassigned | Unassigned | 243_0 | Clustered | 72 | VC_243_0 | 72 | 0.8089 | 1 | 0.8089 | 0 | 0 | 0 | 1 | Ref |
| MH536822 | Unassigned | Unassigned | Unassigned | 243_0 | Clustered | 72 | VC_243_0 | 72 | 0.8089 | 1 | 0.8089 | 0 | 0 | 0 | 1 | Ref |
| MH536823 | Unassigned | Unassigned | Unassigned | 881_0 | Clustered | 7 | VC_881_0 | 7 | 0.3448 | 1 | 0.3448 | 0 | 0 | 0 | 1 | Ref |
| MH536824 | Unassigned | Unassigned | Unassigned | 789_0 | Clustered | 34 | VC_789_0 | 34 | 0.9167 | 1 | 0.9167 | 0 | 0 | 0 | 1 | Ref |
| MH536825 | Unassigned | Unassigned | Unassigned | 532_0 | Clustered | 75 | VC_532_0 | 75 | 0.9393 | 1 | 0.9393 | 0 | 0 | 0 | 1 | Ref |
| MH536827 | Unassigned | Unassigned | Unassigned | 571_7 | Clustered | 28 | VC_571_7 | 28 | 0.1651 | 1 | 0.1651 | 0 | 0 | 0 | 0.8519 | Ref |
| MH536828 | Unassigned | Unassigned | Unassigned | 228_0 | Clustered | 22 | VC_228_0 | 22 | 0.7411 | 1 | 0.7411 | 0 | 0 | 0 | 1 | Ref |
| MH536829 | Unassigned | Unassigned | Unassigned | 571_6 | Clustered | 22 | VC_571_6 | 22 | 0.1207 | 1 | 0.1207 | 0 | 0 | 0 | 0.8095 | Ref |
| MH538193 | Unassigned | Unassigned | Unassigned | 913_0 | Clustered | 28 | VC_913_0 | 28 | 0.6206 | 1 | 0.6206 | 0 | 0 | 0 | 0.9841 | Ref |
| MH540083 | Unassigned | Unassigned | Unassigned | 352_0 | Clustered | 31 | VC_352_0 | 31 | 0.9547 | 1 | 0.9547 | 0 | 0 | 0 | 1 | Ref |
| MH542234 | Unassigned | Unassigned | Unassigned | 156_0 | Clustered | 13 | VC_156_0 | 13 | 0.2378 | 1 | 0.2378 | 0 | 0 | 0 | 0.9872 | Ref |
| MH547045 | Unassigned | Unassigned | Unassigned | 45_0 | Clustered | 38 | VC_45_0 | 38 | 0.5785 | 1 | 0.5785 | 0 | 0 | 0 | 0.9659 | Ref |
| MH550421 | Unassigned | Unassigned | Unassigned | 290_88 | Clustered | 2 | VC_290_88 | 2 | 0.0025 | 0.95596547 | 0.0024 | 0 | 0 | 0 | 1 | Ref |
| MH552500 | Unassigned | Unassigned | Unassigned | 691_1 | Clustered | 5 | VC_691_1 | 5 | 0.1605 | 0.99999993 | 0.1605 | 0 | 0 | 0 | 0.8 | Ref |
| MH553563 | Unassigned | Unassigned | Unassigned | 290_52 | Clustered/Singleton | 1 | VC_290_52 | 1 | 0 | 1 | 0 | 0 | 0 | 0 | 1 | Ref |
| MH560358 | Unassigned | Unassigned | Unassigned | 609_0 | Clustered | 27 | VC_609_0 | 27 | 0.9943 | 1 | 0.9943 | 0 | 0 | 0 | 1 | Ref |
| MH571750 | Unassigned | Unassigned | Unassigned | 340_7 | Clustered | 18 | VC_340_7 | 18 | 0.0936 | 1 | 0.0936 | 0 | 0 | 0 | 0.7778 | Ref |
| MH572269 | Unassigned | Unassigned | Unassigned | 411_0 | Clustered | 48 | VC_411_0 | 48 | 0.4808 | 1 | 0.4808 | 0 | 0 | 0 | 1 | Ref |
| MH572274 | Unassigned | Unassigned | Unassigned | 1418_0 | Clustered | 3 | VC_1418_0 | 3 | 1 | 1 | 1 | 0 | 0 | 0 | 1 | Ref |
| MH572275 | Unassigned | Unassigned | Unassigned | 1418_0 | Clustered | 3 | VC_1418_0 | 3 | 1 | 1 | 1 | 0 | 0 | 0 | 1 | Ref |
| MH572276 | Unassigned | Unassigned | Unassigned | 1418_0 | Clustered | 3 | VC_1418_0 | 3 | 1 | 1 | 1 | 0 | 0 | 0 | 1 | Ref |
| MH572277 | Unassigned | Unassigned | Unassigned | 206_0 | Clustered | 28 | VC_206_0 | 28 | 0.2427 | 0.99971175 | 0.2426 | 0 | 0 | 0 | 1 | Ref |
| MH572279 | Unassigned | Unassigned | Unassigned | 412_0 | Clustered | 25 | VC_412_0 | 25 | 0.5642 | 1 | 0.5642 | 0 | 0 | 0 | 1 | Ref |
| MH572280 | Unassigned | Unassigned | Unassigned | 206_0 | Clustered | 28 | VC_206_0 | 28 | 0.2427 | 0.99971175 | 0.2426 | 0 | 0 | 0 | 1 | Ref |
| MH572282 | Unassigned | Unassigned | Unassigned | 206_0 | Clustered | 28 | VC_206_0 | 28 | 0.2427 | 0.99971175 | 0.2426 | 0 | 0 | 0 | 1 | Ref |
| MH572283 | Unassigned | Unassigned | Unassigned | 1028_0 | Clustered | 69 | VC_1028_0 | 69 | 0.8547 | 1 | 0.8547 | 0 | 0 | 0 | 1 | Ref |
| MH572284 | Unassigned | Unassigned | Unassigned | 1028_0 | Clustered | 69 | VC_1028_0 | 69 | 0.8547 | 1 | 0.8547 | 0 | 0 | 0 | 1 | Ref |
| MH572285 | Unassigned | Unassigned | Unassigned | 1028_0 | Clustered | 69 | VC_1028_0 | 69 | 0.8547 | 1 | 0.8547 | 0 | 0 | 0 | 1 | Ref |
| MH572286 | Unassigned | Unassigned | Unassigned | 1028_0 | Clustered | 69 | VC_1028_0 | 69 | 0.8547 | 1 | 0.8547 | 0 | 0 | 0 | 1 | Ref |
| MH572287 | Unassigned | Unassigned | Unassigned | 1126_0 | Clustered | 9 | VC_1126_0 | 9 | 0.6544 | 1 | 0.6544 | 0 | 0 | 0 | 1 | Ref |
| MH572288 | Unassigned | Unassigned | Unassigned | 1126_0 | Clustered | 9 | VC_1126_0 | 9 | 0.6544 | 1 | 0.6544 | 0 | 0 | 0 | 1 | Ref |
| MH572290 | Unassigned | Unassigned | Unassigned | 206_0 | Clustered | 28 | VC_206_0 | 28 | 0.2427 | 0.99971175 | 0.2426 | 0 | 0 | 0 | 1 | Ref |
| MH572291 | Unassigned | Unassigned | Unassigned | 1028_0 | Clustered | 69 | VC_1028_0 | 69 | 0.8547 | 1 | 0.8547 | 0 | 0 | 0 | 1 | Ref |
| MH572292 | Unassigned | Unassigned | Unassigned | 1028_0 | Clustered | 69 | VC_1028_0 | 69 | 0.8547 | 1 | 0.8547 | 0 | 0 | 0 | 1 | Ref |
| MH572293 | Unassigned | Unassigned | Unassigned | 1028_0 | Clustered | 69 | VC_1028_0 | 69 | 0.8547 | 1 | 0.8547 | 0 | 0 | 0 | 1 | Ref |
| MH572294 | Unassigned | Unassigned | Unassigned | 417_0 | Clustered | 20 | VC_417_0 | 20 | 0.6581 | 1 | 0.6581 | 0 | 0 | 0 | 1 | Ref |
| MH572295 | Unassigned | Unassigned | Unassigned | 417_0 | Clustered | 20 | VC_417_0 | 20 | 0.6581 | 1 | 0.6581 | 0 | 0 | 0 | 1 | Ref |
| MH572296 | Unassigned | Unassigned | Unassigned | 417_0 | Clustered | 20 | VC_417_0 | 20 | 0.6581 | 1 | 0.6581 | 0 | 0 | 0 | 1 | Ref |
| MH572299 | Unassigned | Unassigned | Unassigned | 1031_0 | Clustered | 2 | VC_1031_0 | 2 | 0.0513 | 0.93901809 | 0.0482 | 0 | 0 | 0 | 1 | Ref |
| MH572300 | Unassigned | Unassigned | Unassigned | 412_0 | Clustered | 25 | VC_412_0 | 25 | 0.5642 | 1 | 0.5642 | 0 | 0 | 0 | 1 | Ref |
| MH572301 | Unassigned | Unassigned | Unassigned | 412_0 | Clustered | 25 | VC_412_0 | 25 | 0.5642 | 1 | 0.5642 | 0 | 0 | 0 | 1 | Ref |

| | | | | | | | | | | | | | | | | |
|---|---|---|---|---|---|---|---|---|---|---|---|---|---|---|---|---|
| MH572302 | Unassigned | Unassigned | Unassigned | 412_0 | Clustered | 25 | VC_412_0 | 25 | 0.5642 | 1 | 0.5642 | 0 | 0 | 0 | 1 | Ref |
| MH572303 | Unassigned | Unassigned | Unassigned | 412_0 | Clustered | 25 | VC_412_0 | 25 | 0.5642 | 1 | 0.5642 | 0 | 0 | 0 | 1 | Ref |
| MH572305 | Unassigned | Unassigned | Unassigned | 412_0 | Clustered | 25 | VC_412_0 | 25 | 0.5642 | 1 | 0.5642 | 0 | 0 | 0 | 1 | Ref |
| MH572306 | Unassigned | Unassigned | Unassigned | 412_0 | Clustered | 25 | VC_412_0 | 25 | 0.5642 | 1 | 0.5642 | 0 | 0 | 0 | 1 | Ref |
| MH572307 | Unassigned | Unassigned | Unassigned | 412_0 | Clustered | 25 | VC_412_0 | 25 | 0.5642 | 1 | 0.5642 | 0 | 0 | 0 | 1 | Ref |
| MH572308 | Unassigned | Unassigned | Unassigned | 412_0 | Clustered | 25 | VC_412_0 | 25 | 0.5642 | 1 | 0.5642 | 0 | 0 | 0 | 1 | Ref |
| MH572309 | Unassigned | Unassigned | Unassigned | 412_0 | Clustered | 25 | VC_412_0 | 25 | 0.5642 | 1 | 0.5642 | 0 | 0 | 0 | 1 | Ref |
| MH572310 | Unassigned | Unassigned | Unassigned | 412_0 | Clustered | 25 | VC_412_0 | 25 | 0.5642 | 1 | 0.5642 | 0 | 0 | 0 | 1 | Ref |
| MH572311 | Unassigned | Unassigned | Unassigned | 412_0 | Clustered | 25 | VC_412_0 | 25 | 0.5642 | 1 | 0.5642 | 0 | 0 | 0 | 1 | Ref |
| MH572312 | Unassigned | Unassigned | Unassigned | 412_0 | Clustered | 25 | VC_412_0 | 25 | 0.5642 | 1 | 0.5642 | 0 | 0 | 0 | 1 | Ref |
| MH572313 | Unassigned | Unassigned | Unassigned | 412_0 | Clustered | 25 | VC_412_0 | 25 | 0.5642 | 1 | 0.5642 | 0 | 0 | 0 | 1 | Ref |
| MH572314 | Unassigned | Unassigned | Unassigned | 411_0 | Clustered | 48 | VC_411_0 | 48 | 0.4808 | 1 | 0.4808 | 0 | 0 | 0 | 1 | Ref |
| MH572315 | Unassigned | Unassigned | Unassigned | 411_0 | Clustered | 48 | VC_411_0 | 48 | 0.4808 | 1 | 0.4808 | 0 | 0 | 0 | 1 | Ref |
| MH572316 | Unassigned | Unassigned | Unassigned | 411_0 | Clustered | 48 | VC_411_0 | 48 | 0.4808 | 1 | 0.4808 | 0 | 0 | 0 | 1 | Ref |
| MH572317 | Unassigned | Unassigned | Unassigned | 411_0 | Clustered | 48 | VC_411_0 | 48 | 0.4808 | 1 | 0.4808 | 0 | 0 | 0 | 1 | Ref |
| MH572318 | Unassigned | Unassigned | Unassigned | 411_0 | Clustered | 48 | VC_411_0 | 48 | 0.4808 | 1 | 0.4808 | 0 | 0 | 0 | 1 | Ref |
| MH572319 | Unassigned | Unassigned | Unassigned | 411_0 | Clustered | 48 | VC_411_0 | 48 | 0.4808 | 1 | 0.4808 | 0 | 0 | 0 | 1 | Ref |
| MH572320 | Unassigned | Unassigned | Unassigned | 411_0 | Clustered | 48 | VC_411_0 | 48 | 0.4808 | 1 | 0.4808 | 0 | 0 | 0 | 1 | Ref |
| MH572321 | Unassigned | Unassigned | Unassigned | 411_0 | Clustered | 48 | VC_411_0 | 48 | 0.4808 | 1 | 0.4808 | 0 | 0 | 0 | 1 | Ref |
| MH572325 | Unassigned | Unassigned | Unassigned | 411_0 | Clustered | 48 | VC_411_0 | 48 | 0.4808 | 1 | 0.4808 | 0 | 0 | 0 | 1 | Ref |
| MH572326 | Unassigned | Unassigned | Unassigned | 411_0 | Clustered | 48 | VC_411_0 | 48 | 0.4808 | 1 | 0.4808 | 0 | 0 | 0 | 1 | Ref |
| MH572328 | Unassigned | Unassigned | Unassigned | 411_0 | Clustered | 48 | VC_411_0 | 48 | 0.4808 | 1 | 0.4808 | 0 | 0 | 0 | 1 | Ref |
| MH572329 | Unassigned | Unassigned | Unassigned | 411_0 | Clustered | 48 | VC_411_0 | 48 | 0.4808 | 1 | 0.4808 | 0 | 0 | 0 | 1 | Ref |
| MH572330 | Unassigned | Unassigned | Unassigned | 206_0 | Clustered | 28 | VC_206_0 | 28 | 0.2427 | 0.99971175 | 0.2426 | 0 | 0 | 0 | 1 | Ref |
| MH572334 | Unassigned | Unassigned | Unassigned | 206_0 | Clustered | 28 | VC_206_0 | 28 | 0.2427 | 0.99971175 | 0.2426 | 0 | 0 | 0 | 1 | Ref |
| MH572336 | Unassigned | Unassigned | Unassigned | 411_0 | Clustered | 48 | VC_411_0 | 48 | 0.4808 | 1 | 0.4808 | 0 | 0 | 0 | 1 | Ref |
| MH572340 | Unassigned | Unassigned | Unassigned | 414_0 | Clustered/Singleton | 1 | VC_414_0 | 1 | 0 | 1 | 0 | 0 | 0 | 0 | 1 | Ref |
| MH572341 | Unassigned | Unassigned | Unassigned | 207_0 | Clustered | 12 | VC_207_0 | 12 | 0.4465 | 1 | 0.4465 | 0 | 0 | 0 | 1 | Ref |
| MH572342 | Unassigned | Unassigned | Unassigned | 207_0 | Clustered | 12 | VC_207_0 | 12 | 0.4465 | 1 | 0.4465 | 0 | 0 | 0 | 1 | Ref |
| MH572343 | Unassigned | Unassigned | Unassigned | 207_0 | Clustered | 12 | VC_207_0 | 12 | 0.4465 | 1 | 0.4465 | 0 | 0 | 0 | 1 | Ref |
| MH572345 | Unassigned | Unassigned | Unassigned | 412_0 | Clustered | 25 | VC_412_0 | 25 | 0.5642 | 1 | 0.5642 | 0 | 0 | 0 | 1 | Ref |
| MH572347 | Unassigned | Unassigned | Unassigned | 1025_0 | Clustered | 8 | VC_1025_0 | 8 | 0.386 | 1 | 0.386 | 0 | 0 | 0 | 1 | Ref |
| MH572348 | Unassigned | Unassigned | Unassigned | 1025_0 | Clustered | 8 | VC_1025_0 | 8 | 0.386 | 1 | 0.386 | 0 | 0 | 0 | 1 | Ref |
| MH572349 | Unassigned | Unassigned | Unassigned | 1025_0 | Clustered | 8 | VC_1025_0 | 8 | 0.386 | 1 | 0.386 | 0 | 0 | 0 | 1 | Ref |
| MH572351 | Unassigned | Unassigned | Unassigned | 411_0 | Clustered | 48 | VC_411_0 | 48 | 0.4808 | 1 | 0.4808 | 0 | 0 | 0 | 1 | Ref |
| MH572353 | Unassigned | Unassigned | Unassigned | 1419_0 | Clustered | 8 | VC_1419_0 | 8 | 0.8724 | 0.99998222 | 0.8724 | 0 | 0 | 0 | 1 | Ref |
| MH572354 | Unassigned | Unassigned | Unassigned | 1419_0 | Clustered | 8 | VC_1419_0 | 8 | 0.8724 | 0.99998222 | 0.8724 | 0 | 0 | 0 | 1 | Ref |
| MH572355 | Unassigned | Unassigned | Unassigned | 1419_0 | Clustered | 8 | VC_1419_0 | 8 | 0.8724 | 0.99998222 | 0.8724 | 0 | 0 | 0 | 1 | Ref |
| MH572357 | Unassigned | Unassigned | Unassigned | 1419_0 | Clustered | 8 | VC_1419_0 | 8 | 0.8724 | 0.99998222 | 0.8724 | 0 | 0 | 0 | 1 | Ref |
| MH572358 | Unassigned | Unassigned | Unassigned | 1419_0 | Clustered | 8 | VC_1419_0 | 8 | 0.8724 | 0.99998222 | 0.8724 | 0 | 0 | 0 | 1 | Ref |
| MH572360 | Unassigned | Unassigned | Unassigned | 1121_0 | Clustered | 6 | VC_1121_0 | 6 | 0.9299 | 0.89096579 | 0.8285 | 0 | 0 | 0 | 1 | Ref |
| MH572361 | Unassigned | Unassigned | Unassigned | 1121_0 | Clustered | 6 | VC_1121_0 | 6 | 0.9299 | 0.89096579 | 0.8285 | 0 | 0 | 0 | 1 | Ref |
| MH572362 | Unassigned | Unassigned | Unassigned | 1119_0 | Clustered | 48 | VC_1119_0 | 48 | 0.852 | 1 | 0.852 | 0 | 0 | 0 | 1 | Ref |
| MH572364 | Unassigned | Unassigned | Unassigned | 1130_0 | Clustered | 3 | VC_1130_0 | 3 | 0.7736 | 0.82711071 | 0.6399 | 0 | 0 | 0 | 1 | Ref |
| MH572366 | Unassigned | Unassigned | Unassigned | 1124_0 | Clustered | 3 | VC_1124_0 | 3 | 0.6921 | 0.61954664 | 0.4288 | 0 | 0 | 0 | 1 | Ref |
| MH572367 | Unassigned | Unassigned | Unassigned | 1104_0 | Clustered | 44 | VC_1104_0 | 44 | 0.9574 | 0.72818579 | 0.6972 | 0 | 0 | 0 | 1 | Ref |
| MH572368 | Unassigned | Unassigned | Unassigned | 1104_0 | Clustered | 44 | VC_1104_0 | 44 | 0.9574 | 0.72818579 | 0.6972 | 0 | 0 | 0 | 1 | Ref |
| MH572369 | Unassigned | Unassigned | Unassigned | 1369_0 | Clustered | 10 | VC_1369_0 | 10 | 0.6223 | 0.95845085 | 0.5964 | 0 | 0 | 0 | 1 | Ref |
| MH572371 | Unassigned | Unassigned | Unassigned | 1129_0 | Clustered | 5 | VC_1129_0 | 5 | 0.8077 | 0.98759579 | 0.7976 | 0 | 0 | 0 | 1 | Ref |
| MH572373 | Unassigned | Unassigned | Unassigned | 1129_0 | Clustered | 5 | VC_1129_0 | 5 | 0.8077 | 0.98759579 | 0.7976 | 0 | 0 | 0 | 1 | Ref |
| MH572374 | Unassigned | Unassigned | Unassigned | 1387_0 | Clustered | 6 | VC_1387_0 | 6 | 0.5972 | 0.99460389 | 0.5939 | 0 | 0 | 0 | 1 | Ref |
| MH572375 | Unassigned | Unassigned | Unassigned | 1387_0 | Clustered | 6 | VC_1387_0 | 6 | 0.5972 | 0.99460389 | 0.5939 | 0 | 0 | 0 | 1 | Ref |
| MH572376 | Unassigned | Unassigned | Unassigned | 1387_0 | Clustered | 6 | VC_1387_0 | 6 | 0.5972 | 0.99460389 | 0.5939 | 0 | 0 | 0 | 1 | Ref |
| MH572377 | Unassigned | Unassigned | Unassigned | 1104_0 | Clustered | 44 | VC_1104_0 | 44 | 0.9574 | 0.72818579 | 0.6972 | 0 | 0 | 0 | 1 | Ref |
| MH572378 | Unassigned | Unassigned | Unassigned | 1104_0 | Clustered | 44 | VC_1104_0 | 44 | 0.9574 | 0.72818579 | 0.6972 | 0 | 0 | 0 | 1 | Ref |
| MH572379 | Unassigned | Unassigned | Unassigned | 1104_0 | Clustered | 44 | VC_1104_0 | 44 | 0.9574 | 0.72818579 | 0.6972 | 0 | 0 | 0 | 1 | Ref |
| MH572380 | Unassigned | Unassigned | Unassigned | 1129_0 | Clustered | 5 | VC_1129_0 | 5 | 0.8077 | 0.98759579 | 0.7976 | 0 | 0 | 0 | 1 | Ref |
| MH572382 | Unassigned | Unassigned | Unassigned | 1028_0 | Clustered | 69 | VC_1028_0 | 69 | 0.8547 | 1 | 0.8547 | 0 | 0 | 0 | 1 | Ref |
| MH572383 | Unassigned | Unassigned | Unassigned | 1028_0 | Clustered | 69 | VC_1028_0 | 69 | 0.8547 | 1 | 0.8547 | 0 | 0 | 0 | 1 | Ref |
| MH572384 | Unassigned | Unassigned | Unassigned | 417_0 | Clustered | 20 | VC_417_0 | 20 | 0.6581 | 1 | 0.6581 | 0 | 0 | 0 | 1 | Ref |
| MH572385 | Unassigned | Unassigned | Unassigned | 417_0 | Clustered | 20 | VC_417_0 | 20 | 0.6581 | 1 | 0.6581 | 0 | 0 | 0 | 1 | Ref |
| MH572386 | Unassigned | Unassigned | Unassigned | 417_0 | Clustered | 20 | VC_417_0 | 20 | 0.6581 | 1 | 0.6581 | 0 | 0 | 0 | 1 | Ref |
| MH572387 | Unassigned | Unassigned | Unassigned | 417_0 | Clustered | 20 | VC_417_0 | 20 | 0.6581 | 1 | 0.6581 | 0 | 0 | 0 | 1 | Ref |
| MH572388 | Unassigned | Unassigned | Unassigned | 417_0 | Clustered | 20 | VC_417_0 | 20 | 0.6581 | 1 | 0.6581 | 0 | 0 | 0 | 1 | Ref |
| MH572389 | Unassigned | Unassigned | Unassigned | 417_0 | Clustered | 20 | VC_417_0 | 20 | 0.6581 | 1 | 0.6581 | 0 | 0 | 0 | 1 | Ref |
| MH572390 | Unassigned | Unassigned | Unassigned | 417_0 | Clustered | 20 | VC_417_0 | 20 | 0.6581 | 1 | 0.6581 | 0 | 0 | 0 | 1 | Ref |
| MH572391 | Unassigned | Unassigned | Unassigned | 417_0 | Clustered | 20 | VC_417_0 | 20 | 0.6581 | 1 | 0.6581 | 0 | 0 | 0 | 1 | Ref |
| MH572392 | Unassigned | Unassigned | Unassigned | 412_0 | Clustered | 25 | VC_412_0 | 25 | 0.5642 | 1 | 0.5642 | 0 | 0 | 0 | 1 | Ref |
| MH572393 | Unassigned | Unassigned | Unassigned | 412_0 | Clustered | 25 | VC_412_0 | 25 | 0.5642 | 1 | 0.5642 | 0 | 0 | 0 | 1 | Ref |
| MH572394 | Unassigned | Unassigned | Unassigned | 411_0 | Clustered | 48 | VC_411_0 | 48 | 0.4808 | 1 | 0.4808 | 0 | 0 | 0 | 1 | Ref |
| MH572395 | Unassigned | Unassigned | Unassigned | 411_0 | Clustered | 48 | VC_411_0 | 48 | 0.4808 | 1 | 0.4808 | 0 | 0 | 0 | 1 | Ref |
| MH572396 | Unassigned | Unassigned | Unassigned | 411_0 | Clustered | 48 | VC_411_0 | 48 | 0.4808 | 1 | 0.4808 | 0 | 0 | 0 | 1 | Ref |
| MH572397 | Unassigned | Unassigned | Unassigned | 411_0 | Clustered | 48 | VC_411_0 | 48 | 0.4808 | 1 | 0.4808 | 0 | 0 | 0 | 1 | Ref |
| MH572399 | Unassigned | Unassigned | Unassigned | 207_0 | Clustered | 12 | VC_207_0 | 12 | 0.4465 | 1 | 0.4465 | 0 | 0 | 0 | 1 | Ref |
| MH572400 | Unassigned | Unassigned | Unassigned | 207_0 | Clustered | 12 | VC_207_0 | 12 | 0.4465 | 1 | 0.4465 | 0 | 0 | 0 | 1 | Ref |
| MH572401 | Unassigned | Unassigned | Unassigned | 207_0 | Clustered | 12 | VC_207_0 | 12 | 0.4465 | 1 | 0.4465 | 0 | 0 | 0 | 1 | Ref |
| MH572402 | Unassigned | Unassigned | Unassigned | 207_0 | Clustered | 12 | VC_207_0 | 12 | 0.4465 | 1 | 0.4465 | 0 | 0 | 0 | 1 | Ref |
| MH572403 | Unassigned | Unassigned | Unassigned | 207_0 | Clustered | 12 | VC_207_0 | 12 | 0.4465 | 1 | 0.4465 | 0 | 0 | 0 | 1 | Ref |
| MH572404 | Unassigned | Unassigned | Unassigned | 411_0 | Clustered | 48 | VC_411_0 | 48 | 0.4808 | 1 | 0.4808 | 0 | 0 | 0 | 1 | Ref |
| MH572405 | Unassigned | Unassigned | Unassigned | 1025_0 | Clustered | 8 | VC_1025_0 | 8 | 0.386 | 1 | 0.386 | 0 | 0 | 0 | 1 | Ref |
| MH572406 | Unassigned | Unassigned | Unassigned | 412_0 | Clustered | 25 | VC_412_0 | 25 | 0.5642 | 1 | 0.5642 | 0 | 0 | 0 | 1 | Ref |

| | | | | | | | | | | | | | | | | |
|---|---|---|---|---|---|---|---|---|---|---|---|---|---|---|---|---|
| MH572407 | Unassigned | Unassigned | Unassigned | 1419_0 | Clustered | 8 | VC_1419_0 | 8 | 0.8724 | 0.99998222 | 0.8724 | 0 | 0 | 0 | 1 | Ref |
| MH572408 | Unassigned | Unassigned | Unassigned | 1419_0 | Clustered | 8 | VC_1419_0 | 8 | 0.8724 | 0.99998222 | 0.8724 | 0 | 0 | 0 | 1 | Ref |
| MH572409 | Unassigned | Unassigned | Unassigned | 1121_0 | Clustered | 6 | VC_1121_0 | 6 | 0.9299 | 0.89096579 | 0.8285 | 0 | 0 | 0 | 1 | Ref |
| MH572410 | Unassigned | Unassigned | Unassigned | 1119_0 | Clustered | 48 | VC_1119_0 | 48 | 0.852 | 1 | 0.852 | 0 | 0 | 0 | 1 | Ref |
| MH572411 | Unassigned | Unassigned | Unassigned | 1119_0 | Clustered | 48 | VC_1119_0 | 48 | 0.852 | 1 | 0.852 | 0 | 0 | 0 | 1 | Ref |
| MH572412 | Unassigned | Unassigned | Unassigned | 1119_0 | Clustered | 48 | VC_1119_0 | 48 | 0.852 | 1 | 0.852 | 0 | 0 | 0 | 1 | Ref |
| MH572414 | Unassigned | Unassigned | Unassigned | 1387_0 | Clustered | 6 | VC_1387_0 | 6 | 0.5972 | 0.99460389 | 0.5939 | 0 | 0 | 0 | 1 | Ref |
| MH572418 | Unassigned | Unassigned | Unassigned | 1025_0 | Clustered | 8 | VC_1025_0 | 8 | 0.386 | 1 | 0.386 | 0 | 0 | 0 | 1 | Ref |
| MH572419 | Unassigned | Unassigned | Unassigned | 411_0 | Clustered | 48 | VC_411_0 | 48 | 0.4808 | 1 | 0.4808 | 0 | 0 | 0 | 1 | Ref |
| MH572422 | Unassigned | Unassigned | Unassigned | 206_0 | Clustered | 28 | VC_206_0 | 28 | 0.2427 | 0.99971175 | 0.2426 | 0 | 0 | 0 | 1 | Ref |
| MH572423 | Unassigned | Unassigned | Unassigned | 1028_0 | Clustered | 69 | VC_1028_0 | 69 | 0.8547 | 1 | 0.8547 | 0 | 0 | 0 | 1 | Ref |
| MH572424 | Unassigned | Unassigned | Unassigned | 1028_0 | Clustered | 69 | VC_1028_0 | 69 | 0.8547 | 1 | 0.8547 | 0 | 0 | 0 | 1 | Ref |
| MH572425 | Unassigned | Unassigned | Unassigned | 1031_0 | Clustered | 2 | VC_1031_0 | 2 | 0.0513 | 0.93901809 | 0.0482 | 0 | 0 | 0 | 1 | Ref |
| MH572426 | Unassigned | Unassigned | Unassigned | 1032_0 | Clustered | 2 | VC_1032_0 | 2 | 0.0274 | 0.73989325 | 0.0202 | 0 | 0 | 0 | 1 | Ref |
| MH572427 | Unassigned | Unassigned | Unassigned | 1028_0 | Clustered | 69 | VC_1028_0 | 69 | 0.8547 | 1 | 0.8547 | 0 | 0 | 0 | 1 | Ref |
| MH572428 | Unassigned | Unassigned | Unassigned | 1028_0 | Clustered | 69 | VC_1028_0 | 69 | 0.8547 | 1 | 0.8547 | 0 | 0 | 0 | 1 | Ref |
| MH572430 | Unassigned | Unassigned | Unassigned | 417_0 | Clustered | 20 | VC_417_0 | 20 | 0.6581 | 1 | 0.6581 | 0 | 0 | 0 | 1 | Ref |
| MH572431 | Unassigned | Unassigned | Unassigned | 411_0 | Clustered | 48 | VC_411_0 | 48 | 0.4808 | 1 | 0.4808 | 0 | 0 | 0 | 1 | Ref |
| MH572434 | Unassigned | Unassigned | Unassigned | 209_0 | Clustered | 3 | VC_209_0 | 3 | 0.0996 | 0.69690761 | 0.0694 | 0 | 0 | 0 | 1 | Ref |
| MH572435 | Unassigned | Unassigned | Unassigned | 412_0 | Clustered | 25 | VC_412_0 | 25 | 0.5642 | 1 | 0.5642 | 0 | 0 | 0 | 1 | Ref |
| MH572436 | Unassigned | Unassigned | Unassigned | 412_0 | Clustered | 25 | VC_412_0 | 25 | 0.5642 | 1 | 0.5642 | 0 | 0 | 0 | 1 | Ref |
| MH572437 | Unassigned | Unassigned | Unassigned | 412_0 | Clustered | 25 | VC_412_0 | 25 | 0.5642 | 1 | 0.5642 | 0 | 0 | 0 | 1 | Ref |
| MH572438 | Unassigned | Unassigned | Unassigned | 206_0 | Clustered | 28 | VC_206_0 | 28 | 0.2427 | 0.99971175 | 0.2426 | 0 | 0 | 0 | 1 | Ref |
| MH572439 | Unassigned | Unassigned | Unassigned | 411_0 | Clustered | 48 | VC_411_0 | 48 | 0.4808 | 1 | 0.4808 | 0 | 0 | 0 | 1 | Ref |
| MH572441 | Unassigned | Unassigned | Unassigned | 411_0 | Clustered | 48 | VC_411_0 | 48 | 0.4808 | 1 | 0.4808 | 0 | 0 | 0 | 1 | Ref |
| MH572443 | Unassigned | Unassigned | Unassigned | 411_0 | Clustered | 48 | VC_411_0 | 48 | 0.4808 | 1 | 0.4808 | 0 | 0 | 0 | 1 | Ref |
| MH572444 | Unassigned | Unassigned | Unassigned | 206_0 | Clustered | 28 | VC_206_0 | 28 | 0.2427 | 0.99971175 | 0.2426 | 0 | 0 | 0 | 1 | Ref |
| MH572445 | Unassigned | Unassigned | Unassigned | 411_0 | Clustered | 48 | VC_411_0 | 48 | 0.4808 | 1 | 0.4808 | 0 | 0 | 0 | 1 | Ref |
| MH572446 | Unassigned | Unassigned | Unassigned | 411_0 | Clustered | 48 | VC_411_0 | 48 | 0.4808 | 1 | 0.4808 | 0 | 0 | 0 | 1 | Ref |
| MH572448 | Unassigned | Unassigned | Unassigned | 206_0 | Clustered | 28 | VC_206_0 | 28 | 0.2427 | 0.99971175 | 0.2426 | 0 | 0 | 0 | 1 | Ref |
| MH572451 | Unassigned | Unassigned | Unassigned | 411_0 | Clustered | 48 | VC_411_0 | 48 | 0.4808 | 1 | 0.4808 | 0 | 0 | 0 | 1 | Ref |
| MH572452 | Unassigned | Unassigned | Unassigned | 206_0 | Clustered | 28 | VC_206_0 | 28 | 0.2427 | 0.99971175 | 0.2426 | 0 | 0 | 0 | 1 | Ref |
| MH572453 | Unassigned | Unassigned | Unassigned | 207_0 | Clustered | 12 | VC_207_0 | 12 | 0.4465 | 1 | 0.4465 | 0 | 0 | 0 | 1 | Ref |
| MH572454 | Unassigned | Unassigned | Unassigned | 206_0 | Clustered | 28 | VC_206_0 | 28 | 0.2427 | 0.99971175 | 0.2426 | 0 | 0 | 0 | 1 | Ref |
| MH572455 | Unassigned | Unassigned | Unassigned | 1025_0 | Clustered | 8 | VC_1025_0 | 8 | 0.386 | 1 | 0.386 | 0 | 0 | 0 | 1 | Ref |
| MH572457 | Unassigned | Unassigned | Unassigned | 1121_0 | Clustered | 6 | VC_1121_0 | 6 | 0.9299 | 0.89096579 | 0.8285 | 0 | 0 | 0 | 1 | Ref |
| MH572458 | Unassigned | Unassigned | Unassigned | 1130_0 | Clustered | 3 | VC_1130_0 | 3 | 0.7736 | 0.82711071 | 0.6399 | 0 | 0 | 0 | 1 | Ref |
| MH572459 | Unassigned | Unassigned | Unassigned | 1104_0 | Clustered | 44 | VC_1104_0 | 44 | 0.9574 | 0.72818579 | 0.6972 | 0 | 0 | 0 | 1 | Ref |
| MH572460 | Unassigned | Unassigned | Unassigned | 1104_0 | Clustered | 44 | VC_1104_0 | 44 | 0.9574 | 0.72818579 | 0.6972 | 0 | 0 | 0 | 1 | Ref |
| MH572461 | Unassigned | Unassigned | Unassigned | 1104_0 | Clustered | 44 | VC_1104_0 | 44 | 0.9574 | 0.72818579 | 0.6972 | 0 | 0 | 0 | 1 | Ref |
| MH572462 | Unassigned | Unassigned | Unassigned | 1104_0 | Clustered | 44 | VC_1104_0 | 44 | 0.9574 | 0.72818579 | 0.6972 | 0 | 0 | 0 | 1 | Ref |
| MH572463 | Unassigned | Unassigned | Unassigned | 1104_0 | Clustered | 44 | VC_1104_0 | 44 | 0.9574 | 0.72818579 | 0.6972 | 0 | 0 | 0 | 1 | Ref |
| MH572465 | Unassigned | Unassigned | Unassigned | 1104_0 | Clustered | 44 | VC_1104_0 | 44 | 0.9574 | 0.72818579 | 0.6972 | 0 | 0 | 0 | 1 | Ref |
| MH572466 | Unassigned | Unassigned | Unassigned | 1028_0 | Clustered | 69 | VC_1028_0 | 69 | 0.8547 | 1 | 0.8547 | 0 | 0 | 0 | 1 | Ref |
| MH572467 | Unassigned | Unassigned | Unassigned | 1126_0 | Clustered | 9 | VC_1126_0 | 9 | 0.6544 | 1 | 0.6544 | 0 | 0 | 0 | 1 | Ref |
| MH572468 | Unassigned | Unassigned | Unassigned | 1126_0 | Clustered | 9 | VC_1126_0 | 9 | 0.6544 | 1 | 0.6544 | 0 | 0 | 0 | 1 | Ref |
| MH572469 | Unassigned | Unassigned | Unassigned | 412_0 | Clustered | 25 | VC_412_0 | 25 | 0.5642 | 1 | 0.5642 | 0 | 0 | 0 | 1 | Ref |
| MH572470 | Unassigned | Unassigned | Unassigned | 417_0 | Clustered | 20 | VC_417_0 | 20 | 0.6581 | 1 | 0.6581 | 0 | 0 | 0 | 1 | Ref |
| MH572471 | Unassigned | Unassigned | Unassigned | 417_0 | Clustered | 20 | VC_417_0 | 20 | 0.6581 | 1 | 0.6581 | 0 | 0 | 0 | 1 | Ref |
| MH572472 | Unassigned | Unassigned | Unassigned | 417_0 | Clustered | 20 | VC_417_0 | 20 | 0.6581 | 1 | 0.6581 | 0 | 0 | 0 | 1 | Ref |
| MH572473 | Unassigned | Unassigned | Unassigned | 411_0 | Clustered | 48 | VC_411_0 | 48 | 0.4808 | 1 | 0.4808 | 0 | 0 | 0 | 1 | Ref |
| MH572475 | Unassigned | Unassigned | Unassigned | 209_0 | Clustered | 3 | VC_209_0 | 3 | 0.0996 | 0.69690761 | 0.0694 | 0 | 0 | 0 | 1 | Ref |
| MH572479 | Unassigned | Unassigned | Unassigned | 415_0 | Clustered/Singleton | 1 | VC_415_0 | 1 | 0 | 1 | 0 | 0 | 0 | 0 | 1 | Ref |
| MH572480 | Unassigned | Unassigned | Unassigned | 412_0 | Clustered | 25 | VC_412_0 | 25 | 0.5642 | 1 | 0.5642 | 0 | 0 | 0 | 1 | Ref |
| MH572482 | Unassigned | Unassigned | Unassigned | 1121_0 | Clustered | 6 | VC_1121_0 | 6 | 0.9299 | 0.89096579 | 0.8285 | 0 | 0 | 0 | 1 | Ref |
| MH572483 | Unassigned | Unassigned | Unassigned | 1121_0 | Clustered | 6 | VC_1121_0 | 6 | 0.9299 | 0.89096579 | 0.8285 | 0 | 0 | 0 | 1 | Ref |
| MH572484 | Unassigned | Unassigned | Unassigned | 1401_0 | Clustered | 4 | VC_1401_0 | 4 | 1 | 1 | 1 | 0 | 0 | 0 | 1 | Ref |
| MH572485 | Unassigned | Unassigned | Unassigned | 1387_0 | Clustered | 6 | VC_1387_0 | 6 | 0.5972 | 0.99460389 | 0.5939 | 0 | 0 | 0 | 1 | Ref |
| MH572486 | Unassigned | Unassigned | Unassigned | 1119_0 | Clustered | 48 | VC_1119_0 | 48 | 0.852 | 1 | 0.852 | 0 | 0 | 0 | 1 | Ref |
| MH572487 | Unassigned | Unassigned | Unassigned | 411_0 | Clustered | 48 | VC_411_0 | 48 | 0.4808 | 1 | 0.4808 | 0 | 0 | 0 | 1 | Ref |
| MH572489 | Unassigned | Unassigned | Unassigned | 1131_0 | Clustered | 2 | VC_1131_0 | 2 | 0.5449 | 0.5 | 0.2725 | 0 | 0 | 0 | 1 | Ref |
| MH572490 | Unassigned | Unassigned | Unassigned | 1420_0 | Clustered | 6 | VC_1420_0 | 6 | 1 | 1 | 1 | 0 | 0 | 0 | 1 | Ref |
| MH572491 | Unassigned | Unassigned | Unassigned | 1420_0 | Clustered | 6 | VC_1420_0 | 6 | 1 | 1 | 1 | 0 | 0 | 0 | 1 | Ref |
| MH572492 | Unassigned | Unassigned | Unassigned | 411_0 | Clustered | 48 | VC_411_0 | 48 | 0.4808 | 1 | 0.4808 | 0 | 0 | 0 | 1 | Ref |
| MH572493 | Unassigned | Unassigned | Unassigned | 411_0 | Clustered | 48 | VC_411_0 | 48 | 0.4808 | 1 | 0.4808 | 0 | 0 | 0 | 1 | Ref |
| MH572494 | Unassigned | Unassigned | Unassigned | 411_0 | Clustered | 48 | VC_411_0 | 48 | 0.4808 | 1 | 0.4808 | 0 | 0 | 0 | 1 | Ref |
| MH572495 | Unassigned | Unassigned | Unassigned | 1028_0 | Clustered | 69 | VC_1028_0 | 69 | 0.8547 | 1 | 0.8547 | 0 | 0 | 0 | 1 | Ref |
| MH572496 | Unassigned | Unassigned | Unassigned | 1126_0 | Clustered | 9 | VC_1126_0 | 9 | 0.6544 | 1 | 0.6544 | 0 | 0 | 0 | 1 | Ref |
| MH572497 | Unassigned | Unassigned | Unassigned | 1126_0 | Clustered | 9 | VC_1126_0 | 9 | 0.6544 | 1 | 0.6544 | 0 | 0 | 0 | 1 | Ref |
| MH572498 | Unassigned | Unassigned | Unassigned | 1126_0 | Clustered | 9 | VC_1126_0 | 9 | 0.6544 | 1 | 0.6544 | 0 | 0 | 0 | 1 | Ref |
| MH572499 | Unassigned | Unassigned | Unassigned | 1126_0 | Clustered | 9 | VC_1126_0 | 9 | 0.6544 | 1 | 0.6544 | 0 | 0 | 0 | 1 | Ref |
| MH572500 | Unassigned | Unassigned | Unassigned | 1126_0 | Clustered | 9 | VC_1126_0 | 9 | 0.6544 | 1 | 0.6544 | 0 | 0 | 0 | 1 | Ref |
| MH572503 | Unassigned | Unassigned | Unassigned | 411_0 | Clustered | 48 | VC_411_0 | 48 | 0.4808 | 1 | 0.4808 | 0 | 0 | 0 | 1 | Ref |
| MH572504 | Unassigned | Unassigned | Unassigned | 1028_0 | Clustered | 69 | VC_1028_0 | 69 | 0.8547 | 1 | 0.8547 | 0 | 0 | 0 | 1 | Ref |
| MH572505 | Unassigned | Unassigned | Unassigned | 417_0 | Clustered | 20 | VC_417_0 | 20 | 0.6581 | 1 | 0.6581 | 0 | 0 | 0 | 1 | Ref |
| MH572506 | Unassigned | Unassigned | Unassigned | 417_0 | Clustered | 20 | VC_417_0 | 20 | 0.6581 | 1 | 0.6581 | 0 | 0 | 0 | 1 | Ref |
| MH572507 | Unassigned | Unassigned | Unassigned | 417_0 | Clustered | 20 | VC_417_0 | 20 | 0.6581 | 1 | 0.6581 | 0 | 0 | 0 | 1 | Ref |
| MH572508 | Unassigned | Unassigned | Unassigned | 412_0 | Clustered | 25 | VC_412_0 | 25 | 0.5642 | 1 | 0.5642 | 0 | 0 | 0 | 1 | Ref |
| MH572509 | Unassigned | Unassigned | Unassigned | 412_0 | Clustered | 25 | VC_412_0 | 25 | 0.5642 | 1 | 0.5642 | 0 | 0 | 0 | 1 | Ref |
| MH572510 | Unassigned | Unassigned | Unassigned | 411_0 | Clustered | 48 | VC_411_0 | 48 | 0.4808 | 1 | 0.4808 | 0 | 0 | 0 | 1 | Ref |
| MH572511 | Unassigned | Unassigned | Unassigned | 411_0 | Clustered | 48 | VC_411_0 | 48 | 0.4808 | 1 | 0.4808 | 0 | 0 | 0 | 1 | Ref |

| | | | | | | | | | | | | | | | | |
|---|---|---|---|---|---|---|---|---|---|---|---|---|---|---|---|---|
| MH572512 | Unassigned | Unassigned | Unassigned | 416_0 | Clustered/Singleton | 1 | VC_416_0 | 1 | 0 | 1 | 0 | 0 | 0 | 0 | 1 | Ref |
| MH572513 | Unassigned | Unassigned | Unassigned | 207_0 | Clustered | 12 | VC_207_0 | 12 | 0.4465 | 1 | 0.4465 | 0 | 0 | 0 | 1 | Ref |
| MH572514 | Unassigned | Unassigned | Unassigned | 207_0 | Clustered | 12 | VC_207_0 | 12 | 0.4465 | 1 | 0.4465 | 0 | 0 | 0 | 1 | Ref |
| MH572515 | Unassigned | Unassigned | Unassigned | 1025_0 | Clustered | 8 | VC_1025_0 | 8 | 0.386 | 1 | 0.386 | 0 | 0 | 0 | 1 | Ref |
| MH572516 | Unassigned | Unassigned | Unassigned | 1419_0 | Clustered | 8 | VC_1419_0 | 8 | 0.8724 | 0.99998222 | 0.8724 | 0 | 0 | 0 | 1 | Ref |
| MH572517 | Unassigned | Unassigned | Unassigned | 1420_0 | Clustered | 6 | VC_1420_0 | 6 | 1 | 1 | 1 | 0 | 0 | 0 | 1 | Ref |
| MH572518 | Unassigned | Unassigned | Unassigned | 1420_0 | Clustered | 6 | VC_1420_0 | 6 | 1 | 1 | 1 | 0 | 0 | 0 | 1 | Ref |
| MH572519 | Unassigned | Unassigned | Unassigned | 1420_0 | Clustered | 6 | VC_1420_0 | 6 | 1 | 1 | 1 | 0 | 0 | 0 | 1 | Ref |
| MH572520 | Unassigned | Unassigned | Unassigned | 1420_0 | Clustered | 6 | VC_1420_0 | 6 | 1 | 1 | 1 | 0 | 0 | 0 | 1 | Ref |
| MH572521 | Unassigned | Unassigned | Unassigned | 1119_0 | Clustered | 48 | VC_1119_0 | 48 | 0.852 | 1 | 0.852 | 0 | 0 | 0 | 1 | Ref |
| MH572522 | Unassigned | Unassigned | Unassigned | 1387_0 | Clustered | 6 | VC_1387_0 | 6 | 0.5972 | 0.99460389 | 0.5939 | 0 | 0 | 0 | 1 | Ref |
| MH572524 | Unassigned | Unassigned | Unassigned | 1388_0 | Clustered | 4 | VC_1388_0 | 4 | 0.6008 | 0.54407361 | 0.3269 | 0 | 0 | 0 | 1 | Ref |
| MH572525 | Unassigned | Unassigned | Unassigned | 1299_0 | Clustered | 53 | VC_1299_0 | 53 | 0.8666 | 1 | 0.8666 | 0 | 0 | 0 | 1 | Ref |
| MH572526 | Unassigned | Unassigned | Unassigned | 1299_0 | Clustered | 53 | VC_1299_0 | 53 | 0.8666 | 1 | 0.8666 | 0 | 0 | 0 | 1 | Ref |
| MH576948 | Unassigned | Unassigned | Unassigned | 985_0 | Clustered | 38 | VC_985_0 | 38 | 0.9334 | 1 | 0.9334 | 0 | 0 | 0 | 1 | Ref |
| MH576949 | Unassigned | Unassigned | Unassigned | 532_0 | Clustered | 75 | VC_532_0 | 75 | 0.9393 | 1 | 0.9393 | 0 | 0 | 0 | 1 | Ref |
| MH576950 | Unassigned | Unassigned | Unassigned | 985_0 | Clustered | 38 | VC_985_0 | 38 | 0.9334 | 1 | 0.9334 | 0 | 0 | 0 | 1 | Ref |
| MH576951 | Unassigned | Unassigned | Unassigned | 985_0 | Clustered | 38 | VC_985_0 | 38 | 0.9334 | 1 | 0.9334 | 0 | 0 | 0 | 1 | Ref |
| MH576952 | Unassigned | Unassigned | Unassigned | 985_0 | Clustered | 38 | VC_985_0 | 38 | 0.9334 | 1 | 0.9334 | 0 | 0 | 0 | 1 | Ref |
| MH576953 | Unassigned | Unassigned | Unassigned | 571_7 | Clustered | 28 | VC_571_7 | 28 | 0.1651 | 1 | 0.1651 | 0 | 0 | 0 | 0.8519 | Ref |
| MH576954 | Unassigned | Unassigned | Unassigned | 384_4 | Clustered | 5 | VC_384_4 | 5 | 0.0134 | 0.99999971 | 0.0134 | 0 | 0 | 0 | 1 | Ref |
| MH576955 | Unassigned | Unassigned | Unassigned | 985_0 | Clustered | 38 | VC_985_0 | 38 | 0.9334 | 1 | 0.9334 | 0 | 0 | 0 | 1 | Ref |
| MH576956 | Unassigned | Unassigned | Unassigned | 571_1 | Clustered | 4 | VC_571_1 | 4 | 0.0158 | 0.99966394 | 0.0158 | 0 | 0 | 0 | 0.6667 | Ref |
| MH576957 | Unassigned | Unassigned | Unassigned | 985_0 | Clustered | 38 | VC_985_0 | 38 | 0.9334 | 1 | 0.9334 | 0 | 0 | 0 | 1 | Ref |
| MH576958 | Unassigned | Unassigned | Unassigned | 384_13 | Clustered | 33 | VC_384_13 | 33 | 0.1785 | 1 | 0.1785 | 0 | 0 | 0 | 0.8769 | Ref |
| MH576959 | Unassigned | Unassigned | Unassigned | 335_23 | Clustered | 17 | VC_335_23 | 17 | 0.0714 | 1 | 0.0714 | 0 | 0 | 0 | 0.6397 | Ref |
| MH576960 | Unassigned | Unassigned | Unassigned | 789_0 | Clustered | 34 | VC_789_0 | 34 | 0.9167 | 1 | 0.9167 | 0 | 0 | 0 | 1 | Ref |
| MH576961 | Unassigned | Unassigned | Unassigned | 985_0 | Clustered | 38 | VC_985_0 | 38 | 0.9334 | 1 | 0.9334 | 0 | 0 | 0 | 1 | Ref |
| MH576962 | Unassigned | Unassigned | Unassigned | 1199_0 | Clustered | 3 | VC_1199_0 | 3 | 0.3136 | 0.99425123 | 0.3118 | 0 | 0 | 0 | 1 | Ref |
| MH576963 | Unassigned | Unassigned | Unassigned | 1407_0 | Clustered | 78 | VC_1407_0 | 78 | 0.9731 | 1 | 0.9731 | 0 | 0 | 0 | 1 | Ref |
| MH576964 | Unassigned | Unassigned | Unassigned | 1114_0 | Clustered | 20 | VC_1114_0 | 20 | 0.3238 | 1 | 0.3238 | 0 | 0 | 0 | 0.9842 | Ref |
| MH576965 | Unassigned | Unassigned | Unassigned | 1114_0 | Clustered | 20 | VC_1114_0 | 20 | 0.3238 | 1 | 0.3238 | 0 | 0 | 0 | 0.9842 | Ref |
| MH576966 | Unassigned | Unassigned | Unassigned | 245_0 | Clustered | 17 | VC_245_0 | 17 | 0.6081 | 1 | 0.6081 | 0 | 0 | 0 | 1 | Ref |
| MH576967 | Unassigned | Unassigned | Unassigned | 384_4 | Clustered | 5 | VC_384_4 | 5 | 0.0134 | 0.99999971 | 0.0134 | 0 | 0 | 0 | 1 | Ref |
| MH576968 | Unassigned | Unassigned | Unassigned | 1114_0 | Clustered | 20 | VC_1114_0 | 20 | 0.3238 | 1 | 0.3238 | 0 | 0 | 0 | 0.9842 | Ref |
| MH576969 | Unassigned | Unassigned | Unassigned | 1217_0 | Clustered | 41 | VC_1217_0 | 41 | 0.9546 | 1 | 0.9546 | 0 | 0 | 0 | 0.9976 | Ref |
| MH576970 | Unassigned | Unassigned | Unassigned | 384_15 | Clustered | 44 | VC_384_15 | 44 | 0.139 | 1 | 0.139 | 0 | 0 | 0 | 0.7474 | Ref |
| MH576971 | Unassigned | Unassigned | Unassigned | 335_23 | Clustered | 17 | VC_335_23 | 17 | 0.0714 | 1 | 0.0714 | 0 | 0 | 0 | 0.6397 | Ref |
| MH576972 | Unassigned | Unassigned | Unassigned | 590_0 | Clustered | 9 | VC_590_0 | 9 | 0.4952 | 1 | 0.4952 | 0 | 0 | 0 | 1 | Ref |
| MH576974 | Unassigned | Unassigned | Unassigned | 335_23 | Clustered | 17 | VC_335_23 | 17 | 0.0714 | 1 | 0.0714 | 0 | 0 | 0 | 0.6397 | Ref |
| MH576975 | Unassigned | Unassigned | Unassigned | 335_40 | Clustered | 2 | VC_335_40 | 2 | 0.004 | 0.95751617 | 0.0039 | 0 | 0 | 0 | 1 | Ref |
| MH576976 | Unassigned | Unassigned | Unassigned | 896_0 | Clustered | 9 | VC_896_0 | 9 | 0.2604 | 1 | 0.2604 | 0 | 0 | 0 | 1 | Ref |
| MH576977 | Unassigned | Unassigned | Unassigned | 527_0 | Clustered | 37 | VC_527_0 | 37 | 0.9679 | 1 | 0.9679 | 0 | 0 | 0 | 1 | Ref |
| MH579717 | Unassigned | Unassigned | Unassigned | 496_0 | Clustered | 22 | VC_496_0 | 22 | 0.6101 | 1 | 0.6101 | 0 | 0 | 0 | 1 | Ref |
| MH586730 | Unassigned | Unassigned | Unassigned | 1090_0 | Clustered | 8 | VC_1090_0 | 8 | 0.6377 | 1 | 0.6377 | 0 | 0 | 0 | 1 | Ref |
| MH586731 | Unassigned | Unassigned | Unassigned | 340_0 | Clustered | 2 | VC_340_0 | 2 | 0.0051 | 0.9546458 | 0.0048 | 0 | 0 | 0 | 1 | Ref |
| MH587638 | Unassigned | Unassigned | Unassigned | 848_0 | Clustered | 124 | VC_848_0 | 124 | 0.9484 | 1 | 0.9484 | 0 | 0 | 0 | 1 | Ref |
| MH588544 | Unassigned | Unassigned | Unassigned | 1011_3 | Clustered | 2 | VC_1011_3 | 2 | 0.0256 | 0.88402892 | 0.0226 | 0 | 0 | 0 | 1 | Ref |
| MH588545 | Unassigned | Unassigned | Unassigned | 1011_2 | Clustered | 2 | VC_1011_2 | 2 | 0.0273 | 0.95410794 | 0.0261 | 0 | 0 | 0 | 1 | Ref |
| MH590587 | Unassigned | Unassigned | Unassigned | 571_8 | Clustered | 19 | VC_571_8 | 19 | 0.1031 | 1 | 0.1031 | 0 | 0 | 0 | 0.8713 | Ref |
| MH590588 | Unassigned | Unassigned | Unassigned | 384_11 | Clustered | 31 | VC_384_11 | 31 | 0.0926 | 1 | 0.0926 | 0 | 0 | 0 | 0.7011 | Ref |
| MH590589 | Unassigned | Unassigned | Unassigned | 1115_2 | Clustered | 5 | VC_1115_2 | 5 | 0.0998 | 0.99999315 | 0.0998 | 0 | 0 | 0 | 1 | Ref |
| MH590591 | Unassigned | Unassigned | Unassigned | 526_0 | Clustered | 32 | VC_526_0 | 32 | 0.9037 | 1 | 0.9037 | 0 | 0 | 0 | 1 | Ref |
| MH590592 | Unassigned | Unassigned | Unassigned | 577_0 | Clustered | 21 | VC_577_0 | 21 | 0.9908 | 1 | 0.9908 | 0 | 0 | 0 | 1 | Ref |
| MH590593 | Unassigned | Unassigned | Unassigned | 579_22 | Clustered | 4 | VC_579_22 | 4 | 0.0099 | 0.99020449 | 0.0098 | 0 | 0 | 0 | 0.8333 | Ref |
| MH590594 | Unassigned | Unassigned | Unassigned | 384_10 | Clustered/Singleton | 1 | VC_384_10 | 1 | 0 | 1 | 0 | 0 | 0 | 0 | 1 | Ref |
| MH590595 | Unassigned | Unassigned | Unassigned | 335_56 | Clustered | 7 | VC_335_56 | 7 | 0.0242 | 1 | 0.0242 | 0 | 0 | 0 | 0.9048 | Ref |
| MH590596 | Unassigned | Unassigned | Unassigned | 538_78 | Clustered | 2 | VC_538_78 | 2 | 0.008 | 0.95761522 | 0.0076 | 0 | 0 | 0 | 1 | Ref |
| MH590597 | Unassigned | Unassigned | Unassigned | 1114_0 | Clustered | 20 | VC_1114_0 | 20 | 0.3238 | 1 | 0.3238 | 0 | 0 | 0 | 0.9842 | Ref |
| MH590598 | Unassigned | Unassigned | Unassigned | 538_25 | Clustered/Singleton | 1 | VC_538_25 | 1 | 0 | 1 | 0 | 0 | 0 | 0 | 1 | Ref |
| MH590599 | Unassigned | Unassigned | Unassigned | 1114_0 | Clustered | 20 | VC_1114_0 | 20 | 0.3238 | 1 | 0.3238 | 0 | 0 | 0 | 0.9842 | Ref |
| MH590600 | Unassigned | Unassigned | Unassigned | 1415_0 | Clustered | 31 | VC_1415_0 | 31 | 0.8456 | 1 | 0.8456 | 0 | 0 | 0 | 1 | Ref |
| MH590601 | Unassigned | Unassigned | Unassigned | 1015_1 | Clustered | 26 | VC_1015_1 | 26 | 0.6989 | 1 | 0.6989 | 0 | 0 | 0 | 1 | Ref |
| MH590602 | Unassigned | Unassigned | Unassigned | 538_38 | Clustered | 6 | VC_538_38 | 6 | 0.0517 | 1 | 0.0517 | 0 | 0 | 0 | 0.8667 | Ref |
| MH590603 | Unassigned | Unassigned | Unassigned | 1224_1 | Clustered/Singleton | 1 | VC_1224_1 | 1 | 0 | 1 | 0 | 0 | 0 | 0 | 1 | Ref |
| MH590604 | Unassigned | Unassigned | Unassigned | 1203_0 | Clustered | 28 | VC_1203_0 | 28 | 0.4905 | 1 | 0.4905 | 0 | 0 | 0 | 1 | Ref |
| MH590605 | Unassigned | Unassigned | Unassigned | 783_0 | Clustered | 67 | VC_783_0 | 67 | 0.8945 | 1 | 0.8945 | 0 | 0 | 0 | 1 | Ref |
| MH590606 | Unassigned | Unassigned | Unassigned | 1203_0 | Clustered | 28 | VC_1203_0 | 28 | 0.4905 | 1 | 0.4905 | 0 | 0 | 0 | 1 | Ref |
| MH595538 | Unassigned | Unassigned | Unassigned | 358_3 | Clustered/Singleton | 1 | VC_358_3 | 1 | 0 | 1 | 0 | 0 | 0 | 0 | 1 | Ref |
| MH598512 | Unassigned | Unassigned | Unassigned | 852_0 | Clustered | 3 | VC_852_0 | 3 | 0.4886 | 0.99843584 | 0.4878 | 0 | 0 | 0 | 1 | Ref |
| MH598798 | Unassigned | Unassigned | Unassigned | 496_0 | Clustered | 22 | VC_496_0 | 22 | 0.6101 | 1 | 0.6101 | 0 | 0 | 0 | 1 | Ref |
| MH598800 | Unassigned | Unassigned | Unassigned | 496_0 | Clustered | 22 | VC_496_0 | 22 | 0.6101 | 1 | 0.6101 | 0 | 0 | 0 | 1 | Ref |
| MH598801 | Unassigned | Unassigned | Unassigned | 496_0 | Clustered | 22 | VC_496_0 | 22 | 0.6101 | 1 | 0.6101 | 0 | 0 | 0 | 1 | Ref |
| MH598802 | Unassigned | Unassigned | Unassigned | 496_0 | Clustered | 22 | VC_496_0 | 22 | 0.6101 | 1 | 0.6101 | 0 | 0 | 0 | 1 | Ref |
| MH598803 | Unassigned | Unassigned | Unassigned | 496_0 | Clustered | 22 | VC_496_0 | 22 | 0.6101 | 1 | 0.6101 | 0 | 0 | 0 | 1 | Ref |
| MH598804 | Unassigned | Unassigned | Unassigned | 496_0 | Clustered | 22 | VC_496_0 | 22 | 0.6101 | 1 | 0.6101 | 0 | 0 | 0 | 1 | Ref |
| MH598805 | Unassigned | Unassigned | Unassigned | 496_0 | Clustered | 22 | VC_496_0 | 22 | 0.6101 | 1 | 0.6101 | 0 | 0 | 0 | 1 | Ref |
| MH598806 | Unassigned | Unassigned | Unassigned | 496_0 | Clustered | 22 | VC_496_0 | 22 | 0.6101 | 1 | 0.6101 | 0 | 0 | 0 | 1 | Ref |
| MH598807 | Unassigned | Unassigned | Unassigned | 496_0 | Clustered | 22 | VC_496_0 | 22 | 0.6101 | 1 | 0.6101 | 0 | 0 | 0 | 1 | Ref |
| MH599087 | Unassigned | Unassigned | Unassigned | 911_0 | Clustered | 20 | VC_911_0 | 20 | 0.9915 | 1 | 0.9915 | 0 | 0 | 0 | 1 | Ref |
| MH606185 | Unassigned | Unassigned | Unassigned | 87_5 | Clustered/Singleton | 1 | VC_87_5 | 1 | 0 | 1 | 0 | 0 | 0 | 0 | 1 | Ref |

| | | | | | | | | | | | | | | | | |
|---|---|---|---|---|---|---|---|---|---|---|---|---|---|---|---|---|
| MH616963 | Unassigned | Unassigned | Unassigned | 691_0 | Clustered | 18 | VC_691_0 | 18 | 0.6865 | 1 | 0.6865 | 0 | 0 | 0 | 0.9477 | Ref |
| MH618488 | Unassigned | Unassigned | Unassigned | 110_0 | Clustered | 33 | VC_110_0 | 33 | 0.9488 | 1 | 0.9488 | 0 | 0 | 0 | 1 | Ref |
| MH629685 | Unassigned | Unassigned | Unassigned | 446_16 | Clustered/Singleton | 1 | VC_446_16 | 1 | 0 | 1 | 0 | 0 | 0 | 0 | 1 | Ref |
| MH631453 | Unassigned | Unassigned | Unassigned | 611_0 | Clustered | 50 | VC_611_0 | 50 | 0.7306 | 1 | 0.7306 | 0 | 0 | 0 | 1 | Ref |
| MH631454 | Unassigned | Unassigned | Unassigned | 199_19 | Clustered | 12 | VC_199_19 | 12 | 0.0651 | 1 | 0.0651 | 0 | 0 | 0 | 0.8636 | Ref |
| MH632117 | Unassigned | Unassigned | Unassigned | 335_43 | Clustered | 4 | VC_335_43 | 4 | 0.014 | 0.99995965 | 0.014 | 0 | 0 | 0 | 1 | Ref |
| MH632118 | Unassigned | Unassigned | Unassigned | 335_28 | Clustered | 2 | VC_335_28 | 2 | 0.0044 | 0.95750955 | 0.0042 | 0 | 0 | 0 | 1 | Ref |
| MH632119 | Unassigned | Unassigned | Unassigned | 538_93 | Clustered | 2 | VC_538_93 | 2 | 0.0103 | 0.95762079 | 0.0099 | 0 | 0 | 0 | 1 | Ref |
| MH633484 | Unassigned | Unassigned | Unassigned | 972_0 | Clustered | 99 | VC_972_0 | 99 | 0.9113 | 1 | 0.9113 | 0 | 0 | 0 | 0.9928 | Ref |
| MH633485 | Unassigned | Unassigned | Unassigned | 972_0 | Clustered | 99 | VC_972_0 | 99 | 0.9113 | 1 | 0.9113 | 0 | 0 | 0 | 0.9928 | Ref |
| MH633486 | Unassigned | Unassigned | Unassigned | 972_0 | Clustered | 99 | VC_972_0 | 99 | 0.9113 | 1 | 0.9113 | 0 | 0 | 0 | 0.9928 | Ref |
| MH633487 | Unassigned | Unassigned | Unassigned | 972_0 | Clustered | 99 | VC_972_0 | 99 | 0.9113 | 1 | 0.9113 | 0 | 0 | 0 | 0.9928 | Ref |
| MH638294 | Unassigned | Unassigned | Unassigned | 168_0 | Clustered | 18 | VC_168_0 | 18 | 0.7394 | 1 | 0.7394 | 0 | 0 | 0 | 0.8627 | Ref |
| MH638309 | Unassigned | Unassigned | Unassigned | 195_3 | Clustered | 9 | VC_195_3 | 9 | 0.1955 | 1 | 0.1955 | 0 | 0 | 0 | 0.8611 | Ref |
| MH638310 | Unassigned | Unassigned | Unassigned | 912_0 | Clustered | 10 | VC_912_0 | 10 | 0.1994 | 1 | 0.1994 | 0 | 0 | 0 | 1 | Ref |
| MH638311 | Unassigned | Unassigned | Unassigned | 913_0 | Clustered | 28 | VC_913_0 | 28 | 0.6206 | 1 | 0.6206 | 0 | 0 | 0 | 0.9841 | Ref |
| MH638312 | Unassigned | Unassigned | Unassigned | 195_3 | Clustered | 9 | VC_195_3 | 9 | 0.1955 | 1 | 0.1955 | 0 | 0 | 0 | 0.8611 | Ref |
| MH643777 | Unassigned | Unassigned | Unassigned | 185_0 | Clustered | 53 | VC_185_0 | 53 | 0.9153 | 1 | 0.9153 | 0 | 0 | 0 | 1 | Ref |
| MH643778 | Unassigned | Unassigned | Unassigned | 185_0 | Clustered | 53 | VC_185_0 | 53 | 0.9153 | 1 | 0.9153 | 0 | 0 | 0 | 1 | Ref |
| MH645904 | Unassigned | Unassigned | Unassigned | 1008_0 | Clustered | 6 | VC_1008_0 | 6 | 0.2856 | 1 | 0.2856 | 0 | 0 | 0 | 1 | Ref |
| MH651166 | Unassigned | Unassigned | Unassigned | 1136_0 | Clustered | 40 | VC_1136_0 | 40 | 0.8746 | 1 | 0.8746 | 0 | 0 | 0 | 1 | Ref |
| MH651167 | Unassigned | Unassigned | Unassigned | 701_0 | Clustered | 16 | VC_701_0 | 16 | 0.2957 | 1 | 0.2957 | 0 | 0 | 0 | 0.9167 | Ref |
| MH651168 | Unassigned | Unassigned | Unassigned | 1136_0 | Clustered | 40 | VC_1136_0 | 40 | 0.8746 | 1 | 0.8746 | 0 | 0 | 0 | 1 | Ref |
| MH651169 | Unassigned | Unassigned | Unassigned | 538_45 | Clustered | 7 | VC_538_45 | 7 | 0.0632 | 1 | 0.0632 | 0 | 0 | 0 | 0.8095 | Ref |
| MH651170 | Unassigned | Unassigned | Unassigned | 535_1 | Clustered | 108 | VC_535_1 | 108 | 0.8479 | 1 | 0.8479 | 0 | 0 | 0 | 0.9955 | Ref |
| MH651171 | Unassigned | Unassigned | Unassigned | 245_0 | Clustered | 17 | VC_245_0 | 17 | 0.6081 | 1 | 0.6081 | 0 | 0 | 0 | 1 | Ref |
| MH651172 | Unassigned | Unassigned | Unassigned | 1115_2 | Clustered | 5 | VC_1115_2 | 5 | 0.0998 | 0.99999315 | 0.0998 | 0 | 0 | 0 | 1 | Ref |
| MH651174 | Unassigned | Unassigned | Unassigned | 571_6 | Clustered | 22 | VC_571_6 | 22 | 0.1207 | 1 | 0.1207 | 0 | 0 | 0 | 0.8095 | Ref |
| MH651175 | Unassigned | Unassigned | Unassigned | 384_16 | Clustered | 42 | VC_384_16 | 42 | 0.134 | 1 | 0.134 | 0 | 0 | 0 | 0.7282 | Ref |
| MH651176 | Unassigned | Unassigned | Unassigned | 578_1 | Clustered | 11 | VC_578_1 | 11 | 0.4919 | 1 | 0.4919 | 0 | 0 | 0 | 0.7818 | Ref |
| MH651177 | Unassigned | Unassigned | Unassigned | 384_4 | Clustered | 5 | VC_384_4 | 5 | 0.0134 | 0.99999971 | 0.0134 | 0 | 0 | 0 | 1 | Ref |
| MH651178 | Unassigned | Unassigned | Unassigned | 538_32 | Clustered/Singleton | 1 | VC_538_32 | 1 | 0 | 1 | 0 | 0 | 0 | 0 | 1 | Ref |
| MH651179 | Unassigned | Unassigned | Unassigned | 532_0 | Clustered | 75 | VC_532_0 | 75 | 0.9393 | 1 | 0.9393 | 0 | 0 | 0 | 1 | Ref |
| MH651180 | Unassigned | Unassigned | Unassigned | 335_27 | Clustered | 6 | VC_335_27 | 6 | 0.0266 | 1 | 0.0266 | 0 | 0 | 0 | 0.8 | Ref |
| MH651181 | Unassigned | Unassigned | Unassigned | 1407_0 | Clustered | 78 | VC_1407_0 | 78 | 0.9731 | 1 | 0.9731 | 0 | 0 | 0 | 1 | Ref |
| MH651182 | Unassigned | Unassigned | Unassigned | 919_0 | Clustered | 3 | VC_919_0 | 3 | 0.3486 | 0.99831298 | 0.348 | 0 | 0 | 0 | 1 | Ref |
| MH651183 | Unassigned | Unassigned | Unassigned | 538_41 | Clustered | 2 | VC_538_41 | 2 | 0.0138 | 0.95761457 | 0.0132 | 0 | 0 | 0 | 1 | Ref |
| MH651184 | Unassigned | Unassigned | Unassigned | 384_15 | Clustered | 44 | VC_384_15 | 44 | 0.139 | 1 | 0.139 | 0 | 0 | 0 | 0.7474 | Ref |
| MH651185 | Unassigned | Unassigned | Unassigned | 578_1 | Clustered | 11 | VC_578_1 | 11 | 0.4919 | 1 | 0.4919 | 0 | 0 | 0 | 0.7818 | Ref |
| MH651186 | Unassigned | Unassigned | Unassigned | 384_16 | Clustered | 42 | VC_384_16 | 42 | 0.134 | 1 | 0.134 | 0 | 0 | 0 | 0.7282 | Ref |
| MH651188 | Unassigned | Unassigned | Unassigned | 538_80 | Clustered | 3 | VC_538_80 | 3 | 0.0291 | 0.99859904 | 0.0291 | 0 | 0 | 0 | 1 | Ref |
| MH651189 | Unassigned | Unassigned | Unassigned | 419_0 | Clustered | 11 | VC_419_0 | 11 | 0.9465 | 1 | 0.9465 | 0 | 0 | 0 | 1 | Ref |
| MH651190 | Unassigned | Unassigned | Unassigned | 1015_1 | Clustered | 26 | VC_1015_1 | 26 | 0.6989 | 1 | 0.6989 | 0 | 0 | 0 | 1 | Ref |
| MH669000 | Unassigned | Unassigned | Unassigned | 1138_0 | Clustered | 16 | VC_1138_0 | 16 | 0.4566 | 1 | 0.4566 | 0 | 0 | 0 | 1 | Ref |
| MH669001 | Unassigned | Unassigned | Unassigned | 538_21 | Clustered/Singleton | 1 | VC_538_21 | 1 | 0 | 1 | 0 | 0 | 0 | 0 | 1 | Ref |
| MH669002 | Unassigned | Unassigned | Unassigned | 571_6 | Clustered | 22 | VC_571_6 | 22 | 0.1207 | 1 | 0.1207 | 0 | 0 | 0 | 0.8095 | Ref |
| MH669003 | Unassigned | Unassigned | Unassigned | 538_80 | Clustered | 3 | VC_538_80 | 3 | 0.0291 | 0.99859904 | 0.0291 | 0 | 0 | 0 | 1 | Ref |
| MH669004 | Unassigned | Unassigned | Unassigned | 1015_1 | Clustered | 26 | VC_1015_1 | 26 | 0.6989 | 1 | 0.6989 | 0 | 0 | 0 | 1 | Ref |
| MH669006 | Unassigned | Unassigned | Unassigned | 901_0 | Clustered | 31 | VC_901_0 | 31 | 0.8235 | 1 | 0.8235 | 0 | 0 | 0 | 0.8194 | Ref |
| MH669007 | Unassigned | Unassigned | Unassigned | 789_0 | Clustered | 34 | VC_789_0 | 34 | 0.9167 | 1 | 0.9167 | 0 | 0 | 0 | 1 | Ref |
| MH669008 | Unassigned | Unassigned | Unassigned | 579_50 | Clustered | 15 | VC_579_50 | 15 | 0.0467 | 1 | 0.0467 | 0 | 0 | 0 | 0.9238 | Ref |
| MH669011 | Unassigned | Unassigned | Unassigned | 335_44 | Clustered | 3 | VC_335_44 | 3 | 0.0157 | 0.99859171 | 0.0157 | 0 | 0 | 0 | 1 | Ref |
| MH669012 | Unassigned | Unassigned | Unassigned | 538_73 | Clustered | 2 | VC_538_73 | 2 | 0.0107 | 0.95762743 | 0.0102 | 0 | 0 | 0 | 1 | Ref |
| MH669013 | Unassigned | Unassigned | Unassigned | 789_0 | Clustered | 34 | VC_789_0 | 34 | 0.9167 | 1 | 0.9167 | 0 | 0 | 0 | 1 | Ref |
| MH669014 | Unassigned | Unassigned | Unassigned | 538_69 | Clustered/Singleton | 1 | VC_538_69 | 1 | 0 | 1 | 0 | 0 | 0 | 0 | 1 | Ref |
| MH669015 | Unassigned | Unassigned | Unassigned | 1219_0 | Clustered | 24 | VC_1219_0 | 24 | 0.9519 | 1 | 0.9519 | 0 | 0 | 0 | 1 | Ref |
| MH669016 | Unassigned | Unassigned | Unassigned | 1015_1 | Clustered | 26 | VC_1015_1 | 26 | 0.6989 | 1 | 0.6989 | 0 | 0 | 0 | 1 | Ref |
| MH669017 | Unassigned | Unassigned | Unassigned | 566_4 | Clustered | 16 | VC_566_4 | 16 | 0.2517 | 0.9999998 | 0.2517 | 0 | 0 | 0 | 0.9417 | Ref |
| MH669274 | Unassigned | Unassigned | Unassigned | 801_0 | Clustered | 37 | VC_801_0 | 37 | 0.5672 | 1 | 0.5672 | 0 | 0 | 0 | 1 | Ref |
| MH674343 | Unassigned | Unassigned | Unassigned | 240_0 | Clustered | 2 | VC_240_0 | 2 | 0.7151 | 0.72985431 | 0.5219 | 0 | 0 | 0 | 1 | Ref |
| MH675552 | Unassigned | Unassigned | Unassigned | 1054_1 | Clustered | 8 | VC_1054_1 | 8 | 0.3937 | 1 | 0.3937 | 0 | 0 | 0 | 0.9286 | Ref |
| MH675927 | Unassigned | Unassigned | Unassigned | 872_0 | Clustered | 28 | VC_872_0 | 28 | 0.7887 | 1 | 0.7887 | 0 | 0 | 0 | 1 | Ref |
| MH684921 | Unassigned | Unassigned | Unassigned | 71_0 | Clustered | 27 | VC_71_0 | 27 | 0.5964 | 1 | 0.5964 | 0 | 0 | 0 | 0.9943 | Ref |
| MH688040 | Unassigned | Unassigned | Unassigned | 135_22 | Clustered | 13 | VC_135_22 | 13 | 0.0795 | 1 | 0.0795 | 0 | 0 | 0 | 0.8205 | Ref |
| MH688453 | Unassigned | Unassigned | Unassigned | 972_0 | Clustered | 99 | VC_972_0 | 99 | 0.9113 | 1 | 0.9113 | 0 | 0 | 0 | 0.9928 | Ref |
| MH697575 | Unassigned | Unassigned | Unassigned | 335_54 | Clustered | 5 | VC_335_54 | 5 | 0.0147 | 0.99999078 | 0.0147 | 0 | 0 | 0 | 0.7 | Ref |
| MH697576 | Unassigned | Unassigned | Unassigned | 539_0 | Clustered | 37 | VC_539_0 | 37 | 0.911 | 1 | 0.911 | 0 | 0 | 0 | 1 | Ref |
| MH697577 | Unassigned | Unassigned | Unassigned | 834_0 | Clustered | 18 | VC_834_0 | 18 | 0.9116 | 1 | 0.9116 | 0 | 0 | 0 | 1 | Ref |
| MH697578 | Unassigned | Unassigned | Unassigned | 243_0 | Clustered | 72 | VC_243_0 | 72 | 0.8089 | 1 | 0.8089 | 0 | 0 | 0 | 1 | Ref |
| MH697579 | Unassigned | Unassigned | Unassigned | 579_73 | Clustered | 2 | VC_579_73 | 2 | 0.0033 | 0.82635115 | 0.0027 | 0 | 0 | 0 | 1 | Ref |
| MH697580 | Unassigned | Unassigned | Unassigned | 419_0 | Clustered | 11 | VC_419_0 | 11 | 0.9465 | 1 | 0.9465 | 0 | 0 | 0 | 1 | Ref |
| MH697581 | Unassigned | Unassigned | Unassigned | 335_35 | Clustered | 2 | VC_335_35 | 2 | 0.004 | 0.95574341 | 0.0038 | 0 | 0 | 0 | 1 | Ref |
| MH697582 | Unassigned | Unassigned | Unassigned | 566_4 | Clustered | 16 | VC_566_4 | 16 | 0.2517 | 0.9999998 | 0.2517 | 0 | 0 | 0 | 0.9417 | Ref |
| MH697583 | Unassigned | Unassigned | Unassigned | 566_4 | Clustered | 16 | VC_566_4 | 16 | 0.2517 | 0.9999998 | 0.2517 | 0 | 0 | 0 | 0.9417 | Ref |
| MH697584 | Unassigned | Unassigned | Unassigned | 526_0 | Clustered | 32 | VC_526_0 | 32 | 0.9037 | 1 | 0.9037 | 0 | 0 | 0 | 1 | Ref |
| MH697585 | Unassigned | Unassigned | Unassigned | 539_0 | Clustered | 37 | VC_539_0 | 37 | 0.911 | 1 | 0.911 | 0 | 0 | 0 | 1 | Ref |
| MH697586 | Unassigned | Unassigned | Unassigned | 579_20 | Clustered | 17 | VC_579_20 | 17 | 0.0538 | 1 | 0.0538 | 0 | 0 | 0 | 0.9926 | Ref |
| MH697587 | Unassigned | Unassigned | Unassigned | 785_1 | Clustered | 9 | VC_785_1 | 9 | 0.3679 | 1 | 0.3679 | 0 | 0 | 0 | 1 | Ref |
| MH697588 | Unassigned | Unassigned | Unassigned | 535_1 | Clustered | 108 | VC_535_1 | 108 | 0.8479 | 1 | 0.8479 | 0 | 0 | 0 | 0.9955 | Ref |
| MH697590 | Unassigned | Unassigned | Unassigned | 579_20 | Clustered | 17 | VC_579_20 | 17 | 0.0538 | 1 | 0.0538 | 0 | 0 | 0 | 0.9926 | Ref |
| MH697591 | Unassigned | Unassigned | Unassigned | 223_6 | Clustered | 42 | VC_223_6 | 42 | 0.4717 | 1 | 0.4717 | 0 | 0 | 0 | 0.8351 | Ref |

| | | | | | | | | | | | | | | | | |
|---|---|---|---|---|---|---|---|---|---|---|---|---|---|---|---|---|
| MH697592 | Unassigned | Unassigned | Unassigned | 245_0 | Clustered | 17 | VC_245_0 | 17 | 0.6081 | 1 | 0.6081 | 0 | 0 | 0 | 1 | Ref |
| MH697593 | Unassigned | Unassigned | Unassigned | 539_0 | Clustered | 37 | VC_539_0 | 37 | 0.911 | 1 | 0.911 | 0 | 0 | 0 | 1 | Ref |
| MH707430 | Unassigned | Unassigned | Unassigned | 1422_0 | Clustered | 5 | VC_1422_0 | 5 | 1 | 1 | 1 | 0 | 0 | 0 | 1 | Ref |
| MH709120 | Unassigned | Unassigned | Unassigned | 135_17 | Clustered/Singleton | 1 | VC_135_17 | 1 | 0 | 1 | 0 | 0 | 0 | 0 | 1 | Ref |
| MH709121 | Unassigned | Unassigned | Unassigned | 135_10 | Clustered | 10 | VC_135_10 | 10 | 0.0522 | 1 | 0.0522 | 0 | 0 | 0 | 1 | Ref |
| MH713599 | Unassigned | Unassigned | Unassigned | 510_1 | Clustered | 20 | VC_510_1 | 20 | 0.4172 | 1 | 0.4172 | 0 | 0 | 0 | 1 | Ref |
| MH717096 | Unassigned | Unassigned | Unassigned | 32_1 | Clustered | 16 | VC_32_1 | 16 | 0.1962 | 1 | 0.1962 | 0 | 0 | 0 | 0.8333 | Ref |
| MH717097 | Unassigned | Unassigned | Unassigned | 475_0 | Clustered | 96 | VC_475_0 | 96 | 0.9303 | 1 | 0.9303 | 0 | 0 | 0 | 0.9575 | Ref |
| MH717098 | Unassigned | Unassigned | Unassigned | 405_0 | Clustered | 126 | VC_405_0 | 126 | 0.9614 | 1 | 0.9614 | 0 | 0 | 0 | 1 | Ref |
| MH717099 | Unassigned | Unassigned | Unassigned | 405_0 | Clustered | 126 | VC_405_0 | 126 | 0.9614 | 1 | 0.9614 | 0 | 0 | 0 | 1 | Ref |
| MH717709 | Unassigned | Unassigned | Unassigned | 398_0 | Clustered | 51 | VC_398_0 | 51 | 0.8741 | 1 | 0.8741 | 0 | 0 | 0 | 1 | Ref |
| MH719189 | Unassigned | Unassigned | Unassigned | 192_0 | Clustered | 22 | VC_192_0 | 22 | 0.6582 | 1 | 0.6582 | 0 | 0 | 0 | 1 | Ref |
| MH719190 | Unassigned | Unassigned | Unassigned | 192_0 | Clustered | 22 | VC_192_0 | 22 | 0.6582 | 1 | 0.6582 | 0 | 0 | 0 | 1 | Ref |
| MH719191 | Unassigned | Unassigned | Unassigned | 192_0 | Clustered | 22 | VC_192_0 | 22 | 0.6582 | 1 | 0.6582 | 0 | 0 | 0 | 1 | Ref |
| MH719192 | Unassigned | Unassigned | Unassigned | 192_0 | Clustered | 22 | VC_192_0 | 22 | 0.6582 | 1 | 0.6582 | 0 | 0 | 0 | 1 | Ref |
| MH719193 | Unassigned | Unassigned | Unassigned | 192_0 | Clustered | 22 | VC_192_0 | 22 | 0.6582 | 1 | 0.6582 | 0 | 0 | 0 | 1 | Ref |
| MH719194 | Unassigned | Unassigned | Unassigned | 192_0 | Clustered | 22 | VC_192_0 | 22 | 0.6582 | 1 | 0.6582 | 0 | 0 | 0 | 1 | Ref |
| MH719195 | Unassigned | Unassigned | Unassigned | 192_0 | Clustered | 22 | VC_192_0 | 22 | 0.6582 | 1 | 0.6582 | 0 | 0 | 0 | 1 | Ref |
| MH725810 | Unassigned | Unassigned | Unassigned | 77_0 | Clustered | 37 | VC_77_0 | 37 | 0.9392 | 1 | 0.9392 | 0 | 0 | 0 | 1 | Ref |
| MH727543 | Unassigned | Unassigned | Unassigned | 579_2 | Clustered | 2 | VC_579_2 | 2 | 0.0032 | 0.63743142 | 0.002 | 0 | 0 | 0 | 1 | Ref |
| MH727544 | Unassigned | Unassigned | Unassigned | 243_0 | Clustered | 72 | VC_243_0 | 72 | 0.8089 | 1 | 0.8089 | 0 | 0 | 0 | 1 | Ref |
| MH727546 | Unassigned | Unassigned | Unassigned | 526_0 | Clustered | 32 | VC_526_0 | 32 | 0.9037 | 1 | 0.9037 | 0 | 0 | 0 | 1 | Ref |
| MH727547 | Unassigned | Unassigned | Unassigned | 789_0 | Clustered | 34 | VC_789_0 | 34 | 0.9167 | 1 | 0.9167 | 0 | 0 | 0 | 1 | Ref |
| MH727548 | Unassigned | Unassigned | Unassigned | 538_5 | Clustered/Singleton | 1 | VC_538_5 | 1 | 0 | 1 | 0 | 0 | 0 | 0 | 1 | Ref |
| MH727549 | Unassigned | Unassigned | Unassigned | 834_0 | Clustered | 18 | VC_834_0 | 18 | 0.9116 | 1 | 0.9116 | 0 | 0 | 0 | 1 | Ref |
| MH727551 | Unassigned | Unassigned | Unassigned | 566_4 | Clustered | 16 | VC_566_4 | 16 | 0.2517 | 0.9999998 | 0.2517 | 0 | 0 | 0 | 0.9417 | Ref |
| MH727552 | Unassigned | Unassigned | Unassigned | 789_0 | Clustered | 34 | VC_789_0 | 34 | 0.9167 | 1 | 0.9167 | 0 | 0 | 0 | 1 | Ref |
| MH727553 | Unassigned | Unassigned | Unassigned | 973_0 | Clustered | 15 | VC_973_0 | 15 | 1 | 0 | 0 | 0 | 0 | 0 | 1 | Ref |
| MH727554 | Unassigned | Unassigned | Unassigned | 532_0 | Clustered | 75 | VC_532_0 | 75 | 0.9393 | 1 | 0.9393 | 0 | 0 | 0 | 1 | Ref |
| MH727555 | Unassigned | Unassigned | Unassigned | 384_16 | Clustered | 42 | VC_384_16 | 42 | 0.134 | 1 | 0.134 | 0 | 0 | 0 | 0.7282 | Ref |
| MH727557 | Unassigned | Unassigned | Unassigned | 571_6 | Clustered | 22 | VC_571_6 | 22 | 0.1207 | 1 | 0.1207 | 0 | 0 | 0 | 0.8095 | Ref |
| MH727558 | Unassigned | Unassigned | Unassigned | 579_56 | Clustered/Singleton | 1 | VC_579_56 | 1 | 0 | 1 | 0 | 0 | 0 | 0 | 1 | Ref |
| MH727559 | Unassigned | Unassigned | Unassigned | 918_0 | Clustered | 16 | VC_918_0 | 16 | 0.2945 | 1 | 0.2945 | 0 | 0 | 0 | 1 | Ref |
| MH727560 | Unassigned | Unassigned | Unassigned | 1243_0 | Clustered | 8 | VC_1243_0 | 8 | 0.8266 | 1 | 0.8266 | 0 | 0 | 0 | 1 | Ref |
| MH727561 | Unassigned | Unassigned | Unassigned | 385_1 | Clustered | 9 | VC_385_1 | 9 | 0.1928 | 1 | 0.1928 | 0 | 0 | 0 | 1 | Ref |
| MH727562 | Unassigned | Unassigned | Unassigned | 973_0 | Clustered | 15 | VC_973_0 | 15 | 1 | 0 | 0 | 0 | 0 | 0 | 1 | Ref |
| MH727563 | Unassigned | Unassigned | Unassigned | 535_1 | Clustered | 108 | VC_535_1 | 108 | 0.8479 | 1 | 0.8479 | 0 | 0 | 0 | 0.9955 | Ref |
| MH727564 | Unassigned | Unassigned | Unassigned | 1114_0 | Clustered | 20 | VC_1114_0 | 20 | 0.3238 | 1 | 0.3238 | 0 | 0 | 0 | 0.9842 | Ref |
| MH727593 | Unassigned | Unassigned | Unassigned | 191_0 | Clustered | 2 | VC_191_0 | 2 | 0.7873 | 0.93668477 | 0.7375 | 0 | 0 | 0 | 1 | Ref |
| MH729818 | Unassigned | Unassigned | Unassigned | 466_0 | Clustered | 66 | VC_466_0 | 66 | 0.9246 | 1 | 0.9246 | 0 | 0 | 0 | 1 | Ref |
| MH729819 | Unassigned | Unassigned | Unassigned | 603_0 | Clustered | 28 | VC_603_0 | 28 | 0.7692 | 1 | 0.7692 | 0 | 0 | 0 | 1 | Ref |
| MH729874 | Unassigned | Unassigned | Unassigned | 509_0 | Clustered | 36 | VC_509_0 | 36 | 0.5291 | 1 | 0.5291 | 0 | 0 | 0 | 0.9714 | Ref |
| MH730557 | Unassigned | Unassigned | Unassigned | 1423_0 | Clustered | 11 | VC_1423_0 | 11 | 1 | 0 | 0 | 0 | 0 | 0 | 1 | Ref |
| MH733496 | Unassigned | Unassigned | Unassigned | 801_0 | Clustered | 37 | VC_801_0 | 37 | 0.5672 | 1 | 0.5672 | 0 | 0 | 0 | 1 | Ref |
| MH744414 | Unassigned | Unassigned | Unassigned | 335_10 | Clustered | 3 | VC_335_10 | 3 | 0.012 | 0.99859169 | 0.012 | 0 | 0 | 0 | 1 | Ref |
| MH744415 | Unassigned | Unassigned | Unassigned | 538_36 | Clustered/Singleton | 1 | VC_538_36 | 1 | 0 | 1 | 0 | 0 | 0 | 0 | 1 | Ref |
| MH744416 | Unassigned | Unassigned | Unassigned | 1407_0 | Clustered | 78 | VC_1407_0 | 78 | 0.9731 | 1 | 0.9731 | 0 | 0 | 0 | 1 | Ref |
| MH744417 | Unassigned | Unassigned | Unassigned | 384_16 | Clustered | 42 | VC_384_16 | 42 | 0.134 | 1 | 0.134 | 0 | 0 | 0 | 0.7282 | Ref |
| MH744418 | Unassigned | Unassigned | Unassigned | 1199_1 | Clustered | 2 | VC_1199_1 | 2 | 0.1356 | 0.9073018 | 0.1231 | 0 | 0 | 0 | 1 | Ref |
| MH744419 | Unassigned | Unassigned | Unassigned | 1117_0 | Clustered | 19 | VC_1117_0 | 19 | 0.9177 | 1 | 0.9177 | 0 | 0 | 0 | 1 | Ref |
| MH744420 | Unassigned | Unassigned | Unassigned | 607_0 | Clustered | 2 | VC_607_0 | 2 | 0.0776 | 0.94661631 | 0.0735 | 0 | 0 | 0 | 1 | Ref |
| MH744421 | Unassigned | Unassigned | Unassigned | 1200_0 | Clustered | 73 | VC_1200_0 | 73 | 0.9825 | 1 | 0.9825 | 0 | 0 | 0 | 1 | Ref |
| MH744422 | Unassigned | Unassigned | Unassigned | 579_52 | Clustered/Singleton | 1 | VC_579_52 | 1 | 0 | 1 | 0 | 0 | 0 | 0 | 1 | Ref |
| MH744423 | Unassigned | Unassigned | Unassigned | 385_2 | Clustered | 5 | VC_385_2 | 5 | 0.0717 | 0.99999997 | 0.0717 | 0 | 0 | 0 | 0.9 | Ref |
| MH744424 | Unassigned | Unassigned | Unassigned | 789_0 | Clustered | 34 | VC_789_0 | 34 | 0.9167 | 1 | 0.9167 | 0 | 0 | 0 | 1 | Ref |
| MH746814 | Unassigned | Unassigned | Unassigned | 715_0 | Clustered | 10 | VC_715_0 | 10 | 0.8045 | 1 | 0.8045 | 0 | 0 | 0 | 1 | Ref |
| MH746817 | Unassigned | Unassigned | Unassigned | 177_0 | Clustered | 8 | VC_177_0 | 8 | 0.5848 | 1 | 0.5848 | 0 | 0 | 0 | 1 | Ref |
| MH751506 | Unassigned | Unassigned | Unassigned | 290_92 | Clustered | 4 | VC_290_92 | 4 | 0.0078 | 0.99995066 | 0.0078 | 0 | 0 | 0 | 1 | Ref |
| MH752385 | Unassigned | Unassigned | Unassigned | 106_0 | Clustered | 6 | VC_106_0 | 6 | 0.8265 | 1 | 0.8265 | 0 | 0 | 0 | 1 | Ref |
| MH752840 | Unassigned | Unassigned | Unassigned | 794_11 | Clustered | 6 | VC_794_11 | 6 | 0.0305 | 0.83830784 | 0.0256 | 0 | 0 | 0 | 0.8 | Ref |
| MH763831 | Unassigned | Unassigned | Unassigned | 714_0 | Clustered | 71 | VC_714_0 | 71 | 0.935 | 1 | 0.935 | 0 | 0 | 0 | 1 | Ref |
| MH779498 | Unassigned | Unassigned | Unassigned | 579_49 | Clustered | 7 | VC_579_49 | 7 | 0.0194 | 0.98914431 | 0.0191 | 0 | 0 | 0 | 0.8571 | Ref |
| MH779499 | Unassigned | Unassigned | Unassigned | 789_0 | Clustered | 34 | VC_789_0 | 34 | 0.9167 | 1 | 0.9167 | 0 | 0 | 0 | 1 | Ref |
| MH779500 | Unassigned | Unassigned | Unassigned | 384_3 | Clustered | 22 | VC_384_3 | 22 | 0.0786 | 1 | 0.0786 | 0 | 0 | 0 | 0.9048 | Ref |
| MH779501 | Unassigned | Unassigned | Unassigned | 789_0 | Clustered | 34 | VC_789_0 | 34 | 0.9167 | 1 | 0.9167 | 0 | 0 | 0 | 1 | Ref |
| MH779502 | Unassigned | Unassigned | Unassigned | 579_54 | Clustered/Singleton | 1 | VC_579_54 | 1 | 0 | 1 | 0 | 0 | 0 | 0 | 1 | Ref |
| MH779503 | Unassigned | Unassigned | Unassigned | 571_6 | Clustered | 22 | VC_571_6 | 22 | 0.1207 | 1 | 0.1207 | 0 | 0 | 0 | 0.8095 | Ref |
| MH779504 | Unassigned | Unassigned | Unassigned | 578_1 | Clustered | 11 | VC_578_1 | 11 | 0.4919 | 1 | 0.4919 | 0 | 0 | 0 | 0.7818 | Ref |
| MH779505 | Unassigned | Unassigned | Unassigned | 783_0 | Clustered | 67 | VC_783_0 | 67 | 0.8945 | 1 | 0.8945 | 0 | 0 | 0 | 1 | Ref |
| MH779506 | Unassigned | Unassigned | Unassigned | 985_0 | Clustered | 38 | VC_985_0 | 38 | 0.9334 | 1 | 0.9334 | 0 | 0 | 0 | 1 | Ref |
| MH779507 | Unassigned | Unassigned | Unassigned | 783_0 | Clustered | 67 | VC_783_0 | 67 | 0.8945 | 1 | 0.8945 | 0 | 0 | 0 | 1 | Ref |
| MH779508 | Unassigned | Unassigned | Unassigned | 789_0 | Clustered | 34 | VC_789_0 | 34 | 0.9167 | 1 | 0.9167 | 0 | 0 | 0 | 1 | Ref |
| MH779509 | Unassigned | Unassigned | Unassigned | 783_0 | Clustered | 67 | VC_783_0 | 67 | 0.8945 | 1 | 0.8945 | 0 | 0 | 0 | 1 | Ref |
| MH779510 | Unassigned | Unassigned | Unassigned | 789_0 | Clustered | 34 | VC_789_0 | 34 | 0.9167 | 1 | 0.9167 | 0 | 0 | 0 | 1 | Ref |
| MH779511 | Unassigned | Unassigned | Unassigned | 917_0 | Clustered | 28 | VC_917_0 | 28 | 0.5465 | 1 | 0.5465 | 0 | 0 | 0 | 1 | Ref |
| MH779512 | Unassigned | Unassigned | Unassigned | 1407_0 | Clustered | 78 | VC_1407_0 | 78 | 0.9731 | 1 | 0.9731 | 0 | 0 | 0 | 1 | Ref |
| MH779513 | Unassigned | Unassigned | Unassigned | 783_0 | Clustered | 67 | VC_783_0 | 67 | 0.8945 | 1 | 0.8945 | 0 | 0 | 0 | 1 | Ref |
| MH779514 | Unassigned | Unassigned | Unassigned | 783_0 | Clustered | 67 | VC_783_0 | 67 | 0.8945 | 1 | 0.8945 | 0 | 0 | 0 | 1 | Ref |
| MH779515 | Unassigned | Unassigned | Unassigned | 783_0 | Clustered | 67 | VC_783_0 | 67 | 0.8945 | 1 | 0.8945 | 0 | 0 | 0 | 1 | Ref |
| MH779516 | Unassigned | Unassigned | Unassigned | 384_16 | Clustered | 42 | VC_384_16 | 42 | 0.134 | 1 | 0.134 | 0 | 0 | 0 | 0.7282 | Ref |
| MH779517 | Unassigned | Unassigned | Unassigned | 230_0 | Clustered | 38 | VC_230_0 | 38 | 0.9076 | 1 | 0.9076 | 0 | 0 | 0 | 1 | Ref |

| | | | | | | | | | | | | | | | | |
|---|---|---|---|---|---|---|---|---|---|---|---|---|---|---|---|---|
| MH779518 | Unassigned | Unassigned | Unassigned | 706_0 | Clustered | 52 | VC_706_0 | 52 | 0.9787 | 1 | 0.9787 | 0 | 0 | 0 | 1 | Ref |
| MH779519 | Unassigned | Unassigned | Unassigned | 706_0 | Clustered | 52 | VC_706_0 | 52 | 0.9787 | 1 | 0.9787 | 0 | 0 | 0 | 1 | Ref |
| MH779520 | Unassigned | Unassigned | Unassigned | 706_0 | Clustered | 52 | VC_706_0 | 52 | 0.9787 | 1 | 0.9787 | 0 | 0 | 0 | 1 | Ref |
| MH779521 | Unassigned | Unassigned | Unassigned | 706_0 | Clustered | 52 | VC_706_0 | 52 | 0.9787 | 1 | 0.9787 | 0 | 0 | 0 | 1 | Ref |
| MH779522 | Unassigned | Unassigned | Unassigned | 706_0 | Clustered | 52 | VC_706_0 | 52 | 0.9787 | 1 | 0.9787 | 0 | 0 | 0 | 1 | Ref |
| MH779523 | Unassigned | Unassigned | Unassigned | 706_0 | Clustered | 52 | VC_706_0 | 52 | 0.9787 | 1 | 0.9787 | 0 | 0 | 0 | 1 | Ref |
| MH779524 | Unassigned | Unassigned | Unassigned | 706_0 | Clustered | 52 | VC_706_0 | 52 | 0.9787 | 1 | 0.9787 | 0 | 0 | 0 | 1 | Ref |
| MH779525 | Unassigned | Unassigned | Unassigned | 706_0 | Clustered | 52 | VC_706_0 | 52 | 0.9787 | 1 | 0.9787 | 0 | 0 | 0 | 1 | Ref |
| MH779526 | Unassigned | Unassigned | Unassigned | 706_0 | Clustered | 52 | VC_706_0 | 52 | 0.9787 | 1 | 0.9787 | 0 | 0 | 0 | 1 | Ref |
| MH779527 | Unassigned | Unassigned | Unassigned | 706_0 | Clustered | 52 | VC_706_0 | 52 | 0.9787 | 1 | 0.9787 | 0 | 0 | 0 | 1 | Ref |
| MH779619 | Unassigned | Unassigned | Unassigned | 405_0 | Clustered | 126 | VC_405_0 | 126 | 0.9614 | 1 | 0.9614 | 0 | 0 | 0 | 1 | Ref |
| MH791395 | Unassigned | Unassigned | Unassigned | 611_0 | Clustered | 50 | VC_611_0 | 50 | 0.7306 | 1 | 0.7306 | 0 | 0 | 0 | 1 | Ref |
| MH791396 | Unassigned | Unassigned | Unassigned | 611_0 | Clustered | 50 | VC_611_0 | 50 | 0.7306 | 1 | 0.7306 | 0 | 0 | 0 | 1 | Ref |
| MH791397 | Unassigned | Unassigned | Unassigned | 94_0 | Clustered | 22 | VC_94_0 | 22 | 0.5602 | 1 | 0.5602 | 0 | 0 | 0 | 1 | Ref |
| MH791398 | Unassigned | Unassigned | Unassigned | 457_4 | Clustered | 10 | VC_457_4 | 10 | 0.3316 | 1 | 0.3316 | 0 | 0 | 0 | 0.9333 | Ref |
| MH791399 | Unassigned | Unassigned | Unassigned | 77_0 | Clustered | 37 | VC_77_0 | 37 | 0.9392 | 1 | 0.9392 | 0 | 0 | 0 | 1 | Ref |
| MH791400 | Unassigned | Unassigned | Unassigned | 341_0 | Clustered | 27 | VC_341_0 | 27 | 0.5058 | 1 | 0.5058 | 0 | 0 | 0 | 0.8661 | Ref |
| MH791401 | Unassigned | Unassigned | Unassigned | 457_0 | Clustered | 7 | VC_457_0 | 7 | 0.2549 | 1 | 0.2549 | 0 | 0 | 0 | 0.7143 | Ref |
| MH791402 | Unassigned | Unassigned | Unassigned | 475_0 | Clustered | 96 | VC_475_0 | 96 | 0.9303 | 1 | 0.9303 | 0 | 0 | 0 | 0.9575 | Ref |
| MH791403 | Unassigned | Unassigned | Unassigned | 172_0 | Clustered | 26 | VC_172_0 | 26 | 0.7909 | 1 | 0.7909 | 0 | 0 | 0 | 1 | Ref |
| MH791404 | Unassigned | Unassigned | Unassigned | 457_4 | Clustered | 10 | VC_457_4 | 10 | 0.3316 | 1 | 0.3316 | 0 | 0 | 0 | 0.9333 | Ref |
| MH791405 | Unassigned | Unassigned | Unassigned | 135_6 | Clustered/Singleton | 1 | VC_135_6 | 1 | 0 | 1 | 0 | 0 | 0 | 0 | 1 | Ref |
| MH791406 | Unassigned | Unassigned | Unassigned | 172_0 | Clustered | 26 | VC_172_0 | 26 | 0.7909 | 1 | 0.7909 | 0 | 0 | 0 | 1 | Ref |
| MH791407 | Unassigned | Unassigned | Unassigned | 457_0 | Clustered | 7 | VC_457_0 | 7 | 0.2549 | 1 | 0.2549 | 0 | 0 | 0 | 0.7143 | Ref |
| MH791408 | Unassigned | Unassigned | Unassigned | 77_0 | Clustered | 37 | VC_77_0 | 37 | 0.9392 | 1 | 0.9392 | 0 | 0 | 0 | 1 | Ref |
| MH791409 | Unassigned | Unassigned | Unassigned | 297_6 | Clustered | 12 | VC_297_6 | 12 | 0.1139 | 1 | 0.1139 | 0 | 0 | 0 | 0.8182 | Ref |
| MH791410 | Unassigned | Unassigned | Unassigned | 475_0 | Clustered | 96 | VC_475_0 | 96 | 0.9303 | 1 | 0.9303 | 0 | 0 | 0 | 0.9575 | Ref |
| MH791411 | Unassigned | Unassigned | Unassigned | 170_11 | Clustered | 10 | VC_170_11 | 10 | 0.1502 | 0.99999797 | 0.1502 | 0 | 0 | 0 | 0.9111 | Ref |
| MH791412 | Unassigned | Unassigned | Unassigned | 475_0 | Clustered | 96 | VC_475_0 | 96 | 0.9303 | 1 | 0.9303 | 0 | 0 | 0 | 0.9575 | Ref |
| MH791413 | Unassigned | Unassigned | Unassigned | 341_0 | Clustered | 27 | VC_341_0 | 27 | 0.5058 | 1 | 0.5058 | 0 | 0 | 0 | 0.8661 | Ref |
| MH791414 | Unassigned | Unassigned | Unassigned | 457_2 | Clustered | 2 | VC_457_2 | 2 | 0.0288 | 0.87707845 | 0.0253 | 0 | 0 | 0 | 1 | Ref |
| MH791415 | Unassigned | Unassigned | Unassigned | 110_0 | Clustered | 33 | VC_110_0 | 33 | 0.9488 | 1 | 0.9488 | 0 | 0 | 0 | 1 | Ref |
| MH791416 | Unassigned | Unassigned | Unassigned | 77_0 | Clustered | 37 | VC_77_0 | 37 | 0.9392 | 1 | 0.9392 | 0 | 0 | 0 | 1 | Ref |
| MH800198 | Unassigned | Unassigned | Unassigned | 715_0 | Clustered | 10 | VC_715_0 | 10 | 0.8045 | 1 | 0.8045 | 0 | 0 | 0 | 1 | Ref |
| MH800199 | Unassigned | Unassigned | Unassigned | 715_0 | Clustered | 10 | VC_715_0 | 10 | 0.8045 | 1 | 0.8045 | 0 | 0 | 0 | 1 | Ref |
| MH800200 | Unassigned | Unassigned | Unassigned | 714_0 | Clustered | 71 | VC_714_0 | 71 | 0.935 | 1 | 0.935 | 0 | 0 | 0 | 1 | Ref |
| MH807810 | Unassigned | Unassigned | Unassigned | 472_0 | Clustered | 5 | VC_472_0 | 5 | 0.1909 | 0.99999997 | 0.1909 | 0 | 0 | 0 | 1 | Ref |
| MH807811 | Unassigned | Unassigned | Unassigned | 1018_0 | Clustered | 38 | VC_1018_0 | 38 | 0.9757 | 1 | 0.9757 | 0 | 0 | 0 | 1 | Ref |
| MH807812 | Unassigned | Unassigned | Unassigned | 133_0 | Clustered | 17 | VC_133_0 | 17 | 0.2654 | 1 | 0.2654 | 0 | 0 | 0 | 1 | Ref |
| MH807813 | Unassigned | Unassigned | Unassigned | 472_0 | Clustered | 5 | VC_472_0 | 5 | 0.1909 | 0.99999997 | 0.1909 | 0 | 0 | 0 | 1 | Ref |
| MH807814 | Unassigned | Unassigned | Unassigned | 1018_0 | Clustered | 38 | VC_1018_0 | 38 | 0.9757 | 1 | 0.9757 | 0 | 0 | 0 | 1 | Ref |
| MH807815 | Unassigned | Unassigned | Unassigned | 472_0 | Clustered | 5 | VC_472_0 | 5 | 0.1909 | 0.99999997 | 0.1909 | 0 | 0 | 0 | 1 | Ref |
| MH807816 | Unassigned | Unassigned | Unassigned | 1018_0 | Clustered | 38 | VC_1018_0 | 38 | 0.9757 | 1 | 0.9757 | 0 | 0 | 0 | 1 | Ref |
| MH807817 | Unassigned | Unassigned | Unassigned | 472_0 | Clustered | 5 | VC_472_0 | 5 | 0.1909 | 0.99999997 | 0.1909 | 0 | 0 | 0 | 1 | Ref |
| MH807818 | Unassigned | Unassigned | Unassigned | 1018_0 | Clustered | 38 | VC_1018_0 | 38 | 0.9757 | 1 | 0.9757 | 0 | 0 | 0 | 1 | Ref |
| MH807819 | Unassigned | Unassigned | Unassigned | 1018_0 | Clustered | 38 | VC_1018_0 | 38 | 0.9757 | 1 | 0.9757 | 0 | 0 | 0 | 1 | Ref |
| MH807820 | Unassigned | Unassigned | Unassigned | 133_0 | Clustered | 17 | VC_133_0 | 17 | 0.2654 | 1 | 0.2654 | 0 | 0 | 0 | 1 | Ref |
| MH809528 | Unassigned | Unassigned | Unassigned | 597_0 | Clustered | 6 | VC_597_0 | 6 | 0.9222 | 0.99999993 | 0.9222 | 0 | 0 | 0 | 1 | Ref |
| MH809529 | Unassigned | Unassigned | Unassigned | 544_0 | Clustered | 6 | VC_544_0 | 6 | 0.4346 | 1 | 0.4346 | 0 | 0 | 0 | 1 | Ref |
| MH809530 | Unassigned | Unassigned | Unassigned | 544_0 | Clustered | 6 | VC_544_0 | 6 | 0.4346 | 1 | 0.4346 | 0 | 0 | 0 | 1 | Ref |
| MH809531 | Unassigned | Unassigned | Unassigned | 544_0 | Clustered | 6 | VC_544_0 | 6 | 0.4346 | 1 | 0.4346 | 0 | 0 | 0 | 1 | Ref |
| MH809534 | Unassigned | Unassigned | Unassigned | 399_0 | Clustered | 49 | VC_399_0 | 49 | 0.7927 | 1 | 0.7927 | 0 | 0 | 0 | 1 | Ref |
| MH809535 | Unassigned | Unassigned | Unassigned | 290_34 | Clustered | 4 | VC_290_34 | 4 | 0.0081 | 0.9997166 | 0.0081 | 0 | 0 | 0 | 1 | Ref |
| MH816848 | Unassigned | Unassigned | Unassigned | 872_0 | Clustered | 28 | VC_872_0 | 28 | 0.7887 | 1 | 0.7887 | 0 | 0 | 0 | 1 | Ref |
| MH816966 | Unassigned | Unassigned | Unassigned | 636_0 | Clustered | 13 | VC_636_0 | 13 | 0.6223 | 1 | 0.6223 | 0 | 0 | 0 | 1 | Ref |
| MH817022 | Unassigned | Unassigned | Unassigned | 843_0 | Clustered | 20 | VC_843_0 | 20 | 0.9621 | 1 | 0.9621 | 0 | 0 | 0 | 1 | Ref |
| MH823906 | Unassigned | Unassigned | Unassigned | 687_0 | Clustered | 21 | VC_687_0 | 21 | 0.3608 | 1 | 0.3608 | 0 | 0 | 0 | 1 | Ref |
| MH825697 | Unassigned | Unassigned | Unassigned | 579_12 | Clustered | 3 | VC_579_12 | 3 | 0.0064 | 0.86841267 | 0.0056 | 0 | 0 | 0 | 1 | Ref |
| MH825698 | Unassigned | Unassigned | Unassigned | 1425_0 | Clustered | 6 | VC_1425_0 | 6 | 0.2364 | 1 | 0.2364 | 0 | 0 | 0 | 1 | Ref |
| MH825699 | Unassigned | Unassigned | Unassigned | 742_0 | Clustered | 11 | VC_742_0 | 11 | 0.5046 | 1 | 0.5046 | 0 | 0 | 0 | 1 | Ref |
| MH825700 | Unassigned | Unassigned | Unassigned | 1407_0 | Clustered | 78 | VC_1407_0 | 78 | 0.9731 | 1 | 0.9731 | 0 | 0 | 0 | 1 | Ref |
| MH825701 | Unassigned | Unassigned | Unassigned | 223_4 | Clustered | 13 | VC_223_4 | 13 | 0.1649 | 1 | 0.1649 | 0 | 0 | 0 | 1 | Ref |
| MH825702 | Unassigned | Unassigned | Unassigned | 384_15 | Clustered | 44 | VC_384_15 | 44 | 0.139 | 1 | 0.139 | 0 | 0 | 0 | 0.7474 | Ref |
| MH825703 | Unassigned | Unassigned | Unassigned | 1116_0 | Clustered | 24 | VC_1116_0 | 24 | 0.8376 | 1 | 0.8376 | 0 | 0 | 0 | 1 | Ref |
| MH825704 | Unassigned | Unassigned | Unassigned | 223_6 | Clustered | 42 | VC_223_6 | 42 | 0.4717 | 1 | 0.4717 | 0 | 0 | 0 | 0.8351 | Ref |
| MH825705 | Unassigned | Unassigned | Unassigned | 384_15 | Clustered | 44 | VC_384_15 | 44 | 0.139 | 1 | 0.139 | 0 | 0 | 0 | 0.7474 | Ref |
| MH825706 | Unassigned | Unassigned | Unassigned | 1416_0 | Clustered | 10 | VC_1416_0 | 10 | 1 | 1 | 1 | 0 | 0 | 0 | 1 | Ref |
| MH825707 | Unassigned | Unassigned | Unassigned | 538_30 | Clustered | 2 | VC_538_30 | 2 | 0.0072 | 0.954469 | 0.0069 | 0 | 0 | 0 | 1 | Ref |
| MH825709 | Unassigned | Unassigned | Unassigned | 223_3 | Clustered | 8 | VC_223_3 | 8 | 0.0699 | 1 | 0.0699 | 0 | 0 | 0 | 0.8571 | Ref |
| MH825710 | Unassigned | Unassigned | Unassigned | 535_1 | Clustered | 108 | VC_535_1 | 108 | 0.8479 | 1 | 0.8479 | 0 | 0 | 0 | 0.9955 | Ref |
| MH825711 | Unassigned | Unassigned | Unassigned | 1204_0 | Clustered | 25 | VC_1204_0 | 25 | 0.7382 | 1 | 0.7382 | 0 | 0 | 0 | 1 | Ref |
| MH825713 | Unassigned | Unassigned | Unassigned | 579_50 | Clustered | 15 | VC_579_50 | 15 | 0.0467 | 1 | 0.0467 | 0 | 0 | 0 | 0.9238 | Ref |
| MH834594 | Unassigned | Unassigned | Unassigned | 986_0 | Clustered | 3 | VC_986_0 | 3 | 0.1032 | 0.99917561 | 0.1032 | 0 | 0 | 0 | 1 | Ref |
| MH834595 | Unassigned | Unassigned | Unassigned | 1207_0 | Clustered | 20 | VC_1207_0 | 20 | 0.9898 | 1 | 0.9898 | 0 | 0 | 0 | 1 | Ref |
| MH834596 | Unassigned | Unassigned | Unassigned | 1287_0 | Clustered | 44 | VC_1287_0 | 44 | 1 | 1 | 1 | 0 | 0 | 0 | 1 | Ref |
| MH834597 | Unassigned | Unassigned | Unassigned | 986_0 | Clustered | 3 | VC_986_0 | 3 | 0.1032 | 0.99917561 | 0.1032 | 0 | 0 | 0 | 1 | Ref |
| MH834598 | Unassigned | Unassigned | Unassigned | 1075_0 | Clustered | 11 | VC_1075_0 | 11 | 0.6562 | 1 | 0.6562 | 0 | 0 | 0 | 0.9273 | Ref |
| MH834599 | Unassigned | Unassigned | Unassigned | 1287_0 | Clustered | 44 | VC_1287_0 | 44 | 1 | 1 | 1 | 0 | 0 | 0 | 1 | Ref |
| MH834600 | Unassigned | Unassigned | Unassigned | 917_0 | Clustered | 28 | VC_917_0 | 28 | 0.5465 | 1 | 0.5465 | 0 | 0 | 0 | 1 | Ref |
| MH834602 | Unassigned | Unassigned | Unassigned | 1287_0 | Clustered | 44 | VC_1287_0 | 44 | 1 | 1 | 1 | 0 | 0 | 0 | 1 | Ref |
| MH834603 | Unassigned | Unassigned | Unassigned | 1074_0 | Clustered | 6 | VC_1074_0 | 6 | 0.4836 | 1 | 0.4836 | 0 | 0 | 0 | 1 | Ref |

| | | | | | | | | | | | | | | | | |
|---|---|---|---|---|---|---|---|---|---|---|---|---|---|---|---|---|
| MH834604 | Unassigned | Unassigned | Unassigned | 1287_0 | Clustered | 44 | VC_1287_0 | 44 | 1 | 1 | 1 | 0 | 0 | 0 | 1 | Ref |
| MH834605 | Unassigned | Unassigned | Unassigned | 1074_0 | Clustered | 6 | VC_1074_0 | 6 | 0.4836 | 1 | 0.4836 | 0 | 0 | 0 | 1 | Ref |
| MH834606 | Unassigned | Unassigned | Unassigned | 1207_0 | Clustered | 20 | VC_1207_0 | 20 | 0.9898 | 1 | 0.9898 | 0 | 0 | 0 | 1 | Ref |
| MH834607 | Unassigned | Unassigned | Unassigned | 1427_0 | Clustered | 6 | VC_1427_0 | 6 | 1 | 1 | 1 | 0 | 0 | 0 | 1 | Ref |
| MH834608 | Unassigned | Unassigned | Unassigned | 1207_0 | Clustered | 20 | VC_1207_0 | 20 | 0.9898 | 1 | 0.9898 | 0 | 0 | 0 | 1 | Ref |
| MH834609 | Unassigned | Unassigned | Unassigned | 1207_0 | Clustered | 20 | VC_1207_0 | 20 | 0.9898 | 1 | 0.9898 | 0 | 0 | 0 | 1 | Ref |
| MH834610 | Unassigned | Unassigned | Unassigned | 1250_0 | Clustered | 29 | VC_1250_0 | 29 | 0.822 | 1 | 0.822 | 0 | 0 | 0 | 1 | Ref |
| MH834611 | Unassigned | Unassigned | Unassigned | 1074_0 | Clustered | 6 | VC_1074_0 | 6 | 0.4836 | 1 | 0.4836 | 0 | 0 | 0 | 1 | Ref |
| MH834612 | Unassigned | Unassigned | Unassigned | 1075_0 | Clustered | 11 | VC_1075_0 | 11 | 0.6562 | 1 | 0.6562 | 0 | 0 | 0 | 0.9273 | Ref |
| MH834613 | Unassigned | Unassigned | Unassigned | 591_0 | Clustered | 4 | VC_591_0 | 4 | 0.1897 | 0.9999859 | 0.1897 | 0 | 0 | 0 | 1 | Ref |
| MH834614 | Unassigned | Unassigned | Unassigned | 1074_0 | Clustered | 6 | VC_1074_0 | 6 | 0.4836 | 1 | 0.4836 | 0 | 0 | 0 | 1 | Ref |
| MH834615 | Unassigned | Unassigned | Unassigned | 585_0 | Clustered | 20 | VC_585_0 | 20 | 0.8376 | 1 | 0.8376 | 0 | 0 | 0 | 1 | Ref |
| MH834616 | Unassigned | Unassigned | Unassigned | 1207_0 | Clustered | 20 | VC_1207_0 | 20 | 0.9898 | 1 | 0.9898 | 0 | 0 | 0 | 1 | Ref |
| MH834617 | Unassigned | Unassigned | Unassigned | 538_45 | Clustered | 7 | VC_538_45 | 7 | 0.0632 | 1 | 0.0632 | 0 | 0 | 0 | 0.8095 | Ref |
| MH834618 | Unassigned | Unassigned | Unassigned | 1207_0 | Clustered | 20 | VC_1207_0 | 20 | 0.9898 | 1 | 0.9898 | 0 | 0 | 0 | 1 | Ref |
| MH834619 | Unassigned | Unassigned | Unassigned | 1250_0 | Clustered | 29 | VC_1250_0 | 29 | 0.822 | 1 | 0.822 | 0 | 0 | 0 | 1 | Ref |
| MH834620 | Unassigned | Unassigned | Unassigned | 1207_0 | Clustered | 20 | VC_1207_0 | 20 | 0.9898 | 1 | 0.9898 | 0 | 0 | 0 | 1 | Ref |
| MH834621 | Unassigned | Unassigned | Unassigned | 1076_0 | Clustered | 6 | VC_1076_0 | 6 | 0.6773 | 1 | 0.6773 | 0 | 0 | 0 | 1 | Ref |
| MH834622 | Unassigned | Unassigned | Unassigned | 1427_0 | Clustered | 6 | VC_1427_0 | 6 | 1 | 1 | 1 | 0 | 0 | 0 | 1 | Ref |
| MH834623 | Unassigned | Unassigned | Unassigned | 1074_0 | Clustered | 6 | VC_1074_0 | 6 | 0.4836 | 1 | 0.4836 | 0 | 0 | 0 | 1 | Ref |
| MH834624 | Unassigned | Unassigned | Unassigned | 1207_0 | Clustered | 20 | VC_1207_0 | 20 | 0.9898 | 1 | 0.9898 | 0 | 0 | 0 | 1 | Ref |
| MH834625 | Unassigned | Unassigned | Unassigned | 1075_0 | Clustered | 11 | VC_1075_0 | 11 | 0.6562 | 1 | 0.6562 | 0 | 0 | 0 | 0.9273 | Ref |
| MH834626 | Unassigned | Unassigned | Unassigned | 1287_0 | Clustered | 44 | VC_1287_0 | 44 | 1 | 1 | 1 | 0 | 0 | 0 | 1 | Ref |
| MH834627 | Unassigned | Unassigned | Unassigned | 1076_0 | Clustered | 6 | VC_1076_0 | 6 | 0.6773 | 1 | 0.6773 | 0 | 0 | 0 | 1 | Ref |
| MH834628 | Unassigned | Unassigned | Unassigned | 986_0 | Clustered | 3 | VC_986_0 | 3 | 0.1032 | 0.99917561 | 0.1032 | 0 | 0 | 0 | 1 | Ref |
| MH834629 | Unassigned | Unassigned | Unassigned | 1250_0 | Clustered | 29 | VC_1250_0 | 29 | 0.822 | 1 | 0.822 | 0 | 0 | 0 | 1 | Ref |
| MH837542 | Unassigned | Unassigned | Unassigned | 309_0 | Clustered | 2 | VC_309_0 | 2 | 0.1813 | 0.95040252 | 0.1723 | 0 | 0 | 0 | 1 | Ref |
| MH837543 | Unassigned | Unassigned | Unassigned | 309_0 | Clustered | 2 | VC_309_0 | 2 | 0.1813 | 0.95040252 | 0.1723 | 0 | 0 | 0 | 1 | Ref |
| MH837626 | Unassigned | Unassigned | Unassigned | 290_159 | Clustered/Singleton | 1 | VC_290_159 | 1 | 0 | 1 | 0 | 0 | 0 | 0 | 1 | Ref |
| MH844528 | Unassigned | Unassigned | Unassigned | 155_8 | Clustered | 66 | VC_155_8 | 66 | 0.4537 | 1 | 0.4537 | 0 | 0 | 0 | 0.8182 | Ref |
| MH844529 | Unassigned | Unassigned | Unassigned | 155_8 | Clustered | 66 | VC_155_8 | 66 | 0.4537 | 1 | 0.4537 | 0 | 0 | 0 | 0.8182 | Ref |
| MH844531 | Unassigned | Unassigned | Unassigned | 972_0 | Clustered | 99 | VC_972_0 | 99 | 0.9113 | 1 | 0.9113 | 0 | 0 | 0 | 0.9928 | Ref |
| MH844558 | Unassigned | Unassigned | Unassigned | 145_0 | Clustered | 6 | VC_145_0 | 6 | 0.2581 | 0.86251824 | 0.2226 | 0 | 0 | 0 | 0.8667 | Ref |
| MH853786 | Unassigned | Unassigned | Unassigned | 713_0 | Clustered | 26 | VC_713_0 | 26 | 0.9358 | 1 | 0.9358 | 0 | 0 | 0 | 1 | Ref |
| MH853787 | Unassigned | Unassigned | Unassigned | 713_0 | Clustered | 26 | VC_713_0 | 26 | 0.9358 | 1 | 0.9358 | 0 | 0 | 0 | 1 | Ref |
| MH853788 | Unassigned | Unassigned | Unassigned | 713_0 | Clustered | 26 | VC_713_0 | 26 | 0.9358 | 1 | 0.9358 | 0 | 0 | 0 | 1 | Ref |
| MH880817 | Unassigned | Unassigned | Unassigned | 94_0 | Clustered | 22 | VC_94_0 | 22 | 0.5602 | 1 | 0.5602 | 0 | 0 | 0 | 1 | Ref |
| MH884508 | Unassigned | Unassigned | Unassigned | 489_4 | Clustered/Singleton | 1 | VC_489_4 | 1 | 0 | 1 | 0 | 0 | 0 | 0 | 1 | Ref |
| MH884511 | Unassigned | Unassigned | Unassigned | 89_2 | Clustered/Singleton | 1 | VC_89_2 | 1 | 0 | 1 | 0 | 0 | 0 | 0 | 1 | Ref |
| MH884512 | Unassigned | Unassigned | Unassigned | 638_0 | Clustered | 2 | VC_638_0 | 2 | 0.0686 | 0.56281647 | 0.0386 | 0 | 0 | 0 | 1 | Ref |
| MH884513 | Unassigned | Unassigned | Unassigned | 102_0 | Clustered | 15 | VC_102_0 | 15 | 0.653 | 1 | 0.653 | 0 | 0 | 0 | 1 | Ref |
| MH884514 | Unassigned | Unassigned | Unassigned | 103_0 | Clustered | 3 | VC_103_0 | 3 | 0.369 | 0.99763908 | 0.3682 | 0 | 0 | 0 | 1 | Ref |
| MH885472 | Unassigned | Unassigned | Unassigned | 113_0 | Clustered | 91 | VC_113_0 | 91 | 0.9584 | 1 | 0.9584 | 0 | 0 | 0 | 1 | Ref |
| MH892350 | Unassigned | Unassigned | Unassigned | 262_0 | Clustered | 14 | VC_262_0 | 14 | 0.2364 | 1 | 0.2364 | 0 | 0 | 0 | 1 | Ref |
| MH892351 | Unassigned | Unassigned | Unassigned | 258_0 | Clustered | 121 | VC_258_0 | 121 | 0.7359 | 1 | 0.7359 | 0 | 0 | 0 | 0.9999 | Ref |
| MH892352 | Unassigned | Unassigned | Unassigned | 258_0 | Clustered | 121 | VC_258_0 | 121 | 0.7359 | 1 | 0.7359 | 0 | 0 | 0 | 0.9999 | Ref |
| MH892353 | Unassigned | Unassigned | Unassigned | 258_0 | Clustered | 121 | VC_258_0 | 121 | 0.7359 | 1 | 0.7359 | 0 | 0 | 0 | 0.9999 | Ref |
| MH892354 | Unassigned | Unassigned | Unassigned | 258_0 | Clustered | 121 | VC_258_0 | 121 | 0.7359 | 1 | 0.7359 | 0 | 0 | 0 | 0.9999 | Ref |
| MH892355 | Unassigned | Unassigned | Unassigned | 261_0 | Clustered | 13 | VC_261_0 | 13 | 0.2024 | 1 | 0.2024 | 0 | 0 | 0 | 1 | Ref |
| MH892356 | Unassigned | Unassigned | Unassigned | 258_0 | Clustered | 121 | VC_258_0 | 121 | 0.7359 | 1 | 0.7359 | 0 | 0 | 0 | 0.9999 | Ref |
| MH892357 | Unassigned | Unassigned | Unassigned | 258_0 | Clustered | 121 | VC_258_0 | 121 | 0.7359 | 1 | 0.7359 | 0 | 0 | 0 | 0.9999 | Ref |
| MH892358 | Unassigned | Unassigned | Unassigned | 258_0 | Clustered | 121 | VC_258_0 | 121 | 0.7359 | 1 | 0.7359 | 0 | 0 | 0 | 0.9999 | Ref |
| MH892359 | Unassigned | Unassigned | Unassigned | 258_0 | Clustered | 121 | VC_258_0 | 121 | 0.7359 | 1 | 0.7359 | 0 | 0 | 0 | 0.9999 | Ref |
| MH892360 | Unassigned | Unassigned | Unassigned | 258_0 | Clustered | 121 | VC_258_0 | 121 | 0.7359 | 1 | 0.7359 | 0 | 0 | 0 | 0.9999 | Ref |
| MH892361 | Unassigned | Unassigned | Unassigned | 258_0 | Clustered | 121 | VC_258_0 | 121 | 0.7359 | 1 | 0.7359 | 0 | 0 | 0 | 0.9999 | Ref |
| MH892362 | Unassigned | Unassigned | Unassigned | 260_0 | Clustered | 46 | VC_260_0 | 46 | 0.5137 | 1 | 0.5137 | 0 | 0 | 0 | 1 | Ref |
| MH892363 | Unassigned | Unassigned | Unassigned | 260_0 | Clustered | 46 | VC_260_0 | 46 | 0.5137 | 1 | 0.5137 | 0 | 0 | 0 | 1 | Ref |
| MH892364 | Unassigned | Unassigned | Unassigned | 260_0 | Clustered | 46 | VC_260_0 | 46 | 0.5137 | 1 | 0.5137 | 0 | 0 | 0 | 1 | Ref |
| MH892365 | Unassigned | Unassigned | Unassigned | 262_0 | Clustered | 14 | VC_262_0 | 14 | 0.2364 | 1 | 0.2364 | 0 | 0 | 0 | 1 | Ref |
| MH892366 | Unassigned | Unassigned | Unassigned | 260_0 | Clustered | 46 | VC_260_0 | 46 | 0.5137 | 1 | 0.5137 | 0 | 0 | 0 | 1 | Ref |
| MH892367 | Unassigned | Unassigned | Unassigned | 261_0 | Clustered | 13 | VC_261_0 | 13 | 0.2024 | 1 | 0.2024 | 0 | 0 | 0 | 1 | Ref |
| MH892368 | Unassigned | Unassigned | Unassigned | 262_0 | Clustered | 14 | VC_262_0 | 14 | 0.2364 | 1 | 0.2364 | 0 | 0 | 0 | 1 | Ref |
| MH892369 | Unassigned | Unassigned | Unassigned | 262_0 | Clustered | 14 | VC_262_0 | 14 | 0.2364 | 1 | 0.2364 | 0 | 0 | 0 | 1 | Ref |
| MH892370 | Unassigned | Unassigned | Unassigned | 262_0 | Clustered | 14 | VC_262_0 | 14 | 0.2364 | 1 | 0.2364 | 0 | 0 | 0 | 1 | Ref |
| MH892371 | Unassigned | Unassigned | Unassigned | 262_0 | Clustered | 14 | VC_262_0 | 14 | 0.2364 | 1 | 0.2364 | 0 | 0 | 0 | 1 | Ref |
| MH892372 | Unassigned | Unassigned | Unassigned | 262_0 | Clustered | 14 | VC_262_0 | 14 | 0.2364 | 1 | 0.2364 | 0 | 0 | 0 | 1 | Ref |
| MH892373 | Unassigned | Unassigned | Unassigned | 261_0 | Clustered | 13 | VC_261_0 | 13 | 0.2024 | 1 | 0.2024 | 0 | 0 | 0 | 1 | Ref |
| MH892374 | Unassigned | Unassigned | Unassigned | 262_0 | Clustered | 14 | VC_262_0 | 14 | 0.2364 | 1 | 0.2364 | 0 | 0 | 0 | 1 | Ref |
| MH892375 | Unassigned | Unassigned | Unassigned | 258_0 | Clustered | 121 | VC_258_0 | 121 | 0.7359 | 1 | 0.7359 | 0 | 0 | 0 | 0.9999 | Ref |
| MH892376 | Unassigned | Unassigned | Unassigned | 260_0 | Clustered | 46 | VC_260_0 | 46 | 0.5137 | 1 | 0.5137 | 0 | 0 | 0 | 1 | Ref |
| MH892377 | Unassigned | Unassigned | Unassigned | 258_0 | Clustered | 121 | VC_258_0 | 121 | 0.7359 | 1 | 0.7359 | 0 | 0 | 0 | 0.9999 | Ref |
| MH892378 | Unassigned | Unassigned | Unassigned | 258_0 | Clustered | 121 | VC_258_0 | 121 | 0.7359 | 1 | 0.7359 | 0 | 0 | 0 | 0.9999 | Ref |
| MH892379 | Unassigned | Unassigned | Unassigned | 258_0 | Clustered | 121 | VC_258_0 | 121 | 0.7359 | 1 | 0.7359 | 0 | 0 | 0 | 0.9999 | Ref |
| MH892380 | Unassigned | Unassigned | Unassigned | 258_0 | Clustered | 121 | VC_258_0 | 121 | 0.7359 | 1 | 0.7359 | 0 | 0 | 0 | 0.9999 | Ref |
| MH892381 | Unassigned | Unassigned | Unassigned | 258_0 | Clustered | 121 | VC_258_0 | 121 | 0.7359 | 1 | 0.7359 | 0 | 0 | 0 | 0.9999 | Ref |
| MH892382 | Unassigned | Unassigned | Unassigned | 258_0 | Clustered | 121 | VC_258_0 | 121 | 0.7359 | 1 | 0.7359 | 0 | 0 | 0 | 0.9999 | Ref |
| MH892383 | Unassigned | Unassigned | Unassigned | 260_0 | Clustered | 46 | VC_260_0 | 46 | 0.5137 | 1 | 0.5137 | 0 | 0 | 0 | 1 | Ref |
| MH892384 | Unassigned | Unassigned | Unassigned | 260_0 | Clustered | 46 | VC_260_0 | 46 | 0.5137 | 1 | 0.5137 | 0 | 0 | 0 | 1 | Ref |
| MH892385 | Unassigned | Unassigned | Unassigned | 260_0 | Clustered | 46 | VC_260_0 | 46 | 0.5137 | 1 | 0.5137 | 0 | 0 | 0 | 1 | Ref |
| MH899585 | Unassigned | Unassigned | Unassigned | 802_0 | Clustered | 8 | VC_802_0 | 8 | 0.5141 | 1 | 0.5141 | 0 | 0 | 0 | 1 | Ref |
| MH910036 | Unassigned | Unassigned | Unassigned | 1075_0 | Clustered | 11 | VC_1075_0 | 11 | 0.6562 | 1 | 0.6562 | 0 | 0 | 0 | 0.9273 | Ref |

| | | | | | | | | | | | | | | | | |
|---|---|---|---|---|---|---|---|---|---|---|---|---|---|---|---|---|
| MH910037 | Unassigned | Unassigned | Unassigned | 1075_0 | Clustered | 11 | VC_1075_0 | 11 | 0.6562 | 1 | 0.6562 | 0 | 0 | 0 | 0.9273 | Ref |
| MH910038 | Unassigned | Unassigned | Unassigned | 244_3 | Clustered | 8 | VC_244_3 | 8 | 0.1086 | 1 | 0.1086 | 0 | 0 | 0 | 1 | Ref |
| MH910039 | Unassigned | Unassigned | Unassigned | 244_3 | Clustered | 8 | VC_244_3 | 8 | 0.1086 | 1 | 0.1086 | 0 | 0 | 0 | 1 | Ref |
| MH910040 | Unassigned | Unassigned | Unassigned | 579_33 | Clustered/Singleton | 1 | VC_579_33 | 1 | 0 | 1 | 0 | 0 | 0 | 0 | 1 | Ref |
| MH910041 | Unassigned | Unassigned | Unassigned | 1075_0 | Clustered | 11 | VC_1075_0 | 11 | 0.6562 | 1 | 0.6562 | 0 | 0 | 0 | 0.9273 | Ref |
| MH910042 | Unassigned | Unassigned | Unassigned | 244_3 | Clustered | 8 | VC_244_3 | 8 | 0.1086 | 1 | 0.1086 | 0 | 0 | 0 | 1 | Ref |
| MH917278 | Unassigned | Unassigned | Unassigned | 595_0 | Clustered | 35 | VC_595_0 | 35 | 0.8809 | 1 | 0.8809 | 0 | 0 | 0 | 1 | Ref |
| MH920362 | Unassigned | Unassigned | Unassigned | 341_0 | Clustered | 27 | VC_341_0 | 27 | 0.5058 | 1 | 0.5058 | 0 | 0 | 0 | 0.8661 | Ref |
| MH920639 | Unassigned | Unassigned | Unassigned | 674_7 | Clustered | 3 | VC_674_7 | 3 | 0.0163 | 0.99856944 | 0.0163 | 0 | 0 | 0 | 1 | Ref |
| MH920640 | Unassigned | Unassigned | Unassigned | 674_7 | Clustered | 3 | VC_674_7 | 3 | 0.0163 | 0.99856944 | 0.0163 | 0 | 0 | 0 | 1 | Ref |
| MH925090 | Unassigned | Unassigned | Unassigned | 911_0 | Clustered | 20 | VC_911_0 | 20 | 0.9915 | 1 | 0.9915 | 0 | 0 | 0 | 1 | Ref |
| MH925091 | Unassigned | Unassigned | Unassigned | 935_2 | Clustered | 7 | VC_935_2 | 7 | 0.2837 | 1 | 0.2837 | 0 | 0 | 0 | 1 | Ref |
| MH925092 | Unassigned | Unassigned | Unassigned | 1091_1 | Clustered | 29 | VC_1091_1 | 29 | 0.4856 | 1 | 0.4856 | 0 | 0 | 0 | 0.9581 | Ref |
| MH925094 | Unassigned | Unassigned | Unassigned | 1008_0 | Clustered | 6 | VC_1008_0 | 6 | 0.2856 | 1 | 0.2856 | 0 | 0 | 0 | 1 | Ref |
| MH926055 | Unassigned | Unassigned | Unassigned | 539_0 | Clustered | 37 | VC_539_0 | 37 | 0.911 | 1 | 0.911 | 0 | 0 | 0 | 1 | Ref |
| MH926056 | Unassigned | Unassigned | Unassigned | 790_0 | Clustered | 8 | VC_790_0 | 8 | 0.6981 | 1 | 0.6981 | 0 | 0 | 0 | 1 | Ref |
| MH926057 | Unassigned | Unassigned | Unassigned | 790_0 | Clustered | 8 | VC_790_0 | 8 | 0.6981 | 1 | 0.6981 | 0 | 0 | 0 | 1 | Ref |
| MH926058 | Unassigned | Unassigned | Unassigned | 241_0 | Clustered | 21 | VC_241_0 | 21 | 0.8342 | 1 | 0.8342 | 0 | 0 | 0 | 1 | Ref |
| MH926059 | Unassigned | Unassigned | Unassigned | 588_0 | Clustered | 8 | VC_588_0 | 8 | 0.5695 | 1 | 0.5695 | 0 | 0 | 0 | 1 | Ref |
| MH926060 | Unassigned | Unassigned | Unassigned | 384_1 | Clustered | 11 | VC_384_1 | 11 | 0.0378 | 1 | 0.0378 | 0 | 0 | 0 | 0.8545 | Ref |
| MH926061 | Unassigned | Unassigned | Unassigned | 1243_0 | Clustered | 8 | VC_1243_0 | 8 | 0.8266 | 1 | 0.8266 | 0 | 0 | 0 | 1 | Ref |
| MH926062 | Unassigned | Unassigned | Unassigned | 577_0 | Clustered | 21 | VC_577_0 | 21 | 0.9908 | 1 | 0.9908 | 0 | 0 | 0 | 1 | Ref |
| MH929319 | Unassigned | Unassigned | Unassigned | 134_1 | Clustered | 4 | VC_134_1 | 4 | 0.0354 | 0.99998777 | 0.0354 | 0 | 0 | 0 | 1 | Ref |
| MH931003 | Unassigned | Unassigned | Unassigned | 1095_0 | Clustered | 12 | VC_1095_0 | 12 | 0.2819 | 0.99837092 | 0.2814 | 0 | 0 | 0 | 1 | Ref |
| MH931004 | Unassigned | Unassigned | Unassigned | 1119_0 | Clustered | 48 | VC_1119_0 | 48 | 0.852 | 1 | 0.852 | 0 | 0 | 0 | 1 | Ref |
| MH937457 | Unassigned | Unassigned | Unassigned | 260_0 | Clustered | 46 | VC_260_0 | 46 | 0.5137 | 1 | 0.5137 | 0 | 0 | 0 | 1 | Ref |
| MH937458 | Unassigned | Unassigned | Unassigned | 258_0 | Clustered | 121 | VC_258_0 | 121 | 0.7359 | 1 | 0.7359 | 0 | 0 | 0 | 0.9999 | Ref |
| MH937459 | Unassigned | Unassigned | Unassigned | 258_0 | Clustered | 121 | VC_258_0 | 121 | 0.7359 | 1 | 0.7359 | 0 | 0 | 0 | 0.9999 | Ref |
| MH937460 | Unassigned | Unassigned | Unassigned | 260_0 | Clustered | 46 | VC_260_0 | 46 | 0.5137 | 1 | 0.5137 | 0 | 0 | 0 | 1 | Ref |
| MH937461 | Unassigned | Unassigned | Unassigned | 258_0 | Clustered | 121 | VC_258_0 | 121 | 0.7359 | 1 | 0.7359 | 0 | 0 | 0 | 0.9999 | Ref |
| MH937462 | Unassigned | Unassigned | Unassigned | 258_0 | Clustered | 121 | VC_258_0 | 121 | 0.7359 | 1 | 0.7359 | 0 | 0 | 0 | 0.9999 | Ref |
| MH937463 | Unassigned | Unassigned | Unassigned | 260_0 | Clustered | 46 | VC_260_0 | 46 | 0.5137 | 1 | 0.5137 | 0 | 0 | 0 | 1 | Ref |
| MH937464 | Unassigned | Unassigned | Unassigned | 260_0 | Clustered | 46 | VC_260_0 | 46 | 0.5137 | 1 | 0.5137 | 0 | 0 | 0 | 1 | Ref |
| MH937465 | Unassigned | Unassigned | Unassigned | 258_0 | Clustered | 121 | VC_258_0 | 121 | 0.7359 | 1 | 0.7359 | 0 | 0 | 0 | 0.9999 | Ref |
| MH937466 | Unassigned | Unassigned | Unassigned | 258_0 | Clustered | 121 | VC_258_0 | 121 | 0.7359 | 1 | 0.7359 | 0 | 0 | 0 | 0.9999 | Ref |
| MH937467 | Unassigned | Unassigned | Unassigned | 258_0 | Clustered | 121 | VC_258_0 | 121 | 0.7359 | 1 | 0.7359 | 0 | 0 | 0 | 0.9999 | Ref |
| MH937468 | Unassigned | Unassigned | Unassigned | 258_0 | Clustered | 121 | VC_258_0 | 121 | 0.7359 | 1 | 0.7359 | 0 | 0 | 0 | 0.9999 | Ref |
| MH937469 | Unassigned | Unassigned | Unassigned | 258_0 | Clustered | 121 | VC_258_0 | 121 | 0.7359 | 1 | 0.7359 | 0 | 0 | 0 | 0.9999 | Ref |
| MH937470 | Unassigned | Unassigned | Unassigned | 258_0 | Clustered | 121 | VC_258_0 | 121 | 0.7359 | 1 | 0.7359 | 0 | 0 | 0 | 0.9999 | Ref |
| MH937471 | Unassigned | Unassigned | Unassigned | 258_0 | Clustered | 121 | VC_258_0 | 121 | 0.7359 | 1 | 0.7359 | 0 | 0 | 0 | 0.9999 | Ref |
| MH937472 | Unassigned | Unassigned | Unassigned | 258_0 | Clustered | 121 | VC_258_0 | 121 | 0.7359 | 1 | 0.7359 | 0 | 0 | 0 | 0.9999 | Ref |
| MH937473 | Unassigned | Unassigned | Unassigned | 260_0 | Clustered | 46 | VC_260_0 | 46 | 0.5137 | 1 | 0.5137 | 0 | 0 | 0 | 1 | Ref |
| MH937474 | Unassigned | Unassigned | Unassigned | 258_0 | Clustered | 121 | VC_258_0 | 121 | 0.7359 | 1 | 0.7359 | 0 | 0 | 0 | 0.9999 | Ref |
| MH937475 | Unassigned | Unassigned | Unassigned | 260_0 | Clustered | 46 | VC_260_0 | 46 | 0.5137 | 1 | 0.5137 | 0 | 0 | 0 | 1 | Ref |
| MH937476 | Unassigned | Unassigned | Unassigned | 260_0 | Clustered | 46 | VC_260_0 | 46 | 0.5137 | 1 | 0.5137 | 0 | 0 | 0 | 1 | Ref |
| MH937477 | Unassigned | Unassigned | Unassigned | 258_0 | Clustered | 121 | VC_258_0 | 121 | 0.7359 | 1 | 0.7359 | 0 | 0 | 0 | 0.9999 | Ref |
| MH937478 | Unassigned | Unassigned | Unassigned | 260_0 | Clustered | 46 | VC_260_0 | 46 | 0.5137 | 1 | 0.5137 | 0 | 0 | 0 | 1 | Ref |
| MH937479 | Unassigned | Unassigned | Unassigned | 260_0 | Clustered | 46 | VC_260_0 | 46 | 0.5137 | 1 | 0.5137 | 0 | 0 | 0 | 1 | Ref |
| MH937480 | Unassigned | Unassigned | Unassigned | 258_0 | Clustered | 121 | VC_258_0 | 121 | 0.7359 | 1 | 0.7359 | 0 | 0 | 0 | 0.9999 | Ref |
| MH937481 | Unassigned | Unassigned | Unassigned | 258_0 | Clustered | 121 | VC_258_0 | 121 | 0.7359 | 1 | 0.7359 | 0 | 0 | 0 | 0.9999 | Ref |
| MH937482 | Unassigned | Unassigned | Unassigned | 258_0 | Clustered | 121 | VC_258_0 | 121 | 0.7359 | 1 | 0.7359 | 0 | 0 | 0 | 0.9999 | Ref |
| MH937483 | Unassigned | Unassigned | Unassigned | 258_0 | Clustered | 121 | VC_258_0 | 121 | 0.7359 | 1 | 0.7359 | 0 | 0 | 0 | 0.9999 | Ref |
| MH937484 | Unassigned | Unassigned | Unassigned | 260_0 | Clustered | 46 | VC_260_0 | 46 | 0.5137 | 1 | 0.5137 | 0 | 0 | 0 | 1 | Ref |
| MH937485 | Unassigned | Unassigned | Unassigned | 258_0 | Clustered | 121 | VC_258_0 | 121 | 0.7359 | 1 | 0.7359 | 0 | 0 | 0 | 0.9999 | Ref |
| MH937486 | Unassigned | Unassigned | Unassigned | 258_0 | Clustered | 121 | VC_258_0 | 121 | 0.7359 | 1 | 0.7359 | 0 | 0 | 0 | 0.9999 | Ref |
| MH937487 | Unassigned | Unassigned | Unassigned | 258_0 | Clustered | 121 | VC_258_0 | 121 | 0.7359 | 1 | 0.7359 | 0 | 0 | 0 | 0.9999 | Ref |
| MH937488 | Unassigned | Unassigned | Unassigned | 258_0 | Clustered | 121 | VC_258_0 | 121 | 0.7359 | 1 | 0.7359 | 0 | 0 | 0 | 0.9999 | Ref |
| MH937489 | Unassigned | Unassigned | Unassigned | 258_0 | Clustered | 121 | VC_258_0 | 121 | 0.7359 | 1 | 0.7359 | 0 | 0 | 0 | 0.9999 | Ref |
| MH937490 | Unassigned | Unassigned | Unassigned | 258_0 | Clustered | 121 | VC_258_0 | 121 | 0.7359 | 1 | 0.7359 | 0 | 0 | 0 | 0.9999 | Ref |
| MH937491 | Unassigned | Unassigned | Unassigned | 258_0 | Clustered | 121 | VC_258_0 | 121 | 0.7359 | 1 | 0.7359 | 0 | 0 | 0 | 0.9999 | Ref |
| MH937492 | Unassigned | Unassigned | Unassigned | 258_0 | Clustered | 121 | VC_258_0 | 121 | 0.7359 | 1 | 0.7359 | 0 | 0 | 0 | 0.9999 | Ref |
| MH937493 | Unassigned | Unassigned | Unassigned | 258_0 | Clustered | 121 | VC_258_0 | 121 | 0.7359 | 1 | 0.7359 | 0 | 0 | 0 | 0.9999 | Ref |
| MH937494 | Unassigned | Unassigned | Unassigned | 260_0 | Clustered | 46 | VC_260_0 | 46 | 0.5137 | 1 | 0.5137 | 0 | 0 | 0 | 1 | Ref |
| MH937495 | Unassigned | Unassigned | Unassigned | 258_0 | Clustered | 121 | VC_258_0 | 121 | 0.7359 | 1 | 0.7359 | 0 | 0 | 0 | 0.9999 | Ref |
| MH937496 | Unassigned | Unassigned | Unassigned | 258_0 | Clustered | 121 | VC_258_0 | 121 | 0.7359 | 1 | 0.7359 | 0 | 0 | 0 | 0.9999 | Ref |
| MH937497 | Unassigned | Unassigned | Unassigned | 258_0 | Clustered | 121 | VC_258_0 | 121 | 0.7359 | 1 | 0.7359 | 0 | 0 | 0 | 0.9999 | Ref |
| MH937498 | Unassigned | Unassigned | Unassigned | 260_0 | Clustered | 46 | VC_260_0 | 46 | 0.5137 | 1 | 0.5137 | 0 | 0 | 0 | 1 | Ref |
| MH937499 | Unassigned | Unassigned | Unassigned | 258_0 | Clustered | 121 | VC_258_0 | 121 | 0.7359 | 1 | 0.7359 | 0 | 0 | 0 | 0.9999 | Ref |
| MH937500 | Unassigned | Unassigned | Unassigned | 258_0 | Clustered | 121 | VC_258_0 | 121 | 0.7359 | 1 | 0.7359 | 0 | 0 | 0 | 0.9999 | Ref |
| MH937501 | Unassigned | Unassigned | Unassigned | 258_0 | Clustered | 121 | VC_258_0 | 121 | 0.7359 | 1 | 0.7359 | 0 | 0 | 0 | 0.9999 | Ref |
| MH937502 | Unassigned | Unassigned | Unassigned | 260_0 | Clustered | 46 | VC_260_0 | 46 | 0.5137 | 1 | 0.5137 | 0 | 0 | 0 | 1 | Ref |
| MH937503 | Unassigned | Unassigned | Unassigned | 260_0 | Clustered | 46 | VC_260_0 | 46 | 0.5137 | 1 | 0.5137 | 0 | 0 | 0 | 1 | Ref |
| MH937504 | Unassigned | Unassigned | Unassigned | 258_0 | Clustered | 121 | VC_258_0 | 121 | 0.7359 | 1 | 0.7359 | 0 | 0 | 0 | 0.9999 | Ref |
| MH937505 | Unassigned | Unassigned | Unassigned | 260_0 | Clustered | 46 | VC_260_0 | 46 | 0.5137 | 1 | 0.5137 | 0 | 0 | 0 | 1 | Ref |
| MH937506 | Unassigned | Unassigned | Unassigned | 258_0 | Clustered | 121 | VC_258_0 | 121 | 0.7359 | 1 | 0.7359 | 0 | 0 | 0 | 0.9999 | Ref |
| MH937507 | Unassigned | Unassigned | Unassigned | 260_0 | Clustered | 46 | VC_260_0 | 46 | 0.5137 | 1 | 0.5137 | 0 | 0 | 0 | 1 | Ref |
| MH937508 | Unassigned | Unassigned | Unassigned | 258_0 | Clustered | 121 | VC_258_0 | 121 | 0.7359 | 1 | 0.7359 | 0 | 0 | 0 | 0.9999 | Ref |
| MH937509 | Unassigned | Unassigned | Unassigned | 260_0 | Clustered | 46 | VC_260_0 | 46 | 0.5137 | 1 | 0.5137 | 0 | 0 | 0 | 1 | Ref |
| MH937510 | Unassigned | Unassigned | Unassigned | 260_0 | Clustered | 46 | VC_260_0 | 46 | 0.5137 | 1 | 0.5137 | 0 | 0 | 0 | 1 | Ref |
| MH937511 | Unassigned | Unassigned | Unassigned | 260_0 | Clustered | 46 | VC_260_0 | 46 | 0.5137 | 1 | 0.5137 | 0 | 0 | 0 | 1 | Ref |
| MH939157 | Unassigned | Unassigned | Unassigned | 1428_0 | Clustered | 2 | VC_1428_0 | 2 | 0.8102 | 0.72985431 | 0.5914 | 0 | 0 | 0 | 1 | Ref |

| | | | | | | | | | | | | | | | | |
|---|---|---|---|---|---|---|---|---|---|---|---|---|---|---|---|---|
| MH940212 | Unassigned | Unassigned | Unassigned | 135_22 | Clustered | 13 | VC_135_22 | 13 | 0.0795 | 1 | 0.0795 | 0 | 0 | 0 | 0.8205 | Ref |
| MH972260 | Unassigned | Unassigned | Unassigned | 98_0 | Clustered | 30 | VC_98_0 | 30 | 0.7175 | 1 | 0.7175 | 0 | 0 | 0 | 1 | Ref |
| MH973661 | Unassigned | Unassigned | Unassigned | 258_0 | Clustered | 121 | VC_258_0 | 121 | 0.7359 | 1 | 0.7359 | 0 | 0 | 0 | 0.9999 | Ref |
| MH973662 | Unassigned | Unassigned | Unassigned | 258_0 | Clustered | 121 | VC_258_0 | 121 | 0.7359 | 1 | 0.7359 | 0 | 0 | 0 | 0.9999 | Ref |
| MH973663 | Unassigned | Unassigned | Unassigned | 261_0 | Clustered | 13 | VC_261_0 | 13 | 0.2024 | 1 | 0.2024 | 0 | 0 | 0 | 1 | Ref |
| MH973725 | Unassigned | Unassigned | Unassigned | 486_0 | Clustered | 47 | VC_486_0 | 47 | 0.9022 | 1 | 0.9022 | 0 | 0 | 0 | 1 | Ref |
| MH976506 | Unassigned | Unassigned | Unassigned | 1136_0 | Clustered | 40 | VC_1136_0 | 40 | 0.8746 | 1 | 0.8746 | 0 | 0 | 0 | 1 | Ref |
| MH976507 | Unassigned | Unassigned | Unassigned | 1136_0 | Clustered | 40 | VC_1136_0 | 40 | 0.8746 | 1 | 0.8746 | 0 | 0 | 0 | 1 | Ref |
| MH976508 | Unassigned | Unassigned | Unassigned | 1136_0 | Clustered | 40 | VC_1136_0 | 40 | 0.8746 | 1 | 0.8746 | 0 | 0 | 0 | 1 | Ref |
| MH976509 | Unassigned | Unassigned | Unassigned | 1136_0 | Clustered | 40 | VC_1136_0 | 40 | 0.8746 | 1 | 0.8746 | 0 | 0 | 0 | 1 | Ref |
| MH976510 | Unassigned | Unassigned | Unassigned | 1136_0 | Clustered | 40 | VC_1136_0 | 40 | 0.8746 | 1 | 0.8746 | 0 | 0 | 0 | 1 | Ref |
| MH976511 | Unassigned | Unassigned | Unassigned | 1136_0 | Clustered | 40 | VC_1136_0 | 40 | 0.8746 | 1 | 0.8746 | 0 | 0 | 0 | 1 | Ref |
| MH976512 | Unassigned | Unassigned | Unassigned | 1136_0 | Clustered | 40 | VC_1136_0 | 40 | 0.8746 | 1 | 0.8746 | 0 | 0 | 0 | 1 | Ref |
| MH976513 | Unassigned | Unassigned | Unassigned | 1136_0 | Clustered | 40 | VC_1136_0 | 40 | 0.8746 | 1 | 0.8746 | 0 | 0 | 0 | 1 | Ref |
| MH976514 | Unassigned | Unassigned | Unassigned | 1136_0 | Clustered | 40 | VC_1136_0 | 40 | 0.8746 | 1 | 0.8746 | 0 | 0 | 0 | 1 | Ref |
| MH976515 | Unassigned | Unassigned | Unassigned | 919_1 | Clustered | 4 | VC_919_1 | 4 | 0.4515 | 0.99998463 | 0.4515 | 0 | 0 | 0 | 1 | Ref |
| MH976516 | Unassigned | Unassigned | Unassigned | 790_0 | Clustered | 8 | VC_790_0 | 8 | 0.6981 | 1 | 0.6981 | 0 | 0 | 0 | 1 | Ref |
| MH976517 | Unassigned | Unassigned | Unassigned | 531_0 | Clustered | 32 | VC_531_0 | 32 | 0.6859 | 1 | 0.6859 | 0 | 0 | 0 | 1 | Ref |
| MH976518 | Unassigned | Unassigned | Unassigned | 1136_0 | Clustered | 40 | VC_1136_0 | 40 | 0.8746 | 1 | 0.8746 | 0 | 0 | 0 | 1 | Ref |
| MH976519 | Unassigned | Unassigned | Unassigned | 1136_0 | Clustered | 40 | VC_1136_0 | 40 | 0.8746 | 1 | 0.8746 | 0 | 0 | 0 | 1 | Ref |
| MH979674 | Unassigned | Unassigned | Unassigned | 434_0 | Clustered | 45 | VC_434_0 | 45 | 0.9479 | 1 | 0.9479 | 0 | 0 | 0 | 1 | Ref |
| MH983004 | Unassigned | Unassigned | Unassigned | 279_0 | Clustered | 11 | VC_279_0 | 11 | 0.4839 | 1 | 0.4839 | 0 | 0 | 0 | 0.8727 | Ref |
| MH992121 | Unassigned | Unassigned | Unassigned | 290_188 | Clustered/Singleton | 1 | VC_290_188 | 1 | 0 | 1 | 0 | 0 | 0 | 0 | 1 | Ref |
| MH992122 | Unassigned | Unassigned | Unassigned | 293_10 | Clustered | 9 | VC_293_10 | 9 | 0.0615 | 0.99999884 | 0.0615 | 0 | 0 | 0 | 0.6667 | Ref |
| MH992123 | Unassigned | Unassigned | Unassigned | 801_0 | Clustered | 37 | VC_801_0 | 37 | 0.5672 | 1 | 0.5672 | 0 | 0 | 0 | 1 | Ref |
| MH992131 | Unassigned | Unassigned | Unassigned | 829_0 | Clustered | 4 | VC_829_0 | 4 | 0.9064 | 0.99761409 | 0.9042 | 0 | 0 | 0 | 1 | Ref |
| MH992154 | Unassigned | Unassigned | Unassigned | 1429_0 | Clustered | 3 | VC_1429_0 | 3 | 1 | 1 | 1 | 0 | 0 | 0 | 1 | Ref |
| MH992155 | Unassigned | Unassigned | Unassigned | 1430_0 | Clustered | 3 | VC_1430_0 | 3 | 1 | 1 | 1 | 0 | 0 | 0 | 1 | Ref |
| MH992156 | Unassigned | Unassigned | Unassigned | 1028_0 | Clustered | 69 | VC_1028_0 | 69 | 0.8547 | 1 | 0.8547 | 0 | 0 | 0 | 1 | Ref |
| MH992157 | Unassigned | Unassigned | Unassigned | 206_0 | Clustered | 28 | VC_206_0 | 28 | 0.2427 | 0.99971175 | 0.2426 | 0 | 0 | 0 | 1 | Ref |
| MH992158 | Unassigned | Unassigned | Unassigned | 338_0 | Clustered | 12 | VC_338_0 | 12 | 0.8919 | 1 | 0.8919 | 0 | 0 | 0 | 1 | Ref |
| MH992159 | Unassigned | Unassigned | Unassigned | 1402_0 | Clustered | 7 | VC_1402_0 | 7 | 0.9174 | 0.99772305 | 0.9153 | 0 | 0 | 0 | 1 | Ref |
| MH992160 | Unassigned | Unassigned | Unassigned | 338_0 | Clustered | 12 | VC_338_0 | 12 | 0.8919 | 1 | 0.8919 | 0 | 0 | 0 | 1 | Ref |
| MH992161 | Unassigned | Unassigned | Unassigned | 1028_0 | Clustered | 69 | VC_1028_0 | 69 | 0.8547 | 1 | 0.8547 | 0 | 0 | 0 | 1 | Ref |
| MH992163 | Unassigned | Unassigned | Unassigned | 1119_0 | Clustered | 48 | VC_1119_0 | 48 | 0.852 | 1 | 0.852 | 0 | 0 | 0 | 1 | Ref |
| MH992164 | Unassigned | Unassigned | Unassigned | 1119_0 | Clustered | 48 | VC_1119_0 | 48 | 0.852 | 1 | 0.852 | 0 | 0 | 0 | 1 | Ref |
| MH992165 | Unassigned | Unassigned | Unassigned | 1108_0 | Clustered | 52 | VC_1108_0 | 52 | 0.7674 | 1 | 0.7674 | 0 | 0 | 0 | 1 | Ref |
| MH992167 | Unassigned | Unassigned | Unassigned | 1432_0 | Clustered | 2 | VC_1432_0 | 2 | 1 | 1 | 1 | 0 | 0 | 0 | 1 | Ref |
| MH992169 | Unassigned | Unassigned | Unassigned | 1030_0 | Clustered/Singleton | 1 | VC_1030_0 | 1 | 0 | 1 | 0 | 0 | 0 | 0 | 1 | Ref |
| MH992170 | Unassigned | Unassigned | Unassigned | 1101_0 | Clustered | 3 | VC_1101_0 | 3 | 0.3918 | 0.9839946 | 0.3856 | 0 | 0 | 0 | 1 | Ref |
| MH992171 | Unassigned | Unassigned | Unassigned | 1433_0 | Clustered | 2 | VC_1433_0 | 2 | 1 | 1 | 1 | 0 | 0 | 0 | 1 | Ref |
| MH992173 | Unassigned | Unassigned | Unassigned | 1388_0 | Clustered | 4 | VC_1388_0 | 4 | 0.6008 | 0.54407361 | 0.3269 | 0 | 0 | 0 | 1 | Ref |
| MH992174 | Unassigned | Unassigned | Unassigned | 206_0 | Clustered | 28 | VC_206_0 | 28 | 0.2427 | 0.99971175 | 0.2426 | 0 | 0 | 0 | 1 | Ref |
| MH992175 | Unassigned | Unassigned | Unassigned | 1402_0 | Clustered | 7 | VC_1402_0 | 7 | 0.9174 | 0.99772305 | 0.9153 | 0 | 0 | 0 | 1 | Ref |
| MH992177 | Unassigned | Unassigned | Unassigned | 338_0 | Clustered | 12 | VC_338_0 | 12 | 0.8919 | 1 | 0.8919 | 0 | 0 | 0 | 1 | Ref |
| MH992178 | Unassigned | Unassigned | Unassigned | 338_0 | Clustered | 12 | VC_338_0 | 12 | 0.8919 | 1 | 0.8919 | 0 | 0 | 0 | 1 | Ref |
| MH992180 | Unassigned | Unassigned | Unassigned | 1104_0 | Clustered | 44 | VC_1104_0 | 44 | 0.9574 | 0.72818579 | 0.6972 | 0 | 0 | 0 | 1 | Ref |
| MH992181 | Unassigned | Unassigned | Unassigned | 208_0 | Clustered/Singleton | 1 | VC_208_0 | 1 | 0 | 1 | 0 | 0 | 0 | 0 | 1 | Ref |
| MH992182 | Unassigned | Unassigned | Unassigned | 206_0 | Clustered | 28 | VC_206_0 | 28 | 0.2427 | 0.99971175 | 0.2426 | 0 | 0 | 0 | 1 | Ref |
| MH992184 | Unassigned | Unassigned | Unassigned | 1028_0 | Clustered | 69 | VC_1028_0 | 69 | 0.8547 | 1 | 0.8547 | 0 | 0 | 0 | 1 | Ref |
| MH992185 | Unassigned | Unassigned | Unassigned | 1028_0 | Clustered | 69 | VC_1028_0 | 69 | 0.8547 | 1 | 0.8547 | 0 | 0 | 0 | 1 | Ref |
| MH992189 | Unassigned | Unassigned | Unassigned | 338_0 | Clustered | 12 | VC_338_0 | 12 | 0.8919 | 1 | 0.8919 | 0 | 0 | 0 | 1 | Ref |
| MH992191 | Unassigned | Unassigned | Unassigned | 338_0 | Clustered | 12 | VC_338_0 | 12 | 0.8919 | 1 | 0.8919 | 0 | 0 | 0 | 1 | Ref |
| MH992192 | Unassigned | Unassigned | Unassigned | 1119_0 | Clustered | 48 | VC_1119_0 | 48 | 0.852 | 1 | 0.852 | 0 | 0 | 0 | 1 | Ref |
| MH992193 | Unassigned | Unassigned | Unassigned | 1101_0 | Clustered | 3 | VC_1101_0 | 3 | 0.3918 | 0.9839946 | 0.3856 | 0 | 0 | 0 | 1 | Ref |
| MH992194 | Unassigned | Unassigned | Unassigned | 338_0 | Clustered | 12 | VC_338_0 | 12 | 0.8919 | 1 | 0.8919 | 0 | 0 | 0 | 1 | Ref |
| MH992195 | Unassigned | Unassigned | Unassigned | 411_0 | Clustered | 48 | VC_411_0 | 48 | 0.4808 | 1 | 0.4808 | 0 | 0 | 0 | 1 | Ref |
| MH992196 | Unassigned | Unassigned | Unassigned | 1430_0 | Clustered | 3 | VC_1430_0 | 3 | 1 | 1 | 1 | 0 | 0 | 0 | 1 | Ref |
| MH992197 | Unassigned | Unassigned | Unassigned | 338_0 | Clustered | 12 | VC_338_0 | 12 | 0.8919 | 1 | 0.8919 | 0 | 0 | 0 | 1 | Ref |
| MH992199 | Unassigned | Unassigned | Unassigned | 1119_0 | Clustered | 48 | VC_1119_0 | 48 | 0.852 | 1 | 0.852 | 0 | 0 | 0 | 1 | Ref |
| MH992200 | Unassigned | Unassigned | Unassigned | 1402_0 | Clustered | 7 | VC_1402_0 | 7 | 0.9174 | 0.99772305 | 0.9153 | 0 | 0 | 0 | 1 | Ref |
| MH992202 | Unassigned | Unassigned | Unassigned | 1119_0 | Clustered | 48 | VC_1119_0 | 48 | 0.852 | 1 | 0.852 | 0 | 0 | 0 | 1 | Ref |
| MH992205 | Unassigned | Unassigned | Unassigned | 1430_0 | Clustered | 3 | VC_1430_0 | 3 | 1 | 1 | 1 | 0 | 0 | 0 | 1 | Ref |
| MH992206 | Unassigned | Unassigned | Unassigned | 1119_0 | Clustered | 48 | VC_1119_0 | 48 | 0.852 | 1 | 0.852 | 0 | 0 | 0 | 1 | Ref |
| MH992207 | Unassigned | Unassigned | Unassigned | 338_0 | Clustered | 12 | VC_338_0 | 12 | 0.8919 | 1 | 0.8919 | 0 | 0 | 0 | 1 | Ref |
| MH992208 | Unassigned | Unassigned | Unassigned | 1119_0 | Clustered | 48 | VC_1119_0 | 48 | 0.852 | 1 | 0.852 | 0 | 0 | 0 | 1 | Ref |
| MH992210 | Unassigned | Unassigned | Unassigned | 206_0 | Clustered | 28 | VC_206_0 | 28 | 0.2427 | 0.99971175 | 0.2426 | 0 | 0 | 0 | 1 | Ref |
| MH992211 | Unassigned | Unassigned | Unassigned | 411_0 | Clustered | 48 | VC_411_0 | 48 | 0.4808 | 1 | 0.4808 | 0 | 0 | 0 | 1 | Ref |
| MH992213 | Unassigned | Unassigned | Unassigned | 1028_0 | Clustered | 69 | VC_1028_0 | 69 | 0.8547 | 1 | 0.8547 | 0 | 0 | 0 | 1 | Ref |
| MH992214 | Unassigned | Unassigned | Unassigned | 1119_0 | Clustered | 48 | VC_1119_0 | 48 | 0.852 | 1 | 0.852 | 0 | 0 | 0 | 1 | Ref |
| MH992215 | Unassigned | Unassigned | Unassigned | 1119_0 | Clustered | 48 | VC_1119_0 | 48 | 0.852 | 1 | 0.852 | 0 | 0 | 0 | 1 | Ref |
| MH992216 | Unassigned | Unassigned | Unassigned | 1119_0 | Clustered | 48 | VC_1119_0 | 48 | 0.852 | 1 | 0.852 | 0 | 0 | 0 | 1 | Ref |
| MH992217 | Unassigned | Unassigned | Unassigned | 1119_0 | Clustered | 48 | VC_1119_0 | 48 | 0.852 | 1 | 0.852 | 0 | 0 | 0 | 1 | Ref |
| MH992218 | Unassigned | Unassigned | Unassigned | 338_0 | Clustered | 12 | VC_338_0 | 12 | 0.8919 | 1 | 0.8919 | 0 | 0 | 0 | 1 | Ref |
| MH992219 | Unassigned | Unassigned | Unassigned | 1119_0 | Clustered | 48 | VC_1119_0 | 48 | 0.852 | 1 | 0.852 | 0 | 0 | 0 | 1 | Ref |
| MH992220 | Unassigned | Unassigned | Unassigned | 1119_0 | Clustered | 48 | VC_1119_0 | 48 | 0.852 | 1 | 0.852 | 0 | 0 | 0 | 1 | Ref |
| MH992222 | Unassigned | Unassigned | Unassigned | 1119_0 | Clustered | 48 | VC_1119_0 | 48 | 0.852 | 1 | 0.852 | 0 | 0 | 0 | 1 | Ref |
| MH992509 | Unassigned | Unassigned | Unassigned | 654_0 | Clustered | 115 | VC_654_0 | 115 | 0.7303 | 1 | 0.7303 | 0 | 0 | 0 | 0.8764 | Ref |
| MH992510 | Unassigned | Unassigned | Unassigned | 290_129 | Clustered/Singleton | 1 | VC_290_129 | 1 | 0 | 1 | 0 | 0 | 0 | 0 | 1 | Ref |
| MH992513 | Unassigned | Unassigned | Unassigned | 907_0 | Clustered | 32 | VC_907_0 | 32 | 0.9964 | 1 | 0.9964 | 0 | 0 | 0 | 1 | Ref |
| MH999279 | Unassigned | Unassigned | Unassigned | 928_0 | Clustered | 6 | VC_928_0 | 6 | 0.4065 | 1 | 0.4065 | 0 | 0 | 0 | 1 | Ref |

| | | | | | | | | | | | | | | | | |
|---|---|---|---|---|---|---|---|---|---|---|---|---|---|---|---|---|
| MH999280 | Unassigned | Unassigned | Unassigned | 839_0 | Clustered | 8 | VC_839_0 | 8 | 0.9889 | 1 | 0.9889 | 0 | 0 | 0 | 1 | Ref |
| MK002701 | Unassigned | Unassigned | Unassigned | 364_0 | Clustered | 3 | VC_364_0 | 3 | 0.729 | 0.98867275 | 0.7207 | 0 | 0 | 0 | 1 | Ref |
| MK005300 | Unassigned | Unassigned | Unassigned | 199_20 | Clustered/Singleton | 1 | VC_199_20 | 1 | 0 | 1 | 0 | 0 | 0 | 0 | 1 | Ref |
| MK016491 | Unassigned | Unassigned | Unassigned | 571_5 | Clustered | 2 | VC_571_5 | 2 | 0.0054 | 0.95548062 | 0.0052 | 0 | 0 | 0 | 1 | Ref |
| MK016492 | Unassigned | Unassigned | Unassigned | 543_0 | Clustered | 25 | VC_543_0 | 25 | 0.4584 | 1 | 0.4584 | 0 | 0 | 0 | 1 | Ref |
| MK016494 | Unassigned | Unassigned | Unassigned | 538_31 | Clustered | 11 | VC_538_31 | 11 | 0.0894 | 1 | 0.0894 | 0 | 0 | 0 | 0.6 | Ref |
| MK016495 | Unassigned | Unassigned | Unassigned | 1202_0 | Clustered | 10 | VC_1202_0 | 10 | 0.5679 | 1 | 0.5679 | 0 | 0 | 0 | 1 | Ref |
| MK016496 | Unassigned | Unassigned | Unassigned | 538_77 | Clustered | 3 | VC_538_77 | 3 | 0.0216 | 0.99859911 | 0.0216 | 0 | 0 | 0 | 1 | Ref |
| MK016497 | Unassigned | Unassigned | Unassigned | 1202_0 | Clustered | 10 | VC_1202_0 | 10 | 0.5679 | 1 | 0.5679 | 0 | 0 | 0 | 1 | Ref |
| MK016498 | Unassigned | Unassigned | Unassigned | 571_5 | Clustered | 2 | VC_571_5 | 2 | 0.0054 | 0.95548062 | 0.0052 | 0 | 0 | 0 | 1 | Ref |
| MK016499 | Unassigned | Unassigned | Unassigned | 537_0 | Clustered | 39 | VC_537_0 | 39 | 0.8148 | 1 | 0.8148 | 0 | 0 | 0 | 1 | Ref |
| MK016501 | Unassigned | Unassigned | Unassigned | 583_0 | Clustered | 2 | VC_583_0 | 2 | 0.0713 | 0.956426 | 0.0682 | 0 | 0 | 0 | 1 | Ref |
| MK016502 | Unassigned | Unassigned | Unassigned | 571_0 | Clustered | 16 | VC_571_0 | 16 | 0.091 | 1 | 0.091 | 0 | 0 | 0 | 0.6667 | Ref |
| MK016503 | Unassigned | Unassigned | Unassigned | 243_0 | Clustered | 72 | VC_243_0 | 72 | 0.8089 | 1 | 0.8089 | 0 | 0 | 0 | 1 | Ref |
| MK016504 | Unassigned | Unassigned | Unassigned | 538_90 | Clustered/Singleton | 1 | VC_538_90 | 1 | 0 | 1 | 0 | 0 | 0 | 0 | 1 | Ref |
| MK016662 | Unassigned | Unassigned | Unassigned | 352_0 | Clustered | 31 | VC_352_0 | 31 | 0.9547 | 1 | 0.9547 | 0 | 0 | 0 | 1 | Ref |
| MK016664 | Unassigned | Unassigned | Unassigned | 449_1 | Clustered/Singleton | 1 | VC_449_1 | 1 | 0 | 1 | 0 | 0 | 0 | 0 | 1 | Ref |
| MK017819 | Unassigned | Unassigned | Unassigned | 1060_0 | Clustered | 4 | VC_1060_0 | 4 | 0.875 | 0.99993415 | 0.8749 | 0 | 0 | 0 | 1 | Ref |
| MK017820 | Unassigned | Unassigned | Unassigned | 930_0 | Clustered | 4 | VC_930_0 | 4 | 0.2568 | 0.99997246 | 0.2568 | 0 | 0 | 0 | 1 | Ref |
| MK024806 | Unassigned | Unassigned | Unassigned | 601_3 | Clustered | 12 | VC_601_3 | 12 | 0.2339 | 1 | 0.2339 | 0 | 0 | 0 | 0.8636 | Ref |
| MK033136 | Unassigned | Unassigned | Unassigned | 465_0 | Clustered | 80 | VC_465_0 | 80 | 0.9988 | 1 | 0.9988 | 0 | 0 | 0 | 1 | Ref |
| MK033137 | Unassigned | Unassigned | Unassigned | 132_0 | Clustered | 19 | VC_132_0 | 19 | 0.6051 | 1 | 0.6051 | 0 | 0 | 0 | 1 | Ref |
| MK034952 | Unassigned | Unassigned | Unassigned | 2_0 | Clustered | 4 | VC_2_0 | 4 | 0.13 | 0.99998694 | 0.13 | 0 | 0 | 0 | 1 | Ref |
| MK047717 | Unassigned | Unassigned | Unassigned | 293_15 | Clustered | 2 | VC_293_15 | 2 | 0.0073 | 0.75626308 | 0.0055 | 0 | 0 | 0 | 1 | Ref |
| MK047718 | Unassigned | Unassigned | Unassigned | 293_17 | Clustered | 8 | VC_293_17 | 8 | 0.0534 | 0.99997879 | 0.0534 | 0 | 0 | 0 | 0.6071 | Ref |
| MK050846 | Unassigned | Unassigned | Unassigned | 199_32 | Clustered | 12 | VC_199_32 | 12 | 0.0676 | 1 | 0.0676 | 0 | 0 | 0 | 0.7576 | Ref |
| MK053931 | Unassigned | Unassigned | Unassigned | 907_0 | Clustered | 32 | VC_907_0 | 32 | 0.9964 | 1 | 0.9964 | 0 | 0 | 0 | 1 | Ref |
| MK054204 | Unassigned | Unassigned | Unassigned | 618_0 | Clustered | 50 | VC_618_0 | 50 | 0.9989 | 1 | 0.9989 | 0 | 0 | 0 | 1 | Ref |
| MK054205 | Unassigned | Unassigned | Unassigned | 618_0 | Clustered | 50 | VC_618_0 | 50 | 0.9989 | 1 | 0.9989 | 0 | 0 | 0 | 1 | Ref |
| MK054206 | Unassigned | Unassigned | Unassigned | 618_0 | Clustered | 50 | VC_618_0 | 50 | 0.9989 | 1 | 0.9989 | 0 | 0 | 0 | 1 | Ref |
| MK054207 | Unassigned | Unassigned | Unassigned | 618_0 | Clustered | 50 | VC_618_0 | 50 | 0.9989 | 1 | 0.9989 | 0 | 0 | 0 | 1 | Ref |
| MK054208 | Unassigned | Unassigned | Unassigned | 618_0 | Clustered | 50 | VC_618_0 | 50 | 0.9989 | 1 | 0.9989 | 0 | 0 | 0 | 1 | Ref |
| MK054209 | Unassigned | Unassigned | Unassigned | 618_0 | Clustered | 50 | VC_618_0 | 50 | 0.9989 | 1 | 0.9989 | 0 | 0 | 0 | 1 | Ref |
| MK054210 | Unassigned | Unassigned | Unassigned | 618_0 | Clustered | 50 | VC_618_0 | 50 | 0.9989 | 1 | 0.9989 | 0 | 0 | 0 | 1 | Ref |
| MK054211 | Unassigned | Unassigned | Unassigned | 618_0 | Clustered | 50 | VC_618_0 | 50 | 0.9989 | 1 | 0.9989 | 0 | 0 | 0 | 1 | Ref |
| MK054212 | Unassigned | Unassigned | Unassigned | 618_0 | Clustered | 50 | VC_618_0 | 50 | 0.9989 | 1 | 0.9989 | 0 | 0 | 0 | 1 | Ref |
| MK054213 | Unassigned | Unassigned | Unassigned | 618_0 | Clustered | 50 | VC_618_0 | 50 | 0.9989 | 1 | 0.9989 | 0 | 0 | 0 | 1 | Ref |
| MK054214 | Unassigned | Unassigned | Unassigned | 618_0 | Clustered | 50 | VC_618_0 | 50 | 0.9989 | 1 | 0.9989 | 0 | 0 | 0 | 1 | Ref |
| MK054215 | Unassigned | Unassigned | Unassigned | 618_0 | Clustered | 50 | VC_618_0 | 50 | 0.9989 | 1 | 0.9989 | 0 | 0 | 0 | 1 | Ref |
| MK054216 | Unassigned | Unassigned | Unassigned | 618_0 | Clustered | 50 | VC_618_0 | 50 | 0.9989 | 1 | 0.9989 | 0 | 0 | 0 | 1 | Ref |
| MK054217 | Unassigned | Unassigned | Unassigned | 618_0 | Clustered | 50 | VC_618_0 | 50 | 0.9989 | 1 | 0.9989 | 0 | 0 | 0 | 1 | Ref |
| MK054218 | Unassigned | Unassigned | Unassigned | 618_0 | Clustered | 50 | VC_618_0 | 50 | 0.9989 | 1 | 0.9989 | 0 | 0 | 0 | 1 | Ref |
| MK054219 | Unassigned | Unassigned | Unassigned | 618_0 | Clustered | 50 | VC_618_0 | 50 | 0.9989 | 1 | 0.9989 | 0 | 0 | 0 | 1 | Ref |
| MK054220 | Unassigned | Unassigned | Unassigned | 618_0 | Clustered | 50 | VC_618_0 | 50 | 0.9989 | 1 | 0.9989 | 0 | 0 | 0 | 1 | Ref |
| MK054221 | Unassigned | Unassigned | Unassigned | 618_0 | Clustered | 50 | VC_618_0 | 50 | 0.9989 | 1 | 0.9989 | 0 | 0 | 0 | 1 | Ref |
| MK054222 | Unassigned | Unassigned | Unassigned | 618_0 | Clustered | 50 | VC_618_0 | 50 | 0.9989 | 1 | 0.9989 | 0 | 0 | 0 | 1 | Ref |
| MK054223 | Unassigned | Unassigned | Unassigned | 618_0 | Clustered | 50 | VC_618_0 | 50 | 0.9989 | 1 | 0.9989 | 0 | 0 | 0 | 1 | Ref |
| MK054224 | Unassigned | Unassigned | Unassigned | 618_0 | Clustered | 50 | VC_618_0 | 50 | 0.9989 | 1 | 0.9989 | 0 | 0 | 0 | 1 | Ref |
| MK054225 | Unassigned | Unassigned | Unassigned | 618_0 | Clustered | 50 | VC_618_0 | 50 | 0.9989 | 1 | 0.9989 | 0 | 0 | 0 | 1 | Ref |
| MK054226 | Unassigned | Unassigned | Unassigned | 618_0 | Clustered | 50 | VC_618_0 | 50 | 0.9989 | 1 | 0.9989 | 0 | 0 | 0 | 1 | Ref |
| MK054227 | Unassigned | Unassigned | Unassigned | 618_0 | Clustered | 50 | VC_618_0 | 50 | 0.9989 | 1 | 0.9989 | 0 | 0 | 0 | 1 | Ref |
| MK054228 | Unassigned | Unassigned | Unassigned | 618_0 | Clustered | 50 | VC_618_0 | 50 | 0.9989 | 1 | 0.9989 | 0 | 0 | 0 | 1 | Ref |
| MK054229 | Unassigned | Unassigned | Unassigned | 618_0 | Clustered | 50 | VC_618_0 | 50 | 0.9989 | 1 | 0.9989 | 0 | 0 | 0 | 1 | Ref |
| MK054230 | Unassigned | Unassigned | Unassigned | 618_0 | Clustered | 50 | VC_618_0 | 50 | 0.9989 | 1 | 0.9989 | 0 | 0 | 0 | 1 | Ref |
| MK054231 | Unassigned | Unassigned | Unassigned | 618_0 | Clustered | 50 | VC_618_0 | 50 | 0.9989 | 1 | 0.9989 | 0 | 0 | 0 | 1 | Ref |
| MK054232 | Unassigned | Unassigned | Unassigned | 618_0 | Clustered | 50 | VC_618_0 | 50 | 0.9989 | 1 | 0.9989 | 0 | 0 | 0 | 1 | Ref |
| MK054233 | Unassigned | Unassigned | Unassigned | 618_0 | Clustered | 50 | VC_618_0 | 50 | 0.9989 | 1 | 0.9989 | 0 | 0 | 0 | 1 | Ref |
| MK054234 | Unassigned | Unassigned | Unassigned | 618_0 | Clustered | 50 | VC_618_0 | 50 | 0.9989 | 1 | 0.9989 | 0 | 0 | 0 | 1 | Ref |
| MK054235 | Unassigned | Unassigned | Unassigned | 618_0 | Clustered | 50 | VC_618_0 | 50 | 0.9989 | 1 | 0.9989 | 0 | 0 | 0 | 1 | Ref |
| MK054236 | Unassigned | Unassigned | Unassigned | 618_0 | Clustered | 50 | VC_618_0 | 50 | 0.9989 | 1 | 0.9989 | 0 | 0 | 0 | 1 | Ref |
| MK054237 | Unassigned | Unassigned | Unassigned | 618_0 | Clustered | 50 | VC_618_0 | 50 | 0.9989 | 1 | 0.9989 | 0 | 0 | 0 | 1 | Ref |
| MK059749 | Unassigned | Unassigned | Unassigned | 385_2 | Clustered | 5 | VC_385_2 | 5 | 0.0717 | 0.99999997 | 0.0717 | 0 | 0 | 0 | 0.9 | Ref |
| MK061407 | Unassigned | Unassigned | Unassigned | 973_0 | Clustered | 15 | VC_973_0 | 15 | 1 | 0 | 0 | 0 | 0 | 0 | 1 | Ref |
| MK061408 | Unassigned | Unassigned | Unassigned | 223_4 | Clustered | 13 | VC_223_4 | 13 | 0.1649 | 1 | 0.1649 | 0 | 0 | 0 | 1 | Ref |
| MK061409 | Unassigned | Unassigned | Unassigned | 232_0 | Clustered | 32 | VC_232_0 | 32 | 0.8471 | 1 | 0.8471 | 0 | 0 | 0 | 0.996 | Ref |
| MK061410 | Unassigned | Unassigned | Unassigned | 1218_0 | Clustered | 14 | VC_1218_0 | 14 | 0.7811 | 1 | 0.7811 | 0 | 0 | 0 | 0.989 | Ref |
| MK061411 | Unassigned | Unassigned | Unassigned | 526_0 | Clustered | 32 | VC_526_0 | 32 | 0.9037 | 1 | 0.9037 | 0 | 0 | 0 | 1 | Ref |
| MK061412 | Unassigned | Unassigned | Unassigned | 1115_3 | Clustered | 6 | VC_1115_3 | 6 | 0.1298 | 0.99999994 | 0.1298 | 0 | 0 | 0 | 0.8667 | Ref |
| MK061413 | Unassigned | Unassigned | Unassigned | 1250_0 | Clustered | 29 | VC_1250_0 | 29 | 0.822 | 1 | 0.822 | 0 | 0 | 0 | 1 | Ref |
| MK061414 | Unassigned | Unassigned | Unassigned | 527_0 | Clustered | 37 | VC_527_0 | 37 | 0.9679 | 1 | 0.9679 | 0 | 0 | 0 | 1 | Ref |
| MK061415 | Unassigned | Unassigned | Unassigned | 335_51 | Clustered | 3 | VC_335_51 | 3 | 0.008 | 0.99634936 | 0.008 | 0 | 0 | 0 | 0.6667 | Ref |
| MK064562 | Unassigned | Unassigned | Unassigned | 1417_0 | Clustered | 3 | VC_1417_0 | 3 | 0.9824 | 0.9595722 | 0.9427 | 0 | 0 | 0 | 1 | Ref |
| MK064563 | Unassigned | Unassigned | Unassigned | 1417_0 | Clustered | 3 | VC_1417_0 | 3 | 0.9824 | 0.9595722 | 0.9427 | 0 | 0 | 0 | 1 | Ref |
| MK064564 | Unassigned | Unassigned | Unassigned | 362_0 | Clustered | 2 | VC_362_0 | 2 | 0.3217 | 0.93865445 | 0.3019 | 0 | 0 | 0 | 1 | Ref |
| MK064565 | Unassigned | Unassigned | Unassigned | 360_0 | Clustered | 22 | VC_360_0 | 22 | 0.96 | 1 | 0.96 | 0 | 0 | 0 | 1 | Ref |
| MK069403 | Unassigned | Unassigned | Unassigned | 691_0 | Clustered | 18 | VC_691_0 | 18 | 0.6865 | 1 | 0.6865 | 0 | 0 | 0 | 0.9477 | Ref |
| MK071653 | Unassigned | Unassigned | Unassigned | 1428_0 | Clustered | 2 | VC_1428_0 | 2 | 0.8102 | 0.72985431 | 0.5914 | 0 | 0 | 0 | 1 | Ref |
| MK075001 | Unassigned | Unassigned | Unassigned | 15_1 | Clustered | 9 | VC_15_1 | 9 | 0.1817 | 1 | 0.1817 | 0 | 0 | 0 | 0.9167 | Ref |
| MK075002 | Unassigned | Unassigned | Unassigned | 15_0 | Clustered | 3 | VC_15_0 | 3 | 0.0477 | 0.99853676 | 0.0476 | 0 | 0 | 0 | 1 | Ref |
| MK075003 | Unassigned | Unassigned | Unassigned | 11_0 | Clustered | 19 | VC_11_0 | 19 | 0.7544 | 1 | 0.7544 | 0 | 0 | 0 | 1 | Ref |
| MK075004 | Unassigned | Unassigned | Unassigned | 15_1 | Clustered | 9 | VC_15_1 | 9 | 0.1817 | 1 | 0.1817 | 0 | 0 | 0 | 0.9167 | Ref |

| | | | | | | | | | | | | | | | | |
|---|---|---|---|---|---|---|---|---|---|---|---|---|---|---|---|---|
| MK075006 | Unassigned | Unassigned | Unassigned | 11_0 | Clustered | 19 | VC_11_0 | 19 | 0.7544 | 1 | 0.7544 | 0 | 0 | 0 | 1 | Ref |
| MK080312 | Unassigned | Unassigned | Unassigned | 1275_0 | Clustered | 4 | VC_1275_0 | 4 | 0.747 | 0.9991311 | 0.7463 | 0 | 0 | 0 | 1 | Ref |
| MK084630 | Unassigned | Unassigned | Unassigned | 843_0 | Clustered | 20 | VC_843_0 | 20 | 0.9621 | 1 | 0.9621 | 0 | 0 | 0 | 1 | Ref |
| MK085976 | Unassigned | Unassigned | Unassigned | 719_0 | Clustered | 16 | VC_719_0 | 16 | 0.728 | 1 | 0.728 | 0 | 0 | 0 | 1 | Ref |
| MK088078 | Unassigned | Unassigned | Unassigned | 193_0 | Clustered | 3 | VC_193_0 | 3 | 0.4439 | 0.97148006 | 0.4313 | 0 | 0 | 0 | 1 | Ref |
| MK089780 | Unassigned | Unassigned | Unassigned | 714_0 | Clustered | 71 | VC_714_0 | 71 | 0.935 | 1 | 0.935 | 0 | 0 | 0 | 1 | Ref |
| MK095192 | Unassigned | Unassigned | Unassigned | 1018_0 | Clustered | 38 | VC_1018_0 | 38 | 0.9757 | 1 | 0.9757 | 0 | 0 | 0 | 1 | Ref |
| MK095193 | Unassigned | Unassigned | Unassigned | 1018_0 | Clustered | 38 | VC_1018_0 | 38 | 0.9757 | 1 | 0.9757 | 0 | 0 | 0 | 1 | Ref |
| MK095194 | Unassigned | Unassigned | Unassigned | 1018_0 | Clustered | 38 | VC_1018_0 | 38 | 0.9757 | 1 | 0.9757 | 0 | 0 | 0 | 1 | Ref |
| MK095195 | Unassigned | Unassigned | Unassigned | 1018_0 | Clustered | 38 | VC_1018_0 | 38 | 0.9757 | 1 | 0.9757 | 0 | 0 | 0 | 1 | Ref |
| MK095196 | Unassigned | Unassigned | Unassigned | 1018_0 | Clustered | 38 | VC_1018_0 | 38 | 0.9757 | 1 | 0.9757 | 0 | 0 | 0 | 1 | Ref |
| MK095197 | Unassigned | Unassigned | Unassigned | 1018_0 | Clustered | 38 | VC_1018_0 | 38 | 0.9757 | 1 | 0.9757 | 0 | 0 | 0 | 1 | Ref |
| MK095198 | Unassigned | Unassigned | Unassigned | 1018_0 | Clustered | 38 | VC_1018_0 | 38 | 0.9757 | 1 | 0.9757 | 0 | 0 | 0 | 1 | Ref |
| MK095199 | Unassigned | Unassigned | Unassigned | 1018_0 | Clustered | 38 | VC_1018_0 | 38 | 0.9757 | 1 | 0.9757 | 0 | 0 | 0 | 1 | Ref |
| MK095200 | Unassigned | Unassigned | Unassigned | 1018_0 | Clustered | 38 | VC_1018_0 | 38 | 0.9757 | 1 | 0.9757 | 0 | 0 | 0 | 1 | Ref |
| MK095201 | Unassigned | Unassigned | Unassigned | 1018_0 | Clustered | 38 | VC_1018_0 | 38 | 0.9757 | 1 | 0.9757 | 0 | 0 | 0 | 1 | Ref |
| MK095202 | Unassigned | Unassigned | Unassigned | 1018_0 | Clustered | 38 | VC_1018_0 | 38 | 0.9757 | 1 | 0.9757 | 0 | 0 | 0 | 1 | Ref |
| MK095203 | Unassigned | Unassigned | Unassigned | 1018_0 | Clustered | 38 | VC_1018_0 | 38 | 0.9757 | 1 | 0.9757 | 0 | 0 | 0 | 1 | Ref |
| MK095204 | Unassigned | Unassigned | Unassigned | 1018_0 | Clustered | 38 | VC_1018_0 | 38 | 0.9757 | 1 | 0.9757 | 0 | 0 | 0 | 1 | Ref |
| MK095205 | Unassigned | Unassigned | Unassigned | 1018_0 | Clustered | 38 | VC_1018_0 | 38 | 0.9757 | 1 | 0.9757 | 0 | 0 | 0 | 1 | Ref |
| MK095206 | Unassigned | Unassigned | Unassigned | 1018_0 | Clustered | 38 | VC_1018_0 | 38 | 0.9757 | 1 | 0.9757 | 0 | 0 | 0 | 1 | Ref |
| MK095207 | Unassigned | Unassigned | Unassigned | 1018_0 | Clustered | 38 | VC_1018_0 | 38 | 0.9757 | 1 | 0.9757 | 0 | 0 | 0 | 1 | Ref |
| MK095208 | Unassigned | Unassigned | Unassigned | 1018_0 | Clustered | 38 | VC_1018_0 | 38 | 0.9757 | 1 | 0.9757 | 0 | 0 | 0 | 1 | Ref |
| MK095209 | Unassigned | Unassigned | Unassigned | 1018_0 | Clustered | 38 | VC_1018_0 | 38 | 0.9757 | 1 | 0.9757 | 0 | 0 | 0 | 1 | Ref |
| MK095210 | Unassigned | Unassigned | Unassigned | 1018_0 | Clustered | 38 | VC_1018_0 | 38 | 0.9757 | 1 | 0.9757 | 0 | 0 | 0 | 1 | Ref |
| MK095211 | Unassigned | Unassigned | Unassigned | 1018_0 | Clustered | 38 | VC_1018_0 | 38 | 0.9757 | 1 | 0.9757 | 0 | 0 | 0 | 1 | Ref |
| MK097141 | Unassigned | Unassigned | Unassigned | 661_2 | Clustered | 6 | VC_661_2 | 6 | 0.1674 | 1 | 0.1674 | 0 | 0 | 0 | 1 | Ref |
| MK105819 | Unassigned | Unassigned | Unassigned | 369_0 | Clustered | 2 | VC_369_0 | 2 | 0.1984 | 0.93211095 | 0.185 | 0 | 0 | 0 | 1 | Ref |
| MK105855 | Unassigned | Unassigned | Unassigned | 721_0 | Clustered | 11 | VC_721_0 | 11 | 0.8846 | 1 | 0.8846 | 0 | 0 | 0 | 1 | Ref |
| MK112526 | Unassigned | Unassigned | Unassigned | 1200_0 | Clustered | 73 | VC_1200_0 | 73 | 0.9825 | 1 | 0.9825 | 0 | 0 | 0 | 1 | Ref |
| MK112527 | Unassigned | Unassigned | Unassigned | 384_15 | Clustered | 44 | VC_384_15 | 44 | 0.139 | 1 | 0.139 | 0 | 0 | 0 | 0.7474 | Ref |
| MK112528 | Unassigned | Unassigned | Unassigned | 1200_0 | Clustered | 73 | VC_1200_0 | 73 | 0.9825 | 1 | 0.9825 | 0 | 0 | 0 | 1 | Ref |
| MK112529 | Unassigned | Unassigned | Unassigned | 1200_0 | Clustered | 73 | VC_1200_0 | 73 | 0.9825 | 1 | 0.9825 | 0 | 0 | 0 | 1 | Ref |
| MK112530 | Unassigned | Unassigned | Unassigned | 335_36 | Clustered | 2 | VC_335_36 | 2 | 0.0036 | 0.94071129 | 0.0034 | 0 | 0 | 0 | 1 | Ref |
| MK112531 | Unassigned | Unassigned | Unassigned | 1200_0 | Clustered | 73 | VC_1200_0 | 73 | 0.9825 | 1 | 0.9825 | 0 | 0 | 0 | 1 | Ref |
| MK112532 | Unassigned | Unassigned | Unassigned | 335_33 | Clustered | 4 | VC_335_33 | 4 | 0.0148 | 0.99998565 | 0.0148 | 0 | 0 | 0 | 1 | Ref |
| MK112533 | Unassigned | Unassigned | Unassigned | 579_51 | Clustered/Singleton | 1 | VC_579_51 | 1 | 0 | 1 | 0 | 0 | 0 | 0 | 1 | Ref |
| MK112534 | Unassigned | Unassigned | Unassigned | 579_10 | Clustered/Singleton | 1 | VC_579_10 | 1 | 0 | 1 | 0 | 0 | 0 | 0 | 1 | Ref |
| MK112535 | Unassigned | Unassigned | Unassigned | 1200_0 | Clustered | 73 | VC_1200_0 | 73 | 0.9825 | 1 | 0.9825 | 0 | 0 | 0 | 1 | Ref |
| MK112536 | Unassigned | Unassigned | Unassigned | 384_15 | Clustered | 44 | VC_384_15 | 44 | 0.139 | 1 | 0.139 | 0 | 0 | 0 | 0.7474 | Ref |
| MK112537 | Unassigned | Unassigned | Unassigned | 243_0 | Clustered | 72 | VC_243_0 | 72 | 0.8089 | 1 | 0.8089 | 0 | 0 | 0 | 1 | Ref |
| MK112538 | Unassigned | Unassigned | Unassigned | 1200_0 | Clustered | 73 | VC_1200_0 | 73 | 0.9825 | 1 | 0.9825 | 0 | 0 | 0 | 1 | Ref |
| MK112539 | Unassigned | Unassigned | Unassigned | 384_11 | Clustered | 31 | VC_384_11 | 31 | 0.0926 | 1 | 0.0926 | 0 | 0 | 0 | 0.7011 | Ref |
| MK112540 | Unassigned | Unassigned | Unassigned | 335_42 | Clustered | 2 | VC_335_42 | 2 | 0.0039 | 0.95008941 | 0.0037 | 0 | 0 | 0 | 1 | Ref |
| MK112541 | Unassigned | Unassigned | Unassigned | 579_47 | Clustered | 3 | VC_579_47 | 3 | 0.0064 | 0.78059765 | 0.005 | 0 | 0 | 0 | 1 | Ref |
| MK112542 | Unassigned | Unassigned | Unassigned | 384_16 | Clustered | 42 | VC_384_16 | 42 | 0.134 | 1 | 0.134 | 0 | 0 | 0 | 0.7282 | Ref |
| MK112543 | Unassigned | Unassigned | Unassigned | 532_0 | Clustered | 75 | VC_532_0 | 75 | 0.9393 | 1 | 0.9393 | 0 | 0 | 0 | 1 | Ref |
| MK112544 | Unassigned | Unassigned | Unassigned | 384_12 | Clustered | 4 | VC_384_12 | 4 | 0.0125 | 0.99998491 | 0.0125 | 0 | 0 | 0 | 0.8333 | Ref |
| MK112545 | Unassigned | Unassigned | Unassigned | 579_47 | Clustered | 3 | VC_579_47 | 3 | 0.0064 | 0.78059765 | 0.005 | 0 | 0 | 0 | 1 | Ref |
| MK112546 | Unassigned | Unassigned | Unassigned | 384_11 | Clustered | 31 | VC_384_11 | 31 | 0.0926 | 1 | 0.0926 | 0 | 0 | 0 | 0.7011 | Ref |
| MK112547 | Unassigned | Unassigned | Unassigned | 543_0 | Clustered | 25 | VC_543_0 | 25 | 0.4584 | 1 | 0.4584 | 0 | 0 | 0 | 1 | Ref |
| MK112548 | Unassigned | Unassigned | Unassigned | 579_47 | Clustered | 3 | VC_579_47 | 3 | 0.0064 | 0.78059765 | 0.005 | 0 | 0 | 0 | 1 | Ref |
| MK112549 | Unassigned | Unassigned | Unassigned | 579_34 | Clustered/Singleton | 1 | VC_579_34 | 1 | 0 | 1 | 0 | 0 | 0 | 0 | 1 | Ref |
| MK112550 | Unassigned | Unassigned | Unassigned | 1278_0 | Clustered | 19 | VC_1278_0 | 19 | 1 | 1 | 1 | 0 | 0 | 0 | 1 | Ref |
| MK112551 | Unassigned | Unassigned | Unassigned | 384_11 | Clustered | 31 | VC_384_11 | 31 | 0.0926 | 1 | 0.0926 | 0 | 0 | 0 | 0.7011 | Ref |
| MK112552 | Unassigned | Unassigned | Unassigned | 384_2 | Clustered | 8 | VC_384_2 | 8 | 0.0232 | 1 | 0.0232 | 0 | 0 | 0 | 0.8571 | Ref |
| MK112553 | Unassigned | Unassigned | Unassigned | 384_16 | Clustered | 42 | VC_384_16 | 42 | 0.134 | 1 | 0.134 | 0 | 0 | 0 | 0.7282 | Ref |
| MK112554 | Unassigned | Unassigned | Unassigned | 579_49 | Clustered | 7 | VC_579_49 | 7 | 0.0194 | 0.98914431 | 0.0191 | 0 | 0 | 0 | 0.8571 | Ref |
| MK112555 | Unassigned | Unassigned | Unassigned | 384_15 | Clustered | 44 | VC_384_15 | 44 | 0.139 | 1 | 0.139 | 0 | 0 | 0 | 0.7474 | Ref |
| MK112556 | Unassigned | Unassigned | Unassigned | 538_74 | Clustered | 2 | VC_538_74 | 2 | 0.0075 | 0.95760586 | 0.0072 | 0 | 0 | 0 | 1 | Ref |
| MK112901 | Unassigned | Unassigned | Unassigned | 475_0 | Clustered | 96 | VC_475_0 | 96 | 0.9303 | 1 | 0.9303 | 0 | 0 | 0 | 0.9575 | Ref |
| MK125140 | Unassigned | Unassigned | Unassigned | 711_0 | Clustered | 48 | VC_711_0 | 48 | 0.8217 | 1 | 0.8217 | 0 | 0 | 0 | 1 | Ref |
| MK125141 | Unassigned | Unassigned | Unassigned | 721_0 | Clustered | 11 | VC_721_0 | 11 | 0.8846 | 1 | 0.8846 | 0 | 0 | 0 | 1 | Ref |
| MK134560 | Unassigned | Unassigned | Unassigned | 848_0 | Clustered | 124 | VC_848_0 | 124 | 0.9484 | 1 | 0.9484 | 0 | 0 | 0 | 1 | Ref |
| MK135468 | Unassigned | Unassigned | Unassigned | 113_0 | Clustered | 91 | VC_113_0 | 91 | 0.9584 | 1 | 0.9584 | 0 | 0 | 0 | 1 | Ref |
| MK138526 | Unassigned | Unassigned | Unassigned | 77_0 | Clustered | 37 | VC_77_0 | 37 | 0.9392 | 1 | 0.9392 | 0 | 0 | 0 | 1 | Ref |
| MK138527 | Unassigned | Unassigned | Unassigned | 859_0 | Clustered | 67 | VC_859_0 | 67 | 0.9622 | 1 | 0.9622 | 0 | 0 | 0 | 1 | Ref |
| MK144667 | Unassigned | Unassigned | Unassigned | 185_0 | Clustered | 53 | VC_185_0 | 53 | 0.9153 | 1 | 0.9153 | 0 | 0 | 0 | 1 | Ref |
| MK165087 | Unassigned | Unassigned | Unassigned | 737_0 | Clustered | 13 | VC_737_0 | 13 | 0.5083 | 1 | 0.5083 | 0 | 0 | 0 | 1 | Ref |
| MK165657 | Unassigned | Unassigned | Unassigned | 766_0 | Clustered | 40 | VC_766_0 | 40 | 0.7511 | 1 | 0.7511 | 0 | 0 | 0 | 1 | Ref |
| MK165658 | Unassigned | Unassigned | Unassigned | 766_0 | Clustered | 40 | VC_766_0 | 40 | 0.7511 | 1 | 0.7511 | 0 | 0 | 0 | 1 | Ref |
| MK170160 | Unassigned | Unassigned | Unassigned | 715_0 | Clustered | 10 | VC_715_0 | 10 | 0.8045 | 1 | 0.8045 | 0 | 0 | 0 | 1 | Ref |
| MK170446 | Unassigned | Unassigned | Unassigned | 972_0 | Clustered | 99 | VC_972_0 | 99 | 0.9113 | 1 | 0.9113 | 0 | 0 | 0 | 0.9928 | Ref |
| MK202159 | Unassigned | Unassigned | Unassigned | 261_0 | Clustered | 13 | VC_261_0 | 13 | 0.2024 | 1 | 0.2024 | 0 | 0 | 0 | 1 | Ref |
| MK202160 | Unassigned | Unassigned | Unassigned | 261_0 | Clustered | 13 | VC_261_0 | 13 | 0.2024 | 1 | 0.2024 | 0 | 0 | 0 | 1 | Ref |
| MK202161 | Unassigned | Unassigned | Unassigned | 261_0 | Clustered | 13 | VC_261_0 | 13 | 0.2024 | 1 | 0.2024 | 0 | 0 | 0 | 1 | Ref |
| MK203841 | Unassigned | Unassigned | Unassigned | 848_0 | Clustered | 124 | VC_848_0 | 124 | 0.9484 | 1 | 0.9484 | 0 | 0 | 0 | 1 | Ref |
| MK211557 | Unassigned | Unassigned | Unassigned | 12_2 | Clustered | 19 | VC_12_2 | 19 | 0.2009 | 1 | 0.2009 | 0 | 0 | 0 | 1 | Ref |
| MK213795 | Unassigned | Unassigned | Unassigned | 322_0 | Clustered | 12 | VC_322_0 | 12 | 0.8924 | 1 | 0.8924 | 0 | 0 | 0 | 1 | Ref |
| MK213796 | Unassigned | Unassigned | Unassigned | 595_0 | Clustered | 35 | VC_595_0 | 35 | 0.8809 | 1 | 0.8809 | 0 | 0 | 0 | 1 | Ref |
| MK214385 | Unassigned | Unassigned | Unassigned | 560_0 | Clustered | 15 | VC_560_0 | 15 | 0.2846 | 1 | 0.2846 | 0 | 0 | 0 | 1 | Ref |

| | | | | | | | | | | | | | | | | |
|---|---|---|---|---|---|---|---|---|---|---|---|---|---|---|---|---|
| MK215646 | Unassigned | Unassigned | Unassigned | 912_0 | Clustered | 10 | VC_912_0 | 10 | 0.1994 | 1 | 0.1994 | 0 | 0 | 0 | 1 | Ref |
| MK224497 | Unassigned | Unassigned | Unassigned | 241_0 | Clustered | 21 | VC_241_0 | 21 | 0.8342 | 1 | 0.8342 | 0 | 0 | 0 | 1 | Ref |
| MK224498 | Unassigned | Unassigned | Unassigned | 77_0 | Clustered | 37 | VC_77_0 | 37 | 0.9392 | 1 | 0.9392 | 0 | 0 | 0 | 1 | Ref |
| MK224592 | Unassigned | Unassigned | Unassigned | 538_95 | Clustered/Singleton | 1 | VC_538_95 | 1 | 0 | 1 | 0 | 0 | 0 | 0 | 1 | Ref |
| MK234886 | Unassigned | Unassigned | Unassigned | 297_5 | Clustered | 14 | VC_297_5 | 14 | 0.141 | 1 | 0.141 | 0 | 0 | 0 | 0.9231 | Ref |
| MK238400 | Unassigned | Unassigned | Unassigned | 691_0 | Clustered | 18 | VC_691_0 | 18 | 0.6865 | 1 | 0.6865 | 0 | 0 | 0 | 0.9477 | Ref |
| MK240351 | Unassigned | Unassigned | Unassigned | 510_0 | Clustered | 2 | VC_510_0 | 2 | 0.0158 | 0.67065552 | 0.0106 | 0 | 0 | 0 | 1 | Ref |
| MK240575 | Unassigned | Unassigned | Unassigned | 1414_0 | Clustered | 2 | VC_1414_0 | 2 | 1 | 1 | 1 | 0 | 0 | 0 | 1 | Ref |
| MK249126 | Unassigned | Unassigned | Unassigned | 654_0 | Clustered | 115 | VC_654_0 | 115 | 0.7303 | 1 | 0.7303 | 0 | 0 | 0 | 0.8764 | Ref |
| MK249127 | Unassigned | Unassigned | Unassigned | 1108_0 | Clustered | 52 | VC_1108_0 | 52 | 0.7674 | 1 | 0.7674 | 0 | 0 | 0 | 1 | Ref |
| MK249128 | Unassigned | Unassigned | Unassigned | 1108_0 | Clustered | 52 | VC_1108_0 | 52 | 0.7674 | 1 | 0.7674 | 0 | 0 | 0 | 1 | Ref |
| MK249129 | Unassigned | Unassigned | Unassigned | 1108_0 | Clustered | 52 | VC_1108_0 | 52 | 0.7674 | 1 | 0.7674 | 0 | 0 | 0 | 1 | Ref |
| MK249131 | Unassigned | Unassigned | Unassigned | 1033_0 | Clustered | 21 | VC_1033_0 | 21 | 0.8433 | 0.99999998 | 0.8433 | 0 | 0 | 0 | 1 | Ref |
| MK249132 | Unassigned | Unassigned | Unassigned | 1109_0 | Clustered | 2 | VC_1109_0 | 2 | 0.0119 | 0.85807772 | 0.0102 | 0 | 0 | 0 | 1 | Ref |
| MK249133 | Unassigned | Unassigned | Unassigned | 1104_0 | Clustered | 44 | VC_1104_0 | 44 | 0.9574 | 0.72818579 | 0.6972 | 0 | 0 | 0 | 1 | Ref |
| MK249135 | Unassigned | Unassigned | Unassigned | 1027_0 | Clustered/Singleton | 1 | VC_1027_0 | 1 | 0 | 1 | 0 | 0 | 0 | 0 | 1 | Ref |
| MK249138 | Unassigned | Unassigned | Unassigned | 1104_0 | Clustered | 44 | VC_1104_0 | 44 | 0.9574 | 0.72818579 | 0.6972 | 0 | 0 | 0 | 1 | Ref |
| MK249139 | Unassigned | Unassigned | Unassigned | 1108_0 | Clustered | 52 | VC_1108_0 | 52 | 0.7674 | 1 | 0.7674 | 0 | 0 | 0 | 1 | Ref |
| MK249141 | Unassigned | Unassigned | Unassigned | 1028_0 | Clustered | 69 | VC_1028_0 | 69 | 0.8547 | 1 | 0.8547 | 0 | 0 | 0 | 1 | Ref |
| MK249143 | Unassigned | Unassigned | Unassigned | 411_0 | Clustered | 48 | VC_411_0 | 48 | 0.4808 | 1 | 0.4808 | 0 | 0 | 0 | 1 | Ref |
| MK249144 | Unassigned | Unassigned | Unassigned | 1033_0 | Clustered | 21 | VC_1033_0 | 21 | 0.8433 | 0.99999998 | 0.8433 | 0 | 0 | 0 | 1 | Ref |
| MK249147 | Unassigned | Unassigned | Unassigned | 1028_0 | Clustered | 69 | VC_1028_0 | 69 | 0.8547 | 1 | 0.8547 | 0 | 0 | 0 | 1 | Ref |
| MK249148 | Unassigned | Unassigned | Unassigned | 1322_0 | Clustered | 6 | VC_1322_0 | 6 | 0.7063 | 0.92055725 | 0.6502 | 0 | 0 | 0 | 1 | Ref |
| MK249149 | Unassigned | Unassigned | Unassigned | 1108_0 | Clustered | 52 | VC_1108_0 | 52 | 0.7674 | 1 | 0.7674 | 0 | 0 | 0 | 1 | Ref |
| MK249150 | Unassigned | Unassigned | Unassigned | 1108_0 | Clustered | 52 | VC_1108_0 | 52 | 0.7674 | 1 | 0.7674 | 0 | 0 | 0 | 1 | Ref |
| MK249151 | Unassigned | Unassigned | Unassigned | 411_0 | Clustered | 48 | VC_411_0 | 48 | 0.4808 | 1 | 0.4808 | 0 | 0 | 0 | 1 | Ref |
| MK249153 | Unassigned | Unassigned | Unassigned | 1108_0 | Clustered | 52 | VC_1108_0 | 52 | 0.7674 | 1 | 0.7674 | 0 | 0 | 0 | 1 | Ref |
| MK249154 | Unassigned | Unassigned | Unassigned | 1108_0 | Clustered | 52 | VC_1108_0 | 52 | 0.7674 | 1 | 0.7674 | 0 | 0 | 0 | 1 | Ref |
| MK249155 | Unassigned | Unassigned | Unassigned | 1108_0 | Clustered | 52 | VC_1108_0 | 52 | 0.7674 | 1 | 0.7674 | 0 | 0 | 0 | 1 | Ref |
| MK249156 | Unassigned | Unassigned | Unassigned | 1033_0 | Clustered | 21 | VC_1033_0 | 21 | 0.8433 | 0.99999998 | 0.8433 | 0 | 0 | 0 | 1 | Ref |
| MK249158 | Unassigned | Unassigned | Unassigned | 1108_0 | Clustered | 52 | VC_1108_0 | 52 | 0.7674 | 1 | 0.7674 | 0 | 0 | 0 | 1 | Ref |
| MK249159 | Unassigned | Unassigned | Unassigned | 1033_0 | Clustered | 21 | VC_1033_0 | 21 | 0.8433 | 0.99999998 | 0.8433 | 0 | 0 | 0 | 1 | Ref |
| MK249160 | Unassigned | Unassigned | Unassigned | 1033_0 | Clustered | 21 | VC_1033_0 | 21 | 0.8433 | 0.99999998 | 0.8433 | 0 | 0 | 0 | 1 | Ref |
| MK249162 | Unassigned | Unassigned | Unassigned | 1108_0 | Clustered | 52 | VC_1108_0 | 52 | 0.7674 | 1 | 0.7674 | 0 | 0 | 0 | 1 | Ref |
| MK249163 | Unassigned | Unassigned | Unassigned | 1108_0 | Clustered | 52 | VC_1108_0 | 52 | 0.7674 | 1 | 0.7674 | 0 | 0 | 0 | 1 | Ref |
| MK249165 | Unassigned | Unassigned | Unassigned | 1028_0 | Clustered | 69 | VC_1028_0 | 69 | 0.8547 | 1 | 0.8547 | 0 | 0 | 0 | 1 | Ref |
| MK249166 | Unassigned | Unassigned | Unassigned | 1108_0 | Clustered | 52 | VC_1108_0 | 52 | 0.7674 | 1 | 0.7674 | 0 | 0 | 0 | 1 | Ref |
| MK249167 | Unassigned | Unassigned | Unassigned | 1033_0 | Clustered | 21 | VC_1033_0 | 21 | 0.8433 | 0.99999998 | 0.8433 | 0 | 0 | 0 | 1 | Ref |
| MK249168 | Unassigned | Unassigned | Unassigned | 1108_0 | Clustered | 52 | VC_1108_0 | 52 | 0.7674 | 1 | 0.7674 | 0 | 0 | 0 | 1 | Ref |
| MK249174 | Unassigned | Unassigned | Unassigned | 1108_0 | Clustered | 52 | VC_1108_0 | 52 | 0.7674 | 1 | 0.7674 | 0 | 0 | 0 | 1 | Ref |
| MK249176 | Unassigned | Unassigned | Unassigned | 1108_0 | Clustered | 52 | VC_1108_0 | 52 | 0.7674 | 1 | 0.7674 | 0 | 0 | 0 | 1 | Ref |
| MK249178 | Unassigned | Unassigned | Unassigned | 1108_0 | Clustered | 52 | VC_1108_0 | 52 | 0.7674 | 1 | 0.7674 | 0 | 0 | 0 | 1 | Ref |
| MK249179 | Unassigned | Unassigned | Unassigned | 1033_0 | Clustered | 21 | VC_1033_0 | 21 | 0.8433 | 0.99999998 | 0.8433 | 0 | 0 | 0 | 1 | Ref |
| MK249180 | Unassigned | Unassigned | Unassigned | 1108_0 | Clustered | 52 | VC_1108_0 | 52 | 0.7674 | 1 | 0.7674 | 0 | 0 | 0 | 1 | Ref |
| MK249181 | Unassigned | Unassigned | Unassigned | 1033_0 | Clustered | 21 | VC_1033_0 | 21 | 0.8433 | 0.99999998 | 0.8433 | 0 | 0 | 0 | 1 | Ref |
| MK249185 | Unassigned | Unassigned | Unassigned | 1028_0 | Clustered | 69 | VC_1028_0 | 69 | 0.8547 | 1 | 0.8547 | 0 | 0 | 0 | 1 | Ref |
| MK249188 | Unassigned | Unassigned | Unassigned | 1108_0 | Clustered | 52 | VC_1108_0 | 52 | 0.7674 | 1 | 0.7674 | 0 | 0 | 0 | 1 | Ref |
| MK249189 | Unassigned | Unassigned | Unassigned | 1032_0 | Clustered | 2 | VC_1032_0 | 2 | 0.0274 | 0.73989325 | 0.0202 | 0 | 0 | 0 | 1 | Ref |
| MK249190 | Unassigned | Unassigned | Unassigned | 1028_0 | Clustered | 69 | VC_1028_0 | 69 | 0.8547 | 1 | 0.8547 | 0 | 0 | 0 | 1 | Ref |
| MK249191 | Unassigned | Unassigned | Unassigned | 1110_0 | Clustered | 2 | VC_1110_0 | 2 | 0.0455 | 0.92554266 | 0.0422 | 0 | 0 | 0 | 1 | Ref |
| MK249192 | Unassigned | Unassigned | Unassigned | 1108_0 | Clustered | 52 | VC_1108_0 | 52 | 0.7674 | 1 | 0.7674 | 0 | 0 | 0 | 1 | Ref |
| MK249194 | Unassigned | Unassigned | Unassigned | 1108_0 | Clustered | 52 | VC_1108_0 | 52 | 0.7674 | 1 | 0.7674 | 0 | 0 | 0 | 1 | Ref |
| MK249195 | Unassigned | Unassigned | Unassigned | 1108_0 | Clustered | 52 | VC_1108_0 | 52 | 0.7674 | 1 | 0.7674 | 0 | 0 | 0 | 1 | Ref |
| MK249196 | Unassigned | Unassigned | Unassigned | 1108_0 | Clustered | 52 | VC_1108_0 | 52 | 0.7674 | 1 | 0.7674 | 0 | 0 | 0 | 1 | Ref |
| MK249197 | Unassigned | Unassigned | Unassigned | 1028_0 | Clustered | 69 | VC_1028_0 | 69 | 0.8547 | 1 | 0.8547 | 0 | 0 | 0 | 1 | Ref |
| MK249198 | Unassigned | Unassigned | Unassigned | 1108_0 | Clustered | 52 | VC_1108_0 | 52 | 0.7674 | 1 | 0.7674 | 0 | 0 | 0 | 1 | Ref |
| MK249199 | Unassigned | Unassigned | Unassigned | 1104_0 | Clustered | 44 | VC_1104_0 | 44 | 0.9574 | 0.72818579 | 0.6972 | 0 | 0 | 0 | 1 | Ref |
| MK249201 | Unassigned | Unassigned | Unassigned | 1028_0 | Clustered | 69 | VC_1028_0 | 69 | 0.8547 | 1 | 0.8547 | 0 | 0 | 0 | 1 | Ref |
| MK249202 | Unassigned | Unassigned | Unassigned | 1028_0 | Clustered | 69 | VC_1028_0 | 69 | 0.8547 | 1 | 0.8547 | 0 | 0 | 0 | 1 | Ref |
| MK249203 | Unassigned | Unassigned | Unassigned | 1028_0 | Clustered | 69 | VC_1028_0 | 69 | 0.8547 | 1 | 0.8547 | 0 | 0 | 0 | 1 | Ref |
| MK249204 | Unassigned | Unassigned | Unassigned | 1108_0 | Clustered | 52 | VC_1108_0 | 52 | 0.7674 | 1 | 0.7674 | 0 | 0 | 0 | 1 | Ref |
| MK249205 | Unassigned | Unassigned | Unassigned | 1109_0 | Clustered | 2 | VC_1109_0 | 2 | 0.0119 | 0.85807772 | 0.0102 | 0 | 0 | 0 | 1 | Ref |
| MK249207 | Unassigned | Unassigned | Unassigned | 1104_0 | Clustered | 44 | VC_1104_0 | 44 | 0.9574 | 0.72818579 | 0.6972 | 0 | 0 | 0 | 1 | Ref |
| MK249208 | Unassigned | Unassigned | Unassigned | 1104_0 | Clustered | 44 | VC_1104_0 | 44 | 0.9574 | 0.72818579 | 0.6972 | 0 | 0 | 0 | 1 | Ref |
| MK249211 | Unassigned | Unassigned | Unassigned | 1028_0 | Clustered | 69 | VC_1028_0 | 69 | 0.8547 | 1 | 0.8547 | 0 | 0 | 0 | 1 | Ref |
| MK249212 | Unassigned | Unassigned | Unassigned | 1108_0 | Clustered | 52 | VC_1108_0 | 52 | 0.7674 | 1 | 0.7674 | 0 | 0 | 0 | 1 | Ref |
| MK249213 | Unassigned | Unassigned | Unassigned | 1028_0 | Clustered | 69 | VC_1028_0 | 69 | 0.8547 | 1 | 0.8547 | 0 | 0 | 0 | 1 | Ref |
| MK249214 | Unassigned | Unassigned | Unassigned | 1108_0 | Clustered | 52 | VC_1108_0 | 52 | 0.7674 | 1 | 0.7674 | 0 | 0 | 0 | 1 | Ref |
| MK249215 | Unassigned | Unassigned | Unassigned | 1129_0 | Clustered | 5 | VC_1129_0 | 5 | 0.8077 | 0.98759579 | 0.7976 | 0 | 0 | 0 | 1 | Ref |
| MK249216 | Unassigned | Unassigned | Unassigned | 1429_0 | Clustered | 3 | VC_1429_0 | 3 | 1 | 1 | 1 | 0 | 0 | 0 | 1 | Ref |
| MK249217 | Unassigned | Unassigned | Unassigned | 1429_0 | Clustered | 3 | VC_1429_0 | 3 | 1 | 1 | 1 | 0 | 0 | 0 | 1 | Ref |
| MK249218 | Unassigned | Unassigned | Unassigned | 1402_0 | Clustered | 7 | VC_1402_0 | 7 | 0.9174 | 0.99772305 | 0.9153 | 0 | 0 | 0 | 1 | Ref |
| MK249219 | Unassigned | Unassigned | Unassigned | 1108_0 | Clustered | 52 | VC_1108_0 | 52 | 0.7674 | 1 | 0.7674 | 0 | 0 | 0 | 1 | Ref |
| MK249221 | Unassigned | Unassigned | Unassigned | 1028_0 | Clustered | 69 | VC_1028_0 | 69 | 0.8547 | 1 | 0.8547 | 0 | 0 | 0 | 1 | Ref |
| MK249222 | Unassigned | Unassigned | Unassigned | 1111_0 | Clustered | 5 | VC_1111_0 | 5 | 1 | 1 | 1 | 0 | 0 | 0 | 1 | Ref |
| MK249223 | Unassigned | Unassigned | Unassigned | 411_0 | Clustered | 48 | VC_411_0 | 48 | 0.4808 | 1 | 0.4808 | 0 | 0 | 0 | 1 | Ref |
| MK249228 | Unassigned | Unassigned | Unassigned | 1104_0 | Clustered | 44 | VC_1104_0 | 44 | 0.9574 | 0.72818579 | 0.6972 | 0 | 0 | 0 | 1 | Ref |
| MK249229 | Unassigned | Unassigned | Unassigned | 1402_0 | Clustered | 7 | VC_1402_0 | 7 | 0.9174 | 0.99772305 | 0.9153 | 0 | 0 | 0 | 1 | Ref |
| MK249231 | Unassigned | Unassigned | Unassigned | 411_0 | Clustered | 48 | VC_411_0 | 48 | 0.4808 | 1 | 0.4808 | 0 | 0 | 0 | 1 | Ref |
| MK249232 | Unassigned | Unassigned | Unassigned | 1104_0 | Clustered | 44 | VC_1104_0 | 44 | 0.9574 | 0.72818579 | 0.6972 | 0 | 0 | 0 | 1 | Ref |
| MK249233 | Unassigned | Unassigned | Unassigned | 1402_0 | Clustered | 7 | VC_1402_0 | 7 | 0.9174 | 0.99772305 | 0.9153 | 0 | 0 | 0 | 1 | Ref |

| | | | | | | | | | | | | | | | | |
|---|---|---|---|---|---|---|---|---|---|---|---|---|---|---|---|---|
| MK249308 | Unassigned | Unassigned | Unassigned | 611_0 | Clustered | 50 | VC_611_0 | 50 | 0.7306 | 1 | 0.7306 | 0 | 0 | 0 | 1 | Ref |
| MK249872 | Unassigned | Unassigned | Unassigned | 662_0 | Clustered | 2 | VC_662_0 | 2 | 0.1082 | 0.95085347 | 0.1029 | 0 | 0 | 0 | 1 | Ref |
| MK250015 | Unassigned | Unassigned | Unassigned | 1257_2 | Clustered | 4 | VC_1257_2 | 4 | 0.1181 | 0.96596457 | 0.1141 | 0 | 0 | 0 | 1 | Ref |
| MK250016 | Unassigned | Unassigned | Unassigned | 1257_2 | Clustered | 4 | VC_1257_2 | 4 | 0.1181 | 0.96596457 | 0.1141 | 0 | 0 | 0 | 1 | Ref |
| MK250017 | Unassigned | Unassigned | Unassigned | 1257_2 | Clustered | 4 | VC_1257_2 | 4 | 0.1181 | 0.96596457 | 0.1141 | 0 | 0 | 0 | 1 | Ref |
| MK250018 | Unassigned | Unassigned | Unassigned | 1257_2 | Clustered | 4 | VC_1257_2 | 4 | 0.1181 | 0.96596457 | 0.1141 | 0 | 0 | 0 | 1 | Ref |
| MK250019 | Unassigned | Unassigned | Unassigned | 1257_3 | Clustered | 2 | VC_1257_3 | 2 | 0.0365 | 0.74212829 | 0.0271 | 0 | 0 | 0 | 0 | Ref |
| MK250020 | Unassigned | Unassigned | Unassigned | 1257_1 | Clustered | 7 | VC_1257_1 | 7 | 0.2689 | 0.99956545 | 0.2688 | 0 | 0 | 0 | 1 | Ref |
| MK250021 | Unassigned | Unassigned | Unassigned | 1257_0 | Clustered | 2 | VC_1257_0 | 2 | 0.0366 | 0.70502008 | 0.0258 | 0 | 0 | 0 | 1 | Ref |
| MK250022 | Unassigned | Unassigned | Unassigned | 1257_1 | Clustered | 7 | VC_1257_1 | 7 | 0.2689 | 0.99956545 | 0.2688 | 0 | 0 | 0 | 1 | Ref |
| MK250023 | Unassigned | Unassigned | Unassigned | 1257_0 | Clustered | 2 | VC_1257_0 | 2 | 0.0366 | 0.70502008 | 0.0258 | 0 | 0 | 0 | 1 | Ref |
| MK250024 | Unassigned | Unassigned | Unassigned | 1257_1 | Clustered | 7 | VC_1257_1 | 7 | 0.2689 | 0.99956545 | 0.2688 | 0 | 0 | 0 | 1 | Ref |
| MK250025 | Unassigned | Unassigned | Unassigned | 1257_1 | Clustered | 7 | VC_1257_1 | 7 | 0.2689 | 0.99956545 | 0.2688 | 0 | 0 | 0 | 1 | Ref |
| MK250026 | Unassigned | Unassigned | Unassigned | 1257_1 | Clustered | 7 | VC_1257_1 | 7 | 0.2689 | 0.99956545 | 0.2688 | 0 | 0 | 0 | 1 | Ref |
| MK250027 | Unassigned | Unassigned | Unassigned | 1257_1 | Clustered | 7 | VC_1257_1 | 7 | 0.2689 | 0.99956545 | 0.2688 | 0 | 0 | 0 | 1 | Ref |
| MK250028 | Unassigned | Unassigned | Unassigned | 1257_1 | Clustered | 7 | VC_1257_1 | 7 | 0.2689 | 0.99956545 | 0.2688 | 0 | 0 | 0 | 1 | Ref |
| MK250029 | Unassigned | Unassigned | Unassigned | 1257_3 | Clustered | 2 | VC_1257_3 | 2 | 0.0365 | 0.74212829 | 0.0271 | 0 | 0 | 0 | 0 | Ref |
| MK250904 | Unassigned | Unassigned | Unassigned | 155_15 | Clustered | 16 | VC_155_15 | 16 | 0.0779 | 1 | 0.0779 | 0 | 0 | 0 | 0.8083 | Ref |
| MK257719 | Unassigned | Unassigned | Unassigned | 714_0 | Clustered | 71 | VC_714_0 | 71 | 0.935 | 1 | 0.935 | 0 | 0 | 0 | 1 | Ref |
| MK257720 | Unassigned | Unassigned | Unassigned | 714_0 | Clustered | 71 | VC_714_0 | 71 | 0.935 | 1 | 0.935 | 0 | 0 | 0 | 1 | Ref |
| MK257721 | Unassigned | Unassigned | Unassigned | 714_0 | Clustered | 71 | VC_714_0 | 71 | 0.935 | 1 | 0.935 | 0 | 0 | 0 | 1 | Ref |
| MK257722 | Unassigned | Unassigned | Unassigned | 714_0 | Clustered | 71 | VC_714_0 | 71 | 0.935 | 1 | 0.935 | 0 | 0 | 0 | 1 | Ref |
| MK257723 | Unassigned | Unassigned | Unassigned | 714_0 | Clustered | 71 | VC_714_0 | 71 | 0.935 | 1 | 0.935 | 0 | 0 | 0 | 1 | Ref |
| MK257744 | Unassigned | Unassigned | Unassigned | 493_0 | Clustered | 4 | VC_493_0 | 4 | 0.9871 | 0.99748973 | 0.9847 | 0 | 0 | 0 | 1 | Ref |
| MK268344 | Unassigned | Unassigned | Unassigned | 170_0 | Clustered | 3 | VC_170_0 | 3 | 0.0298 | 0.82983726 | 0.0247 | 0 | 0 | 0 | 1 | Ref |
| MK268686 | Unassigned | Unassigned | Unassigned | 93_0 | Clustered | 22 | VC_93_0 | 22 | 0.5285 | 1 | 0.5285 | 0 | 0 | 0 | 1 | Ref |
| MK278859 | Unassigned | Unassigned | Unassigned | 714_0 | Clustered | 71 | VC_714_0 | 71 | 0.935 | 1 | 0.935 | 0 | 0 | 0 | 1 | Ref |
| MK278860 | Unassigned | Unassigned | Unassigned | 510_2 | Clustered | 8 | VC_510_2 | 8 | 0.122 | 0.98786574 | 0.1205 | 0 | 0 | 0 | 1 | Ref |
| MK278861 | Unassigned | Unassigned | Unassigned | 972_0 | Clustered | 99 | VC_972_0 | 99 | 0.9113 | 1 | 0.9113 | 0 | 0 | 0 | 0.9928 | Ref |
| MK279840 | Unassigned | Unassigned | Unassigned | 594_0 | Clustered | 7 | VC_594_0 | 7 | 0.2725 | 1 | 0.2725 | 0 | 0 | 0 | 1 | Ref |
| MK279841 | Unassigned | Unassigned | Unassigned | 743_0 | Clustered | 3 | VC_743_0 | 3 | 0.3955 | 0.99850259 | 0.3949 | 0 | 0 | 0 | 1 | Ref |
| MK279842 | Unassigned | Unassigned | Unassigned | 973_0 | Clustered | 15 | VC_973_0 | 15 | 1 | 0 | 0 | 0 | 0 | 0 | 1 | Ref |
| MK279843 | Unassigned | Unassigned | Unassigned | 384_11 | Clustered | 31 | VC_384_11 | 31 | 0.0926 | 1 | 0.0926 | 0 | 0 | 0 | 0.7011 | Ref |
| MK279844 | Unassigned | Unassigned | Unassigned | 1200_0 | Clustered | 73 | VC_1200_0 | 73 | 0.9825 | 1 | 0.9825 | 0 | 0 | 0 | 1 | Ref |
| MK279845 | Unassigned | Unassigned | Unassigned | 1200_0 | Clustered | 73 | VC_1200_0 | 73 | 0.9825 | 1 | 0.9825 | 0 | 0 | 0 | 1 | Ref |
| MK279846 | Unassigned | Unassigned | Unassigned | 384_16 | Clustered | 42 | VC_384_16 | 42 | 0.134 | 1 | 0.134 | 0 | 0 | 0 | 0.7282 | Ref |
| MK279847 | Unassigned | Unassigned | Unassigned | 1200_0 | Clustered | 73 | VC_1200_0 | 73 | 0.9825 | 1 | 0.9825 | 0 | 0 | 0 | 1 | Ref |
| MK279849 | Unassigned | Unassigned | Unassigned | 566_7 | Clustered | 4 | VC_566_7 | 4 | 0.0409 | 0.99661604 | 0.0408 | 0 | 0 | 0 | 1 | Ref |
| MK279850 | Unassigned | Unassigned | Unassigned | 384_13 | Clustered | 33 | VC_384_13 | 33 | 0.1785 | 1 | 0.1785 | 0 | 0 | 0 | 0.8769 | Ref |
| MK279851 | Unassigned | Unassigned | Unassigned | 1200_0 | Clustered | 73 | VC_1200_0 | 73 | 0.9825 | 1 | 0.9825 | 0 | 0 | 0 | 1 | Ref |
| MK279852 | Unassigned | Unassigned | Unassigned | 384_16 | Clustered | 42 | VC_384_16 | 42 | 0.134 | 1 | 0.134 | 0 | 0 | 0 | 0.7282 | Ref |
| MK279853 | Unassigned | Unassigned | Unassigned | 1139_0 | Clustered | 5 | VC_1139_0 | 5 | 0.8556 | 0.99869977 | 0.8545 | 0 | 0 | 0 | 1 | Ref |
| MK279854 | Unassigned | Unassigned | Unassigned | 1200_0 | Clustered | 73 | VC_1200_0 | 73 | 0.9825 | 1 | 0.9825 | 0 | 0 | 0 | 1 | Ref |
| MK279855 | Unassigned | Unassigned | Unassigned | 384_15 | Clustered | 44 | VC_384_15 | 44 | 0.139 | 1 | 0.139 | 0 | 0 | 0 | 0.7474 | Ref |
| MK279856 | Unassigned | Unassigned | Unassigned | 1200_0 | Clustered | 73 | VC_1200_0 | 73 | 0.9825 | 1 | 0.9825 | 0 | 0 | 0 | 1 | Ref |
| MK279857 | Unassigned | Unassigned | Unassigned | 224_0 | Clustered | 5 | VC_224_0 | 5 | 0.0685 | 0.99999998 | 0.0685 | 0 | 0 | 0 | 1 | Ref |
| MK279858 | Unassigned | Unassigned | Unassigned | 384_13 | Clustered | 33 | VC_384_13 | 33 | 0.1785 | 1 | 0.1785 | 0 | 0 | 0 | 0.8769 | Ref |
| MK279859 | Unassigned | Unassigned | Unassigned | 384_15 | Clustered | 44 | VC_384_15 | 44 | 0.139 | 1 | 0.139 | 0 | 0 | 0 | 0.7474 | Ref |
| MK279860 | Unassigned | Unassigned | Unassigned | 1200_0 | Clustered | 73 | VC_1200_0 | 73 | 0.9825 | 1 | 0.9825 | 0 | 0 | 0 | 1 | Ref |
| MK279861 | Unassigned | Unassigned | Unassigned | 384_15 | Clustered | 44 | VC_384_15 | 44 | 0.139 | 1 | 0.139 | 0 | 0 | 0 | 0.7474 | Ref |
| MK279862 | Unassigned | Unassigned | Unassigned | 1200_0 | Clustered | 73 | VC_1200_0 | 73 | 0.9825 | 1 | 0.9825 | 0 | 0 | 0 | 1 | Ref |
| MK279863 | Unassigned | Unassigned | Unassigned | 1200_0 | Clustered | 73 | VC_1200_0 | 73 | 0.9825 | 1 | 0.9825 | 0 | 0 | 0 | 1 | Ref |
| MK279864 | Unassigned | Unassigned | Unassigned | 384_13 | Clustered | 33 | VC_384_13 | 33 | 0.1785 | 1 | 0.1785 | 0 | 0 | 0 | 0.8769 | Ref |
| MK279865 | Unassigned | Unassigned | Unassigned | 384_11 | Clustered | 31 | VC_384_11 | 31 | 0.0926 | 1 | 0.0926 | 0 | 0 | 0 | 0.7011 | Ref |
| MK279866 | Unassigned | Unassigned | Unassigned | 384_15 | Clustered | 44 | VC_384_15 | 44 | 0.139 | 1 | 0.139 | 0 | 0 | 0 | 0.7474 | Ref |
| MK279867 | Unassigned | Unassigned | Unassigned | 1200_0 | Clustered | 73 | VC_1200_0 | 73 | 0.9825 | 1 | 0.9825 | 0 | 0 | 0 | 1 | Ref |
| MK279868 | Unassigned | Unassigned | Unassigned | 1200_0 | Clustered | 73 | VC_1200_0 | 73 | 0.9825 | 1 | 0.9825 | 0 | 0 | 0 | 1 | Ref |
| MK279869 | Unassigned | Unassigned | Unassigned | 1200_0 | Clustered | 73 | VC_1200_0 | 73 | 0.9825 | 1 | 0.9825 | 0 | 0 | 0 | 1 | Ref |
| MK279870 | Unassigned | Unassigned | Unassigned | 384_11 | Clustered | 31 | VC_384_11 | 31 | 0.0926 | 1 | 0.0926 | 0 | 0 | 0 | 0.7011 | Ref |
| MK279871 | Unassigned | Unassigned | Unassigned | 384_16 | Clustered | 42 | VC_384_16 | 42 | 0.134 | 1 | 0.134 | 0 | 0 | 0 | 0.7282 | Ref |
| MK279872 | Unassigned | Unassigned | Unassigned | 1200_0 | Clustered | 73 | VC_1200_0 | 73 | 0.9825 | 1 | 0.9825 | 0 | 0 | 0 | 1 | Ref |
| MK279873 | Unassigned | Unassigned | Unassigned | 384_11 | Clustered | 31 | VC_384_11 | 31 | 0.0926 | 1 | 0.0926 | 0 | 0 | 0 | 0.7011 | Ref |
| MK279874 | Unassigned | Unassigned | Unassigned | 1200_0 | Clustered | 73 | VC_1200_0 | 73 | 0.9825 | 1 | 0.9825 | 0 | 0 | 0 | 1 | Ref |
| MK279875 | Unassigned | Unassigned | Unassigned | 1200_0 | Clustered | 73 | VC_1200_0 | 73 | 0.9825 | 1 | 0.9825 | 0 | 0 | 0 | 1 | Ref |
| MK279876 | Unassigned | Unassigned | Unassigned | 1200_0 | Clustered | 73 | VC_1200_0 | 73 | 0.9825 | 1 | 0.9825 | 0 | 0 | 0 | 1 | Ref |
| MK279877 | Unassigned | Unassigned | Unassigned | 384_3 | Clustered | 22 | VC_384_3 | 22 | 0.0786 | 1 | 0.0786 | 0 | 0 | 0 | 0.9048 | Ref |
| MK279878 | Unassigned | Unassigned | Unassigned | 384_11 | Clustered | 31 | VC_384_11 | 31 | 0.0926 | 1 | 0.0926 | 0 | 0 | 0 | 0.7011 | Ref |
| MK279879 | Unassigned | Unassigned | Unassigned | 384_15 | Clustered | 44 | VC_384_15 | 44 | 0.139 | 1 | 0.139 | 0 | 0 | 0 | 0.7474 | Ref |
| MK279880 | Unassigned | Unassigned | Unassigned | 1200_0 | Clustered | 73 | VC_1200_0 | 73 | 0.9825 | 1 | 0.9825 | 0 | 0 | 0 | 1 | Ref |
| MK279881 | Unassigned | Unassigned | Unassigned | 384_15 | Clustered | 44 | VC_384_15 | 44 | 0.139 | 1 | 0.139 | 0 | 0 | 0 | 0.7474 | Ref |
| MK279882 | Unassigned | Unassigned | Unassigned | 384_15 | Clustered | 44 | VC_384_15 | 44 | 0.139 | 1 | 0.139 | 0 | 0 | 0 | 0.7474 | Ref |
| MK279883 | Unassigned | Unassigned | Unassigned | 384_13 | Clustered | 33 | VC_384_13 | 33 | 0.1785 | 1 | 0.1785 | 0 | 0 | 0 | 0.8769 | Ref |
| MK279884 | Unassigned | Unassigned | Unassigned | 1200_0 | Clustered | 73 | VC_1200_0 | 73 | 0.9825 | 1 | 0.9825 | 0 | 0 | 0 | 1 | Ref |
| MK279885 | Unassigned | Unassigned | Unassigned | 384_15 | Clustered | 44 | VC_384_15 | 44 | 0.139 | 1 | 0.139 | 0 | 0 | 0 | 0.7474 | Ref |
| MK279886 | Unassigned | Unassigned | Unassigned | 1200_0 | Clustered | 73 | VC_1200_0 | 73 | 0.9825 | 1 | 0.9825 | 0 | 0 | 0 | 1 | Ref |
| MK279887 | Unassigned | Unassigned | Unassigned | 384_15 | Clustered | 44 | VC_384_15 | 44 | 0.139 | 1 | 0.139 | 0 | 0 | 0 | 0.7474 | Ref |
| MK279888 | Unassigned | Unassigned | Unassigned | 384_15 | Clustered | 44 | VC_384_15 | 44 | 0.139 | 1 | 0.139 | 0 | 0 | 0 | 0.7474 | Ref |
| MK279889 | Unassigned | Unassigned | Unassigned | 384_2 | Clustered | 8 | VC_384_2 | 8 | 0.0232 | 1 | 0.0232 | 0 | 0 | 0 | 0.8571 | Ref |
| MK279890 | Unassigned | Unassigned | Unassigned | 384_13 | Clustered | 33 | VC_384_13 | 33 | 0.1785 | 1 | 0.1785 | 0 | 0 | 0 | 0.8769 | Ref |
| MK279891 | Unassigned | Unassigned | Unassigned | 384_13 | Clustered | 33 | VC_384_13 | 33 | 0.1785 | 1 | 0.1785 | 0 | 0 | 0 | 0.8769 | Ref |
| MK279892 | Unassigned | Unassigned | Unassigned | 1200_0 | Clustered | 73 | VC_1200_0 | 73 | 0.9825 | 1 | 0.9825 | 0 | 0 | 0 | 1 | Ref |

| | | | | | | | | | | | | | | | | |
|---|---|---|---|---|---|---|---|---|---|---|---|---|---|---|---|---|
| MK279893 | Unassigned | Unassigned | Unassigned | 989_0 | Clustered | 13 | VC_989_0 | 13 | 0.8622 | 1 | 0.8622 | 0 | 0 | 0 | 1 | Ref |
| MK279894 | Unassigned | Unassigned | Unassigned | 384_11 | Clustered | 31 | VC_384_11 | 31 | 0.0926 | 1 | 0.0926 | 0 | 0 | 0 | 0.7011 | Ref |
| MK279895 | Unassigned | Unassigned | Unassigned | 384_11 | Clustered | 31 | VC_384_11 | 31 | 0.0926 | 1 | 0.0926 | 0 | 0 | 0 | 0.7011 | Ref |
| MK279896 | Unassigned | Unassigned | Unassigned | 1200_0 | Clustered | 73 | VC_1200_0 | 73 | 0.9825 | 1 | 0.9825 | 0 | 0 | 0 | 1 | Ref |
| MK279897 | Unassigned | Unassigned | Unassigned | 384_11 | Clustered | 31 | VC_384_11 | 31 | 0.0926 | 1 | 0.0926 | 0 | 0 | 0 | 0.7011 | Ref |
| MK279898 | Unassigned | Unassigned | Unassigned | 535_0 | Clustered | 18 | VC_535_0 | 18 | 0.1595 | 1 | 0.1595 | 0 | 0 | 0 | 1 | Ref |
| MK279900 | Unassigned | Unassigned | Unassigned | 952_0 | Clustered | 39 | VC_952_0 | 39 | 0.9064 | 1 | 0.9064 | 0 | 0 | 0 | 1 | Ref |
| MK279901 | Unassigned | Unassigned | Unassigned | 1200_0 | Clustered | 73 | VC_1200_0 | 73 | 0.9825 | 1 | 0.9825 | 0 | 0 | 0 | 1 | Ref |
| MK279902 | Unassigned | Unassigned | Unassigned | 535_1 | Clustered | 108 | VC_535_1 | 108 | 0.8479 | 1 | 0.8479 | 0 | 0 | 0 | 0.9955 | Ref |
| MK279903 | Unassigned | Unassigned | Unassigned | 532_0 | Clustered | 75 | VC_532_0 | 75 | 0.9393 | 1 | 0.9393 | 0 | 0 | 0 | 1 | Ref |
| MK279904 | Unassigned | Unassigned | Unassigned | 384_4 | Clustered | 5 | VC_384_4 | 5 | 0.0134 | 0.99999971 | 0.0134 | 0 | 0 | 0 | 1 | Ref |
| MK279906 | Unassigned | Unassigned | Unassigned | 578_1 | Clustered | 11 | VC_578_1 | 11 | 0.4919 | 1 | 0.4919 | 0 | 0 | 0 | 0.7818 | Ref |
| MK279907 | Unassigned | Unassigned | Unassigned | 1200_0 | Clustered | 73 | VC_1200_0 | 73 | 0.9825 | 1 | 0.9825 | 0 | 0 | 0 | 1 | Ref |
| MK279908 | Unassigned | Unassigned | Unassigned | 384_3 | Clustered | 22 | VC_384_3 | 22 | 0.0786 | 1 | 0.0786 | 0 | 0 | 0 | 0.9048 | Ref |
| MK279909 | Unassigned | Unassigned | Unassigned | 586_0 | Clustered | 2 | VC_586_0 | 2 | 0.098 | 0.95623977 | 0.0937 | 0 | 0 | 0 | 1 | Ref |
| MK279910 | Unassigned | Unassigned | Unassigned | 384_1 | Clustered | 11 | VC_384_1 | 11 | 0.0378 | 1 | 0.0378 | 0 | 0 | 0 | 0.8545 | Ref |
| MK279911 | Unassigned | Unassigned | Unassigned | 1216_0 | Clustered | 22 | VC_1216_0 | 22 | 0.4546 | 1 | 0.4546 | 0 | 0 | 0 | 1 | Ref |
| MK279912 | Unassigned | Unassigned | Unassigned | 1216_0 | Clustered | 22 | VC_1216_0 | 22 | 0.4546 | 1 | 0.4546 | 0 | 0 | 0 | 1 | Ref |
| MK279913 | Unassigned | Unassigned | Unassigned | 532_0 | Clustered | 75 | VC_532_0 | 75 | 0.9393 | 1 | 0.9393 | 0 | 0 | 0 | 1 | Ref |
| MK284518 | Unassigned | Unassigned | Unassigned | 532_0 | Clustered | 75 | VC_532_0 | 75 | 0.9393 | 1 | 0.9393 | 0 | 0 | 0 | 1 | Ref |
| MK284519 | Unassigned | Unassigned | Unassigned | 531_0 | Clustered | 32 | VC_531_0 | 32 | 0.6859 | 1 | 0.6859 | 0 | 0 | 0 | 1 | Ref |
| MK284520 | Unassigned | Unassigned | Unassigned | 701_0 | Clustered | 16 | VC_701_0 | 16 | 0.2957 | 1 | 0.2957 | 0 | 0 | 0 | 0.9167 | Ref |
| MK284521 | Unassigned | Unassigned | Unassigned | 1218_0 | Clustered | 14 | VC_1218_0 | 14 | 0.7811 | 1 | 0.7811 | 0 | 0 | 0 | 0.989 | Ref |
| MK284522 | Unassigned | Unassigned | Unassigned | 223_4 | Clustered | 13 | VC_223_4 | 13 | 0.1649 | 1 | 0.1649 | 0 | 0 | 0 | 1 | Ref |
| MK284526 | Unassigned | Unassigned | Unassigned | 843_0 | Clustered | 20 | VC_843_0 | 20 | 0.9621 | 1 | 0.9621 | 0 | 0 | 0 | 1 | Ref |
| MK284527 | Unassigned | Unassigned | Unassigned | 843_0 | Clustered | 20 | VC_843_0 | 20 | 0.9621 | 1 | 0.9621 | 0 | 0 | 0 | 1 | Ref |
| MK284528 | Unassigned | Unassigned | Unassigned | 843_0 | Clustered | 20 | VC_843_0 | 20 | 0.9621 | 1 | 0.9621 | 0 | 0 | 0 | 1 | Ref |
| MK286578 | Unassigned | Unassigned | Unassigned | 611_0 | Clustered | 50 | VC_611_0 | 50 | 0.7306 | 1 | 0.7306 | 0 | 0 | 0 | 1 | Ref |
| MK288021 | Unassigned | Unassigned | Unassigned | 489_8 | Clustered | 2 | VC_489_8 | 2 | 0.0291 | 0.90088011 | 0.0262 | 0 | 0 | 0 | 1 | Ref |
| MK288022 | Unassigned | Unassigned | Unassigned | 1042_0 | Clustered | 8 | VC_1042_0 | 8 | 0.7385 | 1 | 0.7385 | 0 | 0 | 0 | 1 | Ref |
| MK290737 | Unassigned | Unassigned | Unassigned | 342_0 | Clustered | 4 | VC_342_0 | 4 | 0.0875 | 0.99998775 | 0.0875 | 0 | 0 | 0 | 1 | Ref |
| MK290738 | Unassigned | Unassigned | Unassigned | 342_0 | Clustered | 4 | VC_342_0 | 4 | 0.0875 | 0.99998775 | 0.0875 | 0 | 0 | 0 | 1 | Ref |
| MK290764 | Unassigned | Unassigned | Unassigned | 12_0 | Clustered | 21 | VC_12_0 | 21 | 0.2262 | 1 | 0.2262 | 0 | 0 | 0 | 0.9571 | Ref |
| MK291441 | Unassigned | Unassigned | Unassigned | 982_0 | Clustered | 3 | VC_982_0 | 3 | 0.5537 | 0.96587626 | 0.5348 | 0 | 0 | 0 | 1 | Ref |
| MK291442 | Unassigned | Unassigned | Unassigned | 955_0 | Clustered | 2 | VC_955_0 | 2 | 0.2751 | 0.89435023 | 0.2461 | 0 | 0 | 0 | 1 | Ref |
| MK291443 | Unassigned | Unassigned | Unassigned | 982_0 | Clustered | 3 | VC_982_0 | 3 | 0.5537 | 0.96587626 | 0.5348 | 0 | 0 | 0 | 1 | Ref |
| MK291444 | Unassigned | Unassigned | Unassigned | 982_0 | Clustered | 3 | VC_982_0 | 3 | 0.5537 | 0.96587626 | 0.5348 | 0 | 0 | 0 | 1 | Ref |
| MK295203 | Unassigned | Unassigned | Unassigned | 297_7 | Clustered/Singleton | 1 | VC_297_7 | 1 | 0 | 1 | 0 | 0 | 0 | 0 | 1 | Ref |
| MK295204 | Unassigned | Unassigned | Unassigned | 295_2 | Clustered | 2 | VC_295_2 | 2 | 0.0224 | 0.95539992 | 0.0214 | 0 | 0 | 0 | 1 | Ref |
| MK301437 | Unassigned | Unassigned | Unassigned | 210_0 | Clustered | 105 | VC_210_0 | 105 | 0.4076 | 1 | 0.4076 | 0 | 0 | 0 | 0.7766 | Ref |
| MK301442 | Unassigned | Unassigned | Unassigned | 706_0 | Clustered | 52 | VC_706_0 | 52 | 0.9787 | 1 | 0.9787 | 0 | 0 | 0 | 1 | Ref |
| MK301443 | Unassigned | Unassigned | Unassigned | 706_0 | Clustered | 52 | VC_706_0 | 52 | 0.9787 | 1 | 0.9787 | 0 | 0 | 0 | 1 | Ref |
| MK301532 | Unassigned | Unassigned | Unassigned | 465_0 | Clustered | 80 | VC_465_0 | 80 | 0.9988 | 1 | 0.9988 | 0 | 0 | 0 | 1 | Ref |
| MK305884 | Unassigned | Unassigned | Unassigned | 783_0 | Clustered | 67 | VC_783_0 | 67 | 0.8945 | 1 | 0.8945 | 0 | 0 | 0 | 1 | Ref |
| MK305885 | Unassigned | Unassigned | Unassigned | 989_0 | Clustered | 13 | VC_989_0 | 13 | 0.8622 | 1 | 0.8622 | 0 | 0 | 0 | 1 | Ref |
| MK305887 | Unassigned | Unassigned | Unassigned | 974_0 | Clustered | 34 | VC_974_0 | 34 | 0.7953 | 1 | 0.7953 | 0 | 0 | 0 | 1 | Ref |
| MK305888 | Unassigned | Unassigned | Unassigned | 742_0 | Clustered | 11 | VC_742_0 | 11 | 0.5046 | 1 | 0.5046 | 0 | 0 | 0 | 1 | Ref |
| MK305889 | Unassigned | Unassigned | Unassigned | 1219_0 | Clustered | 24 | VC_1219_0 | 24 | 0.9519 | 1 | 0.9519 | 0 | 0 | 0 | 1 | Ref |
| MK305890 | Unassigned | Unassigned | Unassigned | 1436_0 | Clustered | 3 | VC_1436_0 | 3 | 0.9803 | 0.9595722 | 0.9406 | 0 | 0 | 0 | 1 | Ref |
| MK305891 | Unassigned | Unassigned | Unassigned | 885_0 | Clustered | 9 | VC_885_0 | 9 | 0.9403 | 1 | 0.9403 | 0 | 0 | 0 | 1 | Ref |
| MK305892 | Unassigned | Unassigned | Unassigned | 973_0 | Clustered | 15 | VC_973_0 | 15 | 1 | 0 | 0 | 0 | 0 | 0 | 1 | Ref |
| MK305893 | Unassigned | Unassigned | Unassigned | 335_55 | Clustered | 2 | VC_335_55 | 2 | 0.006 | 0.95750995 | 0.0058 | 0 | 0 | 0 | 1 | Ref |
| MK308637 | Unassigned | Unassigned | Unassigned | 1203_0 | Clustered | 28 | VC_1203_0 | 28 | 0.4905 | 1 | 0.4905 | 0 | 0 | 0 | 1 | Ref |
| MK308638 | Unassigned | Unassigned | Unassigned | 1424_0 | Clustered | 31 | VC_1424_0 | 31 | 0.8834 | 1 | 0.8834 | 0 | 0 | 0 | 1 | Ref |
| MK308674 | Unassigned | Unassigned | Unassigned | 358_0 | Clustered | 4 | VC_358_0 | 4 | 0.453 | 0.99997974 | 0.453 | 0 | 0 | 0 | 1 | Ref |
| MK308675 | Unassigned | Unassigned | Unassigned | 358_0 | Clustered | 4 | VC_358_0 | 4 | 0.453 | 0.99997974 | 0.453 | 0 | 0 | 0 | 1 | Ref |
| MK308676 | Unassigned | Unassigned | Unassigned | 358_0 | Clustered | 4 | VC_358_0 | 4 | 0.453 | 0.99997974 | 0.453 | 0 | 0 | 0 | 1 | Ref |
| MK308677 | Unassigned | Unassigned | Unassigned | 358_0 | Clustered | 4 | VC_358_0 | 4 | 0.453 | 0.99997974 | 0.453 | 0 | 0 | 0 | 1 | Ref |
| MK310138 | Unassigned | Unassigned | Unassigned | 244_0 | Clustered | 4 | VC_244_0 | 4 | 0.0426 | 0.99998759 | 0.0426 | 0 | 0 | 0 | 1 | Ref |
| MK310139 | Unassigned | Unassigned | Unassigned | 384_5 | Clustered | 2 | VC_384_5 | 2 | 0.0035 | 0.95771071 | 0.0033 | 0 | 0 | 0 | 1 | Ref |
| MK310140 | Unassigned | Unassigned | Unassigned | 532_0 | Clustered | 75 | VC_532_0 | 75 | 0.9393 | 1 | 0.9393 | 0 | 0 | 0 | 1 | Ref |
| MK310141 | Unassigned | Unassigned | Unassigned | 335_16 | Clustered | 2 | VC_335_16 | 2 | 0.0094 | 0.95751393 | 0.009 | 0 | 0 | 0 | 1 | Ref |
| MK310142 | Unassigned | Unassigned | Unassigned | 335_61 | Clustered | 8 | VC_335_61 | 8 | 0.0394 | 1 | 0.0394 | 0 | 0 | 0 | 0.7857 | Ref |
| MK310143 | Unassigned | Unassigned | Unassigned | 579_32 | Clustered/Singleton | 1 | VC_579_32 | 1 | 0 | 1 | 0 | 0 | 0 | 0 | 1 | Ref |
| MK310145 | Unassigned | Unassigned | Unassigned | 535_1 | Clustered | 108 | VC_535_1 | 108 | 0.8479 | 1 | 0.8479 | 0 | 0 | 0 | 0.9955 | Ref |
| MK310146 | Unassigned | Unassigned | Unassigned | 783_0 | Clustered | 67 | VC_783_0 | 67 | 0.8945 | 1 | 0.8945 | 0 | 0 | 0 | 1 | Ref |
| MK310182 | Unassigned | Unassigned | Unassigned | 405_0 | Clustered | 126 | VC_405_0 | 126 | 0.9614 | 1 | 0.9614 | 0 | 0 | 0 | 1 | Ref |
| MK310183 | Unassigned | Unassigned | Unassigned | 837_0 | Clustered | 12 | VC_837_0 | 12 | 0.2327 | 1 | 0.2327 | 0 | 0 | 0 | 1 | Ref |
| MK310184 | Unassigned | Unassigned | Unassigned | 297_6 | Clustered | 12 | VC_297_6 | 12 | 0.1139 | 1 | 0.1139 | 0 | 0 | 0 | 0.8182 | Ref |
| MK317959 | Unassigned | Unassigned | Unassigned | 81_0 | Clustered | 41 | VC_81_0 | 41 | 0.5815 | 1 | 0.5815 | 0 | 0 | 0 | 0.9805 | Ref |
| MK318076 | Unassigned | Unassigned | Unassigned | 80_7 | Clustered | 33 | VC_80_7 | 33 | 0.2156 | 1 | 0.2156 | 0 | 0 | 0 | 0.8409 | Ref |
| MK318083 | Unassigned | Unassigned | Unassigned | 203_1 | Clustered/Singleton | 1 | VC_203_1 | 1 | 0 | 1 | 0 | 0 | 0 | 0 | 1 | Ref |
| MK327140 | Unassigned | Unassigned | Unassigned | 474_0 | Clustered | 19 | VC_474_0 | 19 | 0.3729 | 1 | 0.3729 | 0 | 0 | 0 | 1 | Ref |
| MK327928 | Unassigned | Unassigned | Unassigned | 290_18 | Clustered/Singleton | 1 | VC_290_18 | 1 | 0 | 1 | 0 | 0 | 0 | 0 | 1 | Ref |
| MK327929 | Unassigned | Unassigned | Unassigned | 290_127 | Clustered/Singleton | 1 | VC_290_127 | 1 | 0 | 1 | 0 | 0 | 0 | 0 | 1 | Ref |
| MK327930 | Unassigned | Unassigned | Unassigned | 794_19 | Clustered | 16 | VC_794_19 | 16 | 0.0975 | 0.99616626 | 0.0971 | 0 | 0 | 0 | 0.8083 | Ref |
| MK327931 | Unassigned | Unassigned | Unassigned | 170_11 | Clustered | 10 | VC_170_11 | 10 | 0.1502 | 0.99999797 | 0.1502 | 0 | 0 | 0 | 0.9111 | Ref |
| MK327932 | Unassigned | Unassigned | Unassigned | 609_0 | Clustered | 27 | VC_609_0 | 27 | 0.9943 | 1 | 0.9943 | 0 | 0 | 0 | 1 | Ref |
| MK327933 | Unassigned | Unassigned | Unassigned | 293_4 | Clustered/Singleton | 1 | VC_293_4 | 1 | 0 | 1 | 0 | 0 | 0 | 0 | 1 | Ref |
| MK327934 | Unassigned | Unassigned | Unassigned | 293_1 | Clustered | 21 | VC_293_1 | 21 | 0.1694 | 1 | 0.1694 | 0 | 0 | 0 | 0.9048 | Ref |
| MK327935 | Unassigned | Unassigned | Unassigned | 609_0 | Clustered | 27 | VC_609_0 | 27 | 0.9943 | 1 | 0.9943 | 0 | 0 | 0 | 1 | Ref |

| | | | | | | | | | | | | | | | | |
|---|---|---|---|---|---|---|---|---|---|---|---|---|---|---|---|---|
| MK327936 | Unassigned | Unassigned | Unassigned | 290_133 | Clustered/Singleton | 1 | VC_290_133 | 1 | 0 | 1 | 0 | 0 | 0 | 0 | 1 | Ref |
| MK327937 | Unassigned | Unassigned | Unassigned | 290_16 | Clustered/Singleton | 1 | VC_290_16 | 1 | 0 | 1 | 0 | 0 | 0 | 0 | 1 | Ref |
| MK327939 | Unassigned | Unassigned | Unassigned | 290_119 | Clustered/Singleton | 1 | VC_290_119 | 1 | 0 | 1 | 0 | 0 | 0 | 0 | 1 | Ref |
| MK327940 | Unassigned | Unassigned | Unassigned | 290_121 | Clustered | 2 | VC_290_121 | 2 | 0.0026 | 0.90673408 | 0.0023 | 0 | 0 | 0 | 1 | Ref |
| MK327941 | Unassigned | Unassigned | Unassigned | 609_0 | Clustered | 27 | VC_609_0 | 27 | 0.9943 | 1 | 0.9943 | 0 | 0 | 0 | 1 | Ref |
| MK327942 | Unassigned | Unassigned | Unassigned | 290_156 | Clustered/Singleton | 1 | VC_290_156 | 1 | 0 | 1 | 0 | 0 | 0 | 0 | 1 | Ref |
| MK327943 | Unassigned | Unassigned | Unassigned | 293_1 | Clustered | 21 | VC_293_1 | 21 | 0.1694 | 1 | 0.1694 | 0 | 0 | 0 | 0.9048 | Ref |
| MK327944 | Unassigned | Unassigned | Unassigned | 290_134 | Clustered/Singleton | 1 | VC_290_134 | 1 | 0 | 1 | 0 | 0 | 0 | 0 | 1 | Ref |
| MK327945 | Unassigned | Unassigned | Unassigned | 290_121 | Clustered | 2 | VC_290_121 | 2 | 0.0026 | 0.90673408 | 0.0023 | 0 | 0 | 0 | 1 | Ref |
| MK327946 | Unassigned | Unassigned | Unassigned | 290_17 | Clustered/Singleton | 1 | VC_290_17 | 1 | 0 | 1 | 0 | 0 | 0 | 0 | 1 | Ref |
| MK327947 | Unassigned | Unassigned | Unassigned | 609_0 | Clustered | 27 | VC_609_0 | 27 | 0.9943 | 1 | 0.9943 | 0 | 0 | 0 | 1 | Ref |
| MK330684 | Unassigned | Unassigned | Unassigned | 907_0 | Clustered | 32 | VC_907_0 | 32 | 0.9964 | 1 | 0.9964 | 0 | 0 | 0 | 1 | Ref |
| MK331930 | Unassigned | Unassigned | Unassigned | 155_8 | Clustered | 66 | VC_155_8 | 66 | 0.4537 | 1 | 0.4537 | 0 | 0 | 0 | 0.8182 | Ref |
| MK331931 | Unassigned | Unassigned | Unassigned | 155_8 | Clustered | 66 | VC_155_8 | 66 | 0.4537 | 1 | 0.4537 | 0 | 0 | 0 | 0.8182 | Ref |
| MK335533 | Unassigned | Unassigned | Unassigned | 595_0 | Clustered | 35 | VC_595_0 | 35 | 0.8809 | 1 | 0.8809 | 0 | 0 | 0 | 1 | Ref |
| MK340760 | Unassigned | Unassigned | Unassigned | 80_9 | Clustered | 2 | VC_80_9 | 2 | 0.0061 | 0.94860001 | 0.0058 | 0 | 0 | 0 | 1 | Ref |
| MK340761 | Unassigned | Unassigned | Unassigned | 77_0 | Clustered | 37 | VC_77_0 | 37 | 0.9392 | 1 | 0.9392 | 0 | 0 | 0 | 1 | Ref |
| MK340941 | Unassigned | Unassigned | Unassigned | 249_0 | Clustered | 2 | VC_249_0 | 2 | 0.0834 | 0.95416588 | 0.0795 | 0 | 0 | 0 | 1 | Ref |
| MK348510 | Unassigned | Unassigned | Unassigned | 8_0 | Clustered | 58 | VC_8_0 | 58 | 0.8285 | 1 | 0.8285 | 0 | 0 | 0 | 0.9946 | Ref |
| MK359299 | Unassigned | Unassigned | Unassigned | 335_23 | Clustered | 17 | VC_335_23 | 17 | 0.0714 | 1 | 0.0714 | 0 | 0 | 0 | 0.6397 | Ref |
| MK359300 | Unassigned | Unassigned | Unassigned | 335_56 | Clustered | 7 | VC_335_56 | 7 | 0.0242 | 1 | 0.0242 | 0 | 0 | 0 | 0.9048 | Ref |
| MK359301 | Unassigned | Unassigned | Unassigned | 535_1 | Clustered | 108 | VC_535_1 | 108 | 0.8479 | 1 | 0.8479 | 0 | 0 | 0 | 0.9955 | Ref |
| MK359302 | Unassigned | Unassigned | Unassigned | 895_0 | Clustered | 25 | VC_895_0 | 25 | 0.5768 | 1 | 0.5768 | 0 | 0 | 0 | 1 | Ref |
| MK359303 | Unassigned | Unassigned | Unassigned | 579_14 | Clustered | 8 | VC_579_14 | 8 | 0.0227 | 0.99904117 | 0.0227 | 0 | 0 | 0 | 0.8214 | Ref |
| MK359304 | Unassigned | Unassigned | Unassigned | 335_56 | Clustered | 7 | VC_335_56 | 7 | 0.0242 | 1 | 0.0242 | 0 | 0 | 0 | 0.9048 | Ref |
| MK359305 | Unassigned | Unassigned | Unassigned | 579_62 | Clustered/Singleton | 1 | VC_579_62 | 1 | 0 | 1 | 0 | 0 | 0 | 0 | 1 | Ref |
| MK359306 | Unassigned | Unassigned | Unassigned | 579_53 | Clustered/Singleton | 1 | VC_579_53 | 1 | 0 | 1 | 0 | 0 | 0 | 0 | 1 | Ref |
| MK359307 | Unassigned | Unassigned | Unassigned | 538_92 | Clustered | 5 | VC_538_92 | 5 | 0.0653 | 0.99999997 | 0.0653 | 0 | 0 | 0 | 1 | Ref |
| MK359308 | Unassigned | Unassigned | Unassigned | 579_77 | Clustered | 3 | VC_579_77 | 3 | 0.0065 | 0.89175063 | 0.0058 | 0 | 0 | 0 | 0.6667 | Ref |
| MK359309 | Unassigned | Unassigned | Unassigned | 571_7 | Clustered | 28 | VC_571_7 | 28 | 0.1651 | 1 | 0.1651 | 0 | 0 | 0 | 0.8519 | Ref |
| MK359310 | Unassigned | Unassigned | Unassigned | 535_0 | Clustered | 18 | VC_535_0 | 18 | 0.1595 | 1 | 0.1595 | 0 | 0 | 0 | 1 | Ref |
| MK359311 | Unassigned | Unassigned | Unassigned | 579_14 | Clustered | 8 | VC_579_14 | 8 | 0.0227 | 0.99904117 | 0.0227 | 0 | 0 | 0 | 0.8214 | Ref |
| MK359312 | Unassigned | Unassigned | Unassigned | 335_49 | Clustered | 17 | VC_335_49 | 17 | 0.0731 | 1 | 0.0731 | 0 | 0 | 0 | 0.7721 | Ref |
| MK359313 | Unassigned | Unassigned | Unassigned | 1216_0 | Clustered | 22 | VC_1216_0 | 22 | 0.4546 | 1 | 0.4546 | 0 | 0 | 0 | 1 | Ref |
| MK359314 | Unassigned | Unassigned | Unassigned | 579_50 | Clustered | 15 | VC_579_50 | 15 | 0.0467 | 1 | 0.0467 | 0 | 0 | 0 | 0.9238 | Ref |
| MK359315 | Unassigned | Unassigned | Unassigned | 538_80 | Clustered | 3 | VC_538_80 | 3 | 0.0291 | 0.99859904 | 0.0291 | 0 | 0 | 0 | 1 | Ref |
| MK359316 | Unassigned | Unassigned | Unassigned | 335_33 | Clustered | 4 | VC_335_33 | 4 | 0.0148 | 0.99998565 | 0.0148 | 0 | 0 | 0 | 1 | Ref |
| MK359317 | Unassigned | Unassigned | Unassigned | 579_59 | Clustered/Singleton | 1 | VC_579_59 | 1 | 0 | 1 | 0 | 0 | 0 | 0 | 1 | Ref |
| MK359318 | Unassigned | Unassigned | Unassigned | 579_61 | Clustered | 2 | VC_579_61 | 2 | 0.0032 | 0.64519808 | 0.002 | 0 | 0 | 0 | 1 | Ref |
| MK359319 | Unassigned | Unassigned | Unassigned | 989_0 | Clustered | 13 | VC_989_0 | 13 | 0.8622 | 1 | 0.8622 | 0 | 0 | 0 | 1 | Ref |
| MK359320 | Unassigned | Unassigned | Unassigned | 571_0 | Clustered | 16 | VC_571_0 | 16 | 0.091 | 1 | 0.091 | 0 | 0 | 0 | 0.6667 | Ref |
| MK359321 | Unassigned | Unassigned | Unassigned | 579_13 | Clustered/Singleton | 1 | VC_579_13 | 1 | 0 | 1 | 0 | 0 | 0 | 0 | 1 | Ref |
| MK359322 | Unassigned | Unassigned | Unassigned | 535_0 | Clustered | 18 | VC_535_0 | 18 | 0.1595 | 1 | 0.1595 | 0 | 0 | 0 | 1 | Ref |
| MK359323 | Unassigned | Unassigned | Unassigned | 535_1 | Clustered | 108 | VC_535_1 | 108 | 0.8479 | 1 | 0.8479 | 0 | 0 | 0 | 0.9955 | Ref |
| MK359324 | Unassigned | Unassigned | Unassigned | 579_9 | Clustered | 3 | VC_579_9 | 3 | 0.0064 | 0.81181703 | 0.0052 | 0 | 0 | 0 | 0.6667 | Ref |
| MK359325 | Unassigned | Unassigned | Unassigned | 535_1 | Clustered | 108 | VC_535_1 | 108 | 0.8479 | 1 | 0.8479 | 0 | 0 | 0 | 0.9955 | Ref |
| MK359326 | Unassigned | Unassigned | Unassigned | 579_20 | Clustered | 17 | VC_579_20 | 17 | 0.0538 | 1 | 0.0538 | 0 | 0 | 0 | 0.9926 | Ref |
| MK359327 | Unassigned | Unassigned | Unassigned | 579_45 | Clustered/Singleton | 1 | VC_579_45 | 1 | 0 | 1 | 0 | 0 | 0 | 0 | 1 | Ref |
| MK359328 | Unassigned | Unassigned | Unassigned | 579_17 | Clustered/Singleton | 1 | VC_579_17 | 1 | 0 | 1 | 0 | 0 | 0 | 0 | 1 | Ref |
| MK359329 | Unassigned | Unassigned | Unassigned | 579_69 | Clustered/Singleton | 1 | VC_579_69 | 1 | 0 | 1 | 0 | 0 | 0 | 0 | 1 | Ref |
| MK359330 | Unassigned | Unassigned | Unassigned | 579_66 | Clustered/Singleton | 1 | VC_579_66 | 1 | 0 | 1 | 0 | 0 | 0 | 0 | 1 | Ref |
| MK359331 | Unassigned | Unassigned | Unassigned | 335_10 | Clustered | 3 | VC_335_10 | 3 | 0.012 | 0.99859169 | 0.012 | 0 | 0 | 0 | 1 | Ref |
| MK359332 | Unassigned | Unassigned | Unassigned | 1114_0 | Clustered | 20 | VC_1114_0 | 20 | 0.3238 | 1 | 0.3238 | 0 | 0 | 0 | 0.9842 | Ref |
| MK359333 | Unassigned | Unassigned | Unassigned | 579_28 | Clustered/Singleton | 1 | VC_579_28 | 1 | 0 | 1 | 0 | 0 | 0 | 0 | 1 | Ref |
| MK359334 | Unassigned | Unassigned | Unassigned | 579_49 | Clustered | 7 | VC_579_49 | 7 | 0.0194 | 0.98914431 | 0.0191 | 0 | 0 | 0 | 0.8571 | Ref |
| MK359335 | Unassigned | Unassigned | Unassigned | 579_20 | Clustered | 17 | VC_579_20 | 17 | 0.0538 | 1 | 0.0538 | 0 | 0 | 0 | 0.9926 | Ref |
| MK359336 | Unassigned | Unassigned | Unassigned | 579_36 | Clustered | 4 | VC_579_36 | 4 | 0.0096 | 0.84559358 | 0.0081 | 0 | 0 | 0 | 1 | Ref |
| MK359337 | Unassigned | Unassigned | Unassigned | 579_43 | Clustered/Singleton | 1 | VC_579_43 | 1 | 0 | 1 | 0 | 0 | 0 | 0 | 1 | Ref |
| MK359338 | Unassigned | Unassigned | Unassigned | 579_20 | Clustered | 17 | VC_579_20 | 17 | 0.0538 | 1 | 0.0538 | 0 | 0 | 0 | 0.9926 | Ref |
| MK359339 | Unassigned | Unassigned | Unassigned | 579_20 | Clustered | 17 | VC_579_20 | 17 | 0.0538 | 1 | 0.0538 | 0 | 0 | 0 | 0.9926 | Ref |
| MK359340 | Unassigned | Unassigned | Unassigned | 535_1 | Clustered | 108 | VC_535_1 | 108 | 0.8479 | 1 | 0.8479 | 0 | 0 | 0 | 0.9955 | Ref |
| MK359341 | Unassigned | Unassigned | Unassigned | 532_0 | Clustered | 75 | VC_532_0 | 75 | 0.9393 | 1 | 0.9393 | 0 | 0 | 0 | 1 | Ref |
| MK359342 | Unassigned | Unassigned | Unassigned | 579_15 | Clustered/Singleton | 1 | VC_579_15 | 1 | 0 | 1 | 0 | 0 | 0 | 0 | 1 | Ref |
| MK359343 | Unassigned | Unassigned | Unassigned | 538_57 | Clustered/Singleton | 1 | VC_538_57 | 1 | 0 | 1 | 0 | 0 | 0 | 0 | 1 | Ref |
| MK359344 | Unassigned | Unassigned | Unassigned | 579_30 | Clustered | 3 | VC_579_30 | 3 | 0.0064 | 0.74796372 | 0.0048 | 0 | 0 | 0 | 1 | Ref |
| MK359345 | Unassigned | Unassigned | Unassigned | 579_44 | Clustered/Singleton | 1 | VC_579_44 | 1 | 0 | 1 | 0 | 0 | 0 | 0 | 1 | Ref |
| MK359346 | Unassigned | Unassigned | Unassigned | 579_71 | Clustered/Singleton | 1 | VC_579_71 | 1 | 0 | 1 | 0 | 0 | 0 | 0 | 1 | Ref |
| MK359347 | Unassigned | Unassigned | Unassigned | 579_20 | Clustered | 17 | VC_579_20 | 17 | 0.0538 | 1 | 0.0538 | 0 | 0 | 0 | 0.9926 | Ref |
| MK359348 | Unassigned | Unassigned | Unassigned | 579_12 | Clustered | 3 | VC_579_12 | 3 | 0.0064 | 0.86841267 | 0.0056 | 0 | 0 | 0 | 1 | Ref |
| MK359349 | Unassigned | Unassigned | Unassigned | 579_46 | Clustered/Singleton | 1 | VC_579_46 | 1 | 0 | 1 | 0 | 0 | 0 | 0 | 1 | Ref |
| MK359350 | Unassigned | Unassigned | Unassigned | 579_64 | Clustered/Singleton | 1 | VC_579_64 | 1 | 0 | 1 | 0 | 0 | 0 | 0 | 1 | Ref |
| MK359351 | Unassigned | Unassigned | Unassigned | 1114_0 | Clustered | 20 | VC_1114_0 | 20 | 0.3238 | 1 | 0.3238 | 0 | 0 | 0 | 0.9842 | Ref |
| MK359352 | Unassigned | Unassigned | Unassigned | 230_0 | Clustered | 38 | VC_230_0 | 38 | 0.9076 | 1 | 0.9076 | 0 | 0 | 0 | 1 | Ref |
| MK359353 | Unassigned | Unassigned | Unassigned | 579_4 | Clustered/Singleton | 1 | VC_579_4 | 1 | 0 | 1 | 0 | 0 | 0 | 0 | 1 | Ref |
| MK359354 | Unassigned | Unassigned | Unassigned | 335_5 | Clustered | 11 | VC_335_5 | 11 | 0.0469 | 1 | 0.0469 | 0 | 0 | 0 | 0.7636 | Ref |
| MK359355 | Unassigned | Unassigned | Unassigned | 579_3 | Clustered/Singleton | 1 | VC_579_3 | 1 | 0 | 1 | 0 | 0 | 0 | 0 | 1 | Ref |
| MK359356 | Unassigned | Unassigned | Unassigned | 535_1 | Clustered | 108 | VC_535_1 | 108 | 0.8479 | 1 | 0.8479 | 0 | 0 | 0 | 0.9955 | Ref |
| MK359357 | Unassigned | Unassigned | Unassigned | 527_0 | Clustered | 37 | VC_527_0 | 37 | 0.9679 | 1 | 0.9679 | 0 | 0 | 0 | 1 | Ref |
| MK359358 | Unassigned | Unassigned | Unassigned | 579_14 | Clustered | 8 | VC_579_14 | 8 | 0.0227 | 0.99904117 | 0.0227 | 0 | 0 | 0 | 0.8214 | Ref |
| MK359359 | Unassigned | Unassigned | Unassigned | 579_73 | Clustered | 2 | VC_579_73 | 2 | 0.0033 | 0.82635115 | 0.0027 | 0 | 0 | 0 | 1 | Ref |
| MK359360 | Unassigned | Unassigned | Unassigned | 230_0 | Clustered | 38 | VC_230_0 | 38 | 0.9076 | 1 | 0.9076 | 0 | 0 | 0 | 1 | Ref |

| | | | | | | | | | | | | | | | | |
|---|---|---|---|---|---|---|---|---|---|---|---|---|---|---|---|---|
| MK359361 | Unassigned | Unassigned | Unassigned | 579_27 | Clustered | 2 | VC_579_27 | 2 | 0.0032 | 0.63743142 | 0.002 | 0 | 0 | 0 | 1 | Ref |
| MK359362 | Unassigned | Unassigned | Unassigned | 579_20 | Clustered | 17 | VC_579_20 | 17 | 0.0538 | 1 | 0.0538 | 0 | 0 | 0 | 0.9926 | Ref |
| MK359363 | Unassigned | Unassigned | Unassigned | 579_36 | Clustered | 4 | VC_579_36 | 4 | 0.0096 | 0.84559358 | 0.0081 | 0 | 0 | 0 | 1 | Ref |
| MK359364 | Unassigned | Unassigned | Unassigned | 579_39 | Clustered | 3 | VC_579_39 | 3 | 0.0063 | 0.72858376 | 0.0046 | 0 | 0 | 0 | 0.6667 | Ref |
| MK359365 | Unassigned | Unassigned | Unassigned | 579_50 | Clustered | 15 | VC_579_50 | 15 | 0.0467 | 1 | 0.0467 | 0 | 0 | 0 | 0.9238 | Ref |
| MK359366 | Unassigned | Unassigned | Unassigned | 579_36 | Clustered | 4 | VC_579_36 | 4 | 0.0096 | 0.84559358 | 0.0081 | 0 | 0 | 0 | 1 | Ref |
| MK359990 | Unassigned | Unassigned | Unassigned | 647_0 | Clustered | 37 | VC_647_0 | 37 | 0.5847 | 1 | 0.5847 | 0 | 0 | 0 | 0.9234 | Ref |
| MK360024 | Unassigned | Unassigned | Unassigned | 711_0 | Clustered | 48 | VC_711_0 | 48 | 0.8217 | 1 | 0.8217 | 0 | 0 | 0 | 1 | Ref |
| MK360025 | Unassigned | Unassigned | Unassigned | 1004_0 | Clustered | 4 | VC_1004_0 | 4 | 0.2949 | 0.99997642 | 0.2949 | 0 | 0 | 0 | 1 | Ref |
| MK370036 | Unassigned | Unassigned | Unassigned | 199_1 | Clustered/Singleton | 1 | VC_199_1 | 1 | 0 | 1 | 0 | 0 | 0 | 0 | 1 | Ref |
| MK372342 | Unassigned | Unassigned | Unassigned | 470_0 | Clustered | 62 | VC_470_0 | 62 | 0.902 | 1 | 0.902 | 0 | 0 | 0 | 1 | Ref |
| MK373770 | Unassigned | Unassigned | Unassigned | 837_1 | Clustered | 24 | VC_837_1 | 24 | 0.5328 | 1 | 0.5328 | 0 | 0 | 0 | 1 | Ref |
| MK373771 | Unassigned | Unassigned | Unassigned | 466_0 | Clustered | 66 | VC_466_0 | 66 | 0.9246 | 1 | 0.9246 | 0 | 0 | 0 | 1 | Ref |
| MK373772 | Unassigned | Unassigned | Unassigned | 1281_0 | Clustered | 2 | VC_1281_0 | 2 | 0.9858 | 0.81445332 | 0.8029 | 0 | 0 | 0 | 1 | Ref |
| MK373773 | Unassigned | Unassigned | Unassigned | 466_0 | Clustered | 66 | VC_466_0 | 66 | 0.9246 | 1 | 0.9246 | 0 | 0 | 0 | 1 | Ref |
| MK373774 | Unassigned | Unassigned | Unassigned | 293_3 | Clustered | 2 | VC_293_3 | 2 | 0.0073 | 0.74885804 | 0.0055 | 0 | 0 | 0 | 1 | Ref |
| MK373775 | Unassigned | Unassigned | Unassigned | 293_3 | Clustered | 2 | VC_293_3 | 2 | 0.0073 | 0.74885804 | 0.0055 | 0 | 0 | 0 | 1 | Ref |
| MK373776 | Unassigned | Unassigned | Unassigned | 636_0 | Clustered | 13 | VC_636_0 | 13 | 0.6223 | 1 | 0.6223 | 0 | 0 | 0 | 1 | Ref |
| MK373777 | Unassigned | Unassigned | Unassigned | 636_0 | Clustered | 13 | VC_636_0 | 13 | 0.6223 | 1 | 0.6223 | 0 | 0 | 0 | 1 | Ref |
| MK373778 | Unassigned | Unassigned | Unassigned | 293_1 | Clustered | 21 | VC_293_1 | 21 | 0.1694 | 1 | 0.1694 | 0 | 0 | 0 | 0.9048 | Ref |
| MK373779 | Unassigned | Unassigned | Unassigned | 290_124 | Clustered/Singleton | 1 | VC_290_124 | 1 | 0 | 1 | 0 | 0 | 0 | 0 | 1 | Ref |
| MK373780 | Unassigned | Unassigned | Unassigned | 794_19 | Clustered | 16 | VC_794_19 | 16 | 0.0975 | 0.99616626 | 0.0971 | 0 | 0 | 0 | 0.8083 | Ref |
| MK373781 | Unassigned | Unassigned | Unassigned | 290_53 | Clustered/Singleton | 1 | VC_290_53 | 1 | 0 | 1 | 0 | 0 | 0 | 0 | 1 | Ref |
| MK373782 | Unassigned | Unassigned | Unassigned | 293_1 | Clustered | 21 | VC_293_1 | 21 | 0.1694 | 1 | 0.1694 | 0 | 0 | 0 | 0.9048 | Ref |
| MK373783 | Unassigned | Unassigned | Unassigned | 134_2 | Clustered | 17 | VC_134_2 | 17 | 0.1715 | 1 | 0.1715 | 0 | 0 | 0 | 0.9853 | Ref |
| MK373784 | Unassigned | Unassigned | Unassigned | 293_1 | Clustered | 21 | VC_293_1 | 21 | 0.1694 | 1 | 0.1694 | 0 | 0 | 0 | 0.9048 | Ref |
| MK373785 | Unassigned | Unassigned | Unassigned | 290_12 | Clustered/Singleton | 1 | VC_290_12 | 1 | 0 | 1 | 0 | 0 | 0 | 0 | 1 | Ref |
| MK373786 | Unassigned | Unassigned | Unassigned | 290_43 | Clustered/Singleton | 1 | VC_290_43 | 1 | 0 | 1 | 0 | 0 | 0 | 0 | 1 | Ref |
| MK373787 | Unassigned | Unassigned | Unassigned | 290_76 | Clustered/Singleton | 1 | VC_290_76 | 1 | 0 | 1 | 0 | 0 | 0 | 0 | 1 | Ref |
| MK373788 | Unassigned | Unassigned | Unassigned | 871_1 | Clustered | 26 | VC_871_1 | 26 | 0.6303 | 1 | 0.6303 | 0 | 0 | 0 | 0.9815 | Ref |
| MK373789 | Unassigned | Unassigned | Unassigned | 859_0 | Clustered | 67 | VC_859_0 | 67 | 0.9622 | 1 | 0.9622 | 0 | 0 | 0 | 1 | Ref |
| MK373790 | Unassigned | Unassigned | Unassigned | 859_0 | Clustered | 67 | VC_859_0 | 67 | 0.9622 | 1 | 0.9622 | 0 | 0 | 0 | 1 | Ref |
| MK373791 | Unassigned | Unassigned | Unassigned | 859_0 | Clustered | 67 | VC_859_0 | 67 | 0.9622 | 1 | 0.9622 | 0 | 0 | 0 | 1 | Ref |
| MK373792 | Unassigned | Unassigned | Unassigned | 198_1 | Clustered/Singleton | 1 | VC_198_1 | 1 | 0 | 1 | 0 | 0 | 0 | 0 | 1 | Ref |
| MK373793 | Unassigned | Unassigned | Unassigned | 470_0 | Clustered | 62 | VC_470_0 | 62 | 0.902 | 1 | 0.902 | 0 | 0 | 0 | 1 | Ref |
| MK373794 | Unassigned | Unassigned | Unassigned | 737_0 | Clustered | 13 | VC_737_0 | 13 | 0.5083 | 1 | 0.5083 | 0 | 0 | 0 | 1 | Ref |
| MK373795 | Unassigned | Unassigned | Unassigned | 737_0 | Clustered | 13 | VC_737_0 | 13 | 0.5083 | 1 | 0.5083 | 0 | 0 | 0 | 1 | Ref |
| MK373796 | Unassigned | Unassigned | Unassigned | 198_9 | Clustered/Singleton | 1 | VC_198_9 | 1 | 0 | 1 | 0 | 0 | 0 | 0 | 1 | Ref |
| MK373797 | Unassigned | Unassigned | Unassigned | 198_2 | Clustered | 2 | VC_198_2 | 2 | 0.0072 | 0.95491601 | 0.0069 | 0 | 0 | 0 | 1 | Ref |
| MK373798 | Unassigned | Unassigned | Unassigned | 975_0 | Clustered | 40 | VC_975_0 | 40 | 0.6888 | 1 | 0.6888 | 0 | 0 | 0 | 1 | Ref |
| MK373799 | Unassigned | Unassigned | Unassigned | 198_2 | Clustered | 2 | VC_198_2 | 2 | 0.0072 | 0.95491601 | 0.0069 | 0 | 0 | 0 | 1 | Ref |
| MK376953 | Unassigned | Unassigned | Unassigned | 898_1 | Clustered | 27 | VC_898_1 | 27 | 0.8894 | 1 | 0.8894 | 0 | 0 | 0 | 1 | Ref |
| MK376954 | Unassigned | Unassigned | Unassigned | 789_0 | Clustered | 34 | VC_789_0 | 34 | 0.9167 | 1 | 0.9167 | 0 | 0 | 0 | 1 | Ref |
| MK376955 | Unassigned | Unassigned | Unassigned | 244_3 | Clustered | 8 | VC_244_3 | 8 | 0.1086 | 1 | 0.1086 | 0 | 0 | 0 | 1 | Ref |
| MK376956 | Unassigned | Unassigned | Unassigned | 1223_1 | Clustered | 10 | VC_1223_1 | 10 | 0.5795 | 1 | 0.5795 | 0 | 0 | 0 | 1 | Ref |
| MK376957 | Unassigned | Unassigned | Unassigned | 898_1 | Clustered | 27 | VC_898_1 | 27 | 0.8894 | 1 | 0.8894 | 0 | 0 | 0 | 1 | Ref |
| MK376958 | Unassigned | Unassigned | Unassigned | 989_0 | Clustered | 13 | VC_989_0 | 13 | 0.8622 | 1 | 0.8622 | 0 | 0 | 0 | 1 | Ref |
| MK376959 | Unassigned | Unassigned | Unassigned | 1223_1 | Clustered | 10 | VC_1223_1 | 10 | 0.5795 | 1 | 0.5795 | 0 | 0 | 0 | 1 | Ref |
| MK376960 | Unassigned | Unassigned | Unassigned | 789_0 | Clustered | 34 | VC_789_0 | 34 | 0.9167 | 1 | 0.9167 | 0 | 0 | 0 | 1 | Ref |
| MK376961 | Unassigned | Unassigned | Unassigned | 578_1 | Clustered | 11 | VC_578_1 | 11 | 0.4919 | 1 | 0.4919 | 0 | 0 | 0 | 0.7818 | Ref |
| MK376962 | Unassigned | Unassigned | Unassigned | 789_0 | Clustered | 34 | VC_789_0 | 34 | 0.9167 | 1 | 0.9167 | 0 | 0 | 0 | 1 | Ref |
| MK376963 | Unassigned | Unassigned | Unassigned | 881_1 | Clustered | 13 | VC_881_1 | 13 | 0.5859 | 1 | 0.5859 | 0 | 0 | 0 | 1 | Ref |
| MK380014 | Unassigned | Unassigned | Unassigned | 466_0 | Clustered | 66 | VC_466_0 | 66 | 0.9246 | 1 | 0.9246 | 0 | 0 | 0 | 1 | Ref |
| MK380015 | Unassigned | Unassigned | Unassigned | 848_0 | Clustered | 124 | VC_848_0 | 124 | 0.9484 | 1 | 0.9484 | 0 | 0 | 0 | 1 | Ref |
| MK380016 | Unassigned | Unassigned | Unassigned | 848_0 | Clustered | 124 | VC_848_0 | 124 | 0.9484 | 1 | 0.9484 | 0 | 0 | 0 | 1 | Ref |
| MK387309 | Unassigned | Unassigned | Unassigned | 663_0 | Clustered | 5 | VC_663_0 | 5 | 0.6645 | 0.99999974 | 0.6645 | 0 | 0 | 0 | 1 | Ref |
| MK387337 | Unassigned | Unassigned | Unassigned | 869_0 | Clustered | 3 | VC_869_0 | 3 | 0.3287 | 0.99831497 | 0.3281 | 0 | 0 | 0 | 1 | Ref |
| MK387869 | Unassigned | Unassigned | Unassigned | 114_0 | Clustered | 8 | VC_114_0 | 8 | 0.2518 | 1 | 0.2518 | 0 | 0 | 0 | 1 | Ref |
| MK388689 | Unassigned | Unassigned | Unassigned | 870_0 | Clustered | 2 | VC_870_0 | 2 | 0.5991 | 0.92357512 | 0.5533 | 0 | 0 | 0 | 1 | Ref |
| MK392363 | Unassigned | Unassigned | Unassigned | 1015_1 | Clustered | 26 | VC_1015_1 | 26 | 0.6989 | 1 | 0.6989 | 0 | 0 | 0 | 1 | Ref |
| MK392364 | Unassigned | Unassigned | Unassigned | 1015_1 | Clustered | 26 | VC_1015_1 | 26 | 0.6989 | 1 | 0.6989 | 0 | 0 | 0 | 1 | Ref |
| MK392365 | Unassigned | Unassigned | Unassigned | 1015_1 | Clustered | 26 | VC_1015_1 | 26 | 0.6989 | 1 | 0.6989 | 0 | 0 | 0 | 1 | Ref |
| MK392366 | Unassigned | Unassigned | Unassigned | 1015_1 | Clustered | 26 | VC_1015_1 | 26 | 0.6989 | 1 | 0.6989 | 0 | 0 | 0 | 1 | Ref |
| MK392367 | Unassigned | Unassigned | Unassigned | 539_0 | Clustered | 37 | VC_539_0 | 37 | 0.911 | 1 | 0.911 | 0 | 0 | 0 | 1 | Ref |
| MK392368 | Unassigned | Unassigned | Unassigned | 1076_0 | Clustered | 6 | VC_1076_0 | 6 | 0.6773 | 1 | 0.6773 | 0 | 0 | 0 | 1 | Ref |
| MK393882 | Unassigned | Unassigned | Unassigned | 199_16 | Clustered | 12 | VC_199_16 | 12 | 0.064 | 1 | 0.064 | 0 | 0 | 0 | 0.9394 | Ref |
| MK408758 | Unassigned | Unassigned | Unassigned | 867_0 | Clustered | 5 | VC_867_0 | 5 | 0.2806 | 0.99999994 | 0.2806 | 0 | 0 | 0 | 1 | Ref |
| MK411746 | Unassigned | Unassigned | Unassigned | 1426_0 | Clustered | 2 | VC_1426_0 | 2 | 0.8068 | 0.92263554 | 0.7444 | 0 | 0 | 0 | 1 | Ref |
| MK416007 | Unassigned | Unassigned | Unassigned | 249_1 | Clustered | 21 | VC_249_1 | 21 | 0.6775 | 1 | 0.6775 | 0 | 0 | 0 | 0.8476 | Ref |
| MK416009 | Unassigned | Unassigned | Unassigned | 6_0 | Clustered | 44 | VC_6_0 | 44 | 0.9068 | 1 | 0.9068 | 0 | 0 | 0 | 1 | Ref |
| MK416010 | Unassigned | Unassigned | Unassigned | 492_0 | Clustered | 20 | VC_492_0 | 20 | 0.7887 | 1 | 0.7887 | 0 | 0 | 0 | 1 | Ref |
| MK416011 | Unassigned | Unassigned | Unassigned | 6_0 | Clustered | 44 | VC_6_0 | 44 | 0.9068 | 1 | 0.9068 | 0 | 0 | 0 | 1 | Ref |
| MK416012 | Unassigned | Unassigned | Unassigned | 6_0 | Clustered | 44 | VC_6_0 | 44 | 0.9068 | 1 | 0.9068 | 0 | 0 | 0 | 1 | Ref |
| MK416013 | Unassigned | Unassigned | Unassigned | 38_0 | Clustered | 4 | VC_38_0 | 4 | 0.1746 | 0.99998341 | 0.1746 | 0 | 0 | 0 | 1 | Ref |
| MK416014 | Unassigned | Unassigned | Unassigned | 251_0 | Clustered | 2 | VC_251_0 | 2 | 0.0633 | 0.95608273 | 0.0605 | 0 | 0 | 0 | 1 | Ref |
| MK416015 | Unassigned | Unassigned | Unassigned | 236_0 | Clustered | 32 | VC_236_0 | 32 | 0.9115 | 1 | 0.9115 | 0 | 0 | 0 | 1 | Ref |
| MK416017 | Unassigned | Unassigned | Unassigned | 252_0 | Clustered | 12 | VC_252_0 | 12 | 0.4075 | 1 | 0.4075 | 0 | 0 | 0 | 1 | Ref |
| MK416018 | Unassigned | Unassigned | Unassigned | 235_0 | Clustered | 60 | VC_235_0 | 60 | 0.9413 | 1 | 0.9413 | 0 | 0 | 0 | 1 | Ref |
| MK416019 | Unassigned | Unassigned | Unassigned | 249_1 | Clustered | 21 | VC_249_1 | 21 | 0.6775 | 1 | 0.6775 | 0 | 0 | 0 | 0.8476 | Ref |
| MK416020 | Unassigned | Unassigned | Unassigned | 6_0 | Clustered | 44 | VC_6_0 | 44 | 0.9068 | 1 | 0.9068 | 0 | 0 | 0 | 1 | Ref |
| MK416022 | Unassigned | Unassigned | Unassigned | 38_0 | Clustered | 4 | VC_38_0 | 4 | 0.1746 | 0.99998341 | 0.1746 | 0 | 0 | 0 | 1 | Ref |

| | | | | | | | | | | | | | | | | |
|---|---|---|---|---|---|---|---|---|---|---|---|---|---|---|---|---|
| MK417514 | Unassigned | Unassigned | Unassigned | 155_8 | Clustered | 66 | VC_155_8 | 66 | 0.4537 | 1 | 0.4537 | 0 | 0 | 0 | 0.8182 | Ref |
| MK417515 | Unassigned | Unassigned | Unassigned | 155_8 | Clustered | 66 | VC_155_8 | 66 | 0.4537 | 1 | 0.4537 | 0 | 0 | 0 | 0.8182 | Ref |
| MK417516 | Unassigned | Unassigned | Unassigned | 155_8 | Clustered | 66 | VC_155_8 | 66 | 0.4537 | 1 | 0.4537 | 0 | 0 | 0 | 0.8182 | Ref |
| MK421971 | Unassigned | Unassigned | Unassigned | 509_0 | Clustered | 36 | VC_509_0 | 36 | 0.5291 | 1 | 0.5291 | 0 | 0 | 0 | 0.9714 | Ref |
| MK422448 | Unassigned | Unassigned | Unassigned | 492_0 | Clustered | 20 | VC_492_0 | 20 | 0.7887 | 1 | 0.7887 | 0 | 0 | 0 | 1 | Ref |
| MK422449 | Unassigned | Unassigned | Unassigned | 6_0 | Clustered | 44 | VC_6_0 | 44 | 0.9068 | 1 | 0.9068 | 0 | 0 | 0 | 1 | Ref |
| MK422451 | Unassigned | Unassigned | Unassigned | 738_0 | Clustered | 9 | VC_738_0 | 9 | 0.7116 | 1 | 0.7116 | 0 | 0 | 0 | 1 | Ref |
| MK422453 | Unassigned | Unassigned | Unassigned | 6_0 | Clustered | 44 | VC_6_0 | 44 | 0.9068 | 1 | 0.9068 | 0 | 0 | 0 | 1 | Ref |
| MK422454 | Unassigned | Unassigned | Unassigned | 6_0 | Clustered | 44 | VC_6_0 | 44 | 0.9068 | 1 | 0.9068 | 0 | 0 | 0 | 1 | Ref |
| MK422455 | Unassigned | Unassigned | Unassigned | 249_1 | Clustered | 21 | VC_249_1 | 21 | 0.6775 | 1 | 0.6775 | 0 | 0 | 0 | 0.8476 | Ref |
| MK424903 | Unassigned | Unassigned | Unassigned | 284_0 | Clustered | 3 | VC_284_0 | 3 | 1 | 1 | 1 | 0 | 0 | 0 | 1 | Ref |
| MK433259 | Unassigned | Unassigned | Unassigned | 901_0 | Clustered | 31 | VC_901_0 | 31 | 0.8235 | 1 | 0.8235 | 0 | 0 | 0 | 0.8194 | Ref |
| MK433260 | Unassigned | Unassigned | Unassigned | 901_0 | Clustered | 31 | VC_901_0 | 31 | 0.8235 | 1 | 0.8235 | 0 | 0 | 0 | 0.8194 | Ref |
| MK433261 | Unassigned | Unassigned | Unassigned | 895_0 | Clustered | 25 | VC_895_0 | 25 | 0.5768 | 1 | 0.5768 | 0 | 0 | 0 | 1 | Ref |
| MK433262 | Unassigned | Unassigned | Unassigned | 571_8 | Clustered | 19 | VC_571_8 | 19 | 0.1031 | 1 | 0.1031 | 0 | 0 | 0 | 0.8713 | Ref |
| MK433263 | Unassigned | Unassigned | Unassigned | 895_0 | Clustered | 25 | VC_895_0 | 25 | 0.5768 | 1 | 0.5768 | 0 | 0 | 0 | 1 | Ref |
| MK433265 | Unassigned | Unassigned | Unassigned | 895_0 | Clustered | 25 | VC_895_0 | 25 | 0.5768 | 1 | 0.5768 | 0 | 0 | 0 | 1 | Ref |
| MK433266 | Unassigned | Unassigned | Unassigned | 386_0 | Clustered | 10 | VC_386_0 | 10 | 0.7454 | 1 | 0.7454 | 0 | 0 | 0 | 1 | Ref |
| MK433267 | Unassigned | Unassigned | Unassigned | 895_0 | Clustered | 25 | VC_895_0 | 25 | 0.5768 | 1 | 0.5768 | 0 | 0 | 0 | 1 | Ref |
| MK433268 | Unassigned | Unassigned | Unassigned | 901_0 | Clustered | 31 | VC_901_0 | 31 | 0.8235 | 1 | 0.8235 | 0 | 0 | 0 | 0.8194 | Ref |
| MK433269 | Unassigned | Unassigned | Unassigned | 901_0 | Clustered | 31 | VC_901_0 | 31 | 0.8235 | 1 | 0.8235 | 0 | 0 | 0 | 0.8194 | Ref |
| MK433270 | Unassigned | Unassigned | Unassigned | 1016_0 | Clustered | 62 | VC_1016_0 | 62 | 0.8638 | 1 | 0.8638 | 0 | 0 | 0 | 0.743 | Ref |
| MK433271 | Unassigned | Unassigned | Unassigned | 1016_0 | Clustered | 62 | VC_1016_0 | 62 | 0.8638 | 1 | 0.8638 | 0 | 0 | 0 | 0.743 | Ref |
| MK433272 | Unassigned | Unassigned | Unassigned | 789_0 | Clustered | 34 | VC_789_0 | 34 | 0.9167 | 1 | 0.9167 | 0 | 0 | 0 | 1 | Ref |
| MK433273 | Unassigned | Unassigned | Unassigned | 532_0 | Clustered | 75 | VC_532_0 | 75 | 0.9393 | 1 | 0.9393 | 0 | 0 | 0 | 1 | Ref |
| MK433274 | Unassigned | Unassigned | Unassigned | 701_0 | Clustered | 16 | VC_701_0 | 16 | 0.2957 | 1 | 0.2957 | 0 | 0 | 0 | 0.9167 | Ref |
| MK433275 | Unassigned | Unassigned | Unassigned | 701_0 | Clustered | 16 | VC_701_0 | 16 | 0.2957 | 1 | 0.2957 | 0 | 0 | 0 | 0.9167 | Ref |
| MK433276 | Unassigned | Unassigned | Unassigned | 1016_0 | Clustered | 62 | VC_1016_0 | 62 | 0.8638 | 1 | 0.8638 | 0 | 0 | 0 | 0.743 | Ref |
| MK433277 | Unassigned | Unassigned | Unassigned | 783_0 | Clustered | 67 | VC_783_0 | 67 | 0.8945 | 1 | 0.8945 | 0 | 0 | 0 | 1 | Ref |
| MK433278 | Unassigned | Unassigned | Unassigned | 1016_0 | Clustered | 62 | VC_1016_0 | 62 | 0.8638 | 1 | 0.8638 | 0 | 0 | 0 | 0.743 | Ref |
| MK433279 | Unassigned | Unassigned | Unassigned | 783_0 | Clustered | 67 | VC_783_0 | 67 | 0.8945 | 1 | 0.8945 | 0 | 0 | 0 | 1 | Ref |
| MK433577 | Unassigned | Unassigned | Unassigned | 6_0 | Clustered | 44 | VC_6_0 | 44 | 0.9068 | 1 | 0.9068 | 0 | 0 | 0 | 1 | Ref |
| MK433578 | Unassigned | Unassigned | Unassigned | 6_0 | Clustered | 44 | VC_6_0 | 44 | 0.9068 | 1 | 0.9068 | 0 | 0 | 0 | 1 | Ref |
| MK433579 | Unassigned | Unassigned | Unassigned | 249_1 | Clustered | 21 | VC_249_1 | 21 | 0.6775 | 1 | 0.6775 | 0 | 0 | 0 | 0.8476 | Ref |
| MK433580 | Unassigned | Unassigned | Unassigned | 6_0 | Clustered | 44 | VC_6_0 | 44 | 0.9068 | 1 | 0.9068 | 0 | 0 | 0 | 1 | Ref |
| MK433581 | Unassigned | Unassigned | Unassigned | 6_0 | Clustered | 44 | VC_6_0 | 44 | 0.9068 | 1 | 0.9068 | 0 | 0 | 0 | 1 | Ref |
| MK433582 | Unassigned | Unassigned | Unassigned | 6_0 | Clustered | 44 | VC_6_0 | 44 | 0.9068 | 1 | 0.9068 | 0 | 0 | 0 | 1 | Ref |
| MK443264 | Unassigned | Unassigned | Unassigned | 77_0 | Clustered | 37 | VC_77_0 | 37 | 0.9392 | 1 | 0.9392 | 0 | 0 | 0 | 1 | Ref |
| MK443265 | Unassigned | Unassigned | Unassigned | 77_1 | Clustered/Singleton | 1 | VC_77_1 | 1 | 0 | 1 | 0 | 0 | 0 | 0 | 1 | Ref |
| MK443266 | Unassigned | Unassigned | Unassigned | 173_0 | Clustered | 3 | VC_173_0 | 3 | 0.6871 | 0.99826575 | 0.6859 | 0 | 0 | 0 | 1 | Ref |
| MK448230 | Unassigned | Unassigned | Unassigned | 249_1 | Clustered | 21 | VC_249_1 | 21 | 0.6775 | 1 | 0.6775 | 0 | 0 | 0 | 0.8476 | Ref |
| MK448231 | Unassigned | Unassigned | Unassigned | 33_0 | Clustered | 9 | VC_33_0 | 9 | 0.3409 | 1 | 0.3409 | 0 | 0 | 0 | 0.6944 | Ref |
| MK448233 | Unassigned | Unassigned | Unassigned | 33_0 | Clustered | 9 | VC_33_0 | 9 | 0.3409 | 1 | 0.3409 | 0 | 0 | 0 | 0.6944 | Ref |
| MK448234 | Unassigned | Unassigned | Unassigned | 38_0 | Clustered | 4 | VC_38_0 | 4 | 0.1746 | 0.99998341 | 0.1746 | 0 | 0 | 0 | 1 | Ref |
| MK448235 | Unassigned | Unassigned | Unassigned | 33_0 | Clustered | 9 | VC_33_0 | 9 | 0.3409 | 1 | 0.3409 | 0 | 0 | 0 | 0.6944 | Ref |
| MK448237 | Unassigned | Unassigned | Unassigned | 38_0 | Clustered | 4 | VC_38_0 | 4 | 0.1746 | 0.99998341 | 0.1746 | 0 | 0 | 0 | 1 | Ref |
| MK448667 | Unassigned | Unassigned | Unassigned | 649_1 | Clustered | 14 | VC_649_1 | 14 | 0.5226 | 1 | 0.5226 | 0 | 0 | 0 | 0.8352 | Ref |
| MK448671 | Unassigned | Unassigned | Unassigned | 425_0 | Clustered | 40 | VC_425_0 | 40 | 0.8399 | 1 | 0.8399 | 0 | 0 | 0 | 0.9974 | Ref |
| MK448672 | Unassigned | Unassigned | Unassigned | 757_0 | Clustered | 5 | VC_757_0 | 5 | 0.0972 | 0.99999635 | 0.0972 | 0 | 0 | 0 | 0.6 | Ref |
| MK448673 | Unassigned | Unassigned | Unassigned | 797_1 | Clustered | 14 | VC_797_1 | 14 | 0.2785 | 1 | 0.2785 | 0 | 0 | 0 | 0.7582 | Ref |
| MK448674 | Unassigned | Unassigned | Unassigned | 647_0 | Clustered | 37 | VC_647_0 | 37 | 0.5847 | 1 | 0.5847 | 0 | 0 | 0 | 0.9234 | Ref |
| MK448675 | Unassigned | Unassigned | Unassigned | 300_3 | Clustered | 19 | VC_300_3 | 19 | 0.2631 | 1 | 0.2631 | 0 | 0 | 0 | 0.8187 | Ref |
| MK448676 | Unassigned | Unassigned | Unassigned | 300_3 | Clustered | 19 | VC_300_3 | 19 | 0.2631 | 1 | 0.2631 | 0 | 0 | 0 | 0.8187 | Ref |
| MK448678 | Unassigned | Unassigned | Unassigned | 795_0 | Clustered | 23 | VC_795_0 | 23 | 0.5497 | 1 | 0.5497 | 0 | 0 | 0 | 1 | Ref |
| MK448680 | Unassigned | Unassigned | Unassigned | 431_0 | Clustered | 5 | VC_431_0 | 5 | 0.2522 | 0.99999997 | 0.2522 | 0 | 0 | 0 | 1 | Ref |
| MK448681 | Unassigned | Unassigned | Unassigned | 653_0 | Clustered | 9 | VC_653_0 | 9 | 0.2315 | 1 | 0.2315 | 0 | 0 | 0 | 0.9722 | Ref |
| MK448683 | Unassigned | Unassigned | Unassigned | 653_0 | Clustered | 9 | VC_653_0 | 9 | 0.2315 | 1 | 0.2315 | 0 | 0 | 0 | 0.9722 | Ref |
| MK448684 | Unassigned | Unassigned | Unassigned | 431_0 | Clustered | 5 | VC_431_0 | 5 | 0.2522 | 0.99999997 | 0.2522 | 0 | 0 | 0 | 1 | Ref |
| MK448686 | Unassigned | Unassigned | Unassigned | 429_0 | Clustered | 14 | VC_429_0 | 14 | 0.2582 | 1 | 0.2582 | 0 | 0 | 0 | 0.989 | Ref |
| MK448688 | Unassigned | Unassigned | Unassigned | 300_3 | Clustered | 19 | VC_300_3 | 19 | 0.2631 | 1 | 0.2631 | 0 | 0 | 0 | 0.8187 | Ref |
| MK448689 | Unassigned | Unassigned | Unassigned | 795_0 | Clustered | 23 | VC_795_0 | 23 | 0.5497 | 1 | 0.5497 | 0 | 0 | 0 | 1 | Ref |
| MK448690 | Unassigned | Unassigned | Unassigned | 300_3 | Clustered | 19 | VC_300_3 | 19 | 0.2631 | 1 | 0.2631 | 0 | 0 | 0 | 0.8187 | Ref |
| MK448692 | Unassigned | Unassigned | Unassigned | 445_0 | Clustered | 9 | VC_445_0 | 9 | 0.1674 | 1 | 0.1674 | 0 | 0 | 0 | 1 | Ref |
| MK448693 | Unassigned | Unassigned | Unassigned | 795_0 | Clustered | 23 | VC_795_0 | 23 | 0.5497 | 1 | 0.5497 | 0 | 0 | 0 | 1 | Ref |
| MK448694 | Unassigned | Unassigned | Unassigned | 300_3 | Clustered | 19 | VC_300_3 | 19 | 0.2631 | 1 | 0.2631 | 0 | 0 | 0 | 0.8187 | Ref |
| MK448696 | Unassigned | Unassigned | Unassigned | 795_0 | Clustered | 23 | VC_795_0 | 23 | 0.5497 | 1 | 0.5497 | 0 | 0 | 0 | 1 | Ref |
| MK448697 | Unassigned | Unassigned | Unassigned | 795_0 | Clustered | 23 | VC_795_0 | 23 | 0.5497 | 1 | 0.5497 | 0 | 0 | 0 | 1 | Ref |
| MK448698 | Unassigned | Unassigned | Unassigned | 431_0 | Clustered | 5 | VC_431_0 | 5 | 0.2522 | 0.99999997 | 0.2522 | 0 | 0 | 0 | 1 | Ref |
| MK448699 | Unassigned | Unassigned | Unassigned | 795_0 | Clustered | 23 | VC_795_0 | 23 | 0.5497 | 1 | 0.5497 | 0 | 0 | 0 | 1 | Ref |
| MK448700 | Unassigned | Unassigned | Unassigned | 649_1 | Clustered | 14 | VC_649_1 | 14 | 0.5226 | 1 | 0.5226 | 0 | 0 | 0 | 0.8352 | Ref |
| MK448702 | Unassigned | Unassigned | Unassigned | 445_0 | Clustered | 9 | VC_445_0 | 9 | 0.1674 | 1 | 0.1674 | 0 | 0 | 0 | 1 | Ref |
| MK448704 | Unassigned | Unassigned | Unassigned | 299_0 | Clustered | 4 | VC_299_0 | 4 | 0.1735 | 0.99998669 | 0.1735 | 0 | 0 | 0 | 1 | Ref |
| MK448706 | Unassigned | Unassigned | Unassigned | 268_0 | Clustered | 10 | VC_268_0 | 10 | 0.1417 | 1 | 0.1417 | 0 | 0 | 0 | 0.9333 | Ref |
| MK448707 | Unassigned | Unassigned | Unassigned | 299_0 | Clustered | 4 | VC_299_0 | 4 | 0.1735 | 0.99998669 | 0.1735 | 0 | 0 | 0 | 1 | Ref |
| MK448708 | Unassigned | Unassigned | Unassigned | 425_0 | Clustered | 40 | VC_425_0 | 40 | 0.8399 | 1 | 0.8399 | 0 | 0 | 0 | 0.9974 | Ref |
| MK448709 | Unassigned | Unassigned | Unassigned | 299_0 | Clustered | 4 | VC_299_0 | 4 | 0.1735 | 0.99998669 | 0.1735 | 0 | 0 | 0 | 1 | Ref |
| MK448710 | Unassigned | Unassigned | Unassigned | 299_0 | Clustered | 4 | VC_299_0 | 4 | 0.1735 | 0.99998669 | 0.1735 | 0 | 0 | 0 | 1 | Ref |
| MK448711 | Unassigned | Unassigned | Unassigned | 445_0 | Clustered | 9 | VC_445_0 | 9 | 0.1674 | 1 | 0.1674 | 0 | 0 | 0 | 1 | Ref |
| MK448714 | Unassigned | Unassigned | Unassigned | 425_0 | Clustered | 40 | VC_425_0 | 40 | 0.8399 | 1 | 0.8399 | 0 | 0 | 0 | 0.9974 | Ref |
| MK448716 | Unassigned | Unassigned | Unassigned | 445_0 | Clustered | 9 | VC_445_0 | 9 | 0.1674 | 1 | 0.1674 | 0 | 0 | 0 | 1 | Ref |
| MK448720 | Unassigned | Unassigned | Unassigned | 649_1 | Clustered | 14 | VC_649_1 | 14 | 0.5226 | 1 | 0.5226 | 0 | 0 | 0 | 0.8352 | Ref |

| | | | | | | | | | | | | | | | | |
|---|---|---|---|---|---|---|---|---|---|---|---|---|---|---|---|---|
| MK448721 | Unassigned | Unassigned | Unassigned | 268_0 | Clustered | 10 | VC_268_0 | 10 | 0.1417 | 1 | 0.1417 | 0 | 0 | 0 | 0.9333 | Ref |
| MK448724 | Unassigned | Unassigned | Unassigned | 445_0 | Clustered | 9 | VC_445_0 | 9 | 0.1674 | 1 | 0.1674 | 0 | 0 | 0 | 1 | Ref |
| MK448725 | Unassigned | Unassigned | Unassigned | 344_0 | Clustered | 30 | VC_344_0 | 30 | 0.523 | 1 | 0.523 | 0 | 0 | 0 | 0.9954 | Ref |
| MK448730 | Unassigned | Unassigned | Unassigned | 445_0 | Clustered | 9 | VC_445_0 | 9 | 0.1674 | 1 | 0.1674 | 0 | 0 | 0 | 1 | Ref |
| MK448732 | Unassigned | Unassigned | Unassigned | 344_0 | Clustered | 30 | VC_344_0 | 30 | 0.523 | 1 | 0.523 | 0 | 0 | 0 | 0.9954 | Ref |
| MK448733 | Unassigned | Unassigned | Unassigned | 426_0 | Clustered | 3 | VC_426_0 | 3 | 0.041 | 0.99840705 | 0.0409 | 0 | 0 | 0 | 1 | Ref |
| MK448735 | Unassigned | Unassigned | Unassigned | 331_2 | Clustered | 7 | VC_331_2 | 7 | 0.1554 | 1 | 0.1554 | 0 | 0 | 0 | 0.9524 | Ref |
| MK448739 | Unassigned | Unassigned | Unassigned | 344_0 | Clustered | 30 | VC_344_0 | 30 | 0.523 | 1 | 0.523 | 0 | 0 | 0 | 0.9954 | Ref |
| MK448740 | Unassigned | Unassigned | Unassigned | 300_2 | Clustered | 2 | VC_300_2 | 2 | 0.0333 | 0.9570047 | 0.0319 | 0 | 0 | 0 | 1 | Ref |
| MK448741 | Unassigned | Unassigned | Unassigned | 647_0 | Clustered | 37 | VC_647_0 | 37 | 0.5847 | 1 | 0.5847 | 0 | 0 | 0 | 0.9234 | Ref |
| MK448743 | Unassigned | Unassigned | Unassigned | 331_2 | Clustered | 7 | VC_331_2 | 7 | 0.1554 | 1 | 0.1554 | 0 | 0 | 0 | 0.9524 | Ref |
| MK448744 | Unassigned | Unassigned | Unassigned | 445_0 | Clustered | 9 | VC_445_0 | 9 | 0.1674 | 1 | 0.1674 | 0 | 0 | 0 | 1 | Ref |
| MK448745 | Unassigned | Unassigned | Unassigned | 445_0 | Clustered | 9 | VC_445_0 | 9 | 0.1674 | 1 | 0.1674 | 0 | 0 | 0 | 1 | Ref |
| MK448750 | Unassigned | Unassigned | Unassigned | 300_0 | Clustered/Singleton | 1 | VC_300_0 | 1 | 0 | 1 | 0 | 0 | 0 | 0 | 1 | Ref |
| MK448752 | Unassigned | Unassigned | Unassigned | 647_0 | Clustered | 37 | VC_647_0 | 37 | 0.5847 | 1 | 0.5847 | 0 | 0 | 0 | 0.9234 | Ref |
| MK448757 | Unassigned | Unassigned | Unassigned | 331_1 | Clustered | 5 | VC_331_1 | 5 | 0.1517 | 0.99999997 | 0.1517 | 0 | 0 | 0 | 1 | Ref |
| MK448758 | Unassigned | Unassigned | Unassigned | 331_1 | Clustered | 5 | VC_331_1 | 5 | 0.1517 | 0.99999997 | 0.1517 | 0 | 0 | 0 | 1 | Ref |
| MK448759 | Unassigned | Unassigned | Unassigned | 331_1 | Clustered | 5 | VC_331_1 | 5 | 0.1517 | 0.99999997 | 0.1517 | 0 | 0 | 0 | 1 | Ref |
| MK448762 | Unassigned | Unassigned | Unassigned | 429_0 | Clustered | 14 | VC_429_0 | 14 | 0.2582 | 1 | 0.2582 | 0 | 0 | 0 | 0.989 | Ref |
| MK448765 | Unassigned | Unassigned | Unassigned | 797_1 | Clustered | 14 | VC_797_1 | 14 | 0.2785 | 1 | 0.2785 | 0 | 0 | 0 | 0.7582 | Ref |
| MK448766 | Unassigned | Unassigned | Unassigned | 795_0 | Clustered | 23 | VC_795_0 | 23 | 0.5497 | 1 | 0.5497 | 0 | 0 | 0 | 1 | Ref |
| MK448769 | Unassigned | Unassigned | Unassigned | 431_0 | Clustered | 5 | VC_431_0 | 5 | 0.2522 | 0.99999997 | 0.2522 | 0 | 0 | 0 | 1 | Ref |
| MK448772 | Unassigned | Unassigned | Unassigned | 300_3 | Clustered | 19 | VC_300_3 | 19 | 0.2631 | 1 | 0.2631 | 0 | 0 | 0 | 0.8187 | Ref |
| MK448775 | Unassigned | Unassigned | Unassigned | 797_1 | Clustered | 14 | VC_797_1 | 14 | 0.2785 | 1 | 0.2785 | 0 | 0 | 0 | 0.7582 | Ref |
| MK448778 | Unassigned | Unassigned | Unassigned | 300_3 | Clustered | 19 | VC_300_3 | 19 | 0.2631 | 1 | 0.2631 | 0 | 0 | 0 | 0.8187 | Ref |
| MK448779 | Unassigned | Unassigned | Unassigned | 795_0 | Clustered | 23 | VC_795_0 | 23 | 0.5497 | 1 | 0.5497 | 0 | 0 | 0 | 1 | Ref |
| MK448782 | Unassigned | Unassigned | Unassigned | 300_3 | Clustered | 19 | VC_300_3 | 19 | 0.2631 | 1 | 0.2631 | 0 | 0 | 0 | 0.8187 | Ref |
| MK448783 | Unassigned | Unassigned | Unassigned | 653_0 | Clustered | 9 | VC_653_0 | 9 | 0.2315 | 1 | 0.2315 | 0 | 0 | 0 | 0.9722 | Ref |
| MK448784 | Unassigned | Unassigned | Unassigned | 795_0 | Clustered | 23 | VC_795_0 | 23 | 0.5497 | 1 | 0.5497 | 0 | 0 | 0 | 1 | Ref |
| MK448788 | Unassigned | Unassigned | Unassigned | 429_0 | Clustered | 14 | VC_429_0 | 14 | 0.2582 | 1 | 0.2582 | 0 | 0 | 0 | 0.989 | Ref |
| MK448789 | Unassigned | Unassigned | Unassigned | 795_0 | Clustered | 23 | VC_795_0 | 23 | 0.5497 | 1 | 0.5497 | 0 | 0 | 0 | 1 | Ref |
| MK448790 | Unassigned | Unassigned | Unassigned | 653_0 | Clustered | 9 | VC_653_0 | 9 | 0.2315 | 1 | 0.2315 | 0 | 0 | 0 | 0.9722 | Ref |
| MK448791 | Unassigned | Unassigned | Unassigned | 429_0 | Clustered | 14 | VC_429_0 | 14 | 0.2582 | 1 | 0.2582 | 0 | 0 | 0 | 0.989 | Ref |
| MK448793 | Unassigned | Unassigned | Unassigned | 429_0 | Clustered | 14 | VC_429_0 | 14 | 0.2582 | 1 | 0.2582 | 0 | 0 | 0 | 0.989 | Ref |
| MK448794 | Unassigned | Unassigned | Unassigned | 797_1 | Clustered | 14 | VC_797_1 | 14 | 0.2785 | 1 | 0.2785 | 0 | 0 | 0 | 0.7582 | Ref |
| MK448795 | Unassigned | Unassigned | Unassigned | 797_1 | Clustered | 14 | VC_797_1 | 14 | 0.2785 | 1 | 0.2785 | 0 | 0 | 0 | 0.7582 | Ref |
| MK448797 | Unassigned | Unassigned | Unassigned | 795_0 | Clustered | 23 | VC_795_0 | 23 | 0.5497 | 1 | 0.5497 | 0 | 0 | 0 | 1 | Ref |
| MK448798 | Unassigned | Unassigned | Unassigned | 653_0 | Clustered | 9 | VC_653_0 | 9 | 0.2315 | 1 | 0.2315 | 0 | 0 | 0 | 0.9722 | Ref |
| MK448804 | Unassigned | Unassigned | Unassigned | 347_0 | Clustered | 6 | VC_347_0 | 6 | 0.3746 | 1 | 0.3746 | 0 | 0 | 0 | 1 | Ref |
| MK448805 | Unassigned | Unassigned | Unassigned | 347_0 | Clustered | 6 | VC_347_0 | 6 | 0.3746 | 1 | 0.3746 | 0 | 0 | 0 | 1 | Ref |
| MK448807 | Unassigned | Unassigned | Unassigned | 347_0 | Clustered | 6 | VC_347_0 | 6 | 0.3746 | 1 | 0.3746 | 0 | 0 | 0 | 1 | Ref |
| MK448808 | Unassigned | Unassigned | Unassigned | 347_0 | Clustered | 6 | VC_347_0 | 6 | 0.3746 | 1 | 0.3746 | 0 | 0 | 0 | 1 | Ref |
| MK448811 | Unassigned | Unassigned | Unassigned | 647_0 | Clustered | 37 | VC_647_0 | 37 | 0.5847 | 1 | 0.5847 | 0 | 0 | 0 | 0.9234 | Ref |
| MK448814 | Unassigned | Unassigned | Unassigned | 268_1 | Clustered | 20 | VC_268_1 | 20 | 0.1906 | 1 | 0.1906 | 0 | 0 | 0 | 0.6789 | Ref |
| MK448815 | Unassigned | Unassigned | Unassigned | 347_0 | Clustered | 6 | VC_347_0 | 6 | 0.3746 | 1 | 0.3746 | 0 | 0 | 0 | 1 | Ref |
| MK448818 | Unassigned | Unassigned | Unassigned | 814_0 | Clustered/Singleton | 1 | VC_814_0 | 1 | 0 | 1 | 0 | 0 | 0 | 0 | 1 | Ref |
| MK448819 | Unassigned | Unassigned | Unassigned | 647_0 | Clustered | 37 | VC_647_0 | 37 | 0.5847 | 1 | 0.5847 | 0 | 0 | 0 | 0.9234 | Ref |
| MK448820 | Unassigned | Unassigned | Unassigned | 426_1 | Clustered/Singleton | 1 | VC_426_1 | 1 | 0 | 1 | 0 | 0 | 0 | 0 | 1 | Ref |
| MK448821 | Unassigned | Unassigned | Unassigned | 331_0 | Clustered | 2 | VC_331_0 | 2 | 0.0289 | 0.95661675 | 0.0276 | 0 | 0 | 0 | 1 | Ref |
| MK448822 | Unassigned | Unassigned | Unassigned | 331_0 | Clustered | 2 | VC_331_0 | 2 | 0.0289 | 0.95661675 | 0.0276 | 0 | 0 | 0 | 1 | Ref |
| MK448824 | Unassigned | Unassigned | Unassigned | 265_0 | Clustered | 3 | VC_265_0 | 3 | 0.1136 | 0.9985864 | 0.1135 | 0 | 0 | 0 | 1 | Ref |
| MK448828 | Unassigned | Unassigned | Unassigned | 647_0 | Clustered | 37 | VC_647_0 | 37 | 0.5847 | 1 | 0.5847 | 0 | 0 | 0 | 0.9234 | Ref |
| MK448830 | Unassigned | Unassigned | Unassigned | 344_0 | Clustered | 30 | VC_344_0 | 30 | 0.523 | 1 | 0.523 | 0 | 0 | 0 | 0.9954 | Ref |
| MK448831 | Unassigned | Unassigned | Unassigned | 425_0 | Clustered | 40 | VC_425_0 | 40 | 0.8399 | 1 | 0.8399 | 0 | 0 | 0 | 0.9974 | Ref |
| MK448832 | Unassigned | Unassigned | Unassigned | 301_0 | Clustered | 2 | VC_301_0 | 2 | 0.0851 | 0.95735478 | 0.0814 | 0 | 0 | 0 | 1 | Ref |
| MK448833 | Unassigned | Unassigned | Unassigned | 653_0 | Clustered | 9 | VC_653_0 | 9 | 0.2315 | 1 | 0.2315 | 0 | 0 | 0 | 0.9722 | Ref |
| MK448834 | Unassigned | Unassigned | Unassigned | 425_0 | Clustered | 40 | VC_425_0 | 40 | 0.8399 | 1 | 0.8399 | 0 | 0 | 0 | 0.9974 | Ref |
| MK448839 | Unassigned | Unassigned | Unassigned | 649_1 | Clustered | 14 | VC_649_1 | 14 | 0.5226 | 1 | 0.5226 | 0 | 0 | 0 | 0.8352 | Ref |
| MK448841 | Unassigned | Unassigned | Unassigned | 649_1 | Clustered | 14 | VC_649_1 | 14 | 0.5226 | 1 | 0.5226 | 0 | 0 | 0 | 0.8352 | Ref |
| MK448842 | Unassigned | Unassigned | Unassigned | 344_0 | Clustered | 30 | VC_344_0 | 30 | 0.523 | 1 | 0.523 | 0 | 0 | 0 | 0.9954 | Ref |
| MK448843 | Unassigned | Unassigned | Unassigned | 649_1 | Clustered | 14 | VC_649_1 | 14 | 0.5226 | 1 | 0.5226 | 0 | 0 | 0 | 0.8352 | Ref |
| MK448849 | Unassigned | Unassigned | Unassigned | 757_0 | Clustered | 5 | VC_757_0 | 5 | 0.0972 | 0.99999635 | 0.0972 | 0 | 0 | 0 | 0.6 | Ref |
| MK448850 | Unassigned | Unassigned | Unassigned | 300_3 | Clustered | 19 | VC_300_3 | 19 | 0.2631 | 1 | 0.2631 | 0 | 0 | 0 | 0.8187 | Ref |
| MK448852 | Unassigned | Unassigned | Unassigned | 795_0 | Clustered | 23 | VC_795_0 | 23 | 0.5497 | 1 | 0.5497 | 0 | 0 | 0 | 1 | Ref |
| MK448854 | Unassigned | Unassigned | Unassigned | 797_0 | Clustered | 3 | VC_797_0 | 3 | 0.036 | 0.99850226 | 0.036 | 0 | 0 | 0 | 1 | Ref |
| MK448858 | Unassigned | Unassigned | Unassigned | 797_1 | Clustered | 14 | VC_797_1 | 14 | 0.2785 | 1 | 0.2785 | 0 | 0 | 0 | 0.7582 | Ref |
| MK448859 | Unassigned | Unassigned | Unassigned | 300_3 | Clustered | 19 | VC_300_3 | 19 | 0.2631 | 1 | 0.2631 | 0 | 0 | 0 | 0.8187 | Ref |
| MK448861 | Unassigned | Unassigned | Unassigned | 425_0 | Clustered | 40 | VC_425_0 | 40 | 0.8399 | 1 | 0.8399 | 0 | 0 | 0 | 0.9974 | Ref |
| MK448862 | Unassigned | Unassigned | Unassigned | 429_0 | Clustered | 14 | VC_429_0 | 14 | 0.2582 | 1 | 0.2582 | 0 | 0 | 0 | 0.989 | Ref |
| MK448864 | Unassigned | Unassigned | Unassigned | 797_1 | Clustered | 14 | VC_797_1 | 14 | 0.2785 | 1 | 0.2785 | 0 | 0 | 0 | 0.7582 | Ref |
| MK448866 | Unassigned | Unassigned | Unassigned | 795_0 | Clustered | 23 | VC_795_0 | 23 | 0.5497 | 1 | 0.5497 | 0 | 0 | 0 | 1 | Ref |
| MK448867 | Unassigned | Unassigned | Unassigned | 795_0 | Clustered | 23 | VC_795_0 | 23 | 0.5497 | 1 | 0.5497 | 0 | 0 | 0 | 1 | Ref |
| MK448868 | Unassigned | Unassigned | Unassigned | 795_0 | Clustered | 23 | VC_795_0 | 23 | 0.5497 | 1 | 0.5497 | 0 | 0 | 0 | 1 | Ref |
| MK448871 | Unassigned | Unassigned | Unassigned | 795_0 | Clustered | 23 | VC_795_0 | 23 | 0.5497 | 1 | 0.5497 | 0 | 0 | 0 | 1 | Ref |
| MK448874 | Unassigned | Unassigned | Unassigned | 425_0 | Clustered | 40 | VC_425_0 | 40 | 0.8399 | 1 | 0.8399 | 0 | 0 | 0 | 0.9974 | Ref |
| MK448875 | Unassigned | Unassigned | Unassigned | 425_0 | Clustered | 40 | VC_425_0 | 40 | 0.8399 | 1 | 0.8399 | 0 | 0 | 0 | 0.9974 | Ref |
| MK448877 | Unassigned | Unassigned | Unassigned | 300_3 | Clustered | 19 | VC_300_3 | 19 | 0.2631 | 1 | 0.2631 | 0 | 0 | 0 | 0.8187 | Ref |
| MK448881 | Unassigned | Unassigned | Unassigned | 268_1 | Clustered | 20 | VC_268_1 | 20 | 0.1906 | 1 | 0.1906 | 0 | 0 | 0 | 0.6789 | Ref |
| MK448882 | Unassigned | Unassigned | Unassigned | 268_1 | Clustered | 20 | VC_268_1 | 20 | 0.1906 | 1 | 0.1906 | 0 | 0 | 0 | 0.6789 | Ref |
| MK448886 | Unassigned | Unassigned | Unassigned | 425_0 | Clustered | 40 | VC_425_0 | 40 | 0.8399 | 1 | 0.8399 | 0 | 0 | 0 | 0.9974 | Ref |
| MK448887 | Unassigned | Unassigned | Unassigned | 425_0 | Clustered | 40 | VC_425_0 | 40 | 0.8399 | 1 | 0.8399 | 0 | 0 | 0 | 0.9974 | Ref |

| | | | | | | | | | | | | | | | | |
|---|---|---|---|---|---|---|---|---|---|---|---|---|---|---|---|---|
| MK448889 | Unassigned | Unassigned | Unassigned | 757_0 | Clustered | 5 | VC_757_0 | 5 | 0.0972 | 0.99999635 | 0.0972 | 0 | 0 | 0 | 0.6 | Ref |
| MK448893 | Unassigned | Unassigned | Unassigned | 331_2 | Clustered | 7 | VC_331_2 | 7 | 0.1554 | 1 | 0.1554 | 0 | 0 | 0 | 0.9524 | Ref |
| MK448894 | Unassigned | Unassigned | Unassigned | 344_0 | Clustered | 30 | VC_344_0 | 30 | 0.523 | 1 | 0.523 | 0 | 0 | 0 | 0.9954 | Ref |
| MK448895 | Unassigned | Unassigned | Unassigned | 431_0 | Clustered | 5 | VC_431_0 | 5 | 0.2522 | 0.99999997 | 0.2522 | 0 | 0 | 0 | 1 | Ref |
| MK448896 | Unassigned | Unassigned | Unassigned | 268_1 | Clustered | 20 | VC_268_1 | 20 | 0.1906 | 1 | 0.1906 | 0 | 0 | 0 | 0.6789 | Ref |
| MK448897 | Unassigned | Unassigned | Unassigned | 425_0 | Clustered | 40 | VC_425_0 | 40 | 0.8399 | 1 | 0.8399 | 0 | 0 | 0 | 0.9974 | Ref |
| MK448898 | Unassigned | Unassigned | Unassigned | 425_0 | Clustered | 40 | VC_425_0 | 40 | 0.8399 | 1 | 0.8399 | 0 | 0 | 0 | 0.9974 | Ref |
| MK448900 | Unassigned | Unassigned | Unassigned | 268_3 | Clustered/Singleton | 1 | VC_268_3 | 1 | 0 | 1 | 0 | 0 | 0 | 0 | 1 | Ref |
| MK448901 | Unassigned | Unassigned | Unassigned | 344_0 | Clustered | 30 | VC_344_0 | 30 | 0.523 | 1 | 0.523 | 0 | 0 | 0 | 0.9954 | Ref |
| MK448903 | Unassigned | Unassigned | Unassigned | 331_2 | Clustered | 7 | VC_331_2 | 7 | 0.1554 | 1 | 0.1554 | 0 | 0 | 0 | 0.9524 | Ref |
| MK448904 | Unassigned | Unassigned | Unassigned | 426_0 | Clustered | 3 | VC_426_0 | 3 | 0.041 | 0.99840705 | 0.0409 | 0 | 0 | 0 | 1 | Ref |
| MK448905 | Unassigned | Unassigned | Unassigned | 425_0 | Clustered | 40 | VC_425_0 | 40 | 0.8399 | 1 | 0.8399 | 0 | 0 | 0 | 0.9974 | Ref |
| MK448909 | Unassigned | Unassigned | Unassigned | 268_1 | Clustered | 20 | VC_268_1 | 20 | 0.1906 | 1 | 0.1906 | 0 | 0 | 0 | 0.6789 | Ref |
| MK448912 | Unassigned | Unassigned | Unassigned | 344_0 | Clustered | 30 | VC_344_0 | 30 | 0.523 | 1 | 0.523 | 0 | 0 | 0 | 0.9954 | Ref |
| MK448914 | Unassigned | Unassigned | Unassigned | 344_0 | Clustered | 30 | VC_344_0 | 30 | 0.523 | 1 | 0.523 | 0 | 0 | 0 | 0.9954 | Ref |
| MK448915 | Unassigned | Unassigned | Unassigned | 331_2 | Clustered | 7 | VC_331_2 | 7 | 0.1554 | 1 | 0.1554 | 0 | 0 | 0 | 0.9524 | Ref |
| MK448917 | Unassigned | Unassigned | Unassigned | 300_2 | Clustered | 2 | VC_300_2 | 2 | 0.0333 | 0.9570047 | 0.0319 | 0 | 0 | 0 | 1 | Ref |
| MK448918 | Unassigned | Unassigned | Unassigned | 344_0 | Clustered | 30 | VC_344_0 | 30 | 0.523 | 1 | 0.523 | 0 | 0 | 0 | 0.9954 | Ref |
| MK448919 | Unassigned | Unassigned | Unassigned | 331_2 | Clustered | 7 | VC_331_2 | 7 | 0.1554 | 1 | 0.1554 | 0 | 0 | 0 | 0.9524 | Ref |
| MK448920 | Unassigned | Unassigned | Unassigned | 344_0 | Clustered | 30 | VC_344_0 | 30 | 0.523 | 1 | 0.523 | 0 | 0 | 0 | 0.9954 | Ref |
| MK448921 | Unassigned | Unassigned | Unassigned | 331_2 | Clustered | 7 | VC_331_2 | 7 | 0.1554 | 1 | 0.1554 | 0 | 0 | 0 | 0.9524 | Ref |
| MK448923 | Unassigned | Unassigned | Unassigned | 445_0 | Clustered | 9 | VC_445_0 | 9 | 0.1674 | 1 | 0.1674 | 0 | 0 | 0 | 1 | Ref |
| MK448925 | Unassigned | Unassigned | Unassigned | 425_0 | Clustered | 40 | VC_425_0 | 40 | 0.8399 | 1 | 0.8399 | 0 | 0 | 0 | 0.9974 | Ref |
| MK448927 | Unassigned | Unassigned | Unassigned | 425_0 | Clustered | 40 | VC_425_0 | 40 | 0.8399 | 1 | 0.8399 | 0 | 0 | 0 | 0.9974 | Ref |
| MK448931 | Unassigned | Unassigned | Unassigned | 300_3 | Clustered | 19 | VC_300_3 | 19 | 0.2631 | 1 | 0.2631 | 0 | 0 | 0 | 0.8187 | Ref |
| MK448933 | Unassigned | Unassigned | Unassigned | 797_0 | Clustered | 3 | VC_797_0 | 3 | 0.036 | 0.99850226 | 0.036 | 0 | 0 | 0 | 1 | Ref |
| MK448934 | Unassigned | Unassigned | Unassigned | 649_1 | Clustered | 14 | VC_649_1 | 14 | 0.5226 | 1 | 0.5226 | 0 | 0 | 0 | 0.8352 | Ref |
| MK448939 | Unassigned | Unassigned | Unassigned | 331_1 | Clustered | 5 | VC_331_1 | 5 | 0.1517 | 0.99999997 | 0.1517 | 0 | 0 | 0 | 1 | Ref |
| MK448941 | Unassigned | Unassigned | Unassigned | 797_1 | Clustered | 14 | VC_797_1 | 14 | 0.2785 | 1 | 0.2785 | 0 | 0 | 0 | 0.7582 | Ref |
| MK448944 | Unassigned | Unassigned | Unassigned | 797_1 | Clustered | 14 | VC_797_1 | 14 | 0.2785 | 1 | 0.2785 | 0 | 0 | 0 | 0.7582 | Ref |
| MK448945 | Unassigned | Unassigned | Unassigned | 797_1 | Clustered | 14 | VC_797_1 | 14 | 0.2785 | 1 | 0.2785 | 0 | 0 | 0 | 0.7582 | Ref |
| MK448946 | Unassigned | Unassigned | Unassigned | 797_1 | Clustered | 14 | VC_797_1 | 14 | 0.2785 | 1 | 0.2785 | 0 | 0 | 0 | 0.7582 | Ref |
| MK448947 | Unassigned | Unassigned | Unassigned | 795_0 | Clustered | 23 | VC_795_0 | 23 | 0.5497 | 1 | 0.5497 | 0 | 0 | 0 | 1 | Ref |
| MK448949 | Unassigned | Unassigned | Unassigned | 429_0 | Clustered | 14 | VC_429_0 | 14 | 0.2582 | 1 | 0.2582 | 0 | 0 | 0 | 0.989 | Ref |
| MK448950 | Unassigned | Unassigned | Unassigned | 797_0 | Clustered | 3 | VC_797_0 | 3 | 0.036 | 0.99850226 | 0.036 | 0 | 0 | 0 | 1 | Ref |
| MK448951 | Unassigned | Unassigned | Unassigned | 429_0 | Clustered | 14 | VC_429_0 | 14 | 0.2582 | 1 | 0.2582 | 0 | 0 | 0 | 0.989 | Ref |
| MK448952 | Unassigned | Unassigned | Unassigned | 795_0 | Clustered | 23 | VC_795_0 | 23 | 0.5497 | 1 | 0.5497 | 0 | 0 | 0 | 1 | Ref |
| MK448953 | Unassigned | Unassigned | Unassigned | 797_1 | Clustered | 14 | VC_797_1 | 14 | 0.2785 | 1 | 0.2785 | 0 | 0 | 0 | 0.7582 | Ref |
| MK448954 | Unassigned | Unassigned | Unassigned | 429_0 | Clustered | 14 | VC_429_0 | 14 | 0.2582 | 1 | 0.2582 | 0 | 0 | 0 | 0.989 | Ref |
| MK448955 | Unassigned | Unassigned | Unassigned | 300_3 | Clustered | 19 | VC_300_3 | 19 | 0.2631 | 1 | 0.2631 | 0 | 0 | 0 | 0.8187 | Ref |
| MK448957 | Unassigned | Unassigned | Unassigned | 797_1 | Clustered | 14 | VC_797_1 | 14 | 0.2785 | 1 | 0.2785 | 0 | 0 | 0 | 0.7582 | Ref |
| MK448958 | Unassigned | Unassigned | Unassigned | 429_0 | Clustered | 14 | VC_429_0 | 14 | 0.2582 | 1 | 0.2582 | 0 | 0 | 0 | 0.989 | Ref |
| MK448959 | Unassigned | Unassigned | Unassigned | 429_0 | Clustered | 14 | VC_429_0 | 14 | 0.2582 | 1 | 0.2582 | 0 | 0 | 0 | 0.989 | Ref |
| MK448960 | Unassigned | Unassigned | Unassigned | 429_0 | Clustered | 14 | VC_429_0 | 14 | 0.2582 | 1 | 0.2582 | 0 | 0 | 0 | 0.989 | Ref |
| MK448961 | Unassigned | Unassigned | Unassigned | 795_0 | Clustered | 23 | VC_795_0 | 23 | 0.5497 | 1 | 0.5497 | 0 | 0 | 0 | 1 | Ref |
| MK448962 | Unassigned | Unassigned | Unassigned | 300_3 | Clustered | 19 | VC_300_3 | 19 | 0.2631 | 1 | 0.2631 | 0 | 0 | 0 | 0.8187 | Ref |
| MK448965 | Unassigned | Unassigned | Unassigned | 429_0 | Clustered | 14 | VC_429_0 | 14 | 0.2582 | 1 | 0.2582 | 0 | 0 | 0 | 0.989 | Ref |
| MK448966 | Unassigned | Unassigned | Unassigned | 300_3 | Clustered | 19 | VC_300_3 | 19 | 0.2631 | 1 | 0.2631 | 0 | 0 | 0 | 0.8187 | Ref |
| MK448967 | Unassigned | Unassigned | Unassigned | 653_0 | Clustered | 9 | VC_653_0 | 9 | 0.2315 | 1 | 0.2315 | 0 | 0 | 0 | 0.9722 | Ref |
| MK448968 | Unassigned | Unassigned | Unassigned | 795_0 | Clustered | 23 | VC_795_0 | 23 | 0.5497 | 1 | 0.5497 | 0 | 0 | 0 | 1 | Ref |
| MK448970 | Unassigned | Unassigned | Unassigned | 797_1 | Clustered | 14 | VC_797_1 | 14 | 0.2785 | 1 | 0.2785 | 0 | 0 | 0 | 0.7582 | Ref |
| MK448973 | Unassigned | Unassigned | Unassigned | 300_3 | Clustered | 19 | VC_300_3 | 19 | 0.2631 | 1 | 0.2631 | 0 | 0 | 0 | 0.8187 | Ref |
| MK448974 | Unassigned | Unassigned | Unassigned | 795_0 | Clustered | 23 | VC_795_0 | 23 | 0.5497 | 1 | 0.5497 | 0 | 0 | 0 | 1 | Ref |
| MK448975 | Unassigned | Unassigned | Unassigned | 300_3 | Clustered | 19 | VC_300_3 | 19 | 0.2631 | 1 | 0.2631 | 0 | 0 | 0 | 0.8187 | Ref |
| MK448976 | Unassigned | Unassigned | Unassigned | 795_0 | Clustered | 23 | VC_795_0 | 23 | 0.5497 | 1 | 0.5497 | 0 | 0 | 0 | 1 | Ref |
| MK448977 | Unassigned | Unassigned | Unassigned | 429_0 | Clustered | 14 | VC_429_0 | 14 | 0.2582 | 1 | 0.2582 | 0 | 0 | 0 | 0.989 | Ref |
| MK448983 | Unassigned | Unassigned | Unassigned | 647_0 | Clustered | 37 | VC_647_0 | 37 | 0.5847 | 1 | 0.5847 | 0 | 0 | 0 | 0.9234 | Ref |
| MK448988 | Unassigned | Unassigned | Unassigned | 268_1 | Clustered | 20 | VC_268_1 | 20 | 0.1906 | 1 | 0.1906 | 0 | 0 | 0 | 0.6789 | Ref |
| MK448989 | Unassigned | Unassigned | Unassigned | 268_1 | Clustered | 20 | VC_268_1 | 20 | 0.1906 | 1 | 0.1906 | 0 | 0 | 0 | 0.6789 | Ref |
| MK448991 | Unassigned | Unassigned | Unassigned | 347_0 | Clustered | 6 | VC_347_0 | 6 | 0.3746 | 1 | 0.3746 | 0 | 0 | 0 | 1 | Ref |
| MK448995 | Unassigned | Unassigned | Unassigned | 300_1 | Clustered/Singleton | 1 | VC_300_1 | 1 | 0 | 1 | 0 | 0 | 0 | 0 | 1 | Ref |
| MK448997 | Unassigned | Unassigned | Unassigned | 649_1 | Clustered | 14 | VC_649_1 | 14 | 0.5226 | 1 | 0.5226 | 0 | 0 | 0 | 0.8352 | Ref |
| MK449000 | Unassigned | Unassigned | Unassigned | 346_0 | Clustered/Singleton | 1 | VC_346_0 | 1 | 0 | 1 | 0 | 0 | 0 | 0 | 1 | Ref |
| MK449002 | Unassigned | Unassigned | Unassigned | 265_0 | Clustered | 3 | VC_265_0 | 3 | 0.1136 | 0.9985864 | 0.1135 | 0 | 0 | 0 | 1 | Ref |
| MK449003 | Unassigned | Unassigned | Unassigned | 265_0 | Clustered | 3 | VC_265_0 | 3 | 0.1136 | 0.9985864 | 0.1135 | 0 | 0 | 0 | 1 | Ref |
| MK449004 | Unassigned | Unassigned | Unassigned | 300_3 | Clustered | 19 | VC_300_3 | 19 | 0.2631 | 1 | 0.2631 | 0 | 0 | 0 | 0.8187 | Ref |
| MK449006 | Unassigned | Unassigned | Unassigned | 425_0 | Clustered | 40 | VC_425_0 | 40 | 0.8399 | 1 | 0.8399 | 0 | 0 | 0 | 0.9974 | Ref |
| MK449008 | Unassigned | Unassigned | Unassigned | 301_0 | Clustered | 2 | VC_301_0 | 2 | 0.0851 | 0.95735478 | 0.0814 | 0 | 0 | 0 | 1 | Ref |
| MK449009 | Unassigned | Unassigned | Unassigned | 425_0 | Clustered | 40 | VC_425_0 | 40 | 0.8399 | 1 | 0.8399 | 0 | 0 | 0 | 0.9974 | Ref |
| MK449010 | Unassigned | Unassigned | Unassigned | 653_0 | Clustered | 9 | VC_653_0 | 9 | 0.2315 | 1 | 0.2315 | 0 | 0 | 0 | 0.9722 | Ref |
| MK450421 | Unassigned | Unassigned | Unassigned | 386_0 | Clustered | 10 | VC_386_0 | 10 | 0.7454 | 1 | 0.7454 | 0 | 0 | 0 | 1 | Ref |
| MK450422 | Unassigned | Unassigned | Unassigned | 579_65 | Clustered | 2 | VC_579_65 | 2 | 0.0032 | 0.60938642 | 0.0019 | 0 | 0 | 0 | 1 | Ref |
| MK450423 | Unassigned | Unassigned | Unassigned | 579_22 | Clustered | 4 | VC_579_22 | 4 | 0.0099 | 0.99020449 | 0.0098 | 0 | 0 | 0 | 0.8333 | Ref |
| MK450424 | Unassigned | Unassigned | Unassigned | 579_41 | Clustered/Singleton | 1 | VC_579_41 | 1 | 0 | 1 | 0 | 0 | 0 | 0 | 1 | Ref |
| MK450425 | Unassigned | Unassigned | Unassigned | 579_57 | Clustered/Singleton | 1 | VC_579_57 | 1 | 0 | 1 | 0 | 0 | 0 | 0 | 1 | Ref |
| MK450426 | Unassigned | Unassigned | Unassigned | 386_0 | Clustered | 10 | VC_386_0 | 10 | 0.7454 | 1 | 0.7454 | 0 | 0 | 0 | 1 | Ref |
| MK450427 | Unassigned | Unassigned | Unassigned | 579_14 | Clustered | 8 | VC_579_14 | 8 | 0.0227 | 0.99904117 | 0.0227 | 0 | 0 | 0 | 0.8214 | Ref |
| MK450428 | Unassigned | Unassigned | Unassigned | 893_1 | Clustered | 20 | VC_893_1 | 20 | 0.4792 | 1 | 0.4792 | 0 | 0 | 0 | 1 | Ref |
| MK450429 | Unassigned | Unassigned | Unassigned | 579_50 | Clustered | 15 | VC_579_50 | 15 | 0.0467 | 1 | 0.0467 | 0 | 0 | 0 | 0.9238 | Ref |
| MK450430 | Unassigned | Unassigned | Unassigned | 893_1 | Clustered | 20 | VC_893_1 | 20 | 0.4792 | 1 | 0.4792 | 0 | 0 | 0 | 1 | Ref |
| MK450431 | Unassigned | Unassigned | Unassigned | 386_0 | Clustered | 10 | VC_386_0 | 10 | 0.7454 | 1 | 0.7454 | 0 | 0 | 0 | 1 | Ref |

| | | | | | | | | | | | | | | | | |
|---|---|---|---|---|---|---|---|---|---|---|---|---|---|---|---|---|
| MK450432 | Unassigned | Unassigned | Unassigned | 579_20 | Clustered | 17 | VC_579_20 | 17 | 0.0538 | 1 | 0.0538 | 0 | 0 | 0 | 0.9926 | Ref |
| MK450433 | Unassigned | Unassigned | Unassigned | 386_0 | Clustered | 10 | VC_386_0 | 10 | 0.7454 | 1 | 0.7454 | 0 | 0 | 0 | 1 | Ref |
| MK450434 | Unassigned | Unassigned | Unassigned | 579_50 | Clustered | 15 | VC_579_50 | 15 | 0.0467 | 1 | 0.0467 | 0 | 0 | 0 | 0.9238 | Ref |
| MK450435 | Unassigned | Unassigned | Unassigned | 893_1 | Clustered | 20 | VC_893_1 | 20 | 0.4792 | 1 | 0.4792 | 0 | 0 | 0 | 1 | Ref |
| MK450436 | Unassigned | Unassigned | Unassigned | 893_1 | Clustered | 20 | VC_893_1 | 20 | 0.4792 | 1 | 0.4792 | 0 | 0 | 0 | 1 | Ref |
| MK450538 | Unassigned | Unassigned | Unassigned | 15_1 | Clustered | 9 | VC_15_1 | 9 | 0.1817 | 1 | 0.1817 | 0 | 0 | 0 | 0.9167 | Ref |
| MK450543 | Unassigned | Unassigned | Unassigned | 364_0 | Clustered | 3 | VC_364_0 | 3 | 0.729 | 0.98867275 | 0.7207 | 0 | 0 | 0 | 1 | Ref |
| MK455769 | Unassigned | Unassigned | Unassigned | 1067_0 | Clustered | 20 | VC_1067_0 | 20 | 0.7109 | 1 | 0.7109 | 0 | 0 | 0 | 1 | Ref |
| MK455770 | Unassigned | Unassigned | Unassigned | 1067_0 | Clustered | 20 | VC_1067_0 | 20 | 0.7109 | 1 | 0.7109 | 0 | 0 | 0 | 1 | Ref |
| MK460245 | Unassigned | Unassigned | Unassigned | 1015_1 | Clustered | 26 | VC_1015_1 | 26 | 0.6989 | 1 | 0.6989 | 0 | 0 | 0 | 1 | Ref |
| MK460246 | Unassigned | Unassigned | Unassigned | 244_3 | Clustered | 8 | VC_244_3 | 8 | 0.1086 | 1 | 0.1086 | 0 | 0 | 0 | 1 | Ref |
| MK460247 | Unassigned | Unassigned | Unassigned | 243_0 | Clustered | 72 | VC_243_0 | 72 | 0.8089 | 1 | 0.8089 | 0 | 0 | 0 | 1 | Ref |
| MK460248 | Unassigned | Unassigned | Unassigned | 1114_3 | Clustered | 16 | VC_1114_3 | 16 | 0.2336 | 1 | 0.2336 | 0 | 0 | 0 | 0.9667 | Ref |
| MK468591 | Unassigned | Unassigned | Unassigned | 232_0 | Clustered | 32 | VC_232_0 | 32 | 0.8471 | 1 | 0.8471 | 0 | 0 | 0 | 0.996 | Ref |
| MK473373 | Unassigned | Unassigned | Unassigned | 1070_0 | Clustered | 3 | VC_1070_0 | 3 | 0.7872 | 0.99542416 | 0.7836 | 0 | 0 | 0 | 1 | Ref |
| MK473382 | Unassigned | Unassigned | Unassigned | 666_0 | Clustered | 11 | VC_666_0 | 11 | 0.5822 | 1 | 0.5822 | 0 | 0 | 0 | 1 | Ref |
| MK474470 | Unassigned | Unassigned | Unassigned | 861_0 | Clustered | 29 | VC_861_0 | 29 | 0.9954 | 1 | 0.9954 | 0 | 0 | 0 | 1 | Ref |
| MK479295 | Unassigned | Unassigned | Unassigned | 560_0 | Clustered | 15 | VC_560_0 | 15 | 0.2846 | 1 | 0.2846 | 0 | 0 | 0 | 1 | Ref |
| MK482688 | Unassigned | Unassigned | Unassigned | 340_7 | Clustered | 18 | VC_340_7 | 18 | 0.0936 | 1 | 0.0936 | 0 | 0 | 0 | 0.7778 | Ref |
| MK482689 | Unassigned | Unassigned | Unassigned | 340_7 | Clustered | 18 | VC_340_7 | 18 | 0.0936 | 1 | 0.0936 | 0 | 0 | 0 | 0.7778 | Ref |
| MK482690 | Unassigned | Unassigned | Unassigned | 340_7 | Clustered | 18 | VC_340_7 | 18 | 0.0936 | 1 | 0.0936 | 0 | 0 | 0 | 0.7778 | Ref |
| MK483107 | Unassigned | Unassigned | Unassigned | 548_0 | Clustered | 9 | VC_548_0 | 9 | 0.8018 | 1 | 0.8018 | 0 | 0 | 0 | 1 | Ref |
| MK494087 | Unassigned | Unassigned | Unassigned | 222_1 | Clustered | 19 | VC_222_1 | 19 | 0.4166 | 1 | 0.4166 | 0 | 0 | 0 | 1 | Ref |
| MK494088 | Unassigned | Unassigned | Unassigned | 535_1 | Clustered | 108 | VC_535_1 | 108 | 0.8479 | 1 | 0.8479 | 0 | 0 | 0 | 0.9955 | Ref |
| MK494089 | Unassigned | Unassigned | Unassigned | 1087_0 | Clustered | 7 | VC_1087_0 | 7 | 0.94 | 1 | 0.94 | 0 | 0 | 0 | 1 | Ref |
| MK494090 | Unassigned | Unassigned | Unassigned | 230_0 | Clustered | 38 | VC_230_0 | 38 | 0.9076 | 1 | 0.9076 | 0 | 0 | 0 | 1 | Ref |
| MK494091 | Unassigned | Unassigned | Unassigned | 974_0 | Clustered | 34 | VC_974_0 | 34 | 0.7953 | 1 | 0.7953 | 0 | 0 | 0 | 1 | Ref |
| MK494093 | Unassigned | Unassigned | Unassigned | 535_1 | Clustered | 108 | VC_535_1 | 108 | 0.8479 | 1 | 0.8479 | 0 | 0 | 0 | 0.9955 | Ref |
| MK494094 | Unassigned | Unassigned | Unassigned | 535_1 | Clustered | 108 | VC_535_1 | 108 | 0.8479 | 1 | 0.8479 | 0 | 0 | 0 | 0.9955 | Ref |
| MK494095 | Unassigned | Unassigned | Unassigned | 834_0 | Clustered | 18 | VC_834_0 | 18 | 0.9116 | 1 | 0.9116 | 0 | 0 | 0 | 1 | Ref |
| MK494096 | Unassigned | Unassigned | Unassigned | 532_0 | Clustered | 75 | VC_532_0 | 75 | 0.9393 | 1 | 0.9393 | 0 | 0 | 0 | 1 | Ref |
| MK494097 | Unassigned | Unassigned | Unassigned | 535_1 | Clustered | 108 | VC_535_1 | 108 | 0.8479 | 1 | 0.8479 | 0 | 0 | 0 | 0.9955 | Ref |
| MK494098 | Unassigned | Unassigned | Unassigned | 896_0 | Clustered | 9 | VC_896_0 | 9 | 0.2604 | 1 | 0.2604 | 0 | 0 | 0 | 1 | Ref |
| MK494099 | Unassigned | Unassigned | Unassigned | 702_0 | Clustered | 2 | VC_702_0 | 2 | 0.2123 | 0.9539194 | 0.2025 | 0 | 0 | 0 | 1 | Ref |
| MK494101 | Unassigned | Unassigned | Unassigned | 535_1 | Clustered | 108 | VC_535_1 | 108 | 0.8479 | 1 | 0.8479 | 0 | 0 | 0 | 0.9955 | Ref |
| MK494102 | Unassigned | Unassigned | Unassigned | 585_0 | Clustered | 20 | VC_585_0 | 20 | 0.8376 | 1 | 0.8376 | 0 | 0 | 0 | 1 | Ref |
| MK494103 | Unassigned | Unassigned | Unassigned | 985_0 | Clustered | 38 | VC_985_0 | 38 | 0.9334 | 1 | 0.9334 | 0 | 0 | 0 | 1 | Ref |
| MK494104 | Unassigned | Unassigned | Unassigned | 384_16 | Clustered | 42 | VC_384_16 | 42 | 0.134 | 1 | 0.134 | 0 | 0 | 0 | 0.7282 | Ref |
| MK494105 | Unassigned | Unassigned | Unassigned | 784_0 | Clustered | 9 | VC_784_0 | 9 | 0.0884 | 1 | 0.0884 | 0 | 0 | 0 | 1 | Ref |
| MK494106 | Unassigned | Unassigned | Unassigned | 893_1 | Clustered | 20 | VC_893_1 | 20 | 0.4792 | 1 | 0.4792 | 0 | 0 | 0 | 1 | Ref |
| MK494107 | Unassigned | Unassigned | Unassigned | 1117_0 | Clustered | 19 | VC_1117_0 | 19 | 0.9177 | 1 | 0.9177 | 0 | 0 | 0 | 1 | Ref |
| MK494108 | Unassigned | Unassigned | Unassigned | 232_0 | Clustered | 32 | VC_232_0 | 32 | 0.8471 | 1 | 0.8471 | 0 | 0 | 0 | 0.996 | Ref |
| MK494109 | Unassigned | Unassigned | Unassigned | 893_1 | Clustered | 20 | VC_893_1 | 20 | 0.4792 | 1 | 0.4792 | 0 | 0 | 0 | 1 | Ref |
| MK494110 | Unassigned | Unassigned | Unassigned | 538_45 | Clustered | 7 | VC_538_45 | 7 | 0.0632 | 1 | 0.0632 | 0 | 0 | 0 | 0.8095 | Ref |
| MK494111 | Unassigned | Unassigned | Unassigned | 985_0 | Clustered | 38 | VC_985_0 | 38 | 0.9334 | 1 | 0.9334 | 0 | 0 | 0 | 1 | Ref |
| MK494112 | Unassigned | Unassigned | Unassigned | 1114_3 | Clustered | 16 | VC_1114_3 | 16 | 0.2336 | 1 | 0.2336 | 0 | 0 | 0 | 0.9667 | Ref |
| MK494113 | Unassigned | Unassigned | Unassigned | 229_0 | Clustered | 5 | VC_229_0 | 5 | 0.0566 | 0.99999997 | 0.0566 | 0 | 0 | 0 | 1 | Ref |
| MK494114 | Unassigned | Unassigned | Unassigned | 525_0 | Clustered | 8 | VC_525_0 | 8 | 0.443 | 1 | 0.443 | 0 | 0 | 0 | 1 | Ref |
| MK494115 | Unassigned | Unassigned | Unassigned | 535_0 | Clustered | 18 | VC_535_0 | 18 | 0.1595 | 1 | 0.1595 | 0 | 0 | 0 | 1 | Ref |
| MK494116 | Unassigned | Unassigned | Unassigned | 335_45 | Clustered | 4 | VC_335_45 | 4 | 0.0143 | 0.99998616 | 0.0143 | 0 | 0 | 0 | 0.8333 | Ref |
| MK494117 | Unassigned | Unassigned | Unassigned | 535_0 | Clustered | 18 | VC_535_0 | 18 | 0.1595 | 1 | 0.1595 | 0 | 0 | 0 | 1 | Ref |
| MK494118 | Unassigned | Unassigned | Unassigned | 538_72 | Clustered/Singleton | 1 | VC_538_72 | 1 | 0 | 1 | 0 | 0 | 0 | 0 | 1 | Ref |
| MK494119 | Unassigned | Unassigned | Unassigned | 244_0 | Clustered | 4 | VC_244_0 | 4 | 0.0426 | 0.99998759 | 0.0426 | 0 | 0 | 0 | 1 | Ref |
| MK494121 | Unassigned | Unassigned | Unassigned | 893_1 | Clustered | 20 | VC_893_1 | 20 | 0.4792 | 1 | 0.4792 | 0 | 0 | 0 | 1 | Ref |
| MK494122 | Unassigned | Unassigned | Unassigned | 537_0 | Clustered | 39 | VC_537_0 | 39 | 0.8148 | 1 | 0.8148 | 0 | 0 | 0 | 1 | Ref |
| MK494123 | Unassigned | Unassigned | Unassigned | 973_0 | Clustered | 15 | VC_973_0 | 15 | 1 | 0 | 0 | 0 | 0 | 0 | 1 | Ref |
| MK494124 | Unassigned | Unassigned | Unassigned | 533_0 | Clustered | 16 | VC_533_0 | 16 | 0.3765 | 1 | 0.3765 | 0 | 0 | 0 | 1 | Ref |
| MK494125 | Unassigned | Unassigned | Unassigned | 535_1 | Clustered | 108 | VC_535_1 | 108 | 0.8479 | 1 | 0.8479 | 0 | 0 | 0 | 0.9955 | Ref |
| MK494126 | Unassigned | Unassigned | Unassigned | 535_1 | Clustered | 108 | VC_535_1 | 108 | 0.8479 | 1 | 0.8479 | 0 | 0 | 0 | 0.9955 | Ref |
| MK494127 | Unassigned | Unassigned | Unassigned | 531_0 | Clustered | 32 | VC_531_0 | 32 | 0.6859 | 1 | 0.6859 | 0 | 0 | 0 | 1 | Ref |
| MK494128 | Unassigned | Unassigned | Unassigned | 532_0 | Clustered | 75 | VC_532_0 | 75 | 0.9393 | 1 | 0.9393 | 0 | 0 | 0 | 1 | Ref |
| MK494129 | Unassigned | Unassigned | Unassigned | 535_0 | Clustered | 18 | VC_535_0 | 18 | 0.1595 | 1 | 0.1595 | 0 | 0 | 0 | 1 | Ref |
| MK494130 | Unassigned | Unassigned | Unassigned | 228_0 | Clustered | 22 | VC_228_0 | 22 | 0.7411 | 1 | 0.7411 | 0 | 0 | 0 | 1 | Ref |
| MK494131 | Unassigned | Unassigned | Unassigned | 525_0 | Clustered | 8 | VC_525_0 | 8 | 0.443 | 1 | 0.443 | 0 | 0 | 0 | 1 | Ref |
| MK494132 | Unassigned | Unassigned | Unassigned | 525_0 | Clustered | 8 | VC_525_0 | 8 | 0.443 | 1 | 0.443 | 0 | 0 | 0 | 1 | Ref |
| MK496679 | Unassigned | Unassigned | Unassigned | 1437_0 | Clustered | 5 | VC_1437_0 | 5 | 0.7338 | 0.52463554 | 0.385 | 0 | 0 | 0 | 1 | Ref |
| MK496680 | Unassigned | Unassigned | Unassigned | 1437_0 | Clustered | 5 | VC_1437_0 | 5 | 0.7338 | 0.52463554 | 0.385 | 0 | 0 | 0 | 1 | Ref |
| MK496681 | Unassigned | Unassigned | Unassigned | 1439_0 | Clustered | 2 | VC_1439_0 | 2 | 0.6463 | 0.86822376 | 0.5611 | 0 | 0 | 0 | 1 | Ref |
| MK496682 | Unassigned | Unassigned | Unassigned | 1432_0 | Clustered | 2 | VC_1432_0 | 2 | 1 | 1 | 1 | 0 | 0 | 0 | 1 | Ref |
| MK496683 | Unassigned | Unassigned | Unassigned | 1440_0 | Clustered | 2 | VC_1440_0 | 2 | 1 | 1 | 1 | 0 | 0 | 0 | 1 | Ref |
| MK496685 | Unassigned | Unassigned | Unassigned | 1322_0 | Clustered | 6 | VC_1322_0 | 6 | 0.7063 | 0.92055725 | 0.6502 | 0 | 0 | 0 | 1 | Ref |
| MK496686 | Unassigned | Unassigned | Unassigned | 1441_0 | Clustered | 2 | VC_1441_0 | 2 | 1 | 1 | 1 | 0 | 0 | 0 | 1 | Ref |
| MK496688 | Unassigned | Unassigned | Unassigned | 1442_0 | Clustered | 3 | VC_1442_0 | 3 | 1 | 1 | 1 | 0 | 0 | 0 | 1 | Ref |
| MK496689 | Unassigned | Unassigned | Unassigned | 1443_0 | Clustered | 3 | VC_1443_0 | 3 | 1 | 0 | 0 | 0 | 0 | 0 | 1 | Ref |
| MK496690 | Unassigned | Unassigned | Unassigned | 1442_0 | Clustered | 3 | VC_1442_0 | 3 | 1 | 1 | 1 | 0 | 0 | 0 | 1 | Ref |
| MK496692 | Unassigned | Unassigned | Unassigned | 1390_0 | Clustered | 2 | VC_1390_0 | 2 | 1 | 1 | 1 | 0 | 0 | 0 | 1 | Ref |
| MK496693 | Unassigned | Unassigned | Unassigned | 1241_0 | Clustered | 13 | VC_1241_0 | 13 | 0.8675 | 0.76044592 | 0.6597 | 0 | 0 | 0 | 1 | Ref |
| MK496694 | Unassigned | Unassigned | Unassigned | 1299_0 | Clustered | 53 | VC_1299_0 | 53 | 0.8666 | 1 | 0.8666 | 0 | 0 | 0 | 1 | Ref |
| MK496696 | Unassigned | Unassigned | Unassigned | 1357_0 | Clustered | 7 | VC_1357_0 | 7 | 1 | 1 | 1 | 0 | 0 | 0 | 1 | Ref |
| MK496697 | Unassigned | Unassigned | Unassigned | 1444_0 | Clustered | 2 | VC_1444_0 | 2 | 1 | 1 | 1 | 0 | 0 | 0 | 1 | Ref |
| MK496698 | Unassigned | Unassigned | Unassigned | 1438_0 | Clustered | 2 | VC_1438_0 | 2 | 0.1773 | 0.73645537 | 0.1306 | 0 | 0 | 0 | 1 | Ref |

| | | | | | | | | | | | | | | | | |
|---|---|---|---|---|---|---|---|---|---|---|---|---|---|---|---|---|
| MK496699 | Unassigned | Unassigned | Unassigned | 1445_0 | Clustered | 2 | VC_1445_0 | 2 | 1 | 1 | 1 | 0 | 0 | 0 | 1 | Ref |
| MK496700 | Unassigned | Unassigned | Unassigned | 1443_0 | Clustered | 3 | VC_1443_0 | 3 | 1 | 0 | 0 | 0 | 0 | 0 | 1 | Ref |
| MK496701 | Unassigned | Unassigned | Unassigned | 1446_0 | Clustered | 2 | VC_1446_0 | 2 | 1 | 1 | 1 | 0 | 0 | 0 | 1 | Ref |
| MK496702 | Unassigned | Unassigned | Unassigned | 1446_0 | Clustered | 2 | VC_1446_0 | 2 | 1 | 1 | 1 | 0 | 0 | 0 | 1 | Ref |
| MK496704 | Unassigned | Unassigned | Unassigned | 1356_0 | Clustered | 4 | VC_1356_0 | 4 | 0.8682 | 0.91151676 | 0.7914 | 0 | 0 | 0 | 1 | Ref |
| MK496710 | Unassigned | Unassigned | Unassigned | 1447_0 | Clustered | 3 | VC_1447_0 | 3 | 1 | 1 | 1 | 0 | 0 | 0 | 1 | Ref |
| MK496711 | Unassigned | Unassigned | Unassigned | 1357_0 | Clustered | 7 | VC_1357_0 | 7 | 1 | 1 | 1 | 0 | 0 | 0 | 1 | Ref |
| MK496712 | Unassigned | Unassigned | Unassigned | 1448_0 | Clustered | 2 | VC_1448_0 | 2 | 1 | 1 | 1 | 0 | 0 | 0 | 1 | Ref |
| MK496714 | Unassigned | Unassigned | Unassigned | 1176_0 | Clustered | 6 | VC_1176_0 | 6 | 0.1252 | 0.96352059 | 0.1206 | 0 | 0 | 0 | 1 | Ref |
| MK496716 | Unassigned | Unassigned | Unassigned | 1449_0 | Clustered | 2 | VC_1449_0 | 2 | 0.6396 | 0.72985431 | 0.4668 | 0 | 0 | 0 | 1 | Ref |
| MK496717 | Unassigned | Unassigned | Unassigned | 1450_0 | Clustered | 2 | VC_1450_0 | 2 | 1 | 1 | 1 | 0 | 0 | 0 | 1 | Ref |
| MK496719 | Unassigned | Unassigned | Unassigned | 1151_0 | Clustered | 2 | VC_1151_0 | 2 | 0.2002 | 0.77749564 | 0.1557 | 0 | 0 | 0 | 1 | Ref |
| MK496720 | Unassigned | Unassigned | Unassigned | 1451_0 | Clustered | 2 | VC_1451_0 | 2 | 1 | 1 | 1 | 0 | 0 | 0 | 1 | Ref |
| MK496721 | Unassigned | Unassigned | Unassigned | 1452_0 | Clustered | 3 | VC_1452_0 | 3 | 1 | 0 | 0 | 0 | 0 | 0 | 1 | Ref |
| MK496723 | Unassigned | Unassigned | Unassigned | 1095_0 | Clustered | 12 | VC_1095_0 | 12 | 0.2819 | 0.99837092 | 0.2814 | 0 | 0 | 0 | 1 | Ref |
| MK496725 | Unassigned | Unassigned | Unassigned | 1453_0 | Clustered | 3 | VC_1453_0 | 3 | 0.7356 | 0.96173875 | 0.7075 | 0 | 0 | 0 | 1 | Ref |
| MK496726 | Unassigned | Unassigned | Unassigned | 1147_0 | Clustered | 6 | VC_1147_0 | 6 | 0.3066 | 0.99999957 | 0.3066 | 0 | 0 | 0 | 1 | Ref |
| MK496729 | Unassigned | Unassigned | Unassigned | 1356_0 | Clustered | 4 | VC_1356_0 | 4 | 0.8682 | 0.91151676 | 0.7914 | 0 | 0 | 0 | 1 | Ref |
| MK496732 | Unassigned | Unassigned | Unassigned | 1352_0 | Clustered | 3 | VC_1352_0 | 3 | 1 | 1 | 1 | 0 | 0 | 0 | 1 | Ref |
| MK496738 | Unassigned | Unassigned | Unassigned | 1151_0 | Clustered | 2 | VC_1151_0 | 2 | 0.2002 | 0.77749564 | 0.1557 | 0 | 0 | 0 | 1 | Ref |
| MK496745 | Unassigned | Unassigned | Unassigned | 1188_0 | Clustered | 3 | VC_1188_0 | 3 | 0.2005 | 0.95837083 | 0.1922 | 0 | 0 | 0 | 1 | Ref |
| MK496746 | Unassigned | Unassigned | Unassigned | 1453_0 | Clustered | 3 | VC_1453_0 | 3 | 0.7356 | 0.96173875 | 0.7075 | 0 | 0 | 0 | 1 | Ref |
| MK496748 | Unassigned | Unassigned | Unassigned | 1454_0 | Clustered | 2 | VC_1454_0 | 2 | 1 | 1 | 1 | 0 | 0 | 0 | 1 | Ref |
| MK496750 | Unassigned | Unassigned | Unassigned | 1455_0 | Clustered | 3 | VC_1455_0 | 3 | 0.8292 | 0.5 | 0.4146 | 0 | 0 | 0 | 1 | Ref |
| MK496751 | Unassigned | Unassigned | Unassigned | 1160_0 | Clustered | 3 | VC_1160_0 | 3 | 0.2567 | 0.98675879 | 0.2533 | 0 | 0 | 0 | 1 | Ref |
| MK496753 | Unassigned | Unassigned | Unassigned | 1456_0 | Clustered | 2 | VC_1456_0 | 2 | 1 | 1 | 1 | 0 | 0 | 0 | 1 | Ref |
| MK496754 | Unassigned | Unassigned | Unassigned | 1160_0 | Clustered | 3 | VC_1160_0 | 3 | 0.2567 | 0.98675879 | 0.2533 | 0 | 0 | 0 | 1 | Ref |
| MK496756 | Unassigned | Unassigned | Unassigned | 1457_0 | Clustered | 2 | VC_1457_0 | 2 | 1 | 1 | 1 | 0 | 0 | 0 | 1 | Ref |
| MK496757 | Unassigned | Unassigned | Unassigned | 1364_0 | Clustered | 6 | VC_1364_0 | 6 | 0.9472 | 0.54366909 | 0.515 | 0 | 0 | 0 | 1 | Ref |
| MK496758 | Unassigned | Unassigned | Unassigned | 1458_0 | Clustered | 2 | VC_1458_0 | 2 | 1 | 1 | 1 | 0 | 0 | 0 | 1 | Ref |
| MK496760 | Unassigned | Unassigned | Unassigned | 1439_0 | Clustered | 2 | VC_1439_0 | 2 | 0.6463 | 0.86822376 | 0.5611 | 0 | 0 | 0 | 1 | Ref |
| MK496761 | Unassigned | Unassigned | Unassigned | 1440_0 | Clustered | 2 | VC_1440_0 | 2 | 1 | 1 | 1 | 0 | 0 | 0 | 1 | Ref |
| MK496764 | Unassigned | Unassigned | Unassigned | 1441_0 | Clustered | 2 | VC_1441_0 | 2 | 1 | 1 | 1 | 0 | 0 | 0 | 1 | Ref |
| MK496765 | Unassigned | Unassigned | Unassigned | 1354_0 | Clustered | 6 | VC_1354_0 | 6 | 0.6609 | 0.99999272 | 0.6609 | 0 | 0 | 0 | 1 | Ref |
| MK496766 | Unassigned | Unassigned | Unassigned | 1442_0 | Clustered | 3 | VC_1442_0 | 3 | 1 | 1 | 1 | 0 | 0 | 0 | 1 | Ref |
| MK496767 | Unassigned | Unassigned | Unassigned | 1447_0 | Clustered | 3 | VC_1447_0 | 3 | 1 | 1 | 1 | 0 | 0 | 0 | 1 | Ref |
| MK496768 | Unassigned | Unassigned | Unassigned | 1447_0 | Clustered | 3 | VC_1447_0 | 3 | 1 | 1 | 1 | 0 | 0 | 0 | 1 | Ref |
| MK496770 | Unassigned | Unassigned | Unassigned | 1459_0 | Clustered | 2 | VC_1459_0 | 2 | 1 | 1 | 1 | 0 | 0 | 0 | 1 | Ref |
| MK496771 | Unassigned | Unassigned | Unassigned | 1459_0 | Clustered | 2 | VC_1459_0 | 2 | 1 | 1 | 1 | 0 | 0 | 0 | 1 | Ref |
| MK496772 | Unassigned | Unassigned | Unassigned | 1438_0 | Clustered | 2 | VC_1438_0 | 2 | 0.1773 | 0.73645537 | 0.1306 | 0 | 0 | 0 | 1 | Ref |
| MK496774 | Unassigned | Unassigned | Unassigned | 1241_0 | Clustered | 13 | VC_1241_0 | 13 | 0.8675 | 0.76044592 | 0.6597 | 0 | 0 | 0 | 1 | Ref |
| MK496775 | Unassigned | Unassigned | Unassigned | 1299_0 | Clustered | 53 | VC_1299_0 | 53 | 0.8666 | 1 | 0.8666 | 0 | 0 | 0 | 1 | Ref |
| MK496776 | Unassigned | Unassigned | Unassigned | 1437_0 | Clustered | 5 | VC_1437_0 | 5 | 0.7338 | 0.52463554 | 0.385 | 0 | 0 | 0 | 1 | Ref |
| MK496779 | Unassigned | Unassigned | Unassigned | 1176_0 | Clustered | 6 | VC_1176_0 | 6 | 0.1252 | 0.96352059 | 0.1206 | 0 | 0 | 0 | 1 | Ref |
| MK496780 | Unassigned | Unassigned | Unassigned | 1444_0 | Clustered | 2 | VC_1444_0 | 2 | 1 | 1 | 1 | 0 | 0 | 0 | 1 | Ref |
| MK496781 | Unassigned | Unassigned | Unassigned | 1452_0 | Clustered | 3 | VC_1452_0 | 3 | 1 | 0 | 0 | 0 | 0 | 0 | 1 | Ref |
| MK496782 | Unassigned | Unassigned | Unassigned | 1356_0 | Clustered | 4 | VC_1356_0 | 4 | 0.8682 | 0.91151676 | 0.7914 | 0 | 0 | 0 | 1 | Ref |
| MK496784 | Unassigned | Unassigned | Unassigned | 1443_0 | Clustered | 3 | VC_1443_0 | 3 | 1 | 0 | 0 | 0 | 0 | 0 | 1 | Ref |
| MK496785 | Unassigned | Unassigned | Unassigned | 1445_0 | Clustered | 2 | VC_1445_0 | 2 | 1 | 1 | 1 | 0 | 0 | 0 | 1 | Ref |
| MK496786 | Unassigned | Unassigned | Unassigned | 1452_0 | Clustered | 3 | VC_1452_0 | 3 | 1 | 0 | 0 | 0 | 0 | 0 | 1 | Ref |
| MK496787 | Unassigned | Unassigned | Unassigned | 1448_0 | Clustered | 2 | VC_1448_0 | 2 | 1 | 1 | 1 | 0 | 0 | 0 | 1 | Ref |
| MK496790 | Unassigned | Unassigned | Unassigned | 1449_0 | Clustered | 2 | VC_1449_0 | 2 | 0.6396 | 0.72985431 | 0.4668 | 0 | 0 | 0 | 1 | Ref |
| MK496792 | Unassigned | Unassigned | Unassigned | 1176_0 | Clustered | 6 | VC_1176_0 | 6 | 0.1252 | 0.96352059 | 0.1206 | 0 | 0 | 0 | 1 | Ref |
| MK496796 | Unassigned | Unassigned | Unassigned | 1451_0 | Clustered | 2 | VC_1451_0 | 2 | 1 | 1 | 1 | 0 | 0 | 0 | 1 | Ref |
| MK496803 | Unassigned | Unassigned | Unassigned | 1176_0 | Clustered | 6 | VC_1176_0 | 6 | 0.1252 | 0.96352059 | 0.1206 | 0 | 0 | 0 | 1 | Ref |
| MK496804 | Unassigned | Unassigned | Unassigned | 1450_0 | Clustered | 2 | VC_1450_0 | 2 | 1 | 1 | 1 | 0 | 0 | 0 | 1 | Ref |
| MK496808 | Unassigned | Unassigned | Unassigned | 1458_0 | Clustered | 2 | VC_1458_0 | 2 | 1 | 1 | 1 | 0 | 0 | 0 | 1 | Ref |
| MK496811 | Unassigned | Unassigned | Unassigned | 1453_0 | Clustered | 3 | VC_1453_0 | 3 | 0.7356 | 0.96173875 | 0.7075 | 0 | 0 | 0 | 1 | Ref |
| MK496812 | Unassigned | Unassigned | Unassigned | 1161_0 | Clustered | 16 | VC_1161_0 | 16 | 0.4834 | 0.99999958 | 0.4834 | 0 | 0 | 0 | 1 | Ref |
| MK496813 | Unassigned | Unassigned | Unassigned | 1161_0 | Clustered | 16 | VC_1161_0 | 16 | 0.4834 | 0.99999958 | 0.4834 | 0 | 0 | 0 | 1 | Ref |
| MK496815 | Unassigned | Unassigned | Unassigned | 1454_0 | Clustered | 2 | VC_1454_0 | 2 | 1 | 1 | 1 | 0 | 0 | 0 | 1 | Ref |
| MK496816 | Unassigned | Unassigned | Unassigned | 1455_0 | Clustered | 3 | VC_1455_0 | 3 | 0.8292 | 0.5 | 0.4146 | 0 | 0 | 0 | 1 | Ref |
| MK496818 | Unassigned | Unassigned | Unassigned | 1456_0 | Clustered | 2 | VC_1456_0 | 2 | 1 | 1 | 1 | 0 | 0 | 0 | 1 | Ref |
| MK496820 | Unassigned | Unassigned | Unassigned | 1460_0 | Clustered | 2 | VC_1460_0 | 2 | 1 | 1 | 1 | 0 | 0 | 0 | 1 | Ref |
| MK496824 | Unassigned | Unassigned | Unassigned | 1457_0 | Clustered | 2 | VC_1457_0 | 2 | 1 | 1 | 1 | 0 | 0 | 0 | 1 | Ref |
| MK496825 | Unassigned | Unassigned | Unassigned | 1028_0 | Clustered | 69 | VC_1028_0 | 69 | 0.8547 | 1 | 0.8547 | 0 | 0 | 0 | 1 | Ref |
| MK501729 | Unassigned | Unassigned | Unassigned | 1217_0 | Clustered | 41 | VC_1217_0 | 41 | 0.9546 | 1 | 0.9546 | 0 | 0 | 0 | 0.9976 | Ref |
| MK501730 | Unassigned | Unassigned | Unassigned | 1217_0 | Clustered | 41 | VC_1217_0 | 41 | 0.9546 | 1 | 0.9546 | 0 | 0 | 0 | 0.9976 | Ref |
| MK504442 | Unassigned | Unassigned | Unassigned | 598_0 | Clustered | 2 | VC_598_0 | 2 | 0.1903 | 0.93875121 | 0.1786 | 0 | 0 | 0 | 1 | Ref |
| MK504443 | Unassigned | Unassigned | Unassigned | 97_0 | Clustered | 3 | VC_97_0 | 3 | 0.2031 | 0.99852916 | 0.2028 | 0 | 0 | 0 | 1 | Ref |
| MK504444 | Unassigned | Unassigned | Unassigned | 544_3 | Clustered/Singleton | 1 | VC_544_3 | 1 | 0 | 1 | 0 | 0 | 0 | 0 | 1 | Ref |
| MK504446 | Unassigned | Unassigned | Unassigned | 97_0 | Clustered | 3 | VC_97_0 | 3 | 0.2031 | 0.99852916 | 0.2028 | 0 | 0 | 0 | 1 | Ref |
| MK509462 | Unassigned | Unassigned | Unassigned | 133_2 | Clustered | 7 | VC_133_2 | 7 | 0.0679 | 1 | 0.0679 | 0 | 0 | 0 | 0.9048 | Ref |
| MK510962 | Unassigned | Unassigned | Unassigned | 764_0 | Clustered | 23 | VC_764_0 | 23 | 0.8194 | 1 | 0.8194 | 0 | 0 | 0 | 1 | Ref |
| MK510963 | Unassigned | Unassigned | Unassigned | 764_0 | Clustered | 23 | VC_764_0 | 23 | 0.8194 | 1 | 0.8194 | 0 | 0 | 0 | 1 | Ref |
| MK510964 | Unassigned | Unassigned | Unassigned | 764_0 | Clustered | 23 | VC_764_0 | 23 | 0.8194 | 1 | 0.8194 | 0 | 0 | 0 | 1 | Ref |
| MK510965 | Unassigned | Unassigned | Unassigned | 764_0 | Clustered | 23 | VC_764_0 | 23 | 0.8194 | 1 | 0.8194 | 0 | 0 | 0 | 1 | Ref |
| MK510966 | Unassigned | Unassigned | Unassigned | 765_0 | Clustered | 12 | VC_765_0 | 12 | 0.6861 | 1 | 0.6861 | 0 | 0 | 0 | 1 | Ref |
| MK510967 | Unassigned | Unassigned | Unassigned | 765_0 | Clustered | 12 | VC_765_0 | 12 | 0.6861 | 1 | 0.6861 | 0 | 0 | 0 | 1 | Ref |
| MK510968 | Unassigned | Unassigned | Unassigned | 764_0 | Clustered | 23 | VC_764_0 | 23 | 0.8194 | 1 | 0.8194 | 0 | 0 | 0 | 1 | Ref |
| MK510969 | Unassigned | Unassigned | Unassigned | 764_0 | Clustered | 23 | VC_764_0 | 23 | 0.8194 | 1 | 0.8194 | 0 | 0 | 0 | 1 | Ref |

| | | | | | | | | | | | | | | | | |
|---|---|---|---|---|---|---|---|---|---|---|---|---|---|---|---|---|
| MK510970 | Unassigned | Unassigned | Unassigned | 764_0 | Clustered | 23 | VC_764_0 | 23 | 0.8194 | 1 | 0.8194 | 0 | 0 | 0 | 1 | Ref |
| MK510971 | Unassigned | Unassigned | Unassigned | 764_0 | Clustered | 23 | VC_764_0 | 23 | 0.8194 | 1 | 0.8194 | 0 | 0 | 0 | 1 | Ref |
| MK510972 | Unassigned | Unassigned | Unassigned | 765_0 | Clustered | 12 | VC_765_0 | 12 | 0.6861 | 1 | 0.6861 | 0 | 0 | 0 | 1 | Ref |
| MK510973 | Unassigned | Unassigned | Unassigned | 765_0 | Clustered | 12 | VC_765_0 | 12 | 0.6861 | 1 | 0.6861 | 0 | 0 | 0 | 1 | Ref |
| MK510974 | Unassigned | Unassigned | Unassigned | 764_0 | Clustered | 23 | VC_764_0 | 23 | 0.8194 | 1 | 0.8194 | 0 | 0 | 0 | 1 | Ref |
| MK510975 | Unassigned | Unassigned | Unassigned | 764_0 | Clustered | 23 | VC_764_0 | 23 | 0.8194 | 1 | 0.8194 | 0 | 0 | 0 | 1 | Ref |
| MK510976 | Unassigned | Unassigned | Unassigned | 764_0 | Clustered | 23 | VC_764_0 | 23 | 0.8194 | 1 | 0.8194 | 0 | 0 | 0 | 1 | Ref |
| MK510977 | Unassigned | Unassigned | Unassigned | 765_0 | Clustered | 12 | VC_765_0 | 12 | 0.6861 | 1 | 0.6861 | 0 | 0 | 0 | 1 | Ref |
| MK510978 | Unassigned | Unassigned | Unassigned | 764_0 | Clustered | 23 | VC_764_0 | 23 | 0.8194 | 1 | 0.8194 | 0 | 0 | 0 | 1 | Ref |
| MK510979 | Unassigned | Unassigned | Unassigned | 765_0 | Clustered | 12 | VC_765_0 | 12 | 0.6861 | 1 | 0.6861 | 0 | 0 | 0 | 1 | Ref |
| MK510980 | Unassigned | Unassigned | Unassigned | 765_0 | Clustered | 12 | VC_765_0 | 12 | 0.6861 | 1 | 0.6861 | 0 | 0 | 0 | 1 | Ref |
| MK510981 | Unassigned | Unassigned | Unassigned | 765_0 | Clustered | 12 | VC_765_0 | 12 | 0.6861 | 1 | 0.6861 | 0 | 0 | 0 | 1 | Ref |
| MK510982 | Unassigned | Unassigned | Unassigned | 765_0 | Clustered | 12 | VC_765_0 | 12 | 0.6861 | 1 | 0.6861 | 0 | 0 | 0 | 1 | Ref |
| MK510983 | Unassigned | Unassigned | Unassigned | 765_0 | Clustered | 12 | VC_765_0 | 12 | 0.6861 | 1 | 0.6861 | 0 | 0 | 0 | 1 | Ref |
| MK510984 | Unassigned | Unassigned | Unassigned | 764_0 | Clustered | 23 | VC_764_0 | 23 | 0.8194 | 1 | 0.8194 | 0 | 0 | 0 | 1 | Ref |
| MK510985 | Unassigned | Unassigned | Unassigned | 764_0 | Clustered | 23 | VC_764_0 | 23 | 0.8194 | 1 | 0.8194 | 0 | 0 | 0 | 1 | Ref |
| MK510986 | Unassigned | Unassigned | Unassigned | 764_0 | Clustered | 23 | VC_764_0 | 23 | 0.8194 | 1 | 0.8194 | 0 | 0 | 0 | 1 | Ref |
| MK510987 | Unassigned | Unassigned | Unassigned | 764_0 | Clustered | 23 | VC_764_0 | 23 | 0.8194 | 1 | 0.8194 | 0 | 0 | 0 | 1 | Ref |
| MK510988 | Unassigned | Unassigned | Unassigned | 764_0 | Clustered | 23 | VC_764_0 | 23 | 0.8194 | 1 | 0.8194 | 0 | 0 | 0 | 1 | Ref |
| MK510989 | Unassigned | Unassigned | Unassigned | 765_0 | Clustered | 12 | VC_765_0 | 12 | 0.6861 | 1 | 0.6861 | 0 | 0 | 0 | 1 | Ref |
| MK510990 | Unassigned | Unassigned | Unassigned | 764_0 | Clustered | 23 | VC_764_0 | 23 | 0.8194 | 1 | 0.8194 | 0 | 0 | 0 | 1 | Ref |
| MK510991 | Unassigned | Unassigned | Unassigned | 765_0 | Clustered | 12 | VC_765_0 | 12 | 0.6861 | 1 | 0.6861 | 0 | 0 | 0 | 1 | Ref |
| MK510992 | Unassigned | Unassigned | Unassigned | 764_0 | Clustered | 23 | VC_764_0 | 23 | 0.8194 | 1 | 0.8194 | 0 | 0 | 0 | 1 | Ref |
| MK510993 | Unassigned | Unassigned | Unassigned | 764_0 | Clustered | 23 | VC_764_0 | 23 | 0.8194 | 1 | 0.8194 | 0 | 0 | 0 | 1 | Ref |
| MK511001 | Unassigned | Unassigned | Unassigned | 304_1 | Clustered | 20 | VC_304_1 | 20 | 0.5632 | 1 | 0.5632 | 0 | 0 | 0 | 0.7684 | Ref |
| MK511002 | Unassigned | Unassigned | Unassigned | 304_1 | Clustered | 20 | VC_304_1 | 20 | 0.5632 | 1 | 0.5632 | 0 | 0 | 0 | 0.7684 | Ref |
| MK511003 | Unassigned | Unassigned | Unassigned | 304_1 | Clustered | 20 | VC_304_1 | 20 | 0.5632 | 1 | 0.5632 | 0 | 0 | 0 | 0.7684 | Ref |
| MK511004 | Unassigned | Unassigned | Unassigned | 304_1 | Clustered | 20 | VC_304_1 | 20 | 0.5632 | 1 | 0.5632 | 0 | 0 | 0 | 0.7684 | Ref |
| MK511005 | Unassigned | Unassigned | Unassigned | 304_1 | Clustered | 20 | VC_304_1 | 20 | 0.5632 | 1 | 0.5632 | 0 | 0 | 0 | 0.7684 | Ref |
| MK511006 | Unassigned | Unassigned | Unassigned | 304_1 | Clustered | 20 | VC_304_1 | 20 | 0.5632 | 1 | 0.5632 | 0 | 0 | 0 | 0.7684 | Ref |
| MK511007 | Unassigned | Unassigned | Unassigned | 304_1 | Clustered | 20 | VC_304_1 | 20 | 0.5632 | 1 | 0.5632 | 0 | 0 | 0 | 0.7684 | Ref |
| MK511008 | Unassigned | Unassigned | Unassigned | 304_1 | Clustered | 20 | VC_304_1 | 20 | 0.5632 | 1 | 0.5632 | 0 | 0 | 0 | 0.7684 | Ref |
| MK511009 | Unassigned | Unassigned | Unassigned | 304_1 | Clustered | 20 | VC_304_1 | 20 | 0.5632 | 1 | 0.5632 | 0 | 0 | 0 | 0.7684 | Ref |
| MK511010 | Unassigned | Unassigned | Unassigned | 304_1 | Clustered | 20 | VC_304_1 | 20 | 0.5632 | 1 | 0.5632 | 0 | 0 | 0 | 0.7684 | Ref |
| MK511011 | Unassigned | Unassigned | Unassigned | 304_1 | Clustered | 20 | VC_304_1 | 20 | 0.5632 | 1 | 0.5632 | 0 | 0 | 0 | 0.7684 | Ref |
| MK511012 | Unassigned | Unassigned | Unassigned | 304_1 | Clustered | 20 | VC_304_1 | 20 | 0.5632 | 1 | 0.5632 | 0 | 0 | 0 | 0.7684 | Ref |
| MK511013 | Unassigned | Unassigned | Unassigned | 304_1 | Clustered | 20 | VC_304_1 | 20 | 0.5632 | 1 | 0.5632 | 0 | 0 | 0 | 0.7684 | Ref |
| MK511014 | Unassigned | Unassigned | Unassigned | 304_1 | Clustered | 20 | VC_304_1 | 20 | 0.5632 | 1 | 0.5632 | 0 | 0 | 0 | 0.7684 | Ref |
| MK511015 | Unassigned | Unassigned | Unassigned | 304_1 | Clustered | 20 | VC_304_1 | 20 | 0.5632 | 1 | 0.5632 | 0 | 0 | 0 | 0.7684 | Ref |
| MK511016 | Unassigned | Unassigned | Unassigned | 304_1 | Clustered | 20 | VC_304_1 | 20 | 0.5632 | 1 | 0.5632 | 0 | 0 | 0 | 0.7684 | Ref |
| MK511017 | Unassigned | Unassigned | Unassigned | 185_0 | Clustered | 53 | VC_185_0 | 53 | 0.9153 | 1 | 0.9153 | 0 | 0 | 0 | 1 | Ref |
| MK511018 | Unassigned | Unassigned | Unassigned | 185_0 | Clustered | 53 | VC_185_0 | 53 | 0.9153 | 1 | 0.9153 | 0 | 0 | 0 | 1 | Ref |
| MK511019 | Unassigned | Unassigned | Unassigned | 185_0 | Clustered | 53 | VC_185_0 | 53 | 0.9153 | 1 | 0.9153 | 0 | 0 | 0 | 1 | Ref |
| MK511020 | Unassigned | Unassigned | Unassigned | 185_0 | Clustered | 53 | VC_185_0 | 53 | 0.9153 | 1 | 0.9153 | 0 | 0 | 0 | 1 | Ref |
| MK511021 | Unassigned | Unassigned | Unassigned | 185_0 | Clustered | 53 | VC_185_0 | 53 | 0.9153 | 1 | 0.9153 | 0 | 0 | 0 | 1 | Ref |
| MK511022 | Unassigned | Unassigned | Unassigned | 185_0 | Clustered | 53 | VC_185_0 | 53 | 0.9153 | 1 | 0.9153 | 0 | 0 | 0 | 1 | Ref |
| MK511023 | Unassigned | Unassigned | Unassigned | 185_0 | Clustered | 53 | VC_185_0 | 53 | 0.9153 | 1 | 0.9153 | 0 | 0 | 0 | 1 | Ref |
| MK511024 | Unassigned | Unassigned | Unassigned | 185_0 | Clustered | 53 | VC_185_0 | 53 | 0.9153 | 1 | 0.9153 | 0 | 0 | 0 | 1 | Ref |
| MK511025 | Unassigned | Unassigned | Unassigned | 185_0 | Clustered | 53 | VC_185_0 | 53 | 0.9153 | 1 | 0.9153 | 0 | 0 | 0 | 1 | Ref |
| MK511026 | Unassigned | Unassigned | Unassigned | 185_0 | Clustered | 53 | VC_185_0 | 53 | 0.9153 | 1 | 0.9153 | 0 | 0 | 0 | 1 | Ref |
| MK511027 | Unassigned | Unassigned | Unassigned | 185_0 | Clustered | 53 | VC_185_0 | 53 | 0.9153 | 1 | 0.9153 | 0 | 0 | 0 | 1 | Ref |
| MK511028 | Unassigned | Unassigned | Unassigned | 185_0 | Clustered | 53 | VC_185_0 | 53 | 0.9153 | 1 | 0.9153 | 0 | 0 | 0 | 1 | Ref |
| MK511029 | Unassigned | Unassigned | Unassigned | 185_0 | Clustered | 53 | VC_185_0 | 53 | 0.9153 | 1 | 0.9153 | 0 | 0 | 0 | 1 | Ref |
| MK511030 | Unassigned | Unassigned | Unassigned | 185_0 | Clustered | 53 | VC_185_0 | 53 | 0.9153 | 1 | 0.9153 | 0 | 0 | 0 | 1 | Ref |
| MK511031 | Unassigned | Unassigned | Unassigned | 185_0 | Clustered | 53 | VC_185_0 | 53 | 0.9153 | 1 | 0.9153 | 0 | 0 | 0 | 1 | Ref |
| MK511032 | Unassigned | Unassigned | Unassigned | 185_0 | Clustered | 53 | VC_185_0 | 53 | 0.9153 | 1 | 0.9153 | 0 | 0 | 0 | 1 | Ref |
| MK511033 | Unassigned | Unassigned | Unassigned | 185_0 | Clustered | 53 | VC_185_0 | 53 | 0.9153 | 1 | 0.9153 | 0 | 0 | 0 | 1 | Ref |
| MK511034 | Unassigned | Unassigned | Unassigned | 185_0 | Clustered | 53 | VC_185_0 | 53 | 0.9153 | 1 | 0.9153 | 0 | 0 | 0 | 1 | Ref |
| MK511035 | Unassigned | Unassigned | Unassigned | 185_0 | Clustered | 53 | VC_185_0 | 53 | 0.9153 | 1 | 0.9153 | 0 | 0 | 0 | 1 | Ref |
| MK511036 | Unassigned | Unassigned | Unassigned | 185_0 | Clustered | 53 | VC_185_0 | 53 | 0.9153 | 1 | 0.9153 | 0 | 0 | 0 | 1 | Ref |
| MK511037 | Unassigned | Unassigned | Unassigned | 185_0 | Clustered | 53 | VC_185_0 | 53 | 0.9153 | 1 | 0.9153 | 0 | 0 | 0 | 1 | Ref |
| MK511038 | Unassigned | Unassigned | Unassigned | 185_0 | Clustered | 53 | VC_185_0 | 53 | 0.9153 | 1 | 0.9153 | 0 | 0 | 0 | 1 | Ref |
| MK511039 | Unassigned | Unassigned | Unassigned | 645_0 | Clustered | 20 | VC_645_0 | 20 | 0.8502 | 1 | 0.8502 | 0 | 0 | 0 | 1 | Ref |
| MK511040 | Unassigned | Unassigned | Unassigned | 645_0 | Clustered | 20 | VC_645_0 | 20 | 0.8502 | 1 | 0.8502 | 0 | 0 | 0 | 1 | Ref |
| MK511041 | Unassigned | Unassigned | Unassigned | 645_0 | Clustered | 20 | VC_645_0 | 20 | 0.8502 | 1 | 0.8502 | 0 | 0 | 0 | 1 | Ref |
| MK511042 | Unassigned | Unassigned | Unassigned | 645_0 | Clustered | 20 | VC_645_0 | 20 | 0.8502 | 1 | 0.8502 | 0 | 0 | 0 | 1 | Ref |
| MK511043 | Unassigned | Unassigned | Unassigned | 645_0 | Clustered | 20 | VC_645_0 | 20 | 0.8502 | 1 | 0.8502 | 0 | 0 | 0 | 1 | Ref |
| MK511044 | Unassigned | Unassigned | Unassigned | 645_0 | Clustered | 20 | VC_645_0 | 20 | 0.8502 | 1 | 0.8502 | 0 | 0 | 0 | 1 | Ref |
| MK511045 | Unassigned | Unassigned | Unassigned | 645_0 | Clustered | 20 | VC_645_0 | 20 | 0.8502 | 1 | 0.8502 | 0 | 0 | 0 | 1 | Ref |
| MK511046 | Unassigned | Unassigned | Unassigned | 645_0 | Clustered | 20 | VC_645_0 | 20 | 0.8502 | 1 | 0.8502 | 0 | 0 | 0 | 1 | Ref |
| MK511047 | Unassigned | Unassigned | Unassigned | 645_0 | Clustered | 20 | VC_645_0 | 20 | 0.8502 | 1 | 0.8502 | 0 | 0 | 0 | 1 | Ref |
| MK511048 | Unassigned | Unassigned | Unassigned | 645_0 | Clustered | 20 | VC_645_0 | 20 | 0.8502 | 1 | 0.8502 | 0 | 0 | 0 | 1 | Ref |
| MK511049 | Unassigned | Unassigned | Unassigned | 645_0 | Clustered | 20 | VC_645_0 | 20 | 0.8502 | 1 | 0.8502 | 0 | 0 | 0 | 1 | Ref |
| MK511050 | Unassigned | Unassigned | Unassigned | 645_0 | Clustered | 20 | VC_645_0 | 20 | 0.8502 | 1 | 0.8502 | 0 | 0 | 0 | 1 | Ref |
| MK511051 | Unassigned | Unassigned | Unassigned | 645_0 | Clustered | 20 | VC_645_0 | 20 | 0.8502 | 1 | 0.8502 | 0 | 0 | 0 | 1 | Ref |
| MK511057 | Unassigned | Unassigned | Unassigned | 192_0 | Clustered | 22 | VC_192_0 | 22 | 0.6582 | 1 | 0.6582 | 0 | 0 | 0 | 1 | Ref |
| MK511058 | Unassigned | Unassigned | Unassigned | 192_0 | Clustered | 22 | VC_192_0 | 22 | 0.6582 | 1 | 0.6582 | 0 | 0 | 0 | 1 | Ref |
| MK511059 | Unassigned | Unassigned | Unassigned | 192_0 | Clustered | 22 | VC_192_0 | 22 | 0.6582 | 1 | 0.6582 | 0 | 0 | 0 | 1 | Ref |
| MK511060 | Unassigned | Unassigned | Unassigned | 192_0 | Clustered | 22 | VC_192_0 | 22 | 0.6582 | 1 | 0.6582 | 0 | 0 | 0 | 1 | Ref |
| MK511061 | Unassigned | Unassigned | Unassigned | 192_0 | Clustered | 22 | VC_192_0 | 22 | 0.6582 | 1 | 0.6582 | 0 | 0 | 0 | 1 | Ref |
| MK511063 | Unassigned | Unassigned | Unassigned | 192_0 | Clustered | 22 | VC_192_0 | 22 | 0.6582 | 1 | 0.6582 | 0 | 0 | 0 | 1 | Ref |

| | | | | | | | | | | | | | | | | |
|---|---|---|---|---|---|---|---|---|---|---|---|---|---|---|---|---|
| MK511064 | Unassigned | Unassigned | Unassigned | 192_0 | Clustered | 22 | VC_192_0 | 22 | 0.6582 | 1 | 0.6582 | 0 | 0 | 0 | 1 | Ref |
| MK511065 | Unassigned | Unassigned | Unassigned | 192_0 | Clustered | 22 | VC_192_0 | 22 | 0.6582 | 1 | 0.6582 | 0 | 0 | 0 | 1 | Ref |
| MK511066 | Unassigned | Unassigned | Unassigned | 2_0 | Clustered | 4 | VC_2_0 | 4 | 0.13 | 0.99998694 | 0.13 | 0 | 0 | 0 | 1 | Ref |
| MK512531 | Unassigned | Unassigned | Unassigned | 184_0 | Clustered | 5 | VC_184_0 | 5 | 0.7721 | 0.99998991 | 0.7721 | 0 | 0 | 0 | 1 | Ref |
| MK514281 | Unassigned | Unassigned | Unassigned | 507_1 | Clustered | 11 | VC_507_1 | 11 | 0.4612 | 1 | 0.4612 | 0 | 0 | 0 | 1 | Ref |
| MK514282 | Unassigned | Unassigned | Unassigned | 994_0 | Clustered | 5 | VC_994_0 | 5 | 0.0495 | 0.99983811 | 0.0494 | 0 | 0 | 0 | 1 | Ref |
| MK514283 | Unassigned | Unassigned | Unassigned | 632_0 | Clustered | 9 | VC_632_0 | 9 | 0.9524 | 1 | 0.9524 | 0 | 0 | 0 | 1 | Ref |
| MK521904 | Unassigned | Unassigned | Unassigned | 972_0 | Clustered | 99 | VC_972_0 | 99 | 0.9113 | 1 | 0.9113 | 0 | 0 | 0 | 0.9928 | Ref |
| MK521905 | Unassigned | Unassigned | Unassigned | 848_0 | Clustered | 124 | VC_848_0 | 124 | 0.9484 | 1 | 0.9484 | 0 | 0 | 0 | 1 | Ref |
| MK521906 | Unassigned | Unassigned | Unassigned | 125_0 | Clustered | 23 | VC_125_0 | 23 | 0.8163 | 1 | 0.8163 | 0 | 0 | 0 | 1 | Ref |
| MK521907 | Unassigned | Unassigned | Unassigned | 200_0 | Clustered | 15 | VC_200_0 | 15 | 0.5004 | 1 | 0.5004 | 0 | 0 | 0 | 1 | Ref |
| MK524176 | Unassigned | Unassigned | Unassigned | 340_18 | Clustered | 7 | VC_340_18 | 7 | 0.0326 | 1 | 0.0326 | 0 | 0 | 0 | 0.8095 | Ref |
| MK524177 | Unassigned | Unassigned | Unassigned | 237_0 | Clustered | 3 | VC_237_0 | 3 | 0.0732 | 0.99355836 | 0.0727 | 0 | 0 | 0 | 0.6667 | Ref |
| MK524178 | Unassigned | Unassigned | Unassigned | 293_11 | Clustered | 3 | VC_293_11 | 3 | 0.0146 | 0.93460284 | 0.0136 | 0 | 0 | 0 | 1 | Ref |
| MK524485 | Unassigned | Unassigned | Unassigned | 241_0 | Clustered | 21 | VC_241_0 | 21 | 0.8342 | 1 | 0.8342 | 0 | 0 | 0 | 1 | Ref |
| MK524486 | Unassigned | Unassigned | Unassigned | 385_0 | Clustered | 24 | VC_385_0 | 24 | 0.5516 | 1 | 0.5516 | 0 | 0 | 0 | 1 | Ref |
| MK524487 | Unassigned | Unassigned | Unassigned | 991_0 | Clustered | 39 | VC_991_0 | 39 | 1 | 1 | 1 | 0 | 0 | 0 | 1 | Ref |
| MK524488 | Unassigned | Unassigned | Unassigned | 241_0 | Clustered | 21 | VC_241_0 | 21 | 0.8342 | 1 | 0.8342 | 0 | 0 | 0 | 1 | Ref |
| MK524489 | Unassigned | Unassigned | Unassigned | 232_0 | Clustered | 32 | VC_232_0 | 32 | 0.8471 | 1 | 0.8471 | 0 | 0 | 0 | 0.996 | Ref |
| MK524490 | Unassigned | Unassigned | Unassigned | 385_1 | Clustered | 9 | VC_385_1 | 9 | 0.1928 | 1 | 0.1928 | 0 | 0 | 0 | 1 | Ref |
| MK524491 | Unassigned | Unassigned | Unassigned | 223_6 | Clustered | 42 | VC_223_6 | 42 | 0.4717 | 1 | 0.4717 | 0 | 0 | 0 | 0.8351 | Ref |
| MK524493 | Unassigned | Unassigned | Unassigned | 783_0 | Clustered | 67 | VC_783_0 | 67 | 0.8945 | 1 | 0.8945 | 0 | 0 | 0 | 1 | Ref |
| MK524494 | Unassigned | Unassigned | Unassigned | 783_0 | Clustered | 67 | VC_783_0 | 67 | 0.8945 | 1 | 0.8945 | 0 | 0 | 0 | 1 | Ref |
| MK524495 | Unassigned | Unassigned | Unassigned | 991_0 | Clustered | 39 | VC_991_0 | 39 | 1 | 1 | 1 | 0 | 0 | 0 | 1 | Ref |
| MK524496 | Unassigned | Unassigned | Unassigned | 531_0 | Clustered | 32 | VC_531_0 | 32 | 0.6859 | 1 | 0.6859 | 0 | 0 | 0 | 1 | Ref |
| MK524497 | Unassigned | Unassigned | Unassigned | 335_21 | Clustered | 5 | VC_335_21 | 5 | 0.0208 | 0.99999976 | 0.0208 | 0 | 0 | 0 | 0.8 | Ref |
| MK524498 | Unassigned | Unassigned | Unassigned | 585_0 | Clustered | 20 | VC_585_0 | 20 | 0.8376 | 1 | 0.8376 | 0 | 0 | 0 | 1 | Ref |
| MK524499 | Unassigned | Unassigned | Unassigned | 335_22 | Clustered/Singleton | 1 | VC_335_22 | 1 | 0 | 1 | 0 | 0 | 0 | 0 | 1 | Ref |
| MK524500 | Unassigned | Unassigned | Unassigned | 539_0 | Clustered | 37 | VC_539_0 | 37 | 0.911 | 1 | 0.911 | 0 | 0 | 0 | 1 | Ref |
| MK524501 | Unassigned | Unassigned | Unassigned | 1223_0 | Clustered | 2 | VC_1223_0 | 2 | 0.0456 | 0.95126432 | 0.0434 | 0 | 0 | 0 | 1 | Ref |
| MK524502 | Unassigned | Unassigned | Unassigned | 1407_0 | Clustered | 78 | VC_1407_0 | 78 | 0.9731 | 1 | 0.9731 | 0 | 0 | 0 | 1 | Ref |
| MK524503 | Unassigned | Unassigned | Unassigned | 538_87 | Clustered/Singleton | 1 | VC_538_87 | 1 | 0 | 1 | 0 | 0 | 0 | 0 | 1 | Ref |
| MK524504 | Unassigned | Unassigned | Unassigned | 566_4 | Clustered | 16 | VC_566_4 | 16 | 0.2517 | 0.9999998 | 0.2517 | 0 | 0 | 0 | 0.9417 | Ref |
| MK524505 | Unassigned | Unassigned | Unassigned | 1226_0 | Clustered | 5 | VC_1226_0 | 5 | 0.6594 | 0.99999982 | 0.6594 | 0 | 0 | 0 | 1 | Ref |
| MK524506 | Unassigned | Unassigned | Unassigned | 335_49 | Clustered | 17 | VC_335_49 | 17 | 0.0731 | 1 | 0.0731 | 0 | 0 | 0 | 0.7721 | Ref |
| MK524507 | Unassigned | Unassigned | Unassigned | 228_0 | Clustered | 22 | VC_228_0 | 22 | 0.7411 | 1 | 0.7411 | 0 | 0 | 0 | 1 | Ref |
| MK524508 | Unassigned | Unassigned | Unassigned | 537_0 | Clustered | 39 | VC_537_0 | 39 | 0.8148 | 1 | 0.8148 | 0 | 0 | 0 | 1 | Ref |
| MK524509 | Unassigned | Unassigned | Unassigned | 539_0 | Clustered | 37 | VC_539_0 | 37 | 0.911 | 1 | 0.911 | 0 | 0 | 0 | 1 | Ref |
| MK524510 | Unassigned | Unassigned | Unassigned | 1415_0 | Clustered | 31 | VC_1415_0 | 31 | 0.8456 | 1 | 0.8456 | 0 | 0 | 0 | 1 | Ref |
| MK524511 | Unassigned | Unassigned | Unassigned | 228_0 | Clustered | 22 | VC_228_0 | 22 | 0.7411 | 1 | 0.7411 | 0 | 0 | 0 | 1 | Ref |
| MK524512 | Unassigned | Unassigned | Unassigned | 384_1 | Clustered | 11 | VC_384_1 | 11 | 0.0378 | 1 | 0.0378 | 0 | 0 | 0 | 0.8545 | Ref |
| MK524513 | Unassigned | Unassigned | Unassigned | 228_0 | Clustered | 22 | VC_228_0 | 22 | 0.7411 | 1 | 0.7411 | 0 | 0 | 0 | 1 | Ref |
| MK524514 | Unassigned | Unassigned | Unassigned | 228_0 | Clustered | 22 | VC_228_0 | 22 | 0.7411 | 1 | 0.7411 | 0 | 0 | 0 | 1 | Ref |
| MK524515 | Unassigned | Unassigned | Unassigned | 539_0 | Clustered | 37 | VC_539_0 | 37 | 0.911 | 1 | 0.911 | 0 | 0 | 0 | 1 | Ref |
| MK524516 | Unassigned | Unassigned | Unassigned | 566_9 | Clustered/Singleton | 1 | VC_566_9 | 1 | 0 | 1 | 0 | 0 | 0 | 0 | 1 | Ref |
| MK524517 | Unassigned | Unassigned | Unassigned | 538_38 | Clustered | 6 | VC_538_38 | 6 | 0.0517 | 1 | 0.0517 | 0 | 0 | 0 | 0.8667 | Ref |
| MK524518 | Unassigned | Unassigned | Unassigned | 539_0 | Clustered | 37 | VC_539_0 | 37 | 0.911 | 1 | 0.911 | 0 | 0 | 0 | 1 | Ref |
| MK524519 | Unassigned | Unassigned | Unassigned | 228_0 | Clustered | 22 | VC_228_0 | 22 | 0.7411 | 1 | 0.7411 | 0 | 0 | 0 | 1 | Ref |
| MK524520 | Unassigned | Unassigned | Unassigned | 539_0 | Clustered | 37 | VC_539_0 | 37 | 0.911 | 1 | 0.911 | 0 | 0 | 0 | 1 | Ref |
| MK524521 | Unassigned | Unassigned | Unassigned | 566_4 | Clustered | 16 | VC_566_4 | 16 | 0.2517 | 0.9999998 | 0.2517 | 0 | 0 | 0 | 0.9417 | Ref |
| MK524522 | Unassigned | Unassigned | Unassigned | 539_0 | Clustered | 37 | VC_539_0 | 37 | 0.911 | 1 | 0.911 | 0 | 0 | 0 | 1 | Ref |
| MK524523 | Unassigned | Unassigned | Unassigned | 335_16 | Clustered | 2 | VC_335_16 | 2 | 0.0094 | 0.95751393 | 0.009 | 0 | 0 | 0 | 1 | Ref |
| MK524524 | Unassigned | Unassigned | Unassigned | 1015_1 | Clustered | 26 | VC_1015_1 | 26 | 0.6989 | 1 | 0.6989 | 0 | 0 | 0 | 1 | Ref |
| MK524525 | Unassigned | Unassigned | Unassigned | 335_39 | Clustered/Singleton | 1 | VC_335_39 | 1 | 0 | 1 | 0 | 0 | 0 | 0 | 1 | Ref |
| MK524526 | Unassigned | Unassigned | Unassigned | 384_1 | Clustered | 11 | VC_384_1 | 11 | 0.0378 | 1 | 0.0378 | 0 | 0 | 0 | 0.8545 | Ref |
| MK524527 | Unassigned | Unassigned | Unassigned | 566_4 | Clustered | 16 | VC_566_4 | 16 | 0.2517 | 0.9999998 | 0.2517 | 0 | 0 | 0 | 0.9417 | Ref |
| MK524528 | Unassigned | Unassigned | Unassigned | 539_0 | Clustered | 37 | VC_539_0 | 37 | 0.911 | 1 | 0.911 | 0 | 0 | 0 | 1 | Ref |
| MK524529 | Unassigned | Unassigned | Unassigned | 566_4 | Clustered | 16 | VC_566_4 | 16 | 0.2517 | 0.9999998 | 0.2517 | 0 | 0 | 0 | 0.9417 | Ref |
| MK524530 | Unassigned | Unassigned | Unassigned | 229_0 | Clustered | 5 | VC_229_0 | 5 | 0.0566 | 0.99999997 | 0.0566 | 0 | 0 | 0 | 1 | Ref |
| MK524531 | Unassigned | Unassigned | Unassigned | 335_49 | Clustered | 17 | VC_335_49 | 17 | 0.0731 | 1 | 0.0731 | 0 | 0 | 0 | 0.7721 | Ref |
| MK542015 | Unassigned | Unassigned | Unassigned | 655_0 | Clustered | 45 | VC_655_0 | 45 | 0.7722 | 1 | 0.7722 | 0 | 0 | 0 | 1 | Ref |
| MK542821 | Unassigned | Unassigned | Unassigned | 405_0 | Clustered | 126 | VC_405_0 | 126 | 0.9614 | 1 | 0.9614 | 0 | 0 | 0 | 1 | Ref |
| MK552140 | Unassigned | Unassigned | Unassigned | 67_0 | Clustered | 2 | VC_67_0 | 2 | 0.194 | 0.95487418 | 0.1852 | 0 | 0 | 0 | 1 | Ref |
| MK552141 | Unassigned | Unassigned | Unassigned | 67_0 | Clustered | 2 | VC_67_0 | 2 | 0.194 | 0.95487418 | 0.1852 | 0 | 0 | 0 | 1 | Ref |
| MK552327 | Unassigned | Unassigned | Unassigned | 356_3 | Clustered/Singleton | 1 | VC_356_3 | 1 | 0 | 1 | 0 | 0 | 0 | 0 | 1 | Ref |
| MK559427 | Unassigned | Unassigned | Unassigned | 585_0 | Clustered | 20 | VC_585_0 | 20 | 0.8376 | 1 | 0.8376 | 0 | 0 | 0 | 1 | Ref |
| MK559428 | Unassigned | Unassigned | Unassigned | 232_0 | Clustered | 32 | VC_232_0 | 32 | 0.8471 | 1 | 0.8471 | 0 | 0 | 0 | 0.996 | Ref |
| MK559429 | Unassigned | Unassigned | Unassigned | 571_6 | Clustered | 22 | VC_571_6 | 22 | 0.1207 | 1 | 0.1207 | 0 | 0 | 0 | 0.8095 | Ref |
| MK559459 | Unassigned | Unassigned | Unassigned | 935_0 | Clustered | 6 | VC_935_0 | 6 | 0.2317 | 1 | 0.2317 | 0 | 0 | 0 | 1 | Ref |
| MK562502 | Unassigned | Unassigned | Unassigned | 341_0 | Clustered | 27 | VC_341_0 | 27 | 0.5058 | 1 | 0.5058 | 0 | 0 | 0 | 0.8661 | Ref |
| MK562503 | Unassigned | Unassigned | Unassigned | 113_0 | Clustered | 91 | VC_113_0 | 91 | 0.9584 | 1 | 0.9584 | 0 | 0 | 0 | 1 | Ref |
| MK562504 | Unassigned | Unassigned | Unassigned | 341_0 | Clustered | 27 | VC_341_0 | 27 | 0.5058 | 1 | 0.5058 | 0 | 0 | 0 | 0.8661 | Ref |
| MK562505 | Unassigned | Unassigned | Unassigned | 341_0 | Clustered | 27 | VC_341_0 | 27 | 0.5058 | 1 | 0.5058 | 0 | 0 | 0 | 0.8661 | Ref |
| MK568062 | Unassigned | Unassigned | Unassigned | 440_0 | Clustered | 33 | VC_440_0 | 33 | 0.992 | 1 | 0.992 | 0 | 0 | 0 | 1 | Ref |
| MK568540 | Unassigned | Unassigned | Unassigned | 549_0 | Clustered | 9 | VC_549_0 | 9 | 0.2597 | 1 | 0.2597 | 0 | 0 | 0 | 1 | Ref |
| MK570053 | Unassigned | Unassigned | Unassigned | 1461_0 | Clustered | 3 | VC_1461_0 | 3 | 0.7758 | 0.9639009 | 0.7478 | 0 | 0 | 0 | 1 | Ref |
| MK570054 | Unassigned | Unassigned | Unassigned | 1461_0 | Clustered | 3 | VC_1461_0 | 3 | 0.7758 | 0.9639009 | 0.7478 | 0 | 0 | 0 | 1 | Ref |
| MK570055 | Unassigned | Unassigned | Unassigned | 1461_0 | Clustered | 3 | VC_1461_0 | 3 | 0.7758 | 0.9639009 | 0.7478 | 0 | 0 | 0 | 1 | Ref |
| MK570225 | Unassigned | Unassigned | Unassigned | 110_0 | Clustered | 33 | VC_110_0 | 33 | 0.9488 | 1 | 0.9488 | 0 | 0 | 0 | 1 | Ref |
| MK573636 | Unassigned | Unassigned | Unassigned | 609_0 | Clustered | 27 | VC_609_0 | 27 | 0.9943 | 1 | 0.9943 | 0 | 0 | 0 | 1 | Ref |
| MK574011 | Unassigned | Unassigned | Unassigned | 119_0 | Clustered | 9 | VC_119_0 | 9 | 0.9977 | 0.98988637 | 0.9876 | 0 | 0 | 0 | 1 | Ref |

| | | | | | | | | | | | | | | | | |
|---|---|---|---|---|---|---|---|---|---|---|---|---|---|---|---|---|
| MK574078 | Unassigned | Unassigned | Unassigned | 766_0 | Clustered | 40 | VC_766_0 | 40 | 0.7511 | 1 | 0.7511 | 0 | 0 | 0 | 1 | Ref |
| MK577502 | Unassigned | Unassigned | Unassigned | 619_0 | Clustered | 3 | VC_619_0 | 3 | 0.054 | 0.97294713 | 0.0526 | 0 | 0 | 0 | 1 | Ref |
| MK578530 | Unassigned | Unassigned | Unassigned | 654_1 | Clustered | 27 | VC_654_1 | 27 | 0.2571 | 1 | 0.2571 | 0 | 0 | 0 | 0.9858 | Ref |
| MK580972 | Unassigned | Unassigned | Unassigned | 524_0 | Clustered | 8 | VC_524_0 | 8 | 0.7365 | 1 | 0.7365 | 0 | 0 | 0 | 1 | Ref |
| MK584893 | Unassigned | Unassigned | Unassigned | 155_8 | Clustered | 66 | VC_155_8 | 66 | 0.4537 | 1 | 0.4537 | 0 | 0 | 0 | 0.8182 | Ref |
| MK598851 | Unassigned | Unassigned | Unassigned | 113_0 | Clustered | 91 | VC_113_0 | 91 | 0.9584 | 1 | 0.9584 | 0 | 0 | 0 | 1 | Ref |
| MK599315 | Unassigned | Unassigned | Unassigned | 356_1 | Clustered | 3 | VC_356_1 | 3 | 0.0359 | 0.93699065 | 0.0336 | 0 | 0 | 0 | 1 | Ref |
| MK599416 | Unassigned | Unassigned | Unassigned | 475_0 | Clustered | 96 | VC_475_0 | 96 | 0.9303 | 1 | 0.9303 | 0 | 0 | 0 | 0.9575 | Ref |
| MK605242 | Unassigned | Unassigned | Unassigned | 1214_0 | Clustered | 6 | VC_1214_0 | 6 | 0.9948 | 0.99999296 | 0.9948 | 0 | 0 | 0 | 1 | Ref |
| MK605243 | Unassigned | Unassigned | Unassigned | 1214_0 | Clustered | 6 | VC_1214_0 | 6 | 0.9948 | 0.99999296 | 0.9948 | 0 | 0 | 0 | 1 | Ref |
| MK605244 | Unassigned | Unassigned | Unassigned | 1214_0 | Clustered | 6 | VC_1214_0 | 6 | 0.9948 | 0.99999296 | 0.9948 | 0 | 0 | 0 | 1 | Ref |
| MK605245 | Unassigned | Unassigned | Unassigned | 1214_0 | Clustered | 6 | VC_1214_0 | 6 | 0.9948 | 0.99999296 | 0.9948 | 0 | 0 | 0 | 1 | Ref |
| MK605246 | Unassigned | Unassigned | Unassigned | 1214_0 | Clustered | 6 | VC_1214_0 | 6 | 0.9948 | 0.99999296 | 0.9948 | 0 | 0 | 0 | 1 | Ref |
| MK608335 | Unassigned | Unassigned | Unassigned | 398_0 | Clustered | 51 | VC_398_0 | 51 | 0.8741 | 1 | 0.8741 | 0 | 0 | 0 | 1 | Ref |
| MK608336 | Unassigned | Unassigned | Unassigned | 1001_0 | Clustered | 3 | VC_1001_0 | 3 | 0.3482 | 0.99841425 | 0.3476 | 0 | 0 | 0 | 1 | Ref |
| MK610268 | Unassigned | Unassigned | Unassigned | 465_0 | Clustered | 80 | VC_465_0 | 80 | 0.9988 | 1 | 0.9988 | 0 | 0 | 0 | 1 | Ref |
| MK613343 | Unassigned | Unassigned | Unassigned | 312_0 | Clustered | 12 | VC_312_0 | 12 | 0.5719 | 1 | 0.5719 | 0 | 0 | 0 | 0.8485 | Ref |
| MK613344 | Unassigned | Unassigned | Unassigned | 312_0 | Clustered | 12 | VC_312_0 | 12 | 0.5719 | 1 | 0.5719 | 0 | 0 | 0 | 0.8485 | Ref |
| MK613345 | Unassigned | Unassigned | Unassigned | 312_0 | Clustered | 12 | VC_312_0 | 12 | 0.5719 | 1 | 0.5719 | 0 | 0 | 0 | 0.8485 | Ref |
| MK613346 | Unassigned | Unassigned | Unassigned | 311_0 | Clustered | 11 | VC_311_0 | 11 | 0.7851 | 1 | 0.7851 | 0 | 0 | 0 | 1 | Ref |
| MK618657 | Unassigned | Unassigned | Unassigned | 972_0 | Clustered | 99 | VC_972_0 | 99 | 0.9113 | 1 | 0.9113 | 0 | 0 | 0 | 0.9928 | Ref |
| MK618658 | Unassigned | Unassigned | Unassigned | 848_0 | Clustered | 124 | VC_848_0 | 124 | 0.9484 | 1 | 0.9484 | 0 | 0 | 0 | 1 | Ref |
| MK618715 | Unassigned | Unassigned | Unassigned | 372_0 | Clustered | 7 | VC_372_0 | 7 | 0.3691 | 1 | 0.3691 | 0 | 0 | 0 | 1 | Ref |
| MK618716 | Unassigned | Unassigned | Unassigned | 12_0 | Clustered | 21 | VC_12_0 | 21 | 0.2262 | 1 | 0.2262 | 0 | 0 | 0 | 0.9571 | Ref |
| MK620893 | Unassigned | Unassigned | Unassigned | 571_7 | Clustered | 28 | VC_571_7 | 28 | 0.1651 | 1 | 0.1651 | 0 | 0 | 0 | 0.8519 | Ref |
| MK620894 | Unassigned | Unassigned | Unassigned | 1199_0 | Clustered | 3 | VC_1199_0 | 3 | 0.3136 | 0.99425123 | 0.3118 | 0 | 0 | 0 | 1 | Ref |
| MK620895 | Unassigned | Unassigned | Unassigned | 1296_0 | Clustered | 82 | VC_1296_0 | 82 | 0.7938 | 1 | 0.7938 | 0 | 0 | 0 | 1 | Ref |
| MK620896 | Unassigned | Unassigned | Unassigned | 1115_0 | Clustered | 2 | VC_1115_0 | 2 | 0.0262 | 0.95117088 | 0.0249 | 0 | 0 | 0 | 1 | Ref |
| MK620897 | Unassigned | Unassigned | Unassigned | 1407_0 | Clustered | 78 | VC_1407_0 | 78 | 0.9731 | 1 | 0.9731 | 0 | 0 | 0 | 1 | Ref |
| MK620898 | Unassigned | Unassigned | Unassigned | 526_0 | Clustered | 32 | VC_526_0 | 32 | 0.9037 | 1 | 0.9037 | 0 | 0 | 0 | 1 | Ref |
| MK620900 | Unassigned | Unassigned | Unassigned | 1436_0 | Clustered | 3 | VC_1436_0 | 3 | 0.9803 | 0.9595722 | 0.9406 | 0 | 0 | 0 | 1 | Ref |
| MK620901 | Unassigned | Unassigned | Unassigned | 1203_0 | Clustered | 28 | VC_1203_0 | 28 | 0.4905 | 1 | 0.4905 | 0 | 0 | 0 | 1 | Ref |
| MK620902 | Unassigned | Unassigned | Unassigned | 1296_0 | Clustered | 82 | VC_1296_0 | 82 | 0.7938 | 1 | 0.7938 | 0 | 0 | 0 | 1 | Ref |
| MK620903 | Unassigned | Unassigned | Unassigned | 1296_0 | Clustered | 82 | VC_1296_0 | 82 | 0.7938 | 1 | 0.7938 | 0 | 0 | 0 | 1 | Ref |
| MK620904 | Unassigned | Unassigned | Unassigned | 1203_0 | Clustered | 28 | VC_1203_0 | 28 | 0.4905 | 1 | 0.4905 | 0 | 0 | 0 | 1 | Ref |
| MK620905 | Unassigned | Unassigned | Unassigned | 1296_0 | Clustered | 82 | VC_1296_0 | 82 | 0.7938 | 1 | 0.7938 | 0 | 0 | 0 | 1 | Ref |
| MK629527 | Unassigned | Unassigned | Unassigned | 760_0 | Clustered | 15 | VC_760_0 | 15 | 1 | 1 | 1 | 0 | 0 | 0 | 1 | Ref |
| MK629528 | Unassigned | Unassigned | Unassigned | 162_0 | Clustered | 12 | VC_162_0 | 12 | 0.9051 | 1 | 0.9051 | 0 | 0 | 0 | 1 | Ref |
| MK630230 | Unassigned | Unassigned | Unassigned | 200_0 | Clustered | 15 | VC_200_0 | 15 | 0.5004 | 1 | 0.5004 | 0 | 0 | 0 | 1 | Ref |
| MK637516 | Unassigned | Unassigned | Unassigned | 1005_0 | Clustered | 3 | VC_1005_0 | 3 | 0.8261 | 0.99765766 | 0.8242 | 0 | 0 | 0 | 1 | Ref |
| MK639187 | Unassigned | Unassigned | Unassigned | 293_13 | Clustered | 10 | VC_293_13 | 10 | 0.0698 | 0.99999931 | 0.0698 | 0 | 0 | 0 | 0.9556 | Ref |
| MK651787 | Unassigned | Unassigned | Unassigned | 721_0 | Clustered | 11 | VC_721_0 | 11 | 0.8846 | 1 | 0.8846 | 0 | 0 | 0 | 1 | Ref |
| MK656892 | Unassigned | Unassigned | Unassigned | 155_8 | Clustered | 66 | VC_155_8 | 66 | 0.4537 | 1 | 0.4537 | 0 | 0 | 0 | 0.8182 | Ref |
| MK658834 | Unassigned | Unassigned | Unassigned | 9_0 | Clustered | 38 | VC_9_0 | 38 | 0.4859 | 1 | 0.4859 | 0 | 0 | 0 | 1 | Ref |
| MK660712 | Unassigned | Unassigned | Unassigned | 984_0 | Clustered | 5 | VC_984_0 | 5 | 0.1708 | 0.99999984 | 0.1708 | 0 | 0 | 0 | 1 | Ref |
| MK660714 | Unassigned | Unassigned | Unassigned | 1200_0 | Clustered | 73 | VC_1200_0 | 73 | 0.9825 | 1 | 0.9825 | 0 | 0 | 0 | 1 | Ref |
| MK672798 | Unassigned | Unassigned | Unassigned | 819_0 | Clustered | 19 | VC_819_0 | 19 | 0.6835 | 1 | 0.6835 | 0 | 0 | 0 | 1 | Ref |
| MK672799 | Unassigned | Unassigned | Unassigned | 306_0 | Clustered | 24 | VC_306_0 | 24 | 0.9902 | 1 | 0.9902 | 0 | 0 | 0 | 1 | Ref |
| MK672800 | Unassigned | Unassigned | Unassigned | 306_0 | Clustered | 24 | VC_306_0 | 24 | 0.9902 | 1 | 0.9902 | 0 | 0 | 0 | 1 | Ref |
| MK672801 | Unassigned | Unassigned | Unassigned | 306_0 | Clustered | 24 | VC_306_0 | 24 | 0.9902 | 1 | 0.9902 | 0 | 0 | 0 | 1 | Ref |
| MK672803 | Unassigned | Unassigned | Unassigned | 306_0 | Clustered | 24 | VC_306_0 | 24 | 0.9902 | 1 | 0.9902 | 0 | 0 | 0 | 1 | Ref |
| MK672804 | Unassigned | Unassigned | Unassigned | 306_0 | Clustered | 24 | VC_306_0 | 24 | 0.9902 | 1 | 0.9902 | 0 | 0 | 0 | 1 | Ref |
| MK673511 | Unassigned | Unassigned | Unassigned | 440_0 | Clustered | 33 | VC_440_0 | 33 | 0.992 | 1 | 0.992 | 0 | 0 | 0 | 1 | Ref |
| MK685667 | Unassigned | Unassigned | Unassigned | 125_0 | Clustered | 23 | VC_125_0 | 23 | 0.8163 | 1 | 0.8163 | 0 | 0 | 0 | 1 | Ref |
| MK685668 | Unassigned | Unassigned | Unassigned | 399_0 | Clustered | 49 | VC_399_0 | 49 | 0.7927 | 1 | 0.7927 | 0 | 0 | 0 | 1 | Ref |
| MK686068 | Unassigned | Unassigned | Unassigned | 386_0 | Clustered | 10 | VC_386_0 | 10 | 0.7454 | 1 | 0.7454 | 0 | 0 | 0 | 1 | Ref |
| MK686069 | Unassigned | Unassigned | Unassigned | 386_0 | Clustered | 10 | VC_386_0 | 10 | 0.7454 | 1 | 0.7454 | 0 | 0 | 0 | 1 | Ref |
| MK686071 | Unassigned | Unassigned | Unassigned | 891_0 | Clustered | 11 | VC_891_0 | 11 | 0.9663 | 1 | 0.9663 | 0 | 0 | 0 | 1 | Ref |
| MK689364 | Unassigned | Unassigned | Unassigned | 456_2 | Clustered | 4 | VC_456_2 | 4 | 0.0833 | 0.99988129 | 0.0833 | 0 | 0 | 0 | 0.8333 | Ref |
| MK693005 | Unassigned | Unassigned | Unassigned | 113_0 | Clustered | 91 | VC_113_0 | 91 | 0.9584 | 1 | 0.9584 | 0 | 0 | 0 | 1 | Ref |
| MK693030 | Unassigned | Unassigned | Unassigned | 93_0 | Clustered | 22 | VC_93_0 | 22 | 0.5285 | 1 | 0.5285 | 0 | 0 | 0 | 1 | Ref |
| MK714353 | Unassigned | Unassigned | Unassigned | 33_0 | Clustered | 9 | VC_33_0 | 9 | 0.3409 | 1 | 0.3409 | 0 | 0 | 0 | 0.6944 | Ref |
| MK719701 | Unassigned | Unassigned | Unassigned | 1462_0 | Clustered | 110 | VC_1462_0 | 110 | 1 | 1 | 1 | 0 | 0 | 0 | 0.9746 | Ref |
| MK719702 | Unassigned | Unassigned | Unassigned | 1462_0 | Clustered | 110 | VC_1462_0 | 110 | 1 | 1 | 1 | 0 | 0 | 0 | 0.9746 | Ref |
| MK719703 | Unassigned | Unassigned | Unassigned | 1462_0 | Clustered | 110 | VC_1462_0 | 110 | 1 | 1 | 1 | 0 | 0 | 0 | 0.9746 | Ref |
| MK719704 | Unassigned | Unassigned | Unassigned | 1462_0 | Clustered | 110 | VC_1462_0 | 110 | 1 | 1 | 1 | 0 | 0 | 0 | 0.9746 | Ref |
| MK719705 | Unassigned | Unassigned | Unassigned | 1462_0 | Clustered | 110 | VC_1462_0 | 110 | 1 | 1 | 1 | 0 | 0 | 0 | 0.9746 | Ref |
| MK719706 | Unassigned | Unassigned | Unassigned | 1462_0 | Clustered | 110 | VC_1462_0 | 110 | 1 | 1 | 1 | 0 | 0 | 0 | 0.9746 | Ref |
| MK719707 | Unassigned | Unassigned | Unassigned | 1462_0 | Clustered | 110 | VC_1462_0 | 110 | 1 | 1 | 1 | 0 | 0 | 0 | 0.9746 | Ref |
| MK719708 | Unassigned | Unassigned | Unassigned | 1462_0 | Clustered | 110 | VC_1462_0 | 110 | 1 | 1 | 1 | 0 | 0 | 0 | 0.9746 | Ref |
| MK719709 | Unassigned | Unassigned | Unassigned | 1462_0 | Clustered | 110 | VC_1462_0 | 110 | 1 | 1 | 1 | 0 | 0 | 0 | 0.9746 | Ref |
| MK719710 | Unassigned | Unassigned | Unassigned | 1462_0 | Clustered | 110 | VC_1462_0 | 110 | 1 | 1 | 1 | 0 | 0 | 0 | 0.9746 | Ref |
| MK719711 | Unassigned | Unassigned | Unassigned | 1462_0 | Clustered | 110 | VC_1462_0 | 110 | 1 | 1 | 1 | 0 | 0 | 0 | 0.9746 | Ref |
| MK719712 | Unassigned | Unassigned | Unassigned | 1462_0 | Clustered | 110 | VC_1462_0 | 110 | 1 | 1 | 1 | 0 | 0 | 0 | 0.9746 | Ref |
| MK719713 | Unassigned | Unassigned | Unassigned | 1462_0 | Clustered | 110 | VC_1462_0 | 110 | 1 | 1 | 1 | 0 | 0 | 0 | 0.9746 | Ref |
| MK719714 | Unassigned | Unassigned | Unassigned | 1462_0 | Clustered | 110 | VC_1462_0 | 110 | 1 | 1 | 1 | 0 | 0 | 0 | 0.9746 | Ref |
| MK719715 | Unassigned | Unassigned | Unassigned | 1462_0 | Clustered | 110 | VC_1462_0 | 110 | 1 | 1 | 1 | 0 | 0 | 0 | 0.9746 | Ref |
| MK719716 | Unassigned | Unassigned | Unassigned | 1462_0 | Clustered | 110 | VC_1462_0 | 110 | 1 | 1 | 1 | 0 | 0 | 0 | 0.9746 | Ref |
| MK719717 | Unassigned | Unassigned | Unassigned | 1462_0 | Clustered | 110 | VC_1462_0 | 110 | 1 | 1 | 1 | 0 | 0 | 0 | 0.9746 | Ref |
| MK719718 | Unassigned | Unassigned | Unassigned | 1462_0 | Clustered | 110 | VC_1462_0 | 110 | 1 | 1 | 1 | 0 | 0 | 0 | 0.9746 | Ref |
| MK719719 | Unassigned | Unassigned | Unassigned | 1462_0 | Clustered | 110 | VC_1462_0 | 110 | 1 | 1 | 1 | 0 | 0 | 0 | 0.9746 | Ref |

| | | | | | | | | | | | | | | | | |
|---|---|---|---|---|---|---|---|---|---|---|---|---|---|---|---|---|
| MK719720 | Unassigned | Unassigned | Unassigned | 1462_0 | Clustered | 110 | VC_1462_0 | 110 | 1 | 1 | 1 | 0 | 0 | 0 | 0.9746 | Ref |
| MK719721 | Unassigned | Unassigned | Unassigned | 1462_0 | Clustered | 110 | VC_1462_0 | 110 | 1 | 1 | 1 | 0 | 0 | 0 | 0.9746 | Ref |
| MK719722 | Unassigned | Unassigned | Unassigned | 1462_0 | Clustered | 110 | VC_1462_0 | 110 | 1 | 1 | 1 | 0 | 0 | 0 | 0.9746 | Ref |
| MK719723 | Unassigned | Unassigned | Unassigned | 1462_0 | Clustered | 110 | VC_1462_0 | 110 | 1 | 1 | 1 | 0 | 0 | 0 | 0.9746 | Ref |
| MK719724 | Unassigned | Unassigned | Unassigned | 1462_0 | Clustered | 110 | VC_1462_0 | 110 | 1 | 1 | 1 | 0 | 0 | 0 | 0.9746 | Ref |
| MK719725 | Unassigned | Unassigned | Unassigned | 1462_0 | Clustered | 110 | VC_1462_0 | 110 | 1 | 1 | 1 | 0 | 0 | 0 | 0.9746 | Ref |
| MK719726 | Unassigned | Unassigned | Unassigned | 1462_0 | Clustered | 110 | VC_1462_0 | 110 | 1 | 1 | 1 | 0 | 0 | 0 | 0.9746 | Ref |
| MK719727 | Unassigned | Unassigned | Unassigned | 1462_0 | Clustered | 110 | VC_1462_0 | 110 | 1 | 1 | 1 | 0 | 0 | 0 | 0.9746 | Ref |
| MK719728 | Unassigned | Unassigned | Unassigned | 1462_0 | Clustered | 110 | VC_1462_0 | 110 | 1 | 1 | 1 | 0 | 0 | 0 | 0.9746 | Ref |
| MK719729 | Unassigned | Unassigned | Unassigned | 1462_0 | Clustered | 110 | VC_1462_0 | 110 | 1 | 1 | 1 | 0 | 0 | 0 | 0.9746 | Ref |
| MK719730 | Unassigned | Unassigned | Unassigned | 1462_0 | Clustered | 110 | VC_1462_0 | 110 | 1 | 1 | 1 | 0 | 0 | 0 | 0.9746 | Ref |
| MK719731 | Unassigned | Unassigned | Unassigned | 1462_0 | Clustered | 110 | VC_1462_0 | 110 | 1 | 1 | 1 | 0 | 0 | 0 | 0.9746 | Ref |
| MK719732 | Unassigned | Unassigned | Unassigned | 1462_0 | Clustered | 110 | VC_1462_0 | 110 | 1 | 1 | 1 | 0 | 0 | 0 | 0.9746 | Ref |
| MK719733 | Unassigned | Unassigned | Unassigned | 1462_0 | Clustered | 110 | VC_1462_0 | 110 | 1 | 1 | 1 | 0 | 0 | 0 | 0.9746 | Ref |
| MK719734 | Unassigned | Unassigned | Unassigned | 1462_0 | Clustered | 110 | VC_1462_0 | 110 | 1 | 1 | 1 | 0 | 0 | 0 | 0.9746 | Ref |
| MK719735 | Unassigned | Unassigned | Unassigned | 1462_0 | Clustered | 110 | VC_1462_0 | 110 | 1 | 1 | 1 | 0 | 0 | 0 | 0.9746 | Ref |
| MK719736 | Unassigned | Unassigned | Unassigned | 1462_0 | Clustered | 110 | VC_1462_0 | 110 | 1 | 1 | 1 | 0 | 0 | 0 | 0.9746 | Ref |
| MK719737 | Unassigned | Unassigned | Unassigned | 1462_0 | Clustered | 110 | VC_1462_0 | 110 | 1 | 1 | 1 | 0 | 0 | 0 | 0.9746 | Ref |
| MK719738 | Unassigned | Unassigned | Unassigned | 1462_0 | Clustered | 110 | VC_1462_0 | 110 | 1 | 1 | 1 | 0 | 0 | 0 | 0.9746 | Ref |
| MK719739 | Unassigned | Unassigned | Unassigned | 1462_0 | Clustered | 110 | VC_1462_0 | 110 | 1 | 1 | 1 | 0 | 0 | 0 | 0.9746 | Ref |
| MK719740 | Unassigned | Unassigned | Unassigned | 1462_0 | Clustered | 110 | VC_1462_0 | 110 | 1 | 1 | 1 | 0 | 0 | 0 | 0.9746 | Ref |
| MK719741 | Unassigned | Unassigned | Unassigned | 1462_0 | Clustered | 110 | VC_1462_0 | 110 | 1 | 1 | 1 | 0 | 0 | 0 | 0.9746 | Ref |
| MK719742 | Unassigned | Unassigned | Unassigned | 1462_0 | Clustered | 110 | VC_1462_0 | 110 | 1 | 1 | 1 | 0 | 0 | 0 | 0.9746 | Ref |
| MK719743 | Unassigned | Unassigned | Unassigned | 1462_0 | Clustered | 110 | VC_1462_0 | 110 | 1 | 1 | 1 | 0 | 0 | 0 | 0.9746 | Ref |
| MK719744 | Unassigned | Unassigned | Unassigned | 1462_0 | Clustered | 110 | VC_1462_0 | 110 | 1 | 1 | 1 | 0 | 0 | 0 | 0.9746 | Ref |
| MK719745 | Unassigned | Unassigned | Unassigned | 1462_0 | Clustered | 110 | VC_1462_0 | 110 | 1 | 1 | 1 | 0 | 0 | 0 | 0.9746 | Ref |
| MK719746 | Unassigned | Unassigned | Unassigned | 1462_0 | Clustered | 110 | VC_1462_0 | 110 | 1 | 1 | 1 | 0 | 0 | 0 | 0.9746 | Ref |
| MK719747 | Unassigned | Unassigned | Unassigned | 1462_0 | Clustered | 110 | VC_1462_0 | 110 | 1 | 1 | 1 | 0 | 0 | 0 | 0.9746 | Ref |
| MK719748 | Unassigned | Unassigned | Unassigned | 1462_0 | Clustered | 110 | VC_1462_0 | 110 | 1 | 1 | 1 | 0 | 0 | 0 | 0.9746 | Ref |
| MK719749 | Unassigned | Unassigned | Unassigned | 1462_0 | Clustered | 110 | VC_1462_0 | 110 | 1 | 1 | 1 | 0 | 0 | 0 | 0.9746 | Ref |
| MK719750 | Unassigned | Unassigned | Unassigned | 1462_0 | Clustered | 110 | VC_1462_0 | 110 | 1 | 1 | 1 | 0 | 0 | 0 | 0.9746 | Ref |
| MK719751 | Unassigned | Unassigned | Unassigned | 1462_0 | Clustered | 110 | VC_1462_0 | 110 | 1 | 1 | 1 | 0 | 0 | 0 | 0.9746 | Ref |
| MK719752 | Unassigned | Unassigned | Unassigned | 1462_0 | Clustered | 110 | VC_1462_0 | 110 | 1 | 1 | 1 | 0 | 0 | 0 | 0.9746 | Ref |
| MK719753 | Unassigned | Unassigned | Unassigned | 1462_0 | Clustered | 110 | VC_1462_0 | 110 | 1 | 1 | 1 | 0 | 0 | 0 | 0.9746 | Ref |
| MK719754 | Unassigned | Unassigned | Unassigned | 1462_0 | Clustered | 110 | VC_1462_0 | 110 | 1 | 1 | 1 | 0 | 0 | 0 | 0.9746 | Ref |
| MK721183 | Unassigned | Unassigned | Unassigned | 1003_0 | Clustered | 16 | VC_1003_0 | 16 | 0.8967 | 1 | 0.8967 | 0 | 0 | 0 | 1 | Ref |
| MK721184 | Unassigned | Unassigned | Unassigned | 1003_0 | Clustered | 16 | VC_1003_0 | 16 | 0.8967 | 1 | 0.8967 | 0 | 0 | 0 | 1 | Ref |
| MK721185 | Unassigned | Unassigned | Unassigned | 1003_0 | Clustered | 16 | VC_1003_0 | 16 | 0.8967 | 1 | 0.8967 | 0 | 0 | 0 | 1 | Ref |
| MK721186 | Unassigned | Unassigned | Unassigned | 711_0 | Clustered | 48 | VC_711_0 | 48 | 0.8217 | 1 | 0.8217 | 0 | 0 | 0 | 1 | Ref |
| MK721187 | Unassigned | Unassigned | Unassigned | 711_0 | Clustered | 48 | VC_711_0 | 48 | 0.8217 | 1 | 0.8217 | 0 | 0 | 0 | 1 | Ref |
| MK721188 | Unassigned | Unassigned | Unassigned | 1003_0 | Clustered | 16 | VC_1003_0 | 16 | 0.8967 | 1 | 0.8967 | 0 | 0 | 0 | 1 | Ref |
| MK721189 | Unassigned | Unassigned | Unassigned | 110_0 | Clustered | 33 | VC_110_0 | 33 | 0.9488 | 1 | 0.9488 | 0 | 0 | 0 | 1 | Ref |
| MK721190 | Unassigned | Unassigned | Unassigned | 711_0 | Clustered | 48 | VC_711_0 | 48 | 0.8217 | 1 | 0.8217 | 0 | 0 | 0 | 1 | Ref |
| MK721191 | Unassigned | Unassigned | Unassigned | 711_0 | Clustered | 48 | VC_711_0 | 48 | 0.8217 | 1 | 0.8217 | 0 | 0 | 0 | 1 | Ref |
| MK721192 | Unassigned | Unassigned | Unassigned | 93_0 | Clustered | 22 | VC_93_0 | 22 | 0.5285 | 1 | 0.5285 | 0 | 0 | 0 | 1 | Ref |
| MK721193 | Unassigned | Unassigned | Unassigned | 1003_0 | Clustered | 16 | VC_1003_0 | 16 | 0.8967 | 1 | 0.8967 | 0 | 0 | 0 | 1 | Ref |
| MK721194 | Unassigned | Unassigned | Unassigned | 110_0 | Clustered | 33 | VC_110_0 | 33 | 0.9488 | 1 | 0.9488 | 0 | 0 | 0 | 1 | Ref |
| MK721195 | Unassigned | Unassigned | Unassigned | 1003_0 | Clustered | 16 | VC_1003_0 | 16 | 0.8967 | 1 | 0.8967 | 0 | 0 | 0 | 1 | Ref |
| MK721196 | Unassigned | Unassigned | Unassigned | 1003_0 | Clustered | 16 | VC_1003_0 | 16 | 0.8967 | 1 | 0.8967 | 0 | 0 | 0 | 1 | Ref |
| MK721197 | Unassigned | Unassigned | Unassigned | 1003_0 | Clustered | 16 | VC_1003_0 | 16 | 0.8967 | 1 | 0.8967 | 0 | 0 | 0 | 1 | Ref |
| MK721198 | Unassigned | Unassigned | Unassigned | 1003_0 | Clustered | 16 | VC_1003_0 | 16 | 0.8967 | 1 | 0.8967 | 0 | 0 | 0 | 1 | Ref |
| MK721199 | Unassigned | Unassigned | Unassigned | 711_0 | Clustered | 48 | VC_711_0 | 48 | 0.8217 | 1 | 0.8217 | 0 | 0 | 0 | 1 | Ref |
| MK721200 | Unassigned | Unassigned | Unassigned | 711_0 | Clustered | 48 | VC_711_0 | 48 | 0.8217 | 1 | 0.8217 | 0 | 0 | 0 | 1 | Ref |
| MK728541 | Unassigned | Unassigned | Unassigned | 859_0 | Clustered | 67 | VC_859_0 | 67 | 0.9622 | 1 | 0.9622 | 0 | 0 | 0 | 1 | Ref |
| MK728824 | Unassigned | Unassigned | Unassigned | 199_19 | Clustered | 12 | VC_199_19 | 12 | 0.0651 | 1 | 0.0651 | 0 | 0 | 0 | 0.8636 | Ref |
| MK728825 | Unassigned | Unassigned | Unassigned | 199_19 | Clustered | 12 | VC_199_19 | 12 | 0.0651 | 1 | 0.0651 | 0 | 0 | 0 | 0.8636 | Ref |
| MK733259 | Unassigned | Unassigned | Unassigned | 47_0 | Clustered | 12 | VC_47_0 | 12 | 0.3571 | 1 | 0.3571 | 0 | 0 | 0 | 1 | Ref |
| MK733260 | Unassigned | Unassigned | Unassigned | 47_0 | Clustered | 12 | VC_47_0 | 12 | 0.3571 | 1 | 0.3571 | 0 | 0 | 0 | 1 | Ref |
| MK733261 | Unassigned | Unassigned | Unassigned | 47_0 | Clustered | 12 | VC_47_0 | 12 | 0.3571 | 1 | 0.3571 | 0 | 0 | 0 | 1 | Ref |
| MK733262 | Unassigned | Unassigned | Unassigned | 288_0 | Clustered | 3 | VC_288_0 | 3 | 0.3571 | 0.99826828 | 0.3565 | 0 | 0 | 0 | 1 | Ref |
| MK733263 | Unassigned | Unassigned | Unassigned | 288_0 | Clustered | 3 | VC_288_0 | 3 | 0.3571 | 0.99826828 | 0.3565 | 0 | 0 | 0 | 1 | Ref |
| MK733264 | Unassigned | Unassigned | Unassigned | 288_0 | Clustered | 3 | VC_288_0 | 3 | 0.3571 | 0.99826828 | 0.3565 | 0 | 0 | 0 | 1 | Ref |
| MK737937 | Unassigned | Unassigned | Unassigned | 655_0 | Clustered | 45 | VC_655_0 | 45 | 0.7722 | 1 | 0.7722 | 0 | 0 | 0 | 1 | Ref |
| MK737941 | Unassigned | Unassigned | Unassigned | 1406_0 | Clustered | 21 | VC_1406_0 | 21 | 0.9836 | 1 | 0.9836 | 0 | 0 | 0 | 1 | Ref |
| MK737942 | Unassigned | Unassigned | Unassigned | 1202_0 | Clustered | 10 | VC_1202_0 | 10 | 0.5679 | 1 | 0.5679 | 0 | 0 | 0 | 1 | Ref |
| MK737943 | Unassigned | Unassigned | Unassigned | 985_0 | Clustered | 38 | VC_985_0 | 38 | 0.9334 | 1 | 0.9334 | 0 | 0 | 0 | 1 | Ref |
| MK737944 | Unassigned | Unassigned | Unassigned | 1411_0 | Clustered | 5 | VC_1411_0 | 5 | 0.5446 | 0.99999985 | 0.5446 | 0 | 0 | 0 | 1 | Ref |
| MK756083 | Unassigned | Unassigned | Unassigned | 944_0 | Clustered | 76 | VC_944_0 | 76 | 0.9789 | 1 | 0.9789 | 0 | 0 | 0 | 1 | Ref |
| MK756084 | Unassigned | Unassigned | Unassigned | 944_0 | Clustered | 76 | VC_944_0 | 76 | 0.9789 | 1 | 0.9789 | 0 | 0 | 0 | 1 | Ref |
| MK756085 | Unassigned | Unassigned | Unassigned | 944_0 | Clustered | 76 | VC_944_0 | 76 | 0.9789 | 1 | 0.9789 | 0 | 0 | 0 | 1 | Ref |
| MK756086 | Unassigned | Unassigned | Unassigned | 944_0 | Clustered | 76 | VC_944_0 | 76 | 0.9789 | 1 | 0.9789 | 0 | 0 | 0 | 1 | Ref |
| MK756087 | Unassigned | Unassigned | Unassigned | 944_0 | Clustered | 76 | VC_944_0 | 76 | 0.9789 | 1 | 0.9789 | 0 | 0 | 0 | 1 | Ref |
| MK756088 | Unassigned | Unassigned | Unassigned | 944_0 | Clustered | 76 | VC_944_0 | 76 | 0.9789 | 1 | 0.9789 | 0 | 0 | 0 | 1 | Ref |
| MK756089 | Unassigned | Unassigned | Unassigned | 944_0 | Clustered | 76 | VC_944_0 | 76 | 0.9789 | 1 | 0.9789 | 0 | 0 | 0 | 1 | Ref |
| MK756090 | Unassigned | Unassigned | Unassigned | 944_0 | Clustered | 76 | VC_944_0 | 76 | 0.9789 | 1 | 0.9789 | 0 | 0 | 0 | 1 | Ref |
| MK756091 | Unassigned | Unassigned | Unassigned | 944_0 | Clustered | 76 | VC_944_0 | 76 | 0.9789 | 1 | 0.9789 | 0 | 0 | 0 | 1 | Ref |
| MK756092 | Unassigned | Unassigned | Unassigned | 944_0 | Clustered | 76 | VC_944_0 | 76 | 0.9789 | 1 | 0.9789 | 0 | 0 | 0 | 1 | Ref |
| MK756093 | Unassigned | Unassigned | Unassigned | 944_0 | Clustered | 76 | VC_944_0 | 76 | 0.9789 | 1 | 0.9789 | 0 | 0 | 0 | 1 | Ref |
| MK756094 | Unassigned | Unassigned | Unassigned | 944_0 | Clustered | 76 | VC_944_0 | 76 | 0.9789 | 1 | 0.9789 | 0 | 0 | 0 | 1 | Ref |
| MK756095 | Unassigned | Unassigned | Unassigned | 944_0 | Clustered | 76 | VC_944_0 | 76 | 0.9789 | 1 | 0.9789 | 0 | 0 | 0 | 1 | Ref |
| MK756096 | Unassigned | Unassigned | Unassigned | 944_0 | Clustered | 76 | VC_944_0 | 76 | 0.9789 | 1 | 0.9789 | 0 | 0 | 0 | 1 | Ref |

| | | | | | | | | | | | | | | | | |
|---|---|---|---|---|---|---|---|---|---|---|---|---|---|---|---|---|
| MK757445 | Unassigned | Unassigned | Unassigned | 571_7 | Clustered | 28 | VC_571_7 | 28 | 0.1651 | 1 | 0.1651 | 0 | 0 | 0 | 0.8519 | Ref |
| MK757446 | Unassigned | Unassigned | Unassigned | 588_0 | Clustered | 8 | VC_588_0 | 8 | 0.5695 | 1 | 0.5695 | 0 | 0 | 0 | 1 | Ref |
| MK757447 | Unassigned | Unassigned | Unassigned | 1296_0 | Clustered | 82 | VC_1296_0 | 82 | 0.7938 | 1 | 0.7938 | 0 | 0 | 0 | 1 | Ref |
| MK757448 | Unassigned | Unassigned | Unassigned | 1427_0 | Clustered | 6 | VC_1427_0 | 6 | 1 | 1 | 1 | 0 | 0 | 0 | 1 | Ref |
| MK757449 | Unassigned | Unassigned | Unassigned | 1424_0 | Clustered | 31 | VC_1424_0 | 31 | 0.8834 | 1 | 0.8834 | 0 | 0 | 0 | 1 | Ref |
| MK759854 | Unassigned | Unassigned | Unassigned | 475_0 | Clustered | 96 | VC_475_0 | 96 | 0.9303 | 1 | 0.9303 | 0 | 0 | 0 | 0.9575 | Ref |
| MK759883 | Unassigned | Unassigned | Unassigned | 654_1 | Clustered | 27 | VC_654_1 | 27 | 0.2571 | 1 | 0.2571 | 0 | 0 | 0 | 0.9858 | Ref |
| MK759884 | Unassigned | Unassigned | Unassigned | 339_0 | Clustered | 11 | VC_339_0 | 11 | 0.4647 | 1 | 0.4647 | 0 | 0 | 0 | 1 | Ref |
| MK759918 | Unassigned | Unassigned | Unassigned | 746_0 | Clustered | 2 | VC_746_0 | 2 | 0.3667 | 0.95002548 | 0.3484 | 0 | 0 | 0 | 1 | Ref |
| MK761195 | Unassigned | Unassigned | Unassigned | 654_0 | Clustered | 115 | VC_654_0 | 115 | 0.7303 | 1 | 0.7303 | 0 | 0 | 0 | 0.8764 | Ref |
| MK761196 | Unassigned | Unassigned | Unassigned | 654_0 | Clustered | 115 | VC_654_0 | 115 | 0.7303 | 1 | 0.7303 | 0 | 0 | 0 | 0.8764 | Ref |
| MK761197 | Unassigned | Unassigned | Unassigned | 654_0 | Clustered | 115 | VC_654_0 | 115 | 0.7303 | 1 | 0.7303 | 0 | 0 | 0 | 0.8764 | Ref |
| MK761198 | Unassigned | Unassigned | Unassigned | 654_0 | Clustered | 115 | VC_654_0 | 115 | 0.7303 | 1 | 0.7303 | 0 | 0 | 0 | 0.8764 | Ref |
| MK761199 | Unassigned | Unassigned | Unassigned | 654_0 | Clustered | 115 | VC_654_0 | 115 | 0.7303 | 1 | 0.7303 | 0 | 0 | 0 | 0.8764 | Ref |
| MK764384 | Unassigned | Unassigned | Unassigned | 98_0 | Clustered | 30 | VC_98_0 | 30 | 0.7175 | 1 | 0.7175 | 0 | 0 | 0 | 1 | Ref |
| MK764436 | Unassigned | Unassigned | Unassigned | 1080_0 | Clustered | 23 | VC_1080_0 | 23 | 0.9603 | 1 | 0.9603 | 0 | 0 | 0 | 1 | Ref |
| MK764437 | Unassigned | Unassigned | Unassigned | 1061_0 | Clustered | 8 | VC_1061_0 | 8 | 0.8379 | 1 | 0.8379 | 0 | 0 | 0 | 1 | Ref |
| MK764438 | Unassigned | Unassigned | Unassigned | 1080_0 | Clustered | 23 | VC_1080_0 | 23 | 0.9603 | 1 | 0.9603 | 0 | 0 | 0 | 1 | Ref |
| MK764439 | Unassigned | Unassigned | Unassigned | 1080_0 | Clustered | 23 | VC_1080_0 | 23 | 0.9603 | 1 | 0.9603 | 0 | 0 | 0 | 1 | Ref |
| MK764440 | Unassigned | Unassigned | Unassigned | 1061_0 | Clustered | 8 | VC_1061_0 | 8 | 0.8379 | 1 | 0.8379 | 0 | 0 | 0 | 1 | Ref |
| MK764441 | Unassigned | Unassigned | Unassigned | 1198_0 | Clustered | 24 | VC_1198_0 | 24 | 1 | 1 | 1 | 0 | 0 | 0 | 1 | Ref |
| MK764442 | Unassigned | Unassigned | Unassigned | 1059_0 | Clustered | 10 | VC_1059_0 | 10 | 0.8794 | 1 | 0.8794 | 0 | 0 | 0 | 1 | Ref |
| MK764443 | Unassigned | Unassigned | Unassigned | 1080_0 | Clustered | 23 | VC_1080_0 | 23 | 0.9603 | 1 | 0.9603 | 0 | 0 | 0 | 1 | Ref |
| MK764444 | Unassigned | Unassigned | Unassigned | 1080_0 | Clustered | 23 | VC_1080_0 | 23 | 0.9603 | 1 | 0.9603 | 0 | 0 | 0 | 1 | Ref |
| MK764445 | Unassigned | Unassigned | Unassigned | 1059_0 | Clustered | 10 | VC_1059_0 | 10 | 0.8794 | 1 | 0.8794 | 0 | 0 | 0 | 1 | Ref |
| MK764446 | Unassigned | Unassigned | Unassigned | 1080_0 | Clustered | 23 | VC_1080_0 | 23 | 0.9603 | 1 | 0.9603 | 0 | 0 | 0 | 1 | Ref |
| MK764447 | Unassigned | Unassigned | Unassigned | 1080_0 | Clustered | 23 | VC_1080_0 | 23 | 0.9603 | 1 | 0.9603 | 0 | 0 | 0 | 1 | Ref |
| MK764448 | Unassigned | Unassigned | Unassigned | 1059_0 | Clustered | 10 | VC_1059_0 | 10 | 0.8794 | 1 | 0.8794 | 0 | 0 | 0 | 1 | Ref |
| MK764449 | Unassigned | Unassigned | Unassigned | 1198_0 | Clustered | 24 | VC_1198_0 | 24 | 1 | 1 | 1 | 0 | 0 | 0 | 1 | Ref |
| MK764450 | Unassigned | Unassigned | Unassigned | 1061_0 | Clustered | 8 | VC_1061_0 | 8 | 0.8379 | 1 | 0.8379 | 0 | 0 | 0 | 1 | Ref |
| MK765551 | Unassigned | Unassigned | Unassigned | 1463_0 | Clustered | 2 | VC_1463_0 | 2 | 1 | 1 | 1 | 0 | 0 | 0 | 1 | Ref |
| MK765552 | Unassigned | Unassigned | Unassigned | 1344_0 | Clustered | 26 | VC_1344_0 | 26 | 0.5437 | 1 | 0.5437 | 0 | 0 | 0 | 1 | Ref |
| MK765553 | Unassigned | Unassigned | Unassigned | 1344_0 | Clustered | 26 | VC_1344_0 | 26 | 0.5437 | 1 | 0.5437 | 0 | 0 | 0 | 1 | Ref |
| MK765555 | Unassigned | Unassigned | Unassigned | 1389_0 | Clustered | 7 | VC_1389_0 | 7 | 0.9155 | 0.99999981 | 0.9155 | 0 | 0 | 0 | 1 | Ref |
| MK765556 | Unassigned | Unassigned | Unassigned | 1344_0 | Clustered | 26 | VC_1344_0 | 26 | 0.5437 | 1 | 0.5437 | 0 | 0 | 0 | 1 | Ref |
| MK765557 | Unassigned | Unassigned | Unassigned | 1267_0 | Clustered | 26 | VC_1267_0 | 26 | 0.9597 | 0.99999998 | 0.9597 | 0 | 0 | 0 | 1 | Ref |
| MK765558 | Unassigned | Unassigned | Unassigned | 1464_0 | Clustered | 9 | VC_1464_0 | 9 | 1 | 0 | 0 | 0 | 0 | 0 | 1 | Ref |
| MK765559 | Unassigned | Unassigned | Unassigned | 1161_0 | Clustered | 16 | VC_1161_0 | 16 | 0.4834 | 0.99999958 | 0.4834 | 0 | 0 | 0 | 1 | Ref |
| MK765560 | Unassigned | Unassigned | Unassigned | 1344_0 | Clustered | 26 | VC_1344_0 | 26 | 0.5437 | 1 | 0.5437 | 0 | 0 | 0 | 1 | Ref |
| MK765561 | Unassigned | Unassigned | Unassigned | 1299_0 | Clustered | 53 | VC_1299_0 | 53 | 0.8666 | 1 | 0.8666 | 0 | 0 | 0 | 1 | Ref |
| MK765562 | Unassigned | Unassigned | Unassigned | 1344_0 | Clustered | 26 | VC_1344_0 | 26 | 0.5437 | 1 | 0.5437 | 0 | 0 | 0 | 1 | Ref |
| MK765563 | Unassigned | Unassigned | Unassigned | 1299_0 | Clustered | 53 | VC_1299_0 | 53 | 0.8666 | 1 | 0.8666 | 0 | 0 | 0 | 1 | Ref |
| MK765564 | Unassigned | Unassigned | Unassigned | 1299_0 | Clustered | 53 | VC_1299_0 | 53 | 0.8666 | 1 | 0.8666 | 0 | 0 | 0 | 1 | Ref |
| MK765565 | Unassigned | Unassigned | Unassigned | 1344_0 | Clustered | 26 | VC_1344_0 | 26 | 0.5437 | 1 | 0.5437 | 0 | 0 | 0 | 1 | Ref |
| MK765567 | Unassigned | Unassigned | Unassigned | 1389_0 | Clustered | 7 | VC_1389_0 | 7 | 0.9155 | 0.99999981 | 0.9155 | 0 | 0 | 0 | 1 | Ref |
| MK765568 | Unassigned | Unassigned | Unassigned | 1299_0 | Clustered | 53 | VC_1299_0 | 53 | 0.8666 | 1 | 0.8666 | 0 | 0 | 0 | 1 | Ref |
| MK765569 | Unassigned | Unassigned | Unassigned | 1299_0 | Clustered | 53 | VC_1299_0 | 53 | 0.8666 | 1 | 0.8666 | 0 | 0 | 0 | 1 | Ref |
| MK765571 | Unassigned | Unassigned | Unassigned | 1346_0 | Clustered | 25 | VC_1346_0 | 25 | 1 | 1 | 1 | 0 | 0 | 0 | 1 | Ref |
| MK765572 | Unassigned | Unassigned | Unassigned | 1339_0 | Clustered | 2 | VC_1339_0 | 2 | 0.0658 | 0.94989008 | 0.0625 | 0 | 0 | 0 | 1 | Ref |
| MK765573 | Unassigned | Unassigned | Unassigned | 1465_0 | Clustered | 2 | VC_1465_0 | 2 | 1 | 1 | 1 | 0 | 0 | 0 | 1 | Ref |
| MK765574 | Unassigned | Unassigned | Unassigned | 1346_0 | Clustered | 25 | VC_1346_0 | 25 | 1 | 1 | 1 | 0 | 0 | 0 | 1 | Ref |
| MK765575 | Unassigned | Unassigned | Unassigned | 1466_0 | Clustered | 2 | VC_1466_0 | 2 | 1 | 1 | 1 | 0 | 0 | 0 | 1 | Ref |
| MK765576 | Unassigned | Unassigned | Unassigned | 1346_0 | Clustered | 25 | VC_1346_0 | 25 | 1 | 1 | 1 | 0 | 0 | 0 | 1 | Ref |
| MK765577 | Unassigned | Unassigned | Unassigned | 1346_0 | Clustered | 25 | VC_1346_0 | 25 | 1 | 1 | 1 | 0 | 0 | 0 | 1 | Ref |
| MK765578 | Unassigned | Unassigned | Unassigned | 1466_0 | Clustered | 2 | VC_1466_0 | 2 | 1 | 1 | 1 | 0 | 0 | 0 | 1 | Ref |
| MK765579 | Unassigned | Unassigned | Unassigned | 1104_0 | Clustered | 44 | VC_1104_0 | 44 | 0.9574 | 0.72818579 | 0.6972 | 0 | 0 | 0 | 1 | Ref |
| MK765580 | Unassigned | Unassigned | Unassigned | 1346_0 | Clustered | 25 | VC_1346_0 | 25 | 1 | 1 | 1 | 0 | 0 | 0 | 1 | Ref |
| MK765581 | Unassigned | Unassigned | Unassigned | 1464_0 | Clustered | 9 | VC_1464_0 | 9 | 1 | 0 | 0 | 0 | 0 | 0 | 1 | Ref |
| MK765582 | Unassigned | Unassigned | Unassigned | 1104_0 | Clustered | 44 | VC_1104_0 | 44 | 0.9574 | 0.72818579 | 0.6972 | 0 | 0 | 0 | 1 | Ref |
| MK765583 | Unassigned | Unassigned | Unassigned | 1369_0 | Clustered | 10 | VC_1369_0 | 10 | 0.6223 | 0.95845085 | 0.5964 | 0 | 0 | 0 | 1 | Ref |
| MK765587 | Unassigned | Unassigned | Unassigned | 1467_0 | Clustered | 14 | VC_1467_0 | 14 | 0.167 | 0.51390354 | 0.0858 | 0 | 0 | 0 | 1 | Ref |
| MK765588 | Unassigned | Unassigned | Unassigned | 1467_0 | Clustered | 14 | VC_1467_0 | 14 | 0.167 | 0.51390354 | 0.0858 | 0 | 0 | 0 | 1 | Ref |
| MK765589 | Unassigned | Unassigned | Unassigned | 1468_0 | Clustered | 28 | VC_1468_0 | 28 | 0.7511 | 1 | 0.7511 | 0 | 0 | 0 | 1 | Ref |
| MK765590 | Unassigned | Unassigned | Unassigned | 1335_0 | Clustered | 13 | VC_1335_0 | 13 | 0.7519 | 1 | 0.7519 | 0 | 0 | 0 | 1 | Ref |
| MK765591 | Unassigned | Unassigned | Unassigned | 1344_0 | Clustered | 26 | VC_1344_0 | 26 | 0.5437 | 1 | 0.5437 | 0 | 0 | 0 | 1 | Ref |
| MK765592 | Unassigned | Unassigned | Unassigned | 1344_0 | Clustered | 26 | VC_1344_0 | 26 | 0.5437 | 1 | 0.5437 | 0 | 0 | 0 | 1 | Ref |
| MK765593 | Unassigned | Unassigned | Unassigned | 1344_0 | Clustered | 26 | VC_1344_0 | 26 | 0.5437 | 1 | 0.5437 | 0 | 0 | 0 | 1 | Ref |
| MK765594 | Unassigned | Unassigned | Unassigned | 1299_0 | Clustered | 53 | VC_1299_0 | 53 | 0.8666 | 1 | 0.8666 | 0 | 0 | 0 | 1 | Ref |
| MK765596 | Unassigned | Unassigned | Unassigned | 1344_0 | Clustered | 26 | VC_1344_0 | 26 | 0.5437 | 1 | 0.5437 | 0 | 0 | 0 | 1 | Ref |
| MK765598 | Unassigned | Unassigned | Unassigned | 1467_0 | Clustered | 14 | VC_1467_0 | 14 | 0.167 | 0.51390354 | 0.0858 | 0 | 0 | 0 | 1 | Ref |
| MK765599 | Unassigned | Unassigned | Unassigned | 1467_0 | Clustered | 14 | VC_1467_0 | 14 | 0.167 | 0.51390354 | 0.0858 | 0 | 0 | 0 | 1 | Ref |
| MK765600 | Unassigned | Unassigned | Unassigned | 1389_0 | Clustered | 7 | VC_1389_0 | 7 | 0.9155 | 0.99999981 | 0.9155 | 0 | 0 | 0 | 1 | Ref |
| MK765601 | Unassigned | Unassigned | Unassigned | 1364_0 | Clustered | 6 | VC_1364_0 | 6 | 0.9472 | 0.54366909 | 0.515 | 0 | 0 | 0 | 1 | Ref |
| MK765602 | Unassigned | Unassigned | Unassigned | 1181_0 | Clustered | 4 | VC_1181_0 | 4 | 0.1325 | 0.65559538 | 0.0869 | 0 | 0 | 0 | 1 | Ref |
| MK765603 | Unassigned | Unassigned | Unassigned | 1161_0 | Clustered | 16 | VC_1161_0 | 16 | 0.4834 | 0.99999958 | 0.4834 | 0 | 0 | 0 | 1 | Ref |
| MK765604 | Unassigned | Unassigned | Unassigned | 1464_0 | Clustered | 9 | VC_1464_0 | 9 | 1 | 0 | 0 | 0 | 0 | 0 | 1 | Ref |
| MK765605 | Unassigned | Unassigned | Unassigned | 1471_0 | Clustered | 2 | VC_1471_0 | 2 | 1 | 1 | 1 | 0 | 0 | 0 | 1 | Ref |
| MK765606 | Unassigned | Unassigned | Unassigned | 1344_0 | Clustered | 26 | VC_1344_0 | 26 | 0.5437 | 1 | 0.5437 | 0 | 0 | 0 | 1 | Ref |
| MK765607 | Unassigned | Unassigned | Unassigned | 1344_0 | Clustered | 26 | VC_1344_0 | 26 | 0.5437 | 1 | 0.5437 | 0 | 0 | 0 | 1 | Ref |
| MK765609 | Unassigned | Unassigned | Unassigned | 1344_0 | Clustered | 26 | VC_1344_0 | 26 | 0.5437 | 1 | 0.5437 | 0 | 0 | 0 | 1 | Ref |
| MK765610 | Unassigned | Unassigned | Unassigned | 1389_0 | Clustered | 7 | VC_1389_0 | 7 | 0.9155 | 0.99999981 | 0.9155 | 0 | 0 | 0 | 1 | Ref |

| | | | | | | | | | | | | | | | | |
|---|---|---|---|---|---|---|---|---|---|---|---|---|---|---|---|---|
| MK765611 | Unassigned | Unassigned | Unassigned | 1344_0 | Clustered | 26 | VC_1344_0 | 26 | 0.5437 | 1 | 0.5437 | 0 | 0 | 0 | 1 | Ref |
| MK765612 | Unassigned | Unassigned | Unassigned | 1344_0 | Clustered | 26 | VC_1344_0 | 26 | 0.5437 | 1 | 0.5437 | 0 | 0 | 0 | 1 | Ref |
| MK765613 | Unassigned | Unassigned | Unassigned | 1344_0 | Clustered | 26 | VC_1344_0 | 26 | 0.5437 | 1 | 0.5437 | 0 | 0 | 0 | 1 | Ref |
| MK765615 | Unassigned | Unassigned | Unassigned | 1344_0 | Clustered | 26 | VC_1344_0 | 26 | 0.5437 | 1 | 0.5437 | 0 | 0 | 0 | 1 | Ref |
| MK765617 | Unassigned | Unassigned | Unassigned | 1389_0 | Clustered | 7 | VC_1389_0 | 7 | 0.9155 | 0.99999981 | 0.9155 | 0 | 0 | 0 | 1 | Ref |
| MK765620 | Unassigned | Unassigned | Unassigned | 1145_0 | Clustered | 164 | VC_1145_0 | 164 | 0.8868 | 1 | 0.8868 | 0 | 0 | 0 | 1 | Ref |
| MK765621 | Unassigned | Unassigned | Unassigned | 1161_0 | Clustered | 16 | VC_1161_0 | 16 | 0.4834 | 0.99999958 | 0.4834 | 0 | 0 | 0 | 1 | Ref |
| MK765622 | Unassigned | Unassigned | Unassigned | 1464_0 | Clustered | 9 | VC_1464_0 | 9 | 1 | 0 | 0 | 0 | 0 | 0 | 1 | Ref |
| MK765623 | Unassigned | Unassigned | Unassigned | 1467_0 | Clustered | 14 | VC_1467_0 | 14 | 0.167 | 0.51390354 | 0.0858 | 0 | 0 | 0 | 1 | Ref |
| MK765625 | Unassigned | Unassigned | Unassigned | 1467_0 | Clustered | 14 | VC_1467_0 | 14 | 0.167 | 0.51390354 | 0.0858 | 0 | 0 | 0 | 1 | Ref |
| MK765626 | Unassigned | Unassigned | Unassigned | 1469_0 | Clustered/Singleton | 1 | VC_1469_0 | 1 | 0 | 1 | 0 | 0 | 0 | 0 | 1 | Ref |
| MK765628 | Unassigned | Unassigned | Unassigned | 1467_0 | Clustered | 14 | VC_1467_0 | 14 | 0.167 | 0.51390354 | 0.0858 | 0 | 0 | 0 | 1 | Ref |
| MK765630 | Unassigned | Unassigned | Unassigned | 1467_0 | Clustered | 14 | VC_1467_0 | 14 | 0.167 | 0.51390354 | 0.0858 | 0 | 0 | 0 | 1 | Ref |
| MK765631 | Unassigned | Unassigned | Unassigned | 1467_0 | Clustered | 14 | VC_1467_0 | 14 | 0.167 | 0.51390354 | 0.0858 | 0 | 0 | 0 | 1 | Ref |
| MK765633 | Unassigned | Unassigned | Unassigned | 1344_0 | Clustered | 26 | VC_1344_0 | 26 | 0.5437 | 1 | 0.5437 | 0 | 0 | 0 | 1 | Ref |
| MK765634 | Unassigned | Unassigned | Unassigned | 1467_0 | Clustered | 14 | VC_1467_0 | 14 | 0.167 | 0.51390354 | 0.0858 | 0 | 0 | 0 | 1 | Ref |
| MK765638 | Unassigned | Unassigned | Unassigned | 1299_0 | Clustered | 53 | VC_1299_0 | 53 | 0.8666 | 1 | 0.8666 | 0 | 0 | 0 | 1 | Ref |
| MK765639 | Unassigned | Unassigned | Unassigned | 1104_0 | Clustered | 44 | VC_1104_0 | 44 | 0.9574 | 0.72818579 | 0.6972 | 0 | 0 | 0 | 1 | Ref |
| MK765640 | Unassigned | Unassigned | Unassigned | 1464_0 | Clustered | 9 | VC_1464_0 | 9 | 1 | 0 | 0 | 0 | 0 | 0 | 1 | Ref |
| MK765641 | Unassigned | Unassigned | Unassigned | 1471_0 | Clustered | 2 | VC_1471_0 | 2 | 1 | 1 | 1 | 0 | 0 | 0 | 1 | Ref |
| MK765642 | Unassigned | Unassigned | Unassigned | 1104_0 | Clustered | 44 | VC_1104_0 | 44 | 0.9574 | 0.72818579 | 0.6972 | 0 | 0 | 0 | 1 | Ref |
| MK765643 | Unassigned | Unassigned | Unassigned | 1104_0 | Clustered | 44 | VC_1104_0 | 44 | 0.9574 | 0.72818579 | 0.6972 | 0 | 0 | 0 | 1 | Ref |
| MK765644 | Unassigned | Unassigned | Unassigned | 1104_0 | Clustered | 44 | VC_1104_0 | 44 | 0.9574 | 0.72818579 | 0.6972 | 0 | 0 | 0 | 1 | Ref |
| MK765645 | Unassigned | Unassigned | Unassigned | 1119_0 | Clustered | 48 | VC_1119_0 | 48 | 0.852 | 1 | 0.852 | 0 | 0 | 0 | 1 | Ref |
| MK765646 | Unassigned | Unassigned | Unassigned | 1104_0 | Clustered | 44 | VC_1104_0 | 44 | 0.9574 | 0.72818579 | 0.6972 | 0 | 0 | 0 | 1 | Ref |
| MK765648 | Unassigned | Unassigned | Unassigned | 1344_0 | Clustered | 26 | VC_1344_0 | 26 | 0.5437 | 1 | 0.5437 | 0 | 0 | 0 | 1 | Ref |
| MK765649 | Unassigned | Unassigned | Unassigned | 1341_0 | Clustered | 4 | VC_1341_0 | 4 | 1 | 1 | 1 | 0 | 0 | 0 | 1 | Ref |
| MK765650 | Unassigned | Unassigned | Unassigned | 1463_0 | Clustered | 2 | VC_1463_0 | 2 | 1 | 1 | 1 | 0 | 0 | 0 | 1 | Ref |
| MK765651 | Unassigned | Unassigned | Unassigned | 1344_0 | Clustered | 26 | VC_1344_0 | 26 | 0.5437 | 1 | 0.5437 | 0 | 0 | 0 | 1 | Ref |
| MK765653 | Unassigned | Unassigned | Unassigned | 1394_0 | Clustered | 4 | VC_1394_0 | 4 | 0.7326 | 0.99899459 | 0.7318 | 0 | 0 | 0 | 1 | Ref |
| MK765654 | Unassigned | Unassigned | Unassigned | 1464_0 | Clustered | 9 | VC_1464_0 | 9 | 1 | 0 | 0 | 0 | 0 | 0 | 1 | Ref |
| MK765655 | Unassigned | Unassigned | Unassigned | 1267_0 | Clustered | 26 | VC_1267_0 | 26 | 0.9597 | 0.99999998 | 0.9597 | 0 | 0 | 0 | 1 | Ref |
| MK765656 | Unassigned | Unassigned | Unassigned | 1464_0 | Clustered | 9 | VC_1464_0 | 9 | 1 | 0 | 0 | 0 | 0 | 0 | 1 | Ref |
| MK765657 | Unassigned | Unassigned | Unassigned | 1344_0 | Clustered | 26 | VC_1344_0 | 26 | 0.5437 | 1 | 0.5437 | 0 | 0 | 0 | 1 | Ref |
| MK765658 | Unassigned | Unassigned | Unassigned | 1344_0 | Clustered | 26 | VC_1344_0 | 26 | 0.5437 | 1 | 0.5437 | 0 | 0 | 0 | 1 | Ref |
| MK765659 | Unassigned | Unassigned | Unassigned | 1389_0 | Clustered | 7 | VC_1389_0 | 7 | 0.9155 | 0.99999981 | 0.9155 | 0 | 0 | 0 | 1 | Ref |
| MK765660 | Unassigned | Unassigned | Unassigned | 1344_0 | Clustered | 26 | VC_1344_0 | 26 | 0.5437 | 1 | 0.5437 | 0 | 0 | 0 | 1 | Ref |
| MK765661 | Unassigned | Unassigned | Unassigned | 1267_0 | Clustered | 26 | VC_1267_0 | 26 | 0.9597 | 0.99999998 | 0.9597 | 0 | 0 | 0 | 1 | Ref |
| MK765662 | Unassigned | Unassigned | Unassigned | 1465_0 | Clustered | 2 | VC_1465_0 | 2 | 1 | 1 | 1 | 0 | 0 | 0 | 1 | Ref |
| MK765663 | Unassigned | Unassigned | Unassigned | 1464_0 | Clustered | 9 | VC_1464_0 | 9 | 1 | 0 | 0 | 0 | 0 | 0 | 1 | Ref |
| MK765664 | Unassigned | Unassigned | Unassigned | 1389_0 | Clustered | 7 | VC_1389_0 | 7 | 0.9155 | 0.99999981 | 0.9155 | 0 | 0 | 0 | 1 | Ref |
| MK765666 | Unassigned | Unassigned | Unassigned | 1344_0 | Clustered | 26 | VC_1344_0 | 26 | 0.5437 | 1 | 0.5437 | 0 | 0 | 0 | 1 | Ref |
| MK765667 | Unassigned | Unassigned | Unassigned | 1267_0 | Clustered | 26 | VC_1267_0 | 26 | 0.9597 | 0.99999998 | 0.9597 | 0 | 0 | 0 | 1 | Ref |
| MK765668 | Unassigned | Unassigned | Unassigned | 1464_0 | Clustered | 9 | VC_1464_0 | 9 | 1 | 0 | 0 | 0 | 0 | 0 | 1 | Ref |
| MK765669 | Unassigned | Unassigned | Unassigned | 1161_0 | Clustered | 16 | VC_1161_0 | 16 | 0.4834 | 0.99999958 | 0.4834 | 0 | 0 | 0 | 1 | Ref |
| MK770409 | Unassigned | Unassigned | Unassigned | 654_0 | Clustered | 115 | VC_654_0 | 115 | 0.7303 | 1 | 0.7303 | 0 | 0 | 0 | 0.8764 | Ref |
| MK770410 | Unassigned | Unassigned | Unassigned | 199_31 | Clustered | 2 | VC_199_31 | 2 | 0.0056 | 0.92139383 | 0.0052 | 0 | 0 | 0 | 1 | Ref |
| MK770411 | Unassigned | Unassigned | Unassigned | 440_0 | Clustered | 33 | VC_440_0 | 33 | 0.992 | 1 | 0.992 | 0 | 0 | 0 | 1 | Ref |
| MK770412 | Unassigned | Unassigned | Unassigned | 339_0 | Clustered | 11 | VC_339_0 | 11 | 0.4647 | 1 | 0.4647 | 0 | 0 | 0 | 1 | Ref |
| MK770413 | Unassigned | Unassigned | Unassigned | 440_0 | Clustered | 33 | VC_440_0 | 33 | 0.992 | 1 | 0.992 | 0 | 0 | 0 | 1 | Ref |
| MK770414 | Unassigned | Unassigned | Unassigned | 30_1 | Clustered | 53 | VC_30_1 | 53 | 0.7151 | 1 | 0.7151 | 0 | 0 | 0 | 0.9325 | Ref |
| MK770415 | Unassigned | Unassigned | Unassigned | 30_1 | Clustered | 53 | VC_30_1 | 53 | 0.7151 | 1 | 0.7151 | 0 | 0 | 0 | 0.9325 | Ref |
| MK773491 | Unassigned | Unassigned | Unassigned | 170_0 | Clustered | 3 | VC_170_0 | 3 | 0.0298 | 0.82983726 | 0.0247 | 0 | 0 | 0 | 1 | Ref |
| MK774614 | Unassigned | Unassigned | Unassigned | 456_4 | Clustered | 2 | VC_456_4 | 2 | 0.0252 | 0.90563532 | 0.0228 | 0 | 0 | 0 | 1 | Ref |
| MK778457 | Unassigned | Unassigned | Unassigned | 604_1 | Clustered | 3 | VC_604_1 | 3 | 0.0311 | 0.99857668 | 0.0311 | 0 | 0 | 0 | 1 | Ref |
| MK779875 | Unassigned | Unassigned | Unassigned | 824_0 | Clustered | 3 | VC_824_0 | 3 | 0.5034 | 0.9984288 | 0.5026 | 0 | 0 | 0 | 1 | Ref |
| MK792752 | Unassigned | Unassigned | Unassigned | 753_0 | Clustered | 26 | VC_753_0 | 26 | 0.9562 | 1 | 0.9562 | 0 | 0 | 0 | 1 | Ref |
| MK795384 | Unassigned | Unassigned | Unassigned | 136_0 | Clustered | 3 | VC_136_0 | 3 | 0.0934 | 0.99858535 | 0.0932 | 0 | 0 | 0 | 1 | Ref |
| MK795385 | Unassigned | Unassigned | Unassigned | 848_0 | Clustered | 124 | VC_848_0 | 124 | 0.9484 | 1 | 0.9484 | 0 | 0 | 0 | 1 | Ref |
| MK796244 | Unassigned | Unassigned | Unassigned | 1007_1 | Clustered | 24 | VC_1007_1 | 24 | 0.7364 | 1 | 0.7364 | 0 | 0 | 0 | 0.8877 | Ref |
| MK796797 | Unassigned | Unassigned | Unassigned | 831_0 | Clustered | 17 | VC_831_0 | 17 | 0.9057 | 1 | 0.9057 | 0 | 0 | 0 | 1 | Ref |
| MK797984 | Unassigned | Unassigned | Unassigned | 171_0 | Clustered | 4 | VC_171_0 | 4 | 0.2204 | 0.99998328 | 0.2204 | 0 | 0 | 0 | 1 | Ref |
| MK798142 | Unassigned | Unassigned | Unassigned | 125_0 | Clustered | 23 | VC_125_0 | 23 | 0.8163 | 1 | 0.8163 | 0 | 0 | 0 | 1 | Ref |
| MK798143 | Unassigned | Unassigned | Unassigned | 65_0 | Clustered | 3 | VC_65_0 | 3 | 0.5621 | 0.99791205 | 0.5609 | 0 | 0 | 0 | 1 | Ref |
| MK798144 | Unassigned | Unassigned | Unassigned | 65_0 | Clustered | 3 | VC_65_0 | 3 | 0.5621 | 0.99791205 | 0.5609 | 0 | 0 | 0 | 1 | Ref |
| MK799650 | Unassigned | Unassigned | Unassigned | 78_0 | Clustered | 20 | VC_78_0 | 20 | 0.9639 | 1 | 0.9639 | 0 | 0 | 0 | 1 | Ref |
| MK799832 | Unassigned | Unassigned | Unassigned | 446_13 | Clustered/Singleton | 1 | VC_446_13 | 1 | 0 | 1 | 0 | 0 | 0 | 0 | 1 | Ref |
| MK800154 | Unassigned | Unassigned | Unassigned | 110_0 | Clustered | 33 | VC_110_0 | 33 | 0.9488 | 1 | 0.9488 | 0 | 0 | 0 | 1 | Ref |
| MK801680 | Unassigned | Unassigned | Unassigned | 8_0 | Clustered | 58 | VC_8_0 | 58 | 0.8285 | 1 | 0.8285 | 0 | 0 | 0 | 0.9946 | Ref |
| MK801681 | Unassigned | Unassigned | Unassigned | 8_0 | Clustered | 58 | VC_8_0 | 58 | 0.8285 | 1 | 0.8285 | 0 | 0 | 0 | 0.9946 | Ref |
| MK801682 | Unassigned | Unassigned | Unassigned | 9_0 | Clustered | 38 | VC_9_0 | 38 | 0.4859 | 1 | 0.4859 | 0 | 0 | 0 | 1 | Ref |
| MK801683 | Unassigned | Unassigned | Unassigned | 9_0 | Clustered | 38 | VC_9_0 | 38 | 0.4859 | 1 | 0.4859 | 0 | 0 | 0 | 1 | Ref |
| MK801721 | Unassigned | Unassigned | Unassigned | 1217_0 | Clustered | 41 | VC_1217_0 | 41 | 0.9546 | 1 | 0.9546 | 0 | 0 | 0 | 0.9976 | Ref |
| MK801722 | Unassigned | Unassigned | Unassigned | 1114_0 | Clustered | 20 | VC_1114_0 | 20 | 0.3238 | 1 | 0.3238 | 0 | 0 | 0 | 0.9842 | Ref |
| MK801724 | Unassigned | Unassigned | Unassigned | 1217_0 | Clustered | 41 | VC_1217_0 | 41 | 0.9546 | 1 | 0.9546 | 0 | 0 | 0 | 0.9976 | Ref |
| MK801725 | Unassigned | Unassigned | Unassigned | 528_0 | Clustered | 12 | VC_528_0 | 12 | 0.3923 | 1 | 0.3923 | 0 | 0 | 0 | 0.9697 | Ref |
| MK801726 | Unassigned | Unassigned | Unassigned | 571_0 | Clustered | 16 | VC_571_0 | 16 | 0.091 | 1 | 0.091 | 0 | 0 | 0 | 0.6667 | Ref |
| MK801727 | Unassigned | Unassigned | Unassigned | 543_0 | Clustered | 25 | VC_543_0 | 25 | 0.4584 | 1 | 0.4584 | 0 | 0 | 0 | 1 | Ref |
| MK801728 | Unassigned | Unassigned | Unassigned | 895_0 | Clustered | 25 | VC_895_0 | 25 | 0.5768 | 1 | 0.5768 | 0 | 0 | 0 | 1 | Ref |
| MK801730 | Unassigned | Unassigned | Unassigned | 1407_0 | Clustered | 78 | VC_1407_0 | 78 | 0.9731 | 1 | 0.9731 | 0 | 0 | 0 | 1 | Ref |
| MK801731 | Unassigned | Unassigned | Unassigned | 1406_0 | Clustered | 21 | VC_1406_0 | 21 | 0.9836 | 1 | 0.9836 | 0 | 0 | 0 | 1 | Ref |

| | | | | | | | | | | | | | | | | |
|---|---|---|---|---|---|---|---|---|---|---|---|---|---|---|---|---|
| MK801732 | Unassigned | Unassigned | Unassigned | 1406_0 | Clustered | 21 | VC_1406_0 | 21 | 0.9836 | 1 | 0.9836 | 0 | 0 | 0 | 1 | Ref |
| MK801733 | Unassigned | Unassigned | Unassigned | 895_0 | Clustered | 25 | VC_895_0 | 25 | 0.5768 | 1 | 0.5768 | 0 | 0 | 0 | 1 | Ref |
| MK801734 | Unassigned | Unassigned | Unassigned | 1216_0 | Clustered | 22 | VC_1216_0 | 22 | 0.4546 | 1 | 0.4546 | 0 | 0 | 0 | 1 | Ref |
| MK801735 | Unassigned | Unassigned | Unassigned | 223_0 | Clustered | 4 | VC_223_0 | 4 | 0.0478 | 0.9999878 | 0.0478 | 0 | 0 | 0 | 1 | Ref |
| MK803322 | Unassigned | Unassigned | Unassigned | 173_0 | Clustered | 3 | VC_173_0 | 3 | 0.6871 | 0.99826575 | 0.6859 | 0 | 0 | 0 | 1 | Ref |
| MK804891 | Unassigned | Unassigned | Unassigned | 380_0 | Clustered | 6 | VC_380_0 | 6 | 0.51 | 1 | 0.51 | 0 | 0 | 0 | 1 | Ref |
| MK804892 | Unassigned | Unassigned | Unassigned | 380_0 | Clustered | 6 | VC_380_0 | 6 | 0.51 | 1 | 0.51 | 0 | 0 | 0 | 1 | Ref |
| MK804893 | Unassigned | Unassigned | Unassigned | 892_0 | Clustered | 2 | VC_892_0 | 2 | 0.0759 | 0.93631468 | 0.0711 | 0 | 0 | 0 | 1 | Ref |
| MK809530 | Unassigned | Unassigned | Unassigned | 199_32 | Clustered | 12 | VC_199_32 | 12 | 0.0676 | 1 | 0.0676 | 0 | 0 | 0 | 0.7576 | Ref |
| MK813938 | Unassigned | Unassigned | Unassigned | 892_1 | Clustered/Singleton | 1 | VC_892_1 | 1 | 0 | 1 | 0 | 0 | 0 | 0 | 1 | Ref |
| MK813939 | Unassigned | Unassigned | Unassigned | 892_0 | Clustered | 2 | VC_892_0 | 2 | 0.0759 | 0.93631468 | 0.0711 | 0 | 0 | 0 | 1 | Ref |
| MK813940 | Unassigned | Unassigned | Unassigned | 656_0 | Clustered | 2 | VC_656_0 | 2 | 0.2731 | 0.95526917 | 0.2609 | 0 | 0 | 0 | 1 | Ref |
| MK813941 | Unassigned | Unassigned | Unassigned | 892_2 | Clustered/Singleton | 1 | VC_892_2 | 1 | 0 | 1 | 0 | 0 | 0 | 0 | 1 | Ref |
| MK814748 | Unassigned | Unassigned | Unassigned | 223_1 | Clustered/Singleton | 1 | VC_223_1 | 1 | 0 | 1 | 0 | 0 | 0 | 0 | 1 | Ref |
| MK814749 | Unassigned | Unassigned | Unassigned | 1200_0 | Clustered | 73 | VC_1200_0 | 73 | 0.9825 | 1 | 0.9825 | 0 | 0 | 0 | 1 | Ref |
| MK814750 | Unassigned | Unassigned | Unassigned | 1226_0 | Clustered | 5 | VC_1226_0 | 5 | 0.6594 | 0.99999982 | 0.6594 | 0 | 0 | 0 | 1 | Ref |
| MK814751 | Unassigned | Unassigned | Unassigned | 526_0 | Clustered | 32 | VC_526_0 | 32 | 0.9037 | 1 | 0.9037 | 0 | 0 | 0 | 1 | Ref |
| MK814752 | Unassigned | Unassigned | Unassigned | 578_1 | Clustered | 11 | VC_578_1 | 11 | 0.4919 | 1 | 0.4919 | 0 | 0 | 0 | 0.7818 | Ref |
| MK814753 | Unassigned | Unassigned | Unassigned | 1287_0 | Clustered | 44 | VC_1287_0 | 44 | 1 | 1 | 1 | 0 | 0 | 0 | 1 | Ref |
| MK814754 | Unassigned | Unassigned | Unassigned | 335_46 | Clustered/Singleton | 1 | VC_335_46 | 1 | 0 | 1 | 0 | 0 | 0 | 0 | 1 | Ref |
| MK814755 | Unassigned | Unassigned | Unassigned | 543_0 | Clustered | 25 | VC_543_0 | 25 | 0.4584 | 1 | 0.4584 | 0 | 0 | 0 | 1 | Ref |
| MK814756 | Unassigned | Unassigned | Unassigned | 899_0 | Clustered | 2 | VC_899_0 | 2 | 0.0476 | 0.95725601 | 0.0456 | 0 | 0 | 0 | 1 | Ref |
| MK814757 | Unassigned | Unassigned | Unassigned | 1217_0 | Clustered | 41 | VC_1217_0 | 41 | 0.9546 | 1 | 0.9546 | 0 | 0 | 0 | 0.9976 | Ref |
| MK814758 | Unassigned | Unassigned | Unassigned | 537_0 | Clustered | 39 | VC_537_0 | 39 | 0.8148 | 1 | 0.8148 | 0 | 0 | 0 | 1 | Ref |
| MK814759 | Unassigned | Unassigned | Unassigned | 1222_0 | Clustered | 3 | VC_1222_0 | 3 | 0.5104 | 0.99818726 | 0.5095 | 0 | 0 | 0 | 1 | Ref |
| MK814760 | Unassigned | Unassigned | Unassigned | 1141_0 | Clustered | 4 | VC_1141_0 | 4 | 0.5416 | 0.99996462 | 0.5416 | 0 | 0 | 0 | 1 | Ref |
| MK814761 | Unassigned | Unassigned | Unassigned | 1219_0 | Clustered | 24 | VC_1219_0 | 24 | 0.9519 | 1 | 0.9519 | 0 | 0 | 0 | 1 | Ref |
| MK814762 | Unassigned | Unassigned | Unassigned | 1203_0 | Clustered | 28 | VC_1203_0 | 28 | 0.4905 | 1 | 0.4905 | 0 | 0 | 0 | 1 | Ref |
| MK816297 | Unassigned | Unassigned | Unassigned | 820_0 | Clustered | 5 | VC_820_0 | 5 | 0.332 | 0.99999997 | 0.332 | 0 | 0 | 0 | 1 | Ref |
| MK817115 | Unassigned | Unassigned | Unassigned | 170_11 | Clustered | 10 | VC_170_11 | 10 | 0.1502 | 0.99999797 | 0.1502 | 0 | 0 | 0 | 0.9111 | Ref |
| MK819239 | Unassigned | Unassigned | Unassigned | 302_0 | Clustered | 2 | VC_302_0 | 2 | 0.0332 | 0.94488015 | 0.0313 | 0 | 0 | 0 | 1 | Ref |
| MK820013 | Unassigned | Unassigned | Unassigned | 1067_0 | Clustered | 20 | VC_1067_0 | 20 | 0.7109 | 1 | 0.7109 | 0 | 0 | 0 | 1 | Ref |
| MK820638 | Unassigned | Unassigned | Unassigned | 335_23 | Clustered | 17 | VC_335_23 | 17 | 0.0714 | 1 | 0.0714 | 0 | 0 | 0 | 0.6397 | Ref |
| MK820639 | Unassigned | Unassigned | Unassigned | 537_0 | Clustered | 39 | VC_537_0 | 39 | 0.8148 | 1 | 0.8148 | 0 | 0 | 0 | 1 | Ref |
| MK820640 | Unassigned | Unassigned | Unassigned | 985_0 | Clustered | 38 | VC_985_0 | 38 | 0.9334 | 1 | 0.9334 | 0 | 0 | 0 | 1 | Ref |
| MK820641 | Unassigned | Unassigned | Unassigned | 701_0 | Clustered | 16 | VC_701_0 | 16 | 0.2957 | 1 | 0.2957 | 0 | 0 | 0 | 0.9167 | Ref |
| MK820642 | Unassigned | Unassigned | Unassigned | 543_0 | Clustered | 25 | VC_543_0 | 25 | 0.4584 | 1 | 0.4584 | 0 | 0 | 0 | 1 | Ref |
| MK837009 | Unassigned | Unassigned | Unassigned | 80_5 | Clustered | 2 | VC_80_5 | 2 | 0.0046 | 0.92781966 | 0.0043 | 0 | 0 | 0 | 1 | Ref |
| MK837010 | Unassigned | Unassigned | Unassigned | 80_6 | Clustered | 11 | VC_80_6 | 11 | 0.0527 | 1 | 0.0527 | 0 | 0 | 0 | 0.7455 | Ref |
| MK837011 | Unassigned | Unassigned | Unassigned | 486_0 | Clustered | 47 | VC_486_0 | 47 | 0.9022 | 1 | 0.9022 | 0 | 0 | 0 | 1 | Ref |
| MK837012 | Unassigned | Unassigned | Unassigned | 486_0 | Clustered | 47 | VC_486_0 | 47 | 0.9022 | 1 | 0.9022 | 0 | 0 | 0 | 1 | Ref |
| MK838107 | Unassigned | Unassigned | Unassigned | 907_0 | Clustered | 32 | VC_907_0 | 32 | 0.9964 | 1 | 0.9964 | 0 | 0 | 0 | 1 | Ref |
| MK838108 | Unassigned | Unassigned | Unassigned | 907_0 | Clustered | 32 | VC_907_0 | 32 | 0.9964 | 1 | 0.9964 | 0 | 0 | 0 | 1 | Ref |
| MK838109 | Unassigned | Unassigned | Unassigned | 907_0 | Clustered | 32 | VC_907_0 | 32 | 0.9964 | 1 | 0.9964 | 0 | 0 | 0 | 1 | Ref |
| MK838110 | Unassigned | Unassigned | Unassigned | 907_0 | Clustered | 32 | VC_907_0 | 32 | 0.9964 | 1 | 0.9964 | 0 | 0 | 0 | 1 | Ref |
| MK838111 | Unassigned | Unassigned | Unassigned | 907_0 | Clustered | 32 | VC_907_0 | 32 | 0.9964 | 1 | 0.9964 | 0 | 0 | 0 | 1 | Ref |
| MK838113 | Unassigned | Unassigned | Unassigned | 617_0 | Clustered | 6 | VC_617_0 | 6 | 0.1792 | 1 | 0.1792 | 0 | 0 | 0 | 1 | Ref |
| MK838116 | Unassigned | Unassigned | Unassigned | 456_0 | Clustered | 4 | VC_456_0 | 4 | 0.0832 | 0.99994226 | 0.0832 | 0 | 0 | 0 | 1 | Ref |
| MK863032 | Unassigned | Unassigned | Unassigned | 801_3 | Clustered | 7 | VC_801_3 | 7 | 0.0764 | 1 | 0.0764 | 0 | 0 | 0 | 0.7143 | Ref |
| MK864263 | Unassigned | Unassigned | Unassigned | 1217_0 | Clustered | 41 | VC_1217_0 | 41 | 0.9546 | 1 | 0.9546 | 0 | 0 | 0 | 0.9976 | Ref |
| MK864264 | Unassigned | Unassigned | Unassigned | 1219_0 | Clustered | 24 | VC_1219_0 | 24 | 0.9519 | 1 | 0.9519 | 0 | 0 | 0 | 1 | Ref |
| MK864265 | Unassigned | Unassigned | Unassigned | 1219_0 | Clustered | 24 | VC_1219_0 | 24 | 0.9519 | 1 | 0.9519 | 0 | 0 | 0 | 1 | Ref |
| MK864266 | Unassigned | Unassigned | Unassigned | 1219_0 | Clustered | 24 | VC_1219_0 | 24 | 0.9519 | 1 | 0.9519 | 0 | 0 | 0 | 1 | Ref |
| MK864267 | Unassigned | Unassigned | Unassigned | 1219_0 | Clustered | 24 | VC_1219_0 | 24 | 0.9519 | 1 | 0.9519 | 0 | 0 | 0 | 1 | Ref |
| MK867354 | Unassigned | Unassigned | Unassigned | 446_5 | Clustered/Singleton | 1 | VC_446_5 | 1 | 0 | 1 | 0 | 0 | 0 | 0 | 1 | Ref |
| MK867835 | Unassigned | Unassigned | Unassigned | 199_27 | Clustered/Singleton | 1 | VC_199_27 | 1 | 0 | 1 | 0 | 0 | 0 | 0 | 1 | Ref |
| MK875791 | Unassigned | Unassigned | Unassigned | 952_0 | Clustered | 39 | VC_952_0 | 39 | 0.9064 | 1 | 0.9064 | 0 | 0 | 0 | 1 | Ref |
| MK875793 | Unassigned | Unassigned | Unassigned | 543_0 | Clustered | 25 | VC_543_0 | 25 | 0.4584 | 1 | 0.4584 | 0 | 0 | 0 | 1 | Ref |
| MK875795 | Unassigned | Unassigned | Unassigned | 952_0 | Clustered | 39 | VC_952_0 | 39 | 0.9064 | 1 | 0.9064 | 0 | 0 | 0 | 1 | Ref |
| MK875796 | Unassigned | Unassigned | Unassigned | 543_0 | Clustered | 25 | VC_543_0 | 25 | 0.4584 | 1 | 0.4584 | 0 | 0 | 0 | 1 | Ref |
| MK875797 | Unassigned | Unassigned | Unassigned | 1216_0 | Clustered | 22 | VC_1216_0 | 22 | 0.4546 | 1 | 0.4546 | 0 | 0 | 0 | 1 | Ref |
| MK875798 | Unassigned | Unassigned | Unassigned | 1415_0 | Clustered | 31 | VC_1415_0 | 31 | 0.8456 | 1 | 0.8456 | 0 | 0 | 0 | 1 | Ref |
| MK875799 | Unassigned | Unassigned | Unassigned | 535_0 | Clustered | 18 | VC_535_0 | 18 | 0.1595 | 1 | 0.1595 | 0 | 0 | 0 | 1 | Ref |
| MK875800 | Unassigned | Unassigned | Unassigned | 532_0 | Clustered | 75 | VC_532_0 | 75 | 0.9393 | 1 | 0.9393 | 0 | 0 | 0 | 1 | Ref |
| MK878895 | Unassigned | Unassigned | Unassigned | 535_1 | Clustered | 108 | VC_535_1 | 108 | 0.8479 | 1 | 0.8479 | 0 | 0 | 0 | 0.9955 | Ref |
| MK878896 | Unassigned | Unassigned | Unassigned | 1217_0 | Clustered | 41 | VC_1217_0 | 41 | 0.9546 | 1 | 0.9546 | 0 | 0 | 0 | 0.9976 | Ref |
| MK878897 | Unassigned | Unassigned | Unassigned | 579_38 | Clustered | 2 | VC_579_38 | 2 | 0.0032 | 0.6354382 | 0.002 | 0 | 0 | 0 | 1 | Ref |
| MK878898 | Unassigned | Unassigned | Unassigned | 543_0 | Clustered | 25 | VC_543_0 | 25 | 0.4584 | 1 | 0.4584 | 0 | 0 | 0 | 1 | Ref |
| MK878899 | Unassigned | Unassigned | Unassigned | 1407_0 | Clustered | 78 | VC_1407_0 | 78 | 0.9731 | 1 | 0.9731 | 0 | 0 | 0 | 1 | Ref |
| MK878900 | Unassigned | Unassigned | Unassigned | 918_0 | Clustered | 16 | VC_918_0 | 16 | 0.2945 | 1 | 0.2945 | 0 | 0 | 0 | 1 | Ref |
| MK878901 | Unassigned | Unassigned | Unassigned | 1218_0 | Clustered | 14 | VC_1218_0 | 14 | 0.7811 | 1 | 0.7811 | 0 | 0 | 0 | 0.989 | Ref |
| MK878903 | Unassigned | Unassigned | Unassigned | 893_1 | Clustered | 20 | VC_893_1 | 20 | 0.4792 | 1 | 0.4792 | 0 | 0 | 0 | 1 | Ref |
| MK878904 | Unassigned | Unassigned | Unassigned | 1424_0 | Clustered | 31 | VC_1424_0 | 31 | 0.8834 | 1 | 0.8834 | 0 | 0 | 0 | 1 | Ref |
| MK878905 | Unassigned | Unassigned | Unassigned | 535_1 | Clustered | 108 | VC_535_1 | 108 | 0.8479 | 1 | 0.8479 | 0 | 0 | 0 | 0.9955 | Ref |
| MK880124 | Unassigned | Unassigned | Unassigned | 1249_0 | Clustered | 8 | VC_1249_0 | 8 | 0.191 | 1 | 0.191 | 0 | 0 | 0 | 1 | Ref |
| MK883717 | Unassigned | Unassigned | Unassigned | 794_4 | Clustered | 4 | VC_794_4 | 4 | 0.0181 | 0.73018303 | 0.0132 | 0 | 0 | 0 | 1 | Ref |
| MK883718 | Unassigned | Unassigned | Unassigned | 794_4 | Clustered | 4 | VC_794_4 | 4 | 0.0181 | 0.73018303 | 0.0132 | 0 | 0 | 0 | 1 | Ref |
| MK886800 | Unassigned | Unassigned | Unassigned | 290_164 | Clustered | 2 | VC_290_164 | 2 | 0.003 | 0.93435121 | 0.0028 | 0 | 0 | 0 | 1 | Ref |
| MK893987 | Unassigned | Unassigned | Unassigned | 685_2 | Clustered/Singleton | 1 | VC_685_2 | 1 | 0 | 1 | 0 | 0 | 0 | 0 | 1 | Ref |
| MK894432 | Unassigned | Unassigned | Unassigned | 1203_0 | Clustered | 28 | VC_1203_0 | 28 | 0.4905 | 1 | 0.4905 | 0 | 0 | 0 | 1 | Ref |
| MK894433 | Unassigned | Unassigned | Unassigned | 230_0 | Clustered | 38 | VC_230_0 | 38 | 0.9076 | 1 | 0.9076 | 0 | 0 | 0 | 1 | Ref |

| | | | | | | | | | | | | | | | | |
|---|---|---|---|---|---|---|---|---|---|---|---|---|---|---|---|---|
| MK894434 | Unassigned | Unassigned | Unassigned | 1407_0 | Clustered | 78 | VC_1407_0 | 78 | 0.9731 | 1 | 0.9731 | 0 | 0 | 0 | 1 | Ref |
| MK894435 | Unassigned | Unassigned | Unassigned | 1424_0 | Clustered | 31 | VC_1424_0 | 31 | 0.8834 | 1 | 0.8834 | 0 | 0 | 0 | 1 | Ref |
| MK894436 | Unassigned | Unassigned | Unassigned | 1141_0 | Clustered | 4 | VC_1141_0 | 4 | 0.5416 | 0.99996462 | 0.5416 | 0 | 0 | 0 | 1 | Ref |
| MK894438 | Unassigned | Unassigned | Unassigned | 1424_0 | Clustered | 31 | VC_1424_0 | 31 | 0.8834 | 1 | 0.8834 | 0 | 0 | 0 | 1 | Ref |
| MK895508 | Unassigned | Unassigned | Unassigned | 1007_1 | Clustered | 24 | VC_1007_1 | 24 | 0.7364 | 1 | 0.7364 | 0 | 0 | 0 | 0.8877 | Ref |
| MK903033 | Unassigned | Unassigned | Unassigned | 98_0 | Clustered | 30 | VC_98_0 | 30 | 0.7175 | 1 | 0.7175 | 0 | 0 | 0 | 1 | Ref |
| MK903277 | Unassigned | Unassigned | Unassigned | 465_0 | Clustered | 80 | VC_465_0 | 80 | 0.9988 | 1 | 0.9988 | 0 | 0 | 0 | 1 | Ref |
| MK903278 | Unassigned | Unassigned | Unassigned | 75_0 | Clustered | 15 | VC_75_0 | 15 | 0.518 | 1 | 0.518 | 0 | 0 | 0 | 1 | Ref |
| MK903279 | Unassigned | Unassigned | Unassigned | 465_0 | Clustered | 80 | VC_465_0 | 80 | 0.9988 | 1 | 0.9988 | 0 | 0 | 0 | 1 | Ref |
| MK903281 | Unassigned | Unassigned | Unassigned | 465_0 | Clustered | 80 | VC_465_0 | 80 | 0.9988 | 1 | 0.9988 | 0 | 0 | 0 | 1 | Ref |
| MK903282 | Unassigned | Unassigned | Unassigned | 636_0 | Clustered | 13 | VC_636_0 | 13 | 0.6223 | 1 | 0.6223 | 0 | 0 | 0 | 1 | Ref |
| MK903728 | Unassigned | Unassigned | Unassigned | 848_0 | Clustered | 124 | VC_848_0 | 124 | 0.9484 | 1 | 0.9484 | 0 | 0 | 0 | 1 | Ref |
| MK907226 | Unassigned | Unassigned | Unassigned | 470_0 | Clustered | 62 | VC_470_0 | 62 | 0.902 | 1 | 0.902 | 0 | 0 | 0 | 1 | Ref |
| MK907227 | Unassigned | Unassigned | Unassigned | 470_0 | Clustered | 62 | VC_470_0 | 62 | 0.902 | 1 | 0.902 | 0 | 0 | 0 | 1 | Ref |
| MK907228 | Unassigned | Unassigned | Unassigned | 470_0 | Clustered | 62 | VC_470_0 | 62 | 0.902 | 1 | 0.902 | 0 | 0 | 0 | 1 | Ref |
| MK907229 | Unassigned | Unassigned | Unassigned | 470_0 | Clustered | 62 | VC_470_0 | 62 | 0.902 | 1 | 0.902 | 0 | 0 | 0 | 1 | Ref |
| MK907230 | Unassigned | Unassigned | Unassigned | 470_0 | Clustered | 62 | VC_470_0 | 62 | 0.902 | 1 | 0.902 | 0 | 0 | 0 | 1 | Ref |
| MK907231 | Unassigned | Unassigned | Unassigned | 470_0 | Clustered | 62 | VC_470_0 | 62 | 0.902 | 1 | 0.902 | 0 | 0 | 0 | 1 | Ref |
| MK907232 | Unassigned | Unassigned | Unassigned | 470_0 | Clustered | 62 | VC_470_0 | 62 | 0.902 | 1 | 0.902 | 0 | 0 | 0 | 1 | Ref |
| MK907233 | Unassigned | Unassigned | Unassigned | 470_0 | Clustered | 62 | VC_470_0 | 62 | 0.902 | 1 | 0.902 | 0 | 0 | 0 | 1 | Ref |
| MK907234 | Unassigned | Unassigned | Unassigned | 470_0 | Clustered | 62 | VC_470_0 | 62 | 0.902 | 1 | 0.902 | 0 | 0 | 0 | 1 | Ref |
| MK907235 | Unassigned | Unassigned | Unassigned | 470_0 | Clustered | 62 | VC_470_0 | 62 | 0.902 | 1 | 0.902 | 0 | 0 | 0 | 1 | Ref |
| MK907236 | Unassigned | Unassigned | Unassigned | 235_0 | Clustered | 60 | VC_235_0 | 60 | 0.9413 | 1 | 0.9413 | 0 | 0 | 0 | 1 | Ref |
| MK907237 | Unassigned | Unassigned | Unassigned | 235_0 | Clustered | 60 | VC_235_0 | 60 | 0.9413 | 1 | 0.9413 | 0 | 0 | 0 | 1 | Ref |
| MK907238 | Unassigned | Unassigned | Unassigned | 235_0 | Clustered | 60 | VC_235_0 | 60 | 0.9413 | 1 | 0.9413 | 0 | 0 | 0 | 1 | Ref |
| MK907239 | Unassigned | Unassigned | Unassigned | 235_0 | Clustered | 60 | VC_235_0 | 60 | 0.9413 | 1 | 0.9413 | 0 | 0 | 0 | 1 | Ref |
| MK907240 | Unassigned | Unassigned | Unassigned | 470_0 | Clustered | 62 | VC_470_0 | 62 | 0.902 | 1 | 0.902 | 0 | 0 | 0 | 1 | Ref |
| MK907241 | Unassigned | Unassigned | Unassigned | 470_0 | Clustered | 62 | VC_470_0 | 62 | 0.902 | 1 | 0.902 | 0 | 0 | 0 | 1 | Ref |
| MK907242 | Unassigned | Unassigned | Unassigned | 470_0 | Clustered | 62 | VC_470_0 | 62 | 0.902 | 1 | 0.902 | 0 | 0 | 0 | 1 | Ref |
| MK907243 | Unassigned | Unassigned | Unassigned | 470_0 | Clustered | 62 | VC_470_0 | 62 | 0.902 | 1 | 0.902 | 0 | 0 | 0 | 1 | Ref |
| MK907244 | Unassigned | Unassigned | Unassigned | 470_0 | Clustered | 62 | VC_470_0 | 62 | 0.902 | 1 | 0.902 | 0 | 0 | 0 | 1 | Ref |
| MK907245 | Unassigned | Unassigned | Unassigned | 470_0 | Clustered | 62 | VC_470_0 | 62 | 0.902 | 1 | 0.902 | 0 | 0 | 0 | 1 | Ref |
| MK907246 | Unassigned | Unassigned | Unassigned | 470_0 | Clustered | 62 | VC_470_0 | 62 | 0.902 | 1 | 0.902 | 0 | 0 | 0 | 1 | Ref |
| MK907247 | Unassigned | Unassigned | Unassigned | 470_0 | Clustered | 62 | VC_470_0 | 62 | 0.902 | 1 | 0.902 | 0 | 0 | 0 | 1 | Ref |
| MK907248 | Unassigned | Unassigned | Unassigned | 470_0 | Clustered | 62 | VC_470_0 | 62 | 0.902 | 1 | 0.902 | 0 | 0 | 0 | 1 | Ref |
| MK907249 | Unassigned | Unassigned | Unassigned | 470_0 | Clustered | 62 | VC_470_0 | 62 | 0.902 | 1 | 0.902 | 0 | 0 | 0 | 1 | Ref |
| MK907250 | Unassigned | Unassigned | Unassigned | 470_0 | Clustered | 62 | VC_470_0 | 62 | 0.902 | 1 | 0.902 | 0 | 0 | 0 | 1 | Ref |
| MK907251 | Unassigned | Unassigned | Unassigned | 470_0 | Clustered | 62 | VC_470_0 | 62 | 0.902 | 1 | 0.902 | 0 | 0 | 0 | 1 | Ref |
| MK907252 | Unassigned | Unassigned | Unassigned | 470_0 | Clustered | 62 | VC_470_0 | 62 | 0.902 | 1 | 0.902 | 0 | 0 | 0 | 1 | Ref |
| MK907253 | Unassigned | Unassigned | Unassigned | 470_0 | Clustered | 62 | VC_470_0 | 62 | 0.902 | 1 | 0.902 | 0 | 0 | 0 | 1 | Ref |
| MK907254 | Unassigned | Unassigned | Unassigned | 470_0 | Clustered | 62 | VC_470_0 | 62 | 0.902 | 1 | 0.902 | 0 | 0 | 0 | 1 | Ref |
| MK907255 | Unassigned | Unassigned | Unassigned | 470_0 | Clustered | 62 | VC_470_0 | 62 | 0.902 | 1 | 0.902 | 0 | 0 | 0 | 1 | Ref |
| MK907256 | Unassigned | Unassigned | Unassigned | 470_0 | Clustered | 62 | VC_470_0 | 62 | 0.902 | 1 | 0.902 | 0 | 0 | 0 | 1 | Ref |
| MK907257 | Unassigned | Unassigned | Unassigned | 470_0 | Clustered | 62 | VC_470_0 | 62 | 0.902 | 1 | 0.902 | 0 | 0 | 0 | 1 | Ref |
| MK907258 | Unassigned | Unassigned | Unassigned | 470_0 | Clustered | 62 | VC_470_0 | 62 | 0.902 | 1 | 0.902 | 0 | 0 | 0 | 1 | Ref |
| MK907259 | Unassigned | Unassigned | Unassigned | 470_0 | Clustered | 62 | VC_470_0 | 62 | 0.902 | 1 | 0.902 | 0 | 0 | 0 | 1 | Ref |
| MK907260 | Unassigned | Unassigned | Unassigned | 470_0 | Clustered | 62 | VC_470_0 | 62 | 0.902 | 1 | 0.902 | 0 | 0 | 0 | 1 | Ref |
| MK907261 | Unassigned | Unassigned | Unassigned | 470_0 | Clustered | 62 | VC_470_0 | 62 | 0.902 | 1 | 0.902 | 0 | 0 | 0 | 1 | Ref |
| MK907262 | Unassigned | Unassigned | Unassigned | 470_0 | Clustered | 62 | VC_470_0 | 62 | 0.902 | 1 | 0.902 | 0 | 0 | 0 | 1 | Ref |
| MK907263 | Unassigned | Unassigned | Unassigned | 470_0 | Clustered | 62 | VC_470_0 | 62 | 0.902 | 1 | 0.902 | 0 | 0 | 0 | 1 | Ref |
| MK907264 | Unassigned | Unassigned | Unassigned | 470_0 | Clustered | 62 | VC_470_0 | 62 | 0.902 | 1 | 0.902 | 0 | 0 | 0 | 1 | Ref |
| MK907265 | Unassigned | Unassigned | Unassigned | 470_0 | Clustered | 62 | VC_470_0 | 62 | 0.902 | 1 | 0.902 | 0 | 0 | 0 | 1 | Ref |
| MK907266 | Unassigned | Unassigned | Unassigned | 470_0 | Clustered | 62 | VC_470_0 | 62 | 0.902 | 1 | 0.902 | 0 | 0 | 0 | 1 | Ref |
| MK907267 | Unassigned | Unassigned | Unassigned | 199_13 | Clustered | 5 | VC_199_13 | 5 | 0.0231 | 0.99999881 | 0.0231 | 0 | 0 | 0 | 1 | Ref |
| MK907268 | Unassigned | Unassigned | Unassigned | 199_13 | Clustered | 5 | VC_199_13 | 5 | 0.0231 | 0.99999881 | 0.0231 | 0 | 0 | 0 | 1 | Ref |
| MK907269 | Unassigned | Unassigned | Unassigned | 199_13 | Clustered | 5 | VC_199_13 | 5 | 0.0231 | 0.99999881 | 0.0231 | 0 | 0 | 0 | 1 | Ref |
| MK907270 | Unassigned | Unassigned | Unassigned | 199_13 | Clustered | 5 | VC_199_13 | 5 | 0.0231 | 0.99999881 | 0.0231 | 0 | 0 | 0 | 1 | Ref |
| MK907271 | Unassigned | Unassigned | Unassigned | 199_13 | Clustered | 5 | VC_199_13 | 5 | 0.0231 | 0.99999881 | 0.0231 | 0 | 0 | 0 | 1 | Ref |
| MK907272 | Unassigned | Unassigned | Unassigned | 470_0 | Clustered | 62 | VC_470_0 | 62 | 0.902 | 1 | 0.902 | 0 | 0 | 0 | 1 | Ref |
| MK907273 | Unassigned | Unassigned | Unassigned | 470_0 | Clustered | 62 | VC_470_0 | 62 | 0.902 | 1 | 0.902 | 0 | 0 | 0 | 1 | Ref |
| MK907274 | Unassigned | Unassigned | Unassigned | 470_0 | Clustered | 62 | VC_470_0 | 62 | 0.902 | 1 | 0.902 | 0 | 0 | 0 | 1 | Ref |
| MK907275 | Unassigned | Unassigned | Unassigned | 470_0 | Clustered | 62 | VC_470_0 | 62 | 0.902 | 1 | 0.902 | 0 | 0 | 0 | 1 | Ref |
| MK907276 | Unassigned | Unassigned | Unassigned | 470_0 | Clustered | 62 | VC_470_0 | 62 | 0.902 | 1 | 0.902 | 0 | 0 | 0 | 1 | Ref |
| MK907285 | Unassigned | Unassigned | Unassigned | 399_0 | Clustered | 49 | VC_399_0 | 49 | 0.7927 | 1 | 0.7927 | 0 | 0 | 0 | 1 | Ref |
| MK907780 | Unassigned | Unassigned | Unassigned | 1007_1 | Clustered | 24 | VC_1007_1 | 24 | 0.7364 | 1 | 0.7364 | 0 | 0 | 0 | 0.8877 | Ref |
| MK919469 | Unassigned | Unassigned | Unassigned | 543_1 | Clustered | 5 | VC_543_1 | 5 | 0.0484 | 0.99999947 | 0.0484 | 0 | 0 | 0 | 0.9 | Ref |
| MK919470 | Unassigned | Unassigned | Unassigned | 1217_0 | Clustered | 41 | VC_1217_0 | 41 | 0.9546 | 1 | 0.9546 | 0 | 0 | 0 | 0.9976 | Ref |
| MK919471 | Unassigned | Unassigned | Unassigned | 1296_0 | Clustered | 82 | VC_1296_0 | 82 | 0.7938 | 1 | 0.7938 | 0 | 0 | 0 | 1 | Ref |
| MK919472 | Unassigned | Unassigned | Unassigned | 223_6 | Clustered | 42 | VC_223_6 | 42 | 0.4717 | 1 | 0.4717 | 0 | 0 | 0 | 0.8351 | Ref |
| MK919473 | Unassigned | Unassigned | Unassigned | 825_0 | Clustered | 17 | VC_825_0 | 17 | 0.8364 | 1 | 0.8364 | 0 | 0 | 0 | 1 | Ref |
| MK919474 | Unassigned | Unassigned | Unassigned | 881_1 | Clustered | 13 | VC_881_1 | 13 | 0.5859 | 1 | 0.5859 | 0 | 0 | 0 | 1 | Ref |
| MK919475 | Unassigned | Unassigned | Unassigned | 783_0 | Clustered | 67 | VC_783_0 | 67 | 0.8945 | 1 | 0.8945 | 0 | 0 | 0 | 1 | Ref |
| MK919476 | Unassigned | Unassigned | Unassigned | 1200_0 | Clustered | 73 | VC_1200_0 | 73 | 0.9825 | 1 | 0.9825 | 0 | 0 | 0 | 1 | Ref |
| MK919477 | Unassigned | Unassigned | Unassigned | 893_1 | Clustered | 20 | VC_893_1 | 20 | 0.4792 | 1 | 0.4792 | 0 | 0 | 0 | 1 | Ref |
| MK919478 | Unassigned | Unassigned | Unassigned | 1225_0 | Clustered | 6 | VC_1225_0 | 6 | 0.9947 | 0.99996974 | 0.9947 | 0 | 0 | 0 | 1 | Ref |
| MK919479 | Unassigned | Unassigned | Unassigned | 1116_0 | Clustered | 24 | VC_1116_0 | 24 | 0.8376 | 1 | 0.8376 | 0 | 0 | 0 | 1 | Ref |
| MK919480 | Unassigned | Unassigned | Unassigned | 537_0 | Clustered | 39 | VC_537_0 | 39 | 0.8148 | 1 | 0.8148 | 0 | 0 | 0 | 1 | Ref |
| MK919481 | Unassigned | Unassigned | Unassigned | 893_1 | Clustered | 20 | VC_893_1 | 20 | 0.4792 | 1 | 0.4792 | 0 | 0 | 0 | 1 | Ref |
| MK919482 | Unassigned | Unassigned | Unassigned | 834_0 | Clustered | 18 | VC_834_0 | 18 | 0.9116 | 1 | 0.9116 | 0 | 0 | 0 | 1 | Ref |
| MK919483 | Unassigned | Unassigned | Unassigned | 543_0 | Clustered | 25 | VC_543_0 | 25 | 0.4584 | 1 | 0.4584 | 0 | 0 | 0 | 1 | Ref |
| MK919484 | Unassigned | Unassigned | Unassigned | 1200_0 | Clustered | 73 | VC_1200_0 | 73 | 0.9825 | 1 | 0.9825 | 0 | 0 | 0 | 1 | Ref |

| | | | | | | | | | | | | | | | | |
|---|---|---|---|---|---|---|---|---|---|---|---|---|---|---|---|---|
| MK922546 | Unassigned | Unassigned | Unassigned | 98_0 | Clustered | 30 | VC_98_0 | 30 | 0.7175 | 1 | 0.7175 | 0 | 0 | 0 | 1 | Ref |
| MK922547 | Unassigned | Unassigned | Unassigned | 98_0 | Clustered | 30 | VC_98_0 | 30 | 0.7175 | 1 | 0.7175 | 0 | 0 | 0 | 1 | Ref |
| MK922548 | Unassigned | Unassigned | Unassigned | 98_0 | Clustered | 30 | VC_98_0 | 30 | 0.7175 | 1 | 0.7175 | 0 | 0 | 0 | 1 | Ref |
| MK929790 | Unassigned | Unassigned | Unassigned | 1472_0 | Clustered | 2 | VC_1472_0 | 2 | 0.8077 | 0.81445332 | 0.6579 | 0 | 0 | 0 | 1 | Ref |
| MK931297 | Unassigned | Unassigned | Unassigned | 801_0 | Clustered | 37 | VC_801_0 | 37 | 0.5672 | 1 | 0.5672 | 0 | 0 | 0 | 1 | Ref |
| MK931438 | Unassigned | Unassigned | Unassigned | 655_0 | Clustered | 45 | VC_655_0 | 45 | 0.7722 | 1 | 0.7722 | 0 | 0 | 0 | 1 | Ref |
| MK931439 | Unassigned | Unassigned | Unassigned | 655_0 | Clustered | 45 | VC_655_0 | 45 | 0.7722 | 1 | 0.7722 | 0 | 0 | 0 | 1 | Ref |
| MK931440 | Unassigned | Unassigned | Unassigned | 655_0 | Clustered | 45 | VC_655_0 | 45 | 0.7722 | 1 | 0.7722 | 0 | 0 | 0 | 1 | Ref |
| MK931441 | Unassigned | Unassigned | Unassigned | 655_0 | Clustered | 45 | VC_655_0 | 45 | 0.7722 | 1 | 0.7722 | 0 | 0 | 0 | 1 | Ref |
| MK931442 | Unassigned | Unassigned | Unassigned | 972_0 | Clustered | 99 | VC_972_0 | 99 | 0.9113 | 1 | 0.9113 | 0 | 0 | 0 | 0.9928 | Ref |
| MK931443 | Unassigned | Unassigned | Unassigned | 972_0 | Clustered | 99 | VC_972_0 | 99 | 0.9113 | 1 | 0.9113 | 0 | 0 | 0 | 0.9928 | Ref |
| MK931444 | Unassigned | Unassigned | Unassigned | 972_0 | Clustered | 99 | VC_972_0 | 99 | 0.9113 | 1 | 0.9113 | 0 | 0 | 0 | 0.9928 | Ref |
| MK931445 | Unassigned | Unassigned | Unassigned | 972_0 | Clustered | 99 | VC_972_0 | 99 | 0.9113 | 1 | 0.9113 | 0 | 0 | 0 | 0.9928 | Ref |
| MK931446 | Unassigned | Unassigned | Unassigned | 654_1 | Clustered | 27 | VC_654_1 | 27 | 0.2571 | 1 | 0.2571 | 0 | 0 | 0 | 0.9858 | Ref |
| MK932884 | Unassigned | Unassigned | Unassigned | 875_0 | Clustered | 2 | VC_875_0 | 2 | 0.3804 | 0.94412959 | 0.3591 | 0 | 0 | 0 | 1 | Ref |
| MK934841 | Unassigned | Unassigned | Unassigned | 192_0 | Clustered | 22 | VC_192_0 | 22 | 0.6582 | 1 | 0.6582 | 0 | 0 | 0 | 1 | Ref |
| MK936475 | Unassigned | Unassigned | Unassigned | 98_0 | Clustered | 30 | VC_98_0 | 30 | 0.7175 | 1 | 0.7175 | 0 | 0 | 0 | 1 | Ref |
| MK936476 | Unassigned | Unassigned | Unassigned | 98_0 | Clustered | 30 | VC_98_0 | 30 | 0.7175 | 1 | 0.7175 | 0 | 0 | 0 | 1 | Ref |
| MK937591 | Unassigned | Unassigned | Unassigned | 1412_0 | Clustered | 7 | VC_1412_0 | 7 | 0.2471 | 1 | 0.2471 | 0 | 0 | 0 | 1 | Ref |
| MK937593 | Unassigned | Unassigned | Unassigned | 571_0 | Clustered | 16 | VC_571_0 | 16 | 0.091 | 1 | 0.091 | 0 | 0 | 0 | 0.6667 | Ref |
| MK937594 | Unassigned | Unassigned | Unassigned | 1136_0 | Clustered | 40 | VC_1136_0 | 40 | 0.8746 | 1 | 0.8746 | 0 | 0 | 0 | 1 | Ref |
| MK937595 | Unassigned | Unassigned | Unassigned | 387_0 | Clustered | 2 | VC_387_0 | 2 | 0.1249 | 0.95576264 | 0.1193 | 0 | 0 | 0 | 1 | Ref |
| MK937596 | Unassigned | Unassigned | Unassigned | 881_1 | Clustered | 13 | VC_881_1 | 13 | 0.5859 | 1 | 0.5859 | 0 | 0 | 0 | 1 | Ref |
| MK937597 | Unassigned | Unassigned | Unassigned | 578_0 | Clustered | 3 | VC_578_0 | 3 | 0.0807 | 0.99853749 | 0.0806 | 0 | 0 | 0 | 0.6667 | Ref |
| MK937598 | Unassigned | Unassigned | Unassigned | 1287_0 | Clustered | 44 | VC_1287_0 | 44 | 1 | 1 | 1 | 0 | 0 | 0 | 1 | Ref |
| MK937599 | Unassigned | Unassigned | Unassigned | 1218_0 | Clustered | 14 | VC_1218_0 | 14 | 0.7811 | 1 | 0.7811 | 0 | 0 | 0 | 0.989 | Ref |
| MK937600 | Unassigned | Unassigned | Unassigned | 917_0 | Clustered | 28 | VC_917_0 | 28 | 0.5465 | 1 | 0.5465 | 0 | 0 | 0 | 1 | Ref |
| MK937601 | Unassigned | Unassigned | Unassigned | 1136_0 | Clustered | 40 | VC_1136_0 | 40 | 0.8746 | 1 | 0.8746 | 0 | 0 | 0 | 1 | Ref |
| MK937602 | Unassigned | Unassigned | Unassigned | 1203_0 | Clustered | 28 | VC_1203_0 | 28 | 0.4905 | 1 | 0.4905 | 0 | 0 | 0 | 1 | Ref |
| MK937603 | Unassigned | Unassigned | Unassigned | 1219_0 | Clustered | 24 | VC_1219_0 | 24 | 0.9519 | 1 | 0.9519 | 0 | 0 | 0 | 1 | Ref |
| MK937604 | Unassigned | Unassigned | Unassigned | 537_0 | Clustered | 39 | VC_537_0 | 39 | 0.8148 | 1 | 0.8148 | 0 | 0 | 0 | 1 | Ref |
| MK937606 | Unassigned | Unassigned | Unassigned | 1412_0 | Clustered | 7 | VC_1412_0 | 7 | 0.2471 | 1 | 0.2471 | 0 | 0 | 0 | 1 | Ref |
| MK937607 | Unassigned | Unassigned | Unassigned | 1138_0 | Clustered | 16 | VC_1138_0 | 16 | 0.4566 | 1 | 0.4566 | 0 | 0 | 0 | 1 | Ref |
| MK937608 | Unassigned | Unassigned | Unassigned | 1412_0 | Clustered | 7 | VC_1412_0 | 7 | 0.2471 | 1 | 0.2471 | 0 | 0 | 0 | 1 | Ref |
| MK937610 | Unassigned | Unassigned | Unassigned | 1287_0 | Clustered | 44 | VC_1287_0 | 44 | 1 | 1 | 1 | 0 | 0 | 0 | 1 | Ref |
| MK937611 | Unassigned | Unassigned | Unassigned | 1138_0 | Clustered | 16 | VC_1138_0 | 16 | 0.4566 | 1 | 0.4566 | 0 | 0 | 0 | 1 | Ref |
| MK937612 | Unassigned | Unassigned | Unassigned | 785_1 | Clustered | 9 | VC_785_1 | 9 | 0.3679 | 1 | 0.3679 | 0 | 0 | 0 | 1 | Ref |
| MK937613 | Unassigned | Unassigned | Unassigned | 1243_0 | Clustered | 8 | VC_1243_0 | 8 | 0.8266 | 1 | 0.8266 | 0 | 0 | 0 | 1 | Ref |
| MK940809 | Unassigned | Unassigned | Unassigned | 8_0 | Clustered | 58 | VC_8_0 | 58 | 0.8285 | 1 | 0.8285 | 0 | 0 | 0 | 0.9946 | Ref |
| MK942064 | Unassigned | Unassigned | Unassigned | 289_0 | Clustered | 2 | VC_289_0 | 2 | 0.219 | 0.95471323 | 0.2091 | 0 | 0 | 0 | 1 | Ref |
| MK947458 | Unassigned | Unassigned | Unassigned | 199_16 | Clustered | 12 | VC_199_16 | 12 | 0.064 | 1 | 0.064 | 0 | 0 | 0 | 0.9394 | Ref |
| MK947459 | Unassigned | Unassigned | Unassigned | 199_17 | Clustered | 4 | VC_199_17 | 4 | 0.0165 | 0.99893269 | 0.0165 | 0 | 0 | 0 | 0.8333 | Ref |
| MK947460 | Unassigned | Unassigned | Unassigned | 475_0 | Clustered | 96 | VC_475_0 | 96 | 0.9303 | 1 | 0.9303 | 0 | 0 | 0 | 0.9575 | Ref |
| MK962626 | Unassigned | Unassigned | Unassigned | 801_1 | Clustered | 11 | VC_801_1 | 11 | 0.1583 | 1 | 0.1583 | 0 | 0 | 0 | 1 | Ref |
| MK962627 | Unassigned | Unassigned | Unassigned | 177_0 | Clustered | 8 | VC_177_0 | 8 | 0.5848 | 1 | 0.5848 | 0 | 0 | 0 | 1 | Ref |
| MK962628 | Unassigned | Unassigned | Unassigned | 163_0 | Clustered | 3 | VC_163_0 | 3 | 0.3713 | 0.99808097 | 0.3706 | 0 | 0 | 0 | 1 | Ref |
| MK962629 | Unassigned | Unassigned | Unassigned | 801_1 | Clustered | 11 | VC_801_1 | 11 | 0.1583 | 1 | 0.1583 | 0 | 0 | 0 | 1 | Ref |
| MK962630 | Unassigned | Unassigned | Unassigned | 801_1 | Clustered | 11 | VC_801_1 | 11 | 0.1583 | 1 | 0.1583 | 0 | 0 | 0 | 1 | Ref |
| MK962631 | Unassigned | Unassigned | Unassigned | 801_1 | Clustered | 11 | VC_801_1 | 11 | 0.1583 | 1 | 0.1583 | 0 | 0 | 0 | 1 | Ref |
| MK962632 | Unassigned | Unassigned | Unassigned | 801_1 | Clustered | 11 | VC_801_1 | 11 | 0.1583 | 1 | 0.1583 | 0 | 0 | 0 | 1 | Ref |
| MK962633 | Unassigned | Unassigned | Unassigned | 177_0 | Clustered | 8 | VC_177_0 | 8 | 0.5848 | 1 | 0.5848 | 0 | 0 | 0 | 1 | Ref |
| MK962634 | Unassigned | Unassigned | Unassigned | 177_0 | Clustered | 8 | VC_177_0 | 8 | 0.5848 | 1 | 0.5848 | 0 | 0 | 0 | 1 | Ref |
| MK962635 | Unassigned | Unassigned | Unassigned | 768_0 | Clustered | 4 | VC_768_0 | 4 | 0.1754 | 0.99998526 | 0.1754 | 0 | 0 | 0 | 1 | Ref |
| MK962636 | Unassigned | Unassigned | Unassigned | 768_0 | Clustered | 4 | VC_768_0 | 4 | 0.1754 | 0.99998526 | 0.1754 | 0 | 0 | 0 | 1 | Ref |
| MK962637 | Unassigned | Unassigned | Unassigned | 768_0 | Clustered | 4 | VC_768_0 | 4 | 0.1754 | 0.99998526 | 0.1754 | 0 | 0 | 0 | 1 | Ref |
| MK962638 | Unassigned | Unassigned | Unassigned | 163_0 | Clustered | 3 | VC_163_0 | 3 | 0.3713 | 0.99808097 | 0.3706 | 0 | 0 | 0 | 1 | Ref |
| MK962639 | Unassigned | Unassigned | Unassigned | 801_1 | Clustered | 11 | VC_801_1 | 11 | 0.1583 | 1 | 0.1583 | 0 | 0 | 0 | 1 | Ref |
| MK962640 | Unassigned | Unassigned | Unassigned | 163_0 | Clustered | 3 | VC_163_0 | 3 | 0.3713 | 0.99808097 | 0.3706 | 0 | 0 | 0 | 1 | Ref |
| MK962641 | Unassigned | Unassigned | Unassigned | 801_1 | Clustered | 11 | VC_801_1 | 11 | 0.1583 | 1 | 0.1583 | 0 | 0 | 0 | 1 | Ref |
| MK962749 | Unassigned | Unassigned | Unassigned | 794_1 | Clustered | 2 | VC_794_1 | 2 | 0.006 | 0.57682361 | 0.0034 | 0 | 0 | 0 | 1 | Ref |
| MK962750 | Unassigned | Unassigned | Unassigned | 290_169 | Clustered/Singleton | 1 | VC_290_169 | 1 | 0 | 1 | 0 | 0 | 0 | 0 | 1 | Ref |
| MK962751 | Unassigned | Unassigned | Unassigned | 976_0 | Clustered | 36 | VC_976_0 | 36 | 0.7833 | 1 | 0.7833 | 0 | 0 | 0 | 1 | Ref |
| MK962752 | Unassigned | Unassigned | Unassigned | 290_120 | Clustered/Singleton | 1 | VC_290_120 | 1 | 0 | 1 | 0 | 0 | 0 | 0 | 1 | Ref |
| MK962753 | Unassigned | Unassigned | Unassigned | 609_0 | Clustered | 27 | VC_609_0 | 27 | 0.9943 | 1 | 0.9943 | 0 | 0 | 0 | 1 | Ref |
| MK962754 | Unassigned | Unassigned | Unassigned | 293_1 | Clustered | 21 | VC_293_1 | 21 | 0.1694 | 1 | 0.1694 | 0 | 0 | 0 | 0.9048 | Ref |
| MK962755 | Unassigned | Unassigned | Unassigned | 290_35 | Clustered/Singleton | 1 | VC_290_35 | 1 | 0 | 1 | 0 | 0 | 0 | 0 | 1 | Ref |
| MK962756 | Unassigned | Unassigned | Unassigned | 293_1 | Clustered | 21 | VC_293_1 | 21 | 0.1694 | 1 | 0.1694 | 0 | 0 | 0 | 0.9048 | Ref |
| MK962757 | Unassigned | Unassigned | Unassigned | 293_1 | Clustered | 21 | VC_293_1 | 21 | 0.1694 | 1 | 0.1694 | 0 | 0 | 0 | 0.9048 | Ref |
| MK962758 | Unassigned | Unassigned | Unassigned | 340_3 | Clustered | 24 | VC_340_3 | 24 | 0.1205 | 1 | 0.1205 | 0 | 0 | 0 | 0.7283 | Ref |
| MK965969 | Unassigned | Unassigned | Unassigned | 340_1 | Clustered | 17 | VC_340_1 | 17 | 0.0863 | 1 | 0.0863 | 0 | 0 | 0 | 0.8235 | Ref |
| MK965970 | Unassigned | Unassigned | Unassigned | 340_1 | Clustered | 17 | VC_340_1 | 17 | 0.0863 | 1 | 0.0863 | 0 | 0 | 0 | 0.8235 | Ref |
| MK967377 | Unassigned | Unassigned | Unassigned | 1218_0 | Clustered | 14 | VC_1218_0 | 14 | 0.7811 | 1 | 0.7811 | 0 | 0 | 0 | 0.989 | Ref |
| MK967378 | Unassigned | Unassigned | Unassigned | 919_0 | Clustered | 3 | VC_919_0 | 3 | 0.3486 | 0.99831298 | 0.348 | 0 | 0 | 0 | 1 | Ref |
| MK967379 | Unassigned | Unassigned | Unassigned | 566_4 | Clustered | 16 | VC_566_4 | 16 | 0.2517 | 0.9999998 | 0.2517 | 0 | 0 | 0 | 0.9417 | Ref |
| MK967381 | Unassigned | Unassigned | Unassigned | 1219_0 | Clustered | 24 | VC_1219_0 | 24 | 0.9519 | 1 | 0.9519 | 0 | 0 | 0 | 1 | Ref |
| MK967382 | Unassigned | Unassigned | Unassigned | 231_0 | Clustered | 19 | VC_231_0 | 19 | 0.9378 | 1 | 0.9378 | 0 | 0 | 0 | 1 | Ref |
| MK967383 | Unassigned | Unassigned | Unassigned | 952_0 | Clustered | 39 | VC_952_0 | 39 | 0.9064 | 1 | 0.9064 | 0 | 0 | 0 | 1 | Ref |
| MK967384 | Unassigned | Unassigned | Unassigned | 543_1 | Clustered | 5 | VC_543_1 | 5 | 0.0484 | 0.99999947 | 0.0484 | 0 | 0 | 0 | 0.9 | Ref |
| MK967385 | Unassigned | Unassigned | Unassigned | 952_0 | Clustered | 39 | VC_952_0 | 39 | 0.9064 | 1 | 0.9064 | 0 | 0 | 0 | 1 | Ref |
| MK967386 | Unassigned | Unassigned | Unassigned | 901_0 | Clustered | 31 | VC_901_0 | 31 | 0.8235 | 1 | 0.8235 | 0 | 0 | 0 | 0.8194 | Ref |
| MK967387 | Unassigned | Unassigned | Unassigned | 335_19 | Clustered | 3 | VC_335_19 | 3 | 0.0107 | 0.99859221 | 0.0107 | 0 | 0 | 0 | 1 | Ref |

| | | | | | | | | | | | | | | | | |
|---|---|---|---|---|---|---|---|---|---|---|---|---|---|---|---|---|
| MK967388 | Unassigned | Unassigned | Unassigned | 1296_0 | Clustered | 82 | VC_1296_0 | 82 | 0.7938 | 1 | 0.7938 | 0 | 0 | 0 | 1 | Ref |
| MK967389 | Unassigned | Unassigned | Unassigned | 1217_0 | Clustered | 41 | VC_1217_0 | 41 | 0.9546 | 1 | 0.9546 | 0 | 0 | 0 | 0.9976 | Ref |
| MK967390 | Unassigned | Unassigned | Unassigned | 895_0 | Clustered | 25 | VC_895_0 | 25 | 0.5768 | 1 | 0.5768 | 0 | 0 | 0 | 1 | Ref |
| MK967391 | Unassigned | Unassigned | Unassigned | 1296_0 | Clustered | 82 | VC_1296_0 | 82 | 0.7938 | 1 | 0.7938 | 0 | 0 | 0 | 1 | Ref |
| MK967392 | Unassigned | Unassigned | Unassigned | 989_0 | Clustered | 13 | VC_989_0 | 13 | 0.8622 | 1 | 0.8622 | 0 | 0 | 0 | 1 | Ref |
| MK967393 | Unassigned | Unassigned | Unassigned | 988_0 | Clustered | 2 | VC_988_0 | 2 | 0.2055 | 0.9488174 | 0.195 | 0 | 0 | 0 | 1 | Ref |
| MK967394 | Unassigned | Unassigned | Unassigned | 1296_0 | Clustered | 82 | VC_1296_0 | 82 | 0.7938 | 1 | 0.7938 | 0 | 0 | 0 | 1 | Ref |
| MK967395 | Unassigned | Unassigned | Unassigned | 223_4 | Clustered | 13 | VC_223_4 | 13 | 0.1649 | 1 | 0.1649 | 0 | 0 | 0 | 1 | Ref |
| MK967396 | Unassigned | Unassigned | Unassigned | 1136_0 | Clustered | 40 | VC_1136_0 | 40 | 0.8746 | 1 | 0.8746 | 0 | 0 | 0 | 1 | Ref |
| MK967397 | Unassigned | Unassigned | Unassigned | 538_94 | Clustered/Singleton | 1 | VC_538_94 | 1 | 0 | 1 | 0 | 0 | 0 | 0 | 1 | Ref |
| MK967398 | Unassigned | Unassigned | Unassigned | 825_0 | Clustered | 17 | VC_825_0 | 17 | 0.8364 | 1 | 0.8364 | 0 | 0 | 0 | 1 | Ref |
| MK967399 | Unassigned | Unassigned | Unassigned | 532_0 | Clustered | 75 | VC_532_0 | 75 | 0.9393 | 1 | 0.9393 | 0 | 0 | 0 | 1 | Ref |
| MK967400 | Unassigned | Unassigned | Unassigned | 1296_0 | Clustered | 82 | VC_1296_0 | 82 | 0.7938 | 1 | 0.7938 | 0 | 0 | 0 | 1 | Ref |
| MK967401 | Unassigned | Unassigned | Unassigned | 335_40 | Clustered | 2 | VC_335_40 | 2 | 0.004 | 0.95751617 | 0.0039 | 0 | 0 | 0 | 1 | Ref |
| MK967402 | Unassigned | Unassigned | Unassigned | 566_4 | Clustered | 16 | VC_566_4 | 16 | 0.2517 | 0.9999998 | 0.2517 | 0 | 0 | 0 | 0.9417 | Ref |
| MK972685 | Unassigned | Unassigned | Unassigned | 30_1 | Clustered | 53 | VC_30_1 | 53 | 0.7151 | 1 | 0.7151 | 0 | 0 | 0 | 0.9325 | Ref |
| MK972686 | Unassigned | Unassigned | Unassigned | 30_1 | Clustered | 53 | VC_30_1 | 53 | 0.7151 | 1 | 0.7151 | 0 | 0 | 0 | 0.9325 | Ref |
| MK972687 | Unassigned | Unassigned | Unassigned | 30_1 | Clustered | 53 | VC_30_1 | 53 | 0.7151 | 1 | 0.7151 | 0 | 0 | 0 | 0.9325 | Ref |
| MK972690 | Unassigned | Unassigned | Unassigned | 135_21 | Clustered/Singleton | 1 | VC_135_21 | 1 | 0 | 1 | 0 | 0 | 0 | 0 | 1 | Ref |
| MK972691 | Unassigned | Unassigned | Unassigned | 654_0 | Clustered | 115 | VC_654_0 | 115 | 0.7303 | 1 | 0.7303 | 0 | 0 | 0 | 0.8764 | Ref |
| MK972692 | Unassigned | Unassigned | Unassigned | 30_1 | Clustered | 53 | VC_30_1 | 53 | 0.7151 | 1 | 0.7151 | 0 | 0 | 0 | 0.9325 | Ref |
| MK972693 | Unassigned | Unassigned | Unassigned | 30_1 | Clustered | 53 | VC_30_1 | 53 | 0.7151 | 1 | 0.7151 | 0 | 0 | 0 | 0.9325 | Ref |
| MK972694 | Unassigned | Unassigned | Unassigned | 30_1 | Clustered | 53 | VC_30_1 | 53 | 0.7151 | 1 | 0.7151 | 0 | 0 | 0 | 0.9325 | Ref |
| MK972698 | Unassigned | Unassigned | Unassigned | 135_48 | Clustered | 2 | VC_135_48 | 2 | 0.0051 | 0.93000885 | 0.0047 | 0 | 0 | 0 | 1 | Ref |
| MK972699 | Unassigned | Unassigned | Unassigned | 135_48 | Clustered | 2 | VC_135_48 | 2 | 0.0051 | 0.93000885 | 0.0047 | 0 | 0 | 0 | 1 | Ref |
| MK972700 | Unassigned | Unassigned | Unassigned | 654_0 | Clustered | 115 | VC_654_0 | 115 | 0.7303 | 1 | 0.7303 | 0 | 0 | 0 | 0.8764 | Ref |
| MK972701 | Unassigned | Unassigned | Unassigned | 654_0 | Clustered | 115 | VC_654_0 | 115 | 0.7303 | 1 | 0.7303 | 0 | 0 | 0 | 0.8764 | Ref |
| MK972702 | Unassigned | Unassigned | Unassigned | 654_0 | Clustered | 115 | VC_654_0 | 115 | 0.7303 | 1 | 0.7303 | 0 | 0 | 0 | 0.8764 | Ref |
| MK972703 | Unassigned | Unassigned | Unassigned | 654_0 | Clustered | 115 | VC_654_0 | 115 | 0.7303 | 1 | 0.7303 | 0 | 0 | 0 | 0.8764 | Ref |
| MK972704 | Unassigned | Unassigned | Unassigned | 199_3 | Clustered | 5 | VC_199_3 | 5 | 0.0229 | 0.99999699 | 0.0229 | 0 | 0 | 0 | 0.8 | Ref |
| MK972705 | Unassigned | Unassigned | Unassigned | 654_0 | Clustered | 115 | VC_654_0 | 115 | 0.7303 | 1 | 0.7303 | 0 | 0 | 0 | 0.8764 | Ref |
| MK972706 | Unassigned | Unassigned | Unassigned | 654_0 | Clustered | 115 | VC_654_0 | 115 | 0.7303 | 1 | 0.7303 | 0 | 0 | 0 | 0.8764 | Ref |
| MK972707 | Unassigned | Unassigned | Unassigned | 199_3 | Clustered | 5 | VC_199_3 | 5 | 0.0229 | 0.99999699 | 0.0229 | 0 | 0 | 0 | 0.8 | Ref |
| MK972708 | Unassigned | Unassigned | Unassigned | 199_31 | Clustered | 2 | VC_199_31 | 2 | 0.0056 | 0.92139383 | 0.0052 | 0 | 0 | 0 | 1 | Ref |
| MK972709 | Unassigned | Unassigned | Unassigned | 199_3 | Clustered | 5 | VC_199_3 | 5 | 0.0229 | 0.99999699 | 0.0229 | 0 | 0 | 0 | 0.8 | Ref |
| MK972710 | Unassigned | Unassigned | Unassigned | 235_0 | Clustered | 60 | VC_235_0 | 60 | 0.9413 | 1 | 0.9413 | 0 | 0 | 0 | 1 | Ref |
| MK972711 | Unassigned | Unassigned | Unassigned | 235_0 | Clustered | 60 | VC_235_0 | 60 | 0.9413 | 1 | 0.9413 | 0 | 0 | 0 | 1 | Ref |
| MK972712 | Unassigned | Unassigned | Unassigned | 5_0 | Clustered | 27 | VC_5_0 | 27 | 0.9599 | 1 | 0.9599 | 0 | 0 | 0 | 1 | Ref |
| MK972713 | Unassigned | Unassigned | Unassigned | 5_0 | Clustered | 27 | VC_5_0 | 27 | 0.9599 | 1 | 0.9599 | 0 | 0 | 0 | 1 | Ref |
| MK972714 | Unassigned | Unassigned | Unassigned | 5_0 | Clustered | 27 | VC_5_0 | 27 | 0.9599 | 1 | 0.9599 | 0 | 0 | 0 | 1 | Ref |
| MK972715 | Unassigned | Unassigned | Unassigned | 198_20 | Clustered | 4 | VC_198_20 | 4 | 0.0227 | 0.99997722 | 0.0227 | 0 | 0 | 0 | 1 | Ref |
| MK972716 | Unassigned | Unassigned | Unassigned | 198_20 | Clustered | 4 | VC_198_20 | 4 | 0.0227 | 0.99997722 | 0.0227 | 0 | 0 | 0 | 1 | Ref |
| MK972717 | Unassigned | Unassigned | Unassigned | 199_16 | Clustered | 12 | VC_199_16 | 12 | 0.064 | 1 | 0.064 | 0 | 0 | 0 | 0.9394 | Ref |
| MK972831 | Unassigned | Unassigned | Unassigned | 595_0 | Clustered | 35 | VC_595_0 | 35 | 0.8809 | 1 | 0.8809 | 0 | 0 | 0 | 1 | Ref |
| MK976036 | Unassigned | Unassigned | Unassigned | 23_0 | Clustered | 11 | VC_23_0 | 11 | 0.5144 | 1 | 0.5144 | 0 | 0 | 0 | 1 | Ref |
| MK977694 | Unassigned | Unassigned | Unassigned | 290_190 | Clustered/Singleton | 1 | VC_290_190 | 1 | 0 | 1 | 0 | 0 | 0 | 0 | 1 | Ref |
| MK977697 | Unassigned | Unassigned | Unassigned | 1296_0 | Clustered | 82 | VC_1296_0 | 82 | 0.7938 | 1 | 0.7938 | 0 | 0 | 0 | 1 | Ref |
| MK977698 | Unassigned | Unassigned | Unassigned | 1415_0 | Clustered | 31 | VC_1415_0 | 31 | 0.8456 | 1 | 0.8456 | 0 | 0 | 0 | 1 | Ref |
| MK977699 | Unassigned | Unassigned | Unassigned | 789_0 | Clustered | 34 | VC_789_0 | 34 | 0.9167 | 1 | 0.9167 | 0 | 0 | 0 | 1 | Ref |
| MK977700 | Unassigned | Unassigned | Unassigned | 790_0 | Clustered | 8 | VC_790_0 | 8 | 0.6981 | 1 | 0.6981 | 0 | 0 | 0 | 1 | Ref |
| MK977701 | Unassigned | Unassigned | Unassigned | 702_1 | Clustered | 2 | VC_702_1 | 2 | 0.2185 | 0.91327915 | 0.1996 | 0 | 0 | 0 | 1 | Ref |
| MK977702 | Unassigned | Unassigned | Unassigned | 1136_0 | Clustered | 40 | VC_1136_0 | 40 | 0.8746 | 1 | 0.8746 | 0 | 0 | 0 | 1 | Ref |
| MK977705 | Unassigned | Unassigned | Unassigned | 1224_0 | Clustered | 4 | VC_1224_0 | 4 | 0.2813 | 0.99997676 | 0.2813 | 0 | 0 | 0 | 1 | Ref |
| MK977706 | Unassigned | Unassigned | Unassigned | 1243_0 | Clustered | 8 | VC_1243_0 | 8 | 0.8266 | 1 | 0.8266 | 0 | 0 | 0 | 1 | Ref |
| MK977708 | Unassigned | Unassigned | Unassigned | 539_0 | Clustered | 37 | VC_539_0 | 37 | 0.911 | 1 | 0.911 | 0 | 0 | 0 | 1 | Ref |
| MK977709 | Unassigned | Unassigned | Unassigned | 541_0 | Clustered/Singleton | 1 | VC_541_0 | 1 | 0 | 1 | 0 | 0 | 0 | 0 | 1 | Ref |
| MK977710 | Unassigned | Unassigned | Unassigned | 1243_0 | Clustered | 8 | VC_1243_0 | 8 | 0.8266 | 1 | 0.8266 | 0 | 0 | 0 | 1 | Ref |
| MK977711 | Unassigned | Unassigned | Unassigned | 1114_3 | Clustered | 16 | VC_1114_3 | 16 | 0.2336 | 1 | 0.2336 | 0 | 0 | 0 | 0.9667 | Ref |
| MK977712 | Unassigned | Unassigned | Unassigned | 1243_0 | Clustered | 8 | VC_1243_0 | 8 | 0.8266 | 1 | 0.8266 | 0 | 0 | 0 | 1 | Ref |
| MK977713 | Unassigned | Unassigned | Unassigned | 232_0 | Clustered | 32 | VC_232_0 | 32 | 0.8471 | 1 | 0.8471 | 0 | 0 | 0 | 0.996 | Ref |
| MK977714 | Unassigned | Unassigned | Unassigned | 1243_0 | Clustered | 8 | VC_1243_0 | 8 | 0.8266 | 1 | 0.8266 | 0 | 0 | 0 | 1 | Ref |
| MK977715 | Unassigned | Unassigned | Unassigned | 1243_0 | Clustered | 8 | VC_1243_0 | 8 | 0.8266 | 1 | 0.8266 | 0 | 0 | 0 | 1 | Ref |
| MK982307 | Unassigned | Unassigned | Unassigned | 711_0 | Clustered | 48 | VC_711_0 | 48 | 0.8217 | 1 | 0.8217 | 0 | 0 | 0 | 1 | Ref |
| MK994515 | Unassigned | Unassigned | Unassigned | 506_1 | Clustered | 8 | VC_506_1 | 8 | 0.2893 | 1 | 0.2893 | 0 | 0 | 0 | 1 | Ref |
| MN010755 | Unassigned | Unassigned | Unassigned | 1407_0 | Clustered | 78 | VC_1407_0 | 78 | 0.9731 | 1 | 0.9731 | 0 | 0 | 0 | 1 | Ref |
| MN010756 | Unassigned | Unassigned | Unassigned | 587_0 | Clustered | 8 | VC_587_0 | 8 | 0.4907 | 1 | 0.4907 | 0 | 0 | 0 | 1 | Ref |
| MN010757 | Unassigned | Unassigned | Unassigned | 537_0 | Clustered | 39 | VC_537_0 | 39 | 0.8148 | 1 | 0.8148 | 0 | 0 | 0 | 1 | Ref |
| MN010758 | Unassigned | Unassigned | Unassigned | 419_0 | Clustered | 11 | VC_419_0 | 11 | 0.9465 | 1 | 0.9465 | 0 | 0 | 0 | 1 | Ref |
| MN010759 | Unassigned | Unassigned | Unassigned | 1407_0 | Clustered | 78 | VC_1407_0 | 78 | 0.9731 | 1 | 0.9731 | 0 | 0 | 0 | 1 | Ref |
| MN010760 | Unassigned | Unassigned | Unassigned | 1219_0 | Clustered | 24 | VC_1219_0 | 24 | 0.9519 | 1 | 0.9519 | 0 | 0 | 0 | 1 | Ref |
| MN010761 | Unassigned | Unassigned | Unassigned | 898_1 | Clustered | 27 | VC_898_1 | 27 | 0.8894 | 1 | 0.8894 | 0 | 0 | 0 | 1 | Ref |
| MN010762 | Unassigned | Unassigned | Unassigned | 1217_0 | Clustered | 41 | VC_1217_0 | 41 | 0.9546 | 1 | 0.9546 | 0 | 0 | 0 | 0.9976 | Ref |
| MN013074 | Unassigned | Unassigned | Unassigned | 398_0 | Clustered | 51 | VC_398_0 | 51 | 0.8741 | 1 | 0.8741 | 0 | 0 | 0 | 1 | Ref |
| MN013075 | Unassigned | Unassigned | Unassigned | 971_0 | Clustered | 40 | VC_971_0 | 40 | 0.6793 | 1 | 0.6793 | 0 | 0 | 0 | 0.9359 | Ref |
| MN013076 | Unassigned | Unassigned | Unassigned | 971_0 | Clustered | 40 | VC_971_0 | 40 | 0.6793 | 1 | 0.6793 | 0 | 0 | 0 | 0.9359 | Ref |
| MN013077 | Unassigned | Unassigned | Unassigned | 873_6 | Clustered | 7 | VC_873_6 | 7 | 0.1635 | 0.99999999 | 0.1635 | 0 | 0 | 0 | 0.9524 | Ref |
| MN013078 | Unassigned | Unassigned | Unassigned | 971_0 | Clustered | 40 | VC_971_0 | 40 | 0.6793 | 1 | 0.6793 | 0 | 0 | 0 | 0.9359 | Ref |
| MN013079 | Unassigned | Unassigned | Unassigned | 971_0 | Clustered | 40 | VC_971_0 | 40 | 0.6793 | 1 | 0.6793 | 0 | 0 | 0 | 0.9359 | Ref |
| MN013080 | Unassigned | Unassigned | Unassigned | 971_0 | Clustered | 40 | VC_971_0 | 40 | 0.6793 | 1 | 0.6793 | 0 | 0 | 0 | 0.9359 | Ref |
| MN013082 | Unassigned | Unassigned | Unassigned | 406_0 | Clustered | 7 | VC_406_0 | 7 | 0.176 | 1 | 0.176 | 0 | 0 | 0 | 1 | Ref |
| MN013083 | Unassigned | Unassigned | Unassigned | 971_0 | Clustered | 40 | VC_971_0 | 40 | 0.6793 | 1 | 0.6793 | 0 | 0 | 0 | 0.9359 | Ref |

| | | | | | | | | | | | | | | | | |
|---|---|---|---|---|---|---|---|---|---|---|---|---|---|---|---|---|
| MN013085 | Unassigned | Unassigned | Unassigned | 406_0 | Clustered | 7 | VC_406_0 | 7 | 0.176 | 1 | 0.176 | 0 | 0 | 0 | 1 | Ref |
| MN013086 | Unassigned | Unassigned | Unassigned | 40_1 | Clustered | 2 | VC_40_1 | 2 | 0.042 | 0.88750846 | 0.0373 | 0 | 0 | 0 | 1 | Ref |
| MN013087 | Unassigned | Unassigned | Unassigned | 971_0 | Clustered | 40 | VC_971_0 | 40 | 0.6793 | 1 | 0.6793 | 0 | 0 | 0 | 0.9359 | Ref |
| MN013088 | Unassigned | Unassigned | Unassigned | 106_0 | Clustered | 6 | VC_106_0 | 6 | 0.8265 | 1 | 0.8265 | 0 | 0 | 0 | 1 | Ref |
| MN013089 | Unassigned | Unassigned | Unassigned | 195_1 | Clustered | 7 | VC_195_1 | 7 | 0.1409 | 1 | 0.1409 | 0 | 0 | 0 | 1 | Ref |
| MN013356 | Unassigned | Unassigned | Unassigned | 77_0 | Clustered | 37 | VC_77_0 | 37 | 0.9392 | 1 | 0.9392 | 0 | 0 | 0 | 1 | Ref |
| MN016939 | Unassigned | Unassigned | Unassigned | 235_0 | Clustered | 60 | VC_235_0 | 60 | 0.9413 | 1 | 0.9413 | 0 | 0 | 0 | 1 | Ref |
| MN018232 | Unassigned | Unassigned | Unassigned | 446_14 | Clustered/Singleton | 1 | VC_446_14 | 1 | 0 | 1 | 0 | 0 | 0 | 0 | 1 | Ref |
| MN019128 | Unassigned | Unassigned | Unassigned | 604_0 | Clustered | 4 | VC_604_0 | 4 | 0.0531 | 0.99998755 | 0.0531 | 0 | 0 | 0 | 1 | Ref |
| MN022785 | Unassigned | Unassigned | Unassigned | 290_130 | Clustered/Singleton | 1 | VC_290_130 | 1 | 0 | 1 | 0 | 0 | 0 | 0 | 1 | Ref |
| MN022787 | Unassigned | Unassigned | Unassigned | 198_14 | Clustered | 3 | VC_198_14 | 3 | 0.0142 | 0.99685589 | 0.0142 | 0 | 0 | 0 | 0.6667 | Ref |
| MN023176 | Unassigned | Unassigned | Unassigned | 886_0 | Clustered | 16 | VC_886_0 | 16 | 0.994 | 1 | 0.994 | 0 | 0 | 0 | 1 | Ref |
| MN023177 | Unassigned | Unassigned | Unassigned | 886_0 | Clustered | 16 | VC_886_0 | 16 | 0.994 | 1 | 0.994 | 0 | 0 | 0 | 1 | Ref |
| MN023178 | Unassigned | Unassigned | Unassigned | 886_0 | Clustered | 16 | VC_886_0 | 16 | 0.994 | 1 | 0.994 | 0 | 0 | 0 | 1 | Ref |
| MN023179 | Unassigned | Unassigned | Unassigned | 886_0 | Clustered | 16 | VC_886_0 | 16 | 0.994 | 1 | 0.994 | 0 | 0 | 0 | 1 | Ref |
| MN023180 | Unassigned | Unassigned | Unassigned | 886_0 | Clustered | 16 | VC_886_0 | 16 | 0.994 | 1 | 0.994 | 0 | 0 | 0 | 1 | Ref |
| MN023181 | Unassigned | Unassigned | Unassigned | 886_0 | Clustered | 16 | VC_886_0 | 16 | 0.994 | 1 | 0.994 | 0 | 0 | 0 | 1 | Ref |
| MN023182 | Unassigned | Unassigned | Unassigned | 886_0 | Clustered | 16 | VC_886_0 | 16 | 0.994 | 1 | 0.994 | 0 | 0 | 0 | 1 | Ref |
| MN023183 | Unassigned | Unassigned | Unassigned | 886_0 | Clustered | 16 | VC_886_0 | 16 | 0.994 | 1 | 0.994 | 0 | 0 | 0 | 1 | Ref |
| MN023184 | Unassigned | Unassigned | Unassigned | 886_0 | Clustered | 16 | VC_886_0 | 16 | 0.994 | 1 | 0.994 | 0 | 0 | 0 | 1 | Ref |
| MN023185 | Unassigned | Unassigned | Unassigned | 886_0 | Clustered | 16 | VC_886_0 | 16 | 0.994 | 1 | 0.994 | 0 | 0 | 0 | 1 | Ref |
| MN023186 | Unassigned | Unassigned | Unassigned | 886_0 | Clustered | 16 | VC_886_0 | 16 | 0.994 | 1 | 0.994 | 0 | 0 | 0 | 1 | Ref |
| MN023187 | Unassigned | Unassigned | Unassigned | 886_0 | Clustered | 16 | VC_886_0 | 16 | 0.994 | 1 | 0.994 | 0 | 0 | 0 | 1 | Ref |
| MN023188 | Unassigned | Unassigned | Unassigned | 886_0 | Clustered | 16 | VC_886_0 | 16 | 0.994 | 1 | 0.994 | 0 | 0 | 0 | 1 | Ref |
| MN023189 | Unassigned | Unassigned | Unassigned | 886_0 | Clustered | 16 | VC_886_0 | 16 | 0.994 | 1 | 0.994 | 0 | 0 | 0 | 1 | Ref |
| MN023190 | Unassigned | Unassigned | Unassigned | 886_0 | Clustered | 16 | VC_886_0 | 16 | 0.994 | 1 | 0.994 | 0 | 0 | 0 | 1 | Ref |
| MN023191 | Unassigned | Unassigned | Unassigned | 886_0 | Clustered | 16 | VC_886_0 | 16 | 0.994 | 1 | 0.994 | 0 | 0 | 0 | 1 | Ref |
| MN026740 | Unassigned | Unassigned | Unassigned | 405_0 | Clustered | 126 | VC_405_0 | 126 | 0.9614 | 1 | 0.9614 | 0 | 0 | 0 | 1 | Ref |
| MN026741 | Unassigned | Unassigned | Unassigned | 198_23 | Clustered | 10 | VC_198_23 | 10 | 0.0697 | 1 | 0.0697 | 0 | 0 | 0 | 0.8444 | Ref |
| MN027503 | Unassigned | Unassigned | Unassigned | 94_0 | Clustered | 22 | VC_94_0 | 22 | 0.5602 | 1 | 0.5602 | 0 | 0 | 0 | 1 | Ref |
| MN028536 | Unassigned | Unassigned | Unassigned | 11_0 | Clustered | 19 | VC_11_0 | 19 | 0.7544 | 1 | 0.7544 | 0 | 0 | 0 | 1 | Ref |
| MN029011 | Unassigned | Unassigned | Unassigned | 820_0 | Clustered | 5 | VC_820_0 | 5 | 0.332 | 0.99999997 | 0.332 | 0 | 0 | 0 | 1 | Ref |
| MN032614 | Unassigned | Unassigned | Unassigned | 456_3 | Clustered | 3 | VC_456_3 | 3 | 0.0523 | 0.99675181 | 0.0521 | 0 | 0 | 0 | 1 | Ref |
| MN038175 | Unassigned | Unassigned | Unassigned | 687_3 | Clustered/Singleton | 1 | VC_687_3 | 1 | 0 | 1 | 0 | 0 | 0 | 0 | 1 | Ref |
| MN038176 | Unassigned | Unassigned | Unassigned | 913_0 | Clustered | 28 | VC_913_0 | 28 | 0.6206 | 1 | 0.6206 | 0 | 0 | 0 | 0.9841 | Ref |
| MN038177 | Unassigned | Unassigned | Unassigned | 636_0 | Clustered | 13 | VC_636_0 | 13 | 0.6223 | 1 | 0.6223 | 0 | 0 | 0 | 1 | Ref |
| MN038178 | Unassigned | Unassigned | Unassigned | 912_1 | Clustered | 12 | VC_912_1 | 12 | 0.2423 | 1 | 0.2423 | 0 | 0 | 0 | 1 | Ref |
| MN038179 | Unassigned | Unassigned | Unassigned | 913_0 | Clustered | 28 | VC_913_0 | 28 | 0.6206 | 1 | 0.6206 | 0 | 0 | 0 | 0.9841 | Ref |
| MN043729 | Unassigned | Unassigned | Unassigned | 87_10 | Clustered/Singleton | 1 | VC_87_10 | 1 | 0 | 1 | 0 | 0 | 0 | 0 | 1 | Ref |
| MN043730 | Unassigned | Unassigned | Unassigned | 87_4 | Clustered | 3 | VC_87_4 | 3 | 0.0292 | 0.98125255 | 0.0286 | 0 | 0 | 0 | 1 | Ref |
| MN045228 | Unassigned | Unassigned | Unassigned | 155_12 | Clustered | 2 | VC_155_12 | 2 | 0.0044 | 0.82943952 | 0.0037 | 0 | 0 | 0 | 1 | Ref |
| MN045229 | Unassigned | Unassigned | Unassigned | 816_0 | Clustered | 35 | VC_816_0 | 35 | 0.9824 | 1 | 0.9824 | 0 | 0 | 0 | 1 | Ref |
| MN045230 | Unassigned | Unassigned | Unassigned | 134_2 | Clustered | 17 | VC_134_2 | 17 | 0.1715 | 1 | 0.1715 | 0 | 0 | 0 | 0.9853 | Ref |
| MN045231 | Unassigned | Unassigned | Unassigned | 837_1 | Clustered | 24 | VC_837_1 | 24 | 0.5328 | 1 | 0.5328 | 0 | 0 | 0 | 1 | Ref |
| MN047438 | Unassigned | Unassigned | Unassigned | 155_8 | Clustered | 66 | VC_155_8 | 66 | 0.4537 | 1 | 0.4537 | 0 | 0 | 0 | 0.8182 | Ref |
| MN047793 | Unassigned | Unassigned | Unassigned | 69_1 | Clustered/Singleton | 1 | VC_69_1 | 1 | 0 | 1 | 0 | 0 | 0 | 0 | 1 | Ref |
| MN052874 | Unassigned | Unassigned | Unassigned | 848_0 | Clustered | 124 | VC_848_0 | 124 | 0.9484 | 1 | 0.9484 | 0 | 0 | 0 | 1 | Ref |
| MN055691 | Unassigned | Unassigned | Unassigned | 975_0 | Clustered | 40 | VC_975_0 | 40 | 0.6888 | 1 | 0.6888 | 0 | 0 | 0 | 1 | Ref |
| MN061582 | Unassigned | Unassigned | Unassigned | 714_0 | Clustered | 71 | VC_714_0 | 71 | 0.935 | 1 | 0.935 | 0 | 0 | 0 | 1 | Ref |
| MN062185 | Unassigned | Unassigned | Unassigned | 548_0 | Clustered | 9 | VC_548_0 | 9 | 0.8018 | 1 | 0.8018 | 0 | 0 | 0 | 1 | Ref |
| MN062186 | Unassigned | Unassigned | Unassigned | 801_3 | Clustered | 7 | VC_801_3 | 7 | 0.0764 | 1 | 0.0764 | 0 | 0 | 0 | 0.7143 | Ref |
| MN062187 | Unassigned | Unassigned | Unassigned | 766_0 | Clustered | 40 | VC_766_0 | 40 | 0.7511 | 1 | 0.7511 | 0 | 0 | 0 | 1 | Ref |
| MN062188 | Unassigned | Unassigned | Unassigned | 610_0 | Clustered | 14 | VC_610_0 | 14 | 0.5159 | 1 | 0.5159 | 0 | 0 | 0 | 1 | Ref |
| MN062701 | Unassigned | Unassigned | Unassigned | 566_4 | Clustered | 16 | VC_566_4 | 16 | 0.2517 | 0.9999998 | 0.2517 | 0 | 0 | 0 | 0.9417 | Ref |
| MN062702 | Unassigned | Unassigned | Unassigned | 1296_0 | Clustered | 82 | VC_1296_0 | 82 | 0.7938 | 1 | 0.7938 | 0 | 0 | 0 | 1 | Ref |
| MN062704 | Unassigned | Unassigned | Unassigned | 1217_0 | Clustered | 41 | VC_1217_0 | 41 | 0.9546 | 1 | 0.9546 | 0 | 0 | 0 | 0.9976 | Ref |
| MN062706 | Unassigned | Unassigned | Unassigned | 531_0 | Clustered | 32 | VC_531_0 | 32 | 0.6859 | 1 | 0.6859 | 0 | 0 | 0 | 1 | Ref |
| MN062707 | Unassigned | Unassigned | Unassigned | 579_77 | Clustered | 3 | VC_579_77 | 3 | 0.0065 | 0.89175063 | 0.0058 | 0 | 0 | 0 | 0.6667 | Ref |
| MN062708 | Unassigned | Unassigned | Unassigned | 224_1 | Clustered/Singleton | 1 | VC_224_1 | 1 | 0 | 1 | 0 | 0 | 0 | 0 | 1 | Ref |
| MN062709 | Unassigned | Unassigned | Unassigned | 1296_0 | Clustered | 82 | VC_1296_0 | 82 | 0.7938 | 1 | 0.7938 | 0 | 0 | 0 | 1 | Ref |
| MN062710 | Unassigned | Unassigned | Unassigned | 335_54 | Clustered | 5 | VC_335_54 | 5 | 0.0147 | 0.99999078 | 0.0147 | 0 | 0 | 0 | 0.7 | Ref |
| MN062711 | Unassigned | Unassigned | Unassigned | 1425_0 | Clustered | 6 | VC_1425_0 | 6 | 0.2364 | 1 | 0.2364 | 0 | 0 | 0 | 1 | Ref |
| MN062712 | Unassigned | Unassigned | Unassigned | 531_0 | Clustered | 32 | VC_531_0 | 32 | 0.6859 | 1 | 0.6859 | 0 | 0 | 0 | 1 | Ref |
| MN062713 | Unassigned | Unassigned | Unassigned | 1296_0 | Clustered | 82 | VC_1296_0 | 82 | 0.7938 | 1 | 0.7938 | 0 | 0 | 0 | 1 | Ref |
| MN062714 | Unassigned | Unassigned | Unassigned | 1287_0 | Clustered | 44 | VC_1287_0 | 44 | 1 | 1 | 1 | 0 | 0 | 0 | 1 | Ref |
| MN062715 | Unassigned | Unassigned | Unassigned | 701_0 | Clustered | 16 | VC_701_0 | 16 | 0.2957 | 1 | 0.2957 | 0 | 0 | 0 | 0.9167 | Ref |
| MN062716 | Unassigned | Unassigned | Unassigned | 952_0 | Clustered | 39 | VC_952_0 | 39 | 0.9064 | 1 | 0.9064 | 0 | 0 | 0 | 1 | Ref |
| MN062717 | Unassigned | Unassigned | Unassigned | 1287_0 | Clustered | 44 | VC_1287_0 | 44 | 1 | 1 | 1 | 0 | 0 | 0 | 1 | Ref |
| MN062718 | Unassigned | Unassigned | Unassigned | 532_0 | Clustered | 75 | VC_532_0 | 75 | 0.9393 | 1 | 0.9393 | 0 | 0 | 0 | 1 | Ref |
| MN062719 | Unassigned | Unassigned | Unassigned | 1415_0 | Clustered | 31 | VC_1415_0 | 31 | 0.8456 | 1 | 0.8456 | 0 | 0 | 0 | 1 | Ref |
| MN065183 | Unassigned | Unassigned | Unassigned | 20_0 | Clustered | 5 | VC_20_0 | 5 | 0.4268 | 0.9999994 | 0.4268 | 0 | 0 | 0 | 1 | Ref |
| MN066127 | Unassigned | Unassigned | Unassigned | 135_22 | Clustered | 13 | VC_135_22 | 13 | 0.0795 | 1 | 0.0795 | 0 | 0 | 0 | 0.8205 | Ref |
| MN067333 | Unassigned | Unassigned | Unassigned | 44_0 | Clustered | 49 | VC_44_0 | 49 | 0.4506 | 1 | 0.4506 | 0 | 0 | 0 | 0.8605 | Ref |
| MN067430 | Unassigned | Unassigned | Unassigned | 465_0 | Clustered | 80 | VC_465_0 | 80 | 0.9988 | 1 | 0.9988 | 0 | 0 | 0 | 1 | Ref |
| MN070121 | Unassigned | Unassigned | Unassigned | 560_0 | Clustered | 15 | VC_560_0 | 15 | 0.2846 | 1 | 0.2846 | 0 | 0 | 0 | 1 | Ref |
| MN078104 | Unassigned | Unassigned | Unassigned | 854_0 | Clustered | 6 | VC_854_0 | 6 | 0.514 | 1 | 0.514 | 0 | 0 | 0 | 1 | Ref |
| MN080711 | Unassigned | Unassigned | Unassigned | 399_0 | Clustered | 49 | VC_399_0 | 49 | 0.7927 | 1 | 0.7927 | 0 | 0 | 0 | 1 | Ref |
| MN082624 | Unassigned | Unassigned | Unassigned | 843_0 | Clustered | 20 | VC_843_0 | 20 | 0.9621 | 1 | 0.9621 | 0 | 0 | 0 | 1 | Ref |
| MN087708 | Unassigned | Unassigned | Unassigned | 508_1 | Clustered | 5 | VC_508_1 | 5 | 0.0439 | 0.99999996 | 0.0439 | 0 | 0 | 0 | 1 | Ref |
| MN090155 | Unassigned | Unassigned | Unassigned | 604_1 | Clustered | 3 | VC_604_1 | 3 | 0.0311 | 0.99857668 | 0.0311 | 0 | 0 | 0 | 1 | Ref |
| MN091625 | Unassigned | Unassigned | Unassigned | 155_5 | Clustered/Singleton | 1 | VC_155_5 | 1 | 0 | 1 | 0 | 0 | 0 | 0 | 1 | Ref |

| | | | | | | | | | | | | | | | | |
|---|---|---|---|---|---|---|---|---|---|---|---|---|---|---|---|---|
| MN091626 | Unassigned | Unassigned | Unassigned | 463_0 | Clustered | 7 | VC_463_0 | 7 | 0.5971 | 1 | 0.5971 | 0 | 0 | 0 | 1 | Ref |
| MN094788 | Unassigned | Unassigned | Unassigned | 67_1 | Clustered/Singleton | 1 | VC_67_1 | 1 | 0 | 1 | 0 | 0 | 0 | 0 | 1 | Ref |
| MN095770 | Unassigned | Unassigned | Unassigned | 204_0 | Clustered | 2 | VC_204_0 | 2 | 0.0214 | 0.95762696 | 0.0205 | 0 | 0 | 0 | 1 | Ref |
| MN095771 | Unassigned | Unassigned | Unassigned | 291_0 | Clustered | 3 | VC_291_0 | 3 | 0.0482 | 0.99860971 | 0.0482 | 0 | 0 | 0 | 1 | Ref |
| MN095772 | Unassigned | Unassigned | Unassigned | 524_0 | Clustered | 8 | VC_524_0 | 8 | 0.7365 | 1 | 0.7365 | 0 | 0 | 0 | 1 | Ref |
| MN096356 | Unassigned | Unassigned | Unassigned | 537_0 | Clustered | 39 | VC_537_0 | 39 | 0.8148 | 1 | 0.8148 | 0 | 0 | 0 | 1 | Ref |
| MN096357 | Unassigned | Unassigned | Unassigned | 1083_0 | Clustered | 4 | VC_1083_0 | 4 | 0.8577 | 0.99997306 | 0.8576 | 0 | 0 | 0 | 1 | Ref |
| MN096358 | Unassigned | Unassigned | Unassigned | 1083_0 | Clustered | 4 | VC_1083_0 | 4 | 0.8577 | 0.99997306 | 0.8576 | 0 | 0 | 0 | 1 | Ref |
| MN096359 | Unassigned | Unassigned | Unassigned | 527_0 | Clustered | 37 | VC_527_0 | 37 | 0.9679 | 1 | 0.9679 | 0 | 0 | 0 | 1 | Ref |
| MN096360 | Unassigned | Unassigned | Unassigned | 527_0 | Clustered | 37 | VC_527_0 | 37 | 0.9679 | 1 | 0.9679 | 0 | 0 | 0 | 1 | Ref |
| MN096361 | Unassigned | Unassigned | Unassigned | 571_0 | Clustered | 16 | VC_571_0 | 16 | 0.091 | 1 | 0.091 | 0 | 0 | 0 | 0.6667 | Ref |
| MN096363 | Unassigned | Unassigned | Unassigned | 897_0 | Clustered | 5 | VC_897_0 | 5 | 0.4183 | 0.99999997 | 0.4183 | 0 | 0 | 0 | 1 | Ref |
| MN096364 | Unassigned | Unassigned | Unassigned | 571_8 | Clustered | 19 | VC_571_8 | 19 | 0.1031 | 1 | 0.1031 | 0 | 0 | 0 | 0.8713 | Ref |
| MN096365 | Unassigned | Unassigned | Unassigned | 1217_0 | Clustered | 41 | VC_1217_0 | 41 | 0.9546 | 1 | 0.9546 | 0 | 0 | 0 | 0.9976 | Ref |
| MN096366 | Unassigned | Unassigned | Unassigned | 384_2 | Clustered | 8 | VC_384_2 | 8 | 0.0232 | 1 | 0.0232 | 0 | 0 | 0 | 0.8571 | Ref |
| MN096367 | Unassigned | Unassigned | Unassigned | 1203_0 | Clustered | 28 | VC_1203_0 | 28 | 0.4905 | 1 | 0.4905 | 0 | 0 | 0 | 1 | Ref |
| MN096368 | Unassigned | Unassigned | Unassigned | 532_0 | Clustered | 75 | VC_532_0 | 75 | 0.9393 | 1 | 0.9393 | 0 | 0 | 0 | 1 | Ref |
| MN096370 | Unassigned | Unassigned | Unassigned | 578_0 | Clustered | 3 | VC_578_0 | 3 | 0.0807 | 0.99853749 | 0.0806 | 0 | 0 | 0 | 0.6667 | Ref |
| MN096371 | Unassigned | Unassigned | Unassigned | 1114_3 | Clustered | 16 | VC_1114_3 | 16 | 0.2336 | 1 | 0.2336 | 0 | 0 | 0 | 0.9667 | Ref |
| MN096372 | Unassigned | Unassigned | Unassigned | 974_0 | Clustered | 34 | VC_974_0 | 34 | 0.7953 | 1 | 0.7953 | 0 | 0 | 0 | 1 | Ref |
| MN096373 | Unassigned | Unassigned | Unassigned | 1016_0 | Clustered | 62 | VC_1016_0 | 62 | 0.8638 | 1 | 0.8638 | 0 | 0 | 0 | 0.743 | Ref |
| MN096374 | Unassigned | Unassigned | Unassigned | 881_1 | Clustered | 13 | VC_881_1 | 13 | 0.5859 | 1 | 0.5859 | 0 | 0 | 0 | 1 | Ref |
| MN096375 | Unassigned | Unassigned | Unassigned | 1016_0 | Clustered | 62 | VC_1016_0 | 62 | 0.8638 | 1 | 0.8638 | 0 | 0 | 0 | 0.743 | Ref |
| MN096376 | Unassigned | Unassigned | Unassigned | 225_0 | Clustered | 3 | VC_225_0 | 3 | 0.0477 | 0.99860979 | 0.0477 | 0 | 0 | 0 | 1 | Ref |
| MN096377 | Unassigned | Unassigned | Unassigned | 918_0 | Clustered | 16 | VC_918_0 | 16 | 0.2945 | 1 | 0.2945 | 0 | 0 | 0 | 1 | Ref |
| MN096378 | Unassigned | Unassigned | Unassigned | 1203_0 | Clustered | 28 | VC_1203_0 | 28 | 0.4905 | 1 | 0.4905 | 0 | 0 | 0 | 1 | Ref |
| MN096379 | Unassigned | Unassigned | Unassigned | 1016_0 | Clustered | 62 | VC_1016_0 | 62 | 0.8638 | 1 | 0.8638 | 0 | 0 | 0 | 0.743 | Ref |
| MN096380 | Unassigned | Unassigned | Unassigned | 917_0 | Clustered | 28 | VC_917_0 | 28 | 0.5465 | 1 | 0.5465 | 0 | 0 | 0 | 1 | Ref |
| MN096381 | Unassigned | Unassigned | Unassigned | 1016_0 | Clustered | 62 | VC_1016_0 | 62 | 0.8638 | 1 | 0.8638 | 0 | 0 | 0 | 0.743 | Ref |
| MN098325 | Unassigned | Unassigned | Unassigned | 98_0 | Clustered | 30 | VC_98_0 | 30 | 0.7175 | 1 | 0.7175 | 0 | 0 | 0 | 1 | Ref |
| MN098326 | Unassigned | Unassigned | Unassigned | 816_0 | Clustered | 35 | VC_816_0 | 35 | 0.9824 | 1 | 0.9824 | 0 | 0 | 0 | 1 | Ref |
| MN098327 | Unassigned | Unassigned | Unassigned | 249_1 | Clustered | 21 | VC_249_1 | 21 | 0.6775 | 1 | 0.6775 | 0 | 0 | 0 | 0.8476 | Ref |
| MN098328 | Unassigned | Unassigned | Unassigned | 524_0 | Clustered | 8 | VC_524_0 | 8 | 0.7365 | 1 | 0.7365 | 0 | 0 | 0 | 1 | Ref |
| MN098329 | Unassigned | Unassigned | Unassigned | 405_0 | Clustered | 126 | VC_405_0 | 126 | 0.9614 | 1 | 0.9614 | 0 | 0 | 0 | 1 | Ref |
| MN101216 | Unassigned | Unassigned | Unassigned | 848_0 | Clustered | 124 | VC_848_0 | 124 | 0.9484 | 1 | 0.9484 | 0 | 0 | 0 | 1 | Ref |
| MN101217 | Unassigned | Unassigned | Unassigned | 113_0 | Clustered | 91 | VC_113_0 | 91 | 0.9584 | 1 | 0.9584 | 0 | 0 | 0 | 1 | Ref |
| MN101218 | Unassigned | Unassigned | Unassigned | 848_0 | Clustered | 124 | VC_848_0 | 124 | 0.9484 | 1 | 0.9484 | 0 | 0 | 0 | 1 | Ref |
| MN101219 | Unassigned | Unassigned | Unassigned | 113_0 | Clustered | 91 | VC_113_0 | 91 | 0.9584 | 1 | 0.9584 | 0 | 0 | 0 | 1 | Ref |
| MN101220 | Unassigned | Unassigned | Unassigned | 113_0 | Clustered | 91 | VC_113_0 | 91 | 0.9584 | 1 | 0.9584 | 0 | 0 | 0 | 1 | Ref |
| MN101221 | Unassigned | Unassigned | Unassigned | 686_2 | Clustered/Singleton | 1 | VC_686_2 | 1 | 0 | 1 | 0 | 0 | 0 | 0 | 1 | Ref |
| MN101222 | Unassigned | Unassigned | Unassigned | 605_0 | Clustered | 3 | VC_605_0 | 3 | 0.0492 | 0.99859933 | 0.0491 | 0 | 0 | 0 | 1 | Ref |
| MN101223 | Unassigned | Unassigned | Unassigned | 686_0 | Clustered | 25 | VC_686_0 | 25 | 0.5501 | 1 | 0.5501 | 0 | 0 | 0 | 0.96 | Ref |
| MN101224 | Unassigned | Unassigned | Unassigned | 686_3 | Clustered/Singleton | 1 | VC_686_3 | 1 | 0 | 1 | 0 | 0 | 0 | 0 | 1 | Ref |
| MN101225 | Unassigned | Unassigned | Unassigned | 509_1 | Clustered/Singleton | 1 | VC_509_1 | 1 | 0 | 1 | 0 | 0 | 0 | 0 | 1 | Ref |
| MN101226 | Unassigned | Unassigned | Unassigned | 509_0 | Clustered | 36 | VC_509_0 | 36 | 0.5291 | 1 | 0.5291 | 0 | 0 | 0 | 0.9714 | Ref |
| MN101227 | Unassigned | Unassigned | Unassigned | 848_0 | Clustered | 124 | VC_848_0 | 124 | 0.9484 | 1 | 0.9484 | 0 | 0 | 0 | 1 | Ref |
| MN101228 | Unassigned | Unassigned | Unassigned | 200_0 | Clustered | 15 | VC_200_0 | 15 | 0.5004 | 1 | 0.5004 | 0 | 0 | 0 | 1 | Ref |
| MN101229 | Unassigned | Unassigned | Unassigned | 509_0 | Clustered | 36 | VC_509_0 | 36 | 0.5291 | 1 | 0.5291 | 0 | 0 | 0 | 0.9714 | Ref |
| MN101230 | Unassigned | Unassigned | Unassigned | 509_0 | Clustered | 36 | VC_509_0 | 36 | 0.5291 | 1 | 0.5291 | 0 | 0 | 0 | 0.9714 | Ref |
| MN102098 | Unassigned | Unassigned | Unassigned | 456_0 | Clustered | 4 | VC_456_0 | 4 | 0.0832 | 0.99994226 | 0.0832 | 0 | 0 | 0 | 1 | Ref |
| MN103542 | Unassigned | Unassigned | Unassigned | 110_0 | Clustered | 33 | VC_110_0 | 33 | 0.9488 | 1 | 0.9488 | 0 | 0 | 0 | 1 | Ref |
| MN103543 | Unassigned | Unassigned | Unassigned | 356_1 | Clustered | 3 | VC_356_1 | 3 | 0.0359 | 0.93699065 | 0.0336 | 0 | 0 | 0 | 1 | Ref |
| MN114082 | Unassigned | Unassigned | Unassigned | 1002_0 | Clustered | 10 | VC_1002_0 | 10 | 0.9987 | 1 | 0.9987 | 0 | 0 | 0 | 1 | Ref |
| MN114083 | Unassigned | Unassigned | Unassigned | 1002_0 | Clustered | 10 | VC_1002_0 | 10 | 0.9987 | 1 | 0.9987 | 0 | 0 | 0 | 1 | Ref |
| MN116494 | Unassigned | Unassigned | Unassigned | 113_0 | Clustered | 91 | VC_113_0 | 91 | 0.9584 | 1 | 0.9584 | 0 | 0 | 0 | 1 | Ref |
| MN117721 | Unassigned | Unassigned | Unassigned | 816_0 | Clustered | 35 | VC_816_0 | 35 | 0.9824 | 1 | 0.9824 | 0 | 0 | 0 | 1 | Ref |
| MN119376 | Unassigned | Unassigned | Unassigned | 1278_0 | Clustered | 19 | VC_1278_0 | 19 | 1 | 1 | 1 | 0 | 0 | 0 | 1 | Ref |
| MN119377 | Unassigned | Unassigned | Unassigned | 335_37 | Clustered | 4 | VC_335_37 | 4 | 0.0112 | 0.99980442 | 0.0112 | 0 | 0 | 0 | 0.6667 | Ref |
| MN119378 | Unassigned | Unassigned | Unassigned | 585_0 | Clustered | 20 | VC_585_0 | 20 | 0.8376 | 1 | 0.8376 | 0 | 0 | 0 | 1 | Ref |
| MN119379 | Unassigned | Unassigned | Unassigned | 335_10 | Clustered | 3 | VC_335_10 | 3 | 0.012 | 0.99859169 | 0.012 | 0 | 0 | 0 | 1 | Ref |
| MN122072 | Unassigned | Unassigned | Unassigned | 816_0 | Clustered | 35 | VC_816_0 | 35 | 0.9824 | 1 | 0.9824 | 0 | 0 | 0 | 1 | Ref |
| MN128593 | Unassigned | Unassigned | Unassigned | 1002_0 | Clustered | 10 | VC_1002_0 | 10 | 0.9987 | 1 | 0.9987 | 0 | 0 | 0 | 1 | Ref |
| MN128594 | Unassigned | Unassigned | Unassigned | 753_0 | Clustered | 26 | VC_753_0 | 26 | 0.9562 | 1 | 0.9562 | 0 | 0 | 0 | 1 | Ref |
| MN131137 | Unassigned | Unassigned | Unassigned | 456_0 | Clustered | 4 | VC_456_0 | 4 | 0.0832 | 0.99994226 | 0.0832 | 0 | 0 | 0 | 1 | Ref |
| MN131141 | Unassigned | Unassigned | Unassigned | 80_7 | Clustered | 33 | VC_80_7 | 33 | 0.2156 | 1 | 0.2156 | 0 | 0 | 0 | 0.8409 | Ref |
| MN131142 | Unassigned | Unassigned | Unassigned | 80_7 | Clustered | 33 | VC_80_7 | 33 | 0.2156 | 1 | 0.2156 | 0 | 0 | 0 | 0.8409 | Ref |
| MN131143 | Unassigned | Unassigned | Unassigned | 80_7 | Clustered | 33 | VC_80_7 | 33 | 0.2156 | 1 | 0.2156 | 0 | 0 | 0 | 0.8409 | Ref |
| MN136198 | Unassigned | Unassigned | Unassigned | 816_0 | Clustered | 35 | VC_816_0 | 35 | 0.9824 | 1 | 0.9824 | 0 | 0 | 0 | 1 | Ref |
| MN148435 | Unassigned | Unassigned | Unassigned | 837_1 | Clustered | 24 | VC_837_1 | 24 | 0.5328 | 1 | 0.5328 | 0 | 0 | 0 | 1 | Ref |
| MN149903 | Unassigned | Unassigned | Unassigned | 848_0 | Clustered | 124 | VC_848_0 | 124 | 0.9484 | 1 | 0.9484 | 0 | 0 | 0 | 1 | Ref |
| MN149904 | Unassigned | Unassigned | Unassigned | 398_0 | Clustered | 51 | VC_398_0 | 51 | 0.8741 | 1 | 0.8741 | 0 | 0 | 0 | 1 | Ref |
| MN150686 | Unassigned | Unassigned | Unassigned | 195_0 | Clustered | 4 | VC_195_0 | 4 | 0.0656 | 0.9999431 | 0.0656 | 0 | 0 | 0 | 1 | Ref |
| MN150710 | Unassigned | Unassigned | Unassigned | 98_0 | Clustered | 30 | VC_98_0 | 30 | 0.7175 | 1 | 0.7175 | 0 | 0 | 0 | 1 | Ref |
| MN153503 | Unassigned | Unassigned | Unassigned | 912_4 | Clustered/Singleton | 1 | VC_912_4 | 1 | 0 | 1 | 0 | 0 | 0 | 0 | 1 | Ref |
| MN153797 | Unassigned | Unassigned | Unassigned | 595_0 | Clustered | 35 | VC_595_0 | 35 | 0.8809 | 1 | 0.8809 | 0 | 0 | 0 | 1 | Ref |
| MN159079 | Unassigned | Unassigned | Unassigned | 456_0 | Clustered | 4 | VC_456_0 | 4 | 0.0832 | 0.99994226 | 0.0832 | 0 | 0 | 0 | 1 | Ref |
| MN161573 | Unassigned | Unassigned | Unassigned | 30_1 | Clustered | 53 | VC_30_1 | 53 | 0.7151 | 1 | 0.7151 | 0 | 0 | 0 | 0.9325 | Ref |
| MN163280 | Unassigned | Unassigned | Unassigned | 200_0 | Clustered | 15 | VC_200_0 | 15 | 0.5004 | 1 | 0.5004 | 0 | 0 | 0 | 1 | Ref |
| MN163281 | Unassigned | Unassigned | Unassigned | 802_0 | Clustered | 8 | VC_802_0 | 8 | 0.5141 | 1 | 0.5141 | 0 | 0 | 0 | 1 | Ref |
| MN164484 | Unassigned | Unassigned | Unassigned | 470_0 | Clustered | 62 | VC_470_0 | 62 | 0.902 | 1 | 0.902 | 0 | 0 | 0 | 1 | Ref |
| MN166083 | Unassigned | Unassigned | Unassigned | 713_0 | Clustered | 26 | VC_713_0 | 26 | 0.9358 | 1 | 0.9358 | 0 | 0 | 0 | 1 | Ref |

| | | | | | | | | | | | | | | | | |
|---|---|---|---|---|---|---|---|---|---|---|---|---|---|---|---|---|
| MN166823 | Unassigned | Unassigned | Unassigned | 249_1 | Clustered | 21 | VC_249_1 | 21 | 0.6775 | 1 | 0.6775 | 0 | 0 | 0 | 0.8476 | Ref |
| MN172529 | Unassigned | Unassigned | Unassigned | 753_0 | Clustered | 26 | VC_753_0 | 26 | 0.9562 | 1 | 0.9562 | 0 | 0 | 0 | 1 | Ref |
| MN175603 | Unassigned | Unassigned | Unassigned | 1415_0 | Clustered | 31 | VC_1415_0 | 31 | 0.8456 | 1 | 0.8456 | 0 | 0 | 0 | 1 | Ref |
| MN175604 | Unassigned | Unassigned | Unassigned | 540_0 | Clustered | 3 | VC_540_0 | 3 | 0.083 | 0.9985266 | 0.0829 | 0 | 0 | 0 | 1 | Ref |
| MN176217 | Unassigned | Unassigned | Unassigned | 636_0 | Clustered | 13 | VC_636_0 | 13 | 0.6223 | 1 | 0.6223 | 0 | 0 | 0 | 1 | Ref |
| MN176219 | Unassigned | Unassigned | Unassigned | 106_0 | Clustered | 6 | VC_106_0 | 6 | 0.8265 | 1 | 0.8265 | 0 | 0 | 0 | 1 | Ref |
| MN176222 | Unassigned | Unassigned | Unassigned | 105_0 | Clustered | 6 | VC_105_0 | 6 | 0.7335 | 1 | 0.7335 | 0 | 0 | 0 | 1 | Ref |
| MN176223 | Unassigned | Unassigned | Unassigned | 105_0 | Clustered | 6 | VC_105_0 | 6 | 0.7335 | 1 | 0.7335 | 0 | 0 | 0 | 1 | Ref |
| MN176224 | Unassigned | Unassigned | Unassigned | 105_0 | Clustered | 6 | VC_105_0 | 6 | 0.7335 | 1 | 0.7335 | 0 | 0 | 0 | 1 | Ref |
| MN176225 | Unassigned | Unassigned | Unassigned | 105_0 | Clustered | 6 | VC_105_0 | 6 | 0.7335 | 1 | 0.7335 | 0 | 0 | 0 | 1 | Ref |
| MN176226 | Unassigned | Unassigned | Unassigned | 105_0 | Clustered | 6 | VC_105_0 | 6 | 0.7335 | 1 | 0.7335 | 0 | 0 | 0 | 1 | Ref |
| MN176227 | Unassigned | Unassigned | Unassigned | 106_0 | Clustered | 6 | VC_106_0 | 6 | 0.8265 | 1 | 0.8265 | 0 | 0 | 0 | 1 | Ref |
| MN176228 | Unassigned | Unassigned | Unassigned | 106_0 | Clustered | 6 | VC_106_0 | 6 | 0.8265 | 1 | 0.8265 | 0 | 0 | 0 | 1 | Ref |
| MN176229 | Unassigned | Unassigned | Unassigned | 105_0 | Clustered | 6 | VC_105_0 | 6 | 0.7335 | 1 | 0.7335 | 0 | 0 | 0 | 1 | Ref |
| MN176572 | Unassigned | Unassigned | Unassigned | 113_0 | Clustered | 91 | VC_113_0 | 91 | 0.9584 | 1 | 0.9584 | 0 | 0 | 0 | 1 | Ref |
| MN176573 | Unassigned | Unassigned | Unassigned | 113_0 | Clustered | 91 | VC_113_0 | 91 | 0.9584 | 1 | 0.9584 | 0 | 0 | 0 | 1 | Ref |
| MN176574 | Unassigned | Unassigned | Unassigned | 848_0 | Clustered | 124 | VC_848_0 | 124 | 0.9484 | 1 | 0.9484 | 0 | 0 | 0 | 1 | Ref |
| MN180248 | Unassigned | Unassigned | Unassigned | 23_0 | Clustered | 11 | VC_23_0 | 11 | 0.5144 | 1 | 0.5144 | 0 | 0 | 0 | 1 | Ref |
| MN180249 | Unassigned | Unassigned | Unassigned | 23_0 | Clustered | 11 | VC_23_0 | 11 | 0.5144 | 1 | 0.5144 | 0 | 0 | 0 | 1 | Ref |
| MN180250 | Unassigned | Unassigned | Unassigned | 23_0 | Clustered | 11 | VC_23_0 | 11 | 0.5144 | 1 | 0.5144 | 0 | 0 | 0 | 1 | Ref |
| MN180251 | Unassigned | Unassigned | Unassigned | 23_0 | Clustered | 11 | VC_23_0 | 11 | 0.5144 | 1 | 0.5144 | 0 | 0 | 0 | 1 | Ref |
| MN180252 | Unassigned | Unassigned | Unassigned | 23_0 | Clustered | 11 | VC_23_0 | 11 | 0.5144 | 1 | 0.5144 | 0 | 0 | 0 | 1 | Ref |
| MN183279 | Unassigned | Unassigned | Unassigned | 1116_0 | Clustered | 24 | VC_1116_0 | 24 | 0.8376 | 1 | 0.8376 | 0 | 0 | 0 | 1 | Ref |
| MN183280 | Unassigned | Unassigned | Unassigned | 579_60 | Clustered/Singleton | 1 | VC_579_60 | 1 | 0 | 1 | 0 | 0 | 0 | 0 | 1 | Ref |
| MN183281 | Unassigned | Unassigned | Unassigned | 1287_0 | Clustered | 44 | VC_1287_0 | 44 | 1 | 1 | 1 | 0 | 0 | 0 | 1 | Ref |
| MN183282 | Unassigned | Unassigned | Unassigned | 1084_0 | Clustered | 2 | VC_1084_0 | 2 | 0.0596 | 0.95667911 | 0.057 | 0 | 0 | 0 | 1 | Ref |
| MN183283 | Unassigned | Unassigned | Unassigned | 1116_0 | Clustered | 24 | VC_1116_0 | 24 | 0.8376 | 1 | 0.8376 | 0 | 0 | 0 | 1 | Ref |
| MN183284 | Unassigned | Unassigned | Unassigned | 1212_0 | Clustered | 21 | VC_1212_0 | 21 | 0.711 | 1 | 0.711 | 0 | 0 | 0 | 1 | Ref |
| MN183285 | Unassigned | Unassigned | Unassigned | 579_18 | Clustered/Singleton | 1 | VC_579_18 | 1 | 0 | 1 | 0 | 0 | 0 | 0 | 1 | Ref |
| MN183286 | Unassigned | Unassigned | Unassigned | 566_4 | Clustered | 16 | VC_566_4 | 16 | 0.2517 | 0.9999998 | 0.2517 | 0 | 0 | 0 | 0.9417 | Ref |
| MN184885 | Unassigned | Unassigned | Unassigned | 408_0 | Clustered | 5 | VC_408_0 | 5 | 0.1003 | 0.99999998 | 0.1003 | 0 | 0 | 0 | 1 | Ref |
| MN184887 | Unassigned | Unassigned | Unassigned | 601_0 | Clustered | 4 | VC_601_0 | 4 | 0.0581 | 0.99996075 | 0.0581 | 0 | 0 | 0 | 1 | Ref |
| MN187550 | Unassigned | Unassigned | Unassigned | 492_0 | Clustered | 20 | VC_492_0 | 20 | 0.7887 | 1 | 0.7887 | 0 | 0 | 0 | 1 | Ref |
| MN187969 | Unassigned | Unassigned | Unassigned | 199_19 | Clustered | 12 | VC_199_19 | 12 | 0.0651 | 1 | 0.0651 | 0 | 0 | 0 | 0.8636 | Ref |
| MN191861 | Unassigned | Unassigned | Unassigned | 71_0 | Clustered | 27 | VC_71_0 | 27 | 0.5964 | 1 | 0.5964 | 0 | 0 | 0 | 0.9943 | Ref |
| MN197465 | Unassigned | Unassigned | Unassigned | 199_10 | Clustered/Singleton | 1 | VC_199_10 | 1 | 0 | 1 | 0 | 0 | 0 | 0 | 1 | Ref |
| MN200777 | Unassigned | Unassigned | Unassigned | 27_0 | Clustered | 4 | VC_27_0 | 4 | 0.7397 | 0.99936153 | 0.7392 | 0 | 0 | 0 | 1 | Ref |
| MN200778 | Unassigned | Unassigned | Unassigned | 27_0 | Clustered | 4 | VC_27_0 | 4 | 0.7397 | 0.99936153 | 0.7392 | 0 | 0 | 0 | 1 | Ref |
| MN204492 | Unassigned | Unassigned | Unassigned | 901_0 | Clustered | 31 | VC_901_0 | 31 | 0.8235 | 1 | 0.8235 | 0 | 0 | 0 | 0.8194 | Ref |
| MN204493 | Unassigned | Unassigned | Unassigned | 953_0 | Clustered | 2 | VC_953_0 | 2 | 0.1642 | 0.95556296 | 0.1569 | 0 | 0 | 0 | 1 | Ref |
| MN204494 | Unassigned | Unassigned | Unassigned | 901_0 | Clustered | 31 | VC_901_0 | 31 | 0.8235 | 1 | 0.8235 | 0 | 0 | 0 | 0.8194 | Ref |
| MN204495 | Unassigned | Unassigned | Unassigned | 901_0 | Clustered | 31 | VC_901_0 | 31 | 0.8235 | 1 | 0.8235 | 0 | 0 | 0 | 0.8194 | Ref |
| MN204496 | Unassigned | Unassigned | Unassigned | 901_0 | Clustered | 31 | VC_901_0 | 31 | 0.8235 | 1 | 0.8235 | 0 | 0 | 0 | 0.8194 | Ref |
| MN204497 | Unassigned | Unassigned | Unassigned | 901_0 | Clustered | 31 | VC_901_0 | 31 | 0.8235 | 1 | 0.8235 | 0 | 0 | 0 | 0.8194 | Ref |
| MN204498 | Unassigned | Unassigned | Unassigned | 1016_0 | Clustered | 62 | VC_1016_0 | 62 | 0.8638 | 1 | 0.8638 | 0 | 0 | 0 | 0.743 | Ref |
| MN204499 | Unassigned | Unassigned | Unassigned | 1117_0 | Clustered | 19 | VC_1117_0 | 19 | 0.9177 | 1 | 0.9177 | 0 | 0 | 0 | 1 | Ref |
| MN204500 | Unassigned | Unassigned | Unassigned | 901_0 | Clustered | 31 | VC_901_0 | 31 | 0.8235 | 1 | 0.8235 | 0 | 0 | 0 | 0.8194 | Ref |
| MN204501 | Unassigned | Unassigned | Unassigned | 898_1 | Clustered | 27 | VC_898_1 | 27 | 0.8894 | 1 | 0.8894 | 0 | 0 | 0 | 1 | Ref |
| MN204503 | Unassigned | Unassigned | Unassigned | 1117_0 | Clustered | 19 | VC_1117_0 | 19 | 0.9177 | 1 | 0.9177 | 0 | 0 | 0 | 1 | Ref |
| MN204504 | Unassigned | Unassigned | Unassigned | 901_0 | Clustered | 31 | VC_901_0 | 31 | 0.8235 | 1 | 0.8235 | 0 | 0 | 0 | 0.8194 | Ref |
| MN212906 | Unassigned | Unassigned | Unassigned | 714_0 | Clustered | 71 | VC_714_0 | 71 | 0.935 | 1 | 0.935 | 0 | 0 | 0 | 1 | Ref |
| MN215888 | Unassigned | Unassigned | Unassigned | 911_0 | Clustered | 20 | VC_911_0 | 20 | 0.9915 | 1 | 0.9915 | 0 | 0 | 0 | 1 | Ref |
| MN218190 | Unassigned | Unassigned | Unassigned | 199_16 | Clustered | 12 | VC_199_16 | 12 | 0.064 | 1 | 0.064 | 0 | 0 | 0 | 0.9394 | Ref |
| MN218191 | Unassigned | Unassigned | Unassigned | 199_16 | Clustered | 12 | VC_199_16 | 12 | 0.064 | 1 | 0.064 | 0 | 0 | 0 | 0.9394 | Ref |
| MN218775 | Unassigned | Unassigned | Unassigned | 465_0 | Clustered | 80 | VC_465_0 | 80 | 0.9988 | 1 | 0.9988 | 0 | 0 | 0 | 1 | Ref |
| MN224564 | Unassigned | Unassigned | Unassigned | 1416_0 | Clustered | 10 | VC_1416_0 | 10 | 1 | 1 | 1 | 0 | 0 | 0 | 1 | Ref |
| MN224565 | Unassigned | Unassigned | Unassigned | 1416_0 | Clustered | 10 | VC_1416_0 | 10 | 1 | 1 | 1 | 0 | 0 | 0 | 1 | Ref |
| MN227145 | Unassigned | Unassigned | Unassigned | 340_11 | Clustered | 15 | VC_340_11 | 15 | 0.079 | 1 | 0.079 | 0 | 0 | 0 | 0.9333 | Ref |
| MN228696 | Unassigned | Unassigned | Unassigned | 805_0 | Clustered | 3 | VC_805_0 | 3 | 0.0632 | 0.99854868 | 0.0631 | 0 | 0 | 0 | 1 | Ref |
| MN234161 | Unassigned | Unassigned | Unassigned | 1217_0 | Clustered | 41 | VC_1217_0 | 41 | 0.9546 | 1 | 0.9546 | 0 | 0 | 0 | 0.9976 | Ref |
| MN234162 | Unassigned | Unassigned | Unassigned | 592_0 | Clustered | 16 | VC_592_0 | 16 | 0.7259 | 1 | 0.7259 | 0 | 0 | 0 | 1 | Ref |
| MN234163 | Unassigned | Unassigned | Unassigned | 1296_0 | Clustered | 82 | VC_1296_0 | 82 | 0.7938 | 1 | 0.7938 | 0 | 0 | 0 | 1 | Ref |
| MN234164 | Unassigned | Unassigned | Unassigned | 882_0 | Clustered | 11 | VC_882_0 | 11 | 0.943 | 1 | 0.943 | 0 | 0 | 0 | 1 | Ref |
| MN234165 | Unassigned | Unassigned | Unassigned | 243_1 | Clustered | 5 | VC_243_1 | 5 | 0.0356 | 0.99999997 | 0.0356 | 0 | 0 | 0 | 1 | Ref |
| MN234166 | Unassigned | Unassigned | Unassigned | 896_0 | Clustered | 9 | VC_896_0 | 9 | 0.2604 | 1 | 0.2604 | 0 | 0 | 0 | 1 | Ref |
| MN234167 | Unassigned | Unassigned | Unassigned | 232_0 | Clustered | 32 | VC_232_0 | 32 | 0.8471 | 1 | 0.8471 | 0 | 0 | 0 | 0.996 | Ref |
| MN234168 | Unassigned | Unassigned | Unassigned | 1204_0 | Clustered | 25 | VC_1204_0 | 25 | 0.7382 | 1 | 0.7382 | 0 | 0 | 0 | 1 | Ref |
| MN234169 | Unassigned | Unassigned | Unassigned | 222_1 | Clustered | 19 | VC_222_1 | 19 | 0.4166 | 1 | 0.4166 | 0 | 0 | 0 | 1 | Ref |
| MN234171 | Unassigned | Unassigned | Unassigned | 385_0 | Clustered | 24 | VC_385_0 | 24 | 0.5516 | 1 | 0.5516 | 0 | 0 | 0 | 1 | Ref |
| MN234174 | Unassigned | Unassigned | Unassigned | 243_1 | Clustered | 5 | VC_243_1 | 5 | 0.0356 | 0.99999997 | 0.0356 | 0 | 0 | 0 | 1 | Ref |
| MN234176 | Unassigned | Unassigned | Unassigned | 244_2 | Clustered | 11 | VC_244_2 | 11 | 0.1403 | 1 | 0.1403 | 0 | 0 | 0 | 0.9818 | Ref |
| MN234177 | Unassigned | Unassigned | Unassigned | 1296_0 | Clustered | 82 | VC_1296_0 | 82 | 0.7938 | 1 | 0.7938 | 0 | 0 | 0 | 1 | Ref |
| MN234178 | Unassigned | Unassigned | Unassigned | 883_0 | Clustered | 5 | VC_883_0 | 5 | 0.3514 | 0.99996062 | 0.3514 | 0 | 0 | 0 | 1 | Ref |
| MN234179 | Unassigned | Unassigned | Unassigned | 1217_0 | Clustered | 41 | VC_1217_0 | 41 | 0.9546 | 1 | 0.9546 | 0 | 0 | 0 | 0.9976 | Ref |
| MN234181 | Unassigned | Unassigned | Unassigned | 1296_0 | Clustered | 82 | VC_1296_0 | 82 | 0.7938 | 1 | 0.7938 | 0 | 0 | 0 | 1 | Ref |
| MN234182 | Unassigned | Unassigned | Unassigned | 243_0 | Clustered | 72 | VC_243_0 | 72 | 0.8089 | 1 | 0.8089 | 0 | 0 | 0 | 1 | Ref |
| MN234183 | Unassigned | Unassigned | Unassigned | 784_0 | Clustered | 9 | VC_784_0 | 9 | 0.0884 | 1 | 0.0884 | 0 | 0 | 0 | 1 | Ref |
| MN234185 | Unassigned | Unassigned | Unassigned | 784_0 | Clustered | 9 | VC_784_0 | 9 | 0.0884 | 1 | 0.0884 | 0 | 0 | 0 | 1 | Ref |
| MN234190 | Unassigned | Unassigned | Unassigned | 592_0 | Clustered | 16 | VC_592_0 | 16 | 0.7259 | 1 | 0.7259 | 0 | 0 | 0 | 1 | Ref |
| MN234192 | Unassigned | Unassigned | Unassigned | 1015_1 | Clustered | 26 | VC_1015_1 | 26 | 0.6989 | 1 | 0.6989 | 0 | 0 | 0 | 1 | Ref |
| MN234193 | Unassigned | Unassigned | Unassigned | 579_36 | Clustered | 4 | VC_579_36 | 4 | 0.0096 | 0.84559358 | 0.0081 | 0 | 0 | 0 | 1 | Ref |

| | | | | | | | | | | | | | | | | |
|---|---|---|---|---|---|---|---|---|---|---|---|---|---|---|---|---|
| MN234194 | Unassigned | Unassigned | Unassigned | 1204_0 | Clustered | 25 | VC_1204_0 | 25 | 0.7382 | 1 | 0.7382 | 0 | 0 | 0 | 1 | Ref |
| MN234195 | Unassigned | Unassigned | Unassigned | 536_0 | Clustered | 6 | VC_536_0 | 6 | 0.129 | 1 | 0.129 | 0 | 0 | 0 | 1 | Ref |
| MN234196 | Unassigned | Unassigned | Unassigned | 528_0 | Clustered | 12 | VC_528_0 | 12 | 0.3923 | 1 | 0.3923 | 0 | 0 | 0 | 0.9697 | Ref |
| MN234197 | Unassigned | Unassigned | Unassigned | 243_0 | Clustered | 72 | VC_243_0 | 72 | 0.8089 | 1 | 0.8089 | 0 | 0 | 0 | 1 | Ref |
| MN234198 | Unassigned | Unassigned | Unassigned | 243_0 | Clustered | 72 | VC_243_0 | 72 | 0.8089 | 1 | 0.8089 | 0 | 0 | 0 | 1 | Ref |
| MN234199 | Unassigned | Unassigned | Unassigned | 244_1 | Clustered | 13 | VC_244_1 | 13 | 0.1766 | 1 | 0.1766 | 0 | 0 | 0 | 0.7308 | Ref |
| MN234200 | Unassigned | Unassigned | Unassigned | 232_0 | Clustered | 32 | VC_232_0 | 32 | 0.8471 | 1 | 0.8471 | 0 | 0 | 0 | 0.996 | Ref |
| MN234201 | Unassigned | Unassigned | Unassigned | 243_1 | Clustered | 5 | VC_243_1 | 5 | 0.0356 | 0.99999997 | 0.0356 | 0 | 0 | 0 | 1 | Ref |
| MN234203 | Unassigned | Unassigned | Unassigned | 223_6 | Clustered | 42 | VC_223_6 | 42 | 0.4717 | 1 | 0.4717 | 0 | 0 | 0 | 0.8351 | Ref |
| MN234204 | Unassigned | Unassigned | Unassigned | 1296_0 | Clustered | 82 | VC_1296_0 | 82 | 0.7938 | 1 | 0.7938 | 0 | 0 | 0 | 1 | Ref |
| MN234205 | Unassigned | Unassigned | Unassigned | 243_0 | Clustered | 72 | VC_243_0 | 72 | 0.8089 | 1 | 0.8089 | 0 | 0 | 0 | 1 | Ref |
| MN234206 | Unassigned | Unassigned | Unassigned | 1142_0 | Clustered | 2 | VC_1142_0 | 2 | 0.258 | 0.94673154 | 0.2442 | 0 | 0 | 0 | 1 | Ref |
| MN234207 | Unassigned | Unassigned | Unassigned | 592_0 | Clustered | 16 | VC_592_0 | 16 | 0.7259 | 1 | 0.7259 | 0 | 0 | 0 | 1 | Ref |
| MN234208 | Unassigned | Unassigned | Unassigned | 1015_1 | Clustered | 26 | VC_1015_1 | 26 | 0.6989 | 1 | 0.6989 | 0 | 0 | 0 | 1 | Ref |
| MN234209 | Unassigned | Unassigned | Unassigned | 232_0 | Clustered | 32 | VC_232_0 | 32 | 0.8471 | 1 | 0.8471 | 0 | 0 | 0 | 0.996 | Ref |
| MN234211 | Unassigned | Unassigned | Unassigned | 1142_0 | Clustered | 2 | VC_1142_0 | 2 | 0.258 | 0.94673154 | 0.2442 | 0 | 0 | 0 | 1 | Ref |
| MN234212 | Unassigned | Unassigned | Unassigned | 335_54 | Clustered | 5 | VC_335_54 | 5 | 0.0147 | 0.99999078 | 0.0147 | 0 | 0 | 0 | 0.7 | Ref |
| MN234213 | Unassigned | Unassigned | Unassigned | 984_0 | Clustered | 5 | VC_984_0 | 5 | 0.1708 | 0.99999984 | 0.1708 | 0 | 0 | 0 | 1 | Ref |
| MN234214 | Unassigned | Unassigned | Unassigned | 1207_0 | Clustered | 20 | VC_1207_0 | 20 | 0.9898 | 1 | 0.9898 | 0 | 0 | 0 | 1 | Ref |
| MN234215 | Unassigned | Unassigned | Unassigned | 952_0 | Clustered | 39 | VC_952_0 | 39 | 0.9064 | 1 | 0.9064 | 0 | 0 | 0 | 1 | Ref |
| MN234217 | Unassigned | Unassigned | Unassigned | 882_0 | Clustered | 11 | VC_882_0 | 11 | 0.943 | 1 | 0.943 | 0 | 0 | 0 | 1 | Ref |
| MN234219 | Unassigned | Unassigned | Unassigned | 784_0 | Clustered | 9 | VC_784_0 | 9 | 0.0884 | 1 | 0.0884 | 0 | 0 | 0 | 1 | Ref |
| MN234220 | Unassigned | Unassigned | Unassigned | 592_0 | Clustered | 16 | VC_592_0 | 16 | 0.7259 | 1 | 0.7259 | 0 | 0 | 0 | 1 | Ref |
| MN234221 | Unassigned | Unassigned | Unassigned | 1204_0 | Clustered | 25 | VC_1204_0 | 25 | 0.7382 | 1 | 0.7382 | 0 | 0 | 0 | 1 | Ref |
| MN234222 | Unassigned | Unassigned | Unassigned | 1296_0 | Clustered | 82 | VC_1296_0 | 82 | 0.7938 | 1 | 0.7938 | 0 | 0 | 0 | 1 | Ref |
| MN234223 | Unassigned | Unassigned | Unassigned | 527_0 | Clustered | 37 | VC_527_0 | 37 | 0.9679 | 1 | 0.9679 | 0 | 0 | 0 | 1 | Ref |
| MN234224 | Unassigned | Unassigned | Unassigned | 882_0 | Clustered | 11 | VC_882_0 | 11 | 0.943 | 1 | 0.943 | 0 | 0 | 0 | 1 | Ref |
| MN234225 | Unassigned | Unassigned | Unassigned | 526_0 | Clustered | 32 | VC_526_0 | 32 | 0.9037 | 1 | 0.9037 | 0 | 0 | 0 | 1 | Ref |
| MN234226 | Unassigned | Unassigned | Unassigned | 243_1 | Clustered | 5 | VC_243_1 | 5 | 0.0356 | 0.99999997 | 0.0356 | 0 | 0 | 0 | 1 | Ref |
| MN234227 | Unassigned | Unassigned | Unassigned | 585_0 | Clustered | 20 | VC_585_0 | 20 | 0.8376 | 1 | 0.8376 | 0 | 0 | 0 | 1 | Ref |
| MN234228 | Unassigned | Unassigned | Unassigned | 917_0 | Clustered | 28 | VC_917_0 | 28 | 0.5465 | 1 | 0.5465 | 0 | 0 | 0 | 1 | Ref |
| MN234230 | Unassigned | Unassigned | Unassigned | 243_0 | Clustered | 72 | VC_243_0 | 72 | 0.8089 | 1 | 0.8089 | 0 | 0 | 0 | 1 | Ref |
| MN234231 | Unassigned | Unassigned | Unassigned | 243_0 | Clustered | 72 | VC_243_0 | 72 | 0.8089 | 1 | 0.8089 | 0 | 0 | 0 | 1 | Ref |
| MN234232 | Unassigned | Unassigned | Unassigned | 592_0 | Clustered | 16 | VC_592_0 | 16 | 0.7259 | 1 | 0.7259 | 0 | 0 | 0 | 1 | Ref |
| MN234233 | Unassigned | Unassigned | Unassigned | 1244_0 | Clustered | 4 | VC_1244_0 | 4 | 0.8666 | 0.9999992 | 0.8666 | 0 | 0 | 0 | 1 | Ref |
| MN234234 | Unassigned | Unassigned | Unassigned | 1117_0 | Clustered | 19 | VC_1117_0 | 19 | 0.9177 | 1 | 0.9177 | 0 | 0 | 0 | 1 | Ref |
| MN234235 | Unassigned | Unassigned | Unassigned | 232_0 | Clustered | 32 | VC_232_0 | 32 | 0.8471 | 1 | 0.8471 | 0 | 0 | 0 | 0.996 | Ref |
| MN234236 | Unassigned | Unassigned | Unassigned | 536_0 | Clustered | 6 | VC_536_0 | 6 | 0.129 | 1 | 0.129 | 0 | 0 | 0 | 1 | Ref |
| MN241318 | Unassigned | Unassigned | Unassigned | 94_0 | Clustered | 22 | VC_94_0 | 22 | 0.5602 | 1 | 0.5602 | 0 | 0 | 0 | 1 | Ref |
| MN252582 | Unassigned | Unassigned | Unassigned | 399_0 | Clustered | 49 | VC_399_0 | 49 | 0.7927 | 1 | 0.7927 | 0 | 0 | 0 | 1 | Ref |
| MN263053 | Unassigned | Unassigned | Unassigned | 184_0 | Clustered | 5 | VC_184_0 | 5 | 0.7721 | 0.99998991 | 0.7721 | 0 | 0 | 0 | 1 | Ref |
| MN270258 | Unassigned | Unassigned | Unassigned | 647_0 | Clustered | 37 | VC_647_0 | 37 | 0.5847 | 1 | 0.5847 | 0 | 0 | 0 | 0.9234 | Ref |
| MN270259 | Unassigned | Unassigned | Unassigned | 647_0 | Clustered | 37 | VC_647_0 | 37 | 0.5847 | 1 | 0.5847 | 0 | 0 | 0 | 0.9234 | Ref |
| MN270260 | Unassigned | Unassigned | Unassigned | 647_0 | Clustered | 37 | VC_647_0 | 37 | 0.5847 | 1 | 0.5847 | 0 | 0 | 0 | 0.9234 | Ref |
| MN270261 | Unassigned | Unassigned | Unassigned | 647_0 | Clustered | 37 | VC_647_0 | 37 | 0.5847 | 1 | 0.5847 | 0 | 0 | 0 | 0.9234 | Ref |
| MN270262 | Unassigned | Unassigned | Unassigned | 647_0 | Clustered | 37 | VC_647_0 | 37 | 0.5847 | 1 | 0.5847 | 0 | 0 | 0 | 0.9234 | Ref |
| MN270263 | Unassigned | Unassigned | Unassigned | 647_0 | Clustered | 37 | VC_647_0 | 37 | 0.5847 | 1 | 0.5847 | 0 | 0 | 0 | 0.9234 | Ref |
| MN270264 | Unassigned | Unassigned | Unassigned | 647_0 | Clustered | 37 | VC_647_0 | 37 | 0.5847 | 1 | 0.5847 | 0 | 0 | 0 | 0.9234 | Ref |
| MN270265 | Unassigned | Unassigned | Unassigned | 647_0 | Clustered | 37 | VC_647_0 | 37 | 0.5847 | 1 | 0.5847 | 0 | 0 | 0 | 0.9234 | Ref |
| MN270266 | Unassigned | Unassigned | Unassigned | 647_0 | Clustered | 37 | VC_647_0 | 37 | 0.5847 | 1 | 0.5847 | 0 | 0 | 0 | 0.9234 | Ref |
| MN270267 | Unassigned | Unassigned | Unassigned | 647_0 | Clustered | 37 | VC_647_0 | 37 | 0.5847 | 1 | 0.5847 | 0 | 0 | 0 | 0.9234 | Ref |
| MN270268 | Unassigned | Unassigned | Unassigned | 647_0 | Clustered | 37 | VC_647_0 | 37 | 0.5847 | 1 | 0.5847 | 0 | 0 | 0 | 0.9234 | Ref |
| MN270269 | Unassigned | Unassigned | Unassigned | 647_0 | Clustered | 37 | VC_647_0 | 37 | 0.5847 | 1 | 0.5847 | 0 | 0 | 0 | 0.9234 | Ref |
| MN270270 | Unassigned | Unassigned | Unassigned | 647_0 | Clustered | 37 | VC_647_0 | 37 | 0.5847 | 1 | 0.5847 | 0 | 0 | 0 | 0.9234 | Ref |
| MN270271 | Unassigned | Unassigned | Unassigned | 647_0 | Clustered | 37 | VC_647_0 | 37 | 0.5847 | 1 | 0.5847 | 0 | 0 | 0 | 0.9234 | Ref |
| MN270272 | Unassigned | Unassigned | Unassigned | 647_0 | Clustered | 37 | VC_647_0 | 37 | 0.5847 | 1 | 0.5847 | 0 | 0 | 0 | 0.9234 | Ref |
| MN270273 | Unassigned | Unassigned | Unassigned | 647_0 | Clustered | 37 | VC_647_0 | 37 | 0.5847 | 1 | 0.5847 | 0 | 0 | 0 | 0.9234 | Ref |
| MN270274 | Unassigned | Unassigned | Unassigned | 647_0 | Clustered | 37 | VC_647_0 | 37 | 0.5847 | 1 | 0.5847 | 0 | 0 | 0 | 0.9234 | Ref |
| MN270275 | Unassigned | Unassigned | Unassigned | 647_0 | Clustered | 37 | VC_647_0 | 37 | 0.5847 | 1 | 0.5847 | 0 | 0 | 0 | 0.9234 | Ref |
| MN270276 | Unassigned | Unassigned | Unassigned | 647_0 | Clustered | 37 | VC_647_0 | 37 | 0.5847 | 1 | 0.5847 | 0 | 0 | 0 | 0.9234 | Ref |
| MN270277 | Unassigned | Unassigned | Unassigned | 647_0 | Clustered | 37 | VC_647_0 | 37 | 0.5847 | 1 | 0.5847 | 0 | 0 | 0 | 0.9234 | Ref |
| MN270278 | Unassigned | Unassigned | Unassigned | 647_0 | Clustered | 37 | VC_647_0 | 37 | 0.5847 | 1 | 0.5847 | 0 | 0 | 0 | 0.9234 | Ref |
| MN270279 | Unassigned | Unassigned | Unassigned | 647_0 | Clustered | 37 | VC_647_0 | 37 | 0.5847 | 1 | 0.5847 | 0 | 0 | 0 | 0.9234 | Ref |
| MN270887 | Unassigned | Unassigned | Unassigned | 1018_0 | Clustered | 38 | VC_1018_0 | 38 | 0.9757 | 1 | 0.9757 | 0 | 0 | 0 | 1 | Ref |
| MN270888 | Unassigned | Unassigned | Unassigned | 1018_0 | Clustered | 38 | VC_1018_0 | 38 | 0.9757 | 1 | 0.9757 | 0 | 0 | 0 | 1 | Ref |
| MN270891 | Unassigned | Unassigned | Unassigned | 342_0 | Clustered | 4 | VC_342_0 | 4 | 0.0875 | 0.99998775 | 0.0875 | 0 | 0 | 0 | 1 | Ref |
| MN270892 | Unassigned | Unassigned | Unassigned | 342_0 | Clustered | 4 | VC_342_0 | 4 | 0.0875 | 0.99998775 | 0.0875 | 0 | 0 | 0 | 1 | Ref |
| MN271656 | Unassigned | Unassigned | Unassigned | 1018_0 | Clustered | 38 | VC_1018_0 | 38 | 0.9757 | 1 | 0.9757 | 0 | 0 | 0 | 1 | Ref |
| MN276049 | Unassigned | Unassigned | Unassigned | 716_0 | Clustered | 2 | VC_716_0 | 2 | 0.1472 | 0.95249915 | 0.1402 | 0 | 0 | 0 | 1 | Ref |
| MN276192 | Unassigned | Unassigned | Unassigned | 579_40 | Clustered/Singleton | 1 | VC_579_40 | 1 | 0 | 1 | 0 | 0 | 0 | 0 | 1 | Ref |
| MN284892 | Unassigned | Unassigned | Unassigned | 1407_0 | Clustered | 78 | VC_1407_0 | 78 | 0.9731 | 1 | 0.9731 | 0 | 0 | 0 | 1 | Ref |
| MN284893 | Unassigned | Unassigned | Unassigned | 385_1 | Clustered | 9 | VC_385_1 | 9 | 0.1928 | 1 | 0.1928 | 0 | 0 | 0 | 1 | Ref |
| MN284894 | Unassigned | Unassigned | Unassigned | 1278_0 | Clustered | 19 | VC_1278_0 | 19 | 1 | 1 | 1 | 0 | 0 | 0 | 1 | Ref |
| MN284895 | Unassigned | Unassigned | Unassigned | 244_3 | Clustered | 8 | VC_244_3 | 8 | 0.1086 | 1 | 0.1086 | 0 | 0 | 0 | 1 | Ref |
| MN284896 | Unassigned | Unassigned | Unassigned | 898_1 | Clustered | 27 | VC_898_1 | 27 | 0.8894 | 1 | 0.8894 | 0 | 0 | 0 | 1 | Ref |
| MN284897 | Unassigned | Unassigned | Unassigned | 825_0 | Clustered | 17 | VC_825_0 | 17 | 0.8364 | 1 | 0.8364 | 0 | 0 | 0 | 1 | Ref |
| MN284898 | Unassigned | Unassigned | Unassigned | 231_0 | Clustered | 19 | VC_231_0 | 19 | 0.9378 | 1 | 0.9378 | 0 | 0 | 0 | 1 | Ref |
| MN284899 | Unassigned | Unassigned | Unassigned | 1016_0 | Clustered | 62 | VC_1016_0 | 62 | 0.8638 | 1 | 0.8638 | 0 | 0 | 0 | 0.743 | Ref |
| MN284900 | Unassigned | Unassigned | Unassigned | 532_0 | Clustered | 75 | VC_532_0 | 75 | 0.9393 | 1 | 0.9393 | 0 | 0 | 0 | 1 | Ref |
| MN284901 | Unassigned | Unassigned | Unassigned | 1203_0 | Clustered | 28 | VC_1203_0 | 28 | 0.4905 | 1 | 0.4905 | 0 | 0 | 0 | 1 | Ref |
| MN284902 | Unassigned | Unassigned | Unassigned | 1204_0 | Clustered | 25 | VC_1204_0 | 25 | 0.7382 | 1 | 0.7382 | 0 | 0 | 0 | 1 | Ref |

| | | | | | | | | | | | | | | | | |
|---|---|---|---|---|---|---|---|---|---|---|---|---|---|---|---|---|
| MN284904 | Unassigned | Unassigned | Unassigned | 898_1 | Clustered | 27 | VC_898_1 | 27 | 0.8894 | 1 | 0.8894 | 0 | 0 | 0 | 1 | Ref |
| MN284905 | Unassigned | Unassigned | Unassigned | 535_0 | Clustered | 18 | VC_535_0 | 18 | 0.1595 | 1 | 0.1595 | 0 | 0 | 0 | 1 | Ref |
| MN284906 | Unassigned | Unassigned | Unassigned | 952_0 | Clustered | 39 | VC_952_0 | 39 | 0.9064 | 1 | 0.9064 | 0 | 0 | 0 | 1 | Ref |
| MN284907 | Unassigned | Unassigned | Unassigned | 1219_0 | Clustered | 24 | VC_1219_0 | 24 | 0.9519 | 1 | 0.9519 | 0 | 0 | 0 | 1 | Ref |
| MN284908 | Unassigned | Unassigned | Unassigned | 585_0 | Clustered | 20 | VC_585_0 | 20 | 0.8376 | 1 | 0.8376 | 0 | 0 | 0 | 1 | Ref |
| MN294712 | Unassigned | Unassigned | Unassigned | 714_0 | Clustered | 71 | VC_714_0 | 71 | 0.935 | 1 | 0.935 | 0 | 0 | 0 | 1 | Ref |
| MN296515 | Unassigned | Unassigned | Unassigned | 595_0 | Clustered | 35 | VC_595_0 | 35 | 0.8809 | 1 | 0.8809 | 0 | 0 | 0 | 1 | Ref |
| MN304941 | Unassigned | Unassigned | Unassigned | 155_15 | Clustered | 16 | VC_155_15 | 16 | 0.0779 | 1 | 0.0779 | 0 | 0 | 0 | 0.8083 | Ref |
| MN310541 | Unassigned | Unassigned | Unassigned | 227_0 | Clustered | 4 | VC_227_0 | 4 | 0.0609 | 0.99998808 | 0.0609 | 0 | 0 | 0 | 1 | Ref |
| MN310542 | Unassigned | Unassigned | Unassigned | 232_0 | Clustered | 32 | VC_232_0 | 32 | 0.8471 | 1 | 0.8471 | 0 | 0 | 0 | 0.996 | Ref |
| MN310543 | Unassigned | Unassigned | Unassigned | 1203_0 | Clustered | 28 | VC_1203_0 | 28 | 0.4905 | 1 | 0.4905 | 0 | 0 | 0 | 1 | Ref |
| MN310544 | Unassigned | Unassigned | Unassigned | 538_75 | Clustered/Singleton | 1 | VC_538_75 | 1 | 0 | 1 | 0 | 0 | 0 | 0 | 1 | Ref |
| MN310545 | Unassigned | Unassigned | Unassigned | 538_82 | Clustered/Singleton | 1 | VC_538_82 | 1 | 0 | 1 | 0 | 0 | 0 | 0 | 1 | Ref |
| MN310546 | Unassigned | Unassigned | Unassigned | 1424_0 | Clustered | 31 | VC_1424_0 | 31 | 0.8834 | 1 | 0.8834 | 0 | 0 | 0 | 1 | Ref |
| MN310547 | Unassigned | Unassigned | Unassigned | 228_0 | Clustered | 22 | VC_228_0 | 22 | 0.7411 | 1 | 0.7411 | 0 | 0 | 0 | 1 | Ref |
| MN310548 | Unassigned | Unassigned | Unassigned | 538_7 | Clustered/Singleton | 1 | VC_538_7 | 1 | 0 | 1 | 0 | 0 | 0 | 0 | 1 | Ref |
| MN310549 | Unassigned | Unassigned | Unassigned | 952_0 | Clustered | 39 | VC_952_0 | 39 | 0.9064 | 1 | 0.9064 | 0 | 0 | 0 | 1 | Ref |
| MN311487 | Unassigned | Unassigned | Unassigned | 1319_0 | Clustered | 2 | VC_1319_0 | 2 | 0.1187 | 0.95613564 | 0.1135 | 0 | 0 | 0 | 1 | Ref |
| MN311488 | Unassigned | Unassigned | Unassigned | 1104_0 | Clustered | 44 | VC_1104_0 | 44 | 0.9574 | 0.72818579 | 0.6972 | 0 | 0 | 0 | 1 | Ref |
| MN311489 | Unassigned | Unassigned | Unassigned | 1104_0 | Clustered | 44 | VC_1104_0 | 44 | 0.9574 | 0.72818579 | 0.6972 | 0 | 0 | 0 | 1 | Ref |
| MN311490 | Unassigned | Unassigned | Unassigned | 1104_0 | Clustered | 44 | VC_1104_0 | 44 | 0.9574 | 0.72818579 | 0.6972 | 0 | 0 | 0 | 1 | Ref |
| MN311491 | Unassigned | Unassigned | Unassigned | 1104_0 | Clustered | 44 | VC_1104_0 | 44 | 0.9574 | 0.72818579 | 0.6972 | 0 | 0 | 0 | 1 | Ref |
| MN311492 | Unassigned | Unassigned | Unassigned | 1319_0 | Clustered | 2 | VC_1319_0 | 2 | 0.1187 | 0.95613564 | 0.1135 | 0 | 0 | 0 | 1 | Ref |
| MN311493 | Unassigned | Unassigned | Unassigned | 1299_0 | Clustered | 53 | VC_1299_0 | 53 | 0.8666 | 1 | 0.8666 | 0 | 0 | 0 | 1 | Ref |
| MN313256 | Unassigned | Unassigned | Unassigned | 663_0 | Clustered | 5 | VC_663_0 | 5 | 0.6645 | 0.99999974 | 0.6645 | 0 | 0 | 0 | 1 | Ref |
| MN316588 | Unassigned | Unassigned | Unassigned | 198_14 | Clustered | 3 | VC_198_14 | 3 | 0.0142 | 0.99685589 | 0.0142 | 0 | 0 | 0 | 0.6667 | Ref |
| MN317029 | Unassigned | Unassigned | Unassigned | 617_0 | Clustered | 6 | VC_617_0 | 6 | 0.1792 | 1 | 0.1792 | 0 | 0 | 0 | 1 | Ref |
| MN329673 | Unassigned | Unassigned | Unassigned | 1136_0 | Clustered | 40 | VC_1136_0 | 40 | 0.8746 | 1 | 0.8746 | 0 | 0 | 0 | 1 | Ref |
| MN329674 | Unassigned | Unassigned | Unassigned | 973_0 | Clustered | 15 | VC_973_0 | 15 | 1 | 0 | 0 | 0 | 0 | 0 | 1 | Ref |
| MN329675 | Unassigned | Unassigned | Unassigned | 1287_0 | Clustered | 44 | VC_1287_0 | 44 | 1 | 1 | 1 | 0 | 0 | 0 | 1 | Ref |
| MN329676 | Unassigned | Unassigned | Unassigned | 973_0 | Clustered | 15 | VC_973_0 | 15 | 1 | 0 | 0 | 0 | 0 | 0 | 1 | Ref |
| MN329677 | Unassigned | Unassigned | Unassigned | 1136_0 | Clustered | 40 | VC_1136_0 | 40 | 0.8746 | 1 | 0.8746 | 0 | 0 | 0 | 1 | Ref |
| MN329678 | Unassigned | Unassigned | Unassigned | 787_0 | Clustered | 132 | VC_787_0 | 132 | 1 | 1 | 1 | 0 | 0 | 0 | 1 | Ref |
| MN329679 | Unassigned | Unassigned | Unassigned | 1407_0 | Clustered | 78 | VC_1407_0 | 78 | 0.9731 | 1 | 0.9731 | 0 | 0 | 0 | 1 | Ref |
| MN334766 | Unassigned | Unassigned | Unassigned | 506_2 | Clustered | 2 | VC_506_2 | 2 | 0.0324 | 0.95511082 | 0.031 | 0 | 0 | 0 | 1 | Ref |
| MN335248 | Unassigned | Unassigned | Unassigned | 183_0 | Clustered | 5 | VC_183_0 | 5 | 0.7449 | 0.99885646 | 0.744 | 0 | 0 | 0 | 1 | Ref |
| MN336261 | Unassigned | Unassigned | Unassigned | 155_8 | Clustered | 66 | VC_155_8 | 66 | 0.4537 | 1 | 0.4537 | 0 | 0 | 0 | 0.8182 | Ref |
| MN336262 | Unassigned | Unassigned | Unassigned | 155_8 | Clustered | 66 | VC_155_8 | 66 | 0.4537 | 1 | 0.4537 | 0 | 0 | 0 | 0.8182 | Ref |
| MN336263 | Unassigned | Unassigned | Unassigned | 155_8 | Clustered | 66 | VC_155_8 | 66 | 0.4537 | 1 | 0.4537 | 0 | 0 | 0 | 0.8182 | Ref |
| MN336264 | Unassigned | Unassigned | Unassigned | 654_0 | Clustered | 115 | VC_654_0 | 115 | 0.7303 | 1 | 0.7303 | 0 | 0 | 0 | 0.8764 | Ref |
| MN336265 | Unassigned | Unassigned | Unassigned | 654_0 | Clustered | 115 | VC_654_0 | 115 | 0.7303 | 1 | 0.7303 | 0 | 0 | 0 | 0.8764 | Ref |
| MN336266 | Unassigned | Unassigned | Unassigned | 654_0 | Clustered | 115 | VC_654_0 | 115 | 0.7303 | 1 | 0.7303 | 0 | 0 | 0 | 0.8764 | Ref |
| MN337573 | Unassigned | Unassigned | Unassigned | 595_0 | Clustered | 35 | VC_595_0 | 35 | 0.8809 | 1 | 0.8809 | 0 | 0 | 0 | 1 | Ref |
| MN337887 | Unassigned | Unassigned | Unassigned | 706_0 | Clustered | 52 | VC_706_0 | 52 | 0.9787 | 1 | 0.9787 | 0 | 0 | 0 | 1 | Ref |
| MN340231 | Unassigned | Unassigned | Unassigned | 32_1 | Clustered | 16 | VC_32_1 | 16 | 0.1962 | 1 | 0.1962 | 0 | 0 | 0 | 0.8333 | Ref |
| MN342150 | Unassigned | Unassigned | Unassigned | 135_22 | Clustered | 13 | VC_135_22 | 13 | 0.0795 | 1 | 0.0795 | 0 | 0 | 0 | 0.8205 | Ref |
| MN342151 | Unassigned | Unassigned | Unassigned | 341_0 | Clustered | 27 | VC_341_0 | 27 | 0.5058 | 1 | 0.5058 | 0 | 0 | 0 | 0.8661 | Ref |
| MN342247 | Unassigned | Unassigned | Unassigned | 595_0 | Clustered | 35 | VC_595_0 | 35 | 0.8809 | 1 | 0.8809 | 0 | 0 | 0 | 1 | Ref |
| MN364664 | Unassigned | Unassigned | Unassigned | 195_4 | Clustered/Singleton | 1 | VC_195_4 | 1 | 0 | 1 | 0 | 0 | 0 | 0 | 1 | Ref |
| MN369737 | Unassigned | Unassigned | Unassigned | 1296_0 | Clustered | 82 | VC_1296_0 | 82 | 0.7938 | 1 | 0.7938 | 0 | 0 | 0 | 1 | Ref |
| MN369738 | Unassigned | Unassigned | Unassigned | 384_14 | Clustered/Singleton | 1 | VC_384_14 | 1 | 0 | 1 | 0 | 0 | 0 | 0 | 1 | Ref |
| MN369739 | Unassigned | Unassigned | Unassigned | 538_17 | Clustered/Singleton | 1 | VC_538_17 | 1 | 0 | 1 | 0 | 0 | 0 | 0 | 1 | Ref |
| MN369740 | Unassigned | Unassigned | Unassigned | 1217_0 | Clustered | 41 | VC_1217_0 | 41 | 0.9546 | 1 | 0.9546 | 0 | 0 | 0 | 0.9976 | Ref |
| MN369741 | Unassigned | Unassigned | Unassigned | 232_0 | Clustered | 32 | VC_232_0 | 32 | 0.8471 | 1 | 0.8471 | 0 | 0 | 0 | 0.996 | Ref |
| MN369742 | Unassigned | Unassigned | Unassigned | 243_0 | Clustered | 72 | VC_243_0 | 72 | 0.8089 | 1 | 0.8089 | 0 | 0 | 0 | 1 | Ref |
| MN369743 | Unassigned | Unassigned | Unassigned | 1114_3 | Clustered | 16 | VC_1114_3 | 16 | 0.2336 | 1 | 0.2336 | 0 | 0 | 0 | 0.9667 | Ref |
| MN369744 | Unassigned | Unassigned | Unassigned | 384_11 | Clustered | 31 | VC_384_11 | 31 | 0.0926 | 1 | 0.0926 | 0 | 0 | 0 | 0.7011 | Ref |
| MN369745 | Unassigned | Unassigned | Unassigned | 1407_0 | Clustered | 78 | VC_1407_0 | 78 | 0.9731 | 1 | 0.9731 | 0 | 0 | 0 | 1 | Ref |
| MN369746 | Unassigned | Unassigned | Unassigned | 243_0 | Clustered | 72 | VC_243_0 | 72 | 0.8089 | 1 | 0.8089 | 0 | 0 | 0 | 1 | Ref |
| MN369747 | Unassigned | Unassigned | Unassigned | 1424_0 | Clustered | 31 | VC_1424_0 | 31 | 0.8834 | 1 | 0.8834 | 0 | 0 | 0 | 1 | Ref |
| MN369748 | Unassigned | Unassigned | Unassigned | 223_0 | Clustered | 4 | VC_223_0 | 4 | 0.0478 | 0.9999878 | 0.0478 | 0 | 0 | 0 | 1 | Ref |
| MN369749 | Unassigned | Unassigned | Unassigned | 538_19 | Clustered | 2 | VC_538_19 | 2 | 0.0097 | 0.95761552 | 0.0093 | 0 | 0 | 0 | 1 | Ref |
| MN369750 | Unassigned | Unassigned | Unassigned | 1114_0 | Clustered | 20 | VC_1114_0 | 20 | 0.3238 | 1 | 0.3238 | 0 | 0 | 0 | 0.9842 | Ref |
| MN369751 | Unassigned | Unassigned | Unassigned | 538_85 | Clustered | 2 | VC_538_85 | 2 | 0.0146 | 0.95762405 | 0.014 | 0 | 0 | 0 | 1 | Ref |
| MN369752 | Unassigned | Unassigned | Unassigned | 538_10 | Clustered/Singleton | 1 | VC_538_10 | 1 | 0 | 1 | 0 | 0 | 0 | 0 | 1 | Ref |
| MN369753 | Unassigned | Unassigned | Unassigned | 789_0 | Clustered | 34 | VC_789_0 | 34 | 0.9167 | 1 | 0.9167 | 0 | 0 | 0 | 1 | Ref |
| MN369754 | Unassigned | Unassigned | Unassigned | 1115_4 | Clustered | 4 | VC_1115_4 | 4 | 0.0733 | 0.99956559 | 0.0732 | 0 | 0 | 0 | 1 | Ref |
| MN369755 | Unassigned | Unassigned | Unassigned | 538_33 | Clustered | 2 | VC_538_33 | 2 | 0.0087 | 0.95761754 | 0.0083 | 0 | 0 | 0 | 1 | Ref |
| MN369756 | Unassigned | Unassigned | Unassigned | 231_0 | Clustered | 19 | VC_231_0 | 19 | 0.9378 | 1 | 0.9378 | 0 | 0 | 0 | 1 | Ref |
| MN369757 | Unassigned | Unassigned | Unassigned | 1114_0 | Clustered | 20 | VC_1114_0 | 20 | 0.3238 | 1 | 0.3238 | 0 | 0 | 0 | 0.9842 | Ref |
| MN369758 | Unassigned | Unassigned | Unassigned | 528_0 | Clustered | 12 | VC_528_0 | 12 | 0.3923 | 1 | 0.3923 | 0 | 0 | 0 | 0.9697 | Ref |
| MN369759 | Unassigned | Unassigned | Unassigned | 385_0 | Clustered | 24 | VC_385_0 | 24 | 0.5516 | 1 | 0.5516 | 0 | 0 | 0 | 1 | Ref |
| MN369760 | Unassigned | Unassigned | Unassigned | 335_50 | Clustered | 2 | VC_335_50 | 2 | 0.0043 | 0.95751115 | 0.0041 | 0 | 0 | 0 | 1 | Ref |
| MN369761 | Unassigned | Unassigned | Unassigned | 241_0 | Clustered | 21 | VC_241_0 | 21 | 0.8342 | 1 | 0.8342 | 0 | 0 | 0 | 1 | Ref |
| MN369762 | Unassigned | Unassigned | Unassigned | 244_2 | Clustered | 11 | VC_244_2 | 11 | 0.1403 | 1 | 0.1403 | 0 | 0 | 0 | 0.9818 | Ref |
| MN369763 | Unassigned | Unassigned | Unassigned | 232_0 | Clustered | 32 | VC_232_0 | 32 | 0.8471 | 1 | 0.8471 | 0 | 0 | 0 | 0.996 | Ref |
| MN369764 | Unassigned | Unassigned | Unassigned | 232_0 | Clustered | 32 | VC_232_0 | 32 | 0.8471 | 1 | 0.8471 | 0 | 0 | 0 | 0.996 | Ref |
| MN369765 | Unassigned | Unassigned | Unassigned | 1202_0 | Clustered | 10 | VC_1202_0 | 10 | 0.5679 | 1 | 0.5679 | 0 | 0 | 0 | 1 | Ref |
| MN369766 | Unassigned | Unassigned | Unassigned | 789_0 | Clustered | 34 | VC_789_0 | 34 | 0.9167 | 1 | 0.9167 | 0 | 0 | 0 | 1 | Ref |
| MN379460 | Unassigned | Unassigned | Unassigned | 123_0 | Clustered | 4 | VC_123_0 | 4 | 0.1802 | 0.99998531 | 0.1802 | 0 | 0 | 0 | 1 | Ref |
| MN379629 | Unassigned | Unassigned | Unassigned | 1473_0 | Clustered | 2 | VC_1473_0 | 2 | 1 | 1 | 1 | 0 | 0 | 0 | 1 | Ref |

| | | | | | | | | | | | | | | | | |
|---|---|---|---|---|---|---|---|---|---|---|---|---|---|---|---|---|
| MN379630 | Unassigned | Unassigned | Unassigned | 1165_0 | Clustered | 10 | VC_1165_0 | 10 | 0.9263 | 1 | 0.9263 | 0 | 0 | 0 | 1 | Ref |
| MN379632 | Unassigned | Unassigned | Unassigned | 1123_0 | Clustered/Singleton | 1 | VC_1123_0 | 1 | 0 | 1 | 0 | 0 | 0 | 0 | 1 | Ref |
| MN379633 | Unassigned | Unassigned | Unassigned | 1095_0 | Clustered | 12 | VC_1095_0 | 12 | 0.2819 | 0.99837092 | 0.2814 | 0 | 0 | 0 | 1 | Ref |
| MN379636 | Unassigned | Unassigned | Unassigned | 1473_0 | Clustered | 2 | VC_1473_0 | 2 | 1 | 1 | 1 | 0 | 0 | 0 | 1 | Ref |
| MN379637 | Unassigned | Unassigned | Unassigned | 1286_0 | Clustered | 16 | VC_1286_0 | 16 | 0.9448 | 1 | 0.9448 | 0 | 0 | 0 | 1 | Ref |
| MN379638 | Unassigned | Unassigned | Unassigned | 1335_0 | Clustered | 13 | VC_1335_0 | 13 | 0.7519 | 1 | 0.7519 | 0 | 0 | 0 | 1 | Ref |
| MN379639 | Unassigned | Unassigned | Unassigned | 1474_0 | Clustered | 2 | VC_1474_0 | 2 | 1 | 1 | 1 | 0 | 0 | 0 | 1 | Ref |
| MN379640 | Unassigned | Unassigned | Unassigned | 1165_0 | Clustered | 10 | VC_1165_0 | 10 | 0.9263 | 1 | 0.9263 | 0 | 0 | 0 | 1 | Ref |
| MN379641 | Unassigned | Unassigned | Unassigned | 1193_0 | Clustered | 2 | VC_1193_0 | 2 | 0.0731 | 0.80562642 | 0.0589 | 0 | 0 | 0 | 1 | Ref |
| MN379643 | Unassigned | Unassigned | Unassigned | 1475_0 | Clustered | 2 | VC_1475_0 | 2 | 1 | 1 | 1 | 0 | 0 | 0 | 1 | Ref |
| MN379645 | Unassigned | Unassigned | Unassigned | 1333_0 | Clustered | 4 | VC_1333_0 | 4 | 1 | 1 | 1 | 0 | 0 | 0 | 1 | Ref |
| MN379646 | Unassigned | Unassigned | Unassigned | 1474_0 | Clustered | 2 | VC_1474_0 | 2 | 1 | 1 | 1 | 0 | 0 | 0 | 1 | Ref |
| MN379647 | Unassigned | Unassigned | Unassigned | 1286_0 | Clustered | 16 | VC_1286_0 | 16 | 0.9448 | 1 | 0.9448 | 0 | 0 | 0 | 1 | Ref |
| MN379650 | Unassigned | Unassigned | Unassigned | 1475_0 | Clustered | 2 | VC_1475_0 | 2 | 1 | 1 | 1 | 0 | 0 | 0 | 1 | Ref |
| MN379651 | Unassigned | Unassigned | Unassigned | 1095_0 | Clustered | 12 | VC_1095_0 | 12 | 0.2819 | 0.99837092 | 0.2814 | 0 | 0 | 0 | 1 | Ref |
| MN379739 | Unassigned | Unassigned | Unassigned | 199_16 | Clustered | 12 | VC_199_16 | 12 | 0.064 | 1 | 0.064 | 0 | 0 | 0 | 0.9394 | Ref |
| MN379740 | Unassigned | Unassigned | Unassigned | 440_0 | Clustered | 33 | VC_440_0 | 33 | 0.992 | 1 | 0.992 | 0 | 0 | 0 | 1 | Ref |
| MN379832 | Unassigned | Unassigned | Unassigned | 972_0 | Clustered | 99 | VC_972_0 | 99 | 0.9113 | 1 | 0.9113 | 0 | 0 | 0 | 0.9928 | Ref |
| MN380459 | Unassigned | Unassigned | Unassigned | 113_0 | Clustered | 91 | VC_113_0 | 91 | 0.9584 | 1 | 0.9584 | 0 | 0 | 0 | 1 | Ref |
| MN384978 | Unassigned | Unassigned | Unassigned | 466_0 | Clustered | 66 | VC_466_0 | 66 | 0.9246 | 1 | 0.9246 | 0 | 0 | 0 | 1 | Ref |
| MN384979 | Unassigned | Unassigned | Unassigned | 135_9 | Clustered | 3 | VC_135_9 | 3 | 0.0098 | 0.98303301 | 0.0097 | 0 | 0 | 0 | 1 | Ref |
| MN385565 | Unassigned | Unassigned | Unassigned | 310_0 | Clustered | 155 | VC_310_0 | 155 | 0.9979 | 1 | 0.9979 | 0 | 0 | 0 | 1 | Ref |
| MN393078 | Unassigned | Unassigned | Unassigned | 654_0 | Clustered | 115 | VC_654_0 | 115 | 0.7303 | 1 | 0.7303 | 0 | 0 | 0 | 0.8764 | Ref |
| MN393079 | Unassigned | Unassigned | Unassigned | 560_0 | Clustered | 15 | VC_560_0 | 15 | 0.2846 | 1 | 0.2846 | 0 | 0 | 0 | 1 | Ref |
| MN393473 | Unassigned | Unassigned | Unassigned | 595_0 | Clustered | 35 | VC_595_0 | 35 | 0.8809 | 1 | 0.8809 | 0 | 0 | 0 | 1 | Ref |
| MN395285 | Unassigned | Unassigned | Unassigned | 972_0 | Clustered | 99 | VC_972_0 | 99 | 0.9113 | 1 | 0.9113 | 0 | 0 | 0 | 0.9928 | Ref |
| MN395286 | Unassigned | Unassigned | Unassigned | 972_0 | Clustered | 99 | VC_972_0 | 99 | 0.9113 | 1 | 0.9113 | 0 | 0 | 0 | 0.9928 | Ref |
| MN395291 | Unassigned | Unassigned | Unassigned | 714_0 | Clustered | 71 | VC_714_0 | 71 | 0.935 | 1 | 0.935 | 0 | 0 | 0 | 1 | Ref |
| MN402506 | Unassigned | Unassigned | Unassigned | 513_1 | Clustered | 41 | VC_513_1 | 41 | 0.3276 | 1 | 0.3276 | 0 | 0 | 0 | 0.911 | Ref |
| MN414250 | Unassigned | Unassigned | Unassigned | 465_0 | Clustered | 80 | VC_465_0 | 80 | 0.9988 | 1 | 0.9988 | 0 | 0 | 0 | 1 | Ref |
| MN417334 | Unassigned | Unassigned | Unassigned | 1060_0 | Clustered | 4 | VC_1060_0 | 4 | 0.875 | 0.99993415 | 0.8749 | 0 | 0 | 0 | 1 | Ref |
| MN419153 | Unassigned | Unassigned | Unassigned | 513_0 | Clustered | 39 | VC_513_0 | 39 | 0.3078 | 1 | 0.3078 | 0 | 0 | 0 | 1 | Ref |
| MN428049 | Unassigned | Unassigned | Unassigned | 526_0 | Clustered | 32 | VC_526_0 | 32 | 0.9037 | 1 | 0.9037 | 0 | 0 | 0 | 1 | Ref |
| MN428050 | Unassigned | Unassigned | Unassigned | 385_0 | Clustered | 24 | VC_385_0 | 24 | 0.5516 | 1 | 0.5516 | 0 | 0 | 0 | 1 | Ref |
| MN428051 | Unassigned | Unassigned | Unassigned | 538_92 | Clustered | 5 | VC_538_92 | 5 | 0.0653 | 0.99999997 | 0.0653 | 0 | 0 | 0 | 1 | Ref |
| MN428052 | Unassigned | Unassigned | Unassigned | 577_0 | Clustered | 21 | VC_577_0 | 21 | 0.9908 | 1 | 0.9908 | 0 | 0 | 0 | 1 | Ref |
| MN428053 | Unassigned | Unassigned | Unassigned | 540_1 | Clustered | 5 | VC_540_1 | 5 | 0.1012 | 0.99999997 | 0.1012 | 0 | 0 | 0 | 0.8 | Ref |
| MN428054 | Unassigned | Unassigned | Unassigned | 335_49 | Clustered | 17 | VC_335_49 | 17 | 0.0731 | 1 | 0.0731 | 0 | 0 | 0 | 0.7721 | Ref |
| MN428056 | Unassigned | Unassigned | Unassigned | 973_0 | Clustered | 15 | VC_973_0 | 15 | 1 | 0 | 0 | 0 | 0 | 0 | 1 | Ref |
| MN428058 | Unassigned | Unassigned | Unassigned | 577_0 | Clustered | 21 | VC_577_0 | 21 | 0.9908 | 1 | 0.9908 | 0 | 0 | 0 | 1 | Ref |
| MN428059 | Unassigned | Unassigned | Unassigned | 571_6 | Clustered | 22 | VC_571_6 | 22 | 0.1207 | 1 | 0.1207 | 0 | 0 | 0 | 0.8095 | Ref |
| MN428060 | Unassigned | Unassigned | Unassigned | 1114_0 | Clustered | 20 | VC_1114_0 | 20 | 0.3238 | 1 | 0.3238 | 0 | 0 | 0 | 0.9842 | Ref |
| MN428061 | Unassigned | Unassigned | Unassigned | 973_0 | Clustered | 15 | VC_973_0 | 15 | 1 | 0 | 0 | 0 | 0 | 0 | 1 | Ref |
| MN428062 | Unassigned | Unassigned | Unassigned | 1407_0 | Clustered | 78 | VC_1407_0 | 78 | 0.9731 | 1 | 0.9731 | 0 | 0 | 0 | 1 | Ref |
| MN428063 | Unassigned | Unassigned | Unassigned | 789_0 | Clustered | 34 | VC_789_0 | 34 | 0.9167 | 1 | 0.9167 | 0 | 0 | 0 | 1 | Ref |
| MN428064 | Unassigned | Unassigned | Unassigned | 1407_0 | Clustered | 78 | VC_1407_0 | 78 | 0.9731 | 1 | 0.9731 | 0 | 0 | 0 | 1 | Ref |
| MN428065 | Unassigned | Unassigned | Unassigned | 579_38 | Clustered | 2 | VC_579_38 | 2 | 0.0032 | 0.6354382 | 0.002 | 0 | 0 | 0 | 1 | Ref |
| MN428066 | Unassigned | Unassigned | Unassigned | 579_20 | Clustered | 17 | VC_579_20 | 17 | 0.0538 | 1 | 0.0538 | 0 | 0 | 0 | 0.9926 | Ref |
| MN432485 | Unassigned | Unassigned | Unassigned | 975_0 | Clustered | 40 | VC_975_0 | 40 | 0.6888 | 1 | 0.6888 | 0 | 0 | 0 | 1 | Ref |
| MN433707 | Unassigned | Unassigned | Unassigned | 714_0 | Clustered | 71 | VC_714_0 | 71 | 0.935 | 1 | 0.935 | 0 | 0 | 0 | 1 | Ref |
| MN434093 | Unassigned | Unassigned | Unassigned | 848_0 | Clustered | 124 | VC_848_0 | 124 | 0.9484 | 1 | 0.9484 | 0 | 0 | 0 | 1 | Ref |
| MN434094 | Unassigned | Unassigned | Unassigned | 200_0 | Clustered | 15 | VC_200_0 | 15 | 0.5004 | 1 | 0.5004 | 0 | 0 | 0 | 1 | Ref |
| MN434095 | Unassigned | Unassigned | Unassigned | 509_0 | Clustered | 36 | VC_509_0 | 36 | 0.5291 | 1 | 0.5291 | 0 | 0 | 0 | 0.9714 | Ref |
| MN434096 | Unassigned | Unassigned | Unassigned | 200_0 | Clustered | 15 | VC_200_0 | 15 | 0.5004 | 1 | 0.5004 | 0 | 0 | 0 | 1 | Ref |
| MN444868 | Unassigned | Unassigned | Unassigned | 384_3 | Clustered | 22 | VC_384_3 | 22 | 0.0786 | 1 | 0.0786 | 0 | 0 | 0 | 0.9048 | Ref |
| MN444869 | Unassigned | Unassigned | Unassigned | 335_6 | Clustered/Singleton | 1 | VC_335_6 | 1 | 0 | 1 | 0 | 0 | 0 | 0 | 1 | Ref |
| MN444870 | Unassigned | Unassigned | Unassigned | 919_1 | Clustered | 4 | VC_919_1 | 4 | 0.4515 | 0.99998463 | 0.4515 | 0 | 0 | 0 | 1 | Ref |
| MN444871 | Unassigned | Unassigned | Unassigned | 952_0 | Clustered | 39 | VC_952_0 | 39 | 0.9064 | 1 | 0.9064 | 0 | 0 | 0 | 1 | Ref |
| MN444872 | Unassigned | Unassigned | Unassigned | 527_0 | Clustered | 37 | VC_527_0 | 37 | 0.9679 | 1 | 0.9679 | 0 | 0 | 0 | 1 | Ref |
| MN444873 | Unassigned | Unassigned | Unassigned | 527_0 | Clustered | 37 | VC_527_0 | 37 | 0.9679 | 1 | 0.9679 | 0 | 0 | 0 | 1 | Ref |
| MN444874 | Unassigned | Unassigned | Unassigned | 895_0 | Clustered | 25 | VC_895_0 | 25 | 0.5768 | 1 | 0.5768 | 0 | 0 | 0 | 1 | Ref |
| MN444875 | Unassigned | Unassigned | Unassigned | 783_0 | Clustered | 67 | VC_783_0 | 67 | 0.8945 | 1 | 0.8945 | 0 | 0 | 0 | 1 | Ref |
| MN444876 | Unassigned | Unassigned | Unassigned | 1114_4 | Clustered/Singleton | 1 | VC_1114_4 | 1 | 0 | 1 | 0 | 0 | 0 | 0 | 1 | Ref |
| MN444877 | Unassigned | Unassigned | Unassigned | 1415_0 | Clustered | 31 | VC_1415_0 | 31 | 0.8456 | 1 | 0.8456 | 0 | 0 | 0 | 1 | Ref |
| MN444878 | Unassigned | Unassigned | Unassigned | 952_0 | Clustered | 39 | VC_952_0 | 39 | 0.9064 | 1 | 0.9064 | 0 | 0 | 0 | 1 | Ref |
| MN445182 | Unassigned | Unassigned | Unassigned | 198_34 | Clustered/Singleton | 1 | VC_198_34 | 1 | 0 | 1 | 0 | 0 | 0 | 0 | 1 | Ref |
| MN445183 | Unassigned | Unassigned | Unassigned | 507_0 | Clustered | 3 | VC_507_0 | 3 | 0.0676 | 0.98259158 | 0.0664 | 0 | 0 | 0 | 1 | Ref |
| MN445184 | Unassigned | Unassigned | Unassigned | 135_42 | Clustered/Singleton | 1 | VC_135_42 | 1 | 0 | 1 | 0 | 0 | 0 | 0 | 1 | Ref |
| MN445185 | Unassigned | Unassigned | Unassigned | 994_5 | Clustered | 19 | VC_994_5 | 19 | 0.2622 | 1 | 0.2622 | 0 | 0 | 0 | 1 | Ref |
| MN450150 | Unassigned | Unassigned | Unassigned | 400_0 | Clustered | 3 | VC_400_0 | 3 | 0.0419 | 0.99861633 | 0.0418 | 0 | 0 | 0 | 1 | Ref |
| MN453779 | Unassigned | Unassigned | Unassigned | 456_3 | Clustered | 3 | VC_456_3 | 3 | 0.0523 | 0.99675181 | 0.0521 | 0 | 0 | 0 | 1 | Ref |
| MN461279 | Unassigned | Unassigned | Unassigned | 377_0 | Clustered | 16 | VC_377_0 | 16 | 0.6424 | 1 | 0.6424 | 0 | 0 | 0 | 1 | Ref |
| MN478375 | Unassigned | Unassigned | Unassigned | 75_0 | Clustered | 15 | VC_75_0 | 15 | 0.518 | 1 | 0.518 | 0 | 0 | 0 | 1 | Ref |
| MN478377 | Unassigned | Unassigned | Unassigned | 200_2 | Clustered/Singleton | 1 | VC_200_2 | 1 | 0 | 1 | 0 | 0 | 0 | 0 | 1 | Ref |
| MN478483 | Unassigned | Unassigned | Unassigned | 134_2 | Clustered | 17 | VC_134_2 | 17 | 0.1715 | 1 | 0.1715 | 0 | 0 | 0 | 0.9853 | Ref |
| MN481365 | Unassigned | Unassigned | Unassigned | 405_0 | Clustered | 126 | VC_405_0 | 126 | 0.9614 | 1 | 0.9614 | 0 | 0 | 0 | 1 | Ref |
| MN481366 | Unassigned | Unassigned | Unassigned | 405_0 | Clustered | 126 | VC_405_0 | 126 | 0.9614 | 1 | 0.9614 | 0 | 0 | 0 | 1 | Ref |
| MN481367 | Unassigned | Unassigned | Unassigned | 340_11 | Clustered | 15 | VC_340_11 | 15 | 0.079 | 1 | 0.079 | 0 | 0 | 0 | 0.9333 | Ref |
| MN484599 | Unassigned | Unassigned | Unassigned | 1114_0 | Clustered | 20 | VC_1114_0 | 20 | 0.3238 | 1 | 0.3238 | 0 | 0 | 0 | 0.9842 | Ref |
| MN484600 | Unassigned | Unassigned | Unassigned | 1207_0 | Clustered | 20 | VC_1207_0 | 20 | 0.9898 | 1 | 0.9898 | 0 | 0 | 0 | 1 | Ref |
| MN484601 | Unassigned | Unassigned | Unassigned | 1141_0 | Clustered | 4 | VC_1141_0 | 4 | 0.5416 | 0.99996462 | 0.5416 | 0 | 0 | 0 | 1 | Ref |

| | | | | | | | | | | | | | | | | |
|---|---|---|---|---|---|---|---|---|---|---|---|---|---|---|---|---|
| MN496305 | Unassigned | Unassigned | Unassigned | 618_0 | Clustered | 50 | VC_618_0 | 50 | 0.9989 | 1 | 0.9989 | 0 | 0 | 0 | 1 | Ref |
| MN496306 | Unassigned | Unassigned | Unassigned | 618_0 | Clustered | 50 | VC_618_0 | 50 | 0.9989 | 1 | 0.9989 | 0 | 0 | 0 | 1 | Ref |
| MN496307 | Unassigned | Unassigned | Unassigned | 618_0 | Clustered | 50 | VC_618_0 | 50 | 0.9989 | 1 | 0.9989 | 0 | 0 | 0 | 1 | Ref |
| MN496308 | Unassigned | Unassigned | Unassigned | 618_0 | Clustered | 50 | VC_618_0 | 50 | 0.9989 | 1 | 0.9989 | 0 | 0 | 0 | 1 | Ref |
| MN497953 | Unassigned | Unassigned | Unassigned | 532_0 | Clustered | 75 | VC_532_0 | 75 | 0.9393 | 1 | 0.9393 | 0 | 0 | 0 | 1 | Ref |
| MN497954 | Unassigned | Unassigned | Unassigned | 1406_0 | Clustered | 21 | VC_1406_0 | 21 | 0.9836 | 1 | 0.9836 | 0 | 0 | 0 | 1 | Ref |
| MN504636 | Unassigned | Unassigned | Unassigned | 479_0 | Clustered | 7 | VC_479_0 | 7 | 0.3294 | 1 | 0.3294 | 0 | 0 | 0 | 1 | Ref |
| MN508614 | Unassigned | Unassigned | Unassigned | 290_0 | Clustered | 2 | VC_290_0 | 2 | 0.0025 | 0.94831837 | 0.0023 | 0 | 0 | 0 | 1 | Ref |
| MN508615 | Unassigned | Unassigned | Unassigned | 837_1 | Clustered | 24 | VC_837_1 | 24 | 0.5328 | 1 | 0.5328 | 0 | 0 | 0 | 1 | Ref |
| MN508616 | Unassigned | Unassigned | Unassigned | 290_110 | Clustered | 2 | VC_290_110 | 2 | 0.0026 | 0.90858225 | 0.0024 | 0 | 0 | 0 | 1 | Ref |
| MN508617 | Unassigned | Unassigned | Unassigned | 290_110 | Clustered | 2 | VC_290_110 | 2 | 0.0026 | 0.90858225 | 0.0024 | 0 | 0 | 0 | 1 | Ref |
| MN508618 | Unassigned | Unassigned | Unassigned | 290_0 | Clustered | 2 | VC_290_0 | 2 | 0.0025 | 0.94831837 | 0.0023 | 0 | 0 | 0 | 1 | Ref |
| MN508619 | Unassigned | Unassigned | Unassigned | 486_0 | Clustered | 47 | VC_486_0 | 47 | 0.9022 | 1 | 0.9022 | 0 | 0 | 0 | 1 | Ref |
| MN508620 | Unassigned | Unassigned | Unassigned | 81_0 | Clustered | 41 | VC_81_0 | 41 | 0.5815 | 1 | 0.5815 | 0 | 0 | 0 | 0.9805 | Ref |
| MN508621 | Unassigned | Unassigned | Unassigned | 687_0 | Clustered | 21 | VC_687_0 | 21 | 0.3608 | 1 | 0.3608 | 0 | 0 | 0 | 1 | Ref |
| MN508622 | Unassigned | Unassigned | Unassigned | 687_0 | Clustered | 21 | VC_687_0 | 21 | 0.3608 | 1 | 0.3608 | 0 | 0 | 0 | 1 | Ref |
| MN508623 | Unassigned | Unassigned | Unassigned | 687_0 | Clustered | 21 | VC_687_0 | 21 | 0.3608 | 1 | 0.3608 | 0 | 0 | 0 | 1 | Ref |
| MN508624 | Unassigned | Unassigned | Unassigned | 687_0 | Clustered | 21 | VC_687_0 | 21 | 0.3608 | 1 | 0.3608 | 0 | 0 | 0 | 1 | Ref |
| MN508817 | Unassigned | Unassigned | Unassigned | 687_2 | Clustered/Singleton | 1 | VC_687_2 | 1 | 0 | 1 | 0 | 0 | 0 | 0 | 1 | Ref |
| MN509793 | Unassigned | Unassigned | Unassigned | 907_0 | Clustered | 32 | VC_907_0 | 32 | 0.9964 | 1 | 0.9964 | 0 | 0 | 0 | 1 | Ref |
| MN510331 | Unassigned | Unassigned | Unassigned | 465_0 | Clustered | 80 | VC_465_0 | 80 | 0.9988 | 1 | 0.9988 | 0 | 0 | 0 | 1 | Ref |
| MN512538 | Unassigned | Unassigned | Unassigned | 366_0 | Clustered | 4 | VC_366_0 | 4 | 0.1731 | 0.99998684 | 0.1731 | 0 | 0 | 0 | 0.8333 | Ref |
| MN516421 | Unassigned | Unassigned | Unassigned | 713_0 | Clustered | 26 | VC_713_0 | 26 | 0.9358 | 1 | 0.9358 | 0 | 0 | 0 | 1 | Ref |
| MN516422 | Unassigned | Unassigned | Unassigned | 713_0 | Clustered | 26 | VC_713_0 | 26 | 0.9358 | 1 | 0.9358 | 0 | 0 | 0 | 1 | Ref |
| MN518893 | Unassigned | Unassigned | Unassigned | 465_0 | Clustered | 80 | VC_465_0 | 80 | 0.9988 | 1 | 0.9988 | 0 | 0 | 0 | 1 | Ref |
| MN518894 | Unassigned | Unassigned | Unassigned | 465_0 | Clustered | 80 | VC_465_0 | 80 | 0.9988 | 1 | 0.9988 | 0 | 0 | 0 | 1 | Ref |
| MN528768 | Unassigned | Unassigned | Unassigned | 552_0 | Clustered | 9 | VC_552_0 | 9 | 1 | 1 | 1 | 0 | 0 | 0 | 1 | Ref |
| MN534316 | Unassigned | Unassigned | Unassigned | 266_0 | Clustered | 13 | VC_266_0 | 13 | 0.3622 | 1 | 0.3622 | 0 | 0 | 0 | 1 | Ref |
| MN534317 | Unassigned | Unassigned | Unassigned | 266_0 | Clustered | 13 | VC_266_0 | 13 | 0.3622 | 1 | 0.3622 | 0 | 0 | 0 | 1 | Ref |
| MN534320 | Unassigned | Unassigned | Unassigned | 268_0 | Clustered | 10 | VC_268_0 | 10 | 0.1417 | 1 | 0.1417 | 0 | 0 | 0 | 0.9333 | Ref |
| MN536023 | Unassigned | Unassigned | Unassigned | 16_0 | Clustered | 12 | VC_16_0 | 12 | 0.6601 | 1 | 0.6601 | 0 | 0 | 0 | 1 | Ref |
| MN536027 | Unassigned | Unassigned | Unassigned | 192_0 | Clustered | 22 | VC_192_0 | 22 | 0.6582 | 1 | 0.6582 | 0 | 0 | 0 | 1 | Ref |
| MN539736 | Unassigned | Unassigned | Unassigned | 155_8 | Clustered | 66 | VC_155_8 | 66 | 0.4537 | 1 | 0.4537 | 0 | 0 | 0 | 0.8182 | Ref |
| MN539737 | Unassigned | Unassigned | Unassigned | 155_8 | Clustered | 66 | VC_155_8 | 66 | 0.4537 | 1 | 0.4537 | 0 | 0 | 0 | 0.8182 | Ref |
| MN539738 | Unassigned | Unassigned | Unassigned | 155_8 | Clustered | 66 | VC_155_8 | 66 | 0.4537 | 1 | 0.4537 | 0 | 0 | 0 | 0.8182 | Ref |
| MN549360 | Unassigned | Unassigned | Unassigned | 1093_3 | Clustered | 5 | VC_1093_3 | 5 | 0.1846 | 0.98656422 | 0.1822 | 0 | 0 | 0 | 1 | Ref |
| MN549361 | Unassigned | Unassigned | Unassigned | 1093_3 | Clustered | 5 | VC_1093_3 | 5 | 0.1846 | 0.98656422 | 0.1822 | 0 | 0 | 0 | 1 | Ref |
| MN552143 | Unassigned | Unassigned | Unassigned | 824_0 | Clustered | 3 | VC_824_0 | 3 | 0.5034 | 0.9984288 | 0.5026 | 0 | 0 | 0 | 1 | Ref |
| MN552144 | Unassigned | Unassigned | Unassigned | 274_0 | Clustered | 7 | VC_274_0 | 7 | 0.3401 | 1 | 0.3401 | 0 | 0 | 0 | 1 | Ref |
| MN552145 | Unassigned | Unassigned | Unassigned | 488_0 | Clustered | 14 | VC_488_0 | 14 | 0.8516 | 1 | 0.8516 | 0 | 0 | 0 | 1 | Ref |
| MN552146 | Unassigned | Unassigned | Unassigned | 888_0 | Clustered | 9 | VC_888_0 | 9 | 0.5251 | 1 | 0.5251 | 0 | 0 | 0 | 1 | Ref |
| MN552147 | Unassigned | Unassigned | Unassigned | 889_0 | Clustered | 13 | VC_889_0 | 13 | 0.6844 | 1 | 0.6844 | 0 | 0 | 0 | 1 | Ref |
| MN552148 | Unassigned | Unassigned | Unassigned | 210_0 | Clustered | 105 | VC_210_0 | 105 | 0.4076 | 1 | 0.4076 | 0 | 0 | 0 | 0.7766 | Ref |
| MN552149 | Unassigned | Unassigned | Unassigned | 210_0 | Clustered | 105 | VC_210_0 | 105 | 0.4076 | 1 | 0.4076 | 0 | 0 | 0 | 0.7766 | Ref |
| MN552150 | Unassigned | Unassigned | Unassigned | 210_0 | Clustered | 105 | VC_210_0 | 105 | 0.4076 | 1 | 0.4076 | 0 | 0 | 0 | 0.7766 | Ref |
| MN553583 | Unassigned | Unassigned | Unassigned | 862_0 | Clustered | 29 | VC_862_0 | 29 | 0.8594 | 1 | 0.8594 | 0 | 0 | 0 | 1 | Ref |
| MN553584 | Unassigned | Unassigned | Unassigned | 862_0 | Clustered | 29 | VC_862_0 | 29 | 0.8594 | 1 | 0.8594 | 0 | 0 | 0 | 1 | Ref |
| MN553585 | Unassigned | Unassigned | Unassigned | 434_0 | Clustered | 45 | VC_434_0 | 45 | 0.9479 | 1 | 0.9479 | 0 | 0 | 0 | 1 | Ref |
| MN553586 | Unassigned | Unassigned | Unassigned | 486_0 | Clustered | 47 | VC_486_0 | 47 | 0.9022 | 1 | 0.9022 | 0 | 0 | 0 | 1 | Ref |
| MN553587 | Unassigned | Unassigned | Unassigned | 434_0 | Clustered | 45 | VC_434_0 | 45 | 0.9479 | 1 | 0.9479 | 0 | 0 | 0 | 1 | Ref |
| MN553588 | Unassigned | Unassigned | Unassigned | 434_0 | Clustered | 45 | VC_434_0 | 45 | 0.9479 | 1 | 0.9479 | 0 | 0 | 0 | 1 | Ref |
| MN553589 | Unassigned | Unassigned | Unassigned | 434_0 | Clustered | 45 | VC_434_0 | 45 | 0.9479 | 1 | 0.9479 | 0 | 0 | 0 | 1 | Ref |
| MN562749 | Unassigned | Unassigned | Unassigned | 486_0 | Clustered | 47 | VC_486_0 | 47 | 0.9022 | 1 | 0.9022 | 0 | 0 | 0 | 1 | Ref |
| MN563783 | Unassigned | Unassigned | Unassigned | 81_0 | Clustered | 41 | VC_81_0 | 41 | 0.5815 | 1 | 0.5815 | 0 | 0 | 0 | 0.9805 | Ref |
| MN563784 | Unassigned | Unassigned | Unassigned | 356_0 | Clustered | 21 | VC_356_0 | 21 | 0.5409 | 1 | 0.5409 | 0 | 0 | 0 | 1 | Ref |
| MN563785 | Unassigned | Unassigned | Unassigned | 77_0 | Clustered | 37 | VC_77_0 | 37 | 0.9392 | 1 | 0.9392 | 0 | 0 | 0 | 1 | Ref |
| MN563793 | Unassigned | Unassigned | Unassigned | 5_0 | Clustered | 27 | VC_5_0 | 27 | 0.9599 | 1 | 0.9599 | 0 | 0 | 0 | 1 | Ref |
| MN564818 | Unassigned | Unassigned | Unassigned | 81_0 | Clustered | 41 | VC_81_0 | 41 | 0.5815 | 1 | 0.5815 | 0 | 0 | 0 | 0.9805 | Ref |
| MN580668 | Unassigned | Unassigned | Unassigned | 290_179 | Clustered/Singleton | 1 | VC_290_179 | 1 | 0 | 1 | 0 | 0 | 0 | 0 | 1 | Ref |
| MN585130 | Unassigned | Unassigned | Unassigned | 848_0 | Clustered | 124 | VC_848_0 | 124 | 0.9484 | 1 | 0.9484 | 0 | 0 | 0 | 1 | Ref |
| MN585963 | Unassigned | Unassigned | Unassigned | 1217_0 | Clustered | 41 | VC_1217_0 | 41 | 0.9546 | 1 | 0.9546 | 0 | 0 | 0 | 0.9976 | Ref |
| MN585964 | Unassigned | Unassigned | Unassigned | 577_0 | Clustered | 21 | VC_577_0 | 21 | 0.9908 | 1 | 0.9908 | 0 | 0 | 0 | 1 | Ref |
| MN585965 | Unassigned | Unassigned | Unassigned | 384_13 | Clustered | 33 | VC_384_13 | 33 | 0.1785 | 1 | 0.1785 | 0 | 0 | 0 | 0.8769 | Ref |
| MN585966 | Unassigned | Unassigned | Unassigned | 540_1 | Clustered | 5 | VC_540_1 | 5 | 0.1012 | 0.99999997 | 0.1012 | 0 | 0 | 0 | 0.8 | Ref |
| MN585967 | Unassigned | Unassigned | Unassigned | 384_1 | Clustered | 11 | VC_384_1 | 11 | 0.0378 | 1 | 0.0378 | 0 | 0 | 0 | 0.8545 | Ref |
| MN585968 | Unassigned | Unassigned | Unassigned | 223_2 | Clustered | 2 | VC_223_2 | 2 | 0.0138 | 0.95744997 | 0.0132 | 0 | 0 | 0 | 1 | Ref |
| MN585969 | Unassigned | Unassigned | Unassigned | 532_0 | Clustered | 75 | VC_532_0 | 75 | 0.9393 | 1 | 0.9393 | 0 | 0 | 0 | 1 | Ref |
| MN585970 | Unassigned | Unassigned | Unassigned | 335_20 | Clustered/Singleton | 1 | VC_335_20 | 1 | 0 | 1 | 0 | 0 | 0 | 0 | 1 | Ref |
| MN585971 | Unassigned | Unassigned | Unassigned | 898_1 | Clustered | 27 | VC_898_1 | 27 | 0.8894 | 1 | 0.8894 | 0 | 0 | 0 | 1 | Ref |
| MN585972 | Unassigned | Unassigned | Unassigned | 1210_0 | Clustered | 7 | VC_1210_0 | 7 | 0.9808 | 0.99995963 | 0.9807 | 0 | 0 | 0 | 1 | Ref |
| MN585973 | Unassigned | Unassigned | Unassigned | 538_31 | Clustered | 11 | VC_538_31 | 11 | 0.0894 | 1 | 0.0894 | 0 | 0 | 0 | 0.6 | Ref |
| MN585974 | Unassigned | Unassigned | Unassigned | 232_0 | Clustered | 32 | VC_232_0 | 32 | 0.8471 | 1 | 0.8471 | 0 | 0 | 0 | 0.996 | Ref |
| MN585975 | Unassigned | Unassigned | Unassigned | 538_89 | Clustered/Singleton | 1 | VC_538_89 | 1 | 0 | 1 | 0 | 0 | 0 | 0 | 1 | Ref |
| MN585976 | Unassigned | Unassigned | Unassigned | 335_24 | Clustered | 3 | VC_335_24 | 3 | 0.009 | 0.99853949 | 0.0089 | 0 | 0 | 0 | 1 | Ref |
| MN585977 | Unassigned | Unassigned | Unassigned | 537_0 | Clustered | 39 | VC_537_0 | 39 | 0.8148 | 1 | 0.8148 | 0 | 0 | 0 | 1 | Ref |
| MN585978 | Unassigned | Unassigned | Unassigned | 232_0 | Clustered | 32 | VC_232_0 | 32 | 0.8471 | 1 | 0.8471 | 0 | 0 | 0 | 0.996 | Ref |
| MN585979 | Unassigned | Unassigned | Unassigned | 232_0 | Clustered | 32 | VC_232_0 | 32 | 0.8471 | 1 | 0.8471 | 0 | 0 | 0 | 0.996 | Ref |
| MN585980 | Unassigned | Unassigned | Unassigned | 1415_0 | Clustered | 31 | VC_1415_0 | 31 | 0.8456 | 1 | 0.8456 | 0 | 0 | 0 | 1 | Ref |
| MN585981 | Unassigned | Unassigned | Unassigned | 223_6 | Clustered | 42 | VC_223_6 | 42 | 0.4717 | 1 | 0.4717 | 0 | 0 | 0 | 0.8351 | Ref |
| MN585982 | Unassigned | Unassigned | Unassigned | 898_1 | Clustered | 27 | VC_898_1 | 27 | 0.8894 | 1 | 0.8894 | 0 | 0 | 0 | 1 | Ref |
| MN585983 | Unassigned | Unassigned | Unassigned | 223_6 | Clustered | 42 | VC_223_6 | 42 | 0.4717 | 1 | 0.4717 | 0 | 0 | 0 | 0.8351 | Ref |

| | | | | | | | | | | | | | | | | |
|---|---|---|---|---|---|---|---|---|---|---|---|---|---|---|---|---|
| MN585984 | Unassigned | Unassigned | Unassigned | 532_0 | Clustered | 75 | VC_532_0 | 75 | 0.9393 | 1 | 0.9393 | 0 | 0 | 0 | 1 | Ref |
| MN585985 | Unassigned | Unassigned | Unassigned | 335_51 | Clustered | 3 | VC_335_51 | 3 | 0.008 | 0.99634936 | 0.008 | 0 | 0 | 0 | 0.6667 | Ref |
| MN585986 | Unassigned | Unassigned | Unassigned | 1415_0 | Clustered | 31 | VC_1415_0 | 31 | 0.8456 | 1 | 0.8456 | 0 | 0 | 0 | 1 | Ref |
| MN585987 | Unassigned | Unassigned | Unassigned | 538_59 | Clustered | 5 | VC_538_59 | 5 | 0.0456 | 0.99999997 | 0.0456 | 0 | 0 | 0 | 1 | Ref |
| MN585988 | Unassigned | Unassigned | Unassigned | 898_1 | Clustered | 27 | VC_898_1 | 27 | 0.8894 | 1 | 0.8894 | 0 | 0 | 0 | 1 | Ref |
| MN585989 | Unassigned | Unassigned | Unassigned | 222_0 | Clustered | 5 | VC_222_0 | 5 | 0.072 | 0.99999997 | 0.072 | 0 | 0 | 0 | 1 | Ref |
| MN585990 | Unassigned | Unassigned | Unassigned | 1407_0 | Clustered | 78 | VC_1407_0 | 78 | 0.9731 | 1 | 0.9731 | 0 | 0 | 0 | 1 | Ref |
| MN585991 | Unassigned | Unassigned | Unassigned | 335_53 | Clustered | 2 | VC_335_53 | 2 | 0.0039 | 0.95663826 | 0.0037 | 0 | 0 | 0 | 1 | Ref |
| MN585992 | Unassigned | Unassigned | Unassigned | 786_0 | Clustered | 4 | VC_786_0 | 4 | 0.3519 | 0.99997469 | 0.3519 | 0 | 0 | 0 | 1 | Ref |
| MN585993 | Unassigned | Unassigned | Unassigned | 591_0 | Clustered | 4 | VC_591_0 | 4 | 0.1897 | 0.9999859 | 0.1897 | 0 | 0 | 0 | 1 | Ref |
| MN585994 | Unassigned | Unassigned | Unassigned | 232_0 | Clustered | 32 | VC_232_0 | 32 | 0.8471 | 1 | 0.8471 | 0 | 0 | 0 | 0.996 | Ref |
| MN585995 | Unassigned | Unassigned | Unassigned | 538_11 | Clustered/Singleton | 1 | VC_538_11 | 1 | 0 | 1 | 0 | 0 | 0 | 0 | 1 | Ref |
| MN585996 | Unassigned | Unassigned | Unassigned | 898_1 | Clustered | 27 | VC_898_1 | 27 | 0.8894 | 1 | 0.8894 | 0 | 0 | 0 | 1 | Ref |
| MN585997 | Unassigned | Unassigned | Unassigned | 335_31 | Clustered | 2 | VC_335_31 | 2 | 0.0036 | 0.94604798 | 0.0034 | 0 | 0 | 0 | 0 | Ref |
| MN585998 | Unassigned | Unassigned | Unassigned | 223_6 | Clustered | 42 | VC_223_6 | 42 | 0.4717 | 1 | 0.4717 | 0 | 0 | 0 | 0.8351 | Ref |
| MN585999 | Unassigned | Unassigned | Unassigned | 335_5 | Clustered | 11 | VC_335_5 | 11 | 0.0469 | 1 | 0.0469 | 0 | 0 | 0 | 0.7636 | Ref |
| MN586000 | Unassigned | Unassigned | Unassigned | 335_49 | Clustered | 17 | VC_335_49 | 17 | 0.0731 | 1 | 0.0731 | 0 | 0 | 0 | 0.7721 | Ref |
| MN586001 | Unassigned | Unassigned | Unassigned | 538_18 | Clustered/Singleton | 1 | VC_538_18 | 1 | 0 | 1 | 0 | 0 | 0 | 0 | 1 | Ref |
| MN586002 | Unassigned | Unassigned | Unassigned | 223_2 | Clustered | 2 | VC_223_2 | 2 | 0.0138 | 0.95744997 | 0.0132 | 0 | 0 | 0 | 1 | Ref |
| MN586003 | Unassigned | Unassigned | Unassigned | 592_0 | Clustered | 16 | VC_592_0 | 16 | 0.7259 | 1 | 0.7259 | 0 | 0 | 0 | 1 | Ref |
| MN586004 | Unassigned | Unassigned | Unassigned | 538_6 | Clustered | 3 | VC_538_6 | 3 | 0.0273 | 0.99859866 | 0.0272 | 0 | 0 | 0 | 1 | Ref |
| MN586005 | Unassigned | Unassigned | Unassigned | 538_59 | Clustered | 5 | VC_538_59 | 5 | 0.0456 | 0.99999997 | 0.0456 | 0 | 0 | 0 | 1 | Ref |
| MN586006 | Unassigned | Unassigned | Unassigned | 228_0 | Clustered | 22 | VC_228_0 | 22 | 0.7411 | 1 | 0.7411 | 0 | 0 | 0 | 1 | Ref |
| MN586007 | Unassigned | Unassigned | Unassigned | 1415_0 | Clustered | 31 | VC_1415_0 | 31 | 0.8456 | 1 | 0.8456 | 0 | 0 | 0 | 1 | Ref |
| MN586008 | Unassigned | Unassigned | Unassigned | 1407_0 | Clustered | 78 | VC_1407_0 | 78 | 0.9731 | 1 | 0.9731 | 0 | 0 | 0 | 1 | Ref |
| MN586009 | Unassigned | Unassigned | Unassigned | 1016_0 | Clustered | 62 | VC_1016_0 | 62 | 0.8638 | 1 | 0.8638 | 0 | 0 | 0 | 0.743 | Ref |
| MN586010 | Unassigned | Unassigned | Unassigned | 1407_0 | Clustered | 78 | VC_1407_0 | 78 | 0.9731 | 1 | 0.9731 | 0 | 0 | 0 | 1 | Ref |
| MN586011 | Unassigned | Unassigned | Unassigned | 538_41 | Clustered | 2 | VC_538_41 | 2 | 0.0138 | 0.95761457 | 0.0132 | 0 | 0 | 0 | 1 | Ref |
| MN586012 | Unassigned | Unassigned | Unassigned | 538_66 | Clustered/Singleton | 1 | VC_538_66 | 1 | 0 | 1 | 0 | 0 | 0 | 0 | 1 | Ref |
| MN586013 | Unassigned | Unassigned | Unassigned | 571_0 | Clustered | 16 | VC_571_0 | 16 | 0.091 | 1 | 0.091 | 0 | 0 | 0 | 0.6667 | Ref |
| MN586014 | Unassigned | Unassigned | Unassigned | 1225_0 | Clustered | 6 | VC_1225_0 | 6 | 0.9947 | 0.99996974 | 0.9947 | 0 | 0 | 0 | 1 | Ref |
| MN586015 | Unassigned | Unassigned | Unassigned | 952_0 | Clustered | 39 | VC_952_0 | 39 | 0.9064 | 1 | 0.9064 | 0 | 0 | 0 | 1 | Ref |
| MN586016 | Unassigned | Unassigned | Unassigned | 974_0 | Clustered | 34 | VC_974_0 | 34 | 0.7953 | 1 | 0.7953 | 0 | 0 | 0 | 1 | Ref |
| MN586017 | Unassigned | Unassigned | Unassigned | 571_7 | Clustered | 28 | VC_571_7 | 28 | 0.1651 | 1 | 0.1651 | 0 | 0 | 0 | 0.8519 | Ref |
| MN586019 | Unassigned | Unassigned | Unassigned | 571_7 | Clustered | 28 | VC_571_7 | 28 | 0.1651 | 1 | 0.1651 | 0 | 0 | 0 | 0.8519 | Ref |
| MN586021 | Unassigned | Unassigned | Unassigned | 1211_0 | Clustered | 3 | VC_1211_0 | 3 | 0.3232 | 0.99834213 | 0.3227 | 0 | 0 | 0 | 1 | Ref |
| MN586022 | Unassigned | Unassigned | Unassigned | 1139_0 | Clustered | 5 | VC_1139_0 | 5 | 0.8556 | 0.99869977 | 0.8545 | 0 | 0 | 0 | 1 | Ref |
| MN586024 | Unassigned | Unassigned | Unassigned | 785_1 | Clustered | 9 | VC_785_1 | 9 | 0.3679 | 1 | 0.3679 | 0 | 0 | 0 | 1 | Ref |
| MN586026 | Unassigned | Unassigned | Unassigned | 1138_0 | Clustered | 16 | VC_1138_0 | 16 | 0.4566 | 1 | 0.4566 | 0 | 0 | 0 | 1 | Ref |
| MN586027 | Unassigned | Unassigned | Unassigned | 1426_0 | Clustered | 2 | VC_1426_0 | 2 | 0.8068 | 0.92263554 | 0.7444 | 0 | 0 | 0 | 1 | Ref |
| MN586028 | Unassigned | Unassigned | Unassigned | 1138_0 | Clustered | 16 | VC_1138_0 | 16 | 0.4566 | 1 | 0.4566 | 0 | 0 | 0 | 1 | Ref |
| MN586029 | Unassigned | Unassigned | Unassigned | 566_1 | Clustered | 2 | VC_566_1 | 2 | 0.0136 | 0.68880023 | 0.0093 | 0 | 0 | 0 | 1 | Ref |
| MN586030 | Unassigned | Unassigned | Unassigned | 917_0 | Clustered | 28 | VC_917_0 | 28 | 0.5465 | 1 | 0.5465 | 0 | 0 | 0 | 1 | Ref |
| MN586031 | Unassigned | Unassigned | Unassigned | 1217_0 | Clustered | 41 | VC_1217_0 | 41 | 0.9546 | 1 | 0.9546 | 0 | 0 | 0 | 0.9976 | Ref |
| MN586032 | Unassigned | Unassigned | Unassigned | 571_2 | Clustered | 17 | VC_571_2 | 17 | 0.0965 | 1 | 0.0965 | 0 | 0 | 0 | 0.9779 | Ref |
| MN586034 | Unassigned | Unassigned | Unassigned | 571_6 | Clustered | 22 | VC_571_6 | 22 | 0.1207 | 1 | 0.1207 | 0 | 0 | 0 | 0.8095 | Ref |
| MN586035 | Unassigned | Unassigned | Unassigned | 571_7 | Clustered | 28 | VC_571_7 | 28 | 0.1651 | 1 | 0.1651 | 0 | 0 | 0 | 0.8519 | Ref |
| MN586036 | Unassigned | Unassigned | Unassigned | 952_0 | Clustered | 39 | VC_952_0 | 39 | 0.9064 | 1 | 0.9064 | 0 | 0 | 0 | 1 | Ref |
| MN586037 | Unassigned | Unassigned | Unassigned | 571_6 | Clustered | 22 | VC_571_6 | 22 | 0.1207 | 1 | 0.1207 | 0 | 0 | 0 | 0.8095 | Ref |
| MN586038 | Unassigned | Unassigned | Unassigned | 533_0 | Clustered | 16 | VC_533_0 | 16 | 0.3765 | 1 | 0.3765 | 0 | 0 | 0 | 1 | Ref |
| MN586039 | Unassigned | Unassigned | Unassigned | 230_0 | Clustered | 38 | VC_230_0 | 38 | 0.9076 | 1 | 0.9076 | 0 | 0 | 0 | 1 | Ref |
| MN586041 | Unassigned | Unassigned | Unassigned | 571_7 | Clustered | 28 | VC_571_7 | 28 | 0.1651 | 1 | 0.1651 | 0 | 0 | 0 | 0.8519 | Ref |
| MN586042 | Unassigned | Unassigned | Unassigned | 1415_0 | Clustered | 31 | VC_1415_0 | 31 | 0.8456 | 1 | 0.8456 | 0 | 0 | 0 | 1 | Ref |
| MN586043 | Unassigned | Unassigned | Unassigned | 571_1 | Clustered | 4 | VC_571_1 | 4 | 0.0158 | 0.99966394 | 0.0158 | 0 | 0 | 0 | 0.6667 | Ref |
| MN586044 | Unassigned | Unassigned | Unassigned | 571_1 | Clustered | 4 | VC_571_1 | 4 | 0.0158 | 0.99966394 | 0.0158 | 0 | 0 | 0 | 0.6667 | Ref |
| MN586045 | Unassigned | Unassigned | Unassigned | 526_0 | Clustered | 32 | VC_526_0 | 32 | 0.9037 | 1 | 0.9037 | 0 | 0 | 0 | 1 | Ref |
| MN586046 | Unassigned | Unassigned | Unassigned | 526_0 | Clustered | 32 | VC_526_0 | 32 | 0.9037 | 1 | 0.9037 | 0 | 0 | 0 | 1 | Ref |
| MN586047 | Unassigned | Unassigned | Unassigned | 1138_0 | Clustered | 16 | VC_1138_0 | 16 | 0.4566 | 1 | 0.4566 | 0 | 0 | 0 | 1 | Ref |
| MN586048 | Unassigned | Unassigned | Unassigned | 532_0 | Clustered | 75 | VC_532_0 | 75 | 0.9393 | 1 | 0.9393 | 0 | 0 | 0 | 1 | Ref |
| MN586049 | Unassigned | Unassigned | Unassigned | 952_0 | Clustered | 39 | VC_952_0 | 39 | 0.9064 | 1 | 0.9064 | 0 | 0 | 0 | 1 | Ref |
| MN586050 | Unassigned | Unassigned | Unassigned | 571_7 | Clustered | 28 | VC_571_7 | 28 | 0.1651 | 1 | 0.1651 | 0 | 0 | 0 | 0.8519 | Ref |
| MN586051 | Unassigned | Unassigned | Unassigned | 571_2 | Clustered | 17 | VC_571_2 | 17 | 0.0965 | 1 | 0.0965 | 0 | 0 | 0 | 0.9779 | Ref |
| MN586052 | Unassigned | Unassigned | Unassigned | 917_0 | Clustered | 28 | VC_917_0 | 28 | 0.5465 | 1 | 0.5465 | 0 | 0 | 0 | 1 | Ref |
| MN586053 | Unassigned | Unassigned | Unassigned | 1221_0 | Clustered | 3 | VC_1221_0 | 3 | 0.7566 | 0.96819872 | 0.7326 | 0 | 0 | 0 | 1 | Ref |
| MN586054 | Unassigned | Unassigned | Unassigned | 901_0 | Clustered | 31 | VC_901_0 | 31 | 0.8235 | 1 | 0.8235 | 0 | 0 | 0 | 0.8194 | Ref |
| MN586055 | Unassigned | Unassigned | Unassigned | 1216_0 | Clustered | 22 | VC_1216_0 | 22 | 0.4546 | 1 | 0.4546 | 0 | 0 | 0 | 1 | Ref |
| MN586056 | Unassigned | Unassigned | Unassigned | 901_0 | Clustered | 31 | VC_901_0 | 31 | 0.8235 | 1 | 0.8235 | 0 | 0 | 0 | 0.8194 | Ref |
| MN586057 | Unassigned | Unassigned | Unassigned | 952_0 | Clustered | 39 | VC_952_0 | 39 | 0.9064 | 1 | 0.9064 | 0 | 0 | 0 | 1 | Ref |
| MN586058 | Unassigned | Unassigned | Unassigned | 384_1 | Clustered | 11 | VC_384_1 | 11 | 0.0378 | 1 | 0.0378 | 0 | 0 | 0 | 0.8545 | Ref |
| MN586059 | Unassigned | Unassigned | Unassigned | 1415_0 | Clustered | 31 | VC_1415_0 | 31 | 0.8456 | 1 | 0.8456 | 0 | 0 | 0 | 1 | Ref |
| MN586060 | Unassigned | Unassigned | Unassigned | 974_0 | Clustered | 34 | VC_974_0 | 34 | 0.7953 | 1 | 0.7953 | 0 | 0 | 0 | 1 | Ref |
| MN586061 | Unassigned | Unassigned | Unassigned | 1200_0 | Clustered | 73 | VC_1200_0 | 73 | 0.9825 | 1 | 0.9825 | 0 | 0 | 0 | 1 | Ref |
| MN586062 | Unassigned | Unassigned | Unassigned | 974_0 | Clustered | 34 | VC_974_0 | 34 | 0.7953 | 1 | 0.7953 | 0 | 0 | 0 | 1 | Ref |
| MN586063 | Unassigned | Unassigned | Unassigned | 531_0 | Clustered | 32 | VC_531_0 | 32 | 0.6859 | 1 | 0.6859 | 0 | 0 | 0 | 1 | Ref |
| MN592896 | Unassigned | Unassigned | Unassigned | 958_0 | Clustered | 3 | VC_958_0 | 3 | 0.2752 | 0.95138727 | 0.2618 | 0 | 0 | 0 | 1 | Ref |
| MN592897 | Unassigned | Unassigned | Unassigned | 303_0 | Clustered | 6 | VC_303_0 | 6 | 0.5341 | 1 | 0.5341 | 0 | 0 | 0 | 1 | Ref |
| MN602045 | Unassigned | Unassigned | Unassigned | 434_0 | Clustered | 45 | VC_434_0 | 45 | 0.9479 | 1 | 0.9479 | 0 | 0 | 0 | 1 | Ref |
| MN602266 | Unassigned | Unassigned | Unassigned | 970_0 | Clustered | 2 | VC_970_0 | 2 | 0.9266 | 0.81445332 | 0.7546 | 0 | 0 | 0 | 1 | Ref |
| MN602267 | Unassigned | Unassigned | Unassigned | 970_0 | Clustered | 2 | VC_970_0 | 2 | 0.9266 | 0.81445332 | 0.7546 | 0 | 0 | 0 | 1 | Ref |
| MN602268 | Unassigned | Unassigned | Unassigned | 1012_1 | Clustered | 2 | VC_1012_1 | 2 | 0.0779 | 0.93977186 | 0.0732 | 0 | 0 | 0 | 1 | Ref |
| MN604053 | Unassigned | Unassigned | Unassigned | 610_0 | Clustered | 14 | VC_610_0 | 14 | 0.5159 | 1 | 0.5159 | 0 | 0 | 0 | 1 | Ref |

| | | | | | | | | | | | | | | | | |
|---|---|---|---|---|---|---|---|---|---|---|---|---|---|---|---|---|
| MN604230 | Unassigned | Unassigned | Unassigned | 148_0 | Clustered | 2 | VC_148_0 | 2 | 0.3861 | 0.94836333 | 0.3662 | 0 | 0 | 0 | 1 | Ref |
| MN604238 | Unassigned | Unassigned | Unassigned | 714_0 | Clustered | 71 | VC_714_0 | 71 | 0.935 | 1 | 0.935 | 0 | 0 | 0 | 1 | Ref |
| MN604239 | Unassigned | Unassigned | Unassigned | 714_0 | Clustered | 71 | VC_714_0 | 71 | 0.935 | 1 | 0.935 | 0 | 0 | 0 | 1 | Ref |
| MN604407 | Unassigned | Unassigned | Unassigned | 339_0 | Clustered | 11 | VC_339_0 | 11 | 0.4647 | 1 | 0.4647 | 0 | 0 | 0 | 1 | Ref |
| MN604698 | Unassigned | Unassigned | Unassigned | 148_0 | Clustered | 2 | VC_148_0 | 2 | 0.3861 | 0.94836333 | 0.3662 | 0 | 0 | 0 | 1 | Ref |
| MN614471 | Unassigned | Unassigned | Unassigned | 714_0 | Clustered | 71 | VC_714_0 | 71 | 0.935 | 1 | 0.935 | 0 | 0 | 0 | 1 | Ref |
| MN615698 | Unassigned | Unassigned | Unassigned | 434_0 | Clustered | 45 | VC_434_0 | 45 | 0.9479 | 1 | 0.9479 | 0 | 0 | 0 | 1 | Ref |
| MN615699 | Unassigned | Unassigned | Unassigned | 434_0 | Clustered | 45 | VC_434_0 | 45 | 0.9479 | 1 | 0.9479 | 0 | 0 | 0 | 1 | Ref |
| MN615700 | Unassigned | Unassigned | Unassigned | 80_7 | Clustered | 33 | VC_80_7 | 33 | 0.2156 | 1 | 0.2156 | 0 | 0 | 0 | 0.8409 | Ref |
| MN615701 | Unassigned | Unassigned | Unassigned | 80_7 | Clustered | 33 | VC_80_7 | 33 | 0.2156 | 1 | 0.2156 | 0 | 0 | 0 | 0.8409 | Ref |
| MN615702 | Unassigned | Unassigned | Unassigned | 80_7 | Clustered | 33 | VC_80_7 | 33 | 0.2156 | 1 | 0.2156 | 0 | 0 | 0 | 0.8409 | Ref |
| MN617773 | Unassigned | Unassigned | Unassigned | 464_0 | Clustered | 16 | VC_464_0 | 16 | 0.9558 | 1 | 0.9558 | 0 | 0 | 0 | 1 | Ref |
| MN617840 | Unassigned | Unassigned | Unassigned | 1296_0 | Clustered | 82 | VC_1296_0 | 82 | 0.7938 | 1 | 0.7938 | 0 | 0 | 0 | 1 | Ref |
| MN617842 | Unassigned | Unassigned | Unassigned | 985_0 | Clustered | 38 | VC_985_0 | 38 | 0.9334 | 1 | 0.9334 | 0 | 0 | 0 | 1 | Ref |
| MN617843 | Unassigned | Unassigned | Unassigned | 385_2 | Clustered | 5 | VC_385_2 | 5 | 0.0717 | 0.99999997 | 0.0717 | 0 | 0 | 0 | 0.9 | Ref |
| MN617844 | Unassigned | Unassigned | Unassigned | 974_0 | Clustered | 34 | VC_974_0 | 34 | 0.7953 | 1 | 0.7953 | 0 | 0 | 0 | 1 | Ref |
| MN617845 | Unassigned | Unassigned | Unassigned | 335_1 | Clustered | 2 | VC_335_1 | 2 | 0.0043 | 0.95663348 | 0.0041 | 0 | 0 | 0 | 1 | Ref |
| MN642089 | Unassigned | Unassigned | Unassigned | 456_1 | Clustered | 3 | VC_456_1 | 3 | 0.053 | 0.99419552 | 0.0527 | 0 | 0 | 0 | 1 | Ref |
| MN648195 | Unassigned | Unassigned | Unassigned | 510_1 | Clustered | 20 | VC_510_1 | 20 | 0.4172 | 1 | 0.4172 | 0 | 0 | 0 | 1 | Ref |
| MN648445 | Unassigned | Unassigned | Unassigned | 293_17 | Clustered | 8 | VC_293_17 | 8 | 0.0534 | 0.99997879 | 0.0534 | 0 | 0 | 0 | 0.6071 | Ref |
| MN651570 | Unassigned | Unassigned | Unassigned | 714_0 | Clustered | 71 | VC_714_0 | 71 | 0.935 | 1 | 0.935 | 0 | 0 | 0 | 1 | Ref |
| MN655998 | Unassigned | Unassigned | Unassigned | 609_0 | Clustered | 27 | VC_609_0 | 27 | 0.9943 | 1 | 0.9943 | 0 | 0 | 0 | 1 | Ref |
| MN655999 | Unassigned | Unassigned | Unassigned | 340_11 | Clustered | 15 | VC_340_11 | 15 | 0.079 | 1 | 0.079 | 0 | 0 | 0 | 0.9333 | Ref |
| MN656993 | Unassigned | Unassigned | Unassigned | 860_0 | Clustered | 9 | VC_860_0 | 9 | 0.7723 | 1 | 0.7723 | 0 | 0 | 0 | 1 | Ref |
| MN662249 | Unassigned | Unassigned | Unassigned | 510_1 | Clustered | 20 | VC_510_1 | 20 | 0.4172 | 1 | 0.4172 | 0 | 0 | 0 | 1 | Ref |
| MN688132 | Unassigned | Unassigned | Unassigned | 36_0 | Clustered | 4 | VC_36_0 | 4 | 0.0597 | 0.99994573 | 0.0597 | 0 | 0 | 0 | 1 | Ref |
| MN689503 | Unassigned | Unassigned | Unassigned | 706_0 | Clustered | 52 | VC_706_0 | 52 | 0.9787 | 1 | 0.9787 | 0 | 0 | 0 | 1 | Ref |
| MN689504 | Unassigned | Unassigned | Unassigned | 706_0 | Clustered | 52 | VC_706_0 | 52 | 0.9787 | 1 | 0.9787 | 0 | 0 | 0 | 1 | Ref |
| MN689505 | Unassigned | Unassigned | Unassigned | 706_0 | Clustered | 52 | VC_706_0 | 52 | 0.9787 | 1 | 0.9787 | 0 | 0 | 0 | 1 | Ref |
| MN689506 | Unassigned | Unassigned | Unassigned | 706_0 | Clustered | 52 | VC_706_0 | 52 | 0.9787 | 1 | 0.9787 | 0 | 0 | 0 | 1 | Ref |
| MN689507 | Unassigned | Unassigned | Unassigned | 706_0 | Clustered | 52 | VC_706_0 | 52 | 0.9787 | 1 | 0.9787 | 0 | 0 | 0 | 1 | Ref |
| MN689508 | Unassigned | Unassigned | Unassigned | 706_0 | Clustered | 52 | VC_706_0 | 52 | 0.9787 | 1 | 0.9787 | 0 | 0 | 0 | 1 | Ref |
| MN689509 | Unassigned | Unassigned | Unassigned | 266_0 | Clustered | 13 | VC_266_0 | 13 | 0.3622 | 1 | 0.3622 | 0 | 0 | 0 | 1 | Ref |
| MN689510 | Unassigned | Unassigned | Unassigned | 706_0 | Clustered | 52 | VC_706_0 | 52 | 0.9787 | 1 | 0.9787 | 0 | 0 | 0 | 1 | Ref |
| MN689511 | Unassigned | Unassigned | Unassigned | 706_0 | Clustered | 52 | VC_706_0 | 52 | 0.9787 | 1 | 0.9787 | 0 | 0 | 0 | 1 | Ref |
| MN689512 | Unassigned | Unassigned | Unassigned | 706_0 | Clustered | 52 | VC_706_0 | 52 | 0.9787 | 1 | 0.9787 | 0 | 0 | 0 | 1 | Ref |
| MN689513 | Unassigned | Unassigned | Unassigned | 706_0 | Clustered | 52 | VC_706_0 | 52 | 0.9787 | 1 | 0.9787 | 0 | 0 | 0 | 1 | Ref |
| MN689514 | Unassigned | Unassigned | Unassigned | 210_0 | Clustered | 105 | VC_210_0 | 105 | 0.4076 | 1 | 0.4076 | 0 | 0 | 0 | 0.7766 | Ref |
| MN689515 | Unassigned | Unassigned | Unassigned | 706_0 | Clustered | 52 | VC_706_0 | 52 | 0.9787 | 1 | 0.9787 | 0 | 0 | 0 | 1 | Ref |
| MN689516 | Unassigned | Unassigned | Unassigned | 266_0 | Clustered | 13 | VC_266_0 | 13 | 0.3622 | 1 | 0.3622 | 0 | 0 | 0 | 1 | Ref |
| MN689517 | Unassigned | Unassigned | Unassigned | 210_0 | Clustered | 105 | VC_210_0 | 105 | 0.4076 | 1 | 0.4076 | 0 | 0 | 0 | 0.7766 | Ref |
| MN689518 | Unassigned | Unassigned | Unassigned | 210_0 | Clustered | 105 | VC_210_0 | 105 | 0.4076 | 1 | 0.4076 | 0 | 0 | 0 | 0.7766 | Ref |
| MN689519 | Unassigned | Unassigned | Unassigned | 210_0 | Clustered | 105 | VC_210_0 | 105 | 0.4076 | 1 | 0.4076 | 0 | 0 | 0 | 0.7766 | Ref |
| MN689520 | Unassigned | Unassigned | Unassigned | 210_0 | Clustered | 105 | VC_210_0 | 105 | 0.4076 | 1 | 0.4076 | 0 | 0 | 0 | 0.7766 | Ref |
| MN689521 | Unassigned | Unassigned | Unassigned | 266_0 | Clustered | 13 | VC_266_0 | 13 | 0.3622 | 1 | 0.3622 | 0 | 0 | 0 | 1 | Ref |
| MN689522 | Unassigned | Unassigned | Unassigned | 210_0 | Clustered | 105 | VC_210_0 | 105 | 0.4076 | 1 | 0.4076 | 0 | 0 | 0 | 0.7766 | Ref |
| MN689523 | Unassigned | Unassigned | Unassigned | 210_0 | Clustered | 105 | VC_210_0 | 105 | 0.4076 | 1 | 0.4076 | 0 | 0 | 0 | 0.7766 | Ref |
| MN689524 | Unassigned | Unassigned | Unassigned | 210_0 | Clustered | 105 | VC_210_0 | 105 | 0.4076 | 1 | 0.4076 | 0 | 0 | 0 | 0.7766 | Ref |
| MN689525 | Unassigned | Unassigned | Unassigned | 210_0 | Clustered | 105 | VC_210_0 | 105 | 0.4076 | 1 | 0.4076 | 0 | 0 | 0 | 0.7766 | Ref |
| MN689526 | Unassigned | Unassigned | Unassigned | 706_0 | Clustered | 52 | VC_706_0 | 52 | 0.9787 | 1 | 0.9787 | 0 | 0 | 0 | 1 | Ref |
| MN689527 | Unassigned | Unassigned | Unassigned | 706_0 | Clustered | 52 | VC_706_0 | 52 | 0.9787 | 1 | 0.9787 | 0 | 0 | 0 | 1 | Ref |
| MN689528 | Unassigned | Unassigned | Unassigned | 706_0 | Clustered | 52 | VC_706_0 | 52 | 0.9787 | 1 | 0.9787 | 0 | 0 | 0 | 1 | Ref |
| MN689529 | Unassigned | Unassigned | Unassigned | 706_0 | Clustered | 52 | VC_706_0 | 52 | 0.9787 | 1 | 0.9787 | 0 | 0 | 0 | 1 | Ref |
| MN689530 | Unassigned | Unassigned | Unassigned | 266_0 | Clustered | 13 | VC_266_0 | 13 | 0.3622 | 1 | 0.3622 | 0 | 0 | 0 | 1 | Ref |
| MN689531 | Unassigned | Unassigned | Unassigned | 706_0 | Clustered | 52 | VC_706_0 | 52 | 0.9787 | 1 | 0.9787 | 0 | 0 | 0 | 1 | Ref |
| MN689532 | Unassigned | Unassigned | Unassigned | 706_0 | Clustered | 52 | VC_706_0 | 52 | 0.9787 | 1 | 0.9787 | 0 | 0 | 0 | 1 | Ref |
| MN689533 | Unassigned | Unassigned | Unassigned | 706_0 | Clustered | 52 | VC_706_0 | 52 | 0.9787 | 1 | 0.9787 | 0 | 0 | 0 | 1 | Ref |
| MN689778 | Unassigned | Unassigned | Unassigned | 125_0 | Clustered | 23 | VC_125_0 | 23 | 0.8163 | 1 | 0.8163 | 0 | 0 | 0 | 1 | Ref |
| MN689779 | Unassigned | Unassigned | Unassigned | 972_0 | Clustered | 99 | VC_972_0 | 99 | 0.9113 | 1 | 0.9113 | 0 | 0 | 0 | 0.9928 | Ref |
| MN690600 | Unassigned | Unassigned | Unassigned | 16_0 | Clustered | 12 | VC_16_0 | 12 | 0.6601 | 1 | 0.6601 | 0 | 0 | 0 | 1 | Ref |
| MN692672 | Unassigned | Unassigned | Unassigned | 434_0 | Clustered | 45 | VC_434_0 | 45 | 0.9479 | 1 | 0.9479 | 0 | 0 | 0 | 1 | Ref |
| MN692673 | Unassigned | Unassigned | Unassigned | 475_0 | Clustered | 96 | VC_475_0 | 96 | 0.9303 | 1 | 0.9303 | 0 | 0 | 0 | 0.9575 | Ref |
| MN695334 | Unassigned | Unassigned | Unassigned | 446_0 | Clustered | 2 | VC_446_0 | 2 | 0.0354 | 0.95681959 | 0.0339 | 0 | 0 | 0 | 1 | Ref |
| MN695335 | Unassigned | Unassigned | Unassigned | 446_0 | Clustered | 2 | VC_446_0 | 2 | 0.0354 | 0.95681959 | 0.0339 | 0 | 0 | 0 | 1 | Ref |
| MN698239 | Unassigned | Unassigned | Unassigned | 484_0 | Clustered | 3 | VC_484_0 | 3 | 0.5436 | 0.99823901 | 0.5427 | 0 | 0 | 0 | 1 | Ref |
| MN698240 | Unassigned | Unassigned | Unassigned | 1038_0 | Clustered | 4 | VC_1038_0 | 4 | 0.8692 | 0.99983781 | 0.8691 | 0 | 0 | 0 | 1 | Ref |
| MN698241 | Unassigned | Unassigned | Unassigned | 484_0 | Clustered | 3 | VC_484_0 | 3 | 0.5436 | 0.99823901 | 0.5427 | 0 | 0 | 0 | 1 | Ref |
| MN698242 | Unassigned | Unassigned | Unassigned | 501_0 | Clustered | 3 | VC_501_0 | 3 | 0.2351 | 0.99844364 | 0.2347 | 0 | 0 | 0 | 1 | Ref |
| MN698243 | Unassigned | Unassigned | Unassigned | 501_0 | Clustered | 3 | VC_501_0 | 3 | 0.2351 | 0.99844364 | 0.2347 | 0 | 0 | 0 | 1 | Ref |
| MN698245 | Unassigned | Unassigned | Unassigned | 1038_0 | Clustered | 4 | VC_1038_0 | 4 | 0.8692 | 0.99983781 | 0.8691 | 0 | 0 | 0 | 1 | Ref |
| MN698246 | Unassigned | Unassigned | Unassigned | 1038_0 | Clustered | 4 | VC_1038_0 | 4 | 0.8692 | 0.99983781 | 0.8691 | 0 | 0 | 0 | 1 | Ref |
| MN698247 | Unassigned | Unassigned | Unassigned | 501_0 | Clustered | 3 | VC_501_0 | 3 | 0.2351 | 0.99844364 | 0.2347 | 0 | 0 | 0 | 1 | Ref |
| MN698248 | Unassigned | Unassigned | Unassigned | 1038_0 | Clustered | 4 | VC_1038_0 | 4 | 0.8692 | 0.99983781 | 0.8691 | 0 | 0 | 0 | 1 | Ref |
| MN703405 | Unassigned | Unassigned | Unassigned | 228_0 | Clustered | 22 | VC_228_0 | 22 | 0.7411 | 1 | 0.7411 | 0 | 0 | 0 | 1 | Ref |
| MN703406 | Unassigned | Unassigned | Unassigned | 917_0 | Clustered | 28 | VC_917_0 | 28 | 0.5465 | 1 | 0.5465 | 0 | 0 | 0 | 1 | Ref |
| MN703407 | Unassigned | Unassigned | Unassigned | 1408_0 | Clustered | 2 | VC_1408_0 | 2 | 0.1101 | 0.95582225 | 0.1053 | 0 | 0 | 0 | 1 | Ref |
| MN703408 | Unassigned | Unassigned | Unassigned | 224_0 | Clustered | 5 | VC_224_0 | 5 | 0.0685 | 0.99999998 | 0.0685 | 0 | 0 | 0 | 1 | Ref |
| MN703409 | Unassigned | Unassigned | Unassigned | 1207_0 | Clustered | 20 | VC_1207_0 | 20 | 0.9898 | 1 | 0.9898 | 0 | 0 | 0 | 1 | Ref |
| MN703410 | Unassigned | Unassigned | Unassigned | 917_0 | Clustered | 28 | VC_917_0 | 28 | 0.5465 | 1 | 0.5465 | 0 | 0 | 0 | 1 | Ref |
| MN703411 | Unassigned | Unassigned | Unassigned | 1117_0 | Clustered | 19 | VC_1117_0 | 19 | 0.9177 | 1 | 0.9177 | 0 | 0 | 0 | 1 | Ref |
| MN703412 | Unassigned | Unassigned | Unassigned | 1204_0 | Clustered | 25 | VC_1204_0 | 25 | 0.7382 | 1 | 0.7382 | 0 | 0 | 0 | 1 | Ref |

| | | | | | | | | | | | | | | | | |
|---|---|---|---|---|---|---|---|---|---|---|---|---|---|---|---|---|
| MN703413 | Unassigned | Unassigned | Unassigned | 1250_0 | Clustered | 29 | VC_1250_0 | 29 | 0.822 | 1 | 0.822 | 0 | 0 | 0 | 1 | Ref |
| MN703414 | Unassigned | Unassigned | Unassigned | 1408_0 | Clustered | 2 | VC_1408_0 | 2 | 0.1101 | 0.95582225 | 0.1053 | 0 | 0 | 0 | 1 | Ref |
| MN703415 | Unassigned | Unassigned | Unassigned | 384_20 | Clustered/Singleton | 1 | VC_384_20 | 1 | 0 | 1 | 0 | 0 | 0 | 0 | 1 | Ref |
| MN709127 | Unassigned | Unassigned | Unassigned | 609_0 | Clustered | 27 | VC_609_0 | 27 | 0.9943 | 1 | 0.9943 | 0 | 0 | 0 | 1 | Ref |
| MN709128 | Unassigned | Unassigned | Unassigned | 510_1 | Clustered | 20 | VC_510_1 | 20 | 0.4172 | 1 | 0.4172 | 0 | 0 | 0 | 1 | Ref |
| MN710383 | Unassigned | Unassigned | Unassigned | 608_0 | Clustered | 2 | VC_608_0 | 2 | 1 | 1 | 1 | 0 | 0 | 0 | 1 | Ref |
| MN715150 | Unassigned | Unassigned | Unassigned | 465_0 | Clustered | 80 | VC_465_0 | 80 | 0.9988 | 1 | 0.9988 | 0 | 0 | 0 | 1 | Ref |
| MN715356 | Unassigned | Unassigned | Unassigned | 837_1 | Clustered | 24 | VC_837_1 | 24 | 0.5328 | 1 | 0.5328 | 0 | 0 | 0 | 1 | Ref |
| MN716856 | Unassigned | Unassigned | Unassigned | 290_131 | Clustered/Singleton | 1 | VC_290_131 | 1 | 0 | 1 | 0 | 0 | 0 | 0 | 1 | Ref |
| MN718463 | Unassigned | Unassigned | Unassigned | 788_0 | Clustered | 2 | VC_788_0 | 2 | 0.2235 | 0.95055313 | 0.2125 | 0 | 0 | 0 | 1 | Ref |
| MN719123 | Unassigned | Unassigned | Unassigned | 17_0 | Clustered | 9 | VC_17_0 | 9 | 0.6954 | 1 | 0.6954 | 0 | 0 | 0 | 1 | Ref |
| MN722429 | Unassigned | Unassigned | Unassigned | 654_0 | Clustered | 115 | VC_654_0 | 115 | 0.7303 | 1 | 0.7303 | 0 | 0 | 0 | 0.8764 | Ref |
| MN723850 | Unassigned | Unassigned | Unassigned | 510_1 | Clustered | 20 | VC_510_1 | 20 | 0.4172 | 1 | 0.4172 | 0 | 0 | 0 | 1 | Ref |
| MN727882 | Unassigned | Unassigned | Unassigned | 102_0 | Clustered | 15 | VC_102_0 | 15 | 0.653 | 1 | 0.653 | 0 | 0 | 0 | 1 | Ref |
| MN728683 | Unassigned | Unassigned | Unassigned | 230_0 | Clustered | 38 | VC_230_0 | 38 | 0.9076 | 1 | 0.9076 | 0 | 0 | 0 | 1 | Ref |
| MN728684 | Unassigned | Unassigned | Unassigned | 535_1 | Clustered | 108 | VC_535_1 | 108 | 0.8479 | 1 | 0.8479 | 0 | 0 | 0 | 0.9955 | Ref |
| MN729595 | Unassigned | Unassigned | Unassigned | 131_0 | Clustered | 43 | VC_131_0 | 43 | 0.8853 | 1 | 0.8853 | 0 | 0 | 0 | 1 | Ref |
| MN729596 | Unassigned | Unassigned | Unassigned | 131_0 | Clustered | 43 | VC_131_0 | 43 | 0.8853 | 1 | 0.8853 | 0 | 0 | 0 | 1 | Ref |
| MN729597 | Unassigned | Unassigned | Unassigned | 131_0 | Clustered | 43 | VC_131_0 | 43 | 0.8853 | 1 | 0.8853 | 0 | 0 | 0 | 1 | Ref |
| MN729598 | Unassigned | Unassigned | Unassigned | 131_0 | Clustered | 43 | VC_131_0 | 43 | 0.8853 | 1 | 0.8853 | 0 | 0 | 0 | 1 | Ref |
| MN729599 | Unassigned | Unassigned | Unassigned | 131_0 | Clustered | 43 | VC_131_0 | 43 | 0.8853 | 1 | 0.8853 | 0 | 0 | 0 | 1 | Ref |
| MN729600 | Unassigned | Unassigned | Unassigned | 131_0 | Clustered | 43 | VC_131_0 | 43 | 0.8853 | 1 | 0.8853 | 0 | 0 | 0 | 1 | Ref |
| MN732867 | Unassigned | Unassigned | Unassigned | 601_0 | Clustered | 4 | VC_601_0 | 4 | 0.0581 | 0.99996075 | 0.0581 | 0 | 0 | 0 | 1 | Ref |
| MN732883 | Unassigned | Unassigned | Unassigned | 510_1 | Clustered | 20 | VC_510_1 | 20 | 0.4172 | 1 | 0.4172 | 0 | 0 | 0 | 1 | Ref |
| MN734437 | Unassigned | Unassigned | Unassigned | 769_0 | Clustered | 3 | VC_769_0 | 3 | 0.0785 | 0.9984602 | 0.0784 | 0 | 0 | 0 | 1 | Ref |
| MN734438 | Unassigned | Unassigned | Unassigned | 769_0 | Clustered | 3 | VC_769_0 | 3 | 0.0785 | 0.9984602 | 0.0784 | 0 | 0 | 0 | 1 | Ref |
| MN734439 | Unassigned | Unassigned | Unassigned | 769_0 | Clustered | 3 | VC_769_0 | 3 | 0.0785 | 0.9984602 | 0.0784 | 0 | 0 | 0 | 1 | Ref |
| MN735429 | Unassigned | Unassigned | Unassigned | 1200_0 | Clustered | 73 | VC_1200_0 | 73 | 0.9825 | 1 | 0.9825 | 0 | 0 | 0 | 1 | Ref |
| MN735430 | Unassigned | Unassigned | Unassigned | 1296_0 | Clustered | 82 | VC_1296_0 | 82 | 0.7938 | 1 | 0.7938 | 0 | 0 | 0 | 1 | Ref |
| MN735431 | Unassigned | Unassigned | Unassigned | 538_12 | Clustered/Singleton | 1 | VC_538_12 | 1 | 0 | 1 | 0 | 0 | 0 | 0 | 1 | Ref |
| MN735432 | Unassigned | Unassigned | Unassigned | 1087_0 | Clustered | 7 | VC_1087_0 | 7 | 0.94 | 1 | 0.94 | 0 | 0 | 0 | 1 | Ref |
| MN735434 | Unassigned | Unassigned | Unassigned | 579_79 | Clustered/Singleton | 1 | VC_579_79 | 1 | 0 | 1 | 0 | 0 | 0 | 0 | 1 | Ref |
| MN735435 | Unassigned | Unassigned | Unassigned | 1296_0 | Clustered | 82 | VC_1296_0 | 82 | 0.7938 | 1 | 0.7938 | 0 | 0 | 0 | 1 | Ref |
| MN737551 | Unassigned | Unassigned | Unassigned | 475_0 | Clustered | 96 | VC_475_0 | 96 | 0.9303 | 1 | 0.9303 | 0 | 0 | 0 | 0.9575 | Ref |
| MN737622 | Unassigned | Unassigned | Unassigned | 240_0 | Clustered | 2 | VC_240_0 | 2 | 0.7151 | 0.72985431 | 0.5219 | 0 | 0 | 0 | 1 | Ref |
| MN746332 | Unassigned | Unassigned | Unassigned | 289_0 | Clustered | 2 | VC_289_0 | 2 | 0.219 | 0.95471323 | 0.2091 | 0 | 0 | 0 | 1 | Ref |
| MN781580 | Unassigned | Unassigned | Unassigned | 290_21 | Clustered/Singleton | 1 | VC_290_21 | 1 | 0 | 1 | 0 | 0 | 0 | 0 | 1 | Ref |
| MN781674 | Unassigned | Unassigned | Unassigned | 976_0 | Clustered | 36 | VC_976_0 | 36 | 0.7833 | 1 | 0.7833 | 0 | 0 | 0 | 1 | Ref |
| MN782535 | Unassigned | Unassigned | Unassigned | 510_1 | Clustered | 20 | VC_510_1 | 20 | 0.4172 | 1 | 0.4172 | 0 | 0 | 0 | 1 | Ref |
| MN783016 | Unassigned | Unassigned | Unassigned | 972_0 | Clustered | 99 | VC_972_0 | 99 | 0.9113 | 1 | 0.9113 | 0 | 0 | 0 | 0.9928 | Ref |
| MN788369 | Unassigned | Unassigned | Unassigned | 133_0 | Clustered | 17 | VC_133_0 | 17 | 0.2654 | 1 | 0.2654 | 0 | 0 | 0 | 1 | Ref |
| MN794000 | Unassigned | Unassigned | Unassigned | 113_0 | Clustered | 91 | VC_113_0 | 91 | 0.9584 | 1 | 0.9584 | 0 | 0 | 0 | 1 | Ref |
| MN794001 | Unassigned | Unassigned | Unassigned | 113_0 | Clustered | 91 | VC_113_0 | 91 | 0.9584 | 1 | 0.9584 | 0 | 0 | 0 | 1 | Ref |
| MN794002 | Unassigned | Unassigned | Unassigned | 113_0 | Clustered | 91 | VC_113_0 | 91 | 0.9584 | 1 | 0.9584 | 0 | 0 | 0 | 1 | Ref |
| MN794003 | Unassigned | Unassigned | Unassigned | 113_0 | Clustered | 91 | VC_113_0 | 91 | 0.9584 | 1 | 0.9584 | 0 | 0 | 0 | 1 | Ref |
| MN794232 | Unassigned | Unassigned | Unassigned | 549_1 | Clustered | 6 | VC_549_1 | 6 | 0.1479 | 0.9998319 | 0.1478 | 0 | 0 | 0 | 1 | Ref |
| MN807240 | Unassigned | Unassigned | Unassigned | 1067_0 | Clustered | 20 | VC_1067_0 | 20 | 0.7109 | 1 | 0.7109 | 0 | 0 | 0 | 1 | Ref |
| MN807246 | Unassigned | Unassigned | Unassigned | 1296_0 | Clustered | 82 | VC_1296_0 | 82 | 0.7938 | 1 | 0.7938 | 0 | 0 | 0 | 1 | Ref |
| MN807247 | Unassigned | Unassigned | Unassigned | 533_0 | Clustered | 16 | VC_533_0 | 16 | 0.3765 | 1 | 0.3765 | 0 | 0 | 0 | 1 | Ref |
| MN807248 | Unassigned | Unassigned | Unassigned | 537_0 | Clustered | 39 | VC_537_0 | 39 | 0.8148 | 1 | 0.8148 | 0 | 0 | 0 | 1 | Ref |
| MN807249 | Unassigned | Unassigned | Unassigned | 537_0 | Clustered | 39 | VC_537_0 | 39 | 0.8148 | 1 | 0.8148 | 0 | 0 | 0 | 1 | Ref |
| MN807250 | Unassigned | Unassigned | Unassigned | 537_0 | Clustered | 39 | VC_537_0 | 39 | 0.8148 | 1 | 0.8148 | 0 | 0 | 0 | 1 | Ref |
| MN807295 | Unassigned | Unassigned | Unassigned | 714_0 | Clustered | 71 | VC_714_0 | 71 | 0.935 | 1 | 0.935 | 0 | 0 | 0 | 1 | Ref |
| MN812203 | Unassigned | Unassigned | Unassigned | 1059_0 | Clustered | 10 | VC_1059_0 | 10 | 0.8794 | 1 | 0.8794 | 0 | 0 | 0 | 1 | Ref |
| MN812204 | Unassigned | Unassigned | Unassigned | 1059_0 | Clustered | 10 | VC_1059_0 | 10 | 0.8794 | 1 | 0.8794 | 0 | 0 | 0 | 1 | Ref |
| MN812205 | Unassigned | Unassigned | Unassigned | 1059_0 | Clustered | 10 | VC_1059_0 | 10 | 0.8794 | 1 | 0.8794 | 0 | 0 | 0 | 1 | Ref |
| MN812206 | Unassigned | Unassigned | Unassigned | 1049_0 | Clustered | 34 | VC_1049_0 | 34 | 0.9937 | 1 | 0.9937 | 0 | 0 | 0 | 1 | Ref |
| MN812207 | Unassigned | Unassigned | Unassigned | 1049_0 | Clustered | 34 | VC_1049_0 | 34 | 0.9937 | 1 | 0.9937 | 0 | 0 | 0 | 1 | Ref |
| MN812208 | Unassigned | Unassigned | Unassigned | 1049_0 | Clustered | 34 | VC_1049_0 | 34 | 0.9937 | 1 | 0.9937 | 0 | 0 | 0 | 1 | Ref |
| MN812209 | Unassigned | Unassigned | Unassigned | 1049_0 | Clustered | 34 | VC_1049_0 | 34 | 0.9937 | 1 | 0.9937 | 0 | 0 | 0 | 1 | Ref |
| MN812210 | Unassigned | Unassigned | Unassigned | 1049_0 | Clustered | 34 | VC_1049_0 | 34 | 0.9937 | 1 | 0.9937 | 0 | 0 | 0 | 1 | Ref |
| MN812212 | Unassigned | Unassigned | Unassigned | 1049_0 | Clustered | 34 | VC_1049_0 | 34 | 0.9937 | 1 | 0.9937 | 0 | 0 | 0 | 1 | Ref |
| MN812213 | Unassigned | Unassigned | Unassigned | 1049_0 | Clustered | 34 | VC_1049_0 | 34 | 0.9937 | 1 | 0.9937 | 0 | 0 | 0 | 1 | Ref |
| MN812214 | Unassigned | Unassigned | Unassigned | 1049_0 | Clustered | 34 | VC_1049_0 | 34 | 0.9937 | 1 | 0.9937 | 0 | 0 | 0 | 1 | Ref |
| MN812215 | Unassigned | Unassigned | Unassigned | 1049_0 | Clustered | 34 | VC_1049_0 | 34 | 0.9937 | 1 | 0.9937 | 0 | 0 | 0 | 1 | Ref |
| MN812216 | Unassigned | Unassigned | Unassigned | 1049_0 | Clustered | 34 | VC_1049_0 | 34 | 0.9937 | 1 | 0.9937 | 0 | 0 | 0 | 1 | Ref |
| MN812217 | Unassigned | Unassigned | Unassigned | 1049_0 | Clustered | 34 | VC_1049_0 | 34 | 0.9937 | 1 | 0.9937 | 0 | 0 | 0 | 1 | Ref |
| MN812218 | Unassigned | Unassigned | Unassigned | 1049_0 | Clustered | 34 | VC_1049_0 | 34 | 0.9937 | 1 | 0.9937 | 0 | 0 | 0 | 1 | Ref |
| MN812219 | Unassigned | Unassigned | Unassigned | 1049_0 | Clustered | 34 | VC_1049_0 | 34 | 0.9937 | 1 | 0.9937 | 0 | 0 | 0 | 1 | Ref |
| MN812220 | Unassigned | Unassigned | Unassigned | 1049_0 | Clustered | 34 | VC_1049_0 | 34 | 0.9937 | 1 | 0.9937 | 0 | 0 | 0 | 1 | Ref |
| MN812221 | Unassigned | Unassigned | Unassigned | 1049_0 | Clustered | 34 | VC_1049_0 | 34 | 0.9937 | 1 | 0.9937 | 0 | 0 | 0 | 1 | Ref |
| MN812222 | Unassigned | Unassigned | Unassigned | 1049_0 | Clustered | 34 | VC_1049_0 | 34 | 0.9937 | 1 | 0.9937 | 0 | 0 | 0 | 1 | Ref |
| MN812223 | Unassigned | Unassigned | Unassigned | 1049_0 | Clustered | 34 | VC_1049_0 | 34 | 0.9937 | 1 | 0.9937 | 0 | 0 | 0 | 1 | Ref |
| MN812224 | Unassigned | Unassigned | Unassigned | 1049_0 | Clustered | 34 | VC_1049_0 | 34 | 0.9937 | 1 | 0.9937 | 0 | 0 | 0 | 1 | Ref |
| MN812225 | Unassigned | Unassigned | Unassigned | 1049_0 | Clustered | 34 | VC_1049_0 | 34 | 0.9937 | 1 | 0.9937 | 0 | 0 | 0 | 1 | Ref |
| MN812226 | Unassigned | Unassigned | Unassigned | 1049_0 | Clustered | 34 | VC_1049_0 | 34 | 0.9937 | 1 | 0.9937 | 0 | 0 | 0 | 1 | Ref |
| MN812227 | Unassigned | Unassigned | Unassigned | 1049_0 | Clustered | 34 | VC_1049_0 | 34 | 0.9937 | 1 | 0.9937 | 0 | 0 | 0 | 1 | Ref |
| MN812228 | Unassigned | Unassigned | Unassigned | 1049_0 | Clustered | 34 | VC_1049_0 | 34 | 0.9937 | 1 | 0.9937 | 0 | 0 | 0 | 1 | Ref |
| MN812229 | Unassigned | Unassigned | Unassigned | 1049_0 | Clustered | 34 | VC_1049_0 | 34 | 0.9937 | 1 | 0.9937 | 0 | 0 | 0 | 1 | Ref |
| MN812230 | Unassigned | Unassigned | Unassigned | 1049_0 | Clustered | 34 | VC_1049_0 | 34 | 0.9937 | 1 | 0.9937 | 0 | 0 | 0 | 1 | Ref |
| MN812231 | Unassigned | Unassigned | Unassigned | 1049_0 | Clustered | 34 | VC_1049_0 | 34 | 0.9937 | 1 | 0.9937 | 0 | 0 | 0 | 1 | Ref |

| | | | | | | | | | | | | | | | | |
|---|---|---|---|---|---|---|---|---|---|---|---|---|---|---|---|---|
| MN812232 | Unassigned | Unassigned | Unassigned | 1049_0 | Clustered | 34 | VC_1049_0 | 34 | 0.9937 | 1 | 0.9937 | 0 | 0 | 0 | 1 | Ref |
| MN812233 | Unassigned | Unassigned | Unassigned | 1049_0 | Clustered | 34 | VC_1049_0 | 34 | 0.9937 | 1 | 0.9937 | 0 | 0 | 0 | 1 | Ref |
| MN812234 | Unassigned | Unassigned | Unassigned | 1049_0 | Clustered | 34 | VC_1049_0 | 34 | 0.9937 | 1 | 0.9937 | 0 | 0 | 0 | 1 | Ref |
| MN812235 | Unassigned | Unassigned | Unassigned | 1049_0 | Clustered | 34 | VC_1049_0 | 34 | 0.9937 | 1 | 0.9937 | 0 | 0 | 0 | 1 | Ref |
| MN812236 | Unassigned | Unassigned | Unassigned | 1049_0 | Clustered | 34 | VC_1049_0 | 34 | 0.9937 | 1 | 0.9937 | 0 | 0 | 0 | 1 | Ref |
| MN812237 | Unassigned | Unassigned | Unassigned | 1049_0 | Clustered | 34 | VC_1049_0 | 34 | 0.9937 | 1 | 0.9937 | 0 | 0 | 0 | 1 | Ref |
| MN812238 | Unassigned | Unassigned | Unassigned | 1049_0 | Clustered | 34 | VC_1049_0 | 34 | 0.9937 | 1 | 0.9937 | 0 | 0 | 0 | 1 | Ref |
| MN812240 | Unassigned | Unassigned | Unassigned | 1049_0 | Clustered | 34 | VC_1049_0 | 34 | 0.9937 | 1 | 0.9937 | 0 | 0 | 0 | 1 | Ref |
| MN812241 | Unassigned | Unassigned | Unassigned | 1049_0 | Clustered | 34 | VC_1049_0 | 34 | 0.9937 | 1 | 0.9937 | 0 | 0 | 0 | 1 | Ref |
| MN812685 | Unassigned | Unassigned | Unassigned | 865_0 | Clustered | 10 | VC_865_0 | 10 | 0.5975 | 1 | 0.5975 | 0 | 0 | 0 | 1 | Ref |
| MN812686 | Unassigned | Unassigned | Unassigned | 465_0 | Clustered | 80 | VC_465_0 | 80 | 0.9988 | 1 | 0.9988 | 0 | 0 | 0 | 1 | Ref |
| MN812687 | Unassigned | Unassigned | Unassigned | 865_0 | Clustered | 10 | VC_865_0 | 10 | 0.5975 | 1 | 0.5975 | 0 | 0 | 0 | 1 | Ref |
| MN812688 | Unassigned | Unassigned | Unassigned | 816_0 | Clustered | 35 | VC_816_0 | 35 | 0.9824 | 1 | 0.9824 | 0 | 0 | 0 | 1 | Ref |
| MN812690 | Unassigned | Unassigned | Unassigned | 1018_0 | Clustered | 38 | VC_1018_0 | 38 | 0.9757 | 1 | 0.9757 | 0 | 0 | 0 | 1 | Ref |
| MN812691 | Unassigned | Unassigned | Unassigned | 865_0 | Clustered | 10 | VC_865_0 | 10 | 0.5975 | 1 | 0.5975 | 0 | 0 | 0 | 1 | Ref |
| MN812692 | Unassigned | Unassigned | Unassigned | 1018_0 | Clustered | 38 | VC_1018_0 | 38 | 0.9757 | 1 | 0.9757 | 0 | 0 | 0 | 1 | Ref |
| MN812693 | Unassigned | Unassigned | Unassigned | 406_0 | Clustered | 7 | VC_406_0 | 7 | 0.176 | 1 | 0.176 | 0 | 0 | 0 | 1 | Ref |
| MN812694 | Unassigned | Unassigned | Unassigned | 79_4 | Clustered/Singleton | 1 | VC_79_4 | 1 | 0 | 1 | 0 | 0 | 0 | 0 | 1 | Ref |
| MN812722 | Unassigned | Unassigned | Unassigned | 366_0 | Clustered | 4 | VC_366_0 | 4 | 0.1731 | 0.99998684 | 0.1731 | 0 | 0 | 0 | 0.8333 | Ref |
| MN813048 | Unassigned | Unassigned | Unassigned | 133_0 | Clustered | 17 | VC_133_0 | 17 | 0.2654 | 1 | 0.2654 | 0 | 0 | 0 | 1 | Ref |
| MN813049 | Unassigned | Unassigned | Unassigned | 133_0 | Clustered | 17 | VC_133_0 | 17 | 0.2654 | 1 | 0.2654 | 0 | 0 | 0 | 1 | Ref |
| MN813050 | Unassigned | Unassigned | Unassigned | 133_0 | Clustered | 17 | VC_133_0 | 17 | 0.2654 | 1 | 0.2654 | 0 | 0 | 0 | 1 | Ref |
| MN813051 | Unassigned | Unassigned | Unassigned | 133_0 | Clustered | 17 | VC_133_0 | 17 | 0.2654 | 1 | 0.2654 | 0 | 0 | 0 | 1 | Ref |
| MN813052 | Unassigned | Unassigned | Unassigned | 133_0 | Clustered | 17 | VC_133_0 | 17 | 0.2654 | 1 | 0.2654 | 0 | 0 | 0 | 1 | Ref |
| MN813053 | Unassigned | Unassigned | Unassigned | 133_0 | Clustered | 17 | VC_133_0 | 17 | 0.2654 | 1 | 0.2654 | 0 | 0 | 0 | 1 | Ref |
| MN813675 | Unassigned | Unassigned | Unassigned | 787_0 | Clustered | 132 | VC_787_0 | 132 | 1 | 1 | 1 | 0 | 0 | 0 | 1 | Ref |
| MN813676 | Unassigned | Unassigned | Unassigned | 1250_0 | Clustered | 29 | VC_1250_0 | 29 | 0.822 | 1 | 0.822 | 0 | 0 | 0 | 1 | Ref |
| MN813677 | Unassigned | Unassigned | Unassigned | 787_0 | Clustered | 132 | VC_787_0 | 132 | 1 | 1 | 1 | 0 | 0 | 0 | 1 | Ref |
| MN813678 | Unassigned | Unassigned | Unassigned | 526_0 | Clustered | 32 | VC_526_0 | 32 | 0.9037 | 1 | 0.9037 | 0 | 0 | 0 | 1 | Ref |
| MN813679 | Unassigned | Unassigned | Unassigned | 787_0 | Clustered | 132 | VC_787_0 | 132 | 1 | 1 | 1 | 0 | 0 | 0 | 1 | Ref |
| MN813680 | Unassigned | Unassigned | Unassigned | 1200_0 | Clustered | 73 | VC_1200_0 | 73 | 0.9825 | 1 | 0.9825 | 0 | 0 | 0 | 1 | Ref |
| MN813682 | Unassigned | Unassigned | Unassigned | 1412_0 | Clustered | 7 | VC_1412_0 | 7 | 0.2471 | 1 | 0.2471 | 0 | 0 | 0 | 1 | Ref |
| MN813683 | Unassigned | Unassigned | Unassigned | 787_0 | Clustered | 132 | VC_787_0 | 132 | 1 | 1 | 1 | 0 | 0 | 0 | 1 | Ref |
| MN813684 | Unassigned | Unassigned | Unassigned | 1412_0 | Clustered | 7 | VC_1412_0 | 7 | 0.2471 | 1 | 0.2471 | 0 | 0 | 0 | 1 | Ref |
| MN813687 | Unassigned | Unassigned | Unassigned | 593_0 | Clustered | 2 | VC_593_0 | 2 | 0.1092 | 0.95639188 | 0.1045 | 0 | 0 | 0 | 1 | Ref |
| MN813688 | Unassigned | Unassigned | Unassigned | 787_0 | Clustered | 132 | VC_787_0 | 132 | 1 | 1 | 1 | 0 | 0 | 0 | 1 | Ref |
| MN813689 | Unassigned | Unassigned | Unassigned | 787_0 | Clustered | 132 | VC_787_0 | 132 | 1 | 1 | 1 | 0 | 0 | 0 | 1 | Ref |
| MN813690 | Unassigned | Unassigned | Unassigned | 787_0 | Clustered | 132 | VC_787_0 | 132 | 1 | 1 | 1 | 0 | 0 | 0 | 1 | Ref |
| MN813691 | Unassigned | Unassigned | Unassigned | 787_0 | Clustered | 132 | VC_787_0 | 132 | 1 | 1 | 1 | 0 | 0 | 0 | 1 | Ref |
| MN813692 | Unassigned | Unassigned | Unassigned | 787_0 | Clustered | 132 | VC_787_0 | 132 | 1 | 1 | 1 | 0 | 0 | 0 | 1 | Ref |
| MN813693 | Unassigned | Unassigned | Unassigned | 385_4 | Clustered | 3 | VC_385_4 | 3 | 0.0329 | 0.99853257 | 0.0329 | 0 | 0 | 0 | 1 | Ref |
| MN813694 | Unassigned | Unassigned | Unassigned | 1218_0 | Clustered | 14 | VC_1218_0 | 14 | 0.7811 | 1 | 0.7811 | 0 | 0 | 0 | 0.989 | Ref |
| MN813695 | Unassigned | Unassigned | Unassigned | 985_0 | Clustered | 38 | VC_985_0 | 38 | 0.9334 | 1 | 0.9334 | 0 | 0 | 0 | 1 | Ref |
| MN813696 | Unassigned | Unassigned | Unassigned | 535_1 | Clustered | 108 | VC_535_1 | 108 | 0.8479 | 1 | 0.8479 | 0 | 0 | 0 | 0.9955 | Ref |
| MN813697 | Unassigned | Unassigned | Unassigned | 535_0 | Clustered | 18 | VC_535_0 | 18 | 0.1595 | 1 | 0.1595 | 0 | 0 | 0 | 1 | Ref |
| MN813698 | Unassigned | Unassigned | Unassigned | 1225_0 | Clustered | 6 | VC_1225_0 | 6 | 0.9947 | 0.99996974 | 0.9947 | 0 | 0 | 0 | 1 | Ref |
| MN813699 | Unassigned | Unassigned | Unassigned | 532_0 | Clustered | 75 | VC_532_0 | 75 | 0.9393 | 1 | 0.9393 | 0 | 0 | 0 | 1 | Ref |
| MN813700 | Unassigned | Unassigned | Unassigned | 335_12 | Clustered/Singleton | 1 | VC_335_12 | 1 | 0 | 1 | 0 | 0 | 0 | 0 | 1 | Ref |
| MN830252 | Unassigned | Unassigned | Unassigned | 280_0 | Clustered | 4 | VC_280_0 | 4 | 0.3169 | 0.99994916 | 0.3169 | 0 | 0 | 0 | 0.8333 | Ref |
| MN830253 | Unassigned | Unassigned | Unassigned | 280_0 | Clustered | 4 | VC_280_0 | 4 | 0.3169 | 0.99994916 | 0.3169 | 0 | 0 | 0 | 0.8333 | Ref |
| MN830254 | Unassigned | Unassigned | Unassigned | 754_0 | Clustered | 2 | VC_754_0 | 2 | 0.7213 | 0.93085693 | 0.6714 | 0 | 0 | 0 | 1 | Ref |
| MN830255 | Unassigned | Unassigned | Unassigned | 754_0 | Clustered | 2 | VC_754_0 | 2 | 0.7213 | 0.93085693 | 0.6714 | 0 | 0 | 0 | 1 | Ref |
| MN830256 | Unassigned | Unassigned | Unassigned | 315_0 | Clustered | 4 | VC_315_0 | 4 | 0.2058 | 0.99995817 | 0.2057 | 0 | 0 | 0 | 1 | Ref |
| MN830257 | Unassigned | Unassigned | Unassigned | 275_0 | Clustered | 6 | VC_275_0 | 6 | 0.6515 | 0.99999994 | 0.6515 | 0 | 0 | 0 | 0.9333 | Ref |
| MN830258 | Unassigned | Unassigned | Unassigned | 279_0 | Clustered | 11 | VC_279_0 | 11 | 0.4839 | 1 | 0.4839 | 0 | 0 | 0 | 0.8727 | Ref |
| MN840485 | Unassigned | Unassigned | Unassigned | 470_0 | Clustered | 62 | VC_470_0 | 62 | 0.902 | 1 | 0.902 | 0 | 0 | 0 | 1 | Ref |
| MN840486 | Unassigned | Unassigned | Unassigned | 610_0 | Clustered | 14 | VC_610_0 | 14 | 0.5159 | 1 | 0.5159 | 0 | 0 | 0 | 1 | Ref |
| MN844877 | Unassigned | Unassigned | Unassigned | 160_0 | Clustered | 4 | VC_160_0 | 4 | 0.1752 | 0.9999526 | 0.1752 | 0 | 0 | 0 | 1 | Ref |
| MN850564 | Unassigned | Unassigned | Unassigned | 340_3 | Clustered | 24 | VC_340_3 | 24 | 0.1205 | 1 | 0.1205 | 0 | 0 | 0 | 0.7283 | Ref |
| MN850565 | Unassigned | Unassigned | Unassigned | 871_1 | Clustered | 26 | VC_871_1 | 26 | 0.6303 | 1 | 0.6303 | 0 | 0 | 0 | 0.9815 | Ref |
| MN850566 | Unassigned | Unassigned | Unassigned | 340_3 | Clustered | 24 | VC_340_3 | 24 | 0.1205 | 1 | 0.1205 | 0 | 0 | 0 | 0.7283 | Ref |
| MN850567 | Unassigned | Unassigned | Unassigned | 975_0 | Clustered | 40 | VC_975_0 | 40 | 0.6888 | 1 | 0.6888 | 0 | 0 | 0 | 1 | Ref |
| MN850568 | Unassigned | Unassigned | Unassigned | 162_0 | Clustered | 12 | VC_162_0 | 12 | 0.9051 | 1 | 0.9051 | 0 | 0 | 0 | 1 | Ref |
| MN850569 | Unassigned | Unassigned | Unassigned | 975_0 | Clustered | 40 | VC_975_0 | 40 | 0.6888 | 1 | 0.6888 | 0 | 0 | 0 | 1 | Ref |
| MN850570 | Unassigned | Unassigned | Unassigned | 162_0 | Clustered | 12 | VC_162_0 | 12 | 0.9051 | 1 | 0.9051 | 0 | 0 | 0 | 1 | Ref |
| MN850571 | Unassigned | Unassigned | Unassigned | 871_1 | Clustered | 26 | VC_871_1 | 26 | 0.6303 | 1 | 0.6303 | 0 | 0 | 0 | 0.9815 | Ref |
| MN850572 | Unassigned | Unassigned | Unassigned | 975_0 | Clustered | 40 | VC_975_0 | 40 | 0.6888 | 1 | 0.6888 | 0 | 0 | 0 | 1 | Ref |
| MN850573 | Unassigned | Unassigned | Unassigned | 872_0 | Clustered | 28 | VC_872_0 | 28 | 0.7887 | 1 | 0.7887 | 0 | 0 | 0 | 1 | Ref |
| MN850574 | Unassigned | Unassigned | Unassigned | 609_0 | Clustered | 27 | VC_609_0 | 27 | 0.9943 | 1 | 0.9943 | 0 | 0 | 0 | 1 | Ref |
| MN850575 | Unassigned | Unassigned | Unassigned | 859_0 | Clustered | 67 | VC_859_0 | 67 | 0.9622 | 1 | 0.9622 | 0 | 0 | 0 | 1 | Ref |
| MN850576 | Unassigned | Unassigned | Unassigned | 794_19 | Clustered | 16 | VC_794_19 | 16 | 0.0975 | 0.99616626 | 0.0971 | 0 | 0 | 0 | 0.8083 | Ref |
| MN850577 | Unassigned | Unassigned | Unassigned | 340_3 | Clustered | 24 | VC_340_3 | 24 | 0.1205 | 1 | 0.1205 | 0 | 0 | 0 | 0.7283 | Ref |
| MN850578 | Unassigned | Unassigned | Unassigned | 794_19 | Clustered | 16 | VC_794_19 | 16 | 0.0975 | 0.99616626 | 0.0971 | 0 | 0 | 0 | 0.8083 | Ref |
| MN850579 | Unassigned | Unassigned | Unassigned | 293_1 | Clustered | 21 | VC_293_1 | 21 | 0.1694 | 1 | 0.1694 | 0 | 0 | 0 | 0.9048 | Ref |
| MN850580 | Unassigned | Unassigned | Unassigned | 340_3 | Clustered | 24 | VC_340_3 | 24 | 0.1205 | 1 | 0.1205 | 0 | 0 | 0 | 0.7283 | Ref |
| MN850581 | Unassigned | Unassigned | Unassigned | 721_0 | Clustered | 11 | VC_721_0 | 11 | 0.8846 | 1 | 0.8846 | 0 | 0 | 0 | 1 | Ref |
| MN850582 | Unassigned | Unassigned | Unassigned | 976_0 | Clustered | 36 | VC_976_0 | 36 | 0.7833 | 1 | 0.7833 | 0 | 0 | 0 | 1 | Ref |
| MN850583 | Unassigned | Unassigned | Unassigned | 162_0 | Clustered | 12 | VC_162_0 | 12 | 0.9051 | 1 | 0.9051 | 0 | 0 | 0 | 1 | Ref |
| MN850584 | Unassigned | Unassigned | Unassigned | 872_0 | Clustered | 28 | VC_872_0 | 28 | 0.7887 | 1 | 0.7887 | 0 | 0 | 0 | 1 | Ref |
| MN850585 | Unassigned | Unassigned | Unassigned | 340_3 | Clustered | 24 | VC_340_3 | 24 | 0.1205 | 1 | 0.1205 | 0 | 0 | 0 | 0.7283 | Ref |
| MN850586 | Unassigned | Unassigned | Unassigned | 976_0 | Clustered | 36 | VC_976_0 | 36 | 0.7833 | 1 | 0.7833 | 0 | 0 | 0 | 1 | Ref |

| | | | | | | | | | | | | | | | | |
|---|---|---|---|---|---|---|---|---|---|---|---|---|---|---|---|---|
| MN850587 | Unassigned | Unassigned | Unassigned | 341_0 | Clustered | 27 | VC_341_0 | 27 | 0.5058 | 1 | 0.5058 | 0 | 0 | 0 | 0.8661 | Ref |
| MN850588 | Unassigned | Unassigned | Unassigned | 721_0 | Clustered | 11 | VC_721_0 | 11 | 0.8846 | 1 | 0.8846 | 0 | 0 | 0 | 1 | Ref |
| MN850589 | Unassigned | Unassigned | Unassigned | 859_0 | Clustered | 67 | VC_859_0 | 67 | 0.9622 | 1 | 0.9622 | 0 | 0 | 0 | 1 | Ref |
| MN850590 | Unassigned | Unassigned | Unassigned | 293_1 | Clustered | 21 | VC_293_1 | 21 | 0.1694 | 1 | 0.1694 | 0 | 0 | 0 | 0.9048 | Ref |
| MN850591 | Unassigned | Unassigned | Unassigned | 975_0 | Clustered | 40 | VC_975_0 | 40 | 0.6888 | 1 | 0.6888 | 0 | 0 | 0 | 1 | Ref |
| MN850592 | Unassigned | Unassigned | Unassigned | 162_0 | Clustered | 12 | VC_162_0 | 12 | 0.9051 | 1 | 0.9051 | 0 | 0 | 0 | 1 | Ref |
| MN850593 | Unassigned | Unassigned | Unassigned | 794_20 | Clustered | 2 | VC_794_20 | 2 | 0.006 | 0.58868587 | 0.0035 | 0 | 0 | 0 | 1 | Ref |
| MN850594 | Unassigned | Unassigned | Unassigned | 816_0 | Clustered | 35 | VC_816_0 | 35 | 0.9824 | 1 | 0.9824 | 0 | 0 | 0 | 1 | Ref |
| MN850595 | Unassigned | Unassigned | Unassigned | 794_19 | Clustered | 16 | VC_794_19 | 16 | 0.0975 | 0.99616626 | 0.0971 | 0 | 0 | 0 | 0.8083 | Ref |
| MN850596 | Unassigned | Unassigned | Unassigned | 976_0 | Clustered | 36 | VC_976_0 | 36 | 0.7833 | 1 | 0.7833 | 0 | 0 | 0 | 1 | Ref |
| MN850597 | Unassigned | Unassigned | Unassigned | 794_19 | Clustered | 16 | VC_794_19 | 16 | 0.0975 | 0.99616626 | 0.0971 | 0 | 0 | 0 | 0.8083 | Ref |
| MN850598 | Unassigned | Unassigned | Unassigned | 871_1 | Clustered | 26 | VC_871_1 | 26 | 0.6303 | 1 | 0.6303 | 0 | 0 | 0 | 0.9815 | Ref |
| MN850600 | Unassigned | Unassigned | Unassigned | 975_0 | Clustered | 40 | VC_975_0 | 40 | 0.6888 | 1 | 0.6888 | 0 | 0 | 0 | 1 | Ref |
| MN850601 | Unassigned | Unassigned | Unassigned | 872_0 | Clustered | 28 | VC_872_0 | 28 | 0.7887 | 1 | 0.7887 | 0 | 0 | 0 | 1 | Ref |
| MN850602 | Unassigned | Unassigned | Unassigned | 975_0 | Clustered | 40 | VC_975_0 | 40 | 0.6888 | 1 | 0.6888 | 0 | 0 | 0 | 1 | Ref |
| MN850603 | Unassigned | Unassigned | Unassigned | 340_3 | Clustered | 24 | VC_340_3 | 24 | 0.1205 | 1 | 0.1205 | 0 | 0 | 0 | 0.7283 | Ref |
| MN850604 | Unassigned | Unassigned | Unassigned | 976_0 | Clustered | 36 | VC_976_0 | 36 | 0.7833 | 1 | 0.7833 | 0 | 0 | 0 | 1 | Ref |
| MN850605 | Unassigned | Unassigned | Unassigned | 340_3 | Clustered | 24 | VC_340_3 | 24 | 0.1205 | 1 | 0.1205 | 0 | 0 | 0 | 0.7283 | Ref |
| MN850606 | Unassigned | Unassigned | Unassigned | 976_0 | Clustered | 36 | VC_976_0 | 36 | 0.7833 | 1 | 0.7833 | 0 | 0 | 0 | 1 | Ref |
| MN850607 | Unassigned | Unassigned | Unassigned | 975_0 | Clustered | 40 | VC_975_0 | 40 | 0.6888 | 1 | 0.6888 | 0 | 0 | 0 | 1 | Ref |
| MN850608 | Unassigned | Unassigned | Unassigned | 162_0 | Clustered | 12 | VC_162_0 | 12 | 0.9051 | 1 | 0.9051 | 0 | 0 | 0 | 1 | Ref |
| MN850609 | Unassigned | Unassigned | Unassigned | 297_6 | Clustered | 12 | VC_297_6 | 12 | 0.1139 | 1 | 0.1139 | 0 | 0 | 0 | 0.8182 | Ref |
| MN850610 | Unassigned | Unassigned | Unassigned | 340_3 | Clustered | 24 | VC_340_3 | 24 | 0.1205 | 1 | 0.1205 | 0 | 0 | 0 | 0.7283 | Ref |
| MN850611 | Unassigned | Unassigned | Unassigned | 340_3 | Clustered | 24 | VC_340_3 | 24 | 0.1205 | 1 | 0.1205 | 0 | 0 | 0 | 0.7283 | Ref |
| MN850612 | Unassigned | Unassigned | Unassigned | 794_19 | Clustered | 16 | VC_794_19 | 16 | 0.0975 | 0.99616626 | 0.0971 | 0 | 0 | 0 | 0.8083 | Ref |
| MN850613 | Unassigned | Unassigned | Unassigned | 976_0 | Clustered | 36 | VC_976_0 | 36 | 0.7833 | 1 | 0.7833 | 0 | 0 | 0 | 1 | Ref |
| MN850614 | Unassigned | Unassigned | Unassigned | 340_3 | Clustered | 24 | VC_340_3 | 24 | 0.1205 | 1 | 0.1205 | 0 | 0 | 0 | 0.7283 | Ref |
| MN850615 | Unassigned | Unassigned | Unassigned | 609_0 | Clustered | 27 | VC_609_0 | 27 | 0.9943 | 1 | 0.9943 | 0 | 0 | 0 | 1 | Ref |
| MN850616 | Unassigned | Unassigned | Unassigned | 654_0 | Clustered | 115 | VC_654_0 | 115 | 0.7303 | 1 | 0.7303 | 0 | 0 | 0 | 0.8764 | Ref |
| MN850617 | Unassigned | Unassigned | Unassigned | 162_0 | Clustered | 12 | VC_162_0 | 12 | 0.9051 | 1 | 0.9051 | 0 | 0 | 0 | 1 | Ref |
| MN850618 | Unassigned | Unassigned | Unassigned | 871_1 | Clustered | 26 | VC_871_1 | 26 | 0.6303 | 1 | 0.6303 | 0 | 0 | 0 | 0.9815 | Ref |
| MN850619 | Unassigned | Unassigned | Unassigned | 340_3 | Clustered | 24 | VC_340_3 | 24 | 0.1205 | 1 | 0.1205 | 0 | 0 | 0 | 0.7283 | Ref |
| MN850620 | Unassigned | Unassigned | Unassigned | 976_0 | Clustered | 36 | VC_976_0 | 36 | 0.7833 | 1 | 0.7833 | 0 | 0 | 0 | 1 | Ref |
| MN850621 | Unassigned | Unassigned | Unassigned | 297_6 | Clustered | 12 | VC_297_6 | 12 | 0.1139 | 1 | 0.1139 | 0 | 0 | 0 | 0.8182 | Ref |
| MN850622 | Unassigned | Unassigned | Unassigned | 293_17 | Clustered | 8 | VC_293_17 | 8 | 0.0534 | 0.99997879 | 0.0534 | 0 | 0 | 0 | 0.6071 | Ref |
| MN850623 | Unassigned | Unassigned | Unassigned | 721_0 | Clustered | 11 | VC_721_0 | 11 | 0.8846 | 1 | 0.8846 | 0 | 0 | 0 | 1 | Ref |
| MN850624 | Unassigned | Unassigned | Unassigned | 162_0 | Clustered | 12 | VC_162_0 | 12 | 0.9051 | 1 | 0.9051 | 0 | 0 | 0 | 1 | Ref |
| MN850625 | Unassigned | Unassigned | Unassigned | 162_0 | Clustered | 12 | VC_162_0 | 12 | 0.9051 | 1 | 0.9051 | 0 | 0 | 0 | 1 | Ref |
| MN850626 | Unassigned | Unassigned | Unassigned | 794_19 | Clustered | 16 | VC_794_19 | 16 | 0.0975 | 0.99616626 | 0.0971 | 0 | 0 | 0 | 0.8083 | Ref |
| MN850627 | Unassigned | Unassigned | Unassigned | 794_19 | Clustered | 16 | VC_794_19 | 16 | 0.0975 | 0.99616626 | 0.0971 | 0 | 0 | 0 | 0.8083 | Ref |
| MN850628 | Unassigned | Unassigned | Unassigned | 859_0 | Clustered | 67 | VC_859_0 | 67 | 0.9622 | 1 | 0.9622 | 0 | 0 | 0 | 1 | Ref |
| MN850629 | Unassigned | Unassigned | Unassigned | 859_0 | Clustered | 67 | VC_859_0 | 67 | 0.9622 | 1 | 0.9622 | 0 | 0 | 0 | 1 | Ref |
| MN850630 | Unassigned | Unassigned | Unassigned | 794_10 | Clustered | 8 | VC_794_10 | 8 | 0.0433 | 0.9088183 | 0.0394 | 0 | 0 | 0 | 0.9286 | Ref |
| MN850631 | Unassigned | Unassigned | Unassigned | 340_2 | Clustered | 2 | VC_340_2 | 2 | 0.0049 | 0.95560086 | 0.0047 | 0 | 0 | 0 | 1 | Ref |
| MN850632 | Unassigned | Unassigned | Unassigned | 872_0 | Clustered | 28 | VC_872_0 | 28 | 0.7887 | 1 | 0.7887 | 0 | 0 | 0 | 1 | Ref |
| MN850633 | Unassigned | Unassigned | Unassigned | 340_3 | Clustered | 24 | VC_340_3 | 24 | 0.1205 | 1 | 0.1205 | 0 | 0 | 0 | 0.7283 | Ref |
| MN850634 | Unassigned | Unassigned | Unassigned | 976_0 | Clustered | 36 | VC_976_0 | 36 | 0.7833 | 1 | 0.7833 | 0 | 0 | 0 | 1 | Ref |
| MN850635 | Unassigned | Unassigned | Unassigned | 340_3 | Clustered | 24 | VC_340_3 | 24 | 0.1205 | 1 | 0.1205 | 0 | 0 | 0 | 0.7283 | Ref |
| MN850636 | Unassigned | Unassigned | Unassigned | 340_3 | Clustered | 24 | VC_340_3 | 24 | 0.1205 | 1 | 0.1205 | 0 | 0 | 0 | 0.7283 | Ref |
| MN850637 | Unassigned | Unassigned | Unassigned | 340_2 | Clustered | 2 | VC_340_2 | 2 | 0.0049 | 0.95560086 | 0.0047 | 0 | 0 | 0 | 1 | Ref |
| MN850638 | Unassigned | Unassigned | Unassigned | 976_0 | Clustered | 36 | VC_976_0 | 36 | 0.7833 | 1 | 0.7833 | 0 | 0 | 0 | 1 | Ref |
| MN850639 | Unassigned | Unassigned | Unassigned | 340_3 | Clustered | 24 | VC_340_3 | 24 | 0.1205 | 1 | 0.1205 | 0 | 0 | 0 | 0.7283 | Ref |
| MN850640 | Unassigned | Unassigned | Unassigned | 975_0 | Clustered | 40 | VC_975_0 | 40 | 0.6888 | 1 | 0.6888 | 0 | 0 | 0 | 1 | Ref |
| MN850641 | Unassigned | Unassigned | Unassigned | 976_0 | Clustered | 36 | VC_976_0 | 36 | 0.7833 | 1 | 0.7833 | 0 | 0 | 0 | 1 | Ref |
| MN850642 | Unassigned | Unassigned | Unassigned | 794_16 | Clustered | 10 | VC_794_16 | 10 | 0.0563 | 0.9411319 | 0.053 | 0 | 0 | 0 | 0.8667 | Ref |
| MN850643 | Unassigned | Unassigned | Unassigned | 976_0 | Clustered | 36 | VC_976_0 | 36 | 0.7833 | 1 | 0.7833 | 0 | 0 | 0 | 1 | Ref |
| MN850644 | Unassigned | Unassigned | Unassigned | 340_3 | Clustered | 24 | VC_340_3 | 24 | 0.1205 | 1 | 0.1205 | 0 | 0 | 0 | 0.7283 | Ref |
| MN850645 | Unassigned | Unassigned | Unassigned | 872_0 | Clustered | 28 | VC_872_0 | 28 | 0.7887 | 1 | 0.7887 | 0 | 0 | 0 | 1 | Ref |
| MN850646 | Unassigned | Unassigned | Unassigned | 794_6 | Clustered | 3 | VC_794_6 | 3 | 0.012 | 0.63979507 | 0.0077 | 0 | 0 | 0 | 1 | Ref |
| MN850647 | Unassigned | Unassigned | Unassigned | 340_3 | Clustered | 24 | VC_340_3 | 24 | 0.1205 | 1 | 0.1205 | 0 | 0 | 0 | 0.7283 | Ref |
| MN850648 | Unassigned | Unassigned | Unassigned | 871_1 | Clustered | 26 | VC_871_1 | 26 | 0.6303 | 1 | 0.6303 | 0 | 0 | 0 | 0.9815 | Ref |
| MN850649 | Unassigned | Unassigned | Unassigned | 794_6 | Clustered | 3 | VC_794_6 | 3 | 0.012 | 0.63979507 | 0.0077 | 0 | 0 | 0 | 1 | Ref |
| MN850650 | Unassigned | Unassigned | Unassigned | 859_0 | Clustered | 67 | VC_859_0 | 67 | 0.9622 | 1 | 0.9622 | 0 | 0 | 0 | 1 | Ref |
| MN850651 | Unassigned | Unassigned | Unassigned | 293_2 | Clustered/Singleton | 1 | VC_293_2 | 1 | 0 | 1 | 0 | 0 | 0 | 0 | 1 | Ref |
| MN854830 | Unassigned | Unassigned | Unassigned | 93_0 | Clustered | 22 | VC_93_0 | 22 | 0.5285 | 1 | 0.5285 | 0 | 0 | 0 | 1 | Ref |
| MN862068 | Unassigned | Unassigned | Unassigned | 481_0 | Clustered | 5 | VC_481_0 | 5 | 0.6783 | 0.99999996 | 0.6783 | 0 | 0 | 0 | 1 | Ref |
| MN862333 | Unassigned | Unassigned | Unassigned | 1028_0 | Clustered | 69 | VC_1028_0 | 69 | 0.8547 | 1 | 0.8547 | 0 | 0 | 0 | 1 | Ref |
| MN862335 | Unassigned | Unassigned | Unassigned | 1028_0 | Clustered | 69 | VC_1028_0 | 69 | 0.8547 | 1 | 0.8547 | 0 | 0 | 0 | 1 | Ref |
| MN862338 | Unassigned | Unassigned | Unassigned | 1108_0 | Clustered | 52 | VC_1108_0 | 52 | 0.7674 | 1 | 0.7674 | 0 | 0 | 0 | 1 | Ref |
| MN862339 | Unassigned | Unassigned | Unassigned | 1029_0 | Clustered | 2 | VC_1029_0 | 2 | 0.0422 | 0.59038153 | 0.0249 | 0 | 0 | 0 | 1 | Ref |
| MN862340 | Unassigned | Unassigned | Unassigned | 1029_0 | Clustered | 2 | VC_1029_0 | 2 | 0.0422 | 0.59038153 | 0.0249 | 0 | 0 | 0 | 1 | Ref |
| MN862341 | Unassigned | Unassigned | Unassigned | 1028_0 | Clustered | 69 | VC_1028_0 | 69 | 0.8547 | 1 | 0.8547 | 0 | 0 | 0 | 1 | Ref |
| MN862343 | Unassigned | Unassigned | Unassigned | 206_0 | Clustered | 28 | VC_206_0 | 28 | 0.2427 | 0.99971175 | 0.2426 | 0 | 0 | 0 | 1 | Ref |
| MN862345 | Unassigned | Unassigned | Unassigned | 205_0 | Clustered | 5 | VC_205_0 | 5 | 0.1325 | 0.84698401 | 0.1122 | 0 | 0 | 0 | 1 | Ref |
| MN862346 | Unassigned | Unassigned | Unassigned | 1119_0 | Clustered | 48 | VC_1119_0 | 48 | 0.852 | 1 | 0.852 | 0 | 0 | 0 | 1 | Ref |
| MN862347 | Unassigned | Unassigned | Unassigned | 1110_0 | Clustered | 2 | VC_1110_0 | 2 | 0.0455 | 0.92554266 | 0.0422 | 0 | 0 | 0 | 1 | Ref |
| MN864145 | Unassigned | Unassigned | Unassigned | 293_6 | Clustered/Singleton | 1 | VC_293_6 | 1 | 0 | 1 | 0 | 0 | 0 | 0 | 1 | Ref |
| MN864865 | Unassigned | Unassigned | Unassigned | 510_2 | Clustered | 8 | VC_510_2 | 8 | 0.122 | 0.98786574 | 0.1205 | 0 | 0 | 0 | 1 | Ref |
| MN871440 | Unassigned | Unassigned | Unassigned | 457_0 | Clustered | 7 | VC_457_0 | 7 | 0.2549 | 1 | 0.2549 | 0 | 0 | 0 | 0.7143 | Ref |
| MN871441 | Unassigned | Unassigned | Unassigned | 457_4 | Clustered | 10 | VC_457_4 | 10 | 0.3316 | 1 | 0.3316 | 0 | 0 | 0 | 0.9333 | Ref |
| MN871442 | Unassigned | Unassigned | Unassigned | 457_4 | Clustered | 10 | VC_457_4 | 10 | 0.3316 | 1 | 0.3316 | 0 | 0 | 0 | 0.9333 | Ref |

| | | | | | | | | | | | | | | | | |
|---|---|---|---|---|---|---|---|---|---|---|---|---|---|---|---|---|
| MN871443 | Unassigned | Unassigned | Unassigned | 110_0 | Clustered | 33 | VC_110_0 | 33 | 0.9488 | 1 | 0.9488 | 0 | 0 | 0 | 1 | Ref |
| MN871444 | Unassigned | Unassigned | Unassigned | 359_0 | Clustered | 3 | VC_359_0 | 3 | 0.1509 | 0.99844728 | 0.1507 | 0 | 0 | 0 | 1 | Ref |
| MN871445 | Unassigned | Unassigned | Unassigned | 359_0 | Clustered | 3 | VC_359_0 | 3 | 0.1509 | 0.99844728 | 0.1507 | 0 | 0 | 0 | 1 | Ref |
| MN871446 | Unassigned | Unassigned | Unassigned | 506_0 | Clustered | 8 | VC_506_0 | 8 | 0.29 | 1 | 0.29 | 0 | 0 | 0 | 1 | Ref |
| MN871447 | Unassigned | Unassigned | Unassigned | 506_0 | Clustered | 8 | VC_506_0 | 8 | 0.29 | 1 | 0.29 | 0 | 0 | 0 | 1 | Ref |
| MN871448 | Unassigned | Unassigned | Unassigned | 506_0 | Clustered | 8 | VC_506_0 | 8 | 0.29 | 1 | 0.29 | 0 | 0 | 0 | 1 | Ref |
| MN871449 | Unassigned | Unassigned | Unassigned | 506_0 | Clustered | 8 | VC_506_0 | 8 | 0.29 | 1 | 0.29 | 0 | 0 | 0 | 1 | Ref |
| MN871450 | Unassigned | Unassigned | Unassigned | 611_0 | Clustered | 50 | VC_611_0 | 50 | 0.7306 | 1 | 0.7306 | 0 | 0 | 0 | 1 | Ref |
| MN871451 | Unassigned | Unassigned | Unassigned | 506_0 | Clustered | 8 | VC_506_0 | 8 | 0.29 | 1 | 0.29 | 0 | 0 | 0 | 1 | Ref |
| MN871452 | Unassigned | Unassigned | Unassigned | 506_0 | Clustered | 8 | VC_506_0 | 8 | 0.29 | 1 | 0.29 | 0 | 0 | 0 | 1 | Ref |
| MN871453 | Unassigned | Unassigned | Unassigned | 506_0 | Clustered | 8 | VC_506_0 | 8 | 0.29 | 1 | 0.29 | 0 | 0 | 0 | 1 | Ref |
| MN871454 | Unassigned | Unassigned | Unassigned | 81_0 | Clustered | 41 | VC_81_0 | 41 | 0.5815 | 1 | 0.5815 | 0 | 0 | 0 | 0.9805 | Ref |
| MN871455 | Unassigned | Unassigned | Unassigned | 434_0 | Clustered | 45 | VC_434_0 | 45 | 0.9479 | 1 | 0.9479 | 0 | 0 | 0 | 1 | Ref |
| MN871456 | Unassigned | Unassigned | Unassigned | 172_0 | Clustered | 26 | VC_172_0 | 26 | 0.7909 | 1 | 0.7909 | 0 | 0 | 0 | 1 | Ref |
| MN871457 | Unassigned | Unassigned | Unassigned | 80_6 | Clustered | 11 | VC_80_6 | 11 | 0.0527 | 1 | 0.0527 | 0 | 0 | 0 | 0.7455 | Ref |
| MN871458 | Unassigned | Unassigned | Unassigned | 80_0 | Clustered | 64 | VC_80_0 | 64 | 0.418 | 1 | 0.418 | 0 | 0 | 0 | 0.7649 | Ref |
| MN871459 | Unassigned | Unassigned | Unassigned | 80_0 | Clustered | 64 | VC_80_0 | 64 | 0.418 | 1 | 0.418 | 0 | 0 | 0 | 0.7649 | Ref |
| MN871460 | Unassigned | Unassigned | Unassigned | 80_6 | Clustered | 11 | VC_80_6 | 11 | 0.0527 | 1 | 0.0527 | 0 | 0 | 0 | 0.7455 | Ref |
| MN871461 | Unassigned | Unassigned | Unassigned | 80_0 | Clustered | 64 | VC_80_0 | 64 | 0.418 | 1 | 0.418 | 0 | 0 | 0 | 0.7649 | Ref |
| MN871462 | Unassigned | Unassigned | Unassigned | 80_0 | Clustered | 64 | VC_80_0 | 64 | 0.418 | 1 | 0.418 | 0 | 0 | 0 | 0.7649 | Ref |
| MN871463 | Unassigned | Unassigned | Unassigned | 172_0 | Clustered | 26 | VC_172_0 | 26 | 0.7909 | 1 | 0.7909 | 0 | 0 | 0 | 1 | Ref |
| MN871464 | Unassigned | Unassigned | Unassigned | 80_0 | Clustered | 64 | VC_80_0 | 64 | 0.418 | 1 | 0.418 | 0 | 0 | 0 | 0.7649 | Ref |
| MN871465 | Unassigned | Unassigned | Unassigned | 80_0 | Clustered | 64 | VC_80_0 | 64 | 0.418 | 1 | 0.418 | 0 | 0 | 0 | 0.7649 | Ref |
| MN871466 | Unassigned | Unassigned | Unassigned | 80_0 | Clustered | 64 | VC_80_0 | 64 | 0.418 | 1 | 0.418 | 0 | 0 | 0 | 0.7649 | Ref |
| MN871467 | Unassigned | Unassigned | Unassigned | 80_6 | Clustered | 11 | VC_80_6 | 11 | 0.0527 | 1 | 0.0527 | 0 | 0 | 0 | 0.7455 | Ref |
| MN871468 | Unassigned | Unassigned | Unassigned | 80_9 | Clustered | 2 | VC_80_9 | 2 | 0.0061 | 0.94860001 | 0.0058 | 0 | 0 | 0 | 1 | Ref |
| MN871469 | Unassigned | Unassigned | Unassigned | 172_0 | Clustered | 26 | VC_172_0 | 26 | 0.7909 | 1 | 0.7909 | 0 | 0 | 0 | 1 | Ref |
| MN871470 | Unassigned | Unassigned | Unassigned | 77_0 | Clustered | 37 | VC_77_0 | 37 | 0.9392 | 1 | 0.9392 | 0 | 0 | 0 | 1 | Ref |
| MN871471 | Unassigned | Unassigned | Unassigned | 820_0 | Clustered | 5 | VC_820_0 | 5 | 0.332 | 0.99999997 | 0.332 | 0 | 0 | 0 | 1 | Ref |
| MN871472 | Unassigned | Unassigned | Unassigned | 820_0 | Clustered | 5 | VC_820_0 | 5 | 0.332 | 0.99999997 | 0.332 | 0 | 0 | 0 | 1 | Ref |
| MN871473 | Unassigned | Unassigned | Unassigned | 80_0 | Clustered | 64 | VC_80_0 | 64 | 0.418 | 1 | 0.418 | 0 | 0 | 0 | 0.7649 | Ref |
| MN871474 | Unassigned | Unassigned | Unassigned | 820_0 | Clustered | 5 | VC_820_0 | 5 | 0.332 | 0.99999997 | 0.332 | 0 | 0 | 0 | 1 | Ref |
| MN871475 | Unassigned | Unassigned | Unassigned | 135_29 | Clustered | 2 | VC_135_29 | 2 | 0.0052 | 0.85639654 | 0.0044 | 0 | 0 | 0 | 1 | Ref |
| MN871476 | Unassigned | Unassigned | Unassigned | 486_0 | Clustered | 47 | VC_486_0 | 47 | 0.9022 | 1 | 0.9022 | 0 | 0 | 0 | 1 | Ref |
| MN871478 | Unassigned | Unassigned | Unassigned | 172_0 | Clustered | 26 | VC_172_0 | 26 | 0.7909 | 1 | 0.7909 | 0 | 0 | 0 | 1 | Ref |
| MN871479 | Unassigned | Unassigned | Unassigned | 80_0 | Clustered | 64 | VC_80_0 | 64 | 0.418 | 1 | 0.418 | 0 | 0 | 0 | 0.7649 | Ref |
| MN871480 | Unassigned | Unassigned | Unassigned | 356_0 | Clustered | 21 | VC_356_0 | 21 | 0.5409 | 1 | 0.5409 | 0 | 0 | 0 | 1 | Ref |
| MN871481 | Unassigned | Unassigned | Unassigned | 77_0 | Clustered | 37 | VC_77_0 | 37 | 0.9392 | 1 | 0.9392 | 0 | 0 | 0 | 1 | Ref |
| MN871482 | Unassigned | Unassigned | Unassigned | 172_0 | Clustered | 26 | VC_172_0 | 26 | 0.7909 | 1 | 0.7909 | 0 | 0 | 0 | 1 | Ref |
| MN871483 | Unassigned | Unassigned | Unassigned | 434_0 | Clustered | 45 | VC_434_0 | 45 | 0.9479 | 1 | 0.9479 | 0 | 0 | 0 | 1 | Ref |
| MN871484 | Unassigned | Unassigned | Unassigned | 486_0 | Clustered | 47 | VC_486_0 | 47 | 0.9022 | 1 | 0.9022 | 0 | 0 | 0 | 1 | Ref |
| MN871485 | Unassigned | Unassigned | Unassigned | 80_0 | Clustered | 64 | VC_80_0 | 64 | 0.418 | 1 | 0.418 | 0 | 0 | 0 | 0.7649 | Ref |
| MN871486 | Unassigned | Unassigned | Unassigned | 172_0 | Clustered | 26 | VC_172_0 | 26 | 0.7909 | 1 | 0.7909 | 0 | 0 | 0 | 1 | Ref |
| MN871487 | Unassigned | Unassigned | Unassigned | 80_0 | Clustered | 64 | VC_80_0 | 64 | 0.418 | 1 | 0.418 | 0 | 0 | 0 | 0.7649 | Ref |
| MN871488 | Unassigned | Unassigned | Unassigned | 77_0 | Clustered | 37 | VC_77_0 | 37 | 0.9392 | 1 | 0.9392 | 0 | 0 | 0 | 1 | Ref |
| MN871489 | Unassigned | Unassigned | Unassigned | 356_0 | Clustered | 21 | VC_356_0 | 21 | 0.5409 | 1 | 0.5409 | 0 | 0 | 0 | 1 | Ref |
| MN871490 | Unassigned | Unassigned | Unassigned | 475_0 | Clustered | 96 | VC_475_0 | 96 | 0.9303 | 1 | 0.9303 | 0 | 0 | 0 | 0.9575 | Ref |
| MN871491 | Unassigned | Unassigned | Unassigned | 135_36 | Clustered | 2 | VC_135_36 | 2 | 0.0051 | 0.86200079 | 0.0044 | 0 | 0 | 0 | 1 | Ref |
| MN871492 | Unassigned | Unassigned | Unassigned | 135_36 | Clustered | 2 | VC_135_36 | 2 | 0.0051 | 0.86200079 | 0.0044 | 0 | 0 | 0 | 1 | Ref |
| MN871493 | Unassigned | Unassigned | Unassigned | 135_37 | Clustered/Singleton | 1 | VC_135_37 | 1 | 0 | 1 | 0 | 0 | 0 | 0 | 1 | Ref |
| MN871494 | Unassigned | Unassigned | Unassigned | 135_12 | Clustered | 2 | VC_135_12 | 2 | 0.005 | 0.8641083 | 0.0044 | 0 | 0 | 0 | 1 | Ref |
| MN871495 | Unassigned | Unassigned | Unassigned | 135_12 | Clustered | 2 | VC_135_12 | 2 | 0.005 | 0.8641083 | 0.0044 | 0 | 0 | 0 | 1 | Ref |
| MN871496 | Unassigned | Unassigned | Unassigned | 135_43 | Clustered | 4 | VC_135_43 | 4 | 0.0149 | 0.99993288 | 0.0149 | 0 | 0 | 0 | 1 | Ref |
| MN871497 | Unassigned | Unassigned | Unassigned | 135_43 | Clustered | 4 | VC_135_43 | 4 | 0.0149 | 0.99993288 | 0.0149 | 0 | 0 | 0 | 1 | Ref |
| MN871498 | Unassigned | Unassigned | Unassigned | 135_43 | Clustered | 4 | VC_135_43 | 4 | 0.0149 | 0.99993288 | 0.0149 | 0 | 0 | 0 | 1 | Ref |
| MN871499 | Unassigned | Unassigned | Unassigned | 135_43 | Clustered | 4 | VC_135_43 | 4 | 0.0149 | 0.99993288 | 0.0149 | 0 | 0 | 0 | 1 | Ref |
| MN871500 | Unassigned | Unassigned | Unassigned | 135_19 | Clustered | 2 | VC_135_19 | 2 | 0.005 | 0.8667049 | 0.0044 | 0 | 0 | 0 | 1 | Ref |
| MN871501 | Unassigned | Unassigned | Unassigned | 135_29 | Clustered | 2 | VC_135_29 | 2 | 0.0052 | 0.85639654 | 0.0044 | 0 | 0 | 0 | 1 | Ref |
| MN871502 | Unassigned | Unassigned | Unassigned | 135_38 | Clustered/Singleton | 1 | VC_135_38 | 1 | 0 | 1 | 0 | 0 | 0 | 0 | 1 | Ref |
| MN871503 | Unassigned | Unassigned | Unassigned | 475_0 | Clustered | 96 | VC_475_0 | 96 | 0.9303 | 1 | 0.9303 | 0 | 0 | 0 | 0.9575 | Ref |
| MN871504 | Unassigned | Unassigned | Unassigned | 135_10 | Clustered | 10 | VC_135_10 | 10 | 0.0522 | 1 | 0.0522 | 0 | 0 | 0 | 1 | Ref |
| MN871505 | Unassigned | Unassigned | Unassigned | 1008_0 | Clustered | 6 | VC_1008_0 | 6 | 0.2856 | 1 | 0.2856 | 0 | 0 | 0 | 1 | Ref |
| MN871506 | Unassigned | Unassigned | Unassigned | 935_2 | Clustered | 7 | VC_935_2 | 7 | 0.2837 | 1 | 0.2837 | 0 | 0 | 0 | 1 | Ref |
| MN871507 | Unassigned | Unassigned | Unassigned | 619_0 | Clustered | 3 | VC_619_0 | 3 | 0.054 | 0.97294713 | 0.0526 | 0 | 0 | 0 | 1 | Ref |
| MN871508 | Unassigned | Unassigned | Unassigned | 80_0 | Clustered | 64 | VC_80_0 | 64 | 0.418 | 1 | 0.418 | 0 | 0 | 0 | 0.7649 | Ref |
| MN871509 | Unassigned | Unassigned | Unassigned | 80_0 | Clustered | 64 | VC_80_0 | 64 | 0.418 | 1 | 0.418 | 0 | 0 | 0 | 0.7649 | Ref |
| MN871510 | Unassigned | Unassigned | Unassigned | 1008_0 | Clustered | 6 | VC_1008_0 | 6 | 0.2856 | 1 | 0.2856 | 0 | 0 | 0 | 1 | Ref |
| MN871511 | Unassigned | Unassigned | Unassigned | 1008_0 | Clustered | 6 | VC_1008_0 | 6 | 0.2856 | 1 | 0.2856 | 0 | 0 | 0 | 1 | Ref |
| MN876841 | Unassigned | Unassigned | Unassigned | 360_0 | Clustered | 22 | VC_360_0 | 22 | 0.96 | 1 | 0.96 | 0 | 0 | 0 | 1 | Ref |
| MN876842 | Unassigned | Unassigned | Unassigned | 360_0 | Clustered | 22 | VC_360_0 | 22 | 0.96 | 1 | 0.96 | 0 | 0 | 0 | 1 | Ref |
| MN876843 | Unassigned | Unassigned | Unassigned | 360_0 | Clustered | 22 | VC_360_0 | 22 | 0.96 | 1 | 0.96 | 0 | 0 | 0 | 1 | Ref |
| MN876845 | Unassigned | Unassigned | Unassigned | 454_0 | Clustered | 4 | VC_454_0 | 4 | 1 | 1 | 1 | 0 | 0 | 0 | 1 | Ref |
| MN882349 | Unassigned | Unassigned | Unassigned | 609_0 | Clustered | 27 | VC_609_0 | 27 | 0.9943 | 1 | 0.9943 | 0 | 0 | 0 | 1 | Ref |
| MN882542 | Unassigned | Unassigned | Unassigned | 340_18 | Clustered | 7 | VC_340_18 | 7 | 0.0326 | 1 | 0.0326 | 0 | 0 | 0 | 0.8095 | Ref |
| MN882610 | Unassigned | Unassigned | Unassigned | 508_1 | Clustered | 5 | VC_508_1 | 5 | 0.0439 | 0.99999996 | 0.0439 | 0 | 0 | 0 | 1 | Ref |
| MN890136 | Unassigned | Unassigned | Unassigned | 736_0 | Clustered | 9 | VC_736_0 | 9 | 1 | 1 | 1 | 0 | 0 | 0 | 1 | Ref |
| MN890137 | Unassigned | Unassigned | Unassigned | 736_0 | Clustered | 9 | VC_736_0 | 9 | 1 | 1 | 1 | 0 | 0 | 0 | 1 | Ref |
| MN890138 | Unassigned | Unassigned | Unassigned | 736_0 | Clustered | 9 | VC_736_0 | 9 | 1 | 1 | 1 | 0 | 0 | 0 | 1 | Ref |
| MN890139 | Unassigned | Unassigned | Unassigned | 736_0 | Clustered | 9 | VC_736_0 | 9 | 1 | 1 | 1 | 0 | 0 | 0 | 1 | Ref |
| MN890140 | Unassigned | Unassigned | Unassigned | 736_0 | Clustered | 9 | VC_736_0 | 9 | 1 | 1 | 1 | 0 | 0 | 0 | 1 | Ref |
| MN890141 | Unassigned | Unassigned | Unassigned | 736_0 | Clustered | 9 | VC_736_0 | 9 | 1 | 1 | 1 | 0 | 0 | 0 | 1 | Ref |

| | | | | | | | | | | | | | | | | |
|---|---|---|---|---|---|---|---|---|---|---|---|---|---|---|---|---|
| MN890142 | Unassigned | Unassigned | Unassigned | 736_0 | Clustered | 9 | VC_736_0 | 9 | 1 | 1 | 1 | 0 | 0 | 0 | 1 | Ref |
| MN890143 | Unassigned | Unassigned | Unassigned | 736_0 | Clustered | 9 | VC_736_0 | 9 | 1 | 1 | 1 | 0 | 0 | 0 | 1 | Ref |
| MN892483 | Unassigned | Unassigned | Unassigned | 1296_0 | Clustered | 82 | VC_1296_0 | 82 | 0.7938 | 1 | 0.7938 | 0 | 0 | 0 | 1 | Ref |
| MN892484 | Unassigned | Unassigned | Unassigned | 537_0 | Clustered | 39 | VC_537_0 | 39 | 0.8148 | 1 | 0.8148 | 0 | 0 | 0 | 1 | Ref |
| MN892486 | Unassigned | Unassigned | Unassigned | 537_0 | Clustered | 39 | VC_537_0 | 39 | 0.8148 | 1 | 0.8148 | 0 | 0 | 0 | 1 | Ref |
| MN892487 | Unassigned | Unassigned | Unassigned | 228_0 | Clustered | 22 | VC_228_0 | 22 | 0.7411 | 1 | 0.7411 | 0 | 0 | 0 | 1 | Ref |
| MN894885 | Unassigned | Unassigned | Unassigned | 609_0 | Clustered | 27 | VC_609_0 | 27 | 0.9943 | 1 | 0.9943 | 0 | 0 | 0 | 1 | Ref |
| MN895434 | Unassigned | Unassigned | Unassigned | 290_39 | Clustered/Singleton | 1 | VC_290_39 | 1 | 0 | 1 | 0 | 0 | 0 | 0 | 1 | Ref |
| MN895435 | Unassigned | Unassigned | Unassigned | 290_51 | Clustered/Singleton | 1 | VC_290_51 | 1 | 0 | 1 | 0 | 0 | 0 | 0 | 1 | Ref |
| MN895436 | Unassigned | Unassigned | Unassigned | 290_48 | Clustered/Singleton | 1 | VC_290_48 | 1 | 0 | 1 | 0 | 0 | 0 | 0 | 1 | Ref |
| MN895437 | Unassigned | Unassigned | Unassigned | 290_31 | Clustered/Singleton | 1 | VC_290_31 | 1 | 0 | 1 | 0 | 0 | 0 | 0 | 1 | Ref |
| MN895438 | Unassigned | Unassigned | Unassigned | 290_44 | Clustered/Singleton | 1 | VC_290_44 | 1 | 0 | 1 | 0 | 0 | 0 | 0 | 1 | Ref |
| MN901520 | Unassigned | Unassigned | Unassigned | 317_0 | Clustered | 33 | VC_317_0 | 33 | 0.8756 | 1 | 0.8756 | 0 | 0 | 0 | 1 | Ref |
| MN901521 | Unassigned | Unassigned | Unassigned | 317_0 | Clustered | 33 | VC_317_0 | 33 | 0.8756 | 1 | 0.8756 | 0 | 0 | 0 | 1 | Ref |
| MN901924 | Unassigned | Unassigned | Unassigned | 434_0 | Clustered | 45 | VC_434_0 | 45 | 0.9479 | 1 | 0.9479 | 0 | 0 | 0 | 1 | Ref |
| MN904502 | Unassigned | Unassigned | Unassigned | 1002_0 | Clustered | 10 | VC_1002_0 | 10 | 0.9987 | 1 | 0.9987 | 0 | 0 | 0 | 1 | Ref |
| MN904510 | Unassigned | Unassigned | Unassigned | 12_2 | Clustered | 19 | VC_12_2 | 19 | 0.2009 | 1 | 0.2009 | 0 | 0 | 0 | 1 | Ref |
| MN905160 | Unassigned | Unassigned | Unassigned | 79_3 | Clustered | 11 | VC_79_3 | 11 | 0.2561 | 1 | 0.2561 | 0 | 0 | 0 | 1 | Ref |
| MN906996 | Unassigned | Unassigned | Unassigned | 862_0 | Clustered | 29 | VC_862_0 | 29 | 0.8594 | 1 | 0.8594 | 0 | 0 | 0 | 1 | Ref |
| MN908683 | Unassigned | Unassigned | Unassigned | 537_0 | Clustered | 39 | VC_537_0 | 39 | 0.8148 | 1 | 0.8148 | 0 | 0 | 0 | 1 | Ref |
| MN908685 | Unassigned | Unassigned | Unassigned | 1143_0 | Clustered | 2 | VC_1143_0 | 2 | 0.1263 | 0.94673154 | 0.1196 | 0 | 0 | 0 | 1 | Ref |
| MN908689 | Unassigned | Unassigned | Unassigned | 1250_0 | Clustered | 29 | VC_1250_0 | 29 | 0.822 | 1 | 0.822 | 0 | 0 | 0 | 1 | Ref |
| MN908690 | Unassigned | Unassigned | Unassigned | 881_1 | Clustered | 13 | VC_881_1 | 13 | 0.5859 | 1 | 0.5859 | 0 | 0 | 0 | 1 | Ref |
| MN908691 | Unassigned | Unassigned | Unassigned | 1224_0 | Clustered | 4 | VC_1224_0 | 4 | 0.2813 | 0.99997676 | 0.2813 | 0 | 0 | 0 | 1 | Ref |
| MN908692 | Unassigned | Unassigned | Unassigned | 243_0 | Clustered | 72 | VC_243_0 | 72 | 0.8089 | 1 | 0.8089 | 0 | 0 | 0 | 1 | Ref |
| MN908693 | Unassigned | Unassigned | Unassigned | 528_0 | Clustered | 12 | VC_528_0 | 12 | 0.3923 | 1 | 0.3923 | 0 | 0 | 0 | 0.9697 | Ref |
| MN927225 | Unassigned | Unassigned | Unassigned | 655_0 | Clustered | 45 | VC_655_0 | 45 | 0.7722 | 1 | 0.7722 | 0 | 0 | 0 | 1 | Ref |
| MN927226 | Unassigned | Unassigned | Unassigned | 655_0 | Clustered | 45 | VC_655_0 | 45 | 0.7722 | 1 | 0.7722 | 0 | 0 | 0 | 1 | Ref |
| MN929097 | Unassigned | Unassigned | Unassigned | 855_0 | Clustered | 12 | VC_855_0 | 12 | 0.6068 | 1 | 0.6068 | 0 | 0 | 0 | 1 | Ref |
| MN935200 | Unassigned | Unassigned | Unassigned | 155_28 | Clustered/Singleton | 1 | VC_155_28 | 1 | 0 | 1 | 0 | 0 | 0 | 0 | 1 | Ref |
| MN937349 | Unassigned | Unassigned | Unassigned | 689_0 | Clustered | 5 | VC_689_0 | 5 | 0.5195 | 0.99999996 | 0.5195 | 0 | 0 | 0 | 1 | Ref |
| MN939408 | Unassigned | Unassigned | Unassigned | 110_0 | Clustered | 33 | VC_110_0 | 33 | 0.9488 | 1 | 0.9488 | 0 | 0 | 0 | 1 | Ref |
| MN939539 | Unassigned | Unassigned | Unassigned | 753_0 | Clustered | 26 | VC_753_0 | 26 | 0.9562 | 1 | 0.9562 | 0 | 0 | 0 | 1 | Ref |
| MN939540 | Unassigned | Unassigned | Unassigned | 753_0 | Clustered | 26 | VC_753_0 | 26 | 0.9562 | 1 | 0.9562 | 0 | 0 | 0 | 1 | Ref |
| MN940411 | Unassigned | Unassigned | Unassigned | 87_11 | Clustered/Singleton | 1 | VC_87_11 | 1 | 0 | 1 | 0 | 0 | 0 | 0 | 1 | Ref |
| MN945899 | Unassigned | Unassigned | Unassigned | 384_16 | Clustered | 42 | VC_384_16 | 42 | 0.134 | 1 | 0.134 | 0 | 0 | 0 | 0.7282 | Ref |
| MN945901 | Unassigned | Unassigned | Unassigned | 244_2 | Clustered | 11 | VC_244_2 | 11 | 0.1403 | 1 | 0.1403 | 0 | 0 | 0 | 0.9818 | Ref |
| MN945902 | Unassigned | Unassigned | Unassigned | 991_0 | Clustered | 39 | VC_991_0 | 39 | 1 | 1 | 1 | 0 | 0 | 0 | 1 | Ref |
| MN945903 | Unassigned | Unassigned | Unassigned | 384_13 | Clustered | 33 | VC_384_13 | 33 | 0.1785 | 1 | 0.1785 | 0 | 0 | 0 | 0.8769 | Ref |
| MN945904 | Unassigned | Unassigned | Unassigned | 241_0 | Clustered | 21 | VC_241_0 | 21 | 0.8342 | 1 | 0.8342 | 0 | 0 | 0 | 1 | Ref |
| MN945905 | Unassigned | Unassigned | Unassigned | 895_0 | Clustered | 25 | VC_895_0 | 25 | 0.5768 | 1 | 0.5768 | 0 | 0 | 0 | 1 | Ref |
| MN953776 | Unassigned | Unassigned | Unassigned | 135_14 | Clustered | 3 | VC_135_14 | 3 | 0.0101 | 0.96146265 | 0.0097 | 0 | 0 | 0 | 1 | Ref |
| MN954399 | Unassigned | Unassigned | Unassigned | 1007_1 | Clustered | 24 | VC_1007_1 | 24 | 0.7364 | 1 | 0.7364 | 0 | 0 | 0 | 0.8877 | Ref |
| MN956514 | Unassigned | Unassigned | Unassigned | 198_25 | Clustered/Singleton | 1 | VC_198_25 | 1 | 0 | 1 | 0 | 0 | 0 | 0 | 1 | Ref |
| MN958086 | Unassigned | Unassigned | Unassigned | 1007_1 | Clustered | 24 | VC_1007_1 | 24 | 0.7364 | 1 | 0.7364 | 0 | 0 | 0 | 0.8877 | Ref |
| MN966730 | Unassigned | Unassigned | Unassigned | 1007_1 | Clustered | 24 | VC_1007_1 | 24 | 0.7364 | 1 | 0.7364 | 0 | 0 | 0 | 0.8877 | Ref |
| MN966731 | Unassigned | Unassigned | Unassigned | 1007_1 | Clustered | 24 | VC_1007_1 | 24 | 0.7364 | 1 | 0.7364 | 0 | 0 | 0 | 0.8877 | Ref |
| MN966732 | Unassigned | Unassigned | Unassigned | 1007_1 | Clustered | 24 | VC_1007_1 | 24 | 0.7364 | 1 | 0.7364 | 0 | 0 | 0 | 0.8877 | Ref |
| MN970211 | Unassigned | Unassigned | Unassigned | 198_13 | Clustered | 4 | VC_198_13 | 4 | 0.0214 | 0.99996706 | 0.0214 | 0 | 0 | 0 | 0.6667 | Ref |
| MN974282 | Unassigned | Unassigned | Unassigned | 861_0 | Clustered | 29 | VC_861_0 | 29 | 0.9954 | 1 | 0.9954 | 0 | 0 | 0 | 1 | Ref |
| MN988459 | Unassigned | Unassigned | Unassigned | 915_0 | Clustered | 2 | VC_915_0 | 2 | 0.1365 | 0.95352335 | 0.1302 | 0 | 0 | 0 | 1 | Ref |
| MN988460 | Unassigned | Unassigned | Unassigned | 1468_0 | Clustered | 28 | VC_1468_0 | 28 | 0.7511 | 1 | 0.7511 | 0 | 0 | 0 | 1 | Ref |
| MN988461 | Unassigned | Unassigned | Unassigned | 1482_0 | Clustered | 4 | VC_1482_0 | 4 | 1 | 0 | 0 | 0 | 0 | 0 | 1 | Ref |
| MN988462 | Unassigned | Unassigned | Unassigned | 1482_0 | Clustered | 4 | VC_1482_0 | 4 | 1 | 0 | 0 | 0 | 0 | 0 | 1 | Ref |
| MN988463 | Unassigned | Unassigned | Unassigned | 1482_0 | Clustered | 4 | VC_1482_0 | 4 | 1 | 0 | 0 | 0 | 0 | 0 | 1 | Ref |
| MN988465 | Unassigned | Unassigned | Unassigned | 482_0 | Clustered | 4 | VC_482_0 | 4 | 0.1424 | 0.99998478 | 0.1424 | 0 | 0 | 0 | 1 | Ref |
| MN988466 | Unassigned | Unassigned | Unassigned | 1020_0 | Clustered | 22 | VC_1020_0 | 22 | 0.895 | 1 | 0.895 | 0 | 0 | 0 | 1 | Ref |
| MN988467 | Unassigned | Unassigned | Unassigned | 1468_0 | Clustered | 28 | VC_1468_0 | 28 | 0.7511 | 1 | 0.7511 | 0 | 0 | 0 | 1 | Ref |
| MN988468 | Unassigned | Unassigned | Unassigned | 1086_0 | Clustered | 2 | VC_1086_0 | 2 | 0.4283 | 0.94398033 | 0.4043 | 0 | 0 | 0 | 1 | Ref |
| MN988469 | Unassigned | Unassigned | Unassigned | 1468_0 | Clustered | 28 | VC_1468_0 | 28 | 0.7511 | 1 | 0.7511 | 0 | 0 | 0 | 1 | Ref |
| MN988470 | Unassigned | Unassigned | Unassigned | 1006_0 | Clustered | 9 | VC_1006_0 | 9 | 0.9673 | 1 | 0.9673 | 0 | 0 | 0 | 1 | Ref |
| MN988471 | Unassigned | Unassigned | Unassigned | 1468_0 | Clustered | 28 | VC_1468_0 | 28 | 0.7511 | 1 | 0.7511 | 0 | 0 | 0 | 1 | Ref |
| MN988472 | Unassigned | Unassigned | Unassigned | 1482_0 | Clustered | 4 | VC_1482_0 | 4 | 1 | 0 | 0 | 0 | 0 | 0 | 1 | Ref |
| MN988473 | Unassigned | Unassigned | Unassigned | 1468_0 | Clustered | 28 | VC_1468_0 | 28 | 0.7511 | 1 | 0.7511 | 0 | 0 | 0 | 1 | Ref |
| MN988474 | Unassigned | Unassigned | Unassigned | 1468_0 | Clustered | 28 | VC_1468_0 | 28 | 0.7511 | 1 | 0.7511 | 0 | 0 | 0 | 1 | Ref |
| MN988475 | Unassigned | Unassigned | Unassigned | 1468_0 | Clustered | 28 | VC_1468_0 | 28 | 0.7511 | 1 | 0.7511 | 0 | 0 | 0 | 1 | Ref |
| MN988478 | Unassigned | Unassigned | Unassigned | 1468_0 | Clustered | 28 | VC_1468_0 | 28 | 0.7511 | 1 | 0.7511 | 0 | 0 | 0 | 1 | Ref |
| MN988479 | Unassigned | Unassigned | Unassigned | 1468_0 | Clustered | 28 | VC_1468_0 | 28 | 0.7511 | 1 | 0.7511 | 0 | 0 | 0 | 1 | Ref |
| MN988480 | Unassigned | Unassigned | Unassigned | 1468_0 | Clustered | 28 | VC_1468_0 | 28 | 0.7511 | 1 | 0.7511 | 0 | 0 | 0 | 1 | Ref |
| MN988481 | Unassigned | Unassigned | Unassigned | 1468_0 | Clustered | 28 | VC_1468_0 | 28 | 0.7511 | 1 | 0.7511 | 0 | 0 | 0 | 1 | Ref |
| MN988482 | Unassigned | Unassigned | Unassigned | 980_0 | Clustered | 11 | VC_980_0 | 11 | 0.7333 | 1 | 0.7333 | 0 | 0 | 0 | 1 | Ref |
| MN988484 | Unassigned | Unassigned | Unassigned | 1006_0 | Clustered | 9 | VC_1006_0 | 9 | 0.9673 | 1 | 0.9673 | 0 | 0 | 0 | 1 | Ref |
| MN988485 | Unassigned | Unassigned | Unassigned | 1483_0 | Clustered | 2 | VC_1483_0 | 2 | 1 | 1 | 1 | 0 | 0 | 0 | 1 | Ref |
| MN988486 | Unassigned | Unassigned | Unassigned | 1093_4 | Clustered | 6 | VC_1093_4 | 6 | 0.2431 | 0.99795925 | 0.2426 | 0 | 0 | 0 | 1 | Ref |
| MN988488 | Unassigned | Unassigned | Unassigned | 1020_0 | Clustered | 22 | VC_1020_0 | 22 | 0.895 | 1 | 0.895 | 0 | 0 | 0 | 1 | Ref |
| MN988489 | Unassigned | Unassigned | Unassigned | 1468_0 | Clustered | 28 | VC_1468_0 | 28 | 0.7511 | 1 | 0.7511 | 0 | 0 | 0 | 1 | Ref |
| MN988490 | Unassigned | Unassigned | Unassigned | 793_0 | Clustered | 2 | VC_793_0 | 2 | 0.7591 | 0.91600907 | 0.6954 | 0 | 0 | 0 | 1 | Ref |
| MN988491 | Unassigned | Unassigned | Unassigned | 1468_0 | Clustered | 28 | VC_1468_0 | 28 | 0.7511 | 1 | 0.7511 | 0 | 0 | 0 | 1 | Ref |
| MN988492 | Unassigned | Unassigned | Unassigned | 1020_0 | Clustered | 22 | VC_1020_0 | 22 | 0.895 | 1 | 0.895 | 0 | 0 | 0 | 1 | Ref |
| MN988493 | Unassigned | Unassigned | Unassigned | 1020_0 | Clustered | 22 | VC_1020_0 | 22 | 0.895 | 1 | 0.895 | 0 | 0 | 0 | 1 | Ref |
| MN988494 | Unassigned | Unassigned | Unassigned | 1468_0 | Clustered | 28 | VC_1468_0 | 28 | 0.7511 | 1 | 0.7511 | 0 | 0 | 0 | 1 | Ref |

| | | | | | | | | | | | | | | | | |
|---|---|---|---|---|---|---|---|---|---|---|---|---|---|---|---|---|
| MN988495 | Unassigned | Unassigned | Unassigned | 1006_0 | Clustered | 9 | VC_1006_0 | 9 | 0.9673 | 1 | 0.9673 | 0 | 0 | 0 | 1 | Ref |
| MN988496 | Unassigned | Unassigned | Unassigned | 792_0 | Clustered | 5 | VC_792_0 | 5 | 0.8313 | 0.99999405 | 0.8313 | 0 | 0 | 0 | 1 | Ref |
| MN988498 | Unassigned | Unassigned | Unassigned | 1468_0 | Clustered | 28 | VC_1468_0 | 28 | 0.7511 | 1 | 0.7511 | 0 | 0 | 0 | 1 | Ref |
| MN988499 | Unassigned | Unassigned | Unassigned | 1468_0 | Clustered | 28 | VC_1468_0 | 28 | 0.7511 | 1 | 0.7511 | 0 | 0 | 0 | 1 | Ref |
| MN988500 | Unassigned | Unassigned | Unassigned | 980_0 | Clustered | 11 | VC_980_0 | 11 | 0.7333 | 1 | 0.7333 | 0 | 0 | 0 | 1 | Ref |
| MN988501 | Unassigned | Unassigned | Unassigned | 980_0 | Clustered | 11 | VC_980_0 | 11 | 0.7333 | 1 | 0.7333 | 0 | 0 | 0 | 1 | Ref |
| MN988502 | Unassigned | Unassigned | Unassigned | 793_0 | Clustered | 2 | VC_793_0 | 2 | 0.7591 | 0.91600907 | 0.6954 | 0 | 0 | 0 | 1 | Ref |
| MN988503 | Unassigned | Unassigned | Unassigned | 1468_0 | Clustered | 28 | VC_1468_0 | 28 | 0.7511 | 1 | 0.7511 | 0 | 0 | 0 | 1 | Ref |
| MN988504 | Unassigned | Unassigned | Unassigned | 1468_0 | Clustered | 28 | VC_1468_0 | 28 | 0.7511 | 1 | 0.7511 | 0 | 0 | 0 | 1 | Ref |
| MN988505 | Unassigned | Unassigned | Unassigned | 739_1 | Clustered | 6 | VC_739_1 | 6 | 0.434 | 1 | 0.434 | 0 | 0 | 0 | 1 | Ref |
| MN988506 | Unassigned | Unassigned | Unassigned | 739_1 | Clustered | 6 | VC_739_1 | 6 | 0.434 | 1 | 0.434 | 0 | 0 | 0 | 1 | Ref |
| MN988507 | Unassigned | Unassigned | Unassigned | 776_0 | Clustered | 3 | VC_776_0 | 3 | 0.4679 | 0.9981936 | 0.4671 | 0 | 0 | 0 | 1 | Ref |
| MN988508 | Unassigned | Unassigned | Unassigned | 980_0 | Clustered | 11 | VC_980_0 | 11 | 0.7333 | 1 | 0.7333 | 0 | 0 | 0 | 1 | Ref |
| MN988509 | Unassigned | Unassigned | Unassigned | 1093_4 | Clustered | 6 | VC_1093_4 | 6 | 0.2431 | 0.99795925 | 0.2426 | 0 | 0 | 0 | 1 | Ref |
| MN988510 | Unassigned | Unassigned | Unassigned | 776_0 | Clustered | 3 | VC_776_0 | 3 | 0.4679 | 0.9981936 | 0.4671 | 0 | 0 | 0 | 1 | Ref |
| MN988511 | Unassigned | Unassigned | Unassigned | 1468_0 | Clustered | 28 | VC_1468_0 | 28 | 0.7511 | 1 | 0.7511 | 0 | 0 | 0 | 1 | Ref |
| MN988512 | Unassigned | Unassigned | Unassigned | 1020_0 | Clustered | 22 | VC_1020_0 | 22 | 0.895 | 1 | 0.895 | 0 | 0 | 0 | 1 | Ref |
| MN988513 | Unassigned | Unassigned | Unassigned | 1020_0 | Clustered | 22 | VC_1020_0 | 22 | 0.895 | 1 | 0.895 | 0 | 0 | 0 | 1 | Ref |
| MN988514 | Unassigned | Unassigned | Unassigned | 1020_0 | Clustered | 22 | VC_1020_0 | 22 | 0.895 | 1 | 0.895 | 0 | 0 | 0 | 1 | Ref |
| MN988515 | Unassigned | Unassigned | Unassigned | 70_0 | Clustered | 5 | VC_70_0 | 5 | 0.9939 | 0.99995803 | 0.9939 | 0 | 0 | 0 | 1 | Ref |
| MN988516 | Unassigned | Unassigned | Unassigned | 1086_0 | Clustered | 2 | VC_1086_0 | 2 | 0.4283 | 0.94398033 | 0.4043 | 0 | 0 | 0 | 1 | Ref |
| MN988518 | Unassigned | Unassigned | Unassigned | 1468_0 | Clustered | 28 | VC_1468_0 | 28 | 0.7511 | 1 | 0.7511 | 0 | 0 | 0 | 1 | Ref |
| MN988520 | Unassigned | Unassigned | Unassigned | 1093_4 | Clustered | 6 | VC_1093_4 | 6 | 0.2431 | 0.99795925 | 0.2426 | 0 | 0 | 0 | 1 | Ref |
| MN988521 | Unassigned | Unassigned | Unassigned | 1196_0 | Clustered | 7 | VC_1196_0 | 7 | 0.6485 | 1 | 0.6485 | 0 | 0 | 0 | 1 | Ref |
| MN988522 | Unassigned | Unassigned | Unassigned | 1196_0 | Clustered | 7 | VC_1196_0 | 7 | 0.6485 | 1 | 0.6485 | 0 | 0 | 0 | 1 | Ref |
| MN988523 | Unassigned | Unassigned | Unassigned | 1196_0 | Clustered | 7 | VC_1196_0 | 7 | 0.6485 | 1 | 0.6485 | 0 | 0 | 0 | 1 | Ref |
| MN988524 | Unassigned | Unassigned | Unassigned | 1196_0 | Clustered | 7 | VC_1196_0 | 7 | 0.6485 | 1 | 0.6485 | 0 | 0 | 0 | 1 | Ref |
| MN988525 | Unassigned | Unassigned | Unassigned | 1196_0 | Clustered | 7 | VC_1196_0 | 7 | 0.6485 | 1 | 0.6485 | 0 | 0 | 0 | 1 | Ref |
| MN988526 | Unassigned | Unassigned | Unassigned | 354_0 | Clustered | 2 | VC_354_0 | 2 | 0.181 | 0.93411407 | 0.1691 | 0 | 0 | 0 | 1 | Ref |
| MN988527 | Unassigned | Unassigned | Unassigned | 776_0 | Clustered | 3 | VC_776_0 | 3 | 0.4679 | 0.9981936 | 0.4671 | 0 | 0 | 0 | 1 | Ref |
| MN988528 | Unassigned | Unassigned | Unassigned | 1020_0 | Clustered | 22 | VC_1020_0 | 22 | 0.895 | 1 | 0.895 | 0 | 0 | 0 | 1 | Ref |
| MN988529 | Unassigned | Unassigned | Unassigned | 1020_0 | Clustered | 22 | VC_1020_0 | 22 | 0.895 | 1 | 0.895 | 0 | 0 | 0 | 1 | Ref |
| MN988530 | Unassigned | Unassigned | Unassigned | 980_0 | Clustered | 11 | VC_980_0 | 11 | 0.7333 | 1 | 0.7333 | 0 | 0 | 0 | 1 | Ref |
| MN988531 | Unassigned | Unassigned | Unassigned | 1006_0 | Clustered | 9 | VC_1006_0 | 9 | 0.9673 | 1 | 0.9673 | 0 | 0 | 0 | 1 | Ref |
| MN988532 | Unassigned | Unassigned | Unassigned | 1093_4 | Clustered | 6 | VC_1093_4 | 6 | 0.2431 | 0.99795925 | 0.2426 | 0 | 0 | 0 | 1 | Ref |
| MN988533 | Unassigned | Unassigned | Unassigned | 939_0 | Clustered | 3 | VC_939_0 | 3 | 0.5055 | 0.9972453 | 0.5041 | 0 | 0 | 0 | 1 | Ref |
| MN988534 | Unassigned | Unassigned | Unassigned | 1093_4 | Clustered | 6 | VC_1093_4 | 6 | 0.2431 | 0.99795925 | 0.2426 | 0 | 0 | 0 | 1 | Ref |
| MN988535 | Unassigned | Unassigned | Unassigned | 70_0 | Clustered | 5 | VC_70_0 | 5 | 0.9939 | 0.99995803 | 0.9939 | 0 | 0 | 0 | 1 | Ref |
| MN988536 | Unassigned | Unassigned | Unassigned | 969_0 | Clustered | 2 | VC_969_0 | 2 | 0.7027 | 0.72985431 | 0.5129 | 0 | 0 | 0 | 1 | Ref |
| MN988537 | Unassigned | Unassigned | Unassigned | 1006_0 | Clustered | 9 | VC_1006_0 | 9 | 0.9673 | 1 | 0.9673 | 0 | 0 | 0 | 1 | Ref |
| MN988538 | Unassigned | Unassigned | Unassigned | 939_0 | Clustered | 3 | VC_939_0 | 3 | 0.5055 | 0.9972453 | 0.5041 | 0 | 0 | 0 | 1 | Ref |
| MN988540 | Unassigned | Unassigned | Unassigned | 70_0 | Clustered | 5 | VC_70_0 | 5 | 0.9939 | 0.99995803 | 0.9939 | 0 | 0 | 0 | 1 | Ref |
| MN988541 | Unassigned | Unassigned | Unassigned | 482_0 | Clustered | 4 | VC_482_0 | 4 | 0.1424 | 0.99998478 | 0.1424 | 0 | 0 | 0 | 1 | Ref |
| MN988543 | Unassigned | Unassigned | Unassigned | 739_1 | Clustered | 6 | VC_739_1 | 6 | 0.434 | 1 | 0.434 | 0 | 0 | 0 | 1 | Ref |
| MN988544 | Unassigned | Unassigned | Unassigned | 1006_0 | Clustered | 9 | VC_1006_0 | 9 | 0.9673 | 1 | 0.9673 | 0 | 0 | 0 | 1 | Ref |
| MN988545 | Unassigned | Unassigned | Unassigned | 980_0 | Clustered | 11 | VC_980_0 | 11 | 0.7333 | 1 | 0.7333 | 0 | 0 | 0 | 1 | Ref |
| MN988546 | Unassigned | Unassigned | Unassigned | 1006_0 | Clustered | 9 | VC_1006_0 | 9 | 0.9673 | 1 | 0.9673 | 0 | 0 | 0 | 1 | Ref |
| MN988547 | Unassigned | Unassigned | Unassigned | 739_1 | Clustered | 6 | VC_739_1 | 6 | 0.434 | 1 | 0.434 | 0 | 0 | 0 | 1 | Ref |
| MN988548 | Unassigned | Unassigned | Unassigned | 739_1 | Clustered | 6 | VC_739_1 | 6 | 0.434 | 1 | 0.434 | 0 | 0 | 0 | 1 | Ref |
| MN988549 | Unassigned | Unassigned | Unassigned | 980_0 | Clustered | 11 | VC_980_0 | 11 | 0.7333 | 1 | 0.7333 | 0 | 0 | 0 | 1 | Ref |
| MN988550 | Unassigned | Unassigned | Unassigned | 1483_0 | Clustered | 2 | VC_1483_0 | 2 | 1 | 1 | 1 | 0 | 0 | 0 | 1 | Ref |
| MN988552 | Unassigned | Unassigned | Unassigned | 1023_0 | Clustered | 3 | VC_1023_0 | 3 | 0.6263 | 0.99819562 | 0.6252 | 0 | 0 | 0 | 1 | Ref |
| MN988553 | Unassigned | Unassigned | Unassigned | 1093_4 | Clustered | 6 | VC_1093_4 | 6 | 0.2431 | 0.99795925 | 0.2426 | 0 | 0 | 0 | 1 | Ref |
| MN988554 | Unassigned | Unassigned | Unassigned | 482_0 | Clustered | 4 | VC_482_0 | 4 | 0.1424 | 0.99998478 | 0.1424 | 0 | 0 | 0 | 1 | Ref |
| MN988556 | Unassigned | Unassigned | Unassigned | 354_0 | Clustered | 2 | VC_354_0 | 2 | 0.181 | 0.93411407 | 0.1691 | 0 | 0 | 0 | 1 | Ref |
| MN988557 | Unassigned | Unassigned | Unassigned | 1006_0 | Clustered | 9 | VC_1006_0 | 9 | 0.9673 | 1 | 0.9673 | 0 | 0 | 0 | 1 | Ref |
| MN988558 | Unassigned | Unassigned | Unassigned | 915_0 | Clustered | 2 | VC_915_0 | 2 | 0.1365 | 0.95352335 | 0.1302 | 0 | 0 | 0 | 1 | Ref |
| MN988559 | Unassigned | Unassigned | Unassigned | 1196_0 | Clustered | 7 | VC_1196_0 | 7 | 0.6485 | 1 | 0.6485 | 0 | 0 | 0 | 1 | Ref |
| MN988560 | Unassigned | Unassigned | Unassigned | 1196_1 | Clustered/Singleton | 1 | VC_1196_1 | 1 | 0 | 1 | 0 | 0 | 0 | 0 | 1 | Ref |
| MN988561 | Unassigned | Unassigned | Unassigned | 1196_0 | Clustered | 7 | VC_1196_0 | 7 | 0.6485 | 1 | 0.6485 | 0 | 0 | 0 | 1 | Ref |
| MN994275 | Unassigned | Unassigned | Unassigned | 1278_0 | Clustered | 19 | VC_1278_0 | 19 | 1 | 1 | 1 | 0 | 0 | 0 | 1 | Ref |
| MN994496 | Unassigned | Unassigned | Unassigned | 198_15 | Clustered | 3 | VC_198_15 | 3 | 0.0144 | 0.99754982 | 0.0144 | 0 | 0 | 0 | 0.6667 | Ref |
| MN994497 | Unassigned | Unassigned | Unassigned | 290_185 | Clustered/Singleton | 1 | VC_290_185 | 1 | 0 | 1 | 0 | 0 | 0 | 0 | 1 | Ref |
| MN994498 | Unassigned | Unassigned | Unassigned | 340_10 | Clustered/Singleton | 1 | VC_340_10 | 1 | 0 | 1 | 0 | 0 | 0 | 0 | 1 | Ref |
| MN994499 | Unassigned | Unassigned | Unassigned | 340_5 | Clustered/Singleton | 1 | VC_340_5 | 1 | 0 | 1 | 0 | 0 | 0 | 0 | 1 | Ref |
| MN994500 | Unassigned | Unassigned | Unassigned | 603_0 | Clustered | 28 | VC_603_0 | 28 | 0.7692 | 1 | 0.7692 | 0 | 0 | 0 | 1 | Ref |
| MN994501 | Unassigned | Unassigned | Unassigned | 199_16 | Clustered | 12 | VC_199_16 | 12 | 0.064 | 1 | 0.064 | 0 | 0 | 0 | 0.9394 | Ref |
| MN994502 | Unassigned | Unassigned | Unassigned | 198_13 | Clustered | 4 | VC_198_13 | 4 | 0.0214 | 0.99996706 | 0.0214 | 0 | 0 | 0 | 0.6667 | Ref |
| MN994503 | Unassigned | Unassigned | Unassigned | 340_8 | Clustered | 2 | VC_340_8 | 2 | 0.0048 | 0.95654014 | 0.0046 | 0 | 0 | 0 | 1 | Ref |
| MN994504 | Unassigned | Unassigned | Unassigned | 198_3 | Clustered/Singleton | 1 | VC_198_3 | 1 | 0 | 1 | 0 | 0 | 0 | 0 | 1 | Ref |
| MN996301 | Unassigned | Unassigned | Unassigned | 166_0 | Clustered | 2 | VC_166_0 | 2 | 0.4832 | 0.94544348 | 0.4568 | 0 | 0 | 0 | 1 | Ref |
| MPU46938 | Unassigned | Unassigned | Unassigned | 574_0 | Clustered | 2 | VC_574_0 | 2 | 0.2216 | 0.9525409 | 0.2111 | 0 | 0 | 0 | 1 | Ref |
| MT001829 | Unassigned | Unassigned | Unassigned | 170_0 | Clustered | 3 | VC_170_0 | 3 | 0.0298 | 0.82983726 | 0.0247 | 0 | 0 | 0 | 1 | Ref |
| MT002875 | Unassigned | Unassigned | Unassigned | 927_0 | Clustered | 5 | VC_927_0 | 5 | 0.4988 | 0.99999981 | 0.4988 | 0 | 0 | 0 | 1 | Ref |
| MT002975 | Unassigned | Unassigned | Unassigned | 908_0 | Clustered | 36 | VC_908_0 | 36 | 0.9943 | 1 | 0.9943 | 0 | 0 | 0 | 1 | Ref |
| MT004791 | Unassigned | Unassigned | Unassigned | 199_5 | Clustered | 6 | VC_199_5 | 6 | 0.0283 | 0.99999992 | 0.0283 | 0 | 0 | 0 | 1 | Ref |
| MT006214 | Unassigned | Unassigned | Unassigned | 691_0 | Clustered | 18 | VC_691_0 | 18 | 0.6865 | 1 | 0.6865 | 0 | 0 | 0 | 0.9477 | Ref |
| MT006233 | Unassigned | Unassigned | Unassigned | 762_0 | Clustered | 4 | VC_762_0 | 4 | 0.4469 | 0.99996485 | 0.4468 | 0 | 0 | 0 | 1 | Ref |
| MT006234 | Unassigned | Unassigned | Unassigned | 1485_0 | Clustered | 3 | VC_1485_0 | 3 | 0.7417 | 0.9595722 | 0.7117 | 0 | 0 | 0 | 1 | Ref |
| MT006235 | Unassigned | Unassigned | Unassigned | 1485_0 | Clustered | 3 | VC_1485_0 | 3 | 0.7417 | 0.9595722 | 0.7117 | 0 | 0 | 0 | 1 | Ref |
| MT006236 | Unassigned | Unassigned | Unassigned | 826_0 | Clustered | 7 | VC_826_0 | 7 | 0.0509 | 0.99909926 | 0.0508 | 0 | 0 | 0 | 1 | Ref |

| | | | | | | | | | | | | | | | | |
|---|---|---|---|---|---|---|---|---|---|---|---|---|---|---|---|---|
| MT006237 | Unassigned | Unassigned | Unassigned | 879_0 | Clustered | 5 | VC_879_0 | 5 | 0.3611 | 0.99976884 | 0.3611 | 0 | 0 | 0 | 1 | Ref |
| MT006238 | Unassigned | Unassigned | Unassigned | 762_0 | Clustered | 4 | VC_762_0 | 4 | 0.4469 | 0.99996485 | 0.4468 | 0 | 0 | 0 | 1 | Ref |
| MT006239 | Unassigned | Unassigned | Unassigned | 762_0 | Clustered | 4 | VC_762_0 | 4 | 0.4469 | 0.99996485 | 0.4468 | 0 | 0 | 0 | 1 | Ref |
| MT006240 | Unassigned | Unassigned | Unassigned | 762_0 | Clustered | 4 | VC_762_0 | 4 | 0.4469 | 0.99996485 | 0.4468 | 0 | 0 | 0 | 1 | Ref |
| MT012302 | Unassigned | Unassigned | Unassigned | 127_0 | Clustered | 17 | VC_127_0 | 17 | 0.9222 | 1 | 0.9222 | 0 | 0 | 0 | 1 | Ref |
| MT012729 | Unassigned | Unassigned | Unassigned | 560_0 | Clustered | 15 | VC_560_0 | 15 | 0.2846 | 1 | 0.2846 | 0 | 0 | 0 | 1 | Ref |
| MT012730 | Unassigned | Unassigned | Unassigned | 440_0 | Clustered | 33 | VC_440_0 | 33 | 0.992 | 1 | 0.992 | 0 | 0 | 0 | 1 | Ref |
| MT013111 | Unassigned | Unassigned | Unassigned | 9_0 | Clustered | 38 | VC_9_0 | 38 | 0.4859 | 1 | 0.4859 | 0 | 0 | 0 | 1 | Ref |
| MT023084 | Unassigned | Unassigned | Unassigned | 596_0 | Clustered | 4 | VC_596_0 | 4 | 0.0604 | 0.99998766 | 0.0604 | 0 | 0 | 0 | 1 | Ref |
| MT024860 | Unassigned | Unassigned | Unassigned | 1287_0 | Clustered | 44 | VC_1287_0 | 44 | 1 | 1 | 1 | 0 | 0 | 0 | 1 | Ref |
| MT024861 | Unassigned | Unassigned | Unassigned | 230_0 | Clustered | 38 | VC_230_0 | 38 | 0.9076 | 1 | 0.9076 | 0 | 0 | 0 | 1 | Ref |
| MT024863 | Unassigned | Unassigned | Unassigned | 1425_0 | Clustered | 6 | VC_1425_0 | 6 | 0.2364 | 1 | 0.2364 | 0 | 0 | 0 | 1 | Ref |
| MT024864 | Unassigned | Unassigned | Unassigned | 1296_0 | Clustered | 82 | VC_1296_0 | 82 | 0.7938 | 1 | 0.7938 | 0 | 0 | 0 | 1 | Ref |
| MT024865 | Unassigned | Unassigned | Unassigned | 1115_1 | Clustered | 2 | VC_1115_1 | 2 | 0.0256 | 0.95128042 | 0.0243 | 0 | 0 | 0 | 1 | Ref |
| MT024866 | Unassigned | Unassigned | Unassigned | 537_0 | Clustered | 39 | VC_537_0 | 39 | 0.8148 | 1 | 0.8148 | 0 | 0 | 0 | 1 | Ref |
| MT024867 | Unassigned | Unassigned | Unassigned | 1427_0 | Clustered | 6 | VC_1427_0 | 6 | 1 | 1 | 1 | 0 | 0 | 0 | 1 | Ref |
| MT024868 | Unassigned | Unassigned | Unassigned | 1116_0 | Clustered | 24 | VC_1116_0 | 24 | 0.8376 | 1 | 0.8376 | 0 | 0 | 0 | 1 | Ref |
| MT024869 | Unassigned | Unassigned | Unassigned | 1250_0 | Clustered | 29 | VC_1250_0 | 29 | 0.822 | 1 | 0.822 | 0 | 0 | 0 | 1 | Ref |
| MT024870 | Unassigned | Unassigned | Unassigned | 1427_0 | Clustered | 6 | VC_1427_0 | 6 | 1 | 1 | 1 | 0 | 0 | 0 | 1 | Ref |
| MT024871 | Unassigned | Unassigned | Unassigned | 1250_0 | Clustered | 29 | VC_1250_0 | 29 | 0.822 | 1 | 0.822 | 0 | 0 | 0 | 1 | Ref |
| MT024872 | Unassigned | Unassigned | Unassigned | 1115_1 | Clustered | 2 | VC_1115_1 | 2 | 0.0256 | 0.95128042 | 0.0243 | 0 | 0 | 0 | 1 | Ref |
| MT028297 | Unassigned | Unassigned | Unassigned | 836_1 | Clustered | 4 | VC_836_1 | 4 | 0.2236 | 0.99998426 | 0.2236 | 0 | 0 | 0 | 1 | Ref |
| MT047590 | Unassigned | Unassigned | Unassigned | 454_0 | Clustered | 4 | VC_454_0 | 4 | 1 | 1 | 1 | 0 | 0 | 0 | 1 | Ref |
| MT066160 | Unassigned | Unassigned | Unassigned | 771_0 | Clustered | 8 | VC_771_0 | 8 | 0.7491 | 1 | 0.7491 | 0 | 0 | 0 | 1 | Ref |
| MT074134 | Unassigned | Unassigned | Unassigned | 1043_0 | Clustered | 19 | VC_1043_0 | 19 | 0.7615 | 1 | 0.7615 | 0 | 0 | 0 | 1 | Ref |
| MT074135 | Unassigned | Unassigned | Unassigned | 1043_0 | Clustered | 19 | VC_1043_0 | 19 | 0.7615 | 1 | 0.7615 | 0 | 0 | 0 | 1 | Ref |
| MT074136 | Unassigned | Unassigned | Unassigned | 1054_1 | Clustered | 8 | VC_1054_1 | 8 | 0.3937 | 1 | 0.3937 | 0 | 0 | 0 | 0.9286 | Ref |
| MT074137 | Unassigned | Unassigned | Unassigned | 1488_0 | Clustered | 6 | VC_1488_0 | 6 | 0.7842 | 1 | 0.7842 | 0 | 0 | 0 | 1 | Ref |
| MT074138 | Unassigned | Unassigned | Unassigned | 1054_1 | Clustered | 8 | VC_1054_1 | 8 | 0.3937 | 1 | 0.3937 | 0 | 0 | 0 | 0.9286 | Ref |
| MT074139 | Unassigned | Unassigned | Unassigned | 1488_0 | Clustered | 6 | VC_1488_0 | 6 | 0.7842 | 1 | 0.7842 | 0 | 0 | 0 | 1 | Ref |
| MT074140 | Unassigned | Unassigned | Unassigned | 1488_0 | Clustered | 6 | VC_1488_0 | 6 | 0.7842 | 1 | 0.7842 | 0 | 0 | 0 | 1 | Ref |
| MT074141 | Unassigned | Unassigned | Unassigned | 1488_0 | Clustered | 6 | VC_1488_0 | 6 | 0.7842 | 1 | 0.7842 | 0 | 0 | 0 | 1 | Ref |
| MT074142 | Unassigned | Unassigned | Unassigned | 1488_0 | Clustered | 6 | VC_1488_0 | 6 | 0.7842 | 1 | 0.7842 | 0 | 0 | 0 | 1 | Ref |
| MT074143 | Unassigned | Unassigned | Unassigned | 1043_0 | Clustered | 19 | VC_1043_0 | 19 | 0.7615 | 1 | 0.7615 | 0 | 0 | 0 | 1 | Ref |
| MT074144 | Unassigned | Unassigned | Unassigned | 1043_0 | Clustered | 19 | VC_1043_0 | 19 | 0.7615 | 1 | 0.7615 | 0 | 0 | 0 | 1 | Ref |
| MT074145 | Unassigned | Unassigned | Unassigned | 1043_0 | Clustered | 19 | VC_1043_0 | 19 | 0.7615 | 1 | 0.7615 | 0 | 0 | 0 | 1 | Ref |
| MT074146 | Unassigned | Unassigned | Unassigned | 1488_0 | Clustered | 6 | VC_1488_0 | 6 | 0.7842 | 1 | 0.7842 | 0 | 0 | 0 | 1 | Ref |
| MT074147 | Unassigned | Unassigned | Unassigned | 1043_0 | Clustered | 19 | VC_1043_0 | 19 | 0.7615 | 1 | 0.7615 | 0 | 0 | 0 | 1 | Ref |
| MT074148 | Unassigned | Unassigned | Unassigned | 1043_0 | Clustered | 19 | VC_1043_0 | 19 | 0.7615 | 1 | 0.7615 | 0 | 0 | 0 | 1 | Ref |
| MT074149 | Unassigned | Unassigned | Unassigned | 1043_0 | Clustered | 19 | VC_1043_0 | 19 | 0.7615 | 1 | 0.7615 | 0 | 0 | 0 | 1 | Ref |
| MT074150 | Unassigned | Unassigned | Unassigned | 1043_0 | Clustered | 19 | VC_1043_0 | 19 | 0.7615 | 1 | 0.7615 | 0 | 0 | 0 | 1 | Ref |
| MT074151 | Unassigned | Unassigned | Unassigned | 1043_0 | Clustered | 19 | VC_1043_0 | 19 | 0.7615 | 1 | 0.7615 | 0 | 0 | 0 | 1 | Ref |
| MT074152 | Unassigned | Unassigned | Unassigned | 1043_0 | Clustered | 19 | VC_1043_0 | 19 | 0.7615 | 1 | 0.7615 | 0 | 0 | 0 | 1 | Ref |
| MT074153 | Unassigned | Unassigned | Unassigned | 1043_0 | Clustered | 19 | VC_1043_0 | 19 | 0.7615 | 1 | 0.7615 | 0 | 0 | 0 | 1 | Ref |
| MT074154 | Unassigned | Unassigned | Unassigned | 1043_0 | Clustered | 19 | VC_1043_0 | 19 | 0.7615 | 1 | 0.7615 | 0 | 0 | 0 | 1 | Ref |
| MT074155 | Unassigned | Unassigned | Unassigned | 1043_0 | Clustered | 19 | VC_1043_0 | 19 | 0.7615 | 1 | 0.7615 | 0 | 0 | 0 | 1 | Ref |
| MT074156 | Unassigned | Unassigned | Unassigned | 1043_0 | Clustered | 19 | VC_1043_0 | 19 | 0.7615 | 1 | 0.7615 | 0 | 0 | 0 | 1 | Ref |
| MT074157 | Unassigned | Unassigned | Unassigned | 1043_0 | Clustered | 19 | VC_1043_0 | 19 | 0.7615 | 1 | 0.7615 | 0 | 0 | 0 | 1 | Ref |
| MT074158 | Unassigned | Unassigned | Unassigned | 1043_0 | Clustered | 19 | VC_1043_0 | 19 | 0.7615 | 1 | 0.7615 | 0 | 0 | 0 | 1 | Ref |
| MT074159 | Unassigned | Unassigned | Unassigned | 1043_0 | Clustered | 19 | VC_1043_0 | 19 | 0.7615 | 1 | 0.7615 | 0 | 0 | 0 | 1 | Ref |
| MT074160 | Unassigned | Unassigned | Unassigned | 1043_0 | Clustered | 19 | VC_1043_0 | 19 | 0.7615 | 1 | 0.7615 | 0 | 0 | 0 | 1 | Ref |
| MT074429 | Unassigned | Unassigned | Unassigned | 1275_0 | Clustered | 4 | VC_1275_0 | 4 | 0.747 | 0.9991311 | 0.7463 | 0 | 0 | 0 | 1 | Ref |
| MT074430 | Unassigned | Unassigned | Unassigned | 654_1 | Clustered | 27 | VC_654_1 | 27 | 0.2571 | 1 | 0.2571 | 0 | 0 | 0 | 0.9858 | Ref |
| MT074431 | Unassigned | Unassigned | Unassigned | 654_0 | Clustered | 115 | VC_654_0 | 115 | 0.7303 | 1 | 0.7303 | 0 | 0 | 0 | 0.8764 | Ref |
| MT074432 | Unassigned | Unassigned | Unassigned | 654_0 | Clustered | 115 | VC_654_0 | 115 | 0.7303 | 1 | 0.7303 | 0 | 0 | 0 | 0.8764 | Ref |
| MT074433 | Unassigned | Unassigned | Unassigned | 975_0 | Clustered | 40 | VC_975_0 | 40 | 0.6888 | 1 | 0.6888 | 0 | 0 | 0 | 1 | Ref |
| MT074434 | Unassigned | Unassigned | Unassigned | 440_0 | Clustered | 33 | VC_440_0 | 33 | 0.992 | 1 | 0.992 | 0 | 0 | 0 | 1 | Ref |
| MT074435 | Unassigned | Unassigned | Unassigned | 440_0 | Clustered | 33 | VC_440_0 | 33 | 0.992 | 1 | 0.992 | 0 | 0 | 0 | 1 | Ref |
| MT074436 | Unassigned | Unassigned | Unassigned | 440_0 | Clustered | 33 | VC_440_0 | 33 | 0.992 | 1 | 0.992 | 0 | 0 | 0 | 1 | Ref |
| MT074437 | Unassigned | Unassigned | Unassigned | 440_0 | Clustered | 33 | VC_440_0 | 33 | 0.992 | 1 | 0.992 | 0 | 0 | 0 | 1 | Ref |
| MT074438 | Unassigned | Unassigned | Unassigned | 440_0 | Clustered | 33 | VC_440_0 | 33 | 0.992 | 1 | 0.992 | 0 | 0 | 0 | 1 | Ref |
| MT074439 | Unassigned | Unassigned | Unassigned | 440_0 | Clustered | 33 | VC_440_0 | 33 | 0.992 | 1 | 0.992 | 0 | 0 | 0 | 1 | Ref |
| MT074440 | Unassigned | Unassigned | Unassigned | 1275_0 | Clustered | 4 | VC_1275_0 | 4 | 0.747 | 0.9991311 | 0.7463 | 0 | 0 | 0 | 1 | Ref |
| MT074441 | Unassigned | Unassigned | Unassigned | 440_0 | Clustered | 33 | VC_440_0 | 33 | 0.992 | 1 | 0.992 | 0 | 0 | 0 | 1 | Ref |
| MT074442 | Unassigned | Unassigned | Unassigned | 440_0 | Clustered | 33 | VC_440_0 | 33 | 0.992 | 1 | 0.992 | 0 | 0 | 0 | 1 | Ref |
| MT074443 | Unassigned | Unassigned | Unassigned | 440_0 | Clustered | 33 | VC_440_0 | 33 | 0.992 | 1 | 0.992 | 0 | 0 | 0 | 1 | Ref |
| MT074444 | Unassigned | Unassigned | Unassigned | 440_0 | Clustered | 33 | VC_440_0 | 33 | 0.992 | 1 | 0.992 | 0 | 0 | 0 | 1 | Ref |
| MT074445 | Unassigned | Unassigned | Unassigned | 440_0 | Clustered | 33 | VC_440_0 | 33 | 0.992 | 1 | 0.992 | 0 | 0 | 0 | 1 | Ref |
| MT074446 | Unassigned | Unassigned | Unassigned | 440_0 | Clustered | 33 | VC_440_0 | 33 | 0.992 | 1 | 0.992 | 0 | 0 | 0 | 1 | Ref |
| MT074447 | Unassigned | Unassigned | Unassigned | 199_30 | Clustered | 2 | VC_199_30 | 2 | 0.006 | 0.95412846 | 0.0057 | 0 | 0 | 0 | 1 | Ref |
| MT074448 | Unassigned | Unassigned | Unassigned | 198_7 | Clustered | 7 | VC_198_7 | 7 | 0.047 | 1 | 0.047 | 0 | 0 | 0 | 1 | Ref |
| MT074449 | Unassigned | Unassigned | Unassigned | 199_32 | Clustered | 12 | VC_199_32 | 12 | 0.0676 | 1 | 0.0676 | 0 | 0 | 0 | 0.7576 | Ref |
| MT074450 | Unassigned | Unassigned | Unassigned | 199_16 | Clustered | 12 | VC_199_16 | 12 | 0.064 | 1 | 0.064 | 0 | 0 | 0 | 0.9394 | Ref |
| MT074451 | Unassigned | Unassigned | Unassigned | 654_0 | Clustered | 115 | VC_654_0 | 115 | 0.7303 | 1 | 0.7303 | 0 | 0 | 0 | 0.8764 | Ref |
| MT074452 | Unassigned | Unassigned | Unassigned | 199_5 | Clustered | 6 | VC_199_5 | 6 | 0.0283 | 0.99999992 | 0.0283 | 0 | 0 | 0 | 1 | Ref |
| MT074453 | Unassigned | Unassigned | Unassigned | 199_3 | Clustered | 5 | VC_199_3 | 5 | 0.0229 | 0.99999699 | 0.0229 | 0 | 0 | 0 | 0.8 | Ref |
| MT074454 | Unassigned | Unassigned | Unassigned | 199_8 | Clustered | 2 | VC_199_8 | 2 | 0.0055 | 0.91325319 | 0.005 | 0 | 0 | 0 | 1 | Ref |
| MT074455 | Unassigned | Unassigned | Unassigned | 199_4 | Clustered | 2 | VC_199_4 | 2 | 0.0055 | 0.88232441 | 0.0048 | 0 | 0 | 0 | 1 | Ref |
| MT074456 | Unassigned | Unassigned | Unassigned | 199_5 | Clustered | 6 | VC_199_5 | 6 | 0.0283 | 0.99999992 | 0.0283 | 0 | 0 | 0 | 1 | Ref |
| MT074457 | Unassigned | Unassigned | Unassigned | 199_3 | Clustered | 5 | VC_199_3 | 5 | 0.0229 | 0.99999699 | 0.0229 | 0 | 0 | 0 | 0.8 | Ref |
| MT074458 | Unassigned | Unassigned | Unassigned | 199_16 | Clustered | 12 | VC_199_16 | 12 | 0.064 | 1 | 0.064 | 0 | 0 | 0 | 0.9394 | Ref |

| | | | | | | | | | | | | | | | | |
|---|---|---|---|---|---|---|---|---|---|---|---|---|---|---|---|---|
| MT074459 | Unassigned | Unassigned | Unassigned | 199_9 | Clustered | 5 | VC_199_9 | 5 | 0.0226 | 0.99999452 | 0.0226 | 0 | 0 | 0 | 0.7 | Ref |
| MT074460 | Unassigned | Unassigned | Unassigned | 199_5 | Clustered | 6 | VC_199_5 | 6 | 0.0283 | 0.99999992 | 0.0283 | 0 | 0 | 0 | 1 | Ref |
| MT074461 | Unassigned | Unassigned | Unassigned | 199_30 | Clustered | 2 | VC_199_30 | 2 | 0.006 | 0.95412846 | 0.0057 | 0 | 0 | 0 | 1 | Ref |
| MT074462 | Unassigned | Unassigned | Unassigned | 654_0 | Clustered | 115 | VC_654_0 | 115 | 0.7303 | 1 | 0.7303 | 0 | 0 | 0 | 0.8764 | Ref |
| MT074463 | Unassigned | Unassigned | Unassigned | 199_5 | Clustered | 6 | VC_199_5 | 6 | 0.0283 | 0.99999992 | 0.0283 | 0 | 0 | 0 | 1 | Ref |
| MT074464 | Unassigned | Unassigned | Unassigned | 199_12 | Clustered/Singleton | 1 | VC_199_12 | 1 | 0 | 1 | 0 | 0 | 0 | 0 | 1 | Ref |
| MT074465 | Unassigned | Unassigned | Unassigned | 199_32 | Clustered | 12 | VC_199_32 | 12 | 0.0676 | 1 | 0.0676 | 0 | 0 | 0 | 0.7576 | Ref |
| MT074466 | Unassigned | Unassigned | Unassigned | 199_9 | Clustered | 5 | VC_199_9 | 5 | 0.0226 | 0.99999452 | 0.0226 | 0 | 0 | 0 | 0.7 | Ref |
| MT074467 | Unassigned | Unassigned | Unassigned | 199_7 | Clustered/Singleton | 1 | VC_199_7 | 1 | 0 | 1 | 0 | 0 | 0 | 0 | 1 | Ref |
| MT074468 | Unassigned | Unassigned | Unassigned | 199_6 | Clustered/Singleton | 1 | VC_199_6 | 1 | 0 | 1 | 0 | 0 | 0 | 0 | 1 | Ref |
| MT074469 | Unassigned | Unassigned | Unassigned | 135_3 | Clustered | 3 | VC_135_3 | 3 | 0.0104 | 0.99060433 | 0.0103 | 0 | 0 | 0 | 1 | Ref |
| MT074470 | Unassigned | Unassigned | Unassigned | 135_23 | Clustered/Singleton | 1 | VC_135_23 | 1 | 0 | 1 | 0 | 0 | 0 | 0 | 1 | Ref |
| MT074471 | Unassigned | Unassigned | Unassigned | 135_45 | Clustered | 2 | VC_135_45 | 2 | 0.0051 | 0.94571064 | 0.0048 | 0 | 0 | 0 | 1 | Ref |
| MT074472 | Unassigned | Unassigned | Unassigned | 135_2 | Clustered/Singleton | 1 | VC_135_2 | 1 | 0 | 1 | 0 | 0 | 0 | 0 | 1 | Ref |
| MT074473 | Unassigned | Unassigned | Unassigned | 654_0 | Clustered | 115 | VC_654_0 | 115 | 0.7303 | 1 | 0.7303 | 0 | 0 | 0 | 0.8764 | Ref |
| MT074474 | Unassigned | Unassigned | Unassigned | 135_41 | Clustered/Singleton | 1 | VC_135_41 | 1 | 0 | 1 | 0 | 0 | 0 | 0 | 1 | Ref |
| MT074475 | Unassigned | Unassigned | Unassigned | 135_19 | Clustered | 2 | VC_135_19 | 2 | 0.005 | 0.8667049 | 0.0044 | 0 | 0 | 0 | 1 | Ref |
| MT074476 | Unassigned | Unassigned | Unassigned | 135_31 | Clustered | 6 | VC_135_31 | 6 | 0.0262 | 0.99999636 | 0.0262 | 0 | 0 | 0 | 1 | Ref |
| MT074477 | Unassigned | Unassigned | Unassigned | 135_8 | Clustered/Singleton | 1 | VC_135_8 | 1 | 0 | 1 | 0 | 0 | 0 | 0 | 1 | Ref |
| MT074478 | Unassigned | Unassigned | Unassigned | 135_39 | Clustered/Singleton | 1 | VC_135_39 | 1 | 0 | 1 | 0 | 0 | 0 | 0 | 1 | Ref |
| MT074479 | Unassigned | Unassigned | Unassigned | 135_47 | Clustered/Singleton | 1 | VC_135_47 | 1 | 0 | 1 | 0 | 0 | 0 | 0 | 1 | Ref |
| MT074480 | Unassigned | Unassigned | Unassigned | 135_45 | Clustered | 2 | VC_135_45 | 2 | 0.0051 | 0.94571064 | 0.0048 | 0 | 0 | 0 | 1 | Ref |
| MT074481 | Unassigned | Unassigned | Unassigned | 654_0 | Clustered | 115 | VC_654_0 | 115 | 0.7303 | 1 | 0.7303 | 0 | 0 | 0 | 0.8764 | Ref |
| MT074482 | Unassigned | Unassigned | Unassigned | 654_0 | Clustered | 115 | VC_654_0 | 115 | 0.7303 | 1 | 0.7303 | 0 | 0 | 0 | 0.8764 | Ref |
| MT074483 | Unassigned | Unassigned | Unassigned | 654_0 | Clustered | 115 | VC_654_0 | 115 | 0.7303 | 1 | 0.7303 | 0 | 0 | 0 | 0.8764 | Ref |
| MT074484 | Unassigned | Unassigned | Unassigned | 654_0 | Clustered | 115 | VC_654_0 | 115 | 0.7303 | 1 | 0.7303 | 0 | 0 | 0 | 0.8764 | Ref |
| MT075871 | Unassigned | Unassigned | Unassigned | 860_0 | Clustered | 9 | VC_860_0 | 9 | 0.7723 | 1 | 0.7723 | 0 | 0 | 0 | 1 | Ref |
| MT078988 | Unassigned | Unassigned | Unassigned | 133_2 | Clustered | 7 | VC_133_2 | 7 | 0.0679 | 1 | 0.0679 | 0 | 0 | 0 | 0.9048 | Ref |
| MT080102 | Unassigned | Unassigned | Unassigned | 862_0 | Clustered | 29 | VC_862_0 | 29 | 0.8594 | 1 | 0.8594 | 0 | 0 | 0 | 1 | Ref |
| MT080103 | Unassigned | Unassigned | Unassigned | 198_29 | Clustered/Singleton | 1 | VC_198_29 | 1 | 0 | 1 | 0 | 0 | 0 | 0 | 1 | Ref |
| MT080582 | Unassigned | Unassigned | Unassigned | 155_11 | Clustered | 4 | VC_155_11 | 4 | 0.0201 | 0.99981618 | 0.0201 | 0 | 0 | 0 | 1 | Ref |
| MT080583 | Unassigned | Unassigned | Unassigned | 155_11 | Clustered | 4 | VC_155_11 | 4 | 0.0201 | 0.99981618 | 0.0201 | 0 | 0 | 0 | 1 | Ref |
| MT080584 | Unassigned | Unassigned | Unassigned | 159_0 | Clustered | 25 | VC_159_0 | 25 | 0.7699 | 1 | 0.7699 | 0 | 0 | 0 | 1 | Ref |
| MT080585 | Unassigned | Unassigned | Unassigned | 155_17 | Clustered | 4 | VC_155_17 | 4 | 0.0129 | 0.99945541 | 0.0129 | 0 | 0 | 0 | 1 | Ref |
| MT080586 | Unassigned | Unassigned | Unassigned | 155_11 | Clustered | 4 | VC_155_11 | 4 | 0.0201 | 0.99981618 | 0.0201 | 0 | 0 | 0 | 1 | Ref |
| MT080587 | Unassigned | Unassigned | Unassigned | 159_0 | Clustered | 25 | VC_159_0 | 25 | 0.7699 | 1 | 0.7699 | 0 | 0 | 0 | 1 | Ref |
| MT080588 | Unassigned | Unassigned | Unassigned | 159_0 | Clustered | 25 | VC_159_0 | 25 | 0.7699 | 1 | 0.7699 | 0 | 0 | 0 | 1 | Ref |
| MT080589 | Unassigned | Unassigned | Unassigned | 155_17 | Clustered | 4 | VC_155_17 | 4 | 0.0129 | 0.99945541 | 0.0129 | 0 | 0 | 0 | 1 | Ref |
| MT080590 | Unassigned | Unassigned | Unassigned | 159_0 | Clustered | 25 | VC_159_0 | 25 | 0.7699 | 1 | 0.7699 | 0 | 0 | 0 | 1 | Ref |
| MT080591 | Unassigned | Unassigned | Unassigned | 155_17 | Clustered | 4 | VC_155_17 | 4 | 0.0129 | 0.99945541 | 0.0129 | 0 | 0 | 0 | 1 | Ref |
| MT080592 | Unassigned | Unassigned | Unassigned | 155_0 | Clustered | 5 | VC_155_0 | 5 | 0.0278 | 0.99999865 | 0.0278 | 0 | 0 | 0 | 1 | Ref |
| MT080593 | Unassigned | Unassigned | Unassigned | 155_22 | Clustered | 2 | VC_155_22 | 2 | 0.0062 | 0.92916562 | 0.0057 | 0 | 0 | 0 | 1 | Ref |
| MT080594 | Unassigned | Unassigned | Unassigned | 155_11 | Clustered | 4 | VC_155_11 | 4 | 0.0201 | 0.99981618 | 0.0201 | 0 | 0 | 0 | 1 | Ref |
| MT080595 | Unassigned | Unassigned | Unassigned | 159_0 | Clustered | 25 | VC_159_0 | 25 | 0.7699 | 1 | 0.7699 | 0 | 0 | 0 | 1 | Ref |
| MT080596 | Unassigned | Unassigned | Unassigned | 155_22 | Clustered | 2 | VC_155_22 | 2 | 0.0062 | 0.92916562 | 0.0057 | 0 | 0 | 0 | 1 | Ref |
| MT080597 | Unassigned | Unassigned | Unassigned | 155_0 | Clustered | 5 | VC_155_0 | 5 | 0.0278 | 0.99999865 | 0.0278 | 0 | 0 | 0 | 1 | Ref |
| MT080598 | Unassigned | Unassigned | Unassigned | 155_0 | Clustered | 5 | VC_155_0 | 5 | 0.0278 | 0.99999865 | 0.0278 | 0 | 0 | 0 | 1 | Ref |
| MT080599 | Unassigned | Unassigned | Unassigned | 155_0 | Clustered | 5 | VC_155_0 | 5 | 0.0278 | 0.99999865 | 0.0278 | 0 | 0 | 0 | 1 | Ref |
| MT080600 | Unassigned | Unassigned | Unassigned | 155_8 | Clustered | 66 | VC_155_8 | 66 | 0.4537 | 1 | 0.4537 | 0 | 0 | 0 | 0.8182 | Ref |
| MT080601 | Unassigned | Unassigned | Unassigned | 155_17 | Clustered | 4 | VC_155_17 | 4 | 0.0129 | 0.99945541 | 0.0129 | 0 | 0 | 0 | 1 | Ref |
| MT080602 | Unassigned | Unassigned | Unassigned | 155_8 | Clustered | 66 | VC_155_8 | 66 | 0.4537 | 1 | 0.4537 | 0 | 0 | 0 | 0.8182 | Ref |
| MT080603 | Unassigned | Unassigned | Unassigned | 155_0 | Clustered | 5 | VC_155_0 | 5 | 0.0278 | 0.99999865 | 0.0278 | 0 | 0 | 0 | 1 | Ref |
| MT084350 | Unassigned | Unassigned | Unassigned | 287_0 | Clustered | 2 | VC_287_0 | 2 | 0.2918 | 0.9525588 | 0.2779 | 0 | 0 | 0 | 1 | Ref |
| MT090077 | Unassigned | Unassigned | Unassigned | 113_0 | Clustered | 91 | VC_113_0 | 91 | 0.9584 | 1 | 0.9584 | 0 | 0 | 0 | 1 | Ref |
| MT094431 | Unassigned | Unassigned | Unassigned | 132_0 | Clustered | 19 | VC_132_0 | 19 | 0.6051 | 1 | 0.6051 | 0 | 0 | 0 | 1 | Ref |
| MT104465 | Unassigned | Unassigned | Unassigned | 131_0 | Clustered | 43 | VC_131_0 | 43 | 0.8853 | 1 | 0.8853 | 0 | 0 | 0 | 1 | Ref |
| MT104466 | Unassigned | Unassigned | Unassigned | 131_0 | Clustered | 43 | VC_131_0 | 43 | 0.8853 | 1 | 0.8853 | 0 | 0 | 0 | 1 | Ref |
| MT104468 | Unassigned | Unassigned | Unassigned | 161_0 | Clustered | 14 | VC_161_0 | 14 | 0.6064 | 1 | 0.6064 | 0 | 0 | 0 | 1 | Ref |
| MT104469 | Unassigned | Unassigned | Unassigned | 161_0 | Clustered | 14 | VC_161_0 | 14 | 0.6064 | 1 | 0.6064 | 0 | 0 | 0 | 1 | Ref |
| MT104470 | Unassigned | Unassigned | Unassigned | 161_0 | Clustered | 14 | VC_161_0 | 14 | 0.6064 | 1 | 0.6064 | 0 | 0 | 0 | 1 | Ref |
| MT104471 | Unassigned | Unassigned | Unassigned | 161_0 | Clustered | 14 | VC_161_0 | 14 | 0.6064 | 1 | 0.6064 | 0 | 0 | 0 | 1 | Ref |
| MT104472 | Unassigned | Unassigned | Unassigned | 161_0 | Clustered | 14 | VC_161_0 | 14 | 0.6064 | 1 | 0.6064 | 0 | 0 | 0 | 1 | Ref |
| MT104473 | Unassigned | Unassigned | Unassigned | 712_0 | Clustered | 5 | VC_712_0 | 5 | 0.828 | 0.99999971 | 0.828 | 0 | 0 | 0 | 1 | Ref |
| MT104475 | Unassigned | Unassigned | Unassigned | 712_0 | Clustered | 5 | VC_712_0 | 5 | 0.828 | 0.99999971 | 0.828 | 0 | 0 | 0 | 1 | Ref |
| MT104476 | Unassigned | Unassigned | Unassigned | 161_0 | Clustered | 14 | VC_161_0 | 14 | 0.6064 | 1 | 0.6064 | 0 | 0 | 0 | 1 | Ref |
| MT104477 | Unassigned | Unassigned | Unassigned | 161_0 | Clustered | 14 | VC_161_0 | 14 | 0.6064 | 1 | 0.6064 | 0 | 0 | 0 | 1 | Ref |
| MT108723 | Unassigned | Unassigned | Unassigned | 486_0 | Clustered | 47 | VC_486_0 | 47 | 0.9022 | 1 | 0.9022 | 0 | 0 | 0 | 1 | Ref |
| MT108724 | Unassigned | Unassigned | Unassigned | 486_0 | Clustered | 47 | VC_486_0 | 47 | 0.9022 | 1 | 0.9022 | 0 | 0 | 0 | 1 | Ref |
[truncated: 1,321,233 more chars]
