# Supplementary material for: Investigating the Human Intestinal DNA Virome and Predicting Disease-Associated Virus–Host Interactions in Severe Myalgic Encephalomyelitis/Chronic Fatigue Syndrome (ME/CFS)
Source: Int J Mol Sci. 2023 Dec 8;24(24):17267. doi: 10.3390/ijms242417267 (PMC10744171; doi:10.3390/ijms242417267)
Supplement: Supplementary file 1 [file ijms-24-17267-s001.zip › Table S1.pdf]

Table S1-1. Cohort and stats of sequences

Cohort features

Sample IDs	Health status	Gender	Age range at time of collection	# Household-matched pair	Collection of clinical metadata	Sample type	Sample source	Bristol stool scale	Sample dry weight (g)	VLP/mL	VLP/g feces	Total DNA (ng)	DNA yield (ng/g feces)	VLP-enriched DNA virome	Bulk whole metagenome (WMS)
S1	Severe ME/CFS	F	56-60	1M	Y	Frozen faeces	Homo sapiens	2	22.25	1.1×10^8	7.1×10^8	2000.0	89.9	Y	Y
S2	SHHC	M	61-65	1C	NA	Frozen faeces	Homo sapiens	7	21.92	1.4×10^8	8.8×10^8	6120.0	279.2	Y	Y
S3	Severe ME/CFS	F	36-40	2M	Y	Frozen faeces	Homo sapiens	6	44.83	2.7×10^8	1.7×10^9	2745.0	61.2	Y	Y
S4	SHHC	M	36-40	2C	NA	Frozen faeces	Homo sapiens	5	37.29	2.1×10^8	1.3×10^9	3852.0	103.3	Y	Y
S5	Severe ME/CFS	F	21-25	3M	Y	Frozen faeces	Homo sapiens	1	20.77	1.2×10^8	7.7×10^8	383.5	18.5	Y	Y
S6	SHHC	F	56-60	3C	NA	Frozen faeces	Homo sapiens	6	19.42	6.2×10^7	3.9×10^8	3657.0	188.3	Y	Y
S7	Severe ME/CFS	F	21-25	4M	Y	Frozen faeces	Homo sapiens	5	20.00	4.0×10^8	2.5×10^9	615.0	30.8	Y	Y
S8	SHHC	M	21-25	4C	NA	Frozen faeces	Homo sapiens	2	20.29	3.2×10^8	2.0×10^9	3733.2	184.0	Y	Y
S9	Severe ME/CFS	F	36-40	5M		Frozen faeces	Homo sapiens		50.00	2.1×10^8	1.3×10^9	600.0	12.0	Y	Y
S10	SHHC	F	61-65	5C		Frozen faeces	Homo sapiens		50.00	2.2×10^8	1.4×10^9	1842.0	36.8	Y	N
S11	Severe ME/CFS	F	21-25	6M		Frozen faeces	Homo sapiens		41.21	4.6×10^8	2.9×10^9	1027.0	24.9	Y	Y
S12	SHHC	F	56-60	6C		Frozen faeces	Homo sapiens		38.17	3.3×10^8	2.1×10^9	945.0	24.8	Y	Y
S13	Severe ME/CFS	F	36-40	9M (Unmatched)	Unrecorded	Frozen faeces	Homo sapiens	Unrecorded	33.62	7.2×10^7	4.5×10^8	4670.0	138.9	Y	N
S14	Severe ME/CFS	F	16-20	7M		Frozen faeces	Homo sapiens		16.37	2.1×10^8	1.3×10^9	488.0	29.8	Y	N
S15	SHHC	F	51-55	7C		Frozen faeces	Homo sapiens		17.42	4.1×10^8	2.6×10^9	5592.0	321.0	Y	N
S16	SHHC	F	66-70	8C		Frozen faeces	Homo sapiens		31.65	1.9×10^8	1.2×10^9	2806.0	88.7	Y	N
S17	Severe ME/CFS	F	41-45	8M		Frozen faeces	Homo sapiens		40.16	4.6×10^8	2.8×10^9	1738.0	43.3	Y	Y
NC1	Blank	NA	NA	NA	NA	Buffer only	NA	NA	NA	0	0	ND	ND	Y	NA
NC2	Blank	NA	NA	NA	NA	Buffer only	NA	NA	NA	0	0	ND	ND	Y	NA
NC3	Blank	NA	NA	NA	NA	Buffer only	NA	NA	NA	0	0	ND	ND	Y	NA

ME/CFS: Myalgic Encephalomyelitis and chronic fatigue syndrome
SHHC: same household healthy control
NC: negative/processing control sample (buffer only)
M: ME/CFS sample
C: SHHC sample
NA: not applicable
ND: not detectable
Y: samples used for either VLP-enriched or WMS analysis or used for both
N: samples not used for WMS analysis

Table S1-2. enriched-vlp-reads and contigs

Sample_IDs	# Raw reads (PE)	# Cleaned reads (PE)	# Total raw contigs	N50 (bp)	Longest contig length (bp)
S1	72345890	35113882	160117	1328	101988
S2	66466528	56868494	21603	1903	269575
S3	210025926	174177994	126065	7721	303660
S4	49280544	47904786	32063	7049	118697
S5	80290420	65733438	143928	10731	474479
S6	81370828	68149392	56204	15510	501646
S7	97834806	81075886	138310	3884	405769
S8	223264658	183020194	205876	2915	522585
S9	84139802	71023532	90503	2228	321418
S10	87299754	73760336	121264	4484	699342
S11	80553640	67000402	103564	3234	251440
S12	100680974	85558566	187492	3525	621377
S13	72438224	60096768	154404	1978	289301
S14	75299764	61978780	112646	4931	361320
S15	40202586	32235928	77515	4759	317833
S16	89664968	74204032	173237	4480	530177
S17	75436218	58989106	174502	3202	335129
Sum	1586595530	1296891516	2079293	83862	6425736
Mean	93329148.8	76287736.0	122311.4	4933.0	377984.0
SD	48857207.3	41056515.8	53541.4	3618.0	163066.2

enriched-vlp-viral genomes

	Merged_UViGs	UViGs_50-100%_completeness_checkv	nr_vOTUs	vOTUs_size_filtration_1kb_cut_off	HQ-vOTUs_50-100%_completeness_checkv
# Viral genomes	311208	2267	192146	54779	1231
# Viral genomes ≥ 1 kb	88434	2267	54779	54779	1231
# Viral genomes ≥ 5 kb	13160	2240	8038	8038	1210
# Viral genomes ≥ 10 kb	5686	2180	3278	2378	1172
N50 (bp)	4064	NA	3694	6580	49258
Longest representative (bp)	289184	289184	289184	289184	289184
Shortest representative (bp)	167	2070	180	1000	2070

nr: non-redundancy
UViG: uncultivated virus genome
vOTU: virus operational taxonomic unit
HQ: high quality
#: quantity or number
NA: not applicable

Table S1-3. wms-reads and contigs

Sample_IDs	# Raw reads (PE)	# Cleaned reads (PE)	# Total raw contigs	N50 (bp)	Longest contig length (bp)
S1	144016818	144000416	368621	3209	593488
S2	148221396	148218256	238143	7203	593489
S3	12026774	12026424	135476	1492	99031
S4	53446050	53444298	164089	2217	415342
S5	186302984	186273684	210113	9964	587364
S6	57739770	57739286	139652	3307	563736
S7	137959804	137959384	240000	4991	606105
S8	90881904	90880674	266924	2693	525861
S9	87344710	87343694	126134	8430	680403
S11	84507078	84502272	321191	2940	302351
S12	140009544	140008922	386774	3041	431188
S17	110272558	110271798	257744	3366	502130
Sum	1252729390	1252669108	2854861	52853	5900488
Mean	104394115.8	104389092.3	237905.1	4404.4	491707.3
SD	49361687.0	49356246.2	88566.7	2683.3	160841.5

wms-viral genomes

	Merged_UViGs	UViGs_50-100%_completeness_checkv	nr_vOTUs	Size_filtration_1kb_cut_off	HQ-vOTUs_50-100%_completeness_checkv
# Viral genomes	261744	961	184317	59753	688
# Viral genomes ≥ 1 kb	82029	961	59753	59753	688
# Viral genomes ≥ 5 kb	11871	923	8708	8708	659
# Viral genomes ≥ 10 kb	4417	891	3227	3227	636
N50 (bp)	3491	NA	3616	5509	62201
Longest representative (bp)	413955	413955	413955	413955	413955
Shortest representative (bp)	193	1992	196	1000	2384

nr: non-redundancy
UViG: uncultivated virus genome
vOTU: virus operational taxonomic unit
HQ: high quality
#: quantity or number
NA: not applicable

WMS-coassembly for bacteriome analysis

	# Contigs	Total length (bp)	Mean of length (bp)	N50 (bp)	Longest length (bp)	Shortest length (bp)
Quantity	774,887	591,013,764	762.71	890	135,997	212

Table S1-4. BLAST_WMS_against_VLP

qseqid	sseqid	pident	qcovs	length	mismatch	gapopen	qstart	send	sstart	send	evalue	bitscore
S2-wms-combined_95987	S14_vs_9162	100	0	29	0	0	10311	10339	47371	47399	2.05E-05	54.7
S2-wms_248327	S8_vs_50431	100	0	29	0	0	5176	5204	23296	23324	1.87E-05	54.7
S2-wms-combined_185494	S15_vf_33505	100	0	33	0	0	28928	28960	5866	5834	2.68E-06	62.1
S1-wms-combined_353757	S14_vs_66139	97.297	0	37	1	0	18939	18975	156473	156437	1.14E-07	63.9
S9-wms-combined_93232	S5_vf_74661	90	0	50	5	0	13333	13382	20063	20014	4.47E-08	65.8
S2-wms-combined_190624	S16_vs_17493	81.818	0	77	14	0	14724	14800	12955	12879	2.35E-08	65.8
S6-wms-combined_41246	S5_vs_65490	81.176	0	85	14	2	34140	34223	27618	27701	9.95E-09	67.6
S2-wms-combined_135139	S16_vs_173096	95.349	0	43	2	0	4942	4984	15558	15516	7.94E-09	69.4
S1-wms_244836	S10_vs_36598	74.375	1	160	41	0	3739	3898	23033	22874	2.14E-09	69.4
S4-wms-combined_118531	S9_vs_90850	77.778	0	135	28	2	31257	31390	2797	2664	6.07E-13	82.4
S2-wms-combined_99446	S11_vs_8456	94.444	0	54	1	1	19347	19398	22391	22338	3.77E-13	82.4
S2-wms-combined_7756	S5_vs_86562	73.953	0	215	52	4	1587	1799	24722	24934	1.54E-13	84.2
S2-wms-combined_48661	S9_vf_19284	100	1	43	0	0	944	986	28724	28766	1.20E-13	80.5
S17-wms-combined_162786	S16_vf_173099	85.542	0	83	11	1	62725	62806	23501	23583	1.03E-13	86.1
S4-wms-combined_113721	S7_vs_111831	93.103	0	58	3	1	18807	18863	3057	3000	7.22E-14	84.2
S2-wms_192667	S5_vs_8125	83.168	0	101	11	5	4216	4313	22124	22027	5.05E-15	87.9
S8-wms-combined_185611	S1_vs_21439	76.536	0	179	34	8	32101	32275	21956	22130	5.24E-16	91.6
S2-wms-combined_52568	S1_vs_155183	93.75	0	64	4	0	40704	40767	1581	1518	1.54E-17	97.1
S9-wms-combined_71647	S14_vs_96286	95.161	0	62	3	0	13236	13297	7771	7710	1.94E-18	99
S7-wms-combined_143218	S12_vf_18902	89.474	1	76	8	0	483	558	7058	7133	1.80E-18	97.1
S11-wms_32691	S5_vf_103206	90.909	0	77	7	0	114865	114941	18187	18263	2.91E-19	104
S1-wms-combined_197401	S16_vs_26078	83.761	0	117	15	3	51590	51706	19779	19667	7.72E-21	108
S2-wms_140933	S4_vf_13353	85.577	0	104	14	1	23828	23930	44241	44138	6.61E-21	108
S17-wms-combined_179363	S12_vs_75098	71.007	3	576	137	26	2605	3168	12004	11447	2.05E-22	111
S1-wms-combined_40115	S5_vs_77593	92.308	3	78	6	0	62	139	98222	98145	2.63E-23	111
S9-wms-combined_89249	S9_vs_90859	91.011	0	89	4	3	20484	20569	24510	24597	6.02E-24	117
S8-wms-combined_40666	S5_vs_46394	89.216	0	102	5	5	44263	44360	8663	8762	3.69E-25	122
S17-wms_106671	S7_vs_40259	72.235	2	425	108	9	6592	7011	2702	3121	1.22E-25	122
S2-wms-combined_248318	S5_vf_105728	76.754	1	228	49	4	822	1047	33418	33643	3.57E-26	124
S2-wms-combined_180640	S3_vs_16825	91.753	1	97	6	2	3254	3349	47899	47994	7.24E-29	134
S2-wms-combined_89742	S11_vs_36748	86.154	1	130	10	5	21057	21184	4352	4229	5.81E-29	134
S1-wms-combined_143459	S17_vs_177203	91.919	1	99	5	1	706	804	19956	19861	2.38E-29	135
S4-wms_107182	S5_vf_29332	80.628	1	191	33	4	3577	3765	22074	22262	1.90E-32	145
S5-wms-combined_94482	S16_vs_32764	86.62	0	142	12	6	54562	54700	42685	42822	1.42E-33	150
S17-wms-combined_146050	S16_vs_32764	79.654	1	231	34	11	4341	4563	42908	42683	7.44E-35	154
S12-wms-combined_147628	S16_vs_26078	91.379	0	116	6	3	10620	10735	19779	19668	4.04E-35	156
S9-wms_64472	S17_vs_133231	85.276	3	163	13	11	144	300	8959	8802	7.67E-37	158
S5-wms-combined_94786	S3_vs_50557	74.783	1	460	98	15	29109	29559	4001	4451	1.01E-45	191
S17-wms-combined_95360	S7_vs_134784	76.574	0	397	66	16	42179	42551	3186	2793	4.97E-46	193
S8-wms_82880	S17_vs_110666	76.923	1	351	77	4	23882	24230	37120	36772	1.43E-47	196
S1-wms-combined_159544	S17_vs_177203	98.23	1	113	2	0	254	366	20342	20230	1.76E-48	198
S2-wms-combined_135154	S7_vs_134784	90	0	160	10	5	33499	33655	594	750	4.28E-49	202
S5-wms-combined_205046	S14_vs_9162	76.903	1	381	74	11	14516	14889	43334	43707	6.10E-50	204
S1-wms-combined_32863	S15_vs_42043	93.706	1	143	7	1	8139	8281	193486	193346	4.73E-53	213
S4-wms-combined_24659	S3_vf_67333	75.67	7	448	101	6	3021	3464	10612	11055	2.13E-54	217
S2-wms_246296	S3_vf_108959	88.205	0	195	23	0	31598	31792	53743	53937	1.24E-58	233
S2-wms-combined_238929	S8_vf_208126	72.355	2	879	213	29	18441	19304	43437	42574	9.55E-64	250
S12-wms-combined_299872	S14_vf_58919	71.527	4	1166	277	49	12153	13292	13146	14282	9.01E-68	263
S8-wms-combined_211119	S8_vf_93976	81.176	1	340	52	11	6911	7241	24527	24863	7.46E-68	263
S4-wms-combined_64632	S8_vs_59736	78.883	1	412	79	6	31134	31540	23239	22831	2.76E-70	272
S12-wms-combined_263377	S16_vs_172749	76.392	1	521	117	5	51866	52383	1363	846	2.21E-71	276
S12-wms_373775	S5_vs_145593	78.641	3	412	88	0	9724	10135	14381	13970	1.91E-71	274
S17-wms-combined_106262	S14_vs_117161	80.585	1	376	63	9	6766	7136	8510	8140	4.61E-73	281
S7-wms-combined_136642	S4_vf_12624	75.916	5	573	124	13	6145	6710	2408	2973	1.99E-73	281
S5-wms-combined_183113	S14_vs_74067	72.756	3	947	236	18	27317	28252	74941	75876	6.49E-79	300
S11-wms_320555	S12_vf_69136	72.246	7	1189	259	43	29883	31036	27457	28609	1.92E-79	302
S11-wms-combined_46816	S3_vs_15450	79.47	2	453	85	7	15653	16101	26767	26319	2.35E-83	315
S2-wms-combined_58121	S3_vs_64424	89.328	1	253	27	0	20668	20920	34002	33750	1.67E-84	318
S12-wms-combined_334806	S6_vs_1146	73.326	1	911	227	13	42974	43876	51895	52797	3.92E-85	322
S2-wms-combined_176376	S16_vs_173079	80.134	1	448	78	11	19202	19643	14178	14620	5.52E-86	324
S2-wms-combined_143737	S10_vs_62776	83.196	1	363	61	0	30942	31304	14020	13658	1.15E-88	333
S7-wms-combined_157434	S16_vs_140709	78.794	1	514	100	9	29926	30435	19561	20069	6.02E-90	337
S12-wms-combined_270839	S9_vs_84046	73.313	2	1008	235	29	4381	5371	28239	27249	7.16E-91	340
S12-wms-combined_148741	S9_vs_84046	73.42	2	997	231	28	39287	40266	27257	28236	1.50E-91	342
S4-wms-combined_51079	S4_vf_12624	78.276	3	580	108	14	11993	12563	2973	2403	3.20E-96	357
S2-wms-combined_5791	S7_vs_16344	72.511	1	1175	301	19	5921	7084	75136	73973	1.44E-96	361
S1-wms-combined_331544	S4_vf_12624	78.448	2	580	107	14	14954	15524	2973	2403	7.32E-98	363
S2-wms-combined_93845	S5_vs_138980	77.424	1	629	132	9	29374	29997	35421	34798	1.22E-98	366
S12-wms-combined_376326	S12_vs_106329	74.734	2	847	201	13	44098	44938	4417	5256	1.00E-98	366
S4-wms-combined_83492	S3_vs_80151	78.415	2	593	114	14	33415	34000	144122	144707	4.92E-101	374
S12-wms-combined_264845	S5_vs_124677	77.607	6	652	136	9	2626	3272	57017	56371	3.27E-105	387
S7-wms-combined_14529	S16_vs_15093	78.737	1	649	120	12	36814	37449	726	83	3.69E-114	418
S12-wms-combined_264077	S5_vs_124677	79.868	2	606	99	21	19690	20285	8948	9540	1.46E-115	422
S7-wms_80255	S3_vf_111893	73.715	6	1187	271	35	10141	11306	22000	23166	7.37E-117	425
S17-wms-combined_225356	S3_vf_99626	75.276	4	1088	228	36	327	1394	25763	24697	1.98E-133	481
S12-wms-combined_52101	S5_vs_112452	74.513	9	1181	277	20	13598	14766	2052	884	8.28E-137	492
S5-wms-combined_178432	S11_vs_98193	78.396	2	773	161	4	2925	3694	279	1048	3.70E-138	497
S17-wms-combined_126774	S1_vf_138157	86.266	1	466	64	0	64011	64476	20571	21036	8.12E-141	507
S8-wms-combined_117281	S16_vs_18293	75.729	4	1063	236	21	5979	7030	27940	28991	2.00E-143	514
S2-wms-combined_246480	S3_vs_127401	82.295	2	610	97	8	24054	24655	24927	24321	1.71E-144	518
S12-wms-combined_223043	S14_vf_58919	72.439	10	1923	452	64	3522	5406	32739	30857	1.87E-152	544
S11-wms-combined_58538	S11_vs_26767	83.533	3	583	96	0	33450	34032	3832	3250	9.21E-153	545
S11-wms_55610	S4_vs_VIRSorter_4959-cat_2	80.16	1	751	140	6	44063	44807	2577	1830	9.02E-155	553
S12-wms-combined_168259	S12_vs_86928	84.22	6	583	76	12	14402	14976	1198	1772	6.18E-155	553

Sorting:
1. evalue: <1E-05
2. bitscore: set score >1000
3. pident: percentage of identical matches: >95% (similarity)
4. qcovs: query coverage per subject: >85% (coverage)
5. Result: 193/600 hits of WMS-HQ-vOTUs (688) are highly identical against 1231 VLP-HQ-vOTUs

S2-wms-combined_101826	S3_vf_8164	84.764	1	571	85	2	22938	23507	12660	13229	2.65E-160	571
S1-wms-combined_120533	S5_vs_124035	80.816	8	735	133	7	6115	6845	7955	8685	1.33E-160	569
S4-wms-combined_57123	S6_vs_46039	74.981	7	1335	293	35	16159	17471	75449	76764	4.39E-162	577
S12-wms_223235	S16_vs_48714	90.805	11	435	40	0	2326	2760	16647	17081	7.74E-165	582
S12-wms-combined_48648	S8_vs_149700	95.418	3	371	16	1	10825	11195	67882	68251	1.31E-166	590
S6-wms-combined_65225	S7_vs_134784	83.645	3	642	101	3	7032	7672	24267	23629	1.26E-169	601
S2-wms-combined_25727	S4_vf_31979	87.199	2	539	58	7	33482	34013	19953	20487	8.75E-170	603
S8-wms_113693	S4_vf_31979	87.546	3	538	56	7	16119	16649	19953	20486	4.93E-173	612
S5-wms_67169	S8_vf_195032	70.787	9	3584	843	175	33149	36636	2326	5801	2.75E-178	630
S1-wms-combined_340558	S8_vs_9114	91.485	2	458	39	0	26876	27333	4427	3970	1.92E-178	630
S9-wms-combined_53991	S9_vs_67792	99.992	100	61938	4	1	1	61938	61937	1	0	1.14E+05
S9-wms-combined_97755	S9_vs_7881	99.95	57	26114	13	0	1	26114	2152	28265	0	48152
S9-wms-combined_33230	S5_vf_8965	95.799	35	6284	259	4	7261	13541	8044	1763	0	10137
S9-wms-combined_2042	S9_vs_88286	100	81	31342	0	0	1	31342	44732	13391	0	57878
S9-wms-combined_124857	S9_vs_50439	100	28	30128	0	0	50939	81066	1	30128	0	55636
S9-wms-combined_28805	S7_vs_119679	92.087	47	13535	987	54	30565	44043	44863	31357	0	18984
S9-wms-combined_109322	S7_vs_48832	83.583	2	2199	340	19	19556	21744	35780	33593	0	2041
S9-wms-combined_3461	S9_vs_90849	99.995	95	36619	2	0	1	36619	36619	1	0	67612
S9-wms-combined_29611	S17_vs_127519	89.171	0	868	89	4	345751	346617	18567	17704	0	1077
S9-wms-combined_25007	S13_vs_23314	74.995	20	4587	990	107	11442	15966	22553	18062	0	1971
S9-wms-combined_3878	S9_vs_90863	100	91	4891	0	0	665	5555	1	4891	0	9033
S9-wms-combined_105462	S5_vf_116300	84.985	2	686	82	16	30046	30714	17730	17049	0	676
S9-wms-combined_20485	S9_vf_90856	100	42	24912	0	0	49881	74792	24912	1	0	46004
S9-wms-combined_9931	S9_vs_90727	100	100	90630	0	0	1	90630	66187	156816	0	1.67E+05
S9-wms-combined_36459	S9_vs_48347	100	99	27978	0	0	1	27978	27978	1	0	51666
S9-wms-combined_105822	S9_vs_41371	79.467	35	4466	862	46	21501	25934	4677	235	0	3116
S9-wms-combined_26410	S11_vs_68151	96.563	96	26502	868	22	427	26896	34866	8376	0	43853
S9-wms-combined_31456	S9_vs_21850	99.995	76	36645	1	1	9789	46433	1	36644	0	67658
S9-wms-combined_37587	S7_vs_557	81.25	1	1040	181	11	4689	5721	22885	21853	0	828
S9-wms-combined_5526	S9_vs_48424	100	74	38027	0	0	1	38027	16762	54788	0	70223
S9-wms-combined_33229	S9_vs_28197	99.996	100	23889	1	0	1	23889	23889	1	0	44110
S9-wms-combined_76274	S5_vs_63924	99.978	51	27377	4	2	10684	38060	35637	8263	0	50521
S9-wms-combined_44402	S5_vs_72532	79.495	29	4394	836	48	2491	6844	45184	40816	0	3062
S9-wms_19025	S10_vf_64371	99.935	100	24556	16	0	26716	51271	59185	34630	0	45258
S9-wms_128232	S9_vf_23901	99.934	100	4540	3	0	1444	5983	1	4540	0	8368
S11-wms-combined_41367	S11_vs_45658	99.997	99	39169	1	0	13	39181	8205	47373	0	72327
S11-wms-combined_193148	S6_vs_39065	98.05	95	7743	69	20	3727	11429	9993	17693	0	13387
S11-wms-combined_179605	S11_vs_26767	89.152	3	719	74	2	21129	21847	1	715	0	893
S11-wms-combined_221310	S7_vs_129858	97.802	59	10375	226	2	22134	32507	21195	10822	0	17895
S11-wms-combined_14636	S11_vs_20225	99.994	99	35471	2	0	468	35938	1	35471	0	65492
S11-wms-combined_83571	S5_vs_79992	99.958	76	30845	1	1	9777	40621	1	30833	0	56877
S11-wms-combined_125176	S7_vf_117697	99.374	37	24610	151	3	1	24608	883	25491	0	44590
S11-wms-combined_304239	S11_vs_40945	99.983	99	29259	5	0	15448	44706	44575	15317	0	54004
S11-wms-combined_194205	S8_vs_149700	92.66	39	5940	430	5	140	6076	56070	50134	0	8549
S11-wms-combined_236214	S7_vs_87279	98.021	23	5912	93	19	75906	81805	40875	46774	0	10248
S11-wms-combined_264050	S8_vf_151384	97.92	51	29368	591	12	46455	75808	1192	30553	0	50830
S11-wms-combined_140009	S3_vs_119771	78.529	12	4541	823	116	40919	45412	9454	13889	0	2844
S11-wms-combined_168048	S7_vs_21521	84.655	3	1349	174	20	3509	4854	3115	1797	0	1314
S11-wms-combined_195425	S11_vs_84848	99.976	100	29097	7	0	1	29097	2550	31646	0	53694
S11-wms-combined_154845	S11_vs_5077	99.996	100	25037	1	0	1	25037	27863	2827	0	46230
S11-wms-combined_17029	S11_vs_79831	99.97	100	30219	9	0	1	30219	30219	1	0	55755
S11-wms-combined_3507	S1_vf_138157	90.858	6	2855	243	3	42288	45142	14189	11353	0	3810
S11-wms-combined_320710	S11_vs_104069	99.981	100	26195	0	3	18122	44314	1	26192	0	48340
S11-wms-combined_101337	S11_vs_82331	99.999	100	78390	1	0	1	78390	14307	92696	0	1.45E+05
S11-wms-combined_73204	S14_vs_74067	99.303	95	64838	398	48	1	64812	125374	190183	0	1.17E+05
S11-wms-combined_226165	S11_vs_98193	100	99	33032	0	0	1	33032	245	33276	0	60999
S11-wms-combined_321938	S11_vs_3277	99.924	84	35683	27	0	4328	40010	36230	548	0	65745
S11-wms-combined_198512	S11_vf_66160	99.931	86	34798	24	0	5708	40505	34800	3	0	64127
S11-wms-combined_282227	S5_vs_8847	81.746	42	3380	567	37	5223	8590	23672	20331	0	2778
S11-wms-combined_267993	S1_vf_158961	76.142	4	3370	692	78	51281	54576	10311	6980	0	1666
S11-wms-combined_74983	S7_vs_134583	98.78	93	24091	289	3	18563	42649	1044	25133	0	42854
S11-wms-combined_116589	S7_vs_28150	80.844	11	2892	502	43	18891	21759	4754	1892	0	2224
S11-wms-combined_48246	S11_vs_1937	99.994	71	34922	2	0	8306	43227	1	34922	0	64478
S11-wms-combined_283220	S11_vs_52577	99.943	88	29970	17	0	4	29973	29970	1	0	55251
S11-wms-combined_172889	S7_vs_119679	88.612	13	3776	375	45	25479	29228	42686	46432	0	4540
S11-wms-combined_117756	S11_vs_104037	100	100	25717	0	0	1	25717	18094	43810	0	47491
S11-wms-combined_49632	S5_vs_69980	74.662	18	3919	844	124	5148	8994	3846	7687	0	1598
S11-wms-combined_63818	S11_vf_70015	99.964	53	53033	19	0	10357	63389	796	53828	0	97829
S11-wms-combined_215723	S10_vs_37694	98.172	35	16031	262	18	11601	27621	20356	4347	0	27952
S11-wms-combined_64392	S12_vs_23796	77.726	16	5841	1162	123	22260	28030	18578	24349	0	3450
S11-wms-combined_92114	S11_vs_36748	99.993	55	27110	1	1	1	27109	32562	5453	0	50050
S11-wms-combined_119215	S5_vs_69980	79.881	37	6377	1101	146	11419	17682	7938	1631	0	4501
S11-wms-combined_299954	S11_vs_104049	99.988	97	16934	2	0	9379	26312	1	16934	0	31261
S11-wms-combined_217790	S12_vs_26336	99.995	74	40062	2	0	1	40062	27031	67092	0	73970
S11-wms-combined_204522	S11_vs_6458	99.974	100	30685	8	0	1	30685	35244	4560	0	56621
S11-wms_303398	S5_vf_21869	89.593	17	1768	169	13	10493	12256	23361	21605	0	2231
S11-wms_14390	S11_vf_33117	99.997	100	38385	1	0	1	38385	14291	52675	0	70879
S11-wms_3116	S7_vf_39724	96.431	81	31210	1004	69	7366	38537	36206	5069	0	51361
S11-wms_87293	S11_vf_104048	100	100	26833	0	0	1	26833	26833	1	0	49552
S11-wms_6203	S11_vf_89309	99.998	100	45102	1	0	1	45102	1	45102	0	83283
S11-wms_330479	S11_vf_104071	99.924	100	31533	0	1	1	31509	31533	1	0	58076
S1-wms-combined_324053	S13_vs_98591	99.924	90	35476	6	1	37	35491	1341	36816	0	65342
S1-wms-combined_394574	S2_vs_7306	99.967	100	41917	11	1	4917	46833	1	41914	0	77325
S1-wms-combined_394628	S12_vs_137069	88.457	91	5371	542	69	3793	9122	5574	10907	0	6412
S1-wms-combined_246923	S5_vs_69083	78.779	12	2686	507	53	4	2652	1955	4614	0	1744
S1-wms-combined_263562	S3_vs_70508	98.849	97	14948	145	19	13188	28128	28635	13708	0	26626

S1-wms-combined_164894	S14_vs_9162	97.934	36	7502	134	16	6365	13857	85064	92553	0	12975
S1-wms-combined_231234	S13_vs_14794	99.807	84	19668	29	1	1	19668	19659	1	0	36101
S1-wms-combined_132934	S11_vs_19892	88.986	59	23615	2412	144	15106	38628	1	23518	0	29024
S1-wms-combined_17651	S12_vs_164056	76.08	24	1806	394	31	5712	7506	1849	71	0	907
S1-wms-combined_330329	S11_vs_3277	89.217	92	26172	2692	105	5646	31762	7537	33633	0	32577
S1-wms-combined_116938	S7_vs_111831	74.418	39	4636	1043	119	9880	14446	28761	33322	0	1858
S1-wms-combined_166392	S1_vs_133577	99.907	100	19346	17	1	1	19346	1	19345	0	35624
S1-wms-combined_118167	S16_vs_18440	91.491	4	2045	162	11	48211	50249	29851	27813	0	2802
S1-wms-combined_331896	S5_vs_124035	74.938	80	2019	427	61	468	2445	7404	5424	0	852
S1-wms-combined_332236	S13_vs_30167	94.773	97	14139	705	21	703	14822	53140	39017	0	21985
S1-wms-combined_151708	S8_vs_132472	76.895	23	3246	635	85	10361	13540	1	3197	0	1733
S1-wms-combined_52708	S7_vs_97737	78.577	23	2796	548	46	16339	19107	53143	50372	0	1797
S1-wms-combined_68998	S7_vs_135322	89.374	62	11067	1113	42	16349	27383	22258	11223	0	13863
S1-wms-combined_252439	S1_vs_91270	99.988	97	16857	2	0	1	16857	16857	1	0	31119
S1-wms-combined_302166	S1_vf_158961	99.905	57	42117	39	1	17428	59543	42248	132	0	77553
S1-wms-combined_285012	S8_vf_170878	85.079	1	630	92	2	76117	76745	3477	4105	0	641
S1-wms-combined_186878	S1_vs_143929	99.982	100	27678	5	0	99	27776	39654	11977	0	51084
S1-wms-combined_153670	S13_vs_90967	97.139	92	2552	69	4	1	2550	1761	4310	0	4305
S1-wms-combined_351889	S1_vs_155183	99.951	96	30748	3	1	1279	32026	1	30736	0	56687
S1-wms-combined_23248	S3_vs_119771	78.475	6	4539	829	110	38167	42660	13889	9454	0	2833
S1-wms-combined_72167	S13_vs_13129	99.973	99	18770	5	0	1	18770	15307	34076	0	34635
S1-wms-combined_304377	S7_vs_87279	97.235	43	10089	274	4	13057	23144	20423	10339	0	17080
S1-wms-combined_57370	S14_vs_117161	96.542	73	9340	313	10	10442	19776	1302	10636	0	15450
S1-wms-combined_41369	S1_vf_138157	80.771	6	2231	395	29	16270	18486	10858	8648	0	1712
S1-wms-combined_156637	S3_vs_127411	93.517	10	833	54	0	2705	3537	11335	12167	0	1240
S1-wms-combined_107766	S16_vs_19968	99.82	99	39354	71	0	166	39519	54417	15064	0	72280
S1-wms-combined_92523	S6_vs_44070	88.065	16	1349	157	2	27795	29141	16641	17987	0	1596
S1-wms-combined_224578	S11_vf_34008	78.275	6	1565	301	32	11163	12710	27010	25468	0	970
S1-wms-combined_357373	S1_vs_100019	99.865	78	2226	2	1	741	2966	1	2225	0	4093
S1-wms-combined_373308	S12_vs_164056	90.88	35	4309	365	23	9116	13405	4300	1	0	5755
S1-wms-combined_374219	S5_vs_117417	96.517	82	12374	426	5	4556	16926	15211	2840	0	20458
S1-wms-combined_227003	S7_vf_76463	83.178	10	2152	335	22	6598	8740	34307	36440	0	1943
S1-wms-combined_227917	S1_vs_133577	99.806	81	14986	27	2	2035	17019	37683	22699	0	27512
S1-wms-combined_212892	S5_vf_145604	84.853	3	647	85	10	580	1219	27596	26956	0	640
S1-wms-combined_131270	S1_vs_23682	99.91	99	36800	9	1	1	36800	7677	44452	0	67752
S1-wms-combined_279492	S6_vs_1146	93.135	17	2389	152	10	5501	7884	13399	15780	0	3493
S1-wms-combined_164485	S8_vs_149700	92.014	8	1152	85	5	12560	13711	67882	69026	0	1611
S1-wms-combined_85225	S7_vs_73280	84.375	9	2016	286	24	27145	29145	26605	24604	0	1951
S1-wms-combined_307323	S3_vs_123834	93.732	22	4403	244	18	6445	10824	11639	7246	0	6573
S1-wms-combined_94988	S5_vs_74199	78.085	13	2318	397	84	15923	18174	20855	23127	0	1363
S1-wms_296850	S16_vf_6102	98.914	96	34893	364	5	1776	36666	34880	1	0	62321
S1-wms_313991	S1_vf_125650	99.92	57	4994	4	0	6014	11007	6751	1758	0	9201
S1-wms_100010	S1_vf_22149	99.84	92	22487	33	2	267	22751	22486	1	0	41323
S1-wms_265285	S9_vf_90871	87.154	86	15242	1829	99	17945	33100	15615	30813	0	17180
S1-wms_185046	S12_vf_55175	79.294	52	3907	683	94	2722	6590	22432	18614	0	2617
S1-wms_102342	S10_vf_120738	98.645	93	17051	230	1	18212	35261	35815	18765	0	30206
S1-wms_4277	S8_vs_134805	87.005	83	21170	2552	157	7374	28453	34530	13470	0	23669
S1-wms_153615	S1_vs_100019	99.963	98	2717	1	0	67	2783	4858	2142	0	5012
S1-wms_236105	S1_vf_138157	99.948	86	19146	10	0	3216	22361	1	19146	0	35301
S1-wms_170115	S4_vf_31682	98.584	89	15257	215	1	15058	30313	18014	2758	0	26976
S1-wms_106115	S13_vf_147396	99.975	100	16296	4	0	10610	26905	35175	18880	0	30071
S1-wms_223620	S8_vf_65522	98.859	91	29009	283	31	1102	30078	1	28993	0	51692
S1-wms_208875	S13_vf_66508	100	100	18901	0	0	1	18901	33222	14322	0	34904
S1-wms_225995	S13_vf_109235	99.907	96	34569	31	1	11727	46295	39090	4523	0	63658
S1-wms_29288	S7_vs_135322	93.961	30	2484	148	2	32341	34823	50304	47822	0	3755
S1-wms_130214	S1_vf_136526	99.994	77	31148	1	1	1	31148	1	31147	0	57507
S1-wms_212940	S11_vf_13033	96.799	98	13994	434	6	11596	25578	26063	12073	0	23348
S1-wms_394571	S1_vf_159951	99.99	100	101244	10	0	1	101244	745	101988	0	1.87E+05
S3-wms-combined_135428	S3_vs_39940	99.972	38	10828	3	0	17503	28330	1	10828	0	19980
S3-wms-combined_73262	S10_vs_58014	82.72	66	8947	1407	120	21376	30263	23818	32684	0	7829
S3-wms-combined_45305	S3_vs_119771	99.99	100	19171	2	0	1	19171	265	19435	0	35392
S3-wms_23958	S3_vf_82571	99.892	72	25955	25	3	9515	35467	1	25954	0	47772
S3-wms_94494	S4_vs_VIRSorter_5698-cat_2	96.64	5	1012	30	4	19338	20347	15959	16968	0	1677
S3-wms_22744	S3_vf_99734	99.886	99	46297	50	2	1562	47857	46295	1	0	85198
S5-wms-combined_26061	S14_vf_92240	98.169	94	12288	213	9	7330	19610	23924	11642	0	21435
S5-wms-combined_161191	S14_vs_117166	99.997	21	30789	1	0	145128	175916	1	30789	0	56852
S5-wms-combined_134381	S13_vs_35816	97.052	52	13296	346	27	61694	74956	24969	11687	0	22340
S5-wms-combined_90315	S5_vf_18461	99.99	29	29821	3	0	29093	58913	29821	1	0	55053
S5-wms-combined_134743	S5_vs_41663	100	59	57172	0	0	40772	97943	57172	1	0	1.06E+05
S5-wms-combined_72410	S5_vs_85065	100	24	33638	0	0	71400	105037	24195	57832	0	62118
S5-wms-combined_206050	S14_vs_96111	99.986	51	29345	4	0	10578	39922	29345	1	0	54168
S5-wms-combined_73301	S14_vs_117153	99.995	100	20824	1	0	1	20824	13941	34764	0	38450
S5-wms-combined_108794	S5_vs_40848	99.999	100	177204	1	0	1	177204	3467	180670	0	3.27E+05
S5-wms-combined_145067	S5_vs_69980	100	72	22516	0	0	3039	25554	22516	1	0	41580
S5-wms-combined_207291	S5_vs_129970	99.997	56	32526	0	1	12401	44926	32525	1	0	60057
S5-wms-combined_29272	S8_vs_149700	93.628	23	5901	361	9	6013	11902	46073	40177	0	8800
S5-wms-combined_29447	S5_vs_69083	100	28	32116	0	0	220	32335	32116	1	0	59308
S5-wms-combined_199135	S5_vs_36605	99.996	100	26478	1	0	1	26478	26478	1	0	48891
S5-wms-combined_83370	S5_vs_72532	99.92	81	41058	33	0	4090	45147	12526	53583	0	75638
S5-wms-combined_83412	S14_vs_115455	99.996	99	24313	1	0	5079	29391	1	24313	0	44893
S5-wms-combined_66017	S5_vs_145581	100	97	23587	0	0	8165	31751	1	23587	0	43558
S5-wms-combined_66662	S5_vs_121411	100	39	36545	0	0	57695	94239	1	36545	0	67486
S5-wms-combined_147993	S5_vs_59798	100	99	16692	0	0	1392	18083	1	16692	0	30825
S5-wms-combined_156329	S5_vs_122618	99.068	2	429	4	0	1	429	17277	17705	0	771
S5-wms-combined_7292	S5_vs_20268	100	100	14771	0	0	1	14771	1	14771	0	27277
S5-wms-combined_59064	S5_vs_131510	99.985	100	117646	18	0	1	117646	169132	51487	0	2.17E+05

S5-wms-combined_175145	S5_vs_109085	99.989	99	26577	0	2	1	26576	26575	1	0	49059
S5-wms-combined_67650	S5_vs_123256	99.997	81	86610	3	0	6355	92964	1	86610	0	1.60E+05
S5-wms-combined_210959	S5_vs_76500	99.998	33	64407	1	0	122519	186925	1	64407	0	1.19E+05
S5-wms-combined_78434	S5_vs_21778	99.996	99	144358	6	0	2087	146444	157096	12739	0	2.67E+05
S5-wms-combined_204782	S6_vs_32886	99.941	99	23879	11	3	9302	33178	14736	38613	0	44016
S5-wms-combined_98202	S5_vs_142195	100	73	50930	0	0	1	50930	50930	1	0	94050
S5-wms-combined_26971	S11_vf_47632	94.477	33	8076	401	31	14024	22071	6	8064	0	12401
S5-wms-combined_179241	S5_vs_74199	100	85	30526	0	0	3467	33992	30526	1	0	56371
S5-wms-combined_117213	S5_vs_63924	99.989	49	27314	2	1	1	27314	8325	35637	0	50422
S5-wms-combined_163165	S5_vs_65490	100	94	30653	0	0	1	30653	4908	35560	0	56606
S5-wms-combined_52741	S5_vs_86562	100	100	23075	0	0	1	23075	3687	26761	0	42612
S5-wms_62679	S7_vf_67910	99.911	96	15743	12	2	1	15742	15742	1	0	28993
S5-wms_10783	S5_vf_103206	99.995	98	18577	1	0	1	18577	1	18577	0	34300
S5-wms_164032	S5_vf_55687	95.161	100	11283	513	25	1	11275	14211	2954	0	17780
S5-wms_49310	S4_vf_11308	97.519	87	8264	184	7	920	9163	604	8866	0	14105
S5-wms_85650	S5_vs_80496	99.979	100	61026	13	0	1	61026	60938	121963	0	1.13E+05
S5-wms_210460	S5_vs_145581	98.301	4	412	7	0	673	1084	22912	22501	0	723
S5-wms_94965	S5_vf_29332	99.88	90	19974	8	2	3989	23947	17638	37610	0	36738
S5-wms_52566	S5_vs_124035	99.49	100	6468	33	0	1	6468	6992	525	0	11762
S7-wms-combined_217226	S7_vs_29595	99.97	83	64326	18	1	1	64326	64325	1	0	1.19E+05
S7-wms-combined_139683	S7_vs_5236	99.983	100	24118	4	0	8605	32722	1	24118	0	44516
S7-wms-combined_71592	S7_vf_13614	100	77	29799	0	0	8953	38751	5020	34818	0	55029
S7-wms-combined_235165	S7_vs_49426	99.997	100	31876	1	0	1	31876	56890	25015	0	58859
S7-wms-combined_225260	S7_vs_13903	100	100	15940	0	0	1	15940	1	15940	0	29436
S7-wms-combined_31068	S5_vs_69980	79.463	8	2196	383	54	1	2167	5636	3480	0	1495
S7-wms-combined_20921	S7_vs_137611	99.998	80	90273	2	0	51998	142270	1	90273	0	1.67E+05
S7-wms-combined_62119	S16_vs_127669	99.376	23	22259	129	8	76099	98351	24833	47087	0	40326
S7-wms-combined_185016	S5_vs_80496	98.562	25	10431	150	0	1	10431	27299	37729	0	18432
S7-wms-combined_174935	S4_vs_VIRSorter_5698-cat_2	97.746	19	8297	184	3	1	8295	198	8493	0	14283
S7-wms-combined_134452	S7_vs_30665	99.99	15	9811	1	0	42397	52207	1	9811	0	18113
S7-wms-combined_134458	S16_vf_6102	74.964	15	6926	1469	199	22348	29143	8485	1695	0	2939
S7-wms-combined_21693	S8_vs_185041	98.306	78	23264	385	8	10483	33741	49913	26654	0	40769
S7-wms-combined_2122	S7_vs_138109	100	76	29629	0	0	12049	41677	29629	1	0	54715
S7-wms-combined_156351	S7_vs_135679	99.964	100	14020	5	0	1	14020	14020	1	0	25863
S7-wms-combined_104872	S3_vs_70508	97.47	91	19839	496	6	8821	28657	37180	57014	0	33850
S7-wms-combined_238231	S11_vf_33117	78.715	1	1043	206	14	65821	66855	36792	37826	0	682
S7-wms-combined_43950	S10_vs_58014	82.625	31	8944	1421	114	31064	39951	32684	23818	0	7784
S7-wms-combined_3697	S7_vs_54322	100	25	49782	0	0	74473	124254	56805	7024	0	91931
S7-wms-combined_157260	S6_vs_39025	82.11	2	2152	365	17	112278	114421	54371	52232	0	1823
S7-wms-combined_74829	S7_vs_134784	100	82	33154	0	0	4293	37446	35044	1891	0	61224
S7-wms-combined_14267	S7_vf_117697	100	40	25491	0	0	26553	52043	1	25491	0	47074
S7-wms-combined_106253	S11_vs_104042	77.93	1	1237	242	20	2198	3407	23479	24711	0	743
S7-wms-combined_239784	S16_vs_136935	86.943	4	942	87	29	20580	21497	26788	27717	0	1026
S7-wms-combined_189250	S7_vs_55526	99.995	67	19404	1	0	15530	34933	1	19404	0	35827
S7-wms-combined_137858	S7_vs_97737	99.938	98	50000	29	2	1	50000	24630	74627	0	92160
S7-wms-combined_5625	S7_vs_11081	99.965	34	40232	14	0	27422	67653	1	40232	0	74217
S7-wms-combined_190281	S7_vs_55526	83.12	1	936	144	11	62131	63064	18481	19404	0	841
S7-wms-combined_26217	S7_vs_138173	99.969	44	42156	12	1	37843	79998	42155	1	0	77774
S7-wms-combined_69024	S14_vs_117153	82.082	21	4370	737	38	2074	6422	5255	9599	0	3690
S7-wms-combined_7205	S7_vs_48708	99.969	88	62174	19	0	600	62773	62174	1	0	1.15E+05
S7-wms-combined_121062	S7_vf_9334	99.983	100	23648	4	0	5355	29002	40566	16919	0	43648
S7-wms-combined_90393	S7_vs_557	100	100	19692	0	0	1	19692	5333	25024	0	36365
S7-wms-combined_111004	S7_vs_135322	99.927	81	35801	14	10	5071	40862	35798	1	0	65957
S7-wms-combined_90728	S7_vs_944	99.974	99	23257	6	0	195	23451	23257	1	0	42915
S7-wms-combined_40779	S17_vs_127519	98.01	69	5076	89	10	14114	19183	23904	18835	0	8804
S7-wms-combined_28138	S7_vs_16344	99.982	92	28325	5	0	2605	30929	28325	1	0	52279
S7-wms-combined_224523	S16_vs_34021	99.325	100	19119	111	1	1	19119	12384	31484	0	34575
S7-wms-combined_163980	S3_vs_89485	99.985	89	20218	3	0	1677	21894	11996	32213	0	37320
S7-wms-combined_72070	S17_vs_117507	93.003	27	6417	415	25	22600	28999	1239	7638	0	9332
S7-wms-combined_147158	S7_vs_73280	99.937	48	19161	11	1	3	19163	19884	39043	0	35316
S7-wms-combined_218379	S7_vs_47050	99.997	39	36988	1	0	1	36988	36988	1	0	68299
S7-wms-combined_107679_0	S7_vs_119679	99.823	59	40003	67	4	1	40001	11155	51155	0	73475
S7-wms-combined_107679_1	S10_vs_106834	79.276	6	1935	375	21	17125	19045	14754	12832	0	1328
S7-wms_113137	S7_vf_39724	100	99	36889	0	0	3	36891	1	36889	0	68122
S7-wms_123954	S17_vf_140653	92.053	49	9664	725	37	2000	11646	20708	11071	0	13551
S7-wms_53338	S7_vf_89668	99.988	99	16883	2	0	1	16883	23	16905	0	31167
S7-wms_157264	S3_vf_7137	99.981	100	15928	3	0	1	15928	1	15928	0	29397
S7-wms_239833	S7_vf_19866	99.994	99	16468	1	0	1	16468	23	16490	0	30406
S7-wms_127801	S7_vf_8332	100	46	31505	0	0	36900	68404	1	31505	0	58179
S7-wms_118607	S16_vs_86116	78.201	55	7404	1399	170	17996	25286	16	7317	0	4532
S7-wms_159432	S7_vf_5439	99.997	100	33004	0	1	1	33004	33142	140	0	60940
S7-wms_200936	S8_vs_134805	93.064	96	16364	1084	41	13221	29561	18185	34520	0	23883
S7-wms_151112	S7_vf_114476	100	38	10873	0	0	1	10873	10873	1	0	20079
S7-wms_244425	S10_vf_36880	97.256	97	27695	731	27	38974	66659	78667	50993	0	46906
S7-wms_244866	S7_vf_27309	99.955	100	26772	5	4	1	26772	26765	1	0	49365
S17-wms-combined_157468	S5_vs_65490	72.956	11	2507	605	61	19550	22015	25978	28452	0	806
S17-wms-combined_233761	S10_vs_14557	93.302	11	1896	127	0	1	1896	61788	59893	0	2798
S17-wms-combined_56006	S17_vs_110666	99.943	73	70350	37	2	12005	82353	1	70348	0	1.30E+05
S17-wms-combined_56061	S5_vs_130294	76.48	12	4898	975	138	101121	105907	26371	31202	0	2499
S17-wms-combined_223748	S17_vs_133231	99.979	100	4859	1	0	1	4859	8659	3801	0	8968
S17-wms-combined_79709	S5_vs_69980	79.583	19	3737	658	86	2955	6641	3544	7225	0	2577
S17-wms-combined_90378	S4_vs_VIRSorter_27316-cat_2	83.569	33	4382	637	69	17148	21485	50311	54653	0	4026
S17-wms-combined_179771	S17_vs_117507	100	60	25659	0	0	738	26396	25659	1	0	47384
S17-wms-combined_2234	S5_vf_122568	92.568	51	15044	998	90	23746	38738	39202	24228	0	21477
S17-wms-combined_258087	S16_vf_36146	96.296	75	15495	520	43	3824	19293	17210	1745	0	25385
S17-wms-combined_136622	S3_vs_81793	95.034	8	745	37	0	25800	26544	52631	53375	0	1171

S17-wms-combined_203701	S8_vs_179208	99.821	81	57584	99	4	1	57582	57582	1	0	1.06E+05
S17-wms-combined_158354	S16_vs_136935	92.101	5	2228	169	7	13137	15360	5816	8040	0	3133
S17-wms-combined_247494	S5_vs_69980	76.493	4	1842	363	56	16453	18256	3440	1631	0	939
S17-wms-combined_126600	S5_vs_7444	82.056	44	3093	506	45	5149	8219	4266	1201	0	2591
S17-wms-combined_171305	S17_vs_43307	100	50	19701	0	0	1	19701	22799	42499	0	36381
S17-wms-combined_248321	S13_vs_35816	98.629	35	18094	242	3	98275	116362	24960	43053	0	32034
S17-wms-combined_193958	S17_vf_97671	100	18	18558	0	0	82525	101082	19897	1340	0	34271
S17-wms-combined_47860	S9_vs_4558	80.256	82	6964	1286	74	48108	55026	44902	37983	0	5160
S17-wms-combined_259811	S4_vs_VIRSorter_30222-cat_2	99.338	64	16308	94	13	1031	17332	87349	71050	0	29505
S17-wms-combined_127688	S7_vf_5439	98.554	95	16534	222	4	29029	45560	34248	50766	0	29192
S17-wms-combined_260343	S8_vf_170878	81.982	40	4268	736	30	22384	26636	26023	30272	0	3591
S17-wms-combined_72837	S14_vf_117147	97.194	88	12294	322	13	16277	28559	15918	3637	0	20770
S17-wms-combined_16309	S16_vs_127905	92.096	4	544	43	0	18098	18641	18595	18052	0	767
S17-wms-combined_185239	S8_vs_125717	79.349	7	1167	227	14	30472	31631	21770	22929	0	808
S17-wms-combined_39687	S14_vs_3251	99.948	100	19402	10	0	1	19402	14745	34146	0	35774
S17-wms-combined_140517	S8_vs_185041	74.877	15	1624	370	33	15158	16762	21996	23600	0	704
S17-wms-combined_106940	S7_vf_5801	89.4	29	2783	281	13	19119	21894	10857	8082	0	3493
S17-wms-combined_186314	S4_vs_VIRSorter_2744-cat_3	84.932	5	1825	218	33	63551	65351	12090	10299	0	1794
S17-wms-combined_130215	S14_vs_30439	99.491	86	18873	89	5	6696	25565	34044	15176	0	34313
S17-wms-combined_75892	S13_vs_148294	81.511	15	2396	392	39	20421	22775	29230	31615	0	1923
S17-wms-combined_175924	S11_vs_52577	75.836	34	5682	1203	136	13419	19005	7373	12979	0	2730
S17-wms-combined_199440	S7_vs_6626	97.629	80	9741	186	24	13840	23569	44403	34697	0	16667
S17-wms-combined_199746	S17_vs_116640	99.976	35	20806	5	0	6812	27617	2	20807	0	38394
S17-wms-combined_66123	S7_vs_83100	99.417	84	10471	61	0	8814	19284	1050	11520	0	18999
S17-wms-combined_9864	S8_vs_149700	79.804	31	4793	909	52	35602	40365	32563	27801	0	3434
S17-wms-combined_253499	S17_vs_157209	99.974	97	42973	2	1	576	43548	43342	379	0	79287
S17-wms_124308	S14_vs_32499	96.585	94	14552	480	13	1	14543	40325	25782	0	24103
S17-wms_235536	S17_vf_21718	99.997	100	30415	1	0	1	30415	949	31363	0	56161
S17-wms_116248	S11_vf_104070	78.573	58	10216	1951	200	4676	14776	12921	23013	0	6519
S17-wms_29082	S11_vf_34224	98.401	74	29840	473	3	11787	41624	29838	1	0	52458
S17-wms_254872	S17_vf_128147	99.92	99	6243	5	0	1	6243	561	6803	0	11502
S17-wms_266511	S17_vf_177199	99.997	100	35820	0	1	1	35820	35819	1	0	66140
S12-wms-combined_321174	S12_vs_188833	99.898	100	16622	17	0	15500	32121	1	16622	0	30601
S12-wms-combined_401379	S12_vs_137069	99.99	100	10338	1	0	1	10338	10338	1	0	19086
S12-wms-combined_401383	S12_vs_183891	99.975	95	4005	1	0	1	4005	4005	1	0	7391
S12-wms-combined_184338	S8_vs_50431	84.462	28	7929	1198	28	37132	45044	10404	2494	0	7786
S12-wms-combined_368462	S8_vs_149700	79.304	31	6610	1266	92	35649	42200	34366	27801	0	4534
S12-wms-combined_385270	S12_vs_75098	100	99	41466	0	0	1	41466	41466	1	0	76574
S12-wms-combined_201125	S12_vf_60224	99.996	24	54526	2	0	165792	220317	1	54526	0	1.01E+05
S12-wms-combined_368562	S5_vs_131510	92.463	77	16798	1206	51	5231	21994	96448	113219	0	23952
S12-wms-combined_351847	S8_vs_4153	98.193	98	12067	206	5	876	12931	1019	13084	0	21065
S12-wms-combined_117628	S3_vs_122706	90.112	1	627	57	3	57937	58559	3332	2707	0	809
S12-wms-combined_17617	S7_vs_6626	96.359	66	15106	499	39	36517	51591	33990	18905	0	24801
S12-wms-combined_51076	S5_vs_21778	80.346	8	2310	432	20	67764	70066	14549	12255	0	1731
S12-wms-combined_151843	S12_vs_48045	99.994	26	32825	2	0	21039	53863	32825	1	0	60606
S12-wms-combined_18287	S7_vs_4956	96.39	43	26818	902	49	45437	72211	35149	8355	0	44100
S12-wms-combined_320262	S9_vs_2799	97.867	73	18327	385	5	1	18325	54267	35945	0	31673
S12-wms-combined_2450	S5_vs_69083	99.981	38	31347	5	1	50592	81938	32120	775	0	57852
S12-wms-combined_320727	S14_vs_74067	99.682	75	52155	161	5	94741	146894	200667	252817	0	95387
S12-wms-combined_371541	S11_vs_79831	95.928	12	4273	156	10	29418	33678	8904	4638	0	6911
S12-wms-combined_120133	S12_vs_99092	100	69	29908	0	0	1	29908	5354	35261	0	55230
S12-wms-combined_71235	S4_vs_VIRSorter_27316-cat_2	76.386	26	3985	826	94	14465	18387	50326	54257	0	2039
S12-wms-combined_189323	S12_vs_23796	99.997	76	63354	2	0	1	63354	1	63354	0	1.17E+05
S12-wms-combined_190114	S2_vs_10289	79.04	29	3769	719	56	9589	13328	11073	14799	0	2518
S12-wms-combined_256299	S12_vs_111286	99.959	92	49063	10	3	4356	53411	49194	135	0	90483
S12-wms-combined_156234	S7_vs_134583	98.778	65	23903	285	5	1	23898	1233	25133	0	42516
S12-wms-combined_323943	S7_vs_135322	80.742	8	1698	308	13	29525	31214	1985	299	0	1306
S12-wms-combined_191570	S12_vs_150812	99.982	85	22142	2	2	3986	26127	36712	14573	0	40865
S12-wms-combined_23517	S1_vs_21439	78.102	4	3046	620	40	21038	24061	23404	26424	0	1886
S12-wms-combined_375343	S11_vs_79831	98.254	17	4182	73	0	21169	25350	18895	23076	0	7319
S12-wms-combined_325418	S8_vs_115958	93.474	50	12029	773	12	22681	34704	81002	93023	0	17854
S12-wms-combined_41160	S12_vs_28661	99.777	95	21498	44	4	483	21978	27374	5879	0	39430
S12-wms-combined_7174	S13_vs_30167	87.921	62	9256	1050	53	1723	10935	36149	26919	0	10837
S12-wms-combined_358010	S12_vs_167155	82.911	2	749	107	16	49810	50551	21451	22185	0	654
S12-wms-combined_91231	S12_vs_99219	99.996	100	49609	2	0	1	49609	51797	2189	0	91600
S12-wms-combined_107942	S11_vs_104064	96.568	99	30917	1012	30	2	30890	26657	57552	0	51169
S12-wms-combined_293354	S12_vs_94042	99.97	92	36599	10	1	3468	40065	2435	39033	0	67523
S12-wms-combined_75733	S12_vf_188846	99.966	100	2940	1	0	1	2940	2722	5661	0	5424
S12-wms-combined_393703	S14_vs_67094	80.254	16	1889	340	29	4196	6068	3379	1508	0	1391
S12-wms-combined_358797	S3_vs_56292	98.859	62	15689	175	4	300	15986	52533	36847	0	27977
S12-wms-combined_25459	S9_vs_2799	81.271	2	1180	202	17	35581	36749	47629	46458	0	937
S12-wms-combined_359190	S8_vs_185041	91.792	45	16338	1289	42	36389	52695	9711	26027	0	22694
S12-wms-combined_259783	S8_vs_206526	99.107	98	33822	294	5	11597	45415	1	33817	0	60778
S12-wms-combined_260036	S4_vf_12614	92.864	3	1107	74	5	2382	3485	1110	6	0	1602
S12-wms-combined_210942	S3_vs_127411	91.155	60	3663	293	20	8095	11750	15600	11962	0	4940
S12-wms-combined_328554	S12_vs_142625	100	100	43415	0	0	1	43415	80692	37278	0	80173
S12-wms-combined_329242	S5_vs_65490	79.266	11	3188	594	52	46056	49208	25978	29133	0	2163
S12-wms-combined_345948	S4_vs_VIRSorter_27316-cat_2	76.242	37	4268	894	104	14581	18785	54536	50326	0	2154
S12-wms-combined_313953	S12_vs_153780	99.998	100	61118	1	0	1	61118	61281	164	0	1.13E+05
S12-wms-combined_245755	S14_vs_117161	83.032	72	8522	1262	144	11240	19670	23993	32421	0	7557
S12-wms-combined_111524	S12_vs_26336	99.994	60	48766	3	0	1	48766	67092	18327	0	90038
S12-wms-combined_297545	S15_vs_3868	99.157	39	23716	195	2	29802	53514	33905	57618	0	42682
S12-wms-combined_129554	S7_vs_6626	75.138	17	4887	1100	94	5629	10441	42769	37924	0	2187
S12-wms-combined_129799	S16_vf_97279	97.633	55	27075	625	11	40343	67411	1	27065	0	46433
S12-wms-combined_282594	S12_vs_86928	99.996	44	26839	1	0	15499	42337	41629	14791	0	49557
S12-wms-combined_264425	S12_vf_79666	99.866	99	41164	42	1	5374	46524	46218	5055	0	75698

S12-wms-combined_299293	S11_vf_84303	74.794	15	4360	949	117	52642	56920	29921	25631	0	1825
S12-wms-combined_98046	S8_vs_149700	78.923	2	1504	285	26	68293	69780	44578	46065	0	992
S12-wms-combined_31761	S6_vs_54606	72.725	11	4462	972	194	20175	24533	11678	7359	0	1271
S12-wms-combined_99704	S14_vs_96111	96.081	51	16278	628	9	15873	32142	1	16276	0	26517
S12-wms-combined_132907	S12_vs_79785	99.565	98	40727	154	7	626	41330	1398	42123	0	74206
S12-wms-combined_250551	S12_vs_141337	99.998	97	40473	1	0	1269	41741	44369	3897	0	74735
S12-wms-combined_368190	S12_vf_16374	99.997	53	59768	2	0	3482	63249	59768	1	0	1.10E+05
S12-wms-combined_157167	S12_vs_164056	99.971	88	3403	1	0	481	3883	6664	3262	0	6279
S12-wms-combined_164656	S12_vs_32669	100	100	24358	0	0	1	24358	1	24358	0	44981
S12-wms_184276	S12_vf_173666	99.935	99	30650	20	0	20	30669	1	30650	0	56490
S12-wms_235125	S12_vf_55175	99.6	100	12497	50	0	11093	23589	1	12497	0	22801
S12-wms_17950	S15_vs_1407	98.983	92	7372	74	1	4499	11870	1706	9076	0	13197
S12-wms_352846	S12_vf_188830	100	100	38917	0	0	28954	67870	1	38917	0	71867
S12-wms_386692	S12_vf_174374	99.945	100	34590	19	0	1	34590	34590	1	0	63771
S12-wms_202803	S8_vf_72773	98.54	94	18358	238	12	10336	28663	23930	42287	0	32389
S12-wms_336780	S12_vs_133070	98.186	8	441	8	0	8957	9397	131663	132103	0	771
S12-wms_193565	S17_vs_110666	79.622	31	16493	2986	298	1	16323	33876	50163	0	11490
S12-wms_161239	S11_vf_104070	81.863	43	4433	745	51	14637	19043	36162	31763	0	3677
S12-wms_44817	S5_vf_110171	93.802	94	11036	601	65	23980	34968	36290	47289	0	16513
S12-wms_179388	S11_vf_84303	97.227	96	21781	577	11	23677	45451	34626	12867	0	36851
S12-wms_12658	S12_vf_69136	99.903	55	32030	30	1	26593	58621	137	32166	0	58975
S12-wms_15700	S3_vf_2186	97.923	99	26005	519	17	1	25994	38190	12196	0	45011
S12-wms_401366	S12_vf_128794	99.96	100	40062	16	0	5029	45090	1	40062	0	73892
S12-wms_401372	S12_vf_188824	100	100	72979	0	0	14932	87910	87909	14931	0	1.35E+05
S2-wms-combined_207832	S3_vf_99734	97.993	92	13603	268	3	29467	43068	23646	10048	0	23603
S2-wms-combined_248329	S2_vs_4368	99.99	66	20545	2	0	11106	31650	20983	439	0	37929
S2-wms-combined_31084	S7_vs_48708	77.385	31	7190	1520	84	11859	18997	57086	49952	0	4172
S2-wms-combined_196783	S7_vs_69630	83.914	11	1579	246	7	28042	29616	6841	8415	0	1502
S2-wms-combined_165833	S14_vf_58919	86.515	68	12621	1602	76	6614	19164	20753	8163	0	13786
S2-wms-combined_62377	S13_vs_152223	99.964	99	60900	10	1	4	60903	60888	1	0	1.12E+05
S2-wms-combined_72725	S11_vs_104049	94.444	46	6750	346	27	22333	29074	6778	13506	0	10360
S2-wms-combined_62519	S4_vs_VIRSorter_4959-cat_2	99.816	41	34265	62	1	7917	42180	34360	96	0	62925
S2-wms-combined_218002	S6_vs_12945	97.878	46	34587	702	27	41654	76226	37406	2838	0	59775
S2-wms-combined_166665	S6_vs_1146	98.199	89	13603	230	11	32492	46086	36531	50126	0	23749
S2-wms-combined_62839	S8_vf_187742	72.201	3	5047	1238	131	16568	21533	57166	62128	0	1395
S2-wms-combined_197577	S3_vs_127445	98.536	98	43028	594	28	21370	64386	27698	70700	0	75935
S2-wms-combined_208559	S9_vs_2799	78.521	8	2542	506	37	33281	35801	15884	18406	0	1633
S2-wms-combined_229529	S13_vf_66508	94.27	1	1431	70	8	7979	9401	35724	34298	0	2178
S2-wms-combined_126134	S11_vs_104049	80.893	12	2240	412	14	6040	8271	9655	7424	0	1751
S2-wms-combined_74387	S16_vs_92150	74.1	5	3278	757	80	36461	39694	29660	26431	0	1266
S2-wms-combined_147065	S11_vs_3277	98.944	21	26693	282	0	103892	130584	30777	4085	0	47731
S2-wms-combined_137119	S7_vs_134583	77.564	25	7898	1614	147	81703	89519	17313	25133	0	4623
S2-wms-combined_116221	S11_vf_47632	98.516	34	17180	252	2	21664	38840	6	17185	0	30310
S2-wms-combined_157934	S6_vs_10314	74.751	2	2210	474	69	73807	75965	7306	9482	0	913
S2-wms-combined_64692	S13_vs_148294	97.681	20	6424	130	15	37911	44330	11277	17685	0	11020
S2-wms-combined_127080	S12_vs_81729	92.694	64	4038	258	20	12280	16307	23582	27592	0	5788
S2-wms-combined_13246	S5_vs_30490	81.887	6	8530	1382	119	218362	226805	16635	8183	0	7042
S2-wms-combined_199384	S12_vs_14467	92.81	19	1808	123	7	15223	17027	15899	14096	0	2612
S2-wms-combined_55495	S1_vs_62409	97.533	82	8997	190	25	2846	11826	1	8981	0	15356
S2-wms-combined_158821	S4_vs_VIRSorter_13488-cat_2	75.842	52	5936	1220	170	11107	16934	537	6366	0	2820
S2-wms-combined_210822	S5_vs_109527	93.541	11	5063	315	10	40354	45409	24761	29818	0	7528
S2-wms-combined_24549	S11_vs_104049	89.92	9	2252	221	3	51988	54235	29939	27690	0	2896
S2-wms-combined_170845	S8_vs_185041	88.87	36	5867	628	21	6928	12776	31885	37744	0	7193
S2-wms-combined_159670	S1_vs_143929	94.342	6	813	44	2	35637	36448	15383	14572	0	1245
S2-wms-combined_181391	S14_vs_3251	95.111	9	3968	186	5	114724	118687	30183	34146	0	6246
S2-wms-combined_200390	S9_vs_88286	86.188	11	7689	1030	21	30584	38255	796	8469	0	8287
S2-wms-combined_233107	S5_vs_65490	98.184	72	10020	163	10	6176	16186	20563	10554	0	17477
S2-wms-combined_57293	S5_vs_130294	77.139	39	4908	941	144	14230	19027	31207	26371	0	2680
S2-wms-combined_191613	S1_vs_145792	91.144	25	5375	414	34	14602	19921	1	5368	0	7232
S2-wms-combined_98888	S4_vs_VIRSorter_3006-cat_1	95.807	38	13499	559	4	86150	99646	49541	36048	0	21786
S2-wms-combined_181849	S7_vs_87279	89.372	3	1289	117	14	6693	7968	28322	29603	0	1604
S2-wms-combined_201205	S16_vs_173096	96.286	66	11200	390	18	18021	29207	30126	18940	0	18354
S2-wms-combined_78670	S14_vf_56130	86.325	41	5031	643	35	26230	31237	9963	4955	0	5437
S2-wms-combined_140996	S5_vf_122568	97.59	20	25930	600	21	113454	139370	15141	41058	0	44398
S2-wms-combined_58563	S9_vs_90851	82.246	43	10730	1707	160	34324	44958	19154	8528	0	9079
S2-wms-combined_174241	S3_vs_119605	93.61	33	13553	834	28	37436	50970	28472	14934	0	20201
S2-wms-combined_110215	S14_vs_9162	79.159	2	1569	301	21	3536	5088	53481	51923	0	1062
S2-wms-combined_122457	S11_vs_104049	88.67	5	2639	288	9	22737	25367	27194	29829	0	3206
S2-wms-combined_244564	S11_vs_42927	72.591	18	4681	1154	112	47032	51648	40109	44724	0	1417
S2-wms-combined_6605	S5_vs_65490	79.266	6	3188	594	52	18271	21423	25978	29133	0	2163
S2-wms-combined_225908	S3_vs_122706	95.209	11	12105	557	19	99704	111799	9501	21591	0	19119
S2-wms-combined_113619	S13_vs_83447	99.941	100	35571	3	3	1	35571	21098	56650	0	65555
S2-wms-combined_227800	S16_vs_34751	86.224	3	980	126	8	1953	2926	1476	500	0	1053
S2-wms-combined_52006	S14_vs_97835	79.167	60	1632	292	32	1097	2711	11	1611	0	1086
S2-wms-combined_4685	S8_vs_132472	92.297	25	3726	265	16	665	4374	3720	1	0	5271
S2-wms-combined_207000	S5_vs_145598	94.742	45	5382	277	6	22354	27732	38361	32983	0	8366
S2-wms_124416	S17_vs_110666	80.22	74	20020	3563	326	38430	58267	30851	50655	0	14665
S2-wms_156439	S5_vf_8965	96.85	60	18033	537	18	211	18231	1784	19797	0	30125
S2-wms_53881	S14_vf_114491	99.975	97	15913	3	1	478	16389	15913	1	0	29362
S2-wms_23562	S2_vf_3356	100	99	13546	0	0	1	13546	13546	1	0	25015
S2-wms_128831	S13_vf_151045	99.112	99	52952	410	45	33370	86290	1	52923	0	95125
S2-wms_128962	S13_vf_11246	99.974	100	72445	19	0	1	72445	72445	1	0	1.34E+05
S2-wms_79304	S2_vf_16829	100	100	17153	0	0	5	17157	17189	37	0	31676
S2-wms_161986	S3_vf_127405	95.487	90	26346	1155	30	2969	29296	36208	9879	0	42034
S2-wms_130687	S16_vs_82116	98.576	77	29631	392	12	1786	31389	66695	37068	0	52353
S2-wms_110761	S1_vf_125650	99.925	99	6707	5	0	1	6707	45	6751	0	12358

S2-wms_6424	S11_vf_33433	97.724	94	19773	428	17	13740	33500	75021	55259	0	34001
S2-wms_245232	S11_vf_10907	78.964	16	2163	390	47	34908	37045	20907	18785	0	1413
S2-wms_248315	S2_vf_21511	99.996	99	47745	1	1	16505	64248	64083	16339	0	88156
S2-wms_248324	S2_vf_21630	100	100	27232	0	0	1	27232	27232	1	0	50289
S4-wms-combined_14411	S4_vs_VIRSorter_6022-circular-cat_1	99.995	100	40647	2	0	2863	43509	1	40647	0	75050
S4-wms-combined_112093	S4_vs_VIRSorter_13488-cat_2	99.987	99	15391	2	0	10467	25857	1	15391	0	28411
S4-wms-combined_42647	S4_vs_VIRSorter_27316-cat_2	77.973	27	4390	861	91	11786	16122	54662	50326	0	2652
S4-wms-combined_22012	S3_vf_99626	99.86	87	23615	32	1	3592	27205	141	23755	0	43425
S4-wms-combined_57963	S11_vf_34224	98.468	22	15864	238	4	65681	81539	29838	13975	0	27944
S4-wms-combined_10758	S4_vs_VIRSorter_13190-cat_2	99.975	71	59082	12	3	24455	83535	1	59080	0	1.09E+05
S4-wms-combined_17284	S4_vs_VIRSorter_16493-cat_2	99.974	54	22682	5	1	32844	55524	40429	17748	0	41851
S4-wms-combined_121978	S8_vs_185041	91.31	24	20414	1628	102	59998	80312	5857	26223	0	27735
S4-wms-combined_25201	S7_vs_86377	84.896	23	7018	1011	44	11812	18802	6996	1	0	7042
S4-wms-combined_47565	S8_vs_148389	97.442	5	782	19	1	3816	4596	10885	10104	0	1332
S4-wms-combined_41490	S5_vs_20500	98.984	75	48236	399	42	6363	54558	1	48185	0	86276
S4-wms_118690	S4_vf_10506	100	100	22121	0	0	1	22121	22121	1	0	40850
S4-wms_161443	S4_vf_30103	99.949	100	15635	1	4	48	15678	1	15632	0	28821
S4-wms_50475	S4_vf_20440	99.973	63	63000	14	1	24181	87180	77	63073	0	1.16E+05
S4-wms_141607	S4_vf_18803	99.905	81	28337	22	2	120	28451	28337	1	0	52174
S4-wms_45128	S4_vf_4891	99.998	98	40481	1	0	1	40481	2650	43130	0	74749
S4-wms_73990	S4_vf_4838	100	100	67580	0	0	1	67580	26145	93724	0	1.25E+05
S4-wms_62635	S4_vf_13353	100	100	28687	0	0	15609	44295	184	28870	0	52975
S6-wms-combined_117756	S6_vs_56496	100	97	27718	0	0	277	27994	27718	1	0	51186
S6-wms-combined_135223	S5_vs_109527	99.999	96	142385	1	1	5529	147913	1	142384	0	2.63E+05
S6-wms-combined_88414	S6_vs_10314	99.928	83	83767	28	16	1	83750	1	83752	0	1.54E+05
S6-wms-combined_745	S13_vs_9438	99.996	70	25922	1	0	10893	36814	1	25922	0	47864
S6-wms-combined_82597	S6_vs_9784	99.948	28	59906	25	2	155325	215227	1	59903	0	1.10E+05
S6-wms-combined_118427	S8_vf_170878	97.052	26	21810	636	5	124794	146597	46644	24836	0	36706
S6-wms-combined_118484	S14_vs_30439	97.081	80	14630	417	8	49	14670	33781	19154	0	24642
S6-wms-combined_72228	S3_vs_17168	77.771	10	3392	658	73	113	3439	51889	55249	0	1999
S6-wms-combined_119138	S6_vs_39025	99.998	93	57387	1	0	4197	61583	1	57387	0	1.06E+05
S6-wms-combined_49909	S6_vs_1146	99.986	100	28186	4	0	1	28186	32607	4422	0	52028
S6-wms-combined_113986	S6_vs_29152	100	100	34147	0	0	1	34147	46560	12414	0	63058
S6-wms-combined_8508	S5_vs_38949	99.943	50	27835	16	0	9269	37103	16281	44115	0	51313
S6-wms-combined_32530	S6_vs_12945	99.989	82	36605	4	0	8194	44798	1	36605	0	67575
S6-wms-combined_126236	S5_vs_124677	99.999	100	69368	1	0	1	69368	73006	3639	0	1.28E+05
S6-wms-combined_50721	S5_vs_79992	99.996	41	54138	2	0	77965	132102	1	54138	0	99963
S6-wms-combined_138710	S5_vs_130294	99.997	97	38590	1	0	1	38590	38590	1	0	71257
S6-wms-combined_56464	S6_vs_53966	100	62	21784	0	0	1	21784	21784	1	0	40228
S6-wms-combined_110950	S5_vs_30490	99.976	99	33429	6	2	16	33443	56501	23074	0	61686
S6-wms-combined_128972	S5_vs_117417	99.584	89	19695	82	0	2181	21875	19695	1	0	35916
S6-wms-combined_91039	S7_vf_117697	99.197	42	25160	199	3	5767	30924	25491	333	0	45340
S6-wms_54578	S4_vs_VIRSorter_6022-circular-cat_1	94.848	62	5648	257	24	7344	12972	25477	19845	0	8787
S6-wms_137591	S6_vf_6314	99.961	56	7600	3	0	335	7934	1	7600	0	14019
S6-wms_138342	S11_vf_104035	100	100	89495	0	0	1	89495	89495	1	0	1.65E+05
S6-wms_140743	S5_vf_145585	99.99	100	47998	5	0	1	47998	47998	1	0	88608
S8-wms-combined_246389	S8_vs_35308	100	99	38635	0	0	2571	41205	41153	2519	0	71346
S8-wms-combined_215102	S3_vs_90341	97.755	49	11091	247	2	42375	53464	33587	22498	0	19101
S8-wms-combined_276204	S8_vs_71212	100	100	21132	0	0	1	21132	19049	40180	0	39024
S8-wms-combined_276211	S8_vs_88013	99.972	100	24611	0	1	17853	42456	42463	17853	0	45402
S8-wms-combined_23583	S8_vs_9114	100	38	40264	0	0	43816	84079	40264	1	0	74354
S8-wms-combined_81597	S8_vs_99362	99.864	100	24282	30	2	1	24279	2538	26819	0	44654
S8-wms-combined_140059	S7_vs_56529	99.991	99	21727	2	0	1	21727	10539	32265	0	40112
S8-wms-combined_186054	S8_vs_149700	99.968	100	53551	17	0	1	53551	15806	69356	0	98796
S8-wms-combined_25607	S8_vs_145173	99.991	100	22179	2	0	20576	42754	1	22179	0	40946
S8-wms-combined_26087	S15_vs_71968	99.404	48	26856	154	4	28179	55030	39396	12543	0	48702
S8-wms-combined_164289	S8_vs_176604	99.964	100	27875	9	1	1	27875	7709	35582	0	51419
S8-wms-combined_72911	S5_vs_10428	94.32	1	581	33	0	30990	31570	23705	23125	0	891
S8-wms-combined_49369	S7_vs_42563	99.488	63	18957	80	2	10181	29120	9868	28824	0	34454
S8-wms-combined_234473	S5_vs_124035	99.045	99	3038	19	8	5	3037	8442	5410	0	5441
S8-wms-combined_74275	S8_vs_148389	100	65	26113	0	0	1	26113	11619	37731	0	48222
S8-wms-combined_270362	S8_vs_9987	99.919	84	49188	36	4	10377	59562	49186	1	0	90608
S8-wms-combined_200168	S7_vs_6626	75.956	6	4891	1066	91	46503	51322	37917	42768	0	2416
S8-wms-combined_97848	S10_vs_58014	87.952	34	6134	713	20	793	6910	6854	12977	0	7210
S8-wms-combined_108917	S8_vs_115958	100	99	69791	0	0	667	70457	137050	67260	0	1.29E+05
S8-wms-combined_225114	S8_vs_179529	99.934	100	51282	28	3	1	51279	52599	1321	0	94507
S8-wms-combined_134098	S8_vs_145173	76.628	13	2088	415	59	7680	9738	36098	34055	0	1085
S8-wms-combined_134284	S8_vs_208113	99.996	100	26767	1	0	212	26978	41644	14878	0	49424
S8-wms-combined_226076	S8_vf_208107	99.99	100	30625	2	1	1	30625	30624	1	0	56536
S8-wms-combined_169835	S8_vs_125717	99.962	100	52956	20	0	1	52956	1	52956	0	97681
S8-wms-combined_239791	S8_vs_72117	99.892	100	21293	6	4	2	21277	11812	33104	0	39177
S8-wms-combined_217866	S8_vs_47265	99.996	100	50262	1	1	42	50303	18417	68677	0	92804
S8-wms-combined_68780	S8_vs_208127	99.971	65	20685	6	0	51979	72663	11808	32492	0	38165
S8-wms-combined_161181	S4_vs_VIRSorter_4959-cat_2	92.664	66	23173	1661	31	8567	31714	97	23255	0	33338
S8-wms-combined_246185	S5_vs_86562	85.968	2	1012	126	12	2	1005	24638	23635	0	1068
S8-wms_270	S8_vf_208115	100	99	3507	0	0	1894	5400	1	3507	0	6477
S8-wms_127191	S8_vf_163760	99.992	100	38123	3	0	21865	59987	1	38123	0	70384
S8-wms_58515	S12_vf_174374	98.718	33	3900	46	4	15400	19297	33777	29880	0	6922
S8-wms_244525	S8_vf_203044	100	100	17376	0	0	1	17376	17388	13	0	32088
S8-wms_152831	S8_vf_101283	99.99	100	28619	3	0	1	28619	29450	832	0	52833
S8-wms_48935	S8_vf_170878	99.98	99	58755	12	0	1	58755	58755	1	0	1.08E+05
S8-wms_190781	S3_vf_127409	98.147	99	14681	264	6	1	14674	23623	38302	0	25597
S8-wms_30498	S8_vs_134805	99.864	80	24933	34	0	6294	31226	37347	12415	0	45855
S8-wms_111056	S13_vf_3728	97.743	87	22729	499	12	20250	42972	19557	42277	0	39118
S8-wms_20528	S8_vf_208126	99.977	100	26226	1	2	1	26226	26221	1	0	48392
S8-wms_57121	S8_vf_93976	99.993	76	26682	2	0	7746	34427	26682	1	0	49262

Table S1-5. Read mapping

Reference genome: VLP-HQ-nr-vOTUs: 1231

Sample	# Cleaned reads (PE)	# Reads mapped to reference nr-vOTUs	Mapping rate (%)	# Unmapped reads	Unmapping rate (%)
S1-vlp	35113882	12741090	36.29%	22372792	63.71%
S2-vlp	56868494	53293863	93.71%	3574631	6.29%
S3-vlp	174177994	120181758	69.00%	53996236	31.00%
S4-vlp	47904786	38373316	80.10%	9531470	19.90%
S5-vlp	65733438	31012444	47.18%	34720994	52.82%
S6-vlp	68149392	55096198	80.85%	13053194	19.15%
S7-vlp	81075886	65132881	80.34%	15943005	19.66%
S8-vlp	183020194	121979288	66.65%	61040906	33.35%
S9-vlp	71023532	59039474	83.13%	11984058	16.87%
S10-vlp	73760336	22080498	29.94%	51679838	70.06%
S11-vlp	67000402	34020860	50.78%	32979542	49.22%
S12-vlp	85558566	39625666	46.31%	45932900	53.69%
S13-vlp	60096768	31551452	52.50%	28545316	47.50%
S14-vlp	61978780	24937001	40.23%	37041779	59.77%
S15-vlp	32235928	11812670	36.64%	20423258	63.36%
S16-vlp	74204032	41380932	55.77%	32823100	44.23%
S17-vlp	58989106	7611856	12.90%	51377250	87.10%
Mean	76287736.24	45286543.94	59.36%	31001192.29	40.63%
SD			22.34%		77.66%
NC1	360240	23180	6.43%	337060	93.57%
NC2	18592	2610	14.04%	15982	85.96%
NC3	111664	16079	14.40%	95585	85.60%

Reference genome: WMS-HQ-nr-vOTUs: 688

Sample	# Cleaned reads (PE)	# Reads mapped to reference nr-vOTUs	Mapping rate (%)	# Unmapped reads	Unmapping rate (%)
S1-wms	144000416	16319573	11.33%	127680843	88.67%
S2-wms	148218256	17114196	11.55%	131104060	88.45%
S3-wms	12026424	1244734	10.35%	10781690	89.65%
S4-wms	53444298	3980159	7.45%	49464139	92.55%
S5-wms	186273684	21308808	11.44%	164964876	88.56%
S6-wms	57739286	7700678	13.34%	50038608	86.66%
S7-wms	137959384	16360994	11.86%	121598390	88.14%
S8-wms	90880674	11462097	12.61%	79418577	87.39%
S9-wms	87343694	6561914	7.51%	80781780	92.49%
S11-wms	84502272	9987117	11.82%	74515155	88.18%
S12-wms	140008922	14878767	10.63%	125130155	89.37%
S17-wms	110271798	11923131	10.81%	98348667	89.19%
Mean	104389092.33	11570180.67	11.08%	92818911.67	88.92%
SD			1.79%		98.21%

Table S1-6. VLP_avg_cov

vOTUs	NC1_vlp	NC2_vlp	NC3_vlp	S1_vlp_ME	S2_vlp_SHHC	S3_vlp_ME	S4_vlp_SHHC	S5_vlp_ME	S6_vlp_SHHC	S7_vlp_ME	S8_vlp_SHHC	S9_vlp_ME	S10_vlp_SHHC	S11_vlp_ME	S12_vlp_SHHC	S13_vlp_ME	S14_vlp_ME	S15_vlp_SHHC	S16_vlp_SHHC	S17_vlp_ME	Status
S17_vf_140653	0	0	0.01	1.05	2.42	12.02	0.08	3.36	1.32	3.32	3.68	0.1	15.18	2.07	4.57	3.36	8.91	6.12	9.62	81.3	Shared
S17_vf_148303	0	0	0	0.39	0.31	0.84	0.11	0.91	1.06	693.64	0.88	0.13	0.93	0.34	2.62	0.31	0.36	0.22	0.92	312.48	ME
S17_vf_67042	0	0	0	0.34	0.42	0.36	0.06	0.11	0.13	0.12	0.55	0.06	0.38	0.06	0.28	0.14	0.12	0.43	5.37	192.46	ME
S17_vf_111931	0.03	0	0	0.57	0.02	0.42	0.18	0.22	0.06	0.27	0.91	0.07	0.25	1.34	0.99	0.37	14.25	0.24	3.34	80.79	ME
S17_vf_97671	0.01	0	0.01	2.15	0.02	0.52	0.55	3.81	0.59	2.91	8.34	2.22	1.48	3.88	3.35	1.29	2.7	3.31	4.67	33.52	ME
S17_vf_46388	0.01	0	0	0.87	0.04	0.63	8.31	0.16	0.16	0.17	0.58	0.12	0.58	0.66	0.43	0.71	0.28	0.45	106.98	222.67	Shared
S17_vf_172132	0	0	0	0.3	0.04	0.36	0.01	0.15	0.22	619.23	0.97	0.08	0.29	0.08	1.42	0.2	0.11	0.16	1.05	288.85	ME
S17_vf_128147	0	0	0	1.65	4.55	24.48	0.08	9.02	1.95	11.6	3.91	0.3	33.05	4.5	8.63	8.03	24.31	14.07	20.81	50.82	Shared
S17_vf_77057	0	0	0.01	0.37	0.68	0.69	0.13	0.39	0.16	395.03	0.77	0.12	0.84	0.39	0.89	0.44	0.35	0.26	0.63	154.25	ME
S17_vf_174033	0	0	0	1.68	0.04	0.64	0.14	0.19	0.12	0.18	0.82	0.13	2.66	0.28	4.86	0.63	0.4	0.47	23.94	55.63	Shared
S17_vf_174203	0	0	0	0.41	0.19	0.87	0.12	0.28	0.27	481.8	1.3	0.13	0.66	0.28	2.03	0.49	0.34	0.18	0.73	343.07	ME
S17_vf_35138	0	0	0	0.85	0.01	0.47	18.34	0.24	0.88	0.21	0.48	0.27	0.91	0.46	1.17	0.23	0.31	0.27	12.9	45.31	ME
S17_vf_79151	0	0	0	0.26	0.02	0.53	0.17	0.27	0.09	107.18	0.8	0.09	1.58	0.1	0.63	0.46	0.31	0.26	0.49	61.23	ME
S17_vf_36452	0	0	0	6.07	1.45	0.37	0.05	0.2	0.06	0.57	0.78	0.16	0.36	0.31	1.16	0.44	116.2	0.28	15.14	563.74	ME
S17_vf_21718	0	0	0	3.24	0.4	19.18	0.88	1.56	1.16	2.82	3.54	7.88	21.12	5.41	7.68	1.36	58.28	1.35	17.12	141.82	ME
S17_vf_81232	0	0	0	0.95	0.08	0.41	36.61	0.25	0.16	134.28	157.96	0.09	1.69	0.43	1.09	0.58	0.34	0.26	0.81	61.74	Shared
S17_vf_177197	0.01	0	0	2.32	2.91	4.75	29.77	2.81	2.63	3.22	21725.43	0.09	0.73	0.38	0.55	506.01	0.33	0.37	1.21	29.14	Shared
S17_vf_177199	0	0	0	0.61	0.01	0.21	0.09	0.09	0.06	0.06	0.97	0.19	0.26	0.51	0.33	0.15	0.22	0.16	15.66	734.13	ME
S17_vf_177200	0.06	0.04	0	312.8	0.06	0.55	0.08	0.22	0.1	0.13	0.55	0.1	0.64	0.13	0.25	34.93	0.19	0.74	107.46	214.04	Shared
S17_vs_138036	0	0	0	0.66	0.03	0.75	0.13	0.25	0.17	0.19	1.07	0.22	0.66	0.84	0.69	0.38	0.38	0.23	6.61	343.02	ME
S17_vs_103009	0.18	0.04	0	711.72	0.15	1.25	0.1	0.39	0.23	0.36	162.59	0.6	0.43	0.29	1.26	233.34	0.42	1.15	343.22	686.05	Shared
S17_vs_177196	0.01	0	0	0.4	0.01	0.24	0.3	0.2	0.27	0.1	1.58	0.12	0.37	0.09	0.29	0.2	0.14	0.24	56.29	110.08	ME
S17_vs_177202	0	0	0	0.96	0.24	0.3	0.07	0.16	0.09	1.17	0.51	0.11	1.11	0.36	0.26	0.24	0.36	4.18	1.74	42.94	ME
S17_vs_177203	0	0	0	0.86	0.05	0.43	0.06	0.26	0.17	126.22	0.41	0.06	0.28	0.35	0.49	0.28	0.79	0.56	0.68	31.24	ME
S17_vs_177210	0.02	0	0	0.92	0.02	10.97	0.01	0.08	0.13	0.13	0.32	0.09	1.01	0.6	0.27	0.4	0.3	0.21	1.81	22.37	ME
S17_vs_133231	0	0	0	0.58	0.04	0.37	0.02	0.29	0.41	0.18	0.63	0.19	0.62	2	1.92	0.71	0.2	0.35	0.88	30.36	ME
S17_vs_52890	0.12	0.01	0	82	1.21	46.76	0.35	8.06	24.86	19.73	54.65	14.73	48.2	16.34	44.7	73.29	26.18	30	74.95	328.2	Shared
S17_vs_157209	0.01	0	0	0.64	0.01	0.67	0.14	0.34	0.23	0.1	2.08	0.58	0.51	0.51	0.73	0.19	0.43	0.51	54.44	1741.89	Shared
S17_vs_31654	0.01	0	0	0.46	0.04	2.48	0.18	0.57	0.19	0.21	1.09	0.12	0.71	0.18	0.76	0.61	0.39	0.77	1.09	32.83	ME
S17_vs_2079	0	0	0	31.29	0.01	39.28	4.91	0.92	1.19	1.49	0.92	0.33	3.08	11.2	1.92	5.02	1.48	45.36	11.37	174.85	Shared
S17_vs_39515	0	0	0	0.33	0.03	1.48	0.54	0.28	0.18	4.57	0.59	0.1	1.79	67.41	0.52	0.35	10.96	0.33	5.24	59.22	ME
S17_vs_127519	0.02	0	0	1.63	0.08	1.72	40.17	10.8	2.89	2.11	0.93	0.23	0.35	1.11	0.7	1.53	33.4	0.51	15.82	97.3	Shared
S17_vs_121041	0	0	0	43.06	0.32	0.87	1.17	4.32	3.85	4.25	2.73	0.43	1.04	0.98	0.35	4.77	4.92	0.48	35.98	55.87	Shared
S17_vs_41845	0	0	0	0.56	0.05	0.38	0.06	0.3	0.17	3.16	0.98	0.12	0.28	0.95	0.28	0.44	1.45	0.27	2.45	44.5	ME
S17_vs_160502	0	0	0	0.87	0.06	1.28	0.35	0.19	0.15	0.91	0.49	0.24	0.75	1.2	0.88	0.55	0.32	0.27	4.1	122.46	ME
S17_vs_116640	0	0	0	0.58	0.02	0.23	0.05	0.16	0.12	0.11	0.86	0.05	0.16	0.09	0.26	0.16	0.28	0.4	0.48	19.9	ME
S17_vs_43307	0	0	0	0.37	0.06	0.38	0.06	0.18	0.06	2.59	3.45	0.06	0.33	3.99	1.79	0.62	0.19	0.19	12.38	79.43	Shared
S17_vs_58025	0	0	0	1.08	1.48	2.54	0.08	1.37	1.1	1.6	10406.98	0.11	0.26	0.37	0.51	0.79	0.25	0.18	0.34	14.16	Shared
S17_vs_117507	0.01	0	0	1.12	0.06	3.79	0.13	5.03	1.76	15.28	6.77	0.11	36.52	1.88	11.9	19.31	20.69	0.31	7.68	40.45	Shared
S17_vs_110666	0.01	0.01	0	1.54	0.1	3.74	1.12	5.72	3.76	3.54	3.89	0.4	0.81	0.69	1.18	0.93	0.86	0.91	3.77	43.31	ME
S17_vs_73830	0	0	0	1.29	0.01	0.5	0.17	0.36	0.19	0.2	0.98	0.12	0.74	0.31	0.7	0.36	0.39	0.43	4.6	114.81	ME
S17_vs_8690	0	0	0	1.01	0.02	0.34	1	0.38	0.45	1.87	1.98	0.06	0.49	17.49	2.3	0.29	0.27	0.28	1.3	55.34	ME
S9_vf_4115	0	0	0.02	0.36	0.06	0.59	0.08	1.66	0.11	0.13	1.46	114.53	0.46	0.27	0.49	0.22	0.46	0.2	0.43	0.71	ME
S9_vf_26805	0	0	0	1.25	0.09	2.14	0.1	0.31	0.88	0.06	0.49	19.85	0.48	0.17	0.26	0.09	1.85	0.22	0.51	0.87	ME
S9_vf_19284	0	0	0.01	10.81	0.03	2.96	0.06	0.75	0.22	0.36	2.08	145.81	0.9	1.69	4.26	2.04	1.28	0.38	1.29	2.98	ME
S9_vf_79919	0	0	0.02	0.39	0.04	0.79	0.1	0.39	0.14	0.37	0.95	225.2	0.78	0.3	0.48	0.36	0.82	0.31	0.81	1.31	ME
S9_vf_64844	0.02	0	0.03	91.44	0.16	34.81	113.08	7.57	1.25	16.99	43.46	440.57	2.03	0.72	1.45	25.1	33.44	0.66	11.31	6.39	Shared
S9_vf_57277	0	0	0.01	10.88	0.16	7.74	0.41	1.79	0.12	1.43	18.19	198.8	0.25	10.07	1.05	2.62	6.2	1.78	15.79	3.33	Shared
S9_vf_34736	0	0	0	0.35	0.42	0.39	0.01	0.27	0.12	0.09	0.2	16.44	0.69	0.53	0.26	0.13	0.24	0.14	0.52	0.71	ME
S9_vf_934	0	0	0	0.2	0	0.37	0	0.14	0.12	0.04	0.23	25.87	0.22	0.02	0.18	0.12	0.25	0.28	0.24	0.41	ME
S9_vf_73311	0	0	0.01	0.48	0.06	0.97	0.16	0.37	0.2	0.2	0.6	226.33	0.8	0.64	0.9	0.57	0.4	0.55	0.53	1.34	ME
S9_vf_23901	0	0	0	1.4	0.44	1.74	0.3	0.18	0.11	0.26	0.41	134.27	1.04	0.45	1.32	0.41	0.26	0.73	0.98	1.11	ME
S9_vf_76769	0	0	0.02	0.41	0.02	0.79	0.1	0.26	0.13	0.13	1.01	384.53	0.89	0.43	3.57	0.33	0.38	18.17	0.49	0.94	Shared
S9_vf_70236	0	0	0	0.61	0.04	0.2	0.02	0.18	0.14	0.14	0.26	175.55	0.23	0.27	0.3	0.31	0.22	0.29	0.27	0.5	ME
S9_vf_13507	0	0	0.01	1.83	0.19	1.42	92.81	1	0.51	0.45	61.57	50.59	9.36	0.62	0.99	6.08	1.47	1.23	1.44	3.03	Shared
S9_vf_51656	0	0	2.04	0.63	0.05	26.98	1825.52	0.39	0.21	0.26	4.8	33667.86	5.02	5.42	5.46	0.42	4.52	2.69	4.37	9.22	Shared
S9_vf_75363	0	0	0	0.55	5.25	0.55	0.05	0.49	0.19	0.22	0.66	20.04	0.98	0.22	0.97	0.24	0.36	0.29	0.65	1.03	ME
S9_vf_15012	0	0	0	0.67	0.02	0.87	0.16	0.31	0.16	0.2	0.78	23.24	0.56	0.46	0.9	0.57	0.59	0.36	0.53	0.78	ME
S9_vf_90847	0	0	0.04	0.62	0.04	1.2	0.19	0.3	0.18	0.21	1.11	629.09	1.01	0.39	0.84	0.63	0.53	0.49	5.54	1.2	ME
S9_vf_90852	0	0	0	0.26	0.01	0.35	0	0.19	0.15	0.09	0.11	29.39	0.46	0.2	0.28	0.01	0.17	0.24	0.32	0.6	ME
S9_vf_90856	0	0	0	0.56	0.03	2.63	0.11	0.73	0.09	0.59	1.14	79.66	0.48	2.03	0.35	0.36	0.32	0.21	1.2	2.4	ME
S9_vf_90866	0	0	0.09	0.87	0.05	3.89	0.1	1.84	0.29	0.22	0.57	554.23	0.61	0.23	0.62	0.84	0.38	0.46	0.71	1.92	ME
S9_vf_90871	0	0	0.01	5.7	0.17	0.24	0.02	0.78	0.09	0.69	3.35	24.66	0.33	4.08	1.87	2.63	0.31	1.47	4.36	1.12	ME
S9_vs_45812	0.01	0	0	0.83	0.03	0.74	0.08	0.65	0.36	0.44	0.66	137.76	0.55	0.27	0.45	0.51	1.09	0.44	0.44	1.16	ME
S9_vs_88286	0	0	0.1	1.62	0.02	2.77	0.12	0.5	0.15	0.19	2.46	1860.08	0.65	0.67	0.74	0.63	2.56	0.44	0.53	1.19	ME
S9_vs_50439	0	0	0.03	6.17	0.29	5.11	1.05	0.6	0.27	8.38	9.22	936.33	0.43	2.8	2.81	1.58	35.92	0.25	1.86	3.77	ME
S9_vs_5676	0	0	0	0.87	0.06	0.62	0.01	0.21	0.13	0.17	0.45	21.91	0.53	0.1	0.34	0.35	0.34	0.23	0.24	0.48	ME
S9_vs_66877	0	0	0	0.81	0.01	0.55	0.14	1.32	1	0.56	0.8	17.82	0.29	0.92	0.28	0.19	0.9	0.23	0.34	0.43	ME
S9_vs_75050	0	0	0.01	0.71	0.04	6.28	0.06	0.33	0.17	0.18	0.84	36.63	0.52	0.22	0.35	0.31	0.36	0.34	2.03	0.96	ME
S9_vs_48424	0	0	0.17	22.71	3.86	3.65	0.06	0.95	1.18	0.8	46.65	3226.36	1.31	0.76	1.99	8.46	0.56	9.14	2.97	3	Shared
S9_vs_90846	0	0	0.01	0.83	0.03	0.47	0.06	10.24	0.46	0.24	0.91	75.25	0.5	0.91	0.35	0.25	0.39	0.39	1.57	0.84	ME
S9_vs_90848	0	0	0	1.2	0.02	1.56	0.18	15.04	15.05	0.81	0.58	206.53	0.94	0.92	1.32	0.54	7.85	0.56	6.32	3.2	Shared
S9_vs_90849	0	0	0.11	1.03	0.03	1.74	0.06	2.4	0.21	0.25	0.54	1905.28	0.86	0.52	0.76	0.46	0.51	0.61	0.62	1.2	ME
S9_vs_90850	0.01	0	0	0.79	0.03	0.45	0.14	0.42	0.2	0.28	0.67	19.14	0.8	0.4	0.25	0.48	0.4	0.43	0.58	0.96	ME
S9_vs_90851	0	0	0.01	2.72	0.12	2.19	0.41	3.77	0.1	1.19	1.04	245.81	0.93	1.05	2.53	0.96	1.2	7.47	1.05	1.81	ME
S9_vs_90857	0.01	0	0	1.05	0.01	0.29	0.09	0.65	0.08	0.28	0.71	18.33	0.54	0.18	0.45	0.48	0.6	0.19	0.3	0.6	ME
S9_vs_90859	0	0	0.05	0.45	0.04	1.49	0.11	2.64	0.22	2.06	1.73	1293.28	0.83	1.73	1.56	0.42	1.52	0.23	0.53	1.23	ME
S9_vs_90860	0.01	0	0	0.65	0.11	0.46	0.1	0.48	0.12	0.96	0.81	21.71	0.47	0.26	0.43	0.43	1.15	0.26	0.99	0.77	ME
S9_vs_90863	0	0	0	0.09	0	0.59	0	0.15	0.11	0.35	0.81	140.2	0.36	0.33	0.19	0.09	0.21	0.14	0.24	0.51	ME
S9_vs_7881	0	0	0	1.21	0.05	1.07	0.11	0.5	0.18	0.6	0.89	190.11	2.12	0.78	0.32	0.61	0.9	0.51	0.78	1.73	ME
S9_vs_4343	0	0	0	30.66	0.01	1.33	0.06	1.77	0.2	0.63	0.55	19.89	1.43	0.39	0.69	3.02	0.69	1.09	5.71	12.28	ME
S9_vs_84046	0.01	0	0.01	0.67	0	0.35	0.09	0.96	0.09	0.25	0.48	73.7	0.36	0.14	0.28	0.5	0.92	0.26	0.45	0.55	ME
S9_vs_19689	0.01	0	0	1.86	0.03	0.48	0.06	0.23	0.2	1.09	0.47	15.12	0.33	0.53	1.17	0.39	0.35	0.16	0.46	1.24	ME
S9_vs_34898	0	0	0	0.45	0.18	0.41	0.05	1.1	1.9	0.88	0.59	17.33	0.62	1.92	0.35	0.25	0.84	0.56	0.27	0.8	ME
S9_vs_4558	0	0	0.01	0.57	0.09	0.37	0.03	1.42	1.81	0.21	0.4	33.09	0.51	0.22	0.29	0.28	0.33	0.31	0.39	4.82	ME
S9_vs_46498	0	0	0	1.25	0.04	0.73	0.11	0.37	0.2	0.73	1.51	61.14	1.07	0.64	0.5	0.35	3.66	0.54	0.61	1.4	ME
S9_vs_27813	0	0	0.02	1.28	2.96	0.58	1.52	7.49	6.63	11.07	1.06	282.15	0.57	2.17	1.31	20.94	2.87	0.34	4.37	0.95	ME
S9_vs_69322	0	0	0	0.97	0.02	0.84	0.16	0.97	0.12	3.36	1.65	15.45	0.6	0.75	0.71	0.57	0.45	0.35	0.38	0.9	ME
S9_vs_58086	0	0	0	3.04	0.02	0.51	0.09	2.66	0.46	0.31	0.7	22.39	1.59	0.52	0.32	0.38	1.18	0.33	0.55	1.63	ME
S9_vs_28197	0	0	0	0.66	0.03	0.42	0.07	0.21	0.21	0.13	0.28	23.53	0.57	13.24	0.44	0.33	0.22	0.27	12.03	0.91	Shared
S9_vs_1966	0	0	0	2.03	0.03	0.49	0.01	2.19	0.12	2.03	0.43	16.73	0.51	0.91	11.06	0.63	1.17	0.73	0.96	2.43	Shared
S9_vs_58402	0	0.01	0.01	2.65	0.09	6.17	0.02	1.88	0.17	2.24	1.17	15.62	0.45	0.21	1.59	0.72	10.04	0.34	0.98	0.68	ME
S9_vs_73764	0	0	0	0.37	0.01	0.33	0.02	0.22	0.06	0.29	0.61	15.58	0.22	0.15	0.25	0.41	0.29	0.15	0.86	0.6	ME
S9_vs_88847	0	0	0.02	0.84	0.29	201.2	1.26	0.23	0.15	0.23	0.51	19.73	0.33	0.1	0.76	0.34	2.88	1.06	0.25	0.34	ME
S9_vs_32588	0.01	0	0.01	0.62	0.06	2.14	0.06	3.06	0.17	11.27	0.32	94.74	0.54	1.11	0.26	0.23	0.68	0.27	1.17	5.03	ME
S9_vs_62803	0	0	0	0.98	0.05	0.46	0.15	0.38	0.19	0.61	0.48	39.43	0.4	0.42	0.4	0.36	0.98	8	0.37	0.9	ME
S9_vs_44331	0.01	0	0	1.07	0.03	0.58	0.44	0.48	0.35	0.24	0.51	23.36	0.73	18.72	0.3	0.34	1.16	0.35	0.66	2.62	ME
S9_vs_55738	0	0	0.01	0.76	0.05	0.47	0.05	0.5	0.26	0.51	0.44	19.83	0.67	1.76	0.33	0.2	34.18	0.16	0.34	0.79	ME
S9_vs_2799	0.01	0	0.01	17.83	10.31	13.56	0.41	29.86	19.66	13.79	134.6	19.68	9.03	7.26	71.37	6.8	1.43	40.41	80.52	11.49	Shared
S9_vs_21850	0	0	0	1.13	0.06	0.96	4.32	0.88	1.83	0.62	1.59	22.64	2.21	0.33	0.36	1.11	0.92	2.57	0.69	1.31	ME
S9_vs_10424	0	0	0	3.08	0.08	3.03	0.64	1.47	1.65	0.74	5.51	15.68	0.41	0.76	0.35	0.49	23.22	7.3	8.88	3.61	ME
S9_vs_63485	0	0	0	0.55	0.01	7.12	0.09	0.2	0.11	0.12	0.48	42.94	0.61	0.21	0.34	0.29	0.32	0.21	0.38	0.69	ME
S9_vs_48240	0	0	0	0.68	0.03	0.34	0.05	0.4	0.21	0.47	0.63	16.97	0.26	0.56	0.27	0.27	0.59	0.23	1.07	0.76	ME
S9_vs_37437	0	0	0	0.65	0.01	3.02	0.06	0.92	0.44	1.21	0.48	14.75	0.38	0.24	0.27	0.25	2.19	0.23	0.47	0.92	ME
S9_vs_48347	0	0	0.32	0.74	0.01	5.22	0.07	1.29	1.67	0.12	0.58	5976.89	1.15	1.32	1.39	0.21	0.96	0.58	0.96	1.86	ME
S9_vs_14235	0.01	0	0.01	0.69	0.01	0.49	0.03	0.68	0.09	1.59	0.5	15.67	0.38	1.08	0.33	0.28	1.15	0.24	0.45	1.13	ME
S9_vs_56354	0.01	0	0.01	0.8	0.5	0.29	0.33	5.1	0.1	0.41	0.88	159.95	0.33	0.71	3.43	0.42	0.32	0.13	0.31	0.44	ME
S9_vs_41371	0	0	0	0.7	0.04	0.96	0.06	0.46	0.16	0.37	1.8	13.88	0.6	0.21	0.27	0.37	0.44	0.39	1.4	0.88	ME
S9_vs_26179	0	0	0	1.85	0.05	3.29	0.09	15.46	7.85	2.91	0.88	12.81	1.3	1.23	0.67	0.55	3.32	0.49	0.89	1.73	ME
S9_vs_75290	0	0	0	0.42	0.04	0.29	0.19	0.14	0.12	0.07	0.4	12.47	0.25	0.2	0.23	0.17	0.23	0.21	0.13	0.4	ME
S9_vs_67792	0.01	0	0	1.16	0.02	0.49	0.08	2.6	0.41	0.95	0.49	25.46	0.78	0.31	0.51	0.32	5.26	0.36	0.81	5.98	ME
S9_vs_83108	0	0	0.01	0.49	0.02	8.19	0.38	0.23	0.12	0.13	0.32	172.97	1.13	0.39	0.5	0.42	0.56	1.41	0.39	0.82	ME
S9_vs_90727	0.01	0	0	1.46	0.16	1.86	0.19	6.01	0.79	3.08	80.01	45.39	1.62	19.12	1	1.12	4.04	0.72	1.61	3.33	Shared

Note
1. The average coverage large than 10 is highlighted in red
2. By setting a threshold value at 10, those having values > 10 in both ME and SHHC samples are considered as "shared".

S9_vs_42609	0	0	0	0.46	0.14	2.22	0.11	0.41	0.29	0.36	2.91	10.91	2.67	2.09	2.12	0.56	1.38	1.03	1.86	2.12	ME
S11_vf_58587	0	0	0	75.99	4.08	1.13	0.01	0.08	0.16	3.51	0.1	0.39	0.2	201.73	0.15	1.48	0.03	0.2	1.18	3.1	ME
S11_vf_13033	0	0.05	0.06	407.99	0.23	904.28	71.32	2.09	2.47	53.45	55.96	1.3	0.71	62.87	204.54	100.64	0.85	22.66	7.9	16.75	Shared
S11_vf_33117	0.01	0	0.01	0.7	0.02	0.3	0.15	1.15	0.61	1.24	0.85	0.25	1.32	35.13	1.39	0.38	1.17	0.22	0.92	0.73	ME
S11_vf_33433	0.01	0	0	0.53	72.15	0.35	0.26	0.19	0.48	0.29	0.43	0.21	0.54	495.64	163.28	0.31	0.33	0.27	0.34	0.83	Shared
S11_vf_66160	0	0	0	1.27	0.03	0.82	0.41	3.44	0.44	3.66	3	1.51	0.74	50.13	6.14	1.86	4.7	2.08	11.29	1.79	Shared
S11_vf_92391	0	0	0	0.79	0.07	0.45	0.08	0.37	3.32	0.26	0.47	1.37	1.46	3997.25	109.94	0.3	1.09	0.45	0.82	2.15	Shared
S11_vf_66522	0	0	0	0.64	0.02	0.15	0.05	0.2	0.09	0.12	0.96	0.07	0.29	20.56	0.5	0.32	0.14	0.14	1.72	0.4	ME
S11_vf_27845	0	0	0	0.55	0.02	0.24	0.03	0.14	0.1	9.38	0.41	0.05	0.29	48.98	13.78	0.17	0.13	0.14	9.01	0.4	Shared
S11_vf_34008	0	0	0	0.64	0.04	2.61	0.04	5.96	0.31	0.17	1.1	0.56	0.42	94.8	0.35	0.32	1.47	0.19	0.26	0.62	ME
S11_vf_34224	0	0	0	11.83	0.05	129.09	18.42	0.36	0.49	51.23	0.83	0.26	3.24	95.97	2.27	1.94	0.17	2.55	0.55	8.75	Shared
S11_vf_99751	0	0	0	0.52	0.19	0.17	0.05	0.17	0.11	0.07	0.24	0.04	0.32	17.62	0.17	0.88	0.21	0.13	0.17	0.4	ME
S11_vf_47632	0.01	0	0	26.75	0.05	5.52	213.94	45.74	15.79	3.06	135.99	0.31	1.1	49.63	60.45	18.99	1.03	93.98	10.84	0.73	Shared
S11_vf_28515	0	0	0	3.45	0.04	13.78	0.09	4.59	0.62	0.2	31.43	0.38	33.06	618	0.65	9.77	4.03	1.41	0.57	1.08	Shared
S11_vf_28645	0	0	0	0.94	0	1.54	0.13	0.21	0.29	0.34	2.78	0.1	8.8	285.18	0.4	2.09	0.35	0.27	0.46	2.75	ME
S11_vf_3790	0	0	0	0.74	0.03	0.95	0.2	0.41	0.26	0.24	1	0.14	1.5	63.95	0.87	0.53	0.75	0.51	0.81	1.38	ME
S11_vf_95067	0	0	0	0.71	0.04	0.27	0.24	0.24	0.41	0.33	0.44	0.15	0.94	289.81	0.45	0.34	0.21	0.29	0.57	0.58	ME
S11_vf_68693	0	0	0	0.67	0.02	0.33	0.01	2.19	6.24	0.48	4.24	0.09	0.54	95.97	0.75	0.28	0.31	0.28	1.09	3.93	ME
S11_vf_10907	0	0	0	1.1	0.21	11.92	0.1	0.28	0.28	0.21	0.95	0.87	0.84	98.05	2.93	0.58	0.64	1	0.96	1.95	ME
S11_vf_89309	0	0	0	0.37	0.04	0.83	0.1	0.87	0.68	0.39	1.34	5.62	0.99	446.47	0.66	0.64	0.66	0.41	1.31	1.13	ME
S11_vf_43997	0	0	0	0.73	0.03	0.56	0.18	0.36	0.26	0.37	0.79	0.14	1.14	89.06	0.79	0.6	0.51	0.44	0.72	1.11	ME
S11_vf_70015	0	0	0	1.07	0.05	6.31	0.08	0.75	0.29	0.35	1.09	0.16	11.97	21.89	1.07	0.63	0.28	0.83	0.8	0.82	Shared
S11_vf_84303	0	0	0	1.41	0.02	0.18	2.51	0.12	0.1	0.11	0.46	0.06	1.62	21.68	6.75	0.84	0.2	0.44	0.42	0.57	ME
S11_vf_104033	0	0	0	0.53	0.01	0.3	0.13	0.17	0.13	0.08	0.43	0.1	0.38	37.56	0.3	0.16	0.19	0.21	0.23	0.45	ME
S11_vf_104034	0	0	0	1.25	0.03	0.23	0.05	0.28	0.24	0.15	16.87	0.08	0.83	15.06	0.62	0.43	0.23	0.22	0.29	0.47	Shared
S11_vf_104035	0.07	0	0	25.78	449.65	25.08	0.08	24634.69	64096.44	14.48	23.84	0.11	31.94	90.58	60.25	12.29	4.5	0.38	0.36	0.61	Shared
S11_vf_104044	0	0	0	1.49	0.02	0.36	1098.23	0.2	0.64	0.35	1.31	0.29	0.85	635.58	274.01	0.23	0.43	0.37	0.44	0.74	Shared
S11_vf_104046	0	0	0	0.39	0.02	0.31	0.05	0.21	0.15	0.14	0.51	0.07	0.61	37.31	0.44	0.25	0.31	0.26	0.35	0.6	ME
S11_vf_104048	0.01	0	0	0.35	0	0.24	0.05	0.19	32.01	0.09	0.26	11.8	8.2	40124.46	7.67	0.19	5.53	3.04	4.64	11.07	Shared
S11_vf_104052	0	0	0	1.13	0.13	0.87	0.09	0.29	0.28	0.25	0.43	0.11	0.65	63.2	0.51	0.26	0.46	0.42	0.47	1.32	ME
S11_vf_104059	0	0	0	0.75	0.08	0.74	0.2	0.36	3.16	4.89	100.86	1.54	2.25	3486.56	2311.55	0.37	1.48	1.07	1.34	2.85	Shared
S11_vf_104062	0	0	0	23.39	0.51	0.42	0.13	0.1	0.13	198.94	0.31	0.07	0.71	25.7	0.4	0.18	0.17	0.21	0.21	0.42	ME
S11_vf_104070	0.01	0	0.04	0.63	0.09	470.25	0.07	0.22	0.21	0.71	0.74	0.54	0.49	80.86	0.44	0.3	0.14	0.23	0.28	1.23	ME
S11_vf_104071	0	0	0	7.08	0.02	6.56	0.02	0.14	2.18	0.06	0.44	0.79	0.8	2705.27	0.77	0.16	0.56	0.41	0.89	1.02	ME
S11_vs_84627	0.01	0	0	2.15	0.03	0.5	0.15	2.7	4.41	0.39	0.89	0.11	0.64	87.4	0.79	0.77	0.55	0.36	0.61	1.02	ME
S11_vs_40945	0	0	0	0.53	0.13	0.29	0.11	0.36	0.34	0.56	0.63	0.09	0.56	92.93	0.63	0.6	0.19	0.26	0.37	0.65	ME
S11_vs_93101	0	0	0.01	0.37	0.03	0.72	0.23	0.29	0.26	0.17	0.98	0.16	2.02	114.51	0.75	0.43	0.36	0.37	0.64	1.19	ME
S11_vs_3006	0	0	0	0.69	0.02	0.31	0.07	0.24	0.14	0.15	0.48	0.1	0.48	24.55	0.23	0.2	0.3	0.21	0.29	0.72	ME
S11_vs_75554	0	0	0	0.86	0	0.41	0.61	0.12	0.99	0.18	0.79	0.22	0.69	923.6	0.55	0.12	0.28	0.37	0.4	0.66	ME
S11_vs_49071	0	0	0	0.79	0.04	0.65	0.15	1.08	0.22	0.28	1	0.17	0.79	51.86	0.59	0.69	0.63	0.38	0.59	2.67	ME
S11_vs_36748	0.01	0	0	0.75	0.04	2.91	0.37	0.68	0.96	0.44	0.92	0.1	0.49	48.96	1.06	0.99	0.49	1.06	4.72	8.17	ME
S11_vs_82368	0	0	0	0.65	0.01	0.52	0.43	0.54	0.52	0.51	0.64	0.47	0.92	350.43	0.7	0.35	1.26	0.62	0.64	1.6	ME
S11_vs_104032	0	0	0	0.61	0.02	0.53	0.05	0.26	1.47	0.18	0.86	1.14	1.2	1546.22	0.93	0.72	0.6	0.36	0.65	1.53	ME
S11_vs_104036	0	0	0	0.59	0.02	0.59	0.12	0.59	1.52	0.11	1.92	0.28	0.87	685.55	0.87	0.36	0.37	0.39	0.56	0.98	ME
S11_vs_104037	0.01	0	0	0.92	0.02	3.06	0.7	0.25	0.31	0.12	0.41	0.11	1.55	203.09	0.44	0.31	0.26	0.32	0.33	0.54	ME
S11_vs_104038	0	0	0	0.87	0.02	0.86	0.28	0.54	0.91	0.6	0.85	0.38	1.32	679.32	1.47	0.58	0.61	0.53	0.83	1.49	ME
S11_vs_104041	0	0	0	2.12	0.05	0.63	0.13	0.37	0.19	0.22	0.59	0.16	2.37	80.68	0.68	1.6	0.3	0.4	0.5	0.93	ME
S11_vs_104042	0	0	0	1.18	0.01	0.88	0.49	0.31	0.26	0.29	1.07	0.27	0.66	79.12	0.38	0.42	0.44	0.64	2.84	1.01	ME
S11_vs_104047	0	0	0	0.7	0.06	0.26	0.06	0.4	0.15	1.56	0.62	0.16	0.22	42.71	0.29	1.29	0.37	0.13	0.26	0.45	ME
S11_vs_104049	0	0	0.01	0.82	0.21	0.71	0.11	1.13	0.2	1.6	0.81	0.33	0.72	32.32	0.5	0.66	1.29	0.22	0.42	0.97	ME
S11_vs_104053	0	0	0	0.39	0.03	1.54	0.12	0.22	0.23	68.04	16.97	0.1	2.4	41.8	10.42	0.32	0.85	0.39	0.89	0.92	Shared
S11_vs_104054	0	0	0	0.41	0.05	0.41	0.05	0.28	0.16	0.16	0.67	0.05	0.53	21.77	1.48	0.25	0.34	0.94	0.3	0.72	ME
S11_vs_104064	0	0	0	1.29	0.03	0.4	0.14	0.35	0.19	5.24	4.94	1.1	16.35	70.19	2362	0.68	1.09	0.44	1.03	6.27	Shared
S11_vs_104069	0.01	0	0	0.4	0.02	4.47	0.28	0.17	1.63	0.26	0.35	0.52	0.59	1710.2	0.51	0.16	0.43	0.33	0.46	0.75	ME
S11_vs_58702	0	0	0	0.4	0.04	0.44	0.06	0.51	0.15	1.1	0.77	0.12	0.43	13.17	0.62	0.25	0.42	0.51	0.75	1.43	ME
S11_vs_45658	0.01	0	0	7.27	0.69	5.09	1.08	5.35	5.11	2.32	4.66	0.76	11.71	113.75	15.65	3.42	6.47	6	8.09	19.49	Shared
S11_vs_84848	0	0	0	5.94	0.1	1.66	1.08	0.89	1.04	0.71	1.01	0.31	3.04	51.26	0.51	10.42	1.05	3.26	1.89	36.75	ME
S11_vs_19892	0.01	0	0	1.13	9.39	0.23	3.98	0.65	0.38	0.92	0.78	2.81	0.32	20.79	1.72	0.4	0.24	0.32	0.39	0.25	ME
S11_vs_6878	0	0	0	0.43	0.03	0.28	0.04	0.45	0.31	0.15	0.4	0.25	0.27	204.29	0.33	0.26	0.4	0.26	0.3	0.72	ME
S11_vs_58930	0	0.01	0	134.63	0.07	6.19	3.05	0.3	0.4	1.98	0.54	0.28	0.67	119.09	18.16	17.66	0.32	111.26	11.05	233.63	Shared
S11_vs_52577	0.02	0	0	1.06	0.06	0.49	0.1	0.65	0.21	0.42	0.91	0.1	0.39	45.87	0.9	0.53	0.42	0.67	1.69	1.69	ME
S11_vs_98193	0.01	0	0	0.63	0.13	0.46	0.03	12.07	2.4	0.23	2.39	0.23	0.74	19.31	4.82	0.38	0.98	0.57	1.05	1.18	ME
S11_vs_20225	0.01	0	0	0.79	0.07	0.56	0.13	1.32	0.28	3.94	2.37	0.18	0.4	206.5	2.17	2.58	0.56	0.21	3.19	0.53	ME
S11_vs_26767	0	0	0	0.71	0.04	0.37	0.18	0.28	0.17	0.2	0.47	0.36	0.56	17.51	0.22	0.19	0.52	0.39	0.41	0.62	ME
S11_vs_79275	0.01	0	0.01	0.82	0.05	0.34	0.04	0.39	0.14	0.16	0.54	0.13	0.26	31.1	0.6	0.49	0.21	0.2	0.34	0.47	ME
S11_vs_13652	0	0	0	0.84	0.03	2.14	0.17	0.36	0.64	0.57	0.6	1.47	0.52	261.61	0.44	0.34	0.59	0.42	0.66	0.99	ME
S11_vs_53675	0	0	0	0.91	0.01	0.47	0.09	1.27	0.2	0.15	0.65	0.29	0.62	19.99	0.98	0.17	0.54	0.4	0.41	1	ME
S11_vs_79831	0.01	0	0	0.48	0.03	0.56	0.08	0.39	0.36	0.14	0.93	0.09	0.84	79.36	6.13	0.38	0.44	0.32	0.81	1.28	ME
S11_vs_1937	0.01	0	0	1.48	0.03	27.31	0.07	0.16	0.1	0.08	23.52	0.06	24.24	13.59	0.25	0.77	0.16	0.14	4.31	9.11	Shared
S11_vs_8456	0	0	0	0.63	0.01	0.52	0.06	0.36	0.22	0.25	0.68	0.1	0.75	38.55	0.76	0.51	0.65	0.28	0.45	1.12	ME
S11_vs_34137	0.01	0	0	0.68	0.03	0.67	0.09	0.34	0.12	1.2	0.53	0.38	0.54	22.22	0.41	0.47	0.48	0.22	1.15	0.76	ME
S11_vs_14882	0	0	0	0.57	0.02	0.35	0.05	0.15	0.1	0.09	0.39	0.1	0.58	14.03	0.4	0.24	0.59	0.39	0.34	0.48	ME
S11_vs_41107	0	0	0	3.79	0.08	1.74	1.98	8.33	0.09	0.54	1.9	0.11	1.04	16.15	1.51	1.18	2.46	1.4	1.8	2.15	ME
S11_vs_67573	0	0	0	0.94	0.02	2.18	0.72	1.09	1.96	1.25	1.08	2.03	0.8	49.97	0.48	0.61	0.52	0.48	0.9	1.68	ME
S11_vs_48109	0	0	0	0.77	0.02	0.78	0.14	0.3	0.95	0.41	1.1	0.42	0.86	899.64	0.89	0.67	0.5	0.37	0.69	1.18	ME
S11_vs_3277	0	0	0.01	16.69	1.18	24.07	0.17	0.95	0.39	3.14	76.54	0.21	2.44	77.96	1.15	8.02	6.05	1.77	6.04	7.16	Shared
S11_vs_55144	0	0	0	0.45	0.02	0.37	0.05	0.29	0.25	1.53	0.72	0.11	1.04	50.56	0.54	0.37	0.32	0.35	0.47	1.06	ME
S11_vs_23188	0	0	0	0.42	0.1	1.07	0.21	0.24	1.17	0.23	0.74	0.39	0.89	1066.6	0.51	0.24	10.22	0.34	0.71	1.33	ME
S11_vs_23357	0.01	0	0.01	1.04	0.26	0.16	0.16	3.65	4.81	4.92	0.34	3.39	0.5	50.16	0.21	0.46	0.56	0.3	0.81	0.64	ME
S11_vs_10172	0	0	0.01	0.82	23.54	0.19	10.21	3.85	0.34	0.7	0.55	0.18	0.55	21.96	1.1	0.46	0.31	0.84	0.6	0.44	Shared
S11_vs_68151	0	0	0	1.22	0.17	4.71	0.05	0.4	0.67	0.31	4.4	213.71	49.89	74.29	2.16	9.17	13.49	3.29	450.57	1.36	Shared
S11_vs_81775	0	0	0	0.48	0.02	0.43	0.08	0.24	0.14	3.38	0.42	0.1	0.56	22.65	0.61	0.27	0.27	0.28	0.33	0.59	ME
S11_vs_68348	0	0	0	0.79	0.03	0.6	0.26	0.43	0.24	0.28	0.59	2.18	1.08	21.88	0.54	0.46	1.12	0.43	0.61	1.22	ME
S11_vs_42927	0	0	0	1.53	0.05	1.53	0.29	8.5	0.53	2.93	2.37	5.81	1	24.23	1.45	2.01	17.76	0.48	2.74	1.86	ME
S11_vs_62573	0	0	0	0.79	0.02	0.34	0.05	0.31	0.13	0.62	0.6	0.08	0.25	45.11	0.43	8.12	0.43	0.24	1.73	0.47	ME
S11_vs_56762	0	0	0	0.78	0.02	0.6	0.02	2.92	0.15	0.16	0.48	0.23	0.51	16.96	0.48	0.21	1.62	0.31	0.36	0.78	ME
S11_vs_82331	0	0	0	2.02	0.03	0.45	0.09	1.03	1.45	1.97	1.9	0.12	0.57	43.74	3.52	1.09	0.4	0.65	1.05	32.63	ME
S11_vs_11267	0	0	0	6.23	13.14	0.21	0.06	3.88	7.12	2.41	1.31	0.69	0.68	111.39	148.85	0.71	0.2	0.24	0.44	0.65	Shared
S11_vs_76784	0.01	0	0	1.77	0	0.23	6.28	0.38	0.3	7.44	1.31	0.17	0.27	35.74	15.9	0.38	0.44	2.56	0.4	3.09	Shared
S11_vs_5077	0.01	0	0.01	0.36	0.01	0.11	0.1	0.17	0.19	0.08	0.3	0.06	0.13	86.16	0.28	0.14	0.17	0.1	0.17	0.27	ME
S11_vs_30739	0	0	0	1.1	0.04	6.28	1.19	2.45	0.42	1.01	1.21	0.07	0.3	15.61	1.35	0.39	0.52	0.16	0.42	0.69	ME
S11_vs_37666	0.01	0	0	0.51	0.05	0.57	0.03	0.33	0.22	0.13	0.48	0.12	0.85	115.53	0.46	0.21	0.99	0.24	0.24	1.41	ME
S11_vs_89911	0	0	0.02	0.78	0.03	0.33	0.12	0.96	0.14	0.72	0.32	0.05	0.24	14.6	0.2	0.18	0.22	0.17	0.31	0.68	ME
S11_vs_11994	0.01	0	0	0.91	0.02	0.3	0.04	3.63	0.11	0.19	4.17	0.42	0.2	29.71	0.42	0.37	0.71	0.2	0.33	2.01	ME
S11_vs_83666	0	0	0	1.2	0.02	0.72	0.16	0.61	0.21	0.49	0.5	0.32	1.1	12.56	0.43	0.24	0.37	0.29	0.64	1.02	ME
S11_vs_38860	0	0	0	0.56	0.04	0.28	0.08	22.36	0.4	1.05	0.57	0.66	1.08	14.9	0.18	0.27	8.39	0.22	0.24	0.96	ME
S11_vs_6458	0	0	0	10.34	0.02	0.45	0.19	20.18	33.65	0.18	1.01	0.12	0.55	21.64	0.59	6.75	0.3	0.38	0.34	0.8	Shared
S13_vf_1770	0	0.05	0	120.75	0	0.19	0.02	0.09	0.1	0.1	0.84	0.06	0.18	0.22	0.31	60.47	0.03	0.25	0.16	0.19	ME
S13_vf_3728	0.03	0.01	0	50.14	41.5	2.21	0.19	1.2	0.98	1.47	7827.32	0.06	1.09	0.31	0.81	189.79	0.35	0.76	0.75	10.84	Shared
S13_vf_4901	0	0	0	7.15	0.03	0.4	0.15	0.41	0.52	0.22	2.74	0.72	0.95	290.64	3.28	227.33	0.46	0.32	9.49	0.82	ME
S13_vf_7670	0.02	0	0	86.37	0.08	0.26	0.03	0.16	0.1	0.1	0.28	0.04	0.28	0.1	0.18	43.63	0.12	0.42	0.26	0.42	ME
S13_vf_11246	6.5	0.12	0.01	830.44	63863.92	11.12	0.08	233.67	552.7	7.78	14	0.17	1.25	1.29	21.23	338.41	65.71	1.67	0.74	0.58	Shared
S13_vf_22175	0	0	0	0.64	0.02	0.25	0.06	0.2	0.09	0.15	0.44	0.06	0.34	0.33	0.32	92.91	0.2	0.17	0.56	0.45	ME
S13_vf_28309	0	0	0	48.94	0.07	0.76	0.49	0.38	0.28	0.28	0.95	0.09	1.12	0.35	1.4	157.09	0.47	0.44	0.56	1.15	ME
S13_vf_38708	0	0	0	1.11	0.04	0.23	0.09	0.33	0.09	0.22	0.39	0.03	0.25	0.35	0.23	61.18	0.32	0.17	1.84	0.42	ME
S13_vf_54201	0.01	0	0	0.72	0.02	0.17	0.14	0.33	0.1	0.12	0.54	0.07	0.23	0.42	0.25	56.72	0.32	0.21	1.16	0.44	ME
S13_vf_56074	0	0	0.01	2.7	0.12	4.49	0.08	0.26	0.18	0.21	0.65	0.06	8.2	0.18	0.33	1129.59	0.22	1.63	3.41	3.97	ME
S13_vf_66508	0	0.01	0	87.55	2.56	28.96	0.15	0.55	0.22	1	1.77	1.36	2.84	1.19	0.75	35.77	0.56	0.64	1.84	1.21	ME
S13_vf_87873	0.04	0.01	0	23.71	36.16	2.85	5.17	29.52	51.16	141.83	1.17	0.07	1.81	16.31	0.35	366.63	0.35	0.18	25.72	0.42	Shared
S13_vf_91189	0	0	0	0.29	0.07	0.35	0.01	0.12	0.3	0.11	0.7	0.08	0.85	0.38	0.56	582.7	0.43	0.24	1.38	0.55	ME
S13_vf_97702	0.02	0	0	116.3	0.02	0.45	0.01	0.17	0.16	13.78	0.71	0.18	0.44	0.32	0.49	72.43	0.23	0.64	75.06	170.13	Shared
S13_vf_109235	0.03	0.08	0	760.94	0.17	0.47	0.09	0.35	0.28	0.54	0.7	0.09	0.26	2.58	0.72	342.22	1.62	2.38	0.73	0.56	ME
S13_vf_131208	0	0	0	14.07	0.04	0.59	0.45	0.21	0.09	0.13	0.53	0.13	0.55	0.25	1.34	24.47	0.48	0.39	2.24	5.58	ME
S13_vf_143422	0	0.01	0	2.47	0.14	0.61	244.14	0.38	0.19	0.92	930.39	0.06	0.55	0.53	0.65	35.62	0.29	0.31	0.49	2.4	Shared
S13_vf_147396	0.03	0.03	0	265.85	0.08	0.68	0.19	0.4	0.32	0.24	0.95	0.15	0.61	0.33	0.6	53.81	0.43	0.78	0.53	0.93	ME

S13_vf_147589	0	0	0	0.68	0.01	0.19	0.04	0.89	0.11	0.08	0.39	0.02	0.5	0.06	0.93	49.25	0.74	0.21	0.23	0.43	ME
S13_vf_150407	0	0	0	30.88	0.02	0.75	0.29	0.12	0.14	0.24	0.34	0.08	0.18	0.08	0.36	40.31	0.08	0.28	0.32	0.2	ME
S13_vf_151045	0.01	0.06	0	577.5	66.99	0.85	0.08	0.44	0.31	0.32	0.71	0.08	2.04	0.28	0.62	267.24	1.42	1.37	1.13	1.72	Shared
S13_vf_151755	0	0	0	0.72	0.12	0.4	0.13	0.22	0.19	0.2	791.16	0.05	0.4	0.12	0.47	59.04	0.1	0.16	0.23	1.61	Shared
S13_vs_12957	0.02	0	0	260.16	1.6	0.31	0.07	0.19	0.09	0.1	0.43	0.1	0.09	0.22	0.18	27.19	0.03	0.28	0.16	0.33	ME
S13_vs_15645	0	0.01	0	36.07	0.5	9.56	16.68	10.99	1.71	23.27	9	0.11	3.36	198.94	0.29	4022.16	46.66	0.26	5.58	0.52	Shared
S13_vs_27462	0	0.01	0	0.74	0.07	0.76	0.19	15.68	0.2	0.23	1	10.19	18941.24	3.11	3.25	230.12	2.9	335.83	5.26	5.25	Shared
S13_vs_148294	0.01	0.01	0	153.07	4.85	1.35	0.08	1.32	2.12	1.03	2.63	4.94	0.35	2.99	0.34	44.11	1.53	0.41	1.53	0.95	ME
S13_vs_149860	0	0.02	0	83.95	0.04	0.32	0.03	0.23	0.1	0.11	0.55	0.03	0.43	0.09	0.23	61.46	0.18	0.39	0.22	0.9	ME
S13_vs_151510	0.01	0	0.01	0.52	0.07	0.76	1.55	3.63	0.17	0.25	1.76	2.63	4266.81	0.8	1.23	64.17	1.03	0.68	1.33	1.58	Shared
S13_vs_9438	0.04	0.03	0	120.15	0.07	0.59	0.1	194.41	296.71	0.33	0.91	0.1	1.01	3.03	0.38	68.58	0.29	0.46	0.4	0.75	Shared
S13_vs_13129	0.01	0.02	0.01	494.99	0.09	0.36	0.09	0.57	0.13	0.29	0.95	0.21	0.34	0.16	0.48	98.91	0.32	0.79	0.38	0.47	ME
S13_vs_14794	0.02	0.09	0	734.08	0.14	0.44	0.07	0.23	0.19	0.18	0.52	0.06	0.31	0.08	0.18	74.81	0.18	1.18	0.22	0.33	ME
S13_vs_23314	0	0	0	1.64	0.1	1.29	0.07	5.33	0.37	4.27	3.36	2.07	1.94	2.35	1.51	230.41	11.21	0.43	5.13	1.34	ME
S13_vs_26756	0.07	0	0	14.2	0.08	1.49	0.16	0.57	0.26	0.36	1.78	0.19	2.1	0.59	24.12	113.68	0.82	0.74	1.28	1.69	Shared
S13_vs_30101	0	0.01	0	10.56	0.08	1.52	0.19	0.56	0.28	0.45	57.74	0.64	4.04	1.86	8.93	78.16	1.36	0.55	25.93	1.58	Shared
S13_vs_30167	0	0	0	13.36	0.57	4.54	0.27	27.14	1.5	15.54	8.83	3.3	6.09	9.61	5.05	181.07	34.54	1.67	15.97	4.98	Shared
S13_vs_33947	0.02	0.01	0.01	1.71	0.05	1.18	1.05	1.67	0.28	1.18	5.37	0.34	1.09	48.95	2.13	100.45	1.25	0.72	2.02	1.04	ME
S13_vs_35816	0.02	0.08	0.01	778.44	0.26	41.56	194.79	19.15	2.6	23.61	90.92	54.11	4.82	2.02	4.31	280.1	65.66	2.67	21.54	10.15	Shared
S13_vs_45488	0	0	0	60.81	0.1	1.44	0.16	27.53	50.38	0.32	2.69	0.23	1.12	13.14	1.31	60.82	0.46	0.65	0.67	1.65	Shared
S13_vs_49889	0	0.03	0	73.87	0.04	0.26	0.04	0.23	0.14	0.13	1.77	0.06	0.43	0.24	0.5	32.82	0.29	0.32	0.73	1.58	ME
S13_vs_57267	0.22	0.02	0	72.1	0.15	1.04	0.22	0.42	0.25	0.25	1.26	0.58	1.46	0.6	0.88	239.75	0.59	0.62	56.71	170.58	Shared
S13_vs_67429	0	0	0	12.95	0.31	1.89	0.17	0.6	0.36	0.36	17.34	0.2	31.67	0.74	52.51	113.38	0.81	0.75	5.57	1.81	Shared
S13_vs_68102	0	0.09	0	473.7	0.06	0.54	0.07	0.42	0.21	0.24	0.76	0.08	0.61	0.23	0.56	192.67	0.39	0.95	0.49	0.9	ME
S13_vs_83447	0.04	0.07	0.02	345.72	67.67	2.6	0.52	6.81	0.61	3.68	79.74	875.16	4.7	1.89	0.62	171.77	1.58	22.5	3.92	1.41	Shared
S13_vs_87108	0	0	0	7.77	1.17	17.11	0.63	0.19	0.1	0.07	0.27	0.05	0.15	0.06	0.31	28.85	0.11	0.29	0.23	0.56	ME
S13_vs_90967	0	0	0	56.85	0.07	0.11	0	0.16	0.09	0.01	0.19	0.09	0.27	0.01	0.13	53.07	0.02	0.23	2.18	10.5	Shared
S13_vs_98591	0.01	0.26	0	2726.02	0.65	1.66	0.08	536.63	794.31	0.97	1.79	0.07	1.19	1.7	0.44	631.9	0.18	4.66	1.1	0.65	Shared
S13_vs_113593	0	0.11	0	1062.8	0.2	0.68	0.02	0.3	0.25	0.33	0.67	0.05	0.36	0.49	0.31	333.41	0.28	1.75	0.68	0.59	ME
S13_vs_114213	0.01	0	0.01	1.56	0.07	0.69	0.3	3.74	0.69	1.71	3.05	1.26	0.74	19.9	1.18	59.21	2.23	0.85	1.55	1.82	ME
S13_vs_121307	0	0	0	47.08	0.02	0.25	0.09	0.65	0.14	0.14	0.61	0.15	0.31	0.12	0.39	25.92	0.37	0.38	0.25	0.65	ME
S13_vs_152223	0.06	0.35	0	3438.14	758.35	1.42	0.05	0.9	0.64	0.82	1.56	0.08	0.44	0.2	0.32	522.1	0.87	4.82	1.06	0.51	Shared
S14_vf_58919	0	0	0	0.86	0.13	0.51	0.17	1.24	0.18	0.27	0.86	0.61	1.35	0.34	0.68	0.4	34.05	0.37	0.63	13.41	ME
S14_vf_44452	0	0	0	0.35	0.09	0.1	0.04	0.11	0.07	1.31	10.4	0.06	0.16	0.04	0.08	0.33	62.68	2.06	0.25	1.92	Shared
S14_vf_37500	0	0	0	2.87	0.35	1.08	0.1	0.28	0.93	0.07	0.18	0.19	0.52	0.35	0.38	0.85	533.38	0.19	0.48	0.36	ME
S14_vf_106156	0	0	0	0.84	0.03	0.37	0.03	0.24	0.09	0.12	0.43	0.08	1.18	0.34	0.43	0.41	39.06	0.28	0.39	1.21	ME
S14_vf_92240	0.01	0	0	2.79	0.21	62.91	31.15	103.93	11.11	16.69	8.59	0.24	2.11	2.94	8.76	1.23	156.96	1.59	15.4	10.45	Shared
S14_vf_85122	0	0	0	0.52	0.03	1.38	0.3	6.85	0.18	0.22	0.79	0.22	0.81	0.21	0.88	0.6	21.95	0.32	0.66	0.82	ME
S14_vf_99344	0.17	0	0	0.45	1.8	0.77	0.09	0.27	0.17	0.21	14.71	0.59	1.1	0.47	1.18	0.24	2505.39	0.72	0.56	1.32	Shared
S14_vf_114491	2.39	0	0	4.4	27996.65	5.29	0.09	2.73	2.81	3.25	6.23	0.08	0.6	0.42	0.59	2.57	29.76	0.33	0.5	0.71	Shared
S14_vf_78139	0	0	0	0.38	0.04	0.23	0.11	3.61	0.07	0.11	0.36	0.07	0.25	0.08	0.19	0.18	31.75	0.17	0.2	0.48	ME
S14_vf_56130	0	0	0	3.12	0.12	1.08	4.47	14.69	0.11	0.81	1.69	0.07	0.58	3.06	1.82	1.58	24.08	4.81	8.13	1.15	ME
S14_vf_93134	0.16	0	0	4.01	1.9	0.32	0.02	0.22	0.18	0.34	0.54	0.55	0.85	0.47	0.96	0.48	3146.77	0.56	1.29	23.02	ME
S14_vf_48775	0	0	0	0.4	0.12	0.22	0.04	0.13	0.07	0.05	0.21	0.07	0.38	0.1	0.43	0.1	187.15	0.81	0.25	0.4	ME
S14_vf_115736	0.02	0	0	7.45	0.4	1.42	0.18	1.13	0.42	1.68	1.61	0.42	1.72	1.31	0.37	2.54	182.26	0.92	1.37	0.8	ME
S14_vf_43340	0	0	0	1.68	3.97	0.46	3.12	0.71	0.91	0.1	0.27	0.09	0.36	1.51	0.98	1.72	64.69	1.82	0.5	1.12	ME
S14_vf_57689	0.04	0	0.01	0.57	0.03	0.17	0.15	0.24	0.13	0.12	0.92	0.1	0.18	0.09	0.25	0.33	24.65	0.15	0.25	0.45	ME
S14_vf_109545	0.01	0	0	1.04	0.01	1.55	0.49	1.95	3.32	0.37	5.56	12.96	0.17	3.03	0.64	0.39	26.76	14.44	3.24	1.78	Shared
S14_vf_72335	0	0	0	0.69	0.07	0.45	0.02	4.08	0.08	0.14	0.44	0.07	0.48	0.42	0.36	0.31	31.27	0.24	0.28	0.58	ME
S14_vf_117147	0	0	0	0.87	0.08	0.34	0.1	0.22	0.11	0.2	1.59	0.2	0.23	0.39	0.32	0.46	196.08	0.4	0.46	3.2	ME
S14_vf_117150	0	0.01	0	2.02	0.25	3.24	0.63	0.23	0.18	0.37	0.6	0.07	3.47	0.6	0.93	0.75	89.41	5.27	4.83	1.1	ME
S14_vf_72953	0	0	0	0.61	0.05	0.54	0.05	1.36	0.13	0.11	0.43	0.08	0.81	0.13	0.39	0.35	33.23	0.31	0.31	0.81	ME
S14_vf_117155	0.1	0.03	0	0.89	1.13	0.18	0.16	0.14	0.11	0.04	0.2	0.48	0.39	0.32	0.47	0.19	2051.33	501.52	0.5	0.95	Shared
S14_vf_117157	0.03	0	0	0.57	0.27	0.31	0.03	0.13	0.12	0.16	0.41	0.21	0.74	58.81	81.6	0.21	394.45	7.22	4.81	0.6	Shared
S14_vf_117162	0.17	0	0	0.72	1.45	0.28	0.06	0.27	0.08	0.09	0.23	0.57	0.51	0.53	0.6	0.12	2756.02	0.45	0.59	0.82	ME
S14_vf_117164	0.01	0	0	0.64	0.15	0.42	0.06	0.78	0.17	0.24	27.52	0.13	0.37	0.17	0.57	1.12	304.75	0.34	0.34	0.52	Shared
S14_vf_51134	0.04	0	0	0.84	0.74	1.97	0.07	0.18	0.05	1.48	2.66	0.27	0.64	0.42	0.43	0.23	1271.13	9.28	3.86	0.76	ME
S14_vs_15228	0.28	0	0	22.18	3.51	10.79	130.56	1222.83	118.36	60.75	4.02	0.98	11.58	97.48	1.02	40.86	5596.85	0.65	0.69	1.51	Shared
S14_vs_88666	0	0	0	0.48	0.07	0.21	0.02	99.56	0.5	1.68	0.44	0.11	1.02	0.26	0.23	1.2	137.87	0.24	0.2	0.36	ME
S14_vs_30439	0	0	0	0.74	0.17	0.79	2.98	61.71	23.95	11.58	0.59	0.28	0.29	1.44	0.73	0.28	96.93	0.27	14.17	5.24	Shared
S14_vs_96286	0	0	0	1.31	0.06	1.26	0.99	14.53	0.36	4.33	0.52	0.25	14.23	0.89	2.48	9.42	26.7	1.4	3.24	1.14	Shared
S14_vs_9676	0.06	0.01	0	91.56	0.53	1.19	0.2	0.4	0.2	0.33	5.68	0.56	2.36	0.33	0.75	38.67	1086.22	0.5	0.7	1.44	ME
S14_vs_3251	0.02	0	0.01	0.63	0.17	0.87	0.04	2.75	0.65	1.36	1.9	0.38	0.3	0.66	0.38	0.41	28.35	0.15	2.39	0.88	ME
S14_vs_26320	0	0	0	1.14	0.17	1.83	0.19	0.48	0.31	0.71	1.21	0.26	1.39	0.9	1.21	0.7	203.27	0.72	1.05	2.48	ME
S14_vs_4887	0	0	0	0.71	0.29	0.77	0.07	3.37	0.35	2.56	0.59	0.11	0.25	0.19	0.34	0.2	276.96	0.15	0.37	1.11	ME
S14_vs_117153	0	0	0	1.34	0.14	0.86	0.14	246.29	0.16	0.55	0.56	0.14	1.12	0.5	0.41	0.53	70.13	0.39	0.84	1.62	ME
S14_vs_117158	0	0	0	0.68	0.07	0.42	0.04	48.07	0.11	0.14	0.59	0.12	0.49	0.23	0.34	0.39	40.17	0.31	28.21	0.56	Shared
S14_vs_117160	0.05	0	0	0.52	0.96	0.75	0.07	0.29	0.2	0.21	0.88	0.32	1.28	0.61	0.78	0.51	1188.97	0.49	0.99	1.98	ME
S14_vs_117161	0.01	0	0	1.32	0.09	0.26	0.14	3.4	5.1	18.97	0.38	0.11	0.77	0.66	5.94	0.29	97.82	0.42	0.51	5.47	ME
S14_vs_117165	0.01	0	0	5.39	0.35	6.44	1.02	0.4	0.51	42.43	1.49	1.36	0.89	4.61	0.6	1.27	94.74	0.46	4.19	1.09	ME
S14_vs_117166	0.05	0	0	2.72	0.33	0.6	0.05	1028.2	59.88	2	0.95	0.23	3.02	0.92	0.34	0.9	279.88	0.19	0.95	0.54	Shared
S14_vs_73301	0	0	0	0.6	0.04	0.47	0.18	7.75	0.17	0.4	0.63	0.08	1.41	0.36	0.24	0.34	31.18	0.38	0.36	0.88	ME
S14_vs_14737	0	0	0	0.74	0.05	0.34	3	10.35	0.16	2.1	1.51	0.09	0.23	0.32	0.36	0.28	26.87	0.94	0.34	1.55	ME
S14_vs_102698	0	0	0	3.03	4.16	6.74	0.16	24.97	1.42	9.72	2.54	3.65	0.36	0.48	0.5	0.47	116.8	0.3	6.41	2.38	ME
S14_vs_66139	0	0	0	3.76	0.89	7.87	0.64	4.09	0.5	16.04	13.13	4.04	3.08	11.32	1.64	1.98	33.47	0.48	4.4	1.89	Shared
S14_vs_66208	0	0	0	26.2	3.33	15.18	0.62	20.6	10.57	6.2	5.2	2.18	285.56	3.33	4.68	45.18	60.59	259.56	18.5	27.08	Shared
S14_vs_632	0.02	0	0	0.78	0.06	0.34	0.16	73.56	0.15	0.54	0.51	3.52	0.42	0.37	0.45	0.35	30.04	0.46	0.3	0.55	ME
S14_vs_110290	0	0	0	0.88	0.11	0.65	0.07	0.69	0.11	0.19	0.39	2.55	1.03	0.39	13.21	0.27	23.82	0.17	0.53	0.59	Shared
S14_vs_59347	0	0	0	0.51	0.04	0.42	0.16	7.49	0.14	0.16	0.42	0.08	0.6	0.66	0.53	0.41	32.89	0.25	0.41	0.7	ME
S14_vs_30108	0	0	0	0.46	0.08	0.49	0.06	4.3	4.78	13.28	0.55	0.62	0.63	5	0.55	0.23	39.79	0.26	1.32	0.87	ME
S14_vs_96111	0.01	0	0	1.21	0.1	0.73	0.04	270.39	0.2	0.72	2.03	0.13	1.14	0.64	1.6	0.83	58.03	0.29	1.56	2	ME
S14_vs_74067	0.02	0.02	0.01	18.15	16.83	99.95	0.55	77.49	11.79	20.57	18.49	1.28	249.01	19.35	64.86	37.15	157.33	172.77	132.97	62.64	Shared
S14_vs_30566	0	0	0	0.86	0.05	0.74	0.07	0.49	0.2	0.32	1.04	0.95	0.96	0.33	1.62	0.53	31.94	0.47	0.55	1.28	ME
S14_vs_66841	0	0	0	0.56	0.03	0.26	0.03	42.44	0.17	4.69	0.91	0.07	0.31	0.44	0.27	0.2	16.53	0.2	13.8	0.44	Shared
S14_vs_112068	0.01	0	0	2.8	0.12	0.48	0.09	0.6	0.6	1.23	0.55	0.63	0.35	0.74	5.77	0.39	36.39	0.24	1.14	1.64	ME
S14_vs_38905	0.01	0	0.01	0.6	0.03	0.37	0.03	35.96	0.18	0.5	0.66	0.12	0.69	1.19	0.21	0.3	32.07	0.18	0.27	0.62	ME
S14_vs_9162	0.1	0	0.04	9.3	0.43	47.4	7.62	30.28	3.78	26.76	20.84	5.97	10.07	12.88	12.04	14.03	69.12	6.62	11.85	44.32	Shared
S14_vs_112252	0.01	0	0	0.86	0.03	0.41	0.03	0.33	0.17	0.24	0.42	0.08	0.78	0.37	0.48	0.44	24.23	0.3	0.66	0.74	ME
S14_vs_112345	0.03	0.01	0	19.3	2.57	51.1	1.71	75.98	13.29	71.56	31.65	9.09	282.17	11.32	31.88	20.48	390.02	62.25	112.75	302.28	Shared
S14_vs_39266	0.01	0	0	3.18	0.42	4.78	0.67	17.68	1.73	19.96	1.71	8.38	3.19	14.42	1.3	1.35	45.11	0.2	9.21	1.31	ME
S14_vs_90934	0.03	0	0.02	1.6	0.34	15.75	1.11	0.54	0.17	0.78	1.01	373.69	1.15	0.2	4.85	0.67	46.16	2.17	1.01	1.7	ME
S14_vs_39797	0	0	0	1.38	0.07	3.64	0.29	43.22	0.56	1	2.53	0.23	1.05	0.8	0.69	0.4	42.97	0.3	0.67	1.02	ME
S14_vs_97835	0	0	0	0.44	0.06	0	0	17.4	0.04	2.59	0.39	0	0.58	0.15	0.42	0.07	33.98	0.37	0.11	0.13	ME
S14_vs_61530	0.01	0	0	0.66	0.04	5.97	0.04	6.26	0.06	0.35	0.54	0.14	0.77	0.38	0.49	0.3	43.3	0.5	2.61	0.41	ME
S14_vs_2870	0.04	0	0	3.56	2.11	1.84	0.17	0.22	0.08	1.03	1.82	0.56	0.53	0.27	0.28	2.85	73.43	1.58	0.31	0.62	ME
S14_vs_32499	0.01	0	0.01	12.43	16.36	70.17	0.77	22.7	8.81	12.94	13.9	1.03	109.04	11.65	21.54	26.39	122.88	67.07	57.45	96.19	Shared
S14_vs_62178	0	0	0	0.84	0.09	1.25	0.07	0.4	0.18	0.21	0.72	0.22	0.55	0.55	0.53	0.28	82.04	0.34	0.55	0.67	ME
S14_vs_25852	0.01	0	0	0.63	0.48	1.36	1.61	32.02	23.61	5.55	7.39	0.17	5.58	6.7	2.09	1.35	43.76	7.49	23.38	13.12	Shared
S14_vs_11510	0.01	0.02	0	4.97	1.03	32.83	1.51	72.15	49.06	10.17	27	1.21	38.42	9	47.14	7.38	75.33	40.67	70.26	13.05	Shared
S14_vs_78164	0	0	0	1.67	0.24	3.33	0.31	0.55	0.74	1.55	2.24	0.6	2.22	0.52	2.48	0.59	32.53	0.66	1.83	4.27	ME
S14_vs_56251	0	0	0	0.64	0.1	0.72	0.15	2.25	0.29	1.37	0.68	0.21	0.7	0.81	0.33	0.21	121.87	0.64	0.51	1.13	ME
S14_vs_70985	0.01	0	0	12.86	0.74	9.75	0.64	10.97	2.41	3.84	4.67	0.94	18.53	2.04	33.32	2.45	48.43	5.32	6.46	5.08	Shared
S14_vs_56410	0.01	0	0	0.87	0.15	0.87	0.34	7.08	0.83	4.64	3.81	0.15	0.3	0.28	0.23	0.31	20.89	0.21	0.58	2.02	ME
S14_vs_100227	0.03	0	0	1.27	0.09	0.48	0.12	4.79	3.83	0.7	0.49	0.65	0.67	6.1	0.35	0.31	55.77	0.35	1.04	0.96	ME
S14_vs_115377	0	0	0	0.28	0.03	0.42	0.08	120.96	0.52	0.35	0.26	0.87	0.55	1.1	0.23	0.23	24.26	0.32	0.61	0.91	ME
S14_vs_93565	0	0	0	0.82	0.07	0.74	0.01	1.38	0.17	1.01	0.94	0.21	0.32	0.43	0.5	2.1	19.48	0.31	0.81	1.04	ME
S14_vs_20193	0.02	0	0	0.62	0.15	0.57	0.08	0.37	0.39	0.57	0.76	0.12	0.72	0.73	0.4	0.28	130.77	0.25	0.41	0.59	ME
S14_vs_115455	0.01	0	0	1.08	0.08	0.32	0.18	97.57	0.38	0.19	0.74	0.09	0.71	1.04	0.4	0.34	55.69	0.3	0.41	0.76	ME
S14_vs_108376	0	0	0	1.21	0.03	1.62	0.16	24.68	0.15	0.7	0.46	0.04	0.33	0.26	1.58	0.33	14.71	0.15	0.32	0.43	ME
S14_vs_79505	0	0	0	0.63	0.06	0.28	0.16	0.16	0.14	0.09	0.33	0.09	0.31	0.14	0.32	0.17	30.74	0.21	0.21	0.49	ME
S14_vs_101482	0	0	0.01	1.08	0.09	1.14	0.56	58.06	0.11	0.31	0.74	0.12	0.32	1.84	0.72	0.48	57.54	0.31	0.29	0.82	ME
S14_vs_67094	0.01	0	0	1.31	0.02	1.24	0.17	24.32	0.22	1.92	1.68	0.61	0.69	0.52	0.66	1.36	14.19	0.63	1.19	1.9	ME
S1_vf_140259	0	0	0	12.11	0	0.19	0	0.02	0.07	0.06	0.25	0.02	0.05	0.06	0.89	7.51	0	0.11	0.12	0.45	ME
S1_vf_28398	0	0.04	0	773.56	0.11	0.38	0.07	0.52	0.21	0.25	0.74	0.08	0.31	0.09	0.33	5.57	0.26	1.27	2.87	6.15	ME
S1_vf_22149	0	0.01	0	39	0.28	0.26	0.23	0.26	0.1	0.18	0.53	0.07	0.4	0.23	0.31	12.07	0.22	0.34	0.31	0.45	ME

S1_vf_136526	0.01	0.01	0	382.86	0.07	0.29	0.13	0.23	0.11	0.35	0.83	0.1	0.42	0.13	0.35	19.44	0.12	1.18	0.27	0.46	ME
S1_vf_50591	0	0	0	71.75	0.05	0.35	0.04	0.68	0.09	0.09	0.17	0.06	0.2	0.09	0.17	29.13	0.24	0.24	0.24	0.37	ME
S1_vf_138157	0	0.01	0	87.61	0.16	1.89	0.13	4.45	0.86	1.21	1188.7	0.21	0.86	0.67	0.77	4.59	4.61	1.66	0.72	4.65	Shared
S1_vf_125650	0.02	0	0	205.81	25.37	0.3	0	0.15	0.13	0.15	0.69	0.04	0.17	0.14	0.26	13.72	0.09	0.31	0.3	0.15	Shared
S1_vf_6272	0.01	0	0	71.71	0.02	0.39	0.01	0.29	0.14	0.15	49.87	0.08	0.28	0.38	0.29	14.3	0.25	1.9	7.32	0.48	Shared
S1_vf_158961	0.01	0.01	0	51.54	0.04	0.35	0.04	0.28	0.11	0.18	0.51	0.07	0.32	0.25	0.37	17.66	0.27	0.33	0.45	0.69	ME
S1_vf_159951	0.01	0.01	0	159.71	3.04	0.23	0.04	0.14	0.13	0.13	4.44	0.03	0.28	0.2	0.26	42.67	0.15	0.4	0.26	0.38	ME
S1_vs_143929	0	0.01	0	58.83	0.09	0.38	0.33	0.34	0.11	0.17	4.09	0.18	4.7	0.21	0.39	5.1	0.24	0.74	0.34	0.58	ME
S1_vs_133577	0	0.01	0	23.23	0.04	1.83	0.42	0.81	0.18	0.86	0.92	0.32	0.44	0.22	0.55	1.94	0.32	0.31	2.68	2.26	ME
S1_vs_21439	0.01	0.01	0	60.41	0.06	0.31	0.02	0.39	0.35	0.27	44.41	0.12	0.66	0.41	0.43	3.44	0.24	0.36	0.32	0.7	Shared
S1_vs_155183	0	0	0	233.25	0.11	0.36	0.06	0.27	0.17	0.21	265.56	0.12	0.86	0.25	2.17	20.94	0.44	0.82	1.14	0.79	Shared
S1_vs_62409	0	0	0	29.05	30.55	0.32	0.02	2.09	2.88	0.44	0.29	0.08	0.77	15.79	192.65	5.19	0.31	0.18	0.21	0.52	Shared
S1_vs_117317	0.01	0.01	0	48.58	0.04	0.31	0.04	0.25	0.13	0.21	1.34	0.05	0.37	0.29	0.41	2.15	0.21	0.48	0.49	0.54	ME
S1_vs_23682	0	0	0	116.29	0.1	1.18	0.08	0.5	0.13	0.54	0.69	0.1	0.65	0.26	0.34	11.3	1.3	0.59	0.42	0.78	ME
S1_vs_3267	0	0	0	22.36	0	20.22	0.01	0.12	0.09	0.03	0.08	0.02	0.2	0.05	0.13	12	0.18	0.06	3.05	0.36	ME
S1_vs_3435	0	0	0	25.84	0	0.27	0	0.28	0.11	0.09	0.23	0.04	1.18	0.17	0.62	5.51	0.37	0.34	0.18	0.74	ME
S1_vs_91270	0	0	0	82.45	0.17	0.22	0.05	0.21	0.14	2.1	5.34	0.03	0.28	0.15	0.34	0.33	0.18	0.28	0.21	0.42	ME
S1_vs_145792	0.01	0.01	0	51.7	0.06	0.32	0.1	0.26	0.1	0.17	0.68	0.08	0.53	0.26	0.33	3	0.24	0.39	1.32	2.14	ME
S1_vs_100019	0	0	0	75.44	0.02	0.37	0.02	0.18	0.07	0.15	0.4	0.02	0.93	0.16	0.18	21.02	0.24	0.33	3.03	7.17	ME
S3_vf_8058	0	0	0.02	0.44	0.05	160.96	0.49	0.22	0.08	0.1	0.28	0.27	0.36	0.17	0.26	0.21	0.23	0.21	0.33	0.59	ME
S3_vf_8164	0	0	0.01	0.97	0.01	35.95	0.04	0.18	0.09	1.76	0.38	0.05	0.45	0.37	1.08	0.2	0.23	2.85	0.56	0.83	ME
S3_vf_111776	0	0	0.33	0.39	0.39	3273.02	0.11	0.52	0.45	0.61	1.46	4.48	0.24	0.19	0.29	0.73	0.29	0.26	0.29	0.26	ME
S3_vf_87896	0	0	0	0.53	0.01	32.36	0.03	0.38	0.1	0.17	0.51	0.11	0.59	0.5	0.67	0.37	1.71	0.26	0.39	0.98	ME
S3_vf_111893	0.19	0	0.07	9.53	0.18	1033.62	0.04	0.22	0.22	0.42	0.67	1.63	0.31	0.25	44.52	1.51	2.7	7.5	9.33	1.5	Shared
S3_vf_96222	0.32	0	0.03	0.81	2.79	394.21	114.11	9.33	3.2	5.23	19.69	6.75	1.13	1.05	1.01	0.39	4242.9	0.71	6.67	355.21	Shared
S3_vf_80287	0.01	0	0.01	0.68	0.01	104.72	0.31	0.17	0.12	0.09	1.84	0.24	1.81	2.24	1.9	0.42	0.23	16.14	0.31	0.52	Shared
S3_vf_56363	0	0	0.05	0.88	0.27	1175.93	12.07	107.94	0.46	0.81	1.55	3.49	2.13	0.98	1.56	1.25	2.49	1.22	1.46	1.32	Shared
S3_vf_33389	0.13	0	0.04	2.19	0.39	432.12	0.04	0.11	0.09	0.23	0.35	648.36	0.39	1.23	0.62	1.62	0.34	0.46	1708.36	143.32	Shared
S3_vf_73597	0	0	0.01	0.72	0.15	29.14	0.2	0.21	0.31	0.27	0.71	0.18	1.07	39.36	0.48	0.75	13.02	1.11	1.33	7.03	ME
S3_vf_25163	0.02	0	0.08	0.35	0.15	760.09	0.08	0.36	0.19	0.27	6.03	0.97	19.11	0.15	26.24	344.95	0.3	0.5	0.7	0.59	Shared
S3_vf_57422	0.02	0	0.13	3.69	0.1	1786.39	11.26	0.28	0.19	0.31	0.48	2.28	0.19	0.41	0.09	0.96	0.15	0.1	0.23	0.36	Shared
S3_vf_17748	2.3	0	2.16	5.6	4.29	24202.18	0.62	6.39	5.72	2553.43	51.27	32.15	1473.23	0.83	4.68	8970.63	0.56	0.51	12.24	1.51	Shared
S3_vf_2186	0	0	0.04	0.57	0.08	316.36	0.13	0.25	0.73	0.72	0.76	0.84	1.06	760.28	554.78	0.45	0.41	0.42	0.53	1.1	Shared
S3_vf_11428	0	0	0.01	0.89	0.04	48.13	29.85	0.33	0.26	0.35	0.53	0.22	0.62	2.67	0.55	0.33	1.33	3.64	14.61	30.54	Shared
S3_vf_18080	0	0	0.02	10.76	4.19	165.31	2.79	0.63	0.32	1.84	3.05	0.61	3.96	11.07	6.31	14.19	97.44	6.01	8.24	18.44	ME
S3_vf_106401	0.01	0	0	0.75	0.12	31.5	2.8	3.39	0.13	1.96	0.66	0.28	2.06	0.86	0.56	0.32	86.78	0.38	1.64	0.99	ME
S3_vf_59078	0	0	0	1.53	3.21	20.01	2.76	0.32	1.64	0.09	0.84	0.05	1.18	0.29	0.74	3.15	0.5	1.14	0.51	0.77	ME
S3_vf_82571	0	0	0.03	0.65	0.03	463.07	0.26	0.14	0.08	0.13	6.98	0.55	8.9	0.07	17.74	0.22	0.15	0.2	1.45	0.59	Shared
S3_vf_35073	0	0	0.02	1.02	0.03	110.94	0.11	0.34	0.24	0.14	0.77	0.23	1.04	0.18	0.55	0.5	0.25	0.38	0.6	0.79	ME
S3_vf_83536	0	0	0.04	0.73	0.04	262.43	0.09	0.3	0.19	0.1	0.64	0.43	0.37	0.11	0.39	0.26	0.12	0.3	0.37	0.73	ME
S3_vf_18829	0.02	0	0.02	0.4	0.05	372.84	10.39	0.18	0.17	0.12	0.46	1.2	6.03	32.06	0.38	0.24	0.28	0.24	0.35	0.64	ME
S3_vf_115304	0.37	0	1.18	2.05	1.37	12099.04	3647.6	1.55	1.72	12.79	3.71	18.38	2.8	2.07	14583.94	1.75	2.78	1.78	1.64	4.02	Shared
S3_vf_67333	0.15	0	0.1	3.76	0.66	1541.92	0.1	0.42	0.3	0.39	5.64	2.04	0.46	0.31	0.65	1.2	0.26	0.33	0.57	0.79	ME
S3_vf_99626	0	0	0.05	0.92	0.12	821.15	254.23	0.32	0.24	0.18	0.78	1.04	0.38	0.22	0.37	0.54	0.25	0.21	0.35	0.67	Shared
S3_vf_44405	0	0	0.02	0.54	0.05	167.94	0.19	0.23	0.39	1.22	31.27	0.38	0.24	3.3	5.03	1.96	6.35	1.55	1.82	0.39	Shared
S3_vf_99734	0.12	0	0.15	8.71	13.41	2005.64	11.08	1.74	0.61	4.23	6.71	7.11	2.03	0.31	105.01	9.7	10.15	0.28	2.73	1.76	Shared
S3_vf_67828	0	0	6.63	10.25	8.57	56345.14	0.03	9.33	9.31	9.73	21.23	119.17	0.23	0.14	2.35	7.03	0.07	0.41	0.35	0.62	Shared
S3_vf_84832	0	0	0	0.62	0.04	24.96	0.02	0.24	0.23	0.21	0.41	0.12	0.5	0.24	0.92	0.37	0.29	0.47	0.49	0.92	ME
S3_vf_61860	0	0	0.09	1.07	0.33	930.09	1.07	6.31	0.42	0.49	1.73	1.51	3.58	0.98	1.56	1.82	16.13	2.92	1.06	1.5	ME
S3_vf_100440	0	0	0	10.41	0.05	19.34	3.53	12.09	0.1	0.32	3.91	0.14	2.84	4.3	6.21	2.62	1.24	1.49	5.52	4.79	ME
S3_vf_108959	0	0	0.04	1.76	0.17	324.47	0.14	0.35	0.26	0.28	0.86	0.53	1.33	0.6	2.26	0.66	0.56	0.5	2.7	1.27	ME
S3_vf_109277	0	0	0	0.64	0	19.79	0.03	0.14	0.09	0.09	2.73	0.1	30.59	3.32	0.25	4.98	0.35	0.2	0.39	1.54	Shared
S3_vf_5525	0.01	0	0	0.55	0	26.8	0.27	0.14	0.06	0.08	0.3	0.11	0.1	0.05	0.15	0.22	0.09	0.13	0.15	0.24	ME
S3_vf_54353	0	0	0	0.27	0.02	91.16	0.24	0.19	0.05	0.06	1.56	0.18	0.19	0.15	0.23	0.19	0.21	0.12	0.13	0.54	ME
S3_vf_54498	0.05	0	0	1.36	0	26.69	0.17	0.37	0.2	0.23	0.35	0.13	0.72	0.11	0.29	0.22	0.25	0.15	0.31	0.6	ME
S3_vf_101591	0	0	0	0.22	0.02	20.46	4.26	0.3	0.36	0.15	0.78	0.11	0.69	0.11	0.21	0.38	0.42	0.55	0.33	1	ME
S3_vf_101611	0	0	0	0.54	0.07	59.39	0.06	0.27	0.15	3.19	0.53	0.15	4.44	0.43	3.26	0.31	0.26	2.9	15.52	0.76	Shared
S3_vf_7137	0	0	0	6.82	10.11	14.73	0.02	9.43	8.14	70748.48	20.55	0.04	0.27	0.08	85.33	6.52	0.11	0.17	0.14	0.4	Shared
S3_vf_22968	0	0	0	1.01	0	62.02	0.21	0.27	0.18	1	0.7	0.15	1.05	0.31	0.74	0.48	0.65	0.31	0.84	1.86	ME
S3_vf_23042	0.27	0	0.04	0.32	3.9	241.41	121.03	12.11	3.17	3.25	16.01	6.5	1.52	1.24	1.57	0.66	5731.35	0.71	4.39	206.65	Shared
S3_vf_70746	0.01	0	0.02	0.99	0.03	106.38	0.56	0.23	0.1	0.09	0.54	0.24	0.29	0.1	0.42	0.43	0.2	0.23	10.27	0.75	Shared
S3_vf_127168	0	0	0.02	0.31	0.19	787.16	0.02	406.81	585.49	0.3	60.8	0.91	0.74	1.11	0.31	0.47	0.29	0.23	0.62	0.78	Shared
S3_vf_127392	0.1	0	0.02	0.61	0.03	85.42	0.13	0.26	0.13	0.13	0.51	0.23	0.33	0.23	0.35	0.22	27.17	1.25	0.22	0.6	ME
S3_vf_127399	0	0	0	0.66	0.03	48.54	0.07	0.16	0.09	0.08	32.24	0.11	0.39	0.31	0.18	2.74	0.13	0.23	0.16	0.38	Shared
S3_vf_127400	0.01	0.01	0.01	18.47	0.09	50.19	0.06	0.5	0.3	0.19	407.57	0.27	1.77	0.36	1.03	2.91	0.22	0.92	0.44	1.14	Shared
S3_vf_127405	0	0	0	2.47	0.06	41.17	0.05	0.23	0.27	0.26	0.55	0.14	5.07	0.66	0.76	1.29	3.99	2.62	5.46	0.8	ME
S3_vf_127406	0.02	0.01	0.03	2.63	0.07	509.53	0.09	0.29	0.16	0.14	0.84	0.79	0.42	0.19	0.42	13.81	0.23	0.63	2.35	5.18	ME
S3_vf_127408	0.01	0	0.03	0.48	0.01	174.14	0.02	0.16	0.08	0.1	0.25	0.2	0.18	0.16	0.21	0.15	0.15	0.1	0.19	0.33	ME
S3_vf_127409	0.01	0	0	1.2	0.05	52.77	0.15	0.28	0.13	0.16	90.06	0.13	0.48	0.47	0.17	0.34	0.35	0.35	0.3	0.82	Shared
S3_vf_127423	0	0	0.26	0.74	0.34	2637.65	0.38	0.86	0.54	0.62	1.56	3.81	1.16	0.32	1.24	0.82	0.51	0.52	0.81	1.12	ME
S3_vf_127426	0	0	0	0.52	0.02	23.12	0.22	0.31	0.37	1.08	0.9	0.19	0.39	0.08	0.24	1.03	0.19	0.27	0.54	2.15	ME
S3_vf_127431	0	0	0	0.26	0	76.75	0.03	0.16	0.12	0.19	0.2	0.07	0.73	0.73	0.43	0.24	0.76	0.17	0.63	0.89	ME
S3_vf_127447	0.01	0	0.03	0.41	0.07	378.85	0.1	0.33	0.22	0.18	51.46	0.59	1.03	0.38	0.55	0.39	0.32	0.55	0.45	0.76	Shared
S3_vf_127450	0	0	0.53	0.81	0.63	5254.48	0.13	1.03	0.76	0.99	2.04	6.63	2.11	0.45	1.25	1.23	0.44	0.33	0.85	1.1	ME
S3_vs_119605	0	0	0.01	3.72	0.23	27.51	0.16	10.14	0.54	7.05	3.94	4.37	1.64	1.69	2.42	10.55	13.87	0.48	6.15	1.32	ME
S3_vs_80151	0.9	0.03	1.41	15.19	1.85	17429.87	3079.55	2.17	2.31	2.39	4.59	22.4	0.74	0.23	0.43	1.93	0.42	0.32	0.46	1.28	Shared
S3_vs_16825	0.19	0	0.18	79.83	35.71	2451.15	0.1	2	0.57	0.59	5.66	3.2	0.64	3.18	0.67	7.69	0.57	0.4	0.62	1.15	Shared
S3_vs_49835	0.06	0	0.22	73.53	0.3	3104.54	0.11	1.2	0.55	1.04	1.37	5	0.53	0.38	0.44	26.02	0.59	0.47	0.57	1.13	ME
S3_vs_89485	0	0	0	0.77	0.28	28.23	0.58	0.57	0.51	2.2	1085	0.12	1.59	0.3	0.48	0.26	0.25	0.51	1.69	2.56	Shared
S3_vs_81793	0.01	0	0.01	1.41	0.02	86.69	0.09	1.64	0.24	0.77	3.47	2.81	1.84	0.44	0.5	1.5	0.57	0.45	0.97	1.86	ME
S3_vs_90331	0.02	0	0.01	1.18	0.03	47.56	0.1	0.39	0.12	1.07	3.05	0.29	2.65	0.17	0.54	0.91	0.31	6.67	12.91	0.97	Shared
S3_vs_90341	0.01	0	0.01	1.31	0.02	108.88	0.07	0.26	0.09	0.19	12.7	0.21	0.38	0.12	0.73	0.97	0.26	0.25	0.87	0.51	Shared
S3_vs_50924	0.02	0	0.03	2.09	0.09	473.77	0.06	0.51	0.78	1.09	0.65	0.84	0.32	0.09	0.48	0.33	0.42	0.19	0.29	1.29	ME
S3_vs_98675	0	0	0.04	0.33	0.14	727.66	0.86	2.92	0.32	0.46	0.91	1.22	2.22	0.49	0.75	0.98	0.86	0.89	0.95	1.57	ME
S3_vs_82895	0.11	0	0.36	21.71	0.53	4629.97	133.2	3.13	0.92	17.33	36.15	5.9	18.17	65.25	0.21	9.29	10.44	0.17	0.2	0.39	Shared
S3_vs_108076	0	0	0.04	0.6	0.13	115.56	0.07	0.35	0.13	0.55	1.14	0.45	1.07	0.41	0.48	0.53	0.74	0.23	1.66	2.72	ME
S3_vs_28402	0	0	0.02	1.28	0.15	268.83	0.05	1.28	0.19	1.43	1.04	3.13	0.69	0.89	1.78	0.36	7.29	0.35	2.48	0.68	ME
S3_vs_127391	0.02	0	0	0.75	0.05	227.16	0.15	0.41	0.09	0.16	0.62	0.37	0.3	0.12	0.23	0.33	1.12	0.16	5.52	0.45	ME
S3_vs_127395	0.01	0	0	0.74	0.02	23.89	0.03	0.26	0.14	0.25	0.54	0.16	0.45	0.49	0.39	0.28	0.34	0.31	0.6	0.9	ME
S3_vs_127397	0	0	0	0.8	0.03	71.5	0.4	0.25	0.15	0.2	5.77	0.18	0.38	0.26	0.46	0.18	0.28	0.38	0.27	0.64	ME
S3_vs_127401	0.01	0	0.01	1.36	0.09	101.21	0.13	0.32	0.18	0.18	1.02	0.32	0.63	0.22	0.32	0.58	2.04	0.53	0.61	4.2	ME
S3_vs_127404	0.25	0	0.1	0.51	0.09	928.71	0.02	0.48	0.32	0.27	0.66	1.29	0.69	0.13	15.26	0.37	0.33	0.27	0.49	0.64	Shared
S3_vs_127410	0.01	0	0.03	0.74	0.02	271.59	0.09	0.51	0.24	0.27	0.46	0.46	3.57	0.35	40.8	0.64	1.81	0.24	0.35	0.65	Shared
S3_vs_127411	0	0	0	4.27	0	49.1	0.23	0.26	0.16	0.17	2.41	0.17	1.46	0.37	1.08	1.22	0.24	1.45	0.45	0.93	ME
S3_vs_127413	0.08	0	0.06	4.07	0.14	1471.15	0.08	0.29	0.24	0.29	0.55	1.73	0.19	0.66	0.15	0.25	0.06	0.15	0.12	0.33	ME
S3_vs_127421	0	0	0.03	1.32	0.24	159.6	0.28	0.58	0.21	0.18	35.77	0.35	0.75	0.31	0.77	0.33	0.57	0.35	0.46	0.88	Shared
S3_vs_127442	0.26	0	0	1.11	0.04	189.54	0.23	0.36	0.19	0.14	0.53	0.22	0.26	0.25	0.45	0.37	0.26	0.3	0.16	0.67	ME
S3_vs_127445	0.16	0.01	0.12	33.26	48.78	1089.34	0.22	0.65	0.68	0.43	1.19	1.4	1.05	0.44	1.03	33.16	0.49	0.6	0.74	1.22	Shared
S3_vs_39940	0.03	0	0	0.77	0.06	46.73	0.01	0.15	0.09	0.19	0.49	0.19	0.39	0.1	0.2	0.25	13.33	0.16	0.22	0.34	ME
S3_vs_119660	0	0	0.02	10.25	0.06	69.41	5.67	5.3	2.07	2.48	1.56	0.54	1.5	3.7	1.31	2.11	4.77	15.64	4.48	34.1	Shared
S3_vs_119771	0	0	0.01	6.76	0.1	65.61	1.19	19.94	0.5	4.88	4.34	3.56	2.78	0.61	1.33	1.77	0.85	0.61	7.12	5.73	ME
S3_vs_72118	0	0	0.01	0.59	0.07	42.82	0.02	0.36	0.16	0.54	0.58	0.14	0.24	2.04	0.43	0.27	0.35	0.2	0.27	0.65	ME
S3_vs_56292	0	0	0	10.73	8.35	117.66	1.72	63.66	33.72	20.56	29.7	2.69	55.65	13.88	50.18	10.56	75.91	92.85	69.35	23.37	Shared
S3_vs_9158	0.01	0.01	0.01	47.88	7.77	61.45	1.67	63.65	25.61	15.23	18.83	3.99	32.22	8.24	11.01	7.43	46.86	25.34	31.23	60.96	Shared
S3_vs_64424	0.05	0	0	12.78	1.01	65.79	3.83	52.81	7.51	7.07	15.81	2.07	78.92	3.76	16.36	12.57	344.79	39.34	55.27	94.93	Shared
S3_vs_1031	0	0	0	0.87	0.08	157.31	0	0.18	0.09	0.74	0.27	0.4	0.21	0.12	0.82	0.25	0.21	0.2	0.22	0.21	ME
S3_vs_73361	0	0	0	4.31	0.02	26.35	0.09	0.8	0.12	0.12	5.55	0.09	1.4	0.08	1.53	4.08	0.25	0.24	1.21	0.38	ME
S3_vs_1143	0	0	0.02	0.42	0.04	184.89	0.1	0.3	0.15	0.15	0.45	0.36	0.57	0.28	0.33	0.25	0.28	0.29	0.3	0.8	ME
S3_vs_17168	0.02	0	0	6.09	0.59	52.98	11.4	10.8	4.88	7	26.21	1.66	2.12	8.28	12.29	2.63	1.33	39.84	9.42	5.22	Shared
S3_vs_25896	0.01	0	0	1.17	0.12	32.3	0.16	17.71	0.32	1.28	0.9	2.83	1.03	0.97	0.64	1.25	13.7	0.61	1.86	1.23	ME
S3_vs_97827	0	0	0.01	1.33	0.18	30.12	0.42	0.92	1.21	2.91	2.95	1.98	3.31	1.79	6.36	0.59	4.69	1.44	3.1	3.87	ME
S3_vs_89898	0.37	0	0.42	2.02	0.61	5505.16	7	23.98	36.18	12.65	1.95	7.5	0.77	1.59	0.6	0.93	0.39	0.37	0.47	0.98	Shared
S3_vs_50557	0.01	0	0.03	0.58	0.07	313.07	0.04	0.29	0.12	0.19	0.51	0.55	0.23	0.22	0.38	0.33	0.18	0.23	0.27	0.66	ME
S3_vs_42569	0.01	0	0.01	1.63	0.1	93.03	7.81	5.43	1.21	6.11	3.49	1.31	1.58	0.77	1.91	1.4	2.98	1.52	2.43	12.19	ME
S3_vs_114271	0.01	0	0	0.55	0.01	25.51	0.31	2.46	0.09	0.12	1.8	0.12	0.22	0.16	0.62	0.45	0.33	0.17	0.23	0.32	ME

S3_vs_51005	0.01	0	0	1.07	0	65.28	0.01	0.08	0.15	0.06	0.08	0.05	0.05	0.01	0.48	0.47	0.03	0	2.37	1.12	ME
S3_vs_51931	0	0	0	0.5	0.11	19.85	0	0.23	0.01	0.34	0.1	0.26	0.08	0.02	0.08	0.15	2.6	0.04	0.1	0.06	ME
S3_vs_122706	0	0	0.01	6.91	0.09	64.11	0.04	0.23	0.08	0.19	0.52	0.16	0.43	0.6	1.52	0.45	0.36	0.27	1.11	1.34	ME
S3_vs_12973	0	0	0	1.44	0.03	27.16	0.08	0.44	0.48	0.17	1.32	0.17	0.57	1.4	0.42	0.64	0.34	0.64	0.45	1.1	ME
S3_vs_12985	0.01	0	0	1.55	0.02	17.97	1.3	4.61	0.17	1.13	2.1	0.12	0.1	6.11	1.09	0.38	1.12	0.68	0.19	0.5	ME
S3_vs_18858	0.01	0	0	1.7	0.06	29.55	0.07	0.3	0.16	0.19	0.49	0.21	0.37	0.19	0.4	0.64	0.27	7.49	1.24	0.97	ME
S3_vs_77156	0	0	0.05	0.82	0.01	19.98	0.03	0.15	0.32	0.13	0.29	0.12	0.57	0.07	0.42	0.04	0.38	0.34	1.66	0.51	ME
S3_vs_123834	0.01	0	0.02	11.31	0.02	44.86	0.02	0.16	0.09	0.09	1.14	0.09	0.15	0.11	0.24	0.45	0.11	0.15	11.21	1.89	Shared
S3_vs_20315	0	0	0.6	1.38	0.75	6057.75	1.87	10.91	0.87	1.17	2.89	8.33	19.52	0.24	1.68	2.78	0.85	20.4	2.97	3.68	Shared
S3_vs_68031	0.03	0	0.02	2.82	0.14	127.71	4.81	123.54	1.48	58.46	4.4	1.31	4.06	1.83	2.97	2.08	2.29	5.2	7.04	14.1	ME
S3_vs_29077	0	0	0	1.05	0.02	42.81	0.22	0.29	0.22	0.5	0.51	0.19	1.29	0.49	0.4	0.41	0.38	0.28	0.34	0.84	ME
S3_vs_45147	0.06	0	0.05	0.54	0.19	564.44	0.1	0.27	0.18	0.15	0.5	4.73	0.41	0.75	0.29	0.32	0.41	5.83	0.47	0.7	ME
S3_vs_62410	0	0	0	0.78	0.03	35.98	0.05	0.37	0.14	4.18	0.64	0.16	0.9	0.57	0.56	0.45	0.44	0.31	0.72	0.65	ME
S3_vs_100785	0	0	0	0.83	0.11	52.51	0.11	1.83	0.19	0.18	0.84	0.27	0.51	1.83	0.39	0.22	0.38	25.16	0.43	0.92	Shared
S3_vs_15450	0.01	0	0	0.62	0.02	39.93	0.08	0.3	0.08	0.14	4.53	0.12	0.27	0.23	0.41	0.55	0.52	0.16	0.25	0.52	ME
S3_vs_54303	0.02	0	0.02	0.79	2.06	45.96	1.21	14.89	0.19	2.67	2.18	0.14	1.12	34.19	21.49	2.8	0.74	0.35	0.79	0.39	Shared
S3_vs_30318	0	0	0.01	0.86	0.03	23.8	0.14	0.33	0.18	0.2	0.64	0.11	1.14	0.49	0.5	0.47	0.48	0.36	0.52	1.16	ME
S3_vs_94577	0.01	0	0.03	1.13	0.13	202.06	0.81	0.57	0.72	0.38	1.8	0.37	3.49	0.5	2.89	0.93	0.65	1.35	1.52	16.91	ME
S3_vs_6604	0	0	0.01	3.2	0.45	58.42	0.61	0.95	1	1.47	11.34	0.63	2.86	1.27	9.48	0.55	1.36	3.18	6.96	11.03	Shared
S3_vs_70093	0.01	0	0	5.76	0.07	18.2	0.44	0.2	0.2	91.85	0.65	0.79	1.15	5.87	0.53	1.48	9.91	0.09	5.34	0.73	ME
S3_vs_70508	0.02	0	0	34.8	26.74	51.55	0.27	26.23	9.87	53.89	203.4	1.51	5.93	2.99	7.35	10.54	18.13	6.37	9	35.79	Shared
S3_vs_39774	0.02	0.01	0.01	7.75	1.11	53.34	0.12	55.68	2.38	8.51	17.42	0.36	259.82	8	62.67	42.86	85.59	211.1	112.13	13.66	Shared
S3_vs_95574	0	0	0.03	0.43	0.03	18.92	0.07	0.42	0.13	0.15	1.36	0.11	1.39	0.71	0.34	0.27	0.27	0.28	0.36	0.66	ME
S3_vs_23194	0.01	0	0	1.44	0.07	48.79	1.49	0.55	0.17	0.25	1.04	17.78	1.16	0.48	0.49	0.76	22.69	0.38	0.81	3.1	ME
S3_vs_127387	0	0	0	0.87	0.38	63.38	0.48	0.51	0.28	1.19	0.9	0.22	1.73	19.43	0.95	1.42	0.66	0.58	1.14	1.29	ME
S3_vs_87728	0.02	0	0.02	4.26	0.09	72.84	13.48	2.75	4.09	7.92	3.56	1.54	1.3	1.27	3.5	1.65	14.9	1.92	5.67	13.1	Shared
S3_vs_40939	0	0.01	0.01	2.41	0.82	39.61	0.2	10.82	5.95	8.18	3.12	0.31	3.09	2.07	2.02	1.46	2.23	4.6	7.75	15.52	ME
S3_vs_117894	0	0	0.01	2.22	0.41	44.48	0.81	0.97	1.3	8.12	14.12	3.33	5.16	1.2	6.63	0.88	7.08	1.39	3.6	7.53	Shared
S5_vf_18461	0	0	0	0.6	0.04	0.42	0.28	12.44	0.48	0.18	0.63	0.1	13.67	0.43	7.91	0.17	0.62	0.19	0.28	0.53	Shared
S5_vf_6506	0	0	0	0.64	0.01	4.79	0.7	35.39	0.25	0.21	0.81	0.13	1.08	0.58	0.91	0.43	1.12	0.34	0.56	0.84	ME
S5_vf_122568	0.01	0	0	3.29	0.36	4.87	2.77	799.91	0.39	0.35	22.35	2.33	1.58	0.36	16.29	1.78	20.3	2.47	0.68	2.92	Shared
S5_vf_134040	0.02	0.01	0	9.52	0.31	58.41	6.08	73.16	3.28	26.43	22.97	3.32	12.65	8.22	19.21	9	8.7	9.59	11.86	27.02	Shared
S5_vf_116300	0	0	0	4.28	0.32	0.71	0.33	40.86	1.36	0.43	1.63	1.32	0.97	0.96	0.7	2.13	1.93	0.38	0.8	2.67	ME
S5_vf_67622	0	0	0	0.5	0.08	0.74	0.14	66.98	128.64	0.44	23.84	0.1	1.13	0.56	1.87	0.5	0.43	0.33	0.48	0.91	Shared
S5_vf_110171	0	0	0	0.57	0.03	0.33	0.37	21.83	0.09	0.42	0.84	0.06	4.38	0.18	4.92	1.04	1.76	0.25	0.51	0.45	ME
S5_vf_55415	0	0	0	0.76	0.39	2.29	0.6	1420.76	1514.05	0.83	8.83	0.07	2.58	2.23	0.73	0.59	0.31	0.3	0.38	0.85	Shared
S5_vf_38011	0.01	0	0	3.22	0.08	0.26	0.01	287.06	251.71	0.51	0.28	0.04	0.72	0.82	0.08	1.38	0.13	0.15	0.18	1.03	Shared
S5_vf_55687	0	0	0.11	0.44	0.26	1016.37	0.05	348.36	17.73	246.4	10.05	1.34	0.72	0.72	0.63	0.3	1.99	0.29	0.32	12.3	Shared
S5_vf_25423	0.37	0	0	0.37	4.78	180.73	242.15	295.25	8.83	18.34	48.76	41.97	2.87	2.88	2.23	1.13	6860.37	0.93	16.58	951.76	Shared
S5_vf_123990	0	0	0	0.53	6.37	0.29	0.05	56.93	82.49	0.14	0.68	0.03	0.47	0.31	0.49	0.41	0.26	0.17	0.23	0.65	Shared
S5_vf_105728	0	0	0	0.63	0.03	0.33	0.06	13.73	0.44	0.39	1.57	0.21	0.48	65.11	0.36	0.19	4.26	0.22	0.28	0.52	ME
S5_vf_8808	0	0	0	0.18	0.02	0.36	0.14	14.19	0.19	0.16	0.47	0.08	0.3	0.66	0.46	0.31	4.2	0.18	0.3	0.7	ME
S5_vf_8965	0	0	0	3.66	1.86	3.41	0.57	51.24	0.63	1.85	4.59	38.56	4.02	0.98	6.39	0.65	4.6	26.38	4.38	2.54	ME
S5_vf_74661	0.02	0	0	0.45	0.04	0.3	0.02	108.37	0.08	0.11	0.54	4.51	0.37	0.13	0.6	0.17	0.17	0.2	0.23	0.5	ME
S5_vf_136076	0	0	0	0.65	0.03	0.48	2.4	19.19	36.18	0.24	1.74	0.1	1.32	0.87	0.36	0.29	0.49	0.31	0.33	0.48	Shared
S5_vf_21869	0	0	0	14.42	7.58	0.73	0.23	63.83	102.34	2.93	2.21	0.53	1.13	82.02	70.77	4.04	2.8	0.24	2.04	0.74	Shared
S5_vf_6032	0	0	0	0.32	0.03	0.12	0.08	64.45	1.92	1.93	6.09	0.02	0.19	0.03	0.04	0.3	8.46	0.08	0.21	3.21	ME
S5_vf_137260	0	0	0	0.51	0.04	0.25	0.01	49.37	52.39	0.09	0.27	0.04	0.21	0.15	0.24	0.32	0.12	0.16	0.27	0.34	Shared
S5_vf_11450	0.01	0	0	0.47	0.19	0.29	0.02	83.61	0.09	0.23	0.49	0.06	0.37	0.15	0.66	0.2	0.15	0.13	0.44	0.41	ME
S5_vf_28061	0	0	0	0.52	0.06	1.33	0.75	370.71	0.21	0.52	0.55	0.16	1	0.63	0.68	0.33	10.64	0.27	0.55	0.66	ME
S5_vf_77701	0	0	0	0.46	0.01	0.6	0.01	29.26	0.12	0.06	0.26	0.02	0.3	0.21	0.28	0.19	0.19	0.23	0.18	0.45	ME
S5_vf_145579	0	0	0	0.85	0.32	1.2	0.18	547.38	1778.42	0.54	1.28	0.09	1.75	2.75	1.99	0.62	0.4	0.36	0.46	0.86	Shared
S5_vf_145583	0	0	0	0.55	0.04	0.81	0.46	198.81	0.18	0.24	0.61	0.12	1.79	0.28	0.63	0.56	2.08	0.38	0.8	1.06	ME
S5_vf_29332	0.02	0	0	0.94	0.03	0.44	0.07	40.01	0.12	0.18	0.53	0.07	0.36	0.08	0.36	0.31	0.27	0.29	0.32	0.42	ME
S5_vf_145585	0	0	0	0.46	0.02	0.38	0.09	44.5	96.5	0.14	0.37	0.08	0.53	0.36	0.26	0.23	0.25	0.27	0.28	0.65	Shared
S5_vf_145586	0	0	0	0.58	0.08	0.34	0.08	114.11	0.18	0.12	0.41	0.29	0.85	0.24	0.67	0.23	3.34	0.22	0.29	0.48	ME
S5_vf_145588	0	0	0	1.55	0.28	1.41	0.19	1139.5	1002.38	0.76	1.29	0.16	3.13	1.89	0.65	0.79	0.67	0.46	0.61	1.21	Shared
S5_vf_145604	0	0	0	1.42	0.08	1.07	14.96	534.39	0.25	0.27	0.87	0.08	1.31	0.88	0.62	0.39	27.22	2.28	8.02	16.6	Shared
S5_vf_103206	0	0	0	3.84	0.09	3.51	0.58	45.06	0.61	4.05	12.03	2.1	2.36	32.08	7.76	6.71	20.63	1.21	3.58	4.39	Shared
S5_vf_60597	0	0	0	4.87	0.05	1.85	0.05	32.45	57.81	0.18	5.95	1.37	0.72	0.44	0.62	1.57	0.36	0.32	1.9	25.36	Shared
S5_vs_36605	0	0	0	0.67	0.17	1.18	0.07	362.28	0.61	1	0.5	0.09	0.7	0.09	0.17	0.16	34.44	0.14	0.35	0.48	ME
S5_vs_122344	0.02	0	0	3.62	0.91	47.06	0.06	166.98	5.86	0.21	1.68	0.22	0.69	0.54	0.41	0.62	24.45	0.29	0.77	1.1	ME
S5_vs_122618	0	0	0	0.57	0.15	0.72	0.1	794.79	0.44	2.61	0.67	0.92	1.65	0.65	0.56	0.32	0.42	0.35	0.79	1.18	ME
S5_vs_97574	0.03	0	0	1.51	0.32	5.97	0.12	500.54	0.34	0.42	15.05	0.25	5.12	21.42	0.82	2.14	377.05	3.55	1.01	1.64	Shared
S5_vs_37659	0	0	0	0.56	0.02	0.37	0.11	93.17	0.12	0.17	0.4	1.31	0.61	0.38	0.35	0.14	5.92	0.17	0.2	0.36	ME
S5_vs_67958	0	0	0	2.62	0.02	0.64	0.11	68.15	0.15	0.6	0.39	0.08	0.4	0.23	0.32	1.28	14.81	0.19	0.35	0.57	ME
S5_vs_68254	0.01	0	0	0.85	0.13	0.61	0.54	548.47	0.29	1.03	0.77	0.13	1.32	0.8	0.47	0.35	0.82	0.43	0.38	0.86	ME
S5_vs_38949	0	0	0	12.57	1.37	0.33	0.1	292.43	296.52	21.7	1.69	0.11	2.99	1.07	0.55	5.43	9.95	0.21	5.12	11.58	Shared
S5_vs_120079	0.02	0	0	8.37	3.93	6.41	0.13	160.73	0.87	0.5	1.83	0.19	0.91	0.42	0.45	0.95	100.46	0.58	0.42	1.01	ME
S5_vs_10428	0.01	0	0	0.41	0.02	0.21	0.16	45.76	0.11	0.23	0.94	0.05	0.37	0.3	0.51	0.28	1.23	0.2	0.5	0.46	ME
S5_vs_136679	0	0	0	0.57	0.03	0.38	0.08	18.81	0.16	0.19	0.33	0.18	0.42	0.28	0.37	0.2	0.4	0.29	0.32	1.18	ME
S5_vs_120584	0	0	0	8.2	0.02	2.5	0.19	44.75	0.28	4.07	33.52	0.18	2.54	0.42	1.47	1.24	0.75	2.87	1.92	2.71	Shared
S5_vs_109085	0.01	0	0	0.93	0.09	0.5	0.13	879.44	0.12	0.31	1.01	0.18	1.32	0.65	0.28	0.27	7.23	0.21	0.36	2.67	ME
S5_vs_121411	0	0	0	1.32	0.03	0.73	0.06	114.52	0.38	0.2	0.62	0.52	0.55	0.41	0.37	0.39	29.1	0.25	0.32	0.79	ME
S5_vs_121419	0	0	0	0.69	0.04	0.75	0.13	95.41	0.14	0.24	0.89	0.1	0.38	0.52	0.55	0.23	15.44	0.34	0.32	0.81	ME
S5_vs_27918	0	0	0	0.52	0.04	0.29	0.04	37.52	0.13	0.35	0.23	0.08	0.25	0.84	0.81	0.19	0.16	0.37	0.22	0.44	ME
S5_vs_145578	0.01	0	0	0.36	0.07	0.42	0.04	79.22	0.3	0.94	1.16	0.59	0.65	6.03	0.25	0.32	18.72	0.23	0.46	1.4	ME
S5_vs_145581	0	0	0	0.9	0.01	0.51	0.11	55	0.14	0.1	0.53	0.08	0.42	0.29	0.4	0.34	5.47	0.38	0.36	0.51	ME
S5_vs_145582	0	0	0	1.12	0.04	0.55	0.08	47.59	0.24	0.86	1.17	0.19	0.6	0.33	0.51	1.31	0.62	0.39	0.5	1.16	ME
S5_vs_145590	0	0	0	0.94	0.03	0.39	0.06	31.05	0.12	0.32	0.42	0.16	0.89	0.17	0.29	0.31	0.87	0.21	0.46	0.66	ME
S5_vs_145591	0	0	0	0.54	0.03	0.23	0.06	22.59	0.13	2.22	0.45	0.07	0.37	1.17	0.28	0.19	21.42	0.2	0.19	0.44	ME
S5_vs_145592	0	0	0	0.94	0.02	0.81	0.13	57.99	0.24	0.53	0.36	0.16	0.43	0.19	1.2	0.2	0.29	0.39	0.3	0.43	ME
S5_vs_145593	0	0	0	1.23	0.03	0.33	0.07	154.2	0.05	0.34	0.39	0.05	0.8	0.18	0.36	1.6	0.51	0.17	0.9	0.79	ME
S5_vs_145597	0	0	0	0.82	0.01	0.21	0.01	40.04	0.4	0.18	0.59	0.04	0.28	0.4	0.19	0.18	2.72	0.28	0.2	0.36	ME
S5_vs_145598	0	0	0	0.72	0.16	0.49	0.25	31.45	0.19	0.2	5.81	0.16	0.66	0.23	0.45	0.25	0.46	0.4	0.55	1.24	ME
S5_vs_145599	0	0	0	0.83	0.03	6.36	0.09	142.64	0.54	0.73	3.65	0.34	4.07	0.84	6.02	1.55	1.33	0.58	4.29	10.91	ME
S5_vs_145601	0	0	0	0.75	0.02	0.61	0.05	68.03	0.6	0.17	0.89	0.16	0.85	0.2	0.26	0.37	9.46	0.2	0.42	0.74	ME
S5_vs_145602	0.01	0	0	0.87	0.06	0.7	0.09	41.27	0.18	0.47	0.5	0.15	0.84	0.18	0.24	0.25	0.43	0.42	0.52	0.72	ME
S5_vs_145605	0.01	0	0	0.9	0.01	0.54	0.01	25.19	0.19	0.8	0.91	0.04	0.24	0.97	0.64	1.46	0.8	0.25	0.34	0.33	ME
S5_vs_145611	0	0	0	0.94	0.07	1.52	0.06	57.07	0.09	0.2	0.97	0.32	0.59	0.54	0.34	0.29	0.27	0.21	0.32	0.69	ME
S5_vs_145633	0	0	0	1.8	0.02	0.23	0.01	30.23	0.1	0.16	1.02	0.06	0.1	0.25	0.37	0.25	0.11	0.2	0.28	0.79	ME
S5_vs_85065	0	0	0	0.75	14.13	2.06	0.08	100.97	0.91	0.49	3.27	0.23	0.77	11.37	0.46	0.44	12.24	1.01	0.56	0.79	Shared
S5_vs_30490	0.01	0	0.01	18.47	0.29	8.04	0.8	152.73	4.93	12.77	2.37	0.54	4.27	0.9	1.5	1.41	0.8	7.66	2.45	5.88	ME
S5_vs_6478	0.01	0	0	0.49	0.03	0.58	0.2	54.02	0.12	0.16	0.64	0.11	0.96	0.25	0.57	0.46	0.37	0.26	0.43	0.99	ME
S5_vs_109527	0	0	0	1.69	0.9	10.06	1.11	83.64	14.78	8.28	5.68	0.43	18.73	2.68	22.19	2.45	16.96	7.54	8.71	8.02	Shared
S5_vs_49011	0.01	0	0	0.45	0.02	0.44	0.08	34.49	0.11	0.15	0.49	0.99	0.63	0.17	0.33	0.34	1.38	0.23	0.36	0.68	ME
S5_vs_43207	0	0	0	0.84	0.02	0.3	0.08	15.39	0.14	0.6	0.51	0.26	0.24	0.37	0.33	0.28	0.73	0.24	0.32	0.63	ME
S5_vs_850	0	0	0	0.74	0.07	0.71	0.07	33.11	0.23	0.36	7.26	0.12	1.36	0.25	1.1	0.44	0.74	0.45	0.7	1.32	ME
S5_vs_116235	0	0	0	8.66	0.01	0.41	0.03	11.4	0.12	0.14	0.33	0.08	0.24	0.17	0.46	3.2	0.16	0.2	0.26	0.59	ME
S5_vs_1338	0	0	0	1.97	0.02	0.69	0.05	16.14	8.72	0.11	0.47	0.07	0.67	1.29	0.94	2.07	1.32	0.64	0.58	0.65	ME
S5_vs_49480	0.01	0	0	1.31	0.01	0.31	0.08	22.25	0.11	0.27	31.42	0.1	0.31	0.65	6.04	0.5	0.37	0.25	0.87	0.6	Shared
S5_vs_13090	0	0	0	0.72	0.02	0.73	0.09	14.11	0.16	0.35	1.42	0.44	0.51	0.56	0.34	0.22	2.74	0.24	0.38	0.72	ME
S5_vs_128398	0	0	0	0.99	0.09	0.31	0.06	83.21	8.29	0.2	0.57	0.27	0.37	0.26	0.34	0.33	53.96	0.19	0.28	0.58	ME
S5_vs_123256	0.03	0	0	9.72	0.62	24.11	0.83	34.58	12.8	7.1	27.1	1.33	3.86	8.11	15.07	3.53	4.2	28.21	12.85	7.46	Shared
S5_vs_13254	0	0	0	0.66	0.08	0.4	0.04	39.52	0.17	0.67	0.73	0.08	1.03	1.21	0.36	0.42	12.8	0.23	0.34	0.78	ME
S5_vs_1572	0	0.01	0	18.52	13.01	2.23	1	36.73	57.93	206.69	1.94	0.06	2.15	21.69	0.56	23.45	0.25	0.19	35.92	0.54	Shared
S5_vs_117319	0.02	0	0	3.05	0.26	32.91	4.06	54.3	2.19	19.28	5.22	1.79	4	1.39	4.98	1.46	6.29	4.16	5.91	19.76	ME
S5_vs_117417	0	0	0	1.08	0.07	1.99	0.12	35.92	4.46	6.39	0.79	0.12	0.43	0.42	0.45	1.09	11	0.27	1.3	1.92	ME
S5_vs_128881	0.01	0	0	3.15	0.24	1.57	1.56	58.06	0.26	1.35	2.49	0.81	0.77	23.95	0.63	0.9	34.71	0.77	26.82	3.66	Shared
S5_vs_86562	0	0	0	0.89	0.1	1.49	0.08	192.6	0.23	1.08	1.76	0.13	0.52	0.17	0.28	0.29	4.14	0.62	1.2	0.61	ME
S5_vs_140946	0	0.02	0	110.82	0.08	2.18	0.65	48.22	1.86	0.6	2.11	0.13	0.63	0.46	0.21	32.87	45.64	1.99	4.4	0.7	ME
S5_vs_8125	0.01	0	0.01	2.1	0.08	5.45	0.54	24.18	0.19	0.24	0.77	0.28	1.73	0.44	0.57	0.53	22.93	0.76	1.06	4.19	ME
S5_vs_124035	0.02	0	0.06	5.82	0.25	9.71	1.9	36.28	1.73	12.4	25.64	4.58	4.32	14.02	7.9	23.18	20.09	2.58	5.13	10.3	Shared
S5_vs_20268	0	0	0	0.76	0.17	0.39	0.11	81.81	7.57	1.26	0.41	0.03	0.68	0.23	6.81	0.36	9.03	0.11	0.46	0.43	ME
S5_vs_79992	0.02	0	0	9.53	0.94	14.65	0.54	51.76	13.56	13.55	28.24	2.6	3.36	5.55	17.17	2.37	6.79	22.05	14.6	7.44	Shared

S5_vs_118168	0	0	0	0.63	0.03	0.27	0.13	52.6	0.18	0.32	0.33	0.68	0.36	0.58	0.22	0.37	4.91	0.16	0.25	0.34	ME
S5_vs_32885	0	0	0	0.46	0.17	0.95	0.1	93.15	0.1	0.2	0.49	0.06	0.36	0.2	0.6	0.2	3.64	0.2	0.36	0.7	ME
S5_vs_2876	0	0	0	1.16	0.03	0.19	0.12	106.91	0.18	0.14	0.51	0.07	0.38	0.52	0.25	0.7	17.34	0.13	0.49	0.87	ME
S5_vs_56083	0	0	0	26.84	0.04	18.79	0.2	20.92	4.44	0.18	271.66	0.17	14.75	4.69	10.92	11.39	1.34	27.92	16.96	0.76	Shared
S5_vs_20500	0.01	0	0	5.43	0.34	4.72	126.01	41.21	9.15	4.59	13.31	0.86	1.43	7.29	6.39	1.71	3.81	7.16	10.95	3.92	Shared
S5_vs_3183	0	0	0	6.23	0.03	0.8	0.03	21.34	0.11	0.82	1.87	0.53	0.69	0.28	0.53	4.64	2.12	0.41	7.74	1.61	ME
S5_vs_141621	0	0	0	6.22	0.06	0.52	0.32	57.99	2.21	0.18	3.31	1.74	1.26	0.68	0.8	2.15	4.1	0.33	1.45	4.16	ME
S5_vs_69083	0.01	0	0	1.14	0.08	0.26	0.09	42.48	0.38	0.13	0.76	0.06	0.91	0.14	5.74	0.31	5.64	0.28	0.48	0.74	ME
S5_vs_51416	0	0	0	0.64	0.02	0.65	0.07	37.61	0.09	1.22	0.4	0.27	0.36	0.34	0.65	0.27	2.92	0.18	0.24	0.74	ME
S5_vs_8847	0	0	0	0.99	0.03	0.69	0.05	28.62	0.25	2.75	1.32	1.42	0.81	1.56	1.15	0.89	4.43	0.25	1.44	0.78	ME
S5_vs_93839	0	0	0.01	0.51	0.04	6.9	0.07	16.49	5.9	0.38	2.12	1.44	1.1	3.23	0.7	0.58	2.54	0.26	37.58	12.38	Shared
S5_vs_3501	0.03	0	0	0.7	0.11	1.24	0.05	68.65	0.78	2.06	1.58	0.28	1.5	3	0.78	1.99	89.76	0.25	0.51	0.97	ME
S5_vs_129970	0.02	0	0	13.03	13.45	2.2	0.08	23.34	1.04	1.9	97.37	0.21	2.26	0.91	5.43	4.97	5.37	1.15	2.29	5.02	Shared
S5_vs_124677	0.03	0	0.02	4.58	0.27	60.23	8.73	91.22	6.62	33.18	9.49	2.01	7.57	1.53	5.56	3.38	6.76	7.06	8.53	30.86	ME
S5_vs_130032	2.16	0.29	0	0.51	0.01	0.24	0.46	16.84	0.1	0.11	0.25	0.42	0.27	0.28	0.26	0.19	0.27	0.3	0.21	0.63	ME
S5_vs_130294	0.02	0	0	6.75	0.27	72.49	8.87	88.77	2.76	15.16	11.21	3.04	8.4	3.9	6.69	4.42	7.97	6.16	10.16	34.56	Shared
S5_vs_80496	0.01	0.01	0	38.3	3.97	30.75	1.12	77.36	38.2	12.96	9.61	4.05	17.1	5.32	9.86	7.02	40.47	24.13	24.5	42.04	Shared
S5_vs_142195	0	0	0	6.52	0.17	13.28	0.75	17.27	11.16	4.07	19.64	0.42	1.48	6.65	16.63	1.42	1.32	33.83	5.63	3.45	Shared
S5_vs_80498	0	0	0	0.61	0.01	0.48	0.05	175.7	0.36	0.66	1.88	0.09	0.62	1.88	0.51	0.19	4.61	0.93	0.38	0.66	ME
S5_vs_130476	0	0	0	1.12	0.04	0.31	0.03	9.93	0.18	0.46	0.29	0.08	0.42	0.45	0.24	0.32	0.39	0.35	0.72	1.32	ME
S5_vs_107105	0	0	0	0.42	0.01	0.18	0.12	14.69	0.85	0.21	0.56	0.06	0.22	0.27	0.35	0.17	2.21	0.18	0.12	0.43	ME
S5_vs_142450	0	0	0	0.36	0.64	1.23	0.1	572.97	2672.03	6.18	1.17	0.04	1.19	5.13	0.24	0.57	0.19	0.15	0.64	0.73	Shared
S5_vs_112452	0	0	0	1.65	0.04	1.75	0.12	26.44	0.14	0.91	0.42	0.1	0.3	0.11	1.39	0.49	15.34	0.22	0.52	0.46	ME
S5_vs_63924	0.02	0.01	0	0.53	0.04	9.97	0.74	31.91	13.61	0.1	1.88	1.68	0.27	0.07	0.31	0.4	29.72	0.19	0.29	0.56	Shared
S5_vs_130958	0	0	0	0.57	0.02	0.2	0.05	13.55	0.08	0.13	0.5	3.39	0.29	0.46	0.22	0.27	5.98	0.19	0.24	0.79	ME
S5_vs_9747	0.02	0.01	0	30.09	0.13	0.63	0.03	270.98	446.34	0.37	2.05	0.07	0.88	7.59	0.69	20.93	0.33	0.41	0.42	0.81	Shared
S5_vs_4737	0.03	0	0	0.92	0.01	0.25	0.06	37.29	0.14	0.34	1.3	0.07	0.35	2.81	0.74	0.55	4.33	0.16	0.51	0.45	ME
S5_vs_80911	0	0	0	0.67	0.11	1.96	1.59	96.76	0.15	2.07	0.61	0.15	1.6	1.69	0.84	0.56	9.21	0.76	0.83	0.9	ME
S5_vs_69980	0.01	0	0	0.88	0.08	2.2	0.89	18.29	1.85	7.15	1.96	0.16	0.54	0.95	0.31	0.52	13.77	0.38	0.98	2.8	ME
S5_vs_21778	0	0	0	0.57	0.03	0.54	0.06	22.24	0.15	0.29	0.72	0.16	0.41	0.56	0.49	1.46	0.92	0.31	0.98	0.75	ME
S5_vs_113386	0.02	0	0	0.86	0.05	0.39	0.26	10.7	0.15	0.14	0.64	0.26	0.58	0.29	2.65	0.21	0.27	0.27	0.43	0.73	ME
S5_vs_95307	0	0	0	0.48	0.03	0.45	0.06	23.67	0.22	0.63	0.4	1.21	1.45	2.07	0.4	0.38	8.45	0.21	0.47	1.2	ME
S5_vs_131510	0.02	0.01	0	63.14	4.27	34.55	4.43	64.14	13.8	13.05	13.08	3.84	23.86	6.87	12.73	4.99	38.44	25.84	18.57	70.22	Shared
S5_vs_46394	0.01	0	0.01	1.53	0.08	1.9	0.26	18.54	0.44	5.02	2.79	0.48	1.15	1.11	1.12	2.31	14	0.81	1	1.73	ME
S5_vs_89540	0	0	0	0.68	0.04	0.38	0.05	43.2	0.13	0.16	0.32	0.08	0.36	0.54	0.52	0.29	0.82	0.24	0.22	0.73	ME
S5_vs_17520	0	0	0	0.58	0.01	0.56	0.06	17.71	0.14	0.58	0.6	0.79	0.49	0.32	0.24	0.17	1.84	0.26	0.27	0.89	ME
S5_vs_137154	0.01	0	0	0.33	0.05	1.05	0.24	45.76	0.28	0.3	1.11	0.1	1.72	0.32	0.63	0.72	0.93	0.48	0.92	0.95	ME
S5_vs_40848	0	0	0	4.04	1.03	10.62	0.86	50.29	4.08	23.77	9.33	4.27	4.09	12.35	1.85	2.22	8.31	0.49	6.12	1.62	ME
S5_vs_28263	0	0	0	0.81	0.07	0.46	0.2	20.75	0.09	0.64	0.76	0.13	0.46	0.32	0.56	0.33	3.39	0.22	0.45	0.94	ME
S5_vs_72532	0.01	0	0	16.28	1.45	12.18	0.32	33.6	5.89	4.12	4.52	5.58	6.14	2.51	3.78	1.73	14.28	5.89	8.27	18.62	ME
S5_vs_41663	0	0	0	0.61	0.02	0.32	0.04	43.3	0.11	0.1	0.41	0.06	0.4	0.85	0.37	0.29	0.33	0.17	0.22	0.51	ME
S5_vs_145026	0	0	0	0.71	1.23	0.34	0.08	58.63	0.08	0.14	0.46	0.06	0.39	0.17	0.16	0.23	5.78	0.16	0.25	0.44	ME
S5_vs_77593	0.04	0	0.01	2.55	0.09	1.44	0.25	35.03	0.32	1.72	5.15	0.37	1.61	1.11	2.98	2.5	2.61	1.47	1.47	1.46	ME
S5_vs_58766	0	0	0	0.41	0.02	0.4	0.16	11.51	0.09	0.25	0.63	0.19	0.62	0.35	0.36	0.23	2.31	0.17	0.24	0.84	ME
S5_vs_58859	0.01	0	0	0.59	0.04	0.6	0.01	25.01	0.16	1.53	0.92	0.09	0.42	0.25	0.49	0.2	22.58	0.23	1.08	0.71	ME
S5_vs_83886	0	0	0	0.7	0.05	0.48	0.08	46.53	0.47	10.53	9.14	0.23	2.43	3.22	0.4	1.17	11.57	0.54	9.36	9.16	ME
S5_vs_138980	0.01	0	0	1.15	0.03	0.89	0.12	16.09	0.24	0.85	2.83	0.45	1.13	2.46	1.98	1.9	3.38	0.52	0.67	1.07	ME
S5_vs_59798	0.03	0	0	4.15	0.09	0.56	0.09	114.49	0.15	0.68	0.81	0.24	1.1	0.47	0.53	0.35	127.16	0.28	0.45	1.27	ME
S5_vs_84574	0.02	0	0	1.2	0.07	11.35	0.36	25.91	0.16	0.32	1	2.98	0.38	0.53	0.48	0.41	8.42	0.49	0.42	0.65	ME
S5_vs_84946	0	0	0	3.08	0.09	4	0.07	60.91	0.12	1.13	0.93	0.95	0.9	0.48	7.93	0.31	37.18	0.79	0.57	1.16	ME
S5_vs_139401	0	0	0	1.25	0.05	0.36	0.19	43.59	0.2	0.59	0.82	0.24	0.44	0.29	0.5	0.48	0.31	0.25	0.29	0.73	ME
S5_vs_60507	0	0	0	0.43	0.03	0.46	0.06	19.6	0.13	0.28	0.54	0.07	0.41	0.37	0.41	0.34	2.55	0.25	0.4	0.98	ME
S5_vs_7444	0	0	0	0.91	0.01	1.42	0.13	27.64	0.09	0.36	1.33	0.08	0.15	0.67	1.06	1.18	14.99	0.14	0.71	0.56	ME
S5_vs_74199	0.01	0	0	3.57	0.1	1.64	0.47	33.91	0.48	5.24	8.29	0.19	2.76	0.65	3.45	2.13	22.33	1.6	0.6	1.53	ME
S5_vs_65490	0	0	0	0.74	0.21	0.31	0.03	19.44	0.19	0.15	4.58	0.11	0.44	3.46	0.61	0.65	0.2	0.2	0.3	0.53	ME
S5_vs_76500	0	0	0	1.14	0.19	5	0.09	93.72	21.82	1.17	2.97	0.5	4.47	2.85	8.08	1.05	9.65	4.51	3.84	6.25	Shared
S7_vf_5439	0.02	0	0.06	3.52	0.38	500.25	7.47	0.89	0.36	1141.77	7.39	2.24	16.63	0.16	3.43	4.26	0.28	24.11	72.7	33.9	Shared
S7_vf_5801	0.06	0.01	0	28.98	7.19	18.91	77.67	2.68	0.72	138.92	14.64	2.44	63.57	8.59	7.78	27.93	1126.64	31.79	52.14	61.77	Shared
S7_vf_5843	0.01	0	0.01	0.67	0	0.29	7.73	0.14	0.06	10.69	0.36	0.03	0.1	0.12	0.21	0.22	1.03	0.14	0.18	0.4	ME
S7_vf_8332	0.01	0	0	1.48	0.09	1.33	0.17	1.39	0.31	28.36	1.99	0.5	2.51	3.3	2.96	2.15	1.29	3.05	1.67	1.73	ME
S7_vf_9334	0	0	0.02	0.52	0.42	192.04	0.07	0.54	0.48	1998.96	0.89	0.39	0.63	0.31	2.75	0.33	0.22	0.15	0.19	0.52	ME
S7_vf_11744	0	0	0	0.28	0.07	0.69	0.11	0.28	0.13	22.37	2.49	0.11	1.55	0.75	1.33	0.64	0.44	0.27	0.44	0.85	ME
S7_vf_13614	0	0	0	3.14	0.26	0.62	0.05	0.35	0.3	1397.29	0.84	0.08	0.28	0.06	1.79	0.85	1.43	12.99	2.26	0.58	Shared
S7_vf_19866	0	0	0	1.81	3.17	3.58	0.11	2.78	2.07	16931.24	5.5	0.08	0.21	0.28	21.87	1.98	4.59	0.18	0.27	0.46	Shared
S7_vf_20129	0	0	0	0.74	0.88	1.44	0	0.79	1.83	7039.56	2.3	0.16	0.44	910.65	8.93	0.75	0.38	0.31	0.32	0.73	ME
S7_vf_27309	0	0.01	0	21.4	0.8	0.75	0.06	0.37	0.33	1840.74	0.93	0.05	0.36	0.11	2.63	0.36	0.13	0.26	0.18	0.32	ME
S7_vf_27437	0	0	0	4.22	9.85	0.3	0.18	0.2	0.09	120.54	0.67	0.08	0.72	13.01	0.7	0.21	206.01	0.35	0.29	1.04	ME
S7_vf_28429	0	0	0	16.2	0.74	21.53	11.9	13	2.92	4020.95	13.98	0.26	5.57	548.46	5.53	27.36	48.29	0.24	0.29	0.54	Shared
S7_vf_29196	0	0	0	7.57	0.19	3.15	0.06	0.19	0.1	16.34	3.26	249.4	0.27	0.28	0.33	2.52	9.68	0.19	0.27	3.63	ME
S7_vf_29941	0.23	0	0.01	0.39	2.69	80.75	65.6	10.68	3.5	39.21	32.53	14.16	1.72	0.92	1.69	1.83	4082.48	0.73	9.63	554.71	Shared
S7_vf_32396	0	0	0	0.39	0.02	0.16	0.05	0.11	0.09	99.68	0.38	2.87	0.19	31.44	0.47	0.95	0.21	0.16	1.66	0.71	ME
S7_vf_32559	0	0	0	0.31	0.07	0.4	0.05	0.18	0.13	661.84	4.22	0.04	0.12	0.05	1.25	0.19	0.2	0.04	0.18	0.31	ME
S7_vf_36627	0	0	0	1.74	0.03	0.39	0.16	0.19	0.13	48.44	0.71	0.1	0.9	0.19	0.46	0.83	0.32	0.31	0.56	0.84	ME
S7_vf_37279	0.02	0	0.01	1.69	0.49	70.4	12.51	1.14	0.25	145.67	0.89	0.34	1.3	16.82	5.73	1.27	29.56	5.31	1.11	53.51	Shared
S7_vf_37293	0	0	0	0.39	0.03	1.14	0.06	0.22	0.18	35.88	0.65	0.08	0.4	5.69	0.36	0.38	0.27	0.28	0.28	0.66	ME
S7_vf_38697	0	0	0	1.37	1.12	1.87	0.08	1.61	0.94	5690.73	2.57	0.13	1.25	0.22	8.16	1.01	0.54	0.44	0.87	1	ME
S7_vf_39724	0.02	0	0.01	4.09	0.05	40.87	1.14	18.14	0.71	153.34	14.82	0.48	6.89	5.36	11.14	3.3	0.69	6.13	4.25	1.4	Shared
S7_vf_49007	0	0	0	0.46	0.01	0.29	0.05	0.14	0.1	23.56	9.8	0.04	0.54	3.54	0.41	0.22	0.25	0.23	0.3	0.51	ME
S7_vf_49485	0.01	0	0	8.96	0.04	0.51	0.1	0.3	0.16	39.56	0.77	0.11	0.81	0.32	0.5	4.1	0.45	0.4	5.41	11.8	ME
S7_vf_60968	0	0	0	0.63	0.09	0.56	0.15	0.8	1.15	11.49	0.53	0.24	0.68	1.11	0.45	0.41	1.46	0.38	0.46	5.93	ME
S7_vf_63894	0	0	0	0.41	0.01	0.28	0.05	0.33	0.15	13.38	0.48	0.04	0.4	2.81	0.44	0.22	1.22	0.1	0.53	0.44	ME
S7_vf_67910	0.01	0	0.01	0.78	0.03	0.44	0.03	2.13	0.13	20.88	2.65	0.05	0.24	5.03	0.28	0.3	0.14	0.22	0.25	0.55	ME
S7_vf_68239	0	0	0	0.38	0.04	0.65	3.01	0.42	0.15	50.21	1.13	0.28	0.92	0.35	0.84	0.47	38.07	0.24	0.82	26.24	ME
S7_vf_68546	0	0	0	0.75	0.04	3.55	0.22	0.23	0.21	19.5	1.28	0.12	0.55	0.78	0.73	0.34	0.28	0.42	0.45	0.75	ME
S7_vf_69348	0	0	0	0.46	0	0.36	0.02	0.15	0.08	33.71	0.37	0.07	0.56	0.42	0.52	0.3	0.27	0.15	0.21	15.55	ME
S7_vf_71527	0	0	0	1.91	0	1.94	0.36	0.56	0.33	15.5	3.44	1.58	2.24	9.79	1.71	1.97	1.65	1.27	4.04	1.52	ME
S7_vf_72493	0	0	0	0.49	0.04	0.5	0.03	0.59	0.19	14.4	0.63	0.05	4.06	0.3	0.51	0.74	0.33	0.35	0.39	6.67	ME
S7_vf_76382	0	0	0	0.33	0.1	0.36	0.04	0.61	0.63	29.71	537.55	0.03	4.53	0.19	0.34	0.79	0.13	0.08	0.21	1.23	Shared
S7_vf_76463	0	0	0	1.2	0.05	0.46	64.69	0.34	0.1	75.82	28.24	0.07	8.94	0.3	0.45	0.33	0.34	0.6	0.81	0.4	Shared
S7_vf_83624	0	0	0	0.43	0.08	0.35	0.15	0.29	0.2	233.03	0.93	0.08	1.07	0.24	0.68	0.36	5.83	0.26	1.42	1.06	ME
S7_vf_84974	0	0	0	0.82	0.03	1.15	0.29	1.07	0.21	18.41	1.18	0.18	0.53	0.24	0.58	0.31	0.48	0.29	0.54	7.49	ME
S7_vf_89197	0	0	0.1	0.94	0.35	1054.06	0.25	137.03	10.87	794.43	12.79	1.5	0.72	0.29	1.54	0.53	2.2	0.4	0.33	15.86	Shared
S7_vf_89668	0	0	0	0.6	0.86	1.34	0.12	0.93	0.82	5858.05	2.19	0.09	0.2	0.07	6.98	0.76	0.13	0.08	0.13	0.35	ME
S7_vf_114476	0.01	0	0	0.72	0.03	0.57	0.03	0.2	0.17	37.11	0.31	0.05	0.47	0.09	0.5	0.34	0.24	0.16	0.3	0.67	ME
S7_vf_117697	0	0	0.03	41.24	8.67	578.02	5.44	446.26	20.72	74.77	4.51	0.83	20.63	17.49	1.42	14.85	16.15	0.79	9.79	17.97	Shared
S7_vf_134023	0	0	0	0.78	0.03	4.35	0.14	0.78	0.23	32.63	0.73	0.09	1.26	1.05	6.66	0.32	0.78	0.3	0.48	0.54	ME
S7_vf_135778	0	0	0	0.32	0	0.24	0.07	0.15	0.07	69.47	0.5	0.06	0.23	0.34	0.35	0.23	0.11	0.14	0.21	0.49	ME
S7_vf_138019	0	0	0	1.1	0.01	6.96	0.04	0.61	0.2	101.28	1.15	0.11	1	0.54	0.29	0.51	0.41	0.24	3	0.85	ME
S7_vf_138069	0	0	0	0.46	0.1	1.1	0.08	41.72	0.13	97.63	0.48	0.1	0.78	0.3	0.86	0.36	0.32	0.27	7.34	0.71	ME
S7_vf_138225	0	0	0	0.44	0.03	0.27	0.09	0.17	0.11	13.95	0.35	0.09	0.31	2.3	0.24	0.15	0.31	0.31	0.26	0.74	ME
S7_vs_557	0	0	0	1.02	0.23	1.17	0.13	0.63	0.66	1443.35	0.91	0.37	1.51	4.07	2.3	1.02	0.62	2.42	1.16	5.98	ME
S7_vs_2599	0	0	0	1.27	0.54	44.38	1.05	0.4	0.23	177.66	3.47	0.19	1.39	1.12	0.7	0.76	20.78	0.4	0.45	2.7	ME
S7_vs_4956	0	0	0	3.6	7.32	0.75	2.77	111.37	0.59	2589.19	2.8	0.25	2.03	3.81	52.99	6.46	5.03	0.21	0.87	0.42	Shared
S7_vs_5236	0.01	0	0	1.02	0.03	3.24	0.77	2.24	0.22	118.14	1.61	0.38	0.25	2.68	3.54	8.43	0.28	1.16	0.48	2.45	ME
S7_vs_27530	0	0	0	0.63	0.06	0.63	0.15	0.64	0.21	25.45	1.83	0.07	0.5	1.63	1.26	3.61	0.47	0.24	2.93	0.56	ME
S7_vs_36656	0	0	0	0.58	0.06	0.32	0.26	0.16	0.09	23.35	0.44	0.05	0.43	0.22	0.4	0.29	0.17	0.17	0.19	0.58	ME
S7_vs_42563	0.01	0	0.02	1.11	0.07	0.51	0.05	0.45	0.14	30.45	10.31	0.15	1.19	0.42	0.71	0.52	0.23	0.34	8.07	0.62	Shared
S7_vs_54322	0	0	0	1.21	0.16	0.53	0.15	0.33	0.21	24.73	6.66	36.15	2.55	0.26	0.74	0.36	0.36	0.99	1.07	1.84	ME
S7_vs_60775	0	0	0	1.19	0.07	2.46	0.02	0.18	0.12	37.18	0.36	0.09	0.4	0.56	0.79	0.37	0.32	0.33	0.36	0.76	ME
S7_vs_65719	0	0	0	0.73	0.02	1.08	0.04	13.67	14.78	63.57	0.26	0.45	0.32	11.66	0.37	0.17	30.26	0.26	4.4	1.88	Shared
S7_vs_94738	0	0	0	7.01	1.37	1.41	0.84	0.72	0.3	44.98	1.96	0.35	1.9	1.4	0.5	2.83	2.88	0.31	2.56	2.06	ME
S7_vs_95276	0	0	0	0.67	0.72	3.11	0.07	3.42	0.2	39.87	6.06	0.05	0.26	0.11	0.24	0.21	13.34	0.14	0.27	1.47	ME
S7_vs_103530	0	0	0	5.8	0.08	76.93	7.73	0.29	0.15	28.17	1.01	14.15	0.43	0.42	0.63	1.15	0.49	0.44	3.92	14.01	ME
S7_vs_129271	0	0	0	0.68	0.04	0.23	0.05	2.11	0.15	84.05	0.35	0.08	3.13	10.09	0.33	1.58	1.11	0.23	0.23	0.51	ME
S7_vs_134391	0	0	0	5.31	0.01	0.15	0	0.15	0.09	36.08	0.55	0.04	0.13	0.22	0.81	1.93	0.16	0.16	0.15	0.31	ME

S7_vs_134784	0	0	0	0.65	0.06	0.4	0.17	0.43	0.26	45.31	0.5	0.06	0.41	0.28	0.39	0.15	0.6	0.33	0.38	0.91	ME
S7_vs_135322	0.01	0	0	1.79	0.02	5.57	0.18	0.25	0.11	58.32	1.1	0.31	3.58	0.13	0.77	0.57	0.16	0.68	42.35	0.84	Shared
S7_vs_135679	0	0	0	1.49	0.02	0.68	0.19	0.43	0.2	36.7	1.5	0.52	1.07	0.13	0.73	0.25	0.32	0.64	0.66	0.94	ME
S7_vs_135828	0	0	0	0.59	0.19	0.84	0.52	0.43	0.4	59.62	0.72	0.17	1.64	127.2	0.76	0.4	0.73	0.52	0.49	1.27	ME
S7_vs_136164	0	0	0	0.87	0.02	0.25	0.05	0.44	0.1	18.85	0.57	0.1	0.22	0.52	0.43	0.24	0.43	0.25	0.3	0.56	ME
S7_vs_137611	0	0	0	0.74	0.07	1.39	0.21	0.35	0.21	73.63	0.65	0.12	1.03	0.43	0.9	0.45	0.51	0.47	0.56	1.06	ME
S7_vs_137915	0	0	0	0.98	0.09	0.75	0.14	0.35	0.22	444.06	11.57	0.29	0.92	0.28	1.16	0.51	0.46	0.45	0.68	1.01	Shared
S7_vs_137998	0	0	0	4.9	0.08	1.07	0.22	0.43	0.19	295.05	0.93	0.15	0.78	0.59	2.29	1	0.49	0.86	28.92	14.8	Shared
S7_vs_138096	0	0	0	0.59	0.08	4.32	0.07	0.25	0.21	315.06	0.49	0.1	0.35	0.28	1.25	0.42	0.24	0.45	0.25	0.54	ME
S7_vs_138109	0	0	0	0.98	0.05	0.97	0.04	0.9	0.2	38.54	0.62	0.11	0.91	0.47	1.61	0.44	1.11	0.45	0.47	1.14	ME
S7_vs_138116	0	0	0	0.41	0.04	0.54	0.02	0.22	0.15	37.91	0.33	0.1	0.75	0.67	0.59	0.29	0.65	0.37	0.36	1.87	ME
S7_vs_138140	0	0	0	0.9	0.02	3.67	0.02	0.42	0.51	51.84	0.64	0.15	0.38	2.72	0.57	0.3	0.29	0.24	0.38	0.86	ME
S7_vs_138173	0	0	0	0.46	0.02	12.01	31.4	0.38	1.19	63.17	7.22	0.08	6.23	0.25	0.31	0.23	0.41	71.73	13.86	16.61	Shared
S7_vs_944	0.01	0	0	0.73	0.02	0.34	0.05	0.17	0.12	12.64	0.87	0.08	0.68	0.2	0.3	0.25	0.31	0.44	0.36	5.48	ME
S7_vs_4114	0	0	0	2.19	0.53	3.07	0.33	0.79	0.1	42.51	34.28	0.75	0.33	3.32	1.5	1.26	3.12	1.1	1.83	1.05	Shared
S7_vs_6626	0.02	0	0	14.11	0.45	3.1	13.52	28.29	8.2	454.41	6.42	4.87	55.93	0.39	1.47	4.86	2.23	7.22	2.65	7.22	Shared
S7_vs_11081	0.01	0.01	0.01	14.35	0.09	12.69	61.95	0.82	0.32	337.04	2.03	0.2	101.57	7.92	2.96	5.66	0.46	97.69	10.35	10.13	Shared
S7_vs_13041	0	0	0	0.56	0.09	1.17	0.18	0.42	0.3	138.06	1.24	0.16	1.42	1.03	1.18	0.57	0.69	0.47	0.7	1.38	ME
S7_vs_13370	0.04	0	0.01	7	0.38	74.41	12.32	68.51	4.04	62.23	11.89	3.87	12.23	2.27	9.68	3.64	12.42	11.3	13.25	53.52	Shared
S7_vs_13799	0	0	0	1.28	0.02	0.44	0.18	2.87	0.21	15.4	0.49	0.3	0.71	0.8	0.58	0.27	1.25	0.35	0.56	7.16	ME
S7_vs_13903	0	0	0	1.32	1.02	2.33	0.12	1.18	0.83	5498.38	2.47	0.31	1.07	0.53	7.11	0.92	0.47	0.15	0.36	0.92	ME
S7_vs_14275	0	0	0	1.35	0.03	0.36	0.14	0.18	0.13	54.87	12.18	0.08	1.18	0.19	0.76	0.96	0.22	0.25	0.46	0.86	Shared
S7_vs_16344	0.02	0	0	11.9	2.77	16.93	2	49.57	4.32	53.76	30.99	10.46	8.39	52.66	4.84	5.94	33.08	1.37	13.28	6.42	Shared
S7_vs_20200	0	0	0	0.98	0.11	0.49	0.05	1	0.22	25.37	4.01	0.2	0.25	0.47	0.29	0.39	1.98	0.35	0.51	2.19	ME
S7_vs_21521	0	0	0	1.43	0.06	3.57	0.27	2.6	0.36	18.82	10.49	2.02	1.98	42.2	6.71	9.78	1.48	1.05	7.34	8.62	Shared
S7_vs_23770	0	0	0	1.73	2.98	4.28	1.31	118.16	4.75	59.79	17.36	0.62	0.59	0.76	1.76	0.51	185.93	0.35	7.34	1.18	Shared
S7_vs_26235	0	0	0	0.54	0.07	0.48	0.1	0.62	0.93	19.03	0.75	0.13	0.27	0.33	0.29	0.35	1.04	0.22	0.43	8.78	ME
S7_vs_26757	0	0	0	5.11	0.53	52.68	8.68	39.96	0.92	48.8	9.3	0.76	9.5	2.53	8.34	2.35	13.52	5.47	12.23	69.11	Shared
S7_vs_28150	0.01	0	0	1.23	0.03	1.72	0.13	1.81	0.24	14.57	0.91	0.79	0.46	0.82	1.54	0.4	0.97	0.33	0.78	1.2	ME
S7_vs_28733	0	0	0	0.69	0.26	0.71	0.05	0.85	0.19	218.26	0.75	30.39	0.52	0.32	0.73	0.27	18.68	0.28	3.55	3.35	ME
S7_vs_29595	0	0	0	0.75	0.11	0.36	0.08	0.28	0.22	603.3	0.53	0.08	0.35	0.28	0.94	0.32	0.16	0.24	0.23	0.57	ME
S7_vs_30640	0	0	0	0.45	0.04	1.01	0.16	0.31	0.19	39.51	0.52	0.31	0.82	0.3	0.91	0.36	0.32	0.32	5.38	23.84	ME
S7_vs_30665	0	0	0	0.64	0.01	0.14	0.12	0.16	0.08	8.01	0.29	0.04	0.11	0.2	0.39	0.12	0.14	0.11	0.15	0.43	ME
S7_vs_38113	0.01	0	0.01	1	0.11	1.2	0.03	0.54	0.17	317.08	0.8	4.09	0.2	0.3	0.53	0.61	0.34	0.31	0.59	0.75	ME
S7_vs_38834	0	0	0	1.77	0.04	1.36	0.03	2.03	0.97	21.45	3.51	0.73	2.62	19.63	0.65	1.26	0.85	1.49	2.64	5.44	ME
S7_vs_40259	0	0	0	0.5	0.01	0.31	0.06	0.26	0.12	17.16	0.51	0.09	0.4	0.38	0.39	0.39	0.42	0.3	0.34	0.7	ME
S7_vs_41098	0	0	0.01	1.65	0.04	11.83	0.07	0.51	0.17	30.73	0.44	14.09	1.13	0.61	7.96	1.41	0.86	2.82	58.68	8.5	Shared
S7_vs_41534	0	0	0	0.63	0.02	0.37	0.16	0.2	0.12	218.38	0.31	0.02	0.61	0.44	0.41	0.19	0.16	0.16	0.18	0.59	ME
S7_vs_43529	0	0	0	1.77	0.08	0.55	0.05	0.24	0.22	20.01	0.54	0.92	0.21	0.58	1.58	0.39	0.66	0.18	0.56	0.75	ME
S7_vs_47050	0	0	0.02	1.33	0.06	173.49	0.22	0.94	0.32	109.84	2.73	0.43	1.49	1.14	2.11	1.5	0.56	0.48	1.06	1.99	ME
S7_vs_48523	0	0	0	8.47	0.26	6.93	2.4	1.51	3.82	36.57	4.78	5.21	1.04	1.16	0.43	2.26	2.32	0.48	2.5	1.53	ME
S7_vs_48708	0	0	0	2.05	0.07	0.77	0.04	0.46	0.29	40.27	1.61	0.23	2.34	0.54	0.56	2.09	0.45	0.5	1.97	3.52	ME
S7_vs_48832	0	0	0	0.84	0.06	1.34	0.14	0.36	0.2	49.75	1.86	0.12	0.46	0.43	0.35	0.38	4.75	0.47	0.84	1.78	ME
S7_vs_49426	0	0	0	3.31	0.1	11.75	0.13	13.08	0.83	28.84	9.17	0.76	7.11	2.86	2.07	2.67	7.77	2.75	15.87	6.5	Shared
S7_vs_50194	0	0	0	0.48	0.05	0.37	0.03	17.91	32.42	92.51	1.13	0.08	0.29	0.14	0.71	0.22	0.22	0.23	0.36	25.03	Shared
S7_vs_51332	0	0	0	5.97	0.89	3.27	0.24	2.3	3.45	15.2	2.59	0.45	7.06	3.06	2.91	1.84	4.94	2.87	2.87	7.81	ME
S7_vs_55031	0	0	0	0.62	0.01	0.21	0.31	0.13	0.07	22.38	1.3	0.04	0.57	0.31	0.42	0.14	0.25	0.11	0.47	5.92	ME
S7_vs_55409	0.01	0	0	0.57	0.04	0.91	0.29	1.28	0.69	40.85	0.95	0.62	0.44	0.66	1.02	0.39	1.61	0.22	0.37	0.47	ME
S7_vs_55526	0.04	0	0	1.44	0.05	0.31	0.43	0.31	0.13	27.32	1.82	0.11	0.74	0.13	1.01	0.3	0.16	0.23	0.34	0.35	ME
S7_vs_56529	0	0	0	1.27	0.16	0.45	0.02	0.35	0.29	121.77	1033.08	0.1	0.32	0.51	0.72	0.37	1.39	0.26	7.72	1.6	Shared
S7_vs_59465	0.01	0	0	3.34	0.03	0.91	0.07	0.32	0.42	37.01	2.29	0.08	0.42	0.14	0.45	1.01	0.23	0.35	0.46	0.37	ME
S7_vs_69630	0.01	0	0	0.93	0.04	0.45	0.17	0.52	0.47	22.28	0.88	0.12	0.44	0.22	0.35	0.41	0.37	0.18	0.33	0.49	ME
S7_vs_70265	0	0	0	0.45	0.06	0.24	0.01	0.21	0.1	334.6	0.43	0.08	0.35	0.29	0.71	0.24	0.17	0.18	0.39	112.96	ME
S7_vs_73280	0.01	0	0	2.54	0.02	0.31	8.65	0.24	0.12	31.55	2.3	0.07	0.44	0.09	0.27	0.89	0.17	0.19	0.25	0.41	ME
S7_vs_78507	0.02	0.01	0	2.16	0.16	4.28	0.67	11.09	0.3	12.49	0.79	5.46	0.34	31.47	0.36	0.68	69.74	1.19	24.67	2.73	Shared
S7_vs_82140	0	0	0	0.36	0.04	0.32	0.08	0.3	0.12	28.56	0.41	0.06	6.65	0.23	0.45	1.06	0.29	2.77	0.36	0.72	ME
S7_vs_83100	0	0	0	1.28	0.04	0.7	1.59	0.83	0.62	28.19	8.42	0.94	0.24	0.64	0.26	0.24	9.75	0.16	1	1.51	ME
S7_vs_83474	0	0	0	0.74	0.43	0.35	0.11	0.29	0.05	11.18	0.89	0.09	0.62	1.19	0.48	0.27	0.53	0.19	1.91	0.25	ME
S7_vs_86377	0	0	0	1.15	0.12	3.17	0.08	0.71	0.17	142.16	0.98	0.71	0.48	17.88	1.16	0.58	0.54	0.49	0.78	1.2	ME
S7_vs_87279	0.01	0	0.01	3.41	0.1	2.39	0.27	0.23	0.13	24.12	1.26	0.12	1.55	1.46	0.97	0.97	0.21	0.27	3.22	10.68	ME
S7_vs_87439	0	0	0	4.74	0.09	11.56	0.78	0.66	0.47	157.71	1.19	1.14	0.76	6.45	0.91	1.29	18.44	0.18	2.99	0.5	ME
S7_vs_90915	0	0	0	0.37	0.02	0.29	0.07	0.44	0.19	22.01	0.43	0.09	0.42	0.28	0.36	0.4	0.72	0.23	0.54	0.59	ME
S7_vs_97737	0.02	0	0	3.99	0.13	27.8	3.77	22.65	2.18	50.57	7.2	2.21	6.14	1.55	5.05	2.76	7.16	6.59	7.9	32.16	ME
S7_vs_100640	0	0	0	1.08	0.01	0.37	0.12	0.2	0.17	11.7	0.49	0.09	0.71	0.51	0.31	0.24	0.86	0.26	0.78	0.97	ME
S7_vs_101751	0.01	0	0	1.36	0.11	0.73	0.15	418.93	1.76	23.3	1.29	0.13	1.26	5.74	1.04	1.3	84.77	0.34	0.41	1.56	ME
S7_vs_107876	0	0	0	0.53	103.56	0.53	0.07	3.6	5.84	149.85	0.5	0.18	0.51	167.8	0.5	0.5	0.5	0.49	51.7	0.74	Shared
S7_vs_111831	0.02	0	0	0.89	0.03	2.58	4.54	0.42	0.09	12.88	0.52	0.22	0.29	0.58	0.49	0.34	0.46	0.73	3.29	8.57	ME
S7_vs_118295	0.01	0	0	0.97	0.11	0.74	0.15	0.8	0.3	10.56	2.72	0.54	0.24	2.25	1.71	0.49	5.98	0.24	1.13	5.57	ME
S7_vs_119492	0	0	0	0.32	0.09	0.43	0.01	29.4	140.96	93.58	0.43	0.09	0.53	9.38	0.5	0.21	0.29	0.21	0.23	0.59	Shared
S7_vs_119679	0	0	0.01	7.73	0.07	12.88	0.47	38.84	9.12	16.41	11.2	1.05	1.09	4.47	4.9	1.46	5.28	15.27	7.11	5.1	Shared
S7_vs_122151	0.01	0	0	1.45	0.02	2.38	0.04	0.27	0.1	16.17	0.3	0.04	0.42	0.2	0.41	0.81	1.09	0.29	2.45	0.41	ME
S7_vs_129858	0	0	0	0.7	0.01	0.23	0.02	0.24	0.06	11.73	0.54	0.06	0.29	1.71	0.35	0.27	0.57	0.16	0.26	0.44	ME
S7_vs_134583	0.01	0	0	1.07	0.04	3.17	2.77	4.96	11.66	54.78	0.95	0.18	0.45	10.38	42.93	0.42	0.22	6.18	0.4	10.41	Shared
S7_vs_72737	0	0	0	7.61	1.61	12.69	1.45	33.33	3.08	59.7	3.41	1.75	5.22	24.19	3.72	4.95	24.47	0.5	9.23	2.06	ME
S16_vf_1037	0	0	0	3.8	0.17	2.59	0.49	0.25	0.16	0.21	0.65	0.36	0.83	0.5	3.71	1.67	0.63	0.46	1251.41	1.05	SHHC
S16_vf_5270	0	0	0	0.61	0.03	0.42	0.09	0.25	0.12	0.13	0.5	0.07	0.47	0.52	0.64	0.43	0.8	0.34	37.62	0.74	SHHC
S16_vf_5809	0	0	0	0.94	0.13	2.11	0.05	0.22	0.14	0.16	0.45	0.1	0.42	0.38	14.88	0.25	0.28	0.24	89.43	0.68	SHHC
S16_vf_6102	0.01	0	0	59.82	0.05	0.36	0.07	0.47	0.21	0.36	19.05	0.15	42.7	0.21	0.63	18.86	0.24	0.43	130.21	0.72	Shared
S16_vf_9270	0	0	0	0.44	0.02	0.51	0.04	0.18	0.13	0.09	0.58	1.87	1.36	1.04	1.28	32.52	1.15	1.01	8658.84	2.6	Shared
S16_vf_12551	0	0	0	5.28	0.02	0.28	0.09	0.16	0.07	0.13	1.31	0.07	0.36	0.22	0.38	0.74	0.23	1.93	183.79	0.34	SHHC
S16_vf_16182	0	0	0	36.02	0.01	0.19	0.25	0.12	0.05	0.06	0.17	0.22	0.14	0.17	0.15	4.97	0.14	0.21	16.63	33.55	Shared
S16_vf_32359	0	0	0	1.53	0.03	0.37	0.08	0.21	0.14	0.25	22.02	0.2	0.3	0.79	0.32	0.59	0.47	0.2	158.96	0.45	SHHC
S16_vf_33218	0.14	0	0	0.8	3.18	83.19	145.5	8.86	2.27	3.4	16.34	7.17	1.36	0.98	1.7	0.47	4310.88	0.82	34.86	2015.52	Shared
S16_vf_36146	0	0	0	5.8	0.1	3.57	1.52	10.21	0.13	0.85	2.46	0.2	1.33	5.19	3.53	1.73	4.05	1.09	11.95	3.88	Shared
S16_vf_52513	0	0	0	2.95	0.05	2.46	0.13	10.85	0.4	1.6	134.66	1.22	1.1	0.69	2.42	3.6	4.53	1.4	10.78	1.56	Shared
S16_vf_84462	0.06	0	0	92.09	0.05	2.86	0.11	0.46	0.46	0.24	2.9	0.15	0.51	0.23	0.65	43.42	0.39	0.51	76.28	163.35	Shared
S16_vf_87190	0	0	0	0.75	0.04	0.72	0.41	0.29	0.23	0.19	0.93	0.14	0.95	0.65	0.85	0.65	1.95	0.52	62.96	1.28	SHHC
S16_vf_90328	0	0	0	1.11	0.71	1.61	0.36	0.94	0.63	0.47	2.27	0.43	2.48	1.05	2.29	2.32	1.06	1.07	43.58	1.9	SHHC
S16_vf_94866	0.03	0	0.1	0.77	0.02	857.11	0.2	0.19	0.16	0.15	0.82	2.18	0.66	0.75	0.9	3.46	0.81	0.55	4513.82	1.38	Shared
S16_vf_96483	0	0	0	13.8	0.03	1.56	0.45	1.36	0.15	0.15	1	0.14	1.06	0.41	0.36	5.55	0.35	0.47	19.58	42.19	Shared
S16_vf_97279	0	0	0	0.61	0.02	0.32	0.08	0.27	0.12	10.31	0.68	0.08	0.43	0.76	69.85	6.08	0.32	0.33	68.64	3.56	Shared
S16_vf_100235	0	0	0	0.48	0.04	0.25	0.08	0.15	0.09	0.1	0.24	0.56	0.58	0.87	0.42	1.89	0.49	0.38	2513.66	0.88	SHHC
S16_vf_100378	0	0	0	0.31	0.02	0.14	0.75	0.16	0.08	0.06	0.31	0.05	0.21	0.03	0.12	0.1	0.07	0.13	27.27	0.21	SHHC
S16_vf_100473	0.01	0.02	0	113.58	2.74	1.29	0.02	0.07	0.08	3.7	0.33	0.33	0.19	6.12	0.16	1.12	0.1	0.21	64.53	6.98	Shared
S16_vf_108554	0	0	0	10.64	1.27	0.45	0.22	0.67	0.18	0.19	0.39	0.11	0.41	0.32	0.37	4.77	0.5	0.32	115.83	0.67	Shared
S16_vf_119834	0	0	0	0.32	0.03	0.2	0.07	0.12	0.06	0.09	0.51	0.04	0.77	0.31	0.33	0.26	0.31	0.13	26.39	0.45	SHHC
S16_vf_147326	0	0	0	0.27	0.02	0.41	0.11	0.19	0.09	0.17	0.3	1.63	1.35	0.93	1.02	4.84	1.03	0.94	5924.87	1.83	SHHC
S16_vf_151450	1.04	0.09	1.99	139.3	2.7	22255.25	0.08	2.97	3.07	4.99	564.94	36.02	4.85	4.18	2349.41	727.54	6.22	1469	30278.91	10.22	Shared
S16_vf_164913	0	0	0	0.68	0.12	1.02	0.15	426.45	644.63	0.9	80.92	0.11	1.45	1.46	0.66	0.75	0.29	0.46	28.65	1.04	Shared
S16_vf_169895	0	0	0	0.38	45.34	0.71	0.02	0.34	0.11	31.75	1.38	0.3	0.86	0.11	0.84	0.85	0.54	0.42	420.9	2.44	Shared
S16_vf_169985	0.01	0	0	0.31	0.03	19.6	0.11	1.92	0.11	0.12	0.4	1.79	2518.18	0.86	36.24	27.3	0.69	0.61	1774.9	1.56	Shared
S16_vf_172897	0.01	0	0	0.42	0.02	0.21	0.06	0.21	0.08	0.12	0.43	2.03	1.19	1.15	1.29	7.31	1.04	0.9	9540.2	2.25	SHHC
S16_vf_173095	0	0	0	0.37	0.05	0.55	0.12	0.4	0.31	0.14	7.74	0.37	1.45	0.52	0.9	1.36	0.62	1.35	998.42	1.24	SHHC
S16_vf_173099	0	0	0	0.67	0.04	0.46	0.36	0.28	0.13	1.01	0.71	0.14	0.46	0.24	0.28	1.73	1.64	0.22	87.37	0.65	SHHC
S16_vf_173109	0	0	0	0.45	0.02	0.22	0.07	0.1	0.05	0.08	0.28	0.07	0.33	0.08	0.28	0.18	0.24	0.17	80.69	0.5	SHHC
S16_vf_173133	0.02	0	0	1.21	0.01	0.27	0.2	0.2	0.07	0.15	0.64	0.06	0.3	0.36	0.21	0.12	0.32	0.12	22.88	0.44	SHHC
S16_vs_15093	0.01	0	0	0.99	0.02	0.29	0.04	0.2	0.12	0.2	0.65	0.68	0.65	0.49	0.74	2.69	0.53	0.43	2969.8	1.22	SHHC
S16_vs_20060	0	0	0	19.77	0.03	0.28	0.04	0.31	0.13	0.17	36.9	0.1	3.65	0.22	0.97	20.59	0.28	0.69	18.2	41	Shared
S16_vs_59697	0	0	0	0.64	0.02	0.46	0.11	0.22	0.17	0.13	0.61	0.11	0.44	0.55	0.37	0.47	0.28	0.3	123.62	1.24	SHHC
S16_vs_75108	0.01	0	0	0.84	0.03	0.43	0.26	1.39	2.62	11.35	3.97	5.69	0.46	0.82	0.67	10.28	0.25	0.22	378.71	0.45	Shared
S16_vs_76823	0	0	0	3.79	0.02	0.31	0.11	0.27	0.13	0.21	30.51	0.1	0.28	0.15	0.17	0.39	0.2	0.21	58.88	0.71	SHHC
S16_vs_78361	0.01	0	0	14.56	0.01	5.66	0.01	0.12	0.05	0.06	1.45	0.06	0.29	0.12	0.24	0.83	0.15	0.17	130.38	2.15	Shared
S16_vs_78429	0	0	0	5.11	0.05	0.4	0.02	0.22	0.13	0.2	113.43	0.06	0.89	0.32	1.07	0.66	0.42	0.33	135.18	0.54	SHHC
S16_vs_127905	0	0	0	19.59	0.16	4.42	0.4	0.61	0.54	0.45	1.03	0.37	2.45	3.03	1.61	17.88	2.17	6.09	53.99	161.61	Shared
S16_vs_172749	0.01	0	0	8.32	0.03	0.19	0.08	0.25	0.09	0.19	0.35	0.07	7.17	0.1	0.28	1.73	0.12	1.18	30.75	0.29	SHHC

S16_vs_173079	0	0	0	9.12	0.04	0.33	0.09	0.25	0.15	0.17	0.54	0.1	2.33	5.88	2.32	2.99	5.33	6.3	33.63	0.78	SHHC
S16_vs_173096	0	0	0.01	14.74	10.39	0.65	0.01	0.26	0.12	0.87	0.65	0.59	0.46	0.73	0.56	3.32	0.51	0.57	2860.57	1.36	Shared
S16_vs_173102	0.2	0	0.18	236.24	0.63	2620.06	162.99	536.59	855.2	6.29	1.73	3.74	1.24	2.2	0.54	37.1	0.33	0.69	76.47	181.93	Shared
S16_vs_173141	0	0	0	0.52	0	0.28	0.06	0.13	0.16	0.04	0.4	0.06	0.11	38.91	0.48	0.14	0.07	0.11	79.75	1	Shared
S16_vs_4662	0	0	0	0.87	0.08	0.49	0.04	0.3	0.15	0.24	0.48	0.13	0.49	0.16	0.32	0.62	0.32	0.16	61.64	0.6	SHHC
S16_vs_10622	0	0	0	1.25	0.02	0.48	0.19	7.09	0.11	1.06	0.4	0.08	0.85	0.28	0.45	0.42	1.29	0.29	28.1	0.64	SHHC
S16_vs_11649	0	0	0	0.26	0.04	0.43	0.11	0.58	0.92	0.13	10.71	0.13	0.54	0.34	0.34	1.32	0.34	0.3	360.76	0.79	SHHC
S16_vs_17493	0	0	0	0.4	0.01	0.37	0.03	30.36	0.21	17.06	11.3	0.11	1.81	4.8	0.3	0.28	2.31	0.2	15.98	4.25	Shared
S16_vs_18293	0	0	0	0.77	0.05	0.39	0.05	0.42	0.09	0.43	0.76	0.11	0.5	0.29	0.79	0.71	0.41	0.26	11.19	0.62	SHHC
S16_vs_18440	0	0	0	1.83	0.07	5.26	1.8	7.29	0.7	0.16	1.28	0.19	0.66	0.2	0.7	0.62	1.49	0.23	57.52	4.04	SHHC
S16_vs_19320	0	0	0.01	0.51	0.01	0.2	0.01	0.26	0.09	0.06	0.47	0.06	0.14	0.26	0.24	4.91	0.17	0.78	16.54	0.37	SHHC
S16_vs_19968	0.01	0.01	0	27.63	10.53	8.85	0.25	12.99	6.35	7.42	43.66	8.45	8.12	2.59	23.32	8.42	1.89	13.36	87.81	13.22	Shared
S16_vs_26078	0	0	0	12.83	0.02	1	0.09	1.22	0.17	1.17	0.97	0.97	0.27	9.83	0.33	2.93	5.24	0.63	334.28	23.25	Shared
S16_vs_32764	0.01	0.01	0	13.34	6.92	16.55	14.25	23.64	14.34	14.23	92.69	14.43	47.44	9.3	45	9.47	41.16	36.16	91.02	13.18	Shared
S16_vs_33281	0.02	0.01	0.04	3.15	0.85	26.38	0.16	3.76	3.8	2.33	21.9	541.44	12.57	1.67	12.22	4.69	0.54	8.82	25.85	20.86	Shared
S16_vs_34021	0.03	0	0	3.92	0.57	2.18	0.12	0.44	0.24	7.13	11.57	3.17	8.7	1.89	1.23	3.99	129.23	3.08	38.41	2.31	Shared
S16_vs_34751	0	0	0	0.53	0.05	0.43	4.17	41.66	39.68	15.11	0.48	0.19	0.21	1.72	0.69	0.4	12.32	0.52	44.94	3.6	Shared
S16_vs_35031	0	0	0	1.75	0.51	1.69	0.12	0.55	0.38	1.12	3.54	0.72	0.53	1.67	0.63	0.54	2.33	0.29	18.71	0.94	SHHC
S16_vs_38863	0	0.01	0	0.46	0.07	5.81	0.13	0.35	0.18	4.11	0.68	0.14	9.29	0.25	1.19	0.78	0.45	9.53	188.36	0.97	SHHC
S16_vs_48714	0	0	0	1.89	0.04	3.26	0.22	0.32	0.24	3.59	6.14	2.52	3.38	5.16	1.76	2	5.59	3.01	29.85	1.9	SHHC
S16_vs_51901	0	0	0	0.72	0.01	0.21	0.07	0.36	0.52	0.09	0.5	0.35	0.37	0.25	0.4	1.08	0.23	0.3	1465.95	0.88	SHHC
S16_vs_54250	0.01	0	0	0.58	0.24	6.8	0.21	1.28	3.43	0.4	3.07	0.31	2.92	1.69	3.15	1.68	1.49	1.37	37.6	2.59	SHHC
S16_vs_54616	0.02	0	0	0.42	0.02	0.27	0.07	0.11	0.06	0.6	1.33	0.06	0.2	0.25	1.95	0.08	0.15	0.17	21.4	0.35	SHHC
S16_vs_64584	0	0	0	1.67	0.02	0.38	1.28	0.3	0.24	0.29	1.25	0.13	3.21	0.25	1.21	1.38	17.93	4.46	25.63	0.51	Shared
S16_vs_80244	0	0.01	0.01	15.36	0.16	26.72	0.43	0.67	0.38	0.59	1.31	0.5	21.69	0.65	1.06	5.31	2.06	1.28	21.57	52.13	Shared
S16_vs_81805	0.01	0	0.01	1.94	0.04	1.27	0.06	1.24	0.21	1.03	0.43	1.54	0.31	0.51	0.4	0.3	6.6	0.2	24.6	0.58	SHHC
S16_vs_82116	0	0	0	7.67	2.95	25.77	1.92	96.92	11.51	162.89	8.64	3.86	12.83	20.5	2.68	3.89	35.92	0.61	42.15	1.92	Shared
S16_vs_86116	0	0	0	1.03	0.02	0.4	0.03	0.24	0.17	2.35	0.25	0.1	0.63	26.92	1.05	0.36	2.5	0.47	35.1	0.95	Shared
S16_vs_89511	0	0	0	7.23	0.17	4.18	0.08	17.71	8.07	6.65	4.38	2.06	7.68	4.26	5.28	5.3	4.01	8.43	20.45	10.7	Shared
S16_vs_92150	0.01	0	0	0.68	0.06	1.1	0.1	0.66	0.09	0.51	0.49	0.06	0.54	0.29	0.21	0.27	2.32	0.23	19.98	0.37	SHHC
S16_vs_101072	0	0	0	1.97	0.05	7.72	0.08	0.33	0.18	0.2	0.3	2.03	0.69	0.14	0.26	0.54	0.3	0.31	12.86	1.06	SHHC
S16_vs_101671	0.02	0	0	1.85	0	19.38	0	0.23	0.08	0.04	0.14	0.02	0.1	0.02	0.14	0.3	0.11	0.13	20.83	0.52	Shared
S16_vs_103726	0	0	0	0.72	0.06	2.29	0.18	2.76	0.44	1.36	2.15	0.81	0.68	0.4	0.38	0.67	0.98	0.62	18.65	1.44	SHHC
S16_vs_107235	0.02	0	0	3.21	0.1	4.5	0.1	0.84	0.52	4.93	8.09	2.7	4.85	6.45	1.32	2.18	6.73	3.11	35.07	2.81	SHHC
S16_vs_116956	0	0	0	2.04	0.28	7.25	0.11	0.37	1.4	0.46	1.02	0.22	0.91	0.46	0.95	0.96	0.38	0.7	17.12	1.83	SHHC
S16_vs_118029	0	0	0	0.6	0.08	1.34	0.14	0.32	0.25	2.55	0.56	0.74	0.77	0.32	0.63	0.51	0.47	0.42	192.8	2.49	SHHC
S16_vs_120149	0	0	0	0.55	0.02	0.56	0.13	0.23	0.16	0.18	0.41	0.07	1.19	0.48	0.69	0.49	0.27	0.34	12.58	0.84	SHHC
S16_vs_120605	0	0	0	0.1	0	0.13	0.55	0.05	0.01	0.04	0.23	0.14	0.21	0.01	0.08	0.25	0.11	0	7.07	0.09	SHHC
S16_vs_120622	0	0	0	0.49	0.01	0.22	0.05	4.36	1.03	0.21	0.32	0.05	0.22	0.14	3.08	0.12	3.63	0.2	68.32	0.64	SHHC
S16_vs_124703	0	0	0	23.37	0.07	0.55	0.02	0.29	0.13	0.81	7.74	0.17	0.49	0.26	0.25	5.06	0.14	0.23	304.41	0.5	Shared
S16_vs_127669	1.09	0.23	1.49	28.34	2.61	18273.7	9.93	5.95	3.88	1575.51	197.36	26.14	200.3	28.03	56.79	880.54	26.06	3867.01	81.62	18.03	Shared
S16_vs_132979	0	0	0.01	1.83	0.11	1.38	2.35	4.98	7.52	1.83	3.25	1.07	1.34	0.12	2.92	0.56	4.12	0.7	11.13	17.85	Shared
S16_vs_136935	0	0	0	15.93	0.2	20.73	6.56	1.92	0.33	4.63	1.58	0.61	7.62	1.94	0.97	4.87	215.52	5.37	35.26	80.05	Shared
S16_vs_137222	0	0	0	0.56	0.04	0.46	0.08	0.24	0.11	0.13	0.45	0.09	0.62	0.26	0.3	0.24	0.32	0.33	41.8	0.98	SHHC
S16_vs_140468	0	0	0	4.71	0.57	18.17	0.07	0.58	4.5	2.11	2.31	0.76	3.62	3.46	2.58	3.12	0.34	2.63	18.01	4.85	Shared
S16_vs_140709	0	0.01	0	55.25	0.16	0.42	0.08	0.25	0.12	0.19	0.88	0.21	0.52	0.64	13.96	7.98	0.34	1	26.85	60.25	Shared
S16_vs_141218	0	0	0	1.79	0.06	2.51	0.29	0.78	0.36	0.45	24.79	1.34	3.84	12.17	3.87	17.15	2.06	2.01	4252.9	3.13	Shared
S16_vs_142561	0.01	0	0.01	1.31	0.22	3.69	126.85	21.76	3.35	1.55	12.44	1.26	1.54	5.57	7.78	0.69	3.14	1.82	26.66	1.82	Shared
S16_vs_165662	0	0	0	1.06	0.55	6.36	0.65	0.6	0.42	0.47	12.41	0.19	11.57	0.69	10.84	3.74	0.87	0.64	158.61	2.22	SHHC
S10_vf_3644	0	0	0.01	0.28	0.02	34.85	0.09	0.44	0.11	0.14	8.29	0.4	378.39	0.21	2.11	30.97	0.24	0.72	0.35	0.65	Shared
S10_vf_18671	0.01	0	0	14.68	12.56	19.6	38.48	17.87	28.51	154.07	1.36	0.26	116.97	16.27	0.48	41.21	0.27	0.21	46.26	0.55	Shared
S10_vf_24791	0.01	0	0	0.53	0.02	0.25	0.1	0.21	0.07	0.08	0.57	0.12	44.39	0.24	0.28	3.4	0.21	0.22	0.23	0.52	SHHC
S10_vf_25241	0	0	0	0.4	0	15.95	0.06	0.04	0.06	0.06	3.92	0.18	65.24	1.38	0.37	8.1	0.13	0.12	0.13	0.62	Shared
S10_vf_36880	0	0.02	1.61	12.2	9.72	35.39	1147.09	10.3	20	45780.42	12600.25	27872.57	1470.28	15403.57	67.99	888.66	6.72	314.96	6.89	28.16	Shared
S10_vf_43964	0	0.02	0	0.85	0.07	4.6	0.98	0.45	0.15	0.18	0.65	0.31	111.8	4.12	0.81	1.66	4.62	237.72	0.91	4.07	SHHC
S10_vf_46328	0	0	0	0.98	0.05	2.69	0.64	0.34	0.1	0.2	0.3	0.24	27.69	0.3	0.37	3.73	0.33	0.49	0.32	0.5	SHHC
S10_vf_47170	0	0	0	2	0.02	12.78	0.04	0.52	0.35	0.55	6.64	0.11	57.87	20.74	0.42	7.96	0.19	0.32	0.45	1.21	Shared
S10_vf_57705	0	0	0	0.49	0.02	0.47	0.04	0.43	0.12	0.11	0.85	0.41	392.42	0.35	1.92	2.9	0.24	4.82	0.41	0.73	SHHC
S10_vf_62230	0	0	0	1.09	0.02	1.9	0.26	0.5	0.19	0.32	1.49	0.18	26.02	0.37	1.45	3.12	0.62	0.47	0.97	1.22	SHHC
S10_vf_64371	0	0	0.35	3.03	0.62	4.13	0.09	5.45	0.1	0.12	0.35	4999.3	1898.08	1.07	1.36	37.99	1.02	0.69	1.51	2.25	Shared
S10_vf_80712	0	0	0	4.57	0.04	18.35	0.1	4.98	0.35	0.3	36.29	0.37	105.84	192.77	1.36	19.18	3.18	0.65	0.77	1.41	Shared
S10_vf_113489	0	0	0	0.51	0.03	0.29	0.07	0.23	0.1	0.15	0.29	0.09	20.76	0.17	0.16	3.46	0.15	0.17	0.15	0.52	SHHC
S10_vf_120738	0	0.01	0.01	22.11	0.07	0.61	0.12	10.41	16.67	0.26	207.35	128.76	26.3	109.75	31.33	16.46	0.35	55.15	17.46	0.87	Shared
S10_vs_43473	0	0	0	1.07	0.02	2.49	0.31	0.49	1.4	0.11	0.55	0.08	98.59	0.24	1.09	0.38	0.69	0.16	0.27	1.3	SHHC
S10_vs_120489	0	0	0	4.64	0.02	0.8	0.14	0.44	0.2	0.3	0.86	0.26	33.21	0.62	0.61	4.41	0.3	0.37	0.99	0.71	SHHC
S10_vs_120541	0	0	0	3.37	0.01	0.47	0.04	0.26	0.1	0.19	7.09	0.06	91.24	0.28	0.7	0.78	0.23	0.27	2.19	0.59	SHHC
S10_vs_120570	0	0	0	1.52	0.2	0.32	0.06	0.2	0.07	0.66	9.32	0.16	41.86	0.27	0.3	0.66	22.38	1.43	2.98	0.87	Shared
S10_vs_2538	0	0	0	0.56	0.03	0.39	0.11	0.44	0.22	0.25	0.68	0.55	227.09	0.16	0.51	28.74	0.27	0.38	1.27	12.95	Shared
S10_vs_14557	0.02	0.01	0.01	13.28	13.2	64.01	0.51	19.14	8.89	10.13	19.76	1	162.3	9.83	14.44	25.83	58.94	50.57	44.96	56.62	Shared
S10_vs_18901	0	0	0	0.9	0.04	0.39	0.03	0.26	0.15	0.16	0.42	0.08	15.97	0.63	0.48	2.33	0.21	0.2	0.51	0.63	SHHC
S10_vs_20400	0.01	0	0	1.83	0	0.14	0.06	0.18	0.08	0.14	25.34	0.05	16.88	0.09	0.32	1.01	0.27	0.12	0.32	0.55	SHHC
S10_vs_26365	0	0	0.01	2.41	0.02	105.56	0.06	0.42	0.37	0.12	50.64	0.39	37.61	0.71	1.28	1.16	0.25	4.39	454.7	0.87	Shared
S10_vs_35854	0	0	0	0.45	0.04	0.71	0.23	0.79	0.14	0.11	0.37	0.37	669.68	3.36	0.49	7.15	2.91	3.79	0.67	11.18	Shared
S10_vs_36407	0	0	0	0.97	0.03	0.99	0.15	1.18	0.09	5.78	6.2	1.03	16.51	1.14	0.73	4.32	1.02	0.32	0.71	1.12	SHHC
S10_vs_36598	0.01	0	0	3.09	0.45	12.79	15.92	0.24	0.15	3.46	2.51	1.52	76.32	1.45	0.45	2.72	13.7	0.39	4.37	21.58	Shared
S10_vs_37379	0	0	0.01	8.61	0.08	84.82	0.08	3.25	0.37	45.21	32.23	0.24	86.49	40.31	0.26	12.39	11.49	0.13	0.21	0.37	Shared
S10_vs_37694	0.01	0.01	0	19.71	8.87	1.13	0.15	1.98	1.13	7.01	92.72	0.09	17.9	12.06	60.16	4.14	0.34	18.05	32.97	1.9	Shared
S10_vs_46819	0.01	0	0.02	1.22	0.25	3.28	0.78	1.49	0.33	4.06	2.34	0.4	37.08	0.25	1.42	5.78	3.53	1.14	1.23	3.24	SHHC
S10_vs_58014	0.01	0.01	0.01	47.32	2.83	26.93	3.46	29.56	30.43	25.11	19	4.85	335.82	4.13	5.16	28.65	63.69	52.31	30.52	26.87	Shared
S10_vs_62776	0	0	0	1.66	0.43	0.73	2.83	0.32	0.18	0.22	0.92	0.16	33.45	1.54	0.71	2.2	29.78	1.21	2.95	3.22	Shared
S10_vs_67471	0	0	0	0.51	0.01	8.55	1.99	0.18	0.09	3.5	5.03	0.1	24.35	0.15	0.23	0.39	0.24	3.98	2.57	3.71	SHHC
S10_vs_71900	0.01	0.01	0	12.17	0.52	2.06	4.38	0.69	0.21	3.37	1.62	2.3	57.91	16.14	0.62	5.27	333.16	5.41	17.06	17.96	Shared
S10_vs_77004	0	0	0	10.41	0.04	0.52	0.16	0.37	0.17	0.14	0.38	0.39	39.17	0.38	0.61	7.15	0.6	0.87	0.5	1.07	Shared
S10_vs_106834	0	0	0	0.42	0.04	0.97	0.09	0.93	0.62	0.68	1.43	0.11	27.05	0.75	1.87	0.95	0.63	2.18	0.79	1.45	SHHC
S10_vs_119298	0	0	0	0.24	0.05	0.35	0.11	1	2.62	0.12	19.05	0.06	18.85	0.23	0.35	1.62	0.41	0.27	3.28	0.9	SHHC
S12_vf_55175	0.01	0	0.02	3.77	0.24	98.23	1.14	0.38	0.15	0.62	0.66	0.33	0.38	4.31	263.29	0.65	0.74	0.29	0.72	1.45	Shared
S12_vf_8173	0	0	0	0.37	0.01	0.45	0.13	0.18	0.14	0.5	0.74	0.18	2.3	0.28	414.49	0.32	0.2	1.24	0.45	0.67	SHHC
S12_vf_173666	0.01	0	0	0.51	0.02	0.22	0.01	0.25	0.12	0.79	0.78	0.07	0.48	12.16	54.65	0.24	0.14	0.24	17.88	0.51	Shared
S12_vf_16374	0	0	0	0.58	0.08	1.19	555.55	0.94	0.43	7.65	3.06	0.17	20.86	40.96	38.18	3.89	2.78	30.2	1.89	12.56	Shared
S12_vf_79666	0	0	0.14	4.75	0.22	1922.78	4.14	0.4	0.34	8.02	0.75	2.72	6	0.69	150.36	0.86	0.21	6.53	20.42	0.66	Shared
S12_vf_48278	0	0	0	0.49	0.02	0.19	0.05	0.1	0.09	0.23	0.23	0.09	0.18	0.16	191.06	0.08	0.17	0.2	0.17	0.27	SHHC
S12_vf_32615	0.08	0.05	0.1	503.98	779.27	1001.4	0.14	0.78	0.57	1.63	59.23	1.67	1.13	0.43	1107.68	1673.58	1.45	50.08	2.78	1.13	Shared
S12_vf_174374	0	0	0	4.63	0.38	79.52	0.74	17.22	0.97	1.22	25.38	0.23	1.15	1.09	411.19	0.8	1.07	0.21	0.75	1.69	Shared
S12_vf_105542	0	0	0	0.54	0.06	0.91	0.23	0.34	0.14	0.43	1.75	0.21	4.44	0.42	208.32	1.39	0.56	0.49	0.96	1.74	SHHC
S12_vf_18902	0.02	0	0	0.69	0.04	15.31	0.42	0.2	0.09	3.72	1.39	0.97	0.81	4.45	4481.17	0.21	0.91	10.7	20.01	1.67	Shared
S12_vf_128794	0	0	0	2.47	0.11	1.84	0.08	0.18	0.12	1.62	0.28	0.34	0.48	1.93	1423.81	0.53	1.49	1.96	0.36	0.76	SHHC
S12_vf_145044	0.03	0.02	0	92.91	5.5	1.77	0.22	0.63	0.32	3.54	1.04	1.02	2.32	0.44	288.09	434.36	0.58	0.63	1.4	2.75	Shared
S12_vf_10887	0	0	0	0.54	0.01	0.32	0.03	0.2	0.11	0.21	0.4	0.07	0.46	0.21	30.28	0.21	0.27	0.21	0.28	0.58	SHHC
S12_vf_176437	0	0	0	0.75	0.03	0.95	0.01	0.24	0.09	1.06	0.4	0.38	0.77	0.37	1383.39	0.8	0.43	0.3	0.56	1.06	SHHC
S12_vf_169796	0	0	0	7.19	0.03	0.52	0.1	0.76	1.21	0.31	4.14	1.02	1.16	601.61	62.04	50.95	0.44	0.74	8.45	0.98	Shared
S12_vf_67909	0	0	0	0.77	0.03	1.03	0.21	0.41	0.24	3.52	0.97	0.36	1.6	0.55	429.93	0.61	0.52	0.53	0.62	2.87	SHHC
S12_vf_60224	0	0	0	2.15	0.18	1.62	0.44	1.26	0.58	0.52	1.27	0.2	3.99	0.66	21.22	0.72	2.11	1.65	1.54	3.98	SHHC
S12_vf_170614	0	0	0	0.37	0.01	0.2	0.01	0.12	0.03	0.6	0.34	0.04	0.34	0.07	27.05	0.16	0.12	0.2	0.14	0.34	SHHC
S12_vf_61299	0	0	0	3.13	0.34	0.59	0.13	0.14	0.11	0.63	0.4	0.41	0.59	0.38	900.45	0.29	0.78	0.28	0.48	1.2	SHHC
S12_vf_69136	0	0	0	0.55	0.01	0.38	0.14	0.22	0.15	0.2	0.37	0.11	0.5	0.4	128.18	0.16	0.39	0.35	0.3	1.07	SHHC
S12_vf_187941	0	0	0	0.48	0.03	0.61	0.59	0.31	0.15	0.24	1.2	0.13	0.81	0.79	43.19	0.44	0.39	0.41	0.47	1.22	SHHC
S12_vf_188819	0	0	0	0.77	0.06	0.84	0.35	0.43	0.23	0.62	1.79	0.21	1.31	0.51	433.5	0.65	0.78	0.63	0.8	1.36	SHHC
S12_vf_188822	0	0	0	0.29	0.03	0.23	0.09	0.23	0.1	0.14	0.25	0.07	0.41	3.69	58.53	0.18	74.58	0.65	0.24	0.39	Shared
S12_vf_188824	0	0	0	0.46	0.03	9.37	0.09	0.58	0.11	2.07	0.43	0.91	605.81	0.61	2504.75	6.52	0.69	1.21	27.06	1.35	SHHC
S12_vf_188827	0	0	0	0.09	0	0	0	0.01	0.03	0.04	0.19	0.01	0.03	0.03	27.32	0.07	0.01	0	0.09	0.18	SHHC
S12_vf_188830	0	0	0	0.58	0.04	0.35	0.01	0.4	0.1	0.26	0.42	0.07	0.23	0.18	58.08	0.37	0.43	0.26	0.45	0.47	SHHC
S12_vf_188838	0	0	0	0.19	0.04	0.39	0.04	0.23	0.05	0.26	0.37	0.01	0.28	0.2	29.35	0.08	0.28	0.51	0.33	0.65	SHHC
S12_vf_188846	0	0	0	2.18	0.01	0.2	0.12	0.25	0.12	0.1	0.35	0.05	0.13	0.12	34.97	0.37	0.15	0.13	0.49	0.82	SHHC
S12_vf_173208	0	0	0	0.55	0.02	155.13	0.14	0.13	0.11	0.12	5.98	0.26	7.59	0.05	33.33	0.27	0.08	0.16	1.22	0.44	Shared
S12_vf_181024	0.01	0	0	0.8	0.02	0.77	0.09	0.22	0.12	0.23	0.72	0.1	4.1	0.21	79.82	0.42	0.35	0.32	1.88	3.44	SHHC

S12_vs_86928	0	0	0	0.81	0.01	0.76	0.1	6.55	0.12	0.31	0.97	0.14	0.75	0.31	93.35	0.22	0.88	0.24	0.36	0.59	SHHC
S12_vs_87088	0.02	0.01	0	3.28	0.12	2.14	8.69	24.26	0.45	2.63	5.29	5.75	2.33	4.96	30.71	1.23	2.22	0.52	4.11	6.64	Shared
S12_vs_79785	0	0	0	1.59	0.02	0.63	0.81	0.53	0.9	0.25	0.42	0.11	0.89	0.26	17.27	0.63	0.22	0.41	99.29	0.64	SHHC
S12_vs_167155	0	0	0	1	0.02	0.4	0.06	0.22	0.12	0.15	0.53	0.08	0.31	0.24	27.07	0.37	0.21	0.37	0.31	0.61	SHHC
S12_vs_1980	0	0	0.01	0.74	0.14	12.39	0.79	1.98	0.36	5.31	23.87	0.25	1.7	1.62	235.58	2.55	3.05	1.1	1.58	2.58	Shared
S12_vs_111815	0	0	0	0.69	0.05	0.68	0.2	0.4	0.16	6.08	0.76	1.52	1.6	3.98	7454.59	0.5	1.57	0.99	1.29	2.8	SHHC
S12_vs_183891	0	0	0	0.13	0.05	0.59	0.02	0.06	0.05	0.7	1.09	9.03	0.14	0.31	276.74	0.01	0.24	0.15	0.13	0.67	SHHC
S12_vs_137069	0	0	0	51.06	0.01	0.25	0.06	0.21	0.1	4.67	0.86	1.5	2.35	0.23	16.64	78.96	0.1	0.29	0.29	0.69	Shared
S12_vs_188820	0	0	0	0.61	0.03	0.6	0.1	0.56	0.23	0.36	2.23	0.15	2.82	0.75	105.91	0.58	1.1	0.34	0.52	0.89	SHHC
S12_vs_188821	0	0	0	1.29	0.07	1.7	0.15	0.36	0.18	0.99	0.74	0.28	0.88	0.45	1058.2	0.59	0.56	0.47	1.01	1.08	SHHC
S12_vs_188823	0	0	0	0.49	0.01	0.47	0.16	0.19	0.07	0.27	0.57	0.11	0.71	0.62	116.17	0.18	0.4	0.3	0.26	1.01	SHHC
S12_vs_188825	0	0	0	1.37	0.04	1.14	0.03	0.22	0.1	1.67	0.45	0.08	0.33	0.25	83.12	0.35	0.19	0.22	10.78	0.54	SHHC
S12_vs_188828	0	0	0	2.44	0.02	0.21	0.02	0.3	0.1	0.49	0.43	0.07	0.42	0.11	101.96	0.79	0.32	0.21	0.25	0.46	SHHC
S12_vs_188832	0	0	0	0.46	0.06	0.66	0.06	0.27	0.31	0.4	1.46	0.1	0.7	2.8	350.41	0.44	0.43	0.38	0.82	1.21	SHHC
S12_vs_188833	0	0	0	0.79	0.02	0.2	0.07	0.19	0.08	0.91	1	0.05	0.39	5.51	20.94	0.16	1.16	0.19	0.38	0.27	SHHC
S12_vs_118154	0	0	0	0.34	0.02	0.26	0.21	0.21	0.16	0.19	0.57	0.1	0.42	0.1	123.61	0.2	0.19	0.27	0.47	0.5	SHHC
S12_vs_8204	0	0	0	0.9	0.04	2.91	0.11	0.3	0.19	14.22	17.3	0.25	0.62	0.48	39.03	0.53	0.37	1.16	0.63	5.84	Shared
S12_vs_71525	0	0	0.02	1.63	0.27	49.45	0.22	0.75	0.63	0.47	15.63	0.16	2.95	1.52	25.32	0.22	0.5	1.54	3.68	5.05	Shared
S12_vs_142625	0.01	0.01	0	4.96	1.84	8.98	0.34	10.92	6.94	4.83	59.51	9.06	8.57	3.22	53.11	2.69	1.32	12.96	26.79	10.36	Shared
S12_vs_48045	0	0	0	0.77	0.02	0.33	0.06	0.13	0.08	0.07	0.31	0.05	0.27	0.13	33.03	0.14	0.14	0.25	0.29	0.36	SHHC
S12_vs_111286	0	0	0	0.7	0.02	0.23	0.08	0.13	0.09	0.23	0.29	0.08	0.33	0.16	147.21	0.24	0.22	0.24	0.23	0.46	SHHC
S12_vs_150812	0	0	0	0.55	0.02	13.33	0.1	0.3	0.15	0.68	0.64	0.17	0.4	0.27	28.13	1.59	0.23	0.17	0.32	0.57	Shared
S12_vs_151292	0.59	0.19	0.74	16.97	4.32	9419.23	4.65	20.61	7.59	620.8	79.79	16.1	111.1	21.16	112.68	463.73	53.18	2343.87	35.87	27.01	Shared
S12_vs_183752	0	0	0	0.95	0.03	5.75	0.15	0.43	0.61	0.41	1.67	0.12	1.63	0.95	62.29	0.86	0.52	0.57	0.78	1.21	SHHC
S12_vs_58148	0.02	0	0	2.21	0.24	1.46	0.06	0.42	0.17	0.89	0.82	0.15	0.78	0.2	367.06	0.49	0.73	0.3	0.49	0.61	SHHC
S12_vs_26336	0.01	0	0	4.33	2.21	5.26	0.21	19.27	14.89	4.18	49.42	5.23	3.41	6.33	33.55	1.91	1.36	28.1	23.07	4.35	Shared
S12_vs_81729	0	0	0	1.2	10.99	0.42	0.12	0.21	0.13	0.32	0.5	0.13	0.48	0.22	150.05	0.42	0.3	0.25	0.58	0.5	SHHC
S12_vs_98175	0	0	0	3.94	0.13	0.33	0.04	0.21	0.11	0.26	1.28	0.13	0.93	2.19	92.57	0.69	0.39	0.29	0.86	2.99	SHHC
S12_vs_58560	0	0	0	0.56	0.01	0.25	0.04	0.21	0.11	0.27	0.6	0.05	0.46	1.39	18.39	0.9	0.34	0.22	0.44	0.38	SHHC
S12_vs_26847	0	0	0	1.3	0.52	1.74	0.07	0.19	0.11	0.36	0.44	0.08	0.57	0.38	267.72	0.3	0.25	0.18	20.29	0.76	SHHC
S12_vs_106329	0	0	0	1.21	0.04	0.52	1.89	2.2	0.8	0.93	12.38	2.09	0.76	2.02	45.96	0.4	1.36	0.51	0.56	1.61	SHHC
S12_vs_74280	0	0	0	0.48	0	32.76	0.17	0.2	0.09	0.32	15.83	0.04	5.05	3.47	27.28	0.09	0.11	0.66	0.2	0.28	Shared
S12_vs_99092	0	0	0	1.14	0.24	2.75	0.08	0.51	0.31	0.28	0.56	0.09	1.03	0.59	21.27	0.54	0.27	0.43	0.96	2.8	SHHC
S12_vs_99219	0	0	0	0.74	0.08	0.74	0.29	0.28	0.17	0.25	0.96	0.19	1.09	11.06	25.72	2.4	1.81	0.48	0.9	5.73	Shared
S12_vs_138261	0	0	0	0.46	0.12	0.3	0.29	0.17	0.25	1.45	3.94	0.09	0.51	130.32	57.83	0.46	7.78	0.23	1.38	11.67	Shared
S12_vs_121913	0	0	0	2.05	0.17	2.4	0.15	3.28	1.25	0.5	4.17	0.12	2.62	4.33	25.51	0.56	1.76	0.88	4.52	4.77	SHHC
S12_vs_153780	0	0	0	0.75	0.02	0.37	0.12	0.27	0.15	2.84	3.08	0.07	0.33	0.81	28.93	0.34	0.23	1.18	0.4	1.19	SHHC
S12_vs_75098	0.01	0	0	6.65	0.04	1.39	0.19	1.73	0.43	0.92	2.22	0.51	1.25	1.2	46.69	3.78	1.28	0.91	3.74	1.64	SHHC
S12_vs_28661	0.01	0	0	1.25	0.01	0.21	0.02	0.23	0.17	0.8	4.16	0.09	0.19	0.18	45.48	2.16	0.2	0.22	0.26	0.54	SHHC
S12_vs_5857	0.01	0	0.01	0.62	0.01	0.19	0.02	0.21	0.08	0.23	0.69	0.08	0.37	0.15	143.52	5.34	0.22	0.74	0.67	0.45	SHHC
S12_vs_92868	0.01	0	0	1.92	0.17	3.02	0.07	459.82	719.49	1.79	0.94	1.19	1.08	6.94	635.59	1.28	19.69	0.71	2.44	1.44	Shared
S12_vs_123620	0	0	0	1.07	0.9	2.51	0.43	0.82	0.33	1.09	86.98	0.44	185.57	0.63	793.42	5.47	2.26	0.8	18.48	2.35	SHHC
S12_vs_93357	0	0	0	0.55	0.02	0.21	0.07	2.01	0.32	0.13	0.79	0.08	0.25	0.35	16.95	0.31	0.97	0.18	0.16	0.41	SHHC
S12_vs_6836	0	0	0	0.64	0.02	1.31	0.23	0.42	0.28	0.39	1.67	0.26	2.26	1.04	30.01	1.3	0.77	5.33	2.82	1.76	SHHC
S12_vs_163990	0	0	0	0.79	0.04	1	0.21	0.25	0.19	0.88	117.21	0.31	0.44	22.06	120.28	0.32	0.26	32.49	0.97	1.1	Shared
S12_vs_164056	0.04	0	0.01	5.79	0.04	1.2	0.11	4.79	0.73	1.35	3.85	0.69	1.29	0.33	16.07	18.32	3.12	0.45	0.91	2.35	Shared
S12_vs_141337	0.01	0	0.01	45.22	4.83	28.02	3.9	28.21	30.37	22.98	15.58	3.17	106.3	5.16	35.45	20.93	47.39	43.01	20.62	19.44	Shared
S12_vs_70241	0	0	0	0.77	0	0.77	0.3	0.1	0.3	0.4	1.31	0.19	1.77	0.35	12.63	0.19	0.18	0.93	0.54	1.09	SHHC
S12_vs_94042	0	0	0.01	14.68	0.02	4.61	0.02	0.39	0.13	7.67	24.58	0.12	2.83	0.27	29.3	7.08	0.22	3.3	10.88	0.84	Shared
S12_vs_14467	0.01	0	0	1.41	0.03	18.63	0.18	0.29	0.2	0.2	0.71	0.15	3.16	2.52	36.66	0.38	2.5	0.68	0.57	0.96	Shared
S12_vs_22853	0	0	0	0.84	0.03	0.45	0.08	0.23	0.14	0.25	0.43	0.06	0.41	0.41	30.86	0.21	0.19	0.3	0.34	0.6	SHHC
S12_vs_133070	0.01	0	0	28.73	0.17	1.29	0.24	0.83	0.26	1.13	3.35	0.74	0.95	4.46	378.54	13.24	0.85	0.56	3.02	1.57	Shared
S12_vs_172379	0.01	0	0	1.04	0.12	1.82	0.24	0.64	0.31	0.64	7.59	0.26	10.09	0.59	155.57	4.24	0.94	0.78	2.23	2.07	SHHC
S12_vs_14927	0	0	0	27.22	0.07	2.65	0.3	0.66	0.38	2.66	1.63	0.73	3.05	2.94	1867.34	92.11	1.23	1.27	1.47	2.08	Shared
S12_vs_23796	0.01	0	0	0.64	0.15	0.74	0.2	6.66	4.16	0.32	2.88	0.31	1.16	1.6	73.56	0.38	0.58	0.71	1.75	9.34	SHHC
S12_vs_32669	0	0	0	0.73	0.03	0.34	0.01	0.2	0.11	0.22	0.64	0.1	0.67	0.31	105.07	0.31	0.25	0.32	0.38	0.6	SHHC
S15_vf_2032	0	0	0	0.61	0.03	1.2	0.11	0.46	0.2	0.19	0.68	0.34	224.84	0.78	0.52	3.16	1.1	104.61	0.63	14.13	Shared
S15_vf_2513	0	0	0	1.49	0.02	7.84	0.12	0.26	0.23	0.24	0.58	0.13	0.65	0.58	0.75	0.5	0.46	93.1	0.65	1.24	SHHC
S15_vf_20644	0	0.01	0	3.13	0.02	0.48	0.07	0.26	0.11	0.15	0.55	0.12	0.42	0.25	0.47	6.8	0.93	259.66	2.52	4.95	SHHC
S15_vf_33505	0.34	0.15	0.38	931.04	2557.69	4083.17	0.14	1.69	1.45	1.91	68.24	5.04	1.17	0.44	77.52	5433.36	3.64	757.13	7.49	1.09	Shared
S15_vf_55639	0.01	0.02	0	0.33	0.01	0.11	0.04	0.09	0.08	0.1	0.15	0.06	0.21	0.25	0.2	0.16	0.32	56.9	0.21	0.48	SHHC
S15_vf_58467	0	0.02	0	0.87	0.07	1.72	0.17	0.83	0.23	0.29	1.01	0.2	2.44	0.58	1.22	0.87	1.01	140.55	0.81	1.22	SHHC
S15_vf_71616	0	0.04	0	0.87	0.01	0.61	0.07	0.23	0.12	0.16	0.5	0.16	12.36	0.21	2.86	0.54	0.25	143.88	0.31	0.73	SHHC
S15_vf_74586	0	0.04	0	1	0.02	0.98	0.08	0.18	0.09	0.12	0.39	0.17	28.89	0.37	2.17	0.4	0.79	561.09	0.35	0.7	SHHC
S15_vf_75924	0	0.11	0	3.55	0.16	0.75	0.2	0.79	0.38	749.04	2.16	0.83	94.05	89.92	1583.12	1.8	2.42	1696.07	43.34	102.84	Shared
S15_vs_1407	0	0	0	0.38	0.02	2.64	2.14	0.17	0.04	0.08	0.23	0.1	0.8	0.5	3.65	0.36	0.31	227.97	0.18	0.49	SHHC
S15_vs_7103	0	0.01	0	1.38	0.19	1.76	1.09	2.05	2.7	1.34	16.12	7.59	0.29	1.09	0.43	0.51	4.88	141.65	3.97	1.88	SHHC
S15_vs_18274	0	0.01	0	0.62	0.02	0.41	0.07	0.27	0.17	0.17	0.53	0.14	0.79	0.28	0.47	0.34	0.48	50.42	0.46	1.04	SHHC
S15_vs_76390	0.01	0.01	0	1.25	0.05	0.68	0.07	0.33	0.22	0.18	0.79	0.21	0.92	0.36	0.81	0.33	0.56	212.18	0.86	0.94	SHHC
S15_vs_3868	0	0.03	0.03	62.19	1.72	423.76	0.08	1.91	1.73	10218.34	10.17	0.99	1.04	342.38	39.1	75.85	15.07	156.23	69.49	73.42	Shared
S15_vs_27510	0	0.02	0	8.34	0.18	1.51	0.3	0.31	0.13	0.41	2.03	0.64	1.6	2.46	3.24	4.41	1.56	242.89	1.27	22.3	Shared
S15_vs_35445	0.08	0.01	0	29.36	0.82	30.59	0.7	53.99	53.42	6.45	80.34	2.66	41.42	5.3	12.63	5.6	42.44	85.39	23.11	35.86	Shared
S15_vs_39804	0	0	0.04	0.67	0.16	603.74	0.7	2.48	0.27	0.35	5.97	0.86	2.06	0.91	1.07	1.74	0.82	56.64	0.95	1.24	Shared
S15_vs_42043	0.01	0	0	11.64	2.02	22.21	0.47	20.65	30.69	2.97	10.76	0.9	25.26	4.89	6.27	3.55	13.01	92.71	10.07	22.99	Shared
S15_vs_43913	0	0	0	0.66	0.05	0.48	1139.83	0.22	0.17	6.53	1.24	0.11	1.11	14.74	5.56	2.22	2.8	54.89	1.57	0.91	Shared
S15_vs_71968	0.01	0.01	0	5.11	0.28	36.97	0.93	7.01	3	3.77	23.07	1	1.3	8.3	8.3	1.35	0.81	35.46	5.97	3.21	Shared
S2_vf_4130	0.29	0.04	0.01	223.63	2531.26	62.27	15.54	0.57	0.43	0.56	1.5	0.22	1.36	0.33	1.52	22.44	2.9	0.71	0.51	0.83	Shared
S2_vf_17850	0	0	0	10.06	42.1	0.46	0.04	0.32	0.11	0.16	0.75	0.08	0.4	0.09	0.41	0.99	2.3	0.23	0.85	18.14	Shared
S2_vf_16829	0	0	0	0.5	230.53	0.45	0.07	0.12	0.11	0.07	0.27	54.21	0.36	0.04	0.13	0.25	0.46	0.16	218.56	17.5	Shared
S2_vf_3356	0	0	0	229.92	138.02	0.46	0.1	0.31	0.18	0.28	0.24	0.06	0.33	0.07	0.29	5.02	0.39	0.74	0.1	0.59	Shared
S2_vf_20910	0	0	0	0.38	38.6	0.48	0.09	0.19	0.15	4.21	0.38	0.08	0.55	5.93	0.61	0.4	0.45	0.3	0.37	2.56	SHHC
S2_vf_4910	0	0	0	0.4	49.06	0.47	0.01	0.22	0.13	0.24	560.33	0.1	1.44	0.11	0.21	0.25	0.35	0.26	0.28	1.28	SHHC
S2_vf_21511	0	0	0	1.19	62.4	25.44	0.13	0.28	0.25	0.26	1.33	0.1	0.74	0.38	0.48	0.45	0.41	0.34	0.63	0.84	Shared
S2_vf_21630	0	0	0	0.61	43.56	0.32	0.05	0.11	0.07	0.1	0.23	0.06	0.31	0.29	0.19	0.17	0.32	0.11	0.41	0.39	SHHC
S2_vs_7306	0.2	0.09	0.01	793.18	1660.82	202.47	221.17	187.58	317.92	112.97	2.53	0.33	1.43	12.69	0.41	58.79	2.28	1.55	24.75	0.51	Shared
S2_vs_4368	0.03	0	0	1.11	27.26	0.74	0.1	0.45	0.16	4.58	0.45	0.06	0.2	0.54	0.89	0.23	0.8	0.22	0.3	0.63	SHHC
S2_vs_10289	0.01	0	0	13.72	37.96	15.06	0.48	6.51	3.11	13.57	3.31	1.43	27.32	3.47	13.84	8.89	31.35	16.06	74.1	14.3	Shared
S4_vf_2524	0.01	0	0	0.32	0	4.97	21.65	0.22	0.1	0.1	0.23	0.01	0.51	0.69	3.18	0.27	0.2	26.26	0.2	0.36	SHHC
S4_vf_4838	0.01	0	0	3	0.2	0.8	8527.63	0.4	0.26	0.69	1380.5	0.08	0.87	1.33	0.66	14.56	0.36	0.32	0.51	2.88	Shared
S4_vf_4891	0	0	0	0.96	0.02	0.46	14.81	0.27	0.17	0.16	33.26	0.22	1.06	0.34	0.39	0.25	0.33	0.35	0.38	0.84	SHHC
S4_vf_5021	0	0	0	0.38	0.01	0.31	212.82	0.15	0.08	0.12	0.4	0.06	1.02	0.61	0.3	0.29	0.16	0.17	0.27	1	SHHC
S4_vf_10506	0.01	0	0	0.7	0.05	0.63	10658.08	0.21	0.14	0.12	57.13	0.09	0.69	0.17	0.51	1.22	0.31	0.39	0.45	0.95	SHHC
S4_vf_11308	0	0	0	0.76	1.81	1.3	12.54	31.21	3.56	1.29	0.95	0.2	0.52	0.49	2.94	0.31	18.02	0.21	3.07	0.61	Shared
S4_vf_12614	0	0	0	0.53	0.03	0.48	76.95	0.23	1.64	0.19	0.56	0.3	0.66	0.25	1.43	0.23	0.37	0.31	1.57	6.64	SHHC
S4_vf_12624	0.01	0	0	0.4	0.05	0.09	116.26	0.17	0.07	1.48	0.36	0.08	0.22	0.21	0.23	0.17	0.35	0.13	0.17	0.25	SHHC
S4_vf_13353	0	0	0	1.15	0.11	1.41	4571.4	0.23	0.16	0.22	0.86	0.39	0.55	0.25	0.7	0.46	0.39	2.87	34.31	72.32	Shared
S4_vf_13627	0	0	0	3.87	0.02	156.33	316.23	0.13	0.15	0.1	0.15	0.3	0.35	0.12	0.1	1.49	0.13	0.2	0.23	0.26	Shared
S4_vf_13759	0	0	0	0.46	0.04	2.3	9.91	0.95	0.31	0.38	2.22	0.35	2.21	0.44	1.43	0.82	0.87	0.98	2.25	1.66	SHHC
S4_vf_13831	0	0	0	0.7	0.29	1.95	1822.83	0.7	0.48	0.71	1226.1	0.29	3.14	2.03	1.52	1.83	0.93	0.61	1.03	3.56	SHHC
S4_vf_16485	0	0	0	0.3	0.02	0.25	50.09	0.17	0.09	0.1	0.37	0.04	0.17	0.23	0.27	0.84	0.18	0.21	0.24	0.66	SHHC
S4_vf_17948	0.01	0	0	0.29	0.02	0.39	133.3	0.1	0.1	0.08	0.92	0.05	0.29	0.11	0.39	0.19	0.21	0.12	0.17	0.51	SHHC
S4_vf_18803	0.01	0	0	0.42	0.02	0.36	795.17	0.16	0.11	0.09	35.83	0.07	0.29	0.24	0.18	5.7	0.18	0.21	0.21	0.7	SHHC
S4_vf_20440	0	0	0	0.57	0.08	0.4	330.61	0.23	0.18	0.22	355.21	0.19	0.68	0.59	0.27	0.25	0.22	0.3	0.36	1.14	SHHC
S4_vf_22219	0.85	0.04	0.98	1.29	1.34	11507.36	465.16	1.6	1.67	1.65	3.42	14.57	0.83	0.67	0.48	1.53	0.37	0.41	0.46	1.02	Shared
S4_vf_26417	0	0	0	1.25	0.89	2.9	730.2	301.02	811.68	6.53	5532.69	34.47	3.69	5.93	6451.22	4.81	2.7	1.73	4202.33	11.76	Shared
S4_vf_27861	0	0	0	0.52	0.03	13.75	239.09	0.19	0.14	0.08	0.36	0.74	47.36	39.2	0.33	0.78	0.22	0.24	0.34	0.61	Shared
S4_vf_28601	0.02	0	0	0.59	0.01	0.16	164.87	0.23	0.07	0.11	0.55	0.06	0.14	0.07	0.37	0.22	0.11	0.19	0.13	0.36	SHHC
S4_vf_30103	0.01	0	0	0.45	0.03	61.17	4852.83	0.18	0.14	0.09	0.32	0.12	0.3	0.04	0.36	0.19	0.1	0.25	0.16	0.34	Shared
S4_vf_31682	0.01	0.01	0.01	81.11	0.03	0.19	24.46	0.25	0.11	0.99	0.37	0.1	5.4	0.37	0.24	16.35	0.18	8.47	0.38	0.63	Shared
S4_vf_31825	0.39	0	0.38	8.45	31.61	4417.99	1212.55	0.66	0.62	0.7	3.55	5.5	0.43	0.39	0.76	0.91	0.22	0.18	0.31	0.51	Shared
S4_vf_31973	0.01	0	0.02	0.64	0.04	189.95	70.01	2.63	3.15	0.31	0.7	134.54	0.93	0.23	22.23	0.53	0.4	0.36	0.48	0.94	Shared
S4_vf_31978	0	0	0	4.05	0.07	1.39	16.78	0.27	0.14	0.19	0.66	0.12	0.8	0.42	3.62	0.82	0.45	0.39	34.2	0.91	SHHC
S4_vf_31979	0.01	0	0	0.63	0.01	0.36	57.2	0.95	1.21	0.11	0.64	0.09	0.37	0.22	0.67	0.26	0.26	0.2	0.26	0.47	SHHC
S4_vf_31981	0	0	0	0.44	0.05	0.85	198.44	0.38	0.22	0.18	0.96	0.1	1.34	0.31	0.77	0.53	0.44	0.42	0.62	0.99	SHHC
S4_vf_31982	0.01	0	0	0.48	0.02	0.19	286.19	0.18	0.08	0.12	0.51	0.04	0.31	0.26	0.43	0.16	0.2	0.2	0.28	0.36	SHHC

S4_vs_VIRSorter_3	0	0	0	3.01	0.11	1.18	24.04	0.9	0.52	0.32	0.79	0.37	1.83	31.86	0.61	0.42	1.65	1.64	1.31	4.85	Shared
S4_vs_VIRSorter_6	0.09	0.01	0.08	44.34	32.83	1215.41	9554.46	561.71	904.83	183.55	4.67	1.57	1.5	21.58	0.62	1.71	0.24	0.23	3.24	0.55	Shared
S4_vs_VIRSorter_3	0	0	0	0.91	0.04	5.06	47.9	0.35	0.18	1.15	0.66	0.46	0.69	1.02	0.37	0.28	0.37	0.41	1.33	1.39	SHHC
S4_vs_VIRSorter_3	0.21	0	0	14.18	4.01	12.56	32.36	73.62	0.44	60.41	25.06	328.01	3.56	0.95	119.35	5.32	5291.67	21.52	196.81	15.42	Shared
S4_vs_VIRSorter_9	1	0	0.7	6.37	1.17	7413.04	758	269.92	404.13	4.81	2.62	10	0.93	1.34	0.41	1.34	0.73	0.37	1.05	2.35	Shared
S4_vs_VIRSorter_1	0	0	0	0.72	0.07	0.41	70.7	0.49	0.23	1.61	3	1.56	0.45	1.2	0.41	1.51	0.51	0.34	0.4	1.23	SHHC
S4_vs_VIRSorter_3	0.01	0	0	1.19	0.31	5.91	115.6	2.8	2.25	3.6	6.29	2.35	0.4	4.15	1.36	0.52	142.76	0.43	1.36	2.16	Shared
S4_vs_VIRSorter_3	0	0	0	5.54	0.36	3.44	33.68	5.93	0.27	3.94	3.47	0.51	1.04	8.88	2.26	1.53	20.92	1.74	30.8	6.49	Shared
S4_vs_VIRSorter_3	0	0	0	1	0.01	0.7	85.36	0.53	0.13	1.23	0.89	0.09	0.6	0.09	0.52	0.32	0.29	0.2	0.31	0.89	SHHC
S4_vs_VIRSorter_7	0.01	0	0	1.02	2.86	1.1	206.57	10.99	9.24	89.57	0.51	1.87	0.42	1.07	0.34	0.41	4.01	0.65	4.92	0.68	Shared
S4_vs_VIRSorter_3	0	0	0	1.37	0.11	0.43	24.12	4.43	0.09	0.18	0.46	0.1	0.32	0.42	0.2	0.41	4.03	0.22	0.25	0.53	SHHC
S4_vs_VIRSorter_3	0	0	0	1.96	0.03	1.22	28.11	0.26	0.09	0.16	0.61	0.11	0.73	0.15	0.38	0.32	0.2	0.19	0.24	0.84	SHHC
S4_vs_VIRSorter_3	0.01	0	0	0.5	0.02	0.28	46.88	0.18	0.11	0.11	0.44	0.09	0.27	0.19	0.25	0.2	15.77	0.21	8.03	0.4	Shared
S4_vs_VIRSorter_3	0	0	0	0.87	0.03	0.2	21.16	0.19	0.05	0.12	9.17	0.06	0.29	0.13	0.25	3.91	0.14	0.2	0.21	0.33	SHHC
S4_vs_VIRSorter_1	0.04	0	0.04	9.31	0.07	359.34	13896.79	0.18	0.16	0.17	0.39	0.58	0.37	0.15	0.3	0.32	0.27	0.21	0.31	0.61	Shared
S4_vs_VIRSorter_3	0	0	0	1.6	0.05	0.95	387.45	13.36	11.82	2.67	2.06	1.45	0.41	57.75	0.64	28.91	2.74	0.38	9.62	8.46	Shared
S4_vs_VIRSorter_2	0.03	0	0.01	10.57	0.17	87.35	35.89	0.45	0.15	0.32	1.69	2.2	1.27	0.42	1.52	3.55	41.43	1.41	1.05	1.93	Shared
S4_vs_VIRSorter_4	0	0	0	0.85	0.03	0.47	60.32	1.81	1.9	0.17	8.2	0.13	2.97	0.37	1.21	0.59	1.2	0.39	0.92	0.77	SHHC
S4_vs_VIRSorter_8	0.02	0	0	1.17	15.98	51.46	21.32	1	0.18	2.59	0.97	0.19	1.46	0.15	145.84	7.03	1.16	0.2	0.65	0.87	Shared
S4_vs_VIRSorter_1	0.01	0	0	1.05	0.04	0.63	10.25	1.03	0.43	6.79	6.59	0.32	0.24	0.62	0.33	0.71	18.33	0.54	1.92	3.3	Shared
S4_vs_VIRSorter_1	0.1	0	0.02	1.11	0.03	157.63	243.84	0.43	0.19	0.15	0.67	0.29	0.26	0.1	0.29	0.39	0.21	0.27	0.42	0.72	Shared
S4_vs_VIRSorter_2	0	0	0	0.61	0.02	3.84	65.84	0.29	0.18	0.25	0.46	0.15	0.5	0.19	0.44	0.3	0.39	0.3	0.39	0.89	SHHC
S4_vs_VIRSorter_5	0.01	0	0.01	0.37	0.03	2.3	166.53	0.22	0.1	2.3	1.31	0.06	0.3	0.23	0.25	0.39	0.71	0.25	0.26	0.55	SHHC
S4_vs_VIRSorter_1	0	0	0	1.41	0.04	1.48	34.5	0.27	0.21	0.31	0.57	0.09	0.45	0.71	0.32	0.4	0.41	0.34	0.63	1.47	SHHC
S4_vs_VIRSorter_1	0.01	0	0	0.78	0.14	0.39	56	0.37	0.19	1.63	1.97	0.32	0.29	0.27	0.4	0.38	1.15	0.21	0.63	0.54	SHHC
S4_vs_VIRSorter_4	0.01	0	0	0.93	0.9	4.87	11.43	1.11	0.3	1.36	16.59	0.9	1.62	17.78	4.06	0.59	1.56	0.26	0.72	9.49	Shared
S4_vs_VIRSorter_1	0	0.01	0	2.82	0.06	0.64	29.13	24.46	21.02	11.02	1.14	1.44	0.63	2.02	1.31	2.11	8.39	1.29	15.07	3.71	Shared
S4_vs_VIRSorter_1	0	0	0	10.23	0.1	5.27	66.64	4.04	1.03	0.15	3.1	1.2	1.04	3.38	0.86	7.36	0.71	1.31	3.56	1.11	Shared
S4_vs_VIRSorter_1	0	0.01	0	1.07	0.05	1.77	18.38	0.69	0.26	8.72	1.36	0.27	2.05	0.85	1.65	0.65	0.59	0.67	1.16	1.36	SHHC
S4_vs_VIRSorter_2	0	0	0	1.68	0.99	0.28	18.11	0.24	0.1	1.43	0.75	0.08	0.61	0.42	5.94	0.64	0.98	0.18	0.3	0.33	SHHC
S4_vs_VIRSorter_4	0	0	0	0.32	0	35.82	661.72	0.2	0.08	2.09	0.4	0.04	0.4	0.9	0.17	0.3	0.33	0.27	0.3	0.46	Shared
S4_vs_VIRSorter_2	0	0	0	0.92	0.01	1	19.91	0.2	0.1	0.08	32.43	0.06	0.45	0.15	0.22	6.79	0.16	0.18	0.16	2.47	SHHC
S6_vf_47238	0	0	0	0.7	0.88	2.39	0.1	1421.63	2105.49	0.72	1.74	0.12	2.45	4.22	0.84	2.48	0.45	0.41	0.52	1	Shared
S6_vf_45228	0	0	0	0.67	0.11	0.51	0.02	2.98	9.28	0.18	0.51	0.08	0.61	0.57	0.4	0.29	0.37	0.33	0.33	1.06	SHHC
S6_vf_45618	0	0	0	3.67	0.09	0.68	6.87	15.87	23.47	1.01	16.23	4.16	0.26	2.63	2.72	0.68	6.69	15.5	6.94	6.93	Shared
S6_vf_24553	1.07	0	0.02	0.5	9.5	0.6	0	52.07	81.09	2.96	0.22	710.84	1.75	1.6	2.19	27.57	16180.59	1.26	13.67	3.56	Shared
S6_vf_3435	0	0	0.03	0.54	0.12	743.78	0.07	318.56	35.84	38.87	5.47	1.32	1.21	0.55	0.9	0.46	1.28	0.25	0.32	11.25	Shared
S6_vf_6314	0	0	0	0.5	0.78	1.41	0.06	2223.86	3214.06	1.08	2.34	0.01	3.4	4.8	0.4	0.78	0.15	0.18	0.22	0.55	Shared
S6_vf_25710	0	0	0	0.6	0.03	0.4	0.08	19.5	181.78	0.16	0.48	0.05	0.4	0.36	0.3	0.26	0.31	0.28	0.28	0.68	Shared
S6_vf_56484	0	0	0	4.09	0.03	1.37	0.04	9.9	25.5	0.16	2.51	0.06	0.91	0.46	40.2	2.22	0.21	0.28	0.2	0.37	SHHC
S6_vf_56486	0	0	0	1.3	0.07	0.39	0.08	38.15	56.22	0.16	3.29	0.08	0.57	0.26	0.43	0.3	0.31	0.2	0.35	0.64	Shared
S6_vs_32886	0.01	0	0	0.65	0.15	0.63	0.31	320.29	62.28	0.47	0.82	0.13	1.77	4.78	2.12	0.48	99.19	0.42	0.58	0.7	Shared
S6_vs_56485	0	0	0	0.52	0.01	0.94	0.04	10.66	14.24	2.06	0.42	0.32	0.34	0.25	0.31	0.14	0.73	0.16	0.34	1.16	Shared
S6_vs_56490	0	0	0	2.23	0.21	1.95	0.09	798.6	350.27	0.7	1.14	0.85	1.91	1.89	1.05	0.77	134.41	0.59	1.27	2.17	Shared
S6_vs_56491	0	0	0	0.63	0.43	0.43	0.17	22.67	39.94	1.01	0.74	3.42	0.7	2	0.37	0.35	0.77	0.55	0.41	0.76	Shared
S6_vs_56492	0	0	0	2.08	0.14	0.52	0.14	13.35	23.19	1.07	0.51	0.54	0.5	0.54	2.6	0.43	1.44	0.24	0.61	1.21	Shared
S6_vs_56493	0	0	0	1.87	0.06	0.33	0.12	6.56	11.19	0.37	0.64	0.07	0.26	3.27	1.15	1.53	7.32	0.23	0.49	0.51	SHHC
S6_vs_56496	0	0	0	0.88	0.02	0.18	0.05	11	27.34	0.09	0.39	0.09	0.38	0.23	0.18	0.26	0.13	0.19	0.22	0.43	Shared
S6_vs_56498	0	0	0	0.64	0.05	0.39	0.07	30.95	55.36	1.54	0.38	0.27	0.4	0.35	0.35	2.06	0.28	0.21	0.28	0.87	Shared
S6_vs_37812	0	0	0	0.99	0.07	2.41	0.07	10.51	18.42	2.95	9.03	1.2	2.05	22.18	4.02	6.27	2.26	0.43	4.2	2.59	Shared
S6_vs_38250	0	0	0	0.64	0.02	0.61	0.07	6.54	13.63	1.64	0.46	0.19	0.55	0.62	0.5	0.39	0.7	0.29	0.46	0.78	SHHC
S6_vs_9784	0.01	0.01	0	11.92	0.91	11.75	1.61	49.75	88.32	3.83	7.45	0.98	18.2	3.43	4.01	2.72	8.96	32	6.21	19.63	Shared
S6_vs_33711	0.01	0	0	0.57	0.01	0.38	0.1	5.22	16.08	0.2	0.4	0.1	0.71	1.77	0.53	0.41	0.42	0.42	5.88	0.76	SHHC
S6_vs_54606	0.01	0	0	7.69	0.22	9.15	3.48	23.83	29.5	10.23	5.75	1.32	9.34	2.38	3.3	7.48	1.66	6.59	14.64	6.08	Shared
S6_vs_29152	0	0	0	6.66	0.19	3.05	0.72	5.33	30.7	4.11	5.04	0.97	5.32	2.08	2.48	5.76	1.06	4.52	7.74	6.13	SHHC
S6_vs_10314	0	0	0	10.37	0.12	4.7	0.56	5.59	32.36	9.29	9.21	1.84	9.43	3.81	2.1	10.47	1.08	8.23	14.83	7.37	Shared
S6_vs_1146	0.01	0.01	0.01	3.42	0.22	27.76	12.27	19.09	12.83	7.85	6.05	4.27	3.07	1.53	18.04	2.19	5.83	2	14.78	25.7	Shared
S6_vs_46039	0.02	0	0	5.82	0.78	5.84	2.02	67.12	61.8	10.42	9.8	1.2	7.71	11.12	5.39	1.84	66.65	11.23	35.08	17.95	Shared
S6_vs_12945	0.01	0	0	5.71	1.15	3.67	0.22	5.05	30.17	2.17	3.67	1.12	6.17	6.86	3.34	5.5	1.6	4.09	9.62	23.37	Shared
S6_vs_34453	0	0	0	1.21	0.07	3.05	1.26	29.6	49.2	0.69	1.19	9.67	1.27	0.53	0.99	0.65	1.29	0.36	2.02	7.07	Shared
S6_vs_15390	0	0	0	0.46	0.03	0.53	0.08	35.48	86.49	0.11	1.92	0.08	0.75	0.54	0.49	1.1	0.22	0.3	1.21	0.71	Shared
S6_vs_39025	0	0	0	7.4	0.25	3.88	0.11	56.3	28.34	6.78	8.7	1.39	8.95	2.87	1.44	7.42	9.27	6.54	13.4	6.71	Shared
S6_vs_46091	0	0	0.02	0.54	0.04	0.66	0.06	7.77	18.53	0.16	0.35	308.61	0.39	0.39	0.36	0.21	0.75	0.23	0.36	0.62	Shared
S6_vs_15813	0.01	0	0	1.83	0.19	9.71	9.3	16.39	25.37	5.57	4.21	3.51	0.42	2.93	0.85	0.68	167.46	0.32	1.04	2.62	Shared
S6_vs_22637	0	0	0	0.42	0.01	0.39	0.04	18.27	17.69	0.23	0.32	0.05	0.24	0.08	0.18	0.09	2.74	0.14	0.27	0.67	Shared
S6_vs_44070	0	0	0	2.44	0.05	0.45	0.04	10.56	17.22	0.43	1.07	0.09	0.57	1.58	0.65	0.41	0.36	0.32	0.54	0.84	Shared
S6_vs_53966	0.01	0	0	1.06	0.08	1.61	0.88	6.18	8	0.33	1.44	0.39	0.55	0.12	1.27	0.3	0.66	0.52	9.49	4.92	SHHC
S6_vs_39065	0.01	0.01	0	63.26	0.1	0.54	0.03	48.09	87.79	0.3	3.59	0.14	0.8	16.49	0.86	46.57	0.31	0.42	0.65	1.53	Shared
S8_vf_43483	0.01	0	0	1.1	0.05	2.93	0.01	10.55	14.89	0.15	389.95	0.13	0.28	0.13	0.41	0.17	0.1	0.18	0.37	0.96	Shared
S8_vf_122206	0	0	0	1.5	0.09	0.96	0.78	0.55	0.27	0.3	217.68	0.47	1.73	0.22	0.65	39.68	0.89	0.62	0.66	1.98	Shared
S8_vf_131009	0	0	0	1.36	0.1	2.32	0.1	0.62	0.43	0.7	337.25	0.15	1.21	0.39	1.31	1.22	2.59	0.7	7.11	1.86	SHHC
S8_vf_87755	0.01	0	0.03	2.53	1.29	238.32	4.29	2.03	1.47	1573.69	107.74	1.04	700.65	0.57	2.92	8540.27	0.54	0.58	11.2	1.35	Shared
S8_vf_174559	0	0	0	1.79	0.17	2.52	0.29	0.71	0.93	0.42	677.4	0.15	1.05	0.28	0.7	26.76	0.93	0.99	0.86	2.47	Shared
S8_vf_183045	0.03	0	0	0.59	0.27	0.89	182.31	0.48	0.51	0.54	2519.1	0.06	0.47	0.27	0.27	385.66	0.26	0.3	0.74	4.35	Shared
S8_vf_166403	0	0	0	0.62	0.04	0.65	0.15	0.52	0.34	1.13	27.32	1.15	0.2	0.31	0.25	0.35	15.02	0.15	0.63	1.52	Shared
S8_vf_79769	0	0	0	0.77	0.04	0.4	3.25	0.26	0.21	0.16	418.11	0.11	0.61	0.25	0.32	20.9	0.23	0.22	0.28	1.28	Shared
S8_vf_192724	0	0	0	0.36	0.1	0.33	0.14	0.23	0.11	0.19	574.78	0.07	0.54	0.45	0.42	17.03	0.29	0.23	0.33	1.48	Shared
S8_vf_131802	0	0	0	0.68	0.67	1.4	0.07	0.73	0.7	0.84	4703.11	0.16	0.91	0.23	0.57	0.78	0.29	0.41	0.41	6.85	SHHC
S8_vf_166677	0.05	0	0	3.2	4.07	6.29	65.39	3.78	3.39	4.55	29416.89	0.2	1.24	0.35	0.82	3.09	0.59	0.53	0.61	39.7	Shared
S8_vf_71214	0.04	0	0	0.31	0.05	0.09	0.01	0.29	0.05	13.56	37.8	0.01	0.23	0.04	0.1	0.18	0.15	0.1	0.15	1.28	Shared
S8_vf_158225	0	0	0	1.79	2.32	4.85	0.25	837.26	1819.65	9.73	13998.91	0.17	3.76	3.43	1.69	2.43	0.71	0.64	1.02	19.94	Shared
S8_vf_54514	0.01	0.03	0	46.65	1.69	2.99	0.96	4.69	0.98	2.02	88.67	0.83	1.28	2.74	0.81	13.71	9.8	0.46	1.93	4.09	Shared
S8_vf_201695	0	0	0	8.77	0.06	2.29	0.06	2.52	4.87	0.12	31.4	0.08	0.37	0.18	0.33	1.55	0.19	0.25	3.6	76.66	Shared
S8_vf_45776	0.27	0	0.02	0.61	3.37	140.59	150.67	16.11	3.93	11.43	1596.88	10.83	1.65	1.7	1.62	1.07	4891.97	0.75	9.89	532.56	Shared
S8_vf_28956	0.01	0	0	14.41	1.66	22.85	1.43	43.06	7.02	7.54	38.31	3.52	121.43	3.26	18.15	15.17	120.65	31.7	48.85	120.22	Shared
S8_vf_132697	0	0	0	3.25	0.64	11.82	0.16	0.58	0.26	0.29	791.25	0.11	0.58	0.44	0.93	0.76	0.36	0.27	0.7	1.87	Shared
S8_vf_106946	0	0	0	2.59	0.09	0.3	0.04	0.14	0.15	0.16	595.57	0.05	0.25	0.31	0.2	0.2	0.17	0.22	0.16	0.87	SHHC
S8_vf_159173	0	0	0	0.53	0.01	0.39	0	0.22	0.17	0.14	141.2	0.04	0.74	0.4	0.17	0.2	0.48	0.33	0.41	0.76	SHHC
S8_vf_55476	0	0	0	3.85	0.3	2.65	1.13	1.73	0.26	1.29	70.76	6.9	1.13	16.33	2.4	1.16	2.47	0.91	15.67	5.9	Shared
S8_vf_3410	0	0	0	0.42	52.33	0.39	0.09	22.26	41.95	0.63	151.05	0.08	0.9	43.75	0.19	0.29	0.38	0.38	16.81	1.01	Shared
S8_vf_72329	0	0	0	10.33	3.29	1.16	0.31	0.49	0.68	384.15	246.76	0.17	0.81	39.49	71.37	3.51	3.32	0.27	1.24	1.14	Shared
S8_vf_11794	0	0	0	1	0.07	0.76	0.14	0.48	0.21	8.7	408.9	0.1	0.44	0.4	0.54	0.44	1.09	0.29	0.48	1.21	SHHC
S8_vf_142700	0	0	0	0.29	0.03	3.71	0	0.12	0.07	0.06	38	0.03	0.19	0.03	0.28	0.21	0.07	0.21	0.2	0.19	SHHC
S8_vf_72773	0.01	0.06	0	827.83	0.13	20.68	0.08	0.53	0.25	0.42	131.86	0.13	1.42	0.28	38.68	338.85	0.94	1.26	0.78	0.92	Shared
S8_vf_203044	0	0	0	1.13	1.65	2.55	0.04	1.63	1.63	2.08	11690.43	0.09	0.48	0.26	0.47	1.55	0.51	0.25	0.31	17.16	Shared
S8_vf_12212	0	0	0	1.49	0.23	1.4	0.13	0.43	0.24	0.37	202.74	0.12	1.97	0.3	165.13	9.85	0.82	0.6	0.91	1.28	SHHC
S8_vf_195032	0	0	0	0.59	0.01	0.26	0.14	0.22	0.09	0.06	25.85	0.06	0.31	0.18	0.37	0.2	0.14	0.2	0.32	0.54	SHHC
S8_vf_151384	0.01	0	0.01	1.42	0.04	0.77	0.16	0.65	0.45	0.89	67.39	0.21	0.59	9.24	1.63	1.56	1.2	0.27	0.86	1.45	SHHC
S8_vf_91033	0	0	0	0.22	0.03	0.21	0	0.24	0.08	0.21	166.79	0.05	0.26	0.31	0.19	0.13	0.37	0.21	0.15	0.67	SHHC
S8_vf_65522	0	0.01	0	13.32	0.02	0.24	0.06	0.12	0.07	8.69	28.98	0.06	0.25	0.29	1.05	5.99	0.1	0.25	0.29	0.41	Shared
S8_vf_134943	0	0	0	0.35	0.03	0.31	0.14	0.21	0.2	6.35	62.14	0.1	0.71	2.94	8.25	0.36	0.2	0.2	0.27	0.8	SHHC
S8_vf_117796	0.11	0	0	1.07	0.12	0.62	0.97	0.47	0.49	0.45	649.37	0.14	0.81	0.42	0.39	155.75	0.4	0.59	0.72	1.68	Shared
S8_vf_13709	0.01	0	0	0.72	0.08	1.36	0.33	0.6	0.36	0.39	173.75	0.2	1.45	0.56	1.42	1.18	0.52	0.64	0.91	1.86	SHHC
S8_vf_13758	0	0	0	1	0.1	1.83	0.25	0.67	0.36	0.4	63.22	0.21	2.55	0.43	3.11	1.02	0.82	0.7	1.12	1.89	SHHC
S8_vf_170430	0.03	0	0	0.4	0.1	0.2	0.04	0.51	0.61	0.08	72.44	0.36	0.22	0.07	0.22	2.86	1.18	1.27	0.14	0.41	SHHC
S8_vf_196670	0	0	0	0.78	0.03	0.37	0	0.17	0.08	10.41	380.74	0.03	0.21	0.15	0.15	0.18	0.19	0.22	0.14	0.81	Shared
S8_vf_161703	0	0	0	0.88	0.01	0.68	0.09	0.33	0.13	0.28	60.5	0.15	1.18	1.7	0.77	0.41	0.91	0.41	1.15	0.98	SHHC
S8_vf_197061	0	0	0	0.83	0.26	0.81	0.42	0.39	0.22	0.47	2027.94	0.11	0.18	0.11	0.32	32.25	0.33	0.21	0.26	2.62	Shared
S8_vf_170878	0.01	0	0	28.58	0.04	4.35	0.23	4.33	4.32	1.5	111.55	0.46	4.52	3.13	3.23	14.8	1.64	9.36	2.05	3	Shared
S8_vf_153696	0	0	0.01	174.48	6.44	2.12	0.05	0.1	0.07	9.98	28.46	14.49	0.12	5.85	0.14	1.8	0.06	0.34	1.74	8.36	Shared
S8_vf_101283	0	0	0	1.06	0.01	2.99	1.1	0.8	0.1	0.67	39.33	0.69	1.39	0.28	2.63	0.83	9.89	0.24	2.93	1.4	SHHC
S8_vf_23955	0	0	0	0.57	0.06	0.5	0.13	0.23	0.16	0.17	362.41	0.12	1.15	0.38	0.36	18.54	0.32	0.28	0.4	1.12	Shared
S8_vf_136235	0	0	0	0.7	0.14	0.39	8.69	0.82	0.18	324.95	427.78	0.08	0.35	0.3	0.88	0.3	0.19	0.27	0.52	0.91	Shared
S8_vf_187742	0	0	0	0.6	0.02	0.31	0.1	0.23	0.13	0.26	55.14	0.05	0.45	0.26	0.34	0.24	0.25	0.22	0.29	0.65	SHHC
S8_vf_49282	0.01	0	0.02	4.81	4.16	248.25	70.57	0.23	0.13	0.17	42.12	0.48	0.76	0.28	0.54	0.46	0.28	0.29	0.42	0.69	Shared

S8_vf_41376	0	0	0	0.52	0.06	0.48	0.1	0.27	0.19	0.18	183.42	0.1	0.74	0.27	0.6	0.3	0.3	0.25	0.63	0.87	SHHC
S8_vf_7243	0	0	0	2.15	0.06	0.12	0.04	0.12	0.04	3	57.64	0.04	0.15	0.25	0.08	0.36	0.25	0.13	0.06	0.3	SHHC
S8_vf_102508	0	0	0	0.31	0.02	0.14	1.03	0.14	0.08	0.26	82.09	0.03	0.17	0.2	0.26	0.15	0.11	0.26	0.16	0.32	SHHC
S8_vf_93976	0	0	0	0.68	0.04	0.39	0	0.28	0.19	0.16	21.78	0.1	0.36	0.14	0.42	0.25	0.29	0.23	0.31	0.79	SHHC
S8_vf_111790	0	0	0	3.67	0.14	1.28	0.76	1.2	1.14	0.7	182.23	12.26	0.25	1.25	0.98	1.15	3.61	13.85	4.58	4.25	Shared
S8_vf_94361	0	0	0	0.52	0	0.22	0.01	0.12	0.12	0.03	14.82	0.02	0.12	0.09	0.16	0.24	0.15	0.23	0.16	0.56	SHHC
S8_vf_16172	0	0	0	0.51	0.02	0.33	0.09	0.11	0.09	3.33	26.22	0.03	0.32	2.22	0.53	0.18	0.1	0.19	0.18	3.32	SHHC
S8_vf_129423	0	0	0	0.54	0.03	0.51	0.02	0.67	0.24	0.49	36.18	0.06	0.65	0.43	0.4	0.33	2.35	0.35	0.63	0.65	SHHC
S8_vf_163760	0	0	0	2.37	3.32	5.39	0.05	3.37	2.99	4.23	28581.73	0.07	3.02	0.22	0.28	2.54	0.25	0.24	0.21	38.7	Shared
S8_vf_147128	0	0	0	0.71	0.09	0.63	0.11	67.37	160.79	0.28	44.28	2.19	1.1	0.43	0.56	2.47	0.37	0.43	0.49	0.94	Shared
S8_vf_173225	0	0	0	0.98	0.03	0.7	0.15	0.32	0.23	0.18	61.09	0.09	0.94	0.34	0.55	8.07	0.41	0.41	0.62	1.27	SHHC
S8_vf_147338	0	0	0	0.69	0.02	1.66	0.13	0.19	0.13	0.12	35.79	0.09	0.6	0.28	0.29	0.51	0.28	0.24	5.74	1.14	SHHC
S8_vf_208102	0	0	0	4.5	0.37	0.88	0.1	0.47	0.36	0.41	2503.78	3.77	0.33	0.18	1.49	0.93	0.25	0.24	0.25	3.8	SHHC
S8_vf_208107	0	0	0	0.92	0.91	1.67	0.05	0.96	1.04	1.04	7513.08	0.05	0.7	0.16	0.26	0.82	0.19	0.18	0.3	10.73	Shared
S8_vf_52050	0	0.03	0	238.46	0.24	0.71	0.06	0.85	1.03	0.25	160.41	0.06	0.59	0.3	0.74	224.42	0.54	0.7	0.69	0.83	Shared
S8_vf_208110	0	0	0	14.26	0.03	0.51	0.14	0.55	0.69	0.2	71.97	0.47	1.38	142.52	1.57	45.8	0.31	0.27	38.81	1	Shared
S8_vf_208111	0	0.01	0	113.14	0.46	1.04	0.07	849.46	2330.71	0.62	45.62	0.03	1.22	3.59	0.18	21.69	0.21	0.35	0.2	0.49	Shared
S8_vf_208115	0	0	0	1.3	0.29	0.81	0.03	0.43	0.19	0.14	722.11	0.14	0.74	0.08	0.3	1.29	0.16	0.3	0.85	2.77	SHHC
S8_vf_208119	0	0	0	0.4	0	1.33	0.6	0.23	0.15	6.38	116.57	0.46	0.21	0.32	0.67	0.13	0.33	0.14	0.46	0.69	SHHC
S8_vf_208120	0	0	0	0.55	0.08	1.75	0.27	0.2	0.26	0.22	495.83	0.09	0.44	36.88	1.47	0.26	0.2	0.26	0.63	1.61	Shared
S8_vf_208126	0.01	0	0	0.72	0.07	0.3	0.1	0.29	0.1	0.22	190.78	0.1	1.51	1.1	4.24	0.4	0.27	0.25	8.76	0.74	SHHC
S8_vf_208146	0	0	0	0.14	0.03	0.63	0.21	0.1	0.06	0.17	137.23	0.02	0.57	0.14	0.11	0.14	0.2	0.3	0.86	0.7	SHHC
S8_vs_182288	0.01	0	0.01	16.44	0.25	94.49	11.17	2.58	0.46	23.79	1673.04	0.17	15.06	43.11	0.21	20.4	14.72	0.2	2.32	2.49	Shared
S8_vs_9114	0	0	0	0.55	0.18	0.87	0.76	0.77	0.28	0.35	1462.33	0.25	0.61	0.19	1.41	0.43	0.52	0.46	0.44	2.6	SHHC
S8_vs_35308	0.01	0	0	5.99	0.64	1.03	0.09	0.79	0.61	0.76	4701.49	0.07	0.99	0.26	1.15	1.13	0.17	0.43	0.6	6.09	SHHC
S8_vs_88013	0	0	0	7.85	9.76	15.16	0.07	8.61	7.87	11.7	82495.66	0.13	3.93	0.23	0.38	6.96	0.17	0.43	0.26	107.13	Shared
S8_vs_9987	0.01	0	0.01	1.59	0.05	51.28	0.09	0.38	0.24	0.18	160.07	0.14	18.72	6.23	0.35	0.68	0.36	0.33	2.58	7.14	Shared
S8_vs_71212	0.01	0	0	1.42	0.14	0.43	0.06	0.42	0.24	0.34	941.1	0.12	0.82	0.1	0.42	0.46	0.26	0.55	0.54	1.96	SHHC
S8_vs_149700	0	0	0	6.56	0.32	16.31	0.66	30.63	3.35	9.84	2297.71	1.8	8.5	2.08	13.89	6.21	8.52	5.12	7.21	21.23	Shared
S8_vs_132359	0	0	0	0.89	0.06	0.43	0.08	0.33	0.2	0.17	42.96	0.12	0.67	0.52	0.47	0.3	0.42	0.54	0.95	0.93	SHHC
S8_vs_193843	0	0	0	0.67	0.05	0.43	0.09	0.26	0.15	0.18	532.17	0.09	0.43	0.91	0.2	0.34	0.17	0.26	0.25	1.27	SHHC
S8_vs_72117	0	0	0	1.52	1.4	2.53	0.01	1.34	1.19	1.65	9893.41	0.07	0.53	0.14	0.31	0.88	0.26	0.27	0.29	13.59	Shared
S8_vs_40014	0.01	0	0	1.03	0.35	7.95	0.77	0.54	0.36	1.32	3008.23	31.7	1.91	0.16	0.21	0.34	0.25	0.42	0.27	5.28	Shared
S8_vs_162053	0	0	0	2.04	1.3	2.67	0.07	1.37	0.83	1.66	8943.74	0.14	0.54	0.36	0.87	1.2	0.28	0.33	0.52	11.51	Shared
S8_vs_59736	0	0	0	1.08	0.08	4.66	0.24	0.5	0.34	0.4	135.66	0.37	1.74	1.19	1.54	16.7	0.71	1.11	1.45	2.5	Shared
S8_vs_51368	0	0	0	0.84	0.05	0.23	0.02	0.15	0.1	0.11	251.81	0.03	0.21	0.21	0.34	0.13	0.11	0.32	7.22	0.73	SHHC
S8_vs_208103	0	0	0	0.48	0.09	1.23	0.08	0.23	0.11	0.15	115.42	0.07	0.64	0.24	0.45	0.29	0.32	0.26	0.31	0.83	SHHC
S8_vs_208113	0	0	0	1.19	0.11	0.46	0.03	0.29	0.16	0.26	504.67	0.07	0.28	0.1	0.51	0.52	0.24	0.19	0.62	1.17	SHHC
S8_vs_208114	0.01	0	0	0.69	0.02	0.4	0.04	0.22	0.12	0.18	119.31	0.07	0.45	0.12	0.3	0.2	0.17	0.24	0.24	0.69	SHHC
S8_vs_208118	0	0	0	2.17	3.14	4.5	0.08	0.19	0.11	0.25	40.57	0.1	0.16	0.96	1.38	0.62	0.27	0.25	0.2	0.58	SHHC
S8_vs_208127	0.01	0.01	0	10.51	0.04	0.26	0.01	0.28	0.1	0.19	27.61	0.06	1.32	2.05	5.31	8.08	0.27	0.26	0.27	0.53	Shared
S8_vs_208129	0	0	0	0.87	0.02	0.64	0.09	0.33	0.16	0.21	33.99	0.13	0.45	0.74	0.44	0.43	0.48	0.3	0.5	1.21	SHHC
S8_vs_147670	0	0	0	0.42	0.01	0.36	0.04	0.23	0.08	0.12	19.15	0.06	0.28	0.11	0.22	2.44	0.22	0.21	0.24	0.63	SHHC
S8_vs_113059	0	0	0	0.71	0	0.16	0.42	0.11	0.05	0.04	16.32	0.03	0.1	0.02	0.15	0.44	0.06	0.2	0.34	0.36	SHHC
S8_vs_182331	0	0.04	0	80.63	0.07	0.64	0.05	20.4	29.42	0.18	26.5	0.1	0.74	0.51	0.38	23.85	0.19	0.34	0.42	0.58	Shared
S8_vs_147697	0.01	0	0	0.88	1.55	14.17	0.81	20.57	29.77	2.61	59.46	1.57	15.22	2.61	9.22	3.13	40.43	40.23	47.64	3.49	Shared
S8_vs_173961	0.01	0	0	0.9	0.15	11.98	0.14	0.35	0.24	0.21	127.79	0.17	0.97	0.57	1.31	0.46	0.6	0.34	0.49	1.11	Shared
S8_vs_139391	0	0	0	1.71	1.1	1.16	0.17	0.33	0.16	39.44	20.42	0.2	0.45	2.91	4.21	0.37	1.45	0.31	1.06	0.92	Shared
S8_vs_148389	0	0	0	0.75	0.05	0.32	0.7	0.21	0.18	0.15	110.53	0.07	0.31	0.11	0.32	0.25	0.18	0.33	0.29	0.71	SHHC
S8_vs_175235	0	0	0	0.47	0.05	0.29	0.05	0.27	0.17	0.16	380.47	0.1	0.19	0.14	0.28	0.24	0.2	0.18	0.22	1.14	SHHC
S8_vs_54183	0.01	0	0.01	7.16	0.06	1.22	0.2	1.27	0.2	0.8	58.27	0.3	0.71	0.47	0.98	1.75	0.8	0.66	1.39	1.05	SHHC
S8_vs_28311	0	0	0.01	0.93	0.65	25.08	0.3	0.53	0.19	1.63	20.79	0.13	57.84	1.24	2.61	2.23	21.04	0.31	2.74	8.31	Shared
S8_vs_132472	0	0	0	3.31	0.05	3.15	0.13	0.94	0.17	0.23	32.76	0.84	1.66	0.87	0.98	2.68	0.32	0.86	0.74	0.87	SHHC
S8_vs_63277	0	0	0	1.22	0.38	2.93	0.12	0.26	0.12	1.96	34.25	0.12	1.26	0.17	0.81	0.41	0.27	0.42	2.17	3.08	SHHC
S8_vs_176604	0	0	0	6.81	0.04	4.39	0.04	0.15	0.1	0.08	122.61	0.08	5.59	0.14	2.17	4.51	0.12	0.2	0.61	0.48	SHHC
S8_vs_176720	0	0	0.01	0.99	0.04	0.24	0.14	0.41	0.14	0.13	45.23	0.13	0.31	0.31	0.37	0.61	0.37	0.27	0.35	0.57	SHHC
S8_vs_142097	0	0	0	0.48	0.19	1.16	0.15	0.54	0.29	0.35	994.31	0.17	0.49	0.54	0.81	0.29	0.36	0.5	0.37	1.91	SHHC
S8_vs_3224	0	0	0	0.51	0.04	0.31	0.1	0.28	0.13	0.18	27.37	0.11	0.81	0.24	0.46	0.37	0.25	0.32	0.38	0.82	SHHC
S8_vs_115958	0	0	0	1.47	0.27	1.63	0.19	3.44	0.37	2.6	123.17	33.93	1.3	14.11	2.02	1.11	2.74	0.44	1.32	1.75	Shared
S8_vs_185041	0	0	0	5.49	0.46	0.61	0.48	0.89	0.18	10.88	69.21	0.23	23.3	0.37	5.25	5.04	1.65	1.12	0.95	1.67	Shared
S8_vs_99362	0.27	0.06	0	242.33	0.1	83.99	16.89	0.97	0.67	12.81	49.36	0.5	2.03	131.64	24.72	60.22	1.17	319.91	173.27	112.53	Shared
S8_vs_125717	0.01	0	0.01	6.09	0.16	3.12	0.3	9.76	1.17	2.19	371.03	1.11	6.5	1.37	16.91	15.47	8.3	2.46	1.63	2.72	Shared
S8_vs_4153	0.05	0	0	5.32	0.49	14.02	0.21	17.18	47.49	13.14	32.64	2.39	31.23	2.07	3.9	6.03	13.98	18.46	6.66	11.67	Shared
S8_vs_47265	0	0	0.01	3.71	0.04	37.2	24.18	2.15	3.77	10.58	38.41	0.42	18.68	0.45	0.55	1.87	3.02	1.85	13.25	3.99	Shared
S8_vs_143608	0	0	0	0.9	0.01	0.27	0.25	0.21	0.11	0.12	27.15	0.07	0.26	0.78	0.64	0.35	0.11	0.26	0.5	0.39	SHHC
S8_vs_134805	0.01	0	0	3.59	0.02	1.18	0.78	1.25	0.99	2.08	39.29	4.01	3.03	6.04	13.8	2.68	0.57	4.52	5.79	16.44	Shared
S8_vs_204260	0	0	0	0.9	0.03	0.46	0.14	0.39	0.18	0.18	202.23	0.06	0.51	0.2	0.42	0.28	0.34	0.24	0.26	1.08	SHHC
S8_vs_4883	0	0	0	1.14	0.07	0.43	0.02	0.2	0.12	0.1	117.37	0.06	0.41	0.38	0.37	0.13	0.33	0.22	0.2	0.82	SHHC
S8_vs_152553	0	0	0	0.39	0.01	0.62	0.1	0.62	0.31	0.18	78.59	0.06	0.48	0.79	0.5	0.24	0.31	0.18	3.37	0.99	SHHC
S8_vs_126582	0	0	0	0.43	0.04	0.75	0.15	0.24	0.16	2.58	80.03	0.06	0.75	0.68	0.44	0.42	0.35	0.28	0.4	0.97	SHHC
S8_vs_196247	0.01	0	0	0.86	0.04	0.38	0.03	0.2	0.13	0.15	53.78	0.11	0.45	0.11	0.81	0.4	0.13	0.3	0.28	0.76	SHHC
S8_vs_5211	0.17	0	0	2.91	0.09	2.73	0.08	8.21	9.31	0.16	40	0.16	0.45	13.34	4.24	1.23	3.23	0.64	0.44	1.17	Shared
S8_vs_179208	0	0	0.01	6.58	1.78	110.5	1.39	46.17	19.74	33.19	146.95	4.8	25.64	13.24	111.3	5.91	21.48	78.21	77.18	44.1	Shared
S8_vs_179529	0	0	0	1.09	0.03	6.36	1.1	3.5	0.17	3.52	34.56	0.64	1.87	0.25	1.03	0.9	0.76	4.09	14.77	3.65	SHHC
S8_vs_205257	0	0	0	7.1	0.28	6.79	0.52	14.87	1.27	5.71	43.48	2.9	4.47	163.84	12.19	15.02	4.42	2.85	5.56	5.89	Shared
S8_vs_145173	0	0	0.01	0.53	0.14	0.27	0.9	0.47	0.12	0.47	42.04	0.18	0.16	3.91	0.43	0.48	1.44	0.28	0.65	2.49	SHHC
S8_vs_110692	0	0	0	0.49	0.04	5.39	0.19	0.12	0.05	0.09	28.54	0.13	0.12	0.22	0.84	0.18	0.15	0.3	0.22	0.48	SHHC
S8_vs_154231	0	0	0	0.58	0.02	0.29	0.04	0.3	0.12	0.26	26.04	0.14	0.85	1.47	0.65	0.35	3.08	0.2	4.5	1.12	SHHC
S8_vs_136922	0	0	0	0.7	0.05	0.41	1.26	0.34	0.18	0.19	77.29	0.13	0.67	0.19	0.55	0.3	0.54	0.36	0.51	1.19	SHHC
S8_vs_102608	0.01	0	0	0.47	0.02	0.26	0.04	0.34	0.08	0.21	214.48	0.08	0.2	0.18	0.2	0.32	0.13	0.17	5.03	0.83	SHHC
S8_vs_206526	0	0.01	0	1.19	0.04	2.75	0.08	0.59	0.83	0.24	78.5	0.09	0.61	0.18	4.21	0.21	7.62	0.15	2.58	28.72	Shared
S8_vs_76852	0	0	0	0.9	0.05	38.97	0.17	0.23	0.16	8.64	46.95	0.1	0.51	1.49	0.57	0.12	0.18	0.59	0.16	0.5	Shared
S8_vs_50431	0.01	0	0	1.24	0.02	0.54	0.06	0.28	0.09	0.11	27.11	0.08	0.2	0.18	0.4	0.15	0.17	0.24	1.04	0.45	SHHC
#Total	0.04	0.01	0.04	28.54	141.38	312.22	102.3	79.82	145.28	170.25	316.92	154.77	53.29	86.9	101.08	80.04	61.71	28.74	106.89	14.41	116.7376 mean
																					87.04014 SD

Table S1-7. WMS_avg_cov

vOTUs	S1_wms_ME	S2_wms_SHHC	S3_wms_ME	S4_wms_SHHC	S5_wms_ME	S6_wms_SHHC	S7_wms_ME	S8_wms_SHHC	S9_wms_ME	S11_wms_ME	S12_wms_SHHC	S17_wms_ME	Status
S9-wms-combined_27311	0.88	0.82	0.11	0.31	1.16	0.2	0.76	0.89	15.21	0.46	1.15	0.62	ME
S9-wms-combined_64277	2.1	1.02	0.23	0.36	2.2	0.25	2.57	0.73	130.53	0.51	1.31	0.94	ME
S9-wms-combined_10990	17.51	57.54	1.45	8.53	12.84	5.41	30.03	6.66	151.11	6.96	24.21	17.38	Shared
S9-wms-combined_53991	28.18	24.59	0.09	0.72	2.81	5.09	37.75	0.74	65.91	0.56	1.16	13.93	Shared
S9-wms-combined_97755	7.8	8.83	0.72	1.77	4.07	1.2	3.26	3.45	93.72	5.09	3.65	3.04	ME
S9-wms-combined_33230	33.59	70.71	1.95	7.52	61.22	3.44	43.15	14.97	277.62	13.51	26.2	22.79	Shared
S9-wms-combined_2042	4.48	6.65	0.49	1.22	3.4	1.1	2.69	3.25	98.02	3.11	3.38	2.29	ME
S9-wms-combined_124857	35.21	50.02	3.13	4.12	39.54	27.05	12.9	31.53	61.99	41.14	25.04	139.43	Shared
S9-wms-combined_28805	8.88	7.24	0.19	1.73	3.67	1.33	6.54	7.46	33.68	3.5	8.91	2.75	ME
S9-wms-combined_71647	0.82	1.8	0.06	0.79	1.22	0.16	1.64	0.44	16.7	0.36	0.72	0.48	ME
S9-wms-combined_93232	1.06	0.99	0.06	0.42	1.37	0.19	1.06	0.63	22.09	0.82	0.81	0.58	ME
S9-wms-combined_109322	13.34	25.83	1.02	8	4.05	7.85	19.06	4.43	151.24	3.49	6.45	13.13	Shared
S9-wms-combined_3461	1.2	1.55	0.11	0.39	1.17	0.37	1.51	0.85	15.71	0.38	1.17	0.82	ME
S9-wms-combined_29611	33.41	39.63	3.03	3.9	31.36	21.19	13.8	24.3	50.12	34.52	22.07	113.99	Shared
S9-wms-combined_25007	6.76	7.85	0.4	1.21	11.39	1.41	14.72	3.18	27.45	10.82	3.53	4.29	ME
S9-wms-combined_3878	0.31	0.7	0.03	0.27	0.34	0.05	0.53	0.32	24.59	0.18	0.72	0.34	ME
S9-wms-combined_89249	6.69	1.71	0.26	1.1	1.04	0.37	2.95	0.75	15.4	1.5	2.87	3.17	ME
S9-wms-combined_105462	1.65	3.79	0.16	1.46	1.29	0.62	1.9	0.67	33.54	0.53	5.87	1.44	ME
S9-wms-combined_20485	3.85	4.93	0.13	2.35	3.77	0.24	2.26	0.76	59.95	0.51	0.82	2.94	ME
S9-wms-combined_9931	3.17	4.91	0.2	1.25	6.02	0.94	3.64	15.44	116.02	1.07	2.26	1.19	Shared
S9-wms-combined_36459	0.82	0.86	0.05	0.5	1.14	2.7	0.92	0.78	16.07	0.39	0.92	0.64	ME
S9-wms-combined_105822	1.52	1.62	0.51	0.67	1.64	0.54	2.63	0.89	36.66	1.6	1.17	2.02	ME
S9-wms-combined_26410	3.18	2.92	0.12	0.92	1.34	1.21	1.56	0.83	29.97	1.01	2.75	1.3	ME
S9-wms-combined_31456	11.35	4.16	0.53	2.46	39.66	5.04	29.1	7.3	165.36	5.08	12.84	9.03	Shared
S9-wms-combined_37587	16.93	40.38	0.95	6.61	29.05	2.39	17.03	5.97	289.89	5.26	18.45	10.28	Shared
S9-wms-combined_5526	135.59	50.51	3.16	2.28	275.21	163.01	89.43	86.71	313.82	102.89	107.44	29.54	Shared
S9-wms-combined_33229	0.83	0.74	0.18	0.51	0.69	0.16	1.05	0.37	296.16	0.22	1.06	0.48	ME
S9-wms-combined_76274	6.5	1.94	8.31	13.41	32.05	26	8.69	2.56	98.67	1.28	2.38	4.51	Shared
S9-wms-combined_44402	20.19	26.47	0.24	0.6	2.05	0.16	1.05	0.45	382.37	0.71	1.59	0.74	Shared
S9-wms-combined_56773	0.73	2.51	0.18	1.2	0.58	0.21	1.02	0.41	147.32	0.2	0.81	0.84	ME
S9-wms-combined_122742	58.05	28.66	5.84	2.85	99.4	33.42	6.72	23.2	75.29	21.13	35.02	16.88	Shared
S9-wms_64472	9.38	10.07	0.72	1.8	4.43	3.08	6.93	1.66	34.5	3.56	5.79	11.1	Shared
S9-wms_104169	1.13	1.55	0.09	0.42	3.55	0.52	0.29	0.54	17.23	0.62	0.26	0.22	ME
S9-wms_19025	0.33	0.79	0.04	0.48	0.39	0.08	0.62	0.19	25.74	0.14	0.44	0.22	ME
S9-wms_114971	0.45	0.6	0.04	0.45	0.41	0.05	0.53	0.18	17.95	0.14	0.33	0.26	ME
S9-wms_10480	0.55	0.35	0.06	0.08	1.31	0.71	0.24	1.68	7.31	0.46	1.47	0.68	ME
S9-wms_128232	0.52	1	0.27	1.41	0.77	0.22	0.68	0.47	26.4	0.19	0.91	0.32	ME
S11-wms-combined_41367	0.84	0.95	0.06	0.29	0.87	1.43	1.64	0.42	0.44	43.76	1.09	1.45	ME
S11-wms-combined_193148	9.1	1.47	0.16	0.24	1.63	60.11	1.38	2.53	0.89	76.13	1.85	1.21	Shared
S11-wms-combined_179605	11.48	11.07	0.83	2.52	10.53	3.98	6.28	6.42	10.36	52.43	6.27	24.47	Shared
S11-wms-combined_221310	1.44	1.06	0.16	0.34	2.44	0.65	9.83	1.74	0.6	23.55	1.53	1	ME
S11-wms-combined_14636	1.96	1.46	0.25	0.64	4.87	0.79	2.08	2.63	0.97	39.06	2.72	1.55	ME
S11-wms-combined_83571	25.22	0.63	0.98	1.86	114.16	51.63	94.59	21.46	1.29	42.44	8.46	0.86	Shared
S11-wms-combined_125176	206.31	34.33	8.66	17.78	388.14	79.59	232.47	115.11	22.31	136.18	80.22	23.9	Shared
S11-wms-combined_304239	1.54	1.32	0.19	0.4	2.38	0.7	1.61	1.41	1.01	44.5	1.54	1.74	ME
S11-wms-combined_194205	5.36	5.68	1	1.08	11.98	1.75	13.5	9.64	3.23	70.88	12.65	2.36	Shared
S11-wms-combined_84326	2.19	1.32	0.2	7.01	2.53	0.74	1.26	2.5	0.77	53.65	2.01	1.71	ME
S11-wms-combined_236214	76.68	10.46	7.55	10.43	7.02	9.79	8.38	35.26	1.74	43.5	25.36	79.22	Shared
S11-wms-combined_264050	38.92	18.29	3.59	10.15	11.36	2.98	14.54	22.33	3.45	43.84	22.9	31.05	Shared
S11-wms-combined_112147	1.07	1.2	0.09	0.64	1.64	0.23	0.76	1.16	0.68	39.39	1.47	0.87	ME
S11-wms-combined_140009	27.63	8.9	4.44	9.92	3.67	2.95	7.92	16.38	1.66	61.48	36.71	97.16	Shared
S11-wms-combined_168048	4.51	1.76	0.23	1.53	1.68	0.45	8.11	4.26	3.39	42.99	12.48	0.76	Shared
S11-wms-combined_195425	5.24	5.39	0.65	9.55	1.38	0.39	1.24	1.11	0.84	21.05	1.52	119.63	ME
S11-wms-combined_112733	2.16	1.55	0.23	13.99	2.97	0.91	1.55	2.15	0.86	106.45	1.99	1.9	Shared
S11-wms-combined_58538	1.08	0.91	0.09	0.22	1.3	0.32	0.66	1	0.72	20.43	0.71	0.79	ME
S11-wms-combined_154845	0.66	0.56	0.09	0.68	1.12	0.21	0.39	0.91	0.52	31.66	0.76	0.47	ME
S11-wms-combined_17029	0.82	0.93	0.08	0.39	0.91	0.31	0.75	0.33	0.51	103.68	2.97	1.23	ME
S11-wms-combined_3507	117.01	25.97	10.46	4.19	328	9.16	28.85	48.94	5.45	100.16	68.16	29.42	Shared
S11-wms-combined_265453	1.41	1.32	0.05	0.12	0.7	1.46	1.04	0.29	1.55	10.46	1.02	1.75	ME
S11-wms-combined_320710	5.44	7.82	0.36	3.26	7.02	2.51	8.95	2.59	5.34	49.99	4.62	3.43	ME
S11-wms-combined_101337	3.31	1.52	0.19	0.49	1.59	0.97	6.92	1.81	1.09	114.79	11.25	19.65	Shared
S11-wms-combined_73204	190.09	107.07	4.65	1.07	430.65	12.36	100.05	18.78	8.16	177.05	122.38	36.99	Shared
S11-wms-combined_46816	6.46	30.95	1.27	14.73	2.15	0.5	1.33	21.2	0.65	56.18	1.72	1.16	Shared
S11-wms-combined_226165	0.84	0.52	0.09	0.19	99.07	8.92	0.35	1.02	0.39	55.44	12.72	0.86	Shared
S11-wms-combined_321938	48.96	74.68	2.03	5.24	23.04	10.54	66.69	49.16	1.67	57.85	39.56	27.22	Shared
S11-wms-combined_198512	23.18	7.26	0.73	1.5	14.79	1.78	12.61	10.66	1.49	43.55	9.83	10.92	Shared
S11-wms-combined_282227	2.41	4.51	0.28	1.38	6.45	0.54	13.05	1.79	6.8	20.91	2.63	2.36	ME
S11-wms-combined_267993	1.78	1.25	0.15	0.26	4.23	0.4	2.01	2.27	0.76	27.61	2.14	1.2	ME
S11-wms-combined_74983	2.12	1.21	0.17	0.72	2.19	18.72	14.09	1.03	0.56	18.95	96.89	143.89	Shared
S11-wms-combined_116589	19.19	23.94	2.28	2.41	19.06	16.54	14.65	19.17	13.79	51.53	25.6	74.52	Shared
S11-wms-combined_48246	1.9	1.25	1.24	1.02	1.2	1.4	2.03	9.22	1.26	85.89	37.53	0.57	Shared
S11-wms-combined_283220	4.06	4.72	0.24	0.33	3.02	2.01	1.52	2.31	1.08	71.02	2.48	1.76	ME
S11-wms-combined_172889	1.92	1.11	24.16	0.56	1.94	0.53	9.44	2.65	1.21	137.2	1.46	1.77	ME
S11-wms-combined_268967	2.48	1.78	0.32	3.12	3.77	0.62	1.22	2.68	1.05	62.44	2.83	1.7	ME
S11-wms-combined_200084	9.02	0.93	0.14	0.49	0.9	0.21	1.23	0.72	0.99	27.55	2	0.68	ME
S11-wms-combined_117756	1.4	0.94	0.27	0.59	1.72	0.48	1.07	1.22	1.72	21.57	1.31	0.85	ME
S11-wms-combined_49632	2.31	2.78	0.43	0.17	2.03	2.87	4.21	1.64	0.9	31.06	4.11	7.39	ME
S11-wms-combined_63818	69.86	23.88	4.89	3.74	298.95	97.54	59.98	60.58	41.81	324.38	138.49	32.26	Shared
S11-wms-combined_215723	291.55	83.24	2.87	2.18	183.18	174.54	103.94	103.06	100.6	101.52	92.54	31.27	Shared
S11-wms-combined_64392	2.46	1.94	0.14	0.43	8.43	2.43	7.09	14.62	0.83	77.15	11.58	1.53	Shared
S11-wms-combined_92114	85.13	37.47	6.97	3.77	342.54	115.61	69.82	69.07	60.4	143.84	144.77	71.81	Shared
S11-wms-combined_119215	3.04	3.99	0.33	0.4	4.55	1.56	1.97	2.8	1.37	29.37	3.11	5.45	ME
S11-wms-combined_120257	17.24	13.23	2.18	1.51	27.36	6.5	6.8	13.6	3.19	31.82	11.46	23.69	Shared
S11-wms-combined_299954	2.12	6.17	0.22	0.69	3.09	1.12	1.87	1.98	0.83	38.28	2.98	6.76	ME

S11-wms-combined_217790	87.87	24.33	2.28	0.73	184.19	183.26	65.46	50.73	64.93	46.56	41.99	16.13	Shared
S11-wms-combined_204522	1.01	0.95	0.06	0.3	0.88	3.4	1.02	0.62	0.69	31.2	1.03	0.49	ME
S11-wms-combined_329493	81.49	50.92	9	2.81	68.88	6.65	18.67	36.5	3.11	48.06	29.17	14.07	Shared
S11-wms_303398	3.13	49.74	0.25	0.42	2.31	8.41	3.17	3.66	0.98	27.4	3.75	8.71	Shared
S11-wms_55610	1.5	19.96	0.22	0.67	1.79	0.5	3.75	1.74	0.45	18.05	1.53	1.58	Shared
S11-wms_14390	2.48	3.11	0.32	0.81	2.45	7.96	4.11	5.34	0.84	494.02	5.61	1.4	ME
S11-wms_3116	50.14	38.44	7.12	5.48	5.42	40.93	7.29	134.78	3.27	73.94	56.6	32.22	Shared
S11-wms_320555	0.47	0.85	0.06	0.39	0.94	0.14	0.84	0.52	0.79	24.92	0.83	0.43	ME
S11-wms_87293	0.53	0.9	0.19	0.64	0.86	0.18	0.6	0.47	0.62	362.52	1.41	0.4	ME
S11-wms_32691	0.95	9.43	0.08	0.43	1.02	0.24	0.93	0.69	0.47	49.82	1.08	0.49	ME
S11-wms_6203	0.67	1.68	0.06	0.59	1.96	0.24	1.14	1.16	2.17	38.97	0.78	0.39	ME
S11-wms_330479	0.97	1.15	2.53	0.32	1.17	0.17	0.94	0.73	0.55	211.64	0.96	0.67	ME
S1-wms-combined_324053	137.39	0.88	0.09	0.49	0.92	8.43	0.87	0.66	0.79	0.33	0.98	0.52	ME
S1-wms-combined_394574	67.22	850.69	0.59	4.18	0.83	2.24	1.22	0.71	0.73	0.85	1.28	0.68	Shared
S1-wms-combined_394628	73.85	0.52	0.09	0.39	0.47	0.23	1.54	0.77	0.32	0.31	1.84	0.36	ME
S1-wms-combined_246923	24.55	0.61	0.11	0.52	0.89	0.65	0.94	0.89	0.46	11.71	9.47	23.95	ME
S1-wms-combined_263562	161.54	530.16	1.47	0.59	1.07	0.25	36.7	33.56	0.69	7.35	10.13	0.66	Shared
S1-wms-combined_164894	70.03	2.63	6.79	3.95	2.84	0.96	1.86	2.92	1.01	1.78	4.5	2.1	ME
S1-wms-combined_231234	39.45	0.52	0.04	0.45	0.75	0.07	0.72	0.34	0.27	0.25	0.37	0.25	ME
S1-wms-combined_231411	37.6	0.95	0.06	0.76	1.01	0.28	0.6	2.81	0.24	0.49	1.13	0.77	ME
S1-wms-combined_132934	66.35	13.33	2.09	2.11	5.35	2.85	6.76	12.06	2.58	17.18	10.31	5.76	Shared
S1-wms-combined_17651	32.66	10.38	0.69	1.11	12.38	0.6	1.49	4.26	0.71	2.61	17.77	3.17	Shared
S1-wms-combined_313851	17.95	0.74	0.04	0.42	0.39	0.13	0.53	0.33	0.64	0.16	0.77	0.47	ME
S1-wms-combined_330329	41.33	1.21	0.23	0.64	0.69	0.16	0.66	1.23	0.58	1.54	0.72	0.64	ME
S1-wms-combined_116938	35.08	10.59	2.93	5.56	5.79	3.25	6.09	9.86	1.49	8.34	11.87	18.62	Shared
S1-wms-combined_248622	60.56	71.71	0.47	1.45	0.42	0.4	7.16	0.49	1.09	0.47	2.2	9.01	Shared
S1-wms-combined_100430	32.25	0.92	3.45	8.49	1.24	0.16	1.02	0.65	1.06	0.35	0.89	15.01	ME
S1-wms-combined_166392	49.52	6.16	2.93	0.64	2.55	0.47	4.19	3.94	0.82	1.07	1.7	22.11	ME
S1-wms-combined_19031	38.38	0.92	0.69	0.43	1.8	1.09	2.1	2.07	0.63	2.24	4.22	1.18	ME
S1-wms-combined_118167	61.2	1.05	0.37	0.62	1.82	11.97	1.55	1.85	0.68	0.77	2.41	1	Shared
S1-wms-combined_331896	55.48	3.34	0.95	1.97	3.21	1.23	3.22	2.25	4.66	0.67	24.02	4.41	Shared
S1-wms-combined_332236	179.6	10.94	2.46	2.86	38.85	3.16	44.43	8.53	2.9	49.35	17.77	5.46	Shared
S1-wms-combined_151708	36.81	1.66	0.6	0.82	1.74	0.27	1.4	9.53	0.92	0.46	2.27	1.44	ME
S1-wms-combined_52643	27.33	13.95	0.08	0.48	1.34	0.24	0.86	0.91	0.8	0.52	1.07	1	Shared
S1-wms-combined_52708	210.91	129.55	16.07	28.68	11.15	18.8	16.15	97.75	3.57	67.73	64.59	203.77	Shared
S1-wms-combined_185336	36.86	1.61	0.34	0.27	2.63	0.13	4.81	1.73	0.32	0.32	1.26	0.26	ME
S1-wms-combined_68998	49.4	2.75	0.98	1.85	1.66	0.83	3.21	2.1	0.9	1.01	3.65	2.44	ME
S1-wms-combined_102801	42.57	62.59	1.59	0.84	8.43	1.77	12.26	7.15	9.23	37.13	41	24.01	Shared
S1-wms-combined_120533	53.35	4.76	1.68	2.55	2.64	2.34	5.16	50.74	3.26	5.23	15.95	8.29	Shared
S1-wms-combined_252439	19.05	2.6	0.04	0.12	0.84	0.12	0.56	0.85	0.47	0.47	1.27	0.64	ME
S1-wms-combined_302166	322.93	4.13	3.07	1.23	6.98	1.38	29.18	112.39	2.48	4.29	91.36	3.67	Shared
S1-wms-combined_285012	61.82	3.09	1.53	1.69	2.68	0.56	2.47	42.07	0.78	1.99	11.92	1.11	Shared
S1-wms-combined_186878	68.99	1.2	0.09	1.18	1.53	0.32	1.45	1	0.88	0.56	0.91	0.61	ME
S1-wms-combined_153670	58.04	0.46	0.07	0.14	0.88	0.32	1.53	0.56	1.27	0.48	0.87	0.59	ME
S1-wms-combined_351889	73.16	1.06	0.28	0.6	1.86	0.49	1.56	18.21	0.64	0.77	2	1.02	Shared
S1-wms-combined_169788	222.7	1.37	0.24	0.32	1.24	3.63	1.16	1.51	1.14	0.52	60.14	1.75	Shared
S1-wms-combined_220022	35.77	1.45	0.2	0.59	1.12	0.3	1.44	3.13	0.79	0.41	1.08	1.51	ME
S1-wms-combined_169879	25.15	10.68	0.87	0.48	0.75	0.33	1.12	2.15	1.23	0.34	6.86	0.82	Shared
S1-wms-combined_23248	228.01	111.2	26.33	28.09	21.24	46.18	26.21	88.81	5.98	71.81	71.84	265.86	Shared
S1-wms-combined_72167	24.04	1.11	0.22	0.27	2.74	1.05	1.15	2.66	1.04	1.32	2.11	1.8	ME
S1-wms-combined_40115	56.99	8.11	1.08	1.96	4.3	0.74	4.97	7.49	2.61	2	8.67	5.64	ME
S1-wms-combined_304377	135	44.35	9.89	15.86	6.81	12.05	14.84	42.54	2.06	27.1	35.59	94.97	Shared
S1-wms-combined_353757	35.85	1.37	0.29	0.36	1.98	0.55	1.08	6.03	0.87	1.08	9.29	1.08	ME
S1-wms-combined_57370	149.94	12.95	1.65	2.01	38.58	6.93	8.01	38.63	3.24	10.69	45.61	12.67	Shared
S1-wms-combined_354899	23.29	3.67	0.19	0.6	2.57	0.59	1.66	1.41	0.65	0.85	1.34	1.38	ME
S1-wms-combined_41369	16.46	1.36	0.14	0.32	1.75	0.47	1.1	1.63	0.73	0.84	1.58	1.05	ME
S1-wms-combined_156637	83.91	6.84	3.71	4.68	27.27	0.33	7.88	28.3	0.94	0.85	34.24	1.26	Shared
S1-wms-combined_107073	14.45	9.57	0.06	0.25	0.49	0.19	0.89	0.41	0.48	0.21	0.61	0.33	ME
S1-wms-combined_107766	281.97	81.17	3.13	1.33	218.14	266.61	95.76	72.37	94.01	71.85	64.22	29.75	Shared
S1-wms-combined_92523	48.16	1.59	0.2	0.73	2.13	8.12	1.5	1.99	1.21	22.93	8.11	1.95	ME
S1-wms-combined_224578	28.88	1.54	0.07	0.42	1.04	0.21	0.95	0.61	0.64	0.73	1.02	0.59	ME
S1-wms-combined_357373	329.29	1.24	0.34	0.3	1.59	0.59	1.46	0.54	1.38	0.24	0.59	2.17	ME
S1-wms-combined_373308	183.22	32.77	8.36	4.21	107.7	19.81	15.33	206.55	7.74	38.47	225.64	41.1	Shared
S1-wms-combined_143459	40.37	0.96	0.6	0.5	2.02	0.75	5.23	7.17	0.78	1.66	1.61	1.06	ME
S1-wms-combined_308848	35.61	112.41	0.16	0.47	8.68	0.36	1.03	7.16	0.51	0.8	1.27	0.94	Shared
S1-wms-combined_159544	32.19	1.23	0.26	0.53	2	0.55	2.52	9.04	0.63	1.94	1.45	0.77	ME
S1-wms-combined_374219	14.66	6.33	3.06	0.49	5.47	3.8	2.8	2.76	1.36	2.73	2.02	21.49	ME
S1-wms-combined_340558	18.09	0.84	0.25	0.3	1.47	0.46	0.83	0.99	0.75	0.29	14.41	0.51	Shared
S1-wms-combined_227003	24.75	0.93	0.4	0.8	1.09	0.27	1.28	1.11	0.7	0.45	2.64	0.57	ME
S1-wms-combined_227917	42.08	3.58	1.85	0.65	2.65	1.18	3.28	2.13	1.14	1.3	2.4	16.6	ME
S1-wms-combined_360285	31.33	1.24	1.24	0.76	3.24	5.68	11.8	13.36	0.86	3.5	2.73	1.01	Shared
S1-wms-combined_212892	45.4	1.01	0.03	0.59	0.38	0.13	0.55	0.27	0.65	0.2	0.82	0.33	ME
S1-wms-combined_131270	20.64	1.11	0.24	0.55	1.84	0.56	1.02	1.5	1	0.71	1.34	1.23	ME
S1-wms-combined_197401	38.77	135.28	0.43	0.79	1.52	0.5	1.19	1.3	0.83	0.56	3.23	1.15	Shared
S1-wms-combined_279492	102.73	57.01	5.68	18.53	11.15	9.09	48.68	33.43	93.77	10.65	73.86	40.72	Shared
S1-wms-combined_164485	63.38	7.77	4.72	6.19	12.23	1.93	4.24	47.32	1.95	4.66	22.19	11.99	Shared
S1-wms-combined_32863	33.44	27.87	1.85	14.37	44.01	6.5	35.09	15.61	9.04	7.48	14.86	15.13	Shared
S1-wms-combined_331544	42.49	4.31	1.99	1.33	2.41	0.76	1.03	1.42	2.1	0.51	1.33	10.99	ME
S1-wms-combined_85225	33.77	3.9	3.67	5.72	4.98	1.59	7.62	1.82	2.9	0.94	1.51	10.69	ME
S1-wms-combined_136097	88.64	17.94	5.3	2.5	18.15	4.73	6.1	30.77	3.8	13.88	24.13	19.88	Shared
S1-wms-combined_307323	38.99	3.98	3.91	1.67	3.63	1.57	2.3	1.63	3.91	0.76	1.23	6.54	ME
S1-wms-combined_94988	200.49	16.14	4.65	3.09	75.76	10.52	11.19	90.2	5.2	19.84	98.07	15.32	Shared
S1-wms_296850	122.94	2.12	0.9	1.01	3.35	1.03	1.63	27.87	1.21	1.74	4.52	2.36	Shared
S1-wms_313991	21.9	8.94	0.09	0.09	0.66	0.07	0.35	0.54	0.35	0.29	0.34	0.26	ME
S1-wms_100010	41.79	12.03	0.13	0.66	1.99	1.06	1.89	2.67	0.63	1.84	2.81	1.15	Shared
S1-wms_265285	27.87	2.41	0.48	0.38	1.62	0.49	1.5	5.09	0.76	4.8	7.25	1.11	ME

S1-wms_185046	15.74	1.06	0.7	0.46	2.66	0.53	1.09	1.92	0.77	2.23	38.38	1.38	Shared
S1-wms_102342	56.58	1.18	0.17	0.69	1.19	1.46	0.84	1.1	0.84	4.74	40.6	1.65	Shared
S1-wms_4277	75.32	2.39	0.39	0.76	9.28	15.71	3.52	9.94	0.52	59.13	21.34	1.11	Shared
S1-wms_153615	283.6	0.83	0.03	0.14	1.59	0.1	0.11	0.17	0.2	0.21	0.4	1.05	ME
S1-wms_236105	79.78	0.83	0.2	0.6	1.09	0.45	0.75	46.85	0.74	0.63	1.76	0.53	Shared
S1-wms_170115	45.28	3.45	1.91	7.75	2.45	3.54	4.26	3.8	1.05	4.42	6.35	2.87	ME
S1-wms_106115	82.7	0.83	0.04	0.44	0.39	0.16	0.61	0.23	0.76	0.16	0.6	0.48	ME
S1-wms_388292	13.07	0.77	0.2	0.35	6.19	0.41	1.06	0.69	0.48	0.47	1.14	0.51	ME
S1-wms_223620	217.43	0.85	0.4	0.53	1.2	0.43	57.97	229.9	0.69	0.58	7.47	0.76	Shared
S1-wms_208875	29.11	0.84	0.75	0.42	0.59	0.22	1.14	0.38	0.45	0.34	0.68	0.33	ME
S1-wms_225995	40.92	1.48	0.08	0.46	1.46	0.44	1.87	1.24	1.01	3.7	1.86	2.19	ME
S1-wms_29288	28.44	2.32	0.46	0.89	1.63	1.41	1.47	1.64	0.6	0.77	1.91	2.34	ME
S1-wms_244836	22.92	75.83	0.18	3.71	8.21	0.11	2.53	1.02	126.99	4.19	8.68	1.75	Shared
S1-wms_130214	27.7	1.03	0.12	0.66	1.4	0.42	1.4	1.75	0.72	0.62	1.22	0.76	ME
S1-wms_212940	75.56	1.17	1.1	6.19	0.78	1.45	1.64	1.28	0.64	2.22	51.19	0.51	Shared
S1-wms_394571	102.54	40.3	0.11	0.35	1.31	0.32	0.74	1.8	0.48	0.5	1.04	0.63	Shared
S1-wms_32878	35.82	1.18	0.21	0.24	1.2	0.2	0.74	1.12	0.59	0.5	0.99	1.4	ME
S3-wms-combined_135428	1.42	0.94	14.82	0.24	2.36	0.76	7.5	3.52	0.58	0.72	1.46	1.07	ME
S3-wms-combined_73262	83.67	35.19	20.67	23.18	5.72	256.28	86.45	178.77	1.36	74.99	39.56	1.37	Shared
S3-wms-combined_45305	181.56	138.03	46.62	8.15	12.96	3.04	95.53	62.1	1.27	8.57	11.99	56.34	Shared
S3-wms_23958	4.91	3.22	18.02	0.56	1.28	0.81	1.28	15.41	0.63	0.63	47.59	0.83	Shared
S3-wms_94494	1.97	1.13	17.03	0.72	2.95	1.25	5.87	2.09	1.23	1.19	2.36	1.61	ME
S3-wms_22744	24.04	14.73	15.25	2.22	3.03	1.71	4.91	3.3	0.8	3.79	45.78	2.76	Shared
S5-wms-combined_26061	10.9	7.89	2.31	66.99	35.3	58.87	53.01	15.46	1.36	15.35	54.14	19.63	Shared
S5-wms-combined_205046	64.47	73.67	8.09	8.93	87.5	58.6	36.88	48.85	45.58	56.26	49.54	184.36	Shared
S5-wms-combined_18356	7.6	1.22	0.11	0.44	24.04	0.52	1.44	0.95	23.81	2.14	1.32	2.86	ME
S5-wms-combined_161191	22.41	16.75	3.12	2.59	41.09	16.65	14.8	18.5	6.54	22.8	14.44	65.17	Shared
S5-wms-combined_134381	137.54	28.79	6.89	9.04	46.04	6.17	9.28	20.52	2.42	31.16	7.17	10.37	Shared
S5-wms-combined_90315	12.53	14.35	0.68	2.35	93.35	7.69	13.81	16.6	5.06	16.73	46.71	9.67	Shared
S5-wms-combined_134743	1.12	2.1	0.12	0.33	42.98	0.27	1.34	1.1	0.88	0.75	1.53	1.03	ME
S5-wms-combined_197186	1.04	1.46	0.08	0.21	28.32	0.22	1.56	0.71	0.93	0.3	0.91	0.61	ME
S5-wms-combined_117095	11.27	5.94	0.64	5.72	38.33	8.9	33.62	27.19	9.4	8.49	5.81	4.42	Shared
S5-wms-combined_72410	15.99	12.78	1.83	1.96	139.03	15.68	8.58	13.85	13.34	17.67	11.06	16.75	Shared
S5-wms-combined_206050	3.24	3.82	0.33	1.12	24.13	1.37	3.14	2.53	8.02	2.32	4.92	8.22	ME
S5-wms-combined_73301	1.05	2.64	0.19	2.8	15.16	0.27	2.54	0.4	0.85	0.44	1.29	3.85	ME
S5-wms-combined_108794	9.42	27.76	1.25	4.7	138.8	10.71	19.28	6.48	17.08	3.11	4.35	1.33	Shared
S5-wms-combined_145067	4.87	3.79	0.71	1.87	23.09	26	3.43	6.91	3.04	10.48	2.33	68.83	Shared
S5-wms-combined_3201	1.09	0.97	0.07	0.34	26.7	0.15	0.66	0.83	0.74	0.37	0.97	0.56	ME
S5-wms-combined_207291	36.25	95.53	0.95	2.99	57.11	2.55	36.84	33.97	18.28	7.09	24.57	11.85	Shared
S5-wms-combined_29272	14.17	3.14	0.77	1.01	30.96	0.91	6.17	8.53	0.76	4.78	8.37	1.58	ME
S5-wms-combined_29447	14.01	5.77	0.86	1.82	114.37	16.78	11.7	40.76	6.67	29.83	34.75	5.98	Shared
S5-wms-combined_199135	2	1.36	0.07	0.51	19.43	0.63	1.17	1.28	0.59	0.41	1.02	1.9	ME
S5-wms-combined_83370	30.5	59.51	1.81	5.09	77.52	6.85	45.72	18.19	37.23	13.93	19.62	32.48	Shared
S5-wms-combined_83412	0.85	0.85	0.06	0.58	17.46	0.25	0.87	0.51	0.98	0.76	0.89	0.62	ME
S5-wms-combined_66017	0.74	1.03	0.09	0.26	28.04	0.24	0.72	0.57	0.87	0.32	0.72	0.45	ME
S5-wms-combined_66662	18.76	11.09	2.07	2.64	159.11	13.52	8.06	13.35	12.5	14.68	11.57	20.55	Shared
S5-wms-combined_147993	1.19	1.18	0.28	0.4	50.13	0.19	0.85	0.5	1.35	0.21	0.7	0.48	ME
S5-wms-combined_156329	0.94	1.1	0.09	0.21	28.92	0.26	1.46	0.62	1.25	0.21	0.83	0.46	ME
S5-wms-combined_7292	0.69	1.2	0.07	0.36	215.01	10.36	1.23	0.4	0.29	0.2	4.49	0.38	Shared
S5-wms-combined_59064	31.44	73.56	7.39	13.59	90.05	8.33	67.21	26.24	46.49	18.04	32.38	215.03	Shared
S5-wms-combined_175145	0.86	1.12	0.08	0.57	19.46	0.26	0.97	0.61	0.6	0.77	0.93	6.06	ME
S5-wms-combined_94482	1.94	2.77	0.24	1.7	60.07	2.03	16.22	1.33	1.62	1.15	3.94	1.4	ME
S5-wms-combined_67650	195.25	13.77	10.17	11.57	365.17	135.73	198.24	136.26	19.76	171.23	81.6	15.68	Shared
S5-wms-combined_94786	7.4	3.92	1.93	1.37	65.83	5.72	8.02	7.92	4.5	8.09	4.95	16.94	ME
S5-wms-combined_210959	84.51	35.3	5.07	6.9	1045.11	226.03	74.14	63.87	54.03	283.75	420.78	48.42	Shared
S5-wms-combined_78434	2.29	2.58	0.14	0.51	28.95	0.58	1.78	1.47	0.86	1.6	2.58	4.95	ME
S5-wms-combined_106597	0.52	0.69	0.03	0.71	42.93	0.17	0.51	0.39	0.58	0.21	0.75	0.49	ME
S5-wms-combined_204782	1.7	1.17	0.07	0.6	29.93	1.03	2.59	0.65	0.69	1	1.38	0.64	ME
S5-wms-combined_98202	58.72	4.18	2.68	4.94	144.73	77.21	79.68	40.05	5.05	44.21	35.64	4.41	Shared
S5-wms-combined_26971	23.03	3.69	1.21	3.34	265.61	7.49	6.53	26.06	2.19	9.31	21.77	5.12	Shared
S5-wms-combined_178432	13.67	6.34	1.1	1.65	947.72	151.73	8.55	18.05	2.87	101.99	210.09	3.93	Shared
S5-wms-combined_179241	132.71	25.84	10.84	3.83	593.91	7.1	46.01	22.14	1.75	84.76	48.65	11.81	Shared
S5-wms-combined_117213	4.44	1.31	7.75	12.06	31.7	28.6	6.37	1.97	96.12	1.08	1.85	2.91	Shared
S5-wms-combined_163165	1.42	8.15	0.1	0.34	27.36	0.34	1.11	3.52	0.54	13.88	2.14	0.89	ME
S5-wms-combined_39793	0.5	0.44	0	0.13	50.98	17.1	0.72	0.77	0.53	0.6	2.5	0.57	Shared
S5-wms-combined_183113	12.02	5.99	0.48	0.74	64.48	7.08	19.6	7.61	4.35	5.81	9.77	3.49	ME
S5-wms-combined_52741	5.08	13.32	0.58	0.4	214.37	1.05	4.32	76.58	0.48	0.59	1.53	0.99	Shared
S5-wms_62679	1.82	0.98	0.21	0.35	41.05	0.73	1.15	1.93	0.76	1.62	1.4	1.83	ME
S5-wms_10783	40.2	41.83	2.41	11.38	68.1	5.75	34.58	27.66	22.72	19.12	34	45.71	Shared
S5-wms_164032	0.42	0.95	0.31	0.18	16.49	0.59	3.11	0.25	0.6	0.18	0.43	0.6	ME
S5-wms_49310	0.65	2.83	0.3	4.79	192.62	11.66	0.61	0.35	0.57	0.19	1.63	0.4	Shared
S5-wms_67169	2.6	1.57	0.57	1.48	75.15	1.92	2.52	2.12	1.34	1.64	1.97	6.02	ME
S5-wms_85650	7.49	23.32	1	9.31	96.43	14.35	18.88	0.81	1.21	2.55	7.93	5.82	Shared
S5-wms_210460	0.57	0.78	0.2	0.18	17.47	0.08	0.7	0.47	0.52	0.14	0.67	0.2	ME
S5-wms_94965	2.54	2	0.28	0.79	22.02	1.79	1.28	2.68	1.42	1.99	2.85	1.65	ME
S5-wms_52566	127.63	98.63	11.97	12.57	134.68	45.18	52.6	103.23	49.08	100.88	110.63	196.39	Shared
S5-wms_123499	0.69	0.36	0.05	0.11	20.53	0.05	1.8	0.18	0.77	0.2	0.67	0.32	ME
S5-wms_212596	0.95	0.77	0.09	0.64	11.31	0.1	0.48	0.32	0.55	0.11	0.23	0.11	ME
S7-wms-combined_217226	1.07	1.46	0.07	0.38	1.53	0.32	43.87	0.83	0.63	0.5	1.28	0.66	ME
S7-wms-combined_139683	6.55	1.75	2.02	0.69	1.76	0.62	24.09	6.75	0.52	7.4	16.69	0.53	Shared
S7-wms-combined_71592	0.82	11.21	3.37	0.42	0.71	0.24	55.11	1.63	1.4	0.26	7.32	0.48	Shared
S7-wms-combined_235165	2.49	3.9	1.49	1.51	2.95	0.7	86.75	2.04	1.43	0.76	3.73	0.74	ME
S7-wms-combined_225260	1.06	1.58	0.07	0.71	0.96	0.15	19.09	0.6	0.89	0.25	1	0.69	ME
S7-wms-combined_31068	5.03	15.06	1.4	3	5.05	9.12	23.87	5.8	2.98	10.05	11.69	67.87	Shared
S7-wms-combined_123085	16.03	25.89	1.04	11.47	4.56	4.4	50.93	5.48	87.84	1.51	5.01	10.38	Shared
S7-wms-combined_20921	3.06	4.31	0.48	3.76	1.24	5.98	46.44	1.61	25.98	0.52	1.65	5.68	ME

S7-wms-combined_62119	356.97	552.92	22.18	15.03	337.24	59.16	1081.3	72.89	17.56	80.49	141.8	307.74	Shared
S7-wms-combined_185016	24.82	59.93	1.09	6.55	42.76	5.17	50.86	16.11	1.17	7.38	8.99	18.58	Shared
S7-wms-combined_174935	7.97	3.01	19.53	11.01	30.36	17.21	35.73	5.87	20.09	2.42	4.41	5.17	Shared
S7-wms-combined_134452	116.78	6.35	0.84	1.41	6.35	0.74	58.32	35.23	1.44	3.44	65.99	1.72	Shared
S7-wms-combined_134458	21.94	13.79	2.7	3.07	13.4	20.66	39.06	26.76	2.26	5.08	14.87	12.49	Shared
S7-wms-combined_21693	6.93	19.59	0.43	0.75	0.85	3.8	26.75	28.82	0.81	1.2	23.51	0.92	Shared
S7-wms-combined_2122	3.72	5.86	0.38	3.71	1.86	2.09	58.72	1.67	22.12	0.85	2.18	3.42	ME
S7-wms-combined_135154	4.04	7.44	0.67	7.92	47.7	8.73	51.96	4.34	1.49	2.7	6.56	13.56	ME
S7-wms-combined_156351	0.79	1.05	0.07	0.73	0.57	0.15	15.21	1.5	0.89	0.27	0.78	0.43	ME
S7-wms-combined_104872	56.18	160.82	0.92	0.66	4.19	0.12	27.14	25.59	0.75	4.06	12.62	0.68	Shared
S7-wms-combined_238231	17.11	17.39	1.32	2.19	26.72	3.39	37.33	8.86	7.69	17.48	11.35	11.38	Shared
S7-wms-combined_43950	60.15	79.2	10.07	23.17	25.64	191.94	217.49	159.54	22.91	79.81	50.41	31.13	Shared
S7-wms-combined_84688	23.27	21.49	2.56	5.23	37.88	22.63	40.28	10.14	19.28	11.85	8.41	53.04	Shared
S7-wms-combined_3669	0.22	0.7	0.03	0.19	2.93	0.05	8.19	0.4	0.54	0.37	0.74	0.35	ME
S7-wms-combined_3697	164.6	63.77	6.39	3.92	415.49	206.31	188.41	136.75	140.07	181.77	196.97	83.41	Shared
S7-wms-combined_157260	15.62	7.37	0.72	12.38	3.75	1.74	36.68	2.19	14.61	1.19	3.17	6.83	Shared
S7-wms-combined_74829	23.9	18.13	0.11	0.91	1.99	4.59	54.79	0.69	12.35	0.49	1.05	10.43	Shared
S7-wms-combined_14267	271.07	84.02	4.53	5.15	405.75	303.54	323.17	105.8	176.48	117.28	111.5	32.68	Shared
S7-wms-combined_157434	19.39	47.4	1.26	6.26	3.76	5.19	33.61	11.99	22.28	4.48	8.92	39.79	Shared
S7-wms-combined_167867	1.63	1.03	8.88	0.75	1.72	0.45	27.72	1.05	0.72	0.6	1.31	1.09	ME
S7-wms-combined_106253	6.08	8.85	0.78	13.96	5.03	1.51	40.44	2.31	15.21	1.7	3.73	8.43	Shared
S7-wms-combined_239784	45.21	143.6	0.73	1.27	6.61	2.67	383.44	5.92	0.85	4.37	12.18	15.23	Shared
S7-wms-combined_14529	119.71	2.73	3.08	0.91	6.92	1.69	67.37	165.33	1.3	3.81	113.51	3.44	Shared
S7-wms-combined_189250	2.02	1.03	0.37	0.48	3.13	0.99	26.1	16.36	0.86	1.33	1.72	1.16	Shared
S7-wms-combined_137858	85.09	26.45	11.87	12.47	6.3	7.96	53.25	34.34	3.26	37.65	34.51	59.74	Shared
S7-wms-combined_169375	1.81	6.09	0.34	5.93	24.8	0.97	120.02	1.12	153.47	1.22	1.38	2.2	ME
S7-wms-combined_5625	285.48	548.82	20.41	21.61	295.16	44.71	1137.17	54.16	22.09	71.61	118.43	270	Shared
S7-wms-combined_190281	2.97	1.92	4.2	12.74	7.54	1.6	34.43	18.37	1.74	3.75	5.17	18.72	Shared
S7-wms-combined_26217	163.1	10.19	9.07	16.58	229.78	98.62	294.28	122.42	15.69	137.3	68.96	23.78	Shared
S7-wms-combined_69024	0.75	0.92	0.08	0.8	2.68	0.7	13.79	0.56	0.84	0.24	1.13	2.02	ME
S7-wms-combined_7205	12.43	29.98	1.42	1.07	1.97	7.59	70.59	3.94	3.94	6.52	4.57	2.26	Shared
S7-wms-combined_121062	0.58	0.92	0.26	0.57	0.54	0.15	15.29	0.31	0.54	0.24	0.35	0.5	ME
S7-wms-combined_90393	0.52	1.45	0.16	0.35	0.96	0.34	152.96	0.39	0.51	0.32	0.97	2.43	ME
S7-wms-combined_111004	25.94	12.6	4.95	5.32	2.82	6.36	33.81	14.75	1.83	6.83	15.04	29.07	Shared
S7-wms-combined_90728	0.7	1.22	0.03	0.4	0.79	0.25	84.78	0.32	0.86	0.22	0.71	3.64	ME
S7-wms-combined_40779	14.03	7.76	0.49	8.96	2.98	7.32	50.03	2.57	1.4	4.09	3.25	274.22	ME
S7-wms-combined_28138	1.46	3.31	0.12	0.8	16.34	1.65	33.88	0.78	1.84	0.58	1.45	0.48	ME
S7-wms-combined_224523	3.5	62.46	0.18	0.68	0.55	0.32	44.28	7.65	0.78	0.27	7.14	0.94	Shared
S7-wms-combined_143218	6.78	3.17	4.32	4.12	11.72	4.39	25.37	3.7	12.6	0.87	4.15	3.47	ME
S7-wms-combined_163980	7.13	3.42	3.23	2.18	25.6	5.61	53.84	145.74	1.47	2.56	7.2	4.79	ME
S7-wms-combined_72070	50.23	122.56	2.6	2.35	73.8	10.15	1008.58	31.38	6.56	10.39	50.7	23.81	Shared
S7-wms-combined_136642	6.32	1.49	3.9	6.17	6.12	2.8	37.06	2.32	7.87	1.03	1.83	3.74	ME
S7-wms-combined_147158	2.83	1.83	6.4	8.48	6.52	3.31	30.74	1.9	10.42	1.06	1.74	1.47	ME
S7-wms-combined_218379	12.28	7.94	4.73	2.59	7.81	1.87	105.24	15.89	5.06	4.66	9.3	7.21	Shared
S7-wms-combined_107679_0	147.71	6.59	7.73	9.72	247.95	120.46	218.08	108.29	13.43	144.76	58.6	11.97	Shared
S7-wms-combined_107679_1	61.09	9.86	1.46	2.29	191.23	35.76	267.27	73.09	6.16	56.58	28.11	11.66	Shared
S7-wms_113137	6.05	18.23	3.73	2.35	4.09	24.15	32.98	109.66	2.17	21.77	34.47	1.13	Shared
S7-wms_123954	0.71	0.5	0.05	0.51	0.71	0.14	22.14	0.94	0.55	0.25	9.77	4.83	ME
S7-wms_53338	0.37	0.46	0.04	0.11	0.43	0.08	37.52	0.46	0.27	0.19	0.35	0.32	ME
S7-wms_157264	0.4	0.33	0.02	0.18	0.88	0.37	337.77	1.53	0.61	1.26	0.61	0.32	ME
S7-wms_239833	0.73	0.77	0.05	0.54	0.65	0.18	62.64	0.79	0.66	0.32	0.61	0.39	ME
S7-wms_127801	7.92	15.3	3.84	1.05	3.04	0.77	48.5	3.31	2.49	1.48	8.22	2.29	Shared
S7-wms_76437	0.69	0.72	0.04	0.46	0.71	0.15	34.12	0.47	0.42	0.22	0.68	0.39	ME
S7-wms_118607	2.22	1.81	0.21	0.46	1.55	11.91	53.61	1.21	0.99	27.04	12.37	0.92	Shared
S7-wms_159432	5.7	1.03	3.48	2.38	0.8	0.28	70.38	0.67	1.36	0.26	1.91	12.24	ME
S7-wms_200936	9.63	0.77	0.17	0.58	0.74	6.77	14.21	19.13	0.26	20.98	4.42	48.81	Shared
S7-wms_151112	0.78	0.87	0.07	0.35	0.45	0.22	46.88	0.41	0.89	0.18	0.52	0.37	ME
S7-wms_80255	0.8	0.71	0.14	0.22	0.87	0.17	15.22	3.78	0.5	0.5	5.05	0.64	ME
S7-wms_244425	0.43	1.02	0.06	1.61	0.61	0.38	126.34	24.01	26.87	21.98	0.66	0.41	Shared
S7-wms_244866	2.63	1.29	0.04	0.36	0.59	0.2	36.86	0.36	0.54	0.13	0.53	0.32	ME
S17-wms-combined_157468	1.8	2.32	0.08	0.67	9.85	9.11	2.81	4.23	1.44	1.33	1.01	28.87	ME
S17-wms-combined_233761	73.12	108.12	5.91	1.31	71.91	9.34	140.11	5.17	1.01	12.17	20.48	127.59	Shared
S17-wms-combined_56006	27.09	15.78	4.15	11.61	6.63	20.75	7.87	30.21	2.23	5.58	18.91	121.26	Shared
S17-wms-combined_56061	41.76	53.97	6.76	13.09	13.05	17.62	11.04	32.53	5.35	33.54	26.86	247.28	Shared
S17-wms-combined_223748	0.7	3.14	0.11	0.28	1.04	1.13	0.99	0.37	0.27	0.46	0.32	20.83	ME
S17-wms-combined_79709	1.88	3.69	0.19	0.29	2.93	1.19	1.96	1.6	0.8	2.8	2.01	35.35	ME
S17-wms-combined_146050	24.07	50.1	4.15	6.3	23.4	8.91	75.22	4.81	7.15	55.77	49.81	139.66	Shared
S17-wms-combined_179363	2.02	1.43	1.16	0.15	2.94	0.27	1.16	1.65	3.98	0.6	1.8	19.18	ME
S17-wms-combined_90378	13.79	4.07	4.43	3.2	3.29	5.03	4.45	6.51	1.13	1.51	4.77	75.72	ME
S17-wms-combined_179771	1.43	3.48	0.27	0.77	26.92	3.13	71.86	4.67	0.78	1.14	16.93	99.72	Shared
S17-wms-combined_2234	19.5	22.02	2.3	1.87	44.16	15.25	9.17	18.63	6.16	31.3	17.41	197.7	Shared
S17-wms-combined_146850	3.1	1.26	0.13	0.21	2.75	0.42	1.46	1.48	0.6	1.32	2.01	27.47	ME
S17-wms-combined_258087	68.39	20.36	4.06	5.94	6.14	5.35	2.63	14.35	0.71	13.34	18.31	37.73	Shared
S17-wms-combined_225356	2.32	0.94	0.37	0.49	4.69	1.44	2.19	2.85	0.81	11.95	2.29	17.64	ME
S17-wms-combined_136622	1.21	1.02	1.02	0.16	1.05	0.16	0.59	5.53	0.64	0.53	0.68	20.83	ME
S17-wms-combined_203701	108.75	27.36	40.02	30.35	1077.68	114.24	721.8	354.77	19.55	256.43	825.9	86.95	Shared
S17-wms-combined_158354	2.83	2.75	0.29	1.13	0.81	0.35	22.41	0.45	0.68	8.29	3.17	58.7	ME
S17-wms-combined_247494	16.87	29.66	1.89	1.5	14.2	8.15	5.52	10.3	3.19	19.58	10.58	109.69	Shared
S17-wms-combined_70983	23.39	38.6	0.74	1.06	0.42	0.42	16.78	0.52	1.12	1.31	4.38	28.61	Shared
S17-wms-combined_126600	16.02	23.12	0.66	1.93	21.23	6.1	13.44	10.14	4.71	27.59	31.35	70.87	Shared
S17-wms-combined_126774	28.51	21.33	4.04	4.53	62.62	12.92	15.43	15.8	6.95	5.68	14.53	89.36	Shared
S17-wms-combined_171305	0.91	1.78	0.04	0.7	2.69	0.31	1.97	3.08	0.58	1.87	2.32	45.34	ME
S17-wms-combined_248321	107.06	9.22	3.22	10.72	6.82	7.86	5.36	34.22	3.49	9.2	6.63	135.68	Shared
S17-wms-combined_193958	89.18	75.17	10.71	12.15	7.44	16.32	11.79	55.92	1.84	40.45	33.75	347.73	Shared
S17-wms-combined_47860	1.11	6.85	0.15	0.64	1.73	1.25	1.3	2.17	0.58	1.45	1.91	38.86	ME
S17-wms-combined_259811	7.17	13.57	1.23	4.85	7.78	10.19	12.41	6.07	3.14	15.14	12.58	83.27	Shared

S17-wms-combined_127688	27.5	9.16	4.41	4.37	3.82	2.27	38.76	10.4	10.22	3.15	16.53	33.03	Shared
S17-wms-combined_260343	5.7	2.45	1.54	0.65	2.82	0.84	14.41	1.85	0.78	3.54	2.81	86.87	ME
S17-wms-combined_72837	10.43	4.12	0.73	3.28	2.28	0.83	6.71	5.04	0.73	3.72	3.9	26.85	ME
S17-wms-combined_16309	0.43	0.89	0.24	0.63	0.48	0.23	0.95	0.28	0.64	0.25	1.09	41.74	ME
S17-wms-combined_106262	4.67	4.41	0.17	0.71	2.19	1.04	4.57	1.62	0.62	3.23	2.37	69.61	ME
S17-wms-combined_185239	40.8	48.8	4.47	4.63	46.77	22.38	18.96	32.13	17.43	93	31.92	143.66	Shared
S17-wms-combined_39687	2.77	1.63	0.22	0.51	2	8.1	2.74	6.68	0.65	3.67	1.7	18.53	ME
S17-wms-combined_140517	0.66	0.99	0.05	0.46	0.64	0.15	1.12	0.34	1.04	0.46	1.34	18.4	ME
S17-wms-combined_106940	102.76	293.64	4.16	10.91	60.65	8.19	117.38	13.12	4.43	17.34	36.84	151.68	Shared
S17-wms-combined_186314	15.51	2.29	4.31	4.45	4.79	2.78	6.56	1.58	6.12	0.68	1.23	26.1	ME
S17-wms-combined_162786	1.5	1.79	0.11	0.43	1.27	0.26	1.55	0.87	1.48	0.51	1.81	74.24	ME
S17-wms-combined_95360	45.23	55.02	0.36	2.27	4.75	11.09	63.36	1.86	40.05	1.39	2.84	27.14	Shared
S17-wms-combined_130215	16.98	9.92	1.34	1.51	14.43	32.22	9.76	7.89	5.61	5.05	9.81	86.19	Shared
S17-wms-combined_75892	6.23	3.47	0.34	0.62	2.2	0.99	2.48	1.7	0.72	1.48	1.71	26.15	ME
S17-wms-combined_30308	0.77	1.12	0.04	0.18	1.02	0.18	0.49	0.53	0.92	0.44	0.62	13.39	ME
S17-wms-combined_175924	6.04	6.62	0.75	2.94	4.31	5.74	3.14	4.72	4.23	4.56	5.88	112.18	ME
S17-wms-combined_199440	64.65	9.55	3	1.49	28.53	8.04	29.93	10.41	7.3	13.7	8.17	55.36	Shared
S17-wms-combined_199746	4.68	3.48	4.17	0.98	46.21	9.37	5.24	17.86	3.26	17.59	11.07	59.5	Shared
S17-wms-combined_66123	7.96	2.09	0.44	2.1	1.8	1.95	5.05	9.51	1.01	2.61	1.3	39.96	ME
S17-wms-combined_9864	3.33	3.8	1.55	0.6	21.25	6.77	16.33	13.57	1.22	8.97	6.62	22.74	Shared
S17-wms-combined_253499	1.02	0.89	0.09	0.96	2	0.52	1.21	1.02	0.94	3.25	1.19	84.49	ME
S17-wms_124308	0.86	1.28	0.03	0.73	0.71	0.25	4.74	0.4	0.87	0.21	0.81	34.03	ME
S17-wms_235536	1.34	1.67	0.14	0.83	0.58	0.42	0.98	0.22	4.95	1.7	1.43	28.56	ME
S17-wms_116248	9.89	3.51	1.77	1.53	2.11	5.51	2.09	14.81	0.78	2.97	11.15	37.17	Shared
S17-wms_106671	2.2	6.34	0.71	2.19	2.71	0.57	1.48	2.8	3.87	8.5	5.07	35.81	ME
S17-wms_29082	8.68	4.79	2.25	4.36	7.44	2.05	4.29	5.37	3.51	10.65	5.87	43.84	ME
S17-wms_254872	0.31	0.36	0.08	0.24	0.32	0.17	0.51	0.47	0.43	0.08	0.51	23.97	ME
S17-wms_266511	0.49	0.58	0.07	0.5	0.66	0.12	0.48	0.4	0.31	0.3	0.43	239.77	ME
S12-wms-combined_321174	5.49	0.65	0.14	0.39	2.05	1.66	6.41	5.37	0.43	14.26	64.58	0.63	Shared
S12-wms-combined_401379	2.05	0.69	0.05	0.24	0.84	0.26	8.74	0.66	0.79	0.34	53.45	0.84	SHHC
S12-wms-combined_401383	0.25	0.87	0	0.19	0.36	0.08	1.04	0.3	0.34	0.06	18.31	0.13	SHHC
S12-wms-combined_334806	27.49	12.14	2.39	1.64	34.01	8.01	7.28	20.46	4.85	16.82	42.22	25.49	Shared
S12-wms-combined_184338	19.14	15.04	2.2	0.9	5.71	3.07	4.4	83.9	3.52	6.26	56.22	15.92	Shared
S12-wms-combined_368462	6.37	4.21	0.35	0.72	5.48	1.48	5.46	28.56	1.04	2.64	46.98	1.58	SHHC
S12-wms-combined_385270	79.58	5.82	0.55	1.27	6.76	1.14	5.37	4.33	3.4	2.87	183.56	4.6	Shared
S12-wms-combined_201125	25.63	39	1.78	14.48	27.7	6.85	54.47	7.02	10.75	12.13	160.23	30.72	Shared
S12-wms-combined_368562	1.64	3.07	3.09	6.27	10.41	10.8	9.81	2.07	0.72	29.85	121.9	302.89	Shared
S12-wms-combined_351847	37.58	11.42	0.07	0.79	0.89	0.15	1.77	24.26	1.09	0.45	48.23	1.03	Shared
S12-wms-combined_117628	112.29	42.16	3.8	6.75	28.05	7.34	16.09	35.62	11.37	18.27	55.09	27.13	Shared
S12-wms-combined_17617	84.11	10.04	7.27	3.66	12.07	8.8	31.73	15.97	3.77	11.57	24.76	25.79	Shared
S12-wms-combined_51076	5.04	2.78	0.38	0.6	4.24	2.28	2.24	3.77	1.16	2.74	65.27	4.08	SHHC
S12-wms-combined_151843	172.69	4.46	4.74	1.39	8.06	1.6	44.47	181.19	3.7	4.19	232.91	4.48	Shared
S12-wms-combined_302876	0.8	1.08	0.3	0.96	0.77	0.17	0.75	0.37	1	0.84	16.4	0.46	SHHC
S12-wms-combined_18287	55.43	35.55	3.9	3.08	50.31	11.25	63.99	33.53	7.61	45.19	68.41	44.32	Shared
S12-wms-combined_52101	1.55	1.17	0.1	0.51	1.41	0.43	1.35	1.18	1.06	0.69	13.11	0.8	SHHC
S12-wms-combined_320262	304.84	92.53	5.41	2.75	507.87	642.36	231.25	158.9	235.24	212.78	202.01	67.25	Shared
S12-wms-combined_2450	5.21	3.65	0.34	1.03	78.22	8.11	5.71	26.27	2.42	13.74	22.36	2.74	Shared
S12-wms-combined_320727	88.64	54.27	5.03	3.3	360.96	11.27	154.8	26.46	3.5	125.87	365.62	32.42	Shared
S12-wms-combined_371541	16.63	10.8	0.47	4.75	10.92	3.24	15.58	2.4	6.44	68.6	78.53	16.01	Shared
S12-wms-combined_120133	11.52	29.88	0.62	3.26	5.81	2.99	8.38	1.87	22.73	1.06	25.37	11.09	Shared
S12-wms-combined_71235	3.14	1.64	0.7	0.54	1.38	0.54	1.04	1.46	0.85	1.12	16.55	1.18	SHHC
S12-wms-combined_189323	50.72	21.42	3.17	6.87	591.03	156.14	39.55	39.58	31.94	222.13	496.19	42.31	Shared
S12-wms-combined_21504	0.8	0.78	0.07	0.15	1.12	0.26	0.92	0.79	0.82	0.4	18.36	0.53	SHHC
S12-wms-combined_190114	645.11	838.25	4.22	1.83	35.52	12.12	52.89	9.45	1.56	14.15	104.29	49.87	Shared
S12-wms-combined_256299	0.79	0.78	0.07	0.73	0.9	0.28	1.06	0.58	0.57	0.39	135.09	0.44	SHHC
S12-wms-combined_156234	55.16	10.1	5.4	4.8	4.12	9.55	8.82	26.44	1.29	10.65	77.91	72.26	Shared
S12-wms-combined_223043	1.99	3.99	0.66	0.79	1.74	0.18	5.62	5.53	1.1	2.04	15.28	8.75	SHHC
S12-wms-combined_323943	1.9	1.27	0.23	1.69	1.8	0.63	1.13	5.37	0.94	0.74	26.94	1.16	SHHC
S12-wms-combined_390985	0.42	0.76	0.08	0.44	0.83	0.06	0.5	0.33	0.25	0.19	32.37	0.25	SHHC
S12-wms-combined_191570	1.17	1.3	0.83	0.7	0.73	0.2	0.97	0.94	0.45	0.5	51.72	0.63	SHHC
S12-wms-combined_23517	116.25	15.61	4.61	2.22	58.24	14.43	9.2	181.15	5.08	20.08	200.92	23.55	Shared
S12-wms-combined_375343	9.6	3.32	0.23	0.66	5.08	2.25	4.75	1.62	3.49	43.71	68.05	8.5	Shared
S12-wms-combined_325418	4.79	1.38	0.11	0.66	1.21	0.48	2	1.54	2.54	1.66	36.36	0.86	SHHC
S12-wms-combined_41160	4.96	2.61	0.27	0.45	1.91	0.79	1.58	42.09	0.99	1.56	34.05	1.52	SHHC
S12-wms-combined_7174	16.93	4.67	2.79	1.3	11.38	4.69	43.87	9.59	2.33	101.92	50.24	2.26	Shared
S12-wms-combined_358010	5.94	5.05	2.67	1.7	3.17	0.65	2.09	7.07	1.11	1.92	41.99	1.71	SHHC
S12-wms-combined_376326	1.39	1.37	1.89	2.45	1.05	0.34	0.87	0.99	0.78	0.48	33.6	0.78	SHHC
S12-wms-combined_91231	0.74	1.03	0.05	1	0.78	0.23	1.06	0.45	0.61	2.86	24.13	0.43	SHHC
S12-wms-combined_107942	1.53	1.2	0.12	0.31	0.64	0.26	3.22	0.53	1.18	13.81	28.91	1.21	Shared
S12-wms-combined_293354	248.26	1.16	3.92	0.36	1.78	0.65	69.44	281.01	0.89	1.02	251.78	1.03	Shared
S12-wms-combined_75733	0.78	0.52	0.26	1.2	1.1	0.17	0.8	0.49	0.59	0.15	25.41	0.42	SHHC
S12-wms-combined_393703	53.59	6.56	1.02	2.2	2.67	2.12	2.02	14.35	32.09	1.5	28.65	5.17	Shared
S12-wms-combined_358797	107.71	9.04	8.8	7.35	173.86	63.18	96.06	65.71	17.14	101.18	99.4	14.97	Shared
S12-wms-combined_25459	143.21	9.6	12.28	12.68	226.39	137.86	141.15	134.69	20.29	153.48	656.64	26.72	Shared
S12-wms-combined_359190	7.58	23.02	0.47	2.56	1.78	1.01	5.4	9.55	6.18	1.77	36.28	2.22	SHHC
S12-wms-combined_259783	1.34	3.74	0.38	0.44	0.84	1.39	0.76	10.53	0.43	1.2	25.72	67.32	Shared
S12-wms-combined_260036	0.79	0.92	0.05	0.75	14.57	0.17	1.4	0.71	1.04	0.27	32.75	2.24	SHHC
S12-wms-combined_210942	70.51	15	9.66	12.92	13.76	6.13	5.51	21.1	2.62	5.34	61.75	14.96	Shared
S12-wms-combined_328554	24.54	13.72	0.96	0.93	77.64	81.14	26.89	30.86	33.07	33.31	89.61	25.22	Shared
S12-wms-combined_329242	14.54	17.58	0.5	2.74	2.7	42.84	2.21	6.67	2.86	1.47	24.31	5.07	Shared
S12-wms-combined_345948	9.04	1.97	1.73	0.95	2.73	3.55	2.63	15.26	0.75	1.96	18.59	3.17	SHHC
S12-wms-combined_313953	1.12	1.09	0.05	0.31	1.11	0.26	8.06	1.28	0.75	0.96	65.33	0.64	SHHC
S12-wms-combined_245755	1.24	1.14	0.08	0.25	1.39	1.03	1.02	1.09	0.43	0.45	18.43	0.62	SHHC
S12-wms-combined_111524	129.73	38.88	8.21	2.69	361.77	210.32	100.25	96.18	86.68	158.42	131.63	78.06	Shared
S12-wms-combined_263377	1.25	1.03	0.12	0.18	1.28	0.33	0.84	1.07	0.71	0.78	27.62	0.8	SHHC
S12-wms-combined_297545	30.79	49.52	2.21	22.63	29.17	7.72	504.61	8.19	13.35	14.62	131.73	36.81	Shared

S12-wms-combined_129554	1.02	0.54	0.37	0.14	1.18	0.27	0.47	4.45	0.36	0.46	17.36	0.48	SHHC
S12-wms-combined_63056	6.21	2.97	0.24	0.51	1.19	0.36	1.11	0.74	0.64	7.91	19	0.89	SHHC
S12-wms-combined_264077	6.62	2.99	0.88	1.41	2.06	1.32	1.71	4.76	1.1	2.03	24.77	6.23	SHHC
S12-wms-combined_129799	85.2	26.97	7.83	5.38	65.94	4.07	9.16	57.32	2.6	21.67	103.02	12.88	Shared
S12-wms-combined_63649	21.19	1.04	0.3	0.18	1.58	0.49	0.93	11.48	0.5	0.71	55.86	0.84	Shared
S12-wms-combined_282594	20.82	51.02	0.79	0.85	117.37	2.02	8.11	102.67	2.01	15.44	84.32	3.82	Shared
S12-wms-combined_264425	5.19	2.41	2.43	5.56	0.97	0.58	2.12	0.9	0.91	0.67	65.24	0.97	SHHC
S12-wms-combined_147628	7.56	3.88	0.51	1.01	6.48	0.63	1.79	8.69	1.07	1.21	69.27	3.5	SHHC
S12-wms-combined_48648	22.48	6.78	2.96	5.71	16.22	1.7	2.79	69.38	3.29	1.78	64.75	11	Shared
S12-wms-combined_264845	43.69	12.56	5.62	10.69	16.84	4.12	5.96	23.14	4.29	13.02	51.55	26.44	Shared
S12-wms-combined_299293	16.35	19.05	2.9	2.64	6.74	1.49	3.88	22.55	1.91	3.49	46.99	6.32	Shared
S12-wms-combined_98046	2.28	1.52	0.15	0.32	1.83	0.69	1.61	3.12	0.78	1.09	24.25	1.16	SHHC
S12-wms-combined_299872	0.58	2.88	0.07	0.21	0.52	0.23	1.12	0.35	0.58	0.29	29.8	2.67	SHHC
S12-wms-combined_148741	30.2	1.9	0.27	0.77	3.18	0.99	1.63	7.36	1.09	1.5	68.04	2.23	Shared
S12-wms-combined_31761	2.85	5.36	0.2	1.1	1.47	1.17	2.82	0.94	4.54	0.67	23.69	1.7	SHHC
S12-wms-combined_99704	2.12	1.32	0.15	0.25	1.74	0.96	2	1.56	5.05	1.2	15.46	4.91	SHHC
S12-wms-combined_132907	12.7	1.06	0.6	0.84	3.81	2.56	2.6	5.8	0.77	3.47	36.15	0.83	Shared
S12-wms-combined_250551	84.11	114.72	0.63	1.55	1.2	0.98	3.83	5.79	0.73	2.95	175.57	1.05	Shared
S12-wms-combined_368190	43.03	58.55	2.34	32.39	24.26	8.22	93.25	12.41	6.59	41.19	163.52	142.13	Shared
S12-wms-combined_117447	0.5	0.55	0.11	0.19	0.4	0.13	0.28	0.6	0.24	0.19	136.97	0.29	SHHC
S12-wms-combined_168259	5.5	12.23	0.31	0.55	26.96	0.65	1.81	23.06	0.93	6.53	58.04	1.7	Shared
S12-wms-combined_270839	28.67	4.78	1.23	0.74	23.71	2.05	3.45	24.28	1.23	5.9	66.11	3.73	Shared
S12-wms-combined_157167	1.95	0.44	0.05	0.04	0.56	0.28	0.31	0.43	0.52	0.17	49.54	0.25	SHHC
S12-wms-combined_164656	1.2	1.39	0.17	0.4	1.17	0.39	1.5	0.93	0.62	0.63	232.33	0.82	SHHC
S12-wms-combined_133608	1.4	2.11	0.01	0.05	0.6	0.15	0.4	0.16	0.1	0.2	13.7	0.32	SHHC
S12-wms_184276	1.3	0.82	0.12	0.18	1.43	0.91	1.38	1.52	0.47	5.32	45.8	0.81	SHHC
S12-wms_235125	2.86	1.92	4.29	1.24	2.15	0.77	1.71	2.33	0.96	2.14	258.65	2.88	SHHC
S12-wms_17950	1.02	1.24	1.77	1.06	2.03	0.91	0.84	1.17	0.37	2.37	18.18	0.48	SHHC
S12-wms_135291	96.22	63.95	3.74	10.41	26.27	7.24	25.99	32.26	46.78	17.22	133.24	63.82	Shared
S12-wms_352846	0.58	1	0.03	0.14	0.82	0.2	0.73	0.4	0.38	0.25	49.7	0.25	SHHC
S12-wms_386692	14.32	5.15	1.46	0.77	13.12	1.57	7.75	20.65	0.42	3.64	387.22	1.51	Shared
S12-wms_202803	62.69	1.96	3.7	0.6	2.97	1.02	2	4.33	1.06	3.21	37.5	5.81	Shared
S12-wms_336780	17.25	1.36	0.07	0.37	0.34	0.19	0.52	0.2	0.74	0.37	32.18	0.4	Shared
S12-wms_373775	37.66	2.75	0.7	1.56	2.43	1.03	2.48	57.33	1.1	0.98	57.82	3.04	Shared
S12-wms_223235	0.64	1.68	4.61	0.34	0.45	0.13	4.34	1.05	1.37	0.19	14.08	0.4	SHHC
S12-wms_193565	1.82	12.37	0.09	0.26	1.09	0.4	0.61	1.93	0.59	0.99	26.5	0.82	SHHC
S12-wms_161239	59.85	17.65	5.92	3.4	114.59	6.5	15.56	34.78	2.32	31.42	50.83	22.06	Shared
S12-wms_44817	0.86	0.74	0.06	0.42	2.33	0.43	0.94	0.94	0.56	3.14	60.19	0.45	SHHC
S12-wms_179388	11.01	2.17	0.14	6.62	1.05	0.35	1.94	0.96	2.2	10.58	18.26	1.2	Shared
S12-wms_12658	2.54	5.21	0.38	0.81	3.02	0.88	20.7	1.02	1.76	5.33	74.96	7.98	Shared
S12-wms_383851	2.5	2.14	0.11	1.08	2.2	0.5	38.15	1.5	0.79	2.36	155.27	1.4	Shared
S12-wms_15700	0.66	1.22	0.15	0.59	0.53	0.14	1.04	0.24	0.66	4.45	26.74	0.31	SHHC
S12-wms_401366	1.3	2	0.07	0.33	0.64	0.23	0.71	0.68	0.62	2.4	43.59	0.45	SHHC
S12-wms_401372	0.53	1.16	0.33	0.72	0.46	0.22	1.2	0.24	0.65	0.33	150.78	0.37	SHHC
S2-wms-combined_207832	2.73	191.02	1.99	1.18	2.28	1.07	6.62	1.53	0.71	4.16	83.41	1.56	SHHC
S2-wms-combined_248318	3.17	26.83	0.16	0.14	2.61	0.5	1.48	10.16	1.26	0.7	3.05	9.99	SHHC
S2-wms-combined_248329	1.68	15.25	0.08	0.24	1.21	0.3	0.85	0.94	0.63	0.51	1.03	0.61	SHHC
S2-wms-combined_31084	1.61	107.67	0.14	0.68	0.98	0.82	1.79	0.51	1.72	0.34	3.93	0.77	SHHC
S2-wms-combined_196783	16.96	32.94	1.93	2.4	23.23	9.54	8.97	11.51	9.51	20.39	12.05	50.68	Shared
S2-wms-combined_165833	3.61	18.15	0.12	0.38	1.86	0.31	3.37	0.54	1.17	0.32	1.06	7.22	SHHC
S2-wms-combined_176376	1.05	15.73	0.1	0.68	0.82	0.67	0.72	1.16	0.78	0.31	1.32	4.31	SHHC
S2-wms-combined_62377	90.91	190.42	0.11	0.34	0.76	0.12	0.74	0.57	0.57	0.36	0.79	0.51	Shared
S2-wms-combined_72725	42.82	88.08	1.36	3.64	50.69	13.6	16.69	43.68	16.03	20.81	24.82	60.26	Shared
S2-wms-combined_135014	27.51	118.6	0.27	0.65	0.99	15.83	63.37	0.88	3.75	0.73	1.05	20.94	Shared
S2-wms-combined_62519	82.49	184.64	6.79	4.5	22.83	7.24	10.37	124.36	4.24	25.77	52.31	57.92	Shared
S2-wms-combined_135139	36.95	109.53	0.71	0.64	154.8	1.16	8.3	133.84	1.18	14.69	37.34	1.66	Shared
S2-wms-combined_218002	15.51	85.74	0.97	3.24	3.04	3.97	13.54	6.83	11.65	4.59	5.16	15.79	Shared
S2-wms-combined_135154	65.72	96.6	0.25	1.3	5.24	11.93	63.2	1.63	41.55	1.05	2.01	26.87	Shared
S2-wms-combined_238929	16.9	38.66	0.88	0.54	2.39	0.47	1.84	6.77	0.96	1.61	7.46	1.35	Shared
S2-wms-combined_93845	10.87	63.06	0.68	0.95	8.07	3.3	2.25	56.36	2.24	3.25	9.56	3.84	Shared
S2-wms-combined_73327	0.98	15.39	0.06	0.78	0.87	0.29	0.79	0.5	2.45	1.01	1.08	1.88	SHHC
S2-wms-combined_52568	14.82	51.74	1.51	1.27	13.85	2.91	3.79	45.49	2.11	6.1	17.17	7.75	Shared
S2-wms-combined_166665	44.11	71.25	7.49	15.99	3.08	39.54	7.09	25.96	3.87	9.64	31.37	95.71	Shared
S2-wms-combined_62839	23.61	57.21	0.14	0.73	0.91	0.34	1.14	0.9	0.97	0.47	1.08	0.78	Shared
S2-wms-combined_197577	4.99	45.19	1.98	0.41	0.65	0.5	0.89	0.27	0.86	0.19	0.58	0.37	SHHC
S2-wms-combined_208559	149.5	56.71	11.07	5.31	447.38	129.12	100.05	134.25	30.78	109.64	136.34	29.6	Shared
S2-wms-combined_229529	46.37	173.2	1.69	12.15	60.36	10.49	58.92	10	48	13.52	21.32	28.14	Shared
S2-wms-combined_115352	0.86	13.12	0.02	0.16	2.34	0.81	0.99	0.88	0.9	0.52	0.77	0.54	SHHC
S2-wms-combined_84608	102.77	58.45	2.24	1.3	1.17	0.3	5.99	2.5	0.63	2.86	1.26	0.72	Shared
S2-wms-combined_126134	2.53	41.42	0.79	1.65	3.06	5.13	6.84	2.57	2.9	3.94	4.29	16.22	Shared
S2-wms-combined_74387	3.29	20.55	0.2	0.52	2.3	0.81	1.91	4.22	1.45	3.06	4.18	2.19	SHHC
S2-wms-combined_230282	2.95	6.46	0.67	0.31	1.6	0.86	0.52	1.42	1.47	1.05	6.88	1.31	SHHC
S2-wms-combined_126440	1.76	24.57	0.16	0.5	0.92	1.14	1.73	0.71	0.95	0.74	1.04	0.45	SHHC
S2-wms-combined_147065	73.55	186.02	1.74	4.77	29.75	18.71	44.45	13.52	19.24	16.34	18.23	26.96	Shared
S2-wms-combined_137119	8.6	53.73	0.68	0.91	9.17	0.82	4.46	5.59	1.31	10.55	12.02	9.05	Shared
S2-wms-combined_85381	2.55	26.34	0.26	0.82	2.08	1.5	6.96	2.39	6.53	7.81	2.92	5.99	SHHC
S2-wms-combined_116221	21.93	20.59	0.34	4.21	4.97	5.08	2.75	20.92	1.31	5.43	22.94	3.65	Shared
S2-wms-combined_95987	0.96	13.03	0.13	0.53	1.66	0.29	0.43	0.61	0.77	0.49	0.84	0.54	SHHC
S2-wms-combined_157934	7.03	66.04	7.26	25	4.18	12.37	6.67	5.01	35.94	2.38	5.45	4.53	Shared
S2-wms-combined_64692	7.21	27.06	0.15	0.62	0.92	0.84	1.35	1.13	0.53	1.39	0.87	1.31	SHHC
S2-wms-combined_127080	3.42	18.12	0.21	0.94	1.45	0.48	1.22	1.63	0.82	1.11	2.48	1.08	SHHC
S2-wms-combined_13246	146.18	134.84	19.07	24.88	12.49	24.53	20.88	76.53	3.61	47.51	59.09	201.25	Shared
S2-wms-combined_199384	4.37	11.65	0.66	0.26	2.65	0.55	1.96	2.33	1.42	2.56	6.88	3.46	SHHC
S2-wms-combined_55495	3.77	77.42	0.14	0.16	1.6	2.57	0.81	0.93	0.75	2.47	3.16	0.85	SHHC
S2-wms-combined_158821	40.98	42.91	0.53	7.26	0.78	1.65	1.11	6.29	0.86	8.26	10.42	0.53	Shared
S2-wms-combined_210822	675.74	819.46	47.34	37.99	1546.97	343.03	1259.36	210.47	78.36	461.06	902.13	82.64	Shared

S2-wms-combined_190624	0.89	19.47	0.04	0.57	0.59	0.2	0.68	0.59	0.79	0.23	0.6	0.55	SHHC
S2-wms-combined_24549	33.26	73.77	2.92	2.61	39.9	9.66	13.23	23.79	6.54	51.36	26.42	65.31	Shared
S2-wms-combined_180640	19.94	49.97	0.32	0.91	0.61	1.1	1.67	0.87	1.39	0.37	1.28	1.03	Shared
S2-wms-combined_170845	6.22	38.05	0.44	1.67	1.4	0.52	5.23	4.3	2.99	0.79	1.77	6.36	SHHC
S2-wms-combined_159670	16.97	19.75	1.3	0.94	30.17	4.03	3.87	7.14	1.19	7.59	9.64	6.73	Shared
S2-wms-combined_181391	19.46	37.87	1.58	2.28	19.92	13.15	7.75	16.44	21.79	19.55	12.73	79.82	Shared
S2-wms-combined_200390	86.75	83.27	6.3	8.73	20.68	12.93	29.15	23.96	86.77	16.47	31.87	41.16	Shared
S2-wms-combined_233107	18.93	23.81	0.32	1.84	43.34	2.52	13.19	10.35	12.37	9.48	6.6	28.57	Shared
S2-wms-combined_57293	211.1	130.77	23.34	48.29	11.22	75.16	31.08	90.99	5.25	34.28	90.33	262.42	Shared
S2-wms-combined_191613	4.68	22.11	0.36	1.04	2.05	2.65	2.03	10.4	2.78	1.71	3.94	11.45	Shared
S2-wms-combined_98888	77.21	95.29	6.67	25.88	48.22	10.26	39.33	30.02	62.95	22.47	45.92	48.81	Shared
S2-wms-combined_181849	106.7	156.85	12.55	16.35	12.41	20.77	17.67	50.45	2.6	35.66	41.89	133.28	Shared
S2-wms-combined_48661	4.91	16.46	0.66	3.08	3.71	1.32	4.29	2.01	6.76	2.02	5.3	3.69	SHHC
S2-wms-combined_201205	39.26	129.16	0.07	0.54	2.54	0.57	1.18	3.68	0.85	0.95	1.62	0.87	Shared
S2-wms-combined_99446	8.66	64.13	0.76	3.98	1.66	1.15	9.12	2.12	9.96	0.81	2.24	5.99	SHHC
S2-wms-combined_25727	17.23	33.23	2.5	2.04	3.23	0.81	1.85	17.25	1	1.36	29.21	1.53	Shared
S2-wms-combined_78670	65.28	63.69	3.6	2.65	50.54	1.97	6.77	18.95	2.79	12.57	42.37	5.87	Shared
S2-wms-combined_58121	128.36	305.21	3.66	26	73.04	39.19	312.64	14.78	7.47	24.13	163.67	155.16	Shared
S2-wms-combined_140996	145.41	164.68	11.51	6.47	64.81	10.48	12.37	198.85	7.57	41.91	70.52	27.8	Shared
S2-wms-combined_89742	12.87	9.59	0.04	0.23	1.12	0.26	0.97	0.42	0.86	0.34	0.68	0.48	Shared
S2-wms-combined_222535	1.99	54.74	0.21	2.22	0.89	0.35	1.57	0.73	3.74	0.7	0.75	0.56	SHHC
S2-wms-combined_58563	15.34	53.86	1.22	1.27	7.93	2.5	2.84	76.05	2.26	4.51	25.41	3.86	Shared
S2-wms-combined_174241	21.33	28.03	0.98	1.01	9.91	0.88	9.87	3.63	3.15	13.38	4.33	2.29	Shared
S2-wms-combined_110215	16.49	22	1.86	3.47	5.42	2.57	6.63	7.37	1.32	5.51	6.26	14.89	Shared
S2-wms-combined_5791	36.66	53.37	3.61	4.86	7.97	2.63	10.46	7.34	25.61	7.68	11.02	10.97	Shared
S2-wms-combined_51446	0.31	10.91	0.05	0.22	0.55	0.12	0.4	0.78	0.43	0.28	0.87	0.31	SHHC
S2-wms-combined_101826	4.04	44.35	2.95	0.5	1.41	0.38	22.13	7.79	1.64	0.53	9.22	0.97	Shared
S2-wms-combined_122457	16.39	65.87	2.11	2.26	12.19	7.3	7.86	10.02	4.18	27.62	10.15	40.34	Shared
S2-wms-combined_244564	26.1	29.87	1.62	3.28	22.73	2.31	18.69	25.28	9.79	7.82	20.18	10.94	Shared
S2-wms-combined_6605	13.06	21.19	0.33	2.47	2.61	40.7	2.25	6.84	3.22	1.91	22.68	6.39	Shared
S2-wms-combined_185494	31.63	75.2	0.12	0.66	2.28	10.86	64.53	0.77	36.7	0.52	1.09	10.94	Shared
S2-wms-combined_132966	1.26	13.87	0.09	0.17	1.05	0.22	1.19	0.69	0.6	0.36	0.81	0.77	SHHC
S2-wms-combined_225908	110.2	36.86	1.52	2.72	12.11	3.09	5.13	12.21	3.38	7.45	36.48	15.06	Shared
S2-wms-combined_246480	1.13	20.47	0.11	0.62	1.07	0.21	0.86	0.52	0.97	0.41	0.98	0.61	SHHC
S2-wms-combined_113619	385.13	171.37	0.29	1.41	4.47	4.78	6	33.94	63.9	1.66	1.34	0.78	Shared
S2-wms-combined_227800	9.61	37.72	1.45	1.57	22.66	15.4	9.74	12.69	14.21	9.75	12.61	28.61	Shared
S2-wms-combined_30750	5.95	31.7	1.4	0.46	0.88	0.31	9.16	3.76	1.08	0.39	6.93	0.64	SHHC
S2-wms-combined_52006	36.84	36.59	3.12	1.91	467.04	1.03	47.05	28.77	0.69	6.04	18.91	5.09	Shared
S2-wms-combined_75144	23.43	87.15	0.25	1.91	26.93	3.47	28.47	1.3	0.65	17.15	7.25	4.74	Shared
S2-wms-combined_181095	3.68	24.55	1.07	2.94	1.38	4.61	0.57	1.43	0.39	1.04	4.33	1.45	SHHC
S2-wms-combined_4685	46.28	165.47	2.25	0.66	1.88	0.56	2.04	103.73	1.12	5.16	10.23	1.48	Shared
S2-wms-combined_143737	65.83	143.91	0.9	1.25	18.22	28.48	31.72	3.16	4.4	4.91	8.51	10.57	Shared
S2-wms-combined_204225	0.38	12.12	0.01	0.05	0.62	0.11	0.42	0.19	1.46	0.62	0.31	0.18	SHHC
S2-wms-combined_7756	35.87	111.72	0.33	0.6	80.53	0.59	4.36	56.65	1.02	6.06	18.44	0.94	Shared
S2-wms-combined_207000	2.81	41.78	1.07	3.56	3.85	1.84	2.15	1.54	6.27	1.07	1.94	1.14	SHHC
S2-wms_124416	48.39	107.07	2.76	6.45	12.45	4.92	13.91	21.29	5.53	10.54	24.47	22.56	Shared
S2-wms_156439	26.94	106.95	0.96	3.75	58.94	3.74	30.63	7.7	215.23	7.35	30.12	11.07	Shared
S2-wms_53881	1.55	3031.13	1.05	1.1	0.63	0.14	0.81	0.78	2.06	1.64	2.23	1.46	SHHC
S2-wms_23562	5.99	20.88	0.07	0.31	0.65	0.12	0.7	0.3	0.86	0.19	0.8	0.33	SHHC
S2-wms_107329	6.24	29.18	0.89	0.97	8.07	3.21	7.11	5.25	1.66	17.42	5.55	19.03	Shared
S2-wms_128831	0.53	118.94	0.17	0.54	0.51	0.16	1.56	0.31	0.7	0.3	0.5	0.51	SHHC
S2-wms_128962	17.34	3364.14	1.16	1.93	0.49	4.1	0.57	0.29	1.53	1.26	2.51	1.42	Shared
S2-wms_140933	11.66	23.86	0.58	2.28	3.22	1.4	4.07	2.54	6.93	2.01	3.58	4.47	Shared
S2-wms_192667	2.7	29.96	0.5	1.08	0.7	3.67	3.96	2.04	1.66	7.38	5.13	0.64	SHHC
S2-wms_79304	0.79	37.08	0.16	0.41	0.59	0.17	0.73	0.58	0.58	0.31	0.64	3.07	SHHC
S2-wms_161986	17.09	66.05	10.37	0.76	1.59	0.78	2.54	3.13	1.63	7.05	10.78	2.09	Shared
S2-wms_79944	15.7	91.4	0.27	0.67	1.11	0.21	1.53	2.46	0.84	28.36	1.64	0.53	Shared
S2-wms_130687	8.7	52.12	1.42	6.39	132.79	8.02	16.1	4.98	6.75	3.65	4.61	1.28	Shared
S2-wms_110761	16.7	8.63	0.03	0.16	0.45	0.13	0.27	0.3	0.62	0.19	0.47	0.08	Shared
S2-wms_6424	0.6	110.94	0.08	0.55	0.65	0.1	0.81	0.3	0.4	8.93	13.43	0.49	SHHC
S2-wms_245232	1.48	90.61	0.22	0.5	1.16	0.31	1.43	1.02	0.98	1.07	5.88	0.91	SHHC
S2-wms_246296	12.95	51.5	0.26	0.61	1.37	0.27	1.56	2.87	0.99	1.01	22.91	0.67	Shared
S2-wms_248315	0.79	76.59	0.09	0.55	0.77	0.24	8.8	0.33	1.14	0.31	0.71	0.41	SHHC
S2-wms_248324	0.89	52.29	0.07	0.16	0.62	0.15	0.82	0.51	0.58	0.31	0.79	0.52	SHHC
S2-wms_248327	1.22	15.32	0.09	0.12	0.82	0.25	0.89	1.49	0.85	0.43	1.23	0.97	SHHC
S4-wms-combined_118531	1.48	1.63	0.94	69.21	1.47	0.33	1.37	1.05	1.38	0.85	2.01	1.06	SHHC
S4-wms-combined_14411	2.9	24.2	0.53	29.22	0.6	3.21	0.78	0.41	0.42	0.23	0.72	0.37	SHHC
S4-wms-combined_112093	10.65	4.99	0.83	20.89	4.4	14.9	1.3	7.66	1.42	9.81	4.41	1.16	Shared
S4-wms-combined_42647	94.37	70.63	9.34	25.99	32.03	37.81	25.96	36.92	39.62	28.27	62.86	58.68	Shared
S4-wms-combined_1246	95.52	21.33	9.38	15.15	72.49	3.87	17.88	37.49	9.37	13.47	31.55	11.4	Shared
S4-wms-combined_22012	4.05	2.45	9.85	42.15	7.15	1.6	5.9	6	1.69	2.73	3.93	8.81	SHHC
S4-wms-combined_57123	1.54	2.02	0.67	1125.01	8.22	0.47	3.84	6.29	0.99	2.77	3.74	1.06	SHHC
S4-wms-combined_64632	1.66	2.34	0.19	17.5	1.99	0.47	5.18	0.85	3.11	0.66	1.34	1.8	SHHC
S4-wms-combined_113721	52.2	29.2	5.59	16.48	5.16	2.12	19.17	22.45	1.63	57.5	29.79	47.69	Shared
S4-wms-combined_57963	5.18	4.13	3.02	70.83	3.28	0.87	3.07	3.01	2.47	3.59	4.62	3.05	SHHC
S4-wms-combined_10758	0.45	0.73	8.88	54.01	0.75	0.23	0.77	0.3	0.7	0.21	0.52	0.38	SHHC
S4-wms-combined_17284	3.76	1.6	7.01	18.47	15.44	9.83	5.61	2.65	39.22	1.43	2.37	2.5	Shared
S4-wms-combined_24659	0.48	0.86	0.36	23.02	0.62	0.18	1.11	0.73	0.62	0.66	0.75	0.4	SHHC
S4-wms-combined_121978	10.95	12.58	0.65	32.94	3.96	1.61	10.1	6.16	18.81	3.67	5.04	9.71	Shared
S4-wms-combined_25201	10.17	15.34	0.75	37.93	6.19	1.92	13.6	3.56	26.28	3.17	5.98	11.06	Shared
S4-wms-combined_47565	0.77	0.72	0.07	49.77	0.99	0.28	0.88	0.89	0.53	0.24	0.75	0.43	SHHC
S4-wms-combined_83492	2.26	1.04	0.24	22.42	0.96	0.21	0.92	0.92	0.56	0.67	1.44	319.19	Shared
S4-wms-combined_41490	131.69	9.41	8.58	20.74	300.61	132.01	130.55	98.94	17.43	166.14	68.44	13.51	Shared
S4-wms-combined_51079	10.89	2.68	10.24	17.17	10.68	5.57	15.02	3.53	14.54	2.6	3.12	6.31	Shared
S4-wms-combined_143879	0.46	0.98	0.21	23.35	0.4	0.17	0.97	0.47	0.86	0.41	0.76	0.34	SHHC
S4-wms_118690	0.46	0.86	0.05	40.73	0.53	0.15	0.76	0.23	1.21	0.14	0.65	0.44	SHHC

S4-wms_161443	0.47	0.47	0.1	21.97	0.56	0.15	0.24	0.43	0.25	0.24	1	0.22	SHHC
S4-wms_50475	4.98	6.79	0.62	589.25	5.46	1.04	7.71	22.18	2.29	2.34	3.68	5.52	SHHC
S4-wms_141607	2.8	2.55	0.64	1303.88	7.37	0.78	3.52	3.69	1.95	2.73	4.6	1.62	SHHC
S4-wms_107182	2.02	1.22	4.8	12.5	2.64	0.6	1.14	4.99	0.6	0.7	1.83	1.98	SHHC
S4-wms_114120	0.45	0.69	0.03	21.49	0.9	0.16	0.81	0.38	0.64	0.26	0.78	0.51	SHHC
S4-wms_45128	0.65	1.02	0.04	36.6	0.96	0.22	1.21	0.37	0.69	0.25	0.81	0.57	SHHC
S4-wms_73990	0.57	2.05	0.05	42.34	0.51	0.13	0.94	2.54	1.02	0.16	0.61	0.43	SHHC
S4-wms_62635	0.88	1.39	0.15	50.56	4.42	0.38	1.13	0.41	2.64	0.31	0.96	0.58	SHHC
S6-wms-combined_117756	1.51	2.75	0.11	0.41	1.41	144.95	2	1.15	0.73	0.57	1.23	0.9	SHHC
S6-wms-combined_41246	44.99	39.92	5.38	3.85	130	91.67	50.33	22.84	35.19	22.05	13.99	122.8	Shared
S6-wms-combined_135223	16.07	40.42	0.61	20.74	114.07	57.53	350.63	5.24	1.83	9.21	84.71	4.37	Shared
S6-wms-combined_88414	37.68	146.74	5.26	6.22	4.1	66.43	26.48	15.96	15.11	22.65	18.97	9.44	Shared
S6-wms-combined_745	7.89	0.91	0.04	0.2	1.09	30.98	1.25	0.69	0.94	0.64	0.82	0.52	SHHC
S6-wms-combined_82597	55.79	100.74	4.48	5.62	50.72	124.93	107.63	14.08	13.29	27.96	49.76	60.79	Shared
S6-wms-combined_65225	0.76	0.89	0.04	0.48	0.5	18.71	0.61	0.55	0.75	0.24	0.65	0.54	SHHC
S6-wms-combined_118427	37.08	43.04	5.85	6.7	50.49	91.87	33.72	30.54	64.47	33.07	24.78	150.71	Shared
S6-wms-combined_118484	5.09	1.78	0.16	1.07	1.5	43.89	7.77	1.06	0.56	1.06	1.77	28.61	Shared
S6-wms-combined_72228	94.37	31.26	2.09	1.62	216.35	240.44	74.68	58.57	75.05	87	105.2	25.65	Shared
S6-wms-combined_119017	9.3	0.9	0.29	0.65	1.98	40.6	2.95	16.29	0.76	3.71	1.63	0.91	SHHC
S6-wms-combined_119138	26.74	94.16	3.61	2.32	7.92	45.28	19.6	9.04	6.3	11.46	15.82	6.19	Shared
S6-wms-combined_49909	6.6	4.69	1.53	2.21	1.83	68.54	1	2.59	0.87	0.92	10.38	7.54	SHHC
S6-wms-combined_113986	15.71	5.12	0.08	0.52	1.04	29.56	1.37	0.6	0.67	0.26	11.4	0.69	Shared
S6-wms-combined_8508	63.84	52.31	6.43	10.47	65.26	104.53	43.36	49.5	49.48	52.31	48.69	253.49	Shared
S6-wms-combined_32530	10.8	55.81	1.14	3.05	2	29.11	5.94	2.7	5.24	10.35	8.22	5.12	Shared
S6-wms-combined_126236	115.95	52.47	26	18.86	18.62	71.74	31.4	57.08	3.05	53.83	49.03	138.45	Shared
S6-wms-combined_50721	234.99	17.33	11.54	14	407.73	181.98	253.18	165.94	33.61	219.07	119.19	24.82	Shared
S6-wms-combined_138710	148.71	61.23	31	13.48	52.49	64.05	22.3	65.23	3.16	43.48	48.08	67.98	Shared
S6-wms-combined_56464	68.38	29.64	10.82	21.13	7.02	73.72	11.03	38.27	2.05	12.47	30.28	116.35	Shared
S6-wms-combined_110950	10.38	2.12	2.38	0.92	16.31	54.98	6.13	4.69	0.95	4.61	5.6	3.72	Shared
S6-wms-combined_128972	2.99	3.67	0.93	0.58	13.9	85.02	9.79	2.38	1.05	2.58	2.44	19.32	Shared
S6-wms-combined_140731	5.83	3.06	0.31	0.6	2.76	40.12	2.83	2.22	4.51	1.1	2.78	2.5	SHHC
S6-wms-combined_91039	84.24	35.79	5.34	4.58	940.77	159.11	67.26	25.76	16.32	259.25	385.74	29.11	Shared
S6-wms_54578	1.09	7.67	0.93	2.97	0.59	28.42	0.77	0.39	0.29	0.26	0.81	0.39	SHHC
S6-wms_137591	1.41	1.45	0.23	0.51	2.66	28.06	1.81	1.15	1.97	0.48	1.02	0.98	SHHC
S6-wms_132588	228.45	147.13	2.8	1.41	2.1	47.58	5.14	8.52	0.83	4.24	25.26	1.03	Shared
S6-wms_138342	0.77	49.16	0.08	0.64	0.44	353.2	0.58	0.3	0.54	0.12	1.22	0.25	SHHC
S6-wms_140743	0.59	0.79	0.04	0.62	0.66	40.59	0.91	0.3	0.62	0.22	0.59	0.33	SHHC
S8-wms-combined_246389	6.51	1.37	0.15	0.55	1.51	0.55	1.11	283.01	0.77	1.03	1.75	1	SHHC
S8-wms-combined_215102	151.81	2.73	57.84	0.66	4.16	1.17	46.93	328.33	1.35	4.3	145.1	2.79	Shared
S8-wms-combined_276204	2.66	1.38	0.14	0.32	1.97	0.72	1.13	273.45	1.06	0.97	1.71	1.35	SHHC
S8-wms-combined_276211	3.98	1	0.22	0.39	1.38	0.43	1.03	251.61	0.8	0.63	2.42	0.73	SHHC
S8-wms-combined_23583	21.88	4.84	1.56	1.19	141.65	20.79	12.2	111.01	6.92	18.47	25.26	5.73	Shared
S8-wms-combined_81597	0.85	1.08	12.35	11.76	2.34	0.56	6.44	47.26	1.02	277.32	32.91	135.84	Shared
S8-wms-combined_219796	1.49	0.87	0.1	0.26	1.76	0.47	0.47	188.07	0.52	0.66	15.86	0.97	SHHC
S8-wms-combined_185611	2.03	2.49	0.31	1	77.25	0.87	10.29	47.36	1.19	16.32	10.23	1.63	Shared
S8-wms-combined_140059	1.58	1.13	0.11	0.29	1.44	0.56	6.25	57.05	0.51	0.5	3.34	0.66	SHHC
S8-wms-combined_186054	23.52	10.16	3.37	2.64	40.29	5.88	19.5	537.53	15.41	24.64	48.92	8.28	Shared
S8-wms-combined_117281	1.8	1.55	0.26	0.53	4	0.84	10.71	52.79	1.44	9.31	7.39	0.84	Shared
S8-wms-combined_25607	2.36	1.91	0.22	0.46	2.8	1.22	2.14	18.45	0.87	2.38	2.53	9	SHHC
S8-wms-combined_48046	7.38	3.48	0.79	0.72	8.31	1.72	3.92	68.56	1.84	3.93	10.5	3.11	SHHC
S8-wms-combined_26087	69.52	2.44	6.74	4.79	111.74	35.26	86.19	119.84	5.04	109.8	22.98	3.61	Shared
S8-wms-combined_164289	3.6	1.21	0.19	0.51	1.23	0.23	1.12	30.17	0.4	0.45	5.43	0.8	SHHC
S8-wms-combined_72911	1.35	0.81	0.09	0.33	3.1	0.39	1.54	44.84	0.53	0.92	2.84	0.68	SHHC
S8-wms-combined_49369	39.47	25.1	6.57	3.72	5.53	3.4	39.33	37.92	1.94	6.26	8.36	23.75	Shared
S8-wms-combined_234473	9.42	19.61	2.7	2.18	30.35	34.84	17.16	15.71	29.4	8.46	6.66	6.72	Shared
S8-wms-combined_211119	0.91	0.7	0.07	0.14	1.06	0.31	0.72	125.94	0.51	0.49	2.5	0.61	SHHC
S8-wms-combined_74275	1.96	2.47	0.28	474.3	3.16	0.58	1.96	80.63	1.92	1.26	1.98	1.38	SHHC
S8-wms-combined_270362	50.84	3.22	4.48	3.73	68.94	31.21	49.39	73.83	6.91	71.84	24.05	6.66	Shared
S8-wms-combined_200168	123.28	46.79	2.67	5.64	37.97	11.66	24.12	131.57	21.81	36.87	33.4	75.66	Shared
S8-wms-combined_97848	6.47	7.6	0.32	1.63	2.24	0.68	1.13	41.22	0.61	1.01	1.96	4.15	SHHC
S8-wms-combined_108917	4.85	4.06	0.31	0.8	9.05	0.78	2.94	169.84	1.71	3.09	10.19	1.39	SHHC
S8-wms-combined_40666	51.57	30.38	4.44	5.38	169.21	9.75	53.87	68.33	47.62	16.15	25.94	165.13	Shared
S8-wms-combined_225114	2.1	1.43	4.13	0.36	1.24	0.55	1.14	60.04	0.61	0.68	6.27	0.92	SHHC
S8-wms-combined_134098	6.67	1.13	0.5	0.31	3.19	0.62	1.14	20.78	0.88	1.3	3.47	1.28	SHHC
S8-wms-combined_180957	5.09	1.66	0.42	0.84	1.49	0.57	1.33	33.98	0.78	8.6	4.36	1.27	SHHC
S8-wms-combined_134284	2.1	1.18	0.18	0.3	1.89	0.87	1.29	22.46	0.84	2.01	1.9	1.75	SHHC
S8-wms-combined_226076	0.6	0.76	0.04	0.17	0.99	0.22	0.89	126.89	0.44	0.32	0.61	0.38	SHHC
S8-wms-combined_146562	1.38	1	0.06	0.16	1.76	0.39	0.69	173.21	0.67	0.62	5.09	0.58	SHHC
S8-wms-combined_169835	137.44	20.16	6.88	3.6	60.54	18.28	10.73	346.07	8.61	23.86	189.44	26.42	Shared
S8-wms-combined_239791	0.72	0.79	0.05	0.13	1.35	0.22	0.81	38.65	0.72	0.3	1.08	0.42	SHHC
S8-wms-combined_217866	1.15	1.12	2.8	8.36	1.5	12.71	2.86	42.65	0.87	0.34	0.88	0.8	SHHC
S8-wms-combined_68780	17.1	18.98	0.92	1.21	4.55	1.69	3.21	129	1.83	22.45	31.65	4.14	Shared
S8-wms-combined_161181	19.13	37.97	2.77	1.95	8.22	3.54	10.1	123.95	1.78	7.9	28.35	125.74	Shared
S8-wms-combined_264711	1.17	0.94	0.17	0.19	1.41	0.41	0.53	15.42	0.33	0.64	1.53	0.94	SHHC
S8-wms-combined_246185	12	39.24	0.12	1.28	40.88	0.35	9.35	149.72	0.38	3.12	9.95	0.7	Shared
S8-wms_270	1.92	6.21	0.07	0.22	0.79	0.09	0.29	40.62	0.39	0.36	1.11	0.36	SHHC
S8-wms_127191	0.72	0.86	0.05	0.36	0.77	0.14	0.62	59.49	0.39	0.26	0.88	0.32	SHHC
S8-wms_58515	53.11	37.25	10.68	7.63	344.14	39.09	35.92	97.72	25.88	163.58	227.58	94.09	Shared
S8-wms_82880	1.37	9.2	0.14	0.48	4.05	1.56	1.09	36.57	0.79	15.08	1.39	2.04	Shared
S8-wms_244525	0.55	0.94	0.03	0.21	0.43	0.13	0.79	28.75	0.55	0.16	0.66	0.3	SHHC
S8-wms_152831	9.66	1.33	3.87	7.71	1.48	0.41	1.39	96.76	0.72	0.9	17.71	2.56	SHHC
S8-wms_48935	42.68	8.39	2.94	2.08	11.69	6.28	3.88	290.5	2.56	7.38	26.77	9.65	Shared
S8-wms_190781	7.91	0.74	1.23	0.62	0.75	0.21	0.66	34.53	0.57	0.75	0.67	0.46	SHHC
S8-wms_30498	2.75	0.73	0.19	0.19	1.06	2.19	1.06	92.76	0.61	7.67	11.51	41.97	Shared
S8-wms_111056	0.84	4.77	0.06	0.48	0.56	0.19	0.79	31.96	0.4	0.14	0.45	0.28	SHHC
S8-wms_113693	2.75	1.2	1.67	0.38	1.71	0.51	1.25	16.71	0.34	0.62	15.97	0.66	SHHC

S8-wms_20528	1.81	1.56	0.24	0.75	2.76	0.56	1.38	33.9	0.75	1.1	18.87	1.27		SHHC
S8-wms_57121	0.7	0.75	0.05	0.25	0.98	0.2	0.93	93.54	0.64	0.33	3.49	0.55		SHHC
#Total	45.75	52.2	3.29	10.86	61.41	23.04	49.76	31.39	16.74	28.29	42.1	32.49	33.11	mean
													17.7441	SD

Table S1-8. Euk_virus_detection-VIRify

VLP:

High_confidence_taxonomy

contig_ID	genus	subfamily	family	order	class
S7_vs_101751			Herelleviridae		Caudoviricetes
S2_vf_20910					
S12_vs_188832					
S5_vs_63924					
S8_vs_206526		Bastillevirinae	Herelleviridae		Caudoviricetes
S9_vs_58402					
S11_vf_43997					
S7_vs_122151		Nymbaxtervirinae			Caudoviricetes
S10_vs_43473					
S3_vs_127391					
S7_vf_27437					
S3_vs_127395					
S9_vs_48424					
S4_vf_12614					
S14_vf_48775					
S15_vf_71616					
S12_vf_173666		Arquatrovirinae			Caudoviricetes
S3_vs_127445					
S5_vs_121419					
S5_vs_67958		Nymbaxtervirinae			Caudoviricetes
S14_vf_37500					
S8_vf_93976					
S3_vs_40939		Tunavirinae	Drexlerviridae		Caudoviricetes
S7_vs_138096					
S9_vf_75363					
S7_vs_11081					
S16_vf_100378		1			
S16_vs_34751					
S1_vf_125650					
S6_vs_33711					
S7_vs_26235		Spounavirinae	Herelleviridae		Caudoviricetes
S8_vs_193843					
S5_vs_27918					
S11_vf_84303|phage-circular		Spounavirinae	Herelleviridae		Caudoviricetes
S16_vs_127669					
S16_vs_32764					
S3_vf_22968					
S7_vs_129858					
S9_vs_10424		Sepvirinae			Caudoviricetes
S10_vs_58014					
S12_vs_14467		Sepvirinae			Caudoviricetes
S3_vf_54498					
S3_vs_12973		Arquatrovirinae			Caudoviricetes
S7_vs_135679		Spounavirinae	Herelleviridae		Caudoviricetes
S3_vf_127406|phage-circular					
S10_vf_120738|phage-circular					
S9_vs_26179					
S8_vs_99362					
S3_vs_95574		Tevenvirinae	Straboviridae		Caudoviricetes
S5_vs_74199					
S16_vs_116956					
S8_vs_152553		Sepvirinae			Caudoviricetes
S13_vf_147589|phage-circular					
S3_vs_30318		Nymbaxtervirinae			Caudoviricetes
S11_vs_104042					
S3_vs_70093					
S9_vs_19689		1	Herelleviridae		Caudoviricetes
S17_vs_157209		Tunavirinae	Drexlerviridae		Caudoviricetes
S6_vs_15813					
S13_vs_49889					
S9_vf_90866|phage-circular					
S16_vf_100235					
S11_vs_56762					
S7_vs_138140		Sepvirinae			Caudoviricetes
S10_vs_67471		Bclasvirinae			Caudoviricetes
S7_vf_32559					
S7_vs_87439					
S4_vf_10506|phage-circular					
S3_vf_67828|phage-circular					
S11_vf_92391|phage-circular					
S3_vf_44405		Arquatrovirinae			Caudoviricetes
S5_vf_6032		Nclasvirinae			Caudoviricetes
S14_vs_117160		Arquatrovirinae			Caudoviricetes
S3_vs_62410		Arquatrovirinae			Caudoviricetes
S9_vs_50439					
S10_vs_77004		Arquatrovirinae			Caudoviricetes
S11_vs_49071			Herelleviridae		Caudoviricetes
S5_vs_20268					
S5_vs_9747		Tevenvirinae	Straboviridae		Caudoviricetes
S14_vs_110290					
S8_vs_176604					
S5_vs_145592		Nymbaxtervirinae			Caudoviricetes
S7_vf_72493					
S16_vs_54250		Arquatrovirinae			Caudoviricetes
S17_vs_58025					
S8_vs_208113					
S13_vs_23314		1	Herelleviridae		Caudoviricetes
S12_vf_128794|phage-circular		Nymbaxtervirinae			Caudoviricetes
S16_vs_80244					
S4_vf_13627					
S8_vf_196670					
S3_vs_49835					
S4_vf_31973|phage-circular					
S15_vs_1407					
S4_vs_VIRSorter_31980-circular-cat_2					
S17_vs_2079					
S17_vs_103009					
S5_vs_136679					
S8_vs_208129					
S8_vs_50431		Tunavirinae	Drexlerviridae		Caudoviricetes
S9_vs_88286		1	1		
S3_vs_127442					

WMS:

High_confidence_taxonomy

contig_ID	genus	subfamily	family	order	class
S5-wms_94965		Arquatrovirinae			Caudoviricetes
S7-wms-combined_235165		1			
S11-wms-combined_299954					
S12-wms_386692|phage-circular		2	Straboviridae		Caudoviricetes
S7-wms-combined_225260					
S7-wms-combined_189250		Arquatrovirinae			Caudoviricetes
S3-wms-combined_73262		Guernseyvirinae			Caudoviricetes
S1-wms-combined_186878		Nymbaxtervirinae			Caudoviricetes
S11-wms_330479|phage-circular					
S1-wms-combined_263562					
S11-wms_14390|phage-circular		Ounavirinae			Caudoviricetes
S2-wms-combined_52568		Spounavirinae	Herelleviridae		Caudoviricetes
S2-wms-combined_159670					
S5-wms-combined_83412					
S17-wms-combined_158354					
S11-wms-combined_41367					
S2-wms_128831|phage-circular					
S1-wms_244836		Tevenvirinae	Straboviridae		Caudoviricetes
S4-wms_62635		Nymbaxtervirinae			Caudoviricetes
S8-wms-combined_276204					
S7-wms_244866|phage-circular					
S9-wms-combined_31456		Guernseyvirinae			Caudoviricetes
S1-wms-combined_224578			Salasmaviridae		Caudoviricetes
S2-wms_140933		Bclasvirinae			Caudoviricetes
S12-wms-combined_376326		Tevenvirinae	Straboviridae		Caudoviricetes
S1-wms_185046					
S5-wms_123499					
S9-wms-combined_2042		Spounavirinae	Herelleviridae		Caudoviricetes
S2-wms-combined_190624					
S12-wms-combined_264425		Nymbaxtervirinae			Caudoviricetes
S7-wms_157264					
S12-wms-combined_390985					
S6-wms_54578					
S11-wms-combined_195425					
S7-wms-combined_121062					
S4-wms-combined_14411					
S5-wms-combined_134743		1	Herelleviridae		Caudoviricetes
S1-wms-combined_354899		Nymbaxtervirinae			Caudoviricetes
S11-wms-combined_117756					
S12-wms_15700					
S1-wms-combined_324053		Tunavirinae	Drexlerviridae		Caudoviricetes
S8-wms-combined_185611					
S17-wms-combined_253499		Tunavirinae	Drexlerviridae		Caudoviricetes
S1-wms_225995		Nymbaxtervirinae			Caudoviricetes
S2-wms-combined_72725		Sepvirinae			Caudoviricetes
S8-wms-combined_72911		1			
S1-wms-combined_394574					
S5-wms-combined_199135		Sepvirinae			Caudoviricetes
S7-wms-combined_104872					
S4-wms_161443					
S8-wms-combined_211119		Nymbaxtervirinae			Caudoviricetes
S8-wms-combined_97848		Guernseyvirinae			Caudoviricetes
S12-wms_401372|phage-circular					
S5-wms-combined_66017		Arquatrovirinae			Caudoviricetes
S8-wms-combined_226076					
S12-wms-combined_256299					
S9-wms-combined_3461		Spounavirinae	Herelleviridae		Caudoviricetes
S12-wms-combined_250551					
S5-wms_49310		Nymbaxtervirinae			Caudoviricetes
S2-wms-combined_246480					
S8-wms-combined_134098					
S7-wms-combined_40779					
S2-wms-combined_197577					
S8-wms_57121					
S12-wms-combined_259783		Bastillevirinae	Herelleviridae		Caudoviricetes
S12-wms_179388		Spounavirinae	Herelleviridae		Caudoviricetes
S6-wms-combined_65225		1	Herelleviridae		Caudoviricetes
S12-wms_44817					
S12-wms_401366|phage-circular		Nymbaxtervirinae			Caudoviricetes
S2-wms-combined_248318		Arquatrovirinae			Caudoviricetes
S7-wms-combined_163980					
S7-wms-combined_156351		Spounavirinae	Herelleviridae		Caudoviricetes
S5-wms_85650		Ounavirinae			Caudoviricetes
S1-wms_100010		Nymbaxtervirinae			Caudoviricetes
S17-wms-combined_225356		1			
S8-wms-combined_140059					
S1-wms_106115					
S5-wms-combined_7292					
S9-wms_128232|phage-circular					
S6-wms-combined_140731		1	Drexlerviridae		Caudoviricetes
S6-wms-combined_110950					
S6-wms-combined_745					
Prophages:					
S12-wms-combined_299293|prophage-2369[illegible]		Spounavirinae	Herelleviridae		Caudoviricetes
S11-wms-combined_193148|prophage-6157:35963					
S2-wms-combined_101826|prophage-5343:44508					
S1-wms-combined_107766|prophage-0:32406					
S7-wms-combined_134452|prophage-25486:63184					
S8-wms-combined_169835|prophage-33527:		Arquatrovirinae			Caudoviricetes
S4-wms-combined_17284|prophage-20324:5		Arquatrovirinae			Caudoviricetes
S11-wms-combined_46816|prophage-0:25833					
S8-wms-combined_48046|prophage-0:24520		Nymbaxtervirinae			Caudoviricetes
S8-wms-combined_49369|prophage-6161:39940					
S5-wms-combined_178432|prophage-475:34		1			
S2-wms_130687|prophage-32092:69405		Nymbaxtervirinae			Caudoviricetes
S5-wms-combined_161191|prophage-133027:176705					
S8-wms-combined_134284|prophage-1693:26955					
S11-wms-combined_92114|prophage-753:26974					
S8-wms-combined_23583|prophage-49044:83941					
S7-wms-combined_5625|prophage-27372:66709					
S5-wms-combined_207291|prophage-13497:57653					

Euk_virus_detection-Kraken2

VLP:		WMS:	
S1_vlp_ME	S2_vlp_SHHC	S1_wms_ME	S2_wms_SHHC
Human gammaherpesvirus 4	NA	Human gammaherpesvirus 4	Human betaherpesvirus 7
Human betaherpesvirus 7		Human papillomavirus type 54	Human gammaherpesvirus 4
Human coronavirus 229E		Human papillomavirus type 32	
human papillomavirus 163		Human papillomavirus type 131	
Human papillomavirus 175		Human papillomavirus type 156	
Human papillomavirus type 34			
Human papillomavirus type 85			
Human parvovirus 4 G1			
S3_vlp_ME	S4_vlp_SHHC	S3_wms_ME	S4_wms_SHHC
Human betaherpesvirus 7	Human gammaherpesvirus 4	Human gammaherpesvirus 4	Torque teno mini virus 10
Human gammaherpesvirus 4	Torque teno mini virus 10	Human betaherpesvirus 5	
Human alphaherpesvirus 3		Human associated cyclovirus 10	
Human mastadenovirus E			
Human coronavirus NL63			
Human parvovirus 4 G1			
human papillomavirus 30			
Anelloviridae_Torque teno mini virus 4			
S5_vlp_ME	S6_vlp_SHHC	S5_wms_ME	S6_wms_SHHC
Human alphaherpesvirus 3	Human papillomavirus 121	Human coronavirus 229E	Human circovirus VS6600022
Human coronavirus 229E			
Human papillomavirus type 112			
S7_vlp_ME	S8_vlp_SHHC	S7_wms_ME	S8_wms_SHHC
Human betaherpesvirus 5	Human gammaherpesvirus 8	Human alphaherpesvirus 1	Human alphaherpesvirus 1
Human betaherpesvirus 6B	Human alphaherpesvirus 3	Human gammaherpesvirus 4	Human betaherpesvirus 7
Human alphaherpesvirus 1	Human alphaherpesvirus 1	Human adenovirus 54	Human gammaherpesvirus 4
Torque teno mini virus 4	Human mastadenovirus D	Human papillomavirus type 16	Human mastadenovirus E
	Human papillomavirus type 85	Human papillomavirus 184	Torque teno mini virus 4
S9_vlp_ME	S10_vlp_SHHC	S9_wms_ME	
Human alphaherpesvirus 3	Human gammaherpesvirus 4	NA	
Human gammaherpesvirus 4	Human mastadenovirus E		
	Human papillomavirus 201		
	Torque teno midi virus 10		
S11_vlp_ME	S12_vlp_SHHC	S11_wms_ME	S12_wms_SHHC
Human betaherpesvirus 6A	Human betaherpesvirus 5	Human alphaherpesvirus 1	Human betaherpesvirus 5
Human betaherpesvirus 6B	Human betaherpesvirus 7	Human gammaherpesvirus 4	Human gammaherpesvirus 4
Human gammaherpesvirus 4	Human smacovirus 1	Human coronavirus 229E	Human mastadenovirus B
Human papillomavirus type 131	Human associated huchismacovirus 1	Torque teno virus 28	Human mastadenovirus C
	Human mastadenovirus B	Torque teno mini virus 1	Human coronavirus 229E
	Torque teno mini virus 4		Human smacovirus 1
			Human associated huchismacovirus 1
S13_vlp_ME			human papillomavirus 103
Human gammaherpesvirus 8			Human papillomavirus type 90
Human lung-associated vientovirus FB			
Torque teno mini virus 4			
S14_vlp_ME	S15_vlp_SHHC		
Human alphaherpesvirus 3	Human alphaherpesvirus 3		
Human papillomavirus 187	Human papillomavirus type 34		
Torque teno mini virus 4	Torque teno mini virus 4		
S17_vlp_ME	S16_vlp_SHHC	S17_wms_ME	
Human gammaherpesvirus 4	Human betaherpesvirus 6B	Human gammaherpesvirus 4	
Human betaherpesvirus 5	Human betaherpesvirus 7	Human adenovirus 54	
Human parvovirus 4 G1	Human papillomavirus type 96	Human coronavirus 229E	
	Human parvovirus 4 G1	Human betaherpesvirus 5	
	Human adenovirus 7		
	Human coronavirus 229E		
	Torque teno mini virus 4		

Only seen in ME samples

S7_vs_95276			
S7_vf_32396			
S16_vs_140709			
S16_vf_173099|phage-circular			
S8_vf_43483	Picovirinae	Salasmaviridae	Caudoviricetes
S7_vf_138069|phage-circular			
S16_vf_1037|phage-circular			
S8_vf_117796	Guernseyvirinae		Caudoviricetes
S8_vs_4883			
S8_vf_153696			
S17_vf_148303			
S3_vs_72118			
S16_vs_124703			
S15_vf_55639			
S8_vf_65522			
S9_vf_70236			
S4_vf_28601			
S8_vf_208107|phage-circular			
S3_vf_70746			
S3_vs_87728			
S8_vf_87755			
S13_vf_147396|phage-circular	Tunavirinae	Drexlerviridae	Caudoviricetes
S14_vs_30108	1		
S2_vf_3356			
S5_vs_83886	1	1	
S16_vs_75108	Spounavirinae	Herelleviridae	Caudoviricetes
S3_vs_90341	Spounavirinae	Herelleviridae	Caudoviricetes
S12_vs_48045	2	Herelleviridae	Caudoviricetes
S16_vs_173102			
S7_vs_103530			
S12_vs_188825			
S10_vf_57705			
S5_vs_145633			
S7_vs_129271	Nymbaxtervirinae		Caudoviricetes
S8_vs_154231	Nymbaxtervirinae		Caudoviricetes
S7_vs_135828			
S4_vf_2524			
S11_vs_79275			
S9_vf_23901|phage-circular			
S5_vs_139401			
S5_vs_130476			
S8_vs_102608	Nymbaxtervirinae		Caudoviricetes
S2_vs_10289			
S8_vf_134943			
S13_vs_121307			
S8_vs_71212			
S4_vf_18803|phage-circular			
S5_vs_129970			
S1_vf_28398			
S8_vf_166403			
S9_vs_45812	Arquatrovirinae		Caudoviricetes
S16_vf_100473			
S14_vf_117164|phage-circular			
S16_vs_140468			
S15_vs_76390	Tunavirinae	Drexlerviridae	Caudoviricetes
S11_vf_58587			
S7_vf_36627			
S12_vs_188833	Arquatrovirinae		Caudoviricetes
S5_vs_4737	Arquatrovirinae		Caudoviricetes
S12_vf_188822|phage-circular			
S4_vf_4891			
S8_vf_208111|phage-circular			
S5_vf_123990	Picovirinae	Salasmaviridae	Caudoviricetes
S3_vs_81793	Twortvirinae	Herelleviridae	Caudoviricetes
S10_vf_18671|phage-circular			
S5_vs_37659	Arquatrovirinae		Caudoviricetes
S12_vs_99219	1	Herelleviridae	Caudoviricetes
S7_vs_14275			
S10_vs_71900	Tunavirinae	Drexlerviridae	Caudoviricetes
S6_vs_56493			
S16_vf_84462|phage-circular	Twortvirinae	Herelleviridae	Caudoviricetes
S7_vs_138116	Sepvirinae		Caudoviricetes
S16_vs_35031			
S1_vf_138157			
S16_vs_76823			
S7_vs_42563			
S1_vs_145792			
S14_vs_112252			
S5_vs_120079	Nymbaxtervirinae		Caudoviricetes
S5_vs_140946	Sepvirinae		Caudoviricetes
S9_vs_63485	Bastillevirinae	Herelleviridae	Caudoviricetes
S7_vf_76463	Nymbaxtervirinae		Caudoviricetes
S17_vs_127519			
S5_vs_850	Nymbaxtervirinae		Caudoviricetes
S10_vs_36598			
S5_vf_74661	Sepvirinae		Caudoviricetes
S12_vf_10887|phage-circular			
S8_vf_208102|phage-circular	Tevenvirinae	Straboviridae	Caudoviricetes
S3_vs_89485			
S15_vf_20644			
S13_vs_113593			
S16_vs_120149			
S1_vf_22149	Nymbaxtervirinae		Caudoviricetes
S4_vs_VIRSorter_16493-cat_2	Arquatrovirinae		Caudoviricetes
S17_vs_43307			
S5_vs_145026			
S16_vf_164913	Picovirinae	Salasmaviridae	Caudoviricetes
S10_vs_18901	Sepvirinae		Caudoviricetes
S7_vf_117697	1	Straboviridae	Caudoviricetes
S5_vf_136076	Nymbaxtervirinae		Caudoviricetes
S8_vs_51368			
S16_vs_17493			
S9_vf_73311|phage-circular	1	Herelleviridae	Caudoviricetes
S11_vs_58930			
S5_vf_77701	Mccleskeyvirinae		Caudoviricetes
S16_vs_10622|phage-circular			
S4_vs_VIRSorter_31196-circular-cat_2			
S3_vs_25896			
S15_vs_27510	Ounavirinae		Caudoviricetes
S10_vs_120489			
S16_vf_108554			
S12_vf_187941	Tunavirinae	Drexlerviridae	Caudoviricetes
S9_vf_26805|phage-circular			
S14_vf_44452			
S3_vs_119660			

S17-wms-combined_136622|prophage-0:14633			
S4-wms-combined_51079|prophage-2266:17963			
S1-wms-combined_331544|prophage-5239:22022			
S5-wms-combined_59064|prophage-31398:77143			
S7-wms-combined_238231|prophage-41175:	1		
S12-wms-combined_334806|prophage-1282:	Tevenvirinae	Straboviridae	Caudoviricetes
S7-wms-combined_43950|prophage-19667:5	Guernseyvirinae		Caudoviricetes
S8-wms-combined_215102|prophage-32414:64368			
S4-wms-combined_64632|prophage-21507:57119			
S8-wms-combined_68780|prophage-22160:7	Ounavirinae		Caudoviricetes
S2-wms-combined_181391|prophage-95287:117194			
S11-wms-combined_179605|prophage-20178:43615			
S9-wms-combined_124857|prophage-20824:82523			
S7-wms-combined_190281|prophage-28470:	Arquatrovirinae		Caudoviricetes
S2-wms-combined_181849|prophage-3700:40213			
S7-wms-combined_14267|prophage-23745:5	Tevenvirinae	Straboviridae	Caudoviricetes
S6-wms-combined_91039|prophage-5888:44	1	Straboviridae	Caudoviricetes

S5_vs_13254				
S4_vf_13831				
S5_vs_117417				
S11_vs_36748				
S8_vf_94361	Gokushovirinae	Microviridae	Petitvirales	Malgrandaviricetes
S4_vs_VIRSorter_15112-cat_2	Nymbaxtervirinae			Caudoviricetes
S11_vs_104032				
S12_vs_121913				
S10_vf_113489				
S9_vf_15012				
S3_vs_77156				
S7_vs_43529				
S9_vf_57277|phage-circular				
S11_vs_89911	Tevenvirinae	Straboviridae		Caudoviricetes
S7_vf_11744				
S7_vf_89668				
S3_vs_127411				
S11_vs_104054	Nymbaxtervirinae			Caudoviricetes
S16_vs_4662				
S14_vs_90934				
S7_vs_55031				
S4_vs_VIRSorter_9913-cat_1				
S8_vs_176720				
S17_vf_77057				
S3_vs_70508				
S3_vs_18858				
S5_vs_1572				
S9_vf_79919|phage-circular	1			
S17_vs_177196				
S5_vs_145591	Nymbaxtervirinae			Caudoviricetes
S7_vs_136164				
S5_vf_110171|phage-circular	Nymbaxtervirinae			Caudoviricetes
S5_vf_28061|phage-circular				
S7_vs_27530	Ounavirinae			Caudoviricetes
S12_vs_26847	Nymbaxtervirinae			Caudoviricetes
S14_vs_61530	Nymbaxtervirinae			Caudoviricetes
S5_vs_113386	Bastillevirinae	Herelleviridae		Caudoviricetes
S8_vs_5211	Arquatrovirinae			Caudoviricetes
S17_vf_172132				
S5_vs_1338				
S16_vf_173109|phage-circular	Mccleskeyvirinae			Caudoviricetes
S16_vf_173133|phage-circular				
S8_vs_72117				
S16_vs_51901				
S11_vs_75554	Picovirinae	Salasmaviridae		Caudoviricetes
S11_vs_93101	Tunavirinae	Drexlerviridae		Caudoviricetes
S8_vf_102508	Nymbaxtervirinae			Caudoviricetes
S16_vs_127905				
S4_vs_VIRSorter_31976-circular-cat_2				
S3_vf_84832				
S9_vf_4115	Klosneuvirinae	Mimiviridae	Imitervirales	Megaviricetes
S5_vf_103206				
S8_vf_183045|phage-circular				
S6_vs_32886				
S5_vf_38011				
S14_vs_101482				
S9_vs_21850	Guernseyvirinae			Caudoviricetes
S5_vf_145586|phage-circular	Sepvirinae			Caudoviricetes
S16_vs_103726	Nymbaxtervirinae			Caudoviricetes
S12_vf_173208				
S8_vs_204260				
S12_vs_28661				
S7_vf_69348				
S14_vs_3251				
S11_vs_8456	Nymbaxtervirinae			Caudoviricetes
S12_vs_151292	Guernseyvirinae			Caudoviricetes
S9_vs_88847				
S3_vs_1031	Arquatrovirinae			Caudoviricetes
S14_vf_114491				
S12_vf_32615|phage-circular	Tevenvirinae	Straboviridae		Caudoviricetes
S4_vs_VIRSorter_13488-cat_2	Arquatrovirinae			Caudoviricetes
S7_vs_30665				
S13_vs_87108				
S6_vs_56492				
S4_vs_VIRSorter_6022-circular-cat_1				
S7_vf_134023|phage-circular				
S12_vs_111815	Tunavirinae	Drexlerviridae		Caudoviricetes
S7_vf_29196	Nymbaxtervirinae			Caudoviricetes
S7_vs_137998				
S5_vs_13090				
S5_vf_116300	Nymbaxtervirinae			Caudoviricetes
S5_vs_122618	1			
S11_vf_95067|phage-circular				
S8_vs_142097				
S8_vf_55476|phage-circular				
S9_vs_4343	1	1		
S5_vf_60597|phage-circular	Twortvirinae	Herelleviridae		Caudoviricetes
S6_vf_24553				
S11_vs_23188				
S17_vs_138036				
S13_vf_87873				
S3_vf_127168	Picovirinae	Salasmaviridae		Caudoviricetes
S3_vs_50924				
S14_vf_117157|phage-circular				
S7_vs_20200	Bastillevirinae	Herelleviridae		Caudoviricetes
S11_vs_14882				
S16_vs_141218				
S1_vs_143929	Nymbaxtervirinae			Caudoviricetes
S9_vs_83108				
S14_vf_109545				
S5_vs_145593	Nymbaxtervirinae			Caudoviricetes
S9_vs_90857	Sepvirinae			Caudoviricetes
S6_vf_25710				
S12_vs_188828				
S5_vs_8847	Bastillevirinae	Herelleviridae		Caudoviricetes
S17_vf_177197|phage-circular				
S10_vs_37379				
S8_vf_79769|phage-circular				
S16_vf_32359				
S7_vs_944				
S16_vf_96483				
S3_vs_73361				
S3_vs_114271	Arquatrovirinae			Caudoviricetes
S16_vs_59697				
S7_vs_54322				

S5_vs_17520			Salasmaviridae			Caudoviricetes
S7_vs_118295						
S11_vs_81775		1				
S11_vf_68693						
S13_vf_109235|phage-circular	Nymbaxtervirinae					Caudoviricetes
S11_vs_98193						
S3_vf_57422						
S16_vf_5809	Nymbaxtervirinae					Caudoviricetes
S12_vf_8173						
S17_vf_79151	Picovirinae		Salasmaviridae			Caudoviricetes
S11_vs_42927		1	Herelleviridae			Caudoviricetes
S13_vf_56074|phage-circular						
S11_vs_55144		1				
S3_vs_28402	Nclasvirinae					Caudoviricetes
S10_vs_36407						
S14_vs_117166	Nclasvirinae					Caudoviricetes
S17_vs_73830	Nclasvirinae					Caudoviricetes
S14_vf_78139		1		1	Pimascovirales	Megaviricetes
S13_vs_152223		1				
S5_vs_121411		1				
S3_vs_127404						
S7_vs_56529						
S10_vs_120541	Spounavirinae		Herelleviridae			Caudoviricetes
S11_vs_84627	Sepvirinae					Caudoviricetes
S12_vs_94042						
S12_vs_138261	Ounavirinae					Caudoviricetes
S3_vf_5525	Arquatrovirinae					Caudoviricetes
S3_vf_108959|phage-circular		1		1		
S8_vf_7243						
S6_vs_22637						
S11_vs_38860		1				
S14_vf_106156	Sepvirinae					Caudoviricetes
S11_vf_66522	Arquatrovirinae					Caudoviricetes
S5_vs_118168	Nymbaxtervirinae					Caudoviricetes
S8_vf_201695	Twortvirinae		Herelleviridae			Caudoviricetes
S13_vf_38708	Ounavirinae					Caudoviricetes
S16_vs_136935						
S7_vs_28150						
S8_vs_132359						
S11_vs_6458						
S12_vs_141337						
S12_vs_188823						
S5_vs_41663		1	Herelleviridae			Caudoviricetes
S3_vs_89898						
S3_vf_127450|phage-circular						
S8_vs_175235						
S11_vs_104038						
S5_vs_145581	Arquatrovirinae					Caudoviricetes
S7_vs_100640						
S13_vs_26756	Guernseyvirinae					Caudoviricetes
S15_vf_2513|phage-circular						
S14_vf_72953	Sepvirinae					Caudoviricetes
S5_vs_128881						
S16_vf_52513						
S8_vf_12212	Arquatrovirinae					Caudoviricetes
S8_vs_9114						
S10_vf_64371|phage-circular						
S10_vs_120570	Nymbaxtervirinae					Caudoviricetes
S12_vf_174374		2	Straboviridae			Caudoviricetes
S11_vs_84848						
S5_vs_56083	Arquatrovirinae					Caudoviricetes
S4_vf_13759						
S14_vs_79505						
S1_vs_3435						
S11_vs_104037						
S11_vs_53675	Picovirinae		Salasmaviridae			Caudoviricetes
S13_vs_83447						
S12_vf_145044						
S4_vf_16485						
S14_vf_99344						
S5_vf_145585|phage-circular						
S11_vs_79831						
S9_vf_19284|phage-circular	Arquatrovirinae					Caudoviricetes
S7_vf_138019|phage-circular						
S12_vf_79666	Nymbaxtervirinae					Caudoviricetes
S1_vs_91270		1	Salasmaviridae			Caudoviricetes
S9_vs_90849	Spounavirinae		Herelleviridae			Caudoviricetes
S7_vs_78507	Bastillevirinae		Herelleviridae			Caudoviricetes
S11_vf_33117|phage-circular	Ounavirinae					Caudoviricetes
S8_vs_3224	Bastillevirinae		Herelleviridae			Caudoviricetes
S8_vf_208146|phage-circular	Gokushovirinae		Microviridae		Petitvirales	Malgrandaviricetes
S4_vf_31978|phage-circular						
S5_vs_28263						
S3_vf_83536						
S14_vs_112068	Sepvirinae					Caudoviricetes
S7_vs_50194						
S13_vf_91189						
S16_vs_173096						
S12_vs_188820	Tunavirinae		Drexlerviridae			Caudoviricetes
S7_vs_55526	Arquatrovirinae					Caudoviricetes
S14_vs_4887						
S3_vs_97827						
S9_vs_69322						
S3_vf_18080		1		1		
S6_vf_6314	Picovirinae		Salasmaviridae			Caudoviricetes
S12_vs_164056						
S9_vs_75290	Picovirinae		Salasmaviridae			Caudoviricetes
S11_vs_3277						
S12_vs_111286						
S9_vs_14235	Sepvirinae					Caudoviricetes
S2_vs_7306						
S7_vs_134391						
S3_vf_2186|phage-circular						
S16_vf_94866						
S16_vs_64584	Arquatrovirinae					Caudoviricetes
S11_vf_27845						
S7_vs_2599						
S13_vs_9438						
S14_vs_38905						
S11_vf_99751						
S6_vs_38250	Nymbaxtervirinae					Caudoviricetes
S3_vf_127423|phage-circular	Dclasvirinae					Caudoviricetes
S8_vf_197061						
S8_vs_40014						
S11_vs_67573	Spounavirinae		Herelleviridae			Caudoviricetes

S5_vs_84574			
S3_vs_39774			
S8_vf_147128			
S7_vf_67910	Arquatrovirinae		Caudoviricetes
Prophages:			
S3_vs_9158|prophage-2555:27661			
S16_vs_34021|prophage-329:31534	Nymbaxtervirinae		Caudoviricetes
S3_vs_64424|prophage-0:35145			
S5_vs_21778|prophage-7234:115733			
S12_vs_26336|prophage-15751:67092	Spounavirinae	Herelleviridae	Caudoviricetes
S8_vf_28956|prophage-122:25469			
S12_vs_23796|prophage-0:38478			
S14_vs_74067|prophage-149012:246138			
S8_vs_125717|prophage-33527:52956	Arquatrovirinae		Caudoviricetes
S6_vs_39065|prophage-7045:41823			
S14_vs_96111|prophage-2661:24193	1	Herelleviridae	Caudoviricetes
S10_vf_80712|prophage-0:96675	Eucampyvirinae		Caudoviricetes
S5_vs_46394|prophage-32694:43444	Arquatrovirinae		Caudoviricetes
S8_vs_205257|prophage-1642:48626			
S13_vs_33947|prophage-0:26846			
S15_vs_35445|prophage-0:15628			
S12_vs_123620|prophage-0:84060			
S10_vf_80712|prophage-105587:116981			
S5_vs_80496|prophage-58056:120254	Ounavirinae		Caudoviricetes
S5_vs_123256|prophage-34087:71380			
S12_vs_172379|prophage-0:134462			
S6_vs_9784|prophage-7737:47646	Ounavirinae		Caudoviricetes
S3_vs_45147|prophage-6666:74355			
S5_vs_76500|prophage-2854:36602			
S5_vs_142195|prophage-0:48595	Spounavirinae	Herelleviridae	Caudoviricetes
S16_vs_107235|prophage-0:30582	Nymbaxtervirinae		Caudoviricetes
S5_vs_30490|prophage-17846:56501			
S9_vf_13507|prophage-32195:121541			
S7_vs_97737|prophage-47165:74627			
S13_vs_67429|prophage-29606:89258			
S16_vs_19968|prophage-21941:54417			
S14_vs_112345|prophage-26622:38263			
S15_vs_42043|prophage-69410:193243			
S5_vs_130294|prophage-21515:43034			
