# Supplementary material for: Investigating the Human Intestinal DNA Virome and Predicting Disease-Associated Virus–Host Interactions in Severe Myalgic Encephalomyelitis/Chronic Fatigue Syndrome (ME/CFS)
Source: Int J Mol Sci. 2023 Dec 8;24(24):17267. doi: 10.3390/ijms242417267 (PMC10744171; doi:10.3390/ijms242417267)
Supplement: Supplementary file 1 [file ijms-24-17267-s001.zip › Table S2.pdf]

Table S2-1. VLP_vOTUs_checkv

contig_id	contig_length	provirus	proviral_length	viral_genes	host_genes	checkv_quality	miuvig_quality	completeness	contamination
S17_extracted_k141_29558	1174	No	NA	0	1	Not-determined	Genome-fragment	NA	0
S9_extracted_k141_55517	1000	No	NA	2	0	Low-quality	Genome-fragment	1.09	0
S17_extracted_k141_73905	4103	No	NA	0	2	Not-determined	Genome-fragment	NA	0
S17_extracted_k141_44341	7337	No	NA	0	5	Not-determined	Genome-fragment	NA	0
S17_extracted_k141_125625	3571	No	NA	0	0	Not-determined	Genome-fragment	NA	0
S17_extracted_k141_140412	1061	No	NA	0	0	Not-determined	Genome-fragment	NA	0
S17_extracted_k141_88694	1059	No	NA	0	2	Not-determined	Genome-fragment	NA	0
S17_extracted_k141_66541	1536	No	NA	0	1	Not-determined	Genome-fragment	NA	0
S14_extracted_k141_60624	1000	No	NA	0	1	Low-quality	Genome-fragment	2.56	0
S17_extracted_k141_14804	1049	No	NA	0	0	Not-determined	Genome-fragment	NA	0
S14_extracted_k141_11153	1000	No	NA	1	0	Low-quality	Genome-fragment	0.76	0
S17_extracted_k141_66548	1930	No	NA	0	1	Not-determined	Genome-fragment	NA	0
S1_vs_k141_123264||full	1000	No	NA	3	0	Low-quality	Genome-fragment	1.61	0
S7_extracted_65780	1000	No	NA	1	0	Low-quality	Genome-fragment	2.56	0
S17_extracted_k141_14823	1010	No	NA	0	0	Not-determined	Genome-fragment	NA	0
S17_extracted_k141_36993	1031	No	NA	0	1	Not-determined	Genome-fragment	NA	0
S7_vs_25620||full	1000	No	NA	2	0	Low-quality	Genome-fragment	1.99	0
S17_extracted_k141_169985	2542	No	NA	0	0	Not-determined	Genome-fragment	NA	0
S17_extracted_k141_51785	6067	No	NA	0	2	Not-determined	Genome-fragment	NA	0
S17_extracted_k141_96125	1301	No	NA	0	1	Not-determined	Genome-fragment	NA	0
S7_vs_48054||full	1000	No	NA	0	0	Low-quality	Genome-fragment	0.65	0
S17_extracted_k141_96132	1343	No	NA	0	3	Not-determined	Genome-fragment	NA	0
S7_vs_16714||lt2gene	1000	No	NA	0	1	Low-quality	Genome-fragment	1.12	0
S10_extracted_25739	1000	No	NA	0	2	Low-quality	Genome-fragment	1.08	0
S17_extracted_k141_118309	1474	No	NA	0	1	Not-determined	Genome-fragment	NA	0
S17_extracted_k141_96141	1262	No	NA	0	0	Not-determined	Genome-fragment	NA	0
S17_extracted_k141_66582	4129	No	NA	0	0	Not-determined	Genome-fragment	NA	0
S17_extracted_k141_29669	3665	No	NA	0	2	Not-determined	Genome-fragment	NA	0
S17_extracted_k141_29672	1006	No	NA	0	1	Not-determined	Genome-fragment	NA	0
S17_extracted_k141_104	4839	No	NA	0	1	Not-determined	Genome-fragment	NA	0
S17_extracted_k141_133078	1049	No	NA	0	1	Not-determined	Genome-fragment	NA	0
S17_extracted_k141_107	7447	No	NA	0	5	Not-determined	Genome-fragment	NA	0
S17_extracted_k141_162641	1132	No	NA	0	0	Not-determined	Genome-fragment	NA	0
S17_extracted_k141_96154	1386	No	NA	0	1	Not-determined	Genome-fragment	NA	0
S8_extracted_k141_192562	1000	No	NA	0	0	Low-quality	Genome-fragment	1.52	0
S17_extracted_k141_29703	2194	No	NA	0	1	Not-determined	Genome-fragment	NA	0
S17_extracted_k141_140474	3147	No	NA	0	3	Not-determined	Genome-fragment	NA	0
S17_extracted_k141_29713	1436	No	NA	0	1	Not-determined	Genome-fragment	NA	0
S17_extracted_k141_66627	1313	No	NA	0	0	Not-determined	Genome-fragment	NA	0
S17_extracted_k141_74031	2593	No	NA	0	0	Not-determined	Genome-fragment	NA	0
S17_extracted_k141_162664	2006	No	NA	0	2	Not-determined	Genome-fragment	NA	0
S17_extracted_k141_44417	1820	No	NA	0	1	Not-determined	Genome-fragment	NA	0
S17_extracted_k141_22232	2815	No	NA	0	1	Not-determined	Genome-fragment	NA	0
S17_extracted_k141_125697	1388	No	NA	0	1	Not-determined	Genome-fragment	NA	0
S17_extracted_k141_74065	1224	No	NA	0	0	Not-determined	Genome-fragment	NA	0
S17_extracted_k141_74068	1372	No	NA	0	0	Not-determined	Genome-fragment	NA	0
S17_extracted_k141_103593	1264	No	NA	0	1	Not-determined	Genome-fragment	NA	0
S8_extracted_k141_88686	1000	No	NA	0	0	Low-quality	Genome-fragment	1.9	0
S17_extracted_k141_103596	1042	No	NA	0	1	Not-determined	Genome-fragment	NA	0
S17_extracted_k141_37084	1005	No	NA	0	1	Not-determined	Genome-fragment	NA	0
S8_vs_k141_143174||full	1000	No	NA	0	0	Low-quality	Genome-fragment	0.39	0
S17_extracted_k141_88810	2336	No	NA	0	0	Not-determined	Genome-fragment	NA	0
S17_extracted_k141_25378	1001	No	NA	1	0	Low-quality	Genome-fragment	2.24	0
S17_extracted_k141_118382	1010	No	NA	0	0	Not-determined	Genome-fragment	NA	0
S17_extracted_k141_103645	1363	No	NA	0	0	Not-determined	Genome-fragment	NA	0
S17_vs_k141_120886||full	1001	No	NA	0	0	Low-quality	Genome-fragment	1.55	0
S17_extracted_k141_155306	1073	No	NA	0	0	Not-determined	Genome-fragment	NA	0
S17_vs_k141_94174||full	1001	No	NA	0	1	Low-quality	Genome-fragment	2.32	0
S17_extracted_k141_147961	4804	No	NA	0	4	Not-determined	Genome-fragment	NA	0
S17_extracted_k141_51926	1543	No	NA	0	0	Not-determined	Genome-fragment	NA	0
S17_extracted_k141_22300	1330	No	NA	0	0	Not-determined	Genome-fragment	NA	0
S17_extracted_k141_37184	1522	No	NA	0	1	Not-determined	Genome-fragment	NA	0
S17_vs_k141_84320||lt2gene	1001	No	NA	2	0	Low-quality	Genome-fragment	1.79	0
S17_extracted_k141_7647	1103	No	NA	0	0	Not-determined	Genome-fragment	NA	0
S17_extracted_k141_37193	1111	No	NA	0	0	Not-determined	Genome-fragment	NA	0
S17_extracted_k141_103677	2068	No	NA	0	0	Not-determined	Genome-fragment	NA	0
S17_extracted_k141_81330	4034	No	NA	0	4	Not-determined	Genome-fragment	NA	0
S17_extracted_k141_140605	1322	No	NA	0	2	Not-determined	Genome-fragment	NA	0
S17_vs_k141_4543||lt2gene	1001	No	NA	1	0	Low-quality	Genome-fragment	1.6	0
S17_extracted_k141_118438	1737	No	NA	0	4	Not-determined	Genome-fragment	NA	0
S17_vs_k141_63804||lt2gene	1001	No	NA	2	0	Low-quality	Genome-fragment	2.46	0
S17_extracted_k141_96379	1323	No	NA	0	1	Not-determined	Genome-fragment	NA	0
S9_vs_k141_16791||full	1001	No	NA	1	0	Low-quality	Genome-fragment	2.2	0
S17_extracted_k141_125784	1616	No	NA	0	2	Not-determined	Genome-fragment	NA	0
S11_vs_k141_51168||full	1001	No	NA	0	0	Low-quality	Genome-fragment	2.21	0
S13_extracted_38304	1001	No	NA	0	1	Low-quality	Genome-fragment	2.36	0
S13_vs_77368||full	1001	No	NA	0	2	Low-quality	Genome-fragment	1.16	0
S17_extracted_k141_103755	3384	No	NA	0	2	Not-determined	Genome-fragment	NA	0
S17_extracted_k141_155401	1201	No	NA	0	1	Not-determined	Genome-fragment	NA	0
S17_extracted_k141_7719	8499	No	NA	0	2	Not-determined	Genome-fragment	NA	0
S17_extracted_k141_96427	5567	No	NA	0	3	Not-determined	Genome-fragment	NA	0
S17_extracted_k141_74143	3989	No	NA	0	2	Not-determined	Genome-fragment	NA	0
S17_extracted_k141_140653	29997	No	NA	9	1	High-quality	High-quality	100	0
S14_vs_k141_103781||full	1001	No	NA	1	0	Low-quality	Genome-fragment	2.52	0
S14_vs_k141_38094||full	1001	No	NA	1	0	Low-quality	Genome-fragment	2.34	0
S3_vs_k141_121456||full	1001	No	NA	0	0	Low-quality	Genome-fragment	1.42	0
S5_vs_k141_12455||full	1001	No	NA	0	1	Low-quality	Genome-fragment	0.84	0
S17_extracted_k141_125851	1538	No	NA	0	1	Not-determined	Genome-fragment	NA	0
S17_extracted_k141_125863	1004	No	NA	0	1	Not-determined	Genome-fragment	NA	0
S17_extracted_k141_118507	4607	No	NA	0	1	Not-determined	Genome-fragment	NA	0
S17_extracted_k141_148103	1806	No	NA	0	2	Not-determined	Genome-fragment	NA	0
S17_extracted_k141_66746	1169	No	NA	0	0	Not-determined	Genome-fragment	NA	0
S17_extracted_k141_7736	10395	No	NA	0	4	Not-determined	Genome-fragment	NA	0
S7_extracted_42821	1001	No	NA	1	0	Low-quality	Genome-fragment	3.7	0
S17_extracted_k141_29997	1014	No	NA	0	1	Not-determined	Genome-fragment	NA	0
S17_extracted_k141_81413	1133	No	NA	0	1	Not-determined	Genome-fragment	NA	0
S17_extracted_k141_125909	2865	No	NA	0	4	Not-determined	Genome-fragment	NA	0
S7_vs_34835||full	1001	No	NA	0	0	Low-quality	Genome-fragment	2.02	0
S17_extracted_k141_140720	1189	No	NA	0	2	Not-determined	Genome-fragment	NA	0
S17_extracted_k141_37341	1057	No	NA	0	0	Not-determined	Genome-fragment	NA	0
S17_extracted_k141_148160	1921	No	NA	0	1	Not-determined	Genome-fragment	NA	0
S17_extracted_k141_170225	1194	No	NA	0	1	Not-determined	Genome-fragment	NA	0
S17_extracted_k141_289	1451	No	NA	0	2	Not-determined	Genome-fragment	NA	0
S17_extracted_k141_125919	3054	No	NA	0	1	Not-determined	Genome-fragment	NA	0
S17_extracted_k141_52092	1025	No	NA	0	1	Not-determined	Genome-fragment	NA	0
S17_extracted_k141_292	1320	No	NA	0	0	Not-determined	Genome-fragment	NA	0
S17_extracted_k141_103850	4010	No	NA	0	2	Not-determined	Genome-fragment	NA	0
S16_vs_54557||full	1001	No	NA	1	0	Low-quality	Genome-fragment	2.23	0
S17_extracted_k141_111083	1216	No	NA	0	0	Not-determined	Genome-fragment	NA	0
S17_extracted_k141_140766	1060	No	NA	0	1	Not-determined	Genome-fragment	NA	0
S10_extracted_36455	1001	No	NA	0	0	Low-quality	Genome-fragment	1.67	0
S17_extracted_k141_308	1380	No	NA	0	1	Not-determined	Genome-fragment	NA	0
S17_extracted_k141_111096	1865	No	NA	0	1	Not-determined	Genome-fragment	NA	0
S10_extracted_121167	1001	No	NA	1	0	Low-quality	Genome-fragment	2.1	0
S15_extracted_35980	1001	No	NA	0	0	Low-quality	Genome-fragment	0.59	0
S17_extracted_k141_59560	1097	No	NA	0	1	Not-determined	Genome-fragment	NA	0
S17_extracted_k141_325	1150	No	NA	0	1	Not-determined	Genome-fragment	NA	0
S17_extracted_k141_103878	4149	No	NA	0	2	Not-determined	Genome-fragment	NA	0
S17_extracted_k141_140802	1355	No	NA	0	0	Not-determined	Genome-fragment	NA	0
S17_extracted_k141_133365	1241	No	NA	0	0	Not-determined	Genome-fragment	NA	0
S17_extracted_k141_103897	1916	No	NA	0	2	Not-determined	Genome-fragment	NA	0
S15_vs_55331||lt2gene	1001	No	NA	1	0	Low-quality	Genome-fragment	1.74	0
S17_extracted_k141_15078	1148	No	NA	0	1	Not-determined	Genome-fragment	NA	0
S17_extracted_k141_162900	1901	No	NA	0	2	Not-determined	Genome-fragment	NA	0
S17_extracted_k141_88970	1004	No	NA	0	0	Not-determined	Genome-fragment	NA	0
S17_extracted_k141_66837	4239	No	NA	0	6	Not-determined	Genome-fragment	NA	0
S17_extracted_k141_74318	1672	No	NA	0	0	Not-determined	Genome-fragment	NA	0
S17_extracted_k141_360	1192	No	NA	0	1	Not-determined	Genome-fragment	NA	0
S17_extracted_k141_103928	3523	No	NA	0	2	Not-determined	Genome-fragment	NA	0
S17_extracted_k141_118620	7750	No	NA	0	3	Not-determined	Genome-fragment	NA	0
S8_vs_k141_207951||full	1001	No	NA	0	0	Low-quality	Genome-fragment	1.7	0
S17_extracted_k141_162911	1228	No	NA	0	0	Not-determined	Genome-fragment	NA	0
S17_extracted_k141_148243	5411	No	NA	0	1	Not-determined	Genome-fragment	NA	0
S17_extracted_k141_103956	1065	No	NA	0	0	Not-determined	Genome-fragment	NA	0
S17_extracted_k141_19145	1002	No	NA	0	0	Low-quality	Genome-fragment	2.19	0
S17_extracted_k141_148250	2661	No	NA	0	3	Not-determined	Genome-fragment	NA	0
S17_extracted_k141_52179	2308	No	NA	0	2	Not-determined	Genome-fragment	NA	0
S17_extracted_k141_22444	1253	No	NA	0	0	Not-determined	Genome-fragment	NA	0
S5_vs_k141_127737||full	1002	No	NA	0	1	Low-quality	Genome-fragment	1.49	0
S17_extracted_k141_375	1698	No	NA	0	0	Not-determined	Genome-fragment	NA	0
S17_extracted_k141_103966	3099	No	NA	0	1	Not-determined	Genome-fragment	NA	0
S17_extracted_k141_52191	13853	No	NA	0	7	Not-determined	Genome-fragment	NA	0
S5_vs_k141_116068||lt2gene	1002	No	NA	1	0	Low-quality	Genome-fragment	2.18	0
S17_extracted_k141_30095	1520	No	NA	0	1	Not-determined	Genome-fragment	NA	0
S17_extracted_k141_52200	3371	No	NA	0	3	Not-determined	Genome-fragment	NA	0
S17_extracted_k141_155499	2647	No	NA	0	1	Not-determined	Genome-fragment	NA	0
S17_extracted_k141_155502	1385	No	NA	0	1	Not-determined	Genome-fragment	NA	0
S17_extracted_k141_89020	4900	No	NA	0	2	Not-determined	Genome-fragment	NA	0
S17_extracted_k141_425	1082	No	NA	0	0	Not-determined	Genome-fragment	NA	0
S17_extracted_k141_140917	1426	No	NA	0	1	Not-determined	Genome-fragment	NA	0
S17_extracted_k141_111208	1004	No	NA	0	0	Not-determined	Genome-fragment	NA	0
S17_extracted_k141_22496	1039	No	NA	0	0	Not-determined	Genome-fragment	NA	0
S7_vs_31350||full	1002	No	NA	0	0	Low-quality	Genome-fragment	3.11	0
S17_extracted_k141_22498	1237	No	NA	0	0	Not-determined	Genome-fragment	NA	0
S17_extracted_k141_170347	2720	No	NA	0	1	Not-determined	Genome-fragment	NA	0
S16_extracted_19899	1002	No	NA	0	0	Low-quality	Genome-fragment	1.75	0
S17_extracted_k141_118725	1244	No	NA	0	0	Not-determined	Genome-fragment	NA	0
S16_extracted_143827	1002	No	NA	0	0	Low-quality	Genome-fragment	0.94	0
S17_extracted_k141_89053	5042	No	NA	0	4	Not-determined	Genome-fragment	NA	0
S17_extracted_k141_148303	15913	No	NA	10	0	Medium-quality	Genome-fragment	70.05	0
S17_extracted_k141_170364	2048	No	NA	0	1	Not-determined	Genome-fragment	NA	0
S17_extracted_k141_81585	1113	No	NA	0	1	Not-determined	Genome-fragment	NA	0
S17_extracted_k141_74430	5357	No	NA	0	3	Not-determined	Genome-fragment	NA	0
S10_extracted_80128	1002	No	NA	1	0	Low-quality	Genome-fragment	0.87	0
S12_extracted_k141_105604	1002	No	NA	1	0	Low-quality	Genome-fragment	0.57	0
S12_extracted_k141_37605	1002	No	NA	1	0	Low-quality	Genome-fragment	0.82	0
S6_vs_k141_52326||lt2gene	1002	No	NA	1	0	Low-quality	Genome-fragment	2.41	0
S17_extracted_k141_22539	1566	No	NA	0	1	Not-determined	Genome-fragment	NA	0
S17_extracted_k141_507	5467	No	NA	0	1	Not-determined	Genome-fragment	NA	0
S8_extracted_k141_138895	1002	No	NA	0	0	Low-quality	Genome-fragment	1.11	0
S17_extracted_k141_75586	1003	No	NA	1	0	Low-quality	Genome-fragment	2.83	0
S17_extracted_k141_52317	1168	No	NA	0	1	Not-determined	Genome-fragment	NA	0

S17_extracted_k141_132283	1003	No	NA	0	1	Low-quality	Genome-fragment		0.56	0
S17_extracted_k141_118801	3245	No	NA	0	4	Not-determined	Genome-fragment	NA		0
S17_extracted_k141_126044	1468	No	NA	0	1	Not-determined	Genome-fragment	NA		0
S17_extracted_k141_111282	1013	No	NA	0	1	Not-determined	Genome-fragment	NA		0
S17_extracted_k141_552	5196	No	NA	0	2	Not-determined	Genome-fragment	NA		0
S17_extracted_k141_8018	5489	No	NA	0	5	Not-determined	Genome-fragment	NA		0
S11_vs_k141_96733||full	1003	No	NA	0	0	Low-quality	Genome-fragment		2.23	0
S17_extracted_k141_133531	1521	No	NA	0	0	Not-determined	Genome-fragment	NA		0
S14_extracted_k141_69284	1003	No	NA	0	0	Low-quality	Genome-fragment		7.2	0
S17_extracted_k141_8050	2822	No	NA	0	3	Not-determined	Genome-fragment	NA		0
S17_extracted_k141_584	3364	No	NA	0	0	Not-determined	Genome-fragment	NA		0
S17_extracted_k141_170448	1537	No	NA	0	0	Not-determined	Genome-fragment	NA		0
S1_extracted_k141_81469	1003	No	NA	0	0	Low-quality	Genome-fragment		0.57	0
S17_extracted_k141_66932	1547	No	NA	0	0	Not-determined	Genome-fragment	NA		0
S17_extracted_k141_44926	1269	No	NA	0	0	Not-determined	Genome-fragment	NA		0
S1_extracted_k141_115287	1003	No	NA	0	1	Low-quality	Genome-fragment		2.44	0
S3_vs_k141_32391||full	1003	No	NA	0	0	Low-quality	Genome-fragment		1.47	0
S3_vs_k141_31771||full	1003	No	NA	0	0	Low-quality	Genome-fragment		0.47	0
S17_extracted_k141_30324	4069	No	NA	0	10	Not-determined	Genome-fragment	NA		0
S17_extracted_k141_59748	4284	No	NA	0	0	Not-determined	Genome-fragment	NA		0
S17_extracted_k141_104146	9281	No	NA	0	4	Not-determined	Genome-fragment	NA		0
S17_extracted_k141_37716	1127	No	NA	0	0	Not-determined	Genome-fragment	NA		0
S5_vs_k141_61075||full	1003	No	NA	0	0	Low-quality	Genome-fragment		1.87	0
S5_vs_k141_56289||full	1003	No	NA	0	0	Low-quality	Genome-fragment		2.52	0
S17_extracted_k141_30343	1381	No	NA	0	1	Not-determined	Genome-fragment	NA		0
S17_extracted_k141_163112	3357	No	NA	0	0	Not-determined	Genome-fragment	NA		0
S17_extracted_k141_155748	1055	No	NA	0	0	Not-determined	Genome-fragment	NA		0
S5_vs_k141_5104||full	1003	No	NA	0	0	Low-quality	Genome-fragment		2.46	0
S7_extracted_134444	1003	No	NA	0	0	Low-quality	Genome-fragment		1.69	0
S17_extracted_k141_163119	3054	No	NA	0	0	Not-determined	Genome-fragment	NA		0
S17_extracted_k141_81651	1298	No	NA	0	1	Not-determined	Genome-fragment	NA		0
S17_extracted_k141_81661	1199	No	NA	0	1	Not-determined	Genome-fragment	NA		0
S17_extracted_k141_118902	1030	No	NA	0	1	Not-determined	Genome-fragment	NA		0
S17_extracted_k141_104215	1034	No	NA	0	1	Not-determined	Genome-fragment	NA		0
S17_extracted_k141_30360	3612	No	NA	0	4	Not-determined	Genome-fragment	NA		0
S7_vs_98447||full	1003	No	NA	1	0	Low-quality	Genome-fragment		2.46	0
S17_extracted_k141_15341	1807	No	NA	0	0	Not-determined	Genome-fragment	NA		0
S17_extracted_k141_74585	1420	No	NA	0	2	Not-determined	Genome-fragment	NA		0
S17_extracted_k141_89166	3533	No	NA	0	3	Not-determined	Genome-fragment	NA		0
S16_extracted_160405	1003	No	NA	0	0	Low-quality	Genome-fragment		1.62	0
S17_extracted_k141_96608	5512	No	NA	0	2	Not-determined	Genome-fragment	NA		0
S17_extracted_k141_148503	1613	No	NA	0	0	Not-determined	Genome-fragment	NA		0
S17_extracted_k141_30375	6432	No	NA	0	0	Not-determined	Genome-fragment	NA		0
S17_extracted_k141_66986	4689	No	NA	0	3	Not-determined	Genome-fragment	NA		0
S17_extracted_k141_104252	1602	No	NA	0	1	Not-determined	Genome-fragment	NA		0
S16_vs_144302||full	1003	No	NA	0	0	Low-quality	Genome-fragment		1.65	0
S17_extracted_k141_163185	1167	No	NA	0	1	Not-determined	Genome-fragment	NA		0
S16_vs_65129||lt2gene	1003	No	NA	1	0	Low-quality	Genome-fragment		1.69	0
S17_extracted_k141_133619	1184	No	NA	0	1	Not-determined	Genome-fragment	NA		0
S17_extracted_k141_148557	1119	No	NA	0	2	Not-determined	Genome-fragment	NA		0
S10_extracted_39139	1003	No	NA	1	0	Low-quality	Genome-fragment		0.41	0
S17_extracted_k141_104300	1884	No	NA	0	0	Not-determined	Genome-fragment	NA		0
S17_extracted_k141_118943	2862	No	NA	0	2	Not-determined	Genome-fragment	NA		0
S17_extracted_k141_705	1588	No	NA	0	1	Not-determined	Genome-fragment	NA		0
S17_extracted_k141_148586	1124	No	NA	0	0	Not-determined	Genome-fragment	NA		0
S17_extracted_k141_30469	1787	No	NA	0	3	Not-determined	Genome-fragment	NA		0
S17_extracted_k141_111470	2103	No	NA	0	2	Not-determined	Genome-fragment	NA		0
S17_extracted_k141_67042	15604	No	NA	7	0	Medium-quality	Genome-fragment		79.04	0
S17_extracted_k141_67045	1420	No	NA	0	2	Not-determined	Genome-fragment	NA		0
S17_extracted_k141_30483	1021	No	NA	0	1	Not-determined	Genome-fragment	NA		0
S12_extracted_k141_59921	1003	No	NA	1	0	Low-quality	Genome-fragment		2.48	0
S17_extracted_k141_45033	1250	No	NA	0	1	Not-determined	Genome-fragment	NA		0
S17_extracted_k141_45037	3760	No	NA	0	4	Not-determined	Genome-fragment	NA		0
S17_extracted_k141_89281	2613	No	NA	0	0	Not-determined	Genome-fragment	NA		0
S12_vs_k141_85026||full	1003	No	NA	0	1	Low-quality	Genome-fragment		0.59	0
S8_extracted_k141_191251	1003	No	NA	0	0	Low-quality	Genome-fragment		1.6	0
S17_extracted_k141_74698	4576	No	NA	0	1	Not-determined	Genome-fragment	NA		0
S8_vs_k141_10426||full	1003	No	NA	1	0	Low-quality	Genome-fragment		2.87	0
S9_vs_k141_25171||full	1004	No	NA	2	0	Low-quality	Genome-fragment		2.84	0
S17_extracted_k141_67107	1966	No	NA	0	3	Not-determined	Genome-fragment	NA		0
S11_extracted_k141_1385	1004	No	NA	0	0	Low-quality	Genome-fragment		1.2	0
S17_extracted_k141_96660	5565	No	NA	0	3	Not-determined	Genome-fragment	NA		0
S17_extracted_k141_52580	1925	No	NA	0	1	Not-determined	Genome-fragment	NA		0
S17_extracted_k141_45090	1474	No	NA	0	1	Not-determined	Genome-fragment	NA		0
S17_extracted_k141_163318	35575	No	NA	0	13	Not-determined	Genome-fragment	NA		0
S17_extracted_k141_723	1330	No	NA	0	1	Not-determined	Genome-fragment	NA		0
S17_extracted_k141_741	4944	No	NA	0	1	Not-determined	Genome-fragment	NA		0
S11_vs_k141_18151||lt2gene	1004	No	NA	1	0	Low-quality	Genome-fragment		1.6	0
S1_extracted_k141_90594	1004	No	NA	0	0	Low-quality	Genome-fragment		1.63	0
S17_extracted_k141_156032	1388	No	NA	0	1	Not-determined	Genome-fragment	NA		0
S1_extracted_k141_62774	1004	No	NA	0	0	Low-quality	Genome-fragment		2.23	0
S3_extracted_k141_40043	1004	No	NA	0	0	Low-quality	Genome-fragment		0.53	0
S17_extracted_k141_52646	2055	No	NA	0	0	Not-determined	Genome-fragment	NA		0
S7_extracted_191	1004	No	NA	3	0	Low-quality	Genome-fragment		3.29	0
S7_vs_118782||full	1004	No	NA	1	0	Low-quality	Genome-fragment		2.16	0
S12_extracted_k141_68470	1004	No	NA	0	0	Low-quality	Genome-fragment		2.8	0
S17_extracted_k141_104502	1996	No	NA	0	4	Not-determined	Genome-fragment	NA		0
S15_vs_38927||lt2gene	1004	No	NA	1	0	Low-quality	Genome-fragment		0.59	0
S8_extracted_k141_101213	1004	No	NA	0	0	Low-quality	Genome-fragment		1.65	0
S17_extracted_k141_52666	1218	No	NA	0	1	Not-determined	Genome-fragment	NA		0
S17_extracted_k141_111561	2200	No	NA	0	1	Not-determined	Genome-fragment	NA		0
S17_extracted_k141_30555	1218	No	NA	0	1	Not-determined	Genome-fragment	NA		0
S17_extracted_k141_170798	1206	No	NA	0	1	Not-determined	Genome-fragment	NA		0
S17_extracted_k141_133740	1116	No	NA	0	1	Not-determined	Genome-fragment	NA		0
S8_extracted_k141_23829	1004	No	NA	0	1	Low-quality	Genome-fragment		0.16	0
S17_extracted_k141_30571	2857	No	NA	0	2	Not-determined	Genome-fragment	NA		0
S17_extracted_k141_813	1333	No	NA	0	2	Not-determined	Genome-fragment	NA		0
S17_extracted_k141_52703	1360	No	NA	0	2	Not-determined	Genome-fragment	NA		0
S17_extracted_k141_22861	1299	No	NA	0	3	Not-determined	Genome-fragment	NA		0
S8_vs_k141_90820||full	1004	No	NA	1	0	Low-quality	Genome-fragment		2.04	0
S8_vs_k141_59297||full	1004	No	NA	0	0	Low-quality	Genome-fragment		2.69	0
S9_extracted_k141_57957	1005	No	NA	1	0	Low-quality	Genome-fragment		1.03	0
S17_extracted_k141_15435	3278	No	NA	0	1	Not-determined	Genome-fragment	NA		0
S17_extracted_k141_89416	1234	No	NA	0	1	Not-determined	Genome-fragment	NA		0
S13_vs_47062||full	1005	No	NA	0	0	Low-quality	Genome-fragment		1.38	0
S17_extracted_k141_52735	2262	No	NA	0	2	Not-determined	Genome-fragment	NA		0
S14_vs_k141_12239||lt2gene	1005	No	NA	1	0	Low-quality	Genome-fragment		2.53	0
S5_extracted_k141_141743	1005	No	NA	0	0	Low-quality	Genome-fragment		2.27	0
S5_extracted_k141_82725	1005	No	NA	1	0	Low-quality	Genome-fragment		3.15	0
S7_extracted_94514	1005	No	NA	1	0	Low-quality	Genome-fragment		1.74	0
S17_extracted_k141_104602	6411	No	NA	0	3	Not-determined	Genome-fragment	NA		0
S17_extracted_k141_163469	1318	No	NA	0	2	Not-determined	Genome-fragment	NA		0
S17_extracted_k141_148795	2365	No	NA	0	2	Not-determined	Genome-fragment	NA		0
S17_extracted_k141_96865	1299	No	NA	0	2	Not-determined	Genome-fragment	NA		0
S17_extracted_k141_52760	1200	No	NA	0	2	Not-determined	Genome-fragment	NA		0
S17_extracted_k141_59974	1491	No	NA	0	0	Not-determined	Genome-fragment	NA		0
S7_extracted_95140	1005	No	NA	1	0	Low-quality	Genome-fragment		2.51	0
S17_extracted_k141_170896	1434	No	NA	0	3	Not-determined	Genome-fragment	NA		0
S7_vs_77460||lt2gene	1005	No	NA	2	0	Low-quality	Genome-fragment		1.64	0
S17_extracted_k141_38122	2556	No	NA	0	0	Not-determined	Genome-fragment	NA		0
S7_vs_130378||lt2gene	1005	No	NA	1	0	Low-quality	Genome-fragment		2.2	0
S17_extracted_k141_67336	1420	No	NA	0	1	Not-determined	Genome-fragment	NA		0
S17_extracted_k141_156182	3292	No	NA	0	3	Not-determined	Genome-fragment	NA		0
S17_extracted_k141_67348	1062	No	NA	0	2	Not-determined	Genome-fragment	NA		0
S17_extracted_k141_148847	1056	No	NA	0	0	Not-determined	Genome-fragment	NA		0
S17_extracted_k141_38135	1366	No	NA	0	0	Not-determined	Genome-fragment	NA		0
S12_vs_k141_18476||full	1005	No	NA	1	0	Low-quality	Genome-fragment		2.58	0
S17_extracted_k141_60005	1031	No	NA	0	0	Not-determined	Genome-fragment	NA		0
S17_extracted_k141_133782	1012	No	NA	0	0	Not-determined	Genome-fragment	NA		0
S17_extracted_k141_917	1345	No	NA	0	0	Not-determined	Genome-fragment	NA		0
S17_extracted_k141_163483	13324	No	NA	0	8	Not-determined	Genome-fragment	NA		0
S12_vs_k141_89895||full	1005	No	NA	1	0	Low-quality	Genome-fragment		1.89	0
S17_extracted_k141_15488	1385	No	NA	0	0	Not-determined	Genome-fragment	NA		0
S17_extracted_k141_81895	1965	No	NA	0	2	Not-determined	Genome-fragment	NA		0
S12_vs_k141_72079||lt2gene	1005	No	NA	1	0	Low-quality	Genome-fragment		2.67	0
S17_extracted_k141_15500	3715	No	NA	0	1	Not-determined	Genome-fragment	NA		0
S8_extracted_k141_194279	1005	No	NA	2	0	Low-quality	Genome-fragment		1.64	0
S17_extracted_k141_60043	3615	No	NA	0	3	Not-determined	Genome-fragment	NA		0
S17_extracted_k141_126375	1153	No	NA	0	1	Not-determined	Genome-fragment	NA		0
S17_extracted_k141_126377	1290	No	NA	0	1	Not-determined	Genome-fragment	NA		0
S17_extracted_k141_52874	1455	No	NA	0	1	Not-determined	Genome-fragment	NA		0
S17_extracted_k141_8399	1895	No	NA	0	0	Not-determined	Genome-fragment	NA		0
S17_extracted_k141_60070	1303	No	NA	0	0	Not-determined	Genome-fragment	NA		0
S17_extracted_k141_22934	1201	No	NA	0	1	Not-determined	Genome-fragment	NA		0
S17_extracted_k141_60072	4027	No	NA	0	8	Not-determined	Genome-fragment	NA		0
S8_extracted_k141_152570	1005	No	NA	1	0	Low-quality	Genome-fragment		2.51	0
S17_extracted_k141_170940	3949	No	NA	0	1	Not-determined	Genome-fragment	NA		0
S17_extracted_k141_126401	1639	No	NA	0	2	Not-determined	Genome-fragment	NA		0
S8_vs_k141_151704||full	1005	No	NA	1	0	Low-quality	Genome-fragment		0.97	0
S17_extracted_k141_45310	2183	No	NA	0	0	Not-determined	Genome-fragment	NA		0
S9_vs_k141_21618||lt2gene	1006	No	NA	1	0	Low-quality	Genome-fragment		3.25	0
S17_extracted_k141_170950	1178	No	NA	0	1	Not-determined	Genome-fragment	NA		0
S17_extracted_k141_104712	1573	No	NA	0	3	Not-determined	Genome-fragment	NA		0
S17_extracted_k141_133842	1459	No	NA	0	1	Not-determined	Genome-fragment	NA		0
S17_extracted_k141_156302	5399	No	NA	0	4	Not-determined	Genome-fragment	NA		0
S17_extracted_k141_89514	4754	No	NA	0	2	Not-determined	Genome-fragment	NA		0
S17_extracted_k141_60111	1054	No	NA	0	1	Not-determined	Genome-fragment	NA		0
S17_extracted_k141_126410	1270	No	NA	0	1	Not-determined	Genome-fragment	NA		0
S17_extracted_k141_148964	1068	No	NA	0	0	Not-determined	Genome-fragment	NA		0
S17_extracted_k141_156322	1261	No	NA	0	1	Not-determined	Genome-fragment	NA		0
S17_extracted_k141_119240	1007	No	NA	0	1	Not-determined	Genome-fragment	NA		0
S17_extracted_k141_30684	1287	No	NA	0	0	Not-determined	Genome-fragment	NA		0
S11_extracted_k141_73186	1006	No	NA	0	1	Low-quality	Genome-fragment		0.93	0
S17_extracted_k141_15592	1389	No	NA	0	2	Not-determined	Genome-fragment	NA		0
S17_extracted_k141_45353	9531	No	NA	0	2	Not-determined	Genome-fragment	NA		0
S17_extracted_k141_1007	4070	No	NA	0	2	Not-determined	Genome-fragment	NA		0
S17_extracted_k141_67418	3510	No	NA	0	2	Not-determined	Genome-fragment	NA		0
S1_extracted_k141_74959	1006	No	NA	1	0	Low-quality	Genome-fragment		2.96	0
S17_extracted_k141_67427	1120	No	NA	0	1	Not-determined	Genome-fragment	NA		0
S17_extracted_k141_67430	1551	No	NA	0	1	Not-determined	Genome-fragment	NA		0
S17_extracted_k141_104758	1573	No	NA	0	2	Not-determined	Genome-fragment	NA		0
S17_extracted_k141_96972	1002	No	NA	0	0	Not-determined	Genome-fragment	NA		0
S1_vs_k141_153330||full	1006	No	NA	1	0	Low-quality	Genome-fragment		1.26	0
S17_extracted_k141_60166	2958	No	NA	0	2	Not-determined	Genome-fragment	NA		0

S17_extracted_k141_96979	6240	No	NA	0	0	Not-determined	Genome-fragment	NA		0
S17_extracted_k141_149025	9454	No	NA	0	2	Not-determined	Genome-fragment	NA		0
S17_extracted_k141_133906	2780	No	NA	0	0	Not-determined	Genome-fragment	NA		0
S17_extracted_k141_133911	1132	No	NA	0	2	Not-determined	Genome-fragment	NA		0
S17_extracted_k141_30735	1668	No	NA	0	1	Not-determined	Genome-fragment	NA		0
S3_extracted_k141_48668	1006	No	NA	2	0	Low-quality	Genome-fragment		2.67	0
S3_vs_k141_49491||full	1006	No	NA	0	0	Low-quality	Genome-fragment		2.08	0
S17_extracted_k141_8537	1286	No	NA	0	3	Not-determined	Genome-fragment	NA		0
S17_extracted_k141_171083	3829	No	NA	0	0	Not-determined	Genome-fragment	NA		0
S3_vs_k141_6461||lt2gene	1006	No	NA	1	0	Low-quality	Genome-fragment		1.98	0
S17_extracted_k141_60192	2372	No	NA	0	1	Not-determined	Genome-fragment	NA		0
S17_extracted_k141_133930	6713	No	NA	0	0	Not-determined	Genome-fragment	NA		0
S5_vs_k141_106119||lt2gene	1006	No	NA	1	0	Low-quality	Genome-fragment		2.17	0
S17_extracted_k141_22989	1709	No	NA	0	2	Not-determined	Genome-fragment	NA		0
S7_extracted_15759	1006	No	NA	0	0	Low-quality	Genome-fragment		2.48	0
S17_extracted_k141_119294	1922	No	NA	0	1	Not-determined	Genome-fragment	NA		0
S17_extracted_k141_119298	1119	No	NA	0	0	Not-determined	Genome-fragment	NA		0
S17_extracted_k141_97040	1776	No	NA	0	1	Not-determined	Genome-fragment	NA		0
S7_vs_73256||full	1006	No	NA	0	0	Low-quality	Genome-fragment		2.27	0
S17_extracted_k141_156416	3077	No	NA	0	1	Not-determined	Genome-fragment	NA		0
S17_extracted_k141_149089	1071	No	NA	0	2	Not-determined	Genome-fragment	NA		0
S7_vs_92999||full	1006	No	NA	0	0	Low-quality	Genome-fragment		2.17	0
S17_extracted_k141_8558	1989	No	NA	0	2	Not-determined	Genome-fragment	NA		0
S17_extracted_k141_81980	1155	No	NA	0	0	Not-determined	Genome-fragment	NA		0
S17_extracted_k141_81983	1138	No	NA	0	1	Not-determined	Genome-fragment	NA		0
S17_extracted_k141_171112	1249	No	NA	0	0	Not-determined	Genome-fragment	NA		0
S17_extracted_k141_119329	3081	No	NA	0	4	Not-determined	Genome-fragment	NA		0
S17_extracted_k141_67505	1015	No	NA	0	1	Not-determined	Genome-fragment	NA		0
S17_extracted_k141_81998	1131	No	NA	0	1	Not-determined	Genome-fragment	NA		0
S17_extracted_k141_38337	1755	No	NA	0	1	Not-determined	Genome-fragment	NA		0
S17_extracted_k141_156478	1850	No	NA	0	0	Not-determined	Genome-fragment	NA		0
S17_extracted_k141_163676	1491	No	NA	0	1	Not-determined	Genome-fragment	NA		0
S17_extracted_k141_8633	1273	No	NA	0	0	Not-determined	Genome-fragment	NA		0
S17_extracted_k141_104907	1049	No	NA	0	1	Not-determined	Genome-fragment	NA		0
S17_extracted_k141_104909	1120	No	NA	0	1	Not-determined	Genome-fragment	NA		0
S17_extracted_k141_60285	3748	No	NA	0	3	Not-determined	Genome-fragment	NA		0
S17_extracted_k141_149148	2401	No	NA	0	2	Not-determined	Genome-fragment	NA		0
S17_extracted_k141_141531	2781	No	NA	0	2	Not-determined	Genome-fragment	NA		0
S17_extracted_k141_23081	1380	No	NA	0	1	Not-determined	Genome-fragment	NA		0
S17_extracted_k141_149160	4262	No	NA	0	1	Not-determined	Genome-fragment	NA		0
S17_extracted_k141_82062	6098	No	NA	0	1	Not-determined	Genome-fragment	NA		0
S17_extracted_k141_141554	2085	No	NA	0	2	Not-determined	Genome-fragment	NA		0
S17_extracted_k141_38390	1513	No	NA	0	1	Not-determined	Genome-fragment	NA		0
S17_extracted_k141_149184	6097	No	NA	0	4	Not-determined	Genome-fragment	NA		0
S17_extracted_k141_53083	1464	No	NA	0	0	Not-determined	Genome-fragment	NA		0
S7_vs_31062||lt2gene	1006	No	NA	1	0	Low-quality	Genome-fragment		2.08	0
S17_extracted_k141_119398	1675	No	NA	0	0	Not-determined	Genome-fragment	NA		0
S17_extracted_k141_60313	4923	No	NA	0	2	Not-determined	Genome-fragment	NA		0
S17_extracted_k141_156536	5414	No	NA	0	3	Not-determined	Genome-fragment	NA		0
S17_extracted_k141_141611	1433	No	NA	0	0	Not-determined	Genome-fragment	NA		0
S17_extracted_k141_111917	1460	No	NA	0	0	Not-determined	Genome-fragment	NA		0
S17_extracted_k141_104992	6446	No	NA	0	13	Not-determined	Genome-fragment	NA		0
S16_vs_70296||full	1006	No	NA	1	0	Low-quality	Genome-fragment		0.65	0
S17_extracted_k141_97187	1090	No	NA	0	2	Not-determined	Genome-fragment	NA		0
S17_extracted_k141_97188	1222	No	NA	0	1	Not-determined	Genome-fragment	NA		0
S17_extracted_k141_111931	17015	No	NA	9	0	Medium-quality	Genome-fragment		78.9	0
S17_extracted_k141_111932	1047	No	NA	0	0	Not-determined	Genome-fragment	NA		0
S17_extracted_k141_67633	1376	No	NA	0	0	Not-determined	Genome-fragment	NA		0
S17_extracted_k141_119454	1275	No	NA	0	2	Not-determined	Genome-fragment	NA		0
S17_extracted_k141_119459	1067	No	NA	0	0	Not-determined	Genome-fragment	NA		0
S17_extracted_k141_1171	3021	No	NA	0	3	Not-determined	Genome-fragment	NA		0
S17_extracted_k141_1173	5366	No	NA	0	4	Not-determined	Genome-fragment	NA		0
S12_extracted_k141_175897	1006	No	NA	0	0	Low-quality	Genome-fragment		2.24	0
S17_extracted_k141_119476	2243	No	NA	0	1	Not-determined	Genome-fragment	NA		0
S17_extracted_k141_15733	1091	No	NA	0	2	Not-determined	Genome-fragment	NA		0
S17_extracted_k141_23181	1186	No	NA	0	1	Not-determined	Genome-fragment	NA		0
S12_vs_k141_14186||full	1006	No	NA	0	0	Low-quality	Genome-fragment		1.65	0
S17_extracted_k141_134107	1604	No	NA	0	1	Not-determined	Genome-fragment	NA		0
S17_extracted_k141_30960	7498	No	NA	0	3	Not-determined	Genome-fragment	NA		0
S17_extracted_k141_134131	1351	No	NA	0	0	Not-determined	Genome-fragment	NA		0
S17_extracted_k141_1208	1847	No	NA	0	1	Not-determined	Genome-fragment	NA		0
S17_extracted_k141_15750	2517	No	NA	0	3	Not-determined	Genome-fragment	NA		0
S17_extracted_k141_1209	1157	No	NA	0	1	Not-determined	Genome-fragment	NA		0
S4_extracted_15922	1006	No	NA	0	0	Low-quality	Genome-fragment		1.98	0
S17_extracted_k141_134144	1486	No	NA	0	1	Not-determined	Genome-fragment	NA		0
S17_extracted_k141_1219	2097	No	NA	0	1	Not-determined	Genome-fragment	NA		0
S17_extracted_k141_134152	3206	No	NA	0	3	Not-determined	Genome-fragment	NA		0
S17_extracted_k141_53244	2677	No	NA	0	7	Not-determined	Genome-fragment	NA		0
S17_extracted_k141_67768	1228	No	NA	0	3	Not-determined	Genome-fragment	NA		0
S17_extracted_k141_149358	5104	No	NA	0	5	Not-determined	Genome-fragment	NA		0
S17_extracted_k141_82160	1578	No	NA	0	0	Not-determined	Genome-fragment	NA		0
S17_extracted_k141_97302	1877	No	NA	0	0	Not-determined	Genome-fragment	NA		0
S17_extracted_k141_8811	3030	No	NA	0	2	Not-determined	Genome-fragment	NA		0
S17_extracted_k141_97304	1690	No	NA	0	0	Not-determined	Genome-fragment	NA		0
S17_extracted_k141_45721	1391	No	NA	0	1	Not-determined	Genome-fragment	NA		0
S17_extracted_k141_97313	2426	No	NA	0	2	Not-determined	Genome-fragment	NA		0
S17_extracted_k141_171354	1664	No	NA	0	1	Not-determined	Genome-fragment	NA		0
S17_extracted_k141_31035	1170	No	NA	0	0	Not-determined	Genome-fragment	NA		0
S4_extracted_28750	1006	No	NA	0	1	Low-quality	Genome-fragment		1.24	0
S17_extracted_k141_67808	1040	No	NA	0	0	Not-determined	Genome-fragment	NA		0
S17_extracted_k141_67809	3656	No	NA	0	2	Not-determined	Genome-fragment	NA		0
S17_extracted_k141_45776	1110	No	NA	0	1	Not-determined	Genome-fragment	NA		0
S17_extracted_k141_105174	2119	No	NA	0	0	Not-determined	Genome-fragment	NA		0
S17_extracted_k141_134213	1121	No	NA	0	0	Not-determined	Genome-fragment	NA		0
S17_extracted_k141_23262	3488	No	NA	0	2	Not-determined	Genome-fragment	NA		0
S17_extracted_k141_1305	1223	No	NA	0	0	Not-determined	Genome-fragment	NA		0
S17_extracted_k141_38610	1128	No	NA	0	1	Not-determined	Genome-fragment	NA		0
S11_extracted_k141_13453	1007	No	NA	1	0	Low-quality	Genome-fragment		2.52	0
S17_extracted_k141_82209	1816	No	NA	0	0	Not-determined	Genome-fragment	NA		0
S14_extracted_k141_64403	1007	No	NA	0	0	Low-quality	Genome-fragment		2.43	0
S17_extracted_k141_1326	4515	No	NA	0	3	Not-determined	Genome-fragment	NA		0
S14_vs_k141_113931||full	1007	No	NA	0	1	Low-quality	Genome-fragment		1.75	0
S14_vs_k141_117104||lt2gene	1007	No	NA	0	0	Low-quality	Genome-fragment		3.75	0
S17_extracted_k141_156690	1215	No	NA	0	0	Not-determined	Genome-fragment	NA		0
S1_vs_k141_93075||full	1007	No	NA	1	0	Low-quality	Genome-fragment		1.7	0
S3_vs_k141_8400||full	1007	No	NA	0	1	Low-quality	Genome-fragment		1.75	0
S17_extracted_k141_90048	1668	No	NA	0	1	Not-determined	Genome-fragment	NA		0
S17_extracted_k141_67842	1020	No	NA	0	1	Not-determined	Genome-fragment	NA		0
S17_extracted_k141_31113	1257	No	NA	0	2	Not-determined	Genome-fragment	NA		0
S17_extracted_k141_31130	1031	No	NA	0	0	Not-determined	Genome-fragment	NA		0
S17_extracted_k141_45870	1058	No	NA	0	1	Not-determined	Genome-fragment	NA		0
S17_extracted_k141_23322	5416	No	NA	0	0	Not-determined	Genome-fragment	NA		0
S17_extracted_k141_112062	9822	No	NA	0	4	Not-determined	Genome-fragment	NA		0
S17_extracted_k141_141820	3748	No	NA	0	2	Not-determined	Genome-fragment	NA		0
S5_vs_k141_40275||lt2gene	1007	No	NA	1	0	Low-quality	Genome-fragment		2.15	0
S17_extracted_k141_163905	1490	No	NA	0	1	Not-determined	Genome-fragment	NA		0
S17_extracted_k141_38722	1330	No	NA	0	0	Not-determined	Genome-fragment	NA		0
S17_extracted_k141_97482	4501	No	NA	0	4	Not-determined	Genome-fragment	NA		0
S17_extracted_k141_53437	1650	No	NA	0	2	Not-determined	Genome-fragment	NA		0
S17_extracted_k141_156749	1027	No	NA	0	1	Not-determined	Genome-fragment	NA		0
S17_extracted_k141_60570	1201	No	NA	0	1	Not-determined	Genome-fragment	NA		0
S7_extracted_39146	1007	No	NA	1	0	Low-quality	Genome-fragment		17.19	0
S17_extracted_k141_45897	1308	No	NA	0	2	Not-determined	Genome-fragment	NA		0
S17_extracted_k141_97497	3585	No	NA	0	2	Not-determined	Genome-fragment	NA		0
S17_extracted_k141_149552	1263	No	NA	0	1	Not-determined	Genome-fragment	NA		0
S7_vs_83089||full	1007	No	NA	1	0	Low-quality	Genome-fragment		2.64	0
S16_vs_41486||full	1007	No	NA	0	0	Low-quality	Genome-fragment		2.75	0
S17_extracted_k141_67882	1492	No	NA	0	0	Not-determined	Genome-fragment	NA		0
S17_extracted_k141_163947	1888	No	NA	0	0	Not-determined	Genome-fragment	NA		0
S17_extracted_k141_75269	2816	No	NA	0	0	Not-determined	Genome-fragment	NA		0
S17_extracted_k141_171533	1139	No	NA	0	4	Not-determined	Genome-fragment	NA		0
S17_extracted_k141_9035	6973	No	NA	0	4	Not-determined	Genome-fragment	NA		0
S16_vs_101805||full	1007	No	NA	1	0	Low-quality	Genome-fragment		1.77	0
S10_extracted_13937	1007	No	NA	0	1	Low-quality	Genome-fragment		2.24	0
S12_vs_k141_71360||full	1007	No	NA	1	0	Low-quality	Genome-fragment		2.12	0
S12_vs_k141_7266||full	1007	No	NA	0	0	Low-quality	Genome-fragment		1.58	0
S12_vs_k141_98405||lt2gene	1007	No	NA	2	0	Low-quality	Genome-fragment		2.72	0
S17_extracted_k141_1436	1998	No	NA	0	4	Not-determined	Genome-fragment	NA		0
S4_extracted_30862	1007	No	NA	1	0	Low-quality	Genome-fragment		3.45	0
S17_extracted_k141_119722	1020	No	NA	0	1	Not-determined	Genome-fragment	NA		0
S17_extracted_k141_88673	1008	No	NA	0	0	Low-quality	Genome-fragment		1.13	0
S17_extracted_k141_90134	1431	No	NA	0	0	Not-determined	Genome-fragment	NA		0
S9_vs_k141_22028||full	1008	No	NA	1	0	Low-quality	Genome-fragment		1.7	0
S17_extracted_k141_156859	2120	No	NA	0	2	Not-determined	Genome-fragment	NA		0
S17_extracted_k141_97592	2255	No	NA	0	0	Not-determined	Genome-fragment	NA		0
S17_extracted_k141_105339	3885	No	NA	0	4	Not-determined	Genome-fragment	NA		0
S13_extracted_96258	1008	No	NA	0	0	Low-quality	Genome-fragment		1.65	0
S17_extracted_k141_90152	6420	No	NA	0	3	Not-determined	Genome-fragment	NA		0
S17_extracted_k141_23427	3217	No	NA	0	0	Not-determined	Genome-fragment	NA		0
S1_extracted_k141_85332	1008	No	NA	0	0	Low-quality	Genome-fragment		2.17	0
S1_vs_k141_45610||full	1008	No	NA	2	0	Low-quality	Genome-fragment		3.67	0
S17_extracted_k141_126712	3710	No	NA	0	1	Not-determined	Genome-fragment	NA		0
S17_extracted_k141_23438	2315	No	NA	0	1	Not-determined	Genome-fragment	NA		0
S3_vs_k141_22444||lt2gene	1008	No	NA	2	0	Low-quality	Genome-fragment		3.52	0
S17_extracted_k141_119783	1005	No	NA	0	0	Not-determined	Genome-fragment	NA		0
S5_extracted_k141_140708	1008	No	NA	0	0	Low-quality	Genome-fragment		2.69	0
S17_extracted_k141_38813	3447	No	NA	0	3	Not-determined	Genome-fragment	NA		0
S17_extracted_k141_60727	1024	No	NA	0	1	Not-determined	Genome-fragment	NA		0
S7_extracted_3121	1008	No	NA	1	0	Low-quality	Genome-fragment		1.92	0
S17_extracted_k141_68016	1886	No	NA	0	2	Not-determined	Genome-fragment	NA		0
S17_extracted_k141_164110	1329	No	NA	0	1	Not-determined	Genome-fragment	NA		0
S17_extracted_k141_97671	19897	No	NA	8	0	Medium-quality	Genome-fragment		55.59	0
S17_extracted_k141_164117	1197	No	NA	0	1	Not-determined	Genome-fragment	NA		0
S7_extracted_133101	1008	No	NA	2	0	Low-quality	Genome-fragment		2.18	0
S7_vs_73002||full	1008	No	NA	1	0	Low-quality	Genome-fragment		0.68	0
S17_extracted_k141_149631	3122	No	NA	0	1	Not-determined	Genome-fragment	NA		0
S17_extracted_k141_46061	2492	No	NA	0	1	Not-determined	Genome-fragment	NA		0
S17_extracted_k141_82450	1260	No	NA	0	0	Not-determined	Genome-fragment	NA		0
S17_extracted_k141_31318	1092	No	NA	0	2	Not-determined	Genome-fragment	NA		0
S17_extracted_k141_112209	1297	No	NA	0	0	Not-determined	Genome-fragment	NA		0
S17_extracted_k141_15988	1082	No	NA	0	1	Not-determined	Genome-fragment	NA		0
S7_vs_35481||lt2gene	1008	No	NA	1	0	Low-quality	Genome-fragment		2.23	0
S17_extracted_k141_126784	3162	No	NA	0	1	Not-determined	Genome-fragment	NA		0
S16_extracted_125396	1008	No	NA	0	0	Low-quality	Genome-fragment		1.84	0

S16_vs_106372||full	1008	No	NA	0	2	Low-quality	Genome-fragment		1.48	0
S10_extracted_105084	1008	No	NA	1	0	Low-quality	Genome-fragment		0.75	0
S10_vs_50884||full	1008	No	NA	1	0	Low-quality	Genome-fragment		2.36	0
S17_extracted_k141_112260	7608	No	NA	0	3	Not-determined	Genome-fragment	NA		0
S17_extracted_k141_112262	1617	No	NA	0	0	Not-determined	Genome-fragment	NA		0
S17_extracted_k141_112263	1416	No	NA	0	0	Not-determined	Genome-fragment	NA		0
S17_extracted_k141_46168	1375	No	NA	0	1	Not-determined	Genome-fragment	NA		0
S12_vs_k141_6004||full	1008	No	NA	0	0	Low-quality	Genome-fragment		2.49	0
S17_extracted_k141_119879	1140	No	NA	0	0	Not-determined	Genome-fragment	NA		0
S12_vs_k141_45981||full	1008	No	NA	1	0	Low-quality	Genome-fragment		1.49	0
S6_extracted_k141_10626	1008	No	NA	0	0	Low-quality	Genome-fragment		2.94	0
S17_extracted_k141_31376	3477	No	NA	0	1	Not-determined	Genome-fragment	NA		0
S17_extracted_k141_16048	1130	No	NA	0	0	Not-determined	Genome-fragment	NA		0
S17_extracted_k141_46200	1300	No	NA	0	1	Not-determined	Genome-fragment	NA		0
S8_vs_k141_46080||full	1008	No	NA	3	0	Low-quality	Genome-fragment		3.28	0
S8_vs_k141_48494||full	1008	No	NA	0	0	Low-quality	Genome-fragment		2.5	0
S17_extracted_k141_173456	1009	No	NA	1	0	Low-quality	Genome-fragment		1.84	0
S17_extracted_k141_23561	10746	No	NA	0	1	Not-determined	Genome-fragment	NA		0
S17_extracted_k141_134536	1381	No	NA	0	2	Not-determined	Genome-fragment	NA		0
S1_vs_k141_65959||full	1009	No	NA	0	0	Low-quality	Genome-fragment		1.12	0
S17_extracted_k141_90199	4659	No	NA	0	4	Not-determined	Genome-fragment	NA		0
S3_vs_k141_115291||lt2gene	1009	No	NA	1	0	Low-quality	Genome-fragment		0.83	0
S17_extracted_k141_75386	1714	No	NA	0	2	Not-determined	Genome-fragment	NA		0
S17_extracted_k141_31400	1138	No	NA	0	2	Not-determined	Genome-fragment	NA		0
S17_extracted_k141_126911	4870	No	NA	0	0	Not-determined	Genome-fragment	NA		0
S5_vs_k141_54724||full	1009	No	NA	2	0	Low-quality	Genome-fragment		3.34	0
S7_extracted_92265	1009	No	NA	1	0	Low-quality	Genome-fragment		3.42	0
S17_extracted_k141_53775	1687	No	NA	0	1	Not-determined	Genome-fragment	NA		0
S12_vs_k141_28258||full	1009	No	NA	1	0	Low-quality	Genome-fragment		1.47	0
S17_extracted_k141_126939	7777	No	NA	0	2	Not-determined	Genome-fragment	NA		0
S17_extracted_k141_75428	3639	No	NA	0	1	Not-determined	Genome-fragment	NA		0
S17_extracted_k141_68163	2015	No	NA	0	0	Not-determined	Genome-fragment	NA		0
S17_extracted_k141_9292	4260	No	NA	0	1	Not-determined	Genome-fragment	NA		0
S17_extracted_k141_164171	2581	No	NA	0	1	Not-determined	Genome-fragment	NA		0
S17_extracted_k141_97860	1118	No	NA	0	0	Not-determined	Genome-fragment	NA		0
S12_vs_k141_100528||full	1009	No	NA	1	0	Low-quality	Genome-fragment		2.99	0
S15_vs_3058||lt2gene	1009	No	NA	1	0	Low-quality	Genome-fragment		2.35	0
S17_extracted_k141_68184	2137	No	NA	0	0	Not-determined	Genome-fragment	NA		0
S17_extracted_k141_126961	4475	No	NA	0	3	Not-determined	Genome-fragment	NA		0
S4_extracted_3078	1009	No	NA	1	0	Low-quality	Genome-fragment		1.87	0
S4_extracted_3732	1009	No	NA	1	0	Low-quality	Genome-fragment		2.46	0
S17_extracted_k141_1715	1771	No	NA	0	1	Not-determined	Genome-fragment	NA		0
S17_extracted_k141_157123	1385	No	NA	0	1	Not-determined	Genome-fragment	NA		0
S17_extracted_k141_1718	3785	No	NA	0	1	Not-determined	Genome-fragment	NA		0
S17_extracted_k141_16139	1199	No	NA	0	0	Not-determined	Genome-fragment	NA		0
S17_extracted_k141_1724	1026	No	NA	0	1	Not-determined	Genome-fragment	NA		0
S17_extracted_k141_75438	2354	No	NA	0	2	Not-determined	Genome-fragment	NA		0
S17_extracted_k141_105667	1420	No	NA	0	2	Not-determined	Genome-fragment	NA		0
S17_extracted_k141_46347	1603	No	NA	0	1	Not-determined	Genome-fragment	NA		0
S17_extracted_k141_16148	1008	No	NA	0	1	Not-determined	Genome-fragment	NA		0
S6_extracted_k141_14830	1009	No	NA	1	0	Low-quality	Genome-fragment		2.21	0
S17_extracted_k141_23738	4355	No	NA	0	0	Not-determined	Genome-fragment	NA		0
S17_extracted_k141_134648	1280	No	NA	0	0	Not-determined	Genome-fragment	NA		0
S6_extracted_k141_7947	1009	No	NA	0	0	Low-quality	Genome-fragment		0.76	0
S17_extracted_k141_6613	1010	No	NA	0	1	Low-quality	Genome-fragment		0.56	0
S17_extracted_k141_171571	1616	No	NA	0	4	Not-determined	Genome-fragment	NA		0
S17_extracted_k141_127008	1838	No	NA	0	1	Not-determined	Genome-fragment	NA		0
S17_extracted_k141_65150	1010	No	NA	0	0	Low-quality	Genome-fragment		0.58	0
S17_extracted_k141_53877	1017	No	NA	0	0	Not-determined	Genome-fragment	NA		0
S17_vs_k141_95253||lt2gene	1010	No	NA	0	0	Low-quality	Genome-fragment		2.1	0
S17_extracted_k141_171579	5947	No	NA	0	5	Not-determined	Genome-fragment	NA		0
S17_extracted_k141_164225	1480	No	NA	0	1	Not-determined	Genome-fragment	NA		0
S17_extracted_k141_31519	1924	No	NA	0	2	Not-determined	Genome-fragment	NA		0
S11_vs_k141_1814||lt2gene	1010	No	NA	1	0	Low-quality	Genome-fragment		2.36	0
S17_extracted_k141_142297	2583	No	NA	0	1	Not-determined	Genome-fragment	NA		0
S17_extracted_k141_46388	23386	No	NA	5	0	Medium-quality	Genome-fragment		52.03	0
S13_extracted_101293	1010	No	NA	0	1	Low-quality	Genome-fragment		1.46	0
S17_extracted_k141_157161	7596	No	NA	0	4	Not-determined	Genome-fragment	NA		0
S14_extracted_k141_26471	1010	No	NA	3	0	Low-quality	Genome-fragment		21.17	0
S17_extracted_k141_149941	3463	No	NA	0	2	Not-determined	Genome-fragment	NA		0
S17_extracted_k141_39078	1655	No	NA	0	1	Not-determined	Genome-fragment	NA		0
S17_extracted_k141_134712	2690	No	NA	0	1	Not-determined	Genome-fragment	NA		0
S3_vs_k141_104545||full	1010	No	NA	0	0	Low-quality	Genome-fragment		2.39	0
S17_extracted_k141_134726	1084	No	NA	0	0	Not-determined	Genome-fragment	NA		0
S5_extracted_k141_115640	1010	No	NA	0	0	Low-quality	Genome-fragment		1.85	0
S17_extracted_k141_60854	1385	No	NA	0	0	Not-determined	Genome-fragment	NA		0
S17_extracted_k141_82734	3208	No	NA	0	1	Not-determined	Genome-fragment	NA		0
S17_extracted_k141_134742	8096	No	NA	0	6	Not-determined	Genome-fragment	NA		0
S17_extracted_k141_46426	1297	No	NA	0	0	Not-determined	Genome-fragment	NA		0
S17_extracted_k141_134749	1792	No	NA	0	1	Not-determined	Genome-fragment	NA		0
S17_extracted_k141_127121	1176	No	NA	0	2	Not-determined	Genome-fragment	NA		0
S17_extracted_k141_60896	1049	No	NA	0	1	Not-determined	Genome-fragment	NA		0
S17_extracted_k141_150016	1186	No	NA	0	0	Not-determined	Genome-fragment	NA		0
S17_extracted_k141_16303	2220	No	NA	0	1	Not-determined	Genome-fragment	NA		0
S17_extracted_k141_68321	2300	No	NA	0	0	Not-determined	Genome-fragment	NA		0
S17_extracted_k141_90329	1021	No	NA	0	1	Not-determined	Genome-fragment	NA		0
S17_extracted_k141_98059	1466	No	NA	0	0	Not-determined	Genome-fragment	NA		0
S17_extracted_k141_75542	3532	No	NA	0	2	Not-determined	Genome-fragment	NA		0
S17_extracted_k141_98071	1190	No	NA	0	1	Not-determined	Genome-fragment	NA		0
S17_extracted_k141_16327	1160	No	NA	0	1	Not-determined	Genome-fragment	NA		0
S17_extracted_k141_134801	1171	No	NA	0	0	Not-determined	Genome-fragment	NA		0
S17_extracted_k141_112498	1078	No	NA	0	1	Not-determined	Genome-fragment	NA		0
S5_extracted_k141_21571	1010	No	NA	0	0	Low-quality	Genome-fragment		2.14	0
S17_extracted_k141_157227	1099	No	NA	0	0	Not-determined	Genome-fragment	NA		0
S17_extracted_k141_142431	2644	No	NA	0	3	Not-determined	Genome-fragment	NA		0
S7_extracted_86679	1010	No	NA	1	0	Low-quality	Genome-fragment		2.33	0
S7_vs_65712||full	1010	No	NA	0	0	Low-quality	Genome-fragment		2.53	0
S17_extracted_k141_150078	3466	No	NA	0	2	Not-determined	Genome-fragment	NA		0
S17_extracted_k141_39241	1044	No	NA	0	1	Not-determined	Genome-fragment	NA		0
S7_vs_130232||full	1010	No	NA	1	0	Low-quality	Genome-fragment		2.74	0
S17_extracted_k141_134855	1869	No	NA	0	0	Not-determined	Genome-fragment	NA		0
S17_extracted_k141_164399	1632	No	NA	0	1	Not-determined	Genome-fragment	NA		0
S17_extracted_k141_157254	1248	No	NA	0	1	Not-determined	Genome-fragment	NA		0
S17_extracted_k141_31661	16460	No	NA	0	8	Not-determined	Genome-fragment	NA		0
S17_extracted_k141_171729	1056	No	NA	0	0	Not-determined	Genome-fragment	NA		0
S17_extracted_k141_98150	1429	No	NA	0	1	Not-determined	Genome-fragment	NA		0
S17_extracted_k141_98151	1051	No	NA	0	1	Not-determined	Genome-fragment	NA		0
S17_extracted_k141_23923	2619	No	NA	0	1	Not-determined	Genome-fragment	NA		0
S12_extracted_k141_185231	1010	No	NA	0	0	Low-quality	Genome-fragment		1.08	0
S17_extracted_k141_68464	1626	No	NA	0	1	Not-determined	Genome-fragment	NA		0
S17_extracted_k141_164458	1129	No	NA	0	1	Not-determined	Genome-fragment	NA		0
S17_extracted_k141_68471	1287	No	NA	0	1	Not-determined	Genome-fragment	NA		0
S17_extracted_k141_61003	6593	No	NA	0	1	Not-determined	Genome-fragment	NA		0
S17_extracted_k141_23943	1300	No	NA	0	0	Not-determined	Genome-fragment	NA		0
S8_extracted_k141_31236	1010	No	NA	1	0	Low-quality	Genome-fragment		1.56	0
S17_extracted_k141_134904	2489	No	NA	0	2	Not-determined	Genome-fragment	NA		0
S17_extracted_k141_46520	1035	No	NA	0	2	Not-determined	Genome-fragment	NA		0
S8_extracted_k141_169864	1010	No	NA	1	0	Low-quality	Genome-fragment		0.68	0
S17_extracted_k141_16384	3560	No	NA	0	2	Not-determined	Genome-fragment	NA		0
S17_extracted_k141_150177	5880	No	NA	0	3	Not-determined	Genome-fragment	NA		0
S17_extracted_k141_39338	1153	No	NA	0	1	Not-determined	Genome-fragment	NA		0
S17_extracted_k141_68498	3540	No	NA	0	3	Not-determined	Genome-fragment	NA		0
S17_extracted_k141_1963	1253	No	NA	0	2	Not-determined	Genome-fragment	NA		0
S17_extracted_k141_1976	1000	No	NA	0	1	Not-determined	Genome-fragment	NA		0
S17_extracted_k141_75666	6339	No	NA	0	2	Not-determined	Genome-fragment	NA		0
S17_extracted_k141_25648	1011	No	NA	1	0	Low-quality	Genome-fragment		1.77	0
S17_extracted_k141_82878	2389	No	NA	0	1	Not-determined	Genome-fragment	NA		0
S11_vs_k141_4498||full	1011	No	NA	1	0	Low-quality	Genome-fragment		2.36	0
S17_extracted_k141_112668	1080	No	NA	0	2	Not-determined	Genome-fragment	NA		0
S17_extracted_k141_2019	7010	No	NA	0	5	Not-determined	Genome-fragment	NA		0
S17_extracted_k141_112678	1222	No	NA	0	0	Not-determined	Genome-fragment	NA		0
S17_extracted_k141_105981	5737	No	NA	0	2	Not-determined	Genome-fragment	NA		0
S17_extracted_k141_75728	1714	No	NA	0	1	Not-determined	Genome-fragment	NA		0
S17_extracted_k141_31795	1059	No	NA	0	0	Not-determined	Genome-fragment	NA		0
S17_extracted_k141_164530	7653	No	NA	0	4	Not-determined	Genome-fragment	NA		0
S17_extracted_k141_127316	7067	No	NA	0	0	Not-determined	Genome-fragment	NA		0
S11_vs_k141_5958||full	1011	No	NA	1	0	Low-quality	Genome-fragment		1.32	0
S17_extracted_k141_106005	1539	No	NA	0	2	Not-determined	Genome-fragment	NA		0
S11_vs_k141_32365||full	1011	No	NA	0	1	Low-quality	Genome-fragment		0.76	0
S14_vs_k141_9507||full	1011	No	NA	0	0	Low-quality	Genome-fragment		2.36	0
S17_extracted_k141_24071	1625	No	NA	0	2	Not-determined	Genome-fragment	NA		0
S17_extracted_k141_39472	1724	No	NA	0	1	Not-determined	Genome-fragment	NA		0
S17_extracted_k141_164578	2901	No	NA	0	2	Not-determined	Genome-fragment	NA		0
S17_extracted_k141_164581	1059	No	NA	0	1	Not-determined	Genome-fragment	NA		0
S17_extracted_k141_164594	1216	No	NA	0	1	Not-determined	Genome-fragment	NA		0
S17_extracted_k141_31845	1511	No	NA	0	4	Not-determined	Genome-fragment	NA		0
S17_extracted_k141_120343	3440	No	NA	0	0	Not-determined	Genome-fragment	NA		0
S1_vs_k141_115896||full	1011	No	NA	2	0	Low-quality	Genome-fragment		2.44	0
S17_extracted_k141_135058	1399	No	NA	0	0	Not-determined	Genome-fragment	NA		0
S17_extracted_k141_39487	7698	No	NA	0	6	Not-determined	Genome-fragment	NA		0
S1_vs_k141_119881||full	1011	No	NA	0	0	Low-quality	Genome-fragment		1.6	0
S17_extracted_k141_112756	1934	No	NA	0	0	Not-determined	Genome-fragment	NA		0
S17_extracted_k141_39495	3196	No	NA	0	3	Not-determined	Genome-fragment	NA		0
S17_extracted_k141_142688	3242	No	NA	0	1	Not-determined	Genome-fragment	NA		0
S17_extracted_k141_75831	1145	No	NA	0	1	Not-determined	Genome-fragment	NA		0
S17_extracted_k141_171818	1090	No	NA	0	1	Not-determined	Genome-fragment	NA		0
S17_extracted_k141_112769	1438	No	NA	0	1	Not-determined	Genome-fragment	NA		0
S5_extracted_k141_122077	1011	No	NA	0	0	Low-quality	Genome-fragment		2.29	0
S17_extracted_k141_46637	1026	No	NA	0	1	Not-determined	Genome-fragment	NA		0
S17_extracted_k141_16566	1005	No	NA	0	1	Not-determined	Genome-fragment	NA		0
S17_extracted_k141_164656	4902	No	NA	0	3	Not-determined	Genome-fragment	NA		0
S17_extracted_k141_75836	7622	No	NA	0	6	Not-determined	Genome-fragment	NA		0
S17_extracted_k141_16590	1143	No	NA	0	1	Not-determined	Genome-fragment	NA		0
S5_vs_k141_22643||lt2gene	1011	No	NA	1	0	Low-quality	Genome-fragment		1.15	0
S17_extracted_k141_90575	2804	No	NA	0	0	Not-determined	Genome-fragment	NA		0
S16_extracted_101286	1011	No	NA	1	0	Low-quality	Genome-fragment		1.69	0
S17_extracted_k141_106163	1382	No	NA	0	2	Not-determined	Genome-fragment	NA		0
S17_extracted_k141_24139	1133	No	NA	0	1	Not-determined	Genome-fragment	NA		0
S17_extracted_k141_75893	1629	No	NA	0	1	Not-determined	Genome-fragment	NA		0
S17_extracted_k141_112823	1975	No	NA	0	2	Not-determined	Genome-fragment	NA		0
S17_extracted_k141_127417	1364	No	NA	0	1	Not-determined	Genome-fragment	NA		0
S17_extracted_k141_46684	3817	No	NA	0	0	Not-determined	Genome-fragment	NA		0
S17_extracted_k141_68752	1547	No	NA	0	0	Not-determined	Genome-fragment	NA		0
S17_extracted_k141_120441	6109	No	NA	0	5	Not-determined	Genome-fragment	NA		0

S17_extracted_k141_112825	10015	No	NA	0	5	Not-determined	Genome-fragment	NA	0
S17_extracted_k141_90603	4504	No	NA	0	2	Not-determined	Genome-fragment	NA	0
S17_extracted_k141_16642	1088	No	NA	0	1	Not-determined	Genome-fragment	NA	0
S17_extracted_k141_24162	1998	No	NA	0	2	Not-determined	Genome-fragment	NA	0
S16_vs_51425||full	1011	No	NA	0	2	Low-quality	Genome-fragment	2.34	0
S16_vs_103149||full	1011	No	NA	0	0	Low-quality	Genome-fragment	0.93	0
S17_extracted_k141_171935	6402	No	NA	0	3	Not-determined	Genome-fragment	NA	0
S17_extracted_k141_75969	1482	No	NA	0	2	Not-determined	Genome-fragment	NA	0
S17_extracted_k141_75978	1323	No	NA	0	2	Not-determined	Genome-fragment	NA	0
S16_vs_122521||full	1011	No	NA	1	0	Low-quality	Genome-fragment	0.55	0
S17_extracted_k141_46758	1003	No	NA	0	1	Not-determined	Genome-fragment	NA	0
S17_extracted_k141_164740	1132	No	NA	0	0	Not-determined	Genome-fragment	NA	0
S16_vs_146223||full	1011	No	NA	0	1	Low-quality	Genome-fragment	1.97	0
S17_extracted_k141_90646	1010	No	NA	0	0	Not-determined	Genome-fragment	NA	0
S12_vs_k141_152159||lt2gene	1011	No	NA	1	0	Low-quality	Genome-fragment	2.36	0
S17_extracted_k141_54308	1479	No	NA	0	2	Not-determined	Genome-fragment	NA	0
S17_extracted_k141_75995	2091	No	NA	0	2	Not-determined	Genome-fragment	NA	0
S17_extracted_k141_157646	4408	No	NA	0	4	Not-determined	Genome-fragment	NA	0
S17_extracted_k141_135123	1944	No	NA	0	1	Not-determined	Genome-fragment	NA	0
S17_extracted_k141_16684	5628	No	NA	0	3	Not-determined	Genome-fragment	NA	0
S2_extracted_k141_18439	1011	No	NA	2	0	Low-quality	Genome-fragment	3.35	0
S17_extracted_k141_164794	1035	No	NA	0	0	Not-determined	Genome-fragment	NA	0
S8_extracted_k141_89738	1011	No	NA	1	0	Low-quality	Genome-fragment	0.65	0
S17_extracted_k141_39662	2852	No	NA	0	2	Not-determined	Genome-fragment	NA	0
S11_extracted_k141_9305	1012	No	NA	1	0	Low-quality	Genome-fragment	2.41	0
S17_extracted_k141_157704	3316	No	NA	0	0	Not-determined	Genome-fragment	NA	0
S17_extracted_k141_61145	1668	No	NA	0	0	Not-determined	Genome-fragment	NA	0
S17_extracted_k141_16727	1262	No	NA	0	1	Not-determined	Genome-fragment	NA	0
S17_extracted_k141_61157	1615	No	NA	0	1	Not-determined	Genome-fragment	NA	0
S13_vs_13096||full	1012	No	NA	0	0	Low-quality	Genome-fragment	2.34	0
S17_extracted_k141_127523	2734	No	NA	0	0	Not-determined	Genome-fragment	NA	0
S17_extracted_k141_150553	5399	No	NA	0	3	Not-determined	Genome-fragment	NA	0
S17_extracted_k141_142879	1431	No	NA	0	1	Not-determined	Genome-fragment	NA	0
S17_extracted_k141_39705	1455	No	NA	0	2	Not-determined	Genome-fragment	NA	0
S17_extracted_k141_112983	1105	No	NA	0	0	Not-determined	Genome-fragment	NA	0
S17_extracted_k141_142888	4620	No	NA	0	0	Not-determined	Genome-fragment	NA	0
S17_extracted_k141_68904	3651	No	NA	0	5	Not-determined	Genome-fragment	NA	0
S14_vs_k141_29841||full	1012	No	NA	1	0	Low-quality	Genome-fragment	3.06	0
S17_extracted_k141_142906	1054	No	NA	0	2	Not-determined	Genome-fragment	NA	0
S17_extracted_k141_112997	3284	No	NA	0	2	Not-determined	Genome-fragment	NA	0
S17_extracted_k141_120642	1289	No	NA	0	0	Not-determined	Genome-fragment	NA	0
S17_extracted_k141_76093	1101	No	NA	0	0	Not-determined	Genome-fragment	NA	0
S14_vs_k141_100260||full	1012	No	NA	0	1	Low-quality	Genome-fragment	0.27	0
S17_extracted_k141_54395	2162	No	NA	0	2	Not-determined	Genome-fragment	NA	0
S17_extracted_k141_83080	3380	No	NA	0	1	Not-determined	Genome-fragment	NA	0
S17_extracted_k141_142940	1161	No	NA	0	1	Not-determined	Genome-fragment	NA	0
S17_extracted_k141_46832	3512	No	NA	0	2	Not-determined	Genome-fragment	NA	0
S14_vs_k141_56602||full	1012	No	NA	1	0	Low-quality	Genome-fragment	2.47	0
S17_extracted_k141_90803	1720	No	NA	0	1	Not-determined	Genome-fragment	NA	0
S17_extracted_k141_32066	4099	No	NA	0	3	Not-determined	Genome-fragment	NA	0
S17_extracted_k141_90804	1640	No	NA	0	1	Not-determined	Genome-fragment	NA	0
S1_vs_k141_50343||full	1012	No	NA	0	1	Low-quality	Genome-fragment	1.89	0
S17_extracted_k141_54411	1411	No	NA	0	1	Not-determined	Genome-fragment	NA	0
S3_extracted_k141_90378	1012	No	NA	0	0	Low-quality	Genome-fragment	1.94	0
S17_extracted_k141_113064	1072	No	NA	0	0	Not-determined	Genome-fragment	NA	0
S17_extracted_k141_68985	1199	No	NA	0	1	Not-determined	Genome-fragment	NA	0
S17_extracted_k141_16789	1185	No	NA	0	0	Not-determined	Genome-fragment	NA	0
S17_extracted_k141_61271	3904	No	NA	0	3	Not-determined	Genome-fragment	NA	0
S3_extracted_k141_109270	1012	No	NA	1	0	Low-quality	Genome-fragment	2.27	0
S17_extracted_k141_150677	1046	No	NA	0	2	Not-determined	Genome-fragment	NA	0
S17_extracted_k141_113069	1020	No	NA	0	2	Not-determined	Genome-fragment	NA	0
S17_extracted_k141_120721	11346	No	NA	0	5	Not-determined	Genome-fragment	NA	0
S17_extracted_k141_54439	1037	No	NA	0	0	Not-determined	Genome-fragment	NA	0
S17_extracted_k141_32134	1378	No	NA	0	2	Not-determined	Genome-fragment	NA	0
S17_extracted_k141_32137	1266	No	NA	0	0	Not-determined	Genome-fragment	NA	0
S5_vs_k141_100509||lt2gene	1012	No	NA	1	0	Low-quality	Genome-fragment	2.58	0
S17_extracted_k141_135243	1523	No	NA	0	1	Not-determined	Genome-fragment	NA	0
S7_vs_133432||lt2gene	1012	No	NA	1	0	Low-quality	Genome-fragment	0.58	0
S17_extracted_k141_143068	1465	No	NA	0	1	Not-determined	Genome-fragment	NA	0
S17_extracted_k141_113118	5425	No	NA	0	3	Not-determined	Genome-fragment	NA	0
S17_extracted_k141_9981	1510	No	NA	0	0	Not-determined	Genome-fragment	NA	0
S17_extracted_k141_83209	1290	No	NA	0	2	Not-determined	Genome-fragment	NA	0
S17_extracted_k141_46961	1651	No	NA	0	0	Not-determined	Genome-fragment	NA	0
S17_extracted_k141_83214	3812	No	NA	0	0	Not-determined	Genome-fragment	NA	0
S17_extracted_k141_10000	1477	No	NA	0	1	Not-determined	Genome-fragment	NA	0
S17_extracted_k141_32200	2364	No	NA	0	2	Not-determined	Genome-fragment	NA	0
S17_extracted_k141_46981	1092	No	NA	0	0	Not-determined	Genome-fragment	NA	0
S16_extracted_107579	1012	No	NA	0	0	Low-quality	Genome-fragment	1.43	0
S16_vs_19994||full	1012	No	NA	2	0	Low-quality	Genome-fragment	2.89	0
S17_extracted_k141_69039	1324	No	NA	0	1	Not-determined	Genome-fragment	NA	0
S10_vs_74792||full	1012	No	NA	1	0	Low-quality	Genome-fragment	2.14	0
S17_extracted_k141_98675	1462	No	NA	0	1	Not-determined	Genome-fragment	NA	0
S17_extracted_k141_10013	2100	No	NA	0	1	Not-determined	Genome-fragment	NA	0
S12_extracted_k141_71446	1012	No	NA	1	0	Low-quality	Genome-fragment	1.48	0
S17_extracted_k141_143085	4077	No	NA	0	0	Not-determined	Genome-fragment	NA	0
S12_extracted_k141_150701	1012	No	NA	1	0	Low-quality	Genome-fragment	1.54	0
S8_vs_k141_86607||lt2gene	1012	No	NA	1	0	Low-quality	Genome-fragment	2.82	0
S17_extracted_k141_157883	1250	No	NA	0	0	Not-determined	Genome-fragment	NA	0
S17_extracted_k141_47026	11400	No	NA	0	1	Not-determined	Genome-fragment	NA	0
S17_extracted_k141_143144	3266	No	NA	0	0	Not-determined	Genome-fragment	NA	0
S17_extracted_k141_172132	12235	No	NA	4	0	Complete	High-quality	100	0
S17_vs_k141_140709||full	1013	No	NA	0	0	Low-quality	Genome-fragment	0.4	0
S17_extracted_k141_47033	1007	No	NA	0	1	Not-determined	Genome-fragment	NA	0
S11_vs_k141_66539||full	1013	No	NA	0	0	Low-quality	Genome-fragment	0.77	0
S11_vs_k141_28487||full	1013	Yes	863	1	1	Low-quality	Genome-fragment	1.18	14.81
S17_extracted_k141_150808	1882	No	NA	0	1	Not-determined	Genome-fragment	NA	0
S17_extracted_k141_32304	1002	No	NA	0	2	Not-determined	Genome-fragment	NA	0
S17_extracted_k141_164967	4554	No	NA	0	4	Not-determined	Genome-fragment	NA	0
S11_vs_k141_71619||lt2gene	1013	No	NA	1	0	Low-quality	Genome-fragment	2.25	0
S17_extracted_k141_69124	3765	No	NA	0	4	Not-determined	Genome-fragment	NA	0
S17_extracted_k141_83302	1240	No	NA	0	2	Not-determined	Genome-fragment	NA	0
S14_extracted_k141_73544	1013	No	NA	3	0	Low-quality	Genome-fragment	3.31	0
S17_extracted_k141_83315	1480	No	NA	0	2	Not-determined	Genome-fragment	NA	0
S1_extracted_k141_78878	1013	No	NA	0	0	Low-quality	Genome-fragment	2.11	0
S17_extracted_k141_2376	1954	No	NA	0	1	Not-determined	Genome-fragment	NA	0
S17_extracted_k141_98788	1906	No	NA	0	0	Not-determined	Genome-fragment	NA	0
S5_extracted_k141_88667	1013	No	NA	0	0	Low-quality	Genome-fragment	2.6	0
S17_extracted_k141_90996	4952	No	NA	0	1	Not-determined	Genome-fragment	NA	0
S17_extracted_k141_165002	1330	No	NA	0	0	Not-determined	Genome-fragment	NA	0
S17_extracted_k141_98813	1495	No	NA	0	0	Not-determined	Genome-fragment	NA	0
S17_extracted_k141_2395	1005	No	NA	0	0	Not-determined	Genome-fragment	NA	0
S17_extracted_k141_10112	1458	No	NA	0	0	Not-determined	Genome-fragment	NA	0
S5_extracted_k141_60155	1013	No	NA	1	0	Low-quality	Genome-fragment	0.7	0
S5_vs_k141_107997||full	1013	No	NA	0	1	Low-quality	Genome-fragment	1.01	0
S17_extracted_k141_165022	9596	No	NA	0	3	Not-determined	Genome-fragment	NA	0
S17_extracted_k141_16927	1061	No	NA	0	1	Not-determined	Genome-fragment	NA	0
S17_extracted_k141_165026	2038	No	NA	0	1	Not-determined	Genome-fragment	NA	0
S7_vs_66815||full	1013	No	NA	0	0	Low-quality	Genome-fragment	2.39	0
S17_extracted_k141_10128	1611	No	NA	0	1	Not-determined	Genome-fragment	NA	0
S17_extracted_k141_113155	1289	No	NA	0	1	Not-determined	Genome-fragment	NA	0
S17_extracted_k141_165029	5378	No	NA	0	4	Not-determined	Genome-fragment	NA	0
S17_extracted_k141_16942	1037	No	NA	0	1	Not-determined	Genome-fragment	NA	0
S17_extracted_k141_98843	3684	No	NA	0	1	Not-determined	Genome-fragment	NA	0
S17_extracted_k141_135444	5253	No	NA	0	1	Not-determined	Genome-fragment	NA	0
S17_extracted_k141_24488	3787	No	NA	0	3	Not-determined	Genome-fragment	NA	0
S17_extracted_k141_158002	1059	No	NA	0	0	Not-determined	Genome-fragment	NA	0
S17_extracted_k141_113174	2910	No	NA	0	1	Not-determined	Genome-fragment	NA	0
S17_extracted_k141_16972	1038	No	NA	0	1	Not-determined	Genome-fragment	NA	0
S17_extracted_k141_16983	1031	No	NA	0	0	Not-determined	Genome-fragment	NA	0
S16_vs_46507||full	1013	No	NA	1	0	Low-quality	Genome-fragment	2.04	0
S17_extracted_k141_2410	1154	No	NA	0	1	Not-determined	Genome-fragment	NA	0
S17_extracted_k141_32419	1018	No	NA	0	0	Not-determined	Genome-fragment	NA	0
S16_vs_111247||full	1013	No	NA	0	1	Low-quality	Genome-fragment	0.99	0
S12_vs_k141_126925||full	1013	No	NA	1	0	Low-quality	Genome-fragment	19.07	0
S17_extracted_k141_143283	1895	No	NA	0	0	Not-determined	Genome-fragment	NA	0
S17_extracted_k141_127876	1045	No	NA	0	1	Not-determined	Genome-fragment	NA	0
S17_extracted_k141_32453	4714	No	NA	0	3	Not-determined	Genome-fragment	NA	0
S4_extracted_25533	1013	No	NA	0	0	Low-quality	Genome-fragment	2.92	0
S17_extracted_k141_135506	3610	No	NA	0	1	Not-determined	Genome-fragment	NA	0
S17_extracted_k141_32463	1153	No	NA	0	0	Not-determined	Genome-fragment	NA	0
S17_extracted_k141_2484	1037	No	NA	0	1	Not-determined	Genome-fragment	NA	0
S8_extracted_k141_45221	1013	No	NA	0	1	Low-quality	Genome-fragment	0.66	0
S17_extracted_k141_47224	1960	No	NA	0	0	Not-determined	Genome-fragment	NA	0
S17_extracted_k141_69260	1154	No	NA	0	1	Not-determined	Genome-fragment	NA	0
S17_extracted_k141_135538	4846	No	NA	0	0	Not-determined	Genome-fragment	NA	0
S17_extracted_k141_83367	1160	No	NA	0	1	Not-determined	Genome-fragment	NA	0
S17_extracted_k141_32516	6058	No	NA	0	3	Not-determined	Genome-fragment	NA	0
S8_extracted_k141_22478	1013	No	NA	1	0	Low-quality	Genome-fragment	3.34	0
S17_extracted_k141_91199	4540	No	NA	0	1	Not-determined	Genome-fragment	NA	0
S17_extracted_k141_172347	1148	No	NA	0	1	Not-determined	Genome-fragment	NA	0
S17_extracted_k141_113245	1447	No	NA	0	0	Not-determined	Genome-fragment	NA	0
S17_extracted_k141_61513	2746	No	NA	0	0	Not-determined	Genome-fragment	NA	0
S17_extracted_k141_61514	1402	No	NA	0	2	Not-determined	Genome-fragment	NA	0
S17_extracted_k141_113249	1358	No	NA	0	0	Not-determined	Genome-fragment	NA	0
S17_extracted_k141_17092	3545	No	NA	0	1	Not-determined	Genome-fragment	NA	0
S17_extracted_k141_165077	11958	No	NA	0	8	Not-determined	Genome-fragment	NA	0
S17_extracted_k141_120951	3892	No	NA	0	6	Not-determined	Genome-fragment	NA	0
S17_extracted_k141_172356	4512	No	NA	0	2	Not-determined	Genome-fragment	NA	0
S17_extracted_k141_10229	1273	No	NA	0	1	Not-determined	Genome-fragment	NA	0
S8_extracted_k141_137129	1013	No	NA	1	0	Low-quality	Genome-fragment	2.13	0
S17_extracted_k141_143343	1513	No	NA	0	1	Not-determined	Genome-fragment	NA	0
S8_extracted_k141_16453	1013	No	NA	0	0	Low-quality	Genome-fragment	5.18	0
S17_extracted_k141_127961	1214	No	NA	0	2	Not-determined	Genome-fragment	NA	0
S17_extracted_k141_76534	1341	No	NA	0	1	Not-determined	Genome-fragment	NA	0
S17_extracted_k141_106687	4094	No	NA	0	1	Not-determined	Genome-fragment	NA	0
S17_extracted_k141_76540	7313	No	NA	0	2	Not-determined	Genome-fragment	NA	0
S17_extracted_k141_17133	1115	No	NA	0	0	Not-determined	Genome-fragment	NA	0
S17_extracted_k141_83414	1035	No	NA	0	2	Not-determined	Genome-fragment	NA	0
S17_extracted_k141_113298	3675	No	NA	0	1	Not-determined	Genome-fragment	NA	0
S17_extracted_k141_47309	1083	No	NA	0	2	Not-determined	Genome-fragment	NA	0
S17_extracted_k141_76550	1621	No	NA	0	2	Not-determined	Genome-fragment	NA	0
S17_extracted_k141_158159	1424	No	NA	0	1	Not-determined	Genome-fragment	NA	0
S8_vs_k141_80673||lt2gene	1013	No	NA	1	0	Low-quality	Genome-fragment	1.88	0
S17_extracted_k141_17153	1127	No	NA	0	0	Not-determined	Genome-fragment	NA	0

S17_extracted_k141_7908	1014	No	NA	3	0	Low-quality	Genome-fragment		3.35	0
S17_extracted_k141_91274	2283	No	NA	0	2	Not-determined	Genome-fragment	NA		0
S17_extracted_k141_118052	1014	No	NA	0	1	Low-quality	Genome-fragment		0.93	0
S17_extracted_k141_32628	1223	No	NA	0	1	Not-determined	Genome-fragment	NA		0
S17_extracted_k141_135608	1099	No	NA	0	2	Not-determined	Genome-fragment	NA		0
S11_vs_k141_23045||full	1014	No	NA	1	0	Low-quality	Genome-fragment		1.32	0
S17_extracted_k141_91289	1165	No	NA	0	0	Not-determined	Genome-fragment	NA		0
S17_extracted_k141_40252	2010	No	NA	0	1	Not-determined	Genome-fragment	NA		0
S11_vs_k141_82621||full	1014	No	NA	0	0	Low-quality	Genome-fragment		2.25	0
S17_extracted_k141_24669	1202	No	NA	0	1	Not-determined	Genome-fragment	NA		0
S13_vs_112381||full	1014	No	NA	1	0	Low-quality	Genome-fragment		2.16	0
S14_vs_k141_12464||full	1014	No	NA	0	0	Low-quality	Genome-fragment		1.83	0
S17_extracted_k141_61606	1373	No	NA	0	2	Not-determined	Genome-fragment	NA		0
S17_extracted_k141_32660	2855	No	NA	0	1	Not-determined	Genome-fragment	NA		0
S17_extracted_k141_121035	1515	No	NA	0	2	Not-determined	Genome-fragment	NA		0
S1_extracted_k141_70908	1014	No	NA	0	0	Low-quality	Genome-fragment		1.99	0
S17_extracted_k141_40279	1228	No	NA	0	0	Not-determined	Genome-fragment	NA		0
S1_vs_k141_82849||lt2gene	1014	No	NA	1	0	Low-quality	Genome-fragment		17.19	0
S17_extracted_k141_47398	3755	No	NA	0	3	Not-determined	Genome-fragment	NA		0
S5_vs_k141_98664||full	1014	No	NA	0	1	Low-quality	Genome-fragment		2.48	0
S17_extracted_k141_47402	1107	No	NA	0	0	Not-determined	Genome-fragment	NA		0
S17_extracted_k141_47404	1052	No	NA	0	0	Not-determined	Genome-fragment	NA		0
S7_extracted_119169	1014	No	NA	0	1	Low-quality	Genome-fragment		7.27	0
S17_extracted_k141_10277	3441	No	NA	0	0	Not-determined	Genome-fragment	NA		0
S16_vs_136002||full	1014	No	NA	1	0	Low-quality	Genome-fragment		1.97	0
S17_extracted_k141_99085	2671	No	NA	0	0	Not-determined	Genome-fragment	NA		0
S17_extracted_k141_135686	1500	No	NA	0	1	Not-determined	Genome-fragment	NA		0
S17_extracted_k141_165257	1879	No	NA	0	1	Not-determined	Genome-fragment	NA		0
S16_vs_138358||lt2gene	1014	No	NA	1	0	Low-quality	Genome-fragment		2.98	0
S17_extracted_k141_69460	1043	No	NA	0	0	Not-determined	Genome-fragment	NA		0
S17_extracted_k141_17257	1242	No	NA	0	0	Not-determined	Genome-fragment	NA		0
S17_extracted_k141_10316	1125	No	NA	0	2	Not-determined	Genome-fragment	NA		0
S10_vs_74415||full	1014	No	NA	0	0	Low-quality	Genome-fragment		1.08	0
S17_extracted_k141_69478	1293	No	NA	0	2	Not-determined	Genome-fragment	NA		0
S15_vs_62008||full	1014	No	NA	0	0	Low-quality	Genome-fragment		2	0
S17_extracted_k141_17280	4205	No	NA	0	0	Not-determined	Genome-fragment	NA		0
S17_extracted_k141_61693	4137	No	NA	0	2	Not-determined	Genome-fragment	NA		0
S17_extracted_k141_24776	7985	No	NA	0	1	Not-determined	Genome-fragment	NA		0
S8_extracted_k141_11951	1014	No	NA	0	0	Low-quality	Genome-fragment		2.02	0
S17_extracted_k141_47492	1358	No	NA	0	1	Not-determined	Genome-fragment	NA		0
S17_extracted_k141_40343	1523	No	NA	0	2	Not-determined	Genome-fragment	NA		0
S17_extracted_k141_44548	1015	No	NA	0	0	Low-quality	Genome-fragment		1.97	0
S17_extracted_k141_10341	1409	No	NA	0	2	Not-determined	Genome-fragment	NA		0
S17_extracted_k141_128147	7401	No	NA	3	0	High-quality	High-quality		100	0
S17_extracted_k141_54729	1120	No	NA	0	1	Not-determined	Genome-fragment	NA		0
S17_extracted_k141_106936	1177	No	NA	0	0	Not-determined	Genome-fragment	NA		0
S17_extracted_k141_47545	1996	No	NA	0	1	Not-determined	Genome-fragment	NA		0
S17_extracted_k141_106942	1635	No	NA	0	0	Not-determined	Genome-fragment	NA		0
S9_extracted_k141_79590	1015	No	NA	0	0	Low-quality	Genome-fragment		0.63	0
S17_extracted_k141_76763	1268	No	NA	0	1	Not-determined	Genome-fragment	NA		0
S17_extracted_k141_69562	2039	No	NA	0	1	Not-determined	Genome-fragment	NA		0
S9_vs_k141_72672||lt2gene	1015	No	NA	1	0	Low-quality	Genome-fragment		2.78	0
S17_extracted_k141_32701	1122	No	NA	0	1	Not-determined	Genome-fragment	NA		0
S11_extracted_k141_55902	1015	No	NA	1	0	Low-quality	Genome-fragment		2.25	0
S14_extracted_k141_59712	1015	No	NA	1	0	Low-quality	Genome-fragment		0.7	0
S14_extracted_k141_67217	1015	No	NA	1	0	Low-quality	Genome-fragment		0.65	0
S17_extracted_k141_2831	1035	No	NA	0	1	Not-determined	Genome-fragment	NA		0
S17_extracted_k141_99239	6540	No	NA	0	3	Not-determined	Genome-fragment	NA		0
S17_extracted_k141_10394	2525	No	NA	0	1	Not-determined	Genome-fragment	NA		0
S17_extracted_k141_172666	2833	No	NA	0	1	Not-determined	Genome-fragment	NA		0
S14_vs_k141_58507||full	1015	No	NA	3	0	Low-quality	Genome-fragment		2.83	0
S17_extracted_k141_143621	1108	No	NA	0	0	Not-determined	Genome-fragment	NA		0
S1_vs_k141_56077||full	1015	No	NA	0	0	Low-quality	Genome-fragment		1.74	0
S3_extracted_k141_78412	1015	No	NA	1	0	Low-quality	Genome-fragment		1.52	0
S17_extracted_k141_24859	3966	No	NA	0	2	Not-determined	Genome-fragment	NA		0
S3_vs_k141_11246||full	1015	No	NA	0	0	Low-quality	Genome-fragment		0.59	0
S17_extracted_k141_113535	7478	No	NA	0	2	Not-determined	Genome-fragment	NA		0
S17_extracted_k141_135812	1045	No	NA	0	0	Not-determined	Genome-fragment	NA		0
S17_extracted_k141_69627	1153	No	NA	0	1	Not-determined	Genome-fragment	NA		0
S17_extracted_k141_69630	1234	No	NA	0	2	Not-determined	Genome-fragment	NA		0
S5_vs_k141_75564||full	1015	No	NA	2	0	Low-quality	Genome-fragment		2.64	0
S17_extracted_k141_135816	3116	No	NA	0	0	Not-determined	Genome-fragment	NA		0
S17_extracted_k141_165441	1006	No	NA	0	2	Not-determined	Genome-fragment	NA		0
S17_extracted_k141_54836	2122	No	NA	0	0	Not-determined	Genome-fragment	NA		0
S17_extracted_k141_121114	3025	No	NA	0	5	Not-determined	Genome-fragment	NA		0
S7_extracted_69180	1015	No	NA	0	2	Low-quality	Genome-fragment		0.66	0
S17_extracted_k141_47622	3169	No	NA	0	2	Not-determined	Genome-fragment	NA		0
S17_extracted_k141_91488	1507	No	NA	0	2	Not-determined	Genome-fragment	NA		0
S10_vs_27874||full	1015	No	NA	0	0	Low-quality	Genome-fragment		2.06	0
S10_vs_60781||full	1015	No	NA	1	0	Low-quality	Genome-fragment		2.87	0
S17_extracted_k141_113568	5332	No	NA	0	3	Not-determined	Genome-fragment	NA		0
S10_vs_101949||full	1015	No	NA	0	2	Low-quality	Genome-fragment		1.53	0
S12_vs_k141_80353||full	1015	No	NA	0	0	Low-quality	Genome-fragment		0.72	0
S17_extracted_k141_135834	4929	No	NA	0	7	Not-determined	Genome-fragment	NA		0
S17_extracted_k141_91503	1152	No	NA	0	1	Not-determined	Genome-fragment	NA		0
S12_vs_k141_170246||lt2gene	1015	No	NA	1	0	Low-quality	Genome-fragment		0.7	0
S15_vs_23235||full	1015	No	NA	0	0	Low-quality	Genome-fragment		2.34	0
S17_extracted_k141_121138	1016	No	NA	0	2	Not-determined	Genome-fragment	NA		0
S17_extracted_k141_2905	7170	No	NA	0	3	Not-determined	Genome-fragment	NA		0
S17_extracted_k141_121144	1793	No	NA	0	0	Not-determined	Genome-fragment	NA		0
S17_extracted_k141_143670	1060	No	NA	0	1	Not-determined	Genome-fragment	NA		0
S8_extracted_k141_78982	1015	No	NA	0	0	Low-quality	Genome-fragment		2.86	0
S17_extracted_k141_172729	3728	No	NA	0	0	Not-determined	Genome-fragment	NA		0
S17_extracted_k141_135861	6935	No	NA	0	1	Not-determined	Genome-fragment	NA		0
S17_extracted_k141_40500	1405	No	NA	0	0	Not-determined	Genome-fragment	NA		0
S17_extracted_k141_32767	3777	No	NA	0	6	Not-determined	Genome-fragment	NA		0
S17_extracted_k141_24929	1085	No	NA	0	1	Not-determined	Genome-fragment	NA		0
S17_extracted_k141_2939	1011	No	NA	0	0	Not-determined	Genome-fragment	NA		0
S17_extracted_k141_143697	1100	No	NA	0	2	Not-determined	Genome-fragment	NA		0
S8_extracted_k141_202132	1015	No	NA	0	1	Low-quality	Genome-fragment		0.67	0
S17_vs_k141_75192||full	1016	No	NA	0	0	Low-quality	Genome-fragment		1.49	0
S17_extracted_k141_17492	1101	No	NA	0	2	Not-determined	Genome-fragment	NA		0
S17_extracted_k141_83608	1476	No	NA	0	1	Not-determined	Genome-fragment	NA		0
S17_extracted_k141_47714	1288	No	NA	0	0	Not-determined	Genome-fragment	NA		0
S17_extracted_k141_40555	1994	No	NA	0	3	Not-determined	Genome-fragment	NA		0
S17_extracted_k141_2962	3808	No	NA	0	0	Not-determined	Genome-fragment	NA		0
S17_extracted_k141_61932	1309	No	NA	0	1	Not-determined	Genome-fragment	NA		0
S17_extracted_k141_151366	1260	No	NA	0	0	Not-determined	Genome-fragment	NA		0
S17_extracted_k141_128373	2165	No	NA	0	2	Not-determined	Genome-fragment	NA		0
S17_extracted_k141_10597	3342	No	NA	0	2	Not-determined	Genome-fragment	NA		0
S17_extracted_k141_47768	6459	No	NA	0	4	Not-determined	Genome-fragment	NA		0
S17_vs_k141_50491||full	1016	No	NA	0	1	Low-quality	Genome-fragment		2.41	0
S17_extracted_k141_128382	3376	No	NA	0	2	Not-determined	Genome-fragment	NA		0
S17_extracted_k141_76967	1998	No	NA	0	0	Not-determined	Genome-fragment	NA		0
S17_extracted_k141_91626	1145	No	NA	0	2	Not-determined	Genome-fragment	NA		0
S17_extracted_k141_113722	4181	No	NA	0	2	Not-determined	Genome-fragment	NA		0
S9_vs_k141_16260||full	1016	No	NA	0	0	Low-quality	Genome-fragment		0.72	0
S17_extracted_k141_3013	5275	No	NA	0	2	Not-determined	Genome-fragment	NA		0
S17_extracted_k141_99451	1607	No	NA	0	0	Not-determined	Genome-fragment	NA		0
S17_extracted_k141_158439	1314	No	NA	0	0	Not-determined	Genome-fragment	NA		0
S17_extracted_k141_128444	1122	No	NA	0	0	Not-determined	Genome-fragment	NA		0
S14_vs_k141_44112||full	1016	No	NA	0	0	Low-quality	Genome-fragment		1.46	0
S17_extracted_k141_121302	7557	No	NA	0	1	Not-determined	Genome-fragment	NA		0
S14_vs_k141_372||full	1016	No	NA	1	0	Low-quality	Genome-fragment		0.54	0
S17_extracted_k141_55010	4476	No	NA	0	4	Not-determined	Genome-fragment	NA		0
S14_vs_k141_17749||full	1016	No	NA	0	2	Low-quality	Genome-fragment		1.68	0
S17_extracted_k141_69794	2184	No	NA	0	2	Not-determined	Genome-fragment	NA		0
S17_extracted_k141_69801	1458	No	NA	0	2	Not-determined	Genome-fragment	NA		0
S14_vs_k141_46140||lt2gene	1016	No	NA	1	0	Low-quality	Genome-fragment		2.51	0
S17_extracted_k141_77057	15807	No	NA	7	0	Medium-quality	Genome-fragment		52.24	0
S17_extracted_k141_32939	1427	No	NA	0	0	Not-determined	Genome-fragment	NA		0
S17_extracted_k141_135978	3792	No	NA	0	2	Not-determined	Genome-fragment	NA		0
S3_extracted_k141_57908	1016	No	NA	0	0	Low-quality	Genome-fragment		1.7	0
S17_extracted_k141_128487	2506	No	NA	0	2	Not-determined	Genome-fragment	NA		0
S3_extracted_k141_55518	1016	No	NA	0	0	Low-quality	Genome-fragment		2.15	0
S5_extracted_k141_70065	1016	No	NA	0	0	Low-quality	Genome-fragment		1.98	0
S17_extracted_k141_107260	6587	No	NA	0	3	Not-determined	Genome-fragment	NA		0
S17_extracted_k141_165659	1064	No	NA	0	1	Not-determined	Genome-fragment	NA		0
S17_extracted_k141_47869	1987	No	NA	0	1	Not-determined	Genome-fragment	NA		0
S5_vs_k141_103797||full	1016	No	NA	0	0	Low-quality	Genome-fragment		1.81	0
S17_extracted_k141_47870	1338	No	NA	0	2	Not-determined	Genome-fragment	NA		0
S17_extracted_k141_158539	3191	No	NA	0	0	Not-determined	Genome-fragment	NA		0
S17_extracted_k141_10738	1024	No	NA	0	0	Not-determined	Genome-fragment	NA		0
S5_vs_k141_134553||full	1016	No	NA	1	0	Low-quality	Genome-fragment		2.31	0
S17_extracted_k141_17718	1135	No	NA	0	0	Not-determined	Genome-fragment	NA		0
S17_extracted_k141_55069	1018	No	NA	0	1	Not-determined	Genome-fragment	NA		0
S17_extracted_k141_32987	1249	No	NA	0	2	Not-determined	Genome-fragment	NA		0
S17_extracted_k141_17727	1594	No	NA	0	2	Not-determined	Genome-fragment	NA		0
S17_extracted_k141_33001	3096	No	NA	0	1	Not-determined	Genome-fragment	NA		0
S17_extracted_k141_10757	1285	No	NA	0	1	Not-determined	Genome-fragment	NA		0
S17_extracted_k141_121380	3699	No	NA	0	3	Not-determined	Genome-fragment	NA		0
S5_vs_k141_77059||full	1016	No	NA	0	1	Low-quality	Genome-fragment		0.46	0
S17_extracted_k141_3164	1759	No	NA	0	0	Not-determined	Genome-fragment	NA		0
S17_extracted_k141_128556	1321	No	NA	0	0	Not-determined	Genome-fragment	NA		0
S17_extracted_k141_128558	1726	No	NA	0	0	Not-determined	Genome-fragment	NA		0
S7_extracted_29856	1016	No	NA	0	2	Low-quality	Genome-fragment		2.2	0
S17_extracted_k141_172914	1607	No	NA	0	1	Not-determined	Genome-fragment	NA		0
S17_extracted_k141_143895	2396	No	NA	0	1	Not-determined	Genome-fragment	NA		0
S7_extracted_64257	1016	No	NA	0	0	Low-quality	Genome-fragment		4.61	0
S17_extracted_k141_10794	1241	No	NA	0	0	Not-determined	Genome-fragment	NA		0
S17_extracted_k141_136061	1249	No	NA	0	1	Not-determined	Genome-fragment	NA		0
S7_extracted_88438	1016	No	NA	0	0	Low-quality	Genome-fragment		0.68	0
S17_extracted_k141_143923	1216	No	NA	0	0	Not-determined	Genome-fragment	NA		0
S7_vs_32497||lt2gene	1016	No	NA	1	0	Low-quality	Genome-fragment		2.37	0
S16_vs_122382||full	1016	No	NA	0	1	Low-quality	Genome-fragment		1.58	0
S17_extracted_k141_69937	1106	No	NA	0	1	Not-determined	Genome-fragment	NA		0
S17_extracted_k141_113927	2719	No	NA	0	1	Not-determined	Genome-fragment	NA		0
S16_vs_160899||full	1016	No	NA	0	0	Low-quality	Genome-fragment		0.61	0
S16_vs_93496||lt2gene	1016	No	NA	2	0	Low-quality	Genome-fragment		0.54	0
S17_extracted_k141_25170	1037	No	NA	0	0	Not-determined	Genome-fragment	NA		0
S17_extracted_k141_121476	3880	No	NA	0	3	Not-determined	Genome-fragment	NA		0
S17_extracted_k141_40811	3801	No	NA	0	2	Not-determined	Genome-fragment	NA		0
S17_extracted_k141_33135	2388	No	NA	0	3	Not-determined	Genome-fragment	NA		0
S17_extracted_k141_91849	3376	No	NA	0	2	Not-determined	Genome-fragment	NA		0

S17_extracted_k141_77220	1578	No	NA	0	1	Not-determined	Genome-fragment	NA		0
S17_extracted_k141_33161	3477	No	NA	0	1	Not-determined	Genome-fragment	NA		0
S17_extracted_k141_48012	1020	No	NA	0	1	Not-determined	Genome-fragment	NA		0
S17_extracted_k141_113987	5044	No	NA	0	2	Not-determined	Genome-fragment	NA		0
S17_extracted_k141_165831	2474	No	NA	0	2	Not-determined	Genome-fragment	NA		0
S17_extracted_k141_151557	1198	No	NA	0	2	Not-determined	Genome-fragment	NA		0
S17_extracted_k141_128640	2274	No	NA	0	0	Not-determined	Genome-fragment	NA		0
S17_extracted_k141_55267	1105	No	NA	0	1	Not-determined	Genome-fragment	NA		0
S17_extracted_k141_121530	1345	No	NA	0	0	Not-determined	Genome-fragment	NA		0
S17_extracted_k141_158699	3250	No	NA	0	2	Not-determined	Genome-fragment	NA		0
S17_extracted_k141_77273	1049	No	NA	0	1	Not-determined	Genome-fragment	NA		0
S10_vs_25912||full	1016	No	NA	0	0	Low-quality	Genome-fragment		2.08	0
S17_extracted_k141_136204	1779	No	NA	0	2	Not-determined	Genome-fragment	NA		0
S17_extracted_k141_70028	1043	No	NA	0	0	Not-determined	Genome-fragment	NA		0
S17_extracted_k141_33217	1928	No	NA	0	0	Not-determined	Genome-fragment	NA		0
S17_extracted_k141_3375	1432	No	NA	0	1	Not-determined	Genome-fragment	NA		0
S17_extracted_k141_121563	3364	No	NA	0	3	Not-determined	Genome-fragment	NA		0
S17_extracted_k141_10893	1486	No	NA	0	1	Not-determined	Genome-fragment	NA		0
S17_extracted_k141_3401	1451	No	NA	0	1	Not-determined	Genome-fragment	NA		0
S17_extracted_k141_70076	1142	No	NA	0	1	Not-determined	Genome-fragment	NA		0
S12_vs_k141_173318||full	1016	No	NA	0	0	Low-quality	Genome-fragment		1.05	0
S17_extracted_k141_40918	5283	No	NA	0	1	Not-determined	Genome-fragment	NA		0
S17_extracted_k141_33288	1062	No	NA	0	2	Not-determined	Genome-fragment	NA		0
S17_extracted_k141_77323	1808	No	NA	0	0	Not-determined	Genome-fragment	NA		0
S2_vs_k141_11559||full	1016	No	NA	0	0	Low-quality	Genome-fragment		1.06	0
S17_extracted_k141_77328	1997	No	NA	0	0	Not-determined	Genome-fragment	NA		0
S17_extracted_k141_173117	1128	No	NA	0	0	Not-determined	Genome-fragment	NA		0
S17_extracted_k141_173126	1278	No	NA	0	0	Not-determined	Genome-fragment	NA		0
S17_extracted_k141_107508	1689	No	NA	0	2	Not-determined	Genome-fragment	NA		0
S17_extracted_k141_151625	1600	No	NA	0	0	Not-determined	Genome-fragment	NA		0
S17_extracted_k141_70139	1571	No	NA	0	1	Not-determined	Genome-fragment	NA		0
S4_extracted_2119	1016	No	NA	3	0	Low-quality	Genome-fragment		3.34	0
S17_extracted_k141_84003	1570	No	NA	0	1	Not-determined	Genome-fragment	NA		0
S17_extracted_k141_55390	1780	No	NA	0	1	Not-determined	Genome-fragment	NA		0
S4_extracted_6477	1016	No	NA	0	1	Low-quality	Genome-fragment		0.42	0
S17_extracted_k141_173166	1176	No	NA	0	0	Not-determined	Genome-fragment	NA		0
S17_extracted_k141_25328	3020	No	NA	0	2	Not-determined	Genome-fragment	NA		0
S17_extracted_k141_55396	16910	No	NA	0	4	Not-determined	Genome-fragment	NA		0
S17_extracted_k141_158886	1008	No	NA	0	1	Not-determined	Genome-fragment	NA		0
S17_extracted_k141_40991	2237	No	NA	0	2	Not-determined	Genome-fragment	NA		0
S17_extracted_k141_165929	1379	No	NA	0	1	Not-determined	Genome-fragment	NA		0
S17_extracted_k141_62315	2959	No	NA	0	7	Not-determined	Genome-fragment	NA		0
S17_extracted_k141_165933	4553	No	NA	0	3	Not-determined	Genome-fragment	NA		0
S17_extracted_k141_3523	1523	No	NA	0	0	Not-determined	Genome-fragment	NA		0
S17_extracted_k141_33368	1125	No	NA	0	0	Not-determined	Genome-fragment	NA		0
S17_extracted_k141_99845	1597	No	NA	0	1	Not-determined	Genome-fragment	NA		0
S17_extracted_k141_33381	1163	No	NA	0	1	Not-determined	Genome-fragment	NA		0
S4_extracted_19463	1016	No	NA	0	0	Low-quality	Genome-fragment		2.38	0
S17_extracted_k141_144117	1177	No	NA	0	0	Not-determined	Genome-fragment	NA		0
S17_extracted_k141_70226	1189	No	NA	0	1	Not-determined	Genome-fragment	NA		0
S17_extracted_k141_84070	3578	No	NA	0	1	Not-determined	Genome-fragment	NA		0
S17_extracted_k141_165954	1653	No	NA	0	1	Not-determined	Genome-fragment	NA		0
S6_extracted_k141_8874	1016	No	NA	1	0	Low-quality	Genome-fragment		1.72	0
S8_extracted_k141_132247	1016	No	NA	0	1	Low-quality	Genome-fragment		1.64	0
S17_extracted_k141_33391	1326	No	NA	0	1	Not-determined	Genome-fragment	NA		0
S17_extracted_k141_25400	4240	No	NA	0	3	Not-determined	Genome-fragment	NA		0
S17_extracted_k141_107589	2012	No	NA	0	1	Not-determined	Genome-fragment	NA		0
S17_extracted_k141_128878	10585	No	NA	0	4	Not-determined	Genome-fragment	NA		0
S17_extracted_k141_48217	2437	No	NA	0	2	Not-determined	Genome-fragment	NA		0
S8_vs_k141_142926||full	1016	No	NA	0	0	Low-quality	Genome-fragment		1.61	0
S17_extracted_k141_84121	1087	No	NA	0	1	Not-determined	Genome-fragment	NA		0
S17_extracted_k141_84134	2528	No	NA	0	3	Not-determined	Genome-fragment	NA		0
S17_extracted_k141_70293	1099	No	NA	0	0	Not-determined	Genome-fragment	NA		0
S17_extracted_k141_121683	1161	No	NA	0	3	Not-determined	Genome-fragment	NA		0
S17_extracted_k141_152383	1017	No	NA	0	0	Low-quality	Genome-fragment		0.77	0
S17_extracted_k141_18135	1064	No	NA	0	0	Not-determined	Genome-fragment	NA		0
S17_extracted_k141_33437	2938	No	NA	0	2	Not-determined	Genome-fragment	NA		0
S17_extracted_k141_25455	1264	No	NA	0	0	Not-determined	Genome-fragment	NA		0
S17_extracted_k141_128926	3925	No	NA	0	1	Not-determined	Genome-fragment	NA		0
S17_extracted_k141_173334	1384	No	NA	0	2	Not-determined	Genome-fragment	NA		0
S17_extracted_k141_70310	1055	No	NA	0	0	Not-determined	Genome-fragment	NA		0
S13_vs_146034||full	1017	No	NA	0	0	Low-quality	Genome-fragment		2.57	0
S17_extracted_k141_77525	1604	No	NA	0	3	Not-determined	Genome-fragment	NA		0
S17_extracted_k141_77533	1114	No	NA	0	1	Not-determined	Genome-fragment	NA		0
S14_extracted_k141_78717	1017	No	NA	1	0	Low-quality	Genome-fragment		3.28	0
S17_extracted_k141_114242	1137	No	NA	0	1	Not-determined	Genome-fragment	NA		0
S17_extracted_k141_33520	1424	No	NA	0	1	Not-determined	Genome-fragment	NA		0
S14_vs_k141_110747||lt2gene	1017	No	NA	2	0	Low-quality	Genome-fragment		3.61	0
S17_extracted_k141_173369	1307	No	NA	0	0	Not-determined	Genome-fragment	NA		0
S3_extracted_k141_61548	1017	No	NA	0	0	Low-quality	Genome-fragment		0.16	0
S3_vs_k141_55024||full	1017	No	NA	0	0	Low-quality	Genome-fragment		1.94	0
S7_extracted_5818	1017	No	NA	1	0	Low-quality	Genome-fragment		2.16	0
S7_extracted_30681	1017	No	NA	1	0	Low-quality	Genome-fragment		3.69	0
S17_extracted_k141_70372	1413	No	NA	0	2	Not-determined	Genome-fragment	NA		0
S17_extracted_k141_129014	1414	No	NA	0	0	Not-determined	Genome-fragment	NA		0
S17_extracted_k141_151843	5026	No	NA	0	1	Not-determined	Genome-fragment	NA		0
S17_extracted_k141_121747	3509	No	NA	0	2	Not-determined	Genome-fragment	NA		0
S17_extracted_k141_92201	1297	No	NA	0	0	Not-determined	Genome-fragment	NA		0
S17_extracted_k141_159105	1102	No	NA	0	1	Not-determined	Genome-fragment	NA		0
S7_vs_114805||full	1017	No	NA	0	0	Low-quality	Genome-fragment		1.63	0
S17_extracted_k141_70392	2292	No	NA	0	2	Not-determined	Genome-fragment	NA		0
S17_extracted_k141_33592	3767	No	NA	0	2	Not-determined	Genome-fragment	NA		0
S17_extracted_k141_92217	1387	No	NA	0	1	Not-determined	Genome-fragment	NA		0
S16_extracted_129288	1017	No	NA	0	0	Low-quality	Genome-fragment		0.62	0
S17_extracted_k141_3670	3561	No	NA	0	1	Not-determined	Genome-fragment	NA		0
S16_extracted_160631	1017	No	NA	0	1	Low-quality	Genome-fragment		0.57	0
S17_extracted_k141_151890	2813	No	NA	0	1	Not-determined	Genome-fragment	NA		0
S16_vs_132201||full	1017	No	NA	1	0	Low-quality	Genome-fragment		2.48	0
S17_extracted_k141_144308	1249	No	NA	0	0	Not-determined	Genome-fragment	NA		0
S12_extracted_k141_110294	1017	No	NA	0	1	Low-quality	Genome-fragment		1.13	0
S17_extracted_k141_159139	2725	No	NA	0	2	Not-determined	Genome-fragment	NA		0
S17_extracted_k141_151900	1403	No	NA	0	2	Not-determined	Genome-fragment	NA		0
S17_extracted_k141_70425	3577	No	NA	0	2	Not-determined	Genome-fragment	NA		0
S17_extracted_k141_144326	2585	No	NA	0	1	Not-determined	Genome-fragment	NA		0
S17_extracted_k141_55617	3882	No	NA	0	1	Not-determined	Genome-fragment	NA		0
S17_extracted_k141_33629	3467	No	NA	0	3	Not-determined	Genome-fragment	NA		0
S8_vs_k141_174691||full	1017	No	NA	0	0	Low-quality	Genome-fragment		1.69	0
S17_extracted_k141_159152	3688	No	NA	0	2	Not-determined	Genome-fragment	NA		0
S17_extracted_k141_121777	7909	No	NA	0	3	Not-determined	Genome-fragment	NA		0
S17_extracted_k141_18303	2121	No	NA	0	1	Not-determined	Genome-fragment	NA		0
S17_extracted_k141_55628	1373	No	NA	0	1	Not-determined	Genome-fragment	NA		0
S17_extracted_k141_41187	1815	No	NA	0	2	Not-determined	Genome-fragment	NA		0
S17_vs_k141_8319||full	1018	No	NA	0	1	Low-quality	Genome-fragment		0.64	0
S17_extracted_k141_151919	5444	No	NA	0	1	Not-determined	Genome-fragment	NA		0
S17_extracted_k141_159155	1775	No	NA	0	1	Not-determined	Genome-fragment	NA		0
S17_extracted_k141_70499	1300	No	NA	0	1	Not-determined	Genome-fragment	NA		0
S17_extracted_k141_55675	1477	No	NA	0	1	Not-determined	Genome-fragment	NA		0
S17_extracted_k141_77679	4591	No	NA	0	3	Not-determined	Genome-fragment	NA		0
S17_extracted_k141_41246	1024	No	NA	0	1	Not-determined	Genome-fragment	NA		0
S17_extracted_k141_151972	1597	No	NA	0	2	Not-determined	Genome-fragment	NA		0
S17_extracted_k141_41255	1256	No	NA	0	1	Not-determined	Genome-fragment	NA		0
S17_extracted_k141_151974	3863	No	NA	0	0	Not-determined	Genome-fragment	NA		0
S17_extracted_k141_166185	2188	No	NA	0	0	Not-determined	Genome-fragment	NA		0
S9_vs_k141_17230||full	1018	No	NA	0	1	Low-quality	Genome-fragment		1.79	0
S9_vs_k141_42901||lt2gene	1018	No	NA	1	0	Low-quality	Genome-fragment		3.05	0
S17_extracted_k141_114499	1191	No	NA	0	1	Not-determined	Genome-fragment	NA		0
S17_extracted_k141_18388	1764	No	NA	0	3	Not-determined	Genome-fragment	NA		0
S13_vs_94563||full	1018	No	NA	1	0	Low-quality	Genome-fragment		1.75	0
S17_extracted_k141_70504	4613	No	NA	0	3	Not-determined	Genome-fragment	NA		0
S17_extracted_k141_41272	1562	No	NA	0	1	Not-determined	Genome-fragment	NA		0
S14_extracted_k141_22997	1018	No	NA	0	1	Low-quality	Genome-fragment		0.5	0
S17_extracted_k141_159180	1500	No	NA	0	1	Not-determined	Genome-fragment	NA		0
S17_extracted_k141_121844	1196	No	NA	0	0	Not-determined	Genome-fragment	NA		0
S17_extracted_k141_100049	6778	No	NA	0	3	Not-determined	Genome-fragment	NA		0
S17_extracted_k141_114505	16352	No	NA	0	6	Not-determined	Genome-fragment	NA		0
S17_extracted_k141_18413	1010	No	NA	0	0	Not-determined	Genome-fragment	NA		0
S17_extracted_k141_55722	1582	No	NA	0	2	Not-determined	Genome-fragment	NA		0
S17_extracted_k141_114515	1429	No	NA	0	1	Not-determined	Genome-fragment	NA		0
S17_extracted_k141_84286	3873	No	NA	0	5	Not-determined	Genome-fragment	NA		0
S17_extracted_k141_84294	1664	No	NA	0	0	Not-determined	Genome-fragment	NA		0
S14_vs_k141_55241||lt2gene	1018	No	NA	1	0	Low-quality	Genome-fragment		3.35	0
S17_extracted_k141_152017	2283	No	NA	0	2	Not-determined	Genome-fragment	NA		0
S1_vs_k141_56108||full	1018	No	NA	1	0	Low-quality	Genome-fragment		1.35	0
S17_extracted_k141_84311	1334	No	NA	0	1	Not-determined	Genome-fragment	NA		0
S17_extracted_k141_33780	1803	No	NA	0	1	Not-determined	Genome-fragment	NA		0
S17_extracted_k141_18479	1076	No	NA	0	1	Not-determined	Genome-fragment	NA		0
S17_extracted_k141_114570	1003	No	NA	0	1	Not-determined	Genome-fragment	NA		0
S17_extracted_k141_100097	7039	No	NA	0	2	Not-determined	Genome-fragment	NA		0
S3_vs_k141_50542||full	1018	No	NA	0	0	Low-quality	Genome-fragment		2.28	0
S17_extracted_k141_55784	3466	No	NA	0	7	Not-determined	Genome-fragment	NA		0
S17_extracted_k141_84332	3408	No	NA	0	1	Not-determined	Genome-fragment	NA		0
S17_extracted_k141_84337	1633	No	NA	0	2	Not-determined	Genome-fragment	NA		0
S17_extracted_k141_18495	1079	No	NA	0	1	Not-determined	Genome-fragment	NA		0
S7_vs_133144||full	1018	No	NA	0	0	Low-quality	Genome-fragment		1.96	0
S17_extracted_k141_33825	1127	No	NA	0	1	Not-determined	Genome-fragment	NA		0
S17_extracted_k141_173569	3479	No	NA	0	0	Not-determined	Genome-fragment	NA		0
S17_extracted_k141_55803	3557	No	NA	0	1	Not-determined	Genome-fragment	NA		0
S7_vs_30888||lt2gene	1018	No	NA	1	0	Low-quality	Genome-fragment		1.93	0
S17_extracted_k141_33845	1100	No	NA	0	1	Not-determined	Genome-fragment	NA		0
S16_extracted_159849	1018	No	NA	0	1	Low-quality	Genome-fragment		0.6	0
S17_extracted_k141_92331	1009	No	NA	0	1	Not-determined	Genome-fragment	NA		0
S17_extracted_k141_25577	1009	No	NA	0	0	Not-determined	Genome-fragment	NA		0
S17_extracted_k141_136622	1124	No	NA	0	2	Not-determined	Genome-fragment	NA		0
S17_extracted_k141_70605	1675	No	NA	0	2	Not-determined	Genome-fragment	NA		0
S10_vs_93951||full	1018	No	NA	0	1	Low-quality	Genome-fragment		1.82	0
S6_extracted_k141_37264	1018	No	NA	0	0	Low-quality	Genome-fragment		1.66	0
S17_extracted_k141_3956	1714	No	NA	0	3	Not-determined	Genome-fragment	NA		0
S17_extracted_k141_33860	7715	No	NA	0	1	Not-determined	Genome-fragment	NA		0
S17_extracted_k141_136632	2614	No	NA	0	2	Not-determined	Genome-fragment	NA		0
S8_vs_k141_70241||full	1018	No	NA	0	0	Low-quality	Genome-fragment		2.28	0
S17_extracted_k141_41360	1620	No	NA	0	2	Not-determined	Genome-fragment	NA		0
S17_extracted_k141_77883	1465	No	NA	0	2	Not-determined	Genome-fragment	NA		0
S17_extracted_k141_100206	1398	No	NA	0	0	Not-determined	Genome-fragment	NA		0
S17_extracted_k141_121975	1898	No	NA	0	1	Not-determined	Genome-fragment	NA		0

S17_extracted_k141_129375	3356	No	NA	0	1	Not-determined	Genome-fragment	NA		0
S17_extracted_k141_84413	1805	No	NA	0	1	Not-determined	Genome-fragment	NA		0
S17_extracted_k141_70616	2902	No	NA	0	2	Not-determined	Genome-fragment	NA		0
S8_vs_k141_62050||full	1018	No	NA	0	0	Low-quality	Genome-fragment		0.65	0
S17_extracted_k141_70626	1014	No	NA	0	0	Not-determined	Genome-fragment	NA		0
S17_extracted_k141_11341	2920	No	NA	0	0	Not-determined	Genome-fragment	NA		0
S17_extracted_k141_121990	3078	No	NA	0	3	Not-determined	Genome-fragment	NA		0
S17_extracted_k141_121993	1674	No	NA	0	0	Not-determined	Genome-fragment	NA		0
S17_extracted_k141_107974	1214	No	NA	0	1	Not-determined	Genome-fragment	NA		0
S17_vs_k141_15486||full	1019	No	NA	0	0	Low-quality	Genome-fragment		2.1	0
S17_extracted_k141_70670	3458	No	NA	0	3	Not-determined	Genome-fragment	NA		0
S17_extracted_k141_108003	1027	No	NA	0	1	Not-determined	Genome-fragment	NA		0
S17_extracted_k141_41478	1185	No	NA	0	1	Not-determined	Genome-fragment	NA		0
S17_extracted_k141_114748	2158	No	NA	0	0	Not-determined	Genome-fragment	NA		0
S17_extracted_k141_122046	1397	No	NA	0	0	Not-determined	Genome-fragment	NA		0
S17_extracted_k141_129448	2705	No	NA	0	2	Not-determined	Genome-fragment	NA		0
S17_extracted_k141_48660	1613	No	NA	0	2	Not-determined	Genome-fragment	NA		0
S9_extracted_k141_16829	1019	No	NA	1	0	Low-quality	Genome-fragment		2.3	0
S17_extracted_k141_84464	1017	No	NA	0	1	Not-determined	Genome-fragment	NA		0
S17_extracted_k141_108045	4890	No	NA	0	3	Not-determined	Genome-fragment	NA		0
S17_extracted_k141_166462	1002	No	NA	0	2	Not-determined	Genome-fragment	NA		0
S17_extracted_k141_33976	1508	No	NA	0	1	Not-determined	Genome-fragment	NA		0
S17_extracted_k141_152240	1699	No	NA	0	0	Not-determined	Genome-fragment	NA		0
S17_extracted_k141_159350	1825	No	NA	0	0	Not-determined	Genome-fragment	NA		0
S9_extracted_k141_62960	1019	No	NA	0	0	Low-quality	Genome-fragment		1.8	0
S17_extracted_k141_84494	5641	No	NA	0	2	Not-determined	Genome-fragment	NA		0
S17_extracted_k141_48696	5706	No	NA	0	1	Not-determined	Genome-fragment	NA		0
S9_vs_k141_60422||full	1019	No	NA	0	0	Low-quality	Genome-fragment		0.48	0
S11_vs_k141_3274||full	1019	No	NA	0	0	Low-quality	Genome-fragment		1.71	0
S17_extracted_k141_78031	1090	No	NA	0	0	Not-determined	Genome-fragment	NA		0
S17_extracted_k141_41565	8027	No	NA	0	4	Not-determined	Genome-fragment	NA		0
S17_extracted_k141_55981	1136	No	NA	0	0	Not-determined	Genome-fragment	NA		0
S17_extracted_k141_78033	1408	No	NA	0	1	Not-determined	Genome-fragment	NA		0
S17_extracted_k141_48744	1153	No	NA	0	0	Not-determined	Genome-fragment	NA		0
S11_vs_k141_65707||lt2gene	1019	No	NA	0	0	Low-quality	Genome-fragment		2.1	0
S17_extracted_k141_92577	1241	No	NA	0	1	Not-determined	Genome-fragment	NA		0
S13_vs_92484||full	1019	No	NA	0	2	Low-quality	Genome-fragment		0.74	0
S17_extracted_k141_11431	1077	No	NA	0	0	Not-determined	Genome-fragment	NA		0
S14_extracted_k141_25193	1019	No	NA	1	0	Low-quality	Genome-fragment		2.64	0
S14_vs_k141_31919||full	1019	No	NA	1	0	Low-quality	Genome-fragment		0.63	0
S17_extracted_k141_18702	2776	No	NA	0	0	Not-determined	Genome-fragment	NA		0
S17_extracted_k141_56062	1137	No	NA	0	1	Not-determined	Genome-fragment	NA		0
S1_vs_k141_93429||full	1019	No	NA	1	0	Low-quality	Genome-fragment		2.05	0
S17_extracted_k141_114940	1190	No	NA	0	1	Not-determined	Genome-fragment	NA		0
S17_extracted_k141_108191	4006	No	NA	0	3	Not-determined	Genome-fragment	NA		0
S17_extracted_k141_25818	2345	No	NA	0	0	Not-determined	Genome-fragment	NA		0
S17_extracted_k141_152385	4905	No	NA	0	0	Not-determined	Genome-fragment	NA		0
S17_extracted_k141_108205	1197	No	NA	0	0	Not-determined	Genome-fragment	NA		0
S17_extracted_k141_11455	1066	No	NA	0	0	Not-determined	Genome-fragment	NA		0
S17_extracted_k141_41661	1053	No	NA	0	0	Not-determined	Genome-fragment	NA		0
S17_extracted_k141_129614	1176	No	NA	0	1	Not-determined	Genome-fragment	NA		0
S3_extracted_k141_96842	1019	No	NA	1	0	Low-quality	Genome-fragment		2.39	0
S17_extracted_k141_136915	1843	No	NA	0	0	Not-determined	Genome-fragment	NA		0
S17_extracted_k141_78175	1066	No	NA	0	0	Not-determined	Genome-fragment	NA		0
S17_extracted_k141_84590	4227	No	NA	0	2	Not-determined	Genome-fragment	NA		0
S17_extracted_k141_100418	7107	No	NA	0	2	Not-determined	Genome-fragment	NA		0
S17_extracted_k141_84594	2347	No	NA	0	0	Not-determined	Genome-fragment	NA		0
S3_vs_k141_97574||full	1019	No	NA	1	0	Low-quality	Genome-fragment		1.89	0
S17_extracted_k141_56109	1564	No	NA	0	1	Not-determined	Genome-fragment	NA		0
S17_extracted_k141_166613	3350	No	NA	0	2	Not-determined	Genome-fragment	NA		0
S17_extracted_k141_56119	3163	No	NA	0	3	Not-determined	Genome-fragment	NA		0
S17_extracted_k141_100447	4151	No	NA	0	2	Not-determined	Genome-fragment	NA		0
S17_extracted_k141_11506	1517	No	NA	0	1	Not-determined	Genome-fragment	NA		0
S5_vs_k141_136639||full	1019	No	NA	0	0	Low-quality	Genome-fragment		1.48	0
S17_extracted_k141_92738	1217	No	NA	0	2	Not-determined	Genome-fragment	NA		0
S17_extracted_k141_115036	5012	No	NA	0	2	Not-determined	Genome-fragment	NA		0
S17_extracted_k141_136954	3960	No	NA	0	3	Not-determined	Genome-fragment	NA		0
S17_extracted_k141_159511	5767	No	NA	0	1	Not-determined	Genome-fragment	NA		0
S17_extracted_k141_11567	1008	No	NA	0	1	Not-determined	Genome-fragment	NA		0
S17_extracted_k141_34173	3326	No	NA	0	2	Not-determined	Genome-fragment	NA		0
S5_vs_k141_65532||full	1019	No	NA	0	2	Low-quality	Genome-fragment		0.77	0
S17_extracted_k141_4110	1290	No	NA	0	1	Not-determined	Genome-fragment	NA		0
S17_extracted_k141_136971	1659	No	NA	0	1	Not-determined	Genome-fragment	NA		0
S17_extracted_k141_115062	1140	No	NA	0	3	Not-determined	Genome-fragment	NA		0
S17_extracted_k141_173835	1146	No	NA	0	1	Not-determined	Genome-fragment	NA		0
S7_extracted_106978	1019	No	NA	1	0	Low-quality	Genome-fragment		4.65	0
S7_extracted_121645	1019	No	NA	0	0	Low-quality	Genome-fragment		2.34	0
S17_extracted_k141_166693	4304	No	NA	0	0	Not-determined	Genome-fragment	NA		0
S17_extracted_k141_78293	4700	No	NA	0	3	Not-determined	Genome-fragment	NA		0
S17_extracted_k141_129722	1301	No	NA	0	0	Not-determined	Genome-fragment	NA		0
S17_extracted_k141_122195	1664	No	NA	0	0	Not-determined	Genome-fragment	NA		0
S17_extracted_k141_173861	1914	No	NA	0	3	Not-determined	Genome-fragment	NA		0
S17_extracted_k141_137026	1078	No	NA	0	1	Not-determined	Genome-fragment	NA		0
S17_extracted_k141_84714	1308	No	NA	0	0	Not-determined	Genome-fragment	NA		0
S7_vs_98038||full	1019	No	NA	0	0	Low-quality	Genome-fragment		1.84	0
S17_extracted_k141_159570	3278	No	NA	0	1	Not-determined	Genome-fragment	NA		0
S17_extracted_k141_108432	1642	No	NA	0	1	Not-determined	Genome-fragment	NA		0
S17_extracted_k141_108435	1050	No	NA	0	1	Not-determined	Genome-fragment	NA		0
S17_extracted_k141_159583	1645	No	NA	0	1	Not-determined	Genome-fragment	NA		0
S7_vs_121638||full	1019	No	NA	1	0	Low-quality	Genome-fragment		1.72	0
S17_extracted_k141_62564	1472	No	NA	0	1	Not-determined	Genome-fragment	NA		0
S16_vs_1853||full	1019	No	NA	0	0	Low-quality	Genome-fragment		2.83	0
S17_extracted_k141_159598	1222	No	NA	0	0	Not-determined	Genome-fragment	NA		0
S17_extracted_k141_152617	2026	No	NA	0	3	Not-determined	Genome-fragment	NA		0
S17_extracted_k141_122260	1226	No	NA	0	1	Not-determined	Genome-fragment	NA		0
S17_extracted_k141_144763	6846	No	NA	0	1	Not-determined	Genome-fragment	NA		0
S16_vs_63209||full	1019	No	NA	0	0	Low-quality	Genome-fragment		2.25	0
S17_extracted_k141_144770	1203	No	NA	0	1	Not-determined	Genome-fragment	NA		0
S17_extracted_k141_108480	5645	No	NA	0	3	Not-determined	Genome-fragment	NA		0
S17_extracted_k141_56341	1041	No	NA	0	0	Not-determined	Genome-fragment	NA		0
S16_vs_88157||full	1019	No	NA	1	0	Low-quality	Genome-fragment		2.71	0
S17_extracted_k141_173941	1861	No	NA	0	2	Not-determined	Genome-fragment	NA		0
S17_extracted_k141_159635	1181	No	NA	0	1	Not-determined	Genome-fragment	NA		0
S16_vs_130520||lt2gene	1019	No	NA	2	0	Low-quality	Genome-fragment		2.94	0
S17_extracted_k141_129831	1222	No	NA	0	0	Not-determined	Genome-fragment	NA		0
S17_extracted_k141_144787	4528	No	NA	0	0	Not-determined	Genome-fragment	NA		0
S17_extracted_k141_84809	11189	No	NA	0	7	Not-determined	Genome-fragment	NA		0
S17_extracted_k141_41892	1123	No	NA	0	1	Not-determined	Genome-fragment	NA		0
S17_extracted_k141_166828	1906	No	NA	0	2	Not-determined	Genome-fragment	NA		0
S17_extracted_k141_100641	1167	No	NA	0	0	Not-determined	Genome-fragment	NA		0
S10_vs_89019||lt2gene	1019	No	NA	1	0	Low-quality	Genome-fragment		2.45	0
S17_extracted_k141_129875	1037	No	NA	0	0	Not-determined	Genome-fragment	NA		0
S17_extracted_k141_26098	2415	No	NA	0	0	Not-determined	Genome-fragment	NA		0
S17_extracted_k141_174012	1002	No	NA	0	0	Not-determined	Genome-fragment	NA		0
S17_extracted_k141_92883	4649	No	NA	0	4	Not-determined	Genome-fragment	NA		0
S17_extracted_k141_100683	1081	No	NA	0	0	Not-determined	Genome-fragment	NA		0
S17_extracted_k141_49151	1283	No	NA	0	1	Not-determined	Genome-fragment	NA		0
S12_vs_k141_79674||full	1019	No	NA	1	0	Low-quality	Genome-fragment		0.55	0
S17_extracted_k141_49168	1315	No	NA	0	1	Not-determined	Genome-fragment	NA		0
S17_extracted_k141_41950	1423	No	NA	0	2	Not-determined	Genome-fragment	NA		0
S17_extracted_k141_92916	1118	No	NA	0	2	Not-determined	Genome-fragment	NA		0
S17_extracted_k141_92921	1845	No	NA	0	1	Not-determined	Genome-fragment	NA		0
S17_extracted_k141_144851	1082	No	NA	0	0	Not-determined	Genome-fragment	NA		0
S17_extracted_k141_92929	1516	No	NA	0	1	Not-determined	Genome-fragment	NA		0
S17_extracted_k141_78454	1673	No	NA	0	0	Not-determined	Genome-fragment	NA		0
S17_extracted_k141_102405	1020	No	NA	0	0	Low-quality	Genome-fragment		2.85	0
S17_extracted_k141_137198	3205	No	NA	0	2	Not-determined	Genome-fragment	NA		0
S17_extracted_k141_36283	1020	No	NA	1	0	Low-quality	Genome-fragment		0.99	0
S17_extracted_k141_78468	6906	No	NA	0	0	Not-determined	Genome-fragment	NA		0
S11_extracted_k141_57199	1020	No	NA	0	0	Low-quality	Genome-fragment		1.41	0
S17_extracted_k141_62763	1014	No	NA	0	2	Not-determined	Genome-fragment	NA		0
S17_extracted_k141_4347	10420	No	NA	0	8	Not-determined	Genome-fragment	NA		0
S11_vs_k141_92472||full	1020	No	NA	0	0	Low-quality	Genome-fragment		2.43	0
S17_extracted_k141_84922	1028	No	NA	0	1	Not-determined	Genome-fragment	NA		0
S1_vs_k141_147361||full	1020	No	NA	1	0	Low-quality	Genome-fragment		2.64	0
S5_extracted_k141_105375	1020	No	NA	0	0	Low-quality	Genome-fragment		0.92	0
S17_extracted_k141_84939	1083	No	NA	0	1	Not-determined	Genome-fragment	NA		0
S17_extracted_k141_174033	64565	No	NA	16	1	Medium-quality	Genome-fragment		59.33	0
S17_extracted_k141_84947	1227	No	NA	0	0	Not-determined	Genome-fragment	NA		0
S17_extracted_k141_115377	1087	No	NA	0	1	Not-determined	Genome-fragment	NA		0
S17_extracted_k141_42054	2528	No	NA	0	2	Not-determined	Genome-fragment	NA		0
S17_extracted_k141_84962	1055	No	NA	0	1	Not-determined	Genome-fragment	NA		0
S17_extracted_k141_144952	1774	No	NA	0	1	Not-determined	Genome-fragment	NA		0
S17_extracted_k141_108584	1212	No	NA	0	1	Not-determined	Genome-fragment	NA		0
S17_extracted_k141_144966	1261	No	NA	0	0	Not-determined	Genome-fragment	NA		0
S17_extracted_k141_49335	1053	No	NA	0	0	Not-determined	Genome-fragment	NA		0
S17_extracted_k141_167051	1004	No	NA	0	1	Not-determined	Genome-fragment	NA		0
S17_extracted_k141_49344	1033	No	NA	0	2	Not-determined	Genome-fragment	NA		0
S17_extracted_k141_174097	1181	No	NA	0	0	Not-determined	Genome-fragment	NA		0
S17_extracted_k141_42095	1370	No	NA	0	0	Not-determined	Genome-fragment	NA		0
S5_vs_k141_108586||full	1020	No	NA	0	0	Low-quality	Genome-fragment		2.58	0
S7_extracted_45581	1020	No	NA	0	0	Low-quality	Genome-fragment		1.11	0
S17_extracted_k141_34488	1186	No	NA	0	1	Not-determined	Genome-fragment	NA		0
S17_extracted_k141_122306	1156	No	NA	0	0	Not-determined	Genome-fragment	NA		0
S17_extracted_k141_93005	2471	No	NA	0	1	Not-determined	Genome-fragment	NA		0
S17_extracted_k141_115467	5213	No	NA	0	2	Not-determined	Genome-fragment	NA		0
S17_extracted_k141_71267	8655	No	NA	0	6	Not-determined	Genome-fragment	NA		0
S7_vs_23436||full	1020	No	NA	0	0	Low-quality	Genome-fragment		1.55	0
S17_extracted_k141_122314	2184	No	NA	0	0	Not-determined	Genome-fragment	NA		0
S17_extracted_k141_85013	1872	No	NA	0	1	Not-determined	Genome-fragment	NA		0
S8_extracted_k141_40363	1020	No	NA	0	0	Low-quality	Genome-fragment		1.44	0
S17_extracted_k141_62877	2974	No	NA	0	1	Not-determined	Genome-fragment	NA		0
S13_extracted_132059	1021	No	NA	0	0	Low-quality	Genome-fragment		0.53	0
S17_extracted_k141_122338	1084	No	NA	0	2	Not-determined	Genome-fragment	NA		0
S14_extracted_k141_58377	1021	No	NA	1	0	Low-quality	Genome-fragment		1.56	0
S17_extracted_k141_152904	2360	No	NA	0	2	Not-determined	Genome-fragment	NA		0
S14_vs_k141_91904||full	1021	No	NA	1	0	Low-quality	Genome-fragment		2.42	0
S17_extracted_k141_78619	2795	No	NA	0	1	Not-determined	Genome-fragment	NA		0
S17_extracted_k141_115507	11242	No	NA	0	3	Not-determined	Genome-fragment	NA		0
S17_extracted_k141_85027	8138	No	NA	0	2	Not-determined	Genome-fragment	NA		0
S17_extracted_k141_56673	3416	No	NA	0	3	Not-determined	Genome-fragment	NA		0
S17_extracted_k141_115510	3787	No	NA	0	3	Not-determined	Genome-fragment	NA		0
S17_extracted_k141_115514	1228	No	NA	0	0	Not-determined	Genome-fragment	NA		0
S17_extracted_k141_4469	1108	No	NA	0	1	Not-determined	Genome-fragment	NA		0

S17_extracted_k141_174179	1840	No	NA	0	0	Not-determined	Genome-fragment	NA		0
S17_extracted_k141_71328	1367	No	NA	0	0	Not-determined	Genome-fragment	NA		0
S1_extracted_k141_17220	1021	No	NA	0	0	Low-quality	Genome-fragment		0.68	0
S17_extracted_k141_78638	4491	No	NA	0	5	Not-determined	Genome-fragment	NA		0
S1_extracted_k141_99067	1021	No	NA	2	0	Low-quality	Genome-fragment		3.37	0
S17_extracted_k141_159953	1236	No	NA	0	0	Not-determined	Genome-fragment	NA		0
S17_extracted_k141_78648	1478	No	NA	0	1	Not-determined	Genome-fragment	NA		0
S17_extracted_k141_145085	3503	No	NA	0	6	Not-determined	Genome-fragment	NA		0
S17_extracted_k141_78653	1258	No	NA	0	1	Not-determined	Genome-fragment	NA		0
S1_vs_k141_100352||full	1021	No	NA	0	0	Low-quality	Genome-fragment		1.75	0
S17_extracted_k141_167125	20847	No	NA	0	6	Not-determined	Genome-fragment	NA		0
S3_vs_k141_32295||full	1021	No	NA	1	0	Low-quality	Genome-fragment		2.32	0
S17_extracted_k141_56713	1143	No	NA	0	1	Not-determined	Genome-fragment	NA		0
S17_extracted_k141_167138	3494	No	NA	0	0	Not-determined	Genome-fragment	NA		0
S5_vs_k141_37784||full	1021	No	NA	1	0	Low-quality	Genome-fragment		1.32	0
S17_extracted_k141_93124	2907	No	NA	0	2	Not-determined	Genome-fragment	NA		0
S5_vs_k141_81888||full	1021	No	NA	0	0	Low-quality	Genome-fragment		2.44	0
S17_extracted_k141_4530	1228	No	NA	0	2	Not-determined	Genome-fragment	NA		0
S7_extracted_66854	1021	No	NA	0	0	Low-quality	Genome-fragment		0.73	0
S17_extracted_k141_56733	4393	No	NA	0	3	Not-determined	Genome-fragment	NA		0
S17_extracted_k141_115604	1214	No	NA	0	1	Not-determined	Genome-fragment	NA		0
S17_extracted_k141_71384	1506	No	NA	0	1	Not-determined	Genome-fragment	NA		0
S16_extracted_69330	1021	No	NA	0	1	Low-quality	Genome-fragment		0.51	0
S17_extracted_k141_115634	1395	No	NA	0	1	Not-determined	Genome-fragment	NA		0
S17_extracted_k141_63000	2200	No	NA	0	2	Not-determined	Genome-fragment	NA		0
S17_extracted_k141_49467	2073	No	NA	0	4	Not-determined	Genome-fragment	NA		0
S17_extracted_k141_174203	15880	No	NA	7	0	Medium-quality	Genome-fragment		69.95	0
S17_extracted_k141_145112	1021	No	NA	0	1	Not-determined	Genome-fragment	NA		0
S17_extracted_k141_153017	1014	No	NA	0	0	Not-determined	Genome-fragment	NA		0
S17_extracted_k141_167206	7256	No	NA	0	3	Not-determined	Genome-fragment	NA		0
S17_extracted_k141_145129	2119	No	NA	0	1	Not-determined	Genome-fragment	NA		0
S17_extracted_k141_167220	1062	No	NA	0	1	Not-determined	Genome-fragment	NA		0
S17_extracted_k141_63035	1222	No	NA	0	1	Not-determined	Genome-fragment	NA		0
S17_extracted_k141_159998	1036	No	NA	0	1	Not-determined	Genome-fragment	NA		0
S17_extracted_k141_93214	1054	No	NA	0	0	Not-determined	Genome-fragment	NA		0
S17_extracted_k141_167232	3491	No	NA	0	5	Not-determined	Genome-fragment	NA		0
S10_vs_87292||full	1021	No	NA	1	0	Low-quality	Genome-fragment		1.89	0
S17_extracted_k141_71472	1671	No	NA	0	1	Not-determined	Genome-fragment	NA		0
S17_extracted_k141_153065	1018	No	NA	0	1	Not-determined	Genome-fragment	NA		0
S12_extracted_k141_61873	1021	No	NA	1	0	Low-quality	Genome-fragment		0.92	0
S12_vs_k141_102461||full	1021	No	NA	0	0	Low-quality	Genome-fragment		1.44	0
S17_extracted_k141_12066	1224	No	NA	0	1	Not-determined	Genome-fragment	NA		0
S17_extracted_k141_160051	1014	No	NA	0	1	Not-determined	Genome-fragment	NA		0
S17_extracted_k141_93236	1885	No	NA	0	2	Not-determined	Genome-fragment	NA		0
S17_extracted_k141_71492	2668	No	NA	0	2	Not-determined	Genome-fragment	NA		0
S17_extracted_k141_93237	1075	No	NA	0	1	Not-determined	Genome-fragment	NA		0
S12_vs_k141_40143||full	1021	No	NA	1	0	Low-quality	Genome-fragment		1.24	0
S17_extracted_k141_34757	1185	No	NA	0	0	Not-determined	Genome-fragment	NA		0
S17_extracted_k141_71513	1041	No	NA	0	1	Not-determined	Genome-fragment	NA		0
S17_extracted_k141_12086	1696	No	NA	0	1	Not-determined	Genome-fragment	NA		0
S17_extracted_k141_63094	1415	No	NA	0	0	Not-determined	Genome-fragment	NA		0
S17_extracted_k141_101086	1216	No	NA	0	1	Not-determined	Genome-fragment	NA		0
S17_extracted_k141_122498	3120	No	NA	0	3	Not-determined	Genome-fragment	NA		0
S17_extracted_k141_42349	9842	No	NA	0	1	Not-determined	Genome-fragment	NA		0
S17_extracted_k141_56815	1049	No	NA	0	1	Not-determined	Genome-fragment	NA		0
S17_extracted_k141_4688	1770	No	NA	0	1	Not-determined	Genome-fragment	NA		0
S12_vs_k141_68619||full	1021	No	NA	0	1	Low-quality	Genome-fragment		0.46	0
S17_extracted_k141_160077	3092	No	NA	0	2	Not-determined	Genome-fragment	NA		0
S17_extracted_k141_174309	1809	No	NA	0	0	Not-determined	Genome-fragment	NA		0
S15_vs_71291||full	1021	No	NA	0	0	Low-quality	Genome-fragment		2.26	0
S17_extracted_k141_115757	1197	No	NA	0	1	Not-determined	Genome-fragment	NA		0
S17_extracted_k141_160092	1277	No	NA	0	1	Not-determined	Genome-fragment	NA		0
S17_extracted_k141_115761	3590	No	NA	0	2	Not-determined	Genome-fragment	NA		0
S8_extracted_k141_127009	1021	No	NA	1	0	Low-quality	Genome-fragment		1.99	0
S17_extracted_k141_145224	1206	No	NA	0	0	Not-determined	Genome-fragment	NA		0
S17_extracted_k141_108849	5439	No	NA	0	4	Not-determined	Genome-fragment	NA		0
S8_extracted_k141_199463	1021	No	NA	0	0	Low-quality	Genome-fragment		1.75	0
S17_extracted_k141_4730	4117	No	NA	0	0	Not-determined	Genome-fragment	NA		0
S17_extracted_k141_108869	3207	No	NA	0	2	Not-determined	Genome-fragment	NA		0
S8_vs_k141_113446||full	1021	No	NA	0	0	Low-quality	Genome-fragment		2.24	0
S9_vs_k141_68576||lt2gene	1022	No	NA	1	0	Low-quality	Genome-fragment		2.23	0
S11_extracted_k141_59756	1022	No	NA	0	1	Low-quality	Genome-fragment		1.48	0
S17_extracted_k141_93304	2823	No	NA	0	2	Not-determined	Genome-fragment	NA		0
S17_extracted_k141_115794	1160	No	NA	0	1	Not-determined	Genome-fragment	NA		0
S17_extracted_k141_34874	1143	No	NA	0	2	Not-determined	Genome-fragment	NA		0
S17_extracted_k141_145278	1382	No	NA	0	0	Not-determined	Genome-fragment	NA		0
S17_extracted_k141_63152	1037	No	NA	0	2	Not-determined	Genome-fragment	NA		0
S17_extracted_k141_85313	2121	No	NA	0	0	Not-determined	Genome-fragment	NA		0
S17_extracted_k141_42411	1404	No	NA	0	1	Not-determined	Genome-fragment	NA		0
S17_extracted_k141_160126	5830	No	NA	0	2	Not-determined	Genome-fragment	NA		0
S17_extracted_k141_71607	1067	No	NA	0	0	Not-determined	Genome-fragment	NA		0
S17_extracted_k141_4809	1236	No	NA	0	1	Not-determined	Genome-fragment	NA		0
S11_extracted_k141_41572	1022	No	NA	1	0	Low-quality	Genome-fragment		2.58	0
S17_extracted_k141_93360	1168	No	NA	0	0	Not-determined	Genome-fragment	NA		0
S17_extracted_k141_4817	4757	No	NA	0	4	Not-determined	Genome-fragment	NA		0
S11_extracted_k141_29503	1022	No	NA	0	0	Low-quality	Genome-fragment		0.81	0
S17_extracted_k141_108948	3059	No	NA	0	1	Not-determined	Genome-fragment	NA		0
S17_extracted_k141_160167	2228	No	NA	0	1	Not-determined	Genome-fragment	NA		0
S11_extracted_k141_36098	1022	No	NA	2	0	Low-quality	Genome-fragment		2.73	0
S17_extracted_k141_78886	3179	No	NA	0	2	Not-determined	Genome-fragment	NA		0
S17_extracted_k141_153269	1305	No	NA	0	1	Not-determined	Genome-fragment	NA		0
S17_extracted_k141_108976	2629	No	NA	0	1	Not-determined	Genome-fragment	NA		0
S17_extracted_k141_130155	1992	No	NA	0	0	Not-determined	Genome-fragment	NA		0
S17_extracted_k141_78914	1858	No	NA	0	0	Not-determined	Genome-fragment	NA		0
S17_extracted_k141_34992	6009	No	NA	0	2	Not-determined	Genome-fragment	NA		0
S17_extracted_k141_160234	1538	No	NA	0	1	Not-determined	Genome-fragment	NA		0
S17_extracted_k141_137848	1016	No	NA	0	0	Not-determined	Genome-fragment	NA		0
S17_extracted_k141_19384	1529	No	NA	0	0	Not-determined	Genome-fragment	NA		0
S17_extracted_k141_78922	1067	No	NA	0	1	Not-determined	Genome-fragment	NA		0
S11_vs_k141_102647||lt2gene	1022	No	NA	1	0	Low-quality	Genome-fragment		2.22	0
S17_extracted_k141_130193	1088	No	NA	0	2	Not-determined	Genome-fragment	NA		0
S13_extracted_133631	1022	No	NA	0	1	Low-quality	Genome-fragment		2.36	0
S17_extracted_k141_101225	3195	No	NA	0	2	Not-determined	Genome-fragment	NA		0
S17_extracted_k141_85442	3639	No	NA	0	2	Not-determined	Genome-fragment	NA		0
S17_extracted_k141_153303	1283	No	NA	0	2	Not-determined	Genome-fragment	NA		0
S17_extracted_k141_63314	2685	No	NA	0	1	Not-determined	Genome-fragment	NA		0
S17_extracted_k141_122731	1137	No	NA	0	0	Not-determined	Genome-fragment	NA		0
S17_extracted_k141_71693	1130	No	NA	0	1	Not-determined	Genome-fragment	NA		0
S1_extracted_k141_4778	1022	No	NA	1	0	Low-quality	Genome-fragment		2.15	0
S3_extracted_k141_83648	1022	No	NA	1	0	Low-quality	Genome-fragment		3.01	0
S17_extracted_k141_71704	1548	No	NA	0	0	Not-determined	Genome-fragment	NA		0
S17_extracted_k141_35077	2807	No	NA	0	1	Not-determined	Genome-fragment	NA		0
S17_extracted_k141_130222	1566	No	NA	0	1	Not-determined	Genome-fragment	NA		0
S17_extracted_k141_78988	2027	No	NA	0	2	Not-determined	Genome-fragment	NA		0
S17_extracted_k141_71711	1325	No	NA	0	2	Not-determined	Genome-fragment	NA		0
S17_extracted_k141_12207	1466	No	NA	0	1	Not-determined	Genome-fragment	NA		0
S17_extracted_k141_167495	1175	No	NA	0	1	Not-determined	Genome-fragment	NA		0
S17_extracted_k141_174408	3448	No	NA	0	0	Not-determined	Genome-fragment	NA		0
S17_extracted_k141_26560	1197	No	NA	0	0	Not-determined	Genome-fragment	NA		0
S17_extracted_k141_153361	1177	No	NA	0	1	Not-determined	Genome-fragment	NA		0
S17_extracted_k141_4977	1502	No	NA	0	1	Not-determined	Genome-fragment	NA		0
S17_extracted_k141_122793	1670	No	NA	0	1	Not-determined	Genome-fragment	NA		0
S17_extracted_k141_93510	2056	No	NA	0	2	Not-determined	Genome-fragment	NA		0
S17_extracted_k141_19459	2664	No	NA	0	2	Not-determined	Genome-fragment	NA		0
S17_extracted_k141_145430	1276	No	NA	0	2	Not-determined	Genome-fragment	NA		0
S17_extracted_k141_19468	3328	No	NA	0	1	Not-determined	Genome-fragment	NA		0
S17_extracted_k141_26597	2445	No	NA	0	1	Not-determined	Genome-fragment	NA		0
S17_extracted_k141_160321	1367	No	NA	0	0	Not-determined	Genome-fragment	NA		0
S3_vs_k141_54124||full	1022	No	NA	0	0	Low-quality	Genome-fragment		2.58	0
S7_vs_55100||lt2gene	1022	No	NA	1	0	Low-quality	Genome-fragment		0.78	0
S17_extracted_k141_145513	1236	No	NA	0	0	Not-determined	Genome-fragment	NA		0
S7_vs_127549||lt2gene	1022	No	NA	1	0	Low-quality	Genome-fragment		1.35	0
S17_extracted_k141_71821	2293	No	NA	0	1	Not-determined	Genome-fragment	NA		0
S16_extracted_36899	1022	No	NA	1	0	Low-quality	Genome-fragment		3.11	0
S17_extracted_k141_5049	1024	No	NA	0	1	Not-determined	Genome-fragment	NA		0
S17_extracted_k141_35138	52045	No	NA	12	4	Medium-quality	Genome-fragment		73.5	0
S17_extracted_k141_153455	1235	No	NA	0	0	Not-determined	Genome-fragment	NA		0
S17_extracted_k141_109121	4422	No	NA	0	4	Not-determined	Genome-fragment	NA		0
S17_extracted_k141_63391	1656	No	NA	0	1	Not-determined	Genome-fragment	NA		0
S17_extracted_k141_85587	1183	No	NA	0	1	Not-determined	Genome-fragment	NA		0
S17_extracted_k141_42638	2759	No	NA	0	0	Not-determined	Genome-fragment	NA		0
S17_extracted_k141_116099	1453	No	NA	0	2	Not-determined	Genome-fragment	NA		0
S17_extracted_k141_137987	1541	No	NA	0	1	Not-determined	Genome-fragment	NA		0
S17_extracted_k141_116102	1777	No	NA	0	1	Not-determined	Genome-fragment	NA		0
S17_extracted_k141_71823	30287	No	NA	0	5	Not-determined	Genome-fragment	NA		0
S17_extracted_k141_122899	5609	No	NA	0	4	Not-determined	Genome-fragment	NA		0
S17_extracted_k141_49785	1498	No	NA	0	2	Not-determined	Genome-fragment	NA		0
S17_extracted_k141_85621	1595	No	NA	0	1	Not-determined	Genome-fragment	NA		0
S17_extracted_k141_49788	2174	No	NA	0	1	Not-determined	Genome-fragment	NA		0
S16_extracted_124054	1022	No	NA	0	0	Low-quality	Genome-fragment		2.41	0
S17_extracted_k141_85627	1192	No	NA	0	0	Not-determined	Genome-fragment	NA		0
S16_vs_92380||full	1022	No	NA	0	1	Low-quality	Genome-fragment		1.98	0
S16_vs_151457||full	1022	No	NA	1	0	Low-quality	Genome-fragment		2.58	0
S17_extracted_k141_12283	1799	No	NA	0	1	Not-determined	Genome-fragment	NA		0
S17_extracted_k141_71848	1818	No	NA	0	0	Not-determined	Genome-fragment	NA		0
S17_extracted_k141_167607	1286	No	NA	0	1	Not-determined	Genome-fragment	NA		0
S12_vs_k141_21427||full	1022	No	NA	1	0	Low-quality	Genome-fragment		3.16	0
S17_extracted_k141_160438	1291	No	NA	0	0	Not-determined	Genome-fragment	NA		0
S17_extracted_k141_130426	1023	No	NA	0	0	Low-quality	Genome-fragment		0.61	0
S17_extracted_k141_160440	1422	No	NA	0	0	Not-determined	Genome-fragment	NA		0
S17_extracted_k141_63436	1018	No	NA	0	1	Not-determined	Genome-fragment	NA		0
S17_extracted_k141_138035	2981	No	NA	0	2	Not-determined	Genome-fragment	NA		0
S17_extracted_k141_35233	4097	No	NA	0	1	Not-determined	Genome-fragment	NA		0
S17_extracted_k141_57240	1041	No	NA	0	0	Not-determined	Genome-fragment	NA		0
S17_extracted_k141_5184	1466	No	NA	0	1	Not-determined	Genome-fragment	NA		0
S17_extracted_k141_42724	1492	No	NA	0	1	Not-determined	Genome-fragment	NA		0
S17_extracted_k141_12340	2806	No	NA	0	0	Not-determined	Genome-fragment	NA		0
S17_extracted_k141_109248	1302	No	NA	0	2	Not-determined	Genome-fragment	NA		0
S17_extracted_k141_79151	16088	No	NA	7	0	Medium-quality	Genome-fragment		74.35	0
S17_vs_k141_112633||full	1023	No	NA	0	0	Low-quality	Genome-fragment		3.08	0
S17_extracted_k141_167640	1053	No	NA	0	1	Not-determined	Genome-fragment	NA		0
S17_extracted_k141_71934	1268	No	NA	0	1	Not-determined	Genome-fragment	NA		0
S17_extracted_k141_116219	1645	No	NA	0	1	Not-determined	Genome-fragment	NA		0
S17_extracted_k141_85721	1394	No	NA	0	1	Not-determined	Genome-fragment	NA		0

S17_vs_k141_32155||full	1023	No	NA	0	0	Low-quality	Genome-fragment		2.21	0
S17_vs_k141_65098||full	1023	No	NA	1	0	Low-quality	Genome-fragment		0.69	0
S13_extracted_10615	1023	No	NA	0	1	Low-quality	Genome-fragment		1.04	0
S17_extracted_k141_49919	3868	No	NA	0	1	Not-determined	Genome-fragment	NA		0
S17_extracted_k141_35372	1253	No	NA	0	0	Not-determined	Genome-fragment	NA		0
S1_extracted_k141_66855	1023	No	NA	0	0	Low-quality	Genome-fragment		2.81	0
S17_extracted_k141_138125	4655	No	NA	0	2	Not-determined	Genome-fragment	NA		0
S17_extracted_k141_145631	2065	No	NA	0	1	Not-determined	Genome-fragment	NA		0
S17_extracted_k141_138133	2809	No	NA	0	1	Not-determined	Genome-fragment	NA		0
S17_extracted_k141_35398	1267	No	NA	0	1	Not-determined	Genome-fragment	NA		0
S17_extracted_k141_145639	1451	No	NA	0	0	Not-determined	Genome-fragment	NA		0
S1_vs_k141_47231||full	1023	No	NA	1	0	Low-quality	Genome-fragment		1.96	0
S1_vs_k141_135196||full	1023	No	NA	1	0	Low-quality	Genome-fragment		2.59	0
S17_extracted_k141_42808	6582	No	NA	0	4	Not-determined	Genome-fragment	NA		0
S17_extracted_k141_123074	1268	No	NA	0	1	Not-determined	Genome-fragment	NA		0
S17_extracted_k141_5242	1908	No	NA	0	0	Not-determined	Genome-fragment	NA		0
S17_extracted_k141_174610	1951	No	NA	0	1	Not-determined	Genome-fragment	NA		0
S17_extracted_k141_167758	6353	No	NA	0	0	Not-determined	Genome-fragment	NA		0
S17_extracted_k141_72047	1139	No	NA	0	1	Not-determined	Genome-fragment	NA		0
S17_extracted_k141_160567	1018	No	NA	0	1	Not-determined	Genome-fragment	NA		0
S17_extracted_k141_101527	1264	No	NA	0	0	Not-determined	Genome-fragment	NA		0
S17_extracted_k141_19710	1957	No	NA	0	1	Not-determined	Genome-fragment	NA		0
S17_extracted_k141_123096	4945	No	NA	0	1	Not-determined	Genome-fragment	NA		0
S1_vs_k141_57091||lt2gene	1023	No	NA	2	0	Low-quality	Genome-fragment		1.89	0
S17_extracted_k141_138181	1385	No	NA	0	0	Not-determined	Genome-fragment	NA		0
S17_extracted_k141_101547	1258	No	NA	0	2	Not-determined	Genome-fragment	NA		0
S3_vs_k141_14819||lt2gene	1023	No	NA	1	0	Low-quality	Genome-fragment		2.16	0
S17_extracted_k141_160587	3122	No	NA	0	3	Not-determined	Genome-fragment	NA		0
S17_extracted_k141_167794	1695	No	NA	0	1	Not-determined	Genome-fragment	NA		0
S7_extracted_97000	1023	No	NA	0	0	Low-quality	Genome-fragment		2.32	0
S17_extracted_k141_79300	1417	No	NA	0	2	Not-determined	Genome-fragment	NA		0
S17_extracted_k141_85874	1211	No	NA	0	1	Not-determined	Genome-fragment	NA		0
S17_extracted_k141_12520	1462	No	NA	0	1	Not-determined	Genome-fragment	NA		0
S17_extracted_k141_116382	1032	No	NA	0	1	Not-determined	Genome-fragment	NA		0
S17_extracted_k141_85887	1234	No	NA	0	2	Not-determined	Genome-fragment	NA		0
S17_extracted_k141_130553	1237	No	NA	0	2	Not-determined	Genome-fragment	NA		0
S7_extracted_132721	1023	No	NA	0	0	Low-quality	Genome-fragment		0.67	0
S17_extracted_k141_26778	2884	No	NA	0	1	Not-determined	Genome-fragment	NA		0
S17_extracted_k141_145709	1221	No	NA	0	1	Not-determined	Genome-fragment	NA		0
S17_extracted_k141_50088	1163	No	NA	0	2	Not-determined	Genome-fragment	NA		0
S16_extracted_10527	1023	No	NA	0	1	Low-quality	Genome-fragment		1.19	0
S16_extracted_93305	1023	No	NA	0	0	Low-quality	Genome-fragment		1.58	0
S17_extracted_k141_109495	1059	No	NA	0	0	Not-determined	Genome-fragment	NA		0
S17_extracted_k141_174696	4708	No	NA	0	5	Not-determined	Genome-fragment	NA		0
S17_extracted_k141_79353	2385	No	NA	0	2	Not-determined	Genome-fragment	NA		0
S17_extracted_k141_93760	1281	No	NA	0	0	Not-determined	Genome-fragment	NA		0
S17_extracted_k141_101626	2231	No	NA	0	2	Not-determined	Genome-fragment	NA		0
S17_extracted_k141_12557	4083	No	NA	0	4	Not-determined	Genome-fragment	NA		0
S17_extracted_k141_79368	3369	No	NA	0	0	Not-determined	Genome-fragment	NA		0
S16_vs_126196||full	1023	No	NA	2	0	Low-quality	Genome-fragment		2.55	0
S17_extracted_k141_123173	4266	No	NA	0	4	Not-determined	Genome-fragment	NA		0
S17_extracted_k141_101649	2629	No	NA	0	1	Not-determined	Genome-fragment	NA		0
S17_extracted_k141_174712	1184	No	NA	0	0	Not-determined	Genome-fragment	NA		0
S17_extracted_k141_109530	1139	No	NA	0	2	Not-determined	Genome-fragment	NA		0
S6_vs_k141_26265||lt2gene	1023	No	NA	1	0	Low-quality	Genome-fragment		16.77	0
S17_extracted_k141_85940	1061	No	NA	0	1	Not-determined	Genome-fragment	NA		0
S17_extracted_k141_50147	1503	No	NA	0	2	Not-determined	Genome-fragment	NA		0
S17_extracted_k141_93781	3917	No	NA	0	0	Not-determined	Genome-fragment	NA		0
S17_extracted_k141_123210	2743	No	NA	0	0	Not-determined	Genome-fragment	NA		0
S17_extracted_k141_123211	3243	No	NA	0	1	Not-determined	Genome-fragment	NA		0
S8_vs_k141_85083||full	1023	No	NA	0	0	Low-quality	Genome-fragment		1.47	0
S17_extracted_k141_35498	1109	No	NA	0	1	Not-determined	Genome-fragment	NA		0
S17_extracted_k141_145807	1046	No	NA	0	1	Not-determined	Genome-fragment	NA		0
S17_extracted_k141_160715	1964	No	NA	0	4	Not-determined	Genome-fragment	NA		0
S17_extracted_k141_93817	1035	No	NA	0	0	Not-determined	Genome-fragment	NA		0
S17_extracted_k141_123237	3814	No	NA	0	2	Not-determined	Genome-fragment	NA		0
S17_extracted_k141_145831	2348	No	NA	0	0	Not-determined	Genome-fragment	NA		0
S17_extracted_k141_93838	1244	No	NA	0	1	Not-determined	Genome-fragment	NA		0
S17_extracted_k141_93848	1525	No	NA	0	1	Not-determined	Genome-fragment	NA		0
S17_extracted_k141_116509	1519	No	NA	0	1	Not-determined	Genome-fragment	NA		0
S17_extracted_k141_174787	1201	No	NA	0	1	Not-determined	Genome-fragment	NA		0
S17_extracted_k141_167953	3701	No	NA	0	1	Not-determined	Genome-fragment	NA		0
S17_extracted_k141_93869	1126	No	NA	0	0	Not-determined	Genome-fragment	NA		0
S17_extracted_k141_35569	1508	No	NA	0	1	Not-determined	Genome-fragment	NA		0
S17_extracted_k141_50241	1260	No	NA	0	2	Not-determined	Genome-fragment	NA		0
S17_extracted_k141_22641	1024	No	NA	0	1	Low-quality	Genome-fragment		0.18	0
S17_extracted_k141_72176	2648	No	NA	0	0	Not-determined	Genome-fragment	NA		0
S17_extracted_k141_116566	1109	No	NA	0	1	Not-determined	Genome-fragment	NA		0
S17_extracted_k141_160793	1024	No	NA	0	1	Not-determined	Genome-fragment	NA		0
S17_extracted_k141_123311	2840	No	NA	0	1	Not-determined	Genome-fragment	NA		0
S17_extracted_k141_138322	5763	No	NA	0	0	Not-determined	Genome-fragment	NA		0
S17_extracted_k141_116583	4446	No	NA	0	0	Not-determined	Genome-fragment	NA		0
S17_extracted_k141_5550	14093	No	NA	0	5	Not-determined	Genome-fragment	NA		0
S17_extracted_k141_101824	4054	No	NA	0	2	Not-determined	Genome-fragment	NA		0
S17_extracted_k141_168020	5103	No	NA	0	3	Not-determined	Genome-fragment	NA		0
S17_extracted_k141_35629	1272	No	NA	0	1	Not-determined	Genome-fragment	NA		0
S17_extracted_k141_145924	1256	No	NA	0	2	Not-determined	Genome-fragment	NA		0
S17_extracted_k141_63845	1702	No	NA	0	2	Not-determined	Genome-fragment	NA		0
S17_vs_k141_79109||lt2gene	1024	No	NA	1	0	Low-quality	Genome-fragment		2.2	0
S17_extracted_k141_93932	3892	No	NA	0	4	Not-determined	Genome-fragment	NA		0
S17_extracted_k141_63850	3560	No	NA	0	1	Not-determined	Genome-fragment	NA		0
S17_extracted_k141_109722	1606	No	NA	0	3	Not-determined	Genome-fragment	NA		0
S9_extracted_k141_58529	1024	No	NA	0	0	Low-quality	Genome-fragment		1.86	0
S11_vs_k141_72045||full	1024	No	NA	0	1	Low-quality	Genome-fragment		1.31	0
S17_extracted_k141_153861	1266	No	NA	0	0	Not-determined	Genome-fragment	NA		0
S17_extracted_k141_109750	1174	No	NA	0	1	Not-determined	Genome-fragment	NA		0
S17_extracted_k141_109758	1773	No	NA	0	1	Not-determined	Genome-fragment	NA		0
S17_extracted_k141_174871	1032	No	NA	0	1	Not-determined	Genome-fragment	NA		0
S17_extracted_k141_50334	4996	No	NA	0	0	Not-determined	Genome-fragment	NA		0
S17_extracted_k141_79551	1307	No	NA	0	0	Not-determined	Genome-fragment	NA		0
S17_extracted_k141_26952	1403	No	NA	0	0	Not-determined	Genome-fragment	NA		0
S17_extracted_k141_19800	1312	No	NA	0	0	Not-determined	Genome-fragment	NA		0
S17_extracted_k141_43116	1198	No	NA	0	1	Not-determined	Genome-fragment	NA		0
S17_extracted_k141_101899	5103	No	NA	0	1	Not-determined	Genome-fragment	NA		0
S17_extracted_k141_109789	1626	No	NA	0	1	Not-determined	Genome-fragment	NA		0
S17_extracted_k141_109809	1180	No	NA	0	1	Not-determined	Genome-fragment	NA		0
S17_extracted_k141_101935	1853	No	NA	0	0	Not-determined	Genome-fragment	NA		0
S17_extracted_k141_109820	2827	No	NA	0	2	Not-determined	Genome-fragment	NA		0
S14_extracted_k141_45701	1024	No	NA	1	0	Low-quality	Genome-fragment		2.65	0
S14_extracted_k141_46220	1024	No	NA	4	0	Low-quality	Genome-fragment		3.33	0
S17_extracted_k141_12742	7424	No	NA	0	3	Not-determined	Genome-fragment	NA		0
S17_extracted_k141_109870	1558	No	NA	0	2	Not-determined	Genome-fragment	NA		0
S1_extracted_k141_150258	1024	No	NA	0	0	Low-quality	Genome-fragment		1.59	0
S17_extracted_k141_5674	1513	No	NA	0	2	Not-determined	Genome-fragment	NA		0
S17_extracted_k141_146046	1590	No	NA	0	0	Not-determined	Genome-fragment	NA		0
S5_extracted_k141_129437	1024	No	NA	1	0	Low-quality	Genome-fragment		2.24	0
S17_extracted_k141_146058	2209	No	NA	0	1	Not-determined	Genome-fragment	NA		0
S17_extracted_k141_50442	1438	No	NA	0	2	Not-determined	Genome-fragment	NA		0
S17_extracted_k141_5700	3286	No	NA	0	1	Not-determined	Genome-fragment	NA		0
S17_extracted_k141_79672	1052	No	NA	0	0	Not-determined	Genome-fragment	NA		0
S17_extracted_k141_5703	1013	No	NA	0	0	Not-determined	Genome-fragment	NA		0
S17_extracted_k141_168215	1237	No	NA	0	2	Not-determined	Genome-fragment	NA		0
S17_extracted_k141_130763	1475	No	NA	0	1	Not-determined	Genome-fragment	NA		0
S17_extracted_k141_109923	1050	No	NA	0	1	Not-determined	Genome-fragment	NA		0
S5_extracted_k141_99822	1024	No	NA	3	0	Low-quality	Genome-fragment		2.51	0
S17_extracted_k141_116791	2664	No	NA	0	0	Not-determined	Genome-fragment	NA		0
S17_extracted_k141_161084	1053	No	NA	0	0	Not-determined	Genome-fragment	NA		0
S5_extracted_k141_96907	1024	No	NA	2	0	Low-quality	Genome-fragment		3.03	0
S17_extracted_k141_27137	1560	No	NA	0	0	Not-determined	Genome-fragment	NA		0
S17_extracted_k141_116812	1082	No	NA	0	1	Not-determined	Genome-fragment	NA		0
S16_extracted_106038	1024	No	NA	0	2	Low-quality	Genome-fragment		0.86	0
S10_vs_10839||full	1024	No	NA	0	1	Low-quality	Genome-fragment		2.73	0
S17_extracted_k141_19933	2256	No	NA	0	1	Not-determined	Genome-fragment	NA		0
S10_vs_43001||full	1024	No	NA	0	0	Low-quality	Genome-fragment		2.49	0
S17_extracted_k141_154045	1043	No	NA	0	0	Not-determined	Genome-fragment	NA		0
S17_extracted_k141_146135	3232	No	NA	0	0	Not-determined	Genome-fragment	NA		0
S12_vs_k141_178351||full	1024	No	NA	0	1	Low-quality	Genome-fragment		1.79	0
S17_extracted_k141_64032	3053	No	NA	0	0	Not-determined	Genome-fragment	NA		0
S15_extracted_68588	1024	No	NA	0	0	Low-quality	Genome-fragment		1.71	0
S17_extracted_k141_35899	1014	No	NA	0	1	Not-determined	Genome-fragment	NA		0
S17_extracted_k141_43284	1037	No	NA	0	0	Not-determined	Genome-fragment	NA		0
S17_extracted_k141_12878	1825	No	NA	0	1	Not-determined	Genome-fragment	NA		0
S9_vs_k141_4173||full	1025	No	NA	1	0	Low-quality	Genome-fragment		2.07	0
S17_extracted_k141_19964	1177	No	NA	0	0	Not-determined	Genome-fragment	NA		0
S17_extracted_k141_94035	1100	No	NA	0	0	Not-determined	Genome-fragment	NA		0
S17_extracted_k141_5787	2546	No	NA	0	1	Not-determined	Genome-fragment	NA		0
S17_extracted_k141_123589	2127	No	NA	0	0	Not-determined	Genome-fragment	NA		0
S17_extracted_k141_50547	4522	No	NA	0	5	Not-determined	Genome-fragment	NA		0
S17_extracted_k141_138584	2142	No	NA	0	0	Not-determined	Genome-fragment	NA		0
S17_extracted_k141_72472	3888	No	NA	0	0	Not-determined	Genome-fragment	NA		0
S17_extracted_k141_94062	1143	No	NA	0	0	Not-determined	Genome-fragment	NA		0
S17_extracted_k141_94069	3995	No	NA	0	2	Not-determined	Genome-fragment	NA		0
S17_extracted_k141_50562	3183	No	NA	0	2	Not-determined	Genome-fragment	NA		0
S13_extracted_84529	1025	No	NA	0	0	Low-quality	Genome-fragment		1.39	0
S17_extracted_k141_138623	1024	No	NA	0	3	Not-determined	Genome-fragment	NA		0
S17_extracted_k141_27212	1287	No	NA	0	2	Not-determined	Genome-fragment	NA		0
S17_extracted_k141_138629	1034	No	NA	0	0	Not-determined	Genome-fragment	NA		0
S13_extracted_153322	1025	No	NA	0	0	Low-quality	Genome-fragment		1.67	0
S17_extracted_k141_116926	1065	No	NA	0	1	Not-determined	Genome-fragment	NA		0
S17_extracted_k141_20038	3319	No	NA	0	2	Not-determined	Genome-fragment	NA		0
S17_extracted_k141_79826	2349	No	NA	0	0	Not-determined	Genome-fragment	NA		0
S17_extracted_k141_27243	3280	No	NA	0	1	Not-determined	Genome-fragment	NA		0
S17_extracted_k141_110079	1362	No	NA	0	0	Not-determined	Genome-fragment	NA		0
S17_extracted_k141_27245	1114	No	NA	0	1	Not-determined	Genome-fragment	NA		0
S17_extracted_k141_5881	2567	No	NA	0	0	Not-determined	Genome-fragment	NA		0
S17_extracted_k141_20049	2734	No	NA	0	0	Not-determined	Genome-fragment	NA		0
S17_extracted_k141_102087	1343	No	NA	0	1	Not-determined	Genome-fragment	NA		0
S14_extracted_k141_41194	1025	No	NA	1	0	Low-quality	Genome-fragment		5.3	0
S14_vs_k141_58635||full	1025	No	NA	0	0	Low-quality	Genome-fragment		2.93	0
S17_extracted_k141_94160	1097	No	NA	0	0	Not-determined	Genome-fragment	NA		0
S17_extracted_k141_64165	3167	No	NA	0	3	Not-determined	Genome-fragment	NA		0
S17_extracted_k141_123697	1515	No	NA	0	1	Not-determined	Genome-fragment	NA		0
S17_extracted_k141_161283	2646	No	NA	0	0	Not-determined	Genome-fragment	NA		0
S14_vs_k141_96915||lt2gene	1025	No	NA	1	0	Low-quality	Genome-fragment		3.28	0

S17_extracted_k141_154263	1352	No	NA	0	2	Not-determined	Genome-fragment	NA		0
S17_extracted_k141_175063	1431	No	NA	0	0	Not-determined	Genome-fragment	NA		0
S17_extracted_k141_168275	1022	No	NA	0	0	Not-determined	Genome-fragment	NA		0
S17_extracted_k141_168286	1557	No	NA	0	0	Not-determined	Genome-fragment	NA		0
S17_extracted_k141_175085	1030	No	NA	0	1	Not-determined	Genome-fragment	NA		0
S17_extracted_k141_168287	2763	No	NA	0	1	Not-determined	Genome-fragment	NA		0
S17_extracted_k141_57844	1068	No	NA	0	0	Not-determined	Genome-fragment	NA		0
S14_vs_k141_36386||lt2gene	1025	No	NA	1	0	Low-quality	Genome-fragment		1.81	0
S1_vs_k141_41498||full	1025	No	NA	0	0	Low-quality	Genome-fragment		1.94	0
S17_extracted_k141_72564	1935	No	NA	0	3	Not-determined	Genome-fragment	NA		0
S17_extracted_k141_36051	1411	No	NA	0	2	Not-determined	Genome-fragment	NA		0
S17_extracted_k141_72586	1436	No	NA	0	0	Not-determined	Genome-fragment	NA		0
S17_extracted_k141_138782	1144	No	NA	0	0	Not-determined	Genome-fragment	NA		0
S17_extracted_k141_154387	3189	No	NA	0	5	Not-determined	Genome-fragment	NA		0
S17_extracted_k141_168332	3728	No	NA	0	1	Not-determined	Genome-fragment	NA		0
S17_extracted_k141_168339	1509	No	NA	0	1	Not-determined	Genome-fragment	NA		0
S1_vs_k141_111277||full	1025	No	NA	2	0	Low-quality	Genome-fragment		2.13	0
S3_extracted_k141_68770	1025	No	NA	0	0	Low-quality	Genome-fragment		1.66	0
S3_vs_k141_57446||full	1025	No	NA	0	0	Low-quality	Genome-fragment		2.3	0
S3_vs_k141_127211||full	1025	No	NA	1	0	Low-quality	Genome-fragment		2.82	0
S17_extracted_k141_20175	3704	No	NA	0	1	Not-determined	Genome-fragment	NA		0
S17_extracted_k141_168350	1055	No	NA	0	0	Not-determined	Genome-fragment	NA		0
S17_extracted_k141_110238	4343	No	NA	0	1	Not-determined	Genome-fragment	NA		0
S17_extracted_k141_102269	1546	No	NA	0	1	Not-determined	Genome-fragment	NA		0
S17_extracted_k141_94273	1228	No	NA	0	0	Not-determined	Genome-fragment	NA		0
S17_extracted_k141_175125	1623	No	NA	0	1	Not-determined	Genome-fragment	NA		0
S17_extracted_k141_131114	1110	No	NA	0	1	Not-determined	Genome-fragment	NA		0
S5_vs_k141_92007||full	1025	No	NA	2	0	Low-quality	Genome-fragment		2.45	0
S17_extracted_k141_57939	3337	No	NA	0	2	Not-determined	Genome-fragment	NA		0
S17_extracted_k141_131125	1092	No	NA	0	1	Not-determined	Genome-fragment	NA		0
S17_extracted_k141_161442	1132	No	NA	0	2	Not-determined	Genome-fragment	NA		0
S17_extracted_k141_168377	3402	No	NA	0	2	Not-determined	Genome-fragment	NA		0
S5_vs_k141_49799||full	1025	No	NA	1	0	Low-quality	Genome-fragment		1.68	0
S17_extracted_k141_168389	1035	No	NA	0	1	Not-determined	Genome-fragment	NA		0
S5_vs_k141_111120||full	1025	No	NA	0	1	Low-quality	Genome-fragment		0.53	0
S5_vs_k141_89412||lt2gene	1025	No	NA	0	0	Low-quality	Genome-fragment		2.47	0
S7_extracted_109321	1025	No	NA	0	0	Low-quality	Genome-fragment		2.45	0
S17_extracted_k141_146470	1722	No	NA	0	2	Not-determined	Genome-fragment	NA		0
S17_extracted_k141_64375	1241	No	NA	0	2	Not-determined	Genome-fragment	NA		0
S17_extracted_k141_154472	1598	No	NA	0	1	Not-determined	Genome-fragment	NA		0
S7_vs_102924||full	1025	No	NA	1	0	Low-quality	Genome-fragment		2.51	0
S17_extracted_k141_57998	2203	No	NA	0	2	Not-determined	Genome-fragment	NA		0
S17_extracted_k141_58004	1710	No	NA	0	1	Not-determined	Genome-fragment	NA		0
S17_extracted_k141_117222	1251	No	NA	0	1	Not-determined	Genome-fragment	NA		0
S17_extracted_k141_86630	3129	No	NA	0	0	Not-determined	Genome-fragment	NA		0
S17_extracted_k141_146556	1435	No	NA	0	1	Not-determined	Genome-fragment	NA		0
S17_extracted_k141_117234	1065	No	NA	0	1	Not-determined	Genome-fragment	NA		0
S7_vs_112255||lt2gene	1025	No	NA	1	0	Low-quality	Genome-fragment		2.91	0
S16_extracted_85231	1025	No	NA	2	0	Low-quality	Genome-fragment		1.71	0
S17_extracted_k141_138964	1070	No	NA	0	0	Not-determined	Genome-fragment	NA		0
S16_extracted_170284	1025	No	NA	0	0	Low-quality	Genome-fragment		1.04	0
S17_extracted_k141_80015	5180	No	NA	0	0	Not-determined	Genome-fragment	NA		0
S10_extracted_68437	1025	No	NA	0	0	Low-quality	Genome-fragment		2.05	0
S17_extracted_k141_6209	1004	No	NA	0	0	Not-determined	Genome-fragment	NA		0
S10_vs_19377||lt2gene	1025	No	NA	2	0	Low-quality	Genome-fragment		2.74	0
S12_extracted_k141_119354	1025	No	NA	0	2	Low-quality	Genome-fragment		0.86	0
S12_extracted_k141_108233	1025	No	NA	0	1	Low-quality	Genome-fragment		1.29	0
S17_extracted_k141_64497	1035	No	NA	0	0	Not-determined	Genome-fragment	NA		0
S17_extracted_k141_131207	2820	No	NA	0	0	Not-determined	Genome-fragment	NA		0
S17_extracted_k141_110438	1148	No	NA	0	2	Not-determined	Genome-fragment	NA		0
S12_vs_k141_130491||full	1025	No	NA	1	0	Low-quality	Genome-fragment		2.3	0
S17_extracted_k141_36213	1301	No	NA	0	2	Not-determined	Genome-fragment	NA		0
S6_extracted_k141_52909	1025	No	NA	1	0	Low-quality	Genome-fragment		2.08	0
S8_vs_k141_166455||full	1025	No	NA	0	1	Low-quality	Genome-fragment		1.75	0
S8_vs_k141_63960||full	1025	No	NA	0	0	Low-quality	Genome-fragment		0.3	0
S17_extracted_k141_124018	12016	No	NA	0	5	Not-determined	Genome-fragment	NA		0
S17_extracted_k141_175310	1016	No	NA	0	0	Not-determined	Genome-fragment	NA		0
S17_extracted_k141_131244	1060	No	NA	0	2	Not-determined	Genome-fragment	NA		0
S17_extracted_k141_43623	1194	No	NA	0	0	Not-determined	Genome-fragment	NA		0
S9_extracted_k141_21561	1026	No	NA	0	1	Low-quality	Genome-fragment		2.44	0
S17_extracted_k141_154660	2658	No	NA	0	1	Not-determined	Genome-fragment	NA		0
S9_extracted_k141_78037	1026	No	NA	0	0	Low-quality	Genome-fragment		1.74	0
S17_extracted_k141_58076	3809	No	NA	0	0	Not-determined	Genome-fragment	NA		0
S17_extracted_k141_94388	1398	No	NA	0	0	Not-determined	Genome-fragment	NA		0
S17_extracted_k141_131286	1191	No	NA	0	1	Not-determined	Genome-fragment	NA		0
S17_extracted_k141_58091	1438	No	NA	0	1	Not-determined	Genome-fragment	NA		0
S17_extracted_k141_72773	2132	No	NA	0	1	Not-determined	Genome-fragment	NA		0
S17_extracted_k141_154670	5497	No	NA	0	2	Not-determined	Genome-fragment	NA		0
S17_extracted_k141_36263	1440	No	NA	0	1	Not-determined	Genome-fragment	NA		0
S13_vs_61038||full	1026	No	NA	0	0	Low-quality	Genome-fragment		1.1	0
S17_extracted_k141_80139	3585	No	NA	0	2	Not-determined	Genome-fragment	NA		0
S17_extracted_k141_86783	1310	No	NA	0	0	Not-determined	Genome-fragment	NA		0
S13_vs_103022||full	1026	No	NA	0	0	Low-quality	Genome-fragment		0.47	0
S14_vs_k141_19805||lt2gene	1026	No	NA	1	0	Low-quality	Genome-fragment		2.64	0
S1_extracted_k141_122728	1026	No	NA	0	0	Low-quality	Genome-fragment		1.64	0
S17_extracted_k141_131327	5392	No	NA	0	4	Not-determined	Genome-fragment	NA		0
S17_extracted_k141_27691	1942	No	NA	0	0	Not-determined	Genome-fragment	NA		0
S17_extracted_k141_102538	1115	No	NA	0	1	Not-determined	Genome-fragment	NA		0
S17_extracted_k141_175389	1837	No	NA	0	1	Not-determined	Genome-fragment	NA		0
S17_extracted_k141_131345	1077	No	NA	0	0	Not-determined	Genome-fragment	NA		0
S1_vs_k141_16284||full	1026	No	NA	1	0	Low-quality	Genome-fragment		1.6	0
S17_extracted_k141_43710	1152	No	NA	0	0	Not-determined	Genome-fragment	NA		0
S1_vs_k141_77944||lt2gene	1026	No	NA	1	0	Low-quality	Genome-fragment		1.62	0
S17_extracted_k141_6355	1539	No	NA	0	1	Not-determined	Genome-fragment	NA		0
S17_extracted_k141_13382	2338	No	NA	0	1	Not-determined	Genome-fragment	NA		0
S3_extracted_k141_43795	1026	No	NA	0	0	Low-quality	Genome-fragment		1.93	0
S17_extracted_k141_110556	2783	No	NA	0	1	Not-determined	Genome-fragment	NA		0
S17_extracted_k141_43725	2052	No	NA	0	0	Not-determined	Genome-fragment	NA		0
S17_extracted_k141_86873	1609	No	NA	0	1	Not-determined	Genome-fragment	NA		0
S17_extracted_k141_175434	1372	No	NA	0	2	Not-determined	Genome-fragment	NA		0
S17_extracted_k141_27734	1296	No	NA	0	1	Not-determined	Genome-fragment	NA		0
S3_vs_k141_110104||full	1026	No	NA	3	0	Low-quality	Genome-fragment		1.91	0
S17_extracted_k141_175449	3423	No	NA	0	1	Not-determined	Genome-fragment	NA		0
S17_extracted_k141_175451	1114	No	NA	0	0	Not-determined	Genome-fragment	NA		0
S17_extracted_k141_72918	1637	No	NA	0	3	Not-determined	Genome-fragment	NA		0
S5_vs_k141_43122||full	1026	No	NA	1	0	Low-quality	Genome-fragment		2.27	0
S5_vs_k141_2888||full	1026	No	NA	2	0	Low-quality	Genome-fragment		2.4	0
S17_extracted_k141_175465	1085	No	NA	0	1	Not-determined	Genome-fragment	NA		0
S17_extracted_k141_154761	7711	No	NA	0	1	Not-determined	Genome-fragment	NA		0
S17_extracted_k141_20525	3895	No	NA	0	1	Not-determined	Genome-fragment	NA		0
S5_vs_k141_127141||full	1026	No	NA	2	0	Low-quality	Genome-fragment		2.24	0
S17_extracted_k141_117503	1025	No	NA	0	2	Not-determined	Genome-fragment	NA		0
S17_extracted_k141_20531	1215	No	NA	0	2	Not-determined	Genome-fragment	NA		0
S17_extracted_k141_36367	8162	No	NA	0	4	Not-determined	Genome-fragment	NA		0
S5_vs_k141_81744||full	1026	No	NA	0	0	Low-quality	Genome-fragment		1.73	0
S17_extracted_k141_58235	1074	No	NA	0	1	Not-determined	Genome-fragment	NA		0
S17_extracted_k141_131451	1442	No	NA	0	0	Not-determined	Genome-fragment	NA		0
S7_extracted_100664	1026	No	NA	0	0	Low-quality	Genome-fragment		2.1	0
S17_extracted_k141_139165	1154	No	NA	0	1	Not-determined	Genome-fragment	NA		0
S17_extracted_k141_58242	1163	No	NA	0	1	Not-determined	Genome-fragment	NA		0
S17_extracted_k141_94486	2038	No	NA	0	0	Not-determined	Genome-fragment	NA		0
S17_extracted_k141_13500	1870	No	NA	0	1	Not-determined	Genome-fragment	NA		0
S17_extracted_k141_102671	1843	No	NA	0	1	Not-determined	Genome-fragment	NA		0
S17_extracted_k141_51008	1144	No	NA	0	2	Not-determined	Genome-fragment	NA		0
S7_vs_84670||full	1026	No	NA	0	0	Low-quality	Genome-fragment		2.06	0
S17_extracted_k141_110623	3281	No	NA	0	0	Not-determined	Genome-fragment	NA		0
S17_extracted_k141_94502	1004	No	NA	0	0	Not-determined	Genome-fragment	NA		0
S8_vs_k141_123577||full	1026	No	NA	0	1	Low-quality	Genome-fragment		1.7	0
S9_vs_k141_2681||full	1027	No	NA	1	0	Low-quality	Genome-fragment		2.31	0
S17_extracted_k141_146926	1409	No	NA	0	0	Not-determined	Genome-fragment	NA		0
S17_extracted_k141_80356	1392	No	NA	0	0	Not-determined	Genome-fragment	NA		0
S14_vs_k141_56805||full	1027	No	NA	0	0	Low-quality	Genome-fragment		0.58	0
S17_extracted_k141_146940	1607	No	NA	0	1	Not-determined	Genome-fragment	NA		0
S17_extracted_k141_51045	1331	No	NA	0	1	Not-determined	Genome-fragment	NA		0
S17_extracted_k141_117540	1039	No	NA	0	1	Not-determined	Genome-fragment	NA		0
S17_extracted_k141_124273	1638	No	NA	0	1	Not-determined	Genome-fragment	NA		0
S17_extracted_k141_87000	2873	No	NA	0	4	Not-determined	Genome-fragment	NA		0
S17_extracted_k141_27845	1112	No	NA	0	0	Not-determined	Genome-fragment	NA		0
S17_extracted_k141_13557	3770	No	NA	0	3	Not-determined	Genome-fragment	NA		0
S17_extracted_k141_161865	1794	No	NA	0	1	Not-determined	Genome-fragment	NA		0
S17_extracted_k141_43877	1496	No	NA	0	1	Not-determined	Genome-fragment	NA		0
S17_extracted_k141_131534	1342	No	NA	0	1	Not-determined	Genome-fragment	NA		0
S1_vs_k141_16329||full	1027	No	NA	0	0	Low-quality	Genome-fragment		0.65	0
S17_extracted_k141_80423	1038	No	NA	0	1	Not-determined	Genome-fragment	NA		0
S17_extracted_k141_168672	3573	No	NA	0	1	Not-determined	Genome-fragment	NA		0
S17_extracted_k141_80450	2353	No	NA	0	1	Not-determined	Genome-fragment	NA		0
S17_extracted_k141_102805	1719	No	NA	0	0	Not-determined	Genome-fragment	NA		0
S17_extracted_k141_124346	2132	No	NA	0	2	Not-determined	Genome-fragment	NA		0
S17_extracted_k141_124349	4929	No	NA	0	2	Not-determined	Genome-fragment	NA		0
S17_extracted_k141_36452	16135	No	NA	6	0	Medium-quality	Genome-fragment		68.35	0
S17_extracted_k141_6569	2452	No	NA	0	1	Not-determined	Genome-fragment	NA		0
S17_extracted_k141_6572	3094	No	NA	0	2	Not-determined	Genome-fragment	NA		0
S1_vs_k141_11615||lt2gene	1027	No	NA	1	0	Low-quality	Genome-fragment		2.28	0
S17_extracted_k141_27929	1067	No	NA	0	1	Not-determined	Genome-fragment	NA		0
S16_vs_156030||full	1027	No	NA	1	0	Low-quality	Genome-fragment		1.55	0
S17_extracted_k141_154921	4661	No	NA	0	0	Not-determined	Genome-fragment	NA		0
S17_extracted_k141_154934	1792	No	NA	0	0	Not-determined	Genome-fragment	NA		0
S17_extracted_k141_80488	2353	No	NA	0	1	Not-determined	Genome-fragment	NA		0
S10_vs_14075||full	1027	No	NA	0	0	Low-quality	Genome-fragment		1.79	0
S17_extracted_k141_80497	1575	No	NA	0	1	Not-determined	Genome-fragment	NA		0
S17_extracted_k141_110684	4391	No	NA	0	3	Not-determined	Genome-fragment	NA		0
S17_extracted_k141_147144	1004	No	NA	0	0	Not-determined	Genome-fragment	NA		0
S17_extracted_k141_154972	5060	No	NA	0	3	Not-determined	Genome-fragment	NA		0
S17_extracted_k141_139277	9827	No	NA	0	2	Not-determined	Genome-fragment	NA		0
S17_extracted_k141_6603	1068	No	NA	0	1	Not-determined	Genome-fragment	NA		0
S17_extracted_k141_51206	1439	No	NA	0	0	Not-determined	Genome-fragment	NA		0
S17_extracted_k141_175610	4223	No	NA	0	2	Not-determined	Genome-fragment	NA		0
S10_vs_14796||full	1027	No	NA	1	0	Low-quality	Genome-fragment		2.44	0
S12_vs_k141_84258||full	1027	No	NA	0	1	Low-quality	Genome-fragment		2.43	0
S17_extracted_k141_139298	1442	No	NA	0	0	Not-determined	Genome-fragment	NA		0
S17_extracted_k141_175628	1863	No	NA	0	0	Not-determined	Genome-fragment	NA		0
S17_extracted_k141_131642	3201	No	NA	0	1	Not-determined	Genome-fragment	NA		0
S17_extracted_k141_87111	20056	No	NA	0	4	Not-determined	Genome-fragment	NA		0
S17_extracted_k141_44022	2379	No	NA	0	2	Not-determined	Genome-fragment	NA		0

S17_extracted_k141_175638	1182	No	NA	0	0	Not-determined	Genome-fragment	NA		0
S17_extracted_k141_155009	1187	No	NA	0	1	Not-determined	Genome-fragment	NA		0
S17_extracted_k141_175646	1020	No	NA	0	1	Not-determined	Genome-fragment	NA		0
S17_extracted_k141_6631	1255	No	NA	0	1	Not-determined	Genome-fragment	NA		0
S17_extracted_k141_73167	1263	No	NA	0	0	Not-determined	Genome-fragment	NA		0
S17_extracted_k141_162022	1536	No	NA	0	0	Not-determined	Genome-fragment	NA		0
S12_vs_k141_7731||full	1027	No	NA	1	0	Low-quality	Genome-fragment		2.37	0
S17_extracted_k141_175670	1030	No	NA	0	1	Not-determined	Genome-fragment	NA		0
S2_extracted_k141_17221	1027	No	NA	1	0	Low-quality	Genome-fragment		2.56	0
S17_extracted_k141_73175	1107	No	NA	0	0	Not-determined	Genome-fragment	NA		0
S17_extracted_k141_80581	1280	No	NA	0	1	Not-determined	Genome-fragment	NA		0
S17_extracted_k141_155047	1038	No	NA	0	1	Not-determined	Genome-fragment	NA		0
S8_vs_k141_92066||full	1027	No	NA	0	1	Low-quality	Genome-fragment		0.53	0
S17_extracted_k141_51284	1038	No	NA	0	1	Not-determined	Genome-fragment	NA		0
S17_extracted_k141_55693	1028	No	NA	0	1	Low-quality	Genome-fragment		3.46	0
S17_extracted_k141_139373	1012	No	NA	0	0	Not-determined	Genome-fragment	NA		0
S17_extracted_k141_93125	1028	No	NA	4	0	Low-quality	Genome-fragment		3.39	0
S17_extracted_k141_168859	1702	No	NA	0	0	Not-determined	Genome-fragment	NA		0
S17_extracted_k141_80629	1125	No	NA	0	1	Not-determined	Genome-fragment	NA		0
S17_extracted_k141_44060	2502	No	NA	0	1	Not-determined	Genome-fragment	NA		0
S17_extracted_k141_124442	1590	No	NA	0	0	Not-determined	Genome-fragment	NA		0
S17_extracted_k141_94666	4014	No	NA	0	2	Not-determined	Genome-fragment	NA		0
S17_extracted_k141_87208	3553	No	NA	0	0	Not-determined	Genome-fragment	NA		0
S17_extracted_k141_124456	1064	No	NA	0	1	Not-determined	Genome-fragment	NA		0
S17_extracted_k141_13772	1177	No	NA	0	1	Not-determined	Genome-fragment	NA		0
S17_extracted_k141_73250	1326	No	NA	0	3	Not-determined	Genome-fragment	NA		0
S11_vs_k141_71077||full	1028	No	NA	1	0	Low-quality	Genome-fragment		2.2	0
S14_vs_k141_115822||full	1028	No	NA	0	2	Low-quality	Genome-fragment		2.46	0
S17_extracted_k141_131755	1790	No	NA	0	4	Not-determined	Genome-fragment	NA		0
S17_extracted_k141_28031	1043	No	NA	0	0	Not-determined	Genome-fragment	NA		0
S17_extracted_k141_6720	1323	No	NA	0	2	Not-determined	Genome-fragment	NA		0
S17_extracted_k141_13792	2995	No	NA	0	3	Not-determined	Genome-fragment	NA		0
S17_extracted_k141_51337	2430	No	NA	0	0	Not-determined	Genome-fragment	NA		0
S17_extracted_k141_44102	1160	No	NA	0	0	Not-determined	Genome-fragment	NA		0
S17_extracted_k141_73279	3533	No	NA	0	4	Not-determined	Genome-fragment	NA		0
S17_extracted_k141_20705	1171	No	NA	0	1	Not-determined	Genome-fragment	NA		0
S17_extracted_k141_147278	1052	No	NA	0	1	Not-determined	Genome-fragment	NA		0
S17_extracted_k141_58523	1161	No	NA	0	0	Not-determined	Genome-fragment	NA		0
S17_extracted_k141_147284	1225	No	NA	0	3	Not-determined	Genome-fragment	NA		0
S1_vs_k141_134818||full	1028	No	NA	0	0	Low-quality	Genome-fragment		2.5	0
S17_extracted_k141_28055	1854	No	NA	0	3	Not-determined	Genome-fragment	NA		0
S17_extracted_k141_139438	1533	No	NA	0	2	Not-determined	Genome-fragment	NA		0
S17_extracted_k141_131808	1204	No	NA	0	1	Not-determined	Genome-fragment	NA		0
S1_vs_k141_77523||full	1028	No	NA	0	0	Low-quality	Genome-fragment		1.26	0
S1_vs_k141_104936||full	1028	No	NA	1	0	Low-quality	Genome-fragment		2.63	0
S1_vs_k141_102248||lt2gene	1028	No	NA	1	0	Low-quality	Genome-fragment		2.14	0
S17_extracted_k141_168908	1259	No	NA	0	1	Not-determined	Genome-fragment	NA		0
S17_extracted_k141_139473	1057	No	NA	0	2	Not-determined	Genome-fragment	NA		0
S17_extracted_k141_73322	4041	No	NA	0	5	Not-determined	Genome-fragment	NA		0
S17_extracted_k141_64655	5584	No	NA	0	2	Not-determined	Genome-fragment	NA		0
S17_extracted_k141_6794	1289	No	NA	0	2	Not-determined	Genome-fragment	NA		0
S5_vs_k141_18882||full	1028	No	NA	0	0	Low-quality	Genome-fragment		2.64	0
S17_extracted_k141_51384	1174	No	NA	0	0	Not-determined	Genome-fragment	NA		0
S17_extracted_k141_20776	1283	No	NA	0	1	Not-determined	Genome-fragment	NA		0
S17_extracted_k141_87332	1524	No	NA	0	0	Not-determined	Genome-fragment	NA		0
S17_extracted_k141_44156	10711	No	NA	0	6	Not-determined	Genome-fragment	NA		0
S17_extracted_k141_162120	1185	No	NA	0	1	Not-determined	Genome-fragment	NA		0
S17_extracted_k141_51393	2332	No	NA	0	1	Not-determined	Genome-fragment	NA		0
S17_extracted_k141_20794	1065	No	NA	0	0	Not-determined	Genome-fragment	NA		0
S17_extracted_k141_6807	5633	No	NA	0	2	Not-determined	Genome-fragment	NA		0
S17_extracted_k141_80659	1667	No	NA	0	1	Not-determined	Genome-fragment	NA		0
S17_extracted_k141_168960	1921	No	NA	0	1	Not-determined	Genome-fragment	NA		0
S7_extracted_514	1028	No	NA	1	0	Low-quality	Genome-fragment		4.66	0
S7_vs_18537||full	1028	No	NA	0	0	Low-quality	Genome-fragment		2.42	0
S17_extracted_k141_94823	1453	No	NA	0	1	Not-determined	Genome-fragment	NA		0
S17_extracted_k141_175834	2232	No	NA	0	1	Not-determined	Genome-fragment	NA		0
S17_extracted_k141_64712	1438	No	NA	0	0	Not-determined	Genome-fragment	NA		0
S17_extracted_k141_87397	3167	No	NA	0	1	Not-determined	Genome-fragment	NA		0
S17_extracted_k141_51451	1397	No	NA	0	1	Not-determined	Genome-fragment	NA		0
S17_extracted_k141_162180	1141	No	NA	0	1	Not-determined	Genome-fragment	NA		0
S17_extracted_k141_169034	1024	No	NA	0	0	Not-determined	Genome-fragment	NA		0
S17_extracted_k141_131935	1862	No	NA	0	2	Not-determined	Genome-fragment	NA		0
S17_extracted_k141_13885	5568	No	NA	0	3	Not-determined	Genome-fragment	NA		0
S16_vs_23301||full	1028	No	NA	0	0	Low-quality	Genome-fragment		0.63	0
S17_extracted_k141_162200	1175	No	NA	0	1	Not-determined	Genome-fragment	NA		0
S17_extracted_k141_64767	1682	No	NA	0	1	Not-determined	Genome-fragment	NA		0
S10_vs_119900||full	1028	No	NA	0	0	Low-quality	Genome-fragment		1.98	0
S17_extracted_k141_175895	1203	No	NA	0	0	Not-determined	Genome-fragment	NA		0
S17_extracted_k141_80767	1617	No	NA	0	2	Not-determined	Genome-fragment	NA		0
S12_extracted_k141_180636	1028	No	NA	0	1	Low-quality	Genome-fragment		0.66	0
S17_extracted_k141_175970	1389	No	NA	0	0	Not-determined	Genome-fragment	NA		0
S12_vs_k141_164692||full	1028	No	NA	0	2	Low-quality	Genome-fragment		0.73	0
S17_extracted_k141_13941	3223	No	NA	0	3	Not-determined	Genome-fragment	NA		0
S8_extracted_k141_32456	1028	No	NA	0	0	Low-quality	Genome-fragment		2.3	0
S17_extracted_k141_162287	3896	No	NA	0	2	Not-determined	Genome-fragment	NA		0
S11_vs_k141_88844||full	1029	No	NA	0	1	Low-quality	Genome-fragment		0.58	0
S17_extracted_k141_13948	1377	No	NA	0	2	Not-determined	Genome-fragment	NA		0
S17_extracted_k141_51566	2063	No	NA	0	0	Not-determined	Genome-fragment	NA		0
S3_extracted_k141_107231	1029	No	NA	0	0	Low-quality	Genome-fragment		1.02	0
S17_extracted_k141_139626	1352	No	NA	0	0	Not-determined	Genome-fragment	NA		0
S3_vs_k141_34511||full	1029	No	NA	0	0	Low-quality	Genome-fragment		2.06	0
S3_vs_k141_37161||full	1029	No	NA	0	1	Low-quality	Genome-fragment		0.69	0
S5_extracted_k141_137102	1029	No	NA	0	0	Low-quality	Genome-fragment		2.48	0
S17_extracted_k141_64800	1114	No	NA	0	1	Not-determined	Genome-fragment	NA		0
S17_extracted_k141_162326	1564	No	NA	0	2	Not-determined	Genome-fragment	NA		0
S17_extracted_k141_51601	7838	No	NA	0	3	Not-determined	Genome-fragment	NA		0
S17_extracted_k141_80858	3602	No	NA	0	0	Not-determined	Genome-fragment	NA		0
S17_extracted_k141_21028	3249	No	NA	0	2	Not-determined	Genome-fragment	NA		0
S17_extracted_k141_132117	6192	No	NA	0	1	Not-determined	Genome-fragment	NA		0
S17_extracted_k141_132123	1067	No	NA	0	0	Not-determined	Genome-fragment	NA		0
S17_extracted_k141_64847	1235	No	NA	0	2	Not-determined	Genome-fragment	NA		0
S17_extracted_k141_169182	1145	No	NA	0	1	Not-determined	Genome-fragment	NA		0
S17_extracted_k141_176045	1006	No	NA	0	0	Not-determined	Genome-fragment	NA		0
S17_extracted_k141_73623	1879	No	NA	0	0	Not-determined	Genome-fragment	NA		0
S17_extracted_k141_51634	1350	No	NA	0	1	Not-determined	Genome-fragment	NA		0
S17_extracted_k141_147636	1818	No	NA	0	2	Not-determined	Genome-fragment	NA		0
S17_extracted_k141_169208	1925	No	NA	0	1	Not-determined	Genome-fragment	NA		0
S17_extracted_k141_7049	1385	No	NA	0	2	Not-determined	Genome-fragment	NA		0
NC1_vf_k141_1129	1029	No	NA	0	1	Low-quality	Genome-fragment		1.3	0
S17_extracted_k141_28399	1119	No	NA	0	1	Not-determined	Genome-fragment	NA		0
S17_extracted_k141_51658	1047	No	NA	0	1	Not-determined	Genome-fragment	NA		0
S17_extracted_k141_58670	1519	No	NA	0	1	Not-determined	Genome-fragment	NA		0
S17_extracted_k141_7082	1090	No	NA	0	1	Not-determined	Genome-fragment	NA		0
S16_extracted_35204	1029	No	NA	1	0	Low-quality	Genome-fragment		2.05	0
S17_extracted_k141_64909	1086	No	NA	0	0	Not-determined	Genome-fragment	NA		0
S17_extracted_k141_103373	3193	No	NA	0	4	Not-determined	Genome-fragment	NA		0
S17_extracted_k141_95010	2119	No	NA	0	1	Not-determined	Genome-fragment	NA		0
S16_vs_84209||lt2gene	1029	No	NA	0	0	Low-quality	Genome-fragment		2.33	0
S17_extracted_k141_95011	1225	No	NA	0	0	Not-determined	Genome-fragment	NA		0
S17_extracted_k141_28424	1147	No	NA	0	1	Not-determined	Genome-fragment	NA		0
S17_extracted_k141_124776	1395	No	NA	0	1	Not-determined	Genome-fragment	NA		0
S17_extracted_k141_87633	1769	No	NA	0	1	Not-determined	Genome-fragment	NA		0
S17_extracted_k141_87634	2007	No	NA	0	0	Not-determined	Genome-fragment	NA		0
S17_extracted_k141_73684	3401	No	NA	0	3	Not-determined	Genome-fragment	NA		0
S17_extracted_k141_7133	1103	No	NA	0	1	Not-determined	Genome-fragment	NA		0
S12_vs_k141_148654||full	1029	No	NA	0	0	Low-quality	Genome-fragment		0.75	0
S8_vs_k141_103993||full	1029	No	NA	0	1	Low-quality	Genome-fragment		0.62	0
S17_extracted_k141_21170	3056	No	NA	0	2	Not-determined	Genome-fragment	NA		0
S17_extracted_k141_21171	1305	No	NA	0	1	Not-determined	Genome-fragment	NA		0
S17_extracted_k141_162470	1801	No	NA	0	1	Not-determined	Genome-fragment	NA		0
S17_extracted_k141_73695	1534	No	NA	0	0	Not-determined	Genome-fragment	NA		0
S17_extracted_k141_36814	3372	No	NA	0	2	Not-determined	Genome-fragment	NA		0
S17_vs_k141_79878||full	1030	No	NA	0	0	Low-quality	Genome-fragment		1.32	0
S17_extracted_k141_169309	1092	No	NA	0	2	Not-determined	Genome-fragment	NA		0
S17_extracted_k141_28487	4927	No	NA	0	2	Not-determined	Genome-fragment	NA		0
S17_extracted_k141_28488	1091	No	NA	0	1	Not-determined	Genome-fragment	NA		0
S9_extracted_k141_51257	1030	No	NA	0	0	Low-quality	Genome-fragment		1.93	0
S17_extracted_k141_118070	3217	No	NA	0	0	Not-determined	Genome-fragment	NA		0
S17_extracted_k141_169321	3501	No	NA	0	2	Not-determined	Genome-fragment	NA		0
S17_extracted_k141_169344	1068	No	NA	0	0	Not-determined	Genome-fragment	NA		0
S9_vs_k141_2174||lt2gene	1030	No	NA	1	0	Low-quality	Genome-fragment		3.05	0
S17_extracted_k141_81071	2062	No	NA	0	2	Not-determined	Genome-fragment	NA		0
S17_extracted_k141_176200	5838	No	NA	0	4	Not-determined	Genome-fragment	NA		0
S14_extracted_k141_55052	1030	No	NA	0	0	Low-quality	Genome-fragment		3.23	0
S1_extracted_k141_20971	1030	No	NA	0	0	Low-quality	Genome-fragment		3.12	0
S3_extracted_k141_81932	1030	No	NA	0	1	Low-quality	Genome-fragment		0.45	0
S17_extracted_k141_169395	1029	No	NA	0	1	Not-determined	Genome-fragment	NA		0
S3_vs_k141_114453||full	1030	No	NA	0	0	Low-quality	Genome-fragment		1.13	0
S17_extracted_k141_95131	1521	No	NA	0	1	Not-determined	Genome-fragment	NA		0
S5_extracted_k141_39353	1030	No	NA	0	0	Low-quality	Genome-fragment		1.5	0
S17_extracted_k141_176268	1106	No	NA	0	2	Not-determined	Genome-fragment	NA		0
S17_extracted_k141_176270	1171	No	NA	0	1	Not-determined	Genome-fragment	NA		0
S17_extracted_k141_73849	2792	No	NA	0	2	Not-determined	Genome-fragment	NA		0
S17_extracted_k141_169445	1856	No	NA	0	3	Not-determined	Genome-fragment	NA		0
S17_extracted_k141_124959	1503	No	NA	0	0	Not-determined	Genome-fragment	NA		0
S17_extracted_k141_124971	1126	No	NA	0	0	Not-determined	Genome-fragment	NA		0
S5_vs_k141_74671||lt2gene	1030	No	NA	1	0	Low-quality	Genome-fragment		0.64	0
S17_extracted_k141_36907	1642	No	NA	0	2	Not-determined	Genome-fragment	NA		0
S17_extracted_k141_81160	2268	No	NA	0	1	Not-determined	Genome-fragment	NA		0
S17_extracted_k141_65154	10424	No	NA	0	3	Not-determined	Genome-fragment	NA		0
S16_extracted_9538	1030	No	NA	1	0	Low-quality	Genome-fragment		1.39	0
S17_extracted_k141_7335	3164	No	NA	0	0	Not-determined	Genome-fragment	NA		0
S16_extracted_75305	1030	No	NA	0	1	Low-quality	Genome-fragment		1.84	0
S17_extracted_k141_169522	1532	No	NA	0	3	Not-determined	Genome-fragment	NA		0
S17_extracted_k141_169529	1678	No	NA	0	0	Not-determined	Genome-fragment	NA		0
S17_extracted_k141_176330	4147	No	NA	0	1	Not-determined	Genome-fragment	NA		0
S17_extracted_k141_21505	1108	No	NA	0	0	Not-determined	Genome-fragment	NA		0
S16_vs_27996||full	1030	No	NA	2	0	Low-quality	Genome-fragment		2.2	0
S17_extracted_k141_169550	1006	No	NA	0	1	Not-determined	Genome-fragment	NA		0
S17_extracted_k141_81214	4662	No	NA	0	0	Not-determined	Genome-fragment	NA		0
S17_extracted_k141_65272	1111	No	NA	0	1	Not-determined	Genome-fragment	NA		0
S17_extracted_k141_14304	1215	No	NA	0	0	Not-determined	Genome-fragment	NA		0
S17_extracted_k141_14331	1294	No	NA	0	0	Not-determined	Genome-fragment	NA		0
S17_extracted_k141_132526	1022	No	NA	0	1	Not-determined	Genome-fragment	NA		0

S17_extracted_k141_81226	1338	No	NA	0	0	Not-determined	Genome-fragment	NA		0
S17_extracted_k141_65321	8451	No	NA	0	3	Not-determined	Genome-fragment	NA		0
S17_extracted_k141_176441	3900	No	NA	0	2	Not-determined	Genome-fragment	NA		0
S17_extracted_k141_95354	3319	No	NA	0	2	Not-determined	Genome-fragment	NA		0
S17_extracted_k141_169722	1573	No	NA	0	2	Not-determined	Genome-fragment	NA		0
S17_extracted_k141_169731	7446	No	NA	0	4	Not-determined	Genome-fragment	NA		0
S17_extracted_k141_169737	1043	No	NA	0	0	Not-determined	Genome-fragment	NA		0
S17_extracted_k141_28883	1010	No	NA	0	0	Not-determined	Genome-fragment	NA		0
S17_extracted_k141_7367	5528	No	NA	0	1	Not-determined	Genome-fragment	NA		0
S17_extracted_k141_132648	3640	No	NA	0	2	Not-determined	Genome-fragment	NA		0
S10_vs_1259||full	1030	No	NA	0	0	Low-quality	Genome-fragment		1.75	0
S12_extracted_k141_130268	1030	No	NA	1	0	Low-quality	Genome-fragment		2.34	0
S12_vs_k141_119888||full	1030	No	NA	0	0	Low-quality	Genome-fragment		2.14	0
S17_extracted_k141_14436	4219	No	NA	0	4	Not-determined	Genome-fragment	NA		0
S17_extracted_k141_14439	1365	No	NA	0	3	Not-determined	Genome-fragment	NA		0
S17_extracted_k141_59045	3456	No	NA	0	1	Not-determined	Genome-fragment	NA		0
S17_extracted_k141_140087	1319	No	NA	0	1	Not-determined	Genome-fragment	NA		0
S17_extracted_k141_65423	1317	No	NA	0	1	Not-determined	Genome-fragment	NA		0
S17_extracted_k141_65430	1468	No	NA	0	0	Not-determined	Genome-fragment	NA		0
S4_extracted_10627	1030	No	NA	5	0	Low-quality	Genome-fragment		3.38	0
S17_extracted_k141_132696	3778	No	NA	0	1	Not-determined	Genome-fragment	NA		0
S17_extracted_k141_28958	1826	No	NA	0	1	Not-determined	Genome-fragment	NA		0
S17_extracted_k141_14477	1040	No	NA	0	1	Not-determined	Genome-fragment	NA		0
S17_extracted_k141_21718	32371	No	NA	0	6	High-quality	High-quality		93.35	0
S17_extracted_k141_59082	2196	No	NA	0	1	Not-determined	Genome-fragment	NA		0
S17_extracted_k141_88117	1159	No	NA	0	1	Not-determined	Genome-fragment	NA		0
S17_extracted_k141_81232	102314	No	NA	16	1	Complete	High-quality		100	0
S17_extracted_k141_132711	2332	No	NA	0	2	Not-determined	Genome-fragment	NA		0
S17_extracted_k141_65455	9112	No	NA	0	7	Not-determined	Genome-fragment	NA		0
S17_extracted_k141_95413	6729	No	NA	0	2	Not-determined	Genome-fragment	NA		0
S17_extracted_k141_140121	1089	No	NA	0	0	Not-determined	Genome-fragment	NA		0
S17_extracted_k141_81250	7892	No	NA	0	3	Not-determined	Genome-fragment	NA		0
S17_extracted_k141_176593	2473	No	NA	0	6	Not-determined	Genome-fragment	NA		0
S17_extracted_k141_81275	1629	No	NA	0	1	Not-determined	Genome-fragment	NA		0
S17_extracted_k141_95462	1057	No	NA	0	1	Not-determined	Genome-fragment	NA		0
S17_extracted_k141_88186	3488	No	NA	0	1	Not-determined	Genome-fragment	NA		0
S8_vs_k141_92992||full	1030	No	NA	0	2	Low-quality	Genome-fragment		1.66	0
S17_extracted_k141_125242	2674	No	NA	0	1	Not-determined	Genome-fragment	NA		0
S17_extracted_k141_125256	1575	No	NA	0	0	Not-determined	Genome-fragment	NA		0
S17_extracted_k141_21787	4162	No	NA	0	3	Not-determined	Genome-fragment	NA		0
S17_extracted_k141_176614	1476	No	NA	0	1	Not-determined	Genome-fragment	NA		0
S17_extracted_k141_132802	1353	No	NA	0	2	Not-determined	Genome-fragment	NA		0
S17_extracted_k141_125285	1170	No	NA	0	2	Not-determined	Genome-fragment	NA		0
S17_extracted_k141_176627	1233	No	NA	0	1	Not-determined	Genome-fragment	NA		0
S17_extracted_k141_65628	1324	No	NA	0	0	Not-determined	Genome-fragment	NA		0
S17_extracted_k141_14643	3840	No	NA	0	3	Not-determined	Genome-fragment	NA		0
S17_extracted_k141_176672	4516	No	NA	0	3	Not-determined	Genome-fragment	NA		0
S17_extracted_k141_95633	1150	No	NA	0	0	Not-determined	Genome-fragment	NA		0
S17_extracted_k141_14666	1108	No	NA	0	1	Not-determined	Genome-fragment	NA		0
S17_extracted_k141_176703	2247	No	NA	0	1	Not-determined	Genome-fragment	NA		0
S17_extracted_k141_14675	1279	No	NA	0	0	Not-determined	Genome-fragment	NA		0
S17_extracted_k141_125411	1364	No	NA	0	0	Not-determined	Genome-fragment	NA		0
S8_vs_k141_14440||lt2gene	1030	No	NA	1	0	Low-quality	Genome-fragment		2.11	0
S17_extracted_k141_65715	1055	No	NA	0	0	Not-determined	Genome-fragment	NA		0
S17_extracted_k141_132991	1243	No	NA	0	0	Not-determined	Genome-fragment	NA		0
S17_extracted_k141_95777	2160	No	NA	0	1	Not-determined	Genome-fragment	NA		0
S17_extracted_k141_29234	1089	No	NA	0	0	Not-determined	Genome-fragment	NA		0
S17_extracted_k141_125509	1144	No	NA	0	2	Not-determined	Genome-fragment	NA		0
S17_extracted_k141_88431	1393	No	NA	0	0	Not-determined	Genome-fragment	NA		0
S17_extracted_k141_14772	2905	No	NA	0	1	Not-determined	Genome-fragment	NA		0
S17_extracted_k141_65791	1447	No	NA	0	1	Not-determined	Genome-fragment	NA		0
S17_extracted_k141_65806	1115	No	NA	0	2	Not-determined	Genome-fragment	NA		0
S17_extracted_k141_125580	3895	No	NA	0	3	Not-determined	Genome-fragment	NA		0
S17_extracted_k141_32900	1031	No	NA	2	0	Low-quality	Genome-fragment		3.28	0
S17_extracted_k141_65846	1160	No	NA	0	2	Not-determined	Genome-fragment	NA		0
S17_extracted_k141_29319	1088	No	NA	0	3	Not-determined	Genome-fragment	NA		0
S17_extracted_k141_29370	1967	No	NA	0	0	Not-determined	Genome-fragment	NA		0
S17_extracted_k141_65935	3810	No	NA	0	3	Not-determined	Genome-fragment	NA		0
S17_vs_k141_2314||full	1031	No	NA	1	0	Low-quality	Genome-fragment		1.53	0
S17_vs_k141_71471||full	1031	No	NA	0	0	Low-quality	Genome-fragment		0.76	0
S17_extracted_k141_95856	2648	No	NA	0	2	Not-determined	Genome-fragment	NA		0
S17_extracted_k141_176976	2829	No	NA	0	2	Not-determined	Genome-fragment	NA		0
S17_extracted_k141_29447	3467	No	NA	0	2	Not-determined	Genome-fragment	NA		0
S9_vs_k141_48669||full	1031	No	NA	0	0	Low-quality	Genome-fragment		2.29	0
S17_extracted_k141_88604	1197	No	NA	0	1	Not-determined	Genome-fragment	NA		0
S17_extracted_k141_95918	3279	No	NA	0	4	Not-determined	Genome-fragment	NA		0
S17_extracted_k141_177070	1599	No	NA	0	1	Not-determined	Genome-fragment	NA		0
S17_extracted_k141_177088	1172	No	NA	0	0	Not-determined	Genome-fragment	NA		0
S11_extracted_k141_99962	1031	No	NA	1	0	Low-quality	Genome-fragment		0.3	0
S17_extracted_k141_29528	10475	No	NA	0	1	Not-determined	Genome-fragment	NA		0
S17_extracted_k141_29533	4147	No	NA	0	1	Not-determined	Genome-fragment	NA		0
S17_extracted_k141_177147	1105	No	NA	0	0	Not-determined	Genome-fragment	NA		0
S14_vs_k141_34526||lt2gene	1031	No	NA	0	0	Low-quality	Genome-fragment		2.44	0
S1_vs_k141_102446||full	1031	No	NA	0	0	Low-quality	Genome-fragment		0.63	0
S17_extracted_k141_177178	3265	No	NA	0	1	Not-determined	Genome-fragment	NA		0
S17_extracted_k141_177183	1046	No	NA	0	2	Not-determined	Genome-fragment	NA		0
S3_extracted_k141_88430	1031	No	NA	1	0	Low-quality	Genome-fragment		2.79	0
S17_extracted_k141_177197	53491	No	NA	12	0	Complete	High-quality		100	0
S17_extracted_k141_177199	40033	No	NA	44	0	Complete	High-quality		100	0
S17_extracted_k141_177200	36624	No	NA	10	1	Complete	High-quality		100	0
S17_extracted_k141_66356	1042	No	NA	0	1	Not-determined	Genome-fragment	NA		0
S17_extracted_k141_66357	1110	No	NA	0	0	Not-determined	Genome-fragment	NA		0
S17_extracted_k141_177221	3089	No	NA	0	0	Not-determined	Genome-fragment	NA		0
S17_extracted_k141_177254	2664	No	NA	0	1	Not-determined	Genome-fragment	NA		0
S17_vs_k141_15243||full	5390	No	NA	0	0	Not-determined	Genome-fragment	NA		0
S17_vs_k141_153490||full	2132	No	NA	0	0	Not-determined	Genome-fragment	NA		0
S17_vs_k141_138036||full	20778	No	NA	5	0	High-quality	High-quality		98.12	0
S17_vs_k141_57885||full	2732	No	NA	0	2	Not-determined	Genome-fragment	NA		0
S17_vs_k141_103009||full	43347	No	NA	12	0	High-quality	High-quality		100	0
S17_vs_k141_169681||full	5594	No	NA	0	0	Not-determined	Genome-fragment	NA		0
S17_vs_k141_177196||full	38554	No	NA	8	0	High-quality	High-quality		96.54	0
S17_vs_k141_177202||full	39636	No	NA	21	3	High-quality	High-quality		99.91	0
S17_vs_k141_177203||full	49241	No	NA	29	0	High-quality	High-quality		100	0
S17_vs_k141_177210||full	4760	No	NA	4	0	High-quality	High-quality		99.66	0
S17_vs_k141_66506||full	2430	No	NA	0	0	Not-determined	Genome-fragment	NA		0
S17_vs_k141_133010||full	4330	No	NA	0	1	Not-determined	Genome-fragment	NA		0
S17_vs_k141_118249||full	2249	No	NA	0	1	Not-determined	Genome-fragment	NA		0
S17_vs_k141_29590||full	2096	No	NA	0	1	Not-determined	Genome-fragment	NA		0
S3_extracted_k141_34431	1031	No	NA	1	0	Low-quality	Genome-fragment		4.71	0
S17_vs_k141_125634||full	1315	No	NA	0	0	Not-determined	Genome-fragment	NA		0
S3_extracted_k141_14196	1031	No	NA	0	0	Low-quality	Genome-fragment		0.56	0
S17_vs_k141_125657||full	1266	No	NA	0	2	Not-determined	Genome-fragment	NA		0
S17_vs_k141_110876||full	2699	No	NA	0	2	Not-determined	Genome-fragment	NA		0
S3_vs_k141_9509||lt2gene	1031	No	NA	2	0	Low-quality	Genome-fragment		2.78	0
S7_vs_101812||full	1031	No	NA	0	0	Low-quality	Genome-fragment		2.84	0
S16_vs_45294||full	1031	No	NA	0	0	Low-quality	Genome-fragment		2.27	0
S17_vs_k141_44366||full	1833	No	NA	0	0	Not-determined	Genome-fragment	NA		0
S17_vs_k141_110905||full	2324	No	NA	0	0	Not-determined	Genome-fragment	NA		0
S17_vs_k141_118290||full	2266	No	NA	0	0	Not-determined	Genome-fragment	NA		0
S17_vs_k141_44386||full	1915	No	NA	0	2	Not-determined	Genome-fragment	NA		0
S17_vs_k141_162600||full	2860	No	NA	0	2	Not-determined	Genome-fragment	NA		0
S17_vs_k141_37008||full	2609	No	NA	0	1	Not-determined	Genome-fragment	NA		0
S16_vs_93228||full	1031	No	NA	0	0	Low-quality	Genome-fragment		1.79	0
S17_vs_k141_118308||full	5224	No	NA	0	2	Not-determined	Genome-fragment	NA		0
S10_vs_103738||full	1031	No	NA	0	0	Low-quality	Genome-fragment		1.89	0
S12_extracted_k141_166225	1031	No	NA	0	0	Low-quality	Genome-fragment		0.46	0
S17_vs_k141_37017||full	2459	No	NA	0	1	Not-determined	Genome-fragment	NA		0
S17_vs_k141_59208||full	2343	No	NA	0	2	Not-determined	Genome-fragment	NA		0
S17_vs_k141_133074||full	8311	No	NA	0	2	Not-determined	Genome-fragment	NA		0
S17_vs_k141_140464||full	1526	No	NA	0	2	Not-determined	Genome-fragment	NA		0
S12_extracted_k141_163889	1031	No	NA	2	0	Low-quality	Genome-fragment		3.4	0
S17_vs_k141_162643||full	1911	No	NA	0	1	Not-determined	Genome-fragment	NA		0
S8_extracted_k141_177771	1031	No	NA	0	0	Low-quality	Genome-fragment		2.38	0
S17_vs_k141_114||full	2489	No	NA	0	1	Not-determined	Genome-fragment	NA		0
S17_vs_k141_59236||full	1251	No	NA	0	1	Not-determined	Genome-fragment	NA		0
S17_vs_k141_51825||full	11791	No	NA	0	9	Not-determined	Genome-fragment	NA		0
S9_extracted_k141_42856	1032	No	NA	0	0	Low-quality	Genome-fragment		2.08	0
S9_vs_k141_35615||lt2gene	1032	No	NA	1	0	Low-quality	Genome-fragment		1.4	0
S13_vs_149911||full	1032	No	NA	1	0	Low-quality	Genome-fragment		21.7	0
S14_extracted_k141_77896	1032	No	NA	0	0	Low-quality	Genome-fragment		2.68	0
S17_vs_k141_14886||full	1083	No	NA	0	1	Not-determined	Genome-fragment	NA		0
S14_vs_k141_60220||full	1032	No	NA	2	0	Low-quality	Genome-fragment		3.6	0
S17_vs_k141_22238||full	7215	No	NA	0	1	Not-determined	Genome-fragment	NA		0
S14_vs_k141_17440||full	1032	No	NA	3	0	Low-quality	Genome-fragment		3.47	0
S17_vs_k141_96230||full	4419	No	NA	0	1	Not-determined	Genome-fragment	NA		0
S1_vs_k141_81813||full	1032	No	NA	0	0	Low-quality	Genome-fragment		1.21	0
S17_vs_k141_66672||full	2052	No	NA	0	2	Not-determined	Genome-fragment	NA		0
S17_vs_k141_14905||full	1795	No	NA	0	0	Not-determined	Genome-fragment	NA		0
S17_vs_k141_59312||full	12086	No	NA	0	7	Not-determined	Genome-fragment	NA		0
S17_vs_k141_118363||full	1531	No	NA	0	1	Not-determined	Genome-fragment	NA		0
S17_vs_k141_59322||full	6478	No	NA	0	5	Not-determined	Genome-fragment	NA		0
S16_vs_79314||full	1032	No	NA	1	0	Low-quality	Genome-fragment		2.46	0
S10_vs_25512||full	1032	No	NA	0	0	Low-quality	Genome-fragment		2.68	0
S10_vs_114072||full	1032	No	NA	1	0	Low-quality	Genome-fragment		1.61	0
S17_vs_k141_133190||full	4538	No	NA	0	4	Not-determined	Genome-fragment	NA		0
S15_extracted_66954	1032	No	NA	1	0	Low-quality	Genome-fragment		2.29	0
S17_vs_k141_44495||full	4223	No	NA	0	2	Not-determined	Genome-fragment	NA		0
S17_vs_k141_44496||full	1062	No	NA	0	0	Not-determined	Genome-fragment	NA		0
S17_vs_k141_118426||full	3192	No	NA	0	1	Not-determined	Genome-fragment	NA		0
S15_vs_4966||full	1032	No	NA	0	0	Low-quality	Genome-fragment		1.08	0
S4_extracted_26255	1032	No	NA	1	0	Low-quality	Genome-fragment		1.23	0
S8_extracted_k141_166508	1032	No	NA	0	0	Low-quality	Genome-fragment		1.56	0
S17_vs_k141_140615||full	2666	No	NA	0	1	Not-determined	Genome-fragment	NA		0
S17_vs_k141_7690||full	2197	No	NA	0	1	Not-determined	Genome-fragment	NA		0
S8_extracted_k141_64495	1032	No	NA	0	1	Low-quality	Genome-fragment		1.01	0
S17_vs_k141_148004||full	1614	No	NA	0	2	Not-determined	Genome-fragment	NA		0
S17_vs_k141_133231||full	8959	No	NA	1	1	High-quality	High-quality		100	0
S17_vs_k141_96376||full	1431	No	NA	0	1	Not-determined	Genome-fragment	NA		0
S17_vs_k141_162783||full	1087	No	NA	0	1	Not-determined	Genome-fragment	NA		0
S17_vs_k141_44534||full	4606	No	NA	0	2	Not-determined	Genome-fragment	NA		0
S8_extracted_k141_178935	1032	No	NA	1	0	Low-quality	Genome-fragment		0.71	0
S17_vs_k141_7712||full	1074	No	NA	0	2	Not-determined	Genome-fragment	NA		0
S17_vs_k141_51947||full	2410	No	NA	0	2	Not-determined	Genome-fragment	NA		0
S17_vs_k141_155372||full	3447	No	NA	0	0	Not-determined	Genome-fragment	NA		0

S17_vs_k141_148060||full	2757	No	NA	0	2	Not-determined	Genome-fragment	NA		0
S17_vs_k141_96422||full	2373	No	NA	0	1	Not-determined	Genome-fragment	NA		0
S8_extracted_k141_49818	1032	No	NA	0	0	Low-quality	Genome-fragment		2.23	0
S17_vs_k141_155407||full	1437	No	NA	0	0	Not-determined	Genome-fragment	NA		0
S8_extracted_k141_188970	1032	No	NA	1	0	Low-quality	Genome-fragment		2.63	0
S8_vs_k141_122003||lt2gene	1032	No	NA	2	0	Low-quality	Genome-fragment		4.35	0
S17_vs_k141_78761||lt2gene	1033	No	NA	1	0	Low-quality	Genome-fragment		2.53	0
S17_vs_k141_88906||full	1671	No	NA	0	2	Not-determined	Genome-fragment	NA		0
S11_vs_k141_72026||full	1033	No	NA	0	0	Low-quality	Genome-fragment		1.84	0
S11_vs_k141_81891||full	1033	No	NA	2	0	Low-quality	Genome-fragment		2.06	0
S17_vs_k141_88918||full	2262	No	NA	0	2	Not-determined	Genome-fragment	NA		0
S17_vs_k141_37289||full	14637	No	NA	0	8	Not-determined	Genome-fragment	NA		0
S14_vs_k141_111590||full	1033	No	NA	4	0	Low-quality	Genome-fragment		3.4	0
S14_vs_k141_54425||full	1033	No	NA	1	0	Low-quality	Genome-fragment		1.37	0
S17_vs_k141_125870||full	2208	No	NA	0	1	Not-determined	Genome-fragment	NA		0
S7_extracted_70704	1033	No	NA	1	0	Low-quality	Genome-fragment		4.7	0
S7_vs_90197||full	1033	No	NA	0	0	Low-quality	Genome-fragment		2.44	0
S12_vs_k141_184547||lt2gene	1033	No	NA	0	0	Low-quality	Genome-fragment		2.51	0
S17_vs_k141_118515||full	1368	No	NA	0	1	Not-determined	Genome-fragment	NA		0
S15_vs_42295||full	1033	No	NA	0	0	Low-quality	Genome-fragment		1.17	0
S17_vs_k141_96476||full	1476	No	NA	0	2	Not-determined	Genome-fragment	NA		0
S17_vs_k141_111014||full	3315	No	NA	0	0	Not-determined	Genome-fragment	NA		0
S17_vs_k141_111016||full	3779	No	NA	0	0	Not-determined	Genome-fragment	NA		0
S17_vs_k141_162831||full	1300	No	NA	0	0	Not-determined	Genome-fragment	NA		0
S15_vs_25639||lt2gene	1033	No	NA	1	0	Low-quality	Genome-fragment		2.5	0
S17_vs_k141_103828||full	3215	No	NA	0	1	Not-determined	Genome-fragment	NA		0
S17_vs_k141_74215||full	4938	No	NA	0	1	Not-determined	Genome-fragment	NA		0
S4_extracted_18796	1033	No	NA	3	0	Low-quality	Genome-fragment		3.39	0
S17_vs_k141_264||full	3583	No	NA	0	2	Not-determined	Genome-fragment	NA		0
S17_vs_k141_44697||full	4440	No	NA	0	2	Not-determined	Genome-fragment	NA		0
S17_vs_k141_22356||full	1661	No	NA	0	1	Not-determined	Genome-fragment	NA		0
S17_vs_k141_148164||full	1279	No	NA	0	2	Not-determined	Genome-fragment	NA		0
S17_vs_k141_30007||full	15806	No	NA	0	9	Not-determined	Genome-fragment	NA		0
S17_vs_k141_74255||full	1745	No	NA	0	2	Not-determined	Genome-fragment	NA		0
S4_extracted_27105	1033	No	NA	0	0	Low-quality	Genome-fragment		2.23	0
S6_vs_k141_27931||full	1033	No	NA	1	0	Low-quality	Genome-fragment		2.03	0
S6_vs_k141_8807||lt2gene	1033	No	NA	1	0	Low-quality	Genome-fragment		2.15	0
S17_vs_k141_66802||full	2597	No	NA	0	3	Not-determined	Genome-fragment	NA		0
S17_vs_k141_96529||full	5117	No	NA	0	4	Not-determined	Genome-fragment	NA		0
S17_vs_k141_170279||full	4897	No	NA	0	1	Not-determined	Genome-fragment	NA		0
S6_vs_k141_11697||lt2gene	1033	No	NA	0	0	Low-quality	Genome-fragment		1.54	0
S8_vs_k141_560||full	1033	No	NA	0	0	Low-quality	Genome-fragment		2.9	0
S17_extracted_k141_13152	1034	No	NA	0	2	Low-quality	Genome-fragment		0.47	0
S17_vs_k141_170284||full	3856	No	NA	0	3	Not-determined	Genome-fragment	NA		0
S17_vs_k141_7807||full	2117	No	NA	0	2	Not-determined	Genome-fragment	NA		0
S17_vs_k141_88942||full	3207	No	NA	0	1	Not-determined	Genome-fragment	NA		0
S17_vs_k141_137428||full	1034	No	NA	0	0	Low-quality	Genome-fragment		0.19	0
S17_vs_k141_118579||full	5765	No	NA	0	4	Not-determined	Genome-fragment	NA		0
S17_vs_k141_139021||full	1034	No	NA	0	0	Low-quality	Genome-fragment		2.25	0
S17_vs_k141_162880||full	13996	No	NA	0	1	Not-determined	Genome-fragment	NA		0
S17_vs_k141_66827||full	12355	No	NA	0	9	Not-determined	Genome-fragment	NA		0
S13_extracted_102950	1034	No	NA	1	0	Low-quality	Genome-fragment		1.65	0
S17_vs_k141_133375||full	1453	No	NA	0	2	Not-determined	Genome-fragment	NA		0
S17_vs_k141_111162||full	1272	No	NA	0	1	Not-determined	Genome-fragment	NA		0
S5_vs_k141_97065||lt2gene	1034	No	NA	0	0	Low-quality	Genome-fragment		2.87	0
S7_vs_109229||full	1034	No	NA	1	0	Low-quality	Genome-fragment		2.66	0
S17_vs_k141_59625||full	1898	No	NA	0	0	Not-determined	Genome-fragment	NA		0
S17_vs_k141_88979||full	5535	No	NA	0	3	Not-determined	Genome-fragment	NA		0
S17_vs_k141_59631||full	5677	No	NA	0	4	Not-determined	Genome-fragment	NA		0
S17_vs_k141_81521||full	1398	No	NA	0	1	Not-determined	Genome-fragment	NA		0
S16_extracted_44689	1034	No	NA	0	0	Low-quality	Genome-fragment		1.47	0
S17_vs_k141_81545||full	1235	No	NA	0	0	Not-determined	Genome-fragment	NA		0
S17_vs_k141_37491||full	1230	No	NA	0	2	Not-determined	Genome-fragment	NA		0
S17_vs_k141_89008||full	1083	No	NA	0	0	Not-determined	Genome-fragment	NA		0
S17_vs_k141_15131||full	9190	No	NA	0	4	Not-determined	Genome-fragment	NA		0
S17_vs_k141_388||full	5427	No	NA	0	4	Not-determined	Genome-fragment	NA		0
S10_vs_57582||lt2gene	1034	No	NA	1	0	Low-quality	Genome-fragment		1.91	0
S17_vs_k141_170328||full	1587	No	NA	0	0	Not-determined	Genome-fragment	NA		0
S10_vs_90514||lt2gene	1034	No	NA	1	0	Low-quality	Genome-fragment		1.96	0
S17_vs_k141_89026||full	1591	No	NA	0	1	Not-determined	Genome-fragment	NA		0
S17_vs_k141_426||full	1360	No	NA	0	2	Not-determined	Genome-fragment	NA		0
S17_vs_k141_15165||full	4870	No	NA	0	3	Not-determined	Genome-fragment	NA		0
S17_vs_k141_59674||full	2207	No	NA	0	0	Not-determined	Genome-fragment	NA		0
S12_vs_k141_68140||full	1034	No	NA	0	1	Low-quality	Genome-fragment		1.46	0
S17_vs_k141_59678||full	1981	No	NA	0	0	Not-determined	Genome-fragment	NA		0
S17_vs_k141_170343||full	2585	No	NA	0	2	Not-determined	Genome-fragment	NA		0
S2_extracted_k141_4190	1034	No	NA	2	0	Low-quality	Genome-fragment		3.41	0
S8_extracted_k141_75293	1034	No	NA	0	0	Low-quality	Genome-fragment		1.9	0
S17_vs_k141_30134||full	3887	No	NA	0	2	Not-determined	Genome-fragment	NA		0
S17_vs_k141_118719||full	4658	No	NA	0	3	Not-determined	Genome-fragment	NA		0
S11_extracted_k141_8711	1035	No	NA	0	0	Low-quality	Genome-fragment		1.82	0
S17_vs_k141_81581||full	1738	No	NA	0	0	Not-determined	Genome-fragment	NA		0
S17_vs_k141_37565||full	1305	No	NA	0	2	Not-determined	Genome-fragment	NA		0
S17_vs_k141_111224||full	1023	No	NA	0	1	Not-determined	Genome-fragment	NA		0
S11_vs_k141_53263||full	1035	No	NA	1	0	Low-quality	Genome-fragment		2.65	0
S13_vs_7485||full	1035	No	NA	0	1	Low-quality	Genome-fragment		1.06	0
S17_vs_k141_22540||full	3282	No	NA	0	4	Not-determined	Genome-fragment	NA		0
S13_vs_117100||full	1035	No	NA	0	0	Low-quality	Genome-fragment		2.41	0
S17_vs_k141_37608||full	1414	No	NA	0	1	Not-determined	Genome-fragment	NA		0
S14_extracted_k141_88184	1035	No	NA	2	0	Low-quality	Genome-fragment		3.41	0
S17_vs_k141_37615||full	1415	No	NA	0	0	Not-determined	Genome-fragment	NA		0
S17_vs_k141_30203||full	2878	No	NA	0	0	Not-determined	Genome-fragment	NA		0
S14_extracted_k141_82427	1035	No	NA	0	0	Low-quality	Genome-fragment		1.73	0
S5_extracted_k141_93770	1035	No	NA	0	0	Low-quality	Genome-fragment		1.14	0
S17_vs_k141_59724||full	1424	No	NA	0	0	Not-determined	Genome-fragment	NA		0
S17_vs_k141_22563||full	1319	No	NA	0	1	Not-determined	Genome-fragment	NA		0
S17_vs_k141_170402||full	8713	No	NA	0	4	Not-determined	Genome-fragment	NA		0
S17_vs_k141_133494||full	3890	No	NA	0	3	Not-determined	Genome-fragment	NA		0
S5_vs_k141_113653||full	1035	No	NA	1	0	Low-quality	Genome-fragment		2.15	0
S17_vs_k141_74485||full	1713	No	NA	0	0	Not-determined	Genome-fragment	NA		0
S7_extracted_82460	1035	No	NA	0	0	Low-quality	Genome-fragment		2.43	0
S17_vs_k141_52328||full	1512	No	NA	0	2	Not-determined	Genome-fragment	NA		0
S7_vs_41805||full	1035	No	NA	2	0	Low-quality	Genome-fragment		2.4	0
S16_vs_99444||full	1035	No	NA	0	0	Low-quality	Genome-fragment		1.69	0
S17_vs_k141_15274||full	3396	No	NA	0	1	Not-determined	Genome-fragment	NA		0
S17_vs_k141_118812||full	1821	No	NA	0	2	Not-determined	Genome-fragment	NA		0
S17_vs_k141_74490||full	4592	No	NA	0	1	Not-determined	Genome-fragment	NA		0
S16_vs_115067||full	1035	No	NA	2	0	Low-quality	Genome-fragment		2.2	0
S12_extracted_k141_157587	1035	No	NA	3	0	Low-quality	Genome-fragment		1.73	0
S17_vs_k141_59727||full	1099	No	NA	0	0	Not-determined	Genome-fragment	NA		0
S17_vs_k141_126079||full	3072	No	NA	0	0	Not-determined	Genome-fragment	NA		0
S12_vs_k141_31762||full	1035	No	NA	0	0	Low-quality	Genome-fragment		1.85	0
S12_vs_k141_33397||full	1035	No	NA	0	1	Low-quality	Genome-fragment		1.63	0
S12_vs_k141_36721||full	1035	No	NA	0	2	Low-quality	Genome-fragment		0.61	0
S8_vs_k141_183136||full	1035	No	NA	0	1	Low-quality	Genome-fragment		1.35	0
S8_vs_k141_204171||full	1035	No	NA	0	0	Low-quality	Genome-fragment		1.21	0
S17_vs_k141_59733||full	2405	No	NA	0	1	Not-determined	Genome-fragment	NA		0
S17_vs_k141_44934||full	1045	No	NA	0	0	Not-determined	Genome-fragment	NA		0
S17_vs_k141_37695||full	1460	No	NA	0	1	Not-determined	Genome-fragment	NA		0
S17_extracted_k141_146837	1036	No	NA	0	0	Low-quality	Genome-fragment		0.61	0
S17_vs_k141_59854||lt2gene	1036	No	NA	2	0	Low-quality	Genome-fragment		1.13	0
S17_vs_k141_126112||full	15658	No	NA	0	6	Not-determined	Genome-fragment	NA		0
S17_vs_k141_59749||full	2986	No	NA	0	2	Not-determined	Genome-fragment	NA		0
S17_vs_k141_118864||full	2914	No	NA	0	1	Not-determined	Genome-fragment	NA		0
S17_vs_k141_111354||full	16003	No	NA	0	11	Not-determined	Genome-fragment	NA		0
S17_vs_k141_96574||full	1246	No	NA	0	1	Not-determined	Genome-fragment	NA		0
S17_vs_k141_104163||full	2182	No	NA	0	1	Not-determined	Genome-fragment	NA		0
S11_extracted_k141_63694	1036	No	NA	2	0	Low-quality	Genome-fragment		2.37	0
S17_vs_k141_52426||full	1509	No	NA	0	1	Not-determined	Genome-fragment	NA		0
S13_extracted_96722	1036	No	NA	0	1	Low-quality	Genome-fragment		0.68	0
S1_vs_k141_118722||full	1036	No	NA	0	1	Low-quality	Genome-fragment		0.61	0
S17_vs_k141_52436||full	4325	No	NA	0	1	Not-determined	Genome-fragment	NA		0
S17_vs_k141_111380||full	1959	No	NA	0	0	Not-determined	Genome-fragment	NA		0
S1_vs_k141_75675||lt2gene	1036	No	NA	1	0	Low-quality	Genome-fragment		2.32	0
S3_vs_k141_111793||full	1036	No	NA	0	0	Low-quality	Genome-fragment		1.7	0
S17_vs_k141_163142||full	2572	No	NA	0	0	Not-determined	Genome-fragment	NA		0
S17_vs_k141_74580||full	2668	No	NA	0	1	Not-determined	Genome-fragment	NA		0
S17_vs_k141_44971||full	3168	No	NA	0	1	Not-determined	Genome-fragment	NA		0
S17_vs_k141_66970||full	15458	No	NA	0	6	Not-determined	Genome-fragment	NA		0
S17_vs_k141_96600||full	2886	No	NA	0	3	Not-determined	Genome-fragment	NA		0
S5_extracted_k141_112411	1036	No	NA	1	0	Low-quality	Genome-fragment		2.97	0
S7_vs_97356||full	1036	No	NA	1	0	Low-quality	Genome-fragment		2.66	0
S7_vs_110152||full	1036	No	NA	0	0	Low-quality	Genome-fragment		2.56	0
S17_vs_k141_670||full	4148	No	NA	0	3	Not-determined	Genome-fragment	NA		0
S16_extracted_13761	1036	No	NA	0	0	Low-quality	Genome-fragment		2.29	0
S17_vs_k141_89213||full	5475	No	NA	0	1	Not-determined	Genome-fragment	NA		0
S17_vs_k141_104257||full	1318	No	NA	0	2	Not-determined	Genome-fragment	NA		0
S17_vs_k141_163176||full	2486	No	NA	0	2	Not-determined	Genome-fragment	NA		0
S16_extracted_20180	1036	No	NA	0	1	Low-quality	Genome-fragment		0.93	0
S17_vs_k141_8088||full	4121	No	NA	0	2	Not-determined	Genome-fragment	NA		0
S16_extracted_45454	1036	No	NA	0	2	Low-quality	Genome-fragment		0.52	0
S16_vs_104261||full	1036	No	NA	1	0	Low-quality	Genome-fragment		2.33	0
S17_vs_k141_81707||full	2582	No	NA	0	2	Not-determined	Genome-fragment	NA		0
S17_vs_k141_111445||full	4150	No	NA	0	2	Not-determined	Genome-fragment	NA		0
S17_vs_k141_89241||full	2214	No	NA	0	1	Not-determined	Genome-fragment	NA		0
S10_vs_24170||full	1036	No	NA	1	0	Low-quality	Genome-fragment		2.47	0
S10_vs_75932||lt2gene	1036	No	NA	1	0	Low-quality	Genome-fragment		7.03	0
S17_vs_k141_81728||full	2419	No	NA	0	3	Not-determined	Genome-fragment	NA		0
S15_vs_51849||full	1036	No	NA	0	0	Low-quality	Genome-fragment		1.82	0
S17_vs_k141_45041||full	2580	No	NA	0	3	Not-determined	Genome-fragment	NA		0
S17_vs_k141_141192||full	6080	No	NA	0	0	Not-determined	Genome-fragment	NA		0
S8_vs_k141_191926||full	1036	No	NA	0	0	Low-quality	Genome-fragment		1.03	0
S17_vs_k141_52538||full	2743	No	NA	0	2	Not-determined	Genome-fragment	NA		0
S11_vs_k141_92349||full	1037	No	NA	0	1	Low-quality	Genome-fragment		0.8	0
S17_vs_k141_126231||full	7927	No	NA	0	2	Not-determined	Genome-fragment	NA		0
S13_vs_142655||full	1037	No	NA	1	0	Low-quality	Genome-fragment		1.2	0
S17_vs_k141_118977||full	1482	No	NA	0	1	Not-determined	Genome-fragment	NA		0
S17_vs_k141_37924||full	1096	No	NA	0	2	Not-determined	Genome-fragment	NA		0
S14_extracted_k141_84194	1037	No	NA	0	0	Low-quality	Genome-fragment		2.39	0
S17_vs_k141_74723||full	2516	No	NA	0	0	Not-determined	Genome-fragment	NA		0
S17_vs_k141_170699||full	2957	No	NA	0	0	Not-determined	Genome-fragment	NA		0
S17_vs_k141_133634||full	3935	No	NA	0	0	Not-determined	Genome-fragment	NA		0
S17_vs_k141_96669||full	2852	No	NA	0	2	Not-determined	Genome-fragment	NA		0
S17_vs_k141_155977||full	1844	No	NA	0	2	Not-determined	Genome-fragment	NA		0

S14_extracted_k141_42593	1037	No	NA	0	1	Low-quality	Genome-fragment		0.77	0
S1_extracted_k141_94305	1037	No	NA	1	0	Low-quality	Genome-fragment		0.72	0
S17_vs_k141_22742||full	1070	No	NA	0	0	Not-determined	Genome-fragment	NA		0
S1_vs_k141_113507||full	1037	No	NA	0	0	Low-quality	Genome-fragment		2.43	0
S17_vs_k141_89339||full	5239	No	NA	0	2	Not-determined	Genome-fragment	NA		0
S1_vs_k141_141024||full	1037	No	NA	0	0	Low-quality	Genome-fragment		2.63	0
S1_vs_k141_51460||full	1037	No	NA	1	0	Low-quality	Genome-fragment		2.67	0
S17_vs_k141_67155||full	1462	No	NA	0	1	Not-determined	Genome-fragment	NA		0
S17_vs_k141_111539||full	2041	No	NA	0	1	Not-determined	Genome-fragment	NA		0
S17_vs_k141_170747||full	3684	No	NA	0	2	Not-determined	Genome-fragment	NA		0
S17_vs_k141_104488||full	3402	No	NA	0	2	Not-determined	Genome-fragment	NA		0
S5_vs_k141_15272||lt2gene	1037	No	NA	1	0	Low-quality	Genome-fragment		3.02	0
S7_extracted_2254	1037	No	NA	0	0	Low-quality	Genome-fragment		1.98	0
S17_vs_k141_170767||full	1966	No	NA	0	0	Not-determined	Genome-fragment	NA		0
S17_vs_k141_163359||full	1184	No	NA	0	1	Not-determined	Genome-fragment	NA		0
S17_vs_k141_45115||full	4011	No	NA	0	3	Not-determined	Genome-fragment	NA		0
S7_extracted_19799	1037	No	NA	2	0	Low-quality	Genome-fragment		2.35	0
S17_vs_k141_148714||full	2233	No	NA	0	2	Not-determined	Genome-fragment	NA		0
S17_vs_k141_104552||full	2893	No	NA	0	1	Not-determined	Genome-fragment	NA		0
S17_vs_k141_89379||full	1801	No	NA	0	0	Not-determined	Genome-fragment	NA		0
S17_vs_k141_89384||full	1661	No	NA	0	1	Not-determined	Genome-fragment	NA		0
S17_vs_k141_8288||full	3779	No	NA	0	3	Not-determined	Genome-fragment	NA		0
S10_vs_14817||full	1037	No	NA	1	0	Low-quality	Genome-fragment		2.39	0
S17_vs_k141_163418||full	1331	No	NA	0	0	Not-determined	Genome-fragment	NA		0
S17_vs_k141_170830||full	1721	No	NA	0	1	Not-determined	Genome-fragment	NA		0
S12_vs_k141_42432||full	1037	No	NA	1	0	Low-quality	Genome-fragment		2.48	0
S12_vs_k141_3566||full	1037	No	NA	1	0	Low-quality	Genome-fragment		2.2	0
S17_vs_k141_170840||full	1732	No	NA	0	1	Not-determined	Genome-fragment	NA		0
S17_vs_k141_38033||full	2229	No	NA	0	2	Not-determined	Genome-fragment	NA		0
S17_vs_k141_96831||full	4298	No	NA	0	4	Not-determined	Genome-fragment	NA		0
S17_vs_k141_104572||full	2681	No	NA	0	1	Not-determined	Genome-fragment	NA		0
S17_vs_k141_126346||full	1681	No	NA	0	1	Not-determined	Genome-fragment	NA		0
S2_vs_k141_11866||full	1037	No	NA	1	0	Low-quality	Genome-fragment		2.53	0
S17_vs_k141_45179||full	4107	No	NA	0	3	Not-determined	Genome-fragment	NA		0
S6_extracted_k141_17310	1037	No	NA	1	0	Low-quality	Genome-fragment		2.69	0
S8_extracted_k141_61347	1037	No	NA	0	1	Low-quality	Genome-fragment		0.65	0
S8_extracted_k141_28656	1037	No	NA	0	0	Low-quality	Genome-fragment		2.21	0
S8_extracted_k141_155679	1037	No	NA	1	0	Low-quality	Genome-fragment		2.04	0
S8_vs_k141_38085||full	1037	No	NA	1	0	Low-quality	Genome-fragment		2.74	0
S9_vs_k141_68053||full	1038	No	NA	0	0	Low-quality	Genome-fragment		2.74	0
S9_vs_k141_68274||lt2gene	1038	No	NA	1	0	Low-quality	Genome-fragment		2.37	0
S17_vs_k141_52789||full	2778	No	NA	0	0	Not-determined	Genome-fragment	NA		0
S17_vs_k141_148834||full	1784	No	NA	0	0	Not-determined	Genome-fragment	NA		0
S17_vs_k141_52806||full	2949	No	NA	0	1	Not-determined	Genome-fragment	NA		0
S17_vs_k141_111618||full	3060	No	NA	0	0	Not-determined	Genome-fragment	NA		0
S17_vs_k141_133785||full	3328	No	NA	0	4	Not-determined	Genome-fragment	NA		0
S17_vs_k141_67359||full	1056	No	NA	0	0	Not-determined	Genome-fragment	NA		0
S17_vs_k141_96922||full	4978	No	NA	0	6	Not-determined	Genome-fragment	NA		0
S11_vs_k141_68451||full	1038	No	NA	1	0	Low-quality	Genome-fragment		2.28	0
S17_vs_k141_67361||full	3174	No	NA	0	3	Not-determined	Genome-fragment	NA		0
S17_vs_k141_52838||full	1603	No	NA	0	0	Not-determined	Genome-fragment	NA		0
S11_vs_k141_95781||full	1038	No	NA	0	0	Low-quality	Genome-fragment		1.47	0
S17_vs_k141_81899||full	1971	No	NA	0	3	Not-determined	Genome-fragment	NA		0
S17_vs_k141_22912||full	2795	No	NA	0	0	Not-determined	Genome-fragment	NA		0
S17_vs_k141_111663||full	1316	No	NA	0	1	Not-determined	Genome-fragment	NA		0
S13_vs_110212||full	1038	No	NA	3	0	Low-quality	Genome-fragment		2.7	0
S14_extracted_k141_31515	1038	No	NA	4	0	Low-quality	Genome-fragment		3.43	0
S17_vs_k141_30648||full	2061	No	NA	0	0	Not-determined	Genome-fragment	NA		0
S17_vs_k141_8424||full	4529	No	NA	0	2	Not-determined	Genome-fragment	NA		0
S1_extracted_k141_119134	1038	No	NA	2	0	Low-quality	Genome-fragment		1.52	0
S1_vs_k141_155040||lt2gene	1038	No	NA	1	0	Low-quality	Genome-fragment		2.22	0
S3_extracted_k141_65325	1038	No	NA	0	1	Low-quality	Genome-fragment		1.06	0
S17_vs_k141_45336||full	1927	No	NA	0	2	Not-determined	Genome-fragment	NA		0
S16_vs_92419||full	1038	No	NA	1	0	Low-quality	Genome-fragment		2.64	0
S17_vs_k141_984||full	18858	No	NA	0	11	Not-determined	Genome-fragment	NA		0
S12_vs_k141_155264||full	1038	No	NA	1	0	Low-quality	Genome-fragment		2.28	0
S17_vs_k141_141371||full	1398	No	NA	0	0	Not-determined	Genome-fragment	NA		0
S15_extracted_24635	1038	No	NA	0	1	Low-quality	Genome-fragment		0.45	0
S8_vs_k141_203659||full	1038	No	NA	0	0	Low-quality	Genome-fragment		2.19	0
S17_vs_k141_52890||full	60210	No	NA	0	14	Medium-quality	Genome-fragment		63.54	0
S17_vs_k141_81929||full	7371	No	NA	1	2	Not-determined	Genome-fragment	NA		0
S8_vs_k141_25231||full	1038	No	NA	0	0	Low-quality	Genome-fragment		2.19	0
S17_vs_k141_119269||full	5736	No	NA	0	1	Not-determined	Genome-fragment	NA		0
S17_vs_k141_156356||full	6580	No	NA	0	1	Not-determined	Genome-fragment	NA		0
S17_vs_k141_8497||full	2292	No	NA	0	0	Not-determined	Genome-fragment	NA		0
S17_vs_k141_111739||full	1176	No	NA	0	0	Not-determined	Genome-fragment	NA		0
S17_vs_k141_81943||full	2051	No	NA	0	1	Not-determined	Genome-fragment	NA		0
S17_vs_k141_96991||full	2727	No	NA	0	2	Not-determined	Genome-fragment	NA		0
S14_extracted_k141_77244	1039	No	NA	2	0	Low-quality	Genome-fragment		3.11	0
S14_extracted_k141_21771	1039	No	NA	0	1	Low-quality	Genome-fragment		0.29	0
S14_vs_k141_100590||full	1039	No	NA	1	0	Low-quality	Genome-fragment		2.69	0
S3_extracted_k141_30189	1039	No	NA	0	0	Low-quality	Genome-fragment		1.78	0
S17_vs_k141_38278||full	1134	No	NA	0	0	Not-determined	Genome-fragment	NA		0
S17_vs_k141_104812||full	1030	No	NA	0	1	Not-determined	Genome-fragment	NA		0
S17_vs_k141_81960||full	4779	No	NA	0	2	Not-determined	Genome-fragment	NA		0
S17_vs_k141_156425||full	1620	No	NA	0	2	Not-determined	Genome-fragment	NA		0
S17_vs_k141_104840||full	2046	No	NA	0	0	Not-determined	Genome-fragment	NA		0
S17_vs_k141_97048||full	1478	No	NA	0	0	Not-determined	Genome-fragment	NA		0
S17_vs_k141_1050||full	3656	No	NA	0	1	Not-determined	Genome-fragment	NA		0
S17_vs_k141_104847||full	1258	No	NA	0	0	Not-determined	Genome-fragment	NA		0
S17_vs_k141_111843||full	1471	No	NA	0	0	Not-determined	Genome-fragment	NA		0
S17_vs_k141_163659||full	1883	No	NA	0	1	Not-determined	Genome-fragment	NA		0
S5_extracted_k141_68264	1039	No	NA	2	0	Low-quality	Genome-fragment		3.01	0
S17_vs_k141_111855||full	3234	No	NA	0	0	Not-determined	Genome-fragment	NA		0
S17_vs_k141_119353||full	1123	No	NA	0	2	Not-determined	Genome-fragment	NA		0
S5_vs_k141_69955||lt2gene	1039	No	NA	1	0	Low-quality	Genome-fragment		0.93	0
S17_vs_k141_119367||full	1485	No	NA	0	0	Not-determined	Genome-fragment	NA		0
S16_extracted_19515	1039	No	NA	0	0	Low-quality	Genome-fragment		2.45	0
S16_vs_2499||full	1039	No	NA	0	0	Low-quality	Genome-fragment		0.68	0
S17_vs_k141_163680||full	1254	No	NA	0	0	Not-determined	Genome-fragment	NA		0
S17_vs_k141_1087||full	9719	No	NA	0	4	Not-determined	Genome-fragment	NA		0
S17_vs_k141_111875||full	2270	No	NA	0	2	Not-determined	Genome-fragment	NA		0
S17_vs_k141_8631||full	3209	No	NA	0	0	Not-determined	Genome-fragment	NA		0
S17_vs_k141_149144||full	4107	No	NA	0	2	Not-determined	Genome-fragment	NA		0
S17_vs_k141_111889||full	2311	No	NA	0	0	Not-determined	Genome-fragment	NA		0
S17_vs_k141_67579||full	1260	No	NA	0	0	Not-determined	Genome-fragment	NA		0
S16_vs_93314||full	1039	No	NA	0	0	Low-quality	Genome-fragment		2.52	0
S16_vs_54357||lt2gene	1039	No	NA	1	0	Low-quality	Genome-fragment		2.37	0
S17_vs_k141_171198||full	6378	No	NA	0	5	Not-determined	Genome-fragment	NA		0
S17_vs_k141_60305||full	1505	No	NA	0	1	Not-determined	Genome-fragment	NA		0
S10_vs_42914||full	1039	No	NA	0	0	Low-quality	Genome-fragment		2.17	0
S17_vs_k141_171212||full	1111	No	NA	0	2	Not-determined	Genome-fragment	NA		0
S17_vs_k141_53092||full	4196	No	NA	0	4	Not-determined	Genome-fragment	NA		0
S17_vs_k141_38429||full	2968	No	NA	0	0	Not-determined	Genome-fragment	NA		0
S10_vs_98456||full	1039	No	NA	0	0	Low-quality	Genome-fragment		2.53	0
S10_vs_42217||lt2gene	1039	No	NA	2	0	Low-quality	Genome-fragment		2.24	0
S17_vs_k141_163733||full	2210	No	NA	0	2	Not-determined	Genome-fragment	NA		0
S17_vs_k141_74975||full	2686	No	NA	0	0	Not-determined	Genome-fragment	NA		0
S12_vs_k141_37844||full	1039	No	NA	0	0	Low-quality	Genome-fragment		2.99	0
S12_vs_k141_62762||full	1039	No	NA	0	0	Low-quality	Genome-fragment		2.15	0
S17_vs_k141_119426||full	3378	No	NA	0	0	Not-determined	Genome-fragment	NA		0
S6_extracted_k141_9328	1039	No	NA	0	2	Low-quality	Genome-fragment		0.47	0
S17_vs_k141_163760||full	1833	No	NA	0	1	Not-determined	Genome-fragment	NA		0
S17_vs_k141_119449||full	1087	No	NA	0	0	Not-determined	Genome-fragment	NA		0
S8_extracted_k141_143743	1039	No	NA	0	0	Low-quality	Genome-fragment		1.67	0
S17_vs_k141_1160||full	1261	No	NA	0	0	Not-determined	Genome-fragment	NA		0
S8_extracted_k141_49111	1039	No	NA	0	1	Low-quality	Genome-fragment		1.73	0
S17_vs_k141_23145||full	1616	No	NA	0	0	Not-determined	Genome-fragment	NA		0
S8_vs_k141_87414||full	1039	No	NA	0	1	Low-quality	Genome-fragment		3.32	0
S8_vs_k141_141295||lt2gene	1039	No	NA	1	0	Low-quality	Genome-fragment		3.34	0
S17_vs_k141_45678||full	1410	No	NA	0	2	Not-determined	Genome-fragment	NA		0
S17_vs_k141_67686||full	1381	No	NA	0	1	Not-determined	Genome-fragment	NA		0
S17_vs_k141_141684||full	2505	No	NA	0	2	Not-determined	Genome-fragment	NA		0
S17_vs_k141_89829||full	1547	No	NA	0	0	Not-determined	Genome-fragment	NA		0
S17_vs_k141_141701||full	4755	No	NA	0	2	Not-determined	Genome-fragment	NA		0
S8_vs_k141_158478||lt2gene	1039	No	NA	1	0	Low-quality	Genome-fragment		1.73	0
S9_vs_k141_66290||full	1040	No	NA	0	0	Low-quality	Genome-fragment		1.68	0
S17_vs_k141_134110||full	4589	No	NA	0	3	Not-determined	Genome-fragment	NA		0
S17_vs_k141_171294||full	3024	No	NA	0	1	Not-determined	Genome-fragment	NA		0
S11_extracted_k141_37886	1040	No	NA	1	0	Low-quality	Genome-fragment		0.74	0
S11_vs_k141_57753||lt2gene	1040	No	NA	1	0	Low-quality	Genome-fragment		2.75	0
S17_vs_k141_111982||full	1405	No	NA	0	0	Not-determined	Genome-fragment	NA		0
S17_vs_k141_1206||full	1418	No	NA	0	0	Not-determined	Genome-fragment	NA		0
S17_vs_k141_8760||full	1371	No	NA	0	0	Not-determined	Genome-fragment	NA		0
S17_vs_k141_53227||full	3000	No	NA	0	1	Not-determined	Genome-fragment	NA		0
S5_extracted_k141_16013	1040	No	NA	0	0	Low-quality	Genome-fragment		1.7	0
S17_vs_k141_97282||full	1357	No	NA	0	0	Not-determined	Genome-fragment	NA		0
S17_vs_k141_8774||full	3827	No	NA	0	1	Not-determined	Genome-fragment	NA		0
S17_vs_k141_171307||full	4725	No	NA	0	1	Not-determined	Genome-fragment	NA		0
S17_vs_k141_23201||full	2501	No	NA	0	0	Not-determined	Genome-fragment	NA		0
S5_extracted_k141_59429	1040	No	NA	2	0	Low-quality	Genome-fragment		2.16	0
S17_vs_k141_1240||full	1474	No	NA	0	0	Not-determined	Genome-fragment	NA		0
S5_vs_k141_37589||full	1040	No	NA	0	0	Low-quality	Genome-fragment		2.83	0
S7_vs_71164||lt2gene	1040	No	NA	0	0	Low-quality	Genome-fragment		1.78	0
S15_vs_4097||full	1040	No	NA	1	0	Low-quality	Genome-fragment		3.52	0
S17_vs_k141_75118||full	8203	No	NA	0	6	Not-determined	Genome-fragment	NA		0
S8_vs_k141_135352||lt2gene	1040	No	NA	1	0	Low-quality	Genome-fragment		2.35	0
S17_vs_k141_134176||full	4021	No	NA	0	2	Not-determined	Genome-fragment	NA		0
S17_vs_k141_31041||full	1965	No	NA	0	0	Not-determined	Genome-fragment	NA		0
S9_vs_k141_2527||full	1041	No	NA	2	0	Low-quality	Genome-fragment		2.53	0
S17_vs_k141_1276||full	1265	No	NA	0	0	Not-determined	Genome-fragment	NA		0
S11_vs_k141_86434||lt2gene	1041	No	NA	0	0	Low-quality	Genome-fragment		2.47	0
S17_vs_k141_89992||full	3705	No	NA	0	3	Not-determined	Genome-fragment	NA		0
S17_vs_k141_53310||full	1747	No	NA	0	0	Not-determined	Genome-fragment	NA		0
S17_vs_k141_171396||full	2818	No	NA	0	2	Not-determined	Genome-fragment	NA		0
S11_vs_k141_51727||lt2gene	1041	No	NA	1	0	Low-quality	Genome-fragment		2.37	0
S17_vs_k141_156670||full	2928	No	NA	0	1	Not-determined	Genome-fragment	NA		0
S17_vs_k141_134221||full	1238	No	NA	0	1	Not-determined	Genome-fragment	NA		0
S17_vs_k141_141732||full	33997	No	NA	0	6	Not-determined	Genome-fragment	NA		0
S17_vs_k141_97406||full	2281	No	NA	0	2	Not-determined	Genome-fragment	NA		0
S1_extracted_k141_66750	1041	No	NA	0	0	Low-quality	Genome-fragment		2.68	0
S17_vs_k141_90033||full	2987	No	NA	0	1	Not-determined	Genome-fragment	NA		0
S17_vs_k141_31099||full	1649	No	NA	0	0	Not-determined	Genome-fragment	NA		0
S1_vs_k141_38562||lt2gene	1041	No	NA	1	0	Low-quality	Genome-fragment		3.57	0

S17_vs_k141_97431||full	3513	No	NA	0	2	Not-determined	Genome-fragment	NA		0
S3_extracted_k141_60602	1041	No	NA	0	0	Low-quality	Genome-fragment		0.37	0
S3_vs_k141_105337||full	1041	No	NA	0	1	Low-quality	Genome-fragment		1.54	0
S5_vs_k141_102865||lt2gene	1041	No	NA	1	0	Low-quality	Genome-fragment		2.53	0
S17_vs_k141_171449||full	3180	No	NA	0	1	Not-determined	Genome-fragment	NA		0
S16_vs_101034||full	1041	No	NA	1	0	Low-quality	Genome-fragment		1.54	0
S17_vs_k141_75187||full	1609	No	NA	0	2	Not-determined	Genome-fragment	NA		0
S10_extracted_16727	1041	No	NA	0	0	Low-quality	Genome-fragment		1.27	0
S17_vs_k141_126592||full	1863	No	NA	0	1	Not-determined	Genome-fragment	NA		0
S12_vs_k141_146545||full	1041	No	NA	1	0	Low-quality	Genome-fragment		2.35	0
S12_vs_k141_164809||full	1041	No	NA	0	0	Low-quality	Genome-fragment		0.97	0
S17_vs_k141_38710||full	2166	No	NA	0	1	Not-determined	Genome-fragment	NA		0
S17_vs_k141_75205||full	6192	No	NA	0	4	Not-determined	Genome-fragment	NA		0
S17_vs_k141_23341||full	3963	No	NA	0	4	Not-determined	Genome-fragment	NA		0
S15_vs_2427||lt2gene	1041	No	NA	1	0	Low-quality	Genome-fragment		2.87	0
S15_vs_46359||lt2gene	1041	No	NA	1	0	Low-quality	Genome-fragment		19.19	0
S6_vs_k141_38021||lt2gene	1041	No	NA	1	0	Low-quality	Genome-fragment		2.42	0
S8_extracted_k141_16237	1041	No	NA	0	0	Low-quality	Genome-fragment		1.85	0
S17_vs_k141_38734||full	1589	No	NA	0	0	Not-determined	Genome-fragment	NA		0
S11_vs_k141_69969||lt2gene	1042	No	NA	1	0	Low-quality	Genome-fragment		3.14	0
S14_extracted_k141_93236	1042	No	NA	1	0	Low-quality	Genome-fragment		0.55	0
S17_vs_k141_53461||full	1071	No	NA	0	0	Not-determined	Genome-fragment	NA		0
S17_vs_k141_75256||full	1617	No	NA	0	0	Not-determined	Genome-fragment	NA		0
S17_vs_k141_141847||full	2225	No	NA	0	2	Not-determined	Genome-fragment	NA		0
S14_vs_k141_13263||full	1042	No	NA	2	0	Low-quality	Genome-fragment		3.17	0
S5_vs_k141_24818||lt2gene	1042	No	NA	1	0	Low-quality	Genome-fragment		2.3	0
S17_vs_k141_9036||full	1202	No	NA	0	1	Not-determined	Genome-fragment	NA		0
S17_vs_k141_105280||full	4616	No	NA	0	2	Not-determined	Genome-fragment	NA		0
S17_vs_k141_67887||full	2009	No	NA	0	2	Not-determined	Genome-fragment	NA		0
S17_vs_k141_156808||full	18506	No	NA	0	10	Not-determined	Genome-fragment	NA		0
S17_vs_k141_163977||full	2884	No	NA	0	1	Not-determined	Genome-fragment	NA		0
S17_vs_k141_163987||full	2373	No	NA	0	1	Not-determined	Genome-fragment	NA		0
S16_extracted_79229	1042	No	NA	0	2	Low-quality	Genome-fragment		1.87	0
S16_extracted_129768	1042	No	NA	0	1	Low-quality	Genome-fragment		1	0
S16_vs_22423||full	1042	No	NA	1	0	Low-quality	Genome-fragment		0.67	0
S17_vs_k141_97596||full	2313	No	NA	0	1	Not-determined	Genome-fragment	NA		0
S17_vs_k141_105343||full	1543	No	NA	0	1	Not-determined	Genome-fragment	NA		0
S12_extracted_k141_131550	1042	No	NA	0	0	Low-quality	Genome-fragment		2.85	0
S8_extracted_k141_54561	1042	No	NA	0	2	Low-quality	Genome-fragment		1.08	0
S17_vs_k141_15928||full	2623	No	NA	0	2	Not-determined	Genome-fragment	NA		0
S17_vs_k141_53561||full	1203	No	NA	0	1	Not-determined	Genome-fragment	NA		0
S8_vs_k141_194644||full	1042	No	NA	0	0	Low-quality	Genome-fragment		2.48	0
S17_vs_k141_37906||lt2gene	1043	No	NA	2	0	Low-quality	Genome-fragment		2.18	0
S13_vs_61342||full	1043	No	NA	0	0	Low-quality	Genome-fragment		2.28	0
S13_vs_98618||full	1043	No	NA	0	0	Low-quality	Genome-fragment		0.63	0
S17_vs_k141_82367||full	3224	No	NA	0	2	Not-determined	Genome-fragment	NA		0
S17_vs_k141_90192||full	4884	No	NA	0	4	Not-determined	Genome-fragment	NA		0
S17_vs_k141_68010||full	1396	No	NA	0	2	Not-determined	Genome-fragment	NA		0
S17_vs_k141_142025||full	1908	No	NA	0	1	Not-determined	Genome-fragment	NA		0
S17_vs_k141_105412||full	1441	No	NA	0	2	Not-determined	Genome-fragment	NA		0
S17_vs_k141_82403||full	1940	No	NA	0	1	Not-determined	Genome-fragment	NA		0
S14_vs_k141_74444||full	1043	No	NA	0	1	Low-quality	Genome-fragment		0.76	0
S17_vs_k141_53601||full	3089	No	NA	0	1	Not-determined	Genome-fragment	NA		0
S14_vs_k141_62284||lt2gene	1043	No	NA	1	0	Low-quality	Genome-fragment		2.54	0
S17_vs_k141_68037||full	3109	No	NA	0	2	Not-determined	Genome-fragment	NA		0
S1_vs_k141_44502||full	1043	No	NA	1	0	Low-quality	Genome-fragment		0.63	0
S17_vs_k141_53605||full	2061	No	NA	0	0	Not-determined	Genome-fragment	NA		0
S17_vs_k141_53617||full	2393	No	NA	0	3	Not-determined	Genome-fragment	NA		0
S17_vs_k141_82435||full	3118	No	NA	0	2	Not-determined	Genome-fragment	NA		0
S17_vs_k141_156938||full	1098	No	NA	0	1	Not-determined	Genome-fragment	NA		0
S3_extracted_k141_49070	1043	No	NA	0	0	Low-quality	Genome-fragment		1.83	0
S3_extracted_k141_97202	1043	No	NA	0	0	Low-quality	Genome-fragment		1.59	0
S17_vs_k141_105470||full	2182	No	NA	0	2	Not-determined	Genome-fragment	NA		0
S17_vs_k141_23496||full	2285	No	NA	0	2	Not-determined	Genome-fragment	NA		0
S17_vs_k141_38878||full	2096	No	NA	0	0	Not-determined	Genome-fragment	NA		0
S5_vs_k141_121435||full	1043	No	NA	0	0	Low-quality	Genome-fragment		1.61	0
S17_vs_k141_9210||full	2151	No	NA	0	1	Not-determined	Genome-fragment	NA		0
S17_vs_k141_97755||full	2629	No	NA	0	1	Not-determined	Genome-fragment	NA		0
S5_vs_k141_33466||full	1043	No	NA	0	0	Low-quality	Genome-fragment		3.03	0
S7_extracted_86450	1043	No	NA	3	0	Low-quality	Genome-fragment		3.37	0
S17_vs_k141_82499||full	5107	No	NA	0	2	Not-determined	Genome-fragment	NA		0
S7_extracted_108755	1043	No	NA	2	0	Low-quality	Genome-fragment		1.73	0
S17_vs_k141_60786||full	2383	No	NA	0	3	Not-determined	Genome-fragment	NA		0
S17_vs_k141_134513||full	1727	No	NA	0	1	Not-determined	Genome-fragment	NA		0
S17_vs_k141_46188||full	1203	No	NA	0	1	Not-determined	Genome-fragment	NA		0
S7_vs_45525||full	1043	No	NA	1	0	Low-quality	Genome-fragment		2.73	0
S17_vs_k141_126869||full	2904	No	NA	0	1	Not-determined	Genome-fragment	NA		0
S17_vs_k141_1621||full	1167	No	NA	0	0	Not-determined	Genome-fragment	NA		0
S10_extracted_106271	1043	No	NA	0	0	Low-quality	Genome-fragment		2.4	0
S17_vs_k141_16054||full	1799	No	NA	0	0	Not-determined	Genome-fragment	NA		0
S10_extracted_121228	1043	No	NA	2	0	Low-quality	Genome-fragment		3.44	0
S17_vs_k141_75384||full	1620	No	NA	0	0	Not-determined	Genome-fragment	NA		0
S12_extracted_k141_133254	1043	No	NA	0	0	Low-quality	Genome-fragment		1.66	0
S15_vs_616||full	1043	No	NA	0	1	Low-quality	Genome-fragment		2.26	0
S6_vs_k141_6492||full	1043	No	NA	1	0	Low-quality	Genome-fragment		1.96	0
S17_vs_k141_31409||full	1746	No	NA	0	2	Not-determined	Genome-fragment	NA		0
S8_vs_k141_147154||full	1043	No	NA	0	0	Low-quality	Genome-fragment		2.14	0
S8_vs_k141_121783||lt2gene	1043	No	NA	1	0	Low-quality	Genome-fragment		2.69	0
S17_vs_k141_75406||full	2421	No	NA	0	1	Not-determined	Genome-fragment	NA		0
S14_extracted_k141_89399	1044	No	NA	1	0	Low-quality	Genome-fragment		3.44	0
S17_vs_k141_31426||full	2076	No	NA	0	1	Not-determined	Genome-fragment	NA		0
S17_vs_k141_38955||full	2053	No	NA	0	0	Not-determined	Genome-fragment	NA		0
S17_vs_k141_142202||full	1727	No	NA	0	0	Not-determined	Genome-fragment	NA		0
S17_vs_k141_164175||full	9143	No	NA	0	2	Not-determined	Genome-fragment	NA		0
S17_vs_k141_68201||full	1509	No	NA	0	0	Not-determined	Genome-fragment	NA		0
S1_extracted_k141_27060	1044	No	NA	0	0	Low-quality	Genome-fragment		0.59	0
S17_vs_k141_97887||full	1075	No	NA	0	1	Not-determined	Genome-fragment	NA		0
S17_vs_k141_82601||full	10815	No	NA	0	3	Not-determined	Genome-fragment	NA		0
S17_vs_k141_126974||full	1613	No	NA	0	0	Not-determined	Genome-fragment	NA		0
S17_vs_k141_75441||full	1211	No	NA	0	0	Not-determined	Genome-fragment	NA		0
S1_extracted_k141_2421	1044	No	NA	0	0	Low-quality	Genome-fragment		1.06	0
S1_vs_k141_123222||full	1044	No	NA	0	0	Low-quality	Genome-fragment		1.3	0
S1_vs_k141_63395||lt2gene	1044	No	NA	1	0	Low-quality	Genome-fragment		2.77	0
S5_vs_k141_5563||full	1044	No	NA	0	1	Low-quality	Genome-fragment		0.89	0
S17_vs_k141_120044||full	2432	No	NA	0	0	Not-determined	Genome-fragment	NA		0
S7_vs_25695||full	1044	No	NA	0	0	Low-quality	Genome-fragment		2.18	0
S16_extracted_95025	1044	No	NA	0	0	Low-quality	Genome-fragment		1.78	0
S10_extracted_114375	1044	No	NA	1	0	Low-quality	Genome-fragment		0.45	0
S17_vs_k141_60805||full	3558	No	NA	0	1	Not-determined	Genome-fragment	NA		0
S10_vs_54546||full	1044	No	NA	1	0	Low-quality	Genome-fragment		1.78	0
S12_extracted_k141_84821	1044	No	NA	0	1	Low-quality	Genome-fragment		2.97	0
S17_vs_k141_68264||full	1509	No	NA	0	0	Not-determined	Genome-fragment	NA		0
S15_vs_26781||full	1044	No	NA	0	0	Low-quality	Genome-fragment		2.17	0
S17_vs_k141_164233||full	2012	No	NA	0	0	Not-determined	Genome-fragment	NA		0
S6_vs_k141_16440||full	1044	No	NA	0	0	Low-quality	Genome-fragment		2.28	0
S17_vs_k141_82692||full	2752	No	NA	0	2	Not-determined	Genome-fragment	NA		0
S17_vs_k141_16228||full	1589	No	NA	0	0	Not-determined	Genome-fragment	NA		0
S17_vs_k141_164253||full	1661	No	NA	0	2	Not-determined	Genome-fragment	NA		0
S8_extracted_k141_15708	1044	No	NA	0	1	Low-quality	Genome-fragment		3.33	0
S17_vs_k141_82714||full	1389	No	NA	0	1	Not-determined	Genome-fragment	NA		0
S17_extracted_k141_90452	1045	No	NA	1	0	Low-quality	Genome-fragment		1.35	0
S17_vs_k141_23832||full	1497	No	NA	0	1	Not-determined	Genome-fragment	NA		0
S17_vs_k141_60847||full	9520	No	NA	0	5	Not-determined	Genome-fragment	NA		0
S17_vs_k141_164262||full	2816	No	NA	0	0	Not-determined	Genome-fragment	NA		0
S17_vs_k141_60861||full	2435	No	NA	0	0	Not-determined	Genome-fragment	NA		0
S9_extracted_k141_24091	1045	No	NA	0	1	Low-quality	Genome-fragment		0.07	0
S17_vs_k141_23842||full	5450	No	NA	0	2	Not-determined	Genome-fragment	NA		0
S9_vs_k141_76100||lt2gene	1045	No	NA	1	0	Low-quality	Genome-fragment		2.39	0
S17_vs_k141_23845||full	2983	No	NA	0	0	Not-determined	Genome-fragment	NA		0
S17_vs_k141_75506||full	2179	No	NA	0	1	Not-determined	Genome-fragment	NA		0
S14_vs_k141_19064||full	1045	No	NA	3	0	Low-quality	Genome-fragment		3.14	0
S3_extracted_k141_53225	1045	No	NA	0	0	Low-quality	Genome-fragment		3.35	0
S17_vs_k141_120199||full	1512	No	NA	0	0	Not-determined	Genome-fragment	NA		0
S17_vs_k141_127139||full	3352	No	NA	0	1	Not-determined	Genome-fragment	NA		0
S5_extracted_k141_136375	1045	No	NA	0	0	Low-quality	Genome-fragment		1.38	0
S17_vs_k141_120216||full	3257	No	NA	0	1	Not-determined	Genome-fragment	NA		0
S5_extracted_k141_127117	1045	No	NA	0	0	Low-quality	Genome-fragment		2.91	0
S17_vs_k141_157209||full	43342	No	NA	13	0	High-quality	High-quality		95.84	0
S17_vs_k141_1851||full	3456	No	NA	0	0	Not-determined	Genome-fragment	NA		0
S17_vs_k141_16330||full	3470	No	NA	0	0	Not-determined	Genome-fragment	NA		0
S17_vs_k141_23889||full	2613	No	NA	0	1	Not-determined	Genome-fragment	NA		0
S17_vs_k141_60940||full	1761	No	NA	0	2	Not-determined	Genome-fragment	NA		0
S5_extracted_k141_76939	1045	No	NA	1	0	Low-quality	Genome-fragment		1.38	0
S7_extracted_16454	1045	No	NA	0	1	Low-quality	Genome-fragment		1.75	0
S17_vs_k141_112524||full	2434	No	NA	0	1	Not-determined	Genome-fragment	NA		0
S17_vs_k141_31654||full	24962	No	NA	18	0	Medium-quality	Genome-fragment		66.18	0
S17_vs_k141_54041||full	2856	No	NA	0	0	Not-determined	Genome-fragment	NA		0
S17_vs_k141_82770||full	3259	No	NA	0	0	Not-determined	Genome-fragment	NA		0
S7_extracted_129735	1045	No	NA	1	0	Low-quality	Genome-fragment		2.65	0
S17_vs_k141_82775||full	1519	No	NA	0	1	Not-determined	Genome-fragment	NA		0
S17_vs_k141_112544||full	1514	No	NA	0	1	Not-determined	Genome-fragment	NA		0
S17_vs_k141_171722||full	3411	No	NA	0	3	Not-determined	Genome-fragment	NA		0
S17_vs_k141_127200||full	2057	No	NA	0	0	Not-determined	Genome-fragment	NA		0
S7_vs_93782||full	1045	No	NA	2	0	Low-quality	Genome-fragment		2.52	0
S16_extracted_109140	1045	No	NA	0	0	Low-quality	Genome-fragment		2.27	0
S16_vs_9175||lt2gene	1045	No	NA	1	0	Low-quality	Genome-fragment		3.54	0
S17_vs_k141_150136||full	2043	No	NA	0	0	Not-determined	Genome-fragment	NA		0
S10_vs_33835||full	1045	No	NA	0	0	Low-quality	Genome-fragment		2.77	0
S17_vs_k141_171756||full	3545	No	NA	0	0	Not-determined	Genome-fragment	NA		0
S12_extracted_k141_187883	1045	No	NA	0	1	Low-quality	Genome-fragment		3.34	0
S17_vs_k141_134911||full	4231	No	NA	0	3	Not-determined	Genome-fragment	NA		0
S12_vs_k141_90388||lt2gene	1045	No	NA	1	0	Low-quality	Genome-fragment		1.84	0
S17_vs_k141_112617||full	1094	No	NA	0	0	Not-determined	Genome-fragment	NA		0
S17_vs_k141_9459||full	2150	No	NA	0	0	Not-determined	Genome-fragment	NA		0
S17_vs_k141_150181||full	2655	No	NA	0	2	Not-determined	Genome-fragment	NA		0
S6_extracted_k141_30897	1045	No	NA	0	0	Low-quality	Genome-fragment		2.73	0
S6_extracted_k141_8320	1045	No	NA	0	1	Low-quality	Genome-fragment		3.51	0
S17_vs_k141_98183||full	1557	No	NA	0	1	Not-determined	Genome-fragment	NA		0
S17_vs_k141_134954||full	1666	No	NA	0	1	Not-determined	Genome-fragment	NA		0
S6_extracted_k141_27937	1045	No	NA	0	0	Low-quality	Genome-fragment		1.11	0
S17_vs_k141_31721||full	1253	No	NA	0	1	Not-determined	Genome-fragment	NA		0
S17_vs_k141_98200||full	1647	No	NA	0	0	Not-determined	Genome-fragment	NA		0
S8_extracted_k141_192876	1045	No	NA	0	1	Low-quality	Genome-fragment		1.21	0
S8_vs_k141_108783||full	1045	No	NA	1	0	Low-quality	Genome-fragment		1.99	0

<table>
<tr><th></th><th></th><th></th><th></th><th></th><th></th><th></th><th></th><th></th><th></th><th></th></tr>
<tr><td>S17_extracted_k141_124887</td><td>1046</td><td>No</td><td>NA</td><td>0</td><td>1</td><td>Low-quality</td><td>Genome-fragment</td><td></td><td>0.51</td><td>0</td></tr>
<tr><td>S9_vs_k141_77381||full</td><td>1046</td><td>No</td><td>NA</td><td>0</td><td>0</td><td>Low-quality</td><td>Genome-fragment</td><td></td><td>2.41</td><td>0</td></tr>
<tr><td>S14_extracted_k141_105691</td><td>1046</td><td>No</td><td>NA</td><td>0</td><td>0</td><td>Low-quality</td><td>Genome-fragment</td><td></td><td>1.77</td><td>0</td></tr>
<tr><td>S17_vs_k141_150216||full</td><td>2771</td><td>No</td><td>NA</td><td>0</td><td>1</td><td>Not-determined</td><td>Genome-fragment</td><td>NA</td><td></td><td>0</td></tr>
<tr><td>S14_vs_k141_12538||full</td><td>1046</td><td>No</td><td>NA</td><td>0</td><td>1</td><td>Low-quality</td><td>Genome-fragment</td><td></td><td>0.94</td><td>0</td></tr>
<tr><td>S1_extracted_k141_156687</td><td>1046</td><td>No</td><td>NA</td><td>0</td><td>0</td><td>Low-quality</td><td>Genome-fragment</td><td></td><td>2.46</td><td>0</td></tr>
<tr><td>S17_vs_k141_75711||full</td><td>7915</td><td>No</td><td>NA</td><td>0</td><td>1</td><td>Not-determined</td><td>Genome-fragment</td><td>NA</td><td></td><td>0</td></tr>
<tr><td>S3_extracted_k141_10146</td><td>1046</td><td>No</td><td>NA</td><td>0</td><td>0</td><td>Low-quality</td><td>Genome-fragment</td><td></td><td>2.27</td><td>0</td></tr>
<tr><td>S5_vs_k141_121607||lt2gene</td><td>1046</td><td>No</td><td>NA</td><td>1</td><td>0</td><td>Low-quality</td><td>Genome-fragment</td><td></td><td>2.61</td><td>0</td></tr>
<tr><td>S7_vs_13668||lt2gene</td><td>1046</td><td>No</td><td>NA</td><td>1</td><td>0</td><td>Low-quality</td><td>Genome-fragment</td><td></td><td>2.59</td><td>0</td></tr>
<tr><td>S17_vs_k141_134992||full</td><td>6478</td><td>No</td><td>NA</td><td>0</td><td>4</td><td>Not-determined</td><td>Genome-fragment</td><td>NA</td><td></td><td>0</td></tr>
<tr><td>S16_vs_35640||full</td><td>1046</td><td>No</td><td>NA</td><td>0</td><td>0</td><td>Low-quality</td><td>Genome-fragment</td><td></td><td>2.67</td><td>0</td></tr>
<tr><td>S12_extracted_k141_96362</td><td>1046</td><td>No</td><td>NA</td><td>0</td><td>0</td><td>Low-quality</td><td>Genome-fragment</td><td></td><td>2.25</td><td>0</td></tr>
<tr><td>S12_extracted_k141_13937</td><td>1046</td><td>No</td><td>NA</td><td>0</td><td>0</td><td>Low-quality</td><td>Genome-fragment</td><td></td><td>2.29</td><td>0</td></tr>
<tr><td>S17_vs_k141_150251||full</td><td>5468</td><td>No</td><td>NA</td><td>0</td><td>0</td><td>Not-determined</td><td>Genome-fragment</td><td>NA</td><td></td><td>0</td></tr>
<tr><td>S15_extracted_22433</td><td>1046</td><td>No</td><td>NA</td><td>1</td><td>0</td><td>Low-quality</td><td>Genome-fragment</td><td></td><td>2.32</td><td>0</td></tr>
<tr><td>S17_vs_k141_39421||full</td><td>2606</td><td>No</td><td>NA</td><td>0</td><td>0</td><td>Not-determined</td><td>Genome-fragment</td><td>NA</td><td></td><td>0</td></tr>
<tr><td>S17_vs_k141_68589||full</td><td>6833</td><td>No</td><td>NA</td><td>0</td><td>4</td><td>Not-determined</td><td>Genome-fragment</td><td>NA</td><td></td><td>0</td></tr>
<tr><td>S17_vs_k141_2044||full</td><td>1051</td><td>No</td><td>NA</td><td>0</td><td>0</td><td>Not-determined</td><td>Genome-fragment</td><td>NA</td><td></td><td>0</td></tr>
<tr><td>S15_vs_11101||full</td><td>1046</td><td>No</td><td>NA</td><td>2</td><td>0</td><td>Low-quality</td><td>Genome-fragment</td><td></td><td>2.46</td><td>0</td></tr>
<tr><td>S17_vs_k141_127333||full</td><td>3969</td><td>No</td><td>NA</td><td>0</td><td>2</td><td>Not-determined</td><td>Genome-fragment</td><td>NA</td><td></td><td>0</td></tr>
<tr><td>S17_vs_k141_75757||full</td><td>2423</td><td>No</td><td>NA</td><td>0</td><td>2</td><td>Not-determined</td><td>Genome-fragment</td><td>NA</td><td></td><td>0</td></tr>
<tr><td>S6_vs_k141_14134||full</td><td>1046</td><td>No</td><td>NA</td><td>0</td><td>0</td><td>Low-quality</td><td>Genome-fragment</td><td></td><td>1.71</td><td>0</td></tr>
<tr><td>S17_vs_k141_90499||full</td><td>2176</td><td>No</td><td>NA</td><td>0</td><td>0</td><td>Not-determined</td><td>Genome-fragment</td><td>NA</td><td></td><td>0</td></tr>
<tr><td>S6_vs_k141_49090||full</td><td>1046</td><td>No</td><td>NA</td><td>0</td><td>0</td><td>Low-quality</td><td>Genome-fragment</td><td></td><td>1.47</td><td>0</td></tr>
<tr><td>S17_vs_k141_9602||full</td><td>1252</td><td>No</td><td>NA</td><td>0</td><td>1</td><td>Not-determined</td><td>Genome-fragment</td><td>NA</td><td></td><td>0</td></tr>
<tr><td>S17_vs_k141_39473||full</td><td>1412</td><td>No</td><td>NA</td><td>0</td><td>1</td><td>Not-determined</td><td>Genome-fragment</td><td>NA</td><td></td><td>0</td></tr>
<tr><td>S17_vs_k141_68628||full</td><td>1904</td><td>No</td><td>NA</td><td>0</td><td>1</td><td>Not-determined</td><td>Genome-fragment</td><td>NA</td><td></td><td>0</td></tr>
<tr><td>S17_vs_k141_164589||full</td><td>3344</td><td>No</td><td>NA</td><td>0</td><td>1</td><td>Not-determined</td><td>Genome-fragment</td><td>NA</td><td></td><td>0</td></tr>
<tr><td>S8_vs_k141_18937||full</td><td>1046</td><td>No</td><td>NA</td><td>1</td><td>0</td><td>Low-quality</td><td>Genome-fragment</td><td></td><td>1.8</td><td>0</td></tr>
<tr><td>S17_vs_k141_24083||full</td><td>1032</td><td>No</td><td>NA</td><td>0</td><td>0</td><td>Not-determined</td><td>Genome-fragment</td><td>NA</td><td></td><td>0</td></tr>
<tr><td>S17_vs_k141_54212||full</td><td>2860</td><td>No</td><td>NA</td><td>0</td><td>2</td><td>Not-determined</td><td>Genome-fragment</td><td>NA</td><td></td><td>0</td></tr>
<tr><td>S17_vs_k141_68641||full</td><td>2059</td><td>No</td><td>NA</td><td>0</td><td>1</td><td>Not-determined</td><td>Genome-fragment</td><td>NA</td><td></td><td>0</td></tr>
<tr><td>S17_vs_k141_2079||full</td><td>25077</td><td>No</td><td>NA</td><td>2</td><td>2</td><td>Medium-quality</td><td>Genome-fragment</td><td></td><td>58.55</td><td>0</td></tr>
<tr><td>S17_vs_k141_135064||full</td><td>1484</td><td>No</td><td>NA</td><td>0</td><td>2</td><td>Not-determined</td><td>Genome-fragment</td><td>NA</td><td></td><td>0</td></tr>
<tr><td>S8_vs_k141_74921||full</td><td>1046</td><td>No</td><td>NA</td><td>0</td><td>0</td><td>Low-quality</td><td>Genome-fragment</td><td></td><td>1.97</td><td>0</td></tr>
<tr><td>S17_vs_k141_150372||full</td><td>1126</td><td>No</td><td>NA</td><td>0</td><td>0</td><td>Not-determined</td><td>Genome-fragment</td><td>NA</td><td></td><td>0</td></tr>
<tr><td>S17_vs_k141_164625||full</td><td>4132</td><td>No</td><td>NA</td><td>0</td><td>0</td><td>Not-determined</td><td>Genome-fragment</td><td>NA</td><td></td><td>0</td></tr>
<tr><td>S17_vs_k141_16552||full</td><td>2146</td><td>No</td><td>NA</td><td>0</td><td>0</td><td>Not-determined</td><td>Genome-fragment</td><td>NA</td><td></td><td>0</td></tr>
<tr><td>S17_vs_k141_39496||full</td><td>12869</td><td>No</td><td>NA</td><td>1</td><td>2</td><td>Not-determined</td><td>Genome-fragment</td><td>NA</td><td></td><td>0</td></tr>
<tr><td>S17_extracted_k141_9003</td><td>1047</td><td>No</td><td>NA</td><td>1</td><td>0</td><td>Low-quality</td><td>Genome-fragment</td><td></td><td>1.65</td><td>0</td></tr>
<tr><td>S17_extracted_k141_34746</td><td>1047</td><td>No</td><td>NA</td><td>1</td><td>0</td><td>Low-quality</td><td>Genome-fragment</td><td></td><td>2.48</td><td>0</td></tr>
<tr><td>S9_extracted_k141_36682</td><td>1047</td><td>No</td><td>NA</td><td>0</td><td>0</td><td>Low-quality</td><td>Genome-fragment</td><td></td><td>0.6</td><td>0</td></tr>
<tr><td>S17_vs_k141_68706||full</td><td>1097</td><td>No</td><td>NA</td><td>0</td><td>1</td><td>Not-determined</td><td>Genome-fragment</td><td>NA</td><td></td><td>0</td></tr>
<tr><td>S17_vs_k141_157510||full</td><td>1668</td><td>No</td><td>NA</td><td>0</td><td>1</td><td>Not-determined</td><td>Genome-fragment</td><td>NA</td><td></td><td>0</td></tr>
<tr><td>S9_vs_k141_87941||full</td><td>1047</td><td>No</td><td>NA</td><td>0</td><td>1</td><td>Low-quality</td><td>Genome-fragment</td><td></td><td>49.34</td><td>0</td></tr>
<tr><td>S17_vs_k141_150412||full</td><td>1903</td><td>No</td><td>NA</td><td>0</td><td>2</td><td>Not-determined</td><td>Genome-fragment</td><td>NA</td><td></td><td>0</td></tr>
<tr><td>S11_extracted_k141_25118</td><td>1047</td><td>No</td><td>NA</td><td>0</td><td>0</td><td>Low-quality</td><td>Genome-fragment</td><td></td><td>2.48</td><td>0</td></tr>
<tr><td>S17_vs_k141_157531||full</td><td>4333</td><td>No</td><td>NA</td><td>0</td><td>0</td><td>Not-determined</td><td>Genome-fragment</td><td>NA</td><td></td><td>0</td></tr>
<tr><td>S17_vs_k141_171894||full</td><td>2904</td><td>No</td><td>NA</td><td>0</td><td>1</td><td>Not-determined</td><td>Genome-fragment</td><td>NA</td><td></td><td>0</td></tr>
<tr><td>S17_vs_k141_39515||full</td><td>36323</td><td>No</td><td>NA</td><td>4</td><td>1</td><td>Medium-quality</td><td>Genome-fragment</td><td></td><td>58.53</td><td>0</td></tr>
<tr><td>S17_vs_k141_171906||full</td><td>5839</td><td>No</td><td>NA</td><td>0</td><td>4</td><td>Not-determined</td><td>Genome-fragment</td><td>NA</td><td></td><td>0</td></tr>
<tr><td>S17_vs_k141_150440||full</td><td>1873</td><td>No</td><td>NA</td><td>0</td><td>0</td><td>Not-determined</td><td>Genome-fragment</td><td>NA</td><td></td><td>0</td></tr>
<tr><td>S17_vs_k141_171912||full</td><td>1604</td><td>No</td><td>NA</td><td>0</td><td>1</td><td>Not-determined</td><td>Genome-fragment</td><td>NA</td><td></td><td>0</td></tr>
<tr><td>S17_vs_k141_68775||full</td><td>2942</td><td>No</td><td>NA</td><td>0</td><td>2</td><td>Not-determined</td><td>Genome-fragment</td><td>NA</td><td></td><td>0</td></tr>
<tr><td>S13_extracted_48175</td><td>1047</td><td>No</td><td>NA</td><td>0</td><td>1</td><td>Low-quality</td><td>Genome-fragment</td><td></td><td>0.48</td><td>0</td></tr>
<tr><td>S14_vs_k141_2639||full</td><td>1047</td><td>No</td><td>NA</td><td>0</td><td>0</td><td>Low-quality</td><td>Genome-fragment</td><td></td><td>2.63</td><td>0</td></tr>
<tr><td>S17_vs_k141_24165||full</td><td>1715</td><td>No</td><td>NA</td><td>0</td><td>1</td><td>Not-determined</td><td>Genome-fragment</td><td>NA</td><td></td><td>0</td></tr>
<tr><td>S17_vs_k141_112863||full</td><td>1042</td><td>No</td><td>NA</td><td>0</td><td>1</td><td>Not-determined</td><td>Genome-fragment</td><td>NA</td><td></td><td>0</td></tr>
<tr><td>S3_extracted_k141_19230</td><td>1047</td><td>No</td><td>NA</td><td>0</td><td>0</td><td>Low-quality</td><td>Genome-fragment</td><td></td><td>2.22</td><td>0</td></tr>
<tr><td>S17_vs_k141_61049||full</td><td>2524</td><td>No</td><td>NA</td><td>0</td><td>2</td><td>Not-determined</td><td>Genome-fragment</td><td>NA</td><td></td><td>0</td></tr>
<tr><td>S17_vs_k141_171944||full</td><td>2454</td><td>No</td><td>NA</td><td>0</td><td>1</td><td>Not-determined</td><td>Genome-fragment</td><td>NA</td><td></td><td>0</td></tr>
<tr><td>S17_vs_k141_90642||full</td><td>2686</td><td>No</td><td>NA</td><td>0</td><td>0</td><td>Not-determined</td><td>Genome-fragment</td><td>NA</td><td></td><td>0</td></tr>
<tr><td>S3_vs_k141_40918||lt2gene</td><td>1047</td><td>No</td><td>NA</td><td>2</td><td>0</td><td>Low-quality</td><td>Genome-fragment</td><td></td><td>1.71</td><td>0</td></tr>
<tr><td>S17_vs_k141_61079||full</td><td>1048</td><td>No</td><td>NA</td><td>0</td><td>1</td><td>Not-determined</td><td>Genome-fragment</td><td>NA</td><td></td><td>0</td></tr>
<tr><td>S7_vs_57199||full</td><td>1047</td><td>No</td><td>NA</td><td>0</td><td>0</td><td>Low-quality</td><td>Genome-fragment</td><td></td><td>2.32</td><td>0</td></tr>
<tr><td>S17_vs_k141_24204||full</td><td>3060</td><td>No</td><td>NA</td><td>0</td><td>1</td><td>Not-determined</td><td>Genome-fragment</td><td>NA</td><td></td><td>0</td></tr>
<tr><td>S17_vs_k141_82943||full</td><td>3063</td><td>No</td><td>NA</td><td>0</td><td>3</td><td>Not-determined</td><td>Genome-fragment</td><td>NA</td><td></td><td>0</td></tr>
<tr><td>S16_extracted_143699</td><td>1047</td><td>No</td><td>NA</td><td>0</td><td>1</td><td>Low-quality</td><td>Genome-fragment</td><td></td><td>1.87</td><td>0</td></tr>
<tr><td>S17_vs_k141_120527||full</td><td>1479</td><td>No</td><td>NA</td><td>0</td><td>1</td><td>Not-determined</td><td>Genome-fragment</td><td>NA</td><td></td><td>0</td></tr>
<tr><td>S17_vs_k141_61104||full</td><td>1741</td><td>No</td><td>NA</td><td>0</td><td>0</td><td>Not-determined</td><td>Genome-fragment</td><td>NA</td><td></td><td>0</td></tr>
<tr><td>S10_vs_38074||full</td><td>1047</td><td>No</td><td>NA</td><td>1</td><td>0</td><td>Low-quality</td><td>Genome-fragment</td><td></td><td>2.02</td><td>0</td></tr>
<tr><td>S12_extracted_k141_98567</td><td>1047</td><td>No</td><td>NA</td><td>1</td><td>0</td><td>Low-quality</td><td>Genome-fragment</td><td></td><td>3.08</td><td>0</td></tr>
<tr><td>S17_vs_k141_39651||full</td><td>1222</td><td>No</td><td>NA</td><td>0</td><td>1</td><td>Not-determined</td><td>Genome-fragment</td><td>NA</td><td></td><td>0</td></tr>
<tr><td>S17_vs_k141_68824||full</td><td>4822</td><td>No</td><td>NA</td><td>0</td><td>1</td><td>Not-determined</td><td>Genome-fragment</td><td>NA</td><td></td><td>0</td></tr>
<tr><td>S12_vs_k141_165443||full</td><td>1047</td><td>No</td><td>NA</td><td>0</td><td>1</td><td>Low-quality</td><td>Genome-fragment</td><td></td><td>0.5</td><td>0</td></tr>
<tr><td>S17_vs_k141_142838||full</td><td>2281</td><td>No</td><td>NA</td><td>0</td><td>3</td><td>Not-determined</td><td>Genome-fragment</td><td>NA</td><td></td><td>0</td></tr>
<tr><td>S4_extracted_25211</td><td>1047</td><td>No</td><td>NA</td><td>0</td><td>1</td><td>Low-quality</td><td>Genome-fragment</td><td></td><td>3.43</td><td>0</td></tr>
<tr><td>S17_vs_k141_90714||full</td><td>4701</td><td>No</td><td>NA</td><td>0</td><td>7</td><td>Not-determined</td><td>Genome-fragment</td><td>NA</td><td></td><td>0</td></tr>
<tr><td>S6_vs_k141_43922||full</td><td>1047</td><td>No</td><td>NA</td><td>0</td><td>0</td><td>Low-quality</td><td>Genome-fragment</td><td></td><td>1.74</td><td>0</td></tr>
<tr><td>S17_vs_k141_32000||full</td><td>1399</td><td>No</td><td>NA</td><td>0</td><td>0</td><td>Not-determined</td><td>Genome-fragment</td><td>NA</td><td></td><td>0</td></tr>
<tr><td>S8_extracted_k141_70115</td><td>1047</td><td>No</td><td>NA</td><td>2</td><td>0</td><td>Low-quality</td><td>Genome-fragment</td><td></td><td>1.6</td><td>0</td></tr>
<tr><td>S8_extracted_k141_49668</td><td>1047</td><td>No</td><td>NA</td><td>0</td><td>1</td><td>Low-quality</td><td>Genome-fragment</td><td></td><td>0.59</td><td>0</td></tr>
<tr><td>S17_vs_k141_54340||full</td><td>1404</td><td>No</td><td>NA</td><td>0</td><td>0</td><td>Not-determined</td><td>Genome-fragment</td><td>NA</td><td></td><td>0</td></tr>
<tr><td>S8_vs_k141_105928||full</td><td>1047</td><td>No</td><td>NA</td><td>0</td><td>0</td><td>Low-quality</td><td>Genome-fragment</td><td></td><td>1.05</td><td>0</td></tr>
<tr><td>S8_vs_k141_147124||full</td><td>1047</td><td>No</td><td>NA</td><td>2</td><td>0</td><td>Low-quality</td><td>Genome-fragment</td><td></td><td>2.72</td><td>0</td></tr>
<tr><td>S17_vs_k141_76048||full</td><td>13221</td><td>No</td><td>NA</td><td>0</td><td>4</td><td>Not-determined</td><td>Genome-fragment</td><td>NA</td><td></td><td>0</td></tr>
<tr><td>S17_vs_k141_127519||full</td><td>31736</td><td>No</td><td>NA</td><td>7</td><td>0</td><td>Medium-quality</td><td>Genome-fragment</td><td></td><td>72.13</td><td>0</td></tr>
<tr><td>S8_vs_k141_179216||lt2gene</td><td>1047</td><td>No</td><td>NA</td><td>1</td><td>0</td><td>Low-quality</td><td>Genome-fragment</td><td></td><td>0.6</td><td>0</td></tr>
<tr><td>S17_vs_k141_127533||full</td><td>1164</td><td>No</td><td>NA</td><td>0</td><td>1</td><td>Not-determined</td><td>Genome-fragment</td><td>NA</td><td></td><td>0</td></tr>
<tr><td>S17_vs_k141_24273||full</td><td>1048</td><td>No</td><td>NA</td><td>0</td><td>1</td><td>Low-quality</td><td>Genome-fragment</td><td></td><td>2.11</td><td>0</td></tr>
<tr><td>S11_extracted_k141_65743</td><td>1048</td><td>No</td><td>NA</td><td>0</td><td>0</td><td>Low-quality</td><td>Genome-fragment</td><td></td><td>1.33</td><td>0</td></tr>
<tr><td>S17_vs_k141_157729||full</td><td>3582</td><td>No</td><td>NA</td><td>0</td><td>1</td><td>Not-determined</td><td>Genome-fragment</td><td>NA</td><td></td><td>0</td></tr>
<tr><td>S11_vs_k141_98032||full</td><td>1048</td><td>No</td><td>NA</td><td>0</td><td>1</td><td>Low-quality</td><td>Genome-fragment</td><td></td><td>0.47</td><td>0</td></tr>
<tr><td>S11_vs_k141_8141||full</td><td>1048</td><td>No</td><td>NA</td><td>0</td><td>2</td><td>Low-quality</td><td>Genome-fragment</td><td></td><td>1.52</td><td>0</td></tr>
<tr><td>S17_vs_k141_157755||full</td><td>1895</td><td>No</td><td>NA</td><td>0</td><td>2</td><td>Not-determined</td><td>Genome-fragment</td><td>NA</td><td></td><td>0</td></tr>
<tr><td>S17_vs_k141_24311||full</td><td>2588</td><td>No</td><td>NA</td><td>0</td><td>0</td><td>Not-determined</td><td>Genome-fragment</td><td>NA</td><td></td><td>0</td></tr>
<tr><td>S13_extracted_45611</td><td>1048</td><td>No</td><td>NA</td><td>1</td><td>0</td><td>Low-quality</td><td>Genome-fragment</td><td></td><td>2.17</td><td>0</td></tr>
<tr><td>S14_extracted_k141_105021</td><td>1048</td><td>No</td><td>NA</td><td>1</td><td>0</td><td>Low-quality</td><td>Genome-fragment</td><td></td><td>3.81</td><td>0</td></tr>
<tr><td>S3_extracted_k141_19782</td><td>1048</td><td>No</td><td>NA</td><td>1</td><td>0</td><td>Low-quality</td><td>Genome-fragment</td><td></td><td>2.84</td><td>0</td></tr>
<tr><td>S3_vs_k141_123138||full</td><td>1048</td><td>No</td><td>NA</td><td>1</td><td>0</td><td>Low-quality</td><td>Genome-fragment</td><td></td><td>1.92</td><td>0</td></tr>
<tr><td>S3_vs_k141_69775||lt2gene</td><td>1048</td><td>No</td><td>NA</td><td>1</td><td>0</td><td>Low-quality</td><td>Genome-fragment</td><td></td><td>0.72</td><td>0</td></tr>
<tr><td>S5_extracted_k141_134057</td><td>1048</td><td>No</td><td>NA</td><td>2</td><td>0</td><td>Low-quality</td><td>Genome-fragment</td><td></td><td>1.41</td><td>0</td></tr>
<tr><td>S5_vs_k141_27260||lt2gene</td><td>1048</td><td>No</td><td>NA</td><td>1</td><td>0</td><td>Low-quality</td><td>Genome-fragment</td><td></td><td>1.5</td><td>0</td></tr>
<tr><td>S7_vs_39072||full</td><td>1048</td><td>No</td><td>NA</td><td>1</td><td>0</td><td>Low-quality</td><td>Genome-fragment</td><td></td><td>1.98</td><td>0</td></tr>
<tr><td>S16_extracted_83952</td><td>1048</td><td>No</td><td>NA</td><td>0</td><td>0</td><td>Low-quality</td><td>Genome-fragment</td><td></td><td>1.97</td><td>0</td></tr>
<tr><td>S17_vs_k141_16767||full</td><td>2779</td><td>No</td><td>NA</td><td>0</td><td>0</td><td>Not-determined</td><td>Genome-fragment</td><td>NA</td><td></td><td>0</td></tr>
<tr><td>S17_vs_k141_46857||full</td><td>2195</td><td>No</td><td>NA</td><td>0</td><td>1</td><td>Not-determined</td><td>Genome-fragment</td><td>NA</td><td></td><td>0</td></tr>
<tr><td>S16_vs_173119||full</td><td>1048</td><td>No</td><td>NA</td><td>1</td><td>0</td><td>Low-quality</td><td>Genome-fragment</td><td></td><td>0.93</td><td>0</td></tr>
<tr><td>S16_vs_65962||full</td><td>1048</td><td>No</td><td>NA</td><td>0</td><td>1</td><td>Low-quality</td><td>Genome-fragment</td><td></td><td>1.83</td><td>0</td></tr>
<tr><td>S16_vs_141177||full</td><td>1048</td><td>No</td><td>NA</td><td>0</td><td>1</td><td>Low-quality</td><td>Genome-fragment</td><td></td><td>2.06</td><td>0</td></tr>
<tr><td>S10_vs_94607||full</td><td>1048</td><td>No</td><td>NA</td><td>0</td><td>1</td><td>Low-quality</td><td>Genome-fragment</td><td></td><td>2.26</td><td>0</td></tr>
<tr><td>S17_vs_k141_113061||full</td><td>3152</td><td>No</td><td>NA</td><td>0</td><td>3</td><td>Not-determined</td><td>Genome-fragment</td><td>NA</td><td></td><td>0</td></tr>
<tr><td>S10_vs_115859||full</td><td>1048</td><td>No</td><td>NA</td><td>0</td><td>1</td><td>Low-quality</td><td>Genome-fragment</td><td></td><td>3.98</td><td>0</td></tr>
<tr><td>S10_vs_40352||lt2gene</td><td>1048</td><td>No</td><td>NA</td><td>1</td><td>0</td><td>Low-quality</td><td>Genome-fragment</td><td></td><td>2.14</td><td>0</td></tr>
<tr><td>S17_vs_k141_46917||full</td><td>1773</td><td>No</td><td>NA</td><td>0</td><td>1</td><td>Not-determined</td><td>Genome-fragment</td><td>NA</td><td></td><td>0</td></tr>
<tr><td>S17_vs_k141_2270||full</td><td>1181</td><td>No</td><td>NA</td><td>0</td><td>0</td><td>Not-determined</td><td>Genome-fragment</td><td>NA</td><td></td><td>0</td></tr>
<tr><td>S15_vs_42177||lt2gene</td><td>1048</td><td>No</td><td>NA</td><td>1</td><td>0</td><td>Low-quality</td><td>Genome-fragment</td><td></td><td>19.33</td><td>0</td></tr>
<tr><td>S17_vs_k141_127663||full</td><td>1358</td><td>No</td><td>NA</td><td>0</td><td>1</td><td>Not-determined</td><td>Genome-fragment</td><td>NA</td><td></td><td>0</td></tr>
<tr><td>S17_vs_k141_135251||full</td><td>1088</td><td>No</td><td>NA</td><td>0</td><td>2</td><td>Not-determined</td><td>Genome-fragment</td><td>NA</td><td></td><td>0</td></tr>
<tr><td>S8_vs_k141_46095||full</td><td>1048</td><td>No</td><td>NA</td><td>0</td><td>0</td><td>Low-quality</td><td>Genome-fragment</td><td></td><td>2.48</td><td>0</td></tr>
<tr><td>S9_extracted_k141_75804</td><td>1049</td><td>No</td><td>NA</td><td>1</td><td>0</td><td>Low-quality</td><td>Genome-fragment</td><td></td><td>2.24</td><td>0</td></tr>
<tr><td>S17_vs_k141_54483||full</td><td>1063</td><td>No</td><td>NA</td><td>0</td><td>1</td><td>Not-determined</td><td>Genome-fragment</td><td>NA</td><td></td><td>0</td></tr>
<tr><td>S17_vs_k141_98661||full</td><td>3663</td><td>No</td><td>NA</td><td>0</td><td>3</td><td>Not-determined</td><td>Genome-fragment</td><td>NA</td><td></td><td>0</td></tr>
<tr><td>S9_vs_k141_88850||full</td><td>1049</td><td>No</td><td>NA</td><td>0</td><td>0</td><td>Low-quality</td><td>Genome-fragment</td><td></td><td>2.36</td><td>0</td></tr>
<tr><td>S17_vs_k141_76223||full</td><td>2293</td><td>No</td><td>NA</td><td>0</td><td>2</td><td>Not-determined</td><td>Genome-fragment</td><td>NA</td><td></td><td>0</td></tr>
<tr><td>S17_vs_k141_90885||full</td><td>16374</td><td>No</td><td>NA</td><td>0</td><td>7</td><td>Not-determined</td><td>Genome-fragment</td><td>NA</td><td></td><td>0</td></tr>
<tr><td>S1_extracted_k141_7128</td><td>1049</td><td>No</td><td>NA</td><td>0</td><td>1</td><td>Low-quality</td><td>Genome-fragment</td><td></td><td>2.05</td><td>0</td></tr>
<tr><td>S3_vs_k141_120317||full</td><td>1049</td><td>No</td><td>NA</td><td>0</td><td>0</td><td>Low-quality</td><td>Genome-fragment</td><td></td><td>0.6</td><td>0</td></tr>
<tr><td>S3_vs_k141_123189||lt2gene</td><td>1049</td><td>No</td><td>NA</td><td>1</td><td>0</td><td>Low-quality</td><td>Genome-fragment</td><td></td><td>1.67</td><td>0</td></tr>
<tr><td>S5_vs_k141_129802||full</td><td>1049</td><td>No</td><td>NA</td><td>1</td><td>0</td><td>Low-quality</td><td>Genome-fragment</td><td></td><td>2.55</td><td>0</td></tr>
<tr><td>S7_extracted_72280</td><td>1049</td><td>No</td><td>NA</td><td>0</td><td>0</td><td>Low-quality</td><td>Genome-fragment</td><td></td><td>1.94</td><td>0</td></tr>
<tr><td>S7_extracted_98467</td><td>1049</td><td>No</td><td>NA</td><td>1</td><td>0</td><td>Low-quality</td><td>Genome-fragment</td><td></td><td>16.58</td><td>0</td></tr>
<tr><td>S17_vs_k141_90924||full</td><td>3914</td><td>No</td><td>NA</td><td>0</td><td>3</td><td>Not-determined</td><td>Genome-fragment</td><td>NA</td><td></td><td>0</td></tr>
<tr><td>S17_vs_k141_10067||full</td><td>1313</td><td>No</td><td>NA</td><td>0</td><td>2</td><td>Not-determined</td><td>Genome-fragment</td><td>NA</td><td></td><td>0</td></tr>
<tr><td>S16_vs_152872||full</td><td>1049</td><td>No</td><td>NA</td><td>0</td><td>1</td><td>Low-quality</td><td>Genome-fragment</td><td></td><td>1.41</td><td>0</td></tr>
<tr><td>S17_vs_k141_24394||full</td><td>1376</td><td>No</td><td>NA</td><td>0</td><td>1</td><td>Not-determined</td><td>Genome-fragment</td><td>NA</td><td></td><td>0</td></tr>
<tr><td>S12_vs_k141_173976||full</td><td>1049</td><td>No</td><td>NA</td><td>0</td><td>0</td><td>Low-quality</td><td>Genome-fragment</td><td></td><td>2.11</td><td>0</td></tr>
<tr><td>S15_extracted_67508</td><td>1049</td><td>No</td><td>NA</td><td>0</td><td>1</td><td>Low-quality</td><td>Genome-fragment</td><td></td><td>0.99</td><td>0</td></tr>
<tr><td>S17_vs_k141_54578||full</td><td>1640</td><td>No</td><td>NA</td><td>0</td><td>0</td><td>Not-determined</td><td>Genome-fragment</td><td>NA</td><td></td><td>0</td></tr>
<tr><td>S17_vs_k141_157905||full</td><td>1856</td><td>No</td><td>NA</td><td>0</td><td>0</td><td>Not-determined</td><td>Genome-fragment</td><td>NA</td><td></td><td>0</td></tr>
<tr><td>S17_vs_k141_90970||full</td><td>1703</td><td>No</td><td>NA</td><td>0</td><td>1</td><td>Not-determined</td><td>Genome-fragment</td><td>NA</td><td></td><td>0</td></tr>
<tr><td>S17_vs_k141_47043||full</td><td>2708</td><td>No</td><td>NA</td><td>0</td><td>0</td><td>Not-determined</td><td>Genome-fragment</td><td>NA</td><td></td><td>0</td></tr>
<tr><td>S17_extracted_k141_70719</td><td>1050</td><td>No</td><td>NA</td><td>0</td><td>0</td><td>Low-quality</td><td>Genome-fragment</td><td></td><td>0.72</td><td>0</td></tr>
<tr><td>S17_vs_k141_2369||full</td><td>2195</td><td>No</td><td>NA</td><td>0</td><td>0</td><td>Not-determined</td><td>Genome-fragment</td><td>NA</td><td></td><td>0</td></tr>
<tr><td>S17_extracted_k141_34391</td><td>1050</td><td>No</td><td>NA</td><td>0</td><td>0</td><td>Low-quality</td><td>Genome-fragment</td><td></td><td>2.48</td><td>0</td></tr>
<tr><td>S17_vs_k141_106209||full</td><td>1050</td><td>No</td><td>NA</td><td>0</td><td>0</td><td>Low-quality</td><td>Genome-fragment</td><td></td><td>0.44</td><td>0</td></tr>
<tr><td>S17_vs_k141_157957||full</td><td>2826</td><td>No</td><td>NA</td><td>0</td><td>0</td><td>Not-determined</td><td>Genome-fragment</td><td>NA</td><td></td><td>0</td></tr>
<tr><td>S17_vs_k141_120805||full</td><td>3394</td><td>No</td><td>NA</td><td>0</td><td>0</td><td>Not-determined</td><td>Genome-fragment</td><td>NA</td><td></td><td>0</td></tr>
<tr><td>S17_vs_k141_47991||full</td><td>1050</td><td>No</td><td>NA</td><td>0</td><td>0</td><td>Low-quality</td><td>Genome-fragment</td><td></td><td>1.42</td><td>0</td></tr>
<tr><td>S17_vs_k141_10139||full</td><td>1110</td><td>No</td><td>NA</td><td>0</td><td>1</td><td>Not-determined</td><td>Genome-fragment</td><td>NA</td><td></td><td>0</td></tr>
<tr><td>S17_vs_k141_165035||full</td><td>4736</td><td>No</td><td>NA</td><td>0</td><td>3</td><td>Not-determined</td><td>Genome-fragment</td><td>NA</td><td></td><td>0</td></tr>
<tr><td>S9_vs_k141_960||lt2gene</td><td>1050</td><td>No</td><td>NA</td><td>1</td><td>0</td><td>Low-quality</td><td>Genome-fragment</td><td></td><td>2.86</td><td>0</td></tr>
<tr><td>S17_vs_k141_113165||full</td><td>1623</td><td>No</td><td>NA</td><td>0</td><td>0</td><td>Not-determined</td><td>Genome-fragment</td><td>NA</td><td></td><td>0</td></tr>
<tr><td>S17_vs_k141_47126||full</td><td>8421</td><td>No</td><td>NA</td><td>0</td><td>2</td><td>Not-determined</td><td>Genome-fragment</td><td>NA</td><td></td><td>0</td></tr>
<tr><td>S11_vs_k141_87074||full</td><td>1050</td><td>No</td><td>NA</td><td>0</td><td>0</td><td>Low-quality</td><td>Genome-fragment</td><td></td><td>2.05</td><td>0</td></tr>
<tr><td>S1_extracted_k141_27617</td><td>1050</td><td>No</td><td>NA</td><td>0</td><td>0</td><td>Low-quality</td><td>Genome-fragment</td><td></td><td>1.99</td><td>0</td></tr>
<tr><td>S3_vs_k141_39819||full</td><td>1050</td><td>No</td><td>NA</td><td>0</td><td>0</td><td>Low-quality</td><td>Genome-fragment</td><td></td><td>2.13</td><td>0</td></tr>
<tr><td>S17_vs_k141_127837||full</td><td>2039</td><td>No</td><td>NA</td><td>0</td><td>1</td><td>Not-determined</td><td>Genome-fragment</td><td>NA</td><td></td><td>0</td></tr>
<tr><td>S17_vs_k141_32414||full</td><td>1992</td><td>No</td><td>NA</td><td>0</td><td>2</td><td>Not-determined</td><td>Genome-fragment</td><td>NA</td><td></td><td>0</td></tr>
<tr><td>S5_extracted_k141_105048</td><td>1050</td><td>No</td><td>NA</td><td>0</td><td>0</td><td>Low-quality</td><td>Genome-fragment</td><td></td><td>2.62</td><td>0</td></tr>
<tr><td>S5_extracted_k141_120259</td><td>1050</td><td>No</td><td>NA</td><td>1</td><td>0</td><td>Low-quality</td><td>Genome-fragment</td><td></td><td>1.82</td><td>0</td></tr>
<tr><td>S7_vs_121810||lt2gene</td><td>1050</td><td>No</td><td>NA</td><td>1</td><td>0</td><td>Low-quality</td><td>Genome-fragment</td><td></td><td>1.21</td><td>0</td></tr>
<tr><td>S7_vs_134063||lt2gene</td><td>1050</td><td>No</td><td>NA</td><td>1</td><td>0</td><td>Low-quality</td><td>Genome-fragment</td><td></td><td>2.31</td><td>0</td></tr>
<tr><td>S16_vs_84572||full</td><td>1050</td><td>No</td><td>NA</td><td>0</td><td>0</td><td>Low-quality</td><td>Genome-fragment</td><td></td><td>1.31</td><td>0</td></tr>
<tr><td>S17_vs_k141_91135||full</td><td>2476</td><td>No</td><td>NA</td><td>0</td><td>0</td><td>Not-determined</td><td>Genome-fragment</td><td>NA</td><td></td><td>0</td></tr>
<tr><td>S10_extracted_71736</td><td>1050</td><td>No</td><td>NA</td><td>0</td><td>0</td><td>Low-quality</td><td>Genome-fragment</td><td></td><td>2.09</td><td>0</td></tr>
<tr><td>S12_vs_k141_80313||lt2gene</td><td>1050</td><td>No</td><td>NA</td><td>1</td><td>0</td><td>Low-quality</td><td>Genome-fragment</td><td></td><td>3.12</td><td>0</td></tr>
<tr><td>S2_extracted_k141_18125</td><td>1050</td><td>No</td><td>NA</td><td>0</td><td>0</td><td>Low-quality</td><td>Genome-fragment</td><td></td><td>2.4</td><td>0</td></tr>
<tr><td>S8_extracted_k141_146915</td><td>1050</td><td>No</td><td>NA</td><td>0</td><td>1</td><td>Low-quality</td><td>Genome-fragment</td><td></td><td>0.61</td><td>0</td></tr>
<tr><td>S17_vs_k141_32472||full</td><td>5255</td><td>No</td><td>NA</td><td>0</td><td>3</td><td>Not-determined</td><td>Genome-fragment</td><td>NA</td><td></td><td>0</td></tr>
<tr><td>S17_vs_k141_150947||full</td><td>2708</td><td>No</td><td>NA</td><td>0</td><td>0</td><td>Not-determined</td><td>Genome-fragment</td><td>NA</td><td></td><td>0</td></tr>
<tr><td>S11_extracted_k141_37806</td><td>1051</td><td>No</td><td>NA</td><td>0</td><td>0</td><td>Low-quality</td><td>Genome-fragment</td><td></td><td>1.56</td><td>0</td></tr>
<tr><td>S13_extracted_131425</td><td>1051</td><td>No</td><td>NA</td><td>0</td><td>0</td><td>Low-quality</td><td>Genome-fragment</td><td></td><td>0.78</td><td>0</td></tr>
</table>

S17_vs_k141_98933||full	6340	No	NA	0	3	Not-determined	Genome-fragment	NA		0
S17_vs_k141_143306||full	1648	No	NA	0	0	Not-determined	Genome-fragment	NA		0
S17_vs_k141_24563||full	2125	No	NA	0	2	Not-determined	Genome-fragment	NA		0
S17_vs_k141_40130||full	2891	No	NA	0	1	Not-determined	Genome-fragment	NA		0
S13_extracted_137445	1051	No	NA	0	1	Low-quality	Genome-fragment		0.11	0
S1_extracted_k141_99973	1051	No	NA	0	1	Low-quality	Genome-fragment		0.94	0
S1_vs_k141_91764||full	1051	No	NA	0	0	Low-quality	Genome-fragment		0.35	0
S17_vs_k141_69269||full	1848	No	NA	0	1	Not-determined	Genome-fragment	NA		0
S17_vs_k141_83373||full	4616	No	NA	0	2	Not-determined	Genome-fragment	NA		0
S3_vs_k141_14890||full	1051	No	NA	0	1	Low-quality	Genome-fragment		1.3	0
S16_vs_133838||full	1051	No	NA	0	0	Low-quality	Genome-fragment		2.94	0
S10_extracted_104661	1051	No	NA	0	0	Low-quality	Genome-fragment		2.36	0
S17_vs_k141_61543||full	1451	No	NA	0	0	Not-determined	Genome-fragment	NA		0
S17_vs_k141_32568||full	1025	No	NA	0	0	Not-determined	Genome-fragment	NA		0
S17_vs_k141_17137||full	2634	No	NA	0	1	Not-determined	Genome-fragment	NA		0
S17_vs_k141_47316||full	2542	No	NA	0	2	Not-determined	Genome-fragment	NA		0
S10_vs_84954||lt2gene	1051	No	NA	1	0	Low-quality	Genome-fragment		2.31	0
S17_vs_k141_32599||full	1896	No	NA	0	1	Not-determined	Genome-fragment	NA		0
S8_extracted_k141_64148	1051	No	NA	0	2	Low-quality	Genome-fragment		0.8	0
S17_vs_k141_2625||full	2967	No	NA	0	1	Not-determined	Genome-fragment	NA		0
S8_vs_k141_78705||full	1051	No	NA	0	0	Low-quality	Genome-fragment		0.91	0
S8_vs_k141_98624||lt2gene	1051	No	NA	1	0	Low-quality	Genome-fragment		2.31	0
S17_vs_k141_83471||full	1144	No	NA	0	0	Not-determined	Genome-fragment	NA		0
S17_vs_k141_32647||full	1601	No	NA	0	0	Not-determined	Genome-fragment	NA		0
S17_vs_k141_99040||full	2676	No	NA	0	3	Not-determined	Genome-fragment	NA		0
S17_vs_k141_106787||full	1594	No	NA	0	0	Not-determined	Genome-fragment	NA		0
S9_extracted_k141_53740	1052	No	NA	0	1	Low-quality	Genome-fragment		0.29	0
S17_vs_k141_76609||full	1941	No	NA	0	2	Not-determined	Genome-fragment	NA		0
S17_vs_k141_113337||full	1090	No	NA	0	0	Not-determined	Genome-fragment	NA		0
S13_vs_33660||lt2gene	1052	No	NA	1	0	Low-quality	Genome-fragment		2.21	0
S17_vs_k141_61609||full	3164	No	NA	0	0	Not-determined	Genome-fragment	NA		0
S17_vs_k141_40268||full	4471	No	NA	0	0	Not-determined	Genome-fragment	NA		0
S5_vs_k141_119230||full	1052	No	NA	0	0	Low-quality	Genome-fragment		3.01	0
S17_vs_k141_69420||full	3055	No	NA	0	3	Not-determined	Genome-fragment	NA		0
S17_vs_k141_106822||full	1326	No	NA	0	1	Not-determined	Genome-fragment	NA		0
S17_vs_k141_113378||full	3339	No	NA	0	0	Not-determined	Genome-fragment	NA		0
S7_extracted_21812	1052	No	NA	0	0	Low-quality	Genome-fragment		2.21	0
S7_vs_66685||full	1052	No	NA	0	1	Low-quality	Genome-fragment		0.98	0
S7_vs_130592||full	1052	No	NA	0	0	Low-quality	Genome-fragment		2.41	0
S17_vs_k141_54682||full	4092	No	NA	0	5	Not-determined	Genome-fragment	NA		0
S17_vs_k141_113389||full	11255	No	NA	0	3	Not-determined	Genome-fragment	NA		0
S17_vs_k141_128103||full	12626	No	NA	0	1	Not-determined	Genome-fragment	NA		0
S17_vs_k141_10304||full	2538	No	NA	0	1	Not-determined	Genome-fragment	NA		0
S17_vs_k141_121041||full	34329	No	NA	19	0	Medium-quality	Genome-fragment		72.74	0
S16_extracted_41482	1052	No	NA	1	0	Low-quality	Genome-fragment		0.96	0
S12_extracted_k141_16034	1052	No	NA	0	1	Low-quality	Genome-fragment		0.47	0
S17_vs_k141_135708||full	3275	No	NA	0	2	Not-determined	Genome-fragment	NA		0
S17_vs_k141_10324||full	2223	No	NA	0	2	Not-determined	Genome-fragment	NA		0
S12_extracted_k141_137914	1052	No	NA	1	0	Low-quality	Genome-fragment		1.06	0
S15_extracted_12472	1052	No	NA	0	0	Low-quality	Genome-fragment		0.61	0
S17_vs_k141_97347||full	1053	No	NA	0	2	Low-quality	Genome-fragment		1.77	0
S17_vs_k141_83550||full	1152	No	NA	0	1	Not-determined	Genome-fragment	NA		0
S17_vs_k141_61720||full	2718	No	NA	0	0	Not-determined	Genome-fragment	NA		0
S17_vs_k141_17307||full	1374	No	NA	0	2	Not-determined	Genome-fragment	NA		0
S11_vs_k141_34808||full	1053	No	NA	2	0	Low-quality	Genome-fragment		1.93	0
S17_vs_k141_61726||full	2777	No	NA	0	2	Not-determined	Genome-fragment	NA		0
S11_vs_k141_12685||full	1053	No	NA	0	0	Low-quality	Genome-fragment		2.92	0
S17_vs_k141_113436||full	1861	No	NA	0	1	Not-determined	Genome-fragment	NA		0
S14_extracted_k141_29162	1053	No	NA	2	0	Low-quality	Genome-fragment		2.37	0
S17_vs_k141_61752||full	3289	No	NA	0	1	Not-determined	Genome-fragment	NA		0
S17_vs_k141_165348||full	3266	No	NA	0	3	Not-determined	Genome-fragment	NA		0
S17_vs_k141_61765||full	1157	No	NA	0	1	Not-determined	Genome-fragment	NA		0
S17_vs_k141_47560||full	1961	No	NA	0	0	Not-determined	Genome-fragment	NA		0
S17_vs_k141_135783||full	1733	No	NA	0	0	Not-determined	Genome-fragment	NA		0
S3_vs_k141_35594||lt2gene	1053	No	NA	0	0	Low-quality	Genome-fragment		1.78	0
S3_vs_k141_4879||lt2gene	1053	No	NA	1	0	Low-quality	Genome-fragment		2.39	0
S17_vs_k141_32731||full	1295	No	NA	0	1	Not-determined	Genome-fragment	NA		0
S17_vs_k141_99249||full	1729	No	NA	0	2	Not-determined	Genome-fragment	NA		0
S5_vs_k141_88980||full	1053	No	NA	0	0	Low-quality	Genome-fragment		1.77	0
S7_vs_39023||full	1053	No	NA	1	0	Low-quality	Genome-fragment		2.72	0
S16_vs_74218||full	1053	No	NA	1	0	Low-quality	Genome-fragment		1.59	0
S10_extracted_21761	1053	No	NA	0	0	Low-quality	Genome-fragment		0.97	0
S17_vs_k141_121107||full	2013	No	NA	0	2	Not-determined	Genome-fragment	NA		0
S17_vs_k141_135822||full	2265	No	NA	0	1	Not-determined	Genome-fragment	NA		0
S17_vs_k141_143643||full	18147	No	NA	0	9	Not-determined	Genome-fragment	NA		0
S17_vs_k141_121119||full	7296	No	NA	0	1	Not-determined	Genome-fragment	NA		0
S17_vs_k141_40477||full	1189	No	NA	0	2	Not-determined	Genome-fragment	NA		0
S12_vs_k141_175421||full	1053	No	NA	0	0	Low-quality	Genome-fragment		0.75	0
S17_vs_k141_143657||full	2675	No	NA	0	3	Not-determined	Genome-fragment	NA		0
S15_vs_60020||lt2gene	1053	No	NA	0	0	Low-quality	Genome-fragment		2.39	0
S2_extracted_k141_597	1053	No	NA	0	0	Low-quality	Genome-fragment		16.92	0
S17_vs_k141_143665||full	2460	No	NA	0	2	Not-determined	Genome-fragment	NA		0
S17_vs_k141_99346||full	1104	No	NA	0	2	Not-determined	Genome-fragment	NA		0
S17_vs_k141_107017||full	5514	No	NA	0	5	Not-determined	Genome-fragment	NA		0
S17_vs_k141_91533||full	1938	No	NA	0	1	Not-determined	Genome-fragment	NA		0
S17_vs_k141_128287||full	1063	No	NA	0	1	Not-determined	Genome-fragment	NA		0
S8_vs_k141_60654||full	1053	No	NA	0	0	Low-quality	Genome-fragment		2.36	0
S17_vs_k141_7831||full	1054	No	NA	0	0	Low-quality	Genome-fragment		2.44	0
S17_vs_k141_26285||full	1054	No	NA	0	0	Low-quality	Genome-fragment		2	0
S14_extracted_k141_110487	1054	No	NA	2	0	Low-quality	Genome-fragment		2.11	0
S17_vs_k141_24943||full	4386	No	NA	0	3	Not-determined	Genome-fragment	NA		0
S17_vs_k141_158356||full	1323	No	NA	0	1	Not-determined	Genome-fragment	NA		0
S14_extracted_k141_97949	1054	No	NA	2	0	Low-quality	Genome-fragment		3.12	0
S17_vs_k141_17493||full	1585	No	NA	0	0	Not-determined	Genome-fragment	NA		0
S17_vs_k141_113670||full	1844	No	NA	0	2	Not-determined	Genome-fragment	NA		0
S14_extracted_k141_71120	1054	No	NA	1	0	Low-quality	Genome-fragment		1.89	0
S17_vs_k141_24989||full	3139	No	NA	0	3	Not-determined	Genome-fragment	NA		0
S1_extracted_k141_85452	1054	No	NA	0	0	Low-quality	Genome-fragment		1.09	0
S1_extracted_k141_119607	1054	No	NA	0	0	Low-quality	Genome-fragment		1.05	0
S1_vs_k141_92661||full	1054	No	NA	0	1	Low-quality	Genome-fragment		1.65	0
S3_vs_k141_63101||full	1054	No	NA	1	0	Low-quality	Genome-fragment		1.96	0
S17_vs_k141_113746||full	1142	No	NA	0	2	Not-determined	Genome-fragment	NA		0
S17_vs_k141_3053||full	1501	No	NA	0	0	Not-determined	Genome-fragment	NA		0
S16_extracted_89665	1054	No	NA	1	0	Low-quality	Genome-fragment		1.41	0
S16_vs_24263||full	1054	No	NA	0	0	Low-quality	Genome-fragment		1.59	0
S17_vs_k141_158480||full	1625	No	NA	0	0	Not-determined	Genome-fragment	NA		0
S17_vs_k141_107231||full	2096	No	NA	0	2	Not-determined	Genome-fragment	NA		0
S10_extracted_14471	1054	No	NA	0	0	Low-quality	Genome-fragment		1.04	0
S10_vs_106229||full	1054	No	NA	0	0	Low-quality	Genome-fragment		2.25	0
S12_extracted_k141_126369	1054	No	NA	0	1	Low-quality	Genome-fragment		2.97	0
S12_extracted_k141_27289	1054	No	NA	0	0	Low-quality	Genome-fragment		1.27	0
S17_vs_k141_62032||full	2884	No	NA	0	2	Not-determined	Genome-fragment	NA		0
S6_extracted_k141_40989	1054	No	NA	1	0	Low-quality	Genome-fragment		2.26	0
S17_vs_k141_3109||full	3171	No	NA	0	2	Not-determined	Genome-fragment	NA		0
S8_extracted_k141_133252	1054	No	NA	0	1	Low-quality	Genome-fragment		0.67	0
S17_vs_k141_121345||full	5794	No	NA	0	4	Not-determined	Genome-fragment	NA		0
S17_vs_k141_10723||full	4730	No	NA	0	1	Not-determined	Genome-fragment	NA		0
S8_vs_k141_202469||full	1054	No	NA	0	0	Low-quality	Genome-fragment		2.34	0
S17_extracted_k141_10115	1055	No	NA	0	0	Low-quality	Genome-fragment		1.73	0
S9_extracted_k141_30789	1055	No	NA	0	2	Low-quality	Genome-fragment		0.58	0
S17_vs_k141_69850||full	5944	No	NA	0	0	Not-determined	Genome-fragment	NA		0
S11_extracted_k141_18389	1055	No	NA	0	0	Low-quality	Genome-fragment		2.49	0
S17_vs_k141_25100||full	4648	No	NA	0	0	Not-determined	Genome-fragment	NA		0
S17_vs_k141_128520||full	3502	No	NA	0	3	Not-determined	Genome-fragment	NA		0
S13_extracted_153455	1055	No	NA	0	0	Low-quality	Genome-fragment		1.6	0
S17_vs_k141_151453||full	3291	No	NA	0	2	Not-determined	Genome-fragment	NA		0
S1_vs_k141_87697||full	1055	No	NA	0	0	Low-quality	Genome-fragment		1.79	0
S17_vs_k141_107298||full	1176	No	NA	0	1	Not-determined	Genome-fragment	NA		0
S17_vs_k141_25117||full	1214	No	NA	0	2	Not-determined	Genome-fragment	NA		0
S17_vs_k141_107307||full	1198	No	NA	0	1	Not-determined	Genome-fragment	NA		0
S17_vs_k141_25121||full	1609	No	NA	0	0	Not-determined	Genome-fragment	NA		0
S17_vs_k141_128574||full	1377	No	NA	0	1	Not-determined	Genome-fragment	NA		0
S3_vs_k141_58041||full	1055	No	NA	0	1	Low-quality	Genome-fragment		1.74	0
S5_vs_k141_50144||full	1055	No	NA	1	0	Low-quality	Genome-fragment		1.75	0
S17_vs_k141_172925||full	4853	No	NA	0	3	Not-determined	Genome-fragment	NA		0
S17_vs_k141_136057||full	8893	No	NA	0	2	Not-determined	Genome-fragment	NA		0
S17_vs_k141_10796||full	2327	No	NA	0	2	Not-determined	Genome-fragment	NA		0
S17_vs_k141_3183||full	1127	No	NA	0	0	Not-determined	Genome-fragment	NA		0
S5_vs_k141_69264||full	1055	No	NA	1	0	Low-quality	Genome-fragment		1.58	0
S17_vs_k141_33113||full	1262	No	NA	0	2	Not-determined	Genome-fragment	NA		0
S17_vs_k141_91805||full	2989	No	NA	0	1	Not-determined	Genome-fragment	NA		0
S17_vs_k141_17787||full	6708	No	NA	0	5	Not-determined	Genome-fragment	NA		0
S7_vs_75734||lt2gene	1055	No	NA	0	0	Low-quality	Genome-fragment		2.84	0
S16_vs_2073||full	1055	No	NA	0	0	Low-quality	Genome-fragment		2.06	0
S16_vs_40286||full	1055	No	NA	0	0	Low-quality	Genome-fragment		2.21	0
S12_extracted_k141_135897	1055	No	NA	0	1	Low-quality	Genome-fragment		0.25	0
S12_vs_k141_155319||full	1055	No	NA	0	0	Low-quality	Genome-fragment		1.86	0
S17_vs_k141_48007||full	1359	No	NA	0	2	Not-determined	Genome-fragment	NA		0
S8_extracted_k141_110601	1055	No	NA	0	0	Low-quality	Genome-fragment		1.92	0
S17_vs_k141_113983||full	2211	No	NA	0	0	Not-determined	Genome-fragment	NA		0
S17_vs_k141_128661||full	1133	No	NA	0	1	Not-determined	Genome-fragment	NA		0
S17_vs_k141_55292||full	4166	No	NA	0	3	Not-determined	Genome-fragment	NA		0
S17_vs_k141_10860||full	1082	No	NA	0	2	Not-determined	Genome-fragment	NA		0
S17_vs_k141_144023||full	1517	No	NA	0	1	Not-determined	Genome-fragment	NA		0
S8_vs_k141_150814||full	1055	No	NA	0	1	Low-quality	Genome-fragment		1.53	0
S8_vs_k141_118838||full	1055	No	NA	0	1	Low-quality	Genome-fragment		1.02	0
S17_vs_k141_121542||full	1823	No	NA	0	1	Not-determined	Genome-fragment	NA		0
S8_vs_k141_180044||full	1055	No	NA	0	2	Low-quality	Genome-fragment		2.38	0
S8_vs_k141_14823||full	1055	No	NA	0	0	Low-quality	Genome-fragment		2.35	0
S17_vs_k141_77305||full	3723	No	NA	0	1	Not-determined	Genome-fragment	NA		0
S17_extracted_k141_172645	1056	No	NA	0	0	Low-quality	Genome-fragment		2.7	0
S17_vs_k141_48093||full	1082	No	NA	0	0	Not-determined	Genome-fragment	NA		0
S17_vs_k141_99736||full	1215	No	NA	0	1	Not-determined	Genome-fragment	NA		0
S17_vs_k141_3415||full	2339	No	NA	0	3	Not-determined	Genome-fragment	NA		0
S17_vs_k141_151576||full	3618	No	NA	0	2	Not-determined	Genome-fragment	NA		0
S17_vs_k141_114072||full	2054	No	NA	0	0	Not-determined	Genome-fragment	NA		0
S17_extracted_k141_48131	1056	No	NA	0	1	Low-quality	Genome-fragment		0.28	0
S17_extracted_k141_64033	1056	No	NA	1	0	Low-quality	Genome-fragment		1.94	0
S17_vs_k141_128784||full	1423	No	NA	0	0	Not-determined	Genome-fragment	NA		0
S9_extracted_k141_42461	1056	No	NA	0	1	Low-quality	Genome-fragment		0.37	0
S17_vs_k141_70145||full	2275	No	NA	0	2	Not-determined	Genome-fragment	NA		0
S9_extracted_k141_76907	1056	No	NA	0	2	Low-quality	Genome-fragment		0.89	0
S9_vs_k141_11803||lt2gene	1056	No	NA	2	0	Low-quality	Genome-fragment		2.75	0
S11_vs_k141_89126||lt2gene	1056	No	NA	1	0	Low-quality	Genome-fragment		2.28	0

S13_vs_126828||full	1056	No	NA	0	0	Low-quality	Genome-fragment		0.62	0
S17_vs_k141_99815||full	1613	No	NA	0	1	Not-determined	Genome-fragment	NA		0
S17_vs_k141_77403||full	1033	No	NA	0	2	Not-determined	Genome-fragment	NA		0
S17_vs_k141_25322||full	1392	No	NA	0	0	Not-determined	Genome-fragment	NA		0
S17_vs_k141_33342||full	1516	No	NA	0	1	Not-determined	Genome-fragment	NA		0
S17_vs_k141_40965||full	2693	No	NA	0	1	Not-determined	Genome-fragment	NA		0
S17_vs_k141_18017||full	2038	No	NA	0	1	Not-determined	Genome-fragment	NA		0
S17_vs_k141_158876||full	2418	No	NA	0	0	Not-determined	Genome-fragment	NA		0
S17_vs_k141_128820||full	2482	No	NA	0	0	Not-determined	Genome-fragment	NA		0
S17_vs_k141_121617||full	1423	No	NA	0	0	Not-determined	Genome-fragment	NA		0
S14_extracted_k141_76193	1056	No	NA	0	1	Low-quality	Genome-fragment		0.71	0
S17_vs_k141_158911||full	1660	No	NA	0	0	Not-determined	Genome-fragment	NA		0
S17_vs_k141_25377||full	2754	No	NA	0	1	Not-determined	Genome-fragment	NA		0
S14_vs_k141_43337||full	1056	No	NA	0	2	Low-quality	Genome-fragment		0.62	0
S14_vs_k141_36175||lt2gene	1056	No	NA	1	0	Low-quality	Genome-fragment		1.7	0
S17_vs_k141_25387||full	1054	No	NA	0	1	Not-determined	Genome-fragment	NA		0
S17_vs_k141_114181||full	1436	No	NA	0	1	Not-determined	Genome-fragment	NA		0
S1_extracted_k141_102343	1056	No	NA	0	0	Low-quality	Genome-fragment		0.69	0
S17_vs_k141_48200||full	1320	No	NA	0	0	Not-determined	Genome-fragment	NA		0
S3_extracted_k141_117065	1056	No	NA	3	0	Low-quality	Genome-fragment		3.48	0
S3_vs_k141_969||full	1056	No	NA	1	0	Low-quality	Genome-fragment		1.73	0
S3_vs_k141_87651||lt2gene	1056	No	NA	1	0	Low-quality	Genome-fragment		0.95	0
S7_vs_117612||full	1056	No	NA	0	0	Low-quality	Genome-fragment		3.01	0
S17_vs_k141_62378||full	1672	No	NA	0	0	Not-determined	Genome-fragment	NA		0
S17_vs_k141_62385||full	1104	No	NA	0	1	Not-determined	Genome-fragment	NA		0
S7_vs_71477||lt2gene	1056	No	NA	0	1	Low-quality	Genome-fragment		1.4	0
S17_vs_k141_173285||full	1092	No	NA	0	1	Not-determined	Genome-fragment	NA		0
S10_vs_108870||full	1056	No	NA	0	1	Low-quality	Genome-fragment		0.66	0
S17_vs_k141_62399||full	4343	No	NA	0	1	Not-determined	Genome-fragment	NA		0
S17_vs_k141_55472||full	1039	No	NA	0	0	Not-determined	Genome-fragment	NA		0
S17_vs_k141_173300||full	4591	No	NA	0	3	Not-determined	Genome-fragment	NA		0
S17_vs_k141_62406||full	3543	No	NA	0	2	Not-determined	Genome-fragment	NA		0
S17_vs_k141_33450||full	1879	No	NA	0	2	Not-determined	Genome-fragment	NA		0
S17_vs_k141_48255||full	2180	No	NA	0	0	Not-determined	Genome-fragment	NA		0
S17_vs_k141_25462||full	3423	No	NA	0	0	Not-determined	Genome-fragment	NA		0
S17_vs_k141_62429||full	1545	No	NA	0	1	Not-determined	Genome-fragment	NA		0
S17_vs_k141_136436||full	7964	No	NA	0	1	Not-determined	Genome-fragment	NA		0
S12_vs_k141_88342||full	1056	No	NA	1	0	Low-quality	Genome-fragment		1.9	0
S17_vs_k141_165992||full	1114	No	NA	0	1	Not-determined	Genome-fragment	NA		0
S17_vs_k141_165993||full	1729	No	NA	0	1	Not-determined	Genome-fragment	NA		0
S12_vs_k141_46813||full	1056	No	NA	0	1	Low-quality	Genome-fragment		1.92	0
S17_vs_k141_84197||full	2189	No	NA	0	0	Not-determined	Genome-fragment	NA		0
S15_extracted_66338	1056	No	NA	0	1	Low-quality	Genome-fragment		0.74	0
S17_vs_k141_92127||full	1269	No	NA	0	0	Not-determined	Genome-fragment	NA		0
S17_vs_k141_114220||full	21659	No	NA	0	17	Not-determined	Genome-fragment	NA		0
S15_vs_25502||full	1056	No	NA	0	0	Low-quality	Genome-fragment		0.83	0
S17_vs_k141_166017||full	3894	No	NA	0	1	Not-determined	Genome-fragment	NA		0
S17_vs_k141_92130||full	3412	No	NA	0	3	Not-determined	Genome-fragment	NA		0
S17_vs_k141_48290||full	1751	No	NA	0	0	Not-determined	Genome-fragment	NA		0
S17_vs_k141_3566||full	2839	No	NA	0	1	Not-determined	Genome-fragment	NA		0
S8_vs_k141_81267||full	1056	No	NA	1	0	Low-quality	Genome-fragment		2.56	0
S17_extracted_k141_82324	1057	No	NA	2	0	Low-quality	Genome-fragment		3.47	0
S17_vs_k141_84214||full	6444	No	NA	0	3	Not-determined	Genome-fragment	NA		0
S17_vs_k141_9983||full	1057	No	NA	1	0	Low-quality	Genome-fragment		2.03	0
S17_vs_k141_11011||full	15698	No	NA	0	11	Not-determined	Genome-fragment	NA		0
S17_vs_k141_26407||full	1057	No	NA	0	0	Low-quality	Genome-fragment		0.82	0
S17_vs_k141_55538||full	1415	No	NA	0	0	Not-determined	Genome-fragment	NA		0
S17_vs_k141_41099||full	1045	No	NA	0	1	Not-determined	Genome-fragment	NA		0
S17_vs_k141_128985||full	2353	No	NA	0	0	Not-determined	Genome-fragment	NA		0
S17_vs_k141_128992||full	2016	No	NA	0	1	Not-determined	Genome-fragment	NA		0
S9_extracted_k141_78433	1057	No	NA	3	0	Low-quality	Genome-fragment		1.7	0
S17_vs_k141_3609||full	2404	No	NA	0	0	Not-determined	Genome-fragment	NA		0
S17_vs_k141_166050||full	3224	No	NA	0	0	Not-determined	Genome-fragment	NA		0
S17_vs_k141_114311||full	1986	No	NA	0	0	Not-determined	Genome-fragment	NA		0
S17_vs_k141_151849||full	1615	No	NA	0	1	Not-determined	Genome-fragment	NA		0
S11_vs_k141_33160||full	1057	No	NA	0	0	Low-quality	Genome-fragment		2.1	0
S11_vs_k141_51279||lt2gene	1057	No	NA	1	0	Low-quality	Genome-fragment		2.59	0
S13_extracted_27958	1057	No	NA	0	1	Low-quality	Genome-fragment		1.22	0
S13_vs_29156||full	1057	No	NA	0	1	Low-quality	Genome-fragment		1.88	0
S17_vs_k141_121763||full	1941	No	NA	0	1	Not-determined	Genome-fragment	NA		0
S17_vs_k141_114365||full	1255	No	NA	0	1	Not-determined	Genome-fragment	NA		0
S17_vs_k141_114367||full	1604	No	NA	0	1	Not-determined	Genome-fragment	NA		0
S13_vs_49000||full	1057	No	NA	0	0	Low-quality	Genome-fragment		0.94	0
S17_vs_k141_62501||full	4229	No	NA	0	3	Not-determined	Genome-fragment	NA		0
S1_vs_k141_82686||full	1057	No	NA	1	0	Low-quality	Genome-fragment		0.68	0
S17_vs_k141_18300||full	3334	No	NA	0	0	Not-determined	Genome-fragment	NA		0
S17_vs_k141_3728||full	1385	No	NA	0	1	Not-determined	Genome-fragment	NA		0
S3_extracted_k141_116160	1057	No	NA	0	0	Low-quality	Genome-fragment		1.81	0
S17_vs_k141_99979||full	1155	No	NA	0	0	Not-determined	Genome-fragment	NA		0
S17_vs_k141_11165||full	1257	No	NA	0	0	Not-determined	Genome-fragment	NA		0
S3_vs_k141_78643||full	1057	No	NA	0	0	Low-quality	Genome-fragment		1.32	0
S17_vs_k141_166157||full	2140	No	NA	0	1	Not-determined	Genome-fragment	NA		0
S17_vs_k141_33677||full	3377	No	NA	0	3	Not-determined	Genome-fragment	NA		0
S5_vs_k141_130524||full	1057	No	NA	0	0	Low-quality	Genome-fragment		2.46	0
S5_vs_k141_5887||full	1057	No	NA	1	0	Low-quality	Genome-fragment		0.61	0
S7_vs_110557||lt2gene	1057	No	NA	1	0	Low-quality	Genome-fragment		2.46	0
S17_vs_k141_11187||full	1751	No	NA	0	0	Not-determined	Genome-fragment	NA		0
S17_vs_k141_3771||full	1151	No	NA	0	0	Not-determined	Genome-fragment	NA		0
S16_extracted_90187	1057	No	NA	0	0	Low-quality	Genome-fragment		2.76	0
S16_extracted_105478	1057	No	NA	0	0	Low-quality	Genome-fragment		1.68	0
S16_vs_66644||full	1057	No	NA	1	0	Low-quality	Genome-fragment		1.95	0
S17_vs_k141_3780||full	2161	No	NA	0	1	Not-determined	Genome-fragment	NA		0
S17_vs_k141_77718||full	1437	No	NA	0	0	Not-determined	Genome-fragment	NA		0
S16_vs_106899||full	1057	No	NA	0	0	Low-quality	Genome-fragment		1.88	0
S16_vs_115489||full	1057	No	NA	1	0	Low-quality	Genome-fragment		1.37	0
S17_vs_k141_41300||full	2037	No	NA	0	1	Not-determined	Genome-fragment	NA		0
S16_vs_143020||full	1057	No	NA	0	0	Low-quality	Genome-fragment		1.34	0
S10_vs_20372||full	1057	No	NA	1	0	Low-quality	Genome-fragment		1.55	0
S17_vs_k141_143965||full	1058	No	NA	0	1	Low-quality	Genome-fragment		2.28	0
S17_vs_k141_136524||full	1246	No	NA	0	0	Not-determined	Genome-fragment	NA		0
S9_extracted_k141_52850	1058	No	NA	2	0	Low-quality	Genome-fragment		3.49	0
S17_vs_k141_25550||full	2296	No	NA	0	2	Not-determined	Genome-fragment	NA		0
S17_vs_k141_77779||full	1269	No	NA	0	1	Not-determined	Genome-fragment	NA		0
S17_vs_k141_33791||full	1739	No	NA	0	0	Not-determined	Genome-fragment	NA		0
S9_vs_k141_80611||full	1058	No	NA	1	0	Low-quality	Genome-fragment		1.87	0
S17_vs_k141_107832||full	1344	No	NA	0	0	Not-determined	Genome-fragment	NA		0
S9_vs_k141_83938||lt2gene	1058	No	NA	1	0	Low-quality	Genome-fragment		25.42	0
S17_vs_k141_77818||full	4201	No	NA	0	3	Not-determined	Genome-fragment	NA		0
S14_extracted_k141_50271	1058	No	NA	0	1	Low-quality	Genome-fragment		2.33	0
S14_vs_k141_55203||lt2gene	1058	No	NA	1	0	Low-quality	Genome-fragment		1.55	0
S17_vs_k141_152063||full	1205	No	NA	0	1	Not-determined	Genome-fragment	NA		0
S14_vs_k141_33399||lt2gene	1058	No	NA	1	0	Low-quality	Genome-fragment		2.46	0
S17_vs_k141_18502||full	1370	No	NA	0	2	Not-determined	Genome-fragment	NA		0
S16_extracted_113958	1058	No	NA	0	0	Low-quality	Genome-fragment		1.79	0
S16_extracted_162756	1058	No	NA	0	1	Low-quality	Genome-fragment		0.61	0
S17_vs_k141_152065||full	7120	No	NA	0	4	Not-determined	Genome-fragment	NA		0
S16_vs_123264||full	1058	No	NA	1	0	Low-quality	Genome-fragment		3.1	0
S17_vs_k141_166296||full	2673	No	NA	0	4	Not-determined	Genome-fragment	NA		0
S17_vs_k141_77836||full	2510	No	NA	0	1	Not-determined	Genome-fragment	NA		0
S10_extracted_85023	1058	No	NA	0	0	Low-quality	Genome-fragment		2.58	0
S12_extracted_k141_28338	1058	No	NA	2	0	Low-quality	Genome-fragment		3.02	0
S17_vs_k141_55828||full	1914	No	NA	0	1	Not-determined	Genome-fragment	NA		0
S12_extracted_k141_123574	1058	No	NA	0	0	Low-quality	Genome-fragment		0.4	0
S15_extracted_41870	1058	No	NA	1	0	Low-quality	Genome-fragment		1.71	0
S17_vs_k141_18509||full	26539	No	NA	0	7	Not-determined	Genome-fragment	NA		0
S17_vs_k141_121950||full	2302	No	NA	0	0	Not-determined	Genome-fragment	NA		0
S17_vs_k141_33879||full	4685	No	NA	0	2	Not-determined	Genome-fragment	NA		0
S17_vs_k141_114668||full	2270	No	NA	0	0	Not-determined	Genome-fragment	NA		0
S15_vs_71951||full	1058	No	NA	0	1	Low-quality	Genome-fragment		1.59	0
S17_vs_k141_48618||full	3009	No	NA	0	0	Not-determined	Genome-fragment	NA		0
S8_vs_k141_27511||full	1058	No	NA	1	0	Low-quality	Genome-fragment		2.79	0
S17_vs_k141_11352||full	2840	No	NA	0	1	Not-determined	Genome-fragment	NA		0
S17_vs_k141_100230||full	1213	No	NA	0	0	Not-determined	Genome-fragment	NA		0
S17_extracted_k141_16716	1059	No	NA	0	0	Low-quality	Genome-fragment		0.75	0
S17_vs_k141_100238||full	1523	No	NA	0	1	Not-determined	Genome-fragment	NA		0
S17_vs_k141_70672||full	3505	No	NA	0	0	Not-determined	Genome-fragment	NA		0
S17_extracted_k141_47771	1059	No	NA	0	0	Low-quality	Genome-fragment		1.67	0
S9_extracted_k141_31862	1059	No	NA	1	0	Low-quality	Genome-fragment		2.26	0
S17_vs_k141_41492||full	1012	No	NA	0	1	Not-determined	Genome-fragment	NA		0
S17_vs_k141_77993||full	1730	No	NA	0	1	Not-determined	Genome-fragment	NA		0
S17_vs_k141_136760||full	5057	No	NA	0	1	Not-determined	Genome-fragment	NA		0
S17_vs_k141_41534||full	1789	No	NA	0	2	Not-determined	Genome-fragment	NA		0
S11_extracted_k141_16131	1059	No	NA	0	0	Low-quality	Genome-fragment		1.42	0
S14_extracted_k141_58365	1059	No	NA	1	0	Low-quality	Genome-fragment		3.51	0
S17_vs_k141_129488||full	2481	No	NA	0	2	Not-determined	Genome-fragment	NA		0
S17_vs_k141_122091||full	2210	No	NA	0	2	Not-determined	Genome-fragment	NA		0
S1_vs_k141_19827||full	1059	No	NA	0	0	Low-quality	Genome-fragment		1.61	0
S3_extracted_k141_47994	1059	No	NA	0	0	Low-quality	Genome-fragment		0.49	0
S17_vs_k141_41597||full	2911	No	NA	0	0	Not-determined	Genome-fragment	NA		0
S17_vs_k141_56009||full	2064	No	NA	0	1	Not-determined	Genome-fragment	NA		0
S17_vs_k141_56012||full	2054	No	NA	0	0	Not-determined	Genome-fragment	NA		0
S3_extracted_k141_101747	1059	No	NA	0	0	Low-quality	Genome-fragment		0.79	0
S17_vs_k141_11420||full	1115	No	NA	0	0	Not-determined	Genome-fragment	NA		0
S17_vs_k141_114902||full	1026	No	NA	0	1	Not-determined	Genome-fragment	NA		0
S17_vs_k141_84558||full	9526	No	NA	0	4	Not-determined	Genome-fragment	NA		0
S17_vs_k141_166574||full	1214	No	NA	0	1	Not-determined	Genome-fragment	NA		0
S7_vs_87497||full	1059	No	NA	0	1	Low-quality	Genome-fragment		0.37	0
S17_vs_k141_122165||full	1761	No	NA	0	2	Not-determined	Genome-fragment	NA		0
S7_vs_99160||full	1059	No	NA	0	1	Low-quality	Genome-fragment		0.59	0
S17_vs_k141_56074||full	2540	No	NA	0	3	Not-determined	Genome-fragment	NA		0
S7_vs_100394||full	1059	No	NA	0	0	Low-quality	Genome-fragment		2.59	0
S16_vs_24146||full	1059	No	NA	0	2	Low-quality	Genome-fragment		1.61	0
S17_vs_k141_166588||full	1099	No	NA	0	1	Not-determined	Genome-fragment	NA		0
S17_vs_k141_78154||full	1459	No	NA	0	1	Not-determined	Genome-fragment	NA		0
S17_vs_k141_41689||full	1334	No	NA	0	0	Not-determined	Genome-fragment	NA		0
S17_vs_k141_70836||full	2928	No	NA	0	2	Not-determined	Genome-fragment	NA		0
S16_vs_50583||full	1059	No	NA	0	0	Low-quality	Genome-fragment		1.78	0
S17_vs_k141_144583||full	2042	No	NA	0	0	Not-determined	Genome-fragment	NA		0
S10_vs_46279||full	1059	No	NA	0	0	Low-quality	Genome-fragment		1.62	0
S17_vs_k141_78194||full	1259	No	NA	0	0	Not-determined	Genome-fragment	NA		0
S17_vs_k141_18793||full	2728	No	NA	1	0	Not-determined	Genome-fragment	NA		0
S17_vs_k141_132360||full	1060	No	NA	0	0	Low-quality	Genome-fragment		1.96	0
S17_vs_k141_144593||full	3236	No	NA	0	0	Not-determined	Genome-fragment	NA		0
S9_extracted_k141_31318	1060	No	NA	0	0	Low-quality	Genome-fragment		1.92	0

S9_vs_k141_40976||full	1060	No	NA	2	0	Low-quality	Genome-fragment		2.91	0
S17_vs_k141_152455||full	2020	No	NA	0	0	Not-determined	Genome-fragment	NA		0
S17_vs_k141_166626||full	1687	No	NA	0	0	Not-determined	Genome-fragment	NA		0
S17_vs_k141_173767||full	1935	No	NA	0	0	Not-determined	Genome-fragment	NA		0
S13_extracted_70542	1060	No	NA	1	0	Low-quality	Genome-fragment		2.8	0
S1_extracted_k141_101237	1060	No	NA	0	0	Low-quality	Genome-fragment		0.5	0
S1_vs_k141_102432||full	1060	No	NA	0	0	Low-quality	Genome-fragment		1.22	0
S17_vs_k141_70893||full	2090	No	NA	0	1	Not-determined	Genome-fragment	NA		0
S1_vs_k141_114456||lt2gene	1060	No	NA	1	0	Low-quality	Genome-fragment		1.49	0
S17_vs_k141_152479||full	2283	No	NA	0	0	Not-determined	Genome-fragment	NA		0
S17_vs_k141_11547||full	1685	No	NA	0	1	Not-determined	Genome-fragment	NA		0
S17_vs_k141_100478||full	12154	No	NA	0	7	Not-determined	Genome-fragment	NA		0
S17_vs_k141_129667||full	2348	No	NA	0	0	Not-determined	Genome-fragment	NA		0
S3_vs_k141_60206||full	1060	No	NA	3	0	Low-quality	Genome-fragment		1.74	0
S17_vs_k141_100503||full	1569	No	NA	0	2	Not-determined	Genome-fragment	NA		0
S17_vs_k141_56185||full	1224	No	NA	0	0	Not-determined	Genome-fragment	NA		0
S17_vs_k141_129687||full	7736	No	NA	0	8	Not-determined	Genome-fragment	NA		0
S17_vs_k141_136968||full	2828	No	NA	0	0	Not-determined	Genome-fragment	NA		0
S3_vs_k141_110495||full	1060	No	NA	1	0	Low-quality	Genome-fragment		1.53	0
S17_vs_k141_92770||full	1169	No	NA	0	0	Not-determined	Genome-fragment	NA		0
S17_vs_k141_25940||full	5251	No	NA	0	0	Not-determined	Genome-fragment	NA		0
S17_vs_k141_25947||full	2172	No	NA	0	2	Not-determined	Genome-fragment	NA		0
S5_extracted_k141_93184	1060	No	NA	1	0	Low-quality	Genome-fragment		2.86	0
S17_vs_k141_108372||full	8279	No	NA	0	2	Not-determined	Genome-fragment	NA		0
S17_vs_k141_129715||full	2864	No	NA	0	2	Not-determined	Genome-fragment	NA		0
S5_vs_k141_129934||full	1060	No	NA	0	1	Low-quality	Genome-fragment		0.78	0
S17_vs_k141_56227||full	1672	No	NA	0	1	Not-determined	Genome-fragment	NA		0
S17_vs_k141_11577||full	1758	No	NA	0	1	Not-determined	Genome-fragment	NA		0
S17_vs_k141_122194||full	5526	No	NA	0	4	Not-determined	Genome-fragment	NA		0
S5_vs_k141_96637||full	1060	No	NA	1	0	Low-quality	Genome-fragment		0.67	0
S7_vs_39076||full	1060	No	NA	1	0	Low-quality	Genome-fragment		2.55	0
S7_vs_59447||full	1060	No	NA	0	1	Low-quality	Genome-fragment		1.71	0
S17_vs_k141_144720||full	2616	No	NA	0	2	Not-determined	Genome-fragment	NA		0
S17_vs_k141_137039||full	1086	No	NA	0	1	Not-determined	Genome-fragment	NA		0
S17_vs_k141_84733||full	5566	No	NA	0	2	Not-determined	Genome-fragment	NA		0
S7_vs_70066||lt2gene	1060	No	NA	1	0	Low-quality	Genome-fragment		2.65	0
S7_vs_82530||lt2gene	1060	No	NA	1	0	Low-quality	Genome-fragment		2.61	0
S17_vs_k141_166752||full	1515	No	NA	0	1	Not-determined	Genome-fragment	NA		0
S17_vs_k141_173917||full	2462	No	NA	0	2	Not-determined	Genome-fragment	NA		0
S17_vs_k141_71032||full	1199	No	NA	0	1	Not-determined	Genome-fragment	NA		0
S16_extracted_33684	1060	No	NA	0	1	Low-quality	Genome-fragment		0.49	0
S17_vs_k141_34293||full	2097	No	NA	0	2	Not-determined	Genome-fragment	NA		0
S17_vs_k141_41845||full	37248	No	NA	18	2	High-quality	High-quality		100	0
S17_vs_k141_115136||full	1429	No	NA	0	2	Not-determined	Genome-fragment	NA		0
S17_vs_k141_137088||full	4342	No	NA	0	0	Not-determined	Genome-fragment	NA		0
S16_vs_109843||full	1060	No	NA	2	0	Low-quality	Genome-fragment		3.13	0
S16_vs_168338||lt2gene	1060	No	NA	1	0	Low-quality	Genome-fragment		2.5	0
S17_vs_k141_152663||full	2239	No	NA	0	2	Not-determined	Genome-fragment	NA		0
S10_vs_68327||lt2gene	1060	No	NA	1	0	Low-quality	Genome-fragment		1.96	0
S17_vs_k141_62635||full	2053	No	NA	0	1	Not-determined	Genome-fragment	NA		0
S12_extracted_k141_2159	1060	No	NA	2	0	Low-quality	Genome-fragment		4.88	0
S9_extracted_k141_68277	1061	No	NA	0	0	Low-quality	Genome-fragment		0.64	0
S17_vs_k141_115200||full	2063	No	NA	0	2	Not-determined	Genome-fragment	NA		0
S11_vs_k141_86309||full	1061	No	NA	0	1	Low-quality	Genome-fragment		1.21	0
S11_vs_k141_57563||full	1061	No	NA	1	0	Low-quality	Genome-fragment		1.26	0
S1_extracted_k141_59913	1061	No	NA	0	0	Low-quality	Genome-fragment		2.87	0
S17_vs_k141_129862||full	3228	No	NA	0	1	Not-determined	Genome-fragment	NA		0
S5_vs_k141_28825||full	1061	No	NA	1	0	Low-quality	Genome-fragment		2.38	0
S7_extracted_15686	1061	No	NA	1	0	Low-quality	Genome-fragment		2.58	0
S7_vs_20871||full	1061	No	NA	1	0	Low-quality	Genome-fragment		2.19	0
S7_vs_72855||full	1061	No	NA	1	0	Low-quality	Genome-fragment		2.08	0
S17_vs_k141_56377||full	3619	No	NA	0	3	Not-determined	Genome-fragment	NA		0
S7_vs_45559||lt2gene	1061	No	NA	1	0	Low-quality	Genome-fragment		0.73	0
S17_vs_k141_144822||full	3614	No	NA	0	0	Not-determined	Genome-fragment	NA		0
S17_vs_k141_115262||full	1327	No	NA	0	1	Not-determined	Genome-fragment	NA		0
S17_vs_k141_108523||full	5831	No	NA	0	1	Not-determined	Genome-fragment	NA		0
S16_extracted_43610	1061	No	NA	0	1	Low-quality	Genome-fragment		0.4	0
S17_vs_k141_56458||full	1803	No	NA	0	0	Not-determined	Genome-fragment	NA		0
S17_vs_k141_92955||full	2738	No	NA	0	1	Not-determined	Genome-fragment	NA		0
S17_vs_k141_159801||full	1111	No	NA	0	0	Not-determined	Genome-fragment	NA		0
S16_extracted_83869	1061	No	NA	0	0	Low-quality	Genome-fragment		2.66	0
S17_vs_k141_26183||full	1364	No	NA	0	2	Not-determined	Genome-fragment	NA		0
S16_extracted_107625	1061	No	NA	0	0	Low-quality	Genome-fragment		0.2	0
S17_vs_k141_159812||full	4734	No	NA	0	4	Not-determined	Genome-fragment	NA		0
S17_vs_k141_49247||full	1208	No	NA	0	0	Not-determined	Genome-fragment	NA		0
S17_vs_k141_115327||full	2522	No	NA	0	2	Not-determined	Genome-fragment	NA		0
S17_vs_k141_71191||full	2031	No	NA	0	1	Not-determined	Genome-fragment	NA		0
S16_vs_75436||full	1061	No	NA	0	1	Low-quality	Genome-fragment		0.55	0
S10_vs_802||full	1061	No	NA	0	0	Low-quality	Genome-fragment		2.88	0
S17_vs_k141_62814||full	1771	No	NA	0	0	Not-determined	Genome-fragment	NA		0
S10_vs_110892||full	1061	No	NA	0	1	Low-quality	Genome-fragment		1.33	0
S17_vs_k141_166992||full	2328	No	NA	0	3	Not-determined	Genome-fragment	NA		0
S17_vs_k141_137270||full	4843	No	NA	0	4	Not-determined	Genome-fragment	NA		0
S12_extracted_k141_57161	1061	No	NA	0	0	Low-quality	Genome-fragment		11.67	0
S17_vs_k141_34431||full	2138	No	NA	0	3	Not-determined	Genome-fragment	NA		0
S17_vs_k141_11809||full	2491	No	NA	0	0	Not-determined	Genome-fragment	NA		0
S8_vs_k141_48105||full	1061	No	NA	1	0	Low-quality	Genome-fragment		2.42	0
S17_extracted_k141_112107	1062	No	NA	0	1	Low-quality	Genome-fragment		0.6	0
S17_vs_k141_159857||full	2442	No	NA	0	1	Not-determined	Genome-fragment	NA		0
S17_vs_k141_55170||lt2gene	1062	No	NA	2	0	Low-quality	Genome-fragment		3.12	0
S9_vs_k141_87514||lt2gene	1062	No	NA	1	0	Low-quality	Genome-fragment		2.32	0
S17_vs_k141_26239||full	3663	No	NA	0	1	Not-determined	Genome-fragment	NA		0
S17_vs_k141_26244||full	2700	No	NA	0	2	Not-determined	Genome-fragment	NA		0
S17_vs_k141_122281||full	4063	No	NA	0	2	Not-determined	Genome-fragment	NA		0
S17_vs_k141_174098||full	1684	No	NA	0	1	Not-determined	Genome-fragment	NA		0
S13_vs_90249||full	1062	No	NA	0	0	Low-quality	Genome-fragment		2.01	0
S1_vs_k141_48476||full	1062	No	NA	1	0	Low-quality	Genome-fragment		2.6	0
S17_vs_k141_56626||full	2387	No	NA	0	2	Not-determined	Genome-fragment	NA		0
S17_vs_k141_137360||full	2525	No	NA	0	2	Not-determined	Genome-fragment	NA		0
S5_vs_k141_116341||full	1062	No	NA	1	0	Low-quality	Genome-fragment		1.76	0
S7_extracted_108397	1062	No	NA	0	0	Low-quality	Genome-fragment		2.53	0
S17_vs_k141_19135||full	3363	No	NA	0	2	Not-determined	Genome-fragment	NA		0
S7_vs_66491||full	1062	No	NA	0	0	Low-quality	Genome-fragment		2.53	0
S17_vs_k141_108639||full	9342	No	NA	0	3	Not-determined	Genome-fragment	NA		0
S17_vs_k141_174134||full	6085	No	NA	0	1	Not-determined	Genome-fragment	NA		0
S16_vs_105984||full	1062	No	NA	0	1	Low-quality	Genome-fragment		0.83	0
S17_vs_k141_167094||full	2063	No	NA	0	0	Not-determined	Genome-fragment	NA		0
S10_vs_78267||full	1062	No	NA	0	0	Low-quality	Genome-fragment		2.42	0
S12_extracted_k141_34227	1062	No	NA	1	0	Low-quality	Genome-fragment		0.59	0
S17_vs_k141_19181||full	1126	No	NA	0	1	Not-determined	Genome-fragment	NA		0
S17_vs_k141_34565||full	1023	No	NA	0	1	Not-determined	Genome-fragment	NA		0
S17_vs_k141_159949||full	1216	No	NA	0	0	Not-determined	Genome-fragment	NA		0
S8_extracted_k141_140561	1062	No	NA	0	0	Low-quality	Genome-fragment		2.28	0
S17_vs_k141_26315||full	1521	No	NA	0	2	Not-determined	Genome-fragment	NA		0
S17_vs_k141_71342||full	3702	No	NA	0	2	Not-determined	Genome-fragment	NA		0
S17_vs_k141_26316||full	3004	No	NA	0	2	Not-determined	Genome-fragment	NA		0
S17_vs_k141_42214||full	2026	No	NA	0	2	Not-determined	Genome-fragment	NA		0
S17_vs_k141_121365||full	1063	No	NA	0	0	Low-quality	Genome-fragment		1.67	0
S11_extracted_k141_57362	1063	No	NA	0	0	Low-quality	Genome-fragment		1.87	0
S14_vs_k141_39877||full	1063	No	NA	1	0	Low-quality	Genome-fragment		2.21	0
S17_vs_k141_167139||full	1557	No	NA	0	1	Not-determined	Genome-fragment	NA		0
S1_extracted_k141_89111	1063	No	NA	0	0	Low-quality	Genome-fragment		1.74	0
S17_vs_k141_62957||full	4368	No	NA	0	2	Not-determined	Genome-fragment	NA		0
S1_vs_k141_42880||full	1063	No	NA	0	0	Low-quality	Genome-fragment		2.48	0
S17_vs_k141_71375||full	2068	No	NA	0	2	Not-determined	Genome-fragment	NA		0
S17_vs_k141_49424||full	1129	No	NA	0	1	Not-determined	Genome-fragment	NA		0
S17_vs_k141_137479||full	1690	No	NA	0	2	Not-determined	Genome-fragment	NA		0
S17_vs_k141_93151||full	1160	No	NA	0	2	Not-determined	Genome-fragment	NA		0
S1_vs_k141_112167||lt2gene	1063	No	NA	2	0	Low-quality	Genome-fragment		1.35	0
S3_extracted_k141_9341	1063	No	NA	0	0	Low-quality	Genome-fragment		0.85	0
S17_vs_k141_4590||full	4596	No	NA	0	0	Not-determined	Genome-fragment	NA		0
S3_vs_k141_98704||full	1063	No	NA	0	0	Low-quality	Genome-fragment		1.59	0
S5_extracted_k141_40126	1063	No	NA	0	0	Low-quality	Genome-fragment		1.46	0
S17_vs_k141_85133||full	3137	No	NA	0	3	Not-determined	Genome-fragment	NA		0
S5_vs_k141_124638||full	1063	No	NA	0	0	Low-quality	Genome-fragment		1.93	0
S7_vs_119250||full	1063	No	NA	0	2	Low-quality	Genome-fragment		0.28	0
S17_vs_k141_71467||full	3446	No	NA	0	1	Not-determined	Genome-fragment	NA		0
S17_vs_k141_137587||full	1465	No	NA	0	0	Not-determined	Genome-fragment	NA		0
S17_vs_k141_160037||full	1951	No	NA	0	2	Not-determined	Genome-fragment	NA		0
S16_vs_86635||lt2gene	1063	No	NA	1	0	Low-quality	Genome-fragment		2.81	0
S17_vs_k141_101066||full	2238	No	NA	0	2	Not-determined	Genome-fragment	NA		0
S17_vs_k141_160045||full	1814	No	NA	0	0	Not-determined	Genome-fragment	NA		0
S10_extracted_6449	1063	No	NA	0	0	Low-quality	Genome-fragment		0.47	0
S15_extracted_24958	1063	No	NA	1	0	Low-quality	Genome-fragment		2.47	0
S17_vs_k141_167258||full	1438	No	NA	0	1	Not-determined	Genome-fragment	NA		0
S17_vs_k141_4674||full	1816	No	NA	0	2	Not-determined	Genome-fragment	NA		0
S17_vs_k141_85178||full	7811	No	NA	0	6	Not-determined	Genome-fragment	NA		0
S17_vs_k141_4687||full	1374	No	NA	0	0	Not-determined	Genome-fragment	NA		0
S8_vs_k141_153443||lt2gene	1063	No	NA	1	0	Low-quality	Genome-fragment		2.49	0
S17_vs_k141_12091||full	6399	No	NA	0	1	Not-determined	Genome-fragment	NA		0
S9_vs_k141_25597||full	1064	No	NA	1	0	Low-quality	Genome-fragment		3.48	0
S17_vs_k141_42371||full	1821	No	NA	0	0	Not-determined	Genome-fragment	NA		0
S17_vs_k141_42384||full	2308	No	NA	0	2	Not-determined	Genome-fragment	NA		0
S17_vs_k141_137690||full	2143	No	NA	0	2	Not-determined	Genome-fragment	NA		0
S17_vs_k141_78836||full	3282	No	NA	0	1	Not-determined	Genome-fragment	NA		0
S17_vs_k141_160115||full	3771	No	NA	0	0	Not-determined	Genome-fragment	NA		0
S17_vs_k141_160117||full	1162	No	NA	0	2	Not-determined	Genome-fragment	NA		0
S9_vs_k141_82559||full	1064	No	NA	0	0	Low-quality	Genome-fragment		2.46	0
S17_vs_k141_115796||full	2215	No	NA	0	1	Not-determined	Genome-fragment	NA		0
S13_vs_7876||full	1064	No	NA	1	0	Low-quality	Genome-fragment		3.02	0
S17_vs_k141_71587||full	2310	No	NA	0	1	Not-determined	Genome-fragment	NA		0
S14_extracted_k141_105208	1064	No	NA	2	0	Low-quality	Genome-fragment		2.06	0
S14_vs_k141_96618||full	1064	No	NA	1	0	Low-quality	Genome-fragment		2.65	0
S17_vs_k141_56916||full	1292	No	NA	0	0	Not-determined	Genome-fragment	NA		0
S1_vs_k141_73850||lt2gene	1064	No	NA	1	0	Low-quality	Genome-fragment		2.53	0
S17_vs_k141_101146||full	1872	No	NA	0	1	Not-determined	Genome-fragment	NA		0
S3_extracted_k141_75647	1064	No	NA	0	0	Low-quality	Genome-fragment		2.29	0
S3_vs_k141_52985||full	1064	No	NA	0	0	Low-quality	Genome-fragment		2.55	0
S17_vs_k141_93338||full	5920	No	NA	0	4	Not-determined	Genome-fragment	NA		0
S17_vs_k141_49634||full	4770	No	NA	0	1	Not-determined	Genome-fragment	NA		0
S5_vs_k141_73100||full	1064	No	NA	1	0	Low-quality	Genome-fragment		1.78	0
S17_vs_k141_130110||full	1861	No	NA	0	1	Not-determined	Genome-fragment	NA		0
S17_vs_k141_115847||full	2568	No	NA	0	1	Not-determined	Genome-fragment	NA		0
S10_vs_95893||full	1064	No	NA	0	0	Low-quality	Genome-fragment		0.87	0

S17_vs_k141_78864||full	2355	No	NA	0	3	Not-determined	Genome-fragment	NA		0
S10_vs_119001||full	1064	No	NA	0	1	Low-quality	Genome-fragment		2.17	0
S17_vs_k141_78870||full	3318	No	NA	0	0	Not-determined	Genome-fragment	NA		0
S17_vs_k141_56953||full	1748	No	NA	0	1	Not-determined	Genome-fragment	NA		0
S17_vs_k141_56964||full	2597	No	NA	0	2	Not-determined	Genome-fragment	NA		0
S17_vs_k141_4851||full	3066	No	NA	0	1	Not-determined	Genome-fragment	NA		0
S17_vs_k141_85402||full	2977	No	NA	0	0	Not-determined	Genome-fragment	NA		0
S17_vs_k141_26500||full	2234	No	NA	0	2	Not-determined	Genome-fragment	NA		0
S17_vs_k141_93411||full	1319	No	NA	0	1	Not-determined	Genome-fragment	NA		0
S17_vs_k141_49675||full	1868	No	NA	0	2	Not-determined	Genome-fragment	NA		0
S17_vs_k141_130189||full	1974	No	NA	0	1	Not-determined	Genome-fragment	NA		0
S2_vs_k141_19992||full	1064	No	NA	2	0	Low-quality	Genome-fragment		3.01	0
S17_vs_k141_42503||full	1738	No	NA	0	1	Not-determined	Genome-fragment	NA		0
S17_vs_k141_35008||full	1842	No	NA	0	2	Not-determined	Genome-fragment	NA		0
S17_vs_k141_42515||full	1107	No	NA	0	1	Not-determined	Genome-fragment	NA		0
S17_vs_k141_101233||full	3555	No	NA	0	0	Not-determined	Genome-fragment	NA		0
S17_vs_k141_153305||full	3910	No	NA	0	2	Not-determined	Genome-fragment	NA		0
S17_vs_k141_78957||full	1589	No	NA	0	2	Not-determined	Genome-fragment	NA		0
S17_vs_k141_35065||full	2941	No	NA	0	2	Not-determined	Genome-fragment	NA		0
S8_vs_k141_174498||full	1064	No	NA	0	0	Low-quality	Genome-fragment		2.47	0
S17_vs_k141_78973||full	1260	No	NA	0	0	Not-determined	Genome-fragment	NA		0
S17_vs_k141_115976||full	1370	No	NA	0	0	Not-determined	Genome-fragment	NA		0
S17_extracted_k141_160862	1065	No	NA	0	0	Low-quality	Genome-fragment		1.49	0
S17_vs_k141_45223||full	1065	No	NA	0	1	Low-quality	Genome-fragment		0.75	0
S17_vs_k141_167510||full	1486	No	NA	0	1	Not-determined	Genome-fragment	NA		0
S9_extracted_k141_49929	1065	No	NA	0	0	Low-quality	Genome-fragment		0.62	0
S17_vs_k141_109097||full	3015	No	NA	0	3	Not-determined	Genome-fragment	NA		0
S17_vs_k141_137910||full	1052	No	NA	0	1	Not-determined	Genome-fragment	NA		0
S11_extracted_k141_56870	1065	No	NA	0	2	Low-quality	Genome-fragment		0.63	0
S17_vs_k141_63375||full	2084	No	NA	0	2	Not-determined	Genome-fragment	NA		0
S17_vs_k141_71772||full	2322	No	NA	0	0	Not-determined	Genome-fragment	NA		0
S17_vs_k141_4982||full	4719	No	NA	0	1	Not-determined	Genome-fragment	NA		0
S14_extracted_k141_73676	1065	No	NA	0	0	Low-quality	Genome-fragment		1.63	0
S17_vs_k141_145479||full	2766	No	NA	0	1	Not-determined	Genome-fragment	NA		0
S17_vs_k141_19499||full	2051	No	NA	0	0	Not-determined	Genome-fragment	NA		0
S17_vs_k141_145497||full	1172	No	NA	0	1	Not-determined	Genome-fragment	NA		0
S17_vs_k141_19527||full	1944	No	NA	0	0	Not-determined	Genome-fragment	NA		0
S17_vs_k141_49723||full	2250	No	NA	0	2	Not-determined	Genome-fragment	NA		0
S17_vs_k141_122864||full	1361	No	NA	0	3	Not-determined	Genome-fragment	NA		0
S1_extracted_k141_64402	1065	No	NA	0	0	Low-quality	Genome-fragment		1.53	0
S1_extracted_k141_71491	1065	No	NA	1	0	Low-quality	Genome-fragment		2.22	0
S17_vs_k141_42661||full	1258	No	NA	0	1	Not-determined	Genome-fragment	NA		0
S17_vs_k141_138008||full	1831	No	NA	0	1	Not-determined	Genome-fragment	NA		0
S17_vs_k141_5130||full	2653	No	NA	0	2	Not-determined	Genome-fragment	NA		0
S17_vs_k141_174479||full	6977	No	NA	0	4	Not-determined	Genome-fragment	NA		0
S17_vs_k141_138013||full	1629	No	NA	0	0	Not-determined	Genome-fragment	NA		0
S17_vs_k141_167601||full	1289	No	NA	0	2	Not-determined	Genome-fragment	NA		0
S17_vs_k141_116129||full	1519	No	NA	0	0	Not-determined	Genome-fragment	NA		0
S17_vs_k141_167606||full	2900	No	NA	0	2	Not-determined	Genome-fragment	NA		0
S17_vs_k141_138021||full	2427	No	NA	0	2	Not-determined	Genome-fragment	NA		0
S17_vs_k141_71864||full	2659	No	NA	0	0	Not-determined	Genome-fragment	NA		0
S1_vs_k141_94459||lt2gene	1065	No	NA	1	0	Low-quality	Genome-fragment		3.12	0
S17_vs_k141_57234||full	4523	No	NA	0	1	Not-determined	Genome-fragment	NA		0
S17_vs_k141_42715||full	1310	No	NA	0	0	Not-determined	Genome-fragment	NA		0
S17_vs_k141_49836||full	1996	No	NA	0	0	Not-determined	Genome-fragment	NA		0
S3_extracted_k141_51822	1065	No	NA	0	0	Low-quality	Genome-fragment		0.7	0
S17_vs_k141_85673||full	1094	No	NA	0	0	Not-determined	Genome-fragment	NA		0
S7_extracted_35615	1065	No	NA	0	0	Low-quality	Genome-fragment		1.93	0
S7_vs_52987||full	1065	No	NA	0	1	Low-quality	Genome-fragment		1.82	0
S7_vs_61176||full	1065	No	NA	0	0	Low-quality	Genome-fragment		1.75	0
S7_vs_124975||full	1065	No	NA	0	0	Low-quality	Genome-fragment		1.29	0
S16_extracted_142915	1065	No	NA	0	0	Low-quality	Genome-fragment		1.92	0
S16_vs_28960||full	1065	No	NA	0	0	Low-quality	Genome-fragment		0.56	0
S17_vs_k141_153564||full	1415	No	NA	0	2	Not-determined	Genome-fragment	NA		0
S17_vs_k141_42746||full	4467	No	NA	0	4	Not-determined	Genome-fragment	NA		0
S16_vs_121844||full	1065	No	NA	0	0	Low-quality	Genome-fragment		2.55	0
S16_vs_171006||full	1065	No	NA	1	0	Low-quality	Genome-fragment		2.09	0
S12_vs_k141_83854||lt2gene	1065	No	NA	1	0	Low-quality	Genome-fragment		2.11	0
S17_vs_k141_35299||full	1482	No	NA	0	1	Not-determined	Genome-fragment	NA		0
S17_extracted_k141_14995	1066	No	NA	1	0	Low-quality	Genome-fragment		2.98	0
S17_vs_k141_63511||full	3493	No	NA	0	1	Not-determined	Genome-fragment	NA		0
S17_vs_k141_116247||full	1066	No	NA	0	0	Not-determined	Genome-fragment	NA		0
S17_extracted_k141_51481	1066	No	NA	0	1	Low-quality	Genome-fragment		0.32	0
S17_vs_k141_85735||full	1376	No	NA	0	0	Not-determined	Genome-fragment	NA		0
S17_vs_k141_31268||lt2gene	1066	No	NA	0	0	Low-quality	Genome-fragment		1.93	0
S17_vs_k141_71972||full	3832	No	NA	0	1	Not-determined	Genome-fragment	NA		0
S17_vs_k141_160502||full	37084	No	NA	6	1	Medium-quality	Genome-fragment		63.41	0
S11_extracted_k141_42082	1066	No	NA	0	0	Low-quality	Genome-fragment		0.96	0
S13_extracted_154021	1066	No	NA	0	0	Low-quality	Genome-fragment		2.06	0
S14_extracted_k141_111522	1066	No	NA	0	0	Low-quality	Genome-fragment		1.95	0
S14_vs_k141_39961||full	1066	No	NA	2	0	Low-quality	Genome-fragment		2.95	0
S17_vs_k141_123050||full	1605	No	NA	0	1	Not-determined	Genome-fragment	NA		0
S17_vs_k141_63567||full	1566	No	NA	0	0	Not-determined	Genome-fragment	NA		0
S1_vs_k141_12121||full	1066	No	NA	1	0	Low-quality	Genome-fragment		1.34	0
S17_vs_k141_123077||full	1135	No	NA	0	1	Not-determined	Genome-fragment	NA		0
S17_vs_k141_130470||full	1370	No	NA	0	2	Not-determined	Genome-fragment	NA		0
S1_vs_k141_106049||full	1066	No	NA	2	0	Low-quality	Genome-fragment		2.47	0
S17_vs_k141_174617||full	4836	No	NA	0	2	Not-determined	Genome-fragment	NA		0
S5_extracted_k141_78054	1066	No	NA	0	1	Low-quality	Genome-fragment		1.18	0
S17_vs_k141_26758||full	2873	No	NA	0	2	Not-determined	Genome-fragment	NA		0
S17_vs_k141_79284||full	5362	No	NA	0	4	Not-determined	Genome-fragment	NA		0
S5_vs_k141_50748||full	1066	No	NA	1	0	Low-quality	Genome-fragment		3.04	0
S5_vs_k141_9380||full	1066	No	NA	1	0	Low-quality	Genome-fragment		1.76	0
S17_vs_k141_160590||full	1167	No	NA	0	1	Not-determined	Genome-fragment	NA		0
S7_vs_57064||full	1066	No	NA	1	0	Low-quality	Genome-fragment		2.64	0
S17_vs_k141_145688||full	2180	No	NA	0	3	Not-determined	Genome-fragment	NA		0
S17_vs_k141_42860||full	3909	No	NA	0	0	Not-determined	Genome-fragment	NA		0
S7_vs_96792||full	1066	No	NA	0	0	Low-quality	Genome-fragment		2.37	0
S17_vs_k141_26774||full	5536	No	NA	0	2	Not-determined	Genome-fragment	NA		0
S17_vs_k141_12521||full	8202	No	NA	0	3	Not-determined	Genome-fragment	NA		0
S17_vs_k141_12528||full	1368	No	NA	0	2	Not-determined	Genome-fragment	NA		0
S17_vs_k141_153613||full	2518	No	NA	0	3	Not-determined	Genome-fragment	NA		0
S17_vs_k141_12536||full	2088	No	NA	0	0	Not-determined	Genome-fragment	NA		0
S17_vs_k141_85914||full	1963	No	NA	0	2	Not-determined	Genome-fragment	NA		0
S7_vs_20194||lt2gene	1066	No	NA	1	0	Low-quality	Genome-fragment		2.45	0
S16_extracted_15520	1066	No	NA	0	0	Low-quality	Genome-fragment		1.92	0
S17_vs_k141_93770||full	4223	No	NA	0	2	Not-determined	Genome-fragment	NA		0
S10_extracted_40093	1066	No	NA	0	2	Low-quality	Genome-fragment		0.57	0
S17_vs_k141_5401||full	2562	No	NA	0	1	Not-determined	Genome-fragment	NA		0
S10_extracted_57163	1066	No	NA	2	0	Low-quality	Genome-fragment		0.62	0
S17_vs_k141_160676||full	3039	No	NA	0	2	Not-determined	Genome-fragment	NA		0
S12_extracted_k141_299	1066	No	NA	0	0	Low-quality	Genome-fragment		2.45	0
S17_vs_k141_123195||full	1709	No	NA	0	3	Not-determined	Genome-fragment	NA		0
S12_vs_k141_169357||full	1066	No	NA	2	0	Low-quality	Genome-fragment		2.52	0
S17_vs_k141_109558||full	3109	No	NA	0	1	Not-determined	Genome-fragment	NA		0
S6_extracted_k141_42682	1066	No	NA	0	0	Low-quality	Genome-fragment		1.45	0
S17_vs_k141_145811||full	2683	No	NA	0	0	Not-determined	Genome-fragment	NA		0
S8_vs_k141_21776||lt2gene	1066	No	NA	1	0	Low-quality	Genome-fragment		0.57	0
S17_vs_k141_72104||full	3372	No	NA	0	2	Not-determined	Genome-fragment	NA		0
S17_extracted_k141_52075	1067	No	NA	0	0	Low-quality	Genome-fragment		1.72	0
S17_vs_k141_85962||full	11079	No	NA	0	5	Not-determined	Genome-fragment	NA		0
S17_extracted_k141_76021	1067	No	NA	0	0	Low-quality	Genome-fragment		0.81	0
S17_vs_k141_160741||full	1537	No	NA	0	2	Not-determined	Genome-fragment	NA		0
S17_vs_k141_155014||full	1067	No	NA	0	0	Low-quality	Genome-fragment		2.79	0
S17_vs_k141_174752||full	8637	No	NA	0	2	Not-determined	Genome-fragment	NA		0
S17_vs_k141_174758||full	1602	No	NA	0	0	Not-determined	Genome-fragment	NA		0
S9_vs_k141_14419||full	1067	No	NA	1	0	Low-quality	Genome-fragment		0.69	0
S17_vs_k141_109611||full	5287	No	NA	0	3	Not-determined	Genome-fragment	NA		0
S17_vs_k141_12665||full	2292	No	NA	0	2	Not-determined	Genome-fragment	NA		0
S17_vs_k141_79436||full	1143	No	NA	0	1	Not-determined	Genome-fragment	NA		0
S17_vs_k141_93855||full	4428	No	NA	0	1	Not-determined	Genome-fragment	NA		0
S14_vs_k141_13086||full	1067	No	NA	1	0	Low-quality	Genome-fragment		1.86	0
S1_vs_k141_108436||full	1067	No	NA	0	0	Low-quality	Genome-fragment		0.89	0
S17_vs_k141_174798||full	1919	No	NA	0	1	Not-determined	Genome-fragment	NA		0
S1_vs_k141_152021||lt2gene	1067	No	NA	0	0	Low-quality	Genome-fragment		2.64	0
S3_vs_k141_33587||full	1067	No	NA	0	0	Low-quality	Genome-fragment		2.67	0
S3_vs_k141_28622||full	1067	No	NA	0	0	Low-quality	Genome-fragment		1.36	0
S17_vs_k141_138306||full	3504	No	NA	0	2	Not-determined	Genome-fragment	NA		0
S5_vs_k141_13926||full	1067	No	NA	0	0	Low-quality	Genome-fragment		2.14	0
S5_vs_k141_18839||lt2gene	1067	No	NA	1	0	Low-quality	Genome-fragment		2.48	0
S17_vs_k141_138320||full	3006	No	NA	0	2	Not-determined	Genome-fragment	NA		0
S17_vs_k141_35594||full	3539	No	NA	0	4	Not-determined	Genome-fragment	NA		0
S7_extracted_71002	1067	No	NA	1	0	Low-quality	Genome-fragment		1.96	0
S7_vs_103637||full	1067	No	NA	0	0	Low-quality	Genome-fragment		2.58	0
S17_vs_k141_160844||full	2077	No	NA	0	1	Not-determined	Genome-fragment	NA		0
S7_vs_117667||full	1067	No	NA	0	0	Low-quality	Genome-fragment		2.57	0
S16_extracted_5952	1067	No	NA	0	0	Low-quality	Genome-fragment		2.01	0
S17_vs_k141_116634||full	1597	No	NA	0	1	Not-determined	Genome-fragment	NA		0
S17_vs_k141_168049||full	2405	No	NA	0	2	Not-determined	Genome-fragment	NA		0
S17_vs_k141_57559||full	2144	No	NA	0	0	Not-determined	Genome-fragment	NA		0
S10_extracted_6298	1067	No	NA	0	1	Low-quality	Genome-fragment		1.43	0
S17_vs_k141_26945||full	1026	No	NA	0	1	Not-determined	Genome-fragment	NA		0
S12_vs_k141_157599||full	1067	No	NA	1	0	Low-quality	Genome-fragment		1.06	0
S17_vs_k141_116640||full	20807	No	NA	10	0	Medium-quality	Genome-fragment		53.37	0
S17_vs_k141_153891||full	1420	No	NA	0	2	Not-determined	Genome-fragment	NA		0
S12_vs_k141_32262||lt2gene	1067	No	NA	1	0	Low-quality	Genome-fragment		2.33	0
S8_extracted_k141_24192	1067	No	NA	0	0	Low-quality	Genome-fragment		1.62	0
S17_vs_k141_12692||full	2752	No	NA	0	2	Not-determined	Genome-fragment	NA		0
S9_extracted_k141_10150	1068	No	NA	1	0	Low-quality	Genome-fragment		1.7	0
S17_vs_k141_19843||full	2329	No	NA	0	2	Not-determined	Genome-fragment	NA		0
S17_vs_k141_79589||full	3051	No	NA	0	2	Not-determined	Genome-fragment	NA		0
S11_vs_k141_70769||full	1068	No	NA	1	0	Low-quality	Genome-fragment		2.41	0
S17_vs_k141_130684||full	2502	No	NA	0	0	Not-determined	Genome-fragment	NA		0
S17_vs_k141_43165||full	1485	No	NA	0	0	Not-determined	Genome-fragment	NA		0
S17_vs_k141_79599||full	2260	No	NA	0	2	Not-determined	Genome-fragment	NA		0
S17_vs_k141_35766||full	3011	No	NA	0	3	Not-determined	Genome-fragment	NA		0
S17_vs_k141_168159||full	1861	No	NA	0	0	Not-determined	Genome-fragment	NA		0
S13_extracted_97585	1068	No	NA	0	0	Low-quality	Genome-fragment		2.43	0
S13_vs_77||full	1068	No	NA	0	1	Low-quality	Genome-fragment		0.67	0
S17_vs_k141_35783||full	2658	No	NA	0	1	Not-determined	Genome-fragment	NA		0
S17_vs_k141_161002||full	1008	No	NA	0	1	Not-determined	Genome-fragment	NA		0
S17_vs_k141_50403||full	2220	No	NA	0	0	Not-determined	Genome-fragment	NA		0
S17_vs_k141_79628||full	3350	No	NA	0	1	Not-determined	Genome-fragment	NA		0
S13_vs_23914||full	1068	No	NA	0	0	Low-quality	Genome-fragment		1.39	0

S17_vs_k141_130721||full	2637	No	NA	0	2	Not-determined	Genome-fragment	NA		0
S17_vs_k141_174936||full	3030	No	NA	0	1	Not-determined	Genome-fragment	NA		0
S13_vs_28431||full	1068	No	NA	0	0	Low-quality	Genome-fragment		1.99	0
S14_vs_k141_85495||full	1068	No	NA	0	0	Low-quality	Genome-fragment		6.24	0
S17_vs_k141_93957||full	2029	No	NA	0	2	Not-determined	Genome-fragment	NA		0
S14_vs_k141_63984||full	1068	No	NA	1	0	Low-quality	Genome-fragment		2.18	0
S17_vs_k141_161038||full	1714	No	NA	0	3	Not-determined	Genome-fragment	NA		0
S17_vs_k141_57650||full	2168	No	NA	0	0	Not-determined	Genome-fragment	NA		0
S14_vs_k141_90761||lt2gene	1068	No	NA	1	0	Low-quality	Genome-fragment		2.77	0
S1_vs_k141_55346||full	1068	No	NA	0	1	Low-quality	Genome-fragment		2.08	0
S1_vs_k141_134739||lt2gene	1068	No	NA	1	0	Low-quality	Genome-fragment		2.23	0
S17_vs_k141_27108||full	1309	No	NA	0	0	Not-determined	Genome-fragment	NA		0
S3_extracted_k141_45575	1068	No	NA	0	0	Low-quality	Genome-fragment		1.58	0
S3_vs_k141_124683||full	1068	No	NA	2	0	Low-quality	Genome-fragment		1.73	0
S5_vs_k141_133756||full	1068	No	NA	1	0	Low-quality	Genome-fragment		1.77	0
S17_vs_k141_5725||full	1733	No	NA	0	0	Not-determined	Genome-fragment	NA		0
S17_vs_k141_63999||full	1478	No	NA	0	2	Not-determined	Genome-fragment	NA		0
S5_vs_k141_117809||full	1068	No	NA	0	0	Low-quality	Genome-fragment		2.68	0
S5_vs_k141_43255||lt2gene	1068	No	NA	2	0	Low-quality	Genome-fragment		3.29	0
S17_vs_k141_161090||full	1804	No	NA	0	0	Not-determined	Genome-fragment	NA		0
S7_vs_30328||full	1068	No	NA	2	0	Low-quality	Genome-fragment		2.56	0
S7_vs_136428||full	1068	No	NA	0	0	Low-quality	Genome-fragment		2.27	0
S16_vs_9347||full	1068	No	NA	0	0	Low-quality	Genome-fragment		1.93	0
S16_vs_90939||full	1068	No	NA	1	0	Low-quality	Genome-fragment		2.48	0
S12_vs_k141_95253||full	1068	No	NA	0	0	Low-quality	Genome-fragment		1.99	0
S12_vs_k141_125410||full	1068	No	NA	0	0	Low-quality	Genome-fragment		2.94	0
S17_vs_k141_5753||full	2907	No	NA	0	1	Not-determined	Genome-fragment	NA		0
S6_extracted_k141_37223	1068	No	NA	0	1	Low-quality	Genome-fragment		0.34	0
S6_extracted_k141_51098	1068	No	NA	0	0	Low-quality	Genome-fragment		2.3	0
S17_vs_k141_123573||full	1088	No	NA	0	2	Not-determined	Genome-fragment	NA		0
S6_vs_k141_56155||full	1068	No	NA	0	0	Low-quality	Genome-fragment		3.05	0
S17_vs_k141_123582||full	4011	No	NA	0	2	Not-determined	Genome-fragment	NA		0
S17_vs_k141_12881||full	3280	No	NA	0	0	Not-determined	Genome-fragment	NA		0
S17_vs_k141_138575||full	1560	No	NA	0	1	Not-determined	Genome-fragment	NA		0
S17_vs_k141_43300||full	1255	No	NA	0	0	Not-determined	Genome-fragment	NA		0
S17_vs_k141_19974||full	1570	No	NA	0	0	Not-determined	Genome-fragment	NA		0
S11_vs_k141_95490||full	1069	No	NA	0	0	Low-quality	Genome-fragment		1.88	0
S11_vs_k141_36482||lt2gene	1069	No	NA	2	0	Low-quality	Genome-fragment		1.3	0
S17_vs_k141_168235||full	57169	No	NA	0	10	Not-determined	Genome-fragment	NA		0
S17_vs_k141_27183||full	1632	No	NA	0	0	Not-determined	Genome-fragment	NA		0
S13_vs_153118||full	1069	No	NA	0	3	Low-quality	Genome-fragment		0.33	0
S14_extracted_k141_94958	1069	No	NA	1	0	Low-quality	Genome-fragment		2.56	0
S17_vs_k141_50578||full	1234	No	NA	0	0	Not-determined	Genome-fragment	NA		0
S14_vs_k141_53370||full	1069	No	NA	0	0	Low-quality	Genome-fragment		2.77	0
S17_vs_k141_174992||full	1414	No	NA	0	0	Not-determined	Genome-fragment	NA		0
S17_vs_k141_130920||full	1370	No	NA	0	2	Not-determined	Genome-fragment	NA		0
S17_vs_k141_138634||full	1695	No	NA	0	2	Not-determined	Genome-fragment	NA		0
S17_vs_k141_102068||full	16475	No	NA	0	2	Not-determined	Genome-fragment	NA		0
S17_vs_k141_110068||full	4540	No	NA	0	2	Not-determined	Genome-fragment	NA		0
S17_vs_k141_86370||full	3666	No	NA	0	3	Not-determined	Genome-fragment	NA		0
S1_vs_k141_42858||full	1069	No	NA	0	2	Low-quality	Genome-fragment		0.68	0
S17_vs_k141_86377||full	1716	No	NA	0	0	Not-determined	Genome-fragment	NA		0
S17_vs_k141_43307||full	42499	No	NA	5	1	Medium-quality	Genome-fragment		66.94	0
S5_vs_k141_67019||full	1069	No	NA	2	0	Low-quality	Genome-fragment		2.44	0
S16_extracted_125168	1069	No	NA	0	0	Low-quality	Genome-fragment		1.23	0
S17_vs_k141_35957||full	3203	No	NA	0	0	Not-determined	Genome-fragment	NA		0
S17_vs_k141_154186||full	4674	No	NA	0	1	Not-determined	Genome-fragment	NA		0
S17_vs_k141_57762||full	5559	No	NA	0	2	Not-determined	Genome-fragment	NA		0
S17_vs_k141_110100||full	1024	No	NA	0	0	Not-determined	Genome-fragment	NA		0
S16_vs_14820||lt2gene	1069	No	NA	2	0	Low-quality	Genome-fragment		2.24	0
S17_vs_k141_27285||full	3403	No	NA	0	2	Not-determined	Genome-fragment	NA		0
S17_vs_k141_35986||full	3275	No	NA	0	1	Not-determined	Genome-fragment	NA		0
S17_vs_k141_123703||full	1880	No	NA	0	2	Not-determined	Genome-fragment	NA		0
S17_vs_k141_79861||full	3497	No	NA	0	1	Not-determined	Genome-fragment	NA		0
S17_vs_k141_64184||full	1015	No	NA	0	0	Not-determined	Genome-fragment	NA		0
S17_vs_k141_146279||full	4454	No	NA	0	3	Not-determined	Genome-fragment	NA		0
S17_vs_k141_161306||full	1357	No	NA	0	2	Not-determined	Genome-fragment	NA		0
S17_vs_k141_146291||full	3160	No	NA	0	2	Not-determined	Genome-fragment	NA		0
S17_vs_k141_79875||full	5986	No	NA	0	2	Not-determined	Genome-fragment	NA		0
S10_vs_10905||lt2gene	1069	No	NA	1	0	Low-quality	Genome-fragment		1.42	0
S17_vs_k141_146308||full	2362	No	NA	0	0	Not-determined	Genome-fragment	NA		0
S12_extracted_k141_122422	1069	No	NA	0	0	Low-quality	Genome-fragment		1.02	0
S17_vs_k141_102193||full	2735	No	NA	0	1	Not-determined	Genome-fragment	NA		0
S17_vs_k141_131017||full	1644	No	NA	0	2	Not-determined	Genome-fragment	NA		0
S17_vs_k141_161338||full	2199	No	NA	0	1	Not-determined	Genome-fragment	NA		0
S17_vs_k141_146333||full	2058	No	NA	0	1	Not-determined	Genome-fragment	NA		0
S17_vs_k141_50691||full	1413	No	NA	0	2	Not-determined	Genome-fragment	NA		0
S12_vs_k141_141597||full	1069	No	NA	1	0	Low-quality	Genome-fragment		2.36	0
S17_vs_k141_146356||full	2160	No	NA	0	3	Not-determined	Genome-fragment	NA		0
S15_vs_47120||lt2gene	1069	No	NA	0	0	Low-quality	Genome-fragment		1.67	0
S17_vs_k141_146384||full	1048	No	NA	0	1	Not-determined	Genome-fragment	NA		0
S17_vs_k141_72599||full	2644	No	NA	0	1	Not-determined	Genome-fragment	NA		0
S17_vs_k141_8258||lt2gene	1070	No	NA	2	0	Low-quality	Genome-fragment		2.07	0
S17_vs_k141_86500||full	2110	No	NA	0	2	Not-determined	Genome-fragment	NA		0
S17_vs_k141_161388||full	1838	No	NA	0	0	Not-determined	Genome-fragment	NA		0
S9_vs_k141_5041||full	1070	No	NA	0	0	Low-quality	Genome-fragment		1.25	0
S17_vs_k141_43451||full	1906	No	NA	0	1	Not-determined	Genome-fragment	NA		0
S11_extracted_k141_97615	1070	No	NA	0	1	Low-quality	Genome-fragment		0.34	0
S13_extracted_5611	1070	No	NA	0	1	Low-quality	Genome-fragment		0.29	0
S17_vs_k141_102301||full	5022	No	NA	0	3	Not-determined	Genome-fragment	NA		0
S14_extracted_k141_54336	1070	No	NA	0	0	Low-quality	Genome-fragment		2.06	0
S17_vs_k141_102315||full	2003	No	NA	0	0	Not-determined	Genome-fragment	NA		0
S17_vs_k141_64350||full	1708	No	NA	0	1	Not-determined	Genome-fragment	NA		0
S17_vs_k141_110303||full	1796	No	NA	0	2	Not-determined	Genome-fragment	NA		0
S17_vs_k141_13155||full	1065	No	NA	0	0	Not-determined	Genome-fragment	NA		0
S14_extracted_k141_83943	1070	No	NA	0	0	Low-quality	Genome-fragment		5.61	0
S17_vs_k141_138876||full	4152	No	NA	0	3	Not-determined	Genome-fragment	NA		0
S14_vs_k141_112058||lt2gene	1070	No	NA	1	0	Low-quality	Genome-fragment		1.8	0
S5_vs_k141_84901||full	1070	No	NA	0	1	Low-quality	Genome-fragment		2.77	0
S17_vs_k141_161481||full	1262	No	NA	0	0	Not-determined	Genome-fragment	NA		0
S17_vs_k141_57995||full	5499	No	NA	0	1	Not-determined	Genome-fragment	NA		0
S7_extracted_61998	1070	No	NA	1	0	Low-quality	Genome-fragment		2	0
S17_vs_k141_131168||full	2367	No	NA	0	1	Not-determined	Genome-fragment	NA		0
S17_vs_k141_168442||full	7584	No	NA	0	4	Not-determined	Genome-fragment	NA		0
S17_vs_k141_86622||full	1213	No	NA	0	1	Not-determined	Genome-fragment	NA		0
S17_vs_k141_20302||full	1403	No	NA	0	0	Not-determined	Genome-fragment	NA		0
S17_vs_k141_154524||full	2078	No	NA	0	0	Not-determined	Genome-fragment	NA		0
S17_vs_k141_146564||full	1235	No	NA	0	0	Not-determined	Genome-fragment	NA		0
S17_vs_k141_131194||full	1888	No	NA	0	3	Not-determined	Genome-fragment	NA		0
S17_vs_k141_64447||full	3164	No	NA	0	3	Not-determined	Genome-fragment	NA		0
S7_vs_106088||full	1070	No	NA	1	0	Low-quality	Genome-fragment		1.99	0
S16_extracted_101339	1070	No	NA	0	0	Low-quality	Genome-fragment		1.77	0
S17_vs_k141_102370||full	1139	No	NA	0	1	Not-determined	Genome-fragment	NA		0
S17_vs_k141_175234||full	2654	No	NA	0	1	Not-determined	Genome-fragment	NA		0
S17_vs_k141_20317||full	15455	No	NA	0	11	Not-determined	Genome-fragment	NA		0
S16_extracted_104121	1070	No	NA	1	0	Low-quality	Genome-fragment		2.53	0
S17_vs_k141_110426||full	1985	No	NA	0	2	Not-determined	Genome-fragment	NA		0
S17_vs_k141_58025||full	12945	No	NA	5	0	High-quality	High-quality		94.49	0
S17_vs_k141_102412||full	1321	No	NA	0	0	Not-determined	Genome-fragment	NA		0
S16_vs_98242||full	1070	No	NA	0	0	Low-quality	Genome-fragment		0.67	0
S10_extracted_71334	1070	No	NA	0	0	Low-quality	Genome-fragment		1.33	0
S17_vs_k141_36188||full	1817	No	NA	0	0	Not-determined	Genome-fragment	NA		0
S17_vs_k141_27588||full	2801	No	NA	0	3	Not-determined	Genome-fragment	NA		0
S17_vs_k141_110435||full	1042	No	NA	0	2	Not-determined	Genome-fragment	NA		0
S10_extracted_91113	1070	No	NA	0	2	Low-quality	Genome-fragment		1.47	0
S17_vs_k141_64516||full	1113	No	NA	0	1	Not-determined	Genome-fragment	NA		0
S17_vs_k141_161600||full	1471	No	NA	0	1	Not-determined	Genome-fragment	NA		0
S17_vs_k141_175302||full	1342	No	NA	0	1	Not-determined	Genome-fragment	NA		0
S17_vs_k141_117355||full	2667	No	NA	0	0	Not-determined	Genome-fragment	NA		0
S17_vs_k141_64543||full	9122	No	NA	0	4	Not-determined	Genome-fragment	NA		0
S10_vs_61861||full	1070	No	NA	0	0	Low-quality	Genome-fragment		1.66	0
S17_vs_k141_6263||full	5531	No	NA	0	1	Not-determined	Genome-fragment	NA		0
S12_vs_k141_19710||full	1070	No	NA	0	0	Low-quality	Genome-fragment		2.49	0
S17_vs_k141_94401||full	1616	No	NA	0	1	Not-determined	Genome-fragment	NA		0
S12_vs_k141_94784||lt2gene	1070	No	NA	1	0	Low-quality	Genome-fragment		2.41	0
S17_vs_k141_64602||full	1934	No	NA	0	2	Not-determined	Genome-fragment	NA		0
S17_vs_k141_64611||full	1679	No	NA	0	1	Not-determined	Genome-fragment	NA		0
S4_extracted_31063	1070	No	NA	0	0	Low-quality	Genome-fragment		1.18	0
S17_vs_k141_50896||full	2502	No	NA	0	2	Not-determined	Genome-fragment	NA		0
S17_vs_k141_175417||full	1183	No	NA	0	0	Not-determined	Genome-fragment	NA		0
S17_vs_k141_154719||full	1631	No	NA	0	2	Not-determined	Genome-fragment	NA		0
S17_vs_k141_72874||full	1060	No	NA	0	0	Not-determined	Genome-fragment	NA		0
S8_extracted_k141_36618	1070	No	NA	0	1	Low-quality	Genome-fragment		3.42	0
S17_vs_k141_20493||full	1320	No	NA	0	2	Not-determined	Genome-fragment	NA		0
S8_vs_k141_37439||full	1070	No	NA	0	0	Low-quality	Genome-fragment		0.66	0
S17_vs_k141_154736||full	3675	No	NA	0	3	Not-determined	Genome-fragment	NA		0
S17_vs_k141_85751||full	1071	No	NA	0	1	Low-quality	Genome-fragment		1.29	0
S17_vs_k141_168494||full	1946	No	NA	0	1	Not-determined	Genome-fragment	NA		0
S13_vs_31337||full	1071	No	NA	0	0	Low-quality	Genome-fragment		1.81	0
S1_extracted_k141_97017	1071	No	NA	0	0	Low-quality	Genome-fragment		0.72	0
S17_vs_k141_110565||full	2561	No	NA	0	4	Not-determined	Genome-fragment	NA		0
S17_vs_k141_154742||full	2553	No	NA	0	2	Not-determined	Genome-fragment	NA		0
S1_extracted_k141_79737	1071	No	NA	0	1	Low-quality	Genome-fragment		0.97	0
S17_vs_k141_168507||full	1617	No	NA	0	2	Not-determined	Genome-fragment	NA		0
S7_extracted_75779	1071	No	NA	0	0	Low-quality	Genome-fragment		2.67	0
S17_vs_k141_86903||full	2562	No	NA	0	3	Not-determined	Genome-fragment	NA		0
S7_vs_29590||full	1071	No	NA	1	0	Low-quality	Genome-fragment		2.44	0
S16_extracted_142393	1071	No	NA	0	0	Low-quality	Genome-fragment		1.79	0
S17_vs_k141_161749||full	1437	No	NA	0	1	Not-determined	Genome-fragment	NA		0
S17_vs_k141_131428||full	2094	No	NA	0	2	Not-determined	Genome-fragment	NA		0
S16_vs_124728||lt2gene	1071	No	NA	2	0	Low-quality	Genome-fragment		2.68	0
S10_vs_55809||full	1071	No	NA	0	0	Low-quality	Genome-fragment		2.1	0
S17_vs_k141_175502||full	1337	No	NA	0	0	Not-determined	Genome-fragment	NA		0
S17_vs_k141_139161||full	1819	No	NA	0	1	Not-determined	Genome-fragment	NA		0
S17_vs_k141_20542||full	2070	No	NA	0	1	Not-determined	Genome-fragment	NA		0
S17_vs_k141_168565||full	1354	No	NA	0	2	Not-determined	Genome-fragment	NA		0
S17_vs_k141_154785||full	7721	No	NA	0	2	Not-determined	Genome-fragment	NA		0
S17_vs_k141_110614||full	1986	No	NA	0	0	Not-determined	Genome-fragment	NA		0
S17_vs_k141_117507||full	25659	No	NA	1	1	Medium-quality	Genome-fragment		58.11	0
S10_vs_92079||full	1071	No	NA	0	0	Low-quality	Genome-fragment		2.16	0
S17_vs_k141_80348||full	1578	No	NA	0	1	Not-determined	Genome-fragment	NA		0
S10_vs_53381||lt2gene	1071	No	NA	0	0	Low-quality	Genome-fragment		2.57	0
S17_vs_k141_20580||full	3576	No	NA	0	2	Not-determined	Genome-fragment	NA		0
S12_extracted_k141_136650	1071	No	NA	0	0	Low-quality	Genome-fragment		0.47	0

S17_vs_k141_73008||full	1541	No	NA	0	1	Not-determined	Genome-fragment	NA	0
S17_vs_k141_36418||full	1089	No	NA	0	1	Not-determined	Genome-fragment	NA	0
S15_extracted_76136	1071	No	NA	0	1	Low-quality	Genome-fragment	3.63	0
S17_vs_k141_36437||full	5135	No	NA	0	0	Not-determined	Genome-fragment	NA	0
S17_vs_k141_62546||full	1072	No	NA	0	0	Low-quality	Genome-fragment	2.5	0
S17_vs_k141_58303||full	1788	No	NA	0	0	Not-determined	Genome-fragment	NA	0
S17_vs_k141_161889||full	1088	No	NA	0	1	Not-determined	Genome-fragment	NA	0
S17_vs_k141_58306||full	1324	No	NA	0	0	Not-determined	Genome-fragment	NA	0
S17_vs_k141_124322||full	1515	No	NA	0	1	Not-determined	Genome-fragment	NA	0
S11_extracted_k141_60911	1072	No	NA	1	0	Low-quality	Genome-fragment	2.26	0
S17_vs_k141_58318||full	2399	No	NA	0	2	Not-determined	Genome-fragment	NA	0
S14_extracted_k141_25949	1072	No	NA	2	0	Low-quality	Genome-fragment	2.52	0
S1_vs_k141_43311||full	1072	No	NA	1	0	Low-quality	Genome-fragment	2.8	0
S1_vs_k141_158460||full	1072	No	NA	0	0	Low-quality	Genome-fragment	2.54	0
S3_extracted_k141_112047	1072	No	NA	0	0	Low-quality	Genome-fragment	1.36	0
S17_vs_k141_168702||full	1441	No	NA	0	1	Not-determined	Genome-fragment	NA	0
S17_vs_k141_147095||full	1533	No	NA	0	1	Not-determined	Genome-fragment	NA	0
S17_vs_k141_110666||full	70348	Yes	66114	29	4	High-quality	High-quality	100	6.02
S17_vs_k141_51172||full	2156	No	NA	0	1	Not-determined	Genome-fragment	NA	0
S3_extracted_k141_63290	1072	No	NA	0	0	Low-quality	Genome-fragment	0.81	0
S3_vs_k141_33429||full	1072	No	NA	0	0	Low-quality	Genome-fragment	0.77	0
S17_vs_k141_43968||full	1150	No	NA	0	1	Not-determined	Genome-fragment	NA	0
S3_vs_k141_78199||full	1072	No	NA	1	0	Low-quality	Genome-fragment	2.44	0
S17_vs_k141_175574||full	1460	No	NA	0	1	Not-determined	Genome-fragment	NA	0
S17_vs_k141_94612||full	2214	No	NA	0	0	Not-determined	Genome-fragment	NA	0
S17_vs_k141_80501||full	2034	No	NA	0	1	Not-determined	Genome-fragment	NA	0
S3_vs_k141_102351||full	1072	No	NA	0	0	Low-quality	Genome-fragment	2.44	0
S17_vs_k141_175591||full	2267	No	NA	0	1	Not-determined	Genome-fragment	NA	0
S17_vs_k141_73104||full	1848	No	NA	0	2	Not-determined	Genome-fragment	NA	0
S17_vs_k141_124387||full	1432	No	NA	0	0	Not-determined	Genome-fragment	NA	0
S5_extracted_k141_9123	1072	No	NA	1	0	Low-quality	Genome-fragment	2.86	0
S17_vs_k141_168769||full	2432	No	NA	0	1	Not-determined	Genome-fragment	NA	0
S5_extracted_k141_136936	1072	No	NA	0	0	Low-quality	Genome-fragment	2.73	0
S5_vs_k141_20889||full	1072	No	NA	0	0	Low-quality	Genome-fragment	1.3	0
S17_vs_k141_44024||full	1803	No	NA	0	1	Not-determined	Genome-fragment	NA	0
S5_vs_k141_35187||lt2gene	1072	No	NA	1	0	Low-quality	Genome-fragment	2.64	0
S17_vs_k141_6635||full	1330	No	NA	0	0	Not-determined	Genome-fragment	NA	0
S17_vs_k141_147198||full	3174	No	NA	0	3	Not-determined	Genome-fragment	NA	0
S7_vs_14679||full	1072	No	NA	0	0	Low-quality	Genome-fragment	2.36	0
S17_vs_k141_147203||full	1722	No	NA	0	0	Not-determined	Genome-fragment	NA	0
S17_vs_k141_73174||full	2806	No	NA	0	2	Not-determined	Genome-fragment	NA	0
S17_vs_k141_131679||full	1049	No	NA	0	2	Not-determined	Genome-fragment	NA	0
S17_vs_k141_139339||full	2499	No	NA	0	1	Not-determined	Genome-fragment	NA	0
S7_vs_73401||full	1072	No	NA	0	0	Low-quality	Genome-fragment	2.15	0
S17_vs_k141_51265||full	1680	No	NA	0	1	Not-determined	Genome-fragment	NA	0
S17_vs_k141_168824||full	1848	No	NA	0	2	Not-determined	Genome-fragment	NA	0
S16_extracted_22507	1072	No	NA	0	0	Low-quality	Genome-fragment	0.7	0
S17_vs_k141_80606||full	1026	No	NA	0	1	Not-determined	Genome-fragment	NA	0
S17_vs_k141_51291||full	7510	No	NA	0	7	Not-determined	Genome-fragment	NA	0
S17_vs_k141_131733||full	2932	No	NA	0	3	Not-determined	Genome-fragment	NA	0
S17_vs_k141_87206||full	1915	No	NA	0	2	Not-determined	Genome-fragment	NA	0
S17_vs_k141_6697||full	3115	No	NA	0	2	Not-determined	Genome-fragment	NA	0
S16_vs_93993||full	1072	No	NA	0	0	Low-quality	Genome-fragment	3.2	0
S17_vs_k141_73257||full	1007	No	NA	0	0	Not-determined	Genome-fragment	NA	0
S17_vs_k141_80638||full	2092	No	NA	0	2	Not-determined	Genome-fragment	NA	0
S17_vs_k141_117707||full	1958	No	NA	0	1	Not-determined	Genome-fragment	NA	0
S17_vs_k141_44076||full	3167	No	NA	0	0	Not-determined	Genome-fragment	NA	0
S17_vs_k141_51330||full	1547	No	NA	0	0	Not-determined	Genome-fragment	NA	0
S10_vs_33091||lt2gene	1072	No	NA	1	0	Low-quality	Genome-fragment	2.29	0
S12_vs_k141_175954||full	1072	No	NA	0	1	Low-quality	Genome-fragment	1.82	0
S17_vs_k141_139423||full	1624	No	NA	0	0	Not-determined	Genome-fragment	NA	0
S17_vs_k141_73288||full	1067	No	NA	0	0	Not-determined	Genome-fragment	NA	0
S15_extracted_58187	1072	No	NA	1	0	Low-quality	Genome-fragment	1.21	0
S17_vs_k141_44103||full	8521	No	NA	0	3	Not-determined	Genome-fragment	NA	0
S6_vs_k141_6681||full	1072	No	NA	0	0	Low-quality	Genome-fragment	2.05	0
S8_vs_k141_8904||full	1072	No	NA	1	0	Low-quality	Genome-fragment	0.67	0
S8_vs_k141_104000||full	1072	No	NA	0	1	Low-quality	Genome-fragment	0.3	0
S17_vs_k141_168898||full	7403	No	NA	0	2	Not-determined	Genome-fragment	NA	0
S17_vs_k141_13823||full	1715	No	NA	0	2	Not-determined	Genome-fragment	NA	0
S17_vs_k141_139463||full	1506	No	NA	0	1	Not-determined	Genome-fragment	NA	0
S17_vs_k141_110805||full	1880	No	NA	0	1	Not-determined	Genome-fragment	NA	0
S17_vs_k141_73330||full	3568	No	NA	0	3	Not-determined	Genome-fragment	NA	0
S17_vs_k141_20768||full	2161	No	NA	0	1	Not-determined	Genome-fragment	NA	0
S17_extracted_k141_138867	1073	No	NA	0	1	Low-quality	Genome-fragment	0.4	0
S17_vs_k141_64236||lt2gene	1073	No	NA	0	0	Low-quality	Genome-fragment	2.6	0
S9_extracted_k141_49587	1073	No	NA	1	0	Low-quality	Genome-fragment	2.91	0
S17_vs_k141_64684||full	1741	No	NA	0	0	Not-determined	Genome-fragment	NA	0
S9_vs_k141_26177||lt2gene	1073	No	NA	1	0	Low-quality	Genome-fragment	1.57	0
S13_vs_15121||full	1073	No	NA	0	2	Low-quality	Genome-fragment	1.56	0
S17_vs_k141_117802||full	2878	No	NA	0	1	Not-determined	Genome-fragment	NA	0
S17_vs_k141_169010||full	1509	No	NA	0	1	Not-determined	Genome-fragment	NA	0
S17_vs_k141_117830||full	3089	No	NA	0	2	Not-determined	Genome-fragment	NA	0
S17_vs_k141_131917||full	1628	No	NA	0	2	Not-determined	Genome-fragment	NA	0
S14_vs_k141_62476||full	1073	No	NA	0	0	Low-quality	Genome-fragment	2.75	0
S17_vs_k141_80727||full	1386	No	NA	0	1	Not-determined	Genome-fragment	NA	0
S17_vs_k141_51471||full	1623	No	NA	0	1	Not-determined	Genome-fragment	NA	0
S17_vs_k141_103150||full	4415	No	NA	0	1	Not-determined	Genome-fragment	NA	0
S1_vs_k141_15803||full	1073	No	NA	0	1	Low-quality	Genome-fragment	0.45	0
S17_vs_k141_117862||full	1685	No	NA	0	1	Not-determined	Genome-fragment	NA	0
S17_vs_k141_131974||full	1371	No	NA	0	2	Not-determined	Genome-fragment	NA	0
S1_vs_k141_16026||lt2gene	1073	No	NA	0	0	Low-quality	Genome-fragment	2.13	0
S3_extracted_k141_90201	1073	No	NA	0	0	Low-quality	Genome-fragment	0.92	0
S3_vs_k141_101249||full	1073	No	NA	0	0	Low-quality	Genome-fragment	2.69	0
S5_extracted_k141_47797	1073	No	NA	2	0	Low-quality	Genome-fragment	1.67	0
S5_vs_k141_17854||full	1073	No	NA	1	0	Low-quality	Genome-fragment	2.94	0
S17_vs_k141_169079||full	1043	No	NA	0	1	Not-determined	Genome-fragment	NA	0
S17_vs_k141_117885||full	4158	No	NA	0	3	Not-determined	Genome-fragment	NA	0
S17_vs_k141_13929||full	2290	No	NA	0	0	Not-determined	Genome-fragment	NA	0
S17_vs_k141_132049||full	2229	No	NA	0	3	Not-determined	Genome-fragment	NA	0
S17_vs_k141_13953||full	2247	No	NA	0	2	Not-determined	Genome-fragment	NA	0
S17_vs_k141_94924||full	1608	No	NA	0	1	Not-determined	Genome-fragment	NA	0
S17_vs_k141_162305||full	1272	No	NA	0	1	Not-determined	Genome-fragment	NA	0
S17_vs_k141_162310||full	2392	No	NA	0	2	Not-determined	Genome-fragment	NA	0
S17_vs_k141_28327||full	3221	No	NA	0	1	Not-determined	Genome-fragment	NA	0
S5_vs_k141_6797||lt2gene	1073	No	NA	1	0	Low-quality	Genome-fragment	1.65	0
S17_vs_k141_162319||full	1554	No	NA	0	2	Not-determined	Genome-fragment	NA	0
S7_extracted_15893	1073	No	NA	0	0	Low-quality	Genome-fragment	1.34	0
S17_vs_k141_87548||full	14884	No	NA	0	2	Not-determined	Genome-fragment	NA	0
S17_vs_k141_103311||full	1314	No	NA	0	1	Not-determined	Genome-fragment	NA	0
S17_vs_k141_73585||full	7545	No	NA	0	3	Not-determined	Genome-fragment	NA	0
S7_extracted_63375	1073	No	NA	1	0	Low-quality	Genome-fragment	1.61	0
S17_vs_k141_64834||full	2313	No	NA	0	1	Not-determined	Genome-fragment	NA	0
S17_vs_k141_176033||full	2894	No	NA	0	1	Not-determined	Genome-fragment	NA	0
S17_vs_k141_6999||full	1106	No	NA	0	2	Not-determined	Genome-fragment	NA	0
S7_extracted_113821	1073	No	NA	0	1	Low-quality	Genome-fragment	0.36	0
S17_vs_k141_7008||full	1644	No	NA	0	2	Not-determined	Genome-fragment	NA	0
S16_extracted_155746	1073	No	NA	0	0	Low-quality	Genome-fragment	1.73	0
S17_vs_k141_117991||full	15700	No	NA	0	7	Not-determined	Genome-fragment	NA	0
S16_vs_139847||lt2gene	1073	No	NA	1	0	Low-quality	Genome-fragment	1.57	0
S12_vs_k141_70947||full	1073	No	NA	0	1	Low-quality	Genome-fragment	0.24	0
S17_vs_k141_139660||full	1319	No	NA	0	1	Not-determined	Genome-fragment	NA	0
S17_vs_k141_169230||full	2341	No	NA	0	1	Not-determined	Genome-fragment	NA	0
S17_vs_k141_124738||full	1058	No	NA	0	0	Not-determined	Genome-fragment	NA	0
S6_vs_k141_1511||full	1073	No	NA	0	0	Low-quality	Genome-fragment	2.63	0
S17_vs_k141_21113||full	3178	No	NA	0	1	Not-determined	Genome-fragment	NA	0
S8_extracted_k141_31700	1073	No	NA	0	2	Low-quality	Genome-fragment	0.56	0
S17_vs_k141_36792||full	3641	No	NA	0	4	Not-determined	Genome-fragment	NA	0
S17_vs_k141_103398||full	3245	No	NA	0	2	Not-determined	Genome-fragment	NA	0
S17_vs_k141_7140||full	3504	No	NA	0	4	Not-determined	Genome-fragment	NA	0
S8_vs_k141_143907||full	1073	No	NA	0	2	Low-quality	Genome-fragment	0.34	0
S17_vs_k141_64955||full	3060	No	NA	0	2	Not-determined	Genome-fragment	NA	0
S9_extracted_k141_49826	1074	No	NA	0	0	Low-quality	Genome-fragment	0.45	0
S11_vs_k141_38106||lt2gene	1074	No	NA	1	0	Low-quality	Genome-fragment	0.65	0
S17_vs_k141_7189||full	2483	No	NA	0	2	Not-determined	Genome-fragment	NA	0
S17_vs_k141_58752||full	1467	No	NA	0	0	Not-determined	Genome-fragment	NA	0
S17_vs_k141_21232||full	3069	No	NA	0	0	Not-determined	Genome-fragment	NA	0
S14_extracted_k141_79415	1074	No	NA	0	1	Low-quality	Genome-fragment	0.7	0
S17_vs_k141_64984||full	1444	No	NA	0	0	Not-determined	Genome-fragment	NA	0
S14_vs_k141_3519||full	1074	No	NA	0	0	Low-quality	Genome-fragment	2.66	0
S1_extracted_k141_127786	1074	No	NA	0	0	Low-quality	Genome-fragment	1.99	0
S5_vs_k141_17337||full	1074	No	NA	0	0	Low-quality	Genome-fragment	2.29	0
S5_vs_k141_111665||lt2gene	1074	No	NA	0	2	Low-quality	Genome-fragment	1.92	0
S7_vs_25223||full	1074	No	NA	0	0	Low-quality	Genome-fragment	1.09	0
S7_vs_116076||lt2gene	1074	No	NA	1	0	Low-quality	Genome-fragment	1.59	0
S10_vs_114234||full	1074	No	NA	0	1	Low-quality	Genome-fragment	3.15	0
S17_vs_k141_14197||full	2462	No	NA	0	2	Not-determined	Genome-fragment	NA	0
S17_vs_k141_124906||full	3165	No	NA	0	0	Not-determined	Genome-fragment	NA	0
S17_vs_k141_176255||full	1776	No	NA	0	1	Not-determined	Genome-fragment	NA	0
S10_vs_23815||lt2gene	1074	Yes	476	1	1	Low-quality	Genome-fragment	0.84	55.68
S12_extracted_k141_123376	1074	No	NA	1	0	Low-quality	Genome-fragment	0.74	0
S17_vs_k141_73830||full	32605	No	NA	11	0	High-quality	High-quality	94.89	0
S8_extracted_k141_68565	1074	No	NA	1	0	Low-quality	Genome-fragment	1.85	0
S17_extracted_k141_37667	1075	No	NA	0	1	Low-quality	Genome-fragment	0.66	0
S13_vs_53444||lt2gene	1075	No	NA	1	0	Low-quality	Genome-fragment	0.87	0
S17_vs_k141_169440||full	3772	No	NA	0	3	Not-determined	Genome-fragment	NA	0
S17_vs_k141_124939||full	1109	No	NA	0	1	Not-determined	Genome-fragment	NA	0
S17_vs_k141_81137||full	3786	No	NA	0	2	Not-determined	Genome-fragment	NA	0
S14_extracted_k141_13289	1075	No	NA	4	0	Low-quality	Genome-fragment	3.55	0
S14_vs_k141_39284||lt2gene	1075	No	NA	1	0	Low-quality	Genome-fragment	2.85	0
S14_vs_k141_10185||lt2gene	1075	No	NA	1	0	Low-quality	Genome-fragment	2.29	0
S5_extracted_k141_94815	1075	No	NA	0	0	Low-quality	Genome-fragment	2.7	0
S5_vs_k141_127394||full	1075	No	NA	0	0	Low-quality	Genome-fragment	49.27	0
S17_vs_k141_95200||full	4727	No	NA	0	3	Not-determined	Genome-fragment	NA	0
S5_vs_k141_12913||lt2gene	1075	No	NA	2	0	Low-quality	Genome-fragment	2.06	0
S17_vs_k141_81202||full	7113	No	NA	0	1	Not-determined	Genome-fragment	NA	0
S17_vs_k141_36924||full	1478	No	NA	0	1	Not-determined	Genome-fragment	NA	0
S17_vs_k141_58920||full	1520	No	NA	0	2	Not-determined	Genome-fragment	NA	0
S5_vs_k141_136154||lt2gene	1075	No	NA	1	0	Low-quality	Genome-fragment	0.65	0
S17_vs_k141_21523||full	2431	No	NA	0	2	Not-determined	Genome-fragment	NA	0
S17_vs_k141_124990||full	2515	No	NA	0	1	Not-determined	Genome-fragment	NA	0
S17_vs_k141_176388||full	3007	No	NA	0	1	Not-determined	Genome-fragment	NA	0
S17_vs_k141_125004||full	1602	No	NA	0	0	Not-determined	Genome-fragment	NA	0
S17_vs_k141_14290||full	1158	No	NA	0	1	Not-determined	Genome-fragment	NA	0
S17_vs_k141_21577||full	1830	No	NA	0	0	Not-determined	Genome-fragment	NA	0

S16_vs_507||full	1075	No	NA	0	0	Low-quality	Genome-fragment		0.66	0
S17_vs_k141_65286||full	1871	No	NA	0	2	Not-determined	Genome-fragment	NA		0
S17_vs_k141_58946||full	6748	No	NA	0	7	Not-determined	Genome-fragment	NA		0
S17_vs_k141_14365||full	2194	No	NA	0	0	Not-determined	Genome-fragment	NA		0
S10_vs_29910||full	1075	No	NA	1	0	Low-quality	Genome-fragment		0.95	0
S17_vs_k141_7360||full	6605	No	NA	0	4	Not-determined	Genome-fragment	NA		0
S17_vs_k141_14373||full	2105	No	NA	0	3	Not-determined	Genome-fragment	NA		0
S17_vs_k141_21652||full	2817	No	NA	0	2	Not-determined	Genome-fragment	NA		0
S17_vs_k141_14405||full	1414	No	NA	0	0	Not-determined	Genome-fragment	NA		0
S17_vs_k141_58999||full	3428	No	NA	0	3	Not-determined	Genome-fragment	NA		0
S12_extracted_k141_114046	1075	No	NA	1	0	Low-quality	Genome-fragment		0.92	0
S17_vs_k141_169786||full	1560	No	NA	0	0	Not-determined	Genome-fragment	NA		0
S17_vs_k141_59022||full	2525	No	NA	0	0	Not-determined	Genome-fragment	NA		0
S12_vs_k141_29223||full	1075	No	NA	0	0	Low-quality	Genome-fragment		2.62	0
S17_vs_k141_28919||full	2779	No	NA	0	2	Not-determined	Genome-fragment	NA		0
S12_vs_k141_85119||full	1075	No	NA	2	0	Low-quality	Genome-fragment		2.58	0
S17_vs_k141_95372||full	3364	No	NA	0	1	Not-determined	Genome-fragment	NA		0
S8_extracted_k141_176416	1075	No	NA	0	1	Low-quality	Genome-fragment		0.58	0
S17_vs_k141_14510||full	3092	No	NA	0	1	Not-determined	Genome-fragment	NA		0
S17_vs_k141_169875||full	1757	No	NA	0	3	Not-determined	Genome-fragment	NA		0
S17_vs_k141_81255||full	1693	No	NA	0	1	Not-determined	Genome-fragment	NA		0
S17_vs_k141_65485||full	10873	No	NA	0	2	Not-determined	Genome-fragment	NA		0
S17_vs_k141_65502||full	1067	No	NA	0	0	Not-determined	Genome-fragment	NA		0
S17_vs_k141_169921||full	2614	No	NA	0	1	Not-determined	Genome-fragment	NA		0
S17_vs_k141_88218||full	1047	No	NA	0	1	Not-determined	Genome-fragment	NA		0
S17_vs_k141_14569||full	2864	No	NA	0	1	Not-determined	Genome-fragment	NA		0
S11_vs_k141_13482||full	1076	No	NA	0	1	Low-quality	Genome-fragment		0.36	0
S17_vs_k141_21790||full	4254	No	NA	0	2	Not-determined	Genome-fragment	NA		0
S13_extracted_91984	1076	No	NA	0	2	Low-quality	Genome-fragment		2.4	0
S13_vs_96206||full	1076	No	NA	0	0	Low-quality	Genome-fragment		3.38	0
S1_extracted_k141_131374	1076	No	NA	1	0	Low-quality	Genome-fragment		0.7	0
S17_vs_k141_125312||full	1275	No	NA	0	0	Not-determined	Genome-fragment	NA		0
S3_vs_k141_21642||full	1076	No	NA	1	0	Low-quality	Genome-fragment		3.13	0
S17_vs_k141_176686||full	1672	No	NA	0	0	Not-determined	Genome-fragment	NA		0
S5_extracted_k141_45881	1076	No	NA	1	0	Low-quality	Genome-fragment		3.13	0
S17_vs_k141_132895||full	1282	No	NA	0	1	Not-determined	Genome-fragment	NA		0
S7_extracted_15476	1076	No	NA	0	0	Low-quality	Genome-fragment		0.91	0
S17_vs_k141_125458||full	5928	No	NA	0	5	Not-determined	Genome-fragment	NA		0
S17_vs_k141_65746||full	1409	No	NA	0	3	Not-determined	Genome-fragment	NA		0
S7_extracted_34758	1076	No	NA	1	0	Low-quality	Genome-fragment		0.82	0
S17_vs_k141_125556||full	1602	No	NA	0	2	Not-determined	Genome-fragment	NA		0
S17_vs_k141_21993||full	5187	No	NA	0	1	Not-determined	Genome-fragment	NA		0
S7_vs_105065||full	1076	No	NA	1	0	Low-quality	Genome-fragment		3.13	0
S17_vs_k141_65882||full	1940	No	NA	0	2	Not-determined	Genome-fragment	NA		0
S17_vs_k141_88522||full	1850	No	NA	0	1	Not-determined	Genome-fragment	NA		0
S12_extracted_k141_63888	1076	No	NA	1	0	Low-quality	Genome-fragment		0.8	0
S17_vs_k141_95855||full	1922	No	NA	0	1	Not-determined	Genome-fragment	NA		0
S12_extracted_k141_125692	1076	No	NA	0	1	Low-quality	Genome-fragment		0.33	0
S17_vs_k141_95866||full	3044	No	NA	0	1	Not-determined	Genome-fragment	NA		0
S12_vs_k141_51695||full	1076	No	NA	0	0	Low-quality	Genome-fragment		2.4	0
S12_vs_k141_119048||lt2gene	1076	No	NA	1	0	Low-quality	Genome-fragment		2.15	0
S15_vs_12954||lt2gene	1076	No	NA	1	0	Low-quality	Genome-fragment		2.82	0
S17_vs_k141_88632||full	2378	No	NA	0	3	Not-determined	Genome-fragment	NA		0
S17_vs_k141_22071||full	1071	No	NA	0	1	Not-determined	Genome-fragment	NA		0
S8_extracted_k141_3812	1076	No	NA	1	0	Low-quality	Genome-fragment		3.34	0
S8_extracted_k141_151538	1076	No	NA	0	0	Low-quality	Genome-fragment		2.47	0
S8_extracted_k141_169697	1076	No	NA	0	0	Low-quality	Genome-fragment		1.64	0
S17_vs_k141_95960||full	10489	No	NA	0	0	Not-determined	Genome-fragment	NA		0
S17_vs_k141_170180||full	1077	No	NA	2	0	Low-quality	Genome-fragment		2.57	0
S17_vs_k141_177118||full	2563	No	NA	0	2	Not-determined	Genome-fragment	NA		0
S9_extracted_k141_67479	1077	No	NA	0	0	Low-quality	Genome-fragment		2.6	0
S17_vs_k141_177134||full	2939	No	NA	0	2	Not-determined	Genome-fragment	NA		0
S9_extracted_k141_52138	1077	No	NA	0	1	Low-quality	Genome-fragment		1.97	0
S14_vs_k141_45479||full	1077	No	NA	0	0	Low-quality	Genome-fragment		6.12	0
S17_vs_k141_66121||full	1567	No	NA	0	0	Not-determined	Genome-fragment	NA		0
S17_vs_k141_66298||full	2012	No	NA	0	0	Not-determined	Genome-fragment	NA		0
S17_vs_k141_66330||full	3080	No	NA	0	2	Not-determined	Genome-fragment	NA		0
S17_vs_k141_66388||full	5961	No	NA	0	2	Not-determined	Genome-fragment	NA		0
S17_vs_k141_147886||0_partial	13305	No	NA	0	2	Not-determined	Genome-fragment	NA		0
S17_vs_k141_141233||0_partial	15752	No	NA	0	3	Not-determined	Genome-fragment	NA		0
S17_vs_k141_45389||0_partial	13282	No	NA	0	7	Not-determined	Genome-fragment	NA		0
S17_vs_k141_8690||0_partial	17288	No	NA	1	4	High-quality	High-quality		92.42	0
S17_vs_k141_82185||0_partial	12363	No	NA	0	6	Not-determined	Genome-fragment	NA		0
S14_vs_k141_57566||full	1077	No	NA	0	0	Low-quality	Genome-fragment		2.56	0
S1_extracted_k141_138855	1077	No	NA	0	0	Low-quality	Genome-fragment		0.81	0
S17_vs_k141_91924||0_partial	11124	No	NA	0	2	Not-determined	Genome-fragment	NA		0
S17_vs_k141_11405||0_partial	17913	No	NA	0	7	Not-determined	Genome-fragment	NA		0
S17_vs_k141_62552||0_partial	2111	No	NA	0	0	Not-determined	Genome-fragment	NA		0
S3_vs_k141_96475||full	1077	No	NA	0	0	Low-quality	Genome-fragment		2.16	0
S17_vs_k141_139583||0_partial	11782	No	NA	0	4	Not-determined	Genome-fragment	NA		0
S3_vs_k141_123845||full	1077	No	NA	2	0	Low-quality	Genome-fragment		0.68	0
S17_vs_k141_88824||lt2gene	1808	No	NA	0	0	Not-determined	Genome-fragment	NA		0
S5_vs_k141_134347||full	1077	No	NA	0	0	Low-quality	Genome-fragment		2.72	0
S5_vs_k141_118117||full	1077	No	NA	0	0	Low-quality	Genome-fragment		2.15	0
S17_vs_k141_81505||lt2gene	1189	No	NA	0	0	Not-determined	Genome-fragment	NA		0
S7_extracted_34772	1077	No	NA	0	0	Low-quality	Genome-fragment		2.44	0
S17_vs_k141_155556||lt2gene	1068	No	NA	0	0	Not-determined	Genome-fragment	NA		0
S12_vs_k141_152342||full	1077	No	NA	1	0	Low-quality	Genome-fragment		2.33	0
S17_vs_k141_133585||lt2gene	1338	No	NA	0	1	Not-determined	Genome-fragment	NA		0
S12_vs_k141_47872||lt2gene	1077	No	NA	0	0	Low-quality	Genome-fragment		2.56	0
S15_extracted_28290	1077	No	NA	0	0	Low-quality	Genome-fragment		2.5	0
S8_extracted_k141_153948	1077	No	NA	0	0	Low-quality	Genome-fragment		2.6	0
S17_vs_k141_45087||lt2gene	1325	No	NA	0	0	Not-determined	Genome-fragment	NA		0
S8_vs_k141_130323||full	1077	No	NA	0	0	Low-quality	Genome-fragment		1.13	0
S8_vs_k141_48748||full	1077	No	NA	1	0	Low-quality	Genome-fragment		2.62	0
S17_vs_k141_141291||lt2gene	1622	No	NA	0	0	Not-determined	Genome-fragment	NA		0
S17_vs_k141_96898||lt2gene	2521	No	NA	0	0	Not-determined	Genome-fragment	NA		0
S17_vs_k141_89542||lt2gene	1435	No	NA	0	0	Not-determined	Genome-fragment	NA		0
S17_vs_k141_119364||lt2gene	1558	No	NA	0	0	Not-determined	Genome-fragment	NA		0
S17_extracted_k141_10740	1078	No	NA	0	0	Low-quality	Genome-fragment		1.07	0
S17_vs_k141_23394||lt2gene	1702	No	NA	0	0	Not-determined	Genome-fragment	NA		0
S9_vs_k141_25616||full	1078	No	NA	2	0	Low-quality	Genome-fragment		1.67	0
S17_vs_k141_157074||lt2gene	1475	No	NA	0	0	Not-determined	Genome-fragment	NA		0
S11_extracted_k141_47459	1078	No	NA	1	0	Low-quality	Genome-fragment		2.43	0
S3_extracted_k141_24052	1078	No	NA	0	1	Low-quality	Genome-fragment		0.31	0
S3_vs_k141_102208||full	1078	No	NA	0	0	Low-quality	Genome-fragment		1.58	0
S17_vs_k141_134997||lt2gene	1221	No	NA	0	0	Not-determined	Genome-fragment	NA		0
S5_extracted_k141_51723	1078	No	NA	0	0	Low-quality	Genome-fragment		2.38	0
S5_extracted_k141_107656	1078	No	NA	0	0	Low-quality	Genome-fragment		1.84	0
S5_vs_k141_134055||full	1078	No	NA	0	0	Low-quality	Genome-fragment		0.63	0
S5_vs_k141_107266||lt2gene	1078	No	NA	2	0	Low-quality	Genome-fragment		1.48	0
S7_vs_71434||full	1078	No	NA	2	0	Low-quality	Genome-fragment		1.94	0
S16_vs_102575||full	1078	No	NA	0	1	Low-quality	Genome-fragment		0.77	0
S10_vs_26620||full	1078	No	NA	0	0	Low-quality	Genome-fragment		2.62	0
S12_vs_k141_140503||full	1078	No	NA	0	0	Low-quality	Genome-fragment		1.53	0
S17_vs_k141_54925||lt2gene	1471	No	NA	0	1	Not-determined	Genome-fragment	NA		0
S6_vs_k141_27339||full	1078	No	NA	1	0	Low-quality	Genome-fragment		2.39	0
S6_vs_k141_54547||lt2gene	1078	No	NA	1	0	Low-quality	Genome-fragment		2.69	0
S8_extracted_k141_23517	1078	No	NA	1	0	Low-quality	Genome-fragment		0.68	0
S8_vs_k141_202906||full	1078	No	NA	0	2	Low-quality	Genome-fragment		0.28	0
S8_vs_k141_143483||full	1078	No	NA	0	1	Low-quality	Genome-fragment		1.97	0
S17_vs_k141_33057||lt2gene	1078	No	NA	0	0	Not-determined	Genome-fragment	NA		0
S17_extracted_k141_104258	1079	No	NA	3	0	Low-quality	Genome-fragment		3.56	0
S17_vs_k141_176443||full	1079	No	NA	2	0	Low-quality	Genome-fragment		1.77	0
S1_vs_k141_140546||lt2gene	1079	No	NA	1	0	Low-quality	Genome-fragment		0.75	0
S5_vs_k141_102881||full	1079	No	NA	0	0	Low-quality	Genome-fragment		2.62	0
S17_vs_k141_128746||lt2gene	2097	No	NA	0	0	Not-determined	Genome-fragment	NA		0
S7_extracted_76500	1079	No	NA	1	0	Low-quality	Genome-fragment		2.75	0
S17_vs_k141_62516||lt2gene	1161	No	NA	0	0	Not-determined	Genome-fragment	NA		0
S7_extracted_127658	1079	No	NA	0	0	Low-quality	Genome-fragment		1.17	0
S7_vs_61050||full	1079	No	NA	0	0	Low-quality	Genome-fragment		2.86	0
S16_vs_158565||full	1079	No	NA	2	0	Low-quality	Genome-fragment		1.32	0
S17_vs_k141_77956||lt2gene	1295	No	NA	0	0	Not-determined	Genome-fragment	NA		0
S10_extracted_17814	1079	No	NA	0	1	Low-quality	Genome-fragment		2.53	0
S10_extracted_61604	1079	No	NA	1	0	Low-quality	Genome-fragment		3.85	0
S10_vs_53249||full	1079	No	NA	0	0	Low-quality	Genome-fragment		2.39	0
S12_vs_k141_159175||full	1079	No	NA	0	1	Low-quality	Genome-fragment		1.24	0
S15_vs_54942||full	1079	No	NA	0	0	Low-quality	Genome-fragment		2.32	0
S8_extracted_k141_142835	1079	No	NA	1	0	Low-quality	Genome-fragment		1.87	0
S17_vs_k141_27129||lt2gene	1080	No	NA	2	0	Low-quality	Genome-fragment		0.88	0
S17_vs_k141_153143||lt2gene	1025	No	NA	0	0	Not-determined	Genome-fragment	NA		0
S11_extracted_k141_22553	1080	No	NA	0	0	Low-quality	Genome-fragment		2.52	0
S11_vs_k141_32582||full	1080	No	NA	0	0	Low-quality	Genome-fragment		1.06	0
S11_vs_k141_43895||full	1080	No	NA	1	0	Low-quality	Genome-fragment		2.72	0
S17_vs_k141_160591||lt2gene	2291	No	NA	0	2	Not-determined	Genome-fragment	NA		0
S17_vs_k141_5455||lt2gene	3605	No	NA	0	1	Not-determined	Genome-fragment	NA		0
S13_extracted_95417	1080	No	NA	0	0	Low-quality	Genome-fragment		1.12	0
S1_extracted_k141_32200	1080	No	NA	1	0	Low-quality	Genome-fragment		2.98	0
S1_vs_k141_101898||full	1080	No	NA	0	0	Low-quality	Genome-fragment		2.25	0
S3_extracted_k141_38625	1080	No	NA	1	0	Low-quality	Genome-fragment		0.66	0
S17_vs_k141_168156||lt2gene	1693	No	NA	0	0	Not-determined	Genome-fragment	NA		0
S3_vs_k141_4095||full	1080	No	NA	0	0	Low-quality	Genome-fragment		2.19	0
S5_extracted_k141_93304	1080	No	NA	2	0	Low-quality	Genome-fragment		1.91	0
S16_vs_71457||full	1080	No	NA	0	0	Low-quality	Genome-fragment		2.04	0
S16_vs_129382||full	1080	No	NA	0	1	Low-quality	Genome-fragment		0.99	0
S12_vs_k141_24915||full	1080	No	NA	1	0	Low-quality	Genome-fragment		2.46	0
S17_vs_k141_138810||lt2gene	1558	No	NA	0	1	Not-determined	Genome-fragment	NA		0
S15_extracted_31831	1080	No	NA	0	0	Low-quality	Genome-fragment		0.65	0
S17_vs_k141_57967||lt2gene	1634	No	NA	0	1	Not-determined	Genome-fragment	NA		0
S8_extracted_k141_58987	1080	No	NA	2	0	Low-quality	Genome-fragment		1.79	0
S8_extracted_k141_171887	1080	No	NA	1	0	Low-quality	Genome-fragment		1.79	0
S17_vs_k141_51073||lt2gene	1165	No	NA	0	1	Not-determined	Genome-fragment	NA		0
S17_vs_k141_96710||full	1081	No	NA	0	0	Low-quality	Genome-fragment		2.72	0
S17_vs_k141_13703||lt2gene	1514	No	NA	0	0	Not-determined	Genome-fragment	NA		0
S17_vs_k141_152734||full	1081	No	NA	0	0	Low-quality	Genome-fragment		2.39	0
S13_vs_45543||full	1081	No	NA	1	0	Low-quality	Genome-fragment		2.89	0
S17_vs_k141_80879||lt2gene	1004	No	NA	0	0	Not-determined	Genome-fragment	NA		0
S1_extracted_k141_158236	1081	No	NA	1	0	Low-quality	Genome-fragment		0.71	0
S3_extracted_k141_46748	1081	No	NA	1	0	Low-quality	Genome-fragment		2.65	0
S5_vs_k141_113577||lt2gene	1081	No	NA	1	0	Low-quality	Genome-fragment		2.83	0
S9_extracted_k141_15162	1230	No	NA	0	1	Not-determined	Genome-fragment	NA		0
S9_extracted_k141_45474	8137	No	NA	0	3	Not-determined	Genome-fragment	NA		0
S9_extracted_k141_79586	1738	No	NA	0	0	Not-determined	Genome-fragment	NA		0
S9_extracted_k141_87166	1231	No	NA	0	1	Not-determined	Genome-fragment	NA		0

S7_extracted_93724	1081	No	NA		0	0	Low-quality	Genome-fragment		0.96	0
S9_extracted_k141_11404	1253	No	NA		0	0	Not-determined	Genome-fragment	NA		0
S9_extracted_k141_83380	4599	No	NA		0	0	Not-determined	Genome-fragment	NA		0
S7_vs_91304||full	1081	No	NA		2	0	Low-quality	Genome-fragment		2.25	0
S12_vs_k141_43679||full	1081	No	NA		0	0	Low-quality	Genome-fragment		1.57	0
S9_extracted_k141_34148	1119	No	NA		0	0	Not-determined	Genome-fragment	NA		0
S12_vs_k141_37254||full	1081	No	NA		0	0	Low-quality	Genome-fragment		0.61	0
S9_extracted_k141_75808	1444	No	NA		0	1	Not-determined	Genome-fragment	NA		0
S9_extracted_k141_34159	1607	No	NA		0	0	Not-determined	Genome-fragment	NA		0
S8_extracted_k141_29239	1081	No	NA		0	1	Low-quality	Genome-fragment		0.27	0
S9_extracted_k141_87232	1512	No	NA		0	1	Not-determined	Genome-fragment	NA		0
S8_extracted_k141_127447	1081	No	NA		0	1	Low-quality	Genome-fragment		2.02	0
S8_vs_k141_4137||full	1081	No	NA		0	0	Low-quality	Genome-fragment		2.54	0
S9_extracted_k141_22831	1266	No	NA		0	0	Not-determined	Genome-fragment	NA		0
S9_extracted_k141_37942	7031	No	NA		0	13	Not-determined	Genome-fragment	NA		0
S17_extracted_k141_147931	1082	No	NA		0	0	Low-quality	Genome-fragment		1.86	0
S9_extracted_k141_37951	1447	No	NA		0	0	Not-determined	Genome-fragment	NA		0
S9_extracted_k141_75883	1008	No	NA		0	1	Not-determined	Genome-fragment	NA		0
S9_extracted_k141_83471	1494	No	NA		0	0	Not-determined	Genome-fragment	NA		0
S9_extracted_k141_53150	1751	No	NA		0	1	Not-determined	Genome-fragment	NA		0
S9_extracted_k141_53172	1119	No	NA		0	0	Not-determined	Genome-fragment	NA		0
S17_extracted_k141_4694	1082	No	NA		0	0	Low-quality	Genome-fragment		2.11	0
S17_vs_k141_4142||full	1082	No	NA		0	0	Low-quality	Genome-fragment		0.75	0
S9_extracted_k141_75905	1120	No	NA		0	3	Not-determined	Genome-fragment	NA		0
S9_extracted_k141_75906	1872	No	NA		0	0	Not-determined	Genome-fragment	NA		0
S9_extracted_k141_22903	1896	No	NA		0	2	Not-determined	Genome-fragment	NA		0
S9_extracted_k141_83536	1607	No	NA		0	0	Not-determined	Genome-fragment	NA		0
S14_extracted_k141_58947	1082	No	NA		3	0	Low-quality	Genome-fragment		3.33	0
S14_extracted_k141_72534	1082	No	NA		1	0	Low-quality	Genome-fragment		0.68	0
S1_vs_k141_125924||full	1082	No	NA		0	2	Low-quality	Genome-fragment		0.43	0
S3_vs_k141_77715||full	1082	No	NA		1	0	Low-quality	Genome-fragment		2.85	0
S5_extracted_k141_49161	1082	No	NA		0	0	Low-quality	Genome-fragment		1.92	0
S9_extracted_k141_79752	4531	No	NA		0	1	Not-determined	Genome-fragment	NA		0
S9_extracted_k141_45612	1161	No	NA		0	1	Not-determined	Genome-fragment	NA		0
S9_extracted_k141_34362	1050	No	NA		0	1	Not-determined	Genome-fragment	NA		0
S9_extracted_k141_19155	2404	No	NA		0	1	Not-determined	Genome-fragment	NA		0
S5_vs_k141_84736||full	1082	No	NA		0	2	Low-quality	Genome-fragment		1.23	0
S7_extracted_113858	1082	No	NA		0	1	Low-quality	Genome-fragment		1.66	0
S9_extracted_k141_7714	1769	No	NA		0	1	Not-determined	Genome-fragment	NA		0
S9_extracted_k141_34421	1339	No	NA		0	2	Not-determined	Genome-fragment	NA		0
S9_extracted_k141_64618	1987	No	NA		0	2	Not-determined	Genome-fragment	NA		0
S9_extracted_k141_11648	4594	No	NA		0	2	Not-determined	Genome-fragment	NA		0
S9_extracted_k141_68384	3758	No	NA		0	0	Not-determined	Genome-fragment	NA		0
S16_extracted_10876	1082	No	NA		0	1	Low-quality	Genome-fragment		1.87	0
S9_extracted_k141_64631	1008	No	NA		0	2	Not-determined	Genome-fragment	NA		0
S9_extracted_k141_53360	1042	No	NA		0	0	Not-determined	Genome-fragment	NA		0
S9_extracted_k141_49425	1162	No	NA		0	1	Not-determined	Genome-fragment	NA		0
S16_vs_151113||lt2gene	1082	No	NA		1	0	Low-quality	Genome-fragment		3.34	0
S10_vs_20268||full	1082	No	NA		0	0	Low-quality	Genome-fragment		2.4	0
S9_extracted_k141_19272	1051	No	NA		0	1	Not-determined	Genome-fragment	NA		0
S12_extracted_k141_90926	1082	No	NA		0	0	Low-quality	Genome-fragment		2.03	0
S9_extracted_k141_68444	1032	No	NA		0	1	Not-determined	Genome-fragment	NA		0
S9_extracted_k141_26772	1290	No	NA		0	0	Not-determined	Genome-fragment	NA		0
S12_vs_k141_163893||full	1082	No	NA		0	0	Low-quality	Genome-fragment		2.8	0
S9_extracted_k141_57068	1118	No	NA		0	1	Not-determined	Genome-fragment	NA		0
S9_extracted_k141_4115	33438	No	NA		33	0	Medium-quality	Genome-fragment		86.27	0
S9_extracted_k141_38261	11379	No	NA		0	3	Not-determined	Genome-fragment	NA		0
S9_extracted_k141_26805	6114	No	NA		3	0	Complete	High-quality		100	0
S9_extracted_k141_38286	1280	No	NA		0	1	Not-determined	Genome-fragment	NA		0
S9_extracted_k141_15519	1111	No	NA		0	1	Not-determined	Genome-fragment	NA		0
S9_extracted_k141_4137	1085	No	NA		0	0	Not-determined	Genome-fragment	NA		0
S9_extracted_k141_87556	2982	No	NA		0	0	Not-determined	Genome-fragment	NA		0
S9_extracted_k141_42048	1848	No	NA		0	1	Not-determined	Genome-fragment	NA		0
S9_extracted_k141_11720	1105	No	NA		0	1	Not-determined	Genome-fragment	NA		0
S9_extracted_k141_87566	1379	No	NA		0	2	Not-determined	Genome-fragment	NA		0
S9_extracted_k141_15545	3035	No	NA		0	1	Not-determined	Genome-fragment	NA		0
S9_extracted_k141_19284	41326	No	NA		28	1	Complete	High-quality		100	0
S9_extracted_k141_79919	34073	No	NA		23	0	Complete	High-quality		100	0
S8_extracted_k141_4008	1082	No	NA		0	1	Low-quality	Genome-fragment		0.67	0
S9_extracted_k141_68558	1138	No	NA		0	0	Not-determined	Genome-fragment	NA		0
S9_extracted_k141_72340	3672	No	NA		0	3	Not-determined	Genome-fragment	NA		0
S17_vs_k141_66907||full	1083	Yes		750	1	1	Low-quality	Genome-fragment		0.47	30.75
S9_extracted_k141_68561	4316	No	NA		0	2	Not-determined	Genome-fragment	NA		0
S9_extracted_k141_30776	1030	No	NA		0	1	Not-determined	Genome-fragment	NA		0
S9_extracted_k141_465	1076	No	NA		0	0	Not-determined	Genome-fragment	NA		0
S13_vs_92212||full	1083	No	NA		0	0	Low-quality	Genome-fragment		2.79	0
S14_vs_k141_43092||full	1083	No	NA		0	0	Low-quality	Genome-fragment		2.78	0
S3_extracted_k141_55244	1083	No	NA		0	0	Low-quality	Genome-fragment		2.07	0
S3_vs_k141_114930||full	1083	No	NA		0	0	Low-quality	Genome-fragment		2.13	0
S3_vs_k141_52678||full	1083	No	NA		0	2	Low-quality	Genome-fragment		0.29	0
S9_extracted_k141_49616	1796	No	NA		0	3	Not-determined	Genome-fragment	NA		0
S9_extracted_k141_7886	1062	No	NA		0	1	Not-determined	Genome-fragment	NA		0
S9_extracted_k141_61027	5332	No	NA		0	1	Not-determined	Genome-fragment	NA		0
S3_vs_k141_55184||full	1083	No	NA		0	0	Low-quality	Genome-fragment		2.78	0
S16_extracted_115106	1083	No	NA		0	0	Low-quality	Genome-fragment		1.19	0
S9_extracted_k141_79979	1494	No	NA		0	1	Not-determined	Genome-fragment	NA		0
S10_extracted_117308	1083	No	NA		0	1	Low-quality	Genome-fragment		2.17	0
S2_vs_k141_13749||full	1083	No	NA		0	0	Low-quality	Genome-fragment		2.99	0
S9_extracted_k141_42166	5560	No	NA		0	1	Not-determined	Genome-fragment	NA		0
S2_vs_k141_7008||lt2gene	1083	No	NA		1	0	Low-quality	Genome-fragment		2.88	0
S9_extracted_k141_79995	1043	No	NA		0	0	Not-determined	Genome-fragment	NA		0
S9_extracted_k141_546	1374	No	NA		0	1	Not-determined	Genome-fragment	NA		0
S9_extracted_k141_26970	1062	No	NA		0	0	Not-determined	Genome-fragment	NA		0
S9_extracted_k141_15693	4140	No	NA		0	1	Not-determined	Genome-fragment	NA		0
S8_extracted_k141_166420	1083	No	NA		0	0	Low-quality	Genome-fragment		1.75	0
S8_vs_k141_104812||full	1083	No	NA		0	1	Low-quality	Genome-fragment		0.15	0
S9_extracted_k141_76134	2323	No	NA		0	1	Not-determined	Genome-fragment	NA		0
S17_vs_k141_164243||full	1084	No	NA		0	0	Low-quality	Genome-fragment		2.78	0
S9_extracted_k141_61094	1036	No	NA		0	1	Not-determined	Genome-fragment	NA		0
S17_vs_k141_135548||full	1084	No	NA		0	0	Low-quality	Genome-fragment		0.85	0
S9_vs_k141_9220||full	1084	No	NA		1	0	Low-quality	Genome-fragment		2.61	0
S11_extracted_k141_49316	1084	No	NA		0	0	Low-quality	Genome-fragment		2.51	0
S9_extracted_k141_80043	1126	No	NA		0	2	Not-determined	Genome-fragment	NA		0
S9_extracted_k141_72451	1737	No	NA		0	1	Not-determined	Genome-fragment	NA		0
S9_extracted_k141_83857	1662	No	NA		0	0	Not-determined	Genome-fragment	NA		0
S9_extracted_k141_27023	1346	No	NA		0	1	Not-determined	Genome-fragment	NA		0
S9_extracted_k141_87720	1279	No	NA		0	0	Not-determined	Genome-fragment	NA		0
S9_extracted_k141_8008	1339	No	NA		0	0	Not-determined	Genome-fragment	NA		0
S9_extracted_k141_42252	1586	No	NA		0	0	Not-determined	Genome-fragment	NA		0
S1_vs_k141_81303||full	1084	No	NA		0	0	Low-quality	Genome-fragment		2.64	0
S1_vs_k141_88472||full	1084	No	NA		0	0	Low-quality	Genome-fragment		1.79	0
S9_extracted_k141_618	1994	No	NA		0	1	Not-determined	Genome-fragment	NA		0
S3_extracted_k141_92112	1084	No	NA		0	0	Low-quality	Genome-fragment		3.03	0
S9_extracted_k141_80081	1028	No	NA		0	0	Not-determined	Genome-fragment	NA		0
S3_vs_k141_48797||full	1084	No	NA		1	0	Low-quality	Genome-fragment		1.38	0
S9_extracted_k141_87778	3280	No	NA		0	2	Not-determined	Genome-fragment	NA		0
S9_extracted_k141_53606	1038	No	NA		0	0	Not-determined	Genome-fragment	NA		0
S3_vs_k141_105027||full	1084	No	NA		1	0	Low-quality	Genome-fragment		2.31	0
S9_extracted_k141_34660	1263	No	NA		0	0	Not-determined	Genome-fragment	NA		0
S9_extracted_k141_68624	1163	No	NA		0	1	Not-determined	Genome-fragment	NA		0
S5_extracted_k141_20412	1084	No	NA		1	0	Low-quality	Genome-fragment		2.65	0
S9_extracted_k141_53641	1160	No	NA		0	0	Not-determined	Genome-fragment	NA		0
S9_extracted_k141_80145	1047	No	NA		0	1	Not-determined	Genome-fragment	NA		0
S9_extracted_k141_64844	93233	No	NA		42	1	High-quality	High-quality		100	0
S9_extracted_k141_8094	1279	No	NA		0	0	Not-determined	Genome-fragment	NA		0
S9_extracted_k141_45930	1535	No	NA		0	0	Not-determined	Genome-fragment	NA		0
S5_vs_k141_36770||full	1084	No	NA		1	0	Low-quality	Genome-fragment		1.97	0
S9_extracted_k141_57277	56189	No	NA		21	0	Complete	High-quality		100	0
S9_extracted_k141_87867	1339	No	NA		0	0	Not-determined	Genome-fragment	NA		0
S9_extracted_k141_12085	1104	No	NA		0	0	Not-determined	Genome-fragment	NA		0
S9_extracted_k141_15874	1579	No	NA		0	0	Not-determined	Genome-fragment	NA		0
S5_vs_k141_56588||full	1084	No	NA		1	0	Low-quality	Genome-fragment		2.46	0
S7_vs_1280||full	1084	No	NA		0	0	Low-quality	Genome-fragment		2.27	0
S9_extracted_k141_64939	5390	No	NA		0	1	Not-determined	Genome-fragment	NA		0
S9_extracted_k141_34736	13774	No	NA		8	0	Medium-quality	Genome-fragment		62.9	0
S7_vs_128244||lt2gene	1084	No	NA		1	0	Low-quality	Genome-fragment		1.29	0
S16_extracted_147087	1084	No	NA		2	0	Low-quality	Genome-fragment		1.98	0
S9_extracted_k141_53786	1510	No	NA		0	0	Not-determined	Genome-fragment	NA		0
S9_extracted_k141_64976	1596	No	NA		0	0	Not-determined	Genome-fragment	NA		0
S9_extracted_k141_15949	2000	No	NA		0	0	Not-determined	Genome-fragment	NA		0
S9_extracted_k141_68770	2433	No	NA		0	3	Not-determined	Genome-fragment	NA		0
S9_extracted_k141_23394	1283	No	NA		0	1	Not-determined	Genome-fragment	NA		0
S16_vs_114605||full	1084	No	NA		1	0	Low-quality	Genome-fragment		2.98	0
S9_extracted_k141_57339	2674	No	NA		0	1	Not-determined	Genome-fragment	NA		0
S16_vs_111946||lt2gene	1084	No	NA		2	0	Low-quality	Genome-fragment		1.52	0
S12_vs_k141_50836||full	1084	No	NA		1	0	Low-quality	Genome-fragment		2.53	0
S9_extracted_k141_27298	2254	No	NA		0	1	Not-determined	Genome-fragment	NA		0
S9_extracted_k141_27304	1015	No	NA		0	1	Not-determined	Genome-fragment	NA		0
S9_extracted_k141_53821	1663	No	NA		0	0	Not-determined	Genome-fragment	NA		0
S9_extracted_k141_49765	3353	No	NA		0	3	Not-determined	Genome-fragment	NA		0
S15_extracted_72892	1084	No	NA		1	0	Low-quality	Genome-fragment		3.11	0
S8_extracted_k141_66518	1084	No	NA		0	1	Low-quality	Genome-fragment		0.66	0
S9_extracted_k141_737	1500	No	NA		0	0	Not-determined	Genome-fragment	NA		0
S9_extracted_k141_84074	1049	No	NA		0	0	Not-determined	Genome-fragment	NA		0
S9_extracted_k141_80362	1232	No	NA		0	1	Not-determined	Genome-fragment	NA		0
S9_extracted_k141_76394	1226	No	NA		0	1	Not-determined	Genome-fragment	NA		0
S9_extracted_k141_34831	1682	No	NA		0	1	Not-determined	Genome-fragment	NA		0
S14_extracted_k141_4674	1085	No	NA		1	0	Low-quality	Genome-fragment		0.5	0
S9_extracted_k141_46144	1593	No	NA		0	1	Not-determined	Genome-fragment	NA		0
S1_vs_k141_26151||lt2gene	1085	No	NA		2	0	Low-quality	Genome-fragment		3.81	0
S5_extracted_k141_15954	1085	No	NA		0	0	Low-quality	Genome-fragment		0.39	0
S7_vs_106147||lt2gene	1085	No	NA		1	0	Low-quality	Genome-fragment		1.38	0
S9_extracted_k141_23500	1103	No	NA		0	0	Not-determined	Genome-fragment	NA		0
S9_extracted_k141_4544	2738	No	NA		0	0	Not-determined	Genome-fragment	NA		0
S16_vs_57192||full	1085	No	NA		1	0	Low-quality	Genome-fragment		1.6	0
S12_vs_k141_185004||full	1085	No	NA		0	0	Low-quality	Genome-fragment		1.75	0
S4_extracted_8626	1085	No	NA		0	0	Low-quality	Genome-fragment		2.26	0
S9_extracted_k141_53940	1178	No	NA		0	1	Not-determined	Genome-fragment	NA		0
S9_extracted_k141_65093	1222	No	NA		0	1	Not-determined	Genome-fragment	NA		0
S9_extracted_k141_813	3334	No	NA		0	2	Not-determined	Genome-fragment	NA		0
S8_extracted_k141_159645	1085	No	NA		0	1	Low-quality	Genome-fragment		0.77	0
S8_vs_k141_200203||full	1085	No	NA		1	0	Low-quality	Genome-fragment		1.87	0
S8_vs_k141_29377||full	1085	No	NA		0	0	Low-quality	Genome-fragment		1.06	0
S17_extracted_k141_173337	1086	No	NA		0	0	Low-quality	Genome-fragment		3.45	0

S9_extracted_k141_80448	3552	No	NA	0	0	Not-determined	Genome-fragment	NA		0
S17_vs_k141_12842||lt2gene	1086	No	NA	1	0	Low-quality	Genome-fragment		1.78	0
S9_extracted_k141_12317	1213	No	NA	0	0	Not-determined	Genome-fragment	NA		0
S9_extracted_k141_61530	1135	No	NA	0	1	Not-determined	Genome-fragment	NA		0
S9_extracted_k141_57492	3971	No	NA	0	1	Not-determined	Genome-fragment	NA		0
S9_extracted_k141_79709	1086	No	NA	1	0	Low-quality	Genome-fragment		18.23	0
S9_extracted_k141_23571	18101	No	NA	0	4	Not-determined	Genome-fragment	NA		0
S11_vs_k141_40461||full	1086	No	NA	0	0	Low-quality	Genome-fragment		2.03	0
S9_extracted_k141_16167	1003	No	NA	0	1	Not-determined	Genome-fragment	NA		0
S13_extracted_80374	1086	No	NA	0	1	Low-quality	Genome-fragment		0.19	0
S13_extracted_116309	1086	No	NA	0	0	Low-quality	Genome-fragment		17.09	0
S13_extracted_148066	1086	No	NA	0	0	Low-quality	Genome-fragment		1.92	0
S9_extracted_k141_72894	3500	No	NA	0	1	Not-determined	Genome-fragment	NA		0
S9_extracted_k141_4600	1366	No	NA	0	0	Not-determined	Genome-fragment	NA		0
S9_extracted_k141_38992	1726	No	NA	0	1	Not-determined	Genome-fragment	NA		0
S9_extracted_k141_34996	1397	No	NA	0	0	Not-determined	Genome-fragment	NA		0
S9_extracted_k141_57529	3813	No	NA	0	4	Not-determined	Genome-fragment	NA		0
S9_extracted_k141_35001	1138	No	NA	0	0	Not-determined	Genome-fragment	NA		0
S9_extracted_k141_72908	1091	No	NA	0	0	Not-determined	Genome-fragment	NA		0
S14_vs_k141_19955||full	1086	No	NA	0	0	Low-quality	Genome-fragment		3.13	0
S9_extracted_k141_39012	1024	No	NA	0	1	Not-determined	Genome-fragment	NA		0
S9_extracted_k141_934	9848	No	NA	8	0	Medium-quality	Genome-fragment		62.07	0
S14_vs_k141_63281||full	1086	No	NA	0	0	Low-quality	Genome-fragment		2.56	0
S9_extracted_k141_31391	1081	No	NA	0	1	Not-determined	Genome-fragment	NA		0
S9_extracted_k141_954	1653	No	NA	0	0	Not-determined	Genome-fragment	NA		0
S7_vs_84049||lt2gene	1086	No	NA	2	0	Low-quality	Genome-fragment		2.37	0
S16_vs_71180||full	1086	No	NA	0	1	Low-quality	Genome-fragment		0.43	0
S9_extracted_k141_57599	1130	No	NA	0	0	Not-determined	Genome-fragment	NA		0
S9_extracted_k141_72985	1232	No	NA	0	1	Not-determined	Genome-fragment	NA		0
S12_extracted_k141_48450	1086	No	NA	1	0	Low-quality	Genome-fragment		17.71	0
S9_extracted_k141_8251	1523	No	NA	0	1	Not-determined	Genome-fragment	NA		0
S15_vs_17886||full	1086	No	NA	0	0	Low-quality	Genome-fragment		2.25	0
S6_extracted_k141_32873	1086	No	NA	1	0	Low-quality	Genome-fragment		1.54	0
S9_extracted_k141_19850	1469	No	NA	0	0	Not-determined	Genome-fragment	NA		0
S9_extracted_k141_8262	3644	No	NA	0	1	Not-determined	Genome-fragment	NA		0
S9_extracted_k141_65289	1316	No	NA	0	0	Not-determined	Genome-fragment	NA		0
S9_extracted_k141_76513	4827	No	NA	0	4	Not-determined	Genome-fragment	NA		0
S17_vs_k141_84815||full	1087	No	NA	1	0	Low-quality	Genome-fragment		3.04	0
S9_extracted_k141_35133	1139	No	NA	0	1	Not-determined	Genome-fragment	NA		0
S13_extracted_91904	1087	No	NA	1	0	Low-quality	Genome-fragment		3.72	0
S9_extracted_k141_12527	4188	No	NA	0	1	Not-determined	Genome-fragment	NA		0
S14_extracted_k141_24179	1087	No	NA	0	0	Low-quality	Genome-fragment		1.5	0
S9_extracted_k141_16432	1024	No	NA	0	0	Not-determined	Genome-fragment	NA		0
S9_extracted_k141_54257	1410	No	NA	0	1	Not-determined	Genome-fragment	NA		0
S9_extracted_k141_1071	4979	No	NA	0	0	Not-determined	Genome-fragment	NA		0
S14_vs_k141_5340||full	1087	No	NA	0	0	Low-quality	Genome-fragment		2.34	0
S9_extracted_k141_42841	1165	No	NA	0	0	Not-determined	Genome-fragment	NA		0
S14_vs_k141_59037||lt2gene	1087	No	NA	1	0	Low-quality	Genome-fragment		2.51	0
S9_extracted_k141_19957	1155	No	NA	0	1	Not-determined	Genome-fragment	NA		0
S3_vs_k141_102413||lt2gene	1087	No	NA	1	0	Low-quality	Genome-fragment		1.67	0
S9_extracted_k141_8366	1940	No	NA	0	1	Not-determined	Genome-fragment	NA		0
S9_extracted_k141_42865	1233	No	NA	0	1	Not-determined	Genome-fragment	NA		0
S5_extracted_k141_13214	1087	No	NA	1	0	Low-quality	Genome-fragment		4.96	0
S9_extracted_k141_1131	1065	No	NA	0	0	Not-determined	Genome-fragment	NA		0
S9_extracted_k141_84250	3064	No	NA	0	3	Not-determined	Genome-fragment	NA		0
S5_vs_k141_88287||lt2gene	1087	No	NA	1	0	Low-quality	Genome-fragment		2.69	0
S9_extracted_k141_39221	1909	No	NA	0	2	Not-determined	Genome-fragment	NA		0
S9_extracted_k141_61670	5794	No	NA	0	1	Not-determined	Genome-fragment	NA		0
S7_extracted_26937	1087	No	NA	1	0	Low-quality	Genome-fragment		17.06	0
S16_extracted_63489	1087	No	NA	0	0	Low-quality	Genome-fragment		2.64	0
S9_extracted_k141_57740	1107	No	NA	0	0	Not-determined	Genome-fragment	NA		0
S9_extracted_k141_12647	1990	No	NA	0	0	Not-determined	Genome-fragment	NA		0
S9_extracted_k141_27660	1149	No	NA	0	1	Not-determined	Genome-fragment	NA		0
S16_vs_158279||full	1087	No	NA	0	0	Low-quality	Genome-fragment		2.22	0
S9_extracted_k141_4863	1032	No	NA	0	0	Not-determined	Genome-fragment	NA		0
S9_extracted_k141_80519	1118	No	NA	0	0	Not-determined	Genome-fragment	NA		0
S9_extracted_k141_35300	3671	No	NA	0	1	Not-determined	Genome-fragment	NA		0
S10_vs_22617||lt2gene	1087	No	NA	1	0	Low-quality	Genome-fragment		2.18	0
S9_extracted_k141_12677	3406	No	NA	0	8	Not-determined	Genome-fragment	NA		0
S12_extracted_k141_59832	1087	No	NA	0	0	Low-quality	Genome-fragment		1.72	0
S9_extracted_k141_35345	1412	No	NA	0	2	Not-determined	Genome-fragment	NA		0
S4_extracted_18450	1087	No	NA	1	0	Low-quality	Genome-fragment		1.29	0
S9_extracted_k141_1247	3784	No	NA	0	3	Not-determined	Genome-fragment	NA		0
S9_extracted_k141_20115	1118	No	NA	0	0	Not-determined	Genome-fragment	NA		0
S9_extracted_k141_61772	1589	No	NA	0	1	Not-determined	Genome-fragment	NA		0
S8_vs_k141_99936||full	1087	No	NA	2	0	Low-quality	Genome-fragment		1.13	0
S17_vs_k141_68634||full	1088	No	NA	1	0	Low-quality	Genome-fragment		2.32	0
S9_extracted_k141_27161	1088	No	NA	0	0	Low-quality	Genome-fragment		1.38	0
S9_vs_k141_23134||lt2gene	1088	No	NA	1	0	Low-quality	Genome-fragment		3.03	0
S9_extracted_k141_46626	1083	No	NA	0	0	Not-determined	Genome-fragment	NA		0
S9_extracted_k141_57833	1245	No	NA	0	0	Not-determined	Genome-fragment	NA		0
S9_extracted_k141_39417	1310	No	NA	0	0	Not-determined	Genome-fragment	NA		0
S9_extracted_k141_31740	1324	No	NA	0	0	Not-determined	Genome-fragment	NA		0
S11_vs_k141_2603||lt2gene	1088	No	NA	1	0	Low-quality	Genome-fragment		2.45	0
S14_extracted_k141_82213	1088	No	NA	1	0	Low-quality	Genome-fragment		0.6	0
S9_extracted_k141_31753	1987	No	NA	0	1	Not-determined	Genome-fragment	NA		0
S14_extracted_k141_91780	1088	No	NA	0	1	Low-quality	Genome-fragment		0.26	0
S9_extracted_k141_88564	1185	No	NA	0	1	Not-determined	Genome-fragment	NA		0
S9_extracted_k141_73311	38973	No	NA	26	0	Medium-quality	Genome-fragment		70.19	0
S9_extracted_k141_31788	1152	No	NA	0	0	Not-determined	Genome-fragment	NA		0
S9_extracted_k141_23893	1602	No	NA	0	1	Not-determined	Genome-fragment	NA		0
S1_extracted_k141_41842	1088	No	NA	0	0	Low-quality	Genome-fragment		1.8	0
S9_extracted_k141_31819	1213	No	NA	0	1	Not-determined	Genome-fragment	NA		0
S1_extracted_k141_25626	1088	No	NA	0	0	Low-quality	Genome-fragment		2.62	0
S9_extracted_k141_23901	5984	No	NA	3	0	Complete	High-quality		100	0
S1_extracted_k141_52827	1088	No	NA	0	1	Low-quality	Genome-fragment		0.66	0
S9_extracted_k141_5131	1796	No	NA	0	2	Not-determined	Genome-fragment	NA		0
S1_vs_k141_53713||full	1088	No	NA	0	1	Low-quality	Genome-fragment		1.02	0
S1_vs_k141_33705||full	1088	No	NA	1	0	Low-quality	Genome-fragment		2.73	0
S9_extracted_k141_20288	1212	No	NA	0	1	Not-determined	Genome-fragment	NA		0
S3_extracted_k141_66984	1088	No	NA	1	0	Low-quality	Genome-fragment		2	0
S9_extracted_k141_16668	1376	No	NA	0	1	Not-determined	Genome-fragment	NA		0
S3_vs_k141_73430||full	1088	No	NA	2	0	Low-quality	Genome-fragment		2.25	0
S3_vs_k141_74728||full	1088	No	NA	1	0	Low-quality	Genome-fragment		2.45	0
S9_extracted_k141_54426	1088	No	NA	0	0	Not-determined	Genome-fragment	NA		0
S5_vs_k141_6335||full	1088	No	NA	0	0	Low-quality	Genome-fragment		1.26	0
S9_extracted_k141_69324	1056	No	NA	0	0	Not-determined	Genome-fragment	NA		0
S5_vs_k141_37682||full	1088	No	NA	0	1	Low-quality	Genome-fragment		0.75	0
S9_extracted_k141_58032	1108	No	NA	0	0	Not-determined	Genome-fragment	NA		0
S9_extracted_k141_46788	1152	No	NA	0	1	Not-determined	Genome-fragment	NA		0
S9_extracted_k141_80851	1252	No	NA	0	0	Not-determined	Genome-fragment	NA		0
S9_extracted_k141_76769	98250	No	NA	14	2	High-quality	High-quality		97.31	0
S5_vs_k141_35018||lt2gene	1088	No	NA	2	0	Low-quality	Genome-fragment		1.72	0
S7_extracted_84334	1088	No	NA	0	0	Low-quality	Genome-fragment		0.59	0
S9_extracted_k141_69385	1019	No	NA	0	1	Not-determined	Genome-fragment	NA		0
S9_extracted_k141_76779	2860	No	NA	0	0	Not-determined	Genome-fragment	NA		0
S9_extracted_k141_76783	1312	No	NA	0	1	Not-determined	Genome-fragment	NA		0
S7_vs_14857||full	1088	No	NA	0	0	Low-quality	Genome-fragment		0.74	0
S7_vs_47775||full	1088	No	NA	2	0	Low-quality	Genome-fragment		2.81	0
S9_extracted_k141_5316	1047	No	NA	0	1	Not-determined	Genome-fragment	NA		0
S9_extracted_k141_65780	1086	No	NA	0	1	Not-determined	Genome-fragment	NA		0
S9_extracted_k141_39537	1349	No	NA	0	0	Not-determined	Genome-fragment	NA		0
S10_extracted_18947	1088	No	NA	0	0	Low-quality	Genome-fragment		0.64	0
S9_extracted_k141_20447	1121	No	NA	0	1	Not-determined	Genome-fragment	NA		0
S2_extracted_k141_11048	1088	No	NA	0	0	Low-quality	Genome-fragment		1.13	0
S8_extracted_k141_63358	1088	No	NA	0	0	Low-quality	Genome-fragment		1.87	0
S8_extracted_k141_111334	1088	No	NA	1	0	Low-quality	Genome-fragment		2.58	0
S9_vs_k141_83371||full	1089	No	NA	2	0	Low-quality	Genome-fragment		2.69	0
S9_extracted_k141_32126	2334	No	NA	0	1	Not-determined	Genome-fragment	NA		0
S9_extracted_k141_20491	1409	No	NA	0	1	Not-determined	Genome-fragment	NA		0
S9_extracted_k141_28093	1178	No	NA	0	1	Not-determined	Genome-fragment	NA		0
S9_extracted_k141_73520	1346	No	NA	0	3	Not-determined	Genome-fragment	NA		0
S9_extracted_k141_35749	1357	No	NA	0	2	Not-determined	Genome-fragment	NA		0
S9_extracted_k141_28105	1143	No	NA	0	1	Not-determined	Genome-fragment	NA		0
S11_extracted_k141_22454	1089	No	NA	0	0	Low-quality	Genome-fragment		0.83	0
S11_vs_k141_86436||lt2gene	1089	No	NA	1	0	Low-quality	Genome-fragment		2.52	0
S14_vs_k141_81224||full	1089	No	NA	3	0	Low-quality	Genome-fragment		3.59	0
S9_extracted_k141_32180	1605	No	NA	0	2	Not-determined	Genome-fragment	NA		0
S14_vs_k141_44606||full	1089	No	NA	0	0	Low-quality	Genome-fragment		1.51	0
S9_extracted_k141_32195	1839	No	NA	0	1	Not-determined	Genome-fragment	NA		0
S9_extracted_k141_16931	1551	No	NA	0	2	Not-determined	Genome-fragment	NA		0
S9_extracted_k141_46824	1071	No	NA	0	2	Not-determined	Genome-fragment	NA		0
S9_extracted_k141_5431	4359	No	NA	0	1	Not-determined	Genome-fragment	NA		0
S14_vs_k141_18923||lt2gene	1089	No	NA	1	0	Low-quality	Genome-fragment		1.59	0
S1_vs_k141_106704||full	1089	No	NA	1	0	Low-quality	Genome-fragment		2.09	0
S3_vs_k141_39815||full	1089	No	NA	0	0	Low-quality	Genome-fragment		1.93	0
S5_extracted_k141_139293	1089	No	NA	0	0	Low-quality	Genome-fragment		1.93	0
S5_vs_k141_117021||lt2gene	1089	No	NA	2	0	Low-quality	Genome-fragment		3.15	0
S9_extracted_k141_12952	3697	No	NA	0	1	Not-determined	Genome-fragment	NA		0
S9_extracted_k141_32250	1856	No	NA	0	1	Not-determined	Genome-fragment	NA		0
S7_vs_21835||full	1089	No	NA	0	1	Low-quality	Genome-fragment		2.54	0
S9_extracted_k141_54703	1857	No	NA	0	0	Not-determined	Genome-fragment	NA		0
S9_extracted_k141_12961	1347	No	NA	0	1	Not-determined	Genome-fragment	NA		0
S7_vs_91359||full	1089	No	NA	1	0	Low-quality	Genome-fragment		1.59	0
S9_extracted_k141_39684	1198	No	NA	0	0	Not-determined	Genome-fragment	NA		0
S9_extracted_k141_69601	2134	No	NA	0	5	Not-determined	Genome-fragment	NA		0
S9_extracted_k141_17030	1425	No	NA	0	0	Not-determined	Genome-fragment	NA		0
S7_vs_131933||full	1089	No	NA	1	0	Low-quality	Genome-fragment		2.38	0
S9_extracted_k141_28205	1353	No	NA	0	0	Not-determined	Genome-fragment	NA		0
S9_extracted_k141_76974	1317	No	NA	0	0	Not-determined	Genome-fragment	NA		0
S9_extracted_k141_35860	1587	No	NA	0	3	Not-determined	Genome-fragment	NA		0
S10_vs_68920||full	1089	No	NA	0	0	Low-quality	Genome-fragment		1.95	0
S9_extracted_k141_5516	1055	No	NA	0	1	Not-determined	Genome-fragment	NA		0
S8_extracted_k141_84226	1089	No	NA	0	1	Low-quality	Genome-fragment		2.43	0
S8_vs_k141_86922||full	1089	No	NA	0	0	Low-quality	Genome-fragment		1.86	0
S8_vs_k141_94645||full	1089	No	NA	0	0	Low-quality	Genome-fragment		1.17	0
S9_extracted_k141_69697	1415	No	NA	0	0	Not-determined	Genome-fragment	NA		0
S1_extracted_k141_66744	1090	No	NA	0	0	Low-quality	Genome-fragment		0.62	0
S1_vs_k141_154793||full	1090	No	NA	1	0	Low-quality	Genome-fragment		1.81	0
S9_extracted_k141_28299	6409	No	NA	0	2	Not-determined	Genome-fragment	NA		0
S9_extracted_k141_32322	3137	No	NA	0	2	Not-determined	Genome-fragment	NA		0
S9_extracted_k141_28300	1138	No	NA	0	1	Not-determined	Genome-fragment	NA		0
S1_vs_k141_98940||full	1090	No	NA	1	0	Low-quality	Genome-fragment		2.9	0
S9_extracted_k141_58259	2127	No	NA	0	1	Not-determined	Genome-fragment	NA		0

S9_extracted_k141_9136	1429	No	NA		0	1	Not-determined	Genome-fragment	NA		0
S9_extracted_k141_81186	1262	No	NA		0	1	Not-determined	Genome-fragment	NA		0
S9_extracted_k141_28347	1001	No	NA		0	1	Not-determined	Genome-fragment	NA		0
S3_extracted_k141_104533	1090	No	NA		0	1	Low-quality	Genome-fragment		1.98	0
S9_extracted_k141_17185	3341	No	NA		0	0	Not-determined	Genome-fragment	NA		0
S9_extracted_k141_81215	3209	No	NA		0	2	Not-determined	Genome-fragment	NA		0
S9_extracted_k141_1891	1675	No	NA		0	0	Not-determined	Genome-fragment	NA		0
S9_extracted_k141_5661	1421	No	NA		0	0	Not-determined	Genome-fragment	NA		0
S9_extracted_k141_20707	1316	No	NA		0	0	Not-determined	Genome-fragment	NA		0
S5_vs_k141_7647||lt2gene	1090	No	NA		1	0	Low-quality	Genome-fragment		1.83	0
S7_vs_56769||full	1090	No	NA		1	0	Low-quality	Genome-fragment		2.71	0
S7_vs_130938||full	1090	No	NA		0	0	Low-quality	Genome-fragment		1.87	0
S16_extracted_12819	1090	No	NA		1	0	Low-quality	Genome-fragment		22.84	0
S6_vs_k141_51975||full	1090	No	NA		1	0	Low-quality	Genome-fragment		2.84	0
S8_vs_k141_26094||full	1090	No	NA		0	0	Low-quality	Genome-fragment		2.58	0
S9_extracted_k141_43672	1195	No	NA		0	1	Not-determined	Genome-fragment	NA		0
S9_extracted_k141_58362	1473	No	NA		0	1	Not-determined	Genome-fragment	NA		0
S8_vs_k141_207170||full	1090	No	NA		0	0	Low-quality	Genome-fragment		2.45	0
S9_extracted_k141_65917	5221	No	NA		0	2	Not-determined	Genome-fragment	NA		0
S9_extracted_k141_43686	3599	No	NA		0	0	Not-determined	Genome-fragment	NA		0
S9_extracted_k141_24523	1936	No	NA		0	2	Not-determined	Genome-fragment	NA		0
S9_extracted_k141_73675	1711	No	NA		0	0	Not-determined	Genome-fragment	NA		0
S9_extracted_k141_43710	1328	No	NA		0	0	Not-determined	Genome-fragment	NA		0
S9_vs_k141_88793||full	1091	No	NA		0	0	Low-quality	Genome-fragment		2.58	0
S9_extracted_k141_1965	1319	No	NA		0	0	Not-determined	Genome-fragment	NA		0
S9_extracted_k141_20781	1010	No	NA		0	0	Not-determined	Genome-fragment	NA		0
S1_extracted_k141_81236	1091	No	NA		0	1	Low-quality	Genome-fragment		0.52	0
S9_extracted_k141_13186	1026	No	NA		0	0	Not-determined	Genome-fragment	NA		0
S1_vs_k141_121073||full	1091	No	NA		0	0	Low-quality	Genome-fragment		2.31	0
S9_extracted_k141_9190	1123	No	NA		0	0	Not-determined	Genome-fragment	NA		0
S1_vs_k141_53319||full	1091	No	NA		1	0	Low-quality	Genome-fragment		2.73	0
S9_extracted_k141_32388	1336	No	NA		0	0	Not-determined	Genome-fragment	NA		0
S9_extracted_k141_39992	1085	No	NA		0	2	Not-determined	Genome-fragment	NA		0
S9_extracted_k141_73708	1206	No	NA		0	1	Not-determined	Genome-fragment	NA		0
S5_extracted_k141_22457	1091	No	NA		2	0	Low-quality	Genome-fragment		2.46	0
S9_extracted_k141_5709	1769	No	NA		0	1	Not-determined	Genome-fragment	NA		0
S9_extracted_k141_81333	3956	No	NA		0	0	Not-determined	Genome-fragment	NA		0
S5_vs_k141_138270||full	1091	No	NA		0	1	Low-quality	Genome-fragment		1.48	0
S9_extracted_k141_88842	1211	No	NA		0	1	Not-determined	Genome-fragment	NA		0
S9_extracted_k141_69945	3312	No	NA		0	2	Not-determined	Genome-fragment	NA		0
S9_extracted_k141_47102	1964	No	NA		0	0	Not-determined	Genome-fragment	NA		0
S9_extracted_k141_20844	1130	No	NA		0	0	Not-determined	Genome-fragment	NA		0
S9_extracted_k141_81345	3301	No	NA		0	0	Not-determined	Genome-fragment	NA		0
S7_vs_9310||full	1091	No	NA		0	0	Low-quality	Genome-fragment		2.16	0
S9_extracted_k141_58446	5423	No	NA		0	0	Not-determined	Genome-fragment	NA		0
S9_extracted_k141_77294	1358	No	NA		0	1	Not-determined	Genome-fragment	NA		0
S9_extracted_k141_17375	1179	No	NA		0	0	Not-determined	Genome-fragment	NA		0
S9_extracted_k141_55106	1365	No	NA		0	0	Not-determined	Genome-fragment	NA		0
S9_extracted_k141_24658	1393	No	NA		0	1	Not-determined	Genome-fragment	NA		0
S9_extracted_k141_20880	1664	No	NA		0	0	Not-determined	Genome-fragment	NA		0
S16_extracted_39772	1091	No	NA		0	0	Low-quality	Genome-fragment		2.32	0
S9_extracted_k141_32477	1281	No	NA		0	0	Not-determined	Genome-fragment	NA		0
S10_vs_95651||full	1091	No	NA		0	0	Low-quality	Genome-fragment		3.22	0
S9_extracted_k141_77343	1117	No	NA		0	0	Not-determined	Genome-fragment	NA		0
S9_extracted_k141_24691	1155	No	NA		0	1	Not-determined	Genome-fragment	NA		0
S12_vs_k141_28671||full	1091	No	NA		0	0	Low-quality	Genome-fragment		2.33	0
S12_vs_k141_154828||full	1091	No	NA		0	2	Low-quality	Genome-fragment		1.47	0
S9_extracted_k141_13319	1419	No	NA		0	0	Not-determined	Genome-fragment	NA		0
S9_extracted_k141_32500	2362	No	NA		0	0	Not-determined	Genome-fragment	NA		0
S9_extracted_k141_55146	1491	No	NA		0	0	Not-determined	Genome-fragment	NA		0
S8_vs_k141_53768||lt2gene	1091	No	NA		1	0	Low-quality	Genome-fragment		1.82	0
S17_extracted_k141_19736	1092	No	NA		0	1	Low-quality	Genome-fragment		0.47	0
S9_extracted_k141_88874	1602	No	NA		0	0	Not-determined	Genome-fragment	NA		0
S9_extracted_k141_24707	1280	No	NA		0	1	Not-determined	Genome-fragment	NA		0
S9_extracted_k141_66045	1228	No	NA		0	1	Not-determined	Genome-fragment	NA		0
S9_extracted_k141_5826	1143	No	NA		0	2	Not-determined	Genome-fragment	NA		0
S17_vs_k141_42995||lt2gene	1092	No	NA		0	0	Low-quality	Genome-fragment		2.54	0
S11_vs_k141_78395||full	1092	No	NA		1	0	Low-quality	Genome-fragment		0.58	0
S9_extracted_k141_47228	1779	No	NA		0	1	Not-determined	Genome-fragment	NA		0
S9_extracted_k141_17441	1874	No	NA		0	0	Not-determined	Genome-fragment	NA		0
S11_vs_k141_87340||full	1092	No	NA		0	0	Low-quality	Genome-fragment		2.24	0
S9_extracted_k141_24749	3918	No	NA		0	0	Not-determined	Genome-fragment	NA		0
S11_vs_k141_30414||full	1092	No	NA		0	0	Low-quality	Genome-fragment		2.77	0
S9_extracted_k141_43907	1720	No	NA		0	1	Not-determined	Genome-fragment	NA		0
S14_vs_k141_110103||full	1092	No	NA		2	0	Low-quality	Genome-fragment		3.53	0
S5_extracted_k141_110159	1092	No	NA		3	0	Low-quality	Genome-fragment		3.26	0
S9_extracted_k141_28639	1107	No	NA		0	2	Not-determined	Genome-fragment	NA		0
S9_extracted_k141_36220	1134	No	NA		0	2	Not-determined	Genome-fragment	NA		0
S16_vs_70414||full	1092	No	NA		1	0	Low-quality	Genome-fragment		1.83	0
S9_extracted_k141_47276	1116	No	NA		0	1	Not-determined	Genome-fragment	NA		0
S9_extracted_k141_55187	4387	No	NA		0	1	Not-determined	Genome-fragment	NA		0
S16_vs_97670||full	1092	No	NA		1	0	Low-quality	Genome-fragment		2.2	0
S16_vs_156329||lt2gene	1092	No	NA		1	0	Low-quality	Genome-fragment		1.95	0
S10_extracted_8952	1092	No	NA		0	0	Low-quality	Genome-fragment		2.29	0
S12_vs_k141_166976||full	1092	No	NA		1	0	Low-quality	Genome-fragment		2.65	0
S12_vs_k141_21664||full	1092	No	NA		1	0	Low-quality	Genome-fragment		1.76	0
S15_extracted_13793	1092	No	NA		0	0	Low-quality	Genome-fragment		0.75	0
S9_extracted_k141_2108	1667	No	NA		0	1	Not-determined	Genome-fragment	NA		0
S2_extracted_k141_14227	1092	No	NA		1	0	Low-quality	Genome-fragment		1.77	0
S11_vs_k141_75722||full	1093	No	NA		1	0	Low-quality	Genome-fragment		1.64	0
S9_extracted_k141_66139	1152	No	NA		0	0	Not-determined	Genome-fragment	NA		0
S9_extracted_k141_66159	1780	No	NA		0	1	Not-determined	Genome-fragment	NA		0
S9_extracted_k141_89028	1195	No	NA		0	1	Not-determined	Genome-fragment	NA		0
S9_extracted_k141_13504	1489	No	NA		0	1	Not-determined	Genome-fragment	NA		0
S9_extracted_k141_40283	1253	No	NA		0	1	Not-determined	Genome-fragment	NA		0
S9_extracted_k141_5957	3815	No	NA		0	0	Not-determined	Genome-fragment	NA		0
S13_extracted_82067	1093	No	NA		0	0	Low-quality	Genome-fragment		1.69	0
S9_extracted_k141_5963	2157	No	NA		0	1	Not-determined	Genome-fragment	NA		0
S9_extracted_k141_24881	1492	No	NA		0	1	Not-determined	Genome-fragment	NA		0
S9_extracted_k141_28771	2714	No	NA		0	0	Not-determined	Genome-fragment	NA		0
S9_extracted_k141_81547	1188	No	NA		0	1	Not-determined	Genome-fragment	NA		0
S14_vs_k141_104029||full	1093	No	NA		0	0	Low-quality	Genome-fragment		1.77	0
S14_vs_k141_31367||full	1093	No	NA		1	0	Low-quality	Genome-fragment		2.6	0
S9_extracted_k141_28794	1055	No	NA		0	0	Not-determined	Genome-fragment	NA		0
S1_extracted_k141_86381	1093	No	NA		0	1	Low-quality	Genome-fragment		2.6	0
S9_extracted_k141_36375	3496	No	NA		0	3	Not-determined	Genome-fragment	NA		0
S9_extracted_k141_44101	2428	No	NA		0	0	Not-determined	Genome-fragment	NA		0
S9_extracted_k141_2221	2081	No	NA		0	1	Not-determined	Genome-fragment	NA		0
S9_extracted_k141_85234	1514	No	NA		0	0	Not-determined	Genome-fragment	NA		0
S9_extracted_k141_62736	1353	No	NA		0	0	Not-determined	Genome-fragment	NA		0
S9_extracted_k141_81631	1180	No	NA		0	2	Not-determined	Genome-fragment	NA		0
S9_extracted_k141_89107	3673	No	NA		0	0	Not-determined	Genome-fragment	NA		0
S1_vs_k141_85869||lt2gene	1093	No	NA		2	0	Low-quality	Genome-fragment		2.26	0
S9_extracted_k141_66213	1204	No	NA		0	0	Not-determined	Genome-fragment	NA		0
S9_extracted_k141_36433	1069	No	NA		0	0	Not-determined	Genome-fragment	NA		0
S9_extracted_k141_9600	1771	No	NA		0	1	Not-determined	Genome-fragment	NA		0
S3_extracted_k141_25462	1093	No	NA		1	0	Low-quality	Genome-fragment		1.26	0
S9_extracted_k141_36461	1451	No	NA		0	0	Not-determined	Genome-fragment	NA		0
S9_extracted_k141_70236	15768	No	NA		10	0	Medium-quality	Genome-fragment		67.84	0
S9_extracted_k141_74038	2447	No	NA		0	1	Not-determined	Genome-fragment	NA		0
S9_extracted_k141_28923	1049	No	NA		0	1	Not-determined	Genome-fragment	NA		0
S3_vs_k141_27693||full	1093	No	NA		0	0	Low-quality	Genome-fragment		1.37	0
S16_extracted_168173	1093	No	NA		1	0	Low-quality	Genome-fragment		1	0
S16_vs_44425||full	1093	No	NA		1	0	Low-quality	Genome-fragment		2.26	0
S9_extracted_k141_81682	1003	No	NA		0	0	Not-determined	Genome-fragment	NA		0
S16_vs_108796||full	1093	No	NA		1	0	Low-quality	Genome-fragment		1.76	0
S9_extracted_k141_55400	1430	No	NA		0	0	Not-determined	Genome-fragment	NA		0
S9_extracted_k141_9674	1550	No	NA		0	1	Not-determined	Genome-fragment	NA		0
S9_extracted_k141_66331	1145	No	NA		0	0	Not-determined	Genome-fragment	NA		0
S16_vs_129364||full	1093	No	NA		0	0	Low-quality	Genome-fragment		1.48	0
S9_extracted_k141_50888	13484	No	NA		0	4	Not-determined	Genome-fragment	NA		0
S9_extracted_k141_17723	1078	No	NA		0	1	Not-determined	Genome-fragment	NA		0
S9_extracted_k141_74117	2399	No	NA		0	4	Not-determined	Genome-fragment	NA		0
S9_extracted_k141_36576	1313	No	NA		0	0	Not-determined	Genome-fragment	NA		0
S9_extracted_k141_9718	1058	No	NA		0	1	Not-determined	Genome-fragment	NA		0
S6_vs_k141_9436||full	1093	No	NA		0	0	Low-quality	Genome-fragment		2.79	0
S9_extracted_k141_58822	3940	No	NA		0	1	Not-determined	Genome-fragment	NA		0
S8_vs_k141_199717||full	1093	No	NA		0	1	Low-quality	Genome-fragment		0.48	0
S9_extracted_k141_9741	1061	No	NA		0	1	Not-determined	Genome-fragment	NA		0
S9_extracted_k141_58845	1357	No	NA		0	2	Not-determined	Genome-fragment	NA		0
S9_extracted_k141_21337	1338	No	NA		0	1	Not-determined	Genome-fragment	NA		0
S9_extracted_k141_81753	1096	No	NA		0	0	Not-determined	Genome-fragment	NA		0
S9_extracted_k141_36623	2760	No	NA		0	0	Not-determined	Genome-fragment	NA		0
S8_vs_k141_20591||full	1093	No	NA		0	0	Low-quality	Genome-fragment		0.63	0
S9_extracted_k141_55474	4966	No	NA		0	1	Not-determined	Genome-fragment	NA		0
S9_vs_k141_84055||lt2gene	1094	No	NA		2	0	Low-quality	Genome-fragment		1.63	0
S13_extracted_940	1094	No	NA		0	0	Low-quality	Genome-fragment		0.66	0
S13_extracted_144109	1094	No	NA		0	0	Low-quality	Genome-fragment		0.51	0
S9_extracted_k141_13507	231917	Yes		109327	18	73	Complete	High-quality		100	52.86
S14_extracted_k141_70514	1094	No	NA		1	0	Low-quality	Genome-fragment		2.5	0
S1_vs_k141_29331||full	1094	No	NA		0	1	Low-quality	Genome-fragment		0.84	0
S1_vs_k141_57328||full	1094	No	NA		0	2	Low-quality	Genome-fragment		0.39	0
S1_vs_k141_106644||full	1094	No	NA		1	0	Low-quality	Genome-fragment		1.78	0
S9_extracted_k141_36665	1460	No	NA		0	2	Not-determined	Genome-fragment	NA		0
S9_extracted_k141_9790	1867	No	NA		0	0	Not-determined	Genome-fragment	NA		0
S3_vs_k141_106322||full	1094	No	NA		3	0	Low-quality	Genome-fragment		1.81	0
S9_extracted_k141_21444	2754	No	NA		0	2	Not-determined	Genome-fragment	NA		0
S9_extracted_k141_50939	1055	No	NA		0	1	Not-determined	Genome-fragment	NA		0
S9_extracted_k141_6287	6831	No	NA		0	4	Not-determined	Genome-fragment	NA		0
S9_extracted_k141_25217	1127	No	NA		0	1	Not-determined	Genome-fragment	NA		0
S5_extracted_k141_71637	1094	No	NA		2	0	Low-quality	Genome-fragment		2.63	0
S9_extracted_k141_47539	1078	No	NA		0	1	Not-determined	Genome-fragment	NA		0
S9_extracted_k141_58958	1108	No	NA		0	1	Not-determined	Genome-fragment	NA		0
S7_extracted_129409	1094	No	NA		0	0	Low-quality	Genome-fragment		0.65	0
S16_extracted_34442	1094	No	NA		0	1	Low-quality	Genome-fragment		1.5	0
S9_extracted_k141_36717	1516	No	NA		0	1	Not-determined	Genome-fragment	NA		0
S9_extracted_k141_6319	3370	No	NA		0	2	Not-determined	Genome-fragment	NA		0
S9_extracted_k141_25234	25733	No	NA		0	11	Not-determined	Genome-fragment	NA		0
S9_extracted_k141_47602	1959	No	NA		0	0	Not-determined	Genome-fragment	NA		0
S9_extracted_k141_36771	1366	No	NA		0	0	Not-determined	Genome-fragment	NA		0
S9_extracted_k141_2574	1220	No	NA		0	3	Not-determined	Genome-fragment	NA		0
S16_vs_153524||full	1094	No	NA		1	0	Low-quality	Genome-fragment		2.95	0
S9_extracted_k141_32827	1033	No	NA		0	0	Not-determined	Genome-fragment	NA		0
S9_extracted_k141_29205	2373	No	NA		0	1	Not-determined	Genome-fragment	NA		0

S9_extracted_k141_66576	1168	No	NA	0	0	Not-determined	Genome-fragment	NA		0
S9_extracted_k141_29215	3433	No	NA	0	2	Not-determined	Genome-fragment	NA		0
S10_extracted_78510	1094	No	NA	1	0	Low-quality	Genome-fragment		1.74	0
S9_extracted_k141_59039	5248	No	NA	0	5	Not-determined	Genome-fragment	NA		0
S9_extracted_k141_9947	1097	No	NA	0	1	Not-determined	Genome-fragment	NA		0
S10_vs_77518||lt2gene	1094	No	NA	1	0	Low-quality	Genome-fragment		1.14	0
S12_extracted_k141_101704	1094	No	NA	0	1	Low-quality	Genome-fragment		0.5	0
S9_extracted_k141_66585	1352	No	NA	0	2	Not-determined	Genome-fragment	NA		0
S9_extracted_k141_62967	3466	No	NA	0	5	Not-determined	Genome-fragment	NA		0
S9_extracted_k141_6410	1435	No	NA	0	1	Not-determined	Genome-fragment	NA		0
S9_extracted_k141_74265	4099	No	NA	0	4	Not-determined	Genome-fragment	NA		0
S9_extracted_k141_89246	1182	No	NA	0	2	Not-determined	Genome-fragment	NA		0
S9_extracted_k141_59098	1030	No	NA	0	0	Not-determined	Genome-fragment	NA		0
S9_extracted_k141_13711	3319	No	NA	0	1	Not-determined	Genome-fragment	NA		0
S9_extracted_k141_89291	1323	No	NA	0	1	Not-determined	Genome-fragment	NA		0
S9_extracted_k141_2641	1480	No	NA	0	0	Not-determined	Genome-fragment	NA		0
S12_vs_k141_50154||full	1094	No	NA	2	0	Low-quality	Genome-fragment		2.8	0
S9_extracted_k141_32859	1187	No	NA	0	0	Not-determined	Genome-fragment	NA		0
S9_extracted_k141_47706	1327	No	NA	0	0	Not-determined	Genome-fragment	NA		0
S9_extracted_k141_85602	2301	No	NA	0	1	Not-determined	Genome-fragment	NA		0
S8_extracted_k141_55590	1094	No	NA	0	0	Low-quality	Genome-fragment		2.38	0
S9_extracted_k141_13735	1554	No	NA	0	1	Not-determined	Genome-fragment	NA		0
S8_vs_k141_4029||full	1094	No	NA	0	0	Low-quality	Genome-fragment		3.04	0
S8_vs_k141_180306||full	1094	No	NA	0	1	Low-quality	Genome-fragment		1.99	0
S17_extracted_k141_94913	1095	No	NA	0	0	Low-quality	Genome-fragment		0.69	0
S17_vs_k141_91736||lt2gene	1095	No	NA	1	0	Low-quality	Genome-fragment		1.85	0
S9_vs_k141_56398||full	1095	No	NA	0	0	Low-quality	Genome-fragment		1.99	0
S9_extracted_k141_25316	1406	No	NA	0	1	Not-determined	Genome-fragment	NA		0
S9_extracted_k141_32924	1528	No	NA	0	0	Not-determined	Genome-fragment	NA		0
S14_vs_k141_51100||full	1095	No	NA	0	0	Low-quality	Genome-fragment		2.02	0
S9_extracted_k141_10085	2405	No	NA	0	1	Not-determined	Genome-fragment	NA		0
S9_extracted_k141_85681	1045	No	NA	0	1	Not-determined	Genome-fragment	NA		0
S9_extracted_k141_10116	1232	No	NA	0	3	Not-determined	Genome-fragment	NA		0
S9_extracted_k141_74407	3092	No	NA	0	4	Not-determined	Genome-fragment	NA		0
S9_extracted_k141_29344	1160	No	NA	0	0	Not-determined	Genome-fragment	NA		0
S3_extracted_k141_48901	1095	No	NA	0	0	Low-quality	Genome-fragment		3.12	0
S3_vs_k141_75860||lt2gene	1095	No	NA	1	0	Low-quality	Genome-fragment		2.78	0
S9_extracted_k141_55756	1507	No	NA	0	2	Not-determined	Genome-fragment	NA		0
S5_extracted_k141_39607	1095	No	NA	0	0	Low-quality	Genome-fragment		1.83	0
S9_extracted_k141_44594	1641	No	NA	0	0	Not-determined	Genome-fragment	NA		0
S9_extracted_k141_47766	1108	No	NA	0	1	Not-determined	Genome-fragment	NA		0
S9_extracted_k141_29415	1261	No	NA	0	0	Not-determined	Genome-fragment	NA		0
S9_extracted_k141_36980	2754	No	NA	0	1	Not-determined	Genome-fragment	NA		0
S5_vs_k141_63688||full	1095	No	NA	0	0	Low-quality	Genome-fragment		2.85	0
S7_vs_38680||full	1095	No	NA	2	0	Low-quality	Genome-fragment		2.46	0
S9_extracted_k141_33051	1473	No	NA	0	0	Not-determined	Genome-fragment	NA		0
S9_extracted_k141_51061	2998	No	NA	0	0	Not-determined	Genome-fragment	NA		0
S16_extracted_120938	1095	No	NA	2	0	Low-quality	Genome-fragment		2.49	0
S9_extracted_k141_6640	1420	No	NA	0	1	Not-determined	Genome-fragment	NA		0
S9_extracted_k141_70503	1561	No	NA	0	0	Not-determined	Genome-fragment	NA		0
S12_vs_k141_180863||full	1095	No	NA	1	0	Low-quality	Genome-fragment		2.04	0
S9_extracted_k141_47843	2390	No	NA	0	0	Not-determined	Genome-fragment	NA		0
S9_extracted_k141_66782	1131	No	NA	0	0	Not-determined	Genome-fragment	NA		0
S9_extracted_k141_55835	1529	No	NA	0	1	Not-determined	Genome-fragment	NA		0
S9_extracted_k141_82136	2293	No	NA	0	0	Not-determined	Genome-fragment	NA		0
S15_vs_5851||full	1095	No	NA	0	1	Low-quality	Genome-fragment		0.4	0
S6_vs_k141_31308||full	1095	No	NA	0	0	Low-quality	Genome-fragment		2.66	0
S8_extracted_k141_109502	1095	No	NA	0	0	Low-quality	Genome-fragment		0.61	0
S17_extracted_k141_82822	1096	No	NA	0	0	Low-quality	Genome-fragment		2.1	0
S9_extracted_k141_89440	1524	No	NA	0	0	Not-determined	Genome-fragment	NA		0
S11_extracted_k141_78443	1096	No	NA	2	0	Low-quality	Genome-fragment		3.61	0
S9_extracted_k141_85864	4282	No	NA	0	0	Not-determined	Genome-fragment	NA		0
S11_vs_k141_19836||full	1096	No	NA	0	0	Low-quality	Genome-fragment		1.84	0
S9_extracted_k141_14006	1045	No	NA	0	1	Not-determined	Genome-fragment	NA		0
S9_extracted_k141_89460	1507	No	NA	0	0	Not-determined	Genome-fragment	NA		0
S9_extracted_k141_25537	1408	No	NA	0	1	Not-determined	Genome-fragment	NA		0
S13_extracted_105258	1096	No	NA	0	0	Low-quality	Genome-fragment		0.69	0
S9_extracted_k141_66867	1053	No	NA	0	0	Not-determined	Genome-fragment	NA		0
S9_extracted_k141_59321	2315	No	NA	0	0	Not-determined	Genome-fragment	NA		0
S9_extracted_k141_2837	1124	No	NA	0	0	Not-determined	Genome-fragment	NA		0
S9_extracted_k141_89493	1836	No	NA	0	1	Not-determined	Genome-fragment	NA		0
S1_vs_k141_60808||lt2gene	1096	No	NA	1	0	Low-quality	Genome-fragment		3.62	0
S9_extracted_k141_10323	8828	No	NA	0	1	Not-determined	Genome-fragment	NA		0
S5_vs_k141_41401||full	1096	No	NA	0	0	Low-quality	Genome-fragment		0.57	0
S7_extracted_26600	1096	No	NA	1	0	Low-quality	Genome-fragment		0.47	0
S9_extracted_k141_59331	2093	No	NA	0	2	Not-determined	Genome-fragment	NA		0
S9_extracted_k141_37116	1134	No	NA	0	1	Not-determined	Genome-fragment	NA		0
S7_vs_129330||full	1096	No	NA	0	0	Low-quality	Genome-fragment		2.68	0
S9_extracted_k141_89560	1148	No	NA	0	1	Not-determined	Genome-fragment	NA		0
S9_extracted_k141_6729	1076	No	NA	0	1	Not-determined	Genome-fragment	NA		0
S9_extracted_k141_59366	1628	No	NA	0	1	Not-determined	Genome-fragment	NA		0
S9_extracted_k141_40903	1896	No	NA	0	2	Not-determined	Genome-fragment	NA		0
S7_vs_77724||lt2gene	1096	No	NA	1	0	Low-quality	Genome-fragment		2.92	0
S9_extracted_k141_18227	11201	No	NA	0	5	Not-determined	Genome-fragment	NA		0
S9_extracted_k141_55939	1380	No	NA	0	0	Not-determined	Genome-fragment	NA		0
S16_extracted_10465	1096	No	NA	0	1	Low-quality	Genome-fragment		0.82	0
S9_extracted_k141_85975	1604	No	NA	0	0	Not-determined	Genome-fragment	NA		0
S9_extracted_k141_55955	1229	No	NA	0	0	Not-determined	Genome-fragment	NA		0
S10_extracted_80304	1096	No	NA	0	0	Low-quality	Genome-fragment		0.57	0
S9_extracted_k141_89596	4963	No	NA	0	2	Not-determined	Genome-fragment	NA		0
S9_extracted_k141_2932	1163	No	NA	0	1	Not-determined	Genome-fragment	NA		0
S9_extracted_k141_74514	4153	No	NA	0	2	Not-determined	Genome-fragment	NA		0
S10_vs_4693||full	1096	No	NA	0	0	Low-quality	Genome-fragment		2.48	0
S9_extracted_k141_25637	1166	No	NA	0	2	Not-determined	Genome-fragment	NA		0
S9_extracted_k141_44783	1613	No	NA	0	3	Not-determined	Genome-fragment	NA		0
S12_extracted_k141_80715	1096	No	NA	0	0	Low-quality	Genome-fragment		1.91	0
S9_extracted_k141_33323	7178	No	NA	0	3	Not-determined	Genome-fragment	NA		0
S9_extracted_k141_59423	1141	No	NA	0	0	Not-determined	Genome-fragment	NA		0
S9_extracted_k141_10446	1321	No	NA	0	1	Not-determined	Genome-fragment	NA		0
S12_extracted_k141_11943	1096	No	NA	1	0	Low-quality	Genome-fragment		1.54	0
S12_extracted_k141_68087	1096	No	NA	0	0	Low-quality	Genome-fragment		3.43	0
S9_extracted_k141_18346	2750	No	NA	0	0	Not-determined	Genome-fragment	NA		0
S9_extracted_k141_86070	1129	No	NA	0	1	Not-determined	Genome-fragment	NA		0
S9_extracted_k141_51350	2103	No	NA	0	0	Not-determined	Genome-fragment	NA		0
S9_extracted_k141_82302	1008	No	NA	0	1	Not-determined	Genome-fragment	NA		0
S9_extracted_k141_66986	1477	No	NA	0	1	Not-determined	Genome-fragment	NA		0
S9_extracted_k141_82314	1602	No	NA	0	0	Not-determined	Genome-fragment	NA		0
S9_extracted_k141_82326	1000	No	NA	0	0	Not-determined	Genome-fragment	NA		0
S15_extracted_63749	1096	No	NA	2	0	Low-quality	Genome-fragment		3.62	0
S6_vs_k141_44388||full	1096	No	NA	0	0	Low-quality	Genome-fragment		1.73	0
S9_extracted_k141_56109	1038	No	NA	0	0	Not-determined	Genome-fragment	NA		0
S9_extracted_k141_67031	1050	No	NA	0	0	Not-determined	Genome-fragment	NA		0
S8_extracted_k141_168335	1096	No	NA	0	0	Low-quality	Genome-fragment		1.67	0
S9_extracted_k141_70768	1091	No	NA	0	0	Not-determined	Genome-fragment	NA		0
S8_vs_k141_27888||lt2gene	1096	No	NA	1	0	Low-quality	Genome-fragment		2.89	0
S9_extracted_k141_44893	1038	No	NA	0	1	Not-determined	Genome-fragment	NA		0
S9_extracted_k141_74678	1237	No	NA	0	1	Not-determined	Genome-fragment	NA		0
S9_vs_k141_31572||full	1097	No	NA	2	0	Low-quality	Genome-fragment		2.34	0
S9_extracted_k141_48204	1048	No	NA	0	0	Not-determined	Genome-fragment	NA		0
S9_extracted_k141_25827	2303	No	NA	0	1	Not-determined	Genome-fragment	NA		0
S9_vs_k141_63319||full	1097	No	NA	1	0	Low-quality	Genome-fragment		3	0
S9_extracted_k141_10606	1531	No	NA	0	1	Not-determined	Genome-fragment	NA		0
S9_extracted_k141_67104	2332	No	NA	0	0	Not-determined	Genome-fragment	NA		0
S9_extracted_k141_74734	1311	No	NA	0	2	Not-determined	Genome-fragment	NA		0
S9_extracted_k141_18510	1223	No	NA	0	1	Not-determined	Genome-fragment	NA		0
S9_extracted_k141_78266	1660	No	NA	0	1	Not-determined	Genome-fragment	NA		0
S9_extracted_k141_37419	1079	No	NA	0	0	Not-determined	Genome-fragment	NA		0
S13_extracted_108627	1097	No	NA	0	2	Low-quality	Genome-fragment		0.11	0
S9_extracted_k141_25902	1332	No	NA	0	0	Not-determined	Genome-fragment	NA		0
S9_extracted_k141_14135	1011	No	NA	0	0	Not-determined	Genome-fragment	NA		0
S9_extracted_k141_48248	1441	No	NA	0	2	Not-determined	Genome-fragment	NA		0
S1_extracted_k141_123605	1097	No	NA	0	3	Low-quality	Genome-fragment		2.05	0
S1_vs_k141_128139||full	1097	No	NA	0	3	Low-quality	Genome-fragment		0.67	0
S9_extracted_k141_89883	1250	No	NA	0	2	Not-determined	Genome-fragment	NA		0
S1_vs_k141_61111||full	1097	No	NA	0	1	Low-quality	Genome-fragment		1.37	0
S9_extracted_k141_56230	3090	No	NA	0	2	Not-determined	Genome-fragment	NA		0
S1_vs_k141_62162||full	1097	No	NA	0	1	Low-quality	Genome-fragment		1.98	0
S9_extracted_k141_45037	1857	No	NA	0	0	Not-determined	Genome-fragment	NA		0
S9_extracted_k141_29952	1036	No	NA	0	0	Not-determined	Genome-fragment	NA		0
S1_vs_k141_111676||full	1097	No	NA	0	0	Low-quality	Genome-fragment		0.47	0
S3_extracted_k141_44113	1097	No	NA	1	0	Low-quality	Genome-fragment		2.72	0
S9_extracted_k141_70957	1486	No	NA	0	1	Not-determined	Genome-fragment	NA		0
S9_extracted_k141_25973	1010	No	NA	0	0	Not-determined	Genome-fragment	NA		0
S9_extracted_k141_41244	1260	No	NA	0	1	Not-determined	Genome-fragment	NA		0
S9_extracted_k141_25985	3145	No	NA	0	7	Not-determined	Genome-fragment	NA		0
S3_vs_k141_47838||full	1097	No	NA	0	3	Low-quality	Genome-fragment		0.46	0
S9_extracted_k141_22208	2890	No	NA	0	0	Not-determined	Genome-fragment	NA		0
S5_extracted_k141_110548	1097	No	NA	1	0	Low-quality	Genome-fragment		1.73	0
S9_extracted_k141_30069	1269	No	NA	0	0	Not-determined	Genome-fragment	NA		0
S5_vs_k141_4044||full	1097	No	NA	0	0	Low-quality	Genome-fragment		3.2	0
S5_vs_k141_96767||full	1097	No	NA	0	1	Low-quality	Genome-fragment		1.42	0
S9_extracted_k141_59802	1572	No	NA	0	2	Not-determined	Genome-fragment	NA		0
S9_extracted_k141_51517	2672	No	NA	0	0	Not-determined	Genome-fragment	NA		0
S9_extracted_k141_22292	3271	No	NA	0	1	Not-determined	Genome-fragment	NA		0
S7_extracted_66657	1097	No	NA	0	0	Low-quality	Genome-fragment		1.88	0
S16_vs_88094||full	1097	No	NA	0	0	Low-quality	Genome-fragment		1.39	0
S9_extracted_k141_78467	1280	No	NA	0	1	Not-determined	Genome-fragment	NA		0
S10_vs_56023||full	1097	No	NA	0	1	Low-quality	Genome-fragment		0.44	0
S9_extracted_k141_89994	3696	No	NA	0	4	Not-determined	Genome-fragment	NA		0
S9_extracted_k141_41351	1516	No	NA	0	0	Not-determined	Genome-fragment	NA		0
S10_vs_81581||full	1097	No	NA	0	0	Low-quality	Genome-fragment		2.68	0
S12_extracted_k141_156880	1097	No	NA	0	2	Low-quality	Genome-fragment		0.95	0
S9_extracted_k141_26086	4294	No	NA	0	0	Not-determined	Genome-fragment	NA		0
S9_extracted_k141_14270	4183	No	NA	0	1	Not-determined	Genome-fragment	NA		0
S15_vs_53867||full	1097	No	NA	0	0	Low-quality	Genome-fragment		2.04	0
S9_extracted_k141_90019	3373	No	NA	0	2	Not-determined	Genome-fragment	NA		0
S4_extracted_2088	1097	No	NA	4	0	Low-quality	Genome-fragment		3.62	0
S9_extracted_k141_26118	1335	No	NA	0	0	Not-determined	Genome-fragment	NA		0
S9_extracted_k141_26125	1232	No	NA	0	0	Not-determined	Genome-fragment	NA		0
S9_extracted_k141_67316	1519	No	NA	0	0	Not-determined	Genome-fragment	NA		0
S8_vs_k141_183208||full	1097	No	NA	0	0	Low-quality	Genome-fragment		1.79	0
S9_extracted_k141_67326	1259	No	NA	0	0	Not-determined	Genome-fragment	NA		0
S9_extracted_k141_51641	1004	No	NA	0	2	Not-determined	Genome-fragment	NA		0
S9_extracted_k141_75056	1150	No	NA	0	2	Not-determined	Genome-fragment	NA		0

S9_extracted_k141_14345	4486	No	NA	0	4	Not-determined	Genome-fragment	NA		0
S9_extracted_k141_30226	4981	No	NA	0	7	Not-determined	Genome-fragment	NA		0
S8_vs_k141_23594||full	1097	No	NA	0	0	Low-quality	Genome-fragment		0.7	0
S9_extracted_k141_30228	1207	No	NA	0	1	Not-determined	Genome-fragment	NA		0
S8_vs_k141_50893||full	1097	No	NA	1	0	Low-quality	Genome-fragment		3.72	0
S17_extracted_k141_84151	1098	No	NA	0	1	Low-quality	Genome-fragment		1.3	0
S9_extracted_k141_3412	1411	No	NA	0	1	Not-determined	Genome-fragment	NA		0
S9_extracted_k141_78642	1126	No	NA	0	0	Not-determined	Genome-fragment	NA		0
S9_extracted_k141_33829	1031	No	NA	0	0	Not-determined	Genome-fragment	NA		0
S9_extracted_k141_56487	1288	No	NA	0	2	Not-determined	Genome-fragment	NA		0
S9_extracted_k141_56492	1284	No	NA	0	1	Not-determined	Genome-fragment	NA		0
S9_extracted_k141_45370	2473	No	NA	0	2	Not-determined	Genome-fragment	NA		0
S17_extracted_k141_102256	1098	No	NA	0	0	Low-quality	Genome-fragment		1.17	0
S9_extracted_k141_67438	2000	No	NA	0	1	Not-determined	Genome-fragment	NA		0
S9_extracted_k141_3435	1002	No	NA	0	1	Not-determined	Genome-fragment	NA		0
S9_extracted_k141_67454	1903	No	NA	0	0	Not-determined	Genome-fragment	NA		0
S9_extracted_k141_22480	1092	No	NA	0	1	Not-determined	Genome-fragment	NA		0
S9_extracted_k141_86490	1150	No	NA	0	1	Not-determined	Genome-fragment	NA		0
S9_extracted_k141_90195	1259	No	NA	0	0	Not-determined	Genome-fragment	NA		0
S9_extracted_k141_67476	1634	No	NA	0	0	Not-determined	Genome-fragment	NA		0
S14_extracted_k141_60170	1098	No	NA	0	0	Low-quality	Genome-fragment		2.3	0
S14_vs_k141_105888||lt2gene	1098	No	NA	1	0	Low-quality	Genome-fragment		1.2	0
S9_extracted_k141_86527	1035	No	NA	0	0	Not-determined	Genome-fragment	NA		0
S1_vs_k141_28261||full	1098	No	NA	0	0	Low-quality	Genome-fragment		3.28	0
S1_vs_k141_145089||full	1098	No	NA	1	0	Low-quality	Genome-fragment		2.54	0
S3_vs_k141_39268||full	1098	No	NA	1	0	Low-quality	Genome-fragment		1.75	0
S9_extracted_k141_67530	11239	No	NA	0	1	Not-determined	Genome-fragment	NA		0
S9_extracted_k141_51656	128272	No	NA	12	0	Complete	High-quality		100	0
S9_extracted_k141_75235	1699	No	NA	0	1	Not-determined	Genome-fragment	NA		0
S9_extracted_k141_56596	1883	No	NA	0	1	Not-determined	Genome-fragment	NA		0
S5_extracted_k141_121134	1098	No	NA	1	0	Low-quality	Genome-fragment		2.94	0
S9_extracted_k141_56605	1353	No	NA	0	0	Not-determined	Genome-fragment	NA		0
S9_extracted_k141_86572	3424	No	NA	0	2	Not-determined	Genome-fragment	NA		0
S5_extracted_k141_137369	1098	No	NA	0	0	Low-quality	Genome-fragment		0.36	0
S7_extracted_127051	1098	No	NA	1	0	Low-quality	Genome-fragment		2.39	0
S9_extracted_k141_22636	2174	No	NA	0	0	Not-determined	Genome-fragment	NA		0
S9_extracted_k141_3532	1285	No	NA	0	0	Not-determined	Genome-fragment	NA		0
S9_extracted_k141_56633	3319	No	NA	0	1	Not-determined	Genome-fragment	NA		0
S9_extracted_k141_60099	1110	No	NA	0	0	Not-determined	Genome-fragment	NA		0
S16_extracted_136437	1098	No	NA	0	1	Low-quality	Genome-fragment		2.75	0
S9_extracted_k141_48578	1502	No	NA	0	0	Not-determined	Genome-fragment	NA		0
S9_extracted_k141_56645	1484	No	NA	0	0	Not-determined	Genome-fragment	NA		0
S9_extracted_k141_11138	3105	No	NA	0	2	Not-determined	Genome-fragment	NA		0
S12_vs_k141_150035||full	1098	No	NA	0	0	Low-quality	Genome-fragment		4.41	0
S12_vs_k141_97001||full	1098	No	NA	0	0	Low-quality	Genome-fragment		3.06	0
S9_extracted_k141_60119	1375	No	NA	0	2	Not-determined	Genome-fragment	NA		0
S9_extracted_k141_56671	1006	No	NA	0	1	Not-determined	Genome-fragment	NA		0
S9_extracted_k141_3575	4004	No	NA	0	2	Not-determined	Genome-fragment	NA		0
S12_vs_k141_141236||lt2gene	1098	No	NA	1	0	Low-quality	Genome-fragment		2.63	0
S8_extracted_k141_27949	1098	No	NA	0	0	Low-quality	Genome-fragment		1.09	0
S8_vs_k141_84928||full	1098	No	NA	0	0	Low-quality	Genome-fragment		1.96	0
S9_extracted_k141_48607	1272	No	NA	0	0	Not-determined	Genome-fragment	NA		0
S9_extracted_k141_78974	1142	No	NA	0	0	Not-determined	Genome-fragment	NA		0
S9_extracted_k141_71609	1107	No	NA	0	0	Not-determined	Genome-fragment	NA		0
S9_extracted_k141_75363	12595	No	NA	7	1	Medium-quality	Genome-fragment		53.41	0
S17_vs_k141_36429||lt2gene	1099	No	NA	1	0	Low-quality	Genome-fragment		19.85	0
S9_vs_k141_20745||full	1099	No	NA	0	0	Low-quality	Genome-fragment		1.03	0
S11_vs_k141_102790||full	1099	No	NA	0	0	Low-quality	Genome-fragment		1.33	0
S9_extracted_k141_56791	1364	No	NA	0	1	Not-determined	Genome-fragment	NA		0
S9_extracted_k141_82808	1046	No	NA	0	0	Not-determined	Genome-fragment	NA		0
S9_extracted_k141_11316	1025	No	NA	0	0	Not-determined	Genome-fragment	NA		0
S9_extracted_k141_3716	1591	No	NA	0	1	Not-determined	Genome-fragment	NA		0
S3_extracted_k141_60395	1099	No	NA	0	0	Low-quality	Genome-fragment		2.17	0
S5_vs_k141_30480||full	1099	No	NA	1	0	Low-quality	Genome-fragment		2.42	0
S5_vs_k141_18084||full	1099	No	NA	0	0	Low-quality	Genome-fragment		1.47	0
S9_extracted_k141_86860	2634	No	NA	0	7	Not-determined	Genome-fragment	NA		0
S7_vs_1637||full	1099	No	NA	1	0	Low-quality	Genome-fragment		1.22	0
S9_extracted_k141_60357	1184	No	NA	0	2	Not-determined	Genome-fragment	NA		0
S9_extracted_k141_75457	1058	No	NA	0	1	Not-determined	Genome-fragment	NA		0
S9_extracted_k141_79101	1043	No	NA	0	0	Not-determined	Genome-fragment	NA		0
S7_vs_112865||full	1099	No	NA	1	0	Low-quality	Genome-fragment		2.8	0
S9_extracted_k141_82928	1209	No	NA	0	2	Not-determined	Genome-fragment	NA		0
S16_extracted_122732	1099	No	NA	1	0	Low-quality	Genome-fragment		1.73	0
S16_extracted_129711	1099	No	NA	0	0	Low-quality	Genome-fragment		4.31	0
S9_extracted_k141_48824	3182	No	NA	0	0	Not-determined	Genome-fragment	NA		0
S9_extracted_k141_71753	1169	No	NA	0	1	Not-determined	Genome-fragment	NA		0
S12_vs_k141_65584||full	1099	No	NA	0	0	Low-quality	Genome-fragment		0.95	0
S8_extracted_k141_19081	1099	No	NA	0	1	Low-quality	Genome-fragment		2.12	0
S9_extracted_k141_14958	1097	No	NA	0	1	Not-determined	Genome-fragment	NA		0
S9_extracted_k141_83016	2056	No	NA	0	0	Not-determined	Genome-fragment	NA		0
S8_vs_k141_147359||full	1099	No	NA	0	0	Low-quality	Genome-fragment		0.6	0
S9_extracted_k141_75608	1733	No	NA	0	2	Not-determined	Genome-fragment	NA		0
S9_extracted_k141_90548	3857	No	NA	0	2	Not-determined	Genome-fragment	NA		0
S9_extracted_k141_67910	1406	No	NA	0	0	Not-determined	Genome-fragment	NA		0
S14_vs_k141_99173||full	1100	No	NA	1	0	Low-quality	Genome-fragment		0.67	0
S9_extracted_k141_83062	1135	No	NA	0	1	Not-determined	Genome-fragment	NA		0
S1_extracted_k141_156811	1100	No	NA	1	0	Low-quality	Genome-fragment		2.36	0
S9_extracted_k141_79269	3026	No	NA	0	2	Not-determined	Genome-fragment	NA		0
S1_extracted_k141_39224	1100	No	NA	0	0	Low-quality	Genome-fragment		1.81	0
S3_extracted_k141_76931	1100	No	NA	0	0	Low-quality	Genome-fragment		1.24	0
S9_extracted_k141_15012	32097	No	NA	30	0	High-quality	High-quality		99.61	0
S3_vs_k141_98659||full	1100	No	NA	0	1	Low-quality	Genome-fragment		0.77	0
S5_vs_k141_24718||full	1100	No	NA	1	0	Low-quality	Genome-fragment		0.67	0
S9_extracted_k141_64018	1220	No	NA	0	0	Not-determined	Genome-fragment	NA		0
S5_vs_k141_13116||full	1100	No	NA	0	0	Low-quality	Genome-fragment		1.7	0
S9_extracted_k141_75753	1126	No	NA	0	0	Not-determined	Genome-fragment	NA		0
S5_vs_k141_32349||full	1100	No	NA	2	0	Low-quality	Genome-fragment		2.56	0
S7_vs_98994||full	1100	No	NA	1	0	Low-quality	Genome-fragment		2.7	0
S9_extracted_k141_68095	2518	No	NA	0	2	Not-determined	Genome-fragment	NA		0
S9_extracted_k141_68100	7276	No	NA	0	5	Not-determined	Genome-fragment	NA		0
S9_extracted_k141_83140	1466	No	NA	0	0	Not-determined	Genome-fragment	NA		0
S7_vs_99918||full	1100	No	NA	0	1	Low-quality	Genome-fragment		2.11	0
S16_extracted_19198	1100	No	NA	0	0	Low-quality	Genome-fragment		2.54	0
S16_extracted_121252	1100	No	NA	0	1	Low-quality	Genome-fragment		0.3	0
S10_vs_48301||full	1100	No	NA	1	0	Low-quality	Genome-fragment		1.37	0
S9_extracted_k141_64183	1032	No	NA	0	0	Not-determined	Genome-fragment	NA		0
S9_extracted_k141_83236	1073	No	NA	0	0	Not-determined	Genome-fragment	NA		0
S9_extracted_k141_79553	1274	No	NA	0	1	Not-determined	Genome-fragment	NA		0
S8_extracted_k141_35395	1100	No	NA	0	1	Low-quality	Genome-fragment		2.35	0
S9_extracted_k141_64246	1689	No	NA	0	1	Not-determined	Genome-fragment	NA		0
S9_extracted_k141_83314	1133	No	NA	0	1	Not-determined	Genome-fragment	NA		0
S9_extracted_k141_64257	1130	No	NA	0	1	Not-determined	Genome-fragment	NA		0
S9_extracted_k141_83353	1501	No	NA	0	0	Not-determined	Genome-fragment	NA		0
S9_extracted_k141_49239	1499	No	NA	0	1	Not-determined	Genome-fragment	NA		0
S8_vs_k141_202435||lt2gene	1100	No	NA	1	0	Low-quality	Genome-fragment		0.71	0
S9_extracted_k141_90808	1399	No	NA	0	1	Not-determined	Genome-fragment	NA		0
S9_extracted_k141_52611	1424	No	NA	0	1	Not-determined	Genome-fragment	NA		0
S9_extracted_k141_90839	1011	No	NA	0	0	Not-determined	Genome-fragment	NA		0
S9_extracted_k141_52629	2197	No	NA	0	1	Not-determined	Genome-fragment	NA		0
S17_extracted_k141_50502	1101	No	NA	2	0	Low-quality	Genome-fragment		2.87	0
S9_extracted_k141_52713	1192	No	NA	0	1	Not-determined	Genome-fragment	NA		0
S9_extracted_k141_52828	2215	No	NA	0	0	Not-determined	Genome-fragment	NA		0
S17_vs_k141_177075||full	1101	No	NA	2	0	Low-quality	Genome-fragment		2.8	0
S9_extracted_k141_52880	1131	No	NA	0	1	Not-determined	Genome-fragment	NA		0
S9_extracted_k141_52938	1147	No	NA	0	1	Not-determined	Genome-fragment	NA		0
S9_extracted_k141_90847	148940	No	NA	39	5	Complete	High-quality		100	0
S9_extracted_k141_53030	1299	No	NA	0	0	Not-determined	Genome-fragment	NA		0
S9_extracted_k141_90852	5449	No	NA	0	0	Complete	High-quality		100	0
S14_extracted_k141_49984	1101	No	NA	0	0	Low-quality	Genome-fragment		0.12	0
S9_extracted_k141_90856	38132	No	NA	36	0	Complete	High-quality		100	0
S9_extracted_k141_90866	9389	No	NA	6	0	Complete	High-quality		100	0
S9_extracted_k141_90871	36948	No	NA	26	0	Complete	High-quality		100	0
S9_vs_k141_45812||full	36359	No	NA	13	2	Medium-quality	Genome-fragment		69.64	0
S9_vs_k141_49943||full	6495	No	NA	0	0	Not-determined	Genome-fragment	NA		0
S9_vs_k141_88286||full	46223	No	NA	12	1	High-quality	High-quality		100	0
S9_vs_k141_50439||full	56327	No	NA	21	1	High-quality	High-quality		100	0
S1_vs_k141_143883||full	1101	No	NA	1	0	Low-quality	Genome-fragment		1.77	0
S9_vs_k141_5676||full	34189	No	NA	17	0	Complete	High-quality		100	0
S9_vs_k141_66877||full	36296	No	NA	14	0	High-quality	High-quality		94.24	0
S9_vs_k141_75050||full	32777	No	NA	14	2	High-quality	High-quality		100	0
S9_vs_k141_48424||full	54788	No	NA	15	4	High-quality	High-quality		97.96	0
S9_vs_k141_90846||full	38274	No	NA	21	1	High-quality	High-quality		93.75	0
S9_vs_k141_90848||full	42668	No	NA	19	1	Medium-quality	Genome-fragment		79.27	0
S9_vs_k141_90849||full	40590	No	NA	10	0	High-quality	High-quality		100	0
S9_vs_k141_90850||full	38814	No	NA	21	0	High-quality	High-quality		98.71	0
S9_vs_k141_90851||full	45923	No	NA	30	0	High-quality	High-quality		100	0
S9_vs_k141_90857||full	37664	No	NA	8	0	High-quality	High-quality		96.18	0
S9_vs_k141_90859||full	44116	No	NA	30	4	High-quality	High-quality		100	0
S9_vs_k141_90860||full	28818	No	NA	20	1	High-quality	High-quality		95.78	0
S9_vs_k141_90862||full	2114	No	NA	0	0	Not-determined	Genome-fragment	NA		0
S9_vs_k141_90863||full	5218	No	NA	1	0	High-quality	High-quality		94.97	0
S3_vs_k141_104758||full	1101	No	NA	1	0	Low-quality	Genome-fragment		2.09	0
S5_vs_k141_53809||full	1101	No	NA	1	0	Low-quality	Genome-fragment		2.06	0
S5_vs_k141_132815||full	1101	No	NA	1	0	Low-quality	Genome-fragment		1.78	0
S5_vs_k141_58172||full	1101	No	NA	1	0	Low-quality	Genome-fragment		2	0
S9_vs_k141_90894||full	2663	No	NA	0	0	Not-determined	Genome-fragment	NA		0
S16_extracted_148976	1101	No	NA	0	2	Low-quality	Genome-fragment		0.84	0
S12_extracted_k141_105606	1101	No	NA	1	0	Low-quality	Genome-fragment		0.76	0
S12_vs_k141_173460||full	1101	No	NA	1	0	Low-quality	Genome-fragment		2.45	0
S12_vs_k141_186590||full	1101	No	NA	0	1	Low-quality	Genome-fragment		1.39	0
S9_vs_k141_3810||full	1527	No	NA	0	1	Not-determined	Genome-fragment	NA		0
S15_vs_17990||full	1101	No	NA	0	0	Low-quality	Genome-fragment		2.62	0
S9_vs_k141_53086||full	3341	No	NA	0	0	Not-determined	Genome-fragment	NA		0
S9_vs_k141_87184||full	2429	No	NA	0	2	Not-determined	Genome-fragment	NA		0
S9_vs_k141_22773||full	6884	No	NA	0	2	Not-determined	Genome-fragment	NA		0
S9_vs_k141_34149||full	1372	No	NA	0	0	Not-determined	Genome-fragment	NA		0
S17_vs_k141_71306||full	1102	No	NA	0	1	Low-quality	Genome-fragment		1.71	0
S11_vs_k141_76168||full	1102	No	NA	2	0	Low-quality	Genome-fragment		1.66	0
S9_vs_k141_83413||full	1331	No	NA	0	0	Not-determined	Genome-fragment	NA		0
S11_vs_k141_84279||full	1102	No	NA	0	0	Low-quality	Genome-fragment		2.65	0
S13_extracted_8580	1102	No	NA	0	0	Low-quality	Genome-fragment		2.15	0
S9_vs_k141_53092||full	3603	No	NA	0	1	Not-determined	Genome-fragment	NA		0
S9_vs_k141_30347||full	2258	No	NA	0	1	Not-determined	Genome-fragment	NA		0
S14_vs_k141_33050||full	1102	No	NA	0	0	Low-quality	Genome-fragment		3.13	0

S1_vs_k141_137903||full	1102	No	NA	2	0	Low-quality	Genome-fragment		1.82	0
S9_vs_k141_34191||full	2271	No	NA	0	0	Not-determined	Genome-fragment	NA		0
S1_vs_k141_125730||lt2gene	1102	No	NA	1	0	Low-quality	Genome-fragment		2.47	0
S9_vs_k141_22811||full	2443	No	NA	0	1	Not-determined	Genome-fragment	NA		0
S7_extracted_61465	1102	No	NA	0	0	Low-quality	Genome-fragment		2.04	0
S9_vs_k141_15247||full	4948	No	NA	0	3	Not-determined	Genome-fragment	NA		0
S9_vs_k141_18991||full	25513	No	NA	0	16	Not-determined	Genome-fragment	NA		0
S9_vs_k141_7655||full	1540	No	NA	0	0	Not-determined	Genome-fragment	NA		0
S9_vs_k141_22823||full	1298	No	NA	0	0	Not-determined	Genome-fragment	NA		0
S9_vs_k141_83457||full	1517	No	NA	0	0	Not-determined	Genome-fragment	NA		0
S9_vs_k141_64510||full	1350	No	NA	0	0	Not-determined	Genome-fragment	NA		0
S9_vs_k141_22825||full	2802	No	NA	0	0	Not-determined	Genome-fragment	NA		0
S9_vs_k141_68270||full	1498	No	NA	0	1	Not-determined	Genome-fragment	NA		0
S9_vs_k141_60696||full	2402	No	NA	0	1	Not-determined	Genome-fragment	NA		0
S9_vs_k141_83469||full	3457	No	NA	0	3	Not-determined	Genome-fragment	NA		0
S16_extracted_66528	1102	No	NA	1	0	Low-quality	Genome-fragment		3.52	0
S10_vs_48234||full	1102	No	NA	0	1	Low-quality	Genome-fragment		0.74	0
S4_extracted_22682	1102	No	NA	2	0	Low-quality	Genome-fragment		2.99	0
S9_vs_k141_97||full	2246	No	NA	0	0	Not-determined	Genome-fragment	NA		0
S8_extracted_k141_93720	1102	No	NA	0	2	Low-quality	Genome-fragment		3.63	0
S17_extracted_k141_134380	1103	No	NA	0	1	Low-quality	Genome-fragment		0.49	0
S11_vs_k141_80990||full	1103	No	NA	0	0	Low-quality	Genome-fragment		1.51	0
S9_vs_k141_19064||full	4826	No	NA	0	3	Not-determined	Genome-fragment	NA		0
S9_vs_k141_79697||full	1411	No	NA	0	0	Not-determined	Genome-fragment	NA		0
S9_vs_k141_11522||full	2557	No	NA	0	3	Not-determined	Genome-fragment	NA		0
S11_vs_k141_4988||lt2gene	1103	No	NA	2	0	Low-quality	Genome-fragment		2.45	0
S13_vs_21737||full	1103	No	NA	0	1	Low-quality	Genome-fragment		2.51	0
S1_extracted_k141_20215	1103	No	NA	1	0	Low-quality	Genome-fragment		2.17	0
S1_vs_k141_83346||lt2gene	1103	No	NA	1	0	Low-quality	Genome-fragment		2.19	0
S9_vs_k141_68298||full	1833	No	NA	0	2	Not-determined	Genome-fragment	NA		0
S3_vs_k141_82950||lt2gene	1103	No	NA	1	0	Low-quality	Genome-fragment		0.93	0
S9_vs_k141_45576||full	2493	No	NA	0	2	Not-determined	Genome-fragment	NA		0
S5_vs_k141_128832||full	1103	No	NA	2	0	Low-quality	Genome-fragment		1.34	0
S9_vs_k141_34333||full	1059	No	NA	0	1	Not-determined	Genome-fragment	NA		0
S5_vs_k141_93374||lt2gene	1103	No	NA	1	0	Low-quality	Genome-fragment		2.09	0
S7_extracted_132271	1103	No	NA	0	0	Low-quality	Genome-fragment		2.91	0
S9_vs_k141_30556||full	3672	No	NA	0	3	Not-determined	Genome-fragment	NA		0
S16_vs_39452||full	1103	No	NA	0	0	Low-quality	Genome-fragment		1.89	0
S9_vs_k141_11606||full	1342	No	NA	0	0	Not-determined	Genome-fragment	NA		0
S10_extracted_34872	1103	No	NA	0	1	Low-quality	Genome-fragment		0.7	0
S9_vs_k141_34388||full	1016	No	NA	0	0	Not-determined	Genome-fragment	NA		0
S10_vs_51142||full	1103	No	NA	0	0	Low-quality	Genome-fragment		1.58	0
S9_vs_k141_45651||full	1568	No	NA	0	0	Not-determined	Genome-fragment	NA		0
S12_vs_k141_58178||full	1103	No	NA	0	0	Low-quality	Genome-fragment		0.64	0
S9_vs_k141_57003||full	3482	No	NA	0	2	Not-determined	Genome-fragment	NA		0
S9_vs_k141_4043||full	4732	No	NA	0	2	Not-determined	Genome-fragment	NA		0
S9_vs_k141_49380||full	2405	No	NA	0	0	Not-determined	Genome-fragment	NA		0
S9_vs_k141_68371||full	1104	No	NA	0	1	Not-determined	Genome-fragment	NA		0
S9_vs_k141_83574||full	3376	No	NA	0	1	Not-determined	Genome-fragment	NA		0
S2_vs_k141_2873||lt2gene	1103	No	NA	1	0	Low-quality	Genome-fragment		2.65	0
S6_extracted_k141_38690	1103	No	NA	0	3	Low-quality	Genome-fragment		0.31	0
S6_vs_k141_49137||full	1103	No	NA	0	1	Low-quality	Genome-fragment		0.58	0
S9_vs_k141_34461||full	1172	No	NA	0	1	Not-determined	Genome-fragment	NA		0
S9_vs_k141_322||full	2056	No	NA	0	0	Not-determined	Genome-fragment	NA		0
S8_vs_k141_45946||full	1103	No	NA	0	0	Low-quality	Genome-fragment		1.64	0
S8_vs_k141_3882||full	1103	No	NA	0	0	Low-quality	Genome-fragment		2.04	0
S17_extracted_k141_62101	1104	No	NA	0	0	Low-quality	Genome-fragment		0.19	0
S9_vs_k141_38192||full	2521	No	NA	0	1	Not-determined	Genome-fragment	NA		0
S9_vs_k141_41933||full	1628	No	NA	0	0	Not-determined	Genome-fragment	NA		0
S9_vs_k141_68415||full	1387	No	NA	0	0	Not-determined	Genome-fragment	NA		0
S17_extracted_k141_87196	1104	No	NA	0	0	Low-quality	Genome-fragment		0.18	0
S9_vs_k141_38198||full	1854	No	NA	0	1	Not-determined	Genome-fragment	NA		0
S9_vs_k141_87452||full	9268	No	NA	0	3	Not-determined	Genome-fragment	NA		0
S9_vs_k141_64671||full	1260	No	NA	0	2	Not-determined	Genome-fragment	NA		0
S9_extracted_k141_76468	1104	No	NA	0	1	Low-quality	Genome-fragment		2.02	0
S9_vs_k141_57044||full	4309	No	NA	0	0	Not-determined	Genome-fragment	NA		0
S9_extracted_k141_37590	1104	No	NA	0	0	Low-quality	Genome-fragment		1.1	0
S9_vs_k141_3373||full	1104	No	NA	0	0	Low-quality	Genome-fragment		2.61	0
S9_vs_k141_47797||lt2gene	1104	No	NA	2	0	Low-quality	Genome-fragment		2.54	0
S9_vs_k141_57053||full	1275	No	NA	0	0	Not-determined	Genome-fragment	NA		0
S11_extracted_k141_64746	1104	No	NA	1	0	Low-quality	Genome-fragment		2.66	0
S13_vs_140372||full	1104	No	NA	0	0	Low-quality	Genome-fragment		2.46	0
S14_extracted_k141_52132	1104	No	NA	0	0	Low-quality	Genome-fragment		3.54	0
S9_vs_k141_49443||full	1031	No	NA	0	1	Not-determined	Genome-fragment	NA		0
S1_vs_k141_136180||full	1104	No	NA	2	0	Low-quality	Genome-fragment		2.08	0
S7_extracted_42177	1104	No	NA	0	0	Low-quality	Genome-fragment		1.56	0
S9_vs_k141_38250||full	2884	No	NA	0	1	Not-determined	Genome-fragment	NA		0
S7_vs_115857||lt2gene	1104	No	NA	2	0	Low-quality	Genome-fragment		2.73	0
S9_vs_k141_60867||full	2934	No	NA	0	3	Not-determined	Genome-fragment	NA		0
S12_extracted_k141_117961	1104	No	NA	0	0	Low-quality	Genome-fragment		1.1	0
S9_vs_k141_23088||full	1717	No	NA	0	1	Not-determined	Genome-fragment	NA		0
S12_vs_k141_154112||full	1104	No	NA	0	0	Low-quality	Genome-fragment		1.97	0
S2_extracted_k141_14184	1104	No	NA	0	1	Low-quality	Genome-fragment		0.49	0
S6_vs_k141_862||full	1104	No	NA	0	0	Low-quality	Genome-fragment		3.08	0
S8_vs_k141_4171||full	1104	No	NA	0	0	Low-quality	Genome-fragment		0.68	0
S17_extracted_k141_119617	1105	No	NA	1	0	Low-quality	Genome-fragment		5.04	0
S9_vs_k141_15509||full	2005	No	NA	0	1	Not-determined	Genome-fragment	NA		0
S9_vs_k141_7834||full	1920	No	NA	0	2	Not-determined	Genome-fragment	NA		0
S17_vs_k141_93970||full	1105	No	NA	0	1	Low-quality	Genome-fragment		0.86	0
S9_vs_k141_64753||full	1328	No	NA	0	2	Not-determined	Genome-fragment	NA		0
S9_extracted_k141_28637	1105	No	NA	1	0	Low-quality	Genome-fragment		2.83	0
S9_vs_k141_83685||full	1071	No	NA	0	1	Not-determined	Genome-fragment	NA		0
S9_vs_k141_55364||full	1105	No	NA	1	0	Low-quality	Genome-fragment		1.49	0
S11_extracted_k141_58544	1105	No	NA	1	0	Low-quality	Genome-fragment		2.01	0
S13_extracted_2292	1105	No	NA	1	0	Low-quality	Genome-fragment		1.84	0
S14_vs_k141_68324||full	1105	No	NA	0	1	Low-quality	Genome-fragment		1.7	0
S3_extracted_k141_74441	1105	No	NA	1	0	Low-quality	Genome-fragment		1.87	0
S9_vs_k141_64797||full	4492	No	NA	0	3	Not-determined	Genome-fragment	NA		0
S9_vs_k141_30768||full	3203	No	NA	0	0	Not-determined	Genome-fragment	NA		0
S5_vs_k141_114618||full	1105	No	NA	2	0	Low-quality	Genome-fragment		2.93	0
S7_extracted_2837	1105	No	NA	4	0	Low-quality	Genome-fragment		3.64	0
S9_vs_k141_463||full	1196	No	NA	0	0	Not-determined	Genome-fragment	NA		0
S9_vs_k141_76015||full	1502	No	NA	0	1	Not-determined	Genome-fragment	NA		0
S7_vs_83481||lt2gene	1105	No	NA	1	0	Low-quality	Genome-fragment		3.19	0
S16_extracted_127802	1105	No	NA	1	0	Low-quality	Genome-fragment		1.97	0
S16_vs_72966||full	1105	No	NA	0	0	Low-quality	Genome-fragment		0.64	0
S16_vs_95188||full	1105	No	NA	0	0	Low-quality	Genome-fragment		0.67	0
S9_vs_k141_76029||full	3635	No	NA	0	4	Not-determined	Genome-fragment	NA		0
S9_vs_k141_492||full	3802	No	NA	0	0	Not-determined	Genome-fragment	NA		0
S12_extracted_k141_115834	1105	No	NA	1	0	Low-quality	Genome-fragment		1.53	0
S12_extracted_k141_21917	1105	No	NA	1	0	Low-quality	Genome-fragment		2.07	0
S9_vs_k141_7881||full	28302	No	NA	23	0	Medium-quality	Genome-fragment		61.82	0
S9_vs_k141_61025||full	1701	No	NA	0	2	Not-determined	Genome-fragment	NA		0
S6_extracted_k141_39793	1105	No	NA	0	0	Low-quality	Genome-fragment		0.41	0
S8_vs_k141_112073||full	1105	No	NA	0	1	Low-quality	Genome-fragment		2.01	0
S8_vs_k141_107188||lt2gene	1105	No	NA	1	0	Low-quality	Genome-fragment		1.61	0
S9_vs_k141_53442||full	1190	No	NA	0	0	Not-determined	Genome-fragment	NA		0
S9_vs_k141_72385||full	2757	No	NA	0	0	Not-determined	Genome-fragment	NA		0
S9_vs_k141_23184||full	1027	No	NA	0	0	Not-determined	Genome-fragment	NA		0
S9_vs_k141_15647||full	1381	No	NA	0	0	Not-determined	Genome-fragment	NA		0
S17_vs_k141_106261||full	1106	No	NA	5	0	Low-quality	Genome-fragment		3.65	0
S11_extracted_k141_32274	1106	No	NA	0	0	Low-quality	Genome-fragment		1.71	0
S9_vs_k141_19367||full	1103	No	NA	0	2	Not-determined	Genome-fragment	NA		0
S9_vs_k141_26934||full	1804	No	NA	0	0	Not-determined	Genome-fragment	NA		0
S14_extracted_k141_7451	1106	No	NA	2	0	Low-quality	Genome-fragment		3.64	0
S9_vs_k141_34601||full	1093	No	NA	0	1	Not-determined	Genome-fragment	NA		0
S1_extracted_k141_157185	1106	No	NA	1	0	Low-quality	Genome-fragment		2.06	0
S1_vs_k141_1116||full	1106	No	NA	0	0	Low-quality	Genome-fragment		0.92	0
S9_vs_k141_11881||full	1047	No	NA	0	0	Not-determined	Genome-fragment	NA		0
S1_vs_k141_72928||full	1106	No	NA	0	0	Low-quality	Genome-fragment		1.95	0
S9_vs_k141_72431||full	3372	No	NA	0	1	Not-determined	Genome-fragment	NA		0
S9_vs_k141_61077||full	1639	No	NA	0	0	Not-determined	Genome-fragment	NA		0
S9_vs_k141_80026||full	1601	No	NA	0	0	Not-determined	Genome-fragment	NA		0
S9_vs_k141_30850||full	1620	No	NA	0	2	Not-determined	Genome-fragment	NA		0
S5_vs_k141_67181||lt2gene	1106	No	NA	1	0	Low-quality	Genome-fragment		2.46	0
S7_extracted_28574	1106	No	NA	0	0	Low-quality	Genome-fragment		1.27	0
S7_vs_49187||full	1106	No	NA	2	0	Low-quality	Genome-fragment		2.77	0
S7_vs_83840||full	1106	No	NA	0	1	Low-quality	Genome-fragment		0.98	0
S9_vs_k141_7993||full	1170	No	NA	0	1	Not-determined	Genome-fragment	NA		0
S9_vs_k141_30888||full	1675	No	NA	0	0	Not-determined	Genome-fragment	NA		0
S7_vs_85287||full	1106	No	NA	0	0	Low-quality	Genome-fragment		1.09	0
S16_extracted_25375	1106	No	NA	0	0	Low-quality	Genome-fragment		0.64	0
S9_vs_k141_38472||full	10302	No	NA	0	5	Not-determined	Genome-fragment	NA		0
S16_extracted_80254	1106	No	NA	0	0	Low-quality	Genome-fragment		2.58	0
S16_vs_104245||lt2gene	1106	No	NA	1	0	Low-quality	Genome-fragment		2.8	0
S10_extracted_86464	1106	No	NA	0	2	Low-quality	Genome-fragment		2.89	0
S17_extracted_k141_29393	1107	No	NA	0	1	Low-quality	Genome-fragment		0.37	0
S17_vs_k141_99620||full	1107	No	NA	0	0	Low-quality	Genome-fragment		1.33	0
S17_vs_k141_17958||full	1107	No	NA	0	0	Low-quality	Genome-fragment		2.13	0
S14_extracted_k141_45144	1107	No	NA	4	0	Low-quality	Genome-fragment		3.65	0
S9_vs_k141_8062||full	2135	No	NA	0	1	Not-determined	Genome-fragment	NA		0
S14_extracted_k141_46476	1107	No	NA	0	0	Low-quality	Genome-fragment		8.72	0
S5_extracted_k141_120159	1107	No	NA	3	0	Low-quality	Genome-fragment		3.65	0
S9_vs_k141_30963||full	1579	No	NA	0	1	Not-determined	Genome-fragment	NA		0
S9_vs_k141_61223||full	1110	No	NA	0	0	Not-determined	Genome-fragment	NA		0
S7_vs_78050||full	1107	No	NA	0	0	Low-quality	Genome-fragment		2.86	0
S9_vs_k141_45865||full	2976	No	NA	0	0	Not-determined	Genome-fragment	NA		0
S16_extracted_72062	1107	No	NA	0	0	Low-quality	Genome-fragment		2.28	0
S9_vs_k141_4343||full	37691	No	NA	8	0	High-quality	High-quality		95.71	0
S16_extracted_90695	1107	No	NA	0	0	Low-quality	Genome-fragment		0.81	0
S16_vs_20451||full	1107	No	NA	0	1	Low-quality	Genome-fragment		1.86	0
S9_vs_k141_72581||full	3001	No	NA	0	1	Not-determined	Genome-fragment	NA		0
S9_vs_k141_34682||full	2453	No	NA	0	0	Not-determined	Genome-fragment	NA		0
S10_extracted_11953	1107	No	NA	0	0	Low-quality	Genome-fragment		2.4	0
S10_vs_82978||full	1107	No	NA	0	1	Low-quality	Genome-fragment		0.2	0
S9_vs_k141_15835||full	1184	No	NA	0	2	Not-determined	Genome-fragment	NA		0
S9_vs_k141_68658||full	1481	No	NA	0	1	Not-determined	Genome-fragment	NA		0
S12_extracted_k141_99775	1107	No	NA	1	0	Low-quality	Genome-fragment		2.52	0
S12_extracted_k141_131011	1107	No	NA	0	1	Low-quality	Genome-fragment		0.72	0
S8_extracted_k141_62215	1107	No	NA	0	1	Low-quality	Genome-fragment		0.2	0
S9_vs_k141_61287||full	5030	No	NA	0	3	Not-determined	Genome-fragment	NA		0
S9_vs_k141_4360||full	1267	No	NA	0	0	Not-determined	Genome-fragment	NA		0
S17_extracted_k141_90930	1108	No	NA	0	0	Low-quality	Genome-fragment		2.17	0
S17_vs_k141_132777||full	1108	No	NA	1	0	Low-quality	Genome-fragment		2.03	0

S17_vs_k141_128416||lt2gene	1108	No	NA	1	0	Low-quality	Genome-fragment	2.64	0
S9_vs_k141_23349||full	1781	No	NA	0	1	Not-determined	Genome-fragment	NA	0
S9_vs_k141_80211||full	12108	No	NA	0	0	Not-determined	Genome-fragment	NA	0
S11_extracted_k141_86790	1108	No	NA	0	1	Low-quality	Genome-fragment	0.99	0
S9_vs_k141_61326||full	1810	No	NA	0	0	Not-determined	Genome-fragment	NA	0
S13_extracted_38147	1108	No	NA	0	1	Low-quality	Genome-fragment	1.1	0
S14_extracted_k141_101954	1108	No	NA	2	0	Low-quality	Genome-fragment	3.66	0
S9_vs_k141_87923||full	3500	No	NA	0	3	Not-determined	Genome-fragment	NA	0
S1_vs_k141_1371||full	1108	No	NA	3	0	Low-quality	Genome-fragment	2.44	0
S1_vs_k141_6030||full	1108	No	NA	1	0	Low-quality	Genome-fragment	2.28	0
S9_vs_k141_84011||full	2228	No	NA	0	1	Not-determined	Genome-fragment	NA	0
S3_vs_k141_17758||full	1108	No	NA	0	0	Low-quality	Genome-fragment	2.02	0
S3_vs_k141_106606||full	1108	No	NA	1	0	Low-quality	Genome-fragment	1.99	0
S5_extracted_k141_62752	1108	No	NA	1	0	Low-quality	Genome-fragment	2.06	0
S5_vs_k141_55093||full	1108	No	NA	1	0	Low-quality	Genome-fragment	1.64	0
S9_vs_k141_27274||full	7496	No	NA	0	3	Not-determined	Genome-fragment	NA	0
S5_vs_k141_138218||full	1108	No	NA	1	0	Low-quality	Genome-fragment	2.15	0
S9_vs_k141_4422||full	1031	No	NA	0	0	Not-determined	Genome-fragment	NA	0
S7_vs_25343||full	1108	No	NA	0	0	Low-quality	Genome-fragment	2.7	0
S9_vs_k141_53808||full	4644	No	NA	0	2	Not-determined	Genome-fragment	NA	0
S16_extracted_71420	1108	No	NA	0	0	Low-quality	Genome-fragment	2.21	0
S10_extracted_95679	1108	No	NA	0	0	Low-quality	Genome-fragment	2.07	0
S9_vs_k141_80322||full	1564	No	NA	0	0	Not-determined	Genome-fragment	NA	0
S10_vs_78830||full	1108	No	NA	2	0	Low-quality	Genome-fragment	1.38	0
S12_vs_k141_87824||full	1108	No	NA	0	0	Low-quality	Genome-fragment	1.92	0
S9_vs_k141_57345||full	1385	No	NA	0	0	Not-determined	Genome-fragment	NA	0
S11_extracted_k141_65833	1109	No	NA	0	0	Low-quality	Genome-fragment	0.72	0
S9_vs_k141_61358||full	1048	No	NA	0	0	Not-determined	Genome-fragment	NA	0
S9_vs_k141_72712||full	1214	No	NA	0	1	Not-determined	Genome-fragment	NA	0
S9_vs_k141_68803||full	2539	No	NA	0	1	Not-determined	Genome-fragment	NA	0
S11_extracted_k141_44061	1109	No	NA	2	0	Low-quality	Genome-fragment	3.66	0
S11_vs_k141_8963||full	1109	No	NA	0	0	Low-quality	Genome-fragment	1	0
S14_vs_k141_1212||full	1109	No	NA	1	0	Low-quality	Genome-fragment	3.01	0
S1_extracted_k141_149014	1109	No	NA	1	0	Low-quality	Genome-fragment	1.84	0
S1_vs_k141_127040||full	1109	No	NA	1	0	Low-quality	Genome-fragment	1.76	0
S9_vs_k141_27315||full	1300	No	NA	0	1	Not-determined	Genome-fragment	NA	0
S9_vs_k141_84046||full	29842	No	NA	14	1	High-quality	High-quality	93.21	0
S7_extracted_7212	1109	No	NA	0	0	Low-quality	Genome-fragment	0.71	0
S9_vs_k141_49763||full	1493	No	NA	0	1	Not-determined	Genome-fragment	NA	0
S9_vs_k141_76374||full	4193	No	NA	0	2	Not-determined	Genome-fragment	NA	0
S9_vs_k141_57388||full	2324	No	NA	0	2	Not-determined	Genome-fragment	NA	0
S16_extracted_86065	1109	No	NA	1	0	Low-quality	Genome-fragment	2.87	0
S9_vs_k141_65082||full	1340	No	NA	0	2	Not-determined	Genome-fragment	NA	0
S16_extracted_163305	1109	No	NA	0	0	Low-quality	Genome-fragment	0.7	0
S9_vs_k141_23450||full	10166	No	NA	0	4	Not-determined	Genome-fragment	NA	0
S16_vs_113778||full	1109	No	NA	1	0	Low-quality	Genome-fragment	2.32	0
S16_vs_143661||lt2gene	1109	No	NA	1	0	Low-quality	Genome-fragment	1.89	0
S9_vs_k141_87990||full	16416	No	NA	0	12	Not-determined	Genome-fragment	NA	0
S9_vs_k141_23454||full	1044	No	NA	0	2	Not-determined	Genome-fragment	NA	0
S12_vs_k141_110403||full	1109	No	NA	1	0	Low-quality	Genome-fragment	1.98	0
S9_vs_k141_38799||full	1961	No	NA	0	2	Not-determined	Genome-fragment	NA	0
S9_vs_k141_19649||full	1458	No	NA	0	0	Not-determined	Genome-fragment	NA	0
S9_vs_k141_34830||full	1695	No	NA	0	1	Not-determined	Genome-fragment	NA	0
S9_vs_k141_46141||full	1265	No	NA	0	0	Not-determined	Genome-fragment	NA	0
S9_vs_k141_49807||full	1073	No	NA	0	0	Not-determined	Genome-fragment	NA	0
S9_vs_k141_57424||full	2742	No	NA	0	0	Not-determined	Genome-fragment	NA	0
S8_vs_k141_93135||full	1109	Yes	656	1	1	Low-quality	Genome-fragment	0.42	40.85
S9_extracted_k141_23839	1110	No	NA	0	0	Low-quality	Genome-fragment	1.85	0
S9_vs_k141_4441||full	1110	No	NA	0	0	Low-quality	Genome-fragment	2.19	0
S13_vs_98422||full	1110	No	NA	1	0	Low-quality	Genome-fragment	1.92	0
S9_vs_k141_16064||full	1021	No	NA	0	1	Not-determined	Genome-fragment	NA	0
S9_vs_k141_46171||full	2740	No	NA	0	2	Not-determined	Genome-fragment	NA	0
S9_vs_k141_38853||full	2292	No	NA	0	0	Not-determined	Genome-fragment	NA	0
S9_vs_k141_23518||full	1352	No	NA	0	1	Not-determined	Genome-fragment	NA	0
S14_extracted_k141_10015	1110	No	NA	0	0	Low-quality	Genome-fragment	1.59	0
S1_vs_k141_15744||lt2gene	1110	No	NA	1	0	Low-quality	Genome-fragment	2.43	0
S5_extracted_k141_61372	1110	No	NA	0	1	Low-quality	Genome-fragment	0.74	0
S7_extracted_121283	1110	No	NA	0	0	Low-quality	Genome-fragment	0.7	0
S7_extracted_126006	1110	No	NA	0	0	Low-quality	Genome-fragment	1.71	0
S7_vs_47980||full	1110	No	NA	0	0	Low-quality	Genome-fragment	2.33	0
S7_vs_89428||full	1110	No	NA	0	1	Low-quality	Genome-fragment	0.3	0
S9_vs_k141_19689||full	28742	No	NA	11	1	Medium-quality	Genome-fragment	62.32	0
S7_vs_118831||full	1110	No	NA	0	0	Low-quality	Genome-fragment	1.45	0
S10_extracted_32826	1110	No	NA	0	1	Low-quality	Genome-fragment	0.33	0
S9_vs_k141_61504||full	2310	No	NA	0	2	Not-determined	Genome-fragment	NA	0
S9_vs_k141_65132||full	1662	No	NA	0	1	Not-determined	Genome-fragment	NA	0
S9_vs_k141_27348||full	1187	No	NA	0	0	Not-determined	Genome-fragment	NA	0
S9_vs_k141_42619||full	1494	No	NA	0	1	Not-determined	Genome-fragment	NA	0
S12_vs_k141_118277||full	1110	No	NA	1	0	Low-quality	Genome-fragment	1.76	0
S9_vs_k141_12307||full	3099	No	NA	0	4	Not-determined	Genome-fragment	NA	0
S9_vs_k141_34898||full	31642	No	NA	25	0	Medium-quality	Genome-fragment	78.72	0
S9_vs_k141_72853||full	1486	No	NA	0	0	Not-determined	Genome-fragment	NA	0
S9_vs_k141_65155||full	1349	No	NA	0	0	Not-determined	Genome-fragment	NA	0
S9_vs_k141_61525||full	6375	No	NA	0	6	Not-determined	Genome-fragment	NA	0
S12_vs_k141_53502||full	1110	No	NA	1	0	Low-quality	Genome-fragment	2.17	0
S9_vs_k141_38942||full	1617	No	NA	0	1	Not-determined	Genome-fragment	NA	0
S6_extracted_k141_7331	1110	No	NA	0	0	Low-quality	Genome-fragment	1.15	0
S9_vs_k141_4558||full	61044	No	NA	32	0	High-quality	High-quality	96.55	0
S8_extracted_k141_36514	1110	No	NA	0	0	Low-quality	Genome-fragment	0.55	0
S9_vs_k141_31299||full	2184	No	NA	0	0	Not-determined	Genome-fragment	NA	0
S8_vs_k141_57242||full	1110	No	NA	1	0	Low-quality	Genome-fragment	1.84	0
S14_extracted_k141_1057	1111	No	NA	1	0	Low-quality	Genome-fragment	2.9	0
S9_vs_k141_68847||full	1551	No	NA	0	0	Not-determined	Genome-fragment	NA	0
S3_extracted_k141_84807	1111	No	NA	0	0	Low-quality	Genome-fragment	1.8	0
S3_vs_k141_24177||full	1111	No	NA	0	0	Low-quality	Genome-fragment	1.06	0
S3_vs_k141_15830||full	1111	No	NA	0	0	Low-quality	Genome-fragment	0.91	0
S5_extracted_k141_25424	1111	No	NA	1	0	Low-quality	Genome-fragment	2.25	0
S9_vs_k141_65203||full	1661	No	NA	0	0	Not-determined	Genome-fragment	NA	0
S9_vs_k141_46337||full	3419	No	NA	0	0	Not-determined	Genome-fragment	NA	0
S7_extracted_130720	1111	No	NA	0	0	Low-quality	Genome-fragment	1.98	0
S9_vs_k141_42715||full	3388	No	NA	0	2	Not-determined	Genome-fragment	NA	0
S7_vs_84606||full	1111	No	NA	0	0	Low-quality	Genome-fragment	2.46	0
S7_vs_86235||full	1111	No	NA	0	0	Low-quality	Genome-fragment	1.52	0
S16_extracted_82636	1111	No	NA	1	0	Low-quality	Genome-fragment	23.29	0
S16_extracted_109468	1111	No	NA	1	0	Low-quality	Genome-fragment	1.39	0
S16_vs_12247||full	1111	No	NA	1	0	Low-quality	Genome-fragment	0.77	0
S16_vs_105559||full	1111	No	NA	1	0	Low-quality	Genome-fragment	1.95	0
S10_extracted_26796	1111	No	NA	1	0	Low-quality	Genome-fragment	2.79	0
S10_vs_50717||full	1111	No	NA	0	0	Low-quality	Genome-fragment	2.4	0
S9_vs_k141_35043||full	1014	No	NA	0	2	Not-determined	Genome-fragment	NA	0
S12_vs_k141_153035||full	1111	No	NA	1	0	Low-quality	Genome-fragment	0.56	0
S9_vs_k141_16270||full	3386	No	NA	0	1	Not-determined	Genome-fragment	NA	0
S12_vs_k141_69652||full	1111	No	NA	2	0	Low-quality	Genome-fragment	3.11	0
S15_vs_59162||full	1111	No	NA	3	0	Low-quality	Genome-fragment	2.91	0
S9_vs_k141_54131||full	2687	No	NA	0	0	Not-determined	Genome-fragment	NA	0
S9_vs_k141_27444||full	5218	No	NA	0	5	Not-determined	Genome-fragment	NA	0
S8_extracted_k141_130510	1111	No	NA	2	0	Low-quality	Genome-fragment	0.6	0
S8_extracted_k141_129608	1111	No	NA	0	1	Low-quality	Genome-fragment	0.88	0
S17_vs_k141_167005||full	1112	No	NA	1	0	Low-quality	Genome-fragment	2.17	0
S9_vs_k141_19846||full	1199	No	NA	0	1	Not-determined	Genome-fragment	NA	0
S9_vs_k141_41002||lt2gene	1112	No	NA	1	0	Low-quality	Genome-fragment	2.57	0
S11_vs_k141_35350||full	1112	No	NA	1	0	Low-quality	Genome-fragment	1.68	0
S13_vs_149361||full	1112	No	NA	0	0	Low-quality	Genome-fragment	0.97	0
S1_extracted_k141_138468	1112	No	NA	0	0	Low-quality	Genome-fragment	1.94	0
S1_vs_k141_25360||full	1112	No	NA	2	0	Low-quality	Genome-fragment	2.56	0
S5_extracted_k141_145507	1112	No	NA	2	0	Low-quality	Genome-fragment	2.89	0
S10_vs_7700||full	1112	No	NA	0	0	Low-quality	Genome-fragment	2.05	0
S12_extracted_k141_151512	1112	No	NA	0	0	Low-quality	Genome-fragment	1.96	0
S9_vs_k141_23643||full	1785	No	NA	0	1	Not-determined	Genome-fragment	NA	0
S12_extracted_k141_42555	1112	No	NA	2	0	Low-quality	Genome-fragment	1.18	0
S12_extracted_k141_132308	1112	No	NA	1	0	Low-quality	Genome-fragment	2.45	0
S15_extracted_70105	1112	No	NA	2	0	Low-quality	Genome-fragment	1.89	0
S9_vs_k141_57660||full	1696	No	NA	0	1	Not-determined	Genome-fragment	NA	0
S9_vs_k141_19872||full	1604	No	NA	0	0	Not-determined	Genome-fragment	NA	0
S9_vs_k141_27506||full	3442	No	NA	0	3	Not-determined	Genome-fragment	NA	0
S8_extracted_k141_72612	1112	No	NA	0	1	Low-quality	Genome-fragment	0.6	0
S8_vs_k141_151134||full	1112	No	NA	2	0	Low-quality	Genome-fragment	2.68	0
S9_vs_k141_50152||full	1630	No	NA	0	0	Not-determined	Genome-fragment	NA	0
S9_vs_k141_39152||full	1316	No	NA	0	1	Not-determined	Genome-fragment	NA	0
S9_vs_k141_54219||full	2213	No	NA	0	0	Not-determined	Genome-fragment	NA	0
S9_vs_k141_27562||full	1058	No	NA	0	0	Not-determined	Genome-fragment	NA	0
S9_vs_k141_50157||full	2113	No	NA	0	2	Not-determined	Genome-fragment	NA	0
S17_extracted_k141_16517	1113	No	NA	2	0	Low-quality	Genome-fragment	2.51	0
S9_vs_k141_68947||full	1674	No	NA	0	0	Not-determined	Genome-fragment	NA	0
S17_vs_k141_126985||full	1113	No	NA	0	0	Low-quality	Genome-fragment	0.67	0
S14_vs_k141_19021||lt2gene	1113	No	NA	1	0	Low-quality	Genome-fragment	1.79	0
S9_vs_k141_68949||full	2898	No	NA	0	0	Not-determined	Genome-fragment	NA	0
S9_vs_k141_57702||full	1022	No	NA	0	0	Not-determined	Genome-fragment	NA	0
S3_vs_k141_56466||full	1113	No	NA	1	0	Low-quality	Genome-fragment	2.13	0
S3_vs_k141_120696||full	1113	No	NA	1	0	Low-quality	Genome-fragment	0.72	0
S9_vs_k141_84214||full	1903	No	NA	0	1	Not-determined	Genome-fragment	NA	0
S9_vs_k141_1092||full	1625	No	NA	0	0	Not-determined	Genome-fragment	NA	0
S9_vs_k141_88318||full	1454	No	NA	0	1	Not-determined	Genome-fragment	NA	0
S3_vs_k141_13077||lt2gene	1113	No	NA	1	0	Low-quality	Genome-fragment	1.81	0
S5_vs_k141_66159||full	1113	No	NA	0	0	Low-quality	Genome-fragment	2.84	0
S7_vs_34176||full	1113	No	NA	0	1	Low-quality	Genome-fragment	0.87	0
S9_vs_k141_46498||full	20897	Yes	17942	12	4	Medium-quality	Genome-fragment	50.53	14.14
S12_extracted_k141_48581	1113	No	NA	1	0	Low-quality	Genome-fragment	1.43	0
S12_extracted_k141_180519	1113	No	NA	0	0	Low-quality	Genome-fragment	1.71	0
S9_vs_k141_4824||full	10095	No	NA	0	1	Not-determined	Genome-fragment	NA	0
S9_vs_k141_46511||full	1930	No	NA	0	2	Not-determined	Genome-fragment	NA	0
S8_extracted_k141_145937	1113	No	NA	0	0	Low-quality	Genome-fragment	1.52	0
S9_vs_k141_1123||full	1430	No	NA	0	1	Not-determined	Genome-fragment	NA	0
S1_vs_k141_154006||full	1114	No	NA	0	0	Low-quality	Genome-fragment	2.21	0
S3_vs_k141_88247||lt2gene	1114	No	NA	1	0	Low-quality	Genome-fragment	3.1	0
S9_vs_k141_88353||full	2480	No	NA	0	2	Not-determined	Genome-fragment	NA	0
S9_vs_k141_50232||full	1520	No	NA	0	1	Not-determined	Genome-fragment	NA	0
S5_extracted_k141_123651	1114	No	NA	2	0	Low-quality	Genome-fragment	3.34	0
S9_vs_k141_16516||full	1291	No	NA	0	1	Not-determined	Genome-fragment	NA	0
S5_vs_k141_32662||full	1114	No	NA	2	0	Low-quality	Genome-fragment	2.78	0
S5_vs_k141_130955||full	1114	No	NA	0	1	Low-quality	Genome-fragment	0.37	0
S9_vs_k141_1163||full	3827	No	NA	0	1	Not-determined	Genome-fragment	NA	0
S5_vs_k141_40078||full	1114	No	NA	1	0	Low-quality	Genome-fragment	1.99	0
S16_vs_27176||full	1114	No	NA	1	0	Low-quality	Genome-fragment	3.07	0

S12_vs_k141_145808||full	1114	No	NA	0	0	Low-quality	Genome-fragment		2.17	0
S9_vs_k141_57763||full	1000	No	NA	0	1	Not-determined	Genome-fragment	NA		0
S9_vs_k141_88382||full	2419	No	NA	0	0	Not-determined	Genome-fragment	NA		0
S6_vs_k141_49362||lt2gene	1114	No	NA	1	0	Low-quality	Genome-fragment		2.47	0
S8_extracted_k141_123306	1114	No	NA	0	1	Low-quality	Genome-fragment		0.62	0
S9_vs_k141_12660||full	2932	No	NA	0	1	Not-determined	Genome-fragment	NA		0
S8_vs_k141_177801||lt2gene	1114	No	NA	1	0	Low-quality	Genome-fragment		2.02	0
S9_vs_k141_61710||full	2013	No	NA	0	0	Not-determined	Genome-fragment	NA		0
S9_vs_k141_20047||full	1466	No	NA	0	0	Not-determined	Genome-fragment	NA		0
S9_vs_k141_57781||full	1721	No	NA	0	2	Not-determined	Genome-fragment	NA		0
S17_vs_k141_111351||full	1115	No	NA	0	0	Low-quality	Genome-fragment		2.83	0
S9_vs_k141_16562||full	3295	No	NA	0	1	Not-determined	Genome-fragment	NA		0
S9_extracted_k141_82932	1115	No	NA	0	1	Low-quality	Genome-fragment		0.48	0
S1_extracted_k141_155062	1115	No	NA	0	0	Low-quality	Genome-fragment		0.63	0
S1_extracted_k141_65053	1115	No	NA	0	2	Low-quality	Genome-fragment		2.13	0
S9_vs_k141_61739||full	1068	No	NA	0	1	Not-determined	Genome-fragment	NA		0
S3_vs_k141_48341||full	1115	No	NA	0	0	Low-quality	Genome-fragment		1.62	0
S9_vs_k141_54410||full	3249	No	NA	0	1	Not-determined	Genome-fragment	NA		0
S5_vs_k141_24728||full	1115	No	NA	0	0	Low-quality	Genome-fragment		2.81	0
S9_vs_k141_27764||full	1154	No	NA	0	1	Not-determined	Genome-fragment	NA		0
S7_vs_96531||full	1115	No	NA	0	0	Low-quality	Genome-fragment		2.03	0
S9_vs_k141_35366||full	1361	No	NA	0	0	Not-determined	Genome-fragment	NA		0
S9_vs_k141_80599||full	2506	No	NA	0	2	Not-determined	Genome-fragment	NA		0
S9_vs_k141_65448||full	2858	No	NA	0	0	Not-determined	Genome-fragment	NA		0
S16_extracted_66421	1115	No	NA	0	0	Low-quality	Genome-fragment		2.23	0
S12_extracted_k141_137235	1115	No	NA	0	1	Low-quality	Genome-fragment		0.27	0
S12_extracted_k141_98660	1115	No	NA	0	0	Low-quality	Genome-fragment		1.8	0
S8_vs_k141_187193||full	1115	No	NA	0	0	Low-quality	Genome-fragment		2.3	0
S8_vs_k141_49241||full	1115	No	NA	1	0	Low-quality	Genome-fragment		2.62	0
S9_vs_k141_69154||full	4501	No	NA	0	1	Not-determined	Genome-fragment	NA		0
S17_vs_k141_161343||lt2gene	1116	No	NA	2	0	Low-quality	Genome-fragment		2.57	0
S9_vs_k141_21629||full	1116	No	NA	2	0	Low-quality	Genome-fragment		2.78	0
S1_vs_k141_41443||full	1116	No	NA	0	0	Low-quality	Genome-fragment		1.96	0
S9_vs_k141_35410||full	1059	No	NA	0	1	Not-determined	Genome-fragment	NA		0
S9_vs_k141_23801||full	1451	No	NA	0	0	Not-determined	Genome-fragment	NA		0
S9_vs_k141_39399||full	1102	No	NA	0	0	Not-determined	Genome-fragment	NA		0
S3_extracted_k141_80886	1116	No	NA	1	0	Low-quality	Genome-fragment		0.83	0
S3_extracted_k141_74088	1116	No	NA	1	0	Low-quality	Genome-fragment		1.96	0
S9_vs_k141_43089||full	2699	No	NA	0	1	Not-determined	Genome-fragment	NA		0
S9_vs_k141_69207||full	1103	No	NA	0	0	Not-determined	Genome-fragment	NA		0
S10_vs_10326||full	1116	No	NA	0	0	Low-quality	Genome-fragment		0.98	0
S10_vs_103190||full	1116	No	NA	3	0	Low-quality	Genome-fragment		7.55	0
S9_vs_k141_27813||full	43436	No	NA	17	1	High-quality	High-quality		96.78	0
S12_extracted_k141_100684	1116	No	NA	0	1	Low-quality	Genome-fragment		1.35	0
S9_vs_k141_88561||full	1717	No	NA	0	0	Not-determined	Genome-fragment	NA		0
S9_vs_k141_57881||full	2890	No	NA	0	0	Not-determined	Genome-fragment	NA		0
S9_vs_k141_76712||full	1296	No	NA	0	1	Not-determined	Genome-fragment	NA		0
S9_vs_k141_5055||full	1699	No	NA	0	0	Not-determined	Genome-fragment	NA		0
S12_vs_k141_183287||full	1116	No	NA	0	0	Low-quality	Genome-fragment		1.64	0
S9_vs_k141_88571||full	3357	No	NA	0	3	Not-determined	Genome-fragment	NA		0
S9_vs_k141_5070||full	2620	No	NA	0	3	Not-determined	Genome-fragment	NA		0
S8_extracted_k141_31065	1116	No	NA	0	0	Low-quality	Genome-fragment		2.56	0
S8_extracted_k141_137145	1116	No	NA	0	1	Low-quality	Genome-fragment		1.73	0
S8_vs_k141_49103||lt2gene	1116	No	NA	1	0	Low-quality	Genome-fragment		1.97	0
S9_vs_k141_1400||full	1399	No	NA	0	0	Not-determined	Genome-fragment	NA		0
S17_extracted_k141_169811	1117	No	NA	1	0	Low-quality	Genome-fragment		1.89	0
S9_vs_k141_23914||full	1498	No	NA	0	0	Not-determined	Genome-fragment	NA		0
S9_vs_k141_1407||full	2816	No	NA	0	1	Not-determined	Genome-fragment	NA		0
S9_vs_k141_39459||full	1632	No	NA	0	1	Not-determined	Genome-fragment	NA		0
S11_vs_k141_65106||lt2gene	1117	No	NA	1	0	Low-quality	Genome-fragment		2.71	0
S1_extracted_k141_94498	1117	No	NA	0	0	Low-quality	Genome-fragment		2.72	0
S5_vs_k141_7309||full	1117	No	NA	0	0	Low-quality	Genome-fragment		1.07	0
S9_vs_k141_23945||full	2418	No	NA	0	1	Not-determined	Genome-fragment	NA		0
S9_vs_k141_20284||full	1192	No	NA	0	1	Not-determined	Genome-fragment	NA		0
S5_vs_k141_71386||full	1117	No	NA	1	0	Low-quality	Genome-fragment		3.04	0
S5_vs_k141_94334||lt2gene	1117	No	NA	1	0	Low-quality	Genome-fragment		2.85	0
S9_vs_k141_43200||full	1301	No	NA	0	1	Not-determined	Genome-fragment	NA		0
S9_vs_k141_23985||full	2717	No	NA	0	0	Not-determined	Genome-fragment	NA		0
S7_vs_79136||full	1117	No	NA	1	0	Low-quality	Genome-fragment		2.52	0
S16_extracted_143432	1117	No	NA	0	2	Low-quality	Genome-fragment		0.72	0
S16_vs_29224||full	1117	No	NA	1	0	Low-quality	Genome-fragment		0.71	0
S12_extracted_k141_31809	1117	No	NA	0	0	Low-quality	Genome-fragment		1.53	0
S9_vs_k141_69322||full	27451	No	NA	3	0	Medium-quality	Genome-fragment		68.73	0
S12_extracted_k141_39912	1117	No	NA	0	0	Low-quality	Genome-fragment		2.37	0
S9_vs_k141_84512||full	2150	No	NA	0	0	Not-determined	Genome-fragment	NA		0
S9_vs_k141_43255||full	1148	No	NA	0	0	Not-determined	Genome-fragment	NA		0
S12_extracted_k141_151507	1117	No	NA	0	1	Low-quality	Genome-fragment		1.14	0
S17_vs_k141_74821||full	1118	No	NA	0	0	Low-quality	Genome-fragment		2.04	0
S1_vs_k141_131912||full	1118	No	NA	0	0	Low-quality	Genome-fragment		1.47	0
S9_vs_k141_1531||full	3372	No	NA	0	2	Not-determined	Genome-fragment	NA		0
S3_vs_k141_37891||full	1118	No	NA	1	0	Low-quality	Genome-fragment		3.44	0
S7_extracted_84298	1118	No	NA	0	0	Low-quality	Genome-fragment		1.43	0
S7_vs_43857||full	1118	No	NA	0	0	Low-quality	Genome-fragment		0.87	0
S9_vs_k141_54512||full	3125	No	NA	0	0	Not-determined	Genome-fragment	NA		0
S7_vs_131256||lt2gene	1118	No	NA	1	0	Low-quality	Genome-fragment		2.5	0
S16_vs_128966||full	1118	No	NA	1	0	Low-quality	Genome-fragment		1.57	0
S9_vs_k141_65752||full	2477	No	NA	0	0	Not-determined	Genome-fragment	NA		0
S6_vs_k141_20143||full	1118	No	NA	0	0	Low-quality	Genome-fragment		3.12	0
S9_vs_k141_8782||full	1515	No	NA	0	0	Not-determined	Genome-fragment	NA		0
S9_vs_k141_65771||full	2824	No	NA	0	1	Not-determined	Genome-fragment	NA		0
S9_vs_k141_76801||full	1703	No	NA	0	1	Not-determined	Genome-fragment	NA		0
S9_vs_k141_8811||full	1040	No	NA	0	1	Not-determined	Genome-fragment	NA		0
S9_vs_k141_28017||full	1910	No	NA	0	0	Not-determined	Genome-fragment	NA		0
S8_vs_k141_151875||full	1118	No	NA	1	0	Low-quality	Genome-fragment		0.8	0
S11_extracted_k141_53197	1119	No	NA	0	0	Low-quality	Genome-fragment		2.33	0
S9_vs_k141_80939||full	2812	No	NA	0	2	Not-determined	Genome-fragment	NA		0
S9_vs_k141_28050||full	1485	No	NA	0	0	Not-determined	Genome-fragment	NA		0
S9_vs_k141_69466||full	3517	No	NA	0	2	Not-determined	Genome-fragment	NA		0
S11_vs_k141_1953||full	1119	No	NA	0	0	Low-quality	Genome-fragment		2.53	0
S9_vs_k141_62110||full	2179	No	NA	0	1	Not-determined	Genome-fragment	NA		0
S14_extracted_k141_64504	1119	No	NA	0	1	Low-quality	Genome-fragment		0.54	0
S9_vs_k141_80965||full	1423	No	NA	0	0	Not-determined	Genome-fragment	NA		0
S3_vs_k141_28434||full	1119	No	NA	0	0	Low-quality	Genome-fragment		2.67	0
S3_vs_k141_86726||full	1119	No	NA	1	0	Low-quality	Genome-fragment		0.45	0
S3_vs_k141_86985||lt2gene	1119	No	NA	1	0	Low-quality	Genome-fragment		2.93	0
S9_vs_k141_73523||full	2790	No	NA	0	1	Not-determined	Genome-fragment	NA		0
S5_vs_k141_6038||full	1119	No	NA	2	0	Low-quality	Genome-fragment		2.47	0
S7_vs_102789||full	1119	No	NA	1	0	Low-quality	Genome-fragment		3.07	0
S16_vs_4203||full	1119	No	NA	0	1	Low-quality	Genome-fragment		0.58	0
S16_vs_150929||full	1119	No	NA	1	0	Low-quality	Genome-fragment		2.86	0
S10_extracted_40388	1119	No	NA	0	0	Low-quality	Genome-fragment		1.78	0
S9_vs_k141_54656||full	1558	No	NA	0	0	Not-determined	Genome-fragment	NA		0
S12_extracted_k141_5959	1119	No	NA	0	1	Low-quality	Genome-fragment		1.49	0
S9_vs_k141_54660||full	1831	No	NA	0	0	Not-determined	Genome-fragment	NA		0
S12_vs_k141_165004||full	1119	No	NA	1	0	Low-quality	Genome-fragment		2.02	0
S8_extracted_k141_86993	1119	No	NA	0	1	Low-quality	Genome-fragment		2.88	0
S17_extracted_k141_73525	1120	No	NA	0	0	Low-quality	Genome-fragment		1.19	0
S9_vs_k141_16933||full	4431	No	NA	0	2	Not-determined	Genome-fragment	NA		0
S9_vs_k141_58086||full	27326	No	NA	12	2	Medium-quality	Genome-fragment		77.03	0
S9_vs_k141_8553||full	1120	No	NA	2	0	Low-quality	Genome-fragment		2.5	0
S13_vs_8166||full	1120	No	NA	0	1	Low-quality	Genome-fragment		0.44	0
S14_vs_k141_39309||lt2gene	1120	No	NA	1	0	Low-quality	Genome-fragment		1.64	0
S14_vs_k141_19519||lt2gene	1120	No	NA	1	0	Low-quality	Genome-fragment		2.29	0
S9_vs_k141_39674||full	1290	No	NA	0	1	Not-determined	Genome-fragment	NA		0
S9_vs_k141_35808||full	3513	No	NA	0	1	Not-determined	Genome-fragment	NA		0
S3_extracted_k141_124092	1120	No	NA	0	0	Low-quality	Genome-fragment		1.19	0
S9_vs_k141_84678||full	1991	No	NA	0	2	Not-determined	Genome-fragment	NA		0
S10_extracted_21573	1120	No	NA	0	0	Low-quality	Genome-fragment		0.5	0
S12_extracted_k141_124236	1120	No	NA	0	1	Low-quality	Genome-fragment		3.04	0
S9_vs_k141_35820||full	4800	No	NA	0	2	Not-determined	Genome-fragment	NA		0
S15_vs_45881||full	1120	No	NA	0	0	Low-quality	Genome-fragment		0.45	0
S9_vs_k141_35830||full	1271	No	NA	0	1	Not-determined	Genome-fragment	NA		0
S9_vs_k141_28197||full	27879	No	NA	10	0	Medium-quality	Genome-fragment		74.59	0
S15_vs_70494||full	1120	No	NA	0	3	Low-quality	Genome-fragment		1.85	0
S9_vs_k141_1748||full	1704	No	NA	0	1	Not-determined	Genome-fragment	NA		0
S9_vs_k141_20616||full	1812	No	NA	0	1	Not-determined	Genome-fragment	NA		0
S9_vs_k141_35857||full	1403	No	NA	0	1	Not-determined	Genome-fragment	NA		0
S8_extracted_k141_198848	1120	No	NA	0	0	Low-quality	Genome-fragment		2.8	0
S9_vs_k141_54753||full	1321	No	NA	0	0	Not-determined	Genome-fragment	NA		0
S9_vs_k141_65837||full	1297	No	NA	0	0	Not-determined	Genome-fragment	NA		0
S9_vs_k141_43488||full	2065	No	NA	0	1	Not-determined	Genome-fragment	NA		0
S17_extracted_k141_76555	1121	No	NA	0	0	Low-quality	Genome-fragment		2.12	0
S9_vs_k141_39742||full	2342	No	NA	0	1	Not-determined	Genome-fragment	NA		0
S9_vs_k141_81100||full	4207	No	NA	0	2	Not-determined	Genome-fragment	NA		0
S9_vs_k141_46866||full	2827	No	NA	0	1	Not-determined	Genome-fragment	NA		0
S17_vs_k141_146081||full	1121	No	NA	0	2	Low-quality	Genome-fragment		0.88	0
S9_vs_k141_58197||full	1946	No	NA	0	1	Not-determined	Genome-fragment	NA		0
S9_extracted_k141_17367	1121	No	NA	0	0	Low-quality	Genome-fragment		2.32	0
S9_vs_k141_46875||full	2309	No	NA	0	1	Not-determined	Genome-fragment	NA		0
S11_vs_k141_87086||full	1121	No	NA	0	1	Low-quality	Genome-fragment		0.64	0
S9_vs_k141_77037||full	1693	No	NA	0	0	Not-determined	Genome-fragment	NA		0
S11_vs_k141_95559||full	1121	No	NA	1	0	Low-quality	Genome-fragment		1.97	0
S13_vs_61906||full	1121	No	NA	0	0	Low-quality	Genome-fragment		2.56	0
S14_vs_k141_46494||full	1121	No	NA	1	0	Low-quality	Genome-fragment		2.69	0
S14_vs_k141_60212||lt2gene	1121	No	NA	1	0	Low-quality	Genome-fragment		1.64	0
S9_vs_k141_46908||full	1193	No	NA	0	0	Not-determined	Genome-fragment	NA		0
S9_vs_k141_62311||full	3021	No	NA	0	3	Not-determined	Genome-fragment	NA		0
S1_extracted_k141_158632	1121	No	NA	1	0	Low-quality	Genome-fragment		1.87	0
S3_extracted_k141_41132	1121	No	NA	1	0	Low-quality	Genome-fragment		1.44	0
S9_vs_k141_17140||full	2130	No	NA	0	2	Not-determined	Genome-fragment	NA		0
S3_vs_k141_122188||full	1121	No	NA	0	0	Low-quality	Genome-fragment		1.61	0
S7_extracted_5108	1121	No	NA	2	0	Low-quality	Genome-fragment		3.7	0
S7_vs_102738||full	1121	No	NA	0	0	Low-quality	Genome-fragment		1.94	0
S7_vs_54853||lt2gene	1121	No	NA	1	0	Low-quality	Genome-fragment		1.64	0
S9_vs_k141_5600||full	2302	No	NA	0	0	Not-determined	Genome-fragment	NA		0
S9_vs_k141_62334||full	1009	No	NA	0	0	Not-determined	Genome-fragment	NA		0
S16_extracted_15380	1121	No	NA	0	0	Low-quality	Genome-fragment		1.9	0
S9_vs_k141_54878||full	2947	No	NA	0	2	Not-determined	Genome-fragment	NA		0
S16_extracted_86079	1121	No	NA	1	0	Low-quality	Genome-fragment		3.48	0
S9_vs_k141_24425||full	1804	No	NA	0	1	Not-determined	Genome-fragment	NA		0
S10_vs_88078||full	1121	No	NA	0	0	Low-quality	Genome-fragment		2.23	0
S12_vs_k141_46009||full	1121	No	NA	0	1	Low-quality	Genome-fragment		0.36	0
S9_vs_k141_62353||full	2087	No	NA	0	0	Not-determined	Genome-fragment	NA		0
S9_vs_k141_17175||full	2248	No	NA	0	0	Not-determined	Genome-fragment	NA		0

S4_extracted_13057	1121	No	NA	0	0	Low-quality	Genome-fragment		1.06	0
S8_extracted_k141_152045	1121	No	NA	0	0	Low-quality	Genome-fragment		0.66	0
S17_vs_k141_149950||full	1122	No	NA	0	0	Low-quality	Genome-fragment		1.67	0
S9_vs_k141_81209||full	1203	No	NA	0	2	Not-determined	Genome-fragment	NA		0
S1_vs_k141_102255||full	1122	No	NA	0	0	Low-quality	Genome-fragment		2.06	0
S9_vs_k141_46927||full	2539	No	NA	0	1	Not-determined	Genome-fragment	NA		0
S1_vs_k141_32263||full	1122	No	NA	1	0	Low-quality	Genome-fragment		1.83	0
S3_extracted_k141_106953	1122	No	NA	0	0	Low-quality	Genome-fragment		1.87	0
S5_extracted_k141_144238	1122	No	NA	1	0	Low-quality	Genome-fragment		2.32	0
S9_vs_k141_24472||full	4571	No	NA	0	3	Not-determined	Genome-fragment	NA		0
S7_extracted_10877	1122	No	NA	1	0	Low-quality	Genome-fragment		3.62	0
S9_vs_k141_1894||full	3001	No	NA	0	0	Not-determined	Genome-fragment	NA		0
S7_vs_21364||full	1122	No	NA	1	0	Low-quality	Genome-fragment		2.23	0
S16_vs_56427||lt2gene	1122	No	NA	1	0	Low-quality	Genome-fragment		3.29	0
S9_vs_k141_62410||full	3130	No	NA	0	1	Not-determined	Genome-fragment	NA		0
S10_vs_61189||full	1122	No	NA	0	1	Low-quality	Genome-fragment		2.4	0
S9_vs_k141_65910||full	1709	No	NA	0	2	Not-determined	Genome-fragment	NA		0
S10_vs_79667||full	1122	No	NA	0	0	Low-quality	Genome-fragment		3.04	0
S9_vs_k141_28450||full	1747	No	NA	0	0	Not-determined	Genome-fragment	NA		0
S9_vs_k141_1930||full	1187	No	NA	0	0	Not-determined	Genome-fragment	NA		0
S12_extracted_k141_53204	1122	No	NA	1	0	Low-quality	Genome-fragment		2.51	0
S9_vs_k141_81246||full	4676	No	NA	0	4	Not-determined	Genome-fragment	NA		0
S8_extracted_k141_12309	1122	No	NA	1	0	Low-quality	Genome-fragment		2.15	0
S9_vs_k141_77195||full	2418	No	NA	0	0	Not-determined	Genome-fragment	NA		0
S9_vs_k141_65934||full	1137	No	NA	0	0	Not-determined	Genome-fragment	NA		0
S8_vs_k141_155728||full	1122	No	NA	1	0	Low-quality	Genome-fragment		1.14	0
S17_vs_k141_116428||full	1123	No	NA	0	0	Low-quality	Genome-fragment		2.97	0
S9_vs_k141_24546||full	2567	No	NA	0	2	Not-determined	Genome-fragment	NA		0
S17_vs_k141_109864||full	1123	No	NA	0	0	Low-quality	Genome-fragment		1.3	0
S11_vs_k141_22162||full	1123	No	NA	0	0	Low-quality	Genome-fragment		2.2	0
S9_vs_k141_9176||full	1601	No	NA	0	0	Not-determined	Genome-fragment	NA		0
S9_vs_k141_17303||full	2811	No	NA	0	2	Not-determined	Genome-fragment	NA		0
S14_extracted_k141_73218	1123	No	NA	1	0	Low-quality	Genome-fragment		1.99	0
S9_vs_k141_88789||full	3032	No	NA	0	1	Not-determined	Genome-fragment	NA		0
S14_vs_k141_70484||full	1123	No	NA	0	0	Low-quality	Genome-fragment		2.58	0
S5_vs_k141_92942||full	1123	No	NA	2	0	Low-quality	Genome-fragment		2.81	0
S7_extracted_92695	1123	No	NA	0	0	Low-quality	Genome-fragment		1.44	0
S15_vs_62041||full	1123	No	NA	1	0	Low-quality	Genome-fragment		2.16	0
S6_vs_k141_8983||full	1123	No	NA	0	1	Low-quality	Genome-fragment		1.64	0
S17_vs_k141_112893||full	1124	No	NA	0	2	Low-quality	Genome-fragment		0.38	0
S9_vs_k141_1966||full	26960	No	NA	17	1	Medium-quality	Genome-fragment		75.45	0
S9_vs_k141_58402||full	22074	No	NA	8	0	Medium-quality	Genome-fragment		56.74	0
S9_vs_k141_90785||full	1124	No	NA	2	0	Low-quality	Genome-fragment		1.86	0
S9_vs_k141_55072||full	1894	No	NA	0	2	Not-determined	Genome-fragment	NA		0
S9_vs_k141_9250||full	2508	No	NA	0	2	Not-determined	Genome-fragment	NA		0
S11_vs_k141_50521||lt2gene	1124	No	NA	1	0	Low-quality	Genome-fragment		1.7	0
S9_vs_k141_36121||full	4057	No	NA	0	0	Not-determined	Genome-fragment	NA		0
S14_vs_k141_24499||full	1124	No	NA	0	0	Low-quality	Genome-fragment		2.39	0
S9_vs_k141_20869||full	6576	No	NA	0	6	Not-determined	Genome-fragment	NA		0
S1_extracted_k141_48823	1124	No	NA	0	0	Low-quality	Genome-fragment		1.08	0
S9_vs_k141_81370||full	2392	No	NA	0	2	Not-determined	Genome-fragment	NA		0
S3_extracted_k141_124043	1124	No	NA	1	0	Low-quality	Genome-fragment		0.32	0
S9_vs_k141_36142||full	2166	No	NA	0	0	Not-determined	Genome-fragment	NA		0
S5_extracted_k141_113719	1124	No	NA	0	0	Low-quality	Genome-fragment		3.36	0
S9_vs_k141_73764||full	25774	No	NA	18	0	Medium-quality	Genome-fragment		78.08	0
S16_vs_170524||full	1124	No	NA	0	0	Low-quality	Genome-fragment		2.3	0
S9_vs_k141_88847||full	33348	No	NA	9	0	Medium-quality	Genome-fragment		88.26	0
S16_vs_139341||lt2gene	1124	No	NA	1	0	Low-quality	Genome-fragment		3.08	0
S10_extracted_112729	1124	No	NA	1	0	Low-quality	Genome-fragment		1.04	0
S12_vs_k141_21250||full	1124	No	NA	0	0	Low-quality	Genome-fragment		1	0
S9_vs_k141_43844||full	1574	No	NA	0	0	Not-determined	Genome-fragment	NA		0
S9_vs_k141_36162||full	4153	No	NA	0	3	Not-determined	Genome-fragment	NA		0
S12_vs_k141_61261||lt2gene	1124	No	NA	2	0	Low-quality	Genome-fragment		2.76	0
S6_vs_k141_48732||full	1124	No	NA	0	0	Low-quality	Genome-fragment		2.37	0
S17_extracted_k141_51822	1125	No	NA	1	0	Low-quality	Genome-fragment		1.69	0
S9_vs_k141_62557||full	1146	No	NA	0	2	Not-determined	Genome-fragment	NA		0
S9_vs_k141_40115||full	1737	No	NA	0	1	Not-determined	Genome-fragment	NA		0
S17_vs_k141_122952||full	1125	No	NA	0	1	Low-quality	Genome-fragment		2.11	0
S9_vs_k141_62572||full	2174	No	NA	0	0	Not-determined	Genome-fragment	NA		0
S9_vs_k141_34976||full	1125	No	NA	1	0	Low-quality	Genome-fragment		2.04	0
S9_vs_k141_13377||full	2188	No	NA	0	1	Not-determined	Genome-fragment	NA		0
S9_vs_k141_13380||full	1265	No	NA	0	0	Not-determined	Genome-fragment	NA		0
S11_vs_k141_71841||lt2gene	1125	No	NA	1	0	Low-quality	Genome-fragment		2.76	0
S14_vs_k141_24264||full	1125	No	NA	0	0	Low-quality	Genome-fragment		2.14	0
S14_vs_k141_29137||full	1125	No	NA	0	0	Low-quality	Genome-fragment		2.63	0
S3_extracted_k141_94321	1125	No	NA	0	0	Low-quality	Genome-fragment		2.68	0
S5_extracted_k141_109354	1125	No	NA	0	1	Low-quality	Genome-fragment		0.35	0
S5_vs_k141_73531||full	1125	No	NA	1	0	Low-quality	Genome-fragment		2.46	0
S7_vs_46212||full	1125	No	NA	1	0	Low-quality	Genome-fragment		3.21	0
S16_vs_16529||full	1125	No	NA	0	0	Low-quality	Genome-fragment		2.25	0
S9_vs_k141_9442||full	1218	No	NA	0	1	Not-determined	Genome-fragment	NA		0
S16_vs_158820||full	1125	No	NA	0	0	Low-quality	Genome-fragment		2.68	0
S9_vs_k141_58596||full	1859	No	NA	0	1	Not-determined	Genome-fragment	NA		0
S10_extracted_41297	1125	No	NA	0	0	Low-quality	Genome-fragment		2.51	0
S10_vs_1314||lt2gene	1125	No	NA	1	0	Low-quality	Genome-fragment		2.46	0
S6_vs_k141_13941||full	1125	No	NA	2	0	Low-quality	Genome-fragment		2.61	0
S17_vs_k141_111698||full	1126	No	NA	0	1	Low-quality	Genome-fragment		0.87	0
S9_vs_k141_88997||full	2085	No	NA	0	2	Not-determined	Genome-fragment	NA		0
S17_vs_k141_151045||lt2gene	1126	No	NA	1	0	Low-quality	Genome-fragment		2.97	0
S11_vs_k141_95814||full	1126	No	NA	1	0	Low-quality	Genome-fragment		2.71	0
S9_vs_k141_66152||full	3204	No	NA	0	1	Not-determined	Genome-fragment	NA		0
S9_vs_k141_17578||full	1803	No	NA	0	0	Not-determined	Genome-fragment	NA		0
S13_vs_138363||full	1126	No	NA	0	0	Low-quality	Genome-fragment		1.33	0
S9_vs_k141_13487||full	1165	No	NA	0	0	Not-determined	Genome-fragment	NA		0
S9_vs_k141_36290||full	1556	No	NA	0	0	Not-determined	Genome-fragment	NA		0
S14_vs_k141_30519||full	1126	No	NA	1	0	Low-quality	Genome-fragment		1.8	0
S9_vs_k141_28749||full	2895	No	NA	0	3	Not-determined	Genome-fragment	NA		0
S9_vs_k141_9508||full	1892	No	NA	0	2	Not-determined	Genome-fragment	NA		0
S1_vs_k141_12021||full	1126	No	NA	0	0	Low-quality	Genome-fragment		2.94	0
S9_vs_k141_21058||full	1246	No	NA	0	1	Not-determined	Genome-fragment	NA		0
S3_vs_k141_82536||full	1126	No	NA	2	0	Low-quality	Genome-fragment		0.86	0
S3_vs_k141_7149||full	1126	No	NA	0	0	Low-quality	Genome-fragment		2.56	0
S5_extracted_k141_12803	1126	No	NA	0	0	Low-quality	Genome-fragment		2.95	0
S9_vs_k141_32588||full	54215	No	NA	27	1	Medium-quality	Genome-fragment		84.63	0
S5_vs_k141_138904||full	1126	No	NA	0	0	Low-quality	Genome-fragment		1.95	0
S16_vs_141421||lt2gene	1126	No	NA	1	0	Low-quality	Genome-fragment		3.23	0
S9_vs_k141_81572||full	1019	No	NA	0	1	Not-determined	Genome-fragment	NA		0
S12_vs_k141_142803||full	1126	No	NA	0	0	Low-quality	Genome-fragment		1.65	0
S9_vs_k141_44103||full	2280	No	NA	0	0	Not-determined	Genome-fragment	NA		0
S9_vs_k141_89083||full	7414	No	NA	0	4	Not-determined	Genome-fragment	NA		0
S9_vs_k141_36392||full	5703	No	NA	0	2	Not-determined	Genome-fragment	NA		0
S9_vs_k141_2231||full	1421	No	NA	0	1	Not-determined	Genome-fragment	NA		0
S12_vs_k141_168638||full	1126	No	NA	0	0	Low-quality	Genome-fragment		3.07	0
S9_vs_k141_2235||full	1239	No	NA	0	2	Not-determined	Genome-fragment	NA		0
S9_vs_k141_62746||full	4484	No	NA	0	2	Not-determined	Genome-fragment	NA		0
S4_extracted_8746	1126	No	NA	0	0	Low-quality	Genome-fragment		2.9	0
S9_vs_k141_81633||full	1762	No	NA	0	2	Not-determined	Genome-fragment	NA		0
S8_vs_k141_201210||full	1126	No	NA	2	0	Low-quality	Genome-fragment		3.28	0
S17_extracted_k141_52658	1127	No	NA	1	0	Low-quality	Genome-fragment		3.61	0
S17_vs_k141_13519||full	1127	No	NA	0	2	Low-quality	Genome-fragment		2.52	0
S9_vs_k141_8020||full	1127	No	NA	0	0	Low-quality	Genome-fragment		1.34	0
S9_vs_k141_73990||full	2245	No	NA	0	2	Not-determined	Genome-fragment	NA		0
S11_extracted_k141_87614	1127	No	NA	0	0	Low-quality	Genome-fragment		1.86	0
S9_vs_k141_85272||full	1760	No	NA	0	0	Not-determined	Genome-fragment	NA		0
S11_vs_k141_9702||full	1127	No	NA	1	0	Low-quality	Genome-fragment		3.3	0
S1_extracted_k141_113854	1127	No	NA	1	0	Low-quality	Genome-fragment		1.75	0
S9_vs_k141_9612||full	1504	No	NA	0	0	Not-determined	Genome-fragment	NA		0
S1_extracted_k141_75703	1127	No	NA	0	0	Low-quality	Genome-fragment		0.56	0
S9_vs_k141_40397||full	2863	No	NA	0	0	Not-determined	Genome-fragment	NA		0
S1_extracted_k141_30075	1127	No	NA	0	0	Low-quality	Genome-fragment		1.91	0
S1_extracted_k141_106666	1127	No	NA	0	0	Low-quality	Genome-fragment		2.38	0
S3_extracted_k141_102509	1127	No	NA	0	1	Low-quality	Genome-fragment		0.44	0
S5_vs_k141_142054||full	1127	No	NA	2	0	Low-quality	Genome-fragment		3.71	0
S5_vs_k141_32237||lt2gene	1127	No	NA	2	0	Low-quality	Genome-fragment		1.92	0
S7_vs_8367||full	1127	No	NA	0	1	Low-quality	Genome-fragment		0.37	0
S16_vs_50226||lt2gene	1127	No	NA	1	0	Low-quality	Genome-fragment		0.7	0
S12_vs_k141_116123||full	1127	No	NA	0	0	Low-quality	Genome-fragment		2.47	0
S12_vs_k141_155768||full	1127	No	NA	1	0	Low-quality	Genome-fragment		2.45	0
S9_vs_k141_21276||full	6754	No	NA	0	0	Not-determined	Genome-fragment	NA		0
S9_vs_k141_2346||full	1720	No	NA	0	0	Not-determined	Genome-fragment	NA		0
S12_vs_k141_15250||full	1127	No	NA	2	0	Low-quality	Genome-fragment		2.43	0
S9_vs_k141_28986||full	5065	No	NA	0	0	Not-determined	Genome-fragment	NA		0
S17_vs_k141_174782||full	1128	No	NA	0	0	Low-quality	Genome-fragment		2.67	0
S9_vs_k141_40475||full	3413	No	NA	0	0	Not-determined	Genome-fragment	NA		0
S17_vs_k141_14222||full	1128	No	NA	1	0	Low-quality	Genome-fragment		1.81	0
S9_vs_k141_55416||full	1091	No	NA	0	0	Not-determined	Genome-fragment	NA		0
S14_extracted_k141_15526	1128	No	NA	0	1	Low-quality	Genome-fragment		2.56	0
S9_vs_k141_55422||full	2145	No	NA	0	3	Not-determined	Genome-fragment	NA		0
S9_vs_k141_58805||full	7465	No	NA	0	0	Not-determined	Genome-fragment	NA		0
S3_extracted_k141_88101	1128	No	NA	0	0	Low-quality	Genome-fragment		2.28	0
S5_extracted_k141_35633	1128	No	NA	2	0	Low-quality	Genome-fragment		3.03	0
S9_vs_k141_47405||full	1555	No	NA	0	0	Not-determined	Genome-fragment	NA		0
S5_vs_k141_24027||lt2gene	1128	No	NA	2	0	Low-quality	Genome-fragment		2.33	0
S7_extracted_137933	1128	No	NA	1	0	Low-quality	Genome-fragment		4.25	0
S9_vs_k141_81734||full	1253	No	NA	0	2	Not-determined	Genome-fragment	NA		0
S9_vs_k141_55437||full	6960	No	NA	0	2	Not-determined	Genome-fragment	NA		0
S16_extracted_91343	1128	No	NA	1	0	Low-quality	Genome-fragment		0.94	0
S9_vs_k141_21334||full	2389	No	NA	0	1	Not-determined	Genome-fragment	NA		0
S16_vs_8061||full	1128	No	NA	2	0	Low-quality	Genome-fragment		2.65	0
S9_vs_k141_6187||full	3967	No	NA	0	1	Not-determined	Genome-fragment	NA		0
S16_vs_169690||full	1128	No	NA	0	0	Low-quality	Genome-fragment		3	0
S9_vs_k141_62803||full	35651	No	NA	21	0	High-quality	High-quality		92.44	0
S9_vs_k141_55481||full	1772	No	NA	0	2	Not-determined	Genome-fragment	NA		0
S16_vs_138041||lt2gene	1128	No	NA	2	0	Low-quality	Genome-fragment		2.89	0
S10_vs_6587||full	1128	No	NA	0	0	Low-quality	Genome-fragment		2.88	0
S9_vs_k141_36646||full	1075	No	NA	0	1	Not-determined	Genome-fragment	NA		0
S12_extracted_k141_96113	1128	No	NA	2	0	Low-quality	Genome-fragment		1.02	0
S9_vs_k141_62810||full	8469	No	NA	0	1	Not-determined	Genome-fragment	NA		0
S8_vs_k141_33646||full	1128	No	NA	0	0	Low-quality	Genome-fragment		3.16	0
S17_vs_k141_155591||lt2gene	1129	No	NA	1	0	Low-quality	Genome-fragment		3.3	0
S9_extracted_k141_67775	1129	No	NA	0	1	Low-quality	Genome-fragment		0.43	0
S9_vs_k141_62818||full	1541	No	NA	0	0	Not-determined	Genome-fragment	NA		0
S9_vs_k141_74184||full	2382	No	NA	0	2	Not-determined	Genome-fragment	NA		0
S9_vs_k141_87652||lt2gene	1129	No	NA	1	0	Low-quality	Genome-fragment		2.41	0

<table>
<tr><td>S11_vs_k141_33782||lt2gene</td><td>1129</td><td>No</td><td>NA</td><td>1</td><td>0</td><td>Low-quality</td><td>Genome-fragment</td><td></td><td>3.49</td><td>0</td></tr>
<tr><td>S14_extracted_k141_34036</td><td>1129</td><td>No</td><td>NA</td><td>0</td><td>0</td><td>Low-quality</td><td>Genome-fragment</td><td></td><td>1.71</td><td>0</td></tr>
<tr><td>S1_extracted_k141_28410</td><td>1129</td><td>No</td><td>NA</td><td>0</td><td>0</td><td>Low-quality</td><td>Genome-fragment</td><td></td><td>2.03</td><td>0</td></tr>
<tr><td>S9_vs_k141_55518||full</td><td>4802</td><td>No</td><td>NA</td><td>0</td><td>4</td><td>Not-determined</td><td>Genome-fragment</td><td>NA</td><td></td><td>0</td></tr>
<tr><td>S9_vs_k141_66463||full</td><td>1371</td><td>No</td><td>NA</td><td>0</td><td>1</td><td>Not-determined</td><td>Genome-fragment</td><td>NA</td><td></td><td>0</td></tr>
<tr><td>S9_vs_k141_77579||full</td><td>1799</td><td>No</td><td>NA</td><td>0</td><td>2</td><td>Not-determined</td><td>Genome-fragment</td><td>NA</td><td></td><td>0</td></tr>
<tr><td>S9_vs_k141_85472||full</td><td>1704</td><td>No</td><td>NA</td><td>0</td><td>2</td><td>Not-determined</td><td>Genome-fragment</td><td>NA</td><td></td><td>0</td></tr>
<tr><td>S1_vs_k141_157935||lt2gene</td><td>1129</td><td>No</td><td>NA</td><td>1</td><td>0</td><td>Low-quality</td><td>Genome-fragment</td><td></td><td>2.84</td><td>0</td></tr>
<tr><td>S9_vs_k141_55540||full</td><td>2285</td><td>No</td><td>NA</td><td>0</td><td>2</td><td>Not-determined</td><td>Genome-fragment</td><td>NA</td><td></td><td>0</td></tr>
<tr><td>S9_vs_k141_74227||full</td><td>6647</td><td>No</td><td>NA</td><td>0</td><td>3</td><td>Not-determined</td><td>Genome-fragment</td><td>NA</td><td></td><td>0</td></tr>
<tr><td>S3_extracted_k141_97477</td><td>1129</td><td>No</td><td>NA</td><td>1</td><td>0</td><td>Low-quality</td><td>Genome-fragment</td><td></td><td>1.87</td><td>0</td></tr>
<tr><td>S5_extracted_k141_122939</td><td>1129</td><td>No</td><td>NA</td><td>0</td><td>0</td><td>Low-quality</td><td>Genome-fragment</td><td></td><td>2.3</td><td>0</td></tr>
<tr><td>S5_vs_k141_19878||full</td><td>1129</td><td>No</td><td>NA</td><td>0</td><td>1</td><td>Low-quality</td><td>Genome-fragment</td><td></td><td>1.72</td><td>0</td></tr>
<tr><td>S9_vs_k141_32768||full</td><td>2633</td><td>No</td><td>NA</td><td>0</td><td>0</td><td>Not-determined</td><td>Genome-fragment</td><td>NA</td><td></td><td>0</td></tr>
<tr><td>S9_vs_k141_44331||full</td><td>29758</td><td>No</td><td>NA</td><td>18</td><td>1</td><td>Medium-quality</td><td>Genome-fragment</td><td></td><td>71.77</td><td>0</td></tr>
<tr><td>S5_vs_k141_9143||full</td><td>1129</td><td>No</td><td>NA</td><td>0</td><td>0</td><td>Low-quality</td><td>Genome-fragment</td><td></td><td>2.37</td><td>0</td></tr>
<tr><td>S16_vs_131016||lt2gene</td><td>1129</td><td>No</td><td>NA</td><td>0</td><td>0</td><td>Low-quality</td><td>Genome-fragment</td><td></td><td>2.67</td><td>0</td></tr>
<tr><td>S10_vs_65988||full</td><td>1129</td><td>No</td><td>NA</td><td>0</td><td>1</td><td>Low-quality</td><td>Genome-fragment</td><td></td><td>2.76</td><td>0</td></tr>
<tr><td>S9_vs_k141_77604||full</td><td>1279</td><td>No</td><td>NA</td><td>0</td><td>0</td><td>Not-determined</td><td>Genome-fragment</td><td>NA</td><td></td><td>0</td></tr>
<tr><td>S10_vs_633||lt2gene</td><td>1129</td><td>No</td><td>NA</td><td>1</td><td>0</td><td>Low-quality</td><td>Genome-fragment</td><td></td><td>2.04</td><td>0</td></tr>
<tr><td>S10_vs_45215||lt2gene</td><td>1129</td><td>No</td><td>NA</td><td>1</td><td>0</td><td>Low-quality</td><td>Genome-fragment</td><td></td><td>2.62</td><td>0</td></tr>
<tr><td>S12_vs_k141_88623||full</td><td>1129</td><td>No</td><td>NA</td><td>0</td><td>0</td><td>Low-quality</td><td>Genome-fragment</td><td></td><td>2.62</td><td>0</td></tr>
<tr><td>S12_vs_k141_94397||lt2gene</td><td>1129</td><td>No</td><td>NA</td><td>1</td><td>0</td><td>Low-quality</td><td>Genome-fragment</td><td></td><td>1.23</td><td>0</td></tr>
<tr><td>S15_vs_75206||full</td><td>1129</td><td>No</td><td>NA</td><td>1</td><td>0</td><td>Low-quality</td><td>Genome-fragment</td><td></td><td>3.87</td><td>0</td></tr>
<tr><td>S8_vs_k141_104888||full</td><td>1129</td><td>No</td><td>NA</td><td>1</td><td>0</td><td>Low-quality</td><td>Genome-fragment</td><td></td><td>1.39</td><td>0</td></tr>
<tr><td>S8_vs_k141_93129||lt2gene</td><td>1129</td><td>No</td><td>NA</td><td>1</td><td>0</td><td>Low-quality</td><td>Genome-fragment</td><td></td><td>2.93</td><td>0</td></tr>
<tr><td>S9_extracted_k141_86591</td><td>1130</td><td>No</td><td>NA</td><td>2</td><td>0</td><td>Low-quality</td><td>Genome-fragment</td><td></td><td>3.73</td><td>0</td></tr>
<tr><td>S13_extracted_32821</td><td>1130</td><td>No</td><td>NA</td><td>0</td><td>1</td><td>Low-quality</td><td>Genome-fragment</td><td></td><td>1.91</td><td>0</td></tr>
<tr><td>S9_vs_k141_50979||full</td><td>1788</td><td>No</td><td>NA</td><td>0</td><td>2</td><td>Not-determined</td><td>Genome-fragment</td><td>NA</td><td></td><td>0</td></tr>
<tr><td>S9_vs_k141_40606||full</td><td>1481</td><td>No</td><td>NA</td><td>0</td><td>4</td><td>Not-determined</td><td>Genome-fragment</td><td>NA</td><td></td><td>0</td></tr>
<tr><td>S9_vs_k141_17865||full</td><td>8606</td><td>No</td><td>NA</td><td>0</td><td>6</td><td>Not-determined</td><td>Genome-fragment</td><td>NA</td><td></td><td>0</td></tr>
<tr><td>S9_vs_k141_77639||full</td><td>3496</td><td>No</td><td>NA</td><td>0</td><td>4</td><td>Not-determined</td><td>Genome-fragment</td><td>NA</td><td></td><td>0</td></tr>
<tr><td>S9_vs_k141_59008||full</td><td>1502</td><td>No</td><td>NA</td><td>0</td><td>2</td><td>Not-determined</td><td>Genome-fragment</td><td>NA</td><td></td><td>0</td></tr>
<tr><td>S9_vs_k141_29176||full</td><td>2685</td><td>No</td><td>NA</td><td>0</td><td>3</td><td>Not-determined</td><td>Genome-fragment</td><td>NA</td><td></td><td>0</td></tr>
<tr><td>S13_vs_123526||full</td><td>1130</td><td>No</td><td>NA</td><td>2</td><td>0</td><td>Low-quality</td><td>Genome-fragment</td><td></td><td>2.4</td><td>0</td></tr>
<tr><td>S9_vs_k141_89205||full</td><td>2853</td><td>No</td><td>NA</td><td>0</td><td>2</td><td>Not-determined</td><td>Genome-fragment</td><td>NA</td><td></td><td>0</td></tr>
<tr><td>S9_vs_k141_77658||full</td><td>9388</td><td>No</td><td>NA</td><td>0</td><td>1</td><td>Not-determined</td><td>Genome-fragment</td><td>NA</td><td></td><td>0</td></tr>
<tr><td>S9_vs_k141_47631||full</td><td>1754</td><td>No</td><td>NA</td><td>0</td><td>0</td><td>Not-determined</td><td>Genome-fragment</td><td>NA</td><td></td><td>0</td></tr>
<tr><td>S5_vs_k141_5904||full</td><td>1130</td><td>No</td><td>NA</td><td>1</td><td>0</td><td>Low-quality</td><td>Genome-fragment</td><td></td><td>2.55</td><td>0</td></tr>
<tr><td>S9_vs_k141_9939||full</td><td>1994</td><td>No</td><td>NA</td><td>0</td><td>1</td><td>Not-determined</td><td>Genome-fragment</td><td>NA</td><td></td><td>0</td></tr>
<tr><td>S9_vs_k141_74250||full</td><td>1012</td><td>No</td><td>NA</td><td>0</td><td>1</td><td>Not-determined</td><td>Genome-fragment</td><td>NA</td><td></td><td>0</td></tr>
<tr><td>S7_vs_25330||full</td><td>1130</td><td>No</td><td>NA</td><td>0</td><td>1</td><td>Low-quality</td><td>Genome-fragment</td><td></td><td>2.33</td><td>0</td></tr>
<tr><td>S9_vs_k141_47634||full</td><td>1683</td><td>No</td><td>NA</td><td>0</td><td>0</td><td>Not-determined</td><td>Genome-fragment</td><td>NA</td><td></td><td>0</td></tr>
<tr><td>S2_vs_k141_18296||full</td><td>1130</td><td>No</td><td>NA</td><td>1</td><td>0</td><td>Low-quality</td><td>Genome-fragment</td><td></td><td>1.11</td><td>0</td></tr>
<tr><td>S9_vs_k141_2600||full</td><td>3220</td><td>No</td><td>NA</td><td>0</td><td>1</td><td>Not-determined</td><td>Genome-fragment</td><td>NA</td><td></td><td>0</td></tr>
<tr><td>S2_vs_k141_17325||full</td><td>1130</td><td>No</td><td>NA</td><td>0</td><td>0</td><td>Low-quality</td><td>Genome-fragment</td><td></td><td>2.11</td><td>0</td></tr>
<tr><td>S9_vs_k141_74262||full</td><td>1019</td><td>No</td><td>NA</td><td>0</td><td>0</td><td>Not-determined</td><td>Genome-fragment</td><td>NA</td><td></td><td>0</td></tr>
<tr><td>S9_vs_k141_85541||full</td><td>9159</td><td>No</td><td>NA</td><td>0</td><td>9</td><td>Not-determined</td><td>Genome-fragment</td><td>NA</td><td></td><td>0</td></tr>
<tr><td>S8_extracted_k141_47816</td><td>1130</td><td>No</td><td>NA</td><td>0</td><td>1</td><td>Low-quality</td><td>Genome-fragment</td><td></td><td>3.61</td><td>0</td></tr>
<tr><td>S8_vs_k141_96104||full</td><td>1130</td><td>No</td><td>NA</td><td>0</td><td>1</td><td>Low-quality</td><td>Genome-fragment</td><td></td><td>1.48</td><td>0</td></tr>
<tr><td>S9_vs_k141_44434||full</td><td>1117</td><td>No</td><td>NA</td><td>0</td><td>0</td><td>Not-determined</td><td>Genome-fragment</td><td>NA</td><td></td><td>0</td></tr>
<tr><td>S9_vs_k141_70504||lt2gene</td><td>1131</td><td>No</td><td>NA</td><td>1</td><td>0</td><td>Low-quality</td><td>Genome-fragment</td><td></td><td>2.6</td><td>0</td></tr>
<tr><td>S9_vs_k141_81934||full</td><td>3894</td><td>No</td><td>NA</td><td>0</td><td>4</td><td>Not-determined</td><td>Genome-fragment</td><td>NA</td><td></td><td>0</td></tr>
<tr><td>S9_vs_k141_67723||lt2gene</td><td>1131</td><td>No</td><td>NA</td><td>1</td><td>0</td><td>Low-quality</td><td>Genome-fragment</td><td></td><td>1.78</td><td>0</td></tr>
<tr><td>S11_vs_k141_93520||full</td><td>1131</td><td>No</td><td>NA</td><td>1</td><td>0</td><td>Low-quality</td><td>Genome-fragment</td><td></td><td>2.01</td><td>0</td></tr>
<tr><td>S9_vs_k141_17948||full</td><td>1377</td><td>No</td><td>NA</td><td>0</td><td>1</td><td>Not-determined</td><td>Genome-fragment</td><td>NA</td><td></td><td>0</td></tr>
<tr><td>S9_vs_k141_10015||full</td><td>1215</td><td>No</td><td>NA</td><td>0</td><td>0</td><td>Not-determined</td><td>Genome-fragment</td><td>NA</td><td></td><td>0</td></tr>
<tr><td>S9_vs_k141_59105||full</td><td>3312</td><td>No</td><td>NA</td><td>0</td><td>2</td><td>Not-determined</td><td>Genome-fragment</td><td>NA</td><td></td><td>0</td></tr>
<tr><td>S11_vs_k141_58881||lt2gene</td><td>1131</td><td>No</td><td>NA</td><td>1</td><td>0</td><td>Low-quality</td><td>Genome-fragment</td><td></td><td>1.99</td><td>0</td></tr>
<tr><td>S9_vs_k141_13727||full</td><td>1944</td><td>No</td><td>NA</td><td>0</td><td>2</td><td>Not-determined</td><td>Genome-fragment</td><td>NA</td><td></td><td>0</td></tr>
<tr><td>S9_vs_k141_74336||full</td><td>1776</td><td>No</td><td>NA</td><td>0</td><td>1</td><td>Not-determined</td><td>Genome-fragment</td><td>NA</td><td></td><td>0</td></tr>
<tr><td>S13_extracted_69943</td><td>1131</td><td>No</td><td>NA</td><td>0</td><td>2</td><td>Low-quality</td><td>Genome-fragment</td><td></td><td>0.54</td><td>0</td></tr>
<tr><td>S3_vs_k141_90406||full</td><td>1131</td><td>No</td><td>NA</td><td>0</td><td>0</td><td>Low-quality</td><td>Genome-fragment</td><td></td><td>2.56</td><td>0</td></tr>
<tr><td>S5_extracted_k141_117369</td><td>1131</td><td>No</td><td>NA</td><td>2</td><td>0</td><td>Low-quality</td><td>Genome-fragment</td><td></td><td>5.16</td><td>0</td></tr>
<tr><td>S5_vs_k141_141546||full</td><td>1131</td><td>No</td><td>NA</td><td>0</td><td>0</td><td>Low-quality</td><td>Genome-fragment</td><td></td><td>0.65</td><td>0</td></tr>
<tr><td>S9_vs_k141_32888||full</td><td>4622</td><td>No</td><td>NA</td><td>0</td><td>2</td><td>Not-determined</td><td>Genome-fragment</td><td>NA</td><td></td><td>0</td></tr>
<tr><td>S9_vs_k141_10064||full</td><td>1191</td><td>No</td><td>NA</td><td>0</td><td>1</td><td>Not-determined</td><td>Genome-fragment</td><td>NA</td><td></td><td>0</td></tr>
<tr><td>S9_vs_k141_17983||full</td><td>1848</td><td>No</td><td>NA</td><td>0</td><td>0</td><td>Not-determined</td><td>Genome-fragment</td><td>NA</td><td></td><td>0</td></tr>
<tr><td>S5_vs_k141_3366||full</td><td>1131</td><td>No</td><td>NA</td><td>3</td><td>0</td><td>Low-quality</td><td>Genome-fragment</td><td></td><td>2.57</td><td>0</td></tr>
<tr><td>S5_vs_k141_22754||lt2gene</td><td>1131</td><td>No</td><td>NA</td><td>1</td><td>0</td><td>Low-quality</td><td>Genome-fragment</td><td></td><td>0.65</td><td>0</td></tr>
<tr><td>S16_extracted_51965</td><td>1131</td><td>No</td><td>NA</td><td>0</td><td>0</td><td>Low-quality</td><td>Genome-fragment</td><td></td><td>1.04</td><td>0</td></tr>
<tr><td>S12_vs_k141_21127||lt2gene</td><td>1131</td><td>No</td><td>NA</td><td>2</td><td>0</td><td>Low-quality</td><td>Genome-fragment</td><td></td><td>1.37</td><td>0</td></tr>
<tr><td>S6_extracted_k141_45552</td><td>1131</td><td>No</td><td>NA</td><td>1</td><td>0</td><td>Low-quality</td><td>Genome-fragment</td><td></td><td>2.51</td><td>0</td></tr>
<tr><td>S8_extracted_k141_18075</td><td>1131</td><td>No</td><td>NA</td><td>0</td><td>0</td><td>Low-quality</td><td>Genome-fragment</td><td></td><td>2.98</td><td>0</td></tr>
<tr><td>S8_extracted_k141_54877</td><td>1131</td><td>No</td><td>NA</td><td>0</td><td>0</td><td>Low-quality</td><td>Genome-fragment</td><td></td><td>2.13</td><td>0</td></tr>
<tr><td>S8_extracted_k141_136583</td><td>1131</td><td>No</td><td>NA</td><td>0</td><td>0</td><td>Low-quality</td><td>Genome-fragment</td><td></td><td>1.76</td><td>0</td></tr>
<tr><td>S9_vs_k141_32919||full</td><td>1773</td><td>No</td><td>NA</td><td>0</td><td>1</td><td>Not-determined</td><td>Genome-fragment</td><td>NA</td><td></td><td>0</td></tr>
<tr><td>S9_vs_k141_70348||full</td><td>2120</td><td>No</td><td>NA</td><td>0</td><td>0</td><td>Not-determined</td><td>Genome-fragment</td><td>NA</td><td></td><td>0</td></tr>
<tr><td>S9_vs_k141_63089||full</td><td>1811</td><td>No</td><td>NA</td><td>0</td><td>0</td><td>Not-determined</td><td>Genome-fragment</td><td>NA</td><td></td><td>0</td></tr>
<tr><td>S8_extracted_k141_68324</td><td>1131</td><td>No</td><td>NA</td><td>0</td><td>0</td><td>Low-quality</td><td>Genome-fragment</td><td></td><td>2.33</td><td>0</td></tr>
<tr><td>S9_vs_k141_66635||full</td><td>1560</td><td>No</td><td>NA</td><td>0</td><td>1</td><td>Not-determined</td><td>Genome-fragment</td><td>NA</td><td></td><td>0</td></tr>
<tr><td>S8_vs_k141_253||full</td><td>1131</td><td>No</td><td>NA</td><td>0</td><td>0</td><td>Low-quality</td><td>Genome-fragment</td><td></td><td>1.78</td><td>0</td></tr>
<tr><td>S9_vs_k141_18020||full</td><td>1251</td><td>No</td><td>NA</td><td>0</td><td>0</td><td>Not-determined</td><td>Genome-fragment</td><td>NA</td><td></td><td>0</td></tr>
<tr><td>S9_vs_k141_29319||full</td><td>1397</td><td>No</td><td>NA</td><td>0</td><td>0</td><td>Not-determined</td><td>Genome-fragment</td><td>NA</td><td></td><td>0</td></tr>
<tr><td>S17_extracted_k141_16774</td><td>1132</td><td>No</td><td>NA</td><td>1</td><td>0</td><td>Low-quality</td><td>Genome-fragment</td><td></td><td>2.7</td><td>0</td></tr>
<tr><td>S17_extracted_k141_83384</td><td>1132</td><td>No</td><td>NA</td><td>0</td><td>0</td><td>Low-quality</td><td>Genome-fragment</td><td></td><td>0.92</td><td>0</td></tr>
<tr><td>S9_vs_k141_89328||full</td><td>1543</td><td>No</td><td>NA</td><td>0</td><td>0</td><td>Not-determined</td><td>Genome-fragment</td><td>NA</td><td></td><td>0</td></tr>
<tr><td>S17_extracted_k141_113570</td><td>1132</td><td>No</td><td>NA</td><td>0</td><td>1</td><td>Low-quality</td><td>Genome-fragment</td><td></td><td>0.5</td><td>0</td></tr>
<tr><td>S17_vs_k141_125948||lt2gene</td><td>1132</td><td>No</td><td>NA</td><td>1</td><td>0</td><td>Low-quality</td><td>Genome-fragment</td><td></td><td>3.08</td><td>0</td></tr>
<tr><td>S9_vs_k141_10117||full</td><td>1303</td><td>No</td><td>NA</td><td>0</td><td>0</td><td>Not-determined</td><td>Genome-fragment</td><td>NA</td><td></td><td>0</td></tr>
<tr><td>S9_vs_k141_2713||full</td><td>12132</td><td>No</td><td>NA</td><td>0</td><td>5</td><td>Not-determined</td><td>Genome-fragment</td><td>NA</td><td></td><td>0</td></tr>
<tr><td>S9_vs_k141_10119||full</td><td>1021</td><td>No</td><td>NA</td><td>0</td><td>1</td><td>Not-determined</td><td>Genome-fragment</td><td>NA</td><td></td><td>0</td></tr>
<tr><td>S9_vs_k141_21713||full</td><td>2116</td><td>No</td><td>NA</td><td>0</td><td>2</td><td>Not-determined</td><td>Genome-fragment</td><td>NA</td><td></td><td>0</td></tr>
<tr><td>S9_vs_k141_13824||full</td><td>1070</td><td>No</td><td>NA</td><td>0</td><td>0</td><td>Not-determined</td><td>Genome-fragment</td><td>NA</td><td></td><td>0</td></tr>
<tr><td>S9_vs_k141_63127||full</td><td>5787</td><td>No</td><td>NA</td><td>0</td><td>3</td><td>Not-determined</td><td>Genome-fragment</td><td>NA</td><td></td><td>0</td></tr>
<tr><td>S11_vs_k141_69975||lt2gene</td><td>1132</td><td>No</td><td>NA</td><td>1</td><td>0</td><td>Low-quality</td><td>Genome-fragment</td><td></td><td>1.98</td><td>0</td></tr>
<tr><td>S9_vs_k141_77830||full</td><td>8617</td><td>No</td><td>NA</td><td>0</td><td>2</td><td>Not-determined</td><td>Genome-fragment</td><td>NA</td><td></td><td>0</td></tr>
<tr><td>S14_extracted_k141_103280</td><td>1132</td><td>No</td><td>NA</td><td>3</td><td>0</td><td>Low-quality</td><td>Genome-fragment</td><td></td><td>2.83</td><td>0</td></tr>
<tr><td>S9_vs_k141_55738||full</td><td>43096</td><td>No</td><td>NA</td><td>18</td><td>1</td><td>Medium-quality</td><td>Genome-fragment</td><td></td><td>80.9</td><td>0</td></tr>
<tr><td>S9_vs_k141_6555||full</td><td>1165</td><td>No</td><td>NA</td><td>0</td><td>0</td><td>Not-determined</td><td>Genome-fragment</td><td>NA</td><td></td><td>0</td></tr>
<tr><td>S9_vs_k141_77844||full</td><td>1912</td><td>No</td><td>NA</td><td>0</td><td>1</td><td>Not-determined</td><td>Genome-fragment</td><td>NA</td><td></td><td>0</td></tr>
<tr><td>S14_vs_k141_1043||full</td><td>1132</td><td>No</td><td>NA</td><td>0</td><td>0</td><td>Low-quality</td><td>Genome-fragment</td><td></td><td>2.19</td><td>0</td></tr>
<tr><td>S1_vs_k141_77820||full</td><td>1132</td><td>No</td><td>NA</td><td>0</td><td>0</td><td>Low-quality</td><td>Genome-fragment</td><td></td><td>4.5</td><td>0</td></tr>
<tr><td>S1_vs_k141_25683||full</td><td>1132</td><td>No</td><td>NA</td><td>0</td><td>0</td><td>Low-quality</td><td>Genome-fragment</td><td></td><td>0.78</td><td>0</td></tr>
<tr><td>S7_vs_126972||lt2gene</td><td>1132</td><td>No</td><td>NA</td><td>1</td><td>0</td><td>Low-quality</td><td>Genome-fragment</td><td></td><td>3.3</td><td>0</td></tr>
<tr><td>S16_vs_58698||full</td><td>1132</td><td>No</td><td>NA</td><td>0</td><td>0</td><td>Low-quality</td><td>Genome-fragment</td><td></td><td>1.21</td><td>0</td></tr>
<tr><td>S16_vs_153118||full</td><td>1132</td><td>No</td><td>NA</td><td>1</td><td>0</td><td>Low-quality</td><td>Genome-fragment</td><td></td><td>2.45</td><td>0</td></tr>
<tr><td>S9_extracted_k141_75258</td><td>1133</td><td>No</td><td>NA</td><td>0</td><td>1</td><td>Low-quality</td><td>Genome-fragment</td><td></td><td>1.55</td><td>0</td></tr>
<tr><td>S9_vs_k141_18113||full</td><td>1934</td><td>No</td><td>NA</td><td>0</td><td>3</td><td>Not-determined</td><td>Genome-fragment</td><td>NA</td><td></td><td>0</td></tr>
<tr><td>S9_vs_k141_88190||lt2gene</td><td>1133</td><td>No</td><td>NA</td><td>1</td><td>0</td><td>Low-quality</td><td>Genome-fragment</td><td></td><td>3.15</td><td>0</td></tr>
<tr><td>S11_extracted_k141_5403</td><td>1133</td><td>No</td><td>NA</td><td>0</td><td>0</td><td>Low-quality</td><td>Genome-fragment</td><td></td><td>3.89</td><td>0</td></tr>
<tr><td>S9_vs_k141_13880||full</td><td>2243</td><td>No</td><td>NA</td><td>0</td><td>2</td><td>Not-determined</td><td>Genome-fragment</td><td>NA</td><td></td><td>0</td></tr>
<tr><td>S13_vs_81330||full</td><td>1133</td><td>No</td><td>NA</td><td>1</td><td>0</td><td>Low-quality</td><td>Genome-fragment</td><td></td><td>1.94</td><td>0</td></tr>
<tr><td>S9_vs_k141_37010||full</td><td>1330</td><td>No</td><td>NA</td><td>0</td><td>0</td><td>Not-determined</td><td>Genome-fragment</td><td>NA</td><td></td><td>0</td></tr>
<tr><td>S9_vs_k141_47813||full</td><td>3961</td><td>No</td><td>NA</td><td>0</td><td>2</td><td>Not-determined</td><td>Genome-fragment</td><td>NA</td><td></td><td>0</td></tr>
<tr><td>S3_extracted_k141_104139</td><td>1133</td><td>No</td><td>NA</td><td>1</td><td>0</td><td>Low-quality</td><td>Genome-fragment</td><td></td><td>3.35</td><td>0</td></tr>
<tr><td>S3_vs_k141_110177||full</td><td>1133</td><td>No</td><td>NA</td><td>0</td><td>0</td><td>Low-quality</td><td>Genome-fragment</td><td></td><td>3.22</td><td>0</td></tr>
<tr><td>S5_extracted_k141_20921</td><td>1133</td><td>No</td><td>NA</td><td>0</td><td>0</td><td>Low-quality</td><td>Genome-fragment</td><td></td><td>0.91</td><td>0</td></tr>
<tr><td>S5_vs_k141_117424||full</td><td>1133</td><td>No</td><td>NA</td><td>0</td><td>1</td><td>Low-quality</td><td>Genome-fragment</td><td></td><td>2.51</td><td>0</td></tr>
<tr><td>S9_vs_k141_6633||full</td><td>1443</td><td>No</td><td>NA</td><td>0</td><td>0</td><td>Not-determined</td><td>Genome-fragment</td><td>NA</td><td></td><td>0</td></tr>
<tr><td>S5_vs_k141_84609||full</td><td>1133</td><td>No</td><td>NA</td><td>1</td><td>0</td><td>Low-quality</td><td>Genome-fragment</td><td></td><td>2.22</td><td>0</td></tr>
<tr><td>S12_vs_k141_6706||full</td><td>1133</td><td>No</td><td>NA</td><td>0</td><td>0</td><td>Low-quality</td><td>Genome-fragment</td><td></td><td>3.04</td><td>0</td></tr>
<tr><td>S9_vs_k141_21806||full</td><td>1579</td><td>No</td><td>NA</td><td>0</td><td>1</td><td>Not-determined</td><td>Genome-fragment</td><td>NA</td><td></td><td>0</td></tr>
<tr><td>S12_vs_k141_23078||full</td><td>1133</td><td>No</td><td>NA</td><td>0</td><td>0</td><td>Low-quality</td><td>Genome-fragment</td><td></td><td>1.78</td><td>0</td></tr>
<tr><td>S15_vs_69194||full</td><td>1133</td><td>No</td><td>NA</td><td>0</td><td>1</td><td>Low-quality</td><td>Genome-fragment</td><td></td><td>3.62</td><td>0</td></tr>
<tr><td>S8_vs_k141_187155||full</td><td>1133</td><td>No</td><td>NA</td><td>1</td><td>0</td><td>Low-quality</td><td>Genome-fragment</td><td></td><td>2.28</td><td>0</td></tr>
<tr><td>S9_vs_k141_10243||full</td><td>2339</td><td>No</td><td>NA</td><td>0</td><td>0</td><td>Not-determined</td><td>Genome-fragment</td><td>NA</td><td></td><td>0</td></tr>
<tr><td>S9_vs_k141_10246||full</td><td>2410</td><td>No</td><td>NA</td><td>0</td><td>3</td><td>Not-determined</td><td>Genome-fragment</td><td>NA</td><td></td><td>0</td></tr>
<tr><td>S17_extracted_k141_91804</td><td>1134</td><td>No</td><td>NA</td><td>0</td><td>2</td><td>Low-quality</td><td>Genome-fragment</td><td></td><td>2.02</td><td>0</td></tr>
<tr><td>S9_extracted_k141_61570</td><td>1134</td><td>No</td><td>NA</td><td>3</td><td>0</td><td>Low-quality</td><td>Genome-fragment</td><td></td><td>3.74</td><td>0</td></tr>
<tr><td>S9_vs_k141_31151||full</td><td>1134</td><td>No</td><td>NA</td><td>1</td><td>0</td><td>Low-quality</td><td>Genome-fragment</td><td></td><td>2.18</td><td>0</td></tr>
<tr><td>S9_vs_k141_40767||full</td><td>2151</td><td>No</td><td>NA</td><td>0</td><td>1</td><td>Not-determined</td><td>Genome-fragment</td><td>NA</td><td></td><td>0</td></tr>
<tr><td>S9_vs_k141_21848||full</td><td>1596</td><td>No</td><td>NA</td><td>0</td><td>0</td><td>Not-determined</td><td>Genome-fragment</td><td>NA</td><td></td><td>0</td></tr>
<tr><td>S9_vs_k141_44251||full</td><td>1134</td><td>No</td><td>NA</td><td>0</td><td>0</td><td>Low-quality</td><td>Genome-fragment</td><td></td><td>2.87</td><td>0</td></tr>
<tr><td>S9_vs_k141_33105||full</td><td>1163</td><td>No</td><td>NA</td><td>0</td><td>0</td><td>Not-determined</td><td>Genome-fragment</td><td>NA</td><td></td><td>0</td></tr>
<tr><td>S13_vs_34092||full</td><td>1134</td><td>No</td><td>NA</td><td>2</td><td>0</td><td>Low-quality</td><td>Genome-fragment</td><td></td><td>2.78</td><td>0</td></tr>
<tr><td>S9_vs_k141_40778||full</td><td>1502</td><td>No</td><td>NA</td><td>0</td><td>0</td><td>Not-determined</td><td>Genome-fragment</td><td>NA</td><td></td><td>0</td></tr>
<tr><td>S9_vs_k141_33115||full</td><td>1258</td><td>No</td><td>NA</td><td>0</td><td>2</td><td>Not-determined</td><td>Genome-fragment</td><td>NA</td><td></td><td>0</td></tr>
<tr><td>S9_vs_k141_63264||full</td><td>1475</td><td>No</td><td>NA</td><td>0</td><td>1</td><td>Not-determined</td><td>Genome-fragment</td><td>NA</td><td></td><td>0</td></tr>
<tr><td>S1_vs_k141_121762||full</td><td>1134</td><td>No</td><td>NA</td><td>0</td><td>0</td><td>Low-quality</td><td>Genome-fragment</td><td></td><td>2.48</td><td>0</td></tr>
<tr><td>S1_vs_k141_154910||lt2gene</td><td>1134</td><td>No</td><td>NA</td><td>1</td><td>0</td><td>Low-quality</td><td>Genome-fragment</td><td></td><td>1.76</td><td>0</td></tr>
<tr><td>S3_vs_k141_19074||full</td><td>1134</td><td>No</td><td>NA</td><td>0</td><td>0</td><td>Low-quality</td><td>Genome-fragment</td><td></td><td>2.7</td><td>0</td></tr>
<tr><td>S9_vs_k141_2799||full</td><td>60546</td><td>No</td><td>NA</td><td>3</td><td>33</td><td>High-quality</td><td>High-quality</td><td></td><td>90</td><td>0</td></tr>
<tr><td>S9_vs_k141_44663||full</td><td>1854</td><td>No</td><td>NA</td><td>0</td><td>1</td><td>Not-determined</td><td>Genome-fragment</td><td>NA</td><td></td><td>0</td></tr>
<tr><td>S5_extracted_k141_40093</td><td>1134</td><td>No</td><td>NA</td><td>1</td><td>0</td><td>Low-quality</td><td>Genome-fragment</td><td></td><td>2.61</td><td>0</td></tr>
<tr><td>S9_vs_k141_21850||full</td><td>37209</td><td>No</td><td>NA</td><td>9</td><td>2</td><td>Medium-quality</td><td>Genome-fragment</td><td></td><td>77.19</td><td>0</td></tr>
<tr><td>S10_vs_97719||full</td><td>1134</td><td>No</td><td>NA</td><td>1</td><td>0</td><td>Low-quality</td><td>Genome-fragment</td><td></td><td>2.06</td><td>0</td></tr>
<tr><td>S9_vs_k141_63304||full</td><td>1015</td><td>No</td><td>NA</td><td>0</td><td>2</td><td>Not-determined</td><td>Genome-fragment</td><td>NA</td><td></td><td>0</td></tr>
<tr><td>S15_vs_20366||full</td><td>1134</td><td>No</td><td>NA</td><td>1</td><td>0</td><td>Low-quality</td><td>Genome-fragment</td><td></td><td>2.22</td><td>0</td></tr>
<tr><td>S6_vs_k141_5703||full</td><td>1134</td><td>No</td><td>NA</td><td>0</td><td>0</td><td>Low-quality</td><td>Genome-fragment</td><td></td><td>1.83</td><td>0</td></tr>
<tr><td>S8_extracted_k141_112622</td><td>1134</td><td>No</td><td>NA</td><td>3</td><td>0</td><td>Low-quality</td><td>Genome-fragment</td><td></td><td>3.53</td><td>0</td></tr>
<tr><td>S17_vs_k141_120018||lt2gene</td><td>1135</td><td>No</td><td>NA</td><td>0</td><td>0</td><td>Low-quality</td><td>Genome-fragment</td><td></td><td>3.01</td><td>0</td></tr>
<tr><td>S9_extracted_k141_17626</td><td>1135</td><td>No</td><td>NA</td><td>1</td><td>0</td><td>Low-quality</td><td>Genome-fragment</td><td></td><td>2.78</td><td>0</td></tr>
<tr><td>S9_extracted_k141_60322</td><td>1135</td><td>No</td><td>NA</td><td>2</td><td>0</td><td>Low-quality</td><td>Genome-fragment</td><td></td><td>2.08</td><td>0</td></tr>
<tr><td>S11_extracted_k141_58025</td><td>1135</td><td>No</td><td>NA</td><td>0</td><td>3</td><td>Low-quality</td><td>Genome-fragment</td><td></td><td>0.33</td><td>0</td></tr>
<tr><td>S11_vs_k141_34497||full</td><td>1135</td><td>No</td><td>NA</td><td>0</td><td>0</td><td>Low-quality</td><td>Genome-fragment</td><td></td><td>2.21</td><td>0</td></tr>
<tr><td>S3_extracted_k141_79745</td><td>1135</td><td>No</td><td>NA</td><td>0</td><td>1</td><td>Low-quality</td><td>Genome-fragment</td><td></td><td>0.57</td><td>0</td></tr>
<tr><td>S3_vs_k141_55016||full</td><td>1135</td><td>No</td><td>NA</td><td>0</td><td>0</td><td>Low-quality</td><td>Genome-fragment</td><td></td><td>2</td><td>0</td></tr>
<tr><td>S9_vs_k141_6726||full</td><td>1601</td><td>No</td><td>NA</td><td>0</td><td>2</td><td>Not-determined</td><td>Genome-fragment</td><td>NA</td><td></td><td>0</td></tr>
<tr><td>S5_vs_k141_34717||full</td><td>1135</td><td>No</td><td>NA</td><td>1</td><td>0</td><td>Low-quality</td><td>Genome-fragment</td><td></td><td>0.58</td><td>0</td></tr>
<tr><td>S7_vs_128173||full</td><td>1135</td><td>No</td><td>NA</td><td>0</td><td>0</td><td>Low-quality</td><td>Genome-fragment</td><td></td><td>3.01</td><td>0</td></tr>
<tr><td>S9_vs_k141_48006||full</td><td>1970</td><td>No</td><td>NA</td><td>0</td><td>1</td><td>Not-determined</td><td>Genome-fragment</td><td>NA</td><td></td><td>0</td></tr>
<tr><td>S9_vs_k141_18228||full</td><td>1808</td><td>No</td><td>NA</td><td>0</td><td>3</td><td>Not-determined</td><td>Genome-fragment</td><td>NA</td><td></td><td>0</td></tr>
<tr><td>S9_vs_k141_70641||full</td><td>1927</td><td>No</td><td>NA</td><td>0</td><td>1</td><td>Not-determined</td><td>Genome-fragment</td><td>NA</td><td></td><td>0</td></tr>
<tr><td>S16_vs_8265||full</td><td>1135</td><td>No</td><td>NA</td><td>0</td><td>0</td><td>Low-quality</td><td>Genome-fragment</td><td></td><td>2.43</td><td>0</td></tr>
<tr><td>S16_vs_63762||full</td><td>1135</td><td>No</td><td>NA</td><td>0</td><td>0</td><td>Low-quality</td><td>Genome-fragment</td><td></td><td>2.62</td><td>0</td></tr>
<tr><td>S9_vs_k141_85961||full</td><td>2724</td><td>No</td><td>NA</td><td>0</td><td>2</td><td>Not-determined</td><td>Genome-fragment</td><td>NA</td><td></td><td>0</td></tr>
<tr><td>S16_vs_139405||full</td><td>1135</td><td>No</td><td>NA</td><td>2</td><td>0</td><td>Low-quality</td><td>Genome-fragment</td><td></td><td>2.5</td><td>0</td></tr>
<tr><td>S9_vs_k141_70662||full</td><td>2372</td><td>No</td><td>NA</td><td>0</td><td>0</td><td>Not-determined</td><td>Genome-fragment</td><td>NA</td><td></td><td>0</td></tr>
<tr><td>S9_vs_k141_25607||full</td><td>1426</td><td>No</td><td>NA</td><td>0</td><td>2</td><td>Not-determined</td><td>Genome-fragment</td><td>NA</td><td></td><td>0</td></tr>
<tr><td>S12_extracted_k141_152017</td><td>1135</td><td>No</td><td>NA</td><td>1</td><td>0</td><td>Low-quality</td><td>Genome-fragment</td><td></td><td>2.55</td><td>0</td></tr>
<tr><td>S8_extracted_k141_3883</td><td>1135</td><td>No</td><td>NA</td><td>0</td><td>2</td><td>Low-quality</td><td>Genome-fragment</td><td></td><td>0.76</td><td>0</td></tr>
<tr><td>S17_extracted_k141_36566</td><td>1136</td><td>No</td><td>NA</td><td>3</td><td>0</td><td>Low-quality</td><td>Genome-fragment</td><td></td><td>3.74</td><td>0</td></tr>
<tr><td>S17_vs_k141_74407||full</td><td>1136</td><td>No</td><td>NA</td><td>0</td><td>0</td><td>Low-quality</td><td>Genome-fragment</td><td></td><td>3.01</td><td>0</td></tr>
</table>

S17_vs_k141_100148||full	1136	No	NA	2	0	Low-quality	Genome-fragment		2.74	0
S17_vs_k141_20204||full	1136	No	NA	0	0	Low-quality	Genome-fragment		1.16	0
S11_vs_k141_23751||full	1136	No	NA	0	0	Low-quality	Genome-fragment		3.3	0
S9_vs_k141_44770||full	1446	No	NA	0	1	Not-determined	Genome-fragment	NA		0
S9_vs_k141_48067||full	1514	No	NA	0	2	Not-determined	Genome-fragment	NA		0
S9_vs_k141_37191||full	1543	No	NA	0	2	Not-determined	Genome-fragment	NA		0
S9_vs_k141_29653||full	2771	No	NA	0	1	Not-determined	Genome-fragment	NA		0
S9_vs_k141_59411||full	2227	No	NA	0	3	Not-determined	Genome-fragment	NA		0
S13_extracted_149942	1136	No	NA	0	1	Low-quality	Genome-fragment		2.57	0
S9_vs_k141_10424||full	23985	No	NA	5	3	Medium-quality	Genome-fragment		56.13	0
S9_vs_k141_48082||full	1592	No	NA	0	2	Not-determined	Genome-fragment	NA		0
S9_vs_k141_37200||full	7178	No	NA	0	4	Not-determined	Genome-fragment	NA		0
S9_vs_k141_40959||full	1827	No	NA	0	0	Not-determined	Genome-fragment	NA		0
S9_vs_k141_2970||full	2323	No	NA	0	2	Not-determined	Genome-fragment	NA		0
S13_vs_136969||full	1136	No	NA	0	1	Low-quality	Genome-fragment		0.87	0
S1_vs_k141_106864||full	1136	No	NA	1	0	Low-quality	Genome-fragment		2.08	0
S3_extracted_k141_13677	1136	No	NA	0	0	Low-quality	Genome-fragment		1.9	0
S5_vs_k141_92314||full	1136	No	NA	0	0	Low-quality	Genome-fragment		2.25	0
S5_vs_k141_25687||full	1136	No	NA	0	0	Low-quality	Genome-fragment		2.11	0
S7_vs_118689||full	1136	No	NA	0	0	Low-quality	Genome-fragment		2.65	0
S9_vs_k141_25699||full	4342	No	NA	0	1	Not-determined	Genome-fragment	NA		0
S7_vs_122559||full	1136	No	NA	0	1	Low-quality	Genome-fragment		2.44	0
S9_vs_k141_40992||full	1427	No	NA	0	0	Not-determined	Genome-fragment	NA		0
S9_vs_k141_33375||full	2634	No	NA	0	0	Not-determined	Genome-fragment	NA		0
S9_vs_k141_56047||full	1638	No	NA	0	3	Not-determined	Genome-fragment	NA		0
S9_vs_k141_51348||full	1318	No	NA	0	1	Not-determined	Genome-fragment	NA		0
S16_extracted_60206	1136	No	NA	1	0	Low-quality	Genome-fragment		2.95	0
S10_extracted_44082	1136	No	NA	0	1	Low-quality	Genome-fragment		0.73	0
S10_vs_116684||full	1136	No	NA	1	0	Low-quality	Genome-fragment		2.09	0
S9_vs_k141_22020||full	1592	No	NA	0	1	Not-determined	Genome-fragment	NA		0
S17_extracted_k141_173374	1137	No	NA	0	1	Low-quality	Genome-fragment		0.49	0
S9_vs_k141_79949||lt2gene	1137	No	NA	2	0	Low-quality	Genome-fragment		2.64	0
S9_vs_k141_89698||full	1683	No	NA	0	2	Not-determined	Genome-fragment	NA		0
S9_vs_k141_41007||full	2345	No	NA	1	0	Not-determined	Genome-fragment	NA		0
S1_extracted_k141_92214	1137	No	NA	0	1	Low-quality	Genome-fragment		6.77	0
S9_vs_k141_41009||full	5828	No	NA	0	3	Not-determined	Genome-fragment	NA		0
S9_vs_k141_56089||full	2615	No	NA	0	2	Not-determined	Genome-fragment	NA		0
S3_extracted_k141_106306	1137	No	NA	0	1	Low-quality	Genome-fragment		0.5	0
S9_vs_k141_59549||full	1510	No	NA	0	1	Not-determined	Genome-fragment	NA		0
S9_vs_k141_18424||full	1805	No	NA	0	1	Not-determined	Genome-fragment	NA		0
S3_extracted_k141_71125	1137	No	NA	0	1	Low-quality	Genome-fragment		0.65	0
S9_vs_k141_74653||full	1985	No	NA	0	2	Not-determined	Genome-fragment	NA		0
S9_vs_k141_63485||full	37897	No	NA	22	2	High-quality	High-quality		93.66	0
S3_vs_k141_3351||lt2gene	1137	No	NA	1	0	Low-quality	Genome-fragment		2.66	0
S3_vs_k141_44483||lt2gene	1137	No	NA	1	0	Low-quality	Genome-fragment		1.79	0
S9_vs_k141_37315||full	3807	No	NA	0	2	Not-determined	Genome-fragment	NA		0
S9_vs_k141_22070||full	3659	No	NA	0	1	Not-determined	Genome-fragment	NA		0
S9_vs_k141_67052||full	1923	No	NA	0	0	Not-determined	Genome-fragment	NA		0
S9_vs_k141_25816||full	6412	No	NA	0	0	Not-determined	Genome-fragment	NA		0
S9_vs_k141_14089||full	2051	No	NA	0	0	Not-determined	Genome-fragment	NA		0
S9_vs_k141_33457||full	1798	No	NA	0	1	Not-determined	Genome-fragment	NA		0
S9_vs_k141_67059||full	2275	No	NA	0	2	Not-determined	Genome-fragment	NA		0
S5_extracted_k141_33956	1137	No	NA	1	0	Low-quality	Genome-fragment		3.53	0
S9_vs_k141_56154||full	1360	No	NA	0	1	Not-determined	Genome-fragment	NA		0
S9_vs_k141_14098||full	3743	No	NA	0	1	Not-determined	Genome-fragment	NA		0
S9_vs_k141_74705||full	1095	No	NA	0	0	Not-determined	Genome-fragment	NA		0
S9_vs_k141_89776||full	4216	No	NA	0	1	Not-determined	Genome-fragment	NA		0
S5_vs_k141_93278||full	1137	No	NA	0	0	Low-quality	Genome-fragment		2.43	0
S7_extracted_125208	1137	No	NA	0	0	Low-quality	Genome-fragment		3.11	0
S7_vs_53135||full	1137	No	NA	0	0	Low-quality	Genome-fragment		1.91	0
S10_vs_52426||full	1137	No	NA	0	0	Low-quality	Genome-fragment		2.86	0
S12_extracted_k141_122333	1137	No	NA	0	1	Low-quality	Genome-fragment		1.23	0
S9_vs_k141_25853||full	2102	No	NA	0	1	Not-determined	Genome-fragment	NA		0
S9_vs_k141_70838||full	4529	No	NA	0	2	Not-determined	Genome-fragment	NA		0
S9_vs_k141_63560||full	2973	No	NA	0	2	Not-determined	Genome-fragment	NA		0
S9_vs_k141_56170||full	2794	No	NA	0	3	Not-determined	Genome-fragment	NA		0
S9_vs_k141_78246||full	2400	No	NA	0	1	Not-determined	Genome-fragment	NA		0
S9_vs_k141_59640||full	1908	No	NA	0	0	Not-determined	Genome-fragment	NA		0
S12_vs_k141_52758||full	1137	No	NA	2	0	Low-quality	Genome-fragment		1.29	0
S9_vs_k141_86218||full	4083	No	NA	0	7	Not-determined	Genome-fragment	NA		0
S9_vs_k141_22123||full	1458	No	NA	0	1	Not-determined	Genome-fragment	NA		0
S8_vs_k141_49040||full	1137	No	NA	0	2	Low-quality	Genome-fragment		2.39	0
S9_vs_k141_62172||full	1138	No	NA	3	0	Low-quality	Genome-fragment		1.91	0
S11_vs_k141_48803||full	1138	No	NA	1	0	Low-quality	Genome-fragment		2.62	0
S11_vs_k141_71244||full	1138	No	NA	0	0	Low-quality	Genome-fragment		0.67	0
S9_vs_k141_48240||full	33693	No	NA	12	0	Medium-quality	Genome-fragment		81.63	0
S9_vs_k141_22136||full	2939	No	NA	0	1	Not-determined	Genome-fragment	NA		0
S9_vs_k141_10676||full	1937	No	NA	0	2	Not-determined	Genome-fragment	NA		0
S9_vs_k141_41157||full	2817	No	NA	0	1	Not-determined	Genome-fragment	NA		0
S9_vs_k141_7060||full	1613	No	NA	0	1	Not-determined	Genome-fragment	NA		0
S9_vs_k141_18577||full	4323	No	NA	0	0	Not-determined	Genome-fragment	NA		0
S9_vs_k141_56234||full	6567	No	NA	0	0	Not-determined	Genome-fragment	NA		0
S9_vs_k141_14166||full	3122	No	NA	0	0	Not-determined	Genome-fragment	NA		0
S9_vs_k141_59709||full	3039	No	NA	0	2	Not-determined	Genome-fragment	NA		0
S13_extracted_81669	1138	No	NA	0	0	Low-quality	Genome-fragment		1.86	0
S9_vs_k141_70952||full	2393	No	NA	0	1	Not-determined	Genome-fragment	NA		0
S14_vs_k141_64558||full	1138	No	NA	1	0	Low-quality	Genome-fragment		2.61	0
S3_extracted_k141_32085	1138	No	NA	0	1	Low-quality	Genome-fragment		0.64	0
S9_vs_k141_10703||full	3368	No	NA	0	1	Not-determined	Genome-fragment	NA		0
S5_vs_k141_64903||full	1138	No	NA	0	0	Low-quality	Genome-fragment		2.18	0
S9_vs_k141_89926||full	1815	No	NA	0	2	Not-determined	Genome-fragment	NA		0
S5_vs_k141_76730||full	1138	No	NA	0	1	Low-quality	Genome-fragment		0.35	0
S7_extracted_65908	1138	No	NA	1	0	Low-quality	Genome-fragment		2.63	0
S7_vs_115295||full	1138	No	NA	2	0	Low-quality	Genome-fragment		1.78	0
S9_vs_k141_48309||full	1039	No	NA	0	0	Not-determined	Genome-fragment	NA		0
S7_vs_130930||full	1138	No	NA	2	0	Low-quality	Genome-fragment		3.13	0
S9_vs_k141_33625||full	1839	No	NA	0	1	Not-determined	Genome-fragment	NA		0
S9_vs_k141_3173||full	1320	No	NA	0	2	Not-determined	Genome-fragment	NA		0
S4_extracted_19944	1138	No	NA	0	0	Low-quality	Genome-fragment		2.27	0
S4_extracted_30757	1138	No	NA	3	0	Low-quality	Genome-fragment		3.76	0
S9_vs_k141_30030||full	3073	No	NA	0	3	Not-determined	Genome-fragment	NA		0
S9_vs_k141_48325||full	8794	No	NA	0	2	Not-determined	Genome-fragment	NA		0
S9_vs_k141_59760||full	1549	No	NA	0	1	Not-determined	Genome-fragment	NA		0
S9_vs_k141_90037||full	1139	No	NA	0	0	Low-quality	Genome-fragment		1.85	0
S9_vs_k141_37437||full	41219	No	NA	20	1	High-quality	High-quality		98.78	0
S9_vs_k141_30075||full	2621	No	NA	0	1	Not-determined	Genome-fragment	NA		0
S9_vs_k141_3241||full	1192	No	NA	0	0	Not-determined	Genome-fragment	NA		0
S11_extracted_k141_3884	1139	No	NA	0	0	Low-quality	Genome-fragment		2.38	0
S9_vs_k141_71037||full	2512	No	NA	0	2	Not-determined	Genome-fragment	NA		0
S9_vs_k141_37449||full	1798	No	NA	0	1	Not-determined	Genome-fragment	NA		0
S5_extracted_k141_32384	1139	No	NA	1	0	Low-quality	Genome-fragment		2.05	0
S16_vs_44927||full	1139	No	NA	1	0	Low-quality	Genome-fragment		3.14	0
S9_vs_k141_89957||full	1976	No	NA	0	4	Not-determined	Genome-fragment	NA		0
S12_extracted_k141_5387	1139	No	NA	0	0	Low-quality	Genome-fragment		2.21	0
S12_vs_k141_101040||full	1139	No	NA	0	0	Low-quality	Genome-fragment		0.61	0
S9_vs_k141_48347||full	35042	No	NA	21	0	High-quality	High-quality		100	0
S12_vs_k141_125694||full	1139	No	NA	1	0	Low-quality	Genome-fragment		2.86	0
S9_vs_k141_18744||full	3213	No	NA	0	2	Not-determined	Genome-fragment	NA		0
S9_vs_k141_14235||full	21474	No	NA	5	2	Medium-quality	Genome-fragment		57.29	0
S9_vs_k141_51532||full	1569	No	NA	0	2	Not-determined	Genome-fragment	NA		0
S12_vs_k141_15562||full	1139	No	NA	0	0	Low-quality	Genome-fragment		2.01	0
S11_vs_k141_74885||full	1140	No	NA	0	0	Low-quality	Genome-fragment		0.4	0
S9_vs_k141_51543||full	2126	No	NA	0	3	Not-determined	Genome-fragment	NA		0
S9_vs_k141_86383||full	1326	No	NA	0	0	Not-determined	Genome-fragment	NA		0
S11_vs_k141_88753||full	1140	No	NA	2	0	Low-quality	Genome-fragment		3.9	0
S9_vs_k141_10867||full	1479	No	NA	0	0	Not-determined	Genome-fragment	NA		0
S9_vs_k141_41361||full	2627	No	NA	0	0	Not-determined	Genome-fragment	NA		0
S9_vs_k141_56354||full	44039	No	NA	17	2	High-quality	High-quality		98.12	0
S11_vs_k141_64489||lt2gene	1140	No	NA	2	0	Low-quality	Genome-fragment		3.28	0
S14_extracted_k141_110710	1140	No	NA	0	0	Low-quality	Genome-fragment		1.97	0
S14_extracted_k141_7073	1140	No	NA	0	1	Low-quality	Genome-fragment		1.21	0
S9_vs_k141_67291||full	1594	No	NA	0	2	Not-determined	Genome-fragment	NA		0
S14_vs_k141_40567||full	1140	No	NA	2	0	Low-quality	Genome-fragment		3.28	0
S1_extracted_k141_119646	1140	No	NA	0	0	Low-quality	Genome-fragment		1.79	0
S1_vs_k141_157295||lt2gene	1140	No	NA	1	0	Low-quality	Genome-fragment		0.66	0
S3_vs_k141_48255||full	1140	No	NA	1	0	Low-quality	Genome-fragment		0.5	0
S5_extracted_k141_122802	1140	No	NA	1	0	Low-quality	Genome-fragment		3.86	0
S9_vs_k141_41371||full	25415	No	NA	21	0	Medium-quality	Genome-fragment		58.18	0
S5_extracted_k141_104979	1140	No	NA	2	0	Low-quality	Genome-fragment		1.64	0
S5_vs_k141_115528||full	1140	No	NA	2	0	Low-quality	Genome-fragment		2.24	0
S9_vs_k141_59919||full	1641	No	NA	0	1	Not-determined	Genome-fragment	NA		0
S5_vs_k141_128205||lt2gene	1140	No	NA	2	0	Low-quality	Genome-fragment		2.45	0
S9_vs_k141_41397||full	4342	No	NA	0	0	Not-determined	Genome-fragment	NA		0
S9_vs_k141_37560||full	4684	No	NA	0	2	Not-determined	Genome-fragment	NA		0
S7_vs_45577||full	1140	No	NA	0	1	Low-quality	Genome-fragment		0.44	0
S9_vs_k141_71174||full	1108	No	NA	0	0	Not-determined	Genome-fragment	NA		0
S9_vs_k141_78576||full	1205	No	NA	0	0	Not-determined	Genome-fragment	NA		0
S7_vs_85470||full	1140	No	NA	0	0	Low-quality	Genome-fragment		2.22	0
S10_vs_7250||full	1140	No	NA	0	0	Low-quality	Genome-fragment		0.62	0
S4_extracted_21185	1140	No	NA	2	0	Low-quality	Genome-fragment		2.3	0
S9_vs_k141_56427||full	3567	No	NA	0	0	Not-determined	Genome-fragment	NA		0
S8_vs_k141_122922||full	1140	No	NA	0	3	Low-quality	Genome-fragment		1.59	0
S9_vs_k141_14352||full	1618	No	NA	0	1	Not-determined	Genome-fragment	NA		0
S9_vs_k141_78619||full	1717	No	NA	0	1	Not-determined	Genome-fragment	NA		0
S11_vs_k141_506||full	1141	No	NA	0	0	Low-quality	Genome-fragment		1.89	0
S9_vs_k141_30243||full	1921	No	NA	0	1	Not-determined	Genome-fragment	NA		0
S11_vs_k141_46607||full	1141	No	NA	1	0	Low-quality	Genome-fragment		1.98	0
S9_vs_k141_75109||full	1744	No	NA	0	0	Not-determined	Genome-fragment	NA		0
S11_vs_k141_88227||full	1141	No	NA	1	0	Low-quality	Genome-fragment		2.67	0
S9_vs_k141_26179||full	23328	No	NA	16	1	Medium-quality	Genome-fragment		68.67	0
S13_extracted_27313	1141	No	NA	0	0	Low-quality	Genome-fragment		2.06	0
S3_vs_k141_21646||full	1141	No	NA	0	0	Low-quality	Genome-fragment		1.95	0
S3_vs_k141_6333||lt2gene	1141	No	NA	2	0	Low-quality	Genome-fragment		2.77	0
S9_vs_k141_3417||full	1347	No	NA	0	1	Not-determined	Genome-fragment	NA		0
S5_vs_k141_100771||full	1141	No	NA	1	0	Low-quality	Genome-fragment		2.66	0
S9_vs_k141_67414||full	1011	No	NA	0	1	Not-determined	Genome-fragment	NA		0
S7_vs_14867||full	1141	No	NA	0	1	Low-quality	Genome-fragment		0.7	0
S16_extracted_85965	1141	No	NA	1	0	Low-quality	Genome-fragment		2.43	0
S9_vs_k141_41462||full	1525	No	NA	0	0	Not-determined	Genome-fragment	NA		0
S9_vs_k141_67419||full	3255	No	NA	0	2	Not-determined	Genome-fragment	NA		0
S16_extracted_119077	1141	No	NA	3	0	Low-quality	Genome-fragment		2.42	0
S16_vs_131369||full	1141	No	NA	1	0	Low-quality	Genome-fragment		2.57	0
S9_vs_k141_41467||full	1921	No	NA	0	0	Not-determined	Genome-fragment	NA		0
S12_extracted_k141_143058	1141	No	NA	0	0	Low-quality	Genome-fragment		2.41	0

S12_vs_k141_112448||full	1141	No	NA	0	0	Low-quality	Genome-fragment	0.84	0
S15_vs_17424||full	1141	No	NA	0	0	Low-quality	Genome-fragment	1.34	0
S9_vs_k141_14450||full	2476	No	NA	0	2	Not-determined	Genome-fragment	NA	0
S6_vs_k141_13956||full	1141	No	NA	2	0	Low-quality	Genome-fragment	2.9	0
S8_vs_k141_26228||full	1141	No	NA	0	0	Low-quality	Genome-fragment	2.59	0
S9_vs_k141_90185||full	1085	No	NA	0	0	Not-determined	Genome-fragment	NA	0
S17_extracted_k141_139099	1142	No	NA	0	0	Low-quality	Genome-fragment	0.57	0
S9_vs_k141_11044||full	1063	No	NA	0	0	Not-determined	Genome-fragment	NA	0
S9_vs_k141_75168||full	1467	No	NA	0	0	Not-determined	Genome-fragment	NA	0
S17_extracted_k141_95097	1142	No	NA	0	1	Low-quality	Genome-fragment	2.26	0
S9_vs_k141_75175||full	1093	No	NA	0	2	Not-determined	Genome-fragment	NA	0
S9_vs_k141_3467||full	2177	No	NA	0	1	Not-determined	Genome-fragment	NA	0
S9_vs_k141_78689||full	1098	No	NA	0	0	Not-determined	Genome-fragment	NA	0
S9_vs_k141_7440||full	1321	No	NA	0	1	Not-determined	Genome-fragment	NA	0
S9_vs_k141_75195||full	2839	No	NA	0	2	Not-determined	Genome-fragment	NA	0
S13_extracted_9908	1142	No	NA	0	0	Low-quality	Genome-fragment	2.05	0
S9_vs_k141_56556||full	1197	No	NA	0	2	Not-determined	Genome-fragment	NA	0
S13_extracted_145962	1142	No	NA	0	0	Low-quality	Genome-fragment	2.47	0
S13_vs_133156||lt2gene	1142	No	NA	1	0	Low-quality	Genome-fragment	2.19	0
S9_vs_k141_26324||full	1969	No	NA	0	1	Not-determined	Genome-fragment	NA	0
S14_extracted_k141_10151	1142	No	NA	2	0	Low-quality	Genome-fragment	3.75	0
S9_vs_k141_67538||full	2639	No	NA	0	1	Not-determined	Genome-fragment	NA	0
S1_vs_k141_158659||full	1142	No	NA	0	0	Low-quality	Genome-fragment	0.74	0
S3_vs_k141_58133||full	1142	No	NA	0	0	Low-quality	Genome-fragment	2.36	0
S9_vs_k141_51676||full	2849	No	NA	0	1	Not-determined	Genome-fragment	NA	0
S5_extracted_k141_137757	1142	No	NA	0	1	Low-quality	Genome-fragment	0.43	0
S9_vs_k141_78779||full	1306	No	NA	0	2	Not-determined	Genome-fragment	NA	0
S9_vs_k141_37768||full	5737	No	NA	0	0	Not-determined	Genome-fragment	NA	0
S9_vs_k141_48538||full	1402	No	NA	0	3	Not-determined	Genome-fragment	NA	0
S7_extracted_674	1142	No	NA	1	0	Low-quality	Genome-fragment	2.72	0
S9_vs_k141_78796||full	3577	No	NA	0	3	Not-determined	Genome-fragment	NA	0
S7_vs_13867||full	1142	No	NA	2	0	Low-quality	Genome-fragment	2.5	0
S7_vs_42483||full	1142	No	NA	1	0	Low-quality	Genome-fragment	2.35	0
S7_vs_74338||lt2gene	1142	No	NA	1	0	Low-quality	Genome-fragment	0.44	0
S9_vs_k141_3557||full	2130	No	NA	0	0	Not-determined	Genome-fragment	NA	0
S9_vs_k141_75290||full	12648	No	NA	6	0	High-quality	High-quality	92.6	0
S9_vs_k141_14662||full	2418	No	NA	0	1	Not-determined	Genome-fragment	NA	0
S16_extracted_58727	1142	No	NA	0	1	Low-quality	Genome-fragment	1.94	0
S9_vs_k141_90304||full	3180	No	NA	0	3	Not-determined	Genome-fragment	NA	0
S16_vs_14664||full	1142	No	NA	0	1	Low-quality	Genome-fragment	0.82	0
S10_extracted_17657	1142	No	NA	0	0	Low-quality	Genome-fragment	2.11	0
S9_vs_k141_51796||full	2447	No	NA	0	2	Not-determined	Genome-fragment	NA	0
S10_extracted_104192	1142	No	NA	0	1	Low-quality	Genome-fragment	1.57	0
S12_vs_k141_49689||full	1142	No	NA	0	0	Low-quality	Genome-fragment	2.7	0
S9_vs_k141_67662||full	1693	No	NA	0	0	Not-determined	Genome-fragment	NA	0
S9_vs_k141_14698||full	3810	No	NA	0	3	Not-determined	Genome-fragment	NA	0
S12_vs_k141_28971||full	1142	No	NA	0	0	Low-quality	Genome-fragment	1.43	0
S9_vs_k141_51847||full	3948	No	NA	0	1	Not-determined	Genome-fragment	NA	0
S9_vs_k141_67686||full	1662	No	NA	0	0	Not-determined	Genome-fragment	NA	0
S12_vs_k141_101059||full	1142	No	NA	0	0	Low-quality	Genome-fragment	2.46	0
S17_vs_k141_10063||full	1143	No	NA	1	0	Low-quality	Genome-fragment	1.32	0
S9_vs_k141_46315||full	1143	No	NA	3	0	Low-quality	Genome-fragment	2.05	0
S9_vs_k141_67696||full	1223	No	NA	0	0	Not-determined	Genome-fragment	NA	0
S9_vs_k141_14734||full	3251	No	NA	0	2	Not-determined	Genome-fragment	NA	0
S9_vs_k141_56747||full	2542	No	NA	0	0	Not-determined	Genome-fragment	NA	0
S11_vs_k141_54093||full	1143	No	NA	0	0	Low-quality	Genome-fragment	2.23	0
S9_vs_k141_60256||full	1825	No	NA	0	0	Not-determined	Genome-fragment	NA	0
S11_vs_k141_74297||full	1143	No	NA	0	0	Low-quality	Genome-fragment	1.12	0
S9_vs_k141_3678||full	1004	No	NA	0	1	Not-determined	Genome-fragment	NA	0
S1_extracted_k141_81508	1143	No	NA	1	0	Low-quality	Genome-fragment	1.42	0
S1_extracted_k141_159237	1143	No	NA	0	0	Low-quality	Genome-fragment	1.43	0
S9_vs_k141_48692||full	1272	No	NA	0	0	Not-determined	Genome-fragment	NA	0
S9_vs_k141_78993||full	3810	No	NA	0	4	Not-determined	Genome-fragment	NA	0
S9_vs_k141_63732||full	2535	No	NA	0	2	Not-determined	Genome-fragment	NA	0
S9_vs_k141_75406||full	7706	No	NA	0	8	Not-determined	Genome-fragment	NA	0
S5_extracted_k141_107443	1143	No	NA	1	0	Low-quality	Genome-fragment	2.94	0
S9_vs_k141_11339||full	1242	No	NA	0	0	Not-determined	Genome-fragment	NA	0
S9_vs_k141_71681||full	3964	No	NA	0	1	Not-determined	Genome-fragment	NA	0
S5_vs_k141_51455||full	1143	No	NA	0	0	Low-quality	Genome-fragment	0.39	0
S7_vs_55124||full	1143	No	NA	0	1	Low-quality	Genome-fragment	0.95	0
S9_vs_k141_52069||full	2367	No	NA	0	0	Not-determined	Genome-fragment	NA	0
S9_vs_k141_63810||full	2147	No	NA	0	2	Not-determined	Genome-fragment	NA	0
S9_vs_k141_52089||full	1576	No	NA	0	1	Not-determined	Genome-fragment	NA	0
S9_vs_k141_86892||full	2008	No	NA	0	0	Not-determined	Genome-fragment	NA	0
S7_vs_133452||lt2gene	1143	No	NA	2	0	Low-quality	Genome-fragment	1.63	0
S9_vs_k141_67792||full	72275	Yes	39401	26	18	Complete	High-quality	100	45.48
S9_vs_k141_90476||full	1030	No	NA	0	0	Not-determined	Genome-fragment	NA	0
S9_vs_k141_71754||full	1222	No	NA	0	1	Not-determined	Genome-fragment	NA	0
S10_extracted_13057	1143	No	NA	0	1	Low-quality	Genome-fragment	0.31	0
S9_vs_k141_71756||full	1667	No	NA	0	1	Not-determined	Genome-fragment	NA	0
S9_vs_k141_82948||full	2515	No	NA	0	0	Not-determined	Genome-fragment	NA	0
S9_vs_k141_86943||full	4274	No	NA	0	1	Not-determined	Genome-fragment	NA	0
S9_vs_k141_71761||full	1652	No	NA	0	1	Not-determined	Genome-fragment	NA	0
S9_vs_k141_82962||full	1878	No	NA	0	1	Not-determined	Genome-fragment	NA	0
S10_extracted_48853	1143	No	NA	0	0	Low-quality	Genome-fragment	1.42	0
S9_vs_k141_67844||full	3582	No	NA	0	2	Not-determined	Genome-fragment	NA	0
S10_vs_82184||lt2gene	1143	No	NA	2	0	Low-quality	Genome-fragment	3.02	0
S9_vs_k141_63859||full	1979	No	NA	0	0	Not-determined	Genome-fragment	NA	0
S9_vs_k141_48874||full	2126	No	NA	0	0	Not-determined	Genome-fragment	NA	0
S12_vs_k141_176760||lt2gene	1143	No	NA	1	0	Low-quality	Genome-fragment	2.16	0
S15_extracted_41530	1143	No	NA	0	0	Low-quality	Genome-fragment	1.23	0
S9_vs_k141_71837||full	1358	No	NA	0	1	Not-determined	Genome-fragment	NA	0
S2_vs_k141_255||full	1143	No	NA	0	1	Low-quality	Genome-fragment	0.49	0
S9_vs_k141_14967||full	3863	No	NA	0	3	Not-determined	Genome-fragment	NA	0
S9_vs_k141_79217||full	1208	No	NA	0	2	Not-determined	Genome-fragment	NA	0
S17_extracted_k141_117086	1144	No	NA	0	0	Low-quality	Genome-fragment	2.99	0
S9_vs_k141_9712||full	1144	No	NA	2	0	Low-quality	Genome-fragment	2.63	0
S9_vs_k141_34092||full	1647	No	NA	0	0	Not-determined	Genome-fragment	NA	0
S9_vs_k141_52222||full	3291	No	NA	0	0	Not-determined	Genome-fragment	NA	0
S9_vs_k141_15014||full	2014	No	NA	0	2	Not-determined	Genome-fragment	NA	0
S11_extracted_k141_38071	1144	No	NA	0	0	Low-quality	Genome-fragment	3.05	0
S13_vs_22969||full	1144	No	NA	0	0	Low-quality	Genome-fragment	2.85	0
S9_vs_k141_68005||full	2933	No	NA	0	1	Not-determined	Genome-fragment	NA	0
S13_vs_68772||full	1144	No	NA	0	0	Low-quality	Genome-fragment	2	0
S9_vs_k141_64004||full	1613	No	NA	0	2	Not-determined	Genome-fragment	NA	0
S9_vs_k141_60430||full	1505	No	NA	0	1	Not-determined	Genome-fragment	NA	0
S13_vs_104546||full	1144	No	NA	0	0	Low-quality	Genome-fragment	2.79	0
S1_vs_k141_85486||lt2gene	1144	No	NA	2	0	Low-quality	Genome-fragment	2.81	0
S3_vs_k141_95685||full	1144	No	NA	3	0	Low-quality	Genome-fragment	3.75	0
S5_extracted_k141_93878	1144	No	NA	1	0	Low-quality	Genome-fragment	1.81	0
S7_vs_33958||lt2gene	1144	No	NA	1	0	Low-quality	Genome-fragment	2.94	0
S9_vs_k141_49063||full	1896	No	NA	0	1	Not-determined	Genome-fragment	NA	0
S10_vs_58081||full	1144	No	NA	0	0	Low-quality	Genome-fragment	1.98	0
S10_vs_75924||lt2gene	1144	No	NA	1	0	Low-quality	Genome-fragment	3.07	0
S9_vs_k141_83108||full	88175	No	NA	16	1	High-quality	High-quality	100	0
S9_vs_k141_83137||full	1010	No	NA	0	0	Not-determined	Genome-fragment	NA	0
S9_vs_k141_52280||full	5521	No	NA	0	3	Not-determined	Genome-fragment	NA	0
S12_extracted_k141_46902	1144	No	NA	1	0	Low-quality	Genome-fragment	2.75	0
S8_vs_k141_111140||full	1144	No	NA	0	1	Low-quality	Genome-fragment	2.46	0
S17_extracted_k141_156129	1145	No	NA	1	0	Low-quality	Genome-fragment	2.07	0
S17_extracted_k141_62048	1145	No	NA	2	0	Low-quality	Genome-fragment	2.69	0
S11_vs_k141_83167||full	1145	No	NA	0	0	Low-quality	Genome-fragment	3.15	0
S14_extracted_k141_7749	1145	No	NA	1	0	Low-quality	Genome-fragment	3.78	0
S14_extracted_k141_9687	1145	No	NA	3	0	Low-quality	Genome-fragment	3.74	0
S9_vs_k141_49152||full	2871	No	NA	0	1	Not-determined	Genome-fragment	NA	0
S14_extracted_k141_76355	1145	No	NA	0	0	Low-quality	Genome-fragment	2.43	0
S1_extracted_k141_17172	1145	No	NA	0	1	Low-quality	Genome-fragment	1.95	0
S9_vs_k141_83290||full	2163	No	NA	0	0	Not-determined	Genome-fragment	NA	0
S3_vs_k141_2162||full	1145	No	NA	0	0	Low-quality	Genome-fragment	2.16	0
S9_vs_k141_49246||full	1053	No	NA	0	2	Not-determined	Genome-fragment	NA	0
S9_vs_k141_90727||full	173711	Yes	66238	26	68	High-quality	High-quality	97.83	61.87
S9_vs_k141_52543||full	1797	No	NA	0	0	Not-determined	Genome-fragment	NA	0
S5_extracted_k141_103184	1145	No	NA	0	1	Low-quality	Genome-fragment	2.68	0
S16_extracted_58709	1145	No	NA	1	0	Low-quality	Genome-fragment	2.5	0
S9_vs_k141_52757||full	1526	No	NA	0	0	Not-determined	Genome-fragment	NA	0
S16_vs_41073||full	1145	No	NA	0	0	Low-quality	Genome-fragment	2.86	0
S12_extracted_k141_181715	1145	No	NA	0	1	Low-quality	Genome-fragment	1.48	0
S12_extracted_k141_72947	1145	No	NA	3	0	Low-quality	Genome-fragment	3.78	0
S9_vs_k141_16723||full	1146	No	NA	0	1	Low-quality	Genome-fragment	0.59	0
S9_vs_k141_23649||0_partial	18591	No	NA	0	1	Not-determined	Genome-fragment	NA	0
S9_vs_k141_55642||lt2gene	1146	No	NA	1	0	Low-quality	Genome-fragment	2.83	0
S1_extracted_k141_119796	1146	No	NA	1	0	Low-quality	Genome-fragment	1.92	0
S1_extracted_k141_106755	1146	No	NA	0	0	Low-quality	Genome-fragment	2.17	0
S1_vs_k141_48609||full	1146	No	NA	0	0	Low-quality	Genome-fragment	1.87	0
S5_extracted_k141_53674	1146	No	NA	0	0	Low-quality	Genome-fragment	1.41	0
S9_vs_k141_38095||lt2gene	1878	No	NA	0	0	Not-determined	Genome-fragment	NA	0
S5_vs_k141_68374||full	1146	No	NA	1	0	Low-quality	Genome-fragment	3.06	0
S5_vs_k141_73428||lt2gene	1146	No	NA	1	0	Low-quality	Genome-fragment	2.68	0
S16_extracted_29876	1146	No	NA	1	0	Low-quality	Genome-fragment	2.9	0
S16_extracted_122610	1146	No	NA	2	0	Low-quality	Genome-fragment	2.54	0
S16_extracted_152200	1146	No	NA	0	0	Low-quality	Genome-fragment	1.81	0
S16_vs_30547||full	1146	No	NA	0	0	Low-quality	Genome-fragment	0.55	0
S15_extracted_45120	1146	No	NA	0	1	Low-quality	Genome-fragment	1.58	0
S8_extracted_k141_73088	1146	No	NA	1	0	Low-quality	Genome-fragment	0.62	0
S8_extracted_k141_127981	1146	No	NA	0	0	Low-quality	Genome-fragment	1.68	0
S8_vs_k141_105702||full	1146	No	NA	0	0	Low-quality	Genome-fragment	2.8	0
S17_vs_k141_24566||full	1147	No	NA	0	1	Low-quality	Genome-fragment	0.37	0
S11_extracted_k141_12492	1147	No	NA	1	0	Low-quality	Genome-fragment	2.56	0
S11_vs_k141_898||full	1147	No	NA	1	0	Low-quality	Genome-fragment	2.68	0
S11_vs_k141_54717||full	1147	No	NA	1	0	Low-quality	Genome-fragment	2.65	0
S9_vs_k141_42609||lt2gene	2243	No	NA	1	0	Medium-quality	Genome-fragment	53.9	0
S14_vs_k141_106703||full	1147	No	NA	4	0	Low-quality	Genome-fragment	2.99	0
S9_vs_k141_31261||lt2gene	1197	No	NA	0	0	Not-determined	Genome-fragment	NA	0
S14_vs_k141_13631||lt2gene	1147	No	NA	0	0	Low-quality	Genome-fragment	1.88	0
S3_extracted_k141_33312	1147	No	NA	1	0	Low-quality	Genome-fragment	2.64	0
S7_vs_50932||full	1147	No	NA	0	0	Low-quality	Genome-fragment	2.81	0
S7_vs_90137||full	1147	No	NA	2	0	Low-quality	Genome-fragment	2.47	0
S9_vs_k141_27450||lt2gene	1002	No	NA	0	0	Not-determined	Genome-fragment	NA	0
S9_vs_k141_35128||lt2gene	1910	No	NA	0	0	Not-determined	Genome-fragment	NA	0
S16_extracted_89805	1147	No	NA	0	0	Low-quality	Genome-fragment	1.81	0
S9_vs_k141_46489||lt2gene	1824	No	NA	0	0	Not-determined	Genome-fragment	NA	0
S16_extracted_132968	1147	No	NA	0	1	Low-quality	Genome-fragment	2.21	0
S16_vs_23055||lt2gene	1147	No	NA	0	0	Low-quality	Genome-fragment	2.77	0
S10_extracted_48292	1147	No	NA	0	1	Low-quality	Genome-fragment	2.73	0

S9_vs_k141_16515||lt2gene	1254	No	NA	0	0	Not-determined	Genome-fragment	NA		0
S10_vs_52161||full	1147	No	NA	1	0	Low-quality	Genome-fragment		1.67	0
S8_vs_k141_143740||full	1147	No	NA	1	0	Low-quality	Genome-fragment		3.05	0
S8_vs_k141_154279||full	1147	No	NA	1	0	Low-quality	Genome-fragment		2.12	0
S17_vs_k141_27246||full	1148	No	NA	2	0	Low-quality	Genome-fragment		2.11	0
S9_vs_k141_30531||full	1148	No	NA	2	0	Low-quality	Genome-fragment		0.91	0
S3_vs_k141_9681||full	1148	No	NA	1	0	Low-quality	Genome-fragment		1.88	0
S5_extracted_k141_38231	1148	No	NA	3	0	Low-quality	Genome-fragment		3.77	0
S7_extracted_111877	1148	No	NA	0	0	Low-quality	Genome-fragment		2.47	0
S9_vs_k141_28254||lt2gene	1863	No	NA	0	0	Not-determined	Genome-fragment	NA		0
S16_vs_35774||lt2gene	1148	No	NA	1	0	Low-quality	Genome-fragment		2.76	0
S9_vs_k141_35931||lt2gene	2363	No	NA	0	1	Not-determined	Genome-fragment	NA		0
S12_vs_k141_124732||full	1148	No	NA	1	0	Low-quality	Genome-fragment		1.89	0
S2_vs_k141_15593||full	1148	No	NA	0	0	Low-quality	Genome-fragment		2.01	0
S9_vs_k141_62430||lt2gene	1184	No	NA	0	0	Not-determined	Genome-fragment	NA		0
S8_vs_k141_189104||full	1148	No	NA	0	0	Low-quality	Genome-fragment		0.55	0
S11_vs_k141_56219||full	1149	No	NA	1	0	Low-quality	Genome-fragment		8.51	0
S13_extracted_47576	1149	No	NA	1	0	Low-quality	Genome-fragment		3.18	0
S14_extracted_k141_114742	1149	No	NA	0	1	Low-quality	Genome-fragment		0.33	0
S1_extracted_k141_21632	1149	No	NA	0	0	Low-quality	Genome-fragment		0.72	0
S1_vs_k141_110286||full	1149	No	NA	0	0	Low-quality	Genome-fragment		1.75	0
S3_extracted_k141_10466	1149	No	NA	2	0	Low-quality	Genome-fragment		2.01	0
S7_extracted_109515	1149	No	NA	0	0	Low-quality	Genome-fragment		1.76	0
S7_vs_84701||full	1149	No	NA	0	0	Low-quality	Genome-fragment		2.08	0
S16_extracted_75336	1149	No	NA	1	0	Low-quality	Genome-fragment		2.74	0
S16_vs_144209||full	1149	No	NA	1	0	Low-quality	Genome-fragment		0.66	0
S16_vs_164770||full	1149	No	NA	0	0	Low-quality	Genome-fragment		2.43	0
S10_extracted_72212	1149	No	NA	1	0	Low-quality	Genome-fragment		2.01	0
S4_extracted_2012	1149	No	NA	1	0	Low-quality	Genome-fragment		2.1	0
S8_extracted_k141_110426	1149	No	NA	0	3	Low-quality	Genome-fragment		1.02	0
S8_vs_k141_60392||full	1149	No	NA	0	1	Low-quality	Genome-fragment		0.77	0
S17_vs_k141_80458||full	1150	No	NA	0	0	Low-quality	Genome-fragment		0.82	0
S17_vs_k141_28362||full	1150	No	NA	0	0	Low-quality	Genome-fragment		0.85	0
S11_extracted_k141_99225	1150	No	NA	1	0	Low-quality	Genome-fragment		1.86	0
S13_vs_87198||full	1150	No	NA	0	0	Low-quality	Genome-fragment		0.97	0
S14_vs_k141_88714||full	1150	No	NA	0	0	Low-quality	Genome-fragment		0.37	0
S5_vs_k141_24321||lt2gene	1150	No	NA	2	0	Low-quality	Genome-fragment		3.38	0
S7_extracted_27797	1150	No	NA	0	2	Low-quality	Genome-fragment		0.69	0
S7_vs_37168||full	1150	No	NA	1	0	Low-quality	Genome-fragment		3.34	0
S16_vs_68||full	1150	No	NA	1	0	Low-quality	Genome-fragment		2.28	0
S16_vs_152325||full	1150	No	NA	0	0	Low-quality	Genome-fragment		1.07	0
S10_vs_104152||full	1150	No	NA	0	0	Low-quality	Genome-fragment		1.88	0
S10_vs_87428||lt2gene	1150	No	NA	1	0	Low-quality	Genome-fragment		2.66	0
S8_extracted_k141_29890	1150	No	NA	0	0	Low-quality	Genome-fragment		2.99	0
S8_vs_k141_167776||full	1150	No	NA	2	0	Low-quality	Genome-fragment		1.9	0
S9_extracted_k141_16518	1151	No	NA	2	0	Low-quality	Genome-fragment		2.42	0
S9_vs_k141_20273||full	1151	No	NA	0	0	Low-quality	Genome-fragment		1.52	0
S9_vs_k141_85064||full	1151	No	NA	2	0	Low-quality	Genome-fragment		1.71	0
S11_extracted_k141_71708	1151	No	NA	0	0	Low-quality	Genome-fragment		1.96	0
S11_extracted_k141_65509	1151	No	NA	2	0	Low-quality	Genome-fragment		2.83	0
S9_vs_k141_52163||lt2gene	1272	No	NA	0	0	Not-determined	Genome-fragment	NA		0
S9_vs_k141_75650||lt2gene	1377	No	NA	0	0	Not-determined	Genome-fragment	NA		0
S11_vs_k141_89411||full	1151	No	NA	1	0	Low-quality	Genome-fragment		0.79	0
S9_vs_k141_15103||lt2gene	1056	No	NA	0	0	Not-determined	Genome-fragment	NA		0
S13_vs_137798||full	1151	No	NA	0	0	Low-quality	Genome-fragment		0.72	0
S14_extracted_k141_46672	1151	No	NA	2	0	Low-quality	Genome-fragment		2.95	0
S1_vs_k141_7927||full	1151	No	NA	1	0	Low-quality	Genome-fragment		1.51	0
S11_extracted_k141_91116	2262	No	NA	0	0	Not-determined	Genome-fragment	NA		0
S5_vs_k141_54589||full	1151	No	NA	0	0	Low-quality	Genome-fragment		1.86	0
S11_extracted_k141_71598	1001	No	NA	0	0	Not-determined	Genome-fragment	NA		0
S11_extracted_k141_65124	1440	No	NA	0	1	Not-determined	Genome-fragment	NA		0
S11_extracted_k141_19546	5780	No	NA	0	3	Not-determined	Genome-fragment	NA		0
S11_extracted_k141_58587	16434	No	NA	6	0	Medium-quality	Genome-fragment		74.1	0
S11_extracted_k141_45605	4665	No	NA	0	1	Not-determined	Genome-fragment	NA		0
S11_extracted_k141_97663	1041	No	NA	0	1	Not-determined	Genome-fragment	NA		0
S11_extracted_k141_52127	1056	No	NA	0	1	Not-determined	Genome-fragment	NA		0
S11_extracted_k141_71663	1488	No	NA	0	1	Not-determined	Genome-fragment	NA		0
S11_extracted_k141_32596	1899	No	NA	0	0	Not-determined	Genome-fragment	NA		0
S11_extracted_k141_97	2740	No	NA	0	2	Not-determined	Genome-fragment	NA		0
S11_extracted_k141_13033	60444	No	NA	35	1	High-quality	High-quality		100	0
S11_extracted_k141_58676	1049	No	NA	0	1	Not-determined	Genome-fragment	NA		0
S7_extracted_26222	1151	No	NA	1	0	Low-quality	Genome-fragment		2.92	0
S16_extracted_140387	1151	No	NA	1	0	Low-quality	Genome-fragment		0.97	0
S16_vs_160053||full	1151	No	NA	1	0	Low-quality	Genome-fragment		2.3	0
S12_extracted_k141_16621	1151	No	NA	0	0	Low-quality	Genome-fragment		1.85	0
S11_extracted_k141_52218	1456	No	NA	0	1	Not-determined	Genome-fragment	NA		0
S11_extracted_k141_32645	4659	No	NA	0	2	Not-determined	Genome-fragment	NA		0
S12_vs_k141_166537||full	1151	No	NA	0	0	Low-quality	Genome-fragment		2.29	0
S11_extracted_k141_65285	1010	No	NA	0	0	Not-determined	Genome-fragment	NA		0
S12_vs_k141_104741||full	1151	No	NA	0	0	Low-quality	Genome-fragment		1.57	0
S17_extracted_k141_108623	1152	No	NA	0	1	Low-quality	Genome-fragment		2.52	0
S17_vs_k141_163292||lt2gene	1152	No	NA	1	0	Low-quality	Genome-fragment		2.09	0
S11_extracted_k141_91332	1343	No	NA	0	1	Not-determined	Genome-fragment	NA		0
S9_extracted_k141_86863	1152	No	NA	2	0	Low-quality	Genome-fragment		2.22	0
S1_extracted_k141_61101	1152	No	NA	0	1	Low-quality	Genome-fragment		1.14	0
S3_extracted_k141_11592	1152	No	NA	0	0	Low-quality	Genome-fragment		2.76	0
S5_vs_k141_100476||full	1152	No	NA	0	0	Low-quality	Genome-fragment		1.99	0
S11_extracted_k141_78244	1414	No	NA	0	2	Not-determined	Genome-fragment	NA		0
S11_extracted_k141_32744	3750	No	NA	0	1	Not-determined	Genome-fragment	NA		0
S11_extracted_k141_39225	1409	No	NA	0	1	Not-determined	Genome-fragment	NA		0
S7_vs_32211||full	1152	No	NA	0	0	Low-quality	Genome-fragment		2.58	0
S10_vs_76504||full	1152	No	NA	0	1	Low-quality	Genome-fragment		1.35	0
S12_vs_k141_137101||full	1152	No	NA	0	2	Low-quality	Genome-fragment		0.62	0
S11_extracted_k141_58780	1026	No	NA	0	0	Not-determined	Genome-fragment	NA		0
S11_extracted_k141_52371	3167	No	NA	0	1	Not-determined	Genome-fragment	NA		0
S11_extracted_k141_32786	7443	No	NA	0	3	Not-determined	Genome-fragment	NA		0
S11_extracted_k141_407	1472	No	NA	0	0	Not-determined	Genome-fragment	NA		0
S6_extracted_k141_41535	1152	No	NA	0	1	Low-quality	Genome-fragment		0.27	0
S11_extracted_k141_417	1761	No	NA	0	1	Not-determined	Genome-fragment	NA		0
S11_extracted_k141_45673	1052	No	NA	0	0	Not-determined	Genome-fragment	NA		0
S11_extracted_k141_78350	1012	No	NA	0	0	Not-determined	Genome-fragment	NA		0
S11_extracted_k141_39277	1658	No	NA	0	0	Not-determined	Genome-fragment	NA		0
S11_extracted_k141_65366	1302	No	NA	0	2	Not-determined	Genome-fragment	NA		0
S11_extracted_k141_45707	7039	No	NA	0	0	Not-determined	Genome-fragment	NA		0
S11_extracted_k141_58799	1722	No	NA	0	1	Not-determined	Genome-fragment	NA		0
S11_extracted_k141_91455	1020	No	NA	0	2	Not-determined	Genome-fragment	NA		0
S11_extracted_k141_6724	2253	No	NA	0	1	Not-determined	Genome-fragment	NA		0
S11_extracted_k141_58810	2027	No	NA	0	0	Not-determined	Genome-fragment	NA		0
S17_vs_k141_68913||full	1153	No	NA	0	1	Low-quality	Genome-fragment		2.69	0
S9_vs_k141_23189||full	1153	No	NA	2	0	Low-quality	Genome-fragment		2.51	0
S11_extracted_k141_78408	1305	No	NA	0	2	Not-determined	Genome-fragment	NA		0
S11_extracted_k141_39325	2033	No	NA	0	3	Not-determined	Genome-fragment	NA		0
S14_extracted_k141_92527	1153	No	NA	0	0	Low-quality	Genome-fragment		0.92	0
S1_vs_k141_100373||full	1153	No	NA	1	0	Low-quality	Genome-fragment		1.58	0
S11_extracted_k141_19840	1194	No	NA	0	1	Not-determined	Genome-fragment	NA		0
S11_extracted_k141_84982	1589	No	NA	0	0	Not-determined	Genome-fragment	NA		0
S3_extracted_k141_9318	1153	No	NA	0	0	Low-quality	Genome-fragment		1.75	0
S11_extracted_k141_91514	1247	No	NA	0	1	Not-determined	Genome-fragment	NA		0
S11_extracted_k141_26374	1612	No	NA	0	0	Not-determined	Genome-fragment	NA		0
S11_extracted_k141_45751	1271	No	NA	0	0	Not-determined	Genome-fragment	NA		0
S3_extracted_k141_35499	1153	No	NA	0	0	Low-quality	Genome-fragment		0.65	0
S11_extracted_k141_91528	1647	No	NA	0	0	Not-determined	Genome-fragment	NA		0
S3_vs_k141_78702||lt2gene	1153	No	NA	1	0	Low-quality	Genome-fragment		0.74	0
S11_extracted_k141_19884	3116	No	NA	0	2	Not-determined	Genome-fragment	NA		0
S11_extracted_k141_26403	1610	No	NA	0	0	Not-determined	Genome-fragment	NA		0
S5_extracted_k141_287	1153	No	NA	1	0	Low-quality	Genome-fragment		3.35	0
S11_extracted_k141_39400	2668	No	NA	0	2	Not-determined	Genome-fragment	NA		0
S11_extracted_k141_85051	1304	No	NA	0	0	Not-determined	Genome-fragment	NA		0
S11_extracted_k141_78504	3191	No	NA	0	2	Not-determined	Genome-fragment	NA		0
S11_extracted_k141_6824	4654	No	NA	0	0	Not-determined	Genome-fragment	NA		0
S11_extracted_k141_45810	1336	No	NA	0	0	Not-determined	Genome-fragment	NA		0
S7_vs_90963||full	1153	No	NA	1	0	Low-quality	Genome-fragment		2.12	0
S11_extracted_k141_6846	1202	No	NA	0	1	Not-determined	Genome-fragment	NA		0
S16_extracted_168110	1153	No	NA	0	0	Low-quality	Genome-fragment		3.04	0
S16_vs_54099||full	1153	No	NA	1	0	Low-quality	Genome-fragment		2.36	0
S16_vs_130652||lt2gene	1153	No	NA	1	0	Low-quality	Genome-fragment		0.8	0
S11_extracted_k141_633	1329	No	NA	0	0	Not-determined	Genome-fragment	NA		0
S11_extracted_k141_78593	1461	No	NA	0	2	Not-determined	Genome-fragment	NA		0
S11_extracted_k141_71993	1219	No	NA	0	3	Not-determined	Genome-fragment	NA		0
S10_extracted_67249	1153	No	NA	0	0	Low-quality	Genome-fragment		1.45	0
S10_vs_66379||full	1153	No	NA	1	0	Low-quality	Genome-fragment		2.36	0
S11_extracted_k141_13236	7845	No	NA	0	5	Not-determined	Genome-fragment	NA		0
S11_extracted_k141_45926	2077	No	NA	0	0	Not-determined	Genome-fragment	NA		0
S12_vs_k141_112426||full	1153	No	NA	0	1	Low-quality	Genome-fragment		0.67	0
S6_extracted_k141_38142	1153	No	NA	0	1	Low-quality	Genome-fragment		2.54	0
S17_vs_k141_156772||full	1154	No	NA	0	0	Low-quality	Genome-fragment		2.32	0
S11_extracted_k141_45939	3349	No	NA	0	2	Not-determined	Genome-fragment	NA		0
S11_extracted_k141_39557	1289	No	NA	0	0	Not-determined	Genome-fragment	NA		0
S11_extracted_k141_66113	1154	No	NA	2	0	Low-quality	Genome-fragment		2.03	0
S11_vs_k141_15787||full	1154	No	NA	0	0	Low-quality	Genome-fragment		2.7	0
S13_extracted_37506	1154	No	NA	0	2	Low-quality	Genome-fragment		0.6	0
S11_extracted_k141_703	1047	No	NA	0	0	Not-determined	Genome-fragment	NA		0
S5_vs_k141_33345||full	1154	No	NA	0	0	Low-quality	Genome-fragment		1.25	0
S11_extracted_k141_85156	4011	No	NA	0	0	Not-determined	Genome-fragment	NA		0
S11_extracted_k141_85163	1792	No	NA	0	1	Not-determined	Genome-fragment	NA		0
S16_extracted_121096	1154	No	NA	0	2	Low-quality	Genome-fragment		0.35	0
S10_vs_85836||full	1154	No	NA	0	0	Low-quality	Genome-fragment		2.51	0
S11_extracted_k141_52609	1017	No	NA	0	1	Not-determined	Genome-fragment	NA		0
S11_extracted_k141_78714	1192	No	NA	0	2	Not-determined	Genome-fragment	NA		0
S11_extracted_k141_78717	1030	No	NA	0	0	Not-determined	Genome-fragment	NA		0
S11_extracted_k141_98202	1137	No	NA	0	0	Not-determined	Genome-fragment	NA		0
S2_extracted_k141_16985	1154	No	NA	1	0	Low-quality	Genome-fragment		0.72	0
S8_vs_k141_91626||full	1154	No	NA	0	0	Low-quality	Genome-fragment		2.83	0
S8_vs_k141_40278||full	1154	No	NA	1	0	Low-quality	Genome-fragment		0.7	0
S11_extracted_k141_78761	3234	No	NA	0	3	Not-determined	Genome-fragment	NA		0
S17_extracted_k141_77325	1155	No	NA	3	0	Low-quality	Genome-fragment		3.81	0
S11_extracted_k141_13413	3231	No	NA	0	0	Not-determined	Genome-fragment	NA		0
S11_extracted_k141_13418	2000	No	NA	0	1	Not-determined	Genome-fragment	NA		0
S17_vs_k141_68082||full	1155	No	NA	0	0	Low-quality	Genome-fragment		2.64	0
S11_extracted_k141_39676	1432	No	NA	0	1	Not-determined	Genome-fragment	NA		0
S11_extracted_k141_58999	1094	No	NA	0	1	Not-determined	Genome-fragment	NA		0
S11_extracted_k141_33117	52675	No	NA	16	2	Complete	High-quality		100	0
S9_vs_k141_86426||lt2gene	1155	No	NA	0	0	Low-quality	Genome-fragment		2.02	0
S14_vs_k141_93273||full	1155	No	NA	0	0	Low-quality	Genome-fragment		2.84	0

S11_extracted_k141_842	1929	No	NA	0	2	Not-determined	Genome-fragment	NA		0
S1_vs_k141_39887||full	1155	No	NA	0	1	Low-quality	Genome-fragment		1.75	0
S3_vs_k141_91146||full	1155	No	NA	2	0	Low-quality	Genome-fragment		1.45	0
S11_extracted_k141_65695	2244	No	NA	0	0	Not-determined	Genome-fragment	NA		0
S5_vs_k141_133720||lt2gene	1155	No	NA	0	0	Low-quality	Genome-fragment		3.22	0
S11_extracted_k141_59071	2869	No	NA	0	1	Not-determined	Genome-fragment	NA		0
S7_extracted_12927	1155	No	NA	0	1	Low-quality	Genome-fragment		2.07	0
S11_extracted_k141_13559	3022	No	NA	0	0	Not-determined	Genome-fragment	NA		0
S7_vs_47519||full	1155	No	NA	0	0	Low-quality	Genome-fragment		1.12	0
S11_extracted_k141_33253	10572	No	NA	0	2	Not-determined	Genome-fragment	NA		0
S11_extracted_k141_46264	1206	No	NA	0	1	Not-determined	Genome-fragment	NA		0
S11_extracted_k141_947	4657	No	NA	0	0	Not-determined	Genome-fragment	NA		0
S16_extracted_169289	1155	No	NA	0	2	Low-quality	Genome-fragment		2.06	0
S11_extracted_k141_7050	4088	No	NA	0	2	Not-determined	Genome-fragment	NA		0
S11_extracted_k141_85456	1042	No	NA	0	1	Not-determined	Genome-fragment	NA		0
S16_vs_124138||full	1155	No	NA	0	0	Low-quality	Genome-fragment		1.31	0
S10_vs_103769||full	1155	No	NA	1	0	Low-quality	Genome-fragment		2.28	0
S12_extracted_k141_53375	1155	No	NA	0	0	Low-quality	Genome-fragment		3.12	0
S11_extracted_k141_39837	2372	No	NA	0	1	Not-determined	Genome-fragment	NA		0
S11_extracted_k141_46334	1142	No	NA	0	0	Not-determined	Genome-fragment	NA		0
S11_extracted_k141_46338	1720	No	NA	0	2	Not-determined	Genome-fragment	NA		0
S11_extracted_k141_65804	2192	No	NA	0	0	Not-determined	Genome-fragment	NA		0
S11_extracted_k141_65814	1078	No	NA	0	0	Not-determined	Genome-fragment	NA		0
S12_vs_k141_18455||full	1155	No	NA	0	1	Low-quality	Genome-fragment		2.59	0
S11_extracted_k141_46370	1132	No	NA	0	2	Not-determined	Genome-fragment	NA		0
S12_vs_k141_75208||full	1155	No	NA	0	0	Low-quality	Genome-fragment		2.35	0
S11_extracted_k141_1059	2401	No	NA	0	0	Not-determined	Genome-fragment	NA		0
S11_extracted_k141_92052	1020	No	NA	0	1	Not-determined	Genome-fragment	NA		0
S11_extracted_k141_52862	3191	No	NA	0	1	Not-determined	Genome-fragment	NA		0
S11_extracted_k141_1069	7194	No	NA	0	5	Not-determined	Genome-fragment	NA		0
S11_extracted_k141_33410	3095	No	NA	0	1	Not-determined	Genome-fragment	NA		0
S12_vs_k141_52642||lt2gene	1155	No	NA	1	0	Low-quality	Genome-fragment		0.63	0
S11_extracted_k141_98610	1354	No	NA	0	1	Not-determined	Genome-fragment	NA		0
S8_extracted_k141_84220	1155	No	NA	0	1	Low-quality	Genome-fragment		2.58	0
S8_vs_k141_62168||full	1155	No	NA	0	1	Low-quality	Genome-fragment		1.98	0
S11_extracted_k141_98636	1002	No	NA	0	0	Not-determined	Genome-fragment	NA		0
S11_extracted_k141_85620	1309	No	NA	0	2	Not-determined	Genome-fragment	NA		0
S8_vs_k141_142732||full	1155	No	NA	0	2	Low-quality	Genome-fragment		0.77	0
S11_extracted_k141_46429	1319	No	NA	0	2	Not-determined	Genome-fragment	NA		0
S11_extracted_k141_85629	2803	No	NA	0	0	Not-determined	Genome-fragment	NA		0
S11_extracted_k141_52958	4896	No	NA	0	2	Not-determined	Genome-fragment	NA		0
S11_extracted_k141_7239	1171	No	NA	0	1	Not-determined	Genome-fragment	NA		0
S8_vs_k141_127699||full	1155	No	NA	0	0	Low-quality	Genome-fragment		1.84	0
S11_extracted_k141_7248	1145	No	NA	0	0	Not-determined	Genome-fragment	NA		0
S11_extracted_k141_65889	1223	No	NA	0	0	Not-determined	Genome-fragment	NA		0
S11_extracted_k141_52977	1258	No	NA	0	1	Not-determined	Genome-fragment	NA		0
S8_vs_k141_120135||full	1155	No	NA	1	0	Low-quality	Genome-fragment		2.94	0
S11_extracted_k141_79209	1733	No	NA	0	3	Not-determined	Genome-fragment	NA		0
S11_extracted_k141_33433	77933	No	NA	16	1	Complete	High-quality		100	0
S11_extracted_k141_46482	1006	No	NA	0	1	Not-determined	Genome-fragment	NA		0
S11_extracted_k141_59366	3910	No	NA	0	1	Not-determined	Genome-fragment	NA		0
S17_extracted_k141_69129	1156	No	NA	0	3	Low-quality	Genome-fragment		2.61	0
S11_extracted_k141_7335	3375	No	NA	0	2	Not-determined	Genome-fragment	NA		0
S11_extracted_k141_40007	1338	No	NA	0	0	Not-determined	Genome-fragment	NA		0
S17_vs_k141_19595||full	1156	No	NA	0	0	Low-quality	Genome-fragment		1.15	0
S11_extracted_k141_66001	5907	No	NA	0	5	Not-determined	Genome-fragment	NA		0
S11_extracted_k141_72691	1070	No	NA	0	1	Not-determined	Genome-fragment	NA		0
S11_extracted_k141_92196	2226	No	NA	0	3	Not-determined	Genome-fragment	NA		0
S11_extracted_k141_79267	1219	No	NA	0	2	Not-determined	Genome-fragment	NA		0
S11_extracted_k141_7400	1653	No	NA	0	0	Not-determined	Genome-fragment	NA		0
S11_extracted_k141_53099	2880	No	NA	0	1	Not-determined	Genome-fragment	NA		0
S11_extracted_k141_53110	3815	No	NA	0	4	Not-determined	Genome-fragment	NA		0
S11_extracted_k141_27020	1222	No	NA	0	1	Not-determined	Genome-fragment	NA		0
S11_extracted_k141_7442	12033	No	NA	0	4	Not-determined	Genome-fragment	NA		0
S11_extracted_k141_53165	1758	No	NA	0	2	Not-determined	Genome-fragment	NA		0
S11_extracted_k141_53186	4689	No	NA	0	1	Not-determined	Genome-fragment	NA		0
S17_vs_k141_50957||full	1156	No	NA	0	0	Low-quality	Genome-fragment		2.47	0
S11_extracted_k141_79342	1259	No	NA	0	1	Not-determined	Genome-fragment	NA		0
S11_extracted_k141_40129	2977	No	NA	0	1	Not-determined	Genome-fragment	NA		0
S9_vs_k141_38100||full	1156	No	NA	0	0	Low-quality	Genome-fragment		0.67	0
S11_extracted_k141_33661	1113	No	NA	0	1	Not-determined	Genome-fragment	NA		0
S11_extracted_k141_66148	1299	No	NA	0	1	Not-determined	Genome-fragment	NA		0
S9_vs_k141_89126||full	1156	No	NA	1	0	Low-quality	Genome-fragment		3.05	0
S11_extracted_k141_7534	4233	No	NA	0	2	Not-determined	Genome-fragment	NA		0
S11_extracted_k141_7536	1716	No	NA	0	1	Not-determined	Genome-fragment	NA		0
S9_vs_k141_72840||lt2gene	1156	No	NA	1	0	Low-quality	Genome-fragment		2.66	0
S11_extracted_k141_1317	1151	No	NA	0	3	Not-determined	Genome-fragment	NA		0
S11_extracted_k141_55358	1156	No	NA	1	0	Low-quality	Genome-fragment		0.79	0
S11_extracted_k141_53287	1191	No	NA	0	0	Not-determined	Genome-fragment	NA		0
S13_vs_141710||full	1156	No	NA	0	1	Low-quality	Genome-fragment		2	0
S14_extracted_k141_104017	1156	No	NA	1	0	Low-quality	Genome-fragment		2.71	0
S11_extracted_k141_66160	56970	No	NA	13	1	High-quality	High-quality		100	0
S1_vs_k141_144315||full	1156	No	NA	0	0	Low-quality	Genome-fragment		2.94	0
S11_extracted_k141_20813	1902	No	NA	0	2	Not-determined	Genome-fragment	NA		0
S11_extracted_k141_92319	2152	No	NA	0	1	Not-determined	Genome-fragment	NA		0
S11_extracted_k141_72985	1054	No	NA	0	0	Not-determined	Genome-fragment	NA		0
S11_extracted_k141_59708	1080	No	NA	0	0	Not-determined	Genome-fragment	NA		0
S3_vs_k141_88852||full	1156	No	NA	0	1	Low-quality	Genome-fragment		1.52	0
S3_vs_k141_31033||full	1156	No	NA	0	1	Low-quality	Genome-fragment		0.68	0
S5_vs_k141_458||lt2gene	1156	No	NA	1	0	Low-quality	Genome-fragment		1.96	0
S7_extracted_8118	1156	No	NA	0	0	Low-quality	Genome-fragment		1.13	0
S11_extracted_k141_20878	1001	No	NA	0	2	Not-determined	Genome-fragment	NA		0
S11_extracted_k141_27236	1002	No	NA	0	1	Not-determined	Genome-fragment	NA		0
S11_extracted_k141_40221	1468	No	NA	0	1	Not-determined	Genome-fragment	NA		0
S11_extracted_k141_27241	1866	No	NA	0	1	Not-determined	Genome-fragment	NA		0
S16_vs_24111||full	1156	No	NA	0	0	Low-quality	Genome-fragment		1.25	0
S11_extracted_k141_1494	1064	No	NA	0	1	Not-determined	Genome-fragment	NA		0
S11_extracted_k141_86087	4605	No	NA	0	2	Not-determined	Genome-fragment	NA		0
S11_extracted_k141_33774	2045	No	NA	0	0	Not-determined	Genome-fragment	NA		0
S11_extracted_k141_92391	15893	No	NA	7	0	Medium-quality	Genome-fragment		52.52	0
S11_extracted_k141_20907	1597	No	NA	0	0	Not-determined	Genome-fragment	NA		0
S11_extracted_k141_92396	1328	No	NA	0	0	Not-determined	Genome-fragment	NA		0
S12_vs_k141_1138||full	1156	No	NA	0	0	Low-quality	Genome-fragment		1.97	0
S11_extracted_k141_79449	1032	No	NA	0	1	Not-determined	Genome-fragment	NA		0
S11_extracted_k141_46742	5120	No	NA	0	0	Not-determined	Genome-fragment	NA		0
S12_vs_k141_1548||full	1156	No	NA	0	0	Low-quality	Genome-fragment		1.38	0
S11_extracted_k141_59774	1165	No	NA	0	1	Not-determined	Genome-fragment	NA		0
S15_extracted_53618	1156	No	NA	0	0	Low-quality	Genome-fragment		0.82	0
S4_extracted_9057	1156	No	NA	0	0	Low-quality	Genome-fragment		1.22	0
S11_extracted_k141_27297	1425	No	NA	0	2	Not-determined	Genome-fragment	NA		0
S8_vs_k141_9370||lt2gene	1156	No	NA	1	0	Low-quality	Genome-fragment		1.95	0
S11_extracted_k141_46758	1182	No	NA	0	0	Not-determined	Genome-fragment	NA		0
S11_extracted_k141_99014	1989	No	NA	0	1	Not-determined	Genome-fragment	NA		0
S11_extracted_k141_73154	4914	No	NA	0	3	Not-determined	Genome-fragment	NA		0
S17_vs_k141_170932||full	1157	No	NA	1	0	Low-quality	Genome-fragment		0.82	0
S11_extracted_k141_100340	1157	No	NA	1	0	Low-quality	Genome-fragment		0.81	0
S11_extracted_k141_1605	1038	No	NA	0	0	Not-determined	Genome-fragment	NA		0
S11_extracted_k141_27335	1249	No	NA	0	0	Not-determined	Genome-fragment	NA		0
S11_vs_k141_774||full	1157	No	NA	1	0	Low-quality	Genome-fragment		7.36	0
S11_vs_k141_74087||full	1157	No	NA	0	0	Low-quality	Genome-fragment		2.29	0
S11_extracted_k141_99045	3480	No	NA	0	1	Not-determined	Genome-fragment	NA		0
S11_extracted_k141_14040	3195	No	NA	0	2	Not-determined	Genome-fragment	NA		0
S11_extracted_k141_86211	4231	No	NA	0	1	Not-determined	Genome-fragment	NA		0
S13_extracted_115423	1157	No	NA	0	1	Low-quality	Genome-fragment		0.51	0
S11_extracted_k141_14072	1455	No	NA	0	2	Not-determined	Genome-fragment	NA		0
S11_extracted_k141_27391	2140	No	NA	0	0	Not-determined	Genome-fragment	NA		0
S11_extracted_k141_27395	1026	No	NA	0	0	Not-determined	Genome-fragment	NA		0
S3_vs_k141_20908||full	1157	No	NA	0	1	Low-quality	Genome-fragment		2.21	0
S11_extracted_k141_21034	2249	No	NA	0	2	Not-determined	Genome-fragment	NA		0
S11_extracted_k141_53524	1237	No	NA	0	1	Not-determined	Genome-fragment	NA		0
S11_extracted_k141_21059	1014	No	NA	0	0	Not-determined	Genome-fragment	NA		0
S11_extracted_k141_86314	1739	No	NA	0	1	Not-determined	Genome-fragment	NA		0
S11_extracted_k141_99068	3554	No	NA	0	0	Not-determined	Genome-fragment	NA		0
S7_extracted_63068	1157	No	NA	0	1	Low-quality	Genome-fragment		2.34	0
S11_extracted_k141_33868	1996	No	NA	0	1	Not-determined	Genome-fragment	NA		0
S16_vs_102148||full	1157	No	NA	0	1	Low-quality	Genome-fragment		1.73	0
S11_extracted_k141_53586	1723	No	NA	0	0	Not-determined	Genome-fragment	NA		0
S11_extracted_k141_53591	6063	No	NA	0	2	Not-determined	Genome-fragment	NA		0
S11_extracted_k141_46871	4490	No	NA	0	1	Not-determined	Genome-fragment	NA		0
S11_extracted_k141_14176	1669	No	NA	0	1	Not-determined	Genome-fragment	NA		0
S11_extracted_k141_66409	1005	No	NA	0	0	Not-determined	Genome-fragment	NA		0
S11_extracted_k141_40437	1008	No	NA	0	0	Not-determined	Genome-fragment	NA		0
S11_extracted_k141_7978	1084	No	NA	0	1	Not-determined	Genome-fragment	NA		0
S16_vs_149086||full	1157	No	NA	0	1	Low-quality	Genome-fragment		1.29	0
S11_extracted_k141_7990	1291	No	NA	0	0	Not-determined	Genome-fragment	NA		0
S11_extracted_k141_21136	1342	No	NA	0	1	Not-determined	Genome-fragment	NA		0
S12_vs_k141_25892||full	1157	No	NA	0	0	Low-quality	Genome-fragment		2.68	0
S15_extracted_58179	1157	No	NA	1	0	Low-quality	Genome-fragment		2.67	0
S11_extracted_k141_66456	1122	No	NA	0	1	Not-determined	Genome-fragment	NA		0
S11_extracted_k141_21148	1433	No	NA	0	0	Not-determined	Genome-fragment	NA		0
S8_extracted_k141_158982	1157	No	NA	0	1	Low-quality	Genome-fragment		0.9	0
S11_extracted_k141_27614	1621	No	NA	0	2	Not-determined	Genome-fragment	NA		0
S11_extracted_k141_99134	3626	No	NA	0	5	Not-determined	Genome-fragment	NA		0
S11_extracted_k141_8030	1367	No	NA	0	2	Not-determined	Genome-fragment	NA		0
S11_extracted_k141_46975	4628	No	NA	0	3	Not-determined	Genome-fragment	NA		0
S11_extracted_k141_46978	2729	No	NA	0	1	Not-determined	Genome-fragment	NA		0
S8_extracted_k141_15754	1157	No	NA	1	0	Low-quality	Genome-fragment		1.06	0
S11_extracted_k141_8076	1204	No	NA	0	1	Not-determined	Genome-fragment	NA		0
S17_vs_k141_107758||full	1158	No	NA	2	0	Low-quality	Genome-fragment		2.09	0
S11_vs_k141_46942||lt2gene	1158	No	NA	1	0	Low-quality	Genome-fragment		2.91	0
S11_vs_k141_2540||lt2gene	1158	No	NA	1	0	Low-quality	Genome-fragment		1.38	0
S11_extracted_k141_99201	1147	No	NA	0	0	Not-determined	Genome-fragment	NA		0
S11_extracted_k141_53689	2158	No	NA	0	0	Not-determined	Genome-fragment	NA		0
S11_extracted_k141_66522	27814	No	NA	20	0	High-quality	High-quality		92.74	0
S11_extracted_k141_8167	1061	No	NA	0	1	Not-determined	Genome-fragment	NA		0
S1_extracted_k141_151569	1158	No	NA	0	2	Low-quality	Genome-fragment		0.87	0
S11_extracted_k141_92575	1046	No	NA	0	1	Not-determined	Genome-fragment	NA		0
S11_extracted_k141_53719	2053	No	NA	0	0	Not-determined	Genome-fragment	NA		0
S11_extracted_k141_27751	4200	No	NA	0	0	Not-determined	Genome-fragment	NA		0
S11_extracted_k141_40471	1793	No	NA	0	1	Not-determined	Genome-fragment	NA		0
S11_extracted_k141_14426	1611	No	NA	0	1	Not-determined	Genome-fragment	NA		0
S3_extracted_k141_123490	1158	No	NA	0	0	Low-quality	Genome-fragment		2.39	0
S7_vs_32454||full	1158	No	NA	0	0	Low-quality	Genome-fragment		2.77	0
S16_extracted_79347	1158	No	NA	0	0	Low-quality	Genome-fragment		2.14	0

S16_extracted_107975	1158	No	NA	0	0	Low-quality	Genome-fragment		0.56	0
S11_extracted_k141_53758	1024	No	NA	0	1	Not-determined	Genome-fragment	NA		0
S11_extracted_k141_47124	3784	No	NA	0	3	Not-determined	Genome-fragment	NA		0
S11_extracted_k141_40509	1419	No	NA	0	1	Not-determined	Genome-fragment	NA		0
S11_extracted_k141_99255	1210	No	NA	0	1	Not-determined	Genome-fragment	NA		0
S11_extracted_k141_60072	13530	No	NA	0	3	Not-determined	Genome-fragment	NA		0
S11_extracted_k141_27813	1190	No	NA	0	1	Not-determined	Genome-fragment	NA		0
S11_extracted_k141_40546	1513	No	NA	0	0	Not-determined	Genome-fragment	NA		0
S12_vs_k141_40531||full	1158	No	NA	0	0	Low-quality	Genome-fragment		2.87	0
S12_vs_k141_81249||full	1158	No	NA	0	1	Low-quality	Genome-fragment		0.3	0
S11_extracted_k141_21396	4666	No	NA	0	0	Not-determined	Genome-fragment	NA		0
S2_extracted_k141_14126	1158	No	NA	0	0	Low-quality	Genome-fragment		1.21	0
S11_extracted_k141_66655	1439	No	NA	0	0	Not-determined	Genome-fragment	NA		0
S11_extracted_k141_53833	1290	No	NA	0	1	Not-determined	Genome-fragment	NA		0
S11_extracted_k141_47144	1378	No	NA	0	1	Not-determined	Genome-fragment	NA		0
S2_vs_k141_18637||full	1158	No	NA	0	0	Low-quality	Genome-fragment		1.7	0
S4_extracted_31092	1158	No	NA	1	0	Low-quality	Genome-fragment		2.06	0
S11_extracted_k141_47177	1300	No	NA	0	1	Not-determined	Genome-fragment	NA		0
S11_extracted_k141_99342	1035	No	NA	0	2	Not-determined	Genome-fragment	NA		0
S11_extracted_k141_33919	2717	No	NA	0	3	Not-determined	Genome-fragment	NA		0
S11_extracted_k141_53889	4381	No	NA	0	2	Not-determined	Genome-fragment	NA		0
S11_extracted_k141_27845	60409	No	NA	8	0	High-quality	High-quality		95.09	0
S8_vs_k141_180324||full	1158	No	NA	0	0	Low-quality	Genome-fragment		1.58	0
S11_extracted_k141_47215	2802	No	NA	0	0	Not-determined	Genome-fragment	NA		0
S11_extracted_k141_92779	1129	No	NA	0	0	Not-determined	Genome-fragment	NA		0
S17_extracted_k141_60079	1159	No	NA	0	2	Low-quality	Genome-fragment		1.43	0
S11_extracted_k141_86572	1317	No	NA	0	1	Not-determined	Genome-fragment	NA		0
S9_vs_k141_84680||full	1159	No	NA	0	0	Low-quality	Genome-fragment		0.67	0
S9_vs_k141_65710||lt2gene	1159	No	NA	1	0	Low-quality	Genome-fragment		3.01	0
S11_extracted_k141_8373	1135	No	NA	0	0	Not-determined	Genome-fragment	NA		0
S11_extracted_k141_73606	7868	No	NA	0	2	Not-determined	Genome-fragment	NA		0
S11_extracted_k141_21555	1239	No	NA	0	0	Not-determined	Genome-fragment	NA		0
S14_extracted_k141_48525	1159	No	NA	0	1	Low-quality	Genome-fragment		0.39	0
S11_extracted_k141_2087	3565	No	NA	0	0	Not-determined	Genome-fragment	NA		0
S1_vs_k141_72493||full	1159	No	NA	0	1	Low-quality	Genome-fragment		1.1	0
S11_extracted_k141_40691	1159	No	NA	0	1	Not-determined	Genome-fragment	NA		0
S11_extracted_k141_53988	1857	No	NA	0	1	Not-determined	Genome-fragment	NA		0
S5_vs_k141_68194||full	1159	No	NA	0	0	Low-quality	Genome-fragment		2.44	0
S11_extracted_k141_92796	1983	No	NA	0	1	Not-determined	Genome-fragment	NA		0
S11_extracted_k141_54012	1680	No	NA	0	2	Not-determined	Genome-fragment	NA		0
S11_extracted_k141_54014	1650	No	NA	0	1	Not-determined	Genome-fragment	NA		0
S11_extracted_k141_8430	1454	No	NA	0	1	Not-determined	Genome-fragment	NA		0
S11_extracted_k141_40715	1597	No	NA	0	1	Not-determined	Genome-fragment	NA		0
S11_extracted_k141_34008	27571	No	NA	12	0	Medium-quality	Genome-fragment		57.97	0
S11_extracted_k141_86695	5588	No	NA	0	1	Not-determined	Genome-fragment	NA		0
S11_extracted_k141_86707	1466	No	NA	0	0	Not-determined	Genome-fragment	NA		0
S11_extracted_k141_14595	1068	No	NA	0	0	Not-determined	Genome-fragment	NA		0
S7_extracted_13529	1159	No	NA	1	0	Low-quality	Genome-fragment		2.67	0
S11_extracted_k141_40783	1140	No	NA	0	1	Not-determined	Genome-fragment	NA		0
S11_extracted_k141_27953	5256	No	NA	0	3	Not-determined	Genome-fragment	NA		0
S7_vs_39541||lt2gene	1159	No	NA	1	0	Low-quality	Genome-fragment		2.92	0
S11_extracted_k141_73693	1120	No	NA	0	1	Not-determined	Genome-fragment	NA		0
S11_extracted_k141_86736	3513	No	NA	0	0	Not-determined	Genome-fragment	NA		0
S12_extracted_k141_145385	1159	No	NA	1	0	Low-quality	Genome-fragment		2.73	0
S11_extracted_k141_99562	1789	No	NA	0	2	Not-determined	Genome-fragment	NA		0
S11_extracted_k141_92887	5261	No	NA	0	5	Not-determined	Genome-fragment	NA		0
S11_extracted_k141_99565	1028	No	NA	0	1	Not-determined	Genome-fragment	NA		0
S11_extracted_k141_99578	1047	No	NA	0	2	Not-determined	Genome-fragment	NA		0
S11_extracted_k141_34082	1218	No	NA	0	0	Not-determined	Genome-fragment	NA		0
S11_extracted_k141_8512	1378	No	NA	0	0	Not-determined	Genome-fragment	NA		0
S11_extracted_k141_73735	1311	No	NA	0	1	Not-determined	Genome-fragment	NA		0
S11_extracted_k141_21656	1209	No	NA	0	1	Not-determined	Genome-fragment	NA		0
S12_vs_k141_15638||full	1159	No	NA	1	0	Low-quality	Genome-fragment		2.08	0
S12_vs_k141_150129||lt2gene	1159	No	NA	2	0	Low-quality	Genome-fragment		1.43	0
S11_extracted_k141_34112	3707	No	NA	0	2	Not-determined	Genome-fragment	NA		0
S11_extracted_k141_54156	1725	No	NA	0	1	Not-determined	Genome-fragment	NA		0
S11_extracted_k141_73794	1004	No	NA	0	0	Not-determined	Genome-fragment	NA		0
S15_extracted_36576	1159	No	NA	0	2	Low-quality	Genome-fragment		0.88	0
S11_extracted_k141_99619	1403	No	NA	0	0	Not-determined	Genome-fragment	NA		0
S11_extracted_k141_21727	1151	No	NA	0	1	Not-determined	Genome-fragment	NA		0
S11_extracted_k141_99652	1659	No	NA	0	1	Not-determined	Genome-fragment	NA		0
S6_vs_k141_32329||full	1159	No	NA	0	0	Low-quality	Genome-fragment		2.54	0
S8_extracted_k141_45249	1159	No	NA	0	0	Low-quality	Genome-fragment		0.57	0
S8_extracted_k141_68741	1159	No	NA	1	0	Low-quality	Genome-fragment		2.63	0
S11_extracted_k141_66801	3201	No	NA	0	1	Not-determined	Genome-fragment	NA		0
S11_extracted_k141_99734	1475	No	NA	0	1	Not-determined	Genome-fragment	NA		0
S11_extracted_k141_8654	1063	No	NA	0	1	Not-determined	Genome-fragment	NA		0
S11_extracted_k141_34224	35904	No	NA	26	0	Complete	High-quality		100	0
S8_vs_k141_146534||full	1159	No	NA	0	0	Low-quality	Genome-fragment		2.77	0
S11_extracted_k141_80275	3120	No	NA	0	2	Not-determined	Genome-fragment	NA		0
S17_extracted_k141_140779	1160	No	NA	0	2	Low-quality	Genome-fragment		2.07	0
S11_extracted_k141_54299	1392	No	NA	0	0	Not-determined	Genome-fragment	NA		0
S11_extracted_k141_73937	19632	No	NA	0	8	Not-determined	Genome-fragment	NA		0
S11_extracted_k141_8699	1143	No	NA	0	1	Not-determined	Genome-fragment	NA		0
S11_extracted_k141_60438	1538	No	NA	0	0	Not-determined	Genome-fragment	NA		0
S17_extracted_k141_33599	1160	No	NA	1	0	Low-quality	Genome-fragment		3.44	0
S14_extracted_k141_9779	1160	No	NA	2	0	Low-quality	Genome-fragment		3.83	0
S11_extracted_k141_2441	18509	No	NA	0	5	Not-determined	Genome-fragment	NA		0
S11_extracted_k141_54327	1344	No	NA	0	0	Not-determined	Genome-fragment	NA		0
S11_extracted_k141_28186	1238	No	NA	0	0	Not-determined	Genome-fragment	NA		0
S1_vs_k141_132280||full	1160	No	NA	1	0	Low-quality	Genome-fragment		1.98	0
S11_extracted_k141_99751	72288	No	NA	19	1	Medium-quality	Genome-fragment		65.25	0
S11_extracted_k141_66851	1209	No	NA	0	0	Not-determined	Genome-fragment	NA		0
S11_extracted_k141_28195	2999	No	NA	0	4	Not-determined	Genome-fragment	NA		0
S11_extracted_k141_87121	5231	No	NA	0	4	Not-determined	Genome-fragment	NA		0
S11_extracted_k141_54381	1326	No	NA	0	0	Not-determined	Genome-fragment	NA		0
S11_extracted_k141_87138	1160	No	NA	0	0	Not-determined	Genome-fragment	NA		0
S11_extracted_k141_66887	1519	No	NA	0	1	Not-determined	Genome-fragment	NA		0
S11_extracted_k141_34329	1487	No	NA	0	1	Not-determined	Genome-fragment	NA		0
S11_extracted_k141_14916	2889	No	NA	0	0	Not-determined	Genome-fragment	NA		0
S3_vs_k141_96306||full	1160	No	NA	1	0	Low-quality	Genome-fragment		2.64	0
S3_vs_k141_111023||full	1160	No	NA	0	0	Low-quality	Genome-fragment		2.39	0
S11_extracted_k141_54393	17935	No	NA	0	2	Not-determined	Genome-fragment	NA		0
S5_vs_k141_41793||lt2gene	1160	No	NA	1	0	Low-quality	Genome-fragment		2.82	0
S7_extracted_72734	1160	No	NA	2	0	Low-quality	Genome-fragment		2.47	0
S11_extracted_k141_8810	1111	No	NA	0	0	Not-determined	Genome-fragment	NA		0
S11_extracted_k141_54442	1332	No	NA	0	1	Not-determined	Genome-fragment	NA		0
S11_extracted_k141_21967	11502	No	NA	0	0	Not-determined	Genome-fragment	NA		0
S7_vs_100695||full	1160	No	NA	1	0	Low-quality	Genome-fragment		0.65	0
S11_extracted_k141_34408	1367	No	NA	0	0	Not-determined	Genome-fragment	NA		0
S12_vs_k141_160188||full	1160	No	NA	0	0	Low-quality	Genome-fragment		2.24	0
S11_extracted_k141_41251	1359	No	NA	0	1	Not-determined	Genome-fragment	NA		0
S11_extracted_k141_54483	1132	No	NA	0	0	Not-determined	Genome-fragment	NA		0
S11_extracted_k141_2582	1417	No	NA	0	0	Not-determined	Genome-fragment	NA		0
S11_extracted_k141_87231	1358	No	NA	0	1	Not-determined	Genome-fragment	NA		0
S11_extracted_k141_54504	1833	No	NA	0	0	Not-determined	Genome-fragment	NA		0
S12_vs_k141_61885||full	1160	No	NA	0	0	Low-quality	Genome-fragment		2.92	0
S11_extracted_k141_15068	1812	No	NA	0	2	Not-determined	Genome-fragment	NA		0
S11_extracted_k141_67017	1571	No	NA	0	0	Not-determined	Genome-fragment	NA		0
S8_extracted_k141_69731	1160	No	NA	0	0	Low-quality	Genome-fragment		1.41	0
S11_extracted_k141_99943	4382	No	NA	0	3	Not-determined	Genome-fragment	NA		0
S11_extracted_k141_67030	6158	No	NA	0	3	Not-determined	Genome-fragment	NA		0
S8_extracted_k141_197521	1160	No	NA	0	1	Low-quality	Genome-fragment		0.34	0
S17_extracted_k141_122098	1161	No	NA	0	1	Low-quality	Genome-fragment		2.47	0
S9_vs_k141_76359||full	1161	No	NA	1	0	Low-quality	Genome-fragment		2.01	0
S11_extracted_k141_67055	1404	No	NA	0	0	Not-determined	Genome-fragment	NA		0
S13_vs_144237||full	1161	No	NA	1	0	Low-quality	Genome-fragment		1.13	0
S11_extracted_k141_93354	2089	No	NA	0	4	Not-determined	Genome-fragment	NA		0
S11_extracted_k141_93355	1019	No	NA	0	0	Not-determined	Genome-fragment	NA		0
S11_extracted_k141_34501	1936	No	NA	0	0	Not-determined	Genome-fragment	NA		0
S14_vs_k141_71621||full	1161	No	NA	0	0	Low-quality	Genome-fragment		2.02	0
S11_extracted_k141_74200	1479	No	NA	0	0	Not-determined	Genome-fragment	NA		0
S1_extracted_k141_44115	1161	No	NA	0	1	Low-quality	Genome-fragment		2	0
S11_extracted_k141_8946	5632	No	NA	0	3	Not-determined	Genome-fragment	NA		0
S5_extracted_k141_124763	1161	No	NA	0	0	Low-quality	Genome-fragment		1.44	0
S11_extracted_k141_15133	1099	No	NA	0	0	Not-determined	Genome-fragment	NA		0
S11_extracted_k141_54611	1128	No	NA	0	1	Not-determined	Genome-fragment	NA		0
S5_extracted_k141_22198	1161	No	NA	2	0	Low-quality	Genome-fragment		5.39	0
S11_extracted_k141_15178	1176	No	NA	0	1	Not-determined	Genome-fragment	NA		0
S5_extracted_k141_22212	1161	No	NA	2	0	Low-quality	Genome-fragment		5.39	0
S11_extracted_k141_93425	3437	No	NA	0	2	Not-determined	Genome-fragment	NA		0
S5_vs_k141_99473||full	1161	No	NA	0	0	Low-quality	Genome-fragment		1.86	0
S11_extracted_k141_47632	32534	No	NA	17	1	Complete	High-quality		100	0
S11_extracted_k141_47634	1114	No	NA	0	0	Not-determined	Genome-fragment	NA		0
S5_vs_k141_39675||full	1161	No	NA	1	0	Low-quality	Genome-fragment		2.85	0
S5_vs_k141_18210||full	1161	No	NA	0	0	Low-quality	Genome-fragment		2.23	0
S11_extracted_k141_80531	3414	No	NA	0	2	Not-determined	Genome-fragment	NA		0
S7_extracted_33864	1161	No	NA	1	0	Low-quality	Genome-fragment		3.93	0
S7_vs_9317||full	1161	No	NA	0	0	Low-quality	Genome-fragment		1.09	0
S11_extracted_k141_22099	1751	No	NA	0	2	Not-determined	Genome-fragment	NA		0
S7_vs_15860||full	1161	No	NA	0	3	Low-quality	Genome-fragment		1.8	0
S11_extracted_k141_100052	3506	No	NA	0	2	Not-determined	Genome-fragment	NA		0
S11_extracted_k141_15271	1055	No	NA	0	0	Not-determined	Genome-fragment	NA		0
S11_extracted_k141_2744	1585	No	NA	0	0	Not-determined	Genome-fragment	NA		0
S11_extracted_k141_9068	1003	No	NA	0	0	Not-determined	Genome-fragment	NA		0
S11_extracted_k141_87488	2110	No	NA	0	1	Not-determined	Genome-fragment	NA		0
S11_extracted_k141_100104	1110	No	NA	0	0	Not-determined	Genome-fragment	NA		0
S12_extracted_k141_125042	1161	No	NA	2	0	Low-quality	Genome-fragment		2.78	0
S12_extracted_k141_70755	1161	No	NA	0	1	Low-quality	Genome-fragment		0.28	0
S11_extracted_k141_9110	2243	No	NA	0	0	Not-determined	Genome-fragment	NA		0
S11_extracted_k141_80571	1025	No	NA	0	1	Not-determined	Genome-fragment	NA		0
S12_vs_k141_136873||full	1161	No	NA	1	0	Low-quality	Genome-fragment		2.79	0
S11_extracted_k141_41493	1085	No	NA	0	1	Not-determined	Genome-fragment	NA		0
S8_extracted_k141_28176	1161	No	NA	1	0	Low-quality	Genome-fragment		2.67	0
S11_extracted_k141_15359	1924	No	NA	0	1	Not-determined	Genome-fragment	NA		0
S11_extracted_k141_22215	3327	No	NA	0	4	Not-determined	Genome-fragment	NA		0
S11_extracted_k141_93559	2791	No	NA	0	3	Not-determined	Genome-fragment	NA		0
S11_extracted_k141_80669	3257	No	NA	0	0	Not-determined	Genome-fragment	NA		0
S17_vs_k141_74577||full	1162	No	NA	0	0	Low-quality	Genome-fragment		2.2	0
S17_vs_k141_128358||full	1162	No	NA	3	0	Low-quality	Genome-fragment		1.76	0
S11_extracted_k141_67169	1002	No	NA	0	1	Not-determined	Genome-fragment	NA		0
S11_extracted_k141_9180	1558	No	NA	0	3	Not-determined	Genome-fragment	NA		0
S11_extracted_k141_28515	98716	No	NA	26	0	Medium-quality	Genome-fragment		61.32	0
S11_extracted_k141_15367	1572	No	NA	0	2	Not-determined	Genome-fragment	NA		0

S13_extracted_7335	1162	No	NA		1	0	Low-quality	Genome-fragment		0.73	0
S11_extracted_k141_47793	1019	No	NA		0	0	Not-determined	Genome-fragment	NA		0
S14_extracted_k141_95700	1162	No	NA		0	1	Low-quality	Genome-fragment		0.31	0
S14_extracted_k141_65315	1162	No	NA		0	0	Low-quality	Genome-fragment		1.19	0
S14_vs_k141_47975||lt2gene	1162	No	NA		1	0	Low-quality	Genome-fragment		2.66	0
S11_extracted_k141_2943	1054	No	NA		0	1	Not-determined	Genome-fragment	NA		0
S3_extracted_k141_83847	1162	No	NA		1	0	Low-quality	Genome-fragment		0.8	0
S3_vs_k141_56186||lt2gene	1162	No	NA		1	0	Low-quality	Genome-fragment		2.26	0
S11_extracted_k141_67213	1319	No	NA		0	0	Not-determined	Genome-fragment	NA		0
S11_extracted_k141_93644	1002	No	NA		0	1	Not-determined	Genome-fragment	NA		0
S11_extracted_k141_41654	1076	No	NA		0	1	Not-determined	Genome-fragment	NA		0
S5_extracted_k141_124961	1162	No	NA		1	0	Low-quality	Genome-fragment		1.32	0
S5_vs_k141_9582||lt2gene	1162	No	NA		1	0	Low-quality	Genome-fragment		0.96	0
S7_vs_92253||full	1162	No	NA		0	1	Low-quality	Genome-fragment		2.31	0
S11_extracted_k141_93708	4288	No	NA		0	1	Not-determined	Genome-fragment	NA		0
S11_extracted_k141_9286	1295	No	NA		0	1	Not-determined	Genome-fragment	NA		0
S11_extracted_k141_15442	4063	No	NA		0	2	Not-determined	Genome-fragment	NA		0
S7_vs_132828||full	1162	No	NA		0	0	Low-quality	Genome-fragment		1.66	0
S16_vs_162622||full	1162	No	NA		1	0	Low-quality	Genome-fragment		2.8	0
S11_extracted_k141_22355	1246	No	NA		0	1	Not-determined	Genome-fragment	NA		0
S11_extracted_k141_74598	1052	No	NA		0	1	Not-determined	Genome-fragment	NA		0
S10_extracted_61977	1162	No	NA		0	1	Low-quality	Genome-fragment		0.7	0
S10_vs_69703||full	1162	No	NA		0	0	Low-quality	Genome-fragment		1.88	0
S11_extracted_k141_2982	1984	No	NA		0	1	Not-determined	Genome-fragment	NA		0
S11_extracted_k141_60896	9398	No	NA		0	1	Not-determined	Genome-fragment	NA		0
S10_vs_57753||lt2gene	1162	No	NA		2	0	Low-quality	Genome-fragment		2.79	0
S11_extracted_k141_80743	1557	No	NA		0	1	Not-determined	Genome-fragment	NA		0
S15_vs_77043||lt2gene	1162	No	NA		1	0	Low-quality	Genome-fragment		2.28	0
S8_vs_k141_192440||lt2gene	1162	No	NA		2	0	Low-quality	Genome-fragment		3.4	0
S11_extracted_k141_39502	1163	No	NA		0	0	Low-quality	Genome-fragment		2.95	0
S11_extracted_k141_34754	1022	No	NA		0	0	Not-determined	Genome-fragment	NA		0
S11_extracted_k141_67326	1505	No	NA		0	2	Not-determined	Genome-fragment	NA		0
S11_extracted_k141_74663	2597	No	NA		0	1	Not-determined	Genome-fragment	NA		0
S1_extracted_k141_939	1163	No	NA		1	0	Low-quality	Genome-fragment		1.8	0
S1_vs_k141_47516||full	1163	No	NA		0	0	Low-quality	Genome-fragment		2.17	0
S11_extracted_k141_60983	4343	No	NA		0	1	Not-determined	Genome-fragment	NA		0
S11_extracted_k141_9391	3538	No	NA		0	0	Not-determined	Genome-fragment	NA		0
S1_vs_k141_94420||full	1163	Yes		639	1	1	Low-quality	Genome-fragment		0.97	45.06
S3_extracted_k141_119931	1163	No	NA		3	0	Low-quality	Genome-fragment		3.84	0
S11_extracted_k141_28570	1451	No	NA		0	2	Not-determined	Genome-fragment	NA		0
S11_extracted_k141_54768	1688	No	NA		0	2	Not-determined	Genome-fragment	NA		0
S11_extracted_k141_34776	1050	No	NA		0	1	Not-determined	Genome-fragment	NA		0
S11_extracted_k141_48017	1441	No	NA		0	2	Not-determined	Genome-fragment	NA		0
S11_extracted_k141_34783	3491	No	NA		0	2	Not-determined	Genome-fragment	NA		0
S3_extracted_k141_37377	1163	No	NA		1	0	Low-quality	Genome-fragment		1.7	0
S3_vs_k141_45294||full	1163	No	NA		0	0	Low-quality	Genome-fragment		2.76	0
S3_vs_k141_45418||full	1163	No	NA		0	0	Low-quality	Genome-fragment		3.16	0
S7_extracted_129296	1163	No	NA		0	0	Low-quality	Genome-fragment		2.03	0
S16_extracted_64690	1163	No	NA		0	1	Low-quality	Genome-fragment		1.15	0
S11_extracted_k141_28645	11934	No	NA		0	1	Complete	High-quality		100	0
S11_extracted_k141_61113	3404	No	NA		0	0	Not-determined	Genome-fragment	NA		0
S11_extracted_k141_67506	1577	No	NA		0	0	Not-determined	Genome-fragment	NA		0
S16_extracted_142390	1163	No	NA		0	0	Low-quality	Genome-fragment		2.74	0
S11_extracted_k141_3165	1201	No	NA		0	0	Not-determined	Genome-fragment	NA		0
S10_extracted_102502	1163	No	NA		0	0	Low-quality	Genome-fragment		0.89	0
S11_extracted_k141_15628	1024	No	NA		0	0	Not-determined	Genome-fragment	NA		0
S11_extracted_k141_22620	1484	No	NA		0	0	Not-determined	Genome-fragment	NA		0
S11_extracted_k141_61128	1115	No	NA		0	0	Not-determined	Genome-fragment	NA		0
S11_extracted_k141_48078	2214	No	NA		0	2	Not-determined	Genome-fragment	NA		0
S10_vs_52934||lt2gene	1163	No	NA		1	0	Low-quality	Genome-fragment		2.85	0
S11_extracted_k141_41885	1144	No	NA		0	0	Not-determined	Genome-fragment	NA		0
S11_extracted_k141_22638	1250	No	NA		0	1	Not-determined	Genome-fragment	NA		0
S11_extracted_k141_100498	1293	No	NA		0	1	Not-determined	Genome-fragment	NA		0
S8_vs_k141_166516||full	1163	No	NA		0	0	Low-quality	Genome-fragment		1.41	0
S11_extracted_k141_9502	1063	No	NA		0	1	Not-determined	Genome-fragment	NA		0
S17_vs_k141_76264||full	1164	No	NA		0	1	Low-quality	Genome-fragment		0.72	0
S11_extracted_k141_61175	1159	No	NA		0	1	Not-determined	Genome-fragment	NA		0
S11_vs_k141_7825||full	1164	No	NA		2	0	Low-quality	Genome-fragment		2.87	0
S11_extracted_k141_28701	4323	No	NA		0	0	Not-determined	Genome-fragment	NA		0
S11_extracted_k141_61176	3129	No	NA		0	0	Not-determined	Genome-fragment	NA		0
S11_vs_k141_70856||full	1164	No	NA		0	0	Low-quality	Genome-fragment		2.12	0
S11_extracted_k141_100558	3356	No	NA		0	1	Not-determined	Genome-fragment	NA		0
S11_extracted_k141_9540	3839	No	NA		0	4	Not-determined	Genome-fragment	NA		0
S14_extracted_k141_89493	1164	No	NA		2	0	Low-quality	Genome-fragment		3.79	0
S11_extracted_k141_41935	1137	No	NA		0	1	Not-determined	Genome-fragment	NA		0
S14_vs_k141_44322||full	1164	No	NA		1	0	Low-quality	Genome-fragment		1.29	0
S11_extracted_k141_74806	5375	No	NA		0	2	Not-determined	Genome-fragment	NA		0
S5_vs_k141_62654||full	1164	No	NA		1	0	Low-quality	Genome-fragment		2.61	0
S11_extracted_k141_34949	1220	No	NA		0	2	Not-determined	Genome-fragment	NA		0
S7_vs_76151||full	1164	No	NA		1	0	Low-quality	Genome-fragment		3.76	0
S12_extracted_k141_6339	1164	No	NA		0	1	Low-quality	Genome-fragment		3.72	0
S11_extracted_k141_81064	1188	No	NA		0	2	Not-determined	Genome-fragment	NA		0
S11_extracted_k141_22740	5571	No	NA		0	3	Not-determined	Genome-fragment	NA		0
S11_extracted_k141_67616	1163	No	NA		0	0	Not-determined	Genome-fragment	NA		0
S6_vs_k141_18142||full	1164	No	NA		2	0	Low-quality	Genome-fragment		1.72	0
S6_vs_k141_11455||full	1164	No	NA		1	0	Low-quality	Genome-fragment		2.62	0
S11_extracted_k141_35028	3567	No	NA		0	2	Not-determined	Genome-fragment	NA		0
S11_extracted_k141_80303	1165	No	NA		2	0	Low-quality	Genome-fragment		0.88	0
S11_extracted_k141_94162	1066	No	NA		0	0	Not-determined	Genome-fragment	NA		0
S11_extracted_k141_60868	1165	No	NA		1	0	Low-quality	Genome-fragment		2.88	0
S11_extracted_k141_22844	1832	No	NA		0	2	Not-determined	Genome-fragment	NA		0
S11_vs_k141_67640||lt2gene	1165	No	NA		1	0	Low-quality	Genome-fragment		2.86	0
S11_extracted_k141_48199	6111	No	NA		0	0	Not-determined	Genome-fragment	NA		0
S14_vs_k141_26906||full	1165	Yes		724	1	1	Low-quality	Genome-fragment		0.44	37.85
S11_extracted_k141_75038	1033	No	NA		0	0	Not-determined	Genome-fragment	NA		0
S11_extracted_k141_35140	2562	No	NA		0	1	Not-determined	Genome-fragment	NA		0
S1_extracted_k141_64061	1165	No	NA		0	0	Low-quality	Genome-fragment		2.61	0
S11_extracted_k141_15990	1117	No	NA		0	0	Not-determined	Genome-fragment	NA		0
S1_vs_k141_38478||full	1165	No	NA		2	0	Low-quality	Genome-fragment		4.24	0
S3_extracted_k141_24682	1165	No	NA		0	0	Low-quality	Genome-fragment		4.04	0
S11_extracted_k141_42165	1488	No	NA		0	3	Not-determined	Genome-fragment	NA		0
S11_extracted_k141_28957	1020	No	NA		0	0	Not-determined	Genome-fragment	NA		0
S11_extracted_k141_3379	3481	No	NA		0	0	Not-determined	Genome-fragment	NA		0
S11_extracted_k141_94319	1407	No	NA		0	2	Not-determined	Genome-fragment	NA		0
S5_extracted_k141_17309	1165	No	NA		3	0	Low-quality	Genome-fragment		2.57	0
S11_extracted_k141_100864	1045	No	NA		0	1	Not-determined	Genome-fragment	NA		0
S11_extracted_k141_48327	1068	No	NA		0	0	Not-determined	Genome-fragment	NA		0
S11_extracted_k141_61393	1981	No	NA		0	0	Not-determined	Genome-fragment	NA		0
S11_extracted_k141_61405	1830	No	NA		0	0	Not-determined	Genome-fragment	NA		0
S11_extracted_k141_94343	3252	No	NA		0	0	Not-determined	Genome-fragment	NA		0
S11_extracted_k141_94351	1342	No	NA		0	1	Not-determined	Genome-fragment	NA		0
S11_extracted_k141_48355	1798	No	NA		0	1	Not-determined	Genome-fragment	NA		0
S11_extracted_k141_94357	2454	No	NA		0	3	Not-determined	Genome-fragment	NA		0
S7_vs_129641||full	1165	No	NA		1	0	Low-quality	Genome-fragment		2.82	0
S10_vs_116827||lt2gene	1165	No	NA		0	0	Low-quality	Genome-fragment		2.43	0
S11_extracted_k141_81190	1503	No	NA		0	1	Not-determined	Genome-fragment	NA		0
S11_extracted_k141_61439	1179	No	NA		0	2	Not-determined	Genome-fragment	NA		0
S15_extracted_34945	1165	No	NA		0	1	Low-quality	Genome-fragment		0.43	0
S8_extracted_k141_102429	1165	No	NA		0	2	Low-quality	Genome-fragment		2.24	0
S11_extracted_k141_3469	1088	No	NA		0	0	Not-determined	Genome-fragment	NA		0
S11_extracted_k141_16111	1257	No	NA		0	0	Not-determined	Genome-fragment	NA		0
S11_extracted_k141_67826	1436	No	NA		0	0	Not-determined	Genome-fragment	NA		0
S11_extracted_k141_75172	3634	No	NA		0	3	Not-determined	Genome-fragment	NA		0
S11_extracted_k141_87976	1325	No	NA		0	1	Not-determined	Genome-fragment	NA		0
S11_extracted_k141_43257	1166	No	NA		1	0	Low-quality	Genome-fragment		2.77	0
S13_extracted_124335	1166	No	NA		0	1	Low-quality	Genome-fragment		4.34	0
S11_extracted_k141_87985	1080	No	NA		0	0	Not-determined	Genome-fragment	NA		0
S11_extracted_k141_81229	1781	No	NA		0	1	Not-determined	Genome-fragment	NA		0
S14_vs_k141_8785||full	1166	No	NA		0	1	Low-quality	Genome-fragment		0.52	0
S5_vs_k141_140910||full	1166	No	NA		1	0	Low-quality	Genome-fragment		2.92	0
S5_vs_k141_118882||full	1166	No	NA		0	0	Low-quality	Genome-fragment		2.28	0
S11_extracted_k141_75208	1008	No	NA		0	1	Not-determined	Genome-fragment	NA		0
S5_vs_k141_142700||full	1166	No	NA		1	0	Low-quality	Genome-fragment		2.69	0
S11_extracted_k141_3531	1574	No	NA		0	0	Not-determined	Genome-fragment	NA		0
S5_vs_k141_18230||lt2gene	1166	No	NA		1	0	Low-quality	Genome-fragment		1.76	0
S11_extracted_k141_75229	1263	No	NA		0	1	Not-determined	Genome-fragment	NA		0
S11_extracted_k141_35418	1548	No	NA		0	2	Not-determined	Genome-fragment	NA		0
S7_extracted_64967	1166	No	NA		1	0	Low-quality	Genome-fragment		2.46	0
S11_extracted_k141_55302	5347	No	NA		0	4	Not-determined	Genome-fragment	NA		0
S11_extracted_k141_55320	2533	No	NA		0	0	Not-determined	Genome-fragment	NA		0
S7_vs_15522||full	1166	No	NA		2	0	Low-quality	Genome-fragment		2.31	0
S11_extracted_k141_61544	1130	No	NA		0	1	Not-determined	Genome-fragment	NA		0
S16_extracted_136147	1166	No	NA		0	0	Low-quality	Genome-fragment		2.65	0
S16_vs_91033||full	1166	No	NA		2	0	Low-quality	Genome-fragment		2.5	0
S11_extracted_k141_67898	1096	No	NA		0	0	Not-determined	Genome-fragment	NA		0
S11_extracted_k141_67911	3271	No	NA		0	2	Not-determined	Genome-fragment	NA		0
S11_extracted_k141_75295	1069	No	NA		0	3	Not-determined	Genome-fragment	NA		0
S16_vs_77365||lt2gene	1166	No	NA		1	0	Low-quality	Genome-fragment		2.65	0
S11_extracted_k141_29237	1063	No	NA		0	1	Not-determined	Genome-fragment	NA		0
S11_extracted_k141_42430	1893	No	NA		0	2	Not-determined	Genome-fragment	NA		0
S11_extracted_k141_23217	6302	No	NA		0	5	Not-determined	Genome-fragment	NA		0
S10_extracted_50937	1166	No	NA		3	0	Low-quality	Genome-fragment		3.85	0
S11_extracted_k141_75326	1067	No	NA		0	0	Not-determined	Genome-fragment	NA		0
S11_extracted_k141_101025	1950	No	NA		0	1	Not-determined	Genome-fragment	NA		0
S10_vs_37673||lt2gene	1166	No	NA		1	0	Low-quality	Genome-fragment		2.27	0
S15_vs_69876||full	1166	No	NA		0	0	Low-quality	Genome-fragment		0.62	0
S11_extracted_k141_101042	1757	No	NA		0	0	Not-determined	Genome-fragment	NA		0
S11_extracted_k141_61631	1010	No	NA		0	0	Not-determined	Genome-fragment	NA		0
S11_extracted_k141_23250	7789	No	NA		0	8	Not-determined	Genome-fragment	NA		0
S11_extracted_k141_35598	1004	No	NA		0	2	Not-determined	Genome-fragment	NA		0
S11_extracted_k141_16321	4331	No	NA		0	3	Not-determined	Genome-fragment	NA		0
S6_vs_k141_14507||lt2gene	1166	No	NA		1	0	Low-quality	Genome-fragment		2.48	0
S11_extracted_k141_10111	4023	No	NA		0	1	Not-determined	Genome-fragment	NA		0
S11_extracted_k141_29282	1977	No	NA		0	1	Not-determined	Genome-fragment	NA		0
S8_extracted_k141_73933	1166	No	NA		0	1	Low-quality	Genome-fragment		0.83	0
S3_vs_k141_76013||full	1167	No	NA		1	0	Low-quality	Genome-fragment		0.73	0
S11_extracted_k141_23318	1008	No	NA		0	1	Not-determined	Genome-fragment	NA		0
S11_extracted_k141_29301	1444	No	NA		0	0	Not-determined	Genome-fragment	NA		0
S16_extracted_51039	1167	No	NA		0	1	Low-quality	Genome-fragment		1.45	0
S11_extracted_k141_68072	1454	No	NA		0	2	Not-determined	Genome-fragment	NA		0
S11_extracted_k141_10152	2776	No	NA		0	2	Not-determined	Genome-fragment	NA		0
S11_extracted_k141_68083	1046	No	NA		0	1	Not-determined	Genome-fragment	NA		0
S16_vs_30852||lt2gene	1167	No	NA		1	0	Low-quality	Genome-fragment		2.48	0
S11_extracted_k141_42585	6244	No	NA		0	0	Not-determined	Genome-fragment	NA		0

S10_vs_21459||full	1167	No	NA		1	0	Low-quality	Genome-fragment		3.15	0
S11_extracted_k141_42598	1367	No	NA		0	0	Not-determined	Genome-fragment	NA		0
S11_extracted_k141_75543	1372	No	NA		0	0	Not-determined	Genome-fragment	NA		0
S11_extracted_k141_75547	1273	No	NA		0	0	Not-determined	Genome-fragment	NA		0
S10_vs_75992||full	1167	No	NA		0	0	Low-quality	Genome-fragment		2.75	0
S12_extracted_k141_22308	1167	No	NA		2	0	Low-quality	Genome-fragment		2.49	0
S11_extracted_k141_55601	1059	No	NA		0	0	Not-determined	Genome-fragment	NA		0
S11_extracted_k141_23377	1182	No	NA		0	0	Not-determined	Genome-fragment	NA		0
S11_extracted_k141_61748	3808	No	NA		0	0	Not-determined	Genome-fragment	NA		0
S11_extracted_k141_61755	1036	No	NA		0	1	Not-determined	Genome-fragment	NA		0
S11_extracted_k141_75559	1986	No	NA		0	0	Not-determined	Genome-fragment	NA		0
S12_vs_k141_144705||full	1167	No	NA		0	0	Low-quality	Genome-fragment		3.13	0
S11_extracted_k141_75583	1013	No	NA		0	1	Not-determined	Genome-fragment	NA		0
S11_extracted_k141_88266	1416	No	NA		0	0	Not-determined	Genome-fragment	NA		0
S11_extracted_k141_23415	1069	No	NA		0	1	Not-determined	Genome-fragment	NA		0
S8_vs_k141_67392||full	1167	No	NA		0	0	Low-quality	Genome-fragment		1.58	0
S11_extracted_k141_88299	3732	No	NA		0	1	Not-determined	Genome-fragment	NA		0
S11_extracted_k141_81557	1042	No	NA		0	0	Not-determined	Genome-fragment	NA		0
S11_extracted_k141_101316	1061	No	NA		0	1	Not-determined	Genome-fragment	NA		0
S11_extracted_k141_88349	1326	No	NA		0	0	Not-determined	Genome-fragment	NA		0
S17_vs_k141_3810||full	1168	No	NA		0	0	Low-quality	Genome-fragment		2.8	0
S11_extracted_k141_55736	1087	No	NA		0	1	Not-determined	Genome-fragment	NA		0
S11_extracted_k141_94853	1213	No	NA		0	0	Not-determined	Genome-fragment	NA		0
S17_vs_k141_94801||full	1168	No	NA		1	0	Low-quality	Genome-fragment		0.82	0
S11_extracted_k141_55780	3055	No	NA		0	2	Not-determined	Genome-fragment	NA		0
S11_extracted_k141_61920	1007	No	NA		0	1	Not-determined	Genome-fragment	NA		0
S11_extracted_k141_55786	1106	No	NA		0	1	Not-determined	Genome-fragment	NA		0
S11_extracted_k141_101360	1032	No	NA		0	0	Not-determined	Genome-fragment	NA		0
S11_extracted_k141_23503	3158	No	NA		0	5	Not-determined	Genome-fragment	NA		0
S11_extracted_k141_61974	3132	No	NA		0	2	Not-determined	Genome-fragment	NA		0
S11_extracted_k141_55840	1022	No	NA		0	1	Not-determined	Genome-fragment	NA		0
S11_extracted_k141_91862	1168	No	NA		1	0	Low-quality	Genome-fragment		2.98	0
S11_extracted_k141_94948	1174	No	NA		0	2	Not-determined	Genome-fragment	NA		0
S1_vs_k141_154695||full	1168	No	NA		0	0	Low-quality	Genome-fragment		5.96	0
S3_extracted_k141_71523	1168	No	NA		0	0	Low-quality	Genome-fragment		2.22	0
S11_extracted_k141_81653	1419	No	NA		0	0	Not-determined	Genome-fragment	NA		0
S11_extracted_k141_42847	1025	No	NA		0	0	Not-determined	Genome-fragment	NA		0
S3_vs_k141_33513||full	1168	No	NA		0	0	Low-quality	Genome-fragment		2.57	0
S3_vs_k141_17945||full	1168	No	NA		0	0	Low-quality	Genome-fragment		2.32	0
S11_extracted_k141_36030	1387	No	NA		0	1	Not-determined	Genome-fragment	NA		0
S11_extracted_k141_29549	3102	No	NA		0	2	Not-determined	Genome-fragment	NA		0
S11_extracted_k141_81702	1004	No	NA		0	0	Not-determined	Genome-fragment	NA		0
S11_extracted_k141_88513	2190	No	NA		0	1	Not-determined	Genome-fragment	NA		0
S11_extracted_k141_3790	65685	No	NA		16	2	Medium-quality	Genome-fragment		69.9	0
S16_vs_27899||full	1168	No	NA		0	0	Low-quality	Genome-fragment		2.59	0
S16_vs_169854||full	1168	No	NA		0	1	Low-quality	Genome-fragment		0.97	0
S16_vs_31322||lt2gene	1168	No	NA		1	0	Low-quality	Genome-fragment		3.46	0
S11_extracted_k141_3822	1462	No	NA		0	0	Not-determined	Genome-fragment	NA		0
S10_extracted_53462	1168	No	NA		0	0	Low-quality	Genome-fragment		2.07	0
S10_vs_76289||full	1168	No	NA		0	0	Low-quality	Genome-fragment		1.47	0
S11_extracted_k141_88588	1155	No	NA		0	1	Not-determined	Genome-fragment	NA		0
S11_extracted_k141_62133	1472	No	NA		0	1	Not-determined	Genome-fragment	NA		0
S17_vs_k141_88760||full	1169	No	NA		0	0	Low-quality	Genome-fragment		4.11	0
S17_vs_k141_172922||lt2gene	1169	No	NA		0	0	Low-quality	Genome-fragment		2.66	0
S11_extracted_k141_16657	3140	No	NA		0	1	Not-determined	Genome-fragment	NA		0
S11_extracted_k141_88618	1336	No	NA		0	1	Not-determined	Genome-fragment	NA		0
S9_extracted_k141_44920	1169	No	NA		0	0	Low-quality	Genome-fragment		1.95	0
S11_extracted_k141_103070	1169	No	NA		4	0	Low-quality	Genome-fragment		3.86	0
S11_extracted_k141_56049	1070	No	NA		0	0	Not-determined	Genome-fragment	NA		0
S11_extracted_k141_36148	5666	No	NA		0	1	Not-determined	Genome-fragment	NA		0
S11_extracted_k141_29631	3215	No	NA		0	3	Not-determined	Genome-fragment	NA		0
S11_extracted_k141_29642	1685	No	NA		0	1	Not-determined	Genome-fragment	NA		0
S11_extracted_k141_62195	1016	No	NA		0	1	Not-determined	Genome-fragment	NA		0
S11_extracted_k141_36182	1189	No	NA		0	1	Not-determined	Genome-fragment	NA		0
S11_extracted_k141_42929	1221	No	NA		0	0	Not-determined	Genome-fragment	NA		0
S11_extracted_k141_23731	1095	No	NA		0	1	Not-determined	Genome-fragment	NA		0
S11_vs_k141_100876||full	1169	No	NA		1	0	Low-quality	Genome-fragment		3.07	0
S11_extracted_k141_36218	3377	No	NA		0	2	Not-determined	Genome-fragment	NA		0
S11_extracted_k141_36240	1244	No	NA		0	0	Not-determined	Genome-fragment	NA		0
S11_extracted_k141_75911	3629	No	NA		0	4	Not-determined	Genome-fragment	NA		0
S11_extracted_k141_95067	46485	No	NA		12	0	Complete	High-quality		100	0
S13_vs_115006||full	1169	No	NA		0	0	Low-quality	Genome-fragment		2.59	0
S11_extracted_k141_23838	1474	No	NA		0	1	Not-determined	Genome-fragment	NA		0
S14_vs_k141_55512||full	1169	No	NA		0	0	Low-quality	Genome-fragment		2.2	0
S14_vs_k141_72419||full	1169	Yes		657	1	2	Low-quality	Genome-fragment		0.93	43.8
S11_extracted_k141_43097	1746	No	NA		0	2	Not-determined	Genome-fragment	NA		0
S3_extracted_k141_121262	1169	No	NA		0	1	Low-quality	Genome-fragment		1.17	0
S11_extracted_k141_56262	3992	No	NA		0	2	Not-determined	Genome-fragment	NA		0
S11_extracted_k141_36352	1573	No	NA		0	1	Not-determined	Genome-fragment	NA		0
S3_extracted_k141_62064	1169	No	NA		2	0	Low-quality	Genome-fragment		0.69	0
S11_extracted_k141_81971	1261	No	NA		0	2	Not-determined	Genome-fragment	NA		0
S11_extracted_k141_75936	1458	No	NA		0	0	Not-determined	Genome-fragment	NA		0
S11_extracted_k141_4121	1171	No	NA		0	1	Not-determined	Genome-fragment	NA		0
S5_vs_k141_22647||full	1169	No	NA		0	0	Low-quality	Genome-fragment		0.5	0
S11_extracted_k141_29745	1380	No	NA		0	0	Not-determined	Genome-fragment	NA		0
S11_extracted_k141_43191	1206	No	NA		0	1	Not-determined	Genome-fragment	NA		0
S11_extracted_k141_49085	3490	No	NA		0	4	Not-determined	Genome-fragment	NA		0
S11_extracted_k141_36434	1514	No	NA		0	1	Not-determined	Genome-fragment	NA		0
S11_extracted_k141_24027	1184	No	NA		0	1	Not-determined	Genome-fragment	NA		0
S11_extracted_k141_29763	3116	No	NA		0	3	Not-determined	Genome-fragment	NA		0
S7_vs_87313||full	1169	No	NA		1	0	Low-quality	Genome-fragment		2.16	0
S7_vs_71643||lt2gene	1169	No	NA		1	0	Low-quality	Genome-fragment		1.31	0
S11_extracted_k141_36446	2100	No	NA		0	0	Not-determined	Genome-fragment	NA		0
S16_extracted_41996	1169	No	NA		0	1	Low-quality	Genome-fragment		0.77	0
S11_extracted_k141_56401	1831	No	NA		0	2	Not-determined	Genome-fragment	NA		0
S11_extracted_k141_49107	3077	No	NA		0	0	Not-determined	Genome-fragment	NA		0
S11_extracted_k141_62498	1860	No	NA		0	0	Not-determined	Genome-fragment	NA		0
S11_extracted_k141_56426	3100	No	NA		0	3	Not-determined	Genome-fragment	NA		0
S11_extracted_k141_43229	1808	No	NA		0	0	Not-determined	Genome-fragment	NA		0
S11_extracted_k141_95288	1414	No	NA		0	2	Not-determined	Genome-fragment	NA		0
S16_vs_23825||full	1169	No	NA		0	1	Low-quality	Genome-fragment		2.2	0
S10_extracted_9431	1169	No	NA		2	0	Low-quality	Genome-fragment		3.35	0
S11_extracted_k141_95313	1994	No	NA		0	2	Not-determined	Genome-fragment	NA		0
S11_extracted_k141_101831	4961	No	NA		0	0	Not-determined	Genome-fragment	NA		0
S10_vs_6013||full	1169	No	NA		1	0	Low-quality	Genome-fragment		1.96	0
S12_vs_k141_146033||full	1169	No	NA		0	0	Low-quality	Genome-fragment		0.64	0
S11_extracted_k141_76016	1151	No	NA		0	0	Not-determined	Genome-fragment	NA		0
S15_vs_67512||lt2gene	1169	No	NA		0	0	Low-quality	Genome-fragment		2.88	0
S11_extracted_k141_88939	1720	No	NA		0	1	Not-determined	Genome-fragment	NA		0
S11_extracted_k141_43300	1969	No	NA		0	0	Not-determined	Genome-fragment	NA		0
S11_extracted_k141_17017	1452	No	NA		0	1	Not-determined	Genome-fragment	NA		0
S11_extracted_k141_88980	2110	No	NA		0	2	Not-determined	Genome-fragment	NA		0
S8_vs_k141_121670||full	1169	No	NA		0	0	Low-quality	Genome-fragment		2.46	0
S11_extracted_k141_36596	1172	No	NA		0	0	Not-determined	Genome-fragment	NA		0
S8_vs_k141_167797||full	1169	No	NA		2	0	Low-quality	Genome-fragment		1.92	0
S11_extracted_k141_101916	3198	No	NA		0	2	Not-determined	Genome-fragment	NA		0
S11_extracted_k141_68693	40422	No	NA		10	0	Medium-quality	Genome-fragment		67.28	0
S11_extracted_k141_68697	1391	No	NA		0	1	Not-determined	Genome-fragment	NA		0
S11_extracted_k141_82163	4572	No	NA		0	0	Not-determined	Genome-fragment	NA		0
S11_extracted_k141_101974	2218	No	NA		0	0	Not-determined	Genome-fragment	NA		0
S11_extracted_k141_24295	3213	No	NA		0	1	Not-determined	Genome-fragment	NA		0
S11_extracted_k141_17133	1044	No	NA		0	0	Not-determined	Genome-fragment	NA		0
S11_extracted_k141_29970	2353	No	NA		0	2	Not-determined	Genome-fragment	NA		0
S8_vs_k141_173292||full	1169	No	NA		3	0	Low-quality	Genome-fragment		3.16	0
S11_extracted_k141_62662	1065	No	NA		0	1	Not-determined	Genome-fragment	NA		0
S11_extracted_k141_17162	1047	No	NA		0	0	Not-determined	Genome-fragment	NA		0
S11_extracted_k141_36741	1117	No	NA		0	1	Not-determined	Genome-fragment	NA		0
S11_extracted_k141_10907	37668	No	NA		24	0	Complete	High-quality		100	0
S17_vs_k141_160863||full	1170	No	NA		0	0	Low-quality	Genome-fragment		2.04	0
S9_extracted_k141_4929	1170	No	NA		0	0	Low-quality	Genome-fragment		2.37	0
S11_extracted_k141_62703	1582	No	NA		0	2	Not-determined	Genome-fragment	NA		0
S11_extracted_k141_62754	1378	No	NA		0	1	Not-determined	Genome-fragment	NA		0
S11_vs_k141_9562||full	1170	Yes		276	1	1	Low-quality	Genome-fragment		0.71	76.41
S11_extracted_k141_24442	2377	No	NA		0	0	Not-determined	Genome-fragment	NA		0
S11_extracted_k141_10977	1176	No	NA		0	1	Not-determined	Genome-fragment	NA		0
S11_extracted_k141_82286	1747	No	NA		0	0	Not-determined	Genome-fragment	NA		0
S13_vs_51444||full	1170	No	NA		0	0	Low-quality	Genome-fragment		3.13	0
S11_extracted_k141_24522	1004	No	NA		0	0	Not-determined	Genome-fragment	NA		0
S11_extracted_k141_49370	1036	No	NA		0	1	Not-determined	Genome-fragment	NA		0
S11_extracted_k141_95700	2538	No	NA		0	2	Not-determined	Genome-fragment	NA		0
S11_extracted_k141_82301	3138	No	NA		0	0	Not-determined	Genome-fragment	NA		0
S11_extracted_k141_24568	1024	No	NA		0	0	Not-determined	Genome-fragment	NA		0
S13_vs_105436||full	1170	No	NA		0	0	Low-quality	Genome-fragment		1.32	0
S11_extracted_k141_95795	1759	No	NA		0	0	Not-determined	Genome-fragment	NA		0
S11_extracted_k141_24655	1017	No	NA		0	1	Not-determined	Genome-fragment	NA		0
S11_extracted_k141_49474	1123	No	NA		0	1	Not-determined	Genome-fragment	NA		0
S11_extracted_k141_76214	2256	No	NA		0	0	Not-determined	Genome-fragment	NA		0
S11_extracted_k141_49488	3298	No	NA		0	2	Not-determined	Genome-fragment	NA		0
S1_vs_k141_52643||lt2gene	1170	No	NA		1	0	Low-quality	Genome-fragment		3.2	0
S11_extracted_k141_68972	1666	No	NA		0	0	Not-determined	Genome-fragment	NA		0
S11_extracted_k141_17399	1485	No	NA		0	0	Not-determined	Genome-fragment	NA		0
S11_extracted_k141_24701	1005	No	NA		0	0	Not-determined	Genome-fragment	NA		0
S11_extracted_k141_56775	1215	No	NA		0	1	Not-determined	Genome-fragment	NA		0
S11_extracted_k141_102325	1043	No	NA		0	0	Not-determined	Genome-fragment	NA		0
S7_vs_76401||full	1170	No	NA		0	1	Low-quality	Genome-fragment		0.8	0
S7_vs_97361||full	1170	No	NA		0	0	Low-quality	Genome-fragment		3.02	0
S11_extracted_k141_36986	1000	No	NA		0	2	Not-determined	Genome-fragment	NA		0
S11_extracted_k141_76315	1072	No	NA		0	1	Not-determined	Genome-fragment	NA		0
S6_vs_k141_19391||full	1170	No	NA		0	0	Low-quality	Genome-fragment		2.76	0
S11_extracted_k141_82359	4084	No	NA		0	3	Not-determined	Genome-fragment	NA		0
S11_extracted_k141_49566	3229	No	NA		0	1	Not-determined	Genome-fragment	NA		0
S11_extracted_k141_95962	3031	No	NA		0	0	Not-determined	Genome-fragment	NA		0
S11_extracted_k141_49593	3023	No	NA		0	2	Not-determined	Genome-fragment	NA		0
S11_extracted_k141_56861	5414	No	NA		0	1	Not-determined	Genome-fragment	NA		0
S8_vs_k141_166468||full	1170	No	NA		1	0	Low-quality	Genome-fragment		0.09	0
S11_extracted_k141_30255	1075	No	NA		0	1	Not-determined	Genome-fragment	NA		0
S8_vs_k141_180878||full	1170	No	NA		0	0	Low-quality	Genome-fragment		2.24	0
S11_extracted_k141_37096	11660	No	NA		0	3	Not-determined	Genome-fragment	NA		0
S11_extracted_k141_63060	1517	No	NA		0	0	Not-determined	Genome-fragment	NA		0
S11_extracted_k141_96002	1367	No	NA		0	0	Not-determined	Genome-fragment	NA		0
S11_extracted_k141_89309	46753	No	NA		1	9	Medium-quality	Genome-fragment		52.48	0
S14_extracted_k141_759	1171	No	NA		0	0	Low-quality	Genome-fragment		1.69	0
S1_extracted_k141_133667	1171	No	NA		0	0	Low-quality	Genome-fragment		0.67	0
S1_vs_k141_146673||full	1171	No	NA		1	0	Low-quality	Genome-fragment		2.27	0

S11_extracted_k141_82422	1239	No	NA		0	0	Not-determined	Genome-fragment	NA		0
S1_vs_k141_69091||lt2gene	1171	No	NA		1	0	Low-quality	Genome-fragment		0.89	0
S3_extracted_k141_111730	1171	No	NA		0	2	Low-quality	Genome-fragment		0.72	0
S11_extracted_k141_102467	1369	No	NA		0	2	Not-determined	Genome-fragment	NA		0
S11_extracted_k141_56972	3541	No	NA		0	2	Not-determined	Genome-fragment	NA		0
S11_extracted_k141_63109	2306	No	NA		0	0	Not-determined	Genome-fragment	NA		0
S3_extracted_k141_10428	1171	No	NA		1	0	Low-quality	Genome-fragment		2.85	0
S11_extracted_k141_63112	3493	No	NA		0	1	Not-determined	Genome-fragment	NA		0
S11_extracted_k141_102494	1035	No	NA		0	1	Not-determined	Genome-fragment	NA		0
S3_vs_k141_40935||lt2gene	1171	No	NA		0	0	Low-quality	Genome-fragment		3.06	0
S11_extracted_k141_43784	1566	No	NA		0	1	Not-determined	Genome-fragment	NA		0
S11_extracted_k141_30406	1054	No	NA		0	1	Not-determined	Genome-fragment	NA		0
S11_extracted_k141_43815	3915	No	NA		0	0	Not-determined	Genome-fragment	NA		0
S16_vs_28247||full	1171	No	NA		0	0	Low-quality	Genome-fragment		3.27	0
S11_extracted_k141_4900	1353	No	NA		0	0	Not-determined	Genome-fragment	NA		0
S11_extracted_k141_57079	1101	No	NA		0	1	Not-determined	Genome-fragment	NA		0
S11_extracted_k141_30434	1064	No	NA		0	1	Not-determined	Genome-fragment	NA		0
S11_extracted_k141_17679	2114	No	NA		0	1	Not-determined	Genome-fragment	NA		0
S12_extracted_k141_126875	1171	No	NA		0	1	Low-quality	Genome-fragment		0.33	0
S11_extracted_k141_4957	3205	No	NA		0	2	Not-determined	Genome-fragment	NA		0
S12_vs_k141_94302||full	1171	Yes		442	1	1	Low-quality	Genome-fragment		0.69	62.25
S11_extracted_k141_25034	1024	No	NA		0	1	Not-determined	Genome-fragment	NA		0
S12_vs_k141_14935||full	1171	No	NA		0	0	Low-quality	Genome-fragment		1.86	0
S11_extracted_k141_37313	2299	No	NA		0	3	Not-determined	Genome-fragment	NA		0
S3_extracted_k141_94968	1172	No	NA		0	0	Low-quality	Genome-fragment		3.46	0
S5_vs_k141_140154||full	1172	No	NA		0	0	Low-quality	Genome-fragment		2.7	0
S5_vs_k141_137565||full	1172	No	NA		1	0	Low-quality	Genome-fragment		2.05	0
S11_extracted_k141_37339	1991	No	NA		0	0	Not-determined	Genome-fragment	NA		0
S5_vs_k141_110443||lt2gene	1172	No	NA		1	0	Low-quality	Genome-fragment		1.33	0
S11_extracted_k141_49943	1035	No	NA		0	0	Not-determined	Genome-fragment	NA		0
S11_extracted_k141_82626	1398	No	NA		0	2	Not-determined	Genome-fragment	NA		0
S16_vs_163537||full	1172	No	NA		4	0	Low-quality	Genome-fragment		2.91	0
S11_extracted_k141_11431	1696	No	NA		0	1	Not-determined	Genome-fragment	NA		0
S11_extracted_k141_57263	2874	No	NA		0	0	Not-determined	Genome-fragment	NA		0
S10_vs_80342||full	1172	No	NA		1	0	Low-quality	Genome-fragment		0.88	0
S11_extracted_k141_49977	5269	No	NA		0	3	Not-determined	Genome-fragment	NA		0
S15_vs_24092||full	1172	No	NA		2	0	Low-quality	Genome-fragment		3.44	0
S11_extracted_k141_17813	1868	No	NA		0	1	Not-determined	Genome-fragment	NA		0
S8_extracted_k141_45495	1172	No	NA		0	0	Low-quality	Genome-fragment		0.74	0
S11_extracted_k141_69409	1381	No	NA		0	0	Not-determined	Genome-fragment	NA		0
S8_extracted_k141_199134	1172	No	NA		1	0	Low-quality	Genome-fragment		2.61	0
S8_vs_k141_36357||full	1172	No	NA		0	1	Low-quality	Genome-fragment		0.47	0
S8_vs_k141_75737||full	1172	No	NA		0	0	Low-quality	Genome-fragment		0.74	0
S17_extracted_k141_69656	1173	No	NA		0	0	Low-quality	Genome-fragment		2.71	0
S11_extracted_k141_11503	4617	No	NA		0	4	Not-determined	Genome-fragment	NA		0
S17_extracted_k141_55024	1173	No	NA		0	1	Low-quality	Genome-fragment		0.24	0
S11_extracted_k141_43997	59939	No	NA		5	0	Medium-quality	Genome-fragment		65.91	0
S11_extracted_k141_44020	1095	No	NA		0	0	Not-determined	Genome-fragment	NA		0
S17_vs_k141_141043||full	1173	No	NA		1	0	Low-quality	Genome-fragment		1.95	0
S17_vs_k141_71525||lt2gene	1173	No	NA		2	0	Low-quality	Genome-fragment		2.99	0
S9_vs_k141_63331||full	1173	No	NA		0	3	Low-quality	Genome-fragment		0.34	0
S11_extracted_k141_89587	1438	No	NA		0	3	Not-determined	Genome-fragment	NA		0
S1_vs_k141_149876||full	1173	No	NA		1	0	Low-quality	Genome-fragment		3	0
S11_extracted_k141_11565	1354	No	NA		0	1	Not-determined	Genome-fragment	NA		0
S3_extracted_k141_119425	1173	No	NA		0	0	Low-quality	Genome-fragment		2.42	0
S5_extracted_k141_136215	1173	No	NA		1	0	Low-quality	Genome-fragment		1.83	0
S11_extracted_k141_17961	1465	No	NA		0	2	Not-determined	Genome-fragment	NA		0
S11_extracted_k141_96576	1442	No	NA		0	1	Not-determined	Genome-fragment	NA		0
S5_extracted_k141_82676	1173	No	NA		1	0	Low-quality	Genome-fragment		2.67	0
S5_vs_k141_2165||full	1173	No	NA		0	0	Low-quality	Genome-fragment		3.46	0
S11_extracted_k141_57448	5582	No	NA		0	4	Not-determined	Genome-fragment	NA		0
S5_vs_k141_61906||lt2gene	1173	No	NA		0	0	Low-quality	Genome-fragment		2.09	0
S11_extracted_k141_96619	1790	No	NA		0	1	Not-determined	Genome-fragment	NA		0
S11_extracted_k141_76823	1242	No	NA		0	1	Not-determined	Genome-fragment	NA		0
S7_vs_72508||full	1173	No	NA		2	0	Low-quality	Genome-fragment		4.42	0
S11_extracted_k141_5139	5470	No	NA		0	3	Not-determined	Genome-fragment	NA		0
S16_vs_30253||full	1173	No	NA		0	0	Low-quality	Genome-fragment		1.81	0
S11_extracted_k141_69584	5002	No	NA		0	1	Not-determined	Genome-fragment	NA		0
S11_extracted_k141_44177	20533	No	NA		0	1	Not-determined	Genome-fragment	NA		0
S16_vs_159757||full	1173	No	NA		3	0	Low-quality	Genome-fragment		1.94	0
S10_vs_110434||lt2gene	1173	No	NA		0	0	Low-quality	Genome-fragment		2.79	0
S11_extracted_k141_25366	1001	No	NA		0	2	Not-determined	Genome-fragment	NA		0
S11_extracted_k141_63427	2791	No	NA		0	2	Not-determined	Genome-fragment	NA		0
S11_extracted_k141_37722	4253	No	NA		0	4	Not-determined	Genome-fragment	NA		0
S11_extracted_k141_63439	1041	No	NA		0	0	Not-determined	Genome-fragment	NA		0
S12_vs_k141_113261||full	1173	No	NA		0	0	Low-quality	Genome-fragment		3.44	0
S11_extracted_k141_44266	1320	No	NA		0	2	Not-determined	Genome-fragment	NA		0
S4_extracted_25281	1173	No	NA		2	0	Low-quality	Genome-fragment		1.94	0
S11_extracted_k141_44276	2507	No	NA		0	2	Not-determined	Genome-fragment	NA		0
S11_extracted_k141_76969	1272	No	NA		0	0	Not-determined	Genome-fragment	NA		0
S8_vs_k141_1021||full	1173	No	NA		0	2	Low-quality	Genome-fragment		0.67	0
S11_extracted_k141_37758	3043	No	NA		0	0	Not-determined	Genome-fragment	NA		0
S11_extracted_k141_69710	1094	No	NA		0	1	Not-determined	Genome-fragment	NA		0
S11_extracted_k141_89716	1037	No	NA		0	1	Not-determined	Genome-fragment	NA		0
S11_extracted_k141_96735	1289	No	NA		0	1	Not-determined	Genome-fragment	NA		0
S11_extracted_k141_83018	1668	No	NA		0	1	Not-determined	Genome-fragment	NA		0
S8_vs_k141_197901||full	1173	No	NA		1	0	Low-quality	Genome-fragment		2.79	0
S11_extracted_k141_63524	1399	No	NA		0	0	Not-determined	Genome-fragment	NA		0
S11_extracted_k141_69748	2236	No	NA		0	0	Not-determined	Genome-fragment	NA		0
S11_extracted_k141_50116	1298	No	NA		0	2	Not-determined	Genome-fragment	NA		0
S8_vs_k141_155736||full	1173	No	NA		0	1	Low-quality	Genome-fragment		1.47	0
S11_extracted_k141_25448	1808	No	NA		0	0	Not-determined	Genome-fragment	NA		0
S11_extracted_k141_96761	1542	No	NA		0	1	Not-determined	Genome-fragment	NA		0
S9_extracted_k141_83057	1174	No	NA		1	0	Low-quality	Genome-fragment		2.37	0
S11_vs_k141_60470||full	1174	No	NA		0	0	Low-quality	Genome-fragment		2.48	0
S11_extracted_k141_31004	1001	No	NA		0	1	Not-determined	Genome-fragment	NA		0
S11_extracted_k141_18183	1708	No	NA		0	1	Not-determined	Genome-fragment	NA		0
S14_extracted_k141_47656	1174	No	NA		0	0	Low-quality	Genome-fragment		2.36	0
S11_extracted_k141_5328	1394	No	NA		0	0	Not-determined	Genome-fragment	NA		0
S11_extracted_k141_96786	4251	No	NA		0	1	Not-determined	Genome-fragment	NA		0
S11_extracted_k141_96795	1921	No	NA		0	1	Not-determined	Genome-fragment	NA		0
S11_extracted_k141_89785	5915	No	NA		0	4	Not-determined	Genome-fragment	NA		0
S3_extracted_k141_119239	1174	No	NA		0	1	Low-quality	Genome-fragment		0.38	0
S3_vs_k141_112316||full	1174	No	NA		0	0	Low-quality	Genome-fragment		2.03	0
S11_extracted_k141_37887	2052	No	NA		0	2	Not-determined	Genome-fragment	NA		0
S11_extracted_k141_18207	3188	No	NA		0	3	Not-determined	Genome-fragment	NA		0
S11_extracted_k141_5345	1077	No	NA		0	1	Not-determined	Genome-fragment	NA		0
S11_extracted_k141_11726	1745	No	NA		0	1	Not-determined	Genome-fragment	NA		0
S11_extracted_k141_57619	1581	No	NA		0	0	Not-determined	Genome-fragment	NA		0
S11_extracted_k141_63649	2324	No	NA		0	1	Not-determined	Genome-fragment	NA		0
S11_extracted_k141_69850	1299	No	NA		0	0	Not-determined	Genome-fragment	NA		0
S11_extracted_k141_5388	1283	No	NA		0	1	Not-determined	Genome-fragment	NA		0
S11_extracted_k141_89818	2175	No	NA		0	1	Not-determined	Genome-fragment	NA		0
S5_extracted_k141_117229	1174	No	NA		0	0	Low-quality	Genome-fragment		1.71	0
S11_extracted_k141_25547	10782	No	NA		0	0	Not-determined	Genome-fragment	NA		0
S11_extracted_k141_5400	2890	No	NA		0	3	Not-determined	Genome-fragment	NA		0
S7_extracted_86406	1174	No	NA		1	0	Low-quality	Genome-fragment		2.94	0
S11_extracted_k141_96871	1110	No	NA		0	1	Not-determined	Genome-fragment	NA		0
S11_extracted_k141_57691	1754	No	NA		0	2	Not-determined	Genome-fragment	NA		0
S7_vs_12235||full	1174	No	NA		0	0	Low-quality	Genome-fragment		2.29	0
S7_vs_60872||full	1174	No	NA		1	0	Low-quality	Genome-fragment		2.51	0
S11_extracted_k141_83187	1476	No	NA		0	0	Not-determined	Genome-fragment	NA		0
S11_extracted_k141_63734	3939	No	NA		0	3	Not-determined	Genome-fragment	NA		0
S11_extracted_k141_50275	1377	No	NA		0	1	Not-determined	Genome-fragment	NA		0
S11_extracted_k141_37938	1041	No	NA		0	0	Not-determined	Genome-fragment	NA		0
S16_vs_61736||lt2gene	1174	No	NA		1	0	Low-quality	Genome-fragment		2.18	0
S11_extracted_k141_25609	1043	No	NA		0	0	Not-determined	Genome-fragment	NA		0
S11_extracted_k141_31176	1161	No	NA		0	1	Not-determined	Genome-fragment	NA		0
S11_extracted_k141_77180	1381	No	NA		0	1	Not-determined	Genome-fragment	NA		0
S11_extracted_k141_57741	1168	No	NA		0	2	Not-determined	Genome-fragment	NA		0
S12_extracted_k141_135775	1174	No	NA		1	0	Low-quality	Genome-fragment		2.9	0
S12_extracted_k141_153379	1174	No	NA		1	0	Low-quality	Genome-fragment		2.71	0
S15_extracted_63938	1174	No	NA		0	1	Low-quality	Genome-fragment		0.43	0
S11_extracted_k141_44508	1173	No	NA		0	1	Not-determined	Genome-fragment	NA		0
S4_extracted_24138	1174	No	NA		0	0	Low-quality	Genome-fragment		2.84	0
S11_extracted_k141_44510	3984	No	NA		0	3	Not-determined	Genome-fragment	NA		0
S11_extracted_k141_102921	1056	No	NA		0	0	Not-determined	Genome-fragment	NA		0
S11_extracted_k141_18344	1251	No	NA		0	0	Not-determined	Genome-fragment	NA		0
S8_extracted_k141_203326	1174	No	NA		0	0	Low-quality	Genome-fragment		2.74	0
S11_extracted_k141_44541	2340	No	NA		0	1	Not-determined	Genome-fragment	NA		0
S11_extracted_k141_11852	1148	No	NA		0	0	Not-determined	Genome-fragment	NA		0
S8_vs_k141_59170||full	1174	No	NA		1	0	Low-quality	Genome-fragment		2.01	0
S9_vs_k141_85361||full	1175	No	NA		0	0	Low-quality	Genome-fragment		2.95	0
S11_vs_k141_15163||full	1175	No	NA		0	0	Low-quality	Genome-fragment		2.73	0
S11_extracted_k141_11872	4519	No	NA		0	3	Not-determined	Genome-fragment	NA		0
S11_extracted_k141_44573	1469	No	NA		0	2	Not-determined	Genome-fragment	NA		0
S14_extracted_k141_84342	1175	No	NA		3	0	Low-quality	Genome-fragment		1.96	0
S11_extracted_k141_97010	1225	No	NA		0	2	Not-determined	Genome-fragment	NA		0
S11_extracted_k141_97016	4653	No	NA		0	0	Not-determined	Genome-fragment	NA		0
S1_vs_k141_133659||full	1175	No	NA		1	0	Low-quality	Genome-fragment		3.38	0
S11_extracted_k141_38086	1830	No	NA		0	1	Not-determined	Genome-fragment	NA		0
S11_extracted_k141_5617	1856	No	NA		0	2	Not-determined	Genome-fragment	NA		0
S11_extracted_k141_5618	1186	No	NA		0	1	Not-determined	Genome-fragment	NA		0
S11_extracted_k141_18431	3727	No	NA		0	1	Not-determined	Genome-fragment	NA		0
S11_extracted_k141_31312	1881	No	NA		0	1	Not-determined	Genome-fragment	NA		0
S3_extracted_k141_120358	1175	No	NA		0	0	Low-quality	Genome-fragment		0.68	0
S11_extracted_k141_31315	1372	No	NA		0	2	Not-determined	Genome-fragment	NA		0
S3_vs_k141_37046||lt2gene	1175	No	NA		1	0	Low-quality	Genome-fragment		0.81	0
S11_extracted_k141_97064	3434	No	NA		0	1	Not-determined	Genome-fragment	NA		0
S11_extracted_k141_83379	1036	No	NA		0	0	Not-determined	Genome-fragment	NA		0
S11_extracted_k141_50465	1503	No	NA		0	1	Not-determined	Genome-fragment	NA		0
S11_extracted_k141_5673	1077	No	NA		0	1	Not-determined	Genome-fragment	NA		0
S5_extracted_k141_19576	1175	No	NA		1	0	Low-quality	Genome-fragment		3.26	0
S11_extracted_k141_44712	2398	No	NA		0	2	Not-determined	Genome-fragment	NA		0
S11_extracted_k141_70015	53828	No	NA		14	3	High-quality	High-quality		92.95	0
S11_extracted_k141_50487	12577	No	NA		0	2	Not-determined	Genome-fragment	NA		0
S11_extracted_k141_25779	1383	No	NA		0	1	Not-determined	Genome-fragment	NA		0
S11_extracted_k141_18539	3172	No	NA		0	2	Not-determined	Genome-fragment	NA		0
S11_extracted_k141_31409	1546	No	NA		0	2	Not-determined	Genome-fragment	NA		0
S11_extracted_k141_63984	3479	No	NA		0	3	Not-determined	Genome-fragment	NA		0
S11_extracted_k141_90071	1073	No	NA		0	1	Not-determined	Genome-fragment	NA		0
S7_extracted_69815	1175	No	NA		0	0	Low-quality	Genome-fragment		7.1	0
S11_extracted_k141_38233	2270	No	NA		0	0	Not-determined	Genome-fragment	NA		0
S11_extracted_k141_12032	3322	No	NA		0	1	Not-determined	Genome-fragment	NA		0

S11_extracted_k141_57792	1115	No	NA	0	3	Not-determined	Genome-fragment	NA		0
S11_extracted_k141_44795	6103	No	NA	0	3	Not-determined	Genome-fragment	NA		0
S11_extracted_k141_5778	4623	No	NA	0	1	Not-determined	Genome-fragment	NA		0
S11_extracted_k141_57834	5233	No	NA	0	2	Not-determined	Genome-fragment	NA		0
S11_extracted_k141_12093	1002	No	NA	0	0	Not-determined	Genome-fragment	NA		0
S11_extracted_k141_83570	1174	No	NA	0	0	Not-determined	Genome-fragment	NA		0
S11_extracted_k141_57848	1040	No	NA	0	0	Not-determined	Genome-fragment	NA		0
S16_vs_13649||full	1175	No	NA	0	0	Low-quality	Genome-fragment		1.81	0
S11_extracted_k141_50584	1266	No	NA	0	1	Not-determined	Genome-fragment	NA		0
S11_extracted_k141_83579	1037	No	NA	0	0	Not-determined	Genome-fragment	NA		0
S11_extracted_k141_25896	1729	No	NA	0	2	Not-determined	Genome-fragment	NA		0
S11_extracted_k141_57853	5314	No	NA	0	3	Not-determined	Genome-fragment	NA		0
S11_extracted_k141_50602	1339	No	NA	0	0	Not-determined	Genome-fragment	NA		0
S16_vs_154245||lt2gene	1175	No	NA	1	0	Low-quality	Genome-fragment		1.72	0
S11_extracted_k141_70164	1310	No	NA	0	2	Not-determined	Genome-fragment	NA		0
S12_extracted_k141_93756	1175	No	NA	0	0	Low-quality	Genome-fragment		2.79	0
S12_vs_k141_40229||lt2gene	1175	No	NA	2	0	Low-quality	Genome-fragment		1.65	0
S11_extracted_k141_18719	2230	No	NA	0	1	Not-determined	Genome-fragment	NA		0
S11_extracted_k141_57892	1469	No	NA	0	1	Not-determined	Genome-fragment	NA		0
S11_extracted_k141_90228	1336	No	NA	0	1	Not-determined	Genome-fragment	NA		0
S11_extracted_k141_70198	1480	No	NA	0	0	Not-determined	Genome-fragment	NA		0
S11_extracted_k141_12156	1573	No	NA	0	0	Not-determined	Genome-fragment	NA		0
S8_extracted_k141_150071	1175	No	NA	0	0	Low-quality	Genome-fragment		4.08	0
S11_extracted_k141_50660	1083	No	NA	0	0	Not-determined	Genome-fragment	NA		0
S8_extracted_k141_124435	1175	No	NA	0	1	Low-quality	Genome-fragment		4.38	0
S8_vs_k141_13439||full	1175	No	NA	1	0	Low-quality	Genome-fragment		2.56	0
S11_extracted_k141_64141	1021	No	NA	0	0	Not-determined	Genome-fragment	NA		0
S11_extracted_k141_97222	1425	No	NA	0	0	Not-determined	Genome-fragment	NA		0
S11_extracted_k141_38370	1765	No	NA	0	0	Not-determined	Genome-fragment	NA		0
S11_extracted_k141_97227	1500	No	NA	0	1	Not-determined	Genome-fragment	NA		0
S11_extracted_k141_97239	1157	No	NA	0	0	Not-determined	Genome-fragment	NA		0
S11_extracted_k141_70231	1521	No	NA	0	1	Not-determined	Genome-fragment	NA		0
S9_vs_k141_9709||full	1176	No	NA	0	1	Low-quality	Genome-fragment		3.76	0
S5_extracted_k141_129726	1176	No	NA	0	0	Low-quality	Genome-fragment		3.2	0
S5_extracted_k141_96865	1176	No	NA	2	0	Low-quality	Genome-fragment		3.41	0
S11_extracted_k141_18852	1221	No	NA	0	2	Not-determined	Genome-fragment	NA		0
S11_extracted_k141_103213	1958	No	NA	0	0	Not-determined	Genome-fragment	NA		0
S5_vs_k141_39654||full	1176	No	NA	1	0	Low-quality	Genome-fragment		2.2	0
S5_vs_k141_61214||lt2gene	1176	No	NA	1	0	Low-quality	Genome-fragment		3.37	0
S7_extracted_95070	1176	No	NA	1	0	Low-quality	Genome-fragment		2.5	0
S7_vs_106314||full	1176	No	NA	1	0	Low-quality	Genome-fragment		1.08	0
S7_vs_136216||full	1176	No	NA	0	0	Low-quality	Genome-fragment		2.24	0
S16_vs_55382||full	1176	No	NA	0	1	Low-quality	Genome-fragment		2.68	0
S11_extracted_k141_12334	1041	No	NA	0	1	Not-determined	Genome-fragment	NA		0
S10_extracted_98687	1176	No	NA	0	1	Low-quality	Genome-fragment		0.38	0
S11_extracted_k141_90359	1332	No	NA	0	0	Not-determined	Genome-fragment	NA		0
S11_extracted_k141_12339	1026	No	NA	0	1	Not-determined	Genome-fragment	NA		0
S11_extracted_k141_64290	1582	No	NA	0	1	Not-determined	Genome-fragment	NA		0
S11_extracted_k141_5987	3464	No	NA	0	0	Not-determined	Genome-fragment	NA		0
S11_extracted_k141_5997	5679	No	NA	0	7	Not-determined	Genome-fragment	NA		0
S12_extracted_k141_21515	1176	No	NA	0	0	Low-quality	Genome-fragment		2.27	0
S12_vs_k141_897||lt2gene	1176	No	NA	1	0	Low-quality	Genome-fragment		3.12	0
S6_vs_k141_33822||full	1176	No	NA	0	0	Low-quality	Genome-fragment		2.25	0
S8_extracted_k141_95934	1176	No	NA	0	0	Low-quality	Genome-fragment		1.97	0
S17_vs_k141_9374||full	1177	No	NA	1	0	Low-quality	Genome-fragment		2.58	0
S11_extracted_k141_77763	1034	No	NA	0	0	Not-determined	Genome-fragment	NA		0
S11_extracted_k141_6030	1459	No	NA	0	0	Not-determined	Genome-fragment	NA		0
S11_extracted_k141_97386	1015	No	NA	0	1	Not-determined	Genome-fragment	NA		0
S11_extracted_k141_45012	1848	No	NA	0	0	Not-determined	Genome-fragment	NA		0
S11_extracted_k141_103284	2526	No	NA	0	2	Not-determined	Genome-fragment	NA		0
S11_extracted_k141_90457	1429	No	NA	0	0	Not-determined	Genome-fragment	NA		0
S11_extracted_k141_14023	1177	No	NA	0	2	Low-quality	Genome-fragment		0.47	0
S11_extracted_k141_12430	1005	No	NA	0	0	Not-determined	Genome-fragment	NA		0
S11_extracted_k141_83856	2747	No	NA	0	2	Not-determined	Genome-fragment	NA		0
S11_extracted_k141_64342	1093	No	NA	0	0	Not-determined	Genome-fragment	NA		0
S11_extracted_k141_50840	1006	No	NA	0	0	Not-determined	Genome-fragment	NA		0
S11_extracted_k141_103299	1334	No	NA	0	0	Not-determined	Genome-fragment	NA		0
S11_extracted_k141_6088	5241	No	NA	0	3	Not-determined	Genome-fragment	NA		0
S14_extracted_k141_89723	1177	No	NA	2	0	Low-quality	Genome-fragment		3.88	0
S11_extracted_k141_31776	4997	No	NA	0	4	Not-determined	Genome-fragment	NA		0
S11_extracted_k141_83896	1305	No	NA	0	0	Not-determined	Genome-fragment	NA		0
S3_vs_k141_105526||full	1177	No	NA	0	0	Low-quality	Genome-fragment		3.01	0
S11_extracted_k141_103346	1159	No	NA	0	2	Not-determined	Genome-fragment	NA		0
S11_extracted_k141_31806	1930	No	NA	0	0	Not-determined	Genome-fragment	NA		0
S11_extracted_k141_31810	3188	No	NA	0	0	Not-determined	Genome-fragment	NA		0
S5_extracted_k141_99842	1177	No	NA	1	0	Low-quality	Genome-fragment		3.12	0
S11_extracted_k141_12534	1380	No	NA	0	1	Not-determined	Genome-fragment	NA		0
S5_vs_k141_47536||full	1177	No	NA	0	0	Low-quality	Genome-fragment		1.63	0
S7_vs_36556||full	1177	No	NA	1	0	Low-quality	Genome-fragment		2.72	0
S7_vs_125564||full	1177	No	NA	1	0	Low-quality	Genome-fragment		2.97	0
S11_extracted_k141_12586	1312	No	NA	0	1	Not-determined	Genome-fragment	NA		0
S11_extracted_k141_64478	1174	No	NA	0	1	Not-determined	Genome-fragment	NA		0
S16_vs_54462||full	1177	No	NA	2	0	Low-quality	Genome-fragment		2.74	0
S16_vs_95575||full	1177	No	NA	2	0	Low-quality	Genome-fragment		2.65	0
S11_extracted_k141_51062	7054	No	NA	0	1	Not-determined	Genome-fragment	NA		0
S11_extracted_k141_103387	1218	No	NA	0	0	Not-determined	Genome-fragment	NA		0
S10_extracted_31884	1177	No	NA	2	0	Low-quality	Genome-fragment		3.7	0
S6_vs_k141_36677||full	1177	No	NA	2	0	Low-quality	Genome-fragment		2.57	0
S8_vs_k141_1243||full	1177	No	NA	0	0	Low-quality	Genome-fragment		2.28	0
S11_extracted_k141_84088	1044	No	NA	0	1	Not-determined	Genome-fragment	NA		0
S11_extracted_k141_84092	1210	No	NA	0	0	Not-determined	Genome-fragment	NA		0
S11_extracted_k141_64548	1053	No	NA	0	1	Not-determined	Genome-fragment	NA		0
S11_extracted_k141_84107	1261	No	NA	0	2	Not-determined	Genome-fragment	NA		0
S11_extracted_k141_38632	1021	No	NA	0	0	Not-determined	Genome-fragment	NA		0
S8_vs_k141_70093||lt2gene	1177	No	NA	1	0	Low-quality	Genome-fragment		3.2	0
S17_vs_k141_85955||full	1178	No	NA	0	0	Low-quality	Genome-fragment		2.9	0
S11_vs_k141_57710||full	1178	No	NA	0	3	Low-quality	Genome-fragment		0.75	0
S3_vs_k141_42597||full	1178	No	NA	1	0	Low-quality	Genome-fragment		1.98	0
S11_extracted_k141_6217	1181	No	NA	0	1	Not-determined	Genome-fragment	NA		0
S11_extracted_k141_31956	1869	No	NA	0	0	Not-determined	Genome-fragment	NA		0
S11_extracted_k141_45384	1234	No	NA	0	1	Not-determined	Genome-fragment	NA		0
S11_extracted_k141_45411	1508	No	NA	0	1	Not-determined	Genome-fragment	NA		0
S3_vs_k141_119446||full	1178	No	NA	1	0	Low-quality	Genome-fragment		0.76	0
S11_extracted_k141_84172	2976	No	NA	0	2	Not-determined	Genome-fragment	NA		0
S5_extracted_k141_81525	1178	No	NA	1	0	Low-quality	Genome-fragment		2.56	0
S11_extracted_k141_84225	1090	No	NA	0	1	Not-determined	Genome-fragment	NA		0
S5_vs_k141_136774||full	1178	No	NA	1	0	Low-quality	Genome-fragment		2.06	0
S11_extracted_k141_64662	1129	No	NA	0	1	Not-determined	Genome-fragment	NA		0
S11_extracted_k141_38795	3286	No	NA	0	3	Not-determined	Genome-fragment	NA		0
S5_vs_k141_11353||full	1178	No	NA	1	0	Low-quality	Genome-fragment		2.68	0
S7_extracted_97172	1178	No	NA	1	0	Low-quality	Genome-fragment		1.4	0
S11_extracted_k141_64684	3253	No	NA	0	2	Not-determined	Genome-fragment	NA		0
S7_vs_52502||full	1178	No	NA	0	0	Low-quality	Genome-fragment		2.94	0
S7_vs_63781||full	1178	No	NA	0	0	Low-quality	Genome-fragment		1.3	0
S11_extracted_k141_70640	2242	No	NA	0	1	Not-determined	Genome-fragment	NA		0
S11_extracted_k141_64711	1164	No	NA	0	1	Not-determined	Genome-fragment	NA		0
S11_extracted_k141_70645	1082	No	NA	0	1	Not-determined	Genome-fragment	NA		0
S11_extracted_k141_58452	1062	No	NA	0	1	Not-determined	Genome-fragment	NA		0
S10_vs_2091||full	1178	No	NA	0	0	Low-quality	Genome-fragment		2.27	0
S11_extracted_k141_58473	1268	No	NA	0	0	Not-determined	Genome-fragment	NA		0
S10_vs_108072||full	1178	No	NA	0	0	Low-quality	Genome-fragment		2.8	0
S10_vs_18541||lt2gene	1178	No	NA	0	0	Low-quality	Genome-fragment		2.2	0
S11_extracted_k141_6372	2542	No	NA	0	0	Not-determined	Genome-fragment	NA		0
S11_extracted_k141_58502	1157	No	NA	0	1	Not-determined	Genome-fragment	NA		0
S11_extracted_k141_103619	1018	No	NA	0	0	Not-determined	Genome-fragment	NA		0
S11_extracted_k141_90866	1666	No	NA	0	1	Not-determined	Genome-fragment	NA		0
S11_extracted_k141_6409	2941	No	NA	0	2	Not-determined	Genome-fragment	NA		0
S12_vs_k141_166114||full	1178	No	NA	2	0	Low-quality	Genome-fragment		3.05	0
S11_extracted_k141_84303	45395	No	NA	15	0	Complete	High-quality		100	0
S12_vs_k141_112914||full	1178	No	NA	1	0	Low-quality	Genome-fragment		2.71	0
S11_extracted_k141_51350	1941	No	NA	0	1	Not-determined	Genome-fragment	NA		0
S12_vs_k141_137656||full	1178	No	NA	0	0	Low-quality	Genome-fragment		3.63	0
S15_extracted_41729	1178	No	NA	0	1	Low-quality	Genome-fragment		1.16	0
S11_extracted_k141_84373	2609	No	NA	0	1	Not-determined	Genome-fragment	NA		0
S11_extracted_k141_45513	1229	No	NA	0	2	Not-determined	Genome-fragment	NA		0
S11_extracted_k141_38929	1479	No	NA	0	0	Not-determined	Genome-fragment	NA		0
S8_extracted_k141_138842	1178	No	NA	1	0	Low-quality	Genome-fragment		1.96	0
S11_extracted_k141_103696	5294	No	NA	0	3	Not-determined	Genome-fragment	NA		0
S11_extracted_k141_70820	1391	No	NA	0	0	Not-determined	Genome-fragment	NA		0
S11_extracted_k141_103699	4593	No	NA	0	4	Not-determined	Genome-fragment	NA		0
S11_extracted_k141_38998	1205	No	NA	0	1	Not-determined	Genome-fragment	NA		0
S11_extracted_k141_19520	1825	No	NA	0	3	Not-determined	Genome-fragment	NA		0
S11_extracted_k141_51476	5408	No	NA	0	4	Not-determined	Genome-fragment	NA		0
S11_extracted_k141_64913	1116	No	NA	0	0	Not-determined	Genome-fragment	NA		0
S11_extracted_k141_64947	1196	No	NA	0	0	Not-determined	Genome-fragment	NA		0
S11_extracted_k141_51561	1192	No	NA	0	1	Not-determined	Genome-fragment	NA		0
S11_extracted_k141_71006	3753	No	NA	0	1	Not-determined	Genome-fragment	NA		0
S11_extracted_k141_51616	1428	No	NA	0	2	Not-determined	Genome-fragment	NA		0
S11_extracted_k141_65041	1664	No	NA	0	1	Not-determined	Genome-fragment	NA		0
S11_extracted_k141_103935	1056	No	NA	0	0	Not-determined	Genome-fragment	NA		0
S11_extracted_k141_103938	3772	No	NA	0	1	Not-determined	Genome-fragment	NA		0
S8_vs_k141_44125||full	1178	No	NA	0	0	Low-quality	Genome-fragment		2.01	0
S11_extracted_k141_103954	1054	No	NA	0	1	Not-determined	Genome-fragment	NA		0
S8_vs_k141_13179||full	1178	No	NA	0	0	Low-quality	Genome-fragment		2.56	0
S8_vs_k141_153066||lt2gene	1178	No	NA	1	0	Low-quality	Genome-fragment		2.82	0
S9_extracted_k141_54963	1179	No	NA	1	0	Low-quality	Genome-fragment		2.52	0
S11_extracted_k141_71168	1061	No	NA	0	1	Not-determined	Genome-fragment	NA		0
S14_extracted_k141_41150	1179	No	NA	0	0	Low-quality	Genome-fragment		2.09	0
S14_vs_k141_49072||full	1179	No	NA	0	0	Low-quality	Genome-fragment		2.2	0
S11_extracted_k141_51793	2814	No	NA	0	0	Not-determined	Genome-fragment	NA		0
S11_extracted_k141_32332	1100	No	NA	0	0	Not-determined	Genome-fragment	NA		0
S14_vs_k141_65877||lt2gene	1179	No	NA	1	0	Low-quality	Genome-fragment		1.98	0
S11_extracted_k141_71383	5505	No	NA	0	3	Not-determined	Genome-fragment	NA		0
S11_extracted_k141_32476	1166	No	NA	0	1	Not-determined	Genome-fragment	NA		0
S11_extracted_k141_104033	106247	No	NA	18	4	Complete	High-quality		100	0
S11_extracted_k141_32502	1131	No	NA	0	0	Not-determined	Genome-fragment	NA		0
S11_extracted_k141_104034	40797	No	NA	18	3	Complete	High-quality		100	0
S11_extracted_k141_71463	1721	No	NA	0	0	Not-determined	Genome-fragment	NA		0
S11_extracted_k141_104035	99625	No	NA	15	2	Complete	High-quality		100	0
S11_extracted_k141_104044	100812	No	NA	22	0	Complete	High-quality		100	0
S11_extracted_k141_104046	164379	No	NA	17	4	Complete	High-quality		100	0
S11_extracted_k141_104048	37508	No	NA	9	0	Complete	High-quality		100	0
S11_extracted_k141_104052	40792	No	NA	22	0	Complete	High-quality		100	0
S11_extracted_k141_104059	91418	No	NA	8	1	Complete	High-quality		100	0

S11_extracted_k141_104062	34888	No	NA		12	0	Complete	High-quality		100	0
S11_extracted_k141_104070	36162	No	NA		27	0	Complete	High-quality		100	0
S11_extracted_k141_104071	36582	No	NA		8	0	Complete	High-quality		100	0
S11_vs_k141_84627||full	39772	No	NA		10	0	High-quality	High-quality		100	0
S1_extracted_k141_40235	1179	No	NA		0	1	Low-quality	Genome-fragment		0.31	0
S11_vs_k141_20912||full	2051	No	NA		0	0	Not-determined	Genome-fragment	NA		0
S11_vs_k141_40945||full	44575	No	NA		17	0	High-quality	High-quality		99.31	0
S11_vs_k141_93101||full	23434	No	NA		7	0	High-quality	High-quality		99.44	0
S11_vs_k141_3006||full	32415	No	NA		17	0	Medium-quality	Genome-fragment		88.09	0
S11_vs_k141_75554||full	16322	No	NA		7	0	Medium-quality	Genome-fragment		83.95	0
S11_vs_k141_49071||full	89156	No	NA		29	0	High-quality	High-quality		100	0
S11_vs_k141_36748||full	46177	No	NA		14	1	High-quality	High-quality		100	0
S11_vs_k141_82368||full	45487	No	NA		22	0	High-quality	High-quality		100	0
S11_vs_k141_104032||full	44004	No	NA		23	2	High-quality	High-quality		100	0
S11_vs_k141_104036||full	48912	No	NA		15	1	High-quality	High-quality		96.17	0
S11_vs_k141_104037||full	54218	No	NA		15	0	High-quality	High-quality		100	0
S11_vs_k141_104038||full	34262	No	NA		22	0	Medium-quality	Genome-fragment		89.66	0
S11_vs_k141_104041||full	36201	No	NA		16	2	High-quality	High-quality		100	0
S11_vs_k141_104042||full	37290	Yes		35553	18	2	Medium-quality	Genome-fragment		89.78	4.66
S11_vs_k141_104047||full	33359	Yes		30866	19	2	Medium-quality	Genome-fragment		85.83	7.47
S11_vs_k141_104049||full	38479	No	NA		13	0	High-quality	High-quality		100	0
S3_extracted_k141_49619	1179	No	NA		0	0	Low-quality	Genome-fragment		0.77	0
S3_extracted_k141_84800	1179	No	NA		0	0	Low-quality	Genome-fragment		2.04	0
S11_vs_k141_104053||full	13498	No	NA		6	0	High-quality	High-quality		99.05	0
S11_vs_k141_104054||full	29886	No	NA		14	0	Medium-quality	Genome-fragment		74.75	0
S11_vs_k141_104061||full	15739	No	NA		0	3	Not-determined	Genome-fragment	NA		0
S11_vs_k141_104064||full	58041	No	NA		35	0	High-quality	High-quality		96.25	0
S11_vs_k141_104069||full	44378	No	NA		7	0	High-quality	High-quality		99.41	0
S3_vs_k141_43810||full	1179	No	NA		0	1	Low-quality	Genome-fragment		1.46	0
S5_extracted_k141_62607	1179	No	NA		1	0	Low-quality	Genome-fragment		1.76	0
S5_extracted_k141_27629	1179	No	NA		1	0	Low-quality	Genome-fragment		2.91	0
S11_vs_k141_97632||full	3846	No	NA		0	0	Not-determined	Genome-fragment	NA		0
S7_vs_106547||lt2gene	1179	No	NA		1	0	Low-quality	Genome-fragment		3.44	0
S11_vs_k141_52091||full	1570	No	NA		0	0	Not-determined	Genome-fragment	NA		0
S11_vs_k141_38||full	1170	No	NA		0	0	Not-determined	Genome-fragment	NA		0
S11_vs_k141_26054||full	2834	No	NA		0	0	Not-determined	Genome-fragment	NA		0
S7_vs_110702||lt2gene	1179	No	NA		2	0	Low-quality	Genome-fragment		3.94	0
S16_extracted_67340	1179	No	NA		1	0	Low-quality	Genome-fragment		3.14	0
S16_vs_68739||full	1179	No	NA		0	0	Low-quality	Genome-fragment		2.84	0
S10_vs_3709||full	1179	No	NA		0	0	Low-quality	Genome-fragment		2.7	0
S11_vs_k141_19558||full	1174	No	NA		0	1	Not-determined	Genome-fragment	NA		0
S11_vs_k141_78148||full	6383	No	NA		0	3	Not-determined	Genome-fragment	NA		0
S10_vs_108096||full	1179	No	NA		0	0	Low-quality	Genome-fragment		2.07	0
S11_vs_k141_39092||full	1911	No	NA		0	2	Not-determined	Genome-fragment	NA		0
S11_vs_k141_89||full	1511	No	NA		0	0	Not-determined	Genome-fragment	NA		0
S11_vs_k141_58652||full	1387	No	NA		0	1	Not-determined	Genome-fragment	NA		0
S11_vs_k141_58656||full	2995	No	NA		0	2	Not-determined	Genome-fragment	NA		0
S12_vs_k141_13669||full	1179	No	NA		0	0	Low-quality	Genome-fragment		2.43	0
S4_extracted_3543	1179	No	NA		2	0	Low-quality	Genome-fragment		3.88	0
S11_vs_k141_97678||full	1483	No	NA		0	0	Not-determined	Genome-fragment	NA		0
S11_vs_k141_97680||full	4205	No	NA		0	2	Not-determined	Genome-fragment	NA		0
S11_vs_k141_97686||full	5948	No	NA		0	5	Not-determined	Genome-fragment	NA		0
S11_vs_k141_32630||full	1482	No	NA		0	0	Not-determined	Genome-fragment	NA		0
S11_vs_k141_141||full	2228	No	NA		0	0	Not-determined	Genome-fragment	NA		0
S4_extracted_15274	1179	No	NA		2	0	Low-quality	Genome-fragment		3.75	0
S11_vs_k141_164||full	1960	No	NA		0	0	Not-determined	Genome-fragment	NA		0
S11_vs_k141_52268||full	1298	No	NA		0	2	Not-determined	Genome-fragment	NA		0
S8_extracted_k141_195776	1179	No	NA		0	0	Low-quality	Genome-fragment		2.97	0
S11_vs_k141_198||full	1435	No	NA		0	1	Not-determined	Genome-fragment	NA		0
S8_vs_k141_77053||full	1179	No	NA		0	0	Low-quality	Genome-fragment		1.62	0
S9_extracted_k141_76326	1180	No	NA		2	0	Low-quality	Genome-fragment		1.96	0
S11_vs_k141_78168||full	1789	No	NA		0	0	Not-determined	Genome-fragment	NA		0
S11_vs_k141_241||full	2577	No	NA		0	2	Not-determined	Genome-fragment	NA		0
S9_vs_k141_16353||full	1180	No	NA		1	0	Low-quality	Genome-fragment		1.94	0
S9_vs_k141_89494||full	1180	No	NA		1	0	Low-quality	Genome-fragment		1.69	0
S11_vs_k141_91307||full	1605	No	NA		0	1	Not-determined	Genome-fragment	NA		0
S13_vs_19606||full	1180	No	NA		0	0	Low-quality	Genome-fragment		2.26	0
S14_vs_k141_71229||full	1180	No	NA		0	0	Low-quality	Genome-fragment		2.6	0
S11_vs_k141_78182||full	1379	No	NA		0	0	Not-determined	Genome-fragment	NA		0
S11_vs_k141_58702||full	22627	No	NA		22	0	Medium-quality	Genome-fragment		50.16	0
S5_vs_k141_99160||full	1180	No	NA		0	0	Low-quality	Genome-fragment		2.98	0
S7_vs_38683||full	1180	No	NA		0	1	Low-quality	Genome-fragment		3.79	0
S16_vs_50070||full	1180	No	NA		1	0	Low-quality	Genome-fragment		3.43	0
S11_vs_k141_91331||full	3980	No	NA		0	2	Not-determined	Genome-fragment	NA		0
S16_vs_74182||full	1180	No	NA		4	0	Low-quality	Genome-fragment		2.74	0
S12_vs_k141_57041||full	1180	No	NA		0	0	Low-quality	Genome-fragment		0.62	0
S12_vs_k141_105454||full	1180	No	NA		2	0	Low-quality	Genome-fragment		3.37	0
S11_vs_k141_71754||full	1501	No	NA		0	2	Not-determined	Genome-fragment	NA		0
S11_vs_k141_39217||full	8026	No	NA		0	4	Not-determined	Genome-fragment	NA		0
S12_vs_k141_77407||full	1180	No	NA		0	0	Low-quality	Genome-fragment		0.92	0
S11_vs_k141_84824||full	1517	No	NA		0	1	Not-determined	Genome-fragment	NA		0
S11_vs_k141_6632||full	2719	No	NA		0	2	Not-determined	Genome-fragment	NA		0
S11_vs_k141_65304||full	1578	No	NA		0	2	Not-determined	Genome-fragment	NA		0
S11_vs_k141_78305||full	2042	No	NA		0	0	Not-determined	Genome-fragment	NA		0
S15_extracted_46552	1180	No	NA		0	1	Low-quality	Genome-fragment		0.51	0
S11_vs_k141_65307||full	3715	No	NA		0	2	Not-determined	Genome-fragment	NA		0
S4_extracted_30904	1180	No	NA		1	0	Low-quality	Genome-fragment		3.62	0
S11_vs_k141_58776||full	6054	No	NA		0	0	Not-determined	Genome-fragment	NA		0
S11_vs_k141_45658||full	173195	Yes		44246	12	97	Medium-quality	Genome-fragment		68.29	74.45
S8_extracted_k141_117554	1180	No	NA		1	0	Low-quality	Genome-fragment		0.94	0
S11_vs_k141_84848||full	41635	No	NA		7	4	High-quality	High-quality		97.21	0
S8_extracted_k141_189188	1180	No	NA		0	0	Low-quality	Genome-fragment		0.73	0
S9_extracted_k141_56314	1181	No	NA		0	0	Low-quality	Genome-fragment		1.33	0
S11_vs_k141_75246||full	1181	No	NA		0	0	Low-quality	Genome-fragment		2.01	0
S13_vs_149550||full	1181	No	NA		0	0	Low-quality	Genome-fragment		2.28	0
S11_vs_k141_32832||full	9578	No	NA		0	1	Not-determined	Genome-fragment	NA		0
S11_vs_k141_45708||full	3235	No	NA		0	2	Not-determined	Genome-fragment	NA		0
S14_vs_k141_84436||full	1181	No	NA		1	0	Low-quality	Genome-fragment		1.37	0
S11_vs_k141_39279||full	11329	No	NA		0	6	Not-determined	Genome-fragment	NA		0
S11_vs_k141_84917||full	1039	No	NA		0	1	Not-determined	Genome-fragment	NA		0
S14_vs_k141_55408||lt2gene	1181	No	NA		1	0	Low-quality	Genome-fragment		3.03	0
S3_vs_k141_48729||lt2gene	1181	No	NA		1	0	Low-quality	Genome-fragment		2.75	0
S3_vs_k141_18921||lt2gene	1181	No	NA		1	0	Low-quality	Genome-fragment		2.72	0
S5_vs_k141_127576||full	1181	No	NA		0	0	Low-quality	Genome-fragment		3.31	0
S5_vs_k141_144078||full	1181	No	NA		1	0	Low-quality	Genome-fragment		1.48	0
S11_vs_k141_97971||full	1274	No	NA		0	2	Not-determined	Genome-fragment	NA		0
S7_vs_80234||full	1181	No	NA		1	0	Low-quality	Genome-fragment		2.67	0
S16_vs_130283||full	1181	No	NA		0	0	Low-quality	Genome-fragment		2.54	0
S11_vs_k141_6776||full	1283	No	NA		0	0	Not-determined	Genome-fragment	NA		0
S11_vs_k141_65387||full	1118	No	NA		0	1	Not-determined	Genome-fragment	NA		0
S11_vs_k141_522||full	1458	No	NA		0	0	Not-determined	Genome-fragment	NA		0
S10_extracted_27010	1181	No	NA		0	0	Low-quality	Genome-fragment		0.48	0
S11_vs_k141_71899||full	1384	No	NA		0	1	Not-determined	Genome-fragment	NA		0
S11_vs_k141_52430||full	1413	No	NA		0	0	Not-determined	Genome-fragment	NA		0
S11_vs_k141_26383||full	4496	No	NA		0	0	Not-determined	Genome-fragment	NA		0
S10_extracted_29788	1181	No	NA		0	0	Low-quality	Genome-fragment		1.09	0
S10_vs_3849||full	1181	No	NA		0	1	Low-quality	Genome-fragment		2.88	0
S12_extracted_k141_37863	1181	No	NA		0	0	Low-quality	Genome-fragment		3.07	0
S12_vs_k141_21771||lt2gene	1181	No	NA		1	0	Low-quality	Genome-fragment		2.79	0
S6_vs_k141_47337||full	1181	No	NA		0	0	Low-quality	Genome-fragment		2.71	0
S9_extracted_k141_39952	1182	No	NA		1	0	Low-quality	Genome-fragment		2.77	0
S9_extracted_k141_90161	1182	No	NA		0	0	Low-quality	Genome-fragment		2.96	0
S11_vs_k141_19892||full	31610	No	NA		14	1	Medium-quality	Genome-fragment		70.43	0
S11_vs_k141_78527||full	1440	No	NA		0	1	Not-determined	Genome-fragment	NA		0
S13_extracted_147053	1182	No	NA		1	0	Low-quality	Genome-fragment		0.82	0
S11_vs_k141_78534||full	2945	No	NA		0	1	Not-determined	Genome-fragment	NA		0
S14_extracted_k141_30302	1182	No	NA		2	0	Low-quality	Genome-fragment		3.9	0
S11_vs_k141_98028||full	4940	No	NA		0	1	Not-determined	Genome-fragment	NA		0
S14_extracted_k141_56841	1182	No	NA		2	0	Low-quality	Genome-fragment		1.97	0
S11_vs_k141_32947||full	3095	No	NA		0	1	Not-determined	Genome-fragment	NA		0
S1_extracted_k141_2888	1182	No	NA		2	0	Low-quality	Genome-fragment		2.12	0
S11_vs_k141_32954||full	6115	No	NA		0	2	Not-determined	Genome-fragment	NA		0
S3_extracted_k141_89126	1182	No	NA		0	0	Low-quality	Genome-fragment		0.67	0
S3_vs_k141_91791||full	1182	No	NA		0	0	Low-quality	Genome-fragment		2.15	0
S5_vs_k141_43053||full	1182	No	NA		1	0	Low-quality	Genome-fragment		2.72	0
S5_vs_k141_73724||full	1182	No	NA		1	0	Low-quality	Genome-fragment		3.41	0
S5_vs_k141_78804||full	1182	No	NA		0	0	Low-quality	Genome-fragment		2.7	0
S11_vs_k141_52494||full	10446	No	NA		0	3	Not-determined	Genome-fragment	NA		0
S5_vs_k141_45282||lt2gene	1182	No	NA		1	0	Low-quality	Genome-fragment		2.69	0
S16_vs_40552||lt2gene	1182	No	NA		1	0	Low-quality	Genome-fragment		1.92	0
S11_vs_k141_6878||full	42579	No	NA		23	2	High-quality	High-quality		97.62	0
S11_vs_k141_58930||full	23499	No	NA		6	3	Medium-quality	Genome-fragment		55.51	0
S11_vs_k141_98098||full	3879	No	NA		0	2	Not-determined	Genome-fragment	NA		0
S16_vs_113871||lt2gene	1182	No	NA		2	0	Low-quality	Genome-fragment		2.42	0
S11_vs_k141_13239||full	1246	No	NA		0	0	Not-determined	Genome-fragment	NA		0
S16_vs_120355||lt2gene	1182	No	NA		1	0	Low-quality	Genome-fragment		0.81	0
S10_vs_8751||full	1182	No	NA		0	0	Low-quality	Genome-fragment		2.02	0
S4_extracted_26909	1182	No	NA		3	0	Low-quality	Genome-fragment		3.83	0
S8_vs_k141_68947||lt2gene	1182	No	NA		1	0	Low-quality	Genome-fragment		2.05	0
S9_extracted_k141_24390	1183	No	NA		1	0	Low-quality	Genome-fragment		2.89	0
S11_vs_k141_78682||full	1764	No	NA		0	2	Not-determined	Genome-fragment	NA		0
S9_extracted_k141_40858	1183	No	NA		0	1	Low-quality	Genome-fragment		0.32	0
S11_vs_k141_85116||full	1784	No	NA		0	3	Not-determined	Genome-fragment	NA		0
S11_vs_k141_5224||full	1183	No	NA		0	0	Low-quality	Genome-fragment		0.61	0
S13_extracted_152511	1183	No	NA		0	1	Low-quality	Genome-fragment		0.52	0
S14_extracted_k141_58953	1183	No	NA		0	0	Low-quality	Genome-fragment		1.4	0
S11_vs_k141_72109||full	4146	No	NA		0	3	Not-determined	Genome-fragment	NA		0
S1_extracted_k141_104424	1183	No	NA		0	1	Low-quality	Genome-fragment		1.42	0
S11_vs_k141_52577||full	31692	No	NA		15	1	Medium-quality	Genome-fragment		88.54	0
S3_extracted_k141_75140	1183	No	NA		0	0	Low-quality	Genome-fragment		2.81	0
S11_vs_k141_20079||full	6325	No	NA		0	0	Not-determined	Genome-fragment	NA		0
S11_vs_k141_72161||full	2047	No	NA		0	1	Not-determined	Genome-fragment	NA		0
S5_vs_k141_11909||full	1183	No	NA		0	0	Low-quality	Genome-fragment		2.57	0
S11_vs_k141_91702||full	1569	No	NA		0	1	Not-determined	Genome-fragment	NA		0
S5_vs_k141_68699||lt2gene	1183	No	NA		2	0	Low-quality	Genome-fragment		3.3	0
S7_vs_4944||full	1183	No	NA		2	0	Low-quality	Genome-fragment		2.15	0
S7_vs_45447||full	1183	No	NA		1	0	Low-quality	Genome-fragment		2.46	0
S11_vs_k141_98193||full	34086	No	NA		8	2	Medium-quality	Genome-fragment		80.53	0
S11_vs_k141_98194||full	2531	No	NA		0	2	Not-determined	Genome-fragment	NA		0
S16_vs_24630||full	1183	No	NA		0	0	Low-quality	Genome-fragment		2.24	0
S11_vs_k141_78726||full	4258	No	NA		0	0	Not-determined	Genome-fragment	NA		0
S10_vs_81126||full	1183	No	NA		0	2	Low-quality	Genome-fragment		3.78	0
S17_extracted_k141_78152	1184	No	NA		1	0	Low-quality	Genome-fragment		1.9	0
S17_extracted_k141_93659	1184	No	NA		1	0	Low-quality	Genome-fragment		3.01	0

S11_extracted_k141_103628	1184	No	NA	0	0	Low-quality	Genome-fragment		2.59	0
S11_vs_k141_7012||full	1020	No	NA	0	0	Not-determined	Genome-fragment	NA		0
S13_extracted_21061	1184	No	NA	0	0	Low-quality	Genome-fragment		1.18	0
S14_vs_k141_12224||full	1184	No	NA	2	0	Low-quality	Genome-fragment		2.76	0
S11_vs_k141_39684||full	1446	No	NA	0	2	Not-determined	Genome-fragment	NA		0
S3_vs_k141_81839||full	1184	No	NA	0	0	Low-quality	Genome-fragment		0.89	0
S5_extracted_k141_137803	1184	No	NA	0	0	Low-quality	Genome-fragment		0.56	0
S5_vs_k141_37301||lt2gene	1184	No	NA	1	0	Low-quality	Genome-fragment		3.01	0
S7_extracted_50670	1184	No	NA	0	1	Low-quality	Genome-fragment		0.32	0
S7_vs_24685||full	1184	No	NA	1	0	Low-quality	Genome-fragment		3.37	0
S16_vs_61831||full	1184	No	NA	0	0	Low-quality	Genome-fragment		1.62	0
S16_vs_70465||full	1184	No	NA	0	0	Low-quality	Genome-fragment		2.13	0
S10_extracted_114582	1184	No	NA	0	1	Low-quality	Genome-fragment		2.38	0
S10_vs_101445||full	1184	No	NA	2	0	Low-quality	Genome-fragment		3.65	0
S8_extracted_k141_185375	1184	No	NA	1	0	Low-quality	Genome-fragment		0.65	0
S11_vs_k141_13488||full	4117	No	NA	0	3	Not-determined	Genome-fragment	NA		0
S8_vs_k141_104154||full	1184	No	NA	1	0	Low-quality	Genome-fragment		0.45	0
S17_vs_k141_138528||full	1185	No	NA	3	0	Low-quality	Genome-fragment		5.4	0
S11_vs_k141_20225||full	36396	No	NA	20	0	High-quality	High-quality		94.18	0
S11_vs_k141_46148||full	3225	No	NA	0	1	Not-determined	Genome-fragment	NA		0
S14_vs_k141_84329||full	1185	No	NA	0	0	Low-quality	Genome-fragment		3.03	0
S11_vs_k141_65688||full	1028	No	NA	0	0	Not-determined	Genome-fragment	NA		0
S1_vs_k141_96189||full	1185	No	NA	1	0	Low-quality	Genome-fragment		2.95	0
S5_vs_k141_68210||full	1185	No	NA	1	0	Low-quality	Genome-fragment		3.22	0
S11_vs_k141_91868||full	4655	No	NA	0	5	Not-determined	Genome-fragment	NA		0
S5_vs_k141_134998||full	1185	No	NA	0	0	Low-quality	Genome-fragment		2	0
S11_vs_k141_78885||full	2349	No	NA	0	3	Not-determined	Genome-fragment	NA		0
S7_extracted_74842	1185	No	NA	0	1	Low-quality	Genome-fragment		1.3	0
S11_vs_k141_78888||full	6125	No	NA	0	5	Not-determined	Genome-fragment	NA		0
S7_vs_58799||lt2gene	1185	No	NA	1	0	Low-quality	Genome-fragment		4.01	0
S10_extracted_1440	1185	No	NA	1	0	Low-quality	Genome-fragment		2.9	0
S10_extracted_71399	1185	No	NA	0	0	Low-quality	Genome-fragment		1.22	0
S12_extracted_k141_20737	1185	No	NA	1	0	Low-quality	Genome-fragment		1.93	0
S12_extracted_k141_22664	1185	No	NA	0	0	Low-quality	Genome-fragment		2.15	0
S11_vs_k141_26767||full	24781	No	NA	22	0	Medium-quality	Genome-fragment		56.42	0
S11_vs_k141_85405||full	1500	No	NA	0	1	Not-determined	Genome-fragment	NA		0
S11_vs_k141_20321||full	6360	No	NA	0	0	Not-determined	Genome-fragment	NA		0
S11_vs_k141_85411||full	1250	No	NA	0	1	Not-determined	Genome-fragment	NA		0
S11_vs_k141_46258||full	6617	No	NA	0	8	Not-determined	Genome-fragment	NA		0
S12_extracted_k141_38280	1185	No	NA	1	0	Low-quality	Genome-fragment		1.41	0
S12_vs_k141_65050||full	1185	No	NA	2	0	Low-quality	Genome-fragment		2.68	0
S15_vs_208||full	1185	No	NA	1	0	Low-quality	Genome-fragment		2.43	0
S11_vs_k141_39758||full	6229	No	NA	0	4	Not-determined	Genome-fragment	NA		0
S11_vs_k141_965||full	1768	No	NA	0	0	Not-determined	Genome-fragment	NA		0
S4_extracted_4201	1185	No	NA	2	0	Low-quality	Genome-fragment		3.22	0
S11_vs_k141_26824||full	2034	No	NA	0	1	Not-determined	Genome-fragment	NA		0
S8_extracted_k141_79984	1185	No	NA	1	0	Low-quality	Genome-fragment		2.86	0
S8_extracted_k141_144545	1185	No	NA	0	0	Low-quality	Genome-fragment		0.33	0
S11_vs_k141_52777||full	6559	No	NA	0	3	Not-determined	Genome-fragment	NA		0
S8_extracted_k141_127308	1185	No	NA	0	0	Low-quality	Genome-fragment		2.27	0
S11_vs_k141_65767||full	4200	No	NA	0	0	Not-determined	Genome-fragment	NA		0
S8_vs_k141_69494||full	1185	No	NA	0	2	Low-quality	Genome-fragment		0.31	0
S17_vs_k141_134599||full	1186	No	NA	0	0	Low-quality	Genome-fragment		0.69	0
S14_extracted_k141_64368	1186	No	NA	3	0	Low-quality	Genome-fragment		3.92	0
S1_vs_k141_85948||full	1186	No	NA	0	0	Low-quality	Genome-fragment		2.8	0
S11_vs_k141_85463||full	1083	No	NA	0	2	Not-determined	Genome-fragment	NA		0
S11_vs_k141_79013||full	2418	No	NA	0	0	Not-determined	Genome-fragment	NA		0
S3_vs_k141_10946||full	1186	No	NA	0	0	Low-quality	Genome-fragment		2.74	0
S3_vs_k141_60165||full	1186	No	NA	2	0	Low-quality	Genome-fragment		2.6	0
S5_extracted_k141_23080	1186	No	NA	1	0	Low-quality	Genome-fragment		1.95	0
S5_vs_k141_74629||full	1186	No	NA	0	0	Low-quality	Genome-fragment		2.78	0
S7_extracted_9509	1186	No	NA	0	1	Low-quality	Genome-fragment		0.23	0
S11_vs_k141_85502||full	1487	No	NA	0	0	Not-determined	Genome-fragment	NA		0
S16_vs_17937||full	1186	No	NA	0	0	Low-quality	Genome-fragment		0.87	0
S11_vs_k141_98521||full	2633	No	NA	0	0	Not-determined	Genome-fragment	NA		0
S12_extracted_k141_88724	1186	No	NA	0	0	Low-quality	Genome-fragment		0.8	0
S11_vs_k141_65830||full	3231	No	NA	0	2	Not-determined	Genome-fragment	NA		0
S2_extracted_k141_2522	1186	No	NA	0	1	Low-quality	Genome-fragment		1.53	0
S11_vs_k141_46373||full	2650	No	NA	0	0	Not-determined	Genome-fragment	NA		0
S8_extracted_k141_81977	1186	No	NA	1	0	Low-quality	Genome-fragment		3.83	0
S8_extracted_k141_51630	1186	No	NA	0	1	Low-quality	Genome-fragment		0.39	0
S11_vs_k141_79090||full	1197	No	NA	0	2	Not-determined	Genome-fragment	NA		0
S11_vs_k141_59235||full	3991	No	NA	0	3	Not-determined	Genome-fragment	NA		0
S11_vs_k141_20449||full	6871	No	NA	0	3	Not-determined	Genome-fragment	NA		0
S8_vs_k141_154981||full	1186	No	NA	1	0	Low-quality	Genome-fragment		1.55	0
S8_vs_k141_172236||lt2gene	1186	No	NA	1	0	Low-quality	Genome-fragment		2.39	0
S11_vs_k141_59245||full	1158	No	NA	0	2	Not-determined	Genome-fragment	NA		0
S11_vs_k141_52907||full	2121	No	NA	0	0	Not-determined	Genome-fragment	NA		0
S11_vs_k141_72554||full	4244	No	NA	0	3	Not-determined	Genome-fragment	NA		0
S11_vs_k141_7188||full	1579	No	NA	0	0	Not-determined	Genome-fragment	NA		0
S17_extracted_k141_111789	1187	No	NA	4	0	Low-quality	Genome-fragment		5.42	0
S11_vs_k141_52920||full	1762	No	NA	0	0	Not-determined	Genome-fragment	NA		0
S17_extracted_k141_166294	1187	No	NA	0	0	Low-quality	Genome-fragment		0.69	0
S17_vs_k141_31399||full	1187	No	NA	0	0	Low-quality	Genome-fragment		1.07	0
S17_vs_k141_151357||lt2gene	1187	No	NA	1	0	Low-quality	Genome-fragment		1.55	0
S11_vs_k141_92124||full	1227	No	NA	0	1	Not-determined	Genome-fragment	NA		0
S11_vs_k141_46452||full	1006	No	NA	0	1	Not-determined	Genome-fragment	NA		0
S14_vs_k141_53726||full	1187	No	NA	0	0	Low-quality	Genome-fragment		3.08	0
S1_vs_k141_67432||full	1187	No	NA	1	0	Low-quality	Genome-fragment		2.05	0
S3_vs_k141_63788||full	1187	No	NA	4	0	Low-quality	Genome-fragment		3.91	0
S5_vs_k141_99637||full	1187	No	NA	0	1	Low-quality	Genome-fragment		2.38	0
S7_vs_38451||full	1187	No	NA	1	0	Low-quality	Genome-fragment		0.28	0
S11_vs_k141_39961||full	1839	No	NA	0	0	Not-determined	Genome-fragment	NA		0
S11_vs_k141_72617||full	4667	No	NA	0	4	Not-determined	Genome-fragment	NA		0
S11_vs_k141_72636||full	1804	No	NA	0	0	Not-determined	Genome-fragment	NA		0
S11_vs_k141_72639||full	1176	No	NA	0	2	Not-determined	Genome-fragment	NA		0
S7_vs_86635||full	1187	No	NA	1	0	Low-quality	Genome-fragment		2.75	0
S11_vs_k141_33476||full	1406	No	NA	0	1	Not-determined	Genome-fragment	NA		0
S7_vs_96974||lt2gene	1187	No	NA	1	0	Low-quality	Genome-fragment		1.94	0
S12_extracted_k141_182681	1187	No	NA	0	1	Low-quality	Genome-fragment		3.03	0
S12_extracted_k141_156797	1187	No	NA	0	0	Low-quality	Genome-fragment		0.84	0
S8_extracted_k141_101923	1187	No	NA	0	0	Low-quality	Genome-fragment		1.81	0
S8_extracted_k141_51254	1187	No	NA	2	0	Low-quality	Genome-fragment		1.58	0
S11_vs_k141_92188||full	2499	No	NA	0	1	Not-determined	Genome-fragment	NA		0
S11_vs_k141_79242||full	1442	No	NA	0	0	Not-determined	Genome-fragment	NA		0
S11_vs_k141_13608||full	1520	No	NA	0	0	Not-determined	Genome-fragment	NA		0
S17_extracted_k141_94680	1188	No	NA	0	1	Low-quality	Genome-fragment		0.91	0
S9_extracted_k141_34801	1188	No	NA	1	0	Low-quality	Genome-fragment		2.51	0
S11_vs_k141_72718||full	2084	No	NA	0	1	Not-determined	Genome-fragment	NA		0
S9_extracted_k141_43750	1188	No	NA	0	0	Low-quality	Genome-fragment		0.68	0
S14_extracted_k141_113768	1188	No	NA	0	0	Low-quality	Genome-fragment		3.16	0
S11_vs_k141_66035||full	1472	No	NA	0	1	Not-determined	Genome-fragment	NA		0
S14_vs_k141_21522||lt2gene	1188	No	NA	1	0	Low-quality	Genome-fragment		2.25	0
S3_extracted_k141_77597	1188	No	NA	2	0	Low-quality	Genome-fragment		3.42	0
S11_vs_k141_66046||full	3435	No	NA	0	1	Not-determined	Genome-fragment	NA		0
S3_vs_k141_9337||lt2gene	1188	No	NA	2	0	Low-quality	Genome-fragment		1.98	0
S11_vs_k141_72738||full	7722	No	NA	0	3	Not-determined	Genome-fragment	NA		0
S5_vs_k141_108212||full	1188	No	NA	0	1	Low-quality	Genome-fragment		0.71	0
S7_extracted_82107	1188	No	NA	0	0	Low-quality	Genome-fragment		2.86	0
S11_vs_k141_79275||full	35525	No	NA	8	3	High-quality	High-quality		91.26	0
S11_vs_k141_13652||full	23079	No	NA	18	1	Medium-quality	Genome-fragment		63.85	0
S7_vs_23210||full	1188	No	NA	0	0	Low-quality	Genome-fragment		2.85	0
S16_extracted_80512	1188	No	NA	0	0	Low-quality	Genome-fragment		2.6	0
S16_extracted_94149	1188	No	NA	0	0	Low-quality	Genome-fragment		1.21	0
S11_vs_k141_27052||full	1404	No	NA	0	1	Not-determined	Genome-fragment	NA		0
S11_vs_k141_1270||full	3222	No	NA	0	2	Not-determined	Genome-fragment	NA		0
S16_vs_58525||full	1188	No	NA	0	0	Low-quality	Genome-fragment		2.87	0
S12_extracted_k141_140314	1188	No	NA	0	1	Low-quality	Genome-fragment		1.06	0
S11_vs_k141_20687||full	3996	No	NA	0	2	Not-determined	Genome-fragment	NA		0
S12_vs_k141_69120||full	1188	No	NA	0	0	Low-quality	Genome-fragment		3.79	0
S4_extracted_5677	1188	No	NA	2	0	Low-quality	Genome-fragment		3.92	0
S6_extracted_k141_30838	1188	No	NA	0	1	Low-quality	Genome-fragment		0.73	0
S8_extracted_k141_106538	1188	No	NA	0	1	Low-quality	Genome-fragment		1.86	0
S11_vs_k141_40119||full	5231	No	NA	0	3	Not-determined	Genome-fragment	NA		0
S8_extracted_k141_71536	1188	No	NA	0	0	Low-quality	Genome-fragment		2.82	0
S8_extracted_k141_4533	1188	No	NA	2	0	Low-quality	Genome-fragment		2.11	0
S11_vs_k141_13693||full	6355	No	NA	0	1	Not-determined	Genome-fragment	NA		0
S11_vs_k141_98793||full	1600	No	NA	0	0	Not-determined	Genome-fragment	NA		0
S17_extracted_k141_127838	1189	No	NA	0	1	Low-quality	Genome-fragment		0.83	0
S11_vs_k141_91879||lt2gene	1189	No	NA	1	0	Low-quality	Genome-fragment		18.32	0
S14_extracted_k141_67432	1189	No	NA	0	1	Low-quality	Genome-fragment		1.25	0
S11_vs_k141_7511||full	1510	No	NA	0	0	Not-determined	Genome-fragment	NA		0
S16_vs_48620||full	1189	No	NA	0	1	Low-quality	Genome-fragment		2.38	0
S16_vs_74430||full	1189	No	NA	1	0	Low-quality	Genome-fragment		0.8	0
S12_extracted_k141_144315	1189	No	NA	0	0	Low-quality	Genome-fragment		0.87	0
S6_vs_k141_4871||full	1189	No	NA	0	0	Low-quality	Genome-fragment		0.71	0
S9_vs_k141_31082||full	1190	No	NA	0	0	Low-quality	Genome-fragment		2.92	0
S11_vs_k141_13767||full	1914	No	NA	0	1	Not-determined	Genome-fragment	NA		0
S13_extracted_66471	1190	No	NA	0	0	Low-quality	Genome-fragment		2.49	0
S14_extracted_k141_30633	1190	No	NA	0	0	Low-quality	Genome-fragment		1.8	0
S1_extracted_k141_131566	1190	No	NA	0	0	Low-quality	Genome-fragment		2.56	0
S1_extracted_k141_139543	1190	No	NA	0	0	Low-quality	Genome-fragment		2.86	0
S3_extracted_k141_20024	1190	No	NA	2	0	Low-quality	Genome-fragment		3.19	0
S3_vs_k141_94117||full	1190	No	NA	0	0	Low-quality	Genome-fragment		2.6	0
S11_vs_k141_1393||full	1236	No	NA	0	0	Not-determined	Genome-fragment	NA		0
S5_extracted_k141_3517	1190	No	NA	2	0	Low-quality	Genome-fragment		3.16	0
S7_extracted_99074	1190	No	NA	0	0	Low-quality	Genome-fragment		0.53	0
S11_vs_k141_59667||full	2662	No	NA	0	0	Not-determined	Genome-fragment	NA		0
S16_extracted_32260	1190	No	NA	0	0	Low-quality	Genome-fragment		1.64	0
S11_vs_k141_59693||full	2632	No	NA	0	0	Not-determined	Genome-fragment	NA		0
S16_vs_133152||full	1190	No	NA	1	0	Low-quality	Genome-fragment		1.48	0
S10_vs_34362||full	1190	No	NA	0	0	Low-quality	Genome-fragment		1.93	0
S10_vs_66847||full	1190	No	NA	0	0	Low-quality	Genome-fragment		1.51	0
S10_vs_77325||full	1190	No	NA	0	0	Low-quality	Genome-fragment		2.2	0
S8_extracted_k141_78162	1190	No	NA	0	2	Low-quality	Genome-fragment		0.59	0
S8_vs_k141_196615||full	1190	No	NA	0	2	Low-quality	Genome-fragment		0.35	0
S9_extracted_k141_83091	1191	No	NA	0	0	Low-quality	Genome-fragment		1.71	0
S9_vs_k141_2321||lt2gene	1191	No	NA	1	0	Low-quality	Genome-fragment		3.04	0
S11_vs_k141_1493||full	2438	No	NA	0	1	Not-determined	Genome-fragment	NA		0
S11_vs_k141_66225||full	2448	No	NA	0	0	Not-determined	Genome-fragment	NA		0
S11_vs_k141_66230||full	1175	No	NA	0	0	Not-determined	Genome-fragment	NA		0
S11_vs_k141_1511||full	2739	No	NA	0	0	Not-determined	Genome-fragment	NA		0
S13_vs_97140||full	1191	Yes	378	1	1	Low-quality	Genome-fragment		0.91	68.26
S14_extracted_k141_81119	1191	No	NA	5	0	Low-quality	Genome-fragment		3.92	0

S1_extracted_k141_965	1191	No	NA		0	0	Low-quality	Genome-fragment		2.14	0
S1_vs_k141_99502||full	1191	No	NA		0	0	Low-quality	Genome-fragment		2.23	0
S3_vs_k141_104097||full	1191	No	NA		0	0	Low-quality	Genome-fragment		3.12	0
S11_vs_k141_86123||full	3390	No	NA		0	1	Not-determined	Genome-fragment	NA		0
S11_vs_k141_46770||full	2725	No	NA		0	1	Not-determined	Genome-fragment	NA		0
S5_extracted_k141_122928	1191	No	NA		0	1	Low-quality	Genome-fragment		0.4	0
S11_vs_k141_13996||full	1462	No	NA		0	1	Not-determined	Genome-fragment	NA		0
S11_vs_k141_59813||full	2836	No	NA		0	1	Not-determined	Genome-fragment	NA		0
S11_vs_k141_73172||full	6391	No	NA		0	0	Not-determined	Genome-fragment	NA		0
S11_vs_k141_66326||full	1626	No	NA		0	3	Not-determined	Genome-fragment	NA		0
S5_extracted_k141_78386	1191	No	NA		0	0	Low-quality	Genome-fragment		2.78	0
S16_extracted_123553	1191	No	NA		0	0	Low-quality	Genome-fragment		1.57	0
S16_vs_89394||full	1191	No	NA		0	0	Low-quality	Genome-fragment		3.05	0
S11_vs_k141_86204||full	4969	No	NA		0	2	Not-determined	Genome-fragment	NA		0
S16_vs_154841||full	1191	No	NA		0	0	Low-quality	Genome-fragment		2.4	0
S11_vs_k141_79545||full	5080	No	NA		0	3	Not-determined	Genome-fragment	NA		0
S12_vs_k141_20764||full	1191	No	NA		0	0	Low-quality	Genome-fragment		2.27	0
S11_vs_k141_27397||full	1767	No	NA		0	1	Not-determined	Genome-fragment	NA		0
S8_extracted_k141_154703	1191	No	NA		2	0	Low-quality	Genome-fragment		6.09	0
S8_vs_k141_18408||full	1191	No	NA		2	0	Low-quality	Genome-fragment		2.32	0
S11_vs_k141_27404||full	2695	No	NA		0	0	Not-determined	Genome-fragment	NA		0
S17_extracted_k141_171577	1192	No	NA		1	0	Low-quality	Genome-fragment		3.2	0
S17_extracted_k141_64912	1192	No	NA		0	0	Low-quality	Genome-fragment		2.64	0
S11_vs_k141_51872||full	1192	No	NA		1	0	Low-quality	Genome-fragment		2.8	0
S14_extracted_k141_95824	1192	No	NA		0	0	Low-quality	Genome-fragment		3.68	0
S11_vs_k141_86317||full	6858	No	NA		0	1	Not-determined	Genome-fragment	NA		0
S14_extracted_k141_50131	1192	No	NA		0	0	Low-quality	Genome-fragment		2.78	0
S5_vs_k141_101905||full	1192	No	NA		0	0	Low-quality	Genome-fragment		3.18	0
S11_vs_k141_73247||full	2646	No	NA		0	2	Not-determined	Genome-fragment	NA		0
S5_vs_k141_131114||lt2gene	1192	No	NA		1	0	Low-quality	Genome-fragment		2.61	0
S7_vs_62320||full	1192	No	NA		1	0	Low-quality	Genome-fragment		1.99	0
S11_vs_k141_40397||full	1978	No	NA		0	2	Not-determined	Genome-fragment	NA		0
S11_vs_k141_1731||full	1203	No	NA		0	2	Not-determined	Genome-fragment	NA		0
S10_vs_62678||full	1192	No	NA		0	1	Low-quality	Genome-fragment		1.55	0
S11_vs_k141_99088||full	1254	No	NA		0	0	Not-determined	Genome-fragment	NA		0
S6_vs_k141_54231||full	1192	No	NA		0	0	Low-quality	Genome-fragment		0.78	0
S11_vs_k141_40421||full	2754	No	NA		0	1	Not-determined	Genome-fragment	NA		0
S11_vs_k141_66411||full	1880	No	NA		0	0	Not-determined	Genome-fragment	NA		0
S11_vs_k141_14202||full	1121	No	NA		0	0	Not-determined	Genome-fragment	NA		0
S8_extracted_k141_61476	1192	No	NA		0	0	Low-quality	Genome-fragment		3.09	0
S11_vs_k141_21120||full	2540	No	NA		0	1	Not-determined	Genome-fragment	NA		0
S8_vs_k141_132707||full	1192	No	NA		0	0	Low-quality	Genome-fragment		2.62	0
S11_vs_k141_46940||full	3866	No	NA		0	0	Not-determined	Genome-fragment	NA		0
S11_vs_k141_73316||full	2989	No	NA		0	2	Not-determined	Genome-fragment	NA		0
S11_vs_k141_93090||lt2gene	1193	No	NA		1	0	Low-quality	Genome-fragment		2.12	0
S11_vs_k141_79712||full	1603	No	NA		0	1	Not-determined	Genome-fragment	NA		0
S11_vs_k141_21189||full	1599	No	NA		0	0	Not-determined	Genome-fragment	NA		0
S11_vs_k141_99152||full	1242	No	NA		0	0	Not-determined	Genome-fragment	NA		0
S14_extracted_k141_97583	1193	No	NA		1	0	Low-quality	Genome-fragment		4.11	0
S11_vs_k141_79756||full	1939	No	NA		0	2	Not-determined	Genome-fragment	NA		0
S14_vs_k141_19691||full	1193	No	NA		0	0	Low-quality	Genome-fragment		3.01	0
S3_extracted_k141_25389	1193	No	NA		2	0	Low-quality	Genome-fragment		3.8	0
S11_vs_k141_14357||full	2431	No	NA		0	1	Not-determined	Genome-fragment	NA		0
S11_vs_k141_1899||full	5275	No	NA		0	4	Not-determined	Genome-fragment	NA		0
S3_extracted_k141_103569	1193	No	NA		1	0	Low-quality	Genome-fragment		0.78	0
S3_vs_k141_118817||full	1193	No	NA		1	0	Low-quality	Genome-fragment		2.67	0
S11_vs_k141_53675||full	20682	No	NA		9	2	Medium-quality	Genome-fragment		57.33	0
S11_vs_k141_27717||full	1172	No	NA		0	0	Not-determined	Genome-fragment	NA		0
S11_vs_k141_14383||full	1752	No	NA		0	1	Not-determined	Genome-fragment	NA		0
S11_vs_k141_21281||full	1985	No	NA		0	1	Not-determined	Genome-fragment	NA		0
S11_vs_k141_53702||full	1891	No	NA		0	1	Not-determined	Genome-fragment	NA		0
S5_extracted_k141_4128	1193	No	NA		0	0	Low-quality	Genome-fragment		2.39	0
S5_extracted_k141_64239	1193	No	NA		2	0	Low-quality	Genome-fragment		0.62	0
S5_vs_k141_93698||full	1193	No	NA		0	0	Low-quality	Genome-fragment		2.24	0
S7_vs_109186||full	1193	No	NA		0	0	Low-quality	Genome-fragment		2.85	0
S7_vs_137050||lt2gene	1193	No	NA		1	0	Low-quality	Genome-fragment		2.13	0
S16_extracted_91382	1193	No	NA		0	0	Low-quality	Genome-fragment		0.87	0
S11_vs_k141_60051||full	2152	No	NA		0	1	Not-determined	Genome-fragment	NA		0
S10_extracted_50806	1193	No	NA		1	0	Low-quality	Genome-fragment		2.58	0
S12_extracted_k141_159636	1193	No	NA		0	0	Low-quality	Genome-fragment		2.73	0
S15_vs_7036||lt2gene	1193	No	NA		1	0	Low-quality	Genome-fragment		1.9	0
S11_vs_k141_47131||full	1286	No	NA		0	0	Not-determined	Genome-fragment	NA		0
S11_vs_k141_79831||full	40938	No	NA		6	3	Medium-quality	Genome-fragment		72.66	0
S8_extracted_k141_46933	1193	No	NA		1	0	Low-quality	Genome-fragment		1.21	0
S11_vs_k141_1937||full	35578	Yes		32094	5	3	Medium-quality	Genome-fragment		55.12	9.79
S8_vs_k141_147582||full	1193	No	NA		0	1	Low-quality	Genome-fragment		1.06	0
S17_vs_k141_153854||lt2gene	1194	No	NA		0	0	Low-quality	Genome-fragment		2.78	0
S17_vs_k141_176021||lt2gene	1194	No	NA		1	0	Low-quality	Genome-fragment		3.75	0
S11_vs_k141_21394||full	8478	No	NA		0	5	Not-determined	Genome-fragment	NA		0
S11_vs_k141_64419||full	1194	No	NA		1	0	Low-quality	Genome-fragment		2.97	0
S14_extracted_k141_83317	1194	No	NA		1	0	Low-quality	Genome-fragment		2.18	0
S11_vs_k141_53818||full	2374	No	NA		0	0	Not-determined	Genome-fragment	NA		0
S11_vs_k141_66652||full	3285	No	NA		0	1	Not-determined	Genome-fragment	NA		0
S11_vs_k141_60106||full	1680	No	NA		0	0	Not-determined	Genome-fragment	NA		0
S3_extracted_k141_110651	1194	No	NA		1	0	Low-quality	Genome-fragment		0.85	0
S11_vs_k141_1976||full	3017	No	NA		0	2	Not-determined	Genome-fragment	NA		0
S5_vs_k141_44105||lt2gene	1194	No	NA		2	0	Low-quality	Genome-fragment		1.74	0
S11_vs_k141_73489||full	2445	No	NA		0	3	Not-determined	Genome-fragment	NA		0
S11_vs_k141_66673||full	5522	No	NA		0	3	Not-determined	Genome-fragment	NA		0
S11_vs_k141_86484||full	3508	No	NA		0	2	Not-determined	Genome-fragment	NA		0
S16_extracted_52725	1194	No	NA		0	0	Low-quality	Genome-fragment		2.91	0
S11_vs_k141_99330||full	2707	No	NA		0	1	Not-determined	Genome-fragment	NA		0
S11_vs_k141_53878||full	1038	No	NA		0	0	Not-determined	Genome-fragment	NA		0
S11_vs_k141_92722||full	4325	No	NA		0	2	Not-determined	Genome-fragment	NA		0
S11_vs_k141_47188||full	2317	No	NA		0	1	Not-determined	Genome-fragment	NA		0
S16_extracted_57774	1194	No	NA		0	0	Low-quality	Genome-fragment		0.59	0
S11_vs_k141_53886||full	4505	No	NA		0	0	Not-determined	Genome-fragment	NA		0
S11_vs_k141_21461||full	4894	No	NA		0	0	Not-determined	Genome-fragment	NA		0
S10_extracted_102235	1194	No	NA		0	0	Low-quality	Genome-fragment		2.02	0
S11_vs_k141_2034||full	1493	No	NA		0	1	Not-determined	Genome-fragment	NA		0
S12_extracted_k141_114951	1194	No	NA		0	0	Low-quality	Genome-fragment		1.07	0
S11_vs_k141_33950||full	2392	No	NA		0	2	Not-determined	Genome-fragment	NA		0
S11_vs_k141_79921||full	1300	No	NA		0	0	Not-determined	Genome-fragment	NA		0
S11_vs_k141_92769||full	1220	No	NA		0	0	Not-determined	Genome-fragment	NA		0
S12_vs_k141_64858||lt2gene	1194	No	NA		1	0	Low-quality	Genome-fragment		2.29	0
S11_vs_k141_73576||full	1293	No	NA		0	1	Not-determined	Genome-fragment	NA		0
S15_extracted_37891	1194	No	NA		1	0	Low-quality	Genome-fragment		1.49	0
S11_vs_k141_86583||full	1848	No	NA		0	0	Not-determined	Genome-fragment	NA		0
S8_extracted_k141_177800	1194	No	NA		0	2	Low-quality	Genome-fragment		0.69	0
S17_vs_k141_156381||full	1195	No	NA		0	0	Low-quality	Genome-fragment		1.7	0
S11_vs_k141_21552||full	4212	No	NA		0	2	Not-determined	Genome-fragment	NA		0
S11_vs_k141_86617||full	4112	No	NA		0	2	Not-determined	Genome-fragment	NA		0
S17_vs_k141_127297||full	1195	No	NA		1	0	Low-quality	Genome-fragment		1.38	0
S11_vs_k141_86626||full	1996	No	NA		0	2	Not-determined	Genome-fragment	NA		0
S11_vs_k141_54007||full	1790	No	NA		0	0	Not-determined	Genome-fragment	NA		0
S9_extracted_k141_88072	1195	No	NA		0	0	Low-quality	Genome-fragment		2.29	0
S11_vs_k141_23719||full	1195	No	NA		2	0	Low-quality	Genome-fragment		2.37	0
S13_extracted_122087	1195	No	NA		0	1	Low-quality	Genome-fragment		0.29	0
S13_vs_16317||full	1195	No	NA		0	0	Low-quality	Genome-fragment		1.99	0
S13_vs_16798||full	1195	No	NA		0	1	Low-quality	Genome-fragment		3.1	0
S14_vs_k141_55971||full	1195	No	NA		1	0	Low-quality	Genome-fragment		0.89	0
S3_vs_k141_59012||full	1195	No	NA		2	0	Low-quality	Genome-fragment		3.4	0
S11_vs_k141_73621||full	1524	No	NA		0	1	Not-determined	Genome-fragment	NA		0
S5_vs_k141_97034||full	1195	No	NA		0	0	Low-quality	Genome-fragment		0.77	0
S5_vs_k141_45148||lt2gene	1195	No	NA		1	0	Low-quality	Genome-fragment		2.6	0
S11_vs_k141_86682||full	1098	No	NA		0	0	Not-determined	Genome-fragment	NA		0
S11_vs_k141_80057||full	6788	No	NA		0	2	Not-determined	Genome-fragment	NA		0
S11_vs_k141_99505||full	3263	No	NA		0	4	Not-determined	Genome-fragment	NA		0
S11_vs_k141_40762||full	2864	No	NA		0	3	Not-determined	Genome-fragment	NA		0
S7_extracted_76225	1195	No	NA		0	0	Low-quality	Genome-fragment		1.8	0
S11_vs_k141_92845||full	1288	No	NA		0	0	Not-determined	Genome-fragment	NA		0
S7_vs_46246||full	1195	No	NA		0	1	Low-quality	Genome-fragment		0.4	0
S10_vs_37263||lt2gene	1195	No	NA		1	0	Low-quality	Genome-fragment		1.75	0
S11_vs_k141_8456||full	36244	No	NA		19	0	High-quality	High-quality		98.52	0
S11_vs_k141_14610||full	2102	No	NA		0	3	Not-determined	Genome-fragment	NA		0
S12_extracted_k141_67938	1195	No	NA		0	1	Low-quality	Genome-fragment		2.7	0
S12_vs_k141_39535||full	1195	No	NA		0	2	Low-quality	Genome-fragment		1.1	0
S12_vs_k141_51935||full	1195	No	NA		2	0	Low-quality	Genome-fragment		3.37	0
S11_vs_k141_2226||full	1458	No	NA		0	0	Not-determined	Genome-fragment	NA		0
S11_vs_k141_14655||full	1957	No	NA		0	0	Not-determined	Genome-fragment	NA		0
S11_vs_k141_8498||full	2540	No	NA		0	0	Not-determined	Genome-fragment	NA		0
S11_vs_k141_92917||full	2788	No	NA		0	1	Not-determined	Genome-fragment	NA		0
S11_vs_k141_40869||full	11467	No	NA		0	3	Not-determined	Genome-fragment	NA		0
S11_vs_k141_34100||full	3751	No	NA		0	0	Not-determined	Genome-fragment	NA		0
S11_vs_k141_86785||full	1612	No	NA		0	1	Not-determined	Genome-fragment	NA		0
S8_vs_k141_184279||full	1195	No	NA		0	2	Low-quality	Genome-fragment		0.35	0
S11_vs_k141_86792||full	1085	No	NA		0	1	Not-determined	Genome-fragment	NA		0
S17_vs_k141_159026||full	1196	No	NA		0	1	Low-quality	Genome-fragment		2.21	0
S11_extracted_k141_8986	1196	No	NA		2	0	Low-quality	Genome-fragment		1.93	0
S11_vs_k141_14723||full	1439	No	NA		0	0	Not-determined	Genome-fragment	NA		0
S11_vs_k141_40939||full	4008	No	NA		0	3	Not-determined	Genome-fragment	NA		0
S13_vs_16730||full	1196	No	NA		0	0	Low-quality	Genome-fragment		2.33	0
S11_vs_k141_86849||full	1507	No	NA		0	0	Not-determined	Genome-fragment	NA		0
S3_extracted_k141_79465	1196	No	NA		4	0	Low-quality	Genome-fragment		3.27	0
S3_vs_k141_59728||full	1196	No	NA		0	0	Low-quality	Genome-fragment		1.28	0
S5_extracted_k141_67576	1196	No	NA		1	0	Low-quality	Genome-fragment		2.3	0
S5_vs_k141_109565||full	1196	No	NA		0	0	Low-quality	Genome-fragment		2.51	0
S11_vs_k141_34137||full	35941	No	NA		18	0	High-quality	High-quality		100	0
S16_extracted_123987	1196	No	NA		0	1	Low-quality	Genome-fragment		0.75	0
S16_vs_20735||full	1196	No	NA		2	0	Low-quality	Genome-fragment		1.9	0
S11_vs_k141_86885||full	1638	No	NA		0	0	Not-determined	Genome-fragment	NA		0
S16_vs_81322||full	1196	No	NA		0	0	Low-quality	Genome-fragment		1.46	0
S16_vs_153413||full	1196	No	NA		0	0	Low-quality	Genome-fragment		2.79	0
S12_extracted_k141_48502	1196	No	NA		0	0	Low-quality	Genome-fragment		1.87	0
S11_vs_k141_60347||full	12444	No	NA		0	6	Not-determined	Genome-fragment	NA		0
S15_vs_31640||full	1196	No	NA		0	0	Low-quality	Genome-fragment		1.96	0
S11_vs_k141_21765||full	6186	No	NA		0	1	Not-determined	Genome-fragment	NA		0
S11_vs_k141_86938||full	1002	No	NA		0	0	Not-determined	Genome-fragment	NA		0
S11_vs_k141_73858||full	2205	No	NA		0	2	Not-determined	Genome-fragment	NA		0
S11_vs_k141_21782||full	1073	No	NA		0	0	Not-determined	Genome-fragment	NA		0
S6_vs_k141_17099||full	1196	No	NA		2	0	Low-quality	Genome-fragment		2.74	0
S8_extracted_k141_181481	1196	No	NA		1	0	Low-quality	Genome-fragment		1.08	0
S8_extracted_k141_34286	1196	No	NA		0	0	Low-quality	Genome-fragment		2.82	0
S11_vs_k141_28075||full	4381	No	NA		0	2	Not-determined	Genome-fragment	NA		0

S8_vs_k141_89209||full	1196	No	NA	0	0	Low-quality	Genome-fragment		1.47	0
S9_vs_k141_37532||full	1197	No	NA	0	0	Low-quality	Genome-fragment		0.76	0
S11_vs_k141_73902||full	1286	No	NA	0	0	Not-determined	Genome-fragment	NA		0
S13_vs_27135||full	1197	No	NA	1	0	Low-quality	Genome-fragment		2.4	0
S14_extracted_k141_752	1197	No	NA	5	0	Low-quality	Genome-fragment		3.94	0
S11_vs_k141_2427||full	2363	No	NA	0	1	Not-determined	Genome-fragment	NA		0
S14_extracted_k141_22560	1197	No	NA	2	0	Low-quality	Genome-fragment		3.95	0
S14_extracted_k141_9403	1197	No	NA	1	0	Low-quality	Genome-fragment		3.83	0
S5_extracted_k141_22260	1197	No	NA	0	0	Low-quality	Genome-fragment		1.56	0
S11_vs_k141_21871||full	2329	No	NA	0	1	Not-determined	Genome-fragment	NA		0
S7_extracted_8646	1197	No	NA	1	0	Low-quality	Genome-fragment		3.65	0
S7_extracted_25302	1197	No	NA	0	0	Low-quality	Genome-fragment		1.87	0
S11_vs_k141_93139||full	1470	No	NA	0	0	Not-determined	Genome-fragment	NA		0
S7_vs_108610||full	1197	No	NA	0	1	Low-quality	Genome-fragment		0.69	0
S7_vs_119473||full	1197	No	NA	1	0	Low-quality	Genome-fragment		3.07	0
S16_vs_159890||full	1197	No	NA	0	0	Low-quality	Genome-fragment		0.59	0
S11_vs_k141_14882||full	38803	No	NA	9	0	Medium-quality	Genome-fragment		64.08	0
S12_extracted_k141_42999	1197	No	NA	0	1	Low-quality	Genome-fragment		0.49	0
S11_vs_k141_34301||full	1855	No	NA	0	2	Not-determined	Genome-fragment	NA		0
S11_vs_k141_41107||full	37369	No	NA	31	0	Medium-quality	Genome-fragment		81.54	0
S11_vs_k141_8752||full	3137	No	NA	0	2	Not-determined	Genome-fragment	NA		0
S11_vs_k141_41131||full	3159	No	NA	0	0	Not-determined	Genome-fragment	NA		0
S11_vs_k141_80399||full	1938	No	NA	0	0	Not-determined	Genome-fragment	NA		0
S11_vs_k141_93204||full	2320	No	NA	0	2	Not-determined	Genome-fragment	NA		0
S6_vs_k141_45493||full	1197	No	NA	0	1	Low-quality	Genome-fragment		1.29	0
S8_vs_k141_98928||full	1197	No	NA	0	1	Low-quality	Genome-fragment		1.24	0
S8_vs_k141_203715||lt2gene	1197	No	NA	1	0	Low-quality	Genome-fragment		1.75	0
S11_vs_k141_47490||full	1179	No	NA	0	2	Not-determined	Genome-fragment	NA		0
S17_vs_k141_93394||lt2gene	1198	No	NA	1	0	Low-quality	Genome-fragment		3.23	0
S11_vs_k141_80417||full	3607	No	NA	0	1	Not-determined	Genome-fragment	NA		0
S9_extracted_k141_36525	1198	No	NA	0	1	Low-quality	Genome-fragment		0.95	0
S11_vs_k141_97757||full	1198	No	NA	0	0	Low-quality	Genome-fragment		0.73	0
S11_vs_k141_93269||full	1542	No	NA	0	0	Not-determined	Genome-fragment	NA		0
S5_vs_k141_86223||full	1198	No	NA	3	0	Low-quality	Genome-fragment		2.94	0
S11_vs_k141_15052||full	1590	No	NA	0	2	Not-determined	Genome-fragment	NA		0
S11_vs_k141_87244||full	1549	No	NA	0	3	Not-determined	Genome-fragment	NA		0
S11_vs_k141_47561||full	3902	No	NA	0	0	Not-determined	Genome-fragment	NA		0
S7_extracted_15649	1198	No	NA	1	0	Low-quality	Genome-fragment		2.27	0
S11_vs_k141_8884||full	4177	No	NA	0	4	Not-determined	Genome-fragment	NA		0
S16_extracted_131919	1198	No	NA	0	0	Low-quality	Genome-fragment		0.93	0
S16_extracted_149231	1198	No	NA	1	0	Low-quality	Genome-fragment		2.03	0
S11_vs_k141_60540||full	1163	No	NA	0	0	Not-determined	Genome-fragment	NA		0
S16_vs_48369||full	1198	No	NA	3	0	Low-quality	Genome-fragment		2.28	0
S16_vs_68179||full	1198	No	NA	1	0	Low-quality	Genome-fragment		1.99	0
S16_vs_99345||lt2gene	1198	No	NA	1	0	Low-quality	Genome-fragment		0.64	0
S11_vs_k141_67052||full	1187	No	NA	0	1	Not-determined	Genome-fragment	NA		0
S10_extracted_90086	1198	No	NA	0	0	Low-quality	Genome-fragment		0.9	0
S12_extracted_k141_106885	1198	No	NA	0	0	Low-quality	Genome-fragment		1.87	0
S12_vs_k141_113484||full	1198	No	NA	1	0	Low-quality	Genome-fragment		2.09	0
S15_vs_4774||full	1198	No	NA	1	0	Low-quality	Genome-fragment		2.05	0
S11_vs_k141_22001||full	3752	No	NA	0	2	Not-determined	Genome-fragment	NA		0
S8_vs_k141_72213||full	1198	No	NA	0	1	Low-quality	Genome-fragment		1.9	0
S8_vs_k141_16012||full	1198	No	NA	1	0	Low-quality	Genome-fragment		2.19	0
S11_extracted_k141_95392	1199	No	NA	1	0	Low-quality	Genome-fragment		27.31	0
S13_vs_36497||full	1199	No	NA	0	1	Low-quality	Genome-fragment		2.96	0
S1_extracted_k141_30281	1199	No	NA	1	0	Low-quality	Genome-fragment		2.87	0
S1_extracted_k141_111936	1199	No	NA	0	0	Low-quality	Genome-fragment		1.33	0
S1_vs_k141_81723||full	1199	No	NA	0	0	Low-quality	Genome-fragment		0.93	0
S3_vs_k141_46577||lt2gene	1199	No	NA	1	0	Low-quality	Genome-fragment		3.55	0
S11_vs_k141_54639||full	1771	No	NA	0	3	Not-determined	Genome-fragment	NA		0
S5_vs_k141_101688||full	1199	No	NA	2	0	Low-quality	Genome-fragment		3.2	0
S7_vs_22199||full	1199	No	NA	2	0	Low-quality	Genome-fragment		3.02	0
S16_extracted_86975	1199	No	NA	1	0	Low-quality	Genome-fragment		1.52	0
S11_vs_k141_15209||full	2716	No	NA	0	0	Not-determined	Genome-fragment	NA		0
S6_vs_k141_33835||full	1199	No	NA	0	0	Low-quality	Genome-fragment		2.29	0
S8_vs_k141_167435||full	1199	No	NA	0	1	Low-quality	Genome-fragment		1.48	0
S8_vs_k141_181366||full	1199	No	NA	0	0	Low-quality	Genome-fragment		2.83	0
S17_extracted_k141_34567	1200	No	NA	0	0	Low-quality	Genome-fragment		21.71	0
S9_extracted_k141_40631	1200	No	NA	0	1	Low-quality	Genome-fragment		2.22	0
S11_vs_k141_47673||full	1708	No	NA	0	2	Not-determined	Genome-fragment	NA		0
S11_vs_k141_41441||full	3712	No	NA	0	1	Not-determined	Genome-fragment	NA		0
S11_vs_k141_80539||full	1102	No	NA	0	0	Not-determined	Genome-fragment	NA		0
S11_vs_k141_28479||full	1767	No	NA	0	0	Not-determined	Genome-fragment	NA		0
S9_extracted_k141_79492	1200	No	NA	1	0	Low-quality	Genome-fragment		2.69	0
S11_vs_k141_27118||lt2gene	1200	No	NA	0	0	Low-quality	Genome-fragment		2.66	0
S3_vs_k141_83057||full	1200	No	NA	0	0	Low-quality	Genome-fragment		2.39	0
S11_vs_k141_34539||full	1116	No	NA	0	0	Not-determined	Genome-fragment	NA		0
S11_vs_k141_34548||full	1053	No	NA	0	2	Not-determined	Genome-fragment	NA		0
S3_vs_k141_86703||full	1200	No	NA	0	0	Low-quality	Genome-fragment		1.37	0
S11_vs_k141_22135||full	1131	No	NA	0	0	Not-determined	Genome-fragment	NA		0
S5_vs_k141_71295||full	1200	No	NA	1	0	Low-quality	Genome-fragment		2.79	0
S11_vs_k141_47723||full	1432	No	NA	0	0	Not-determined	Genome-fragment	NA		0
S7_extracted_32817	1200	No	NA	0	0	Low-quality	Genome-fragment		2.04	0
S7_vs_82705||full	1200	No	NA	1	0	Low-quality	Genome-fragment		1.8	0
S7_vs_117526||lt2gene	1200	No	NA	1	0	Low-quality	Genome-fragment		1.95	0
S2_extracted_k141_14342	1200	No	NA	0	0	Low-quality	Genome-fragment		1.05	0
S11_vs_k141_67157||full	6496	No	NA	0	2	Not-determined	Genome-fragment	NA		0
S6_extracted_k141_27333	1200	No	NA	2	0	Low-quality	Genome-fragment		1.7	0
S11_vs_k141_15311||full	3279	No	NA	0	0	Not-determined	Genome-fragment	NA		0
S11_vs_k141_87524||full	1990	No	NA	0	0	Not-determined	Genome-fragment	NA		0
S11_vs_k141_34610||full	3769	No	NA	0	0	Not-determined	Genome-fragment	NA		0
S8_extracted_k141_202276	1200	No	NA	0	2	Low-quality	Genome-fragment		0.2	0
S8_extracted_k141_39293	1200	No	NA	0	1	Low-quality	Genome-fragment		0.56	0
S8_vs_k141_34708||full	1200	No	NA	1	0	Low-quality	Genome-fragment		2.79	0
S11_vs_k141_15330||full	1208	No	NA	0	1	Not-determined	Genome-fragment	NA		0
S11_vs_k141_2839||full	3916	No	NA	0	1	Not-determined	Genome-fragment	NA		0
S9_extracted_k141_58746	1201	No	NA	1	0	Low-quality	Genome-fragment		1.8	0
S11_vs_k141_87570||full	2305	No	NA	0	1	Not-determined	Genome-fragment	NA		0
S11_vs_k141_60756||full	2003	No	NA	0	0	Not-determined	Genome-fragment	NA		0
S11_vs_k141_34659||full	2829	No	NA	0	0	Not-determined	Genome-fragment	NA		0
S13_extracted_65857	1201	No	NA	0	1	Low-quality	Genome-fragment		1.23	0
S11_vs_k141_74430||full	2198	No	NA	0	0	Not-determined	Genome-fragment	NA		0
S13_vs_76971||full	1201	No	NA	0	0	Low-quality	Genome-fragment		2.35	0
S1_extracted_k141_37481	1201	No	NA	0	0	Low-quality	Genome-fragment		2.97	0
S1_vs_k141_142003||full	1201	No	NA	0	0	Low-quality	Genome-fragment		2.46	0
S1_vs_k141_72705||full	1201	No	NA	2	0	Low-quality	Genome-fragment		1.97	0
S3_vs_k141_59969||full	1201	No	NA	0	0	Low-quality	Genome-fragment		1.52	0
S11_vs_k141_74510||full	1419	No	NA	0	1	Not-determined	Genome-fragment	NA		0
S7_extracted_34197	1201	No	NA	1	0	Low-quality	Genome-fragment		0.93	0
S11_vs_k141_93671||full	4595	No	NA	0	4	Not-determined	Genome-fragment	NA		0
S7_extracted_81790	1201	No	NA	0	0	Low-quality	Genome-fragment		2.62	0
S16_vs_52685||full	1201	No	NA	0	2	Low-quality	Genome-fragment		0.53	0
S11_vs_k141_9270||full	1662	No	NA	0	2	Not-determined	Genome-fragment	NA		0
S12_extracted_k141_150820	1201	No	NA	0	0	Low-quality	Genome-fragment		2.3	0
S11_vs_k141_47874||full	3713	No	NA	0	0	Not-determined	Genome-fragment	NA		0
S12_extracted_k141_104477	1201	No	NA	0	0	Low-quality	Genome-fragment		1.56	0
S11_vs_k141_60867||full	3384	No	NA	0	0	Not-determined	Genome-fragment	NA		0
S8_extracted_k141_150419	1201	No	NA	0	1	Low-quality	Genome-fragment		0.32	0
S11_vs_k141_54666||full	1050	No	NA	0	0	Not-determined	Genome-fragment	NA		0
S17_vs_k141_110316||full	1202	No	NA	0	0	Low-quality	Genome-fragment		2.41	0
S13_extracted_122232	1202	No	NA	0	0	Low-quality	Genome-fragment		0.67	0
S13_extracted_124805	1202	No	NA	0	1	Low-quality	Genome-fragment		1.94	0
S13_vs_86639||full	1202	No	NA	0	0	Low-quality	Genome-fragment		0.89	0
S1_vs_k141_117465||full	1202	No	NA	0	0	Low-quality	Genome-fragment		1.94	0
S5_extracted_k141_38525	1202	No	NA	1	0	Low-quality	Genome-fragment		3.43	0
S7_vs_49512||full	1202	No	NA	0	0	Low-quality	Genome-fragment		1.36	0
S11_vs_k141_34729||full	2123	No	NA	0	1	Not-determined	Genome-fragment	NA		0
S11_vs_k141_74619||full	2587	No	NA	0	4	Not-determined	Genome-fragment	NA		0
S11_vs_k141_54710||full	2150	No	NA	0	0	Not-determined	Genome-fragment	NA		0
S16_extracted_150035	1202	No	NA	4	0	Low-quality	Genome-fragment		3.95	0
S16_vs_67684||full	1202	No	NA	0	2	Low-quality	Genome-fragment		2.55	0
S10_vs_103901||full	1202	No	NA	0	0	Low-quality	Genome-fragment		2.91	0
S12_vs_k141_184717||full	1202	No	NA	0	0	Low-quality	Genome-fragment		2.62	0
S6_extracted_k141_54222	1202	No	NA	0	0	Low-quality	Genome-fragment		3.27	0
S17_vs_k141_109594||full	1203	No	NA	0	0	Low-quality	Genome-fragment		1.46	0
S11_vs_k141_22418||full	1030	No	NA	0	1	Not-determined	Genome-fragment	NA		0
S11_vs_k141_22422||full	5427	No	NA	0	1	Not-determined	Genome-fragment	NA		0
S9_extracted_k141_74800	1203	No	NA	0	0	Low-quality	Genome-fragment		2.92	0
S11_vs_k141_93825||full	1865	No	NA	0	0	Not-determined	Genome-fragment	NA		0
S9_vs_k141_34821||full	1203	No	NA	1	0	Low-quality	Genome-fragment		2.8	0
S11_vs_k141_67390||full	1584	No	NA	0	2	Not-determined	Genome-fragment	NA		0
S5_vs_k141_36823||lt2gene	1203	No	NA	2	0	Low-quality	Genome-fragment		2.99	0
S7_vs_39647||full	1203	No	NA	1	0	Low-quality	Genome-fragment		2.91	0
S7_vs_66305||full	1203	No	NA	1	0	Low-quality	Genome-fragment		2.8	0
S11_vs_k141_15548||full	9412	No	NA	0	5	Not-determined	Genome-fragment	NA		0
S11_vs_k141_80866||full	1272	No	NA	0	1	Not-determined	Genome-fragment	NA		0
S16_vs_680||full	1203	No	NA	1	0	Low-quality	Genome-fragment		2.06	0
S11_vs_k141_54773||full	2932	No	NA	0	1	Not-determined	Genome-fragment	NA		0
S10_extracted_35230	1203	No	NA	0	0	Low-quality	Genome-fragment		2.91	0
S15_extracted_1224	1203	No	NA	0	0	Low-quality	Genome-fragment		2.24	0
S11_vs_k141_41802||full	1759	No	NA	0	1	Not-determined	Genome-fragment	NA		0
S11_vs_k141_34803||full	2090	No	NA	0	1	Not-determined	Genome-fragment	NA		0
S8_vs_k141_150718||lt2gene	1203	No	NA	1	0	Low-quality	Genome-fragment		6.16	0
S11_vs_k141_28617||full	3051	No	NA	0	3	Not-determined	Genome-fragment	NA		0
S9_vs_k141_76079||full	1204	No	NA	1	0	Low-quality	Genome-fragment		2.28	0
S13_extracted_58191	1204	No	NA	0	1	Low-quality	Genome-fragment		0.73	0
S14_vs_k141_6236||full	1204	No	NA	1	0	Low-quality	Genome-fragment		1.72	0
S1_extracted_k141_132168	1204	No	NA	1	0	Low-quality	Genome-fragment		3.31	0
S11_vs_k141_9442||full	2004	No	NA	0	1	Not-determined	Genome-fragment	NA		0
S11_vs_k141_34852||full	2827	No	NA	0	3	Not-determined	Genome-fragment	NA		0
S5_vs_k141_117266||full	1204	No	NA	0	2	Low-quality	Genome-fragment		0.81	0
S11_vs_k141_9471||full	1245	No	NA	0	0	Not-determined	Genome-fragment	NA		0
S7_extracted_16758	1204	No	NA	1	0	Low-quality	Genome-fragment		2.82	0
S11_vs_k141_28678||full	2260	No	NA	0	2	Not-determined	Genome-fragment	NA		0
S7_vs_82854||full	1204	No	NA	0	0	Low-quality	Genome-fragment		2.78	0
S16_extracted_163916	1204	No	NA	0	1	Low-quality	Genome-fragment		2.84	0
S11_vs_k141_61162||full	1922	No	NA	0	0	Not-determined	Genome-fragment	NA		0
S11_vs_k141_93995||full	3366	No	NA	0	0	Not-determined	Genome-fragment	NA		0
S16_vs_167354||full	1204	No	NA	0	0	Low-quality	Genome-fragment		2.83	0
S6_extracted_k141_51171	1204	No	NA	0	0	Low-quality	Genome-fragment		1.09	0
S8_extracted_k141_113654	1204	No	NA	0	1	Low-quality	Genome-fragment		0.91	0
S11_vs_k141_34927||full	6373	No	NA	0	1	Not-determined	Genome-fragment	NA		0
S8_vs_k141_178110||full	1204	No	NA	0	0	Low-quality	Genome-fragment		2.76	0
S11_vs_k141_34931||full	3374	No	NA	0	1	Not-determined	Genome-fragment	NA		0

S11_vs_k141_41934||full	2364	No	NA		0	0	Not-determined	Genome-fragment	NA		0
S11_vs_k141_67573||full	27945	No	NA		3	1	Medium-quality	Genome-fragment		72.1	0
S17_vs_k141_132155||full	1205	No	NA		0	0	Low-quality	Genome-fragment		1.92	0
S9_extracted_k141_75802	1205	No	NA		1	0	Low-quality	Genome-fragment		1.72	0
S14_extracted_k141_47615	1205	No	NA		3	0	Low-quality	Genome-fragment		3.97	0
S14_extracted_k141_35945	1205	No	NA		2	0	Low-quality	Genome-fragment		3.97	0
S7_vs_111176||full	1205	No	NA		1	0	Low-quality	Genome-fragment		3.12	0
S11_vs_k141_54914||full	2054	No	NA		0	0	Not-determined	Genome-fragment	NA		0
S16_vs_161606||full	1205	No	NA		0	0	Low-quality	Genome-fragment		2.44	0
S10_extracted_56940	1205	No	NA		0	0	Low-quality	Genome-fragment		1.09	0
S11_vs_k141_34954||full	3579	No	NA		0	3	Not-determined	Genome-fragment	NA		0
S12_extracted_k141_93397	1205	No	NA		0	0	Low-quality	Genome-fragment		0.97	0
S12_vs_k141_167480||full	1205	No	NA		1	0	Low-quality	Genome-fragment		1.98	0
S11_vs_k141_34964||full	4930	No	NA		0	0	Not-determined	Genome-fragment	NA		0
S11_vs_k141_48109||full	65738	Yes		32360	17	25	Medium-quality	Genome-fragment		65.76	50.77
S12_vs_k141_51985||full	1205	No	NA		1	0	Low-quality	Genome-fragment		2.2	0
S11_vs_k141_54965||full	1661	No	NA		0	1	Not-determined	Genome-fragment	NA		0
S2_extracted_k141_7975	1205	No	NA		2	0	Low-quality	Genome-fragment		2.74	0
S11_vs_k141_3277||full	37231	Yes		29695	7	5	Medium-quality	Genome-fragment		69.33	20.24
S8_vs_k141_72217||full	1205	No	NA		1	0	Low-quality	Genome-fragment		2.33	0
S8_vs_k141_103742||full	1205	No	NA		0	0	Low-quality	Genome-fragment		2.04	0
S11_vs_k141_3292||full	3162	No	NA		0	3	Not-determined	Genome-fragment	NA		0
S17_extracted_k141_76287	1206	No	NA		1	0	Low-quality	Genome-fragment		2.9	0
S17_extracted_k141_55556	1206	No	NA		0	1	Low-quality	Genome-fragment		0.67	0
S11_vs_k141_81101||full	4926	No	NA		0	2	Not-determined	Genome-fragment	NA		0
S9_vs_k141_58556||full	1206	No	NA		0	0	Low-quality	Genome-fragment		1.28	0
S14_extracted_k141_65722	1206	No	NA		0	1	Low-quality	Genome-fragment		0.34	0
S11_vs_k141_55019||full	5611	No	NA		0	0	Not-determined	Genome-fragment	NA		0
S1_vs_k141_140529||full	1206	No	NA		3	0	Low-quality	Genome-fragment		2.8	0
S11_vs_k141_67662||full	2458	No	NA		0	1	Not-determined	Genome-fragment	NA		0
S3_vs_k141_122779||full	1206	No	NA		0	0	Low-quality	Genome-fragment		0.75	0
S11_vs_k141_42031||full	1719	No	NA		0	1	Not-determined	Genome-fragment	NA		0
S5_vs_k141_38520||full	1206	No	NA		0	0	Low-quality	Genome-fragment		3.14	0
S10_extracted_71855	1206	No	NA		0	1	Low-quality	Genome-fragment		0.29	0
S10_extracted_118980	1206	No	NA		0	0	Low-quality	Genome-fragment		2.6	0
S11_vs_k141_48187||full	2683	No	NA		0	2	Not-determined	Genome-fragment	NA		0
S4_extracted_31423	1206	No	NA		1	0	Low-quality	Genome-fragment		2.82	0
S8_extracted_k141_112826	1206	Yes		319	1	1	Low-quality	Genome-fragment		0.34	73.55
S9_vs_k141_45412||full	1207	No	NA		2	0	Low-quality	Genome-fragment		2.52	0
S14_vs_k141_48569||full	1207	No	NA		1	0	Low-quality	Genome-fragment		1.86	0
S14_vs_k141_78858||full	1207	No	NA		0	0	Low-quality	Genome-fragment		3.18	0
S5_extracted_k141_93723	1207	No	NA		1	0	Low-quality	Genome-fragment		3.51	0
S11_vs_k141_9725||full	11703	No	NA		0	6	Not-determined	Genome-fragment	NA		0
S11_vs_k141_100710||full	8113	No	NA		0	4	Not-determined	Genome-fragment	NA		0
S11_vs_k141_22912||full	2086	No	NA		0	1	Not-determined	Genome-fragment	NA		0
S7_extracted_123646	1207	No	NA		1	0	Low-quality	Genome-fragment		2.68	0
S11_vs_k141_42137||full	4849	No	NA		0	3	Not-determined	Genome-fragment	NA		0
S11_vs_k141_15985||full	2236	No	NA		0	1	Not-determined	Genome-fragment	NA		0
S16_extracted_109158	1207	No	NA		0	1	Low-quality	Genome-fragment		1.48	0
S16_vs_3734||full	1207	No	NA		1	0	Low-quality	Genome-fragment		0.81	0
S12_vs_k141_16234||full	1207	No	NA		1	0	Low-quality	Genome-fragment		2.87	0
S6_vs_k141_18467||full	1207	No	NA		0	0	Low-quality	Genome-fragment		3.51	0
S8_extracted_k141_61698	1207	No	NA		1	0	Low-quality	Genome-fragment		1.79	0
S8_vs_k141_5222||full	1207	No	NA		1	0	Low-quality	Genome-fragment		1.92	0
S11_vs_k141_16017||full	1835	No	NA		0	2	Not-determined	Genome-fragment	NA		0
S11_vs_k141_55144||full	39745	No	NA		25	0	High-quality	High-quality		99.69	0
S11_vs_k141_28962||full	2589	No	NA		0	2	Not-determined	Genome-fragment	NA		0
S11_vs_k141_61383||full	1757	No	NA		0	0	Not-determined	Genome-fragment	NA		0
S11_vs_k141_3377||full	1976	No	NA		0	1	Not-determined	Genome-fragment	NA		0
S11_vs_k141_16034||full	2220	No	NA		0	0	Not-determined	Genome-fragment	NA		0
S17_extracted_k141_165749	1208	No	NA		0	0	Low-quality	Genome-fragment		1.16	0
S11_vs_k141_48312||full	2966	No	NA		0	3	Not-determined	Genome-fragment	NA		0
S11_vs_k141_81150||full	7979	No	NA		0	0	Not-determined	Genome-fragment	NA		0
S9_extracted_k141_8890	1208	No	NA		1	0	Low-quality	Genome-fragment		1.17	0
S14_vs_k141_77836||full	1208	No	NA		0	0	Low-quality	Genome-fragment		3.47	0
S11_vs_k141_48339||full	2997	No	NA		0	1	Not-determined	Genome-fragment	NA		0
S1_extracted_k141_47787	1208	No	NA		0	0	Low-quality	Genome-fragment		0.23	0
S3_vs_k141_50966||lt2gene	1208	No	NA		1	0	Low-quality	Genome-fragment		2.56	0
S11_vs_k141_81185||full	1255	No	NA		0	0	Not-determined	Genome-fragment	NA		0
S7_vs_62483||full	1208	No	NA		1	0	Low-quality	Genome-fragment		2.93	0
S16_extracted_35649	1208	No	NA		1	0	Low-quality	Genome-fragment		2.18	0
S11_vs_k141_3444||full	1493	No	NA		0	0	Not-determined	Genome-fragment	NA		0
S11_vs_k141_81192||full	7035	No	NA		0	1	Not-determined	Genome-fragment	NA		0
S11_vs_k141_29066||full	2269	No	NA		0	2	Not-determined	Genome-fragment	NA		0
S16_vs_116486||full	1208	No	NA		0	1	Low-quality	Genome-fragment		2.35	0
S10_extracted_67002	1208	No	NA		0	1	Low-quality	Genome-fragment		2.88	0
S12_extracted_k141_138258	1208	No	NA		1	0	Low-quality	Genome-fragment		3.01	0
S11_vs_k141_29080||full	4324	No	NA		0	4	Not-determined	Genome-fragment	NA		0
S2_vs_k141_13401||full	1208	No	NA		0	0	Low-quality	Genome-fragment		1.76	0
S8_vs_k141_27776||full	1208	No	NA		1	0	Low-quality	Genome-fragment		1.46	0
S17_vs_k141_12513||full	1209	No	NA		0	1	Low-quality	Genome-fragment		3.34	0
S11_vs_k141_3484||full	1658	No	NA		0	3	Not-determined	Genome-fragment	NA		0
S14_vs_k141_111915||full	1209	No	NA		0	0	Low-quality	Genome-fragment		3.33	0
S11_vs_k141_100886||full	2998	No	NA		0	1	Not-determined	Genome-fragment	NA		0
S11_vs_k141_55246||full	2740	No	NA		0	1	Not-determined	Genome-fragment	NA		0
S14_vs_k141_107471||full	1209	No	NA		1	0	Low-quality	Genome-fragment		2.84	0
S1_extracted_k141_69012	1209	No	NA		0	2	Low-quality	Genome-fragment		0.88	0
S11_vs_k141_100894||full	1819	No	NA		0	0	Not-determined	Genome-fragment	NA		0
S11_vs_k141_87998||full	1530	No	NA		0	0	Not-determined	Genome-fragment	NA		0
S11_vs_k141_67858||full	3259	No	NA		0	2	Not-determined	Genome-fragment	NA		0
S11_vs_k141_23153||full	1492	No	NA		0	0	Not-determined	Genome-fragment	NA		0
S1_extracted_k141_42763	1209	No	NA		0	1	Low-quality	Genome-fragment		0.67	0
S5_vs_k141_78070||full	1209	No	NA		3	0	Low-quality	Genome-fragment		2.65	0
S11_vs_k141_61510||full	2350	No	NA		0	1	Not-determined	Genome-fragment	NA		0
S16_vs_30111||full	1209	No	NA		0	0	Low-quality	Genome-fragment		0.7	0
S17_extracted_k141_4554	1210	No	NA		0	0	Low-quality	Genome-fragment		1.96	0
S17_vs_k141_72261||lt2gene	1210	No	NA		1	0	Low-quality	Genome-fragment		1.36	0
S11_vs_k141_30436||full	1210	No	NA		0	0	Low-quality	Genome-fragment		2.6	0
S11_vs_k141_75241||full	1073	No	NA		0	1	Not-determined	Genome-fragment	NA		0
S13_vs_19701||full	1210	No	NA		0	1	Low-quality	Genome-fragment		2.09	0
S1_extracted_k141_103466	1210	No	NA		1	0	Low-quality	Genome-fragment		1.23	0
S11_vs_k141_61540||full	1819	No	NA		0	1	Not-determined	Genome-fragment	NA		0
S3_vs_k141_60629||full	1210	No	NA		1	0	Low-quality	Genome-fragment		2.64	0
S11_vs_k141_55331||full	2157	No	NA		0	2	Not-determined	Genome-fragment	NA		0
S11_vs_k141_61541||full	5499	No	NA		0	2	Not-determined	Genome-fragment	NA		0
S5_vs_k141_55446||full	1210	No	NA		1	0	Low-quality	Genome-fragment		1.95	0
S11_vs_k141_61552||full	1071	No	NA		0	1	Not-determined	Genome-fragment	NA		0
S5_vs_k141_55117||lt2gene	1210	No	NA		1	0	Low-quality	Genome-fragment		2.63	0
S7_vs_21167||full	1210	No	NA		1	0	Low-quality	Genome-fragment		2.66	0
S11_vs_k141_81327||full	3131	No	NA		0	1	Not-determined	Genome-fragment	NA		0
S11_vs_k141_23188||full	35761	No	NA		12	3	Medium-quality	Genome-fragment		60.13	0
S11_vs_k141_94430||full	1372	No	NA		0	0	Not-determined	Genome-fragment	NA		0
S11_vs_k141_29249||full	1306	No	NA		0	0	Not-determined	Genome-fragment	NA		0
S7_vs_27795||full	1210	No	NA		0	0	Low-quality	Genome-fragment		2.77	0
S7_vs_101993||full	1210	No	NA		1	0	Low-quality	Genome-fragment		2.43	0
S11_vs_k141_88095||full	2792	No	NA		0	2	Not-determined	Genome-fragment	NA		0
S11_vs_k141_88097||full	1063	No	NA		0	1	Not-determined	Genome-fragment	NA		0
S16_extracted_104099	1210	No	NA		1	0	Low-quality	Genome-fragment		2.83	0
S16_vs_45027||full	1210	No	NA		0	0	Low-quality	Genome-fragment		2.38	0
S11_vs_k141_35575||full	5637	No	NA		0	1	Not-determined	Genome-fragment	NA		0
S10_vs_29888||full	1210	No	NA		1	0	Low-quality	Genome-fragment		2.41	0
S12_extracted_k141_40616	1210	No	NA		1	0	Low-quality	Genome-fragment		1.9	0
S11_vs_k141_10103||full	1419	No	NA		0	0	Not-determined	Genome-fragment	NA		0
S12_vs_k141_112154||full	1210	No	NA		0	0	Low-quality	Genome-fragment		3.4	0
S12_vs_k141_112170||full	1210	No	NA		0	0	Low-quality	Genome-fragment		3.51	0
S15_extracted_19045	1210	No	NA		0	0	Low-quality	Genome-fragment		0.7	0
S11_vs_k141_61652||full	3331	No	NA		0	3	Not-determined	Genome-fragment	NA		0
S8_extracted_k141_161671	1210	No	NA		0	0	Low-quality	Genome-fragment		2.57	0
S8_vs_k141_116185||full	1210	No	NA		1	0	Low-quality	Genome-fragment		2.84	0
S8_vs_k141_134442||full	1210	No	NA		0	0	Low-quality	Genome-fragment		2.68	0
S11_vs_k141_68056||full	4842	No	NA		0	1	Not-determined	Genome-fragment	NA		0
S8_vs_k141_190236||full	1210	No	NA		0	1	Low-quality	Genome-fragment		0.98	0
S13_vs_12876||full	1211	No	NA		0	2	Low-quality	Genome-fragment		0.45	0
S14_extracted_k141_66794	1211	No	NA		0	0	Low-quality	Genome-fragment		1.98	0
S14_vs_k141_59387||full	1211	No	NA		1	0	Low-quality	Genome-fragment		1.52	0
S14_vs_k141_15197||lt2gene	1211	No	NA		1	0	Low-quality	Genome-fragment		3.27	0
S14_vs_k141_81713||lt2gene	1211	No	NA		1	0	Low-quality	Genome-fragment		4.05	0
S1_extracted_k141_116521	1211	No	NA		0	0	Low-quality	Genome-fragment		1.05	0
S11_vs_k141_35706||full	1694	No	NA		0	0	Not-determined	Genome-fragment	NA		0
S11_vs_k141_68099||full	2656	No	NA		0	0	Not-determined	Genome-fragment	NA		0
S11_vs_k141_75542||full	4094	No	NA		0	1	Not-determined	Genome-fragment	NA		0
S11_vs_k141_42616||full	2352	No	NA		0	1	Not-determined	Genome-fragment	NA		0
S11_vs_k141_23357||full	43390	No	NA		17	1	High-quality	High-quality		96.67	0
S11_vs_k141_10172||full	43295	No	NA		16	1	High-quality	High-quality		96.46	0
S11_vs_k141_10181||full	1394	No	NA		0	1	Not-determined	Genome-fragment	NA		0
S3_extracted_k141_98052	1211	No	NA		1	0	Low-quality	Genome-fragment		3.06	0
S11_vs_k141_23381||full	2152	No	NA		0	0	Not-determined	Genome-fragment	NA		0
S3_vs_k141_1468||full	1211	No	NA		0	0	Low-quality	Genome-fragment		3.31	0
S16_vs_129304||lt2gene	1211	No	NA		1	0	Low-quality	Genome-fragment		1.8	0
S11_vs_k141_29335||full	2880	No	NA		0	0	Not-determined	Genome-fragment	NA		0
S2_vs_k141_15364||full	1211	No	NA		1	0	Low-quality	Genome-fragment		2.59	0
S8_extracted_k141_69284	1211	No	NA		3	0	Low-quality	Genome-fragment		2.08	0
S11_vs_k141_75576||full	5089	No	NA		0	4	Not-determined	Genome-fragment	NA		0
S11_vs_k141_3569||full	7783	No	NA		0	6	Not-determined	Genome-fragment	NA		0
S8_vs_k141_10279||full	1211	No	NA		0	1	Low-quality	Genome-fragment		2.25	0
S17_extracted_k141_45967	1212	No	NA		1	0	Low-quality	Genome-fragment		5.52	0
S11_vs_k141_94765||full	1877	No	NA		0	1	Not-determined	Genome-fragment	NA		0
S17_extracted_k141_18984	1212	No	NA		0	2	Low-quality	Genome-fragment		0.33	0
S9_vs_k141_15310||full	1212	No	NA		0	0	Low-quality	Genome-fragment		2.8	0
S11_vs_k141_81513||full	2094	No	NA		0	1	Not-determined	Genome-fragment	NA		0
S9_vs_k141_87322||full	1212	No	NA		2	0	Low-quality	Genome-fragment		2.56	0
S9_vs_k141_34909||full	1212	No	NA		0	0	Low-quality	Genome-fragment		2.76	0
S11_vs_k141_10276||full	1117	No	NA		0	2	Not-determined	Genome-fragment	NA		0
S11_vs_k141_35855||full	8706	No	NA		0	0	Not-determined	Genome-fragment	NA		0
S11_extracted_k141_46908	1212	No	NA		0	1	Low-quality	Genome-fragment		0.22	0
S11_vs_k141_94808||full	1612	No	NA		0	0	Not-determined	Genome-fragment	NA		0
S11_vs_k141_88318||full	1275	No	NA		0	1	Not-determined	Genome-fragment	NA		0
S14_vs_k141_24184||full	1212	No	NA		1	0	Low-quality	Genome-fragment		2.59	0
S14_vs_k141_15268||lt2gene	1212	No	NA		1	0	Low-quality	Genome-fragment		19.86	0
S3_extracted_k141_3370	1212	No	NA		0	0	Low-quality	Genome-fragment		3.47	0
S5_extracted_k141_115210	1212	No	NA		1	0	Low-quality	Genome-fragment		1.45	0
S11_vs_k141_42677||full	1491	No	NA		0	2	Not-determined	Genome-fragment	NA		0
S16_extracted_102535	1212	No	NA		1	0	Low-quality	Genome-fragment		3.08	0
S16_vs_19092||full	1212	No	NA		1	0	Low-quality	Genome-fragment		2	0

S11_vs_k141_55768||full	2974	No	NA		0	3	Not-determined	Genome-fragment	NA		0
S11_vs_k141_94868||full	4952	No	NA		0	3	Not-determined	Genome-fragment	NA		0
S11_vs_k141_68151||full	61398	No	NA		32	1	High-quality	High-quality		100	0
S10_vs_116625||full	1212	No	NA		0	2	Low-quality	Genome-fragment		0.83	0
S11_vs_k141_35952||full	1840	No	NA		0	0	Not-determined	Genome-fragment	NA		0
S10_vs_96162||lt2gene	1212	No	NA		1	0	Low-quality	Genome-fragment		3.11	0
S12_vs_k141_183141||full	1212	No	NA		1	0	Low-quality	Genome-fragment		0.82	0
S15_vs_11321||full	1212	No	NA		0	0	Low-quality	Genome-fragment		2.7	0
S6_vs_k141_7563||full	1212	No	NA		1	0	Low-quality	Genome-fragment		2.73	0
S11_vs_k141_101401||full	1526	No	NA		0	0	Not-determined	Genome-fragment	NA		0
S8_vs_k141_101777||lt2gene	1212	No	NA		0	0	Low-quality	Genome-fragment		2.01	0
S17_extracted_k141_86508	1213	No	NA		2	0	Low-quality	Genome-fragment		4	0
S13_extracted_27511	1213	No	NA		1	0	Low-quality	Genome-fragment		2.05	0
S11_vs_k141_3771||full	1729	No	NA		0	0	Not-determined	Genome-fragment	NA		0
S11_vs_k141_94925||full	2984	No	NA		0	3	Not-determined	Genome-fragment	NA		0
S14_vs_k141_10132||full	1213	No	NA		0	0	Low-quality	Genome-fragment		3.04	0
S1_vs_k141_33401||full	1213	No	NA		1	0	Low-quality	Genome-fragment		2.41	0
S5_extracted_k141_84257	1213	No	NA		0	0	Low-quality	Genome-fragment		1.64	0
S7_vs_5511||full	1213	No	NA		0	0	Low-quality	Genome-fragment		2.94	0
S7_vs_68390||full	1213	No	NA		0	0	Low-quality	Genome-fragment		2.43	0
S11_vs_k141_55875||full	2427	No	NA		0	2	Not-determined	Genome-fragment	NA		0
S7_vs_105602||lt2gene	1213	No	NA		1	0	Low-quality	Genome-fragment		2.31	0
S16_vs_97470||full	1213	No	NA		1	0	Low-quality	Genome-fragment		2.41	0
S16_vs_152168||full	1213	No	NA		2	0	Low-quality	Genome-fragment		3.39	0
S11_vs_k141_101466||full	1232	No	NA		0	0	Not-determined	Genome-fragment	NA		0
S11_vs_k141_29529||full	1614	No	NA		0	1	Not-determined	Genome-fragment	NA		0
S10_vs_7558||full	1213	No	NA		1	0	Low-quality	Genome-fragment		2.83	0
S11_vs_k141_36043||full	1901	No	NA		0	0	Not-determined	Genome-fragment	NA		0
S10_vs_31861||full	1213	No	NA		0	0	Low-quality	Genome-fragment		1.16	0
S10_vs_44453||full	1213	No	NA		2	0	Low-quality	Genome-fragment		2.97	0
S11_vs_k141_101501||full	3316	No	NA		0	1	Not-determined	Genome-fragment	NA		0
S12_vs_k141_58472||full	1213	No	NA		1	0	Low-quality	Genome-fragment		2.43	0
S11_vs_k141_101525||full	1051	No	NA		0	1	Not-determined	Genome-fragment	NA		0
S6_vs_k141_24983||full	1213	No	NA		0	1	Low-quality	Genome-fragment		0.24	0
S8_extracted_k141_61520	1213	No	NA		3	0	Low-quality	Genome-fragment		3.91	0
S17_vs_k141_175888||full	1214	No	NA		0	1	Low-quality	Genome-fragment		3.41	0
S9_vs_k141_60276||full	1214	No	NA		0	2	Low-quality	Genome-fragment		1.03	0
S11_extracted_k141_101587	1214	No	NA		0	1	Low-quality	Genome-fragment		0.61	0
S11_vs_k141_75850||full	1254	No	NA		0	1	Not-determined	Genome-fragment	NA		0
S11_vs_k141_8098||full	1214	No	NA		1	0	Low-quality	Genome-fragment		3.05	0
S11_vs_k141_3836||full	3311	No	NA		0	1	Not-determined	Genome-fragment	NA		0
S11_vs_k141_62127||full	1933	No	NA		0	1	Not-determined	Genome-fragment	NA		0
S11_vs_k141_48807||full	1214	No	NA		1	0	Low-quality	Genome-fragment		3.04	0
S11_vs_k141_36105||full	1840	No	NA		0	0	Not-determined	Genome-fragment	NA		0
S13_extracted_91847	1214	No	NA		1	0	Low-quality	Genome-fragment		2.89	0
S11_vs_k141_42921||full	4413	No	NA		0	2	Not-determined	Genome-fragment	NA		0
S11_vs_k141_56002||full	1297	No	NA		0	0	Not-determined	Genome-fragment	NA		0
S11_vs_k141_16667||full	2976	No	NA		0	0	Not-determined	Genome-fragment	NA		0
S13_vs_57190||full	1214	No	NA		0	1	Low-quality	Genome-fragment		0.39	0
S11_vs_k141_88633||full	1875	No	NA		0	1	Not-determined	Genome-fragment	NA		0
S11_vs_k141_56021||full	1243	No	NA		0	1	Not-determined	Genome-fragment	NA		0
S13_vs_65421||full	1214	No	NA		0	0	Low-quality	Genome-fragment		2.33	0
S13_vs_76182||full	1214	No	NA		0	0	Low-quality	Genome-fragment		1.47	0
S1_vs_k141_64238||full	1214	No	NA		1	0	Low-quality	Genome-fragment		21.96	0
S11_vs_k141_88667||full	1708	No	NA		0	1	Not-determined	Genome-fragment	NA		0
S5_vs_k141_30472||full	1214	No	NA		2	0	Low-quality	Genome-fragment		2.92	0
S7_extracted_122436	1214	No	NA		0	3	Low-quality	Genome-fragment		0.32	0
S7_vs_58149||full	1214	No	NA		1	0	Low-quality	Genome-fragment		0.95	0
S11_vs_k141_3914||full	1311	No	NA		0	1	Not-determined	Genome-fragment	NA		0
S11_vs_k141_48936||full	1139	No	NA		0	1	Not-determined	Genome-fragment	NA		0
S11_vs_k141_88692||full	4608	No	NA		0	4	Not-determined	Genome-fragment	NA		0
S11_vs_k141_81775||full	36215	No	NA		17	2	High-quality	High-quality		93	0
S7_vs_87422||full	1214	No	NA		2	0	Low-quality	Genome-fragment		2.64	0
S11_vs_k141_3934||full	4465	No	NA		0	0	Not-determined	Genome-fragment	NA		0
S11_vs_k141_68348||full	41562	No	NA		26	2	High-quality	High-quality		100	0
S11_vs_k141_42927||full	56290	Yes		48437	23	6	Medium-quality	Genome-fragment		87.04	13.95
S11_vs_k141_23724||full	1307	No	NA		0	1	Not-determined	Genome-fragment	NA		0
S11_vs_k141_68355||full	3816	No	NA		0	0	Not-determined	Genome-fragment	NA		0
S7_vs_94461||full	1214	No	NA		0	0	Low-quality	Genome-fragment		2.58	0
S16_extracted_20791	1214	No	NA		1	0	Low-quality	Genome-fragment		0.74	0
S11_vs_k141_42950||full	3259	No	NA		0	0	Not-determined	Genome-fragment	NA		0
S11_vs_k141_42953||full	4654	No	NA		0	2	Not-determined	Genome-fragment	NA		0
S16_extracted_129151	1214	No	NA		0	0	Low-quality	Genome-fragment		2.46	0
S10_extracted_86686	1214	No	NA		1	0	Low-quality	Genome-fragment		0.64	0
S12_extracted_k141_1337	1214	No	NA		0	0	Low-quality	Genome-fragment		2.59	0
S15_vs_68187||lt2gene	1214	No	NA		1	0	Low-quality	Genome-fragment		3.98	0
S11_vs_k141_49002||full	1761	No	NA		0	1	Not-determined	Genome-fragment	NA		0
S11_vs_k141_62260||full	1272	No	NA		0	1	Not-determined	Genome-fragment	NA		0
S8_extracted_k141_23257	1214	No	NA		0	0	Low-quality	Genome-fragment		1.54	0
S11_vs_k141_62262||full	1091	No	NA		0	0	Not-determined	Genome-fragment	NA		0
S8_vs_k141_74976||full	1214	No	NA		0	2	Low-quality	Genome-fragment		1.03	0
S11_extracted_k141_80965	1215	No	NA		1	0	Low-quality	Genome-fragment		2.96	0
S14_vs_k141_60258||full	1215	No	NA		0	0	Low-quality	Genome-fragment		0.8	0
S14_vs_k141_27046||full	1215	No	NA		3	0	Low-quality	Genome-fragment		2.18	0
S3_extracted_k141_89808	1215	No	NA		0	0	Low-quality	Genome-fragment		0.87	0
S3_extracted_k141_94477	1215	No	NA		0	1	Low-quality	Genome-fragment		0.3	0
S11_vs_k141_68455||full	1021	No	NA		0	2	Not-determined	Genome-fragment	NA		0
S5_vs_k141_35022||full	1215	No	NA		0	0	Low-quality	Genome-fragment		3.15	0
S5_vs_k141_5739||full	1215	No	NA		0	1	Low-quality	Genome-fragment		0.52	0
S7_vs_107802||lt2gene	1215	No	NA		1	0	Low-quality	Genome-fragment		2.6	0
S10_vs_99694||full	1215	No	NA		0	0	Low-quality	Genome-fragment		2.66	0
S12_vs_k141_2524||full	1215	No	NA		2	0	Low-quality	Genome-fragment		2.89	0
S12_vs_k141_11139||full	1215	No	NA		0	0	Low-quality	Genome-fragment		2.53	0
S11_vs_k141_36288||full	1104	No	NA		0	1	Not-determined	Genome-fragment	NA		0
S4_extracted_7510	1215	No	NA		1	0	Low-quality	Genome-fragment		2.22	0
S11_vs_k141_56210||full	5955	No	NA		0	2	Not-determined	Genome-fragment	NA		0
S11_vs_k141_4040||full	1593	No	NA		0	0	Not-determined	Genome-fragment	NA		0
S6_extracted_k141_17562	1215	No	NA		0	0	Low-quality	Genome-fragment		0.85	0
S8_extracted_k141_162271	1215	No	NA		0	1	Low-quality	Genome-fragment		0.74	0
S11_vs_k141_23923||full	1334	No	NA		0	1	Not-determined	Genome-fragment	NA		0
S11_vs_k141_23924||full	4713	No	NA		0	2	Not-determined	Genome-fragment	NA		0
S11_vs_k141_62365||full	2238	No	NA		0	2	Not-determined	Genome-fragment	NA		0
S11_vs_k141_81966||full	1403	No	NA		0	1	Not-determined	Genome-fragment	NA		0
S8_extracted_k141_76440	1215	No	NA		0	0	Low-quality	Genome-fragment		1.42	0
S9_vs_k141_9652||lt2gene	1216	No	NA		1	0	Low-quality	Genome-fragment		2.22	0
S11_extracted_k141_23760	1216	No	NA		0	0	Low-quality	Genome-fragment		1.96	0
S11_vs_k141_29683||full	2642	No	NA		0	2	Not-determined	Genome-fragment	NA		0
S11_vs_k141_23943||full	1978	No	NA		0	0	Not-determined	Genome-fragment	NA		0
S11_vs_k141_4104||full	11761	No	NA		0	1	Not-determined	Genome-fragment	NA		0
S1_extracted_k141_9364	1216	No	NA		1	0	Low-quality	Genome-fragment		0.6	0
S3_vs_k141_8361||full	1216	No	NA		0	0	Low-quality	Genome-fragment		3.05	0
S5_vs_k141_73890||full	1216	No	NA		1	0	Low-quality	Genome-fragment		2.77	0
S7_extracted_4228	1216	No	NA		0	0	Low-quality	Genome-fragment		0.77	0
S7_vs_19896||full	1216	No	NA		1	0	Low-quality	Genome-fragment		3	0
S11_vs_k141_10613||full	3376	No	NA		0	3	Not-determined	Genome-fragment	NA		0
S16_vs_11222||full	1216	Yes		682	1	1	Low-quality	Genome-fragment		1.4	43.91
S16_vs_102206||full	1216	No	NA		0	0	Low-quality	Genome-fragment		1.34	0
S11_vs_k141_49082||full	2753	No	NA		0	1	Not-determined	Genome-fragment	NA		0
S16_vs_90509||lt2gene	1216	No	NA		1	0	Low-quality	Genome-fragment		2.95	0
S8_vs_k141_32227||full	1216	No	NA		1	0	Low-quality	Genome-fragment		2.66	0
S8_vs_k141_101750||full	1216	No	NA		0	0	Low-quality	Genome-fragment		0.83	0
S11_vs_k141_29765||full	1207	No	NA		0	0	Not-determined	Genome-fragment	NA		0
S11_vs_k141_62486||full	2490	No	NA		0	1	Not-determined	Genome-fragment	NA		0
S17_vs_k141_35265||full	1217	No	NA		0	1	Low-quality	Genome-fragment		0.73	0
S14_extracted_k141_33479	1217	No	NA		0	0	Low-quality	Genome-fragment		2.38	0
S1_vs_k141_19729||lt2gene	1217	No	NA		1	0	Low-quality	Genome-fragment		2.56	0
S11_vs_k141_88890||full	2360	No	NA		0	1	Not-determined	Genome-fragment	NA		0
S3_vs_k141_124544||lt2gene	1217	No	NA		2	0	Low-quality	Genome-fragment		2.52	0
S7_vs_9156||full	1217	No	NA		2	0	Low-quality	Genome-fragment		3.62	0
S11_vs_k141_24067||full	1103	No	NA		0	0	Not-determined	Genome-fragment	NA		0
S16_extracted_122863	1217	No	NA		0	0	Low-quality	Genome-fragment		2.45	0
S11_vs_k141_36484||full	3995	No	NA		0	2	Not-determined	Genome-fragment	NA		0
S11_vs_k141_24082||full	3699	No	NA		0	2	Not-determined	Genome-fragment	NA		0
S16_vs_41545||full	1217	No	NA		0	0	Low-quality	Genome-fragment		2.32	0
S16_vs_122421||full	1217	No	NA		0	1	Low-quality	Genome-fragment		3.17	0
S12_vs_k141_162161||lt2gene	1217	No	NA		1	0	Low-quality	Genome-fragment		1.87	0
S11_vs_k141_29831||full	6592	No	NA		0	2	Not-determined	Genome-fragment	NA		0
S6_vs_k141_13824||full	1217	No	NA		0	0	Low-quality	Genome-fragment		2.68	0
S11_vs_k141_56481||full	2106	No	NA		0	0	Not-determined	Genome-fragment	NA		0
S11_vs_k141_4195||full	14081	No	NA		0	2	Not-determined	Genome-fragment	NA		0
S17_vs_k141_50768||full	1218	No	NA		0	0	Low-quality	Genome-fragment		1.88	0
S9_vs_k141_3054||lt2gene	1218	No	NA		1	0	Low-quality	Genome-fragment		3.19	0
S11_vs_k141_36577||full	1871	No	NA		0	1	Not-determined	Genome-fragment	NA		0
S11_vs_k141_36578||full	1590	No	NA		0	1	Not-determined	Genome-fragment	NA		0
S11_vs_k141_1445||full	1218	No	NA		1	0	Low-quality	Genome-fragment		2.57	0
S13_extracted_41207	1218	No	NA		0	0	Low-quality	Genome-fragment		1.26	0
S11_vs_k141_101884||full	1152	No	NA		0	1	Not-determined	Genome-fragment	NA		0
S14_vs_k141_68794||full	1218	No	NA		0	1	Low-quality	Genome-fragment		2.01	0
S14_vs_k141_80373||full	1218	No	NA		0	1	Low-quality	Genome-fragment		0.63	0
S11_vs_k141_62573||full	54058	No	NA		13	2	High-quality	High-quality		99.29	0
S14_vs_k141_9377||lt2gene	1218	No	NA		0	0	Low-quality	Genome-fragment		2.08	0
S1_vs_k141_78568||full	1218	No	NA		0	0	Low-quality	Genome-fragment		2.72	0
S11_vs_k141_101924||full	1029	No	NA		0	0	Not-determined	Genome-fragment	NA		0
S5_vs_k141_143327||full	1218	No	NA		0	0	Low-quality	Genome-fragment		2.84	0
S11_vs_k141_10842||full	1475	No	NA		0	1	Not-determined	Genome-fragment	NA		0
S5_vs_k141_11043||full	1218	No	NA		0	1	Low-quality	Genome-fragment		1.41	0
S5_vs_k141_101845||full	1218	No	NA		2	0	Low-quality	Genome-fragment		2.81	0
S16_vs_32133||full	1218	No	NA		1	0	Low-quality	Genome-fragment		2.85	0
S11_vs_k141_82176||full	2924	No	NA		0	0	Not-determined	Genome-fragment	NA		0
S16_vs_51655||full	1218	No	NA		0	0	Low-quality	Genome-fragment		3.59	0
S16_vs_91369||full	1218	No	NA		1	0	Low-quality	Genome-fragment		2.01	0
S16_vs_122429||full	1218	No	NA		0	2	Low-quality	Genome-fragment		1.27	0
S11_vs_k141_43389||full	1431	No	NA		0	3	Not-determined	Genome-fragment	NA		0
S12_vs_k141_90339||full	1218	No	NA		1	0	Low-quality	Genome-fragment		2.47	0
S6_vs_k141_537||full	1218	No	NA		0	1	Low-quality	Genome-fragment		0.75	0
S11_vs_k141_10901||full	1428	No	NA		0	0	Not-determined	Genome-fragment	NA		0
S11_vs_k141_43404||full	1935	No	NA		0	1	Not-determined	Genome-fragment	NA		0
S9_vs_k141_69175||lt2gene	1219	No	NA		0	0	Low-quality	Genome-fragment		2.33	0
S11_vs_k141_17154||full	1847	No	NA		0	1	Not-determined	Genome-fragment	NA		0
S11_vs_k141_4371||full	1019	No	NA		0	0	Not-determined	Genome-fragment	NA		0
S11_extracted_k141_102695	1219	No	NA		2	0	Low-quality	Genome-fragment		2.69	0
S11_vs_k141_62673||full	4574	No	NA		0	1	Not-determined	Genome-fragment	NA		0
S11_vs_k141_17187||full	1844	No	NA		0	2	Not-determined	Genome-fragment	NA		0
S14_vs_k141_61880||lt2gene	1219	No	NA		1	0	Low-quality	Genome-fragment		2.83	0

S11_vs_k141_43449||full	8786	No	NA		0	5	Not-determined	Genome-fragment	NA		0
S1_extracted_k141_10420	1219	No	NA		1	0	Low-quality	Genome-fragment		3.3	0
S3_vs_k141_109021||full	1219	No	NA		0	0	Low-quality	Genome-fragment		2.5	0
S7_vs_129180||lt2gene	1219	No	NA		1	0	Low-quality	Genome-fragment		0.38	0
S11_vs_k141_49275||full	2126	No	NA		0	1	Not-determined	Genome-fragment	NA		0
S11_vs_k141_10924||full	4257	No	NA		0	0	Not-determined	Genome-fragment	NA		0
S16_vs_140160||full	1219	Yes		532	1	1	Low-quality	Genome-fragment		0.6	56.36
S10_extracted_61490	1219	Yes		786	3	1	Low-quality	Genome-fragment		2.4	35.52
S10_vs_84224||full	1219	No	NA		1	0	Low-quality	Genome-fragment		1.47	0
S12_vs_k141_10097||full	1219	No	NA		3	0	Low-quality	Genome-fragment		1.99	0
S8_vs_k141_112174||full	1219	No	NA		0	0	Low-quality	Genome-fragment		2.27	0
S11_vs_k141_62773||full	1351	No	NA		0	0	Not-determined	Genome-fragment	NA		0
S17_extracted_k141_117251	1220	No	NA		0	0	Low-quality	Genome-fragment		1.61	0
S11_vs_k141_68842||full	1948	No	NA		0	1	Not-determined	Genome-fragment	NA		0
S11_vs_k141_43482||full	1135	No	NA		0	0	Not-determined	Genome-fragment	NA		0
S11_vs_k141_49362||full	8365	No	NA		0	6	Not-determined	Genome-fragment	NA		0
S17_vs_k141_7868||full	1220	No	NA		1	0	Low-quality	Genome-fragment		2.86	0
S17_vs_k141_3436||lt2gene	1220	No	NA		2	0	Low-quality	Genome-fragment		3.11	0
S9_vs_k141_16329||full	1220	No	NA		0	0	Low-quality	Genome-fragment		3.63	0
S11_vs_k141_82863||full	1220	No	NA		0	0	Low-quality	Genome-fragment		3.07	0
S13_vs_112026||full	1220	No	NA		0	0	Low-quality	Genome-fragment		0.8	0
S14_vs_k141_23295||full	1220	No	NA		1	0	Low-quality	Genome-fragment		2.88	0
S5_vs_k141_9459||lt2gene	1220	No	NA		1	0	Low-quality	Genome-fragment		0.84	0
S11_vs_k141_43533||full	1191	No	NA		0	0	Not-determined	Genome-fragment	NA		0
S11_vs_k141_68913||full	5864	No	NA		0	2	Not-determined	Genome-fragment	NA		0
S5_vs_k141_88384||lt2gene	1220	No	NA		1	0	Low-quality	Genome-fragment		2.53	0
S7_extracted_36307	1220	No	NA		0	0	Low-quality	Genome-fragment		10.67	0
S11_vs_k141_43557||full	1689	No	NA		0	1	Not-determined	Genome-fragment	NA		0
S7_vs_22225||lt2gene	1220	No	NA		1	0	Low-quality	Genome-fragment		2.83	0
S15_extracted_952	1220	No	NA		0	0	Low-quality	Genome-fragment		0.71	0
S4_extracted_4499	1220	No	NA		3	0	Low-quality	Genome-fragment		4.04	0
S6_vs_k141_25592||full	1220	No	NA		0	0	Low-quality	Genome-fragment		0.77	0
S11_vs_k141_43567||full	1422	No	NA		0	0	Not-determined	Genome-fragment	NA		0
S8_extracted_k141_69420	1220	No	NA		1	0	Low-quality	Genome-fragment		0.74	0
S11_vs_k141_49455||full	2150	No	NA		0	2	Not-determined	Genome-fragment	NA		0
S11_vs_k141_36895||full	4309	No	NA		0	3	Not-determined	Genome-fragment	NA		0
S8_vs_k141_154938||full	1220	No	NA		0	0	Low-quality	Genome-fragment		3.44	0
S9_vs_k141_59855||full	1221	No	NA		0	0	Low-quality	Genome-fragment		2.53	0
S7_vs_39984||full	1221	No	NA		0	0	Low-quality	Genome-fragment		2.97	0
S7_vs_78585||full	1221	No	NA		2	0	Low-quality	Genome-fragment		3.1	0
S11_vs_k141_62884||full	4096	No	NA		0	0	Not-determined	Genome-fragment	NA		0
S7_vs_133793||full	1221	No	NA		0	0	Low-quality	Genome-fragment		2.96	0
S11_vs_k141_95849||full	2455	No	NA		0	0	Not-determined	Genome-fragment	NA		0
S16_vs_114919||lt2gene	1221	No	NA		2	0	Low-quality	Genome-fragment		2.74	0
S12_vs_k141_125345||lt2gene	1221	No	NA		1	0	Low-quality	Genome-fragment		3.71	0
S17_vs_k141_107860||full	1222	No	NA		0	1	Low-quality	Genome-fragment		0.53	0
S11_vs_k141_84565||full	1222	No	NA		0	0	Low-quality	Genome-fragment		2.42	0
S11_vs_k141_56762||full	27623	No	NA		6	2	Medium-quality	Genome-fragment		70.15	0
S14_extracted_k141_22810	1222	No	NA		1	0	Low-quality	Genome-fragment		2.71	0
S14_vs_k141_85743||full	1222	No	NA		0	0	Low-quality	Genome-fragment		2.83	0
S11_vs_k141_95908||full	1515	No	NA		0	2	Not-determined	Genome-fragment	NA		0
S11_vs_k141_36967||full	4853	No	NA		0	2	Not-determined	Genome-fragment	NA		0
S11_vs_k141_82331||full	97795	Yes		37251	16	31	Complete	High-quality		100	61.91
S14_vs_k141_100565||full	1222	No	NA		2	0	Low-quality	Genome-fragment		3.28	0
S3_extracted_k141_40058	1222	No	NA		0	1	Low-quality	Genome-fragment		0.86	0
S3_vs_k141_70453||full	1222	No	NA		0	0	Low-quality	Genome-fragment		3.8	0
S3_vs_k141_68252||lt2gene	1222	No	NA		0	0	Low-quality	Genome-fragment		2.13	0
S5_vs_k141_7298||full	1222	No	NA		0	0	Low-quality	Genome-fragment		2.47	0
S7_vs_86065||lt2gene	1222	No	NA		1	0	Low-quality	Genome-fragment		19.34	0
S16_extracted_2449	1222	No	NA		2	0	Low-quality	Genome-fragment		2.91	0
S11_vs_k141_95982||full	1542	No	NA		0	1	Not-determined	Genome-fragment	NA		0
S10_vs_51300||full	1222	No	NA		0	1	Low-quality	Genome-fragment		2.17	0
S12_extracted_k141_158287	1222	No	NA		0	0	Low-quality	Genome-fragment		3.15	0
S11_vs_k141_30253||full	2281	No	NA		0	0	Not-determined	Genome-fragment	NA		0
S12_vs_k141_33280||full	1222	No	NA		0	2	Low-quality	Genome-fragment		2.23	0
S11_vs_k141_24817||full	8558	No	NA		0	3	Not-determined	Genome-fragment	NA		0
S8_extracted_k141_127452	1222	No	NA		1	0	Low-quality	Genome-fragment		2.97	0
S11_vs_k141_4776||full	2359	No	NA		0	3	Not-determined	Genome-fragment	NA		0
S9_vs_k141_90716||full	1223	No	NA		0	0	Low-quality	Genome-fragment		0.75	0
S11_vs_k141_102435||full	1731	No	NA		0	0	Not-determined	Genome-fragment	NA		0
S5_extracted_k141_6992	1223	No	NA		0	1	Low-quality	Genome-fragment		0.3	0
S5_extracted_k141_39032	1223	No	NA		1	0	Low-quality	Genome-fragment		2.03	0
S11_vs_k141_89320||full	2657	No	NA		0	1	Not-determined	Genome-fragment	NA		0
S5_vs_k141_141280||full	1223	No	NA		0	0	Low-quality	Genome-fragment		2.86	0
S11_vs_k141_96031||full	2428	No	NA		0	2	Not-determined	Genome-fragment	NA		0
S7_vs_97560||full	1223	No	NA		0	0	Low-quality	Genome-fragment		2.86	0
S16_extracted_21084	1223	No	NA		0	0	Low-quality	Genome-fragment		2.59	0
S11_vs_k141_96048||full	1962	No	NA		0	0	Not-determined	Genome-fragment	NA		0
S10_extracted_70242	1223	No	NA		0	0	Low-quality	Genome-fragment		2.13	0
S11_vs_k141_96059||full	4942	No	NA		0	5	Not-determined	Genome-fragment	NA		0
S10_vs_87408||lt2gene	1223	No	NA		0	0	Low-quality	Genome-fragment		2.92	0
S12_vs_k141_166154||lt2gene	1223	No	NA		0	1	Low-quality	Genome-fragment		1.39	0
S11_vs_k141_37171||full	2068	No	NA		0	0	Not-determined	Genome-fragment	NA		0
S11_vs_k141_4842||full	5747	No	NA		0	2	Not-determined	Genome-fragment	NA		0
S9_extracted_k141_22052	1224	No	NA		0	1	Low-quality	Genome-fragment		2.09	0
S11_vs_k141_56994||full	1346	No	NA		0	1	Not-determined	Genome-fragment	NA		0
S11_vs_k141_56995||full	2553	No	NA		0	0	Not-determined	Genome-fragment	NA		0
S11_vs_k141_3840||full	1224	No	NA		1	0	Low-quality	Genome-fragment		2.38	0
S11_vs_k141_4868||full	1571	No	NA		0	0	Not-determined	Genome-fragment	NA		0
S11_vs_k141_23369||lt2gene	1224	No	NA		2	0	Low-quality	Genome-fragment		3.16	0
S11_vs_k141_37193||full	1203	No	NA		0	2	Not-determined	Genome-fragment	NA		0
S11_vs_k141_69191||full	2372	No	NA		0	1	Not-determined	Genome-fragment	NA		0
S11_vs_k141_82477||full	1424	No	NA		0	0	Not-determined	Genome-fragment	NA		0
S13_extracted_33151	1224	No	NA		0	1	Low-quality	Genome-fragment		0.8	0
S14_extracted_k141_13264	1224	No	NA		0	0	Low-quality	Genome-fragment		2.12	0
S11_vs_k141_17611||full	2345	No	NA		0	0	Not-determined	Genome-fragment	NA		0
S14_vs_k141_94290||full	1224	No	NA		0	0	Low-quality	Genome-fragment		2.84	0
S14_vs_k141_30837||lt2gene	1224	No	NA		1	0	Low-quality	Genome-fragment		3.14	0
S11_vs_k141_89403||full	1617	No	NA		0	0	Not-determined	Genome-fragment	NA		0
S14_vs_k141_76474||lt2gene	1224	No	NA		2	0	Low-quality	Genome-fragment		2.89	0
S1_extracted_k141_109621	1224	No	NA		1	0	Low-quality	Genome-fragment		0.66	0
S11_vs_k141_17628||full	1499	No	NA		0	0	Not-determined	Genome-fragment	NA		0
S5_extracted_k141_33384	1224	No	NA		2	0	Low-quality	Genome-fragment		3.59	0
S5_vs_k141_132720||full	1224	No	NA		0	0	Low-quality	Genome-fragment		4.3	0
S11_vs_k141_11267||full	18789	No	NA		13	2	Medium-quality	Genome-fragment		53.56	0
S11_vs_k141_17649||full	1971	No	NA		0	0	Not-determined	Genome-fragment	NA		0
S16_extracted_16127	1224	No	NA		2	0	Low-quality	Genome-fragment		3.29	0
S16_vs_37872||full	1224	No	NA		3	0	Low-quality	Genome-fragment		3.49	0
S10_vs_110997||full	1224	No	NA		0	0	Low-quality	Genome-fragment		2.9	0
S11_vs_k141_49813||full	1709	No	NA		0	2	Not-determined	Genome-fragment	NA		0
S12_vs_k141_142966||full	1224	No	NA		0	3	Low-quality	Genome-fragment		0.31	0
S12_vs_k141_19188||full	1224	No	NA		0	0	Low-quality	Genome-fragment		3.47	0
S12_vs_k141_51157||full	1224	No	NA		1	0	Low-quality	Genome-fragment		2.27	0
S6_extracted_k141_17865	1224	No	NA		0	1	Low-quality	Genome-fragment		0.81	0
S6_extracted_k141_22681	1224	No	NA		0	1	Low-quality	Genome-fragment		2.66	0
S8_extracted_k141_57635	1224	No	NA		0	1	Low-quality	Genome-fragment		1.09	0
S11_vs_k141_37293||full	1747	No	NA		0	0	Not-determined	Genome-fragment	NA		0
S11_vs_k141_11205||full	1225	No	NA		2	0	Low-quality	Genome-fragment		2.85	0
S13_vs_26799||full	1225	No	NA		0	1	Low-quality	Genome-fragment		2.29	0
S13_vs_91521||full	1225	No	NA		0	0	Low-quality	Genome-fragment		1.28	0
S14_extracted_k141_52289	1225	No	NA		1	0	Low-quality	Genome-fragment		3.2	0
S14_extracted_k141_34433	1225	No	NA		2	0	Low-quality	Genome-fragment		2.72	0
S11_vs_k141_96269||full	1044	No	NA		0	0	Not-determined	Genome-fragment	NA		0
S14_vs_k141_86365||full	1225	No	NA		0	0	Low-quality	Genome-fragment		2.29	0
S5_extracted_k141_108995	1225	No	NA		0	0	Low-quality	Genome-fragment		0.9	0
S11_vs_k141_37345||full	1269	No	NA		0	0	Not-determined	Genome-fragment	NA		0
S5_vs_k141_113291||full	1225	No	NA		0	1	Low-quality	Genome-fragment		2.08	0
S11_vs_k141_82619||full	1827	No	NA		0	1	Not-determined	Genome-fragment	NA		0
S7_extracted_11633	1225	No	NA		0	0	Low-quality	Genome-fragment		1.94	0
S7_vs_22404||full	1225	No	NA		3	0	Low-quality	Genome-fragment		2.77	0
S7_vs_31669||full	1225	No	NA		0	0	Low-quality	Genome-fragment		2.57	0
S11_vs_k141_76655||full	8533	No	NA		0	6	Not-determined	Genome-fragment	NA		0
S7_vs_118160||lt2gene	1225	No	NA		1	0	Low-quality	Genome-fragment		2.84	0
S16_vs_52841||full	1225	No	NA		0	1	Low-quality	Genome-fragment		0.96	0
S10_vs_82234||full	1225	No	NA		2	0	Low-quality	Genome-fragment		2.66	0
S11_vs_k141_11437||full	1055	No	NA		0	0	Not-determined	Genome-fragment	NA		0
S11_vs_k141_17808||full	1776	No	NA		0	0	Not-determined	Genome-fragment	NA		0
S11_vs_k141_25132||full	8110	No	NA		0	3	Not-determined	Genome-fragment	NA		0
S11_vs_k141_30549||full	1473	No	NA		0	0	Not-determined	Genome-fragment	NA		0
S8_extracted_k141_50638	1225	No	NA		1	0	Low-quality	Genome-fragment		3.71	0
S11_vs_k141_57283||full	2179	No	NA		0	2	Not-determined	Genome-fragment	NA		0
S11_vs_k141_63229||full	1615	No	NA		0	1	Not-determined	Genome-fragment	NA		0
S11_vs_k141_82705||full	2661	No	NA		0	0	Not-determined	Genome-fragment	NA		0
S8_vs_k141_17338||full	1225	No	NA		1	0	Low-quality	Genome-fragment		2.43	0
S11_vs_k141_69425||full	2356	No	NA		0	0	Not-determined	Genome-fragment	NA		0
S17_vs_k141_155691||full	1226	No	NA		0	0	Low-quality	Genome-fragment		2.59	0
S11_extracted_k141_73351	1226	No	NA		1	0	Low-quality	Genome-fragment		2.23	0
S11_extracted_k141_29494	1226	No	NA		0	1	Low-quality	Genome-fragment		0.56	0
S11_vs_k141_5037||full	1847	No	NA		0	2	Not-determined	Genome-fragment	NA		0
S11_vs_k141_11512||full	3908	No	NA		0	0	Not-determined	Genome-fragment	NA		0
S11_vs_k141_76745||full	1724	No	NA		0	1	Not-determined	Genome-fragment	NA		0
S13_vs_38685||full	1226	No	NA		0	2	Low-quality	Genome-fragment		0.7	0
S11_vs_k141_63264||full	1935	No	NA		0	2	Not-determined	Genome-fragment	NA		0
S14_extracted_k141_22460	1226	No	NA		4	0	Low-quality	Genome-fragment		1.98	0
S14_extracted_k141_47784	1226	No	NA		1	0	Low-quality	Genome-fragment		2.72	0
S11_vs_k141_30652||full	2531	No	NA		0	2	Not-determined	Genome-fragment	NA		0
S14_vs_k141_16732||full	1226	No	NA		1	0	Low-quality	Genome-fragment		1.99	0
S1_extracted_k141_35181	1226	No	NA		0	1	Low-quality	Genome-fragment		0.14	0
S1_vs_k141_3778||full	1226	No	NA		0	1	Low-quality	Genome-fragment		1.11	0
S11_vs_k141_76784||full	34647	No	NA		20	2	High-quality	High-quality		99.4	0
S11_vs_k141_17934||full	3338	No	NA		0	0	Not-determined	Genome-fragment	NA		0
S11_vs_k141_11563||full	3943	No	NA		0	2	Not-determined	Genome-fragment	NA		0
S11_vs_k141_17935||full	4841	No	NA		0	5	Not-determined	Genome-fragment	NA		0
S11_vs_k141_37561||full	2630	No	NA		0	2	Not-determined	Genome-fragment	NA		0
S5_vs_k141_135346||full	1226	No	NA		1	0	Low-quality	Genome-fragment		2.43	0
S11_vs_k141_57410||full	2300	No	NA		0	0	Not-determined	Genome-fragment	NA		0
S11_vs_k141_25206||full	2010	No	NA		0	1	Not-determined	Genome-fragment	NA		0
S11_vs_k141_37581||full	3140	No	NA		0	0	Not-determined	Genome-fragment	NA		0
S11_vs_k141_5077||full	31125	No	NA		24	0	High-quality	High-quality		100	0
S7_vs_72170||full	1226	No	NA		0	0	Low-quality	Genome-fragment		3.61	0
S11_vs_k141_82825||full	2412	No	NA		0	4	Not-determined	Genome-fragment	NA		0
S7_vs_113329||full	1226	No	NA		0	1	Low-quality	Genome-fragment		1.93	0
S11_vs_k141_30739||full	21433	No	NA		7	1	Medium-quality	Genome-fragment		66.44	0
S12_extracted_k141_107482	1226	No	NA		1	0	Low-quality	Genome-fragment		0.7	0

S12_vs_k141_174156||full	1226	No	NA		1	0	Low-quality	Genome-fragment		2.3	0
S12_vs_k141_136286||full	1226	No	NA		0	0	Low-quality	Genome-fragment		3.83	0
S12_vs_k141_58496||full	1226	No	NA		0	0	Low-quality	Genome-fragment		3.04	0
S11_vs_k141_30764||full	1235	No	NA		0	0	Not-determined	Genome-fragment	NA		0
S12_vs_k141_125306||full	1226	No	NA		0	0	Low-quality	Genome-fragment		1.98	0
S11_vs_k141_69566||full	1407	No	NA		0	0	Not-determined	Genome-fragment	NA		0
S6_vs_k141_9901||full	1226	No	NA		0	1	Low-quality	Genome-fragment		0.96	0
S6_vs_k141_31519||full	1226	No	NA		0	0	Low-quality	Genome-fragment		0.81	0
S8_extracted_k141_139112	1226	No	NA		0	0	Low-quality	Genome-fragment		2.48	0
S11_vs_k141_5140||full	1113	No	NA		0	0	Not-determined	Genome-fragment	NA		0
S8_vs_k141_207254||full	1226	No	NA		0	0	Low-quality	Genome-fragment		2.63	0
S13_vs_115283||full	1227	No	NA		0	1	Low-quality	Genome-fragment		1.3	0
S11_vs_k141_25347||full	1140	No	NA		0	1	Not-determined	Genome-fragment	NA		0
S11_vs_k141_82944||full	1390	No	NA		0	2	Not-determined	Genome-fragment	NA		0
S11_vs_k141_37666||full	13735	No	NA		7	0	Medium-quality	Genome-fragment		60.5	0
S3_extracted_k141_69732	1227	No	NA		0	0	Low-quality	Genome-fragment		1.55	0
S3_extracted_k141_95557	1227	No	NA		0	0	Low-quality	Genome-fragment		1.64	0
S11_vs_k141_76900||full	2217	No	NA		0	2	Not-determined	Genome-fragment	NA		0
S11_vs_k141_44221||full	3661	No	NA		0	0	Not-determined	Genome-fragment	NA		0
S11_vs_k141_69626||full	1297	No	NA		0	1	Not-determined	Genome-fragment	NA		0
S5_extracted_k141_45716	1227	No	NA		1	0	Low-quality	Genome-fragment		0.82	0
S16_vs_61187||full	1227	No	NA		0	0	Low-quality	Genome-fragment		2.78	0
S2_extracted_k141_13623	1227	No	NA		2	0	Low-quality	Genome-fragment		4.05	0
S4_extracted_7205	1227	No	NA		0	2	Low-quality	Genome-fragment		0.42	0
S11_vs_k141_44255||full	1780	No	NA		0	1	Not-determined	Genome-fragment	NA		0
S6_extracted_k141_51808	1227	No	NA		0	1	Low-quality	Genome-fragment		0.41	0
S8_extracted_k141_115441	1227	No	NA		0	0	Low-quality	Genome-fragment		0.95	0
S17_extracted_k141_94303	1228	No	NA		0	0	Low-quality	Genome-fragment		0.32	0
S9_extracted_k141_37832	1228	No	NA		1	0	Low-quality	Genome-fragment		2.24	0
S9_vs_k141_89331||full	1228	No	NA		0	1	Low-quality	Genome-fragment		2.18	0
S9_vs_k141_47758||full	1228	No	NA		0	2	Low-quality	Genome-fragment		0.69	0
S11_extracted_k141_53942	1228	No	NA		1	0	Low-quality	Genome-fragment		2.7	0
S14_extracted_k141_66112	1228	No	NA		1	0	Low-quality	Genome-fragment		9.11	0
S11_vs_k141_69740||full	1310	No	NA		0	1	Not-determined	Genome-fragment	NA		0
S1_vs_k141_133999||full	1228	No	NA		1	0	Low-quality	Genome-fragment		2.91	0
S1_vs_k141_72470||full	1228	No	NA		0	1	Low-quality	Genome-fragment		1.25	0
S11_vs_k141_50108||full	8747	No	NA		0	7	Not-determined	Genome-fragment	NA		0
S3_vs_k141_73375||full	1228	No	NA		3	0	Low-quality	Genome-fragment		2.02	0
S3_vs_k141_104851||full	1228	No	NA		0	0	Low-quality	Genome-fragment		2.15	0
S5_vs_k141_58948||full	1228	No	NA		1	0	Low-quality	Genome-fragment		3.1	0
S11_vs_k141_25447||full	1107	No	NA		0	0	Not-determined	Genome-fragment	NA		0
S7_extracted_123190	1228	No	NA		0	0	Low-quality	Genome-fragment		1.65	0
S7_vs_75108||full	1228	No	NA		1	0	Low-quality	Genome-fragment		2.54	0
S7_vs_86528||full	1228	No	NA		0	1	Low-quality	Genome-fragment		0.86	0
S16_extracted_5441	1228	No	NA		0	1	Low-quality	Genome-fragment		3.8	0
S11_vs_k141_63563||full	1104	No	NA		0	0	Not-determined	Genome-fragment	NA		0
S16_extracted_32823	1228	No	NA		4	0	Low-quality	Genome-fragment		3.31	0
S12_extracted_k141_130123	1228	No	NA		0	3	Low-quality	Genome-fragment		0.32	0
S12_extracted_k141_170907	1228	No	NA		0	1	Low-quality	Genome-fragment		0.76	0
S12_vs_k141_147023||full	1228	No	NA		0	0	Low-quality	Genome-fragment		2.93	0
S11_vs_k141_69816||full	3190	No	NA		0	0	Not-determined	Genome-fragment	NA		0
S11_vs_k141_25485||full	1338	No	NA		0	0	Not-determined	Genome-fragment	NA		0
S11_vs_k141_77087||full	2359	No	NA		0	0	Not-determined	Genome-fragment	NA		0
S15_extracted_70730	1228	No	NA		0	0	Low-quality	Genome-fragment		2.73	0
S6_extracted_k141_40405	1228	No	NA		1	0	Low-quality	Genome-fragment		3.46	0
S11_vs_k141_18191||full	7289	No	NA		0	4	Not-determined	Genome-fragment	NA		0
S11_vs_k141_77101||full	3383	No	NA		0	0	Not-determined	Genome-fragment	NA		0
S11_vs_k141_44383||full	5070	No	NA		0	1	Not-determined	Genome-fragment	NA		0
S11_vs_k141_37881||full	2675	No	NA		0	0	Not-determined	Genome-fragment	NA		0
S8_vs_k141_53007||full	1228	No	NA		0	0	Low-quality	Genome-fragment		2.03	0
S11_vs_k141_77117||full	9045	No	NA		0	7	Not-determined	Genome-fragment	NA		0
S11_vs_k141_77067||full	1229	No	NA		0	2	Low-quality	Genome-fragment		0.37	0
S11_vs_k141_50200||full	4454	No	NA		0	0	Not-determined	Genome-fragment	NA		0
S13_vs_130289||full	1229	No	NA		0	0	Low-quality	Genome-fragment		2.36	0
S11_vs_k141_102841||full	1649	No	NA		0	0	Not-determined	Genome-fragment	NA		0
S11_vs_k141_31087||full	2171	No	NA		0	1	Not-determined	Genome-fragment	NA		0
S11_vs_k141_57664||full	1652	No	NA		0	2	Not-determined	Genome-fragment	NA		0
S1_extracted_k141_90407	1229	No	NA		0	1	Low-quality	Genome-fragment		0.86	0
S1_vs_k141_104488||full	1229	No	NA		1	0	Low-quality	Genome-fragment		0.53	0
S5_extracted_k141_68693	1229	No	NA		0	0	Low-quality	Genome-fragment		3.27	0
S5_vs_k141_8004||full	1229	No	NA		0	0	Low-quality	Genome-fragment		2.5	0
S7_vs_86975||lt2gene	1229	No	NA		1	0	Low-quality	Genome-fragment		3.29	0
S11_vs_k141_50227||full	1013	No	NA		0	0	Not-determined	Genome-fragment	NA		0
S16_extracted_171065	1229	No	NA		0	0	Low-quality	Genome-fragment		0.76	0
S16_vs_167261||full	1229	No	NA		0	0	Low-quality	Genome-fragment		0.71	0
S11_vs_k141_96864||full	1137	No	NA		0	1	Not-determined	Genome-fragment	NA		0
S10_extracted_26754	1229	No	NA		0	0	Low-quality	Genome-fragment		0.94	0
S11_vs_k141_96876||full	2907	No	NA		0	1	Not-determined	Genome-fragment	NA		0
S11_vs_k141_18256||full	1229	No	NA		0	1	Not-determined	Genome-fragment	NA		0
S10_vs_117586||full	1229	No	NA		0	0	Low-quality	Genome-fragment		2.74	0
S11_vs_k141_69897||full	6612	No	NA		0	1	Not-determined	Genome-fragment	NA		0
S12_vs_k141_137900||full	1229	No	NA		0	0	Low-quality	Genome-fragment		1.53	0
S12_vs_k141_139452||full	1229	No	NA		1	0	Low-quality	Genome-fragment		2.9	0
S11_vs_k141_31140||full	7306	No	NA		0	1	Not-determined	Genome-fragment	NA		0
S6_extracted_k141_46334	1229	No	NA		0	1	Low-quality	Genome-fragment		1.35	0
S8_extracted_k141_99578	1229	No	NA		0	1	Low-quality	Genome-fragment		1.19	0
S11_vs_k141_37929||full	3496	No	NA		0	3	Not-determined	Genome-fragment	NA		0
S11_vs_k141_57720||full	2092	No	NA		0	1	Not-determined	Genome-fragment	NA		0
S11_vs_k141_69925||full	1313	No	NA		0	0	Not-determined	Genome-fragment	NA		0
S8_vs_k141_70713||full	1229	No	NA		0	0	Low-quality	Genome-fragment		2.35	0
S11_vs_k141_63756||full	2659	No	NA		0	3	Not-determined	Genome-fragment	NA		0
S9_extracted_k141_58587	1230	No	NA		1	0	Low-quality	Genome-fragment		0.72	0
S11_vs_k141_44489||full	2230	No	NA		0	0	Not-determined	Genome-fragment	NA		0
S9_vs_k141_45602||full	1230	No	NA		2	0	Low-quality	Genome-fragment		2.88	0
S11_extracted_k141_60766	1230	No	NA		1	0	Low-quality	Genome-fragment		1.8	0
S11_vs_k141_44506||full	4736	No	NA		0	3	Not-determined	Genome-fragment	NA		0
S11_vs_k141_31201||full	8735	No	NA		0	3	Not-determined	Genome-fragment	NA		0
S14_vs_k141_6521||full	1230	No	NA		0	1	Low-quality	Genome-fragment		2.14	0
S1_extracted_k141_130935	1230	No	NA		0	1	Low-quality	Genome-fragment		0.65	0
S3_extracted_k141_55063	1230	No	NA		0	0	Low-quality	Genome-fragment		2.51	0
S11_vs_k141_63825||full	1365	No	NA		0	0	Not-determined	Genome-fragment	NA		0
S11_vs_k141_50337||full	5860	No	NA		0	4	Not-determined	Genome-fragment	NA		0
S11_vs_k141_11850||full	2031	No	NA		0	0	Not-determined	Genome-fragment	NA		0
S7_extracted_137464	1230	No	NA		0	1	Low-quality	Genome-fragment		2.81	0
S7_vs_5041||full	1230	No	NA		1	0	Low-quality	Genome-fragment		0.6	0
S11_vs_k141_38049||full	1508	No	NA		0	2	Not-determined	Genome-fragment	NA		0
S11_vs_k141_38054||full	4709	No	NA		0	1	Not-determined	Genome-fragment	NA		0
S12_extracted_k141_47410	1230	No	NA		0	1	Low-quality	Genome-fragment		0.8	0
S11_vs_k141_69953||full	1017	No	NA		0	1	Not-determined	Genome-fragment	NA		0
S6_extracted_k141_14751	1230	No	NA		0	2	Low-quality	Genome-fragment		0.5	0
S6_vs_k141_19489||full	1230	No	NA		0	0	Low-quality	Genome-fragment		4.23	0
S6_vs_k141_32703||full	1230	No	NA		1	0	Low-quality	Genome-fragment		2.37	0
S11_vs_k141_83298||full	1402	No	NA		0	0	Not-determined	Genome-fragment	NA		0
S11_vs_k141_89911||full	22236	No	NA		13	0	Medium-quality	Genome-fragment		52.7	0
S8_extracted_k141_124191	1230	No	NA		1	0	Low-quality	Genome-fragment		1.79	0
S11_vs_k141_83299||full	7677	No	NA		0	2	Not-determined	Genome-fragment	NA		0
S11_vs_k141_11918||full	5619	No	NA		0	4	Not-determined	Genome-fragment	NA		0
S14_vs_k141_111631||full	1231	No	NA		0	0	Low-quality	Genome-fragment		0.83	0
S11_vs_k141_97035||full	2454	No	NA		0	1	Not-determined	Genome-fragment	NA		0
S1_extracted_k141_66689	1231	No	NA		0	0	Low-quality	Genome-fragment		1.24	0
S11_vs_k141_83349||full	1278	No	NA		0	2	Not-determined	Genome-fragment	NA		0
S3_extracted_k141_43974	1231	No	NA		0	0	Low-quality	Genome-fragment		2.83	0
S5_extracted_k141_121720	1231	No	NA		1	0	Low-quality	Genome-fragment		3.56	0
S11_vs_k141_77381||full	1467	No	NA		0	2	Not-determined	Genome-fragment	NA		0
S11_vs_k141_90010||full	1668	No	NA		0	0	Not-determined	Genome-fragment	NA		0
S5_vs_k141_144667||full	1231	No	NA		0	2	Low-quality	Genome-fragment		1.38	0
S11_vs_k141_83417||full	2904	No	NA		0	1	Not-determined	Genome-fragment	NA		0
S11_vs_k141_31372||full	2596	No	NA		0	0	Not-determined	Genome-fragment	NA		0
S11_vs_k141_97071||full	19839	No	NA		0	8	Not-determined	Genome-fragment	NA		0
S11_vs_k141_25790||full	1684	No	NA		0	2	Not-determined	Genome-fragment	NA		0
S11_vs_k141_83455||full	1386	No	NA		0	0	Not-determined	Genome-fragment	NA		0
S11_vs_k141_25793||full	1932	No	NA		0	0	Not-determined	Genome-fragment	NA		0
S7_vs_87621||full	1231	No	NA		1	0	Low-quality	Genome-fragment		3.06	0
S11_vs_k141_11994||full	54490	No	NA		23	1	High-quality	High-quality		98.71	0
S11_vs_k141_50530||full	1647	No	NA		0	0	Not-determined	Genome-fragment	NA		0
S11_vs_k141_90076||full	3761	No	NA		0	1	Not-determined	Genome-fragment	NA		0
S11_vs_k141_57778||full	2256	No	NA		0	2	Not-determined	Genome-fragment	NA		0
S7_vs_109694||full	1231	No	NA		0	1	Low-quality	Genome-fragment		2.7	0
S16_extracted_20661	1231	No	NA		0	0	Low-quality	Genome-fragment		3.43	0
S16_extracted_144695	1231	No	NA		0	2	Low-quality	Genome-fragment		2.38	0
S11_vs_k141_38239||full	2043	No	NA		0	3	Not-determined	Genome-fragment	NA		0
S11_vs_k141_18596||full	1347	No	NA		0	2	Not-determined	Genome-fragment	NA		0
S16_vs_71434||full	1231	No	NA		0	0	Low-quality	Genome-fragment		0.75	0
S10_vs_25691||lt2gene	1231	No	NA		1	0	Low-quality	Genome-fragment		0.5	0
S11_vs_k141_57809||full	1354	No	NA		0	0	Not-determined	Genome-fragment	NA		0
S12_extracted_k141_106748	1231	No	NA		0	1	Low-quality	Genome-fragment		2.21	0
S12_vs_k141_69372||full	1231	No	NA		0	1	Low-quality	Genome-fragment		2.64	0
S11_vs_k141_25858||full	1371	No	NA		0	1	Not-determined	Genome-fragment	NA		0
S15_vs_5425||full	1231	No	NA		0	1	Low-quality	Genome-fragment		2.05	0
S8_extracted_k141_79399	1231	No	NA		2	0	Low-quality	Genome-fragment		2.22	0
S8_extracted_k141_53834	1231	No	NA		3	0	Low-quality	Genome-fragment		4.06	0
S11_vs_k141_5792||full	1547	No	NA		0	2	Not-determined	Genome-fragment	NA		0
S13_extracted_68291	1232	No	NA		1	0	Low-quality	Genome-fragment		3.31	0
S1_vs_k141_130107||full	1232	Yes		630	1	1	Low-quality	Genome-fragment		0.78	48.86
S11_vs_k141_57847||full	2032	No	NA		0	0	Not-determined	Genome-fragment	NA		0
S11_vs_k141_5809||full	1452	No	NA		0	0	Not-determined	Genome-fragment	NA		0
S11_vs_k141_77528||full	1665	No	NA		0	0	Not-determined	Genome-fragment	NA		0
S5_vs_k141_137467||full	1232	No	NA		0	0	Low-quality	Genome-fragment		2.02	0
S11_vs_k141_31477||full	4484	No	NA		0	3	Not-determined	Genome-fragment	NA		0
S11_vs_k141_31479||full	1137	No	NA		0	0	Not-determined	Genome-fragment	NA		0
S11_vs_k141_64093||full	2811	No	NA		0	2	Not-determined	Genome-fragment	NA		0
S11_vs_k141_64109||full	2007	No	NA		0	2	Not-determined	Genome-fragment	NA		0
S5_vs_k141_144420||full	1232	No	NA		1	0	Low-quality	Genome-fragment		2.32	0
S7_vs_131367||full	1232	No	NA		0	0	Low-quality	Genome-fragment		3.5	0
S16_vs_52643||full	1232	No	NA		0	0	Low-quality	Genome-fragment		0.81	0
S11_vs_k141_18728||full	1420	No	NA		0	0	Not-determined	Genome-fragment	NA		0
S11_vs_k141_44822||full	1269	No	NA		0	2	Not-determined	Genome-fragment	NA		0
S11_vs_k141_70211||full	1808	No	NA		0	0	Not-determined	Genome-fragment	NA		0
S8_vs_k141_66351||full	1232	No	NA		0	0	Low-quality	Genome-fragment		1.91	0
S17_vs_k141_172720||lt2gene	1233	No	NA		1	0	Low-quality	Genome-fragment		2.33	0
S9_vs_k141_70216||full	1233	No	NA		1	0	Low-quality	Genome-fragment		0.46	0
S11_vs_k141_26012||full	1580	No	NA		0	1	Not-determined	Genome-fragment	NA		0
S14_vs_k141_55410||full	1233	No	NA		2	0	Low-quality	Genome-fragment		2.39	0
S11_vs_k141_70227||full	1241	No	NA		0	0	Not-determined	Genome-fragment	NA		0

S11_vs_k141_12207||full	2734	No	NA	0	4	Not-determined	Genome-fragment	NA	0
S11_vs_k141_31557||full	2341	No	NA	0	2	Not-determined	Genome-fragment	NA	0
S11_vs_k141_103170||full	3988	No	NA	0	1	Not-determined	Genome-fragment	NA	0
S1_vs_k141_54295||full	1233	No	NA	1	0	Low-quality	Genome-fragment	2.73	0
S1_vs_k141_57817||full	1233	No	NA	0	0	Low-quality	Genome-fragment	3.05	0
S11_vs_k141_83666||full	24312	No	NA	18	0	Medium-quality	Genome-fragment	60.55	0
S3_extracted_k141_17374	1233	No	NA	1	0	Low-quality	Genome-fragment	0.85	0
S11_vs_k141_64232||full	1107	No	NA	0	1	Not-determined	Genome-fragment	NA	0
S11_vs_k141_70263||full	4163	No	NA	0	0	Not-determined	Genome-fragment	NA	0
S11_vs_k141_90335||full	9662	No	NA	0	10	Not-determined	Genome-fragment	NA	0
S16_extracted_24279	1233	No	NA	0	0	Low-quality	Genome-fragment	1.89	0
S11_vs_k141_77704||full	1516	No	NA	0	0	Not-determined	Genome-fragment	NA	0
S16_extracted_46016	1233	No	NA	1	0	Low-quality	Genome-fragment	2.19	0
S11_vs_k141_103252||full	1200	No	NA	0	1	Not-determined	Genome-fragment	NA	0
S11_vs_k141_77734||full	1915	No	NA	0	2	Not-determined	Genome-fragment	NA	0
S16_extracted_139978	1233	No	NA	1	0	Low-quality	Genome-fragment	2.1	0
S12_vs_k141_176805||full	1233	No	NA	1	0	Low-quality	Genome-fragment	3.05	0
S12_vs_k141_160554||lt2gene	1233	No	NA	1	0	Low-quality	Genome-fragment	2.71	0
S17_extracted_k141_16240	1234	No	NA	0	2	Low-quality	Genome-fragment	1.73	0
S11_vs_k141_103273||full	1743	No	NA	0	1	Not-determined	Genome-fragment	NA	0
S9_vs_k141_80834||full	1234	No	NA	0	2	Low-quality	Genome-fragment	0.74	0
S11_vs_k141_33683||full	1234	No	NA	0	0	Low-quality	Genome-fragment	3.01	0
S11_vs_k141_64329||full	2790	No	NA	0	2	Not-determined	Genome-fragment	NA	0
S13_vs_28539||full	1234	No	NA	0	0	Low-quality	Genome-fragment	3.04	0
S14_extracted_k141_97064	1234	No	NA	0	1	Low-quality	Genome-fragment	2.7	0
S11_vs_k141_97429||full	1487	No	NA	0	1	Not-determined	Genome-fragment	NA	0
S14_extracted_k141_84338	1234	No	NA	1	0	Low-quality	Genome-fragment	2.44	0
S1_extracted_k141_79606	1234	No	NA	0	2	Low-quality	Genome-fragment	0.48	0
S3_vs_k141_53025||full	1234	No	NA	0	0	Low-quality	Genome-fragment	1.9	0
S11_vs_k141_70434||full	2303	No	NA	0	2	Not-determined	Genome-fragment	NA	0
S11_vs_k141_90510||full	3345	No	NA	0	0	Not-determined	Genome-fragment	NA	0
S11_vs_k141_12483||full	8822	No	NA	0	8	Not-determined	Genome-fragment	NA	0
S5_vs_k141_11282||full	1234	No	NA	1	0	Low-quality	Genome-fragment	2.27	0
S7_extracted_689	1234	No	NA	0	1	Low-quality	Genome-fragment	0.13	0
S7_vs_45576||full	1234	No	NA	0	0	Low-quality	Genome-fragment	2.97	0
S16_extracted_22487	1234	No	NA	0	0	Low-quality	Genome-fragment	2.61	0
S16_vs_134475||full	1234	No	NA	1	0	Low-quality	Genome-fragment	2.18	0
S11_vs_k141_45120||full	3148	No	NA	0	1	Not-determined	Genome-fragment	NA	0
S12_extracted_k141_30055	1234	No	NA	2	0	Low-quality	Genome-fragment	2.1	0
S15_vs_28877||full	1234	No	NA	0	1	Low-quality	Genome-fragment	2.17	0
S6_vs_k141_41379||lt2gene	1234	No	NA	0	0	Low-quality	Genome-fragment	2.89	0
S8_extracted_k141_3486	1234	No	NA	0	0	Low-quality	Genome-fragment	1.67	0
S11_vs_k141_83997||full	1706	No	NA	0	0	Not-determined	Genome-fragment	NA	0
S17_vs_k141_128268||full	1235	No	NA	0	0	Low-quality	Genome-fragment	2.39	0
S11_vs_k141_97591||full	2207	No	NA	0	3	Not-determined	Genome-fragment	NA	0
S9_extracted_k141_50310	1235	No	NA	0	0	Low-quality	Genome-fragment	3.29	0
S11_vs_k141_45223||full	1705	No	NA	0	2	Not-determined	Genome-fragment	NA	0
S11_vs_k141_51044||full	1453	No	NA	0	2	Not-determined	Genome-fragment	NA	0
S11_vs_k141_51057||full	5975	No	NA	0	4	Not-determined	Genome-fragment	NA	0
S14_extracted_k141_96922	1235	No	NA	4	0	Low-quality	Genome-fragment	4.06	0
S11_vs_k141_103384||full	2468	No	NA	0	0	Not-determined	Genome-fragment	NA	0
S11_vs_k141_58291||full	3276	No	NA	0	0	Not-determined	Genome-fragment	NA	0
S1_vs_k141_21973||full	1235	No	NA	1	0	Low-quality	Genome-fragment	2.58	0
S7_extracted_134409	1235	No	NA	0	0	Low-quality	Genome-fragment	1.29	0
S7_vs_125290||full	1235	No	NA	0	0	Low-quality	Genome-fragment	2.06	0
S10_vs_74752||full	1235	No	NA	1	0	Low-quality	Genome-fragment	3.33	0
S12_extracted_k141_49937	1235	No	NA	1	0	Low-quality	Genome-fragment	0.67	0
S11_vs_k141_90650||full	3140	No	NA	0	1	Not-determined	Genome-fragment	NA	0
S12_extracted_k141_61254	1235	No	NA	0	0	Low-quality	Genome-fragment	1.43	0
S12_vs_k141_91977||full	1235	No	NA	0	0	Low-quality	Genome-fragment	2.82	0
S12_vs_k141_93061||full	1235	No	NA	0	1	Low-quality	Genome-fragment	3.4	0
S12_vs_k141_82246||lt2gene	1235	No	NA	1	0	Low-quality	Genome-fragment	3.21	0
S11_vs_k141_38700||full	2384	No	NA	0	2	Not-determined	Genome-fragment	NA	0
S8_vs_k141_17906||full	1235	No	NA	0	1	Low-quality	Genome-fragment	2.75	0
S11_vs_k141_6246||full	1946	No	NA	0	0	Not-determined	Genome-fragment	NA	0
S11_vs_k141_51161||full	9355	No	NA	0	4	Not-determined	Genome-fragment	NA	0
S8_vs_k141_207072||full	1235	No	NA	0	0	Low-quality	Genome-fragment	8.55	0
S11_vs_k141_70533||full	1218	No	NA	0	1	Not-determined	Genome-fragment	NA	0
S11_extracted_k141_48429	1236	No	NA	3	0	Low-quality	Genome-fragment	3.31	0
S11_vs_k141_86521||lt2gene	1236	No	NA	2	0	Low-quality	Genome-fragment	2.82	0
S11_vs_k141_38773||full	3397	No	NA	0	2	Not-determined	Genome-fragment	NA	0
S11_vs_k141_64642||full	1604	No	NA	0	0	Not-determined	Genome-fragment	NA	0
S13_extracted_2627	1236	No	NA	0	0	Low-quality	Genome-fragment	0.77	0
S11_vs_k141_64657||full	2196	No	NA	0	1	Not-determined	Genome-fragment	NA	0
S13_extracted_109394	1236	No	NA	0	0	Low-quality	Genome-fragment	2.52	0
S1_extracted_k141_67804	1236	No	NA	0	1	Low-quality	Genome-fragment	1.52	0
S11_vs_k141_103546||full	1957	No	NA	0	0	Not-determined	Genome-fragment	NA	0
S11_vs_k141_6328||full	10738	No	NA	0	5	Not-determined	Genome-fragment	NA	0
S5_vs_k141_122165||full	1236	No	NA	1	0	Low-quality	Genome-fragment	3.61	0
S11_vs_k141_84269||full	7459	No	NA	0	0	Not-determined	Genome-fragment	NA	0
S11_vs_k141_64715||full	1673	No	NA	0	0	Not-determined	Genome-fragment	NA	0
S7_vs_34056||full	1236	No	NA	0	0	Low-quality	Genome-fragment	2.13	0
S16_extracted_94711	1236	No	NA	0	0	Low-quality	Genome-fragment	0.24	0
S10_extracted_3306	1236	No	NA	0	0	Low-quality	Genome-fragment	2.68	0
S11_vs_k141_19361||full	2505	No	NA	0	1	Not-determined	Genome-fragment	NA	0
S11_vs_k141_90835||full	1818	No	NA	0	1	Not-determined	Genome-fragment	NA	0
S10_extracted_50801	1236	No	NA	0	0	Low-quality	Genome-fragment	2.71	0
S11_vs_k141_38860||full	39451	No	NA	21	0	High-quality	High-quality	100	0
S12_extracted_k141_47018	1236	No	NA	0	0	Low-quality	Genome-fragment	2.29	0
S12_vs_k141_156013||full	1236	No	NA	3	0	Low-quality	Genome-fragment	2.96	0
S11_vs_k141_32067||full	1541	No	NA	0	2	Not-determined	Genome-fragment	NA	0
S11_vs_k141_51342||full	2431	No	NA	0	2	Not-determined	Genome-fragment	NA	0
S12_vs_k141_86325||full	1236	No	NA	0	1	Low-quality	Genome-fragment	1.96	0
S15_extracted_14150	1236	No	NA	0	1	Low-quality	Genome-fragment	0.82	0
S8_extracted_k141_141653	1236	No	NA	0	1	Low-quality	Genome-fragment	1.4	0
S8_vs_k141_124572||full	1236	No	NA	1	0	Low-quality	Genome-fragment	1.06	0
S8_vs_k141_47209||lt2gene	1236	No	NA	1	0	Low-quality	Genome-fragment	2.86	0
S11_vs_k141_6458||full	42936	Yes	37546	12	2	Medium-quality	Genome-fragment	81.42	12.55
S17_vs_k141_119243||full	1237	No	NA	0	0	Low-quality	Genome-fragment	3.07	0
S11_vs_k141_103698||full	2943	No	NA	0	1	Not-determined	Genome-fragment	NA	0
S17_vs_k141_41027||full	1237	No	NA	2	0	Low-quality	Genome-fragment	2.21	0
S11_vs_k141_51428||full	1929	No	NA	0	1	Not-determined	Genome-fragment	NA	0
S11_vs_k141_246||lt2gene	1237	No	NA	2	0	Low-quality	Genome-fragment	1.72	0
S11_vs_k141_39010||full	1750	No	NA	0	0	Not-determined	Genome-fragment	NA	0
S14_vs_k141_92469||full	1237	No	NA	0	0	Low-quality	Genome-fragment	2.68	0
S11_vs_k141_70860||full	1261	No	NA	0	0	Not-determined	Genome-fragment	NA	0
S3_vs_k141_37261||full	1237	No	NA	2	0	Low-quality	Genome-fragment	3.1	0
S11_vs_k141_39036||full	5425	No	NA	0	2	Not-determined	Genome-fragment	NA	0
S11_vs_k141_70895||full	3632	No	NA	0	1	Not-determined	Genome-fragment	NA	0
S3_vs_k141_21529||full	1237	No	NA	1	0	Low-quality	Genome-fragment	2.41	0
S11_vs_k141_91047||full	1917	No	NA	0	2	Not-determined	Genome-fragment	NA	0
S7_vs_8737||lt2gene	1237	No	NA	2	0	Low-quality	Genome-fragment	3.11	0
S11_vs_k141_91058||full	1866	No	NA	0	2	Not-determined	Genome-fragment	NA	0
S11_vs_k141_103809||full	2276	No	NA	0	1	Not-determined	Genome-fragment	NA	0
S11_vs_k141_70991||full	3237	No	NA	0	1	Not-determined	Genome-fragment	NA	0
S7_vs_75987||lt2gene	1237	No	NA	1	0	Low-quality	Genome-fragment	2.94	0
S16_extracted_70387	1237	No	NA	0	0	Low-quality	Genome-fragment	1.18	0
S11_vs_k141_91106||full	1940	No	NA	0	2	Not-determined	Genome-fragment	NA	0
S4_extracted_23361	1237	No	NA	2	0	Low-quality	Genome-fragment	4.07	0
S11_vs_k141_32189||full	2949	No	NA	0	2	Not-determined	Genome-fragment	NA	0
S14_vs_k141_45506||full	1238	No	NA	2	0	Low-quality	Genome-fragment	2.66	0
S11_vs_k141_84480||full	1541	No	NA	0	0	Not-determined	Genome-fragment	NA	0
S11_vs_k141_51614||full	1430	No	NA	0	1	Not-determined	Genome-fragment	NA	0
S11_vs_k141_103885||full	1800	No	NA	0	1	Not-determined	Genome-fragment	NA	0
S1_extracted_k141_135120	1238	No	NA	1	0	Low-quality	Genome-fragment	0.83	0
S11_vs_k141_51642||full	2465	No	NA	0	1	Not-determined	Genome-fragment	NA	0
S1_vs_k141_49297||full	1238	No	NA	0	1	Low-quality	Genome-fragment	2.4	0
S1_vs_k141_148305||full	1238	No	NA	0	0	Low-quality	Genome-fragment	0.86	0
S11_vs_k141_71085||full	1687	No	NA	0	2	Not-determined	Genome-fragment	NA	0
S1_vs_k141_144137||lt2gene	1238	No	NA	1	0	Low-quality	Genome-fragment	0.85	0
S11_vs_k141_71090||full	1326	No	NA	0	1	Not-determined	Genome-fragment	NA	0
S3_extracted_k141_101749	1238	No	NA	0	0	Low-quality	Genome-fragment	0.76	0
S5_extracted_k141_141965	1238	No	NA	2	0	Low-quality	Genome-fragment	2.71	0
S7_vs_100120||full	1238	No	NA	0	0	Low-quality	Genome-fragment	2.28	0
S7_vs_105515||full	1238	No	NA	1	0	Low-quality	Genome-fragment	2.79	0
S11_vs_k141_71208||full	2112	No	NA	0	1	Not-determined	Genome-fragment	NA	0
S16_extracted_43350	1238	No	NA	1	0	Low-quality	Genome-fragment	3.84	0
S11_vs_k141_71262||full	2913	No	NA	0	1	Not-determined	Genome-fragment	NA	0
S16_vs_33377||full	1238	No	NA	0	0	Low-quality	Genome-fragment	2.6	0
S10_extracted_18727	1238	No	NA	0	0	Low-quality	Genome-fragment	1.65	0
S11_vs_k141_32438||full	1900	No	NA	0	0	Not-determined	Genome-fragment	NA	0
S12_vs_k141_178716||full	1238	No	NA	2	0	Low-quality	Genome-fragment	1.99	0
S11_vs_k141_71365||full	1373	No	NA	0	1	Not-determined	Genome-fragment	NA	0
S15_vs_12115||full	1238	No	NA	1	0	Low-quality	Genome-fragment	2.72	0
S11_vs_k141_92442||0_partial	15242	No	NA	0	7	Not-determined	Genome-fragment	NA	0
S11_vs_k141_2527||0_partial	10385	No	NA	0	8	Not-determined	Genome-fragment	NA	0
S8_vs_k141_61814||full	1238	No	NA	1	0	Low-quality	Genome-fragment	2.97	0
S8_vs_k141_8216||full	1238	No	NA	0	0	Low-quality	Genome-fragment	3.81	0
S17_extracted_k141_105503	1239	No	NA	0	0	Low-quality	Genome-fragment	2.33	0
S9_extracted_k141_64137	1239	No	NA	0	1	Low-quality	Genome-fragment	0.48	0
S11_vs_k141_64544||0_partial	12817	No	NA	0	6	Not-determined	Genome-fragment	NA	0
S13_vs_31328||full	1239	No	NA	0	0	Low-quality	Genome-fragment	0.76	0
S13_vs_12091||lt2gene	1239	No	NA	1	0	Low-quality	Genome-fragment	3.55	0
S3_vs_k141_58424||lt2gene	1239	No	NA	1	0	Low-quality	Genome-fragment	2.63	0
S5_vs_k141_66264||full	1239	No	NA	0	0	Low-quality	Genome-fragment	2.49	0
S5_vs_k141_41331||lt2gene	1239	No	NA	1	0	Low-quality	Genome-fragment	2.04	0
S15_vs_42528||full	1239	No	NA	0	0	Low-quality	Genome-fragment	2.02	0
S11_vs_k141_32963||lt2gene	1001	No	NA	0	0	Not-determined	Genome-fragment	NA	0
S6_vs_k141_53387||full	1239	No	NA	0	0	Low-quality	Genome-fragment	3.02	0
S8_extracted_k141_167048	1239	No	NA	0	1	Low-quality	Genome-fragment	0.66	0
S8_extracted_k141_32148	1239	No	NA	0	0	Low-quality	Genome-fragment	2.89	0
S17_extracted_k141_90748	1240	No	NA	0	0	Low-quality	Genome-fragment	3	0
S17_vs_k141_125277||lt2gene	1240	No	NA	2	0	Low-quality	Genome-fragment	3.11	0
S11_extracted_k141_42162	1240	No	NA	0	0	Low-quality	Genome-fragment	0.81	0
S11_vs_k141_86737||full	1240	No	NA	1	0	Low-quality	Genome-fragment	0.67	0
S11_vs_k141_34107||full	1240	No	NA	0	0	Low-quality	Genome-fragment	2.22	0
S11_vs_k141_33431||lt2gene	2048	No	NA	0	1	Not-determined	Genome-fragment	NA	0
S13_extracted_30379	1240	No	NA	0	0	Low-quality	Genome-fragment	3.7	0
S13_extracted_46447	1240	No	NA	0	1	Low-quality	Genome-fragment	0.78	0
S14_vs_k141_99770||lt2gene	1240	No	NA	1	0	Low-quality	Genome-fragment	2.85	0
S1_vs_k141_84960||full	1240	No	NA	1	0	Low-quality	Genome-fragment	0.35	0
S11_vs_k141_59570||lt2gene	1352	No	NA	0	0	Not-determined	Genome-fragment	NA	0
S1_vs_k141_19116||full	1240	No	NA	0	0	Low-quality	Genome-fragment	2.74	0
S3_extracted_k141_16330	1240	No	NA	0	0	Low-quality	Genome-fragment	1.94	0

S5_extracted_k141_89611	1240	No	NA	0	1	Low-quality	Genome-fragment		3.17	0
S12_extracted_k141_49159	1240	No	NA	3	0	Low-quality	Genome-fragment		2.75	0
S15_extracted_72823	1240	No	NA	0	1	Low-quality	Genome-fragment		0.7	0
S2_extracted_k141_16713	1240	No	NA	2	0	Low-quality	Genome-fragment		4.09	0
S8_extracted_k141_70924	1240	No	NA	0	2	Low-quality	Genome-fragment		3.96	0
S13_vs_130515||full	1241	No	NA	1	0	Low-quality	Genome-fragment		0.8	0
S11_vs_k141_79633||lt2gene	2239	No	NA	0	0	Not-determined	Genome-fragment	NA		0
S14_extracted_k141_86540	1241	No	NA	1	0	Low-quality	Genome-fragment		1.56	0
S16_extracted_28551	1241	No	NA	1	0	Low-quality	Genome-fragment		2.79	0
S16_vs_18703||full	1241	No	NA	0	1	Low-quality	Genome-fragment		2.01	0
S10_extracted_5422	1241	No	NA	2	0	Low-quality	Genome-fragment		3.18	0
S12_vs_k141_42601||full	1241	No	NA	1	0	Low-quality	Genome-fragment		3.05	0
S8_vs_k141_165190||full	1241	No	NA	1	0	Low-quality	Genome-fragment		0.8	0
S11_extracted_k141_79420	1242	No	NA	0	2	Low-quality	Genome-fragment		2.77	0
S11_vs_k141_58174||full	1242	No	NA	0	0	Low-quality	Genome-fragment		3.08	0
S11_vs_k141_54000||lt2gene	1170	No	NA	0	0	Not-determined	Genome-fragment	NA		0
S11_vs_k141_8425||lt2gene	1104	No	NA	0	0	Not-determined	Genome-fragment	NA		0
S14_vs_k141_23890||full	1242	No	NA	0	0	Low-quality	Genome-fragment		2.36	0
S14_vs_k141_18459||full	1242	No	NA	0	0	Low-quality	Genome-fragment		1.48	0
S14_vs_k141_55003||full	1242	No	NA	0	0	Low-quality	Genome-fragment		3.18	0
S14_vs_k141_55608||lt2gene	1242	No	NA	2	0	Low-quality	Genome-fragment		3.68	0
S3_extracted_k141_70218	1242	No	NA	1	0	Low-quality	Genome-fragment		3.14	0
S5_vs_k141_126040||full	1242	No	NA	0	0	Low-quality	Genome-fragment		2.07	0
S5_vs_k141_53769||full	1242	No	NA	1	0	Low-quality	Genome-fragment		2.25	0
S11_vs_k141_87368||lt2gene	2836	No	NA	0	0	Not-determined	Genome-fragment	NA		0
S11_vs_k141_80521||lt2gene	1323	No	NA	0	0	Not-determined	Genome-fragment	NA		0
S5_vs_k141_50774||lt2gene	1242	No	NA	1	0	Low-quality	Genome-fragment		1.81	0
S7_extracted_119757	1242	No	NA	0	1	Low-quality	Genome-fragment		0.48	0
S7_vs_23029||full	1242	No	NA	1	0	Low-quality	Genome-fragment		2.93	0
S16_extracted_84914	1242	No	NA	0	0	Low-quality	Genome-fragment		5.1	0
S12_vs_k141_131199||full	1242	No	NA	1	0	Low-quality	Genome-fragment		0.67	0
S8_vs_k141_116731||full	1242	No	NA	3	0	Low-quality	Genome-fragment		2.08	0
S8_vs_k141_50089||full	1242	No	NA	3	0	Low-quality	Genome-fragment		3.12	0
S11_vs_k141_28987||lt2gene	2732	No	NA	0	1	Not-determined	Genome-fragment	NA		0
S17_vs_k141_87837||lt2gene	1243	No	NA	0	0	Low-quality	Genome-fragment		2.4	0
S11_vs_k141_65342||full	1243	No	NA	0	0	Low-quality	Genome-fragment		3.71	0
S13_vs_8351||full	1243	No	NA	0	0	Low-quality	Genome-fragment		2.95	0
S14_extracted_k141_5373	1243	No	NA	1	0	Low-quality	Genome-fragment		7.35	0
S14_vs_k141_86814||full	1243	No	NA	1	0	Low-quality	Genome-fragment		0.8	0
S5_vs_k141_120462||full	1243	No	NA	1	0	Low-quality	Genome-fragment		0.53	0
S16_extracted_105999	1243	No	NA	0	0	Low-quality	Genome-fragment		0.9	0
S16_vs_113140||lt2gene	1243	No	NA	1	0	Low-quality	Genome-fragment		1.68	0
S15_vs_35113||full	1243	No	NA	0	0	Low-quality	Genome-fragment		2.24	0
S11_vs_k141_29797||lt2gene	2426	No	NA	0	1	Not-determined	Genome-fragment	NA		0
S15_vs_29426||lt2gene	1243	No	NA	1	0	Low-quality	Genome-fragment		0.62	0
S11_vs_k141_49195||lt2gene	1383	No	NA	0	2	Not-determined	Genome-fragment	NA		0
S11_vs_k141_49235||lt2gene	1583	No	NA	0	1	Not-determined	Genome-fragment	NA		0
S11_vs_k141_49323||lt2gene	1202	No	NA	0	0	Not-determined	Genome-fragment	NA		0
S2_vs_k141_10117||full	1243	No	NA	1	0	Low-quality	Genome-fragment		2.99	0
S8_extracted_k141_3969	1243	No	NA	0	0	Low-quality	Genome-fragment		2.89	0
S8_extracted_k141_111184	1243	No	NA	1	0	Low-quality	Genome-fragment		2.99	0
S8_vs_k141_188628||lt2gene	1243	No	NA	1	0	Low-quality	Genome-fragment		2.27	0
S17_extracted_k141_123545	1244	No	NA	0	1	Low-quality	Genome-fragment		2.17	0
S9_extracted_k141_44224	1244	No	NA	0	0	Low-quality	Genome-fragment		1.9	0
S11_extracted_k141_84135	1244	No	NA	2	0	Low-quality	Genome-fragment		3.07	0
S11_vs_k141_68876||full	1244	No	NA	1	0	Low-quality	Genome-fragment		1.11	0
S11_vs_k141_30788||full	1244	No	NA	0	0	Low-quality	Genome-fragment		2.71	0
S11_vs_k141_49946||lt2gene	1267	No	NA	0	0	Not-determined	Genome-fragment	NA		0
S11_vs_k141_51127||full	1244	No	NA	0	0	Low-quality	Genome-fragment		0.63	0
S13_extracted_86527	1244	No	NA	0	1	Low-quality	Genome-fragment		0.12	0
S1_extracted_k141_60162	1244	No	NA	1	0	Low-quality	Genome-fragment		2.83	0
S1_extracted_k141_39340	1244	No	NA	0	1	Low-quality	Genome-fragment		0.81	0
S11_vs_k141_37902||lt2gene	1084	No	NA	0	0	Not-determined	Genome-fragment	NA		0
S3_extracted_k141_55295	1244	No	NA	0	0	Low-quality	Genome-fragment		1.51	0
S5_vs_k141_29198||lt2gene	1244	No	NA	1	0	Low-quality	Genome-fragment		1.84	0
S7_vs_70898||full	1244	No	NA	1	0	Low-quality	Genome-fragment		2.65	0
S7_vs_6651||lt2gene	1244	No	NA	1	0	Low-quality	Genome-fragment		3.62	0
S16_vs_148696||lt2gene	1244	No	NA	1	0	Low-quality	Genome-fragment		3.27	0
S17_extracted_k141_115320	1245	No	NA	2	0	Low-quality	Genome-fragment		5.68	0
S17_vs_k141_164283||full	1245	No	NA	2	0	Low-quality	Genome-fragment		2.8	0
S17_vs_k141_124979||full	1245	No	NA	0	3	Low-quality	Genome-fragment		0.51	0
S11_extracted_k141_65788	1245	No	NA	0	0	Low-quality	Genome-fragment		0.48	0
S11_vs_k141_103736||full	1245	No	NA	0	0	Low-quality	Genome-fragment		3.13	0
S14_vs_k141_88706||full	1245	No	NA	0	0	Low-quality	Genome-fragment		2.51	0
S14_vs_k141_14394||full	1245	No	NA	0	0	Low-quality	Genome-fragment		2.38	0
S1_vs_k141_81243||full	1245	No	NA	0	2	Low-quality	Genome-fragment		1.69	0
S11_vs_k141_38829||lt2gene	1137	No	NA	0	0	Not-determined	Genome-fragment	NA		0
S3_vs_k141_11953||full	1245	No	NA	2	0	Low-quality	Genome-fragment		2.68	0
S3_vs_k141_54117||full	1245	No	NA	2	0	Low-quality	Genome-fragment		2.58	0
S5_extracted_k141_115611	1245	No	NA	1	0	Low-quality	Genome-fragment		2.3	0
S5_extracted_k141_123730	1245	No	NA	1	0	Low-quality	Genome-fragment		0.95	0
S11_vs_k141_51732||lt2gene	1672	No	NA	0	0	Not-determined	Genome-fragment	NA		0
S11_vs_k141_32329||lt2gene	1050	No	NA	0	0	Not-determined	Genome-fragment	NA		0
S5_vs_k141_135196||full	1245	No	NA	0	1	Low-quality	Genome-fragment		2.34	0
S5_vs_k141_45135||lt2gene	1245	No	NA	1	0	Low-quality	Genome-fragment		2.18	0
S7_extracted_7544	1245	No	NA	1	0	Low-quality	Genome-fragment		2.34	0
S13_extracted_601	1023	No	NA	0	2	Not-determined	Genome-fragment	NA		0
S13_extracted_712	1156	No	NA	0	0	Not-determined	Genome-fragment	NA		0
S13_extracted_758	1342	No	NA	0	1	Not-determined	Genome-fragment	NA		0
S7_extracted_9316	1245	No	NA	0	0	Low-quality	Genome-fragment		0.28	0
S7_extracted_81635	1245	No	NA	0	1	Low-quality	Genome-fragment		4.15	0
S13_extracted_966	1345	No	NA	0	0	Not-determined	Genome-fragment	NA		0
S13_extracted_1137	1045	No	NA	0	0	Not-determined	Genome-fragment	NA		0
S6_vs_k141_52459||full	1245	No	NA	0	2	Low-quality	Genome-fragment		2.77	0
S13_extracted_1546	1311	No	NA	0	1	Not-determined	Genome-fragment	NA		0
S13_extracted_1750	1789	No	NA	0	2	Not-determined	Genome-fragment	NA		0
S13_extracted_1770	12617	No	NA	4	0	High-quality	High-quality		100	0
S13_extracted_1820	1432	No	NA	0	0	Not-determined	Genome-fragment	NA		0
S13_extracted_2054	1291	No	NA	0	0	Not-determined	Genome-fragment	NA		0
S13_extracted_2135	1182	No	NA	0	1	Not-determined	Genome-fragment	NA		0
S13_extracted_2158	1144	No	NA	0	0	Not-determined	Genome-fragment	NA		0
S13_extracted_2229	1337	No	NA	0	0	Not-determined	Genome-fragment	NA		0
S17_extracted_k141_176089	1246	No	NA	1	0	Low-quality	Genome-fragment		2.28	0
S13_extracted_2348	2157	No	NA	0	0	Not-determined	Genome-fragment	NA		0
S13_extracted_2436	2073	No	NA	0	1	Not-determined	Genome-fragment	NA		0
S13_extracted_2463	2074	No	NA	0	0	Not-determined	Genome-fragment	NA		0
S13_extracted_2522	3804	No	NA	0	1	Not-determined	Genome-fragment	NA		0
S13_extracted_2524	15055	No	NA	0	10	Not-determined	Genome-fragment	NA		0
S17_vs_k141_102707||full	1246	No	NA	0	0	Low-quality	Genome-fragment		0.86	0
S13_extracted_2772	2651	No	NA	0	6	Not-determined	Genome-fragment	NA		0
S13_extracted_3092	2021	No	NA	0	2	Not-determined	Genome-fragment	NA		0
S13_extracted_3140	1386	No	NA	0	1	Not-determined	Genome-fragment	NA		0
S13_extracted_3645	3970	No	NA	0	5	Not-determined	Genome-fragment	NA		0
S13_extracted_3728	93098	No	NA	6	0	Complete	High-quality		100	0
S13_extracted_3819	1139	No	NA	0	1	Not-determined	Genome-fragment	NA		0
S13_extracted_3860	1467	No	NA	0	1	Not-determined	Genome-fragment	NA		0
S13_extracted_4181	3410	No	NA	0	1	Not-determined	Genome-fragment	NA		0
S14_vs_k141_16362||lt2gene	1246	No	NA	1	0	Low-quality	Genome-fragment		1.86	0
S13_extracted_4314	3653	No	NA	0	1	Not-determined	Genome-fragment	NA		0
S13_extracted_4416	3047	No	NA	0	1	Not-determined	Genome-fragment	NA		0
S13_extracted_4553	1049	No	NA	0	1	Not-determined	Genome-fragment	NA		0
S13_extracted_4598	4754	No	NA	0	1	Not-determined	Genome-fragment	NA		0
S13_extracted_4610	9974	No	NA	0	6	Not-determined	Genome-fragment	NA		0
S13_extracted_4717	1117	No	NA	0	1	Not-determined	Genome-fragment	NA		0
S13_extracted_4752	14343	No	NA	0	4	Not-determined	Genome-fragment	NA		0
S13_extracted_4771	1189	No	NA	0	0	Not-determined	Genome-fragment	NA		0
S13_extracted_4798	1117	No	NA	0	1	Not-determined	Genome-fragment	NA		0
S13_extracted_4831	1116	No	NA	0	0	Not-determined	Genome-fragment	NA		0
S13_extracted_4901	95651	No	NA	20	3	Medium-quality	Genome-fragment		70.74	0
S3_extracted_k141_19519	1246	No	NA	2	0	Low-quality	Genome-fragment		2.24	0
S13_extracted_5117	1707	No	NA	0	2	Not-determined	Genome-fragment	NA		0
S13_extracted_5203	1143	No	NA	0	0	Not-determined	Genome-fragment	NA		0
S13_extracted_5273	1032	No	NA	0	0	Not-determined	Genome-fragment	NA		0
S13_extracted_5409	1020	No	NA	0	1	Not-determined	Genome-fragment	NA		0
S5_vs_k141_142076||full	1246	No	NA	1	0	Low-quality	Genome-fragment		2.69	0
S13_extracted_5726	1048	No	NA	0	0	Not-determined	Genome-fragment	NA		0
S13_extracted_5768	1337	No	NA	0	0	Not-determined	Genome-fragment	NA		0
S13_extracted_5905	5593	No	NA	0	1	Not-determined	Genome-fragment	NA		0
S13_extracted_5977	2307	No	NA	0	1	Not-determined	Genome-fragment	NA		0
S13_extracted_6036	1061	No	NA	0	1	Not-determined	Genome-fragment	NA		0
S13_extracted_6085	1826	No	NA	0	1	Not-determined	Genome-fragment	NA		0
S13_extracted_6387	1041	No	NA	0	1	Not-determined	Genome-fragment	NA		0
S7_vs_12884||full	1246	No	NA	0	0	Low-quality	Genome-fragment		2.4	0
S13_extracted_6782	1179	No	NA	0	2	Not-determined	Genome-fragment	NA		0
S13_extracted_6940	2133	No	NA	0	0	Not-determined	Genome-fragment	NA		0
S13_extracted_6962	4397	No	NA	0	3	Not-determined	Genome-fragment	NA		0
S13_extracted_7323	3586	No	NA	0	2	Not-determined	Genome-fragment	NA		0
S7_vs_117406||full	1246	No	NA	0	0	Low-quality	Genome-fragment		2.9	0
S13_extracted_7588	1201	No	NA	0	0	Not-determined	Genome-fragment	NA		0
S13_extracted_7604	1293	No	NA	0	1	Not-determined	Genome-fragment	NA		0
S13_extracted_7636	1307	No	NA	0	1	Not-determined	Genome-fragment	NA		0
S13_extracted_7670	67724	No	NA	8	0	High-quality	High-quality		96.7	0
S13_extracted_7728	1122	No	NA	0	1	Not-determined	Genome-fragment	NA		0
S13_extracted_7780	1170	No	NA	0	2	Not-determined	Genome-fragment	NA		0
S13_extracted_7884	1078	No	NA	0	2	Not-determined	Genome-fragment	NA		0
S13_extracted_7952	1753	No	NA	0	0	Not-determined	Genome-fragment	NA		0
S13_extracted_8039	1146	No	NA	0	1	Not-determined	Genome-fragment	NA		0
S13_extracted_8102	1177	No	NA	0	1	Not-determined	Genome-fragment	NA		0
S13_extracted_8239	1345	No	NA	0	2	Not-determined	Genome-fragment	NA		0
S13_extracted_8285	3496	No	NA	0	0	Not-determined	Genome-fragment	NA		0
S10_vs_12494||lt2gene	1246	No	NA	1	0	Low-quality	Genome-fragment		2.87	0
S12_extracted_k141_182053	1246	No	NA	1	0	Low-quality	Genome-fragment		2.55	0
S13_extracted_8496	2524	No	NA	0	1	Not-determined	Genome-fragment	NA		0
S13_extracted_8551	1235	No	NA	0	1	Not-determined	Genome-fragment	NA		0
S12_extracted_k141_145945	1246	No	NA	0	2	Low-quality	Genome-fragment		0.79	0
S13_extracted_8583	2182	No	NA	0	0	Not-determined	Genome-fragment	NA		0
S13_extracted_8646	1170	No	NA	0	1	Not-determined	Genome-fragment	NA		0
S13_extracted_8731	1003	No	NA	0	1	Not-determined	Genome-fragment	NA		0
S13_extracted_8754	5140	No	NA	0	3	Not-determined	Genome-fragment	NA		0
S13_extracted_8761	2942	No	NA	0	1	Not-determined	Genome-fragment	NA		0
S13_extracted_8857	1319	No	NA	0	3	Not-determined	Genome-fragment	NA		0
S13_extracted_8859	3135	No	NA	0	1	Not-determined	Genome-fragment	NA		0
S13_extracted_9024	5188	No	NA	0	1	Not-determined	Genome-fragment	NA		0
S13_extracted_9094	1145	No	NA	0	1	Not-determined	Genome-fragment	NA		0

S13_extracted_9170	1383	No	NA	0	0	Not-determined	Genome-fragment	NA		0
S13_extracted_9294	1245	No	NA	0	3	Not-determined	Genome-fragment	NA		0
S13_extracted_9296	2045	No	NA	0	0	Not-determined	Genome-fragment	NA		0
S13_extracted_9324	1023	No	NA	0	1	Not-determined	Genome-fragment	NA		0
S13_extracted_9372	1081	No	NA	0	0	Not-determined	Genome-fragment	NA		0
S12_vs_k141_56737||full	1246	No	NA	0	2	Low-quality	Genome-fragment		1.18	0
S13_extracted_9429	1767	No	NA	0	0	Not-determined	Genome-fragment	NA		0
S13_extracted_9537	1150	No	NA	0	1	Not-determined	Genome-fragment	NA		0
S13_extracted_9545	1184	No	NA	0	1	Not-determined	Genome-fragment	NA		0
S13_extracted_9648	1730	No	NA	0	1	Not-determined	Genome-fragment	NA		0
S13_extracted_9811	1154	No	NA	0	0	Not-determined	Genome-fragment	NA		0
S13_extracted_9884	1566	No	NA	0	1	Not-determined	Genome-fragment	NA		0
S15_vs_76957||full	1246	No	NA	0	0	Low-quality	Genome-fragment		3.23	0
S13_extracted_9993	1156	No	NA	0	2	Not-determined	Genome-fragment	NA		0
S13_extracted_10119	1010	No	NA	0	2	Not-determined	Genome-fragment	NA		0
S13_extracted_10127	17386	No	NA	0	17	Not-determined	Genome-fragment	NA		0
S6_vs_k141_38699||full	1246	No	NA	1	0	Low-quality	Genome-fragment		2.05	0
S13_extracted_10480	1994	No	NA	0	1	Not-determined	Genome-fragment	NA		0
S8_extracted_k141_122770	1246	No	NA	0	1	Low-quality	Genome-fragment		0.38	0
S13_extracted_10557	1568	No	NA	0	0	Not-determined	Genome-fragment	NA		0
S8_vs_k141_173478||full	1246	No	NA	1	0	Low-quality	Genome-fragment		2.87	0
S9_vs_k141_56843||full	1247	No	NA	2	0	Low-quality	Genome-fragment		2.95	0
S13_extracted_10620	1190	No	NA	0	2	Not-determined	Genome-fragment	NA		0
S13_extracted_10715	1349	No	NA	0	1	Not-determined	Genome-fragment	NA		0
S13_extracted_10859	1907	No	NA	0	2	Not-determined	Genome-fragment	NA		0
S9_vs_k141_44260||full	1247	No	NA	0	0	Low-quality	Genome-fragment		2.45	0
S13_extracted_11193	1715	No	NA	0	2	Not-determined	Genome-fragment	NA		0
S13_extracted_11246	97342	No	NA	15	2	Complete	High-quality		100	0
S13_extracted_11316	1263	No	NA	0	1	Not-determined	Genome-fragment	NA		0
S9_vs_k141_21080||lt2gene	1247	No	NA	1	0	Low-quality	Genome-fragment		3.36	0
S13_extracted_11639	1329	No	NA	0	1	Not-determined	Genome-fragment	NA		0
S14_extracted_k141_55813	1247	No	NA	0	0	Low-quality	Genome-fragment		3.12	0
S13_extracted_11763	2640	No	NA	0	3	Not-determined	Genome-fragment	NA		0
S13_extracted_11877	3266	No	NA	0	1	Not-determined	Genome-fragment	NA		0
S14_vs_k141_33797||full	1247	No	NA	1	0	Low-quality	Genome-fragment		2.22	0
S14_vs_k141_13391||lt2gene	1247	No	NA	2	0	Low-quality	Genome-fragment		2.92	0
S13_extracted_12007	1036	No	NA	0	0	Not-determined	Genome-fragment	NA		0
S13_extracted_12080	1299	No	NA	0	2	Not-determined	Genome-fragment	NA		0
S13_extracted_12088	2331	No	NA	0	0	Not-determined	Genome-fragment	NA		0
S5_vs_k141_9564||full	1247	No	NA	0	0	Low-quality	Genome-fragment		2.94	0
S13_extracted_12447	1468	No	NA	0	0	Not-determined	Genome-fragment	NA		0
S13_extracted_12589	1046	No	NA	0	1	Not-determined	Genome-fragment	NA		0
S13_extracted_12623	1017	No	NA	0	1	Not-determined	Genome-fragment	NA		0
S13_extracted_12796	1198	No	NA	0	2	Not-determined	Genome-fragment	NA		0
S13_extracted_12862	1241	No	NA	0	2	Not-determined	Genome-fragment	NA		0
S13_extracted_13010	1425	No	NA	0	1	Not-determined	Genome-fragment	NA		0
S5_vs_k141_65089||full	1247	No	NA	0	0	Low-quality	Genome-fragment		2.17	0
S13_extracted_13219	1050	No	NA	0	0	Not-determined	Genome-fragment	NA		0
S13_extracted_13245	1947	No	NA	0	0	Not-determined	Genome-fragment	NA		0
S13_extracted_13537	1309	No	NA	0	2	Not-determined	Genome-fragment	NA		0
S13_extracted_13607	1565	No	NA	0	0	Not-determined	Genome-fragment	NA		0
S13_extracted_13729	2211	No	NA	0	1	Not-determined	Genome-fragment	NA		0
S13_extracted_13949	2626	No	NA	0	1	Not-determined	Genome-fragment	NA		0
S13_extracted_14074	1873	No	NA	0	0	Not-determined	Genome-fragment	NA		0
S5_vs_k141_60458||full	1247	No	NA	2	0	Low-quality	Genome-fragment		2.78	0
S13_extracted_14328	1189	No	NA	0	0	Not-determined	Genome-fragment	NA		0
S7_extracted_103359	1247	No	NA	0	0	Low-quality	Genome-fragment		1.82	0
S7_vs_88133||lt2gene	1247	No	NA	1	0	Low-quality	Genome-fragment		0.71	0
S16_vs_25467||full	1247	No	NA	0	2	Low-quality	Genome-fragment		0.67	0
S13_extracted_14765	2005	No	NA	0	1	Not-determined	Genome-fragment	NA		0
S16_vs_77497||full	1247	No	NA	0	0	Low-quality	Genome-fragment		1.48	0
S13_extracted_14929	1076	No	NA	0	0	Not-determined	Genome-fragment	NA		0
S12_vs_k141_183487||lt2gene	1247	No	NA	1	0	Low-quality	Genome-fragment		2.91	0
S13_extracted_15054	2783	No	NA	0	0	Not-determined	Genome-fragment	NA		0
S13_extracted_15194	3033	No	NA	0	6	Not-determined	Genome-fragment	NA		0
S6_vs_k141_52358||full	1247	No	NA	0	0	Low-quality	Genome-fragment		3.28	0
S13_extracted_15300	1773	No	NA	0	1	Not-determined	Genome-fragment	NA		0
S13_extracted_15353	1314	No	NA	0	2	Not-determined	Genome-fragment	NA		0
S13_extracted_15426	1501	No	NA	0	0	Not-determined	Genome-fragment	NA		0
S13_extracted_15463	1776	No	NA	0	2	Not-determined	Genome-fragment	NA		0
S8_extracted_k141_18612	1247	No	NA	0	1	Low-quality	Genome-fragment		3.98	0
S13_extracted_15684	1439	No	NA	0	1	Not-determined	Genome-fragment	NA		0
S13_extracted_15729	1058	No	NA	0	1	Not-determined	Genome-fragment	NA		0
S13_extracted_15817	2390	No	NA	0	0	Not-determined	Genome-fragment	NA		0
S13_extracted_15910	1203	No	NA	0	1	Not-determined	Genome-fragment	NA		0
S13_extracted_16030	1324	No	NA	0	1	Not-determined	Genome-fragment	NA		0
S13_extracted_16073	1239	No	NA	0	1	Not-determined	Genome-fragment	NA		0
S13_extracted_16144	1175	No	NA	0	1	Not-determined	Genome-fragment	NA		0
S13_extracted_16281	1719	No	NA	0	0	Not-determined	Genome-fragment	NA		0
S13_extracted_16327	1255	No	NA	0	0	Not-determined	Genome-fragment	NA		0
S13_extracted_16419	1063	No	NA	0	0	Not-determined	Genome-fragment	NA		0
S13_extracted_16719	1754	No	NA	0	1	Not-determined	Genome-fragment	NA		0
S13_extracted_16913	1375	No	NA	0	1	Not-determined	Genome-fragment	NA		0
S13_extracted_17085	1096	No	NA	0	1	Not-determined	Genome-fragment	NA		0
S13_extracted_17099	1021	No	NA	0	1	Not-determined	Genome-fragment	NA		0
S8_extracted_k141_197950	1247	No	NA	0	0	Low-quality	Genome-fragment		3.84	0
S13_extracted_17191	2180	No	NA	0	2	Not-determined	Genome-fragment	NA		0
S13_extracted_17355	2178	No	NA	0	0	Not-determined	Genome-fragment	NA		0
S13_extracted_17422	5395	No	NA	0	2	Not-determined	Genome-fragment	NA		0
S17_vs_k141_133632||full	1248	No	NA	1	0	Low-quality	Genome-fragment		1.91	0
S13_extracted_17699	1058	No	NA	0	1	Not-determined	Genome-fragment	NA		0
S13_extracted_17733	2216	No	NA	0	1	Not-determined	Genome-fragment	NA		0
S13_extracted_17833	4738	No	NA	0	4	Not-determined	Genome-fragment	NA		0
S13_extracted_17878	1123	No	NA	0	1	Not-determined	Genome-fragment	NA		0
S13_extracted_17981	1061	No	NA	0	0	Not-determined	Genome-fragment	NA		0
S9_vs_k141_21094||full	1248	No	NA	0	0	Low-quality	Genome-fragment		2.41	0
S13_extracted_18325	1525	No	NA	0	2	Not-determined	Genome-fragment	NA		0
S13_extracted_18384	1255	No	NA	0	1	Not-determined	Genome-fragment	NA		0
S13_extracted_18461	1187	No	NA	0	1	Not-determined	Genome-fragment	NA		0
S13_extracted_18564	1230	No	NA	0	1	Not-determined	Genome-fragment	NA		0
S13_extracted_18606	1240	No	NA	0	1	Not-determined	Genome-fragment	NA		0
S9_vs_k141_6112||lt2gene	1248	No	NA	1	0	Low-quality	Genome-fragment		2.81	0
S13_extracted_18724	3333	No	NA	0	1	Not-determined	Genome-fragment	NA		0
S13_extracted_18811	1607	No	NA	0	2	Not-determined	Genome-fragment	NA		0
S13_extracted_18863	1418	No	NA	0	1	Not-determined	Genome-fragment	NA		0
S14_vs_k141_31350||full	1248	No	NA	0	0	Low-quality	Genome-fragment		2.58	0
S13_extracted_19021	1130	No	NA	0	1	Not-determined	Genome-fragment	NA		0
S13_extracted_19051	1677	No	NA	0	2	Not-determined	Genome-fragment	NA		0
S13_extracted_19114	4320	No	NA	0	3	Not-determined	Genome-fragment	NA		0
S13_extracted_19187	2371	No	NA	0	0	Not-determined	Genome-fragment	NA		0
S13_extracted_19236	1243	No	NA	0	2	Not-determined	Genome-fragment	NA		0
S13_extracted_19243	4864	No	NA	0	3	Not-determined	Genome-fragment	NA		0
S13_extracted_19343	4820	No	NA	0	0	Not-determined	Genome-fragment	NA		0
S13_extracted_19414	1460	No	NA	0	0	Not-determined	Genome-fragment	NA		0
S14_vs_k141_10823||full	1248	No	NA	0	1	Low-quality	Genome-fragment		1.61	0
S13_extracted_19586	1573	No	NA	0	0	Not-determined	Genome-fragment	NA		0
S13_extracted_19630	1595	No	NA	0	2	Not-determined	Genome-fragment	NA		0
S13_extracted_19825	1031	No	NA	0	1	Not-determined	Genome-fragment	NA		0
S13_extracted_20386	3735	No	NA	0	3	Not-determined	Genome-fragment	NA		0
S1_vs_k141_32986||full	1248	No	NA	2	0	Low-quality	Genome-fragment		2.22	0
S13_extracted_20450	1061	No	NA	0	1	Not-determined	Genome-fragment	NA		0
S13_extracted_20573	1157	No	NA	0	0	Not-determined	Genome-fragment	NA		0
S13_extracted_20633	6616	No	NA	0	5	Not-determined	Genome-fragment	NA		0
S13_extracted_20669	1463	No	NA	0	0	Not-determined	Genome-fragment	NA		0
S13_extracted_20741	3969	No	NA	0	2	Not-determined	Genome-fragment	NA		0
S13_extracted_20840	1488	No	NA	0	0	Not-determined	Genome-fragment	NA		0
S13_extracted_20954	1086	No	NA	0	2	Not-determined	Genome-fragment	NA		0
S13_extracted_21012	1595	No	NA	0	2	Not-determined	Genome-fragment	NA		0
S5_vs_k141_72943||full	1248	No	NA	0	1	Low-quality	Genome-fragment		1.84	0
S13_extracted_21128	2712	No	NA	0	2	Not-determined	Genome-fragment	NA		0
S13_extracted_21174	1627	No	NA	0	0	Not-determined	Genome-fragment	NA		0
S5_vs_k141_19175||full	1248	No	NA	0	1	Low-quality	Genome-fragment		1.82	0
S13_extracted_21343	1053	No	NA	0	0	Not-determined	Genome-fragment	NA		0
S5_vs_k141_76162||full	1248	No	NA	1	0	Low-quality	Genome-fragment		2.99	0
S5_vs_k141_52073||lt2gene	1248	No	NA	1	0	Low-quality	Genome-fragment		0.76	0
S13_extracted_21692	1060	No	NA	0	1	Not-determined	Genome-fragment	NA		0
S13_extracted_21694	1666	No	NA	0	0	Not-determined	Genome-fragment	NA		0
S13_extracted_21772	1352	No	NA	0	5	Not-determined	Genome-fragment	NA		0
S13_extracted_21779	1185	No	NA	0	0	Not-determined	Genome-fragment	NA		0
S7_vs_5767||full	1248	No	NA	0	1	Low-quality	Genome-fragment		2.96	0
S13_extracted_21949	13143	No	NA	0	0	Not-determined	Genome-fragment	NA		0
S13_extracted_21997	1376	No	NA	0	0	Not-determined	Genome-fragment	NA		0
S13_extracted_22175	36043	No	NA	17	0	High-quality	High-quality		100	0
S13_extracted_22186	7085	No	NA	0	5	Not-determined	Genome-fragment	NA		0
S7_vs_108664||full	1248	No	NA	0	1	Low-quality	Genome-fragment		1	0
S13_extracted_22368	1273	No	NA	0	1	Not-determined	Genome-fragment	NA		0
S13_extracted_22384	2330	No	NA	0	2	Not-determined	Genome-fragment	NA		0
S7_vs_111735||full	1248	No	NA	0	0	Low-quality	Genome-fragment		2.93	0
S7_vs_89927||lt2gene	1248	No	NA	0	0	Low-quality	Genome-fragment		2.48	0
S13_extracted_23268	1222	No	NA	0	0	Not-determined	Genome-fragment	NA		0
S13_extracted_23350	3802	No	NA	0	1	Not-determined	Genome-fragment	NA		0
S13_extracted_23357	3039	No	NA	0	2	Not-determined	Genome-fragment	NA		0
S13_extracted_23452	2219	No	NA	0	0	Not-determined	Genome-fragment	NA		0
S13_extracted_23474	1060	No	NA	0	0	Not-determined	Genome-fragment	NA		0
S13_extracted_23488	5970	No	NA	0	12	Not-determined	Genome-fragment	NA		0
S6_vs_k141_12491||full	1248	No	NA	0	0	Low-quality	Genome-fragment		2.93	0
S13_extracted_23533	1026	No	NA	0	1	Not-determined	Genome-fragment	NA		0
S13_extracted_23579	1165	No	NA	0	1	Not-determined	Genome-fragment	NA		0
S13_extracted_23593	1067	No	NA	0	0	Not-determined	Genome-fragment	NA		0
S13_extracted_23641	3120	No	NA	0	0	Not-determined	Genome-fragment	NA		0
S8_vs_k141_150679||full	1248	No	NA	0	0	Low-quality	Genome-fragment		2.86	0
S13_extracted_23724	1326	No	NA	0	0	Not-determined	Genome-fragment	NA		0
S13_extracted_23744	1129	No	NA	0	1	Not-determined	Genome-fragment	NA		0
S13_extracted_23803	1856	No	NA	0	1	Not-determined	Genome-fragment	NA		0
S13_extracted_24071	1039	No	NA	0	0	Not-determined	Genome-fragment	NA		0
S13_extracted_24236	3946	No	NA	0	3	Not-determined	Genome-fragment	NA		0
S13_extracted_24273	1452	No	NA	0	1	Not-determined	Genome-fragment	NA		0
S13_extracted_24337	1179	No	NA	0	0	Not-determined	Genome-fragment	NA		0
S13_extracted_24418	1714	No	NA	0	1	Not-determined	Genome-fragment	NA		0
S13_extracted_24524	1215	No	NA	0	1	Not-determined	Genome-fragment	NA		0
S13_extracted_24765	1207	No	NA	0	1	Not-determined	Genome-fragment	NA		0
S13_extracted_24881	1125	No	NA	0	1	Not-determined	Genome-fragment	NA		0
S13_extracted_24995	11236	No	NA	0	1	Not-determined	Genome-fragment	NA		0
S13_extracted_25047	3179	No	NA	0	3	Not-determined	Genome-fragment	NA		0

S13_extracted_25103	1034	No	NA		0	1	Not-determined	Genome-fragment	NA		0
S13_extracted_25244	1239	No	NA		0	1	Not-determined	Genome-fragment	NA		0
S13_extracted_25483	1106	No	NA		0	0	Not-determined	Genome-fragment	NA		0
S13_extracted_25506	3382	No	NA		0	1	Not-determined	Genome-fragment	NA		0
S13_extracted_25553	2329	No	NA		0	0	Not-determined	Genome-fragment	NA		0
S13_extracted_25693	1018	No	NA		0	2	Not-determined	Genome-fragment	NA		0
S13_extracted_26011	1109	No	NA		0	0	Not-determined	Genome-fragment	NA		0
S13_extracted_26115	4882	No	NA		0	2	Not-determined	Genome-fragment	NA		0
S8_vs_k141_74180||full	1248	No	NA		1	0	Low-quality	Genome-fragment		2.85	0
S13_extracted_26492	3932	No	NA		0	1	Not-determined	Genome-fragment	NA		0
S13_extracted_26536	1750	No	NA		0	1	Not-determined	Genome-fragment	NA		0
S13_extracted_26634	1031	No	NA		0	1	Not-determined	Genome-fragment	NA		0
S17_vs_k141_18271||full	1249	No	NA		0	0	Low-quality	Genome-fragment		2.11	0
S13_extracted_26805	1043	No	NA		0	0	Not-determined	Genome-fragment	NA		0
S13_extracted_26904	1406	No	NA		0	1	Not-determined	Genome-fragment	NA		0
S13_extracted_27027	1775	No	NA		0	0	Not-determined	Genome-fragment	NA		0
S13_extracted_27042	1448	No	NA		0	1	Not-determined	Genome-fragment	NA		0
S13_extracted_70987	1249	No	NA		0	1	Low-quality	Genome-fragment		3.03	0
S13_extracted_27161	3156	No	NA		0	1	Not-determined	Genome-fragment	NA		0
S13_extracted_27184	1091	No	NA		0	0	Not-determined	Genome-fragment	NA		0
S13_vs_145058||full	1249	No	NA		0	0	Low-quality	Genome-fragment		2.69	0
S13_extracted_27347	2637	No	NA		0	2	Not-determined	Genome-fragment	NA		0
S13_extracted_27390	1228	No	NA		0	0	Not-determined	Genome-fragment	NA		0
S13_vs_51561||lt2gene	1249	No	NA		2	0	Low-quality	Genome-fragment		2.49	0
S14_vs_k141_109225||lt2gene	1249	No	NA		1	0	Low-quality	Genome-fragment		2.92	0
S13_extracted_27542	1070	No	NA		0	2	Not-determined	Genome-fragment	NA		0
S13_extracted_27648	6378	No	NA		0	2	Not-determined	Genome-fragment	NA		0
S3_extracted_k141_63420	1249	No	NA		0	0	Low-quality	Genome-fragment		2.08	0
S3_vs_k141_41718||lt2gene	1249	No	NA		1	0	Low-quality	Genome-fragment		3.02	0
S13_extracted_28309	15508	No	NA		7	0	Medium-quality	Genome-fragment		66.4	0
S7_vs_34948||full	1249	No	NA		2	0	Low-quality	Genome-fragment		2.82	0
S7_vs_113193||full	1249	No	NA		0	0	Low-quality	Genome-fragment		3.17	0
S13_extracted_28773	1021	No	NA		0	0	Not-determined	Genome-fragment	NA		0
S13_extracted_28790	2692	No	NA		0	2	Not-determined	Genome-fragment	NA		0
S13_extracted_29085	1131	No	NA		0	0	Not-determined	Genome-fragment	NA		0
S13_extracted_29503	5147	No	NA		0	2	Not-determined	Genome-fragment	NA		0
S13_extracted_29559	1175	No	NA		0	3	Not-determined	Genome-fragment	NA		0
S10_extracted_66597	1249	No	NA		1	0	Low-quality	Genome-fragment		1.19	0
S13_extracted_29706	1132	No	NA		0	1	Not-determined	Genome-fragment	NA		0
S10_vs_49323||full	1249	No	NA		0	0	Low-quality	Genome-fragment		2.41	0
S13_extracted_30218	1463	No	NA		0	0	Not-determined	Genome-fragment	NA		0
S13_extracted_30219	1012	No	NA		0	0	Not-determined	Genome-fragment	NA		0
S13_extracted_30353	5700	No	NA		0	5	Not-determined	Genome-fragment	NA		0
S12_extracted_k141_173554	1249	No	NA		1	0	Low-quality	Genome-fragment		3.4	0
S13_extracted_30394	1105	No	NA		0	1	Not-determined	Genome-fragment	NA		0
S6_vs_k141_980||full	1249	No	NA		3	0	Low-quality	Genome-fragment		3.59	0
S13_extracted_30465	1879	No	NA		0	0	Not-determined	Genome-fragment	NA		0
S13_extracted_30474	1529	No	NA		0	2	Not-determined	Genome-fragment	NA		0
S13_extracted_30500	1230	No	NA		0	1	Not-determined	Genome-fragment	NA		0
S13_extracted_30599	1190	No	NA		0	1	Not-determined	Genome-fragment	NA		0
S17_vs_k141_112565||full	1250	No	NA		0	0	Low-quality	Genome-fragment		2.11	0
S13_extracted_31218	10922	No	NA		0	3	Not-determined	Genome-fragment	NA		0
S9_vs_k141_88324||full	1250	No	NA		1	0	Low-quality	Genome-fragment		2	0
S11_vs_k141_51995||lt2gene	1250	No	NA		1	0	Low-quality	Genome-fragment		1.83	0
S13_extracted_31537	1395	No	NA		0	0	Not-determined	Genome-fragment	NA		0
S13_extracted_31848	2339	No	NA		0	1	Not-determined	Genome-fragment	NA		0
S13_extracted_31967	1090	No	NA		0	1	Not-determined	Genome-fragment	NA		0
S13_extracted_32040	2150	No	NA		0	1	Not-determined	Genome-fragment	NA		0
S13_extracted_32063	1624	No	NA		0	0	Not-determined	Genome-fragment	NA		0
S13_extracted_32097	1064	No	NA		0	1	Not-determined	Genome-fragment	NA		0
S13_extracted_32198	1722	No	NA		0	0	Not-determined	Genome-fragment	NA		0
S13_extracted_32207	2109	No	NA		0	0	Not-determined	Genome-fragment	NA		0
S13_extracted_110816	1250	No	NA		0	1	Low-quality	Genome-fragment		1.69	0
S13_extracted_32344	1184	No	NA		0	1	Not-determined	Genome-fragment	NA		0
S14_extracted_k141_109332	1250	No	NA		1	0	Low-quality	Genome-fragment		1.83	0
S13_extracted_32475	1029	No	NA		0	1	Not-determined	Genome-fragment	NA		0
S13_extracted_32523	1553	No	NA		0	0	Not-determined	Genome-fragment	NA		0
S13_extracted_32554	1152	No	NA		0	1	Not-determined	Genome-fragment	NA		0
S13_extracted_32708	3516	No	NA		0	1	Not-determined	Genome-fragment	NA		0
S1_vs_k141_62792||full	1250	No	NA		1	0	Low-quality	Genome-fragment		2.08	0
S13_extracted_32838	1131	No	NA		0	2	Not-determined	Genome-fragment	NA		0
S13_extracted_32847	11215	No	NA		0	7	Not-determined	Genome-fragment	NA		0
S1_vs_k141_155973||full	1250	No	NA		0	0	Low-quality	Genome-fragment		5.24	0
S3_extracted_k141_73121	1250	No	NA		0	0	Low-quality	Genome-fragment		1.32	0
S7_vs_58436||lt2gene	1250	No	NA		1	0	Low-quality	Genome-fragment		1.66	0
S13_extracted_33317	1487	No	NA		0	2	Not-determined	Genome-fragment	NA		0
S13_extracted_33420	1609	No	NA		0	2	Not-determined	Genome-fragment	NA		0
S15_extracted_51952	1250	No	NA		0	0	Low-quality	Genome-fragment		1.5	0
S13_extracted_33794	1144	No	NA		0	1	Not-determined	Genome-fragment	NA		0
S13_extracted_33914	1612	No	NA		0	2	Not-determined	Genome-fragment	NA		0
S13_extracted_34105	1775	No	NA		0	1	Not-determined	Genome-fragment	NA		0
S13_extracted_34215	1077	No	NA		0	1	Not-determined	Genome-fragment	NA		0
S13_extracted_34235	1225	No	NA		0	1	Not-determined	Genome-fragment	NA		0
S13_extracted_34315	2375	No	NA		0	1	Not-determined	Genome-fragment	NA		0
S13_extracted_34514	2057	No	NA		0	1	Not-determined	Genome-fragment	NA		0
S13_extracted_34683	4310	No	NA		0	1	Not-determined	Genome-fragment	NA		0
S13_extracted_34837	1168	No	NA		0	2	Not-determined	Genome-fragment	NA		0
S13_extracted_34912	1126	No	NA		0	0	Not-determined	Genome-fragment	NA		0
S13_extracted_35054	1045	No	NA		0	1	Not-determined	Genome-fragment	NA		0
S13_extracted_35308	1268	No	NA		0	0	Not-determined	Genome-fragment	NA		0
S13_extracted_35315	1821	No	NA		0	0	Not-determined	Genome-fragment	NA		0
S13_extracted_35402	1236	No	NA		0	1	Not-determined	Genome-fragment	NA		0
S13_extracted_35686	3014	No	NA		0	2	Not-determined	Genome-fragment	NA		0
S13_extracted_35800	1386	No	NA		0	0	Not-determined	Genome-fragment	NA		0
S13_extracted_35844	1285	No	NA		0	1	Not-determined	Genome-fragment	NA		0
S13_extracted_36096	10981	No	NA		0	2	Not-determined	Genome-fragment	NA		0
S13_extracted_36156	1113	No	NA		0	1	Not-determined	Genome-fragment	NA		0
S13_extracted_36200	1056	No	NA		0	2	Not-determined	Genome-fragment	NA		0
S13_extracted_36452	4534	No	NA		0	5	Not-determined	Genome-fragment	NA		0
S13_extracted_36508	2731	No	NA		0	1	Not-determined	Genome-fragment	NA		0
S13_extracted_37036	1013	No	NA		0	2	Not-determined	Genome-fragment	NA		0
S9_vs_k141_54104||full	1251	No	NA		0	0	Low-quality	Genome-fragment		2.75	0
S13_extracted_37164	2685	No	NA		0	1	Not-determined	Genome-fragment	NA		0
S13_extracted_37293	1423	No	NA		0	1	Not-determined	Genome-fragment	NA		0
S11_vs_k141_27891||full	1251	No	NA		0	0	Low-quality	Genome-fragment		3.3	0
S13_extracted_37722	1185	No	NA		0	1	Not-determined	Genome-fragment	NA		0
S3_vs_k141_115593||full	1251	No	NA		0	0	Low-quality	Genome-fragment		1.48	0
S13_extracted_38143	1199	No	NA		0	1	Not-determined	Genome-fragment	NA		0
S3_vs_k141_6607||full	1251	No	NA		1	0	Low-quality	Genome-fragment		3.75	0
S5_extracted_k141_76625	1251	No	NA		0	0	Low-quality	Genome-fragment		2.5	0
S7_vs_28158||full	1251	No	NA		0	0	Low-quality	Genome-fragment		0.91	0
S7_vs_80674||full	1251	No	NA		2	0	Low-quality	Genome-fragment		2.84	0
S16_extracted_91732	1251	No	NA		2	0	Low-quality	Genome-fragment		2.85	0
S13_extracted_38708	29135	No	NA		16	1	Medium-quality	Genome-fragment		53.17	0
S13_extracted_38984	1075	No	NA		0	0	Not-determined	Genome-fragment	NA		0
S13_extracted_39069	2066	No	NA		0	1	Not-determined	Genome-fragment	NA		0
S10_extracted_108721	1251	No	NA		0	1	Low-quality	Genome-fragment		0.22	0
S13_extracted_39196	1478	No	NA		0	1	Not-determined	Genome-fragment	NA		0
S8_vs_k141_166224||full	1251	No	NA		0	1	Low-quality	Genome-fragment		1.41	0
S13_extracted_39531	1490	No	NA		0	0	Not-determined	Genome-fragment	NA		0
S13_extracted_39533	1416	No	NA		0	0	Not-determined	Genome-fragment	NA		0
S13_extracted_39622	1153	No	NA		0	1	Not-determined	Genome-fragment	NA		0
S13_extracted_39908	2135	No	NA		0	0	Not-determined	Genome-fragment	NA		0
S8_vs_k141_49063||full	1251	No	NA		0	1	Low-quality	Genome-fragment		0.89	0
S13_extracted_40220	1432	No	NA		0	1	Not-determined	Genome-fragment	NA		0
S17_extracted_k141_174688	1252	No	NA		0	1	Low-quality	Genome-fragment		1.12	0
S13_extracted_40432	1647	No	NA		0	2	Not-determined	Genome-fragment	NA		0
S13_extracted_40443	1132	No	NA		0	0	Not-determined	Genome-fragment	NA		0
S13_extracted_40791	1478	No	NA		0	0	Not-determined	Genome-fragment	NA		0
S13_extracted_41076	1379	No	NA		0	1	Not-determined	Genome-fragment	NA		0
S17_vs_k141_82881||full	1252	No	NA		0	0	Low-quality	Genome-fragment		0.86	0
S9_vs_k141_68296||full	1252	No	NA		1	0	Low-quality	Genome-fragment		3.49	0
S13_extracted_41273	1579	No	NA		0	0	Not-determined	Genome-fragment	NA		0
S13_extracted_41311	1216	No	NA		0	0	Not-determined	Genome-fragment	NA		0
S13_extracted_41404	1084	No	NA		0	1	Not-determined	Genome-fragment	NA		0
S13_extracted_41514	2207	No	NA		0	0	Not-determined	Genome-fragment	NA		0
S13_extracted_41842	3240	No	NA		0	2	Not-determined	Genome-fragment	NA		0
S9_vs_k141_65499||full	1252	No	NA		0	1	Low-quality	Genome-fragment		2.53	0
S13_extracted_41980	4238	No	NA		0	3	Not-determined	Genome-fragment	NA		0
S13_extracted_42250	2041	No	NA		0	1	Not-determined	Genome-fragment	NA		0
S13_extracted_42434	2831	No	NA		0	1	Not-determined	Genome-fragment	NA		0
S13_extracted_42450	1322	No	NA		0	1	Not-determined	Genome-fragment	NA		0
S13_extracted_42655	1037	No	NA		0	1	Not-determined	Genome-fragment	NA		0
S11_vs_k141_89027||full	1252	No	NA		0	0	Low-quality	Genome-fragment		3.65	0
S13_extracted_43171	1480	No	NA		0	1	Not-determined	Genome-fragment	NA		0
S11_vs_k141_49270||full	1252	No	NA		0	2	Low-quality	Genome-fragment		0.56	0
S7_extracted_73218	1252	No	NA		0	0	Low-quality	Genome-fragment		2.6	0
S13_extracted_43858	1405	No	NA		0	1	Not-determined	Genome-fragment	NA		0
S13_extracted_43871	1852	No	NA		0	0	Not-determined	Genome-fragment	NA		0
S13_extracted_43889	3568	No	NA		0	2	Not-determined	Genome-fragment	NA		0
S13_extracted_44034	1428	No	NA		0	1	Not-determined	Genome-fragment	NA		0
S13_extracted_44078	2275	No	NA		0	0	Not-determined	Genome-fragment	NA		0
S7_vs_14671||full	1252	No	NA		1	0	Low-quality	Genome-fragment		3.26	0
S13_extracted_44381	3548	No	NA		0	3	Not-determined	Genome-fragment	NA		0
S13_extracted_44394	3855	No	NA		0	1	Not-determined	Genome-fragment	NA		0
S16_extracted_104425	1252	No	NA		0	0	Low-quality	Genome-fragment		3.28	0
S13_extracted_44585	1065	No	NA		0	0	Not-determined	Genome-fragment	NA		0
S13_extracted_44822	1270	No	NA		0	1	Not-determined	Genome-fragment	NA		0
S13_extracted_44879	1058	No	NA		0	0	Not-determined	Genome-fragment	NA		0
S10_extracted_19554	1252	No	NA		1	0	Low-quality	Genome-fragment		2.53	0
S13_extracted_44944	1776	No	NA		0	2	Not-determined	Genome-fragment	NA		0
S13_extracted_45035	1185	No	NA		0	1	Not-determined	Genome-fragment	NA		0
S13_extracted_45039	1645	No	NA		0	2	Not-determined	Genome-fragment	NA		0
S13_extracted_45087	1595	No	NA		0	2	Not-determined	Genome-fragment	NA		0
S13_extracted_45330	1918	No	NA		0	1	Not-determined	Genome-fragment	NA		0
S13_extracted_45386	1703	No	NA		0	2	Not-determined	Genome-fragment	NA		0
S13_extracted_45424	1399	No	NA		0	1	Not-determined	Genome-fragment	NA		0
S12_extracted_k141_41441	1252	Yes		506	1	1	Low-quality	Genome-fragment		1.55	59.58
S12_extracted_k141_105976	1252	No	NA		1	0	Low-quality	Genome-fragment		2.66	0
S13_extracted_45646	1111	No	NA		0	2	Not-determined	Genome-fragment	NA		0
S8_extracted_k141_35181	1252	No	NA		0	0	Low-quality	Genome-fragment		2.98	0
S13_extracted_45790	1350	No	NA		0	0	Not-determined	Genome-fragment	NA		0
S13_extracted_45811	1723	No	NA		0	3	Not-determined	Genome-fragment	NA		0
S13_extracted_45898	1288	No	NA		0	1	Not-determined	Genome-fragment	NA		0

S13_extracted_45986	1298	No	NA	0	1	Not-determined	Genome-fragment	NA		0
S13_extracted_45999	1032	No	NA	0	1	Not-determined	Genome-fragment	NA		0
S8_extracted_k141_123126	1252	No	NA	1	0	Low-quality	Genome-fragment		0.97	0
S13_extracted_46568	1462	No	NA	0	0	Not-determined	Genome-fragment	NA		0
S13_extracted_46617	1133	No	NA	0	2	Not-determined	Genome-fragment	NA		0
S13_extracted_46767	5986	No	NA	0	4	Not-determined	Genome-fragment	NA		0
S13_extracted_46865	3211	No	NA	0	2	Not-determined	Genome-fragment	NA		0
S13_extracted_46957	1601	No	NA	0	1	Not-determined	Genome-fragment	NA		0
S13_extracted_46985	1457	No	NA	0	0	Not-determined	Genome-fragment	NA		0
S13_extracted_47343	4457	No	NA	0	1	Not-determined	Genome-fragment	NA		0
S9_vs_k141_47816||full	1253	No	NA	0	0	Low-quality	Genome-fragment		1.94	0
S13_extracted_47662	1814	No	NA	0	2	Not-determined	Genome-fragment	NA		0
S13_extracted_47937	2752	No	NA	0	4	Not-determined	Genome-fragment	NA		0
S14_extracted_k141_53246	1253	No	NA	0	1	Low-quality	Genome-fragment		1.81	0
S13_extracted_47994	4534	No	NA	0	2	Not-determined	Genome-fragment	NA		0
S13_extracted_48067	3220	No	NA	0	1	Not-determined	Genome-fragment	NA		0
S13_extracted_48100	1064	No	NA	0	2	Not-determined	Genome-fragment	NA		0
S13_extracted_48122	1468	No	NA	0	1	Not-determined	Genome-fragment	NA		0
S5_extracted_k141_138377	1253	No	NA	2	0	Low-quality	Genome-fragment		3.04	0
S13_extracted_48359	3225	No	NA	0	1	Not-determined	Genome-fragment	NA		0
S13_extracted_48416	1281	No	NA	0	0	Not-determined	Genome-fragment	NA		0
S13_extracted_48447	1035	No	NA	0	0	Not-determined	Genome-fragment	NA		0
S7_vs_60415||full	1253	No	NA	1	0	Low-quality	Genome-fragment		1.93	0
S13_extracted_48635	3757	No	NA	0	2	Not-determined	Genome-fragment	NA		0
S16_vs_16200||full	1253	No	NA	0	0	Low-quality	Genome-fragment		2.66	0
S13_extracted_49501	1212	No	NA	0	1	Not-determined	Genome-fragment	NA		0
S16_vs_58752||lt2gene	1253	No	NA	1	0	Low-quality	Genome-fragment		2.04	0
S10_extracted_94592	1253	No	NA	1	0	Low-quality	Genome-fragment		1.43	0
S13_extracted_50140	1231	No	NA	0	1	Not-determined	Genome-fragment	NA		0
S13_extracted_50510	1074	No	NA	0	0	Not-determined	Genome-fragment	NA		0
S13_extracted_50625	1153	No	NA	0	1	Not-determined	Genome-fragment	NA		0
S12_vs_k141_143702||full	1253	No	NA	0	2	Low-quality	Genome-fragment		0.42	0
S13_extracted_50864	3774	No	NA	0	1	Not-determined	Genome-fragment	NA		0
S13_extracted_50892	1348	No	NA	0	1	Not-determined	Genome-fragment	NA		0
S13_extracted_51115	1580	No	NA	0	1	Not-determined	Genome-fragment	NA		0
S13_extracted_51149	1082	No	NA	0	1	Not-determined	Genome-fragment	NA		0
S13_extracted_51168	1330	No	NA	0	0	Not-determined	Genome-fragment	NA		0
S13_extracted_51365	1825	No	NA	0	0	Not-determined	Genome-fragment	NA		0
S13_extracted_51417	2711	No	NA	0	0	Not-determined	Genome-fragment	NA		0
S13_extracted_51592	1410	No	NA	0	2	Not-determined	Genome-fragment	NA		0
S13_extracted_51902	4871	No	NA	0	4	Not-determined	Genome-fragment	NA		0
S13_extracted_52128	1556	No	NA	0	1	Not-determined	Genome-fragment	NA		0
S13_extracted_52421	3062	No	NA	0	3	Not-determined	Genome-fragment	NA		0
S13_extracted_52450	1376	No	NA	0	0	Not-determined	Genome-fragment	NA		0
S13_extracted_52546	3435	No	NA	0	5	Not-determined	Genome-fragment	NA		0
S13_extracted_52597	1168	No	NA	0	1	Not-determined	Genome-fragment	NA		0
S13_extracted_52694	1031	No	NA	0	0	Not-determined	Genome-fragment	NA		0
S13_extracted_52697	3591	No	NA	0	0	Not-determined	Genome-fragment	NA		0
S13_extracted_52722	1744	No	NA	0	0	Not-determined	Genome-fragment	NA		0
S13_extracted_52798	1071	No	NA	0	1	Not-determined	Genome-fragment	NA		0
S13_extracted_52824	1393	No	NA	0	0	Not-determined	Genome-fragment	NA		0
S13_extracted_53023	1647	No	NA	0	1	Not-determined	Genome-fragment	NA		0
S13_extracted_53177	1403	No	NA	0	1	Not-determined	Genome-fragment	NA		0
S13_extracted_53284	3273	No	NA	0	1	Not-determined	Genome-fragment	NA		0
S13_extracted_53341	9067	No	NA	0	2	Not-determined	Genome-fragment	NA		0
S13_extracted_53383	1222	No	NA	0	2	Not-determined	Genome-fragment	NA		0
S13_extracted_53405	1110	No	NA	0	2	Not-determined	Genome-fragment	NA		0
S13_extracted_53407	3218	No	NA	0	0	Not-determined	Genome-fragment	NA		0
S13_extracted_53527	1020	No	NA	0	2	Not-determined	Genome-fragment	NA		0
S13_extracted_53633	5163	No	NA	0	2	Not-determined	Genome-fragment	NA		0
S2_extracted_k141_14500	1253	No	NA	0	0	Low-quality	Genome-fragment		0.59	0
S13_extracted_53649	1052	No	NA	0	0	Not-determined	Genome-fragment	NA		0
S13_extracted_53729	1082	No	NA	0	0	Not-determined	Genome-fragment	NA		0
S13_extracted_53842	2803	No	NA	0	2	Not-determined	Genome-fragment	NA		0
S13_extracted_53926	1792	No	NA	0	2	Not-determined	Genome-fragment	NA		0
S13_extracted_53959	2407	No	NA	0	0	Not-determined	Genome-fragment	NA		0
S13_extracted_54201	37389	No	NA	13	1	Medium-quality	Genome-fragment		64.56	0
S13_extracted_54371	1318	No	NA	0	1	Not-determined	Genome-fragment	NA		0
S8_vs_k141_159407||full	1253	No	NA	0	0	Low-quality	Genome-fragment		1.08	0
S13_extracted_54541	3266	No	NA	0	2	Not-determined	Genome-fragment	NA		0
S13_extracted_54653	1847	No	NA	0	4	Not-determined	Genome-fragment	NA		0
S8_vs_k141_188622||full	1253	No	NA	0	1	Low-quality	Genome-fragment		2.12	0
S13_extracted_54882	2652	No	NA	0	3	Not-determined	Genome-fragment	NA		0
S13_extracted_55148	1299	No	NA	0	1	Not-determined	Genome-fragment	NA		0
S13_extracted_55592	1564	No	NA	0	0	Not-determined	Genome-fragment	NA		0
S13_extracted_55632	1152	No	NA	0	0	Not-determined	Genome-fragment	NA		0
S13_extracted_55637	1275	No	NA	0	1	Not-determined	Genome-fragment	NA		0
S13_extracted_55653	5110	No	NA	0	3	Not-determined	Genome-fragment	NA		0
S13_extracted_55711	3024	No	NA	0	0	Not-determined	Genome-fragment	NA		0
S13_extracted_55802	3058	No	NA	0	3	Not-determined	Genome-fragment	NA		0
S13_extracted_55962	1192	No	NA	0	2	Not-determined	Genome-fragment	NA		0
S13_extracted_56074	77817	No	NA	16	0	Complete	High-quality		100	0
S13_extracted_56100	1722	No	NA	0	1	Not-determined	Genome-fragment	NA		0
S13_extracted_56229	1036	No	NA	0	1	Not-determined	Genome-fragment	NA		0
S13_extracted_56790	1399	No	NA	0	0	Not-determined	Genome-fragment	NA		0
S13_extracted_56873	4377	No	NA	0	1	Not-determined	Genome-fragment	NA		0
S13_vs_138714||full	1254	No	NA	0	1	Low-quality	Genome-fragment		2.97	0
S13_extracted_57051	1588	No	NA	0	1	Not-determined	Genome-fragment	NA		0
S13_extracted_57234	1107	No	NA	0	0	Not-determined	Genome-fragment	NA		0
S13_extracted_57276	1277	No	NA	0	0	Not-determined	Genome-fragment	NA		0
S13_extracted_57330	1169	No	NA	0	1	Not-determined	Genome-fragment	NA		0
S13_extracted_57766	3631	No	NA	0	3	Not-determined	Genome-fragment	NA		0
S13_extracted_57881	1753	No	NA	0	1	Not-determined	Genome-fragment	NA		0
S13_extracted_57910	1509	No	NA	0	0	Not-determined	Genome-fragment	NA		0
S13_extracted_57965	1348	No	NA	0	1	Not-determined	Genome-fragment	NA		0
S14_extracted_k141_88861	1254	No	NA	0	0	Low-quality	Genome-fragment		1.62	0
S1_extracted_k141_89491	1254	No	NA	0	0	Low-quality	Genome-fragment		1.25	0
S1_extracted_k141_136946	1254	No	NA	0	1	Low-quality	Genome-fragment		0.69	0
S13_extracted_58207	1612	No	NA	0	2	Not-determined	Genome-fragment	NA		0
S13_extracted_58274	1173	No	NA	0	1	Not-determined	Genome-fragment	NA		0
S13_extracted_58303	1202	No	NA	0	1	Not-determined	Genome-fragment	NA		0
S13_extracted_58351	1578	No	NA	0	1	Not-determined	Genome-fragment	NA		0
S13_extracted_58449	1562	No	NA	0	2	Not-determined	Genome-fragment	NA		0
S13_extracted_58466	1417	No	NA	0	1	Not-determined	Genome-fragment	NA		0
S13_extracted_58581	1502	No	NA	0	1	Not-determined	Genome-fragment	NA		0
S3_extracted_k141_86816	1254	No	NA	1	0	Low-quality	Genome-fragment		0.27	0
S13_extracted_58862	3312	No	NA	0	2	Not-determined	Genome-fragment	NA		0
S13_extracted_58878	1097	No	NA	0	1	Not-determined	Genome-fragment	NA		0
S13_extracted_58986	1614	No	NA	0	2	Not-determined	Genome-fragment	NA		0
S13_extracted_59075	1223	No	NA	0	0	Not-determined	Genome-fragment	NA		0
S5_extracted_k141_64679	1254	No	NA	2	0	Low-quality	Genome-fragment		3.53	0
S13_extracted_59450	1292	No	NA	0	0	Not-determined	Genome-fragment	NA		0
S13_extracted_59570	1010	No	NA	0	1	Not-determined	Genome-fragment	NA		0
S5_vs_k141_94871||full	1254	No	NA	1	0	Low-quality	Genome-fragment		3.24	0
S13_extracted_59894	3378	No	NA	0	1	Not-determined	Genome-fragment	NA		0
S13_extracted_59944	1131	No	NA	0	1	Not-determined	Genome-fragment	NA		0
S13_extracted_60194	3130	No	NA	0	1	Not-determined	Genome-fragment	NA		0
S13_extracted_60250	1402	No	NA	0	0	Not-determined	Genome-fragment	NA		0
S13_extracted_60487	5241	No	NA	0	4	Not-determined	Genome-fragment	NA		0
S13_extracted_60600	3724	No	NA	0	3	Not-determined	Genome-fragment	NA		0
S13_extracted_60925	5318	No	NA	0	5	Not-determined	Genome-fragment	NA		0
S5_vs_k141_102115||full	1254	No	NA	1	0	Low-quality	Genome-fragment		2.21	0
S13_extracted_61109	1604	No	NA	0	2	Not-determined	Genome-fragment	NA		0
S13_extracted_61328	1046	No	NA	0	1	Not-determined	Genome-fragment	NA		0
S13_extracted_61378	1693	No	NA	0	2	Not-determined	Genome-fragment	NA		0
S7_extracted_80519	1254	No	NA	2	0	Low-quality	Genome-fragment		0.48	0
S13_extracted_61475	1511	No	NA	0	1	Not-determined	Genome-fragment	NA		0
S13_extracted_61504	1324	No	NA	0	1	Not-determined	Genome-fragment	NA		0
S13_extracted_61518	1447	No	NA	0	0	Not-determined	Genome-fragment	NA		0
S13_extracted_61613	1263	No	NA	0	1	Not-determined	Genome-fragment	NA		0
S16_extracted_123255	1254	No	NA	0	0	Low-quality	Genome-fragment		2.16	0
S13_extracted_61719	1943	No	NA	0	2	Not-determined	Genome-fragment	NA		0
S13_extracted_61886	1473	No	NA	0	0	Not-determined	Genome-fragment	NA		0
S13_extracted_61904	1026	No	NA	0	1	Not-determined	Genome-fragment	NA		0
S13_extracted_62001	1678	No	NA	0	1	Not-determined	Genome-fragment	NA		0
S13_extracted_62006	1256	No	NA	0	1	Not-determined	Genome-fragment	NA		0
S13_extracted_62048	1338	No	NA	0	1	Not-determined	Genome-fragment	NA		0
S13_extracted_62189	1311	No	NA	0	0	Not-determined	Genome-fragment	NA		0
S13_extracted_62262	1201	No	NA	0	0	Not-determined	Genome-fragment	NA		0
S13_extracted_62321	1693	No	NA	0	1	Not-determined	Genome-fragment	NA		0
S13_extracted_62411	1858	No	NA	0	1	Not-determined	Genome-fragment	NA		0
S13_extracted_62432	3547	No	NA	0	0	Not-determined	Genome-fragment	NA		0
S16_extracted_162747	1254	No	NA	1	0	Low-quality	Genome-fragment		2.31	0
S10_vs_71029||full	1254	No	NA	0	2	Low-quality	Genome-fragment		0.75	0
S13_extracted_62500	1042	No	NA	0	0	Not-determined	Genome-fragment	NA		0
S13_extracted_62746	1392	No	NA	0	0	Not-determined	Genome-fragment	NA		0
S13_extracted_62752	1223	No	NA	0	1	Not-determined	Genome-fragment	NA		0
S13_extracted_62783	1641	No	NA	0	1	Not-determined	Genome-fragment	NA		0
S13_extracted_62858	1997	No	NA	0	2	Not-determined	Genome-fragment	NA		0
S13_extracted_62907	2312	No	NA	0	1	Not-determined	Genome-fragment	NA		0
S13_extracted_63300	1243	No	NA	0	1	Not-determined	Genome-fragment	NA		0
S13_extracted_63395	4027	No	NA	0	2	Not-determined	Genome-fragment	NA		0
S13_extracted_63587	1837	No	NA	0	0	Not-determined	Genome-fragment	NA		0
S13_extracted_63723	1271	No	NA	0	0	Not-determined	Genome-fragment	NA		0
S12_extracted_k141_138942	1254	No	NA	0	1	Low-quality	Genome-fragment		0.68	0
S13_extracted_64182	1292	No	NA	0	1	Not-determined	Genome-fragment	NA		0
S13_extracted_64298	3333	No	NA	0	3	Not-determined	Genome-fragment	NA		0
S13_extracted_64323	1255	No	NA	0	0	Not-determined	Genome-fragment	NA		0
S13_extracted_64360	1255	No	NA	0	1	Not-determined	Genome-fragment	NA		0
S12_extracted_k141_85877	1254	No	NA	0	0	Low-quality	Genome-fragment		2.96	0
S13_extracted_64596	1954	No	NA	0	1	Not-determined	Genome-fragment	NA		0
S13_extracted_64664	2467	No	NA	0	1	Not-determined	Genome-fragment	NA		0
S13_extracted_64872	1211	No	NA	0	1	Not-determined	Genome-fragment	NA		0
S13_extracted_64877	1119	No	NA	0	1	Not-determined	Genome-fragment	NA		0
S13_extracted_65108	1320	No	NA	0	0	Not-determined	Genome-fragment	NA		0
S13_extracted_65151	1415	No	NA	0	1	Not-determined	Genome-fragment	NA		0
S13_extracted_65300	1325	No	NA	0	0	Not-determined	Genome-fragment	NA		0
S13_extracted_65333	1322	No	NA	0	1	Not-determined	Genome-fragment	NA		0
S13_extracted_65400	1338	No	NA	0	0	Not-determined	Genome-fragment	NA		0
S13_extracted_65465	1202	No	NA	0	1	Not-determined	Genome-fragment	NA		0
S12_vs_k141_67797||full	1254	No	NA	0	1	Low-quality	Genome-fragment		2.82	0
S6_extracted_k141_3017	1254	No	NA	2	0	Low-quality	Genome-fragment		2.39	0
S9_extracted_k141_1383	1255	No	NA	0	1	Low-quality	Genome-fragment		0.58	0
S13_extracted_66039	2070	No	NA	0	1	Not-determined	Genome-fragment	NA		0

S13_extracted_66170	1172	No	NA		0	0	Not-determined	Genome-fragment	NA		0
S13_vs_38192||full	1255	No	NA		0	0	Low-quality	Genome-fragment		2.22	0
S13_extracted_66348	3470	No	NA		0	2	Not-determined	Genome-fragment	NA		0
S13_extracted_66365	7357	No	NA		0	3	Not-determined	Genome-fragment	NA		0
S14_extracted_k141_83046	1255	No	NA		2	0	Low-quality	Genome-fragment		2.85	0
S13_extracted_66508	38005	No	NA		5	0	High-quality	High-quality		100	0
S13_extracted_66738	1091	No	NA		0	2	Not-determined	Genome-fragment	NA		0
S13_extracted_66791	1410	No	NA		0	0	Not-determined	Genome-fragment	NA		0
S13_extracted_66808	3321	No	NA		0	1	Not-determined	Genome-fragment	NA		0
S13_extracted_67092	3525	No	NA		0	0	Not-determined	Genome-fragment	NA		0
S13_extracted_67095	1044	No	NA		0	1	Not-determined	Genome-fragment	NA		0
S13_extracted_67785	1490	No	NA		0	1	Not-determined	Genome-fragment	NA		0
S13_extracted_68135	1244	No	NA		0	1	Not-determined	Genome-fragment	NA		0
S13_extracted_68234	1054	No	NA		0	0	Not-determined	Genome-fragment	NA		0
S1_extracted_k141_76531	1255	No	NA		0	1	Low-quality	Genome-fragment		0.34	0
S5_extracted_k141_130708	1255	No	NA		1	0	Low-quality	Genome-fragment		2.71	0
S13_extracted_68715	1846	No	NA		0	0	Not-determined	Genome-fragment	NA		0
S13_extracted_69063	3885	No	NA		0	1	Not-determined	Genome-fragment	NA		0
S13_extracted_69155	1748	No	NA		0	0	Not-determined	Genome-fragment	NA		0
S13_extracted_69250	6052	No	NA		0	1	Not-determined	Genome-fragment	NA		0
S5_vs_k141_10744||full	1255	No	NA		0	0	Low-quality	Genome-fragment		3.22	0
S13_extracted_69467	3270	No	NA		0	4	Not-determined	Genome-fragment	NA		0
S13_extracted_69784	1145	No	NA		0	1	Not-determined	Genome-fragment	NA		0
S13_extracted_69789	1020	No	NA		0	0	Not-determined	Genome-fragment	NA		0
S13_extracted_69796	1153	No	NA		0	0	Not-determined	Genome-fragment	NA		0
S13_extracted_69822	2053	No	NA		0	0	Not-determined	Genome-fragment	NA		0
S13_extracted_69886	1795	No	NA		0	0	Not-determined	Genome-fragment	NA		0
S7_vs_86323||full	1255	No	NA		1	0	Low-quality	Genome-fragment		3.03	0
S13_extracted_69978	1971	No	NA		0	2	Not-determined	Genome-fragment	NA		0
S13_extracted_70081	1005	No	NA		0	0	Not-determined	Genome-fragment	NA		0
S13_extracted_70190	1329	No	NA		0	2	Not-determined	Genome-fragment	NA		0
S13_extracted_70223	1837	No	NA		0	1	Not-determined	Genome-fragment	NA		0
S13_extracted_70237	1080	No	NA		0	0	Not-determined	Genome-fragment	NA		0
S13_extracted_70293	1063	No	NA		0	0	Not-determined	Genome-fragment	NA		0
S13_extracted_70399	1690	No	NA		0	0	Not-determined	Genome-fragment	NA		0
S13_extracted_70486	1177	No	NA		0	3	Not-determined	Genome-fragment	NA		0
S16_vs_153164||lt2gene	1255	No	NA		2	0	Low-quality	Genome-fragment		2.86	0
S13_extracted_70630	1091	No	NA		0	0	Not-determined	Genome-fragment	NA		0
S13_extracted_70825	1334	No	NA		0	1	Not-determined	Genome-fragment	NA		0
S12_vs_k141_119282||full	1255	Yes		980	1	1	Low-quality	Genome-fragment		2.15	21.91
S13_extracted_71043	2094	No	NA		0	0	Not-determined	Genome-fragment	NA		0
S13_extracted_71066	1359	No	NA		0	1	Not-determined	Genome-fragment	NA		0
S13_extracted_71130	1049	No	NA		0	1	Not-determined	Genome-fragment	NA		0
S13_extracted_71544	1666	No	NA		0	1	Not-determined	Genome-fragment	NA		0
S13_extracted_71753	1440	No	NA		0	0	Not-determined	Genome-fragment	NA		0
S13_extracted_71802	3467	No	NA		0	3	Not-determined	Genome-fragment	NA		0
S13_extracted_71960	1603	No	NA		0	0	Not-determined	Genome-fragment	NA		0
S13_extracted_71990	5240	No	NA		0	4	Not-determined	Genome-fragment	NA		0
S8_vs_k141_73332||full	1255	No	NA		0	1	Low-quality	Genome-fragment		3.13	0
S13_extracted_72193	3930	No	NA		0	7	Not-determined	Genome-fragment	NA		0
S13_extracted_72233	1865	No	NA		0	0	Not-determined	Genome-fragment	NA		0
S13_extracted_72390	1347	No	NA		0	4	Not-determined	Genome-fragment	NA		0
S13_extracted_72772	1153	No	NA		0	1	Not-determined	Genome-fragment	NA		0
S13_extracted_72858	1372	No	NA		0	2	Not-determined	Genome-fragment	NA		0
S13_extracted_72894	1689	No	NA		0	2	Not-determined	Genome-fragment	NA		0
S13_extracted_73357	1647	No	NA		0	0	Not-determined	Genome-fragment	NA		0
S17_extracted_k141_98381	1256	No	NA		0	0	Low-quality	Genome-fragment		0.77	0
S13_extracted_73777	1107	No	NA		0	0	Not-determined	Genome-fragment	NA		0
S13_extracted_73779	2479	No	NA		0	1	Not-determined	Genome-fragment	NA		0
S13_extracted_73788	1321	No	NA		0	0	Not-determined	Genome-fragment	NA		0
S13_extracted_73971	5982	No	NA		0	2	Not-determined	Genome-fragment	NA		0
S13_extracted_73973	1926	No	NA		0	0	Not-determined	Genome-fragment	NA		0
S14_extracted_k141_67265	1256	No	NA		0	0	Low-quality	Genome-fragment		1.53	0
S13_extracted_74648	1302	No	NA		0	0	Not-determined	Genome-fragment	NA		0
S13_extracted_74771	1002	No	NA		0	0	Not-determined	Genome-fragment	NA		0
S13_extracted_75153	1262	No	NA		0	2	Not-determined	Genome-fragment	NA		0
S13_extracted_75230	2133	No	NA		0	2	Not-determined	Genome-fragment	NA		0
S13_extracted_75400	1099	No	NA		0	1	Not-determined	Genome-fragment	NA		0
S13_extracted_75499	1021	No	NA		0	0	Not-determined	Genome-fragment	NA		0
S13_extracted_75593	3184	No	NA		0	0	Not-determined	Genome-fragment	NA		0
S13_extracted_75606	1172	No	NA		0	1	Not-determined	Genome-fragment	NA		0
S13_extracted_75610	1009	No	NA		0	0	Not-determined	Genome-fragment	NA		0
S13_extracted_75783	3144	No	NA		0	1	Not-determined	Genome-fragment	NA		0
S13_extracted_76319	1133	No	NA		0	1	Not-determined	Genome-fragment	NA		0
S13_extracted_76364	1536	No	NA		0	0	Not-determined	Genome-fragment	NA		0
S13_extracted_76418	1782	No	NA		0	2	Not-determined	Genome-fragment	NA		0
S13_extracted_76436	1002	No	NA		0	1	Not-determined	Genome-fragment	NA		0
S13_extracted_76684	2420	No	NA		0	1	Not-determined	Genome-fragment	NA		0
S13_extracted_77042	1567	No	NA		0	0	Not-determined	Genome-fragment	NA		0
S14_extracted_k141_57446	1256	No	NA		1	0	Low-quality	Genome-fragment		0.71	0
S14_vs_k141_15907||full	1256	No	NA		1	0	Low-quality	Genome-fragment		2.08	0
S13_extracted_77819	4150	No	NA		0	1	Not-determined	Genome-fragment	NA		0
S13_extracted_77923	1396	No	NA		0	0	Not-determined	Genome-fragment	NA		0
S13_extracted_77947	1612	No	NA		0	1	Not-determined	Genome-fragment	NA		0
S13_extracted_78009	4094	No	NA		0	3	Not-determined	Genome-fragment	NA		0
S13_extracted_78277	1030	No	NA		0	2	Not-determined	Genome-fragment	NA		0
S14_vs_k141_70375||full	1256	No	NA		1	0	Low-quality	Genome-fragment		2.8	0
S13_extracted_78386	8729	No	NA		0	7	Not-determined	Genome-fragment	NA		0
S13_extracted_78436	1079	No	NA		0	1	Not-determined	Genome-fragment	NA		0
S13_extracted_78675	2265	No	NA		0	2	Not-determined	Genome-fragment	NA		0
S13_extracted_79092	1368	No	NA		0	1	Not-determined	Genome-fragment	NA		0
S13_extracted_79107	2094	No	NA		0	3	Not-determined	Genome-fragment	NA		0
S13_extracted_79215	1290	No	NA		0	1	Not-determined	Genome-fragment	NA		0
S1_extracted_k141_32196	1256	No	NA		0	0	Low-quality	Genome-fragment		0.93	0
S13_extracted_79450	14684	No	NA		0	10	Not-determined	Genome-fragment	NA		0
S13_extracted_79483	1133	No	NA		0	0	Not-determined	Genome-fragment	NA		0
S1_vs_k141_95489||full	1256	No	NA		0	1	Low-quality	Genome-fragment		0.39	0
S13_extracted_79527	2353	No	NA		0	0	Not-determined	Genome-fragment	NA		0
S13_extracted_79555	1188	No	NA		0	0	Not-determined	Genome-fragment	NA		0
S13_extracted_79633	1108	No	NA		0	0	Not-determined	Genome-fragment	NA		0
S13_extracted_79689	3765	No	NA		0	1	Not-determined	Genome-fragment	NA		0
S13_extracted_79713	1118	No	NA		0	1	Not-determined	Genome-fragment	NA		0
S3_vs_k141_111667||full	1256	No	NA		2	0	Low-quality	Genome-fragment		2.87	0
S13_extracted_79898	1227	No	NA		0	1	Not-determined	Genome-fragment	NA		0
S13_extracted_80065	1370	No	NA		0	1	Not-determined	Genome-fragment	NA		0
S13_extracted_80238	3478	No	NA		0	0	Not-determined	Genome-fragment	NA		0
S5_vs_k141_75054||full	1256	No	NA		0	0	Low-quality	Genome-fragment		2.91	0
S13_extracted_80378	1127	No	NA		0	0	Not-determined	Genome-fragment	NA		0
S13_extracted_80626	2278	No	NA		0	0	Not-determined	Genome-fragment	NA		0
S13_extracted_80794	9918	No	NA		0	3	Not-determined	Genome-fragment	NA		0
S13_extracted_80933	1742	No	NA		0	0	Not-determined	Genome-fragment	NA		0
S13_extracted_81124	1155	No	NA		0	0	Not-determined	Genome-fragment	NA		0
S13_extracted_81283	1831	No	NA		0	1	Not-determined	Genome-fragment	NA		0
S13_extracted_81406	4038	No	NA		0	1	Not-determined	Genome-fragment	NA		0
S13_extracted_81471	1257	No	NA		0	1	Not-determined	Genome-fragment	NA		0
S13_extracted_81557	1176	No	NA		0	1	Not-determined	Genome-fragment	NA		0
S13_extracted_81658	1117	No	NA		0	0	Not-determined	Genome-fragment	NA		0
S7_vs_102374||full	1256	No	NA		0	0	Low-quality	Genome-fragment		2.12	0
S13_extracted_81703	1008	No	NA		0	1	Not-determined	Genome-fragment	NA		0
S13_extracted_81707	1327	No	NA		0	1	Not-determined	Genome-fragment	NA		0
S13_extracted_81765	2046	No	NA		0	0	Not-determined	Genome-fragment	NA		0
S13_extracted_81891	2797	No	NA		0	1	Not-determined	Genome-fragment	NA		0
S16_extracted_125527	1256	No	NA		0	0	Low-quality	Genome-fragment		1.07	0
S13_extracted_82089	1443	No	NA		0	2	Not-determined	Genome-fragment	NA		0
S13_extracted_82254	1147	No	NA		0	0	Not-determined	Genome-fragment	NA		0
S13_extracted_82326	1387	No	NA		0	0	Not-determined	Genome-fragment	NA		0
S13_extracted_82348	1417	No	NA		0	1	Not-determined	Genome-fragment	NA		0
S13_extracted_82355	1202	No	NA		0	1	Not-determined	Genome-fragment	NA		0
S13_extracted_82368	1117	No	NA		0	1	Not-determined	Genome-fragment	NA		0
S16_vs_10984||full	1256	No	NA		0	1	Low-quality	Genome-fragment		0.47	0
S13_extracted_82762	1795	No	NA		0	1	Not-determined	Genome-fragment	NA		0
S13_extracted_83038	1140	No	NA		0	0	Not-determined	Genome-fragment	NA		0
S13_extracted_83303	1066	No	NA		0	0	Not-determined	Genome-fragment	NA		0
S13_extracted_83540	1248	No	NA		0	1	Not-determined	Genome-fragment	NA		0
S13_extracted_83747	1231	No	NA		0	0	Not-determined	Genome-fragment	NA		0
S13_extracted_83803	1064	No	NA		0	0	Not-determined	Genome-fragment	NA		0
S13_extracted_83808	1687	No	NA		0	0	Not-determined	Genome-fragment	NA		0
S13_extracted_83847	1535	No	NA		0	0	Not-determined	Genome-fragment	NA		0
S13_extracted_83872	1164	No	NA		0	1	Not-determined	Genome-fragment	NA		0
S13_extracted_83932	1131	No	NA		0	0	Not-determined	Genome-fragment	NA		0
S13_extracted_83959	1244	No	NA		0	1	Not-determined	Genome-fragment	NA		0
S16_vs_55970||full	1256	No	NA		0	0	Low-quality	Genome-fragment		0.9	0
S13_extracted_84102	1378	No	NA		0	0	Not-determined	Genome-fragment	NA		0
S16_vs_156640||lt2gene	1256	No	NA		1	0	Low-quality	Genome-fragment		3.65	0
S13_extracted_84223	6089	No	NA		0	2	Not-determined	Genome-fragment	NA		0
S13_extracted_84372	4350	No	NA		0	1	Not-determined	Genome-fragment	NA		0
S13_extracted_84409	1085	No	NA		0	1	Not-determined	Genome-fragment	NA		0
S13_extracted_84419	1020	No	NA		0	1	Not-determined	Genome-fragment	NA		0
S12_extracted_k141_44254	1256	No	NA		0	0	Low-quality	Genome-fragment		1.03	0
S13_extracted_84544	1032	No	NA		0	0	Not-determined	Genome-fragment	NA		0
S13_extracted_84688	1075	No	NA		0	0	Not-determined	Genome-fragment	NA		0
S13_extracted_84721	1650	No	NA		0	2	Not-determined	Genome-fragment	NA		0
S13_extracted_84829	1003	No	NA		0	0	Not-determined	Genome-fragment	NA		0
S12_vs_k141_38565||full	1256	No	NA		0	0	Low-quality	Genome-fragment		2.24	0
S13_extracted_84866	1501	No	NA		0	0	Not-determined	Genome-fragment	NA		0
S8_extracted_k141_98228	1256	No	NA		0	0	Low-quality	Genome-fragment		0.98	0
S13_extracted_85171	1032	No	NA		0	0	Not-determined	Genome-fragment	NA		0
S13_extracted_85281	1713	No	NA		0	1	Not-determined	Genome-fragment	NA		0
S13_extracted_85475	1448	No	NA		0	1	Not-determined	Genome-fragment	NA		0
S13_extracted_85525	2055	No	NA		0	3	Not-determined	Genome-fragment	NA		0
S13_extracted_85624	1352	No	NA		0	0	Not-determined	Genome-fragment	NA		0
S13_extracted_85626	1082	No	NA		0	0	Not-determined	Genome-fragment	NA		0
S13_extracted_85946	1924	No	NA		0	0	Not-determined	Genome-fragment	NA		0
S13_extracted_86064	1012	No	NA		0	0	Not-determined	Genome-fragment	NA		0
S13_extracted_86088	1092	No	NA		0	1	Not-determined	Genome-fragment	NA		0
S13_extracted_86178	3244	No	NA		0	0	Not-determined	Genome-fragment	NA		0
S13_extracted_86475	1452	No	NA		0	1	Not-determined	Genome-fragment	NA		0
S8_extracted_k141_31011	1256	No	NA		2	0	Low-quality	Genome-fragment		2.16	0
S13_extracted_86916	1102	No	NA		0	1	Not-determined	Genome-fragment	NA		0
S13_extracted_86955	1408	No	NA		0	0	Not-determined	Genome-fragment	NA		0
S13_extracted_87022	1131	No	NA		0	2	Not-determined	Genome-fragment	NA		0
S13_extracted_87437	1079	No	NA		0	1	Not-determined	Genome-fragment	NA		0
S8_vs_k141_47687||full	1256	No	NA		0	1	Low-quality	Genome-fragment		1.71	0
S8_vs_k141_7155||lt2gene	1256	No	NA		1	0	Low-quality	Genome-fragment		2.75	0

S13_extracted_87601	1735	No	NA	0	1	Not-determined	Genome-fragment	NA		0
S13_extracted_87873	38348	No	NA	10	1	Medium-quality	Genome-fragment		84.63	0
S13_extracted_87952	1120	No	NA	0	1	Not-determined	Genome-fragment	NA		0
S9_vs_k141_4192||full	1257	No	NA	2	0	Low-quality	Genome-fragment		2.97	0
S13_extracted_88021	1249	No	NA	0	1	Not-determined	Genome-fragment	NA		0
S14_vs_k141_73757||full	1257	No	NA	1	0	Low-quality	Genome-fragment		2.17	0
S13_extracted_88235	1015	No	NA	0	0	Not-determined	Genome-fragment	NA		0
S14_vs_k141_95921||full	1257	No	NA	0	0	Low-quality	Genome-fragment		1.18	0
S13_extracted_88479	1582	No	NA	0	1	Not-determined	Genome-fragment	NA		0
S13_extracted_88871	2213	No	NA	0	0	Not-determined	Genome-fragment	NA		0
S1_vs_k141_98270||full	1257	No	NA	1	0	Low-quality	Genome-fragment		2.91	0
S13_extracted_89182	1633	No	NA	0	0	Not-determined	Genome-fragment	NA		0
S13_extracted_89247	3819	No	NA	0	1	Not-determined	Genome-fragment	NA		0
S13_extracted_89262	4236	No	NA	0	1	Not-determined	Genome-fragment	NA		0
S13_extracted_89409	2277	No	NA	0	1	Not-determined	Genome-fragment	NA		0
S13_extracted_89412	1133	No	NA	0	1	Not-determined	Genome-fragment	NA		0
S13_extracted_89432	3569	No	NA	0	3	Not-determined	Genome-fragment	NA		0
S13_extracted_89476	1034	No	NA	0	0	Not-determined	Genome-fragment	NA		0
S5_vs_k141_71156||full	1257	No	NA	0	0	Low-quality	Genome-fragment		2.64	0
S5_vs_k141_102939||full	1257	No	NA	0	0	Low-quality	Genome-fragment		1.53	0
S13_extracted_90239	1502	No	NA	0	2	Not-determined	Genome-fragment	NA		0
S5_vs_k141_50056||lt2gene	1257	No	NA	1	0	Low-quality	Genome-fragment		2.83	0
S13_extracted_90364	2349	No	NA	0	2	Not-determined	Genome-fragment	NA		0
S13_extracted_90794	1062	No	NA	0	1	Not-determined	Genome-fragment	NA		0
S13_extracted_90904	4356	No	NA	0	1	Not-determined	Genome-fragment	NA		0
S13_extracted_91133	1037	No	NA	0	2	Not-determined	Genome-fragment	NA		0
S13_extracted_91189	13057	No	NA	4	0	High-quality	High-quality		95.48	0
S13_extracted_91229	1327	No	NA	0	1	Not-determined	Genome-fragment	NA		0
S13_extracted_91252	1014	No	NA	0	1	Not-determined	Genome-fragment	NA		0
S13_extracted_91576	1175	No	NA	0	1	Not-determined	Genome-fragment	NA		0
S13_extracted_91625	1351	No	NA	0	1	Not-determined	Genome-fragment	NA		0
S13_extracted_91653	3776	No	NA	0	2	Not-determined	Genome-fragment	NA		0
S13_extracted_91710	1089	No	NA	0	1	Not-determined	Genome-fragment	NA		0
S13_extracted_91789	1044	No	NA	0	1	Not-determined	Genome-fragment	NA		0
S7_extracted_40879	1257	No	NA	1	0	Low-quality	Genome-fragment		0.87	0
S7_vs_10926||full	1257	No	NA	0	0	Low-quality	Genome-fragment		3.25	0
S13_extracted_91939	3015	No	NA	0	2	Not-determined	Genome-fragment	NA		0
S10_extracted_20110	1257	No	NA	0	0	Low-quality	Genome-fragment		2.58	0
S13_extracted_91986	1381	No	NA	0	1	Not-determined	Genome-fragment	NA		0
S13_extracted_92008	1292	No	NA	0	0	Not-determined	Genome-fragment	NA		0
S13_extracted_92143	8774	No	NA	0	2	Not-determined	Genome-fragment	NA		0
S13_extracted_92159	1194	No	NA	0	0	Not-determined	Genome-fragment	NA		0
S10_extracted_54661	1257	No	NA	0	1	Low-quality	Genome-fragment		0.44	0
S13_extracted_92401	1993	No	NA	0	1	Not-determined	Genome-fragment	NA		0
S13_extracted_92448	1371	No	NA	0	2	Not-determined	Genome-fragment	NA		0
S13_extracted_92452	1415	No	NA	0	1	Not-determined	Genome-fragment	NA		0
S13_extracted_92525	5294	No	NA	0	0	Not-determined	Genome-fragment	NA		0
S13_extracted_92555	1098	No	NA	0	1	Not-determined	Genome-fragment	NA		0
S12_extracted_k141_168808	1257	No	NA	1	0	Low-quality	Genome-fragment		3.23	0
S13_extracted_92813	1223	No	NA	0	0	Not-determined	Genome-fragment	NA		0
S13_extracted_92924	1347	No	NA	0	1	Not-determined	Genome-fragment	NA		0
S13_extracted_93038	1246	No	NA	0	0	Not-determined	Genome-fragment	NA		0
S13_extracted_93060	1735	No	NA	0	6	Not-determined	Genome-fragment	NA		0
S13_extracted_93220	1029	No	NA	0	0	Not-determined	Genome-fragment	NA		0
S12_vs_k141_166606||full	1257	No	NA	1	0	Low-quality	Genome-fragment		2.43	0
S13_extracted_93329	1082	No	NA	0	0	Not-determined	Genome-fragment	NA		0
S12_vs_k141_82904||lt2gene	1257	No	NA	1	0	Low-quality	Genome-fragment		2.09	0
S15_vs_57503||full	1257	No	NA	3	0	Low-quality	Genome-fragment		3.15	0
S13_extracted_93976	1453	No	NA	0	1	Not-determined	Genome-fragment	NA		0
S13_extracted_94041	2304	No	NA	0	0	Not-determined	Genome-fragment	NA		0
S13_extracted_94050	1054	No	NA	0	1	Not-determined	Genome-fragment	NA		0
S6_vs_k141_35194||full	1257	No	NA	0	0	Low-quality	Genome-fragment		1.76	0
S13_extracted_94221	1640	No	NA	0	1	Not-determined	Genome-fragment	NA		0
S13_extracted_94349	1168	No	NA	0	0	Not-determined	Genome-fragment	NA		0
S13_extracted_94403	2386	No	NA	0	1	Not-determined	Genome-fragment	NA		0
S8_vs_k141_177881||full	1257	No	NA	0	1	Low-quality	Genome-fragment		0.92	0
S13_extracted_94529	1028	No	NA	0	1	Not-determined	Genome-fragment	NA		0
S13_extracted_94588	1123	No	NA	0	0	Not-determined	Genome-fragment	NA		0
S13_extracted_94618	5304	No	NA	0	3	Not-determined	Genome-fragment	NA		0
S13_extracted_94854	1439	No	NA	0	3	Not-determined	Genome-fragment	NA		0
S13_extracted_94945	7259	No	NA	0	1	Not-determined	Genome-fragment	NA		0
S8_vs_k141_68909||full	1257	No	NA	0	0	Low-quality	Genome-fragment		1.85	0
S13_extracted_95036	1011	No	NA	0	2	Not-determined	Genome-fragment	NA		0
S13_extracted_95107	1056	No	NA	0	0	Not-determined	Genome-fragment	NA		0
S14_extracted_k141_9601	1258	No	NA	0	0	Low-quality	Genome-fragment		3.67	0
S14_vs_k141_102235||full	1258	No	NA	0	0	Low-quality	Genome-fragment		3.2	0
S13_extracted_95548	1401	No	NA	0	2	Not-determined	Genome-fragment	NA		0
S1_vs_k141_150050||full	1258	No	NA	0	0	Low-quality	Genome-fragment		2.81	0
S13_extracted_95868	1108	No	NA	0	2	Not-determined	Genome-fragment	NA		0
S13_extracted_96205	1640	No	NA	0	2	Not-determined	Genome-fragment	NA		0
S3_extracted_k141_7336	1258	No	NA	0	0	Low-quality	Genome-fragment		2.05	0
S13_extracted_96326	6011	No	NA	0	2	Not-determined	Genome-fragment	NA		0
S13_extracted_96646	1012	No	NA	0	0	Not-determined	Genome-fragment	NA		0
S3_vs_k141_96451||full	1258	No	NA	0	0	Low-quality	Genome-fragment		3.37	0
S13_extracted_96889	1009	No	NA	0	0	Not-determined	Genome-fragment	NA		0
S13_extracted_97175	4831	No	NA	0	3	Not-determined	Genome-fragment	NA		0
S13_extracted_97386	1080	No	NA	0	0	Not-determined	Genome-fragment	NA		0
S13_extracted_97520	1095	No	NA	0	1	Not-determined	Genome-fragment	NA		0
S5_extracted_k141_110972	1258	No	NA	0	0	Low-quality	Genome-fragment		1.98	0
S13_extracted_97612	1324	No	NA	0	0	Not-determined	Genome-fragment	NA		0
S5_extracted_k141_136558	1258	No	NA	0	0	Low-quality	Genome-fragment		0.62	0
S13_extracted_97678	1017	No	NA	0	0	Not-determined	Genome-fragment	NA		0
S13_extracted_97702	16409	No	NA	7	0	Complete	High-quality		100	0
S13_extracted_97950	2297	No	NA	0	2	Not-determined	Genome-fragment	NA		0
S13_extracted_98168	1023	No	NA	0	1	Not-determined	Genome-fragment	NA		0
S13_extracted_98187	4579	No	NA	0	4	Not-determined	Genome-fragment	NA		0
S13_extracted_98335	1343	No	NA	0	1	Not-determined	Genome-fragment	NA		0
S5_vs_k141_118645||full	1258	No	NA	0	0	Low-quality	Genome-fragment		2.36	0
S13_extracted_98478	1079	No	NA	0	1	Not-determined	Genome-fragment	NA		0
S13_extracted_98499	1392	No	NA	0	1	Not-determined	Genome-fragment	NA		0
S13_extracted_99191	1186	No	NA	0	1	Not-determined	Genome-fragment	NA		0
S13_extracted_99397	4582	No	NA	0	3	Not-determined	Genome-fragment	NA		0
S13_extracted_99469	3884	No	NA	0	2	Not-determined	Genome-fragment	NA		0
S13_extracted_99590	4465	No	NA	0	1	Not-determined	Genome-fragment	NA		0
S13_extracted_99745	1562	No	NA	0	1	Not-determined	Genome-fragment	NA		0
S13_extracted_99858	3420	No	NA	0	2	Not-determined	Genome-fragment	NA		0
S13_extracted_99965	1594	No	NA	0	2	Not-determined	Genome-fragment	NA		0
S13_extracted_100155	1743	No	NA	0	3	Not-determined	Genome-fragment	NA		0
S13_extracted_100161	1126	No	NA	0	0	Not-determined	Genome-fragment	NA		0
S13_extracted_100335	2389	No	NA	0	1	Not-determined	Genome-fragment	NA		0
S13_extracted_100565	1669	No	NA	0	1	Not-determined	Genome-fragment	NA		0
S13_extracted_100584	2490	No	NA	0	1	Not-determined	Genome-fragment	NA		0
S7_extracted_53064	1258	No	NA	1	0	Low-quality	Genome-fragment		4.15	0
S13_extracted_101065	4529	No	NA	0	2	Not-determined	Genome-fragment	NA		0
S13_extracted_101153	3006	No	NA	0	2	Not-determined	Genome-fragment	NA		0
S16_vs_50425||full	1258	No	NA	1	0	Low-quality	Genome-fragment		2.15	0
S16_vs_126823||full	1258	No	NA	0	1	Low-quality	Genome-fragment		0.76	0
S13_extracted_101568	1221	No	NA	0	1	Not-determined	Genome-fragment	NA		0
S13_extracted_101593	1077	No	NA	0	1	Not-determined	Genome-fragment	NA		0
S13_extracted_101654	1162	No	NA	0	0	Not-determined	Genome-fragment	NA		0
S13_extracted_101739	2211	No	NA	0	2	Not-determined	Genome-fragment	NA		0
S13_extracted_101961	5952	No	NA	0	5	Not-determined	Genome-fragment	NA		0
S13_extracted_102007	1034	No	NA	0	0	Not-determined	Genome-fragment	NA		0
S13_extracted_102027	3246	No	NA	0	1	Not-determined	Genome-fragment	NA		0
S13_extracted_102212	1072	No	NA	0	0	Not-determined	Genome-fragment	NA		0
S13_extracted_102321	1136	No	NA	0	2	Not-determined	Genome-fragment	NA		0
S13_extracted_102363	1159	No	NA	0	0	Not-determined	Genome-fragment	NA		0
S13_extracted_102587	1393	No	NA	0	0	Not-determined	Genome-fragment	NA		0
S16_vs_164949||lt2gene	1258	No	NA	1	0	Low-quality	Genome-fragment		2.55	0
S13_extracted_102752	1163	No	NA	0	1	Not-determined	Genome-fragment	NA		0
S10_vs_92896||full	1258	No	NA	0	2	Low-quality	Genome-fragment		0.67	0
S13_extracted_103174	1007	No	NA	0	0	Not-determined	Genome-fragment	NA		0
S13_extracted_103185	3318	No	NA	0	0	Not-determined	Genome-fragment	NA		0
S13_extracted_103332	1116	No	NA	0	1	Not-determined	Genome-fragment	NA		0
S13_extracted_103635	5862	No	NA	0	0	Not-determined	Genome-fragment	NA		0
S13_extracted_103867	1288	No	NA	0	0	Not-determined	Genome-fragment	NA		0
S13_extracted_104032	1196	No	NA	0	1	Not-determined	Genome-fragment	NA		0
S12_extracted_k141_17430	1258	No	NA	0	1	Low-quality	Genome-fragment		0.3	0
S15_extracted_70581	1258	No	NA	0	2	Low-quality	Genome-fragment		3.3	0
S13_extracted_104352	1775	No	NA	0	1	Not-determined	Genome-fragment	NA		0
S13_extracted_104525	1177	No	NA	0	0	Not-determined	Genome-fragment	NA		0
S13_extracted_104575	1228	No	NA	0	1	Not-determined	Genome-fragment	NA		0
S9_vs_k141_42419||full	1259	No	NA	0	0	Low-quality	Genome-fragment		2.45	0
S13_extracted_104907	1160	No	NA	0	1	Not-determined	Genome-fragment	NA		0
S13_extracted_105049	1054	No	NA	0	1	Not-determined	Genome-fragment	NA		0
S13_extracted_105223	1037	No	NA	0	1	Not-determined	Genome-fragment	NA		0
S11_extracted_k141_41554	1259	No	NA	0	0	Low-quality	Genome-fragment		3.09	0
S13_extracted_105328	1759	No	NA	0	1	Not-determined	Genome-fragment	NA		0
S13_extracted_105449	7609	No	NA	0	4	Not-determined	Genome-fragment	NA		0
S14_vs_k141_46413||full	1259	No	NA	2	0	Low-quality	Genome-fragment		2.16	0
S13_extracted_105480	1180	No	NA	0	1	Not-determined	Genome-fragment	NA		0
S13_extracted_105611	1205	No	NA	0	0	Not-determined	Genome-fragment	NA		0
S13_extracted_105726	1318	No	NA	0	1	Not-determined	Genome-fragment	NA		0
S13_extracted_105790	1056	No	NA	0	0	Not-determined	Genome-fragment	NA		0
S13_extracted_105992	1353	No	NA	0	0	Not-determined	Genome-fragment	NA		0
S13_extracted_106111	1466	No	NA	0	0	Not-determined	Genome-fragment	NA		0
S14_vs_k141_63340||full	1259	No	NA	0	0	Low-quality	Genome-fragment		3.4	0
S13_extracted_106340	1593	No	NA	0	1	Not-determined	Genome-fragment	NA		0
S13_extracted_106464	1383	No	NA	0	1	Not-determined	Genome-fragment	NA		0
S13_extracted_106617	1019	No	NA	0	1	Not-determined	Genome-fragment	NA		0
S13_extracted_106814	1437	No	NA	0	0	Not-determined	Genome-fragment	NA		0
S13_extracted_106856	1496	No	NA	0	0	Not-determined	Genome-fragment	NA		0
S13_extracted_107014	1501	No	NA	0	1	Not-determined	Genome-fragment	NA		0
S13_extracted_107182	1904	No	NA	0	1	Not-determined	Genome-fragment	NA		0
S13_extracted_107471	1083	No	NA	0	0	Not-determined	Genome-fragment	NA		0
S13_extracted_107618	1383	No	NA	0	2	Not-determined	Genome-fragment	NA		0
S1_extracted_k141_123403	1259	No	NA	0	0	Low-quality	Genome-fragment		1.88	0
S1_vs_k141_5403||full	1259	No	NA	0	0	Low-quality	Genome-fragment		1.96	0
S13_extracted_108098	1072	No	NA	0	1	Not-determined	Genome-fragment	NA		0
S13_extracted_108177	1167	No	NA	0	0	Not-determined	Genome-fragment	NA		0
S13_extracted_108231	1364	No	NA	0	1	Not-determined	Genome-fragment	NA		0
S13_extracted_108272	1012	No	NA	0	0	Not-determined	Genome-fragment	NA		0
S13_extracted_108288	1746	No	NA	0	1	Not-determined	Genome-fragment	NA		0
S7_vs_99556||full	1259	No	NA	2	0	Low-quality	Genome-fragment		3.13	0

S16_vs_51659||full	1259	No	NA	1	0	Low-quality	Genome-fragment		2.32	0
S13_extracted_108473	3128	No	NA	0	0	Not-determined	Genome-fragment	NA		0
S13_extracted_108559	1009	No	NA	0	0	Not-determined	Genome-fragment	NA		0
S10_vs_68985||full	1259	No	NA	0	0	Low-quality	Genome-fragment		2.36	0
S13_extracted_108714	1650	No	NA	0	3	Not-determined	Genome-fragment	NA		0
S12_extracted_k141_136256	1259	No	NA	0	0	Low-quality	Genome-fragment		2.18	0
S12_vs_k141_167761||lt2gene	1259	No	NA	1	0	Low-quality	Genome-fragment		2.06	0
S13_extracted_108857	1105	No	NA	0	1	Not-determined	Genome-fragment	NA		0
S13_extracted_108877	1477	No	NA	0	1	Not-determined	Genome-fragment	NA		0
S13_extracted_108898	1154	No	NA	0	1	Not-determined	Genome-fragment	NA		0
S13_extracted_108970	1426	No	NA	0	1	Not-determined	Genome-fragment	NA		0
S13_extracted_109133	1458	No	NA	0	2	Not-determined	Genome-fragment	NA		0
S13_extracted_109235	45453	No	NA	14	0	Complete	High-quality		100	0
S8_extracted_k141_131161	1259	No	NA	0	1	Low-quality	Genome-fragment		0.77	0
S8_extracted_k141_207108	1259	No	NA	2	0	Low-quality	Genome-fragment		4.15	0
S13_extracted_109446	1296	No	NA	0	1	Not-determined	Genome-fragment	NA		0
S13_extracted_109458	1335	No	NA	0	0	Not-determined	Genome-fragment	NA		0
S13_extracted_109577	1369	No	NA	0	0	Not-determined	Genome-fragment	NA		0
S11_vs_k141_93602||full	1260	No	NA	0	0	Low-quality	Genome-fragment		2	0
S13_extracted_109825	1069	No	NA	0	0	Not-determined	Genome-fragment	NA		0
S13_extracted_109869	1065	No	NA	0	1	Not-determined	Genome-fragment	NA		0
S11_vs_k141_83183||lt2gene	1260	No	NA	1	0	Low-quality	Genome-fragment		1.67	0
S13_extracted_110219	1202	No	NA	0	1	Not-determined	Genome-fragment	NA		0
S13_extracted_110313	1442	No	NA	0	1	Not-determined	Genome-fragment	NA		0
S13_extracted_110345	1562	No	NA	0	1	Not-determined	Genome-fragment	NA		0
S13_extracted_110462	3453	No	NA	0	4	Not-determined	Genome-fragment	NA		0
S13_extracted_110593	1133	No	NA	0	1	Not-determined	Genome-fragment	NA		0
S13_extracted_110670	2840	No	NA	0	3	Not-determined	Genome-fragment	NA		0
S13_extracted_110701	1341	No	NA	0	1	Not-determined	Genome-fragment	NA		0
S13_extracted_110710	1688	No	NA	0	1	Not-determined	Genome-fragment	NA		0
S13_extracted_110747	1218	No	NA	0	1	Not-determined	Genome-fragment	NA		0
S14_extracted_k141_47197	1260	No	NA	6	0	Low-quality	Genome-fragment		4.17	0
S13_extracted_110833	1072	No	NA	0	1	Not-determined	Genome-fragment	NA		0
S13_extracted_110855	1010	No	NA	0	0	Not-determined	Genome-fragment	NA		0
S13_extracted_110911	2311	No	NA	0	1	Not-determined	Genome-fragment	NA		0
S13_extracted_111050	1077	No	NA	0	2	Not-determined	Genome-fragment	NA		0
S14_vs_k141_92583||full	1260	No	NA	0	0	Low-quality	Genome-fragment		1.71	0
S13_extracted_111207	1363	No	NA	0	2	Not-determined	Genome-fragment	NA		0
S13_extracted_111234	3561	No	NA	0	1	Not-determined	Genome-fragment	NA		0
S13_extracted_111334	1693	No	NA	0	0	Not-determined	Genome-fragment	NA		0
S1_extracted_k141_23259	1260	No	NA	0	0	Low-quality	Genome-fragment		2.2	0
S13_extracted_111443	1367	No	NA	0	0	Not-determined	Genome-fragment	NA		0
S3_extracted_k141_42441	1260	No	NA	0	0	Low-quality	Genome-fragment		0.69	0
S13_extracted_111860	1489	No	NA	0	1	Not-determined	Genome-fragment	NA		0
S13_extracted_111876	3461	No	NA	0	1	Not-determined	Genome-fragment	NA		0
S13_extracted_111914	14845	No	NA	0	28	Not-determined	Genome-fragment	NA		0
S13_extracted_112004	1150	No	NA	0	1	Not-determined	Genome-fragment	NA		0
S13_extracted_112155	1295	No	NA	0	0	Not-determined	Genome-fragment	NA		0
S13_extracted_112178	1383	No	NA	0	1	Not-determined	Genome-fragment	NA		0
S13_extracted_112423	1927	No	NA	0	1	Not-determined	Genome-fragment	NA		0
S13_extracted_112484	2391	No	NA	0	0	Not-determined	Genome-fragment	NA		0
S13_extracted_112561	1101	No	NA	0	1	Not-determined	Genome-fragment	NA		0
S13_extracted_112625	1121	No	NA	0	2	Not-determined	Genome-fragment	NA		0
S13_extracted_112953	1132	No	NA	0	1	Not-determined	Genome-fragment	NA		0
S13_extracted_113063	1805	No	NA	0	1	Not-determined	Genome-fragment	NA		0
S13_extracted_113237	1598	No	NA	0	0	Not-determined	Genome-fragment	NA		0
S13_extracted_113577	1239	No	NA	0	2	Not-determined	Genome-fragment	NA		0
S13_extracted_114078	1024	No	NA	0	0	Not-determined	Genome-fragment	NA		0
S13_extracted_114193	1192	No	NA	0	1	Not-determined	Genome-fragment	NA		0
S13_extracted_114421	1906	No	NA	0	1	Not-determined	Genome-fragment	NA		0
S13_extracted_114496	1535	No	NA	0	1	Not-determined	Genome-fragment	NA		0
S13_extracted_114766	3934	No	NA	0	1	Not-determined	Genome-fragment	NA		0
S13_extracted_114935	1405	No	NA	0	0	Not-determined	Genome-fragment	NA		0
S13_extracted_115021	7179	No	NA	0	5	Not-determined	Genome-fragment	NA		0
S13_extracted_115047	1016	No	NA	0	0	Not-determined	Genome-fragment	NA		0
S13_extracted_115081	1057	No	NA	0	0	Not-determined	Genome-fragment	NA		0
S13_extracted_115173	1414	No	NA	0	1	Not-determined	Genome-fragment	NA		0
S13_extracted_115373	4139	No	NA	0	0	Not-determined	Genome-fragment	NA		0
S13_extracted_115391	4833	No	NA	0	0	Not-determined	Genome-fragment	NA		0
S3_vs_k141_104569||lt2gene	1260	No	NA	2	0	Low-quality	Genome-fragment		3.07	0
S13_extracted_115743	2103	No	NA	0	0	Not-determined	Genome-fragment	NA		0
S13_extracted_115818	3692	No	NA	0	2	Not-determined	Genome-fragment	NA		0
S5_extracted_k141_100800	1260	No	NA	0	0	Low-quality	Genome-fragment		3.54	0
S13_extracted_116190	1491	No	NA	0	0	Not-determined	Genome-fragment	NA		0
S5_vs_k141_126304||full	1260	No	NA	1	0	Low-quality	Genome-fragment		2.92	0
S13_extracted_116383	2144	No	NA	0	3	Not-determined	Genome-fragment	NA		0
S13_extracted_116533	1102	No	NA	0	0	Not-determined	Genome-fragment	NA		0
S13_extracted_116538	7022	No	NA	0	5	Not-determined	Genome-fragment	NA		0
S13_extracted_116830	1668	No	NA	0	0	Not-determined	Genome-fragment	NA		0
S13_extracted_116840	2494	No	NA	0	2	Not-determined	Genome-fragment	NA		0
S13_extracted_116935	1563	No	NA	0	1	Not-determined	Genome-fragment	NA		0
S13_extracted_117035	2114	No	NA	0	1	Not-determined	Genome-fragment	NA		0
S5_vs_k141_46839||full	1260	No	NA	0	0	Low-quality	Genome-fragment		3.17	0
S13_extracted_117093	1293	No	NA	0	1	Not-determined	Genome-fragment	NA		0
S13_extracted_117109	3553	No	NA	0	3	Not-determined	Genome-fragment	NA		0
S13_extracted_117175	1535	No	NA	0	2	Not-determined	Genome-fragment	NA		0
S13_extracted_117237	7084	No	NA	0	2	Not-determined	Genome-fragment	NA		0
S13_extracted_117292	3362	No	NA	0	1	Not-determined	Genome-fragment	NA		0
S13_extracted_117327	9600	No	NA	0	6	Not-determined	Genome-fragment	NA		0
S13_extracted_117364	3460	No	NA	0	2	Not-determined	Genome-fragment	NA		0
S13_extracted_117530	1351	No	NA	0	2	Not-determined	Genome-fragment	NA		0
S13_extracted_117580	1035	No	NA	0	1	Not-determined	Genome-fragment	NA		0
S7_vs_74294||full	1260	No	NA	1	0	Low-quality	Genome-fragment		0.88	0
S13_extracted_118092	1001	No	NA	0	0	Not-determined	Genome-fragment	NA		0
S13_extracted_118458	3076	No	NA	0	2	Not-determined	Genome-fragment	NA		0
S13_extracted_118613	2072	No	NA	0	0	Not-determined	Genome-fragment	NA		0
S13_extracted_118713	1757	No	NA	0	0	Not-determined	Genome-fragment	NA		0
S13_extracted_118920	1023	No	NA	0	1	Not-determined	Genome-fragment	NA		0
S13_extracted_119018	3380	No	NA	0	0	Not-determined	Genome-fragment	NA		0
S13_extracted_119101	1179	No	NA	0	1	Not-determined	Genome-fragment	NA		0
S13_extracted_119154	1232	No	NA	0	1	Not-determined	Genome-fragment	NA		0
S13_extracted_119240	1196	No	NA	0	1	Not-determined	Genome-fragment	NA		0
S13_extracted_119295	1061	No	NA	0	0	Not-determined	Genome-fragment	NA		0
S13_extracted_119320	1271	No	NA	0	0	Not-determined	Genome-fragment	NA		0
S13_extracted_119397	4766	No	NA	0	3	Not-determined	Genome-fragment	NA		0
S13_extracted_119588	6426	No	NA	0	5	Not-determined	Genome-fragment	NA		0
S13_extracted_119595	1246	No	NA	0	1	Not-determined	Genome-fragment	NA		0
S13_extracted_119750	1195	No	NA	0	1	Not-determined	Genome-fragment	NA		0
S7_vs_79226||full	1260	No	NA	1	0	Low-quality	Genome-fragment		2.17	0
S13_extracted_119949	1295	No	NA	0	0	Not-determined	Genome-fragment	NA		0
S13_extracted_119994	8479	No	NA	0	2	Not-determined	Genome-fragment	NA		0
S13_extracted_120223	1309	No	NA	0	1	Not-determined	Genome-fragment	NA		0
S13_extracted_120241	2435	No	NA	0	3	Not-determined	Genome-fragment	NA		0
S13_extracted_120273	1214	No	NA	0	1	Not-determined	Genome-fragment	NA		0
S13_extracted_120391	1243	No	NA	0	1	Not-determined	Genome-fragment	NA		0
S13_extracted_120399	1002	No	NA	0	2	Not-determined	Genome-fragment	NA		0
S13_extracted_120426	1157	No	NA	0	1	Not-determined	Genome-fragment	NA		0
S13_extracted_120429	1676	No	NA	0	1	Not-determined	Genome-fragment	NA		0
S13_extracted_120732	1774	No	NA	0	1	Not-determined	Genome-fragment	NA		0
S13_extracted_120984	1588	No	NA	0	1	Not-determined	Genome-fragment	NA		0
S16_extracted_26284	1260	No	NA	0	1	Low-quality	Genome-fragment		2.54	0
S13_extracted_121167	1101	No	NA	0	1	Not-determined	Genome-fragment	NA		0
S16_extracted_111925	1260	No	NA	1	0	Low-quality	Genome-fragment		3.29	0
S13_extracted_121265	1000	No	NA	0	1	Not-determined	Genome-fragment	NA		0
S13_extracted_121388	1049	No	NA	0	0	Not-determined	Genome-fragment	NA		0
S13_extracted_121431	1330	No	NA	0	0	Not-determined	Genome-fragment	NA		0
S10_extracted_43997	1260	No	NA	0	0	Low-quality	Genome-fragment		1.49	0
S10_extracted_98594	1260	No	NA	0	0	Low-quality	Genome-fragment		0.62	0
S10_vs_52359||full	1260	No	NA	0	0	Low-quality	Genome-fragment		2.6	0
S13_extracted_122210	7410	No	NA	0	7	Not-determined	Genome-fragment	NA		0
S6_vs_k141_25105||full	1260	No	NA	0	0	Low-quality	Genome-fragment		2.18	0
S13_extracted_122392	5301	No	NA	0	0	Not-determined	Genome-fragment	NA		0
S6_vs_k141_4465||full	1260	No	NA	0	0	Low-quality	Genome-fragment		2.09	0
S13_extracted_122580	3684	No	NA	0	0	Not-determined	Genome-fragment	NA		0
S13_extracted_122585	1490	No	NA	0	1	Not-determined	Genome-fragment	NA		0
S13_extracted_122617	2298	No	NA	0	1	Not-determined	Genome-fragment	NA		0
S13_extracted_122673	1277	No	NA	0	0	Not-determined	Genome-fragment	NA		0
S13_extracted_122804	1285	No	NA	0	0	Not-determined	Genome-fragment	NA		0
S13_extracted_122837	6382	No	NA	0	5	Not-determined	Genome-fragment	NA		0
S13_extracted_123025	2544	No	NA	0	2	Not-determined	Genome-fragment	NA		0
S13_extracted_123203	1444	No	NA	0	0	Not-determined	Genome-fragment	NA		0
S6_vs_k141_33116||lt2gene	1260	No	NA	1	0	Low-quality	Genome-fragment		2.95	0
S13_extracted_123302	1019	No	NA	0	0	Not-determined	Genome-fragment	NA		0
S13_extracted_123367	4054	No	NA	0	2	Not-determined	Genome-fragment	NA		0
S13_extracted_123375	2221	No	NA	0	0	Not-determined	Genome-fragment	NA		0
S13_extracted_123462	1186	No	NA	0	1	Not-determined	Genome-fragment	NA		0
S13_extracted_123490	1267	No	NA	0	1	Not-determined	Genome-fragment	NA		0
S13_extracted_123496	1253	No	NA	0	2	Not-determined	Genome-fragment	NA		0
S11_vs_k141_20007||full	1261	No	NA	0	1	Low-quality	Genome-fragment		0.6	0
S13_extracted_123554	2306	No	NA	0	0	Not-determined	Genome-fragment	NA		0
S13_extracted_123567	1487	No	NA	0	0	Not-determined	Genome-fragment	NA		0
S13_extracted_123865	1405	No	NA	0	0	Not-determined	Genome-fragment	NA		0
S13_extracted_124304	1408	No	NA	0	2	Not-determined	Genome-fragment	NA		0
S11_vs_k141_92655||full	1261	No	NA	0	0	Low-quality	Genome-fragment		0.71	0
S13_extracted_124357	1102	No	NA	0	0	Not-determined	Genome-fragment	NA		0
S13_extracted_124500	1455	No	NA	0	1	Not-determined	Genome-fragment	NA		0
S13_extracted_124706	1325	No	NA	0	0	Not-determined	Genome-fragment	NA		0
S13_extracted_124759	1267	No	NA	0	2	Not-determined	Genome-fragment	NA		0
S14_vs_k141_67520||full	1261	No	NA	1	0	Low-quality	Genome-fragment		2.75	0
S13_extracted_124842	2079	No	NA	0	0	Not-determined	Genome-fragment	NA		0
S14_vs_k141_99477||full	1261	No	NA	0	0	Low-quality	Genome-fragment		1.66	0
S1_vs_k141_122886||full	1261	No	NA	0	0	Low-quality	Genome-fragment		3.22	0
S13_extracted_125105	1016	No	NA	0	1	Not-determined	Genome-fragment	NA		0
S13_extracted_125190	1573	No	NA	0	1	Not-determined	Genome-fragment	NA		0
S13_extracted_125265	1145	No	NA	0	2	Not-determined	Genome-fragment	NA		0
S13_extracted_125326	3593	No	NA	0	4	Not-determined	Genome-fragment	NA		0
S13_extracted_125352	1104	No	NA	0	1	Not-determined	Genome-fragment	NA		0
S13_extracted_125372	1824	No	NA	0	1	Not-determined	Genome-fragment	NA		0
S13_extracted_125449	1815	No	NA	0	0	Not-determined	Genome-fragment	NA		0
S13_extracted_125461	1091	No	NA	0	2	Not-determined	Genome-fragment	NA		0
S13_extracted_125516	1987	No	NA	0	1	Not-determined	Genome-fragment	NA		0
S13_extracted_125519	1048	No	NA	0	0	Not-determined	Genome-fragment	NA		0
S13_extracted_125527	1028	No	NA	0	2	Not-determined	Genome-fragment	NA		0
S13_extracted_125695	1361	No	NA	0	0	Not-determined	Genome-fragment	NA		0

S13_extracted_125726	1671	No	NA		0	0	Not-determined	Genome-fragment	NA		0
S1_vs_k141_12843||full	1261	No	NA		2	0	Low-quality	Genome-fragment		2.18	0
S3_vs_k141_41611||full	1261	No	NA		0	1	Low-quality	Genome-fragment		0.72	0
S13_extracted_125908	1009	No	NA		0	1	Not-determined	Genome-fragment	NA		0
S13_extracted_125952	1184	No	NA		0	1	Not-determined	Genome-fragment	NA		0
S13_extracted_126032	1196	No	NA		0	1	Not-determined	Genome-fragment	NA		0
S13_extracted_126042	1269	No	NA		0	1	Not-determined	Genome-fragment	NA		0
S13_extracted_126106	4377	No	NA		0	2	Not-determined	Genome-fragment	NA		0
S13_extracted_126274	1932	No	NA		0	1	Not-determined	Genome-fragment	NA		0
S13_extracted_126350	1536	No	NA		0	1	Not-determined	Genome-fragment	NA		0
S13_extracted_126429	1028	No	NA		0	0	Not-determined	Genome-fragment	NA		0
S13_extracted_126444	2384	No	NA		0	1	Not-determined	Genome-fragment	NA		0
S13_extracted_126505	1381	No	NA		0	2	Not-determined	Genome-fragment	NA		0
S13_extracted_126603	1404	No	NA		0	1	Not-determined	Genome-fragment	NA		0
S13_extracted_126620	1403	No	NA		0	0	Not-determined	Genome-fragment	NA		0
S13_extracted_126693	1036	No	NA		0	1	Not-determined	Genome-fragment	NA		0
S13_extracted_126766	2095	No	NA		0	0	Not-determined	Genome-fragment	NA		0
S13_extracted_126801	1493	No	NA		0	2	Not-determined	Genome-fragment	NA		0
S3_vs_k141_27410||full	1261	No	NA		0	0	Low-quality	Genome-fragment		2.19	0
S5_vs_k141_58438||lt2gene	1261	No	NA		1	0	Low-quality	Genome-fragment		3.33	0
S13_extracted_127020	2210	No	NA		0	1	Not-determined	Genome-fragment	NA		0
S13_extracted_127028	3392	No	NA		0	3	Not-determined	Genome-fragment	NA		0
S13_extracted_127078	1183	No	NA		0	1	Not-determined	Genome-fragment	NA		0
S13_extracted_127286	1853	No	NA		0	0	Not-determined	Genome-fragment	NA		0
S13_extracted_127376	1770	No	NA		0	2	Not-determined	Genome-fragment	NA		0
S13_extracted_127402	1044	No	NA		0	0	Not-determined	Genome-fragment	NA		0
S13_extracted_127556	1042	No	NA		0	1	Not-determined	Genome-fragment	NA		0
S13_extracted_127744	2245	No	NA		0	2	Not-determined	Genome-fragment	NA		0
S13_extracted_128030	1485	No	NA		0	2	Not-determined	Genome-fragment	NA		0
S13_extracted_128194	1920	No	NA		0	1	Not-determined	Genome-fragment	NA		0
S13_extracted_128222	3446	No	NA		0	2	Not-determined	Genome-fragment	NA		0
S13_extracted_128254	1348	No	NA		0	4	Not-determined	Genome-fragment	NA		0
S7_vs_16087||full	1261	No	NA		0	0	Low-quality	Genome-fragment		1.85	0
S13_extracted_128572	6755	No	NA		0	5	Not-determined	Genome-fragment	NA		0
S13_extracted_128736	6841	No	NA		0	5	Not-determined	Genome-fragment	NA		0
S13_extracted_128812	2280	No	NA		0	2	Not-determined	Genome-fragment	NA		0
S13_extracted_128983	2570	No	NA		0	2	Not-determined	Genome-fragment	NA		0
S7_vs_54071||full	1261	No	NA		0	0	Low-quality	Genome-fragment		3.01	0
S13_extracted_129213	1043	No	NA		0	1	Not-determined	Genome-fragment	NA		0
S13_extracted_129313	1008	No	NA		0	0	Not-determined	Genome-fragment	NA		0
S13_extracted_129534	6670	No	NA		0	4	Not-determined	Genome-fragment	NA		0
S13_extracted_129666	4721	No	NA		0	9	Not-determined	Genome-fragment	NA		0
S13_extracted_129729	1606	No	NA		0	3	Not-determined	Genome-fragment	NA		0
S13_extracted_129941	2449	No	NA		0	5	Not-determined	Genome-fragment	NA		0
S13_extracted_130013	3785	No	NA		0	1	Not-determined	Genome-fragment	NA		0
S13_extracted_130272	1127	No	NA		0	0	Not-determined	Genome-fragment	NA		0
S13_extracted_130299	3852	No	NA		0	1	Not-determined	Genome-fragment	NA		0
S13_extracted_130331	9236	No	NA		0	6	Not-determined	Genome-fragment	NA		0
S13_extracted_130594	1182	No	NA		0	1	Not-determined	Genome-fragment	NA		0
S13_extracted_130640	1124	No	NA		0	0	Not-determined	Genome-fragment	NA		0
S13_extracted_130670	1745	No	NA		0	1	Not-determined	Genome-fragment	NA		0
S13_extracted_130688	1072	No	NA		0	1	Not-determined	Genome-fragment	NA		0
S13_extracted_130760	2001	No	NA		0	0	Not-determined	Genome-fragment	NA		0
S13_extracted_130968	1041	No	NA		0	1	Not-determined	Genome-fragment	NA		0
S13_extracted_131208	13280	No	NA		8	0	Medium-quality	Genome-fragment		64.98	0
S13_extracted_131280	1180	No	NA		0	0	Not-determined	Genome-fragment	NA		0
S13_extracted_131313	1016	No	NA		0	1	Not-determined	Genome-fragment	NA		0
S12_vs_k141_21577||full	1261	No	NA		0	0	Low-quality	Genome-fragment		1.81	0
S12_vs_k141_47559||lt2gene	1261	No	NA		1	0	Low-quality	Genome-fragment		2.12	0
S6_vs_k141_45266||full	1261	No	NA		0	0	Low-quality	Genome-fragment		3.23	0
S13_extracted_131479	1423	No	NA		0	1	Not-determined	Genome-fragment	NA		0
S6_vs_k141_54481||full	1261	No	NA		0	1	Low-quality	Genome-fragment		1.57	0
S13_extracted_131633	3054	No	NA		0	2	Not-determined	Genome-fragment	NA		0
S13_extracted_131650	1076	No	NA		0	1	Not-determined	Genome-fragment	NA		0
S13_extracted_131686	1741	No	NA		0	2	Not-determined	Genome-fragment	NA		0
S13_extracted_131708	3127	No	NA		0	1	Not-determined	Genome-fragment	NA		0
S13_extracted_131854	3813	No	NA		0	4	Not-determined	Genome-fragment	NA		0
S13_extracted_131861	21298	No	NA		0	4	Not-determined	Genome-fragment	NA		0
S13_extracted_131889	1720	No	NA		0	1	Not-determined	Genome-fragment	NA		0
S13_extracted_132020	1163	No	NA		0	1	Not-determined	Genome-fragment	NA		0
S13_extracted_132022	1143	No	NA		0	1	Not-determined	Genome-fragment	NA		0
S8_extracted_k141_155948	1261	No	NA		0	1	Low-quality	Genome-fragment		2.5	0
S8_vs_k141_5668||full	1261	No	NA		0	0	Low-quality	Genome-fragment		3.41	0
S17_vs_k141_78851||full	1262	No	NA		0	1	Low-quality	Genome-fragment		0.59	0
S13_extracted_132198	1021	No	NA		0	0	Not-determined	Genome-fragment	NA		0
S13_extracted_132372	1220	No	NA		0	1	Not-determined	Genome-fragment	NA		0
S13_extracted_132407	1199	No	NA		0	0	Not-determined	Genome-fragment	NA		0
S17_vs_k141_177009||full	1262	No	NA		0	2	Low-quality	Genome-fragment		0.59	0
S13_extracted_132643	1514	No	NA		0	2	Not-determined	Genome-fragment	NA		0
S13_extracted_133001	1298	No	NA		0	1	Not-determined	Genome-fragment	NA		0
S13_extracted_133136	5641	No	NA		0	0	Not-determined	Genome-fragment	NA		0
S9_vs_k141_47657||full	1262	No	NA		0	1	Low-quality	Genome-fragment		1.63	0
S13_extracted_133211	1009	No	NA		0	0	Not-determined	Genome-fragment	NA		0
S11_extracted_k141_58714	1262	No	NA		0	0	Low-quality	Genome-fragment		2.05	0
S13_extracted_133621	1305	No	NA		0	1	Not-determined	Genome-fragment	NA		0
S13_extracted_122158	1262	No	NA		0	0	Low-quality	Genome-fragment		0.96	0
S14_extracted_k141_5033	1262	No	NA		1	0	Low-quality	Genome-fragment		2.82	0
S13_extracted_134065	1982	No	NA		0	1	Not-determined	Genome-fragment	NA		0
S3_vs_k141_111350||full	1262	No	NA		0	0	Low-quality	Genome-fragment		2.55	0
S3_vs_k141_52350||lt2gene	1262	No	NA		1	0	Low-quality	Genome-fragment		2.45	0
S13_extracted_134641	2928	No	NA		0	0	Not-determined	Genome-fragment	NA		0
S13_extracted_134691	3308	No	NA		0	1	Not-determined	Genome-fragment	NA		0
S13_extracted_134726	1227	No	NA		0	1	Not-determined	Genome-fragment	NA		0
S13_extracted_134794	1742	No	NA		0	0	Not-determined	Genome-fragment	NA		0
S13_extracted_134844	1562	No	NA		0	2	Not-determined	Genome-fragment	NA		0
S13_extracted_135000	1414	No	NA		0	0	Not-determined	Genome-fragment	NA		0
S13_extracted_135009	1051	No	NA		0	2	Not-determined	Genome-fragment	NA		0
S13_extracted_135426	1963	No	NA		0	1	Not-determined	Genome-fragment	NA		0
S5_vs_k141_74521||lt2gene	1262	No	NA		1	0	Low-quality	Genome-fragment		2.6	0
S13_extracted_135562	1036	No	NA		0	1	Not-determined	Genome-fragment	NA		0
S13_extracted_135658	1214	No	NA		0	2	Not-determined	Genome-fragment	NA		0
S13_extracted_135823	1146	No	NA		0	0	Not-determined	Genome-fragment	NA		0
S13_extracted_135824	1374	No	NA		0	2	Not-determined	Genome-fragment	NA		0
S13_extracted_135899	1788	No	NA		0	1	Not-determined	Genome-fragment	NA		0
S13_extracted_135900	1302	No	NA		0	0	Not-determined	Genome-fragment	NA		0
S13_extracted_135901	1125	No	NA		0	0	Not-determined	Genome-fragment	NA		0
S13_extracted_135938	1089	No	NA		0	0	Not-determined	Genome-fragment	NA		0
S13_extracted_136077	1424	No	NA		0	0	Not-determined	Genome-fragment	NA		0
S13_extracted_136142	1090	No	NA		0	0	Not-determined	Genome-fragment	NA		0
S13_extracted_136171	1764	No	NA		0	0	Not-determined	Genome-fragment	NA		0
S13_extracted_136186	1203	No	NA		0	1	Not-determined	Genome-fragment	NA		0
S7_vs_60829||full	1262	No	NA		0	0	Low-quality	Genome-fragment		3.55	0
S13_extracted_136508	1334	No	NA		0	1	Not-determined	Genome-fragment	NA		0
S13_extracted_136806	1561	No	NA		0	1	Not-determined	Genome-fragment	NA		0
S8_vs_k141_158171||full	1262	No	NA		0	0	Low-quality	Genome-fragment		0.81	0
S8_vs_k141_66903||full	1262	No	NA		0	0	Low-quality	Genome-fragment		1.09	0
S13_extracted_137194	1252	No	NA		0	2	Not-determined	Genome-fragment	NA		0
S13_extracted_137426	1371	No	NA		0	1	Not-determined	Genome-fragment	NA		0
S14_vs_k141_89202||full	1263	No	NA		0	0	Low-quality	Genome-fragment		2.86	0
S13_extracted_137634	1121	No	NA		0	1	Not-determined	Genome-fragment	NA		0
S14_vs_k141_68930||full	1263	No	NA		0	0	Low-quality	Genome-fragment		2.28	0
S13_extracted_138116	1779	No	NA		0	0	Not-determined	Genome-fragment	NA		0
S1_vs_k141_21038||full	1263	No	NA		0	0	Low-quality	Genome-fragment		2.8	0
S13_extracted_138157	1327	No	NA		0	0	Not-determined	Genome-fragment	NA		0
S13_extracted_138200	3163	No	NA		0	1	Not-determined	Genome-fragment	NA		0
S13_extracted_138272	1155	No	NA		0	0	Not-determined	Genome-fragment	NA		0
S13_extracted_138457	4326	No	NA		0	2	Not-determined	Genome-fragment	NA		0
S13_extracted_138583	1521	No	NA		0	0	Not-determined	Genome-fragment	NA		0
S13_extracted_138701	1028	No	NA		0	2	Not-determined	Genome-fragment	NA		0
S13_extracted_139314	1027	No	NA		0	3	Not-determined	Genome-fragment	NA		0
S13_extracted_139332	1567	No	NA		0	1	Not-determined	Genome-fragment	NA		0
S13_extracted_139453	1241	No	NA		0	0	Not-determined	Genome-fragment	NA		0
S13_extracted_139550	1497	No	NA		0	2	Not-determined	Genome-fragment	NA		0
S3_vs_k141_122910||full	1263	No	NA		0	0	Low-quality	Genome-fragment		1.7	0
S5_extracted_k141_143162	1263	No	NA		0	0	Low-quality	Genome-fragment		3.16	0
S13_extracted_139924	1856	No	NA		0	1	Not-determined	Genome-fragment	NA		0
S13_extracted_140062	1399	No	NA		0	1	Not-determined	Genome-fragment	NA		0
S13_extracted_140136	9760	No	NA		0	2	Not-determined	Genome-fragment	NA		0
S13_extracted_140199	1105	No	NA		0	1	Not-determined	Genome-fragment	NA		0
S13_extracted_140461	1144	No	NA		0	0	Not-determined	Genome-fragment	NA		0
S13_extracted_140611	1087	No	NA		0	1	Not-determined	Genome-fragment	NA		0
S13_extracted_140647	1075	No	NA		0	0	Not-determined	Genome-fragment	NA		0
S13_extracted_140697	4199	No	NA		0	3	Not-determined	Genome-fragment	NA		0
S5_extracted_k141_66409	1263	No	NA		1	0	Low-quality	Genome-fragment		2.1	0
S13_extracted_140954	4667	No	NA		0	1	Not-determined	Genome-fragment	NA		0
S5_vs_k141_80850||full	1263	No	NA		0	0	Low-quality	Genome-fragment		2.36	0
S13_extracted_141115	1397	No	NA		0	1	Not-determined	Genome-fragment	NA		0
S7_extracted_119109	1263	No	NA		0	1	Low-quality	Genome-fragment		4.21	0
S13_extracted_141133	1287	No	NA		0	0	Not-determined	Genome-fragment	NA		0
S13_extracted_141184	1149	No	NA		0	0	Not-determined	Genome-fragment	NA		0
S16_extracted_142073	1263	No	NA		0	0	Low-quality	Genome-fragment		2.29	0
S17_extracted_k141_76082	1264	No	NA		0	1	Low-quality	Genome-fragment		0.59	0
S13_extracted_141355	1387	No	NA		0	2	Not-determined	Genome-fragment	NA		0
S13_extracted_141394	1164	No	NA		0	0	Not-determined	Genome-fragment	NA		0
S13_extracted_141397	1184	No	NA		0	1	Not-determined	Genome-fragment	NA		0
S13_extracted_141444	1107	No	NA		0	0	Not-determined	Genome-fragment	NA		0
S9_vs_k141_12870||full	1264	No	NA		2	0	Low-quality	Genome-fragment		3.59	0
S13_extracted_141793	1273	No	NA		0	1	Not-determined	Genome-fragment	NA		0
S11_vs_k141_42027||full	1264	No	NA		1	0	Low-quality	Genome-fragment		3.33	0
S13_extracted_142191	1055	No	NA		0	1	Not-determined	Genome-fragment	NA		0
S13_extracted_142563	1445	No	NA		0	1	Not-determined	Genome-fragment	NA		0
S13_extracted_142922	1269	No	NA		0	1	Not-determined	Genome-fragment	NA		0
S13_extracted_143029	1029	No	NA		0	2	Not-determined	Genome-fragment	NA		0
S13_extracted_143121	1446	No	NA		0	0	Not-determined	Genome-fragment	NA		0
S13_extracted_143129	1643	No	NA		0	1	Not-determined	Genome-fragment	NA		0
S13_extracted_143335	1694	No	NA		0	1	Not-determined	Genome-fragment	NA		0
S13_extracted_143377	1305	Yes		670	1	1	Not-determined	Genome-fragment	NA		48.66
S13_extracted_143402	1079	No	NA		0	1	Not-determined	Genome-fragment	NA		0
S13_extracted_143422	54390	No	NA		8	0	Medium-quality	Genome-fragment		54.31	0
S13_extracted_143435	1198	No	NA		0	1	Not-determined	Genome-fragment	NA		0
S13_extracted_143536	1018	No	NA		0	1	Not-determined	Genome-fragment	NA		0
S13_extracted_143599	1673	No	NA		0	6	Not-determined	Genome-fragment	NA		0
S13_extracted_143633	4901	No	NA		0	2	Not-determined	Genome-fragment	NA		0
S13_extracted_143838	2295	No	NA		0	2	Not-determined	Genome-fragment	NA		0

S13_extracted_143946	1121	No	NA	0	1	Not-determined	Genome-fragment	NA	0
S13_extracted_144048	1692	No	NA	0	0	Not-determined	Genome-fragment	NA	0
S13_vs_9701||full	1264	No	NA	0	0	Low-quality	Genome-fragment	1.08	0
S13_extracted_144132	3181	No	NA	0	4	Not-determined	Genome-fragment	NA	0
S1_vs_k141_130024||full	1264	No	NA	0	0	Low-quality	Genome-fragment	3.47	0
S13_extracted_144367	3494	No	NA	0	4	Not-determined	Genome-fragment	NA	0
S13_extracted_144578	1770	No	NA	0	0	Not-determined	Genome-fragment	NA	0
S13_extracted_144777	1583	No	NA	0	1	Not-determined	Genome-fragment	NA	0
S13_extracted_144902	1480	No	NA	0	2	Not-determined	Genome-fragment	NA	0
S13_extracted_144920	9511	No	NA	0	3	Not-determined	Genome-fragment	NA	0
S1_vs_k141_50975||full	1264	No	NA	1	0	Low-quality	Genome-fragment	2.36	0
S13_extracted_145254	2069	No	NA	0	0	Not-determined	Genome-fragment	NA	0
S13_extracted_145421	1959	No	NA	0	2	Not-determined	Genome-fragment	NA	0
S13_extracted_145573	2452	No	NA	0	1	Not-determined	Genome-fragment	NA	0
S13_extracted_145746	1119	No	NA	0	1	Not-determined	Genome-fragment	NA	0
S13_extracted_145859	1002	No	NA	0	1	Not-determined	Genome-fragment	NA	0
S13_extracted_145870	1452	No	NA	0	1	Not-determined	Genome-fragment	NA	0
S13_extracted_145932	1380	No	NA	0	1	Not-determined	Genome-fragment	NA	0
S3_extracted_k141_78558	1264	No	NA	1	0	Low-quality	Genome-fragment	3.16	0
S13_extracted_146229	1708	No	NA	0	1	Not-determined	Genome-fragment	NA	0
S13_extracted_146385	2155	No	NA	0	0	Not-determined	Genome-fragment	NA	0
S13_extracted_146437	1026	No	NA	0	1	Not-determined	Genome-fragment	NA	0
S13_extracted_146449	1003	No	NA	0	0	Not-determined	Genome-fragment	NA	0
S13_extracted_146453	2273	No	NA	0	2	Not-determined	Genome-fragment	NA	0
S13_extracted_146550	1138	No	NA	0	0	Not-determined	Genome-fragment	NA	0
S13_extracted_146731	1505	No	NA	0	2	Not-determined	Genome-fragment	NA	0
S13_extracted_146803	3541	No	NA	0	0	Not-determined	Genome-fragment	NA	0
S13_extracted_146808	1054	No	NA	0	0	Not-determined	Genome-fragment	NA	0
S13_extracted_146971	1005	No	NA	0	0	Not-determined	Genome-fragment	NA	0
S5_extracted_k141_136897	1264	No	NA	0	0	Low-quality	Genome-fragment	2.96	0
S13_extracted_147094	1268	No	NA	0	0	Not-determined	Genome-fragment	NA	0
S7_vs_68218||full	1264	No	NA	0	0	Low-quality	Genome-fragment	3.26	0
S7_vs_63225||lt2gene	1264	No	NA	1	0	Low-quality	Genome-fragment	2.11	0
S13_extracted_147343	1182	No	NA	0	1	Not-determined	Genome-fragment	NA	0
S13_extracted_147385	3535	No	NA	0	0	Not-determined	Genome-fragment	NA	0
S13_extracted_147396	35175	No	NA	13	0	Complete	High-quality	100	0
S13_extracted_147469	1249	No	NA	0	1	Not-determined	Genome-fragment	NA	0
S16_extracted_144183	1264	No	NA	1	0	Low-quality	Genome-fragment	3.1	0
S16_vs_91731||full	1264	No	NA	0	0	Low-quality	Genome-fragment	2.09	0
S13_extracted_147589	11817	No	NA	2	1	Complete	High-quality	100	0
S13_extracted_147593	1755	No	NA	0	0	Not-determined	Genome-fragment	NA	0
S13_extracted_147649	2010	No	NA	0	1	Not-determined	Genome-fragment	NA	0
S13_extracted_147660	1048	No	NA	0	1	Not-determined	Genome-fragment	NA	0
S13_extracted_147900	3661	No	NA	0	0	Not-determined	Genome-fragment	NA	0
S13_extracted_148054	3081	No	NA	0	0	Not-determined	Genome-fragment	NA	0
S16_vs_139864||full	1264	No	NA	0	0	Low-quality	Genome-fragment	0.32	0
S13_extracted_148190	1009	No	NA	0	1	Not-determined	Genome-fragment	NA	0
S16_vs_45769||lt2gene	1264	No	NA	1	0	Low-quality	Genome-fragment	0.87	0
S13_extracted_148387	1006	No	NA	0	0	Not-determined	Genome-fragment	NA	0
S13_extracted_148631	1138	No	NA	0	0	Not-determined	Genome-fragment	NA	0
S13_extracted_148898	1061	No	NA	0	2	Not-determined	Genome-fragment	NA	0
S13_extracted_148949	1074	No	NA	0	0	Not-determined	Genome-fragment	NA	0
S13_extracted_148978	1349	No	NA	0	1	Not-determined	Genome-fragment	NA	0
S13_extracted_148979	1082	No	NA	0	0	Not-determined	Genome-fragment	NA	0
S12_vs_k141_118784||full	1264	No	NA	0	0	Low-quality	Genome-fragment	2.78	0
S13_extracted_149267	1437	No	NA	0	0	Not-determined	Genome-fragment	NA	0
S17_extracted_k141_135066	1265	No	NA	0	0	Low-quality	Genome-fragment	1.89	0
S13_extracted_149436	1027	No	NA	0	0	Not-determined	Genome-fragment	NA	0
S9_vs_k141_79703||full	1265	No	NA	0	0	Low-quality	Genome-fragment	2.53	0
S13_extracted_11748	1265	No	NA	1	0	Low-quality	Genome-fragment	3	0
S13_extracted_149888	1192	No	NA	0	1	Not-determined	Genome-fragment	NA	0
S13_extracted_149903	1155	No	NA	0	1	Not-determined	Genome-fragment	NA	0
S14_extracted_k141_54825	1265	No	NA	1	0	Low-quality	Genome-fragment	0.87	0
S13_extracted_150246	1380	No	NA	0	1	Not-determined	Genome-fragment	NA	0
S14_extracted_k141_76885	1265	No	NA	2	0	Low-quality	Genome-fragment	4.23	0
S13_extracted_150317	3751	No	NA	0	0	Not-determined	Genome-fragment	NA	0
S13_extracted_150407	5403	No	NA	0	0	Complete	High-quality	100	0
S13_extracted_150506	1176	No	NA	0	1	Not-determined	Genome-fragment	NA	0
S13_extracted_150691	1090	No	NA	0	1	Not-determined	Genome-fragment	NA	0
S13_extracted_150779	1615	No	NA	0	1	Not-determined	Genome-fragment	NA	0
S1_vs_k141_140360||full	1265	No	NA	0	0	Low-quality	Genome-fragment	2.14	0
S13_extracted_151045	86139	No	NA	17	2	Complete	High-quality	100	0
S13_extracted_151086	1639	No	NA	0	1	Not-determined	Genome-fragment	NA	0
S13_extracted_151312	1060	No	NA	0	0	Not-determined	Genome-fragment	NA	0
S13_extracted_151344	1368	No	NA	0	0	Not-determined	Genome-fragment	NA	0
S13_extracted_151589	1298	No	NA	0	1	Not-determined	Genome-fragment	NA	0
S13_extracted_151697	5076	No	NA	0	0	Not-determined	Genome-fragment	NA	0
S13_extracted_151755	43394	No	NA	28	0	Complete	High-quality	100	0
S13_extracted_151847	6800	No	NA	0	3	Not-determined	Genome-fragment	NA	0
S13_extracted_151901	1286	No	NA	0	0	Not-determined	Genome-fragment	NA	0
S13_extracted_152065	1094	No	NA	0	1	Not-determined	Genome-fragment	NA	0
S3_extracted_k141_121667	1265	No	NA	0	1	Low-quality	Genome-fragment	2.16	0
S3_extracted_k141_2484	1265	No	NA	1	0	Low-quality	Genome-fragment	0.87	0
S13_extracted_152250	1056	No	NA	0	1	Not-determined	Genome-fragment	NA	0
S13_extracted_152340	1834	No	NA	0	1	Not-determined	Genome-fragment	NA	0
S13_extracted_152369	1114	No	NA	0	1	Not-determined	Genome-fragment	NA	0
S13_extracted_152435	1256	No	NA	0	0	Not-determined	Genome-fragment	NA	0
S3_extracted_k141_109451	1265	No	NA	0	0	Low-quality	Genome-fragment	1.02	0
S13_extracted_152536	1752	No	NA	0	0	Not-determined	Genome-fragment	NA	0
S13_extracted_152747	1057	No	NA	0	1	Not-determined	Genome-fragment	NA	0
S3_extracted_k141_39040	1265	No	NA	0	0	Low-quality	Genome-fragment	1.63	0
S13_extracted_153057	3638	No	NA	0	2	Not-determined	Genome-fragment	NA	0
S13_extracted_153076	1003	No	NA	0	0	Not-determined	Genome-fragment	NA	0
S5_vs_k141_132565||full	1265	No	NA	0	0	Low-quality	Genome-fragment	2.58	0
S13_extracted_153288	1190	No	NA	0	1	Not-determined	Genome-fragment	NA	0
S7_vs_4807||full	1265	No	NA	0	1	Low-quality	Genome-fragment	0.71	0
S13_extracted_153390	1145	No	NA	0	2	Not-determined	Genome-fragment	NA	0
S16_vs_126000||full	1265	No	NA	0	0	Low-quality	Genome-fragment	2.27	0
S13_extracted_153526	1151	No	NA	0	1	Not-determined	Genome-fragment	NA	0
S13_extracted_153577	1260	No	NA	0	0	Not-determined	Genome-fragment	NA	0
S13_extracted_153612	1133	No	NA	0	2	Not-determined	Genome-fragment	NA	0
S13_extracted_153622	1280	No	NA	0	1	Not-determined	Genome-fragment	NA	0
S13_extracted_153657	1833	No	NA	0	1	Not-determined	Genome-fragment	NA	0
S10_extracted_113182	1265	No	NA	1	0	Low-quality	Genome-fragment	3.12	0
S13_extracted_153743	1174	No	NA	0	2	Not-determined	Genome-fragment	NA	0
S10_vs_16269||full	1265	No	NA	0	0	Low-quality	Genome-fragment	2.74	0
S13_extracted_154047	1362	No	NA	0	0	Not-determined	Genome-fragment	NA	0
S13_extracted_154130	1192	No	NA	0	0	Not-determined	Genome-fragment	NA	0
S13_extracted_154245	1186	No	NA	0	0	Not-determined	Genome-fragment	NA	0
S13_vs_9029||full	6727	No	NA	0	3	Not-determined	Genome-fragment	NA	0
S13_vs_12957||full	15580	No	NA	8	0	Medium-quality	Genome-fragment	51	0
S13_vs_15645||full	153823	No	NA	18	4	High-quality	High-quality	100	0
S13_vs_27462||full	65150	No	NA	27	4	Medium-quality	Genome-fragment	82.87	0
S13_vs_28316||full	3119	No	NA	0	3	Not-determined	Genome-fragment	NA	0
S13_vs_148294||full	43488	No	NA	26	2	High-quality	High-quality	100	0
S13_vs_149860||full	19576	No	NA	8	0	High-quality	High-quality	100	0
S13_vs_150477||full	8781	No	NA	0	3	Not-determined	Genome-fragment	NA	0
S13_vs_151510||full	97768	No	NA	22	0	High-quality	High-quality	98.51	0
S6_vs_k141_31212||full	1265	No	NA	1	0	Low-quality	Genome-fragment	2.06	0
S13_vs_152||full	1242	No	NA	0	2	Not-determined	Genome-fragment	NA	0
S17_extracted_k141_34243	1266	No	NA	4	0	Low-quality	Genome-fragment	4.16	0
S13_vs_502||full	1557	No	NA	0	0	Not-determined	Genome-fragment	NA	0
S13_vs_587||full	1337	No	NA	0	1	Not-determined	Genome-fragment	NA	0
S13_vs_702||full	2780	No	NA	0	0	Not-determined	Genome-fragment	NA	0
S13_vs_763||full	2724	No	NA	0	0	Not-determined	Genome-fragment	NA	0
S13_vs_837||full	2915	No	NA	0	1	Not-determined	Genome-fragment	NA	0
S13_vs_845||full	3137	No	NA	0	4	Not-determined	Genome-fragment	NA	0
S13_vs_852||full	1813	No	NA	0	1	Not-determined	Genome-fragment	NA	0
S13_vs_1092||full	3269	No	NA	0	1	Not-determined	Genome-fragment	NA	0
S13_vs_1200||full	4539	No	NA	0	0	Not-determined	Genome-fragment	NA	0
S13_vs_1676||full	6275	No	NA	0	4	Not-determined	Genome-fragment	NA	0
S17_vs_k141_252||full	1266	No	NA	0	0	Low-quality	Genome-fragment	2.79	0
S13_vs_2215||full	1171	No	NA	0	0	Not-determined	Genome-fragment	NA	0
S13_vs_2276||full	1216	No	NA	0	0	Not-determined	Genome-fragment	NA	0
S13_vs_2380||full	1505	No	NA	0	1	Not-determined	Genome-fragment	NA	0
S13_vs_2416||full	2071	No	NA	0	1	Not-determined	Genome-fragment	NA	0
S9_vs_k141_32504||full	1266	No	NA	0	0	Low-quality	Genome-fragment	2.79	0
S11_vs_k141_1000||full	1266	No	NA	0	0	Low-quality	Genome-fragment	2.49	0
S3_extracted_k141_109899	1266	No	NA	1	0	Low-quality	Genome-fragment	1.5	0
S13_vs_2841||full	2224	No	NA	0	0	Not-determined	Genome-fragment	NA	0
S3_extracted_k141_38782	1266	No	NA	0	0	Low-quality	Genome-fragment	2.11	0
S13_vs_3423||full	2061	No	NA	0	0	Not-determined	Genome-fragment	NA	0
S13_vs_3488||full	1676	No	NA	0	1	Not-determined	Genome-fragment	NA	0
S5_extracted_k141_132812	1266	No	NA	0	1	Low-quality	Genome-fragment	2.75	0
S13_vs_3666||full	1569	No	NA	0	1	Not-determined	Genome-fragment	NA	0
S13_vs_3823||full	1310	No	NA	0	1	Not-determined	Genome-fragment	NA	0
S13_vs_3899||full	1676	No	NA	0	2	Not-determined	Genome-fragment	NA	0
S13_vs_3910||full	71167	No	NA	0	18	Not-determined	Genome-fragment	NA	0
S7_vs_8492||full	1266	No	NA	2	0	Low-quality	Genome-fragment	1.73	0
S7_vs_40647||lt2gene	1266	Yes	660	1	1	Low-quality	Genome-fragment	0.31	47.87
S10_extracted_44117	1266	No	NA	0	0	Low-quality	Genome-fragment	1.51	0
S13_vs_4534||full	2588	No	NA	0	1	Not-determined	Genome-fragment	NA	0
S12_extracted_k141_3019	1266	No	NA	1	0	Low-quality	Genome-fragment	2.83	0
S13_vs_4729||full	2577	No	NA	0	1	Not-determined	Genome-fragment	NA	0
S13_vs_4837||full	5053	No	NA	0	2	Not-determined	Genome-fragment	NA	0
S13_vs_4910||full	2336	No	NA	0	2	Not-determined	Genome-fragment	NA	0
S13_vs_4968||full	1120	No	NA	0	1	Not-determined	Genome-fragment	NA	0
S2_extracted_k141_6638	1266	No	NA	0	0	Low-quality	Genome-fragment	1.25	0
S13_vs_5336||full	2313	No	NA	0	2	Not-determined	Genome-fragment	NA	0
S13_vs_5350||full	2473	No	NA	0	1	Not-determined	Genome-fragment	NA	0
S13_vs_5352||full	1290	No	NA	0	0	Not-determined	Genome-fragment	NA	0
S13_vs_5404||full	1243	No	NA	0	2	Not-determined	Genome-fragment	NA	0
S8_extracted_k141_1829	1266	No	NA	0	1	Low-quality	Genome-fragment	2.1	0
S8_extracted_k141_182051	1266	No	NA	1	0	Low-quality	Genome-fragment	0.87	0
S17_vs_k141_160787||full	1267	No	NA	0	0	Low-quality	Genome-fragment	2.53	0
S13_vs_5898||full	6773	No	NA	0	2	Not-determined	Genome-fragment	NA	0
S9_extracted_k141_53222	1267	No	NA	3	0	Low-quality	Genome-fragment	4.18	0
S11_extracted_k141_8934	1267	No	NA	1	0	Low-quality	Genome-fragment	2.51	0
S13_vs_6287||full	2182	No	NA	0	2	Not-determined	Genome-fragment	NA	0
S1_extracted_k141_154107	1267	No	NA	0	0	Low-quality	Genome-fragment	2.52	0
S13_vs_6366||full	1734	No	NA	0	3	Not-determined	Genome-fragment	NA	0
S13_vs_6385||full	1870	No	NA	0	1	Not-determined	Genome-fragment	NA	0
S5_vs_k141_55972||full	1267	No	NA	0	1	Low-quality	Genome-fragment	1.89	0
S13_vs_6558||full	1757	No	NA	0	1	Not-determined	Genome-fragment	NA	0

S13_vs_6769||full	1674	No	NA	0	1	Not-determined	Genome-fragment	NA		0
S13_vs_6794||full	6292	No	NA	0	5	Not-determined	Genome-fragment	NA		0
S7_vs_13386||full	1267	No	NA	1	0	Low-quality	Genome-fragment		2.36	0
S10_extracted_1275	1267	No	NA	1	0	Low-quality	Genome-fragment		7.05	0
S13_vs_7574||full	4813	No	NA	0	2	Not-determined	Genome-fragment	NA		0
S13_vs_7581||full	1471	No	NA	0	0	Not-determined	Genome-fragment	NA		0
S13_vs_7659||full	2718	No	NA	0	0	Not-determined	Genome-fragment	NA		0
S10_vs_29865||full	1267	No	NA	1	0	Low-quality	Genome-fragment		1.82	0
S13_vs_7762||full	1117	No	NA	0	1	Not-determined	Genome-fragment	NA		0
S13_vs_7863||full	1658	No	NA	0	0	Not-determined	Genome-fragment	NA		0
S15_vs_14821||lt2gene	1267	No	NA	2	0	Low-quality	Genome-fragment		3.36	0
S13_vs_7911||full	1196	No	NA	0	0	Not-determined	Genome-fragment	NA		0
S13_vs_7963||full	3195	No	NA	0	1	Not-determined	Genome-fragment	NA		0
S13_vs_7970||full	1100	No	NA	0	0	Not-determined	Genome-fragment	NA		0
S13_vs_8070||full	3385	No	NA	0	1	Not-determined	Genome-fragment	NA		0
S8_vs_k141_48567||full	1267	No	NA	1	0	Low-quality	Genome-fragment		4.34	0
S13_vs_8259||full	1671	No	NA	0	1	Not-determined	Genome-fragment	NA		0
S13_vs_8262||full	5396	No	NA	0	1	Not-determined	Genome-fragment	NA		0
S17_vs_k141_84245||full	1268	No	NA	2	0	Low-quality	Genome-fragment		2.26	0
S13_extracted_94955	1268	No	NA	0	0	Low-quality	Genome-fragment		2.38	0
S14_extracted_k141_66485	1268	No	NA	4	0	Low-quality	Genome-fragment		4.18	0
S13_vs_8785||full	1604	No	NA	0	0	Not-determined	Genome-fragment	NA		0
S13_vs_8788||full	1256	No	NA	0	2	Not-determined	Genome-fragment	NA		0
S13_vs_8817||full	1618	No	NA	0	1	Not-determined	Genome-fragment	NA		0
S14_vs_k141_15080||full	1268	No	NA	0	0	Low-quality	Genome-fragment		0.75	0
S1_vs_k141_18524||full	1268	No	NA	0	0	Low-quality	Genome-fragment		0.72	0
S13_vs_9055||full	4684	No	NA	0	0	Not-determined	Genome-fragment	NA		0
S13_vs_9066||full	2726	No	NA	0	0	Not-determined	Genome-fragment	NA		0
S13_vs_9438||full	32269	Yes	27620	8	3	Medium-quality	Genome-fragment		68.61	14.41
S13_vs_9631||full	2059	No	NA	0	2	Not-determined	Genome-fragment	NA		0
S13_vs_9653||full	2171	No	NA	0	2	Not-determined	Genome-fragment	NA		0
S3_extracted_k141_60767	1268	No	NA	0	0	Low-quality	Genome-fragment		2.91	0
S13_vs_9751||full	2512	No	NA	0	0	Not-determined	Genome-fragment	NA		0
S13_vs_9791||full	2136	No	NA	0	2	Not-determined	Genome-fragment	NA		0
S5_vs_k141_34111||full	1268	No	NA	0	1	Low-quality	Genome-fragment		2.19	0
S13_vs_9896||full	2767	No	NA	0	3	Not-determined	Genome-fragment	NA		0
S13_vs_10001||full	1313	No	NA	0	1	Not-determined	Genome-fragment	NA		0
S13_vs_10334||full	1585	No	NA	0	1	Not-determined	Genome-fragment	NA		0
S7_vs_101554||full	1268	No	NA	0	0	Low-quality	Genome-fragment		2.61	0
S13_vs_10518||full	1370	No	NA	0	0	Not-determined	Genome-fragment	NA		0
S13_vs_10995||full	1638	No	NA	0	1	Not-determined	Genome-fragment	NA		0
S13_vs_11099||full	1683	No	NA	0	1	Not-determined	Genome-fragment	NA		0
S16_extracted_109420	1268	No	NA	0	0	Low-quality	Genome-fragment		0.61	0
S13_vs_11653||full	1935	No	NA	0	1	Not-determined	Genome-fragment	NA		0
S16_vs_97611||full	1268	No	NA	0	1	Low-quality	Genome-fragment		3.5	0
S13_vs_11809||full	1258	No	NA	0	0	Not-determined	Genome-fragment	NA		0
S13_vs_11917||full	1280	No	NA	0	1	Not-determined	Genome-fragment	NA		0
S13_vs_12015||full	3839	No	NA	0	3	Not-determined	Genome-fragment	NA		0
S13_vs_12037||full	1234	No	NA	0	2	Not-determined	Genome-fragment	NA		0
S13_vs_12198||full	2353	No	NA	0	2	Not-determined	Genome-fragment	NA		0
S13_vs_12215||full	1148	No	NA	0	0	Not-determined	Genome-fragment	NA		0
S12_extracted_k141_92007	1268	No	NA	0	1	Low-quality	Genome-fragment		0.91	0
S12_vs_k141_5711||full	1268	No	NA	1	0	Low-quality	Genome-fragment		3.21	0
S13_vs_12406||full	1870	No	NA	0	0	Not-determined	Genome-fragment	NA		0
S13_vs_12724||full	3972	No	NA	0	1	Not-determined	Genome-fragment	NA		0
S12_vs_k141_163011||full	1268	No	NA	0	0	Low-quality	Genome-fragment		2.35	0
S13_vs_12867||full	1608	No	NA	0	1	Not-determined	Genome-fragment	NA		0
S6_vs_k141_24085||lt2gene	1268	No	NA	1	0	Low-quality	Genome-fragment		3.25	0
S8_extracted_k141_124153	1268	No	NA	0	0	Low-quality	Genome-fragment		1.92	0
S13_vs_13129||full	34076	No	NA	13	1	High-quality	High-quality		99.76	0
S13_vs_13498||full	1021	No	NA	0	2	Not-determined	Genome-fragment	NA		0
S11_vs_k141_19919||full	1269	No	NA	1	0	Low-quality	Genome-fragment		1.07	0
S13_vs_13710||full	1196	No	NA	0	1	Not-determined	Genome-fragment	NA		0
S13_vs_13825||full	4706	No	NA	0	1	Not-determined	Genome-fragment	NA		0
S11_vs_k141_26580||full	1269	No	NA	0	0	Low-quality	Genome-fragment		3.35	0
S13_vs_14086||full	1467	No	NA	0	1	Not-determined	Genome-fragment	NA		0
S13_vs_14117||full	3311	No	NA	0	0	Not-determined	Genome-fragment	NA		0
S13_vs_14207||full	1665	No	NA	0	0	Not-determined	Genome-fragment	NA		0
S13_vs_88285||lt2gene	1269	No	NA	2	0	Low-quality	Genome-fragment		27.17	0
S13_vs_14701||full	2400	No	NA	0	0	Not-determined	Genome-fragment	NA		0
S13_vs_14774||full	8676	No	NA	0	1	Not-determined	Genome-fragment	NA		0
S13_vs_14794||full	36336	No	NA	1	0	Medium-quality	Genome-fragment		88.55	0
S7_vs_7907||lt2gene	1269	No	NA	1	0	Low-quality	Genome-fragment		2.65	0
S13_vs_14981||full	3969	No	NA	0	2	Not-determined	Genome-fragment	NA		0
S16_extracted_6210	1269	No	NA	0	0	Low-quality	Genome-fragment		1.54	0
S12_vs_k141_130005||full	1269	No	NA	0	0	Low-quality	Genome-fragment		0.83	0
S13_vs_15162||full	1392	No	NA	0	0	Not-determined	Genome-fragment	NA		0
S6_vs_k141_22443||full	1269	No	NA	0	0	Low-quality	Genome-fragment		0.81	0
S13_vs_15459||full	1287	No	NA	0	0	Not-determined	Genome-fragment	NA		0
S13_vs_15502||full	4554	No	NA	0	4	Not-determined	Genome-fragment	NA		0
S13_vs_15781||full	1119	No	NA	0	2	Not-determined	Genome-fragment	NA		0
S13_vs_15802||full	2613	No	NA	0	2	Not-determined	Genome-fragment	NA		0
S13_vs_16063||full	1371	No	NA	0	2	Not-determined	Genome-fragment	NA		0
S13_vs_16168||full	8163	No	NA	0	1	Not-determined	Genome-fragment	NA		0
S17_vs_k141_44916||full	1270	No	NA	1	0	Low-quality	Genome-fragment		1.9	0
S13_vs_16282||full	1653	No	NA	0	1	Not-determined	Genome-fragment	NA		0
S17_vs_k141_42127||full	1270	No	NA	0	0	Low-quality	Genome-fragment		1.84	0
S9_extracted_k141_41203	1270	No	NA	2	0	Low-quality	Genome-fragment		4.17	0
S13_vs_16572||full	1519	No	NA	0	0	Not-determined	Genome-fragment	NA		0
S11_vs_k141_72248||full	1270	No	NA	0	0	Low-quality	Genome-fragment		1.52	0
S14_extracted_k141_108111	1270	No	NA	1	0	Low-quality	Genome-fragment		2.78	0
S3_extracted_k141_33605	1270	No	NA	0	0	Low-quality	Genome-fragment		1.22	0
S3_extracted_k141_105613	1270	No	NA	2	0	Low-quality	Genome-fragment		2.87	0
S3_vs_k141_101610||full	1270	No	NA	1	0	Low-quality	Genome-fragment		1.84	0
S13_vs_17289||full	4552	No	NA	0	0	Not-determined	Genome-fragment	NA		0
S3_vs_k141_110559||lt2gene	1270	No	NA	0	1	Low-quality	Genome-fragment		3.62	0
S7_vs_91455||full	1270	No	NA	0	0	Low-quality	Genome-fragment		2.44	0
S13_vs_17713||full	1909	No	NA	0	0	Not-determined	Genome-fragment	NA		0
S16_extracted_63446	1270	No	NA	1	0	Low-quality	Genome-fragment		2.89	0
S13_vs_18143||full	1296	No	NA	0	1	Not-determined	Genome-fragment	NA		0
S13_vs_18161||full	1236	No	NA	0	0	Not-determined	Genome-fragment	NA		0
S16_extracted_113782	1270	No	NA	0	2	Low-quality	Genome-fragment		3.32	0
S13_vs_18217||full	1330	No	NA	0	0	Not-determined	Genome-fragment	NA		0
S13_vs_18318||full	1932	No	NA	0	0	Not-determined	Genome-fragment	NA		0
S13_vs_18429||full	5542	No	NA	0	1	Not-determined	Genome-fragment	NA		0
S13_vs_18535||full	1503	No	NA	0	2	Not-determined	Genome-fragment	NA		0
S10_vs_68477||full	1270	No	NA	0	1	Low-quality	Genome-fragment		0.94	0
S13_vs_18613||full	4301	No	NA	0	2	Not-determined	Genome-fragment	NA		0
S13_vs_18693||full	3367	No	NA	0	0	Not-determined	Genome-fragment	NA		0
S13_vs_18744||full	1399	No	NA	0	0	Not-determined	Genome-fragment	NA		0
S6_vs_k141_54578||full	1270	No	NA	0	0	Low-quality	Genome-fragment		1.71	0
S6_vs_k141_53968||lt2gene	1270	No	NA	2	0	Low-quality	Genome-fragment		1.82	0
S13_vs_19256||full	1004	No	NA	0	1	Not-determined	Genome-fragment	NA		0
S9_extracted_k141_57744	1271	No	NA	0	0	Low-quality	Genome-fragment		4.32	0
S9_vs_k141_45526||full	1271	No	NA	4	0	Low-quality	Genome-fragment		3.09	0
S11_vs_k141_5456||full	1271	No	NA	0	1	Low-quality	Genome-fragment		3.49	0
S13_vs_19692||full	3107	No	NA	0	3	Not-determined	Genome-fragment	NA		0
S14_extracted_k141_114032	1271	No	NA	0	1	Low-quality	Genome-fragment		0.69	0
S13_vs_19784||full	1529	No	NA	0	2	Not-determined	Genome-fragment	NA		0
S13_vs_19808||full	35526	No	NA	0	22	Not-determined	Genome-fragment	NA		0
S13_vs_19886||full	1874	No	NA	0	1	Not-determined	Genome-fragment	NA		0
S13_vs_19953||full	1895	No	NA	0	2	Not-determined	Genome-fragment	NA		0
S13_vs_20101||full	2539	No	NA	0	3	Not-determined	Genome-fragment	NA		0
S14_vs_k141_2009||full	1271	No	NA	0	0	Low-quality	Genome-fragment		2.94	0
S13_vs_20167||full	4851	No	NA	0	4	Not-determined	Genome-fragment	NA		0
S13_vs_20171||full	1095	No	NA	0	0	Not-determined	Genome-fragment	NA		0
S1_extracted_k141_8547	1271	No	NA	0	1	Low-quality	Genome-fragment		0.84	0
S13_vs_20301||full	3358	No	NA	0	2	Not-determined	Genome-fragment	NA		0
S5_extracted_k141_19161	1271	No	NA	0	0	Low-quality	Genome-fragment		1.61	0
S13_vs_20790||full	1686	No	NA	0	2	Not-determined	Genome-fragment	NA		0
S13_vs_21614||full	1892	No	NA	0	1	Not-determined	Genome-fragment	NA		0
S13_vs_21657||full	2544	No	NA	0	0	Not-determined	Genome-fragment	NA		0
S5_vs_k141_44843||full	1271	No	NA	0	0	Low-quality	Genome-fragment		0.38	0
S13_vs_21844||full	1085	No	NA	0	0	Not-determined	Genome-fragment	NA		0
S13_vs_21924||full	4702	No	NA	0	3	Not-determined	Genome-fragment	NA		0
S13_vs_22455||full	1831	No	NA	0	0	Not-determined	Genome-fragment	NA		0
S13_vs_22476||full	2178	No	NA	0	0	Not-determined	Genome-fragment	NA		0
S5_vs_k141_9038||full	1271	No	NA	0	0	Low-quality	Genome-fragment		0.99	0
S13_vs_22748||full	1645	No	NA	0	1	Not-determined	Genome-fragment	NA		0
S7_extracted_17219	1271	No	NA	2	0	Low-quality	Genome-fragment		2.86	0
S13_vs_22995||full	1520	No	NA	0	2	Not-determined	Genome-fragment	NA		0
S13_vs_23014||full	2064	No	NA	0	2	Not-determined	Genome-fragment	NA		0
S13_vs_23211||full	1416	No	NA	0	1	Not-determined	Genome-fragment	NA		0
S13_vs_23314||full	52988	Yes	45151	25	7	High-quality	High-quality		95.28	14.79
S7_extracted_42931	1271	No	NA	0	0	Low-quality	Genome-fragment		3.04	0
S7_vs_88727||full	1271	No	NA	2	0	Low-quality	Genome-fragment		2.59	0
S13_vs_23467||full	1353	No	NA	0	0	Not-determined	Genome-fragment	NA		0
S7_vs_98924||full	1271	No	NA	0	0	Low-quality	Genome-fragment		2.89	0
S7_vs_106000||lt2gene	1271	No	NA	2	0	Low-quality	Genome-fragment		2.61	0
S13_vs_24002||full	2185	No	NA	0	1	Not-determined	Genome-fragment	NA		0
S16_extracted_79390	1271	No	NA	4	0	Low-quality	Genome-fragment		4.19	0
S10_extracted_51127	1271	No	NA	0	1	Low-quality	Genome-fragment		2.28	0
S10_extracted_53454	1271	No	NA	1	0	Low-quality	Genome-fragment		2.18	0
S10_extracted_89129	1271	No	NA	1	0	Low-quality	Genome-fragment		2.14	0
S12_extracted_k141_117343	1271	No	NA	0	0	Low-quality	Genome-fragment		2.38	0
S13_vs_24914||full	1471	No	NA	0	0	Not-determined	Genome-fragment	NA		0
S13_vs_24994||full	1445	No	NA	0	0	Not-determined	Genome-fragment	NA		0
S13_vs_25004||full	2759	No	NA	0	1	Not-determined	Genome-fragment	NA		0
S13_vs_25035||full	1841	No	NA	0	0	Not-determined	Genome-fragment	NA		0
S15_extracted_6432	1271	No	NA	0	0	Low-quality	Genome-fragment		2.81	0
S13_vs_25266||full	2297	No	NA	0	0	Not-determined	Genome-fragment	NA		0
S13_vs_25271||full	1400	No	NA	0	0	Not-determined	Genome-fragment	NA		0
S13_vs_25595||full	1751	No	NA	0	0	Not-determined	Genome-fragment	NA		0
S13_vs_25599||full	2907	No	NA	0	2	Not-determined	Genome-fragment	NA		0
S13_vs_25675||full	1438	No	NA	0	1	Not-determined	Genome-fragment	NA		0
S13_vs_25687||full	3831	No	NA	0	3	Not-determined	Genome-fragment	NA		0
S13_vs_25708||full	3030	No	NA	0	1	Not-determined	Genome-fragment	NA		0
S13_vs_25862||full	7705	No	NA	0	2	Not-determined	Genome-fragment	NA		0
S13_vs_25894||full	2449	No	NA	0	2	Not-determined	Genome-fragment	NA		0
S9_vs_k141_7134||full	1272	No	NA	0	0	Low-quality	Genome-fragment		2.37	0
S13_vs_26057||full	1450	No	NA	0	1	Not-determined	Genome-fragment	NA		0
S13_vs_26127||full	1802	No	NA	0	0	Not-determined	Genome-fragment	NA		0
S14_extracted_k141_77215	1272	No	NA	0	1	Low-quality	Genome-fragment		1.39	0
S13_vs_26756||full	48783	Yes	36490	15	8	High-quality	High-quality		99.69	25.2

S14_vs_k141_41343||lt2gene	1272	No	NA	1	0	Low-quality	Genome-fragment	30.57	0
S13_vs_26900||full	2817	No	NA	0	2	Not-determined	Genome-fragment	NA	0
S13_vs_26909||full	2375	No	NA	0	2	Not-determined	Genome-fragment	NA	0
S13_vs_27107||full	3604	No	NA	0	0	Not-determined	Genome-fragment	NA	0
S13_vs_27115||full	1120	No	NA	0	1	Not-determined	Genome-fragment	NA	0
S3_extracted_k141_105316	1272	No	NA	0	0	Low-quality	Genome-fragment	2.9	0
S3_extracted_k141_106628	1272	No	NA	0	0	Low-quality	Genome-fragment	3.62	0
S13_vs_27427||full	4793	No	NA	0	0	Not-determined	Genome-fragment	NA	0
S13_vs_27534||full	1131	No	NA	0	0	Not-determined	Genome-fragment	NA	0
S13_vs_27539||full	3445	No	NA	0	2	Not-determined	Genome-fragment	NA	0
S13_vs_27659||full	1448	No	NA	0	0	Not-determined	Genome-fragment	NA	0
S13_vs_27698||full	7904	No	NA	0	4	Not-determined	Genome-fragment	NA	0
S7_extracted_97666	1272	No	NA	0	1	Low-quality	Genome-fragment	2.53	0
S7_vs_68816||lt2gene	1272	No	NA	1	0	Low-quality	Genome-fragment	5.68	0
S16_extracted_41443	1272	No	NA	0	1	Low-quality	Genome-fragment	0.2	0
S16_vs_18269||full	1272	No	NA	1	0	Low-quality	Genome-fragment	2.41	0
S13_vs_29089||full	2228	No	NA	0	2	Not-determined	Genome-fragment	NA	0
S10_vs_56444||full	1272	No	NA	0	0	Low-quality	Genome-fragment	0.93	0
S6_vs_k141_25731||lt2gene	1272	No	NA	1	0	Low-quality	Genome-fragment	3.65	0
S13_vs_29425||full	1430	No	NA	0	0	Not-determined	Genome-fragment	NA	0
S8_extracted_k141_5867	1272	No	NA	2	0	Low-quality	Genome-fragment	3.5	0
S13_vs_29974||full	1729	No	NA	0	0	Not-determined	Genome-fragment	NA	0
S13_vs_29985||full	8122	No	NA	0	5	Not-determined	Genome-fragment	NA	0
S13_vs_30080||full	1967	No	NA	0	1	Not-determined	Genome-fragment	NA	0
S13_vs_30101||full	113152	Yes	36858	23	27	Medium-quality	Genome-fragment	77.39	67.43
S13_vs_30167||full	62048	Yes	54104	25	9	High-quality	High-quality	100	12.8
S13_vs_30241||full	6511	No	NA	0	1	Not-determined	Genome-fragment	NA	0
S13_vs_30515||full	2537	No	NA	0	2	Not-determined	Genome-fragment	NA	0
S17_extracted_k141_36613	1273	No	NA	1	0	Low-quality	Genome-fragment	2.33	0
S13_vs_30709||full	2376	No	NA	0	1	Not-determined	Genome-fragment	NA	0
S13_vs_31033||full	1197	No	NA	0	0	Not-determined	Genome-fragment	NA	0
S13_vs_31065||full	1808	No	NA	0	1	Not-determined	Genome-fragment	NA	0
S9_extracted_k141_10271	1273	No	NA	2	0	Low-quality	Genome-fragment	1.8	0
S14_extracted_k141_112565	1273	No	NA	0	1	Low-quality	Genome-fragment	2.83	0
S13_vs_31463||full	5566	No	NA	0	4	Not-determined	Genome-fragment	NA	0
S1_vs_k141_10607||full	1273	No	NA	1	0	Low-quality	Genome-fragment	3.01	0
S13_vs_31824||full	3787	No	NA	0	1	Not-determined	Genome-fragment	NA	0
S13_vs_32037||full	2702	No	NA	0	1	Not-determined	Genome-fragment	NA	0
S3_extracted_k141_19562	1273	No	NA	0	1	Low-quality	Genome-fragment	0.95	0
S13_vs_32150||full	2088	No	NA	0	3	Not-determined	Genome-fragment	NA	0
S13_vs_32488||full	1283	No	NA	0	1	Not-determined	Genome-fragment	NA	0
S3_vs_k141_87595||full	1273	No	NA	0	0	Low-quality	Genome-fragment	1.61	0
S5_vs_k141_118041||full	1273	No	NA	2	0	Low-quality	Genome-fragment	2.71	0
S13_vs_33145||full	6257	No	NA	0	1	Not-determined	Genome-fragment	NA	0
S13_vs_33417||full	1719	No	NA	0	1	Not-determined	Genome-fragment	NA	0
S13_vs_33558||full	2852	No	NA	0	0	Not-determined	Genome-fragment	NA	0
S13_vs_33645||full	1705	No	NA	0	1	Not-determined	Genome-fragment	NA	0
S5_vs_k141_77180||full	1273	No	NA	0	1	Low-quality	Genome-fragment	0.55	0
S7_extracted_112181	1273	No	NA	1	0	Low-quality	Genome-fragment	2.71	0
S7_vs_47861||full	1273	No	NA	0	0	Low-quality	Genome-fragment	0.88	0
S13_vs_33947||full	86062	Yes	28387	5	43	Medium-quality	Genome-fragment	70.13	67.02
S7_vs_99015||full	1273	No	NA	0	2	Low-quality	Genome-fragment	0.68	0
S16_vs_21212||full	1273	No	NA	0	0	Low-quality	Genome-fragment	0.81	0
S13_vs_34111||full	2130	No	NA	0	2	Not-determined	Genome-fragment	NA	0
S16_vs_115477||full	1273	No	NA	0	0	Low-quality	Genome-fragment	2.46	0
S13_vs_34344||full	1650	No	NA	0	0	Not-determined	Genome-fragment	NA	0
S12_vs_k141_171020||full	1273	No	NA	0	0	Low-quality	Genome-fragment	3.51	0
S13_vs_34512||full	2406	No	NA	0	1	Not-determined	Genome-fragment	NA	0
S13_vs_34627||full	1243	No	NA	0	0	Not-determined	Genome-fragment	NA	0
S12_vs_k141_56846||lt2gene	1273	No	NA	0	0	Low-quality	Genome-fragment	2.28	0
S2_vs_k141_13092||full	1273	No	NA	1	0	Low-quality	Genome-fragment	3.24	0
S13_vs_35130||full	1807	No	NA	0	0	Not-determined	Genome-fragment	NA	0
S6_vs_k141_39180||full	1273	No	NA	0	0	Low-quality	Genome-fragment	1.92	0
S13_vs_35347||full	1027	No	NA	0	0	Not-determined	Genome-fragment	NA	0
S13_vs_35464||full	1080	No	NA	0	3	Not-determined	Genome-fragment	NA	0
S8_extracted_k141_139681	1273	No	NA	0	0	Low-quality	Genome-fragment	4.05	0
S8_extracted_k141_186517	1273	No	NA	0	1	Low-quality	Genome-fragment	0.73	0
S13_vs_35816||full	57224	Yes	42012	22	6	Complete	High-quality	100	26.58
S13_vs_36251||full	1342	No	NA	0	1	Not-determined	Genome-fragment	NA	0
S9_vs_k141_13557||full	1274	No	NA	2	0	Low-quality	Genome-fragment	2.68	0
S11_vs_k141_31199||full	1274	No	NA	0	0	Low-quality	Genome-fragment	0.86	0
S13_vs_36460||full	1409	No	NA	0	1	Not-determined	Genome-fragment	NA	0
S11_vs_k141_89650||lt2gene	1274	No	NA	1	0	Low-quality	Genome-fragment	1.13	0
S14_vs_k141_41042||lt2gene	1274	No	NA	2	0	Low-quality	Genome-fragment	2.9	0
S5_extracted_k141_67469	1274	No	NA	0	0	Low-quality	Genome-fragment	0.75	0
S13_vs_36932||full	1837	No	NA	0	1	Not-determined	Genome-fragment	NA	0
S13_vs_37340||full	1639	No	NA	0	0	Not-determined	Genome-fragment	NA	0
S16_extracted_48525	1274	No	NA	2	0	Low-quality	Genome-fragment	2.04	0
S16_vs_82032||full	1274	No	NA	0	0	Low-quality	Genome-fragment	1.84	0
S16_vs_142672||full	1274	No	NA	0	0	Low-quality	Genome-fragment	3.03	0
S13_vs_37853||full	2622	No	NA	0	0	Not-determined	Genome-fragment	NA	0
S13_vs_38096||full	1629	No	NA	0	1	Not-determined	Genome-fragment	NA	0
S13_vs_38153||full	6669	No	NA	0	5	Not-determined	Genome-fragment	NA	0
S13_vs_38187||full	2343	No	NA	0	1	Not-determined	Genome-fragment	NA	0
S12_vs_k141_66813||full	1274	No	NA	0	0	Low-quality	Genome-fragment	0.87	0
S12_vs_k141_170128||full	1274	No	NA	0	2	Low-quality	Genome-fragment	1.22	0
S13_vs_38579||full	2034	No	NA	0	1	Not-determined	Genome-fragment	NA	0
S6_extracted_k141_8670	1274	No	NA	0	0	Low-quality	Genome-fragment	1.54	0
S13_vs_38664||full	4569	No	NA	0	3	Not-determined	Genome-fragment	NA	0
S8_vs_k141_125699||full	1274	No	NA	2	0	Low-quality	Genome-fragment	2.1	0
S8_vs_k141_179418||full	1274	No	NA	0	0	Low-quality	Genome-fragment	2.47	0
S13_vs_38724||full	1820	No	NA	0	0	Not-determined	Genome-fragment	NA	0
S13_vs_39245||full	1204	No	NA	0	1	Not-determined	Genome-fragment	NA	0
S17_extracted_k141_134651	1275	No	NA	0	1	Low-quality	Genome-fragment	3.22	0
S13_vs_39536||full	2782	No	NA	0	1	Not-determined	Genome-fragment	NA	0
S14_vs_k141_18853||full	1275	No	NA	1	0	Low-quality	Genome-fragment	3	0
S13_vs_39702||full	2408	No	NA	0	1	Not-determined	Genome-fragment	NA	0
S13_vs_39800||full	1161	No	NA	0	1	Not-determined	Genome-fragment	NA	0
S13_vs_39884||full	1523	No	NA	0	0	Not-determined	Genome-fragment	NA	0
S13_vs_39954||full	2284	No	NA	0	1	Not-determined	Genome-fragment	NA	0
S13_vs_40008||full	3203	No	NA	0	1	Not-determined	Genome-fragment	NA	0
S13_vs_40043||full	1300	No	NA	0	2	Not-determined	Genome-fragment	NA	0
S1_extracted_k141_154648	1275	No	NA	1	0	Low-quality	Genome-fragment	0.82	0
S1_extracted_k141_56493	1275	No	NA	0	0	Low-quality	Genome-fragment	2.99	0
S13_vs_40526||full	1707	No	NA	0	1	Not-determined	Genome-fragment	NA	0
S3_vs_k141_90167||full	1275	No	NA	0	0	Low-quality	Genome-fragment	1.43	0
S13_vs_40645||full	3012	No	NA	0	2	Not-determined	Genome-fragment	NA	0
S5_vs_k141_30851||full	1275	No	NA	0	0	Low-quality	Genome-fragment	3.51	0
S5_vs_k141_96935||full	1275	No	NA	0	0	Low-quality	Genome-fragment	2.19	0
S10_extracted_8195	1275	No	NA	0	0	Low-quality	Genome-fragment	3.09	0
S12_extracted_k141_10514	1275	No	NA	0	0	Low-quality	Genome-fragment	2.71	0
S15_vs_10023||full	1275	No	NA	1	0	Low-quality	Genome-fragment	3.14	0
S13_vs_41418||full	1717	No	NA	0	2	Not-determined	Genome-fragment	NA	0
S13_vs_42028||full	1761	No	NA	0	2	Not-determined	Genome-fragment	NA	0
S13_vs_42165||full	1523	No	NA	0	0	Not-determined	Genome-fragment	NA	0
S6_vs_k141_48769||full	1275	No	NA	0	0	Low-quality	Genome-fragment	0.81	0
S8_extracted_k141_111337	1275	No	NA	2	0	Low-quality	Genome-fragment	3.13	0
S13_vs_42345||full	2140	No	NA	0	0	Not-determined	Genome-fragment	NA	0
S8_vs_k141_52595||full	1275	No	NA	0	4	Low-quality	Genome-fragment	0.4	0
S13_vs_42582||full	1524	No	NA	0	2	Not-determined	Genome-fragment	NA	0
S13_vs_43414||full	1721	No	NA	0	2	Not-determined	Genome-fragment	NA	0
S13_vs_43648||full	1269	No	NA	0	1	Not-determined	Genome-fragment	NA	0
S13_vs_43828||full	1312	No	NA	0	1	Not-determined	Genome-fragment	NA	0
S17_extracted_k141_116055	1276	No	NA	0	0	Low-quality	Genome-fragment	1.72	0
S13_vs_43920||full	1413	No	NA	0	2	Not-determined	Genome-fragment	NA	0
S17_vs_k141_131420||lt2gene	1276	No	NA	1	0	Low-quality	Genome-fragment	2.59	0
S11_vs_k141_84220||lt2gene	1276	No	NA	1	0	Low-quality	Genome-fragment	3.25	0
S14_vs_k141_59883||full	1276	No	NA	0	0	Low-quality	Genome-fragment	2.36	0
S13_vs_44629||full	2155	No	NA	0	2	Not-determined	Genome-fragment	NA	0
S1_vs_k141_68252||full	1276	No	NA	0	0	Low-quality	Genome-fragment	0.73	0
S13_vs_44939||full	1392	No	NA	0	0	Not-determined	Genome-fragment	NA	0
S5_extracted_k141_82592	1276	No	NA	2	0	Low-quality	Genome-fragment	1.83	0
S13_vs_45268||full	2449	No	NA	0	0	Not-determined	Genome-fragment	NA	0
S13_vs_45282||full	2098	No	NA	0	1	Not-determined	Genome-fragment	NA	0
S13_vs_45320||full	1116	No	NA	0	0	Not-determined	Genome-fragment	NA	0
S13_vs_45335||full	3068	No	NA	0	0	Not-determined	Genome-fragment	NA	0
S13_vs_45460||full	1747	No	NA	0	2	Not-determined	Genome-fragment	NA	0
S13_vs_45488||full	124456	Yes	35117	26	44	Medium-quality	Genome-fragment	75.61	71.78
S5_extracted_k141_29300	1276	No	NA	0	0	Low-quality	Genome-fragment	3.53	0
S7_vs_13001||full	1276	No	NA	0	1	Low-quality	Genome-fragment	1.96	0
S13_vs_45600||full	2417	No	NA	0	1	Not-determined	Genome-fragment	NA	0
S13_vs_45609||full	3544	No	NA	0	1	Not-determined	Genome-fragment	NA	0
S13_vs_45671||full	1477	No	NA	0	1	Not-determined	Genome-fragment	NA	0
S12_vs_k141_175093||full	1276	No	NA	0	0	Low-quality	Genome-fragment	1.94	0
S13_vs_45868||full	1425	No	NA	0	2	Not-determined	Genome-fragment	NA	0
S12_vs_k141_115965||full	1276	No	NA	0	1	Low-quality	Genome-fragment	2.75	0
S12_vs_k141_53847||full	1276	No	NA	0	1	Low-quality	Genome-fragment	1.88	0
S8_vs_k141_74946||full	1276	No	NA	0	2	Low-quality	Genome-fragment	0.66	0
S17_vs_k141_52998||full	1277	No	NA	1	0	Low-quality	Genome-fragment	2.76	0
S13_vs_46348||full	12354	No	NA	0	3	Not-determined	Genome-fragment	NA	0
S13_vs_46551||full	6163	No	NA	0	4	Not-determined	Genome-fragment	NA	0
S11_vs_k141_68188||lt2gene	1277	No	NA	1	0	Low-quality	Genome-fragment	1.67	0
S13_vs_46672||full	1695	No	NA	0	0	Not-determined	Genome-fragment	NA	0
S14_vs_k141_65229||full	1277	No	NA	0	0	Low-quality	Genome-fragment	2.71	0
S1_vs_k141_17207||full	1277	No	NA	0	0	Low-quality	Genome-fragment	2.52	0
S5_vs_k141_10793||full	1277	No	NA	1	0	Low-quality	Genome-fragment	2.58	0
S13_vs_47612||full	2840	No	NA	0	3	Not-determined	Genome-fragment	NA	0
S13_vs_47615||full	2539	No	NA	0	2	Not-determined	Genome-fragment	NA	0
S13_vs_47999||full	1691	No	NA	0	1	Not-determined	Genome-fragment	NA	0
S13_vs_48224||full	1011	No	NA	0	1	Not-determined	Genome-fragment	NA	0
S13_vs_48345||full	1749	No	NA	0	0	Not-determined	Genome-fragment	NA	0
S13_vs_48471||full	4607	No	NA	0	1	Not-determined	Genome-fragment	NA	0
S7_extracted_57944	1277	No	NA	2	0	Low-quality	Genome-fragment	5.83	0
S13_vs_48790||full	1626	No	NA	0	2	Not-determined	Genome-fragment	NA	0
S13_vs_48932||full	1558	No	NA	0	2	Not-determined	Genome-fragment	NA	0
S7_vs_55379||full	1277	No	NA	0	0	Low-quality	Genome-fragment	2.78	0
S13_vs_49513||full	3808	No	NA	0	0	Not-determined	Genome-fragment	NA	0
S16_extracted_122042	1277	No	NA	0	1	Low-quality	Genome-fragment	3.6	0
S13_vs_49675||full	2956	No	NA	0	3	Not-determined	Genome-fragment	NA	0
S13_vs_49823||full	1056	No	NA	0	0	Not-determined	Genome-fragment	NA	0
S13_vs_49889||full	31718	No	NA	12	0	Medium-quality	Genome-fragment	72.11	0
S16_vs_116913||full	1277	No	NA	1	0	Low-quality	Genome-fragment	3.74	0
S13_vs_50451||full	6090	No	NA	0	1	Not-determined	Genome-fragment	NA	0
S13_vs_50693||full	2330	No	NA	0	0	Not-determined	Genome-fragment	NA	0

S10_vs_34060||full	1277	No	NA	0	0	Low-quality	Genome-fragment	10.64	0
S12_vs_k141_640||full	1277	No	NA	0	0	Low-quality	Genome-fragment	3.17	0
S4_extracted_757	1277	No	NA	4	0	Low-quality	Genome-fragment	4.23	0
S6_vs_k141_6942||lt2gene	1277	No	NA	1	0	Low-quality	Genome-fragment	2.86	0
S13_vs_51939||full	2594	No	NA	0	3	Not-determined	Genome-fragment	NA	0
S8_vs_k141_86756||full	1277	No	NA	0	0	Low-quality	Genome-fragment	1.61	0
S9_vs_k141_68651||full	1278	No	NA	0	0	Low-quality	Genome-fragment	1.71	0
S11_extracted_k141_95412	1278	No	NA	0	0	Low-quality	Genome-fragment	2.02	0
S11_vs_k141_30184||full	1278	No	NA	0	1	Low-quality	Genome-fragment	2.19	0
S14_vs_k141_4504||full	1278	No	NA	0	1	Low-quality	Genome-fragment	2.17	0
S3_extracted_k141_111379	1278	No	NA	0	0	Low-quality	Genome-fragment	0.9	0
S13_vs_52652||full	1933	No	NA	0	0	Not-determined	Genome-fragment	NA	0
S13_vs_52666||full	3156	No	NA	0	1	Not-determined	Genome-fragment	NA	0
S13_vs_52912||full	6979	No	NA	0	5	Not-determined	Genome-fragment	NA	0
S13_vs_52924||full	1822	No	NA	0	2	Not-determined	Genome-fragment	NA	0
S5_vs_k141_56350||full	1278	No	NA	0	0	Low-quality	Genome-fragment	2.85	0
S10_extracted_86478	1278	No	NA	0	2	Low-quality	Genome-fragment	0.59	0
S13_vs_53676||full	2043	No	NA	0	0	Not-determined	Genome-fragment	NA	0
S10_vs_38500||full	1278	No	NA	0	2	Low-quality	Genome-fragment	2.18	0
S13_vs_53857||full	1058	No	NA	0	1	Not-determined	Genome-fragment	NA	0
S13_vs_54076||full	6304	No	NA	0	0	Not-determined	Genome-fragment	NA	0
S13_vs_54183||full	2025	No	NA	0	0	Not-determined	Genome-fragment	NA	0
S12_extracted_k141_126683	1278	No	NA	1	0	Low-quality	Genome-fragment	2.9	0
S13_vs_54602||full	1656	No	NA	0	0	Not-determined	Genome-fragment	NA	0
S13_vs_54826||full	1288	No	NA	0	1	Not-determined	Genome-fragment	NA	0
S13_vs_54920||full	1507	No	NA	0	3	Not-determined	Genome-fragment	NA	0
S13_vs_55038||full	1012	No	NA	0	0	Not-determined	Genome-fragment	NA	0
S12_vs_k141_119160||full	1278	No	NA	1	0	Low-quality	Genome-fragment	1.3	0
S13_vs_55658||full	1059	No	NA	0	0	Not-determined	Genome-fragment	NA	0
S13_vs_55660||full	2230	No	NA	0	0	Not-determined	Genome-fragment	NA	0
S6_vs_k141_48708||full	1278	No	NA	2	0	Low-quality	Genome-fragment	2.14	0
S13_vs_27729||lt2gene	1279	No	NA	1	0	Low-quality	Genome-fragment	9.37	0
S14_extracted_k141_23002	1279	No	NA	1	0	Low-quality	Genome-fragment	0.49	0
S13_vs_55906||full	1038	No	NA	0	1	Not-determined	Genome-fragment	NA	0
S1_vs_k141_103529||full	1279	No	NA	0	0	Low-quality	Genome-fragment	2.64	0
S13_vs_55997||full	7716	No	NA	0	6	Not-determined	Genome-fragment	NA	0
S10_extracted_93894	1279	No	NA	0	0	Low-quality	Genome-fragment	3.01	0
S13_vs_56260||full	3422	No	NA	0	2	Not-determined	Genome-fragment	NA	0
S13_vs_56327||full	1089	No	NA	0	1	Not-determined	Genome-fragment	NA	0
S10_vs_41166||full	1279	No	NA	0	0	Low-quality	Genome-fragment	0.56	0
S12_vs_k141_123229||full	1279	No	NA	0	0	Low-quality	Genome-fragment	2.42	0
S13_vs_56606||full	1028	No	NA	0	0	Not-determined	Genome-fragment	NA	0
S8_vs_k141_73029||full	1279	No	NA	0	0	Low-quality	Genome-fragment	2.83	0
S8_vs_k141_91532||full	1279	No	NA	0	0	Low-quality	Genome-fragment	2.71	0
S9_vs_k141_19154||full	1280	No	NA	0	0	Low-quality	Genome-fragment	2	0
S9_vs_k141_82472||lt2gene	1280	No	NA	1	0	Low-quality	Genome-fragment	3.15	0
S13_vs_57079||full	2091	No	NA	0	2	Not-determined	Genome-fragment	NA	0
S13_vs_57133||full	5135	No	NA	0	3	Not-determined	Genome-fragment	NA	0
S13_vs_57144||full	2467	No	NA	0	2	Not-determined	Genome-fragment	NA	0
S14_vs_k141_94380||full	1280	No	NA	4	0	Low-quality	Genome-fragment	2.64	0
S13_vs_57251||full	1178	No	NA	0	0	Not-determined	Genome-fragment	NA	0
S13_vs_57267||full	289184	No	NA	24	9	Medium-quality	Genome-fragment	72.12	0
S13_vs_57417||full	1880	No	NA	0	0	Not-determined	Genome-fragment	NA	0
S14_vs_k141_13370||full	1280	No	NA	0	1	Low-quality	Genome-fragment	2.91	0
S13_vs_57889||full	1107	No	NA	0	0	Not-determined	Genome-fragment	NA	0
S13_vs_58129||full	1686	No	NA	0	0	Not-determined	Genome-fragment	NA	0
S13_vs_58230||full	3969	No	NA	0	1	Not-determined	Genome-fragment	NA	0
S13_vs_58609||full	1051	No	NA	0	0	Not-determined	Genome-fragment	NA	0
S13_vs_58699||full	2595	No	NA	0	1	Not-determined	Genome-fragment	NA	0
S13_vs_58864||full	1296	No	NA	0	1	Not-determined	Genome-fragment	NA	0
S1_vs_k141_22927||full	1280	No	NA	0	0	Low-quality	Genome-fragment	1.42	0
S13_vs_58904||full	10659	No	NA	0	7	Not-determined	Genome-fragment	NA	0
S1_vs_k141_25391||full	1280	No	NA	0	0	Low-quality	Genome-fragment	2.89	0
S13_vs_59103||full	1615	No	NA	0	1	Not-determined	Genome-fragment	NA	0
S13_vs_59225||full	3306	No	NA	0	2	Not-determined	Genome-fragment	NA	0
S3_extracted_k141_68139	1280	No	NA	0	0	Low-quality	Genome-fragment	3.07	0
S13_vs_59490||full	8533	No	NA	0	0	Not-determined	Genome-fragment	NA	0
S13_vs_59547||full	1549	No	NA	0	1	Not-determined	Genome-fragment	NA	0
S13_vs_59961||full	2007	No	NA	0	1	Not-determined	Genome-fragment	NA	0
S13_vs_59975||full	1570	No	NA	0	0	Not-determined	Genome-fragment	NA	0
S13_vs_60281||full	1249	No	NA	0	2	Not-determined	Genome-fragment	NA	0
S13_vs_60368||full	2153	No	NA	0	2	Not-determined	Genome-fragment	NA	0
S13_vs_60467||full	1121	No	NA	0	1	Not-determined	Genome-fragment	NA	0
S13_vs_60534||full	4786	No	NA	0	0	Not-determined	Genome-fragment	NA	0
S13_vs_60535||full	1589	No	NA	0	0	Not-determined	Genome-fragment	NA	0
S3_vs_k141_4460||full	1280	No	NA	0	0	Low-quality	Genome-fragment	3.45	0
S5_vs_k141_68558||full	1280	No	NA	0	0	Low-quality	Genome-fragment	1.97	0
S13_vs_60997||full	1281	No	NA	0	1	Not-determined	Genome-fragment	NA	0
S7_extracted_110715	1280	No	NA	0	0	Low-quality	Genome-fragment	3.03	0
S7_vs_23211||full	1280	No	NA	0	0	Low-quality	Genome-fragment	0.89	0
S13_vs_61225||full	3354	No	NA	0	3	Not-determined	Genome-fragment	NA	0
S13_vs_61262||full	5859	No	NA	0	2	Not-determined	Genome-fragment	NA	0
S7_vs_134278||lt2gene	1280	No	NA	1	0	Low-quality	Genome-fragment	2.09	0
S16_vs_5266||full	1280	No	NA	0	0	Low-quality	Genome-fragment	0.71	0
S13_vs_61677||full	2595	No	NA	0	1	Not-determined	Genome-fragment	NA	0
S16_vs_53255||full	1280	No	NA	0	0	Low-quality	Genome-fragment	2.35	0
S13_vs_62031||full	1518	No	NA	0	1	Not-determined	Genome-fragment	NA	0
S12_extracted_k141_6775	1280	No	NA	0	0	Low-quality	Genome-fragment	1.31	0
S13_vs_62142||full	32085	No	NA	0	8	Not-determined	Genome-fragment	NA	0
S13_vs_62243||full	1976	No	NA	0	0	Not-determined	Genome-fragment	NA	0
S13_vs_62489||full	1832	No	NA	0	0	Not-determined	Genome-fragment	NA	0
S13_vs_62728||full	2197	No	NA	0	1	Not-determined	Genome-fragment	NA	0
S12_extracted_k141_21779	1280	No	NA	0	1	Low-quality	Genome-fragment	0.84	0
S12_vs_k141_142876||full	1280	No	NA	1	0	Low-quality	Genome-fragment	3.06	0
S13_vs_63080||full	2295	No	NA	0	2	Not-determined	Genome-fragment	NA	0
S6_vs_k141_24207||full	1280	No	NA	0	1	Low-quality	Genome-fragment	2.08	0
S13_vs_63425||full	2104	No	NA	0	0	Not-determined	Genome-fragment	NA	0
S13_vs_63455||full	4154	No	NA	0	2	Not-determined	Genome-fragment	NA	0
S13_vs_63794||full	2820	No	NA	0	4	Not-determined	Genome-fragment	NA	0
S13_vs_63833||full	1280	No	NA	0	1	Not-determined	Genome-fragment	NA	0
S8_vs_k141_11646||lt2gene	1280	No	NA	2	0	Low-quality	Genome-fragment	3.16	0
S13_vs_63949||full	1893	No	NA	0	1	Not-determined	Genome-fragment	NA	0
S13_vs_64129||full	1127	No	NA	0	0	Not-determined	Genome-fragment	NA	0
S17_vs_k141_162569||full	1281	No	NA	0	0	Low-quality	Genome-fragment	3.07	0
S13_vs_64380||full	1357	No	NA	0	0	Not-determined	Genome-fragment	NA	0
S13_vs_64381||full	1173	No	NA	0	1	Not-determined	Genome-fragment	NA	0
S13_vs_64514||full	1255	No	NA	0	1	Not-determined	Genome-fragment	NA	0
S13_vs_64701||full	1746	No	NA	0	0	Not-determined	Genome-fragment	NA	0
S13_vs_64745||full	11958	No	NA	0	6	Not-determined	Genome-fragment	NA	0
S17_vs_k141_153266||lt2gene	1281	No	NA	1	0	Low-quality	Genome-fragment	3.05	0
S13_extracted_66306	1281	No	NA	2	0	Low-quality	Genome-fragment	3.36	0
S13_vs_65215||full	3526	No	NA	0	3	Not-determined	Genome-fragment	NA	0
S14_extracted_k141_15501	1281	No	NA	2	0	Low-quality	Genome-fragment	4.23	0
S14_extracted_k141_82287	1281	No	NA	1	0	Low-quality	Genome-fragment	4.22	0
S13_vs_65782||full	1128	No	NA	0	1	Not-determined	Genome-fragment	NA	0
S13_vs_65800||full	3219	No	NA	0	0	Not-determined	Genome-fragment	NA	0
S13_vs_65904||full	5998	No	NA	0	0	Not-determined	Genome-fragment	NA	0
S1_vs_k141_155635||full	1281	No	NA	0	1	Low-quality	Genome-fragment	0.75	0
S3_extracted_k141_94221	1281	No	NA	0	0	Low-quality	Genome-fragment	1.23	0
S5_extracted_k141_59290	1281	No	NA	0	0	Low-quality	Genome-fragment	5.85	0
S13_vs_66485||full	1368	No	NA	0	1	Not-determined	Genome-fragment	NA	0
S5_vs_k141_38715||full	1281	No	NA	0	0	Low-quality	Genome-fragment	3.39	0
S7_extracted_42135	1281	No	NA	0	0	Low-quality	Genome-fragment	2.83	0
S7_extracted_129772	1281	No	NA	1	0	Low-quality	Genome-fragment	0.93	0
S13_vs_67201||full	1663	No	NA	0	0	Not-determined	Genome-fragment	NA	0
S7_vs_79045||full	1281	No	NA	0	0	Low-quality	Genome-fragment	1.75	0
S13_vs_67277||full	1275	No	NA	0	0	Not-determined	Genome-fragment	NA	0
S13_vs_67429||full	91295	Yes	41498	17	24	High-quality	High-quality	100	54.55
S13_vs_67502||full	6834	No	NA	0	5	Not-determined	Genome-fragment	NA	0
S13_vs_67604||full	1527	No	NA	0	2	Not-determined	Genome-fragment	NA	0
S16_vs_40708||full	1281	No	NA	1	0	Low-quality	Genome-fragment	2.73	0
S13_vs_67787||full	1424	No	NA	0	1	Not-determined	Genome-fragment	NA	0
S13_vs_67875||full	1039	No	NA	0	0	Not-determined	Genome-fragment	NA	0
S13_vs_68102||full	15847	No	NA	8	0	Medium-quality	Genome-fragment	73.21	0
S13_vs_68142||full	2602	No	NA	0	2	Not-determined	Genome-fragment	NA	0
S13_vs_68179||full	1865	No	NA	0	3	Not-determined	Genome-fragment	NA	0
S16_vs_27331||lt2gene	1281	Yes	670	1	1	Low-quality	Genome-fragment	1.11	47.7
S12_vs_k141_64935||full	1281	No	NA	0	0	Low-quality	Genome-fragment	2.47	0
S13_vs_68492||full	1821	No	NA	0	3	Not-determined	Genome-fragment	NA	0
S13_vs_68615||full	1126	No	NA	0	1	Not-determined	Genome-fragment	NA	0
S13_vs_68630||full	1761	No	NA	0	1	Not-determined	Genome-fragment	NA	0
S13_vs_68703||full	1857	No	NA	0	0	Not-determined	Genome-fragment	NA	0
S13_vs_68752||full	2679	No	NA	0	1	Not-determined	Genome-fragment	NA	0
S6_vs_k141_36418||full	1281	No	NA	1	0	Low-quality	Genome-fragment	2.86	0
S17_extracted_k141_141142	1282	No	NA	0	0	Low-quality	Genome-fragment	2.03	0
S13_vs_68813||full	2305	No	NA	0	0	Not-determined	Genome-fragment	NA	0
S13_vs_68986||full	4011	No	NA	0	1	Not-determined	Genome-fragment	NA	0
S13_vs_69027||full	2527	No	NA	0	4	Not-determined	Genome-fragment	NA	0
S9_vs_k141_82496||full	1282	No	NA	0	0	Low-quality	Genome-fragment	2.32	0
S13_vs_69186||full	2670	No	NA	0	2	Not-determined	Genome-fragment	NA	0
S11_vs_k141_35949||full	1282	No	NA	0	1	Low-quality	Genome-fragment	0.39	0
S13_vs_69722||full	1121	No	NA	0	0	Not-determined	Genome-fragment	NA	0
S1_vs_k141_64244||full	1282	No	NA	1	0	Low-quality	Genome-fragment	0.94	0
S13_vs_69959||full	1767	No	NA	0	1	Not-determined	Genome-fragment	NA	0
S13_vs_69998||full	1203	No	NA	0	1	Not-determined	Genome-fragment	NA	0
S13_vs_70063||full	1103	No	NA	0	1	Not-determined	Genome-fragment	NA	0
S5_extracted_k141_69147	1282	No	NA	0	0	Low-quality	Genome-fragment	3.17	0
S13_vs_70347||full	4307	No	NA	0	1	Not-determined	Genome-fragment	NA	0
S5_vs_k141_104527||lt2gene	1282	No	NA	1	0	Low-quality	Genome-fragment	3.52	0
S16_extracted_132659	1282	No	NA	0	1	Low-quality	Genome-fragment	1.65	0
S13_vs_71060||full	1388	No	NA	0	1	Not-determined	Genome-fragment	NA	0
S13_vs_71086||full	1311	No	NA	0	2	Not-determined	Genome-fragment	NA	0
S16_vs_38174||full	1282	No	NA	0	2	Low-quality	Genome-fragment	0.37	0
S13_vs_71319||full	1296	No	NA	0	2	Not-determined	Genome-fragment	NA	0
S13_vs_71448||full	1226	No	NA	0	0	Not-determined	Genome-fragment	NA	0
S13_vs_71450||full	2701	No	NA	0	2	Not-determined	Genome-fragment	NA	0
S13_vs_71476||full	1263	No	NA	0	1	Not-determined	Genome-fragment	NA	0
S13_vs_71522||full	1917	No	NA	0	0	Not-determined	Genome-fragment	NA	0
S13_vs_71851||full	1745	No	NA	0	3	Not-determined	Genome-fragment	NA	0
S15_extracted_52124	1282	No	NA	0	0	Low-quality	Genome-fragment	0.88	0
S15_vs_5582||full	1282	No	NA	0	0	Low-quality	Genome-fragment	3.15	0
S13_vs_72212||full	3714	No	NA	0	3	Not-determined	Genome-fragment	NA	0
S13_vs_72276||full	2562	No	NA	0	0	Not-determined	Genome-fragment	NA	0
S13_vs_72637||full	2218	No	NA	0	2	Not-determined	Genome-fragment	NA	0

S13_vs_72654||full	2035	No	NA	0	2	Not-determined	Genome-fragment	NA		0
S13_vs_72668||full	1954	No	NA	0	1	Not-determined	Genome-fragment	NA		0
S13_vs_72848||full	1109	No	NA	0	1	Not-determined	Genome-fragment	NA		0
S13_vs_72996||full	1123	No	NA	0	0	Not-determined	Genome-fragment	NA		0
S13_vs_73159||full	3773	No	NA	0	2	Not-determined	Genome-fragment	NA		0
S4_extracted_14061	1282	No	NA	1	0	Low-quality	Genome-fragment		4.23	0
S13_vs_73333||full	1654	No	NA	0	0	Not-determined	Genome-fragment	NA		0
S8_vs_k141_70989||full	1282	No	NA	2	0	Low-quality	Genome-fragment		2.86	0
S13_vs_73418||full	12113	No	NA	0	6	Not-determined	Genome-fragment	NA		0
S13_vs_73517||full	1238	No	NA	0	0	Not-determined	Genome-fragment	NA		0
S13_vs_73658||full	2266	No	NA	0	2	Not-determined	Genome-fragment	NA		0
S13_vs_73837||full	1701	No	NA	0	0	Not-determined	Genome-fragment	NA		0
S13_vs_73855||full	1794	No	NA	0	0	Not-determined	Genome-fragment	NA		0
S13_vs_73864||full	2323	No	NA	0	3	Not-determined	Genome-fragment	NA		0
S13_vs_73911||full	1870	No	NA	0	2	Not-determined	Genome-fragment	NA		0
S17_vs_k141_95044||full	1283	No	NA	0	0	Low-quality	Genome-fragment		1.86	0
S13_vs_74518||full	1246	No	NA	0	3	Not-determined	Genome-fragment	NA		0
S13_vs_74542||full	1222	No	NA	0	0	Not-determined	Genome-fragment	NA		0
S13_vs_74580||full	1755	No	NA	0	0	Not-determined	Genome-fragment	NA		0
S9_vs_k141_72767||full	1283	No	NA	0	0	Low-quality	Genome-fragment		2.63	0
S13_vs_74849||full	1180	No	NA	0	1	Not-determined	Genome-fragment	NA		0
S11_extracted_k141_37737	1283	No	NA	1	0	Low-quality	Genome-fragment		3.2	0
S13_vs_74971||full	1256	No	NA	0	1	Not-determined	Genome-fragment	NA		0
S13_vs_75012||full	1617	No	NA	0	0	Not-determined	Genome-fragment	NA		0
S14_vs_k141_10958||full	1283	No	NA	0	0	Low-quality	Genome-fragment		2.11	0
S13_vs_75369||full	1534	No	NA	0	1	Not-determined	Genome-fragment	NA		0
S7_vs_79482||full	1283	No	NA	1	0	Low-quality	Genome-fragment		3.49	0
S13_vs_75681||full	2457	No	NA	0	0	Not-determined	Genome-fragment	NA		0
S13_vs_75695||full	2239	No	NA	0	3	Not-determined	Genome-fragment	NA		0
S16_extracted_50777	1283	No	NA	0	1	Low-quality	Genome-fragment		1.56	0
S13_vs_75867||full	2155	No	NA	0	1	Not-determined	Genome-fragment	NA		0
S13_vs_76004||full	1613	No	NA	0	0	Not-determined	Genome-fragment	NA		0
S13_vs_76115||full	2506	No	NA	0	0	Not-determined	Genome-fragment	NA		0
S6_vs_k141_42820||full	1283	No	NA	1	0	Low-quality	Genome-fragment		3.21	0
S13_vs_76193||full	3550	No	NA	0	1	Not-determined	Genome-fragment	NA		0
S8_extracted_k141_51330	1283	No	NA	2	0	Low-quality	Genome-fragment		2.83	0
S17_vs_k141_71998||full	1284	No	NA	0	2	Low-quality	Genome-fragment		0.45	0
S13_vs_76573||full	1219	No	NA	0	1	Not-determined	Genome-fragment	NA		0
S9_vs_k141_84577||full	1284	No	NA	2	0	Low-quality	Genome-fragment		3.28	0
S13_vs_76934||full	1842	No	NA	0	3	Not-determined	Genome-fragment	NA		0
S13_vs_76950||full	2058	No	NA	0	3	Not-determined	Genome-fragment	NA		0
S11_vs_k141_101017||full	1284	No	NA	1	0	Low-quality	Genome-fragment		3.37	0
S13_vs_76980||full	3115	No	NA	0	3	Not-determined	Genome-fragment	NA		0
S13_vs_77128||full	1561	No	NA	0	0	Not-determined	Genome-fragment	NA		0
S14_extracted_k141_102871	1284	No	NA	0	0	Low-quality	Genome-fragment		1.54	0
S14_extracted_k141_52055	1284	No	NA	1	0	Low-quality	Genome-fragment		1.88	0
S13_vs_77370||full	1424	No	NA	0	2	Not-determined	Genome-fragment	NA		0
S13_vs_77981||full	20756	No	NA	0	13	Not-determined	Genome-fragment	NA		0
S13_vs_78017||full	2517	No	NA	0	0	Not-determined	Genome-fragment	NA		0
S13_vs_78620||full	1780	No	NA	0	2	Not-determined	Genome-fragment	NA		0
S13_vs_78659||full	1301	No	NA	0	1	Not-determined	Genome-fragment	NA		0
S13_vs_78895||full	1172	No	NA	0	0	Not-determined	Genome-fragment	NA		0
S13_vs_79793||full	1219	No	NA	0	2	Not-determined	Genome-fragment	NA		0
S14_extracted_k141_11983	1284	No	NA	0	0	Low-quality	Genome-fragment		1.29	0
S13_vs_79966||full	5152	No	NA	0	1	Not-determined	Genome-fragment	NA		0
S13_vs_80078||full	1659	No	NA	0	1	Not-determined	Genome-fragment	NA		0
S13_vs_80446||full	1971	No	NA	0	0	Not-determined	Genome-fragment	NA		0
S13_vs_80456||full	1646	No	NA	0	0	Not-determined	Genome-fragment	NA		0
S13_vs_80570||full	2050	No	NA	0	2	Not-determined	Genome-fragment	NA		0
S13_vs_80579||full	3982	No	NA	0	3	Not-determined	Genome-fragment	NA		0
S13_vs_80645||full	2428	No	NA	0	0	Not-determined	Genome-fragment	NA		0
S14_extracted_k141_56808	1284	No	NA	0	0	Low-quality	Genome-fragment		2.87	0
S13_vs_80839||full	1368	No	NA	0	0	Not-determined	Genome-fragment	NA		0
S14_vs_k141_107498||full	1284	No	NA	0	2	Low-quality	Genome-fragment		1.59	0
S13_vs_81140||full	10596	No	NA	0	1	Not-determined	Genome-fragment	NA		0
S13_vs_81222||full	3992	No	NA	0	0	Not-determined	Genome-fragment	NA		0
S1_extracted_k141_56901	1284	No	NA	2	0	Low-quality	Genome-fragment		2.88	0
S13_vs_81364||full	2131	No	NA	0	1	Not-determined	Genome-fragment	NA		0
S13_vs_81494||full	2502	No	NA	0	1	Not-determined	Genome-fragment	NA		0
S7_vs_95122||full	1284	No	NA	0	0	Low-quality	Genome-fragment		2.86	0
S13_vs_81789||full	2508	No	NA	0	1	Not-determined	Genome-fragment	NA		0
S16_extracted_14634	1284	No	NA	0	0	Low-quality	Genome-fragment		2.14	0
S13_vs_82039||full	2238	No	NA	0	0	Not-determined	Genome-fragment	NA		0
S13_vs_82277||full	1259	No	NA	0	2	Not-determined	Genome-fragment	NA		0
S13_vs_82498||full	4264	No	NA	0	2	Not-determined	Genome-fragment	NA		0
S13_vs_82520||full	2505	No	NA	0	1	Not-determined	Genome-fragment	NA		0
S13_vs_83104||full	1136	No	NA	0	1	Not-determined	Genome-fragment	NA		0
S13_vs_83228||full	2576	No	NA	0	2	Not-determined	Genome-fragment	NA		0
S10_vs_20402||full	1284	No	NA	0	1	Low-quality	Genome-fragment		2.84	0
S13_vs_83326||full	2083	No	NA	0	1	Not-determined	Genome-fragment	NA		0
S10_vs_27043||full	1284	No	NA	1	0	Low-quality	Genome-fragment		1.9	0
S13_vs_83404||full	1719	No	NA	0	0	Not-determined	Genome-fragment	NA		0
S13_vs_83447||full	56650	No	NA	16	4	High-quality	High-quality		100	0
S12_vs_k141_187991||full	1284	No	NA	1	0	Low-quality	Genome-fragment		3	0
S13_vs_83622||full	1779	No	NA	0	0	Not-determined	Genome-fragment	NA		0
S13_vs_83809||full	1711	No	NA	0	0	Not-determined	Genome-fragment	NA		0
S13_vs_84187||full	1969	No	NA	0	0	Not-determined	Genome-fragment	NA		0
S13_vs_84458||full	1386	No	NA	0	0	Not-determined	Genome-fragment	NA		0
S13_vs_84539||full	2149	No	NA	0	3	Not-determined	Genome-fragment	NA		0
S4_extracted_6170	1284	No	NA	0	1	Low-quality	Genome-fragment		1.62	0
S8_extracted_k141_54823	1284	No	NA	0	1	Low-quality	Genome-fragment		0.96	0
S8_extracted_k141_49838	1284	No	NA	0	0	Low-quality	Genome-fragment		1.19	0
S13_vs_85204||full	1840	No	NA	0	0	Not-determined	Genome-fragment	NA		0
S13_vs_85252||full	1262	No	NA	0	0	Not-determined	Genome-fragment	NA		0
S13_vs_85254||full	2127	No	NA	0	2	Not-determined	Genome-fragment	NA		0
S8_vs_k141_105554||full	1284	No	NA	0	0	Low-quality	Genome-fragment		3.08	0
S17_vs_k141_11988||full	1285	No	NA	1	0	Low-quality	Genome-fragment		2.2	0
S13_vs_85535||full	19277	No	NA	0	11	Not-determined	Genome-fragment	NA		0
S13_vs_85647||full	1206	No	NA	0	0	Not-determined	Genome-fragment	NA		0
S9_vs_k141_75327||lt2gene	1285	No	NA	1	0	Low-quality	Genome-fragment		2.86	0
S13_vs_85853||full	1588	No	NA	0	2	Not-determined	Genome-fragment	NA		0
S13_vs_85895||full	2221	No	NA	0	0	Not-determined	Genome-fragment	NA		0
S11_extracted_k141_12025	1285	No	NA	2	0	Low-quality	Genome-fragment		3.17	0
S13_vs_86100||full	1320	No	NA	0	0	Not-determined	Genome-fragment	NA		0
S11_vs_k141_88175||full	1285	No	NA	1	0	Low-quality	Genome-fragment		2.21	0
S13_vs_86212||full	1859	No	NA	0	0	Not-determined	Genome-fragment	NA		0
S13_vs_86222||full	1736	No	NA	0	1	Not-determined	Genome-fragment	NA		0
S13_vs_86251||full	1452	No	NA	0	0	Not-determined	Genome-fragment	NA		0
S11_vs_k141_79894||lt2gene	1285	No	NA	1	0	Low-quality	Genome-fragment		1.18	0
S13_extracted_108812	1285	No	NA	1	0	Low-quality	Genome-fragment		0.82	0
S13_vs_86796||full	2442	No	NA	0	1	Not-determined	Genome-fragment	NA		0
S1_vs_k141_138039||full	1285	No	NA	0	1	Low-quality	Genome-fragment		0.34	0
S13_vs_86872||full	1389	No	NA	0	2	Not-determined	Genome-fragment	NA		0
S13_vs_87084||full	1231	No	NA	0	1	Not-determined	Genome-fragment	NA		0
S13_vs_87108||full	10612	No	NA	3	0	Medium-quality	Genome-fragment		77.8	0
S3_vs_k141_76574||full	1285	No	NA	0	0	Low-quality	Genome-fragment		2.46	0
S13_vs_87197||full	2147	No	NA	0	0	Not-determined	Genome-fragment	NA		0
S3_vs_k141_117145||full	1285	No	NA	0	0	Low-quality	Genome-fragment		0.87	0
S13_vs_87204||full	5785	No	NA	0	1	Not-determined	Genome-fragment	NA		0
S5_extracted_k141_122911	1285	No	NA	0	1	Low-quality	Genome-fragment		0.84	0
S13_vs_87782||full	1064	No	NA	0	1	Not-determined	Genome-fragment	NA		0
S5_vs_k141_141907||full	1285	No	NA	0	1	Low-quality	Genome-fragment		2.25	0
S16_extracted_27364	1285	No	NA	1	0	Low-quality	Genome-fragment		2.38	0
S13_vs_87981||full	1128	No	NA	0	0	Not-determined	Genome-fragment	NA		0
S13_vs_88012||full	1296	No	NA	0	0	Not-determined	Genome-fragment	NA		0
S13_vs_88059||full	2900	No	NA	0	1	Not-determined	Genome-fragment	NA		0
S13_vs_88097||full	1737	No	NA	0	3	Not-determined	Genome-fragment	NA		0
S13_vs_88101||full	1253	No	NA	0	1	Not-determined	Genome-fragment	NA		0
S13_vs_88103||full	3441	No	NA	0	0	Not-determined	Genome-fragment	NA		0
S13_vs_88234||full	1011	No	NA	0	0	Not-determined	Genome-fragment	NA		0
S16_extracted_101202	1285	No	NA	0	0	Low-quality	Genome-fragment		1.14	0
S10_extracted_37968	1285	No	NA	0	0	Low-quality	Genome-fragment		0.75	0
S13_vs_88573||full	1529	No	NA	0	2	Not-determined	Genome-fragment	NA		0
S13_vs_88674||full	3292	No	NA	0	1	Not-determined	Genome-fragment	NA		0
S13_vs_88907||full	1169	No	NA	0	1	Not-determined	Genome-fragment	NA		0
S13_vs_88917||full	1484	No	NA	0	0	Not-determined	Genome-fragment	NA		0
S13_vs_89299||full	2154	No	NA	0	1	Not-determined	Genome-fragment	NA		0
S13_vs_89656||full	6197	No	NA	0	3	Not-determined	Genome-fragment	NA		0
S13_vs_89871||full	4373	No	NA	0	2	Not-determined	Genome-fragment	NA		0
S13_vs_90223||full	1074	No	NA	0	1	Not-determined	Genome-fragment	NA		0
S10_vs_118236||full	1285	No	NA	1	0	Low-quality	Genome-fragment		3.3	0
S2_extracted_k141_5390	1285	No	NA	0	2	Low-quality	Genome-fragment		1.09	0
S13_vs_90280||full	1610	No	NA	0	0	Not-determined	Genome-fragment	NA		0
S13_vs_90430||full	2930	No	NA	0	2	Not-determined	Genome-fragment	NA		0
S13_vs_90441||full	1457	No	NA	0	0	Not-determined	Genome-fragment	NA		0
S13_vs_90489||full	1751	No	NA	0	0	Not-determined	Genome-fragment	NA		0
S13_vs_90500||full	3490	No	NA	0	3	Not-determined	Genome-fragment	NA		0
S8_vs_k141_138678||full	1285	No	NA	1	0	Low-quality	Genome-fragment		2.97	0
S9_vs_k141_61351||lt2gene	1286	No	NA	1	0	Low-quality	Genome-fragment		2.44	0
S13_vs_90967||full	4316	No	NA	1	0	Medium-quality	Genome-fragment		78.07	0
S13_vs_91158||full	1446	No	NA	0	1	Not-determined	Genome-fragment	NA		0
S11_extracted_k141_45261	1286	No	NA	0	0	Low-quality	Genome-fragment		1.63	0
S13_vs_91417||full	6246	No	NA	0	2	Not-determined	Genome-fragment	NA		0
S11_vs_k141_22246||full	1286	No	NA	2	0	Low-quality	Genome-fragment		2.98	0
S13_vs_91633||full	2211	No	NA	0	1	Not-determined	Genome-fragment	NA		0
S13_vs_92136||full	1399	No	NA	0	0	Not-determined	Genome-fragment	NA		0
S13_vs_152907||full	1286	No	NA	0	1	Low-quality	Genome-fragment		0.83	0
S13_vs_92264||full	1952	No	NA	0	1	Not-determined	Genome-fragment	NA		0
S1_vs_k141_114212||full	1286	No	NA	0	0	Low-quality	Genome-fragment		2.82	0
S13_vs_92307||full	1348	No	NA	0	1	Not-determined	Genome-fragment	NA		0
S3_vs_k141_35958||lt2gene	1286	No	NA	1	0	Low-quality	Genome-fragment		2.16	0
S5_vs_k141_51254||lt2gene	1286	No	NA	1	0	Low-quality	Genome-fragment		1.53	0
S7_extracted_115588	1286	No	NA	1	0	Low-quality	Genome-fragment		4.59	0
S13_vs_92693||full	1634	No	NA	0	2	Not-determined	Genome-fragment	NA		0
S7_vs_46396||lt2gene	1286	No	NA	2	0	Low-quality	Genome-fragment		2.97	0
S13_vs_92846||full	2098	No	NA	0	0	Not-determined	Genome-fragment	NA		0
S7_vs_81246||lt2gene	1286	No	NA	0	0	Low-quality	Genome-fragment		3.27	0
S13_vs_93081||full	1228	No	NA	0	1	Not-determined	Genome-fragment	NA		0
S13_vs_93182||full	1368	No	NA	0	1	Not-determined	Genome-fragment	NA		0
S13_vs_93326||full	1589	No	NA	0	0	Not-determined	Genome-fragment	NA		0
S16_vs_117654||full	1286	No	NA	0	0	Low-quality	Genome-fragment		1.24	0
S13_vs_93466||full	2981	No	NA	0	0	Not-determined	Genome-fragment	NA		0
S12_extracted_k141_96472	1286	No	NA	0	0	Low-quality	Genome-fragment		2.05	0
S12_extracted_k141_136339	1286	No	NA	2	0	Low-quality	Genome-fragment		2.1	0

S13_vs_93782||full	2343	No	NA	0	3	Not-determined	Genome-fragment	NA	0
S8_extracted_k141_183915	1286	No	NA	0	0	Low-quality	Genome-fragment	2.63	0
S8_extracted_k141_178362	1286	No	NA	0	0	Low-quality	Genome-fragment	1.74	0
S13_vs_94233||full	2290	No	NA	0	3	Not-determined	Genome-fragment	NA	0
S17_extracted_k141_74432	1287	No	NA	0	1	Low-quality	Genome-fragment	0.84	0
S13_vs_94522||full	1744	No	NA	0	0	Not-determined	Genome-fragment	NA	0
S11_extracted_k141_89684	1287	No	NA	0	0	Low-quality	Genome-fragment	2.31	0
S13_extracted_108364	1287	No	NA	0	1	Low-quality	Genome-fragment	0.95	0
S14_vs_k141_76106||full	1287	No	NA	1	0	Low-quality	Genome-fragment	3.56	0
S13_vs_94899||full	1743	No	NA	0	1	Not-determined	Genome-fragment	NA	0
S13_vs_94981||full	5188	No	NA	0	3	Not-determined	Genome-fragment	NA	0
S3_vs_k141_118553||full	1287	No	NA	0	0	Low-quality	Genome-fragment	3.34	0
S13_vs_95217||full	1558	No	NA	0	2	Not-determined	Genome-fragment	NA	0
S13_vs_95293||full	2221	No	NA	0	1	Not-determined	Genome-fragment	NA	0
S13_vs_95520||full	1201	No	NA	0	0	Not-determined	Genome-fragment	NA	0
S5_extracted_k141_69756	1287	No	NA	1	0	Low-quality	Genome-fragment	2.33	0
S7_vs_6672||full	1287	No	NA	2	0	Low-quality	Genome-fragment	2.88	0
S16_extracted_28204	1287	No	NA	1	0	Low-quality	Genome-fragment	3.16	0
S16_extracted_75737	1287	No	NA	1	0	Low-quality	Genome-fragment	3.21	0
S13_vs_96242||full	2606	No	NA	0	2	Not-determined	Genome-fragment	NA	0
S16_vs_44219||full	1287	No	NA	0	0	Low-quality	Genome-fragment	1.74	0
S10_extracted_64265	1287	No	NA	0	0	Low-quality	Genome-fragment	3.19	0
S10_vs_54100||full	1287	No	NA	1	0	Low-quality	Genome-fragment	2.72	0
S13_vs_97142||full	4751	No	NA	0	1	Not-determined	Genome-fragment	NA	0
S4_extracted_15117	1287	No	NA	1	0	Low-quality	Genome-fragment	2.13	0
S13_vs_97247||full	6062	No	NA	0	5	Not-determined	Genome-fragment	NA	0
S13_vs_97313||full	1310	No	NA	0	1	Not-determined	Genome-fragment	NA	0
S8_extracted_k141_191341	1287	No	NA	0	0	Low-quality	Genome-fragment	1.38	0
S17_extracted_k141_15290	1288	No	NA	0	0	Low-quality	Genome-fragment	2.31	0
S9_vs_k141_1288||full	1288	No	NA	0	1	Low-quality	Genome-fragment	3.01	0
S13_vs_97832||full	1372	No	NA	0	1	Not-determined	Genome-fragment	NA	0
S11_extracted_k141_30481	1288	No	NA	1	0	Low-quality	Genome-fragment	3.24	0
S13_vs_98160||full	1125	No	NA	0	0	Not-determined	Genome-fragment	NA	0
S13_vs_67734||full	1288	No	NA	0	1	Low-quality	Genome-fragment	0.91	0
S1_vs_k141_159562||full	1288	No	NA	0	0	Low-quality	Genome-fragment	0.86	0
S5_vs_k141_322||full	1288	No	NA	0	1	Low-quality	Genome-fragment	1.1	0
S13_vs_98591||full	36816	No	NA	21	0	Medium-quality	Genome-fragment	85.63	0
S16_vs_44766||full	1288	No	NA	2	0	Low-quality	Genome-fragment	2.52	0
S6_extracted_k141_36680	1288	No	NA	1	0	Low-quality	Genome-fragment	2.2	0
S6_vs_k141_708||full	1288	No	NA	0	0	Low-quality	Genome-fragment	0.99	0
S17_vs_k141_47807||full	1289	No	NA	0	0	Low-quality	Genome-fragment	3.13	0
S13_vs_98880||full	1254	No	NA	0	0	Not-determined	Genome-fragment	NA	0
S13_vs_98886||full	1136	No	NA	0	0	Not-determined	Genome-fragment	NA	0
S9_vs_k141_66802||full	1289	No	NA	0	0	Low-quality	Genome-fragment	1.72	0
S13_vs_98986||full	5752	No	NA	0	4	Not-determined	Genome-fragment	NA	0
S9_vs_k141_51475||lt2gene	1289	No	NA	1	0	Low-quality	Genome-fragment	2.17	0
S11_vs_k141_32783||full	1289	No	NA	0	0	Low-quality	Genome-fragment	3.47	0
S13_vs_17846||full	1289	No	NA	1	0	Low-quality	Genome-fragment	2.05	0
S13_vs_99539||full	1370	No	NA	0	0	Not-determined	Genome-fragment	NA	0
S1_vs_k141_104821||full	1289	No	NA	0	0	Low-quality	Genome-fragment	1.82	0
S1_vs_k141_16554||lt2gene	1289	No	NA	1	0	Low-quality	Genome-fragment	0.89	0
S7_extracted_7051	1289	No	NA	0	0	Low-quality	Genome-fragment	3.13	0
S10_extracted_90424	1289	No	NA	0	1	Low-quality	Genome-fragment	1.63	0
S13_vs_100013||full	2521	No	NA	0	1	Not-determined	Genome-fragment	NA	0
S13_vs_100077||full	16410	No	NA	0	7	Not-determined	Genome-fragment	NA	0
S10_vs_4849||lt2gene	1289	No	NA	1	0	Low-quality	Genome-fragment	1.71	0
S13_vs_100367||full	2131	No	NA	0	2	Not-determined	Genome-fragment	NA	0
S13_vs_100371||full	2696	No	NA	0	0	Not-determined	Genome-fragment	NA	0
S13_vs_100468||full	2141	No	NA	0	0	Not-determined	Genome-fragment	NA	0
S13_vs_100559||full	3871	No	NA	0	1	Not-determined	Genome-fragment	NA	0
S13_vs_100631||full	3266	No	NA	0	0	Not-determined	Genome-fragment	NA	0
S13_vs_100690||full	3484	No	NA	0	2	Not-determined	Genome-fragment	NA	0
S12_vs_k141_116559||full	1289	No	NA	0	0	Low-quality	Genome-fragment	2.33	0
S8_extracted_k141_123850	1289	No	NA	0	0	Low-quality	Genome-fragment	2.34	0
S13_vs_101541||full	1097	No	NA	0	0	Not-determined	Genome-fragment	NA	0
S8_extracted_k141_11415	1289	No	NA	0	1	Low-quality	Genome-fragment	0.85	0
S13_vs_102146||full	1036	No	NA	0	0	Not-determined	Genome-fragment	NA	0
S13_vs_102287||full	8212	No	NA	0	8	Not-determined	Genome-fragment	NA	0
S13_vs_102356||full	3628	No	NA	0	0	Not-determined	Genome-fragment	NA	0
S13_vs_102884||full	4274	No	NA	0	0	Not-determined	Genome-fragment	NA	0
S13_vs_102899||full	1378	No	NA	0	0	Not-determined	Genome-fragment	NA	0
S13_vs_103000||full	1147	No	NA	0	0	Not-determined	Genome-fragment	NA	0
S17_extracted_k141_118112	1290	No	NA	0	1	Low-quality	Genome-fragment	2.21	0
S13_vs_103161||full	2251	No	NA	0	0	Not-determined	Genome-fragment	NA	0
S13_vs_103278||full	1165	No	NA	0	0	Not-determined	Genome-fragment	NA	0
S13_vs_103322||full	1014	No	NA	0	2	Not-determined	Genome-fragment	NA	0
S13_vs_103595||full	1842	No	NA	0	2	Not-determined	Genome-fragment	NA	0
S13_vs_103751||full	1992	No	NA	0	0	Not-determined	Genome-fragment	NA	0
S9_extracted_k141_29218	1290	No	NA	0	0	Low-quality	Genome-fragment	0.77	0
S9_vs_k141_2355||lt2gene	1290	No	NA	1	0	Low-quality	Genome-fragment	0.8	0
S13_vs_104198||full	1369	No	NA	0	0	Not-determined	Genome-fragment	NA	0
S13_vs_104367||full	5223	No	NA	0	0	Not-determined	Genome-fragment	NA	0
S13_vs_104383||full	6492	No	NA	0	3	Not-determined	Genome-fragment	NA	0
S14_extracted_k141_72646	1290	No	NA	0	0	Low-quality	Genome-fragment	0.21	0
S13_vs_104418||full	1132	No	NA	0	1	Not-determined	Genome-fragment	NA	0
S13_vs_104517||full	1065	No	NA	0	1	Not-determined	Genome-fragment	NA	0
S14_vs_k141_52739||full	1290	No	NA	3	0	Low-quality	Genome-fragment	3.55	0
S13_vs_104606||full	1419	No	NA	0	2	Not-determined	Genome-fragment	NA	0
S14_vs_k141_13520||full	1290	No	NA	0	0	Low-quality	Genome-fragment	2.64	0
S13_vs_104940||full	2344	No	NA	0	1	Not-determined	Genome-fragment	NA	0
S13_vs_104946||full	1006	No	NA	0	0	Not-determined	Genome-fragment	NA	0
S13_vs_105097||full	2626	No	NA	0	2	Not-determined	Genome-fragment	NA	0
S13_vs_105337||full	2850	No	NA	0	2	Not-determined	Genome-fragment	NA	0
S5_extracted_k141_88410	1290	No	NA	0	0	Low-quality	Genome-fragment	2.22	0
S5_vs_k141_17308||full	1290	No	NA	0	0	Low-quality	Genome-fragment	0.92	0
S7_extracted_29858	1290	No	NA	1	0	Low-quality	Genome-fragment	1.73	0
S13_vs_105491||full	4169	No	NA	0	2	Not-determined	Genome-fragment	NA	0
S13_vs_105758||full	2284	No	NA	0	1	Not-determined	Genome-fragment	NA	0
S13_vs_105886||full	4455	No	NA	0	3	Not-determined	Genome-fragment	NA	0
S7_extracted_126646	1290	No	NA	2	0	Low-quality	Genome-fragment	9.56	0
S13_vs_105974||full	1148	No	NA	0	0	Not-determined	Genome-fragment	NA	0
S13_vs_106129||full	1878	No	NA	0	2	Not-determined	Genome-fragment	NA	0
S7_vs_5428||full	1290	No	NA	1	0	Low-quality	Genome-fragment	2.3	0
S13_vs_106474||full	1262	No	NA	0	0	Not-determined	Genome-fragment	NA	0
S13_vs_106504||full	3345	No	NA	0	3	Not-determined	Genome-fragment	NA	0
S13_vs_106667||full	2997	No	NA	0	1	Not-determined	Genome-fragment	NA	0
S13_vs_107105||full	1192	No	NA	0	1	Not-determined	Genome-fragment	NA	0
S13_vs_107223||full	1733	No	NA	0	2	Not-determined	Genome-fragment	NA	0
S13_vs_107405||full	2804	No	NA	0	2	Not-determined	Genome-fragment	NA	0
S13_vs_107465||full	1375	No	NA	0	0	Not-determined	Genome-fragment	NA	0
S7_vs_52309||full	1290	No	NA	0	0	Low-quality	Genome-fragment	2.21	0
S7_vs_40830||lt2gene	1290	No	NA	1	0	Low-quality	Genome-fragment	3.03	0
S13_vs_107719||full	1437	No	NA	0	1	Not-determined	Genome-fragment	NA	0
S13_vs_107727||full	1423	No	NA	0	0	Not-determined	Genome-fragment	NA	0
S13_vs_107963||full	5570	No	NA	0	1	Not-determined	Genome-fragment	NA	0
S13_vs_107990||full	1577	No	NA	0	2	Not-determined	Genome-fragment	NA	0
S13_vs_108031||full	2148	No	NA	0	0	Not-determined	Genome-fragment	NA	0
S13_vs_108047||full	1096	No	NA	0	0	Not-determined	Genome-fragment	NA	0
S13_vs_108069||full	4513	No	NA	0	5	Not-determined	Genome-fragment	NA	0
S13_vs_108120||full	1447	No	NA	0	2	Not-determined	Genome-fragment	NA	0
S13_vs_108252||full	7266	No	NA	0	4	Not-determined	Genome-fragment	NA	0
S10_extracted_79366	1290	No	NA	0	0	Low-quality	Genome-fragment	3.26	0
S13_vs_108381||full	2328	No	NA	0	1	Not-determined	Genome-fragment	NA	0
S13_vs_108445||full	1389	No	NA	0	0	Not-determined	Genome-fragment	NA	0
S13_vs_108463||full	1954	No	NA	0	1	Not-determined	Genome-fragment	NA	0
S8_vs_k141_199772||full	1290	No	NA	2	0	Low-quality	Genome-fragment	3.01	0
S8_vs_k141_188276||full	1290	No	NA	0	0	Low-quality	Genome-fragment	2.93	0
S13_vs_109160||full	2588	No	NA	0	3	Not-determined	Genome-fragment	NA	0
S17_vs_k141_150732||full	1291	No	NA	2	0	Low-quality	Genome-fragment	2.76	0
S13_vs_109515||full	1863	No	NA	0	1	Not-determined	Genome-fragment	NA	0
S13_vs_109661||full	3021	No	NA	0	0	Not-determined	Genome-fragment	NA	0
S13_vs_110059||full	2899	No	NA	0	3	Not-determined	Genome-fragment	NA	0
S9_vs_k141_22626||full	1291	No	NA	0	0	Low-quality	Genome-fragment	2.05	0
S13_vs_110572||full	1798	No	NA	0	1	Not-determined	Genome-fragment	NA	0
S13_vs_110622||full	1771	No	NA	0	2	Not-determined	Genome-fragment	NA	0
S13_vs_110673||full	6112	No	NA	0	3	Not-determined	Genome-fragment	NA	0
S13_vs_110741||full	3263	No	NA	0	1	Not-determined	Genome-fragment	NA	0
S13_vs_110818||full	1634	No	NA	0	1	Not-determined	Genome-fragment	NA	0
S13_extracted_45787	1291	No	NA	0	1	Low-quality	Genome-fragment	0.4	0
S13_vs_111037||full	2328	No	NA	0	2	Not-determined	Genome-fragment	NA	0
S13_vs_111098||full	3576	No	NA	0	0	Not-determined	Genome-fragment	NA	0
S13_vs_111128||full	1931	No	NA	0	2	Not-determined	Genome-fragment	NA	0
S13_extracted_141214	1291	No	NA	1	0	Low-quality	Genome-fragment	2.15	0
S13_vs_35761||full	1291	No	NA	0	1	Low-quality	Genome-fragment	2.25	0
S13_vs_111398||full	1271	No	NA	0	1	Not-determined	Genome-fragment	NA	0
S13_vs_111863||full	1183	No	NA	0	2	Not-determined	Genome-fragment	NA	0
S13_vs_133097||full	1291	No	NA	0	0	Low-quality	Genome-fragment	2.71	0
S1_extracted_k141_100640	1291	No	NA	2	0	Low-quality	Genome-fragment	3.81	0
S3_extracted_k141_38349	1291	No	NA	1	0	Low-quality	Genome-fragment	0.45	0
S5_vs_k141_32344||full	1291	No	NA	0	0	Low-quality	Genome-fragment	2.92	0
S5_vs_k141_30110||full	1291	No	NA	0	1	Low-quality	Genome-fragment	0.95	0
S13_vs_112463||full	2925	No	NA	0	1	Not-determined	Genome-fragment	NA	0
S13_vs_112470||full	1103	No	NA	0	0	Not-determined	Genome-fragment	NA	0
S13_vs_112701||full	1685	No	NA	0	1	Not-determined	Genome-fragment	NA	0
S13_vs_112817||full	2759	No	NA	0	1	Not-determined	Genome-fragment	NA	0
S13_vs_112820||full	2031	No	NA	0	1	Not-determined	Genome-fragment	NA	0
S7_extracted_125837	1291	No	NA	2	0	Low-quality	Genome-fragment	4.48	0
S13_vs_113085||full	1789	No	NA	0	2	Not-determined	Genome-fragment	NA	0
S13_vs_113185||full	1179	No	NA	0	0	Not-determined	Genome-fragment	NA	0
S7_vs_29759||full	1291	No	NA	0	0	Low-quality	Genome-fragment	2.74	0
S7_vs_37556||full	1291	No	NA	0	1	Low-quality	Genome-fragment	3.03	0
S7_vs_80048||full	1291	No	NA	1	0	Low-quality	Genome-fragment	0.73	0
S13_vs_113536||full	5702	No	NA	0	0	Not-determined	Genome-fragment	NA	0
S13_vs_113593||full	56024	No	NA	34	1	High-quality	High-quality	98.71	0
S10_vs_48135||full	1291	No	NA	0	0	Low-quality	Genome-fragment	2.68	0
S13_vs_114070||full	1215	No	NA	0	2	Not-determined	Genome-fragment	NA	0
S8_extracted_k141_201120	1291	No	NA	0	1	Low-quality	Genome-fragment	1.37	0
S13_vs_114213||full	34608	Yes	28952	11	6	Medium-quality	Genome-fragment	58.7	16.34
S13_vs_114471||full	2625	No	NA	0	0	Not-determined	Genome-fragment	NA	0
S13_vs_114648||full	2630	No	NA	0	2	Not-determined	Genome-fragment	NA	0
S8_extracted_k141_126903	1291	No	NA	0	1	Low-quality	Genome-fragment	1.74	0
S13_vs_114874||full	1279	No	NA	0	1	Not-determined	Genome-fragment	NA	0
S13_vs_114899||full	1384	No	NA	0	1	Not-determined	Genome-fragment	NA	0
S13_vs_114958||full	6369	No	NA	0	4	Not-determined	Genome-fragment	NA	0

S8_extracted_k141_155630	1291	No	NA	0	0	Low-quality	Genome-fragment		2.12	0
S13_vs_115136||full	4632	No	NA	0	4	Not-determined	Genome-fragment	NA		0
S8_vs_k141_177024||full	1291	No	NA	0	0	Low-quality	Genome-fragment		0.97	0
S13_vs_115285||full	1073	No	NA	0	1	Not-determined	Genome-fragment	NA		0
S13_vs_115310||full	1895	No	NA	0	0	Not-determined	Genome-fragment	NA		0
S13_vs_115584||full	2170	No	NA	0	0	Not-determined	Genome-fragment	NA		0
S13_vs_115675||full	2048	No	NA	0	1	Not-determined	Genome-fragment	NA		0
S13_vs_115730||full	1766	No	NA	0	1	Not-determined	Genome-fragment	NA		0
S11_vs_k141_64128||full	1292	No	NA	0	0	Low-quality	Genome-fragment		0.63	0
S13_vs_116049||full	3425	No	NA	0	3	Not-determined	Genome-fragment	NA		0
S13_vs_116102||full	1401	No	NA	0	0	Not-determined	Genome-fragment	NA		0
S14_vs_k141_68605||full	1292	No	NA	1	0	Low-quality	Genome-fragment		0.74	0
S13_vs_116294||full	1699	No	NA	0	0	Not-determined	Genome-fragment	NA		0
S14_vs_k141_56199||full	1292	No	NA	0	0	Low-quality	Genome-fragment		3.91	0
S14_vs_k141_64889||full	1292	No	NA	1	0	Low-quality	Genome-fragment		2.77	0
S13_vs_117422||full	1512	No	NA	0	1	Not-determined	Genome-fragment	NA		0
S13_vs_117683||full	2387	No	NA	0	2	Not-determined	Genome-fragment	NA		0
S1_vs_k141_78912||full	1292	No	NA	1	0	Low-quality	Genome-fragment		2.54	0
S5_vs_k141_4462||full	1292	No	NA	0	0	Low-quality	Genome-fragment		3.67	0
S5_vs_k141_53234||lt2gene	1292	No	NA	1	0	Low-quality	Genome-fragment		2.97	0
S13_vs_118442||full	5123	No	NA	0	4	Not-determined	Genome-fragment	NA		0
S13_vs_118461||full	3154	No	NA	0	3	Not-determined	Genome-fragment	NA		0
S13_vs_118462||full	1551	No	NA	0	2	Not-determined	Genome-fragment	NA		0
S7_extracted_59357	1292	No	NA	1	0	Low-quality	Genome-fragment		2.26	0
S7_extracted_79538	1292	No	NA	0	0	Low-quality	Genome-fragment		2.33	0
S13_vs_119066||full	1615	No	NA	0	1	Not-determined	Genome-fragment	NA		0
S13_vs_119122||full	4839	No	NA	0	3	Not-determined	Genome-fragment	NA		0
S13_vs_119144||full	1050	No	NA	0	2	Not-determined	Genome-fragment	NA		0
S13_vs_119401||full	1538	No	NA	0	1	Not-determined	Genome-fragment	NA		0
S7_vs_35639||full	1292	No	NA	1	0	Low-quality	Genome-fragment		2.73	0
S7_vs_106077||full	1292	No	NA	0	0	Low-quality	Genome-fragment		3.34	0
S16_vs_166188||full	1292	No	NA	0	0	Low-quality	Genome-fragment		2.01	0
S12_extracted_k141_126570	1292	No	NA	0	1	Low-quality	Genome-fragment		1.21	0
S13_vs_120386||full	1763	No	NA	0	0	Not-determined	Genome-fragment	NA		0
S13_vs_120521||full	2684	No	NA	0	1	Not-determined	Genome-fragment	NA		0
S13_vs_120725||full	2144	No	NA	0	2	Not-determined	Genome-fragment	NA		0
S13_vs_120787||full	1346	No	NA	0	2	Not-determined	Genome-fragment	NA		0
S13_vs_120793||full	2757	No	NA	0	3	Not-determined	Genome-fragment	NA		0
S8_vs_k141_246||full	1292	No	NA	0	1	Low-quality	Genome-fragment		2.25	0
S13_vs_120975||full	1928	No	NA	0	1	Not-determined	Genome-fragment	NA		0
S8_vs_k141_31266||full	1292	No	NA	1	0	Low-quality	Genome-fragment		3.17	0
S13_extracted_137915	1293	No	NA	0	0	Low-quality	Genome-fragment		2.14	0
S13_vs_121307||full	20430	No	NA	3	1	Medium-quality	Genome-fragment		53.16	0
S13_vs_121392||full	1571	No	NA	0	1	Not-determined	Genome-fragment	NA		0
S13_vs_121608||full	3029	No	NA	0	1	Not-determined	Genome-fragment	NA		0
S13_extracted_152131	1293	No	NA	0	1	Low-quality	Genome-fragment		2.59	0
S1_vs_k141_6465||full	1293	No	NA	0	0	Low-quality	Genome-fragment		2.86	0
S13_vs_122124||full	3545	No	NA	0	1	Not-determined	Genome-fragment	NA		0
S13_vs_122581||full	2752	No	NA	0	2	Not-determined	Genome-fragment	NA		0
S13_vs_122600||full	2803	No	NA	0	3	Not-determined	Genome-fragment	NA		0
S13_vs_122910||full	2212	No	NA	0	0	Not-determined	Genome-fragment	NA		0
S3_extracted_k141_10869	1293	No	NA	0	1	Low-quality	Genome-fragment		1.09	0
S13_vs_123058||full	2879	No	NA	0	3	Not-determined	Genome-fragment	NA		0
S5_vs_k141_96618||full	1293	No	NA	0	0	Low-quality	Genome-fragment		3.4	0
S13_vs_123251||full	1026	No	NA	0	2	Not-determined	Genome-fragment	NA		0
S13_vs_123253||full	1819	No	NA	0	1	Not-determined	Genome-fragment	NA		0
S13_vs_123304||full	1973	No	NA	0	0	Not-determined	Genome-fragment	NA		0
S13_vs_123486||full	1724	No	NA	0	1	Not-determined	Genome-fragment	NA		0
S13_vs_123495||full	1868	No	NA	0	0	Not-determined	Genome-fragment	NA		0
S7_extracted_14239	1293	No	NA	2	0	Low-quality	Genome-fragment		2.31	0
S13_vs_123651||full	5432	No	NA	0	1	Not-determined	Genome-fragment	NA		0
S7_vs_135405||full	1293	No	NA	1	0	Low-quality	Genome-fragment		3.76	0
S13_vs_123987||full	2301	No	NA	0	3	Not-determined	Genome-fragment	NA		0
S13_vs_124264||full	1420	No	NA	0	0	Not-determined	Genome-fragment	NA		0
S16_extracted_114416	1293	No	NA	0	1	Low-quality	Genome-fragment		2.77	0
S8_extracted_k141_26516	1293	No	NA	0	0	Low-quality	Genome-fragment		0.97	0
S13_vs_124622||full	1614	No	NA	0	0	Not-determined	Genome-fragment	NA		0
S13_vs_124830||full	4025	No	NA	0	3	Not-determined	Genome-fragment	NA		0
S13_vs_124929||full	1697	No	NA	0	0	Not-determined	Genome-fragment	NA		0
S13_vs_125459||full	1356	No	NA	0	1	Not-determined	Genome-fragment	NA		0
S8_vs_k141_94479||full	1293	No	NA	2	0	Low-quality	Genome-fragment		2.3	0
S13_vs_126016||full	1340	No	NA	0	0	Not-determined	Genome-fragment	NA		0
S13_vs_126059||full	3127	No	NA	0	3	Not-determined	Genome-fragment	NA		0
S13_vs_126245||full	2569	No	NA	0	2	Not-determined	Genome-fragment	NA		0
S13_vs_126340||full	1261	No	NA	0	2	Not-determined	Genome-fragment	NA		0
S11_extracted_k141_96330	1294	No	NA	0	0	Low-quality	Genome-fragment		2.68	0
S13_vs_91288||full	1294	No	NA	0	0	Low-quality	Genome-fragment		0.92	0
S13_vs_126858||full	1379	No	NA	0	2	Not-determined	Genome-fragment	NA		0
S13_vs_126870||full	2884	No	NA	0	1	Not-determined	Genome-fragment	NA		0
S14_extracted_k141_93334	1294	No	NA	3	0	Low-quality	Genome-fragment		2.15	0
S13_vs_127009||full	1088	No	NA	0	0	Not-determined	Genome-fragment	NA		0
S13_vs_127088||full	4864	No	NA	0	0	Not-determined	Genome-fragment	NA		0
S13_vs_127196||full	2079	No	NA	0	0	Not-determined	Genome-fragment	NA		0
S13_vs_127354||full	1124	No	NA	0	0	Not-determined	Genome-fragment	NA		0
S13_vs_127603||full	6598	No	NA	0	4	Not-determined	Genome-fragment	NA		0
S14_vs_k141_12821||full	1294	No	NA	1	0	Low-quality	Genome-fragment		4.16	0
S14_vs_k141_56878||full	1294	No	NA	0	0	Low-quality	Genome-fragment		1.37	0
S5_vs_k141_87135||full	1294	No	NA	0	2	Low-quality	Genome-fragment		0.43	0
S13_vs_127862||full	2861	No	NA	0	0	Not-determined	Genome-fragment	NA		0
S5_vs_k141_137066||full	1294	No	NA	0	1	Low-quality	Genome-fragment		0.81	0
S7_vs_63696||full	1294	No	NA	0	0	Low-quality	Genome-fragment		1.08	0
S7_vs_122511||full	1294	No	NA	0	0	Low-quality	Genome-fragment		2.66	0
S13_vs_128358||full	2319	No	NA	0	1	Not-determined	Genome-fragment	NA		0
S16_extracted_158911	1294	No	NA	1	0	Low-quality	Genome-fragment		3.08	0
S13_vs_128552||full	1433	No	NA	0	1	Not-determined	Genome-fragment	NA		0
S13_vs_128637||full	3131	No	NA	0	1	Not-determined	Genome-fragment	NA		0
S13_vs_128781||full	3637	No	NA	0	1	Not-determined	Genome-fragment	NA		0
S13_vs_129132||full	1509	No	NA	0	0	Not-determined	Genome-fragment	NA		0
S16_vs_130471||full	1294	No	NA	0	0	Low-quality	Genome-fragment		2.09	0
S8_vs_k141_107910||full	1294	No	NA	2	0	Low-quality	Genome-fragment		2.73	0
S13_vs_129367||full	2078	No	NA	0	3	Not-determined	Genome-fragment	NA		0
S13_vs_129410||full	1471	No	NA	0	1	Not-determined	Genome-fragment	NA		0
S13_vs_129498||full	1829	No	NA	0	1	Not-determined	Genome-fragment	NA		0
S13_vs_129602||full	2446	No	NA	0	1	Not-determined	Genome-fragment	NA		0
S13_vs_129705||full	1110	No	NA	0	1	Not-determined	Genome-fragment	NA		0
S17_vs_k141_138596||full	1295	No	NA	0	0	Low-quality	Genome-fragment		0.88	0
S13_vs_130179||full	1167	No	NA	0	1	Not-determined	Genome-fragment	NA		0
S9_vs_k141_69232||full	1295	No	NA	1	0	Low-quality	Genome-fragment		3.18	0
S13_vs_130445||full	1163	No	NA	0	1	Not-determined	Genome-fragment	NA		0
S11_extracted_k141_11699	1295	No	NA	0	1	Low-quality	Genome-fragment		0.68	0
S13_vs_130713||full	2238	No	NA	0	0	Not-determined	Genome-fragment	NA		0
S13_vs_130925||full	6001	No	NA	0	4	Not-determined	Genome-fragment	NA		0
S13_vs_130939||full	1559	No	NA	0	0	Not-determined	Genome-fragment	NA		0
S11_vs_k141_103680||lt2gene	1295	No	NA	1	0	Low-quality	Genome-fragment		2.45	0
S13_vs_131191||full	2851	No	NA	0	2	Not-determined	Genome-fragment	NA		0
S14_extracted_k141_95604	1295	No	NA	2	0	Low-quality	Genome-fragment		4.55	0
S13_vs_131227||full	1535	No	NA	0	1	Not-determined	Genome-fragment	NA		0
S5_extracted_k141_830	1295	No	NA	1	0	Low-quality	Genome-fragment		3.12	0
S5_extracted_k141_138969	1295	No	NA	0	0	Low-quality	Genome-fragment		2.37	0
S13_vs_131689||full	6448	No	NA	0	2	Not-determined	Genome-fragment	NA		0
S13_vs_131724||full	1281	No	NA	0	2	Not-determined	Genome-fragment	NA		0
S5_vs_k141_26013||full	1295	No	NA	1	0	Low-quality	Genome-fragment		3.56	0
S13_vs_132105||full	3775	No	NA	0	1	Not-determined	Genome-fragment	NA		0
S13_vs_132182||full	1740	No	NA	0	3	Not-determined	Genome-fragment	NA		0
S5_vs_k141_59388||full	1295	No	NA	0	1	Low-quality	Genome-fragment		3.74	0
S12_vs_k141_41682||full	1295	No	NA	0	0	Low-quality	Genome-fragment		2.07	0
S15_extracted_38900	1295	No	NA	0	1	Low-quality	Genome-fragment		0.81	0
S6_extracted_k141_20615	1295	No	NA	1	0	Low-quality	Genome-fragment		2.18	0
S9_vs_k141_73603||full	1296	No	NA	1	0	Low-quality	Genome-fragment		2.04	0
S13_vs_133300||full	5077	No	NA	0	2	Not-determined	Genome-fragment	NA		0
S13_vs_133375||full	1159	No	NA	0	0	Not-determined	Genome-fragment	NA		0
S9_vs_k141_30565||lt2gene	1296	No	NA	2	0	Low-quality	Genome-fragment		3.16	0
S9_vs_k141_82546||lt2gene	1296	No	NA	2	0	Low-quality	Genome-fragment		2.16	0
S13_vs_133917||full	1423	No	NA	0	1	Not-determined	Genome-fragment	NA		0
S13_vs_134004||full	1281	No	NA	0	0	Not-determined	Genome-fragment	NA		0
S13_vs_134115||full	1931	No	NA	0	3	Not-determined	Genome-fragment	NA		0
S11_extracted_k141_92098	1296	No	NA	2	0	Low-quality	Genome-fragment		2.91	0
S13_vs_54555||full	1296	No	NA	2	0	Low-quality	Genome-fragment		2.31	0
S13_vs_135018||full	1917	No	NA	0	0	Not-determined	Genome-fragment	NA		0
S13_vs_64160||full	1296	No	NA	0	2	Low-quality	Genome-fragment		0.49	0
S13_vs_135119||full	1119	No	NA	0	0	Not-determined	Genome-fragment	NA		0
S1_extracted_k141_8143	1296	No	NA	0	0	Low-quality	Genome-fragment		1.32	0
S13_vs_135200||full	2217	No	NA	0	1	Not-determined	Genome-fragment	NA		0
S13_vs_135507||full	2766	No	NA	0	1	Not-determined	Genome-fragment	NA		0
S13_vs_135750||full	1962	No	NA	0	0	Not-determined	Genome-fragment	NA		0
S13_vs_136187||full	1837	No	NA	0	0	Not-determined	Genome-fragment	NA		0
S13_vs_136271||full	2450	No	NA	0	0	Not-determined	Genome-fragment	NA		0
S13_vs_136290||full	1007	No	NA	0	1	Not-determined	Genome-fragment	NA		0
S13_vs_136502||full	9223	No	NA	0	1	Not-determined	Genome-fragment	NA		0
S13_vs_136561||full	2403	No	NA	0	1	Not-determined	Genome-fragment	NA		0
S13_vs_136583||full	1517	No	NA	0	2	Not-determined	Genome-fragment	NA		0
S13_vs_136824||full	1341	No	NA	0	0	Not-determined	Genome-fragment	NA		0
S13_vs_136914||full	1152	No	NA	0	1	Not-determined	Genome-fragment	NA		0
S7_vs_72006||lt2gene	1296	No	NA	0	0	Low-quality	Genome-fragment		3.19	0
S13_vs_137086||full	2919	No	NA	0	1	Not-determined	Genome-fragment	NA		0
S7_vs_104014||lt2gene	1296	No	NA	1	0	Low-quality	Genome-fragment		3.24	0
S13_vs_137288||full	2043	No	NA	0	0	Not-determined	Genome-fragment	NA		0
S13_vs_137365||full	1741	No	NA	0	1	Not-determined	Genome-fragment	NA		0
S16_extracted_165043	1296	No	NA	0	0	Low-quality	Genome-fragment		2.04	0
S13_vs_137452||full	1103	No	NA	0	1	Not-determined	Genome-fragment	NA		0
S13_vs_137587||full	2297	No	NA	0	2	Not-determined	Genome-fragment	NA		0
S16_vs_640||full	1296	No	NA	1	0	Low-quality	Genome-fragment		2.06	0
S13_vs_137913||full	2266	No	NA	0	1	Not-determined	Genome-fragment	NA		0
S13_vs_138152||full	1009	No	NA	0	0	Not-determined	Genome-fragment	NA		0
S13_vs_138161||full	2903	No	NA	0	4	Not-determined	Genome-fragment	NA		0
S10_vs_79821||full	1296	No	NA	0	0	Low-quality	Genome-fragment		2.45	0
S12_extracted_k141_54397	1296	No	NA	0	0	Low-quality	Genome-fragment		3.86	0
S13_vs_138801||full	2015	No	NA	0	3	Not-determined	Genome-fragment	NA		0
S13_vs_138810||full	2994	No	NA	0	0	Not-determined	Genome-fragment	NA		0
S13_vs_138825||full	7682	No	NA	0	2	Not-determined	Genome-fragment	NA		0
S13_vs_138862||full	1916	No	NA	0	1	Not-determined	Genome-fragment	NA		0
S8_extracted_k141_53368	1296	No	NA	0	0	Low-quality	Genome-fragment		1.97	0
S13_vs_139091||full	6761	No	NA	0	1	Not-determined	Genome-fragment	NA		0
S13_vs_139181||full	1397	No	NA	0	0	Not-determined	Genome-fragment	NA		0

S8_vs_k141_48294||full	1296	No	NA		0	1	Low-quality	Genome-fragment		2.9	0
S13_vs_139693||full	3276	No	NA		0	3	Not-determined	Genome-fragment	NA		0
S13_vs_139750||full	1306	No	NA		0	0	Not-determined	Genome-fragment	NA		0
S9_extracted_k141_18881	1297	No	NA		0	1	Low-quality	Genome-fragment		0.91	0
S13_vs_140391||full	2551	No	NA		0	1	Not-determined	Genome-fragment	NA		0
S13_vs_140564||full	3593	No	NA		0	1	Not-determined	Genome-fragment	NA		0
S3_extracted_k141_36240	1297	No	NA		0	0	Low-quality	Genome-fragment		0.8	0
S13_vs_141050||full	1811	No	NA		0	1	Not-determined	Genome-fragment	NA		0
S13_vs_141153||full	1057	No	NA		0	1	Not-determined	Genome-fragment	NA		0
S3_extracted_k141_83902	1297	No	NA		0	0	Low-quality	Genome-fragment		2.17	0
S13_vs_141329||full	15942	No	NA		0	3	Not-determined	Genome-fragment	NA		0
S10_vs_106645||full	1297	No	NA		0	0	Low-quality	Genome-fragment		0.36	0
S13_vs_141580||full	2873	No	NA		0	2	Not-determined	Genome-fragment	NA		0
S12_extracted_k141_47014	1297	No	NA		1	0	Low-quality	Genome-fragment		2.34	0
S13_vs_141772||full	1636	No	NA		0	2	Not-determined	Genome-fragment	NA		0
S13_vs_141825||full	1282	No	NA		0	0	Not-determined	Genome-fragment	NA		0
S13_vs_141933||full	5929	No	NA		0	2	Not-determined	Genome-fragment	NA		0
S13_vs_142082||full	1376	No	NA		0	0	Not-determined	Genome-fragment	NA		0
S13_vs_142214||full	1067	No	NA		0	1	Not-determined	Genome-fragment	NA		0
S13_vs_142359||full	2493	No	NA		0	2	Not-determined	Genome-fragment	NA		0
S13_vs_142485||full	1039	No	NA		0	1	Not-determined	Genome-fragment	NA		0
S4_extracted_30620	1297	No	NA		2	0	Low-quality	Genome-fragment		2.3	0
S6_vs_k141_25903||full	1297	No	NA		0	0	Low-quality	Genome-fragment		3.72	0
S13_vs_142740||full	1422	No	NA		0	0	Not-determined	Genome-fragment	NA		0
S13_vs_142808||full	1888	No	NA		0	0	Not-determined	Genome-fragment	NA		0
S13_vs_142865||full	11419	No	NA		0	4	Not-determined	Genome-fragment	NA		0
S13_vs_142913||full	1331	No	NA		0	0	Not-determined	Genome-fragment	NA		0
S13_vs_143030||full	5790	No	NA		0	1	Not-determined	Genome-fragment	NA		0
S13_vs_143068||full	1367	No	NA		0	1	Not-determined	Genome-fragment	NA		0
S8_extracted_k141_14442	1297	No	NA		0	0	Low-quality	Genome-fragment		2.47	0
S13_vs_143304||full	2356	No	NA		0	2	Not-determined	Genome-fragment	NA		0
S13_vs_143385||full	2261	No	NA		0	2	Not-determined	Genome-fragment	NA		0
S17_extracted_k141_76243	1298	No	NA		0	0	Low-quality	Genome-fragment		0.81	0
S13_vs_143739||full	1131	No	NA		0	2	Not-determined	Genome-fragment	NA		0
S17_extracted_k141_41339	1298	No	NA		3	0	Low-quality	Genome-fragment		4.26	0
S9_vs_k141_14415||full	1298	No	NA		1	0	Low-quality	Genome-fragment		2.4	0
S13_vs_144384||full	1268	No	NA		0	1	Not-determined	Genome-fragment	NA		0
S11_vs_k141_30313||full	1298	No	NA		1	0	Low-quality	Genome-fragment		2.18	0
S13_vs_144572||full	1290	No	NA		0	0	Not-determined	Genome-fragment	NA		0
S13_extracted_8430	1298	No	NA		0	0	Low-quality	Genome-fragment		1.44	0
S13_vs_144645||full	1897	No	NA		0	1	Not-determined	Genome-fragment	NA		0
S13_vs_144853||full	2097	No	NA		0	0	Not-determined	Genome-fragment	NA		0
S13_vs_145001||full	1051	No	NA		0	0	Not-determined	Genome-fragment	NA		0
S3_extracted_k141_35828	1298	No	NA		3	0	Low-quality	Genome-fragment		2.13	0
S13_vs_145278||full	2773	No	NA		0	6	Not-determined	Genome-fragment	NA		0
S13_vs_145288||full	1285	No	NA		0	2	Not-determined	Genome-fragment	NA		0
S13_vs_145546||full	1772	No	NA		0	2	Not-determined	Genome-fragment	NA		0
S13_vs_145594||full	4475	No	NA		0	0	Not-determined	Genome-fragment	NA		0
S3_vs_k141_119885||full	1298	No	NA		0	1	Low-quality	Genome-fragment		2.69	0
S7_extracted_40420	1298	No	NA		1	0	Low-quality	Genome-fragment		3.25	0
S7_vs_40293||lt2gene	1298	No	NA		1	0	Low-quality	Genome-fragment		1.72	0
S13_vs_146187||full	3181	No	NA		0	2	Not-determined	Genome-fragment	NA		0
S12_vs_k141_66844||full	1298	No	NA		1	0	Low-quality	Genome-fragment		2.78	0
S13_vs_146579||full	1767	No	NA		0	2	Not-determined	Genome-fragment	NA		0
S13_vs_146666||full	1983	No	NA		0	1	Not-determined	Genome-fragment	NA		0
S13_vs_146761||full	1275	No	NA		0	1	Not-determined	Genome-fragment	NA		0
S12_vs_k141_44116||lt2gene	1298	No	NA		1	0	Low-quality	Genome-fragment		3.47	0
S13_vs_147159||full	1158	No	NA		0	3	Not-determined	Genome-fragment	NA		0
S13_vs_147712||full	1412	No	NA		0	1	Not-determined	Genome-fragment	NA		0
S8_vs_k141_177812||full	1298	Yes		539	1	1	Low-quality	Genome-fragment		0.23	58.47
S13_vs_148446||full	30296	No	NA		0	10	Not-determined	Genome-fragment	NA		0
S13_vs_148495||full	2561	No	NA		0	3	Not-determined	Genome-fragment	NA		0
S13_vs_148622||full	3284	No	NA		0	2	Not-determined	Genome-fragment	NA		0
S13_vs_148648||full	3188	No	NA		0	2	Not-determined	Genome-fragment	NA		0
S13_vs_148952||full	1391	No	NA		0	0	Not-determined	Genome-fragment	NA		0
S13_vs_149085||full	3786	No	NA		0	4	Not-determined	Genome-fragment	NA		0
S13_vs_149345||full	1010	No	NA		0	1	Not-determined	Genome-fragment	NA		0
S8_vs_k141_15244||full	1298	No	NA		0	1	Low-quality	Genome-fragment		1.3	0
S17_vs_k141_57247||full	1299	No	NA		3	0	Low-quality	Genome-fragment		3.12	0
S13_vs_149596||full	1630	No	NA		0	3	Not-determined	Genome-fragment	NA		0
S13_vs_149597||full	1013	No	NA		0	0	Not-determined	Genome-fragment	NA		0
S11_extracted_k141_55583	1299	No	NA		0	0	Low-quality	Genome-fragment		1.82	0
S11_vs_k141_93299||full	1299	No	NA		0	1	Low-quality	Genome-fragment		2.62	0
S13_vs_150073||full	1649	No	NA		0	1	Not-determined	Genome-fragment	NA		0
S13_vs_150216||full	1067	No	NA		0	1	Not-determined	Genome-fragment	NA		0
S13_vs_150252||full	1403	No	NA		0	1	Not-determined	Genome-fragment	NA		0
S13_vs_150379||full	4654	No	NA		0	0	Not-determined	Genome-fragment	NA		0
S14_extracted_k141_81689	1299	No	NA		3	0	Low-quality	Genome-fragment		4.38	0
S13_vs_150977||full	2355	No	NA		0	0	Not-determined	Genome-fragment	NA		0
S13_vs_151150||full	1841	No	NA		0	2	Not-determined	Genome-fragment	NA		0
S13_vs_151217||full	1415	No	NA		0	0	Not-determined	Genome-fragment	NA		0
S1_extracted_k141_33147	1299	No	NA		0	0	Low-quality	Genome-fragment		0.83	0
S13_vs_151601||full	2576	No	NA		0	0	Not-determined	Genome-fragment	NA		0
S5_extracted_k141_139114	1299	No	NA		2	0	Low-quality	Genome-fragment		3	0
S13_vs_152214||full	1370	No	NA		0	1	Not-determined	Genome-fragment	NA		0
S13_vs_152223||full	60897	No	NA		33	0	High-quality	High-quality		100	0
S13_vs_152287||full	2421	No	NA		0	3	Not-determined	Genome-fragment	NA		0
S13_vs_152414||full	3507	No	NA		0	0	Not-determined	Genome-fragment	NA		0
S13_vs_152436||full	1509	No	NA		0	2	Not-determined	Genome-fragment	NA		0
S13_vs_152697||full	1946	No	NA		0	1	Not-determined	Genome-fragment	NA		0
S13_vs_152718||full	1459	No	NA		0	0	Not-determined	Genome-fragment	NA		0
S13_vs_152750||full	6397	No	NA		0	4	Not-determined	Genome-fragment	NA		0
S7_vs_100659||full	1299	No	NA		3	0	Low-quality	Genome-fragment		2.95	0
S7_vs_119853||full	1299	No	NA		1	0	Low-quality	Genome-fragment		2.81	0
S16_vs_55817||full	1299	No	NA		0	0	Low-quality	Genome-fragment		0.81	0
S13_vs_153321||full	2323	No	NA		0	2	Not-determined	Genome-fragment	NA		0
S13_vs_153332||full	2938	No	NA		0	1	Not-determined	Genome-fragment	NA		0
S13_vs_153584||full	5104	No	NA		0	5	Not-determined	Genome-fragment	NA		0
S10_vs_100998||full	1299	No	NA		0	0	Low-quality	Genome-fragment		1.39	0
S13_vs_153906||full	1972	No	NA		0	2	Not-determined	Genome-fragment	NA		0
S13_vs_153952||full	3090	No	NA		0	2	Not-determined	Genome-fragment	NA		0
S13_vs_154019||full	1741	No	NA		0	2	Not-determined	Genome-fragment	NA		0
S8_extracted_k141_97465	1299	No	NA		0	0	Low-quality	Genome-fragment		1.39	0
S13_vs_154229||full	1227	No	NA		0	0	Not-determined	Genome-fragment	NA		0
S8_extracted_k141_198187	1299	No	NA		0	1	Low-quality	Genome-fragment		1.99	0
S13_vs_22523||0_partial	12564	No	NA		0	4	Not-determined	Genome-fragment	NA		0
S8_extracted_k141_164548	1299	No	NA		1	0	Low-quality	Genome-fragment		0.89	0
S8_vs_k141_169169||full	1299	No	NA		2	0	Low-quality	Genome-fragment		3.39	0
S14_vs_k141_17462||lt2gene	1300	No	NA		0	0	Low-quality	Genome-fragment		2.45	0
S1_extracted_k141_140219	1300	No	NA		2	0	Low-quality	Genome-fragment		2.93	0
S1_extracted_k141_116946	1300	No	NA		1	0	Low-quality	Genome-fragment		3.03	0
S3_extracted_k141_73210	1300	No	NA		3	0	Low-quality	Genome-fragment		4.27	0
S13_vs_123344||0_partial	11899	No	NA		0	9	Not-determined	Genome-fragment	NA		0
S5_vs_k141_53705||full	1300	No	NA		0	0	Low-quality	Genome-fragment		2.73	0
S16_vs_51807||full	1300	No	NA		0	0	Low-quality	Genome-fragment		0.75	0
S13_vs_149741||0_partial	14767	No	NA		0	5	Not-determined	Genome-fragment	NA		0
S16_vs_141221||lt2gene	1300	No	NA		1	0	Low-quality	Genome-fragment		2.18	0
S15_vs_17827||full	1300	No	NA		0	0	Low-quality	Genome-fragment		0.8	0
S8_vs_k141_40527||full	1300	No	NA		0	0	Low-quality	Genome-fragment		2.96	0
S13_vs_11769||lt2gene	1002	No	NA		0	0	Not-determined	Genome-fragment	NA		0
S9_extracted_k141_54530	1301	No	NA		0	3	Low-quality	Genome-fragment		1.11	0
S13_vs_92628||full	1301	No	NA		1	0	Low-quality	Genome-fragment		9.68	0
S13_vs_21278||lt2gene	1071	No	NA		0	0	Not-determined	Genome-fragment	NA		0
S3_extracted_k141_59911	1301	No	NA		1	0	Low-quality	Genome-fragment		0.66	0
S5_vs_k141_86782||full	1301	No	NA		0	1	Low-quality	Genome-fragment		3.18	0
S7_extracted_43073	1301	No	NA		1	0	Low-quality	Genome-fragment		4.42	0
S13_vs_31411||lt2gene	2111	No	NA		0	0	Not-determined	Genome-fragment	NA		0
S16_vs_27638||full	1301	No	NA		0	0	Low-quality	Genome-fragment		2.36	0
S12_vs_k141_107193||full	1301	No	NA		2	0	Low-quality	Genome-fragment		3.71	0
S13_vs_36246||lt2gene	1475	No	NA		0	0	Not-determined	Genome-fragment	NA		0
S13_vs_48407||lt2gene	1067	No	NA		0	1	Not-determined	Genome-fragment	NA		0
S13_vs_49985||lt2gene	1211	No	NA		0	0	Not-determined	Genome-fragment	NA		0
S15_extracted_17672	1301	No	NA		0	2	Low-quality	Genome-fragment		1.6	0
S15_extracted_25053	1301	No	NA		0	0	Low-quality	Genome-fragment		1.62	0
S13_vs_57461||lt2gene	1208	No	NA		0	0	Not-determined	Genome-fragment	NA		0
S13_vs_59104||lt2gene	1504	No	NA		0	0	Not-determined	Genome-fragment	NA		0
S13_vs_60714||lt2gene	1303	No	NA		0	0	Not-determined	Genome-fragment	NA		0
S13_vs_64050||lt2gene	2430	No	NA		0	0	Not-determined	Genome-fragment	NA		0
S13_vs_64338||lt2gene	1013	No	NA		0	0	Not-determined	Genome-fragment	NA		0
S15_vs_21198||full	1301	No	NA		0	1	Low-quality	Genome-fragment		3	0
S13_vs_71256||lt2gene	1958	No	NA		0	0	Not-determined	Genome-fragment	NA		0
S6_vs_k141_46151||full	1301	No	NA		0	0	Low-quality	Genome-fragment		4.06	0
S8_vs_k141_105795||full	1301	No	NA		0	0	Low-quality	Genome-fragment		2.02	0
S8_vs_k141_40743||full	1301	No	NA		1	0	Low-quality	Genome-fragment		1.63	0
S13_vs_94682||lt2gene	1120	No	NA		0	0	Not-determined	Genome-fragment	NA		0
S11_vs_k141_79319||lt2gene	1302	No	NA		1	0	Low-quality	Genome-fragment		0.9	0
S13_extracted_40051	1302	No	NA		0	1	Low-quality	Genome-fragment		0.35	0
S16_extracted_155875	1302	No	NA		1	0	Low-quality	Genome-fragment		2.19	0
S13_vs_112434||lt2gene	1189	No	NA		0	0	Not-determined	Genome-fragment	NA		0
S15_extracted_51745	1302	No	NA		1	0	Low-quality	Genome-fragment		2.82	0
S6_extracted_k141_45904	1302	No	NA		0	0	Low-quality	Genome-fragment		2.4	0
S13_vs_124194||lt2gene	1133	No	NA		0	0	Not-determined	Genome-fragment	NA		0
S6_vs_k141_54648||full	1302	No	NA		0	0	Low-quality	Genome-fragment		2.88	0
S13_vs_124659||lt2gene	1383	No	NA		0	0	Not-determined	Genome-fragment	NA		0
S8_vs_k141_5836||full	1302	No	NA		0	1	Low-quality	Genome-fragment		1.84	0
S13_vs_135988||lt2gene	1465	No	NA		0	0	Not-determined	Genome-fragment	NA		0
S13_vs_138220||lt2gene	1021	No	NA		0	0	Not-determined	Genome-fragment	NA		0
S13_vs_145476||lt2gene	1158	No	NA		0	0	Not-determined	Genome-fragment	NA		0
S9_vs_k141_11129||full	1303	No	NA		0	0	Low-quality	Genome-fragment		2.1	0
S14_extracted_k141_36657	3239	No	NA		0	2	Not-determined	Genome-fragment	NA		0
S1_vs_k141_57969||full	1303	No	NA		1	0	Low-quality	Genome-fragment		3.87	0
S5_vs_k141_86000||full	1303	No	NA		2	0	Low-quality	Genome-fragment		3.55	0
S14_extracted_k141_36727	1212	No	NA		0	1	Not-determined	Genome-fragment	NA		0
S5_vs_k141_41702||lt2gene	1303	No	NA		1	0	Low-quality	Genome-fragment		3.8	0
S16_extracted_84472	1303	No	NA		0	0	Low-quality	Genome-fragment		1.39	0
S10_vs_57416||full	1303	No	NA		0	0	Low-quality	Genome-fragment		2.99	0
S14_extracted_k141_22061	1788	No	NA		0	1	Not-determined	Genome-fragment	NA		0
S14_extracted_k141_120	1809	No	NA		0	2	Not-determined	Genome-fragment	NA		0
S6_extracted_k141_8420	1303	No	NA		0	0	Low-quality	Genome-fragment		3.23	0
S6_vs_k141_52354||full	1303	No	NA		0	0	Low-quality	Genome-fragment		3	0
S11_extracted_k141_63477	1304	No	NA		0	0	Low-quality	Genome-fragment		3.2	0
S14_vs_k141_16003||lt2gene	1304	No	NA		1	0	Low-quality	Genome-fragment		2.1	0
S14_vs_k141_91573||lt2gene	1304	No	NA		1	0	Low-quality	Genome-fragment		2.33	0
S1_vs_k141_13388||full	1304	No	NA		0	0	Low-quality	Genome-fragment		2.98	0

S14_extracted_k141_36795	1039	No	NA	0	0	Not-determined	Genome-fragment	NA	0
S7_vs_28972||full	1304	No	NA	0	0	Low-quality	Genome-fragment	3.06	0
S7_vs_31206||full	1304	No	NA	1	0	Low-quality	Genome-fragment	2.93	0
S7_vs_74001||full	1304	No	NA	1	0	Low-quality	Genome-fragment	1.57	0
S12_vs_k141_17957||full	1304	Yes	414	1	1	Low-quality	Genome-fragment	0.96	68.25
S14_extracted_k141_102690	1069	No	NA	0	1	Not-determined	Genome-fragment	NA	0
S15_extracted_44290	1304	No	NA	1	0	Low-quality	Genome-fragment	1.37	0
S14_extracted_k141_44139	1329	No	NA	0	1	Not-determined	Genome-fragment	NA	0
S14_extracted_k141_185	2056	No	NA	0	0	Not-determined	Genome-fragment	NA	0
S15_extracted_45304	1304	No	NA	0	1	Low-quality	Genome-fragment	1.47	0
S8_extracted_k141_196969	1304	No	NA	0	1	Low-quality	Genome-fragment	0.81	0
S8_vs_k141_30867||lt2gene	1304	No	NA	1	0	Low-quality	Genome-fragment	1.91	0
S14_extracted_k141_22148	3080	No	NA	0	1	Not-determined	Genome-fragment	NA	0
S14_extracted_k141_110024	1225	No	NA	0	2	Not-determined	Genome-fragment	NA	0
S14_extracted_k141_51465	1863	No	NA	0	2	Not-determined	Genome-fragment	NA	0
S17_extracted_k141_102259	1305	No	NA	0	0	Low-quality	Genome-fragment	1.96	0
S17_vs_k141_137101||full	1305	No	NA	0	0	Low-quality	Genome-fragment	2.44	0
S9_vs_k141_2425||full	1305	No	NA	0	0	Low-quality	Genome-fragment	1.59	0
S14_extracted_k141_7526	3042	No	NA	0	0	Not-determined	Genome-fragment	NA	0
S14_extracted_k141_14815	5011	No	NA	0	2	Not-determined	Genome-fragment	NA	0
S14_extracted_k141_29530	7915	No	NA	0	4	Not-determined	Genome-fragment	NA	0
S14_extracted_k141_95430	1128	No	NA	0	1	Not-determined	Genome-fragment	NA	0
S13_vs_14802||full	1305	No	NA	0	1	Low-quality	Genome-fragment	1.33	0
S14_extracted_k141_26238	1305	No	NA	4	0	Low-quality	Genome-fragment	4.3	0
S14_extracted_k141_36905	4092	No	NA	0	2	Not-determined	Genome-fragment	NA	0
S14_extracted_k141_58879	1049	No	NA	0	1	Not-determined	Genome-fragment	NA	0
S14_extracted_k141_49714	1305	No	NA	2	0	Low-quality	Genome-fragment	2.03	0
S14_extracted_k141_29537	1021	No	NA	0	1	Not-determined	Genome-fragment	NA	0
S14_extracted_k141_51552	1471	No	NA	0	1	Not-determined	Genome-fragment	NA	0
S14_extracted_k141_7612	15544	No	NA	0	9	Not-determined	Genome-fragment	NA	0
S3_extracted_k141_120184	1305	No	NA	0	1	Low-quality	Genome-fragment	2.61	0
S14_extracted_k141_29569	1239	No	NA	0	1	Not-determined	Genome-fragment	NA	0
S5_vs_k141_22359||full	1305	No	NA	0	1	Low-quality	Genome-fragment	1.43	0
S14_extracted_k141_95520	4407	No	NA	0	1	Not-determined	Genome-fragment	NA	0
S5_vs_k141_103035||full	1305	No	NA	0	0	Low-quality	Genome-fragment	1.9	0
S14_extracted_k141_102768	1621	No	NA	0	1	Not-determined	Genome-fragment	NA	0
S14_extracted_k141_58919	36704	No	NA	17	0	High-quality	High-quality	100	0
S14_extracted_k141_359	3192	No	NA	0	3	Not-determined	Genome-fragment	NA	0
S14_extracted_k141_22259	1723	No	NA	0	0	Not-determined	Genome-fragment	NA	0
S5_vs_k141_135674||lt2gene	1305	No	NA	0	1	Low-quality	Genome-fragment	2.51	0
S7_extracted_25897	1305	No	NA	2	0	Low-quality	Genome-fragment	4.53	0
S7_extracted_114900	1305	No	NA	0	0	Low-quality	Genome-fragment	3.26	0
S6_vs_k141_46149||full	1305	No	NA	2	0	Low-quality	Genome-fragment	0.99	0
S8_extracted_k141_95775	1305	No	NA	1	0	Low-quality	Genome-fragment	2.01	0
S11_extracted_k141_59769	1306	No	NA	0	0	Low-quality	Genome-fragment	3.21	0
S11_vs_k141_6594||full	1306	No	NA	0	0	Low-quality	Genome-fragment	1.23	0
S1_vs_k141_132692||full	1306	No	NA	0	0	Low-quality	Genome-fragment	3.32	0
S14_extracted_k141_66179	1577	No	NA	0	1	Not-determined	Genome-fragment	NA	0
S14_extracted_k141_59002	3793	No	NA	0	3	Not-determined	Genome-fragment	NA	0
S1_vs_k141_125994||full	1306	No	NA	0	1	Low-quality	Genome-fragment	2.43	0
S3_vs_k141_55947||lt2gene	1306	No	NA	1	0	Low-quality	Genome-fragment	2.37	0
S5_extracted_k141_47500	1306	No	NA	0	0	Low-quality	Genome-fragment	2.67	0
S14_extracted_k141_95598	1663	No	NA	0	2	Not-determined	Genome-fragment	NA	0
S5_vs_k141_98396||full	1306	No	NA	0	1	Low-quality	Genome-fragment	2.26	0
S14_extracted_k141_88250	1009	No	NA	0	1	Not-determined	Genome-fragment	NA	0
S14_extracted_k141_102882	2026	No	NA	0	2	Not-determined	Genome-fragment	NA	0
S14_extracted_k141_59018	1377	No	NA	0	1	Not-determined	Genome-fragment	NA	0
S14_extracted_k141_14989	1515	No	NA	0	2	Not-determined	Genome-fragment	NA	0
S14_extracted_k141_80935	2433	No	NA	0	2	Not-determined	Genome-fragment	NA	0
S14_extracted_k141_88269	1016	No	NA	0	0	Not-determined	Genome-fragment	NA	0
S14_extracted_k141_110231	1177	No	NA	0	0	Not-determined	Genome-fragment	NA	0
S7_extracted_20297	1306	No	NA	0	1	Low-quality	Genome-fragment	0.56	0
S14_extracted_k141_88291	1037	No	NA	0	1	Not-determined	Genome-fragment	NA	0
S14_extracted_k141_44255	1392	No	NA	0	0	Not-determined	Genome-fragment	NA	0
S14_extracted_k141_44263	1174	No	NA	0	0	Not-determined	Genome-fragment	NA	0
S7_vs_6860||full	1306	No	NA	0	1	Low-quality	Genome-fragment	0.96	0
S14_extracted_k141_545	1074	No	NA	0	0	Not-determined	Genome-fragment	NA	0
S14_extracted_k141_59081	1552	No	NA	0	2	Not-determined	Genome-fragment	NA	0
S10_vs_77029||full	1306	No	NA	4	0	Low-quality	Genome-fragment	2.43	0
S12_extracted_k141_161737	1306	No	NA	0	1	Low-quality	Genome-fragment	3.07	0
S14_extracted_k141_59083	5551	No	NA	0	5	Not-determined	Genome-fragment	NA	0
S14_extracted_k141_102954	4906	No	NA	0	2	Not-determined	Genome-fragment	NA	0
S12_vs_k141_170049||full	1306	No	NA	0	0	Low-quality	Genome-fragment	2.2	0
S9_extracted_k141_13318	1307	No	NA	1	0	Low-quality	Genome-fragment	2.63	0
S3_vs_k141_1497||full	1307	No	NA	0	0	Low-quality	Genome-fragment	1.65	0
S14_extracted_k141_102984	1263	No	NA	0	1	Not-determined	Genome-fragment	NA	0
S14_extracted_k141_612	2510	No	NA	0	7	Not-determined	Genome-fragment	NA	0
S5_vs_k141_136056||full	1307	No	NA	0	1	Low-quality	Genome-fragment	1.78	0
S14_extracted_k141_73540	1278	No	NA	0	0	Not-determined	Genome-fragment	NA	0
S5_vs_k141_21625||full	1307	No	NA	0	0	Low-quality	Genome-fragment	2.35	0
S14_extracted_k141_44343	3324	No	NA	0	6	Not-determined	Genome-fragment	NA	0
S5_vs_k141_22886||full	1307	No	NA	1	0	Low-quality	Genome-fragment	3.18	0
S5_vs_k141_71528||full	1307	No	NA	1	0	Low-quality	Genome-fragment	2.39	0
S14_extracted_k141_110287	1312	No	NA	0	2	Not-determined	Genome-fragment	NA	0
S14_extracted_k141_15074	1069	No	NA	0	0	Not-determined	Genome-fragment	NA	0
S7_vs_44914||full	1307	No	NA	1	0	Low-quality	Genome-fragment	2.08	0
S14_extracted_k141_103085	3143	No	NA	0	0	Not-determined	Genome-fragment	NA	0
S12_vs_k141_99338||full	1307	No	NA	0	0	Low-quality	Genome-fragment	0.95	0
S6_vs_k141_18819||full	1307	No	NA	3	0	Low-quality	Genome-fragment	3.69	0
S14_extracted_k141_44364	1323	No	NA	0	0	Not-determined	Genome-fragment	NA	0
S8_vs_k141_18419||full	1307	No	NA	0	0	Low-quality	Genome-fragment	2.07	0
S8_vs_k141_124229||full	1307	No	NA	1	0	Low-quality	Genome-fragment	2.08	0
S17_vs_k141_108507||full	1308	No	NA	0	1	Low-quality	Genome-fragment	0.94	0
S14_extracted_k141_37228	2297	No	NA	0	0	Not-determined	Genome-fragment	NA	0
S14_extracted_k141_22398	3993	No	NA	0	0	Not-determined	Genome-fragment	NA	0
S14_extracted_k141_95844	2922	No	NA	0	2	Not-determined	Genome-fragment	NA	0
S17_vs_k141_28907||full	1308	No	NA	0	0	Low-quality	Genome-fragment	3.79	0
S14_extracted_k141_22405	1632	No	NA	0	0	Not-determined	Genome-fragment	NA	0
S11_vs_k141_104103||full	1308	No	NA	1	0	Low-quality	Genome-fragment	0.43	0
S3_vs_k141_95167||full	1308	No	NA	0	0	Low-quality	Genome-fragment	2.92	0
S14_extracted_k141_37243	4105	No	NA	0	4	Not-determined	Genome-fragment	NA	0
S14_extracted_k141_44388	1995	No	NA	0	1	Not-determined	Genome-fragment	NA	0
S14_extracted_k141_29876	2183	No	NA	0	2	Not-determined	Genome-fragment	NA	0
S7_vs_21781||full	1308	No	NA	1	0	Low-quality	Genome-fragment	3.35	0
S16_extracted_163112	1308	No	NA	1	0	Low-quality	Genome-fragment	2	0
S10_vs_51888||full	1308	No	NA	0	0	Low-quality	Genome-fragment	1.04	0
S14_extracted_k141_81128	1269	No	NA	0	1	Not-determined	Genome-fragment	NA	0
S14_extracted_k141_95871	1075	No	NA	0	1	Not-determined	Genome-fragment	NA	0
S12_vs_k141_55696||full	1308	No	NA	0	0	Low-quality	Genome-fragment	2.12	0
S14_extracted_k141_73643	3685	No	NA	0	0	Not-determined	Genome-fragment	NA	0
S14_extracted_k141_88499	5417	No	NA	0	6	Not-determined	Genome-fragment	NA	0
S12_vs_k141_82084||full	1308	No	NA	0	0	Low-quality	Genome-fragment	1.11	0
S14_extracted_k141_103154	5450	No	NA	0	0	Not-determined	Genome-fragment	NA	0
S12_vs_k141_60776||full	1308	No	NA	0	0	Low-quality	Genome-fragment	1.18	0
S12_vs_k141_37196||full	1308	No	NA	1	0	Low-quality	Genome-fragment	3.17	0
S15_vs_1706||full	1308	No	NA	0	0	Low-quality	Genome-fragment	3.46	0
S14_extracted_k141_7878	2152	No	NA	0	1	Not-determined	Genome-fragment	NA	0
S8_extracted_k141_195952	1308	No	NA	0	0	Low-quality	Genome-fragment	3.13	0
S14_extracted_k141_59210	1995	No	NA	0	3	Not-determined	Genome-fragment	NA	0
S14_extracted_k141_59214	4267	No	NA	0	3	Not-determined	Genome-fragment	NA	0
S14_extracted_k141_44452	31164	No	NA	16	0	High-quality	High-quality	99.12	0
S17_extracted_k141_138048	1309	No	NA	0	0	Low-quality	Genome-fragment	5.86	0
S9_vs_k141_63617||full	1309	No	NA	2	0	Low-quality	Genome-fragment	1.93	0
S3_vs_k141_40548||full	1309	No	NA	0	1	Low-quality	Genome-fragment	3.67	0
S5_vs_k141_129684||full	1309	No	NA	0	0	Low-quality	Genome-fragment	2.26	0
S14_extracted_k141_770	1252	No	NA	0	1	Not-determined	Genome-fragment	NA	0
S16_extracted_142589	1309	No	NA	1	0	Low-quality	Genome-fragment	2.48	0
S14_extracted_k141_110439	3250	No	NA	0	1	Not-determined	Genome-fragment	NA	0
S14_extracted_k141_66399	15983	No	NA	0	2	Not-determined	Genome-fragment	NA	0
S16_vs_115313||full	1309	No	NA	0	0	Low-quality	Genome-fragment	1.99	0
S14_extracted_k141_51983	1938	No	NA	0	3	Not-determined	Genome-fragment	NA	0
S12_extracted_k141_97987	1309	No	NA	0	0	Low-quality	Genome-fragment	1.66	0
S12_vs_k141_120502||full	1309	No	NA	0	1	Low-quality	Genome-fragment	1.92	0
S14_extracted_k141_110464	1891	No	NA	0	2	Not-determined	Genome-fragment	NA	0
S14_extracted_k141_110468	1341	No	NA	0	2	Not-determined	Genome-fragment	NA	0
S14_extracted_k141_59294	16909	No	NA	0	4	Not-determined	Genome-fragment	NA	0
S12_vs_k141_149370||full	1309	No	NA	0	0	Low-quality	Genome-fragment	1.06	0
S8_extracted_k141_167303	1309	No	NA	1	0	Low-quality	Genome-fragment	2.75	0
S14_extracted_k141_52011	19473	No	NA	0	7	Not-determined	Genome-fragment	NA	0
S8_extracted_k141_89444	1309	No	NA	2	0	Low-quality	Genome-fragment	1.78	0
S8_extracted_k141_142212	1309	No	NA	0	2	Low-quality	Genome-fragment	0.87	0
S14_extracted_k141_44513	1374	No	NA	0	1	Not-determined	Genome-fragment	NA	0
S8_extracted_k141_121126	1309	No	NA	0	0	Low-quality	Genome-fragment	4.04	0
S14_extracted_k141_22541	1272	No	NA	0	1	Not-determined	Genome-fragment	NA	0
S14_extracted_k141_110513	4889	No	NA	0	2	Not-determined	Genome-fragment	NA	0
S17_vs_k141_49114||full	1310	No	NA	2	0	Low-quality	Genome-fragment	3.06	0
S14_extracted_k141_831	1268	No	NA	0	1	Not-determined	Genome-fragment	NA	0
S14_extracted_k141_30031	3647	No	NA	0	2	Not-determined	Genome-fragment	NA	0
S14_extracted_k141_52056	4360	No	NA	0	1	Not-determined	Genome-fragment	NA	0
S14_extracted_k141_52114	4614	No	NA	0	0	Not-determined	Genome-fragment	NA	0
S14_extracted_k141_37467	2627	No	NA	0	2	Not-determined	Genome-fragment	NA	0
S14_extracted_k141_73803	3754	No	NA	0	3	Not-determined	Genome-fragment	NA	0
S14_extracted_k141_30074	1478	No	NA	0	0	Not-determined	Genome-fragment	NA	0
S9_vs_k141_35544||full	1310	No	NA	2	0	Low-quality	Genome-fragment	3.2	0
S14_extracted_k141_15293	1373	No	NA	0	1	Not-determined	Genome-fragment	NA	0
S14_vs_k141_84162||full	1310	No	NA	2	0	Low-quality	Genome-fragment	2.37	0
S14_extracted_k141_37500	5961	No	NA	2	0	High-quality	High-quality	97.65	0
S1_extracted_k141_119212	1310	No	NA	0	0	Low-quality	Genome-fragment	1.33	0
S14_extracted_k141_869	1038	No	NA	0	1	Not-determined	Genome-fragment	NA	0
S14_extracted_k141_37513	3440	No	NA	0	0	Not-determined	Genome-fragment	NA	0
S5_vs_k141_129240||full	1310	No	NA	2	0	Low-quality	Genome-fragment	2.76	0
S5_vs_k141_50476||lt2gene	1310	No	NA	1	0	Low-quality	Genome-fragment	1.84	0
S7_extracted_75574	1310	No	NA	0	0	Low-quality	Genome-fragment	2.72	0
S16_vs_15401||full	1310	No	NA	1	0	Low-quality	Genome-fragment	2.89	0
S14_extracted_k141_103285	5607	No	NA	0	3	Not-determined	Genome-fragment	NA	0
S14_extracted_k141_103308	1541	No	NA	0	1	Not-determined	Genome-fragment	NA	0
S16_vs_117110||full	1310	No	NA	0	1	Low-quality	Genome-fragment	3.56	0
S14_extracted_k141_920	1095	No	NA	0	1	Not-determined	Genome-fragment	NA	0
S12_vs_k141_72718||full	1310	No	NA	1	0	Low-quality	Genome-fragment	3.18	0
S15_extracted_7794	1310	No	NA	1	0	Low-quality	Genome-fragment	3.84	0
S6_vs_k141_18032||full	1310	No	NA	0	2	Low-quality	Genome-fragment	2.11	0
S6_vs_k141_34549||full	1310	No	NA	0	0	Low-quality	Genome-fragment	2.51	0
S8_extracted_k141_147986	1310	No	NA	0	0	Low-quality	Genome-fragment	4.68	0
S8_vs_k141_50616||full	1310	No	NA	0	2	Low-quality	Genome-fragment	3	0

S17_extracted_k141_19202	1311	No	NA	2	0	Low-quality	Genome-fragment		4.16	0
S17_vs_k141_58513||full	1311	No	NA	0	1	Low-quality	Genome-fragment		1.14	0
S11_vs_k141_10661||full	1311	No	NA	0	1	Low-quality	Genome-fragment		0.83	0
S14_extracted_k141_52297	1474	No	NA	0	0	Not-determined	Genome-fragment	NA		0
S13_extracted_28634	1311	No	NA	2	0	Low-quality	Genome-fragment		2.49	0
S14_extracted_k141_968	7070	No	NA	0	2	Not-determined	Genome-fragment	NA		0
S14_extracted_k141_53794	1311	No	NA	3	0	Low-quality	Genome-fragment		4.33	0
S3_extracted_k141_92703	1311	No	NA	0	1	Low-quality	Genome-fragment		0.34	0
S16_vs_115322||full	1311	No	NA	1	0	Low-quality	Genome-fragment		3.83	0
S14_extracted_k141_59503	3918	No	NA	0	0	Not-determined	Genome-fragment	NA		0
S12_extracted_k141_33500	1311	No	NA	0	0	Low-quality	Genome-fragment		3.05	0
S14_extracted_k141_22685	1305	No	NA	0	0	Not-determined	Genome-fragment	NA		0
S14_extracted_k141_44726	2309	No	NA	0	0	Not-determined	Genome-fragment	NA		0
S15_vs_50440||full	1311	No	NA	0	0	Low-quality	Genome-fragment		0.89	0
S8_vs_k141_3951||full	1311	No	NA	0	0	Low-quality	Genome-fragment		0.4	0
S14_extracted_k141_110722	5514	No	NA	0	2	Not-determined	Genome-fragment	NA		0
S14_extracted_k141_74063	1125	No	NA	0	0	Not-determined	Genome-fragment	NA		0
S14_extracted_k141_103457	1499	No	NA	0	1	Not-determined	Genome-fragment	NA		0
S14_vs_k141_88892||full	1312	No	NA	3	0	Low-quality	Genome-fragment		4.33	0
S14_extracted_k141_95984	1300	No	NA	0	0	Not-determined	Genome-fragment	NA		0
S1_extracted_k141_67911	1312	No	NA	0	1	Low-quality	Genome-fragment		2.57	0
S3_vs_k141_99797||full	1312	No	NA	0	0	Low-quality	Genome-fragment		2.81	0
S14_extracted_k141_88879	3493	No	NA	0	2	Not-determined	Genome-fragment	NA		0
S14_extracted_k141_30256	1004	No	NA	0	0	Not-determined	Genome-fragment	NA		0
S16_extracted_72925	1312	No	NA	0	0	Low-quality	Genome-fragment		2.23	0
S14_extracted_k141_44777	1144	No	NA	0	0	Not-determined	Genome-fragment	NA		0
S16_vs_4218||full	1312	No	NA	0	0	Low-quality	Genome-fragment		1.01	0
S10_extracted_47430	1312	No	NA	1	0	Low-quality	Genome-fragment		3.06	0
S14_extracted_k141_110756	3692	No	NA	0	2	Not-determined	Genome-fragment	NA		0
S10_extracted_82493	1312	No	NA	0	0	Low-quality	Genome-fragment		1.88	0
S12_extracted_k141_163068	1312	No	NA	0	1	Low-quality	Genome-fragment		3.35	0
S12_vs_k141_126376||full	1312	No	NA	0	3	Low-quality	Genome-fragment		2.02	0
S12_vs_k141_142138||full	1312	No	NA	0	0	Low-quality	Genome-fragment		3.27	0
S4_extracted_6192	1312	No	NA	3	0	Low-quality	Genome-fragment		4.26	0
S14_extracted_k141_44805	1097	No	NA	0	0	Not-determined	Genome-fragment	NA		0
S14_extracted_k141_66584	3254	No	NA	0	0	Not-determined	Genome-fragment	NA		0
S14_extracted_k141_110832	8404	No	NA	0	4	Not-determined	Genome-fragment	NA		0
S14_extracted_k141_103602	1185	No	NA	0	0	Not-determined	Genome-fragment	NA		0
S6_vs_k141_32332||full	1312	No	NA	0	0	Low-quality	Genome-fragment		0.93	0
S8_vs_k141_33139||lt2gene	1312	No	NA	1	0	Low-quality	Genome-fragment		2.8	0
S17_extracted_k141_171820	1313	No	NA	0	0	Low-quality	Genome-fragment		2.31	0
S1_extracted_k141_138870	1313	No	NA	0	0	Low-quality	Genome-fragment		2.02	0
S14_extracted_k141_96087	2968	No	NA	0	1	Not-determined	Genome-fragment	NA		0
S1_vs_k141_119558||full	1313	No	NA	0	0	Low-quality	Genome-fragment		1.7	0
S3_vs_k141_122730||full	1313	No	NA	0	5	Low-quality	Genome-fragment		0.39	0
S14_extracted_k141_1147	5130	No	NA	0	1	Not-determined	Genome-fragment	NA		0
S14_extracted_k141_103671	1030	No	NA	0	0	Not-determined	Genome-fragment	NA		0
S5_extracted_k141_101653	1313	No	NA	2	0	Low-quality	Genome-fragment		2.24	0
S5_vs_k141_57043||full	1313	No	NA	1	0	Low-quality	Genome-fragment		3.49	0
S14_extracted_k141_89006	4108	No	NA	0	2	Not-determined	Genome-fragment	NA		0
S5_vs_k141_43343||lt2gene	1313	No	NA	1	0	Low-quality	Genome-fragment		1.92	0
S7_extracted_85081	1313	No	NA	0	0	Low-quality	Genome-fragment		2.47	0
S14_extracted_k141_96112	1032	No	NA	0	0	Not-determined	Genome-fragment	NA		0
S7_vs_34954||full	1313	No	NA	2	0	Low-quality	Genome-fragment		0.76	0
S7_vs_117717||full	1313	No	NA	0	0	Low-quality	Genome-fragment		3.09	0
S14_extracted_k141_22844	4594	No	NA	0	2	Not-determined	Genome-fragment	NA		0
S7_vs_130315||full	1313	No	NA	0	0	Low-quality	Genome-fragment		0.89	0
S10_vs_114248||full	1313	No	NA	0	0	Low-quality	Genome-fragment		3.43	0
S12_vs_k141_1601||full	1313	No	NA	0	0	Low-quality	Genome-fragment		1.52	0
S14_extracted_k141_59699	3882	No	NA	0	1	Not-determined	Genome-fragment	NA		0
S2_extracted_k141_5288	1313	No	NA	1	0	Low-quality	Genome-fragment		3.36	0
S6_vs_k141_22807||full	1313	No	NA	1	0	Low-quality	Genome-fragment		3.29	0
S14_extracted_k141_1223	1010	No	NA	0	0	Not-determined	Genome-fragment	NA		0
S14_extracted_k141_44932	2774	No	NA	0	2	Not-determined	Genome-fragment	NA		0
S8_vs_k141_152270||full	1313	No	NA	0	0	Low-quality	Genome-fragment		3.36	0
S13_vs_84613||full	1314	No	NA	0	0	Low-quality	Genome-fragment		1.74	0
S14_vs_k141_90494||full	1314	No	NA	1	0	Low-quality	Genome-fragment		3.55	0
S3_extracted_k141_50768	1314	No	NA	2	0	Low-quality	Genome-fragment		3.26	0
S3_vs_k141_97692||full	1314	No	NA	0	0	Low-quality	Genome-fragment		0.9	0
S5_vs_k141_139041||full	1314	No	NA	0	0	Low-quality	Genome-fragment		3.6	0
S14_extracted_k141_15803	1113	No	NA	0	1	Not-determined	Genome-fragment	NA		0
S14_extracted_k141_66689	1322	No	NA	0	1	Not-determined	Genome-fragment	NA		0
S5_vs_k141_37093||lt2gene	1314	No	NA	2	0	Low-quality	Genome-fragment		3.44	0
S16_vs_119293||full	1314	No	NA	0	1	Low-quality	Genome-fragment		1.39	0
S14_extracted_k141_30537	6067	No	NA	0	5	Not-determined	Genome-fragment	NA		0
S14_extracted_k141_74213	1021	No	NA	0	0	Not-determined	Genome-fragment	NA		0
S14_extracted_k141_89129	5241	No	NA	0	5	Not-determined	Genome-fragment	NA		0
S15_extracted_54875	1314	No	NA	1	0	Low-quality	Genome-fragment		2.91	0
S15_vs_21341||full	1314	No	NA	0	0	Low-quality	Genome-fragment		2.86	0
S2_extracted_k141_12410	1314	No	NA	0	0	Low-quality	Genome-fragment		1.37	0
S14_extracted_k141_81646	2214	No	NA	0	1	Not-determined	Genome-fragment	NA		0
S14_extracted_k141_66702	2616	No	NA	0	0	Not-determined	Genome-fragment	NA		0
S8_vs_k141_182519||lt2gene	1314	No	NA	1	0	Low-quality	Genome-fragment		2.29	0
S14_extracted_k141_66713	1093	No	NA	0	0	Not-determined	Genome-fragment	NA		0
S14_extracted_k141_8436	1771	No	NA	0	0	Not-determined	Genome-fragment	NA		0
S14_extracted_k141_74225	3044	No	NA	0	4	Not-determined	Genome-fragment	NA		0
S14_extracted_k141_81680	1352	No	NA	0	2	Not-determined	Genome-fragment	NA		0
S8_vs_k141_5131||lt2gene	1314	No	NA	1	0	Low-quality	Genome-fragment		0.51	0
S17_extracted_k141_61894	1315	No	NA	2	0	Low-quality	Genome-fragment		6	0
S14_extracted_k141_89176	1130	No	NA	0	0	Not-determined	Genome-fragment	NA		0
S14_extracted_k141_22988	1262	No	NA	0	1	Not-determined	Genome-fragment	NA		0
S14_extracted_k141_74270	1002	No	NA	0	0	Not-determined	Genome-fragment	NA		0
S9_vs_k141_82145||lt2gene	1315	No	NA	1	0	Low-quality	Genome-fragment		1.85	0
S11_extracted_k141_55613	1315	No	NA	0	0	Low-quality	Genome-fragment		2.77	0
S14_extracted_k141_81729	4095	No	NA	0	3	Not-determined	Genome-fragment	NA		0
S14_vs_k141_99593||full	1315	No	NA	2	0	Low-quality	Genome-fragment		2.82	0
S14_vs_k141_79868||full	1315	No	NA	2	0	Low-quality	Genome-fragment		3.16	0
S14_extracted_k141_8467	6853	No	NA	0	1	Not-determined	Genome-fragment	NA		0
S3_vs_k141_60616||full	1315	No	NA	1	0	Low-quality	Genome-fragment		2.82	0
S14_extracted_k141_111238	1674	No	NA	0	2	Not-determined	Genome-fragment	NA		0
S14_extracted_k141_59845	6195	No	NA	0	2	Not-determined	Genome-fragment	NA		0
S14_extracted_k141_15976	1735	No	NA	0	2	Not-determined	Genome-fragment	NA		0
S14_extracted_k141_23057	1299	No	NA	0	0	Not-determined	Genome-fragment	NA		0
S14_extracted_k141_81814	1304	No	NA	0	0	Not-determined	Genome-fragment	NA		0
S14_extracted_k141_52548	1063	No	NA	0	1	Not-determined	Genome-fragment	NA		0
S3_vs_k141_45925||full	1315	No	NA	1	0	Low-quality	Genome-fragment		2.15	0
S14_extracted_k141_66796	9524	No	NA	0	7	Not-determined	Genome-fragment	NA		0
S14_extracted_k141_111271	2245	No	NA	0	0	Not-determined	Genome-fragment	NA		0
S7_vs_119932||full	1315	No	NA	1	0	Low-quality	Genome-fragment		0.51	0
S16_extracted_41013	1315	No	NA	1	0	Low-quality	Genome-fragment		2.2	0
S16_vs_46186||full	1315	No	NA	1	0	Low-quality	Genome-fragment		2.2	0
S16_vs_134788||full	1315	No	NA	0	1	Low-quality	Genome-fragment		1.79	0
S10_vs_40126||full	1315	No	NA	1	0	Low-quality	Genome-fragment		0.69	0
S12_vs_k141_181388||full	1315	No	NA	0	2	Low-quality	Genome-fragment		0.71	0
S15_vs_35563||full	1315	No	NA	2	0	Low-quality	Genome-fragment		3.08	0
S14_extracted_k141_96265	1128	No	NA	0	0	Not-determined	Genome-fragment	NA		0
S14_extracted_k141_104059	3118	No	NA	0	0	Not-determined	Genome-fragment	NA		0
S6_vs_k141_56015||lt2gene	1315	No	NA	1	0	Low-quality	Genome-fragment		3.1	0
S8_vs_k141_58818||lt2gene	1315	No	NA	1	0	Low-quality	Genome-fragment		4.81	0
S14_extracted_k141_30721	10765	No	NA	0	0	Not-determined	Genome-fragment	NA		0
S14_extracted_k141_1265	3459	No	NA	0	3	Not-determined	Genome-fragment	NA		0
S14_extracted_k141_8598	1008	No	NA	0	1	Not-determined	Genome-fragment	NA		0
S9_vs_k141_90036||full	1316	No	NA	0	0	Low-quality	Genome-fragment		1.98	0
S14_extracted_k141_81943	5207	No	NA	0	2	Not-determined	Genome-fragment	NA		0
S14_extracted_k141_8630	1076	No	NA	0	1	Not-determined	Genome-fragment	NA		0
S11_vs_k141_28463||full	1316	No	NA	0	0	Low-quality	Genome-fragment		2.46	0
S14_extracted_k141_16123	1726	No	NA	0	2	Not-determined	Genome-fragment	NA		0
S14_extracted_k141_111391	3001	No	NA	0	1	Not-determined	Genome-fragment	NA		0
S11_vs_k141_56916||full	1316	No	NA	0	1	Low-quality	Genome-fragment		3.55	0
S14_extracted_k141_82210	1316	No	NA	4	0	Low-quality	Genome-fragment		4.34	0
S14_extracted_k141_96349	1036	No	NA	0	0	Not-determined	Genome-fragment	NA		0
S14_extracted_k141_23250	1591	No	NA	0	1	Not-determined	Genome-fragment	NA		0
S14_vs_k141_90418||full	1316	No	NA	4	0	Low-quality	Genome-fragment		2.16	0
S1_vs_k141_56918||full	1316	No	NA	2	0	Low-quality	Genome-fragment		3.63	0
S14_extracted_k141_66956	1434	No	NA	0	2	Not-determined	Genome-fragment	NA		0
S14_extracted_k141_60073	3245	No	NA	0	3	Not-determined	Genome-fragment	NA		0
S1_vs_k141_84319||lt2gene	1316	No	NA	1	0	Low-quality	Genome-fragment		1.74	0
S5_extracted_k141_55843	1316	No	NA	0	0	Low-quality	Genome-fragment		2.46	0
S5_vs_k141_60873||full	1316	No	NA	2	0	Low-quality	Genome-fragment		1.73	0
S5_vs_k141_18792||full	1316	No	NA	0	0	Low-quality	Genome-fragment		2.25	0
S14_extracted_k141_66996	3672	No	NA	0	2	Not-determined	Genome-fragment	NA		0
S5_vs_k141_19134||lt2gene	1316	No	NA	2	0	Low-quality	Genome-fragment		2.57	0
S16_vs_79629||full	1316	No	NA	1	0	Low-quality	Genome-fragment		2.63	0
S14_extracted_k141_52790	1315	No	NA	0	1	Not-determined	Genome-fragment	NA		0
S14_extracted_k141_52799	1239	No	NA	0	1	Not-determined	Genome-fragment	NA		0
S12_vs_k141_112074||full	1316	No	NA	0	0	Low-quality	Genome-fragment		3.2	0
S12_vs_k141_43773||lt2gene	1316	No	NA	1	0	Low-quality	Genome-fragment		2.12	0
S6_vs_k141_50666||lt2gene	1316	No	NA	2	0	Low-quality	Genome-fragment		2.88	0
S8_extracted_k141_46864	1316	No	NA	0	1	Low-quality	Genome-fragment		3.83	0
S17_extracted_k141_95487	1317	No	NA	1	0	Low-quality	Genome-fragment		0.77	0
S14_extracted_k141_38277	1137	No	NA	0	1	Not-determined	Genome-fragment	NA		0
S14_extracted_k141_96446	1307	No	NA	0	2	Not-determined	Genome-fragment	NA		0
S14_extracted_k141_52830	3044	No	NA	0	1	Not-determined	Genome-fragment	NA		0
S17_vs_k141_127535||full	1317	No	NA	0	0	Low-quality	Genome-fragment		2.08	0
S14_extracted_k141_38309	2187	No	NA	0	1	Not-determined	Genome-fragment	NA		0
S3_vs_k141_28779||full	1317	No	NA	0	0	Low-quality	Genome-fragment		2.13	0
S14_extracted_k141_60222	1087	No	NA	0	1	Not-determined	Genome-fragment	NA		0
S14_extracted_k141_89472	1286	No	NA	0	2	Not-determined	Genome-fragment	NA		0
S16_vs_124981||full	1317	No	NA	0	0	Low-quality	Genome-fragment		2.34	0
S16_vs_74591||lt2gene	1317	No	NA	0	0	Low-quality	Genome-fragment		2.89	0
S10_extracted_76897	1317	No	NA	0	0	Low-quality	Genome-fragment		9.73	0
S14_extracted_k141_60283	2226	No	NA	0	1	Not-determined	Genome-fragment	NA		0
S14_extracted_k141_8901	1658	No	NA	0	1	Not-determined	Genome-fragment	NA		0
S12_extracted_k141_166559	1317	No	NA	0	2	Low-quality	Genome-fragment		3.07	0
S8_extracted_k141_79229	1317	No	NA	0	0	Low-quality	Genome-fragment		0.24	0
S14_extracted_k141_89511	1991	No	NA	0	2	Not-determined	Genome-fragment	NA		0
S14_extracted_k141_104417	3813	No	NA	0	4	Not-determined	Genome-fragment	NA		0
S8_vs_k141_64176||full	1317	No	NA	2	0	Low-quality	Genome-fragment		2.14	0
S17_vs_k141_111001||full	1318	No	NA	1	0	Low-quality	Genome-fragment		2.67	0
S14_extracted_k141_16392	1036	No	NA	0	0	Not-determined	Genome-fragment	NA		0
S14_extracted_k141_8927	27261	No	NA	0	5	Not-determined	Genome-fragment	NA		0
S13_vs_105438||full	1318	No	NA	0	1	Low-quality	Genome-fragment		1.63	0
S14_extracted_k141_111792	6647	No	NA	0	3	Not-determined	Genome-fragment	NA		0
S14_extracted_k141_14866	1318	No	NA	4	0	Low-quality	Genome-fragment		4.35	0

S14_extracted_k141_74724	3216	No	NA	0	2	Not-determined	Genome-fragment	NA		0
S14_vs_k141_86850||full	1318	No	NA	0	1	Low-quality	Genome-fragment		0.95	0
S3_extracted_k141_104992	1318	No	NA	0	0	Low-quality	Genome-fragment		0.86	0
S3_vs_k141_64324||full	1318	No	NA	1	0	Low-quality	Genome-fragment		1.88	0
S14_extracted_k141_1515	1279	No	NA	0	1	Not-determined	Genome-fragment	NA		0
S3_vs_k141_108476||lt2gene	1318	No	NA	1	0	Low-quality	Genome-fragment		1.68	0
S5_vs_k141_95556||full	1318	No	NA	0	1	Low-quality	Genome-fragment		2.2	0
S7_vs_46134||full	1318	No	NA	1	0	Low-quality	Genome-fragment		2.02	0
S14_extracted_k141_38470	1629	No	NA	0	0	Not-determined	Genome-fragment	NA		0
S14_extracted_k141_74768	1690	No	NA	0	0	Not-determined	Genome-fragment	NA		0
S14_extracted_k141_104530	1082	No	NA	0	0	Not-determined	Genome-fragment	NA		0
S14_extracted_k141_74780	1087	No	NA	0	1	Not-determined	Genome-fragment	NA		0
S14_extracted_k141_31099	1019	No	NA	0	1	Not-determined	Genome-fragment	NA		0
S7_vs_81343||full	1318	No	NA	1	0	Low-quality	Genome-fragment		2.25	0
S7_vs_74253||lt2gene	1318	No	NA	1	0	Low-quality	Genome-fragment		3.73	0
S14_extracted_k141_1539	1091	No	NA	0	0	Not-determined	Genome-fragment	NA		0
S14_extracted_k141_38501	1146	No	NA	0	1	Not-determined	Genome-fragment	NA		0
S14_extracted_k141_82271	1503	No	NA	0	1	Not-determined	Genome-fragment	NA		0
S16_vs_39803||full	1318	No	NA	0	1	Low-quality	Genome-fragment		2.08	0
S16_vs_123704||full	1318	No	NA	0	1	Low-quality	Genome-fragment		2.32	0
S14_extracted_k141_111875	1004	No	NA	0	0	Not-determined	Genome-fragment	NA		0
S13_extracted_63849	1319	No	NA	0	1	Low-quality	Genome-fragment		1.25	0
S14_extracted_k141_53022	1778	No	NA	0	2	Not-determined	Genome-fragment	NA		0
S14_extracted_k141_67575	1319	No	NA	2	0	Low-quality	Genome-fragment		4.34	0
S14_vs_k141_111951||full	1319	No	NA	4	0	Low-quality	Genome-fragment		4.33	0
S1_extracted_k141_125844	1319	No	NA	0	1	Low-quality	Genome-fragment		0.93	0
S15_extracted_14624	1319	No	NA	2	0	Low-quality	Genome-fragment		2.94	0
S14_extracted_k141_31158	2002	No	NA	0	2	Not-determined	Genome-fragment	NA		0
S8_vs_k141_66979||full	1319	No	NA	1	0	Low-quality	Genome-fragment		2.14	0
S14_extracted_k141_74961	1118	No	NA	0	0	Not-determined	Genome-fragment	NA		0
S14_extracted_k141_1622	1035	No	NA	0	1	Not-determined	Genome-fragment	NA		0
S14_extracted_k141_104679	1312	No	NA	0	0	Not-determined	Genome-fragment	NA		0
S14_vs_k141_81134||lt2gene	1320	No	NA	1	0	Low-quality	Genome-fragment		4.04	0
S14_extracted_k141_1648	1077	No	NA	0	1	Not-determined	Genome-fragment	NA		0
S1_extracted_k141_155679	1320	No	NA	1	0	Low-quality	Genome-fragment		1.32	0
S14_extracted_k141_104691	1062	No	NA	0	0	Not-determined	Genome-fragment	NA		0
S1_extracted_k141_59699	1320	No	NA	1	0	Low-quality	Genome-fragment		2.22	0
S14_extracted_k141_8940	1836	No	NA	0	1	Not-determined	Genome-fragment	NA		0
S14_extracted_k141_38611	1374	No	NA	0	0	Not-determined	Genome-fragment	NA		0
S1_extracted_k141_60061	1320	No	NA	0	1	Low-quality	Genome-fragment		3.72	0
S14_extracted_k141_96781	1201	No	NA	0	1	Not-determined	Genome-fragment	NA		0
S1_vs_k141_107954||full	1320	No	NA	1	0	Low-quality	Genome-fragment		2.34	0
S14_extracted_k141_104708	3584	No	NA	0	1	Not-determined	Genome-fragment	NA		0
S14_extracted_k141_96802	3107	No	NA	0	2	Not-determined	Genome-fragment	NA		0
S3_extracted_k141_78924	1320	No	NA	0	1	Low-quality	Genome-fragment		2.61	0
S14_extracted_k141_82400	2898	No	NA	0	1	Not-determined	Genome-fragment	NA		0
S14_extracted_k141_8957	1163	No	NA	0	1	Not-determined	Genome-fragment	NA		0
S3_vs_k141_117904||full	1320	No	NA	0	0	Low-quality	Genome-fragment		0.58	0
S14_extracted_k141_31233	1778	No	NA	0	1	Not-determined	Genome-fragment	NA		0
S14_extracted_k141_45366	1647	No	NA	0	0	Not-determined	Genome-fragment	NA		0
S10_extracted_54035	1320	No	NA	1	0	Low-quality	Genome-fragment		0.54	0
S8_extracted_k141_81983	1320	No	NA	0	2	Low-quality	Genome-fragment		1.88	0
S14_extracted_k141_53183	1317	No	NA	0	3	Not-determined	Genome-fragment	NA		0
S17_vs_k141_158721||lt2gene	1321	No	NA	1	0	Low-quality	Genome-fragment		1.93	0
S11_extracted_k141_65649	1321	No	NA	0	2	Low-quality	Genome-fragment		0.83	0
S14_extracted_k141_75076	1108	No	NA	0	1	Not-determined	Genome-fragment	NA		0
S14_extracted_k141_45391	8902	No	NA	0	4	Not-determined	Genome-fragment	NA		0
S14_extracted_k141_60691	3424	No	NA	0	2	Not-determined	Genome-fragment	NA		0
S14_extracted_k141_82455	1026	No	NA	0	1	Not-determined	Genome-fragment	NA		0
S14_extracted_k141_31311	1398	No	NA	0	3	Not-determined	Genome-fragment	NA		0
S11_vs_k141_44550||full	1321	No	NA	3	0	Low-quality	Genome-fragment		2.18	0
S14_extracted_k141_82467	1084	No	NA	0	1	Not-determined	Genome-fragment	NA		0
S14_extracted_k141_38726	1305	No	NA	0	1	Not-determined	Genome-fragment	NA		0
S14_extracted_k141_22200	1321	No	NA	1	0	Low-quality	Genome-fragment		0.69	0
S14_extracted_k141_45447	1534	No	NA	0	0	Not-determined	Genome-fragment	NA		0
S14_extracted_k141_31343	2128	No	NA	0	1	Not-determined	Genome-fragment	NA		0
S1_vs_k141_44768||full	1321	No	NA	0	1	Low-quality	Genome-fragment		1.1	0
S5_vs_k141_68623||full	1321	No	NA	0	1	Low-quality	Genome-fragment		1.63	0
S14_extracted_k141_60763	6623	No	NA	0	6	Not-determined	Genome-fragment	NA		0
S14_extracted_k141_23748	1188	No	NA	0	0	Not-determined	Genome-fragment	NA		0
S5_vs_k141_15854||full	1321	No	NA	0	0	Low-quality	Genome-fragment		2.62	0
S14_extracted_k141_38851	1491	No	NA	0	1	Not-determined	Genome-fragment	NA		0
S5_vs_k141_36342||full	1321	No	NA	1	0	Low-quality	Genome-fragment		0.89	0
S16_extracted_36088	1321	No	NA	0	0	Low-quality	Genome-fragment		3.17	0
S16_vs_33023||full	1321	No	NA	0	0	Low-quality	Genome-fragment		0.32	0
S14_extracted_k141_53416	1213	No	NA	0	0	Not-determined	Genome-fragment	NA		0
S14_extracted_k141_53417	1036	No	NA	0	0	Not-determined	Genome-fragment	NA		0
S14_extracted_k141_104954	1227	No	NA	0	1	Not-determined	Genome-fragment	NA		0
S14_extracted_k141_67690	3398	No	NA	0	0	Not-determined	Genome-fragment	NA		0
S12_vs_k141_126208||full	1321	No	NA	0	2	Low-quality	Genome-fragment		1.71	0
S14_extracted_k141_38960	1167	No	NA	0	1	Not-determined	Genome-fragment	NA		0
S14_extracted_k141_67732	4357	No	NA	0	2	Not-determined	Genome-fragment	NA		0
S14_extracted_k141_112173	1189	No	NA	0	2	Not-determined	Genome-fragment	NA		0
S15_vs_16156||full	1321	No	NA	0	0	Low-quality	Genome-fragment		2.37	0
S15_vs_65294||full	1321	No	NA	0	0	Low-quality	Genome-fragment		1.5	0
S14_extracted_k141_105002	1249	No	NA	0	1	Not-determined	Genome-fragment	NA		0
S14_extracted_k141_82736	5689	No	NA	0	2	Not-determined	Genome-fragment	NA		0
S8_vs_k141_155412||lt2gene	1321	No	NA	1	0	Low-quality	Genome-fragment		0.71	0
S17_vs_k141_164906||full	1322	No	NA	1	0	Low-quality	Genome-fragment		1.12	0
S17_vs_k141_51377||full	1322	No	NA	0	1	Low-quality	Genome-fragment		2.77	0
S9_extracted_k141_31622	1322	No	NA	1	0	Low-quality	Genome-fragment		2.95	0
S14_extracted_k141_105058	3059	No	NA	0	0	Not-determined	Genome-fragment	NA		0
S9_vs_k141_6541||full	1322	No	NA	0	0	Low-quality	Genome-fragment		2.73	0
S14_extracted_k141_75300	1174	No	NA	0	1	Not-determined	Genome-fragment	NA		0
S14_extracted_k141_75303	1164	No	NA	0	3	Not-determined	Genome-fragment	NA		0
S14_extracted_k141_31437	1076	No	NA	0	0	Not-determined	Genome-fragment	NA		0
S13_vs_68786||full	1322	No	NA	0	0	Low-quality	Genome-fragment		2.35	0
S14_extracted_k141_82770	1135	No	NA	0	1	Not-determined	Genome-fragment	NA		0
S14_extracted_k141_31441	1954	No	NA	0	1	Not-determined	Genome-fragment	NA		0
S14_vs_k141_82046||full	1322	No	NA	0	0	Low-quality	Genome-fragment		2.81	0
S14_vs_k141_35185||full	1322	No	NA	0	0	Low-quality	Genome-fragment		1.84	0
S1_extracted_k141_140797	1322	No	NA	0	2	Low-quality	Genome-fragment		0.46	0
S5_vs_k141_25542||lt2gene	1322	No	NA	1	0	Low-quality	Genome-fragment		2.68	0
S14_extracted_k141_1927	6101	No	NA	0	3	Not-determined	Genome-fragment	NA		0
S14_extracted_k141_31444	13377	No	NA	0	5	Not-determined	Genome-fragment	NA		0
S5_vs_k141_58617||lt2gene	1322	No	NA	1	0	Low-quality	Genome-fragment		0.82	0
S14_extracted_k141_90110	3828	No	NA	0	1	Not-determined	Genome-fragment	NA		0
S12_vs_k141_63426||full	1322	No	NA	0	1	Low-quality	Genome-fragment		0.83	0
S12_vs_k141_87776||full	1322	No	NA	1	0	Low-quality	Genome-fragment		3.09	0
S8_vs_k141_157830||full	1322	No	NA	2	0	Low-quality	Genome-fragment		3.22	0
S14_extracted_k141_75365	1530	No	NA	0	3	Not-determined	Genome-fragment	NA		0
S8_vs_k141_5716||full	1322	No	NA	0	2	Low-quality	Genome-fragment		1.55	0
S14_extracted_k141_53605	2061	No	NA	0	1	Not-determined	Genome-fragment	NA		0
S14_extracted_k141_112258	1778	No	NA	0	2	Not-determined	Genome-fragment	NA		0
S14_extracted_k141_67918	3793	No	NA	0	1	Not-determined	Genome-fragment	NA		0
S17_extracted_k141_41175	1323	No	NA	0	0	Low-quality	Genome-fragment		1.32	0
S11_vs_k141_72422||full	1323	No	NA	0	0	Low-quality	Genome-fragment		2.9	0
S14_extracted_k141_24079	3164	No	NA	0	1	Not-determined	Genome-fragment	NA		0
S14_extracted_k141_97067	1126	No	NA	0	0	Not-determined	Genome-fragment	NA		0
S14_extracted_k141_16969	2222	No	NA	0	2	Not-determined	Genome-fragment	NA		0
S13_vs_33032||full	1323	No	NA	1	0	Low-quality	Genome-fragment		1.02	0
S14_extracted_k141_7832	1323	No	NA	3	0	Low-quality	Genome-fragment		4.34	0
S14_extracted_k141_53687	3574	No	NA	0	8	Not-determined	Genome-fragment	NA		0
S14_extracted_k141_82897	1206	No	NA	0	1	Not-determined	Genome-fragment	NA		0
S14_extracted_k141_82904	1253	No	NA	0	1	Not-determined	Genome-fragment	NA		0
S14_vs_k141_47996||full	1323	No	NA	1	0	Low-quality	Genome-fragment		2.09	0
S14_extracted_k141_17002	1517	No	NA	0	1	Not-determined	Genome-fragment	NA		0
S14_extracted_k141_24114	3461	No	NA	0	2	Not-determined	Genome-fragment	NA		0
S14_vs_k141_7973||lt2gene	1323	No	NA	2	0	Low-quality	Genome-fragment		0.94	0
S5_vs_k141_2004||full	1323	No	NA	2	0	Low-quality	Genome-fragment		3.43	0
S14_extracted_k141_97184	3694	No	NA	0	2	Not-determined	Genome-fragment	NA		0
S14_extracted_k141_82953	3044	No	NA	0	0	Not-determined	Genome-fragment	NA		0
S14_extracted_k141_60910	1086	No	NA	0	0	Not-determined	Genome-fragment	NA		0
S5_vs_k141_60502||full	1323	No	NA	0	0	Low-quality	Genome-fragment		3.02	0
S14_extracted_k141_97193	1206	No	NA	0	0	Not-determined	Genome-fragment	NA		0
S7_extracted_115796	1323	No	NA	0	0	Low-quality	Genome-fragment		2.95	0
S14_extracted_k141_90260	3109	No	NA	0	2	Not-determined	Genome-fragment	NA		0
S16_extracted_162941	1323	No	NA	0	0	Low-quality	Genome-fragment		3.28	0
S8_extracted_k141_184555	1323	No	NA	0	0	Low-quality	Genome-fragment		4.2	0
S14_extracted_k141_17041	3953	No	NA	0	2	Not-determined	Genome-fragment	NA		0
S14_extracted_k141_83010	2188	No	NA	0	3	Not-determined	Genome-fragment	NA		0
S14_extracted_k141_17083	1095	No	NA	0	0	Not-determined	Genome-fragment	NA		0
S8_vs_k141_52145||full	1323	No	NA	0	1	Low-quality	Genome-fragment		1.49	0
S14_extracted_k141_112398	1142	No	NA	0	1	Not-determined	Genome-fragment	NA		0
S17_extracted_k141_117296	1324	No	NA	1	0	Low-quality	Genome-fragment		0.11	0
S14_extracted_k141_90268	1152	No	NA	0	0	Not-determined	Genome-fragment	NA		0
S9_vs_k141_66609||full	1324	No	NA	1	0	Low-quality	Genome-fragment		3.81	0
S9_vs_k141_74947||full	1324	No	NA	0	0	Low-quality	Genome-fragment		2.42	0
S11_vs_k141_69717||full	1324	No	NA	1	0	Low-quality	Genome-fragment		0.78	0
S14_extracted_k141_90305	1716	No	NA	0	1	Not-determined	Genome-fragment	NA		0
S14_extracted_k141_9557	1140	No	NA	0	2	Not-determined	Genome-fragment	NA		0
S11_vs_k141_34404||lt2gene	1324	No	NA	2	0	Low-quality	Genome-fragment		3.35	0
S13_vs_133143||full	1324	No	NA	2	0	Low-quality	Genome-fragment		3.41	0
S14_vs_k141_84218||full	1324	No	NA	0	0	Low-quality	Genome-fragment		2.79	0
S1_vs_k141_7354||full	1324	No	NA	0	0	Low-quality	Genome-fragment		0.8	0
S14_extracted_k141_31717	1099	No	NA	0	2	Not-determined	Genome-fragment	NA		0
S5_vs_k141_65718||full	1324	No	NA	1	0	Low-quality	Genome-fragment		2.12	0
S14_extracted_k141_97357	1057	No	NA	0	1	Not-determined	Genome-fragment	NA		0
S14_extracted_k141_61147	1017	No	NA	0	0	Not-determined	Genome-fragment	NA		0
S14_extracted_k141_105461	4381	No	NA	0	0	Not-determined	Genome-fragment	NA		0
S7_vs_5077||full	1324	No	NA	1	0	Low-quality	Genome-fragment		2.52	0
S14_extracted_k141_68215	1920	No	NA	0	0	Not-determined	Genome-fragment	NA		0
S16_vs_169807||full	1324	No	NA	2	0	Low-quality	Genome-fragment		3.09	0
S14_extracted_k141_61182	1089	No	NA	0	1	Not-determined	Genome-fragment	NA		0
S14_extracted_k141_68229	1136	No	NA	0	0	Not-determined	Genome-fragment	NA		0
S10_extracted_58799	1324	No	NA	0	0	Low-quality	Genome-fragment		1.86	0
S12_vs_k141_28552||full	1324	No	NA	0	0	Low-quality	Genome-fragment		4.63	0
S14_extracted_k141_17277	1169	No	NA	0	1	Not-determined	Genome-fragment	NA		0
S14_extracted_k141_112586	1811	No	NA	0	1	Not-determined	Genome-fragment	NA		0
S14_extracted_k141_75894	1103	No	NA	0	0	Not-determined	Genome-fragment	NA		0
S14_extracted_k141_24254	12666	No	NA	0	1	Not-determined	Genome-fragment	NA		0
S14_extracted_k141_31817	3613	No	NA	0	4	Not-determined	Genome-fragment	NA		0
S15_extracted_41353	1324	No	NA	0	0	Low-quality	Genome-fragment		2.83	0
S14_extracted_k141_31824	1265	No	NA	0	0	Not-determined	Genome-fragment	NA		0

S2_extracted_k141_2535	1324	No	NA	2	0	Low-quality	Genome-fragment		4.82	0
S17_vs_k141_60781||full	1325	No	NA	0	0	Low-quality	Genome-fragment		3.16	0
S14_extracted_k141_24315	1176	No	NA	0	0	Not-determined	Genome-fragment	NA		0
S14_extracted_k141_112623	1439	No	NA	0	0	Not-determined	Genome-fragment	NA		0
S14_extracted_k141_97461	5020	No	NA	0	0	Not-determined	Genome-fragment	NA		0
S14_extracted_k141_105582	1189	No	NA	0	0	Not-determined	Genome-fragment	NA		0
S17_vs_k141_135069||full	1325	No	NA	0	0	Low-quality	Genome-fragment		2.79	0
S14_extracted_k141_24349	1861	No	NA	0	1	Not-determined	Genome-fragment	NA		0
S14_extracted_k141_31851	13966	No	NA	0	5	Not-determined	Genome-fragment	NA		0
S14_extracted_k141_76012	2222	No	NA	0	1	Not-determined	Genome-fragment	NA		0
S17_vs_k141_139750||full	1325	No	NA	0	0	Low-quality	Genome-fragment		3.51	0
S17_vs_k141_38488||lt2gene	1325	No	NA	1	0	Low-quality	Genome-fragment		2.42	0
S14_extracted_k141_83263	3852	No	NA	0	3	Not-determined	Genome-fragment	NA		0
S14_extracted_k141_31916	1167	No	NA	0	2	Not-determined	Genome-fragment	NA		0
S11_vs_k141_30155||full	1325	No	NA	0	0	Low-quality	Genome-fragment		2.06	0
S14_extracted_k141_68491	1605	No	NA	0	1	Not-determined	Genome-fragment	NA		0
S13_vs_4371||full	1325	No	NA	0	1	Low-quality	Genome-fragment		0.79	0
S14_vs_k141_76814||full	1325	No	NA	0	0	Low-quality	Genome-fragment		2.64	0
S1_vs_k141_81277||lt2gene	1325	No	NA	0	0	Low-quality	Genome-fragment		3.61	0
S14_extracted_k141_39524	4455	No	NA	0	0	Not-determined	Genome-fragment	NA		0
S5_extracted_k141_123004	1325	No	NA	0	1	Low-quality	Genome-fragment		0.63	0
S14_extracted_k141_112686	1572	No	NA	0	0	Not-determined	Genome-fragment	NA		0
S7_extracted_76682	1325	No	NA	0	0	Low-quality	Genome-fragment		1.91	0
S14_extracted_k141_76109	1366	No	NA	0	1	Not-determined	Genome-fragment	NA		0
S14_extracted_k141_2471	6585	No	NA	0	2	Not-determined	Genome-fragment	NA		0
S7_vs_52331||full	1325	No	NA	1	0	Low-quality	Genome-fragment		1.98	0
S7_vs_92878||lt2gene	1325	No	NA	1	0	Low-quality	Genome-fragment		2.93	0
S14_extracted_k141_54118	1328	No	NA	0	1	Not-determined	Genome-fragment	NA		0
S16_vs_161848||lt2gene	1325	No	NA	1	0	Low-quality	Genome-fragment		0.51	0
S14_extracted_k141_97635	2199	No	NA	0	0	Not-determined	Genome-fragment	NA		0
S14_extracted_k141_54171	4154	No	NA	0	1	Not-determined	Genome-fragment	NA		0
S14_extracted_k141_68566	7027	No	NA	0	4	Not-determined	Genome-fragment	NA		0
S10_extracted_98339	1325	No	NA	0	0	Low-quality	Genome-fragment		2.42	0
S14_extracted_k141_97654	3624	No	NA	0	2	Not-determined	Genome-fragment	NA		0
S6_extracted_k141_13275	1325	No	NA	0	0	Low-quality	Genome-fragment		2.47	0
S8_extracted_k141_112733	1325	No	NA	0	1	Low-quality	Genome-fragment		0.38	0
S14_extracted_k141_9897	1312	No	NA	0	1	Not-determined	Genome-fragment	NA		0
S8_vs_k141_196761||full	1325	No	NA	1	0	Low-quality	Genome-fragment		3.19	0
S14_extracted_k141_90781	1220	No	NA	0	0	Not-determined	Genome-fragment	NA		0
S8_vs_k141_100981||full	1325	No	NA	0	0	Low-quality	Genome-fragment		3.07	0
S8_vs_k141_205417||full	1325	No	NA	2	0	Low-quality	Genome-fragment		3.18	0
S9_extracted_k141_5634	1326	No	NA	2	0	Low-quality	Genome-fragment		2.02	0
S9_vs_k141_69304||full	1326	No	NA	0	1	Low-quality	Genome-fragment		0.63	0
S14_extracted_k141_2591	1075	No	NA	0	1	Not-determined	Genome-fragment	NA		0
S14_extracted_k141_81252	1326	No	NA	2	0	Low-quality	Genome-fragment		4.36	0
S14_vs_k141_62872||full	1326	No	NA	3	0	Low-quality	Genome-fragment		3.41	0
S14_extracted_k141_83439	3399	No	NA	0	3	Not-determined	Genome-fragment	NA		0
S14_extracted_k141_9988	4885	No	NA	0	2	Not-determined	Genome-fragment	NA		0
S14_vs_k141_12141||lt2gene	1326	No	NA	1	0	Low-quality	Genome-fragment		2.17	0
S14_extracted_k141_32110	1164	No	NA	0	0	Not-determined	Genome-fragment	NA		0
S1_vs_k141_72545||full	1326	No	NA	0	0	Low-quality	Genome-fragment		3.29	0
S7_extracted_35119	1326	No	NA	0	1	Low-quality	Genome-fragment		1	0
S14_extracted_k141_54344	1041	No	NA	0	1	Not-determined	Genome-fragment	NA		0
S14_extracted_k141_32142	3086	No	NA	0	0	Not-determined	Genome-fragment	NA		0
S14_extracted_k141_61371	1369	No	NA	0	1	Not-determined	Genome-fragment	NA		0
S14_extracted_k141_54357	1678	No	NA	0	0	Not-determined	Genome-fragment	NA		0
S14_extracted_k141_68679	1180	No	NA	0	1	Not-determined	Genome-fragment	NA		0
S7_vs_134704||full	1326	No	NA	0	1	Low-quality	Genome-fragment		0.93	0
S14_extracted_k141_24621	1020	No	NA	0	0	Not-determined	Genome-fragment	NA		0
S14_extracted_k141_68699	1392	No	NA	0	1	Not-determined	Genome-fragment	NA		0
S14_extracted_k141_39753	1147	No	NA	0	0	Not-determined	Genome-fragment	NA		0
S14_extracted_k141_10028	1872	No	NA	0	0	Not-determined	Genome-fragment	NA		0
S7_vs_135584||lt2gene	1326	No	NA	2	0	Low-quality	Genome-fragment		2.66	0
S16_vs_65755||full	1326	No	NA	0	1	Low-quality	Genome-fragment		2.79	0
S14_extracted_k141_76366	1217	No	NA	0	1	Not-determined	Genome-fragment	NA		0
S14_extracted_k141_61448	1097	No	NA	0	1	Not-determined	Genome-fragment	NA		0
S16_vs_3909||lt2gene	1326	No	NA	0	0	Low-quality	Genome-fragment		3.67	0
S14_extracted_k141_46380	1369	No	NA	0	1	Not-determined	Genome-fragment	NA		0
S14_extracted_k141_2640	1206	No	NA	0	1	Not-determined	Genome-fragment	NA		0
S14_extracted_k141_83574	3061	No	NA	0	0	Not-determined	Genome-fragment	NA		0
S16_vs_6204||lt2gene	1326	No	NA	1	0	Low-quality	Genome-fragment		0.92	0
S10_extracted_17256	1326	No	NA	1	0	Low-quality	Genome-fragment		0.91	0
S14_extracted_k141_83593	3568	No	NA	0	0	Not-determined	Genome-fragment	NA		0
S14_extracted_k141_68748	3107	No	NA	0	3	Not-determined	Genome-fragment	NA		0
S14_extracted_k141_105895	1070	No	NA	0	0	Not-determined	Genome-fragment	NA		0
S14_extracted_k141_39817	1318	No	NA	0	0	Not-determined	Genome-fragment	NA		0
S12_vs_k141_150592||full	1326	No	NA	3	0	Low-quality	Genome-fragment		3.54	0
S14_extracted_k141_76464	2303	No	NA	0	2	Not-determined	Genome-fragment	NA		0
S17_extracted_k141_4720	1327	No	NA	1	0	Low-quality	Genome-fragment		2.7	0
S14_extracted_k141_32295	2490	No	NA	0	0	Not-determined	Genome-fragment	NA		0
S14_extracted_k141_76480	1420	No	NA	0	1	Not-determined	Genome-fragment	NA		0
S14_extracted_k141_54508	6837	No	NA	0	5	Not-determined	Genome-fragment	NA		0
S17_vs_k141_129691||full	1327	No	NA	0	0	Low-quality	Genome-fragment		2.57	0
S14_extracted_k141_76491	4131	No	NA	0	4	Not-determined	Genome-fragment	NA		0
S9_extracted_k141_20317	1327	No	NA	4	0	Low-quality	Genome-fragment		4.35	0
S14_extracted_k141_68855	1238	No	NA	0	1	Not-determined	Genome-fragment	NA		0
S13_vs_98603||full	1327	No	NA	0	0	Low-quality	Genome-fragment		1.86	0
S14_extracted_k141_2758	1387	No	NA	0	0	Not-determined	Genome-fragment	NA		0
S14_extracted_k141_68862	1730	No	NA	0	1	Not-determined	Genome-fragment	NA		0
S14_vs_k141_44677||full	1327	No	NA	2	0	Low-quality	Genome-fragment		2.94	0
S14_extracted_k141_68882	2765	No	NA	0	1	Not-determined	Genome-fragment	NA		0
S14_extracted_k141_54590	1126	No	NA	0	1	Not-determined	Genome-fragment	NA		0
S14_extracted_k141_2792	3545	No	NA	0	2	Not-determined	Genome-fragment	NA		0
S14_extracted_k141_105991	1511	No	NA	0	0	Not-determined	Genome-fragment	NA		0
S14_extracted_k141_39919	1044	No	NA	0	1	Not-determined	Genome-fragment	NA		0
S1_vs_k141_44939||full	1327	No	NA	2	0	Low-quality	Genome-fragment		2.48	0
S14_extracted_k141_68913	4025	No	NA	0	1	Not-determined	Genome-fragment	NA		0
S14_extracted_k141_83735	1395	No	NA	0	1	Not-determined	Genome-fragment	NA		0
S14_extracted_k141_97887	1238	No	NA	0	1	Not-determined	Genome-fragment	NA		0
S1_vs_k141_25685||lt2gene	1327	No	NA	1	0	Low-quality	Genome-fragment		2.86	0
S3_extracted_k141_90402	1327	No	NA	1	0	Low-quality	Genome-fragment		2.43	0
S14_extracted_k141_91032	2114	No	NA	0	2	Not-determined	Genome-fragment	NA		0
S14_extracted_k141_106055	1156	No	NA	0	1	Not-determined	Genome-fragment	NA		0
S14_extracted_k141_83790	1995	No	NA	0	0	Not-determined	Genome-fragment	NA		0
S14_extracted_k141_46606	1393	No	NA	0	0	Not-determined	Genome-fragment	NA		0
S3_extracted_k141_90524	1327	No	NA	0	0	Low-quality	Genome-fragment		2.22	0
S14_extracted_k141_76587	3525	No	NA	0	2	Not-determined	Genome-fragment	NA		0
S16_vs_61095||full	1327	No	NA	0	2	Low-quality	Genome-fragment		1.13	0
S14_extracted_k141_10311	10317	No	NA	0	6	Not-determined	Genome-fragment	NA		0
S14_extracted_k141_76606	6166	No	NA	0	1	Not-determined	Genome-fragment	NA		0
S10_extracted_114219	1327	No	NA	1	0	Low-quality	Genome-fragment		2.52	0
S12_vs_k141_85514||full	1327	No	NA	1	0	Low-quality	Genome-fragment		2.1	0
S14_extracted_k141_24893	1188	No	NA	0	0	Not-determined	Genome-fragment	NA		0
S14_extracted_k141_68996	1491	No	NA	0	1	Not-determined	Genome-fragment	NA		0
S8_vs_k141_150677||full	1327	No	NA	0	0	Low-quality	Genome-fragment		0.55	0
S14_extracted_k141_76699	1044	No	NA	0	1	Not-determined	Genome-fragment	NA		0
S17_vs_k141_54314||full	1328	No	NA	0	0	Low-quality	Genome-fragment		1.19	0
S14_extracted_k141_98032	3079	No	NA	0	0	Not-determined	Genome-fragment	NA		0
S13_vs_132887||full	1328	No	NA	0	1	Low-quality	Genome-fragment		2.17	0
S14_extracted_k141_76707	1517	No	NA	0	0	Not-determined	Genome-fragment	NA		0
S13_vs_99840||lt2gene	1328	No	NA	2	0	Low-quality	Genome-fragment		2.18	0
S1_extracted_k141_21219	1328	No	NA	0	0	Low-quality	Genome-fragment		2.19	0
S14_extracted_k141_91205	1098	No	NA	0	1	Not-determined	Genome-fragment	NA		0
S14_extracted_k141_2883	1235	No	NA	0	0	Not-determined	Genome-fragment	NA		0
S14_extracted_k141_113149	1084	No	NA	0	1	Not-determined	Genome-fragment	NA		0
S3_extracted_k141_42206	1328	No	NA	1	0	Low-quality	Genome-fragment		0.47	0
S14_extracted_k141_106156	31621	No	NA	26	0	Medium-quality	Genome-fragment		86.11	0
S14_extracted_k141_106157	1224	No	NA	0	2	Not-determined	Genome-fragment	NA		0
S14_extracted_k141_98082	1145	No	NA	0	1	Not-determined	Genome-fragment	NA		0
S14_extracted_k141_69104	1119	No	NA	0	1	Not-determined	Genome-fragment	NA		0
S14_extracted_k141_32520	1061	No	NA	0	2	Not-determined	Genome-fragment	NA		0
S5_vs_k141_9591||full	1328	No	NA	1	0	Low-quality	Genome-fragment		3.06	0
S14_extracted_k141_98105	11803	No	NA	0	2	Not-determined	Genome-fragment	NA		0
S7_extracted_108211	1328	No	NA	0	0	Low-quality	Genome-fragment		2.4	0
S14_extracted_k141_24983	1077	No	NA	0	0	Not-determined	Genome-fragment	NA		0
S16_vs_7893||full	1328	No	NA	0	1	Low-quality	Genome-fragment		1.01	0
S12_extracted_k141_89421	1328	No	NA	0	1	Low-quality	Genome-fragment		0.88	0
S14_extracted_k141_106205	2557	No	NA	0	2	Not-determined	Genome-fragment	NA		0
S14_extracted_k141_69156	1021	No	NA	0	1	Not-determined	Genome-fragment	NA		0
S14_extracted_k141_25012	1655	No	NA	0	1	Not-determined	Genome-fragment	NA		0
S14_extracted_k141_106220	1718	No	NA	0	1	Not-determined	Genome-fragment	NA		0
S12_vs_k141_166283||full	1328	No	NA	0	0	Low-quality	Genome-fragment		1.96	0
S12_vs_k141_98207||full	1328	No	NA	2	0	Low-quality	Genome-fragment		0.71	0
S14_extracted_k141_40243	1245	No	NA	0	2	Not-determined	Genome-fragment	NA		0
S14_extracted_k141_91334	3214	No	NA	0	0	Not-determined	Genome-fragment	NA		0
S12_vs_k141_8819||lt2gene	1328	No	NA	1	0	Low-quality	Genome-fragment		0.92	0
S9_extracted_k141_13237	1329	No	NA	0	0	Low-quality	Genome-fragment		1.9	0
S14_extracted_k141_69191	3685	No	NA	0	2	Not-determined	Genome-fragment	NA		0
S5_vs_k141_33957||full	1329	No	NA	3	0	Low-quality	Genome-fragment		3.51	0
S5_vs_k141_83158||full	1329	No	NA	0	0	Low-quality	Genome-fragment		3.66	0
S16_vs_43249||full	1329	No	NA	2	0	Low-quality	Genome-fragment		3.44	0
S14_extracted_k141_113325	4853	No	NA	0	2	Not-determined	Genome-fragment	NA		0
S10_vs_523||full	1329	No	NA	0	0	Low-quality	Genome-fragment		3.18	0
S12_vs_k141_6379||full	1329	No	NA	0	2	Low-quality	Genome-fragment		1.84	0
S4_extracted_11230	1329	No	NA	3	0	Low-quality	Genome-fragment		4.38	0
S14_extracted_k141_25101	1061	No	NA	0	1	Not-determined	Genome-fragment	NA		0
S14_extracted_k141_25108	1486	No	NA	0	1	Not-determined	Genome-fragment	NA		0
S6_extracted_k141_21738	1329	No	NA	4	0	Low-quality	Genome-fragment		4.37	0
S14_extracted_k141_18146	2067	No	NA	0	2	Not-determined	Genome-fragment	NA		0
S6_vs_k141_43398||full	1329	No	NA	0	3	Low-quality	Genome-fragment		0.4	0
S14_extracted_k141_46921	1783	No	NA	0	1	Not-determined	Genome-fragment	NA		0
S14_extracted_k141_54871	1080	No	NA	0	0	Not-determined	Genome-fragment	NA		0
S14_extracted_k141_69319	3078	No	NA	0	0	Not-determined	Genome-fragment	NA		0
S14_extracted_k141_3152	1098	No	NA	0	1	Not-determined	Genome-fragment	NA		0
S6_vs_k141_49402||full	1329	No	NA	1	0	Low-quality	Genome-fragment		2.17	0
S17_vs_k141_113869||full	1330	No	NA	0	1	Low-quality	Genome-fragment		0.95	0
S14_extracted_k141_10581	1238	No	NA	0	1	Not-determined	Genome-fragment	NA		0
S9_vs_k141_15823||full	1330	No	NA	0	2	Low-quality	Genome-fragment		0.67	0
S14_extracted_k141_106328	1372	No	NA	0	0	Not-determined	Genome-fragment	NA		0
S9_vs_k141_20494||full	1330	No	NA	0	0	Low-quality	Genome-fragment		2.89	0
S14_extracted_k141_98350	1050	No	NA	0	1	Not-determined	Genome-fragment	NA		0
S9_vs_k141_62200||full	1330	No	NA	0	1	Low-quality	Genome-fragment		0.91	0
S11_extracted_k141_5957	1330	No	NA	0	1	Low-quality	Genome-fragment		3.5	0
S14_extracted_k141_69419	7561	No	NA	0	4	Not-determined	Genome-fragment	NA		0

S11_vs_k141_85368||full	1330	No	NA	3	0	Low-quality	Genome-fragment		3.05	0
S14_extracted_k141_69420	5878	No	NA	0	5	Not-determined	Genome-fragment	NA		0
S14_extracted_k141_18245	2048	No	NA	0	1	Not-determined	Genome-fragment	NA		0
S11_vs_k141_70606||full	1330	No	NA	1	0	Low-quality	Genome-fragment		2.06	0
S14_extracted_k141_84240	1888	No	NA	0	2	Not-determined	Genome-fragment	NA		0
S1_vs_k141_145672||full	1330	No	NA	0	1	Low-quality	Genome-fragment		2.83	0
S14_extracted_k141_3246	15693	No	NA	0	7	Not-determined	Genome-fragment	NA		0
S5_vs_k141_67965||full	1330	No	NA	0	0	Low-quality	Genome-fragment		1.01	0
S14_extracted_k141_69446	1108	No	NA	0	0	Not-determined	Genome-fragment	NA		0
S14_extracted_k141_10662	1926	No	NA	0	0	Not-determined	Genome-fragment	NA		0
S14_extracted_k141_98415	1090	No	NA	0	0	Not-determined	Genome-fragment	NA		0
S14_extracted_k141_47035	6427	No	NA	0	4	Not-determined	Genome-fragment	NA		0
S14_extracted_k141_25250	1733	No	NA	0	1	Not-determined	Genome-fragment	NA		0
S7_vs_82191||full	1330	No	NA	0	0	Low-quality	Genome-fragment		2.83	0
S14_extracted_k141_47059	1238	No	NA	0	1	Not-determined	Genome-fragment	NA		0
S14_extracted_k141_32805	1054	No	NA	0	0	Not-determined	Genome-fragment	NA		0
S14_extracted_k141_32808	1859	No	NA	0	1	Not-determined	Genome-fragment	NA		0
S14_extracted_k141_3252	3478	No	NA	0	2	Not-determined	Genome-fragment	NA		0
S14_extracted_k141_40424	1497	No	NA	0	1	Not-determined	Genome-fragment	NA		0
S10_extracted_78265	1330	No	NA	0	1	Low-quality	Genome-fragment		0.63	0
S10_vs_95461||lt2gene	1330	No	NA	0	0	Low-quality	Genome-fragment		3.09	0
S14_extracted_k141_3277	1089	No	NA	0	1	Not-determined	Genome-fragment	NA		0
S14_extracted_k141_47141	1251	No	NA	0	1	Not-determined	Genome-fragment	NA		0
S14_extracted_k141_69503	2668	No	NA	0	1	Not-determined	Genome-fragment	NA		0
S14_extracted_k141_18371	2275	No	NA	0	1	Not-determined	Genome-fragment	NA		0
S14_extracted_k141_61913	1320	No	NA	0	1	Not-determined	Genome-fragment	NA		0
S14_extracted_k141_113596	1231	No	NA	0	3	Not-determined	Genome-fragment	NA		0
S12_vs_k141_153269||lt2gene	1330	No	NA	1	0	Low-quality	Genome-fragment		2.95	0
S8_vs_k141_71950||full	1330	No	NA	0	1	Low-quality	Genome-fragment		2.24	0
S14_extracted_k141_18392	1106	No	NA	0	0	Not-determined	Genome-fragment	NA		0
S14_extracted_k141_98502	1895	No	NA	0	1	Not-determined	Genome-fragment	NA		0
S14_extracted_k141_10776	5036	No	NA	0	4	Not-determined	Genome-fragment	NA		0
S17_extracted_k141_19858	1331	No	NA	2	0	Low-quality	Genome-fragment		2.9	0
S17_vs_k141_124452||full	1331	No	NA	0	0	Low-quality	Genome-fragment		1.36	0
S9_vs_k141_30094||full	1331	No	NA	0	0	Low-quality	Genome-fragment		1.58	0
S14_extracted_k141_10796	1050	No	NA	0	1	Not-determined	Genome-fragment	NA		0
S11_vs_k141_96321||full	1331	No	NA	1	0	Low-quality	Genome-fragment		3.27	0
S14_extracted_k141_53017	1331	No	NA	1	0	Low-quality	Genome-fragment		3.57	0
S14_extracted_k141_91701	1007	No	NA	0	2	Not-determined	Genome-fragment	NA		0
S14_extracted_k141_10819	1003	No	NA	0	1	Not-determined	Genome-fragment	NA		0
S14_extracted_k141_61962	1013	No	NA	0	0	Not-determined	Genome-fragment	NA		0
S14_extracted_k141_47248	1549	No	NA	0	1	Not-determined	Genome-fragment	NA		0
S14_extracted_k141_32918	1655	No	NA	0	2	Not-determined	Genome-fragment	NA		0
S14_extracted_k141_69583	2247	No	NA	0	1	Not-determined	Genome-fragment	NA		0
S14_vs_k141_25710||full	1331	No	NA	0	0	Low-quality	Genome-fragment		2.31	0
S14_extracted_k141_18461	2065	No	NA	0	2	Not-determined	Genome-fragment	NA		0
S14_extracted_k141_18465	1129	No	NA	0	1	Not-determined	Genome-fragment	NA		0
S14_extracted_k141_113680	1906	No	NA	0	0	Not-determined	Genome-fragment	NA		0
S7_vs_82078||full	1331	No	NA	2	0	Low-quality	Genome-fragment		3.39	0
S14_extracted_k141_55018	1843	No	NA	0	2	Not-determined	Genome-fragment	NA		0
S14_extracted_k141_10903	1048	No	NA	0	2	Not-determined	Genome-fragment	NA		0
S7_vs_90049||full	1331	No	NA	0	0	Low-quality	Genome-fragment		2.29	0
S10_extracted_94973	1331	No	NA	0	0	Low-quality	Genome-fragment		2.85	0
S14_extracted_k141_84472	1631	No	NA	0	0	Not-determined	Genome-fragment	NA		0
S12_extracted_k141_143394	1331	No	NA	0	0	Low-quality	Genome-fragment		1.44	0
S14_extracted_k141_10946	1046	No	NA	0	1	Not-determined	Genome-fragment	NA		0
S12_vs_k141_15086||full	1331	No	NA	1	0	Low-quality	Genome-fragment		2.56	0
S8_vs_k141_124259||full	1331	No	NA	2	0	Low-quality	Genome-fragment		2.27	0
S14_extracted_k141_113802	1132	No	NA	0	0	Not-determined	Genome-fragment	NA		0
S9_vs_k141_82359||full	1332	No	NA	0	0	Low-quality	Genome-fragment		1.89	0
S14_extracted_k141_113808	1113	No	NA	0	0	Not-determined	Genome-fragment	NA		0
S14_extracted_k141_106620	1635	No	NA	0	0	Not-determined	Genome-fragment	NA		0
S1_extracted_k141_94667	1332	No	NA	0	2	Low-quality	Genome-fragment		2.26	0
S14_extracted_k141_33021	1728	No	NA	0	1	Not-determined	Genome-fragment	NA		0
S14_extracted_k141_91868	1210	No	NA	0	0	Not-determined	Genome-fragment	NA		0
S14_extracted_k141_40735	1973	No	NA	0	3	Not-determined	Genome-fragment	NA		0
S14_extracted_k141_47394	1011	No	NA	0	2	Not-determined	Genome-fragment	NA		0
S14_extracted_k141_33032	1049	No	NA	0	1	Not-determined	Genome-fragment	NA		0
S1_vs_k141_123503||full	1332	No	NA	2	0	Low-quality	Genome-fragment		2.27	0
S7_vs_70070||full	1332	No	NA	0	0	Low-quality	Genome-fragment		0.76	0
S12_vs_k141_95934||full	1332	No	NA	0	0	Low-quality	Genome-fragment		0.79	0
S14_extracted_k141_47440	1241	No	NA	0	0	Not-determined	Genome-fragment	NA		0
S8_vs_k141_193229||full	1332	No	NA	0	0	Low-quality	Genome-fragment		2.86	0
S14_extracted_k141_106687	1390	No	NA	0	1	Not-determined	Genome-fragment	NA		0
S14_extracted_k141_77123	1533	No	NA	0	2	Not-determined	Genome-fragment	NA		0
S8_vs_k141_53754||lt2gene	1332	No	NA	1	0	Low-quality	Genome-fragment		3.07	0
S17_vs_k141_143922||full	1333	No	NA	1	0	Low-quality	Genome-fragment		2.91	0
S14_extracted_k141_47474	6913	No	NA	0	5	Not-determined	Genome-fragment	NA		0
S9_extracted_k141_90844	1333	No	NA	0	0	Low-quality	Genome-fragment		3.46	0
S14_extracted_k141_113926	1035	No	NA	0	0	Not-determined	Genome-fragment	NA		0
S11_extracted_k141_30320	1333	No	NA	0	0	Low-quality	Genome-fragment		2.5	0
S14_extracted_k141_106751	1054	No	NA	0	2	Not-determined	Genome-fragment	NA		0
S14_extracted_k141_47510	1484	No	NA	0	1	Not-determined	Genome-fragment	NA		0
S14_extracted_k141_62219	3024	No	NA	0	3	Not-determined	Genome-fragment	NA		0
S14_extracted_k141_47523	2522	No	NA	0	2	Not-determined	Genome-fragment	NA		0
S14_extracted_k141_3572	3647	No	NA	0	2	Not-determined	Genome-fragment	NA		0
S11_vs_k141_63050||full	1333	No	NA	0	0	Low-quality	Genome-fragment		2.35	0
S11_vs_k141_56622||lt2gene	1333	No	NA	1	0	Low-quality	Genome-fragment		3.05	0
S14_extracted_k141_113960	1013	No	NA	0	0	Not-determined	Genome-fragment	NA		0
S14_extracted_k141_59472	1333	No	NA	2	0	Low-quality	Genome-fragment		4.4	0
S14_extracted_k141_92004	3841	No	NA	0	8	Not-determined	Genome-fragment	NA		0
S1_extracted_k141_127828	1333	No	NA	1	0	Low-quality	Genome-fragment		2.96	0
S14_extracted_k141_11223	5104	No	NA	0	0	Not-determined	Genome-fragment	NA		0
S1_vs_k141_154342||full	1333	No	NA	0	0	Low-quality	Genome-fragment		2.9	0
S14_extracted_k141_77256	1235	No	NA	0	1	Not-determined	Genome-fragment	NA		0
S14_extracted_k141_18746	1461	No	NA	0	0	Not-determined	Genome-fragment	NA		0
S14_extracted_k141_25680	3738	No	NA	0	3	Not-determined	Genome-fragment	NA		0
S1_vs_k141_66033||full	1333	No	NA	2	0	Low-quality	Genome-fragment		3.27	0
S3_vs_k141_101199||full	1333	No	NA	0	0	Low-quality	Genome-fragment		2.45	0
S14_extracted_k141_18794	1086	No	NA	0	0	Not-determined	Genome-fragment	NA		0
S5_extracted_k141_21896	1333	No	NA	1	0	Low-quality	Genome-fragment		3.48	0
S5_vs_k141_41513||full	1333	No	NA	2	0	Low-quality	Genome-fragment		3.07	0
S14_extracted_k141_92092	5347	No	NA	0	5	Not-determined	Genome-fragment	NA		0
S7_vs_41724||full	1333	No	NA	0	1	Low-quality	Genome-fragment		2.71	0
S14_extracted_k141_98914	1734	No	NA	0	1	Not-determined	Genome-fragment	NA		0
S14_extracted_k141_11298	1688	No	NA	0	1	Not-determined	Genome-fragment	NA		0
S10_extracted_20902	1333	No	NA	0	0	Low-quality	Genome-fragment		0.62	0
S10_vs_8936||full	1333	No	NA	0	0	Low-quality	Genome-fragment		3.62	0
S14_extracted_k141_25736	1195	No	NA	0	1	Not-determined	Genome-fragment	NA		0
S10_vs_111703||full	1333	No	NA	1	0	Low-quality	Genome-fragment		3.28	0
S12_vs_k141_45331||full	1333	No	NA	0	0	Low-quality	Genome-fragment		1.52	0
S14_extracted_k141_18951	1088	No	NA	0	1	Not-determined	Genome-fragment	NA		0
S12_vs_k141_109600||full	1333	No	NA	0	1	Low-quality	Genome-fragment		0.79	0
S14_extracted_k141_98981	1565	No	NA	0	0	Not-determined	Genome-fragment	NA		0
S14_extracted_k141_11409	3564	No	NA	0	2	Not-determined	Genome-fragment	NA		0
S14_extracted_k141_77487	4141	No	NA	0	0	Not-determined	Genome-fragment	NA		0
S15_extracted_29627	1333	No	NA	1	0	Low-quality	Genome-fragment		1.07	0
S14_extracted_k141_47592	5359	No	NA	0	3	Not-determined	Genome-fragment	NA		0
S14_extracted_k141_92240	34251	No	NA	15	2	High-quality	High-quality		98.36	0
S14_extracted_k141_41141	1232	No	NA	0	1	Not-determined	Genome-fragment	NA		0
S14_extracted_k141_3905	1147	No	NA	0	0	Not-determined	Genome-fragment	NA		0
S11_extracted_k141_41325	1334	No	NA	1	0	Low-quality	Genome-fragment		2.06	0
S14_extracted_k141_40659	1334	No	NA	1	0	Low-quality	Genome-fragment		3.44	0
S14_extracted_k141_93973	1334	No	NA	0	0	Low-quality	Genome-fragment		2.11	0
S14_extracted_k141_62370	1084	No	NA	0	1	Not-determined	Genome-fragment	NA		0
S14_extracted_k141_19075	1411	No	NA	0	1	Not-determined	Genome-fragment	NA		0
S14_vs_k141_109833||full	1334	No	NA	0	0	Low-quality	Genome-fragment		2.11	0
S1_vs_k141_43802||full	1334	No	NA	0	0	Low-quality	Genome-fragment		1.62	0
S14_extracted_k141_41188	1461	No	NA	0	0	Not-determined	Genome-fragment	NA		0
S14_extracted_k141_11491	2946	No	NA	0	1	Not-determined	Genome-fragment	NA		0
S1_vs_k141_65375||full	1334	No	NA	0	0	Low-quality	Genome-fragment		0.95	0
S14_extracted_k141_11495	1368	No	NA	0	1	Not-determined	Genome-fragment	NA		0
S14_extracted_k141_11496	1522	No	NA	0	1	Not-determined	Genome-fragment	NA		0
S14_extracted_k141_33433	1167	No	NA	0	1	Not-determined	Genome-fragment	NA		0
S3_extracted_k141_44617	1334	No	NA	0	0	Low-quality	Genome-fragment		0.92	0
S14_extracted_k141_99109	6380	No	NA	0	3	Not-determined	Genome-fragment	NA		0
S14_extracted_k141_62475	1002	No	NA	0	1	Not-determined	Genome-fragment	NA		0
S14_extracted_k141_85020	7582	No	NA	0	0	Not-determined	Genome-fragment	NA		0
S3_vs_k141_28149||full	1334	No	NA	0	1	Low-quality	Genome-fragment		0.71	0
S5_extracted_k141_59529	1334	No	NA	1	0	Low-quality	Genome-fragment		4.06	0
S14_extracted_k141_99127	1028	No	NA	0	1	Not-determined	Genome-fragment	NA		0
S5_vs_k141_47804||full	1334	No	NA	1	0	Low-quality	Genome-fragment		0.86	0
S14_extracted_k141_4031	1118	No	NA	0	2	Not-determined	Genome-fragment	NA		0
S7_vs_27154||full	1334	No	NA	0	0	Low-quality	Genome-fragment		3.15	0
S14_extracted_k141_33501	1686	No	NA	0	2	Not-determined	Genome-fragment	NA		0
S14_extracted_k141_99138	1133	No	NA	0	1	Not-determined	Genome-fragment	NA		0
S14_extracted_k141_114328	4159	No	NA	0	2	Not-determined	Genome-fragment	NA		0
S14_extracted_k141_4043	2663	No	NA	0	0	Not-determined	Genome-fragment	NA		0
S14_extracted_k141_92354	1604	No	NA	0	0	Not-determined	Genome-fragment	NA		0
S8_vs_k141_97665||full	1334	No	NA	0	1	Low-quality	Genome-fragment		0.88	0
S9_extracted_k141_43205	1335	No	NA	0	0	Low-quality	Genome-fragment		3.59	0
S9_vs_k141_14726||lt2gene	1335	No	NA	0	0	Low-quality	Genome-fragment		1.7	0
S14_extracted_k141_33556	1870	No	NA	0	0	Not-determined	Genome-fragment	NA		0
S11_extracted_k141_97061	1335	No	NA	0	1	Low-quality	Genome-fragment		1.07	0
S14_extracted_k141_70201	2125	No	NA	0	1	Not-determined	Genome-fragment	NA		0
S14_extracted_k141_62589	1100	No	NA	0	0	Not-determined	Genome-fragment	NA		0
S14_extracted_k141_81207	1335	No	NA	2	0	Low-quality	Genome-fragment		4.4	0
S14_extracted_k141_70216	1024	No	NA	0	0	Not-determined	Genome-fragment	NA		0
S14_extracted_k141_114373	3886	No	NA	0	2	Not-determined	Genome-fragment	NA		0
S14_extracted_k141_77711	1929	No	NA	0	0	Not-determined	Genome-fragment	NA		0
S3_extracted_k141_6103	1335	No	NA	0	1	Low-quality	Genome-fragment		2.29	0
S14_extracted_k141_25939	1967	No	NA	0	0	Not-determined	Genome-fragment	NA		0
S14_extracted_k141_11631	1814	No	NA	0	2	Not-determined	Genome-fragment	NA		0
S3_vs_k141_82265||full	1335	No	NA	0	1	Low-quality	Genome-fragment		0.94	0
S14_extracted_k141_19383	1753	No	NA	0	0	Not-determined	Genome-fragment	NA		0
S14_extracted_k141_85122	32993	No	NA	19	0	Medium-quality	Genome-fragment		79.58	0
S14_extracted_k141_62647	1196	No	NA	0	1	Not-determined	Genome-fragment	NA		0
S14_extracted_k141_107177	1031	No	NA	0	1	Not-determined	Genome-fragment	NA		0
S14_extracted_k141_62679	1334	No	NA	0	0	Not-determined	Genome-fragment	NA		0
S14_extracted_k141_92498	1342	No	NA	0	0	Not-determined	Genome-fragment	NA		0
S14_extracted_k141_41423	1123	No	NA	0	1	Not-determined	Genome-fragment	NA		0
S14_extracted_k141_19444	1035	No	NA	0	1	Not-determined	Genome-fragment	NA		0
S14_extracted_k141_41455	1314	No	NA	0	0	Not-determined	Genome-fragment	NA		0
S5_vs_k141_34246||lt2gene	1335	No	NA	2	0	Low-quality	Genome-fragment		5.78	0

S14_extracted_k141_26000	11615	No	NA		0	3	Not-determined	Genome-fragment	NA		0
S14_extracted_k141_33647	1180	No	NA		0	0	Not-determined	Genome-fragment	NA		0
S12_extracted_k141_13845	1335	No	NA		2	0	Low-quality	Genome-fragment		3.02	0
S14_extracted_k141_99344	15862	No	NA		8	0	Medium-quality	Genome-fragment		66.98	0
S12_extracted_k141_155969	1335	No	NA		0	1	Low-quality	Genome-fragment		0.31	0
S14_extracted_k141_99366	4930	No	NA		0	4	Not-determined	Genome-fragment	NA		0
S12_vs_k141_124699||full	1335	No	NA		1	0	Low-quality	Genome-fragment		3.67	0
S14_extracted_k141_114491	15913	No	NA		7	0	Medium-quality	Genome-fragment		68.55	0
S8_extracted_k141_90167	1335	No	NA		0	0	Low-quality	Genome-fragment		2.03	0
S8_extracted_k141_66557	1335	No	NA		1	0	Low-quality	Genome-fragment		2.38	0
S14_extracted_k141_55819	1183	No	NA		0	2	Not-determined	Genome-fragment	NA		0
S14_extracted_k141_99411	3646	No	NA		0	0	Not-determined	Genome-fragment	NA		0
S17_vs_k141_16748||full	1336	No	NA		1	0	Low-quality	Genome-fragment		3.31	0
S14_extracted_k141_92593	2587	No	NA		0	0	Not-determined	Genome-fragment	NA		0
S9_extracted_k141_71797	1336	No	NA		0	0	Low-quality	Genome-fragment		0.81	0
S14_extracted_k141_4242	1389	No	NA		0	1	Not-determined	Genome-fragment	NA		0
S14_extracted_k141_70368	3037	No	NA		0	3	Not-determined	Genome-fragment	NA		0
S14_extracted_k141_19580	1236	No	NA		0	2	Not-determined	Genome-fragment	NA		0
S14_extracted_k141_55885	2981	No	NA		0	1	Not-determined	Genome-fragment	NA		0
S9_vs_k141_75216||full	1336	No	NA		0	1	Low-quality	Genome-fragment		3.39	0
S11_vs_k141_70130||full	1336	No	NA		0	0	Low-quality	Genome-fragment		2.4	0
S14_extracted_k141_4266	5455	No	NA		0	1	Not-determined	Genome-fragment	NA		0
S14_extracted_k141_47883	1115	No	NA		0	1	Not-determined	Genome-fragment	NA		0
S14_extracted_k141_70435	1082	No	NA		0	0	Not-determined	Genome-fragment	NA		0
S14_extracted_k141_107398	1430	No	NA		0	1	Not-determined	Genome-fragment	NA		0
S11_vs_k141_365||lt2gene	1336	No	NA		1	0	Low-quality	Genome-fragment		3.89	0
S14_extracted_k141_47942	1283	No	NA		0	0	Not-determined	Genome-fragment	NA		0
S14_extracted_k141_4306	1105	No	NA		0	0	Not-determined	Genome-fragment	NA		0
S14_extracted_k141_86188	1336	No	NA		5	0	Low-quality	Genome-fragment		4.41	0
S1_extracted_k141_152750	1336	No	NA		0	0	Low-quality	Genome-fragment		2.69	0
S5_extracted_k141_143719	1336	No	NA		2	0	Low-quality	Genome-fragment		3.88	0
S14_extracted_k141_47961	1332	No	NA		0	1	Not-determined	Genome-fragment	NA		0
S14_extracted_k141_19642	1296	No	NA		0	3	Not-determined	Genome-fragment	NA		0
S14_extracted_k141_114686	1005	No	NA		0	1	Not-determined	Genome-fragment	NA		0
S7_vs_87919||full	1336	No	NA		1	0	Low-quality	Genome-fragment		3.1	0
S10_extracted_23266	1336	No	NA		0	1	Low-quality	Genome-fragment		0.47	0
S4_extracted_31525	1336	No	NA		5	0	Low-quality	Genome-fragment		4.41	0
S8_vs_k141_55239||full	1336	No	NA		0	2	Low-quality	Genome-fragment		0.5	0
S17_vs_k141_160861||full	1337	No	NA		0	3	Low-quality	Genome-fragment		0.82	0
S9_extracted_k141_54700	1337	No	NA		0	0	Low-quality	Genome-fragment		0.77	0
S11_vs_k141_2151||lt2gene	1337	No	NA		1	0	Low-quality	Genome-fragment		0.92	0
S14_extracted_k141_19753	2474	No	NA		0	1	Not-determined	Genome-fragment	NA		0
S14_extracted_k141_63009	1016	No	NA		0	1	Not-determined	Genome-fragment	NA		0
S11_vs_k141_32115||lt2gene	1337	No	NA		1	0	Low-quality	Genome-fragment		2.65	0
S14_vs_k141_85880||full	1337	No	NA		0	0	Low-quality	Genome-fragment		0.77	0
S14_vs_k141_100233||full	1337	No	NA		0	1	Low-quality	Genome-fragment		2.7	0
S3_vs_k141_98361||full	1337	No	NA		2	0	Low-quality	Genome-fragment		3.3	0
S14_extracted_k141_78138	6833	No	NA		0	3	Not-determined	Genome-fragment	NA		0
S14_extracted_k141_99647	1903	No	NA		0	2	Not-determined	Genome-fragment	NA		0
S7_extracted_78555	1337	No	NA		0	0	Low-quality	Genome-fragment		4.37	0
S14_extracted_k141_70715	1256	No	NA		0	1	Not-determined	Genome-fragment	NA		0
S14_extracted_k141_85442	6385	No	NA		0	1	Not-determined	Genome-fragment	NA		0
S7_vs_62064||full	1337	No	NA		0	2	Low-quality	Genome-fragment		0.5	0
S14_extracted_k141_107595	1089	No	NA		0	0	Not-determined	Genome-fragment	NA		0
S14_extracted_k141_33937	2098	No	NA		0	0	Not-determined	Genome-fragment	NA		0
S14_extracted_k141_78139	53948	Yes		50221	30	2	High-quality	High-quality		93.75	6.91
S7_vs_131218||full	1337	No	NA		1	0	Low-quality	Genome-fragment		3.1	0
S14_extracted_k141_33974	1620	No	NA		0	1	Not-determined	Genome-fragment	NA		0
S16_vs_18403||full	1337	No	NA		0	3	Low-quality	Genome-fragment		0.63	0
S16_vs_96575||full	1337	No	NA		0	1	Low-quality	Genome-fragment		0.92	0
S10_vs_38501||full	1337	No	NA		0	0	Low-quality	Genome-fragment		1.51	0
S12_extracted_k141_54063	1337	No	NA		1	0	Low-quality	Genome-fragment		2.68	0
S6_extracted_k141_48550	1337	No	NA		1	0	Low-quality	Genome-fragment		3.4	0
S14_extracted_k141_19914	1145	No	NA		0	1	Not-determined	Genome-fragment	NA		0
S14_extracted_k141_107712	3196	No	NA		0	2	Not-determined	Genome-fragment	NA		0
S14_extracted_k141_19928	1194	No	NA		0	0	Not-determined	Genome-fragment	NA		0
S14_extracted_k141_107723	1689	No	NA		0	2	Not-determined	Genome-fragment	NA		0
S14_extracted_k141_63195	1321	No	NA		0	1	Not-determined	Genome-fragment	NA		0
S8_vs_k141_134666||full	1337	No	NA		0	0	Low-quality	Genome-fragment		2.69	0
S8_vs_k141_57369||full	1337	No	NA		0	0	Low-quality	Genome-fragment		3.53	0
S14_extracted_k141_56130	46683	No	NA		31	0	High-quality	High-quality		100	0
S9_extracted_k141_68583	1338	No	NA		0	0	Low-quality	Genome-fragment		2.05	0
S11_vs_k141_93031||full	1338	No	NA		0	0	Low-quality	Genome-fragment		3.81	0
S14_extracted_k141_41930	1121	No	NA		0	1	Not-determined	Genome-fragment	NA		0
S13_vs_8881||full	1338	No	NA		0	2	Low-quality	Genome-fragment		0.47	0
S14_extracted_k141_93078	7888	No	NA		0	4	Not-determined	Genome-fragment	NA		0
S13_vs_8915||full	1338	No	NA		0	2	Low-quality	Genome-fragment		2.09	0
S14_extracted_k141_41984	3037	No	NA		0	1	Not-determined	Genome-fragment	NA		0
S14_extracted_k141_93134	15540	No	NA		6	0	Medium-quality	Genome-fragment		65.86	0
S14_extracted_k141_107789	1050	No	NA		0	1	Not-determined	Genome-fragment	NA		0
S3_vs_k141_23258||full	1338	No	NA		0	0	Low-quality	Genome-fragment		2.63	0
S14_extracted_k141_56170	1858	No	NA		0	1	Not-determined	Genome-fragment	NA		0
S14_extracted_k141_114964	4653	No	NA		0	3	Not-determined	Genome-fragment	NA		0
S14_extracted_k141_99815	1208	No	NA		0	1	Not-determined	Genome-fragment	NA		0
S7_vs_32732||full	1338	No	NA		1	0	Low-quality	Genome-fragment		2.33	0
S7_vs_69113||full	1338	No	NA		1	0	Low-quality	Genome-fragment		3.55	0
S14_extracted_k141_85720	1145	No	NA		0	2	Not-determined	Genome-fragment	NA		0
S14_extracted_k141_114981	1084	No	NA		0	0	Not-determined	Genome-fragment	NA		0
S7_vs_109696||full	1338	No	NA		1	0	Low-quality	Genome-fragment		3.32	0
S14_extracted_k141_42063	1068	No	NA		0	2	Not-determined	Genome-fragment	NA		0
S7_vs_92612||lt2gene	1338	No	NA		0	1	Low-quality	Genome-fragment		1.57	0
S14_extracted_k141_99886	1949	No	NA		0	0	Not-determined	Genome-fragment	NA		0
S14_extracted_k141_48325	1005	No	NA		0	0	Not-determined	Genome-fragment	NA		0
S14_extracted_k141_4834	1303	No	NA		0	0	Not-determined	Genome-fragment	NA		0
S14_extracted_k141_34150	1752	No	NA		0	0	Not-determined	Genome-fragment	NA		0
S16_extracted_118634	1338	No	NA		0	0	Low-quality	Genome-fragment		2.19	0
S8_extracted_k141_63621	1338	No	NA		0	0	Low-quality	Genome-fragment		2.09	0
S14_extracted_k141_78302	1100	No	NA		0	1	Not-determined	Genome-fragment	NA		0
S14_extracted_k141_26450	1067	No	NA		0	1	Not-determined	Genome-fragment	NA		0
S8_vs_k141_192648||full	1338	No	NA		1	0	Low-quality	Genome-fragment		3.08	0
S14_extracted_k141_93264	1062	No	NA		0	1	Not-determined	Genome-fragment	NA		0
S17_vs_k141_141040||full	1339	No	NA		0	0	Low-quality	Genome-fragment		1.12	0
S14_extracted_k141_93279	4544	No	NA		0	3	Not-determined	Genome-fragment	NA		0
S14_extracted_k141_115093	1428	No	NA		0	0	Not-determined	Genome-fragment	NA		0
S14_extracted_k141_107921	1127	No	NA		0	1	Not-determined	Genome-fragment	NA		0
S9_vs_k141_25426||full	1339	No	NA		0	0	Low-quality	Genome-fragment		1.79	0
S14_extracted_k141_107934	1571	No	NA		0	2	Not-determined	Genome-fragment	NA		0
S11_extracted_k141_30599	1339	No	NA		1	0	Low-quality	Genome-fragment		2.85	0
S1_vs_k141_21666||full	1339	No	NA		1	0	Low-quality	Genome-fragment		2.31	0
S1_vs_k141_149452||lt2gene	1339	No	NA		1	0	Low-quality	Genome-fragment		1.75	0
S5_vs_k141_94404||full	1339	No	NA		0	0	Low-quality	Genome-fragment		3.09	0
S14_extracted_k141_115149	3692	No	NA		0	1	Not-determined	Genome-fragment	NA		0
S14_extracted_k141_78441	1053	No	NA		0	1	Not-determined	Genome-fragment	NA		0
S14_extracted_k141_4942	2893	No	NA		0	0	Not-determined	Genome-fragment	NA		0
S7_vs_128301||full	1339	No	NA		0	0	Low-quality	Genome-fragment		3.33	0
S14_extracted_k141_34309	1042	No	NA		0	2	Not-determined	Genome-fragment	NA		0
S14_extracted_k141_78486	1234	No	NA		0	2	Not-determined	Genome-fragment	NA		0
S14_extracted_k141_48453	1837	No	NA		0	1	Not-determined	Genome-fragment	NA		0
S10_extracted_32872	1339	No	NA		0	1	Low-quality	Genome-fragment		1.33	0
S10_extracted_85362	1339	No	NA		0	0	Low-quality	Genome-fragment		3.06	0
S15_extracted_43484	1339	No	NA		1	0	Low-quality	Genome-fragment		3.38	0
S17_extracted_k141_130326	1340	No	NA		0	1	Low-quality	Genome-fragment		9.57	0
S17_vs_k141_47490||full	1340	No	NA		1	0	Low-quality	Genome-fragment		1.2	0
S14_extracted_k141_56439	4099	No	NA		0	1	Not-determined	Genome-fragment	NA		0
S14_extracted_k141_71097	1201	No	NA		0	2	Not-determined	Genome-fragment	NA		0
S14_extracted_k141_71106	1150	No	NA		0	1	Not-determined	Genome-fragment	NA		0
S14_extracted_k141_63590	1190	No	NA		0	3	Not-determined	Genome-fragment	NA		0
S9_vs_k141_17873||full	1340	No	NA		1	0	Low-quality	Genome-fragment		3.65	0
S11_vs_k141_91805||lt2gene	1340	No	NA		2	0	Low-quality	Genome-fragment		3.29	0
S14_extracted_k141_115269	2062	No	NA		0	1	Not-determined	Genome-fragment	NA		0
S14_extracted_k141_20023	1091	No	NA		0	1	Not-determined	Genome-fragment	NA		0
S14_extracted_k141_26627	1319	No	NA		0	3	Not-determined	Genome-fragment	NA		0
S14_extracted_k141_31947	1340	No	NA		4	0	Low-quality	Genome-fragment		4.42	0
S14_extracted_k141_63633	1090	No	NA		0	1	Not-determined	Genome-fragment	NA		0
S14_extracted_k141_78634	1219	No	NA		0	1	Not-determined	Genome-fragment	NA		0
S14_extracted_k141_48582	4530	No	NA		0	1	Not-determined	Genome-fragment	NA		0
S14_extracted_k141_85902	2237	No	NA		0	0	Not-determined	Genome-fragment	NA		0
S14_vs_k141_82119||full	1340	No	NA		0	0	Low-quality	Genome-fragment		1.56	0
S14_extracted_k141_100232	6510	No	NA		0	1	Not-determined	Genome-fragment	NA		0
S14_vs_k141_49925||lt2gene	1340	No	NA		1	0	Low-quality	Genome-fragment		2.93	0
S14_extracted_k141_63692	1171	No	NA		0	1	Not-determined	Genome-fragment	NA		0
S14_extracted_k141_63693	1236	No	NA		0	1	Not-determined	Genome-fragment	NA		0
S14_extracted_k141_108145	1184	No	NA		0	0	Not-determined	Genome-fragment	NA		0
S1_vs_k141_92702||full	1340	No	NA		0	0	Low-quality	Genome-fragment		4.56	0
S14_extracted_k141_63726	2676	No	NA		0	2	Not-determined	Genome-fragment	NA		0
S3_extracted_k141_89675	1340	No	NA		0	0	Low-quality	Genome-fragment		0.79	0
S14_extracted_k141_115379	4325	No	NA		0	2	Not-determined	Genome-fragment	NA		0
S3_vs_k141_74446||full	1340	No	NA		4	0	Low-quality	Genome-fragment		3.09	0
S14_extracted_k141_48696	1223	No	NA		0	2	Not-determined	Genome-fragment	NA		0
S14_extracted_k141_86022	1351	No	NA		0	1	Not-determined	Genome-fragment	NA		0
S14_extracted_k141_5123	1651	No	NA		0	1	Not-determined	Genome-fragment	NA		0
S14_extracted_k141_100331	4305	No	NA		0	6	Not-determined	Genome-fragment	NA		0
S14_extracted_k141_100339	2318	No	NA		0	2	Not-determined	Genome-fragment	NA		0
S7_vs_134898||full	1340	No	NA		1	0	Low-quality	Genome-fragment		2	0
S7_vs_117919||lt2gene	1340	No	NA		1	0	Low-quality	Genome-fragment		2.62	0
S14_extracted_k141_42605	1118	No	NA		0	1	Not-determined	Genome-fragment	NA		0
S14_extracted_k141_42614	1139	No	NA		0	1	Not-determined	Genome-fragment	NA		0
S16_extracted_28900	1340	No	NA		2	0	Low-quality	Genome-fragment		2.22	0
S14_extracted_k141_34693	2179	No	NA		0	0	Not-determined	Genome-fragment	NA		0
S16_vs_58431||full	1340	No	NA		0	0	Low-quality	Genome-fragment		2.48	0
S14_extracted_k141_100431	1134	No	NA		0	0	Not-determined	Genome-fragment	NA		0
S14_extracted_k141_34715	1533	No	NA		0	2	Not-determined	Genome-fragment	NA		0
S14_extracted_k141_12570	1054	No	NA		0	0	Not-determined	Genome-fragment	NA		0
S14_extracted_k141_26904	1001	No	NA		0	1	Not-determined	Genome-fragment	NA		0
S14_extracted_k141_20278	1069	No	NA		0	0	Not-determined	Genome-fragment	NA		0
S14_extracted_k141_26931	3111	No	NA		0	1	Not-determined	Genome-fragment	NA		0
S14_extracted_k141_56681	1532	No	NA		0	1	Not-determined	Genome-fragment	NA		0
S10_vs_87486||lt2gene	1340	No	NA		2	0	Low-quality	Genome-fragment		2.38	0
S14_extracted_k141_48775	36317	No	NA		9	0	Medium-quality	Genome-fragment		60.96	0
S14_extracted_k141_86189	7440	No	NA		0	4	Not-determined	Genome-fragment	NA		0
S14_extracted_k141_20320	1130	No	NA		0	0	Not-determined	Genome-fragment	NA		0
S12_vs_k141_21369||lt2gene	1340	No	NA		1	0	Low-quality	Genome-fragment		3.04	0
S2_vs_k141_10032||full	1340	No	NA		0	1	Low-quality	Genome-fragment		3.13	0
S8_vs_k141_81846||full	1340	No	NA		1	0	Low-quality	Genome-fragment		2.8	0

S11_vs_k141_42928||lt2gene	1341	No	NA	1	0	Low-quality	Genome-fragment		3.26	0
S3_extracted_k141_58222	1341	No	NA	2	0	Low-quality	Genome-fragment		3.88	0
S14_extracted_k141_115516	1430	No	NA	0	1	Not-determined	Genome-fragment	NA		0
S14_extracted_k141_71465	1069	No	NA	0	1	Not-determined	Genome-fragment	NA		0
S14_extracted_k141_34850	3283	No	NA	0	1	Not-determined	Genome-fragment	NA		0
S5_vs_k141_51996||full	1341	No	NA	1	0	Low-quality	Genome-fragment		2.39	0
S7_extracted_87481	1341	No	NA	3	0	Low-quality	Genome-fragment		6.12	0
S14_extracted_k141_100571	1365	No	NA	0	1	Not-determined	Genome-fragment	NA		0
S7_vs_23402||full	1341	No	NA	0	1	Low-quality	Genome-fragment		1.35	0
S16_vs_29714||full	1341	No	NA	0	0	Low-quality	Genome-fragment		3.19	0
S14_extracted_k141_48853	1161	No	NA	0	0	Not-determined	Genome-fragment	NA		0
S14_extracted_k141_12710	1195	No	NA	0	1	Not-determined	Genome-fragment	NA		0
S14_extracted_k141_48872	1260	No	NA	0	0	Not-determined	Genome-fragment	NA		0
S16_vs_158116||lt2gene	1341	No	NA	1	0	Low-quality	Genome-fragment		3.09	0
S10_vs_51114||full	1341	No	NA	0	0	Low-quality	Genome-fragment		1.01	0
S14_extracted_k141_108397	1944	No	NA	0	2	Not-determined	Genome-fragment	NA		0
S10_vs_69176||full	1341	No	NA	0	0	Low-quality	Genome-fragment		3.1	0
S14_extracted_k141_34967	5414	No	NA	0	5	Not-determined	Genome-fragment	NA		0
S12_extracted_k141_103393	1341	No	NA	0	2	Low-quality	Genome-fragment		0.43	0
S12_extracted_k141_5094	1341	No	NA	0	0	Low-quality	Genome-fragment		3.15	0
S12_vs_k141_63255||full	1341	No	NA	0	2	Low-quality	Genome-fragment		1.11	0
S14_extracted_k141_20496	1028	No	NA	0	0	Not-determined	Genome-fragment	NA		0
S14_extracted_k141_78836	1257	No	NA	0	1	Not-determined	Genome-fragment	NA		0
S14_extracted_k141_71669	1535	No	NA	0	2	Not-determined	Genome-fragment	NA		0
S14_extracted_k141_12895	1098	No	NA	0	0	Not-determined	Genome-fragment	NA		0
S14_extracted_k141_49049	11402	No	NA	0	5	Not-determined	Genome-fragment	NA		0
S14_extracted_k141_71686	1284	No	NA	0	0	Not-determined	Genome-fragment	NA		0
S14_extracted_k141_78877	1267	No	NA	0	1	Not-determined	Genome-fragment	NA		0
S14_extracted_k141_43040	1651	No	NA	0	4	Not-determined	Genome-fragment	NA		0
S14_extracted_k141_64096	1046	No	NA	0	2	Not-determined	Genome-fragment	NA		0
S12_vs_k141_171775||full	1341	No	NA	0	0	Low-quality	Genome-fragment		2.52	0
S15_extracted_55764	1341	No	NA	0	0	Low-quality	Genome-fragment		2.18	0
S14_extracted_k141_108556	3793	No	NA	0	2	Not-determined	Genome-fragment	NA		0
S14_extracted_k141_12989	1655	No	NA	0	0	Not-determined	Genome-fragment	NA		0
S17_extracted_k141_58485	1342	No	NA	0	0	Low-quality	Genome-fragment		0.88	0
S14_extracted_k141_86483	5439	No	NA	0	1	Not-determined	Genome-fragment	NA		0
S14_extracted_k141_115736	42416	No	NA	19	0	Complete	High-quality		100	0
S14_extracted_k141_27277	2083	No	NA	0	3	Not-determined	Genome-fragment	NA		0
S14_extracted_k141_5570	1063	No	NA	0	1	Not-determined	Genome-fragment	NA		0
S14_extracted_k141_13035	4885	No	NA	0	3	Not-determined	Genome-fragment	NA		0
S9_vs_k141_32350||full	1342	No	NA	1	0	Low-quality	Genome-fragment		2.76	0
S14_extracted_k141_43182	1458	No	NA	0	0	Not-determined	Genome-fragment	NA		0
S14_extracted_k141_108632	1202	No	NA	0	1	Not-determined	Genome-fragment	NA		0
S11_vs_k141_7711||full	1342	No	NA	1	0	Low-quality	Genome-fragment		3.25	0
S14_extracted_k141_43184	1096	No	NA	0	0	Not-determined	Genome-fragment	NA		0
S14_extracted_k141_108648	1630	No	NA	0	0	Not-determined	Genome-fragment	NA		0
S14_extracted_k141_78958	2157	No	NA	0	1	Not-determined	Genome-fragment	NA		0
S14_extracted_k141_20684	6711	No	NA	0	2	Not-determined	Genome-fragment	NA		0
S14_extracted_k141_20694	4549	No	NA	0	1	Not-determined	Genome-fragment	NA		0
S14_extracted_k141_108686	3267	No	NA	0	3	Not-determined	Genome-fragment	NA		0
S14_extracted_k141_71887	3669	No	NA	0	2	Not-determined	Genome-fragment	NA		0
S13_vs_142648||full	1342	No	NA	0	1	Low-quality	Genome-fragment		1.67	0
S14_extracted_k141_43235	1092	No	NA	0	1	Not-determined	Genome-fragment	NA		0
S14_extracted_k141_49103	1793	No	NA	0	0	Not-determined	Genome-fragment	NA		0
S14_extracted_k141_78989	1241	No	NA	0	1	Not-determined	Genome-fragment	NA		0
S14_extracted_k141_57011	1211	No	NA	0	0	Not-determined	Genome-fragment	NA		0
S14_extracted_k141_100082	1342	No	NA	2	0	Low-quality	Genome-fragment		0.73	0
S14_extracted_k141_64230	3991	No	NA	0	2	Not-determined	Genome-fragment	NA		0
S14_extracted_k141_49195	1359	No	NA	0	1	Not-determined	Genome-fragment	NA		0
S4_extracted_7231	1342	No	NA	0	0	Low-quality	Genome-fragment		3.44	0
S14_extracted_k141_108837	1081	No	NA	0	0	Not-determined	Genome-fragment	NA		0
S14_extracted_k141_5774	1041	No	NA	0	0	Not-determined	Genome-fragment	NA		0
S14_extracted_k141_108851	1141	No	NA	0	1	Not-determined	Genome-fragment	NA		0
S8_vs_k141_185532||full	1342	No	NA	0	0	Low-quality	Genome-fragment		1.4	0
S14_extracted_k141_5788	1163	No	NA	0	1	Not-determined	Genome-fragment	NA		0
S9_vs_k141_18480||lt2gene	1343	No	NA	2	0	Low-quality	Genome-fragment		3.77	0
S13_vs_139321||full	1343	No	NA	0	1	Low-quality	Genome-fragment		3.03	0
S1_vs_k141_27995||full	1343	No	NA	0	0	Low-quality	Genome-fragment		2.7	0
S14_extracted_k141_43340	6047	No	NA	3	0	High-quality	High-quality		97.55	0
S14_extracted_k141_101065	1069	No	NA	0	2	Not-determined	Genome-fragment	NA		0
S14_extracted_k141_64288	1654	No	NA	0	1	Not-determined	Genome-fragment	NA		0
S14_extracted_k141_79107	1583	No	NA	0	1	Not-determined	Genome-fragment	NA		0
S3_extracted_k141_112626	1343	No	NA	1	0	Low-quality	Genome-fragment		0.96	0
S3_vs_k141_110122||full	1343	No	NA	3	0	Low-quality	Genome-fragment		3.24	0
S14_extracted_k141_35347	1355	No	NA	0	1	Not-determined	Genome-fragment	NA		0
S14_extracted_k141_57142	1397	No	NA	0	0	Not-determined	Genome-fragment	NA		0
S14_extracted_k141_79124	5693	No	NA	0	4	Not-determined	Genome-fragment	NA		0
S14_extracted_k141_13197	1234	No	NA	0	1	Not-determined	Genome-fragment	NA		0
S14_extracted_k141_5843	1182	No	NA	0	1	Not-determined	Genome-fragment	NA		0
S14_extracted_k141_108936	1454	No	NA	0	1	Not-determined	Genome-fragment	NA		0
S14_extracted_k141_35366	1363	No	NA	0	2	Not-determined	Genome-fragment	NA		0
S5_extracted_k141_49886	1343	No	NA	1	0	Low-quality	Genome-fragment		3.35	0
S7_vs_73531||full	1343	No	NA	1	0	Low-quality	Genome-fragment		2.75	0
S14_extracted_k141_5869	1100	No	NA	0	2	Not-determined	Genome-fragment	NA		0
S14_extracted_k141_27638	1131	No	NA	0	0	Not-determined	Genome-fragment	NA		0
S14_extracted_k141_79177	1524	No	NA	0	1	Not-determined	Genome-fragment	NA		0
S7_vs_130683||lt2gene	1343	No	NA	2	0	Low-quality	Genome-fragment		3.61	0
S16_extracted_120016	1343	No	NA	1	0	Low-quality	Genome-fragment		0.54	0
S14_extracted_k141_86870	7486	No	NA	0	4	Not-determined	Genome-fragment	NA		0
S14_extracted_k141_57240	2280	No	NA	0	0	Not-determined	Genome-fragment	NA		0
S16_vs_111582||full	1343	No	NA	0	0	Low-quality	Genome-fragment		3.06	0
S12_vs_k141_152211||full	1343	No	NA	0	0	Low-quality	Genome-fragment		19.54	0
S8_vs_k141_161771||full	1343	No	NA	0	0	Low-quality	Genome-fragment		3.34	0
S14_extracted_k141_86942	1043	No	NA	0	0	Not-determined	Genome-fragment	NA		0
S14_extracted_k141_21002	1712	No	NA	0	0	Not-determined	Genome-fragment	NA		0
S17_vs_k141_81020||full	1344	No	NA	0	0	Low-quality	Genome-fragment		2.51	0
S14_extracted_k141_101201	1023	No	NA	0	1	Not-determined	Genome-fragment	NA		0
S9_vs_k141_20008||full	1344	No	NA	0	3	Low-quality	Genome-fragment		0.73	0
S14_extracted_k141_79275	3188	No	NA	0	1	Not-determined	Genome-fragment	NA		0
S9_vs_k141_83160||lt2gene	1344	No	NA	1	0	Low-quality	Genome-fragment		2.93	0
S14_extracted_k141_79278	1497	No	NA	0	0	Not-determined	Genome-fragment	NA		0
S14_extracted_k141_27715	3368	No	NA	0	2	Not-determined	Genome-fragment	NA		0
S14_extracted_k141_101223	3346	No	NA	0	2	Not-determined	Genome-fragment	NA		0
S11_vs_k141_65792||full	1344	No	NA	0	0	Low-quality	Genome-fragment		1.92	0
S14_extracted_k141_109060	1217	No	NA	0	0	Not-determined	Genome-fragment	NA		0
S14_extracted_k141_87043	3019	No	NA	0	2	Not-determined	Genome-fragment	NA		0
S14_extracted_k141_79335	2684	No	NA	0	0	Not-determined	Genome-fragment	NA		0
S14_extracted_k141_64390	1172	No	NA	0	0	Not-determined	Genome-fragment	NA		0
S14_extracted_k141_72101	2953	No	NA	0	1	Not-determined	Genome-fragment	NA		0
S14_extracted_k141_94537	1609	No	NA	0	4	Not-determined	Genome-fragment	NA		0
S14_extracted_k141_94547	2351	No	NA	0	0	Not-determined	Genome-fragment	NA		0
S14_extracted_k141_35619	1138	No	NA	0	1	Not-determined	Genome-fragment	NA		0
S13_vs_129291||full	1344	No	NA	0	0	Low-quality	Genome-fragment		2.16	0
S14_extracted_k141_94568	1483	No	NA	0	1	Not-determined	Genome-fragment	NA		0
S1_extracted_k141_150898	1344	No	NA	1	0	Low-quality	Genome-fragment		1.97	0
S3_extracted_k141_46539	1344	No	NA	2	0	Low-quality	Genome-fragment		26.13	0
S14_extracted_k141_64419	1364	No	NA	0	1	Not-determined	Genome-fragment	NA		0
S3_vs_k141_23265||full	1344	No	NA	0	0	Low-quality	Genome-fragment		1.99	0
S14_extracted_k141_64425	1142	No	NA	0	2	Not-determined	Genome-fragment	NA		0
S5_extracted_k141_111068	1344	No	NA	0	0	Low-quality	Genome-fragment		1.02	0
S14_extracted_k141_94602	4564	No	NA	0	3	Not-determined	Genome-fragment	NA		0
S14_extracted_k141_6137	1697	No	NA	0	1	Not-determined	Genome-fragment	NA		0
S5_vs_k141_70073||full	1344	No	NA	0	0	Low-quality	Genome-fragment		2.1	0
S14_extracted_k141_101376	1167	No	NA	0	0	Not-determined	Genome-fragment	NA		0
S14_extracted_k141_57452	1045	No	NA	0	1	Not-determined	Genome-fragment	NA		0
S7_vs_18238||lt2gene	1344	No	NA	2	0	Low-quality	Genome-fragment		3.08	0
S14_extracted_k141_27933	3495	No	NA	0	2	Not-determined	Genome-fragment	NA		0
S14_extracted_k141_49689	2605	No	NA	0	0	Not-determined	Genome-fragment	NA		0
S12_vs_k141_166953||full	1344	No	NA	0	0	Low-quality	Genome-fragment		0.9	0
S17_extracted_k141_38287	1345	No	NA	0	1	Low-quality	Genome-fragment		1.2	0
S11_extracted_k141_73665	1345	No	NA	0	0	Low-quality	Genome-fragment		2.36	0
S11_extracted_k141_69904	1345	No	NA	0	0	Low-quality	Genome-fragment		1.29	0
S14_extracted_k141_94719	1179	No	NA	0	1	Not-determined	Genome-fragment	NA		0
S14_extracted_k141_13495	1471	No	NA	0	1	Not-determined	Genome-fragment	NA		0
S14_extracted_k141_72253	3055	No	NA	0	2	Not-determined	Genome-fragment	NA		0
S14_extracted_k141_87276	1015	No	NA	0	1	Not-determined	Genome-fragment	NA		0
S14_extracted_k141_94750	1396	No	NA	0	0	Not-determined	Genome-fragment	NA		0
S14_extracted_k141_43787	1242	No	NA	0	2	Not-determined	Genome-fragment	NA		0
S14_extracted_k141_21265	1028	No	NA	0	0	Not-determined	Genome-fragment	NA		0
S14_extracted_k141_35829	20156	No	NA	0	4	Not-determined	Genome-fragment	NA		0
S7_vs_134439||full	1345	No	NA	0	0	Low-quality	Genome-fragment		3.72	0
S12_extracted_k141_150016	1345	No	NA	0	1	Low-quality	Genome-fragment		3.05	0
S14_extracted_k141_57629	1299	No	NA	0	0	Not-determined	Genome-fragment	NA		0
S12_extracted_k141_159644	1345	No	NA	0	2	Low-quality	Genome-fragment		0.88	0
S12_vs_k141_24636||full	1345	No	NA	0	0	Low-quality	Genome-fragment		0.92	0
S14_extracted_k141_94800	1212	No	NA	0	0	Not-determined	Genome-fragment	NA		0
S14_extracted_k141_94815	1090	No	NA	0	0	Not-determined	Genome-fragment	NA		0
S12_vs_k141_35422||full	1345	No	NA	0	1	Low-quality	Genome-fragment		0.6	0
S15_vs_10652||full	1345	No	NA	0	1	Low-quality	Genome-fragment		0.58	0
S14_extracted_k141_72309	1219	No	NA	0	0	Not-determined	Genome-fragment	NA		0
S14_extracted_k141_116541	1012	No	NA	0	1	Not-determined	Genome-fragment	NA		0
S14_extracted_k141_64639	1014	No	NA	0	0	Not-determined	Genome-fragment	NA		0
S8_vs_k141_176045||full	1345	No	NA	0	0	Low-quality	Genome-fragment		1.43	0
S8_vs_k141_21875||full	1345	No	NA	0	0	Low-quality	Genome-fragment		2.17	0
S9_vs_k141_54286||full	1346	No	NA	1	0	Low-quality	Genome-fragment		2.65	0
S14_extracted_k141_109431	1046	No	NA	0	0	Not-determined	Genome-fragment	NA		0
S14_extracted_k141_6450	1979	No	NA	0	0	Not-determined	Genome-fragment	NA		0
S14_extracted_k141_57689	34453	No	NA	12	0	Medium-quality	Genome-fragment		60.77	0
S9_vs_k141_69014||full	1346	No	NA	0	0	Low-quality	Genome-fragment		2.97	0
S14_extracted_k141_109467	1110	No	NA	0	0	Not-determined	Genome-fragment	NA		0
S14_extracted_k141_109480	3843	No	NA	0	2	Not-determined	Genome-fragment	NA		0
S11_vs_k141_37137||full	1346	No	NA	1	0	Low-quality	Genome-fragment		3.18	0
S14_extracted_k141_36016	1698	No	NA	0	0	Not-determined	Genome-fragment	NA		0
S14_extracted_k141_6532	3348	No	NA	0	0	Not-determined	Genome-fragment	NA		0
S14_extracted_k141_57830	8089	No	NA	0	0	Not-determined	Genome-fragment	NA		0
S14_extracted_k141_109545	26025	No	NA	5	1	Medium-quality	Genome-fragment		60.65	0
S14_extracted_k141_101686	2436	No	NA	0	1	Not-determined	Genome-fragment	NA		0
S14_vs_k141_73558||lt2gene	1346	No	NA	1	0	Low-quality	Genome-fragment		3.96	0
S3_extracted_k141_23050	1346	No	NA	1	0	Low-quality	Genome-fragment		2.95	0
S3_vs_k141_71340||lt2gene	1346	No	NA	1	0	Low-quality	Genome-fragment		3.55	0
S14_extracted_k141_72335	43571	No	NA	19	0	High-quality	High-quality		100	0
S5_vs_k141_23703||lt2gene	1346	No	NA	1	0	Low-quality	Genome-fragment		1.97	0
S14_extracted_k141_36118	5322	No	NA	0	3	Not-determined	Genome-fragment	NA		0
S14_extracted_k141_116747	1043	No	NA	0	0	Not-determined	Genome-fragment	NA		0

S14_extracted_k141_6638	1109	No	NA	0	2	Not-determined	Genome-fragment	NA		0
S7_extracted_30359	1346	No	NA	1	0	Low-quality	Genome-fragment		1.97	0
S14_extracted_k141_109721	5728	No	NA	0	4	Not-determined	Genome-fragment	NA		0
S7_extracted_57893	1346	No	NA	0	0	Low-quality	Genome-fragment		3.64	0
S14_extracted_k141_109753	1146	No	NA	0	0	Not-determined	Genome-fragment	NA		0
S7_vs_82712||full	1346	No	NA	1	0	Low-quality	Genome-fragment		3.14	0
S14_extracted_k141_57993	1356	No	NA	0	0	Not-determined	Genome-fragment	NA		0
S16_extracted_6990	1346	No	NA	0	0	Low-quality	Genome-fragment		2.78	0
S14_extracted_k141_80055	1535	No	NA	0	0	Not-determined	Genome-fragment	NA		0
S14_extracted_k141_6730	1117	No	NA	0	1	Not-determined	Genome-fragment	NA		0
S16_vs_91880||lt2gene	1346	No	NA	1	0	Low-quality	Genome-fragment		1.78	0
S14_extracted_k141_101865	1972	No	NA	0	0	Not-determined	Genome-fragment	NA		0
S14_extracted_k141_6741	1156	No	NA	0	0	Not-determined	Genome-fragment	NA		0
S10_extracted_46005	1346	No	NA	0	3	Low-quality	Genome-fragment		0.96	0
S14_extracted_k141_109814	1065	No	NA	0	1	Not-determined	Genome-fragment	NA		0
S14_extracted_k141_109817	1648	No	NA	0	2	Not-determined	Genome-fragment	NA		0
S14_extracted_k141_80088	3610	No	NA	0	3	Not-determined	Genome-fragment	NA		0
S10_vs_103886||full	1346	No	NA	0	0	Low-quality	Genome-fragment		2.54	0
S12_vs_k141_83422||full	1346	No	NA	0	0	Low-quality	Genome-fragment		2.6	0
S14_extracted_k141_101880	1950	No	NA	0	1	Not-determined	Genome-fragment	NA		0
S6_extracted_k141_27256	1346	No	NA	0	2	Low-quality	Genome-fragment		1.14	0
S17_vs_k141_62665||full	1347	No	NA	0	0	Low-quality	Genome-fragment		1.86	0
S11_vs_k141_32282||full	1347	No	NA	0	0	Low-quality	Genome-fragment		3.09	0
S14_extracted_k141_116900	1114	No	NA	0	0	Not-determined	Genome-fragment	NA		0
S14_extracted_k141_32599	1347	No	NA	1	0	Low-quality	Genome-fragment		0.91	0
S7_vs_45990||full	1347	No	NA	0	0	Low-quality	Genome-fragment		3.06	0
S14_extracted_k141_116951	1672	No	NA	0	0	Not-determined	Genome-fragment	NA		0
S14_extracted_k141_36332	1858	No	NA	0	1	Not-determined	Genome-fragment	NA		0
S14_extracted_k141_21833	1896	No	NA	0	0	Not-determined	Genome-fragment	NA		0
S16_extracted_22217	1347	No	NA	0	0	Low-quality	Genome-fragment		4.2	0
S14_extracted_k141_50256	1864	No	NA	0	1	Not-determined	Genome-fragment	NA		0
S10_vs_90902||lt2gene	1347	No	NA	1	0	Low-quality	Genome-fragment		3.32	0
S15_extracted_9761	1347	No	NA	1	0	Low-quality	Genome-fragment		0.81	0
S2_vs_k141_879||lt2gene	1347	No	NA	1	0	Low-quality	Genome-fragment		3.13	0
S8_extracted_k141_174696	1347	No	NA	0	0	Low-quality	Genome-fragment		1.07	0
S14_extracted_k141_58246	2107	No	NA	0	3	Not-determined	Genome-fragment	NA		0
S14_extracted_k141_6879	1603	No	NA	0	0	Not-determined	Genome-fragment	NA		0
S14_extracted_k141_21932	3016	No	NA	0	1	Not-determined	Genome-fragment	NA		0
S14_extracted_k141_80214	3820	No	NA	0	2	Not-determined	Genome-fragment	NA		0
S14_extracted_k141_80216	1172	No	NA	0	1	Not-determined	Genome-fragment	NA		0
S14_extracted_k141_65153	3547	No	NA	0	1	Not-determined	Genome-fragment	NA		0
S17_extracted_k141_164718	1348	No	NA	0	0	Low-quality	Genome-fragment		2.76	0
S14_extracted_k141_65173	1079	No	NA	0	0	Not-determined	Genome-fragment	NA		0
S14_extracted_k141_102028	1078	No	NA	0	0	Not-determined	Genome-fragment	NA		0
S17_vs_k141_78481||full	1348	No	NA	1	0	Low-quality	Genome-fragment		3.27	0
S14_extracted_k141_117135	1300	No	NA	0	0	Not-determined	Genome-fragment	NA		0
S14_extracted_k141_14179	5264	No	NA	0	2	Not-determined	Genome-fragment	NA		0
S14_extracted_k141_58353	2968	No	NA	0	1	Not-determined	Genome-fragment	NA		0
S9_vs_k141_8420||full	1348	No	NA	2	0	Low-quality	Genome-fragment		2.39	0
S14_extracted_k141_28518	1041	No	NA	0	2	Not-determined	Genome-fragment	NA		0
S11_vs_k141_79995||lt2gene	1348	No	NA	2	0	Low-quality	Genome-fragment		3.51	0
S1_vs_k141_48655||lt2gene	1348	No	NA	1	0	Low-quality	Genome-fragment		3.32	0
S14_extracted_k141_117147	35338	No	NA	16	0	Complete	High-quality		100	0
S7_vs_127316||full	1348	No	NA	0	0	Low-quality	Genome-fragment		0.77	0
S16_extracted_45096	1348	No	NA	1	0	Low-quality	Genome-fragment		3.35	0
S14_extracted_k141_72909	2652	No	NA	0	0	Not-determined	Genome-fragment	NA		0
S16_vs_37694||full	1348	No	NA	1	0	Low-quality	Genome-fragment		0.8	0
S6_extracted_k141_27934	1348	No	NA	0	0	Low-quality	Genome-fragment		1.74	0
S14_extracted_k141_28591	1494	No	NA	0	1	Not-determined	Genome-fragment	NA		0
S14_extracted_k141_102190	1204	No	NA	0	1	Not-determined	Genome-fragment	NA		0
S14_extracted_k141_7100	1141	No	NA	0	1	Not-determined	Genome-fragment	NA		0
S14_extracted_k141_117150	39147	No	NA	17	1	Complete	High-quality		100	0
S17_vs_k141_3366||full	1349	No	NA	0	0	Low-quality	Genome-fragment		0.97	0
S14_extracted_k141_72953	38044	No	NA	26	1	High-quality	High-quality		98.41	0
S14_extracted_k141_50595	1169	No	NA	0	0	Not-determined	Genome-fragment	NA		0
S14_extracted_k141_72990	1011	No	NA	0	0	Not-determined	Genome-fragment	NA		0
S14_extracted_k141_58520	1186	No	NA	0	0	Not-determined	Genome-fragment	NA		0
S14_extracted_k141_65431	3366	No	NA	0	0	Not-determined	Genome-fragment	NA		0
S14_extracted_k141_28683	1962	No	NA	0	2	Not-determined	Genome-fragment	NA		0
S14_extracted_k141_73050	1158	No	NA	0	0	Not-determined	Genome-fragment	NA		0
S14_extracted_k141_80473	1865	No	NA	0	1	Not-determined	Genome-fragment	NA		0
S14_extracted_k141_7262	5959	No	NA	0	3	Not-determined	Genome-fragment	NA		0
S14_extracted_k141_7265	1478	No	NA	0	0	Not-determined	Genome-fragment	NA		0
S14_extracted_k141_28740	1182	No	NA	0	0	Not-determined	Genome-fragment	NA		0
S14_extracted_k141_65495	1331	No	NA	0	0	Not-determined	Genome-fragment	NA		0
S14_extracted_k141_17329	1349	No	NA	0	0	Low-quality	Genome-fragment		3.15	0
S14_extracted_k141_80532	2600	No	NA	0	2	Not-determined	Genome-fragment	NA		0
S14_extracted_k141_107562	1349	No	NA	1	0	Low-quality	Genome-fragment		3.1	0
S14_vs_k141_104005||full	1349	No	NA	2	0	Low-quality	Genome-fragment		3.47	0
S14_extracted_k141_102427	1282	No	NA	0	0	Not-determined	Genome-fragment	NA		0
S14_extracted_k141_117155	38997	No	NA	27	0	Medium-quality	Genome-fragment		65.14	0
S14_extracted_k141_50778	1051	No	NA	0	2	Not-determined	Genome-fragment	NA		0
S14_extracted_k141_73196	1454	No	NA	0	3	Not-determined	Genome-fragment	NA		0
S3_vs_k141_120725||full	1349	No	NA	0	0	Low-quality	Genome-fragment		2.06	0
S14_extracted_k141_28910	1126	No	NA	0	0	Not-determined	Genome-fragment	NA		0
S14_extracted_k141_117157	59976	No	NA	9	0	Complete	High-quality		100	0
S14_extracted_k141_73262	3969	No	NA	0	3	Not-determined	Genome-fragment	NA		0
S14_extracted_k141_28966	1397	No	NA	0	2	Not-determined	Genome-fragment	NA		0
S5_vs_k141_38199||full	1349	No	NA	1	0	Low-quality	Genome-fragment		2.88	0
S14_extracted_k141_65676	1233	No	NA	0	1	Not-determined	Genome-fragment	NA		0
S5_vs_k141_145346||full	1349	No	NA	1	0	Low-quality	Genome-fragment		0.4	0
S14_extracted_k141_117162	36948	No	NA	11	1	Complete	High-quality		100	0
S7_vs_69467||lt2gene	1349	No	NA	2	0	Low-quality	Genome-fragment		4.34	0
S14_extracted_k141_117164	14258	No	NA	6	0	Medium-quality	Genome-fragment		65.51	0
S14_extracted_k141_51134	33359	No	NA	24	1	Complete	High-quality		100	0
S14_extracted_k141_65861	3397	No	NA	0	2	Not-determined	Genome-fragment	NA		0
S16_extracted_10659	1349	No	NA	0	0	Low-quality	Genome-fragment		1.4	0
S14_extracted_k141_117194	1393	No	NA	0	0	Not-determined	Genome-fragment	NA		0
S14_vs_k141_15228||full	150458	No	NA	13	5	High-quality	High-quality		97.82	0
S14_vs_k141_88666||full	36753	No	NA	17	1	High-quality	High-quality		90.08	0
S14_vs_k141_30439||full	34044	No	NA	16	0	High-quality	High-quality		99.24	0
S14_vs_k141_96286||full	30986	No	NA	16	0	High-quality	High-quality		91.93	0
S14_vs_k141_9676||full	42662	No	NA	12	0	High-quality	High-quality		100	0
S14_vs_k141_69166||full	4148	No	NA	0	2	Not-determined	Genome-fragment	NA		0
S14_vs_k141_3251||full	34146	No	NA	14	2	High-quality	High-quality		100	0
S14_vs_k141_26320||full	172200	No	NA	33	6	High-quality	High-quality		100	0
S14_vs_k141_4887||full	40549	No	NA	7	2	High-quality	High-quality		97.58	0
S14_vs_k141_42401||full	2574	No	NA	0	0	Not-determined	Genome-fragment	NA		0
S14_vs_k141_94397||full	5962	No	NA	0	0	Not-determined	Genome-fragment	NA		0
S16_vs_129987||lt2gene	1349	No	NA	1	0	Low-quality	Genome-fragment		2.52	0
S14_vs_k141_117153||full	37231	No	NA	13	0	High-quality	High-quality		100	0
S10_extracted_2042	1349	No	NA	0	0	Low-quality	Genome-fragment		4.09	0
S14_vs_k141_117158||full	40845	No	NA	23	2	High-quality	High-quality		93.32	0
S14_vs_k141_117160||full	34792	No	NA	24	1	Medium-quality	Genome-fragment		89.27	0
S14_vs_k141_117161||full	35177	No	NA	18	0	High-quality	High-quality		99.84	0
S14_vs_k141_117163||full	14322	No	NA	0	0	Not-determined	Genome-fragment	NA		0
S14_vs_k141_117165||full	40247	No	NA	8	0	High-quality	High-quality		100	0
S14_vs_k141_117166||full	38784	No	NA	18	2	High-quality	High-quality		94.06	0
S14_vs_k141_117171||full	2414	No	NA	0	0	Not-determined	Genome-fragment	NA		0
S14_vs_k141_7331||full	2679	No	NA	0	1	Not-determined	Genome-fragment	NA		0
S10_extracted_9812	1349	No	NA	0	0	Low-quality	Genome-fragment		1.82	0
S14_vs_k141_29315||full	1729	No	NA	0	2	Not-determined	Genome-fragment	NA		0
S14_vs_k141_22006||full	1509	No	NA	0	2	Not-determined	Genome-fragment	NA		0
S14_vs_k141_43992||full	3614	No	NA	0	1	Not-determined	Genome-fragment	NA		0
S14_vs_k141_65985||full	2717	No	NA	0	2	Not-determined	Genome-fragment	NA		0
S14_vs_k141_44020||full	2139	No	NA	0	1	Not-determined	Genome-fragment	NA		0
S14_vs_k141_95278||full	1316	No	NA	0	0	Not-determined	Genome-fragment	NA		0
S12_extracted_k141_35360	1349	No	NA	0	0	Low-quality	Genome-fragment		2.55	0
S8_extracted_k141_14313	1349	No	NA	1	0	Low-quality	Genome-fragment		1.97	0
S14_vs_k141_29356||full	2981	No	NA	0	2	Not-determined	Genome-fragment	NA		0
S14_vs_k141_7403||full	1459	No	NA	0	1	Not-determined	Genome-fragment	NA		0
S14_vs_k141_87956||full	2397	No	NA	0	1	Not-determined	Genome-fragment	NA		0
S14_vs_k141_73301||full	29973	No	NA	17	0	Medium-quality	Genome-fragment		71.49	0
S14_vs_k141_36754||full	1784	No	NA	0	1	Not-determined	Genome-fragment	NA		0
S14_vs_k141_29398||full	1185	No	NA	0	1	Not-determined	Genome-fragment	NA		0
S11_extracted_k141_52622	1350	No	NA	0	1	Low-quality	Genome-fragment		0.41	0
S14_vs_k141_14737||full	35673	No	NA	16	1	High-quality	High-quality		98.83	0
S11_vs_k141_43808||full	1350	No	NA	2	0	Low-quality	Genome-fragment		3.64	0
S7_vs_99743||full	1350	No	NA	0	0	Low-quality	Genome-fragment		2.75	0
S16_vs_119454||full	1350	No	NA	0	0	Low-quality	Genome-fragment		2.73	0
S16_vs_121129||full	1350	No	NA	0	2	Low-quality	Genome-fragment		0.38	0
S14_vs_k141_36807||full	3398	No	NA	0	5	Not-determined	Genome-fragment	NA		0
S10_vs_11356||full	1350	No	NA	0	1	Low-quality	Genome-fragment		1.83	0
S14_vs_k141_7465||full	2883	No	NA	0	0	Not-determined	Genome-fragment	NA		0
S12_vs_k141_77072||full	1350	No	NA	0	0	Low-quality	Genome-fragment		2.64	0
S4_extracted_7875	1350	No	NA	1	0	Low-quality	Genome-fragment		4.45	0
S11_vs_k141_81040||lt2gene	1351	No	NA	1	0	Low-quality	Genome-fragment		3.56	0
S14_vs_k141_183||full	11611	No	NA	0	4	Not-determined	Genome-fragment	NA		0
S14_vs_k141_95415||full	1125	No	NA	0	0	Not-determined	Genome-fragment	NA		0
S14_vs_k141_102698||full	22313	No	NA	15	0	Medium-quality	Genome-fragment		66.53	0
S13_vs_69345||full	1351	No	NA	1	0	Low-quality	Genome-fragment		2.75	0
S14_vs_k141_51463||full	3038	No	NA	0	2	Not-determined	Genome-fragment	NA		0
S14_extracted_k141_113114	1351	No	NA	0	1	Low-quality	Genome-fragment		0.23	0
S14_vs_k141_58815||full	11138	No	NA	0	3	Not-determined	Genome-fragment	NA		0
S14_vs_k141_102726||full	5385	No	NA	0	3	Not-determined	Genome-fragment	NA		0
S14_vs_k141_246||full	1072	No	NA	0	0	Not-determined	Genome-fragment	NA		0
S14_vs_k141_47251||full	1351	No	NA	0	1	Low-quality	Genome-fragment		3.5	0
S3_vs_k141_49962||full	1351	No	NA	1	0	Low-quality	Genome-fragment		3.37	0
S7_vs_132893||full	1351	No	NA	1	0	Low-quality	Genome-fragment		3.33	0
S14_vs_k141_58870||full	4165	No	NA	0	1	Not-determined	Genome-fragment	NA		0
S12_extracted_k141_38943	1351	No	NA	0	1	Low-quality	Genome-fragment		3.25	0
S14_vs_k141_80799||full	2854	No	NA	0	2	Not-determined	Genome-fragment	NA		0
S14_vs_k141_51515||full	1891	No	NA	0	1	Not-determined	Genome-fragment	NA		0
S12_vs_k141_138448||full	1351	No	NA	1	0	Low-quality	Genome-fragment		3.24	0
S14_vs_k141_80815||full	2192	No	NA	0	1	Not-determined	Genome-fragment	NA		0
S14_vs_k141_7605||full	3759	No	NA	0	1	Not-determined	Genome-fragment	NA		0
S2_extracted_k141_8635	1351	No	NA	1	0	Low-quality	Genome-fragment		3.16	0
S17_vs_k141_32974||full	1352	No	NA	0	0	Low-quality	Genome-fragment		2.29	0
S14_vs_k141_102744||full	3274	No	NA	0	0	Not-determined	Genome-fragment	NA		0
S14_extracted_k141_2227	1352	No	NA	5	0	Low-quality	Genome-fragment		4.47	0
S14_extracted_k141_71607	1352	No	NA	0	0	Low-quality	Genome-fragment		3.14	0
S14_vs_k141_80831||full	9744	No	NA	0	8	Not-determined	Genome-fragment	NA		0
S14_vs_k141_10901||full	1352	No	NA	0	0	Low-quality	Genome-fragment		2.16	0
S14_vs_k141_26487||full	1352	No	NA	0	0	Low-quality	Genome-fragment		0.77	0

S14_vs_k141_93590||full	1352	No	NA	0	0	Low-quality	Genome-fragment	1.74	0
S14_vs_k141_88156||full	1778	No	NA	0	0	Not-determined	Genome-fragment	NA	0
S14_vs_k141_29595||full	3392	No	NA	0	4	Not-determined	Genome-fragment	NA	0
S14_vs_k141_7635||full	7248	No	NA	0	1	Not-determined	Genome-fragment	NA	0
S14_vs_k141_102804||full	1730	No	NA	0	0	Not-determined	Genome-fragment	NA	0
S3_extracted_k141_108191	1352	No	NA	2	0	Low-quality	Genome-fragment	2.63	0
S3_vs_k141_115556||full	1352	No	NA	1	0	Low-quality	Genome-fragment	2.52	0
S3_vs_k141_103361||full	1352	No	NA	1	0	Low-quality	Genome-fragment	0.75	0
S7_vs_36722||full	1352	No	NA	0	2	Low-quality	Genome-fragment	1.87	0
S14_vs_k141_66139||full	198450	Yes	56174	26	89	Medium-quality	Genome-fragment	82.23	71.69
S14_vs_k141_22271||full	4976	No	NA	0	3	Not-determined	Genome-fragment	NA	0
S7_vs_83683||full	1352	No	NA	1	0	Low-quality	Genome-fragment	2.16	0
S14_vs_k141_7683||full	6337	No	NA	0	0	Not-determined	Genome-fragment	NA	0
S8_extracted_k141_183956	1352	No	NA	0	0	Low-quality	Genome-fragment	1.6	0
S9_extracted_k141_41983	1353	No	NA	1	0	Low-quality	Genome-fragment	1.54	0
S11_vs_k141_61010||full	1353	No	NA	0	0	Low-quality	Genome-fragment	1.79	0
S14_vs_k141_51657||full	1692	No	NA	0	2	Not-determined	Genome-fragment	NA	0
S14_vs_k141_51676||full	3457	No	NA	0	1	Not-determined	Genome-fragment	NA	0
S14_vs_k141_80917||full	1866	No	NA	0	2	Not-determined	Genome-fragment	NA	0
S14_vs_k141_14985||full	7969	No	NA	0	5	Not-determined	Genome-fragment	NA	0
S14_vs_k141_491||full	1685	No	NA	0	0	Not-determined	Genome-fragment	NA	0
S14_vs_k141_44218||full	1578	No	NA	0	1	Not-determined	Genome-fragment	NA	0
S14_vs_k141_102917||full	5647	No	NA	0	2	Not-determined	Genome-fragment	NA	0
S11_vs_k141_15578||full	1353	No	NA	1	0	Low-quality	Genome-fragment	3.38	0
S14_vs_k141_88282||full	1914	No	NA	0	0	Not-determined	Genome-fragment	NA	0
S14_vs_k141_547||full	1476	No	NA	0	0	Not-determined	Genome-fragment	NA	0
S14_vs_k141_66208||full	67174	Yes	48001	14	10	Complete	High-quality	100	28.54
S13_extracted_28117	1353	No	NA	0	1	Low-quality	Genome-fragment	2.26	0
S13_extracted_39161	1353	No	NA	0	0	Low-quality	Genome-fragment	1.4	0
S1_vs_k141_126895||full	1353	No	NA	0	0	Low-quality	Genome-fragment	2.59	0
S14_vs_k141_29751||full	1144	No	NA	0	2	Not-determined	Genome-fragment	NA	0
S1_vs_k141_152456||full	1353	No	NA	1	0	Low-quality	Genome-fragment	2.26	0
S1_vs_k141_41883||lt2gene	1353	No	NA	0	0	Low-quality	Genome-fragment	2.31	0
S3_extracted_k141_96516	1353	No	NA	0	1	Low-quality	Genome-fragment	2.66	0
S3_extracted_k141_82331	1353	No	NA	0	0	Low-quality	Genome-fragment	2.02	0
S14_vs_k141_44323||full	3074	No	NA	0	1	Not-determined	Genome-fragment	NA	0
S3_vs_k141_111919||full	1353	No	NA	0	0	Low-quality	Genome-fragment	2.25	0
S14_vs_k141_103003||full	8979	No	NA	0	6	Not-determined	Genome-fragment	NA	0
S5_extracted_k141_35285	1353	No	NA	0	2	Low-quality	Genome-fragment	0.65	0
S14_vs_k141_29805||full	1440	No	NA	0	0	Not-determined	Genome-fragment	NA	0
S5_vs_k141_78886||full	1353	No	NA	0	0	Low-quality	Genome-fragment	3.08	0
S10_extracted_7419	1353	No	NA	1	0	Low-quality	Genome-fragment	1.25	0
S10_vs_1307||full	1353	No	NA	0	0	Low-quality	Genome-fragment	2.49	0
S10_vs_23387||full	1353	No	NA	0	0	Low-quality	Genome-fragment	2.68	0
S14_vs_k141_29833||full	12367	No	NA	0	3	Not-determined	Genome-fragment	NA	0
S14_vs_k141_37199||full	1114	No	NA	0	0	Not-determined	Genome-fragment	NA	0
S12_vs_k141_122661||full	1353	No	NA	0	2	Low-quality	Genome-fragment	3.13	0
S8_extracted_k141_111370	1353	No	NA	1	0	Low-quality	Genome-fragment	2.74	0
S14_vs_k141_632||full	37447	No	NA	33	1	Medium-quality	Genome-fragment	83.64	0
S14_vs_k141_110290||full	33612	No	NA	22	1	High-quality	High-quality	99.36	0
S14_vs_k141_7771||full	1350	No	NA	0	0	Not-determined	Genome-fragment	NA	0
S17_vs_k141_54149||full	1354	No	NA	1	0	Low-quality	Genome-fragment	3.17	0
S14_vs_k141_95835||full	3057	No	NA	0	2	Not-determined	Genome-fragment	NA	0
S14_extracted_k141_92119	1354	No	NA	1	0	Low-quality	Genome-fragment	4.37	0
S16_extracted_163388	1354	No	NA	0	0	Low-quality	Genome-fragment	2.23	0
S14_vs_k141_88409||full	6371	No	NA	0	2	Not-determined	Genome-fragment	NA	0
S8_vs_k141_171538||full	1354	No	NA	3	0	Low-quality	Genome-fragment	2.43	0
S14_vs_k141_7808||full	1252	No	NA	0	1	Not-determined	Genome-fragment	NA	0
S17_extracted_k141_75034	1355	No	NA	0	1	Low-quality	Genome-fragment	0.88	0
S14_vs_k141_44383||full	15296	No	NA	0	5	Not-determined	Genome-fragment	NA	0
S17_extracted_k141_27867	1355	No	NA	0	0	Low-quality	Genome-fragment	4.19	0
S9_vs_k141_53017||full	1355	No	NA	0	0	Low-quality	Genome-fragment	3.38	0
S11_vs_k141_77053||full	1355	No	NA	1	0	Low-quality	Genome-fragment	3.99	0
S14_vs_k141_44424||full	1599	No	NA	0	2	Not-determined	Genome-fragment	NA	0
S14_extracted_k141_81858	1355	No	NA	0	0	Low-quality	Genome-fragment	3.01	0
S14_vs_k141_88484||full	2285	No	NA	0	1	Not-determined	Genome-fragment	NA	0
S14_extracted_k141_77655	1355	No	NA	4	0	Low-quality	Genome-fragment	4.47	0
S3_vs_k141_57587||full	1355	No	NA	0	0	Low-quality	Genome-fragment	3.84	0
S5_vs_k141_138674||lt2gene	1355	No	NA	1	0	Low-quality	Genome-fragment	2.35	0
S7_vs_70866||lt2gene	1355	No	NA	2	0	Low-quality	Genome-fragment	3.68	0
S16_vs_54005||full	1355	No	NA	0	0	Low-quality	Genome-fragment	1.87	0
S14_vs_k141_51883||full	2205	No	NA	0	0	Not-determined	Genome-fragment	NA	0
S12_extracted_k141_178625	1355	No	NA	0	0	Low-quality	Genome-fragment	3.29	0
S12_vs_k141_167253||full	1355	No	NA	0	0	Low-quality	Genome-fragment	2.67	0
S4_extracted_22209	1355	No	NA	1	0	Low-quality	Genome-fragment	2.98	0
S8_vs_k141_144063||full	1355	No	NA	0	0	Low-quality	Genome-fragment	0.75	0
S8_vs_k141_50777||full	1355	No	NA	0	0	Low-quality	Genome-fragment	1.93	0
S17_vs_k141_90330||full	1356	No	NA	0	0	Low-quality	Genome-fragment	3.02	0
S17_vs_k141_6827||full	1356	No	NA	0	0	Low-quality	Genome-fragment	2.21	0
S14_vs_k141_110424||full	2404	No	NA	0	1	Not-determined	Genome-fragment	NA	0
S11_extracted_k141_67110	1356	No	NA	0	0	Low-quality	Genome-fragment	2.14	0
S11_vs_k141_93905||full	1356	No	NA	0	0	Low-quality	Genome-fragment	2.26	0
S14_vs_k141_59292||full	1957	No	NA	0	3	Not-determined	Genome-fragment	NA	0
S11_vs_k141_97178||full	1356	No	NA	0	0	Low-quality	Genome-fragment	2.37	0
S14_vs_k141_66412||full	1301	No	NA	0	1	Not-determined	Genome-fragment	NA	0
S3_vs_k141_93773||full	1356	No	NA	1	0	Low-quality	Genome-fragment	3.1	0
S14_vs_k141_59300||full	1107	No	NA	0	0	Not-determined	Genome-fragment	NA	0
S16_vs_123110||lt2gene	1356	No	NA	1	0	Low-quality	Genome-fragment	3.8	0
S14_vs_k141_818||full	1657	No	NA	0	2	Not-determined	Genome-fragment	NA	0
S14_vs_k141_110493||full	3090	No	NA	0	0	Not-determined	Genome-fragment	NA	0
S10_extracted_5550	1356	No	NA	0	0	Low-quality	Genome-fragment	1.26	0
S14_vs_k141_88639||full	1549	No	NA	0	2	Not-determined	Genome-fragment	NA	0
S14_vs_k141_73784||full	2348	No	NA	0	2	Not-determined	Genome-fragment	NA	0
S14_vs_k141_110522||full	2727	No	NA	0	2	Not-determined	Genome-fragment	NA	0
S10_vs_24272||full	1356	No	NA	1	0	Low-quality	Genome-fragment	2.46	0
S14_vs_k141_30032||full	1717	No	NA	0	0	Not-determined	Genome-fragment	NA	0
S14_vs_k141_52058||full	1146	No	NA	0	1	Not-determined	Genome-fragment	NA	0
S14_vs_k141_15237||full	3935	No	NA	0	2	Not-determined	Genome-fragment	NA	0
S14_vs_k141_44531||full	18080	No	NA	0	12	Not-determined	Genome-fragment	NA	0
S12_extracted_k141_50389	1356	No	NA	0	2	Low-quality	Genome-fragment	0.88	0
S8_vs_k141_109433||full	1356	No	NA	0	0	Low-quality	Genome-fragment	3.65	0
S9_vs_k141_6602||full	1357	No	NA	1	0	Low-quality	Genome-fragment	0.98	0
S11_extracted_k141_3220	1357	No	NA	2	0	Low-quality	Genome-fragment	1.14	0
S14_vs_k141_8024||full	4821	No	NA	0	0	Not-determined	Genome-fragment	NA	0
S14_vs_k141_52133||full	2018	No	NA	0	1	Not-determined	Genome-fragment	NA	0
S11_vs_k141_28871||lt2gene	1357	No	NA	1	0	Low-quality	Genome-fragment	2.28	0
S14_vs_k141_88695||full	2964	No	NA	0	3	Not-determined	Genome-fragment	NA	0
S14_vs_k141_59347||full	36660	No	NA	15	1	High-quality	High-quality	95.27	0
S14_extracted_k141_22878	1357	No	NA	2	0	Low-quality	Genome-fragment	4.48	0
S14_vs_k141_89450||full	1357	No	NA	2	0	Low-quality	Genome-fragment	3.22	0
S14_vs_k141_73857||full	1511	No	NA	0	0	Not-determined	Genome-fragment	NA	0
S14_vs_k141_73862||full	1171	No	NA	0	0	Not-determined	Genome-fragment	NA	0
S14_vs_k141_37522||full	3825	No	NA	0	1	Not-determined	Genome-fragment	NA	0
S14_vs_k141_30108||full	28701	No	NA	9	1	Medium-quality	Genome-fragment	66.39	0
S1_extracted_k141_55607	1357	No	NA	1	0	Low-quality	Genome-fragment	3.43	0
S3_vs_k141_76999||lt2gene	1357	No	NA	1	0	Low-quality	Genome-fragment	3.41	0
S14_vs_k141_15354||full	2887	No	NA	0	0	Not-determined	Genome-fragment	NA	0
S14_vs_k141_52160||full	28351	No	NA	0	5	Not-determined	Genome-fragment	NA	0
S14_vs_k141_52177||full	6451	No	NA	0	2	Not-determined	Genome-fragment	NA	0
S16_vs_924||full	1357	No	NA	0	0	Low-quality	Genome-fragment	2.16	0
S12_vs_k141_4461||full	1357	No	NA	0	1	Low-quality	Genome-fragment	0.6	0
S12_vs_k141_172835||full	1357	No	NA	1	0	Low-quality	Genome-fragment	2	0
S15_extracted_57319	1357	No	NA	0	0	Low-quality	Genome-fragment	3.27	0
S8_extracted_k141_165629	1357	No	NA	1	0	Low-quality	Genome-fragment	2.55	0
S14_vs_k141_8104||full	1164	No	NA	0	1	Not-determined	Genome-fragment	NA	0
S14_vs_k141_110634||full	4559	No	NA	0	3	Not-determined	Genome-fragment	NA	0
S8_vs_k141_144151||full	1357	No	NA	0	2	Low-quality	Genome-fragment	0.72	0
S14_vs_k141_88767||full	1752	No	NA	0	2	Not-determined	Genome-fragment	NA	0
S14_vs_k141_30152||full	12244	No	NA	0	1	Not-determined	Genome-fragment	NA	0
S17_vs_k141_93825||full	1358	No	NA	0	0	Low-quality	Genome-fragment	2.98	0
S9_extracted_k141_87125	1358	No	NA	1	0	Low-quality	Genome-fragment	1.46	0
S11_vs_k141_11234||full	1358	No	NA	1	0	Low-quality	Genome-fragment	2.01	0
S14_vs_k141_103381||full	1246	No	NA	0	0	Not-determined	Genome-fragment	NA	0
S14_vs_k141_37607||full	14720	No	NA	0	5	Not-determined	Genome-fragment	NA	0
S3_extracted_k141_112240	1358	No	NA	0	1	Low-quality	Genome-fragment	2.14	0
S14_vs_k141_15427||full	15353	No	NA	0	0	Not-determined	Genome-fragment	NA	0
S5_vs_k141_122580||full	1358	No	NA	0	0	Low-quality	Genome-fragment	3.07	0
S14_vs_k141_44661||full	5538	No	NA	0	2	Not-determined	Genome-fragment	NA	0
S5_vs_k141_44964||full	1358	No	NA	2	0	Low-quality	Genome-fragment	1.89	0
S5_vs_k141_107597||full	1358	No	NA	0	2	Low-quality	Genome-fragment	1.84	0
S5_vs_k141_71837||lt2gene	1358	No	NA	1	0	Low-quality	Genome-fragment	3.7	0
S14_vs_k141_15457||full	7828	No	NA	0	6	Not-determined	Genome-fragment	NA	0
S14_vs_k141_1001||full	2856	No	NA	0	1	Not-determined	Genome-fragment	NA	0
S16_vs_120819||lt2gene	1358	No	NA	1	0	Low-quality	Genome-fragment	2.67	0
S14_vs_k141_110714||full	3424	No	NA	0	0	Not-determined	Genome-fragment	NA	0
S12_vs_k141_40558||full	1358	No	NA	0	0	Low-quality	Genome-fragment	2.12	0
S14_vs_k141_103450||full	1959	No	NA	0	1	Not-determined	Genome-fragment	NA	0
S4_extracted_20267	1358	No	NA	0	0	Low-quality	Genome-fragment	3.31	0
S6_vs_k141_46420||full	1358	No	NA	0	0	Low-quality	Genome-fragment	2.12	0
S9_extracted_k141_60163	1359	No	NA	0	1	Low-quality	Genome-fragment	0.78	0
S13_vs_16671||full	1359	No	NA	1	0	Low-quality	Genome-fragment	3.5	0
S14_vs_k141_52314||full	2335	No	NA	0	2	Not-determined	Genome-fragment	NA	0
S14_vs_k141_1037||full	1969	No	NA	0	2	Not-determined	Genome-fragment	NA	0
S14_vs_k141_52317||full	1742	No	NA	0	3	Not-determined	Genome-fragment	NA	0
S14_extracted_k141_86741	1359	No	NA	2	0	Low-quality	Genome-fragment	3.1	0
S7_extracted_66914	1359	No	NA	0	3	Low-quality	Genome-fragment	0.36	0
S14_vs_k141_59556||full	1289	No	NA	0	0	Not-determined	Genome-fragment	NA	0
S7_vs_95006||full	1359	No	NA	1	0	Low-quality	Genome-fragment	3.36	0
S14_vs_k141_52352||full	3814	No	NA	0	3	Not-determined	Genome-fragment	NA	0
S12_vs_k141_17503||full	1359	No	NA	0	1	Low-quality	Genome-fragment	1.87	0
S14_vs_k141_15541||full	1642	No	NA	0	2	Not-determined	Genome-fragment	NA	0
S12_vs_k141_28176||full	1359	No	NA	0	0	Low-quality	Genome-fragment	3.75	0
S14_vs_k141_52360||full	1084	No	NA	0	0	Not-determined	Genome-fragment	NA	0
S14_vs_k141_44798||full	1522	No	NA	0	1	Not-determined	Genome-fragment	NA	0
S2_vs_k141_6123||full	1359	No	NA	0	0	Low-quality	Genome-fragment	2.2	0
S14_vs_k141_1071||full	3191	No	NA	0	1	Not-determined	Genome-fragment	NA	0
S14_vs_k141_37733||full	2075	No	NA	0	1	Not-determined	Genome-fragment	NA	0
S14_vs_k141_81430||full	1371	No	NA	0	0	Not-determined	Genome-fragment	NA	0
S14_vs_k141_103549||full	1247	No	NA	0	0	Not-determined	Genome-fragment	NA	0
S14_vs_k141_1083||full	4929	No	NA	0	2	Not-determined	Genome-fragment	NA	0
S14_vs_k141_66590||full	1266	No	NA	0	1	Not-determined	Genome-fragment	NA	0
S14_vs_k141_30336||full	20033	No	NA	0	10	Not-determined	Genome-fragment	NA	0
S8_extracted_k141_35610	1359	No	NA	0	2	Low-quality	Genome-fragment	0.41	0
S8_vs_k141_72768||full	1359	No	NA	1	0	Low-quality	Genome-fragment	3.1	0

S8_vs_k141_146296||full	1359	No	NA		0	0	Low-quality	Genome-fragment		1.45	0
S9_vs_k141_4798||lt2gene	1360	No	NA		0	0	Low-quality	Genome-fragment		1.55	0
S13_extracted_94146	1360	No	NA		0	0	Low-quality	Genome-fragment		7.36	0
S14_vs_k141_103625||full	1891	No	NA		0	2	Not-determined	Genome-fragment	NA		0
S13_vs_85916||full	1360	No	NA		0	0	Low-quality	Genome-fragment		0.75	0
S7_vs_92108||full	1360	No	NA		0	0	Low-quality	Genome-fragment		3.49	0
S7_vs_60639||lt2gene	1360	No	NA		1	0	Low-quality	Genome-fragment		1.25	0
S15_extracted_32737	1360	No	NA		1	0	Low-quality	Genome-fragment		1.29	0
S17_vs_k141_135125||lt2gene	1361	No	NA		1	0	Low-quality	Genome-fragment		2.87	0
S14_vs_k141_8212||full	1156	No	NA		0	1	Not-determined	Genome-fragment	NA		0
S14_vs_k141_52429||full	1836	No	NA		0	3	Not-determined	Genome-fragment	NA		0
S9_vs_k141_835||full	1361	No	NA		0	1	Low-quality	Genome-fragment		1.77	0
S14_vs_k141_103700||full	1403	No	NA		0	0	Not-determined	Genome-fragment	NA		0
S14_vs_k141_96111||full	29345	Yes		26132	19	5	Medium-quality	Genome-fragment		56.04	10.95
S14_vs_k141_103805||full	1361	No	NA		1	0	Low-quality	Genome-fragment		3.23	0
S1_extracted_k141_150655	1361	No	NA		2	0	Low-quality	Genome-fragment		3.03	0
S14_vs_k141_59667||full	2508	No	NA		0	0	Not-determined	Genome-fragment	NA		0
S1_vs_k141_40202||full	1361	No	NA		1	0	Low-quality	Genome-fragment		2.27	0
S5_extracted_k141_106398	1361	No	NA		0	0	Low-quality	Genome-fragment		0.89	0
S14_vs_k141_59695||full	3871	No	NA		0	3	Not-determined	Genome-fragment	NA		0
S7_extracted_77819	1361	No	NA		3	0	Low-quality	Genome-fragment		6.21	0
S14_vs_k141_89023||full	1475	No	NA		0	0	Not-determined	Genome-fragment	NA		0
S7_vs_8555||full	1361	No	NA		1	0	Low-quality	Genome-fragment		3.05	0
S16_extracted_172999	1361	No	NA		0	1	Low-quality	Genome-fragment		1.63	0
S10_extracted_69050	1361	No	NA		0	0	Low-quality	Genome-fragment		3.5	0
S14_vs_k141_74067||full	252822	Yes		59702	13	94	Medium-quality	Genome-fragment		51.55	76.39
S14_vs_k141_111017||full	2698	No	NA		0	2	Not-determined	Genome-fragment	NA		0
S14_vs_k141_1221||full	1249	No	NA		0	2	Not-determined	Genome-fragment	NA		0
S14_vs_k141_1231||full	1373	No	NA		0	0	Not-determined	Genome-fragment	NA		0
S10_extracted_105209	1361	No	NA		0	1	Low-quality	Genome-fragment		0.63	0
S12_extracted_k141_113589	1361	No	NA		0	1	Low-quality	Genome-fragment		0.47	0
S14_vs_k141_66662||full	1152	No	NA		0	0	Not-determined	Genome-fragment	NA		0
S14_vs_k141_37896||full	1013	No	NA		0	0	Not-determined	Genome-fragment	NA		0
S14_vs_k141_59756||full	2239	No	NA		0	0	Not-determined	Genome-fragment	NA		0
S8_extracted_k141_178962	1361	No	NA		0	0	Low-quality	Genome-fragment		2.62	0
S14_vs_k141_15757||full	2033	No	NA		0	1	Not-determined	Genome-fragment	NA		0
S8_extracted_k141_128463	1361	No	NA		1	0	Low-quality	Genome-fragment		3.8	0
S14_vs_k141_111050||full	1066	No	NA		0	0	Not-determined	Genome-fragment	NA		0
S17_extracted_k141_94173	1362	No	NA		0	1	Low-quality	Genome-fragment		0.16	0
S14_vs_k141_37935||full	4930	No	NA		0	2	Not-determined	Genome-fragment	NA		0
S11_extracted_k141_96207	1362	No	NA		4	0	Low-quality	Genome-fragment		3.66	0
S13_vs_36254||full	1362	No	NA		1	0	Low-quality	Genome-fragment		1.86	0
S14_extracted_k141_30313	1362	No	NA		2	0	Low-quality	Genome-fragment		2.27	0
S14_vs_k141_52461||full	4562	No	NA		0	3	Not-determined	Genome-fragment	NA		0
S14_vs_k141_56985||full	1362	No	NA		1	0	Low-quality	Genome-fragment		2.49	0
S1_vs_k141_6753||full	1362	No	NA		0	0	Low-quality	Genome-fragment		3.65	0
S14_vs_k141_30566||full	34961	No	NA		27	0	Medium-quality	Genome-fragment		87.84	0
S14_vs_k141_59802||full	4600	No	NA		0	1	Not-determined	Genome-fragment	NA		0
S14_vs_k141_15896||full	3422	No	NA		0	1	Not-determined	Genome-fragment	NA		0
S3_extracted_k141_14732	1362	No	NA		0	0	Low-quality	Genome-fragment		1.99	0
S5_extracted_k141_90290	1362	No	NA		0	1	Low-quality	Genome-fragment		2.11	0
S5_vs_k141_133407||full	1362	No	NA		0	5	Low-quality	Genome-fragment		0.4	0
S14_vs_k141_45054||full	3586	No	NA		0	2	Not-determined	Genome-fragment	NA		0
S14_vs_k141_8459||full	1120	No	NA		0	2	Not-determined	Genome-fragment	NA		0
S7_vs_128067||full	1362	No	NA		0	0	Low-quality	Genome-fragment		3.12	0
S14_vs_k141_45073||full	1523	No	NA		0	1	Not-determined	Genome-fragment	NA		0
S16_vs_32918||full	1362	No	NA		0	0	Low-quality	Genome-fragment		2.21	0
S14_vs_k141_89208||full	1752	No	NA		0	1	Not-determined	Genome-fragment	NA		0
S14_vs_k141_15965||full	1809	No	NA		0	0	Not-determined	Genome-fragment	NA		0
S14_vs_k141_66757||full	2330	No	NA		0	2	Not-determined	Genome-fragment	NA		0
S14_vs_k141_74328||full	1897	No	NA		0	0	Not-determined	Genome-fragment	NA		0
S14_vs_k141_66768||full	5433	No	NA		0	4	Not-determined	Genome-fragment	NA		0
S10_vs_39248||lt2gene	1362	No	NA		1	0	Low-quality	Genome-fragment		3.61	0
S12_extracted_k141_86511	1362	No	NA		0	0	Low-quality	Genome-fragment		0.89	0
S6_extracted_k141_5407	1362	No	NA		1	0	Low-quality	Genome-fragment		4.61	0
S9_extracted_k141_76465	1363	No	NA		4	0	Low-quality	Genome-fragment		3.92	0
S14_vs_k141_96194||full	1472	No	NA		0	1	Not-determined	Genome-fragment	NA		0
S14_vs_k141_89271||full	1485	No	NA		0	2	Not-determined	Genome-fragment	NA		0
S9_extracted_k141_51293	1363	No	NA		1	0	Low-quality	Genome-fragment		3.58	0
S1_vs_k141_60681||full	1363	No	NA		0	0	Low-quality	Genome-fragment		4.02	0
S3_vs_k141_74111||full	1363	No	NA		1	0	Low-quality	Genome-fragment		1.73	0
S14_vs_k141_38083||full	1370	No	NA		0	0	Not-determined	Genome-fragment	NA		0
S14_vs_k141_23115||full	3392	No	NA		0	2	Not-determined	Genome-fragment	NA		0
S3_vs_k141_13350||full	1363	No	NA		2	0	Low-quality	Genome-fragment		3.6	0
S16_vs_125137||full	1363	No	NA		0	0	Low-quality	Genome-fragment		2.58	0
S10_vs_109406||full	1363	No	NA		0	0	Low-quality	Genome-fragment		1.52	0
S8_extracted_k141_104269	1363	No	NA		0	0	Low-quality	Genome-fragment		1.37	0
S9_vs_k141_8467||lt2gene	1364	No	NA		2	0	Low-quality	Genome-fragment		2.95	0
S13_vs_153832||full	1364	No	NA		0	1	Low-quality	Genome-fragment		0.97	0
S14_vs_k141_89302||full	1020	No	NA		0	0	Not-determined	Genome-fragment	NA		0
S14_vs_k141_73517||lt2gene	1364	No	NA		1	0	Low-quality	Genome-fragment		3.05	0
S1_extracted_k141_116595	1364	No	NA		2	0	Low-quality	Genome-fragment		3.42	0
S14_vs_k141_104053||full	3454	No	NA		0	2	Not-determined	Genome-fragment	NA		0
S14_vs_k141_66841||full	19282	No	NA		13	0	Medium-quality	Genome-fragment		55	0
S14_vs_k141_23188||full	5216	No	NA		0	1	Not-determined	Genome-fragment	NA		0
S14_vs_k141_38162||full	1044	No	NA		0	0	Not-determined	Genome-fragment	NA		0
S14_vs_k141_23208||full	1302	No	NA		0	1	Not-determined	Genome-fragment	NA		0
S1_vs_k141_115769||full	1364	No	NA		0	1	Low-quality	Genome-fragment		2.08	0
S14_vs_k141_96317||full	2505	No	NA		0	0	Not-determined	Genome-fragment	NA		0
S14_vs_k141_38182||full	3429	No	NA		0	2	Not-determined	Genome-fragment	NA		0
S14_vs_k141_111399||full	3680	No	NA		0	0	Not-determined	Genome-fragment	NA		0
S3_extracted_k141_94417	1364	No	NA		0	1	Low-quality	Genome-fragment		2.26	0
S5_vs_k141_87119||full	1364	No	NA		0	1	Low-quality	Genome-fragment		0.87	0
S14_vs_k141_23227||full	1100	No	NA		0	0	Not-determined	Genome-fragment	NA		0
S16_vs_68659||full	1364	No	NA		0	0	Low-quality	Genome-fragment		2.39	0
S12_vs_k141_82128||full	1364	No	NA		0	0	Low-quality	Genome-fragment		3.63	0
S14_vs_k141_60036||full	14901	No	NA		0	10	Not-determined	Genome-fragment	NA		0
S4_extracted_13994	1364	No	NA		1	0	Low-quality	Genome-fragment		5.02	0
S17_extracted_k141_167426	1365	No	NA		0	1	Low-quality	Genome-fragment		0.94	0
S9_vs_k141_58080||full	1365	No	NA		0	0	Low-quality	Genome-fragment		0.79	0
S11_vs_k141_63115||lt2gene	1365	No	NA		1	0	Low-quality	Genome-fragment		3.18	0
S14_vs_k141_66951||full	1516	No	NA		0	0	Not-determined	Genome-fragment	NA		0
S14_vs_k141_52679||full	1075	No	NA		0	1	Not-determined	Genome-fragment	NA		0
S14_extracted_k141_30507	1365	No	NA		2	0	Low-quality	Genome-fragment		3.59	0
S14_vs_k141_60066||full	1870	No	NA		0	1	Not-determined	Genome-fragment	NA		0
S14_vs_k141_96358||full	2894	No	NA		0	3	Not-determined	Genome-fragment	NA		0
S14_extracted_k141_18318	1365	No	NA		3	0	Low-quality	Genome-fragment		4.51	0
S14_vs_k141_96364||full	2604	No	NA		0	2	Not-determined	Genome-fragment	NA		0
S3_extracted_k141_6734	1365	No	NA		1	0	Low-quality	Genome-fragment		2.21	0
S7_extracted_80286	1365	No	NA		1	0	Low-quality	Genome-fragment		0.79	0
S7_vs_5751||lt2gene	1365	No	NA		2	0	Low-quality	Genome-fragment		3.22	0
S16_vs_66753||full	1365	No	NA		1	0	Low-quality	Genome-fragment		3.82	0
S10_extracted_2684	1365	No	NA		3	0	Low-quality	Genome-fragment		4.82	0
S14_vs_k141_8783||full	1326	No	NA		0	2	Not-determined	Genome-fragment	NA		0
S14_vs_k141_52800||full	1674	No	NA		0	1	Not-determined	Genome-fragment	NA		0
S12_extracted_k141_148408	1365	No	NA		2	0	Low-quality	Genome-fragment		4.21	0
S14_vs_k141_16241||full	6920	No	NA		0	4	Not-determined	Genome-fragment	NA		0
S3_vs_k141_8908||full	1366	No	NA		2	0	Low-quality	Genome-fragment		3.61	0
S5_extracted_k141_140681	1366	No	NA		1	0	Low-quality	Genome-fragment		0.92	0
S14_vs_k141_52814||full	2134	No	NA		0	2	Not-determined	Genome-fragment	NA		0
S14_vs_k141_30852||full	1841	No	NA		0	0	Not-determined	Genome-fragment	NA		0
S7_extracted_97012	1366	No	NA		0	0	Low-quality	Genome-fragment		6.23	0
S16_vs_10205||full	1366	No	NA		1	0	Low-quality	Genome-fragment		3.57	0
S16_vs_150937||full	1366	No	NA		0	2	Low-quality	Genome-fragment		0.5	0
S14_vs_k141_8818||full	2981	No	NA		0	1	Not-determined	Genome-fragment	NA		0
S10_vs_98692||full	1366	No	NA		1	0	Low-quality	Genome-fragment		2.94	0
S12_vs_k141_157172||full	1366	No	NA		1	0	Low-quality	Genome-fragment		10.28	0
S8_extracted_k141_186486	1366	No	NA		2	0	Low-quality	Genome-fragment		3.71	0
S17_vs_k141_15652||full	1367	No	NA		0	0	Low-quality	Genome-fragment		3.27	0
S14_vs_k141_74599||full	1384	No	NA		0	0	Not-determined	Genome-fragment	NA		0
S14_vs_k141_1419||full	2561	No	NA		0	0	Not-determined	Genome-fragment	NA		0
S14_vs_k141_30904||full	4240	No	NA		0	2	Not-determined	Genome-fragment	NA		0
S17_vs_k141_97077||full	1367	No	NA		0	0	Low-quality	Genome-fragment		2.94	0
S14_vs_k141_111617||full	1770	No	NA		0	1	Not-determined	Genome-fragment	NA		0
S14_vs_k141_104343||full	2671	No	NA		0	1	Not-determined	Genome-fragment	NA		0
S11_vs_k141_80040||full	1367	No	NA		2	0	Low-quality	Genome-fragment		2.69	0
S13_extracted_44086	1367	No	NA		0	0	Low-quality	Genome-fragment		3.06	0
S14_vs_k141_96492||full	1910	No	NA		0	3	Not-determined	Genome-fragment	NA		0
S14_vs_k141_38328||full	1982	No	NA		0	0	Not-determined	Genome-fragment	NA		0
S14_extracted_k141_106805	1367	No	NA		0	1	Low-quality	Genome-fragment		0.38	0
S14_vs_k141_1441||full	6365	No	NA		0	3	Not-determined	Genome-fragment	NA		0
S14_vs_k141_61906||full	1367	No	NA		0	0	Low-quality	Genome-fragment		2.13	0
S14_vs_k141_23457||full	3017	No	NA		0	2	Not-determined	Genome-fragment	NA		0
S1_vs_k141_138013||full	1367	No	NA		0	1	Low-quality	Genome-fragment		1.86	0
S14_vs_k141_104402||full	1784	No	NA		0	2	Not-determined	Genome-fragment	NA		0
S1_vs_k141_38942||full	1367	No	NA		1	0	Low-quality	Genome-fragment		2.83	0
S14_vs_k141_96557||full	2897	No	NA		0	1	Not-determined	Genome-fragment	NA		0
S14_vs_k141_52947||full	2733	No	NA		0	1	Not-determined	Genome-fragment	NA		0
S5_vs_k141_135898||full	1367	No	NA		0	0	Low-quality	Genome-fragment		2.9	0
S5_vs_k141_28040||full	1367	No	NA		0	0	Low-quality	Genome-fragment		3.17	0
S14_vs_k141_74643||full	9713	No	NA		0	5	Not-determined	Genome-fragment	NA		0
S14_vs_k141_82148||full	2850	No	NA		0	2	Not-determined	Genome-fragment	NA		0
S7_extracted_3752	1367	No	NA		2	0	Low-quality	Genome-fragment		4.5	0
S7_vs_34735||full	1367	No	NA		0	0	Low-quality	Genome-fragment		3.03	0
S14_vs_k141_74671||full	1453	No	NA		0	0	Not-determined	Genome-fragment	NA		0
S7_vs_39428||lt2gene	1367	No	NA		2	0	Low-quality	Genome-fragment		3.59	0
S16_extracted_10036	1367	No	NA		0	0	Low-quality	Genome-fragment		2.4	0
S14_vs_k141_111766||full	2192	No	NA		0	0	Not-determined	Genome-fragment	NA		0
S16_vs_107837||full	1367	No	NA		1	0	Low-quality	Genome-fragment		2.19	0
S16_vs_128627||full	1367	No	NA		2	0	Low-quality	Genome-fragment		2.2	0
S17_extracted_k141_56342	1368	No	NA		0	0	Low-quality	Genome-fragment		2.28	0
S14_vs_k141_8929||full	1124	No	NA		0	1	Not-determined	Genome-fragment	NA		0
S17_vs_k141_134548||full	1368	No	NA		1	0	Low-quality	Genome-fragment		2.91	0
S9_extracted_k141_89500	1368	No	NA		0	4	Low-quality	Genome-fragment		0.39	0
S14_vs_k141_82187||full	2753	No	NA		0	4	Not-determined	Genome-fragment	NA		0
S9_vs_k141_26568||full	1368	No	NA		0	0	Low-quality	Genome-fragment		2.65	0
S9_vs_k141_4541||full	1368	No	NA		2	0	Low-quality	Genome-fragment		2.99	0
S14_vs_k141_82211||full	3090	No	NA		0	1	Not-determined	Genome-fragment	NA		0
S14_vs_k141_23497||full	3535	No	NA		0	0	Not-determined	Genome-fragment	NA		0
S14_vs_k141_96674||full	5446	No	NA		0	2	Not-determined	Genome-fragment	NA		0
S11_vs_k141_37237||full	1368	No	NA		2	0	Low-quality	Genome-fragment		3.36	0
S13_extracted_109353	1368	No	NA		0	2	Low-quality	Genome-fragment		0.24	0
S14_vs_k141_89620||full	5152	No	NA		0	0	Not-determined	Genome-fragment	NA		0
S14_vs_k141_67200||full	1097	No	NA		0	0	Not-determined	Genome-fragment	NA		0

<table>
<tr><td>S14_vs_k141_89631||full</td><td>2205</td><td>No</td><td>NA</td><td></td><td>0</td><td>3</td><td>Not-determined</td><td>Genome-fragment</td><td>NA</td><td></td><td>0</td></tr>
<tr><td>S14_vs_k141_38480||full</td><td>8999</td><td>No</td><td>NA</td><td></td><td>0</td><td>5</td><td>Not-determined</td><td>Genome-fragment</td><td>NA</td><td></td><td>0</td></tr>
<tr><td>S13_vs_6013||full</td><td>1368</td><td>No</td><td>NA</td><td></td><td>1</td><td>0</td><td>Low-quality</td><td>Genome-fragment</td><td></td><td>1.59</td><td>0</td></tr>
<tr><td>S14_vs_k141_49820||full</td><td>1368</td><td>No</td><td>NA</td><td></td><td>0</td><td>0</td><td>Low-quality</td><td>Genome-fragment</td><td></td><td>0.7</td><td>0</td></tr>
<tr><td>S14_vs_k141_16523||full</td><td>1420</td><td>No</td><td>NA</td><td></td><td>0</td><td>1</td><td>Not-determined</td><td>Genome-fragment</td><td>NA</td><td></td><td>0</td></tr>
<tr><td>S1_extracted_k141_145077</td><td>1368</td><td>No</td><td>NA</td><td></td><td>1</td><td>0</td><td>Low-quality</td><td>Genome-fragment</td><td></td><td>1.38</td><td>0</td></tr>
<tr><td>S5_vs_k141_54348||full</td><td>1368</td><td>No</td><td>NA</td><td></td><td>0</td><td>0</td><td>Low-quality</td><td>Genome-fragment</td><td></td><td>3.56</td><td>0</td></tr>
<tr><td>S5_vs_k141_11145||full</td><td>1368</td><td>No</td><td>NA</td><td></td><td>1</td><td>0</td><td>Low-quality</td><td>Genome-fragment</td><td></td><td>1.53</td><td>0</td></tr>
<tr><td>S5_vs_k141_77176||full</td><td>1368</td><td>No</td><td>NA</td><td></td><td>1</td><td>0</td><td>Low-quality</td><td>Genome-fragment</td><td></td><td>3.2</td><td>0</td></tr>
<tr><td>S14_vs_k141_53034||full</td><td>3242</td><td>No</td><td>NA</td><td></td><td>0</td><td>2</td><td>Not-determined</td><td>Genome-fragment</td><td>NA</td><td></td><td>0</td></tr>
<tr><td>S16_vs_1124||full</td><td>1368</td><td>No</td><td>NA</td><td></td><td>0</td><td>0</td><td>Low-quality</td><td>Genome-fragment</td><td></td><td>3.76</td><td>0</td></tr>
<tr><td>S16_vs_43334||full</td><td>1368</td><td>No</td><td>NA</td><td></td><td>0</td><td>2</td><td>Low-quality</td><td>Genome-fragment</td><td></td><td>2.18</td><td>0</td></tr>
<tr><td>S14_vs_k141_111927||full</td><td>1361</td><td>No</td><td>NA</td><td></td><td>0</td><td>0</td><td>Not-determined</td><td>Genome-fragment</td><td>NA</td><td></td><td>0</td></tr>
<tr><td>S14_vs_k141_16562||full</td><td>5592</td><td>No</td><td>NA</td><td></td><td>0</td><td>1</td><td>Not-determined</td><td>Genome-fragment</td><td>NA</td><td></td><td>0</td></tr>
<tr><td>S14_vs_k141_67324||full</td><td>1290</td><td>No</td><td>NA</td><td></td><td>0</td><td>1</td><td>Not-determined</td><td>Genome-fragment</td><td>NA</td><td></td><td>0</td></tr>
<tr><td>S14_vs_k141_31134||full</td><td>1625</td><td>No</td><td>NA</td><td></td><td>0</td><td>1</td><td>Not-determined</td><td>Genome-fragment</td><td>NA</td><td></td><td>0</td></tr>
<tr><td>S10_extracted_48765</td><td>1368</td><td>No</td><td>NA</td><td></td><td>1</td><td>0</td><td>Low-quality</td><td>Genome-fragment</td><td></td><td>2.98</td><td>0</td></tr>
<tr><td>S14_vs_k141_74925||full</td><td>4250</td><td>No</td><td>NA</td><td></td><td>0</td><td>1</td><td>Not-determined</td><td>Genome-fragment</td><td>NA</td><td></td><td>0</td></tr>
<tr><td>S14_vs_k141_31145||full</td><td>2043</td><td>No</td><td>NA</td><td></td><td>0</td><td>1</td><td>Not-determined</td><td>Genome-fragment</td><td>NA</td><td></td><td>0</td></tr>
<tr><td>S14_vs_k141_60507||full</td><td>1221</td><td>No</td><td>NA</td><td></td><td>0</td><td>2</td><td>Not-determined</td><td>Genome-fragment</td><td>NA</td><td></td><td>0</td></tr>
<tr><td>S10_vs_23883||full</td><td>1368</td><td>No</td><td>NA</td><td></td><td>0</td><td>1</td><td>Low-quality</td><td>Genome-fragment</td><td></td><td>0.71</td><td>0</td></tr>
<tr><td>S14_vs_k141_53096||full</td><td>1354</td><td>No</td><td>NA</td><td></td><td>0</td><td>0</td><td>Not-determined</td><td>Genome-fragment</td><td>NA</td><td></td><td>0</td></tr>
<tr><td>S14_vs_k141_82321||full</td><td>1187</td><td>No</td><td>NA</td><td></td><td>0</td><td>1</td><td>Not-determined</td><td>Genome-fragment</td><td>NA</td><td></td><td>0</td></tr>
<tr><td>S14_vs_k141_38581||full</td><td>4784</td><td>No</td><td>NA</td><td></td><td>0</td><td>0</td><td>Not-determined</td><td>Genome-fragment</td><td>NA</td><td></td><td>0</td></tr>
<tr><td>S14_vs_k141_67402||full</td><td>3438</td><td>No</td><td>NA</td><td></td><td>0</td><td>1</td><td>Not-determined</td><td>Genome-fragment</td><td>NA</td><td></td><td>0</td></tr>
<tr><td>S8_extracted_k141_53806</td><td>1368</td><td>No</td><td>NA</td><td></td><td>0</td><td>1</td><td>Low-quality</td><td>Genome-fragment</td><td></td><td>2.5</td><td>0</td></tr>
<tr><td>S11_extracted_k141_98114</td><td>1369</td><td>No</td><td>NA</td><td></td><td>0</td><td>1</td><td>Low-quality</td><td>Genome-fragment</td><td></td><td>3.56</td><td>0</td></tr>
<tr><td>S14_vs_k141_38607||full</td><td>1196</td><td>No</td><td>NA</td><td></td><td>0</td><td>1</td><td>Not-determined</td><td>Genome-fragment</td><td>NA</td><td></td><td>0</td></tr>
<tr><td>S14_vs_k141_46088||full</td><td>1369</td><td>No</td><td>NA</td><td></td><td>0</td><td>0</td><td>Low-quality</td><td>Genome-fragment</td><td></td><td>2.23</td><td>0</td></tr>
<tr><td>S14_vs_k141_31199||full</td><td>8760</td><td>No</td><td>NA</td><td></td><td>0</td><td>6</td><td>Not-determined</td><td>Genome-fragment</td><td>NA</td><td></td><td>0</td></tr>
<tr><td>S14_vs_k141_89794||full</td><td>1059</td><td>No</td><td>NA</td><td></td><td>0</td><td>0</td><td>Not-determined</td><td>Genome-fragment</td><td>NA</td><td></td><td>0</td></tr>
<tr><td>S14_vs_k141_5845||lt2gene</td><td>1369</td><td>No</td><td>NA</td><td></td><td>2</td><td>0</td><td>Low-quality</td><td>Genome-fragment</td><td></td><td>3.31</td><td>0</td></tr>
<tr><td>S14_vs_k141_75004||full</td><td>1291</td><td>No</td><td>NA</td><td></td><td>0</td><td>1</td><td>Not-determined</td><td>Genome-fragment</td><td>NA</td><td></td><td>0</td></tr>
<tr><td>S14_vs_k141_67439||full</td><td>1640</td><td>No</td><td>NA</td><td></td><td>0</td><td>0</td><td>Not-determined</td><td>Genome-fragment</td><td>NA</td><td></td><td>0</td></tr>
<tr><td>S3_vs_k141_72110||full</td><td>1369</td><td>No</td><td>NA</td><td></td><td>0</td><td>1</td><td>Low-quality</td><td>Genome-fragment</td><td></td><td>1.39</td><td>0</td></tr>
<tr><td>S14_vs_k141_104703||full</td><td>1010</td><td>No</td><td>NA</td><td></td><td>0</td><td>1</td><td>Not-determined</td><td>Genome-fragment</td><td>NA</td><td></td><td>0</td></tr>
<tr><td>S3_vs_k141_37197||full</td><td>1369</td><td>No</td><td>NA</td><td></td><td>0</td><td>1</td><td>Low-quality</td><td>Genome-fragment</td><td></td><td>1.86</td><td>0</td></tr>
<tr><td>S7_vs_86461||full</td><td>1369</td><td>No</td><td>NA</td><td></td><td>0</td><td>1</td><td>Low-quality</td><td>Genome-fragment</td><td></td><td>0.63</td><td>0</td></tr>
<tr><td>S14_vs_k141_31221||full</td><td>1946</td><td>No</td><td>NA</td><td></td><td>0</td><td>1</td><td>Not-determined</td><td>Genome-fragment</td><td>NA</td><td></td><td>0</td></tr>
<tr><td>S16_extracted_38579</td><td>1369</td><td>No</td><td>NA</td><td></td><td>0</td><td>2</td><td>Low-quality</td><td>Genome-fragment</td><td></td><td>1.08</td><td>0</td></tr>
<tr><td>S14_vs_k141_75014||full</td><td>20775</td><td>No</td><td>NA</td><td></td><td>0</td><td>10</td><td>Not-determined</td><td>Genome-fragment</td><td>NA</td><td></td><td>0</td></tr>
<tr><td>S16_vs_95076||full</td><td>1369</td><td>No</td><td>NA</td><td></td><td>0</td><td>0</td><td>Low-quality</td><td>Genome-fragment</td><td></td><td>3.19</td><td>0</td></tr>
<tr><td>S14_vs_k141_82401||full</td><td>1330</td><td>No</td><td>NA</td><td></td><td>0</td><td>2</td><td>Not-determined</td><td>Genome-fragment</td><td>NA</td><td></td><td>0</td></tr>
<tr><td>S14_vs_k141_31231||full</td><td>2687</td><td>No</td><td>NA</td><td></td><td>0</td><td>3</td><td>Not-determined</td><td>Genome-fragment</td><td>NA</td><td></td><td>0</td></tr>
<tr><td>S10_vs_84953||full</td><td>1369</td><td>No</td><td>NA</td><td></td><td>0</td><td>0</td><td>Low-quality</td><td>Genome-fragment</td><td></td><td>1.88</td><td>0</td></tr>
<tr><td>S14_vs_k141_45387||full</td><td>2232</td><td>No</td><td>NA</td><td></td><td>0</td><td>3</td><td>Not-determined</td><td>Genome-fragment</td><td>NA</td><td></td><td>0</td></tr>
<tr><td>S14_vs_k141_16694||full</td><td>2897</td><td>No</td><td>NA</td><td></td><td>0</td><td>1</td><td>Not-determined</td><td>Genome-fragment</td><td>NA</td><td></td><td>0</td></tr>
<tr><td>S12_vs_k141_146677||lt2gene</td><td>1369</td><td>No</td><td>NA</td><td></td><td>1</td><td>0</td><td>Low-quality</td><td>Genome-fragment</td><td></td><td>1.7</td><td>0</td></tr>
<tr><td>S8_vs_k141_139715||full</td><td>1369</td><td>No</td><td>NA</td><td></td><td>1</td><td>0</td><td>Low-quality</td><td>Genome-fragment</td><td></td><td>1.64</td><td>0</td></tr>
<tr><td>S14_vs_k141_31314||full</td><td>1682</td><td>No</td><td>NA</td><td></td><td>0</td><td>0</td><td>Not-determined</td><td>Genome-fragment</td><td>NA</td><td></td><td>0</td></tr>
<tr><td>S14_vs_k141_23710||full</td><td>2952</td><td>No</td><td>NA</td><td></td><td>0</td><td>2</td><td>Not-determined</td><td>Genome-fragment</td><td>NA</td><td></td><td>0</td></tr>
<tr><td>S14_vs_k141_112068||full</td><td>44297</td><td>No</td><td>NA</td><td></td><td>16</td><td>0</td><td>High-quality</td><td>High-quality</td><td></td><td>99.28</td><td>0</td></tr>
<tr><td>S17_vs_k141_97177||full</td><td>1370</td><td>No</td><td>NA</td><td></td><td>1</td><td>0</td><td>Low-quality</td><td>Genome-fragment</td><td></td><td>2.23</td><td>0</td></tr>
<tr><td>S14_vs_k141_16759||full</td><td>1007</td><td>No</td><td>NA</td><td></td><td>0</td><td>0</td><td>Not-determined</td><td>Genome-fragment</td><td>NA</td><td></td><td>0</td></tr>
<tr><td>S14_vs_k141_16763||full</td><td>1605</td><td>No</td><td>NA</td><td></td><td>0</td><td>0</td><td>Not-determined</td><td>Genome-fragment</td><td>NA</td><td></td><td>0</td></tr>
<tr><td>S9_extracted_k141_60412</td><td>1370</td><td>No</td><td>NA</td><td></td><td>2</td><td>0</td><td>Low-quality</td><td>Genome-fragment</td><td></td><td>2.28</td><td>0</td></tr>
<tr><td>S14_vs_k141_53298||full</td><td>2001</td><td>No</td><td>NA</td><td></td><td>0</td><td>3</td><td>Not-determined</td><td>Genome-fragment</td><td>NA</td><td></td><td>0</td></tr>
<tr><td>S14_vs_k141_67541||full</td><td>3281</td><td>No</td><td>NA</td><td></td><td>0</td><td>1</td><td>Not-determined</td><td>Genome-fragment</td><td>NA</td><td></td><td>0</td></tr>
<tr><td>S9_vs_k141_774||full</td><td>1370</td><td>No</td><td>NA</td><td></td><td>0</td><td>0</td><td>Low-quality</td><td>Genome-fragment</td><td></td><td>2.44</td><td>0</td></tr>
<tr><td>S11_vs_k141_94531||full</td><td>1370</td><td>No</td><td>NA</td><td></td><td>0</td><td>1</td><td>Low-quality</td><td>Genome-fragment</td><td></td><td>1.98</td><td>0</td></tr>
<tr><td>S1_vs_k141_113503||full</td><td>1370</td><td>No</td><td>NA</td><td></td><td>1</td><td>0</td><td>Low-quality</td><td>Genome-fragment</td><td></td><td>1.84</td><td>0</td></tr>
<tr><td>S3_extracted_k141_45311</td><td>1370</td><td>No</td><td>NA</td><td></td><td>0</td><td>0</td><td>Low-quality</td><td>Genome-fragment</td><td></td><td>1.95</td><td>0</td></tr>
<tr><td>S5_vs_k141_1297||full</td><td>1370</td><td>No</td><td>NA</td><td></td><td>0</td><td>0</td><td>Low-quality</td><td>Genome-fragment</td><td></td><td>2.21</td><td>0</td></tr>
<tr><td>S14_vs_k141_104835||full</td><td>1006</td><td>No</td><td>NA</td><td></td><td>0</td><td>0</td><td>Not-determined</td><td>Genome-fragment</td><td>NA</td><td></td><td>0</td></tr>
<tr><td>S14_vs_k141_53311||full</td><td>2002</td><td>No</td><td>NA</td><td></td><td>0</td><td>3</td><td>Not-determined</td><td>Genome-fragment</td><td>NA</td><td></td><td>0</td></tr>
<tr><td>S14_vs_k141_104841||full</td><td>1965</td><td>No</td><td>NA</td><td></td><td>0</td><td>0</td><td>Not-determined</td><td>Genome-fragment</td><td>NA</td><td></td><td>0</td></tr>
<tr><td>S7_extracted_100935</td><td>1370</td><td>No</td><td>NA</td><td></td><td>0</td><td>0</td><td>Low-quality</td><td>Genome-fragment</td><td></td><td>2.22</td><td>0</td></tr>
<tr><td>S7_vs_88748||lt2gene</td><td>1370</td><td>No</td><td>NA</td><td></td><td>1</td><td>0</td><td>Low-quality</td><td>Genome-fragment</td><td></td><td>3.54</td><td>0</td></tr>
<tr><td>S16_vs_108365||full</td><td>1370</td><td>No</td><td>NA</td><td></td><td>0</td><td>0</td><td>Low-quality</td><td>Genome-fragment</td><td></td><td>3.33</td><td>0</td></tr>
<tr><td>S12_vs_k141_31787||full</td><td>1370</td><td>No</td><td>NA</td><td></td><td>1</td><td>0</td><td>Low-quality</td><td>Genome-fragment</td><td></td><td>0.86</td><td>0</td></tr>
<tr><td>S6_extracted_k141_52258</td><td>1370</td><td>No</td><td>NA</td><td></td><td>0</td><td>0</td><td>Low-quality</td><td>Genome-fragment</td><td></td><td>0.52</td><td>0</td></tr>
<tr><td>S14_vs_k141_67601||full</td><td>3822</td><td>No</td><td>NA</td><td></td><td>0</td><td>2</td><td>Not-determined</td><td>Genome-fragment</td><td>NA</td><td></td><td>0</td></tr>
<tr><td>S8_extracted_k141_122766</td><td>1370</td><td>No</td><td>NA</td><td></td><td>1</td><td>0</td><td>Low-quality</td><td>Genome-fragment</td><td></td><td>2</td><td>0</td></tr>
<tr><td>S8_extracted_k141_149318</td><td>1370</td><td>No</td><td>NA</td><td></td><td>0</td><td>0</td><td>Low-quality</td><td>Genome-fragment</td><td></td><td>0.87</td><td>0</td></tr>
<tr><td>S17_vs_k141_144567||full</td><td>1371</td><td>No</td><td>NA</td><td></td><td>1</td><td>0</td><td>Low-quality</td><td>Genome-fragment</td><td></td><td>2.96</td><td>0</td></tr>
<tr><td>S14_vs_k141_45534||full</td><td>2778</td><td>No</td><td>NA</td><td></td><td>0</td><td>1</td><td>Not-determined</td><td>Genome-fragment</td><td>NA</td><td></td><td>0</td></tr>
<tr><td>S14_vs_k141_38905||full</td><td>29314</td><td>No</td><td>NA</td><td></td><td>12</td><td>1</td><td>Medium-quality</td><td>Genome-fragment</td><td></td><td>72.3</td><td>0</td></tr>
<tr><td>S11_vs_k141_52317||full</td><td>1371</td><td>No</td><td>NA</td><td></td><td>0</td><td>0</td><td>Low-quality</td><td>Genome-fragment</td><td></td><td>3.61</td><td>0</td></tr>
<tr><td>S14_vs_k141_23805||full</td><td>1706</td><td>No</td><td>NA</td><td></td><td>0</td><td>2</td><td>Not-determined</td><td>Genome-fragment</td><td>NA</td><td></td><td>0</td></tr>
<tr><td>S14_vs_k141_53419||full</td><td>2993</td><td>No</td><td>NA</td><td></td><td>0</td><td>4</td><td>Not-determined</td><td>Genome-fragment</td><td>NA</td><td></td><td>0</td></tr>
<tr><td>S14_vs_k141_1828||full</td><td>1316</td><td>No</td><td>NA</td><td></td><td>0</td><td>0</td><td>Not-determined</td><td>Genome-fragment</td><td>NA</td><td></td><td>0</td></tr>
<tr><td>S14_extracted_k141_21403</td><td>1371</td><td>No</td><td>NA</td><td></td><td>2</td><td>0</td><td>Low-quality</td><td>Genome-fragment</td><td></td><td>4.54</td><td>0</td></tr>
<tr><td>S1_extracted_k141_102536</td><td>1371</td><td>No</td><td>NA</td><td></td><td>0</td><td>0</td><td>Low-quality</td><td>Genome-fragment</td><td></td><td>1.39</td><td>0</td></tr>
<tr><td>S5_vs_k141_97188||full</td><td>1371</td><td>No</td><td>NA</td><td></td><td>0</td><td>0</td><td>Low-quality</td><td>Genome-fragment</td><td></td><td>2.7</td><td>0</td></tr>
<tr><td>S7_extracted_67454</td><td>1371</td><td>No</td><td>NA</td><td></td><td>2</td><td>0</td><td>Low-quality</td><td>Genome-fragment</td><td></td><td>3.12</td><td>0</td></tr>
<tr><td>S14_vs_k141_67670||full</td><td>2697</td><td>No</td><td>NA</td><td></td><td>0</td><td>0</td><td>Not-determined</td><td>Genome-fragment</td><td>NA</td><td></td><td>0</td></tr>
<tr><td>S7_vs_12292||full</td><td>1371</td><td>No</td><td>NA</td><td></td><td>0</td><td>0</td><td>Low-quality</td><td>Genome-fragment</td><td></td><td>3.2</td><td>0</td></tr>
<tr><td>S14_vs_k141_45610||full</td><td>2650</td><td>No</td><td>NA</td><td></td><td>0</td><td>0</td><td>Not-determined</td><td>Genome-fragment</td><td>NA</td><td></td><td>0</td></tr>
<tr><td>S7_vs_32131||lt2gene</td><td>1371</td><td>No</td><td>NA</td><td></td><td>1</td><td>0</td><td>Low-quality</td><td>Genome-fragment</td><td></td><td>3.09</td><td>0</td></tr>
<tr><td>S12_extracted_k141_58716</td><td>1371</td><td>No</td><td>NA</td><td></td><td>0</td><td>0</td><td>Low-quality</td><td>Genome-fragment</td><td></td><td>2.2</td><td>0</td></tr>
<tr><td>S14_vs_k141_9162||full</td><td>102634</td><td>Yes</td><td></td><td>20846</td><td>16</td><td>57</td><td>Medium-quality</td><td>Genome-fragment</td><td></td><td>60.28</td><td>79.69</td></tr>
<tr><td>S14_vs_k141_67727||full</td><td>1070</td><td>No</td><td>NA</td><td></td><td>0</td><td>1</td><td>Not-determined</td><td>Genome-fragment</td><td>NA</td><td></td><td>0</td></tr>
<tr><td>S6_vs_k141_38247||full</td><td>1371</td><td>No</td><td>NA</td><td></td><td>0</td><td>0</td><td>Low-quality</td><td>Genome-fragment</td><td></td><td>2.26</td><td>0</td></tr>
<tr><td>S8_vs_k141_156209||full</td><td>1371</td><td>No</td><td>NA</td><td></td><td>0</td><td>2</td><td>Low-quality</td><td>Genome-fragment</td><td></td><td>2.75</td><td>0</td></tr>
<tr><td>S14_vs_k141_112176||full</td><td>1077</td><td>No</td><td>NA</td><td></td><td>0</td><td>1</td><td>Not-determined</td><td>Genome-fragment</td><td>NA</td><td></td><td>0</td></tr>
<tr><td>S14_vs_k141_53490||full</td><td>7848</td><td>No</td><td>NA</td><td></td><td>0</td><td>4</td><td>Not-determined</td><td>Genome-fragment</td><td>NA</td><td></td><td>0</td></tr>
<tr><td>S8_vs_k141_141534||full</td><td>1371</td><td>No</td><td>NA</td><td></td><td>0</td><td>0</td><td>Low-quality</td><td>Genome-fragment</td><td></td><td>3.09</td><td>0</td></tr>
<tr><td>S9_vs_k141_81303||full</td><td>1372</td><td>No</td><td>NA</td><td></td><td>2</td><td>0</td><td>Low-quality</td><td>Genome-fragment</td><td></td><td>3.36</td><td>0</td></tr>
<tr><td>S14_extracted_k141_116897</td><td>1372</td><td>No</td><td>NA</td><td></td><td>0</td><td>0</td><td>Low-quality</td><td>Genome-fragment</td><td></td><td>2.9</td><td>0</td></tr>
<tr><td>S14_vs_k141_11785||lt2gene</td><td>1372</td><td>No</td><td>NA</td><td></td><td>0</td><td>0</td><td>Low-quality</td><td>Genome-fragment</td><td></td><td>3.49</td><td>0</td></tr>
<tr><td>S1_vs_k141_85411||full</td><td>1372</td><td>No</td><td>NA</td><td></td><td>0</td><td>2</td><td>Low-quality</td><td>Genome-fragment</td><td></td><td>1.83</td><td>0</td></tr>
<tr><td>S5_vs_k141_124024||full</td><td>1372</td><td>No</td><td>NA</td><td></td><td>0</td><td>0</td><td>Low-quality</td><td>Genome-fragment</td><td></td><td>2.91</td><td>0</td></tr>
<tr><td>S5_vs_k141_11792||full</td><td>1372</td><td>No</td><td>NA</td><td></td><td>0</td><td>0</td><td>Low-quality</td><td>Genome-fragment</td><td></td><td>2.29</td><td>0</td></tr>
<tr><td>S14_vs_k141_96959||full</td><td>5171</td><td>No</td><td>NA</td><td></td><td>0</td><td>0</td><td>Not-determined</td><td>Genome-fragment</td><td>NA</td><td></td><td>0</td></tr>
<tr><td>S14_vs_k141_105116||full</td><td>2820</td><td>No</td><td>NA</td><td></td><td>0</td><td>2</td><td>Not-determined</td><td>Genome-fragment</td><td>NA</td><td></td><td>0</td></tr>
<tr><td>S14_vs_k141_53567||full</td><td>5575</td><td>No</td><td>NA</td><td></td><td>0</td><td>3</td><td>Not-determined</td><td>Genome-fragment</td><td>NA</td><td></td><td>0</td></tr>
<tr><td>S16_extracted_66212</td><td>1372</td><td>No</td><td>NA</td><td></td><td>1</td><td>0</td><td>Low-quality</td><td>Genome-fragment</td><td></td><td>2</td><td>0</td></tr>
<tr><td>S14_vs_k141_90123||full</td><td>6521</td><td>No</td><td>NA</td><td></td><td>0</td><td>1</td><td>Not-determined</td><td>Genome-fragment</td><td>NA</td><td></td><td>0</td></tr>
<tr><td>S14_vs_k141_9286||full</td><td>3885</td><td>No</td><td>NA</td><td></td><td>0</td><td>5</td><td>Not-determined</td><td>Genome-fragment</td><td>NA</td><td></td><td>0</td></tr>
<tr><td>S16_extracted_113444</td><td>1372</td><td>No</td><td>NA</td><td></td><td>0</td><td>0</td><td>Low-quality</td><td>Genome-fragment</td><td></td><td>2.6</td><td>0</td></tr>
<tr><td>S14_vs_k141_112252||full</td><td>22565</td><td>No</td><td>NA</td><td></td><td>11</td><td>0</td><td>Medium-quality</td><td>Genome-fragment</td><td></td><td>67.86</td><td>0</td></tr>
<tr><td>S16_extracted_153405</td><td>1372</td><td>No</td><td>NA</td><td></td><td>0</td><td>0</td><td>Low-quality</td><td>Genome-fragment</td><td></td><td>2.15</td><td>0</td></tr>
<tr><td>S16_vs_47675||full</td><td>1372</td><td>No</td><td>NA</td><td></td><td>0</td><td>0</td><td>Low-quality</td><td>Genome-fragment</td><td></td><td>2.13</td><td>0</td></tr>
<tr><td>S16_vs_55725||full</td><td>1372</td><td>No</td><td>NA</td><td></td><td>2</td><td>0</td><td>Low-quality</td><td>Genome-fragment</td><td></td><td>2.73</td><td>0</td></tr>
<tr><td>S16_vs_94098||full</td><td>1372</td><td>No</td><td>NA</td><td></td><td>0</td><td>0</td><td>Low-quality</td><td>Genome-fragment</td><td></td><td>1.95</td><td>0</td></tr>
<tr><td>S14_vs_k141_60862||full</td><td>1411</td><td>No</td><td>NA</td><td></td><td>0</td><td>2</td><td>Not-determined</td><td>Genome-fragment</td><td>NA</td><td></td><td>0</td></tr>
<tr><td>S14_vs_k141_31464||full</td><td>2955</td><td>No</td><td>NA</td><td></td><td>0</td><td>1</td><td>Not-determined</td><td>Genome-fragment</td><td>NA</td><td></td><td>0</td></tr>
<tr><td>S14_vs_k141_53611||full</td><td>3930</td><td>No</td><td>NA</td><td></td><td>0</td><td>1</td><td>Not-determined</td><td>Genome-fragment</td><td>NA</td><td></td><td>0</td></tr>
<tr><td>S10_extracted_57122</td><td>1372</td><td>No</td><td>NA</td><td></td><td>0</td><td>1</td><td>Low-quality</td><td>Genome-fragment</td><td></td><td>2.31</td><td>0</td></tr>
<tr><td>S14_vs_k141_75419||full</td><td>2364</td><td>No</td><td>NA</td><td></td><td>0</td><td>2</td><td>Not-determined</td><td>Genome-fragment</td><td>NA</td><td></td><td>0</td></tr>
<tr><td>S10_vs_41650||full</td><td>1372</td><td>No</td><td>NA</td><td></td><td>1</td><td>0</td><td>Low-quality</td><td>Genome-fragment</td><td></td><td>1.79</td><td>0</td></tr>
<tr><td>S10_vs_78305||lt2gene</td><td>1372</td><td>No</td><td>NA</td><td></td><td>1</td><td>0</td><td>Low-quality</td><td>Genome-fragment</td><td></td><td>2</td><td>0</td></tr>
<tr><td>S14_vs_k141_60904||full</td><td>1796</td><td>No</td><td>NA</td><td></td><td>0</td><td>0</td><td>Not-determined</td><td>Genome-fragment</td><td>NA</td><td></td><td>0</td></tr>
<tr><td>S15_vs_16308||full</td><td>1372</td><td>No</td><td>NA</td><td></td><td>0</td><td>0</td><td>Low-quality</td><td>Genome-fragment</td><td></td><td>2.49</td><td>0</td></tr>
<tr><td>S6_extracted_k141_25186</td><td>1372</td><td>No</td><td>NA</td><td></td><td>0</td><td>0</td><td>Low-quality</td><td>Genome-fragment</td><td></td><td>0.48</td><td>0</td></tr>
<tr><td>S8_extracted_k141_7244</td><td>1372</td><td>No</td><td>NA</td><td></td><td>1</td><td>0</td><td>Low-quality</td><td>Genome-fragment</td><td></td><td>0.89</td><td>0</td></tr>
<tr><td>S14_vs_k141_75475||full</td><td>1739</td><td>No</td><td>NA</td><td></td><td>0</td><td>0</td><td>Not-determined</td><td>Genome-fragment</td><td>NA</td><td></td><td>0</td></tr>
<tr><td>S14_vs_k141_9413||full</td><td>2307</td><td>No</td><td>NA</td><td></td><td>0</td><td>1</td><td>Not-determined</td><td>Genome-fragment</td><td>NA</td><td></td><td>0</td></tr>
<tr><td>S14_vs_k141_53688||full</td><td>11920</td><td>No</td><td>NA</td><td></td><td>0</td><td>1</td><td>Not-determined</td><td>Genome-fragment</td><td>NA</td><td></td><td>0</td></tr>
<tr><td>S17_extracted_k141_109331</td><td>1373</td><td>No</td><td>NA</td><td></td><td>1</td><td>0</td><td>Low-quality</td><td>Genome-fragment</td><td></td><td>0.79</td><td>0</td></tr>
<tr><td>S17_vs_k141_109540||full</td><td>1373</td><td>No</td><td>NA</td><td></td><td>0</td><td>1</td><td>Low-quality</td><td>Genome-fragment</td><td></td><td>4.68</td><td>0</td></tr>
<tr><td>S9_extracted_k141_24236</td><td>1373</td><td>No</td><td>NA</td><td></td><td>2</td><td>0</td><td>Low-quality</td><td>Genome-fragment</td><td></td><td>3.3</td><td>0</td></tr>
<tr><td>S9_vs_k141_83412||full</td><td>1373</td><td>No</td><td>NA</td><td></td><td>0</td><td>0</td><td>Low-quality</td><td>Genome-fragment</td><td></td><td>2.82</td><td>0</td></tr>
<tr><td>S14_vs_k141_24123||full</td><td>1185</td><td>No</td><td>NA</td><td></td><td>0</td><td>0</td><td>Not-determined</td><td>Genome-fragment</td><td>NA</td><td></td><td>0</td></tr>
<tr><td>S14_vs_k141_53731||full</td><td>1606</td><td>No</td><td>NA</td><td></td><td>0</td><td>0</td><td>Not-determined</td><td>Genome-fragment</td><td>NA</td><td></td><td>0</td></tr>
<tr><td>S14_vs_k141_2016||full</td><td>9685</td><td>No</td><td>NA</td><td></td><td>0</td><td>5</td><td>Not-determined</td><td>Genome-fragment</td><td>NA</td><td></td><td>0</td></tr>
<tr><td>S11_extracted_k141_83742</td><td>1373</td><td>No</td><td>NA</td><td></td><td>0</td><td>0</td><td>Low-quality</td><td>Genome-fragment</td><td></td><td>3.45</td><td>0</td></tr>
<tr><td>S14_vs_k141_60930||full</td><td>2090</td><td>No</td><td>NA</td><td></td><td>0</td><td>1</td><td>Not-determined</td><td>Genome-fragment</td><td>NA</td><td></td><td>0</td></tr>
<tr><td>S13_extracted_141093</td><td>1373</td><td>No</td><td>NA</td><td></td><td>0</td><td>0</td><td>Low-quality</td><td>Genome-fragment</td><td></td><td>1.67</td><td>0</td></tr>
<tr><td>S14_vs_k141_32426||lt2gene</td><td>1373</td><td>No</td><td>NA</td><td></td><td>1</td><td>0</td><td>Low-quality</td><td>Genome-fragment</td><td></td><td>1.93</td><td>0</td></tr>
<tr><td>S14_vs_k141_2037||full</td><td>3989</td><td>No</td><td>NA</td><td></td><td>0</td><td>3</td><td>Not-determined</td><td>Genome-fragment</td><td>NA</td><td></td><td>0</td></tr>
<tr><td>S3_vs_k141_103988||full</td><td>1373</td><td>No</td><td>NA</td><td></td><td>1</td><td>0</td><td>Low-quality</td><td>Genome-fragment</td><td></td><td>0.92</td><td>0</td></tr>
<tr><td>S14_vs_k141_90264||full</td><td>3293</td><td>No</td><td>NA</td><td></td><td>0</td><td>1</td><td>Not-determined</td><td>Genome-fragment</td><td>NA</td><td></td><td>0</td></tr>
<tr><td>S3_vs_k141_98974||full</td><td>1373</td><td>No</td><td>NA</td><td></td><td>0</td><td>0</td><td>Low-quality</td><td>Genome-fragment</td><td></td><td>2.97</td><td>0</td></tr>
<tr><td>S14_vs_k141_2069||full</td><td>2524</td><td>No</td><td>NA</td><td></td><td>0</td><td>1</td><td>Not-determined</td><td>Genome-fragment</td><td>NA</td><td></td><td>0</td></tr>
<tr><td>S14_vs_k141_9453||full</td><td>6415</td><td>No</td><td>NA</td><td></td><td>0</td><td>4</td><td>Not-determined</td><td>Genome-fragment</td><td>NA</td><td></td><td>0</td></tr>
<tr><td>S3_vs_k141_116147||full</td><td>1373</td><td>No</td><td>NA</td><td></td><td>0</td><td>0</td><td>Low-quality</td><td>Genome-fragment</td><td></td><td>2.51</td><td>0</td></tr>
<tr><td>S14_vs_k141_24191||full</td><td>1410</td><td>No</td><td>NA</td><td></td><td>0</td><td>1</td><td>Not-determined</td><td>Genome-fragment</td><td>NA</td><td></td><td>0</td></tr>
<tr><td>S14_vs_k141_17049||full</td><td>4522</td><td>No</td><td>NA</td><td></td><td>0</td><td>3</td><td>Not-determined</td><td>Genome-fragment</td><td>NA</td><td></td><td>0</td></tr>
<tr><td>S14_vs_k141_112345||full</td><td>38263</td><td>No</td><td>NA</td><td></td><td>1</td><td>17</td><td>Medium-quality</td><td>Genome-fragment</td><td></td><td>71.14</td><td>0</td></tr>
<tr><td>S14_vs_k141_105287||full</td><td>1628</td><td>No</td><td>NA</td><td></td><td>0</td><td>1</td><td>Not-determined</td><td>Genome-fragment</td><td>NA</td><td></td><td>0</td></tr>
<tr><td>S14_vs_k141_39266||full</td><td>37426</td><td>Yes</td><td></td><td>26099</td><td>12</td><td>6</td><td>Medium-quality</td><td>Genome-fragment</td><td></td><td>62.52</td><td>30.27</td></tr>
<tr><td>S14_vs_k141_9464||full</td><td>2304</td><td>No</td><td>NA</td><td></td><td>0</td><td>2</td><td>Not-determined</td><td>Genome-fragment</td><td>NA</td><td></td><td>0</td></tr>
<tr><td>S14_vs_k141_2115||full</td><td>2479</td><td>No</td><td>NA</td><td></td><td>0</td><td>0</td><td>Not-determined</td><td>Genome-fragment</td><td>NA</td><td></td><td>0</td></tr>
<tr><td>S7_extracted_75915</td><td>1373</td><td>No</td><td>NA</td><td></td><td>3</td><td>0</td><td>Low-quality</td><td>Genome-fragment</td><td></td><td>3.03</td><td>0</td></tr>
<tr><td>S16_extracted_10843</td><td>1373</td><td>No</td><td>NA</td><td></td><td>0</td><td>0</td><td>Low-quality</td><td>Genome-fragment</td><td></td><td>4.21</td><td>0</td></tr>
<tr><td>S16_vs_54432||full</td><td>1373</td><td>No</td><td>NA</td><td></td><td>0</td><td>1</td><td>Low-quality</td><td>Genome-fragment</td><td></td><td>1.13</td><td>0</td></tr>
<tr><td>S14_vs_k141_31623||full</td><td>1488</td><td>No</td><td>NA</td><td></td><td>0</td><td>0</td><td>Not-determined</td><td>Genome-fragment</td><td>NA</td><td></td><td>0</td></tr>
<tr><td>S12_extracted_k141_158381</td><td>1373</td><td>No</td><td>NA</td><td></td><td>0</td><td>0</td><td>Low-quality</td><td>Genome-fragment</td><td></td><td>3.36</td><td>0</td></tr>
<tr><td>S14_vs_k141_97256||full</td><td>2134</td><td>No</td><td>NA</td><td></td><td>0</td><td>0</td><td>Not-determined</td><td>Genome-fragment</td><td>NA</td><td></td><td>0</td></tr>
<tr><td>S14_vs_k141_17121||full</td><td>2752</td><td>No</td><td>NA</td><td></td><td>0</td><td>0</td><td>Not-determined</td><td>Genome-fragment</td><td>NA</td><td></td><td>0</td></tr>
<tr><td>S6_extracted_k141_36604</td><td>1373</td><td>No</td><td>NA</td><td></td><td>0</td><td>1</td><td>Low-quality</td><td>Genome-fragment</td><td></td><td>0.45</td><td>0</td></tr>
<tr><td>S6_vs_k141_14364||full</td><td>1373</td><td>No</td><td>NA</td><td></td><td>1</td><td>0</td><td>Low-quality</td><td>Genome-fragment</td><td></td><td>0.85</td><td>0</td></tr>
<tr><td>S14_vs_k141_2191||full</td><td>1952</td><td>No</td><td>NA</td><td></td><td>0</td><td>0</td><td>Not-determined</td><td>Genome-fragment</td><td>NA</td><td></td><td>0</td></tr>
<tr><td>S8_vs_k141_105291||full</td><td>1373</td><td>No</td><td>NA</td><td></td><td>0</td><td>0</td><td>Low-quality</td><td>Genome-fragment</td><td></td><td>3.1</td><td>0</td></tr>
<tr><td>S14_vs_k141_75686||full</td><td>13410</td><td>No</td><td>NA</td><td></td><td>0</td><td>2</td><td>Not-determined</td><td>Genome-fragment</td><td>NA</td><td></td><td>0</td></tr>
<tr><td>S14_vs_k141_9574||full</td><td>2241</td><td>No</td><td>NA</td><td></td><td>0</td><td>0</td><td>Not-determined</td><td>Genome-fragment</td><td>NA</td><td></td><td>0</td></tr>
<tr><td>S17_extracted_k141_119139</td><td>1374</td><td>No</td><td>NA</td><td></td><td>3</td><td>0</td><td>Low-quality</td><td>Genome-fragment</td><td></td><td>2.3</td><td>0</td></tr>
<tr><td>S17_vs_k141_167981||full</td><td>1374</td><td>No</td><td>NA</td><td></td><td>0</td><td>0</td><td>Low-quality</td><td>Genome-fragment</td><td></td><td>1.63</td><td>0</td></tr>
<tr><td>S9_extracted_k141_65481</td><td>1374</td><td>No</td><td>NA</td><td></td><td>2</td><td>0</td><td>Low-quality</td><td>Genome-fragment</td><td></td><td>4.09</td><td>0</td></tr>
<tr><td>S9_vs_k141_79846||full</td><td>1374</td><td>No</td><td>NA</td><td></td><td>1</td><td>0</td><td>Low-quality</td><td>Genome-fragment</td><td></td><td>3.56</td><td>0</td></tr>
<tr><td>S9_vs_k141_81230||full</td><td>1374</td><td>No</td><td>NA</td><td></td><td>0</td><td>0</td><td>Low-quality</td><td>Genome-fragment</td><td></td><td>26.63</td><td>0</td></tr>
<tr><td>S14_vs_k141_83117||full</td><td>2419</td><td>No</td><td>NA</td><td></td><td>0</td><td>0</td><td>Not-determined</td><td>Genome-fragment</td><td>NA</td><td></td><td>0</td></tr>
<tr><td>S14_extracted_k141_45456</td><td>1374</td><td>No</td><td>NA</td><td></td><td>2</td><td>0</td><td>Low-quality</td><td>Genome-fragment</td><td></td><td>4.42</td><td>0</td></tr>
</table>

S14_vs_k141_97324||full	1882	No	NA		0	0	Not-determined	Genome-fragment	NA		0
S14_vs_k141_111011||full	1374	No	NA		0	0	Low-quality	Genome-fragment		3	0
S14_vs_k141_112522||full	2080	No	NA		0	1	Not-determined	Genome-fragment	NA		0
S14_vs_k141_61114||full	1126	No	NA		0	1	Not-determined	Genome-fragment	NA		0
S14_vs_k141_31725||full	4332	No	NA		0	2	Not-determined	Genome-fragment	NA		0
S14_vs_k141_97349||full	5142	No	NA		0	1	Not-determined	Genome-fragment	NA		0
S14_vs_k141_70246||full	1374	No	NA		1	0	Low-quality	Genome-fragment		2.75	0
S3_extracted_k141_73281	1374	No	NA		1	0	Low-quality	Genome-fragment		2.21	0
S14_vs_k141_97352||full	2232	No	NA		0	0	Not-determined	Genome-fragment	NA		0
S14_vs_k141_31732||full	1951	No	NA		0	2	Not-determined	Genome-fragment	NA		0
S16_vs_170074||full	1374	No	NA		1	0	Low-quality	Genome-fragment		0.8	0
S10_vs_24438||lt2gene	1374	No	NA		1	0	Low-quality	Genome-fragment		2.6	0
S14_vs_k141_75808||full	5698	No	NA		0	3	Not-determined	Genome-fragment	NA		0
S2_extracted_k141_11527	1374	No	NA		1	0	Low-quality	Genome-fragment		4.53	0
S14_vs_k141_97371||full	2114	No	NA		0	2	Not-determined	Genome-fragment	NA		0
S8_vs_k141_176661||full	1374	No	NA		0	0	Low-quality	Genome-fragment		3.03	0
S14_vs_k141_53854||full	9579	No	NA		0	6	Not-determined	Genome-fragment	NA		0
S14_vs_k141_53857||full	2562	No	NA		0	3	Not-determined	Genome-fragment	NA		0
S14_vs_k141_31768||full	1004	No	NA		0	0	Not-determined	Genome-fragment	NA		0
S1_vs_k141_32753||full	1375	No	NA		0	1	Low-quality	Genome-fragment		0.86	0
S1_vs_k141_5446||full	1375	No	NA		0	1	Low-quality	Genome-fragment		1.35	0
S14_vs_k141_17287||full	4161	No	NA		0	3	Not-determined	Genome-fragment	NA		0
S3_vs_k141_97863||full	1375	No	NA		2	0	Low-quality	Genome-fragment		2.78	0
S5_vs_k141_72878||full	1375	No	NA		0	0	Low-quality	Genome-fragment		1.17	0
S5_vs_k141_109895||full	1375	No	NA		2	0	Low-quality	Genome-fragment		2.87	0
S5_vs_k141_81338||lt2gene	1375	No	NA		1	0	Low-quality	Genome-fragment		2.76	0
S16_vs_43957||full	1375	No	NA		0	0	Low-quality	Genome-fragment		2.87	0
S14_vs_k141_31811||full	5106	No	NA		0	1	Not-determined	Genome-fragment	NA		0
S16_vs_110218||full	1375	No	NA		3	0	Low-quality	Genome-fragment		2.24	0
S14_vs_k141_2294||full	1221	No	NA		0	0	Not-determined	Genome-fragment	NA		0
S10_vs_54538||full	1375	No	NA		0	1	Low-quality	Genome-fragment		2.33	0
S10_vs_114138||lt2gene	1375	No	NA		1	0	Low-quality	Genome-fragment		1.81	0
S14_vs_k141_97429||full	3446	No	NA		0	1	Not-determined	Genome-fragment	NA		0
S14_vs_k141_9702||full	3884	No	NA		0	0	Not-determined	Genome-fragment	NA		0
S14_vs_k141_9704||full	1377	No	NA		0	0	Not-determined	Genome-fragment	NA		0
S14_vs_k141_105536||full	1101	No	NA		0	1	Not-determined	Genome-fragment	NA		0
S14_vs_k141_68318||full	2322	No	NA		0	1	Not-determined	Genome-fragment	NA		0
S14_vs_k141_105549||full	1169	No	NA		0	1	Not-determined	Genome-fragment	NA		0
S12_vs_k141_17673||full	1375	No	NA		1	0	Low-quality	Genome-fragment		3.26	0
S14_vs_k141_75965||full	2251	No	NA		0	1	Not-determined	Genome-fragment	NA		0
S14_vs_k141_75967||full	1242	No	NA		0	1	Not-determined	Genome-fragment	NA		0
S2_vs_k141_5055||lt2gene	1375	No	NA		1	0	Low-quality	Genome-fragment		2.6	0
S14_vs_k141_31846||full	34007	No	NA		0	16	Not-determined	Genome-fragment	NA		0
S4_extracted_6710	1375	No	NA		0	1	Low-quality	Genome-fragment		3.25	0
S14_vs_k141_31848||full	2062	No	NA		0	2	Not-determined	Genome-fragment	NA		0
S8_vs_k141_19850||full	1375	No	NA		0	0	Low-quality	Genome-fragment		3.19	0
S8_vs_k141_72513||full	1375	No	NA		0	1	Low-quality	Genome-fragment		2.02	0
S9_vs_k141_16185||lt2gene	1376	No	NA		1	0	Low-quality	Genome-fragment		1.26	0
S13_extracted_11453	1376	No	NA		2	0	Low-quality	Genome-fragment		2.31	0
S14_vs_k141_68407||full	1705	No	NA		0	0	Not-determined	Genome-fragment	NA		0
S14_vs_k141_24356||full	2308	No	NA		0	1	Not-determined	Genome-fragment	NA		0
S14_vs_k141_54000||full	1450	No	NA		0	2	Not-determined	Genome-fragment	NA		0
S14_vs_k141_60518||full	1376	No	NA		0	0	Low-quality	Genome-fragment		3.47	0
S14_vs_k141_49403||full	1376	No	NA		1	0	Low-quality	Genome-fragment		2.09	0
S1_vs_k141_146922||full	1376	No	NA		0	0	Low-quality	Genome-fragment		2.81	0
S14_vs_k141_24380||full	1635	No	NA		0	1	Not-determined	Genome-fragment	NA		0
S1_vs_k141_119898||full	1376	No	NA		1	0	Low-quality	Genome-fragment		0.87	0
S14_vs_k141_2383||full	2456	No	NA		0	1	Not-determined	Genome-fragment	NA		0
S14_vs_k141_68453||full	1130	No	NA		0	0	Not-determined	Genome-fragment	NA		0
S5_vs_k141_53758||full	1376	No	NA		0	0	Low-quality	Genome-fragment		1.81	0
S14_vs_k141_31917||full	7512	No	NA		0	6	Not-determined	Genome-fragment	NA		0
S5_vs_k141_89901||full	1376	No	NA		0	0	Low-quality	Genome-fragment		2.98	0
S5_vs_k141_66618||full	1376	No	NA		1	0	Low-quality	Genome-fragment		3.1	0
S14_vs_k141_68470||full	6116	No	NA		0	7	Not-determined	Genome-fragment	NA		0
S16_vs_107426||full	1376	No	NA		0	1	Low-quality	Genome-fragment		1.93	0
S16_vs_107916||full	1376	No	NA		3	0	Low-quality	Genome-fragment		3.22	0
S10_vs_20087||full	1376	No	NA		0	0	Low-quality	Genome-fragment		3.84	0
S12_extracted_k141_98132	1376	No	NA		0	1	Low-quality	Genome-fragment		1.42	0
S12_vs_k141_71122||full	1376	No	NA		0	0	Low-quality	Genome-fragment		2.34	0
S14_vs_k141_105634||full	2820	No	NA		0	0	Not-determined	Genome-fragment	NA		0
S11_extracted_k141_16151	1377	No	NA		0	0	Low-quality	Genome-fragment		1.74	0
S11_extracted_k141_90601	1377	No	NA		0	0	Low-quality	Genome-fragment		1.54	0
S14_vs_k141_83347||full	3204	No	NA		0	2	Not-determined	Genome-fragment	NA		0
S14_extracted_k141_70783	1377	No	NA		0	0	Low-quality	Genome-fragment		3.58	0
S14_vs_k141_105672||full	1755	No	NA		0	0	Not-determined	Genome-fragment	NA		0
S14_vs_k141_61302||full	1366	No	NA		0	1	Not-determined	Genome-fragment	NA		0
S14_vs_k141_2523||full	2604	No	NA		0	2	Not-determined	Genome-fragment	NA		0
S14_vs_k141_83374||full	1167	No	NA		0	0	Not-determined	Genome-fragment	NA		0
S14_vs_k141_97664||full	1798	No	NA		0	0	Not-determined	Genome-fragment	NA		0
S14_vs_k141_39602||full	1517	No	NA		0	2	Not-determined	Genome-fragment	NA		0
S14_vs_k141_32497||full	1377	No	NA		0	0	Low-quality	Genome-fragment		2.4	0
S1_extracted_k141_137327	1377	No	NA		1	0	Low-quality	Genome-fragment		2.15	0
S3_vs_k141_114478||full	1377	No	NA		1	0	Low-quality	Genome-fragment		2.57	0
S5_extracted_k141_11534	1377	No	NA		0	0	Low-quality	Genome-fragment		0.52	0
S5_vs_k141_18763||lt2gene	1377	No	NA		2	0	Low-quality	Genome-fragment		3.19	0
S5_vs_k141_143057||lt2gene	1377	No	NA		2	0	Low-quality	Genome-fragment		2.96	0
S7_vs_28955||full	1377	No	NA		0	0	Low-quality	Genome-fragment		0.75	0
S14_vs_k141_32051||full	1199	No	NA		0	0	Not-determined	Genome-fragment	NA		0
S16_vs_170351||full	1377	No	NA		1	0	Low-quality	Genome-fragment		2.88	0
S12_extracted_k141_154730	1377	No	NA		0	0	Low-quality	Genome-fragment		1.96	0
S14_vs_k141_112800||full	1117	No	NA		0	2	Not-determined	Genome-fragment	NA		0
S14_vs_k141_105739||full	1273	No	NA		0	0	Not-determined	Genome-fragment	NA		0
S14_vs_k141_17574||full	1959	No	NA		0	2	Not-determined	Genome-fragment	NA		0
S12_vs_k141_81336||full	1377	No	NA		2	0	Low-quality	Genome-fragment		3.02	0
S12_vs_k141_184260||full	1377	No	NA		0	3	Low-quality	Genome-fragment		1.83	0
S12_vs_k141_90736||full	1377	No	NA		0	0	Low-quality	Genome-fragment		3.45	0
S15_vs_63955||full	1377	No	NA		0	0	Low-quality	Genome-fragment		3.03	0
S8_extracted_k141_18023	1377	No	NA		2	0	Low-quality	Genome-fragment		21.6	0
S8_extracted_k141_177074	1377	No	NA		1	0	Low-quality	Genome-fragment		1.99	0
S17_vs_k141_50495||full	1378	No	NA		0	1	Low-quality	Genome-fragment		0.84	0
S14_vs_k141_83488||full	3065	No	NA		0	2	Not-determined	Genome-fragment	NA		0
S9_extracted_k141_37803	1378	No	NA		0	1	Low-quality	Genome-fragment		2.42	0
S14_vs_k141_68670||full	1498	No	NA		0	1	Not-determined	Genome-fragment	NA		0
S14_vs_k141_54361||full	5517	No	NA		0	4	Not-determined	Genome-fragment	NA		0
S11_vs_k141_43115||full	1378	No	NA		1	0	Low-quality	Genome-fragment		2.75	0
S14_vs_k141_17652||full	1193	No	NA		0	0	Not-determined	Genome-fragment	NA		0
S13_vs_100308||full	1378	No	NA		2	0	Low-quality	Genome-fragment		2.3	0
S14_vs_k141_90910||full	2032	No	NA		0	0	Not-determined	Genome-fragment	NA		0
S14_vs_k141_61400||full	2394	No	NA		0	2	Not-determined	Genome-fragment	NA		0
S14_vs_k141_61420||full	1443	No	NA		0	1	Not-determined	Genome-fragment	NA		0
S14_vs_k141_76342||full	3893	No	NA		0	2	Not-determined	Genome-fragment	NA		0
S14_vs_k141_17668||full	5466	No	NA		0	0	Not-determined	Genome-fragment	NA		0
S14_vs_k141_39784||full	5300	No	NA		0	3	Not-determined	Genome-fragment	NA		0
S14_vs_k141_61445||full	1783	No	NA		0	1	Not-determined	Genome-fragment	NA		0
S1_vs_k141_105741||full	1378	No	NA		0	2	Low-quality	Genome-fragment		0.67	0
S14_vs_k141_17695||full	1995	No	NA		0	3	Not-determined	Genome-fragment	NA		0
S1_vs_k141_25700||lt2gene	1378	No	NA		1	0	Low-quality	Genome-fragment		2.26	0
S3_vs_k141_19591||full	1378	No	NA		0	0	Low-quality	Genome-fragment		2.15	0
S14_vs_k141_90934||full	37471	No	NA		5	1	High-quality	High-quality		98.98	0
S5_extracted_k141_145298	1378	No	NA		0	0	Low-quality	Genome-fragment		2.86	0
S14_vs_k141_105866||full	3237	No	NA		0	4	Not-determined	Genome-fragment	NA		0
S5_vs_k141_97230||full	1378	No	NA		1	0	Low-quality	Genome-fragment		3.44	0
S5_vs_k141_67333||full	1378	No	NA		1	0	Low-quality	Genome-fragment		3.67	0
S5_vs_k141_94272||lt2gene	1378	No	NA		1	0	Low-quality	Genome-fragment		2.01	0
S14_vs_k141_112964||full	2506	No	NA		0	2	Not-determined	Genome-fragment	NA		0
S14_vs_k141_61487||full	2173	No	NA		0	1	Not-determined	Genome-fragment	NA		0
S14_vs_k141_39797||full	41367	No	NA		20	1	High-quality	High-quality		95.9	0
S16_vs_54472||full	1378	No	NA		0	1	Low-quality	Genome-fragment		1.87	0
S16_vs_77919||full	1378	No	NA		1	0	Low-quality	Genome-fragment		3.26	0
S14_vs_k141_68777||full	1174	No	NA		0	1	Not-determined	Genome-fragment	NA		0
S16_vs_116915||full	1378	No	NA		1	0	Low-quality	Genome-fragment		1.24	0
S12_vs_k141_178102||full	1378	Yes		1069	1	1	Low-quality	Genome-fragment		1.77	22.42
S15_vs_40212||lt2gene	1378	No	NA		1	0	Low-quality	Genome-fragment		3.18	0
S14_vs_k141_112976||full	11171	No	NA		0	9	Not-determined	Genome-fragment	NA		0
S4_extracted_8487	1378	No	NA		1	0	Low-quality	Genome-fragment		3.47	0
S14_vs_k141_105918||full	1250	No	NA		0	0	Not-determined	Genome-fragment	NA		0
S14_vs_k141_68824||full	1225	No	NA		0	0	Not-determined	Genome-fragment	NA		0
S14_vs_k141_54526||full	8733	No	NA		0	8	Not-determined	Genome-fragment	NA		0
S14_vs_k141_39857||full	12636	No	NA		0	6	Not-determined	Genome-fragment	NA		0
S9_extracted_k141_10196	1379	No	NA		0	0	Low-quality	Genome-fragment		1.81	0
S11_extracted_k141_84920	1379	No	NA		2	0	Low-quality	Genome-fragment		21.85	0
S13_extracted_132163	1379	No	NA		1	0	Low-quality	Genome-fragment		3.73	0
S14_vs_k141_68858||full	3209	No	NA		0	1	Not-determined	Genome-fragment	NA		0
S3_vs_k141_109339||full	1379	No	NA		1	0	Low-quality	Genome-fragment		2.82	0
S14_vs_k141_54569||full	2671	No	NA		0	2	Not-determined	Genome-fragment	NA		0
S14_vs_k141_91001||full	1185	No	NA		0	0	Not-determined	Genome-fragment	NA		0
S16_vs_4414||lt2gene	1379	No	NA		2	0	Low-quality	Genome-fragment		3.46	0
S14_vs_k141_97835||full	2070	No	NA		1	0	Medium-quality	Genome-fragment		60.76	0
S10_extracted_8693	1379	No	NA		0	1	Low-quality	Genome-fragment		2.45	0
S6_extracted_k141_22610	1379	No	NA		0	1	Low-quality	Genome-fragment		0.46	0
S14_vs_k141_24772||full	1042	No	NA		0	0	Not-determined	Genome-fragment	NA		0
S8_extracted_k141_32252	1379	No	NA		0	1	Low-quality	Genome-fragment		0.86	0
S17_vs_k141_144224||full	1380	No	NA		5	0	Low-quality	Genome-fragment		3.17	0
S9_vs_k141_49330||full	1380	No	NA		0	1	Low-quality	Genome-fragment		2.37	0
S1_extracted_k141_23277	1380	No	NA		0	0	Low-quality	Genome-fragment		0.79	0
S1_vs_k141_95975||full	1380	No	NA		0	0	Low-quality	Genome-fragment		3.44	0
S14_vs_k141_24800||full	1099	No	NA		0	2	Not-determined	Genome-fragment	NA		0
S14_vs_k141_46577||full	2198	No	NA		0	1	Not-determined	Genome-fragment	NA		0
S3_extracted_k141_108605	1380	No	NA		0	0	Low-quality	Genome-fragment		2.23	0
S5_vs_k141_135332||full	1380	No	NA		0	0	Low-quality	Genome-fragment		2.47	0
S5_vs_k141_88284||full	1380	No	NA		1	0	Low-quality	Genome-fragment		2.67	0
S5_vs_k141_34900||full	1380	Yes		711	1	1	Low-quality	Genome-fragment		0.42	48.48
S14_vs_k141_46599||full	1732	No	NA		0	2	Not-determined	Genome-fragment	NA		0
S16_extracted_71116	1380	No	NA		0	0	Low-quality	Genome-fragment		2.27	0
S16_vs_2248||full	1380	No	NA		0	0	Low-quality	Genome-fragment		1.26	0
S10_extracted_30512	1380	No	NA		0	0	Low-quality	Genome-fragment		3	0
S8_vs_k141_145874||full	1380	No	NA		0	0	Low-quality	Genome-fragment		1.39	0
S8_vs_k141_156036||full	1380	No	NA		0	2	Low-quality	Genome-fragment		1.79	0
S14_vs_k141_17836||full	6466	No	NA		0	3	Not-determined	Genome-fragment	NA		0
S17_vs_k141_52467||full	1381	No	NA		2	0	Low-quality	Genome-fragment		4.06	0
S14_vs_k141_91116||full	2208	No	NA		0	4	Not-determined	Genome-fragment	NA		0
S14_vs_k141_10330||full	2034	No	NA		0	3	Not-determined	Genome-fragment	NA		0
S14_vs_k141_83863||full	6807	No	NA		0	0	Not-determined	Genome-fragment	NA		0
S17_vs_k141_153054||full	1381	No	NA		1	0	Low-quality	Genome-fragment		1.63	0

S14_vs_k141_61529||full	1407	No	NA		0	2	Not-determined	Genome-fragment	NA		0
S14_vs_k141_83875||full	5690	No	NA		0	4	Not-determined	Genome-fragment	NA		0
S14_vs_k141_68974||full	2394	No	NA		0	1	Not-determined	Genome-fragment	NA		0
S14_extracted_k141_116320	1381	No	NA		0	0	Low-quality	Genome-fragment		2.35	0
S14_vs_k141_113085||full	12500	No	NA		0	5	Not-determined	Genome-fragment	NA		0
S14_vs_k141_46693||full	3578	No	NA		0	1	Not-determined	Genome-fragment	NA		0
S14_vs_k141_61530||full	30159	No	NA		15	0	Medium-quality	Genome-fragment		89.33	0
S14_vs_k141_2870||full	88314	Yes		46524	30	19	Medium-quality	Genome-fragment		86.01	47.32
S14_vs_k141_24922||full	1973	No	NA		0	0	Not-determined	Genome-fragment	NA		0
S14_vs_k141_106136||full	1920	No	NA		0	1	Not-determined	Genome-fragment	NA		0
S14_vs_k141_17906||full	2916	No	NA		0	1	Not-determined	Genome-fragment	NA		0
S14_vs_k141_107252||full	1381	No	NA		1	0	Low-quality	Genome-fragment		2.95	0
S14_vs_k141_11792||full	1381	No	NA		0	0	Low-quality	Genome-fragment		3.16	0
S14_vs_k141_76700||full	3812	No	NA		0	5	Not-determined	Genome-fragment	NA		0
S1_extracted_k141_108885	1381	No	NA		0	0	Low-quality	Genome-fragment		2.58	0
S14_vs_k141_32499||full	78663	Yes		27007	11	38	Complete	High-quality		100	65.67
S14_vs_k141_24969||full	1705	No	NA		0	0	Not-determined	Genome-fragment	NA		0
S14_vs_k141_61629||full	1599	No	NA		0	0	Not-determined	Genome-fragment	NA		0
S14_vs_k141_32522||full	1856	No	NA		0	3	Not-determined	Genome-fragment	NA		0
S14_vs_k141_113169||full	1691	No	NA		0	0	Not-determined	Genome-fragment	NA		0
S1_vs_k141_98109||full	1381	No	NA		1	0	Low-quality	Genome-fragment		3.92	0
S14_vs_k141_61662||full	1136	No	NA		0	1	Not-determined	Genome-fragment	NA		0
S14_vs_k141_61668||full	1581	No	NA		0	1	Not-determined	Genome-fragment	NA		0
S3_vs_k141_1664||full	1381	No	NA		0	0	Low-quality	Genome-fragment		2.68	0
S7_vs_47615||full	1381	No	NA		3	0	Low-quality	Genome-fragment		3.82	0
S7_vs_69477||full	1381	No	NA		1	0	Low-quality	Genome-fragment		2.72	0
S14_vs_k141_113226||full	1637	No	NA		0	2	Not-determined	Genome-fragment	NA		0
S14_vs_k141_113227||full	1621	No	NA		0	2	Not-determined	Genome-fragment	NA		0
S7_vs_70385||full	1381	No	NA		0	0	Low-quality	Genome-fragment		0.76	0
S7_vs_87594||full	1381	No	NA		0	0	Low-quality	Genome-fragment		0.87	0
S14_vs_k141_91308||full	1840	No	NA		0	1	Not-determined	Genome-fragment	NA		0
S14_vs_k141_83976||full	1404	No	NA		0	0	Not-determined	Genome-fragment	NA		0
S14_vs_k141_98150||full	1205	No	NA		0	0	Not-determined	Genome-fragment	NA		0
S14_vs_k141_113258||full	2176	No	NA		0	2	Not-determined	Genome-fragment	NA		0
S7_vs_105916||full	1381	No	NA		0	0	Low-quality	Genome-fragment		3.62	0
S14_vs_k141_83996||full	1589	No	NA		0	1	Not-determined	Genome-fragment	NA		0
S9_vs_k141_14951||full	1382	No	NA		2	0	Low-quality	Genome-fragment		3.11	0
S14_vs_k141_69183||full	6075	No	NA		0	2	Not-determined	Genome-fragment	NA		0
S14_vs_k141_69184||full	3022	No	NA		0	3	Not-determined	Genome-fragment	NA		0
S14_vs_k141_98169||full	3743	No	NA		0	2	Not-determined	Genome-fragment	NA		0
S14_extracted_k141_78974	1382	No	NA		0	2	Low-quality	Genome-fragment		1.02	0
S14_vs_k141_18066||full	6339	No	NA		0	4	Not-determined	Genome-fragment	NA		0
S1_extracted_k141_132069	1382	No	NA		1	0	Low-quality	Genome-fragment		3.48	0
S14_vs_k141_106240||full	3450	No	NA		0	2	Not-determined	Genome-fragment	NA		0
S14_vs_k141_46812||full	1449	No	NA		0	1	Not-determined	Genome-fragment	NA		0
S14_vs_k141_106248||full	1253	No	NA		0	0	Not-determined	Genome-fragment	NA		0
S1_vs_k141_42951||full	1382	No	NA		0	0	Low-quality	Genome-fragment		3.05	0
S14_vs_k141_91404||full	1322	No	NA		0	1	Not-determined	Genome-fragment	NA		0
S14_vs_k141_76874||full	2340	No	NA		0	1	Not-determined	Genome-fragment	NA		0
S14_vs_k141_69262||full	1839	No	NA		0	2	Not-determined	Genome-fragment	NA		0
S3_extracted_k141_84137	1382	No	NA		0	0	Low-quality	Genome-fragment		0.71	0
S3_vs_k141_51278||full	1382	No	NA		1	0	Low-quality	Genome-fragment		2.25	0
S7_extracted_19795	1382	No	NA		0	0	Low-quality	Genome-fragment		2.17	0
S14_vs_k141_25088||full	1510	No	NA		0	1	Not-determined	Genome-fragment	NA		0
S14_vs_k141_10527||full	17551	No	NA		0	8	Not-determined	Genome-fragment	NA		0
S14_vs_k141_76938||full	2518	No	NA		0	1	Not-determined	Genome-fragment	NA		0
S14_vs_k141_84126||full	1471	No	NA		0	0	Not-determined	Genome-fragment	NA		0
S14_vs_k141_113384||full	4977	No	NA		0	0	Not-determined	Genome-fragment	NA		0
S14_vs_k141_54846||full	8729	No	NA		0	4	Not-determined	Genome-fragment	NA		0
S14_vs_k141_98261||full	3306	No	NA		0	2	Not-determined	Genome-fragment	NA		0
S14_vs_k141_3122||full	2469	No	NA		0	3	Not-determined	Genome-fragment	NA		0
S14_vs_k141_76981||full	1449	No	NA		0	1	Not-determined	Genome-fragment	NA		0
S14_vs_k141_76994||full	1802	No	NA		0	0	Not-determined	Genome-fragment	NA		0
S14_vs_k141_46941||full	2061	No	NA		0	1	Not-determined	Genome-fragment	NA		0
S7_vs_7281||full	1382	No	NA		1	0	Low-quality	Genome-fragment		2.08	0
S16_extracted_75195	1382	No	NA		0	3	Low-quality	Genome-fragment		0.52	0
S10_vs_73191||full	1382	No	NA		0	1	Low-quality	Genome-fragment		1.83	0
S14_vs_k141_10582||full	2481	No	NA		0	2	Not-determined	Genome-fragment	NA		0
S12_vs_k141_148868||lt2gene	1382	No	NA		2	0	Low-quality	Genome-fragment		2.23	0
S17_extracted_k141_13938	1383	No	NA		3	0	Low-quality	Genome-fragment		3.71	0
S9_vs_k141_5696||full	1383	No	NA		0	0	Low-quality	Genome-fragment		3.12	0
S11_vs_k141_101500||full	1383	No	NA		0	0	Low-quality	Genome-fragment		3.31	0
S11_vs_k141_24944||full	1383	No	NA		1	0	Low-quality	Genome-fragment		2.96	0
S14_vs_k141_113456||full	2705	No	NA		0	1	Not-determined	Genome-fragment	NA		0
S13_vs_81796||full	1383	No	NA		0	0	Low-quality	Genome-fragment		1.61	0
S14_vs_k141_91543||full	1765	No	NA		0	3	Not-determined	Genome-fragment	NA		0
S5_vs_k141_53533||full	1383	No	NA		0	0	Low-quality	Genome-fragment		3.13	0
S7_vs_12553||full	1383	No	NA		0	0	Low-quality	Genome-fragment		2.24	0
S7_vs_46650||full	1383	No	NA		0	2	Low-quality	Genome-fragment		1.68	0
S14_vs_k141_3209||full	1530	No	NA		0	1	Not-determined	Genome-fragment	NA		0
S14_vs_k141_25213||full	2637	No	NA		0	0	Not-determined	Genome-fragment	NA		0
S7_vs_62906||full	1383	No	NA		0	0	Low-quality	Genome-fragment		2.53	0
S14_vs_k141_32748||full	1064	No	NA		0	0	Not-determined	Genome-fragment	NA		0
S14_vs_k141_47026||full	1189	No	NA		0	1	Not-determined	Genome-fragment	NA		0
S10_extracted_82233	1383	No	NA		0	0	Low-quality	Genome-fragment		0.85	0
S14_vs_k141_25239||full	1687	No	NA		0	2	Not-determined	Genome-fragment	NA		0
S10_vs_5252||full	1383	No	NA		1	0	Low-quality	Genome-fragment		2.26	0
S6_vs_k141_7338||full	1383	No	NA		1	0	Low-quality	Genome-fragment		3.59	0
S6_vs_k141_3053||full	1383	No	NA		0	0	Low-quality	Genome-fragment		2.27	0
S8_vs_k141_206885||full	1383	No	NA		0	1	Low-quality	Genome-fragment		2.41	0
S14_vs_k141_84289||full	2031	No	NA		0	0	Not-determined	Genome-fragment	NA		0
S14_vs_k141_18333||full	1463	No	NA		0	1	Not-determined	Genome-fragment	NA		0
S17_vs_k141_37189||full	1384	No	NA		0	0	Low-quality	Genome-fragment		0.8	0
S14_vs_k141_32799||full	5606	No	NA		0	4	Not-determined	Genome-fragment	NA		0
S14_vs_k141_25269||full	4579	No	NA		0	4	Not-determined	Genome-fragment	NA		0
S14_vs_k141_54951||full	1405	No	NA		0	0	Not-determined	Genome-fragment	NA		0
S9_vs_k141_71291||full	1384	No	NA		1	0	Low-quality	Genome-fragment		2.92	0
S14_vs_k141_10693||full	1831	No	NA		0	1	Not-determined	Genome-fragment	NA		0
S11_extracted_k141_29156	1384	No	NA		0	0	Low-quality	Genome-fragment		1.16	0
S14_vs_k141_10729||full	7461	No	NA		0	8	Not-determined	Genome-fragment	NA		0
S13_vs_36916||full	1384	No	NA		0	0	Low-quality	Genome-fragment		1.74	0
S14_vs_k141_40471||full	1940	No	NA		0	2	Not-determined	Genome-fragment	NA		0
S13_vs_70694||full	1384	No	NA		1	0	Low-quality	Genome-fragment		2.66	0
S3_extracted_k141_28889	1384	No	NA		0	2	Low-quality	Genome-fragment		0.96	0
S14_vs_k141_106466||full	4349	No	NA		0	2	Not-determined	Genome-fragment	NA		0
S14_vs_k141_113588||full	1728	No	NA		0	0	Not-determined	Genome-fragment	NA		0
S14_vs_k141_69519||full	2019	No	NA		0	3	Not-determined	Genome-fragment	NA		0
S3_vs_k141_68959||full	1384	No	NA		0	1	Low-quality	Genome-fragment		1.11	0
S5_vs_k141_49702||full	1384	No	NA		0	0	Low-quality	Genome-fragment		0.87	0
S5_vs_k141_129266||full	1384	No	NA		1	0	Low-quality	Genome-fragment		4.25	0
S14_vs_k141_98500||full	5742	No	NA		0	1	Not-determined	Genome-fragment	NA		0
S14_vs_k141_84332||full	5215	No	NA		0	0	Not-determined	Genome-fragment	NA		0
S7_extracted_137936	1384	No	NA		2	0	Low-quality	Genome-fragment		2.86	0
S16_vs_172299||full	1384	No	NA		0	0	Low-quality	Genome-fragment		3.53	0
S10_vs_37395||full	1384	No	NA		1	0	Low-quality	Genome-fragment		2.55	0
S12_vs_k141_66690||full	1384	No	NA		0	1	Low-quality	Genome-fragment		0.33	0
S12_vs_k141_171019||full	1384	No	NA		1	0	Low-quality	Genome-fragment		3.04	0
S2_vs_k141_1248||full	1384	No	NA		0	0	Low-quality	Genome-fragment		2.39	0
S6_vs_k141_4356||full	1384	No	NA		0	2	Low-quality	Genome-fragment		0.41	0
S17_extracted_k141_98763	1385	No	NA		0	1	Low-quality	Genome-fragment		0.75	0
S13_vs_146182||full	1385	No	NA		0	4	Low-quality	Genome-fragment		0.41	0
S14_vs_k141_47250||full	6014	No	NA		0	4	Not-determined	Genome-fragment	NA		0
S7_extracted_73242	1385	No	NA		0	1	Low-quality	Genome-fragment		0.68	0
S14_vs_k141_98559||full	2459	No	NA		0	2	Not-determined	Genome-fragment	NA		0
S8_vs_k141_61696||full	1385	No	NA		1	0	Low-quality	Genome-fragment		3.87	0
S14_vs_k141_18451||full	2063	No	NA		0	0	Not-determined	Genome-fragment	NA		0
S8_vs_k141_111418||full	1385	No	NA		2	0	Low-quality	Genome-fragment		2.53	0
S17_vs_k141_35792||lt2gene	1386	No	NA		1	0	Low-quality	Genome-fragment		2.43	0
S9_vs_k141_30841||full	1386	No	NA		0	0	Low-quality	Genome-fragment		2.87	0
S14_vs_k141_32922||full	1890	No	NA		0	1	Not-determined	Genome-fragment	NA		0
S14_vs_k141_47259||full	1261	No	NA		0	0	Not-determined	Genome-fragment	NA		0
S11_vs_k141_65836||full	1386	No	NA		0	2	Low-quality	Genome-fragment		0.56	0
S14_vs_k141_47260||full	4701	No	NA		0	2	Not-determined	Genome-fragment	NA		0
S11_vs_k141_33677||full	1386	No	NA		1	0	Low-quality	Genome-fragment		4.15	0
S14_extracted_k141_65050	1386	No	NA		2	0	Low-quality	Genome-fragment		4.57	0
S5_vs_k141_76806||full	1386	No	NA		1	0	Low-quality	Genome-fragment		2.07	0
S7_extracted_134303	1386	No	NA		0	0	Low-quality	Genome-fragment		4.2	0
S14_vs_k141_10866||full	2313	No	NA		0	3	Not-determined	Genome-fragment	NA		0
S7_vs_59453||full	1386	No	NA		1	0	Low-quality	Genome-fragment		2.73	0
S7_vs_79004||full	1386	No	NA		1	0	Low-quality	Genome-fragment		0.85	0
S7_vs_88274||full	1386	No	NA		1	0	Low-quality	Genome-fragment		2.26	0
S14_vs_k141_32970||full	4939	No	NA		0	1	Not-determined	Genome-fragment	NA		0
S7_vs_41681||lt2gene	1386	No	NA		1	0	Low-quality	Genome-fragment		1.28	0
S16_extracted_30145	1386	No	NA		0	0	Low-quality	Genome-fragment		3.23	0
S8_vs_k141_26077||full	1386	No	NA		1	0	Low-quality	Genome-fragment		1.59	0
S11_vs_k141_91648||lt2gene	1387	No	NA		1	0	Low-quality	Genome-fragment		1.3	0
S1_vs_k141_58506||full	1387	No	NA		0	0	Low-quality	Genome-fragment		2.13	0
S1_vs_k141_71541||full	1387	No	NA		1	0	Low-quality	Genome-fragment		4.54	0
S5_vs_k141_52984||full	1387	No	NA		3	0	Low-quality	Genome-fragment		3.17	0
S16_extracted_125584	1387	No	NA		0	0	Low-quality	Genome-fragment		2.59	0
S14_vs_k141_69659||full	2454	No	NA		0	1	Not-determined	Genome-fragment	NA		0
S16_vs_45824||full	1387	No	NA		0	0	Low-quality	Genome-fragment		0.91	0
S16_vs_107866||full	1387	No	NA		0	0	Low-quality	Genome-fragment		2.33	0
S12_extracted_k141_155104	1387	No	NA		0	0	Low-quality	Genome-fragment		2.5	0
S14_vs_k141_10942||full	2020	No	NA		0	2	Not-determined	Genome-fragment	NA		0
S14_vs_k141_69695||full	2972	No	NA		0	0	Not-determined	Genome-fragment	NA		0
S14_vs_k141_106576||full	3111	No	NA		0	0	Not-determined	Genome-fragment	NA		0
S11_extracted_k141_61902	1388	No	NA		0	0	Low-quality	Genome-fragment		2.04	0
S14_vs_k141_98666||full	3074	No	NA		0	1	Not-determined	Genome-fragment	NA		0
S14_vs_k141_84519||full	2180	No	NA		0	1	Not-determined	Genome-fragment	NA		0
S11_vs_k141_55272||full	1388	No	NA		0	2	Low-quality	Genome-fragment		0.42	0
S11_vs_k141_89019||full	1388	No	NA		3	0	Low-quality	Genome-fragment		2.3	0
S14_vs_k141_98685||full	6174	No	NA		0	5	Not-determined	Genome-fragment	NA		0
S14_vs_k141_106635||full	3014	No	NA		0	0	Not-determined	Genome-fragment	NA		0
S13_vs_87859||full	1388	No	NA		1	0	Low-quality	Genome-fragment		3.81	0
S14_extracted_k141_92591	1388	No	NA		1	0	Low-quality	Genome-fragment		0.86	0
S14_vs_k141_7991||full	1388	No	NA		2	0	Low-quality	Genome-fragment		3.29	0
S14_vs_k141_47392||full	2444	No	NA		0	3	Not-determined	Genome-fragment	NA		0
S14_vs_k141_34827||lt2gene	1388	No	NA		1	0	Low-quality	Genome-fragment		3.29	0
S3_extracted_k141_26197	1388	No	NA		0	1	Low-quality	Genome-fragment		1.76	0
S14_vs_k141_3453||full	2470	No	NA		0	1	Not-determined	Genome-fragment	NA		0
S14_vs_k141_69758||full	4571	No	NA		0	2	Not-determined	Genome-fragment	NA		0
S14_vs_k141_33079||full	3922	No	NA		0	2	Not-determined	Genome-fragment	NA		0
S14_vs_k141_55119||full	1554	No	NA		0	1	Not-determined	Genome-fragment	NA		0

S14_vs_k141_40812||full	2053	No	NA		0	2	Not-determined	Genome-fragment	NA		0
S14_vs_k141_18636||full	1562	No	NA		0	1	Not-determined	Genome-fragment	NA		0
S5_vs_k141_116016||full	1388	No	NA		0	0	Low-quality	Genome-fragment		4.03	0
S14_vs_k141_106697||full	1636	No	NA		0	0	Not-determined	Genome-fragment	NA		0
S5_vs_k141_117133||full	1388	No	NA		2	0	Low-quality	Genome-fragment		4.16	0
S16_extracted_103824	1388	No	NA		1	0	Low-quality	Genome-fragment		3.83	0
S14_vs_k141_106711||full	1255	No	NA		0	0	Not-determined	Genome-fragment	NA		0
S14_vs_k141_69847||full	1356	No	NA		0	3	Not-determined	Genome-fragment	NA		0
S16_vs_125892||full	1388	No	NA		1	0	Low-quality	Genome-fragment		0.91	0
S16_vs_130550||full	1388	No	NA		2	0	Low-quality	Genome-fragment		3.95	0
S10_extracted_36677	1388	No	NA		1	0	Low-quality	Genome-fragment		1.23	0
S14_vs_k141_69864||full	2237	No	NA		0	3	Not-determined	Genome-fragment	NA		0
S2_vs_k141_20480||full	1388	No	NA		0	2	Low-quality	Genome-fragment		2.32	0
S14_vs_k141_77148||full	2324	No	NA		0	3	Not-determined	Genome-fragment	NA		0
S14_vs_k141_62178||full	32459	No	NA		20	2	High-quality	High-quality		96.78	0
S8_extracted_k141_31199	1388	No	NA		0	0	Low-quality	Genome-fragment		0.75	0
S14_vs_k141_69870||full	3892	No	NA		0	3	Not-determined	Genome-fragment	NA		0
S9_vs_k141_44698||full	1389	No	NA		0	0	Low-quality	Genome-fragment		3.35	0
S14_vs_k141_47488||full	1058	No	NA		0	0	Not-determined	Genome-fragment	NA		0
S14_vs_k141_33133||full	1087	No	NA		0	1	Not-determined	Genome-fragment	NA		0
S14_vs_k141_40918||full	1059	No	NA		0	0	Not-determined	Genome-fragment	NA		0
S14_extracted_k141_95333	1389	No	NA		0	0	Low-quality	Genome-fragment		2.37	0
S14_vs_k141_30990||full	1389	No	NA		1	0	Low-quality	Genome-fragment		3.12	0
S14_vs_k141_84654||full	7602	No	NA		0	2	Not-determined	Genome-fragment	NA		0
S3_vs_k141_88962||full	1389	No	NA		1	0	Low-quality	Genome-fragment		3.03	0
S3_vs_k141_127030||full	1389	No	NA		0	0	Low-quality	Genome-fragment		1.14	0
S5_vs_k141_91956||full	1389	No	NA		0	0	Low-quality	Genome-fragment		1.48	0
S14_vs_k141_55267||full	10555	No	NA		0	6	Not-determined	Genome-fragment	NA		0
S5_vs_k141_99062||full	1389	No	NA		1	0	Low-quality	Genome-fragment		1.33	0
S14_vs_k141_18734||full	1782	No	NA		0	0	Not-determined	Genome-fragment	NA		0
S14_vs_k141_3620||full	2231	No	NA		0	1	Not-determined	Genome-fragment	NA		0
S16_vs_14716||full	1389	No	NA		2	0	Low-quality	Genome-fragment		3.24	0
S16_vs_76194||full	1389	No	NA		0	0	Low-quality	Genome-fragment		2.33	0
S14_vs_k141_113984||full	2105	No	NA		0	2	Not-determined	Genome-fragment	NA		0
S8_extracted_k141_201328	1389	No	NA		0	2	Low-quality	Genome-fragment		0.32	0
S17_vs_k141_149532||full	1390	No	NA		1	0	Low-quality	Genome-fragment		3.39	0
S14_vs_k141_77304||full	2322	No	NA		0	1	Not-determined	Genome-fragment	NA		0
S14_vs_k141_114022||full	1080	No	NA		0	1	Not-determined	Genome-fragment	NA		0
S17_vs_k141_24527||full	1390	No	NA		0	0	Low-quality	Genome-fragment		3.53	0
S14_vs_k141_92076||full	1165	No	NA		0	0	Not-determined	Genome-fragment	NA		0
S14_vs_k141_98909||full	1749	No	NA		0	2	Not-determined	Genome-fragment	NA		0
S9_vs_k141_64690||full	1390	No	NA		1	0	Low-quality	Genome-fragment		3.53	0
S7_extracted_74414	1390	No	NA		2	0	Low-quality	Genome-fragment		4.88	0
S14_vs_k141_18845||full	2772	No	NA		0	3	Not-determined	Genome-fragment	NA		0
S7_vs_25008||full	1390	No	NA		0	0	Low-quality	Genome-fragment		2.63	0
S10_vs_4214||full	1390	No	NA		0	0	Low-quality	Genome-fragment		2.56	0
S12_extracted_k141_156391	1390	No	NA		0	1	Low-quality	Genome-fragment		0.4	0
S14_vs_k141_25741||full	1456	No	NA		0	0	Not-determined	Genome-fragment	NA		0
S12_vs_k141_100679||full	1390	No	NA		1	0	Low-quality	Genome-fragment		3.18	0
S14_vs_k141_33280||full	1445	No	NA		0	0	Not-determined	Genome-fragment	NA		0
S4_extracted_7057	1390	No	NA		0	1	Low-quality	Genome-fragment		1.18	0
S14_vs_k141_55376||full	1101	No	NA		0	1	Not-determined	Genome-fragment	NA		0
S14_vs_k141_18962||full	1969	No	NA		0	1	Not-determined	Genome-fragment	NA		0
S14_vs_k141_98965||full	3251	No	NA		0	0	Not-determined	Genome-fragment	NA		0
S14_vs_k141_55407||full	6005	No	NA		0	1	Not-determined	Genome-fragment	NA		0
S8_vs_k141_32596||full	1390	No	NA		1	0	Low-quality	Genome-fragment		2.57	0
S14_vs_k141_92222||full	2602	No	NA		0	2	Not-determined	Genome-fragment	NA		0
S17_extracted_k141_91998	1391	No	NA		0	0	Low-quality	Genome-fragment		0.97	0
S14_vs_k141_106971||full	1839	No	NA		0	0	Not-determined	Genome-fragment	NA		0
S14_vs_k141_99003||full	6352	No	NA		0	3	Not-determined	Genome-fragment	NA		0
S17_extracted_k141_85169	1391	No	NA		0	1	Low-quality	Genome-fragment		1.33	0
S14_vs_k141_99022||full	3870	No	NA		0	0	Not-determined	Genome-fragment	NA		0
S17_vs_k141_49192||lt2gene	1391	No	NA		2	0	Low-quality	Genome-fragment		3.24	0
S11_vs_k141_32019||full	1391	No	NA		0	0	Low-quality	Genome-fragment		3	0
S14_vs_k141_69959||full	1334	No	NA		0	2	Not-determined	Genome-fragment	NA		0
S13_vs_42233||full	1391	No	NA		0	0	Low-quality	Genome-fragment		2.16	0
S14_vs_k141_55498||full	5183	No	NA		0	3	Not-determined	Genome-fragment	NA		0
S14_vs_k141_99040||full	2478	No	NA		0	0	Not-determined	Genome-fragment	NA		0
S14_vs_k141_55502||full	2524	No	NA		0	2	Not-determined	Genome-fragment	NA		0
S14_vs_k141_55503||full	1302	No	NA		0	2	Not-determined	Genome-fragment	NA		0
S14_vs_k141_42462||full	1391	No	NA		1	0	Low-quality	Genome-fragment		3.12	0
S14_vs_k141_59523||lt2gene	1391	No	NA		1	0	Low-quality	Genome-fragment		2.63	0
S14_vs_k141_99048||full	3089	No	NA		0	2	Not-determined	Genome-fragment	NA		0
S14_vs_k141_54815||lt2gene	1391	No	NA		1	0	Low-quality	Genome-fragment		3.31	0
S3_vs_k141_81564||full	1391	No	NA		2	0	Low-quality	Genome-fragment		3.88	0
S5_vs_k141_61841||full	1391	Yes		667	1	1	Low-quality	Genome-fragment		1.16	52.05
S5_vs_k141_106711||full	1391	No	NA		0	0	Low-quality	Genome-fragment		1.15	0
S7_vs_85762||full	1391	No	NA		0	0	Low-quality	Genome-fragment		3.01	0
S14_vs_k141_41178||full	5267	No	NA		0	2	Not-determined	Genome-fragment	NA		0
S10_vs_41304||full	1391	No	NA		0	0	Low-quality	Genome-fragment		2.7	0
S14_vs_k141_3973||full	3779	No	NA		0	3	Not-determined	Genome-fragment	NA		0
S14_vs_k141_70031||full	1742	No	NA		0	0	Not-determined	Genome-fragment	NA		0
S10_vs_39275||lt2gene	1391	No	NA		0	0	Low-quality	Genome-fragment		1.9	0
S14_vs_k141_19163||full	2168	No	NA		0	1	Not-determined	Genome-fragment	NA		0
S8_extracted_k141_161109	1391	No	NA		1	0	Low-quality	Genome-fragment		2.8	0
S14_vs_k141_85005||full	13517	No	NA		0	4	Not-determined	Genome-fragment	NA		0
S14_vs_k141_19175||full	2075	No	NA		0	1	Not-determined	Genome-fragment	NA		0
S8_vs_k141_164079||full	1391	No	NA		0	0	Low-quality	Genome-fragment		3.32	0
S14_vs_k141_25852||full	107963	No	NA		2	29	Medium-quality	Genome-fragment		69.62	0
S13_extracted_136965	1392	No	NA		2	0	Low-quality	Genome-fragment		3.46	0
S14_vs_k141_36772||full	1392	No	NA		0	0	Low-quality	Genome-fragment		3.39	0
S14_vs_k141_57506||full	1392	No	NA		0	0	Low-quality	Genome-fragment		3.47	0
S14_vs_k141_92342||full	2953	No	NA		0	0	Not-determined	Genome-fragment	NA		0
S1_vs_k141_58112||full	1392	No	NA		1	0	Low-quality	Genome-fragment		29.78	0
S14_vs_k141_25877||full	2507	No	NA		0	0	Not-determined	Genome-fragment	NA		0
S14_vs_k141_19223||full	1561	No	NA		0	0	Not-determined	Genome-fragment	NA		0
S1_vs_k141_34393||lt2gene	1392	No	NA		1	0	Low-quality	Genome-fragment		3.88	0
S14_vs_k141_11510||full	66098	No	NA		1	19	Medium-quality	Genome-fragment		58.68	0
S3_extracted_k141_79286	1392	No	NA		0	0	Low-quality	Genome-fragment		1.47	0
S14_vs_k141_4037||full	4235	No	NA		0	1	Not-determined	Genome-fragment	NA		0
S14_vs_k141_11522||full	1867	No	NA		0	2	Not-determined	Genome-fragment	NA		0
S14_vs_k141_85078||full	2209	No	NA		0	2	Not-determined	Genome-fragment	NA		0
S5_extracted_k141_87538	1392	No	NA		3	0	Low-quality	Genome-fragment		4.13	0
S5_extracted_k141_130972	1392	No	NA		1	0	Low-quality	Genome-fragment		3.82	0
S14_vs_k141_33541||full	5493	No	NA		0	4	Not-determined	Genome-fragment	NA		0
S7_vs_84737||lt2gene	1392	No	NA		1	0	Low-quality	Genome-fragment		3.16	0
S14_vs_k141_4097||full	1472	No	NA		0	1	Not-determined	Genome-fragment	NA		0
S10_vs_89160||full	1392	No	NA		1	0	Low-quality	Genome-fragment		3.73	0
S14_vs_k141_62577||full	1294	No	NA		0	0	Not-determined	Genome-fragment	NA		0
S14_vs_k141_19329||full	5498	No	NA		0	1	Not-determined	Genome-fragment	NA		0
S8_extracted_k141_86084	1392	No	NA		0	1	Low-quality	Genome-fragment		1.1	0
S14_vs_k141_41354||full	2674	No	NA		0	2	Not-determined	Genome-fragment	NA		0
S9_vs_k141_55411||lt2gene	1393	No	NA		1	0	Low-quality	Genome-fragment		2.24	0
S14_vs_k141_25933||full	5352	No	NA		0	2	Not-determined	Genome-fragment	NA		0
S1_vs_k141_89819||full	1393	No	NA		0	2	Low-quality	Genome-fragment		1.89	0
S14_vs_k141_92465||full	1666	No	NA		0	0	Not-determined	Genome-fragment	NA		0
S5_extracted_k141_120953	1393	No	NA		1	0	Low-quality	Genome-fragment		0.41	0
S7_extracted_71777	1393	No	NA		0	1	Low-quality	Genome-fragment		0.98	0
S7_vs_47193||lt2gene	1393	No	NA		1	0	Low-quality	Genome-fragment		4.5	0
S16_extracted_107656	1393	No	NA		0	0	Low-quality	Genome-fragment		1.17	0
S14_vs_k141_41420||full	1560	No	NA		0	0	Not-determined	Genome-fragment	NA		0
S14_vs_k141_114422||full	3118	No	NA		0	1	Not-determined	Genome-fragment	NA		0
S14_vs_k141_41422||full	5436	No	NA		0	4	Not-determined	Genome-fragment	NA		0
S16_vs_24186||full	1393	No	NA		1	0	Low-quality	Genome-fragment		3.16	0
S14_vs_k141_19445||full	1634	No	NA		0	2	Not-determined	Genome-fragment	NA		0
S14_vs_k141_41437||full	3743	No	NA		0	3	Not-determined	Genome-fragment	NA		0
S10_vs_51363||full	1393	No	NA		1	0	Low-quality	Genome-fragment		1.2	0
S14_vs_k141_92517||full	2876	No	NA		0	2	Not-determined	Genome-fragment	NA		0
S6_vs_k141_40758||full	1393	No	NA		0	0	Low-quality	Genome-fragment		2.36	0
S8_extracted_k141_72173	1393	No	NA		0	0	Low-quality	Genome-fragment		1.26	0
S8_vs_k141_3475||full	1393	No	NA		0	1	Low-quality	Genome-fragment		0.92	0
S9_vs_k141_12174||full	1394	No	NA		0	0	Low-quality	Genome-fragment		1.01	0
S1_extracted_k141_52848	1394	No	NA		1	0	Low-quality	Genome-fragment		2.99	0
S1_vs_k141_107800||full	1394	No	NA		0	1	Low-quality	Genome-fragment		2.57	0
S3_vs_k141_100339||full	1394	No	NA		0	0	Low-quality	Genome-fragment		2.28	0
S14_vs_k141_62734||full	1270	No	NA		0	0	Not-determined	Genome-fragment	NA		0
S5_extracted_k141_107956	1394	No	NA		0	0	Low-quality	Genome-fragment		0.95	0
S14_vs_k141_19517||full	1113	No	NA		0	0	Not-determined	Genome-fragment	NA		0
S5_vs_k141_98819||full	1394	No	NA		0	0	Low-quality	Genome-fragment		2.41	0
S14_vs_k141_62743||full	5683	No	NA		0	4	Not-determined	Genome-fragment	NA		0
S7_vs_83540||full	1394	No	NA		0	0	Low-quality	Genome-fragment		3.12	0
S14_vs_k141_114499||full	1098	No	NA		0	1	Not-determined	Genome-fragment	NA		0
S14_vs_k141_4191||full	2115	No	NA		0	0	Not-determined	Genome-fragment	NA		0
S14_vs_k141_62771||full	1042	No	NA		0	0	Not-determined	Genome-fragment	NA		0
S7_vs_132921||full	1394	No	NA		1	0	Low-quality	Genome-fragment		3.01	0
S10_extracted_14017	1394	No	NA		0	0	Low-quality	Genome-fragment		3.11	0
S12_extracted_k141_104870	1394	No	NA		0	0	Low-quality	Genome-fragment		2.25	0
S8_extracted_k141_118988	1394	No	NA		1	0	Low-quality	Genome-fragment		2.45	0
S14_vs_k141_85269||full	1040	No	NA		0	1	Not-determined	Genome-fragment	NA		0
S14_vs_k141_4253||full	1855	No	NA		0	2	Not-determined	Genome-fragment	NA		0
S8_vs_k141_46417||full	1394	No	NA		0	0	Low-quality	Genome-fragment		3.11	0
S17_vs_k141_148982||full	1395	No	NA		0	2	Low-quality	Genome-fragment		0.98	0
S17_vs_k141_154910||full	1395	No	NA		2	0	Low-quality	Genome-fragment		2.15	0
S14_vs_k141_55884||full	2308	No	NA		0	2	Not-determined	Genome-fragment	NA		0
S11_vs_k141_22903||full	1395	No	NA		0	2	Low-quality	Genome-fragment		0.63	0
S14_vs_k141_19598||full	1829	No	NA		0	0	Not-determined	Genome-fragment	NA		0
S14_vs_k141_4291||full	1672	No	NA		0	0	Not-determined	Genome-fragment	NA		0
S14_extracted_k141_44872	1395	No	NA		4	0	Low-quality	Genome-fragment		4.51	0
S14_vs_k141_114690||lt2gene	1395	No	NA		2	0	Low-quality	Genome-fragment		3.37	0
S1_vs_k141_38014||full	1395	No	NA		1	0	Low-quality	Genome-fragment		3.24	0
S3_extracted_k141_59311	1395	No	NA		0	0	Low-quality	Genome-fragment		5.19	0
S14_vs_k141_33682||full	2448	No	NA		0	1	Not-determined	Genome-fragment	NA		0
S14_vs_k141_11660||full	1885	No	NA		0	1	Not-determined	Genome-fragment	NA		0
S3_vs_k141_83117||full	1395	No	NA		1	0	Low-quality	Genome-fragment		2.73	0
S7_extracted_59193	1395	No	NA		0	1	Low-quality	Genome-fragment		2.39	0
S14_vs_k141_70455||full	2464	No	NA		0	0	Not-determined	Genome-fragment	NA		0
S7_extracted_108526	1395	Yes		326	1	1	Low-quality	Genome-fragment		0.7	76.63
S8_vs_k141_14240||lt2gene	1395	No	NA		1	0	Low-quality	Genome-fragment		1.57	0
S14_vs_k141_114648||full	3604	No	NA		0	3	Not-determined	Genome-fragment	NA		0
S14_vs_k141_64335||full	1396	No	NA		0	0	Low-quality	Genome-fragment		2.98	0
S14_vs_k141_33707||full	1013	No	NA		0	0	Not-determined	Genome-fragment	NA		0
S14_vs_k141_4342||full	1943	No	NA		0	0	Not-determined	Genome-fragment	NA		0
S3_vs_k141_82510||full	1396	No	NA		1	0	Low-quality	Genome-fragment		3.39	0
S14_vs_k141_114657||full	4493	No	NA		0	1	Not-determined	Genome-fragment	NA		0

S16_extracted_154831	1396	No	NA		0	2	Low-quality	Genome-fragment		3.94	0
S14_vs_k141_33719||full	2099	No	NA		0	1	Not-determined	Genome-fragment	NA		0
S10_vs_101297||lt2gene	1396	No	NA		2	0	Low-quality	Genome-fragment		2.4	0
S14_vs_k141_26380||full	1397	No	NA		0	0	Low-quality	Genome-fragment		4.05	0
S14_vs_k141_107472||full	4976	No	NA		0	4	Not-determined	Genome-fragment	NA		0
S14_vs_k141_85373||full	1468	No	NA		0	0	Not-determined	Genome-fragment	NA		0
S14_vs_k141_92797||full	1356	No	NA		0	0	Not-determined	Genome-fragment	NA		0
S1_vs_k141_155456||lt2gene	1397	No	NA		1	0	Low-quality	Genome-fragment		3.06	0
S3_extracted_k141_91242	1397	No	NA		2	0	Low-quality	Genome-fragment		2.29	0
S5_extracted_k141_114417	1397	No	NA		0	0	Low-quality	Genome-fragment		3.38	0
S14_vs_k141_92821||full	12227	No	NA		0	2	Not-determined	Genome-fragment	NA		0
S14_vs_k141_26180||full	3982	No	NA		0	2	Not-determined	Genome-fragment	NA		0
S7_extracted_137347	1397	No	NA		1	0	Low-quality	Genome-fragment		2.43	0
S14_vs_k141_70579||full	1289	No	NA		0	0	Not-determined	Genome-fragment	NA		0
S16_vs_75404||full	1397	No	NA		2	0	Low-quality	Genome-fragment		2.99	0
S14_vs_k141_78078||full	5167	No	NA		0	3	Not-determined	Genome-fragment	NA		0
S12_vs_k141_63513||full	1397	No	NA		0	0	Low-quality	Genome-fragment		2.45	0
S4_extracted_25293	1397	No	NA		0	2	Low-quality	Genome-fragment		0.78	0
S14_vs_k141_26208||full	2870	No	NA		0	2	Not-determined	Genome-fragment	NA		0
S8_vs_k141_888||full	1397	No	NA		0	1	Low-quality	Genome-fragment		3.23	0
S14_vs_k141_19724||full	1917	No	NA		0	1	Not-determined	Genome-fragment	NA		0
S17_vs_k141_96326||full	1398	No	NA		0	0	Low-quality	Genome-fragment		2.11	0
S14_extracted_k141_79927	1398	No	NA		1	0	Low-quality	Genome-fragment		2.13	0
S1_vs_k141_120906||full	1398	No	NA		1	0	Low-quality	Genome-fragment		3.97	0
S14_vs_k141_85424||full	1455	No	NA		0	0	Not-determined	Genome-fragment	NA		0
S1_vs_k141_141061||full	1398	No	NA		0	0	Low-quality	Genome-fragment		3.46	0
S14_vs_k141_107540||full	1834	No	NA		0	0	Not-determined	Genome-fragment	NA		0
S14_vs_k141_41743||full	1184	No	NA		0	1	Not-determined	Genome-fragment	NA		0
S1_vs_k141_2797||full	1398	No	NA		0	0	Low-quality	Genome-fragment		2.96	0
S14_vs_k141_107544||full	5718	No	NA		0	4	Not-determined	Genome-fragment	NA		0
S3_extracted_k141_115575	1398	No	NA		2	0	Low-quality	Genome-fragment		4.61	0
S3_vs_k141_35725||full	1398	No	NA		1	0	Low-quality	Genome-fragment		0.46	0
S14_vs_k141_19787||full	1806	No	NA		0	0	Not-determined	Genome-fragment	NA		0
S14_vs_k141_26283||full	2362	No	NA		0	2	Not-determined	Genome-fragment	NA		0
S14_vs_k141_107564||full	2340	No	NA		0	2	Not-determined	Genome-fragment	NA		0
S7_vs_92674||full	1398	No	NA		0	0	Low-quality	Genome-fragment		3.8	0
S16_extracted_123900	1398	No	NA		0	1	Low-quality	Genome-fragment		2.64	0
S14_vs_k141_107573||full	1168	No	NA		0	0	Not-determined	Genome-fragment	NA		0
S6_vs_k141_11680||full	1398	No	NA		0	2	Low-quality	Genome-fragment		0.44	0
S8_vs_k141_97942||lt2gene	1398	No	NA		1	0	Low-quality	Genome-fragment		3.27	0
S17_extracted_k141_98213	1399	No	NA		1	0	Low-quality	Genome-fragment		2.34	0
S17_extracted_k141_110641	1399	No	NA		0	0	Low-quality	Genome-fragment		3.52	0
S14_vs_k141_107611||full	1403	No	NA		0	1	Not-determined	Genome-fragment	NA		0
S14_vs_k141_41831||full	1573	No	NA		0	2	Not-determined	Genome-fragment	NA		0
S9_vs_k141_3349||full	1399	No	NA		0	1	Low-quality	Genome-fragment		3.32	0
S14_vs_k141_85509||full	1445	No	NA		0	0	Not-determined	Genome-fragment	NA		0
S11_extracted_k141_19923	1399	No	NA		0	0	Low-quality	Genome-fragment		4.09	0
S14_vs_k141_112796||full	1399	No	NA		1	0	Low-quality	Genome-fragment		3.57	0
S14_vs_k141_70801||full	2350	No	NA		0	2	Not-determined	Genome-fragment	NA		0
S1_extracted_k141_54785	1399	No	NA		1	0	Low-quality	Genome-fragment		2.84	0
S14_vs_k141_107669||full	4491	No	NA		0	4	Not-determined	Genome-fragment	NA		0
S14_vs_k141_70810||full	2275	No	NA		0	1	Not-determined	Genome-fragment	NA		0
S14_vs_k141_41869||full	1392	No	NA		0	0	Not-determined	Genome-fragment	NA		0
S14_vs_k141_114886||full	1230	No	NA		0	0	Not-determined	Genome-fragment	NA		0
S3_extracted_k141_73391	1399	No	NA		0	0	Low-quality	Genome-fragment		2.31	0
S14_vs_k141_78159||full	1525	No	NA		0	1	Not-determined	Genome-fragment	NA		0
S5_vs_k141_25872||full	1399	No	NA		0	1	Low-quality	Genome-fragment		1.91	0
S14_vs_k141_85558||full	2362	No	NA		0	2	Not-determined	Genome-fragment	NA		0
S14_vs_k141_34001||full	3147	No	NA		0	2	Not-determined	Genome-fragment	NA		0
S14_vs_k141_41888||full	1174	No	NA		0	1	Not-determined	Genome-fragment	NA		0
S14_vs_k141_4604||full	1300	No	NA		0	1	Not-determined	Genome-fragment	NA		0
S14_vs_k141_4607||full	3109	No	NA		0	3	Not-determined	Genome-fragment	NA		0
S14_vs_k141_85617||full	6246	No	NA		0	0	Not-determined	Genome-fragment	NA		0
S5_vs_k141_15035||full	1399	No	NA		1	0	Low-quality	Genome-fragment		3.67	0
S14_vs_k141_78164||full	38093	Yes		33442	8	6	Medium-quality	Genome-fragment		78.08	12.21
S14_vs_k141_19952||full	2360	No	NA		0	1	Not-determined	Genome-fragment	NA		0
S12_extracted_k141_37641	1399	No	NA		1	0	Low-quality	Genome-fragment		5.15	0
S12_vs_k141_180556||full	1399	No	NA		1	0	Low-quality	Genome-fragment		2.45	0
S14_vs_k141_89424||full	1400	No	NA		1	0	Low-quality	Genome-fragment		2.59	0
S5_extracted_k141_51130	1400	No	NA		0	0	Low-quality	Genome-fragment		2.47	0
S14_vs_k141_48196||full	2244	No	NA		0	0	Not-determined	Genome-fragment	NA		0
S5_vs_k141_18831||full	1400	No	NA		0	1	Low-quality	Genome-fragment		1.07	0
S14_vs_k141_114951||full	1129	No	NA		0	0	Not-determined	Genome-fragment	NA		0
S5_vs_k141_71824||lt2gene	1400	No	NA		2	0	Low-quality	Genome-fragment		3.81	0
S14_vs_k141_78218||full	3306	No	NA		0	1	Not-determined	Genome-fragment	NA		0
S14_vs_k141_26348||full	1470	No	NA		0	1	Not-determined	Genome-fragment	NA		0
S14_vs_k141_26351||full	3103	No	NA		0	3	Not-determined	Genome-fragment	NA		0
S14_vs_k141_4745||full	3236	No	NA		0	0	Not-determined	Genome-fragment	NA		0
S16_vs_583||full	1400	No	NA		0	0	Low-quality	Genome-fragment		1.01	0
S16_vs_87424||full	1400	No	NA		1	0	Low-quality	Genome-fragment		2.7	0
S16_vs_150908||full	1400	No	NA		1	0	Low-quality	Genome-fragment		2.47	0
S14_vs_k141_99849||full	2765	No	NA		0	1	Not-determined	Genome-fragment	NA		0
S14_vs_k141_12004||full	5072	No	NA		0	0	Not-determined	Genome-fragment	NA		0
S12_vs_k141_33631||full	1400	No	NA		2	0	Low-quality	Genome-fragment		2.6	0
S4_extracted_15963	1400	No	NA		0	0	Low-quality	Genome-fragment		3.51	0
S6_vs_k141_24720||full	1400	No	NA		1	0	Low-quality	Genome-fragment		1.7	0
S14_vs_k141_42052||full	1004	No	NA		0	1	Not-determined	Genome-fragment	NA		0
S14_vs_k141_4799||full	3945	No	NA		0	2	Not-determined	Genome-fragment	NA		0
S8_vs_k141_137626||full	1400	No	NA		1	0	Low-quality	Genome-fragment		2.76	0
S14_vs_k141_85759||full	5913	No	NA		0	4	Not-determined	Genome-fragment	NA		0
S14_vs_k141_93211||full	1920	No	NA		0	0	Not-determined	Genome-fragment	NA		0
S14_vs_k141_99908||full	1210	No	NA		0	3	Not-determined	Genome-fragment	NA		0
S14_vs_k141_107829||full	2104	No	NA		0	1	Not-determined	Genome-fragment	NA		0
S14_vs_k141_4847||full	2572	No	NA		0	2	Not-determined	Genome-fragment	NA		0
S14_vs_k141_78282||full	2080	No	NA		0	0	Not-determined	Genome-fragment	NA		0
S14_vs_k141_78296||full	1384	No	NA		0	0	Not-determined	Genome-fragment	NA		0
S14_vs_k141_93237||full	1219	No	NA		0	0	Not-determined	Genome-fragment	NA		0
S17_vs_k141_173398||full	1401	No	NA		1	0	Low-quality	Genome-fragment		1.57	0
S9_vs_k141_47537||full	1401	No	NA		0	1	Low-quality	Genome-fragment		2.9	0
S14_vs_k141_12087||full	1496	No	NA		0	1	Not-determined	Genome-fragment	NA		0
S13_vs_4321||full	1401	No	NA		1	0	Low-quality	Genome-fragment		0.63	0
S14_vs_k141_99951||full	1343	No	NA		0	3	Not-determined	Genome-fragment	NA		0
S14_vs_k141_53659||full	1401	No	NA		0	1	Low-quality	Genome-fragment		3.01	0
S3_extracted_k141_113642	1401	No	NA		2	0	Low-quality	Genome-fragment		2.22	0
S5_vs_k141_11718||full	1401	No	NA		0	1	Low-quality	Genome-fragment		0.43	0
S14_vs_k141_56251||full	31100	No	NA		18	1	Medium-quality	Genome-fragment		75.74	0
S14_vs_k141_115062||full	1735	No	NA		0	0	Not-determined	Genome-fragment	NA		0
S14_vs_k141_56261||full	2466	No	NA		0	1	Not-determined	Genome-fragment	NA		0
S14_vs_k141_115075||full	1248	No	NA		0	0	Not-determined	Genome-fragment	NA		0
S14_vs_k141_70985||full	158000	No	NA		4	46	Medium-quality	Genome-fragment		77.59	0
S7_vs_854||full	1401	No	NA		0	1	Low-quality	Genome-fragment		2.45	0
S7_vs_44556||full	1401	No	NA		0	0	Low-quality	Genome-fragment		2.42	0
S14_vs_k141_93284||full	1281	No	NA		0	1	Not-determined	Genome-fragment	NA		0
S14_vs_k141_63402||full	1196	No	NA		0	1	Not-determined	Genome-fragment	NA		0
S10_vs_25876||lt2gene	1401	No	NA		1	0	Low-quality	Genome-fragment		3.12	0
S15_extracted_59383	1401	No	NA		0	1	Low-quality	Genome-fragment		0.54	0
S8_extracted_k141_206020	1401	No	NA		0	2	Low-quality	Genome-fragment		3.24	0
S14_vs_k141_42195||full	2371	No	NA		0	1	Not-determined	Genome-fragment	NA		0
S8_vs_k141_43913||lt2gene	1401	No	NA		1	0	Low-quality	Genome-fragment		3.47	0
S14_vs_k141_93354||full	2137	No	NA		0	2	Not-determined	Genome-fragment	NA		0
S14_vs_k141_48416||full	5289	No	NA		0	3	Not-determined	Genome-fragment	NA		0
S14_vs_k141_12167||full	1275	No	NA		0	0	Not-determined	Genome-fragment	NA		0
S14_vs_k141_71051||full	1241	No	NA		0	2	Not-determined	Genome-fragment	NA		0
S3_extracted_k141_64860	1402	No	NA		3	0	Low-quality	Genome-fragment		6.4	0
S14_vs_k141_34300||full	4816	No	NA		0	3	Not-determined	Genome-fragment	NA		0
S12_vs_k141_140580||full	1402	No	NA		0	0	Low-quality	Genome-fragment		3.6	0
S12_vs_k141_59957||lt2gene	1402	No	NA		1	0	Low-quality	Genome-fragment		3.35	0
S15_vs_70553||full	1402	No	NA		0	0	Low-quality	Genome-fragment		2.21	0
S17_extracted_k141_135821	1403	No	NA		4	0	Low-quality	Genome-fragment		4.6	0
S3_vs_k141_48910||full	1403	No	NA		0	0	Low-quality	Genome-fragment		1.75	0
S3_vs_k141_22838||full	1403	No	NA		0	1	Low-quality	Genome-fragment		2.06	0
S5_vs_k141_72607||full	1403	No	NA		0	1	Low-quality	Genome-fragment		1.91	0
S14_vs_k141_115202||full	1132	No	NA		0	1	Not-determined	Genome-fragment	NA		0
S16_vs_117760||full	1403	No	NA		0	2	Low-quality	Genome-fragment		1.16	0
S14_vs_k141_26522||full	6819	No	NA		0	4	Not-determined	Genome-fragment	NA		0
S16_vs_146828||full	1403	No	NA		0	0	Low-quality	Genome-fragment		3.39	0
S14_vs_k141_63514||full	1201	No	NA		0	0	Not-determined	Genome-fragment	NA		0
S10_extracted_12866	1403	No	NA		1	0	Low-quality	Genome-fragment		1.12	0
S14_vs_k141_56410||full	19996	No	NA		7	0	Medium-quality	Genome-fragment		50.21	0
S14_vs_k141_78500||full	1635	No	NA		0	0	Not-determined	Genome-fragment	NA		0
S12_vs_k141_176841||full	1403	No	NA		0	1	Low-quality	Genome-fragment		0.99	0
S14_extracted_k141_11450	1404	No	NA		2	0	Low-quality	Genome-fragment		2.34	0
S14_vs_k141_108029||full	3548	No	NA		0	4	Not-determined	Genome-fragment	NA		0
S14_vs_k141_12216||full	6583	No	NA		0	5	Not-determined	Genome-fragment	NA		0
S1_extracted_k141_60373	1404	No	NA		0	0	Low-quality	Genome-fragment		1.02	0
S1_extracted_k141_42742	1404	No	NA		0	2	Low-quality	Genome-fragment		0.57	0
S14_vs_k141_108054||full	7289	No	NA		0	4	Not-determined	Genome-fragment	NA		0
S14_vs_k141_63578||full	1325	No	NA		0	1	Not-determined	Genome-fragment	NA		0
S1_vs_k141_63380||lt2gene	1404	No	NA		2	0	Low-quality	Genome-fragment		3.44	0
S3_vs_k141_87816||full	1404	No	NA		1	0	Low-quality	Genome-fragment		6.41	0
S14_vs_k141_42386||full	2102	No	NA		0	0	Not-determined	Genome-fragment	NA		0
S14_vs_k141_5085||full	1142	No	NA		0	2	Not-determined	Genome-fragment	NA		0
S3_vs_k141_25561||lt2gene	1404	No	NA		1	0	Low-quality	Genome-fragment		2.66	0
S5_extracted_k141_85074	1404	No	NA		2	0	Low-quality	Genome-fragment		4.48	0
S14_vs_k141_78589||full	1220	No	NA		0	0	Not-determined	Genome-fragment	NA		0
S5_vs_k141_99881||full	1404	No	NA		1	0	Low-quality	Genome-fragment		1.74	0
S14_vs_k141_26585||full	1106	No	NA		0	1	Not-determined	Genome-fragment	NA		0
S5_vs_k141_106625||lt2gene	1404	No	NA		2	0	Low-quality	Genome-fragment		2.59	0
S7_vs_48363||lt2gene	1404	No	NA		1	0	Low-quality	Genome-fragment		0.87	0
NC3_vf_k141_707	1404	No	NA		0	0	Low-quality	Genome-fragment		2.76	0
S16_vs_129366||full	1404	No	NA		0	0	Low-quality	Genome-fragment		1.2	0
S16_vs_164497||full	1404	No	NA		1	0	Low-quality	Genome-fragment		3.24	0
S14_vs_k141_100227||full	24160	No	NA		19	1	Medium-quality	Genome-fragment		56.88	0
S12_extracted_k141_52512	1404	No	NA		2	0	Low-quality	Genome-fragment		2.34	0
S12_vs_k141_130395||full	1404	No	NA		0	0	Low-quality	Genome-fragment		3.33	0
S4_extracted_10611	1404	No	NA		0	0	Low-quality	Genome-fragment		1.34	0
S14_vs_k141_115370||full	2045	No	NA		0	1	Not-determined	Genome-fragment	NA		0
S14_vs_k141_42466||full	1978	No	NA		0	0	Not-determined	Genome-fragment	NA		0
S8_vs_k141_49092||full	1404	No	NA		1	0	Low-quality	Genome-fragment		3.19	0
S14_vs_k141_85948||full	1467	No	NA		0	1	Not-determined	Genome-fragment	NA		0
S14_vs_k141_85950||full	1369	No	NA		0	0	Not-determined	Genome-fragment	NA		0
S17_vs_k141_44782||full	1405	No	NA		0	0	Low-quality	Genome-fragment		3.09	0
S14_vs_k141_42489||full	4768	No	NA		0	5	Not-determined	Genome-fragment	NA		0
S11_vs_k141_90718||full	1405	No	NA		0	0	Low-quality	Genome-fragment		3.72	0

S13_vs_149929||full	1405	No	NA	0	0	Low-quality	Genome-fragment		3.55	0
S14_vs_k141_100017||full	1405	No	NA	1	0	Low-quality	Genome-fragment		3.11	0
S14_vs_k141_108159||full	1381	No	NA	0	0	Not-determined	Genome-fragment	NA		0
S14_vs_k141_115377||full	24316	No	NA	10	0	Medium-quality	Genome-fragment		53.35	0
S1_extracted_k141_126854	1405	No	NA	0	0	Low-quality	Genome-fragment		2.45	0
S14_vs_k141_34548||full	4626	No	NA	0	4	Not-determined	Genome-fragment	NA		0
S1_extracted_k141_119293	1405	No	NA	0	0	Low-quality	Genome-fragment		0.96	0
S3_vs_k141_104964||full	1405	No	NA	0	0	Low-quality	Genome-fragment		3.53	0
S14_vs_k141_63741||full	2056	No	NA	0	2	Not-determined	Genome-fragment	NA		0
S3_vs_k141_91808||full	1405	No	NA	2	0	Low-quality	Genome-fragment		0.71	0
S3_vs_k141_92178||lt2gene	1405	No	NA	1	0	Low-quality	Genome-fragment		2.36	0
S14_vs_k141_34568||full	1452	No	NA	0	0	Not-determined	Genome-fragment	NA		0
S7_extracted_57687	1405	No	NA	0	0	Low-quality	Genome-fragment		2.35	0
S14_vs_k141_34583||full	2716	No	NA	0	2	Not-determined	Genome-fragment	NA		0
S14_vs_k141_26768||full	8432	No	NA	0	5	Not-determined	Genome-fragment	NA		0
S14_vs_k141_108193||full	2805	No	NA	0	0	Not-determined	Genome-fragment	NA		0
S16_vs_171681||full	1405	No	NA	0	1	Low-quality	Genome-fragment		1.06	0
S10_vs_87897||full	1405	No	NA	1	0	Low-quality	Genome-fragment		1.77	0
S14_vs_k141_42577||full	12726	No	NA	0	11	Not-determined	Genome-fragment	NA		0
S4_extracted_28323	1405	No	NA	0	0	Low-quality	Genome-fragment		2.12	0
S14_vs_k141_108218||full	1290	No	NA	0	0	Not-determined	Genome-fragment	NA		0
S8_vs_k141_155891||full	1405	No	NA	0	0	Low-quality	Genome-fragment		4.17	0
S14_vs_k141_93565||full	16641	No	NA	8	0	Medium-quality	Genome-fragment		70.27	0
S17_vs_k141_78281||full	1406	No	NA	1	0	Low-quality	Genome-fragment		0.11	0
S14_vs_k141_71397||full	3157	No	NA	0	1	Not-determined	Genome-fragment	NA		0
S14_extracted_k141_44157	1406	No	NA	2	0	Low-quality	Genome-fragment		1.51	0
S14_vs_k141_56642||full	1310	No	NA	0	0	Not-determined	Genome-fragment	NA		0
S1_vs_k141_151255||full	1406	No	NA	0	1	Low-quality	Genome-fragment		0.82	0
S14_vs_k141_56643||full	1542	No	NA	0	0	Not-determined	Genome-fragment	NA		0
S14_vs_k141_12542||full	2130	No	NA	0	0	Not-determined	Genome-fragment	NA		0
S14_vs_k141_20193||full	48326	No	NA	22	1	High-quality	High-quality		100	0
S14_vs_k141_26872||full	2316	No	NA	0	0	Not-determined	Genome-fragment	NA		0
S3_vs_k141_42453||full	1406	No	NA	3	0	Low-quality	Genome-fragment		4.65	0
S14_vs_k141_34725||full	1219	No	NA	0	0	Not-determined	Genome-fragment	NA		0
S14_vs_k141_42705||full	7484	No	NA	0	5	Not-determined	Genome-fragment	NA		0
S5_extracted_k141_38634	1406	No	NA	0	0	Low-quality	Genome-fragment		4.14	0
S5_vs_k141_85486||lt2gene	1406	No	NA	2	0	Low-quality	Genome-fragment		2.49	0
S7_vs_55740||full	1406	No	NA	0	1	Low-quality	Genome-fragment		1.64	0
S14_vs_k141_42718||full	1167	No	NA	0	0	Not-determined	Genome-fragment	NA		0
S14_vs_k141_100470||full	1226	No	NA	0	0	Not-determined	Genome-fragment	NA		0
S14_vs_k141_34768||full	2455	No	NA	0	2	Not-determined	Genome-fragment	NA		0
S14_vs_k141_93661||full	5972	No	NA	0	3	Not-determined	Genome-fragment	NA		0
S7_vs_137273||full	1406	No	NA	0	1	Low-quality	Genome-fragment		0.44	0
S12_extracted_k141_161611	1406	No	NA	0	0	Low-quality	Genome-fragment		3.54	0
S12_vs_k141_143949||full	1406	No	NA	0	3	Low-quality	Genome-fragment		2.05	0
S14_vs_k141_115455||full	40864	No	NA	15	1	Medium-quality	Genome-fragment		67.27	0
S14_vs_k141_100488||full	2151	No	NA	0	2	Not-determined	Genome-fragment	NA		0
S8_extracted_k141_69444	1406	No	NA	0	2	Low-quality	Genome-fragment		0.91	0
S8_vs_k141_109649||full	1406	No	NA	0	0	Low-quality	Genome-fragment		3.09	0
S14_vs_k141_71438||full	1169	No	NA	0	1	Not-determined	Genome-fragment	NA		0
S14_vs_k141_5339||full	2460	No	NA	0	3	Not-determined	Genome-fragment	NA		0
S11_extracted_k141_30556	1407	No	NA	1	0	Low-quality	Genome-fragment		3.45	0
S14_vs_k141_26993||full	1270	No	NA	0	1	Not-determined	Genome-fragment	NA		0
S14_vs_k141_56733||full	2777	No	NA	0	3	Not-determined	Genome-fragment	NA		0
S14_extracted_k141_6679	1407	No	NA	1	0	Low-quality	Genome-fragment		1.52	0
S1_extracted_k141_157233	1407	No	NA	1	0	Low-quality	Genome-fragment		2.67	0
S14_vs_k141_108337||full	1043	No	NA	0	0	Not-determined	Genome-fragment	NA		0
S5_extracted_k141_111514	1407	No	NA	0	0	Low-quality	Genome-fragment		2.42	0
S14_vs_k141_12662||full	2278	No	NA	0	0	Not-determined	Genome-fragment	NA		0
S7_vs_119069||lt2gene	1407	No	NA	1	0	Low-quality	Genome-fragment		0.97	0
S16_extracted_136490	1407	No	NA	0	0	Low-quality	Genome-fragment		2.96	0
S10_vs_35382||full	1407	No	NA	0	1	Low-quality	Genome-fragment		1.17	0
S12_vs_k141_40346||full	1407	No	NA	0	0	Low-quality	Genome-fragment		2.53	0
S8_vs_k141_95861||full	1407	No	NA	0	0	Low-quality	Genome-fragment		1.74	0
S13_vs_52568||full	1408	No	NA	0	0	Low-quality	Genome-fragment		2.8	0
S14_vs_k141_583||full	1408	No	NA	0	2	Low-quality	Genome-fragment		1.52	0
S14_vs_k141_34899||full	1878	No	NA	0	1	Not-determined	Genome-fragment	NA		0
S1_vs_k141_103446||full	1408	No	NA	1	0	Low-quality	Genome-fragment		2.79	0
S14_vs_k141_27049||full	6269	No	NA	0	3	Not-determined	Genome-fragment	NA		0
S14_vs_k141_108376||full	19905	No	NA	9	1	Medium-quality	Genome-fragment		55	0
S7_vs_99448||full	1408	No	NA	1	0	Low-quality	Genome-fragment		4.32	0
S7_vs_131214||lt2gene	1408	No	NA	1	0	Low-quality	Genome-fragment		4.65	0
S16_vs_130658||lt2gene	1408	No	NA	1	0	Low-quality	Genome-fragment		2.45	0
S14_vs_k141_78735||full	5404	No	NA	0	2	Not-determined	Genome-fragment	NA		0
S14_vs_k141_27062||full	1623	No	NA	0	3	Not-determined	Genome-fragment	NA		0
S10_vs_75252||full	1408	No	NA	0	2	Low-quality	Genome-fragment		0.69	0
S14_vs_k141_108396||full	1311	No	NA	0	1	Not-determined	Genome-fragment	NA		0
S14_vs_k141_48894||full	1024	No	NA	0	0	Not-determined	Genome-fragment	NA		0
S12_vs_k141_105313||lt2gene	1408	No	NA	1	0	Low-quality	Genome-fragment		2.93	0
S14_vs_k141_34934||full	1159	No	NA	0	0	Not-determined	Genome-fragment	NA		0
S17_vs_k141_89510||full	1409	No	NA	0	0	Low-quality	Genome-fragment		2	0
S9_extracted_k141_76644	1409	No	NA	0	2	Low-quality	Genome-fragment		0.71	0
S14_vs_k141_5418||full	1797	No	NA	0	3	Not-determined	Genome-fragment	NA		0
S14_vs_k141_63981||full	1651	No	NA	0	1	Not-determined	Genome-fragment	NA		0
S11_vs_k141_93428||full	1409	No	NA	0	0	Low-quality	Genome-fragment		3.22	0
S14_vs_k141_65827||full	1409	No	NA	2	0	Low-quality	Genome-fragment		4.41	0
S14_vs_k141_56834||full	1466	No	NA	0	2	Not-determined	Genome-fragment	NA		0
S14_vs_k141_78789||full	1389	No	NA	0	1	Not-determined	Genome-fragment	NA		0
S3_vs_k141_84863||full	1409	No	NA	0	0	Low-quality	Genome-fragment		0.83	0
S14_vs_k141_12788||full	1568	No	NA	0	2	Not-determined	Genome-fragment	NA		0
S3_vs_k141_20480||lt2gene	1409	No	NA	1	0	Low-quality	Genome-fragment		3.35	0
S14_vs_k141_20437||full	1511	No	NA	0	1	Not-determined	Genome-fragment	NA		0
S14_vs_k141_86335||full	5924	No	NA	0	4	Not-determined	Genome-fragment	NA		0
S14_vs_k141_27153||full	1640	No	NA	0	1	Not-determined	Genome-fragment	NA		0
S5_vs_k141_19190||lt2gene	1409	No	NA	1	0	Low-quality	Genome-fragment		2.06	0
S16_extracted_1005	1409	No	NA	1	0	Low-quality	Genome-fragment		4.07	0
S16_extracted_80526	1409	No	NA	2	0	Low-quality	Genome-fragment		4.39	0
S14_vs_k141_42940||full	2953	No	NA	0	2	Not-determined	Genome-fragment	NA		0
S16_vs_98547||full	1409	No	NA	1	0	Low-quality	Genome-fragment		0.87	0
S12_vs_k141_17400||lt2gene	1409	No	NA	1	0	Low-quality	Genome-fragment		0.97	0
S14_vs_k141_71631||full	2207	No	NA	0	1	Not-determined	Genome-fragment	NA		0
S8_extracted_k141_196984	1409	No	NA	1	0	Low-quality	Genome-fragment		22.1	0
S9_vs_k141_6056||full	1410	No	NA	0	1	Low-quality	Genome-fragment		3.07	0
S14_vs_k141_12855||full	1006	No	NA	0	0	Not-determined	Genome-fragment	NA		0
S13_extracted_95667	1410	No	NA	2	0	Low-quality	Genome-fragment		2.31	0
S14_vs_k141_12862||full	2615	No	NA	0	1	Not-determined	Genome-fragment	NA		0
S14_vs_k141_12868||full	3275	No	NA	0	3	Not-determined	Genome-fragment	NA		0
S14_vs_k141_78833||full	2621	No	NA	0	2	Not-determined	Genome-fragment	NA		0
S14_vs_k141_71653||full	2524	No	NA	0	0	Not-determined	Genome-fragment	NA		0
S5_extracted_k141_61455	1410	No	NA	1	0	Low-quality	Genome-fragment		1.59	0
S7_vs_118181||full	1410	No	NA	0	1	Low-quality	Genome-fragment		3.43	0
S14_vs_k141_49023||full	10696	No	NA	0	7	Not-determined	Genome-fragment	NA		0
S14_vs_k141_86421||full	2387	No	NA	0	2	Not-determined	Genome-fragment	NA		0
S14_vs_k141_27178||full	2103	No	NA	0	4	Not-determined	Genome-fragment	NA		0
S7_vs_120441||full	1410	No	NA	0	0	Low-quality	Genome-fragment		3.07	0
S14_vs_k141_27179||full	3105	No	NA	0	1	Not-determined	Genome-fragment	NA		0
S14_vs_k141_71678||full	2819	No	NA	0	4	Not-determined	Genome-fragment	NA		0
S14_vs_k141_27195||full	1277	No	NA	0	1	Not-determined	Genome-fragment	NA		0
S8_vs_k141_176717||full	1410	No	NA	0	0	Low-quality	Genome-fragment		4.5	0
S14_vs_k141_12914||full	1472	No	NA	0	0	Not-determined	Genome-fragment	NA		0
S14_vs_k141_93937||full	2891	No	NA	0	1	Not-determined	Genome-fragment	NA		0
S14_vs_k141_78884||full	1133	No	NA	0	0	Not-determined	Genome-fragment	NA		0
S9_vs_k141_17991||full	1411	No	NA	0	0	Low-quality	Genome-fragment		4.5	0
S11_extracted_k141_62570	1411	No	NA	0	1	Low-quality	Genome-fragment		2.82	0
S14_vs_k141_13626||full	1411	No	NA	2	0	Low-quality	Genome-fragment		1.3	0
S1_vs_k141_43629||full	1411	No	NA	3	0	Low-quality	Genome-fragment		3.66	0
S14_vs_k141_27241||full	2940	No	NA	0	3	Not-determined	Genome-fragment	NA		0
S3_vs_k141_100542||lt2gene	1411	No	NA	1	0	Low-quality	Genome-fragment		3.21	0
S14_vs_k141_108553||full	1194	No	NA	0	0	Not-determined	Genome-fragment	NA		0
S5_vs_k141_136814||full	1411	No	NA	2	0	Low-quality	Genome-fragment		3.17	0
S16_extracted_103142	1411	No	NA	0	0	Low-quality	Genome-fragment		1.95	0
S16_vs_134070||full	1411	No	NA	0	1	Low-quality	Genome-fragment		1	0
S14_vs_k141_43112||full	1521	No	NA	0	1	Not-determined	Genome-fragment	NA		0
S14_vs_k141_108570||full	4484	No	NA	0	6	Not-determined	Genome-fragment	NA		0
S10_vs_21324||full	1411	No	NA	2	0	Low-quality	Genome-fragment		2.26	0
S14_vs_k141_27279||full	7875	No	NA	0	5	Not-determined	Genome-fragment	NA		0
S10_vs_28166||full	1411	No	NA	0	0	Low-quality	Genome-fragment		2.94	0
S14_vs_k141_35144||full	1243	No	NA	0	1	Not-determined	Genome-fragment	NA		0
S10_vs_77663||full	1411	No	NA	1	0	Low-quality	Genome-fragment		2.19	0
S6_vs_k141_52038||full	1411	No	NA	0	2	Low-quality	Genome-fragment		0.83	0
S14_vs_k141_56989||full	1458	No	NA	0	0	Not-determined	Genome-fragment	NA		0
S6_vs_k141_26403||full	1411	No	NA	0	1	Low-quality	Genome-fragment		0.6	0
S11_vs_k141_52479||full	1412	No	NA	0	1	Low-quality	Genome-fragment		1.08	0
S11_vs_k141_70817||full	1412	No	NA	0	0	Low-quality	Genome-fragment		1.08	0
S14_vs_k141_20673||full	3315	No	NA	0	1	Not-determined	Genome-fragment	NA		0
S1_extracted_k141_68132	1412	No	NA	0	0	Low-quality	Genome-fragment		1.45	0
S14_vs_k141_43189||full	1556	No	NA	0	1	Not-determined	Genome-fragment	NA		0
S14_vs_k141_35204||full	2783	No	NA	0	4	Not-determined	Genome-fragment	NA		0
S1_extracted_k141_18605	1412	No	NA	0	1	Low-quality	Genome-fragment		3.09	0
S14_vs_k141_5616||full	2681	No	NA	0	2	Not-determined	Genome-fragment	NA		0
S1_vs_k141_16591||full	1412	No	NA	0	1	Low-quality	Genome-fragment		0.81	0
S7_vs_92812||full	1412	No	NA	1	0	Low-quality	Genome-fragment		3.58	0
S7_vs_94296||full	1412	No	NA	1	0	Low-quality	Genome-fragment		2.84	0
S14_vs_k141_64161||full	1362	No	NA	0	0	Not-determined	Genome-fragment	NA		0
S7_vs_109090||full	1412	No	NA	0	1	Low-quality	Genome-fragment		3.39	0
S14_vs_k141_43225||full	2028	No	NA	0	2	Not-determined	Genome-fragment	NA		0
S14_vs_k141_78979||full	2092	No	NA	0	1	Not-determined	Genome-fragment	NA		0
S16_vs_109211||full	1412	No	NA	1	0	Low-quality	Genome-fragment		1	0
S10_extracted_103947	1412	No	NA	0	1	Low-quality	Genome-fragment		3.98	0
S14_vs_k141_78984||full	12703	No	NA	0	8	Not-determined	Genome-fragment	NA		0
S14_vs_k141_100965||full	1539	No	NA	0	0	Not-determined	Genome-fragment	NA		0
S12_vs_k141_13851||full	1412	No	NA	0	2	Low-quality	Genome-fragment		0.45	0
S3_vs_k141_3225||lt2gene	1413	No	NA	2	0	Low-quality	Genome-fragment		3.13	0
S14_vs_k141_64199||full	1278	No	NA	0	1	Not-determined	Genome-fragment	NA		0
S5_vs_k141_87956||lt2gene	1413	No	NA	2	0	Low-quality	Genome-fragment		3.34	0
S14_vs_k141_27446||full	2485	No	NA	0	2	Not-determined	Genome-fragment	NA		0
S7_vs_3284||full	1413	No	NA	1	0	Low-quality	Genome-fragment		2.58	0
S7_vs_75071||full	1413	No	NA	0	0	Low-quality	Genome-fragment		3.32	0
S14_vs_k141_20836||full	1414	No	NA	0	2	Not-determined	Genome-fragment	NA		0
S2_extracted_k141_3536	1413	No	NA	0	1	Low-quality	Genome-fragment		0.79	0
S14_vs_k141_94273||full	1871	No	NA	0	2	Not-determined	Genome-fragment	NA		0
S6_vs_k141_43211||full	1413	No	NA	2	0	Low-quality	Genome-fragment		2.01	0
S14_vs_k141_20849||full	1418	No	NA	0	0	Not-determined	Genome-fragment	NA		0
S8_extracted_k141_101026	1413	No	NA	0	1	Low-quality	Genome-fragment		0.87	0

S8_vs_k141_112230||full	1413	No	NA	1	0	Low-quality	Genome-fragment		3.22	0
S11_extracted_k141_6979	1414	No	NA	0	0	Low-quality	Genome-fragment		3.64	0
S1_vs_k141_980||lt2gene	1414	No	NA	2	0	Low-quality	Genome-fragment		2.6	0
S10_vs_20219||full	1414	No	NA	0	0	Low-quality	Genome-fragment		3.36	0
S14_vs_k141_94320||full	3415	No	NA	0	2	Not-determined	Genome-fragment	NA		0
S14_vs_k141_35348||full	3970	No	NA	0	4	Not-determined	Genome-fragment	NA		0
S12_extracted_k141_959	1414	No	NA	0	0	Low-quality	Genome-fragment		2.79	0
S12_extracted_k141_187394	1414	No	NA	0	0	Low-quality	Genome-fragment		2.23	0
S15_extracted_23267	1414	No	NA	0	0	Low-quality	Genome-fragment		0.84	0
S14_vs_k141_101105||full	2766	No	NA	0	2	Not-determined	Genome-fragment	NA		0
S15_vs_5155||full	1414	No	NA	1	0	Low-quality	Genome-fragment		2.76	0
S8_vs_k141_122027||full	1414	No	NA	0	0	Low-quality	Genome-fragment		3.3	0
S8_vs_k141_151320||full	1414	No	NA	1	0	Low-quality	Genome-fragment		2.47	0
S11_vs_k141_16282||full	1415	No	NA	0	0	Low-quality	Genome-fragment		0.8	0
S1_extracted_k141_36038	1415	No	NA	0	1	Low-quality	Genome-fragment		0.24	0
S5_extracted_k141_8403	1415	No	NA	0	1	Low-quality	Genome-fragment		2.98	0
S14_vs_k141_35410||full	1147	No	NA	0	1	Not-determined	Genome-fragment	NA		0
S14_vs_k141_5887||full	3883	No	NA	0	2	Not-determined	Genome-fragment	NA		0
S5_vs_k141_61261||lt2gene	1415	No	NA	1	0	Low-quality	Genome-fragment		2.77	0
S16_extracted_160726	1415	No	NA	0	0	Low-quality	Genome-fragment		2.42	0
S14_vs_k141_49309||full	1234	No	NA	0	1	Not-determined	Genome-fragment	NA		0
S16_vs_13456||full	1415	No	NA	0	0	Low-quality	Genome-fragment		1.84	0
S16_vs_159084||full	1415	No	NA	0	1	Low-quality	Genome-fragment		0.87	0
S10_vs_34981||full	1415	No	NA	0	0	Low-quality	Genome-fragment		3.11	0
S14_vs_k141_13248||full	2233	No	NA	0	2	Not-determined	Genome-fragment	NA		0
S14_vs_k141_94393||full	1553	No	NA	0	0	Not-determined	Genome-fragment	NA		0
S14_vs_k141_35463||full	2960	No	NA	0	2	Not-determined	Genome-fragment	NA		0
S17_vs_k141_142064||full	1416	No	NA	0	1	Low-quality	Genome-fragment		1.81	0
S14_vs_k141_101172||full	2607	No	NA	0	1	Not-determined	Genome-fragment	NA		0
S14_vs_k141_94409||full	2076	No	NA	0	0	Not-determined	Genome-fragment	NA		0
S14_vs_k141_57266||full	1911	No	NA	0	0	Not-determined	Genome-fragment	NA		0
S14_vs_k141_13306||full	4614	No	NA	0	1	Not-determined	Genome-fragment	NA		0
S9_vs_k141_79683||lt2gene	1416	No	NA	1	0	Low-quality	Genome-fragment		3.26	0
S14_vs_k141_13310||full	6460	No	NA	0	1	Not-determined	Genome-fragment	NA		0
S14_extracted_k141_96924	1416	No	NA	0	0	Low-quality	Genome-fragment		1.98	0
S14_vs_k141_21021||full	1139	No	NA	0	1	Not-determined	Genome-fragment	NA		0
S14_vs_k141_101211||full	1118	No	NA	0	0	Not-determined	Genome-fragment	NA		0
S3_vs_k141_70307||full	1416	No	NA	0	2	Low-quality	Genome-fragment		0.91	0
S5_vs_k141_33952||full	1416	No	NA	1	0	Low-quality	Genome-fragment		3.59	0
S14_vs_k141_94458||full	2680	No	NA	0	1	Not-determined	Genome-fragment	NA		0
S12_extracted_k141_65880	1416	No	NA	1	0	Low-quality	Genome-fragment		0.87	0
S12_vs_k141_7898||full	1416	No	NA	0	1	Low-quality	Genome-fragment		2.92	0
S14_vs_k141_57342||full	1175	No	NA	0	0	Not-determined	Genome-fragment	NA		0
S12_vs_k141_37086||full	1416	No	NA	0	1	Low-quality	Genome-fragment		1.66	0
S14_vs_k141_5990||full	1389	No	NA	0	1	Not-determined	Genome-fragment	NA		0
S14_vs_k141_94475||full	2316	No	NA	0	2	Not-determined	Genome-fragment	NA		0
S8_extracted_k141_112695	1416	No	NA	0	1	Low-quality	Genome-fragment		0.32	0
S8_vs_k141_107404||lt2gene	1416	No	NA	2	0	Low-quality	Genome-fragment		4.33	0
S14_vs_k141_21040||full	1712	No	NA	0	1	Not-determined	Genome-fragment	NA		0
S17_extracted_k141_163096	1417	No	NA	0	1	Low-quality	Genome-fragment		2.59	0
S14_vs_k141_101256||full	1364	No	NA	0	0	Not-determined	Genome-fragment	NA		0
S9_vs_k141_70418||full	1417	No	NA	1	0	Low-quality	Genome-fragment		3.24	0
S11_vs_k141_46549||full	1417	No	NA	0	0	Low-quality	Genome-fragment		1.28	0
S14_vs_k141_8711||full	1417	No	NA	0	0	Low-quality	Genome-fragment		1.99	0
S14_vs_k141_72072||full	2635	No	NA	0	0	Not-determined	Genome-fragment	NA		0
S5_extracted_k141_133017	1417	No	NA	4	0	Low-quality	Genome-fragment		3.84	0
S14_vs_k141_64389||full	13951	No	NA	0	4	Not-determined	Genome-fragment	NA		0
S14_vs_k141_94519||full	1490	No	NA	0	0	Not-determined	Genome-fragment	NA		0
S14_vs_k141_57396||full	1746	No	NA	0	1	Not-determined	Genome-fragment	NA		0
S5_vs_k141_4647||full	1417	No	NA	2	0	Low-quality	Genome-fragment		2.44	0
S16_vs_69945||full	1417	No	NA	0	0	Low-quality	Genome-fragment		2.32	0
S10_vs_14888||full	1417	No	NA	0	0	Low-quality	Genome-fragment		2.78	0
S14_vs_k141_87100||full	4035	No	NA	0	4	Not-determined	Genome-fragment	NA		0
S12_extracted_k141_186779	1417	No	NA	0	0	Low-quality	Genome-fragment		2.92	0
S4_extracted_27892	1417	No	NA	0	0	Low-quality	Genome-fragment		3.26	0
S14_vs_k141_79397||full	3669	No	NA	0	1	Not-determined	Genome-fragment	NA		0
S14_vs_k141_116265||full	1196	No	NA	0	1	Not-determined	Genome-fragment	NA		0
S14_vs_k141_101324||full	1009	No	NA	0	0	Not-determined	Genome-fragment	NA		0
S8_extracted_k141_113378	1417	No	NA	0	2	Low-quality	Genome-fragment		0.39	0
S14_vs_k141_94577||full	1677	No	NA	0	2	Not-determined	Genome-fragment	NA		0
S14_vs_k141_6095||full	1538	No	NA	0	0	Not-determined	Genome-fragment	NA		0
S8_extracted_k141_51683	1417	No	NA	0	0	Low-quality	Genome-fragment		3.28	0
S14_vs_k141_27862||full	1188	No	NA	0	0	Not-determined	Genome-fragment	NA		0
S14_vs_k141_27867||full	2101	No	NA	0	3	Not-determined	Genome-fragment	NA		0
S14_vs_k141_109181||full	1136	No	NA	0	0	Not-determined	Genome-fragment	NA		0
S8_vs_k141_80750||full	1417	No	NA	2	0	Low-quality	Genome-fragment		3.23	0
S14_vs_k141_116327||full	1323	No	NA	0	1	Not-determined	Genome-fragment	NA		0
S9_extracted_k141_12981	1418	No	NA	1	0	Low-quality	Genome-fragment		3.5	0
S14_vs_k141_49637||full	1821	No	NA	0	0	Not-determined	Genome-fragment	NA		0
S14_vs_k141_116336||full	1553	No	NA	0	0	Not-determined	Genome-fragment	NA		0
S9_vs_k141_62021||full	1418	No	NA	0	0	Low-quality	Genome-fragment		2.48	0
S14_vs_k141_57454||full	2204	No	NA	0	3	Not-determined	Genome-fragment	NA		0
S13_vs_35207||full	1418	No	NA	1	0	Low-quality	Genome-fragment		2.83	0
S14_vs_k141_57458||full	1308	No	NA	0	1	Not-determined	Genome-fragment	NA		0
S14_vs_k141_72204||full	2908	No	NA	0	1	Not-determined	Genome-fragment	NA		0
S13_vs_86462||full	1418	No	NA	0	0	Low-quality	Genome-fragment		3.12	0
S14_vs_k141_94660||full	1009	No	NA	0	0	Not-determined	Genome-fragment	NA		0
S14_vs_k141_116376||full	1353	No	NA	0	2	Not-determined	Genome-fragment	NA		0
S14_vs_k141_13430||full	2591	No	NA	0	1	Not-determined	Genome-fragment	NA		0
S14_vs_k141_35029||full	1418	No	NA	3	0	Low-quality	Genome-fragment		3.7	0
S14_vs_k141_27920||full	3203	No	NA	0	2	Not-determined	Genome-fragment	NA		0
S14_vs_k141_87221||full	3754	No	NA	0	5	Not-determined	Genome-fragment	NA		0
S14_vs_k141_87627||full	1418	No	NA	1	0	Low-quality	Genome-fragment		3.05	0
S14_vs_k141_27925||full	1477	No	NA	0	0	Not-determined	Genome-fragment	NA		0
S14_vs_k141_64472||full	2223	No	NA	0	2	Not-determined	Genome-fragment	NA		0
S14_vs_k141_13436||full	1184	No	NA	0	0	Not-determined	Genome-fragment	NA		0
S14_vs_k141_94673||full	2357	No	NA	0	2	Not-determined	Genome-fragment	NA		0
S14_vs_k141_79505||full	40116	No	NA	7	3	Medium-quality	Genome-fragment		89.03	0
S14_vs_k141_14658||lt2gene	1418	No	NA	2	0	Low-quality	Genome-fragment		3.01	0
S14_vs_k141_13451||full	2692	No	NA	0	0	Not-determined	Genome-fragment	NA		0
S14_vs_k141_87250||full	2419	No	NA	0	3	Not-determined	Genome-fragment	NA		0
S3_extracted_k141_59130	1418	No	NA	0	2	Low-quality	Genome-fragment		0.96	0
S14_vs_k141_72219||full	1426	No	NA	0	0	Not-determined	Genome-fragment	NA		0
S14_vs_k141_49704||full	1683	No	NA	0	1	Not-determined	Genome-fragment	NA		0
S7_extracted_66072	1418	No	NA	0	0	Low-quality	Genome-fragment		2.94	0
S14_vs_k141_21224||full	6635	No	NA	0	4	Not-determined	Genome-fragment	NA		0
S14_vs_k141_57540||full	2759	No	NA	0	2	Not-determined	Genome-fragment	NA		0
S7_vs_73472||full	1418	No	NA	1	0	Low-quality	Genome-fragment		2.57	0
S14_vs_k141_27951||full	16144	No	NA	0	7	Not-determined	Genome-fragment	NA		0
S7_vs_98220||full	1418	No	NA	0	0	Low-quality	Genome-fragment		1.08	0
S12_vs_k141_112996||full	1418	No	NA	0	0	Low-quality	Genome-fragment		1.76	0
S14_vs_k141_87280||full	1427	No	NA	0	0	Not-determined	Genome-fragment	NA		0
S14_vs_k141_35822||full	1493	No	NA	0	0	Not-determined	Genome-fragment	NA		0
S15_vs_16090||lt2gene	1418	No	NA	1	0	Low-quality	Genome-fragment		2.28	0
S6_vs_k141_10674||full	1418	No	NA	1	0	Low-quality	Genome-fragment		2.4	0
S14_vs_k141_109316||full	14598	No	NA	0	5	Not-determined	Genome-fragment	NA		0
S14_vs_k141_21278||full	4498	No	NA	0	2	Not-determined	Genome-fragment	NA		0
S14_vs_k141_13533||full	2306	No	NA	0	0	Not-determined	Genome-fragment	NA		0
S14_vs_k141_79558||full	1870	No	NA	0	0	Not-determined	Genome-fragment	NA		0
S14_vs_k141_101482||full	20333	No	NA	3	1	Medium-quality	Genome-fragment		54.32	0
S14_vs_k141_94798||full	1992	No	NA	0	1	Not-determined	Genome-fragment	NA		0
S14_vs_k141_94804||full	2031	No	NA	0	2	Not-determined	Genome-fragment	NA		0
S8_vs_k141_128199||full	1418	No	NA	2	0	Low-quality	Genome-fragment		2.39	0
S17_vs_k141_165235||full	1419	No	NA	0	3	Low-quality	Genome-fragment		0.44	0
S14_vs_k141_28016||full	5302	No	NA	0	3	Not-determined	Genome-fragment	NA		0
S14_vs_k141_72292||full	1989	No	NA	0	1	Not-determined	Genome-fragment	NA		0
S14_vs_k141_79637||full	1758	No	NA	0	1	Not-determined	Genome-fragment	NA		0
S9_extracted_k141_61511	1419	No	NA	0	1	Low-quality	Genome-fragment		0.93	0
S14_vs_k141_79638||full	2363	No	NA	0	1	Not-determined	Genome-fragment	NA		0
S9_vs_k141_30624||full	1419	No	NA	1	0	Low-quality	Genome-fragment		3.56	0
S11_vs_k141_92066||lt2gene	1419	No	NA	2	0	Low-quality	Genome-fragment		3.23	0
S14_vs_k141_43890||full	1927	No	NA	0	2	Not-determined	Genome-fragment	NA		0
S14_vs_k141_94829||full	1682	No	NA	0	1	Not-determined	Genome-fragment	NA		0
S14_vs_k141_6417||full	2930	No	NA	0	3	Not-determined	Genome-fragment	NA		0
S11_vs_k141_9767||lt2gene	1419	No	NA	1	0	Low-quality	Genome-fragment		3.22	0
S14_vs_k141_43908||full	2231	No	NA	0	3	Not-determined	Genome-fragment	NA		0
S5_vs_k141_53577||full	1419	No	NA	0	0	Low-quality	Genome-fragment		2.27	0
S7_vs_1468||full	1419	No	NA	0	1	Low-quality	Genome-fragment		2.02	0
S12_extracted_k141_70529	1419	No	NA	1	0	Low-quality	Genome-fragment		4.13	0
S2_extracted_k141_17149	1419	No	NA	1	0	Low-quality	Genome-fragment		1.48	0
S4_extracted_28836	1419	No	NA	2	0	Low-quality	Genome-fragment		2.24	0
S14_vs_k141_87448||full	7482	No	NA	0	3	Not-determined	Genome-fragment	NA		0
S14_vs_k141_28118||full	4479	No	NA	0	1	Not-determined	Genome-fragment	NA		0
S9_vs_k141_88781||full	1420	No	NA	3	0	Low-quality	Genome-fragment		3.25	0
S5_vs_k141_119001||full	1420	No	NA	2	0	Low-quality	Genome-fragment		3.64	0
S7_vs_93072||full	1420	No	NA	1	0	Low-quality	Genome-fragment		2.56	0
S16_vs_39531||full	1420	No	NA	0	0	Low-quality	Genome-fragment		1.05	0
S14_vs_k141_21476||full	1577	No	NA	0	0	Not-determined	Genome-fragment	NA		0
S10_extracted_55869	1420	No	NA	1	0	Low-quality	Genome-fragment		3.15	0
S14_vs_k141_21484||full	2366	No	NA	0	1	Not-determined	Genome-fragment	NA		0
S14_vs_k141_109503||full	4521	No	NA	0	2	Not-determined	Genome-fragment	NA		0
S14_vs_k141_28145||full	2842	No	NA	0	0	Not-determined	Genome-fragment	NA		0
S10_vs_8778||full	1420	No	NA	1	0	Low-quality	Genome-fragment		4.06	0
S12_extracted_k141_17655	1420	No	NA	0	2	Low-quality	Genome-fragment		3.26	0
S12_extracted_k141_147964	1420	No	NA	0	0	Low-quality	Genome-fragment		2.27	0
S14_vs_k141_13706||full	1800	No	NA	0	0	Not-determined	Genome-fragment	NA		0
S14_vs_k141_49927||full	1234	No	NA	0	0	Not-determined	Genome-fragment	NA		0
S12_vs_k141_27326||full	1420	No	NA	0	0	Low-quality	Genome-fragment		2.65	0
S12_vs_k141_116501||lt2gene	1420	No	NA	1	0	Low-quality	Genome-fragment		2.07	0
S14_vs_k141_116693||full	1978	No	NA	0	2	Not-determined	Genome-fragment	NA		0
S15_extracted_71598	1420	No	NA	0	0	Low-quality	Genome-fragment		3.46	0
S14_vs_k141_57853||full	2028	No	NA	0	1	Not-determined	Genome-fragment	NA		0
S14_vs_k141_79875||full	1818	No	NA	0	0	Not-determined	Genome-fragment	NA		0
S14_vs_k141_109568||full	2434	No	NA	0	0	Not-determined	Genome-fragment	NA		0
S14_vs_k141_109571||full	1991	No	NA	0	2	Not-determined	Genome-fragment	NA		0
S14_vs_k141_36087||full	1433	No	NA	0	0	Not-determined	Genome-fragment	NA		0
S14_vs_k141_116718||full	13281	No	NA	0	13	Not-determined	Genome-fragment	NA		0
S14_vs_k141_64836||full	1644	No	NA	0	0	Not-determined	Genome-fragment	NA		0
S14_vs_k141_57926||full	2036	No	NA	0	1	Not-determined	Genome-fragment	NA		0
S15_vs_73875||lt2gene	1420	No	NA	1	0	Low-quality	Genome-fragment		3.16	0
S14_vs_k141_109674||full	2282	No	NA	0	1	Not-determined	Genome-fragment	NA		0
S8_extracted_k141_202403	1420	No	NA	0	0	Low-quality	Genome-fragment		0.75	0
S8_vs_k141_147736||full	1420	No	NA	2	0	Low-quality	Genome-fragment		3.17	0
S14_vs_k141_13852||full	1470	No	NA	0	0	Not-determined	Genome-fragment	NA		0

S14_vs_k141_116805||full	1806	No	NA	0	0	Not-determined	Genome-fragment	NA	0
S8_vs_k141_31178||full	1420	No	NA	0	0	Low-quality	Genome-fragment	4.27	0
S14_vs_k141_101829||full	1501	No	NA	0	0	Not-determined	Genome-fragment	NA	0
S14_vs_k141_13877||full	1748	No	NA	0	3	Not-determined	Genome-fragment	NA	0
S14_vs_k141_21677||full	3026	No	NA	0	1	Not-determined	Genome-fragment	NA	0
S8_vs_k141_196674||full	1420	No	NA	0	0	Low-quality	Genome-fragment	1.95	0
S14_vs_k141_80027||full	2750	No	NA	0	0	Not-determined	Genome-fragment	NA	0
S14_vs_k141_101843||full	3513	No	NA	0	1	Not-determined	Genome-fragment	NA	0
S14_vs_k141_80033||full	1014	No	NA	0	1	Not-determined	Genome-fragment	NA	0
S14_vs_k141_109770||full	2208	No	NA	0	1	Not-determined	Genome-fragment	NA	0
S14_vs_k141_95159||full	1160	No	NA	0	0	Not-determined	Genome-fragment	NA	0
S14_vs_k141_58026||full	1037	No	NA	0	1	Not-determined	Genome-fragment	NA	0
S14_vs_k141_87517||full	1507	No	NA	0	0	Not-determined	Genome-fragment	NA	0
S13_vs_4655||full	1421	No	NA	2	0	Low-quality	Genome-fragment	3.81	0
S14_vs_k141_21729||full	2295	No	NA	0	1	Not-determined	Genome-fragment	NA	0
S14_vs_k141_64981||full	2193	No	NA	0	0	Not-determined	Genome-fragment	NA	0
S14_vs_k141_72511||full	1765	No	NA	0	0	Not-determined	Genome-fragment	NA	0
S7_vs_123439||full	1421	No	NA	0	0	Low-quality	Genome-fragment	2.54	0
S14_vs_k141_109830||full	4423	No	NA	0	0	Not-determined	Genome-fragment	NA	0
S12_vs_k141_42674||full	1421	No	NA	0	0	Low-quality	Genome-fragment	3.13	0
S17_vs_k141_143883||lt2gene	1422	No	NA	0	0	Low-quality	Genome-fragment	2.95	0
S14_vs_k141_21774||full	2981	No	NA	0	0	Not-determined	Genome-fragment	NA	0
S14_vs_k141_6767||full	6666	No	NA	0	3	Not-determined	Genome-fragment	NA	0
S14_vs_k141_21796||full	2075	No	NA	0	0	Not-determined	Genome-fragment	NA	0
S14_vs_k141_65030||full	2336	No	NA	0	0	Not-determined	Genome-fragment	NA	0
S14_vs_k141_116910||full	1192	No	NA	0	0	Not-determined	Genome-fragment	NA	0
S9_vs_k141_18750||full	1422	No	NA	1	0	Low-quality	Genome-fragment	2.45	0
S11_vs_k141_52063||lt2gene	1422	No	NA	1	0	Low-quality	Genome-fragment	4.14	0
S14_vs_k141_83076||full	1422	No	NA	0	0	Low-quality	Genome-fragment	1.89	0
S14_vs_k141_58114||full	1547	No	NA	0	2	Not-determined	Genome-fragment	NA	0
S14_vs_k141_21811||full	1999	No	NA	0	0	Not-determined	Genome-fragment	NA	0
S3_vs_k141_108196||full	1422	No	NA	0	0	Low-quality	Genome-fragment	2.58	0
S14_vs_k141_21824||full	1000	No	NA	0	1	Not-determined	Genome-fragment	NA	0
S14_vs_k141_65072||full	14656	No	NA	0	7	Not-determined	Genome-fragment	NA	0
S7_vs_34597||full	1422	No	NA	0	0	Low-quality	Genome-fragment	4.29	0
S14_vs_k141_36336||full	1760	No	NA	0	1	Not-determined	Genome-fragment	NA	0
S14_vs_k141_21843||full	3139	No	NA	0	1	Not-determined	Genome-fragment	NA	0
S7_vs_79578||full	1422	No	NA	0	2	Low-quality	Genome-fragment	1.09	0
S14_vs_k141_87657||full	1018	No	NA	0	0	Not-determined	Genome-fragment	NA	0
S16_extracted_18907	1422	No	NA	0	0	Low-quality	Genome-fragment	0.92	0
S14_vs_k141_87661||full	3308	No	NA	0	3	Not-determined	Genome-fragment	NA	0
S16_extracted_32536	1422	No	NA	0	0	Low-quality	Genome-fragment	2.66	0
S16_vs_59042||full	1422	No	NA	0	0	Low-quality	Genome-fragment	1.14	0
S14_vs_k141_58206||full	2088	No	NA	0	1	Not-determined	Genome-fragment	NA	0
S16_vs_129772||full	1422	No	NA	1	0	Low-quality	Genome-fragment	3.46	0
S14_vs_k141_21892||full	1873	No	NA	0	2	Not-determined	Genome-fragment	NA	0
S14_vs_k141_36382||full	4637	No	NA	0	4	Not-determined	Genome-fragment	NA	0
S10_vs_91843||full	1422	No	NA	0	0	Low-quality	Genome-fragment	1.73	0
S14_vs_k141_14074||full	1465	No	NA	0	0	Not-determined	Genome-fragment	NA	0
S14_vs_k141_87711||full	2312	No	NA	0	2	Not-determined	Genome-fragment	NA	0
S12_extracted_k141_169593	1422	No	NA	1	0	Low-quality	Genome-fragment	2.9	0
S12_vs_k141_51315||full	1422	No	NA	1	0	Low-quality	Genome-fragment	3.09	0
S14_vs_k141_21935||full	2402	No	NA	0	2	Not-determined	Genome-fragment	NA	0
S2_extracted_k141_10568	1422	No	NA	2	0	Low-quality	Genome-fragment	4.67	0
S14_extracted_k141_88333	1423	No	NA	3	0	Low-quality	Genome-fragment	3.87	0
S14_vs_k141_36482||full	1533	No	NA	0	2	Not-determined	Genome-fragment	NA	0
S14_vs_k141_75667||full	1423	No	NA	1	0	Low-quality	Genome-fragment	2.38	0
S14_vs_k141_21978||full	1408	No	NA	0	0	Not-determined	Genome-fragment	NA	0
S14_vs_k141_11455||full	1423	No	NA	0	0	Low-quality	Genome-fragment	3.39	0
S14_vs_k141_117106||full	1089	No	NA	0	1	Not-determined	Genome-fragment	NA	0
S1_extracted_k141_22986	1423	No	NA	1	0	Low-quality	Genome-fragment	1.47	0
S5_extracted_k141_92035	1423	No	NA	1	0	Low-quality	Genome-fragment	3.13	0
S5_vs_k141_81489||lt2gene	1423	No	NA	1	0	Low-quality	Genome-fragment	0.88	0
S7_extracted_62238	1423	No	NA	3	0	Low-quality	Genome-fragment	6.49	0
S7_vs_42284||full	1423	No	NA	0	0	Low-quality	Genome-fragment	3.81	0
S14_vs_k141_6981||full	9629	No	NA	0	4	Not-determined	Genome-fragment	NA	0
S16_vs_5293||full	1423	No	NA	0	0	Low-quality	Genome-fragment	3.79	0
S16_vs_115968||full	1423	No	NA	0	0	Low-quality	Genome-fragment	3.07	0
S14_vs_k141_65232||full	1570	No	NA	0	2	Not-determined	Genome-fragment	NA	0
S14_vs_k141_65248||full	2434	No	NA	0	0	Not-determined	Genome-fragment	NA	0
S14_vs_k141_58376||full	1425	No	NA	0	2	Not-determined	Genome-fragment	NA	0
S14_vs_k141_87827||full	1072	No	NA	0	1	Not-determined	Genome-fragment	NA	0
S14_vs_k141_80342||full	2389	No	NA	0	2	Not-determined	Genome-fragment	NA	0
S8_vs_k141_180752||full	1423	No	NA	0	0	Low-quality	Genome-fragment	4.93	0
S14_vs_k141_80362||full	2241	No	NA	0	2	Not-determined	Genome-fragment	NA	0
S14_vs_k141_58392||full	1151	No	NA	0	0	Not-determined	Genome-fragment	NA	0
S8_vs_k141_195028||lt2gene	1423	No	NA	1	0	Low-quality	Genome-fragment	3.14	0
S17_vs_k141_155672||full	1424	No	NA	0	0	Low-quality	Genome-fragment	1.52	0
S17_vs_k141_113735||full	1424	No	NA	2	0	Low-quality	Genome-fragment	3.88	0
S13_vs_87153||full	1424	No	NA	0	0	Low-quality	Genome-fragment	1.51	0
S14_vs_k141_36632||full	4294	No	NA	0	1	Not-determined	Genome-fragment	NA	0
S14_vs_k141_50531||full	1023	No	NA	0	1	Not-determined	Genome-fragment	NA	0
S14_vs_k141_102181||full	1086	No	NA	0	0	Not-determined	Genome-fragment	NA	0
S14_vs_k141_21980||full	1424	No	NA	0	0	Low-quality	Genome-fragment	3.03	0
S14_vs_k141_14355||full	1911	No	NA	0	1	Not-determined	Genome-fragment	NA	0
S14_vs_k141_7109||full	3087	No	NA	0	2	Not-determined	Genome-fragment	NA	0
S1_extracted_k141_4241	1424	No	NA	1	0	Low-quality	Genome-fragment	1.98	0
S14_vs_k141_50587||full	1196	No	NA	0	0	Not-determined	Genome-fragment	NA	0
S14_vs_k141_80415||full	2354	No	NA	0	1	Not-determined	Genome-fragment	NA	0
S1_vs_k141_68338||full	1424	No	NA	2	0	Low-quality	Genome-fragment	1.79	0
S1_vs_k141_96483||full	1424	No	NA	1	0	Low-quality	Genome-fragment	3.37	0
S14_vs_k141_58459||full	7263	No	NA	0	2	Not-determined	Genome-fragment	NA	0
S14_vs_k141_80431||full	6489	No	NA	0	3	Not-determined	Genome-fragment	NA	0
S14_vs_k141_14403||full	1488	No	NA	0	0	Not-determined	Genome-fragment	NA	0
S1_vs_k141_151943||full	1424	No	NA	0	1	Low-quality	Genome-fragment	1.02	0
S14_vs_k141_7200||full	1013	No	NA	0	1	Not-determined	Genome-fragment	NA	0
S3_vs_k141_19344||full	1424	No	NA	1	0	Low-quality	Genome-fragment	2.53	0
S14_vs_k141_102300||full	1063	No	NA	0	1	Not-determined	Genome-fragment	NA	0
S3_vs_k141_20785||full	1424	No	NA	1	0	Low-quality	Genome-fragment	3.21	0
S14_vs_k141_73014||full	4861	No	NA	0	2	Not-determined	Genome-fragment	NA	0
S14_vs_k141_80468||full	1627	No	NA	0	0	Not-determined	Genome-fragment	NA	0
S14_vs_k141_73040||full	2739	No	NA	0	2	Not-determined	Genome-fragment	NA	0
S14_vs_k141_7229||full	8865	No	NA	0	3	Not-determined	Genome-fragment	NA	0
S14_vs_k141_7242||full	3085	No	NA	0	1	Not-determined	Genome-fragment	NA	0
S7_extracted_76059	1424	No	NA	0	0	Low-quality	Genome-fragment	3.06	0
S14_vs_k141_65452||full	5696	No	NA	0	4	Not-determined	Genome-fragment	NA	0
S7_vs_117621||full	1424	No	NA	2	0	Low-quality	Genome-fragment	4.57	0
S16_vs_60511||full	1424	No	NA	1	0	Low-quality	Genome-fragment	2.84	0
S14_vs_k141_28727||full	1591	No	NA	0	3	Not-determined	Genome-fragment	NA	0
S14_vs_k141_73106||full	1466	No	NA	0	0	Not-determined	Genome-fragment	NA	0
S14_vs_k141_65483||full	1939	No	NA	0	0	Not-determined	Genome-fragment	NA	0
S14_vs_k141_14468||full	1884	No	NA	0	0	Not-determined	Genome-fragment	NA	0
S14_vs_k141_73126||full	6496	No	NA	0	5	Not-determined	Genome-fragment	NA	0
S14_vs_k141_80588||full	1012	No	NA	0	2	Not-determined	Genome-fragment	NA	0
S14_vs_k141_73214||full	2863	No	NA	0	0	Not-determined	Genome-fragment	NA	0
S16_vs_60737||full	1424	No	NA	1	0	Low-quality	Genome-fragment	1.7	0
S14_vs_k141_50825||full	1938	No	NA	0	0	Not-determined	Genome-fragment	NA	0
S16_vs_93409||full	1424	No	NA	0	0	Low-quality	Genome-fragment	3.52	0
S13_vs_17205||full	1425	No	NA	2	0	Low-quality	Genome-fragment	3.03	0
S14_vs_k141_65541||full	2930	No	NA	0	1	Not-determined	Genome-fragment	NA	0
S14_extracted_k141_39426	1425	No	NA	1	0	Low-quality	Genome-fragment	3.19	0
S14_vs_k141_73261||full	2802	No	NA	0	1	Not-determined	Genome-fragment	NA	0
S3_vs_k141_123094||full	1425	No	NA	0	0	Low-quality	Genome-fragment	2.69	0
S14_vs_k141_65561||full	2077	No	NA	0	0	Not-determined	Genome-fragment	NA	0
S14_vs_k141_50896||full	2163	No	NA	0	2	Not-determined	Genome-fragment	NA	0
S5_extracted_k141_110720	1425	No	NA	0	1	Low-quality	Genome-fragment	0.36	0
S14_vs_k141_65616||full	3895	No	NA	0	4	Not-determined	Genome-fragment	NA	0
S14_vs_k141_29064||full	1316	No	NA	0	0	Not-determined	Genome-fragment	NA	0
S14_vs_k141_51027||full	3425	No	NA	0	2	Not-determined	Genome-fragment	NA	0
S14_vs_k141_51066||full	5970	No	NA	0	1	Not-determined	Genome-fragment	NA	0
S14_vs_k141_65725||full	1507	No	NA	0	0	Not-determined	Genome-fragment	NA	0
S5_extracted_k141_28354	1425	No	NA	0	0	Low-quality	Genome-fragment	3.27	0
S16_vs_19722||full	1425	No	NA	0	0	Low-quality	Genome-fragment	4.16	0
S16_vs_159873||full	1425	No	NA	2	0	Low-quality	Genome-fragment	3.72	0
S14_vs_k141_65751||full	4417	No	NA	0	0	Not-determined	Genome-fragment	NA	0
S12_vs_k141_55253||full	1425	No	NA	0	0	Low-quality	Genome-fragment	3.38	0
S12_vs_k141_174427||full	1425	No	NA	0	2	Low-quality	Genome-fragment	2.55	0
S14_vs_k141_51211||full	6463	No	NA	0	4	Not-determined	Genome-fragment	NA	0
S14_vs_k141_117151||0_partial	13849	No	NA	0	2	Not-determined	Genome-fragment	NA	0
S17_extracted_k141_85360	1426	No	NA	5	0	Low-quality	Genome-fragment	4.7	0
S14_vs_k141_38028||0_partial	15429	No	NA	0	4	Not-determined	Genome-fragment	NA	0
S14_vs_k141_30677||0_partial	25495	No	NA	0	15	Not-determined	Genome-fragment	NA	0
S14_vs_k141_16287||0_partial	14321	No	NA	0	6	Not-determined	Genome-fragment	NA	0
S14_vs_k141_67094||0_partial	6045	Yes	4638	1	1	High-quality	High-quality	100	23.28
S11_vs_k141_55081||lt2gene	1426	No	NA	1	0	Low-quality	Genome-fragment	2.8	0
S13_extracted_23524	1426	No	NA	0	1	Low-quality	Genome-fragment	0.53	0
S13_vs_44406||full	1426	No	NA	0	0	Low-quality	Genome-fragment	3.14	0
S1_vs_k141_25974||full	1426	No	NA	0	0	Low-quality	Genome-fragment	12	0
S14_vs_k141_60822||0_partial	18485	No	NA	0	7	Not-determined	Genome-fragment	NA	0
S3_vs_k141_42834||full	1426	No	NA	1	0	Low-quality	Genome-fragment	2.29	0
S5_vs_k141_66177||full	1426	No	NA	0	0	Low-quality	Genome-fragment	3.28	0
S5_vs_k141_31477||lt2gene	1426	No	NA	1	0	Low-quality	Genome-fragment	2.08	0
S14_vs_k141_26120||0_partial	11041	No	NA	0	6	Not-determined	Genome-fragment	NA	0
S16_vs_7257||full	1426	No	NA	0	2	Low-quality	Genome-fragment	1.29	0
S16_vs_44207||full	1426	No	NA	1	0	Low-quality	Genome-fragment	1.75	0
S2_extracted_k141_6717	1426	No	NA	0	0	Low-quality	Genome-fragment	3.84	0
S6_vs_k141_52014||full	1426	No	NA	2	0	Low-quality	Genome-fragment	2.46	0
S17_extracted_k141_37232	1427	No	NA	0	0	Low-quality	Genome-fragment	2.5	0
S9_vs_k141_77679||full	1427	No	NA	0	2	Low-quality	Genome-fragment	0.88	0
S11_vs_k141_75029||full	1427	No	NA	0	0	Low-quality	Genome-fragment	3.65	0
S5_vs_k141_109623||full	1427	No	NA	1	0	Low-quality	Genome-fragment	2.3	0
S7_extracted_60171	1427	No	NA	0	0	Low-quality	Genome-fragment	2.6	0
S7_vs_105297||full	1427	No	NA	0	1	Low-quality	Genome-fragment	1	0
S7_vs_85004||lt2gene	1427	No	NA	2	0	Low-quality	Genome-fragment	4.39	0
S12_extracted_k141_117659	1427	No	NA	0	1	Low-quality	Genome-fragment	1.08	0
S12_vs_k141_104921||full	1427	No	NA	1	0	Low-quality	Genome-fragment	4.07	0
S8_vs_k141_60316||full	1427	No	NA	0	0	Low-quality	Genome-fragment	2.67	0
S14_vs_k141_59339||lt2gene	1837	No	NA	0	0	Not-determined	Genome-fragment	NA	0
S13_vs_143108||full	1428	No	NA	0	2	Low-quality	Genome-fragment	0.76	0
S3_vs_k141_108516||full	1428	No	NA	0	1	Low-quality	Genome-fragment	0.72	0
S3_vs_k141_117689||full	1428	No	NA	1	0	Low-quality	Genome-fragment	2.5	0
S10_vs_61922||full	1428	No	NA	1	0	Low-quality	Genome-fragment	3.51	0
S12_extracted_k141_99150	1428	No	NA	0	1	Low-quality	Genome-fragment	2.37	0
S12_vs_k141_132899||full	1428	No	NA	0	0	Low-quality	Genome-fragment	2.1	0

S8_vs_k141_66755||full	1428	No	NA		0	0	Low-quality	Genome-fragment		2.6	0
S9_vs_k141_44652||full	1429	Yes		379	1	1	Low-quality	Genome-fragment		0.9	73.48
S3_vs_k141_3913||full	1429	No	NA		0	0	Low-quality	Genome-fragment		0.95	0
S3_vs_k141_109214||lt2gene	1429	No	NA		1	0	Low-quality	Genome-fragment		3.62	0
S7_extracted_33677	1429	No	NA		1	0	Low-quality	Genome-fragment		6.53	0
S7_extracted_102255	1429	No	NA		0	0	Low-quality	Genome-fragment		4.67	0
S7_vs_83346||full	1429	No	NA		2	0	Low-quality	Genome-fragment		3.11	0
S12_vs_k141_24543||full	1429	No	NA		0	2	Low-quality	Genome-fragment		0.75	0
S17_extracted_k141_165705	1430	No	NA		0	1	Low-quality	Genome-fragment		1.01	0
S9_vs_k141_54614||full	1430	No	NA		2	0	Low-quality	Genome-fragment		3.34	0
S14_extracted_k141_88566	1430	No	NA		0	0	Low-quality	Genome-fragment		5.32	0
S3_vs_k141_3899||full	1430	No	NA		0	0	Low-quality	Genome-fragment		1	0
S14_vs_k141_67210||lt2gene	1339	No	NA		0	0	Not-determined	Genome-fragment	NA		0
S3_vs_k141_125568||full	1430	No	NA		0	0	Low-quality	Genome-fragment		1.6	0
S7_vs_35002||full	1430	No	NA		0	0	Low-quality	Genome-fragment		3.43	0
S16_extracted_22925	1430	No	NA		0	0	Low-quality	Genome-fragment		3.76	0
S16_extracted_170909	1430	No	NA		0	0	Low-quality	Genome-fragment		3	0
S14_vs_k141_67707||lt2gene	4712	No	NA		0	0	Not-determined	Genome-fragment	NA		0
S16_vs_50084||full	1430	No	NA		0	1	Low-quality	Genome-fragment		0.8	0
S10_extracted_46177	1430	No	NA		0	0	Low-quality	Genome-fragment		2.59	0
S12_extracted_k141_161796	1430	No	NA		0	1	Low-quality	Genome-fragment		0.68	0
S15_vs_3527||full	1430	No	NA		1	0	Low-quality	Genome-fragment		1.8	0
S9_vs_k141_67241||full	1431	No	NA		1	0	Low-quality	Genome-fragment		3.05	0
S11_vs_k141_101665||lt2gene	1431	No	NA		1	0	Low-quality	Genome-fragment		0.99	0
S13_vs_144639||full	1431	No	NA		0	0	Low-quality	Genome-fragment		3.41	0
S14_vs_k141_112103||full	1431	No	NA		3	0	Low-quality	Genome-fragment		4.73	0
S14_vs_k141_18114||full	1431	No	NA		0	0	Low-quality	Genome-fragment		2.53	0
S1_vs_k141_79379||lt2gene	1431	No	NA		1	0	Low-quality	Genome-fragment		2.3	0
S5_vs_k141_7718||full	1431	No	NA		1	0	Low-quality	Genome-fragment		2.84	0
S7_vs_118718||full	1431	No	NA		1	0	Low-quality	Genome-fragment		3.7	0
S7_vs_123161||full	1431	No	NA		0	0	Low-quality	Genome-fragment		3.51	0
S16_extracted_73274	1431	No	NA		1	0	Low-quality	Genome-fragment		3.08	0
S16_vs_50761||full	1431	No	NA		2	0	Low-quality	Genome-fragment		3.27	0
S8_vs_k141_74813||full	1431	No	NA		0	0	Low-quality	Genome-fragment		1.67	0
S14_extracted_k141_36625	1432	No	NA		0	0	Low-quality	Genome-fragment		1.35	0
S5_vs_k141_8357||full	1432	No	NA		0	0	Low-quality	Genome-fragment		4.17	0
S5_vs_k141_107406||full	1432	No	NA		0	0	Low-quality	Genome-fragment		2.8	0
S7_vs_82404||full	1432	No	NA		0	0	Low-quality	Genome-fragment		0.98	0
S16_extracted_101237	1432	No	NA		0	1	Low-quality	Genome-fragment		2.89	0
S16_extracted_158794	1432	No	NA		0	0	Low-quality	Genome-fragment		2.42	0
S12_vs_k141_7130||full	1432	No	NA		1	0	Low-quality	Genome-fragment		2.03	0
S4_extracted_16200	1432	No	NA		5	0	Low-quality	Genome-fragment		4.67	0
S9_extracted_k141_66054	1433	No	NA		0	0	Low-quality	Genome-fragment		2.84	0
S13_vs_141576||full	1433	No	NA		0	0	Low-quality	Genome-fragment		3.65	0
S1_vs_k141_77181||full	1433	Yes		898	1	1	Low-quality	Genome-fragment		2.3	37.33
S3_vs_k141_107948||full	1433	No	NA		2	0	Low-quality	Genome-fragment		1.2	0
S5_vs_k141_78086||full	1433	No	NA		0	0	Low-quality	Genome-fragment		2.53	0
S16_extracted_13418	1433	No	NA		2	0	Low-quality	Genome-fragment		4.45	0
S4_extracted_21280	1433	No	NA		5	0	Low-quality	Genome-fragment		4.71	0
S8_vs_k141_60079||full	1433	No	NA		0	1	Low-quality	Genome-fragment		0.37	0
S17_extracted_k141_123675	1434	No	NA		1	0	Low-quality	Genome-fragment		3.91	0
S5_extracted_k141_94703	1434	No	NA		0	1	Low-quality	Genome-fragment		1.71	0
S5_vs_k141_83416||lt2gene	1434	No	NA		1	0	Low-quality	Genome-fragment		3.94	0
S7_vs_38725||full	1434	No	NA		1	0	Low-quality	Genome-fragment		0.79	0
S12_vs_k141_49045||full	1434	No	NA		0	0	Low-quality	Genome-fragment		2.73	0
S15_vs_5944||full	1434	No	NA		0	2	Low-quality	Genome-fragment		4.58	0
S17_vs_k141_89251||full	1435	No	NA		0	1	Low-quality	Genome-fragment		0.83	0
S14_vs_k141_26461||lt2gene	1200	No	NA		0	0	Not-determined	Genome-fragment	NA		0
S1_vs_k141_7093||full	1435	No	NA		3	0	Low-quality	Genome-fragment		4.12	0
S3_extracted_k141_42343	1435	No	NA		1	0	Low-quality	Genome-fragment		0.76	0
S14_vs_k141_63534||lt2gene	2038	No	NA		0	0	Not-determined	Genome-fragment	NA		0
S7_vs_115992||full	1435	No	NA		0	0	Low-quality	Genome-fragment		0.9	0
S16_vs_9176||full	1435	No	NA		0	0	Low-quality	Genome-fragment		1.83	0
S16_vs_156424||full	1435	No	NA		0	1	Low-quality	Genome-fragment		0.94	0
S10_extracted_92748	1435	No	NA		0	1	Low-quality	Genome-fragment		0.6	0
S8_vs_k141_104667||full	1435	No	NA		0	0	Low-quality	Genome-fragment		2.56	0
S17_vs_k141_1478||full	1436	No	NA		2	0	Low-quality	Genome-fragment		3.29	0
S13_vs_8704||full	1436	No	NA		0	0	Low-quality	Genome-fragment		3.33	0
S14_extracted_k141_98245	1436	No	NA		1	0	Low-quality	Genome-fragment		4.63	0
S14_vs_k141_20441||lt2gene	1178	No	NA		0	0	Not-determined	Genome-fragment	NA		0
S1_vs_k141_144510||full	1436	No	NA		0	1	Low-quality	Genome-fragment		0.74	0
S14_vs_k141_64191||lt2gene	1154	No	NA		0	0	Not-determined	Genome-fragment	NA		0
S1_vs_k141_150907||full	1436	No	NA		2	0	Low-quality	Genome-fragment		5.22	0
S3_vs_k141_108237||full	1436	No	NA		0	0	Low-quality	Genome-fragment		2.85	0
S5_vs_k141_2329||full	1436	No	NA		2	0	Low-quality	Genome-fragment		3.01	0
S16_vs_121940||lt2gene	1436	No	NA		1	0	Low-quality	Genome-fragment		3.76	0
S10_vs_43817||full	1436	No	NA		0	1	Low-quality	Genome-fragment		0.7	0
S12_extracted_k141_161887	1436	No	NA		0	0	Low-quality	Genome-fragment		3.61	0
S12_vs_k141_109500||full	1436	No	NA		1	0	Low-quality	Genome-fragment		3.13	0
S15_extracted_44337	1436	No	NA		0	1	Low-quality	Genome-fragment		2.43	0
S6_vs_k141_4303||full	1436	No	NA		0	0	Low-quality	Genome-fragment		1.95	0
S8_vs_k141_30046||full	1436	No	NA		0	1	Low-quality	Genome-fragment		1.24	0
S11_vs_k141_35988||full	1437	No	NA		2	0	Low-quality	Genome-fragment		2.02	0
S14_vs_k141_36244||lt2gene	1025	No	NA		0	0	Not-determined	Genome-fragment	NA		0
S14_vs_k141_111025||full	1437	No	NA		2	0	Low-quality	Genome-fragment		3.37	0
S1_extracted_k141_101922	1437	No	NA		0	0	Low-quality	Genome-fragment		1.47	0
S1_vs_k141_148884||full	1437	No	NA		2	0	Low-quality	Genome-fragment		3.38	0
S14_vs_k141_14458||lt2gene	1943	No	NA		0	0	Not-determined	Genome-fragment	NA		0
S3_vs_k141_5035||full	1437	No	NA		0	0	Low-quality	Genome-fragment		3.38	0
S5_extracted_k141_123207	1437	No	NA		2	0	Low-quality	Genome-fragment		3.92	0
S1_extracted_k141_46726	1126	No	NA		0	1	Not-determined	Genome-fragment	NA		0
S1_extracted_k141_153522	1268	No	NA		0	0	Not-determined	Genome-fragment	NA		0
S1_extracted_k141_106803	1128	No	NA		0	0	Not-determined	Genome-fragment	NA		0
S1_extracted_k141_26703	1267	No	NA		0	0	Not-determined	Genome-fragment	NA		0
S1_extracted_k141_53406	1236	No	NA		0	2	Not-determined	Genome-fragment	NA		0
S1_extracted_k141_46732	1166	No	NA		0	2	Not-determined	Genome-fragment	NA		0
S5_vs_k141_101072||full	1437	No	NA		2	0	Low-quality	Genome-fragment		7.43	0
S1_extracted_k141_126830	4924	No	NA		0	0	Not-determined	Genome-fragment	NA		0
S1_extracted_k141_133514	1100	No	NA		0	1	Not-determined	Genome-fragment	NA		0
S16_vs_125793||full	1437	No	NA		0	2	Low-quality	Genome-fragment		2.14	0
S17_vs_k141_154783||full	1438	No	NA		0	0	Low-quality	Genome-fragment		3.52	0
S1_extracted_k141_6699	3534	No	NA		0	2	Not-determined	Genome-fragment	NA		0
S1_extracted_k141_33400	1206	No	NA		0	1	Not-determined	Genome-fragment	NA		0
S1_extracted_k141_113502	1205	No	NA		0	2	Not-determined	Genome-fragment	NA		0
S9_vs_k141_89728||full	1438	No	NA		0	0	Low-quality	Genome-fragment		1.81	0
S11_extracted_k141_18373	1438	No	NA		1	0	Low-quality	Genome-fragment		3.4	0
S1_extracted_k141_20067	1292	No	NA		0	0	Not-determined	Genome-fragment	NA		0
S11_vs_k141_92299||full	1438	No	NA		1	0	Low-quality	Genome-fragment		3.16	0
S1_extracted_k141_13382	1600	No	NA		0	3	Not-determined	Genome-fragment	NA		0
S1_extracted_k141_13401	1087	No	NA		0	1	Not-determined	Genome-fragment	NA		0
S14_vs_k141_30643||lt2gene	1438	No	NA		1	0	Low-quality	Genome-fragment		3.06	0
S3_extracted_k141_114656	1438	No	NA		1	0	Low-quality	Genome-fragment		24.51	0
S1_extracted_k141_140259	2466	No	NA		3	0	Medium-quality	Genome-fragment		51.91	0
S1_extracted_k141_26760	2324	No	NA		0	0	Not-determined	Genome-fragment	NA		0
S1_extracted_k141_46821	2387	No	NA		0	0	Not-determined	Genome-fragment	NA		0
S1_extracted_k141_100203	1384	No	NA		0	1	Not-determined	Genome-fragment	NA		0
S1_extracted_k141_120258	1322	No	NA		0	0	Not-determined	Genome-fragment	NA		0
S1_extracted_k141_106910	1012	No	NA		0	1	Not-determined	Genome-fragment	NA		0
S1_extracted_k141_100211	1666	No	NA		0	1	Not-determined	Genome-fragment	NA		0
S1_extracted_k141_126961	1264	No	NA		0	0	Not-determined	Genome-fragment	NA		0
S5_extracted_k141_67375	1438	No	NA		2	0	Low-quality	Genome-fragment		4.23	0
S5_extracted_k141_19587	1438	No	NA		1	0	Low-quality	Genome-fragment		3.94	0
S5_vs_k141_73680||full	1438	No	NA		0	2	Low-quality	Genome-fragment		3.69	0
S16_extracted_62706	1438	No	NA		2	0	Low-quality	Genome-fragment		3.17	0
S1_extracted_k141_73557	1300	No	NA		0	2	Not-determined	Genome-fragment	NA		0
S1_extracted_k141_106936	1527	No	NA		0	1	Not-determined	Genome-fragment	NA		0
S1_extracted_k141_73570	3091	No	NA		0	1	Not-determined	Genome-fragment	NA		0
S1_extracted_k141_73579	4803	No	NA		0	1	Not-determined	Genome-fragment	NA		0
S1_extracted_k141_127028	3209	No	NA		0	2	Not-determined	Genome-fragment	NA		0
S1_extracted_k141_100291	1023	No	NA		0	1	Not-determined	Genome-fragment	NA		0
S1_extracted_k141_13542	2462	No	NA		0	2	Not-determined	Genome-fragment	NA		0
S16_extracted_89129	1438	No	NA		0	0	Low-quality	Genome-fragment		1.76	0
S16_vs_18571||full	1438	No	NA		0	0	Low-quality	Genome-fragment		1.97	0
S16_vs_39567||full	1438	No	NA		1	0	Low-quality	Genome-fragment		2.63	0
S17_extracted_k141_61861	1439	No	NA		0	1	Low-quality	Genome-fragment		2.99	0
S1_extracted_k141_20241	4076	No	NA		0	4	Not-determined	Genome-fragment	NA		0
S1_extracted_k141_73636	1206	No	NA		0	0	Not-determined	Genome-fragment	NA		0
S11_vs_k141_97274||full	1439	No	NA		0	0	Low-quality	Genome-fragment		3.32	0
S14_extracted_k141_44110	1439	No	NA		4	0	Low-quality	Genome-fragment		4.74	0
S1_extracted_k141_140439	2130	No	NA		0	1	Not-determined	Genome-fragment	NA		0
S14_extracted_k141_63168	1439	No	NA		3	0	Low-quality	Genome-fragment		4.75	0
S1_extracted_k141_120440	1092	No	NA		0	0	Not-determined	Genome-fragment	NA		0
S1_extracted_k141_113725	2410	No	NA		0	2	Not-determined	Genome-fragment	NA		0
S1_extracted_k141_73708	2319	No	NA		0	0	Not-determined	Genome-fragment	NA		0
S14_vs_k141_52468||lt2gene	1439	Yes		1367	1	1	Low-quality	Genome-fragment		2.3	5
S1_extracted_k141_60197	1604	No	NA		0	1	Not-determined	Genome-fragment	NA		0
S1_extracted_k141_127165	2463	No	NA		0	1	Not-determined	Genome-fragment	NA		0
S1_extracted_k141_13666	1251	No	NA		0	1	Not-determined	Genome-fragment	NA		0
S1_extracted_k141_93783	1867	No	NA		0	0	Not-determined	Genome-fragment	NA		0
S1_extracted_k141_147046	3490	No	NA		0	3	Not-determined	Genome-fragment	NA		0
S1_extracted_k141_13673	1141	No	NA		0	3	Not-determined	Genome-fragment	NA		0
S1_vs_k141_25702||full	1439	No	NA		0	0	Low-quality	Genome-fragment		3.31	0
S1_extracted_k141_6982	1105	No	NA		0	1	Not-determined	Genome-fragment	NA		0
S1_extracted_k141_33663	1630	No	NA		0	1	Not-determined	Genome-fragment	NA		0
S1_extracted_k141_140482	4672	No	NA		0	2	Not-determined	Genome-fragment	NA		0
S1_extracted_k141_120487	1453	No	NA		0	1	Not-determined	Genome-fragment	NA		0
S1_extracted_k141_93825	1789	No	NA		0	1	Not-determined	Genome-fragment	NA		0
S1_extracted_k141_20355	1100	No	NA		0	1	Not-determined	Genome-fragment	NA		0
S1_extracted_k141_40386	1613	No	NA		0	1	Not-determined	Genome-fragment	NA		0
S1_vs_k141_119856||full	1439	No	NA		1	0	Low-quality	Genome-fragment		2.34	0
S1_extracted_k141_127227	1392	No	NA		0	1	Not-determined	Genome-fragment	NA		0
S1_extracted_k141_40413	1015	No	NA		0	0	Not-determined	Genome-fragment	NA		0
S1_extracted_k141_53800	4903	No	NA		0	3	Not-determined	Genome-fragment	NA		0
S5_vs_k141_31262||full	1439	No	NA		1	0	Low-quality	Genome-fragment		2.98	0
S5_vs_k141_11753||full	1439	No	NA		0	0	Low-quality	Genome-fragment		3.76	0
S1_extracted_k141_80522	1180	No	NA		0	0	Not-determined	Genome-fragment	NA		0
S1_extracted_k141_80527	1140	No	NA		0	0	Not-determined	Genome-fragment	NA		0
S1_extracted_k141_67072	3468	No	NA		0	1	Not-determined	Genome-fragment	NA		0
S1_extracted_k141_100506	1348	No	NA		0	1	Not-determined	Genome-fragment	NA		0
S10_extracted_48978	1439	No	NA		0	0	Low-quality	Genome-fragment		3.49	0
S8_vs_k141_11565||full	1439	No	NA		0	2	Low-quality	Genome-fragment		1.99	0
S1_extracted_k141_47096	1625	No	NA		0	2	Not-determined	Genome-fragment	NA		0
S1_extracted_k141_140552	1016	No	NA		0	0	Not-determined	Genome-fragment	NA		0
S9_vs_k141_15743||full	1440	No	NA		0	1	Low-quality	Genome-fragment		1.08	0

S1_extracted_k141_20439	1073	No	NA	0	0	Not-determined	Genome-fragment	NA		0
S11_vs_k141_80699||full	1440	No	NA	0	0	Low-quality	Genome-fragment		2.88	0
S1_extracted_k141_40502	1047	No	NA	0	0	Not-determined	Genome-fragment	NA		0
S13_extracted_147113	1440	No	NA	0	0	Low-quality	Genome-fragment		3.25	0
S1_extracted_k141_87219	2129	No	NA	0	1	Not-determined	Genome-fragment	NA		0
S1_extracted_k141_40512	1102	No	NA	0	1	Not-determined	Genome-fragment	NA		0
S13_vs_33845||full	1440	No	NA	1	0	Low-quality	Genome-fragment		2.96	0
S1_extracted_k141_100568	1121	No	NA	0	0	Not-determined	Genome-fragment	NA		0
S3_extracted_k141_56162	1440	No	NA	0	0	Low-quality	Genome-fragment		0.16	0
S1_extracted_k141_33757	4440	No	NA	0	2	Not-determined	Genome-fragment	NA		0
S1_extracted_k141_40537	1021	No	NA	0	2	Not-determined	Genome-fragment	NA		0
S1_extracted_k141_140595	1393	No	NA	0	1	Not-determined	Genome-fragment	NA		0
S5_vs_k141_82749||full	1440	No	NA	1	0	Low-quality	Genome-fragment		3.79	0
S1_extracted_k141_93964	3190	No	NA	0	2	Not-determined	Genome-fragment	NA		0
S1_extracted_k141_13880	1332	No	NA	0	1	Not-determined	Genome-fragment	NA		0
S1_extracted_k141_100617	1129	No	NA	0	1	Not-determined	Genome-fragment	NA		0
S1_extracted_k141_100634	1236	No	NA	0	0	Not-determined	Genome-fragment	NA		0
S7_vs_19766||full	1440	No	NA	0	1	Low-quality	Genome-fragment		3.95	0
S1_extracted_k141_80655	2049	No	NA	0	2	Not-determined	Genome-fragment	NA		0
S7_vs_77869||full	1440	No	NA	0	0	Low-quality	Genome-fragment		1	0
S7_vs_108708||full	1440	No	NA	0	0	Low-quality	Genome-fragment		6.05	0
S1_extracted_k141_33823	1533	No	NA	0	1	Not-determined	Genome-fragment	NA		0
S1_extracted_k141_47209	1311	No	NA	0	0	Not-determined	Genome-fragment	NA		0
S1_extracted_k141_47213	1006	No	NA	0	1	Not-determined	Genome-fragment	NA		0
S10_extracted_82450	1440	No	NA	1	0	Low-quality	Genome-fragment		0.87	0
S10_vs_4550||full	1440	No	NA	0	0	Low-quality	Genome-fragment		3.19	0
S1_extracted_k141_140687	1392	No	NA	0	1	Not-determined	Genome-fragment	NA		0
S1_extracted_k141_127382	2424	No	NA	0	0	Not-determined	Genome-fragment	NA		0
S1_extracted_k141_87334	1098	No	NA	0	0	Not-determined	Genome-fragment	NA		0
S1_extracted_k141_27179	1576	No	NA	0	1	Not-determined	Genome-fragment	NA		0
S1_extracted_k141_153805	1098	No	NA	0	1	Not-determined	Genome-fragment	NA		0
S1_extracted_k141_47235	1769	No	NA	0	0	Not-determined	Genome-fragment	NA		0
S17_extracted_k141_36973	1441	No	NA	0	0	Low-quality	Genome-fragment		1.63	0
S17_extracted_k141_102814	1441	No	NA	2	0	Low-quality	Genome-fragment		3.89	0
S1_extracted_k141_515	1306	No	NA	0	0	Not-determined	Genome-fragment	NA		0
S1_extracted_k141_67265	3116	No	NA	0	1	Not-determined	Genome-fragment	NA		0
S1_extracted_k141_120721	1538	No	NA	0	1	Not-determined	Genome-fragment	NA		0
S1_extracted_k141_153849	2877	No	NA	0	3	Not-determined	Genome-fragment	NA		0
S9_vs_k141_20673||full	1441	No	NA	0	0	Low-quality	Genome-fragment		0.94	0
S1_extracted_k141_54075	1032	No	NA	0	1	Not-determined	Genome-fragment	NA		0
S9_vs_k141_90644||lt2gene	1441	No	NA	1	0	Low-quality	Genome-fragment		4.19	0
S1_extracted_k141_60393	2924	No	NA	0	1	Not-determined	Genome-fragment	NA		0
S1_extracted_k141_100782	1753	No	NA	0	2	Not-determined	Genome-fragment	NA		0
S1_vs_k141_21174||full	1441	No	NA	0	0	Low-quality	Genome-fragment		2.74	0
S1_extracted_k141_114080	1659	No	NA	0	0	Not-determined	Genome-fragment	NA		0
S1_extracted_k141_27307	1008	No	NA	0	0	Not-determined	Genome-fragment	NA		0
S1_extracted_k141_120788	3346	No	NA	0	1	Not-determined	Genome-fragment	NA		0
S1_extracted_k141_592	1152	No	NA	0	1	Not-determined	Genome-fragment	NA		0
S1_extracted_k141_87453	4621	No	NA	0	3	Not-determined	Genome-fragment	NA		0
S1_vs_k141_136616||full	1441	No	NA	2	0	Low-quality	Genome-fragment		3.64	0
S1_vs_k141_123842||full	1441	No	NA	1	0	Low-quality	Genome-fragment		3.26	0
S1_extracted_k141_107380	1077	No	NA	0	1	Not-determined	Genome-fragment	NA		0
S5_vs_k141_18561||full	1441	No	NA	1	0	Low-quality	Genome-fragment		2.14	0
S5_vs_k141_83482||full	1441	No	NA	2	0	Low-quality	Genome-fragment		3.25	0
S7_vs_64118||full	1441	No	NA	2	0	Low-quality	Genome-fragment		3.25	0
S7_vs_76569||full	1441	No	NA	0	3	Low-quality	Genome-fragment		1.27	0
S1_extracted_k141_153901	7780	No	NA	0	1	Not-determined	Genome-fragment	NA		0
S1_extracted_k141_14183	1375	No	NA	0	1	Not-determined	Genome-fragment	NA		0
S1_extracted_k141_54197	1457	No	NA	0	1	Not-determined	Genome-fragment	NA		0
S7_vs_101433||full	1441	No	NA	1	0	Low-quality	Genome-fragment		2.81	0
S7_vs_104572||full	1441	No	NA	1	0	Low-quality	Genome-fragment		6.94	0
S10_vs_53281||full	1441	No	NA	0	3	Low-quality	Genome-fragment		1.36	0
S1_extracted_k141_54263	2039	No	NA	0	2	Not-determined	Genome-fragment	NA		0
S1_extracted_k141_134174	1532	No	NA	0	1	Not-determined	Genome-fragment	NA		0
S1_extracted_k141_54265	2313	No	NA	0	1	Not-determined	Genome-fragment	NA		0
S12_vs_k141_20920||full	1441	No	NA	0	0	Low-quality	Genome-fragment		3.5	0
S1_extracted_k141_153933	1205	No	NA	0	1	Not-determined	Genome-fragment	NA		0
S1_extracted_k141_27440	1308	No	NA	0	0	Not-determined	Genome-fragment	NA		0
S17_vs_k141_48858||full	1442	No	NA	1	0	Low-quality	Genome-fragment		1.17	0
S1_extracted_k141_127660	3198	No	NA	0	2	Not-determined	Genome-fragment	NA		0
S1_extracted_k141_107461	1112	No	NA	0	1	Not-determined	Genome-fragment	NA		0
S1_extracted_k141_80946	1001	No	NA	0	1	Not-determined	Genome-fragment	NA		0
S1_extracted_k141_47532	1879	No	NA	0	1	Not-determined	Genome-fragment	NA		0
S17_vs_k141_57056||full	1442	No	NA	0	0	Low-quality	Genome-fragment		2.39	0
S1_extracted_k141_120876	1038	No	NA	0	2	Not-determined	Genome-fragment	NA		0
S9_vs_k141_25680||full	1442	No	NA	1	0	Low-quality	Genome-fragment		3.38	0
S1_extracted_k141_34081	1253	No	NA	0	1	Not-determined	Genome-fragment	NA		0
S14_vs_k141_22824||full	1442	No	NA	0	0	Low-quality	Genome-fragment		1.93	0
S1_extracted_k141_14333	1293	No	NA	0	1	Not-determined	Genome-fragment	NA		0
S1_extracted_k141_140979	1253	No	NA	0	2	Not-determined	Genome-fragment	NA		0
S5_vs_k141_61748||full	1442	No	NA	0	0	Low-quality	Genome-fragment		3.66	0
S1_extracted_k141_114228	1232	No	NA	0	0	Not-determined	Genome-fragment	NA		0
S1_extracted_k141_54331	1343	No	NA	0	1	Not-determined	Genome-fragment	NA		0
S1_extracted_k141_60562	1277	No	NA	0	1	Not-determined	Genome-fragment	NA		0
S1_extracted_k141_101038	1285	No	NA	0	0	Not-determined	Genome-fragment	NA		0
S7_vs_37||full	1442	No	NA	1	0	Low-quality	Genome-fragment		1.9	0
S1_extracted_k141_134241	1273	No	NA	0	0	Not-determined	Genome-fragment	NA		0
S1_extracted_k141_54350	3080	No	NA	0	0	Not-determined	Genome-fragment	NA		0
S7_vs_21596||full	1442	No	NA	1	0	Low-quality	Genome-fragment		2.25	0
S1_extracted_k141_127747	1002	No	NA	0	1	Not-determined	Genome-fragment	NA		0
S1_extracted_k141_87710	1089	No	NA	0	1	Not-determined	Genome-fragment	NA		0
S1_extracted_k141_47616	2664	No	NA	0	1	Not-determined	Genome-fragment	NA		0
S1_extracted_k141_822	2483	No	NA	0	2	Not-determined	Genome-fragment	NA		0
S1_extracted_k141_147277	2558	No	NA	0	1	Not-determined	Genome-fragment	NA		0
S16_vs_43755||full	1442	No	NA	0	0	Low-quality	Genome-fragment		0.54	0
S1_extracted_k141_141072	1519	No	NA	0	2	Not-determined	Genome-fragment	NA		0
S12_extracted_k141_14459	1442	No	NA	1	0	Low-quality	Genome-fragment		2.35	0
S1_extracted_k141_81100	1127	No	NA	0	0	Not-determined	Genome-fragment	NA		0
S1_extracted_k141_81104	3095	No	NA	0	2	Not-determined	Genome-fragment	NA		0
S1_extracted_k141_141094	1524	No	NA	0	1	Not-determined	Genome-fragment	NA		0
S1_extracted_k141_101114	1558	No	NA	0	1	Not-determined	Genome-fragment	NA		0
S1_extracted_k141_94451	1087	No	NA	0	0	Not-determined	Genome-fragment	NA		0
S12_vs_k141_96886||full	1442	No	NA	0	0	Low-quality	Genome-fragment		2.9	0
S1_extracted_k141_34186	1004	No	NA	0	1	Not-determined	Genome-fragment	NA		0
S1_extracted_k141_60631	3157	No	NA	0	2	Not-determined	Genome-fragment	NA		0
S1_extracted_k141_20965	1103	No	NA	0	0	Not-determined	Genome-fragment	NA		0
S8_vs_k141_202372||full	1442	No	NA	1	0	Low-quality	Genome-fragment		2.39	0
S11_vs_k141_92838||full	1443	No	NA	0	1	Low-quality	Genome-fragment		2.09	0
S1_extracted_k141_107620	1046	No	NA	0	1	Not-determined	Genome-fragment	NA		0
S1_extracted_k141_101144	1224	No	NA	0	2	Not-determined	Genome-fragment	NA		0
S14_vs_k141_90845||full	1443	No	NA	0	0	Low-quality	Genome-fragment		3.2	0
S1_extracted_k141_34212	1105	No	NA	0	1	Not-determined	Genome-fragment	NA		0
S14_vs_k141_12316||lt2gene	1443	No	NA	1	0	Low-quality	Genome-fragment		1.89	0
S1_extracted_k141_141154	1376	No	NA	0	0	Not-determined	Genome-fragment	NA		0
S1_vs_k141_131439||lt2gene	1443	No	NA	1	0	Low-quality	Genome-fragment		4.04	0
S1_extracted_k141_20994	3135	No	NA	0	1	Not-determined	Genome-fragment	NA		0
S3_vs_k141_17658||full	1443	No	NA	0	0	Low-quality	Genome-fragment		0.93	0
S1_extracted_k141_114369	1086	No	NA	0	1	Not-determined	Genome-fragment	NA		0
S1_extracted_k141_87781	3170	No	NA	0	1	Not-determined	Genome-fragment	NA		0
S1_extracted_k141_134370	1445	No	NA	0	0	Not-determined	Genome-fragment	NA		0
S1_extracted_k141_47711	3043	No	NA	0	0	Not-determined	Genome-fragment	NA		0
S1_extracted_k141_14615	1241	No	NA	0	1	Not-determined	Genome-fragment	NA		0
S1_extracted_k141_27657	1225	No	NA	0	1	Not-determined	Genome-fragment	NA		0
S1_extracted_k141_147318	1237	No	NA	0	0	Not-determined	Genome-fragment	NA		0
S1_extracted_k141_14623	3459	No	NA	0	0	Not-determined	Genome-fragment	NA		0
S5_extracted_k141_137822	1443	No	NA	1	0	Low-quality	Genome-fragment		6.57	0
S1_extracted_k141_14637	1442	No	NA	0	0	Not-determined	Genome-fragment	NA		0
S1_extracted_k141_74440	1506	No	NA	0	1	Not-determined	Genome-fragment	NA		0
S5_vs_k141_125077||lt2gene	1443	No	NA	1	0	Low-quality	Genome-fragment		3.44	0
S7_vs_101303||full	1443	No	NA	0	0	Low-quality	Genome-fragment		4.19	0
S16_extracted_118392	1443	No	NA	0	1	Low-quality	Genome-fragment		0.24	0
S1_extracted_k141_127906	1233	No	NA	0	1	Not-determined	Genome-fragment	NA		0
S6_vs_k141_21228||full	1443	No	NA	0	0	Low-quality	Genome-fragment		1.89	0
S1_extracted_k141_134447	6467	No	NA	0	3	Not-determined	Genome-fragment	NA		0
S1_extracted_k141_141249	1067	No	NA	0	0	Not-determined	Genome-fragment	NA		0
S17_extracted_k141_102842	1444	No	NA	2	0	Low-quality	Genome-fragment		2.04	0
S9_vs_k141_73048||full	1444	No	NA	2	0	Low-quality	Genome-fragment		2.5	0
S1_extracted_k141_114465	1045	No	NA	0	1	Not-determined	Genome-fragment	NA		0
S3_vs_k141_49283||full	1444	No	NA	0	1	Low-quality	Genome-fragment		0.63	0
S1_extracted_k141_47800	1062	No	NA	0	2	Not-determined	Genome-fragment	NA		0
S1_extracted_k141_41244	1486	No	NA	0	1	Not-determined	Genome-fragment	NA		0
S3_vs_k141_92000||full	1444	No	NA	1	0	Low-quality	Genome-fragment		1.03	0
S5_vs_k141_68364||full	1444	No	NA	1	0	Low-quality	Genome-fragment		3.47	0
S1_extracted_k141_147347	1214	No	NA	0	1	Not-determined	Genome-fragment	NA		0
S1_extracted_k141_101268	1027	No	NA	0	1	Not-determined	Genome-fragment	NA		0
S1_extracted_k141_953	1808	No	NA	0	1	Not-determined	Genome-fragment	NA		0
S5_vs_k141_71530||full	1444	No	NA	0	0	Low-quality	Genome-fragment		3.73	0
S5_vs_k141_58314||full	1444	No	NA	0	0	Low-quality	Genome-fragment		2.66	0
S1_extracted_k141_41260	3465	No	NA	0	1	Not-determined	Genome-fragment	NA		0
S7_vs_118432||full	1444	No	NA	2	0	Low-quality	Genome-fragment		1.75	0
S10_vs_37280||full	1444	No	NA	1	0	Low-quality	Genome-fragment		2.49	0
S1_extracted_k141_27748	3990	No	NA	0	2	Not-determined	Genome-fragment	NA		0
S1_extracted_k141_966	1018	No	NA	0	0	Not-determined	Genome-fragment	NA		0
S1_extracted_k141_141293	1037	No	NA	0	2	Not-determined	Genome-fragment	NA		0
S1_extracted_k141_94656	1004	No	NA	0	1	Not-determined	Genome-fragment	NA		0
S2_vs_k141_5131||lt2gene	1444	No	NA	1	0	Low-quality	Genome-fragment		3.53	0
S6_vs_k141_24342||full	1444	No	NA	0	0	Low-quality	Genome-fragment		0.84	0
S6_vs_k141_3378||full	1444	No	NA	4	0	Low-quality	Genome-fragment		2.95	0
S1_extracted_k141_54574	1015	No	NA	0	1	Not-determined	Genome-fragment	NA		0
S1_extracted_k141_54579	1411	No	NA	0	1	Not-determined	Genome-fragment	NA		0
S1_extracted_k141_81320	4631	No	NA	0	2	Not-determined	Genome-fragment	NA		0
S1_extracted_k141_141315	1102	No	NA	0	1	Not-determined	Genome-fragment	NA		0
S1_extracted_k141_134524	3159	No	NA	0	1	Not-determined	Genome-fragment	NA		0
S1_extracted_k141_47875	1388	No	NA	0	0	Not-determined	Genome-fragment	NA		0
S1_extracted_k141_41313	3619	No	NA	0	3	Not-determined	Genome-fragment	NA		0
S1_extracted_k141_94697	3328	No	NA	0	0	Not-determined	Genome-fragment	NA		0
S1_extracted_k141_21189	1281	No	NA	0	1	Not-determined	Genome-fragment	NA		0
S1_extracted_k141_154202	3124	No	NA	0	2	Not-determined	Genome-fragment	NA		0
S1_extracted_k141_101369	1662	No	NA	0	0	Not-determined	Genome-fragment	NA		0
S1_extracted_k141_94715	3042	No	NA	0	2	Not-determined	Genome-fragment	NA		0
S1_extracted_k141_101376	2034	No	NA	0	1	Not-determined	Genome-fragment	NA		0
S8_vs_k141_166915||full	1444	No	NA	0	0	Low-quality	Genome-fragment		3.5	0
S17_extracted_k141_94363	1445	No	NA	0	2	Low-quality	Genome-fragment		0.41	0
S1_extracted_k141_128035	1220	No	NA	0	1	Not-determined	Genome-fragment	NA		0

S1_extracted_k141_94726	1067	No	NA	0	1	Not-determined	Genome-fragment	NA		0
S1_extracted_k141_67892	1502	No	NA	0	2	Not-determined	Genome-fragment	NA		0
S1_extracted_k141_94735	1190	No	NA	0	1	Not-determined	Genome-fragment	NA		0
S17_vs_k141_145686||full	1445	No	NA	0	0	Low-quality	Genome-fragment		1.02	0
S9_extracted_k141_82418	1445	No	NA	4	0	Low-quality	Genome-fragment		4.77	0
S1_extracted_k141_107924	1207	No	NA	0	0	Not-determined	Genome-fragment	NA		0
S1_extracted_k141_21250	1012	No	NA	0	0	Not-determined	Genome-fragment	NA		0
S1_extracted_k141_101426	1010	No	NA	0	1	Not-determined	Genome-fragment	NA		0
S13_extracted_59751	1445	No	NA	0	1	Low-quality	Genome-fragment		0.8	0
S14_extracted_k141_95559	1445	No	NA	3	0	Low-quality	Genome-fragment		4.76	0
S14_extracted_k141_106355	1445	No	NA	0	0	Low-quality	Genome-fragment		2.64	0
S14_vs_k141_3196||full	1445	No	NA	0	0	Low-quality	Genome-fragment		1.58	0
S1_extracted_k141_67948	1193	No	NA	0	1	Not-determined	Genome-fragment	NA		0
S1_vs_k141_48454||full	1445	No	NA	1	0	Low-quality	Genome-fragment		0.94	0
S1_extracted_k141_114627	1032	No	NA	0	1	Not-determined	Genome-fragment	NA		0
S1_extracted_k141_107958	2640	No	NA	0	2	Not-determined	Genome-fragment	NA		0
S1_extracted_k141_81431	4981	No	NA	0	0	Not-determined	Genome-fragment	NA		0
S5_vs_k141_114577||full	1445	No	NA	0	0	Low-quality	Genome-fragment		3.07	0
S1_extracted_k141_67966	1166	No	NA	0	1	Not-determined	Genome-fragment	NA		0
S1_extracted_k141_14940	1111	No	NA	0	1	Not-determined	Genome-fragment	NA		0
S1_extracted_k141_60828	4008	No	NA	0	2	Not-determined	Genome-fragment	NA		0
S16_extracted_146369	1445	No	NA	0	0	Low-quality	Genome-fragment		3.64	0
S1_extracted_k141_88092	1319	No	NA	0	1	Not-determined	Genome-fragment	NA		0
S1_extracted_k141_121238	1900	No	NA	0	0	Not-determined	Genome-fragment	NA		0
S1_extracted_k141_41463	1095	No	NA	0	1	Not-determined	Genome-fragment	NA		0
S10_vs_81305||full	1445	No	NA	0	0	Low-quality	Genome-fragment		2.48	0
S1_extracted_k141_8103	1132	No	NA	0	3	Not-determined	Genome-fragment	NA		0
S1_extracted_k141_147441	1644	No	NA	0	1	Not-determined	Genome-fragment	NA		0
S15_extracted_56155	1445	No	NA	1	0	Low-quality	Genome-fragment		3.15	0
S8_extracted_k141_131413	1445	No	NA	3	0	Low-quality	Genome-fragment		4.76	0
S9_vs_k141_11271||full	1446	No	NA	2	0	Low-quality	Genome-fragment		3.85	0
S1_extracted_k141_154283	1589	No	NA	0	0	Not-determined	Genome-fragment	NA		0
S1_extracted_k141_108033	1919	No	NA	0	2	Not-determined	Genome-fragment	NA		0
S1_vs_k141_5688||full	1446	No	NA	0	0	Low-quality	Genome-fragment		1.14	0
S1_extracted_k141_121270	1457	No	NA	0	0	Not-determined	Genome-fragment	NA		0
S3_extracted_k141_97385	1446	No	NA	0	0	Low-quality	Genome-fragment		1.13	0
S3_vs_k141_103772||full	1446	No	NA	0	2	Low-quality	Genome-fragment		0.81	0
S1_extracted_k141_94880	1423	No	NA	0	0	Not-determined	Genome-fragment	NA		0
S3_vs_k141_8308||full	1446	No	NA	0	2	Low-quality	Genome-fragment		3.34	0
S1_extracted_k141_68084	1122	No	NA	0	2	Not-determined	Genome-fragment	NA		0
S7_vs_19803||full	1446	No	NA	0	2	Low-quality	Genome-fragment		1.14	0
S16_extracted_11764	1446	No	NA	0	3	Low-quality	Genome-fragment		0.47	0
S12_vs_k141_171152||full	1446	No	NA	1	0	Low-quality	Genome-fragment		3.73	0
S1_extracted_k141_141498	1078	No	NA	0	1	Not-determined	Genome-fragment	NA		0
S11_vs_k141_24625||full	1447	No	NA	0	1	Low-quality	Genome-fragment		2.22	0
S14_extracted_k141_17134	1447	No	NA	1	0	Low-quality	Genome-fragment		4.6	0
S1_extracted_k141_28075	1285	No	NA	0	0	Not-determined	Genome-fragment	NA		0
S1_extracted_k141_141510	4322	No	NA	0	4	Not-determined	Genome-fragment	NA		0
S14_extracted_k141_40953	1447	No	NA	0	1	Low-quality	Genome-fragment		1.66	0
S1_extracted_k141_114828	7077	No	NA	0	2	Not-determined	Genome-fragment	NA		0
S1_extracted_k141_147489	1621	No	NA	0	0	Not-determined	Genome-fragment	NA		0
S1_extracted_k141_1188	1029	No	NA	0	0	Not-determined	Genome-fragment	NA		0
S1_extracted_k141_74844	1515	No	NA	0	2	Not-determined	Genome-fragment	NA		0
S14_vs_k141_6642||lt2gene	1447	No	NA	2	0	Low-quality	Genome-fragment		3.04	0
S1_extracted_k141_74858	1002	No	NA	0	0	Not-determined	Genome-fragment	NA		0
S5_vs_k141_117816||full	1447	No	NA	2	0	Low-quality	Genome-fragment		3.32	0
S16_extracted_133238	1447	No	NA	4	0	Low-quality	Genome-fragment		4.75	0
S1_extracted_k141_95017	1231	No	NA	0	2	Not-determined	Genome-fragment	NA		0
S16_vs_31282||full	1447	No	NA	2	0	Low-quality	Genome-fragment		3.79	0
S1_extracted_k141_147516	1124	No	NA	0	1	Not-determined	Genome-fragment	NA		0
S1_extracted_k141_54886	1008	No	NA	0	1	Not-determined	Genome-fragment	NA		0
S10_vs_92493||full	1447	No	NA	0	2	Low-quality	Genome-fragment		1.08	0
S1_extracted_k141_1259	3368	No	NA	0	0	Not-determined	Genome-fragment	NA		0
S1_extracted_k141_95036	1064	No	NA	0	1	Not-determined	Genome-fragment	NA		0
S10_vs_119860||lt2gene	1447	No	NA	1	0	Low-quality	Genome-fragment		2.43	0
S1_extracted_k141_147526	1170	No	NA	0	1	Not-determined	Genome-fragment	NA		0
S6_vs_k141_19919||full	1447	No	NA	0	0	Low-quality	Genome-fragment		2.8	0
S1_extracted_k141_81649	1236	No	NA	0	0	Not-determined	Genome-fragment	NA		0
S1_extracted_k141_101740	1050	No	NA	0	0	Not-determined	Genome-fragment	NA		0
S1_extracted_k141_8333	3161	No	NA	0	1	Not-determined	Genome-fragment	NA		0
S8_extracted_k141_44652	1447	No	NA	0	0	Low-quality	Genome-fragment		3.19	0
S8_vs_k141_138125||full	1447	No	NA	0	0	Low-quality	Genome-fragment		2.99	0
S1_extracted_k141_48301	1153	No	NA	0	1	Not-determined	Genome-fragment	NA		0
S1_extracted_k141_28222	1731	No	NA	0	1	Not-determined	Genome-fragment	NA		0
S1_extracted_k141_41677	1199	No	NA	0	1	Not-determined	Genome-fragment	NA		0
S8_vs_k141_207778||full	1447	No	NA	0	1	Low-quality	Genome-fragment		1.72	0
S1_extracted_k141_88319	1142	No	NA	0	0	Not-determined	Genome-fragment	NA		0
S1_extracted_k141_81711	1066	No	NA	0	0	Not-determined	Genome-fragment	NA		0
S1_extracted_k141_15273	1139	No	NA	0	0	Not-determined	Genome-fragment	NA		0
S1_extracted_k141_55012	1054	No	NA	0	1	Not-determined	Genome-fragment	NA		0
S1_extracted_k141_68301	1591	No	NA	0	1	Not-determined	Genome-fragment	NA		0
S1_extracted_k141_21607	1264	No	NA	0	0	Not-determined	Genome-fragment	NA		0
S1_extracted_k141_147574	2406	No	NA	0	1	Not-determined	Genome-fragment	NA		0
S17_extracted_k141_61364	1448	No	NA	0	0	Low-quality	Genome-fragment		1.47	0
S1_extracted_k141_75103	1177	No	NA	0	1	Not-determined	Genome-fragment	NA		0
S1_extracted_k141_68343	1152	No	NA	0	1	Not-determined	Genome-fragment	NA		0
S1_extracted_k141_147578	5782	No	NA	0	1	Not-determined	Genome-fragment	NA		0
S9_vs_k141_45889||full	1448	No	NA	2	0	Low-quality	Genome-fragment		2.26	0
S1_extracted_k141_95168	1320	No	NA	0	1	Not-determined	Genome-fragment	NA		0
S1_extracted_k141_66394	1448	No	NA	0	0	Low-quality	Genome-fragment		1.47	0
S1_extracted_k141_34779	2133	No	NA	0	1	Not-determined	Genome-fragment	NA		0
S1_extracted_k141_28375	1135	No	NA	0	1	Not-determined	Genome-fragment	NA		0
S1_extracted_k141_95205	1126	No	NA	0	2	Not-determined	Genome-fragment	NA		0
S1_extracted_k141_88413	1225	No	NA	0	2	Not-determined	Genome-fragment	NA		0
S1_extracted_k141_101877	1000	No	NA	0	0	Not-determined	Genome-fragment	NA		0
S1_extracted_k141_135081	1613	No	NA	0	1	Not-determined	Genome-fragment	NA		0
S1_extracted_k141_21695	1552	No	NA	0	2	Not-determined	Genome-fragment	NA		0
S1_extracted_k141_154570	1837	No	NA	0	1	Not-determined	Genome-fragment	NA		0
S1_vs_k141_105720||full	1448	No	NA	0	1	Low-quality	Genome-fragment		1.02	0
S5_vs_k141_13759||full	1448	No	NA	3	0	Low-quality	Genome-fragment		4.53	0
S16_vs_32137||full	1448	No	NA	2	0	Low-quality	Genome-fragment		3.09	0
S1_extracted_k141_8521	1053	No	NA	0	0	Not-determined	Genome-fragment	NA		0
S1_extracted_k141_95225	3571	No	NA	0	4	Not-determined	Genome-fragment	NA		0
S1_extracted_k141_28398	28234	No	NA	10	0	Medium-quality	Genome-fragment		61.16	0
S16_vs_38779||full	1448	No	NA	1	0	Low-quality	Genome-fragment		3.52	0
S8_vs_k141_193282||full	1448	No	NA	0	1	Low-quality	Genome-fragment		1.68	0
S8_vs_k141_153029||full	1448	No	NA	1	0	Low-quality	Genome-fragment		1	0
S8_vs_k141_205520||full	1448	No	NA	2	0	Low-quality	Genome-fragment		3	0
S8_vs_k141_111510||full	1448	No	NA	0	0	Low-quality	Genome-fragment		2.01	0
S8_vs_k141_103165||lt2gene	1448	No	NA	1	0	Low-quality	Genome-fragment		2.15	0
S1_extracted_k141_21719	1083	No	NA	0	1	Not-determined	Genome-fragment	NA		0
S1_extracted_k141_68461	1175	No	NA	0	0	Not-determined	Genome-fragment	NA		0
S11_vs_k141_81053||full	1449	No	NA	0	0	Low-quality	Genome-fragment		3.6	0
S1_extracted_k141_108445	2027	No	NA	0	1	Not-determined	Genome-fragment	NA		0
S1_extracted_k141_81934	4695	No	NA	0	1	Not-determined	Genome-fragment	NA		0
S1_extracted_k141_48565	2287	No	NA	0	0	Not-determined	Genome-fragment	NA		0
S1_extracted_k141_147632	4887	No	NA	0	2	Not-determined	Genome-fragment	NA		0
S1_extracted_k141_88510	3252	No	NA	0	3	Not-determined	Genome-fragment	NA		0
S1_extracted_k141_15508	1154	No	NA	0	0	Not-determined	Genome-fragment	NA		0
S13_vs_128473||full	1449	No	NA	0	0	Low-quality	Genome-fragment		2.4	0
S1_extracted_k141_154623	6139	No	NA	0	4	Not-determined	Genome-fragment	NA		0
S13_vs_139016||full	1449	No	NA	0	0	Low-quality	Genome-fragment		3.39	0
S1_extracted_k141_28489	1396	No	NA	0	0	Not-determined	Genome-fragment	NA		0
S1_extracted_k141_75237	1294	No	NA	0	1	Not-determined	Genome-fragment	NA		0
S1_extracted_k141_41958	1098	No	NA	0	1	Not-determined	Genome-fragment	NA		0
S14_extracted_k141_63361	1449	No	NA	3	0	Low-quality	Genome-fragment		2.96	0
S1_extracted_k141_81992	1872	No	NA	0	0	Not-determined	Genome-fragment	NA		0
S1_extracted_k141_141955	1529	No	NA	0	3	Not-determined	Genome-fragment	NA		0
S1_extracted_k141_102022	3835	No	NA	0	1	Not-determined	Genome-fragment	NA		0
S1_extracted_k141_68564	1025	No	NA	0	1	Not-determined	Genome-fragment	NA		0
S14_vs_k141_85100||full	1449	No	NA	0	3	Low-quality	Genome-fragment		0.65	0
S1_extracted_k141_115281	1208	No	NA	0	0	Not-determined	Genome-fragment	NA		0
S1_extracted_k141_34916	1370	No	NA	0	1	Not-determined	Genome-fragment	NA		0
S1_vs_k141_43354||full	1449	No	NA	1	0	Low-quality	Genome-fragment		3.77	0
S1_extracted_k141_48647	1043	No	NA	0	1	Not-determined	Genome-fragment	NA		0
S1_extracted_k141_15611	4104	No	NA	0	1	Not-determined	Genome-fragment	NA		0
S1_extracted_k141_28540	1010	No	NA	0	0	Not-determined	Genome-fragment	NA		0
S1_extracted_k141_128717	1871	No	NA	0	1	Not-determined	Genome-fragment	NA		0
S1_extracted_k141_1564	1335	No	NA	0	0	Not-determined	Genome-fragment	NA		0
S1_extracted_k141_34932	1355	No	NA	0	0	Not-determined	Genome-fragment	NA		0
S1_extracted_k141_21844	1174	No	NA	0	0	Not-determined	Genome-fragment	NA		0
S1_extracted_k141_135283	1303	No	NA	0	0	Not-determined	Genome-fragment	NA		0
S1_extracted_k141_61093	1279	No	NA	0	2	Not-determined	Genome-fragment	NA		0
S1_extracted_k141_1582	4432	No	NA	0	3	Not-determined	Genome-fragment	NA		0
S1_extracted_k141_95423	1337	No	NA	0	1	Not-determined	Genome-fragment	NA		0
S1_extracted_k141_34969	1404	No	NA	0	0	Not-determined	Genome-fragment	NA		0
S5_vs_k141_110760||lt2gene	1449	No	NA	1	0	Low-quality	Genome-fragment		3.9	0
S1_extracted_k141_121822	2917	No	NA	0	1	Not-determined	Genome-fragment	NA		0
S1_extracted_k141_142055	1260	No	NA	0	0	Not-determined	Genome-fragment	NA		0
S1_extracted_k141_82098	2055	No	NA	0	2	Not-determined	Genome-fragment	NA		0
S1_extracted_k141_42065	1248	No	NA	0	1	Not-determined	Genome-fragment	NA		0
S16_vs_75800||full	1449	No	NA	0	0	Low-quality	Genome-fragment		3.29	0
S1_extracted_k141_88704	3182	No	NA	0	1	Not-determined	Genome-fragment	NA		0
S1_extracted_k141_68680	1144	No	NA	0	1	Not-determined	Genome-fragment	NA		0
S1_extracted_k141_61136	1391	No	NA	0	1	Not-determined	Genome-fragment	NA		0
S1_extracted_k141_68699	1225	No	NA	0	1	Not-determined	Genome-fragment	NA		0
S1_extracted_k141_135391	8825	No	NA	0	3	Not-determined	Genome-fragment	NA		0
S10_vs_69281||full	1449	No	NA	0	1	Low-quality	Genome-fragment		0.89	0
S1_extracted_k141_21932	3638	No	NA	0	1	Not-determined	Genome-fragment	NA		0
S12_vs_k141_16455||full	1449	No	NA	0	1	Low-quality	Genome-fragment		2.02	0
S1_extracted_k141_75451	1607	No	NA	0	1	Not-determined	Genome-fragment	NA		0
S8_extracted_k141_24435	1449	No	NA	1	0	Low-quality	Genome-fragment		2.46	0
S1_extracted_k141_55475	3175	No	NA	0	2	Not-determined	Genome-fragment	NA		0
S8_vs_k141_144187||full	1449	No	NA	1	0	Low-quality	Genome-fragment		3.22	0
S9_extracted_k141_67795	1450	No	NA	2	0	Low-quality	Genome-fragment		3.86	0
S1_extracted_k141_82207	2774	No	NA	0	1	Not-determined	Genome-fragment	NA		0
S1_extracted_k141_8819	3185	No	NA	0	1	Not-determined	Genome-fragment	NA		0
S1_extracted_k141_135466	1290	No	NA	0	1	Not-determined	Genome-fragment	NA		0
S1_extracted_k141_48855	1002	No	NA	0	1	Not-determined	Genome-fragment	NA		0
S1_extracted_k141_88804	1045	No	NA	0	1	Not-determined	Genome-fragment	NA		0
S1_extracted_k141_128913	1002	No	NA	0	1	Not-determined	Genome-fragment	NA		0
S1_extracted_k141_42196	1742	No	NA	0	1	Not-determined	Genome-fragment	NA		0
S1_extracted_k141_22047	1021	No	NA	0	0	Not-determined	Genome-fragment	NA		0
S11_vs_k141_59745||full	1450	No	NA	1	0	Low-quality	Genome-fragment		3.42	0

S1_extracted_k141_42214	2194	No	NA	0	0	Not-determined	Genome-fragment	NA		0
S1_extracted_k141_61257	1310	No	NA	0	0	Not-determined	Genome-fragment	NA		0
S1_extracted_k141_8884	1135	No	NA	0	0	Not-determined	Genome-fragment	NA		0
S3_vs_k141_2522||full	1450	No	NA	2	0	Low-quality	Genome-fragment		2.52	0
S1_extracted_k141_108820	1148	No	NA	0	0	Not-determined	Genome-fragment	NA		0
S5_extracted_k141_89722	1450	No	NA	3	0	Low-quality	Genome-fragment		4.75	0
S1_extracted_k141_128998	1045	No	NA	0	1	Not-determined	Genome-fragment	NA		0
S7_vs_109915||full	1450	No	NA	1	0	Low-quality	Genome-fragment		2.54	0
S12_vs_k141_135131||lt2gene	1450	No	NA	1	0	Low-quality	Genome-fragment		3.11	0
S1_extracted_k141_95707	2500	No	NA	0	2	Not-determined	Genome-fragment	NA		0
S15_vs_15419||full	1450	No	NA	0	1	Low-quality	Genome-fragment		2.85	0
S1_extracted_k141_135557	2844	No	NA	0	2	Not-determined	Genome-fragment	NA		0
S1_extracted_k141_75630	2568	No	NA	0	0	Not-determined	Genome-fragment	NA		0
S1_extracted_k141_135573	1168	No	NA	0	1	Not-determined	Genome-fragment	NA		0
S1_extracted_k141_108884	1323	No	NA	0	1	Not-determined	Genome-fragment	NA		0
S4_extracted_10849	1450	No	NA	2	0	Low-quality	Genome-fragment		4.77	0
S4_extracted_12542	1450	No	NA	2	0	Low-quality	Genome-fragment		4.78	0
S1_extracted_k141_61296	1461	No	NA	0	0	Not-determined	Genome-fragment	NA		0
S1_extracted_k141_115648	1224	No	NA	0	1	Not-determined	Genome-fragment	NA		0
S17_extracted_k141_158510	1451	No	NA	0	0	Low-quality	Genome-fragment		2.87	0
S1_extracted_k141_16044	3449	No	NA	0	1	Not-determined	Genome-fragment	NA		0
S1_extracted_k141_95759	1220	No	NA	0	1	Not-determined	Genome-fragment	NA		0
S1_extracted_k141_122178	1080	No	NA	0	1	Not-determined	Genome-fragment	NA		0
S1_extracted_k141_16052	1062	No	NA	0	1	Not-determined	Genome-fragment	NA		0
S1_extracted_k141_16056	1213	No	NA	0	0	Not-determined	Genome-fragment	NA		0
S17_extracted_k141_48468	1451	No	NA	0	0	Low-quality	Genome-fragment		1.96	0
S1_extracted_k141_55657	1413	No	NA	0	1	Not-determined	Genome-fragment	NA		0
S1_extracted_k141_115694	1016	No	NA	0	1	Not-determined	Genome-fragment	NA		0
S1_extracted_k141_108930	1100	No	NA	0	1	Not-determined	Genome-fragment	NA		0
S9_extracted_k141_81429	1451	No	NA	0	0	Low-quality	Genome-fragment		1.66	0
S1_extracted_k141_75689	1140	No	NA	0	1	Not-determined	Genome-fragment	NA		0
S1_extracted_k141_22149	42111	No	NA	9	0	High-quality	High-quality		91.64	0
S9_vs_k141_49470||full	1451	No	NA	0	1	Low-quality	Genome-fragment		2.23	0
S1_extracted_k141_154951	1193	No	NA	0	0	Not-determined	Genome-fragment	NA		0
S1_extracted_k141_108949	3616	No	NA	0	2	Not-determined	Genome-fragment	NA		0
S1_extracted_k141_68949	8689	No	NA	0	18	Not-determined	Genome-fragment	NA		0
S14_vs_k141_114582||full	1451	No	NA	0	0	Low-quality	Genome-fragment		3.4	0
S1_extracted_k141_82457	1004	No	NA	0	0	Not-determined	Genome-fragment	NA		0
S1_extracted_k141_75735	4761	No	NA	0	1	Not-determined	Genome-fragment	NA		0
S1_extracted_k141_75746	1454	No	NA	0	1	Not-determined	Genome-fragment	NA		0
S1_extracted_k141_115759	1194	No	NA	0	1	Not-determined	Genome-fragment	NA		0
S5_extracted_k141_45159	1451	No	NA	0	0	Low-quality	Genome-fragment		5.17	0
S1_extracted_k141_122279	3177	No	NA	0	1	Not-determined	Genome-fragment	NA		0
S1_extracted_k141_69000	1358	No	NA	0	0	Not-determined	Genome-fragment	NA		0
S12_vs_k141_163464||lt2gene	1451	No	NA	2	0	Low-quality	Genome-fragment		4.23	0
S2_extracted_k141_5097	1451	No	NA	1	0	Low-quality	Genome-fragment		1.43	0
S8_vs_k141_160091||full	1451	No	NA	0	1	Low-quality	Genome-fragment		1.14	0
S17_extracted_k141_134432	1452	No	NA	1	0	Low-quality	Genome-fragment		2.4	0
S11_vs_k141_45741||full	1452	No	NA	0	0	Low-quality	Genome-fragment		3.08	0
S1_extracted_k141_29055	1317	No	NA	0	0	Not-determined	Genome-fragment	NA		0
S1_vs_k141_125595||full	1452	No	NA	0	1	Low-quality	Genome-fragment		3.1	0
S5_extracted_k141_19858	1452	No	NA	1	0	Low-quality	Genome-fragment		2.79	0
S1_extracted_k141_115845	1012	No	NA	0	1	Not-determined	Genome-fragment	NA		0
S1_extracted_k141_29064	1081	No	NA	0	1	Not-determined	Genome-fragment	NA		0
S1_extracted_k141_42513	2938	No	NA	0	2	Not-determined	Genome-fragment	NA		0
S1_extracted_k141_16230	1248	No	NA	0	1	Not-determined	Genome-fragment	NA		0
S1_extracted_k141_95960	1221	No	NA	0	1	Not-determined	Genome-fragment	NA		0
S7_vs_26865||full	1452	No	NA	0	1	Low-quality	Genome-fragment		1.81	0
S1_extracted_k141_142484	1120	No	NA	0	0	Not-determined	Genome-fragment	NA		0
S16_extracted_170077	1452	No	NA	0	0	Low-quality	Genome-fragment		2.43	0
S1_extracted_k141_42533	1102	No	NA	0	1	Not-determined	Genome-fragment	NA		0
S10_vs_115054||full	1452	No	NA	0	1	Low-quality	Genome-fragment		0.52	0
S1_extracted_k141_9195	1340	No	NA	0	2	Not-determined	Genome-fragment	NA		0
S1_extracted_k141_16245	1468	No	NA	0	0	Not-determined	Genome-fragment	NA		0
S1_extracted_k141_29103	1273	No	NA	0	0	Not-determined	Genome-fragment	NA		0
S1_extracted_k141_42555	4632	No	NA	0	5	Not-determined	Genome-fragment	NA		0
S1_extracted_k141_9224	1302	No	NA	0	1	Not-determined	Genome-fragment	NA		0
S1_extracted_k141_147938	2391	No	NA	0	1	Not-determined	Genome-fragment	NA		0
S1_extracted_k141_42576	1165	No	NA	0	1	Not-determined	Genome-fragment	NA		0
S12_extracted_k141_123490	1452	No	NA	0	0	Low-quality	Genome-fragment		2.66	0
S1_extracted_k141_155049	1616	No	NA	0	1	Not-determined	Genome-fragment	NA		0
S1_extracted_k141_75888	1058	No	NA	0	0	Not-determined	Genome-fragment	NA		0
S4_extracted_8680	1452	No	NA	0	0	Low-quality	Genome-fragment		2.44	0
S17_vs_k141_50321||full	1453	No	NA	0	1	Low-quality	Genome-fragment		1.97	0
S1_extracted_k141_16353	1476	No	NA	0	2	Not-determined	Genome-fragment	NA		0
S1_extracted_k141_96071	1491	No	NA	0	1	Not-determined	Genome-fragment	NA		0
S17_vs_k141_142206||lt2gene	1453	No	NA	1	0	Low-quality	Genome-fragment		3.78	0
S14_extracted_k141_54153	1453	No	NA	1	0	Low-quality	Genome-fragment		2.24	0
S1_extracted_k141_35409	1071	No	NA	0	1	Not-determined	Genome-fragment	NA		0
S3_vs_k141_45230||full	1453	No	NA	1	0	Low-quality	Genome-fragment		4.02	0
S7_vs_78210||full	1453	No	NA	0	0	Low-quality	Genome-fragment		3.73	0
S1_extracted_k141_142633	1053	No	NA	0	1	Not-determined	Genome-fragment	NA		0
S1_extracted_k141_82718	1078	No	NA	0	0	Not-determined	Genome-fragment	NA		0
S16_vs_125296||full	1453	No	NA	1	0	Low-quality	Genome-fragment		0.94	0
S16_vs_144420||lt2gene	1453	No	NA	2	0	Low-quality	Genome-fragment		4.52	0
S1_extracted_k141_61560	1350	No	NA	0	0	Not-determined	Genome-fragment	NA		0
S1_extracted_k141_61563	1051	No	NA	0	0	Not-determined	Genome-fragment	NA		0
S1_extracted_k141_142647	3583	No	NA	0	0	Not-determined	Genome-fragment	NA		0
S1_extracted_k141_96125	1062	No	NA	0	0	Not-determined	Genome-fragment	NA		0
S1_extracted_k141_49391	1164	No	NA	0	1	Not-determined	Genome-fragment	NA		0
S1_extracted_k141_82765	1135	No	NA	0	2	Not-determined	Genome-fragment	NA		0
S1_extracted_k141_135963	1358	No	NA	0	2	Not-determined	Genome-fragment	NA		0
S6_vs_k141_42379||full	1453	No	NA	0	2	Low-quality	Genome-fragment		1.35	0
S1_extracted_k141_89262	1117	No	NA	0	1	Not-determined	Genome-fragment	NA		0
S8_vs_k141_4557||full	1453	No	NA	0	0	Low-quality	Genome-fragment		2.92	0
S9_vs_k141_73088||full	1454	No	NA	0	0	Low-quality	Genome-fragment		1.47	0
S7_extracted_21155	1454	No	NA	0	0	Low-quality	Genome-fragment		2.71	0
S7_vs_114936||full	1454	No	NA	0	0	Low-quality	Genome-fragment		3.31	0
S1_extracted_k141_135985	2078	No	NA	0	0	Not-determined	Genome-fragment	NA		0
S1_extracted_k141_116098	1204	No	NA	0	1	Not-determined	Genome-fragment	NA		0
S16_vs_95190||full	1454	No	NA	0	0	Low-quality	Genome-fragment		1.85	0
S1_extracted_k141_129462	3149	No	NA	0	1	Not-determined	Genome-fragment	NA		0
S10_vs_38948||full	1454	No	NA	0	0	Low-quality	Genome-fragment		2.55	0
S10_vs_42752||full	1454	No	NA	0	0	Low-quality	Genome-fragment		1.81	0
S1_extracted_k141_22491	1652	No	NA	0	1	Not-determined	Genome-fragment	NA		0
S8_extracted_k141_176338	1454	No	NA	0	0	Low-quality	Genome-fragment		0.95	0
S1_extracted_k141_122563	2311	No	NA	0	1	Not-determined	Genome-fragment	NA		0
S1_extracted_k141_82852	5260	No	NA	0	3	Not-determined	Genome-fragment	NA		0
S1_extracted_k141_16536	1266	No	NA	0	0	Not-determined	Genome-fragment	NA		0
S8_vs_k141_1278||full	1454	No	NA	1	0	Low-quality	Genome-fragment		2.87	0
S1_extracted_k141_116191	3158	No	NA	0	3	Not-determined	Genome-fragment	NA		0
S1_extracted_k141_96240	3214	No	NA	0	1	Not-determined	Genome-fragment	NA		0
S1_extracted_k141_16567	7933	No	NA	0	3	Not-determined	Genome-fragment	NA		0
S1_extracted_k141_29446	1095	No	NA	0	1	Not-determined	Genome-fragment	NA		0
S1_extracted_k141_148035	1163	No	NA	0	1	Not-determined	Genome-fragment	NA		0
S1_extracted_k141_29459	1316	No	NA	0	2	Not-determined	Genome-fragment	NA		0
S1_extracted_k141_35579	1254	No	NA	0	0	Not-determined	Genome-fragment	NA		0
S17_extracted_k141_81467	1455	No	NA	0	1	Low-quality	Genome-fragment		0.24	0
S1_extracted_k141_89384	1030	No	NA	0	1	Not-determined	Genome-fragment	NA		0
S1_extracted_k141_129574	3585	No	NA	0	2	Not-determined	Genome-fragment	NA		0
S1_extracted_k141_136113	10754	No	NA	0	1	Not-determined	Genome-fragment	NA		0
S1_extracted_k141_22608	1128	No	NA	0	1	Not-determined	Genome-fragment	NA		0
S1_extracted_k141_96286	2133	No	NA	0	5	Not-determined	Genome-fragment	NA		0
S1_extracted_k141_76174	1097	No	NA	0	2	Not-determined	Genome-fragment	NA		0
S1_extracted_k141_142833	10106	No	NA	0	2	Not-determined	Genome-fragment	NA		0
S1_extracted_k141_9562	1001	No	NA	0	1	Not-determined	Genome-fragment	NA		0
S1_extracted_k141_69400	3726	No	NA	0	0	Not-determined	Genome-fragment	NA		0
S1_extracted_k141_82994	2897	No	NA	0	1	Not-determined	Genome-fragment	NA		0
S1_extracted_k141_42929	1535	No	NA	0	0	Not-determined	Genome-fragment	NA		0
S1_extracted_k141_102997	1042	No	NA	0	0	Not-determined	Genome-fragment	NA		0
S1_extracted_k141_116313	6847	No	NA	0	2	Not-determined	Genome-fragment	NA		0
S17_vs_k141_71883||full	1455	No	NA	0	2	Low-quality	Genome-fragment		3.36	0
S17_vs_k141_83829||lt2gene	1455	No	NA	2	0	Low-quality	Genome-fragment		3.78	0
S3_extracted_k141_22767	1455	No	NA	0	0	Low-quality	Genome-fragment		1.39	0
S10_extracted_8897	1455	No	NA	1	0	Low-quality	Genome-fragment		2.77	0
S1_extracted_k141_109471	3326	No	NA	0	1	Not-determined	Genome-fragment	NA		0
S12_extracted_k141_28710	1455	No	NA	0	0	Low-quality	Genome-fragment		1.6	0
S1_extracted_k141_116358	1128	No	NA	0	2	Not-determined	Genome-fragment	NA		0
S12_extracted_k141_86373	1455	No	NA	1	0	Low-quality	Genome-fragment		2.59	0
S6_vs_k141_41848||full	1455	No	NA	2	0	Low-quality	Genome-fragment		1.96	0
S8_vs_k141_14467||full	1455	No	NA	1	0	Low-quality	Genome-fragment		3.85	0
S17_vs_k141_126480||full	1456	No	NA	1	0	Low-quality	Genome-fragment		2.48	0
S1_extracted_k141_69520	1693	No	NA	0	0	Not-determined	Genome-fragment	NA		0
S1_extracted_k141_89557	1094	No	NA	0	1	Not-determined	Genome-fragment	NA		0
S1_extracted_k141_22729	1492	No	NA	0	1	Not-determined	Genome-fragment	NA		0
S1_extracted_k141_16789	2245	No	NA	0	1	Not-determined	Genome-fragment	NA		0
S1_extracted_k141_2434	1248	No	NA	0	1	Not-determined	Genome-fragment	NA		0
S1_extracted_k141_142898	6791	No	NA	0	4	Not-determined	Genome-fragment	NA		0
S1_extracted_k141_96440	1638	No	NA	0	1	Not-determined	Genome-fragment	NA		0
S1_extracted_k141_109567	1466	No	NA	0	1	Not-determined	Genome-fragment	NA		0
S17_vs_k141_86785||lt2gene	1456	No	NA	1	0	Low-quality	Genome-fragment		2.45	0
S1_extracted_k141_22753	4084	No	NA	0	1	Not-determined	Genome-fragment	NA		0
S9_vs_k141_73181||lt2gene	1456	No	NA	1	0	Low-quality	Genome-fragment		3.63	0
S1_extracted_k141_68197	1456	No	NA	0	0	Low-quality	Genome-fragment		2.84	0
S1_extracted_k141_16839	1449	No	NA	0	0	Not-determined	Genome-fragment	NA		0
S1_extracted_k141_136336	1018	No	NA	0	1	Not-determined	Genome-fragment	NA		0
S3_extracted_k141_24725	1456	No	NA	0	0	Low-quality	Genome-fragment		0.71	0
S1_extracted_k141_9763	1226	No	NA	0	1	Not-determined	Genome-fragment	NA		0
S1_extracted_k141_116484	1715	No	NA	0	1	Not-determined	Genome-fragment	NA		0
S5_vs_k141_2878||full	1456	No	NA	1	0	Low-quality	Genome-fragment		2.68	0
S1_extracted_k141_89646	2992	No	NA	0	1	Not-determined	Genome-fragment	NA		0
S7_vs_104590||full	1456	No	NA	1	0	Low-quality	Genome-fragment		3.53	0
S16_vs_18749||full	1456	No	NA	0	0	Low-quality	Genome-fragment		4.17	0
S1_extracted_k141_103225	1243	No	NA	0	1	Not-determined	Genome-fragment	NA		0
S1_extracted_k141_56380	3848	No	NA	0	0	Not-determined	Genome-fragment	NA		0
S1_extracted_k141_9800	2539	No	NA	0	2	Not-determined	Genome-fragment	NA		0
S16_vs_113652||full	1456	No	NA	0	0	Low-quality	Genome-fragment		2.98	0
S1_extracted_k141_69626	2807	No	NA	0	2	Not-determined	Genome-fragment	NA		0
S1_extracted_k141_89674	1542	No	NA	0	2	Not-determined	Genome-fragment	NA		0
S1_extracted_k141_109673	1324	No	NA	0	1	Not-determined	Genome-fragment	NA		0
S1_extracted_k141_122911	1072	No	NA	0	0	Not-determined	Genome-fragment	NA		0
S1_extracted_k141_22869	1038	No	NA	0	1	Not-determined	Genome-fragment	NA		0
S1_extracted_k141_9828	1696	No	NA	0	0	Not-determined	Genome-fragment	NA		0
S1_extracted_k141_89686	1240	No	NA	0	1	Not-determined	Genome-fragment	NA		0

S1_extracted_k141_16933	1890	No	NA	0	1	Not-determined	Genome-fragment	NA		0
S13_extracted_14545	1457	No	NA	0	1	Low-quality	Genome-fragment		2.82	0
S1_extracted_k141_107387	1457	No	NA	0	0	Low-quality	Genome-fragment		3.94	0
S1_extracted_k141_76457	2165	No	NA	0	1	Not-determined	Genome-fragment	NA		0
S1_extracted_k141_49928	2295	No	NA	0	3	Not-determined	Genome-fragment	NA		0
S1_extracted_k141_148170	1797	No	NA	0	1	Not-determined	Genome-fragment	NA		0
S1_extracted_k141_136427	1058	No	NA	0	0	Not-determined	Genome-fragment	NA		0
S1_extracted_k141_49939	1129	No	NA	0	1	Not-determined	Genome-fragment	NA		0
S1_extracted_k141_143049	3404	No	NA	0	3	Not-determined	Genome-fragment	NA		0
S1_extracted_k141_158229	1457	No	NA	0	0	Low-quality	Genome-fragment		2.54	0
S3_vs_k141_116586||full	1457	No	NA	0	0	Low-quality	Genome-fragment		1.32	0
S1_extracted_k141_83249	1682	No	NA	0	0	Not-determined	Genome-fragment	NA		0
S1_extracted_k141_129928	1078	No	NA	0	0	Not-determined	Genome-fragment	NA		0
S5_vs_k141_1043||full	1457	No	NA	1	0	Low-quality	Genome-fragment		2.16	0
S16_extracted_210	1457	No	NA	0	2	Low-quality	Genome-fragment		0.46	0
S16_extracted_125389	1457	No	NA	0	1	Low-quality	Genome-fragment		0.61	0
S1_extracted_k141_155378	4100	No	NA	0	1	Not-determined	Genome-fragment	NA		0
S10_vs_110152||full	1457	No	NA	1	0	Low-quality	Genome-fragment		3.29	0
S1_extracted_k141_103361	1266	No	NA	0	2	Not-determined	Genome-fragment	NA		0
S15_extracted_13212	1457	No	NA	0	0	Low-quality	Genome-fragment		3.52	0
S1_extracted_k141_29861	1061	No	NA	0	1	Not-determined	Genome-fragment	NA		0
S8_extracted_k141_165040	1457	No	NA	0	0	Low-quality	Genome-fragment		4.15	0
S8_extracted_k141_127845	1457	No	NA	0	0	Low-quality	Genome-fragment		1.37	0
S1_extracted_k141_148262	3046	No	NA	0	1	Not-determined	Genome-fragment	NA		0
S1_extracted_k141_29877	3715	No	NA	0	2	Not-determined	Genome-fragment	NA		0
S1_extracted_k141_69755	1815	No	NA	0	1	Not-determined	Genome-fragment	NA		0
S1_extracted_k141_103436	2678	No	NA	0	2	Not-determined	Genome-fragment	NA		0
S1_extracted_k141_130079	1017	No	NA	0	0	Not-determined	Genome-fragment	NA		0
S1_extracted_k141_83406	1046	No	NA	0	0	Not-determined	Genome-fragment	NA		0
S8_vs_k141_121910||full	1457	No	NA	0	0	Low-quality	Genome-fragment		2.72	0
S17_vs_k141_22275||full	1458	No	NA	0	1	Low-quality	Genome-fragment		1.17	0
S9_extracted_k141_87916	1458	No	NA	0	2	Low-quality	Genome-fragment		0.95	0
S1_extracted_k141_96814	1225	No	NA	0	0	Not-determined	Genome-fragment	NA		0
S1_extracted_k141_136526	31147	No	NA	12	0	Medium-quality	Genome-fragment		73.92	0
S1_extracted_k141_56618	1843	No	NA	0	2	Not-determined	Genome-fragment	NA		0
S3_extracted_k141_89338	1458	No	NA	0	1	Low-quality	Genome-fragment		1.85	0
S1_extracted_k141_83421	1721	No	NA	0	1	Not-determined	Genome-fragment	NA		0
S1_extracted_k141_23016	1129	No	NA	0	1	Not-determined	Genome-fragment	NA		0
S1_extracted_k141_96829	4581	No	NA	0	2	Not-determined	Genome-fragment	NA		0
S1_extracted_k141_56635	1333	No	NA	0	1	Not-determined	Genome-fragment	NA		0
S1_extracted_k141_143225	1119	No	NA	0	1	Not-determined	Genome-fragment	NA		0
S1_extracted_k141_23029	1189	No	NA	0	0	Not-determined	Genome-fragment	NA		0
S1_extracted_k141_143228	1400	No	NA	0	0	Not-determined	Genome-fragment	NA		0
S1_extracted_k141_83448	1198	No	NA	0	1	Not-determined	Genome-fragment	NA		0
S1_extracted_k141_10087	1293	No	NA	0	0	Not-determined	Genome-fragment	NA		0
S5_extracted_k141_80449	1458	No	NA	0	0	Low-quality	Genome-fragment		2.88	0
S1_extracted_k141_69812	1503	No	NA	0	0	Not-determined	Genome-fragment	NA		0
NC3_vf_k141_2069	1458	No	NA	0	0	Low-quality	Genome-fragment		2.27	0
S1_extracted_k141_56686	1162	No	NA	0	0	Not-determined	Genome-fragment	NA		0
S10_vs_39050||full	1458	No	NA	0	0	Low-quality	Genome-fragment		2.86	0
S12_extracted_k141_28707	1458	No	NA	0	0	Low-quality	Genome-fragment		3.32	0
S12_extracted_k141_116611	1458	No	NA	0	0	Low-quality	Genome-fragment		1.5	0
S8_extracted_k141_133117	1458	No	NA	1	0	Low-quality	Genome-fragment		3.89	0
S8_vs_k141_178635||full	1458	No	NA	0	0	Low-quality	Genome-fragment		3.66	0
S1_extracted_k141_103548	1066	No	NA	0	0	Not-determined	Genome-fragment	NA		0
S17_vs_k141_155589||full	1459	No	NA	2	0	Low-quality	Genome-fragment		3.57	0
S1_extracted_k141_110052	1049	No	NA	0	1	Not-determined	Genome-fragment	NA		0
S1_extracted_k141_69838	2215	No	NA	0	1	Not-determined	Genome-fragment	NA		0
S1_extracted_k141_106818	1459	No	NA	0	0	Low-quality	Genome-fragment		1.47	0
S1_extracted_k141_69839	1060	No	NA	0	1	Not-determined	Genome-fragment	NA		0
S5_vs_k141_17501||full	1459	No	NA	0	1	Low-quality	Genome-fragment		1.15	0
S1_extracted_k141_90008	1201	No	NA	0	1	Not-determined	Genome-fragment	NA		0
S1_extracted_k141_50288	1767	No	NA	0	1	Not-determined	Genome-fragment	NA		0
S16_vs_37247||full	1459	No	NA	0	0	Low-quality	Genome-fragment		2.57	0
S10_extracted_54021	1459	No	NA	0	0	Low-quality	Genome-fragment		4.39	0
S1_extracted_k141_17258	1182	No	NA	0	0	Not-determined	Genome-fragment	NA		0
S1_extracted_k141_103612	2326	No	NA	0	1	Not-determined	Genome-fragment	NA		0
S6_vs_k141_17079||full	1459	No	NA	0	0	Low-quality	Genome-fragment		3.73	0
S1_extracted_k141_43548	1228	No	NA	0	3	Not-determined	Genome-fragment	NA		0
S8_extracted_k141_123889	1459	No	NA	0	1	Low-quality	Genome-fragment		1.44	0
S1_extracted_k141_155550	1282	No	NA	0	1	Not-determined	Genome-fragment	NA		0
S8_vs_k141_180109||full	1459	No	NA	2	0	Low-quality	Genome-fragment		4.64	0
S11_vs_k141_53711||full	1460	No	NA	0	0	Low-quality	Genome-fragment		2.59	0
S1_extracted_k141_62237	1727	No	NA	0	0	Not-determined	Genome-fragment	NA		0
S1_extracted_k141_17302	1559	No	NA	0	1	Not-determined	Genome-fragment	NA		0
S1_extracted_k141_2838	1528	No	NA	0	1	Not-determined	Genome-fragment	NA		0
S1_extracted_k141_123326	4263	No	NA	0	2	Not-determined	Genome-fragment	NA		0
S1_extracted_k141_90062	1041	No	NA	0	3	Not-determined	Genome-fragment	NA		0
S1_extracted_k141_116985	1042	No	NA	0	1	Not-determined	Genome-fragment	NA		0
S1_extracted_k141_36130	7262	No	NA	0	1	Not-determined	Genome-fragment	NA		0
S13_extracted_835	1460	No	NA	0	1	Low-quality	Genome-fragment		0.27	0
S1_extracted_k141_143400	1410	No	NA	0	2	Not-determined	Genome-fragment	NA		0
S13_extracted_29615	1460	No	NA	0	0	Low-quality	Genome-fragment		1.51	0
S13_extracted_32269	1460	No	NA	0	0	Low-quality	Genome-fragment		0.78	0
S1_extracted_k141_130252	3536	No	NA	0	0	Not-determined	Genome-fragment	NA		0
S1_extracted_k141_123368	1190	No	NA	0	0	Not-determined	Genome-fragment	NA		0
S13_vs_79868||full	1460	No	NA	0	0	Low-quality	Genome-fragment		2.46	0
S13_vs_81708||full	1460	No	NA	0	0	Low-quality	Genome-fragment		0.79	0
S1_extracted_k141_136793	3930	No	NA	0	2	Not-determined	Genome-fragment	NA		0
S1_extracted_k141_117040	1065	No	NA	0	1	Not-determined	Genome-fragment	NA		0
S1_vs_k141_37656||full	1460	No	NA	1	0	Low-quality	Genome-fragment		3.44	0
S1_extracted_k141_69917	1404	No	NA	0	2	Not-determined	Genome-fragment	NA		0
S5_vs_k141_94120||full	1460	No	NA	0	2	Low-quality	Genome-fragment		1.41	0
S1_extracted_k141_110192	1386	No	NA	0	1	Not-determined	Genome-fragment	NA		0
S1_extracted_k141_148424	1009	No	NA	0	0	Not-determined	Genome-fragment	NA		0
S5_vs_k141_21134||full	1460	No	NA	0	0	Low-quality	Genome-fragment		1.81	0
S1_extracted_k141_50380	1010	No	NA	0	3	Not-determined	Genome-fragment	NA		0
S1_extracted_k141_36172	1684	No	NA	0	2	Not-determined	Genome-fragment	NA		0
S1_extracted_k141_83763	1342	No	NA	0	1	Not-determined	Genome-fragment	NA		0
S5_vs_k141_104079||lt2gene	1460	No	NA	1	0	Low-quality	Genome-fragment		4.07	0
S1_extracted_k141_136867	3269	No	NA	0	1	Not-determined	Genome-fragment	NA		0
S1_extracted_k141_62357	3259	No	NA	0	1	Not-determined	Genome-fragment	NA		0
S5_vs_k141_113275||lt2gene	1460	No	NA	1	0	Low-quality	Genome-fragment		1.01	0
S1_extracted_k141_148488	1183	No	NA	0	0	Not-determined	Genome-fragment	NA		0
S1_extracted_k141_57012	1058	No	NA	0	1	Not-determined	Genome-fragment	NA		0
S16_vs_62086||full	1460	No	NA	0	1	Low-quality	Genome-fragment		0.88	0
S1_extracted_k141_103761	1542	No	NA	0	1	Not-determined	Genome-fragment	NA		0
S16_vs_143001||full	1460	No	NA	0	1	Low-quality	Genome-fragment		2.99	0
S10_vs_27376||lt2gene	1460	No	NA	2	0	Low-quality	Genome-fragment		3.65	0
S1_extracted_k141_43732	1607	No	NA	0	2	Not-determined	Genome-fragment	NA		0
S1_extracted_k141_97169	4458	No	NA	0	0	Not-determined	Genome-fragment	NA		0
S12_extracted_k141_47931	1460	No	NA	0	3	Low-quality	Genome-fragment		0.43	0
S12_vs_k141_917||full	1460	No	NA	0	0	Low-quality	Genome-fragment		3.69	0
S1_extracted_k141_43750	1158	No	NA	0	1	Not-determined	Genome-fragment	NA		0
S12_vs_k141_146903||full	1460	No	NA	0	0	Low-quality	Genome-fragment		3.83	0
S12_vs_k141_86205||full	1460	No	NA	0	1	Low-quality	Genome-fragment		2.28	0
S1_extracted_k141_43755	1150	No	NA	0	1	Not-determined	Genome-fragment	NA		0
S1_extracted_k141_90242	1243	No	NA	0	1	Not-determined	Genome-fragment	NA		0
S8_vs_k141_151754||full	1460	No	NA	0	0	Low-quality	Genome-fragment		1.74	0
S17_extracted_k141_76622	1461	No	NA	0	0	Low-quality	Genome-fragment		1.52	0
S14_vs_k141_71691||full	1461	No	NA	0	1	Low-quality	Genome-fragment		0.39	0
S3_extracted_k141_80188	1461	No	NA	0	0	Low-quality	Genome-fragment		1	0
S3_extracted_k141_912	1461	No	NA	1	0	Low-quality	Genome-fragment		3.35	0
S1_extracted_k141_97255	1022	No	NA	0	1	Not-determined	Genome-fragment	NA		0
S1_extracted_k141_148545	1523	No	NA	0	1	Not-determined	Genome-fragment	NA		0
S1_extracted_k141_110384	2237	No	NA	0	2	Not-determined	Genome-fragment	NA		0
S1_extracted_k141_77132	4630	No	NA	0	1	Not-determined	Genome-fragment	NA		0
S16_extracted_121181	1461	No	NA	0	0	Low-quality	Genome-fragment		2.42	0
S1_extracted_k141_30421	1215	No	NA	0	0	Not-determined	Genome-fragment	NA		0
S1_extracted_k141_155721	1001	No	NA	0	0	Not-determined	Genome-fragment	NA		0
S1_extracted_k141_77142	1012	No	NA	0	2	Not-determined	Genome-fragment	NA		0
S1_extracted_k141_103879	1181	No	NA	0	1	Not-determined	Genome-fragment	NA		0
S1_extracted_k141_123597	1402	No	NA	0	0	Not-determined	Genome-fragment	NA		0
S1_extracted_k141_10553	3606	No	NA	0	2	Not-determined	Genome-fragment	NA		0
S1_extracted_k141_155727	3198	No	NA	0	1	Not-determined	Genome-fragment	NA		0
S1_extracted_k141_23533	1046	No	NA	0	1	Not-determined	Genome-fragment	NA		0
S1_extracted_k141_50591	21026	No	NA	13	0	Medium-quality	Genome-fragment		54.77	0
S1_extracted_k141_83910	1017	No	NA	0	1	Not-determined	Genome-fragment	NA		0
S16_vs_19706||lt2gene	1461	No	NA	1	0	Low-quality	Genome-fragment		3.8	0
S1_extracted_k141_143598	3049	No	NA	0	2	Not-determined	Genome-fragment	NA		0
S1_extracted_k141_110433	1182	No	NA	0	1	Not-determined	Genome-fragment	NA		0
S1_extracted_k141_57143	2641	No	NA	0	2	Not-determined	Genome-fragment	NA		0
S1_extracted_k141_10578	1168	No	NA	0	2	Not-determined	Genome-fragment	NA		0
S1_extracted_k141_83927	1077	No	NA	0	0	Not-determined	Genome-fragment	NA		0
S10_vs_27420||lt2gene	1461	No	NA	0	0	Low-quality	Genome-fragment		3.84	0
S12_vs_k141_24348||full	1461	No	NA	0	0	Low-quality	Genome-fragment		2.55	0
S8_extracted_k141_21967	1461	No	NA	0	0	Low-quality	Genome-fragment		0.98	0
S1_extracted_k141_43878	1229	No	NA	0	1	Not-determined	Genome-fragment	NA		0
S1_extracted_k141_3007	6093	No	NA	0	2	Not-determined	Genome-fragment	NA		0
S8_vs_k141_41771||full	1461	No	NA	1	0	Low-quality	Genome-fragment		28.46	0
S17_vs_k141_18398||full	1462	No	NA	1	0	Low-quality	Genome-fragment		3.92	0
S1_extracted_k141_148585	3370	No	NA	0	1	Not-determined	Genome-fragment	NA		0
S1_extracted_k141_143642	1156	No	NA	0	1	Not-determined	Genome-fragment	NA		0
S1_extracted_k141_43913	1014	No	NA	0	1	Not-determined	Genome-fragment	NA		0
S1_extracted_k141_30523	1281	No	NA	0	0	Not-determined	Genome-fragment	NA		0
S1_extracted_k141_70073	3036	No	NA	0	1	Not-determined	Genome-fragment	NA		0
S1_extracted_k141_57208	1304	No	NA	0	2	Not-determined	Genome-fragment	NA		0
S1_extracted_k141_137145	1120	No	NA	0	3	Not-determined	Genome-fragment	NA		0
S1_extracted_k141_97392	1083	No	NA	0	0	Not-determined	Genome-fragment	NA		0
S1_extracted_k141_97399	1473	No	NA	0	1	Not-determined	Genome-fragment	NA		0
S9_extracted_k141_24225	1462	No	NA	0	0	Low-quality	Genome-fragment		2.44	0
S1_extracted_k141_50698	3528	No	NA	0	1	Not-determined	Genome-fragment	NA		0
S1_extracted_k141_50699	1396	No	NA	0	0	Not-determined	Genome-fragment	NA		0
S11_vs_k141_73889||full	1462	No	NA	0	0	Low-quality	Genome-fragment		1.13	0
S1_extracted_k141_117322	2463	No	NA	0	1	Not-determined	Genome-fragment	NA		0
S1_extracted_k141_84012	1124	No	NA	0	1	Not-determined	Genome-fragment	NA		0
S1_extracted_k141_50719	2488	No	NA	0	6	Not-determined	Genome-fragment	NA		0
S14_vs_k141_28905||full	1462	No	NA	2	0	Low-quality	Genome-fragment		4.35	0
S1_extracted_k141_155788	1737	No	NA	0	3	Not-determined	Genome-fragment	NA		0
S1_extracted_k141_123695	1033	No	NA	0	1	Not-determined	Genome-fragment	NA		0
S3_extracted_k141_34872	1462	No	NA	0	1	Low-quality	Genome-fragment		4	0
S5_extracted_k141_25102	1462	No	NA	1	0	Low-quality	Genome-fragment		6.67	0
S1_extracted_k141_155791	1472	No	NA	0	0	Not-determined	Genome-fragment	NA		0
S1_extracted_k141_84068	1879	No	NA	0	4	Not-determined	Genome-fragment	NA		0

S7_vs_421||lt2gene	1462	No	NA	2	0	Low-quality	Genome-fragment		3.5	0
S16_vs_81252||full	1462	No	NA	1	0	Low-quality	Genome-fragment		2.52	0
S1_extracted_k141_17738	1089	No	NA	0	1	Not-determined	Genome-fragment	NA		0
S1_extracted_k141_123727	1190	No	NA	0	2	Not-determined	Genome-fragment	NA		0
S2_extracted_k141_834	1462	No	NA	3	0	Low-quality	Genome-fragment		4.82	0
S1_extracted_k141_97528	2819	No	NA	0	1	Not-determined	Genome-fragment	NA		0
S8_extracted_k141_5234	1462	No	NA	0	1	Low-quality	Genome-fragment		0.73	0
S1_extracted_k141_30690	1262	No	NA	0	1	Not-determined	Genome-fragment	NA		0
S1_extracted_k141_137267	3726	No	NA	0	0	Not-determined	Genome-fragment	NA		0
S1_extracted_k141_110651	1309	No	NA	0	1	Not-determined	Genome-fragment	NA		0
S1_extracted_k141_110652	1023	No	NA	0	1	Not-determined	Genome-fragment	NA		0
S1_extracted_k141_137269	1115	No	NA	0	0	Not-determined	Genome-fragment	NA		0
S8_extracted_k141_121484	1462	No	NA	0	0	Low-quality	Genome-fragment		3.48	0
S8_vs_k141_57316||full	1462	No	NA	0	2	Low-quality	Genome-fragment		0.39	0
S17_vs_k141_107707||full	1463	No	NA	0	0	Low-quality	Genome-fragment		1.88	0
S9_vs_k141_27356||full	1463	No	NA	0	0	Low-quality	Genome-fragment		0.7	0
S1_extracted_k141_57367	1357	No	NA	0	1	Not-determined	Genome-fragment	NA		0
S1_extracted_k141_90621	1224	No	NA	0	2	Not-determined	Genome-fragment	NA		0
S14_vs_k141_46095||full	1463	No	NA	0	0	Low-quality	Genome-fragment		2.45	0
S1_extracted_k141_62742	1270	No	NA	0	1	Not-determined	Genome-fragment	NA		0
S14_vs_k141_11819||full	1463	No	NA	0	1	Low-quality	Genome-fragment		1.91	0
S1_extracted_k141_116718	1463	No	NA	0	0	Low-quality	Genome-fragment		2.37	0
S3_vs_k141_76750||full	1463	No	NA	2	0	Low-quality	Genome-fragment		3.23	0
S3_vs_k141_93734||full	1463	No	NA	1	0	Low-quality	Genome-fragment		1.52	0
S1_extracted_k141_143862	2154	No	NA	0	3	Not-determined	Genome-fragment	NA		0
S5_vs_k141_112100||full	1463	No	NA	0	1	Low-quality	Genome-fragment		0.98	0
S1_extracted_k141_17871	1164	No	NA	0	0	Not-determined	Genome-fragment	NA		0
S1_extracted_k141_57459	1823	No	NA	0	2	Not-determined	Genome-fragment	NA		0
S1_extracted_k141_44184	1128	No	NA	0	1	Not-determined	Genome-fragment	NA		0
S1_extracted_k141_97644	1097	No	NA	0	1	Not-determined	Genome-fragment	NA		0
S1_extracted_k141_84213	1022	No	NA	0	1	Not-determined	Genome-fragment	NA		0
S5_vs_k141_29351||full	1463	No	NA	0	0	Low-quality	Genome-fragment		2.83	0
S1_extracted_k141_137412	1337	No	NA	0	2	Not-determined	Genome-fragment	NA		0
S1_extracted_k141_57488	1135	No	NA	0	0	Not-determined	Genome-fragment	NA		0
S1_extracted_k141_130847	1836	No	NA	0	2	Not-determined	Genome-fragment	NA		0
S1_extracted_k141_30848	1442	No	NA	0	1	Not-determined	Genome-fragment	NA		0
S1_extracted_k141_62808	1089	No	NA	0	1	Not-determined	Genome-fragment	NA		0
S1_extracted_k141_143927	1929	No	NA	0	1	Not-determined	Genome-fragment	NA		0
S1_extracted_k141_104236	1517	No	NA	0	1	Not-determined	Genome-fragment	NA		0
S16_vs_140370||full	1463	No	NA	0	0	Low-quality	Genome-fragment		3.49	0
S12_vs_k141_116997||full	1463	No	NA	1	0	Low-quality	Genome-fragment		3.29	0
S1_extracted_k141_110833	3494	No	NA	0	1	Not-determined	Genome-fragment	NA		0
S1_extracted_k141_117635	1302	No	NA	0	1	Not-determined	Genome-fragment	NA		0
S1_extracted_k141_117647	1003	No	NA	0	2	Not-determined	Genome-fragment	NA		0
S6_vs_k141_25409||full	1463	No	NA	0	0	Low-quality	Genome-fragment		3.17	0
S1_extracted_k141_90788	1299	No	NA	0	0	Not-determined	Genome-fragment	NA		0
S17_vs_k141_46016||full	1464	No	NA	0	0	Low-quality	Genome-fragment		2.85	0
S1_extracted_k141_117689	1085	No	NA	0	1	Not-determined	Genome-fragment	NA		0
S9_extracted_k141_81991	1464	No	NA	0	0	Low-quality	Genome-fragment		3.6	0
S9_vs_k141_61809||full	1464	No	NA	2	0	Low-quality	Genome-fragment		3.52	0
S1_extracted_k141_62923	2083	No	NA	0	1	Not-determined	Genome-fragment	NA		0
S9_vs_k141_70343||full	1464	No	NA	4	0	Low-quality	Genome-fragment		4.18	0
S1_extracted_k141_51122	1038	No	NA	0	2	Not-determined	Genome-fragment	NA		0
S1_extracted_k141_18041	1391	No	NA	0	0	Not-determined	Genome-fragment	NA		0
S1_extracted_k141_130965	2947	No	NA	0	1	Not-determined	Genome-fragment	NA		0
S1_extracted_k141_90852	1229	No	NA	0	1	Not-determined	Genome-fragment	NA		0
S1_extracted_k141_18056	1030	No	NA	0	1	Not-determined	Genome-fragment	NA		0
S1_extracted_k141_90858	1915	No	NA	0	1	Not-determined	Genome-fragment	NA		0
S1_extracted_k141_62954	1052	No	NA	0	0	Not-determined	Genome-fragment	NA		0
S1_extracted_k141_130991	1524	No	NA	0	4	Not-determined	Genome-fragment	NA		0
S1_extracted_k141_51145	1040	No	NA	0	1	Not-determined	Genome-fragment	NA		0
S1_extracted_k141_131012	1943	No	NA	0	1	Not-determined	Genome-fragment	NA		0
S1_extracted_k141_3369	1440	No	NA	0	1	Not-determined	Genome-fragment	NA		0
S1_extracted_k141_155941	1872	No	NA	0	1	Not-determined	Genome-fragment	NA		0
S13_vs_68533||lt2gene	1464	No	NA	0	0	Low-quality	Genome-fragment		3.79	0
S14_vs_k141_15307||full	1464	No	NA	1	0	Low-quality	Genome-fragment		3.7	0
S1_extracted_k141_117780	1699	No	NA	0	2	Not-determined	Genome-fragment	NA		0
S14_vs_k141_78100||full	1464	No	NA	1	0	Low-quality	Genome-fragment		2.65	0
S1_extracted_k141_57591	3064	No	NA	0	0	Not-determined	Genome-fragment	NA		0
S1_extracted_k141_31058	1016	No	NA	0	2	Not-determined	Genome-fragment	NA		0
S5_extracted_k141_61990	1464	No	NA	0	0	Low-quality	Genome-fragment		3.13	0
S5_vs_k141_84600||full	1464	No	NA	0	0	Low-quality	Genome-fragment		1.99	0
S7_vs_77082||full	1464	No	NA	0	2	Low-quality	Genome-fragment		0.79	0
S16_vs_156482||full	1464	No	NA	1	0	Low-quality	Genome-fragment		3.1	0
S1_extracted_k141_97869	1250	No	NA	0	1	Not-determined	Genome-fragment	NA		0
S1_extracted_k141_36772	3064	No	NA	0	2	Not-determined	Genome-fragment	NA		0
S12_vs_k141_73916||full	1464	No	NA	2	0	Low-quality	Genome-fragment		3.46	0
S1_extracted_k141_90967	1619	No	NA	0	1	Not-determined	Genome-fragment	NA		0
S8_extracted_k141_145824	1464	No	NA	1	0	Low-quality	Genome-fragment		2.35	0
S1_extracted_k141_36794	1005	No	NA	0	0	Not-determined	Genome-fragment	NA		0
S1_extracted_k141_104449	4635	No	NA	0	13	Not-determined	Genome-fragment	NA		0
S9_vs_k141_7102||full	1465	No	NA	2	0	Low-quality	Genome-fragment		3.32	0
S1_extracted_k141_44479	1052	No	NA	0	1	Not-determined	Genome-fragment	NA		0
S1_extracted_k141_111091	1204	No	NA	0	1	Not-determined	Genome-fragment	NA		0
S1_extracted_k141_36840	1078	No	NA	0	2	Not-determined	Genome-fragment	NA		0
S1_extracted_k141_57667	2232	No	NA	0	1	Not-determined	Genome-fragment	NA		0
S1_extracted_k141_137696	1257	No	NA	0	2	Not-determined	Genome-fragment	NA		0
S1_extracted_k141_31125	1041	No	NA	0	2	Not-determined	Genome-fragment	NA		0
S1_extracted_k141_44512	2608	No	NA	0	5	Not-determined	Genome-fragment	NA		0
S1_extracted_k141_31142	3297	No	NA	0	1	Not-determined	Genome-fragment	NA		0
S1_extracted_k141_77822	1114	No	NA	0	0	Not-determined	Genome-fragment	NA		0
S1_extracted_k141_51325	1143	No	NA	0	0	Not-determined	Genome-fragment	NA		0
S1_extracted_k141_117889	1162	No	NA	0	1	Not-determined	Genome-fragment	NA		0
S1_extracted_k141_104531	1974	No	NA	0	0	Not-determined	Genome-fragment	NA		0
S1_extracted_k141_51368	1185	No	NA	0	1	Not-determined	Genome-fragment	NA		0
S1_extracted_k141_131203	1000	No	NA	0	2	Not-determined	Genome-fragment	NA		0
S1_extracted_k141_91099	1320	No	NA	0	0	Not-determined	Genome-fragment	NA		0
S1_extracted_k141_131220	1912	No	NA	0	3	Not-determined	Genome-fragment	NA		0
S11_vs_k141_76120||full	1465	No	NA	0	0	Low-quality	Genome-fragment		3.68	0
S1_extracted_k141_97996	1365	No	NA	0	1	Not-determined	Genome-fragment	NA		0
S1_extracted_k141_70551	1803	No	NA	0	0	Not-determined	Genome-fragment	NA		0
S1_extracted_k141_70556	1273	No	NA	0	1	Not-determined	Genome-fragment	NA		0
S1_extracted_k141_18319	1045	No	NA	0	0	Not-determined	Genome-fragment	NA		0
S1_extracted_k141_36973	1016	No	NA	0	0	Not-determined	Genome-fragment	NA		0
S1_extracted_k141_18346	1098	No	NA	0	1	Not-determined	Genome-fragment	NA		0
S1_extracted_k141_63229	1806	No	NA	0	1	Not-determined	Genome-fragment	NA		0
S1_extracted_k141_70620	2137	No	NA	0	2	Not-determined	Genome-fragment	NA		0
S3_extracted_k141_91295	1465	No	NA	0	1	Low-quality	Genome-fragment		0.25	0
S1_extracted_k141_44663	1338	No	NA	0	1	Not-determined	Genome-fragment	NA		0
S1_extracted_k141_137886	1106	No	NA	0	0	Not-determined	Genome-fragment	NA		0
S1_extracted_k141_31334	2089	No	NA	0	1	Not-determined	Genome-fragment	NA		0
S1_extracted_k141_84663	1473	No	NA	0	1	Not-determined	Genome-fragment	NA		0
S1_extracted_k141_63260	2783	No	NA	0	1	Not-determined	Genome-fragment	NA		0
S1_extracted_k141_104674	1239	No	NA	0	1	Not-determined	Genome-fragment	NA		0
S3_extracted_k141_47223	1465	No	NA	0	0	Low-quality	Genome-fragment		0.25	0
S5_extracted_k141_45943	1465	No	NA	2	0	Low-quality	Genome-fragment		3.41	0
S1_extracted_k141_51544	1038	No	NA	0	0	Not-determined	Genome-fragment	NA		0
S1_extracted_k141_118045	3160	No	NA	0	1	Not-determined	Genome-fragment	NA		0
S1_extracted_k141_24400	1031	No	NA	0	0	Not-determined	Genome-fragment	NA		0
S5_vs_k141_34232||lt2gene	1465	No	NA	2	0	Low-quality	Genome-fragment		3.1	0
S1_extracted_k141_3648	1244	No	NA	0	1	Not-determined	Genome-fragment	NA		0
S1_extracted_k141_137981	1493	No	NA	0	1	Not-determined	Genome-fragment	NA		0
S1_extracted_k141_124386	1550	No	NA	0	1	Not-determined	Genome-fragment	NA		0
S1_extracted_k141_137997	1842	No	NA	0	1	Not-determined	Genome-fragment	NA		0
S1_extracted_k141_111367	1210	No	NA	0	1	Not-determined	Genome-fragment	NA		0
S1_extracted_k141_78107	1087	No	NA	0	1	Not-determined	Genome-fragment	NA		0
S7_vs_105216||full	1465	No	NA	0	1	Low-quality	Genome-fragment		2.1	0
S1_extracted_k141_148981	1468	No	NA	0	0	Not-determined	Genome-fragment	NA		0
S16_vs_49373||full	1465	No	NA	0	0	Low-quality	Genome-fragment		3.02	0
S1_extracted_k141_138042	3254	No	NA	0	1	Not-determined	Genome-fragment	NA		0
S1_extracted_k141_91354	1385	No	NA	0	0	Not-determined	Genome-fragment	NA		0
S1_extracted_k141_118183	2201	No	NA	0	1	Not-determined	Genome-fragment	NA		0
S10_vs_52443||full	1465	No	NA	2	0	Low-quality	Genome-fragment		3.45	0
S1_extracted_k141_58053	1428	No	NA	0	0	Not-determined	Genome-fragment	NA		0
S1_extracted_k141_111439	3365	No	NA	0	0	Not-determined	Genome-fragment	NA		0
S1_extracted_k141_51683	2151	No	NA	0	3	Not-determined	Genome-fragment	NA		0
S1_extracted_k141_78185	1262	No	NA	0	1	Not-determined	Genome-fragment	NA		0
S10_vs_68299||full	1465	No	NA	1	0	Low-quality	Genome-fragment		4.67	0
S1_extracted_k141_84840	7118	No	NA	0	3	Not-determined	Genome-fragment	NA		0
S1_extracted_k141_24596	1332	No	NA	0	2	Not-determined	Genome-fragment	NA		0
S12_vs_k141_19554||full	1465	No	NA	0	0	Low-quality	Genome-fragment		4.08	0
S1_extracted_k141_149006	1193	No	NA	0	0	Not-determined	Genome-fragment	NA		0
S15_vs_33688||lt2gene	1465	No	NA	2	0	Low-quality	Genome-fragment		3.32	0
S8_extracted_k141_53232	1465	No	NA	0	1	Low-quality	Genome-fragment		0.37	0
S1_extracted_k141_98311	1229	No	NA	0	1	Not-determined	Genome-fragment	NA		0
S8_vs_k141_140236||lt2gene	1465	No	NA	1	0	Low-quality	Genome-fragment		3.78	0
S1_extracted_k141_149033	1471	No	NA	0	1	Not-determined	Genome-fragment	NA		0
S11_extracted_k141_48520	1466	No	NA	0	2	Low-quality	Genome-fragment		1.91	0
S1_extracted_k141_149037	1222	No	NA	0	2	Not-determined	Genome-fragment	NA		0
S1_extracted_k141_78265	1006	No	NA	0	1	Not-determined	Genome-fragment	NA		0
S1_extracted_k141_11757	1007	No	NA	0	1	Not-determined	Genome-fragment	NA		0
S11_vs_k141_16997||full	1466	No	NA	1	0	Low-quality	Genome-fragment		3.92	0
S1_extracted_k141_111574	6376	No	NA	0	4	Not-determined	Genome-fragment	NA		0
S1_extracted_k141_156225	3096	No	NA	0	2	Not-determined	Genome-fragment	NA		0
S1_extracted_k141_3849	1124	No	NA	0	0	Not-determined	Genome-fragment	NA		0
S1_extracted_k141_58167	1194	No	NA	0	1	Not-determined	Genome-fragment	NA		0
S1_extracted_k141_111588	1588	No	NA	0	0	Not-determined	Genome-fragment	NA		0
S1_extracted_k141_131662	1318	No	NA	0	1	Not-determined	Genome-fragment	NA		0
S1_extracted_k141_44983	1040	No	NA	0	0	Not-determined	Genome-fragment	NA		0
S1_extracted_k141_138157	22249	No	NA	13	0	Medium-quality	Genome-fragment		50.23	0
S1_extracted_k141_18718	7642	No	NA	0	6	Not-determined	Genome-fragment	NA		0
S1_extracted_k141_37250	2337	No	NA	0	2	Not-determined	Genome-fragment	NA		0
S1_extracted_k141_118331	1078	No	NA	0	1	Not-determined	Genome-fragment	NA		0
S1_extracted_k141_144633	2427	No	NA	0	1	Not-determined	Genome-fragment	NA		0
S1_extracted_k141_91596	1016	No	NA	0	1	Not-determined	Genome-fragment	NA		0
S11_vs_k141_39884||lt2gene	1466	No	NA	1	0	Low-quality	Genome-fragment		4.56	0
S1_extracted_k141_24772	1951	No	NA	0	1	Not-determined	Genome-fragment	NA		0
S1_extracted_k141_3889	1217	No	NA	0	0	Not-determined	Genome-fragment	NA		0
S1_extracted_k141_78362	1265	No	NA	0	2	Not-determined	Genome-fragment	NA		0
S1_extracted_k141_138196	1681	No	NA	0	3	Not-determined	Genome-fragment	NA		0
S14_extracted_k141_15503	1466	No	NA	1	0	Low-quality	Genome-fragment		4.64	0
S1_extracted_k141_37313	1028	No	NA	0	0	Not-determined	Genome-fragment	NA		0
S1_extracted_k141_63682	1063	No	NA	0	2	Not-determined	Genome-fragment	NA		0
S1_extracted_k141_78386	1247	No	NA	0	1	Not-determined	Genome-fragment	NA		0

S1_extracted_k141_78388	1448	No	NA	0	1	Not-determined	Genome-fragment	NA		0
S1_extracted_k141_149159	1612	No	NA	0	0	Not-determined	Genome-fragment	NA		0
S1_extracted_k141_37327	1003	No	NA	0	0	Not-determined	Genome-fragment	NA		0
S3_extracted_k141_4270	1466	No	NA	2	0	Low-quality	Genome-fragment		3.21	0
S1_extracted_k141_11934	1319	No	NA	0	0	Not-determined	Genome-fragment	NA		0
S1_extracted_k141_3972	1231	No	NA	0	1	Not-determined	Genome-fragment	NA		0
S5_vs_k141_211||full	1466	No	NA	0	1	Low-quality	Genome-fragment		0.76	0
S5_vs_k141_2572||full	1466	No	NA	0	1	Low-quality	Genome-fragment		0.44	0
S1_extracted_k141_31728	1294	No	NA	0	0	Not-determined	Genome-fragment	NA		0
S1_extracted_k141_85104	1270	No	NA	0	1	Not-determined	Genome-fragment	NA		0
S15_extracted_66367	1466	No	NA	0	0	Low-quality	Genome-fragment		2.38	0
S1_extracted_k141_118488	1121	No	NA	0	1	Not-determined	Genome-fragment	NA		0
S1_extracted_k141_144809	1124	No	NA	0	1	Not-determined	Genome-fragment	NA		0
S1_extracted_k141_63804	1369	No	NA	0	1	Not-determined	Genome-fragment	NA		0
S1_extracted_k141_24941	1063	No	NA	0	2	Not-determined	Genome-fragment	NA		0
S1_extracted_k141_52024	3853	No	NA	0	0	Not-determined	Genome-fragment	NA		0
S1_extracted_k141_78538	1126	No	NA	0	3	Not-determined	Genome-fragment	NA		0
S1_extracted_k141_18971	3369	No	NA	0	1	Not-determined	Genome-fragment	NA		0
S1_extracted_k141_52038	1189	No	NA	0	0	Not-determined	Genome-fragment	NA		0
S1_extracted_k141_52049	2445	No	NA	0	2	Not-determined	Genome-fragment	NA		0
S1_extracted_k141_58419	1028	No	NA	0	1	Not-determined	Genome-fragment	NA		0
S1_extracted_k141_149230	2043	No	NA	0	1	Not-determined	Genome-fragment	NA		0
S1_extracted_k141_24978	1473	No	NA	0	0	Not-determined	Genome-fragment	NA		0
S6_extracted_k141_51308	1466	No	NA	0	0	Low-quality	Genome-fragment		2.47	0
S1_extracted_k141_111848	2320	No	NA	0	1	Not-determined	Genome-fragment	NA		0
S1_extracted_k141_31822	1453	No	NA	0	2	Not-determined	Genome-fragment	NA		0
S1_extracted_k141_58440	3443	No	NA	0	0	Not-determined	Genome-fragment	NA		0
S1_extracted_k141_52080	1271	No	NA	0	1	Not-determined	Genome-fragment	NA		0
S1_extracted_k141_138338	1089	No	NA	0	1	Not-determined	Genome-fragment	NA		0
S1_extracted_k141_12072	1642	No	NA	0	1	Not-determined	Genome-fragment	NA		0
S8_vs_k141_138428||lt2gene	1466	No	NA	1	0	Low-quality	Genome-fragment		1.92	0
S17_vs_k141_127606||full	1467	No	NA	1	0	Low-quality	Genome-fragment		2.52	0
S1_extracted_k141_144915	1163	No	NA	0	0	Not-determined	Genome-fragment	NA		0
S1_extracted_k141_131996	1370	No	NA	0	0	Not-determined	Genome-fragment	NA		0
S1_extracted_k141_91890	1170	No	NA	0	1	Not-determined	Genome-fragment	NA		0
S14_extracted_k141_47220	1467	No	NA	3	0	Low-quality	Genome-fragment		4.84	0
S1_extracted_k141_4143	1762	No	NA	0	1	Not-determined	Genome-fragment	NA		0
S5_vs_k141_107933||full	1467	No	NA	0	1	Low-quality	Genome-fragment		0.68	0
S7_vs_85869||full	1467	No	NA	1	0	Low-quality	Genome-fragment		2.19	0
S1_extracted_k141_25064	1077	No	NA	0	1	Not-determined	Genome-fragment	NA		0
S12_vs_k141_95655||full	1467	No	NA	3	0	Low-quality	Genome-fragment		1.96	0
S1_extracted_k141_98744	2898	No	NA	0	2	Not-determined	Genome-fragment	NA		0
S12_vs_k141_1479||full	1467	No	NA	2	0	Low-quality	Genome-fragment		4.04	0
S1_extracted_k141_85261	2813	No	NA	0	1	Not-determined	Genome-fragment	NA		0
S1_extracted_k141_111915	3872	No	NA	0	3	Not-determined	Genome-fragment	NA		0
S1_extracted_k141_37502	1577	No	NA	0	0	Not-determined	Genome-fragment	NA		0
S12_vs_k141_69563||full	1467	No	NA	0	1	Low-quality	Genome-fragment		4.68	0
S9_vs_k141_72464||full	1468	No	NA	2	0	Low-quality	Genome-fragment		1.97	0
S1_extracted_k141_118666	2738	No	NA	0	1	Not-determined	Genome-fragment	NA		0
S1_extracted_k141_105361	1851	No	NA	0	1	Not-determined	Genome-fragment	NA		0
S14_vs_k141_112128||full	1468	No	NA	0	0	Low-quality	Genome-fragment		3.18	0
S1_extracted_k141_95967	1468	No	NA	0	0	Low-quality	Genome-fragment		2.14	0
S7_extracted_22000	1468	No	NA	0	0	Low-quality	Genome-fragment		2.81	0
S1_extracted_k141_25122	3073	No	NA	0	1	Not-determined	Genome-fragment	NA		0
S1_extracted_k141_138490	2185	No	NA	0	3	Not-determined	Genome-fragment	NA		0
S1_extracted_k141_31945	1295	No	NA	0	1	Not-determined	Genome-fragment	NA		0
S1_extracted_k141_105397	1481	No	NA	0	1	Not-determined	Genome-fragment	NA		0
S15_vs_10868||full	1468	No	NA	0	0	Low-quality	Genome-fragment		3.73	0
S1_extracted_k141_98804	1771	No	NA	0	0	Not-determined	Genome-fragment	NA		0
S17_vs_k141_151310||full	1469	No	NA	1	0	Low-quality	Genome-fragment		3.36	0
S17_vs_k141_139415||full	1469	No	NA	0	2	Low-quality	Genome-fragment		2.83	0
S1_extracted_k141_12221	1327	No	NA	0	2	Not-determined	Genome-fragment	NA		0
S1_extracted_k141_31977	1114	No	NA	0	1	Not-determined	Genome-fragment	NA		0
S1_extracted_k141_118712	1090	No	NA	0	0	Not-determined	Genome-fragment	NA		0
S1_extracted_k141_71050	1719	No	NA	0	1	Not-determined	Genome-fragment	NA		0
S14_vs_k141_56044||full	1469	No	NA	0	1	Low-quality	Genome-fragment		2.77	0
S8_extracted_k141_139205	1469	No	NA	0	1	Low-quality	Genome-fragment		1.67	0
S1_extracted_k141_145030	3532	No	NA	0	3	Not-determined	Genome-fragment	NA		0
S1_extracted_k141_138536	1189	No	NA	0	1	Not-determined	Genome-fragment	NA		0
S1_extracted_k141_45421	1921	No	NA	0	0	Not-determined	Genome-fragment	NA		0
S1_extracted_k141_32015	1011	No	NA	0	1	Not-determined	Genome-fragment	NA		0
S1_extracted_k141_12256	1717	No	NA	0	1	Not-determined	Genome-fragment	NA		0
S8_vs_k141_122457||full	1469	No	NA	0	0	Low-quality	Genome-fragment		3.08	0
S1_extracted_k141_118757	1287	No	NA	0	1	Not-determined	Genome-fragment	NA		0
S8_vs_k141_112621||full	1469	No	NA	0	2	Low-quality	Genome-fragment		0.44	0
S9_vs_k141_4865||full	1470	No	NA	2	0	Low-quality	Genome-fragment		3.19	0
S1_extracted_k141_138575	1233	No	NA	0	0	Not-determined	Genome-fragment	NA		0
S1_extracted_k141_32063	1184	No	NA	0	0	Not-determined	Genome-fragment	NA		0
S1_extracted_k141_118773	1145	No	NA	0	0	Not-determined	Genome-fragment	NA		0
S1_extracted_k141_92082	1053	No	NA	0	0	Not-determined	Genome-fragment	NA		0
S1_extracted_k141_58684	1077	No	NA	0	2	Not-determined	Genome-fragment	NA		0
S11_extracted_k141_68324	1470	No	NA	0	0	Low-quality	Genome-fragment		7.08	0
S1_extracted_k141_138591	3067	No	NA	0	5	Not-determined	Genome-fragment	NA		0
S13_vs_34708||full	1470	No	NA	1	0	Low-quality	Genome-fragment		2.51	0
S1_extracted_k141_145085	1262	No	NA	0	1	Not-determined	Genome-fragment	NA		0
S1_extracted_k141_98878	1408	No	NA	0	0	Not-determined	Genome-fragment	NA		0
S13_vs_27970||lt2gene	1470	No	NA	0	1	Low-quality	Genome-fragment		3.34	0
S1_extracted_k141_112099	1096	No	NA	0	0	Not-determined	Genome-fragment	NA		0
S3_extracted_k141_28089	1470	No	NA	0	1	Low-quality	Genome-fragment		0.74	0
S3_vs_k141_19681||full	1470	No	NA	0	0	Low-quality	Genome-fragment		1.87	0
S10_extracted_11536	1470	No	NA	1	0	Low-quality	Genome-fragment		1.35	0
S1_extracted_k141_32157	1096	No	NA	0	1	Not-determined	Genome-fragment	NA		0
S10_vs_28643||lt2gene	1470	No	NA	1	0	Low-quality	Genome-fragment		3.3	0
S1_extracted_k141_12378	1481	No	NA	0	1	Not-determined	Genome-fragment	NA		0
S12_extracted_k141_175821	1470	No	NA	3	0	Low-quality	Genome-fragment		2.84	0
S1_extracted_k141_12406	1172	No	NA	0	0	Not-determined	Genome-fragment	NA		0
S15_extracted_60755	1470	No	NA	0	1	Low-quality	Genome-fragment		4.15	0
S1_extracted_k141_112147	5829	No	NA	0	2	Not-determined	Genome-fragment	NA		0
S1_extracted_k141_118907	3887	No	NA	0	2	Not-determined	Genome-fragment	NA		0
S1_extracted_k141_32211	1201	No	NA	0	1	Not-determined	Genome-fragment	NA		0
S1_extracted_k141_37651	1590	No	NA	0	1	Not-determined	Genome-fragment	NA		0
S6_vs_k141_27310||full	1470	No	NA	3	0	Low-quality	Genome-fragment		3.66	0
S8_vs_k141_81071||full	1470	No	NA	1	0	Low-quality	Genome-fragment		2.44	0
S1_extracted_k141_105605	2271	No	NA	0	1	Not-determined	Genome-fragment	NA		0
S1_extracted_k141_99022	1046	No	NA	0	0	Not-determined	Genome-fragment	NA		0
S17_vs_k141_68115||full	1471	No	NA	0	0	Low-quality	Genome-fragment		3.26	0
S1_extracted_k141_156493	1540	No	NA	0	1	Not-determined	Genome-fragment	NA		0
S1_extracted_k141_125313	1401	No	NA	0	1	Not-determined	Genome-fragment	NA		0
S1_extracted_k141_145230	1626	No	NA	0	0	Not-determined	Genome-fragment	NA		0
S1_extracted_k141_25397	1599	No	NA	0	1	Not-determined	Genome-fragment	NA		0
S3_vs_k141_83743||full	1471	No	NA	0	2	Low-quality	Genome-fragment		2.37	0
S1_extracted_k141_125321	1733	No	NA	0	1	Not-determined	Genome-fragment	NA		0
S1_extracted_k141_45665	4349	No	NA	0	2	Not-determined	Genome-fragment	NA		0
S1_extracted_k141_138728	1261	No	NA	0	0	Not-determined	Genome-fragment	NA		0
S12_vs_k141_186880||full	1471	No	NA	1	0	Low-quality	Genome-fragment		2.51	0
S1_extracted_k141_64357	1262	No	NA	0	1	Not-determined	Genome-fragment	NA		0
S1_extracted_k141_112259	1228	No	NA	0	2	Not-determined	Genome-fragment	NA		0
S1_extracted_k141_156547	1098	No	NA	0	1	Not-determined	Genome-fragment	NA		0
S1_extracted_k141_99107	1624	No	NA	0	1	Not-determined	Genome-fragment	NA		0
S1_extracted_k141_32343	1591	No	NA	0	2	Not-determined	Genome-fragment	NA		0
S1_extracted_k141_125396	1062	No	NA	0	1	Not-determined	Genome-fragment	NA		0
S1_extracted_k141_25490	2578	No	NA	0	1	Not-determined	Genome-fragment	NA		0
S1_extracted_k141_45764	3065	No	NA	0	1	Not-determined	Genome-fragment	NA		0
S1_extracted_k141_19520	1508	No	NA	0	1	Not-determined	Genome-fragment	NA		0
S1_extracted_k141_119050	3922	No	NA	0	2	Not-determined	Genome-fragment	NA		0
S1_extracted_k141_92360	1143	No	NA	0	1	Not-determined	Genome-fragment	NA		0
S6_extracted_k141_53314	1471	No	NA	2	0	Low-quality	Genome-fragment		3.52	0
S8_vs_k141_185530||full	1471	No	NA	1	0	Low-quality	Genome-fragment		4.4	0
S1_extracted_k141_85754	1007	No	NA	0	0	Not-determined	Genome-fragment	NA		0
S1_extracted_k141_19545	1698	No	NA	0	1	Not-determined	Genome-fragment	NA		0
S17_vs_k141_122245||full	1472	No	NA	0	0	Low-quality	Genome-fragment		3.06	0
S1_extracted_k141_125466	1155	No	NA	0	0	Not-determined	Genome-fragment	NA		0
S9_vs_k141_73597||full	1472	No	NA	2	0	Low-quality	Genome-fragment		3.25	0
S9_vs_k141_7024||full	1472	No	NA	0	0	Low-quality	Genome-fragment		3.99	0
S1_extracted_k141_138858	1518	No	NA	0	1	Not-determined	Genome-fragment	NA		0
S3_vs_k141_52680||full	1472	No	NA	0	0	Low-quality	Genome-fragment		3.91	0
S1_extracted_k141_32448	1707	No	NA	0	1	Not-determined	Genome-fragment	NA		0
S1_extracted_k141_112384	3998	No	NA	0	3	Not-determined	Genome-fragment	NA		0
S5_extracted_k141_44642	1472	No	NA	0	0	Low-quality	Genome-fragment		2.32	0
S1_extracted_k141_132526	1232	No	NA	0	0	Not-determined	Genome-fragment	NA		0
S7_vs_3942||full	1472	No	NA	0	0	Low-quality	Genome-fragment		1.44	0
S7_vs_49690||lt2gene	1472	No	NA	2	0	Low-quality	Genome-fragment		2.52	0
S1_extracted_k141_119127	3274	No	NA	0	3	Not-determined	Genome-fragment	NA		0
S1_extracted_k141_145408	2024	No	NA	0	0	Not-determined	Genome-fragment	NA		0
S1_extracted_k141_25593	1004	No	NA	0	1	Not-determined	Genome-fragment	NA		0
S1_extracted_k141_19636	1605	No	NA	0	1	Not-determined	Genome-fragment	NA		0
S16_vs_121040||full	1472	No	NA	0	1	Low-quality	Genome-fragment		1.69	0
S16_vs_131978||full	1472	No	NA	0	0	Low-quality	Genome-fragment		2.4	0
S1_extracted_k141_99246	1987	No	NA	0	1	Not-determined	Genome-fragment	NA		0
S1_extracted_k141_25606	1329	No	NA	0	2	Not-determined	Genome-fragment	NA		0
S1_extracted_k141_25615	1755	No	NA	0	0	Not-determined	Genome-fragment	NA		0
S2_vs_k141_16349||full	1472	No	NA	0	0	Low-quality	Genome-fragment		1.53	0
S1_extracted_k141_59103	1101	No	NA	0	1	Not-determined	Genome-fragment	NA		0
S1_extracted_k141_19676	3625	No	NA	0	2	Not-determined	Genome-fragment	NA		0
S8_extracted_k141_53382	1472	No	NA	0	2	Low-quality	Genome-fragment		0.49	0
S1_extracted_k141_19693	1090	No	NA	0	1	Not-determined	Genome-fragment	NA		0
S1_extracted_k141_92482	1472	No	NA	0	0	Not-determined	Genome-fragment	NA		0
S1_extracted_k141_138965	3393	No	NA	0	2	Not-determined	Genome-fragment	NA		0
S8_vs_k141_188400||full	1472	No	NA	2	0	Low-quality	Genome-fragment		2.48	0
S1_extracted_k141_37888	1312	No	NA	0	0	Not-determined	Genome-fragment	NA		0
S9_extracted_k141_83735	1473	No	NA	1	0	Low-quality	Genome-fragment		0.73	0
S9_extracted_k141_29961	1473	No	NA	1	0	Low-quality	Genome-fragment		3.39	0
S1_extracted_k141_32580	1148	No	NA	0	1	Not-determined	Genome-fragment	NA		0
S1_extracted_k141_59161	1040	No	NA	0	0	Not-determined	Genome-fragment	NA		0
S9_vs_k141_48179||full	1473	No	NA	0	0	Low-quality	Genome-fragment		2.93	0
S11_vs_k141_95080||full	1473	No	NA	0	0	Low-quality	Genome-fragment		2.75	0
S1_extracted_k141_37910	2215	No	NA	0	1	Not-determined	Genome-fragment	NA		0
S1_extracted_k141_156686	1812	No	NA	0	0	Not-determined	Genome-fragment	NA		0
S13_vs_22681||full	1473	No	NA	0	0	Low-quality	Genome-fragment		2.36	0
S14_extracted_k141_8498	1473	No	NA	2	0	Low-quality	Genome-fragment		4.89	0
S3_vs_k141_96054||full	1473	No	NA	0	0	Low-quality	Genome-fragment		3.23	0
S1_extracted_k141_59182	1870	No	NA	0	2	Not-determined	Genome-fragment	NA		0
S1_extracted_k141_45939	5340	No	NA	0	2	Not-determined	Genome-fragment	NA		0
S5_extracted_k141_16172	1473	No	NA	0	0	Low-quality	Genome-fragment		3.07	0

S10_vs_50093||full	1473	No	NA	2	0	Low-quality	Genome-fragment		3.12	0
S1_extracted_k141_145531	1193	No	NA	0	0	Not-determined	Genome-fragment	NA		0
S12_vs_k141_15170||full	1473	No	NA	2	0	Low-quality	Genome-fragment		2.47	0
S1_extracted_k141_64620	4153	No	NA	0	2	Not-determined	Genome-fragment	NA		0
S1_extracted_k141_12788	1162	No	NA	0	1	Not-determined	Genome-fragment	NA		0
S1_extracted_k141_32637	1249	No	NA	0	0	Not-determined	Genome-fragment	NA		0
S6_vs_k141_232||full	1473	No	NA	1	0	Low-quality	Genome-fragment		1.2	0
S1_extracted_k141_125650	7776	No	NA	3	0	Medium-quality	Genome-fragment		56.76	0
S1_extracted_k141_45982	4331	No	NA	0	1	Not-determined	Genome-fragment	NA		0
S8_vs_k141_138713||full	1473	No	NA	0	1	Low-quality	Genome-fragment		1.68	0
S1_extracted_k141_125673	1087	No	NA	0	1	Not-determined	Genome-fragment	NA		0
S1_extracted_k141_25789	1266	No	NA	0	1	Not-determined	Genome-fragment	NA		0
S1_extracted_k141_79459	1160	No	NA	0	1	Not-determined	Genome-fragment	NA		0
S13_extracted_10330	1474	No	NA	0	0	Low-quality	Genome-fragment		2.29	0
S1_extracted_k141_125709	1135	No	NA	0	1	Not-determined	Genome-fragment	NA		0
S1_extracted_k141_25824	1282	No	NA	0	0	Not-determined	Genome-fragment	NA		0
S14_extracted_k141_67335	1474	No	NA	5	0	Low-quality	Genome-fragment		4.86	0
S14_vs_k141_104719||full	1474	No	NA	1	0	Low-quality	Genome-fragment		3.16	0
S1_extracted_k141_149707	3213	No	NA	0	1	Not-determined	Genome-fragment	NA		0
S1_extracted_k141_132812	1078	No	NA	0	0	Not-determined	Genome-fragment	NA		0
S1_extracted_k141_12899	4211	No	NA	0	4	Not-determined	Genome-fragment	NA		0
S1_extracted_k141_25839	5226	No	NA	0	2	Not-determined	Genome-fragment	NA		0
S3_extracted_k141_64850	1474	No	NA	3	0	Low-quality	Genome-fragment		6.73	0
S1_extracted_k141_106095	1024	No	NA	0	2	Not-determined	Genome-fragment	NA		0
S1_extracted_k141_112623	1511	No	NA	0	1	Not-determined	Genome-fragment	NA		0
S3_vs_k141_303||lt2gene	1474	No	NA	1	0	Low-quality	Genome-fragment		2.7	0
S1_extracted_k141_4747	3279	No	NA	0	1	Not-determined	Genome-fragment	NA		0
S1_extracted_k141_125782	1013	No	NA	0	1	Not-determined	Genome-fragment	NA		0
S1_extracted_k141_59346	13067	No	NA	0	3	Not-determined	Genome-fragment	NA		0
S1_extracted_k141_59355	1764	No	NA	0	1	Not-determined	Genome-fragment	NA		0
S1_extracted_k141_149767	1687	No	NA	0	1	Not-determined	Genome-fragment	NA		0
S5_extracted_k141_97240	1474	No	NA	3	0	Low-quality	Genome-fragment		3.64	0
S1_extracted_k141_99531	1155	No	NA	0	0	Not-determined	Genome-fragment	NA		0
S7_extracted_55836	1474	No	NA	0	0	Low-quality	Genome-fragment		0.88	0
S1_extracted_k141_125817	2090	No	NA	0	0	Not-determined	Genome-fragment	NA		0
S1_extracted_k141_38111	3055	No	NA	0	2	Not-determined	Genome-fragment	NA		0
S1_extracted_k141_32810	1164	No	NA	0	1	Not-determined	Genome-fragment	NA		0
S7_vs_111300||full	1474	No	NA	1	0	Low-quality	Genome-fragment		3.65	0
S7_vs_116651||lt2gene	1474	No	NA	1	0	Low-quality	Genome-fragment		3.11	0
S1_extracted_k141_149820	1006	No	NA	0	1	Not-determined	Genome-fragment	NA		0
S1_extracted_k141_64810	1048	No	NA	0	1	Not-determined	Genome-fragment	NA		0
S1_extracted_k141_145746	1306	No	NA	0	1	Not-determined	Genome-fragment	NA		0
S16_vs_88343||full	1474	No	NA	0	0	Low-quality	Genome-fragment		2.38	0
S12_vs_k141_185295||full	1474	No	NA	2	0	Low-quality	Genome-fragment		3.39	0
S1_extracted_k141_125901	1122	No	NA	0	0	Not-determined	Genome-fragment	NA		0
S12_vs_k141_174162||lt2gene	1474	No	NA	1	0	Low-quality	Genome-fragment		1.38	0
S6_vs_k141_1103||lt2gene	1474	No	NA	0	0	Low-quality	Genome-fragment		3.76	0
S1_extracted_k141_59434	5371	No	NA	0	5	Not-determined	Genome-fragment	NA		0
S8_extracted_k141_175408	1474	No	NA	0	0	Low-quality	Genome-fragment		1.45	0
S1_extracted_k141_149859	2142	No	NA	0	0	Not-determined	Genome-fragment	NA		0
S1_extracted_k141_59437	3184	No	NA	0	2	Not-determined	Genome-fragment	NA		0
S1_extracted_k141_4826	7257	No	NA	0	5	Not-determined	Genome-fragment	NA		0
S1_extracted_k141_112757	1198	No	NA	0	0	Not-determined	Genome-fragment	NA		0
S1_extracted_k141_79650	1228	No	NA	0	1	Not-determined	Genome-fragment	NA		0
S1_extracted_k141_106276	2004	No	NA	0	1	Not-determined	Genome-fragment	NA		0
S1_extracted_k141_106282	1636	No	NA	0	0	Not-determined	Genome-fragment	NA		0
S1_extracted_k141_112790	1240	No	NA	0	1	Not-determined	Genome-fragment	NA		0
S8_vs_k141_157306||full	1474	No	NA	0	0	Low-quality	Genome-fragment		0.88	0
S17_extracted_k141_43609	1475	No	NA	0	0	Low-quality	Genome-fragment		2.02	0
S14_vs_k141_33111||full	1475	No	NA	0	0	Low-quality	Genome-fragment		4.25	0
S1_extracted_k141_88445	1475	No	NA	0	0	Low-quality	Genome-fragment		3.67	0
S1_vs_k141_20466||full	1475	No	NA	0	0	Low-quality	Genome-fragment		1.99	0
S1_extracted_k141_53184	1363	No	NA	0	1	Not-determined	Genome-fragment	NA		0
S1_extracted_k141_92898	1016	No	NA	0	1	Not-determined	Genome-fragment	NA		0
S1_extracted_k141_64935	3263	No	NA	0	1	Not-determined	Genome-fragment	NA		0
S1_extracted_k141_26069	1060	No	NA	0	0	Not-determined	Genome-fragment	NA		0
S1_extracted_k141_4907	1302	No	NA	0	1	Not-determined	Genome-fragment	NA		0
NC1_vf_k141_2039	1475	No	NA	0	0	Low-quality	Genome-fragment		3.13	0
S10_vs_51675||full	1475	No	NA	0	2	Low-quality	Genome-fragment		2.05	0
S1_extracted_k141_38247	1019	No	NA	0	1	Not-determined	Genome-fragment	NA		0
S12_vs_k141_12437||full	1475	No	NA	1	0	Low-quality	Genome-fragment		2.63	0
S12_vs_k141_108751||full	1475	No	NA	0	0	Low-quality	Genome-fragment		1.14	0
S13_vs_63037||full	1476	No	NA	0	0	Low-quality	Genome-fragment		2.31	0
S1_vs_k141_155816||full	1476	No	NA	0	0	Low-quality	Genome-fragment		1.84	0
S1_extracted_k141_13244	1058	No	NA	0	0	Not-determined	Genome-fragment	NA		0
S1_extracted_k141_106403	1036	No	NA	0	1	Not-determined	Genome-fragment	NA		0
S1_extracted_k141_53299	3656	No	NA	0	1	Not-determined	Genome-fragment	NA		0
S1_extracted_k141_119691	1602	No	NA	0	2	Not-determined	Genome-fragment	NA		0
S5_extracted_k141_4794	1476	No	NA	1	0	Low-quality	Genome-fragment		0.85	0
S1_extracted_k141_65027	1005	No	NA	0	0	Not-determined	Genome-fragment	NA		0
NC1_vf_k141_1196	1476	No	NA	0	0	Low-quality	Genome-fragment		3.25	0
S16_extracted_9466	1476	No	NA	1	0	Low-quality	Genome-fragment		3.46	0
S16_extracted_92050	1476	No	NA	0	0	Low-quality	Genome-fragment		1.02	0
S16_vs_17158||full	1476	No	NA	1	0	Low-quality	Genome-fragment		2.35	0
S16_vs_171000||full	1476	No	NA	1	0	Low-quality	Genome-fragment		3.03	0
S1_extracted_k141_86477	2201	No	NA	0	0	Not-determined	Genome-fragment	NA		0
S1_extracted_k141_13349	1874	No	NA	0	2	Not-determined	Genome-fragment	NA		0
S1_extracted_k141_93050	1517	No	NA	0	2	Not-determined	Genome-fragment	NA		0
S15_extracted_23928	1476	No	NA	0	0	Low-quality	Genome-fragment		3.23	0
S8_extracted_k141_75832	1476	No	NA	0	1	Low-quality	Genome-fragment		4.71	0
S1_extracted_k141_26259	1434	No	NA	0	1	Not-determined	Genome-fragment	NA		0
S1_extracted_k141_145937	1593	No	NA	0	1	Not-determined	Genome-fragment	NA		0
S1_extracted_k141_126200	1056	No	NA	0	0	Not-determined	Genome-fragment	NA		0
S17_extracted_k141_12828	1477	No	NA	1	0	Low-quality	Genome-fragment		2.45	0
S11_extracted_k141_68278	1477	No	NA	0	0	Low-quality	Genome-fragment		3.1	0
S11_vs_k141_34288||full	1477	No	NA	1	0	Low-quality	Genome-fragment		4.47	0
S11_vs_k141_14637||lt2gene	1477	No	NA	1	0	Low-quality	Genome-fragment		1.38	0
S11_vs_k141_43555||lt2gene	1477	No	NA	2	0	Low-quality	Genome-fragment		3.83	0
S14_extracted_k141_32728	1477	No	NA	1	0	Low-quality	Genome-fragment		4.94	0
S1_extracted_k141_126210	5383	No	NA	0	2	Not-determined	Genome-fragment	NA		0
S1_extracted_k141_26305	1079	No	NA	0	0	Not-determined	Genome-fragment	NA		0
S1_extracted_k141_133332	1231	No	NA	0	1	Not-determined	Genome-fragment	NA		0
S1_extracted_k141_139654	1683	No	NA	0	1	Not-determined	Genome-fragment	NA		0
S1_extracted_k141_38425	1135	No	NA	0	1	Not-determined	Genome-fragment	NA		0
S1_extracted_k141_139663	1307	No	NA	0	0	Not-determined	Genome-fragment	NA		0
S1_extracted_k141_133352	5618	No	NA	0	3	Not-determined	Genome-fragment	NA		0
S1_extracted_k141_106579	1436	No	NA	0	1	Not-determined	Genome-fragment	NA		0
S1_vs_k141_138411||full	1477	No	NA	0	0	Low-quality	Genome-fragment		1.6	0
S16_vs_153993||full	1477	No	NA	1	0	Low-quality	Genome-fragment		3.37	0
S1_extracted_k141_139681	1530	No	NA	0	1	Not-determined	Genome-fragment	NA		0
S1_extracted_k141_33287	1549	No	NA	0	0	Not-determined	Genome-fragment	NA		0
S1_extracted_k141_93201	1013	No	NA	0	0	Not-determined	Genome-fragment	NA		0
S1_extracted_k141_133393	1320	No	NA	0	1	Not-determined	Genome-fragment	NA		0
S1_extracted_k141_65246	1248	No	NA	0	1	Not-determined	Genome-fragment	NA		0
S1_extracted_k141_26382	1283	No	NA	0	1	Not-determined	Genome-fragment	NA		0
S1_extracted_k141_139732	3395	No	NA	0	1	Not-determined	Genome-fragment	NA		0
S10_vs_96347||full	1477	No	NA	1	0	Low-quality	Genome-fragment		3.97	0
S12_vs_k141_134939||full	1477	No	NA	1	0	Low-quality	Genome-fragment		2.81	0
S1_extracted_k141_93270	1139	No	NA	0	0	Not-determined	Genome-fragment	NA		0
S6_vs_k141_1983||full	1477	No	NA	0	1	Low-quality	Genome-fragment		2.74	0
S1_extracted_k141_119936	1044	No	NA	0	1	Not-determined	Genome-fragment	NA		0
S1_extracted_k141_65312	4007	No	NA	0	2	Not-determined	Genome-fragment	NA		0
S1_extracted_k141_146146	3672	No	NA	0	3	Not-determined	Genome-fragment	NA		0
S8_extracted_k141_160940	1477	No	NA	1	0	Low-quality	Genome-fragment		2.52	0
S1_extracted_k141_126373	2315	No	NA	0	2	Not-determined	Genome-fragment	NA		0
S1_extracted_k141_38561	1082	No	NA	0	1	Not-determined	Genome-fragment	NA		0
S1_extracted_k141_65352	1088	No	NA	0	0	Not-determined	Genome-fragment	NA		0
S1_extracted_k141_93375	1105	No	NA	0	1	Not-determined	Genome-fragment	NA		0
S1_extracted_k141_65356	1188	No	NA	0	0	Not-determined	Genome-fragment	NA		0
S8_extracted_k141_187194	1477	No	NA	0	1	Low-quality	Genome-fragment		2.33	0
S1_extracted_k141_93383	2476	No	NA	0	1	Not-determined	Genome-fragment	NA		0
S1_extracted_k141_71832	1243	No	NA	0	1	Not-determined	Genome-fragment	NA		0
S1_extracted_k141_113310	1328	No	NA	0	1	Not-determined	Genome-fragment	NA		0
S8_vs_k141_113793||full	1477	No	NA	0	1	Low-quality	Genome-fragment		3.17	0
S1_extracted_k141_146170	1046	No	NA	0	1	Not-determined	Genome-fragment	NA		0
S1_extracted_k141_113321	1198	No	NA	0	0	Not-determined	Genome-fragment	NA		0
S11_vs_k141_60028||full	1478	No	NA	0	1	Low-quality	Genome-fragment		2.07	0
S1_extracted_k141_126385	1104	No	NA	0	1	Not-determined	Genome-fragment	NA		0
S1_extracted_k141_120021	1503	No	NA	0	3	Not-determined	Genome-fragment	NA		0
S14_vs_k141_62586||full	1478	No	NA	1	0	Low-quality	Genome-fragment		3.87	0
S1_extracted_k141_120035	1992	No	NA	0	1	Not-determined	Genome-fragment	NA		0
S14_vs_k141_12587||full	1478	No	NA	0	2	Low-quality	Genome-fragment		2.48	0
S1_extracted_k141_150116	2244	No	NA	0	0	Not-determined	Genome-fragment	NA		0
S1_extracted_k141_26545	1144	No	NA	0	0	Not-determined	Genome-fragment	NA		0
S1_extracted_k141_150124	1002	No	NA	0	0	Not-determined	Genome-fragment	NA		0
S1_vs_k141_27448||full	1478	No	NA	0	0	Low-quality	Genome-fragment		3.16	0
S1_extracted_k141_65550	1781	No	NA	0	0	Not-determined	Genome-fragment	NA		0
S1_extracted_k141_146379	1542	No	NA	0	0	Not-determined	Genome-fragment	NA		0
S1_extracted_k141_38665	1926	No	NA	0	0	Not-determined	Genome-fragment	NA		0
S1_extracted_k141_38705	1340	No	NA	0	1	Not-determined	Genome-fragment	NA		0
S1_extracted_k141_5440	1536	No	NA	0	0	Not-determined	Genome-fragment	NA		0
S1_extracted_k141_65660	1104	No	NA	0	0	Not-determined	Genome-fragment	NA		0
S1_extracted_k141_150175	1897	No	NA	0	0	Not-determined	Genome-fragment	NA		0
S1_extracted_k141_38755	1735	No	NA	0	0	Not-determined	Genome-fragment	NA		0
S1_extracted_k141_140114	1055	No	NA	0	1	Not-determined	Genome-fragment	NA		0
S1_extracted_k141_65711	1183	No	NA	0	1	Not-determined	Genome-fragment	NA		0
S5_vs_k141_55372||full	1478	No	NA	0	0	Low-quality	Genome-fragment		2.87	0
S1_extracted_k141_126677	1135	No	NA	0	1	Not-determined	Genome-fragment	NA		0
S1_extracted_k141_65767	1661	No	NA	0	1	Not-determined	Genome-fragment	NA		0
S5_vs_k141_1761||full	1478	No	NA	0	0	Low-quality	Genome-fragment		0.98	0
S1_extracted_k141_5542	3754	No	NA	0	0	Not-determined	Genome-fragment	NA		0
S7_vs_6806||full	1478	No	NA	2	0	Low-quality	Genome-fragment		3.14	0
S7_vs_39792||full	1478	No	NA	0	1	Low-quality	Genome-fragment		1.75	0
S1_extracted_k141_126805	1920	No	NA	0	0	Not-determined	Genome-fragment	NA		0
S16_vs_8497||full	1478	No	NA	0	0	Low-quality	Genome-fragment		3	0
S1_extracted_k141_65912	1834	No	NA	0	0	Not-determined	Genome-fragment	NA		0
S14_vs_k141_37210||lt2gene	1479	No	NA	2	0	Low-quality	Genome-fragment		2.74	0
S3_extracted_k141_81207	1479	No	NA	2	0	Low-quality	Genome-fragment		3.93	0
S1_extracted_k141_146819	1229	No	NA	0	0	Not-determined	Genome-fragment	NA		0
S1_extracted_k141_66029	1317	No	NA	0	1	Not-determined	Genome-fragment	NA		0
S1_extracted_k141_5759	3324	No	NA	0	1	Not-determined	Genome-fragment	NA		0
S1_extracted_k141_157421	1172	No	NA	0	0	Not-determined	Genome-fragment	NA		0
S1_extracted_k141_66193	1001	No	NA	0	0	Not-determined	Genome-fragment	NA		0
S1_extracted_k141_66254	1541	No	NA	0	2	Not-determined	Genome-fragment	NA		0

S3_vs_k141_107387||full	1479	No	NA	0	0	Low-quality	Genome-fragment	1.22	0
S1_extracted_k141_39177	1116	No	NA	0	1	Not-determined	Genome-fragment	NA	0
S1_extracted_k141_66296	3090	No	NA	0	1	Not-determined	Genome-fragment	NA	0
S1_extracted_k141_5947	1703	No	NA	0	0	Not-determined	Genome-fragment	NA	0
S3_vs_k141_49868||lt2gene	1479	No	NA	2	0	Low-quality	Genome-fragment	4.4	0
S1_extracted_k141_66370	1148	No	NA	0	0	Not-determined	Genome-fragment	NA	0
S1_extracted_k141_39238	1177	No	NA	0	0	Not-determined	Genome-fragment	NA	0
S16_vs_72346||full	1479	No	NA	1	0	Low-quality	Genome-fragment	3.75	0
S10_vs_49241||full	1479	No	NA	4	0	Low-quality	Genome-fragment	4.25	0
S10_vs_49267||full	1479	No	NA	0	0	Low-quality	Genome-fragment	3.59	0
S1_extracted_k141_6216	1304	No	NA	0	1	Not-determined	Genome-fragment	NA	0
S1_extracted_k141_66633	1193	No	NA	0	2	Not-determined	Genome-fragment	NA	0
S1_extracted_k141_6231	1352	No	NA	0	1	Not-determined	Genome-fragment	NA	0
S8_vs_k141_141770||full	1479	No	NA	0	0	Low-quality	Genome-fragment	2.51	0
S9_vs_k141_45783||full	1480	No	NA	2	0	Low-quality	Genome-fragment	2.93	0
S1_extracted_k141_6272	40918	No	NA	16	0	Complete	High-quality	100	0
S1_extracted_k141_6300	1029	No	NA	0	0	Not-determined	Genome-fragment	NA	0
S1_extracted_k141_157693	1033	No	NA	0	1	Not-determined	Genome-fragment	NA	0
S11_vs_k141_74333||lt2gene	1480	No	NA	1	0	Low-quality	Genome-fragment	3.98	0
S13_vs_120915||full	1480	No	NA	1	0	Low-quality	Genome-fragment	0.43	0
S1_extracted_k141_157715	1588	No	NA	0	1	Not-determined	Genome-fragment	NA	0
S14_extracted_k141_88403	1480	No	NA	3	0	Low-quality	Genome-fragment	4.87	0
S1_extracted_k141_157816	1418	No	NA	0	1	Not-determined	Genome-fragment	NA	0
S1_extracted_k141_6472	1116	No	NA	0	1	Not-determined	Genome-fragment	NA	0
S1_extracted_k141_39736	3754	No	NA	0	3	Not-determined	Genome-fragment	NA	0
S14_extracted_k141_9082	1480	No	NA	3	0	Low-quality	Genome-fragment	3.86	0
S1_extracted_k141_6578	1163	No	NA	0	0	Not-determined	Genome-fragment	NA	0
S1_extracted_k141_72902	1110	No	NA	0	1	Not-determined	Genome-fragment	NA	0
S14_vs_k141_44211||full	1480	No	NA	0	0	Low-quality	Genome-fragment	2.09	0
S1_extracted_k141_39854	1415	No	NA	0	0	Not-determined	Genome-fragment	NA	0
S14_vs_k141_67318||full	1480	No	NA	0	0	Low-quality	Genome-fragment	3.07	0
S1_extracted_k141_150750	1891	No	NA	0	1	Not-determined	Genome-fragment	NA	0
S1_extracted_k141_158015	1208	No	NA	0	1	Not-determined	Genome-fragment	NA	0
S1_extracted_k141_158033	1365	No	NA	0	1	Not-determined	Genome-fragment	NA	0
S1_extracted_k141_158087	1056	No	NA	0	1	Not-determined	Genome-fragment	NA	0
S1_vs_k141_87155||full	1480	No	NA	0	2	Low-quality	Genome-fragment	2.15	0
S5_extracted_k141_28089	1480	No	NA	1	0	Low-quality	Genome-fragment	2.53	0
S5_vs_k141_32176||full	1480	No	NA	0	0	Low-quality	Genome-fragment	3.72	0
S1_extracted_k141_73094	1035	No	NA	0	1	Not-determined	Genome-fragment	NA	0
S1_extracted_k141_73097	1006	No	NA	0	2	Not-determined	Genome-fragment	NA	0
S16_vs_86909||full	1480	No	NA	0	0	Low-quality	Genome-fragment	1.8	0
S12_vs_k141_152266||full	1480	No	NA	1	0	Low-quality	Genome-fragment	2.45	0
S6_vs_k141_22986||full	1480	No	NA	0	1	Low-quality	Genome-fragment	2.67	0
S1_extracted_k141_73188	1126	No	NA	0	2	Not-determined	Genome-fragment	NA	0
S1_extracted_k141_151027	1028	No	NA	0	0	Not-determined	Genome-fragment	NA	0
S1_extracted_k141_158420	1486	No	NA	0	0	Not-determined	Genome-fragment	NA	0
S1_extracted_k141_158462	1035	No	NA	0	0	Not-determined	Genome-fragment	NA	0
S1_extracted_k141_151183	5309	No	NA	0	1	Not-determined	Genome-fragment	NA	0
S1_extracted_k141_151250	1557	No	NA	0	1	Not-determined	Genome-fragment	NA	0
S1_extracted_k141_158603	2164	No	NA	0	2	Not-determined	Genome-fragment	NA	0
S13_extracted_147516	1481	No	NA	1	0	Low-quality	Genome-fragment	3.67	0
S3_extracted_k141_41336	1481	No	NA	2	0	Low-quality	Genome-fragment	2.82	0
S1_extracted_k141_151391	1101	No	NA	0	1	Not-determined	Genome-fragment	NA	0
S1_extracted_k141_158693	2030	No	NA	0	1	Not-determined	Genome-fragment	NA	0
S1_extracted_k141_151485	1031	No	NA	0	1	Not-determined	Genome-fragment	NA	0
S3_vs_k141_96398||full	1481	No	NA	0	0	Low-quality	Genome-fragment	2.05	0
S1_extracted_k141_158890	1566	No	NA	0	1	Not-determined	Genome-fragment	NA	0
S1_extracted_k141_151678	1227	No	NA	0	0	Not-determined	Genome-fragment	NA	0
S5_extracted_k141_28971	1481	No	NA	1	0	Low-quality	Genome-fragment	0.92	0
S7_vs_63635||full	1481	No	NA	0	1	Low-quality	Genome-fragment	4.73	0
S1_extracted_k141_151780	2082	No	NA	0	2	Not-determined	Genome-fragment	NA	0
S1_extracted_k141_158961	43352	No	NA	9	1	Medium-quality	Genome-fragment	80.9	0
S1_extracted_k141_158985	2752	No	NA	0	1	Not-determined	Genome-fragment	NA	0
S1_extracted_k141_159144	1157	No	NA	0	2	Not-determined	Genome-fragment	NA	0
S16_extracted_64876	1481	No	NA	0	1	Low-quality	Genome-fragment	0.86	0
S1_extracted_k141_152189	2109	No	NA	0	2	Not-determined	Genome-fragment	NA	0
S10_extracted_34939	1481	No	NA	0	1	Low-quality	Genome-fragment	3.98	0
S1_extracted_k141_159322	1771	No	NA	0	1	Not-determined	Genome-fragment	NA	0
S1_extracted_k141_159329	1177	No	NA	0	1	Not-determined	Genome-fragment	NA	0
S1_extracted_k141_159479	1036	No	NA	0	2	Not-determined	Genome-fragment	NA	0
S1_extracted_k141_159524	1279	No	NA	0	1	Not-determined	Genome-fragment	NA	0
S1_extracted_k141_159543	1948	No	NA	0	0	Not-determined	Genome-fragment	NA	0
S12_extracted_k141_40887	1481	No	NA	0	2	Low-quality	Genome-fragment	3.22	0
S1_extracted_k141_152496	1277	No	NA	0	1	Not-determined	Genome-fragment	NA	0
S1_extracted_k141_159589	6712	No	NA	0	2	Not-determined	Genome-fragment	NA	0
S1_extracted_k141_159657	1097	No	NA	0	1	Not-determined	Genome-fragment	NA	0
S15_vs_28280||full	1481	No	NA	0	0	Low-quality	Genome-fragment	3.3	0
S8_extracted_k141_92549	1481	No	NA	0	0	Low-quality	Genome-fragment	1.64	0
S1_extracted_k141_159785	1446	No	NA	0	1	Not-determined	Genome-fragment	NA	0
S1_extracted_k141_159839	1037	No	NA	0	0	Not-determined	Genome-fragment	NA	0
S8_vs_k141_8054||full	1481	No	NA	0	0	Low-quality	Genome-fragment	2.73	0
S1_extracted_k141_153109	7731	No	NA	0	6	Not-determined	Genome-fragment	NA	0
S14_vs_k141_51106||full	1482	No	NA	0	1	Low-quality	Genome-fragment	0.79	0
S1_extracted_k141_159951	101988	No	NA	17	3	Complete	High-quality	100	0
S1_extracted_k141_153189	1685	No	NA	0	1	Not-determined	Genome-fragment	NA	0
S1_extracted_k141_153230	1017	No	NA	0	1	Not-determined	Genome-fragment	NA	0
S1_extracted_k141_153237	1147	No	NA	0	1	Not-determined	Genome-fragment	NA	0
S1_extracted_k141_153397	1027	No	NA	0	2	Not-determined	Genome-fragment	NA	0
S1_extracted_k141_153500	2637	No	NA	0	1	Not-determined	Genome-fragment	NA	0
S1_vs_k141_143929||full	39654	No	NA	14	1	High-quality	High-quality	94.85	0
S1_vs_k141_66751||full	1760	No	NA	0	0	Not-determined	Genome-fragment	NA	0
S1_vs_k141_80114||full	1352	No	NA	0	2	Not-determined	Genome-fragment	NA	0
S1_extracted_k141_31875	1482	No	NA	1	0	Low-quality	Genome-fragment	1.85	0
S1_vs_k141_146858||full	3656	No	NA	0	3	Not-determined	Genome-fragment	NA	0
S3_extracted_k141_36917	1482	No	NA	1	0	Low-quality	Genome-fragment	2.55	0
S1_vs_k141_33403||full	1012	No	NA	0	0	Not-determined	Genome-fragment	NA	0
S3_extracted_k141_28999	1482	No	NA	0	1	Low-quality	Genome-fragment	0.91	0
S5_vs_k141_74854||full	1482	No	NA	1	2	Low-quality	Genome-fragment	0.24	0
S7_vs_26041||full	1482	No	NA	0	2	Low-quality	Genome-fragment	0.47	0
S7_vs_133315||full	1482	No	NA	0	0	Low-quality	Genome-fragment	3.31	0
S1_vs_k141_86808||full	1517	No	NA	0	0	Not-determined	Genome-fragment	NA	0
S1_vs_k141_26723||full	1304	No	NA	0	1	Not-determined	Genome-fragment	NA	0
S1_vs_k141_133544||full	1951	No	NA	0	2	Not-determined	Genome-fragment	NA	0
S1_vs_k141_73449||full	4289	No	NA	0	4	Not-determined	Genome-fragment	NA	0
S1_vs_k141_20064||full	6319	No	NA	0	5	Not-determined	Genome-fragment	NA	0
S16_vs_61133||full	1482	No	NA	0	0	Low-quality	Genome-fragment	2.46	0
S1_vs_k141_113515||full	2691	No	NA	0	1	Not-determined	Genome-fragment	NA	0
S1_vs_k141_120200||full	2133	No	NA	0	1	Not-determined	Genome-fragment	NA	0
S16_vs_121885||full	1482	No	NA	1	0	Low-quality	Genome-fragment	3.23	0
S16_vs_146459||full	1482	No	NA	0	0	Low-quality	Genome-fragment	1.3	0
S10_vs_58897||full	1482	Yes	999	1	1	Low-quality	Genome-fragment	0.69	32.59
S12_extracted_k141_183885	1482	No	NA	0	0	Low-quality	Genome-fragment	1.06	0
S12_extracted_k141_140828	1482	No	NA	0	1	Low-quality	Genome-fragment	0.27	0
S12_extracted_k141_124648	1482	No	NA	1	0	Low-quality	Genome-fragment	1.7	0
S1_vs_k141_110||full	2157	No	NA	0	2	Not-determined	Genome-fragment	NA	0
S1_vs_k141_86859||full	1715	No	NA	0	3	Not-determined	Genome-fragment	NA	0
S1_vs_k141_66832||full	1189	No	NA	0	1	Not-determined	Genome-fragment	NA	0
S8_vs_k141_96715||full	1482	No	NA	1	0	Low-quality	Genome-fragment	1.37	0
S1_vs_k141_66837||full	4083	No	NA	0	0	Not-determined	Genome-fragment	NA	0
S17_vs_k141_44479||full	1483	No	NA	0	1	Low-quality	Genome-fragment	3.6	0
S1_vs_k141_73513||full	2343	No	NA	0	2	Not-determined	Genome-fragment	NA	0
S1_extracted_k141_156374	1483	Yes	1048	1	1	Low-quality	Genome-fragment	2.29	29.33
S1_vs_k141_46837||full	2070	No	NA	0	2	Not-determined	Genome-fragment	NA	0
S1_vs_k141_140306||full	2429	No	NA	0	3	Not-determined	Genome-fragment	NA	0
S1_vs_k141_80245||full	2171	No	NA	0	2	Not-determined	Genome-fragment	NA	0
S1_vs_k141_133577||full	37683	No	NA	17	1	High-quality	High-quality	97.57	0
S1_vs_k141_73542||full	1200	No	NA	0	1	Not-determined	Genome-fragment	NA	0
S7_vs_36793||lt2gene	1483	No	NA	1	0	Low-quality	Genome-fragment	3.59	0
S1_vs_k141_80273||full	1471	No	NA	0	2	Not-determined	Genome-fragment	NA	0
S1_vs_k141_113634||full	1099	No	NA	0	0	Not-determined	Genome-fragment	NA	0
S15_extracted_11555	1483	No	NA	0	2	Low-quality	Genome-fragment	0.67	0
S4_vs_VIRSorter_31698-cat_3	1483	No	NA	1	0	Low-quality	Genome-fragment	3.83	0
S6_vs_k141_36798||full	1483	No	NA	0	0	Low-quality	Genome-fragment	4.69	0
S17_vs_k141_151326||full	1484	No	NA	1	0	Low-quality	Genome-fragment	2.8	0
S1_vs_k141_204||full	1044	No	NA	0	0	Not-determined	Genome-fragment	NA	0
S17_vs_k141_124312||full	1484	No	NA	0	2	Low-quality	Genome-fragment	0.81	0
S17_vs_k141_21395||full	1484	No	NA	2	0	Low-quality	Genome-fragment	3.46	0
S1_vs_k141_20257||full	3067	No	NA	0	0	Not-determined	Genome-fragment	NA	0
S14_vs_k141_20113||full	1484	No	NA	0	0	Low-quality	Genome-fragment	3.19	0
S14_vs_k141_94513||full	1484	No	NA	0	1	Low-quality	Genome-fragment	1.97	0
S1_vs_k141_87042||full	1981	No	NA	0	3	Not-determined	Genome-fragment	NA	0
S10_extracted_8869	1484	No	NA	0	2	Low-quality	Genome-fragment	0.45	0
S10_vs_79283||full	1484	No	NA	1	0	Low-quality	Genome-fragment	3.8	0
S8_vs_k141_175601||full	1484	No	NA	0	0	Low-quality	Genome-fragment	3	0
S1_vs_k141_113719||full	2446	No	NA	0	1	Not-determined	Genome-fragment	NA	0
S1_vs_k141_127131||full	2151	No	NA	0	0	Not-determined	Genome-fragment	NA	0
S1_vs_k141_33617||full	1016	No	NA	0	0	Not-determined	Genome-fragment	NA	0
S1_vs_k141_66989||full	3099	No	NA	0	2	Not-determined	Genome-fragment	NA	0
S1_vs_k141_26890||full	1970	No	NA	0	1	Not-determined	Genome-fragment	NA	0
S8_vs_k141_48652||full	1484	No	NA	0	1	Low-quality	Genome-fragment	0.95	0
S9_extracted_k141_29541	1485	No	NA	2	0	Low-quality	Genome-fragment	4.2	0
S1_vs_k141_66995||full	3974	No	NA	0	2	Not-determined	Genome-fragment	NA	0
S11_vs_k141_88004||full	1485	No	NA	0	0	Low-quality	Genome-fragment	3.61	0
S1_vs_k141_113749||full	1680	No	NA	0	2	Not-determined	Genome-fragment	NA	0
S13_vs_74441||full	1485	No	NA	0	1	Low-quality	Genome-fragment	0.48	0
S1_vs_k141_133731||full	1211	No	NA	0	0	Not-determined	Genome-fragment	NA	0
S1_vs_k141_127197||full	1173	No	NA	0	1	Not-determined	Genome-fragment	NA	0
S14_vs_k141_45208||1_partial	1485	No	NA	1	0	Low-quality	Genome-fragment	33.16	0
S5_extracted_k141_65432	1485	No	NA	1	0	Low-quality	Genome-fragment	2.53	0
S1_vs_k141_120496||full	2292	No	NA	0	1	Not-determined	Genome-fragment	NA	0
S1_vs_k141_140495||full	2649	No	NA	0	2	Not-determined	Genome-fragment	NA	0
S1_vs_k141_350||full	1222	No	NA	0	1	Not-determined	Genome-fragment	NA	0
S1_vs_k141_73761||full	1303	No	NA	0	1	Not-determined	Genome-fragment	NA	0
S5_vs_k141_19719||full	1485	No	NA	0	0	Low-quality	Genome-fragment	1.26	0
S1_vs_k141_33692||full	1777	No	NA	0	1	Not-determined	Genome-fragment	NA	0
S5_vs_k141_16437||full	1485	No	NA	0	0	Low-quality	Genome-fragment	1.72	0
S5_vs_k141_143980||full	1485	No	NA	0	0	Low-quality	Genome-fragment	3.12	0
S10_vs_103735||full	1485	No	NA	1	0	Low-quality	Genome-fragment	1.3	0
S1_vs_k141_13759||full	1060	No	NA	0	1	Not-determined	Genome-fragment	NA	0
S12_vs_k141_136803||full	1485	No	NA	0	0	Low-quality	Genome-fragment	3.04	0
S17_vs_k141_120010||lt2gene	1486	No	NA	1	0	Low-quality	Genome-fragment	2.51	0
S9_vs_k141_75120||full	1486	No	NA	0	1	Low-quality	Genome-fragment	1.31	0
S1_vs_k141_53866||full	3531	No	NA	0	3	Not-determined	Genome-fragment	NA	0
S1_extracted_k141_17701	1486	No	NA	0	0	Low-quality	Genome-fragment	5.18	0

S1_vs_k141_31655||full	1486	No	NA	0	0	Low-quality	Genome-fragment		2.98	0
S3_vs_k141_46246||lt2gene	1486	No	NA	1	0	Low-quality	Genome-fragment		1.03	0
S5_extracted_k141_80626	1486	No	NA	0	0	Low-quality	Genome-fragment		3.52	0
S1_vs_k141_47170||full	1260	No	NA	0	0	Not-determined	Genome-fragment	NA		0
S1_vs_k141_93967||full	2266	No	NA	0	1	Not-determined	Genome-fragment	NA		0
S1_vs_k141_113935||full	2251	No	NA	0	3	Not-determined	Genome-fragment	NA		0
S1_vs_k141_73917||full	1120	No	NA	0	2	Not-determined	Genome-fragment	NA		0
S1_vs_k141_120637||full	2543	No	NA	0	1	Not-determined	Genome-fragment	NA		0
S5_vs_k141_49268||full	1486	No	NA	1	0	Low-quality	Genome-fragment		4	0
S1_vs_k141_47221||full	1159	No	NA	0	1	Not-determined	Genome-fragment	NA		0
S5_vs_k141_59061||full	1486	No	NA	0	0	Low-quality	Genome-fragment		2.6	0
S7_extracted_87035	1486	No	NA	2	0	Low-quality	Genome-fragment		3.84	0
S16_extracted_101826	1486	No	NA	2	0	Low-quality	Genome-fragment		3.45	0
S1_vs_k141_140702||full	2976	No	NA	0	4	Not-determined	Genome-fragment	NA		0
S1_vs_k141_47236||full	2982	No	NA	0	2	Not-determined	Genome-fragment	NA		0
S16_vs_55677||full	1486	No	NA	0	0	Low-quality	Genome-fragment		0.86	0
S15_extracted_42392	1486	No	NA	0	2	Low-quality	Genome-fragment		1.05	0
S1_vs_k141_27200||full	1000	No	NA	0	0	Not-determined	Genome-fragment	NA		0
S1_vs_k141_113975||full	2169	No	NA	0	0	Not-determined	Genome-fragment	NA		0
S15_vs_68973||full	1486	No	NA	1	0	Low-quality	Genome-fragment		1.14	0
S1_vs_k141_20537||full	2182	No	NA	0	2	Not-determined	Genome-fragment	NA		0
S17_extracted_k141_175431	1487	No	NA	1	0	Low-quality	Genome-fragment		3.35	0
S1_vs_k141_87391||full	2513	No	NA	0	1	Not-determined	Genome-fragment	NA		0
S1_vs_k141_114040||full	1586	No	NA	0	1	Not-determined	Genome-fragment	NA		0
S14_vs_k141_4798||full	1487	No	NA	0	1	Low-quality	Genome-fragment		0.75	0
S14_vs_k141_38233||lt2gene	1487	No	NA	1	0	Low-quality	Genome-fragment		3.03	0
S1_vs_k141_120731||full	1059	No	NA	0	1	Not-determined	Genome-fragment	NA		0
S7_vs_2747||full	1487	No	NA	0	0	Low-quality	Genome-fragment		1.73	0
S12_extracted_k141_71340	1487	No	NA	0	0	Low-quality	Genome-fragment		4.58	0
S8_vs_k141_19419||lt2gene	1487	No	NA	1	0	Low-quality	Genome-fragment		1.55	0
S14_extracted_k141_3424	1488	No	NA	0	2	Low-quality	Genome-fragment		3.58	0
S1_vs_k141_40755||full	1300	No	NA	0	0	Not-determined	Genome-fragment	NA		0
S3_vs_k141_68237||full	1488	No	NA	0	0	Low-quality	Genome-fragment		4.11	0
S1_vs_k141_14149||full	2304	No	NA	0	1	Not-determined	Genome-fragment	NA		0
S3_vs_k141_101414||full	1488	No	NA	1	0	Low-quality	Genome-fragment		3.26	0
S1_vs_k141_40790||full	1146	No	NA	0	0	Not-determined	Genome-fragment	NA		0
S1_vs_k141_94172||full	3815	No	NA	0	1	Not-determined	Genome-fragment	NA		0
S1_vs_k141_74138||full	1065	No	NA	0	0	Not-determined	Genome-fragment	NA		0
S3_vs_k141_46507||full	1488	No	NA	2	0	Low-quality	Genome-fragment		1.04	0
S5_vs_k141_98558||full	1488	No	NA	0	0	Low-quality	Genome-fragment		2.72	0
S1_vs_k141_67403||full	2032	No	NA	0	1	Not-determined	Genome-fragment	NA		0
S1_vs_k141_140932||full	2319	No	NA	0	2	Not-determined	Genome-fragment	NA		0
S7_extracted_48945	1488	No	NA	1	0	Low-quality	Genome-fragment		2.76	0
S1_vs_k141_27422||full	2134	No	NA	0	2	Not-determined	Genome-fragment	NA		0
S16_extracted_152695	1488	No	NA	3	0	Low-quality	Genome-fragment		2.56	0
S1_vs_k141_107452||full	3933	No	NA	0	2	Not-determined	Genome-fragment	NA		0
S16_vs_124558||lt2gene	1488	No	NA	1	0	Low-quality	Genome-fragment		1.03	0
S12_vs_k141_72878||full	1488	No	NA	0	0	Low-quality	Genome-fragment		1.41	0
S15_extracted_29185	1488	No	NA	0	0	Low-quality	Genome-fragment		3.33	0
S1_vs_k141_94301||full	2758	No	NA	0	2	Not-determined	Genome-fragment	NA		0
S15_extracted_40218	1488	No	NA	0	1	Low-quality	Genome-fragment		0.99	0
S8_extracted_k141_54909	1488	No	NA	0	1	Low-quality	Genome-fragment		1.02	0
S8_vs_k141_57783||full	1488	No	NA	0	2	Low-quality	Genome-fragment		3.85	0
S1_vs_k141_140995||full	1007	No	NA	0	0	Not-determined	Genome-fragment	NA		0
S11_extracted_k141_103473	1489	No	NA	1	0	Low-quality	Genome-fragment		1.97	0
S11_vs_k141_103627||full	1489	No	NA	0	0	Low-quality	Genome-fragment		1.4	0
S13_vs_92862||full	1489	No	NA	0	1	Low-quality	Genome-fragment		0.98	0
S1_vs_k141_120880||full	1221	No	NA	0	1	Not-determined	Genome-fragment	NA		0
S1_vs_k141_40963||full	2059	No	NA	0	2	Not-determined	Genome-fragment	NA		0
S1_extracted_k141_155271	1489	No	NA	1	0	Low-quality	Genome-fragment		3.79	0
S1_vs_k141_67543||full	1661	No	NA	0	1	Not-determined	Genome-fragment	NA		0
S1_vs_k141_27524||full	3628	No	NA	0	2	Not-determined	Genome-fragment	NA		0
S16_extracted_119163	1489	No	NA	0	1	Low-quality	Genome-fragment		0.8	0
S10_vs_96835||full	1489	No	NA	1	0	Low-quality	Genome-fragment		3.85	0
S12_vs_k141_151634||full	1489	No	NA	0	0	Low-quality	Genome-fragment		3.53	0
S15_extracted_69246	1489	No	NA	0	2	Low-quality	Genome-fragment		0.63	0
S8_extracted_k141_6227	1489	No	NA	0	1	Low-quality	Genome-fragment		3.85	0
S1_vs_k141_20910||full	2810	No	NA	0	2	Not-determined	Genome-fragment	NA		0
S17_extracted_k141_12846	1490	No	NA	1	0	Low-quality	Genome-fragment		2.35	0
S9_vs_k141_39685||full	1490	No	NA	1	0	Low-quality	Genome-fragment		3.61	0
S13_vs_35590||full	1490	No	NA	1	0	Low-quality	Genome-fragment		3.16	0
S1_vs_k141_87711||full	1346	No	NA	0	1	Not-determined	Genome-fragment	NA		0
S13_vs_109253||full	1490	No	NA	0	0	Low-quality	Genome-fragment		2.72	0
S3_vs_k141_39146||full	1490	No	NA	0	0	Low-quality	Genome-fragment		2.7	0
S10_vs_52573||full	1490	No	NA	1	0	Low-quality	Genome-fragment		1.71	0
S12_vs_k141_127056||full	1490	No	NA	0	0	Low-quality	Genome-fragment		0.86	0
S1_vs_k141_60613||full	3502	No	NA	0	1	Not-determined	Genome-fragment	NA		0
S1_vs_k141_114332||full	2050	No	NA	0	0	Not-determined	Genome-fragment	NA		0
S4_extracted_8279	1490	No	NA	0	0	Low-quality	Genome-fragment		1.07	0
S11_vs_k141_77050||full	1491	No	NA	1	0	Low-quality	Genome-fragment		2.8	0
S14_extracted_k141_31563	1491	No	NA	2	0	Low-quality	Genome-fragment		4.85	0
S1_vs_k141_94469||full	1830	No	NA	0	1	Not-determined	Genome-fragment	NA		0
S14_vs_k141_11773||full	1491	No	NA	1	0	Low-quality	Genome-fragment		3.43	0
S1_vs_k141_107613||full	2097	No	NA	0	0	Not-determined	Genome-fragment	NA		0
S1_vs_k141_47819||full	1491	No	NA	1	0	Low-quality	Genome-fragment		3.41	0
S3_extracted_k141_24182	1491	No	NA	0	1	Low-quality	Genome-fragment		1.01	0
S5_vs_k141_14094||lt2gene	1491	No	NA	1	0	Low-quality	Genome-fragment		3.02	0
S7_extracted_19384	1491	No	NA	0	0	Low-quality	Genome-fragment		2.95	0
S16_vs_65239||full	1491	No	NA	0	1	Low-quality	Genome-fragment		3.48	0
S1_vs_k141_896||full	2093	No	NA	0	1	Not-determined	Genome-fragment	NA		0
S1_vs_k141_141202||full	1930	No	NA	0	2	Not-determined	Genome-fragment	NA		0
S16_vs_23137||lt2gene	1491	No	NA	2	0	Low-quality	Genome-fragment		3.27	0
S1_vs_k141_81207||full	6380	No	NA	0	7	Not-determined	Genome-fragment	NA		0
S6_vs_k141_7555||full	1491	No	NA	0	1	Low-quality	Genome-fragment		1.06	0
S1_vs_k141_912||full	8990	No	NA	0	3	Not-determined	Genome-fragment	NA		0
S1_vs_k141_54491||full	1493	No	NA	0	1	Not-determined	Genome-fragment	NA		0
S6_vs_k141_45573||full	1491	No	NA	0	0	Low-quality	Genome-fragment		1.39	0
S8_vs_k141_57429||full	1491	No	NA	0	1	Low-quality	Genome-fragment		0.97	0
S1_vs_k141_21090||full	1042	No	NA	0	0	Not-determined	Genome-fragment	NA		0
S8_vs_k141_180910||full	1491	No	NA	0	0	Low-quality	Genome-fragment		1.62	0
S17_vs_k141_28531||full	1492	No	NA	0	0	Low-quality	Genome-fragment		3.14	0
S9_vs_k141_38768||full	1492	No	NA	1	0	Low-quality	Genome-fragment		3.34	0
S9_vs_k141_62719||full	1492	No	NA	0	0	Low-quality	Genome-fragment		3.14	0
S14_vs_k141_8657||full	1492	No	NA	0	0	Low-quality	Genome-fragment		3.1	0
S5_vs_k141_10183||full	1492	No	NA	1	0	Low-quality	Genome-fragment		2.24	0
S1_vs_k141_94638||full	1940	No	NA	0	0	Not-determined	Genome-fragment	NA		0
S1_vs_k141_101269||full	2134	No	NA	0	0	Not-determined	Genome-fragment	NA		0
S7_vs_299||full	1492	No	NA	1	0	Low-quality	Genome-fragment		2.01	0
S1_vs_k141_47832||full	4015	No	NA	0	1	Not-determined	Genome-fragment	NA		0
S16_vs_59764||full	1492	No	NA	0	1	Low-quality	Genome-fragment		0.77	0
S1_vs_k141_81314||full	1187	No	NA	0	1	Not-determined	Genome-fragment	NA		0
S15_vs_9780||full	1492	No	NA	2	0	Low-quality	Genome-fragment		3.3	0
S8_vs_k141_56168||full	1492	No	NA	1	0	Low-quality	Genome-fragment		0.86	0
S1_vs_k141_41290||full	2854	No	NA	0	0	Not-determined	Genome-fragment	NA		0
S1_vs_k141_141309||full	2304	No	NA	0	1	Not-determined	Genome-fragment	NA		0
S8_vs_k141_146953||full	1492	No	NA	1	0	Low-quality	Genome-fragment		3.33	0
S17_vs_k141_32085||full	1493	No	NA	0	0	Low-quality	Genome-fragment		1.65	0
S1_vs_k141_27788||full	2289	No	NA	0	3	Not-determined	Genome-fragment	NA		0
S11_extracted_k141_37120	1493	No	NA	0	0	Low-quality	Genome-fragment		3.27	0
S11_vs_k141_92210||full	1493	No	NA	1	0	Low-quality	Genome-fragment		3.54	0
S1_vs_k141_41325||full	1244	No	NA	0	1	Not-determined	Genome-fragment	NA		0
S3_vs_k141_126263||full	1493	No	NA	0	0	Low-quality	Genome-fragment		3.7	0
S1_vs_k141_14853||full	1265	No	NA	0	1	Not-determined	Genome-fragment	NA		0
S1_vs_k141_1033||full	2538	No	NA	0	2	Not-determined	Genome-fragment	NA		0
S1_vs_k141_128046||full	7825	No	NA	0	3	Not-determined	Genome-fragment	NA		0
S1_vs_k141_27866||full	1126	No	NA	0	1	Not-determined	Genome-fragment	NA		0
S5_vs_k141_85760||lt2gene	1493	No	NA	2	0	Low-quality	Genome-fragment		2.5	0
S16_vs_134123||full	1493	No	NA	0	0	Low-quality	Genome-fragment		3.62	0
S10_vs_3934||full	1493	No	NA	0	0	Low-quality	Genome-fragment		1.92	0
S6_extracted_k141_46715	1493	No	NA	0	1	Low-quality	Genome-fragment		3.32	0
S1_vs_k141_21263||full	14418	No	NA	0	9	Not-determined	Genome-fragment	NA		0
S6_vs_k141_13215||lt2gene	1493	No	NA	1	0	Low-quality	Genome-fragment		2.51	0
S1_vs_k141_154248||full	1019	No	NA	0	2	Not-determined	Genome-fragment	NA		0
S8_extracted_k141_739	1493	No	NA	0	0	Low-quality	Genome-fragment		4.75	0
S1_vs_k141_48031||full	2285	No	NA	0	0	Not-determined	Genome-fragment	NA		0
S13_extracted_68522	1494	No	NA	0	0	Low-quality	Genome-fragment		2.01	0
S1_vs_k141_54705||full	1928	No	NA	0	1	Not-determined	Genome-fragment	NA		0
S7_vs_40601||full	1494	No	NA	1	0	Low-quality	Genome-fragment		2.02	0
S1_vs_k141_54708||full	5273	No	NA	0	2	Not-determined	Genome-fragment	NA		0
S1_vs_k141_54717||full	1491	No	NA	0	0	Not-determined	Genome-fragment	NA		0
S7_vs_105989||full	1494	No	NA	1	0	Low-quality	Genome-fragment		0.89	0
S1_vs_k141_141428||full	4318	No	NA	0	3	Not-determined	Genome-fragment	NA		0
S16_vs_11046||full	1494	No	NA	0	0	Low-quality	Genome-fragment		2.96	0
S1_vs_k141_41473||full	1684	No	NA	0	0	Not-determined	Genome-fragment	NA		0
S6_vs_k141_5624||full	1494	No	NA	0	0	Low-quality	Genome-fragment		2	0
S8_extracted_k141_76891	1494	No	NA	0	1	Low-quality	Genome-fragment		0.8	0
S8_vs_k141_38068||full	1494	No	NA	0	0	Low-quality	Genome-fragment		3.12	0
S17_vs_k141_49140||full	1495	No	NA	0	0	Low-quality	Genome-fragment		1.95	0
S1_vs_k141_1139||full	2101	No	NA	0	2	Not-determined	Genome-fragment	NA		0
S1_vs_k141_74768||full	3032	No	NA	0	1	Not-determined	Genome-fragment	NA		0
S13_vs_25368||lt2gene	1495	No	NA	1	0	Low-quality	Genome-fragment		3.92	0
S1_vs_k141_81533||full	2360	No	NA	0	2	Not-determined	Genome-fragment	NA		0
S1_vs_k141_88156||full	2489	No	NA	0	0	Not-determined	Genome-fragment	NA		0
S1_vs_k141_101602||full	2085	No	NA	0	1	Not-determined	Genome-fragment	NA		0
S1_vs_k141_137457||full	1495	No	NA	2	0	Low-quality	Genome-fragment		3.95	0
S3_vs_k141_20255||full	1495	No	NA	0	0	Low-quality	Genome-fragment		3.02	0
S5_extracted_k141_140153	1495	No	NA	1	0	Low-quality	Genome-fragment		23.95	0
S1_vs_k141_81575||full	3625	No	NA	0	2	Not-determined	Genome-fragment	NA		0
S5_extracted_k141_121069	1495	No	NA	0	0	Low-quality	Genome-fragment		3.18	0
S13_vs_111195||full	1496	No	NA	0	0	Low-quality	Genome-fragment		3.77	0
S13_vs_110649||lt2gene	1496	No	NA	1	0	Low-quality	Genome-fragment		2.41	0
S1_vs_k141_68137||full	10139	No	NA	0	5	Not-determined	Genome-fragment	NA		0
S1_vs_k141_108132||full	2217	No	NA	0	0	Not-determined	Genome-fragment	NA		0
S1_vs_k141_154366||full	5645	No	NA	0	2	Not-determined	Genome-fragment	NA		0
S1_vs_k141_21439||full	32806	No	NA	15	0	Medium-quality	Genome-fragment		77.41	0
S3_vs_k141_58300||full	1496	No	NA	1	0	Low-quality	Genome-fragment		0.89	0
S12_vs_k141_168499||full	1496	No	NA	2	0	Low-quality	Genome-fragment		3.24	0
S1_vs_k141_54880||full	2549	No	NA	0	1	Not-determined	Genome-fragment	NA		0
S12_vs_k141_30225||full	1496	No	NA	0	0	Low-quality	Genome-fragment		0.74	0
S1_vs_k141_15193||full	1661	No	NA	0	2	Not-determined	Genome-fragment	NA		0
S15_extracted_57194	1496	No	NA	0	1	Low-quality	Genome-fragment		0.25	0
S2_extracted_k141_13597	1496	No	NA	2	0	Low-quality	Genome-fragment		4.94	0
S1_vs_k141_154403||full	2774	No	NA	0	0	Not-determined	Genome-fragment	NA		0
S1_vs_k141_134887||full	4360	No	NA	0	2	Not-determined	Genome-fragment	NA		0

S6_vs_k141_26066||full	1496	No	NA	0	2	Low-quality	Genome-fragment		1.23	0
S1_vs_k141_95065||full	2323	No	NA	0	1	Not-determined	Genome-fragment	NA		0
S6_vs_k141_36924||full	1496	No	NA	0	0	Low-quality	Genome-fragment		2.61	0
S8_extracted_k141_180053	1496	No	NA	0	0	Low-quality	Genome-fragment		0.93	0
S1_vs_k141_101747||full	1505	No	NA	0	0	Not-determined	Genome-fragment	NA		0
S9_extracted_k141_1832	1497	No	NA	0	0	Low-quality	Genome-fragment		2.49	0
S9_vs_k141_65292||full	1497	No	NA	0	0	Low-quality	Genome-fragment		4.37	0
S1_vs_k141_1144||full	1497	No	NA	3	0	Low-quality	Genome-fragment		4.31	0
S1_vs_k141_111313||full	1497	No	NA	1	0	Low-quality	Genome-fragment		4.01	0
S12_vs_k141_14922||full	1497	No	NA	0	1	Low-quality	Genome-fragment		1.22	0
S1_vs_k141_134935||full	1340	No	NA	0	1	Not-determined	Genome-fragment	NA		0
S6_vs_k141_8702||full	1497	No	NA	2	0	Low-quality	Genome-fragment		2.67	0
S8_extracted_k141_174010	1497	No	NA	0	2	Low-quality	Genome-fragment		0.94	0
S1_vs_k141_55011||full	1348	No	NA	0	2	Not-determined	Genome-fragment	NA		0
S8_extracted_k141_192038	1497	No	NA	1	0	Low-quality	Genome-fragment		2.32	0
S9_vs_k141_49234||full	1498	No	NA	1	0	Low-quality	Genome-fragment		3.09	0
S1_vs_k141_55032||full	3120	No	NA	0	1	Not-determined	Genome-fragment	NA		0
S1_vs_k141_108318||full	1653	No	NA	0	0	Not-determined	Genome-fragment	NA		0
S1_vs_k141_128440||full	1047	No	NA	0	1	Not-determined	Genome-fragment	NA		0
S1_vs_k141_41754||full	6172	No	NA	0	2	Not-determined	Genome-fragment	NA		0
S9_vs_k141_52995||full	1498	No	NA	2	0	Low-quality	Genome-fragment		3.2	0
S1_vs_k141_15337||full	6505	No	NA	0	2	Not-determined	Genome-fragment	NA		0
S1_vs_k141_34749||full	2061	No	NA	0	1	Not-determined	Genome-fragment	NA		0
S11_vs_k141_37716||full	1498	No	NA	0	1	Low-quality	Genome-fragment		1.87	0
S14_vs_k141_109894||full	1498	No	NA	0	0	Low-quality	Genome-fragment		2.88	0
S1_vs_k141_121573||full	1491	No	NA	0	1	Not-determined	Genome-fragment	NA		0
S1_vs_k141_48428||full	2078	No	NA	0	2	Not-determined	Genome-fragment	NA		0
S14_vs_k141_28423||full	1498	No	NA	0	0	Low-quality	Genome-fragment		3.38	0
S1_vs_k141_101835||full	1082	No	NA	0	1	Not-determined	Genome-fragment	NA		0
S1_extracted_k141_117652	1498	No	NA	0	1	Low-quality	Genome-fragment		1.96	0
S1_vs_k141_37922||full	1498	No	NA	2	0	Low-quality	Genome-fragment		2.45	0
S5_extracted_k141_131615	1498	No	NA	0	1	Low-quality	Genome-fragment		1.77	0
S1_vs_k141_8461||full	1740	No	NA	0	1	Not-determined	Genome-fragment	NA		0
S10_extracted_15889	1498	No	NA	0	1	Low-quality	Genome-fragment		3.82	0
S17_extracted_k141_82683	1499	No	NA	0	2	Low-quality	Genome-fragment		0.71	0
S9_vs_k141_45469||full	1499	No	NA	0	1	Low-quality	Genome-fragment		1.33	0
S16_vs_109445||full	1499	No	NA	2	0	Low-quality	Genome-fragment		2.83	0
S10_vs_38278||full	1499	No	NA	0	1	Low-quality	Genome-fragment		4.23	0
S11_vs_k141_24819||lt2gene	1500	No	NA	1	0	Low-quality	Genome-fragment		3.79	0
S14_extracted_k141_62978	1500	No	NA	1	0	Low-quality	Genome-fragment		2.52	0
S1_vs_k141_90132||lt2gene	1500	No	NA	1	0	Low-quality	Genome-fragment		0.97	0
S1_vs_k141_15413||full	5143	No	NA	0	1	Not-determined	Genome-fragment	NA		0
S1_vs_k141_75149||full	8778	No	NA	0	6	Not-determined	Genome-fragment	NA		0
S3_vs_k141_80771||full	1500	No	NA	0	0	Low-quality	Genome-fragment		2.64	0
S1_vs_k141_8517||full	1147	No	NA	0	0	Not-determined	Genome-fragment	NA		0
S3_vs_k141_103446||lt2gene	1500	No	NA	2	0	Low-quality	Genome-fragment		1.07	0
S1_vs_k141_15446||full	1306	No	NA	0	1	Not-determined	Genome-fragment	NA		0
S10_vs_4673||full	1500	No	NA	1	0	Low-quality	Genome-fragment		2.88	0
S1_vs_k141_75193||full	2576	No	NA	0	1	Not-determined	Genome-fragment	NA		0
S10_vs_24357||full	1500	No	NA	0	0	Low-quality	Genome-fragment		4.13	0
S1_vs_k141_88467||full	1318	No	NA	0	1	Not-determined	Genome-fragment	NA		0
S1_vs_k141_154596||full	3147	No	NA	0	3	Not-determined	Genome-fragment	NA		0
S12_vs_k141_76139||full	1500	No	NA	0	0	Low-quality	Genome-fragment		2.89	0
S1_vs_k141_154608||full	5978	No	NA	0	6	Not-determined	Genome-fragment	NA		0
S17_vs_k141_129213||lt2gene	1501	No	NA	1	0	Low-quality	Genome-fragment		2.63	0
S1_vs_k141_88512||full	1020	No	NA	0	0	Not-determined	Genome-fragment	NA		0
S1_vs_k141_61059||full	1089	No	NA	0	0	Not-determined	Genome-fragment	NA		0
S11_vs_k141_79681||full	1501	No	NA	0	0	Low-quality	Genome-fragment		3.45	0
S1_vs_k141_8591||full	5231	No	NA	0	3	Not-determined	Genome-fragment	NA		0
S1_vs_k141_68518||full	3310	No	NA	0	2	Not-determined	Genome-fragment	NA		0
S14_vs_k141_15631||full	1501	No	NA	0	0	Low-quality	Genome-fragment		3.71	0
S1_vs_k141_48597||full	1029	No	NA	0	0	Not-determined	Genome-fragment	NA		0
S1_vs_k141_15540||full	2061	No	NA	0	0	Not-determined	Genome-fragment	NA		0
S3_vs_k141_112710||full	1501	No	NA	1	0	Low-quality	Genome-fragment		3.6	0
S1_vs_k141_115256||full	2231	No	NA	0	0	Not-determined	Genome-fragment	NA		0
S5_vs_k141_156||full	1501	No	NA	2	0	Low-quality	Genome-fragment		2.41	0
S1_vs_k141_141964||full	2950	No	NA	0	3	Not-determined	Genome-fragment	NA		0
S7_vs_10561||full	1501	No	NA	0	1	Low-quality	Genome-fragment		1.26	0
S1_vs_k141_21832||full	5391	No	NA	0	6	Not-determined	Genome-fragment	NA		0
S12_extracted_k141_14602	1501	No	NA	1	0	Low-quality	Genome-fragment		3.83	0
S12_vs_k141_91868||full	1501	No	NA	1	0	Low-quality	Genome-fragment		2.9	0
S12_vs_k141_147282||full	1501	No	NA	0	0	Low-quality	Genome-fragment		3.18	0
S12_vs_k141_148474||full	1501	No	NA	0	0	Low-quality	Genome-fragment		0.84	0
S1_vs_k141_61092||full	1014	No	NA	0	0	Not-determined	Genome-fragment	NA		0
S6_vs_k141_49906||full	1501	No	NA	1	0	Low-quality	Genome-fragment		2.62	0
S1_vs_k141_75354||full	1294	No	NA	0	1	Not-determined	Genome-fragment	NA		0
S1_vs_k141_28600||full	2250	No	NA	0	2	Not-determined	Genome-fragment	NA		0
S6_vs_k141_27445||full	1501	No	NA	0	0	Low-quality	Genome-fragment		4.26	0
S9_extracted_k141_70925	1502	No	NA	0	0	Low-quality	Genome-fragment		1.38	0
S9_vs_k141_18162||full	1502	No	NA	0	2	Low-quality	Genome-fragment		0.8	0
S13_vs_34701||full	1502	No	NA	3	0	Low-quality	Genome-fragment		2.46	0
S13_vs_53690||full	1502	No	NA	1	0	Low-quality	Genome-fragment		3.63	0
S1_vs_k141_68889||full	1502	No	NA	1	0	Low-quality	Genome-fragment		4.23	0
S3_vs_k141_126464||full	1502	No	NA	0	0	Low-quality	Genome-fragment		2.73	0
S3_vs_k141_80041||lt2gene	1502	No	NA	2	0	Low-quality	Genome-fragment		3.21	0
S1_vs_k141_75403||full	8872	No	NA	0	5	Not-determined	Genome-fragment	NA		0
S5_extracted_k141_98794	1502	No	NA	1	0	Low-quality	Genome-fragment		4.17	0
S1_vs_k141_68686||full	1291	No	NA	0	3	Not-determined	Genome-fragment	NA		0
S1_vs_k141_8760||full	1073	No	NA	0	0	Not-determined	Genome-fragment	NA		0
S5_vs_k141_138995||lt2gene	1502	No	NA	0	0	Low-quality	Genome-fragment		4.61	0
S7_vs_70290||full	1502	No	NA	0	2	Low-quality	Genome-fragment		0.65	0
S1_vs_k141_48783||full	1206	No	NA	0	1	Not-determined	Genome-fragment	NA		0
S1_vs_k141_21936||full	2382	No	NA	0	1	Not-determined	Genome-fragment	NA		0
S16_extracted_72283	1502	No	NA	0	0	Low-quality	Genome-fragment		2.36	0
S1_vs_k141_102196||full	3843	No	NA	0	5	Not-determined	Genome-fragment	NA		0
S1_vs_k141_48796||full	1422	No	NA	0	1	Not-determined	Genome-fragment	NA		0
S1_vs_k141_82190||full	2823	No	NA	0	2	Not-determined	Genome-fragment	NA		0
S10_extracted_85642	1502	No	NA	0	0	Low-quality	Genome-fragment		2.27	0
S1_vs_k141_1696||full	1403	No	NA	0	1	Not-determined	Genome-fragment	NA		0
S10_vs_40985||full	1502	No	NA	0	0	Low-quality	Genome-fragment		2.78	0
S15_extracted_38566	1502	No	NA	0	1	Low-quality	Genome-fragment		0.56	0
S2_extracted_k141_18690	1502	No	NA	0	1	Low-quality	Genome-fragment		0.61	0
S1_vs_k141_68723||full	2570	No	NA	0	2	Not-determined	Genome-fragment	NA		0
S1_vs_k141_88772||full	1381	No	NA	0	0	Not-determined	Genome-fragment	NA		0
S1_vs_k141_88777||full	4338	No	NA	0	2	Not-determined	Genome-fragment	NA		0
S6_extracted_k141_9626	1502	No	NA	2	0	Low-quality	Genome-fragment		2.78	0
S1_vs_k141_35057||full	1584	No	NA	0	0	Not-determined	Genome-fragment	NA		0
S6_vs_k141_48295||full	1502	No	NA	1	0	Low-quality	Genome-fragment		4.21	0
S1_vs_k141_95592||full	2377	No	NA	0	2	Not-determined	Genome-fragment	NA		0
S1_vs_k141_121990||full	1241	No	NA	0	0	Not-determined	Genome-fragment	NA		0
S8_vs_k141_176184||full	1502	No	NA	1	0	Low-quality	Genome-fragment		3.29	0
S1_vs_k141_75515||full	10470	No	NA	0	2	Not-determined	Genome-fragment	NA		0
S1_vs_k141_82263||full	1090	No	NA	0	1	Not-determined	Genome-fragment	NA		0
S9_vs_k141_82634||full	1503	No	NA	0	0	Low-quality	Genome-fragment		3.95	0
S11_vs_k141_40002||full	1503	No	NA	1	0	Low-quality	Genome-fragment		3.22	0
S1_vs_k141_154851||full	1186	No	NA	0	1	Not-determined	Genome-fragment	NA		0
S1_vs_k141_82307||full	5383	No	NA	0	3	Not-determined	Genome-fragment	NA		0
S1_vs_k141_28822||full	2160	No	NA	0	0	Not-determined	Genome-fragment	NA		0
S1_vs_k141_22105||full	3011	No	NA	0	0	Not-determined	Genome-fragment	NA		0
S1_vs_k141_35134||full	3044	No	NA	0	3	Not-determined	Genome-fragment	NA		0
S11_vs_k141_102210||full	1503	No	NA	1	0	Low-quality	Genome-fragment		3.41	0
S12_extracted_k141_169020	1503	No	NA	0	1	Low-quality	Genome-fragment		0.99	0
S1_vs_k141_8955||full	3002	No	NA	0	1	Not-determined	Genome-fragment	NA		0
S12_vs_k141_238||full	1503	No	NA	3	0	Low-quality	Genome-fragment		1.88	0
S1_vs_k141_42301||full	1758	No	NA	0	1	Not-determined	Genome-fragment	NA		0
S1_vs_k141_35182||full	1350	No	NA	0	0	Not-determined	Genome-fragment	NA		0
S1_vs_k141_16041||full	5415	No	NA	0	2	Not-determined	Genome-fragment	NA		0
S12_vs_k141_14382||full	1503	No	NA	1	0	Low-quality	Genome-fragment		3.69	0
S1_vs_k141_75659||full	4347	No	NA	0	2	Not-determined	Genome-fragment	NA		0
S8_vs_k141_106204||full	1503	No	NA	0	0	Low-quality	Genome-fragment		3.57	0
S8_vs_k141_185701||full	1503	No	NA	0	1	Low-quality	Genome-fragment		1.76	0
S8_vs_k141_60058||full	1503	No	NA	0	1	Low-quality	Genome-fragment		0.36	0
S9_extracted_k141_23833	1504	No	NA	0	1	Low-quality	Genome-fragment		3.11	0
S1_vs_k141_82405||full	3623	No	NA	0	2	Not-determined	Genome-fragment	NA		0
S1_vs_k141_122195||full	1387	No	NA	0	1	Not-determined	Genome-fragment	NA		0
S11_extracted_k141_22197	1504	No	NA	1	0	Low-quality	Genome-fragment		3.59	0
S1_vs_k141_122206||full	3004	No	NA	0	0	Not-determined	Genome-fragment	NA		0
S14_vs_k141_36876||full	1504	No	NA	2	0	Low-quality	Genome-fragment		2.64	0
S14_vs_k141_99299||full	1504	No	NA	2	0	Low-quality	Genome-fragment		3.75	0
S1_vs_k141_16117||full	1110	No	NA	0	0	Not-determined	Genome-fragment	NA		0
S14_vs_k141_62003||lt2gene	1504	No	NA	2	0	Low-quality	Genome-fragment		3.86	0
S16_vs_38912||full	1504	No	NA	0	0	Low-quality	Genome-fragment		4.18	0
S12_vs_k141_161809||full	1504	No	NA	0	0	Low-quality	Genome-fragment		2.21	0
S1_vs_k141_122257||full	1604	No	NA	0	0	Not-determined	Genome-fragment	NA		0
S9_extracted_k141_13348	1505	No	NA	2	0	Low-quality	Genome-fragment		0.86	0
S11_extracted_k141_30889	1505	No	NA	0	0	Low-quality	Genome-fragment		3.65	0
S1_vs_k141_28994||full	10611	No	NA	0	9	Not-determined	Genome-fragment	NA		0
S1_vs_k141_102522||full	2342	No	NA	0	3	Not-determined	Genome-fragment	NA		0
S1_vs_k141_142408||full	3908	No	NA	0	2	Not-determined	Genome-fragment	NA		0
S1_vs_k141_22216||full	8041	No	NA	0	6	Not-determined	Genome-fragment	NA		0
S14_extracted_k141_2730	1505	No	NA	2	0	Low-quality	Genome-fragment		4.83	0
S3_extracted_k141_81982	1505	No	NA	1	0	Low-quality	Genome-fragment		0.9	0
S1_vs_k141_109039||full	1301	No	NA	0	1	Not-determined	Genome-fragment	NA		0
S1_vs_k141_16203||full	2647	No	NA	0	1	Not-determined	Genome-fragment	NA		0
S1_vs_k141_16216||full	1001	No	NA	0	0	Not-determined	Genome-fragment	NA		0
S1_vs_k141_75836||full	1568	No	NA	0	1	Not-determined	Genome-fragment	NA		0
S1_vs_k141_109088||full	3552	No	NA	0	0	Not-determined	Genome-fragment	NA		0
S10_extracted_15917	1505	No	NA	0	2	Low-quality	Genome-fragment		0.48	0
S1_vs_k141_55858||full	1145	No	NA	0	0	Not-determined	Genome-fragment	NA		0
S6_extracted_k141_44267	1505	No	NA	4	0	Low-quality	Genome-fragment		3.78	0
S14_vs_k141_68515||full	1506	No	NA	0	0	Low-quality	Genome-fragment		3.54	0
S14_vs_k141_107992||full	1506	No	NA	3	0	Low-quality	Genome-fragment		3.07	0
S1_vs_k141_109128||full	1302	No	NA	0	0	Not-determined	Genome-fragment	NA		0
S1_extracted_k141_81250	1506	No	NA	1	0	Low-quality	Genome-fragment		1.91	0
S1_vs_k141_155039||full	2093	No	NA	0	2	Not-determined	Genome-fragment	NA		0
S7_vs_58691||full	1506	No	NA	0	0	Low-quality	Genome-fragment		2.29	0
S7_vs_114260||full	1506	No	NA	2	0	Low-quality	Genome-fragment		3.31	0
S12_extracted_k141_24553	1506	No	NA	1	0	Low-quality	Genome-fragment		2.33	0
S1_vs_k141_82604||full	1872	No	NA	0	0	Not-determined	Genome-fragment	NA		0
S1_vs_k141_135847||full	4568	No	NA	0	2	Not-determined	Genome-fragment	NA		0
S1_vs_k141_16300||full	1514	No	NA	0	2	Not-determined	Genome-fragment	NA		0
S1_vs_k141_147943||full	1064	No	NA	0	1	Not-determined	Genome-fragment	NA		0
S1_vs_k141_129321||full	3811	No	NA	0	0	Not-determined	Genome-fragment	NA		0
S1_vs_k141_155058||full	1769	No	NA	0	0	Not-determined	Genome-fragment	NA		0

S12_vs_k141_187265||full	1506	No	NA	0	0	Low-quality	Genome-fragment	2.57	0
S8_extracted_k141_73613	1506	No	NA	0	0	Low-quality	Genome-fragment	1.94	0
S1_vs_k141_82662||full	3142	No	NA	0	1	Not-determined	Genome-fragment	NA	0
S1_vs_k141_102687||full	1891	No	NA	0	2	Not-determined	Genome-fragment	NA	0
S1_vs_k141_22361||full	1904	No	NA	0	2	Not-determined	Genome-fragment	NA	0
S1_vs_k141_89189||full	1346	No	NA	0	0	Not-determined	Genome-fragment	NA	0
S1_vs_k141_22371||full	1896	No	NA	0	0	Not-determined	Genome-fragment	NA	0
S8_vs_k141_179623||full	1506	No	NA	0	0	Low-quality	Genome-fragment	0.5	0
S1_vs_k141_82691||full	1412	No	NA	0	3	Not-determined	Genome-fragment	NA	0
S9_vs_k141_59994||full	1507	No	NA	0	1	Low-quality	Genome-fragment	2.67	0
S14_vs_k141_113117||full	1507	No	NA	1	0	Low-quality	Genome-fragment	4.08	0
S14_vs_k141_67369||lt2gene	1507	No	NA	1	0	Low-quality	Genome-fragment	3.87	0
S1_vs_k141_9343||full	1024	No	NA	0	0	Not-determined	Genome-fragment	NA	0
S1_vs_k141_56020||full	1382	No	NA	0	0	Not-determined	Genome-fragment	NA	0
S1_extracted_k141_50205	1507	No	NA	0	1	Low-quality	Genome-fragment	0.26	0
S5_vs_k141_15042||full	1507	No	NA	0	0	Low-quality	Genome-fragment	3.38	0
S1_vs_k141_69231||full	5411	No	NA	0	1	Not-determined	Genome-fragment	NA	0
S1_vs_k141_109289||full	1094	No	NA	0	1	Not-determined	Genome-fragment	NA	0
S5_vs_k141_99502||full	1507	No	NA	1	0	Low-quality	Genome-fragment	3.4	0
S7_vs_20432||full	1507	No	NA	1	0	Low-quality	Genome-fragment	2.33	0
S10_extracted_83392	1507	No	NA	0	1	Low-quality	Genome-fragment	0.57	0
S15_vs_52164||full	1507	No	NA	0	0	Low-quality	Genome-fragment	5.22	0
S6_vs_k141_35734||full	1507	No	NA	0	2	Low-quality	Genome-fragment	1.13	0
S6_vs_k141_24632||full	1507	No	NA	0	1	Low-quality	Genome-fragment	3.75	0
S1_vs_k141_142716||full	1071	No	NA	0	1	Not-determined	Genome-fragment	NA	0
S1_vs_k141_9403||full	1876	No	NA	0	2	Not-determined	Genome-fragment	NA	0
S1_vs_k141_35492||full	1228	No	NA	0	0	Not-determined	Genome-fragment	NA	0
S17_vs_k141_17544||full	1508	No	NA	0	1	Low-quality	Genome-fragment	1.79	0
S9_extracted_k141_84567	1508	No	NA	2	0	Low-quality	Genome-fragment	3.36	0
S11_vs_k141_1011||full	1508	No	NA	3	0	Low-quality	Genome-fragment	3.41	0
S14_vs_k141_103462||full	1508	No	NA	0	1	Low-quality	Genome-fragment	1.45	0
S1_vs_k141_96192||full	3538	No	NA	0	1	Not-determined	Genome-fragment	NA	0
S3_vs_k141_48981||full	1508	No	NA	2	0	Low-quality	Genome-fragment	3.46	0
S1_vs_k141_76106||full	4018	No	NA	0	2	Not-determined	Genome-fragment	NA	0
S1_vs_k141_56110||full	1206	No	NA	0	0	Not-determined	Genome-fragment	NA	0
S3_vs_k141_30955||full	1508	No	NA	2	0	Low-quality	Genome-fragment	3.98	0
S1_vs_k141_96231||full	1314	No	NA	0	0	Not-determined	Genome-fragment	NA	0
S1_vs_k141_142793||full	3273	No	NA	0	3	Not-determined	Genome-fragment	NA	0
S1_vs_k141_49539||full	2576	No	NA	0	0	Not-determined	Genome-fragment	NA	0
S5_vs_k141_7756||full	1508	No	NA	0	0	Low-quality	Genome-fragment	2.59	0
S16_vs_49456||full	1508	No	NA	0	1	Low-quality	Genome-fragment	1.04	0
S1_vs_k141_2251||full	2079	No	NA	0	0	Not-determined	Genome-fragment	NA	0
S1_vs_k141_155183||full	30775	No	NA	12	0	Medium-quality	Genome-fragment	74.09	0
S10_extracted_95257	1508	No	NA	0	1	Low-quality	Genome-fragment	1.01	0
S8_vs_k141_127191||full	1508	No	NA	0	0	Low-quality	Genome-fragment	3.19	0
S1_vs_k141_49565||full	1146	No	NA	0	0	Not-determined	Genome-fragment	NA	0
S8_vs_k141_207314||full	1508	No	NA	0	1	Low-quality	Genome-fragment	0.92	0
S1_vs_k141_102915||full	1119	No	NA	0	0	Not-determined	Genome-fragment	NA	0
S17_extracted_k141_33778	1509	No	NA	1	0	Low-quality	Genome-fragment	3.38	0
S9_extracted_k141_76527	1509	No	NA	3	0	Low-quality	Genome-fragment	2.32	0
S11_vs_k141_53070||lt2gene	1509	No	NA	1	0	Low-quality	Genome-fragment	1.74	0
S14_extracted_k141_63150	1509	No	NA	1	0	Low-quality	Genome-fragment	1.18	0
S1_vs_k141_42883||full	1064	No	NA	0	0	Not-determined	Genome-fragment	NA	0
S14_vs_k141_102751||full	1509	No	NA	1	0	Low-quality	Genome-fragment	4.42	0
S16_vs_71762||full	1509	No	NA	2	0	Low-quality	Genome-fragment	2.51	0
S10_extracted_15520	1509	No	NA	0	0	Low-quality	Genome-fragment	2.66	0
S1_vs_k141_49621||full	1270	No	NA	0	2	Not-determined	Genome-fragment	NA	0
S1_vs_k141_9574||full	2795	No	NA	0	3	Not-determined	Genome-fragment	NA	0
S1_vs_k141_9575||full	1167	No	NA	0	0	Not-determined	Genome-fragment	NA	0
S12_extracted_k141_119422	1509	No	NA	1	0	Low-quality	Genome-fragment	1.23	0
S8_extracted_k141_14804	1509	No	NA	1	0	Low-quality	Genome-fragment	1.41	0
S1_vs_k141_9610||full	3585	No	NA	0	0	Not-determined	Genome-fragment	NA	0
S1_vs_k141_103022||full	1912	No	NA	0	1	Not-determined	Genome-fragment	NA	0
S8_vs_k141_151332||lt2gene	1509	No	NA	2	0	Low-quality	Genome-fragment	4.17	0
S1_vs_k141_22687||full	2430	No	NA	0	0	Not-determined	Genome-fragment	NA	0
S1_vs_k141_83035||full	1463	No	NA	0	2	Not-determined	Genome-fragment	NA	0
S9_extracted_k141_86608	1510	No	NA	0	0	Low-quality	Genome-fragment	4.28	0
S9_vs_k141_58730||full	1510	No	NA	1	0	Low-quality	Genome-fragment	2.96	0
S14_extracted_k141_24935	1510	No	NA	1	0	Low-quality	Genome-fragment	3.38	0
S1_vs_k141_69490||full	1618	No	NA	0	0	Not-determined	Genome-fragment	NA	0
S3_extracted_k141_60749	1510	No	NA	0	0	Low-quality	Genome-fragment	1.45	0
S3_vs_k141_47443||full	1510	No	NA	0	0	Low-quality	Genome-fragment	2.68	0
S1_vs_k141_22716||full	1264	No	NA	0	1	Not-determined	Genome-fragment	NA	0
S16_vs_40111||full	1510	No	NA	0	1	Low-quality	Genome-fragment	3.59	0
S1_vs_k141_116400||full	2516	No	NA	0	2	Not-determined	Genome-fragment	NA	0
S1_vs_k141_49763||full	3479	No	NA	0	4	Not-determined	Genome-fragment	NA	0
S4_extracted_6674	1510	No	NA	0	2	Low-quality	Genome-fragment	0.8	0
S6_vs_k141_33214||full	1510	No	NA	0	1	Low-quality	Genome-fragment	2.98	0
S17_vs_k141_45968||full	1511	No	NA	1	0	Low-quality	Genome-fragment	3.94	0
S1_vs_k141_122805||full	2984	No	NA	0	0	Not-determined	Genome-fragment	NA	0
S11_extracted_k141_75426	1511	No	NA	0	2	Low-quality	Genome-fragment	2.54	0
S13_extracted_87962	1511	No	NA	0	0	Low-quality	Genome-fragment	0.95	0
S14_extracted_k141_101845	1511	No	NA	0	0	Low-quality	Genome-fragment	28.43	0
S7_vs_42464||full	1511	No	NA	1	0	Low-quality	Genome-fragment	2.4	0
S16_extracted_147795	1511	No	NA	0	0	Low-quality	Genome-fragment	1.78	0
S1_vs_k141_148101||full	1629	No	NA	0	1	Not-determined	Genome-fragment	NA	0
S12_vs_k141_134462||full	1511	No	NA	0	2	Low-quality	Genome-fragment	2.98	0
S1_vs_k141_89615||full	1596	No	NA	0	0	Not-determined	Genome-fragment	NA	0
S2_extracted_k141_16549	1511	No	NA	0	0	Low-quality	Genome-fragment	2.47	0
S11_extracted_k141_66322	1512	No	NA	0	1	Low-quality	Genome-fragment	0.44	0
S11_extracted_k141_34466	1512	No	NA	0	1	Low-quality	Genome-fragment	0.86	0
S1_vs_k141_83124||full	1125	No	NA	0	0	Not-determined	Genome-fragment	NA	0
S11_vs_k141_94560||full	1512	No	NA	0	0	Low-quality	Genome-fragment	2.55	0
S1_vs_k141_61792||full	1716	No	NA	0	2	Not-determined	Genome-fragment	NA	0
S13_vs_477||full	1512	No	NA	1	0	Low-quality	Genome-fragment	3.01	0
S1_vs_k141_35758||full	1313	No	NA	0	2	Not-determined	Genome-fragment	NA	0
S14_extracted_k141_25133	1512	No	NA	4	0	Low-quality	Genome-fragment	4.99	0
S1_vs_k141_134022||lt2gene	1512	No	NA	1	0	Low-quality	Genome-fragment	3.5	0
S1_vs_k141_122871||full	6345	No	NA	0	2	Not-determined	Genome-fragment	NA	0
S1_vs_k141_136355||full	6839	No	NA	0	3	Not-determined	Genome-fragment	NA	0
S1_vs_k141_56370||full	2720	No	NA	0	1	Not-determined	Genome-fragment	NA	0
S1_vs_k141_16880||full	1065	No	NA	0	1	Not-determined	Genome-fragment	NA	0
S5_vs_k141_79812||full	1512	No	NA	1	0	Low-quality	Genome-fragment	2.8	0
S16_vs_80406||full	1512	No	NA	1	0	Low-quality	Genome-fragment	1.67	0
S1_vs_k141_76430||full	1136	No	NA	0	0	Not-determined	Genome-fragment	NA	0
S1_vs_k141_61824||full	3488	No	NA	0	1	Not-determined	Genome-fragment	NA	0
S16_vs_116577||full	1512	No	NA	0	1	Low-quality	Genome-fragment	0.99	0
S1_vs_k141_35773||full	1465	No	NA	0	1	Not-determined	Genome-fragment	NA	0
S10_vs_55748||full	1512	No	NA	0	2	Low-quality	Genome-fragment	0.66	0
S8_vs_k141_85406||full	1512	Yes	1017	1	1	Low-quality	Genome-fragment	2.13	32.74
S17_extracted_k141_106256	1513	No	NA	0	0	Low-quality	Genome-fragment	2.67	0
S1_vs_k141_2565||full	1699	No	NA	0	1	Not-determined	Genome-fragment	NA	0
S1_vs_k141_69668||full	1900	No	NA	0	3	Not-determined	Genome-fragment	NA	0
S17_vs_k141_138661||full	1513	No	NA	2	0	Low-quality	Genome-fragment	2.32	0
S1_vs_k141_89710||full	4505	No	NA	0	1	Not-determined	Genome-fragment	NA	0
S14_extracted_k141_39046	1513	No	NA	3	0	Low-quality	Genome-fragment	4.99	0
S14_extracted_k141_1919	1513	No	NA	0	1	Low-quality	Genome-fragment	0.28	0
S1_vs_k141_61897||full	1454	No	NA	0	0	Not-determined	Genome-fragment	NA	0
S5_extracted_k141_135065	1513	No	NA	0	1	Low-quality	Genome-fragment	0.14	0
S5_vs_k141_90054||full	1513	No	NA	0	0	Low-quality	Genome-fragment	11.18	0
S7_extracted_100045	1513	No	NA	2	0	Low-quality	Genome-fragment	1.09	0
S1_vs_k141_103321||full	4678	No	NA	0	1	Not-determined	Genome-fragment	NA	0
S1_vs_k141_22905||full	2038	No	NA	0	3	Not-determined	Genome-fragment	NA	0
S7_vs_47417||lt2gene	1513	No	NA	2	0	Low-quality	Genome-fragment	3	0
S1_vs_k141_122999||full	1444	No	NA	0	1	Not-determined	Genome-fragment	NA	0
S10_vs_74095||full	1513	No	NA	0	1	Low-quality	Genome-fragment	3.27	0
S1_vs_k141_61947||full	5430	No	NA	0	2	Not-determined	Genome-fragment	NA	0
S11_extracted_k141_41664	1514	No	NA	0	0	Low-quality	Genome-fragment	2.44	0
S1_vs_k141_29833||full	4086	No	NA	0	1	Not-determined	Genome-fragment	NA	0
S13_extracted_131317	1514	No	NA	0	3	Low-quality	Genome-fragment	0.26	0
S1_vs_k141_129979||full	1565	No	NA	0	1	Not-determined	Genome-fragment	NA	0
S13_vs_131210||full	1514	No	NA	0	0	Low-quality	Genome-fragment	2.35	0
S14_vs_k141_25384||full	1514	No	NA	0	0	Low-quality	Genome-fragment	3.54	0
S1_vs_k141_123034||full	1832	No	NA	0	2	Not-determined	Genome-fragment	NA	0
S1_vs_k141_116699||full	1267	No	NA	0	2	Not-determined	Genome-fragment	NA	0
S1_extracted_k141_102363	1514	No	NA	0	1	Low-quality	Genome-fragment	1.11	0
S1_extracted_k141_147906	1514	Yes	1220	1	2	Low-quality	Genome-fragment	2.53	19.42
S1_vs_k141_64779||full	1514	No	NA	1	0	Low-quality	Genome-fragment	3.64	0
S3_extracted_k141_89960	1514	No	NA	1	0	Low-quality	Genome-fragment	2.36	0
S1_vs_k141_62040||full	1807	No	NA	0	2	Not-determined	Genome-fragment	NA	0
S3_vs_k141_108852||full	1514	Yes	680	1	1	Low-quality	Genome-fragment	0.35	55.09
S1_vs_k141_123082||full	2195	No	NA	0	2	Not-determined	Genome-fragment	NA	0
S3_vs_k141_35714||lt2gene	1514	No	NA	1	0	Low-quality	Genome-fragment	3.2	0
S5_extracted_k141_108693	1514	No	NA	0	1	Low-quality	Genome-fragment	0.61	0
S1_vs_k141_62045||full	9875	No	NA	0	4	Not-determined	Genome-fragment	NA	0
S5_extracted_k141_84705	1514	No	NA	0	1	Low-quality	Genome-fragment	1.91	0
S5_vs_k141_69674||lt2gene	1514	No	NA	1	0	Low-quality	Genome-fragment	4.51	0
S1_vs_k141_123094||full	2269	No	NA	0	0	Not-determined	Genome-fragment	NA	0
S7_extracted_14624	1514	No	NA	0	1	Low-quality	Genome-fragment	0.4	0
S1_vs_k141_83426||full	2031	No	NA	0	2	Not-determined	Genome-fragment	NA	0
S7_vs_4708||full	1514	No	NA	1	0	Low-quality	Genome-fragment	2.19	0
S16_vs_8589||full	1514	No	NA	0	0	Low-quality	Genome-fragment	3.42	0
S1_vs_k141_143236||full	2718	No	NA	0	2	Not-determined	Genome-fragment	NA	0
S16_vs_27232||full	1514	No	NA	0	0	Low-quality	Genome-fragment	2.7	0
S16_vs_169915||full	1514	No	NA	1	0	Low-quality	Genome-fragment	3.12	0
S1_vs_k141_2729||full	2271	No	NA	0	0	Not-determined	Genome-fragment	NA	0
S1_vs_k141_29992||full	3026	No	NA	0	1	Not-determined	Genome-fragment	NA	0
S10_vs_107526||full	1514	No	NA	0	0	Low-quality	Genome-fragment	2.87	0
S6_vs_k141_15030||full	1514	No	NA	0	0	Low-quality	Genome-fragment	4.71	0
S1_vs_k141_56700||full	1210	No	NA	0	1	Not-determined	Genome-fragment	NA	0
S8_vs_k141_32265||full	1514	No	NA	0	0	Low-quality	Genome-fragment	2.33	0
S17_vs_k141_59941||lt2gene	1515	No	NA	1	0	Low-quality	Genome-fragment	3.22	0
S11_vs_k141_95401||full	1515	No	NA	1	0	Low-quality	Genome-fragment	3.73	0
S14_extracted_k141_62996	1515	No	NA	1	0	Low-quality	Genome-fragment	2.54	0
S1_vs_k141_96904||full	4371	No	NA	0	0	Not-determined	Genome-fragment	NA	0
S1_vs_k141_56165||full	1515	No	NA	0	0	Low-quality	Genome-fragment	0.63	0
S1_vs_k141_24066||full	1515	No	NA	0	0	Low-quality	Genome-fragment	3.14	0
S1_vs_k141_50243||full	1050	No	NA	0	0	Not-determined	Genome-fragment	NA	0
S5_extracted_k141_95237	1515	No	NA	2	0	Low-quality	Genome-fragment	2.59	0
S10_vs_84692||full	1515	No	NA	0	1	Low-quality	Genome-fragment	1.04	0
S10_vs_121091||full	1515	No	NA	1	0	Low-quality	Genome-fragment	2	0
S1_vs_k141_143302||full	5037	No	NA	0	3	Not-determined	Genome-fragment	NA	0
S1_vs_k141_123250||full	1030	No	NA	0	0	Not-determined	Genome-fragment	NA	0
S12_extracted_k141_91874	1515	No	NA	0	0	Low-quality	Genome-fragment	3	0

S6_vs_k141_36360||full	1515	No	NA	2	0	Low-quality	Genome-fragment		3.04	0
S1_vs_k141_83575||full	1568	No	NA	0	1	Not-determined	Genome-fragment	NA		0
S6_vs_k141_55606||full	1515	No	NA	0	0	Low-quality	Genome-fragment		2.57	0
S8_extracted_k141_65820	1515	No	NA	0	1	Low-quality	Genome-fragment		2.53	0
S1_vs_k141_96972||full	1080	No	NA	0	0	Not-determined	Genome-fragment	NA		0
S1_vs_k141_83594||full	1122	No	NA	0	0	Not-determined	Genome-fragment	NA		0
S1_vs_k141_136718||full	1743	No	NA	0	1	Not-determined	Genome-fragment	NA		0
S8_extracted_k141_137477	1515	No	NA	0	0	Low-quality	Genome-fragment		2.3	0
S1_vs_k141_30131||full	1692	No	NA	0	1	Not-determined	Genome-fragment	NA		0
S17_vs_k141_138439||full	1516	No	NA	1	0	Low-quality	Genome-fragment		3.18	0
S9_vs_k141_35276||full	1516	No	NA	2	0	Low-quality	Genome-fragment		4.48	0
S11_extracted_k141_59809	1516	No	NA	0	0	Low-quality	Genome-fragment		2.41	0
S11_extracted_k141_79842	1516	No	NA	0	0	Low-quality	Genome-fragment		2.4	0
S13_vs_131640||full	1516	No	NA	0	0	Low-quality	Genome-fragment		2.92	0
S1_vs_k141_83677||full	3369	No	NA	0	3	Not-determined	Genome-fragment	NA		0
S14_vs_k141_113430||full	1516	No	NA	2	0	Low-quality	Genome-fragment		4.02	0
S1_vs_k141_136781||full	2173	No	NA	0	2	Not-determined	Genome-fragment	NA		0
S1_vs_k141_90107||full	1816	No	NA	0	3	Not-determined	Genome-fragment	NA		0
S1_vs_k141_9902||full	1516	No	NA	0	1	Low-quality	Genome-fragment		1	0
S1_vs_k141_83704||full	2296	No	NA	0	1	Not-determined	Genome-fragment	NA		0
S5_vs_k141_122627||full	1516	No	NA	0	0	Low-quality	Genome-fragment		3.87	0
S7_extracted_38846	1516	No	NA	0	1	Low-quality	Genome-fragment		1.19	0
S7_vs_37889||full	1516	No	NA	1	0	Low-quality	Genome-fragment		5.99	0
S1_vs_k141_136821||full	1554	No	NA	0	2	Not-determined	Genome-fragment	NA		0
S16_extracted_82201	1516	No	NA	0	0	Low-quality	Genome-fragment		4.18	0
S12_extracted_k141_138605	1516	No	NA	1	0	Low-quality	Genome-fragment		2.22	0
S4_extracted_7485	1516	No	NA	5	0	Low-quality	Genome-fragment		5.01	0
S1_vs_k141_50446||full	2252	No	NA	0	2	Not-determined	Genome-fragment	NA		0
S4_extracted_18990	1516	No	NA	0	0	Low-quality	Genome-fragment		2.15	0
S1_vs_k141_136903||full	2033	No	NA	0	3	Not-determined	Genome-fragment	NA		0
S8_vs_k141_55708||full	1516	No	NA	0	1	Low-quality	Genome-fragment		1.78	0
S1_vs_k141_123489||full	1525	No	NA	0	1	Not-determined	Genome-fragment	NA		0
S1_vs_k141_103765||full	1406	No	NA	0	0	Not-determined	Genome-fragment	NA		0
S1_vs_k141_17466||full	6837	No	NA	0	1	Not-determined	Genome-fragment	NA		0
S17_vs_k141_104025||full	1517	No	NA	0	0	Low-quality	Genome-fragment		3.44	0
S11_extracted_k141_86595	1517	No	NA	0	0	Low-quality	Genome-fragment		3.58	0
S1_vs_k141_23408||full	1247	No	NA	0	2	Not-determined	Genome-fragment	NA		0
S1_vs_k141_62409||full	16910	No	NA	10	0	Medium-quality	Genome-fragment		50	0
S1_vs_k141_90265||full	1958	No	NA	0	2	Not-determined	Genome-fragment	NA		0
S1_vs_k141_136968||full	2037	No	NA	0	1	Not-determined	Genome-fragment	NA		0
S11_vs_k141_42277||full	1517	No	NA	0	0	Low-quality	Genome-fragment		3.64	0
S14_extracted_k141_56246	1517	No	NA	0	1	Low-quality	Genome-fragment		0.66	0
S1_vs_k141_103839||full	2894	No	NA	0	1	Not-determined	Genome-fragment	NA		0
S1_vs_k141_76279||full	1517	No	NA	0	1	Low-quality	Genome-fragment		3.19	0
S1_vs_k141_136983||full	2299	No	NA	0	2	Not-determined	Genome-fragment	NA		0
S1_vs_k141_57082||full	2186	No	NA	0	2	Not-determined	Genome-fragment	NA		0
S1_vs_k141_90305||full	1158	No	NA	0	0	Not-determined	Genome-fragment	NA		0
S3_vs_k141_72060||full	1517	No	NA	0	0	Low-quality	Genome-fragment		3.83	0
S3_vs_k141_90447||full	1517	No	NA	1	0	Low-quality	Genome-fragment		3.39	0
S1_vs_k141_23540||full	2698	No	NA	0	3	Not-determined	Genome-fragment	NA		0
S5_vs_k141_35102||full	1517	No	NA	0	0	Low-quality	Genome-fragment		2.53	0
S7_vs_93904||lt2gene	1517	No	NA	1	0	Low-quality	Genome-fragment		2.13	0
S1_vs_k141_97324||full	5349	No	NA	0	0	Not-determined	Genome-fragment	NA		0
S1_vs_k141_143608||full	1472	No	NA	0	1	Not-determined	Genome-fragment	NA		0
S1_vs_k141_117303||full	1673	No	NA	0	0	Not-determined	Genome-fragment	NA		0
S16_extracted_126825	1517	No	NA	0	0	Low-quality	Genome-fragment		0.99	0
S1_vs_k141_50650||full	1189	No	NA	0	0	Not-determined	Genome-fragment	NA		0
S16_extracted_159367	1517	No	NA	0	0	Low-quality	Genome-fragment		1	0
S16_vs_105783||full	1517	No	NA	0	0	Low-quality	Genome-fragment		2.32	0
S1_vs_k141_97370||full	1332	No	NA	0	1	Not-determined	Genome-fragment	NA		0
S10_vs_112725||full	1517	No	NA	0	0	Low-quality	Genome-fragment		2.54	0
S1_vs_k141_70084||full	2742	No	NA	0	2	Not-determined	Genome-fragment	NA		0
S1_vs_k141_130612||full	1330	No	NA	0	0	Not-determined	Genome-fragment	NA		0
S1_vs_k141_117317||full	33680	No	NA	14	1	Medium-quality	Genome-fragment		81.81	0
S1_vs_k141_84004||full	1200	No	NA	0	1	Not-determined	Genome-fragment	NA		0
S12_extracted_k141_4720	1517	No	NA	0	2	Low-quality	Genome-fragment		1.19	0
S15_vs_50157||full	1517	No	NA	0	0	Low-quality	Genome-fragment		3.3	0
S1_vs_k141_143727||full	1586	No	NA	0	2	Not-determined	Genome-fragment	NA		0
S1_vs_k141_137204||full	5350	No	NA	0	2	Not-determined	Genome-fragment	NA		0
S17_extracted_k141_134575	1518	No	NA	0	2	Low-quality	Genome-fragment		3.34	0
S1_vs_k141_17717||full	1344	No	NA	0	1	Not-determined	Genome-fragment	NA		0
S9_vs_k141_66191||lt2gene	1518	No	NA	2	0	Low-quality	Genome-fragment		3.97	0
S13_extracted_122436	1518	No	NA	0	0	Low-quality	Genome-fragment		2.03	0
S1_vs_k141_62672||full	1864	No	NA	0	1	Not-determined	Genome-fragment	NA		0
S13_extracted_125844	1518	No	NA	1	0	Low-quality	Genome-fragment		0.86	0
S3_extracted_k141_29674	1518	No	NA	1	0	Low-quality	Genome-fragment		3.77	0
S16_vs_28928||full	1518	No	NA	0	0	Low-quality	Genome-fragment		3.15	0
S10_vs_119265||full	1518	No	NA	0	0	Low-quality	Genome-fragment		0.9	0
S12_vs_k141_156235||full	1518	No	NA	0	0	Low-quality	Genome-fragment		3.22	0
S6_vs_k141_26883||full	1518	No	NA	0	2	Low-quality	Genome-fragment		0.43	0
S9_vs_k141_20884||full	1519	No	NA	0	2	Low-quality	Genome-fragment		1.36	0
S13_extracted_141715	1519	No	NA	0	0	Low-quality	Genome-fragment		3.32	0
S1_vs_k141_70228||full	1086	No	NA	0	1	Not-determined	Genome-fragment	NA		0
S1_vs_k141_23682||full	44452	No	NA	18	0	High-quality	High-quality		100	0
S1_vs_k141_30728||full	2108	No	NA	0	2	Not-determined	Genome-fragment	NA		0
S1_vs_k141_99969||full	1519	No	NA	0	0	Low-quality	Genome-fragment		2.05	0
S7_vs_34665||full	1519	No	NA	0	0	Low-quality	Genome-fragment		2.51	0
S1_vs_k141_148660||full	1240	No	NA	0	0	Not-determined	Genome-fragment	NA		0
S7_vs_109847||full	1519	No	NA	1	0	Low-quality	Genome-fragment		2.82	0
S16_vs_144825||full	1519	No	NA	0	0	Low-quality	Genome-fragment		0.81	0
S8_vs_k141_195462||lt2gene	1519	No	NA	1	0	Low-quality	Genome-fragment		1.4	0
S1_vs_k141_3173||full	1220	No	NA	0	0	Not-determined	Genome-fragment	NA		0
S1_vs_k141_104138||full	1486	No	NA	0	2	Not-determined	Genome-fragment	NA		0
S1_vs_k141_10846||full	2422	No	NA	0	2	Not-determined	Genome-fragment	NA		0
S1_vs_k141_36520||full	2897	No	NA	0	2	Not-determined	Genome-fragment	NA		0
S1_vs_k141_143860||full	1510	No	NA	0	1	Not-determined	Genome-fragment	NA		0
S14_extracted_k141_114789	1520	No	NA	0	1	Low-quality	Genome-fragment		3.67	0
S1_vs_k141_17870||full	4203	No	NA	0	3	Not-determined	Genome-fragment	NA		0
S14_vs_k141_116315||full	1520	No	NA	0	0	Low-quality	Genome-fragment		0.87	0
S1_vs_k141_63844||full	1520	No	NA	0	2	Low-quality	Genome-fragment		3.39	0
S3_extracted_k141_62122	1520	No	NA	0	0	Low-quality	Genome-fragment		1	0
S3_vs_k141_113504||full	1520	No	NA	0	2	Low-quality	Genome-fragment		0.58	0
S1_vs_k141_143887||full	1781	No	NA	0	2	Not-determined	Genome-fragment	NA		0
S5_vs_k141_9629||full	1520	No	NA	0	0	Low-quality	Genome-fragment		1.67	0
S7_vs_87071||full	1520	No	NA	2	0	Low-quality	Genome-fragment		3.31	0
S1_vs_k141_110783||full	3131	No	NA	0	4	Not-determined	Genome-fragment	NA		0
S7_vs_96117||lt2gene	1520	No	NA	2	0	Low-quality	Genome-fragment		3.19	0
S16_vs_17592||full	1520	No	NA	0	0	Low-quality	Genome-fragment		3.48	0
S1_vs_k141_104200||full	9414	No	NA	0	7	Not-determined	Genome-fragment	NA		0
S1_vs_k141_97681||full	3520	No	NA	0	4	Not-determined	Genome-fragment	NA		0
S1_vs_k141_77509||full	2709	No	NA	0	1	Not-determined	Genome-fragment	NA		0
S1_vs_k141_36584||full	1304	No	NA	0	2	Not-determined	Genome-fragment	NA		0
S8_vs_k141_168086||full	1520	No	NA	0	1	Low-quality	Genome-fragment		0.93	0
S1_vs_k141_10967||full	3492	No	NA	0	0	Not-determined	Genome-fragment	NA		0
S1_vs_k141_123896||full	2760	No	NA	0	2	Not-determined	Genome-fragment	NA		0
S8_vs_k141_172806||full	1520	No	NA	0	0	Low-quality	Genome-fragment		4.09	0
S14_vs_k141_55848||full	1521	No	NA	2	0	Low-quality	Genome-fragment		3.03	0
S1_vs_k141_3267||full	4887	No	NA	6	0	High-quality	High-quality		100	0
S1_vs_k141_130882||full	4747	No	NA	0	1	Not-determined	Genome-fragment	NA		0
S14_vs_k141_15333||lt2gene	1521	No	NA	1	0	Low-quality	Genome-fragment		2.6	0
S1_vs_k141_10979||full	1150	No	NA	0	1	Not-determined	Genome-fragment	NA		0
S3_extracted_k141_3500	1521	No	NA	1	0	Low-quality	Genome-fragment		2.49	0
S3_extracted_k141_110388	1521	No	NA	0	0	Low-quality	Genome-fragment		4.32	0
S3_vs_k141_35215||lt2gene	1521	No	NA	2	0	Low-quality	Genome-fragment		2.64	0
S3_vs_k141_21523||lt2gene	1521	No	NA	1	0	Low-quality	Genome-fragment		1.05	0
S1_vs_k141_90786||full	2073	No	NA	0	0	Not-determined	Genome-fragment	NA		0
S1_vs_k141_123934||full	1227	No	NA	0	1	Not-determined	Genome-fragment	NA		0
S5_vs_k141_98521||full	1521	No	NA	1	0	Low-quality	Genome-fragment		4.84	0
S5_vs_k141_82157||full	1521	No	NA	0	2	Low-quality	Genome-fragment		0.87	0
S1_vs_k141_84346||full	2157	No	NA	0	0	Not-determined	Genome-fragment	NA		0
S1_vs_k141_123953||full	5516	No	NA	0	2	Not-determined	Genome-fragment	NA		0
S7_extracted_30020	1521	No	NA	1	0	Low-quality	Genome-fragment		3.53	0
S7_vs_105470||full	1521	No	NA	0	0	Low-quality	Genome-fragment		3.42	0
S1_vs_k141_23996||full	1090	No	NA	0	0	Not-determined	Genome-fragment	NA		0
S1_vs_k141_97796||full	3099	No	NA	0	1	Not-determined	Genome-fragment	NA		0
S16_extracted_61554	1521	No	NA	0	1	Low-quality	Genome-fragment		1.01	0
S1_vs_k141_44358||full	3030	No	NA	0	1	Not-determined	Genome-fragment	NA		0
S16_vs_76370||full	1521	No	NA	0	0	Low-quality	Genome-fragment		3.57	0
S1_vs_k141_123963||full	1118	No	NA	0	1	Not-determined	Genome-fragment	NA		0
S15_extracted_33395	1521	No	NA	0	0	Low-quality	Genome-fragment		0.89	0
S8_vs_k141_183495||full	1521	No	NA	0	0	Low-quality	Genome-fragment		2.87	0
S8_vs_k141_37134||full	1521	No	NA	0	0	Low-quality	Genome-fragment		3.55	0
S1_vs_k141_104399||full	2135	No	NA	0	1	Not-determined	Genome-fragment	NA		0
S1_vs_k141_131038||full	2444	No	NA	0	0	Not-determined	Genome-fragment	NA		0
S1_vs_k141_11152||full	1714	No	NA	0	2	Not-determined	Genome-fragment	NA		0
S8_vs_k141_2678||full	1521	No	NA	0	0	Low-quality	Genome-fragment		4.59	0
S1_vs_k141_44436||full	2012	No	NA	0	1	Not-determined	Genome-fragment	NA		0
S17_extracted_k141_129484	1522	No	NA	3	0	Low-quality	Genome-fragment		5.02	0
S1_vs_k141_137646||full	1135	No	NA	0	1	Not-determined	Genome-fragment	NA		0
S1_vs_k141_24058||full	4841	No	NA	0	3	Not-determined	Genome-fragment	NA		0
S17_vs_k141_82166||full	1522	No	NA	0	2	Low-quality	Genome-fragment		2.07	0
S13_vs_3811||lt2gene	1522	No	NA	0	1	Low-quality	Genome-fragment		4.31	0
S1_vs_k141_24074||full	1429	No	NA	0	1	Not-determined	Genome-fragment	NA		0
S1_vs_k141_131103||full	5200	No	NA	0	3	Not-determined	Genome-fragment	NA		0
S1_vs_k141_32423||full	1522	No	NA	0	0	Low-quality	Genome-fragment		2.49	0
S1_vs_k141_3435||full	3839	No	NA	2	0	Medium-quality	Genome-fragment		70.06	0
S1_vs_k141_137685||full	1724	No	NA	0	1	Not-determined	Genome-fragment	NA		0
S3_vs_k141_47056||full	1522	No	NA	1	0	Low-quality	Genome-fragment		3.22	0
S3_vs_k141_10435||lt2gene	1522	No	NA	1	0	Low-quality	Genome-fragment		4.41	0
S1_vs_k141_111093||full	2287	No	NA	0	3	Not-determined	Genome-fragment	NA		0
S16_extracted_118150	1522	No	NA	1	0	Low-quality	Genome-fragment		2.16	0
S1_vs_k141_91019||full	5820	No	NA	0	2	Not-determined	Genome-fragment	NA		0
S16_vs_101681||full	1522	No	NA	0	1	Low-quality	Genome-fragment		1.24	0
S1_vs_k141_36848||full	2960	No	NA	0	1	Not-determined	Genome-fragment	NA		0
S1_vs_k141_77817||full	1058	No	NA	0	0	Not-determined	Genome-fragment	NA		0
S12_vs_k141_130225||full	1522	No	NA	0	0	Low-quality	Genome-fragment		4.26	0
S4_extracted_10388	1522	No	NA	2	0	Low-quality	Genome-fragment		4.13	0
S8_extracted_k141_188978	1522	No	NA	0	1	Low-quality	Genome-fragment		0.31	0
S1_vs_k141_84524||full	1644	No	NA	0	2	Not-determined	Genome-fragment	NA		0
S17_extracted_k141_119911	1523	No	NA	2	0	Low-quality	Genome-fragment		4.91	0
S1_vs_k141_91074||full	3106	No	NA	0	1	Not-determined	Genome-fragment	NA		0
S1_vs_k141_36898||full	1448	No	NA	0	4	Not-determined	Genome-fragment	NA		0
S1_vs_k141_24203||full	1688	No	NA	0	1	Not-determined	Genome-fragment	NA		0
S1_vs_k141_148878||full	1857	No	NA	0	2	Not-determined	Genome-fragment	NA		0

S1_vs_k141_57750||full	1623	No	NA	0	2	Not-determined	Genome-fragment	NA		0
S17_vs_k141_160776||full	1523	No	NA	0	0	Low-quality	Genome-fragment		3.56	0
S11_vs_k141_17483||lt2gene	1523	No	NA	0	0	Low-quality	Genome-fragment		3.45	0
S1_vs_k141_44585||full	1697	No	NA	0	2	Not-determined	Genome-fragment	NA		0
S1_vs_k141_111186||full	4673	No	NA	0	2	Not-determined	Genome-fragment	NA		0
S1_vs_k141_70560||full	2109	No	NA	0	1	Not-determined	Genome-fragment	NA		0
S11_vs_k141_69248||lt2gene	1523	No	NA	1	0	Low-quality	Genome-fragment		2.82	0
S1_vs_k141_18329||full	1737	No	NA	0	1	Not-determined	Genome-fragment	NA		0
S14_vs_k141_90846||full	1523	No	NA	1	0	Low-quality	Genome-fragment		3.24	0
S14_vs_k141_105967||full	1523	No	NA	1	0	Low-quality	Genome-fragment		3.3	0
S3_vs_k141_101397||full	1523	No	NA	1	0	Low-quality	Genome-fragment		3.26	0
S5_vs_k141_17888||full	1523	No	NA	0	2	Low-quality	Genome-fragment		1.03	0
S7_extracted_28804	1523	No	NA	0	1	Low-quality	Genome-fragment		0.49	0
S7_vs_50445||full	1523	No	NA	2	0	Low-quality	Genome-fragment		3.23	0
S16_vs_88394||full	1523	No	NA	0	2	Low-quality	Genome-fragment		1.67	0
S10_extracted_108848	1523	No	NA	1	0	Low-quality	Genome-fragment		0.66	0
S12_extracted_k141_885	1523	No	NA	1	0	Low-quality	Genome-fragment		1.6	0
S12_vs_k141_103781||full	1523	No	NA	0	0	Low-quality	Genome-fragment		3.48	0
S1_vs_k141_18382||full	1430	No	NA	0	1	Not-determined	Genome-fragment	NA		0
S1_vs_k141_144309||full	1532	No	NA	0	0	Not-determined	Genome-fragment	NA		0
S12_vs_k141_86440||lt2gene	1523	No	NA	2	0	Low-quality	Genome-fragment		4.04	0
S1_vs_k141_144333||full	4378	No	NA	0	2	Not-determined	Genome-fragment	NA		0
S1_vs_k141_104662||full	2357	No	NA	0	0	Not-determined	Genome-fragment	NA		0
S17_extracted_k141_74802	1524	No	NA	1	0	Low-quality	Genome-fragment		6.96	0
S1_vs_k141_118032||full	3684	No	NA	0	2	Not-determined	Genome-fragment	NA		0
S1_vs_k141_137897||full	1250	No	NA	0	2	Not-determined	Genome-fragment	NA		0
S1_vs_k141_124297||full	2345	No	NA	0	2	Not-determined	Genome-fragment	NA		0
S17_vs_k141_22103||full	1524	No	NA	1	0	Low-quality	Genome-fragment		0.88	0
S11_vs_k141_80357||full	1524	No	NA	1	0	Low-quality	Genome-fragment		3.5	0
S3_extracted_k141_404	1524	No	NA	1	0	Low-quality	Genome-fragment		2.19	0
S16_extracted_8505	1524	No	NA	0	0	Low-quality	Genome-fragment		2.73	0
S16_vs_116417||full	1524	No	NA	0	3	Low-quality	Genome-fragment		0.8	0
S1_vs_k141_24387||full	2405	No	NA	0	2	Not-determined	Genome-fragment	NA		0
S12_vs_k141_139479||full	1524	No	NA	1	0	Low-quality	Genome-fragment		3.1	0
S8_vs_k141_75379||full	1524	No	NA	0	0	Low-quality	Genome-fragment		1.55	0
S17_extracted_k141_59286	1525	No	NA	1	0	Low-quality	Genome-fragment		2.55	0
S1_vs_k141_31365||full	1116	No	NA	0	1	Not-determined	Genome-fragment	NA		0
S9_vs_k141_2296||full	1525	No	NA	0	0	Low-quality	Genome-fragment		3.4	0
S1_vs_k141_91270||full	18290	No	NA	11	0	Medium-quality	Genome-fragment		53.58	0
S1_vs_k141_37065||full	2009	No	NA	0	2	Not-determined	Genome-fragment	NA		0
S1_vs_k141_78072||full	2707	No	NA	0	1	Not-determined	Genome-fragment	NA		0
S11_vs_k141_64993||full	1525	No	NA	0	0	Low-quality	Genome-fragment		3.39	0
S1_vs_k141_106515||full	1525	No	NA	0	0	Low-quality	Genome-fragment		2.99	0
S5_vs_k141_23192||full	1525	No	NA	3	0	Low-quality	Genome-fragment		4.28	0
S7_extracted_16739	1525	No	NA	0	0	Low-quality	Genome-fragment		3.29	0
S16_extracted_152213	1525	No	NA	0	1	Low-quality	Genome-fragment		0.15	0
S16_vs_116233||full	1525	No	NA	0	0	Low-quality	Genome-fragment		3.73	0
S12_vs_k141_154991||full	1525	No	NA	1	0	Low-quality	Genome-fragment		2.53	0
S1_vs_k141_98193||full	1791	No	NA	0	3	Not-determined	Genome-fragment	NA		0
S6_vs_k141_8754||full	1525	No	NA	0	0	Low-quality	Genome-fragment		4.08	0
S1_vs_k141_156131||full	1377	No	NA	0	1	Not-determined	Genome-fragment	NA		0
S1_vs_k141_98210||full	1599	No	NA	0	0	Not-determined	Genome-fragment	NA		0
S1_vs_k141_3742||full	1401	No	NA	0	0	Not-determined	Genome-fragment	NA		0
S1_vs_k141_111419||full	1431	No	NA	0	2	Not-determined	Genome-fragment	NA		0
S13_extracted_152195	1526	No	NA	0	0	Low-quality	Genome-fragment		1.62	0
S13_vs_56702||full	1526	No	NA	0	1	Low-quality	Genome-fragment		1.46	0
S1_vs_k141_98239||full	1160	No	NA	0	0	Not-determined	Genome-fragment	NA		0
S1_vs_k141_101707||full	1526	No	NA	0	1	Low-quality	Genome-fragment		3.35	0
S1_vs_k141_68634||full	1526	No	NA	0	1	Low-quality	Genome-fragment		0.8	0
S1_vs_k141_69082||full	1526	No	NA	2	0	Low-quality	Genome-fragment		2.51	0
S1_vs_k141_147045||lt2gene	1526	No	NA	1	0	Low-quality	Genome-fragment		3.38	0
S3_extracted_k141_9963	1526	No	NA	0	0	Low-quality	Genome-fragment		2.42	0
S1_vs_k141_37157||full	2008	No	NA	0	0	Not-determined	Genome-fragment	NA		0
S5_vs_k141_49100||full	1526	No	NA	0	0	Low-quality	Genome-fragment		4.29	0
S1_vs_k141_3779||full	1075	No	NA	0	0	Not-determined	Genome-fragment	NA		0
S16_extracted_137895	1526	No	NA	1	0	Low-quality	Genome-fragment		1.82	0
S16_vs_164904||full	1526	No	NA	0	1	Low-quality	Genome-fragment		0.91	0
S11_vs_k141_79492||full	1527	No	NA	0	2	Low-quality	Genome-fragment		1.74	0
S1_vs_k141_98292||full	3963	No	NA	0	1	Not-determined	Genome-fragment	NA		0
S1_vs_k141_144537||full	2849	No	NA	0	1	Not-determined	Genome-fragment	NA		0
S1_extracted_k141_44390	1527	No	NA	1	0	Low-quality	Genome-fragment		3.81	0
S1_vs_k141_18657||full	1865	No	NA	0	4	Not-determined	Genome-fragment	NA		0
S1_vs_k141_42994||full	1527	No	NA	0	1	Low-quality	Genome-fragment		1.49	0
S1_vs_k141_44917||full	4719	No	NA	0	1	Not-determined	Genome-fragment	NA		0
S16_extracted_18151	1527	No	NA	0	1	Low-quality	Genome-fragment		0.73	0
S12_vs_k141_157107||full	1527	No	NA	0	1	Low-quality	Genome-fragment		3.07	0
S6_extracted_k141_32499	1527	No	NA	1	0	Low-quality	Genome-fragment		1.07	0
S6_vs_k141_10755||full	1527	No	NA	0	0	Low-quality	Genome-fragment		2.37	0
S11_vs_k141_28276||full	1528	No	NA	0	0	Low-quality	Genome-fragment		3.52	0
S14_extracted_k141_115988	1528	No	NA	0	0	Low-quality	Genome-fragment		3.17	0
S1_vs_k141_58139||full	3324	No	NA	0	1	Not-determined	Genome-fragment	NA		0
S1_vs_k141_104951||full	4773	No	NA	0	3	Not-determined	Genome-fragment	NA		0
S14_vs_k141_81076||full	1528	No	NA	0	0	Low-quality	Genome-fragment		3.21	0
S1_vs_k141_118326||full	1390	No	NA	0	1	Not-determined	Genome-fragment	NA		0
S1_vs_k141_128880||lt2gene	1528	No	NA	1	0	Low-quality	Genome-fragment		1.43	0
S1_vs_k141_138167||full	2121	No	NA	0	1	Not-determined	Genome-fragment	NA		0
S3_extracted_k141_9243	1528	No	NA	0	0	Low-quality	Genome-fragment		0.85	0
S1_vs_k141_131684||full	5340	No	NA	0	1	Not-determined	Genome-fragment	NA		0
S5_vs_k141_130924||full	1528	No	NA	0	0	Low-quality	Genome-fragment		3.03	0
S7_vs_75579||full	1528	No	NA	0	0	Low-quality	Genome-fragment		4.14	0
S1_vs_k141_11821||full	3180	No	NA	0	3	Not-determined	Genome-fragment	NA		0
S10_vs_67258||full	1528	No	NA	0	0	Low-quality	Genome-fragment		3.34	0
S10_vs_75993||full	1528	No	NA	0	0	Low-quality	Genome-fragment		2.86	0
S12_vs_k141_45635||full	1528	No	NA	0	3	Low-quality	Genome-fragment		0.39	0
S1_vs_k141_45026||full	2038	No	NA	0	4	Not-determined	Genome-fragment	NA		0
S1_vs_k141_31597||full	3115	No	NA	0	3	Not-determined	Genome-fragment	NA		0
S12_vs_k141_45875||full	1528	No	NA	1	0	Low-quality	Genome-fragment		4.36	0
S8_vs_k141_59497||full	1528	No	NA	2	0	Low-quality	Genome-fragment		3.81	0
S17_vs_k141_16695||full	1529	No	NA	0	1	Low-quality	Genome-fragment		1.39	0
S17_vs_k141_138410||full	1529	No	NA	0	1	Low-quality	Genome-fragment		2.08	0
S9_vs_k141_74839||full	1529	No	NA	0	2	Low-quality	Genome-fragment		0.82	0
S11_vs_k141_84368||full	1529	No	NA	1	0	Low-quality	Genome-fragment		3.5	0
S1_vs_k141_18824||full	1178	No	NA	0	0	Not-determined	Genome-fragment	NA		0
S3_extracted_k141_113602	1529	No	NA	0	0	Low-quality	Genome-fragment		1.88	0
S1_vs_k141_91663||full	1023	No	NA	0	0	Not-determined	Genome-fragment	NA		0
S3_vs_k141_119332||full	1529	No	NA	0	1	Low-quality	Genome-fragment		1.02	0
S7_extracted_41259	1529	No	NA	0	0	Low-quality	Genome-fragment		2.73	0
S1_vs_k141_144725||full	4044	No	NA	0	0	Not-determined	Genome-fragment	NA		0
S16_extracted_137676	1529	No	NA	1	0	Low-quality	Genome-fragment		3.48	0
S16_vs_25443||full	1529	No	NA	0	0	Low-quality	Genome-fragment		3.33	0
S16_vs_29840||full	1529	No	NA	0	2	Low-quality	Genome-fragment		0.49	0
S12_vs_k141_30989||full	1529	No	NA	1	0	Low-quality	Genome-fragment		1.09	0
S9_vs_k141_65410||full	1530	No	NA	0	0	Low-quality	Genome-fragment		3.66	0
S11_vs_k141_58568||full	1530	No	NA	0	0	Low-quality	Genome-fragment		3.52	0
S1_vs_k141_144791||full	1052	No	NA	0	1	Not-determined	Genome-fragment	NA		0
S14_vs_k141_89885||full	1530	No	NA	0	1	Low-quality	Genome-fragment		2.5	0
S14_vs_k141_41099||full	1530	No	NA	0	1	Low-quality	Genome-fragment		1.79	0
S1_vs_k141_31749||full	1735	No	NA	0	0	Not-determined	Genome-fragment	NA		0
S1_extracted_k141_113198	1530	No	NA	0	0	Low-quality	Genome-fragment		0.86	0
S1_vs_k141_4042||full	2285	No	NA	0	2	Not-determined	Genome-fragment	NA		0
S3_vs_k141_8859||full	1530	No	NA	1	0	Low-quality	Genome-fragment		1.77	0
S3_vs_k141_65206||full	1530	No	NA	0	1	Low-quality	Genome-fragment		2.08	0
S3_vs_k141_14503||full	1530	No	NA	0	0	Low-quality	Genome-fragment		3.31	0
S7_extracted_28377	1530	No	NA	0	0	Low-quality	Genome-fragment		1.85	0
S1_vs_k141_85180||full	2740	No	NA	0	1	Not-determined	Genome-fragment	NA		0
S7_extracted_52597	1530	No	NA	0	1	Low-quality	Genome-fragment		4.16	0
S16_extracted_117186	1530	No	NA	0	0	Low-quality	Genome-fragment		2.63	0
S10_vs_9087||full	1530	No	NA	0	0	Low-quality	Genome-fragment		1.04	0
S12_extracted_k141_179806	1530	No	NA	0	0	Low-quality	Genome-fragment		3.48	0
S6_vs_k141_8488||full	1530	No	NA	1	0	Low-quality	Genome-fragment		0.84	0
S1_vs_k141_25000||full	1394	No	NA	0	2	Not-determined	Genome-fragment	NA		0
S1_vs_k141_25005||full	1579	No	NA	0	1	Not-determined	Genome-fragment	NA		0
S8_extracted_k141_107519	1530	No	NA	0	1	Low-quality	Genome-fragment		1.42	0
S17_extracted_k141_83451	1531	No	NA	0	1	Low-quality	Genome-fragment		1.42	0
S1_vs_k141_124942||full	3029	No	NA	0	3	Not-determined	Genome-fragment	NA		0
S1_vs_k141_111868||full	4981	No	NA	0	5	Not-determined	Genome-fragment	NA		0
S1_vs_k141_91864||full	1529	No	NA	0	1	Not-determined	Genome-fragment	NA		0
S9_vs_k141_22207||lt2gene	1531	No	NA	1	0	Low-quality	Genome-fragment		2.65	0
S1_vs_k141_19045||full	1136	No	NA	0	0	Not-determined	Genome-fragment	NA		0
S11_extracted_k141_88911	1531	No	NA	0	1	Low-quality	Genome-fragment		1.6	0
S11_extracted_k141_96926	1531	No	NA	2	0	Low-quality	Genome-fragment		24.12	0
S11_vs_k141_3294||full	1531	No	NA	0	0	Low-quality	Genome-fragment		3.43	0
S13_vs_11133||full	1531	No	NA	0	0	Low-quality	Genome-fragment		1.9	0
S1_vs_k141_45325||full	1213	No	NA	0	0	Not-determined	Genome-fragment	NA		0
S14_vs_k141_45687||full	1531	No	NA	0	0	Low-quality	Genome-fragment		3.52	0
S1_extracted_k141_57597	1531	No	NA	0	0	Low-quality	Genome-fragment		0.66	0
S3_extracted_k141_43696	1531	No	NA	3	0	Low-quality	Genome-fragment		7.01	0
S5_vs_k141_43546||full	1531	No	NA	2	0	Low-quality	Genome-fragment		4.15	0
S1_vs_k141_45396||full	6966	No	NA	0	4	Not-determined	Genome-fragment	NA		0
S1_vs_k141_125096||full	7099	No	NA	0	5	Not-determined	Genome-fragment	NA		0
S5_vs_k141_10113||full	1531	No	NA	1	0	Low-quality	Genome-fragment		4.43	0
S16_vs_85405||full	1531	No	NA	0	1	Low-quality	Genome-fragment		2.44	0
S1_vs_k141_32013||full	1094	No	NA	0	1	Not-determined	Genome-fragment	NA		0
S1_vs_k141_145035||full	1721	No	NA	0	0	Not-determined	Genome-fragment	NA		0
S16_vs_139275||lt2gene	1531	No	NA	1	0	Low-quality	Genome-fragment		3.38	0
S1_vs_k141_45422||full	2844	No	NA	0	3	Not-determined	Genome-fragment	NA		0
S8_vs_k141_155582||full	1531	No	NA	0	0	Low-quality	Genome-fragment		1.09	0
S1_vs_k141_105456||full	3382	No	NA	0	2	Not-determined	Genome-fragment	NA		0
S17_extracted_k141_28306	1532	No	NA	3	0	Low-quality	Genome-fragment		6.99	0
S14_extracted_k141_59826	1532	No	NA	1	0	Low-quality	Genome-fragment		3.29	0
S5_vs_k141_101592||full	1532	No	NA	1	0	Low-quality	Genome-fragment		4	0
S1_vs_k141_92090||full	1319	No	NA	0	0	Not-determined	Genome-fragment	NA		0
S1_vs_k141_37576||full	1945	No	NA	0	0	Not-determined	Genome-fragment	NA		0
S16_vs_32086||full	1532	No	NA	1	0	Low-quality	Genome-fragment		3.07	0
S16_vs_157123||full	1532	No	NA	1	0	Low-quality	Genome-fragment		1.1	0
S12_extracted_k141_109633	1532	No	NA	0	1	Low-quality	Genome-fragment		3.32	0
S6_extracted_k141_34497	1532	No	NA	1	0	Low-quality	Genome-fragment		2.85	0
S6_vs_k141_49295||full	1532	No	NA	0	1	Low-quality	Genome-fragment		0.91	0
S8_extracted_k141_173401	1532	No	NA	0	1	Low-quality	Genome-fragment		0.41	0
S1_vs_k141_37617||full	3546	No	NA	0	0	Not-determined	Genome-fragment	NA		0
S1_vs_k141_138637||full	10188	No	NA	0	7	Not-determined	Genome-fragment	NA		0
S1_vs_k141_52363||full	1406	No	NA	0	1	Not-determined	Genome-fragment	NA		0
S9_extracted_k141_28072	1533	No	NA	1	0	Low-quality	Genome-fragment		2.59	0
S11_vs_k141_94232||full	1533	No	NA	0	0	Low-quality	Genome-fragment		2.8	0

S1_vs_k141_56323||full	1533	No	NA	1	0	Low-quality	Genome-fragment		3.52	0
S12_extracted_k141_176886	1533	No	NA	1	0	Low-quality	Genome-fragment		2.67	0
S12_vs_k141_72065||full	1533	No	NA	1	0	Low-quality	Genome-fragment		3.59	0
S1_vs_k141_19378||full	4815	No	NA	0	2	Not-determined	Genome-fragment	NA		0
S12_vs_k141_67553||full	1533	No	NA	0	0	Low-quality	Genome-fragment		2.26	0
S17_vs_k141_50520||full	1534	No	NA	3	0	Low-quality	Genome-fragment		2.61	0
S9_extracted_k141_57432	1534	No	NA	0	0	Low-quality	Genome-fragment		0.29	0
S14_vs_k141_15072||full	1534	No	NA	1	0	Low-quality	Genome-fragment		4.43	0
S1_vs_k141_78976||full	1489	No	NA	0	0	Not-determined	Genome-fragment	NA		0
S1_vs_k141_94096||full	1534	No	NA	0	0	Low-quality	Genome-fragment		3.63	0
S1_vs_k141_138837||full	1534	No	NA	1	0	Low-quality	Genome-fragment		3.93	0
S1_vs_k141_85583||full	1832	No	NA	0	0	Not-determined	Genome-fragment	NA		0
S1_vs_k141_46122||full	1534	No	NA	2	0	Low-quality	Genome-fragment		2.6	0
S3_extracted_k141_56840	1534	No	NA	1	0	Low-quality	Genome-fragment		1.71	0
S5_vs_k141_89116||full	1534	No	NA	0	0	Low-quality	Genome-fragment		46.34	0
S7_extracted_9647	1534	No	NA	0	0	Low-quality	Genome-fragment		2.91	0
S7_vs_47010||full	1534	No	NA	0	1	Low-quality	Genome-fragment		1.62	0
S10_extracted_22518	1534	No	NA	0	0	Low-quality	Genome-fragment		1	0
S1_vs_k141_99061||full	1779	No	NA	0	2	Not-determined	Genome-fragment	NA		0
S1_vs_k141_79019||full	1478	No	NA	0	0	Not-determined	Genome-fragment	NA		0
S17_vs_k141_5749||full	1535	No	NA	0	0	Low-quality	Genome-fragment		3.25	0
S14_extracted_k141_37988	1535	No	NA	0	0	Low-quality	Genome-fragment		2.78	0
S14_extracted_k141_86830	1535	No	NA	5	0	Low-quality	Genome-fragment		5.04	0
S1_vs_k141_124331||full	1535	No	NA	0	0	Low-quality	Genome-fragment		0.76	0
S1_vs_k141_52546||full	4239	No	NA	0	1	Not-determined	Genome-fragment	NA		0
S3_vs_k141_71044||full	1535	No	NA	0	0	Low-quality	Genome-fragment		1.74	0
S1_vs_k141_85684||full	1254	No	NA	0	1	Not-determined	Genome-fragment	NA		0
S1_vs_k141_138796||full	1105	No	NA	0	1	Not-determined	Genome-fragment	NA		0
S1_vs_k141_119028||full	1367	No	NA	0	2	Not-determined	Genome-fragment	NA		0
S5_extracted_k141_74729	1535	No	NA	0	0	Low-quality	Genome-fragment		4.35	0
S5_vs_k141_49890||full	1535	No	NA	0	0	Low-quality	Genome-fragment		3.73	0
S7_vs_28647||full	1535	No	NA	1	0	Low-quality	Genome-fragment		2.31	0
S16_vs_58938||lt2gene	1535	No	NA	1	0	Low-quality	Genome-fragment		3.41	0
S12_vs_k141_62509||full	1535	No	NA	1	0	Low-quality	Genome-fragment		3.85	0
S8_extracted_k141_131124	1535	No	NA	0	0	Low-quality	Genome-fragment		0.81	0
S17_extracted_k141_72980	1536	No	NA	0	0	Low-quality	Genome-fragment		4.88	0
S17_vs_k141_169490||full	1536	No	NA	0	1	Low-quality	Genome-fragment		3.86	0
S1_vs_k141_52600||full	1022	No	NA	0	0	Not-determined	Genome-fragment	NA		0
S1_vs_k141_99158||full	1563	No	NA	0	1	Not-determined	Genome-fragment	NA		0
S1_vs_k141_149979||full	1536	No	NA	1	0	Low-quality	Genome-fragment		3.55	0
S3_extracted_k141_4659	1536	No	NA	0	0	Low-quality	Genome-fragment		0.75	0
S1_vs_k141_25524||full	3461	No	NA	0	2	Not-determined	Genome-fragment	NA		0
S3_vs_k141_120548||full	1536	No	NA	0	1	Low-quality	Genome-fragment		1.48	0
S1_vs_k141_99175||full	2378	No	NA	0	2	Not-determined	Genome-fragment	NA		0
S5_extracted_k141_20966	1536	No	NA	0	0	Low-quality	Genome-fragment		3.37	0
S5_vs_k141_106913||lt2gene	1536	No	NA	1	0	Low-quality	Genome-fragment		3.59	0
S16_extracted_77914	1536	No	NA	0	0	Low-quality	Genome-fragment		1.25	0
S16_vs_9218||full	1536	No	NA	0	0	Low-quality	Genome-fragment		4.12	0
S10_extracted_1923	1536	No	NA	0	0	Low-quality	Genome-fragment		2.74	0
S15_vs_61073||full	1536	No	NA	0	0	Low-quality	Genome-fragment		2.57	0
S1_vs_k141_64468||full	3443	No	NA	0	1	Not-determined	Genome-fragment	NA		0
S1_vs_k141_52685||full	1683	No	NA	0	2	Not-determined	Genome-fragment	NA		0
S9_vs_k141_11713||lt2gene	1537	No	NA	1	0	Low-quality	Genome-fragment		3.5	0
S1_vs_k141_108320||lt2gene	1537	No	NA	2	0	Low-quality	Genome-fragment		3.93	0
S5_vs_k141_88736||full	1537	No	NA	1	0	Low-quality	Genome-fragment		3.43	0
S1_vs_k141_19670||full	1922	No	NA	0	0	Not-determined	Genome-fragment	NA		0
S1_vs_k141_92453||full	1197	No	NA	0	0	Not-determined	Genome-fragment	NA		0
S1_vs_k141_19675||full	1316	No	NA	0	0	Not-determined	Genome-fragment	NA		0
S1_vs_k141_92486||full	1323	No	NA	0	0	Not-determined	Genome-fragment	NA		0
S1_vs_k141_145456||full	1141	No	NA	0	1	Not-determined	Genome-fragment	NA		0
S8_extracted_k141_3736	1537	No	NA	2	0	Low-quality	Genome-fragment		4.99	0
S1_vs_k141_112467||full	1851	No	NA	0	2	Not-determined	Genome-fragment	NA		0
S7_vs_70016||full	1538	No	NA	2	0	Low-quality	Genome-fragment		3.82	0
S1_vs_k141_138973||full	1103	No	NA	0	1	Not-determined	Genome-fragment	NA		0
S16_vs_17468||full	1538	No	NA	1	0	Low-quality	Genome-fragment		3.52	0
S1_vs_k141_25680||full	1357	No	NA	0	0	Not-determined	Genome-fragment	NA		0
S1_vs_k141_105924||full	1341	No	NA	0	0	Not-determined	Genome-fragment	NA		0
S16_vs_53780||lt2gene	1538	No	NA	1	0	Low-quality	Genome-fragment		2.91	0
S10_extracted_18682	1538	No	NA	0	2	Low-quality	Genome-fragment		0.92	0
S1_vs_k141_79353||full	1174	No	NA	0	2	Not-determined	Genome-fragment	NA		0
S1_vs_k141_149633||full	1300	No	NA	0	0	Not-determined	Genome-fragment	NA		0
S12_vs_k141_101417||full	1538	No	NA	0	0	Low-quality	Genome-fragment		1.65	0
S8_vs_k141_106344||full	1538	No	NA	0	0	Low-quality	Genome-fragment		3.38	0
S8_vs_k141_40329||lt2gene	1538	No	NA	1	0	Low-quality	Genome-fragment		0.13	0
S1_vs_k141_112480||full	1024	No	NA	0	1	Not-determined	Genome-fragment	NA		0
S1_vs_k141_105944||full	1809	No	NA	0	1	Not-determined	Genome-fragment	NA		0
S1_vs_k141_145513||full	1550	No	NA	0	1	Not-determined	Genome-fragment	NA		0
S17_extracted_k141_24589	1539	No	NA	0	1	Low-quality	Genome-fragment		4.34	0
S1_vs_k141_4629||full	1761	No	NA	0	1	Not-determined	Genome-fragment	NA		0
S11_vs_k141_26850||full	1539	No	NA	1	0	Low-quality	Genome-fragment		3.51	0
S1_vs_k141_45960||full	2318	No	NA	0	1	Not-determined	Genome-fragment	NA		0
S1_vs_k141_85990||full	2402	No	NA	0	2	Not-determined	Genome-fragment	NA		0
S14_extracted_k141_63198	1539	No	NA	2	0	Low-quality	Genome-fragment		2.68	0
S1_vs_k141_92575||full	2594	No	NA	0	2	Not-determined	Genome-fragment	NA		0
S1_vs_k141_25748||full	2035	No	NA	0	2	Not-determined	Genome-fragment	NA		0
S5_vs_k141_87211||full	1539	No	NA	1	0	Low-quality	Genome-fragment		4.23	0
S5_vs_k141_42370||full	1539	No	NA	1	0	Low-quality	Genome-fragment		2.75	0
S1_vs_k141_32665||full	1817	No	NA	0	1	Not-determined	Genome-fragment	NA		0
S1_vs_k141_37979||full	2650	No	NA	0	0	Not-determined	Genome-fragment	NA		0
S1_vs_k141_86035||full	1142	No	NA	0	1	Not-determined	Genome-fragment	NA		0
S16_vs_56546||full	1539	No	NA	0	1	Low-quality	Genome-fragment		3.08	0
S16_vs_1589||lt2gene	1539	No	NA	0	0	Low-quality	Genome-fragment		4.12	0
S8_extracted_k141_33047	1539	No	NA	0	1	Low-quality	Genome-fragment		0.5	0
S13_vs_13635||lt2gene	1540	No	NA	1	0	Low-quality	Genome-fragment		2.25	0
S1_extracted_k141_108678	1540	No	NA	0	1	Low-quality	Genome-fragment		1.15	0
S1_vs_k141_120320||full	1540	No	NA	2	0	Low-quality	Genome-fragment		4.5	0
S3_extracted_k141_88809	1540	No	NA	1	0	Low-quality	Genome-fragment		3.04	0
S3_extracted_k141_61030	1540	No	NA	0	0	Low-quality	Genome-fragment		2.75	0
S3_vs_k141_91814||full	1540	No	NA	0	0	Low-quality	Genome-fragment		2.44	0
S5_extracted_k141_77617	1540	No	NA	0	1	Low-quality	Genome-fragment		0.75	0
S7_vs_81552||full	1540	No	NA	1	0	Low-quality	Genome-fragment		3.52	0
S7_vs_43903||lt2gene	1540	No	NA	1	0	Low-quality	Genome-fragment		3.35	0
S1_vs_k141_71393||full	1116	No	NA	0	1	Not-determined	Genome-fragment	NA		0
S16_extracted_128427	1540	No	NA	2	0	Low-quality	Genome-fragment		3.48	0
S1_vs_k141_12922||full	1088	No	NA	0	1	Not-determined	Genome-fragment	NA		0
S16_extracted_160888	1540	No	NA	3	0	Low-quality	Genome-fragment		4.07	0
S10_vs_4519||full	1540	No	NA	1	0	Low-quality	Genome-fragment		0.55	0
S8_vs_k141_178128||lt2gene	1540	No	NA	1	0	Low-quality	Genome-fragment		3.37	0
S1_vs_k141_32764||full	6028	No	NA	0	1	Not-determined	Genome-fragment	NA		0
S11_vs_k141_35189||full	1541	No	NA	0	2	Low-quality	Genome-fragment		3.02	0
S13_vs_65024||full	1541	No	NA	0	0	Low-quality	Genome-fragment		2.69	0
S14_vs_k141_50639||full	1541	No	NA	1	0	Low-quality	Genome-fragment		3.84	0
S1_extracted_k141_30753	1541	No	NA	2	0	Low-quality	Genome-fragment		3.92	0
S1_vs_k141_71409||full	2070	No	NA	0	2	Not-determined	Genome-fragment	NA		0
S1_vs_k141_106123||full	1883	No	NA	0	0	Not-determined	Genome-fragment	NA		0
S1_extracted_k141_125607	1541	No	NA	0	1	Low-quality	Genome-fragment		2.63	0
S7_vs_67765||full	1541	No	NA	0	3	Low-quality	Genome-fragment		2.47	0
S1_vs_k141_53070||full	1164	No	NA	0	1	Not-determined	Genome-fragment	NA		0
S7_vs_88588||lt2gene	1541	No	NA	1	0	Low-quality	Genome-fragment		3.77	0
S1_vs_k141_132924||full	2446	No	NA	0	1	Not-determined	Genome-fragment	NA		0
S16_vs_172379||full	1541	No	NA	0	1	Low-quality	Genome-fragment		2.86	0
S10_extracted_74007	1541	No	NA	0	1	Low-quality	Genome-fragment		3.44	0
S1_vs_k141_156821||full	2010	No	NA	0	1	Not-determined	Genome-fragment	NA		0
S10_vs_99459||full	1541	No	NA	2	0	Low-quality	Genome-fragment		3.54	0
S1_vs_k141_79577||full	22821	No	NA	0	13	Not-determined	Genome-fragment	NA		0
S12_vs_k141_179592||full	1541	No	NA	0	0	Low-quality	Genome-fragment		2.8	0
S8_extracted_k141_139122	1541	No	NA	0	0	Low-quality	Genome-fragment		2.45	0
S8_vs_k141_110253||full	1541	No	NA	1	0	Low-quality	Genome-fragment		3.72	0
S9_vs_k141_64095||full	1542	No	NA	0	1	Low-quality	Genome-fragment		2.38	0
S13_extracted_49964	1542	No	NA	0	0	Low-quality	Genome-fragment		0.26	0
S14_extracted_k141_48649	1542	No	NA	0	1	Low-quality	Genome-fragment		0.16	0
S1_vs_k141_4830||full	1623	No	NA	0	0	Not-determined	Genome-fragment	NA		0
S14_vs_k141_116655||full	1542	No	NA	0	0	Low-quality	Genome-fragment		1.16	0
S1_vs_k141_4836||full	1662	No	NA	0	2	Not-determined	Genome-fragment	NA		0
S14_vs_k141_56680||lt2gene	1542	No	NA	1	0	Low-quality	Genome-fragment		3.47	0
S1_vs_k141_79655||full	1190	No	NA	0	1	Not-determined	Genome-fragment	NA		0
S1_vs_k141_46993||full	1542	No	NA	0	1	Low-quality	Genome-fragment		0.7	0
S3_vs_k141_76276||full	1542	No	NA	0	0	Low-quality	Genome-fragment		0.97	0
S7_vs_79139||full	1542	No	NA	1	0	Low-quality	Genome-fragment		4.11	0
S16_extracted_148119	1542	No	NA	0	0	Low-quality	Genome-fragment		2.62	0
S1_vs_k141_112814||full	1506	No	NA	0	2	Not-determined	Genome-fragment	NA		0
S1_vs_k141_139386||full	1262	No	NA	0	2	Not-determined	Genome-fragment	NA		0
S16_vs_22254||full	1542	No	NA	0	0	Low-quality	Genome-fragment		2.62	0
S16_vs_96268||full	1542	No	NA	0	0	Low-quality	Genome-fragment		2.91	0
S10_vs_12626||full	1542	No	NA	0	1	Low-quality	Genome-fragment		2.13	0
S12_extracted_k141_172523	1542	No	NA	0	0	Low-quality	Genome-fragment		2.51	0
S8_vs_k141_117694||full	1542	No	NA	2	0	Low-quality	Genome-fragment		4.22	0
S1_vs_k141_53216||full	1821	No	NA	0	2	Not-determined	Genome-fragment	NA		0
S17_extracted_k141_37459	1543	No	NA	0	1	Low-quality	Genome-fragment		0.98	0
S1_vs_k141_79734||full	1092	No	NA	0	0	Not-determined	Genome-fragment	NA		0
S17_vs_k141_78052||full	1543	No	NA	0	0	Low-quality	Genome-fragment		1.36	0
S1_vs_k141_145792||full	23601	No	NA	1	1	Medium-quality	Genome-fragment		55.35	0
S1_vs_k141_46318||full	2042	No	NA	0	0	Not-determined	Genome-fragment	NA		0
S1_vs_k141_71554||full	1458	No	NA	0	0	Not-determined	Genome-fragment	NA		0
S11_vs_k141_42570||full	1543	No	NA	1	0	Low-quality	Genome-fragment		2.48	0
S1_vs_k141_46339||full	1461	No	NA	0	1	Not-determined	Genome-fragment	NA		0
S1_vs_k141_86382||full	3921	No	NA	0	0	Not-determined	Genome-fragment	NA		0
S1_vs_k141_71567||full	2388	No	NA	0	2	Not-determined	Genome-fragment	NA		0
S13_vs_52326||full	1543	No	NA	0	1	Low-quality	Genome-fragment		1.25	0
S1_vs_k141_13256||full	4005	No	NA	0	0	Not-determined	Genome-fragment	NA		0
S1_vs_k141_92969||full	11064	No	NA	0	11	Not-determined	Genome-fragment	NA		0
S14_vs_k141_65219||full	1543	No	NA	1	0	Low-quality	Genome-fragment		0.91	0
S1_vs_k141_106418||full	1791	No	NA	0	1	Not-determined	Genome-fragment	NA		0
S1_vs_k141_126102||full	2170	No	NA	0	0	Not-determined	Genome-fragment	NA		0
S3_extracted_k141_78393	1543	No	NA	1	0	Low-quality	Genome-fragment		1.98	0
S1_vs_k141_79780||full	2413	No	NA	0	1	Not-determined	Genome-fragment	NA		0
S1_vs_k141_59633||full	2042	No	NA	0	3	Not-determined	Genome-fragment	NA		0
S1_vs_k141_13330||full	1717	No	NA	0	2	Not-determined	Genome-fragment	NA		0
S1_vs_k141_119722||full	2065	No	NA	0	0	Not-determined	Genome-fragment	NA		0
S10_vs_68211||full	1543	No	NA	1	0	Low-quality	Genome-fragment		3.74	0
S12_extracted_k141_138560	1543	No	NA	0	0	Low-quality	Genome-fragment		1.58	0
S15_vs_48389||full	1543	No	NA	2	0	Low-quality	Genome-fragment		3	0
S1_vs_k141_26229||full	1389	No	NA	0	0	Not-determined	Genome-fragment	NA		0
S1_vs_k141_38334||full	1773	No	NA	0	0	Not-determined	Genome-fragment	NA		0

<table>
<tr><td>S6_extracted_k141_47425</td><td>1543</td><td>No</td><td>NA</td><td>0</td><td>0</td><td>Low-quality</td><td>Genome-fragment</td><td>1.9</td><td>0</td></tr>
<tr><td>S14_vs_k141_110034||full</td><td>1544</td><td>No</td><td>NA</td><td>0</td><td>0</td><td>Low-quality</td><td>Genome-fragment</td><td>3.7</td><td>0</td></tr>
<tr><td>S3_vs_k141_3670||full</td><td>1544</td><td>No</td><td>NA</td><td>2</td><td>0</td><td>Low-quality</td><td>Genome-fragment</td><td>2.83</td><td>0</td></tr>
<tr><td>S1_vs_k141_126196||full</td><td>1494</td><td>No</td><td>NA</td><td>0</td><td>0</td><td>Not-determined</td><td>Genome-fragment</td><td>NA</td><td>0</td></tr>
<tr><td>S3_vs_k141_20573||full</td><td>1544</td><td>No</td><td>NA</td><td>1</td><td>0</td><td>Low-quality</td><td>Genome-fragment</td><td>2.84</td><td>0</td></tr>
<tr><td>S7_extracted_23903</td><td>1544</td><td>No</td><td>NA</td><td>0</td><td>0</td><td>Low-quality</td><td>Genome-fragment</td><td>1.01</td><td>0</td></tr>
<tr><td>S7_vs_37851||full</td><td>1544</td><td>No</td><td>NA</td><td>1</td><td>0</td><td>Low-quality</td><td>Genome-fragment</td><td>3.89</td><td>0</td></tr>
<tr><td>S16_vs_123991||full</td><td>1544</td><td>No</td><td>NA</td><td>1</td><td>0</td><td>Low-quality</td><td>Genome-fragment</td><td>1.41</td><td>0</td></tr>
<tr><td>S16_vs_162490||lt2gene</td><td>1544</td><td>No</td><td>NA</td><td>2</td><td>0</td><td>Low-quality</td><td>Genome-fragment</td><td>3.04</td><td>0</td></tr>
<tr><td>S1_vs_k141_93112||full</td><td>2198</td><td>No</td><td>NA</td><td>0</td><td>1</td><td>Not-determined</td><td>Genome-fragment</td><td>NA</td><td>0</td></tr>
<tr><td>S10_extracted_93122</td><td>1544</td><td>No</td><td>NA</td><td>0</td><td>1</td><td>Low-quality</td><td>Genome-fragment</td><td>1.22</td><td>0</td></tr>
<tr><td>S4_extracted_18145</td><td>1544</td><td>No</td><td>NA</td><td>4</td><td>0</td><td>Low-quality</td><td>Genome-fragment</td><td>5.09</td><td>0</td></tr>
<tr><td>S1_vs_k141_65158||full</td><td>1467</td><td>No</td><td>NA</td><td>0</td><td>1</td><td>Not-determined</td><td>Genome-fragment</td><td>NA</td><td>0</td></tr>
<tr><td>S1_vs_k141_26298||full</td><td>4596</td><td>No</td><td>NA</td><td>0</td><td>4</td><td>Not-determined</td><td>Genome-fragment</td><td>NA</td><td>0</td></tr>
<tr><td>S6_extracted_k141_51871</td><td>1544</td><td>No</td><td>NA</td><td>1</td><td>0</td><td>Low-quality</td><td>Genome-fragment</td><td>2.57</td><td>0</td></tr>
<tr><td>S1_vs_k141_119810||full</td><td>3511</td><td>No</td><td>NA</td><td>0</td><td>2</td><td>Not-determined</td><td>Genome-fragment</td><td>NA</td><td>0</td></tr>
<tr><td>S8_vs_k141_176074||full</td><td>1544</td><td>No</td><td>NA</td><td>1</td><td>0</td><td>Low-quality</td><td>Genome-fragment</td><td>2.56</td><td>0</td></tr>
<tr><td>S17_extracted_k141_58229</td><td>1545</td><td>No</td><td>NA</td><td>0</td><td>1</td><td>Low-quality</td><td>Genome-fragment</td><td>0.78</td><td>0</td></tr>
<tr><td>S1_vs_k141_5102||full</td><td>4763</td><td>No</td><td>NA</td><td>0</td><td>2</td><td>Not-determined</td><td>Genome-fragment</td><td>NA</td><td>0</td></tr>
<tr><td>S1_vs_k141_38430||full</td><td>1953</td><td>No</td><td>NA</td><td>0</td><td>1</td><td>Not-determined</td><td>Genome-fragment</td><td>NA</td><td>0</td></tr>
<tr><td>S1_vs_k141_106573||full</td><td>1506</td><td>No</td><td>NA</td><td>0</td><td>2</td><td>Not-determined</td><td>Genome-fragment</td><td>NA</td><td>0</td></tr>
<tr><td>S17_vs_k141_59956||full</td><td>1545</td><td>No</td><td>NA</td><td>0</td><td>0</td><td>Low-quality</td><td>Genome-fragment</td><td>2.93</td><td>0</td></tr>
<tr><td>S9_vs_k141_6639||full</td><td>1545</td><td>No</td><td>NA</td><td>0</td><td>0</td><td>Low-quality</td><td>Genome-fragment</td><td>3.51</td><td>0</td></tr>
<tr><td>S13_vs_130962||full</td><td>1545</td><td>No</td><td>NA</td><td>0</td><td>0</td><td>Low-quality</td><td>Genome-fragment</td><td>1.57</td><td>0</td></tr>
<tr><td>S1_vs_k141_23244||full</td><td>1545</td><td>No</td><td>NA</td><td>0</td><td>0</td><td>Low-quality</td><td>Genome-fragment</td><td>0.88</td><td>0</td></tr>
<tr><td>S1_vs_k141_113156||full</td><td>1419</td><td>No</td><td>NA</td><td>0</td><td>2</td><td>Not-determined</td><td>Genome-fragment</td><td>NA</td><td>0</td></tr>
<tr><td>S3_vs_k141_53027||full</td><td>1545</td><td>No</td><td>NA</td><td>0</td><td>0</td><td>Low-quality</td><td>Genome-fragment</td><td>3.13</td><td>0</td></tr>
<tr><td>S1_vs_k141_59804||full</td><td>1406</td><td>No</td><td>NA</td><td>0</td><td>0</td><td>Not-determined</td><td>Genome-fragment</td><td>NA</td><td>0</td></tr>
<tr><td>S1_vs_k141_100019||full</td><td>4858</td><td>No</td><td>NA</td><td>2</td><td>0</td><td>Medium-quality</td><td>Genome-fragment</td><td>87.87</td><td>0</td></tr>
<tr><td>S5_vs_k141_116379||full</td><td>1545</td><td>No</td><td>NA</td><td>0</td><td>1</td><td>Low-quality</td><td>Genome-fragment</td><td>2.24</td><td>0</td></tr>
<tr><td>S7_vs_25200||full</td><td>1545</td><td>No</td><td>NA</td><td>1</td><td>0</td><td>Low-quality</td><td>Genome-fragment</td><td>3.32</td><td>0</td></tr>
<tr><td>S10_extracted_114349</td><td>1545</td><td>No</td><td>NA</td><td>2</td><td>0</td><td>Low-quality</td><td>Genome-fragment</td><td>0.92</td><td>0</td></tr>
<tr><td>S10_vs_93910||full</td><td>1545</td><td>No</td><td>NA</td><td>0</td><td>0</td><td>Low-quality</td><td>Genome-fragment</td><td>2.81</td><td>0</td></tr>
<tr><td>S1_vs_k141_38485||full</td><td>2010</td><td>No</td><td>NA</td><td>0</td><td>2</td><td>Not-determined</td><td>Genome-fragment</td><td>NA</td><td>0</td></tr>
<tr><td>S1_vs_k141_133422||full</td><td>3144</td><td>No</td><td>NA</td><td>0</td><td>2</td><td>Not-determined</td><td>Genome-fragment</td><td>NA</td><td>0</td></tr>
<tr><td>S12_vs_k141_68486||full</td><td>1545</td><td>No</td><td>NA</td><td>0</td><td>0</td><td>Low-quality</td><td>Genome-fragment</td><td>2.55</td><td>0</td></tr>
<tr><td>S1_vs_k141_26418||full</td><td>2092</td><td>No</td><td>NA</td><td>0</td><td>2</td><td>Not-determined</td><td>Genome-fragment</td><td>NA</td><td>0</td></tr>
<tr><td>S1_vs_k141_26419||full</td><td>1746</td><td>No</td><td>NA</td><td>0</td><td>1</td><td>Not-determined</td><td>Genome-fragment</td><td>NA</td><td>0</td></tr>
<tr><td>S8_vs_k141_157573||full</td><td>1545</td><td>No</td><td>NA</td><td>0</td><td>0</td><td>Low-quality</td><td>Genome-fragment</td><td>5.55</td><td>0</td></tr>
<tr><td>S1_vs_k141_146096||full</td><td>5232</td><td>No</td><td>NA</td><td>0</td><td>3</td><td>Not-determined</td><td>Genome-fragment</td><td>NA</td><td>0</td></tr>
<tr><td>S9_vs_k141_55777||full</td><td>1546</td><td>No</td><td>NA</td><td>0</td><td>0</td><td>Low-quality</td><td>Genome-fragment</td><td>1.3</td><td>0</td></tr>
<tr><td>S11_vs_k141_63940||full</td><td>1546</td><td>No</td><td>NA</td><td>1</td><td>0</td><td>Low-quality</td><td>Genome-fragment</td><td>3.96</td><td>0</td></tr>
<tr><td>S14_extracted_k141_10246</td><td>1546</td><td>No</td><td>NA</td><td>2</td><td>0</td><td>Low-quality</td><td>Genome-fragment</td><td>4.99</td><td>0</td></tr>
<tr><td>S1_vs_k141_46676||full</td><td>3157</td><td>No</td><td>NA</td><td>0</td><td>3</td><td>Not-determined</td><td>Genome-fragment</td><td>NA</td><td>0</td></tr>
<tr><td>S14_vs_k141_75392||full</td><td>1546</td><td>No</td><td>NA</td><td>1</td><td>0</td><td>Low-quality</td><td>Genome-fragment</td><td>2.61</td><td>0</td></tr>
<tr><td>S1_vs_k141_71787||full</td><td>1012</td><td>No</td><td>NA</td><td>0</td><td>1</td><td>Not-determined</td><td>Genome-fragment</td><td>NA</td><td>0</td></tr>
<tr><td>S1_vs_k141_86729||full</td><td>1699</td><td>No</td><td>NA</td><td>0</td><td>1</td><td>Not-determined</td><td>Genome-fragment</td><td>NA</td><td>0</td></tr>
<tr><td>S1_vs_k141_119949||full</td><td>1454</td><td>No</td><td>NA</td><td>0</td><td>0</td><td>Not-determined</td><td>Genome-fragment</td><td>NA</td><td>0</td></tr>
<tr><td>S1_vs_k141_124046||full</td><td>1546</td><td>No</td><td>NA</td><td>0</td><td>0</td><td>Low-quality</td><td>Genome-fragment</td><td>1.08</td><td>0</td></tr>
<tr><td>S1_vs_k141_71799||full</td><td>1179</td><td>No</td><td>NA</td><td>0</td><td>2</td><td>Not-determined</td><td>Genome-fragment</td><td>NA</td><td>0</td></tr>
<tr><td>S7_vs_45494||full</td><td>1546</td><td>No</td><td>NA</td><td>0</td><td>0</td><td>Low-quality</td><td>Genome-fragment</td><td>2.93</td><td>0</td></tr>
<tr><td>S16_extracted_14182</td><td>1546</td><td>No</td><td>NA</td><td>2</td><td>0</td><td>Low-quality</td><td>Genome-fragment</td><td>3.4</td><td>0</td></tr>
<tr><td>S16_vs_119381||full</td><td>1546</td><td>No</td><td>NA</td><td>1</td><td>0</td><td>Low-quality</td><td>Genome-fragment</td><td>3.55</td><td>0</td></tr>
<tr><td>S1_vs_k141_65361||full</td><td>1936</td><td>No</td><td>NA</td><td>0</td><td>2</td><td>Not-determined</td><td>Genome-fragment</td><td>NA</td><td>0</td></tr>
<tr><td>S1_vs_k141_46716||full</td><td>2368</td><td>No</td><td>NA</td><td>0</td><td>0</td><td>Not-determined</td><td>Genome-fragment</td><td>NA</td><td>0</td></tr>
<tr><td>S1_vs_k141_119974||full</td><td>4871</td><td>No</td><td>NA</td><td>0</td><td>4</td><td>Not-determined</td><td>Genome-fragment</td><td>NA</td><td>0</td></tr>
<tr><td>S10_vs_69242||full</td><td>1546</td><td>No</td><td>NA</td><td>2</td><td>0</td><td>Low-quality</td><td>Genome-fragment</td><td>8.61</td><td>0</td></tr>
<tr><td>S1_vs_k141_26455||full</td><td>1262</td><td>No</td><td>NA</td><td>0</td><td>1</td><td>Not-determined</td><td>Genome-fragment</td><td>NA</td><td>0</td></tr>
<tr><td>S12_vs_k141_104093||full</td><td>1546</td><td>No</td><td>NA</td><td>0</td><td>1</td><td>Low-quality</td><td>Genome-fragment</td><td>1.12</td><td>0</td></tr>
<tr><td>S17_vs_k141_149240||full</td><td>1547</td><td>No</td><td>NA</td><td>1</td><td>0</td><td>Low-quality</td><td>Genome-fragment</td><td>3.45</td><td>0</td></tr>
<tr><td>S1_vs_k141_88069||full</td><td>1547</td><td>Yes</td><td>808</td><td>2</td><td>1</td><td>Low-quality</td><td>Genome-fragment</td><td>1.44</td><td>47.77</td></tr>
<tr><td>S10_vs_13145||full</td><td>1547</td><td>No</td><td>NA</td><td>0</td><td>0</td><td>Low-quality</td><td>Genome-fragment</td><td>1.14</td><td>0</td></tr>
<tr><td>S1_vs_k141_26586||full</td><td>2738</td><td>No</td><td>NA</td><td>0</td><td>2</td><td>Not-determined</td><td>Genome-fragment</td><td>NA</td><td>0</td></tr>
<tr><td>S1_vs_k141_146354||full</td><td>1824</td><td>No</td><td>NA</td><td>0</td><td>1</td><td>Not-determined</td><td>Genome-fragment</td><td>NA</td><td>0</td></tr>
<tr><td>S17_extracted_k141_49419</td><td>1548</td><td>No</td><td>NA</td><td>0</td><td>0</td><td>Low-quality</td><td>Genome-fragment</td><td>2.32</td><td>0</td></tr>
<tr><td>S1_vs_k141_26638||full</td><td>1688</td><td>No</td><td>NA</td><td>0</td><td>2</td><td>Not-determined</td><td>Genome-fragment</td><td>NA</td><td>0</td></tr>
<tr><td>S1_vs_k141_5432||full</td><td>3162</td><td>No</td><td>NA</td><td>0</td><td>2</td><td>Not-determined</td><td>Genome-fragment</td><td>NA</td><td>0</td></tr>
<tr><td>S1_vs_k141_146444||full</td><td>2065</td><td>No</td><td>NA</td><td>0</td><td>2</td><td>Not-determined</td><td>Genome-fragment</td><td>NA</td><td>0</td></tr>
<tr><td>S17_vs_k141_31423||full</td><td>1548</td><td>No</td><td>NA</td><td>2</td><td>0</td><td>Low-quality</td><td>Genome-fragment</td><td>3.54</td><td>0</td></tr>
<tr><td>S11_vs_k141_24880||full</td><td>1548</td><td>No</td><td>NA</td><td>1</td><td>0</td><td>Low-quality</td><td>Genome-fragment</td><td>2.61</td><td>0</td></tr>
<tr><td>S14_vs_k141_26346||full</td><td>1548</td><td>No</td><td>NA</td><td>0</td><td>1</td><td>Low-quality</td><td>Genome-fragment</td><td>0.96</td><td>0</td></tr>
<tr><td>S1_vs_k141_140167||full</td><td>1782</td><td>No</td><td>NA</td><td>0</td><td>0</td><td>Not-determined</td><td>Genome-fragment</td><td>NA</td><td>0</td></tr>
<tr><td>S1_vs_k141_5524||full</td><td>1537</td><td>No</td><td>NA</td><td>0</td><td>2</td><td>Not-determined</td><td>Genome-fragment</td><td>NA</td><td>0</td></tr>
<tr><td>S1_vs_k141_38795||full</td><td>2663</td><td>No</td><td>NA</td><td>0</td><td>0</td><td>Not-determined</td><td>Genome-fragment</td><td>NA</td><td>0</td></tr>
<tr><td>S1_vs_k141_47963||lt2gene</td><td>1548</td><td>No</td><td>NA</td><td>1</td><td>0</td><td>Low-quality</td><td>Genome-fragment</td><td>3.64</td><td>0</td></tr>
<tr><td>S5_vs_k141_115747||full</td><td>1548</td><td>No</td><td>NA</td><td>1</td><td>0</td><td>Low-quality</td><td>Genome-fragment</td><td>3.34</td><td>0</td></tr>
<tr><td>S7_extracted_25249</td><td>1548</td><td>No</td><td>NA</td><td>0</td><td>0</td><td>Low-quality</td><td>Genome-fragment</td><td>3.43</td><td>0</td></tr>
<tr><td>S16_vs_12227||lt2gene</td><td>1548</td><td>No</td><td>NA</td><td>1</td><td>0</td><td>Low-quality</td><td>Genome-fragment</td><td>2.53</td><td>0</td></tr>
<tr><td>S1_vs_k141_126755||full</td><td>4255</td><td>No</td><td>NA</td><td>0</td><td>3</td><td>Not-determined</td><td>Genome-fragment</td><td>NA</td><td>0</td></tr>
<tr><td>S10_extracted_57712</td><td>1548</td><td>No</td><td>NA</td><td>0</td><td>1</td><td>Low-quality</td><td>Genome-fragment</td><td>1</td><td>0</td></tr>
<tr><td>S1_vs_k141_146644||full</td><td>2762</td><td>No</td><td>NA</td><td>0</td><td>0</td><td>Not-determined</td><td>Genome-fragment</td><td>NA</td><td>0</td></tr>
<tr><td>S12_vs_k141_156872||full</td><td>1548</td><td>No</td><td>NA</td><td>0</td><td>0</td><td>Low-quality</td><td>Genome-fragment</td><td>3.91</td><td>0</td></tr>
<tr><td>S17_extracted_k141_14811</td><td>1549</td><td>No</td><td>NA</td><td>4</td><td>0</td><td>Low-quality</td><td>Genome-fragment</td><td>4.04</td><td>0</td></tr>
<tr><td>S1_vs_k141_65899||full</td><td>2770</td><td>No</td><td>NA</td><td>0</td><td>0</td><td>Not-determined</td><td>Genome-fragment</td><td>NA</td><td>0</td></tr>
<tr><td>S1_vs_k141_5646||full</td><td>2684</td><td>No</td><td>NA</td><td>0</td><td>2</td><td>Not-determined</td><td>Genome-fragment</td><td>NA</td><td>0</td></tr>
<tr><td>S1_extracted_k141_125670</td><td>1549</td><td>No</td><td>NA</td><td>1</td><td>0</td><td>Low-quality</td><td>Genome-fragment</td><td>4.12</td><td>0</td></tr>
<tr><td>S1_vs_k141_145528||full</td><td>1549</td><td>No</td><td>NA</td><td>0</td><td>0</td><td>Low-quality</td><td>Genome-fragment</td><td>3.65</td><td>0</td></tr>
<tr><td>S3_vs_k141_91390||full</td><td>1549</td><td>No</td><td>NA</td><td>0</td><td>0</td><td>Low-quality</td><td>Genome-fragment</td><td>2.98</td><td>0</td></tr>
<tr><td>S1_vs_k141_146764||full</td><td>3654</td><td>No</td><td>NA</td><td>0</td><td>2</td><td>Not-determined</td><td>Genome-fragment</td><td>NA</td><td>0</td></tr>
<tr><td>S1_vs_k141_157283||full</td><td>1341</td><td>No</td><td>NA</td><td>0</td><td>0</td><td>Not-determined</td><td>Genome-fragment</td><td>NA</td><td>0</td></tr>
<tr><td>S1_vs_k141_65984||full</td><td>2765</td><td>No</td><td>NA</td><td>0</td><td>1</td><td>Not-determined</td><td>Genome-fragment</td><td>NA</td><td>0</td></tr>
<tr><td>S5_extracted_k141_86854</td><td>1549</td><td>No</td><td>NA</td><td>1</td><td>0</td><td>Low-quality</td><td>Genome-fragment</td><td>2.35</td><td>0</td></tr>
<tr><td>S1_vs_k141_66089||full</td><td>1532</td><td>No</td><td>NA</td><td>0</td><td>1</td><td>Not-determined</td><td>Genome-fragment</td><td>NA</td><td>0</td></tr>
<tr><td>S1_vs_k141_66182||full</td><td>1301</td><td>No</td><td>NA</td><td>0</td><td>2</td><td>Not-determined</td><td>Genome-fragment</td><td>NA</td><td>0</td></tr>
<tr><td>S1_vs_k141_39095||full</td><td>2231</td><td>No</td><td>NA</td><td>0</td><td>0</td><td>Not-determined</td><td>Genome-fragment</td><td>NA</td><td>0</td></tr>
<tr><td>S5_extracted_k141_124458</td><td>1549</td><td>No</td><td>NA</td><td>2</td><td>0</td><td>Low-quality</td><td>Genome-fragment</td><td>4.1</td><td>0</td></tr>
<tr><td>S5_vs_k141_123206||full</td><td>1549</td><td>No</td><td>NA</td><td>0</td><td>0</td><td>Low-quality</td><td>Genome-fragment</td><td>3.5</td><td>0</td></tr>
<tr><td>S5_vs_k141_88318||full</td><td>1549</td><td>No</td><td>NA</td><td>1</td><td>0</td><td>Low-quality</td><td>Genome-fragment</td><td>3.82</td><td>0</td></tr>
<tr><td>S1_vs_k141_66340||full</td><td>1146</td><td>No</td><td>NA</td><td>0</td><td>1</td><td>Not-determined</td><td>Genome-fragment</td><td>NA</td><td>0</td></tr>
<tr><td>S1_vs_k141_39244||full</td><td>4216</td><td>No</td><td>NA</td><td>0</td><td>3</td><td>Not-determined</td><td>Genome-fragment</td><td>NA</td><td>0</td></tr>
<tr><td>S7_vs_94006||full</td><td>1549</td><td>No</td><td>NA</td><td>0</td><td>0</td><td>Low-quality</td><td>Genome-fragment</td><td>3.52</td><td>0</td></tr>
<tr><td>S16_vs_19047||full</td><td>1549</td><td>No</td><td>NA</td><td>1</td><td>0</td><td>Low-quality</td><td>Genome-fragment</td><td>3.75</td><td>0</td></tr>
<tr><td>S1_vs_k141_6033||full</td><td>1672</td><td>No</td><td>NA</td><td>0</td><td>0</td><td>Not-determined</td><td>Genome-fragment</td><td>NA</td><td>0</td></tr>
<tr><td>S1_vs_k141_6037||full</td><td>2076</td><td>No</td><td>NA</td><td>0</td><td>1</td><td>Not-determined</td><td>Genome-fragment</td><td>NA</td><td>0</td></tr>
<tr><td>S1_vs_k141_157548||full</td><td>2228</td><td>No</td><td>NA</td><td>0</td><td>1</td><td>Not-determined</td><td>Genome-fragment</td><td>NA</td><td>0</td></tr>
<tr><td>S16_vs_97459||full</td><td>1549</td><td>No</td><td>NA</td><td>0</td><td>1</td><td>Low-quality</td><td>Genome-fragment</td><td>2.23</td><td>0</td></tr>
<tr><td>S12_vs_k141_54209||full</td><td>1549</td><td>No</td><td>NA</td><td>1</td><td>0</td><td>Low-quality</td><td>Genome-fragment</td><td>3.63</td><td>0</td></tr>
<tr><td>S15_vs_56252||full</td><td>1549</td><td>No</td><td>NA</td><td>0</td><td>1</td><td>Low-quality</td><td>Genome-fragment</td><td>2.46</td><td>0</td></tr>
<tr><td>S8_extracted_k141_4173</td><td>1549</td><td>No</td><td>NA</td><td>1</td><td>0</td><td>Low-quality</td><td>Genome-fragment</td><td>2.46</td><td>0</td></tr>
<tr><td>S1_vs_k141_6259||full</td><td>1530</td><td>No</td><td>NA</td><td>0</td><td>1</td><td>Not-determined</td><td>Genome-fragment</td><td>NA</td><td>0</td></tr>
<tr><td>S1_vs_k141_66685||full</td><td>1521</td><td>No</td><td>NA</td><td>0</td><td>0</td><td>Not-determined</td><td>Genome-fragment</td><td>NA</td><td>0</td></tr>
<tr><td>S1_vs_k141_72656||full</td><td>2848</td><td>No</td><td>NA</td><td>0</td><td>3</td><td>Not-determined</td><td>Genome-fragment</td><td>NA</td><td>0</td></tr>
<tr><td>S1_vs_k141_150530||full</td><td>1397</td><td>No</td><td>NA</td><td>0</td><td>2</td><td>Not-determined</td><td>Genome-fragment</td><td>NA</td><td>0</td></tr>
<tr><td>S8_vs_k141_74161||full</td><td>1549</td><td>No</td><td>NA</td><td>0</td><td>0</td><td>Low-quality</td><td>Genome-fragment</td><td>2.46</td><td>0</td></tr>
<tr><td>S1_vs_k141_6281||full</td><td>1211</td><td>No</td><td>NA</td><td>0</td><td>1</td><td>Not-determined</td><td>Genome-fragment</td><td>NA</td><td>0</td></tr>
<tr><td>S17_vs_k141_97588||full</td><td>1550</td><td>No</td><td>NA</td><td>0</td><td>0</td><td>Low-quality</td><td>Genome-fragment</td><td>2.59</td><td>0</td></tr>
<tr><td>S1_vs_k141_72762||full</td><td>1998</td><td>No</td><td>NA</td><td>0</td><td>1</td><td>Not-determined</td><td>Genome-fragment</td><td>NA</td><td>0</td></tr>
<tr><td>S17_vs_k141_147618||full</td><td>1550</td><td>No</td><td>NA</td><td>0</td><td>0</td><td>Low-quality</td><td>Genome-fragment</td><td>0.91</td><td>0</td></tr>
<tr><td>S1_extracted_k141_103614</td><td>1550</td><td>No</td><td>NA</td><td>0</td><td>2</td><td>Low-quality</td><td>Genome-fragment</td><td>0.17</td><td>0</td></tr>
<tr><td>S1_vs_k141_157764||full</td><td>5060</td><td>No</td><td>NA</td><td>0</td><td>2</td><td>Not-determined</td><td>Genome-fragment</td><td>NA</td><td>0</td></tr>
<tr><td>S16_vs_29977||full</td><td>1550</td><td>No</td><td>NA</td><td>0</td><td>0</td><td>Low-quality</td><td>Genome-fragment</td><td>1.81</td><td>0</td></tr>
<tr><td>S16_vs_158572||full</td><td>1550</td><td>No</td><td>NA</td><td>1</td><td>0</td><td>Low-quality</td><td>Genome-fragment</td><td>3.74</td><td>0</td></tr>
<tr><td>S1_vs_k141_39730||full</td><td>1048</td><td>No</td><td>NA</td><td>0</td><td>2</td><td>Not-determined</td><td>Genome-fragment</td><td>NA</td><td>0</td></tr>
<tr><td>S2_vs_k141_17411||full</td><td>1550</td><td>No</td><td>NA</td><td>1</td><td>0</td><td>Low-quality</td><td>Genome-fragment</td><td>3.14</td><td>0</td></tr>
<tr><td>S1_vs_k141_39809||full</td><td>2467</td><td>No</td><td>NA</td><td>0</td><td>3</td><td>Not-determined</td><td>Genome-fragment</td><td>NA</td><td>0</td></tr>
<tr><td>S6_vs_k141_15257||full</td><td>1550</td><td>No</td><td>NA</td><td>0</td><td>0</td><td>Low-quality</td><td>Genome-fragment</td><td>0.91</td><td>0</td></tr>
<tr><td>S1_vs_k141_115119||full</td><td>1551</td><td>No</td><td>NA</td><td>0</td><td>0</td><td>Low-quality</td><td>Genome-fragment</td><td>1.13</td><td>0</td></tr>
<tr><td>S1_vs_k141_39913||full</td><td>1406</td><td>No</td><td>NA</td><td>0</td><td>0</td><td>Not-determined</td><td>Genome-fragment</td><td>NA</td><td>0</td></tr>
<tr><td>S3_vs_k141_16184||full</td><td>1551</td><td>No</td><td>NA</td><td>0</td><td>0</td><td>Low-quality</td><td>Genome-fragment</td><td>2.72</td><td>0</td></tr>
<tr><td>S1_vs_k141_150865||full</td><td>2223</td><td>No</td><td>NA</td><td>0</td><td>2</td><td>Not-determined</td><td>Genome-fragment</td><td>NA</td><td>0</td></tr>
<tr><td>S1_vs_k141_158190||full</td><td>1002</td><td>No</td><td>NA</td><td>0</td><td>1</td><td>Not-determined</td><td>Genome-fragment</td><td>NA</td><td>0</td></tr>
<tr><td>S7_vs_7267||full</td><td>1551</td><td>No</td><td>NA</td><td>0</td><td>0</td><td>Low-quality</td><td>Genome-fragment</td><td>3.86</td><td>0</td></tr>
<tr><td>S1_vs_k141_150919||full</td><td>1655</td><td>No</td><td>NA</td><td>0</td><td>1</td><td>Not-determined</td><td>Genome-fragment</td><td>NA</td><td>0</td></tr>
<tr><td>S1_vs_k141_158362||full</td><td>1251</td><td>No</td><td>NA</td><td>0</td><td>0</td><td>Not-determined</td><td>Genome-fragment</td><td>NA</td><td>0</td></tr>
<tr><td>S7_vs_75084||full</td><td>1551</td><td>No</td><td>NA</td><td>0</td><td>0</td><td>Low-quality</td><td>Genome-fragment</td><td>3.62</td><td>0</td></tr>
<tr><td>S15_vs_45237||lt2gene</td><td>1551</td><td>No</td><td>NA</td><td>1</td><td>0</td><td>Low-quality</td><td>Genome-fragment</td><td>3.48</td><td>0</td></tr>
<tr><td>S1_vs_k141_158492||full</td><td>7107</td><td>No</td><td>NA</td><td>0</td><td>5</td><td>Not-determined</td><td>Genome-fragment</td><td>NA</td><td>0</td></tr>
<tr><td>S1_vs_k141_158493||full</td><td>1129</td><td>No</td><td>NA</td><td>0</td><td>1</td><td>Not-determined</td><td>Genome-fragment</td><td>NA</td><td>0</td></tr>
<tr><td>S4_extracted_4997</td><td>1551</td><td>No</td><td>NA</td><td>2</td><td>0</td><td>Low-quality</td><td>Genome-fragment</td><td>5.1</td><td>0</td></tr>
<tr><td>S4_extracted_13380</td><td>1551</td><td>No</td><td>NA</td><td>0</td><td>0</td><td>Low-quality</td><td>Genome-fragment</td><td>2.12</td><td>0</td></tr>
<tr><td>S6_vs_k141_7352||full</td><td>1551</td><td>No</td><td>NA</td><td>1</td><td>0</td><td>Low-quality</td><td>Genome-fragment</td><td>2.99</td><td>0</td></tr>
<tr><td>S1_vs_k141_151403||full</td><td>5241</td><td>No</td><td>NA</td><td>0</td><td>3</td><td>Not-determined</td><td>Genome-fragment</td><td>NA</td><td>0</td></tr>
<tr><td>S6_vs_k141_11485||full</td><td>1551</td><td>No</td><td>NA</td><td>0</td><td>2</td><td>Low-quality</td><td>Genome-fragment</td><td>3.28</td><td>0</td></tr>
<tr><td>S8_extracted_k141_152231</td><td>1551</td><td>No</td><td>NA</td><td>0</td><td>1</td><td>Low-quality</td><td>Genome-fragment</td><td>0.84</td><td>0</td></tr>
<tr><td>S1_vs_k141_151692||full</td><td>1708</td><td>No</td><td>NA</td><td>0</td><td>0</td><td>Not-determined</td><td>Genome-fragment</td><td>NA</td><td>0</td></tr>
<tr><td>S8_vs_k141_8231||full</td><td>1551</td><td>No</td><td>NA</td><td>1</td><td>0</td><td>Low-quality</td><td>Genome-fragment</td><td>4.81</td><td>0</td></tr>
<tr><td>S1_vs_k141_159004||full</td><td>3978</td><td>No</td><td>NA</td><td>0</td><td>0</td><td>Not-determined</td><td>Genome-fragment</td><td>NA</td><td>0</td></tr>
<tr><td>S1_vs_k141_151840||full</td><td>3139</td><td>No</td><td>NA</td><td>0</td><td>2</td><td>Not-determined</td><td>Genome-fragment</td><td>NA</td><td>0</td></tr>
<tr><td>S1_vs_k141_151883||full</td><td>2246</td><td>No</td><td>NA</td><td>0</td><td>2</td><td>Not-determined</td><td>Genome-fragment</td><td>NA</td><td>0</td></tr>
<tr><td>S8_vs_k141_56314||lt2gene</td><td>1551</td><td>No</td><td>NA</td><td>2</td><td>0</td><td>Low-quality</td><td>Genome-fragment</td><td>1.18</td><td>0</td></tr>
<tr><td>S17_extracted_k141_120249</td><td>1552</td><td>No</td><td>NA</td><td>1</td><td>0</td><td>Low-quality</td><td>Genome-fragment</td><td>2.51</td><td>0</td></tr>
<tr><td>S17_extracted_k141_71464</td><td>1552</td><td>No</td><td>NA</td><td>0</td><td>0</td><td>Low-quality</td><td>Genome-fragment</td><td>1.55</td><td>0</td></tr>
<tr><td>S1_vs_k141_159244||full</td><td>2412</td><td>No</td><td>NA</td><td>0</td><td>2</td><td>Not-determined</td><td>Genome-fragment</td><td>NA</td><td>0</td></tr>
<tr><td>S9_vs_k141_27805||full</td><td>1552</td><td>No</td><td>NA</td><td>2</td><td>0</td><td>Low-quality</td><td>Genome-fragment</td><td>4.31</td><td>0</td></tr>
<tr><td>S1_vs_k141_159281||full</td><td>1530</td><td>No</td><td>NA</td><td>0</td><td>0</td><td>Not-determined</td><td>Genome-fragment</td><td>NA</td><td>0</td></tr>
<tr><td>S11_vs_k141_20453||full</td><td>1552</td><td>No</td><td>NA</td><td>2</td><td>0</td><td>Low-quality</td><td>Genome-fragment</td><td>1.91</td><td>0</td></tr>
<tr><td>S1_vs_k141_106624||full</td><td>1552</td><td>No</td><td>NA</td><td>1</td><td>0</td><td>Low-quality</td><td>Genome-fragment</td><td>3.96</td><td>0</td></tr>
<tr><td>S1_vs_k141_152288||full</td><td>1040</td><td>No</td><td>NA</td><td>0</td><td>0</td><td>Not-determined</td><td>Genome-fragment</td><td>NA</td><td>0</td></tr>
<tr><td>S1_vs_k141_152399||full</td><td>3204</td><td>No</td><td>NA</td><td>0</td><td>1</td><td>Not-determined</td><td>Genome-fragment</td><td>NA</td><td>0</td></tr>
<tr><td>S3_vs_k141_30745||full</td><td>1552</td><td>No</td><td>NA</td><td>0</td><td>1</td><td>Low-quality</td><td>Genome-fragment</td><td>2.27</td><td>0</td></tr>
<tr><td>S3_vs_k141_7652||full</td><td>1552</td><td>No</td><td>NA</td><td>0</td><td>0</td><td>Low-quality</td><td>Genome-fragment</td><td>2.82</td><td>0</td></tr>
<tr><td>S7_extracted_44429</td><td>1552</td><td>No</td><td>NA</td><td>0</td><td>1</td><td>Low-quality</td><td>Genome-fragment</td><td>0.88</td><td>0</td></tr>
<tr><td>S12_extracted_k141_170295</td><td>1552</td><td>No</td><td>NA</td><td>2</td><td>0</td><td>Low-quality</td><td>Genome-fragment</td><td>3.4</td><td>0</td></tr>
<tr><td>S12_vs_k141_165477||lt2gene</td><td>1552</td><td>No</td><td>NA</td><td>1</td><td>0</td><td>Low-quality</td><td>Genome-fragment</td><td>3.77</td><td>0</td></tr>
<tr><td>S1_vs_k141_159736||full</td><td>1139</td><td>No</td><td>NA</td><td>0</td><td>2</td><td>Not-determined</td><td>Genome-fragment</td><td>NA</td><td>0</td></tr>
<tr><td>S1_vs_k141_152864||full</td><td>1201</td><td>No</td><td>NA</td><td>0</td><td>1</td><td>Not-determined</td><td>Genome-fragment</td><td>NA</td><td>0</td></tr>
<tr><td>S1_vs_k141_159927||full</td><td>3184</td><td>No</td><td>NA</td><td>0</td><td>0</td><td>Not-determined</td><td>Genome-fragment</td><td>NA</td><td>0</td></tr>
<tr><td>S8_vs_k141_162534||full</td><td>1552</td><td>No</td><td>NA</td><td>0</td><td>0</td><td>Low-quality</td><td>Genome-fragment</td><td>2.12</td><td>0</td></tr>
<tr><td>S17_vs_k141_133303||full</td><td>1553</td><td>No</td><td>NA</td><td>1</td><td>0</td><td>Low-quality</td><td>Genome-fragment</td><td>3</td><td>0</td></tr>
<tr><td>S1_vs_k141_153340||full</td><td>4479</td><td>No</td><td>NA</td><td>0</td><td>1</td><td>Not-determined</td><td>Genome-fragment</td><td>NA</td><td>0</td></tr>
<tr><td>S1_vs_k141_53468||lt2gene</td><td>1434</td><td>No</td><td>NA</td><td>0</td><td>0</td><td>Not-determined</td><td>Genome-fragment</td><td>NA</td><td>0</td></tr>
<tr><td>S17_vs_k141_15298||full</td><td>1553</td><td>No</td><td>NA</td><td>0</td><td>0</td><td>Low-quality</td><td>Genome-fragment</td><td>1.27</td><td>0</td></tr>
<tr><td>S17_vs_k141_150951||full</td><td>1553</td><td>No</td><td>NA</td><td>0</td><td>0</td><td>Low-quality</td><td>Genome-fragment</td><td>2.14</td><td>0</td></tr>
<tr><td>S11_vs_k141_1973||full</td><td>1553</td><td>No</td><td>NA</td><td>0</td><td>0</td><td>Low-quality</td><td>Genome-fragment</td><td>3.35</td><td>0</td></tr>
</table>

S14_vs_k141_22370||full	1553	No	NA		0	0	Low-quality	Genome-fragment		3.85	0
S1_vs_k141_73975||lt2gene	1142	No	NA		0	0	Not-determined	Genome-fragment	NA		0
S14_vs_k141_30059||full	1553	No	NA		0	2	Low-quality	Genome-fragment		1.12	0
S1_vs_k141_153906||lt2gene	2950	No	NA		0	1	Not-determined	Genome-fragment	NA		0
S14_vs_k141_73802||full	1553	No	NA		0	0	Low-quality	Genome-fragment		3.6	0
S3_extracted_k141_114692	1553	No	NA		1	0	Low-quality	Genome-fragment		3.4	0
S3_vs_k141_22812||full	1553	No	NA		1	0	Low-quality	Genome-fragment		2.42	0
S7_extracted_29742	1553	No	NA		0	2	Low-quality	Genome-fragment		2.88	0
S7_extracted_60692	1553	No	NA		1	0	Low-quality	Genome-fragment		2.93	0
S7_vs_125711||full	1553	No	NA		3	0	Low-quality	Genome-fragment		3.33	0
S16_vs_166471||full	1553	No	NA		0	2	Low-quality	Genome-fragment		0.76	0
S10_vs_67733||lt2gene	1553	No	NA		1	0	Low-quality	Genome-fragment		0.84	0
S12_extracted_k141_146725	1553	No	NA		0	0	Low-quality	Genome-fragment		4.13	0
S9_vs_k141_46896||full	1554	No	NA		2	0	Low-quality	Genome-fragment		4.63	0
S3_vs_k141_75706||full	1554	No	NA		1	0	Low-quality	Genome-fragment		2.73	0
S3_vs_k141_81639||lt2gene	1554	No	NA		1	0	Low-quality	Genome-fragment		1.49	0
S1_vs_k141_48439||lt2gene	1236	No	NA		0	0	Not-determined	Genome-fragment	NA		0
S16_vs_136042||lt2gene	1554	No	NA		1	0	Low-quality	Genome-fragment		4.09	0
S12_vs_k141_163346||full	1554	No	NA		2	0	Low-quality	Genome-fragment		3.96	0
S13_vs_20158||full	1555	No	NA		0	0	Low-quality	Genome-fragment		2.25	0
S7_vs_51535||full	1555	No	NA		0	1	Low-quality	Genome-fragment		1.97	0
S16_vs_129975||full	1555	No	NA		2	0	Low-quality	Genome-fragment		2.38	0
S1_vs_k141_61144||lt2gene	1996	No	NA		0	1	Not-determined	Genome-fragment	NA		0
S17_vs_k141_146824||full	1556	No	NA		0	1	Low-quality	Genome-fragment		3.19	0
S13_vs_24549||full	1556	No	NA		0	2	Low-quality	Genome-fragment		1.41	0
S1_vs_k141_115502||lt2gene	1053	No	NA		0	0	Not-determined	Genome-fragment	NA		0
S13_vs_113751||lt2gene	1556	No	NA		1	0	Low-quality	Genome-fragment		2.95	0
S1_extracted_k141_109462	1556	No	NA		1	0	Low-quality	Genome-fragment		2.53	0
S1_extracted_k141_65477	1556	No	NA		1	0	Low-quality	Genome-fragment		1.57	0
S5_vs_k141_25057||full	1556	No	NA		0	1	Low-quality	Genome-fragment		0.49	0
S5_vs_k141_25827||full	1556	No	NA		0	1	Low-quality	Genome-fragment		1	0
S16_vs_25689||full	1556	No	NA		0	0	Low-quality	Genome-fragment		1.98	0
S16_vs_136724||full	1556	No	NA		0	0	Low-quality	Genome-fragment		2.6	0
S12_extracted_k141_54184	1556	No	NA		2	0	Low-quality	Genome-fragment		3.49	0
S1_vs_k141_55888||lt2gene	2453	No	NA		0	0	Not-determined	Genome-fragment	NA		0
S1_vs_k141_2162||lt2gene	2002	No	NA		0	0	Not-determined	Genome-fragment	NA		0
S12_vs_k141_118308||full	1556	No	NA		1	0	Low-quality	Genome-fragment		4.71	0
S8_vs_k141_199660||lt2gene	1556	No	NA		1	0	Low-quality	Genome-fragment		4.15	0
S1_vs_k141_69370||lt2gene	2020	No	NA		0	0	Not-determined	Genome-fragment	NA		0
S1_vs_k141_96477||lt2gene	2064	No	NA		0	0	Not-determined	Genome-fragment	NA		0
S8_vs_k141_170500||lt2gene	1556	No	NA		1	0	Low-quality	Genome-fragment		3	0
S1_vs_k141_49970||lt2gene	1543	No	NA		0	1	Not-determined	Genome-fragment	NA		0
S17_vs_k141_91677||full	1557	No	NA		0	0	Low-quality	Genome-fragment		2.99	0
S9_vs_k141_57782||full	1557	No	NA		2	0	Low-quality	Genome-fragment		3.83	0
S1_vs_k141_136636||lt2gene	1214	No	NA		0	1	Not-determined	Genome-fragment	NA		0
S9_vs_k141_9103||full	1557	No	NA		0	0	Low-quality	Genome-fragment		3.54	0
S14_extracted_k141_102728	1557	No	NA		3	0	Low-quality	Genome-fragment		5.04	0
S1_vs_k141_30448||lt2gene	1307	No	NA		0	0	Not-determined	Genome-fragment	NA		0
S1_vs_k141_97439||lt2gene	1771	No	NA		0	1	Not-determined	Genome-fragment	NA		0
S1_vs_k141_84240||lt2gene	1023	No	NA		0	1	Not-determined	Genome-fragment	NA		0
S1_vs_k141_155806||full	1557	No	NA		3	0	Low-quality	Genome-fragment		3.26	0
S1_vs_k141_90613||full	1557	No	NA		0	0	Low-quality	Genome-fragment		3.72	0
S3_vs_k141_96818||full	1557	No	NA		0	0	Low-quality	Genome-fragment		2.73	0
S1_vs_k141_51427||lt2gene	1344	No	NA		0	0	Not-determined	Genome-fragment	NA		0
S3_vs_k141_82821||full	1557	No	NA		0	0	Low-quality	Genome-fragment		1.16	0
S1_vs_k141_24465||lt2gene	1333	No	NA		0	0	Not-determined	Genome-fragment	NA		0
S5_vs_k141_130285||full	1557	Yes		802	1	1	Low-quality	Genome-fragment		1.6	48.49
S7_vs_117006||full	1557	No	NA		0	0	Low-quality	Genome-fragment		0.93	0
S16_extracted_121629	1557	No	NA		0	0	Low-quality	Genome-fragment		0.97	0
S12_extracted_k141_75408	1557	No	NA		0	0	Low-quality	Genome-fragment		1.73	0
S8_vs_k141_173289||full	1557	No	NA		2	0	Low-quality	Genome-fragment		4.09	0
S17_vs_k141_124083||full	1558	No	NA		0	2	Low-quality	Genome-fragment		1.75	0
S1_vs_k141_37190||lt2gene	1915	No	NA		0	0	Not-determined	Genome-fragment	NA		0
S17_vs_k141_22026||full	1558	No	NA		0	0	Low-quality	Genome-fragment		3.46	0
S1_vs_k141_144838||lt2gene	1157	No	NA		0	0	Not-determined	Genome-fragment	NA		0
S13_vs_123916||full	1558	No	NA		0	1	Low-quality	Genome-fragment		3.2	0
S1_vs_k141_145087||lt2gene	1162	No	NA		0	0	Not-determined	Genome-fragment	NA		0
S14_vs_k141_61176||full	1558	No	NA		0	0	Low-quality	Genome-fragment		3.6	0
S3_extracted_k141_98639	1558	No	NA		0	0	Low-quality	Genome-fragment		2.45	0
S3_extracted_k141_126731	1558	No	NA		0	0	Low-quality	Genome-fragment		2.37	0
S5_vs_k141_62141||full	1558	No	NA		0	4	Low-quality	Genome-fragment		0.44	0
S16_vs_25681||full	1558	No	NA		0	1	Low-quality	Genome-fragment		0.57	0
S16_vs_59861||full	1558	No	NA		0	2	Low-quality	Genome-fragment		2.88	0
S10_vs_27922||full	1558	No	NA		1	0	Low-quality	Genome-fragment		2.95	0
S10_vs_49307||full	1558	No	NA		0	1	Low-quality	Genome-fragment		2.1	0
S8_extracted_k141_200820	1558	No	NA		0	1	Low-quality	Genome-fragment		3.71	0
S8_extracted_k141_11231	1558	No	NA		0	2	Low-quality	Genome-fragment		1.97	0
S9_vs_k141_43872||full	1559	No	NA		0	0	Low-quality	Genome-fragment		0.9	0
S9_vs_k141_47582||full	1559	No	NA		0	0	Low-quality	Genome-fragment		3.71	0
S1_vs_k141_149817||lt2gene	1085	No	NA		0	0	Not-determined	Genome-fragment	NA		0
S11_extracted_k141_96375	1559	No	NA		1	0	Low-quality	Genome-fragment		3.4	0
S14_vs_k141_81725||full	1559	No	NA		2	0	Low-quality	Genome-fragment		3.3	0
S3_extracted_k141_98820	1559	No	NA		2	0	Low-quality	Genome-fragment		5.14	0
S3_vs_k141_44716||full	1559	No	NA		1	0	Low-quality	Genome-fragment		3.74	0
S1_vs_k141_146363||lt2gene	1120	No	NA		0	0	Not-determined	Genome-fragment	NA		0
S3_vs_k141_22171||full	1559	No	NA		0	1	Low-quality	Genome-fragment		1.84	0
S1_vs_k141_39058||lt2gene	1072	No	NA		0	0	Not-determined	Genome-fragment	NA		0
S1_vs_k141_150696||lt2gene	1661	No	NA		0	0	Not-determined	Genome-fragment	NA		0
S7_vs_87108||full	1559	No	NA		1	0	Low-quality	Genome-fragment		3.14	0
S16_extracted_57523	1559	No	NA		0	0	Low-quality	Genome-fragment		4.22	0
S1_vs_k141_158282||lt2gene	1084	No	NA		0	0	Not-determined	Genome-fragment	NA		0
S16_vs_27759||full	1559	No	NA		0	0	Low-quality	Genome-fragment		2.26	0
S16_vs_136280||full	1559	No	NA		0	0	Low-quality	Genome-fragment		3.18	0
S3_extracted_k141_63761	6864	No	NA		0	1	Not-determined	Genome-fragment	NA		0
S3_extracted_k141_15957	1626	No	NA		0	0	Not-determined	Genome-fragment	NA		0
S3_extracted_k141_71750	1580	No	NA		0	0	Not-determined	Genome-fragment	NA		0
S16_vs_172320||full	1559	No	NA		2	0	Low-quality	Genome-fragment		3.31	0
S3_extracted_k141_63800	7289	No	NA		0	6	Not-determined	Genome-fragment	NA		0
S10_vs_49610||full	1559	No	NA		2	0	Low-quality	Genome-fragment		3.46	0
S15_extracted_64024	1559	No	NA		0	0	Low-quality	Genome-fragment		3.47	0
S3_extracted_k141_111632	3684	No	NA		0	3	Not-determined	Genome-fragment	NA		0
S3_extracted_k141_63814	2579	No	NA		0	1	Not-determined	Genome-fragment	NA		0
S3_extracted_k141_47927	2902	No	NA		0	2	Not-determined	Genome-fragment	NA		0
S6_vs_k141_51812||full	1559	No	NA		0	0	Low-quality	Genome-fragment		2.79	0
S3_extracted_k141_24046	1520	No	NA		0	1	Not-determined	Genome-fragment	NA		0
S3_extracted_k141_63825	1100	No	NA		0	1	Not-determined	Genome-fragment	NA		0
S8_extracted_k141_106759	1559	No	NA		0	2	Low-quality	Genome-fragment		0.9	0
S3_extracted_k141_71819	1666	No	NA		0	2	Not-determined	Genome-fragment	NA		0
S8_vs_k141_155394||full	1559	No	NA		0	0	Low-quality	Genome-fragment		2.65	0
S3_extracted_k141_87692	15849	No	NA		0	7	Not-determined	Genome-fragment	NA		0
S3_extracted_k141_79771	1114	No	NA		0	1	Not-determined	Genome-fragment	NA		0
S3_extracted_k141_31936	1409	No	NA		0	1	Not-determined	Genome-fragment	NA		0
S1_extracted_k141_158613	1560	No	NA		0	0	Low-quality	Genome-fragment		2.23	0
S3_extracted_k141_71851	3053	No	NA		0	1	Not-determined	Genome-fragment	NA		0
S3_vs_k141_63581||full	1560	No	NA		2	0	Low-quality	Genome-fragment		4.03	0
S3_extracted_k141_8058	49145	No	NA		19	0	High-quality	High-quality		100	0
S16_vs_57802||full	1560	No	NA		0	0	Low-quality	Genome-fragment		3.49	0
S3_extracted_k141_119629	1225	No	NA		0	1	Not-determined	Genome-fragment	NA		0
S3_extracted_k141_55923	1550	No	NA		0	1	Not-determined	Genome-fragment	NA		0
S16_vs_127579||full	1560	No	NA		1	0	Low-quality	Genome-fragment		3.18	0
S12_extracted_k141_46816	1560	No	NA		3	0	Low-quality	Genome-fragment		1.02	0
S12_vs_k141_99141||full	1560	No	NA		0	0	Low-quality	Genome-fragment		0.6	0
S3_extracted_k141_24136	1299	No	NA		0	0	Not-determined	Genome-fragment	NA		0
S3_extracted_k141_24139	1299	No	NA		0	0	Not-determined	Genome-fragment	NA		0
S8_vs_k141_89673||full	1560	No	NA		0	0	Low-quality	Genome-fragment		3.01	0
S3_extracted_k141_87730	1617	No	NA		0	1	Not-determined	Genome-fragment	NA		0
S3_extracted_k141_8122	1035	No	NA		0	1	Not-determined	Genome-fragment	NA		0
S3_extracted_k141_8132	1020	No	NA		0	0	Not-determined	Genome-fragment	NA		0
S3_extracted_k141_79795	1490	No	NA		0	3	Not-determined	Genome-fragment	NA		0
S3_extracted_k141_55992	1464	No	NA		0	1	Not-determined	Genome-fragment	NA		0
S3_extracted_k141_154	1278	No	NA		0	0	Not-determined	Genome-fragment	NA		0
S3_extracted_k141_8157	2690	No	NA		0	0	Not-determined	Genome-fragment	NA		0
S3_extracted_k141_31988	5641	No	NA		0	3	Not-determined	Genome-fragment	NA		0
S8_vs_k141_152193||full	1560	No	NA		1	0	Low-quality	Genome-fragment		1.02	0
S17_extracted_k141_104599	1561	No	NA		0	0	Low-quality	Genome-fragment		1.68	0
S3_extracted_k141_56007	1473	No	NA		0	1	Not-determined	Genome-fragment	NA		0
S3_extracted_k141_111741	1041	No	NA		0	1	Not-determined	Genome-fragment	NA		0
S3_extracted_k141_8164	36393	No	NA		18	0	High-quality	High-quality		94.33	0
S3_extracted_k141_95782	4790	No	NA		0	3	Not-determined	Genome-fragment	NA		0
S14_extracted_k141_66092	1561	No	NA		2	0	Low-quality	Genome-fragment		5.15	0
S3_extracted_k141_79891	1205	No	NA		0	1	Not-determined	Genome-fragment	NA		0
S3_extracted_k141_39992	2000	No	NA		0	0	Not-determined	Genome-fragment	NA		0
S14_vs_k141_9817||full	1561	No	NA		1	0	Low-quality	Genome-fragment		3.91	0
S3_extracted_k141_116727	1561	No	NA		0	0	Low-quality	Genome-fragment		1.55	0
S5_vs_k141_68051||full	1561	No	NA		1	0	Low-quality	Genome-fragment		3.04	0
S3_extracted_k141_48189	1190	No	NA		0	1	Not-determined	Genome-fragment	NA		0
S5_vs_k141_113538||full	1561	No	NA		0	0	Low-quality	Genome-fragment		3.58	0
S3_extracted_k141_64013	2177	No	NA		0	1	Not-determined	Genome-fragment	NA		0
S3_extracted_k141_234	6907	No	NA		0	1	Not-determined	Genome-fragment	NA		0
S7_vs_112332||full	1561	No	NA		2	0	Low-quality	Genome-fragment		1.94	0
S3_extracted_k141_56047	3304	No	NA		0	2	Not-determined	Genome-fragment	NA		0
S3_extracted_k141_95866	5603	No	NA		0	2	Not-determined	Genome-fragment	NA		0
S3_extracted_k141_79945	1387	No	NA		0	1	Not-determined	Genome-fragment	NA		0
S16_vs_152420||full	1561	No	NA		0	1	Low-quality	Genome-fragment		1.18	0
S10_vs_63017||full	1561	No	NA		0	1	Low-quality	Genome-fragment		1.4	0
S17_vs_k141_104459||full	1562	No	NA		0	0	Low-quality	Genome-fragment		0.89	0
S3_extracted_k141_40064	1077	No	NA		0	0	Not-determined	Genome-fragment	NA		0
S3_extracted_k141_87845	1329	No	NA		0	1	Not-determined	Genome-fragment	NA		0
S3_extracted_k141_72023	9202	No	NA		0	3	Not-determined	Genome-fragment	NA		0
S3_extracted_k141_72024	1104	No	NA		0	1	Not-determined	Genome-fragment	NA		0
S3_extracted_k141_103806	1420	No	NA		0	1	Not-determined	Genome-fragment	NA		0
S9_vs_k141_26410||full	1562	No	NA		1	0	Low-quality	Genome-fragment		0.92	0
S3_extracted_k141_111776	16294	No	NA		8	0	Medium-quality	Genome-fragment		70.53	0
S3_extracted_k141_119735	7142	No	NA		0	1	Not-determined	Genome-fragment	NA		0
S9_vs_k141_60417||full	1562	No	NA		0	0	Low-quality	Genome-fragment		3.59	0
S3_extracted_k141_87878	2106	No	NA		0	0	Not-determined	Genome-fragment	NA		0
S3_extracted_k141_358	1009	No	NA		0	1	Not-determined	Genome-fragment	NA		0
S11_vs_k141_81191||full	1562	No	NA		1	0	Low-quality	Genome-fragment		3.92	0
S3_extracted_k141_56135	1026	No	NA		0	0	Not-determined	Genome-fragment	NA		0
S3_extracted_k141_72066	1352	No	NA		0	0	Not-determined	Genome-fragment	NA		0
S11_vs_k141_48927||full	1562	No	NA		1	0	Low-quality	Genome-fragment		3.35	0
S13_extracted_33519	1562	No	NA		0	0	Low-quality	Genome-fragment		2.95	0
S3_extracted_k141_24303	1947	No	NA		0	1	Not-determined	Genome-fragment	NA		0
S3_extracted_k141_87896	14143	No	NA		1	1	Medium-quality	Genome-fragment		87.95	0

S14_vs_k141_88862||full	1562	No	NA		1	0	Low-quality	Genome-fragment		3.2	0
S14_vs_k141_35809||full	1562	No	NA		0	1	Low-quality	Genome-fragment		3.59	0
S3_extracted_k141_56214	2303	No	NA		0	1	Not-determined	Genome-fragment	NA		0
S3_extracted_k141_8477	2063	No	NA		0	2	Not-determined	Genome-fragment	NA		0
S3_extracted_k141_103951	1216	No	NA		0	1	Not-determined	Genome-fragment	NA		0
S3_extracted_k141_96116	1030	No	NA		0	2	Not-determined	Genome-fragment	NA		0
S14_vs_k141_108940||lt2gene	1562	No	NA		1	0	Low-quality	Genome-fragment		3.89	0
S3_extracted_k141_80111	1396	No	NA		0	0	Not-determined	Genome-fragment	NA		0
S3_extracted_k141_445	3363	No	NA		0	1	Not-determined	Genome-fragment	NA		0
S3_extracted_k141_32316	1129	No	NA		0	0	Not-determined	Genome-fragment	NA		0
S3_extracted_k141_16146	3358	No	NA		0	0	Not-determined	Genome-fragment	NA		0
S3_extracted_k141_87970	1640	No	NA		0	2	Not-determined	Genome-fragment	NA		0
S3_extracted_k141_111893	34477	No	NA		24	0	Complete	High-quality		100	0
S3_extracted_k141_56240	1071	No	NA		0	1	Not-determined	Genome-fragment	NA		0
S3_vs_k141_78756||full	1562	No	NA		1	0	Low-quality	Genome-fragment		1.81	0
S3_extracted_k141_40291	1436	No	NA		0	1	Not-determined	Genome-fragment	NA		0
S5_vs_k141_38295||full	1562	Yes		921	1	1	Low-quality	Genome-fragment		0.5	41.04
S3_extracted_k141_56251	1073	No	NA		0	1	Not-determined	Genome-fragment	NA		0
S3_extracted_k141_96141	1385	No	NA		0	0	Not-determined	Genome-fragment	NA		0
S3_extracted_k141_64175	12906	No	NA		0	5	Not-determined	Genome-fragment	NA		0
S3_extracted_k141_104028	2963	No	NA		0	0	Not-determined	Genome-fragment	NA		0
S7_vs_8853||full	1562	No	NA		0	0	Low-quality	Genome-fragment		2.75	0
S3_extracted_k141_119940	1020	No	NA		0	1	Not-determined	Genome-fragment	NA		0
S16_vs_152644||full	1562	No	NA		0	1	Low-quality	Genome-fragment		3.36	0
S3_extracted_k141_96156	1671	No	NA		0	2	Not-determined	Genome-fragment	NA		0
S3_extracted_k141_40369	2906	No	NA		0	1	Not-determined	Genome-fragment	NA		0
S3_extracted_k141_119967	1356	No	NA		0	0	Not-determined	Genome-fragment	NA		0
S3_extracted_k141_56261	3569	No	NA		0	1	Not-determined	Genome-fragment	NA		0
S3_extracted_k141_119991	1492	No	NA		0	1	Not-determined	Genome-fragment	NA		0
S3_extracted_k141_104061	4266	No	NA		0	4	Not-determined	Genome-fragment	NA		0
S10_extracted_70720	1562	No	NA		0	1	Low-quality	Genome-fragment		4.39	0
S3_extracted_k141_16170	1077	No	NA		0	0	Not-determined	Genome-fragment	NA		0
S3_extracted_k141_64230	4332	No	NA		0	2	Not-determined	Genome-fragment	NA		0
S3_extracted_k141_72134	1588	No	NA		0	1	Not-determined	Genome-fragment	NA		0
S3_extracted_k141_64248	1301	No	NA		0	0	Not-determined	Genome-fragment	NA		0
S3_extracted_k141_8669	2134	No	NA		0	1	Not-determined	Genome-fragment	NA		0
S3_extracted_k141_72155	1429	No	NA		0	1	Not-determined	Genome-fragment	NA		0
S3_extracted_k141_40391	3241	No	NA		0	0	Not-determined	Genome-fragment	NA		0
S3_extracted_k141_569	1097	No	NA		0	1	Not-determined	Genome-fragment	NA		0
S3_extracted_k141_16193	1240	No	NA		0	0	Not-determined	Genome-fragment	NA		0
S3_extracted_k141_48433	2078	No	NA		0	1	Not-determined	Genome-fragment	NA		0
S3_extracted_k141_587	1073	No	NA		0	1	Not-determined	Genome-fragment	NA		0
S3_extracted_k141_88065	3070	No	NA		0	4	Not-determined	Genome-fragment	NA		0
S3_extracted_k141_32494	1302	No	NA		0	1	Not-determined	Genome-fragment	NA		0
S3_extracted_k141_598	1015	No	NA		0	1	Not-determined	Genome-fragment	NA		0
S17_vs_k141_96090||full	1563	No	NA		0	0	Low-quality	Genome-fragment		2.62	0
S3_extracted_k141_602	4744	No	NA		0	2	Not-determined	Genome-fragment	NA		0
S17_vs_k141_51117||full	1563	No	NA		3	0	Low-quality	Genome-fragment		4.32	0
S14_extracted_k141_88197	1563	No	NA		4	0	Low-quality	Genome-fragment		5.16	0
S3_extracted_k141_104128	1149	No	NA		0	1	Not-determined	Genome-fragment	NA		0
S3_extracted_k141_8789	1107	No	NA		0	0	Not-determined	Genome-fragment	NA		0
S3_extracted_k141_112004	1001	No	NA		0	0	Not-determined	Genome-fragment	NA		0
S14_extracted_k141_68516	1563	No	NA		0	0	Low-quality	Genome-fragment		0.59	0
S3_extracted_k141_112037	1016	No	NA		0	1	Not-determined	Genome-fragment	NA		0
S3_extracted_k141_8836	4175	No	NA		0	0	Not-determined	Genome-fragment	NA		0
S3_extracted_k141_96222	22007	No	NA		31	0	Medium-quality	Genome-fragment		72.48	0
S3_extracted_k141_96223	1084	No	NA		0	1	Not-determined	Genome-fragment	NA		0
S3_extracted_k141_16198	6117	No	NA		0	3	Not-determined	Genome-fragment	NA		0
S3_extracted_k141_32547	2933	No	NA		0	1	Not-determined	Genome-fragment	NA		0
S1_vs_k141_111010||full	1563	No	NA		0	1	Low-quality	Genome-fragment		0.88	0
S3_extracted_k141_88111	1435	No	NA		0	0	Not-determined	Genome-fragment	NA		0
S7_extracted_30034	1563	No	NA		0	0	Low-quality	Genome-fragment		2.14	0
S8_vs_k141_64366||full	1563	No	NA		0	0	Low-quality	Genome-fragment		3.62	0
S8_vs_k141_109353||full	1563	No	NA		0	0	Low-quality	Genome-fragment		22.81	0
S17_extracted_k141_49697	1564	No	NA		1	0	Low-quality	Genome-fragment		3.28	0
S3_extracted_k141_32617	1719	No	NA		0	0	Not-determined	Genome-fragment	NA		0
S3_extracted_k141_728	1880	No	NA		0	1	Not-determined	Genome-fragment	NA		0
S3_extracted_k141_32655	1497	No	NA		0	0	Not-determined	Genome-fragment	NA		0
S3_extracted_k141_64287	1004	No	NA		0	1	Not-determined	Genome-fragment	NA		0
S11_vs_k141_13585||full	1564	No	NA		0	0	Low-quality	Genome-fragment		2.77	0
S3_extracted_k141_8875	1425	No	NA		0	1	Not-determined	Genome-fragment	NA		0
S3_extracted_k141_32677	3380	No	NA		0	1	Not-determined	Genome-fragment	NA		0
S3_extracted_k141_80287	44726	No	NA		31	0	Complete	High-quality		100	0
S3_extracted_k141_80292	1043	No	NA		0	0	Not-determined	Genome-fragment	NA		0
S3_extracted_k141_56363	34937	No	NA		20	1	Medium-quality	Genome-fragment		86.49	0
S3_extracted_k141_80303	1004	No	NA		0	0	Not-determined	Genome-fragment	NA		0
S3_extracted_k141_751	1233	No	NA		0	0	Not-determined	Genome-fragment	NA		0
S11_vs_k141_92310||full	1564	No	NA		0	1	Low-quality	Genome-fragment		3.61	0
S3_extracted_k141_48670	4074	No	NA		0	1	Not-determined	Genome-fragment	NA		0
S3_extracted_k141_48684	2313	No	NA		0	1	Not-determined	Genome-fragment	NA		0
S11_vs_k141_88233||full	1564	No	NA		0	3	Low-quality	Genome-fragment		0.85	0
S3_extracted_k141_56449	1385	No	NA		0	1	Not-determined	Genome-fragment	NA		0
S11_vs_k141_56715||full	1564	No	NA		0	1	Low-quality	Genome-fragment		3.39	0
S14_extracted_k141_106255	1564	No	NA		2	0	Low-quality	Genome-fragment		5.16	0
S14_vs_k141_53833||full	1564	No	NA		2	0	Low-quality	Genome-fragment		3.51	0
S3_extracted_k141_96404	1081	No	NA		0	0	Not-determined	Genome-fragment	NA		0
S3_extracted_k141_104317	1684	No	NA		0	0	Not-determined	Genome-fragment	NA		0
S3_extracted_k141_9030	4413	No	NA		0	1	Not-determined	Genome-fragment	NA		0
S3_extracted_k141_56503	2356	No	NA		0	0	Not-determined	Genome-fragment	NA		0
S16_extracted_37937	1564	No	NA		0	0	Low-quality	Genome-fragment		1.38	0
S3_extracted_k141_16269	4908	No	NA		0	3	Not-determined	Genome-fragment	NA		0
S3_extracted_k141_9046	6983	No	NA		0	2	Not-determined	Genome-fragment	NA		0
S3_extracted_k141_32819	1314	No	NA		0	2	Not-determined	Genome-fragment	NA		0
S3_extracted_k141_9074	2520	No	NA		0	1	Not-determined	Genome-fragment	NA		0
S16_extracted_172017	1564	No	NA		1	0	Low-quality	Genome-fragment		1.44	0
S3_extracted_k141_16328	7310	No	NA		0	6	Not-determined	Genome-fragment	NA		0
S16_vs_136361||full	1564	No	NA		1	0	Low-quality	Genome-fragment		3.78	0
S10_extracted_50108	1564	No	NA		0	1	Low-quality	Genome-fragment		0.66	0
S12_vs_k141_182183||lt2gene	1564	No	NA		1	0	Low-quality	Genome-fragment		2.85	0
S3_extracted_k141_112249	9942	No	NA		0	0	Not-determined	Genome-fragment	NA		0
S3_extracted_k141_56580	1199	No	NA		0	0	Not-determined	Genome-fragment	NA		0
S6_extracted_k141_27722	1564	No	NA		0	1	Low-quality	Genome-fragment		1.65	0
S17_vs_k141_39022||full	1565	No	NA		1	0	Low-quality	Genome-fragment		3.97	0
S3_extracted_k141_9147	5981	No	NA		0	4	Not-determined	Genome-fragment	NA		0
S3_extracted_k141_9154	3014	No	NA		0	3	Not-determined	Genome-fragment	NA		0
S3_extracted_k141_24676	1642	No	NA		0	1	Not-determined	Genome-fragment	NA		0
S11_extracted_k141_13170	1565	No	NA		1	0	Low-quality	Genome-fragment		4.08	0
S3_extracted_k141_80446	1829	No	NA		0	2	Not-determined	Genome-fragment	NA		0
S3_extracted_k141_56613	1147	No	NA		0	1	Not-determined	Genome-fragment	NA		0
S3_extracted_k141_88306	1720	No	NA		0	0	Not-determined	Genome-fragment	NA		0
S3_extracted_k141_80463	2175	No	NA		0	0	Not-determined	Genome-fragment	NA		0
S3_extracted_k141_72769	1442	No	NA		0	0	Not-determined	Genome-fragment	NA		0
S14_extracted_k141_30535	1565	No	NA		1	0	Low-quality	Genome-fragment		5.02	0
S14_extracted_k141_54060	1565	No	NA		0	2	Low-quality	Genome-fragment		1.88	0
S1_vs_k141_12945||full	1565	No	NA		2	0	Low-quality	Genome-fragment		3.47	0
S3_extracted_k141_96592	3391	No	NA		0	0	Not-determined	Genome-fragment	NA		0
S5_vs_k141_106468||full	1565	No	NA		0	1	Low-quality	Genome-fragment		0.58	0
S10_vs_71985||full	1565	No	NA		2	0	Low-quality	Genome-fragment		3.95	0
S3_extracted_k141_56715	1188	No	NA		0	0	Not-determined	Genome-fragment	NA		0
S10_vs_88825||lt2gene	1565	No	NA		1	0	Low-quality	Genome-fragment		0.6	0
S3_extracted_k141_72853	6373	No	NA		0	0	Not-determined	Genome-fragment	NA		0
S12_extracted_k141_169647	1565	No	NA		0	0	Low-quality	Genome-fragment		2.12	0
S3_extracted_k141_56726	14683	No	NA		0	0	Not-determined	Genome-fragment	NA		0
S12_vs_k141_9612||full	1565	No	NA		1	0	Low-quality	Genome-fragment		3.77	0
S15_extracted_49118	1565	No	NA		0	1	Low-quality	Genome-fragment		1.35	0
S4_extracted_684	1565	No	NA		3	0	Low-quality	Genome-fragment		5.16	0
S3_extracted_k141_96685	1169	No	NA		0	1	Not-determined	Genome-fragment	NA		0
S3_extracted_k141_72910	1069	No	NA		0	1	Not-determined	Genome-fragment	NA		0
S3_extracted_k141_9265	3041	No	NA		0	2	Not-determined	Genome-fragment	NA		0
S6_vs_k141_43058||full	1565	No	NA		0	0	Low-quality	Genome-fragment		5.26	0
S3_extracted_k141_72923	4532	No	NA		0	0	Not-determined	Genome-fragment	NA		0
S8_vs_k141_36189||full	1565	No	NA		0	0	Low-quality	Genome-fragment		1.69	0
S3_extracted_k141_72933	3320	No	NA		0	2	Not-determined	Genome-fragment	NA		0
S13_extracted_37066	1566	No	NA		0	1	Low-quality	Genome-fragment		0.67	0
S3_extracted_k141_24780	5675	No	NA		0	4	Not-determined	Genome-fragment	NA		0
S14_vs_k141_2887||lt2gene	1566	No	NA		1	0	Low-quality	Genome-fragment		3.62	0
S3_vs_k141_76849||full	1566	No	NA		1	0	Low-quality	Genome-fragment		2.54	0
S3_extracted_k141_49022	1068	No	NA		0	0	Not-determined	Genome-fragment	NA		0
S7_vs_97206||lt2gene	1566	No	NA		1	0	Low-quality	Genome-fragment		3.63	0
S3_extracted_k141_120181	1350	No	NA		0	1	Not-determined	Genome-fragment	NA		0
S16_vs_150465||full	1566	No	NA		3	0	Low-quality	Genome-fragment		4.65	0
S3_extracted_k141_9291	1236	No	NA		0	1	Not-determined	Genome-fragment	NA		0
S16_vs_164342||full	1566	No	NA		0	2	Low-quality	Genome-fragment		1.23	0
S12_vs_k141_13258||full	1566	No	NA		0	0	Low-quality	Genome-fragment		2.7	0
S12_vs_k141_45622||lt2gene	1566	No	NA		1	0	Low-quality	Genome-fragment		3	0
S3_extracted_k141_88484	1039	No	NA		0	3	Not-determined	Genome-fragment	NA		0
S3_extracted_k141_64507	1184	No	NA		0	0	Not-determined	Genome-fragment	NA		0
S3_extracted_k141_104465	1057	No	NA		0	0	Not-determined	Genome-fragment	NA		0
S17_vs_k141_45987||full	1567	No	NA		2	0	Low-quality	Genome-fragment		2.55	0
S3_extracted_k141_49045	1004	No	NA		0	0	Not-determined	Genome-fragment	NA		0
S17_vs_k141_33819||full	1567	No	NA		2	0	Low-quality	Genome-fragment		3.63	0
S3_extracted_k141_49050	2554	No	NA		0	1	Not-determined	Genome-fragment	NA		0
S11_vs_k141_45974||full	1567	No	NA		0	1	Low-quality	Genome-fragment		2.96	0
S3_extracted_k141_56817	3562	No	NA		0	3	Not-determined	Genome-fragment	NA		0
S14_vs_k141_114806||full	1567	No	NA		1	0	Low-quality	Genome-fragment		5.12	0
S1_extracted_k141_114645	1567	No	NA		0	1	Low-quality	Genome-fragment		1.9	0
S1_vs_k141_75646||full	1567	No	NA		1	0	Low-quality	Genome-fragment		1.81	0
S3_extracted_k141_56843	3455	No	NA		0	5	Not-determined	Genome-fragment	NA		0
S1_vs_k141_48705||lt2gene	1567	No	NA		1	0	Low-quality	Genome-fragment		3.74	0
S3_extracted_k141_56853	4420	No	NA		0	6	Not-determined	Genome-fragment	NA		0
S10_vs_46170||full	1567	No	NA		0	1	Low-quality	Genome-fragment		2.5	0
S3_extracted_k141_24901	3995	No	NA		0	2	Not-determined	Genome-fragment	NA		0
S3_extracted_k141_24920	1019	No	NA		0	1	Not-determined	Genome-fragment	NA		0
S3_extracted_k141_73099	1897	No	NA		0	1	Not-determined	Genome-fragment	NA		0
S3_extracted_k141_9431	1239	No	NA		0	1	Not-determined	Genome-fragment	NA		0
S10_vs_62338||full	1567	No	NA		0	1	Low-quality	Genome-fragment		3.64	0
S12_vs_k141_181741||full	1567	No	NA		0	2	Low-quality	Genome-fragment		5.23	0
S3_extracted_k141_104536	1168	No	NA		0	0	Not-determined	Genome-fragment	NA		0
S3_extracted_k141_104575	2186	No	NA		0	0	Not-determined	Genome-fragment	NA		0
S3_extracted_k141_9481	1071	No	NA		0	0	Not-determined	Genome-fragment	NA		0
S3_extracted_k141_49209	2214	No	NA		0	0	Not-determined	Genome-fragment	NA		0
S3_extracted_k141_16602	1023	No	NA		0	0	Not-determined	Genome-fragment	NA		0
S12_vs_k141_14678||full	1567	No	NA		0	0	Low-quality	Genome-fragment		3.12	0
S3_extracted_k141_73208	10456	No	NA		0	0	Not-determined	Genome-fragment	NA		0

S9_vs_k141_62162||full	1568	No	NA		0	0	Low-quality	Genome-fragment		2.64	0
S3_extracted_k141_96883	1382	No	NA		0	0	Not-determined	Genome-fragment	NA		0
S3_extracted_k141_120266	2060	No	NA		0	3	Not-determined	Genome-fragment	NA		0
S13_vs_99394||full	1568	No	NA		0	0	Low-quality	Genome-fragment		0.95	0
S5_extracted_k141_40840	1568	No	NA		1	0	Low-quality	Genome-fragment		4.53	0
S3_extracted_k141_64602	2167	No	NA		0	1	Not-determined	Genome-fragment	NA		0
S3_extracted_k141_1051	4374	No	NA		0	3	Not-determined	Genome-fragment	NA		0
S7_vs_3626||full	1568	No	NA		1	0	Low-quality	Genome-fragment		2.68	0
S3_extracted_k141_73309	1529	No	NA		0	1	Not-determined	Genome-fragment	NA		0
S7_vs_62647||full	1568	No	NA		1	0	Low-quality	Genome-fragment		3.82	0
S16_extracted_139875	1568	No	NA		0	0	Low-quality	Genome-fragment		1.8	0
S3_extracted_k141_80909	1595	No	NA		0	1	Not-determined	Genome-fragment	NA		0
S16_vs_45200||full	1568	No	NA		0	0	Low-quality	Genome-fragment		2.41	0
S3_extracted_k141_97026	4003	No	NA		0	0	Not-determined	Genome-fragment	NA		0
S3_extracted_k141_40991	1141	No	NA		0	1	Not-determined	Genome-fragment	NA		0
S16_vs_82598||full	1568	No	NA		0	0	Low-quality	Genome-fragment		3.43	0
S3_extracted_k141_64674	7822	No	NA		0	2	Not-determined	Genome-fragment	NA		0
S3_extracted_k141_88683	1372	No	NA		0	0	Not-determined	Genome-fragment	NA		0
S12_vs_k141_133877||full	1568	No	NA		0	0	Low-quality	Genome-fragment		2.41	0
S3_extracted_k141_97053	6198	No	NA		0	3	Not-determined	Genome-fragment	NA		0
S15_vs_51281||full	1568	No	NA		4	0	Low-quality	Genome-fragment		1.95	0
S3_extracted_k141_112476	1473	No	NA		0	0	Not-determined	Genome-fragment	NA		0
S17_vs_k141_70768||lt2gene	1569	Yes		1107	1	1	Low-quality	Genome-fragment		2.94	29.45
S3_extracted_k141_104699	1216	No	NA		0	1	Not-determined	Genome-fragment	NA		0
S11_extracted_k141_21665	1569	No	NA		1	0	Low-quality	Genome-fragment		2.35	0
S13_vs_99716||full	1569	No	NA		1	0	Low-quality	Genome-fragment		1.18	0
S3_extracted_k141_120401	1330	No	NA		0	1	Not-determined	Genome-fragment	NA		0
S5_vs_k141_24287||full	1569	No	NA		0	0	Low-quality	Genome-fragment		3.74	0
S16_vs_131552||full	1569	No	NA		0	0	Low-quality	Genome-fragment		3.56	0
S12_extracted_k141_132728	1569	No	NA		2	0	Low-quality	Genome-fragment		3.44	0
S3_extracted_k141_120409	4421	No	NA		0	3	Not-determined	Genome-fragment	NA		0
S12_vs_k141_29766||full	1569	No	NA		0	1	Low-quality	Genome-fragment		2.55	0
S15_vs_29613||full	1569	No	NA		1	0	Low-quality	Genome-fragment		3.22	0
S3_extracted_k141_64743	4930	No	NA		0	3	Not-determined	Genome-fragment	NA		0
S3_extracted_k141_81118	1028	No	NA		0	0	Not-determined	Genome-fragment	NA		0
S3_extracted_k141_104715	3951	No	NA		0	1	Not-determined	Genome-fragment	NA		0
S3_extracted_k141_33389	17765	No	NA		8	0	High-quality	High-quality		91.08	0
S15_vs_73644||full	1569	No	NA		0	0	Low-quality	Genome-fragment		3.73	0
S6_extracted_k141_39574	1569	No	NA		2	0	Low-quality	Genome-fragment		3.47	0
S8_extracted_k141_131057	1569	No	NA		1	0	Low-quality	Genome-fragment		2.94	0
S8_vs_k141_108852||full	1569	No	NA		0	0	Low-quality	Genome-fragment		3.32	0
S8_vs_k141_153546||full	1569	No	NA		0	0	Low-quality	Genome-fragment		3.3	0
S14_extracted_k141_75158	1570	No	NA		1	0	Low-quality	Genome-fragment		3.58	0
S14_vs_k141_68035||full	1570	No	NA		0	0	Low-quality	Genome-fragment		3.26	0
S3_extracted_k141_112585	2540	No	NA		0	2	Not-determined	Genome-fragment	NA		0
S14_vs_k141_15410||lt2gene	1570	No	NA		0	0	Low-quality	Genome-fragment		1.54	0
S3_extracted_k141_81165	3387	No	NA		0	2	Not-determined	Genome-fragment	NA		0
S1_vs_k141_51445||full	1570	No	NA		0	1	Low-quality	Genome-fragment		0.99	0
S3_vs_k141_95045||full	1570	No	NA		1	0	Low-quality	Genome-fragment		3.76	0
S3_extracted_k141_97131	1033	No	NA		0	0	Not-determined	Genome-fragment	NA		0
S5_vs_k141_44872||full	1570	No	NA		2	0	Low-quality	Genome-fragment		2.6	0
S16_extracted_130686	1570	No	NA		0	0	Low-quality	Genome-fragment		1.99	0
S16_vs_27690||full	1570	No	NA		0	1	Low-quality	Genome-fragment		3.15	0
S12_vs_k141_63288||full	1570	No	NA		1	0	Low-quality	Genome-fragment		4.15	0
S3_extracted_k141_81200	1170	No	NA		0	1	Not-determined	Genome-fragment	NA		0
S17_vs_k141_14875||full	1571	No	NA		0	0	Low-quality	Genome-fragment		3.24	0
S3_extracted_k141_16969	3277	No	NA		0	2	Not-determined	Genome-fragment	NA		0
S3_extracted_k141_16983	1164	No	NA		0	1	Not-determined	Genome-fragment	NA		0
S3_extracted_k141_104799	1343	No	NA		0	0	Not-determined	Genome-fragment	NA		0
S17_vs_k141_30258||full	1571	No	NA		3	0	Low-quality	Genome-fragment		2.7	0
S3_extracted_k141_88868	1951	No	NA		0	0	Not-determined	Genome-fragment	NA		0
S17_vs_k141_165641||full	1571	No	NA		1	0	Low-quality	Genome-fragment		2.88	0
S3_extracted_k141_1335	4084	No	NA		0	2	Not-determined	Genome-fragment	NA		0
S3_extracted_k141_104842	3385	No	NA		0	2	Not-determined	Genome-fragment	NA		0
S3_extracted_k141_41207	3305	No	NA		0	1	Not-determined	Genome-fragment	NA		0
S3_extracted_k141_120562	4356	No	NA		0	2	Not-determined	Genome-fragment	NA		0
S9_vs_k141_52896||full	1571	No	NA		0	0	Low-quality	Genome-fragment		1.88	0
S3_extracted_k141_41221	3771	No	NA		0	1	Not-determined	Genome-fragment	NA		0
S14_vs_k141_68771||full	1571	No	NA		0	0	Low-quality	Genome-fragment		1.38	0
S3_extracted_k141_57197	6541	No	NA		0	3	Not-determined	Genome-fragment	NA		0
S14_vs_k141_54369||lt2gene	1571	No	NA		1	0	Low-quality	Genome-fragment		2.87	0
S3_extracted_k141_57208	5651	No	NA		0	2	Not-determined	Genome-fragment	NA		0
S3_extracted_k141_17011	1421	No	NA		0	0	Not-determined	Genome-fragment	NA		0
S16_vs_6415||full	1571	No	NA		0	0	Low-quality	Genome-fragment		2.6	0
S10_vs_40642||full	1571	No	NA		0	0	Low-quality	Genome-fragment		2.64	0
S3_extracted_k141_64989	3887	No	NA		0	2	Not-determined	Genome-fragment	NA		0
S3_extracted_k141_81302	1078	No	NA		0	2	Not-determined	Genome-fragment	NA		0
S12_extracted_k141_64256	1571	No	NA		2	0	Low-quality	Genome-fragment		3.52	0
S15_vs_31634||full	1571	No	NA		0	1	Low-quality	Genome-fragment		2.77	0
S6_vs_k141_49511||full	1571	No	NA		1	0	Low-quality	Genome-fragment		3.1	0
S3_extracted_k141_73597	57020	No	NA		14	4	Medium-quality	Genome-fragment		82.14	0
S3_extracted_k141_112725	3898	No	NA		0	2	Not-determined	Genome-fragment	NA		0
S3_extracted_k141_17054	1107	No	NA		0	1	Not-determined	Genome-fragment	NA		0
S3_extracted_k141_57274	2988	No	NA		0	2	Not-determined	Genome-fragment	NA		0
S17_extracted_k141_8178	1572	No	NA		0	1	Low-quality	Genome-fragment		0.85	0
S3_extracted_k141_25131	1164	No	NA		0	0	Not-determined	Genome-fragment	NA		0
S3_extracted_k141_41255	4606	No	NA		0	0	Not-determined	Genome-fragment	NA		0
S17_extracted_k141_36927	1572	No	NA		0	0	Low-quality	Genome-fragment		3.26	0
S3_extracted_k141_73718	1306	No	NA		0	0	Not-determined	Genome-fragment	NA		0
S3_extracted_k141_41300	1287	No	NA		0	1	Not-determined	Genome-fragment	NA		0
S3_extracted_k141_73732	1705	No	NA		0	1	Not-determined	Genome-fragment	NA		0
S9_extracted_k141_63775	1572	No	NA		1	0	Low-quality	Genome-fragment		2.56	0
S3_extracted_k141_88934	2723	No	NA		0	0	Not-determined	Genome-fragment	NA		0
S3_extracted_k141_81426	3800	No	NA		0	2	Not-determined	Genome-fragment	NA		0
S3_extracted_k141_65092	1278	No	NA		0	0	Not-determined	Genome-fragment	NA		0
S3_extracted_k141_57359	2182	No	NA		0	0	Not-determined	Genome-fragment	NA		0
S14_extracted_k141_45070	1572	No	NA		1	0	Low-quality	Genome-fragment		1.93	0
S14_extracted_k141_35469	1572	No	NA		1	0	Low-quality	Genome-fragment		5.1	0
S3_extracted_k141_41373	1271	No	NA		0	1	Not-determined	Genome-fragment	NA		0
S3_extracted_k141_49680	1447	No	NA		0	1	Not-determined	Genome-fragment	NA		0
S7_vs_50853||full	1572	No	NA		1	0	Low-quality	Genome-fragment		3.55	0
S3_extracted_k141_57398	1082	No	NA		0	1	Not-determined	Genome-fragment	NA		0
S3_extracted_k141_120690	1337	No	NA		0	1	Not-determined	Genome-fragment	NA		0
S3_extracted_k141_25163	103478	No	NA		16	1	Complete	High-quality		100	0
S3_extracted_k141_25170	1434	No	NA		0	0	Not-determined	Genome-fragment	NA		0
S12_vs_k141_132413||full	1572	No	NA		0	0	Low-quality	Genome-fragment		3.17	0
S3_extracted_k141_57422	15991	No	NA		8	0	Medium-quality	Genome-fragment		70.78	0
S3_extracted_k141_73779	3335	No	NA		0	0	Not-determined	Genome-fragment	NA		0
S3_extracted_k141_49708	1243	No	NA		0	0	Not-determined	Genome-fragment	NA		0
S3_extracted_k141_73784	1008	No	NA		0	0	Not-determined	Genome-fragment	NA		0
S3_extracted_k141_1593	1003	No	NA		0	1	Not-determined	Genome-fragment	NA		0
S3_extracted_k141_97354	1248	No	NA		0	1	Not-determined	Genome-fragment	NA		0
S4_extracted_6598	1572	No	NA		4	0	Low-quality	Genome-fragment		5.19	0
S17_extracted_k141_66881	1573	No	NA		0	0	Low-quality	Genome-fragment		0.29	0
S3_extracted_k141_73855	2670	No	NA		0	0	Not-determined	Genome-fragment	NA		0
S11_vs_k141_22322||full	1573	No	NA		1	0	Low-quality	Genome-fragment		3.59	0
S13_vs_9868||full	1573	No	NA		0	1	Low-quality	Genome-fragment		1.66	0
S3_extracted_k141_47390	1573	No	NA		0	0	Low-quality	Genome-fragment		2.97	0
S7_vs_11752||full	1573	No	NA		1	0	Low-quality	Genome-fragment		4.29	0
S3_extracted_k141_17230	7606	No	NA		0	3	Not-determined	Genome-fragment	NA		0
S3_extracted_k141_73893	1427	No	NA		0	1	Not-determined	Genome-fragment	NA		0
S7_vs_90253||full	1573	No	NA		2	0	Low-quality	Genome-fragment		1.93	0
S3_extracted_k141_112984	1158	No	NA		0	0	Not-determined	Genome-fragment	NA		0
S3_extracted_k141_57481	1168	No	NA		0	0	Not-determined	Genome-fragment	NA		0
S10_vs_18570||full	1573	No	NA		0	0	Low-quality	Genome-fragment		3.23	0
S17_vs_k141_126823||full	1574	No	NA		0	0	Low-quality	Genome-fragment		3.03	0
S3_extracted_k141_120799	1014	No	NA		0	0	Not-determined	Genome-fragment	NA		0
S3_extracted_k141_120817	1485	No	NA		0	2	Not-determined	Genome-fragment	NA		0
S3_extracted_k141_10258	1640	No	NA		0	0	Not-determined	Genome-fragment	NA		0
S3_extracted_k141_17280	1194	No	NA		0	1	Not-determined	Genome-fragment	NA		0
S3_extracted_k141_89209	4682	No	NA		0	1	Not-determined	Genome-fragment	NA		0
S9_vs_k141_74183||lt2gene	1574	No	NA		2	0	Low-quality	Genome-fragment		4.14	0
S7_extracted_118389	1574	No	NA		0	0	Low-quality	Genome-fragment		1.87	0
S16_vs_78756||full	1574	No	NA		2	0	Low-quality	Genome-fragment		3.74	0
S3_extracted_k141_41508	3496	No	NA		0	9	Not-determined	Genome-fragment	NA		0
S3_extracted_k141_17284	2016	No	NA		0	0	Not-determined	Genome-fragment	NA		0
S3_extracted_k141_74010	1174	No	NA		0	2	Not-determined	Genome-fragment	NA		0
S12_vs_k141_142412||full	1574	No	NA		0	1	Low-quality	Genome-fragment		1.83	0
S1_vs_k141_126860||full	1575	No	NA		0	0	Low-quality	Genome-fragment		1.55	0
S7_extracted_99362	1575	No	NA		0	3	Low-quality	Genome-fragment		4.44	0
S16_extracted_140584	1575	No	NA		0	0	Low-quality	Genome-fragment		1.89	0
S10_extracted_31625	1575	No	NA		1	0	Low-quality	Genome-fragment		24.91	0
S10_vs_51880||full	1575	No	NA		0	1	Low-quality	Genome-fragment		0.84	0
S8_vs_k141_154183||lt2gene	1575	No	NA		1	0	Low-quality	Genome-fragment		1.48	0
S3_extracted_k141_49904	1261	No	NA		0	0	Not-determined	Genome-fragment	NA		0
S3_extracted_k141_81538	5357	No	NA		0	2	Not-determined	Genome-fragment	NA		0
S3_extracted_k141_105014	2855	No	NA		0	0	Not-determined	Genome-fragment	NA		0
S3_extracted_k141_113154	1341	No	NA		0	1	Not-determined	Genome-fragment	NA		0
S11_extracted_k141_78694	1576	No	NA		0	0	Low-quality	Genome-fragment		2.35	0
S3_extracted_k141_1751	3119	No	NA		0	0	Not-determined	Genome-fragment	NA		0
S3_extracted_k141_41590	1124	No	NA		0	0	Not-determined	Genome-fragment	NA		0
S3_extracted_k141_81579	2219	No	NA		0	0	Not-determined	Genome-fragment	NA		0
S3_extracted_k141_57677	11276	No	NA		0	7	Not-determined	Genome-fragment	NA		0
S3_extracted_k141_17399	1120	No	NA		0	1	Not-determined	Genome-fragment	NA		0
S13_vs_26034||full	1576	No	NA		0	0	Low-quality	Genome-fragment		3.5	0
S14_vs_k141_111784||full	1576	No	NA		1	0	Low-quality	Genome-fragment		3.12	0
S14_vs_k141_80302||full	1576	No	NA		1	0	Low-quality	Genome-fragment		3.83	0
S5_vs_k141_94805||full	1576	No	NA		0	1	Low-quality	Genome-fragment		0.84	0
S7_extracted_16045	1576	No	NA		0	1	Low-quality	Genome-fragment		1.83	0
S3_extracted_k141_105111	1230	No	NA		0	0	Not-determined	Genome-fragment	NA		0
S10_vs_96780||full	1576	No	NA		0	0	Low-quality	Genome-fragment		3.75	0
S11_vs_k141_26421||full	1577	No	NA		0	0	Low-quality	Genome-fragment		3.94	0
S3_extracted_k141_105131	6126	No	NA		0	3	Not-determined	Genome-fragment	NA		0
S3_extracted_k141_50005	3826	No	NA		0	4	Not-determined	Genome-fragment	NA		0
S3_extracted_k141_25494	1018	No	NA		0	3	Not-determined	Genome-fragment	NA		0
S3_extracted_k141_113254	1232	No	NA		0	0	Not-determined	Genome-fragment	NA		0
S3_extracted_k141_1850	3856	No	NA		0	2	Not-determined	Genome-fragment	NA		0
S11_vs_k141_3750||full	1577	No	NA		0	0	Low-quality	Genome-fragment		2.72	0
S3_extracted_k141_1868	1027	No	NA		0	1	Not-determined	Genome-fragment	NA		0
S3_extracted_k141_105168	1331	No	NA		0	1	Not-determined	Genome-fragment	NA		0
S3_extracted_k141_41703	1333	No	NA		0	1	Not-determined	Genome-fragment	NA		0
S14_extracted_k141_22950	1577	No	NA		2	0	Low-quality	Genome-fragment		4.55	0
S3_extracted_k141_120985	3233	No	NA		0	0	Not-determined	Genome-fragment	NA		0
S3_extracted_k141_105181	2174	No	NA		0	1	Not-determined	Genome-fragment	NA		0

S3_extracted_k141_113276	1032	No	NA		0	2	Not-determined	Genome-fragment	NA		0
S3_extracted_k141_25513	1786	No	NA		0	1	Not-determined	Genome-fragment	NA		0
S3_extracted_k141_33903	4718	No	NA		0	3	Not-determined	Genome-fragment	NA		0
S1_extracted_k141_100237	1577	No	NA		0	1	Low-quality	Genome-fragment		0.49	0
S3_extracted_k141_105869	1577	No	NA		0	1	Low-quality	Genome-fragment		0.97	0
S3_extracted_k141_57780	2820	No	NA		0	0	Not-determined	Genome-fragment	NA		0
S3_extracted_k141_10539	8831	No	NA		0	2	Not-determined	Genome-fragment	NA		0
S3_vs_k141_77469||full	1577	No	NA		0	1	Low-quality	Genome-fragment		1.18	0
S3_extracted_k141_41725	3297	No	NA		0	1	Not-determined	Genome-fragment	NA		0
S7_vs_45166||full	1577	No	NA		0	0	Low-quality	Genome-fragment		0.99	0
S3_extracted_k141_25557	1688	No	NA		0	0	Not-determined	Genome-fragment	NA		0
S3_extracted_k141_57795	4432	No	NA		0	2	Not-determined	Genome-fragment	NA		0
S3_extracted_k141_57797	1234	No	NA		0	1	Not-determined	Genome-fragment	NA		0
S16_vs_64603||full	1577	No	NA		0	0	Low-quality	Genome-fragment		4.29	0
S15_extracted_73591	1577	No	NA		0	1	Low-quality	Genome-fragment		0.27	0
S15_vs_76400||full	1577	No	NA		2	0	Low-quality	Genome-fragment		3.6	0
S3_extracted_k141_74099	1058	No	NA		0	2	Not-determined	Genome-fragment	NA		0
S3_extracted_k141_25593	1593	No	NA		0	1	Not-determined	Genome-fragment	NA		0
S3_extracted_k141_81697	1092	No	NA		0	0	Not-determined	Genome-fragment	NA		0
S3_extracted_k141_34005	4998	No	NA		0	5	Not-determined	Genome-fragment	NA		0
S3_extracted_k141_34009	3154	No	NA		0	0	Not-determined	Genome-fragment	NA		0
S3_extracted_k141_105305	6712	No	NA		0	2	Not-determined	Genome-fragment	NA		0
S8_vs_k141_134720||full	1577	No	NA		0	0	Low-quality	Genome-fragment		3.68	0
S3_extracted_k141_25616	2531	No	NA		0	3	Not-determined	Genome-fragment	NA		0
S11_extracted_k141_78336	1578	No	NA		1	0	Low-quality	Genome-fragment		2.62	0
S3_extracted_k141_81720	1333	No	NA		0	1	Not-determined	Genome-fragment	NA		0
S3_extracted_k141_17487	2711	No	NA		0	4	Not-determined	Genome-fragment	NA		0
S3_extracted_k141_113441	8879	No	NA		0	2	Not-determined	Genome-fragment	NA		0
S13_extracted_133210	1578	No	NA		1	0	Low-quality	Genome-fragment		1.73	0
S1_extracted_k141_153122	1578	No	NA		1	0	Low-quality	Genome-fragment		5.35	0
S3_extracted_k141_121169	1405	No	NA		0	1	Not-determined	Genome-fragment	NA		0
S3_extracted_k141_65384	1035	No	NA		0	1	Not-determined	Genome-fragment	NA		0
S3_extracted_k141_34082	1540	No	NA		0	1	Not-determined	Genome-fragment	NA		0
S3_vs_k141_91974||full	1578	No	NA		0	0	Low-quality	Genome-fragment		2.78	0
S5_vs_k141_49727||full	1578	No	NA		2	0	Low-quality	Genome-fragment		2.42	0
S7_vs_75153||full	1578	No	NA		0	0	Low-quality	Genome-fragment		3.92	0
S3_extracted_k141_97659	5869	No	NA		0	3	Not-determined	Genome-fragment	NA		0
S10_vs_64349||full	1578	No	NA		1	0	Low-quality	Genome-fragment		2.77	0
S12_extracted_k141_124205	1578	No	NA		2	0	Low-quality	Genome-fragment		2.49	0
S3_extracted_k141_89604	1996	No	NA		0	1	Not-determined	Genome-fragment	NA		0
S6_vs_k141_18315||full	1578	No	NA		0	1	Low-quality	Genome-fragment		0.72	0
S8_vs_k141_58560||full	1578	Yes		632	1	1	Low-quality	Genome-fragment		1.11	59.95
S8_vs_k141_112563||full	1578	No	NA		0	0	Low-quality	Genome-fragment		3.07	0
S3_extracted_k141_57903	1428	No	NA		0	2	Not-determined	Genome-fragment	NA		0
S9_extracted_k141_38325	1579	No	NA		2	0	Low-quality	Genome-fragment		3.84	0
S3_extracted_k141_17559	1984	No	NA		0	0	Not-determined	Genome-fragment	NA		0
S13_extracted_5074	1579	No	NA		0	0	Low-quality	Genome-fragment		2.87	0
S13_vs_48542||full	1579	No	NA		0	0	Low-quality	Genome-fragment		1.77	0
S14_vs_k141_103571||full	1579	No	NA		2	0	Low-quality	Genome-fragment		3.68	0
S3_extracted_k141_34249	4827	No	NA		0	0	Not-determined	Genome-fragment	NA		0
S3_extracted_k141_121318	2013	No	NA		0	1	Not-determined	Genome-fragment	NA		0
S3_extracted_k141_34265	1885	No	NA		0	2	Not-determined	Genome-fragment	NA		0
S5_vs_k141_113514||full	1579	No	NA		1	0	Low-quality	Genome-fragment		3.97	0
S3_extracted_k141_17593	1000	No	NA		0	1	Not-determined	Genome-fragment	NA		0
S3_extracted_k141_17594	3747	No	NA		0	7	Not-determined	Genome-fragment	NA		0
S3_extracted_k141_57952	1417	No	NA		0	2	Not-determined	Genome-fragment	NA		0
S3_extracted_k141_17633	1359	No	NA		0	0	Not-determined	Genome-fragment	NA		0
S3_extracted_k141_65555	2342	No	NA		0	1	Not-determined	Genome-fragment	NA		0
S3_extracted_k141_10970	1094	No	NA		0	0	Not-determined	Genome-fragment	NA		0
S7_vs_75148||full	1579	No	NA		0	0	Low-quality	Genome-fragment		2.39	0
S3_extracted_k141_105472	14209	No	NA		0	3	Not-determined	Genome-fragment	NA		0
S12_extracted_k141_186906	1579	No	NA		0	2	Low-quality	Genome-fragment		0.69	0
S3_extracted_k141_97723	3440	No	NA		0	2	Not-determined	Genome-fragment	NA		0
S12_vs_k141_112785||full	1579	No	NA		1	0	Low-quality	Genome-fragment		3.07	0
S3_extracted_k141_121378	7330	No	NA		0	2	Not-determined	Genome-fragment	NA		0
S3_extracted_k141_34339	1090	No	NA		0	1	Not-determined	Genome-fragment	NA		0
S12_vs_k141_160231||full	1579	No	NA		0	1	Low-quality	Genome-fragment		1.1	0
S3_extracted_k141_113607	1043	No	NA		0	0	Not-determined	Genome-fragment	NA		0
S3_extracted_k141_65592	1745	No	NA		0	0	Not-determined	Genome-fragment	NA		0
S11_extracted_k141_4768	1580	No	NA		2	0	Low-quality	Genome-fragment		3.73	0
S3_extracted_k141_89686	1507	No	NA		0	2	Not-determined	Genome-fragment	NA		0
S3_extracted_k141_121400	5972	No	NA		0	7	Not-determined	Genome-fragment	NA		0
S1_vs_k141_34995||full	1580	No	NA		1	0	Low-quality	Genome-fragment		2.73	0
S5_extracted_k141_103482	1580	No	NA		2	0	Low-quality	Genome-fragment		4.08	0
S3_extracted_k141_105531	5403	No	NA		0	3	Not-determined	Genome-fragment	NA		0
S16_vs_79584||lt2gene	1580	No	NA		2	0	Low-quality	Genome-fragment		4.6	0
S16_vs_79860||lt2gene	1580	No	NA		2	0	Low-quality	Genome-fragment		4.6	0
S12_extracted_k141_134134	1580	No	NA		2	0	Low-quality	Genome-fragment		4.15	0
S3_extracted_k141_11040	1100	No	NA		0	0	Not-determined	Genome-fragment	NA		0
S3_extracted_k141_50225	4181	No	NA		0	3	Not-determined	Genome-fragment	NA		0
S3_extracted_k141_50229	1805	No	NA		0	2	Not-determined	Genome-fragment	NA		0
S3_extracted_k141_11057	1165	No	NA		0	0	Not-determined	Genome-fragment	NA		0
S12_extracted_k141_166810	1580	No	NA		0	1	Low-quality	Genome-fragment		0.92	0
S3_extracted_k141_113688	2346	No	NA		0	1	Not-determined	Genome-fragment	NA		0
S3_extracted_k141_65701	1078	No	NA		0	1	Not-determined	Genome-fragment	NA		0
S8_extracted_k141_200404	1580	No	NA		0	1	Low-quality	Genome-fragment		0.47	0
S3_extracted_k141_58115	1144	No	NA		0	1	Not-determined	Genome-fragment	NA		0
S3_extracted_k141_42068	1028	No	NA		0	0	Not-determined	Genome-fragment	NA		0
S8_vs_k141_78583||full	1580	No	NA		1	0	Low-quality	Genome-fragment		3.92	0
S17_vs_k141_158544||full	1581	No	NA		0	0	Low-quality	Genome-fragment		3.87	0
S3_extracted_k141_113743	1021	No	NA		0	0	Not-determined	Genome-fragment	NA		0
S11_vs_k141_67659||full	1581	No	NA		0	0	Low-quality	Genome-fragment		6.7	0
S13_vs_40355||full	1581	No	NA		0	0	Low-quality	Genome-fragment		1.88	0
S14_extracted_k141_66403	1581	No	NA		3	0	Low-quality	Genome-fragment		5.21	0
S3_extracted_k141_81824	3111	No	NA		0	0	Not-determined	Genome-fragment	NA		0
S3_extracted_k141_80649	1581	No	NA		0	0	Low-quality	Genome-fragment		7.21	0
S3_extracted_k141_11156	1691	No	NA		0	1	Not-determined	Genome-fragment	NA		0
S7_vs_123556||full	1581	No	NA		0	0	Low-quality	Genome-fragment		3.15	0
S16_extracted_69261	1581	No	NA		0	0	Low-quality	Genome-fragment		3.68	0
S3_extracted_k141_50256	1633	No	NA		0	0	Not-determined	Genome-fragment	NA		0
S3_extracted_k141_65744	2795	No	NA		0	0	Not-determined	Genome-fragment	NA		0
S14_extracted_k141_109960	1582	No	NA		2	0	Low-quality	Genome-fragment		5.22	0
S14_extracted_k141_15175	1582	No	NA		2	0	Low-quality	Genome-fragment		3.48	0
S3_extracted_k141_25934	1657	No	NA		0	2	Not-determined	Genome-fragment	NA		0
S3_extracted_k141_42108	5897	No	NA		0	1	Not-determined	Genome-fragment	NA		0
S3_extracted_k141_50268	7115	No	NA		0	4	Not-determined	Genome-fragment	NA		0
S3_extracted_k141_11191	1005	No	NA		0	0	Not-determined	Genome-fragment	NA		0
S3_vs_k141_95113||full	1582	No	NA		0	0	Low-quality	Genome-fragment		3.99	0
S12_vs_k141_40043||full	1582	No	NA		0	2	Low-quality	Genome-fragment		3.29	0
S3_extracted_k141_81868	1186	No	NA		0	1	Not-determined	Genome-fragment	NA		0
S3_extracted_k141_65799	3333	No	NA		0	2	Not-determined	Genome-fragment	NA		0
S3_extracted_k141_121460	1866	No	NA		0	0	Not-determined	Genome-fragment	NA		0
S12_vs_k141_19971||full	1582	No	NA		1	0	Low-quality	Genome-fragment		1.88	0
S3_extracted_k141_50328	1204	No	NA		0	2	Not-determined	Genome-fragment	NA		0
S3_extracted_k141_58218	4514	No	NA		0	3	Not-determined	Genome-fragment	NA		0
S6_extracted_k141_26003	1582	No	NA		0	0	Low-quality	Genome-fragment		2.86	0
S3_extracted_k141_65838	1730	No	NA		0	0	Not-determined	Genome-fragment	NA		0
S3_extracted_k141_11295	3831	No	NA		0	1	Not-determined	Genome-fragment	NA		0
S3_extracted_k141_34479	1287	No	NA		0	0	Not-determined	Genome-fragment	NA		0
S8_extracted_k141_56860	1582	No	NA		2	0	Low-quality	Genome-fragment		4.77	0
S9_vs_k141_74607||full	1583	No	NA		2	0	Low-quality	Genome-fragment		3.92	0
S13_vs_111027||lt2gene	1583	No	NA		1	0	Low-quality	Genome-fragment		4.13	0
S14_vs_k141_31088||full	1583	No	NA		1	0	Low-quality	Genome-fragment		3.47	0
S3_extracted_k141_17748	96667	No	NA		12	0	Complete	High-quality		100	0
S3_extracted_k141_113788	5231	No	NA		0	2	Not-determined	Genome-fragment	NA		0
S1_vs_k141_1837||lt2gene	1583	No	NA		1	0	Low-quality	Genome-fragment		3.27	0
S3_extracted_k141_17751	2472	No	NA		0	2	Not-determined	Genome-fragment	NA		0
S3_extracted_k141_2186	55789	No	NA		6	0	Medium-quality	Genome-fragment		77.64	0
S3_extracted_k141_89872	2686	No	NA		0	0	Not-determined	Genome-fragment	NA		0
S3_extracted_k141_50468	3198	No	NA		0	1	Not-determined	Genome-fragment	NA		0
S3_extracted_k141_121579	1395	No	NA		0	0	Not-determined	Genome-fragment	NA		0
S3_extracted_k141_74484	3196	No	NA		0	1	Not-determined	Genome-fragment	NA		0
S3_extracted_k141_113819	2649	No	NA		0	1	Not-determined	Genome-fragment	NA		0
S3_extracted_k141_65930	1385	No	NA		0	0	Not-determined	Genome-fragment	NA		0
S3_extracted_k141_121593	1035	No	NA		0	1	Not-determined	Genome-fragment	NA		0
S3_vs_k141_2878||full	1583	No	NA		1	0	Low-quality	Genome-fragment		2.35	0
S7_vs_74395||full	1583	No	NA		0	0	Low-quality	Genome-fragment		3.64	0
S3_extracted_k141_26015	5582	No	NA		0	3	Not-determined	Genome-fragment	NA		0
S3_extracted_k141_58272	1305	No	NA		0	3	Not-determined	Genome-fragment	NA		0
S16_extracted_120447	1583	No	NA		0	2	Low-quality	Genome-fragment		0.98	0
S16_vs_48478||full	1583	No	NA		1	0	Low-quality	Genome-fragment		3.59	0
S3_extracted_k141_11428	45436	No	NA		16	1	High-quality	High-quality		100	0
S16_vs_119362||full	1583	No	NA		0	2	Low-quality	Genome-fragment		2.65	0
S3_extracted_k141_81915	4764	No	NA		0	1	Not-determined	Genome-fragment	NA		0
S12_vs_k141_8187||full	1583	No	NA		0	1	Low-quality	Genome-fragment		0.42	0
S3_extracted_k141_81935	1743	No	NA		0	1	Not-determined	Genome-fragment	NA		0
S3_extracted_k141_17786	1031	No	NA		0	1	Not-determined	Genome-fragment	NA		0
S3_extracted_k141_42234	1200	No	NA		0	0	Not-determined	Genome-fragment	NA		0
S3_extracted_k141_42245	2622	No	NA		0	0	Not-determined	Genome-fragment	NA		0
S17_extracted_k141_165546	1584	No	NA		0	0	Low-quality	Genome-fragment		2.01	0
S17_vs_k141_142256||full	1584	No	NA		2	0	Low-quality	Genome-fragment		2.44	0
S3_extracted_k141_97935	1661	No	NA		0	0	Not-determined	Genome-fragment	NA		0
S3_extracted_k141_74580	1039	No	NA		0	0	Not-determined	Genome-fragment	NA		0
S3_extracted_k141_26095	1629	No	NA		0	2	Not-determined	Genome-fragment	NA		0
S9_vs_k141_55122||full	1584	No	NA		2	0	Low-quality	Genome-fragment		3.28	0
S14_vs_k141_27716||full	1584	No	NA		0	0	Low-quality	Genome-fragment		2.37	0
S3_extracted_k141_105924	3616	No	NA		0	0	Not-determined	Genome-fragment	NA		0
S3_extracted_k141_42303	3515	No	NA		0	1	Not-determined	Genome-fragment	NA		0
S3_extracted_k141_42307	1856	No	NA		0	1	Not-determined	Genome-fragment	NA		0
S3_extracted_k141_105962	1712	No	NA		0	1	Not-determined	Genome-fragment	NA		0
S1_extracted_k141_22988	1584	No	NA		0	0	Low-quality	Genome-fragment		1.56	0
S1_vs_k141_131929||full	1584	No	NA		1	0	Low-quality	Genome-fragment		2.98	0
S3_extracted_k141_97973	3251	No	NA		0	5	Not-determined	Genome-fragment	NA		0
S3_extracted_k141_83202	1584	No	NA		1	0	Low-quality	Genome-fragment		3.26	0
S3_extracted_k141_2309	1539	No	NA		0	1	Not-determined	Genome-fragment	NA		0
S3_vs_k141_19104||lt2gene	1584	No	NA		2	0	Low-quality	Genome-fragment		1.07	0
S7_extracted_42607	1584	No	NA		0	2	Low-quality	Genome-fragment		0.91	0
S3_extracted_k141_58381	1728	No	NA		0	0	Not-determined	Genome-fragment	NA		0
S7_extracted_118747	1584	No	NA		0	1	Low-quality	Genome-fragment		0.36	0
S3_extracted_k141_74666	1070	No	NA		0	1	Not-determined	Genome-fragment	NA		0
S7_vs_22558||full	1584	No	NA		0	0	Low-quality	Genome-fragment		2.77	0
S3_extracted_k141_50608	5553	No	NA		0	5	Not-determined	Genome-fragment	NA		0
S3_extracted_k141_42430	1714	No	NA		0	0	Not-determined	Genome-fragment	NA		0
S3_extracted_k141_114029	2642	No	NA		0	0	Not-determined	Genome-fragment	NA		0

S3_extracted_k141_17848	1274	No	NA		0	0	Not-determined	Genome-fragment	NA		0
S16_vs_110371||full	1584	No	NA		0	1	Low-quality	Genome-fragment		2.01	0
S3_extracted_k141_11692	3129	No	NA		0	2	Not-determined	Genome-fragment	NA		0
S3_extracted_k141_74707	2689	No	NA		0	1	Not-determined	Genome-fragment	NA		0
S3_extracted_k141_17877	1720	No	NA		0	0	Not-determined	Genome-fragment	NA		0
S12_extracted_k141_54891	1584	No	NA		0	1	Low-quality	Genome-fragment		0.85	0
S12_vs_k141_66576||full	1584	No	NA		1	0	Low-quality	Genome-fragment		2.53	0
S8_extracted_k141_154642	1584	No	NA		0	1	Low-quality	Genome-fragment		2.26	0
S9_vs_k141_34769||full	1585	No	NA		2	0	Low-quality	Genome-fragment		1.94	0
S9_vs_k141_58390||full	1585	No	NA		1	0	Low-quality	Genome-fragment		1.02	0
S1_vs_k141_97950||full	1585	No	NA		1	0	Low-quality	Genome-fragment		2.76	0
S3_extracted_k141_34587	1233	No	NA		0	0	Not-determined	Genome-fragment	NA		0
S3_extracted_k141_58438	3240	No	NA		0	2	Not-determined	Genome-fragment	NA		0
NC1_vf_k141_1922	1585	No	NA		0	0	Low-quality	Genome-fragment		2.26	0
S3_extracted_k141_90146	5479	No	NA		0	3	Not-determined	Genome-fragment	NA		0
S16_vs_139186||full	1585	No	NA		0	0	Low-quality	Genome-fragment		3.57	0
S3_extracted_k141_26326	2954	No	NA		0	2	Not-determined	Genome-fragment	NA		0
S3_extracted_k141_42501	1667	No	NA		0	0	Not-determined	Genome-fragment	NA		0
S3_extracted_k141_106100	1801	No	NA		0	2	Not-determined	Genome-fragment	NA		0
S8_vs_k141_41521||lt2gene	1585	No	NA		2	0	Low-quality	Genome-fragment		4.61	0
S3_extracted_k141_106112	1144	No	NA		0	1	Not-determined	Genome-fragment	NA		0
S9_vs_k141_77539||full	1586	No	NA		0	0	Low-quality	Genome-fragment		3.76	0
S3_extracted_k141_58530	1093	No	NA		0	3	Not-determined	Genome-fragment	NA		0
S14_vs_k141_11784||full	1586	No	NA		0	3	Low-quality	Genome-fragment		0.76	0
S3_extracted_k141_90207	1241	No	NA		0	0	Not-determined	Genome-fragment	NA		0
S3_extracted_k141_50683	3400	No	NA		0	2	Not-determined	Genome-fragment	NA		0
S5_vs_k141_48051||full	1586	No	NA		1	0	Low-quality	Genome-fragment		3.5	0
S3_extracted_k141_2479	1645	No	NA		0	1	Not-determined	Genome-fragment	NA		0
S3_extracted_k141_34687	1168	No	NA		0	1	Not-determined	Genome-fragment	NA		0
S7_vs_75321||full	1586	No	NA		0	0	Low-quality	Genome-fragment		4.25	0
S3_extracted_k141_17976	1376	No	NA		0	0	Not-determined	Genome-fragment	NA		0
S3_extracted_k141_98191	1154	No	NA		0	0	Not-determined	Genome-fragment	NA		0
S7_vs_134529||full	1586	No	NA		0	0	Low-quality	Genome-fragment		4.79	0
S3_extracted_k141_106165	1084	No	NA		0	0	Not-determined	Genome-fragment	NA		0
S10_vs_112309||full	1586	No	NA		0	1	Low-quality	Genome-fragment		0.11	0
S10_vs_119797||full	1586	No	NA		0	1	Low-quality	Genome-fragment		3.74	0
S3_extracted_k141_82322	1238	No	NA		0	3	Not-determined	Genome-fragment	NA		0
S3_extracted_k141_98231	7697	No	NA		0	4	Not-determined	Genome-fragment	NA		0
S3_extracted_k141_106197	1678	No	NA		0	2	Not-determined	Genome-fragment	NA		0
S8_vs_k141_48749||full	1586	No	NA		0	1	Low-quality	Genome-fragment		3.87	0
S17_extracted_k141_162053	1587	No	NA		0	0	Low-quality	Genome-fragment		2.93	0
S3_extracted_k141_26454	1220	No	NA		0	1	Not-determined	Genome-fragment	NA		0
S3_extracted_k141_26461	1250	No	NA		0	0	Not-determined	Genome-fragment	NA		0
S3_extracted_k141_26462	1160	No	NA		0	1	Not-determined	Genome-fragment	NA		0
S3_extracted_k141_11779	1198	No	NA		0	1	Not-determined	Genome-fragment	NA		0
S3_extracted_k141_34731	7492	No	NA		0	6	Not-determined	Genome-fragment	NA		0
S11_vs_k141_46142||full	1587	No	NA		0	0	Low-quality	Genome-fragment		2.87	0
S11_vs_k141_24019||full	1587	No	NA		3	0	Low-quality	Genome-fragment		3.95	0
S14_vs_k141_87850||full	1587	No	NA		0	0	Low-quality	Genome-fragment		2.98	0
S1_vs_k141_58878||full	1587	No	NA		2	0	Low-quality	Genome-fragment		2.69	0
S3_extracted_k141_42636	3335	No	NA		0	1	Not-determined	Genome-fragment	NA		0
S3_extracted_k141_90550	1587	No	NA		0	0	Low-quality	Genome-fragment		0.5	0
S3_vs_k141_114630||full	1587	No	NA		1	0	Low-quality	Genome-fragment		3.18	0
S3_extracted_k141_74915	1745	No	NA		0	2	Not-determined	Genome-fragment	NA		0
S7_vs_42026||full	1587	No	NA		3	0	Low-quality	Genome-fragment		2.98	0
S3_extracted_k141_11826	4132	No	NA		0	4	Not-determined	Genome-fragment	NA		0
S3_extracted_k141_121847	1262	No	NA		0	0	Not-determined	Genome-fragment	NA		0
S3_extracted_k141_106267	2927	No	NA		0	0	Not-determined	Genome-fragment	NA		0
S3_extracted_k141_26547	2641	No	NA		0	1	Not-determined	Genome-fragment	NA		0
S3_extracted_k141_74958	8252	No	NA		0	4	Not-determined	Genome-fragment	NA		0
S3_extracted_k141_74961	2448	No	NA		0	1	Not-determined	Genome-fragment	NA		0
S3_extracted_k141_34812	1128	No	NA		0	1	Not-determined	Genome-fragment	NA		0
S16_extracted_16281	1587	No	NA		0	0	Low-quality	Genome-fragment		2.87	0
S3_extracted_k141_26589	3637	No	NA		0	1	Not-determined	Genome-fragment	NA		0
S12_vs_k141_119180||full	1587	No	NA		2	0	Low-quality	Genome-fragment		3.9	0
S12_vs_k141_3037||full	1587	No	NA		1	0	Low-quality	Genome-fragment		0.8	0
S3_extracted_k141_58845	1499	No	NA		0	1	Not-determined	Genome-fragment	NA		0
S3_extracted_k141_114260	1814	No	NA		0	3	Not-determined	Genome-fragment	NA		0
S3_extracted_k141_11876	1068	No	NA		0	1	Not-determined	Genome-fragment	NA		0
S3_extracted_k141_42759	1245	No	NA		0	2	Not-determined	Genome-fragment	NA		0
S3_extracted_k141_2770	1237	No	NA		0	1	Not-determined	Genome-fragment	NA		0
S3_extracted_k141_18080	46776	No	NA		11	1	Medium-quality	Genome-fragment		84.86	0
S3_extracted_k141_75046	1309	No	NA		0	0	Not-determined	Genome-fragment	NA		0
S3_extracted_k141_34847	1448	No	NA		0	1	Not-determined	Genome-fragment	NA		0
S3_extracted_k141_58913	1193	No	NA		0	0	Not-determined	Genome-fragment	NA		0
S6_vs_k141_36466||full	1587	No	NA		0	1	Low-quality	Genome-fragment		0.78	0
S3_extracted_k141_90380	2387	No	NA		0	1	Not-determined	Genome-fragment	NA		0
S3_extracted_k141_26656	4007	No	NA		0	2	Not-determined	Genome-fragment	NA		0
S3_extracted_k141_106401	39810	Yes		30429	19	6	Medium-quality	Genome-fragment		77.08	23.56
S8_extracted_k141_201475	1587	No	NA		0	0	Low-quality	Genome-fragment		1.33	0
S11_extracted_k141_69475	1588	No	NA		0	2	Low-quality	Genome-fragment		0.79	0
S3_extracted_k141_106405	3769	No	NA		0	3	Not-determined	Genome-fragment	NA		0
S3_extracted_k141_75082	1018	No	NA		0	1	Not-determined	Genome-fragment	NA		0
S1_vs_k141_69816||full	1588	No	NA		0	1	Low-quality	Genome-fragment		3.57	0
S3_extracted_k141_121995	1062	No	NA		0	1	Not-determined	Genome-fragment	NA		0
S1_vs_k141_92278||full	1588	No	NA		0	0	Low-quality	Genome-fragment		3.51	0
S3_extracted_k141_90416	1552	No	NA		0	2	Not-determined	Genome-fragment	NA		0
S3_extracted_k141_11997	6497	No	NA		0	2	Not-determined	Genome-fragment	NA		0
S3_extracted_k141_50987	1097	No	NA		0	1	Not-determined	Genome-fragment	NA		0
S7_vs_32990||full	1588	No	NA		0	1	Low-quality	Genome-fragment		3.02	0
S16_vs_126981||full	1588	No	NA		2	0	Low-quality	Genome-fragment		3.72	0
S12_vs_k141_143470||full	1588	No	NA		0	0	Low-quality	Genome-fragment		3.15	0
S3_extracted_k141_114355	1209	No	NA		0	1	Not-determined	Genome-fragment	NA		0
S3_extracted_k141_114368	3685	No	NA		0	3	Not-determined	Genome-fragment	NA		0
S3_extracted_k141_35008	1217	No	NA		0	0	Not-determined	Genome-fragment	NA		0
S3_extracted_k141_59078	5910	No	NA		2	0	High-quality	High-quality		95.34	0
S3_extracted_k141_82571	25954	No	NA		16	0	Medium-quality	Genome-fragment		80.6	0
S8_extracted_k141_168405	1588	No	NA		1	0	Low-quality	Genome-fragment		3.41	0
S17_vs_k141_86007||full	1589	No	NA		0	1	Low-quality	Genome-fragment		4.15	0
S3_extracted_k141_106526	4408	No	NA		0	4	Not-determined	Genome-fragment	NA		0
S3_extracted_k141_82615	3940	No	NA		0	0	Not-determined	Genome-fragment	NA		0
S7_extracted_43862	1589	No	NA		0	1	Low-quality	Genome-fragment		2.76	0
S3_extracted_k141_98509	1174	No	NA		0	0	Not-determined	Genome-fragment	NA		0
S3_extracted_k141_26817	3136	No	NA		0	2	Not-determined	Genome-fragment	NA		0
S12_vs_k141_87745||full	1589	No	NA		0	0	Low-quality	Genome-fragment		3.13	0
S3_extracted_k141_35073	59386	No	NA		33	0	High-quality	High-quality		97.39	0
S3_extracted_k141_90593	2469	No	NA		0	1	Not-determined	Genome-fragment	NA		0
S3_extracted_k141_12092	1051	No	NA		0	0	Not-determined	Genome-fragment	NA		0
S12_vs_k141_183161||full	1589	No	NA		0	0	Low-quality	Genome-fragment		5.59	0
S12_vs_k141_184491||full	1589	No	NA		0	1	Low-quality	Genome-fragment		1.66	0
S2_extracted_k141_6805	1589	No	NA		0	0	Low-quality	Genome-fragment		1.09	0
S3_extracted_k141_12116	15486	No	NA		0	3	Not-determined	Genome-fragment	NA		0
S3_extracted_k141_106604	5009	No	NA		0	2	Not-determined	Genome-fragment	NA		0
S3_extracted_k141_82741	1591	No	NA		0	2	Not-determined	Genome-fragment	NA		0
S3_extracted_k141_43010	1181	No	NA		0	1	Not-determined	Genome-fragment	NA		0
S3_extracted_k141_75346	1190	No	NA		0	0	Not-determined	Genome-fragment	NA		0
S3_extracted_k141_98588	3573	No	NA		0	1	Not-determined	Genome-fragment	NA		0
S6_vs_k141_9065||full	1589	No	NA		0	1	Low-quality	Genome-fragment		0.97	0
S17_extracted_k141_24655	1590	No	NA		0	2	Low-quality	Genome-fragment		0.75	0
S9_vs_k141_71857||full	1590	Yes		1362	1	1	Low-quality	Genome-fragment		0.83	14.34
S3_extracted_k141_2948	1404	No	NA		0	2	Not-determined	Genome-fragment	NA		0
S13_vs_24178||full	1590	No	NA		0	0	Low-quality	Genome-fragment		3.89	0
S3_extracted_k141_3012	1937	No	NA		0	2	Not-determined	Genome-fragment	NA		0
S3_extracted_k141_75400	1820	No	NA		0	2	Not-determined	Genome-fragment	NA		0
S14_vs_k141_105749||full	1590	No	NA		2	0	Low-quality	Genome-fragment		2.49	0
S3_extracted_k141_3030	1145	No	NA		0	0	Not-determined	Genome-fragment	NA		0
S3_extracted_k141_98647	3590	No	NA		0	3	Not-determined	Genome-fragment	NA		0
S3_extracted_k141_12231	1908	No	NA		0	0	Not-determined	Genome-fragment	NA		0
S16_extracted_24727	1590	No	NA		0	0	Low-quality	Genome-fragment		3.17	0
S3_extracted_k141_90684	10747	No	NA		0	1	Not-determined	Genome-fragment	NA		0
S3_extracted_k141_3050	2362	No	NA		0	1	Not-determined	Genome-fragment	NA		0
S3_extracted_k141_106759	1416	No	NA		0	1	Not-determined	Genome-fragment	NA		0
S16_extracted_128965	1590	No	NA		0	0	Low-quality	Genome-fragment		18.68	0
S3_extracted_k141_75463	1629	No	NA		0	1	Not-determined	Genome-fragment	NA		0
S16_vs_89883||lt2gene	1590	No	NA		2	0	Low-quality	Genome-fragment		5.09	0
S3_extracted_k141_114537	1288	No	NA		0	2	Not-determined	Genome-fragment	NA		0
S3_extracted_k141_106789	5768	No	NA		0	2	Not-determined	Genome-fragment	NA		0
S3_extracted_k141_66373	1303	No	NA		0	1	Not-determined	Genome-fragment	NA		0
S3_extracted_k141_27065	1115	No	NA		0	2	Not-determined	Genome-fragment	NA		0
S3_extracted_k141_75476	1246	No	NA		0	1	Not-determined	Genome-fragment	NA		0
S3_extracted_k141_106805	1171	No	NA		0	0	Not-determined	Genome-fragment	NA		0
S3_extracted_k141_98677	1008	No	NA		0	0	Not-determined	Genome-fragment	NA		0
S3_extracted_k141_12294	1495	No	NA		0	1	Not-determined	Genome-fragment	NA		0
S3_extracted_k141_114576	4815	No	NA		0	0	Not-determined	Genome-fragment	NA		0
S12_extracted_k141_126405	1590	No	NA		0	0	Low-quality	Genome-fragment		2.93	0
S3_extracted_k141_98706	1071	No	NA		0	0	Not-determined	Genome-fragment	NA		0
S13_vs_153132||full	1591	No	NA		0	1	Low-quality	Genome-fragment		0.47	0
S14_extracted_k141_88830	1591	No	NA		2	0	Low-quality	Genome-fragment		5.23	0
S14_extracted_k141_56212	1591	No	NA		2	0	Low-quality	Genome-fragment		3.24	0
S14_vs_k141_20458||full	1591	No	NA		0	0	Low-quality	Genome-fragment		2.84	0
S3_extracted_k141_98759	1079	No	NA		0	0	Not-determined	Genome-fragment	NA		0
S3_extracted_k141_66426	1242	No	NA		0	0	Not-determined	Genome-fragment	NA		0
S3_vs_k141_29033||full	1591	No	NA		4	0	Low-quality	Genome-fragment		2.98	0
S3_extracted_k141_3151	1055	No	NA		0	1	Not-determined	Genome-fragment	NA		0
S3_extracted_k141_98771	3031	No	NA		0	1	Not-determined	Genome-fragment	NA		0
S8_vs_k141_122503||full	1591	No	NA		0	1	Low-quality	Genome-fragment		0.72	0
S3_extracted_k141_59351	1515	No	NA		0	1	Not-determined	Genome-fragment	NA		0
S3_extracted_k141_75625	1889	No	NA		0	1	Not-determined	Genome-fragment	NA		0
S11_vs_k141_54670||full	1592	No	NA		0	0	Low-quality	Genome-fragment		2.82	0
S3_vs_k141_16642||full	1592	No	NA		0	0	Low-quality	Genome-fragment		1.02	0
S5_vs_k141_79537||full	1592	No	NA		0	0	Low-quality	Genome-fragment		1.44	0
S3_extracted_k141_90820	1145	No	NA		0	0	Not-determined	Genome-fragment	NA		0
S3_extracted_k141_122228	1109	No	NA		0	0	Not-determined	Genome-fragment	NA		0
S3_extracted_k141_90833	1421	No	NA		0	0	Not-determined	Genome-fragment	NA		0
S8_extracted_k141_111477	1592	No	NA		0	1	Low-quality	Genome-fragment		1.43	0
S3_extracted_k141_114707	4627	No	NA		0	2	Not-determined	Genome-fragment	NA		0
S9_extracted_k141_39086	1593	No	NA		1	0	Low-quality	Genome-fragment		2.69	0
S3_extracted_k141_106967	15363	No	NA		0	3	Not-determined	Genome-fragment	NA		0
S3_extracted_k141_90858	3983	No	NA		0	0	Not-determined	Genome-fragment	NA		0
S3_extracted_k141_12403	1228	No	NA		0	0	Not-determined	Genome-fragment	NA		0
S3_extracted_k141_98814	1066	No	NA		0	0	Not-determined	Genome-fragment	NA		0
S3_extracted_k141_12413	1160	No	NA		0	0	Not-determined	Genome-fragment	NA		0
S3_extracted_k141_90874	1085	No	NA		0	1	Not-determined	Genome-fragment	NA		0
S3_extracted_k141_35519	1172	No	NA		0	1	Not-determined	Genome-fragment	NA		0

S11_vs_k141_94561||full	1593	No	NA	0	0	Low-quality	Genome-fragment		2.57	0
S3_extracted_k141_106993	1407	No	NA	0	2	Not-determined	Genome-fragment	NA		0
S1_vs_k141_63833||full	1593	No	NA	3	0	Low-quality	Genome-fragment		2.57	0
S3_extracted_k141_59402	1032	No	NA	0	0	Not-determined	Genome-fragment	NA		0
S3_extracted_k141_51543	3007	No	NA	0	2	Not-determined	Genome-fragment	NA		0
S3_extracted_k141_90937	2499	No	NA	0	0	Not-determined	Genome-fragment	NA		0
S3_extracted_k141_35583	1843	No	NA	0	2	Not-determined	Genome-fragment	NA		0
S7_extracted_12531	1593	No	NA	1	0	Low-quality	Genome-fragment		1.09	0
S3_extracted_k141_51574	1436	No	NA	0	1	Not-determined	Genome-fragment	NA		0
S3_extracted_k141_122283	1494	No	NA	0	1	Not-determined	Genome-fragment	NA		0
S3_extracted_k141_82911	1770	No	NA	0	0	Not-determined	Genome-fragment	NA		0
S3_extracted_k141_66508	1220	No	NA	0	1	Not-determined	Genome-fragment	NA		0
S3_extracted_k141_27199	1023	No	NA	0	0	Not-determined	Genome-fragment	NA		0
S3_extracted_k141_51592	1981	No	NA	0	1	Not-determined	Genome-fragment	NA		0
S7_extracted_31035	1593	No	NA	2	0	Low-quality	Genome-fragment		11.57	0
S3_extracted_k141_82940	8311	No	NA	0	4	Not-determined	Genome-fragment	NA		0
S3_extracted_k141_18555	1083	No	NA	0	0	Not-determined	Genome-fragment	NA		0
S3_extracted_k141_12504	3792	No	NA	0	0	Not-determined	Genome-fragment	NA		0
S3_extracted_k141_51628	3598	No	NA	0	3	Not-determined	Genome-fragment	NA		0
S3_extracted_k141_43331	1926	No	NA	0	1	Not-determined	Genome-fragment	NA		0
S3_extracted_k141_35668	1083	No	NA	0	0	Not-determined	Genome-fragment	NA		0
S12_vs_k141_24489||full	1593	No	NA	0	0	Low-quality	Genome-fragment		2.11	0
S6_extracted_k141_160	1593	No	NA	0	1	Low-quality	Genome-fragment		1.29	0
S8_extracted_k141_120043	1593	No	NA	0	3	Low-quality	Genome-fragment		0.45	0
S3_extracted_k141_27318	1383	No	NA	0	1	Not-determined	Genome-fragment	NA		0
S3_extracted_k141_99000	4445	No	NA	0	1	Not-determined	Genome-fragment	NA		0
S3_extracted_k141_91060	5014	No	NA	0	2	Not-determined	Genome-fragment	NA		0
S3_extracted_k141_99050	2754	No	NA	0	4	Not-determined	Genome-fragment	NA		0
S3_extracted_k141_12572	1611	No	NA	0	2	Not-determined	Genome-fragment	NA		0
S3_extracted_k141_18648	2100	No	NA	0	2	Not-determined	Genome-fragment	NA		0
S8_vs_k141_64113||full	1593	No	NA	0	0	Low-quality	Genome-fragment		3.53	0
S17_extracted_k141_76601	1594	No	NA	1	0	Low-quality	Genome-fragment		6.85	0
S17_extracted_k141_88602	1594	No	NA	4	0	Low-quality	Genome-fragment		4.35	0
S3_extracted_k141_12598	7516	No	NA	0	1	Not-determined	Genome-fragment	NA		0
S3_extracted_k141_83068	1311	No	NA	0	1	Not-determined	Genome-fragment	NA		0
S3_extracted_k141_27350	1088	No	NA	0	1	Not-determined	Genome-fragment	NA		0
S3_extracted_k141_3288	1342	No	NA	0	0	Not-determined	Genome-fragment	NA		0
S3_extracted_k141_83077	1135	No	NA	0	0	Not-determined	Genome-fragment	NA		0
S3_extracted_k141_122516	1455	No	NA	0	1	Not-determined	Genome-fragment	NA		0
S17_vs_k141_84002||full	1594	No	NA	1	0	Low-quality	Genome-fragment		2.93	0
S3_extracted_k141_51711	1095	No	NA	0	1	Not-determined	Genome-fragment	NA		0
S9_vs_k141_80704||full	1594	No	NA	0	0	Low-quality	Genome-fragment		3.65	0
S3_extracted_k141_107235	11821	No	NA	0	2	Not-determined	Genome-fragment	NA		0
S9_vs_k141_84831||full	1594	No	NA	0	0	Low-quality	Genome-fragment		3.51	0
S11_extracted_k141_34886	1594	No	NA	0	1	Low-quality	Genome-fragment		4.15	0
S3_extracted_k141_99097	3039	No	NA	0	2	Not-determined	Genome-fragment	NA		0
S3_extracted_k141_107261	10076	No	NA	0	4	Not-determined	Genome-fragment	NA		0
S3_extracted_k141_7979	1594	No	NA	0	0	Low-quality	Genome-fragment		1.19	0
S3_extracted_k141_27433	2161	No	NA	0	2	Not-determined	Genome-fragment	NA		0
S3_extracted_k141_43442	1134	No	NA	0	2	Not-determined	Genome-fragment	NA		0
S3_extracted_k141_122572	1810	No	NA	0	2	Not-determined	Genome-fragment	NA		0
S3_extracted_k141_66696	1195	No	NA	0	0	Not-determined	Genome-fragment	NA		0
S3_extracted_k141_35764	1785	No	NA	0	2	Not-determined	Genome-fragment	NA		0
S5_vs_k141_89026||full	1594	No	NA	0	0	Low-quality	Genome-fragment		2.74	0
S3_extracted_k141_107299	9491	No	NA	0	4	Not-determined	Genome-fragment	NA		0
S3_extracted_k141_91166	1005	No	NA	0	0	Not-determined	Genome-fragment	NA		0
S7_extracted_48754	1594	No	NA	1	0	Low-quality	Genome-fragment		1.18	0
S16_extracted_158733	1594	No	NA	0	1	Low-quality	Genome-fragment		0.43	0
S16_extracted_164579	1594	No	NA	0	0	Low-quality	Genome-fragment		1.46	0
S6_vs_k141_18056||full	1594	No	NA	1	0	Low-quality	Genome-fragment		2.59	0
S8_vs_k141_71575||full	1594	No	NA	1	0	Low-quality	Genome-fragment		4.47	0
S8_vs_k141_178789||full	1594	No	NA	0	0	Low-quality	Genome-fragment		2.61	0
S3_extracted_k141_83256	1096	No	NA	0	0	Not-determined	Genome-fragment	NA		0
S3_extracted_k141_107327	1042	No	NA	0	2	Not-determined	Genome-fragment	NA		0
S3_extracted_k141_59691	5486	No	NA	0	2	Not-determined	Genome-fragment	NA		0
S3_extracted_k141_12744	4616	No	NA	0	1	Not-determined	Genome-fragment	NA		0
S3_extracted_k141_99164	2282	No	NA	0	1	Not-determined	Genome-fragment	NA		0
S3_extracted_k141_83296	3586	No	NA	0	0	Not-determined	Genome-fragment	NA		0
S3_extracted_k141_43531	1264	No	NA	0	1	Not-determined	Genome-fragment	NA		0
S3_extracted_k141_122622	2909	No	NA	0	1	Not-determined	Genome-fragment	NA		0
S3_extracted_k141_43538	1097	No	NA	0	1	Not-determined	Genome-fragment	NA		0
S8_vs_k141_66952||full	1594	No	NA	0	1	Low-quality	Genome-fragment		3.62	0
S11_vs_k141_6190||full	1595	No	NA	1	0	Low-quality	Genome-fragment		3.75	0
S3_extracted_k141_122643	4665	No	NA	0	3	Not-determined	Genome-fragment	NA		0
S3_extracted_k141_12773	1667	No	NA	0	0	Not-determined	Genome-fragment	NA		0
S3_extracted_k141_43554	1136	No	NA	0	0	Not-determined	Genome-fragment	NA		0
S3_extracted_k141_115115	5094	No	NA	0	2	Not-determined	Genome-fragment	NA		0
S3_extracted_k141_12779	3131	No	NA	0	5	Not-determined	Genome-fragment	NA		0
S3_extracted_k141_91272	1331	No	NA	0	1	Not-determined	Genome-fragment	NA		0
S14_vs_k141_7910||full	1595	No	NA	0	0	Low-quality	Genome-fragment		3.06	0
S1_extracted_k141_43554	1595	No	NA	0	0	Low-quality	Genome-fragment		3.45	0
S1_extracted_k141_30484	1595	No	NA	1	0	Low-quality	Genome-fragment		2.69	0
S3_extracted_k141_122696	1093	No	NA	0	0	Not-determined	Genome-fragment	NA		0
S5_vs_k141_40455||full	1595	No	NA	0	2	Low-quality	Genome-fragment		1.83	0
S3_extracted_k141_59818	3223	No	NA	0	1	Not-determined	Genome-fragment	NA		0
S3_extracted_k141_115164	4731	No	NA	0	3	Not-determined	Genome-fragment	NA		0
S3_extracted_k141_91357	6746	No	NA	0	4	Not-determined	Genome-fragment	NA		0
S7_vs_129901||full	1595	No	NA	2	0	Low-quality	Genome-fragment		3.09	0
S3_extracted_k141_3475	1209	No	NA	0	2	Not-determined	Genome-fragment	NA		0
S3_extracted_k141_51971	1558	No	NA	0	1	Not-determined	Genome-fragment	NA		0
S16_vs_133472||full	1595	No	NA	1	0	Low-quality	Genome-fragment		3.87	0
S16_vs_156625||lt2gene	1595	No	NA	2	0	Low-quality	Genome-fragment		4.18	0
S3_extracted_k141_66848	2915	No	NA	0	0	Not-determined	Genome-fragment	NA		0
S12_vs_k141_82831||full	1595	No	NA	1	0	Low-quality	Genome-fragment		2.74	0
S3_extracted_k141_18766	3638	No	NA	0	3	Not-determined	Genome-fragment	NA		0
S6_vs_k141_15196||full	1595	No	NA	0	0	Low-quality	Genome-fragment		4.18	0
S3_extracted_k141_3535	1033	No	NA	0	2	Not-determined	Genome-fragment	NA		0
S3_extracted_k141_122750	2311	No	NA	0	0	Not-determined	Genome-fragment	NA		0
S3_extracted_k141_52051	1069	No	NA	0	0	Not-determined	Genome-fragment	NA		0
S13_vs_5405||lt2gene	1596	No	NA	1	0	Low-quality	Genome-fragment		3.36	0
S3_extracted_k141_52059	4653	No	NA	0	2	Not-determined	Genome-fragment	NA		0
S3_extracted_k141_52060	4530	No	NA	0	1	Not-determined	Genome-fragment	NA		0
S3_extracted_k141_83450	1234	No	NA	0	1	Not-determined	Genome-fragment	NA		0
S1_extracted_k141_106538	1596	No	NA	0	0	Low-quality	Genome-fragment		2.37	0
S5_extracted_k141_140268	1596	No	NA	1	0	Low-quality	Genome-fragment		4.36	0
S5_vs_k141_124891||full	1596	No	NA	1	0	Low-quality	Genome-fragment		3.15	0
S5_vs_k141_76580||full	1596	No	NA	0	1	Low-quality	Genome-fragment		4.78	0
S16_extracted_39964	1596	No	NA	1	0	Low-quality	Genome-fragment		2.41	0
S3_extracted_k141_12893	1156	No	NA	0	0	Not-determined	Genome-fragment	NA		0
S16_vs_33610||full	1596	No	NA	0	0	Low-quality	Genome-fragment		4.44	0
S3_extracted_k141_27642	6009	No	NA	0	6	Not-determined	Genome-fragment	NA		0
S10_extracted_1019	1596	No	NA	0	0	Low-quality	Genome-fragment		3.09	0
S3_extracted_k141_83505	1238	No	NA	0	1	Not-determined	Genome-fragment	NA		0
S12_vs_k141_68273||full	1596	No	NA	1	0	Low-quality	Genome-fragment		2.58	0
S13_vs_45970||full	1597	No	NA	0	2	Low-quality	Genome-fragment		0.72	0
S3_extracted_k141_36008	4645	No	NA	0	0	Not-determined	Genome-fragment	NA		0
S3_extracted_k141_83536	15430	No	NA	7	0	Medium-quality	Genome-fragment		66.36	0
S3_extracted_k141_18829	86825	No	NA	15	2	Complete	High-quality		100	0
S3_extracted_k141_36063	3368	No	NA	0	1	Not-determined	Genome-fragment	NA		0
S13_vs_65500||full	1597	No	NA	0	1	Low-quality	Genome-fragment		1.47	0
S3_extracted_k141_36079	1978	No	NA	0	0	Not-determined	Genome-fragment	NA		0
S3_extracted_k141_83560	3157	No	NA	0	1	Not-determined	Genome-fragment	NA		0
S3_extracted_k141_91556	3824	No	NA	0	2	Not-determined	Genome-fragment	NA		0
S3_extracted_k141_83583	2090	No	NA	0	1	Not-determined	Genome-fragment	NA		0
S3_extracted_k141_91582	2241	No	NA	0	0	Not-determined	Genome-fragment	NA		0
S5_vs_k141_104893||full	1597	No	NA	1	0	Low-quality	Genome-fragment		2.21	0
S3_extracted_k141_115304	100971	No	NA	16	1	Complete	High-quality		100	0
S3_extracted_k141_36093	14516	No	NA	0	3	Not-determined	Genome-fragment	NA		0
S3_extracted_k141_107482	7654	No	NA	0	3	Not-determined	Genome-fragment	NA		0
S3_extracted_k141_60053	2649	No	NA	0	1	Not-determined	Genome-fragment	NA		0
S5_vs_k141_48057||full	1597	No	NA	1	0	Low-quality	Genome-fragment		3.52	0
S3_extracted_k141_60075	3445	No	NA	0	2	Not-determined	Genome-fragment	NA		0
S3_extracted_k141_60085	1667	No	NA	0	1	Not-determined	Genome-fragment	NA		0
S7_vs_45085||full	1597	No	NA	1	0	Low-quality	Genome-fragment		3.89	0
S12_extracted_k141_72777	1597	No	NA	2	0	Low-quality	Genome-fragment		3.79	0
S3_extracted_k141_60095	3335	No	NA	0	2	Not-determined	Genome-fragment	NA		0
S3_extracted_k141_60112	1467	No	NA	0	2	Not-determined	Genome-fragment	NA		0
S3_extracted_k141_76243	1302	No	NA	0	1	Not-determined	Genome-fragment	NA		0
S12_vs_k141_18949||full	1597	No	NA	1	0	Low-quality	Genome-fragment		4.36	0
S3_extracted_k141_13075	4326	No	NA	0	3	Not-determined	Genome-fragment	NA		0
S3_extracted_k141_52386	1063	No	NA	0	1	Not-determined	Genome-fragment	NA		0
S3_extracted_k141_60147	5130	No	NA	0	2	Not-determined	Genome-fragment	NA		0
S2_extracted_k141_13592	1597	No	NA	0	1	Low-quality	Genome-fragment		2.43	0
S3_extracted_k141_52418	1212	No	NA	0	0	Not-determined	Genome-fragment	NA		0
S3_extracted_k141_91677	5510	No	NA	0	1	Not-determined	Genome-fragment	NA		0
S6_extracted_k141_25454	1597	No	NA	1	0	Low-quality	Genome-fragment		3.77	0
S8_extracted_k141_71753	1597	No	NA	0	2	Low-quality	Genome-fragment		0.71	0
S3_extracted_k141_122901	1028	No	NA	0	1	Not-determined	Genome-fragment	NA		0
S3_extracted_k141_107627	1858	No	NA	0	2	Not-determined	Genome-fragment	NA		0
S3_extracted_k141_13154	1014	No	NA	0	0	Not-determined	Genome-fragment	NA		0
S3_extracted_k141_3792	6349	No	NA	0	0	Not-determined	Genome-fragment	NA		0
S3_extracted_k141_52454	6126	No	NA	0	1	Not-determined	Genome-fragment	NA		0
S8_vs_k141_33497||full	1597	No	NA	1	0	Low-quality	Genome-fragment		4.35	0
S3_extracted_k141_60201	4682	No	NA	0	4	Not-determined	Genome-fragment	NA		0
S3_extracted_k141_107662	1266	No	NA	0	1	Not-determined	Genome-fragment	NA		0
S17_extracted_k141_102607	1598	No	NA	2	0	Low-quality	Genome-fragment		4.34	0
S3_extracted_k141_3841	1229	No	NA	0	0	Not-determined	Genome-fragment	NA		0
S17_vs_k141_19327||full	1598	No	NA	0	0	Low-quality	Genome-fragment		1.15	0
S17_vs_k141_9849||lt2gene	1598	No	NA	1	0	Low-quality	Genome-fragment		4.44	0
S14_extracted_k141_70559	1598	No	NA	0	0	Low-quality	Genome-fragment		1.02	0
S3_vs_k141_91154||full	1598	No	NA	0	0	Low-quality	Genome-fragment		3.4	0
S3_vs_k141_76309||full	1598	No	NA	1	0	Low-quality	Genome-fragment		2.85	0
S3_extracted_k141_43921	2123	No	NA	0	1	Not-determined	Genome-fragment	NA		0
S3_extracted_k141_91782	1041	No	NA	0	0	Not-determined	Genome-fragment	NA		0
S5_vs_k141_63291||lt2gene	1598	No	NA	1	0	Low-quality	Genome-fragment		3.58	0
S3_extracted_k141_67143	1018	No	NA	0	0	Not-determined	Genome-fragment	NA		0
S3_extracted_k141_3957	1705	No	NA	0	1	Not-determined	Genome-fragment	NA		0
S3_extracted_k141_91803	2250	No	NA	0	1	Not-determined	Genome-fragment	NA		0
S3_extracted_k141_36248	2671	No	NA	0	2	Not-determined	Genome-fragment	NA		0
S7_vs_65590||full	1598	No	NA	0	0	Low-quality	Genome-fragment		4.37	0
S15_vs_19881||full	1598	No	NA	1	0	Low-quality	Genome-fragment		3.54	0
S15_vs_74723||full	1598	No	NA	0	0	Low-quality	Genome-fragment		1.18	0
S3_extracted_k141_123013	11838	No	NA	0	3	Not-determined	Genome-fragment	NA		0
S3_extracted_k141_107759	4800	No	NA	0	0	Not-determined	Genome-fragment	NA		0

S2_extracted_k141_19419	1598	No	NA	0	0	Low-quality	Genome-fragment		1.66	0
S8_extracted_k141_25157	1598	No	NA	0	0	Low-quality	Genome-fragment		3.76	0
S3_extracted_k141_91865	3274	No	NA	0	2	Not-determined	Genome-fragment	NA		0
S3_extracted_k141_107768	1180	No	NA	0	0	Not-determined	Genome-fragment	NA		0
S8_vs_k141_1943||full	1598	No	NA	0	0	Low-quality	Genome-fragment		18.16	0
S11_vs_k141_32679||full	1599	No	NA	1	0	Low-quality	Genome-fragment		2.31	0
S3_extracted_k141_107799	5072	No	NA	0	2	Not-determined	Genome-fragment	NA		0
S3_extracted_k141_13359	1431	No	NA	0	2	Not-determined	Genome-fragment	NA		0
S3_extracted_k141_19190	13533	No	NA	0	2	Not-determined	Genome-fragment	NA		0
S3_extracted_k141_91875	1129	No	NA	0	1	Not-determined	Genome-fragment	NA		0
S11_vs_k141_24669||full	1599	No	NA	2	0	Low-quality	Genome-fragment		2.4	0
S1_extracted_k141_27994	1599	No	NA	0	1	Low-quality	Genome-fragment		0.6	0
S10_vs_57536||full	1599	No	NA	0	3	Low-quality	Genome-fragment		2.44	0
S3_extracted_k141_27984	5648	No	NA	0	1	Not-determined	Genome-fragment	NA		0
S3_extracted_k141_67270	5219	No	NA	0	0	Not-determined	Genome-fragment	NA		0
S3_extracted_k141_13438	1241	No	NA	0	0	Not-determined	Genome-fragment	NA		0
S10_vs_93700||full	1599	No	NA	1	0	Low-quality	Genome-fragment		3.4	0
S8_vs_k141_24812||full	1599	No	NA	1	0	Low-quality	Genome-fragment		3.78	0
S3_extracted_k141_52607	1152	No	NA	0	0	Not-determined	Genome-fragment	NA		0
S3_extracted_k141_36385	1158	No	NA	0	1	Not-determined	Genome-fragment	NA		0
S3_extracted_k141_4067	6741	No	NA	0	11	Not-determined	Genome-fragment	NA		0
S17_extracted_k141_155914	1600	No	NA	2	0	Low-quality	Genome-fragment		7.25	0
S9_extracted_k141_164	1600	No	NA	0	0	Low-quality	Genome-fragment		2.21	0
S13_vs_98755||full	1600	No	NA	0	0	Low-quality	Genome-fragment		3.98	0
S3_extracted_k141_4082	1751	No	NA	0	1	Not-determined	Genome-fragment	NA		0
S14_extracted_k141_79091	1600	No	NA	0	0	Low-quality	Genome-fragment		3.72	0
S14_vs_k141_13609||full	1600	No	NA	0	2	Low-quality	Genome-fragment		2.08	0
S3_extracted_k141_13510	1749	No	NA	0	1	Not-determined	Genome-fragment	NA		0
S14_vs_k141_104305||lt2gene	1600	No	NA	1	0	Low-quality	Genome-fragment		3.01	0
S3_extracted_k141_60543	4225	No	NA	0	10	Not-determined	Genome-fragment	NA		0
S1_extracted_k141_96105	1600	No	NA	0	0	Low-quality	Genome-fragment		1.72	0
S3_extracted_k141_123230	3562	No	NA	0	2	Not-determined	Genome-fragment	NA		0
S3_extracted_k141_67333	56683	No	NA	14	0	Complete	High-quality		100	0
S3_extracted_k141_60581	2060	No	NA	0	2	Not-determined	Genome-fragment	NA		0
S3_extracted_k141_99567	1116	No	NA	0	0	Not-determined	Genome-fragment	NA		0
S5_extracted_k141_12960	1600	No	NA	0	0	Low-quality	Genome-fragment		1.02	0
S3_extracted_k141_52709	1027	No	NA	0	0	Not-determined	Genome-fragment	NA		0
S3_extracted_k141_4138	1231	No	NA	0	1	Not-determined	Genome-fragment	NA		0
S10_vs_35001||full	1600	No	NA	0	0	Low-quality	Genome-fragment		3.35	0
S3_extracted_k141_44104	7909	No	NA	0	3	Not-determined	Genome-fragment	NA		0
S10_vs_72962||full	1600	No	NA	0	2	Low-quality	Genome-fragment		2.76	0
S10_vs_69130||lt2gene	1600	No	NA	2	0	Low-quality	Genome-fragment		3.5	0
S3_extracted_k141_36425	8709	No	NA	0	0	Not-determined	Genome-fragment	NA		0
S3_extracted_k141_13604	1120	No	NA	0	0	Not-determined	Genome-fragment	NA		0
S3_extracted_k141_123294	1211	No	NA	0	1	Not-determined	Genome-fragment	NA		0
S3_extracted_k141_60660	1566	No	NA	0	2	Not-determined	Genome-fragment	NA		0
S12_vs_k141_178076||full	1600	No	NA	0	0	Low-quality	Genome-fragment		3.89	0
S3_extracted_k141_52767	1305	No	NA	0	0	Not-determined	Genome-fragment	NA		0
S12_vs_k141_171571||full	1600	No	NA	0	0	Low-quality	Genome-fragment		2.08	0
S3_extracted_k141_76729	6698	No	NA	0	3	Not-determined	Genome-fragment	NA		0
S3_extracted_k141_52772	3804	No	NA	0	3	Not-determined	Genome-fragment	NA		0
S3_extracted_k141_123321	2583	No	NA	0	2	Not-determined	Genome-fragment	NA		0
S8_extracted_k141_63596	1600	No	NA	0	0	Low-quality	Genome-fragment		1.14	0
S3_extracted_k141_83839	1914	No	NA	0	6	Not-determined	Genome-fragment	NA		0
S3_extracted_k141_60682	1527	No	NA	0	1	Not-determined	Genome-fragment	NA		0
S9_vs_k141_21238||full	1601	No	NA	0	0	Low-quality	Genome-fragment		4.02	0
S3_extracted_k141_60692	4173	No	NA	0	1	Not-determined	Genome-fragment	NA		0
S3_extracted_k141_115815	1720	No	NA	0	1	Not-determined	Genome-fragment	NA		0
S3_extracted_k141_76762	7576	No	NA	0	3	Not-determined	Genome-fragment	NA		0
S13_vs_4991||full	1601	No	NA	0	0	Low-quality	Genome-fragment		4.43	0
S3_extracted_k141_44145	1881	No	NA	0	0	Not-determined	Genome-fragment	NA		0
S14_extracted_k141_93760	1601	No	NA	3	0	Low-quality	Genome-fragment		3.55	0
S3_extracted_k141_115861	2082	No	NA	0	2	Not-determined	Genome-fragment	NA		0
S3_extracted_k141_99626	39745	No	NA	21	0	Complete	High-quality		100	0
S14_vs_k141_9012||full	1601	No	NA	0	0	Low-quality	Genome-fragment		3.76	0
S3_extracted_k141_67436	1697	No	NA	0	1	Not-determined	Genome-fragment	NA		0
S3_extracted_k141_13673	9090	No	NA	0	5	Not-determined	Genome-fragment	NA		0
S1_vs_k141_49044||full	1601	No	NA	1	0	Low-quality	Genome-fragment		3.47	0
S3_extracted_k141_123409	4145	No	NA	0	0	Not-determined	Genome-fragment	NA		0
S3_extracted_k141_64973	1601	No	NA	0	1	Low-quality	Genome-fragment		1	0
S3_extracted_k141_115869	3204	No	NA	0	3	Not-determined	Genome-fragment	NA		0
S3_vs_k141_119193||full	1601	No	NA	1	0	Low-quality	Genome-fragment		3.84	0
S3_extracted_k141_28171	1540	No	NA	0	3	Not-determined	Genome-fragment	NA		0
S3_extracted_k141_115901	1650	No	NA	0	1	Not-determined	Genome-fragment	NA		0
S5_vs_k141_82186||lt2gene	1601	No	NA	1	0	Low-quality	Genome-fragment		2.34	0
S3_extracted_k141_36485	35347	No	NA	0	13	Not-determined	Genome-fragment	NA		0
S3_extracted_k141_83910	8049	No	NA	0	5	Not-determined	Genome-fragment	NA		0
S10_vs_92518||full	1601	No	NA	0	1	Low-quality	Genome-fragment		2.2	0
S12_vs_k141_170641||full	1601	No	NA	0	0	Low-quality	Genome-fragment		2.88	0
S8_extracted_k141_132078	1601	No	NA	0	0	Low-quality	Genome-fragment		1	0
S9_extracted_k141_6490	1602	No	NA	1	0	Low-quality	Genome-fragment		1.86	0
S3_extracted_k141_108049	1098	No	NA	0	1	Not-determined	Genome-fragment	NA		0
S13_vs_96119||full	1602	No	NA	1	0	Low-quality	Genome-fragment		3.62	0
S3_extracted_k141_67556	1334	No	NA	0	1	Not-determined	Genome-fragment	NA		0
S3_extracted_k141_36509	1626	No	NA	0	1	Not-determined	Genome-fragment	NA		0
S1_extracted_k141_47748	1602	No	NA	0	1	Low-quality	Genome-fragment		0.89	0
S3_extracted_k141_13804	1495	No	NA	0	1	Not-determined	Genome-fragment	NA		0
S3_extracted_k141_92053	4291	No	NA	0	1	Not-determined	Genome-fragment	NA		0
S3_extracted_k141_52947	7764	No	NA	0	3	Not-determined	Genome-fragment	NA		0
S3_extracted_k141_76896	3253	No	NA	0	2	Not-determined	Genome-fragment	NA		0
S3_extracted_k141_36558	5958	No	NA	0	5	Not-determined	Genome-fragment	NA		0
S3_extracted_k141_13827	2098	No	NA	0	0	Not-determined	Genome-fragment	NA		0
S1_extracted_k141_104905	1602	No	NA	0	0	Low-quality	Genome-fragment		3.65	0
S1_vs_k141_42042||full	1602	No	NA	0	0	Low-quality	Genome-fragment		4	0
S3_extracted_k141_4272	1292	No	NA	0	3	Not-determined	Genome-fragment	NA		0
S3_vs_k141_111423||full	1602	No	NA	0	0	Low-quality	Genome-fragment		4.13	0
S16_vs_6261||full	1602	No	NA	0	0	Low-quality	Genome-fragment		3.69	0
S3_extracted_k141_36577	3234	No	NA	0	2	Not-determined	Genome-fragment	NA		0
S3_extracted_k141_19536	1049	No	NA	0	0	Not-determined	Genome-fragment	NA		0
S3_extracted_k141_108122	4204	No	NA	0	3	Not-determined	Genome-fragment	NA		0
S16_vs_53753||full	1602	No	NA	0	0	Low-quality	Genome-fragment		2.93	0
S3_extracted_k141_108127	1297	No	NA	0	0	Not-determined	Genome-fragment	NA		0
S3_extracted_k141_19544	3193	No	NA	0	1	Not-determined	Genome-fragment	NA		0
S10_extracted_27357	1602	No	NA	2	0	Low-quality	Genome-fragment		2.08	0
S3_extracted_k141_44397	4338	No	NA	0	1	Not-determined	Genome-fragment	NA		0
S3_extracted_k141_28306	1000	No	NA	0	0	Not-determined	Genome-fragment	NA		0
S12_extracted_k141_86737	1602	No	NA	0	0	Low-quality	Genome-fragment		2.57	0
S8_vs_k141_86373||full	1602	No	NA	0	1	Low-quality	Genome-fragment		2.25	0
S3_extracted_k141_28309	8674	No	NA	0	4	Not-determined	Genome-fragment	NA		0
S11_extracted_k141_98157	1603	No	NA	0	0	Low-quality	Genome-fragment		3.5	0
S3_extracted_k141_84066	1680	No	NA	0	0	Not-determined	Genome-fragment	NA		0
S3_extracted_k141_53053	3046	No	NA	0	4	Not-determined	Genome-fragment	NA		0
S11_vs_k141_74777||full	1603	No	NA	0	0	Low-quality	Genome-fragment		2.08	0
S14_vs_k141_84166||full	1603	No	NA	0	0	Low-quality	Genome-fragment		1	0
S3_extracted_k141_123575	4915	No	NA	0	3	Not-determined	Genome-fragment	NA		0
S3_extracted_k141_44405	38184	No	NA	26	0	Medium-quality	Genome-fragment		63.68	0
S3_vs_k141_56834||full	1603	No	NA	1	0	Low-quality	Genome-fragment		0.71	0
S3_extracted_k141_19637	3081	No	NA	0	3	Not-determined	Genome-fragment	NA		0
S7_vs_84859||full	1603	No	NA	1	0	Low-quality	Genome-fragment		3.65	0
S3_extracted_k141_53108	3131	No	NA	0	1	Not-determined	Genome-fragment	NA		0
S7_vs_109895||full	1603	No	NA	0	0	Low-quality	Genome-fragment		2.99	0
S3_extracted_k141_4359	2000	No	NA	0	0	Not-determined	Genome-fragment	NA		0
S16_vs_94972||full	1603	No	NA	0	0	Low-quality	Genome-fragment		3.16	0
S10_vs_107588||full	1603	No	NA	0	0	Low-quality	Genome-fragment		0.65	0
S3_extracted_k141_99732	13105	No	NA	0	7	Not-determined	Genome-fragment	NA		0
S17_extracted_k141_44673	1604	No	NA	0	1	Low-quality	Genome-fragment		0.6	0
S3_extracted_k141_84179	1056	No	NA	0	2	Not-determined	Genome-fragment	NA		0
S3_extracted_k141_14060	1066	No	NA	0	1	Not-determined	Genome-fragment	NA		0
S9_vs_k141_2680||full	1604	No	NA	2	0	Low-quality	Genome-fragment		4.19	0
S3_extracted_k141_92218	3182	No	NA	0	0	Not-determined	Genome-fragment	NA		0
S3_extracted_k141_77117	5315	No	NA	0	3	Not-determined	Genome-fragment	NA		0
S3_extracted_k141_99734	46304	No	NA	18	0	High-quality	High-quality		98.26	0
S14_extracted_k141_39622	1604	No	NA	0	1	Low-quality	Genome-fragment		0.85	0
S3_extracted_k141_4406	1185	No	NA	0	1	Not-determined	Genome-fragment	NA		0
S3_extracted_k141_19734	2449	No	NA	0	2	Not-determined	Genome-fragment	NA		0
S3_extracted_k141_99750	3513	No	NA	0	3	Not-determined	Genome-fragment	NA		0
S3_extracted_k141_19767	1070	No	NA	0	1	Not-determined	Genome-fragment	NA		0
S3_extracted_k141_36683	2340	No	NA	0	1	Not-determined	Genome-fragment	NA		0
S3_extracted_k141_28419	1460	No	NA	0	1	Not-determined	Genome-fragment	NA		0
S3_extracted_k141_67772	3582	No	NA	0	2	Not-determined	Genome-fragment	NA		0
S3_extracted_k141_92336	1550	No	NA	0	0	Not-determined	Genome-fragment	NA		0
S3_extracted_k141_67786	3212	No	NA	0	2	Not-determined	Genome-fragment	NA		0
S3_vs_k141_66207||full	1604	No	NA	0	0	Low-quality	Genome-fragment		1.2	0
S3_extracted_k141_44445	1009	No	NA	0	0	Not-determined	Genome-fragment	NA		0
S3_extracted_k141_77224	2318	No	NA	0	0	Not-determined	Genome-fragment	NA		0
S3_extracted_k141_77227	1923	No	NA	0	1	Not-determined	Genome-fragment	NA		0
S3_extracted_k141_28466	1620	No	NA	0	1	Not-determined	Genome-fragment	NA		0
S3_extracted_k141_77230	1600	No	NA	0	1	Not-determined	Genome-fragment	NA		0
S5_vs_k141_28743||full	1604	No	NA	0	0	Low-quality	Genome-fragment		2.08	0
S3_extracted_k141_4440	3503	No	NA	0	3	Not-determined	Genome-fragment	NA		0
S3_extracted_k141_61062	1969	No	NA	0	1	Not-determined	Genome-fragment	NA		0
S16_extracted_114946	1604	No	NA	1	0	Low-quality	Genome-fragment		2.47	0
S16_vs_112982||full	1604	No	NA	1	0	Low-quality	Genome-fragment		0.89	0
S3_extracted_k141_77242	1406	No	NA	0	1	Not-determined	Genome-fragment	NA		0
S16_vs_156641||full	1604	No	NA	0	0	Low-quality	Genome-fragment		2.75	0
S6_extracted_k141_55199	1604	No	NA	0	0	Low-quality	Genome-fragment		2.01	0
S3_extracted_k141_67828	6187	No	NA	2	0	Complete	High-quality		100	0
S6_vs_k141_22524||full	1604	No	NA	4	0	Low-quality	Genome-fragment		4.22	0
S8_extracted_k141_192470	1604	No	NA	0	0	Low-quality	Genome-fragment		0.92	0
S3_extracted_k141_84385	1327	No	NA	0	0	Not-determined	Genome-fragment	NA		0
S17_extracted_k141_61388	1605	No	NA	0	0	Low-quality	Genome-fragment		1.47	0
S17_extracted_k141_100264	1605	No	NA	0	0	Low-quality	Genome-fragment		4.01	0
S3_extracted_k141_123731	2021	No	NA	0	1	Not-determined	Genome-fragment	NA		0
S13_vs_40441||full	1605	No	NA	0	0	Low-quality	Genome-fragment		1.61	0
S14_extracted_k141_62820	1605	No	NA	0	1	Low-quality	Genome-fragment		1.43	0
S3_extracted_k141_92484	1001	No	NA	0	1	Not-determined	Genome-fragment	NA		0
S14_extracted_k141_64579	1605	No	NA	0	0	Low-quality	Genome-fragment		2.68	0
S14_vs_k141_98701||full	1605	No	NA	3	0	Low-quality	Genome-fragment		3.29	0
S1_vs_k141_89447||full	1605	No	NA	0	0	Low-quality	Genome-fragment		3.8	0
S3_extracted_k141_4545	1765	No	NA	0	0	Not-determined	Genome-fragment	NA		0
S3_vs_k141_34524||full	1605	No	NA	0	1	Low-quality	Genome-fragment		1.54	0
S7_vs_38028||full	1605	No	NA	1	0	Low-quality	Genome-fragment		3.55	0
S3_extracted_k141_4574	2056	No	NA	0	1	Not-determined	Genome-fragment	NA		0
S3_extracted_k141_61332	1795	No	NA	0	1	Not-determined	Genome-fragment	NA		0

S3_extracted_k141_116362	3952	No	NA		0	2	Not-determined	Genome-fragment	NA		0
S16_extracted_48641	1605	No	NA		0	0	Low-quality	Genome-fragment		3.9	0
S3_extracted_k141_77346	1081	No	NA		0	2	Not-determined	Genome-fragment	NA		0
S3_extracted_k141_14361	3307	No	NA		0	0	Not-determined	Genome-fragment	NA		0
S3_extracted_k141_99990	2015	No	NA		0	1	Not-determined	Genome-fragment	NA		0
S3_extracted_k141_77365	1944	No	NA		0	1	Not-determined	Genome-fragment	NA		0
S16_vs_138721||full	1605	No	NA		0	0	Low-quality	Genome-fragment		3.42	0
S3_extracted_k141_44555	1662	No	NA		0	0	Not-determined	Genome-fragment	NA		0
S3_extracted_k141_123797	3312	No	NA		0	1	Not-determined	Genome-fragment	NA		0
S3_extracted_k141_67926	1368	No	NA		0	0	Not-determined	Genome-fragment	NA		0
S3_extracted_k141_123799	4080	No	NA		0	1	Not-determined	Genome-fragment	NA		0
S12_vs_k141_114960||full	1605	No	NA		3	0	Low-quality	Genome-fragment		4.68	0
S3_extracted_k141_116400	1278	No	NA		0	1	Not-determined	Genome-fragment	NA		0
S12_vs_k141_148410||full	1605	No	NA		1	0	Low-quality	Genome-fragment		2.22	0
S3_extracted_k141_108459	3113	No	NA		0	2	Not-determined	Genome-fragment	NA		0
S3_extracted_k141_123809	6021	No	NA		0	2	Not-determined	Genome-fragment	NA		0
S3_extracted_k141_84548	2310	No	NA		0	3	Not-determined	Genome-fragment	NA		0
S3_extracted_k141_4618	5458	No	NA		0	4	Not-determined	Genome-fragment	NA		0
S11_vs_k141_7972||full	1606	No	NA		3	0	Low-quality	Genome-fragment		3.52	0
S3_extracted_k141_116442	1005	No	NA		0	2	Not-determined	Genome-fragment	NA		0
S3_extracted_k141_116468	2687	No	NA		0	3	Not-determined	Genome-fragment	NA		0
S3_extracted_k141_67958	2369	No	NA		0	0	Not-determined	Genome-fragment	NA		0
S14_vs_k141_23148||full	1606	No	NA		0	0	Low-quality	Genome-fragment		2.75	0
S3_extracted_k141_20071	1119	No	NA		0	0	Not-determined	Genome-fragment	NA		0
S3_extracted_k141_28704	1670	No	NA		0	2	Not-determined	Genome-fragment	NA		0
S5_vs_k141_81352||full	1606	No	NA		0	0	Low-quality	Genome-fragment		1.05	0
S5_vs_k141_123926||lt2gene	1606	No	NA		1	0	Low-quality	Genome-fragment		2.71	0
S7_vs_86435||full	1606	No	NA		2	0	Low-quality	Genome-fragment		4.7	0
S3_extracted_k141_14465	1010	No	NA		0	0	Not-determined	Genome-fragment	NA		0
S10_vs_115452||full	1606	No	NA		2	0	Low-quality	Genome-fragment		2.69	0
S3_extracted_k141_123878	1845	No	NA		0	2	Not-determined	Genome-fragment	NA		0
S3_extracted_k141_123888	2133	No	NA		0	1	Not-determined	Genome-fragment	NA		0
S3_extracted_k141_36932	1085	No	NA		0	1	Not-determined	Genome-fragment	NA		0
S12_vs_k141_80270||full	1606	No	NA		0	1	Low-quality	Genome-fragment		0.76	0
S3_extracted_k141_123890	1016	No	NA		0	0	Not-determined	Genome-fragment	NA		0
S12_vs_k141_148746||full	1606	No	NA		1	0	Low-quality	Genome-fragment		2.43	0
S6_vs_k141_17960||full	1606	No	NA		0	0	Low-quality	Genome-fragment		3.53	0
S8_vs_k141_78737||full	1606	No	NA		0	1	Low-quality	Genome-fragment		1.3	0
S3_extracted_k141_14489	3162	No	NA		0	1	Not-determined	Genome-fragment	NA		0
S3_extracted_k141_28722	1472	No	NA		0	0	Not-determined	Genome-fragment	NA		0
S8_vs_k141_165916||full	1606	No	NA		0	1	Low-quality	Genome-fragment		1.92	0
S17_extracted_k141_143228	1607	No	NA		1	0	Low-quality	Genome-fragment		2.6	0
S17_vs_k141_136591||full	1607	No	NA		1	0	Low-quality	Genome-fragment		2.4	0
S3_extracted_k141_61564	2115	No	NA		0	3	Not-determined	Genome-fragment	NA		0
S3_extracted_k141_108546	4668	No	NA		0	3	Not-determined	Genome-fragment	NA		0
S9_extracted_k141_12268	1607	No	NA		0	0	Low-quality	Genome-fragment		3.87	0
S11_vs_k141_2150||full	1607	No	NA		0	1	Low-quality	Genome-fragment		1.35	0
S3_extracted_k141_100080	3058	No	NA		0	1	Not-determined	Genome-fragment	NA		0
S3_extracted_k141_61590	2355	No	NA		0	3	Not-determined	Genome-fragment	NA		0
S3_extracted_k141_53501	1065	No	NA		0	0	Not-determined	Genome-fragment	NA		0
S13_vs_88503||full	1607	No	NA		0	1	Low-quality	Genome-fragment		3.45	0
S14_extracted_k141_45652	1607	No	NA		2	0	Low-quality	Genome-fragment		5.31	0
S14_vs_k141_4310||full	1607	No	NA		0	0	Low-quality	Genome-fragment		3.79	0
S3_extracted_k141_84832	12719	No	NA		10	0	Medium-quality	Genome-fragment		54.33	0
S3_extracted_k141_68038	1000	No	NA		0	1	Not-determined	Genome-fragment	NA		0
S3_vs_k141_27821||full	1607	No	NA		1	0	Low-quality	Genome-fragment		2.55	0
S3_extracted_k141_92779	1927	No	NA		0	0	Not-determined	Genome-fragment	NA		0
S3_extracted_k141_124049	1502	No	NA		0	0	Not-determined	Genome-fragment	NA		0
S3_extracted_k141_108597	2034	No	NA		0	2	Not-determined	Genome-fragment	NA		0
S3_extracted_k141_108599	1166	No	NA		0	0	Not-determined	Genome-fragment	NA		0
S3_extracted_k141_68059	1383	No	NA		0	1	Not-determined	Genome-fragment	NA		0
S5_vs_k141_3735||full	1607	No	NA		0	0	Low-quality	Genome-fragment		4.01	0
S16_extracted_29992	1607	No	NA		0	0	Low-quality	Genome-fragment		2.93	0
S3_extracted_k141_124077	1032	No	NA		0	1	Not-determined	Genome-fragment	NA		0
S12_vs_k141_71075||full	1607	No	NA		1	0	Low-quality	Genome-fragment		3.66	0
S6_vs_k141_8216||full	1607	No	NA		1	0	Low-quality	Genome-fragment		3.64	0
S3_extracted_k141_84866	1181	No	NA		0	1	Not-determined	Genome-fragment	NA		0
S3_extracted_k141_14672	1142	No	NA		0	1	Not-determined	Genome-fragment	NA		0
S3_extracted_k141_92798	1146	No	NA		0	1	Not-determined	Genome-fragment	NA		0
S3_extracted_k141_108641	1631	No	NA		0	1	Not-determined	Genome-fragment	NA		0
S8_vs_k141_200146||full	1607	No	NA		1	0	Low-quality	Genome-fragment		3.03	0
S8_vs_k141_146133||full	1607	No	NA		0	1	Low-quality	Genome-fragment		1.55	0
S3_extracted_k141_108662	1105	No	NA		0	1	Not-determined	Genome-fragment	NA		0
S11_vs_k141_22434||full	1608	No	NA		0	0	Low-quality	Genome-fragment		2.57	0
S3_extracted_k141_92844	1635	No	NA		0	1	Not-determined	Genome-fragment	NA		0
S11_vs_k141_36954||full	1608	No	NA		0	0	Low-quality	Genome-fragment		4.62	0
S3_extracted_k141_68157	1295	No	NA		0	0	Not-determined	Genome-fragment	NA		0
S13_vs_141198||full	1608	Yes		1464	1	1	Low-quality	Genome-fragment		3.74	8.96
S3_extracted_k141_4723	1050	No	NA		0	0	Not-determined	Genome-fragment	NA		0
S3_extracted_k141_68210	1243	No	NA		0	0	Not-determined	Genome-fragment	NA		0
S1_extracted_k141_49240	1608	No	NA		1	0	Low-quality	Genome-fragment		3.66	0
S3_extracted_k141_68220	1105	No	NA		0	2	Not-determined	Genome-fragment	NA		0
S1_extracted_k141_118586	1608	No	NA		1	0	Low-quality	Genome-fragment		0.92	0
S3_extracted_k141_92883	1160	No	NA		0	3	Not-determined	Genome-fragment	NA		0
S3_extracted_k141_14796	1344	No	NA		0	1	Not-determined	Genome-fragment	NA		0
S3_extracted_k141_4761	1088	No	NA		0	1	Not-determined	Genome-fragment	NA		0
S1_vs_k141_31294||full	1608	No	NA		0	0	Low-quality	Genome-fragment		1.13	0
S3_extracted_k141_61795	1217	No	NA		0	0	Not-determined	Genome-fragment	NA		0
S7_vs_47569||full	1608	No	NA		0	1	Low-quality	Genome-fragment		1.06	0
S3_extracted_k141_4783	1750	No	NA		0	0	Not-determined	Genome-fragment	NA		0
S16_vs_101328||full	1608	No	NA		0	0	Low-quality	Genome-fragment		0.94	0
S3_extracted_k141_124184	1185	No	NA		0	0	Not-determined	Genome-fragment	NA		0
S3_extracted_k141_37218	7637	No	NA		0	6	Not-determined	Genome-fragment	NA		0
S3_extracted_k141_116704	1262	No	NA		0	0	Not-determined	Genome-fragment	NA		0
S10_vs_76332||full	1608	No	NA		0	1	Low-quality	Genome-fragment		1.09	0
S3_extracted_k141_108841	2315	No	NA		0	2	Not-determined	Genome-fragment	NA		0
S6_vs_k141_23047||full	1608	No	NA		0	0	Low-quality	Genome-fragment		4.16	0
S8_vs_k141_177854||full	1608	No	NA		0	2	Low-quality	Genome-fragment		2.9	0
S3_extracted_k141_14849	5705	No	NA		0	3	Not-determined	Genome-fragment	NA		0
S3_extracted_k141_85052	1561	No	NA		0	1	Not-determined	Genome-fragment	NA		0
S3_extracted_k141_37253	1298	No	NA		0	0	Not-determined	Genome-fragment	NA		0
S3_extracted_k141_77840	1489	No	NA		0	0	Not-determined	Genome-fragment	NA		0
S3_extracted_k141_108897	6123	No	NA		0	3	Not-determined	Genome-fragment	NA		0
S11_vs_k141_60283||full	1609	No	NA		0	0	Low-quality	Genome-fragment		3.76	0
S11_vs_k141_93141||full	1609	No	NA		0	1	Low-quality	Genome-fragment		2.51	0
S3_extracted_k141_61860	67881	No	NA		27	0	Complete	High-quality		100	0
S3_extracted_k141_108936	1102	No	NA		0	0	Not-determined	Genome-fragment	NA		0
S3_extracted_k141_92980	3249	No	NA		0	0	Not-determined	Genome-fragment	NA		0
S3_extracted_k141_68317	1056	No	NA		0	0	Not-determined	Genome-fragment	NA		0
S11_vs_k141_79473||lt2gene	1609	No	NA		1	0	Low-quality	Genome-fragment		3.05	0
S3_extracted_k141_28981	2818	No	NA		0	3	Not-determined	Genome-fragment	NA		0
S3_extracted_k141_85104	1085	No	NA		0	1	Not-determined	Genome-fragment	NA		0
S1_extracted_k141_2973	1609	No	NA		0	0	Low-quality	Genome-fragment		2.77	0
S3_extracted_k141_29008	4575	No	NA		0	2	Not-determined	Genome-fragment	NA		0
S3_extracted_k141_68366	1239	No	NA		0	0	Not-determined	Genome-fragment	NA		0
S1_vs_k141_85839||full	1609	No	NA		0	0	Low-quality	Genome-fragment		4.44	0
S3_extracted_k141_100440	31270	No	NA		24	0	Medium-quality	Genome-fragment		68.24	0
S3_extracted_k141_37312	3304	No	NA		0	3	Not-determined	Genome-fragment	NA		0
S3_extracted_k141_5021	1514	No	NA		0	1	Not-determined	Genome-fragment	NA		0
S3_extracted_k141_108959	61482	No	NA		26	1	Complete	High-quality		100	0
S3_extracted_k141_61941	1720	No	NA		0	0	Not-determined	Genome-fragment	NA		0
S3_extracted_k141_68398	1429	No	NA		0	1	Not-determined	Genome-fragment	NA		0
S3_extracted_k141_61959	4813	No	NA		0	2	Not-determined	Genome-fragment	NA		0
S3_extracted_k141_5042	2385	No	NA		0	1	Not-determined	Genome-fragment	NA		0
S3_extracted_k141_68417	1476	No	NA		0	1	Not-determined	Genome-fragment	NA		0
S3_vs_k141_12119||full	1609	No	NA		0	1	Low-quality	Genome-fragment		1.54	0
S7_vs_113013||full	1609	No	NA		0	0	Low-quality	Genome-fragment		3.79	0
S3_extracted_k141_68442	1051	No	NA		0	0	Not-determined	Genome-fragment	NA		0
S3_extracted_k141_5123	1000	No	NA		0	0	Not-determined	Genome-fragment	NA		0
S7_vs_126743||full	1609	No	NA		0	0	Low-quality	Genome-fragment		1.35	0
S7_vs_61046||lt2gene	1609	No	NA		1	0	Low-quality	Genome-fragment		4.02	0
S16_vs_112078||full	1609	No	NA		2	0	Low-quality	Genome-fragment		4.07	0
S3_extracted_k141_37410	1166	No	NA		0	0	Not-determined	Genome-fragment	NA		0
S16_vs_170711||full	1609	Yes		309	1	1	Low-quality	Genome-fragment		0.5	80.8
S3_extracted_k141_109090	1043	No	NA		0	2	Not-determined	Genome-fragment	NA		0
S10_extracted_42331	1609	No	NA		1	0	Low-quality	Genome-fragment		1.58	0
S3_extracted_k141_68523	1032	No	NA		0	1	Not-determined	Genome-fragment	NA		0
S3_extracted_k141_29099	4741	No	NA		0	1	Not-determined	Genome-fragment	NA		0
S3_extracted_k141_116927	1761	No	NA		0	0	Not-determined	Genome-fragment	NA		0
S3_extracted_k141_78056	3494	No	NA		0	1	Not-determined	Genome-fragment	NA		0
S3_extracted_k141_15003	5303	No	NA		0	1	Not-determined	Genome-fragment	NA		0
S3_extracted_k141_85324	1590	No	NA		0	0	Not-determined	Genome-fragment	NA		0
S3_extracted_k141_109151	3701	No	NA		0	4	Not-determined	Genome-fragment	NA		0
S10_vs_11420||full	1609	No	NA		3	0	Low-quality	Genome-fragment		1.52	0
S3_extracted_k141_15084	4963	No	NA		0	4	Not-determined	Genome-fragment	NA		0
S8_extracted_k141_195304	1609	No	NA		1	0	Low-quality	Genome-fragment		1.03	0
S3_extracted_k141_116976	1341	No	NA		0	1	Not-determined	Genome-fragment	NA		0
S17_vs_k141_123891||full	1610	No	NA		2	0	Low-quality	Genome-fragment		3.35	0
S14_vs_k141_41053||full	1610	No	NA		0	2	Low-quality	Genome-fragment		2.36	0
S1_extracted_k141_120575	1610	No	NA		0	0	Low-quality	Genome-fragment		3.52	0
S3_extracted_k141_68675	1559	No	NA		0	1	Not-determined	Genome-fragment	NA		0
S1_vs_k141_21855||full	1610	No	NA		0	2	Low-quality	Genome-fragment		1.15	0
S3_extracted_k141_37563	1106	No	NA		0	0	Not-determined	Genome-fragment	NA		0
S3_extracted_k141_15218	1326	No	NA		0	0	Not-determined	Genome-fragment	NA		0
S3_extracted_k141_15221	3266	No	NA		0	3	Not-determined	Genome-fragment	NA		0
S8_extracted_k141_41297	1610	No	NA		1	0	Low-quality	Genome-fragment		2.57	0
S3_extracted_k141_124497	1093	No	NA		0	0	Not-determined	Genome-fragment	NA		0
S3_extracted_k141_109277	7138	No	NA		0	0	Medium-quality	Genome-fragment		60.06	0
S8_vs_k141_13980||full	1610	No	NA		0	1	Low-quality	Genome-fragment		1.68	0
S3_extracted_k141_78134	1801	No	NA		0	1	Not-determined	Genome-fragment	NA		0
S8_vs_k141_24603||full	1610	No	NA		0	1	Low-quality	Genome-fragment		1.42	0
S7_vs_43884||full	1611	No	NA		1	0	Low-quality	Genome-fragment		3.9	0
S16_extracted_124353	1611	No	NA		1	0	Low-quality	Genome-fragment		2.73	0
S10_vs_15728||full	1611	No	NA		0	1	Low-quality	Genome-fragment		3.82	0
S3_extracted_k141_20739	1730	No	NA		0	2	Not-determined	Genome-fragment	NA		0
S12_extracted_k141_114703	1611	No	NA		0	0	Low-quality	Genome-fragment		2.38	0
S11_vs_k141_54995||full	1612	No	NA		0	0	Low-quality	Genome-fragment		3.52	0
S14_vs_k141_112401||full	1612	No	NA		0	0	Low-quality	Genome-fragment		3.99	0
S3_extracted_k141_85533	1182	No	NA		0	1	Not-determined	Genome-fragment	NA		0
S3_extracted_k141_20759	10910	No	NA		0	1	Not-determined	Genome-fragment	NA		0
S3_extracted_k141_37639	13259	No	NA		0	1	Not-determined	Genome-fragment	NA		0
S3_extracted_k141_37640	3671	No	NA		0	5	Not-determined	Genome-fragment	NA		0
S14_vs_k141_63729||full	1612	No	NA		1	0	Low-quality	Genome-fragment		3.81	0
S3_extracted_k141_37645	1358	No	NA		0	2	Not-determined	Genome-fragment	NA		0

<table>
<tr><td>S3_extracted_k141_109474</td><td>1158</td><td>No</td><td>NA</td><td>0</td><td>1</td><td>Not-determined</td><td>Genome-fragment</td><td>NA</td><td>0</td></tr>
<tr><td>S1_vs_k141_125111||lt2gene</td><td>1612</td><td>No</td><td>NA</td><td>1</td><td>0</td><td>Low-quality</td><td>Genome-fragment</td><td>3.73</td><td>0</td></tr>
<tr><td>S3_extracted_k141_15318</td><td>1102</td><td>No</td><td>NA</td><td>0</td><td>1</td><td>Not-determined</td><td>Genome-fragment</td><td>NA</td><td>0</td></tr>
<tr><td>S5_extracted_k141_20127</td><td>1612</td><td>No</td><td>NA</td><td>1</td><td>0</td><td>Low-quality</td><td>Genome-fragment</td><td>0.85</td><td>0</td></tr>
<tr><td>S7_vs_112992||full</td><td>1612</td><td>No</td><td>NA</td><td>1</td><td>0</td><td>Low-quality</td><td>Genome-fragment</td><td>3.83</td><td>0</td></tr>
<tr><td>S10_extracted_21376</td><td>1612</td><td>No</td><td>NA</td><td>0</td><td>0</td><td>Low-quality</td><td>Genome-fragment</td><td>2.8</td><td>0</td></tr>
<tr><td>S4_extracted_15002</td><td>1612</td><td>No</td><td>NA</td><td>0</td><td>0</td><td>Low-quality</td><td>Genome-fragment</td><td>2.89</td><td>0</td></tr>
<tr><td>S3_extracted_k141_29410</td><td>2097</td><td>No</td><td>NA</td><td>0</td><td>2</td><td>Not-determined</td><td>Genome-fragment</td><td>NA</td><td>0</td></tr>
<tr><td>S9_vs_k141_59739||full</td><td>1613</td><td>No</td><td>NA</td><td>1</td><td>0</td><td>Low-quality</td><td>Genome-fragment</td><td>0.98</td><td>0</td></tr>
<tr><td>S11_vs_k141_29327||full</td><td>1613</td><td>No</td><td>NA</td><td>1</td><td>0</td><td>Low-quality</td><td>Genome-fragment</td><td>2.96</td><td>0</td></tr>
<tr><td>S3_extracted_k141_78265</td><td>14179</td><td>No</td><td>NA</td><td>0</td><td>2</td><td>Not-determined</td><td>Genome-fragment</td><td>NA</td><td>0</td></tr>
<tr><td>S3_extracted_k141_124596</td><td>1486</td><td>No</td><td>NA</td><td>0</td><td>1</td><td>Not-determined</td><td>Genome-fragment</td><td>NA</td><td>0</td></tr>
<tr><td>S3_extracted_k141_78281</td><td>1241</td><td>No</td><td>NA</td><td>0</td><td>2</td><td>Not-determined</td><td>Genome-fragment</td><td>NA</td><td>0</td></tr>
<tr><td>S1_vs_k141_146884||full</td><td>1613</td><td>No</td><td>NA</td><td>0</td><td>0</td><td>Low-quality</td><td>Genome-fragment</td><td>3.81</td><td>0</td></tr>
<tr><td>S3_extracted_k141_78288</td><td>3002</td><td>No</td><td>NA</td><td>0</td><td>4</td><td>Not-determined</td><td>Genome-fragment</td><td>NA</td><td>0</td></tr>
<tr><td>S3_extracted_k141_117135</td><td>1519</td><td>No</td><td>NA</td><td>0</td><td>2</td><td>Not-determined</td><td>Genome-fragment</td><td>NA</td><td>0</td></tr>
<tr><td>S3_extracted_k141_29480</td><td>2188</td><td>No</td><td>NA</td><td>0</td><td>3</td><td>Not-determined</td><td>Genome-fragment</td><td>NA</td><td>0</td></tr>
<tr><td>S3_extracted_k141_45352</td><td>1835</td><td>No</td><td>NA</td><td>0</td><td>1</td><td>Not-determined</td><td>Genome-fragment</td><td>NA</td><td>0</td></tr>
<tr><td>S3_extracted_k141_15383</td><td>1333</td><td>No</td><td>NA</td><td>0</td><td>0</td><td>Not-determined</td><td>Genome-fragment</td><td>NA</td><td>0</td></tr>
<tr><td>S3_extracted_k141_93406</td><td>2767</td><td>No</td><td>NA</td><td>0</td><td>0</td><td>Not-determined</td><td>Genome-fragment</td><td>NA</td><td>0</td></tr>
<tr><td>S3_extracted_k141_32594</td><td>1613</td><td>No</td><td>NA</td><td>0</td><td>1</td><td>Low-quality</td><td>Genome-fragment</td><td>0.45</td><td>0</td></tr>
<tr><td>S3_extracted_k141_109593</td><td>1159</td><td>No</td><td>NA</td><td>0</td><td>0</td><td>Not-determined</td><td>Genome-fragment</td><td>NA</td><td>0</td></tr>
<tr><td>S3_extracted_k141_38467</td><td>1613</td><td>No</td><td>NA</td><td>0</td><td>1</td><td>Low-quality</td><td>Genome-fragment</td><td>3.71</td><td>0</td></tr>
<tr><td>S5_extracted_k141_67368</td><td>1613</td><td>No</td><td>NA</td><td>0</td><td>0</td><td>Low-quality</td><td>Genome-fragment</td><td>2.88</td><td>0</td></tr>
<tr><td>S3_extracted_k141_109607</td><td>1062</td><td>No</td><td>NA</td><td>0</td><td>0</td><td>Not-determined</td><td>Genome-fragment</td><td>NA</td><td>0</td></tr>
<tr><td>S3_extracted_k141_29514</td><td>7426</td><td>No</td><td>NA</td><td>0</td><td>3</td><td>Not-determined</td><td>Genome-fragment</td><td>NA</td><td>0</td></tr>
<tr><td>S3_extracted_k141_29531</td><td>3323</td><td>No</td><td>NA</td><td>0</td><td>0</td><td>Not-determined</td><td>Genome-fragment</td><td>NA</td><td>0</td></tr>
<tr><td>S7_extracted_13765</td><td>1613</td><td>No</td><td>NA</td><td>1</td><td>0</td><td>Low-quality</td><td>Genome-fragment</td><td>6</td><td>0</td></tr>
<tr><td>S3_extracted_k141_5525</td><td>40068</td><td>No</td><td>NA</td><td>16</td><td>0</td><td>High-quality</td><td>High-quality</td><td>90.61</td><td>0</td></tr>
<tr><td>NC1_vf_k141_2670</td><td>1613</td><td>No</td><td>NA</td><td>0</td><td>1</td><td>Low-quality</td><td>Genome-fragment</td><td>2</td><td>0</td></tr>
<tr><td>S12_vs_k141_24782||full</td><td>1613</td><td>No</td><td>NA</td><td>0</td><td>0</td><td>Low-quality</td><td>Genome-fragment</td><td>3.4</td><td>0</td></tr>
<tr><td>S3_extracted_k141_54185</td><td>2544</td><td>No</td><td>NA</td><td>0</td><td>0</td><td>Not-determined</td><td>Genome-fragment</td><td>NA</td><td>0</td></tr>
<tr><td>S3_extracted_k141_29581</td><td>1189</td><td>No</td><td>NA</td><td>0</td><td>1</td><td>Not-determined</td><td>Genome-fragment</td><td>NA</td><td>0</td></tr>
<tr><td>S3_extracted_k141_117190</td><td>1126</td><td>No</td><td>NA</td><td>0</td><td>0</td><td>Not-determined</td><td>Genome-fragment</td><td>NA</td><td>0</td></tr>
<tr><td>S3_extracted_k141_5569</td><td>1040</td><td>No</td><td>NA</td><td>0</td><td>1</td><td>Not-determined</td><td>Genome-fragment</td><td>NA</td><td>0</td></tr>
<tr><td>S9_extracted_k141_58524</td><td>1614</td><td>No</td><td>NA</td><td>1</td><td>0</td><td>Low-quality</td><td>Genome-fragment</td><td>4.07</td><td>0</td></tr>
<tr><td>S11_vs_k141_78333||full</td><td>1614</td><td>No</td><td>NA</td><td>2</td><td>0</td><td>Low-quality</td><td>Genome-fragment</td><td>4.22</td><td>0</td></tr>
<tr><td>S3_extracted_k141_45497</td><td>1392</td><td>No</td><td>NA</td><td>0</td><td>2</td><td>Not-determined</td><td>Genome-fragment</td><td>NA</td><td>0</td></tr>
<tr><td>S3_extracted_k141_45506</td><td>1008</td><td>No</td><td>NA</td><td>0</td><td>1</td><td>Not-determined</td><td>Genome-fragment</td><td>NA</td><td>0</td></tr>
<tr><td>S5_vs_k141_11307||full</td><td>1614</td><td>Yes</td><td>510</td><td>1</td><td>1</td><td>Low-quality</td><td>Genome-fragment</td><td>0.37</td><td>68.4</td></tr>
<tr><td>S3_extracted_k141_45540</td><td>1156</td><td>No</td><td>NA</td><td>0</td><td>0</td><td>Not-determined</td><td>Genome-fragment</td><td>NA</td><td>0</td></tr>
<tr><td>S12_vs_k141_37836||lt2gene</td><td>1614</td><td>No</td><td>NA</td><td>2</td><td>0</td><td>Low-quality</td><td>Genome-fragment</td><td>4.3</td><td>0</td></tr>
<tr><td>S3_extracted_k141_117272</td><td>1012</td><td>No</td><td>NA</td><td>0</td><td>0</td><td>Not-determined</td><td>Genome-fragment</td><td>NA</td><td>0</td></tr>
<tr><td>S3_extracted_k141_16045</td><td>1615</td><td>No</td><td>NA</td><td>0</td><td>0</td><td>Low-quality</td><td>Genome-fragment</td><td>2.7</td><td>0</td></tr>
<tr><td>S3_extracted_k141_93558</td><td>1367</td><td>No</td><td>NA</td><td>0</td><td>1</td><td>Not-determined</td><td>Genome-fragment</td><td>NA</td><td>0</td></tr>
<tr><td>S5_vs_k141_49783||full</td><td>1615</td><td>No</td><td>NA</td><td>0</td><td>0</td><td>Low-quality</td><td>Genome-fragment</td><td>3.7</td><td>0</td></tr>
<tr><td>S3_extracted_k141_15477</td><td>1058</td><td>No</td><td>NA</td><td>0</td><td>2</td><td>Not-determined</td><td>Genome-fragment</td><td>NA</td><td>0</td></tr>
<tr><td>S3_extracted_k141_5641</td><td>1107</td><td>No</td><td>NA</td><td>0</td><td>2</td><td>Not-determined</td><td>Genome-fragment</td><td>NA</td><td>0</td></tr>
<tr><td>S3_extracted_k141_5642</td><td>1051</td><td>No</td><td>NA</td><td>0</td><td>1</td><td>Not-determined</td><td>Genome-fragment</td><td>NA</td><td>0</td></tr>
<tr><td>S3_extracted_k141_62507</td><td>1922</td><td>No</td><td>NA</td><td>0</td><td>2</td><td>Not-determined</td><td>Genome-fragment</td><td>NA</td><td>0</td></tr>
<tr><td>S3_extracted_k141_5659</td><td>1016</td><td>No</td><td>NA</td><td>0</td><td>0</td><td>Not-determined</td><td>Genome-fragment</td><td>NA</td><td>0</td></tr>
<tr><td>S7_extracted_111242</td><td>1615</td><td>No</td><td>NA</td><td>2</td><td>0</td><td>Low-quality</td><td>Genome-fragment</td><td>3.41</td><td>0</td></tr>
<tr><td>S7_vs_67596||full</td><td>1615</td><td>No</td><td>NA</td><td>0</td><td>2</td><td>Low-quality</td><td>Genome-fragment</td><td>2.74</td><td>0</td></tr>
<tr><td>S7_vs_121348||full</td><td>1615</td><td>No</td><td>NA</td><td>0</td><td>0</td><td>Low-quality</td><td>Genome-fragment</td><td>3.88</td><td>0</td></tr>
<tr><td>S3_extracted_k141_15591</td><td>3038</td><td>No</td><td>NA</td><td>0</td><td>2</td><td>Not-determined</td><td>Genome-fragment</td><td>NA</td><td>0</td></tr>
<tr><td>S3_extracted_k141_85834</td><td>1909</td><td>No</td><td>NA</td><td>0</td><td>0</td><td>Not-determined</td><td>Genome-fragment</td><td>NA</td><td>0</td></tr>
<tr><td>S16_extracted_79515</td><td>1615</td><td>No</td><td>NA</td><td>1</td><td>0</td><td>Low-quality</td><td>Genome-fragment</td><td>2.8</td><td>0</td></tr>
<tr><td>S3_extracted_k141_21052</td><td>1157</td><td>No</td><td>NA</td><td>0</td><td>2</td><td>Not-determined</td><td>Genome-fragment</td><td>NA</td><td>0</td></tr>
<tr><td>S3_extracted_k141_124835</td><td>1754</td><td>No</td><td>NA</td><td>0</td><td>0</td><td>Not-determined</td><td>Genome-fragment</td><td>NA</td><td>0</td></tr>
<tr><td>S3_extracted_k141_69003</td><td>3368</td><td>No</td><td>NA</td><td>0</td><td>1</td><td>Not-determined</td><td>Genome-fragment</td><td>NA</td><td>0</td></tr>
<tr><td>S3_extracted_k141_85852</td><td>10278</td><td>No</td><td>NA</td><td>0</td><td>4</td><td>Not-determined</td><td>Genome-fragment</td><td>NA</td><td>0</td></tr>
<tr><td>S3_extracted_k141_29796</td><td>1029</td><td>No</td><td>NA</td><td>0</td><td>1</td><td>Not-determined</td><td>Genome-fragment</td><td>NA</td><td>0</td></tr>
<tr><td>S3_extracted_k141_78594</td><td>4145</td><td>No</td><td>NA</td><td>0</td><td>3</td><td>Not-determined</td><td>Genome-fragment</td><td>NA</td><td>0</td></tr>
<tr><td>S3_extracted_k141_45761</td><td>3286</td><td>No</td><td>NA</td><td>0</td><td>2</td><td>Not-determined</td><td>Genome-fragment</td><td>NA</td><td>0</td></tr>
<tr><td>S3_extracted_k141_124956</td><td>1052</td><td>No</td><td>NA</td><td>0</td><td>0</td><td>Not-determined</td><td>Genome-fragment</td><td>NA</td><td>0</td></tr>
<tr><td>S16_extracted_126842</td><td>1615</td><td>No</td><td>NA</td><td>1</td><td>0</td><td>Low-quality</td><td>Genome-fragment</td><td>2.6</td><td>0</td></tr>
<tr><td>S3_extracted_k141_21140</td><td>1659</td><td>No</td><td>NA</td><td>0</td><td>2</td><td>Not-determined</td><td>Genome-fragment</td><td>NA</td><td>0</td></tr>
<tr><td>S3_extracted_k141_100917</td><td>3131</td><td>No</td><td>NA</td><td>0</td><td>1</td><td>Not-determined</td><td>Genome-fragment</td><td>NA</td><td>0</td></tr>
<tr><td>S10_extracted_76079</td><td>1615</td><td>No</td><td>NA</td><td>0</td><td>1</td><td>Low-quality</td><td>Genome-fragment</td><td>2.6</td><td>0</td></tr>
<tr><td>S3_extracted_k141_54353</td><td>57601</td><td>No</td><td>NA</td><td>14</td><td>0</td><td>High-quality</td><td>High-quality</td><td>99.5</td><td>0</td></tr>
<tr><td>S3_extracted_k141_45807</td><td>1920</td><td>No</td><td>NA</td><td>0</td><td>3</td><td>Not-determined</td><td>Genome-fragment</td><td>NA</td><td>0</td></tr>
<tr><td>S3_extracted_k141_15721</td><td>1625</td><td>No</td><td>NA</td><td>0</td><td>2</td><td>Not-determined</td><td>Genome-fragment</td><td>NA</td><td>0</td></tr>
<tr><td>S3_extracted_k141_117375</td><td>1854</td><td>No</td><td>NA</td><td>0</td><td>0</td><td>Not-determined</td><td>Genome-fragment</td><td>NA</td><td>0</td></tr>
<tr><td>S3_extracted_k141_109833</td><td>5846</td><td>No</td><td>NA</td><td>0</td><td>5</td><td>Not-determined</td><td>Genome-fragment</td><td>NA</td><td>0</td></tr>
<tr><td>S4_extracted_10417</td><td>1615</td><td>No</td><td>NA</td><td>4</td><td>0</td><td>Low-quality</td><td>Genome-fragment</td><td>5.33</td><td>0</td></tr>
<tr><td>S8_extracted_k141_170307</td><td>1615</td><td>No</td><td>NA</td><td>0</td><td>1</td><td>Low-quality</td><td>Genome-fragment</td><td>1.07</td><td>0</td></tr>
<tr><td>S8_vs_k141_186215||full</td><td>1615</td><td>No</td><td>NA</td><td>0</td><td>1</td><td>Low-quality</td><td>Genome-fragment</td><td>1.88</td><td>0</td></tr>
<tr><td>S3_extracted_k141_21221</td><td>1038</td><td>No</td><td>NA</td><td>0</td><td>1</td><td>Not-determined</td><td>Genome-fragment</td><td>NA</td><td>0</td></tr>
<tr><td>S3_extracted_k141_117389</td><td>3109</td><td>No</td><td>NA</td><td>0</td><td>1</td><td>Not-determined</td><td>Genome-fragment</td><td>NA</td><td>0</td></tr>
<tr><td>S3_extracted_k141_45909</td><td>2226</td><td>No</td><td>NA</td><td>0</td><td>0</td><td>Not-determined</td><td>Genome-fragment</td><td>NA</td><td>0</td></tr>
<tr><td>S3_extracted_k141_15791</td><td>1423</td><td>No</td><td>NA</td><td>0</td><td>2</td><td>Not-determined</td><td>Genome-fragment</td><td>NA</td><td>0</td></tr>
<tr><td>S3_extracted_k141_69051</td><td>2579</td><td>No</td><td>NA</td><td>0</td><td>1</td><td>Not-determined</td><td>Genome-fragment</td><td>NA</td><td>0</td></tr>
<tr><td>S3_extracted_k141_93912</td><td>1712</td><td>No</td><td>NA</td><td>0</td><td>2</td><td>Not-determined</td><td>Genome-fragment</td><td>NA</td><td>0</td></tr>
<tr><td>S3_extracted_k141_54498</td><td>5405</td><td>No</td><td>NA</td><td>2</td><td>0</td><td>High-quality</td><td>High-quality</td><td>93.06</td><td>0</td></tr>
<tr><td>S11_vs_k141_87940||full</td><td>1616</td><td>No</td><td>NA</td><td>0</td><td>1</td><td>Low-quality</td><td>Genome-fragment</td><td>2.74</td><td>0</td></tr>
<tr><td>S7_vs_87048||lt2gene</td><td>1616</td><td>No</td><td>NA</td><td>1</td><td>0</td><td>Low-quality</td><td>Genome-fragment</td><td>1.12</td><td>0</td></tr>
<tr><td>S3_extracted_k141_29950</td><td>1015</td><td>No</td><td>NA</td><td>0</td><td>0</td><td>Not-determined</td><td>Genome-fragment</td><td>NA</td><td>0</td></tr>
<tr><td>S3_extracted_k141_54554</td><td>1135</td><td>No</td><td>NA</td><td>0</td><td>0</td><td>Not-determined</td><td>Genome-fragment</td><td>NA</td><td>0</td></tr>
<tr><td>S16_extracted_48384</td><td>1616</td><td>No</td><td>NA</td><td>0</td><td>1</td><td>Low-quality</td><td>Genome-fragment</td><td>3.61</td><td>0</td></tr>
<tr><td>S16_vs_122887||full</td><td>1616</td><td>No</td><td>NA</td><td>0</td><td>0</td><td>Low-quality</td><td>Genome-fragment</td><td>1.02</td><td>0</td></tr>
<tr><td>S4_extracted_1473</td><td>1616</td><td>No</td><td>NA</td><td>4</td><td>0</td><td>Low-quality</td><td>Genome-fragment</td><td>5.33</td><td>0</td></tr>
<tr><td>S3_extracted_k141_78779</td><td>2224</td><td>No</td><td>NA</td><td>0</td><td>1</td><td>Not-determined</td><td>Genome-fragment</td><td>NA</td><td>0</td></tr>
<tr><td>S6_vs_k141_3203||full</td><td>1616</td><td>No</td><td>NA</td><td>0</td><td>1</td><td>Low-quality</td><td>Genome-fragment</td><td>2.74</td><td>0</td></tr>
<tr><td>S3_extracted_k141_125076</td><td>1801</td><td>No</td><td>NA</td><td>0</td><td>0</td><td>Not-determined</td><td>Genome-fragment</td><td>NA</td><td>0</td></tr>
<tr><td>S3_extracted_k141_69104</td><td>1731</td><td>No</td><td>NA</td><td>0</td><td>1</td><td>Not-determined</td><td>Genome-fragment</td><td>NA</td><td>0</td></tr>
<tr><td>S11_vs_k141_102076||full</td><td>1617</td><td>No</td><td>NA</td><td>1</td><td>0</td><td>Low-quality</td><td>Genome-fragment</td><td>4.02</td><td>0</td></tr>
<tr><td>S3_extracted_k141_78835</td><td>3533</td><td>No</td><td>NA</td><td>0</td><td>3</td><td>Not-determined</td><td>Genome-fragment</td><td>NA</td><td>0</td></tr>
<tr><td>S5_extracted_k141_16198</td><td>1617</td><td>No</td><td>NA</td><td>0</td><td>0</td><td>Low-quality</td><td>Genome-fragment</td><td>2.7</td><td>0</td></tr>
<tr><td>S3_extracted_k141_86319</td><td>1166</td><td>No</td><td>NA</td><td>0</td><td>0</td><td>Not-determined</td><td>Genome-fragment</td><td>NA</td><td>0</td></tr>
<tr><td>S3_extracted_k141_5872</td><td>3290</td><td>No</td><td>NA</td><td>0</td><td>1</td><td>Not-determined</td><td>Genome-fragment</td><td>NA</td><td>0</td></tr>
<tr><td>S5_vs_k141_115581||full</td><td>1617</td><td>No</td><td>NA</td><td>0</td><td>2</td><td>Low-quality</td><td>Genome-fragment</td><td>2.77</td><td>0</td></tr>
<tr><td>S3_extracted_k141_78845</td><td>2339</td><td>No</td><td>NA</td><td>0</td><td>0</td><td>Not-determined</td><td>Genome-fragment</td><td>NA</td><td>0</td></tr>
<tr><td>S5_vs_k141_131726||full</td><td>1617</td><td>No</td><td>NA</td><td>0</td><td>2</td><td>Low-quality</td><td>Genome-fragment</td><td>1.86</td><td>0</td></tr>
<tr><td>S3_extracted_k141_110042</td><td>9816</td><td>No</td><td>NA</td><td>0</td><td>6</td><td>Not-determined</td><td>Genome-fragment</td><td>NA</td><td>0</td></tr>
<tr><td>S3_extracted_k141_110077</td><td>1387</td><td>No</td><td>NA</td><td>0</td><td>1</td><td>Not-determined</td><td>Genome-fragment</td><td>NA</td><td>0</td></tr>
<tr><td>S3_extracted_k141_110094</td><td>1973</td><td>No</td><td>NA</td><td>0</td><td>1</td><td>Not-determined</td><td>Genome-fragment</td><td>NA</td><td>0</td></tr>
<tr><td>S3_extracted_k141_62907</td><td>1115</td><td>No</td><td>NA</td><td>0</td><td>0</td><td>Not-determined</td><td>Genome-fragment</td><td>NA</td><td>0</td></tr>
<tr><td>S7_vs_45206||full</td><td>1617</td><td>No</td><td>NA</td><td>0</td><td>1</td><td>Low-quality</td><td>Genome-fragment</td><td>0.57</td><td>0</td></tr>
<tr><td>S3_extracted_k141_46155</td><td>2718</td><td>No</td><td>NA</td><td>0</td><td>1</td><td>Not-determined</td><td>Genome-fragment</td><td>NA</td><td>0</td></tr>
<tr><td>S3_extracted_k141_78891</td><td>3217</td><td>No</td><td>NA</td><td>0</td><td>1</td><td>Not-determined</td><td>Genome-fragment</td><td>NA</td><td>0</td></tr>
<tr><td>S10_vs_71903||lt2gene</td><td>1617</td><td>No</td><td>NA</td><td>2</td><td>0</td><td>Low-quality</td><td>Genome-fragment</td><td>4.27</td><td>0</td></tr>
<tr><td>S3_extracted_k141_69162</td><td>4947</td><td>No</td><td>NA</td><td>0</td><td>3</td><td>Not-determined</td><td>Genome-fragment</td><td>NA</td><td>0</td></tr>
<tr><td>S6_vs_k141_7116||full</td><td>1617</td><td>No</td><td>NA</td><td>0</td><td>0</td><td>Low-quality</td><td>Genome-fragment</td><td>5.06</td><td>0</td></tr>
<tr><td>S13_vs_121968||full</td><td>1618</td><td>No</td><td>NA</td><td>0</td><td>0</td><td>Low-quality</td><td>Genome-fragment</td><td>0.71</td><td>0</td></tr>
<tr><td>S3_extracted_k141_30206</td><td>1655</td><td>No</td><td>NA</td><td>0</td><td>1</td><td>Not-determined</td><td>Genome-fragment</td><td>NA</td><td>0</td></tr>
<tr><td>S3_extracted_k141_46218</td><td>3112</td><td>No</td><td>NA</td><td>0</td><td>2</td><td>Not-determined</td><td>Genome-fragment</td><td>NA</td><td>0</td></tr>
<tr><td>S3_extracted_k141_38234</td><td>1587</td><td>No</td><td>NA</td><td>0</td><td>1</td><td>Not-determined</td><td>Genome-fragment</td><td>NA</td><td>0</td></tr>
<tr><td>S14_extracted_k141_110058</td><td>1618</td><td>No</td><td>NA</td><td>0</td><td>0</td><td>Low-quality</td><td>Genome-fragment</td><td>3.98</td><td>0</td></tr>
<tr><td>S3_extracted_k141_78953</td><td>1170</td><td>No</td><td>NA</td><td>0</td><td>0</td><td>Not-determined</td><td>Genome-fragment</td><td>NA</td><td>0</td></tr>
<tr><td>S3_extracted_k141_54726</td><td>8662</td><td>No</td><td>NA</td><td>0</td><td>4</td><td>Not-determined</td><td>Genome-fragment</td><td>NA</td><td>0</td></tr>
<tr><td>S14_extracted_k141_37862</td><td>1618</td><td>No</td><td>NA</td><td>6</td><td>0</td><td>Low-quality</td><td>Genome-fragment</td><td>5.34</td><td>0</td></tr>
<tr><td>S1_vs_k141_74319||full</td><td>1618</td><td>No</td><td>NA</td><td>2</td><td>0</td><td>Low-quality</td><td>Genome-fragment</td><td>4.09</td><td>0</td></tr>
<tr><td>S3_extracted_k141_46263</td><td>1374</td><td>No</td><td>NA</td><td>0</td><td>0</td><td>Not-determined</td><td>Genome-fragment</td><td>NA</td><td>0</td></tr>
<tr><td>S3_vs_k141_10496||full</td><td>1618</td><td>No</td><td>NA</td><td>3</td><td>0</td><td>Low-quality</td><td>Genome-fragment</td><td>4.27</td><td>0</td></tr>
<tr><td>S16_extracted_86998</td><td>1618</td><td>No</td><td>NA</td><td>0</td><td>1</td><td>Low-quality</td><td>Genome-fragment</td><td>3.14</td><td>0</td></tr>
<tr><td>S6_vs_k141_16727||full</td><td>1618</td><td>No</td><td>NA</td><td>2</td><td>0</td><td>Low-quality</td><td>Genome-fragment</td><td>4.54</td><td>0</td></tr>
<tr><td>S13_extracted_121042</td><td>1619</td><td>No</td><td>NA</td><td>0</td><td>0</td><td>Low-quality</td><td>Genome-fragment</td><td>3.2</td><td>0</td></tr>
<tr><td>S3_extracted_k141_69216</td><td>1140</td><td>No</td><td>NA</td><td>0</td><td>0</td><td>Not-determined</td><td>Genome-fragment</td><td>NA</td><td>0</td></tr>
<tr><td>S14_vs_k141_101570||full</td><td>1619</td><td>No</td><td>NA</td><td>0</td><td>0</td><td>Low-quality</td><td>Genome-fragment</td><td>1.14</td><td>0</td></tr>
<tr><td>S3_extracted_k141_38348</td><td>2757</td><td>No</td><td>NA</td><td>0</td><td>2</td><td>Not-determined</td><td>Genome-fragment</td><td>NA</td><td>0</td></tr>
<tr><td>S1_vs_k141_2028||full</td><td>1619</td><td>No</td><td>NA</td><td>0</td><td>0</td><td>Low-quality</td><td>Genome-fragment</td><td>1.27</td><td>0</td></tr>
<tr><td>S3_extracted_k141_122731</td><td>1619</td><td>No</td><td>NA</td><td>0</td><td>2</td><td>Low-quality</td><td>Genome-fragment</td><td>0.6</td><td>0</td></tr>
<tr><td>S3_extracted_k141_63125</td><td>1157</td><td>No</td><td>NA</td><td>0</td><td>1</td><td>Not-determined</td><td>Genome-fragment</td><td>NA</td><td>0</td></tr>
<tr><td>S3_extracted_k141_94239</td><td>5601</td><td>No</td><td>NA</td><td>0</td><td>7</td><td>Not-determined</td><td>Genome-fragment</td><td>NA</td><td>0</td></tr>
<tr><td>S3_extracted_k141_54849</td><td>5339</td><td>No</td><td>NA</td><td>0</td><td>4</td><td>Not-determined</td><td>Genome-fragment</td><td>NA</td><td>0</td></tr>
<tr><td>S3_vs_k141_80974||full</td><td>1619</td><td>No</td><td>NA</td><td>1</td><td>0</td><td>Low-quality</td><td>Genome-fragment</td><td>1.74</td><td>0</td></tr>
<tr><td>S3_extracted_k141_125357</td><td>1101</td><td>No</td><td>NA</td><td>0</td><td>1</td><td>Not-determined</td><td>Genome-fragment</td><td>NA</td><td>0</td></tr>
<tr><td>S5_extracted_k141_7560</td><td>1619</td><td>No</td><td>NA</td><td>2</td><td>0</td><td>Low-quality</td><td>Genome-fragment</td><td>4.36</td><td>0</td></tr>
<tr><td>S3_extracted_k141_38389</td><td>1543</td><td>No</td><td>NA</td><td>0</td><td>2</td><td>Not-determined</td><td>Genome-fragment</td><td>NA</td><td>0</td></tr>
<tr><td>S3_extracted_k141_30312</td><td>2002</td><td>No</td><td>NA</td><td>0</td><td>2</td><td>Not-determined</td><td>Genome-fragment</td><td>NA</td><td>0</td></tr>
<tr><td>S3_extracted_k141_110312</td><td>9088</td><td>No</td><td>NA</td><td>0</td><td>1</td><td>Not-determined</td><td>Genome-fragment</td><td>NA</td><td>0</td></tr>
<tr><td>S5_vs_k141_49764||full</td><td>1619</td><td>No</td><td>NA</td><td>0</td><td>0</td><td>Low-quality</td><td>Genome-fragment</td><td>2.45</td><td>0</td></tr>
<tr><td>S10_extracted_25552</td><td>1619</td><td>No</td><td>NA</td><td>1</td><td>0</td><td>Low-quality</td><td>Genome-fragment</td><td>5.14</td><td>0</td></tr>
<tr><td>S3_extracted_k141_6137</td><td>2201</td><td>No</td><td>NA</td><td>0</td><td>2</td><td>Not-determined</td><td>Genome-fragment</td><td>NA</td><td>0</td></tr>
<tr><td>S3_extracted_k141_69304</td><td>1482</td><td>No</td><td>NA</td><td>0</td><td>1</td><td>Not-determined</td><td>Genome-fragment</td><td>NA</td><td>0</td></tr>
<tr><td>S3_extracted_k141_21549</td><td>1145</td><td>No</td><td>NA</td><td>0</td><td>1</td><td>Not-determined</td><td>Genome-fragment</td><td>NA</td><td>0</td></tr>
<tr><td>S10_extracted_107470</td><td>1619</td><td>No</td><td>NA</td><td>1</td><td>0</td><td>Low-quality</td><td>Genome-fragment</td><td>3.08</td><td>0</td></tr>
<tr><td>S15_vs_1056||full</td><td>1619</td><td>No</td><td>NA</td><td>1</td><td>0</td><td>Low-quality</td><td>Genome-fragment</td><td>0.92</td><td>0</td></tr>
<tr><td>S3_extracted_k141_6177</td><td>2203</td><td>No</td><td>NA</td><td>0</td><td>1</td><td>Not-determined</td><td>Genome-fragment</td><td>NA</td><td>0</td></tr>
<tr><td>S3_extracted_k141_110361</td><td>3902</td><td>No</td><td>NA</td><td>0</td><td>2</td><td>Not-determined</td><td>Genome-fragment</td><td>NA</td><td>0</td></tr>
<tr><td>S3_extracted_k141_110372</td><td>2072</td><td>No</td><td>NA</td><td>0</td><td>2</td><td>Not-determined</td><td>Genome-fragment</td><td>NA</td><td>0</td></tr>
<tr><td>S6_vs_k141_9256||full</td><td>1619</td><td>No</td><td>NA</td><td>1</td><td>0</td><td>Low-quality</td><td>Genome-fragment</td><td>3.43</td><td>0</td></tr>
<tr><td>S3_extracted_k141_46419</td><td>4078</td><td>No</td><td>NA</td><td>0</td><td>3</td><td>Not-determined</td><td>Genome-fragment</td><td>NA</td><td>0</td></tr>
<tr><td>S9_vs_k141_36181||full</td><td>1620</td><td>No</td><td>NA</td><td>0</td><td>0</td><td>Low-quality</td><td>Genome-fragment</td><td>3.71</td><td>0</td></tr>
<tr><td>S3_extracted_k141_69389</td><td>1026</td><td>No</td><td>NA</td><td>0</td><td>0</td><td>Not-determined</td><td>Genome-fragment</td><td>NA</td><td>0</td></tr>
<tr><td>S1_vs_k141_74243||full</td><td>1620</td><td>No</td><td>NA</td><td>0</td><td>0</td><td>Low-quality</td><td>Genome-fragment</td><td>0.98</td><td>0</td></tr>
<tr><td>S3_extracted_k141_86745</td><td>1402</td><td>No</td><td>NA</td><td>0</td><td>1</td><td>Not-determined</td><td>Genome-fragment</td><td>NA</td><td>0</td></tr>
<tr><td>S3_extracted_k141_117927</td><td>3007</td><td>No</td><td>NA</td><td>0</td><td>2</td><td>Not-determined</td><td>Genome-fragment</td><td>NA</td><td>0</td></tr>
<tr><td>S3_vs_k141_66384||full</td><td>1620</td><td>No</td><td>NA</td><td>1</td><td>0</td><td>Low-quality</td><td>Genome-fragment</td><td>3.6</td><td>0</td></tr>
<tr><td>S3_extracted_k141_86753</td><td>12294</td><td>No</td><td>NA</td><td>0</td><td>6</td><td>Not-determined</td><td>Genome-fragment</td><td>NA</td><td>0</td></tr>
<tr><td>S7_vs_24874||full</td><td>1620</td><td>No</td><td>NA</td><td>0</td><td>0</td><td>Low-quality</td><td>Genome-fragment</td><td>3.36</td><td>0</td></tr>
<tr><td>S7_vs_107485||full</td><td>1620</td><td>No</td><td>NA</td><td>0</td><td>0</td><td>Low-quality</td><td>Genome-fragment</td><td>2.98</td><td>0</td></tr>
<tr><td>S3_extracted_k141_86766</td><td>1084</td><td>No</td><td>NA</td><td>0</td><td>1</td><td>Not-determined</td><td>Genome-fragment</td><td>NA</td><td>0</td></tr>
<tr><td>S16_extracted_11388</td><td>1620</td><td>No</td><td>NA</td><td>1</td><td>0</td><td>Low-quality</td><td>Genome-fragment</td><td>0.96</td><td>0</td></tr>
<tr><td>S3_extracted_k141_118004</td><td>1531</td><td>No</td><td>NA</td><td>0</td><td>0</td><td>Not-determined</td><td>Genome-fragment</td><td>NA</td><td>0</td></tr>
<tr><td>S3_extracted_k141_125466</td><td>1432</td><td>No</td><td>NA</td><td>0</td><td>0</td><td>Not-determined</td><td>Genome-fragment</td><td>NA</td><td>0</td></tr>
<tr><td>S16_extracted_167250</td><td>1620</td><td>No</td><td>NA</td><td>0</td><td>1</td><td>Low-quality</td><td>Genome-fragment</td><td>2.65</td><td>0</td></tr>
<tr><td>S16_vs_15312||full</td><td>1620</td><td>No</td><td>NA</td><td>0</td><td>0</td><td>Low-quality</td><td>Genome-fragment</td><td>2.44</td><td>0</td></tr>
<tr><td>S12_vs_k141_62044||full</td><td>1620</td><td>No</td><td>NA</td><td>0</td><td>0</td><td>Low-quality</td><td>Genome-fragment</td><td>3.59</td><td>0</td></tr>
<tr><td>S3_extracted_k141_125480</td><td>12884</td><td>No</td><td>NA</td><td>0</td><td>9</td><td>Not-determined</td><td>Genome-fragment</td><td>NA</td><td>0</td></tr>
<tr><td>S2_vs_k141_3534||full</td><td>1620</td><td>No</td><td>NA</td><td>0</td><td>3</td><td>Low-quality</td><td>Genome-fragment</td><td>1.11</td><td>0</td></tr>
<tr><td>S8_vs_k141_183232||full</td><td>1620</td><td>No</td><td>NA</td><td>0</td><td>0</td><td>Low-quality</td><td>Genome-fragment</td><td>3.04</td><td>0</td></tr>
<tr><td>S3_extracted_k141_63326</td><td>1051</td><td>No</td><td>NA</td><td>0</td><td>0</td><td>Not-determined</td><td>Genome-fragment</td><td>NA</td><td>0</td></tr>
<tr><td>S3_extracted_k141_69429</td><td>1301</td><td>No</td><td>NA</td><td>0</td><td>1</td><td>Not-determined</td><td>Genome-fragment</td><td>NA</td><td>0</td></tr>
</table>

S3_extracted_k141_21772	2228	No	NA		0	0	Not-determined	Genome-fragment	NA		0
S17_vs_k141_29696||full	1621	No	NA		0	0	Low-quality	Genome-fragment		1.47	0
S3_extracted_k141_125574	1337	No	NA		0	2	Not-determined	Genome-fragment	NA		0
S17_vs_k141_82360||full	1621	No	NA		0	1	Low-quality	Genome-fragment		2.24	0
S3_extracted_k141_55082	1950	No	NA		0	0	Not-determined	Genome-fragment	NA		0
S17_vs_k141_97928||full	1621	No	NA		0	0	Low-quality	Genome-fragment		1.34	0
S3_extracted_k141_69462	1077	No	NA		0	0	Not-determined	Genome-fragment	NA		0
S3_extracted_k141_125587	2002	No	NA		0	0	Not-determined	Genome-fragment	NA		0
S3_extracted_k141_86820	1040	No	NA		0	0	Not-determined	Genome-fragment	NA		0
S11_extracted_k141_58038	1621	No	NA		0	0	Low-quality	Genome-fragment		1.31	0
S3_extracted_k141_125609	3882	No	NA		0	2	Not-determined	Genome-fragment	NA		0
S3_extracted_k141_86846	1059	No	NA		0	0	Not-determined	Genome-fragment	NA		0
S3_extracted_k141_30516	2939	No	NA		0	2	Not-determined	Genome-fragment	NA		0
S3_extracted_k141_118140	1317	No	NA		0	0	Not-determined	Genome-fragment	NA		0
S11_vs_k141_33094||lt2gene	1621	No	NA		1	0	Low-quality	Genome-fragment		2.81	0
S3_extracted_k141_94424	1371	No	NA		0	2	Not-determined	Genome-fragment	NA		0
S3_extracted_k141_86857	5642	No	NA		0	1	Not-determined	Genome-fragment	NA		0
S3_extracted_k141_79096	4328	No	NA		0	4	Not-determined	Genome-fragment	NA		0
S3_extracted_k141_79104	1042	No	NA		0	1	Not-determined	Genome-fragment	NA		0
S3_vs_k141_119029||full	1621	No	NA		1	0	Low-quality	Genome-fragment		2.96	0
S3_extracted_k141_21909	1328	No	NA		0	0	Not-determined	Genome-fragment	NA		0
S5_extracted_k141_137052	1621	No	NA		0	0	Low-quality	Genome-fragment		1.73	0
S5_vs_k141_117378||full	1621	No	NA		0	0	Low-quality	Genome-fragment		3.58	0
S3_extracted_k141_21931	1086	No	NA		0	0	Not-determined	Genome-fragment	NA		0
S5_vs_k141_111704||full	1621	No	NA		0	1	Low-quality	Genome-fragment		2.98	0
S3_extracted_k141_21949	1570	No	NA		0	0	Not-determined	Genome-fragment	NA		0
S3_extracted_k141_30592	1353	No	NA		0	2	Not-determined	Genome-fragment	NA		0
S5_vs_k141_77080||full	1621	No	NA		1	0	Low-quality	Genome-fragment		3.03	0
S3_extracted_k141_22018	1266	No	NA		0	0	Not-determined	Genome-fragment	NA		0
S3_extracted_k141_6510	1067	No	NA		0	0	Not-determined	Genome-fragment	NA		0
S7_vs_114917||full	1621	No	NA		2	0	Low-quality	Genome-fragment		4.12	0
S3_extracted_k141_94518	1009	No	NA		0	0	Not-determined	Genome-fragment	NA		0
S16_vs_146532||full	1621	Yes		489	1	1	Low-quality	Genome-fragment		2.97	69.83
S1_extracted_k141_121438	1622	No	NA		1	0	Low-quality	Genome-fragment		3.13	0
S3_extracted_k141_6513	3571	No	NA		0	2	Not-determined	Genome-fragment	NA		0
S3_extracted_k141_101591	4782	No	NA		4	0	Medium-quality	Genome-fragment		84.94	0
S3_vs_k141_120316||full	1622	No	NA		0	0	Low-quality	Genome-fragment		3.96	0
S3_extracted_k141_63459	3733	No	NA		0	1	Not-determined	Genome-fragment	NA		0
S3_vs_k141_1518||full	1622	No	NA		0	0	Low-quality	Genome-fragment		2.94	0
S3_extracted_k141_6536	3271	No	NA		0	3	Not-determined	Genome-fragment	NA		0
S3_extracted_k141_125734	1036	No	NA		0	2	Not-determined	Genome-fragment	NA		0
S3_vs_k141_80823||full	1623	No	NA		0	0	Low-quality	Genome-fragment		2.32	0
S3_extracted_k141_110664	4383	No	NA		0	2	Not-determined	Genome-fragment	NA		0
S3_extracted_k141_22087	1426	No	NA		0	1	Not-determined	Genome-fragment	NA		0
S3_vs_k141_107438||full	1623	No	NA		1	0	Low-quality	Genome-fragment		3.71	0
S3_extracted_k141_101597	1016	No	NA		0	0	Not-determined	Genome-fragment	NA		0
S16_vs_169934||full	1623	No	NA		2	0	Low-quality	Genome-fragment		2.8	0
S10_extracted_19900	1623	No	NA		1	0	Low-quality	Genome-fragment		2.46	0
S12_vs_k141_144247||full	1623	Yes		954	1	1	Low-quality	Genome-fragment		2.16	41.22
S3_extracted_k141_94585	5039	No	NA		0	3	Not-determined	Genome-fragment	NA		0
S6_extracted_k141_31420	1623	No	NA		0	0	Low-quality	Genome-fragment		1.02	0
S8_extracted_k141_9837	1623	No	NA		0	1	Low-quality	Genome-fragment		4.88	0
S3_extracted_k141_118320	1386	No	NA		0	0	Not-determined	Genome-fragment	NA		0
S8_vs_k141_61681||full	1623	No	NA		0	1	Low-quality	Genome-fragment		1.6	0
S3_extracted_k141_118322	3128	No	NA		0	2	Not-determined	Genome-fragment	NA		0
S11_extracted_k141_69111	1624	No	NA		0	0	Low-quality	Genome-fragment		1.94	0
S3_extracted_k141_22158	1224	No	NA		0	1	Not-determined	Genome-fragment	NA		0
S3_extracted_k141_101611	40986	No	NA		24	3	Medium-quality	Genome-fragment		73.47	0
S3_extracted_k141_22166	1231	No	NA		0	0	Not-determined	Genome-fragment	NA		0
S3_extracted_k141_79418	1040	No	NA		0	0	Not-determined	Genome-fragment	NA		0
S3_extracted_k141_87113	1062	No	NA		0	1	Not-determined	Genome-fragment	NA		0
S3_extracted_k141_94650	1476	No	NA		0	2	Not-determined	Genome-fragment	NA		0
S14_vs_k141_73280||full	1624	No	NA		0	0	Low-quality	Genome-fragment		4.07	0
S3_vs_k141_37332||full	1624	No	NA		0	0	Low-quality	Genome-fragment		3.02	0
S3_extracted_k141_94659	3925	No	NA		0	0	Not-determined	Genome-fragment	NA		0
S7_vs_96808||full	1624	No	NA		0	0	Low-quality	Genome-fragment		5.19	0
S3_extracted_k141_87133	4199	No	NA		0	2	Not-determined	Genome-fragment	NA		0
S3_extracted_k141_94665	1205	No	NA		0	2	Not-determined	Genome-fragment	NA		0
S3_extracted_k141_46787	1416	No	NA		0	0	Not-determined	Genome-fragment	NA		0
S3_extracted_k141_55262	5684	No	NA		0	1	Not-determined	Genome-fragment	NA		0
S16_extracted_153022	1624	No	NA		1	0	Low-quality	Genome-fragment		4.08	0
S3_extracted_k141_94721	1096	No	NA		0	1	Not-determined	Genome-fragment	NA		0
S3_extracted_k141_6609	1048	No	NA		0	0	Not-determined	Genome-fragment	NA		0
S12_vs_k141_90939||full	1624	No	NA		0	0	Low-quality	Genome-fragment		3.84	0
S8_extracted_k141_153417	1624	No	NA		0	0	Low-quality	Genome-fragment		3.35	0
S8_vs_k141_189191||full	1624	No	NA		0	1	Low-quality	Genome-fragment		2.26	0
S17_vs_k141_135717||full	1625	No	NA		0	0	Low-quality	Genome-fragment		3.49	0
S3_extracted_k141_46863	1621	No	NA		0	1	Not-determined	Genome-fragment	NA		0
S3_extracted_k141_94791	5466	No	NA		0	0	Not-determined	Genome-fragment	NA		0
S3_extracted_k141_38899	1036	No	NA		0	0	Not-determined	Genome-fragment	NA		0
S11_vs_k141_36386||full	1625	No	NA		2	0	Low-quality	Genome-fragment		3.77	0
S3_extracted_k141_81238	1625	No	NA		0	0	Low-quality	Genome-fragment		3.24	0
S16_vs_71631||full	1625	No	NA		2	0	Low-quality	Genome-fragment		0.94	0
S3_extracted_k141_125868	3401	No	NA		0	1	Not-determined	Genome-fragment	NA		0
S3_extracted_k141_118417	1422	No	NA		0	1	Not-determined	Genome-fragment	NA		0
S8_extracted_k141_175243	1625	No	NA		0	0	Low-quality	Genome-fragment		2.18	0
S17_extracted_k141_89237	1626	No	NA		0	1	Low-quality	Genome-fragment		4.15	0
S3_extracted_k141_69921	3347	No	NA		0	2	Not-determined	Genome-fragment	NA		0
S17_extracted_k141_169250	1626	No	NA		1	0	Low-quality	Genome-fragment		2.42	0
S3_extracted_k141_118442	3637	No	NA		0	1	Not-determined	Genome-fragment	NA		0
S3_extracted_k141_94858	4201	No	NA		0	2	Not-determined	Genome-fragment	NA		0
S3_extracted_k141_94861	1694	No	NA		0	0	Not-determined	Genome-fragment	NA		0
S3_extracted_k141_101821	1759	No	NA		0	1	Not-determined	Genome-fragment	NA		0
S3_extracted_k141_125932	2579	No	NA		0	2	Not-determined	Genome-fragment	NA		0
S17_vs_k141_119048||full	1626	No	NA		1	0	Low-quality	Genome-fragment		2.91	0
S3_extracted_k141_63743	1445	No	NA		0	1	Not-determined	Genome-fragment	NA		0
S14_vs_k141_6363||full	1626	No	NA		0	2	Low-quality	Genome-fragment		1.41	0
S3_extracted_k141_55394	1095	No	NA		0	0	Not-determined	Genome-fragment	NA		0
S3_extracted_k141_101853	1214	No	NA		0	0	Not-determined	Genome-fragment	NA		0
S3_extracted_k141_125956	2891	No	NA		0	2	Not-determined	Genome-fragment	NA		0
S3_extracted_k141_55410	1322	No	NA		0	1	Not-determined	Genome-fragment	NA		0
S3_extracted_k141_88471	1626	No	NA		0	0	Low-quality	Genome-fragment		1.24	0
S3_extracted_k141_6775	2236	No	NA		0	1	Not-determined	Genome-fragment	NA		0
S3_extracted_k141_6780	6559	No	NA		0	6	Not-determined	Genome-fragment	NA		0
S3_extracted_k141_70005	1096	No	NA		0	1	Not-determined	Genome-fragment	NA		0
S3_extracted_k141_125983	1444	No	NA		0	1	Not-determined	Genome-fragment	NA		0
S3_vs_k141_4719||full	1626	No	NA		0	0	Low-quality	Genome-fragment		1.79	0
S3_extracted_k141_55454	5054	No	NA		0	4	Not-determined	Genome-fragment	NA		0
S3_extracted_k141_94977	1760	No	NA		0	1	Not-determined	Genome-fragment	NA		0
S5_vs_k141_122530||full	1626	No	NA		1	0	Low-quality	Genome-fragment		3.61	0
S3_extracted_k141_47053	1636	No	NA		0	0	Not-determined	Genome-fragment	NA		0
S3_extracted_k141_94997	4342	No	NA		0	5	Not-determined	Genome-fragment	NA		0
S3_extracted_k141_70054	1289	No	NA		0	1	Not-determined	Genome-fragment	NA		0
S3_extracted_k141_126040	3295	No	NA		0	1	Not-determined	Genome-fragment	NA		0
S5_vs_k141_113385||full	1626	No	NA		0	0	Low-quality	Genome-fragment		3.28	0
S3_extracted_k141_101896	3636	No	NA		0	4	Not-determined	Genome-fragment	NA		0
S7_extracted_86547	1626	No	NA		0	1	Low-quality	Genome-fragment		0.66	0
S3_extracted_k141_118599	1374	No	NA		0	1	Not-determined	Genome-fragment	NA		0
S3_extracted_k141_118603	1025	No	NA		0	0	Not-determined	Genome-fragment	NA		0
S3_extracted_k141_126087	1294	No	NA		0	1	Not-determined	Genome-fragment	NA		0
S3_extracted_k141_95036	3224	No	NA		0	2	Not-determined	Genome-fragment	NA		0
S10_vs_35219||full	1626	No	NA		0	1	Low-quality	Genome-fragment		1.31	0
S3_extracted_k141_39144	1000	No	NA		0	2	Not-determined	Genome-fragment	NA		0
S3_extracted_k141_30972	1376	No	NA		0	1	Not-determined	Genome-fragment	NA		0
S8_vs_k141_43919||full	1626	No	NA		0	1	Low-quality	Genome-fragment		3.59	0
S3_extracted_k141_39181	1019	No	NA		0	2	Not-determined	Genome-fragment	NA		0
S9_extracted_k141_23769	1627	No	NA		1	0	Low-quality	Genome-fragment		27.31	0
S3_extracted_k141_39187	2067	No	NA		0	0	Not-determined	Genome-fragment	NA		0
S3_extracted_k141_87351	3439	No	NA		0	1	Not-determined	Genome-fragment	NA		0
S3_extracted_k141_87357	1041	No	NA		0	0	Not-determined	Genome-fragment	NA		0
S3_extracted_k141_39193	1180	No	NA		0	0	Not-determined	Genome-fragment	NA		0
S3_extracted_k141_118695	1115	No	NA		0	1	Not-determined	Genome-fragment	NA		0
S3_extracted_k141_118710	3034	No	NA		0	2	Not-determined	Genome-fragment	NA		0
S3_extracted_k141_47186	2832	No	NA		0	0	Not-determined	Genome-fragment	NA		0
S14_vs_k141_54938||full	1627	No	NA		1	0	Low-quality	Genome-fragment		5.28	0
S3_extracted_k141_111218	3530	No	NA		0	0	Not-determined	Genome-fragment	NA		0
S3_extracted_k141_126250	1626	No	NA		0	2	Not-determined	Genome-fragment	NA		0
S14_vs_k141_5315||full	1627	No	NA		5	0	Low-quality	Genome-fragment		5.37	0
S3_extracted_k141_70217	1019	No	NA		0	0	Not-determined	Genome-fragment	NA		0
S3_extracted_k141_89656	1627	No	NA		2	0	Low-quality	Genome-fragment		2.85	0
S7_vs_106350||full	1627	No	NA		0	2	Low-quality	Genome-fragment		3.26	0
S3_extracted_k141_87433	1043	No	NA		0	0	Not-determined	Genome-fragment	NA		0
S16_extracted_63641	1627	No	NA		0	1	Low-quality	Genome-fragment		3.5	0
S3_extracted_k141_47258	1283	No	NA		0	1	Not-determined	Genome-fragment	NA		0
S3_extracted_k141_31108	1845	No	NA		0	0	Not-determined	Genome-fragment	NA		0
S3_extracted_k141_87484	1521	No	NA		0	0	Not-determined	Genome-fragment	NA		0
S16_vs_62132||full	1627	No	NA		1	0	Low-quality	Genome-fragment		0.96	0
S3_extracted_k141_111330	2142	No	NA		0	0	Not-determined	Genome-fragment	NA		0
S3_extracted_k141_111334	1151	No	NA		0	1	Not-determined	Genome-fragment	NA		0
S3_extracted_k141_95195	1995	No	NA		0	1	Not-determined	Genome-fragment	NA		0
S16_vs_128797||lt2gene	1627	No	NA		1	0	Low-quality	Genome-fragment		1.25	0
S10_vs_34208||full	1627	No	NA		0	0	Low-quality	Genome-fragment		2.85	0
S12_vs_k141_8287||full	1627	No	NA		2	0	Low-quality	Genome-fragment		4.04	0
S2_vs_k141_21411||full	1627	No	NA		0	0	Low-quality	Genome-fragment		2.41	0
S3_extracted_k141_55665	5952	No	NA		0	4	Not-determined	Genome-fragment	NA		0
[truncated: 3,646,310 more chars]
