# Supplementary material for: Investigating the Human Intestinal DNA Virome and Predicting Disease-Associated Virus–Host Interactions in Severe Myalgic Encephalomyelitis/Chronic Fatigue Syndrome (ME/CFS)
Source: Int J Mol Sci. 2023 Dec 8;24(24):17267. doi: 10.3390/ijms242417267 (PMC10744171; doi:10.3390/ijms242417267)
Supplement: Supplementary file 1 [file ijms-24-17267-s001.zip › Table S3.pdf]

**Table S3-1. WMS_vOTUs_checkv**

| contig_id | contig_length | provirus | proviral_length | viral_genes | host_genes | checkv_quality | miuvig_quality | completeness | contamination |
|---|---|---|---|---|---|---|---|---|---|
| S9-final-viral-combined_k141_18\|\|full | 2748 | No | NA | 0 | 0 | Not-determined | Genome-fragment | NA | 0 |
| S9-final-viral-combined_k141_27311\|\|full | 10561 | No | NA | 0 | 2 | Medium-quality | Genome-fragment | 80.96 | 0 |
| S9-final-viral-combined_k141_61921\|\|full | 3541 | No | NA | 0 | 1 | Not-determined | Genome-fragment | NA | 0 |
| S9-final-viral-combined_k141_70652\|\|lt2gene | 1000 | No | NA | 1 | 0 | Low-quality | Genome-fragment | 1.86 | 0 |
| S9-final-viral-combined_k141_128240\|\|full | 2114 | No | NA | 0 | 0 | Not-determined | Genome-fragment | NA | 0 |
| S9-final-viral-combined_k141_80221\|\|full | 1203 | No | NA | 0 | 0 | Not-determined | Genome-fragment | NA | 0 |
| S11-final-viral-combined_k141_221480\|\|full | 1000 | No | NA | 0 | 1 | Low-quality | Genome-fragment | 2.34 | 0 |
| S1-final-viral-combined_k141_132027\|\|lt2gene | 1000 | No | NA | 1 | 0 | Low-quality | Genome-fragment | 2.77 | 0 |
| S9-final-viral-combined_k141_101618\|\|full | 2377 | No | NA | 0 | 2 | Not-determined | Genome-fragment | NA | 0 |
| S1_k141_254098 | 1000 | No | NA | 0 | 1 | Low-quality | Genome-fragment | 0.55 | 0 |
| S9-final-viral-combined_k141_21414\|\|full | 1549 | No | NA | 0 | 1 | Not-determined | Genome-fragment | NA | 0 |
| S3-final-viral-combined_k141_78230\|\|full | 1000 | No | NA | 0 | 0 | Low-quality | Genome-fragment | 4.52 | 0 |
| S5_k141_49235 | 1000 | No | NA | 0 | 1 | Low-quality | Genome-fragment | 1.35 | 0 |
| S9-final-viral-combined_k141_101645\|\|full | 1618 | No | NA | 0 | 1 | Not-determined | Genome-fragment | NA | 0 |
| S7-final-viral-combined_k141_173981\|\|full | 1000 | No | NA | 0 | 0 | Low-quality | Genome-fragment | 3.17 | 0 |
| S9-final-viral-combined_k141_16080\|\|full | 3423 | No | NA | 0 | 2 | Not-determined | Genome-fragment | NA | 0 |
| S9-final-viral-combined_k141_96317\|\|full | 2087 | No | NA | 0 | 0 | Not-determined | Genome-fragment | NA | 0 |
| S7-final-viral-combined_k141_163338\|\|full | 1000 | No | NA | 0 | 1 | Low-quality | Genome-fragment | 1.44 | 0 |
| S9-final-viral-combined_k141_32130\|\|full | 2477 | No | NA | 0 | 3 | Not-determined | Genome-fragment | NA | 0 |
| S7-final-viral-combined_k141_147972\|\|lt2gene | 1000 | No | NA | 1 | 0 | Low-quality | Genome-fragment | 2.69 | 0 |
| S9-final-viral-combined_k141_42858\|\|full | 3399 | No | NA | 0 | 2 | Not-determined | Genome-fragment | NA | 0 |
| S9-final-viral-combined_k141_16107\|\|full | 4057 | No | NA | 0 | 0 | Not-determined | Genome-fragment | NA | 0 |
| S9-final-viral-combined_k141_26779\|\|full | 2234 | No | NA | 0 | 0 | Not-determined | Genome-fragment | NA | 0 |
| S9-final-viral-combined_k141_74918\|\|full | 2022 | No | NA | 0 | 2 | Not-determined | Genome-fragment | NA | 0 |
| S12-final-viral-combined_k141_312026\|\|full | 1000 | No | NA | 0 | 0 | Low-quality | Genome-fragment | 2.58 | 0 |
| S2-final-viral-combined_k141_9078\|\|full | 1000 | No | NA | 1 | 0 | Low-quality | Genome-fragment | 2.25 | 0 |
| S4-final-viral-combined_k141_24368\|\|full | 1000 | No | NA | 0 | 0 | Low-quality | Genome-fragment | 2.12 | 0 |
| S9-final-viral-combined_k141_86\|\|full | 3102 | No | NA | 0 | 2 | Not-determined | Genome-fragment | NA | 0 |
| S9-final-viral-combined_k141_101673\|\|full | 6045 | No | NA | 0 | 2 | Not-determined | Genome-fragment | NA | 0 |
| S4-final-viral-combined_k141_71788\|\|lt2gene | 1000 | No | NA | 2 | 0 | Low-quality | Genome-fragment | 2.1 | 0 |
| S4_k141_145029 | 1000 | No | NA | 0 | 0 | Low-quality | Genome-fragment | 1.87 | 0 |
| S8-final-viral-combined_k141_82360\|\|lt2gene | 1000 | No | NA | 1 | 0 | Low-quality | Genome-fragment | 2.12 | 0 |
| S9-final-viral-combined_k141_16134\|\|full | 1525 | No | NA | 0 | 2 | Not-determined | Genome-fragment | NA | 0 |
| S9-final-viral-combined_k141_9435\|\|full | 1001 | No | NA | 0 | 0 | Low-quality | Genome-fragment | 1.02 | 0 |
| S9-final-viral-combined_k141_26789\|\|full | 1108 | No | NA | 0 | 1 | Not-determined | Genome-fragment | NA | 0 |
| S9-final-viral-combined_k141_117781\|\|full | 1622 | No | NA | 0 | 0 | Not-determined | Genome-fragment | NA | 0 |
| S9-final-viral-combined_k141_26797\|\|full | 1946 | No | NA | 0 | 1 | Not-determined | Genome-fragment | NA | 0 |
| S9-final-viral-combined_k141_108039\|\|lt2gene | 1001 | No | NA | 1 | 0 | Low-quality | Genome-fragment | 2.34 | 0 |
| S9-final-viral-combined_k141_168\|\|full | 4262 | No | NA | 0 | 3 | Not-determined | Genome-fragment | NA | 0 |
| S11-final-viral-combined_k141_60295\|\|full | 1001 | No | NA | 0 | 0 | Low-quality | Genome-fragment | 2.78 | 0 |
| S11-final-viral-combined_k141_60310\|\|full | 1001 | No | NA | 0 | 0 | Low-quality | Genome-fragment | 2.8 | 0 |
| S1-final-viral-combined_k141_179428\|\|full | 1001 | No | NA | 0 | 0 | Low-quality | Genome-fragment | 1.64 | 0 |
| S9-final-viral-combined_k141_112457\|\|full | 2848 | No | NA | 0 | 2 | Not-determined | Genome-fragment | NA | 0 |
| S9-final-viral-combined_k141_74987\|\|full | 2344 | No | NA | 0 | 3 | Not-determined | Genome-fragment | NA | 0 |
| S9-final-viral-combined_k141_37568\|\|full | 1514 | No | NA | 0 | 0 | Not-determined | Genome-fragment | NA | 0 |
| S5-final-viral-combined_k141_100625\|\|full | 1001 | No | NA | 0 | 0 | Low-quality | Genome-fragment | 1.51 | 0 |
| S7-final-viral-combined_k141_194217\|\|full | 1001 | No | NA | 0 | 0 | Low-quality | Genome-fragment | 2.42 | 0 |
| S9-final-viral-combined_k141_75027\|\|full | 3303 | No | NA | 0 | 3 | Not-determined | Genome-fragment | NA | 0 |
| S9-final-viral-combined_k141_75033\|\|full | 1004 | No | NA | 0 | 0 | Not-determined | Genome-fragment | NA | 0 |
| S7-final-viral-combined_k141_14684\|\|full | 1001 | No | NA | 0 | 1 | Low-quality | Genome-fragment | 0.75 | 0 |
| S7_k141_210522 | 1001 | No | NA | 0 | 0 | Low-quality | Genome-fragment | 1.2 | 0 |
| S9-final-viral-combined_k141_96403\|\|full | 6462 | No | NA | 0 | 4 | Not-determined | Genome-fragment | NA | 0 |
| S9-final-viral-combined_k141_101800\|\|full | 1511 | No | NA | 0 | 0 | Not-determined | Genome-fragment | NA | 0 |
| S17-final-viral-combined_k141_256713\|\|full | 1001 | No | NA | 0 | 0 | Low-quality | Genome-fragment | 2.28 | 0 |
| S17-final-viral-combined_k141_209668\|\|full | 1001 | No | NA | 0 | 0 | Low-quality | Genome-fragment | 2.54 | 0 |
| S17-k141_145617 | 1001 | No | NA | 0 | 0 | Low-quality | Genome-fragment | 1.45 | 0 |
| S17-k141_193417 | 1001 | No | NA | 0 | 0 | Low-quality | Genome-fragment | 2.49 | 0 |
| S12-final-viral-combined_k141_67449\|\|lt2gene | 1001 | No | NA | 1 | 0 | Low-quality | Genome-fragment | 2.36 | 0 |
| S9-final-viral-combined_k141_69628\|\|full | 2855 | No | NA | 0 | 2 | Not-determined | Genome-fragment | NA | 0 |
| S12-final-viral-combined_k141_337949\|\|lt2gene | 1001 | No | NA | 1 | 0 | Low-quality | Genome-fragment | 2.67 | 0 |
| S12_k141_357790 | 1001 | No | NA | 0 | 1 | Low-quality | Genome-fragment | 0.17 | 0 |
| S9-final-viral-combined_k141_85755\|\|full | 4207 | No | NA | 0 | 1 | Not-determined | Genome-fragment | NA | 0 |
| S9-final-viral-combined_k141_69645\|\|full | 3178 | No | NA | 0 | 0 | Not-determined | Genome-fragment | NA | 0 |
| S2_k141_87457 | 1001 | No | NA | 0 | 0 | Low-quality | Genome-fragment | 1.65 | 0 |
| S4-final-viral-combined_k141_24500\|\|full | 1001 | No | NA | 1 | 0 | Low-quality | Genome-fragment | 2.27 | 0 |
| S9-final-viral-combined_k141_43024\|\|full | 1925 | No | NA | 0 | 1 | Not-determined | Genome-fragment | NA | 0 |
| S9-final-viral-combined_k141_287\|\|full | 5659 | No | NA | 0 | 2 | Not-determined | Genome-fragment | NA | 0 |
| S6-final-viral-combined_k141_10749\|\|full | 1001 | No | NA | 0 | 1 | Low-quality | Genome-fragment | 2.35 | 0 |
| S9-final-viral-combined_k141_5476\|\|full | 2190 | No | NA | 0 | 0 | Not-determined | Genome-fragment | NA | 0 |
| S9-final-viral-combined_k141_299\|\|full | 1375 | No | NA | 0 | 1 | Not-determined | Genome-fragment | NA | 0 |
| S9-final-viral-combined_k141_305\|\|full | 3295 | No | NA | 0 | 2 | Not-determined | Genome-fragment | NA | 0 |
| S8-final-viral-combined_k141_37709\|\|full | 1001 | No | NA | 0 | 0 | Low-quality | Genome-fragment | 0.66 | 0 |
| S9-final-viral-combined_k141_26934\|\|full | 2820 | No | NA | 0 | 1 | Not-determined | Genome-fragment | NA | 0 |
| S9-final-viral-combined_k141_32282\|\|full | 1099 | No | NA | 0 | 1 | Not-determined | Genome-fragment | NA | 0 |
| S8_k141_166590 | 1001 | No | NA | 0 | 1 | Low-quality | Genome-fragment | 0.71 | 0 |
| S9-final-viral-combined_k141_32287\|\|full | 2131 | No | NA | 0 | 1 | Not-determined | Genome-fragment | NA | 0 |
| S9-final-viral-combined_k141_32289\|\|full | 1042 | No | NA | 0 | 1 | Not-determined | Genome-fragment | NA | 0 |
| S9-final-viral-combined_k141_123194\|\|full | 1827 | No | NA | 0 | 1 | Not-determined | Genome-fragment | NA | 0 |
| S9-final-viral-combined_k141_48351\|\|full | 2893 | No | NA | 0 | 1 | Not-determined | Genome-fragment | NA | 0 |
| S11-final-viral-combined_k141_10324\|\|full | 1002 | No | NA | 0 | 0 | Low-quality | Genome-fragment | 1.63 | 0 |
| S9-final-viral-combined_k141_16253\|\|full | 2326 | No | NA | 0 | 2 | Not-determined | Genome-fragment | NA | 0 |
| S9-final-viral-combined_k141_117850\|\|full | 4441 | No | NA | 0 | 3 | Not-determined | Genome-fragment | NA | 0 |
| S9-final-viral-combined_k141_64277\|\|full | 95138 | Yes | 90419 | 34 | 12 | High-quality | High-quality | 100 | 4.96 |
| S11_k141_218887 | 1002 | No | NA | 0 | 0 | Low-quality | Genome-fragment | 2.05 | 0 |
| S9-final-viral-combined_k141_80423\|\|full | 1883 | No | NA | 0 | 2 | Not-determined | Genome-fragment | NA | 0 |
| S1-final-viral-combined_k141_238768\|\|full | 1002 | No | NA | 1 | 0 | Low-quality | Genome-fragment | 1.5 | 0 |
| S5-final-viral-combined_k141_187922\|\|full | 1002 | No | NA | 0 | 1 | Low-quality | Genome-fragment | 2.16 | 0 |
| S9-final-viral-combined_k141_27000\|\|full | 3168 | No | NA | 0 | 1 | Not-determined | Genome-fragment | NA | 0 |
| S9-final-viral-combined_k141_101848\|\|full | 3941 | No | NA | 0 | 3 | Not-determined | Genome-fragment | NA | 0 |
| S9-final-viral-combined_k141_27009\|\|full | 5257 | No | NA | 0 | 4 | Not-determined | Genome-fragment | NA | 0 |
| S9-final-viral-combined_k141_96528\|\|full | 1011 | No | NA | 0 | 0 | Not-determined | Genome-fragment | NA | 0 |
| S5-final-viral-combined_k141_26622\|\|full | 1002 | No | NA | 1 | 0 | Low-quality | Genome-fragment | 0.61 | 0 |
| S5_k141_74139 | 1002 | No | NA | 0 | 1 | Low-quality | Genome-fragment | 0.42 | 0 |
| S9-final-viral-combined_k141_21587\|\|full | 1630 | No | NA | 0 | 1 | Not-determined | Genome-fragment | NA | 0 |
| S7_k141_184241 | 1002 | No | NA | 0 | 0 | Low-quality | Genome-fragment | 1.39 | 0 |
| S7_k141_156568 | 1002 | No | NA | 0 | 1 | Low-quality | Genome-fragment | 0.66 | 0 |
| S9-final-viral-combined_k141_80509\|\|full | 2958 | No | NA | 0 | 1 | Not-determined | Genome-fragment | NA | 0 |
| S17-final-viral-combined_k141_2883\|\|full | 1002 | Yes | 504 | 1 | 1 | Low-quality | Genome-fragment | 0.69 | 49.7 |
| S9-final-viral-combined_k141_32337\|\|full | 1910 | No | NA | 0 | 0 | Not-determined | Genome-fragment | NA | 0 |
| S9-final-viral-combined_k141_117949\|\|full | 1389 | No | NA | 0 | 1 | Not-determined | Genome-fragment | NA | 0 |
| S9-final-viral-combined_k141_16354\|\|full | 1703 | No | NA | 0 | 1 | Not-determined | Genome-fragment | NA | 0 |
| S9-final-viral-combined_k141_48425\|\|full | 5789 | No | NA | 0 | 4 | Not-determined | Genome-fragment | NA | 0 |
| S12_k141_390149 | 1002 | No | NA | 2 | 0 | Low-quality | Genome-fragment | 2.2 | 0 |
| S8-final-viral-combined_k141_195215\|\|full | 1002 | No | NA | 0 | 0 | Low-quality | Genome-fragment | 1 | 0 |
| S9-final-viral-combined_k141_37598\|\|full | 2183 | No | NA | 0 | 0 | Not-determined | Genome-fragment | NA | 0 |
| S8_k141_95325 | 1002 | No | NA | 0 | 0 | Low-quality | Genome-fragment | 1.73 | 0 |
| S8_k141_187889 | 1002 | No | NA | 0 | 1 | Low-quality | Genome-fragment | 3.26 | 0 |
| S1-final-viral-combined_k141_151461\|\|full | 1003 | No | NA | 0 | 1 | Low-quality | Genome-fragment | 0.69 | 0 |
| S9-final-viral-combined_k141_48451\|\|full | 2296 | No | NA | 0 | 2 | Not-determined | Genome-fragment | NA | 0 |
| S9-final-viral-combined_k141_64400\|\|full | 3263 | No | NA | 0 | 2 | Not-determined | Genome-fragment | NA | 0 |
| S9-final-viral-combined_k141_85928\|\|full | 2736 | No | NA | 0 | 1 | Not-determined | Genome-fragment | NA | 0 |
| S9-final-viral-combined_k141_112628\|\|full | 1497 | No | NA | 0 | 1 | Not-determined | Genome-fragment | NA | 0 |
| S1-final-viral-combined_k141_16219\|\|lt2gene | 1003 | No | NA | 1 | 0 | Low-quality | Genome-fragment | 2.63 | 0 |
| S3-final-viral-combined_k141_136120\|\|full | 1003 | No | NA | 0 | 0 | Low-quality | Genome-fragment | 3.04 | 0 |
| S9-final-viral-combined_k141_479\|\|full | 2809 | No | NA | 0 | 2 | Not-determined | Genome-fragment | NA | 0 |
| S9-final-viral-combined_k141_117972\|\|full | 1924 | No | NA | 0 | 2 | Not-determined | Genome-fragment | NA | 0 |
| S9-final-viral-combined_k141_27116\|\|full | 6599 | No | NA | 0 | 2 | Not-determined | Genome-fragment | NA | 0 |
| S9-final-viral-combined_k141_59132\|\|full | 1486 | No | NA | 0 | 1 | Not-determined | Genome-fragment | NA | 0 |
| S5_k141_199576 | 1003 | No | NA | 0 | 1 | Low-quality | Genome-fragment | 1.88 | 0 |
| S7-final-viral-combined_k141_66882\|\|lt2gene | 1003 | No | NA | 1 | 0 | Low-quality | Genome-fragment | 2.19 | 0 |
| S17-final-viral-combined_k141_80973\|\|full | 1003 | No | NA | 1 | 0 | Low-quality | Genome-fragment | 6.66 | 0 |
| S9-final-viral-combined_k141_112695\|\|full | 2514 | No | NA | 0 | 1 | Not-determined | Genome-fragment | NA | 0 |
| S9-final-viral-combined_k141_80603\|\|full | 1005 | No | NA | 0 | 0 | Not-determined | Genome-fragment | NA | 0 |
| S9-final-viral-combined_k141_59179\|\|full | 2886 | No | NA | 0 | 4 | Not-determined | Genome-fragment | NA | 0 |
| S9-final-viral-combined_k141_53731\|\|full | 1687 | No | NA | 0 | 3 | Not-determined | Genome-fragment | NA | 0 |
| S9-final-viral-combined_k141_96645\|\|full | 1076 | No | NA | 0 | 1 | Not-determined | Genome-fragment | NA | 0 |
| S9-final-viral-combined_k141_48495\|\|full | 1411 | No | NA | 0 | 0 | Not-determined | Genome-fragment | NA | 0 |
| S12-final-viral-combined_k141_135765\|\|full | 1003 | No | NA | 2 | 0 | Low-quality | Genome-fragment | 1.77 | 0 |
| S12_k141_47120 | 1003 | No | NA | 0 | 0 | Low-quality | Genome-fragment | 0.94 | 0 |
| S9-final-viral-combined_k141_37676\|\|full | 3769 | No | NA | 0 | 2 | Not-determined | Genome-fragment | NA | 0 |
| S9-final-viral-combined_k141_16439\|\|full | 1359 | No | NA | 0 | 3 | Not-determined | Genome-fragment | NA | 0 |
| S9-final-viral-combined_k141_21601\|\|full | 1568 | No | NA | 0 | 0 | Not-determined | Genome-fragment | NA | 0 |
| S2-final-viral-combined_k141_191035\|\|full | 1003 | No | NA | 0 | 0 | Low-quality | Genome-fragment | 1.24 | 0 |
| S2-final-viral-combined_k141_208406\|\|lt2gene | 1003 | No | NA | 1 | 0 | Low-quality | Genome-fragment | 2.55 | 0 |
| S4-final-viral-combined_k141_127725\|\|full | 1003 | No | NA | 1 | 0 | Low-quality | Genome-fragment | 2.47 | 0 |
| S9-final-viral-combined_k141_96686\|\|full | 1116 | No | NA | 0 | 0 | Not-determined | Genome-fragment | NA | 0 |
| S4_k141_28814 | 1003 | No | NA | 0 | 0 | Low-quality | Genome-fragment | 0.75 | 0 |
| S9-final-viral-combined_k141_5572\|\|full | 1160 | No | NA | 0 | 0 | Not-determined | Genome-fragment | NA | 0 |
| S9-final-viral-combined_k141_107113\|\|full | 2137 | No | NA | 0 | 2 | Not-determined | Genome-fragment | NA | 0 |
| S8-final-viral-combined_k141_244332\|\|full | 1003 | No | NA | 0 | 0 | Low-quality | Genome-fragment | 2.39 | 0 |
| S9-final-viral-combined_k141_102119\|\|full | 1892 | No | NA | 0 | 0 | Not-determined | Genome-fragment | NA | 0 |
| S8-final-viral-combined_k141_167785\|\|full | 1003 | No | NA | 0 | 0 | Low-quality | Genome-fragment | 1.79 | 0 |
| S9-final-viral-combined_k141_59247\|\|full | 1630 | No | NA | 0 | 2 | Not-determined | Genome-fragment | NA | 0 |
| S8-final-viral-combined_k141_128674\|\|lt2gene | 1003 | No | NA | 2 | 0 | Low-quality | Genome-fragment | 1.75 | 0 |
| S9-final-viral-combined_k141_21506\|\|lt2gene | 1004 | No | NA | 1 | 0 | Low-quality | Genome-fragment | 2.61 | 0 |
| S9-final-viral-combined_k141_386\|\|lt2gene | 1004 | No | NA | 0 | 1 | Low-quality | Genome-fragment | 1.61 | 0 |
| S9-final-viral-combined_k141_102132\|\|full | 1576 | No | NA | 0 | 1 | Not-determined | Genome-fragment | NA | 0 |
| S9-final-viral-combined_k141_59250\|\|full | 2764 | No | NA | 0 | 2 | Not-determined | Genome-fragment | NA | 0 |
| S9-final-viral-combined_k141_96745\|\|full | 1846 | No | NA | 0 | 0 | Not-determined | Genome-fragment | NA | 0 |
| S11_k141_155867 | 1004 | No | NA | 0 | 0 | Low-quality | Genome-fragment | 2.51 | 0 |
| S9-final-viral-combined_k141_48628\|\|full | 1890 | No | NA | 0 | 2 | Not-determined | Genome-fragment | NA | 0 |
| S1-final-viral-combined_k141_238667\|\|full | 1004 | No | NA | 1 | 0 | Low-quality | Genome-fragment | 0.61 | 0 |
| S1-final-viral-combined_k141_13666\|\|lt2gene | 1004 | No | NA | 1 | 0 | Low-quality | Genome-fragment | 3.21 | 0 |
| S1_k141_242902 | 1004 | No | NA | 0 | 0 | Low-quality | Genome-fragment | 2.36 | 0 |
| S9-final-viral-combined_k141_610\|\|full | 1479 | No | NA | 0 | 0 | Not-determined | Genome-fragment | NA | 0 |
| S9-final-viral-combined_k141_5668\|\|full | 1594 | No | NA | 0 | 0 | Not-determined | Genome-fragment | NA | 0 |
| S1_k141_309685 | 1004 | No | NA | 2 | 0 | Low-quality | Genome-fragment | 2.51 | 0 |
| S9-final-viral-combined_k141_59282\|\|full | 1078 | No | NA | 0 | 0 | Not-determined | Genome-fragment | NA | 0 |
| S9-final-viral-combined_k141_59296\|\|full | 1097 | No | NA | 0 | 2 | Not-determined | Genome-fragment | NA | 0 |
| S9-final-viral-combined_k141_118134\|\|full | 1988 | No | NA | 0 | 2 | Not-determined | Genome-fragment | NA | 0 |
| S9-final-viral-combined_k141_118149\|\|full | 2527 | No | NA | 0 | 3 | Not-determined | Genome-fragment | NA | 0 |
| S9-final-viral-combined_k141_107208\|\|full | 5224 | No | NA | 0 | 0 | Not-determined | Genome-fragment | NA | 0 |
| S3-final-viral-combined_k141_37307\|\|lt2gene | 1004 | No | NA | 1 | 0 | Low-quality | Genome-fragment | 2.29 | 0 |
| S9-final-viral-combined_k141_96864\|\|full | 2790 | No | NA | 0 | 2 | Not-determined | Genome-fragment | NA | 0 |
| S9-final-viral-combined_k141_32383\|\|full | 5423 | No | NA | 0 | 3 | Not-determined | Genome-fragment | NA | 0 |
| S5-final-viral-combined_k141_78615\|\|full | 1004 | No | NA | 0 | 0 | Low-quality | Genome-fragment | 1.9 | 0 |
| S9-final-viral-combined_k141_10972\|\|full | 1531 | No | NA | 0 | 1 | Not-determined | Genome-fragment | NA | 0 |
| S9-final-viral-combined_k141_59345\|\|full | 1827 | No | NA | 0 | 2 | Not-determined | Genome-fragment | NA | 0 |
| S12-final-viral-combined_k141_261345\|\|full | 1004 | No | NA | 0 | 0 | Low-quality | Genome-fragment | 1.06 | 0 |
| S12-final-viral-combined_k141_315632\|\|full | 1004 | No | NA | 1 | 0 | Low-quality | Genome-fragment | 2.19 | 0 |
| S9-final-viral-combined_k141_32398\|\|full | 2574 | No | NA | 0 | 2 | Not-determined | Genome-fragment | NA | 0 |
| S12-final-viral-combined_k141_217859\|\|lt2gene | 1004 | No | NA | 1 | 0 | Low-quality | Genome-fragment | 0.82 | 0 |

| | | | | | | | | | | | |
|---|---|---|---|---|---|---|---|---|---|---|---|
| S9-final-viral-combined_k141_86103\|\|full | 2608 | No | NA | | 0 | 2 | Not-determined | Genome-fragment | NA | | 0 |
| S9-final-viral-combined_k141_702\|\|full | 3727 | No | NA | | 0 | 2 | Not-determined | Genome-fragment | NA | | 0 |
| S9-final-viral-combined_k141_86118\|\|full | 2382 | No | NA | | 0 | 2 | Not-determined | Genome-fragment | NA | | 0 |
| S9-final-viral-combined_k141_37803\|\|full | 1048 | No | NA | | 0 | 1 | Not-determined | Genome-fragment | NA | | 0 |
| S2-final-viral-combined_k141_42687\|\|full | 1004 | No | NA | | 3 | 0 | Low-quality | Genome-fragment | | 1.65 | 0 |
| S2-final-viral-combined_k141_70451\|\|full | 1004 | No | NA | | 0 | 0 | Low-quality | Genome-fragment | | 2.24 | 0 |
| S9-final-viral-combined_k141_102219\|\|full | 1520 | No | NA | | 0 | 1 | Not-determined | Genome-fragment | NA | | 0 |
| S4-final-viral-combined_k141_87892\|\|lt2gene | 1004 | No | NA | | 1 | 0 | Low-quality | Genome-fragment | | 2.64 | 0 |
| S9-final-viral-combined_k141_48768\|\|full | 4645 | No | NA | | 0 | 3 | Not-determined | Genome-fragment | NA | | 0 |
| S8-final-viral-combined_k141_147665\|\|full | 1004 | No | NA | | 0 | 0 | Low-quality | Genome-fragment | | 0.8 | 0 |
| S9-final-viral-combined_k141_108241\|\|full | 1005 | No | NA | | 0 | 2 | Low-quality | Genome-fragment | | 1.84 | 0 |
| S11-final-viral-combined_k141_241219\|\|full | 1005 | No | NA | | 0 | 0 | Low-quality | Genome-fragment | | 0.89 | 0 |
| S9-final-viral-combined_k141_53913\|\|full | 2392 | No | NA | | 0 | 2 | Not-determined | Genome-fragment | NA | | 0 |
| S9-final-viral-combined_k141_118237\|\|full | 2111 | No | NA | | 0 | 1 | Not-determined | Genome-fragment | NA | | 0 |
| S11_k141_257486 | 1005 | No | NA | | 0 | 0 | Low-quality | Genome-fragment | | 2.26 | 0 |
| S9-final-viral-combined_k141_21772\|\|full | 2196 | No | NA | | 0 | 0 | Not-determined | Genome-fragment | NA | | 0 |
| S9-final-viral-combined_k141_16513\|\|full | 1923 | No | NA | | 0 | 2 | Not-determined | Genome-fragment | NA | | 0 |
| S9-final-viral-combined_k141_5767\|\|full | 4900 | No | NA | | 0 | 0 | Not-determined | Genome-fragment | NA | | 0 |
| S9-final-viral-combined_k141_10990\|\|full | 52978 | No | NA | | 1 | 18 | Medium-quality | Genome-fragment | | 80.16 | 0 |
| S5_k141_167266 | 1005 | No | NA | | 0 | 0 | Low-quality | Genome-fragment | | 2.63 | 0 |
| S9-final-viral-combined_k141_27269\|\|full | 2697 | No | NA | | 0 | 1 | Not-determined | Genome-fragment | NA | | 0 |
| S17-final-viral-combined_k141_139274\|\|full | 1005 | No | NA | | 0 | 1 | Low-quality | Genome-fragment | | 0.45 | 0 |
| S9-final-viral-combined_k141_37880\|\|full | 2114 | No | NA | | 0 | 2 | Not-determined | Genome-fragment | NA | | 0 |
| S9-final-viral-combined_k141_27278\|\|full | 2464 | No | NA | | 0 | 1 | Not-determined | Genome-fragment | NA | | 0 |
| S12-final-viral-combined_k141_150834\|\|full | 1005 | No | NA | | 0 | 0 | Low-quality | Genome-fragment | | 2.39 | 0 |
| S12-final-viral-combined_k141_356355\|\|lt2gene | 1005 | No | NA | | 1 | 0 | Low-quality | Genome-fragment | | 0.62 | 0 |
| S9-final-viral-combined_k141_107318\|\|full | 2853 | No | NA | | 0 | 0 | Not-determined | Genome-fragment | NA | | 0 |
| S9-final-viral-combined_k141_16580\|\|full | 2707 | No | NA | | 0 | 0 | Not-determined | Genome-fragment | NA | | 0 |
| S12_k141_160583 | 1005 | No | NA | | 0 | 1 | Low-quality | Genome-fragment | | 1.04 | 0 |
| S9-final-viral-combined_k141_80897\|\|full | 4099 | No | NA | | 0 | 0 | Not-determined | Genome-fragment | NA | | 0 |
| S9-final-viral-combined_k141_102297\|\|full | 2760 | No | NA | | 0 | 2 | Not-determined | Genome-fragment | NA | | 0 |
| S9-final-viral-combined_k141_118292\|\|full | 4656 | No | NA | | 0 | 3 | Not-determined | Genome-fragment | NA | | 0 |
| S9-final-viral-combined_k141_59492\|\|full | 1146 | No | NA | | 0 | 1 | Not-determined | Genome-fragment | NA | | 0 |
| S9-final-viral-combined_k141_112903\|\|full | 2427 | No | NA | | 0 | 2 | Not-determined | Genome-fragment | NA | | 0 |
| S4_k141_132142 | 1005 | No | NA | | 0 | 0 | Low-quality | Genome-fragment | | 2.35 | 0 |
| S9-final-viral-combined_k141_824\|\|full | 2295 | No | NA | | 0 | 1 | Not-determined | Genome-fragment | NA | | 0 |
| S9-final-viral-combined_k141_112916\|\|full | 2348 | No | NA | | 0 | 3 | Not-determined | Genome-fragment | NA | | 0 |
| S9-final-viral-combined_k141_53991\|\|full | 61938 | Yes | | 38377 | 26 | 13 | High-quality | High-quality | | 90.69 | 38.04 |
| S6-final-viral-combined_k141_130583\|\|lt2gene | 1005 | No | NA | | 1 | 0 | Low-quality | Genome-fragment | | 1.32 | 0 |
| S9-final-viral-combined_k141_102344\|\|full | 3777 | No | NA | | 0 | 0 | Not-determined | Genome-fragment | NA | | 0 |
| S9-final-viral-combined_k141_86247\|\|full | 1010 | No | NA | | 0 | 0 | Not-determined | Genome-fragment | NA | | 0 |
| S8-final-viral-combined_k141_120656\|\|full | 1005 | No | NA | | 1 | 0 | Low-quality | Genome-fragment | | 0.55 | 0 |
| S8_k141_234378 | 1005 | No | NA | | 0 | 0 | Low-quality | Genome-fragment | | 0.68 | 0 |
| S9-final-viral-combined_k141_80975\|\|full | 1121 | No | NA | | 0 | 0 | Not-determined | Genome-fragment | NA | | 0 |
| S9-final-viral-combined_k141_898\|\|full | 2197 | No | NA | | 0 | 1 | Not-determined | Genome-fragment | NA | | 0 |
| S9-final-viral-combined_k141_59595\|\|full | 4898 | No | NA | | 0 | 2 | Not-determined | Genome-fragment | NA | | 0 |
| S8_k141_22586 | 1005 | No | NA | | 0 | 0 | Low-quality | Genome-fragment | | 0.64 | 0 |
| S9-final-viral-combined_k141_102361\|\|full | 5078 | No | NA | | 0 | 6 | Not-determined | Genome-fragment | NA | | 0 |
| S9-final-viral-combined_k141_92846\|\|full | 1006 | No | NA | | 0 | 0 | Low-quality | Genome-fragment | | 2.55 | 0 |
| S9_k141_5414 | 1006 | No | NA | | 0 | 0 | Low-quality | Genome-fragment | | 0.56 | 0 |
| S9-final-viral-combined_k141_102368\|\|full | 2183 | No | NA | | 0 | 1 | Not-determined | Genome-fragment | NA | | 0 |
| S9-final-viral-combined_k141_32630\|\|full | 6808 | No | NA | | 0 | 4 | Not-determined | Genome-fragment | NA | | 0 |
| S9-final-viral-combined_k141_916\|\|full | 5764 | No | NA | | 0 | 1 | Not-determined | Genome-fragment | NA | | 0 |
| S9-final-viral-combined_k141_48856\|\|full | 2275 | No | NA | | 0 | 0 | Not-determined | Genome-fragment | NA | | 0 |
| S11_k141_96511 | 1006 | No | NA | | 0 | 0 | Low-quality | Genome-fragment | | 1.69 | 0 |
| S9-final-viral-combined_k141_5932\|\|full | 3430 | No | NA | | 0 | 0 | Not-determined | Genome-fragment | NA | | 0 |
| S9-final-viral-combined_k141_64659\|\|full | 1171 | No | NA | | 0 | 1 | Not-determined | Genome-fragment | NA | | 0 |
| S9-final-viral-combined_k141_5937\|\|full | 1378 | No | NA | | 0 | 0 | Not-determined | Genome-fragment | NA | | 0 |
| S9-final-viral-combined_k141_38008\|\|full | 2032 | No | NA | | 0 | 2 | Not-determined | Genome-fragment | NA | | 0 |
| S9-final-viral-combined_k141_64676\|\|full | 1246 | No | NA | | 0 | 1 | Not-determined | Genome-fragment | NA | | 0 |
| S9-final-viral-combined_k141_97142\|\|full | 7110 | No | NA | | 0 | 5 | Not-determined | Genome-fragment | NA | | 0 |
| S9-final-viral-combined_k141_32669\|\|full | 3323 | No | NA | | 0 | 1 | Not-determined | Genome-fragment | NA | | 0 |
| S1-final-viral-combined_k141_70920\|\|full | 1006 | No | NA | | 0 | 0 | Low-quality | Genome-fragment | | 1.51 | 0 |
| S5_k141_67176 | 1006 | No | NA | | 0 | 0 | Low-quality | Genome-fragment | | 2.06 | 0 |
| S9-final-viral-combined_k141_48891\|\|full | 2210 | No | NA | | 0 | 1 | Not-determined | Genome-fragment | NA | | 0 |
| S7-final-viral-combined_k141_54060\|\|full | 1006 | No | NA | | 0 | 0 | Low-quality | Genome-fragment | | 1.59 | 0 |
| S12-final-viral-combined_k141_9666\|\|full | 1006 | No | NA | | 0 | 0 | Low-quality | Genome-fragment | | 2.07 | 0 |
| S9-final-viral-combined_k141_32690\|\|full | 2110 | No | NA | | 0 | 1 | Not-determined | Genome-fragment | NA | | 0 |
| S9-final-viral-combined_k141_64721\|\|full | 2037 | No | NA | | 0 | 3 | Not-determined | Genome-fragment | NA | | 0 |
| S12-final-viral-combined_k141_199360\|\|full | 1006 | No | NA | | 2 | 0 | Low-quality | Genome-fragment | | 1.78 | 0 |
| S9-final-viral-combined_k141_11101\|\|full | 16618 | No | NA | | 0 | 6 | Not-determined | Genome-fragment | NA | | 0 |
| S9-final-viral-combined_k141_48911\|\|full | 4270 | No | NA | | 0 | 1 | Not-determined | Genome-fragment | NA | | 0 |
| S9-final-viral-combined_k141_81063\|\|full | 1841 | No | NA | | 0 | 2 | Not-determined | Genome-fragment | NA | | 0 |
| S9-final-viral-combined_k141_69955\|\|full | 4458 | No | NA | | 0 | 0 | Not-determined | Genome-fragment | NA | | 0 |
| S9-final-viral-combined_k141_11112\|\|full | 1735 | No | NA | | 0 | 1 | Not-determined | Genome-fragment | NA | | 0 |
| S9-final-viral-combined_k141_91374\|\|full | 1755 | No | NA | | 0 | 1 | Not-determined | Genome-fragment | NA | | 0 |
| S2-final-viral-combined_k141_13601\|\|lt2gene | 1006 | No | NA | | 0 | 0 | Low-quality | Genome-fragment | | 1.54 | 0 |
| S4_k141_62838 | 1006 | No | NA | | 0 | 0 | Low-quality | Genome-fragment | | 2.52 | 0 |
| S4_k141_114413 | 1006 | No | NA | | 0 | 0 | Low-quality | Genome-fragment | | 1.67 | 0 |
| S4_k141_13643 | 1006 | No | NA | | 0 | 0 | Low-quality | Genome-fragment | | 2.26 | 0 |
| S6_k141_76011 | 1006 | No | NA | | 1 | 0 | Low-quality | Genome-fragment | | 2.51 | 0 |
| S9-final-viral-combined_k141_82433\|\|full | 1007 | No | NA | | 2 | 0 | Low-quality | Genome-fragment | | 2.23 | 0 |
| S9-final-viral-combined_k141_107416\|\|full | 2090 | No | NA | | 0 | 1 | Not-determined | Genome-fragment | NA | | 0 |
| S9-final-viral-combined_k141_70007\|\|full | 2867 | No | NA | | 0 | 2 | Not-determined | Genome-fragment | NA | | 0 |
| S9-final-viral-combined_k141_38048\|\|full | 1195 | No | NA | | 0 | 0 | Not-determined | Genome-fragment | NA | | 0 |
| S9-final-viral-combined_k141_11167\|\|full | 1509 | No | NA | | 0 | 2 | Not-determined | Genome-fragment | NA | | 0 |
| S9-final-viral-combined_k141_86397\|\|full | 4826 | No | NA | | 0 | 2 | Not-determined | Genome-fragment | NA | | 0 |
| S9-final-viral-combined_k141_38061\|\|full | 2053 | No | NA | | 0 | 2 | Not-determined | Genome-fragment | NA | | 0 |
| S9-final-viral-combined_k141_48980\|\|full | 4670 | No | NA | | 0 | 5 | Not-determined | Genome-fragment | NA | | 0 |
| S9_k141_25337 | 1007 | No | NA | | 0 | 1 | Low-quality | Genome-fragment | | 2.25 | 0 |
| S9-final-viral-combined_k141_48999\|\|full | 2400 | No | NA | | 0 | 3 | Not-determined | Genome-fragment | NA | | 0 |
| S9-final-viral-combined_k141_27431\|\|full | 1719 | No | NA | | 0 | 0 | Not-determined | Genome-fragment | NA | | 0 |
| S11-final-viral-combined_k141_132281\|\|full | 1007 | No | NA | | 0 | 1 | Low-quality | Genome-fragment | | 0.84 | 0 |
| S9-final-viral-combined_k141_1115\|\|full | 3274 | No | NA | | 0 | 0 | Not-determined | Genome-fragment | NA | | 0 |
| S9-final-viral-combined_k141_64752\|\|full | 4374 | No | NA | | 0 | 5 | Not-determined | Genome-fragment | NA | | 0 |
| S9-final-viral-combined_k141_6012\|\|full | 3639 | No | NA | | 0 | 2 | Not-determined | Genome-fragment | NA | | 0 |
| S1-final-viral-combined_k141_363837\|\|full | 1007 | No | NA | | 0 | 0 | Low-quality | Genome-fragment | | 0.76 | 0 |
| S9-final-viral-combined_k141_27448\|\|full | 3836 | No | NA | | 0 | 3 | Not-determined | Genome-fragment | NA | | 0 |
| S1-final-viral-combined_k141_236843\|\|lt2gene | 1007 | No | NA | | 1 | 0 | Low-quality | Genome-fragment | | 1.79 | 0 |
| S9-final-viral-combined_k141_27471\|\|full | 2939 | No | NA | | 0 | 1 | Not-determined | Genome-fragment | NA | | 0 |
| S9-final-viral-combined_k141_64784\|\|full | 1769 | No | NA | | 0 | 0 | Not-determined | Genome-fragment | NA | | 0 |
| S9-final-viral-combined_k141_64785\|\|full | 1564 | No | NA | | 0 | 0 | Not-determined | Genome-fragment | NA | | 0 |
| S17-final-viral-combined_k141_35307\|\|full | 1007 | No | NA | | 0 | 0 | Low-quality | Genome-fragment | | 0.72 | 0 |
| S9-final-viral-combined_k141_81192\|\|full | 2057 | No | NA | | 0 | 1 | Not-determined | Genome-fragment | NA | | 0 |
| S9-final-viral-combined_k141_54087\|\|full | 3583 | No | NA | | 0 | 3 | Not-determined | Genome-fragment | NA | | 0 |
| S9-final-viral-combined_k141_97219\|\|full | 3835 | No | NA | | 0 | 2 | Not-determined | Genome-fragment | NA | | 0 |
| S9-final-viral-combined_k141_54102\|\|full | 1748 | No | NA | | 0 | 3 | Not-determined | Genome-fragment | NA | | 0 |
| S9-final-viral-combined_k141_38176\|\|full | 1562 | No | NA | | 0 | 1 | Not-determined | Genome-fragment | NA | | 0 |
| S9-final-viral-combined_k141_38183\|\|full | 1405 | No | NA | | 0 | 1 | Not-determined | Genome-fragment | NA | | 0 |
| S9-final-viral-combined_k141_81263\|\|full | 1016 | No | NA | | 0 | 0 | Not-determined | Genome-fragment | NA | | 0 |
| S17-final-viral-combined_k141_5221\|\|full | 1007 | No | NA | | 0 | 0 | Low-quality | Genome-fragment | | 1.96 | 0 |
| S9-final-viral-combined_k141_54136\|\|full | 2200 | No | NA | | 0 | 0 | Not-determined | Genome-fragment | NA | | 0 |
| S9-final-viral-combined_k141_107449\|\|full | 2976 | No | NA | | 0 | 0 | Not-determined | Genome-fragment | NA | | 0 |
| S12-final-viral-combined_k141_390112\|\|full | 1007 | No | NA | | 1 | 0 | Low-quality | Genome-fragment | | 2.52 | 0 |
| S9-final-viral-combined_k141_91559\|\|full | 2029 | No | NA | | 0 | 1 | Not-determined | Genome-fragment | NA | | 0 |
| S9-final-viral-combined_k141_27506\|\|full | 7113 | No | NA | | 0 | 0 | Not-determined | Genome-fragment | NA | | 0 |
| S9-final-viral-combined_k141_1255\|\|full | 1565 | No | NA | | 0 | 1 | Not-determined | Genome-fragment | NA | | 0 |
| S9-final-viral-combined_k141_123614\|\|full | 1022 | No | NA | | 0 | 3 | Not-determined | Genome-fragment | NA | | 0 |
| S9-final-viral-combined_k141_118553\|\|full | 2676 | No | NA | | 0 | 0 | Not-determined | Genome-fragment | NA | | 0 |
| S12_k141_293816 | 1007 | No | NA | | 1 | 0 | Low-quality | Genome-fragment | | 0.54 | 0 |
| S9-final-viral-combined_k141_91580\|\|full | 5090 | No | NA | | 0 | 1 | Not-determined | Genome-fragment | NA | | 0 |
| S9-final-viral-combined_k141_54162\|\|full | 2009 | No | NA | | 0 | 0 | Not-determined | Genome-fragment | NA | | 0 |
| S9-final-viral-combined_k141_91604\|\|full | 6465 | No | NA | | 0 | 6 | Not-determined | Genome-fragment | NA | | 0 |
| S12_k141_176746 | 1007 | Yes | | 376 | 1 | 1 | Low-quality | Genome-fragment | | 1.05 | 62.66 |
| S2_k141_69923 | 1007 | No | NA | | 0 | 2 | Low-quality | Genome-fragment | | 2.36 | 0 |
| S9-final-viral-combined_k141_59785\|\|full | 1475 | No | NA | | 0 | 1 | Not-determined | Genome-fragment | NA | | 0 |
| S9-final-viral-combined_k141_123667\|\|full | 1457 | No | NA | | 0 | 1 | Not-determined | Genome-fragment | NA | | 0 |
| S8-final-viral-combined_k141_94524\|\|full | 1007 | No | NA | | 1 | 0 | Low-quality | Genome-fragment | | 3.24 | 0 |
| S9-final-viral-combined_k141_107528\|\|full | 1534 | No | NA | | 0 | 0 | Not-determined | Genome-fragment | NA | | 0 |
| S9-final-viral-combined_k141_113103\|\|full | 1735 | No | NA | | 0 | 2 | Not-determined | Genome-fragment | NA | | 0 |
| S8-final-viral-combined_k141_112398\|\|lt2gene | 1007 | No | NA | | 1 | 0 | Low-quality | Genome-fragment | | 0.7 | 0 |
| S8_k141_221455 | 1007 | No | NA | | 0 | 1 | Low-quality | Genome-fragment | | 0.61 | 0 |
| S9-final-viral-combined_k141_64845\|\|full | 1340 | No | NA | | 0 | 2 | Not-determined | Genome-fragment | NA | | 0 |
| S9-final-viral-combined_k141_90196\|\|full | 1008 | No | NA | | 0 | 0 | Low-quality | Genome-fragment | | 1.44 | 0 |
| S9-final-viral-combined_k141_86572\|\|full | 2154 | No | NA | | 0 | 1 | Not-determined | Genome-fragment | NA | | 0 |
| S9-final-viral-combined_k141_22091\|\|full | 1425 | No | NA | | 0 | 2 | Not-determined | Genome-fragment | NA | | 0 |
| S9-final-viral-combined_k141_38052\|\|lt2gene | 1008 | No | NA | | 1 | 0 | Low-quality | Genome-fragment | | 2.4 | 0 |
| S9-final-viral-combined_k141_64859\|\|full | 9084 | No | NA | | 0 | 6 | Not-determined | Genome-fragment | NA | | 0 |
| S9-final-viral-combined_k141_86580\|\|full | 2124 | No | NA | | 0 | 3 | Not-determined | Genome-fragment | NA | | 0 |
| S9-final-viral-combined_k141_1334\|\|full | 1560 | No | NA | | 0 | 1 | Not-determined | Genome-fragment | NA | | 0 |
| S11-final-viral-combined_k141_46292\|\|full | 1008 | No | NA | | 0 | 0 | Low-quality | Genome-fragment | | 2.2 | 0 |
| S11-final-viral-combined_k141_78559\|\|full | 1008 | No | NA | | 0 | 1 | Low-quality | Genome-fragment | | 1.88 | 0 |
| S9-final-viral-combined_k141_102631\|\|full | 2780 | No | NA | | 0 | 3 | Not-determined | Genome-fragment | NA | | 0 |
| S9-final-viral-combined_k141_123754\|\|full | 1303 | No | NA | | 0 | 1 | Not-determined | Genome-fragment | NA | | 0 |
| S9-final-viral-combined_k141_91678\|\|full | 2498 | No | NA | | 0 | 0 | Not-determined | Genome-fragment | NA | | 0 |
| S9-final-viral-combined_k141_102639\|\|full | 3116 | No | NA | | 0 | 3 | Not-determined | Genome-fragment | NA | | 0 |
| S9-final-viral-combined_k141_27618\|\|full | 1749 | No | NA | | 0 | 2 | Not-determined | Genome-fragment | NA | | 0 |
| S3-final-viral-combined_k141_116063\|\|lt2gene | 1008 | No | NA | | 1 | 0 | Low-quality | Genome-fragment | | 2.66 | 0 |
| S9-final-viral-combined_k141_64935\|\|full | 1443 | No | NA | | 0 | 0 | Not-determined | Genome-fragment | NA | | 0 |
| S9-final-viral-combined_k141_6108\|\|full | 1243 | No | NA | | 0 | 0 | Not-determined | Genome-fragment | NA | | 0 |
| S7-final-viral-combined_k141_47370\|\|full | 1008 | No | NA | | 1 | 0 | Low-quality | Genome-fragment | | 2.48 | 0 |
| S9-final-viral-combined_k141_64944\|\|full | 3466 | No | NA | | 0 | 4 | Not-determined | Genome-fragment | NA | | 0 |
| S9-final-viral-combined_k141_118657\|\|full | 1782 | No | NA | | 0 | 0 | Not-determined | Genome-fragment | NA | | 0 |
| S7_k141_74505 | 1008 | No | NA | | 0 | 1 | Low-quality | Genome-fragment | | 1.45 | 0 |
| S9-final-viral-combined_k141_11400\|\|full | 1535 | No | NA | | 0 | 0 | Not-determined | Genome-fragment | NA | | 0 |
| S9-final-viral-combined_k141_27687\|\|full | 1024 | No | NA | | 0 | 1 | Not-determined | Genome-fragment | NA | | 0 |
| S9-final-viral-combined_k141_33106\|\|full | 1673 | No | NA | | 0 | 0 | Not-determined | Genome-fragment | NA | | 0 |
| S9-final-viral-combined_k141_59889\|\|full | 1384 | No | NA | | 0 | 1 | Not-determined | Genome-fragment | NA | | 0 |
| S7_k141_108024 | 1008 | No | NA | | 0 | 1 | Low-quality | Genome-fragment | | 2.06 | 0 |
| S7_k141_220303 | 1008 | No | NA | | 0 | 1 | Low-quality | Genome-fragment | | 0.41 | 0 |
| S9-final-viral-combined_k141_22136\|\|full | 2657 | No | NA | | 0 | 1 | Not-determined | Genome-fragment | NA | | 0 |
| S12-final-viral-combined_k141_366295\|\|lt2gene | 1008 | No | NA | | 2 | 0 | Low-quality | Genome-fragment | | 2.07 | 0 |
| S9-final-viral-combined_k141_54363\|\|full | 1061 | No | NA | | 0 | 2 | Not-determined | Genome-fragment | NA | | 0 |
| S2_k141_139658 | 1008 | No | NA | | 0 | 1 | Low-quality | Genome-fragment | | 0.57 | 0 |
| S9-final-viral-combined_k141_33158\|\|full | 2590 | No | NA | | 0 | 4 | Not-determined | Genome-fragment | NA | | 0 |
| S9-final-viral-combined_k141_11423\|\|full | 1776 | No | NA | | 0 | 0 | Not-determined | Genome-fragment | NA | | 0 |
| S4-final-viral-combined_k141_123075\|\|full | 1008 | No | NA | | 0 | 0 | Low-quality | Genome-fragment | | 1.79 | 0 |
| S9-final-viral-combined_k141_11424\|\|full | 2810 | No | NA | | 0 | 0 | Not-determined | Genome-fragment | NA | | 0 |
| S6-final-viral-combined_k141_67902\|\|lt2gene | 1008 | No | NA | | 1 | 0 | Low-quality | Genome-fragment | | 2.13 | 0 |
| S9-final-viral-combined_k141_102708\|\|full | 1836 | No | NA | | 0 | 2 | Not-determined | Genome-fragment | NA | | 0 |
| S11-final-viral-combined_k141_1909\|\|full | 1009 | No | NA | | 0 | 0 | Low-quality | Genome-fragment | | 1.52 | 0 |
| S9-final-viral-combined_k141_11440\|\|full | 1662 | No | NA | | 0 | 0 | Not-determined | Genome-fragment | NA | | 0 |
| S11-final-viral-combined_k141_126314\|\|full | 1009 | No | NA | | 0 | 1 | Low-quality | Genome-fragment | | 2.87 | 0 |
| S11-final-viral-combined_k141_147480\|\|full | 1009 | No | NA | | 1 | 0 | Low-quality | Genome-fragment | | 1.76 | 0 |

| | | | | | | | | | |
|---|---|---|---|---|---|---|---|---|---|
| S1-final-viral-combined_k141_268120\|\|full | 1009 | No | NA | 1 | 0 | Low-quality | Genome-fragment | 2.73 | 0 |
| S5_k141_71145 | 1009 | No | NA | 0 | 0 | Low-quality | Genome-fragment | 0.4 | 0 |
| S12-final-viral-combined_k141_136420\|\|full | 1009 | No | NA | 0 | 0 | Low-quality | Genome-fragment | 2.57 | 0 |
| S12-final-viral-combined_k141_89972\|\|lt2gene | 1009 | No | NA | 2 | 0 | Low-quality | Genome-fragment | 1.43 | 0 |
| S9-final-viral-combined_k141_27794\|\|full | 4228 | No | NA | 0 | 4 | Not-determined | Genome-fragment | NA | 0 |
| S9-final-viral-combined_k141_43624\|\|full | 2884 | No | NA | 0 | 2 | Not-determined | Genome-fragment | NA | 0 |
| S9-final-viral-combined_k141_27824\|\|full | 3150 | No | NA | 0 | 3 | Not-determined | Genome-fragment | NA | 0 |
| S9-final-viral-combined_k141_43635\|\|full | 11936 | No | NA | 0 | 1 | Not-determined | Genome-fragment | NA | 0 |
| S12-final-viral-combined_k141_297935\|\|lt2gene | 1009 | No | NA | 2 | 0 | Low-quality | Genome-fragment | 2.72 | 0 |
| S12_k141_199802 | 1009 | No | NA | 1 | 0 | Low-quality | Genome-fragment | 0.47 | 0 |
| S9-final-viral-combined_k141_75548\|\|full | 6295 | No | NA | 0 | 3 | Not-determined | Genome-fragment | NA | 0 |
| S12_k141_317852 | 1009 | No | NA | 0 | 0 | Low-quality | Genome-fragment | 0.54 | 0 |
| S2-final-viral-combined_k141_125920\|\|full | 1009 | No | NA | 1 | 0 | Low-quality | Genome-fragment | 1.41 | 0 |
| S6-final-viral-combined_k141_38689\|\|full | 1009 | No | NA | 0 | 0 | Low-quality | Genome-fragment | 1.38 | 0 |
| S9-final-viral-combined_k141_16775\|\|full | 2111 | No | NA | 0 | 1 | Not-determined | Genome-fragment | NA | 0 |
| S9-final-viral-combined_k141_86752\|\|full | 2899 | No | NA | 0 | 0 | Not-determined | Genome-fragment | NA | 0 |
| S9-final-viral-combined_k141_91837\|\|full | 2703 | No | NA | 0 | 3 | Not-determined | Genome-fragment | NA | 0 |
| S8_k141_19242 | 1009 | No | NA | 0 | 1 | Low-quality | Genome-fragment | 0.59 | 0 |
| S1_k141_202753 | 1010 | No | NA | 0 | 0 | Low-quality | Genome-fragment | 2.41 | 0 |
| S1_k141_73154 | 1010 | No | NA | 0 | 1 | Low-quality | Genome-fragment | 0.67 | 0 |
| S1_k141_390043 | 1010 | No | NA | 0 | 1 | Low-quality | Genome-fragment | 0.44 | 0 |
| S9-final-viral-combined_k141_102840\|\|full | 1404 | No | NA | 0 | 0 | Not-determined | Genome-fragment | NA | 0 |
| S5-final-viral-combined_k141_45398\|\|full | 1010 | No | NA | 3 | 0 | Low-quality | Genome-fragment | 2.64 | 0 |
| S5_k141_208360 | 1010 | No | NA | 0 | 0 | Low-quality | Genome-fragment | 0.66 | 0 |
| S9-final-viral-combined_k141_97506\|\|full | 4155 | No | NA | 0 | 3 | Not-determined | Genome-fragment | NA | 0 |
| S12_k141_133365 | 1010 | No | NA | 0 | 2 | Low-quality | Genome-fragment | 0.8 | 0 |
| S4-final-viral-combined_k141_22390\|\|full | 1010 | No | NA | 0 | 1 | Low-quality | Genome-fragment | 0.44 | 0 |
| S9-final-viral-combined_k141_43653\|\|full | 2225 | No | NA | 0 | 1 | Not-determined | Genome-fragment | NA | 0 |
| S9-final-viral-combined_k141_22387\|\|full | 2989 | No | NA | 0 | 3 | Not-determined | Genome-fragment | NA | 0 |
| S4_k141_102294 | 1010 | No | NA | 0 | 0 | Low-quality | Genome-fragment | 1.06 | 0 |
| S8_k141_43513 | 1010 | No | NA | 0 | 0 | Low-quality | Genome-fragment | 1.08 | 0 |
| S9-final-viral-combined_k141_70220\|\|full | 1928 | No | NA | 0 | 2 | Not-determined | Genome-fragment | NA | 0 |
| S9-final-viral-combined_k141_127926\|\|full | 1011 | No | NA | 1 | 0 | Low-quality | Genome-fragment | 2.16 | 0 |
| S11-final-viral-combined_k141_266226\|\|full | 1011 | No | NA | 2 | 0 | Low-quality | Genome-fragment | 2.38 | 0 |
| S9-final-viral-combined_k141_22427\|\|full | 1219 | No | NA | 0 | 0 | Not-determined | Genome-fragment | NA | 0 |
| S9-final-viral-combined_k141_11542\|\|full | 2125 | No | NA | 0 | 0 | Not-determined | Genome-fragment | NA | 0 |
| S11-final-viral-combined_k141_260242\|\|full | 1011 | No | NA | 0 | 0 | Low-quality | Genome-fragment | 1.8 | 0 |
| S9-final-viral-combined_k141_22437\|\|full | 1655 | No | NA | 0 | 0 | Not-determined | Genome-fragment | NA | 0 |
| S9-final-viral-combined_k141_6387\|\|full | 2354 | No | NA | 0 | 1 | Not-determined | Genome-fragment | NA | 0 |
| S9-final-viral-combined_k141_75612\|\|full | 1665 | No | NA | 0 | 1 | Not-determined | Genome-fragment | NA | 0 |
| S11-final-viral-combined_k141_312627\|\|lt2gene | 1011 | No | NA | 1 | 0 | Low-quality | Genome-fragment | 2.28 | 0 |
| S9-final-viral-combined_k141_123995\|\|full | 1106 | No | NA | 0 | 1 | Not-determined | Genome-fragment | NA | 0 |
| S11-final-viral-combined_k141_54431\|\|lt2gene | 1011 | No | NA | 1 | 0 | Low-quality | Genome-fragment | 0.65 | 0 |
| S9-final-viral-combined_k141_65132\|\|full | 1728 | No | NA | 0 | 2 | Not-determined | Genome-fragment | NA | 0 |
| S9-final-viral-combined_k141_124013\|\|full | 3148 | No | NA | 0 | 1 | Not-determined | Genome-fragment | NA | 0 |
| S9-final-viral-combined_k141_118836\|\|full | 6761 | No | NA | 0 | 4 | Not-determined | Genome-fragment | NA | 0 |
| S11_k141_19356 | 1011 | No | NA | 0 | 0 | Low-quality | Genome-fragment | 0.7 | 0 |
| S1-final-viral-combined_k141_19176\|\|full | 1011 | No | NA | 0 | 1 | Low-quality | Genome-fragment | 2.38 | 0 |
| S9-final-viral-combined_k141_16859\|\|full | 2757 | No | NA | 0 | 0 | Not-determined | Genome-fragment | NA | 0 |
| S9-final-viral-combined_k141_102893\|\|full | 2884 | No | NA | 0 | 1 | Not-determined | Genome-fragment | NA | 0 |
| S1-final-viral-combined_k141_39556\|\|full | 1011 | No | NA | 0 | 0 | Low-quality | Genome-fragment | 1.93 | 0 |
| S9-final-viral-combined_k141_70344\|\|full | 1724 | No | NA | 0 | 2 | Not-determined | Genome-fragment | NA | 0 |
| S9-final-viral-combined_k141_124050\|\|full | 3234 | No | NA | 0 | 3 | Not-determined | Genome-fragment | NA | 0 |
| S9-final-viral-combined_k141_22475\|\|full | 2857 | No | NA | 0 | 1 | Not-determined | Genome-fragment | NA | 0 |
| S9-final-viral-combined_k141_22476\|\|full | 1912 | No | NA | 0 | 1 | Not-determined | Genome-fragment | NA | 0 |
| S9-final-viral-combined_k141_6416\|\|full | 6509 | No | NA | 0 | 1 | Not-determined | Genome-fragment | NA | 0 |
| S9-final-viral-combined_k141_6434\|\|full | 5896 | No | NA | 0 | 4 | Not-determined | Genome-fragment | NA | 0 |
| S1-final-viral-combined_k141_73347\|\|full | 1011 | No | NA | 1 | 0 | Low-quality | Genome-fragment | 2.19 | 0 |
| S9-final-viral-combined_k141_118884\|\|full | 2150 | No | NA | 0 | 2 | Not-determined | Genome-fragment | NA | 0 |
| S9-final-viral-combined_k141_6448\|\|full | 1203 | No | NA | 0 | 1 | Not-determined | Genome-fragment | NA | 0 |
| S1-final-viral-combined_k141_73801\|\|full | 1011 | No | NA | 0 | 0 | Low-quality | Genome-fragment | 2.59 | 0 |
| S9-final-viral-combined_k141_81619\|\|full | 1677 | No | NA | 0 | 0 | Not-determined | Genome-fragment | NA | 0 |
| S9-final-viral-combined_k141_22494\|\|full | 2907 | No | NA | 0 | 3 | Not-determined | Genome-fragment | NA | 0 |
| S9-final-viral-combined_k141_124081\|\|full | 1809 | No | NA | 0 | 1 | Not-determined | Genome-fragment | NA | 0 |
| S3_k141_21907 | 1011 | No | NA | 0 | 2 | Low-quality | Genome-fragment | 1.57 | 0 |
| S7-final-viral-combined_k141_93462\|\|full | 1011 | No | NA | 3 | 0 | Low-quality | Genome-fragment | 2.21 | 0 |
| S7_k141_60893 | 1011 | No | NA | 2 | 0 | Low-quality | Genome-fragment | 1.94 | 0 |
| S12_k141_179759 | 1011 | No | NA | 2 | 0 | Low-quality | Genome-fragment | 1.54 | 0 |
| S9-final-viral-combined_k141_113286\|\|full | 5219 | No | NA | 0 | 0 | Not-determined | Genome-fragment | NA | 0 |
| S2-final-viral-combined_k141_121831\|\|full | 1011 | No | NA | 0 | 1 | Low-quality | Genome-fragment | 0.74 | 0 |
| S9-final-viral-combined_k141_97550\|\|full | 6576 | No | NA | 0 | 4 | Not-determined | Genome-fragment | NA | 0 |
| S9-final-viral-combined_k141_49339\|\|full | 1607 | No | NA | 0 | 2 | Not-determined | Genome-fragment | NA | 0 |
| S9-final-viral-combined_k141_124119\|\|full | 2659 | No | NA | 0 | 0 | Not-determined | Genome-fragment | NA | 0 |
| S9-final-viral-combined_k141_1700\|\|full | 1288 | No | NA | 0 | 1 | Not-determined | Genome-fragment | NA | 0 |
| S9-final-viral-combined_k141_91982\|\|full | 2373 | No | NA | 0 | 2 | Not-determined | Genome-fragment | NA | 0 |
| S9-final-viral-combined_k141_113325\|\|full | 1661 | No | NA | 0 | 0 | Not-determined | Genome-fragment | NA | 0 |
| S9-final-viral-combined_k141_49367\|\|full | 2232 | No | NA | 0 | 0 | Not-determined | Genome-fragment | NA | 0 |
| S2-final-viral-combined_k141_48877\|\|lt2gene | 1011 | No | NA | 1 | 0 | Low-quality | Genome-fragment | 3.46 | 0 |
| S9-final-viral-combined_k141_113334\|\|full | 2106 | No | NA | 0 | 2 | Not-determined | Genome-fragment | NA | 0 |
| S9-final-viral-combined_k141_81683\|\|full | 1872 | No | NA | 0 | 2 | Not-determined | Genome-fragment | NA | 0 |
| S9-final-viral-combined_k141_54741\|\|full | 3801 | No | NA | 0 | 0 | Not-determined | Genome-fragment | NA | 0 |
| S9-final-viral-combined_k141_60152\|\|full | 1748 | No | NA | 0 | 2 | Not-determined | Genome-fragment | NA | 0 |
| S9-final-viral-combined_k141_81767\|\|full | 1890 | No | NA | 0 | 2 | Not-determined | Genome-fragment | NA | 0 |
| S9-final-viral-combined_k141_43812\|\|full | 2690 | No | NA | 0 | 3 | Not-determined | Genome-fragment | NA | 0 |
| S2_k141_209746 | 1011 | No | NA | 0 | 0 | Low-quality | Genome-fragment | 2.1 | 0 |
| S4_k141_30108 | 1011 | No | NA | 0 | 0 | Low-quality | Genome-fragment | 1.01 | 0 |
| S4_k141_59007 | 1011 | No | NA | 0 | 0 | Low-quality | Genome-fragment | 1.08 | 0 |
| S6_k141_38305 | 1011 | No | NA | 0 | 0 | Low-quality | Genome-fragment | 0.63 | 0 |
| S9-final-viral-combined_k141_107986\|\|full | 1313 | No | NA | 0 | 0 | Not-determined | Genome-fragment | NA | 0 |
| S9-final-viral-combined_k141_41230\|\|lt2gene | 1012 | No | NA | 2 | 0 | Low-quality | Genome-fragment | 2.2 | 0 |
| S9-final-viral-combined_k141_119028\|\|full | 2167 | No | NA | 0 | 2 | Not-determined | Genome-fragment | NA | 0 |
| S11-final-viral-combined_k141_260954\|\|full | 1012 | No | NA | 0 | 0 | Low-quality | Genome-fragment | 2.4 | 0 |
| S1-final-viral-combined_k141_363364\|\|full | 1012 | No | NA | 0 | 1 | Low-quality | Genome-fragment | 0.83 | 0 |
| S9-final-viral-combined_k141_97633\|\|full | 2086 | No | NA | 0 | 2 | Not-determined | Genome-fragment | NA | 0 |
| S9-final-viral-combined_k141_22701\|\|full | 4420 | No | NA | 0 | 3 | Not-determined | Genome-fragment | NA | 0 |
| S9-final-viral-combined_k141_75888\|\|full | 3102 | No | NA | 0 | 2 | Not-determined | Genome-fragment | NA | 0 |
| S9-final-viral-combined_k141_65378\|\|full | 4624 | No | NA | 0 | 1 | Not-determined | Genome-fragment | NA | 0 |
| S1_k141_346007 | 1012 | No | NA | 0 | 1 | Low-quality | Genome-fragment | 0.38 | 0 |
| S9-final-viral-combined_k141_119043\|\|full | 1902 | No | NA | 0 | 2 | Not-determined | Genome-fragment | NA | 0 |
| S1_k141_342765 | 1012 | No | NA | 0 | 1 | Low-quality | Genome-fragment | 0.64 | 0 |
| S9-final-viral-combined_k141_60228\|\|full | 1964 | No | NA | 0 | 2 | Not-determined | Genome-fragment | NA | 0 |
| S3-final-viral-combined_k141_98471\|\|full | 1012 | No | NA | 0 | 0 | Low-quality | Genome-fragment | 1.64 | 0 |
| S5-final-viral-combined_k141_163033\|\|full | 1012 | No | NA | 1 | 0 | Low-quality | Genome-fragment | 1.59 | 0 |
| S9-final-viral-combined_k141_124178\|\|full | 1477 | No | NA | 0 | 0 | Not-determined | Genome-fragment | NA | 0 |
| S9-final-viral-combined_k141_17102\|\|full | 1224 | No | NA | 0 | 0 | Not-determined | Genome-fragment | NA | 0 |
| S5-final-viral-combined_k141_7633\|\|full | 1012 | No | NA | 0 | 0 | Low-quality | Genome-fragment | 0.57 | 0 |
| S9-final-viral-combined_k141_17104\|\|full | 2536 | No | NA | 0 | 3 | Not-determined | Genome-fragment | NA | 0 |
| S9-final-viral-combined_k141_49486\|\|full | 4755 | No | NA | 0 | 0 | Not-determined | Genome-fragment | NA | 0 |
| S9-final-viral-combined_k141_65428\|\|full | 1312 | No | NA | 0 | 2 | Not-determined | Genome-fragment | NA | 0 |
| S5-final-viral-combined_k141_193241\|\|full | 1012 | No | NA | 1 | 0 | Low-quality | Genome-fragment | 3 | 0 |
| S9-final-viral-combined_k141_97696\|\|full | 1615 | No | NA | 0 | 1 | Not-determined | Genome-fragment | NA | 0 |
| S9-final-viral-combined_k141_22758\|\|full | 3388 | No | NA | 0 | 3 | Not-determined | Genome-fragment | NA | 0 |
| S9-final-viral-combined_k141_43917\|\|full | 1622 | No | NA | 0 | 0 | Not-determined | Genome-fragment | NA | 0 |
| S9-final-viral-combined_k141_70577\|\|full | 1813 | No | NA | 0 | 3 | Not-determined | Genome-fragment | NA | 0 |
| S9-final-viral-combined_k141_54870\|\|full | 6477 | No | NA | 0 | 1 | Not-determined | Genome-fragment | NA | 0 |
| S5_k141_24917 | 1012 | No | NA | 0 | 0 | Low-quality | Genome-fragment | 0.57 | 0 |
| S7_k141_4465 | 1012 | No | NA | 0 | 0 | Low-quality | Genome-fragment | 3.05 | 0 |
| S9-final-viral-combined_k141_22768\|\|full | 4193 | No | NA | 0 | 3 | Not-determined | Genome-fragment | NA | 0 |
| S9-final-viral-combined_k141_54874\|\|full | 4928 | No | NA | 0 | 2 | Not-determined | Genome-fragment | NA | 0 |
| S2-final-viral-combined_k141_200740\|\|lt2gene | 1012 | No | NA | 1 | 0 | Low-quality | Genome-fragment | 1.74 | 0 |
| S4-final-viral-combined_k141_132705\|\|full | 1012 | No | NA | 0 | 1 | Low-quality | Genome-fragment | 2.73 | 0 |
| S9-final-viral-combined_k141_124242\|\|full | 2391 | No | NA | 0 | 2 | Not-determined | Genome-fragment | NA | 0 |
| S9-final-viral-combined_k141_108098\|\|full | 11780 | No | NA | 0 | 2 | Not-determined | Genome-fragment | NA | 0 |
| S4-final-viral-combined_k141_155184\|\|full | 1012 | No | NA | 1 | 0 | Low-quality | Genome-fragment | 2.08 | 0 |
| S9-final-viral-combined_k141_60336\|\|full | 1929 | No | NA | 0 | 2 | Not-determined | Genome-fragment | NA | 0 |
| S9-final-viral-combined_k141_28127\|\|full | 1435 | No | NA | 0 | 0 | Not-determined | Genome-fragment | NA | 0 |
| S9-final-viral-combined_k141_38556\|\|full | 1176 | No | NA | 0 | 0 | Not-determined | Genome-fragment | NA | 0 |
| S4_k141_120030 | 1012 | No | NA | 0 | 1 | Low-quality | Genome-fragment | 2.26 | 0 |
| S4_k141_102546 | 1012 | No | NA | 0 | 0 | Low-quality | Genome-fragment | 1.62 | 0 |
| S9-final-viral-combined_k141_28154\|\|full | 3358 | No | NA | 0 | 2 | Not-determined | Genome-fragment | NA | 0 |
| S9-final-viral-combined_k141_60361\|\|full | 1758 | No | NA | 0 | 0 | Not-determined | Genome-fragment | NA | 0 |
| S9-final-viral-combined_k141_113537\|\|full | 1430 | No | NA | 0 | 1 | Not-determined | Genome-fragment | NA | 0 |
| S8-final-viral-combined_k141_71875\|\|lt2gene | 1012 | No | NA | 0 | 2 | Low-quality | Genome-fragment | 0.49 | 0 |
| S8-final-viral-combined_k141_158363\|\|lt2gene | 1012 | No | NA | 2 | 0 | Low-quality | Genome-fragment | 1.62 | 0 |
| S9-final-viral-combined_k141_97755\|\|full | 46121 | Yes | 27506 | 24 | 13 | Medium-quality | Genome-fragment | 60.07 | 40.36 |
| S9-final-viral-combined_k141_124283\|\|full | 1971 | No | NA | 0 | 2 | Not-determined | Genome-fragment | NA | 0 |
| S8_k141_28646 | 1012 | No | NA | 0 | 0 | Low-quality | Genome-fragment | 2.33 | 0 |
| S9-final-viral-combined_k141_70662\|\|full | 2885 | No | NA | 0 | 3 | Not-determined | Genome-fragment | NA | 0 |
| S9-final-viral-combined_k141_28178\|\|full | 2834 | No | NA | 0 | 2 | Not-determined | Genome-fragment | NA | 0 |
| S9-final-viral-combined_k141_124289\|\|full | 5708 | No | NA | 0 | 1 | Not-determined | Genome-fragment | NA | 0 |
| S9-final-viral-combined_k141_65537\|\|full | 3401 | No | NA | 0 | 3 | Not-determined | Genome-fragment | NA | 0 |
| S9-final-viral-combined_k141_42484\|\|full | 1013 | No | NA | 1 | 0 | Low-quality | Genome-fragment | 1.9 | 0 |
| S9-final-viral-combined_k141_6877\|\|full | 3760 | No | NA | 0 | 0 | Not-determined | Genome-fragment | NA | 0 |
| S9-final-viral-combined_k141_14018\|\|lt2gene | 1013 | No | NA | 1 | 0 | Low-quality | Genome-fragment | 2.37 | 0 |
| S9-final-viral-combined_k141_22906\|\|full | 1244 | No | NA | 0 | 1 | Not-determined | Genome-fragment | NA | 0 |
| S1-final-viral-combined_k141_129799\|\|full | 1013 | No | NA | 0 | 2 | Low-quality | Genome-fragment | 0.45 | 0 |
| S9-final-viral-combined_k141_60417\|\|full | 1375 | No | NA | 0 | 3 | Not-determined | Genome-fragment | NA | 0 |
| S9-final-viral-combined_k141_33230\|\|full | 41954 | Yes | 27561 | 11 | 12 | Medium-quality | Genome-fragment | 84.97 | 34.31 |
| S3-final-viral-combined_k141_19862\|\|full | 1013 | Yes | 680 | 1 | 1 | Low-quality | Genome-fragment | 0.23 | 32.87 |
| S9-final-viral-combined_k141_17227\|\|full | 2299 | No | NA | 0 | 1 | Not-determined | Genome-fragment | NA | 0 |
| S3-final-viral-combined_k141_56360\|\|full | 1013 | No | NA | 0 | 0 | Low-quality | Genome-fragment | 0.73 | 0 |
| S9-final-viral-combined_k141_49568\|\|full | 4992 | No | NA | 0 | 2 | Not-determined | Genome-fragment | NA | 0 |
| S9-final-viral-combined_k141_17245\|\|full | 2165 | No | NA | 0 | 0 | Not-determined | Genome-fragment | NA | 0 |
| S7_k141_29961 | 1013 | No | NA | 0 | 0 | Low-quality | Genome-fragment | 0.71 | 0 |
| S9-final-viral-combined_k141_17262\|\|full | 1438 | No | NA | 0 | 1 | Not-determined | Genome-fragment | NA | 0 |
| S9-final-viral-combined_k141_17275\|\|full | 1975 | No | NA | 0 | 0 | Not-determined | Genome-fragment | NA | 0 |
| S9-final-viral-combined_k141_119052\|\|full | 1806 | No | NA | 0 | 0 | Not-determined | Genome-fragment | NA | 0 |
| S17-final-viral-combined_k141_69188\|\|lt2gene | 1013 | No | NA | 1 | 0 | Low-quality | Genome-fragment | 2.32 | 0 |
| S9-final-viral-combined_k141_113626\|\|full | 1595 | No | NA | 0 | 0 | Not-determined | Genome-fragment | NA | 0 |
| S12-final-viral-combined_k141_193111\|\|full | 1013 | No | NA | 0 | 0 | Low-quality | Genome-fragment | 2.8 | 0 |
| S9-final-viral-combined_k141_124315\|\|full | 3192 | No | NA | 0 | 0 | Not-determined | Genome-fragment | NA | 0 |
| S12_k141_322645 | 1013 | No | NA | 0 | 1 | Low-quality | Genome-fragment | 0.81 | 0 |
| S2-final-viral-combined_k141_202436\|\|full | 1013 | No | NA | 0 | 0 | Low-quality | Genome-fragment | 1.43 | 0 |
| S9-final-viral-combined_k141_81877\|\|full | 12933 | No | NA | 0 | 5 | Not-determined | Genome-fragment | NA | 0 |
| S9-final-viral-combined_k141_60466\|\|full | 14246 | No | NA | 0 | 7 | Not-determined | Genome-fragment | NA | 0 |
| S9-final-viral-combined_k141_70788\|\|full | 1294 | No | NA | 0 | 0 | Not-determined | Genome-fragment | NA | 0 |
| S9-final-viral-combined_k141_65634\|\|full | 1696 | No | NA | 0 | 1 | Not-determined | Genome-fragment | NA | 0 |
| S9-final-viral-combined_k141_22993\|\|full | 1812 | No | NA | 0 | 0 | Not-determined | Genome-fragment | NA | 0 |
| S2-final-viral-combined_k141_130641\|\|full | 1013 | No | NA | 1 | 0 | Low-quality | Genome-fragment | 2.96 | 0 |
| S2-final-viral-combined_k141_6256\|\|full | 1013 | No | NA | 0 | 0 | Low-quality | Genome-fragment | 1.34 | 0 |
| S9-final-viral-combined_k141_23007\|\|full | 2337 | No | NA | 0 | 2 | Not-determined | Genome-fragment | NA | 0 |
| S2-final-viral-combined_k141_97382\|\|lt2gene | 1013 | No | NA | 1 | 0 | Low-quality | Genome-fragment | 0.63 | 0 |
| S9-final-viral-combined_k141_49672\|\|full | 14140 | No | NA | 0 | 6 | Not-determined | Genome-fragment | NA | 0 |
| S9-final-viral-combined_k141_103194\|\|full | 1964 | No | NA | 0 | 2 | Not-determined | Genome-fragment | NA | 0 |

| | | | | | | | | | | | |
|---|---|---|---|---|---|---|---|---|---|---|---|
| S2_k141_243481 | 1013 | No | NA | | 0 | 1 | Low-quality | Genome-fragment | | 1.28 | 0 |
| S9-final-viral-combined_k141_103203\|\|full | 2290 | No | NA | | 0 | 1 | Not-determined | Genome-fragment | NA | | 0 |
| S9-final-viral-combined_k141_87023\|\|full | 1960 | No | NA | | 0 | 2 | Not-determined | Genome-fragment | NA | | 0 |
| S11-final-viral-combined_k141_40047\|\|full | 1014 | No | NA | | 0 | 1 | Low-quality | Genome-fragment | | 0.46 | 0 |
| S9-final-viral-combined_k141_65689\|\|full | 1506 | No | NA | | 0 | 1 | Not-determined | Genome-fragment | NA | | 0 |
| S9-final-viral-combined_k141_55042\|\|full | 4244 | No | NA | | 0 | 1 | Not-determined | Genome-fragment | NA | | 0 |
| S9-final-viral-combined_k141_11866\|\|full | 1988 | No | NA | | 0 | 1 | Not-determined | Genome-fragment | NA | | 0 |
| S9-final-viral-combined_k141_87106\|\|full | 1042 | No | NA | | 0 | 0 | Not-determined | Genome-fragment | NA | | 0 |
| S9-final-viral-combined_k141_103271\|\|full | 2102 | No | NA | | 0 | 0 | Not-determined | Genome-fragment | NA | | 0 |
| S9-final-viral-combined_k141_49726\|\|full | 4409 | No | NA | | 0 | 0 | Not-determined | Genome-fragment | NA | | 0 |
| S9-final-viral-combined_k141_17376\|\|full | 2169 | No | NA | | 0 | 2 | Not-determined | Genome-fragment | NA | | 0 |
| S11-final-viral-combined_k141_242373\|\|lt2gene | 1014 | No | NA | | 1 | 0 | Low-quality | Genome-fragment | | 2.77 | 0 |
| S9-final-viral-combined_k141_28314\|\|full | 2234 | No | NA | | 0 | 1 | Not-determined | Genome-fragment | NA | | 0 |
| S11_k141_289981 | 1014 | No | NA | | 0 | 0 | Low-quality | Genome-fragment | | 2.18 | 0 |
| S9-final-viral-combined_k141_28321\|\|full | 2474 | No | NA | | 0 | 2 | Not-determined | Genome-fragment | NA | | 0 |
| S1_k141_271796 | 1014 | No | NA | | 0 | 0 | Low-quality | Genome-fragment | | 0.59 | 0 |
| S5-final-viral-combined_k141_170026\|\|full | 1014 | No | NA | | 0 | 0 | Low-quality | Genome-fragment | | 2 | 0 |
| S5-final-viral-combined_k141_518\|\|lt2gene | 1014 | No | NA | | 0 | 0 | Low-quality | Genome-fragment | | 2.59 | 0 |
| S7-final-viral-combined_k141_67860\|\|full | 1014 | No | NA | | 0 | 2 | Low-quality | Genome-fragment | | 0.32 | 0 |
| S9-final-viral-combined_k141_108227\|\|full | 1768 | No | NA | | 0 | 2 | Not-determined | Genome-fragment | NA | | 0 |
| S9-final-viral-combined_k141_75960\|\|full | 1547 | No | NA | | 0 | 3 | Not-determined | Genome-fragment | NA | | 0 |
| S17-k141_157531 | 1014 | No | NA | | 0 | 0 | Low-quality | Genome-fragment | | 2.07 | 0 |
| S17-k141_86259 | 1014 | No | NA | | 0 | 0 | Low-quality | Genome-fragment | | 1.74 | 0 |
| S12-final-viral-combined_k141_34117\|\|full | 1014 | No | NA | | 1 | 0 | Low-quality | Genome-fragment | | 2.06 | 0 |
| S12-final-viral-combined_k141_321842\|\|full | 1014 | No | NA | | 0 | 0 | Low-quality | Genome-fragment | | 1.71 | 0 |
| S9-final-viral-combined_k141_81967\|\|full | 2416 | No | NA | | 0 | 1 | Not-determined | Genome-fragment | NA | | 0 |
| S12-final-viral-combined_k141_367240\|\|lt2gene | 1014 | No | NA | | 0 | 0 | Low-quality | Genome-fragment | | 2.79 | 0 |
| S2-final-viral-combined_k141_76965\|\|lt2gene | 1014 | No | NA | | 1 | 0 | Low-quality | Genome-fragment | | 2.6 | 0 |
| S9-final-viral-combined_k141_97840\|\|full | 2284 | No | NA | | 0 | 2 | Not-determined | Genome-fragment | NA | | 0 |
| S9-final-viral-combined_k141_119138\|\|full | 19823 | No | NA | | 0 | 11 | Not-determined | Genome-fragment | NA | | 0 |
| S9-final-viral-combined_k141_76013\|\|full | 1603 | No | NA | | 0 | 1 | Not-determined | Genome-fragment | NA | | 0 |
| S9-final-viral-combined_k141_38821\|\|full | 2630 | No | NA | | 0 | 1 | Not-determined | Genome-fragment | NA | | 0 |
| S2-final-viral-combined_k141_80291\|\|lt2gene | 1014 | No | NA | | 1 | 0 | Low-quality | Genome-fragment | | 2.74 | 0 |
| S9-final-viral-combined_k141_108280\|\|full | 1597 | No | NA | | 0 | 0 | Not-determined | Genome-fragment | NA | | 0 |
| S9-final-viral-combined_k141_49846\|\|full | 4402 | No | NA | | 0 | 0 | Not-determined | Genome-fragment | NA | | 0 |
| S9-final-viral-combined_k141_65859\|\|full | 2822 | No | NA | | 0 | 3 | Not-determined | Genome-fragment | NA | | 0 |
| S4-final-viral-combined_k141_103131\|\|full | 1014 | No | NA | | 0 | 1 | Low-quality | Genome-fragment | | 1.48 | 0 |
| S4-final-viral-combined_k141_58989\|\|lt2gene | 1014 | No | NA | | 1 | 0 | Low-quality | Genome-fragment | | 2.28 | 0 |
| S9-final-viral-combined_k141_23081\|\|full | 3304 | No | NA | | 0 | 3 | Not-determined | Genome-fragment | NA | | 0 |
| S6-final-viral-combined_k141_1339\|\|full | 1014 | No | NA | | 0 | 1 | Low-quality | Genome-fragment | | 0.48 | 0 |
| S9-final-viral-combined_k141_65877\|\|full | 1974 | No | NA | | 0 | 2 | Not-determined | Genome-fragment | NA | | 0 |
| S8-final-viral-combined_k141_83451\|\|lt2gene | 1014 | No | NA | | 1 | 0 | Low-quality | Genome-fragment | | 1.78 | 0 |
| S9-final-viral-combined_k141_17525\|\|full | 2422 | No | NA | | 0 | 2 | Not-determined | Genome-fragment | NA | | 0 |
| S8_k141_173134 | 1014 | No | NA | | 0 | 0 | Low-quality | Genome-fragment | | 0.75 | 0 |
| S9-final-viral-combined_k141_60610\|\|full | 2187 | No | NA | | 0 | 1 | Not-determined | Genome-fragment | NA | | 0 |
| S9-final-viral-combined_k141_2042\|\|full | 38861 | Yes | | 31053 | 5 | 5 | Medium-quality | Genome-fragment | | 68.98 | 20.09 |
| S9-final-viral-combined_k141_23130\|\|full | 2086 | No | NA | | 0 | 0 | Not-determined | Genome-fragment | NA | | 0 |
| S9-final-viral-combined_k141_76068\|\|full | 2866 | No | NA | | 0 | 0 | Not-determined | Genome-fragment | NA | | 0 |
| S9-final-viral-combined_k141_85743\|\|full | 1015 | No | NA | | 4 | 0 | Low-quality | Genome-fragment | | 3.19 | 0 |
| S9-final-viral-combined_k141_115400\|\|full | 1015 | No | NA | | 0 | 0 | Low-quality | Genome-fragment | | 0.4 | 0 |
| S9-final-viral-combined_k141_23142\|\|full | 13473 | No | NA | | 0 | 1 | Not-determined | Genome-fragment | NA | | 0 |
| S9-final-viral-combined_k141_49880\|\|full | 1553 | No | NA | | 0 | 2 | Not-determined | Genome-fragment | NA | | 0 |
| S11-final-viral-combined_k141_59514\|\|full | 1015 | No | NA | | 0 | 0 | Low-quality | Genome-fragment | | 1.65 | 0 |
| S9-final-viral-combined_k141_33405\|\|full | 1884 | No | NA | | 0 | 2 | Not-determined | Genome-fragment | NA | | 0 |
| S11_k141_239566 | 1015 | No | NA | | 0 | 0 | Low-quality | Genome-fragment | | 1.67 | 0 |
| S1-final-viral-combined_k141_76605\|\|full | 1015 | No | NA | | 0 | 0 | Low-quality | Genome-fragment | | 0.76 | 0 |
| S1-final-viral-combined_k141_375441\|\|full | 1015 | No | NA | | 0 | 1 | Low-quality | Genome-fragment | | 2.17 | 0 |
| S9-final-viral-combined_k141_33421\|\|full | 1723 | No | NA | | 0 | 3 | Not-determined | Genome-fragment | NA | | 0 |
| S3-final-viral-combined_k141_79746\|\|full | 1015 | No | NA | | 0 | 0 | Low-quality | Genome-fragment | | 2.52 | 0 |
| S9-final-viral-combined_k141_2130\|\|full | 2653 | No | NA | | 0 | 4 | Not-determined | Genome-fragment | NA | | 0 |
| S9-final-viral-combined_k141_124574\|\|full | 1686 | No | NA | | 0 | 3 | Not-determined | Genome-fragment | NA | | 0 |
| S9-final-viral-combined_k141_17589\|\|full | 2283 | No | NA | | 0 | 2 | Not-determined | Genome-fragment | NA | | 0 |
| S9-final-viral-combined_k141_55305\|\|full | 1332 | No | NA | | 0 | 1 | Not-determined | Genome-fragment | NA | | 0 |
| S9-final-viral-combined_k141_17591\|\|full | 4162 | No | NA | | 0 | 0 | Not-determined | Genome-fragment | NA | | 0 |
| S3_k141_12277 | 1015 | No | NA | | 0 | 1 | Low-quality | Genome-fragment | | 2.44 | 0 |
| S9-final-viral-combined_k141_103358\|\|full | 8408 | No | NA | | 0 | 3 | Not-determined | Genome-fragment | NA | | 0 |
| S5-final-viral-combined_k141_135348\|\|full | 1015 | No | NA | | 3 | 0 | Low-quality | Genome-fragment | | 2.57 | 0 |
| S9-final-viral-combined_k141_71022\|\|full | 1018 | No | NA | | 0 | 2 | Not-determined | Genome-fragment | NA | | 0 |
| S9-final-viral-combined_k141_55339\|\|full | 4769 | No | NA | | 0 | 3 | Not-determined | Genome-fragment | NA | | 0 |
| S5-final-viral-combined_k141_79883\|\|lt2gene | 1015 | No | NA | | 1 | 0 | Low-quality | Genome-fragment | | 1.52 | 0 |
| S9-final-viral-combined_k141_55349\|\|full | 2669 | No | NA | | 0 | 1 | Not-determined | Genome-fragment | NA | | 0 |
| S9-final-viral-combined_k141_82135\|\|full | 2734 | No | NA | | 0 | 1 | Not-determined | Genome-fragment | NA | | 0 |
| S9-final-viral-combined_k141_97913\|\|full | 1433 | No | NA | | 0 | 2 | Not-determined | Genome-fragment | NA | | 0 |
| S7-final-viral-combined_k141_220984\|\|full | 1015 | Yes | | 503 | 1 | 1 | Low-quality | Genome-fragment | | 1.4 | 50.44 |
| S17-final-viral-combined_k141_74417\|\|full | 1015 | No | NA | | 0 | 2 | Low-quality | Genome-fragment | | 0.36 | 0 |
| S12-final-viral-combined_k141_25826\|\|full | 1015 | No | NA | | 0 | 0 | Low-quality | Genome-fragment | | 1.66 | 0 |
| S9-final-viral-combined_k141_108379\|\|full | 2556 | No | NA | | 0 | 1 | Not-determined | Genome-fragment | NA | | 0 |
| S9-final-viral-combined_k141_55404\|\|full | 2179 | No | NA | | 0 | 1 | Not-determined | Genome-fragment | NA | | 0 |
| S9-final-viral-combined_k141_7225\|\|full | 1553 | No | NA | | 0 | 1 | Not-determined | Genome-fragment | NA | | 0 |
| S12-final-viral-combined_k141_211435\|\|lt2gene | 1015 | No | NA | | 1 | 0 | Low-quality | Genome-fragment | | 2.56 | 0 |
| S9-final-viral-combined_k141_55416\|\|full | 3682 | No | NA | | 0 | 4 | Not-determined | Genome-fragment | NA | | 0 |
| S9-final-viral-combined_k141_97945\|\|full | 1998 | No | NA | | 0 | 2 | Not-determined | Genome-fragment | NA | | 0 |
| S12_k141_351943 | 1015 | No | NA | | 0 | 0 | Low-quality | Genome-fragment | | 3.86 | 0 |
| S9-final-viral-combined_k141_97948\|\|full | 3083 | No | NA | | 0 | 0 | Not-determined | Genome-fragment | NA | | 0 |
| S9-final-viral-combined_k141_60754\|\|full | 2282 | No | NA | | 0 | 0 | Not-determined | Genome-fragment | NA | | 0 |
| S12_k141_81639 | 1015 | No | NA | | 0 | 0 | Low-quality | Genome-fragment | | 0.55 | 0 |
| S2-final-viral-combined_k141_49613\|\|full | 1015 | No | NA | | 2 | 0 | Low-quality | Genome-fragment | | 1.85 | 0 |
| S9-final-viral-combined_k141_7232\|\|full | 2610 | No | NA | | 0 | 3 | Not-determined | Genome-fragment | NA | | 0 |
| S9-final-viral-combined_k141_82228\|\|full | 1662 | No | NA | | 0 | 0 | Not-determined | Genome-fragment | NA | | 0 |
| S9-final-viral-combined_k141_12108\|\|full | 3730 | No | NA | | 0 | 2 | Not-determined | Genome-fragment | NA | | 0 |
| S8-final-viral-combined_k141_107501\|\|full | 1015 | No | NA | | 1 | 0 | Low-quality | Genome-fragment | | 1.85 | 0 |
| S8-final-viral-combined_k141_271396\|\|full | 1015 | No | NA | | 0 | 0 | Low-quality | Genome-fragment | | 2.2 | 0 |
| S8-final-viral-combined_k141_10271\|\|full | 1015 | No | NA | | 0 | 0 | Low-quality | Genome-fragment | | 0.66 | 0 |
| S11-final-viral-combined_k141_33192\|\|full | 1016 | No | NA | | 1 | 0 | Low-quality | Genome-fragment | | 2.5 | 0 |
| S9-final-viral-combined_k141_17744\|\|full | 2576 | No | NA | | 0 | 2 | Not-determined | Genome-fragment | NA | | 0 |
| S9-final-viral-combined_k141_23303\|\|full | 2894 | No | NA | | 0 | 3 | Not-determined | Genome-fragment | NA | | 0 |
| S11-final-viral-combined_k141_207281\|\|lt2gene | 1016 | No | NA | | 1 | 0 | Low-quality | Genome-fragment | | 1.18 | 0 |
| S9-final-viral-combined_k141_17759\|\|full | 4027 | No | NA | | 0 | 1 | Not-determined | Genome-fragment | NA | | 0 |
| S9-final-viral-combined_k141_12150\|\|full | 4577 | No | NA | | 0 | 4 | Not-determined | Genome-fragment | NA | | 0 |
| S1-final-viral-combined_k141_194280\|\|full | 1016 | No | NA | | 0 | 1 | Low-quality | Genome-fragment | | 15.76 | 0 |
| S9-final-viral-combined_k141_17788\|\|full | 11015 | No | NA | | 0 | 6 | Not-determined | Genome-fragment | NA | | 0 |
| S9-final-viral-combined_k141_17789\|\|full | 2498 | No | NA | | 0 | 1 | Not-determined | Genome-fragment | NA | | 0 |
| S9-final-viral-combined_k141_28427\|\|full | 1199 | No | NA | | 0 | 1 | Not-determined | Genome-fragment | NA | | 0 |
| S9-final-viral-combined_k141_55492\|\|full | 1274 | No | NA | | 0 | 2 | Not-determined | Genome-fragment | NA | | 0 |
| S1_k141_318985 | 1016 | No | NA | | 0 | 1 | Low-quality | Genome-fragment | | 0.71 | 0 |
| S5_k141_144978 | 1016 | No | NA | | 0 | 0 | Low-quality | Genome-fragment | | 7.01 | 0 |
| S9-final-viral-combined_k141_33473\|\|full | 1202 | No | NA | | 0 | 1 | Not-determined | Genome-fragment | NA | | 0 |
| S9-final-viral-combined_k141_55507\|\|full | 1429 | No | NA | | 0 | 0 | Not-determined | Genome-fragment | NA | | 0 |
| S9-final-viral-combined_k141_12205\|\|full | 1542 | No | NA | | 0 | 2 | Not-determined | Genome-fragment | NA | | 0 |
| S9-final-viral-combined_k141_82293\|\|full | 19360 | No | NA | | 0 | 10 | Not-determined | Genome-fragment | NA | | 0 |
| S7-final-viral-combined_k141_143168\|\|full | 1016 | No | NA | | 1 | 0 | Low-quality | Genome-fragment | | 2.6 | 0 |
| S17-final-viral-combined_k141_110883\|\|full | 1016 | No | NA | | 0 | 0 | Low-quality | Genome-fragment | | 1.38 | 0 |
| S9-final-viral-combined_k141_119377\|\|full | 3419 | No | NA | | 0 | 3 | Not-determined | Genome-fragment | NA | | 0 |
| S9-final-viral-combined_k141_87381\|\|full | 3519 | No | NA | | 0 | 2 | Not-determined | Genome-fragment | NA | | 0 |
| S9-final-viral-combined_k141_2348\|\|full | 1215 | No | NA | | 0 | 1 | Not-determined | Genome-fragment | NA | | 0 |
| S9-final-viral-combined_k141_71118\|\|full | 2771 | No | NA | | 0 | 1 | Not-determined | Genome-fragment | NA | | 0 |
| S12-final-viral-combined_k141_240948\|\|full | 1016 | No | NA | | 0 | 1 | Low-quality | Genome-fragment | | 0.75 | 0 |
| S12-final-viral-combined_k141_309841\|\|full | 1016 | No | NA | | 0 | 0 | Low-quality | Genome-fragment | | 2.64 | 0 |
| S9-final-viral-combined_k141_71124\|\|full | 7164 | No | NA | | 0 | 3 | Not-determined | Genome-fragment | NA | | 0 |
| S9-final-viral-combined_k141_55551\|\|full | 2895 | No | NA | | 0 | 2 | Not-determined | Genome-fragment | NA | | 0 |
| S9-final-viral-combined_k141_92671\|\|full | 1647 | No | NA | | 0 | 0 | Not-determined | Genome-fragment | NA | | 0 |
| S12-final-viral-combined_k141_16054\|\|full | 1016 | No | NA | | 0 | 0 | Low-quality | Genome-fragment | | 1.73 | 0 |
| S4_k141_114384 | 1016 | No | NA | | 0 | 0 | Low-quality | Genome-fragment | | 1.26 | 0 |
| S9-final-viral-combined_k141_98074\|\|full | 1374 | No | NA | | 0 | 0 | Not-determined | Genome-fragment | NA | | 0 |
| S9-final-viral-combined_k141_87411\|\|full | 1479 | No | NA | | 0 | 0 | Not-determined | Genome-fragment | NA | | 0 |
| S9-final-viral-combined_k141_13693\|\|full | 1017 | No | NA | | 1 | 0 | Low-quality | Genome-fragment | | 2.19 | 0 |
| S9-final-viral-combined_k141_28569\|\|full | 2666 | No | NA | | 0 | 0 | Not-determined | Genome-fragment | NA | | 0 |
| S9-final-viral-combined_k141_119428\|\|full | 1633 | No | NA | | 0 | 1 | Not-determined | Genome-fragment | NA | | 0 |
| S9-final-viral-combined_k141_79301\|\|lt2gene | 1017 | No | NA | | 1 | 0 | Low-quality | Genome-fragment | | 2.53 | 0 |
| S11-final-viral-combined_k141_89094\|\|full | 1017 | No | NA | | 2 | 0 | Low-quality | Genome-fragment | | 2.5 | 0 |
| S1-final-viral-combined_k141_129920\|\|full | 1017 | No | NA | | 0 | 0 | Low-quality | Genome-fragment | | 1.55 | 0 |
| S9-final-viral-combined_k141_98127\|\|full | 1228 | No | NA | | 0 | 0 | Not-determined | Genome-fragment | NA | | 0 |
| S9-final-viral-combined_k141_60894\|\|full | 3525 | No | NA | | 0 | 1 | Not-determined | Genome-fragment | NA | | 0 |
| S9-final-viral-combined_k141_92731\|\|full | 2954 | No | NA | | 0 | 0 | Not-determined | Genome-fragment | NA | | 0 |
| S1-final-viral-combined_k141_317633\|\|lt2gene | 1017 | No | NA | | 2 | 0 | Low-quality | Genome-fragment | | 1.69 | 0 |
| S9-final-viral-combined_k141_98152\|\|full | 3335 | No | NA | | 0 | 0 | Not-determined | Genome-fragment | NA | | 0 |
| S9-final-viral-combined_k141_71209\|\|full | 3637 | No | NA | | 0 | 2 | Not-determined | Genome-fragment | NA | | 0 |
| S9-final-viral-combined_k141_103556\|\|full | 1043 | No | NA | | 0 | 0 | Not-determined | Genome-fragment | NA | | 0 |
| S9-final-viral-combined_k141_33510\|\|full | 2110 | No | NA | | 0 | 0 | Not-determined | Genome-fragment | NA | | 0 |
| S9-final-viral-combined_k141_103559\|\|full | 8547 | No | NA | | 0 | 6 | Not-determined | Genome-fragment | NA | | 0 |
| S9-final-viral-combined_k141_39169\|\|full | 4786 | No | NA | | 0 | 1 | Not-determined | Genome-fragment | NA | | 0 |
| S5-final-viral-combined_k141_17168\|\|full | 1017 | No | NA | | 1 | 0 | Low-quality | Genome-fragment | | 1.97 | 0 |
| S7-final-viral-combined_k141_151117\|\|full | 1017 | No | NA | | 1 | 0 | Low-quality | Genome-fragment | | 2.58 | 0 |
| S9-final-viral-combined_k141_39183\|\|full | 2033 | No | NA | | 0 | 2 | Not-determined | Genome-fragment | NA | | 0 |
| S17-k141_146064 | 1017 | No | NA | | 0 | 2 | Low-quality | Genome-fragment | | 1.04 | 0 |
| S9-final-viral-combined_k141_66268\|\|full | 4333 | No | NA | | 0 | 2 | Not-determined | Genome-fragment | NA | | 0 |
| S12-final-viral-combined_k141_331711\|\|full | 1017 | No | NA | | 1 | 0 | Low-quality | Genome-fragment | | 2.63 | 0 |
| S9-final-viral-combined_k141_7492\|\|full | 2367 | No | NA | | 0 | 2 | Not-determined | Genome-fragment | NA | | 0 |
| S12_k141_232758 | 1017 | No | NA | | 1 | 0 | Low-quality | Genome-fragment | | 0.73 | 0 |
| S9-final-viral-combined_k141_71254\|\|full | 2761 | No | NA | | 0 | 1 | Not-determined | Genome-fragment | NA | | 0 |
| S2-final-viral-combined_k141_222193\|\|full | 1017 | No | NA | | 1 | 0 | Low-quality | Genome-fragment | | 2.36 | 0 |
| S8-final-viral-combined_k141_91846\|\|full | 1017 | No | NA | | 0 | 0 | Low-quality | Genome-fragment | | 1.67 | 0 |
| S9-final-viral-combined_k141_28657\|\|full | 8326 | No | NA | | 0 | 5 | Not-determined | Genome-fragment | NA | | 0 |
| S9-final-viral-combined_k141_87551\|\|full | 2492 | No | NA | | 0 | 0 | Not-determined | Genome-fragment | NA | | 0 |
| S9-final-viral-combined_k141_28662\|\|full | 1242 | No | NA | | 0 | 0 | Not-determined | Genome-fragment | NA | | 0 |
| S9-final-viral-combined_k141_39253\|\|full | 1416 | No | NA | | 0 | 1 | Not-determined | Genome-fragment | NA | | 0 |
| S9-final-viral-combined_k141_17465\|\|lt2gene | 1018 | No | NA | | 1 | 0 | Low-quality | Genome-fragment | | 2.71 | 0 |
| S9-final-viral-combined_k141_82397\|\|full | 1958 | No | NA | | 0 | 4 | Not-determined | Genome-fragment | NA | | 0 |
| S9-final-viral-combined_k141_103620\|\|full | 1767 | No | NA | | 0 | 1 | Not-determined | Genome-fragment | NA | | 0 |
| S9-final-viral-combined_k141_124733\|\|full | 3145 | No | NA | | 0 | 0 | Not-determined | Genome-fragment | NA | | 0 |
| S9-final-viral-combined_k141_108514\|\|full | 2127 | No | NA | | 0 | 1 | Not-determined | Genome-fragment | NA | | 0 |
| S9-final-viral-combined_k141_71300\|\|full | 1913 | No | NA | | 0 | 1 | Not-determined | Genome-fragment | NA | | 0 |
| S9-final-viral-combined_k141_12394\|\|full | 2714 | No | NA | | 0 | 0 | Not-determined | Genome-fragment | NA | | 0 |
| S9-final-viral-combined_k141_17959\|\|full | 2927 | No | NA | | 0 | 1 | Not-determined | Genome-fragment | NA | | 0 |
| S11_k141_326073 | 1018 | No | NA | | 0 | 1 | Low-quality | Genome-fragment | | 1.28 | 0 |
| S9-final-viral-combined_k141_50388\|\|full | 1238 | No | NA | | 0 | 1 | Not-determined | Genome-fragment | NA | | 0 |
| S5-final-viral-combined_k141_63315\|\|full | 1018 | No | NA | | 0 | 1 | Low-quality | Genome-fragment | | 0.29 | 0 |
| S9-final-viral-combined_k141_87615\|\|full | 3540 | No | NA | | 0 | 2 | Not-determined | Genome-fragment | NA | | 0 |
| S5-final-viral-combined_k141_22930\|\|full | 1018 | No | NA | | 0 | 0 | Low-quality | Genome-fragment | | 1.88 | 0 |
| S5-final-viral-combined_k141_133269\|\|full | 1018 | No | NA | | 0 | 0 | Low-quality | Genome-fragment | | 0.64 | 0 |
| S5-final-viral-combined_k141_26173\|\|full | 1018 | No | NA | | 1 | 0 | Low-quality | Genome-fragment | | 2.25 | 0 |
| S5_k141_21929 | 1018 | No | NA | | 0 | 1 | Low-quality | Genome-fragment | | 2.12 | 0 |
| S7-final-viral-combined_k141_86430\|\|full | 1018 | No | NA | | 0 | 0 | Low-quality | Genome-fragment | | 1.06 | 0 |
| S9-final-viral-combined_k141_119563\|\|full | 1441 | No | NA | | 0 | 1 | Not-determined | Genome-fragment | NA | | 0 |

| | | | | | | | | | |
|---|---|---|---|---|---|---|---|---|---|
| S7-final-viral-combined_k141_244194\|\|full | 1018 | No | NA | 0 | 0 | Low-quality | Genome-fragment | 0.99 | 0 |
| S12-final-viral-combined_k141_1130\|\|full | 1018 | No | NA | 0 | 0 | Low-quality | Genome-fragment | 1.24 | 0 |
| S12-final-viral-combined_k141_337148\|\|lt2gene | 1018 | No | NA | 0 | 0 | Low-quality | Genome-fragment | 6.57 | 0 |
| S12-final-viral-combined_k141_321381\|\|lt2gene | 1018 | No | NA | 1 | 0 | Low-quality | Genome-fragment | 2.2 | 0 |
| S12_k141_125931 | 1018 | No | NA | 1 | 0 | Low-quality | Genome-fragment | 2.42 | 0 |
| S9-final-viral-combined_k141_108599\|\|full | 2865 | No | NA | 0 | 0 | Not-determined | Genome-fragment | NA | 0 |
| S4-final-viral-combined_k141_80850\|\|full | 1018 | No | NA | 0 | 0 | Low-quality | Genome-fragment | 2.65 | 0 |
| S9-final-viral-combined_k141_82481\|\|full | 2666 | No | NA | 0 | 2 | Not-determined | Genome-fragment | NA | 0 |
| S4-final-viral-combined_k141_165078\|\|full | 1018 | No | NA | 0 | 0 | Low-quality | Genome-fragment | 2.25 | 0 |
| S9-final-viral-combined_k141_113920\|\|full | 1257 | No | NA | 0 | 1 | Not-determined | Genome-fragment | NA | 0 |
| S9-final-viral-combined_k141_18095\|\|full | 4771 | No | NA | 0 | 1 | Not-determined | Genome-fragment | NA | 0 |
| S4-final-viral-combined_k141_31960\|\|lt2gene | 1018 | No | NA | 1 | 0 | Low-quality | Genome-fragment | 3.04 | 0 |
| S4_k141_144510 | 1018 | No | NA | 0 | 0 | Low-quality | Genome-fragment | 2.38 | 0 |
| S6-final-viral-combined_k141_124804\|\|full | 1018 | No | NA | 0 | 0 | Low-quality | Genome-fragment | 0.73 | 0 |
| S8_k141_19757 | 1018 | No | NA | 0 | 0 | Low-quality | Genome-fragment | 8.15 | 0 |
| S9-final-viral-combined_k141_108695\|\|full | 2308 | No | NA | 0 | 3 | Not-determined | Genome-fragment | NA | 0 |
| S11-final-viral-combined_k141_22853\|\|lt2gene | 1019 | No | NA | 1 | 0 | Low-quality | Genome-fragment | 1.33 | 0 |
| S1-final-viral-combined_k141_285168\|\|full | 1019 | No | NA | 0 | 0 | Low-quality | Genome-fragment | 2.48 | 0 |
| S9-final-viral-combined_k141_23411\|\|full | 2327 | No | NA | 0 | 3 | Not-determined | Genome-fragment | NA | 0 |
| S1_k141_17327 | 1019 | No | NA | 0 | 0 | Low-quality | Genome-fragment | 1.4 | 0 |
| S3-final-viral-combined_k141_51850\|\|full | 1019 | No | NA | 1 | 0 | Low-quality | Genome-fragment | 2.67 | 0 |
| S3_k141_47395 | 1019 | No | NA | 1 | 0 | Low-quality | Genome-fragment | 2.42 | 0 |
| S9-final-viral-combined_k141_18274\|\|full | 7313 | No | NA | 0 | 0 | Not-determined | Genome-fragment | NA | 0 |
| S9-final-viral-combined_k141_23447\|\|full | 1362 | No | NA | 0 | 0 | Not-determined | Genome-fragment | NA | 0 |
| S9-final-viral-combined_k141_18287\|\|full | 1543 | No | NA | 0 | 1 | Not-determined | Genome-fragment | NA | 0 |
| S7-final-viral-combined_k141_36944\|\|full | 1019 | No | NA | 0 | 0 | Low-quality | Genome-fragment | 0.72 | 0 |
| S9-final-viral-combined_k141_71406\|\|full | 2590 | No | NA | 0 | 1 | Not-determined | Genome-fragment | NA | 0 |
| S9-final-viral-combined_k141_119617\|\|full | 1249 | No | NA | 0 | 0 | Not-determined | Genome-fragment | NA | 0 |
| S17-final-viral-combined_k141_121605\|\|full | 1019 | No | NA | 1 | 0 | Low-quality | Genome-fragment | 1.63 | 0 |
| S9-final-viral-combined_k141_23467\|\|full | 3279 | No | NA | 0 | 3 | Not-determined | Genome-fragment | NA | 0 |
| S12-final-viral-combined_k141_242030\|\|full | 1019 | No | NA | 0 | 1 | Low-quality | Genome-fragment | 0.86 | 0 |
| S9-final-viral-combined_k141_113982\|\|full | 1078 | No | NA | 0 | 0 | Not-determined | Genome-fragment | NA | 0 |
| S9-final-viral-combined_k141_113997\|\|full | 1579 | No | NA | 0 | 0 | Not-determined | Genome-fragment | NA | 0 |
| S9-final-viral-combined_k141_50533\|\|full | 2206 | No | NA | 0 | 1 | Not-determined | Genome-fragment | NA | 0 |
| S12-final-viral-combined_k141_362616\|\|full | 1019 | No | NA | 0 | 0 | Low-quality | Genome-fragment | 2.34 | 0 |
| S9-final-viral-combined_k141_12737\|\|full | 1279 | No | NA | 0 | 1 | Not-determined | Genome-fragment | NA | 0 |
| S12-final-viral-combined_k141_202455\|\|lt2gene | 1019 | No | NA | 1 | 0 | Low-quality | Genome-fragment | 2.93 | 0 |
| S9-final-viral-combined_k141_50568\|\|full | 2223 | No | NA | 0 | 2 | Not-determined | Genome-fragment | NA | 0 |
| S9-final-viral-combined_k141_12746\|\|full | 5276 | No | NA | 0 | 0 | Not-determined | Genome-fragment | NA | 0 |
| S12_k141_70088 | 1019 | No | NA | 1 | 0 | Low-quality | Genome-fragment | 0.55 | 0 |
| S9-final-viral-combined_k141_44505\|\|full | 6443 | No | NA | 0 | 1 | Not-determined | Genome-fragment | NA | 0 |
| S9-final-viral-combined_k141_23526\|\|full | 1166 | No | NA | 0 | 1 | Not-determined | Genome-fragment | NA | 0 |
| S12_k141_63393 | 1019 | No | NA | 0 | 0 | Low-quality | Genome-fragment | 1.87 | 0 |
| S9-final-viral-combined_k141_124857\|\|full | 199218 | Yes | 56670 | 20 | 102 | Medium-quality | Genome-fragment | 65.17 | 71.55 |
| S12_k141_283835 | 1019 | No | NA | 0 | 2 | Low-quality | Genome-fragment | 1.23 | 0 |
| S9-final-viral-combined_k141_124865\|\|full | 7139 | No | NA | 0 | 4 | Not-determined | Genome-fragment | NA | 0 |
| S9-final-viral-combined_k141_55759\|\|full | 1214 | No | NA | 0 | 1 | Not-determined | Genome-fragment | NA | 0 |
| S9-final-viral-combined_k141_12802\|\|full | 1448 | No | NA | 0 | 2 | Not-determined | Genome-fragment | NA | 0 |
| S2-final-viral-combined_k141_182157\|\|full | 1019 | No | NA | 0 | 0 | Low-quality | Genome-fragment | 1.7 | 0 |
| S9-final-viral-combined_k141_124878\|\|full | 2691 | No | NA | 0 | 3 | Not-determined | Genome-fragment | NA | 0 |
| S4-final-viral-combined_k141_162829\|\|full | 1019 | No | NA | 0 | 0 | Low-quality | Genome-fragment | 1.72 | 0 |
| S9-final-viral-combined_k141_108776\|\|full | 5138 | No | NA | 0 | 3 | Not-determined | Genome-fragment | NA | 0 |
| S8-final-viral-combined_k141_243842\|\|lt2gene | 1019 | No | NA | 0 | 0 | Low-quality | Genome-fragment | 2.54 | 0 |
| S9-final-viral-combined_k141_23618\|\|full | 2897 | No | NA | 0 | 3 | Not-determined | Genome-fragment | NA | 0 |
| S9-final-viral-combined_k141_82741\|\|full | 1896 | No | NA | 0 | 1 | Not-determined | Genome-fragment | NA | 0 |
| S9-final-viral-combined_k141_44547\|\|full | 1782 | No | NA | 0 | 0 | Not-determined | Genome-fragment | NA | 0 |
| S8-final-viral-combined_k141_17005\|\|lt2gene | 1019 | No | NA | 1 | 0 | Low-quality | Genome-fragment | 2.37 | 0 |
| S9-final-viral-combined_k141_87838\|\|full | 1146 | No | NA | 0 | 0 | Not-determined | Genome-fragment | NA | 0 |
| S8_k141_135678 | 1019 | No | NA | 2 | 0 | Low-quality | Genome-fragment | 1.94 | 0 |
| S9-final-viral-combined_k141_23622\|\|full | 2001 | No | NA | 0 | 1 | Not-determined | Genome-fragment | NA | 0 |
| S11-final-viral-combined_k141_126019\|\|full | 1020 | No | NA | 0 | 1 | Low-quality | Genome-fragment | 1.6 | 0 |
| S9-final-viral-combined_k141_87857\|\|full | 5892 | No | NA | 0 | 1 | Not-determined | Genome-fragment | NA | 0 |
| S9-final-viral-combined_k141_28805\|\|full | 59739 | Yes | 35476 | 10 | 11 | Medium-quality | Genome-fragment | 67.17 | 40.62 |
| S9-final-viral-combined_k141_7880\|\|full | 4975 | No | NA | 0 | 5 | Not-determined | Genome-fragment | NA | 0 |
| S11-final-viral-combined_k141_104158\|\|lt2gene | 1020 | No | NA | 1 | 0 | Low-quality | Genome-fragment | 1.49 | 0 |
| S9-final-viral-combined_k141_33812\|\|full | 1866 | No | NA | 0 | 1 | Not-determined | Genome-fragment | NA | 0 |
| S9-final-viral-combined_k141_98281\|\|full | 2342 | No | NA | 0 | 1 | Not-determined | Genome-fragment | NA | 0 |
| S1-final-viral-combined_k141_109457\|\|full | 1020 | No | NA | 0 | 1 | Low-quality | Genome-fragment | 3.09 | 0 |
| S9-final-viral-combined_k141_66432\|\|full | 2332 | No | NA | 0 | 1 | Not-determined | Genome-fragment | NA | 0 |
| S5-final-viral-combined_k141_152915\|\|lt2gene | 1020 | No | NA | 1 | 0 | Low-quality | Genome-fragment | 2.71 | 0 |
| S7_k141_95862 | 1020 | No | NA | 0 | 1 | Low-quality | Genome-fragment | 1.4 | 0 |
| S9-final-viral-combined_k141_82840\|\|full | 1405 | No | NA | 0 | 1 | Not-determined | Genome-fragment | NA | 0 |
| S12_k141_6205 | 1020 | No | NA | 0 | 0 | Low-quality | Genome-fragment | 2.17 | 0 |
| S9-final-viral-combined_k141_66447\|\|full | 1112 | No | NA | 0 | 0 | Not-determined | Genome-fragment | NA | 0 |
| S12_k141_211237 | 1020 | No | NA | 0 | 1 | Low-quality | Genome-fragment | 2.66 | 0 |
| S9-final-viral-combined_k141_93068\|\|full | 1734 | No | NA | 0 | 0 | Not-determined | Genome-fragment | NA | 0 |
| S9-final-viral-combined_k141_2765\|\|full | 11811 | No | NA | 0 | 6 | Not-determined | Genome-fragment | NA | 0 |
| S2-final-viral-combined_k141_227005\|\|full | 1020 | No | NA | 1 | 0 | Low-quality | Genome-fragment | 0.75 | 0 |
| S2_k141_202342 | 1020 | No | NA | 0 | 0 | Low-quality | Genome-fragment | 2.43 | 0 |
| S4_k141_70502 | 1020 | No | NA | 0 | 0 | Low-quality | Genome-fragment | 0.51 | 0 |
| S9-final-viral-combined_k141_28888\|\|full | 2973 | No | NA | 0 | 1 | Not-determined | Genome-fragment | NA | 0 |
| S8-final-viral-combined_k141_119409\|\|lt2gene | 1020 | No | NA | 1 | 0 | Low-quality | Genome-fragment | 2.2 | 0 |
| S9-final-viral-combined_k141_33875\|\|full | 1529 | No | NA | 0 | 1 | Not-determined | Genome-fragment | NA | 0 |
| S9-final-viral-combined_k141_28895\|\|full | 1949 | No | NA | 0 | 1 | Not-determined | Genome-fragment | NA | 0 |
| S9-final-viral-combined_k141_119682\|\|full | 1425 | No | NA | 0 | 0 | Not-determined | Genome-fragment | NA | 0 |
| S9-final-viral-combined_k141_82431\|\|full | 1021 | No | NA | 0 | 1 | Low-quality | Genome-fragment | 1.03 | 0 |
| S9-final-viral-combined_k141_50775\|\|full | 1502 | No | NA | 0 | 0 | Not-determined | Genome-fragment | NA | 0 |
| S9-final-viral-combined_k141_125111\|\|full | 3267 | No | NA | 0 | 2 | Not-determined | Genome-fragment | NA | 0 |
| S9-final-viral-combined_k141_95764\|\|full | 1021 | No | NA | 0 | 0 | Low-quality | Genome-fragment | 1.98 | 0 |
| S11-final-viral-combined_k141_192434\|\|lt2gene | 1021 | No | NA | 1 | 0 | Low-quality | Genome-fragment | 1.77 | 0 |
| S1-final-viral-combined_k141_325030\|\|lt2gene | 1021 | No | NA | 1 | 0 | Low-quality | Genome-fragment | 1.72 | 0 |
| S9-final-viral-combined_k141_44647\|\|full | 2059 | No | NA | 0 | 0 | Not-determined | Genome-fragment | NA | 0 |
| S1_k141_112474 | 1021 | No | NA | 0 | 1 | Low-quality | Genome-fragment | 0.32 | 0 |
| S9-final-viral-combined_k141_88033\|\|full | 3145 | No | NA | 0 | 2 | Not-determined | Genome-fragment | NA | 0 |
| S9-final-viral-combined_k141_50813\|\|full | 1040 | No | NA | 0 | 0 | Not-determined | Genome-fragment | NA | 0 |
| S3-final-viral-combined_k141_87050\|\|full | 1021 | No | NA | 0 | 0 | Low-quality | Genome-fragment | 1.28 | 0 |
| S9-final-viral-combined_k141_119698\|\|full | 4567 | No | NA | 0 | 2 | Not-determined | Genome-fragment | NA | 0 |
| S9-final-viral-combined_k141_39711\|\|full | 1126 | No | NA | 0 | 0 | Not-determined | Genome-fragment | NA | 0 |
| S9-final-viral-combined_k141_114104\|\|full | 2300 | No | NA | 0 | 2 | Not-determined | Genome-fragment | NA | 0 |
| S9-final-viral-combined_k141_119729\|\|full | 3328 | No | NA | 0 | 2 | Not-determined | Genome-fragment | NA | 0 |
| S9-final-viral-combined_k141_83057\|\|full | 5541 | No | NA | 0 | 5 | Not-determined | Genome-fragment | NA | 0 |
| S9-final-viral-combined_k141_71647\|\|full | 22390 | No | NA | 11 | 2 | Medium-quality | Genome-fragment | 55.46 | 0 |
| S9-final-viral-combined_k141_18470\|\|full | 1224 | No | NA | 0 | 1 | Not-determined | Genome-fragment | NA | 0 |
| S9-final-viral-combined_k141_8026\|\|full | 2474 | No | NA | 0 | 0 | Not-determined | Genome-fragment | NA | 0 |
| S9-final-viral-combined_k141_50838\|\|full | 6286 | No | NA | 0 | 0 | Not-determined | Genome-fragment | NA | 0 |
| S9-final-viral-combined_k141_8034\|\|full | 1995 | No | NA | 0 | 1 | Not-determined | Genome-fragment | NA | 0 |
| S9-final-viral-combined_k141_88107\|\|full | 2337 | No | NA | 0 | 2 | Not-determined | Genome-fragment | NA | 0 |
| S17-final-viral-combined_k141_163289\|\|full | 1021 | No | NA | 0 | 0 | Low-quality | Genome-fragment | 2.01 | 0 |
| S9-final-viral-combined_k141_66624\|\|full | 1173 | No | NA | 0 | 0 | Not-determined | Genome-fragment | NA | 0 |
| S9-final-viral-combined_k141_18502\|\|full | 1123 | No | NA | 0 | 1 | Not-determined | Genome-fragment | NA | 0 |
| S9-final-viral-combined_k141_88130\|\|full | 1685 | No | NA | 0 | 1 | Not-determined | Genome-fragment | NA | 0 |
| S9-final-viral-combined_k141_18503\|\|full | 1386 | No | NA | 0 | 0 | Not-determined | Genome-fragment | NA | 0 |
| S12-final-viral-combined_k141_10219\|\|full | 1021 | No | NA | 0 | 0 | Low-quality | Genome-fragment | 2.41 | 0 |
| S9-final-viral-combined_k141_66639\|\|full | 4820 | No | NA | 0 | 1 | Not-determined | Genome-fragment | NA | 0 |
| S12_k141_10074 | 1021 | No | NA | 0 | 0 | Low-quality | Genome-fragment | 2.6 | 0 |
| S2_k141_116060 | 1021 | No | NA | 2 | 0 | Low-quality | Genome-fragment | 2.6 | 0 |
| S9-final-viral-combined_k141_18555\|\|full | 3614 | No | NA | 0 | 3 | Not-determined | Genome-fragment | NA | 0 |
| S9-final-viral-combined_k141_29025\|\|full | 1237 | No | NA | 0 | 1 | Not-determined | Genome-fragment | NA | 0 |
| S9-final-viral-combined_k141_56119\|\|full | 2288 | No | NA | 0 | 2 | Not-determined | Genome-fragment | NA | 0 |
| S4-final-viral-combined_k141_45340\|\|full | 1021 | No | NA | 0 | 0 | Low-quality | Genome-fragment | 0.7 | 0 |
| S8-final-viral-combined_k141_133333\|\|full | 1021 | No | NA | 1 | 0 | Low-quality | Genome-fragment | 0.66 | 0 |
| S9-final-viral-combined_k141_83158\|\|full | 2228 | No | NA | 0 | 0 | Not-determined | Genome-fragment | NA | 0 |
| S9-final-viral-combined_k141_18579\|\|full | 1519 | No | NA | 0 | 1 | Not-determined | Genome-fragment | NA | 0 |
| S8_k141_213008 | 1021 | No | NA | 0 | 0 | Low-quality | Genome-fragment | 4.89 | 0 |
| S9-final-viral-combined_k141_18601\|\|full | 1654 | No | NA | 0 | 1 | Not-determined | Genome-fragment | NA | 0 |
| S9-final-viral-combined_k141_33862\|\|lt2gene | 1022 | No | NA | 1 | 0 | Low-quality | Genome-fragment | 2.24 | 0 |
| S9-final-viral-combined_k141_76914\|\|full | 2127 | No | NA | 0 | 0 | Not-determined | Genome-fragment | NA | 0 |
| S9-final-viral-combined_k141_23827\|\|full | 2849 | No | NA | 0 | 1 | Not-determined | Genome-fragment | NA | 0 |
| S9-final-viral-combined_k141_2960\|\|full | 1872 | No | NA | 0 | 1 | Not-determined | Genome-fragment | NA | 0 |
| S9-final-viral-combined_k141_8095\|\|full | 5415 | No | NA | 0 | 1 | Not-determined | Genome-fragment | NA | 0 |
| S9-final-viral-combined_k141_119905\|\|full | 2428 | No | NA | 0 | 3 | Not-determined | Genome-fragment | NA | 0 |
| S9-final-viral-combined_k141_83225\|\|full | 2244 | No | NA | 0 | 1 | Not-determined | Genome-fragment | NA | 0 |
| S9-final-viral-combined_k141_83231\|\|full | 5975 | No | NA | 0 | 6 | Not-determined | Genome-fragment | NA | 0 |
| S1-final-viral-combined_k141_149430\|\|full | 1022 | No | NA | 0 | 1 | Low-quality | Genome-fragment | 2.43 | 0 |
| S9-final-viral-combined_k141_44815\|\|full | 1896 | No | NA | 0 | 2 | Not-determined | Genome-fragment | NA | 0 |
| S9-final-viral-combined_k141_119941\|\|full | 1163 | No | NA | 0 | 0 | Not-determined | Genome-fragment | NA | 0 |
| S9-final-viral-combined_k141_76946\|\|full | 2742 | No | NA | 0 | 2 | Not-determined | Genome-fragment | NA | 0 |
| S9-final-viral-combined_k141_93232\|\|full | 50669 | Yes | 44439 | 33 | 6 | High-quality | High-quality | 100 | 12.3 |
| S9-final-viral-combined_k141_29073\|\|full | 3187 | No | NA | 0 | 3 | Not-determined | Genome-fragment | NA | 0 |
| S1-final-viral-combined_k141_241242\|\|full | 1022 | No | NA | 0 | 0 | Low-quality | Genome-fragment | 1.02 | 0 |
| S1-final-viral-combined_k141_175645\|\|lt2gene | 1022 | No | NA | 1 | 0 | Low-quality | Genome-fragment | 2.78 | 0 |
| S9-final-viral-combined_k141_29084\|\|full | 1338 | No | NA | 0 | 1 | Not-determined | Genome-fragment | NA | 0 |
| S9-final-viral-combined_k141_83270\|\|full | 1178 | No | NA | 0 | 0 | Not-determined | Genome-fragment | NA | 0 |
| S9-final-viral-combined_k141_119966\|\|full | 1066 | No | NA | 0 | 2 | Not-determined | Genome-fragment | NA | 0 |
| S9-final-viral-combined_k141_12969\|\|full | 6441 | No | NA | 0 | 0 | Not-determined | Genome-fragment | NA | 0 |
| S1_k141_160136 | 1022 | No | NA | 0 | 0 | Low-quality | Genome-fragment | 1.71 | 0 |
| S9-final-viral-combined_k141_18698\|\|full | 1753 | No | NA | 0 | 0 | Not-determined | Genome-fragment | NA | 0 |
| S9-final-viral-combined_k141_98450\|\|full | 1192 | No | NA | 0 | 2 | Not-determined | Genome-fragment | NA | 0 |
| S1_k141_391403 | 1022 | No | NA | 0 | 0 | Low-quality | Genome-fragment | 1.25 | 0 |
| S5-final-viral-combined_k141_56158\|\|full | 1022 | No | NA | 3 | 0 | Low-quality | Genome-fragment | 3.2 | 0 |
| S5-final-viral-combined_k141_5641\|\|full | 1022 | No | NA | 0 | 0 | Low-quality | Genome-fragment | 3 | 0 |
| S9-final-viral-combined_k141_18726\|\|full | 2122 | No | NA | 0 | 1 | Not-determined | Genome-fragment | NA | 0 |
| S5-final-viral-combined_k141_67030\|\|full | 1022 | No | NA | 2 | 0 | Low-quality | Genome-fragment | 3.03 | 0 |
| S7-final-viral-combined_k141_151242\|\|full | 1022 | No | NA | 0 | 1 | Low-quality | Genome-fragment | 2.23 | 0 |
| S17-k141_11498 | 1022 | No | NA | 0 | 0 | Low-quality | Genome-fragment | 2.29 | 0 |
| S9-final-viral-combined_k141_18730\|\|full | 1529 | No | NA | 0 | 1 | Not-determined | Genome-fragment | NA | 0 |
| S17-k141_196673 | 1022 | No | NA | 1 | 0 | Low-quality | Genome-fragment | 16.44 | 0 |
| S12-final-viral-combined_k141_89262\|\|full | 1022 | No | NA | 1 | 0 | Low-quality | Genome-fragment | 2.04 | 0 |
| S9-final-viral-combined_k141_18749\|\|full | 3719 | No | NA | 0 | 0 | Not-determined | Genome-fragment | NA | 0 |
| S9-final-viral-combined_k141_50940\|\|full | 3017 | No | NA | 0 | 0 | Not-determined | Genome-fragment | NA | 0 |
| S12-final-viral-combined_k141_279240\|\|full | 1022 | No | NA | 0 | 0 | Low-quality | Genome-fragment | 0.74 | 0 |
| S9-final-viral-combined_k141_114343\|\|full | 2566 | No | NA | 0 | 2 | Not-determined | Genome-fragment | NA | 0 |
| S12-final-viral-combined_k141_164717\|\|full | 1022 | No | NA | 1 | 0 | Low-quality | Genome-fragment | 1.41 | 0 |
| S9-final-viral-combined_k141_114356\|\|full | 1453 | No | NA | 0 | 2 | Not-determined | Genome-fragment | NA | 0 |
| S12_k141_387881 | 1022 | No | NA | 1 | 0 | Low-quality | Genome-fragment | 2.79 | 0 |
| S9-final-viral-combined_k141_108985\|\|full | 1433 | No | NA | 0 | 2 | Not-determined | Genome-fragment | NA | 0 |
| S9-final-viral-combined_k141_61619\|\|full | 2655 | No | NA | 0 | 0 | Not-determined | Genome-fragment | NA | 0 |
| S9-final-viral-combined_k141_93347\|\|full | 1293 | No | NA | 0 | 0 | Not-determined | Genome-fragment | NA | 0 |
| S9-final-viral-combined_k141_83368\|\|full | 2461 | No | NA | 0 | 3 | Not-determined | Genome-fragment | NA | 0 |
| S2-final-viral-combined_k141_72501\|\|lt2gene | 1022 | No | NA | 1 | 0 | Low-quality | Genome-fragment | 2.24 | 0 |
| S2_k141_84139 | 1022 | No | NA | 0 | 0 | Low-quality | Genome-fragment | 1.69 | 0 |
| S8-final-viral-combined_k141_249966\|\|full | 1022 | No | NA | 0 | 0 | Low-quality | Genome-fragment | 2.23 | 0 |
| S11-final-viral-combined_k141_255435\|\|lt2gene | 1023 | No | NA | 1 | 0 | Low-quality | Genome-fragment | 1.44 | 0 |
| S1_k141_237366 | 1023 | No | NA | 1 | 0 | Low-quality | Genome-fragment | 0.94 | 0 |

| | | | | | | | | | | | |
|---|---|---|---|---|---|---|---|---|---|---|---|
| S9-final-viral-combined_k141_120040\|\|full | 1487 | No | NA | | 0 | 1 | Not-determined | Genome-fragment | NA | | 0 |
| S1_k141_129549 | 1023 | No | NA | | 0 | 0 | Low-quality | Genome-fragment | | 0.66 | 0 |
| S1_k141_195477 | 1023 | No | NA | | 0 | 1 | Low-quality | Genome-fragment | | 0.31 | 0 |
| S5-final-viral-combined_k141_39408\|\|full | 1023 | No | NA | | 1 | 0 | Low-quality | Genome-fragment | | 2.14 | 0 |
| S9-final-viral-combined_k141_98520\|\|full | 1402 | No | NA | | 0 | 0 | Not-determined | Genome-fragment | NA | | 0 |
| S5_k141_238 | 1023 | No | NA | | 0 | 1 | Low-quality | Genome-fragment | | 0.53 | 0 |
| S7-final-viral-combined_k141_88405\|\|full | 1023 | No | NA | | 1 | 0 | Low-quality | Genome-fragment | | 2.21 | 0 |
| S17-final-viral-combined_k141_188638\|\|full | 1023 | No | NA | | 0 | 0 | Low-quality | Genome-fragment | | 2.41 | 0 |
| S9-final-viral-combined_k141_24056\|\|full | 3037 | No | NA | | 0 | 1 | Not-determined | Genome-fragment | NA | | 0 |
| S9-final-viral-combined_k141_13151\|\|full | 1249 | No | NA | | 0 | 1 | Not-determined | Genome-fragment | NA | | 0 |
| S9-final-viral-combined_k141_71908\|\|full | 3486 | No | NA | | 0 | 5 | Not-determined | Genome-fragment | NA | | 0 |
| S9-final-viral-combined_k141_8347\|\|full | 1115 | No | NA | | 0 | 1 | Not-determined | Genome-fragment | NA | | 0 |
| S17-final-viral-combined_k141_32009\|\|full | 1023 | No | NA | | 1 | 0 | Low-quality | Genome-fragment | | 2.75 | 0 |
| S17-final-viral-combined_k141_99708\|\|full | 1023 | No | NA | | 0 | 0 | Low-quality | Genome-fragment | | 1.06 | 0 |
| S9-final-viral-combined_k141_45052\|\|full | 5219 | No | NA | | 0 | 5 | Not-determined | Genome-fragment | NA | | 0 |
| S2_k141_43142 | 1023 | No | NA | | 0 | 0 | Low-quality | Genome-fragment | | 1.59 | 0 |
| S4-final-viral-combined_k141_134748\|\|full | 1023 | No | NA | | 1 | 0 | Low-quality | Genome-fragment | | 1.66 | 0 |
| S9-final-viral-combined_k141_13250\|\|full | 2950 | No | NA | | 0 | 1 | Not-determined | Genome-fragment | NA | | 0 |
| S9-final-viral-combined_k141_18893\|\|full | 2593 | No | NA | | 0 | 1 | Not-determined | Genome-fragment | NA | | 0 |
| S9-final-viral-combined_k141_39921\|\|full | 1353 | No | NA | | 0 | 1 | Not-determined | Genome-fragment | NA | | 0 |
| S9-final-viral-combined_k141_61670\|\|full | 1314 | No | NA | | 0 | 2 | Not-determined | Genome-fragment | NA | | 0 |
| S9-final-viral-combined_k141_13280\|\|full | 1198 | No | NA | | 0 | 0 | Not-determined | Genome-fragment | NA | | 0 |
| S9-final-viral-combined_k141_77206\|\|full | 2522 | No | NA | | 0 | 0 | Not-determined | Genome-fragment | NA | | 0 |
| S9-final-viral-combined_k141_45088\|\|full | 1498 | No | NA | | 0 | 1 | Not-determined | Genome-fragment | NA | | 0 |
| S9-final-viral-combined_k141_98595\|\|full | 2015 | No | NA | | 0 | 2 | Not-determined | Genome-fragment | NA | | 0 |
| S4-final-viral-combined_k141_44350\|\|full | 1023 | No | NA | | 0 | 0 | Low-quality | Genome-fragment | | 2.03 | 0 |
| S9-final-viral-combined_k141_18947\|\|full | 1757 | No | NA | | 0 | 0 | Not-determined | Genome-fragment | NA | | 0 |
| S9-final-viral-combined_k141_109090\|\|full | 3918 | No | NA | | 0 | 4 | Not-determined | Genome-fragment | NA | | 0 |
| S9-final-viral-combined_k141_13308\|\|full | 1729 | No | NA | | 0 | 0 | Not-determined | Genome-fragment | NA | | 0 |
| S9-final-viral-combined_k141_45113\|\|full | 1362 | No | NA | | 0 | 0 | Not-determined | Genome-fragment | NA | | 0 |
| S9-final-viral-combined_k141_8440\|\|full | 7592 | No | NA | | 0 | 1 | Not-determined | Genome-fragment | NA | | 0 |
| S9-final-viral-combined_k141_13325\|\|full | 3334 | No | NA | | 0 | 3 | Not-determined | Genome-fragment | NA | | 0 |
| S4-final-viral-combined_k141_156839\|\|full | 1023 | No | NA | | 0 | 2 | Low-quality | Genome-fragment | | 0.56 | 0 |
| S4_k141_114656 | 1023 | No | NA | | 2 | 0 | Low-quality | Genome-fragment | | 1.46 | 0 |
| S6-final-viral-combined_k141_82446\|\|full | 1023 | No | NA | | 0 | 0 | Low-quality | Genome-fragment | | 0.95 | 0 |
| S8-final-viral-combined_k141_20976\|\|full | 1023 | No | NA | | 0 | 0 | Low-quality | Genome-fragment | | 2.3 | 0 |
| S8_k141_173869 | 1023 | No | NA | | 0 | 0 | Low-quality | Genome-fragment | | 2.01 | 0 |
| S9-final-viral-combined_k141_88795\|\|full | 1024 | No | NA | | 1 | 0 | Low-quality | Genome-fragment | | 1.27 | 0 |
| S9_k141_53384 | 1024 | No | NA | | 2 | 0 | Low-quality | Genome-fragment | | 2.66 | 0 |
| S11-final-viral-combined_k141_294408\|\|full | 1024 | No | NA | | 1 | 0 | Low-quality | Genome-fragment | | 1.04 | 0 |
| S11_k141_225185 | 1024 | No | NA | | 0 | 1 | Low-quality | Genome-fragment | | 0.44 | 0 |
| S1-final-viral-combined_k141_247126\|\|full | 1024 | No | NA | | 1 | 0 | Low-quality | Genome-fragment | | 1.73 | 0 |
| S9-final-viral-combined_k141_51131\|\|full | 1876 | No | NA | | 0 | 1 | Not-determined | Genome-fragment | NA | | 0 |
| S3-final-viral-combined_k141_112598\|\|lt2gene | 1024 | No | NA | | 1 | 0 | Low-quality | Genome-fragment | | 2.45 | 0 |
| S9-final-viral-combined_k141_125354\|\|full | 1849 | No | NA | | 0 | 1 | Not-determined | Genome-fragment | NA | | 0 |
| S3-final-viral-combined_k141_136988\|\|lt2gene | 1024 | No | NA | | 1 | 0 | Low-quality | Genome-fragment | | 2.6 | 0 |
| S5-final-viral-combined_k141_4356\|\|full | 1024 | No | NA | | 0 | 0 | Low-quality | Genome-fragment | | 1.76 | 0 |
| S9-final-viral-combined_k141_77343\|\|full | 1293 | No | NA | | 0 | 2 | Not-determined | Genome-fragment | NA | | 0 |
| S9-final-viral-combined_k141_71973\|\|full | 2954 | No | NA | | 0 | 2 | Not-determined | Genome-fragment | NA | | 0 |
| S5-final-viral-combined_k141_92790\|\|full | 1024 | No | NA | | 1 | 0 | Low-quality | Genome-fragment | | 2.2 | 0 |
| S9-final-viral-combined_k141_45238\|\|full | 2265 | No | NA | | 0 | 2 | Not-determined | Genome-fragment | NA | | 0 |
| S5-final-viral-combined_k141_40719\|\|full | 1024 | No | NA | | 0 | 0 | Low-quality | Genome-fragment | | 0.99 | 0 |
| S9-final-viral-combined_k141_77355\|\|full | 5721 | No | NA | | 0 | 0 | Not-determined | Genome-fragment | NA | | 0 |
| S9-final-viral-combined_k141_8506\|\|full | 2385 | No | NA | | 0 | 1 | Not-determined | Genome-fragment | NA | | 0 |
| S9-final-viral-combined_k141_66732\|\|full | 1491 | No | NA | | 0 | 2 | Not-determined | Genome-fragment | NA | | 0 |
| S5-final-viral-combined_k141_69336\|\|lt2gene | 1024 | No | NA | | 1 | 0 | Low-quality | Genome-fragment | | 0.63 | 0 |
| S17-final-viral-combined_k141_207471\|\|full | 1024 | No | NA | | 2 | 0 | Low-quality | Genome-fragment | | 1.81 | 0 |
| S9-final-viral-combined_k141_8514\|\|full | 2468 | No | NA | | 0 | 3 | Not-determined | Genome-fragment | NA | | 0 |
| S17-final-viral-combined_k141_125690\|\|lt2gene | 1024 | No | NA | | 1 | 0 | Low-quality | Genome-fragment | | 2.33 | 0 |
| S9-final-viral-combined_k141_56467\|\|full | 2131 | No | NA | | 0 | 0 | Not-determined | Genome-fragment | NA | | 0 |
| S17-k141_111422 | 1024 | No | NA | | 0 | 0 | Low-quality | Genome-fragment | | 3.52 | 0 |
| S12-final-viral-combined_k141_149637\|\|lt2gene | 1024 | No | NA | | 0 | 2 | Low-quality | Genome-fragment | | 1.1 | 0 |
| S9-final-viral-combined_k141_45266\|\|full | 2688 | No | NA | | 0 | 2 | Not-determined | Genome-fragment | NA | | 0 |
| S9-final-viral-combined_k141_13390\|\|full | 5225 | No | NA | | 0 | 4 | Not-determined | Genome-fragment | NA | | 0 |
| S9-final-viral-combined_k141_66758\|\|full | 2204 | No | NA | | 0 | 2 | Not-determined | Genome-fragment | NA | | 0 |
| S12_k141_374763 | 1024 | No | NA | | 1 | 0 | Low-quality | Genome-fragment | | 3.03 | 0 |
| S9-final-viral-combined_k141_61819\|\|full | 2252 | No | NA | | 0 | 0 | Not-determined | Genome-fragment | NA | | 0 |
| S4-final-viral-combined_k141_152675\|\|full | 1024 | No | NA | | 0 | 1 | Low-quality | Genome-fragment | | 2.06 | 0 |
| S4_k141_50719 | 1024 | No | NA | | 0 | 0 | Low-quality | Genome-fragment | | 8.27 | 0 |
| S8-final-viral-combined_k141_53162\|\|full | 1024 | No | NA | | 0 | 1 | Low-quality | Genome-fragment | | 2.18 | 0 |
| S8_k141_82387 | 1024 | No | NA | | 0 | 1 | Low-quality | Genome-fragment | | 0.77 | 0 |
| S9-final-viral-combined_k141_13432\|\|full | 3168 | No | NA | | 0 | 0 | Not-determined | Genome-fragment | NA | | 0 |
| S9-final-viral-combined_k141_93538\|\|full | 1091 | No | NA | | 0 | 1 | Not-determined | Genome-fragment | NA | | 0 |
| S9-final-viral-combined_k141_77412\|\|full | 1525 | No | NA | | 0 | 0 | Not-determined | Genome-fragment | NA | | 0 |
| S9-final-viral-combined_k141_99856\|\|full | 1025 | No | NA | | 3 | 0 | Low-quality | Genome-fragment | | 2.22 | 0 |
| S9-final-viral-combined_k141_47584\|\|full | 1025 | No | NA | | 0 | 1 | Low-quality | Genome-fragment | | 0.79 | 0 |
| S9-final-viral-combined_k141_19081\|\|full | 2596 | No | NA | | 0 | 1 | Not-determined | Genome-fragment | NA | | 0 |
| S9-final-viral-combined_k141_66822\|\|full | 2123 | No | NA | | 0 | 1 | Not-determined | Genome-fragment | NA | | 0 |
| S11-final-viral-combined_k141_235062\|\|full | 1025 | No | NA | | 0 | 0 | Low-quality | Genome-fragment | | 1.79 | 0 |
| S9-final-viral-combined_k141_29350\|\|full | 1174 | No | NA | | 0 | 2 | Not-determined | Genome-fragment | NA | | 0 |
| S9-final-viral-combined_k141_93553\|\|full | 3376 | No | NA | | 0 | 3 | Not-determined | Genome-fragment | NA | | 0 |
| S11-final-viral-combined_k141_180500\|\|full | 1025 | No | NA | | 0 | 0 | Low-quality | Genome-fragment | | 2.41 | 0 |
| S9-final-viral-combined_k141_93556\|\|full | 1847 | No | NA | | 0 | 2 | Not-determined | Genome-fragment | NA | | 0 |
| S9-final-viral-combined_k141_24316\|\|full | 1096 | No | NA | | 0 | 0 | Not-determined | Genome-fragment | NA | | 0 |
| S9-final-viral-combined_k141_3210\|\|full | 2124 | No | NA | | 0 | 2 | Not-determined | Genome-fragment | NA | | 0 |
| S1-final-viral-combined_k141_162810\|\|full | 1025 | No | NA | | 1 | 0 | Low-quality | Genome-fragment | | 1.9 | 0 |
| S3-final-viral-combined_k141_35604\|\|full | 1025 | No | NA | | 0 | 0 | Low-quality | Genome-fragment | | 1.64 | 0 |
| S9-final-viral-combined_k141_29363\|\|full | 1617 | No | NA | | 0 | 1 | Not-determined | Genome-fragment | NA | | 0 |
| S9-final-viral-combined_k141_56567\|\|full | 2455 | No | NA | | 0 | 2 | Not-determined | Genome-fragment | NA | | 0 |
| S9-final-viral-combined_k141_3232\|\|full | 1003 | No | NA | | 0 | 0 | Not-determined | Genome-fragment | NA | | 0 |
| S9-final-viral-combined_k141_77472\|\|full | 2144 | No | NA | | 0 | 3 | Not-determined | Genome-fragment | NA | | 0 |
| S5-final-viral-combined_k141_78822\|\|lt2gene | 1025 | No | NA | | 1 | 0 | Low-quality | Genome-fragment | | 2.16 | 0 |
| S9-final-viral-combined_k141_3241\|\|full | 1726 | No | NA | | 0 | 0 | Not-determined | Genome-fragment | NA | | 0 |
| S9-final-viral-combined_k141_109158\|\|full | 1410 | No | NA | | 0 | 2 | Not-determined | Genome-fragment | NA | | 0 |
| S9-final-viral-combined_k141_98830\|\|full | 8992 | No | NA | | 0 | 0 | Not-determined | Genome-fragment | NA | | 0 |
| S7-final-viral-combined_k141_196100\|\|full | 1025 | No | NA | | 1 | 0 | Low-quality | Genome-fragment | | 1.98 | 0 |
| S9-final-viral-combined_k141_83693\|\|full | 1497 | No | NA | | 0 | 0 | Not-determined | Genome-fragment | NA | | 0 |
| S9-final-viral-combined_k141_66896\|\|full | 1651 | No | NA | | 0 | 0 | Not-determined | Genome-fragment | NA | | 0 |
| S9-final-viral-combined_k141_77512\|\|full | 2054 | No | NA | | 0 | 2 | Not-determined | Genome-fragment | NA | | 0 |
| S17-final-viral-combined_k141_228466\|\|full | 1025 | No | NA | | 0 | 0 | Low-quality | Genome-fragment | | 1.41 | 0 |
| S9-final-viral-combined_k141_34618\|\|full | 1260 | No | NA | | 0 | 1 | Not-determined | Genome-fragment | NA | | 0 |
| S9-final-viral-combined_k141_40227\|\|full | 14586 | No | NA | | 0 | 2 | Not-determined | Genome-fragment | NA | | 0 |
| S9-final-viral-combined_k141_114849\|\|full | 1294 | No | NA | | 0 | 0 | Not-determined | Genome-fragment | NA | | 0 |
| S9-final-viral-combined_k141_45423\|\|full | 2337 | No | NA | | 0 | 2 | Not-determined | Genome-fragment | NA | | 0 |
| S12-final-viral-combined_k141_93996\|\|full | 1025 | No | NA | | 0 | 1 | Low-quality | Genome-fragment | | 0.66 | 0 |
| S12-final-viral-combined_k141_65288\|\|full | 1025 | No | NA | | 1 | 0 | Low-quality | Genome-fragment | | 2.79 | 0 |
| S12_k141_344875 | 1025 | No | NA | | 0 | 0 | Low-quality | Genome-fragment | | 0.2 | 0 |
| S2-final-viral-combined_k141_127476\|\|full | 1025 | No | NA | | 0 | 2 | Low-quality | Genome-fragment | | 0.48 | 0 |
| S9-final-viral-combined_k141_40235\|\|full | 2632 | No | NA | | 0 | 2 | Not-determined | Genome-fragment | NA | | 0 |
| S9-final-viral-combined_k141_40244\|\|full | 1779 | No | NA | | 0 | 0 | Not-determined | Genome-fragment | NA | | 0 |
| S4_k141_24006 | 1025 | No | NA | | 1 | 0 | Low-quality | Genome-fragment | | 0.51 | 0 |
| S9-final-viral-combined_k141_114890\|\|full | 1523 | No | NA | | 0 | 1 | Not-determined | Genome-fragment | NA | | 0 |
| S9-final-viral-combined_k141_56665\|\|full | 1414 | No | NA | | 0 | 2 | Not-determined | Genome-fragment | NA | | 0 |
| S4_k141_143268 | 1025 | No | NA | | 0 | 0 | Low-quality | Genome-fragment | | 1.85 | 0 |
| S9-final-viral-combined_k141_72175\|\|full | 3340 | No | NA | | 0 | 2 | Not-determined | Genome-fragment | NA | | 0 |
| S4_k141_53134 | 1025 | No | NA | | 0 | 1 | Low-quality | Genome-fragment | | 2.34 | 0 |
| S8-final-viral-combined_k141_26172\|\|full | 1025 | No | NA | | 2 | 0 | Low-quality | Genome-fragment | | 2.73 | 0 |
| S8-final-viral-combined_k141_189837\|\|full | 1025 | No | NA | | 0 | 0 | Low-quality | Genome-fragment | | 0.67 | 0 |
| S9-final-viral-combined_k141_66940\|\|full | 4728 | No | NA | | 0 | 4 | Not-determined | Genome-fragment | NA | | 0 |
| S9-final-viral-combined_k141_56714\|\|full | 1329 | No | NA | | 0 | 1 | Not-determined | Genome-fragment | NA | | 0 |
| S8_k141_204889 | 1025 | No | NA | | 1 | 0 | Low-quality | Genome-fragment | | 1.82 | 0 |
| S9-final-viral-combined_k141_8611\|\|full | 5725 | No | NA | | 0 | 5 | Not-determined | Genome-fragment | NA | | 0 |
| S9-final-viral-combined_k141_56720\|\|full | 2613 | No | NA | | 0 | 3 | Not-determined | Genome-fragment | NA | | 0 |
| S11_k141_112816 | 1026 | No | NA | | 0 | 0 | Low-quality | Genome-fragment | | 2.54 | 0 |
| S9-final-viral-combined_k141_51266\|\|full | 2680 | No | NA | | 0 | 1 | Not-determined | Genome-fragment | NA | | 0 |
| S1-final-viral-combined_k141_22904\|\|full | 1026 | No | NA | | 2 | 0 | Low-quality | Genome-fragment | | 1.75 | 0 |
| S1-final-viral-combined_k141_72382\|\|full | 1026 | No | NA | | 0 | 0 | Low-quality | Genome-fragment | | 0.8 | 0 |
| S9-final-viral-combined_k141_8653\|\|full | 1076 | No | NA | | 0 | 1 | Not-determined | Genome-fragment | NA | | 0 |
| S1-final-viral-combined_k141_393408\|\|full | 1026 | No | NA | | 1 | 0 | Low-quality | Genome-fragment | | 2.31 | 0 |
| S9-final-viral-combined_k141_98965\|\|full | 3473 | No | NA | | 0 | 1 | Not-determined | Genome-fragment | NA | | 0 |
| S7_k141_178289 | 1026 | No | NA | | 0 | 0 | Low-quality | Genome-fragment | | 1.25 | 0 |
| S17-final-viral-combined_k141_203145\|\|full | 1026 | No | NA | | 0 | 0 | Low-quality | Genome-fragment | | 2.11 | 0 |
| S17-final-viral-combined_k141_13519\|\|full | 1026 | No | NA | | 1 | 0 | Low-quality | Genome-fragment | | 2.43 | 0 |
| S17-final-viral-combined_k141_161615\|\|full | 1026 | No | NA | | 1 | 0 | Low-quality | Genome-fragment | | 0.79 | 0 |
| S9-final-viral-combined_k141_120604\|\|full | 1771 | No | NA | | 0 | 1 | Not-determined | Genome-fragment | NA | | 0 |
| S12-final-viral-combined_k141_376490\|\|full | 1026 | Yes | | 336 | 1 | 1 | Low-quality | Genome-fragment | | 0.56 | 67.25 |
| S9-final-viral-combined_k141_114979\|\|full | 8497 | No | NA | | 0 | 6 | Not-determined | Genome-fragment | NA | | 0 |
| S12-final-viral-combined_k141_308974\|\|lt2gene | 1026 | No | NA | | 1 | 0 | Low-quality | Genome-fragment | | 1.97 | 0 |
| S12_k141_307864 | 1026 | No | NA | | 0 | 1 | Low-quality | Genome-fragment | | 2.08 | 0 |
| S12_k141_50028 | 1026 | No | NA | | 1 | 0 | Low-quality | Genome-fragment | | 0.76 | 0 |
| S9-final-viral-combined_k141_83831\|\|full | 2398 | No | NA | | 0 | 2 | Not-determined | Genome-fragment | NA | | 0 |
| S9-final-viral-combined_k141_62019\|\|full | 1471 | No | NA | | 0 | 1 | Not-determined | Genome-fragment | NA | | 0 |
| S9-final-viral-combined_k141_34793\|\|full | 1044 | No | NA | | 0 | 0 | Not-determined | Genome-fragment | NA | | 0 |
| S4-final-viral-combined_k141_14051\|\|full | 1026 | No | NA | | 0 | 0 | Low-quality | Genome-fragment | | 0.99 | 0 |
| S9-final-viral-combined_k141_125608\|\|full | 2340 | No | NA | | 0 | 1 | Not-determined | Genome-fragment | NA | | 0 |
| S9-final-viral-combined_k141_34810\|\|full | 5601 | No | NA | | 0 | 2 | Not-determined | Genome-fragment | NA | | 0 |
| S9-final-viral-combined_k141_88450\|\|full | 4599 | No | NA | | 0 | 3 | Not-determined | Genome-fragment | NA | | 0 |
| S9-final-viral-combined_k141_34821\|\|full | 1281 | No | NA | | 0 | 2 | Not-determined | Genome-fragment | NA | | 0 |
| S9-final-viral-combined_k141_120630\|\|full | 1928 | No | NA | | 0 | 1 | Not-determined | Genome-fragment | NA | | 0 |
| S9-final-viral-combined_k141_13607\|\|full | 13483 | No | NA | | 0 | 5 | Not-determined | Genome-fragment | NA | | 0 |
| S4-final-viral-combined_k141_114151\|\|full | 1026 | No | NA | | 0 | 0 | Low-quality | Genome-fragment | | 1.29 | 0 |
| S4_k141_53745 | 1026 | No | NA | | 0 | 1 | Low-quality | Genome-fragment | | 1.94 | 0 |
| S9-final-viral-combined_k141_83909\|\|full | 1732 | No | NA | | 0 | 2 | Not-determined | Genome-fragment | NA | | 0 |
| S8-final-viral-combined_k141_70284\|\|lt2gene | 1026 | No | NA | | 1 | 0 | Low-quality | Genome-fragment | | 2.72 | 0 |
| S9-final-viral-combined_k141_88497\|\|full | 3192 | No | NA | | 0 | 2 | Not-determined | Genome-fragment | NA | | 0 |
| S9-final-viral-combined_k141_120676\|\|full | 3921 | No | NA | | 0 | 3 | Not-determined | Genome-fragment | NA | | 0 |
| S9_k141_32515 | 1027 | No | NA | | 0 | 0 | Low-quality | Genome-fragment | | 8.41 | 0 |
| S11-final-viral-combined_k141_126059\|\|full | 1027 | No | NA | | 1 | 0 | Low-quality | Genome-fragment | | 2.35 | 0 |
| S9-final-viral-combined_k141_104589\|\|full | 3022 | No | NA | | 0 | 1 | Not-determined | Genome-fragment | NA | | 0 |
| S9-final-viral-combined_k141_125651\|\|full | 2483 | No | NA | | 0 | 3 | Not-determined | Genome-fragment | NA | | 0 |
| S9-final-viral-combined_k141_109300\|\|full | 1489 | No | NA | | 0 | 1 | Not-determined | Genome-fragment | NA | | 0 |
| S9-final-viral-combined_k141_93772\|\|full | 1090 | No | NA | | 0 | 0 | Not-determined | Genome-fragment | NA | | 0 |
| S11-final-viral-combined_k141_78255\|\|lt2gene | 1027 | No | NA | | 1 | 0 | Low-quality | Genome-fragment | | 2.48 | 0 |
| S1-final-viral-combined_k141_148858\|\|full | 1027 | No | NA | | 1 | 0 | Low-quality | Genome-fragment | | 2.45 | 0 |
| S9-final-viral-combined_k141_99059\|\|full | 2339 | No | NA | | 0 | 1 | Not-determined | Genome-fragment | NA | | 0 |
| S5-final-viral-combined_k141_111468\|\|full | 1027 | No | NA | | 0 | 0 | Low-quality | Genome-fragment | | 0.7 | 0 |
| S9-final-viral-combined_k141_99107\|\|full | 2957 | No | NA | | 0 | 3 | Not-determined | Genome-fragment | NA | | 0 |
| S9-final-viral-combined_k141_88518\|\|full | 3216 | No | NA | | 0 | 0 | Not-determined | Genome-fragment | NA | | 0 |
| S9-final-viral-combined_k141_19273\|\|full | 1469 | No | NA | | 0 | 1 | Not-determined | Genome-fragment | NA | | 0 |
| S5-final-viral-combined_k141_52499\|\|full | 1027 | No | NA | | 0 | 0 | Low-quality | Genome-fragment | | 1.95 | 0 |
| S9-final-viral-combined_k141_24557\|\|full | 3704 | No | NA | | 0 | 3 | Not-determined | Genome-fragment | NA | | 0 |
| S7-final-viral-combined_k141_226465\|\|full | 1027 | No | NA | | 0 | 0 | Low-quality | Genome-fragment | | 2.32 | 0 |
| S9-final-viral-combined_k141_115022\|\|full | 3414 | No | NA | | 0 | 2 | Not-determined | Genome-fragment | NA | | 0 |
| S7-final-viral-combined_k141_203658\|\|full | 1027 | No | NA | | 0 | 0 | Low-quality | Genome-fragment | | 2.56 | 0 |

| | | | | | | | | | |
|---|---|---|---|---|---|---|---|---|---|
| S9-final-viral-combined_k141_120727\|\|full | 2149 | No | NA | 0 | 3 | Not-determined | Genome-fragment | NA | 0 |
| S7-final-viral-combined_k141_67453\|\|lt2gene | 1027 | No | NA | 1 | 0 | Low-quality | Genome-fragment | 19.49 | 0 |
| S9-final-viral-combined_k141_40359\|\|full | 1336 | No | NA | 0 | 0 | Not-determined | Genome-fragment | NA | 0 |
| S9-final-viral-combined_k141_34945\|\|full | 1067 | No | NA | 0 | 1 | Not-determined | Genome-fragment | NA | 0 |
| S9-final-viral-combined_k141_56822\|\|full | 1481 | No | NA | 0 | 3 | Not-determined | Genome-fragment | NA | 0 |
| S17-final-viral-combined_k141_93719\|\|full | 1027 | No | NA | 0 | 0 | Low-quality | Genome-fragment | 1.18 | 0 |
| S17-k141_184406 | 1027 | No | NA | 0 | 1 | Low-quality | Genome-fragment | 0.49 | 0 |
| S9-final-viral-combined_k141_88583\|\|full | 2498 | No | NA | 0 | 0 | Not-determined | Genome-fragment | NA | 0 |
| S9-final-viral-combined_k141_51484\|\|full | 1418 | No | NA | 0 | 0 | Not-determined | Genome-fragment | NA | 0 |
| S9-final-viral-combined_k141_88598\|\|full | 2114 | No | NA | 0 | 0 | Not-determined | Genome-fragment | NA | 0 |
| S9-final-viral-combined_k141_56855\|\|full | 2339 | No | NA | 0 | 1 | Not-determined | Genome-fragment | NA | 0 |
| S9-final-viral-combined_k141_77796\|\|full | 1479 | No | NA | 0 | 1 | Not-determined | Genome-fragment | NA | 0 |
| S17-k141_208747 | 1027 | No | NA | 0 | 1 | Low-quality | Genome-fragment | 1.27 | 0 |
| S9-final-viral-combined_k141_88632\|\|full | 1130 | No | NA | 0 | 1 | Not-determined | Genome-fragment | NA | 0 |
| S9-final-viral-combined_k141_109322\|\|full | 103451 | Yes | 43439 | 27 | 51 | High-quality | High-quality | 100 | 58.01 |
| S9-final-viral-combined_k141_51498\|\|full | 2292 | No | NA | 0 | 2 | Not-determined | Genome-fragment | NA | 0 |
| S9-final-viral-combined_k141_125712\|\|full | 5977 | No | NA | 0 | 1 | Not-determined | Genome-fragment | NA | 0 |
| S12-final-viral-combined_k141_71489\|\|full | 1027 | No | NA | 2 | 0 | Low-quality | Genome-fragment | 3.06 | 0 |
| S12-final-viral-combined_k141_310156\|\|full | 1027 | No | NA | 0 | 0 | Low-quality | Genome-fragment | 2.88 | 0 |
| S12-final-viral-combined_k141_363056\|\|full | 1027 | No | NA | 0 | 2 | Low-quality | Genome-fragment | 1.15 | 0 |
| S9-final-viral-combined_k141_40392\|\|full | 2220 | No | NA | 0 | 1 | Not-determined | Genome-fragment | NA | 0 |
| S9-final-viral-combined_k141_120791\|\|full | 1723 | No | NA | 0 | 0 | Not-determined | Genome-fragment | NA | 0 |
| S12_k141_339725 | 1027 | No | NA | 0 | 0 | Low-quality | Genome-fragment | 2.42 | 0 |
| S2-final-viral-combined_k141_29416\|\|full | 1027 | No | NA | 0 | 2 | Low-quality | Genome-fragment | 2.11 | 0 |
| S2_k141_10566 | 1027 | No | NA | 0 | 0 | Low-quality | Genome-fragment | 0.75 | 0 |
| S2_k141_57931 | 1027 | No | NA | 0 | 0 | Low-quality | Genome-fragment | 2.36 | 0 |
| S2_k141_78766 | 1027 | No | NA | 1 | 0 | Low-quality | Genome-fragment | 1.33 | 0 |
| S9-final-viral-combined_k141_120803\|\|full | 1225 | No | NA | 0 | 1 | Not-determined | Genome-fragment | NA | 0 |
| S8-final-viral-combined_k141_137477\|\|lt2gene | 1027 | No | NA | 1 | 0 | Low-quality | Genome-fragment | 2.43 | 0 |
| S9-final-viral-combined_k141_27853\|\|full | 1028 | No | NA | 0 | 0 | Low-quality | Genome-fragment | 1.64 | 0 |
| S9-final-viral-combined_k141_113720\|\|full | 1028 | No | NA | 0 | 0 | Low-quality | Genome-fragment | 2.02 | 0 |
| S9-final-viral-combined_k141_115088\|\|full | 2297 | No | NA | 0 | 2 | Not-determined | Genome-fragment | NA | 0 |
| S9-final-viral-combined_k141_115949\|\|full | 1028 | No | NA | 1 | 0 | Low-quality | Genome-fragment | 0.51 | 0 |
| S9-final-viral-combined_k141_104675\|\|full | 3636 | No | NA | 0 | 2 | Not-determined | Genome-fragment | NA | 0 |
| S9-final-viral-combined_k141_35045\|\|full | 2116 | No | NA | 0 | 2 | Not-determined | Genome-fragment | NA | 0 |
| S1-final-viral-combined_k141_124731\|\|full | 1028 | No | NA | 0 | 0 | Low-quality | Genome-fragment | 1.44 | 0 |
| S3-final-viral-combined_k141_102562\|\|full | 1028 | No | NA | 1 | 0 | Low-quality | Genome-fragment | 2.25 | 0 |
| S5-final-viral-combined_k141_161234\|\|full | 1028 | No | NA | 1 | 0 | Low-quality | Genome-fragment | 0.8 | 0 |
| S5-final-viral-combined_k141_109667\|\|full | 1028 | No | NA | 0 | 0 | Low-quality | Genome-fragment | 3.01 | 0 |
| S9-final-viral-combined_k141_120824\|\|full | 1374 | No | NA | 0 | 2 | Not-determined | Genome-fragment | NA | 0 |
| S5-final-viral-combined_k141_82989\|\|lt2gene | 1028 | No | NA | 1 | 0 | Low-quality | Genome-fragment | 25.27 | 0 |
| S9-final-viral-combined_k141_51604\|\|full | 2810 | No | NA | 0 | 1 | Not-determined | Genome-fragment | NA | 0 |
| S9-final-viral-combined_k141_77844\|\|full | 2883 | No | NA | 0 | 1 | Not-determined | Genome-fragment | NA | 0 |
| S9-final-viral-combined_k141_57010\|\|full | 2456 | No | NA | 0 | 1 | Not-determined | Genome-fragment | NA | 0 |
| S9-final-viral-combined_k141_19464\|\|full | 3623 | No | NA | 0 | 2 | Not-determined | Genome-fragment | NA | 0 |
| S7-final-viral-combined_k141_242528\|\|full | 1028 | No | NA | 0 | 0 | Low-quality | Genome-fragment | 2.03 | 0 |
| S9-final-viral-combined_k141_24651\|\|full | 2147 | No | NA | 0 | 2 | Not-determined | Genome-fragment | NA | 0 |
| S9-final-viral-combined_k141_99255\|\|full | 1762 | No | NA | 0 | 2 | Not-determined | Genome-fragment | NA | 0 |
| S9-final-viral-combined_k141_24657\|\|full | 1612 | No | NA | 0 | 1 | Not-determined | Genome-fragment | NA | 0 |
| S9-final-viral-combined_k141_57026\|\|full | 3537 | No | NA | 0 | 0 | Not-determined | Genome-fragment | NA | 0 |
| S9-final-viral-combined_k141_77868\|\|full | 4251 | No | NA | 0 | 1 | Not-determined | Genome-fragment | NA | 0 |
| S9-final-viral-combined_k141_24661\|\|full | 2066 | No | NA | 0 | 0 | Not-determined | Genome-fragment | NA | 0 |
| S7-final-viral-combined_k141_31118\|\|lt2gene | 1028 | No | NA | 1 | 0 | Low-quality | Genome-fragment | 2.31 | 0 |
| S17-k141_139020 | 1028 | No | NA | 0 | 0 | Low-quality | Genome-fragment | 0.67 | 0 |
| S9-final-viral-combined_k141_35116\|\|full | 1990 | No | NA | 0 | 2 | Not-determined | Genome-fragment | NA | 0 |
| S12-final-viral-combined_k141_372788\|\|full | 1028 | No | NA | 0 | 0 | Low-quality | Genome-fragment | 1.22 | 0 |
| S9-final-viral-combined_k141_45764\|\|full | 2367 | No | NA | 0 | 2 | Not-determined | Genome-fragment | NA | 0 |
| S9-final-viral-combined_k141_62260\|\|full | 1635 | No | NA | 0 | 0 | Not-determined | Genome-fragment | NA | 0 |
| S9-final-viral-combined_k141_3461\|\|full | 41772 | No | NA | 10 | 0 | High-quality | High-quality | 100 | 0 |
| S9-final-viral-combined_k141_8891\|\|full | 1815 | No | NA | 0 | 2 | Not-determined | Genome-fragment | NA | 0 |
| S12-final-viral-combined_k141_211794\|\|full | 1028 | No | NA | 0 | 0 | Low-quality | Genome-fragment | 2.03 | 0 |
| S12_k141_258913 | 1028 | No | NA | 0 | 0 | Low-quality | Genome-fragment | 2.26 | 0 |
| S2-final-viral-combined_k141_196869\|\|full | 1028 | No | NA | 0 | 2 | Low-quality | Genome-fragment | 0.34 | 0 |
| S9-final-viral-combined_k141_45785\|\|full | 1210 | No | NA | 0 | 1 | Not-determined | Genome-fragment | NA | 0 |
| S9-final-viral-combined_k141_40517\|\|full | 1605 | No | NA | 0 | 0 | Not-determined | Genome-fragment | NA | 0 |
| S2-final-viral-combined_k141_86909\|\|full | 1028 | No | NA | 2 | 0 | Low-quality | Genome-fragment | 2.38 | 0 |
| S2-final-viral-combined_k141_221808\|\|lt2gene | 1028 | No | NA | 1 | 0 | Low-quality | Genome-fragment | 0.71 | 0 |
| S4-final-viral-combined_k141_82060\|\|full | 1028 | No | NA | 0 | 0 | Low-quality | Genome-fragment | 0.84 | 0 |
| S9-final-viral-combined_k141_3539\|\|full | 1141 | No | NA | 0 | 0 | Not-determined | Genome-fragment | NA | 0 |
| S8-final-viral-combined_k141_232220\|\|full | 1028 | No | NA | 1 | 0 | Low-quality | Genome-fragment | 1.12 | 0 |
| S9-final-viral-combined_k141_120976\|\|full | 2890 | No | NA | 0 | 2 | Not-determined | Genome-fragment | NA | 0 |
| S8_k141_253371 | 1028 | No | NA | 0 | 1 | Low-quality | Genome-fragment | 1.06 | 0 |
| S9-final-viral-combined_k141_57208\|\|full | 2046 | No | NA | 0 | 0 | Not-determined | Genome-fragment | NA | 0 |
| S11-final-viral-combined_k141_10237\|\|full | 1029 | No | NA | 0 | 0 | Low-quality | Genome-fragment | 2.94 | 0 |
| S9-final-viral-combined_k141_35248\|\|full | 11465 | No | NA | 0 | 4 | Not-determined | Genome-fragment | NA | 0 |
| S11-final-viral-combined_k141_298946\|\|full | 1029 | No | NA | 1 | 0 | Low-quality | Genome-fragment | 1.67 | 0 |
| S9-final-viral-combined_k141_115223\|\|full | 1858 | No | NA | 0 | 1 | Not-determined | Genome-fragment | NA | 0 |
| S9-final-viral-combined_k141_78006\|\|full | 7183 | No | NA | 0 | 1 | Not-determined | Genome-fragment | NA | 0 |
| S11_k141_154141 | 1029 | No | NA | 0 | 1 | Low-quality | Genome-fragment | 0.26 | 0 |
| S9-final-viral-combined_k141_78021\|\|full | 7287 | No | NA | 0 | 7 | Not-determined | Genome-fragment | NA | 0 |
| S9-final-viral-combined_k141_121049\|\|full | 1004 | No | NA | 0 | 1 | Not-determined | Genome-fragment | NA | 0 |
| S9-final-viral-combined_k141_67276\|\|full | 6839 | No | NA | 0 | 4 | Not-determined | Genome-fragment | NA | 0 |
| S11_k141_216465 | 1029 | No | NA | 0 | 1 | Low-quality | Genome-fragment | 0.36 | 0 |
| S1-final-viral-combined_k141_116216\|\|full | 1029 | No | NA | 0 | 0 | Low-quality | Genome-fragment | 2.6 | 0 |
| S9-final-viral-combined_k141_125989\|\|full | 3298 | No | NA | 0 | 2 | Not-determined | Genome-fragment | NA | 0 |
| S1-final-viral-combined_k141_302896\|\|full | 1029 | No | NA | 0 | 0 | Low-quality | Genome-fragment | 2.24 | 0 |
| S9-final-viral-combined_k141_40544\|\|full | 5484 | No | NA | 0 | 6 | Not-determined | Genome-fragment | NA | 0 |
| S1-final-viral-combined_k141_188384\|\|full | 1029 | No | NA | 1 | 0 | Low-quality | Genome-fragment | 2.8 | 0 |
| S5-final-viral-combined_k141_109819\|\|full | 1029 | No | NA | 1 | 0 | Low-quality | Genome-fragment | 2.05 | 0 |
| S9-final-viral-combined_k141_29611\|\|full | 413955 | Yes | 96620 | 35 | 266 | Complete | High-quality | 100 | 76.66 |
| S9-final-viral-combined_k141_62401\|\|full | 2470 | No | NA | 0 | 2 | Not-determined | Genome-fragment | NA | 0 |
| S9-final-viral-combined_k141_3609\|\|full | 1186 | No | NA | 0 | 1 | Not-determined | Genome-fragment | NA | 0 |
| S9-final-viral-combined_k141_62410\|\|full | 2661 | No | NA | 0 | 0 | Not-determined | Genome-fragment | NA | 0 |
| S9-final-viral-combined_k141_29645\|\|full | 3055 | No | NA | 0 | 3 | Not-determined | Genome-fragment | NA | 0 |
| S5-final-viral-combined_k141_124995\|\|lt2gene | 1029 | No | NA | 1 | 0 | Low-quality | Genome-fragment | 2.65 | 0 |
| S5-final-viral-combined_k141_198522\|\|lt2gene | 1029 | No | NA | 2 | 0 | Low-quality | Genome-fragment | 2.25 | 0 |
| S9-final-viral-combined_k141_29664\|\|full | 1679 | No | NA | 0 | 0 | Not-determined | Genome-fragment | NA | 0 |
| S7-final-viral-combined_k141_130863\|\|full | 1029 | No | NA | 1 | 0 | Low-quality | Genome-fragment | 2.34 | 0 |
| S9-final-viral-combined_k141_51949\|\|full | 1262 | No | NA | 0 | 1 | Not-determined | Genome-fragment | NA | 0 |
| S9-final-viral-combined_k141_99483\|\|full | 1502 | No | NA | 0 | 2 | Not-determined | Genome-fragment | NA | 0 |
| S17-k141_254135 | 1029 | No | NA | 0 | 0 | Low-quality | Genome-fragment | 1.72 | 0 |
| S9-final-viral-combined_k141_51956\|\|full | 2782 | No | NA | 0 | 2 | Not-determined | Genome-fragment | NA | 0 |
| S12-final-viral-combined_k141_2349\|\|full | 1029 | No | NA | 0 | 1 | Low-quality | Genome-fragment | 1.62 | 0 |
| S9-final-viral-combined_k141_67383\|\|full | 3089 | No | NA | 0 | 4 | Not-determined | Genome-fragment | NA | 0 |
| S12-final-viral-combined_k141_388047\|\|lt2gene | 1029 | No | NA | 1 | 0 | Low-quality | Genome-fragment | 0.92 | 0 |
| S9-final-viral-combined_k141_40587\|\|full | 1997 | No | NA | 0 | 0 | Not-determined | Genome-fragment | NA | 0 |
| S12-final-viral-combined_k141_92609\|\|lt2gene | 1029 | No | NA | 1 | 0 | Low-quality | Genome-fragment | 2.58 | 0 |
| S9-final-viral-combined_k141_57377\|\|full | 2098 | No | NA | 0 | 2 | Not-determined | Genome-fragment | NA | 0 |
| S9-final-viral-combined_k141_109588\|\|full | 1246 | No | NA | 0 | 1 | Not-determined | Genome-fragment | NA | 0 |
| S9-final-viral-combined_k141_19728\|\|full | 4612 | No | NA | 0 | 2 | Not-determined | Genome-fragment | NA | 0 |
| S12_k141_208204 | 1029 | No | NA | 0 | 1 | Low-quality | Genome-fragment | 1.44 | 0 |
| S2-final-viral-combined_k141_145164\|\|full | 1029 | No | NA | 0 | 0 | Low-quality | Genome-fragment | 1.79 | 0 |
| S9-final-viral-combined_k141_62477\|\|full | 2264 | No | NA | 0 | 1 | Not-determined | Genome-fragment | NA | 0 |
| S2-final-viral-combined_k141_188393\|\|full | 1029 | No | NA | 0 | 0 | Low-quality | Genome-fragment | 1.88 | 0 |
| S9-final-viral-combined_k141_78167\|\|full | 1313 | No | NA | 0 | 1 | Not-determined | Genome-fragment | NA | 0 |
| S2_k141_70349 | 1029 | No | NA | 0 | 0 | Low-quality | Genome-fragment | 0.59 | 0 |
| S9-final-viral-combined_k141_57395\|\|full | 1114 | No | NA | 0 | 0 | Not-determined | Genome-fragment | NA | 0 |
| S2_k141_9348 | 1029 | No | NA | 0 | 1 | Low-quality | Genome-fragment | 0.25 | 0 |
| S9-final-viral-combined_k141_3745\|\|full | 1584 | No | NA | 0 | 0 | Not-determined | Genome-fragment | NA | 0 |
| S4-final-viral-combined_k141_31600\|\|full | 1029 | No | NA | 0 | 1 | Low-quality | Genome-fragment | 0.98 | 0 |
| S6-final-viral-combined_k141_77448\|\|full | 1029 | No | NA | 0 | 3 | Low-quality | Genome-fragment | 0.45 | 0 |
| S9-final-viral-combined_k141_115419\|\|full | 2231 | No | NA | 0 | 2 | Not-determined | Genome-fragment | NA | 0 |
| S9-final-viral-combined_k141_78204\|\|full | 2396 | No | NA | 0 | 1 | Not-determined | Genome-fragment | NA | 0 |
| S9-final-viral-combined_k141_104856\|\|full | 2394 | No | NA | 0 | 1 | Not-determined | Genome-fragment | NA | 0 |
| S9-final-viral-combined_k141_29708\|\|full | 1616 | No | NA | 0 | 1 | Not-determined | Genome-fragment | NA | 0 |
| S6-final-viral-combined_k141_109873\|\|full | 1029 | No | NA | 0 | 0 | Low-quality | Genome-fragment | 1.35 | 0 |
| S11-final-viral-combined_k141_163736\|\|lt2gene | 1030 | No | NA | 0 | 0 | Low-quality | Genome-fragment | 1.68 | 0 |
| S1_k141_94801 | 1030 | No | NA | 0 | 1 | Low-quality | Genome-fragment | 2.18 | 0 |
| S9-final-viral-combined_k141_24997\|\|full | 1094 | No | NA | 0 | 2 | Not-determined | Genome-fragment | NA | 0 |
| S9-final-viral-combined_k141_121242\|\|full | 1408 | No | NA | 0 | 2 | Not-determined | Genome-fragment | NA | 0 |
| S9-final-viral-combined_k141_57429\|\|full | 3154 | No | NA | 0 | 1 | Not-determined | Genome-fragment | NA | 0 |
| S3-final-viral-combined_k141_81623\|\|full | 1030 | No | NA | 1 | 0 | Low-quality | Genome-fragment | 1.66 | 0 |
| S3-final-viral-combined_k141_40795\|\|lt2gene | 1030 | No | NA | 1 | 0 | Low-quality | Genome-fragment | 2.74 | 0 |
| S9-final-viral-combined_k141_72491\|\|full | 1943 | No | NA | 0 | 3 | Not-determined | Genome-fragment | NA | 0 |
| S9-final-viral-combined_k141_109643\|\|full | 3475 | No | NA | 0 | 1 | Not-determined | Genome-fragment | NA | 0 |
| S9-final-viral-combined_k141_25007\|\|full | 30091 | Yes | 27739 | 20 | 5 | Medium-quality | Genome-fragment | 59.16 | 7.82 |
| S7-final-viral-combined_k141_159266\|\|full | 1030 | No | NA | 0 | 1 | Low-quality | Genome-fragment | 0.96 | 0 |
| S9-final-viral-combined_k141_89121\|\|full | 2096 | No | NA | 0 | 0 | Not-determined | Genome-fragment | NA | 0 |
| S7-final-viral-combined_k141_222402\|\|full | 1030 | No | NA | 1 | 0 | Low-quality | Genome-fragment | 1.72 | 0 |
| S9-final-viral-combined_k141_84194\|\|full | 2347 | No | NA | 0 | 0 | Not-determined | Genome-fragment | NA | 0 |
| S17-final-viral-combined_k141_236903\|\|full | 1030 | No | NA | 1 | 0 | Low-quality | Genome-fragment | 3.21 | 0 |
| S9-final-viral-combined_k141_67478\|\|full | 4359 | No | NA | 0 | 2 | Not-determined | Genome-fragment | NA | 0 |
| S9-final-viral-combined_k141_67485\|\|full | 1831 | No | NA | 0 | 0 | Not-determined | Genome-fragment | NA | 0 |
| S17-final-viral-combined_k141_152713\|\|full | 1030 | No | NA | 0 | 1 | Low-quality | Genome-fragment | 0.59 | 0 |
| S9-final-viral-combined_k141_67492\|\|full | 1694 | No | NA | 0 | 2 | Not-determined | Genome-fragment | NA | 0 |
| S9-final-viral-combined_k141_9179\|\|full | 1681 | No | NA | 0 | 0 | Not-determined | Genome-fragment | NA | 0 |
| S17-final-viral-combined_k141_9808\|\|full | 1030 | No | NA | 0 | 0 | Low-quality | Genome-fragment | 2.08 | 0 |
| S9-final-viral-combined_k141_67507\|\|full | 1496 | No | NA | 0 | 2 | Not-determined | Genome-fragment | NA | 0 |
| S9-final-viral-combined_k141_115543\|\|full | 2792 | No | NA | 0 | 1 | Not-determined | Genome-fragment | NA | 0 |
| S17-final-viral-combined_k141_82361\|\|lt2gene | 1030 | No | NA | 0 | 0 | Low-quality | Genome-fragment | 2.46 | 0 |
| S9-final-viral-combined_k141_72581\|\|full | 1259 | No | NA | 0 | 1 | Not-determined | Genome-fragment | NA | 0 |
| S17-k141_79643 | 1030 | No | NA | 2 | 0 | Low-quality | Genome-fragment | 3.4 | 0 |
| S9-final-viral-combined_k141_35415\|\|full | 3936 | No | NA | 0 | 3 | Not-determined | Genome-fragment | NA | 0 |
| S9-final-viral-combined_k141_29789\|\|full | 1917 | No | NA | 0 | 3 | Not-determined | Genome-fragment | NA | 0 |
| S9-final-viral-combined_k141_29806\|\|full | 1885 | No | NA | 0 | 2 | Not-determined | Genome-fragment | NA | 0 |
| S9-final-viral-combined_k141_115564\|\|full | 1022 | No | NA | 0 | 1 | Not-determined | Genome-fragment | NA | 0 |
| S12-final-viral-combined_k141_309457\|\|full | 1030 | No | NA | 2 | 0 | Low-quality | Genome-fragment | 1.79 | 0 |
| S9-final-viral-combined_k141_99643\|\|full | 5489 | No | NA | 0 | 1 | Not-determined | Genome-fragment | NA | 0 |
| S9-final-viral-combined_k141_25065\|\|full | 14748 | No | NA | 0 | 2 | Not-determined | Genome-fragment | NA | 0 |
| S9-final-viral-combined_k141_72601\|\|full | 1878 | No | NA | 0 | 2 | Not-determined | Genome-fragment | NA | 0 |
| S9-final-viral-combined_k141_45959\|\|full | 1449 | No | NA | 0 | 0 | Not-determined | Genome-fragment | NA | 0 |
| S2_k141_245569 | 1030 | No | NA | 0 | 0 | Low-quality | Genome-fragment | 1.24 | 0 |
| S6_k141_35861 | 1030 | No | NA | 0 | 1 | Low-quality | Genome-fragment | 0.18 | 0 |
| S9-final-viral-combined_k141_84249\|\|full | 2785 | No | NA | 0 | 1 | Not-determined | Genome-fragment | NA | 0 |
| S9-final-viral-combined_k141_84250\|\|full | 1440 | No | NA | 0 | 1 | Not-determined | Genome-fragment | NA | 0 |
| S9-final-viral-combined_k141_109714\|\|full | 8846 | No | NA | 0 | 7 | Not-determined | Genome-fragment | NA | 0 |
| S9-final-viral-combined_k141_3878\|\|full | 5555 | No | NA | 1 | 0 | High-quality | High-quality | 100 | 0 |
| S8-final-viral-combined_k141_186169\|\|full | 1030 | No | NA | 0 | 0 | Low-quality | Genome-fragment | 2.47 | 0 |
| S9_k141_75911 | 1031 | No | NA | 0 | 0 | Low-quality | Genome-fragment | 1.37 | 0 |
| S11-final-viral-combined_k141_223346\|\|full | 1031 | No | NA | 0 | 0 | Low-quality | Genome-fragment | 2.51 | 0 |
| S9-final-viral-combined_k141_45998\|\|full | 1751 | No | NA | 0 | 0 | Not-determined | Genome-fragment | NA | 0 |
| S9-final-viral-combined_k141_89249\|\|full | 25111 | No | NA | 12 | 0 | Medium-quality | Genome-fragment | 56.42 | 0 |
| S9-final-viral-combined_k141_46011\|\|full | 2259 | No | NA | 0 | 1 | Not-determined | Genome-fragment | NA | 0 |
| S9-final-viral-combined_k141_67544\|\|full | 1319 | No | NA | 0 | 1 | Not-determined | Genome-fragment | NA | 0 |

| | | | | | | | | | |
|---|---|---|---|---|---|---|---|---|---|
| S9-final-viral-combined_k141_35522\|\|full | 1924 | No | NA | 0 | 0 | Not-determined | Genome-fragment | NA | 0 |
| S9-final-viral-combined_k141_9230\|\|full | 2260 | No | NA | 0 | 1 | Not-determined | Genome-fragment | NA | 0 |
| S9-final-viral-combined_k141_105058\|\|full | 1640 | No | NA | 0 | 3 | Not-determined | Genome-fragment | NA | 0 |
| S11_k141_66707 | 1031 | No | NA | 0 | 0 | Low-quality | Genome-fragment | 1.98 | 0 |
| S9-final-viral-combined_k141_105067\|\|full | 2911 | No | NA | 0 | 0 | Not-determined | Genome-fragment | NA | 0 |
| S1_k141_222392 | 1031 | No | NA | 0 | 1 | Low-quality | Genome-fragment | 0.36 | 0 |
| S9-final-viral-combined_k141_121464\|\|full | 1912 | No | NA | 0 | 2 | Not-determined | Genome-fragment | NA | 0 |
| S9-final-viral-combined_k141_67619\|\|full | 2278 | No | NA | 0 | 3 | Not-determined | Genome-fragment | NA | 0 |
| S5_k141_205857 | 1031 | No | NA | 0 | 1 | Low-quality | Genome-fragment | 2.21 | 0 |
| S9-final-viral-combined_k141_89354\|\|full | 1979 | No | NA | 0 | 2 | Not-determined | Genome-fragment | NA | 0 |
| S9-final-viral-combined_k141_14185\|\|full | 2561 | No | NA | 0 | 2 | Not-determined | Genome-fragment | NA | 0 |
| S17-final-viral-combined_k141_39295\|\|full | 1031 | No | NA | 0 | 2 | Low-quality | Genome-fragment | 0.42 | 0 |
| S9-final-viral-combined_k141_78481\|\|full | 1574 | No | NA | 0 | 1 | Not-determined | Genome-fragment | NA | 0 |
| S9-final-viral-combined_k141_3982\|\|full | 1683 | No | NA | 0 | 0 | Not-determined | Genome-fragment | NA | 0 |
| S9-final-viral-combined_k141_115654\|\|full | 1728 | No | NA | 0 | 2 | Not-determined | Genome-fragment | NA | 0 |
| S9-final-viral-combined_k141_14226\|\|full | 1199 | No | NA | 0 | 2 | Not-determined | Genome-fragment | NA | 0 |
| S9-final-viral-combined_k141_25157\|\|full | 1432 | No | NA | 0 | 1 | Not-determined | Genome-fragment | NA | 0 |
| S9-final-viral-combined_k141_25165\|\|full | 1363 | No | NA | 0 | 2 | Not-determined | Genome-fragment | NA | 0 |
| S9-final-viral-combined_k141_126256\|\|full | 1599 | No | NA | 0 | 0 | Not-determined | Genome-fragment | NA | 0 |
| S9-final-viral-combined_k141_89420\|\|full | 1437 | No | NA | 0 | 2 | Not-determined | Genome-fragment | NA | 0 |
| S9-final-viral-combined_k141_72636\|\|full | 2204 | No | NA | 0 | 1 | Not-determined | Genome-fragment | NA | 0 |
| S9-final-viral-combined_k141_52208\|\|full | 1745 | No | NA | 0 | 1 | Not-determined | Genome-fragment | NA | 0 |
| S17-k141_207077 | 1031 | No | NA | 1 | 0 | Low-quality | Genome-fragment | 1.08 | 0 |
| S12-final-viral-combined_k141_373554\|\|full | 1031 | No | NA | 0 | 1 | Low-quality | Genome-fragment | 0.29 | 0 |
| S9-final-viral-combined_k141_115753\|\|full | 1316 | No | NA | 0 | 1 | Not-determined | Genome-fragment | NA | 0 |
| S9-final-viral-combined_k141_67691\|\|full | 2826 | No | NA | 0 | 1 | Not-determined | Genome-fragment | NA | 0 |
| S9-final-viral-combined_k141_89444\|\|full | 1618 | No | NA | 0 | 0 | Not-determined | Genome-fragment | NA | 0 |
| S9-final-viral-combined_k141_89445\|\|full | 2342 | No | NA | 0 | 3 | Not-determined | Genome-fragment | NA | 0 |
| S12-final-viral-combined_k141_5016\|\|full | 1031 | No | NA | 0 | 1 | Low-quality | Genome-fragment | 1.86 | 0 |
| S9-final-viral-combined_k141_19994\|\|full | 1814 | No | NA | 0 | 1 | Not-determined | Genome-fragment | NA | 0 |
| S9-final-viral-combined_k141_57504\|\|full | 1713 | No | NA | 0 | 0 | Not-determined | Genome-fragment | NA | 0 |
| S9-final-viral-combined_k141_25250\|\|full | 1310 | No | NA | 0 | 1 | Not-determined | Genome-fragment | NA | 0 |
| S9-final-viral-combined_k141_115776\|\|full | 2027 | No | NA | 0 | 0 | Not-determined | Genome-fragment | NA | 0 |
| S9-final-viral-combined_k141_67719\|\|full | 3214 | No | NA | 0 | 5 | Not-determined | Genome-fragment | NA | 0 |
| S9-final-viral-combined_k141_72667\|\|full | 2520 | No | NA | 0 | 1 | Not-determined | Genome-fragment | NA | 0 |
| S12-final-viral-combined_k141_243330\|\|lt2gene | 1031 | No | NA | 1 | 0 | Low-quality | Genome-fragment | 1.79 | 0 |
| S9-final-viral-combined_k141_29914\|\|full | 3512 | No | NA | 0 | 1 | Not-determined | Genome-fragment | NA | 0 |
| S2-final-viral-combined_k141_234290\|\|lt2gene | 1031 | No | NA | 1 | 0 | Low-quality | Genome-fragment | 2.73 | 0 |
| S4-final-viral-combined_k141_114850\|\|full | 1031 | No | NA | 1 | 0 | Low-quality | Genome-fragment | 2.38 | 0 |
| S9-final-viral-combined_k141_29936\|\|full | 1542 | No | NA | 0 | 1 | Not-determined | Genome-fragment | NA | 0 |
| S4_k141_69250 | 1031 | No | NA | 2 | 0 | Low-quality | Genome-fragment | 0.58 | 0 |
| S9-final-viral-combined_k141_9402\|\|full | 2727 | No | NA | 0 | 3 | Not-determined | Genome-fragment | NA | 0 |
| S8_k141_216795 | 1031 | No | NA | 0 | 1 | Low-quality | Genome-fragment | 1.76 | 0 |
| S11_k141_56944 | 1032 | No | NA | 1 | 0 | Low-quality | Genome-fragment | 1.19 | 0 |
| S9-final-viral-combined_k141_126321\|\|full | 1888 | No | NA | 0 | 1 | Not-determined | Genome-fragment | NA | 0 |
| S9-final-viral-combined_k141_35663\|\|full | 1629 | No | NA | 0 | 1 | Not-determined | Genome-fragment | NA | 0 |
| S1-final-viral-combined_k141_318328\|\|full | 1032 | No | NA | 0 | 0 | Low-quality | Genome-fragment | 2.63 | 0 |
| S9-final-viral-combined_k141_25284\|\|full | 9080 | No | NA | 0 | 6 | Not-determined | Genome-fragment | NA | 0 |
| S9-final-viral-combined_k141_121625\|\|full | 2849 | No | NA | 0 | 2 | Not-determined | Genome-fragment | NA | 0 |
| S9-final-viral-combined_k141_9423\|\|full | 4377 | No | NA | 0 | 5 | Not-determined | Genome-fragment | NA | 0 |
| S3-final-viral-combined_k141_7655\|\|lt2gene | 1032 | No | NA | 2 | 0 | Low-quality | Genome-fragment | 2.29 | 0 |
| S9-final-viral-combined_k141_67797\|\|full | 3177 | No | NA | 0 | 2 | Not-determined | Genome-fragment | NA | 0 |
| S9-final-viral-combined_k141_67799\|\|full | 1023 | No | NA | 0 | 0 | Not-determined | Genome-fragment | NA | 0 |
| S9-final-viral-combined_k141_14418\|\|full | 3132 | No | NA | 0 | 0 | Not-determined | Genome-fragment | NA | 0 |
| S9-final-viral-combined_k141_109869\|\|full | 1319 | No | NA | 0 | 1 | Not-determined | Genome-fragment | NA | 0 |
| S5_k141_167550 | 1032 | No | NA | 0 | 0 | Low-quality | Genome-fragment | 0.59 | 0 |
| S9-final-viral-combined_k141_99745\|\|full | 2363 | No | NA | 0 | 1 | Not-determined | Genome-fragment | NA | 0 |
| S12-final-viral-combined_k141_211088\|\|full | 1032 | No | NA | 0 | 0 | Low-quality | Genome-fragment | 2.93 | 0 |
| S9-final-viral-combined_k141_72726\|\|full | 2961 | No | NA | 0 | 0 | Not-determined | Genome-fragment | NA | 0 |
| S9-final-viral-combined_k141_29980\|\|full | 1968 | No | NA | 0 | 0 | Not-determined | Genome-fragment | NA | 0 |
| S12_k141_81584 | 1032 | No | NA | 0 | 0 | Low-quality | Genome-fragment | 0.57 | 0 |
| S9-final-viral-combined_k141_78706\|\|full | 1963 | No | NA | 0 | 1 | Not-determined | Genome-fragment | NA | 0 |
| S2-final-viral-combined_k141_209338\|\|full | 1032 | No | NA | 0 | 1 | Low-quality | Genome-fragment | 2.66 | 0 |
| S2_k141_223394 | 1032 | No | NA | 0 | 0 | Low-quality | Genome-fragment | 0.42 | 0 |
| S4-final-viral-combined_k141_78140\|\|full | 1032 | No | NA | 0 | 1 | Low-quality | Genome-fragment | 1.79 | 0 |
| S4_k141_153945 | 1032 | No | NA | 0 | 0 | Low-quality | Genome-fragment | 1.72 | 0 |
| S8-final-viral-combined_k141_62520\|\|lt2gene | 1032 | No | NA | 1 | 0 | Low-quality | Genome-fragment | 0.66 | 0 |
| S9-final-viral-combined_k141_111231\|\|full | 1033 | No | NA | 0 | 0 | Low-quality | Genome-fragment | 1.86 | 0 |
| S9-final-viral-combined_k141_72770\|\|full | 2037 | No | NA | 0 | 2 | Not-determined | Genome-fragment | NA | 0 |
| S9-final-viral-combined_k141_78736\|\|full | 1795 | No | NA | 0 | 0 | Not-determined | Genome-fragment | NA | 0 |
| S9-final-viral-combined_k141_109925\|\|full | 3081 | No | NA | 0 | 2 | Not-determined | Genome-fragment | NA | 0 |
| S9-final-viral-combined_k141_84378\|\|full | 3000 | No | NA | 0 | 3 | Not-determined | Genome-fragment | NA | 0 |
| S11-final-viral-combined_k141_248292\|\|full | 1033 | No | NA | 0 | 0 | Low-quality | Genome-fragment | 1.46 | 0 |
| S9-final-viral-combined_k141_89517\|\|full | 3137 | No | NA | 0 | 1 | Not-determined | Genome-fragment | NA | 0 |
| S9-final-viral-combined_k141_121710\|\|full | 2094 | No | NA | 0 | 2 | Not-determined | Genome-fragment | NA | 0 |
| S9-final-viral-combined_k141_105128\|\|full | 3298 | No | NA | 0 | 1 | Not-determined | Genome-fragment | NA | 0 |
| S11-final-viral-combined_k141_187345\|\|full | 1033 | No | NA | 0 | 0 | Low-quality | Genome-fragment | 1.04 | 0 |
| S9-final-viral-combined_k141_4194\|\|full | 1180 | No | NA | 0 | 1 | Not-determined | Genome-fragment | NA | 0 |
| S9-final-viral-combined_k141_99845\|\|full | 1943 | No | NA | 0 | 3 | Not-determined | Genome-fragment | NA | 0 |
| S9-final-viral-combined_k141_40869\|\|full | 2998 | No | NA | 0 | 2 | Not-determined | Genome-fragment | NA | 0 |
| S11-final-viral-combined_k141_222996\|\|lt2gene | 1033 | No | NA | 1 | 0 | Low-quality | Genome-fragment | 0.97 | 0 |
| S9-final-viral-combined_k141_72801\|\|full | 5048 | No | NA | 0 | 1 | Not-determined | Genome-fragment | NA | 0 |
| S9-final-viral-combined_k141_121754\|\|full | 1325 | No | NA | 0 | 1 | Not-determined | Genome-fragment | NA | 0 |
| S9-final-viral-combined_k141_30029\|\|full | 6016 | No | NA | 0 | 1 | Not-determined | Genome-fragment | NA | 0 |
| S1-final-viral-combined_k141_251192\|\|full | 1033 | No | NA | 0 | 0 | Low-quality | Genome-fragment | 2.41 | 0 |
| S9-final-viral-combined_k141_9500\|\|full | 2304 | No | NA | 0 | 0 | Not-determined | Genome-fragment | NA | 0 |
| S9-final-viral-combined_k141_67912\|\|full | 1805 | No | NA | 0 | 3 | Not-determined | Genome-fragment | NA | 0 |
| S9-final-viral-combined_k141_126341\|\|full | 9842 | No | NA | 0 | 1 | Not-determined | Genome-fragment | NA | 0 |
| S5_k141_74002 | 1033 | No | NA | 0 | 0 | Low-quality | Genome-fragment | 2.55 | 0 |
| S5_k141_30005 | 1033 | No | NA | 0 | 0 | Low-quality | Genome-fragment | 1.4 | 0 |
| S9-final-viral-combined_k141_30032\|\|full | 6936 | No | NA | 0 | 5 | Not-determined | Genome-fragment | NA | 0 |
| S7-final-viral-combined_k141_113529\|\|full | 1033 | No | NA | 0 | 0 | Low-quality | Genome-fragment | 2.04 | 0 |
| S7-final-viral-combined_k141_159253\|\|lt2gene | 1033 | No | NA | 2 | 0 | Low-quality | Genome-fragment | 2.79 | 0 |
| S7-final-viral-combined_k141_38611\|\|lt2gene | 1033 | No | NA | 1 | 0 | Low-quality | Genome-fragment | 2.15 | 0 |
| S17-final-viral-combined_k141_160006\|\|full | 1033 | No | NA | 1 | 0 | Low-quality | Genome-fragment | 2.98 | 0 |
| S9-final-viral-combined_k141_30041\|\|full | 2292 | No | NA | 0 | 0 | Not-determined | Genome-fragment | NA | 0 |
| S9-final-viral-combined_k141_126365\|\|full | 2768 | No | NA | 0 | 3 | Not-determined | Genome-fragment | NA | 0 |
| S17-final-viral-combined_k141_64213\|\|full | 1033 | No | NA | 3 | 0 | Low-quality | Genome-fragment | 2.85 | 0 |
| S9-final-viral-combined_k141_35736\|\|full | 2578 | No | NA | 0 | 1 | Not-determined | Genome-fragment | NA | 0 |
| S9-final-viral-combined_k141_109995\|\|full | 1552 | No | NA | 0 | 0 | Not-determined | Genome-fragment | NA | 0 |
| S9-final-viral-combined_k141_72832\|\|full | 1078 | No | NA | 0 | 1 | Not-determined | Genome-fragment | NA | 0 |
| S9-final-viral-combined_k141_84493\|\|full | 1027 | No | NA | 0 | 0 | Not-determined | Genome-fragment | NA | 0 |
| S17-final-viral-combined_k141_155344\|\|lt2gene | 1033 | No | NA | 1 | 0 | Low-quality | Genome-fragment | 2.54 | 0 |
| S12-final-viral-combined_k141_308608\|\|full | 1033 | Yes | 423 | 1 | 1 | Low-quality | Genome-fragment | 0.55 | 59.05 |
| S9-final-viral-combined_k141_89587\|\|full | 1160 | No | NA | 0 | 0 | Not-determined | Genome-fragment | NA | 0 |
| S9-final-viral-combined_k141_126389\|\|full | 3647 | No | NA | 0 | 3 | Not-determined | Genome-fragment | NA | 0 |
| S12-final-viral-combined_k141_396656\|\|full | 1033 | No | NA | 2 | 0 | Low-quality | Genome-fragment | 2.26 | 0 |
| S9-final-viral-combined_k141_46188\|\|full | 1074 | No | NA | 0 | 0 | Not-determined | Genome-fragment | NA | 0 |
| S9-final-viral-combined_k141_46203\|\|full | 1787 | No | NA | 0 | 2 | Not-determined | Genome-fragment | NA | 0 |
| S9-final-viral-combined_k141_25440\|\|full | 1477 | No | NA | 0 | 1 | Not-determined | Genome-fragment | NA | 0 |
| S12-final-viral-combined_k141_314659\|\|full | 1033 | No | NA | 2 | 0 | Low-quality | Genome-fragment | 2.63 | 0 |
| S9-final-viral-combined_k141_46214\|\|full | 3186 | No | NA | 0 | 0 | Not-determined | Genome-fragment | NA | 0 |
| S9-final-viral-combined_k141_30124\|\|full | 2057 | No | NA | 0 | 2 | Not-determined | Genome-fragment | NA | 0 |
| S9-final-viral-combined_k141_105178\|\|full | 1494 | No | NA | 0 | 1 | Not-determined | Genome-fragment | NA | 0 |
| S12-final-viral-combined_k141_81324\|\|full | 1033 | No | NA | 0 | 0 | Low-quality | Genome-fragment | 1.76 | 0 |
| S9-final-viral-combined_k141_126467\|\|full | 1942 | No | NA | 0 | 1 | Not-determined | Genome-fragment | NA | 0 |
| S9-final-viral-combined_k141_72891\|\|full | 1643 | No | NA | 0 | 2 | Not-determined | Genome-fragment | NA | 0 |
| S9-final-viral-combined_k141_40988\|\|full | 3104 | No | NA | 0 | 4 | Not-determined | Genome-fragment | NA | 0 |
| S9-final-viral-combined_k141_46260\|\|full | 3289 | No | NA | 0 | 2 | Not-determined | Genome-fragment | NA | 0 |
| S9-final-viral-combined_k141_126480\|\|full | 3020 | No | NA | 0 | 1 | Not-determined | Genome-fragment | NA | 0 |
| S9-final-viral-combined_k141_72902\|\|full | 1451 | No | NA | 0 | 1 | Not-determined | Genome-fragment | NA | 0 |
| S9-final-viral-combined_k141_99943\|\|full | 3100 | No | NA | 0 | 3 | Not-determined | Genome-fragment | NA | 0 |
| S12-final-viral-combined_k141_220662\|\|lt2gene | 1033 | No | NA | 2 | 0 | Low-quality | Genome-fragment | 2.37 | 0 |
| S12_k141_229923 | 1033 | No | NA | 1 | 0 | Low-quality | Genome-fragment | 3.5 | 0 |
| S9-final-viral-combined_k141_30212\|\|full | 2148 | No | NA | 0 | 1 | Not-determined | Genome-fragment | NA | 0 |
| S9-final-viral-combined_k141_78905\|\|full | 1111 | No | NA | 0 | 0 | Not-determined | Genome-fragment | NA | 0 |
| S2-final-viral-combined_k141_117916\|\|full | 1033 | No | NA | 0 | 1 | Low-quality | Genome-fragment | 0.62 | 0 |
| S9-final-viral-combined_k141_41058\|\|full | 1598 | No | NA | 0 | 2 | Not-determined | Genome-fragment | NA | 0 |
| S9-final-viral-combined_k141_99990\|\|full | 2222 | No | NA | 0 | 0 | Not-determined | Genome-fragment | NA | 0 |
| S4-final-viral-combined_k141_12414\|\|full | 1033 | No | NA | 3 | 0 | Low-quality | Genome-fragment | 1.69 | 0 |
| S11-final-viral-combined_k141_92607\|\|full | 1034 | No | NA | 0 | 0 | Low-quality | Genome-fragment | 0.96 | 0 |
| S11-final-viral-combined_k141_147842\|\|full | 1034 | No | NA | 0 | 0 | Low-quality | Genome-fragment | 1.8 | 0 |
| S9-final-viral-combined_k141_100007\|\|full | 3186 | No | NA | 0 | 2 | Not-determined | Genome-fragment | NA | 0 |
| S9-final-viral-combined_k141_72925\|\|full | 1137 | No | NA | 0 | 0 | Not-determined | Genome-fragment | NA | 0 |
| S9-final-viral-combined_k141_121920\|\|full | 1376 | No | NA | 0 | 0 | Not-determined | Genome-fragment | NA | 0 |
| S1-final-viral-combined_k141_217583\|\|lt2gene | 1034 | No | NA | 1 | 0 | Low-quality | Genome-fragment | 2.88 | 0 |
| S9-final-viral-combined_k141_30245\|\|full | 1641 | No | NA | 0 | 2 | Not-determined | Genome-fragment | NA | 0 |
| S9-final-viral-combined_k141_121927\|\|full | 9005 | No | NA | 0 | 2 | Not-determined | Genome-fragment | NA | 0 |
| S1_k141_187950 | 1034 | No | NA | 1 | 0 | Low-quality | Genome-fragment | 2.14 | 0 |
| S3-final-viral-combined_k141_2508\|\|full | 1034 | No | NA | 0 | 0 | Low-quality | Genome-fragment | 2.71 | 0 |
| S9-final-viral-combined_k141_100035\|\|full | 1781 | No | NA | 0 | 2 | Not-determined | Genome-fragment | NA | 0 |
| S9-final-viral-combined_k141_121938\|\|full | 1486 | No | NA | 0 | 0 | Not-determined | Genome-fragment | NA | 0 |
| S9-final-viral-combined_k141_84547\|\|full | 1752 | No | NA | 0 | 1 | Not-determined | Genome-fragment | NA | 0 |
| S9-final-viral-combined_k141_52546\|\|full | 7164 | No | NA | 0 | 3 | Not-determined | Genome-fragment | NA | 0 |
| S9-final-viral-combined_k141_52550\|\|full | 2115 | No | NA | 0 | 0 | Not-determined | Genome-fragment | NA | 0 |
| S9-final-viral-combined_k141_116003\|\|full | 2045 | No | NA | 0 | 1 | Not-determined | Genome-fragment | NA | 0 |
| S9-final-viral-combined_k141_30279\|\|full | 4737 | No | NA | 0 | 3 | Not-determined | Genome-fragment | NA | 0 |
| S9-final-viral-combined_k141_89799\|\|full | 1985 | No | NA | 0 | 2 | Not-determined | Genome-fragment | NA | 0 |
| S9-final-viral-combined_k141_73006\|\|full | 1042 | No | NA | 0 | 1 | Not-determined | Genome-fragment | NA | 0 |
| S9-final-viral-combined_k141_94328\|\|full | 2610 | No | NA | 0 | 0 | Not-determined | Genome-fragment | NA | 0 |
| S9-final-viral-combined_k141_14703\|\|full | 1068 | No | NA | 0 | 1 | Not-determined | Genome-fragment | NA | 0 |
| S9-final-viral-combined_k141_122003\|\|full | 1051 | No | NA | 0 | 0 | Not-determined | Genome-fragment | NA | 0 |
| S9-final-viral-combined_k141_105393\|\|full | 2727 | No | NA | 0 | 0 | Not-determined | Genome-fragment | NA | 0 |
| S9-final-viral-combined_k141_116047\|\|full | 2108 | No | NA | 0 | 0 | Not-determined | Genome-fragment | NA | 0 |
| S3-final-viral-combined_k141_49481\|\|full | 1034 | No | NA | 0 | 0 | Low-quality | Genome-fragment | 0.58 | 0 |
| S9-final-viral-combined_k141_94369\|\|full | 1910 | No | NA | 0 | 1 | Not-determined | Genome-fragment | NA | 0 |
| S9-final-viral-combined_k141_78952\|\|full | 1942 | No | NA | 0 | 0 | Not-determined | Genome-fragment | NA | 0 |
| S3_k141_85285 | 1034 | No | NA | 0 | 0 | Low-quality | Genome-fragment | 1.31 | 0 |
| S5-final-viral-combined_k141_83545\|\|full | 1034 | No | NA | 1 | 0 | Low-quality | Genome-fragment | 1.9 | 0 |
| S5-final-viral-combined_k141_78485\|\|full | 1034 | No | NA | 1 | 0 | Low-quality | Genome-fragment | 2.2 | 0 |
| S9-final-viral-combined_k141_52613\|\|full | 3908 | No | NA | 0 | 0 | Not-determined | Genome-fragment | NA | 0 |
| S9-final-viral-combined_k141_89907\|\|full | 2032 | No | NA | 0 | 1 | Not-determined | Genome-fragment | NA | 0 |
| S9-final-viral-combined_k141_89920\|\|full | 1129 | No | NA | 0 | 2 | Not-determined | Genome-fragment | NA | 0 |
| S5-final-viral-combined_k141_204500\|\|full | 1034 | No | NA | 1 | 0 | Low-quality | Genome-fragment | 2.35 | 0 |
| S9-final-viral-combined_k141_63174\|\|full | 3950 | No | NA | 0 | 3 | Not-determined | Genome-fragment | NA | 0 |
| S9-final-viral-combined_k141_20274\|\|full | 5711 | No | NA | 0 | 2 | Not-determined | Genome-fragment | NA | 0 |
| S9-final-viral-combined_k141_14752\|\|full | 4713 | No | NA | 0 | 6 | Not-determined | Genome-fragment | NA | 0 |
| S9-final-viral-combined_k141_35929\|\|full | 1493 | No | NA | 0 | 2 | Not-determined | Genome-fragment | NA | 0 |
| S9-final-viral-combined_k141_35930\|\|full | 1745 | No | NA | 0 | 2 | Not-determined | Genome-fragment | NA | 0 |
| S9-final-viral-combined_k141_20287\|\|full | 4234 | No | NA | 0 | 1 | Not-determined | Genome-fragment | NA | 0 |
| S7-final-viral-combined_k141_183295\|\|full | 1034 | No | NA | 0 | 0 | Low-quality | Genome-fragment | 2.25 | 0 |
| S7-final-viral-combined_k141_152322\|\|full | 1034 | No | NA | 0 | 0 | Low-quality | Genome-fragment | 1.15 | 0 |
| S17-final-viral-combined_k141_117954\|\|full | 1034 | No | NA | 0 | 0 | Low-quality | Genome-fragment | 2.59 | 0 |
| S9-final-viral-combined_k141_41167\|\|full | 8576 | No | NA | 0 | 3 | Not-determined | Genome-fragment | NA | 0 |

| | | | | | | | | | | | |
|---|---|---|---|---|---|---|---|---|---|---|---|
| S9-final-viral-combined_k141_116120\|\|full | 2234 | No | NA | | 0 | 3 | Not-determined | Genome-fragment | NA | | 0 |
| S17-final-viral-combined_k141_199782\|\|full | 1034 | No | NA | | 0 | 0 | Low-quality | Genome-fragment | | 0.74 | 0 |
| S9-final-viral-combined_k141_46394\|\|full | 1393 | No | NA | | 0 | 0 | Not-determined | Genome-fragment | NA | | 0 |
| S12-final-viral-combined_k141_235769\|\|full | 1034 | No | NA | | 1 | 0 | Low-quality | Genome-fragment | | 2.99 | 0 |
| S9-final-viral-combined_k141_41180\|\|full | 1607 | No | NA | | 0 | 2 | Not-determined | Genome-fragment | NA | | 0 |
| S9-final-viral-combined_k141_14809\|\|full | 2058 | No | NA | | 0 | 1 | Not-determined | Genome-fragment | NA | | 0 |
| S9-final-viral-combined_k141_57820\|\|full | 3230 | No | NA | | 0 | 0 | Not-determined | Genome-fragment | NA | | 0 |
| S9-final-viral-combined_k141_122067\|\|full | 10762 | No | NA | | 0 | 7 | Not-determined | Genome-fragment | NA | | 0 |
| S12-final-viral-combined_k141_21270\|\|full | 1034 | No | NA | | 0 | 1 | Low-quality | Genome-fragment | | 1.27 | 0 |
| S9-final-viral-combined_k141_52671\|\|full | 5771 | No | NA | | 0 | 3 | Not-determined | Genome-fragment | NA | | 0 |
| S9-final-viral-combined_k141_46439\|\|full | 1298 | No | NA | | 0 | 0 | Not-determined | Genome-fragment | NA | | 0 |
| S9-final-viral-combined_k141_35990\|\|full | 2108 | No | NA | | 0 | 1 | Not-determined | Genome-fragment | NA | | 0 |
| S9-final-viral-combined_k141_105462\|\|full | 30714 | No | NA | | 19 | 3 | Medium-quality | Genome-fragment | | 68.9 | 0 |
| S12-final-viral-combined_k141_122318\|\|full | 1034 | No | NA | | 0 | 0 | Low-quality | Genome-fragment | | 0.3 | 0 |
| S2-final-viral-combined_k141_219847\|\|full | 1034 | No | NA | | 0 | 0 | Low-quality | Genome-fragment | | 2.07 | 0 |
| S2-final-viral-combined_k141_107981\|\|full | 1034 | No | NA | | 0 | 2 | Low-quality | Genome-fragment | | 0.31 | 0 |
| S9-final-viral-combined_k141_122084\|\|full | 2786 | No | NA | | 0 | 1 | Not-determined | Genome-fragment | NA | | 0 |
| S9-final-viral-combined_k141_84735\|\|full | 3232 | No | NA | | 0 | 1 | Not-determined | Genome-fragment | NA | | 0 |
| S2-final-viral-combined_k141_202578\|\|full | 1034 | No | NA | | 0 | 0 | Low-quality | Genome-fragment | | 0.74 | 0 |
| S9-final-viral-combined_k141_57844\|\|full | 2487 | No | NA | | 0 | 1 | Not-determined | Genome-fragment | NA | | 0 |
| S8-final-viral-combined_k141_164368\|\|lt2gene | 1034 | No | NA | | 1 | 0 | Low-quality | Genome-fragment | | 0.96 | 0 |
| S9-final-viral-combined_k141_36010\|\|full | 2108 | No | NA | | 0 | 0 | Not-determined | Genome-fragment | NA | | 0 |
| S9-final-viral-combined_k141_57855\|\|full | 2717 | No | NA | | 0 | 1 | Not-determined | Genome-fragment | NA | | 0 |
| S8_k141_114240 | 1034 | No | NA | | 0 | 0 | Low-quality | Genome-fragment | | 1.6 | 0 |
| S11-final-viral-combined_k141_136302\|\|full | 1035 | No | NA | | 1 | 0 | Low-quality | Genome-fragment | | 0.67 | 0 |
| S11_k141_199534 | 1035 | No | NA | | 0 | 1 | Low-quality | Genome-fragment | | 2.12 | 0 |
| S1-final-viral-combined_k141_334103\|\|full | 1035 | No | NA | | 1 | 0 | Low-quality | Genome-fragment | | 1.85 | 0 |
| S9-final-viral-combined_k141_36053\|\|full | 1242 | No | NA | | 0 | 1 | Not-determined | Genome-fragment | NA | | 0 |
| S9-final-viral-combined_k141_110263\|\|full | 2737 | No | NA | | 0 | 0 | Not-determined | Genome-fragment | NA | | 0 |
| S1_k141_268356 | 1035 | No | NA | | 0 | 2 | Low-quality | Genome-fragment | | 0.5 | 0 |
| S9-final-viral-combined_k141_100210\|\|full | 4272 | No | NA | | 0 | 3 | Not-determined | Genome-fragment | NA | | 0 |
| S9-final-viral-combined_k141_94577\|\|full | 2178 | No | NA | | 0 | 2 | Not-determined | Genome-fragment | NA | | 0 |
| S3_k141_31389 | 1035 | No | NA | | 0 | 1 | Low-quality | Genome-fragment | | 0.44 | 0 |
| S9-final-viral-combined_k141_90016\|\|full | 3753 | No | NA | | 0 | 3 | Not-determined | Genome-fragment | NA | | 0 |
| S9-final-viral-combined_k141_46507\|\|full | 2612 | No | NA | | 0 | 2 | Not-determined | Genome-fragment | NA | | 0 |
| S7-final-viral-combined_k141_226848\|\|full | 1035 | No | NA | | 0 | 0 | Low-quality | Genome-fragment | | 1.67 | 0 |
| S9-final-viral-combined_k141_84849\|\|full | 1468 | No | NA | | 0 | 1 | Not-determined | Genome-fragment | NA | | 0 |
| S9-final-viral-combined_k141_41220\|\|full | 1201 | No | NA | | 0 | 1 | Not-determined | Genome-fragment | NA | | 0 |
| S9-final-viral-combined_k141_63412\|\|full | 1018 | No | NA | | 0 | 2 | Not-determined | Genome-fragment | NA | | 0 |
| S12-final-viral-combined_k141_386017\|\|full | 1035 | No | NA | | 0 | 0 | Low-quality | Genome-fragment | | 1.72 | 0 |
| S12-final-viral-combined_k141_95332\|\|full | 1035 | No | NA | | 0 | 0 | Low-quality | Genome-fragment | | 2.21 | 0 |
| S12-final-viral-combined_k141_265465\|\|full | 1035 | No | NA | | 0 | 0 | Low-quality | Genome-fragment | | 2.44 | 0 |
| S9-final-viral-combined_k141_25704\|\|full | 1967 | No | NA | | 0 | 1 | Not-determined | Genome-fragment | NA | | 0 |
| S9-final-viral-combined_k141_25706\|\|full | 1685 | No | NA | | 0 | 1 | Not-determined | Genome-fragment | NA | | 0 |
| S12-final-viral-combined_k141_345613\|\|lt2gene | 1035 | No | NA | | 1 | 0 | Low-quality | Genome-fragment | | 1.88 | 0 |
| S9-final-viral-combined_k141_126620\|\|full | 3278 | No | NA | | 0 | 3 | Not-determined | Genome-fragment | NA | | 0 |
| S2_k141_158887 | 1035 | No | NA | | 1 | 0 | Low-quality | Genome-fragment | | 2.41 | 0 |
| S4_k141_75982 | 1035 | No | NA | | 0 | 0 | Low-quality | Genome-fragment | | 1.01 | 0 |
| S9-final-viral-combined_k141_84913\|\|full | 4199 | No | NA | | 0 | 2 | Not-determined | Genome-fragment | NA | | 0 |
| S4_k141_118283 | 1035 | No | NA | | 0 | 0 | Low-quality | Genome-fragment | | 1.5 | 0 |
| S9-final-viral-combined_k141_57937\|\|full | 2896 | No | NA | | 0 | 2 | Not-determined | Genome-fragment | NA | | 0 |
| S9-final-viral-combined_k141_22965\|\|full | 1036 | No | NA | | 0 | 0 | Low-quality | Genome-fragment | | 0.85 | 0 |
| S9-final-viral-combined_k141_63464\|\|full | 1581 | No | NA | | 0 | 1 | Not-determined | Genome-fragment | NA | | 0 |
| S9-final-viral-combined_k141_9884\|\|full | 1862 | No | NA | | 0 | 2 | Not-determined | Genome-fragment | NA | | 0 |
| S9-final-viral-combined_k141_30435\|\|full | 2066 | No | NA | | 0 | 1 | Not-determined | Genome-fragment | NA | | 0 |
| S9-final-viral-combined_k141_23738\|\|lt2gene | 1036 | No | NA | | 1 | 0 | Low-quality | Genome-fragment | | 0.77 | 0 |
| S11-final-viral-combined_k141_127675\|\|full | 1036 | No | NA | | 0 | 0 | Low-quality | Genome-fragment | | 2.15 | 0 |
| S9-final-viral-combined_k141_79115\|\|full | 2460 | No | NA | | 0 | 0 | Not-determined | Genome-fragment | NA | | 0 |
| S9-final-viral-combined_k141_110324\|\|full | 2398 | No | NA | | 0 | 4 | Not-determined | Genome-fragment | NA | | 0 |
| S9-final-viral-combined_k141_36196\|\|full | 1886 | No | NA | | 0 | 0 | Not-determined | Genome-fragment | NA | | 0 |
| S11-final-viral-combined_k141_247174\|\|full | 1036 | No | NA | | 0 | 0 | Low-quality | Genome-fragment | | 3.14 | 0 |
| S7-final-viral-combined_k141_95159\|\|full | 1036 | No | NA | | 0 | 0 | Low-quality | Genome-fragment | | 1.78 | 0 |
| S7-final-viral-combined_k141_35944\|\|lt2gene | 1036 | No | NA | | 0 | 0 | Low-quality | Genome-fragment | | 1.92 | 0 |
| S9-final-viral-combined_k141_79133\|\|full | 1024 | No | NA | | 0 | 1 | Not-determined | Genome-fragment | NA | | 0 |
| S9-final-viral-combined_k141_30472\|\|full | 4844 | No | NA | | 0 | 3 | Not-determined | Genome-fragment | NA | | 0 |
| S17-final-viral-combined_k141_129024\|\|full | 1036 | No | NA | | 1 | 0 | Low-quality | Genome-fragment | | 2.37 | 0 |
| S12-final-viral-combined_k141_272478\|\|full | 1036 | No | NA | | 0 | 0 | Low-quality | Genome-fragment | | 1.2 | 0 |
| S9-final-viral-combined_k141_41287\|\|full | 2633 | No | NA | | 0 | 3 | Not-determined | Genome-fragment | NA | | 0 |
| S9-final-viral-combined_k141_30483\|\|full | 2109 | No | NA | | 0 | 1 | Not-determined | Genome-fragment | NA | | 0 |
| S9-final-viral-combined_k141_41289\|\|full | 1291 | No | NA | | 0 | 0 | Not-determined | Genome-fragment | NA | | 0 |
| S9-final-viral-combined_k141_9918\|\|full | 1438 | No | NA | | 0 | 2 | Not-determined | Genome-fragment | NA | | 0 |
| S9-final-viral-combined_k141_9929\|\|full | 1874 | No | NA | | 0 | 3 | Not-determined | Genome-fragment | NA | | 0 |
| S9-final-viral-combined_k141_126656\|\|full | 7921 | No | NA | | 0 | 2 | Not-determined | Genome-fragment | NA | | 0 |
| S12-final-viral-combined_k141_215420\|\|full | 1036 | No | NA | | 0 | 0 | Low-quality | Genome-fragment | | 2.56 | 0 |
| S2-final-viral-combined_k141_40943\|\|full | 1036 | No | NA | | 0 | 0 | Low-quality | Genome-fragment | | 0.69 | 0 |
| S2-final-viral-combined_k141_195006\|\|lt2gene | 1036 | No | NA | | 1 | 0 | Low-quality | Genome-fragment | | 2.62 | 0 |
| S9_k141_39619 | 1037 | No | NA | | 0 | 0 | Low-quality | Genome-fragment | | 1.73 | 0 |
| S11-final-viral-combined_k141_77953\|\|full | 1037 | No | NA | | 0 | 1 | Low-quality | Genome-fragment | | 0.45 | 0 |
| S9-final-viral-combined_k141_58051\|\|full | 2115 | No | NA | | 0 | 1 | Not-determined | Genome-fragment | NA | | 0 |
| S11-final-viral-combined_k141_305212\|\|lt2gene | 1037 | No | NA | | 1 | 0 | Low-quality | Genome-fragment | | 2.27 | 0 |
| S9-final-viral-combined_k141_90167\|\|full | 1756 | No | NA | | 0 | 2 | Not-determined | Genome-fragment | NA | | 0 |
| S9-final-viral-combined_k141_94690\|\|full | 7777 | No | NA | | 0 | 6 | Not-determined | Genome-fragment | NA | | 0 |
| S9-final-viral-combined_k141_68596\|\|full | 2570 | No | NA | | 0 | 2 | Not-determined | Genome-fragment | NA | | 0 |
| S9-final-viral-combined_k141_63519\|\|full | 1020 | No | NA | | 0 | 1 | Not-determined | Genome-fragment | NA | | 0 |
| S1-final-viral-combined_k141_347725\|\|full | 1037 | No | NA | | 0 | 0 | Low-quality | Genome-fragment | | 2.14 | 0 |
| S5-final-viral-combined_k141_57499\|\|full | 1037 | No | NA | | 0 | 0 | Low-quality | Genome-fragment | | 1.66 | 0 |
| S7-final-viral-combined_k141_134520\|\|full | 1037 | No | NA | | 0 | 1 | Low-quality | Genome-fragment | | 0.5 | 0 |
| S9-final-viral-combined_k141_126713\|\|full | 1343 | No | NA | | 0 | 1 | Not-determined | Genome-fragment | NA | | 0 |
| S9-final-viral-combined_k141_90185\|\|full | 4470 | No | NA | | 0 | 2 | Not-determined | Genome-fragment | NA | | 0 |
| S7-final-viral-combined_k141_128899\|\|full | 1037 | No | NA | | 1 | 0 | Low-quality | Genome-fragment | | 1.85 | 0 |
| S7_k141_131325 | 1037 | No | NA | | 1 | 0 | Low-quality | Genome-fragment | | 2.36 | 0 |
| S9-final-viral-combined_k141_20485\|\|full | 90295 | Yes | | 38188 | 36 | 45 | Complete | High-quality | | 100 | 57.71 |
| S9-final-viral-combined_k141_122305\|\|full | 1101 | No | NA | | 0 | 1 | Not-determined | Genome-fragment | NA | | 0 |
| S9-final-viral-combined_k141_9931\|\|full | 90630 | Yes | | 43033 | 25 | 16 | Medium-quality | Genome-fragment | | 63.56 | 52.52 |
| S17-final-viral-combined_k141_260489\|\|full | 1037 | No | NA | | 1 | 0 | Low-quality | Genome-fragment | | 0.56 | 0 |
| S17-final-viral-combined_k141_250368\|\|full | 1037 | No | NA | | 1 | 0 | Low-quality | Genome-fragment | | 1.83 | 0 |
| S17-k141_129807 | 1037 | No | NA | | 0 | 0 | Low-quality | Genome-fragment | | 8.1 | 0 |
| S9-final-viral-combined_k141_52901\|\|full | 2274 | No | NA | | 0 | 3 | Not-determined | Genome-fragment | NA | | 0 |
| S17-k141_143946 | 1037 | No | NA | | 0 | 1 | Low-quality | Genome-fragment | | 1.49 | 0 |
| S9-final-viral-combined_k141_4592\|\|full | 5015 | No | NA | | 0 | 5 | Not-determined | Genome-fragment | NA | | 0 |
| S12-final-viral-combined_k141_59656\|\|full | 1037 | No | NA | | 1 | 0 | Low-quality | Genome-fragment | | 2.36 | 0 |
| S2-final-viral-combined_k141_187210\|\|full | 1037 | No | NA | | 0 | 1 | Low-quality | Genome-fragment | | 1.52 | 0 |
| S9-final-viral-combined_k141_52911\|\|full | 1469 | No | NA | | 0 | 0 | Not-determined | Genome-fragment | NA | | 0 |
| S9-final-viral-combined_k141_68636\|\|full | 1051 | No | NA | | 0 | 1 | Not-determined | Genome-fragment | NA | | 0 |
| S9-final-viral-combined_k141_94795\|\|full | 2286 | No | NA | | 0 | 1 | Not-determined | Genome-fragment | NA | | 0 |
| S2-final-viral-combined_k141_222648\|\|full | 1037 | No | NA | | 2 | 0 | Low-quality | Genome-fragment | | 2.57 | 0 |
| S9-final-viral-combined_k141_94800\|\|full | 5033 | No | NA | | 0 | 5 | Not-determined | Genome-fragment | NA | | 0 |
| S9-final-viral-combined_k141_105561\|\|full | 3635 | No | NA | | 0 | 1 | Not-determined | Genome-fragment | NA | | 0 |
| S9-final-viral-combined_k141_41506\|\|full | 2332 | No | NA | | 0 | 0 | Not-determined | Genome-fragment | NA | | 0 |
| S9-final-viral-combined_k141_41507\|\|full | 1394 | No | NA | | 0 | 2 | Not-determined | Genome-fragment | NA | | 0 |
| S9-final-viral-combined_k141_30636\|\|full | 2752 | No | NA | | 0 | 0 | Not-determined | Genome-fragment | NA | | 0 |
| S9-final-viral-combined_k141_116398\|\|full | 5525 | No | NA | | 0 | 2 | Not-determined | Genome-fragment | NA | | 0 |
| S9-final-viral-combined_k141_36299\|\|full | 2607 | No | NA | | 0 | 2 | Not-determined | Genome-fragment | NA | | 0 |
| S9-final-viral-combined_k141_63666\|\|full | 1547 | No | NA | | 0 | 1 | Not-determined | Genome-fragment | NA | | 0 |
| S9-final-viral-combined_k141_58080\|\|full | 1961 | No | NA | | 0 | 2 | Not-determined | Genome-fragment | NA | | 0 |
| S2-final-viral-combined_k141_43047\|\|lt2gene | 1037 | No | NA | | 1 | 0 | Low-quality | Genome-fragment | | 2.63 | 0 |
| S9-final-viral-combined_k141_30672\|\|full | 1974 | No | NA | | 0 | 1 | Not-determined | Genome-fragment | NA | | 0 |
| S8_k141_22263 | 1037 | No | NA | | 0 | 0 | Low-quality | Genome-fragment | | 2.46 | 0 |
| S9-final-viral-combined_k141_20602\|\|full | 1974 | No | NA | | 0 | 2 | Not-determined | Genome-fragment | NA | | 0 |
| S9-final-viral-combined_k141_49194\|\|full | 1038 | No | NA | | 0 | 2 | Low-quality | Genome-fragment | | 0.34 | 0 |
| S9_k141_7196 | 1038 | No | NA | | 0 | 0 | Low-quality | Genome-fragment | | 0.7 | 0 |
| S11-final-viral-combined_k141_21024\|\|full | 1038 | No | NA | | 0 | 0 | Low-quality | Genome-fragment | | 2.1 | 0 |
| S9-final-viral-combined_k141_58120\|\|full | 1547 | No | NA | | 0 | 1 | Not-determined | Genome-fragment | NA | | 0 |
| S11-final-viral-combined_k141_47382\|\|lt2gene | 1038 | No | NA | | 0 | 0 | Low-quality | Genome-fragment | | 1.82 | 0 |
| S9-final-viral-combined_k141_58125\|\|full | 4444 | No | NA | | 0 | 0 | Not-determined | Genome-fragment | NA | | 0 |
| S9-final-viral-combined_k141_52999\|\|full | 2000 | No | NA | | 0 | 2 | Not-determined | Genome-fragment | NA | | 0 |
| S9-final-viral-combined_k141_126758\|\|full | 1646 | No | NA | | 0 | 0 | Not-determined | Genome-fragment | NA | | 0 |
| S11_k141_156595 | 1038 | No | NA | | 1 | 0 | Low-quality | Genome-fragment | | 2.16 | 0 |
| S1-final-viral-combined_k141_84663\|\|full | 1038 | No | NA | | 2 | 0 | Low-quality | Genome-fragment | | 2.62 | 0 |
| S1-final-viral-combined_k141_324147\|\|full | 1038 | No | NA | | 0 | 1 | Low-quality | Genome-fragment | | 0.78 | 0 |
| S9-final-viral-combined_k141_15065\|\|full | 4187 | No | NA | | 0 | 2 | Not-determined | Genome-fragment | NA | | 0 |
| S9-final-viral-combined_k141_126769\|\|full | 4039 | No | NA | | 0 | 0 | Not-determined | Genome-fragment | NA | | 0 |
| S9-final-viral-combined_k141_90372\|\|full | 11598 | No | NA | | 0 | 8 | Not-determined | Genome-fragment | NA | | 0 |
| S9-final-viral-combined_k141_84979\|\|full | 1519 | No | NA | | 0 | 1 | Not-determined | Genome-fragment | NA | | 0 |
| S9-final-viral-combined_k141_100514\|\|full | 1942 | No | NA | | 0 | 0 | Not-determined | Genome-fragment | NA | | 0 |
| S1_k141_319682 | 1038 | No | NA | | 0 | 0 | Low-quality | Genome-fragment | | 0.91 | 0 |
| S9-final-viral-combined_k141_122484\|\|full | 1385 | No | NA | | 0 | 1 | Not-determined | Genome-fragment | NA | | 0 |
| S9-final-viral-combined_k141_4687\|\|full | 2693 | No | NA | | 0 | 1 | Not-determined | Genome-fragment | NA | | 0 |
| S9-final-viral-combined_k141_30743\|\|full | 3177 | No | NA | | 0 | 0 | Not-determined | Genome-fragment | NA | | 0 |
| S9-final-viral-combined_k141_110439\|\|full | 7250 | No | NA | | 0 | 4 | Not-determined | Genome-fragment | NA | | 0 |
| S9-final-viral-combined_k141_73514\|\|full | 2315 | No | NA | | 0 | 2 | Not-determined | Genome-fragment | NA | | 0 |
| S9-final-viral-combined_k141_79515\|\|full | 2070 | No | NA | | 0 | 2 | Not-determined | Genome-fragment | NA | | 0 |
| S9-final-viral-combined_k141_122543\|\|full | 1388 | No | NA | | 0 | 0 | Not-determined | Genome-fragment | NA | | 0 |
| S9-final-viral-combined_k141_10070\|\|full | 7254 | No | NA | | 0 | 2 | Not-determined | Genome-fragment | NA | | 0 |
| S5-final-viral-combined_k141_27739\|\|full | 1038 | No | NA | | 1 | 0 | Low-quality | Genome-fragment | | 2.67 | 0 |
| S9-final-viral-combined_k141_68881\|\|full | 1520 | No | NA | | 0 | 2 | Not-determined | Genome-fragment | NA | | 0 |
| S9-final-viral-combined_k141_63829\|\|full | 12632 | No | NA | | 0 | 7 | Not-determined | Genome-fragment | NA | | 0 |
| S5-final-viral-combined_k141_197102\|\|full | 1038 | No | NA | | 1 | 0 | Low-quality | Genome-fragment | | 3.21 | 0 |
| S9-final-viral-combined_k141_68888\|\|full | 1174 | No | NA | | 0 | 0 | Not-determined | Genome-fragment | NA | | 0 |
| S5-final-viral-combined_k141_188500\|\|full | 1038 | No | NA | | 1 | 0 | Low-quality | Genome-fragment | | 2.58 | 0 |
| S5-final-viral-combined_k141_91704\|\|lt2gene | 1038 | No | NA | | 0 | 0 | Low-quality | Genome-fragment | | 0.64 | 0 |
| S9-final-viral-combined_k141_85059\|\|full | 4571 | No | NA | | 0 | 3 | Not-determined | Genome-fragment | NA | | 0 |
| S5_k141_46987 | 1038 | No | NA | | 0 | 0 | Low-quality | Genome-fragment | | 1.44 | 0 |
| S9-final-viral-combined_k141_79532\|\|full | 6693 | No | NA | | 0 | 1 | Not-determined | Genome-fragment | NA | | 0 |
| S5_k141_4139 | 1038 | No | NA | | 0 | 0 | Low-quality | Genome-fragment | | 7.12 | 0 |
| S9-final-viral-combined_k141_110458\|\|full | 1458 | No | NA | | 0 | 2 | Not-determined | Genome-fragment | NA | | 0 |
| S7_k141_226762 | 1038 | No | NA | | 0 | 1 | Low-quality | Genome-fragment | | 0.9 | 0 |
| S17-final-viral-combined_k141_138139\|\|full | 1038 | No | NA | | 2 | 0 | Low-quality | Genome-fragment | | 2.39 | 0 |
| S9-final-viral-combined_k141_90479\|\|full | 4547 | No | NA | | 0 | 4 | Not-determined | Genome-fragment | NA | | 0 |
| S9-final-viral-combined_k141_36459\|\|full | 29514 | No | NA | | 19 | 0 | Medium-quality | Genome-fragment | | 89.69 | 0 |
| S17-final-viral-combined_k141_152578\|\|full | 1038 | No | NA | | 0 | 0 | Low-quality | Genome-fragment | | 2.45 | 0 |
| S9-final-viral-combined_k141_53137\|\|full | 1298 | No | NA | | 0 | 1 | Not-determined | Genome-fragment | NA | | 0 |
| S9-final-viral-combined_k141_95014\|\|full | 2998 | No | NA | | 0 | 3 | Not-determined | Genome-fragment | NA | | 0 |
| S12-final-viral-combined_k141_159271\|\|full | 1038 | No | NA | | 1 | 0 | Low-quality | Genome-fragment | | 0.63 | 0 |
| S9-final-viral-combined_k141_10141\|\|full | 6594 | No | NA | | 0 | 3 | Not-determined | Genome-fragment | NA | | 0 |
| S9-final-viral-combined_k141_46771\|\|full | 2292 | No | NA | | 0 | 2 | Not-determined | Genome-fragment | NA | | 0 |
| S12-final-viral-combined_k141_32091\|\|lt2gene | 1038 | No | NA | | 1 | 0 | Low-quality | Genome-fragment | | 0.72 | 0 |
| S12_k141_122990 | 1038 | No | NA | | 0 | 1 | Low-quality | Genome-fragment | | 2.5 | 0 |
| S12_k141_224244 | 1038 | No | NA | | 0 | 1 | Low-quality | Genome-fragment | | 0.64 | 0 |
| S9-final-viral-combined_k141_63904\|\|full | 1130 | No | NA | | 0 | 2 | Not-determined | Genome-fragment | NA | | 0 |
| S12_k141_395526 | 1038 | No | NA | | 0 | 0 | Low-quality | Genome-fragment | | 1.83 | 0 |
| S9-final-viral-combined_k141_79607\|\|full | 1252 | No | NA | | 0 | 0 | Not-determined | Genome-fragment | NA | | 0 |
| S9-final-viral-combined_k141_46804\|\|full | 8520 | No | NA | | 0 | 8 | Not-determined | Genome-fragment | NA | | 0 |
| S2-final-viral-combined_k141_220097\|\|full | 1038 | No | NA | | 1 | 0 | Low-quality | Genome-fragment | | 2.64 | 0 |

| | | | | | | | | | | | |
|---|---|---|---|---|---|---|---|---|---|---|---|
| S9-final-viral-combined_k141_36526||full | 1124 | No | NA | | 0 | 1 | Not-determined | Genome-fragment | NA | | 0 |
| S2_k141_243759 | 1038 | No | NA | | 1 | 0 | Low-quality | Genome-fragment | | 1.14 | 0 |
| S4-final-viral-combined_k141_106357||full | 1038 | No | NA | | 0 | 1 | Low-quality | Genome-fragment | | 0.37 | 0 |
| S9-final-viral-combined_k141_100710||full | 1092 | No | NA | | 0 | 1 | Not-determined | Genome-fragment | NA | | 0 |
| S4_k141_49856 | 1038 | No | NA | | 1 | 0 | Low-quality | Genome-fragment | | 1.56 | 0 |
| S9-final-viral-combined_k141_41615||full | 2346 | No | NA | | 0 | 2 | Not-determined | Genome-fragment | NA | | 0 |
| S9-final-viral-combined_k141_116492||full | 1714 | No | NA | | 0 | 2 | Not-determined | Genome-fragment | NA | | 0 |
| S6-final-viral-combined_k141_81723||full | 1038 | No | NA | | 1 | 0 | Low-quality | Genome-fragment | | 1.54 | 0 |
| S9-final-viral-combined_k141_41644||full | 1790 | No | NA | | 0 | 0 | Not-determined | Genome-fragment | NA | | 0 |
| S9-final-viral-combined_k141_110571||full | 3823 | No | NA | | 0 | 2 | Not-determined | Genome-fragment | NA | | 0 |
| S8-final-viral-combined_k141_121582||full | 1038 | No | NA | | 0 | 0 | Low-quality | Genome-fragment | | 1.39 | 0 |
| S9-final-viral-combined_k141_26173||full | 1965 | No | NA | | 0 | 1 | Not-determined | Genome-fragment | NA | | 0 |
| S9-final-viral-combined_k141_10213||full | 1186 | No | NA | | 0 | 1 | Not-determined | Genome-fragment | NA | | 0 |
| S8_k141_24031 | 1038 | No | NA | | 0 | 1 | Low-quality | Genome-fragment | | 2.17 | 0 |
| S9-final-viral-combined_k141_53178||full | 1387 | No | NA | | 0 | 1 | Not-determined | Genome-fragment | NA | | 0 |
| S9-final-viral-combined_k141_95072||full | 2479 | No | NA | | 0 | 2 | Not-determined | Genome-fragment | NA | | 0 |
| S9-final-viral-combined_k141_97083||full | 1039 | No | NA | | 2 | 0 | Low-quality | Genome-fragment | | 2.88 | 0 |
| S9-final-viral-combined_k141_60207||full | 1039 | No | NA | | 0 | 0 | Low-quality | Genome-fragment | | 2.28 | 0 |
| S9-final-viral-combined_k141_88708||full | 1039 | No | NA | | 0 | 0 | Low-quality | Genome-fragment | | 2.23 | 0 |
| S9_k141_30942 | 1039 | No | NA | | 0 | 0 | Low-quality | Genome-fragment | | 0.66 | 0 |
| S9-final-viral-combined_k141_100783||full | 1594 | No | NA | | 0 | 1 | Not-determined | Genome-fragment | NA | | 0 |
| S9-final-viral-combined_k141_69102||full | 1342 | No | NA | | 0 | 2 | Not-determined | Genome-fragment | NA | | 0 |
| S11-final-viral-combined_k141_60999||full | 1039 | No | NA | | 0 | 0 | Low-quality | Genome-fragment | | 0.79 | 0 |
| S9-final-viral-combined_k141_69103||full | 5438 | No | NA | | 0 | 3 | Not-determined | Genome-fragment | NA | | 0 |
| S1-final-viral-combined_k141_150039||full | 1039 | No | NA | | 0 | 0 | Low-quality | Genome-fragment | | 2.42 | 0 |
| S9-final-viral-combined_k141_36694||full | 1421 | No | NA | | 0 | 1 | Not-determined | Genome-fragment | NA | | 0 |
| S1_k141_109164 | 1039 | No | NA | | 0 | 0 | Low-quality | Genome-fragment | | 1.92 | 0 |
| S9-final-viral-combined_k141_126981||full | 4142 | No | NA | | 0 | 2 | Not-determined | Genome-fragment | NA | | 0 |
| S3_k141_81461 | 1039 | No | NA | | 1 | 0 | Low-quality | Genome-fragment | | 1.1 | 0 |
| S7-final-viral-combined_k141_226730||full | 1039 | No | NA | | 0 | 0 | Low-quality | Genome-fragment | | 1.55 | 0 |
| S17-final-viral-combined_k141_210775||full | 1039 | No | NA | | 0 | 0 | Low-quality | Genome-fragment | | 2.38 | 0 |
| S2-final-viral-combined_k141_172383||lt2gene | 1039 | No | NA | | 1 | 0 | Low-quality | Genome-fragment | | 2.25 | 0 |
| S2_k141_241185 | 1039 | No | NA | | 0 | 2 | Low-quality | Genome-fragment | | 0.94 | 0 |
| S8_k141_204886 | 1039 | No | NA | | 0 | 2 | Low-quality | Genome-fragment | | 0.8 | 0 |
| S9-final-viral-combined_k141_19063||lt2gene | 1040 | No | NA | | 1 | 0 | Low-quality | Genome-fragment | | 2.24 | 0 |
| S9-final-viral-combined_k141_4936||full | 2115 | No | NA | | 0 | 0 | Not-determined | Genome-fragment | NA | | 0 |
| S9-final-viral-combined_k141_36747||full | 5815 | No | NA | | 0 | 1 | Not-determined | Genome-fragment | NA | | 0 |
| S1-final-viral-combined_k141_217186||full | 1040 | No | NA | | 0 | 0 | Low-quality | Genome-fragment | | 2.47 | 0 |
| S9-final-viral-combined_k141_53295||full | 1578 | No | NA | | 0 | 1 | Not-determined | Genome-fragment | NA | | 0 |
| S9-final-viral-combined_k141_85328||full | 4066 | No | NA | | 0 | 3 | Not-determined | Genome-fragment | NA | | 0 |
| S9-final-viral-combined_k141_116694||full | 2187 | No | NA | | 0 | 2 | Not-determined | Genome-fragment | NA | | 0 |
| S1_k141_379001 | 1040 | No | NA | | 0 | 1 | Low-quality | Genome-fragment | | 0.5 | 0 |
| S9-final-viral-combined_k141_41802||full | 4608 | No | NA | | 0 | 1 | Not-determined | Genome-fragment | NA | | 0 |
| S9-final-viral-combined_k141_26287||full | 1879 | No | NA | | 0 | 2 | Not-determined | Genome-fragment | NA | | 0 |
| S7_k141_99963 | 1040 | No | NA | | 0 | 1 | Low-quality | Genome-fragment | | 0.16 | 0 |
| S9-final-viral-combined_k141_41818||full | 2621 | No | NA | | 0 | 2 | Not-determined | Genome-fragment | NA | | 0 |
| S17-final-viral-combined_k141_11390||full | 1040 | No | NA | | 2 | 0 | Low-quality | Genome-fragment | | 2.23 | 0 |
| S9-final-viral-combined_k141_90808||full | 1222 | No | NA | | 0 | 1 | Not-determined | Genome-fragment | NA | | 0 |
| S9-final-viral-combined_k141_30979||full | 3417 | No | NA | | 0 | 3 | Not-determined | Genome-fragment | NA | | 0 |
| S9-final-viral-combined_k141_73784||full | 1465 | No | NA | | 0 | 1 | Not-determined | Genome-fragment | NA | | 0 |
| S9-final-viral-combined_k141_90818||full | 1486 | No | NA | | 0 | 0 | Not-determined | Genome-fragment | NA | | 0 |
| S9-final-viral-combined_k141_69175||full | 4213 | No | NA | | 0 | 1 | Not-determined | Genome-fragment | NA | | 0 |
| S9-final-viral-combined_k141_73804||full | 17806 | No | NA | | 0 | 9 | Not-determined | Genome-fragment | NA | | 0 |
| S17-final-viral-combined_k141_105895||full | 1040 | No | NA | | 0 | 0 | Low-quality | Genome-fragment | | 2.04 | 0 |
| S9-final-viral-combined_k141_58366||full | 1277 | No | NA | | 0 | 1 | Not-determined | Genome-fragment | NA | | 0 |
| S9-final-viral-combined_k141_36873||full | 1689 | No | NA | | 0 | 0 | Not-determined | Genome-fragment | NA | | 0 |
| S17-final-viral-combined_k141_150482||lt2gene | 1040 | No | NA | | 0 | 0 | Low-quality | Genome-fragment | | 1.92 | 0 |
| S9-final-viral-combined_k141_85431||full | 3215 | No | NA | | 0 | 1 | Not-determined | Genome-fragment | NA | | 0 |
| S9-final-viral-combined_k141_31057||full | 3222 | No | NA | | 0 | 2 | Not-determined | Genome-fragment | NA | | 0 |
| S9-final-viral-combined_k141_116758||full | 2439 | No | NA | | 0 | 2 | Not-determined | Genome-fragment | NA | | 0 |
| S9-final-viral-combined_k141_85438||full | 2835 | No | NA | | 0 | 2 | Not-determined | Genome-fragment | NA | | 0 |
| S9-final-viral-combined_k141_116765||full | 5298 | No | NA | | 0 | 4 | Not-determined | Genome-fragment | NA | | 0 |
| S9-final-viral-combined_k141_41907||full | 1695 | No | NA | | 0 | 0 | Not-determined | Genome-fragment | NA | | 0 |
| S17-k141_196905 | 1040 | No | NA | | 0 | 0 | Low-quality | Genome-fragment | | 2.29 | 0 |
| S9-final-viral-combined_k141_105822||full | 43006 | No | NA | | 32 | 0 | High-quality | High-quality | | 97.86 | 0 |
| S17-k141_266339 | 1040 | No | NA | | 0 | 1 | Low-quality | Genome-fragment | | 0.33 | 0 |
| S9-final-viral-combined_k141_116787||full | 1193 | No | NA | | 0 | 1 | Not-determined | Genome-fragment | NA | | 0 |
| S9-final-viral-combined_k141_90897||full | 1854 | No | NA | | 0 | 0 | Not-determined | Genome-fragment | NA | | 0 |
| S12-final-viral-combined_k141_220734||full | 1040 | No | NA | | 1 | 0 | Low-quality | Genome-fragment | | 0.66 | 0 |
| S9-final-viral-combined_k141_15463||full | 1189 | No | NA | | 0 | 1 | Not-determined | Genome-fragment | NA | | 0 |
| S12-final-viral-combined_k141_75489||full | 1040 | No | NA | | 3 | 0 | Low-quality | Genome-fragment | | 1.82 | 0 |
| S9-final-viral-combined_k141_69210||full | 2624 | No | NA | | 0 | 2 | Not-determined | Genome-fragment | NA | | 0 |
| S9-final-viral-combined_k141_105854||full | 1308 | No | NA | | 0 | 1 | Not-determined | Genome-fragment | NA | | 0 |
| S12-final-viral-combined_k141_29418||full | 1040 | No | NA | | 0 | 1 | Low-quality | Genome-fragment | | 0.27 | 0 |
| S9-final-viral-combined_k141_53426||full | 1787 | No | NA | | 0 | 2 | Not-determined | Genome-fragment | NA | | 0 |
| S12-final-viral-combined_k141_367961||lt2gene | 1040 | No | NA | | 1 | 0 | Low-quality | Genome-fragment | | 2.52 | 0 |
| S2-final-viral-combined_k141_56058||lt2gene | 1040 | No | NA | | 1 | 0 | Low-quality | Genome-fragment | | 2.21 | 0 |
| S9-final-viral-combined_k141_79889||full | 2416 | No | NA | | 0 | 1 | Not-determined | Genome-fragment | NA | | 0 |
| S9-final-viral-combined_k141_31066||full | 3896 | No | NA | | 0 | 1 | Not-determined | Genome-fragment | NA | | 0 |
| S9-final-viral-combined_k141_93448||full | 1041 | No | NA | | 0 | 0 | Low-quality | Genome-fragment | | 2.63 | 0 |
| S9-final-viral-combined_k141_58458||full | 1893 | No | NA | | 0 | 1 | Not-determined | Genome-fragment | NA | | 0 |
| S9-final-viral-combined_k141_26410||full | 63551 | No | NA | | 33 | 2 | High-quality | High-quality | | 100 | 0 |
| S1-final-viral-combined_k141_3234||lt2gene | 1041 | No | NA | | 1 | 0 | Low-quality | Genome-fragment | | 1.9 | 0 |
| S5-final-viral-combined_k141_155346||full | 1041 | No | NA | | 2 | 0 | Low-quality | Genome-fragment | | 3.07 | 0 |
| S5-final-viral-combined_k141_16432||lt2gene | 1041 | No | NA | | 2 | 0 | Low-quality | Genome-fragment | | 1.5 | 0 |
| S9-final-viral-combined_k141_122746||full | 1987 | No | NA | | 0 | 1 | Not-determined | Genome-fragment | NA | | 0 |
| S9-final-viral-combined_k141_122747||full | 2196 | No | NA | | 0 | 1 | Not-determined | Genome-fragment | NA | | 0 |
| S9-final-viral-combined_k141_101111||full | 1634 | No | NA | | 0 | 1 | Not-determined | Genome-fragment | NA | | 0 |
| S9-final-viral-combined_k141_10362||full | 1328 | No | NA | | 0 | 1 | Not-determined | Genome-fragment | NA | | 0 |
| S9-final-viral-combined_k141_26458||full | 1282 | No | NA | | 0 | 0 | Not-determined | Genome-fragment | NA | | 0 |
| S9-final-viral-combined_k141_15629||full | 2450 | No | NA | | 0 | 2 | Not-determined | Genome-fragment | NA | | 0 |
| S7-final-viral-combined_k141_121472||lt2gene | 1041 | No | NA | | 1 | 0 | Low-quality | Genome-fragment | | 2.19 | 0 |
| S9-final-viral-combined_k141_58515||full | 1459 | No | NA | | 0 | 2 | Not-determined | Genome-fragment | NA | | 0 |
| S12-final-viral-combined_k141_352541||full | 1041 | No | NA | | 0 | 0 | Low-quality | Genome-fragment | | 1.52 | 0 |
| S9-final-viral-combined_k141_37121||full | 1587 | No | NA | | 0 | 0 | Not-determined | Genome-fragment | NA | | 0 |
| S9-final-viral-combined_k141_37126||full | 1601 | No | NA | | 0 | 0 | Not-determined | Genome-fragment | NA | | 0 |
| S9-final-viral-combined_k141_26533||full | 1823 | No | NA | | 0 | 1 | Not-determined | Genome-fragment | NA | | 0 |
| S9-final-viral-combined_k141_101207||full | 1217 | No | NA | | 0 | 1 | Not-determined | Genome-fragment | NA | | 0 |
| S9-final-viral-combined_k141_26559||full | 1948 | No | NA | | 0 | 2 | Not-determined | Genome-fragment | NA | | 0 |
| S9-final-viral-combined_k141_47018||full | 1183 | No | NA | | 0 | 0 | Not-determined | Genome-fragment | NA | | 0 |
| S12_k141_256968 | 1041 | No | NA | | 0 | 2 | Low-quality | Genome-fragment | | 2.12 | 0 |
| S2-final-viral-combined_k141_52837||full | 1041 | No | NA | | 0 | 0 | Low-quality | Genome-fragment | | 1.28 | 0 |
| S2-final-viral-combined_k141_240189||full | 1041 | No | NA | | 0 | 0 | Low-quality | Genome-fragment | | 1.83 | 0 |
| S9-final-viral-combined_k141_127086||full | 1014 | No | NA | | 0 | 1 | Not-determined | Genome-fragment | NA | | 0 |
| S9-final-viral-combined_k141_5130||full | 1336 | No | NA | | 0 | 1 | Not-determined | Genome-fragment | NA | | 0 |
| S9-final-viral-combined_k141_26615||full | 3058 | No | NA | | 0 | 0 | Not-determined | Genome-fragment | NA | | 0 |
| S4_k141_76287 | 1041 | No | NA | | 0 | 1 | Low-quality | Genome-fragment | | 0.14 | 0 |
| S9-final-viral-combined_k141_15729||full | 3159 | No | NA | | 0 | 2 | Not-determined | Genome-fragment | NA | | 0 |
| S11-final-viral-combined_k141_245289||full | 1042 | No | NA | | 0 | 0 | Low-quality | Genome-fragment | | 2.06 | 0 |
| S9-final-viral-combined_k141_74040||full | 5135 | No | NA | | 0 | 2 | Not-determined | Genome-fragment | NA | | 0 |
| S1-final-viral-combined_k141_342141||full | 1042 | No | NA | | 0 | 0 | Low-quality | Genome-fragment | | 2.15 | 0 |
| S9-final-viral-combined_k141_10460||full | 3549 | No | NA | | 0 | 1 | Not-determined | Genome-fragment | NA | | 0 |
| S1-final-viral-combined_k141_146027||full | 1042 | No | NA | | 1 | 0 | Low-quality | Genome-fragment | | 2.36 | 0 |
| S9-final-viral-combined_k141_74063||full | 4092 | No | NA | | 0 | 1 | Not-determined | Genome-fragment | NA | | 0 |
| S9-final-viral-combined_k141_21198||full | 1041 | No | NA | | 0 | 1 | Not-determined | Genome-fragment | NA | | 0 |
| S9-final-viral-combined_k141_15776||full | 2609 | No | NA | | 0 | 3 | Not-determined | Genome-fragment | NA | | 0 |
| S9-final-viral-combined_k141_26652||full | 13994 | No | NA | | 0 | 4 | Not-determined | Genome-fragment | NA | | 0 |
| S9-final-viral-combined_k141_26654||full | 3200 | No | NA | | 0 | 3 | Not-determined | Genome-fragment | NA | | 0 |
| S9-final-viral-combined_k141_26656||full | 1343 | No | NA | | 0 | 0 | Not-determined | Genome-fragment | NA | | 0 |
| S9-final-viral-combined_k141_101270||full | 1105 | No | NA | | 0 | 2 | Not-determined | Genome-fragment | NA | | 0 |
| S9-final-viral-combined_k141_26666||full | 2981 | No | NA | | 0 | 3 | Not-determined | Genome-fragment | NA | | 0 |
| S9-final-viral-combined_k141_58608||full | 4746 | No | NA | | 0 | 1 | Not-determined | Genome-fragment | NA | | 0 |
| S9-final-viral-combined_k141_47096||full | 1379 | No | NA | | 0 | 1 | Not-determined | Genome-fragment | NA | | 0 |
| S9-final-viral-combined_k141_80036||full | 2664 | No | NA | | 0 | 0 | Not-determined | Genome-fragment | NA | | 0 |
| S9-final-viral-combined_k141_95399||full | 1338 | No | NA | | 0 | 0 | Not-determined | Genome-fragment | NA | | 0 |
| S1_k141_38 | 1042 | No | NA | | 0 | 1 | Low-quality | Genome-fragment | | 0.76 | 0 |
| S9-final-viral-combined_k141_74121||full | 2460 | No | NA | | 0 | 1 | Not-determined | Genome-fragment | NA | | 0 |
| S5-final-viral-combined_k141_67484||full | 1042 | No | NA | | 1 | 0 | Low-quality | Genome-fragment | | 0.63 | 0 |
| S9-final-viral-combined_k141_10518||full | 1133 | No | NA | | 0 | 0 | Not-determined | Genome-fragment | NA | | 0 |
| S5-final-viral-combined_k141_64131||lt2gene | 1042 | No | NA | | 1 | 0 | Low-quality | Genome-fragment | | 1.86 | 0 |
| S9-final-viral-combined_k141_37257||full | 2774 | No | NA | | 0 | 4 | Not-determined | Genome-fragment | NA | | 0 |
| S7_k141_80582 | 1042 | No | NA | | 0 | 2 | Low-quality | Genome-fragment | | 0.78 | 0 |
| S9-final-viral-combined_k141_37289||full | 2196 | No | NA | | 0 | 1 | Not-determined | Genome-fragment | NA | | 0 |
| S9-final-viral-combined_k141_31296||full | 1591 | No | NA | | 0 | 2 | Not-determined | Genome-fragment | NA | | 0 |
| S9-final-viral-combined_k141_21301||full | 1444 | No | NA | | 0 | 0 | Not-determined | Genome-fragment | NA | | 0 |
| S17-final-viral-combined_k141_103019||full | 1042 | No | NA | | 0 | 1 | Low-quality | Genome-fragment | | 1.66 | 0 |
| S9-final-viral-combined_k141_21310||full | 6308 | No | NA | | 0 | 3 | Not-determined | Genome-fragment | NA | | 0 |
| S17-final-viral-combined_k141_140102||full | 1042 | No | NA | | 0 | 0 | Low-quality | Genome-fragment | | 2.61 | 0 |
| S9-final-viral-combined_k141_42180||full | 7539 | No | NA | | 0 | 4 | Not-determined | Genome-fragment | NA | | 0 |
| S17-k141_44388 | 1042 | No | NA | | 0 | 0 | Low-quality | Genome-fragment | | 0.77 | 0 |
| S9-final-viral-combined_k141_80110||full | 3636 | No | NA | | 0 | 1 | Not-determined | Genome-fragment | NA | | 0 |
| S9-final-viral-combined_k141_116905||full | 3364 | No | NA | | 0 | 2 | Not-determined | Genome-fragment | NA | | 0 |
| S12-final-viral-combined_k141_3132||full | 1042 | No | NA | | 1 | 0 | Low-quality | Genome-fragment | | 1.73 | 0 |
| S12-final-viral-combined_k141_324970||full | 1042 | No | NA | | 1 | 0 | Low-quality | Genome-fragment | | 1.71 | 0 |
| S9-final-viral-combined_k141_116938||full | 1094 | No | NA | | 0 | 0 | Not-determined | Genome-fragment | NA | | 0 |
| S9-final-viral-combined_k141_42214||full | 5813 | No | NA | | 0 | 0 | Not-determined | Genome-fragment | NA | | 0 |
| S12_k141_343032 | 1042 | No | NA | | 0 | 1 | Low-quality | Genome-fragment | | 0.17 | 0 |
| S12_k141_260513 | 1042 | No | NA | | 0 | 0 | Low-quality | Genome-fragment | | 2.13 | 0 |
| S9-final-viral-combined_k141_47111||full | 1510 | No | NA | | 0 | 1 | Not-determined | Genome-fragment | NA | | 0 |
| S9-final-viral-combined_k141_47115||full | 2687 | No | NA | | 0 | 1 | Not-determined | Genome-fragment | NA | | 0 |
| S9-final-viral-combined_k141_47119||full | 2589 | No | NA | | 0 | 2 | Not-determined | Genome-fragment | NA | | 0 |
| S9-final-viral-combined_k141_127159||full | 1755 | No | NA | | 0 | 1 | Not-determined | Genome-fragment | NA | | 0 |
| S2-final-viral-combined_k141_54858||full | 1042 | No | NA | | 0 | 0 | Low-quality | Genome-fragment | | 2.14 | 0 |
| S2-final-viral-combined_k141_154491||lt2gene | 1042 | No | NA | | 1 | 0 | Low-quality | Genome-fragment | | 2.2 | 0 |
| S9-final-viral-combined_k141_42267||full | 2251 | No | NA | | 0 | 0 | Not-determined | Genome-fragment | NA | | 0 |
| S2_k141_119555 | 1042 | No | NA | | 0 | 0 | Low-quality | Genome-fragment | | 1.09 | 0 |
| S9-final-viral-combined_k141_31425||full | 2543 | No | NA | | 0 | 1 | Not-determined | Genome-fragment | NA | | 0 |
| S4-final-viral-combined_k141_113983||full | 1042 | No | NA | | 0 | 1 | Low-quality | Genome-fragment | | 1.01 | 0 |
| S9-final-viral-combined_k141_31440||full | 4603 | No | NA | | 0 | 1 | Not-determined | Genome-fragment | NA | | 0 |
| S4_k141_147591 | 1042 | No | NA | | 0 | 0 | Low-quality | Genome-fragment | | 1.09 | 0 |
| S4_k141_24386 | 1042 | No | NA | | 0 | 0 | Low-quality | Genome-fragment | | 0.74 | 0 |
| S9-final-viral-combined_k141_117052||full | 1328 | No | NA | | 0 | 0 | Not-determined | Genome-fragment | NA | | 0 |
| S9-final-viral-combined_k141_95550||full | 1423 | No | NA | | 0 | 2 | Not-determined | Genome-fragment | NA | | 0 |
| S9-final-viral-combined_k141_16017||full | 2960 | No | NA | | 0 | 1 | Not-determined | Genome-fragment | NA | | 0 |
| S9-final-viral-combined_k141_117093||full | 3091 | No | NA | | 0 | 0 | Not-determined | Genome-fragment | NA | | 0 |
| S6_k141_65318 | 1042 | No | NA | | 0 | 0 | Low-quality | Genome-fragment | | 2.77 | 0 |
| S9-final-viral-combined_k141_117094||full | 2018 | No | NA | | 0 | 1 | Not-determined | Genome-fragment | NA | | 0 |
| S8_k141_255585 | 1042 | No | NA | | 0 | 0 | Low-quality | Genome-fragment | | 0.68 | 0 |
| S9-final-viral-combined_k141_117124||full | 2120 | No | NA | | 0 | 2 | Not-determined | Genome-fragment | NA | | 0 |
| S9-final-viral-combined_k141_31456||full | 48386 | Yes | | 34943 | 9 | 6 | Medium-quality | Genome-fragment | | 72.49 | 27.78 |
| S9-final-viral-combined_k141_74315||full | 4687 | No | NA | | 0 | 4 | Not-determined | Genome-fragment | NA | | 0 |
| S9-final-viral-combined_k141_101527||full | 2567 | No | NA | | 0 | 2 | Not-determined | Genome-fragment | NA | | 0 |
| S9-final-viral-combined_k141_64950||lt2gene | 1043 | No | NA | | 1 | 0 | Low-quality | Genome-fragment | | 2.23 | 0 |
| S9-final-viral-combined_k141_106161||full | 2404 | No | NA | | 0 | 1 | Not-determined | Genome-fragment | NA | | 0 |

| | | | | | | | | | | | |
|---|---|---|---|---|---|---|---|---|---|---|---|
| S9-final-viral-combined_k141_127273||full | 1035 | No | NA | | 0 | 2 | Not-determined | Genome-fragment | NA | | 0 |
| S9-final-viral-combined_k141_106166||full | 1679 | No | NA | | 0 | 0 | Not-determined | Genome-fragment | NA | | 0 |
| S9-final-viral-combined_k141_117152||full | 1720 | No | NA | | 0 | 2 | Not-determined | Genome-fragment | NA | | 0 |
| S9-final-viral-combined_k141_127281||full | 2522 | No | NA | | 0 | 3 | Not-determined | Genome-fragment | NA | | 0 |
| S9-final-viral-combined_k141_101551||full | 4535 | No | NA | | 0 | 0 | Not-determined | Genome-fragment | NA | | 0 |
| S11-final-viral-combined_k141_42262||full | 1043 | No | NA | | 0 | 0 | Low-quality | Genome-fragment | | 2.29 | 0 |
| S11-final-viral-combined_k141_329087||full | 1043 | No | NA | | 0 | 0 | Low-quality | Genome-fragment | | 2.11 | 0 |
| S11-final-viral-combined_k141_135144||lt2gene | 1043 | No | NA | | 2 | 0 | Low-quality | Genome-fragment | | 1.68 | 0 |
| S1-final-viral-combined_k141_245604||lt2gene | 1043 | No | NA | | 1 | 0 | Low-quality | Genome-fragment | | 20.33 | 0 |
| S5-final-viral-combined_k141_209279||full | 1043 | No | NA | | 0 | 0 | Low-quality | Genome-fragment | | 1.91 | 0 |
| S9-final-viral-combined_k141_95624||full | 3522 | No | NA | | 0 | 1 | Not-determined | Genome-fragment | NA | | 0 |
| S17-final-viral-combined_k141_90077||full | 1043 | No | NA | | 1 | 0 | Low-quality | Genome-fragment | | 2.64 | 0 |
| S17-final-viral-combined_k141_98610||lt2gene | 1043 | No | NA | | 1 | 0 | Low-quality | Genome-fragment | | 0.63 | 0 |
| S9-final-viral-combined_k141_106258||full | 1149 | No | NA | | 0 | 0 | Not-determined | Genome-fragment | NA | | 0 |
| S9-final-viral-combined_k141_101602||full | 2492 | No | NA | | 0 | 1 | Not-determined | Genome-fragment | NA | | 0 |
| S12-final-viral-combined_k141_54795||full | 1043 | No | NA | | 0 | 0 | Low-quality | Genome-fragment | | 2.44 | 0 |
| S9-final-viral-combined_k141_127319||full | 7358 | No | NA | | 0 | 6 | Not-determined | Genome-fragment | NA | | 0 |
| S12-final-viral-combined_k141_229714||full | 1043 | No | NA | | 0 | 1 | Low-quality | Genome-fragment | | 0.65 | 0 |
| S2-final-viral-combined_k141_126249||full | 1043 | No | NA | | 0 | 0 | Low-quality | Genome-fragment | | 0.59 | 0 |
| S9-final-viral-combined_k141_31614||full | 2041 | No | NA | | 0 | 1 | Not-determined | Genome-fragment | NA | | 0 |
| S9-final-viral-combined_k141_42439||full | 1387 | No | NA | | 0 | 1 | Not-determined | Genome-fragment | NA | | 0 |
| S9-final-viral-combined_k141_106308||full | 3315 | No | NA | | 0 | 1 | Not-determined | Genome-fragment | NA | | 0 |
| S9-final-viral-combined_k141_31682||full | 2326 | No | NA | | 0 | 2 | Not-determined | Genome-fragment | NA | | 0 |
| S9-final-viral-combined_k141_110827||full | 3816 | No | NA | | 0 | 2 | Not-determined | Genome-fragment | NA | | 0 |
| S4-final-viral-combined_k141_30678||full | 1043 | No | NA | | 0 | 1 | Low-quality | Genome-fragment | | 0.38 | 0 |
| S9-final-viral-combined_k141_74484||full | 2643 | No | NA | | 0 | 2 | Not-determined | Genome-fragment | NA | | 0 |
| S6-final-viral-combined_k141_125401||full | 1043 | No | NA | | 3 | 0 | Low-quality | Genome-fragment | | 1.67 | 0 |
| S9-final-viral-combined_k141_110846||full | 5478 | No | NA | | 0 | 4 | Not-determined | Genome-fragment | NA | | 0 |
| S9-final-viral-combined_k141_106379||full | 2048 | No | NA | | 0 | 0 | Not-determined | Genome-fragment | NA | | 0 |
| S8-final-viral-combined_k141_237713||lt2gene | 1043 | No | NA | | 1 | 0 | Low-quality | Genome-fragment | | 2.48 | 0 |
| S8_k141_267644 | 1043 | No | NA | | 1 | 0 | Low-quality | Genome-fragment | | 0.75 | 0 |
| S9-final-viral-combined_k141_48028||lt2gene | 1044 | No | NA | | 1 | 0 | Low-quality | Genome-fragment | | 2.27 | 0 |
| S9-final-viral-combined_k141_110875||full | 1505 | No | NA | | 0 | 1 | Not-determined | Genome-fragment | NA | | 0 |
| S9-final-viral-combined_k141_47311||full | 1851 | No | NA | | 0 | 1 | Not-determined | Genome-fragment | NA | | 0 |
| S9-final-viral-combined_k141_74507||full | 3556 | No | NA | | 0 | 1 | Not-determined | Genome-fragment | NA | | 0 |
| S11-final-viral-combined_k141_95743||lt2gene | 1044 | No | NA | | 1 | 0 | Low-quality | Genome-fragment | | 2.67 | 0 |
| S11_k141_310817 | 1044 | No | NA | | 0 | 0 | Low-quality | Genome-fragment | | 1.69 | 0 |
| S9-final-viral-combined_k141_110908||full | 5545 | No | NA | | 0 | 3 | Not-determined | Genome-fragment | NA | | 0 |
| S9-final-viral-combined_k141_47363||full | 1151 | No | NA | | 0 | 1 | Not-determined | Genome-fragment | NA | | 0 |
| S1-final-viral-combined_k141_159859||full | 1044 | No | NA | | 1 | 0 | Low-quality | Genome-fragment | | 2.11 | 0 |
| S5-final-viral-combined_k141_2706||full | 1044 | No | NA | | 1 | 0 | Low-quality | Genome-fragment | | 2.16 | 0 |
| S9-final-viral-combined_k141_117317||full | 1167 | No | NA | | 0 | 0 | Not-determined | Genome-fragment | NA | | 0 |
| S9-final-viral-combined_k141_95825||full | 1052 | No | NA | | 0 | 1 | Not-determined | Genome-fragment | NA | | 0 |
| S5-final-viral-combined_k141_203745||full | 1044 | No | NA | | 0 | 1 | Low-quality | Genome-fragment | | 0.66 | 0 |
| S17-k141_125977 | 1044 | No | NA | | 1 | 0 | Low-quality | Genome-fragment | | 2.1 | 0 |
| S9-final-viral-combined_k141_106504||full | 4219 | No | NA | | 0 | 1 | Not-determined | Genome-fragment | NA | | 0 |
| S12-final-viral-combined_k141_359752||lt2gene | 1044 | No | NA | | 0 | 0 | Low-quality | Genome-fragment | | 2.13 | 0 |
| S2_k141_94686 | 1044 | No | NA | | 0 | 1 | Low-quality | Genome-fragment | | 0.69 | 0 |
| S9-final-viral-combined_k141_117424||full | 2978 | No | NA | | 0 | 1 | Not-determined | Genome-fragment | NA | | 0 |
| S4-final-viral-combined_k141_105304||full | 1044 | No | NA | | 1 | 0 | Low-quality | Genome-fragment | | 0.62 | 0 |
| S9-final-viral-combined_k141_110937||full | 1160 | No | NA | | 0 | 2 | Not-determined | Genome-fragment | NA | | 0 |
| S9-final-viral-combined_k141_95959||full | 2967 | No | NA | | 0 | 1 | Not-determined | Genome-fragment | NA | | 0 |
| S9-final-viral-combined_k141_106570||full | 1029 | No | NA | | 0 | 1 | Not-determined | Genome-fragment | NA | | 0 |
| S9-final-viral-combined_k141_96018||full | 4555 | No | NA | | 0 | 3 | Not-determined | Genome-fragment | NA | | 0 |
| S9-final-viral-combined_k141_106607||full | 3665 | No | NA | | 0 | 3 | Not-determined | Genome-fragment | NA | | 0 |
| S9-final-viral-combined_k141_106617||full | 3274 | No | NA | | 0 | 2 | Not-determined | Genome-fragment | NA | | 0 |
| S9-final-viral-combined_k141_111003||full | 1225 | No | NA | | 0 | 1 | Not-determined | Genome-fragment | NA | | 0 |
| S9-final-viral-combined_k141_127537||full | 3128 | No | NA | | 0 | 2 | Not-determined | Genome-fragment | NA | | 0 |
| S9-final-viral-combined_k141_127540||full | 1113 | No | NA | | 0 | 1 | Not-determined | Genome-fragment | NA | | 0 |
| S9-final-viral-combined_k141_127545||full | 2989 | No | NA | | 0 | 3 | Not-determined | Genome-fragment | NA | | 0 |
| S9-final-viral-combined_k141_106662||full | 2471 | No | NA | | 0 | 2 | Not-determined | Genome-fragment | NA | | 0 |
| S4_k141_73093 | 1044 | No | NA | | 0 | 0 | Low-quality | Genome-fragment | | 1.2 | 0 |
| S9-final-viral-combined_k141_42719||full | 4225 | No | NA | | 0 | 0 | Not-determined | Genome-fragment | NA | | 0 |
| S6-final-viral-combined_k141_104815||full | 1044 | No | NA | | 0 | 1 | Low-quality | Genome-fragment | | 1.7 | 0 |
| S9-final-viral-combined_k141_31834||full | 3549 | No | NA | | 0 | 4 | Not-determined | Genome-fragment | NA | | 0 |
| S9_k141_62479 | 1045 | No | NA | | 0 | 0 | Low-quality | Genome-fragment | | 2.8 | 0 |
| S11-final-viral-combined_k141_62145||lt2gene | 1045 | No | NA | | 1 | 0 | Low-quality | Genome-fragment | | 2.45 | 0 |
| S11-final-viral-combined_k141_301018||lt2gene | 1045 | No | NA | | 1 | 0 | Low-quality | Genome-fragment | | 19.47 | 0 |
| S9-final-viral-combined_k141_31854||full | 2735 | No | NA | | 0 | 3 | Not-determined | Genome-fragment | NA | | 0 |
| S11_k141_255351 | 1045 | No | NA | | 0 | 0 | Low-quality | Genome-fragment | | 0.68 | 0 |
| S1-final-viral-combined_k141_297508||full | 1045 | No | NA | | 1 | 0 | Low-quality | Genome-fragment | | 1.96 | 0 |
| S1-final-viral-combined_k141_335887||full | 1045 | No | NA | | 1 | 0 | Low-quality | Genome-fragment | | 1.8 | 0 |
| S1-final-viral-combined_k141_243642||full | 1045 | No | NA | | 0 | 0 | Low-quality | Genome-fragment | | 2.57 | 0 |
| S9-final-viral-combined_k141_106733||full | 1880 | No | NA | | 0 | 2 | Not-determined | Genome-fragment | NA | | 0 |
| S1-final-viral-combined_k141_111128||full | 1045 | No | NA | | 0 | 0 | Low-quality | Genome-fragment | | 1.76 | 0 |
| S9-final-viral-combined_k141_106751||full | 4826 | No | NA | | 0 | 2 | Not-determined | Genome-fragment | NA | | 0 |
| S5_k141_21553 | 1045 | No | NA | | 0 | 2 | Low-quality | Genome-fragment | | 3.44 | 0 |
| S17-final-viral-combined_k141_214599||full | 1045 | No | NA | | 0 | 0 | Low-quality | Genome-fragment | | 2.93 | 0 |
| S9-final-viral-combined_k141_127771||full | 2179 | No | NA | | 0 | 1 | Not-determined | Genome-fragment | NA | | 0 |
| S9-final-viral-combined_k141_111151||full | 1410 | No | NA | | 0 | 0 | Not-determined | Genome-fragment | NA | | 0 |
| S17-final-viral-combined_k141_195949||full | 1045 | No | NA | | 0 | 0 | Low-quality | Genome-fragment | | 0.78 | 0 |
| S12-final-viral-combined_k141_51000||full | 1045 | No | NA | | 0 | 1 | Low-quality | Genome-fragment | | 1.91 | 0 |
| S12-final-viral-combined_k141_133897||full | 1045 | No | NA | | 1 | 0 | Low-quality | Genome-fragment | | 1.67 | 0 |
| S9-final-viral-combined_k141_111171||full | 1729 | No | NA | | 0 | 2 | Not-determined | Genome-fragment | NA | | 0 |
| S9-final-viral-combined_k141_127812||full | 1005 | No | NA | | 0 | 1 | Not-determined | Genome-fragment | NA | | 0 |
| S12-final-viral-combined_k141_93914||lt2gene | 1045 | No | NA | | 1 | 0 | Low-quality | Genome-fragment | | 2.91 | 0 |
| S9-final-viral-combined_k141_32027||full | 1929 | No | NA | | 0 | 0 | Not-determined | Genome-fragment | NA | | 0 |
| S12_k141_127957 | 1045 | No | NA | | 0 | 0 | Low-quality | Genome-fragment | | 0.57 | 0 |
| S9-final-viral-combined_k141_47774||full | 3253 | No | NA | | 0 | 3 | Not-determined | Genome-fragment | NA | | 0 |
| S9-final-viral-combined_k141_111201||full | 1334 | No | NA | | 0 | 0 | Not-determined | Genome-fragment | NA | | 0 |
| S9-final-viral-combined_k141_32086||full | 1575 | No | NA | | 0 | 1 | Not-determined | Genome-fragment | NA | | 0 |
| S12_k141_96585 | 1045 | No | NA | | 0 | 0 | Low-quality | Genome-fragment | | 2.04 | 0 |
| S2-final-viral-combined_k141_66574||lt2gene | 1045 | No | NA | | 2 | 0 | Low-quality | Genome-fragment | | 2 | 0 |
| S9-final-viral-combined_k141_111243||full | 1673 | No | NA | | 0 | 2 | Not-determined | Genome-fragment | NA | | 0 |
| S2_k141_135819 | 1045 | No | NA | | 0 | 0 | Low-quality | Genome-fragment | | 1.33 | 0 |
| S2_k141_32438 | 1045 | No | NA | | 0 | 0 | Low-quality | Genome-fragment | | 1.75 | 0 |
| S9-final-viral-combined_k141_111336||full | 1995 | No | NA | | 0 | 1 | Not-determined | Genome-fragment | NA | | 0 |
| S4-final-viral-combined_k141_102810||lt2gene | 1045 | No | NA | | 0 | 0 | Low-quality | Genome-fragment | | 2.53 | 0 |
| S9-final-viral-combined_k141_48030||full | 3739 | No | NA | | 0 | 2 | Not-determined | Genome-fragment | NA | | 0 |
| S9-final-viral-combined_k141_111515||full | 2295 | No | NA | | 0 | 0 | Not-determined | Genome-fragment | NA | | 0 |
| S8_k141_214262 | 1045 | No | NA | | 0 | 1 | Low-quality | Genome-fragment | | 2.13 | 0 |
| S9-final-viral-combined_k141_94387||full | 1046 | No | NA | | 0 | 0 | Low-quality | Genome-fragment | | 1.63 | 0 |
| S9_k141_78394 | 1046 | No | NA | | 0 | 0 | Low-quality | Genome-fragment | | 1.75 | 0 |
| S9-final-viral-combined_k141_111791||full | 2896 | No | NA | | 0 | 2 | Not-determined | Genome-fragment | NA | | 0 |
| S9-final-viral-combined_k141_111884||full | 1984 | No | NA | | 0 | 1 | Not-determined | Genome-fragment | NA | | 0 |
| S9-final-viral-combined_k141_111935||full | 3079 | No | NA | | 0 | 2 | Not-determined | Genome-fragment | NA | | 0 |
| S11-final-viral-combined_k141_127570||full | 1046 | No | NA | | 0 | 1 | Low-quality | Genome-fragment | | 0.44 | 0 |
| S9-final-viral-combined_k141_112067||full | 1840 | No | NA | | 0 | 1 | Not-determined | Genome-fragment | NA | | 0 |
| S11-final-viral-combined_k141_259849||full | 1046 | No | NA | | 0 | 1 | Low-quality | Genome-fragment | | 0.76 | 0 |
| S11-final-viral-combined_k141_82237||full | 1046 | No | NA | | 0 | 0 | Low-quality | Genome-fragment | | 2.78 | 0 |
| S9-final-viral-combined_k141_37587||0_partial | 82228 | Yes | | 46493 | 10 | 17 | Medium-quality | Genome-fragment | | 85.36 | 43.46 |
| S9-final-viral-combined_k141_43110||0_partial | 15458 | No | NA | | 0 | 7 | Not-determined | Genome-fragment | NA | | 0 |
| S9-final-viral-combined_k141_5526||0_partial | 51870 | Yes | | 36902 | 7 | 16 | Medium-quality | Genome-fragment | | 66.25 | 28.86 |
| S1-final-viral-combined_k141_94227||full | 1046 | No | NA | | 0 | 0 | Low-quality | Genome-fragment | | 2.06 | 0 |
| S9-final-viral-combined_k141_5696||0_partial | 14816 | No | NA | | 0 | 13 | Not-determined | Genome-fragment | NA | | 0 |
| S1-final-viral-combined_k141_164467||full | 1046 | No | NA | | 1 | 0 | Low-quality | Genome-fragment | | 2.57 | 0 |
| S1_k141_159538 | 1046 | No | NA | | 0 | 0 | Low-quality | Genome-fragment | | 2.54 | 0 |
| S5-final-viral-combined_k141_25072||lt2gene | 1046 | No | NA | | 1 | 0 | Low-quality | Genome-fragment | | 0.56 | 0 |
| S9-final-viral-combined_k141_33229||0_partial | 23889 | No | NA | | 10 | 0 | Medium-quality | Genome-fragment | | 62.78 | 0 |
| S9-final-viral-combined_k141_54941||0_partial | 19411 | No | NA | | 0 | 6 | Not-determined | Genome-fragment | NA | | 0 |
| S9-final-viral-combined_k141_44292||0_partial | 19773 | No | NA | | 0 | 10 | Not-determined | Genome-fragment | NA | | 0 |
| S17-final-viral-combined_k141_200117||full | 1046 | No | NA | | 0 | 1 | Low-quality | Genome-fragment | | 0.54 | 0 |
| S9-final-viral-combined_k141_76274||0_partial | 68824 | Yes | | 31753 | 23 | 20 | Complete | High-quality | | 100 | 53.86 |
| S9-final-viral-combined_k141_23386||0_partial | 21837 | No | NA | | 0 | 9 | Not-determined | Genome-fragment | NA | | 0 |
| S9-final-viral-combined_k141_44402||0_partial | 32331 | No | NA | | 9 | 1 | Medium-quality | Genome-fragment | | 76.65 | 0 |
| S12-final-viral-combined_k141_209813||full | 1046 | No | NA | | 1 | 0 | Low-quality | Genome-fragment | | 1.64 | 0 |
| S9-final-viral-combined_k141_93117||0_partial | 19133 | No | NA | | 0 | 7 | Not-determined | Genome-fragment | NA | | 0 |
| S12-final-viral-combined_k141_378296||full | 1046 | No | NA | | 0 | 0 | Low-quality | Genome-fragment | | 2.12 | 0 |
| S12-final-viral-combined_k141_114797||full | 1046 | No | NA | | 1 | 0 | Low-quality | Genome-fragment | | 3.01 | 0 |
| S12_k141_94395 | 1046 | No | NA | | 0 | 1 | Low-quality | Genome-fragment | | 1.85 | 0 |
| S2-final-viral-combined_k141_198209||full | 1046 | No | NA | | 1 | 0 | Low-quality | Genome-fragment | | 3.01 | 0 |
| S9-final-viral-combined_k141_61898||0_partial | 22191 | No | NA | | 0 | 8 | Not-determined | Genome-fragment | NA | | 0 |
| S9-final-viral-combined_k141_56773||0_partial | 5561 | Yes | | 2977 | 1 | 1 | Medium-quality | Genome-fragment | | 71.54 | 46.47 |
| S2_k141_35532 | 1046 | No | NA | | 0 | 0 | Low-quality | Genome-fragment | | 1.08 | 0 |
| S8_k141_40800 | 1046 | No | NA | | 0 | 0 | Low-quality | Genome-fragment | | 2.84 | 0 |
| S9-final-viral-combined_k141_4767||full | 1047 | No | NA | | 0 | 0 | Low-quality | Genome-fragment | | 8.14 | 0 |
| S11-final-viral-combined_k141_20828||full | 1047 | No | NA | | 1 | 0 | Low-quality | Genome-fragment | | 1.74 | 0 |
| S9-final-viral-combined_k141_19806||0_partial | 11167 | No | NA | | 0 | 6 | Not-determined | Genome-fragment | NA | | 0 |
| S3-final-viral-combined_k141_37833||full | 1047 | No | NA | | 0 | 0 | Low-quality | Genome-fragment | | 2.48 | 0 |
| S3_k141_108175 | 1047 | No | NA | | 0 | 0 | Low-quality | Genome-fragment | | 2.32 | 0 |
| S7-final-viral-combined_k141_44401||full | 1047 | No | NA | | 0 | 0 | Low-quality | Genome-fragment | | 1.79 | 0 |
| S17-final-viral-combined_k141_77806||full | 1047 | No | NA | | 1 | 0 | Low-quality | Genome-fragment | | 2.72 | 0 |
| S9-final-viral-combined_k141_14742||0_partial | 11051 | No | NA | | 0 | 7 | Not-determined | Genome-fragment | NA | | 0 |
| S9-final-viral-combined_k141_9647||0_partial | 12997 | No | NA | | 0 | 5 | Not-determined | Genome-fragment | NA | | 0 |
| S9-final-viral-combined_k141_4518||0_partial | 33577 | No | NA | | 0 | 11 | Not-determined | Genome-fragment | NA | | 0 |
| S9-final-viral-combined_k141_58078||0_partial | 31656 | No | NA | | 0 | 9 | Not-determined | Genome-fragment | NA | | 0 |
| S9-final-viral-combined_k141_63934||0_partial | 19182 | No | NA | | 0 | 9 | Not-determined | Genome-fragment | NA | | 0 |
| S17-final-viral-combined_k141_230779||full | 1047 | No | NA | | 0 | 0 | Low-quality | Genome-fragment | | 1.69 | 0 |
| S9-final-viral-combined_k141_10274||0_partial | 16360 | No | NA | | 0 | 11 | Not-determined | Genome-fragment | NA | | 0 |
| S9-final-viral-combined_k141_122742||0_partial | 24352 | No | NA | | 0 | 4 | Medium-quality | Genome-fragment | | 73.74 | 0 |
| S17-k141_4339 | 1047 | No | NA | | 0 | 0 | Low-quality | Genome-fragment | | 15.95 | 0 |
| S9-final-viral-combined_k141_10665||0_partial | 10566 | No | NA | | 0 | 7 | Not-determined | Genome-fragment | NA | | 0 |
| S9-final-viral-combined_k141_96070||0_partial | 11396 | No | NA | | 0 | 9 | Not-determined | Genome-fragment | NA | | 0 |
| S9-final-viral-combined_k141_106708||0_partial | 13339 | No | NA | | 0 | 8 | Not-determined | Genome-fragment | NA | | 0 |
| S12-final-viral-combined_k141_359392||full | 1047 | No | NA | | 0 | 0 | Low-quality | Genome-fragment | | 2.06 | 0 |
| S2_k141_18565 | 1047 | No | NA | | 0 | 1 | Low-quality | Genome-fragment | | 0.3 | 0 |
| S11-final-viral-combined_k141_248108||lt2gene | 1048 | No | NA | | 1 | 0 | Low-quality | Genome-fragment | | 2.34 | 0 |
| S11-final-viral-combined_k141_182330||lt2gene | 1048 | No | NA | | 1 | 0 | Low-quality | Genome-fragment | | 1.97 | 0 |
| S5_k141_135256 | 1048 | No | NA | | 0 | 0 | Low-quality | Genome-fragment | | 0.81 | 0 |
| S9-final-viral-combined_k141_75345||lt2gene | 2631 | No | NA | | 0 | 0 | Not-determined | Genome-fragment | NA | | 0 |
| S17-final-viral-combined_k141_253535||full | 1048 | No | NA | | 0 | 0 | Low-quality | Genome-fragment | | 1.3 | 0 |
| S8-final-viral-combined_k141_158152||full | 1048 | No | NA | | 2 | 0 | Low-quality | Genome-fragment | | 1.75 | 0 |
| S11_k141_38222 | 1049 | No | NA | | 0 | 0 | Low-quality | Genome-fragment | | 1.42 | 0 |
| S1-final-viral-combined_k141_16500||full | 1049 | No | NA | | 0 | 1 | Low-quality | Genome-fragment | | 2.69 | 0 |
| S9-final-viral-combined_k141_10986||lt2gene | 1368 | No | NA | | 0 | 1 | Not-determined | Genome-fragment | NA | | 0 |
| S1-final-viral-combined_k141_297613||full | 1049 | No | NA | | 0 | 0 | Low-quality | Genome-fragment | | 1.22 | 0 |
| S1-final-viral-combined_k141_111833||full | 1049 | No | NA | | 1 | 0 | Low-quality | Genome-fragment | | 2.03 | 0 |
| S3-final-viral-combined_k141_1865||full | 1049 | No | NA | | 1 | 0 | Low-quality | Genome-fragment | | 1.84 | 0 |
| S9-final-viral-combined_k141_123509||lt2gene | 1585 | No | NA | | 0 | 0 | Not-determined | Genome-fragment | NA | | 0 |
| S5-final-viral-combined_k141_112418||full | 1049 | No | NA | | 0 | 2 | Low-quality | Genome-fragment | | 0.45 | 0 |
| S5-final-viral-combined_k141_147508||lt2gene | 1049 | No | NA | | 2 | 0 | Low-quality | Genome-fragment | | 2.95 | 0 |
| S7-final-viral-combined_k141_159815||full | 1049 | No | NA | | 1 | 0 | Low-quality | Genome-fragment | | 2.02 | 0 |
| S9-final-viral-combined_k141_16684||lt2gene | 3277 | No | NA | | 0 | 1 | Not-determined | Genome-fragment | NA | | 0 |
| S7_k141_33911 | 1049 | No | NA | | 0 | 0 | Low-quality | Genome-fragment | | 1.88 | 0 |

<table>
<tr><td>S17-k141_80826</td><td>1049</td><td>No</td><td>NA</td><td>0</td><td>0</td><td>Low-quality</td><td>Genome-fragment</td><td></td><td>3.23</td><td>0</td></tr>
<tr><td>S17-k141_59189</td><td>1049</td><td>No</td><td>NA</td><td>0</td><td>1</td><td>Low-quality</td><td>Genome-fragment</td><td></td><td>1.86</td><td>0</td></tr>
<tr><td>S17-k141_17225</td><td>1049</td><td>No</td><td>NA</td><td>1</td><td>0</td><td>Low-quality</td><td>Genome-fragment</td><td></td><td>2.01</td><td>0</td></tr>
<tr><td>S12_k141_192265</td><td>1049</td><td>No</td><td>NA</td><td>0</td><td>0</td><td>Low-quality</td><td>Genome-fragment</td><td></td><td>1.43</td><td>0</td></tr>
<tr><td>S2-final-viral-combined_k141_210509||full</td><td>1049</td><td>No</td><td>NA</td><td>0</td><td>0</td><td>Low-quality</td><td>Genome-fragment</td><td></td><td>2.7</td><td>0</td></tr>
<tr><td>S2-final-viral-combined_k141_108558||full</td><td>1049</td><td>No</td><td>NA</td><td>0</td><td>0</td><td>Low-quality</td><td>Genome-fragment</td><td></td><td>2.07</td><td>0</td></tr>
<tr><td>S2-final-viral-combined_k141_108573||full</td><td>1049</td><td>No</td><td>NA</td><td>0</td><td>0</td><td>Low-quality</td><td>Genome-fragment</td><td></td><td>2.08</td><td>0</td></tr>
<tr><td>S2_k141_163296</td><td>1049</td><td>No</td><td>NA</td><td>1</td><td>0</td><td>Low-quality</td><td>Genome-fragment</td><td></td><td>15.7</td><td>0</td></tr>
<tr><td>S4-final-viral-combined_k141_76065||full</td><td>1049</td><td>No</td><td>NA</td><td>0</td><td>0</td><td>Low-quality</td><td>Genome-fragment</td><td></td><td>1.44</td><td>0</td></tr>
<tr><td>S11-final-viral-combined_k141_213662||lt2gene</td><td>1050</td><td>No</td><td>NA</td><td>0</td><td>0</td><td>Low-quality</td><td>Genome-fragment</td><td></td><td>1.95</td><td>0</td></tr>
<tr><td>S11_k141_241660</td><td>1050</td><td>No</td><td>NA</td><td>0</td><td>0</td><td>Low-quality</td><td>Genome-fragment</td><td></td><td>2.62</td><td>0</td></tr>
<tr><td>S11_k141_160266</td><td>1050</td><td>No</td><td>NA</td><td>0</td><td>1</td><td>Low-quality</td><td>Genome-fragment</td><td></td><td>2.07</td><td>0</td></tr>
<tr><td>S1-final-viral-combined_k141_178240||full</td><td>1050</td><td>No</td><td>NA</td><td>1</td><td>0</td><td>Low-quality</td><td>Genome-fragment</td><td></td><td>2.48</td><td>0</td></tr>
<tr><td>S1-final-viral-combined_k141_243219||lt2gene</td><td>1050</td><td>No</td><td>NA</td><td>1</td><td>0</td><td>Low-quality</td><td>Genome-fragment</td><td></td><td>1.74</td><td>0</td></tr>
<tr><td>S3-final-viral-combined_k141_58118||lt2gene</td><td>1050</td><td>No</td><td>NA</td><td>1</td><td>0</td><td>Low-quality</td><td>Genome-fragment</td><td></td><td>0.96</td><td>0</td></tr>
<tr><td>S7_k141_185264</td><td>1050</td><td>No</td><td>NA</td><td>1</td><td>0</td><td>Low-quality</td><td>Genome-fragment</td><td></td><td>2.03</td><td>0</td></tr>
<tr><td>S9-final-viral-combined_k141_23006||lt2gene</td><td>1314</td><td>No</td><td>NA</td><td>0</td><td>0</td><td>Not-determined</td><td>Genome-fragment</td><td>NA</td><td></td><td>0</td></tr>
<tr><td>S17-k141_28237</td><td>1050</td><td>No</td><td>NA</td><td>0</td><td>1</td><td>Low-quality</td><td>Genome-fragment</td><td></td><td>2.75</td><td>0</td></tr>
<tr><td>S12-final-viral-combined_k141_46232||full</td><td>1050</td><td>No</td><td>NA</td><td>1</td><td>0</td><td>Low-quality</td><td>Genome-fragment</td><td></td><td>2.37</td><td>0</td></tr>
<tr><td>S12-final-viral-combined_k141_200616||full</td><td>1050</td><td>No</td><td>NA</td><td>2</td><td>0</td><td>Low-quality</td><td>Genome-fragment</td><td></td><td>2.32</td><td>0</td></tr>
<tr><td>S2-final-viral-combined_k141_207420||full</td><td>1050</td><td>No</td><td>NA</td><td>0</td><td>0</td><td>Low-quality</td><td>Genome-fragment</td><td></td><td>1.89</td><td>0</td></tr>
<tr><td>S6-final-viral-combined_k141_44630||full</td><td>1050</td><td>No</td><td>NA</td><td>0</td><td>0</td><td>Low-quality</td><td>Genome-fragment</td><td></td><td>4.12</td><td>0</td></tr>
<tr><td>S8_k141_69815</td><td>1050</td><td>No</td><td>NA</td><td>1</td><td>0</td><td>Low-quality</td><td>Genome-fragment</td><td></td><td>2.41</td><td>0</td></tr>
<tr><td>S1-final-viral-combined_k141_174246||lt2gene</td><td>1051</td><td>No</td><td>NA</td><td>1</td><td>0</td><td>Low-quality</td><td>Genome-fragment</td><td></td><td>2.68</td><td>0</td></tr>
<tr><td>S5-final-viral-combined_k141_37104||full</td><td>1051</td><td>No</td><td>NA</td><td>1</td><td>0</td><td>Low-quality</td><td>Genome-fragment</td><td></td><td>2.07</td><td>0</td></tr>
<tr><td>S5-final-viral-combined_k141_190157||full</td><td>1051</td><td>No</td><td>NA</td><td>0</td><td>0</td><td>Low-quality</td><td>Genome-fragment</td><td></td><td>1.76</td><td>0</td></tr>
<tr><td>S5-final-viral-combined_k141_35794||lt2gene</td><td>1051</td><td>No</td><td>NA</td><td>0</td><td>1</td><td>Low-quality</td><td>Genome-fragment</td><td></td><td>1.43</td><td>0</td></tr>
<tr><td>S17-k141_262360</td><td>1051</td><td>No</td><td>NA</td><td>0</td><td>0</td><td>Low-quality</td><td>Genome-fragment</td><td></td><td>1.97</td><td>0</td></tr>
<tr><td>S12-final-viral-combined_k141_21768||full</td><td>1051</td><td>No</td><td>NA</td><td>0</td><td>0</td><td>Low-quality</td><td>Genome-fragment</td><td></td><td>1.75</td><td>0</td></tr>
<tr><td>S4-final-viral-combined_k141_25324||full</td><td>1051</td><td>No</td><td>NA</td><td>0</td><td>0</td><td>Low-quality</td><td>Genome-fragment</td><td></td><td>1.62</td><td>0</td></tr>
<tr><td>S8-final-viral-combined_k141_244643||full</td><td>1051</td><td>No</td><td>NA</td><td>0</td><td>0</td><td>Low-quality</td><td>Genome-fragment</td><td></td><td>0.77</td><td>0</td></tr>
<tr><td>S9-final-viral-combined_k141_103726||lt2gene</td><td>1158</td><td>No</td><td>NA</td><td>0</td><td>1</td><td>Not-determined</td><td>Genome-fragment</td><td>NA</td><td></td><td>0</td></tr>
<tr><td>S8-final-viral-combined_k141_54304||full</td><td>1051</td><td>No</td><td>NA</td><td>0</td><td>0</td><td>Low-quality</td><td>Genome-fragment</td><td></td><td>1.44</td><td>0</td></tr>
<tr><td>S8_k141_126400</td><td>1051</td><td>No</td><td>NA</td><td>0</td><td>0</td><td>Low-quality</td><td>Genome-fragment</td><td></td><td>1.74</td><td>0</td></tr>
<tr><td>S9-final-viral-combined_k141_66679||full</td><td>1052</td><td>No</td><td>NA</td><td>0</td><td>0</td><td>Low-quality</td><td>Genome-fragment</td><td></td><td>2.53</td><td>0</td></tr>
<tr><td>S9_k141_31178</td><td>1052</td><td>No</td><td>NA</td><td>1</td><td>0</td><td>Low-quality</td><td>Genome-fragment</td><td></td><td>1.48</td><td>0</td></tr>
<tr><td>S11_k141_196535</td><td>1052</td><td>No</td><td>NA</td><td>0</td><td>0</td><td>Low-quality</td><td>Genome-fragment</td><td></td><td>1.98</td><td>0</td></tr>
<tr><td>S3-final-viral-combined_k141_3448||full</td><td>1052</td><td>No</td><td>NA</td><td>0</td><td>0</td><td>Low-quality</td><td>Genome-fragment</td><td></td><td>1.68</td><td>0</td></tr>
<tr><td>S3-final-viral-combined_k141_4994||full</td><td>1052</td><td>No</td><td>NA</td><td>0</td><td>0</td><td>Low-quality</td><td>Genome-fragment</td><td></td><td>1.3</td><td>0</td></tr>
<tr><td>S5-final-viral-combined_k141_200618||full</td><td>1052</td><td>No</td><td>NA</td><td>1</td><td>0</td><td>Low-quality</td><td>Genome-fragment</td><td></td><td>2.55</td><td>0</td></tr>
<tr><td>S7-final-viral-combined_k141_93489||full</td><td>1052</td><td>No</td><td>NA</td><td>0</td><td>0</td><td>Low-quality</td><td>Genome-fragment</td><td></td><td>1.33</td><td>0</td></tr>
<tr><td>S9-final-viral-combined_k141_93198||lt2gene</td><td>2167</td><td>No</td><td>NA</td><td>0</td><td>0</td><td>Not-determined</td><td>Genome-fragment</td><td>NA</td><td></td><td>0</td></tr>
<tr><td>S7-final-viral-combined_k141_8725||full</td><td>1052</td><td>No</td><td>NA</td><td>0</td><td>0</td><td>Low-quality</td><td>Genome-fragment</td><td></td><td>2.05</td><td>0</td></tr>
<tr><td>S17-final-viral-combined_k141_22787||full</td><td>1052</td><td>No</td><td>NA</td><td>1</td><td>0</td><td>Low-quality</td><td>Genome-fragment</td><td></td><td>3.04</td><td>0</td></tr>
<tr><td>S17-final-viral-combined_k141_58063||full</td><td>1052</td><td>No</td><td>NA</td><td>2</td><td>0</td><td>Low-quality</td><td>Genome-fragment</td><td></td><td>1.9</td><td>0</td></tr>
<tr><td>S17-final-viral-combined_k141_137374||full</td><td>1052</td><td>No</td><td>NA</td><td>0</td><td>0</td><td>Low-quality</td><td>Genome-fragment</td><td></td><td>2.43</td><td>0</td></tr>
<tr><td>S17-final-viral-combined_k141_49364||full</td><td>1052</td><td>No</td><td>NA</td><td>0</td><td>0</td><td>Low-quality</td><td>Genome-fragment</td><td></td><td>2.06</td><td>0</td></tr>
<tr><td>S17-k141_169645</td><td>1052</td><td>No</td><td>NA</td><td>0</td><td>0</td><td>Low-quality</td><td>Genome-fragment</td><td></td><td>1.25</td><td>0</td></tr>
<tr><td>S12_k141_72011</td><td>1052</td><td>No</td><td>NA</td><td>0</td><td>0</td><td>Low-quality</td><td>Genome-fragment</td><td></td><td>2.17</td><td>0</td></tr>
<tr><td>S2_k141_144452</td><td>1052</td><td>No</td><td>NA</td><td>0</td><td>0</td><td>Low-quality</td><td>Genome-fragment</td><td></td><td>0.48</td><td>0</td></tr>
<tr><td>S2_k141_133307</td><td>1052</td><td>No</td><td>NA</td><td>0</td><td>0</td><td>Low-quality</td><td>Genome-fragment</td><td></td><td>2.92</td><td>0</td></tr>
<tr><td>S6-final-viral-combined_k141_30548||full</td><td>1052</td><td>No</td><td>NA</td><td>0</td><td>0</td><td>Low-quality</td><td>Genome-fragment</td><td></td><td>2.94</td><td>0</td></tr>
<tr><td>S8-final-viral-combined_k141_104886||lt2gene</td><td>1052</td><td>No</td><td>NA</td><td>1</td><td>0</td><td>Low-quality</td><td>Genome-fragment</td><td></td><td>2.69</td><td>0</td></tr>
<tr><td>S11-final-viral-combined_k141_308804||full</td><td>1053</td><td>No</td><td>NA</td><td>0</td><td>0</td><td>Low-quality</td><td>Genome-fragment</td><td></td><td>1.99</td><td>0</td></tr>
<tr><td>S11-final-viral-combined_k141_325920||full</td><td>1053</td><td>No</td><td>NA</td><td>0</td><td>0</td><td>Low-quality</td><td>Genome-fragment</td><td></td><td>2.49</td><td>0</td></tr>
<tr><td>S11-final-viral-combined_k141_301515||full</td><td>1053</td><td>No</td><td>NA</td><td>0</td><td>0</td><td>Low-quality</td><td>Genome-fragment</td><td></td><td>2.48</td><td>0</td></tr>
<tr><td>S11_k141_278563</td><td>1053</td><td>No</td><td>NA</td><td>0</td><td>0</td><td>Low-quality</td><td>Genome-fragment</td><td></td><td>1.96</td><td>0</td></tr>
<tr><td>S11_k141_226113</td><td>1053</td><td>No</td><td>NA</td><td>0</td><td>0</td><td>Low-quality</td><td>Genome-fragment</td><td></td><td>2.9</td><td>0</td></tr>
<tr><td>S1-final-viral-combined_k141_151614||full</td><td>1053</td><td>No</td><td>NA</td><td>0</td><td>2</td><td>Low-quality</td><td>Genome-fragment</td><td></td><td>0.78</td><td>0</td></tr>
<tr><td>S1-final-viral-combined_k141_188168||full</td><td>1053</td><td>No</td><td>NA</td><td>0</td><td>0</td><td>Low-quality</td><td>Genome-fragment</td><td></td><td>2.92</td><td>0</td></tr>
<tr><td>S9-final-viral-combined_k141_9291||lt2gene</td><td>1495</td><td>No</td><td>NA</td><td>0</td><td>0</td><td>Not-determined</td><td>Genome-fragment</td><td>NA</td><td></td><td>0</td></tr>
<tr><td>S9-final-viral-combined_k141_46147||lt2gene</td><td>1402</td><td>No</td><td>NA</td><td>0</td><td>1</td><td>Not-determined</td><td>Genome-fragment</td><td>NA</td><td></td><td>0</td></tr>
<tr><td>S1_k141_188431</td><td>1053</td><td>No</td><td>NA</td><td>0</td><td>1</td><td>Low-quality</td><td>Genome-fragment</td><td></td><td>0.49</td><td>0</td></tr>
<tr><td>S5_k141_148552</td><td>1053</td><td>No</td><td>NA</td><td>1</td><td>0</td><td>Low-quality</td><td>Genome-fragment</td><td></td><td>2.26</td><td>0</td></tr>
<tr><td>S7-final-viral-combined_k141_137255||lt2gene</td><td>1053</td><td>No</td><td>NA</td><td>1</td><td>0</td><td>Low-quality</td><td>Genome-fragment</td><td></td><td>1.95</td><td>0</td></tr>
<tr><td>S7-final-viral-combined_k141_130441||lt2gene</td><td>1053</td><td>No</td><td>NA</td><td>2</td><td>0</td><td>Low-quality</td><td>Genome-fragment</td><td></td><td>0.79</td><td>0</td></tr>
<tr><td>S9-final-viral-combined_k141_72790||lt2gene</td><td>1816</td><td>No</td><td>NA</td><td>0</td><td>0</td><td>Not-determined</td><td>Genome-fragment</td><td>NA</td><td></td><td>0</td></tr>
<tr><td>S17-final-viral-combined_k141_240538||full</td><td>1053</td><td>No</td><td>NA</td><td>0</td><td>0</td><td>Low-quality</td><td>Genome-fragment</td><td></td><td>1.82</td><td>0</td></tr>
<tr><td>S12-final-viral-combined_k141_18163||full</td><td>1053</td><td>No</td><td>NA</td><td>0</td><td>1</td><td>Low-quality</td><td>Genome-fragment</td><td></td><td>0.6</td><td>0</td></tr>
<tr><td>S12-final-viral-combined_k141_234346||lt2gene</td><td>1053</td><td>No</td><td>NA</td><td>1</td><td>0</td><td>Low-quality</td><td>Genome-fragment</td><td></td><td>2.15</td><td>0</td></tr>
<tr><td>S2-final-viral-combined_k141_171111||full</td><td>1053</td><td>No</td><td>NA</td><td>1</td><td>0</td><td>Low-quality</td><td>Genome-fragment</td><td></td><td>2.61</td><td>0</td></tr>
<tr><td>S2-final-viral-combined_k141_214785||full</td><td>1053</td><td>No</td><td>NA</td><td>0</td><td>1</td><td>Low-quality</td><td>Genome-fragment</td><td></td><td>0.48</td><td>0</td></tr>
<tr><td>S2-final-viral-combined_k141_170854||lt2gene</td><td>1053</td><td>No</td><td>NA</td><td>1</td><td>0</td><td>Low-quality</td><td>Genome-fragment</td><td></td><td>2.98</td><td>0</td></tr>
<tr><td>S4_k141_115323</td><td>1053</td><td>No</td><td>NA</td><td>0</td><td>0</td><td>Low-quality</td><td>Genome-fragment</td><td></td><td>0.93</td><td>0</td></tr>
<tr><td>S8-final-viral-combined_k141_23687||full</td><td>1053</td><td>No</td><td>NA</td><td>0</td><td>0</td><td>Low-quality</td><td>Genome-fragment</td><td></td><td>0.93</td><td>0</td></tr>
<tr><td>S9-final-viral-combined_k141_52810||lt2gene</td><td>2091</td><td>No</td><td>NA</td><td>0</td><td>0</td><td>Not-determined</td><td>Genome-fragment</td><td>NA</td><td></td><td>0</td></tr>
<tr><td>S8-final-viral-combined_k141_191056||full</td><td>1053</td><td>No</td><td>NA</td><td>0</td><td>0</td><td>Low-quality</td><td>Genome-fragment</td><td></td><td>2.48</td><td>0</td></tr>
<tr><td>S8-final-viral-combined_k141_215251||full</td><td>1053</td><td>No</td><td>NA</td><td>0</td><td>0</td><td>Low-quality</td><td>Genome-fragment</td><td></td><td>2.08</td><td>0</td></tr>
<tr><td>S1-final-viral-combined_k141_387282||full</td><td>1054</td><td>No</td><td>NA</td><td>1</td><td>0</td><td>Low-quality</td><td>Genome-fragment</td><td></td><td>2.48</td><td>0</td></tr>
<tr><td>S1-final-viral-combined_k141_377851||full</td><td>1054</td><td>No</td><td>NA</td><td>0</td><td>0</td><td>Low-quality</td><td>Genome-fragment</td><td></td><td>2.05</td><td>0</td></tr>
<tr><td>S5-final-viral-combined_k141_130839||lt2gene</td><td>1054</td><td>No</td><td>NA</td><td>1</td><td>0</td><td>Low-quality</td><td>Genome-fragment</td><td></td><td>2.44</td><td>0</td></tr>
<tr><td>S17-final-viral-combined_k141_63866||lt2gene</td><td>1054</td><td>No</td><td>NA</td><td>1</td><td>0</td><td>Low-quality</td><td>Genome-fragment</td><td></td><td>1.92</td><td>0</td></tr>
<tr><td>S17-final-viral-combined_k141_241837||lt2gene</td><td>1054</td><td>No</td><td>NA</td><td>1</td><td>0</td><td>Low-quality</td><td>Genome-fragment</td><td></td><td>2.82</td><td>0</td></tr>
<tr><td>S12-final-viral-combined_k141_13200||full</td><td>1054</td><td>No</td><td>NA</td><td>1</td><td>0</td><td>Low-quality</td><td>Genome-fragment</td><td></td><td>2.54</td><td>0</td></tr>
<tr><td>S12_k141_385619</td><td>1054</td><td>No</td><td>NA</td><td>0</td><td>2</td><td>Low-quality</td><td>Genome-fragment</td><td></td><td>2.23</td><td>0</td></tr>
<tr><td>S9-final-viral-combined_k141_58343||lt2gene</td><td>1520</td><td>No</td><td>NA</td><td>0</td><td>0</td><td>Not-determined</td><td>Genome-fragment</td><td>NA</td><td></td><td>0</td></tr>
<tr><td>S4-final-viral-combined_k141_121588||lt2gene</td><td>1054</td><td>No</td><td>NA</td><td>2</td><td>0</td><td>Low-quality</td><td>Genome-fragment</td><td></td><td>0.39</td><td>0</td></tr>
<tr><td>S4_k141_76767</td><td>1054</td><td>No</td><td>NA</td><td>1</td><td>0</td><td>Low-quality</td><td>Genome-fragment</td><td></td><td>1.06</td><td>0</td></tr>
<tr><td>S6-final-viral-combined_k141_99443||full</td><td>1054</td><td>No</td><td>NA</td><td>0</td><td>1</td><td>Low-quality</td><td>Genome-fragment</td><td></td><td>0.47</td><td>0</td></tr>
<tr><td>S6-final-viral-combined_k141_74481||lt2gene</td><td>1054</td><td>No</td><td>NA</td><td>1</td><td>0</td><td>Low-quality</td><td>Genome-fragment</td><td></td><td>3.88</td><td>0</td></tr>
<tr><td>S8-final-viral-combined_k141_202649||full</td><td>1054</td><td>No</td><td>NA</td><td>0</td><td>0</td><td>Low-quality</td><td>Genome-fragment</td><td></td><td>2.29</td><td>0</td></tr>
<tr><td>S8-final-viral-combined_k141_135778||full</td><td>1054</td><td>No</td><td>NA</td><td>0</td><td>1</td><td>Low-quality</td><td>Genome-fragment</td><td></td><td>1.43</td><td>0</td></tr>
<tr><td>S8_k141_152419</td><td>1054</td><td>No</td><td>NA</td><td>1</td><td>0</td><td>Low-quality</td><td>Genome-fragment</td><td></td><td>0.93</td><td>0</td></tr>
<tr><td>S9_k141_112305</td><td>3113</td><td>No</td><td>NA</td><td>0</td><td>0</td><td>Not-determined</td><td>Genome-fragment</td><td>NA</td><td></td><td>0</td></tr>
<tr><td>S9_k141_122993</td><td>8129</td><td>No</td><td>NA</td><td>0</td><td>18</td><td>Not-determined</td><td>Genome-fragment</td><td>NA</td><td></td><td>0</td></tr>
<tr><td>S9_k141_53485</td><td>1440</td><td>No</td><td>NA</td><td>0</td><td>0</td><td>Not-determined</td><td>Genome-fragment</td><td>NA</td><td></td><td>0</td></tr>
<tr><td>S9_k141_106986</td><td>1614</td><td>No</td><td>NA</td><td>0</td><td>0</td><td>Not-determined</td><td>Genome-fragment</td><td>NA</td><td></td><td>0</td></tr>
<tr><td>S9_k141_101639</td><td>1372</td><td>No</td><td>NA</td><td>0</td><td>1</td><td>Not-determined</td><td>Genome-fragment</td><td>NA</td><td></td><td>0</td></tr>
<tr><td>S9_k141_112338</td><td>1072</td><td>No</td><td>NA</td><td>0</td><td>2</td><td>Not-determined</td><td>Genome-fragment</td><td>NA</td><td></td><td>0</td></tr>
<tr><td>S9_k141_10739</td><td>1112</td><td>No</td><td>NA</td><td>0</td><td>0</td><td>Not-determined</td><td>Genome-fragment</td><td>NA</td><td></td><td>0</td></tr>
<tr><td>S11-final-viral-combined_k141_58489||full</td><td>1055</td><td>No</td><td>NA</td><td>0</td><td>0</td><td>Low-quality</td><td>Genome-fragment</td><td></td><td>0.68</td><td>0</td></tr>
<tr><td>S9_k141_64239</td><td>1127</td><td>No</td><td>NA</td><td>0</td><td>0</td><td>Not-determined</td><td>Genome-fragment</td><td>NA</td><td></td><td>0</td></tr>
<tr><td>S9_k141_10765</td><td>2555</td><td>No</td><td>NA</td><td>0</td><td>1</td><td>Not-determined</td><td>Genome-fragment</td><td>NA</td><td></td><td>0</td></tr>
<tr><td>S11-final-viral-combined_k141_91999||full</td><td>1055</td><td>No</td><td>NA</td><td>0</td><td>0</td><td>Low-quality</td><td>Genome-fragment</td><td></td><td>1.5</td><td>0</td></tr>
<tr><td>S9_k141_97</td><td>1094</td><td>No</td><td>NA</td><td>0</td><td>1</td><td>Not-determined</td><td>Genome-fragment</td><td>NA</td><td></td><td>0</td></tr>
<tr><td>S11-final-viral-combined_k141_301696||lt2gene</td><td>1055</td><td>No</td><td>NA</td><td>1</td><td>0</td><td>Low-quality</td><td>Genome-fragment</td><td></td><td>3.01</td><td>0</td></tr>
<tr><td>S1_k141_285035</td><td>1055</td><td>No</td><td>NA</td><td>0</td><td>1</td><td>Low-quality</td><td>Genome-fragment</td><td></td><td>0.1</td><td>0</td></tr>
<tr><td>S9_k141_48218</td><td>3813</td><td>No</td><td>NA</td><td>0</td><td>3</td><td>Not-determined</td><td>Genome-fragment</td><td>NA</td><td></td><td>0</td></tr>
<tr><td>S3-final-viral-combined_k141_62294||full</td><td>1055</td><td>No</td><td>NA</td><td>0</td><td>0</td><td>Low-quality</td><td>Genome-fragment</td><td></td><td>2.39</td><td>0</td></tr>
<tr><td>S5-final-viral-combined_k141_145529||lt2gene</td><td>1055</td><td>No</td><td>NA</td><td>1</td><td>0</td><td>Low-quality</td><td>Genome-fragment</td><td></td><td>3.06</td><td>0</td></tr>
<tr><td>S9_k141_21476</td><td>9252</td><td>No</td><td>NA</td><td>0</td><td>1</td><td>Not-determined</td><td>Genome-fragment</td><td>NA</td><td></td><td>0</td></tr>
<tr><td>S9_k141_37559</td><td>1304</td><td>No</td><td>NA</td><td>0</td><td>0</td><td>Not-determined</td><td>Genome-fragment</td><td>NA</td><td></td><td>0</td></tr>
<tr><td>S9_k141_183</td><td>4319</td><td>No</td><td>NA</td><td>0</td><td>1</td><td>Not-determined</td><td>Genome-fragment</td><td>NA</td><td></td><td>0</td></tr>
<tr><td>S9_k141_74993</td><td>2033</td><td>No</td><td>NA</td><td>0</td><td>1</td><td>Not-determined</td><td>Genome-fragment</td><td>NA</td><td></td><td>0</td></tr>
<tr><td>S9_k141_197</td><td>2199</td><td>No</td><td>NA</td><td>0</td><td>1</td><td>Not-determined</td><td>Genome-fragment</td><td>NA</td><td></td><td>0</td></tr>
<tr><td>S9_k141_112490</td><td>2523</td><td>No</td><td>NA</td><td>0</td><td>1</td><td>Not-determined</td><td>Genome-fragment</td><td>NA</td><td></td><td>0</td></tr>
<tr><td>S9_k141_117800</td><td>1795</td><td>No</td><td>NA</td><td>0</td><td>2</td><td>Not-determined</td><td>Genome-fragment</td><td>NA</td><td></td><td>0</td></tr>
<tr><td>S9_k141_69615</td><td>2008</td><td>No</td><td>NA</td><td>0</td><td>1</td><td>Not-determined</td><td>Genome-fragment</td><td>NA</td><td></td><td>0</td></tr>
<tr><td>S9_k141_32208</td><td>1323</td><td>No</td><td>NA</td><td>0</td><td>0</td><td>Not-determined</td><td>Genome-fragment</td><td>NA</td><td></td><td>0</td></tr>
<tr><td>S5_k141_152706</td><td>1055</td><td>No</td><td>NA</td><td>0</td><td>0</td><td>Low-quality</td><td>Genome-fragment</td><td></td><td>1.38</td><td>0</td></tr>
<tr><td>S7-final-viral-combined_k141_169125||full</td><td>1055</td><td>No</td><td>NA</td><td>0</td><td>1</td><td>Low-quality</td><td>Genome-fragment</td><td></td><td>1.64</td><td>0</td></tr>
<tr><td>S9_k141_267</td><td>3385</td><td>No</td><td>NA</td><td>0</td><td>2</td><td>Not-determined</td><td>Genome-fragment</td><td>NA</td><td></td><td>0</td></tr>
<tr><td>S9_k141_112569</td><td>2011</td><td>No</td><td>NA</td><td>0</td><td>2</td><td>Not-determined</td><td>Genome-fragment</td><td>NA</td><td></td><td>0</td></tr>
<tr><td>S7-final-viral-combined_k141_190750||full</td><td>1055</td><td>No</td><td>NA</td><td>0</td><td>0</td><td>Low-quality</td><td>Genome-fragment</td><td></td><td>2.5</td><td>0</td></tr>
<tr><td>S9_k141_48326</td><td>1248</td><td>No</td><td>NA</td><td>0</td><td>0</td><td>Not-determined</td><td>Genome-fragment</td><td>NA</td><td></td><td>0</td></tr>
<tr><td>S7_k141_5065</td><td>1055</td><td>No</td><td>NA</td><td>0</td><td>1</td><td>Low-quality</td><td>Genome-fragment</td><td></td><td>0.67</td><td>0</td></tr>
<tr><td>S9_k141_117823</td><td>1373</td><td>No</td><td>NA</td><td>0</td><td>1</td><td>Not-determined</td><td>Genome-fragment</td><td>NA</td><td></td><td>0</td></tr>
<tr><td>S17-k141_45507</td><td>1055</td><td>No</td><td>NA</td><td>0</td><td>0</td><td>Low-quality</td><td>Genome-fragment</td><td></td><td>1.64</td><td>0</td></tr>
<tr><td>S9_k141_59026</td><td>1149</td><td>No</td><td>NA</td><td>0</td><td>0</td><td>Not-determined</td><td>Genome-fragment</td><td>NA</td><td></td><td>0</td></tr>
<tr><td>S9_k141_101841</td><td>3731</td><td>No</td><td>NA</td><td>0</td><td>0</td><td>Not-determined</td><td>Genome-fragment</td><td>NA</td><td></td><td>0</td></tr>
<tr><td>S12_k141_225280</td><td>1055</td><td>No</td><td>NA</td><td>1</td><td>0</td><td>Low-quality</td><td>Genome-fragment</td><td></td><td>0.57</td><td>0</td></tr>
<tr><td>S2-final-viral-combined_k141_1668||full</td><td>1055</td><td>No</td><td>NA</td><td>1</td><td>0</td><td>Low-quality</td><td>Genome-fragment</td><td></td><td>1.96</td><td>0</td></tr>
<tr><td>S9_k141_75180</td><td>1973</td><td>No</td><td>NA</td><td>0</td><td>1</td><td>Not-determined</td><td>Genome-fragment</td><td>NA</td><td></td><td>0</td></tr>
<tr><td>S9_k141_101845</td><td>3595</td><td>No</td><td>NA</td><td>0</td><td>2</td><td>Not-determined</td><td>Genome-fragment</td><td>NA</td><td></td><td>0</td></tr>
<tr><td>S9_k141_96501</td><td>1266</td><td>No</td><td>NA</td><td>0</td><td>1</td><td>Not-determined</td><td>Genome-fragment</td><td>NA</td><td></td><td>0</td></tr>
<tr><td>S4-final-viral-combined_k141_75388||full</td><td>1055</td><td>No</td><td>NA</td><td>0</td><td>0</td><td>Low-quality</td><td>Genome-fragment</td><td></td><td>2.52</td><td>0</td></tr>
<tr><td>S8-final-viral-combined_k141_60335||full</td><td>1055</td><td>No</td><td>NA</td><td>1</td><td>0</td><td>Low-quality</td><td>Genome-fragment</td><td></td><td>2.27</td><td>0</td></tr>
<tr><td>S9_k141_27022</td><td>1219</td><td>No</td><td>NA</td><td>0</td><td>0</td><td>Not-determined</td><td>Genome-fragment</td><td>NA</td><td></td><td>0</td></tr>
<tr><td>S9_k141_117902</td><td>2257</td><td>No</td><td>NA</td><td>0</td><td>2</td><td>Not-determined</td><td>Genome-fragment</td><td>NA</td><td></td><td>0</td></tr>
<tr><td>S8-final-viral-combined_k141_182756||lt2gene</td><td>1055</td><td>No</td><td>NA</td><td>1</td><td>0</td><td>Low-quality</td><td>Genome-fragment</td><td></td><td>2.09</td><td>0</td></tr>
<tr><td>S9_k141_101876</td><td>6057</td><td>No</td><td>NA</td><td>0</td><td>10</td><td>Not-determined</td><td>Genome-fragment</td><td>NA</td><td></td><td>0</td></tr>
<tr><td>S9_k141_64387</td><td>1092</td><td>No</td><td>NA</td><td>0</td><td>1</td><td>Not-determined</td><td>Genome-fragment</td><td>NA</td><td></td><td>0</td></tr>
<tr><td>S9_k141_117947</td><td>3656</td><td>No</td><td>NA</td><td>0</td><td>3</td><td>Not-determined</td><td>Genome-fragment</td><td>NA</td><td></td><td>0</td></tr>
<tr><td>S9_k141_85874</td><td>1733</td><td>No</td><td>NA</td><td>0</td><td>2</td><td>Not-determined</td><td>Genome-fragment</td><td>NA</td><td></td><td>0</td></tr>
<tr><td>S9_k141_101966</td><td>3218</td><td>No</td><td>NA</td><td>0</td><td>5</td><td>Not-determined</td><td>Genome-fragment</td><td>NA</td><td></td><td>0</td></tr>
<tr><td>S9_k141_96593</td><td>10757</td><td>No</td><td>NA</td><td>0</td><td>2</td><td>Not-determined</td><td>Genome-fragment</td><td>NA</td><td></td><td>0</td></tr>
<tr><td>S8_k141_14240</td><td>1055</td><td>No</td><td>NA</td><td>0</td><td>0</td><td>Low-quality</td><td>Genome-fragment</td><td></td><td>0.63</td><td>0</td></tr>
<tr><td>S1-final-viral-combined_k141_65890||full</td><td>1056</td><td>No</td><td>NA</td><td>1</td><td>0</td><td>Low-quality</td><td>Genome-fragment</td><td></td><td>0.8</td><td>0</td></tr>
<tr><td>S9_k141_69773</td><td>2475</td><td>No</td><td>NA</td><td>0</td><td>1</td><td>Not-determined</td><td>Genome-fragment</td><td>NA</td><td></td><td>0</td></tr>
<tr><td>S3-final-viral-combined_k141_51566||full</td><td>1056</td><td>No</td><td>NA</td><td>0</td><td>1</td><td>Low-quality</td><td>Genome-fragment</td><td></td><td>0.48</td><td>0</td></tr>
<tr><td>S9_k141_59140</td><td>1614</td><td>No</td><td>NA</td><td>0</td><td>1</td><td>Not-determined</td><td>Genome-fragment</td><td>NA</td><td></td><td>0</td></tr>
<tr><td>S9_k141_102037</td><td>3441</td><td>No</td><td>NA</td><td>0</td><td>2</td><td>Not-determined</td><td>Genome-fragment</td><td>NA</td><td></td><td>0</td></tr>
<tr><td>S9_k141_53692</td><td>9365</td><td>No</td><td>NA</td><td>0</td><td>1</td><td>Not-determined</td><td>Genome-fragment</td><td>NA</td><td></td><td>0</td></tr>
<tr><td>S7-final-viral-combined_k141_227167||full</td><td>1056</td><td>No</td><td>NA</td><td>0</td><td>0</td><td>Low-quality</td><td>Genome-fragment</td><td></td><td>1.9</td><td>0</td></tr>
<tr><td>S9_k141_75318</td><td>3810</td><td>No</td><td>NA</td><td>0</td><td>2</td><td>Not-determined</td><td>Genome-fragment</td><td>NA</td><td></td><td>0</td></tr>
<tr><td>S9_k141_59158</td><td>1200</td><td>No</td><td>NA</td><td>0</td><td>1</td><td>Not-determined</td><td>Genome-fragment</td><td>NA</td><td></td><td>0</td></tr>
<tr><td>S7-final-viral-combined_k141_175244||lt2gene</td><td>1056</td><td>No</td><td>NA</td><td>1</td><td>0</td><td>Low-quality</td><td>Genome-fragment</td><td></td><td>2.29</td><td>0</td></tr>
<tr><td>S9_k141_59165</td><td>1155</td><td>No</td><td>NA</td><td>0</td><td>1</td><td>Not-determined</td><td>Genome-fragment</td><td>NA</td><td></td><td>0</td></tr>
<tr><td>S9_k141_16429</td><td>3574</td><td>No</td><td>NA</td><td>0</td><td>4</td><td>Not-determined</td><td>Genome-fragment</td><td>NA</td><td></td><td>0</td></tr>
<tr><td>S7_k141_36178</td><td>1056</td><td>No</td><td>NA</td><td>0</td><td>0</td><td>Low-quality</td><td>Genome-fragment</td><td></td><td>0.86</td><td>0</td></tr>
<tr><td>S17-final-viral-combined_k141_137215||full</td><td>1056</td><td>No</td><td>NA</td><td>0</td><td>0</td><td>Low-quality</td><td>Genome-fragment</td><td></td><td>2.65</td><td>0</td></tr>
<tr><td>S17-k141_28011</td><td>1056</td><td>No</td><td>NA</td><td>1</td><td>0</td><td>Low-quality</td><td>Genome-fragment</td><td></td><td>2.4</td><td>0</td></tr>
<tr><td>S9_k141_80637</td><td>3724</td><td>No</td><td>NA</td><td>0</td><td>3</td><td>Not-determined</td><td>Genome-fragment</td><td>NA</td><td></td><td>0</td></tr>
<tr><td>S9_k141_37691</td><td>1187</td><td>No</td><td>NA</td><td>0</td><td>0</td><td>Not-determined</td><td>Genome-fragment</td><td>NA</td><td></td><td>0</td></tr>
<tr><td>S9_k141_75361</td><td>6033</td><td>No</td><td>NA</td><td>0</td><td>2</td><td>Not-determined</td><td>Genome-fragment</td><td>NA</td><td></td><td>0</td></tr>
<tr><td>S9_k141_102082</td><td>3832</td><td>No</td><td>NA</td><td>0</td><td>0</td><td>Not-determined</td><td>Genome-fragment</td><td>NA</td><td></td><td>0</td></tr>
<tr><td>S12-final-viral-combined_k141_267922||lt2gene</td><td>1056</td><td>No</td><td>NA</td><td>1</td><td>0</td><td>Low-quality</td><td>Genome-fragment</td><td></td><td>2.75</td><td>0</td></tr>
<tr><td>S4-final-viral-combined_k141_34284||full</td><td>1056</td><td>No</td><td>NA</td><td>0</td><td>0</td><td>Low-quality</td><td>Genome-fragment</td><td></td><td>1.56</td><td>0</td></tr>
<tr><td>S9_k141_64472</td><td>5450</td><td>No</td><td>NA</td><td>1</td><td>0</td><td>High-quality</td><td>High-quality</td><td></td><td>90.91</td><td>0</td></tr>
<tr><td>S4_k141_154182</td><td>1056</td><td>No</td><td>NA</td><td>0</td><td>0</td><td>Low-quality</td><td>Genome-fragment</td><td></td><td>2.36</td><td>0</td></tr>
<tr><td>S9_k141_598</td><td>1054</td><td>No</td><td>NA</td><td>0</td><td>1</td><td>Not-determined</td><td>Genome-fragment</td><td>NA</td><td></td><td>0</td></tr>
<tr><td>S9_k141_96751</td><td>6695</td><td>No</td><td>NA</td><td>0</td><td>3</td><td>Not-determined</td><td>Genome-fragment</td><td>NA</td><td></td><td>0</td></tr>
<tr><td>S9_k141_53853</td><td>2062</td><td>No</td><td>NA</td><td>0</td><td>1</td><td>Not-determined</td><td>Genome-fragment</td><td>NA</td><td></td><td>0</td></tr>
<tr><td>S9_k141_86071</td><td>1022</td><td>No</td><td>NA</td><td>0</td><td>1</td><td>Not-determined</td><td>Genome-fragment</td><td>NA</td><td></td><td>0</td></tr>
</table>

| | | | | | | | | | | | |
|---|---|---|---|---|---|---|---|---|---|---|---|
| S9_k141_59317 | 1501 | No | NA | | 0 | 1 | Not-determined | Genome-fragment | NA | | 0 |
| S9_k141_48692 | 2306 | No | NA | | 0 | 1 | Not-determined | Genome-fragment | NA | | 0 |
| S9_k141_107209 | 4298 | No | NA | | 0 | 1 | Not-determined | Genome-fragment | NA | | 0 |
| S4_k141_2243 | 1056 | No | NA | | 0 | 1 | Low-quality | Genome-fragment | | 2.37 | 0 |
| S9_k141_48699 | 5833 | No | NA | | 0 | 3 | Not-determined | Genome-fragment | NA | | 0 |
| S9_k141_21434 | 1057 | No | NA | | 0 | 0 | Low-quality | Genome-fragment | | 0.97 | 0 |
| S11-final-viral-combined_k141_138489\|\|full | 1057 | No | NA | | 1 | 0 | Low-quality | Genome-fragment | | 1.3 | 0 |
| S9_k141_86102 | 1496 | No | NA | | 0 | 2 | Not-determined | Genome-fragment | NA | | 0 |
| S9_k141_43271 | 2194 | No | NA | | 0 | 1 | Not-determined | Genome-fragment | NA | | 0 |
| S9_k141_37805 | 1106 | No | NA | | 0 | 1 | Not-determined | Genome-fragment | NA | | 0 |
| S9_k141_123378 | 1295 | No | NA | | 0 | 1 | Not-determined | Genome-fragment | NA | | 0 |
| S9_k141_43281 | 1445 | No | NA | | 0 | 0 | Not-determined | Genome-fragment | NA | | 0 |
| S9_k141_32452 | 2186 | No | NA | | 0 | 0 | Not-determined | Genome-fragment | NA | | 0 |
| S11-final-viral-combined_k141_183264\|\|full | 1057 | No | NA | | 0 | 2 | Low-quality | Genome-fragment | | 0.51 | 0 |
| S1-final-viral-combined_k141_134958\|\|full | 1057 | No | NA | | 0 | 0 | Low-quality | Genome-fragment | | 3.17 | 0 |
| S9_k141_37826 | 4171 | No | NA | | 1 | 0 | Not-determined | Genome-fragment | NA | | 0 |
| S9_k141_80781 | 1126 | No | NA | | 0 | 1 | Not-determined | Genome-fragment | NA | | 0 |
| S1_k141_318752 | 1057 | No | NA | | 0 | 1 | Low-quality | Genome-fragment | | 2.77 | 0 |
| S9_k141_86196 | 1498 | No | NA | | 0 | 1 | Not-determined | Genome-fragment | NA | | 0 |
| S9_k141_118238 | 4356 | No | NA | | 0 | 2 | Not-determined | Genome-fragment | NA | | 0 |
| S9_k141_80803 | 2123 | No | NA | | 0 | 0 | Not-determined | Genome-fragment | NA | | 0 |
| S9_k141_107272 | 1775 | No | NA | | 0 | 2 | Not-determined | Genome-fragment | NA | | 0 |
| S9_k141_37869 | 3947 | No | NA | | 0 | 1 | Not-determined | Genome-fragment | NA | | 0 |
| S9_k141_5775 | 1879 | No | NA | | 0 | 1 | Not-determined | Genome-fragment | NA | | 0 |
| S9_k141_80830 | 7294 | No | NA | | 0 | 3 | Not-determined | Genome-fragment | NA | | 0 |
| S9_k141_123395 | 5834 | No | NA | | 0 | 4 | Not-determined | Genome-fragment | NA | | 0 |
| S9_k141_80854 | 4924 | No | NA | | 0 | 2 | Not-determined | Genome-fragment | NA | | 0 |
| S9_k141_789 | 1830 | No | NA | | 0 | 0 | Not-determined | Genome-fragment | NA | | 0 |
| S1_k141_108947 | 1057 | No | NA | | 0 | 0 | Low-quality | Genome-fragment | | 3.45 | 0 |
| S7_k141_209108 | 1057 | No | NA | | 0 | 0 | Low-quality | Genome-fragment | | 5.04 | 0 |
| S7_k141_15667 | 1057 | No | NA | | 0 | 0 | Low-quality | Genome-fragment | | 2.33 | 0 |
| S9_k141_59493 | 1454 | No | NA | | 0 | 0 | Not-determined | Genome-fragment | NA | | 0 |
| S7_k141_224227 | 1057 | No | NA | | 0 | 0 | Low-quality | Genome-fragment | | 0.76 | 0 |
| S9_k141_97062 | 2878 | No | NA | | 0 | 2 | Not-determined | Genome-fragment | NA | | 0 |
| S12-final-viral-combined_k141_35943\|\|lt2gene | 1057 | No | NA | | 1 | 0 | Low-quality | Genome-fragment | | 0.94 | 0 |
| S9_k141_43428 | 1381 | No | NA | | 0 | 0 | Not-determined | Genome-fragment | NA | | 0 |
| S9_k141_37964 | 1108 | No | NA | | 0 | 0 | Not-determined | Genome-fragment | NA | | 0 |
| S9_k141_59591 | 1319 | No | NA | | 0 | 2 | Not-determined | Genome-fragment | NA | | 0 |
| S9_k141_54007 | 2761 | No | NA | | 0 | 1 | Not-determined | Genome-fragment | NA | | 0 |
| S9_k141_86296 | 1078 | No | NA | | 0 | 1 | Not-determined | Genome-fragment | NA | | 0 |
| S12-final-viral-combined_k141_311249\|\|lt2gene | 1057 | No | NA | | 1 | 0 | Low-quality | Genome-fragment | | 2.39 | 0 |
| S2-final-viral-combined_k141_20708\|\|lt2gene | 1057 | No | NA | | 1 | 0 | Low-quality | Genome-fragment | | 1.54 | 0 |
| S9_k141_11080 | 1000 | No | NA | | 0 | 0 | Not-determined | Genome-fragment | NA | | 0 |
| S9_k141_11081 | 1029 | No | NA | | 0 | 1 | Not-determined | Genome-fragment | NA | | 0 |
| S4-final-viral-combined_k141_68479\|\|full | 1057 | No | NA | | 0 | 0 | Low-quality | Genome-fragment | | 2.77 | 0 |
| S9_k141_80996 | 4337 | No | NA | | 0 | 6 | Not-determined | Genome-fragment | NA | | 0 |
| S6_k141_58266 | 1057 | No | NA | | 0 | 2 | Low-quality | Genome-fragment | | 0.37 | 0 |
| S9_k141_123466 | 2023 | No | NA | | 0 | 1 | Not-determined | Genome-fragment | NA | | 0 |
| S9_k141_118421 | 2030 | No | NA | | 0 | 0 | Not-determined | Genome-fragment | NA | | 0 |
| S9_k141_5951 | 5033 | No | NA | | 0 | 0 | Not-determined | Genome-fragment | NA | | 0 |
| S9_k141_59618 | 3377 | No | NA | | 0 | 1 | Not-determined | Genome-fragment | NA | | 0 |
| S9_k141_5959 | 1090 | No | NA | | 0 | 1 | Not-determined | Genome-fragment | NA | | 0 |
| S9_k141_951 | 1641 | No | NA | | 0 | 1 | Not-determined | Genome-fragment | NA | | 0 |
| S9_k141_953 | 2140 | No | NA | | 0 | 0 | Not-determined | Genome-fragment | NA | | 0 |
| S9_k141_958 | 1299 | No | NA | | 0 | 1 | Not-determined | Genome-fragment | NA | | 0 |
| S9_k141_971 | 3933 | No | NA | | 0 | 1 | Not-determined | Genome-fragment | NA | | 0 |
| S9_k141_123492 | 11321 | No | NA | | 0 | 14 | Not-determined | Genome-fragment | NA | | 0 |
| S9_k141_64706 | 4164 | No | NA | | 0 | 0 | Not-determined | Genome-fragment | NA | | 0 |
| S9_k141_11095 | 6723 | No | NA | | 0 | 1 | Not-determined | Genome-fragment | NA | | 0 |
| S8_k141_181469 | 1057 | No | NA | | 0 | 0 | Low-quality | Genome-fragment | | 0.69 | 0 |
| S8_k141_8344 | 1057 | No | NA | | 0 | 0 | Low-quality | Genome-fragment | | 1.06 | 0 |
| S9-final-viral-combined_k141_6290\|\|full | 1058 | No | NA | | 1 | 0 | Low-quality | Genome-fragment | | 3.03 | 0 |
| S9_k141_102486 | 1073 | No | NA | | 0 | 0 | Not-determined | Genome-fragment | NA | | 0 |
| S11-final-viral-combined_k141_65965\|\|full | 1058 | No | NA | | 0 | 1 | Low-quality | Genome-fragment | | 0.55 | 0 |
| S9_k141_91379 | 4998 | No | NA | | 0 | 0 | Not-determined | Genome-fragment | NA | | 0 |
| S9_k141_69979 | 1195 | No | NA | | 0 | 2 | Not-determined | Genome-fragment | NA | | 0 |
| S1-final-viral-combined_k141_142819\|\|full | 1058 | No | NA | | 0 | 2 | Low-quality | Genome-fragment | | 0.38 | 0 |
| S9_k141_118481 | 1331 | No | NA | | 0 | 0 | Not-determined | Genome-fragment | NA | | 0 |
| S1-final-viral-combined_k141_96163\|\|full | 1058 | No | NA | | 2 | 0 | Low-quality | Genome-fragment | | 2.92 | 0 |
| S9_k141_102542 | 3076 | No | NA | | 0 | 2 | Not-determined | Genome-fragment | NA | | 0 |
| S9_k141_123545 | 1038 | No | NA | | 0 | 1 | Not-determined | Genome-fragment | NA | | 0 |
| S1_k141_68868 | 1058 | No | NA | | 0 | 0 | Low-quality | Genome-fragment | | 0.86 | 0 |
| S9_k141_48989 | 1571 | No | NA | | 0 | 1 | Not-determined | Genome-fragment | NA | | 0 |
| S3-final-viral-combined_k141_89943\|\|full | 1058 | No | NA | | 0 | 0 | Low-quality | Genome-fragment | | 2.09 | 0 |
| S3-final-viral-combined_k141_33719\|\|full | 1058 | No | NA | | 0 | 0 | Low-quality | Genome-fragment | | 0.73 | 0 |
| S5-final-viral-combined_k141_201112\|\|full | 1058 | No | NA | | 0 | 1 | Low-quality | Genome-fragment | | 0.58 | 0 |
| S9_k141_27452 | 3548 | No | NA | | 0 | 1 | Not-determined | Genome-fragment | NA | | 0 |
| S9_k141_91499 | 1578 | No | NA | | 0 | 0 | Not-determined | Genome-fragment | NA | | 0 |
| S9_k141_32850 | 1404 | No | NA | | 0 | 0 | Not-determined | Genome-fragment | NA | | 0 |
| S9_k141_38162 | 1172 | No | NA | | 0 | 1 | Not-determined | Genome-fragment | NA | | 0 |
| S9_k141_97227 | 1359 | No | NA | | 0 | 1 | Not-determined | Genome-fragment | NA | | 0 |
| S9_k141_1205 | 1415 | No | NA | | 0 | 1 | Not-determined | Genome-fragment | NA | | 0 |
| S9_k141_38166 | 1652 | No | NA | | 0 | 1 | Not-determined | Genome-fragment | NA | | 0 |
| S9_k141_38172 | 1033 | No | NA | | 0 | 0 | Not-determined | Genome-fragment | NA | | 0 |
| S5-final-viral-combined_k141_76987\|\|full | 1058 | No | NA | | 1 | 0 | Low-quality | Genome-fragment | | 7.84 | 0 |
| S9_k141_22024 | 1575 | No | NA | | 0 | 1 | Not-determined | Genome-fragment | NA | | 0 |
| S9_k141_97251 | 1475 | No | NA | | 0 | 0 | Not-determined | Genome-fragment | NA | | 0 |
| S9_k141_22027 | 4155 | No | NA | | 0 | 1 | Not-determined | Genome-fragment | NA | | 0 |
| S9_k141_22030 | 5466 | No | NA | | 0 | 4 | Not-determined | Genome-fragment | NA | | 0 |
| S9_k141_86492 | 1424 | No | NA | | 0 | 0 | Not-determined | Genome-fragment | NA | | 0 |
| S5-final-viral-combined_k141_68012\|\|full | 1058 | No | NA | | 2 | 0 | Low-quality | Genome-fragment | | 1.79 | 0 |
| S9_k141_81301 | 4909 | No | NA | | 0 | 6 | Not-determined | Genome-fragment | NA | | 0 |
| S9_k141_59749 | 2284 | No | NA | | 0 | 2 | Not-determined | Genome-fragment | NA | | 0 |
| S9_k141_118569 | 5511 | No | NA | | 0 | 3 | Not-determined | Genome-fragment | NA | | 0 |
| S7-final-viral-combined_k141_205129\|\|full | 1058 | No | NA | | 0 | 0 | Low-quality | Genome-fragment | | 2.63 | 0 |
| S9_k141_123637 | 1066 | No | NA | | 0 | 1 | Not-determined | Genome-fragment | NA | | 0 |
| S9_k141_59772 | 1037 | No | NA | | 0 | 0 | Not-determined | Genome-fragment | NA | | 0 |
| S9_k141_123663 | 1024 | No | NA | | 0 | 1 | Not-determined | Genome-fragment | NA | | 0 |
| S9_k141_54171 | 1175 | No | NA | | 0 | 2 | Not-determined | Genome-fragment | NA | | 0 |
| S7-final-viral-combined_k141_238295\|\|full | 1058 | No | NA | | 0 | 0 | Low-quality | Genome-fragment | | 0.58 | 0 |
| S9_k141_123693 | 1430 | No | NA | | 0 | 1 | Not-determined | Genome-fragment | NA | | 0 |
| S9_k141_86539 | 1139 | No | NA | | 0 | 1 | Not-determined | Genome-fragment | NA | | 0 |
| S7-final-viral-combined_k141_118290\|\|full | 1058 | Yes | | 794 | 1 | 1 | Low-quality | Genome-fragment | | 1.11 | 24.95 |
| S7_k141_14923 | 1058 | No | NA | | 0 | 1 | Low-quality | Genome-fragment | | 1.65 | 0 |
| S9_k141_6049 | 1095 | No | NA | | 0 | 1 | Not-determined | Genome-fragment | NA | | 0 |
| S9_k141_1336 | 1152 | No | NA | | 0 | 1 | Not-determined | Genome-fragment | NA | | 0 |
| S9_k141_1339 | 1223 | No | NA | | 0 | 1 | Not-determined | Genome-fragment | NA | | 0 |
| S9_k141_64874 | 1571 | No | NA | | 0 | 1 | Not-determined | Genome-fragment | NA | | 0 |
| S12-final-viral-combined_k141_234699\|\|full | 1058 | No | NA | | 0 | 0 | Low-quality | Genome-fragment | | 2.37 | 0 |
| S12-final-viral-combined_k141_335766\|\|full | 1058 | No | NA | | 0 | 1 | Low-quality | Genome-fragment | | 1.23 | 0 |
| S9_k141_86600 | 1035 | No | NA | | 0 | 4 | Not-determined | Genome-fragment | NA | | 0 |
| S9_k141_54302 | 1355 | No | NA | | 0 | 1 | Not-determined | Genome-fragment | NA | | 0 |
| S12-final-viral-combined_k141_52191\|\|full | 1058 | No | NA | | 0 | 0 | Low-quality | Genome-fragment | | 1.65 | 0 |
| S9_k141_102650 | 3487 | No | NA | | 0 | 1 | Not-determined | Genome-fragment | NA | | 0 |
| S9_k141_33050 | 4061 | No | NA | | 0 | 1 | Not-determined | Genome-fragment | NA | | 0 |
| S9_k141_113139 | 5222 | No | NA | | 0 | 4 | Not-determined | Genome-fragment | NA | | 0 |
| S9_k141_27633 | 1469 | No | NA | | 0 | 1 | Not-determined | Genome-fragment | NA | | 0 |
| S12-final-viral-combined_k141_337974\|\|full | 1058 | No | NA | | 0 | 0 | Low-quality | Genome-fragment | | 2.72 | 0 |
| S9_k141_123793 | 1026 | No | NA | | 0 | 1 | Not-determined | Genome-fragment | NA | | 0 |
| S9_k141_59838 | 1160 | No | NA | | 0 | 1 | Not-determined | Genome-fragment | NA | | 0 |
| S9_k141_118687 | 1578 | No | NA | | 0 | 1 | Not-determined | Genome-fragment | NA | | 0 |
| S9_k141_33128 | 1205 | No | NA | | 0 | 1 | Not-determined | Genome-fragment | NA | | 0 |
| S9_k141_43541 | 1018 | No | NA | | 0 | 1 | Not-determined | Genome-fragment | NA | | 0 |
| S9_k141_59921 | 3731 | No | NA | | 0 | 3 | Not-determined | Genome-fragment | NA | | 0 |
| S9_k141_102682 | 1105 | No | NA | | 0 | 1 | Not-determined | Genome-fragment | NA | | 0 |
| S9_k141_33164 | 1509 | No | NA | | 0 | 1 | Not-determined | Genome-fragment | NA | | 0 |
| S12-final-viral-combined_k141_240745\|\|full | 1058 | No | NA | | 0 | 0 | Low-quality | Genome-fragment | | 2.52 | 0 |
| S6-final-viral-combined_k141_117069\|\|full | 1058 | No | NA | | 0 | 0 | Low-quality | Genome-fragment | | 1.98 | 0 |
| S9_k141_6194 | 5172 | No | NA | | 0 | 2 | Not-determined | Genome-fragment | NA | | 0 |
| S9_k141_11430 | 1026 | No | NA | | 0 | 1 | Not-determined | Genome-fragment | NA | | 0 |
| S8-final-viral-combined_k141_237272\|\|lt2gene | 1058 | No | NA | | 0 | 0 | Low-quality | Genome-fragment | | 39.56 | 0 |
| S9_k141_107701 | 3063 | No | NA | | 0 | 3 | Not-determined | Genome-fragment | NA | | 0 |
| S9-final-viral-combined_k141_127677\|\|full | 1059 | No | NA | | 0 | 0 | Low-quality | Genome-fragment | | 2.36 | 0 |
| S9_k141_16756 | 1246 | No | NA | | 0 | 0 | Not-determined | Genome-fragment | NA | | 0 |
| S9_k141_38310 | 2758 | No | NA | | 0 | 0 | Not-determined | Genome-fragment | NA | | 0 |
| S9_k141_38323 | 1320 | No | NA | | 0 | 1 | Not-determined | Genome-fragment | NA | | 0 |
| S9_k141_43638 | 1385 | No | NA | | 0 | 0 | Not-determined | Genome-fragment | NA | | 0 |
| S9-final-viral-combined_k141_67851\|\|lt2gene | 1059 | No | NA | | 2 | 0 | Low-quality | Genome-fragment | | 1.34 | 0 |
| S9_k141_97479 | 1845 | No | NA | | 0 | 1 | Not-determined | Genome-fragment | NA | | 0 |
| S9_k141_70197 | 1063 | No | NA | | 0 | 1 | Not-determined | Genome-fragment | NA | | 0 |
| S9_k141_54511 | 1728 | No | NA | | 0 | 0 | Not-determined | Genome-fragment | NA | | 0 |
| S11-final-viral-combined_k141_240025\|\|full | 1059 | No | NA | | 1 | 0 | Low-quality | Genome-fragment | | 3.31 | 0 |
| S9_k141_1524 | 1193 | No | NA | | 0 | 2 | Not-determined | Genome-fragment | NA | | 0 |
| S9_k141_123938 | 1264 | No | NA | | 0 | 1 | Not-determined | Genome-fragment | NA | | 0 |
| S9_k141_70231 | 5111 | No | NA | | 0 | 0 | Not-determined | Genome-fragment | NA | | 0 |
| S9_k141_16829 | 2054 | No | NA | | 0 | 2 | Not-determined | Genome-fragment | NA | | 0 |
| S9_k141_123953 | 1610 | No | NA | | 0 | 1 | Not-determined | Genome-fragment | NA | | 0 |
| S9_k141_11551 | 1131 | No | NA | | 0 | 0 | Not-determined | Genome-fragment | NA | | 0 |
| S9_k141_27947 | 1331 | No | NA | | 0 | 0 | Not-determined | Genome-fragment | NA | | 0 |
| S9_k141_65129 | 1092 | No | NA | | 0 | 1 | Not-determined | Genome-fragment | NA | | 0 |
| S9_k141_16843 | 1274 | No | NA | | 0 | 1 | Not-determined | Genome-fragment | NA | | 0 |
| S9_k141_81552 | 1227 | No | NA | | 0 | 1 | Not-determined | Genome-fragment | NA | | 0 |
| S11-final-viral-combined_k141_321788\|\|full | 1059 | No | NA | | 0 | 0 | Low-quality | Genome-fragment | | 2.05 | 0 |
| S9_k141_86814 | 3669 | No | NA | | 0 | 5 | Not-determined | Genome-fragment | NA | | 0 |
| S9_k141_118854 | 1047 | No | NA | | 0 | 1 | Not-determined | Genome-fragment | NA | | 0 |
| S9_k141_113242 | 3263 | No | NA | | 0 | 2 | Not-determined | Genome-fragment | NA | | 0 |
| S11_k141_181159 | 1059 | No | NA | | 0 | 0 | Low-quality | Genome-fragment | | 0.68 | 0 |
| S11_k141_321180 | 1059 | No | NA | | 0 | 0 | Low-quality | Genome-fragment | | 2.75 | 0 |
| S1-final-viral-combined_k141_150710\|\|full | 1059 | No | NA | | 0 | 0 | Low-quality | Genome-fragment | | 0.86 | 0 |
| S9_k141_113259 | 3241 | No | NA | | 0 | 2 | Not-determined | Genome-fragment | NA | | 0 |
| S9_k141_81627 | 2208 | No | NA | | 0 | 1 | Not-determined | Genome-fragment | NA | | 0 |
| S9_k141_54668 | 1029 | No | NA | | 0 | 1 | Not-determined | Genome-fragment | NA | | 0 |
| S9_k141_43722 | 1060 | No | NA | | 0 | 1 | Not-determined | Genome-fragment | NA | | 0 |
| S1-final-viral-combined_k141_276406\|\|full | 1059 | No | NA | | 0 | 1 | Low-quality | Genome-fragment | | 0.52 | 0 |
| S9_k141_113309 | 2001 | No | NA | | 0 | 1 | Not-determined | Genome-fragment | NA | | 0 |
| S5-final-viral-combined_k141_29060\|\|full | 1059 | No | NA | | 1 | 0 | Low-quality | Genome-fragment | | 2.22 | 0 |
| S9_k141_75751 | 1078 | No | NA | | 0 | 1 | Not-determined | Genome-fragment | NA | | 0 |
| S9_k141_75769 | 1066 | No | NA | | 0 | 0 | Not-determined | Genome-fragment | NA | | 0 |
| S9_k141_75781 | 3822 | No | NA | | 0 | 4 | Not-determined | Genome-fragment | NA | | 0 |
| S9_k141_92051 | 3213 | No | NA | | 0 | 3 | Not-determined | Genome-fragment | NA | | 0 |
| S9_k141_75803 | 6719 | No | NA | | 0 | 2 | Not-determined | Genome-fragment | NA | | 0 |
| S9_k141_22608 | 3349 | No | NA | | 0 | 1 | Not-determined | Genome-fragment | NA | | 0 |
| S9_k141_38425 | 3815 | No | NA | | 0 | 1 | Not-determined | Genome-fragment | NA | | 0 |
| S9_k141_60164 | 1056 | No | NA | | 0 | 2 | Not-determined | Genome-fragment | NA | | 0 |

| | | | | | | | | | |
|---|---|---|---|---|---|---|---|---|---|
| S9_k141_1736 | 1085 | No | NA | 0 | 1 | Not-determined | Genome-fragment | NA | 0 |
| S9_k141_1739 | 1336 | No | NA | 0 | 0 | Not-determined | Genome-fragment | NA | 0 |
| S9_k141_70472 | 3335 | No | NA | 0 | 1 | Not-determined | Genome-fragment | NA | 0 |
| S9_k141_22640 | 1045 | No | NA | 0 | 0 | Not-determined | Genome-fragment | NA | 0 |
| S5-final-viral-combined_k141_31776\|\|full | 1059 | No | NA | 1 | 0 | Low-quality | Genome-fragment | 2.2 | 0 |
| S9_k141_92124 | 1041 | No | NA | 0 | 1 | Not-determined | Genome-fragment | NA | 0 |
| S9_k141_86857 | 1307 | No | NA | 0 | 0 | Not-determined | Genome-fragment | NA | 0 |
| S7_k141_218025 | 1059 | No | NA | 0 | 0 | Low-quality | Genome-fragment | 1 | 0 |
| S17-final-viral-combined_k141_196696\|\|full | 1059 | No | NA | 1 | 0 | Low-quality | Genome-fragment | 2.8 | 0 |
| S9_k141_28051 | 1605 | No | NA | 0 | 1 | Not-determined | Genome-fragment | NA | 0 |
| S12-final-viral-combined_k141_43924\|\|full | 1059 | No | NA | 1 | 0 | Low-quality | Genome-fragment | 0.64 | 0 |
| S12_k141_125950 | 1059 | No | NA | 0 | 0 | Low-quality | Genome-fragment | 0.47 | 0 |
| S4_k141_150536 | 1059 | No | NA | 0 | 2 | Low-quality | Genome-fragment | 0.55 | 0 |
| S9_k141_65420 | 1384 | No | NA | 0 | 0 | Not-determined | Genome-fragment | NA | 0 |
| S9_k141_86878 | 28068 | No | NA | 0 | 0 | Not-determined | Genome-fragment | NA | 0 |
| S9_k141_113498 | 1854 | No | NA | 0 | 1 | Not-determined | Genome-fragment | NA | 0 |
| S11-final-viral-combined_k141_99537\|\|full | 1060 | No | NA | 1 | 0 | Low-quality | Genome-fragment | 1.4 | 0 |
| S9_k141_70589 | 1121 | No | NA | 0 | 1 | Not-determined | Genome-fragment | NA | 0 |
| S3_k141_16513 | 1060 | No | NA | 0 | 1 | Low-quality | Genome-fragment | 0.26 | 0 |
| S9_k141_70594 | 1177 | No | NA | 0 | 1 | Not-determined | Genome-fragment | NA | 0 |
| S9_k141_81797 | 1003 | No | NA | 0 | 1 | Not-determined | Genome-fragment | NA | 0 |
| S9_k141_54891 | 2082 | No | NA | 0 | 0 | Not-determined | Genome-fragment | NA | 0 |
| S9_k141_103009 | 1031 | No | NA | 0 | 0 | Not-determined | Genome-fragment | NA | 0 |
| S9_k141_38535 | 3310 | No | NA | 0 | 1 | Not-determined | Genome-fragment | NA | 0 |
| S5-final-viral-combined_k141_171858\|\|full | 1060 | No | NA | 0 | 0 | Low-quality | Genome-fragment | 2.09 | 0 |
| S9_k141_54925 | 1365 | No | NA | 0 | 1 | Not-determined | Genome-fragment | NA | 0 |
| S5-final-viral-combined_k141_162102\|\|lt2gene | 1060 | No | NA | 1 | 0 | Low-quality | Genome-fragment | 2.28 | 0 |
| S9_k141_113532 | 3423 | No | NA | 0 | 2 | Not-determined | Genome-fragment | NA | 0 |
| S9_k141_6819 | 1252 | No | NA | 0 | 1 | Not-determined | Genome-fragment | NA | 0 |
| S9_k141_22868 | 3916 | No | NA | 0 | 1 | Not-determined | Genome-fragment | NA | 0 |
| S9_k141_70683 | 5586 | No | NA | 0 | 0 | Not-determined | Genome-fragment | NA | 0 |
| S9_k141_81841 | 4518 | No | NA | 0 | 1 | Not-determined | Genome-fragment | NA | 0 |
| S9_k141_70750 | 2664 | No | NA | 0 | 2 | Not-determined | Genome-fragment | NA | 0 |
| S9_k141_28235 | 2403 | No | NA | 0 | 0 | Not-determined | Genome-fragment | NA | 0 |
| S9_k141_28236 | 1096 | No | NA | 0 | 1 | Not-determined | Genome-fragment | NA | 0 |
| S9_k141_38578 | 1213 | No | NA | 0 | 0 | Not-determined | Genome-fragment | NA | 0 |
| S9_k141_55012 | 1336 | No | NA | 0 | 0 | Not-determined | Genome-fragment | NA | 0 |
| S7-final-viral-combined_k141_146205\|\|full | 1060 | No | NA | 1 | 0 | Low-quality | Genome-fragment | 2.29 | 0 |
| S17-final-viral-combined_k141_21063\|\|full | 1060 | No | NA | 0 | 1 | Low-quality | Genome-fragment | 0.54 | 0 |
| S9_k141_6983 | 1004 | No | NA | 0 | 0 | Not-determined | Genome-fragment | NA | 0 |
| S9_k141_28275 | 1368 | No | NA | 0 | 0 | Not-determined | Genome-fragment | NA | 0 |
| S9_k141_38657 | 1312 | No | NA | 0 | 1 | Not-determined | Genome-fragment | NA | 0 |
| S9_k141_81948 | 1184 | No | NA | 0 | 1 | Not-determined | Genome-fragment | NA | 0 |
| S9_k141_124410 | 3270 | No | NA | 0 | 3 | Not-determined | Genome-fragment | NA | 0 |
| S9_k141_70808 | 6028 | No | NA | 0 | 5 | Not-determined | Genome-fragment | NA | 0 |
| S9_k141_17342 | 1023 | No | NA | 0 | 1 | Not-determined | Genome-fragment | NA | 0 |
| S9_k141_49731 | 3475 | No | NA | 0 | 2 | Not-determined | Genome-fragment | NA | 0 |
| S9_k141_108215 | 1262 | No | NA | 0 | 1 | Not-determined | Genome-fragment | NA | 0 |
| S9_k141_103303 | 1014 | No | NA | 0 | 1 | Not-determined | Genome-fragment | NA | 0 |
| S9_k141_44188 | 2099 | No | NA | 0 | 1 | Not-determined | Genome-fragment | NA | 0 |
| S9_k141_44189 | 1012 | No | NA | 0 | 1 | Not-determined | Genome-fragment | NA | 0 |
| S9_k141_97816 | 33242 | No | NA | 0 | 5 | Not-determined | Genome-fragment | NA | 0 |
| S9_k141_33372 | 1507 | No | NA | 0 | 1 | Not-determined | Genome-fragment | NA | 0 |
| S9_k141_119113 | 1244 | No | NA | 0 | 1 | Not-determined | Genome-fragment | NA | 0 |
| S9_k141_75989 | 1117 | No | NA | 0 | 0 | Not-determined | Genome-fragment | NA | 0 |
| S9_k141_65768 | 1590 | No | NA | 0 | 0 | Not-determined | Genome-fragment | NA | 0 |
| S9_k141_75990 | 6391 | No | NA | 0 | 3 | Not-determined | Genome-fragment | NA | 0 |
| S9_k141_87187 | 25238 | No | NA | 0 | 31 | Not-determined | Genome-fragment | NA | 0 |
| S12_k141_36777 | 1060 | No | NA | 0 | 1 | Low-quality | Genome-fragment | 2.99 | 0 |
| S9_k141_33392 | 8414 | No | NA | 0 | 8 | Not-determined | Genome-fragment | NA | 0 |
| S9_k141_70929 | 1877 | No | NA | 0 | 2 | Not-determined | Genome-fragment | NA | 0 |
| S9_k141_17501 | 1763 | No | NA | 0 | 0 | Not-determined | Genome-fragment | NA | 0 |
| S9_k141_60586 | 1308 | No | NA | 0 | 1 | Not-determined | Genome-fragment | NA | 0 |
| S9_k141_55198 | 6796 | No | NA | 0 | 1 | Not-determined | Genome-fragment | NA | 0 |
| S9_k141_124517 | 2220 | No | NA | 0 | 0 | Not-determined | Genome-fragment | NA | 0 |
| S9_k141_33397 | 1458 | No | NA | 0 | 1 | Not-determined | Genome-fragment | NA | 0 |
| S12_k141_93685 | 1060 | No | NA | 0 | 0 | Low-quality | Genome-fragment | 0.57 | 0 |
| S2-final-viral-combined_k141_85104\|\|full | 1060 | No | NA | 0 | 0 | Low-quality | Genome-fragment | 1.52 | 0 |
| S9_k141_2097 | 1565 | No | NA | 0 | 2 | Not-determined | Genome-fragment | NA | 0 |
| S2_k141_11508 | 1060 | No | NA | 0 | 0 | Low-quality | Genome-fragment | 2.73 | 0 |
| S9_k141_49893 | 9651 | No | NA | 0 | 0 | Not-determined | Genome-fragment | NA | 0 |
| S9_k141_2112 | 2358 | No | NA | 0 | 2 | Not-determined | Genome-fragment | NA | 0 |
| S2_k141_16178 | 1060 | No | NA | 0 | 0 | Low-quality | Genome-fragment | 2.2 | 0 |
| S9_k141_33426 | 9829 | No | NA | 0 | 2 | Not-determined | Genome-fragment | NA | 0 |
| S9_k141_44300 | 3468 | No | NA | 0 | 1 | Not-determined | Genome-fragment | NA | 0 |
| S9_k141_33437 | 5306 | No | NA | 0 | 2 | Not-determined | Genome-fragment | NA | 0 |
| S9_k141_92533 | 1627 | No | NA | 0 | 1 | Not-determined | Genome-fragment | NA | 0 |
| S2_k141_6263 | 1060 | No | NA | 0 | 1 | Low-quality | Genome-fragment | 0.37 | 0 |
| S4-final-viral-combined_k141_22818\|\|full | 1060 | No | NA | 0 | 2 | Low-quality | Genome-fragment | 1.55 | 0 |
| S9_k141_23223 | 3014 | No | NA | 0 | 1 | Not-determined | Genome-fragment | NA | 0 |
| S9_k141_55340 | 1519 | No | NA | 0 | 0 | Not-determined | Genome-fragment | NA | 0 |
| S9_k141_92567 | 1123 | No | NA | 0 | 0 | Not-determined | Genome-fragment | NA | 0 |
| S9_k141_66026 | 4639 | No | NA | 0 | 3 | Not-determined | Genome-fragment | NA | 0 |
| S9_k141_108358 | 1454 | No | NA | 0 | 0 | Not-determined | Genome-fragment | NA | 0 |
| S9_k141_76180 | 1109 | No | NA | 0 | 0 | Not-determined | Genome-fragment | NA | 0 |
| S4-final-viral-combined_k141_90288\|\|lt2gene | 1060 | No | NA | 1 | 0 | Low-quality | Genome-fragment | 1.72 | 0 |
| S4_k141_156157 | 1060 | No | NA | 0 | 0 | Low-quality | Genome-fragment | 1.05 | 0 |
| S9_k141_12052 | 1238 | No | NA | 0 | 1 | Not-determined | Genome-fragment | NA | 0 |
| S9_k141_60730 | 1210 | No | NA | 0 | 0 | Not-determined | Genome-fragment | NA | 0 |
| S9-final-viral-combined_k141_67516\|\|lt2gene | 1061 | No | NA | 1 | 0 | Low-quality | Genome-fragment | 1.57 | 0 |
| S9_k141_87287 | 1097 | No | NA | 0 | 1 | Not-determined | Genome-fragment | NA | 0 |
| S9_k141_55446 | 2161 | No | NA | 0 | 1 | Not-determined | Genome-fragment | NA | 0 |
| S11-final-viral-combined_k141_170906\|\|full | 1061 | No | NA | 0 | 0 | Low-quality | Genome-fragment | 2.05 | 0 |
| S9_k141_23306 | 2070 | No | NA | 0 | 1 | Not-determined | Genome-fragment | NA | 0 |
| S9_k141_119339 | 6441 | No | NA | 0 | 1 | Not-determined | Genome-fragment | NA | 0 |
| S11_k141_138123 | 1061 | No | NA | 0 | 0 | Low-quality | Genome-fragment | 0.8 | 0 |
| S9_k141_50122 | 1281 | No | NA | 0 | 1 | Not-determined | Genome-fragment | NA | 0 |
| S11_k141_304463 | 1061 | No | NA | 0 | 1 | Low-quality | Genome-fragment | 0.37 | 0 |
| S1-final-viral-combined_k141_229995\|\|full | 1061 | No | NA | 0 | 0 | Low-quality | Genome-fragment | 1.6 | 0 |
| S5_k141_42642 | 1061 | No | NA | 0 | 1 | Low-quality | Genome-fragment | 0.57 | 0 |
| S9_k141_28449 | 1722 | No | NA | 0 | 1 | Not-determined | Genome-fragment | NA | 0 |
| S9_k141_23356 | 1218 | No | NA | 0 | 1 | Not-determined | Genome-fragment | NA | 0 |
| S9_k141_28454 | 1642 | No | NA | 0 | 2 | Not-determined | Genome-fragment | NA | 0 |
| S17-final-viral-combined_k141_182615\|\|full | 1061 | No | NA | 1 | 0 | Low-quality | Genome-fragment | 2.69 | 0 |
| S9_k141_12231 | 1252 | No | NA | 0 | 2 | Not-determined | Genome-fragment | NA | 0 |
| S17-final-viral-combined_k141_141281\|\|full | 1061 | No | NA | 0 | 0 | Low-quality | Genome-fragment | 0.58 | 0 |
| S12-final-viral-combined_k141_173536\|\|lt2gene | 1061 | No | NA | 2 | 0 | Low-quality | Genome-fragment | 2.63 | 0 |
| S2-final-viral-combined_k141_152804\|\|full | 1061 | No | NA | 0 | 0 | Low-quality | Genome-fragment | 2.54 | 0 |
| S2-final-viral-combined_k141_71170\|\|full | 1061 | No | NA | 0 | 0 | Low-quality | Genome-fragment | 2.28 | 0 |
| S9_k141_28581 | 1403 | No | NA | 0 | 0 | Not-determined | Genome-fragment | NA | 0 |
| S9_k141_33527 | 1190 | No | NA | 0 | 1 | Not-determined | Genome-fragment | NA | 0 |
| S2_k141_151235 | 1061 | No | NA | 0 | 0 | Low-quality | Genome-fragment | 1.96 | 0 |
| S9_k141_71225 | 5795 | No | NA | 0 | 1 | Not-determined | Genome-fragment | NA | 0 |
| S4-final-viral-combined_k141_165568\|\|full | 1061 | No | NA | 0 | 0 | Low-quality | Genome-fragment | 1.73 | 0 |
| S9_k141_7489 | 1343 | No | NA | 0 | 0 | Not-determined | Genome-fragment | NA | 0 |
| S4-final-viral-combined_k141_153421\|\|lt2gene | 1061 | No | NA | 1 | 0 | Low-quality | Genome-fragment | 2.54 | 0 |
| S9_k141_60965 | 1507 | No | NA | 0 | 0 | Not-determined | Genome-fragment | NA | 0 |
| S9_k141_66281 | 1274 | No | NA | 0 | 0 | Not-determined | Genome-fragment | NA | 0 |
| S9_k141_12372 | 1234 | No | NA | 0 | 0 | Not-determined | Genome-fragment | NA | 0 |
| S9_k141_2448 | 1359 | No | NA | 0 | 0 | Not-determined | Genome-fragment | NA | 0 |
| S9_k141_17925 | 1426 | No | NA | 0 | 1 | Not-determined | Genome-fragment | NA | 0 |
| S6-final-viral-combined_k141_49802\|\|full | 1061 | No | NA | 0 | 0 | Low-quality | Genome-fragment | 2.29 | 0 |
| S9_k141_39264 | 1526 | No | NA | 0 | 2 | Not-determined | Genome-fragment | NA | 0 |
| S9_k141_119531 | 6014 | No | NA | 0 | 1 | Not-determined | Genome-fragment | NA | 0 |
| S9_k141_87590 | 1378 | No | NA | 0 | 1 | Not-determined | Genome-fragment | NA | 0 |
| S9_k141_12403 | 4026 | No | NA | 0 | 2 | Not-determined | Genome-fragment | NA | 0 |
| S8_k141_110912 | 1061 | No | NA | 0 | 0 | Low-quality | Genome-fragment | 3.21 | 0 |
| S9_k141_92913 | 4464 | No | NA | 0 | 4 | Not-determined | Genome-fragment | NA | 0 |
| S9_k141_12445 | 1652 | No | NA | 0 | 1 | Not-determined | Genome-fragment | NA | 0 |
| S9_k141_66317 | 1309 | No | NA | 0 | 1 | Not-determined | Genome-fragment | NA | 0 |
| S9_k141_61053 | 1475 | No | NA | 0 | 1 | Not-determined | Genome-fragment | NA | 0 |
| S9-final-viral-combined_k141_90970\|\|full | 1062 | No | NA | 0 | 0 | Low-quality | Genome-fragment | 2.64 | 0 |
| S9_k141_12462 | 4158 | No | NA | 0 | 3 | Not-determined | Genome-fragment | NA | 0 |
| S9_k141_119581 | 1793 | No | NA | 0 | 0 | Not-determined | Genome-fragment | NA | 0 |
| S1-final-viral-combined_k141_120326\|\|full | 1062 | No | NA | 0 | 0 | Low-quality | Genome-fragment | 0.81 | 0 |
| S9_k141_98240 | 3086 | No | NA | 0 | 0 | Not-determined | Genome-fragment | NA | 0 |
| S1-final-viral-combined_k141_96959\|\|full | 1062 | No | NA | 0 | 0 | Low-quality | Genome-fragment | 1.82 | 0 |
| S9_k141_76397 | 1702 | No | NA | 0 | 2 | Not-determined | Genome-fragment | NA | 0 |
| S1-final-viral-combined_k141_179759\|\|full | 1062 | No | NA | 0 | 0 | Low-quality | Genome-fragment | 3.44 | 0 |
| S9_k141_76419 | 6893 | No | NA | 0 | 2 | Not-determined | Genome-fragment | NA | 0 |
| S9_k141_50434 | 1127 | No | NA | 0 | 1 | Not-determined | Genome-fragment | NA | 0 |
| S3-final-viral-combined_k141_127141\|\|full | 1062 | No | NA | 0 | 1 | Low-quality | Genome-fragment | 0.63 | 0 |
| S9_k141_18090 | 4913 | No | NA | 0 | 1 | Not-determined | Genome-fragment | NA | 0 |
| S9_k141_82487 | 1602 | No | NA | 0 | 0 | Not-determined | Genome-fragment | NA | 0 |
| S3-final-viral-combined_k141_4667\|\|full | 1062 | No | NA | 0 | 0 | Low-quality | Genome-fragment | 1.92 | 0 |
| S7-final-viral-combined_k141_211396\|\|lt2gene | 1062 | No | NA | 2 | 0 | Low-quality | Genome-fragment | 1.51 | 0 |
| S9_k141_61124 | 1359 | No | NA | 0 | 2 | Not-determined | Genome-fragment | NA | 0 |
| S7-final-viral-combined_k141_7465\|\|lt2gene | 1062 | No | NA | 2 | 0 | Low-quality | Genome-fragment | 2.36 | 0 |
| S9_k141_113957 | 4144 | No | NA | 0 | 0 | Not-determined | Genome-fragment | NA | 0 |
| S9_k141_33732 | 3512 | No | NA | 0 | 3 | Not-determined | Genome-fragment | NA | 0 |
| S9_k141_71360 | 2194 | No | NA | 0 | 1 | Not-determined | Genome-fragment | NA | 0 |
| S17-final-viral-combined_k141_170653\|\|full | 1062 | No | NA | 0 | 0 | Low-quality | Genome-fragment | 2.26 | 0 |
| S9_k141_71370 | 5727 | No | NA | 0 | 1 | Not-determined | Genome-fragment | NA | 0 |
| S9_k141_12629 | 1779 | No | NA | 0 | 3 | Not-determined | Genome-fragment | NA | 0 |
| S9_k141_87685 | 1031 | No | NA | 0 | 0 | Not-determined | Genome-fragment | NA | 0 |
| S9_k141_39420 | 4716 | No | NA | 0 | 1 | Not-determined | Genome-fragment | NA | 0 |
| S17-final-viral-combined_k141_84422\|\|full | 1062 | No | NA | 1 | 0 | Low-quality | Genome-fragment | 2.52 | 0 |
| S9_k141_71400 | 4373 | No | NA | 0 | 4 | Not-determined | Genome-fragment | NA | 0 |
| S9_k141_55641 | 6750 | No | NA | 0 | 3 | Not-determined | Genome-fragment | NA | 0 |
| S9_k141_55661 | 1038 | No | NA | 0 | 2 | Not-determined | Genome-fragment | NA | 0 |
| S9_k141_7677 | 1785 | No | NA | 0 | 1 | Not-determined | Genome-fragment | NA | 0 |
| S17-k141_193759 | 1062 | No | NA | 0 | 1 | Low-quality | Genome-fragment | 0.7 | 0 |
| S9_k141_50511 | 1282 | No | NA | 0 | 2 | Not-determined | Genome-fragment | NA | 0 |
| S9_k141_114004 | 1656 | No | NA | 0 | 0 | Not-determined | Genome-fragment | NA | 0 |
| S9_k141_55681 | 2667 | No | NA | 0 | 0 | Not-determined | Genome-fragment | NA | 0 |
| S9_k141_44490 | 2524 | No | NA | 0 | 0 | Not-determined | Genome-fragment | NA | 0 |
| S12-final-viral-combined_k141_208372\|\|full | 1062 | No | NA | 0 | 1 | Low-quality | Genome-fragment | 1.28 | 0 |
| S9_k141_50588 | 4444 | No | NA | 0 | 5 | Not-determined | Genome-fragment | NA | 0 |
| S9_k141_7733 | 2998 | No | NA | 0 | 0 | Not-determined | Genome-fragment | NA | 0 |
| S9_k141_12789 | 1016 | No | NA | 0 | 0 | Not-determined | Genome-fragment | NA | 0 |
| S9_k141_61267 | 2568 | No | NA | 0 | 1 | Not-determined | Genome-fragment | NA | 0 |
| S12_k141_98457 | 1062 | No | NA | 0 | 0 | Low-quality | Genome-fragment | 1.81 | 0 |
| S9_k141_66373 | 1293 | No | NA | 0 | 2 | Not-determined | Genome-fragment | NA | 0 |
| S9_k141_87792 | 9049 | No | NA | 0 | 8 | Not-determined | Genome-fragment | NA | 0 |

| | | | | | | | | | | |
|---|---|---|---|---|---|---|---|---|---|---|
| S9_k141_108773 | 1038 | No | NA | 0 | 1 | Not-determined | Genome-fragment | NA | | 0 |
| S9_k141_44541 | 1094 | No | NA | 0 | 0 | Not-determined | Genome-fragment | NA | | 0 |
| S9_k141_108787 | 1114 | No | NA | 0 | 0 | Not-determined | Genome-fragment | NA | | 0 |
| S9_k141_61303 | 1064 | No | NA | 0 | 1 | Not-determined | Genome-fragment | NA | | 0 |
| S9_k141_12839 | 1446 | No | NA | 0 | 1 | Not-determined | Genome-fragment | NA | | 0 |
| S9_k141_108798 | 5124 | No | NA | 0 | 0 | Not-determined | Genome-fragment | NA | | 0 |
| S9_k141_124914 | 1182 | No | NA | 0 | 1 | Not-determined | Genome-fragment | NA | | 0 |
| S9_k141_98264 | 9643 | No | NA | 0 | 1 | Not-determined | Genome-fragment | NA | | 0 |
| S9_k141_82766 | 3429 | No | NA | 0 | 3 | Not-determined | Genome-fragment | NA | | 0 |
| S12_k141_33022 | 1062 | No | NA | 1 | 0 | Low-quality | Genome-fragment | | 2.93 | 0 |
| S9_k141_98279 | 1997 | No | NA | 0 | 1 | Not-determined | Genome-fragment | NA | | 0 |
| S4-final-viral-combined_k141_128781\|\|lt2gene | 1062 | No | NA | 1 | 0 | Low-quality | Genome-fragment | | 0.69 | 0 |
| S9_k141_2748 | 3030 | No | NA | 0 | 0 | Not-determined | Genome-fragment | NA | | 0 |
| S9_k141_82844 | 1005 | No | NA | 0 | 0 | Not-determined | Genome-fragment | NA | | 0 |
| S9_k141_2754 | 2974 | No | NA | 0 | 2 | Not-determined | Genome-fragment | NA | | 0 |
| S8-final-viral-combined_k141_32038\|\|full | 1062 | No | NA | 0 | 0 | Low-quality | Genome-fragment | | 1.76 | 0 |
| S9_k141_93069 | 3343 | No | NA | 0 | 2 | Not-determined | Genome-fragment | NA | | 0 |
| S9_k141_7913 | 1643 | No | NA | 0 | 1 | Not-determined | Genome-fragment | NA | | 0 |
| S9_k141_103983 | 1214 | No | NA | 0 | 0 | Not-determined | Genome-fragment | NA | | 0 |
| S9_k141_2790 | 1210 | No | NA | 0 | 1 | Not-determined | Genome-fragment | NA | | 0 |
| S9-final-viral-combined_k141_48284\|\|full | 1063 | No | NA | 1 | 0 | Low-quality | Genome-fragment | | 1.91 | 0 |
| S11-final-viral-combined_k141_162437\|\|full | 1063 | No | NA | 1 | 0 | Low-quality | Genome-fragment | | 2.18 | 0 |
| S9_k141_82893 | 3598 | No | NA | 0 | 4 | Not-determined | Genome-fragment | NA | | 0 |
| S11-final-viral-combined_k141_314483\|\|full | 1063 | No | NA | 0 | 1 | Low-quality | Genome-fragment | | 0.73 | 0 |
| S17-final-viral-combined_k141_256032\|\|full | 1063 | No | NA | 2 | 0 | Low-quality | Genome-fragment | | 2.33 | 0 |
| S9_k141_93095 | 1130 | No | NA | 0 | 3 | Not-determined | Genome-fragment | NA | | 0 |
| S9_k141_87967 | 1378 | No | NA | 0 | 1 | Not-determined | Genome-fragment | NA | | 0 |
| S9_k141_114036 | 2305 | No | NA | 0 | 0 | Not-determined | Genome-fragment | NA | | 0 |
| S17-final-viral-combined_k141_181839\|\|full | 1063 | No | NA | 2 | 1 | Low-quality | Genome-fragment | | 2.46 | 0 |
| S9_k141_23694 | 1062 | No | NA | 0 | 2 | Not-determined | Genome-fragment | NA | | 0 |
| S9_k141_83003 | 1130 | No | NA | 0 | 0 | Not-determined | Genome-fragment | NA | | 0 |
| S9_k141_50811 | 1471 | No | NA | 0 | 1 | Not-determined | Genome-fragment | NA | | 0 |
| S9_k141_55972 | 1862 | No | NA | 0 | 1 | Not-determined | Genome-fragment | NA | | 0 |
| S9_k141_66557 | 4224 | No | NA | 0 | 0 | Not-determined | Genome-fragment | NA | | 0 |
| S9_k141_50826 | 4490 | No | NA | 0 | 3 | Not-determined | Genome-fragment | NA | | 0 |
| S17-k141_186583 | 1063 | No | NA | 0 | 0 | Low-quality | Genome-fragment | | 2.12 | 0 |
| S9_k141_33954 | 2554 | No | NA | 0 | 1 | Not-determined | Genome-fragment | NA | | 0 |
| S9_k141_83062 | 2004 | No | NA | 0 | 1 | Not-determined | Genome-fragment | NA | | 0 |
| S9_k141_61332 | 3472 | No | NA | 0 | 2 | Not-determined | Genome-fragment | NA | | 0 |
| S9_k141_50839 | 1036 | No | NA | 0 | 0 | Not-determined | Genome-fragment | NA | | 0 |
| S9_k141_104090 | 1106 | No | NA | 0 | 1 | Not-determined | Genome-fragment | NA | | 0 |
| S9_k141_2862 | 3386 | No | NA | 0 | 3 | Not-determined | Genome-fragment | NA | | 0 |
| S9_k141_88115 | 1049 | No | NA | 0 | 0 | Not-determined | Genome-fragment | NA | | 0 |
| S9_k141_76822 | 1405 | No | NA | 0 | 0 | Not-determined | Genome-fragment | NA | | 0 |
| S9_k141_98391 | 3054 | No | NA | 0 | 3 | Not-determined | Genome-fragment | NA | | 0 |
| S12-final-viral-combined_k141_24813\|\|full | 1063 | No | NA | 0 | 1 | Low-quality | Genome-fragment | | 1.82 | 0 |
| S9_k141_93181 | 1145 | No | NA | 0 | 0 | Not-determined | Genome-fragment | NA | | 0 |
| S9_k141_104169 | 4074 | No | NA | 0 | 0 | Medium-quality | Genome-fragment | | 69.89 | 0 |
| S9_k141_104188 | 3112 | No | NA | 0 | 0 | Not-determined | Genome-fragment | NA | | 0 |
| S12_k141_211540 | 1063 | No | NA | 0 | 0 | Low-quality | Genome-fragment | | 0.71 | 0 |
| S9_k141_39771 | 1355 | No | NA | 0 | 1 | Not-determined | Genome-fragment | NA | | 0 |
| S9_k141_29045 | 9750 | No | NA | 0 | 17 | Not-determined | Genome-fragment | NA | | 0 |
| S9_k141_12895 | 18327 | No | NA | 0 | 4 | Not-determined | Genome-fragment | NA | | 0 |
| S12_k141_348550 | 1063 | No | NA | 0 | 0 | Low-quality | Genome-fragment | | 1.88 | 0 |
| S9_k141_39785 | 1318 | No | NA | 0 | 0 | Not-determined | Genome-fragment | NA | | 0 |
| S9_k141_56161 | 4176 | No | NA | 0 | 2 | Not-determined | Genome-fragment | NA | | 0 |
| S9_k141_39790 | 1207 | No | NA | 0 | 1 | Not-determined | Genome-fragment | NA | | 0 |
| S9_k141_56162 | 2275 | No | NA | 0 | 1 | Not-determined | Genome-fragment | NA | | 0 |
| S9_k141_12927 | 1263 | No | NA | 0 | 0 | Not-determined | Genome-fragment | NA | | 0 |
| S2-final-viral-combined_k141_180558\|\|full | 1063 | No | NA | 0 | 1 | Low-quality | Genome-fragment | | 1.56 | 0 |
| S9_k141_119951 | 1000 | No | NA | 0 | 0 | Not-determined | Genome-fragment | NA | | 0 |
| S2-final-viral-combined_k141_131353\|\|full | 1063 | No | NA | 0 | 1 | Low-quality | Genome-fragment | | 2.54 | 0 |
| S2_k141_126931 | 1063 | No | NA | 0 | 0 | Low-quality | Genome-fragment | | 1.11 | 0 |
| S9_k141_98412 | 1396 | No | NA | 0 | 1 | Not-determined | Genome-fragment | NA | | 0 |
| S4-final-viral-combined_k141_40094\|\|full | 1063 | No | NA | 1 | 0 | Low-quality | Genome-fragment | | 2.71 | 0 |
| S9_k141_8199 | 3319 | No | NA | 0 | 1 | Not-determined | Genome-fragment | NA | | 0 |
| S8_k141_6037 | 1063 | No | NA | 1 | 0 | Low-quality | Genome-fragment | | 2.42 | 0 |
| S9_k141_71790 | 3250 | No | NA | 0 | 1 | Not-determined | Genome-fragment | NA | | 0 |
| S11-final-viral-combined_k141_264272\|\|lt2gene | 1064 | No | NA | 1 | 0 | Low-quality | Genome-fragment | | 2.62 | 0 |
| S9_k141_3045 | 2063 | No | NA | 0 | 2 | Not-determined | Genome-fragment | NA | | 0 |
| S9_k141_13047 | 6352 | No | NA | 0 | 1 | Not-determined | Genome-fragment | NA | | 0 |
| S9_k141_76995 | 2378 | No | NA | 0 | 1 | Not-determined | Genome-fragment | NA | | 0 |
| S1-final-viral-combined_k141_313304\|\|full | 1064 | No | NA | 1 | 0 | Low-quality | Genome-fragment | | 1.9 | 0 |
| S9_k141_66684 | 1375 | No | NA | 0 | 0 | Not-determined | Genome-fragment | NA | | 0 |
| S9_k141_61598 | 1141 | No | NA | 0 | 0 | Not-determined | Genome-fragment | NA | | 0 |
| S9_k141_8258 | 1117 | No | NA | 0 | 1 | Not-determined | Genome-fragment | NA | | 0 |
| S9_k141_50936 | 1089 | No | NA | 0 | 1 | Not-determined | Genome-fragment | NA | | 0 |
| S9_k141_114339 | 1574 | No | NA | 0 | 1 | Not-determined | Genome-fragment | NA | | 0 |
| S9_k141_50939 | 1108 | No | NA | 0 | 1 | Not-determined | Genome-fragment | NA | | 0 |
| S9_k141_39845 | 1048 | No | NA | 0 | 0 | Not-determined | Genome-fragment | NA | | 0 |
| S1-final-viral-combined_k141_210059\|\|full | 1064 | No | NA | 1 | 0 | Low-quality | Genome-fragment | | 2.32 | 0 |
| S9_k141_23972 | 1432 | No | NA | 0 | 1 | Not-determined | Genome-fragment | NA | | 0 |
| S9_k141_93348 | 1409 | No | NA | 0 | 0 | Not-determined | Genome-fragment | NA | | 0 |
| S9_k141_3089 | 1134 | No | NA | 0 | 1 | Not-determined | Genome-fragment | NA | | 0 |
| S9_k141_34191 | 1154 | No | NA | 0 | 0 | Not-determined | Genome-fragment | NA | | 0 |
| S9_k141_56250 | 3112 | No | NA | 0 | 2 | Not-determined | Genome-fragment | NA | | 0 |
| S9_k141_18785 | 1057 | No | NA | 0 | 1 | Not-determined | Genome-fragment | NA | | 0 |
| S1-final-viral-combined_k141_300325\|\|lt2gene | 1064 | No | NA | 1 | 0 | Low-quality | Genome-fragment | | 2.05 | 0 |
| S9_k141_83401 | 1265 | No | NA | 0 | 1 | Not-determined | Genome-fragment | NA | | 0 |
| S9_k141_8287 | 1213 | No | NA | 0 | 0 | Not-determined | Genome-fragment | NA | | 0 |
| S9_k141_120036 | 1345 | No | NA | 0 | 2 | Not-determined | Genome-fragment | NA | | 0 |
| S9_k141_98502 | 1687 | No | NA | 0 | 4 | Not-determined | Genome-fragment | NA | | 0 |
| S9_k141_77109 | 1283 | No | NA | 0 | 0 | Not-determined | Genome-fragment | NA | | 0 |
| S9_k141_114421 | 1045 | No | NA | 0 | 1 | Not-determined | Genome-fragment | NA | | 0 |
| S9_k141_8313 | 1179 | No | NA | 0 | 0 | Not-determined | Genome-fragment | NA | | 0 |
| S9_k141_18802 | 1867 | No | NA | 0 | 0 | Not-determined | Genome-fragment | NA | | 0 |
| S9_k141_125240 | 1094 | No | NA | 0 | 0 | Not-determined | Genome-fragment | NA | | 0 |
| S9_k141_120085 | 1118 | No | NA | 0 | 1 | Not-determined | Genome-fragment | NA | | 0 |
| S9_k141_29161 | 4733 | No | NA | 0 | 2 | Not-determined | Genome-fragment | NA | | 0 |
| S9_k141_8337 | 2978 | No | NA | 0 | 3 | Not-determined | Genome-fragment | NA | | 0 |
| S9_k141_120117 | 1382 | No | NA | 0 | 0 | Not-determined | Genome-fragment | NA | | 0 |
| S9_k141_120142 | 1322 | No | NA | 0 | 1 | Not-determined | Genome-fragment | NA | | 0 |
| S9_k141_18844 | 5638 | No | NA | 0 | 2 | Not-determined | Genome-fragment | NA | | 0 |
| S9_k141_24126 | 6203 | No | NA | 0 | 1 | Not-determined | Genome-fragment | NA | | 0 |
| S9_k141_13215 | 5801 | No | NA | 0 | 2 | Not-determined | Genome-fragment | NA | | 0 |
| S1_k141_376641 | 1064 | No | NA | 0 | 0 | Low-quality | Genome-fragment | | 2.23 | 0 |
| S9_k141_77185 | 1007 | No | NA | 0 | 1 | Not-determined | Genome-fragment | NA | | 0 |
| S9_k141_13266 | 2229 | No | NA | 0 | 1 | Not-determined | Genome-fragment | NA | | 0 |
| S9_k141_18916 | 1636 | No | NA | 0 | 2 | Not-determined | Genome-fragment | NA | | 0 |
| S9_k141_45093 | 1068 | No | NA | 0 | 0 | Not-determined | Genome-fragment | NA | | 0 |
| S9_k141_29245 | 2208 | No | NA | 0 | 1 | Not-determined | Genome-fragment | NA | | 0 |
| S9_k141_13297 | 1264 | No | NA | 0 | 0 | Not-determined | Genome-fragment | NA | | 0 |
| S9_k141_39962 | 1976 | No | NA | 0 | 1 | Not-determined | Genome-fragment | NA | | 0 |
| S5-final-viral-combined_k141_116021\|\|full | 1064 | No | NA | 1 | 0 | Low-quality | Genome-fragment | | 1.97 | 0 |
| S9_k141_51090 | 2970 | No | NA | 0 | 1 | Not-determined | Genome-fragment | NA | | 0 |
| S9_k141_61735 | 1532 | No | NA | 0 | 0 | Not-determined | Genome-fragment | NA | | 0 |
| S5_k141_1696 | 1064 | No | NA | 0 | 1 | Low-quality | Genome-fragment | | 0.4 | 0 |
| S9_k141_120262 | 1217 | No | NA | 0 | 0 | Not-determined | Genome-fragment | NA | | 0 |
| S9_k141_125363 | 2091 | No | NA | 0 | 4 | Not-determined | Genome-fragment | NA | | 0 |
| S9_k141_24232 | 3251 | No | NA | 0 | 7 | Not-determined | Genome-fragment | NA | | 0 |
| S9_k141_120272 | 11828 | No | NA | 0 | 9 | Not-determined | Genome-fragment | NA | | 0 |
| S9_k141_120278 | 1802 | No | NA | 0 | 3 | Not-determined | Genome-fragment | NA | | 0 |
| S9_k141_40039 | 2964 | No | NA | 0 | 0 | Not-determined | Genome-fragment | NA | | 0 |
| S9_k141_45253 | 1497 | No | NA | 0 | 0 | Not-determined | Genome-fragment | NA | | 0 |
| S9_k141_13379 | 12264 | No | NA | 0 | 1 | Not-determined | Genome-fragment | NA | | 0 |
| S9_k141_19025 | 55093 | No | NA | 9 | 0 | High-quality | High-quality | | 90.22 | 0 |
| S9_k141_24282 | 6325 | No | NA | 0 | 0 | Not-determined | Genome-fragment | NA | | 0 |
| S7-final-viral-combined_k141_53652\|\|full | 1064 | No | NA | 1 | 0 | Low-quality | Genome-fragment | | 2.09 | 0 |
| S9_k141_120313 | 1056 | No | NA | 0 | 0 | Not-determined | Genome-fragment | NA | | 0 |
| S12-final-viral-combined_k141_17272\|\|full | 1064 | No | NA | 1 | 0 | Low-quality | Genome-fragment | | 1.41 | 0 |
| S9_k141_120333 | 1124 | No | NA | 0 | 2 | Not-determined | Genome-fragment | NA | | 0 |
| S12-final-viral-combined_k141_1769\|\|full | 1064 | No | NA | 0 | 2 | Low-quality | Genome-fragment | | 0.31 | 0 |
| S9_k141_125422 | 31732 | No | NA | 0 | 18 | Not-determined | Genome-fragment | NA | | 0 |
| S9_k141_72086 | 1219 | No | NA | 0 | 1 | Not-determined | Genome-fragment | NA | | 0 |
| S12_k141_144277 | 1064 | No | NA | 0 | 0 | Low-quality | Genome-fragment | | 0.56 | 0 |
| S9_k141_45348 | 3273 | No | NA | 0 | 1 | Not-determined | Genome-fragment | NA | | 0 |
| S2-final-viral-combined_k141_129330\|\|full | 1064 | No | NA | 0 | 0 | Low-quality | Genome-fragment | | 2.43 | 0 |
| S9_k141_120427 | 1373 | No | NA | 0 | 1 | Not-determined | Genome-fragment | NA | | 0 |
| S9_k141_93563 | 3155 | No | NA | 0 | 1 | Not-determined | Genome-fragment | NA | | 0 |
| S4-final-viral-combined_k141_117952\|\|full | 1064 | No | NA | 1 | 0 | Low-quality | Genome-fragment | | 1.61 | 0 |
| S9_k141_24348 | 6316 | No | NA | 0 | 2 | Not-determined | Genome-fragment | NA | | 0 |
| S6_k141_52463 | 1064 | No | NA | 0 | 0 | Low-quality | Genome-fragment | | 1.26 | 0 |
| S9-final-viral-combined_k141_122966\|\|full | 1065 | No | NA | 0 | 0 | Low-quality | Genome-fragment | | 2.9 | 0 |
| S9_k141_83667 | 1007 | No | NA | 0 | 1 | Not-determined | Genome-fragment | NA | | 0 |
| S9_k141_40215 | 4880 | No | NA | 0 | 2 | Not-determined | Genome-fragment | NA | | 0 |
| S9_k141_34586 | 2323 | No | NA | 0 | 2 | Not-determined | Genome-fragment | NA | | 0 |
| S9_k141_120458 | 1197 | No | NA | 0 | 1 | Not-determined | Genome-fragment | NA | | 0 |
| S9_k141_34630 | 1159 | No | NA | 0 | 1 | Not-determined | Genome-fragment | NA | | 0 |
| S11-final-viral-combined_k141_87737\|\|full | 1065 | No | NA | 1 | 0 | Low-quality | Genome-fragment | | 2.19 | 0 |
| S9_k141_45466 | 1246 | No | NA | 0 | 0 | Not-determined | Genome-fragment | NA | | 0 |
| S9_k141_120509 | 1258 | No | NA | 0 | 0 | Not-determined | Genome-fragment | NA | | 0 |
| S9_k141_83744 | 1913 | No | NA | 0 | 1 | Not-determined | Genome-fragment | NA | | 0 |
| S9_k141_13537 | 3594 | No | NA | 0 | 0 | Not-determined | Genome-fragment | NA | | 0 |
| S11_k141_317021 | 1065 | No | NA | 1 | 0 | Low-quality | Genome-fragment | | 1.26 | 0 |
| S9_k141_61970 | 1038 | No | NA | 0 | 1 | Not-determined | Genome-fragment | NA | | 0 |
| S9_k141_77570 | 1225 | No | NA | 0 | 0 | Not-determined | Genome-fragment | NA | | 0 |
| S9_k141_93669 | 1688 | No | NA | 0 | 0 | Not-determined | Genome-fragment | NA | | 0 |
| S9_k141_34729 | 1097 | No | NA | 0 | 0 | Not-determined | Genome-fragment | NA | | 0 |
| S9_k141_114967 | 2505 | No | NA | 0 | 2 | Not-determined | Genome-fragment | NA | | 0 |
| S9_k141_61987 | 1941 | No | NA | 0 | 1 | Not-determined | Genome-fragment | NA | | 0 |
| S11_k141_91541 | 1065 | No | NA | 0 | 0 | Low-quality | Genome-fragment | | 1.27 | 0 |
| S9_k141_114971 | 26413 | No | NA | 22 | 2 | Medium-quality | Genome-fragment | | 61.92 | 0 |
| S9_k141_24388 | 5019 | No | NA | 0 | 3 | Not-determined | Genome-fragment | NA | | 0 |
| S9_k141_88387 | 1860 | No | NA | 0 | 1 | Not-determined | Genome-fragment | NA | | 0 |
| S11_k141_259055 | 1065 | No | NA | 0 | 0 | Low-quality | Genome-fragment | | 2.44 | 0 |
| S9_k141_62015 | 1328 | No | NA | 0 | 0 | Not-determined | Genome-fragment | NA | | 0 |
| S9_k141_109222 | 2150 | No | NA | 0 | 1 | Not-determined | Genome-fragment | NA | | 0 |
| S1-final-viral-combined_k141_362148\|\|lt2gene | 1065 | No | NA | 1 | 0 | Low-quality | Genome-fragment | | 2.78 | 0 |
| S1_k141_183006 | 1065 | No | NA | 0 | 1 | Low-quality | Genome-fragment | | 1.78 | 0 |
| S9_k141_125607 | 1485 | No | NA | 0 | 1 | Not-determined | Genome-fragment | NA | | 0 |
| S9_k141_109234 | 3380 | No | NA | 0 | 0 | Not-determined | Genome-fragment | NA | | 0 |
| S9_k141_125616 | 2038 | No | NA | 0 | 2 | Not-determined | Genome-fragment | NA | | 0 |
| S3-final-viral-combined_k141_99924\|\|full | 1065 | No | NA | 0 | 1 | Low-quality | Genome-fragment | | 0.63 | 0 |
| S9_k141_34843 | 4927 | No | NA | 0 | 1 | Not-determined | Genome-fragment | NA | | 0 |

| | | | | | | | | | | |
|---|---|---|---|---|---|---|---|---|---|---|
| S17-final-viral-combined_k141_156754\|\|full | 1065 | No | NA | 1 | 0 | Low-quality | Genome-fragment | | 2.01 | 0 |
| S9_k141_93758 | 9212 | No | NA | 0 | 8 | Not-determined | Genome-fragment | NA | | 0 |
| S9_k141_34892 | 1872 | No | NA | 0 | 2 | Not-determined | Genome-fragment | NA | | 0 |
| S9_k141_104620 | 2587 | No | NA | 0 | 0 | Not-determined | Genome-fragment | NA | | 0 |
| S9_k141_88517 | 1389 | No | NA | 0 | 1 | Not-determined | Genome-fragment | NA | | 0 |
| S9_k141_77712 | 1172 | No | NA | 0 | 0 | Not-determined | Genome-fragment | NA | | 0 |
| S9_k141_120695 | 3989 | No | NA | 0 | 4 | Not-determined | Genome-fragment | NA | | 0 |
| S9_k141_34898 | 1384 | No | NA | 0 | 0 | Not-determined | Genome-fragment | NA | | 0 |
| S9_k141_88526 | 9856 | No | NA | 0 | 9 | Not-determined | Genome-fragment | NA | | 0 |
| S9_k141_72217 | 4695 | No | NA | 0 | 0 | Not-determined | Genome-fragment | NA | | 0 |
| S9_k141_8789 | 2848 | No | NA | 0 | 1 | Not-determined | Genome-fragment | NA | | 0 |
| S17-k141_74923 | 1065 | No | NA | 1 | 0 | Low-quality | Genome-fragment | | 0.67 | 0 |
| S12-final-viral-combined_k141_332320\|\|full | 1065 | No | NA | 0 | 0 | Low-quality | Genome-fragment | | 2.84 | 0 |
| S9_k141_83927 | 1626 | No | NA | 0 | 0 | Not-determined | Genome-fragment | NA | | 0 |
| S9_k141_72260 | 1319 | No | NA | 0 | 1 | Not-determined | Genome-fragment | NA | | 0 |
| S9_k141_88605 | 1093 | No | NA | 0 | 1 | Not-determined | Genome-fragment | NA | | 0 |
| S12_k141_314102 | 1065 | No | NA | 0 | 0 | Low-quality | Genome-fragment | | 0.64 | 0 |
| S2_k141_14561 | 1065 | No | NA | 0 | 1 | Low-quality | Genome-fragment | | 0.35 | 0 |
| S4_k141_29454 | 1065 | No | NA | 0 | 0 | Low-quality | Genome-fragment | | 2.11 | 0 |
| S9_k141_115050 | 1488 | No | NA | 0 | 1 | Not-determined | Genome-fragment | NA | | 0 |
| S9_k141_51500 | 1355 | No | NA | 0 | 0 | Not-determined | Genome-fragment | NA | | 0 |
| S9_k141_45635 | 1393 | No | NA | 0 | 0 | Not-determined | Genome-fragment | NA | | 0 |
| S9_k141_77823 | 1385 | No | NA | 0 | 0 | Not-determined | Genome-fragment | NA | | 0 |
| S9_k141_125773 | 1268 | No | NA | 0 | 1 | Not-determined | Genome-fragment | NA | | 0 |
| S9_k141_24647 | 1005 | No | NA | 0 | 2 | Not-determined | Genome-fragment | NA | | 0 |
| S9_k141_40477 | 1083 | No | NA | 0 | 1 | Not-determined | Genome-fragment | NA | | 0 |
| S9_k141_77857 | 3712 | No | NA | 0 | 0 | Not-determined | Genome-fragment | NA | | 0 |
| S8_k141_1732 | 1065 | No | NA | 1 | 0 | Low-quality | Genome-fragment | | 0.69 | 0 |
| S9-final-viral-combined_k141_41787\|\|full | 1066 | No | NA | 0 | 0 | Low-quality | Genome-fragment | | 2.83 | 0 |
| S9-final-viral-combined_k141_95869\|\|full | 1066 | No | NA | 0 | 0 | Low-quality | Genome-fragment | | 2.03 | 0 |
| S9_k141_104703 | 1473 | No | NA | 0 | 1 | Not-determined | Genome-fragment | NA | | 0 |
| S9_k141_88725 | 1538 | No | NA | 0 | 1 | Not-determined | Genome-fragment | NA | | 0 |
| S9_k141_13878 | 1293 | No | NA | 0 | 1 | Not-determined | Genome-fragment | NA | | 0 |
| S9_k141_83978 | 1564 | No | NA | 0 | 2 | Not-determined | Genome-fragment | NA | | 0 |
| S9_k141_120958 | 1668 | No | NA | 0 | 1 | Not-determined | Genome-fragment | NA | | 0 |
| S11-final-viral-combined_k141_322719\|\|full | 1066 | No | NA | 0 | 0 | Low-quality | Genome-fragment | | 0.75 | 0 |
| S9_k141_62279 | 15206 | No | NA | 0 | 7 | Not-determined | Genome-fragment | NA | | 0 |
| S9_k141_35211 | 1114 | No | NA | 0 | 0 | Not-determined | Genome-fragment | NA | | 0 |
| S9_k141_24745 | 3919 | No | NA | 0 | 1 | Not-determined | Genome-fragment | NA | | 0 |
| S9_k141_8971 | 3000 | No | NA | 0 | 0 | Not-determined | Genome-fragment | NA | | 0 |
| S9_k141_9031 | 2229 | No | NA | 0 | 1 | Not-determined | Genome-fragment | NA | | 0 |
| S1-final-viral-combined_k141_346882\|\|full | 1066 | No | NA | 1 | 0 | Low-quality | Genome-fragment | | 1.91 | 0 |
| S9_k141_35280 | 11818 | No | NA | 0 | 9 | Not-determined | Genome-fragment | NA | | 0 |
| S9_k141_57251 | 15440 | No | NA | 0 | 30 | Not-determined | Genome-fragment | NA | | 0 |
| S9_k141_57274 | 1952 | No | NA | 0 | 2 | Not-determined | Genome-fragment | NA | | 0 |
| S9_k141_78031 | 2178 | No | NA | 0 | 1 | Not-determined | Genome-fragment | NA | | 0 |
| S9_k141_121078 | 1353 | No | NA | 0 | 0 | Not-determined | Genome-fragment | NA | | 0 |
| S9_k141_3581 | 1680 | No | NA | 0 | 0 | Not-determined | Genome-fragment | NA | | 0 |
| S9_k141_67314 | 1559 | No | NA | 0 | 1 | Not-determined | Genome-fragment | NA | | 0 |
| S1-final-viral-combined_k141_19422\|\|full | 1066 | No | NA | 0 | 1 | Low-quality | Genome-fragment | | 1.67 | 0 |
| S9_k141_88971 | 6836 | No | NA | 0 | 1 | Not-determined | Genome-fragment | NA | | 0 |
| S9_k141_3629 | 1051 | No | NA | 0 | 1 | Not-determined | Genome-fragment | NA | | 0 |
| S9_k141_45866 | 4630 | No | NA | 0 | 3 | Not-determined | Genome-fragment | NA | | 0 |
| S1-final-viral-combined_k141_173169\|\|full | 1066 | No | NA | 0 | 0 | Low-quality | Genome-fragment | | 2.66 | 0 |
| S9_k141_72404 | 4699 | No | NA | 0 | 4 | Not-determined | Genome-fragment | NA | | 0 |
| S3-final-viral-combined_k141_69815\|\|full | 1066 | No | NA | 0 | 0 | Low-quality | Genome-fragment | | 1.6 | 0 |
| S9_k141_45892 | 1492 | No | NA | 0 | 1 | Not-determined | Genome-fragment | NA | | 0 |
| S9_k141_109580 | 1349 | No | NA | 0 | 0 | Not-determined | Genome-fragment | NA | | 0 |
| S3_k141_80874 | 1066 | No | NA | 0 | 0 | Low-quality | Genome-fragment | | 1.35 | 0 |
| S5_k141_64072 | 1066 | No | NA | 0 | 1 | Low-quality | Genome-fragment | | 3.54 | 0 |
| S9_k141_3725 | 3217 | No | NA | 0 | 2 | Not-determined | Genome-fragment | NA | | 0 |
| S7_k141_78129 | 1066 | No | NA | 0 | 0 | Low-quality | Genome-fragment | | 2.4 | 0 |
| S9_k141_84138 | 7744 | No | NA | 0 | 1 | Not-determined | Genome-fragment | NA | | 0 |
| S17-final-viral-combined_k141_38771\|\|full | 1066 | No | NA | 1 | 0 | Low-quality | Genome-fragment | | 3.72 | 0 |
| S9_k141_89053 | 1365 | No | NA | 0 | 0 | Not-determined | Genome-fragment | NA | | 0 |
| S12-final-viral-combined_k141_339232\|\|full | 1066 | No | NA | 0 | 0 | Low-quality | Genome-fragment | | 2.94 | 0 |
| S12-final-viral-combined_k141_23361\|\|full | 1066 | No | NA | 0 | 1 | Low-quality | Genome-fragment | | 1.37 | 0 |
| S9_k141_45915 | 2674 | No | NA | 0 | 0 | Not-determined | Genome-fragment | NA | | 0 |
| S9_k141_93843 | 2424 | No | NA | 0 | 2 | Not-determined | Genome-fragment | NA | | 0 |
| S9_k141_121222 | 1697 | No | NA | 0 | 1 | Not-determined | Genome-fragment | NA | | 0 |
| S9_k141_52017 | 2018 | No | NA | 0 | 1 | Not-determined | Genome-fragment | NA | | 0 |
| S9_k141_62516 | 2163 | No | NA | 0 | 1 | Not-determined | Genome-fragment | NA | | 0 |
| S12-final-viral-combined_k141_248808\|\|full | 1066 | No | NA | 0 | 0 | Low-quality | Genome-fragment | | 1.29 | 0 |
| S9_k141_78223 | 1093 | No | NA | 0 | 1 | Not-determined | Genome-fragment | NA | | 0 |
| S9_k141_67426 | 1683 | No | NA | 0 | 0 | Not-determined | Genome-fragment | NA | | 0 |
| S9_k141_72487 | 1203 | No | NA | 0 | 0 | Not-determined | Genome-fragment | NA | | 0 |
| S9_k141_45935 | 2254 | No | NA | 0 | 0 | Not-determined | Genome-fragment | NA | | 0 |
| S9_k141_3814 | 3043 | No | NA | 0 | 2 | Not-determined | Genome-fragment | NA | | 0 |
| S9_k141_45936 | 1197 | No | NA | 0 | 0 | Not-determined | Genome-fragment | NA | | 0 |
| S9_k141_104932 | 2823 | No | NA | 0 | 2 | Not-determined | Genome-fragment | NA | | 0 |
| S12-final-viral-combined_k141_400887\|\|full | 1066 | No | NA | 0 | 0 | Low-quality | Genome-fragment | | 1.2 | 0 |
| S9_k141_40601 | 1429 | No | NA | 0 | 2 | Not-determined | Genome-fragment | NA | | 0 |
| S9_k141_14004 | 6947 | No | NA | 0 | 3 | Not-determined | Genome-fragment | NA | | 0 |
| S12-final-viral-combined_k141_3458\|\|lt2gene | 1066 | No | NA | 0 | 1 | Low-quality | Genome-fragment | | 1.43 | 0 |
| S9_k141_3842 | 22440 | No | NA | 0 | 8 | Not-determined | Genome-fragment | NA | | 0 |
| S9_k141_109676 | 2208 | No | NA | 0 | 2 | Not-determined | Genome-fragment | NA | | 0 |
| S12_k141_122436 | 1066 | No | NA | 0 | 0 | Low-quality | Genome-fragment | | 2.28 | 0 |
| S9_k141_93943 | 4238 | No | NA | 0 | 2 | Not-determined | Genome-fragment | NA | | 0 |
| S9_k141_29785 | 4514 | No | NA | 0 | 9 | Not-determined | Genome-fragment | NA | | 0 |
| S9_k141_115550 | 1162 | No | NA | 0 | 1 | Not-determined | Genome-fragment | NA | | 0 |
| S2-final-viral-combined_k141_48890\|\|full | 1066 | No | NA | 0 | 1 | Low-quality | Genome-fragment | | 0.87 | 0 |
| S9_k141_126104 | 1147 | No | NA | 0 | 1 | Not-determined | Genome-fragment | NA | | 0 |
| S2_k141_83974 | 1066 | No | NA | 1 | 0 | Low-quality | Genome-fragment | | 3.8 | 0 |
| S9_k141_84236 | 22854 | No | NA | 0 | 1 | Not-determined | Genome-fragment | NA | | 0 |
| S9_k141_45964 | 2472 | No | NA | 0 | 1 | Not-determined | Genome-fragment | NA | | 0 |
| S9_k141_3864 | 1129 | No | NA | 0 | 0 | Not-determined | Genome-fragment | NA | | 0 |
| S9_k141_72611 | 6580 | No | NA | 0 | 2 | Not-determined | Genome-fragment | NA | | 0 |
| S9_k141_9222 | 1358 | No | NA | 0 | 2 | Not-determined | Genome-fragment | NA | | 0 |
| S6-final-viral-combined_k141_51172\|\|full | 1066 | No | NA | 0 | 0 | Low-quality | Genome-fragment | | 0.91 | 0 |
| S9_k141_67536 | 6390 | No | NA | 0 | 1 | Not-determined | Genome-fragment | NA | | 0 |
| S9_k141_67537 | 1365 | No | NA | 0 | 1 | Not-determined | Genome-fragment | NA | | 0 |
| S8-final-viral-combined_k141_5303\|\|full | 1066 | No | NA | 2 | 0 | Low-quality | Genome-fragment | | 1.3 | 0 |
| S9-final-viral-combined_k141_87001\|\|full | 1067 | No | NA | 0 | 0 | Low-quality | Genome-fragment | | 2.4 | 0 |
| S9_k141_78416 | 6381 | No | NA | 0 | 9 | Not-determined | Genome-fragment | NA | | 0 |
| S9_k141_67584 | 2197 | No | NA | 0 | 0 | Not-determined | Genome-fragment | NA | | 0 |
| S9_k141_9237 | 1101 | No | NA | 0 | 1 | Not-determined | Genome-fragment | NA | | 0 |
| S9_k141_14170 | 1316 | No | NA | 0 | 1 | Not-determined | Genome-fragment | NA | | 0 |
| S9_k141_115677 | 3425 | No | NA | 0 | 1 | Not-determined | Genome-fragment | NA | | 0 |
| S9_k141_78516 | 1373 | No | NA | 0 | 1 | Not-determined | Genome-fragment | NA | | 0 |
| S9_k141_14246 | 1753 | No | NA | 0 | 1 | Not-determined | Genome-fragment | NA | | 0 |
| S7-final-viral-combined_k141_219231\|\|lt2gene | 1067 | No | NA | 1 | 0 | Low-quality | Genome-fragment | | 2.88 | 0 |
| S9_k141_84314 | 1286 | No | NA | 0 | 0 | Not-determined | Genome-fragment | NA | | 0 |
| S17-final-viral-combined_k141_53502\|\|lt2gene | 1067 | No | NA | 1 | 0 | Low-quality | Genome-fragment | | 2.47 | 0 |
| S9_k141_52237 | 1678 | No | NA | 0 | 1 | Not-determined | Genome-fragment | NA | | 0 |
| S9_k141_57500 | 1967 | No | NA | 0 | 5 | Not-determined | Genome-fragment | NA | | 0 |
| S12-final-viral-combined_k141_362570\|\|full | 1067 | No | NA | 0 | 0 | Low-quality | Genome-fragment | | 1.6 | 0 |
| S9_k141_40736 | 1367 | No | NA | 0 | 1 | Not-determined | Genome-fragment | NA | | 0 |
| S2-final-viral-combined_k141_186346\|\|full | 1067 | No | NA | 0 | 2 | Low-quality | Genome-fragment | | 0.29 | 0 |
| S9_k141_121605 | 1132 | No | NA | 0 | 1 | Not-determined | Genome-fragment | NA | | 0 |
| S9_k141_78631 | 1003 | No | NA | 0 | 0 | Not-determined | Genome-fragment | NA | | 0 |
| S9_k141_9398 | 1248 | No | NA | 0 | 0 | Not-determined | Genome-fragment | NA | | 0 |
| S9_k141_20045 | 1015 | No | NA | 0 | 1 | Not-determined | Genome-fragment | NA | | 0 |
| S9_k141_4059 | 1387 | No | NA | 0 | 0 | Not-determined | Genome-fragment | NA | | 0 |
| S9_k141_14434 | 1523 | No | NA | 0 | 1 | Not-determined | Genome-fragment | NA | | 0 |
| S4-final-viral-combined_k141_139665\|\|full | 1067 | No | NA | 2 | 0 | Low-quality | Genome-fragment | | 2.52 | 0 |
| S9_k141_72715 | 3091 | No | NA | 0 | 0 | Not-determined | Genome-fragment | NA | | 0 |
| S9_k141_14445 | 2176 | No | NA | 0 | 0 | Not-determined | Genome-fragment | NA | | 0 |
| S9_k141_40764 | 1327 | No | NA | 0 | 1 | Not-determined | Genome-fragment | NA | | 0 |
| S9_k141_109894 | 1153 | No | NA | 0 | 1 | Not-determined | Genome-fragment | NA | | 0 |
| S9_k141_14447 | 3643 | No | NA | 0 | 2 | Not-determined | Genome-fragment | NA | | 0 |
| S9_k141_72722 | 1027 | No | NA | 0 | 1 | Not-determined | Genome-fragment | NA | | 0 |
| S4-final-viral-combined_k141_81329\|\|full | 1067 | No | NA | 0 | 0 | Low-quality | Genome-fragment | | 1.65 | 0 |
| S9_k141_14455 | 2037 | No | NA | 0 | 0 | Not-determined | Genome-fragment | NA | | 0 |
| S4-final-viral-combined_k141_34298\|\|full | 1067 | No | NA | 1 | 0 | Low-quality | Genome-fragment | | 3.13 | 0 |
| S9_k141_78718 | 1518 | No | NA | 0 | 2 | Not-determined | Genome-fragment | NA | | 0 |
| S9_k141_52329 | 1100 | No | NA | 0 | 1 | Not-determined | Genome-fragment | NA | | 0 |
| S9_k141_14481 | 1637 | No | NA | 0 | 2 | Not-determined | Genome-fragment | NA | | 0 |
| S9_k141_52338 | 1029 | No | NA | 0 | 0 | Not-determined | Genome-fragment | NA | | 0 |
| S9_k141_78754 | 1391 | No | NA | 0 | 0 | Not-determined | Genome-fragment | NA | | 0 |
| S9_k141_9484 | 1489 | No | NA | 0 | 0 | Not-determined | Genome-fragment | NA | | 0 |
| S9_k141_72798 | 1476 | No | NA | 0 | 1 | Not-determined | Genome-fragment | NA | | 0 |
| S9_k141_99830 | 1745 | No | NA | 0 | 0 | Not-determined | Genome-fragment | NA | | 0 |
| S9-final-viral-combined_k141_101566\|\|lt2gene | 1068 | No | NA | 1 | 0 | Low-quality | Genome-fragment | | 2.49 | 0 |
| S9_k141_20112 | 1103 | No | NA | 0 | 2 | Not-determined | Genome-fragment | NA | | 0 |
| S9_k141_40874 | 4042 | No | NA | 0 | 3 | Not-determined | Genome-fragment | NA | | 0 |
| S9_k141_7495 | 1068 | No | NA | 0 | 0 | Low-quality | Genome-fragment | | 0.88 | 0 |
| S9_k141_25359 | 3472 | No | NA | 0 | 3 | Not-determined | Genome-fragment | NA | | 0 |
| S9_k141_126358 | 2741 | No | NA | 0 | 6 | Not-determined | Genome-fragment | NA | | 0 |
| S9_k141_9516 | 3953 | No | NA | 0 | 7 | Not-determined | Genome-fragment | NA | | 0 |
| S11-final-viral-combined_k141_183284\|\|full | 1068 | No | NA | 0 | 0 | Low-quality | Genome-fragment | | 2.07 | 0 |
| S9_k141_89584 | 3146 | No | NA | 0 | 3 | Not-determined | Genome-fragment | NA | | 0 |
| S9_k141_89597 | 1592 | No | NA | 0 | 0 | Not-determined | Genome-fragment | NA | | 0 |
| S9_k141_94189 | 2523 | No | NA | 0 | 1 | Not-determined | Genome-fragment | NA | | 0 |
| S9_k141_9558 | 2989 | No | NA | 0 | 0 | Not-determined | Genome-fragment | NA | | 0 |
| S9_k141_94199 | 1164 | No | NA | 0 | 1 | Not-determined | Genome-fragment | NA | | 0 |
| S9_k141_25475 | 1461 | No | NA | 0 | 2 | Not-determined | Genome-fragment | NA | | 0 |
| S9_k141_9603 | 1696 | No | NA | 0 | 0 | Not-determined | Genome-fragment | NA | | 0 |
| S9_k141_30129 | 1176 | No | NA | 0 | 1 | Not-determined | Genome-fragment | NA | | 0 |
| S9_k141_78889 | 1605 | No | NA | 0 | 1 | Not-determined | Genome-fragment | NA | | 0 |
| S11-final-viral-combined_k141_61543\|\|full | 1068 | No | NA | 0 | 0 | Low-quality | Genome-fragment | | 2.52 | 0 |
| S9_k141_46255 | 1287 | No | NA | 0 | 1 | Not-determined | Genome-fragment | NA | | 0 |
| S11-final-viral-combined_k141_241328\|\|full | 1068 | No | NA | 1 | 0 | Low-quality | Genome-fragment | | 1.4 | 0 |
| S9_k141_68032 | 1214 | No | NA | 0 | 1 | Not-determined | Genome-fragment | NA | | 0 |
| S11-final-viral-combined_k141_186929\|\|full | 1068 | No | NA | 1 | 0 | Low-quality | Genome-fragment | | 1.63 | 0 |
| S11-final-viral-combined_k141_215863\|\|full | 1068 | No | NA | 0 | 0 | Low-quality | Genome-fragment | | 2.36 | 0 |
| S9_k141_121879 | 7736 | No | NA | 0 | 3 | Not-determined | Genome-fragment | NA | | 0 |
| S9_k141_78899 | 5779 | No | NA | 0 | 6 | Not-determined | Genome-fragment | NA | | 0 |
| S9_k141_121900 | 1019 | No | NA | 0 | 1 | Not-determined | Genome-fragment | NA | | 0 |
| S9_k141_89699 | 2119 | No | NA | 0 | 2 | Not-determined | Genome-fragment | NA | | 0 |
| S9_k141_4292 | 3669 | No | NA | 0 | 1 | Not-determined | Genome-fragment | NA | | 0 |
| S9_k141_20227 | 2265 | No | NA | 0 | 1 | Not-determined | Genome-fragment | NA | | 0 |
| S11-final-viral-combined_k141_269272\|\|lt2gene | 1068 | No | NA | 1 | 0 | Low-quality | Genome-fragment | | 3.19 | 0 |
| S9_k141_25606 | 1002 | No | NA | 0 | 0 | Not-determined | Genome-fragment | NA | | 0 |
| S9_k141_25609 | 3250 | No | NA | 0 | 3 | Not-determined | Genome-fragment | NA | | 0 |

| | | | | | | | | | | |
|---|---|---|---|---|---|---|---|---|---|---|
| S9_k141_100042 | 4220 | No | NA | 0 | 9 | Not-determined | Genome-fragment | NA | | 0 |
| S9_k141_41073 | 8200 | No | NA | 0 | 5 | Not-determined | Genome-fragment | NA | | 0 |
| S9_k141_35853 | 1792 | No | NA | 0 | 1 | Not-determined | Genome-fragment | NA | | 0 |
| S9_k141_73005 | 1012 | No | NA | 0 | 1 | Not-determined | Genome-fragment | NA | | 0 |
| S9_k141_57764 | 1055 | No | NA | 0 | 1 | Not-determined | Genome-fragment | NA | | 0 |
| S9_k141_110142 | 1294 | No | NA | 0 | 1 | Not-determined | Genome-fragment | NA | | 0 |
| S9_k141_126533 | 1083 | No | NA | 0 | 1 | Not-determined | Genome-fragment | NA | | 0 |
| S11_k141_19439 | 1068 | No | NA | 0 | 0 | Low-quality | Genome-fragment | | 0.51 | 0 |
| S1-final-viral-combined_k141_118705\|\|full | 1068 | No | NA | 0 | 0 | Low-quality | Genome-fragment | | 2.47 | 0 |
| S9_k141_94378 | 4509 | No | NA | 0 | 5 | Not-determined | Genome-fragment | NA | | 0 |
| S9_k141_73103 | 1329 | No | NA | 0 | 1 | Not-determined | Genome-fragment | NA | | 0 |
| S7-final-viral-combined_k141_114641\|\|full | 1068 | No | NA | 0 | 1 | Low-quality | Genome-fragment | | 1.9 | 0 |
| S7-final-viral-combined_k141_16034\|\|full | 1068 | No | NA | 0 | 0 | Low-quality | Genome-fragment | | 4.06 | 0 |
| S9_k141_116116 | 1007 | No | NA | 0 | 1 | Not-determined | Genome-fragment | NA | | 0 |
| S7_k141_154351 | 1068 | No | NA | 0 | 0 | Low-quality | Genome-fragment | | 2.29 | 0 |
| S9_k141_73144 | 1018 | No | NA | 0 | 0 | Not-determined | Genome-fragment | NA | | 0 |
| S9_k141_35952 | 11525 | No | NA | 0 | 24 | Not-determined | Genome-fragment | NA | | 0 |
| S9_k141_94481 | 1797 | No | NA | 0 | 1 | Not-determined | Genome-fragment | NA | | 0 |
| S9_k141_9685 | 1081 | No | NA | 0 | 0 | Not-determined | Genome-fragment | NA | | 0 |
| S9_k141_126566 | 2636 | No | NA | 0 | 1 | Not-determined | Genome-fragment | NA | | 0 |
| S9_k141_84727 | 1071 | No | NA | 0 | 2 | Not-determined | Genome-fragment | NA | | 0 |
| S9_k141_9717 | 2121 | No | NA | 0 | 1 | Not-determined | Genome-fragment | NA | | 0 |
| S9_k141_30378 | 1142 | No | NA | 0 | 0 | Not-determined | Genome-fragment | NA | | 0 |
| S17-final-viral-combined_k141_81874\|\|full | 1068 | No | NA | 1 | 0 | Low-quality | Genome-fragment | | 2.52 | 0 |
| S9_k141_57848 | 1073 | No | NA | 0 | 1 | Not-determined | Genome-fragment | NA | | 0 |
| S9_k141_4442 | 2893 | No | NA | 0 | 0 | Not-determined | Genome-fragment | NA | | 0 |
| S9_k141_122107 | 1244 | No | NA | 0 | 0 | Not-determined | Genome-fragment | NA | | 0 |
| S9_k141_94521 | 3163 | No | NA | 0 | 3 | Not-determined | Genome-fragment | NA | | 0 |
| S9_k141_73186 | 1367 | No | NA | 0 | 1 | Not-determined | Genome-fragment | NA | | 0 |
| S9_k141_122119 | 1472 | No | NA | 0 | 2 | Not-determined | Genome-fragment | NA | | 0 |
| S9_k141_63353 | 1017 | No | NA | 0 | 0 | Not-determined | Genome-fragment | NA | | 0 |
| S9_k141_90013 | 1302 | No | NA | 0 | 1 | Not-determined | Genome-fragment | NA | | 0 |
| S9_k141_20372 | 1123 | No | NA | 0 | 0 | Not-determined | Genome-fragment | NA | | 0 |
| S17-final-viral-combined_k141_74577\|\|full | 1068 | No | NA | 0 | 1 | Low-quality | Genome-fragment | | 0.75 | 0 |
| S9_k141_116247 | 3713 | No | NA | 0 | 3 | Not-determined | Genome-fragment | NA | | 0 |
| S9_k141_20377 | 3184 | No | NA | 0 | 0 | Not-determined | Genome-fragment | NA | | 0 |
| S12_k141_228689 | 1068 | No | NA | 0 | 2 | Low-quality | Genome-fragment | | 0.32 | 0 |
| S9_k141_52748 | 1213 | No | NA | 0 | 3 | Not-determined | Genome-fragment | NA | | 0 |
| S9_k141_52759 | 1161 | No | NA | 0 | 1 | Not-determined | Genome-fragment | NA | | 0 |
| S8-final-viral-combined_k141_264432\|\|full | 1068 | No | NA | 0 | 1 | Low-quality | Genome-fragment | | 0.91 | 0 |
| S9_k141_14831 | 1010 | No | NA | 0 | 0 | Not-determined | Genome-fragment | NA | | 0 |
| S9_k141_25687 | 1094 | No | NA | 0 | 0 | Not-determined | Genome-fragment | NA | | 0 |
| S9_k141_90075 | 4065 | No | NA | 0 | 2 | Not-determined | Genome-fragment | NA | | 0 |
| S9-final-viral-combined_k141_36387\|\|full | 1069 | No | NA | 0 | 1 | Low-quality | Genome-fragment | | 8.32 | 0 |
| S9_k141_122207 | 1515 | No | NA | 0 | 2 | Not-determined | Genome-fragment | NA | | 0 |
| S11-final-viral-combined_k141_33341\|\|full | 1069 | No | NA | 1 | 0 | Low-quality | Genome-fragment | | 1.78 | 0 |
| S11-final-viral-combined_k141_219851\|\|full | 1069 | No | NA | 0 | 1 | Low-quality | Genome-fragment | | 0.44 | 0 |
| S9_k141_68476 | 1399 | No | NA | 0 | 0 | Not-determined | Genome-fragment | NA | | 0 |
| S9_k141_84937 | 1140 | No | NA | 0 | 1 | Not-determined | Genome-fragment | NA | | 0 |
| S9_k141_52780 | 2869 | No | NA | 0 | 1 | Not-determined | Genome-fragment | NA | | 0 |
| S1_k141_337552 | 1069 | No | NA | 0 | 1 | Low-quality | Genome-fragment | | 0.18 | 0 |
| S9_k141_110319 | 1003 | No | NA | 0 | 0 | Not-determined | Genome-fragment | NA | | 0 |
| S9_k141_41272 | 1066 | No | NA | 0 | 1 | Not-determined | Genome-fragment | NA | | 0 |
| S9_k141_57964 | 1182 | No | NA | 0 | 0 | Not-determined | Genome-fragment | NA | | 0 |
| S9_k141_79122 | 1195 | No | NA | 0 | 0 | Not-determined | Genome-fragment | NA | | 0 |
| S5-final-viral-combined_k141_211127\|\|full | 1069 | No | NA | 0 | 0 | Low-quality | Genome-fragment | | 1.99 | 0 |
| S5-final-viral-combined_k141_33780\|\|full | 1069 | No | NA | 0 | 1 | Low-quality | Genome-fragment | | 0.91 | 0 |
| S9_k141_25724 | 1058 | No | NA | 0 | 0 | Not-determined | Genome-fragment | NA | | 0 |
| S9_k141_68526 | 3507 | No | NA | 0 | 2 | Not-determined | Genome-fragment | NA | | 0 |
| S5-final-viral-combined_k141_25411\|\|full | 1069 | No | NA | 0 | 0 | Low-quality | Genome-fragment | | 1.87 | 0 |
| S9_k141_68542 | 1072 | No | NA | 0 | 2 | Not-determined | Genome-fragment | NA | | 0 |
| S9_k141_68549 | 1275 | No | NA | 0 | 1 | Not-determined | Genome-fragment | NA | | 0 |
| S9_k141_46627 | 1174 | No | NA | 0 | 1 | Not-determined | Genome-fragment | NA | | 0 |
| S9_k141_73409 | 6462 | No | NA | 0 | 3 | Not-determined | Genome-fragment | NA | | 0 |
| S9_k141_126662 | 1273 | No | NA | 0 | 0 | Not-determined | Genome-fragment | NA | | 0 |
| S5_k141_207017 | 1069 | No | NA | 0 | 0 | Low-quality | Genome-fragment | | 1.79 | 0 |
| S9_k141_122290 | 1118 | No | NA | 0 | 0 | Not-determined | Genome-fragment | NA | | 0 |
| S9_k141_36230 | 1176 | No | NA | 0 | 1 | Not-determined | Genome-fragment | NA | | 0 |
| S9_k141_36238 | 1256 | No | NA | 0 | 1 | Not-determined | Genome-fragment | NA | | 0 |
| S9_k141_116337 | 1603 | No | NA | 0 | 0 | Not-determined | Genome-fragment | NA | | 0 |
| S9_k141_90190 | 5336 | No | NA | 0 | 0 | Not-determined | Genome-fragment | NA | | 0 |
| S5_k141_202930 | 1069 | No | NA | 0 | 1 | Low-quality | Genome-fragment | | 0.5 | 0 |
| S17-final-viral-combined_k141_61342\|\|lt2gene | 1069 | No | NA | 1 | 0 | Low-quality | Genome-fragment | | 2.34 | 0 |
| S12-final-viral-combined_k141_110038\|\|full | 1069 | No | NA | 0 | 0 | Low-quality | Genome-fragment | | 1.69 | 0 |
| S9_k141_4577 | 1421 | No | NA | 0 | 0 | Not-determined | Genome-fragment | NA | | 0 |
| S6-final-viral-combined_k141_21155\|\|full | 1069 | No | NA | 1 | 0 | Low-quality | Genome-fragment | | 1.76 | 0 |
| S9_k141_100349 | 3838 | No | NA | 0 | 3 | Not-determined | Genome-fragment | NA | | 0 |
| S9_k141_52872 | 1218 | No | NA | 0 | 1 | Not-determined | Genome-fragment | NA | | 0 |
| S8_k141_243856 | 1069 | No | NA | 0 | 1 | Low-quality | Genome-fragment | | 0.73 | 0 |
| S9_k141_94761 | 5607 | No | NA | 0 | 5 | Not-determined | Genome-fragment | NA | | 0 |
| S9_k141_4584 | 1161 | No | NA | 0 | 0 | Not-determined | Genome-fragment | NA | | 0 |
| S9_k141_90214 | 3450 | No | NA | 0 | 5 | Not-determined | Genome-fragment | NA | | 0 |
| S9_k141_110408 | 1152 | No | NA | 0 | 0 | Not-determined | Genome-fragment | NA | | 0 |
| S9_k141_122314 | 1169 | No | NA | 0 | 1 | Not-determined | Genome-fragment | NA | | 0 |
| S9_k141_46708 | 4237 | No | NA | 0 | 0 | Not-determined | Genome-fragment | NA | | 0 |
| S9_k141_79332 | 1449 | No | NA | 0 | 0 | Not-determined | Genome-fragment | NA | | 0 |
| S9_k141_36269 | 1069 | No | NA | 0 | 0 | Not-determined | Genome-fragment | NA | | 0 |
| S9_k141_49616 | 1070 | No | NA | 0 | 1 | Low-quality | Genome-fragment | | 2.55 | 0 |
| S11_k141_271466 | 1070 | No | NA | 1 | 0 | Low-quality | Genome-fragment | | 0.62 | 0 |
| S9_k141_79373 | 7864 | No | NA | 0 | 5 | Not-determined | Genome-fragment | NA | | 0 |
| S9_k141_73468 | 2553 | No | NA | 0 | 2 | Not-determined | Genome-fragment | NA | | 0 |
| S9_k141_15040 | 1072 | No | NA | 0 | 0 | Not-determined | Genome-fragment | NA | | 0 |
| S9_k141_20599 | 1502 | No | NA | 0 | 1 | Not-determined | Genome-fragment | NA | | 0 |
| S9_k141_25887 | 6057 | No | NA | 0 | 6 | Not-determined | Genome-fragment | NA | | 0 |
| S9_k141_30689 | 1077 | No | NA | 0 | 1 | Not-determined | Genome-fragment | NA | | 0 |
| S9_k141_20618 | 4564 | No | NA | 0 | 0 | Not-determined | Genome-fragment | NA | | 0 |
| S9_k141_25911 | 1039 | No | NA | 0 | 1 | Not-determined | Genome-fragment | NA | | 0 |
| S9_k141_63720 | 6385 | No | NA | 0 | 4 | Not-determined | Genome-fragment | NA | | 0 |
| S9_k141_126748 | 1460 | No | NA | 0 | 1 | Not-determined | Genome-fragment | NA | | 0 |
| S1-final-viral-combined_k141_104636\|\|full | 1070 | No | NA | 1 | 0 | Low-quality | Genome-fragment | | 1.68 | 0 |
| S9_k141_20655 | 4108 | No | NA | 0 | 0 | Not-determined | Genome-fragment | NA | | 0 |
| S9_k141_126750 | 3047 | No | NA | 0 | 4 | Not-determined | Genome-fragment | NA | | 0 |
| S1-final-viral-combined_k141_125132\|\|full | 1070 | No | NA | 0 | 0 | Low-quality | Genome-fragment | | 0.81 | 0 |
| S9_k141_79460 | 1496 | No | NA | 0 | 0 | Not-determined | Genome-fragment | NA | | 0 |
| S9_k141_84972 | 1733 | No | NA | 0 | 1 | Not-determined | Genome-fragment | NA | | 0 |
| S9_k141_36371 | 1947 | No | NA | 0 | 0 | Not-determined | Genome-fragment | NA | | 0 |
| S9_k141_36377 | 1253 | No | NA | 0 | 0 | Not-determined | Genome-fragment | NA | | 0 |
| S9_k141_58187 | 1576 | No | NA | 0 | 1 | Not-determined | Genome-fragment | NA | | 0 |
| S9_k141_122479 | 5221 | No | NA | 0 | 0 | Not-determined | Genome-fragment | NA | | 0 |
| S1-final-viral-combined_k141_266863\|\|lt2gene | 1070 | No | NA | 1 | 0 | Low-quality | Genome-fragment | | 3.17 | 0 |
| S9_k141_68832 | 6574 | No | NA | 0 | 1 | Not-determined | Genome-fragment | NA | | 0 |
| S9_k141_73523 | 4115 | No | NA | 0 | 0 | Not-determined | Genome-fragment | NA | | 0 |
| S9_k141_94935 | 1209 | No | NA | 0 | 1 | Not-determined | Genome-fragment | NA | | 0 |
| S9_k141_36423 | 3183 | No | NA | 0 | 4 | Not-determined | Genome-fragment | NA | | 0 |
| S9_k141_25999 | 1171 | No | NA | 0 | 0 | Not-determined | Genome-fragment | NA | | 0 |
| S9_k141_53058 | 3310 | No | NA | 0 | 2 | Not-determined | Genome-fragment | NA | | 0 |
| S9_k141_20698 | 1106 | No | NA | 0 | 0 | Not-determined | Genome-fragment | NA | | 0 |
| S9_k141_30756 | 3188 | No | NA | 0 | 2 | Not-determined | Genome-fragment | NA | | 0 |
| S9_k141_4718 | 1392 | No | NA | 0 | 0 | Not-determined | Genome-fragment | NA | | 0 |
| S9_k141_85049 | 1654 | No | NA | 0 | 2 | Not-determined | Genome-fragment | NA | | 0 |
| S9_k141_94970 | 1834 | No | NA | 0 | 2 | Not-determined | Genome-fragment | NA | | 0 |
| S9_k141_94986 | 3938 | No | NA | 0 | 0 | Not-determined | Genome-fragment | NA | | 0 |
| S9_k141_68908 | 1212 | No | NA | 0 | 0 | Not-determined | Genome-fragment | NA | | 0 |
| S1-final-viral-combined_k141_160650\|\|lt2gene | 1070 | No | NA | 1 | 0 | Low-quality | Genome-fragment | | 2.18 | 0 |
| S9_k141_10126 | 2121 | No | NA | 0 | 1 | Not-determined | Genome-fragment | NA | | 0 |
| S9_k141_53114 | 2871 | No | NA | 0 | 1 | Not-determined | Genome-fragment | NA | | 0 |
| S9_k141_94997 | 6999 | No | NA | 0 | 2 | Not-determined | Genome-fragment | NA | | 0 |
| S7-final-viral-combined_k141_136498\|\|full | 1070 | No | NA | 0 | 1 | Low-quality | Genome-fragment | | 2.26 | 0 |
| S9_k141_20764 | 1879 | No | NA | 0 | 1 | Not-determined | Genome-fragment | NA | | 0 |
| S7-final-viral-combined_k141_45468\|\|full | 1070 | No | NA | 0 | 0 | Low-quality | Genome-fragment | | 1.96 | 0 |
| S9_k141_90492 | 3505 | No | NA | 0 | 1 | Not-determined | Genome-fragment | NA | | 0 |
| S9_k141_20777 | 4115 | No | NA | 0 | 2 | Not-determined | Genome-fragment | NA | | 0 |
| S17-final-viral-combined_k141_59998\|\|lt2gene | 1070 | No | NA | 1 | 0 | Low-quality | Genome-fragment | | 0.74 | 0 |
| S17-k141_158541 | 1070 | No | NA | 0 | 1 | Low-quality | Genome-fragment | | 0.75 | 0 |
| S9_k141_41592 | 1001 | No | NA | 0 | 0 | Not-determined | Genome-fragment | NA | | 0 |
| S12-final-viral-combined_k141_153084\|\|lt2gene | 1070 | No | NA | 1 | 0 | Low-quality | Genome-fragment | | 0.74 | 0 |
| S9_k141_15199 | 1036 | No | NA | 0 | 1 | Not-determined | Genome-fragment | NA | | 0 |
| S9_k141_15230 | 9630 | No | NA | 0 | 3 | Not-determined | Genome-fragment | NA | | 0 |
| S9_k141_85198 | 1310 | No | NA | 0 | 1 | Not-determined | Genome-fragment | NA | | 0 |
| S9_k141_110589 | 3615 | No | NA | 0 | 1 | Not-determined | Genome-fragment | NA | | 0 |
| S9_k141_95068 | 1427 | No | NA | 0 | 0 | Not-determined | Genome-fragment | NA | | 0 |
| S9_k141_15250 | 1512 | No | NA | 0 | 1 | Not-determined | Genome-fragment | NA | | 0 |
| S9_k141_73608 | 15847 | No | NA | 0 | 10 | Not-determined | Genome-fragment | NA | | 0 |
| S12-final-viral-combined_k141_375264\|\|lt2gene | 1070 | No | NA | 1 | 0 | Low-quality | Genome-fragment | | 1.79 | 0 |
| S9_k141_20862 | 3613 | No | NA | 0 | 3 | Not-determined | Genome-fragment | NA | | 0 |
| S12_k141_311026 | 1070 | No | NA | 1 | 0 | Low-quality | Genome-fragment | | 1.91 | 0 |
| S9_k141_116571 | 5288 | No | NA | 0 | 3 | Not-determined | Genome-fragment | NA | | 0 |
| S9_k141_95089 | 1355 | No | NA | 0 | 0 | Not-determined | Genome-fragment | NA | | 0 |
| S9_k141_90693 | 1088 | No | NA | 0 | 1 | Not-determined | Genome-fragment | NA | | 0 |
| S9_k141_20883 | 7317 | No | NA | 0 | 5 | Not-determined | Genome-fragment | NA | | 0 |
| S9_k141_126967 | 1338 | No | NA | 0 | 0 | Not-determined | Genome-fragment | NA | | 0 |
| S4-final-viral-combined_k141_160099\|\|full | 1070 | No | NA | 1 | 0 | Low-quality | Genome-fragment | | 1.71 | 0 |
| S4_k141_49365 | 1070 | No | NA | 0 | 0 | Low-quality | Genome-fragment | | 0.99 | 0 |
| S6-final-viral-combined_k141_15389\|\|full | 1070 | No | NA | 2 | 0 | Low-quality | Genome-fragment | | 1.31 | 0 |
| S9_k141_85311 | 3213 | No | NA | 0 | 0 | Not-determined | Genome-fragment | NA | | 0 |
| S9_k141_15358 | 2692 | No | NA | 1 | 0 | Not-determined | Genome-fragment | NA | | 0 |
| S9_k141_73744 | 1578 | No | NA | 0 | 1 | Not-determined | Genome-fragment | NA | | 0 |
| S9_k141_116666 | 3076 | No | NA | 0 | 2 | Not-determined | Genome-fragment | NA | | 0 |
| S9_k141_73750 | 3316 | No | NA | 0 | 2 | Not-determined | Genome-fragment | NA | | 0 |
| S6-final-viral-combined_k141_25954\|\|lt2gene | 1070 | No | NA | 1 | 0 | Low-quality | Genome-fragment | | 2.56 | 0 |
| S9_k141_100905 | 3204 | No | NA | 0 | 2 | Not-determined | Genome-fragment | NA | | 0 |
| S9_k141_101281 | 1071 | No | NA | 0 | 1 | Low-quality | Genome-fragment | | 3.6 | 0 |
| S9_k141_41847 | 1272 | No | NA | 0 | 0 | Not-determined | Genome-fragment | NA | | 0 |
| S9_k141_69184 | 1772 | No | NA | 0 | 1 | Not-determined | Genome-fragment | NA | | 0 |
| S9_k141_36851 | 1183 | No | NA | 0 | 1 | Not-determined | Genome-fragment | NA | | 0 |
| S9_k141_5020 | 1114 | No | NA | 0 | 0 | Not-determined | Genome-fragment | NA | | 0 |
| S9_k141_73819 | 1498 | No | NA | 0 | 1 | Not-determined | Genome-fragment | NA | | 0 |
| S9_k141_116742 | 1540 | No | NA | 0 | 1 | Not-determined | Genome-fragment | NA | | 0 |
| S9_k141_73837 | 2323 | No | NA | 0 | 1 | Not-determined | Genome-fragment | NA | | 0 |
| S9_k141_110702 | 2084 | No | NA | 0 | 2 | Not-determined | Genome-fragment | NA | | 0 |
| S9_k141_41947 | 1269 | No | NA | 0 | 0 | Not-determined | Genome-fragment | NA | | 0 |
| S9_k141_20945 | 10667 | No | NA | 0 | 9 | Not-determined | Genome-fragment | NA | | 0 |
| S9_k141_85471 | 1071 | No | NA | 0 | 0 | Not-determined | Genome-fragment | NA | | 0 |
| S1-final-viral-combined_k141_129997\|\|full | 1071 | No | NA | 0 | 1 | Low-quality | Genome-fragment | | 0.68 | 0 |

| | | | | | | | | | | |
|---|---|---|---|---|---|---|---|---|---|---|
| S9_k141_58405 | 1866 | No | NA | 0 | 0 | Not-determined | Genome-fragment | NA | | 0 |
| S9_k141_53398 | 4849 | No | NA | 0 | 2 | Not-determined | Genome-fragment | NA | | 0 |
| S9_k141_5083 | 1712 | No | NA | 0 | 1 | Not-determined | Genome-fragment | NA | | 0 |
| S9_k141_105843 | 1204 | No | NA | 0 | 2 | Not-determined | Genome-fragment | NA | | 0 |
| S9_k141_10313 | 3213 | No | NA | 0 | 0 | Not-determined | Genome-fragment | NA | | 0 |
| S9_k141_15499 | 5249 | No | NA | 0 | 3 | Not-determined | Genome-fragment | NA | | 0 |
| S9_k141_41969 | 2462 | No | NA | 0 | 0 | Not-determined | Genome-fragment | NA | | 0 |
| S9_k141_10326 | 1100 | No | NA | 0 | 0 | Not-determined | Genome-fragment | NA | | 0 |
| S9_k141_15514 | 1009 | No | NA | 0 | 0 | Not-determined | Genome-fragment | NA | | 0 |
| S9_k141_122739 | 1684 | No | NA | 0 | 1 | Not-determined | Genome-fragment | NA | | 0 |
| S9_k141_31062 | 1170 | No | NA | 0 | 0 | Not-determined | Genome-fragment | NA | | 0 |
| S9_k141_101055 | 2937 | No | NA | 0 | 3 | Not-determined | Genome-fragment | NA | | 0 |
| S9_k141_79893 | 1903 | No | NA | 0 | 2 | Not-determined | Genome-fragment | NA | | 0 |
| S9_k141_95175 | 1471 | No | NA | 0 | 1 | Not-determined | Genome-fragment | NA | | 0 |
| S9_k141_101079 | 3259 | No | NA | 0 | 4 | Not-determined | Genome-fragment | NA | | 0 |
| S9_k141_53450 | 4512 | No | NA | 0 | 2 | Not-determined | Genome-fragment | NA | | 0 |
| S9_k141_21049 | 7788 | No | NA | 0 | 1 | Not-determined | Genome-fragment | NA | | 0 |
| S1-final-viral-combined_k141_392331\|\|full | 1071 | No | NA | 1 | 0 | Low-quality | Genome-fragment | | 1.71 | 0 |
| S3-final-viral-combined_k141_88484\|\|full | 1071 | No | NA | 0 | 1 | Low-quality | Genome-fragment | | 0.9 | 0 |
| S9_k141_69296 | 1166 | No | NA | 0 | 2 | Not-determined | Genome-fragment | NA | | 0 |
| S9_k141_53470 | 1592 | No | NA | 0 | 1 | Not-determined | Genome-fragment | NA | | 0 |
| S9_k141_37086 | 1104 | No | NA | 0 | 0 | Not-determined | Genome-fragment | NA | | 0 |
| S9_k141_79953 | 1583 | No | NA | 0 | 1 | Not-determined | Genome-fragment | NA | | 0 |
| S9_k141_10407 | 2275 | No | NA | 0 | 2 | Not-determined | Genome-fragment | NA | | 0 |
| S9_k141_95241 | 1572 | No | NA | 0 | 1 | Not-determined | Genome-fragment | NA | | 0 |
| S9_k141_79980 | 1233 | No | NA | 0 | 1 | Not-determined | Genome-fragment | NA | | 0 |
| S9_k141_42021 | 3782 | No | NA | 0 | 2 | Not-determined | Genome-fragment | NA | | 0 |
| S9_k141_122809 | 3496 | No | NA | 0 | 4 | Not-determined | Genome-fragment | NA | | 0 |
| S9_k141_37153 | 1175 | No | NA | 0 | 1 | Not-determined | Genome-fragment | NA | | 0 |
| S9_k141_106039 | 4247 | No | NA | 0 | 3 | Not-determined | Genome-fragment | NA | | 0 |
| S3-final-viral-combined_k141_107381\|\|full | 1071 | No | NA | 0 | 1 | Low-quality | Genome-fragment | | 0.87 | 0 |
| S9_k141_26614 | 1174 | No | NA | 0 | 0 | Not-determined | Genome-fragment | NA | | 0 |
| S7_k141_239658 | 1071 | No | NA | 0 | 0 | Low-quality | Genome-fragment | | 1.94 | 0 |
| S9_k141_21161 | 2323 | No | NA | 0 | 1 | Not-determined | Genome-fragment | NA | | 0 |
| S7_k141_182147 | 1071 | No | NA | 1 | 0 | Low-quality | Genome-fragment | | 2.74 | 0 |
| S9_k141_106067 | 3084 | No | NA | 0 | 2 | Not-determined | Genome-fragment | NA | | 0 |
| S12-final-viral-combined_k141_207024\|\|full | 1071 | No | NA | 0 | 0 | Low-quality | Genome-fragment | | 2.45 | 0 |
| S12-final-viral-combined_k141_347575\|\|full | 1071 | No | NA | 1 | 0 | Low-quality | Genome-fragment | | 2.65 | 0 |
| S9_k141_10480 | 3938 | No | NA | 1 | 0 | High-quality | High-quality | | 94.63 | 0 |
| S9_k141_58594 | 1420 | No | NA | 0 | 3 | Not-determined | Genome-fragment | NA | | 0 |
| S9_k141_80008 | 2006 | No | NA | 0 | 2 | Not-determined | Genome-fragment | NA | | 0 |
| S9_k141_69458 | 1210 | No | NA | 0 | 0 | Not-determined | Genome-fragment | NA | | 0 |
| S12-final-viral-combined_k141_203417\|\|lt2gene | 1071 | No | NA | 2 | 0 | Low-quality | Genome-fragment | | 1.77 | 0 |
| S9_k141_69465 | 1512 | No | NA | 0 | 1 | Not-determined | Genome-fragment | NA | | 0 |
| S9_k141_74088 | 1335 | No | NA | 0 | 0 | Not-determined | Genome-fragment | NA | | 0 |
| S12-final-viral-combined_k141_277230\|\|lt2gene | 1071 | No | NA | 1 | 0 | Low-quality | Genome-fragment | | 0.88 | 0 |
| S9_k141_31251 | 1104 | No | NA | 0 | 0 | Not-determined | Genome-fragment | NA | | 0 |
| S2-final-viral-combined_k141_194534\|\|full | 1071 | No | NA | 0 | 0 | Low-quality | Genome-fragment | | 2.87 | 0 |
| S9_k141_15849 | 5805 | No | NA | 0 | 1 | Not-determined | Genome-fragment | NA | | 0 |
| S9_k141_101377 | 6564 | No | NA | 0 | 3 | Not-determined | Genome-fragment | NA | | 0 |
| S9_k141_42187 | 1127 | No | NA | 0 | 1 | Not-determined | Genome-fragment | NA | | 0 |
| S9_k141_80116 | 3701 | No | NA | 0 | 2 | Not-determined | Genome-fragment | NA | | 0 |
| S8_k141_11460 | 1071 | No | NA | 0 | 0 | Low-quality | Genome-fragment | | 0.75 | 0 |
| S9_k141_37339 | 1076 | No | NA | 0 | 0 | Not-determined | Genome-fragment | NA | | 0 |
| S11-final-viral-combined_k141_10708\|\|full | 1072 | No | NA | 0 | 0 | Low-quality | Genome-fragment | | 1.1 | 0 |
| S9_k141_127153 | 1426 | No | NA | 0 | 1 | Not-determined | Genome-fragment | NA | | 0 |
| S9_k141_31404 | 1197 | No | NA | 0 | 3 | Not-determined | Genome-fragment | NA | | 0 |
| S9_k141_10635 | 1015 | No | NA | 0 | 0 | Not-determined | Genome-fragment | NA | | 0 |
| S1-final-viral-combined_k141_7169\|\|full | 1072 | No | NA | 0 | 0 | Low-quality | Genome-fragment | | 2.05 | 0 |
| S9_k141_31434 | 4399 | No | NA | 0 | 2 | Not-determined | Genome-fragment | NA | | 0 |
| S9_k141_95543 | 3372 | No | NA | 0 | 4 | Not-determined | Genome-fragment | NA | | 0 |
| S9_k141_101466 | 1176 | No | NA | 0 | 1 | Not-determined | Genome-fragment | NA | | 0 |
| S3-final-viral-combined_k141_73907\|\|full | 1072 | No | NA | 0 | 0 | Low-quality | Genome-fragment | | 1.01 | 0 |
| S9_k141_127258 | 2589 | No | NA | 0 | 1 | Not-determined | Genome-fragment | NA | | 0 |
| S9_k141_16032 | 2373 | No | NA | 0 | 0 | Not-determined | Genome-fragment | NA | | 0 |
| S9_k141_47196 | 1044 | No | NA | 0 | 0 | Not-determined | Genome-fragment | NA | | 0 |
| S9_k141_101559 | 1337 | No | NA | 0 | 1 | Not-determined | Genome-fragment | NA | | 0 |
| S9_k141_31504 | 1697 | No | NA | 0 | 2 | Not-determined | Genome-fragment | NA | | 0 |
| S9_k141_106243 | 7672 | No | NA | 0 | 6 | Not-determined | Genome-fragment | NA | | 0 |
| S7-final-viral-combined_k141_180497\|\|lt2gene | 1072 | No | NA | 1 | 0 | Low-quality | Genome-fragment | | 2.03 | 0 |
| S9_k141_42452 | 4460 | No | NA | 0 | 1 | Not-determined | Genome-fragment | NA | | 0 |
| S9_k141_31703 | 3531 | No | NA | 0 | 0 | Not-determined | Genome-fragment | NA | | 0 |
| S17-final-viral-combined_k141_187613\|\|full | 1072 | No | NA | 0 | 1 | Low-quality | Genome-fragment | | 0.56 | 0 |
| S17-k141_187897 | 1072 | No | NA | 0 | 1 | Low-quality | Genome-fragment | | 0.6 | 0 |
| S9_k141_106461 | 1175 | No | NA | 0 | 1 | Not-determined | Genome-fragment | NA | | 0 |
| S9_k141_31739 | 3184 | No | NA | 0 | 4 | Not-determined | Genome-fragment | NA | | 0 |
| S9_k141_117390 | 1045 | No | NA | 0 | 1 | Not-determined | Genome-fragment | NA | | 0 |
| S12-final-viral-combined_k141_360696\|\|lt2gene | 1072 | No | NA | 1 | 0 | Low-quality | Genome-fragment | | 0.59 | 0 |
| S9_k141_106520 | 1093 | No | NA | 0 | 0 | Not-determined | Genome-fragment | NA | | 0 |
| S9_k141_106559 | 7978 | No | NA | 0 | 5 | Not-determined | Genome-fragment | NA | | 0 |
| S9_k141_117520 | 1082 | No | NA | 0 | 0 | Not-determined | Genome-fragment | NA | | 0 |
| S9_k141_42648 | 2201 | No | NA | 0 | 1 | Not-determined | Genome-fragment | NA | | 0 |
| S9_k141_110997 | 3617 | No | NA | 0 | 0 | Not-determined | Genome-fragment | NA | | 0 |
| S9_k141_74703 | 4620 | No | NA | 0 | 2 | Not-determined | Genome-fragment | NA | | 0 |
| S9_k141_127536 | 1313 | No | NA | 0 | 1 | Not-determined | Genome-fragment | NA | | 0 |
| S12_k141_155025 | 1072 | No | NA | 0 | 0 | Low-quality | Genome-fragment | | 0.78 | 0 |
| S9_k141_42663 | 2212 | No | NA | 0 | 2 | Not-determined | Genome-fragment | NA | | 0 |
| S9_k141_47550 | 1760 | No | NA | 0 | 0 | Not-determined | Genome-fragment | NA | | 0 |
| S9_k141_127559 | 1324 | No | NA | 0 | 2 | Not-determined | Genome-fragment | NA | | 0 |
| S9_k141_127565 | 1200 | No | NA | 0 | 0 | Not-determined | Genome-fragment | NA | | 0 |
| S2-final-viral-combined_k141_105778\|\|lt2gene | 1072 | No | NA | 1 | 0 | Low-quality | Genome-fragment | | 1.79 | 0 |
| S2_k141_135760 | 1072 | No | NA | 0 | 0 | Low-quality | Genome-fragment | | 1.05 | 0 |
| S9_k141_42707 | 6015 | No | NA | 0 | 1 | Not-determined | Genome-fragment | NA | | 0 |
| S9_k141_47603 | 1343 | No | NA | 0 | 0 | Not-determined | Genome-fragment | NA | | 0 |
| S9_k141_127625 | 1425 | No | NA | 0 | 0 | Not-determined | Genome-fragment | NA | | 0 |
| S4-final-viral-combined_k141_83897\|\|full | 1072 | No | NA | 2 | 0 | Low-quality | Genome-fragment | | 2.97 | 0 |
| S9_k141_31844 | 1111 | No | NA | 0 | 1 | Not-determined | Genome-fragment | NA | | 0 |
| S9_k141_96224 | 1264 | No | NA | 0 | 1 | Not-determined | Genome-fragment | NA | | 0 |
| S4_k141_137455 | 1072 | No | NA | 0 | 1 | Low-quality | Genome-fragment | | 0.65 | 0 |
| S9_k141_32004 | 2893 | No | NA | 0 | 2 | Not-determined | Genome-fragment | NA | | 0 |
| S9_k141_32026 | 5039 | No | NA | 0 | 9 | Not-determined | Genome-fragment | NA | | 0 |
| S9_k141_111192 | 1061 | No | NA | 0 | 1 | Not-determined | Genome-fragment | NA | | 0 |
| S6_k141_28189 | 1072 | No | NA | 0 | 0 | Low-quality | Genome-fragment | | 2.13 | 0 |
| S9_k141_127892 | 1043 | No | NA | 0 | 1 | Not-determined | Genome-fragment | NA | | 0 |
| S9_k141_111207 | 1211 | No | NA | 0 | 0 | Not-determined | Genome-fragment | NA | | 0 |
| S8-final-viral-combined_k141_54946\|\|full | 1072 | No | NA | 1 | 0 | Low-quality | Genome-fragment | | 2.68 | 0 |
| S9_k141_111237 | 3424 | No | NA | 0 | 1 | Not-determined | Genome-fragment | NA | | 0 |
| S9_k141_47867 | 11339 | No | NA | 0 | 0 | Not-determined | Genome-fragment | NA | | 0 |
| S9_k141_47937 | 1795 | No | NA | 0 | 2 | Not-determined | Genome-fragment | NA | | 0 |
| S9_k141_111454 | 1096 | No | NA | 0 | 1 | Not-determined | Genome-fragment | NA | | 0 |
| S9_k141_111488 | 2167 | No | NA | 0 | 1 | Not-determined | Genome-fragment | NA | | 0 |
| S9_k141_111537 | 1522 | No | NA | 0 | 0 | Not-determined | Genome-fragment | NA | | 0 |
| S9_k141_111575 | 1254 | No | NA | 0 | 1 | Not-determined | Genome-fragment | NA | | 0 |
| S11-final-viral-combined_k141_318520\|\|full | 1073 | No | NA | 0 | 1 | Low-quality | Genome-fragment | | 0.33 | 0 |
| S9_k141_48112 | 2968 | No | NA | 0 | 0 | Not-determined | Genome-fragment | NA | | 0 |
| S9_k141_128151 | 1116 | No | NA | 0 | 1 | Not-determined | Genome-fragment | NA | | 0 |
| S9_k141_128198 | 1195 | No | NA | 0 | 0 | Not-determined | Genome-fragment | NA | | 0 |
| S9_k141_128211 | 1203 | No | NA | 0 | 2 | Not-determined | Genome-fragment | NA | | 0 |
| S9_k141_128232 | 5983 | No | NA | 3 | 0 | Complete | High-quality | | 100 | 0 |
| S9_k141_128248 | 3515 | No | NA | 0 | 2 | Not-determined | Genome-fragment | NA | | 0 |
| S11-final-viral-combined_k141_130664\|\|full | 1073 | No | NA | 0 | 0 | Low-quality | Genome-fragment | | 1.09 | 0 |
| S11-final-viral-combined_k141_204529\|\|lt2gene | 1073 | No | NA | 2 | 0 | Low-quality | Genome-fragment | | 2.35 | 0 |
| S1-final-viral-combined_k141_233149\|\|full | 1073 | No | NA | 0 | 2 | Low-quality | Genome-fragment | | 0.95 | 0 |
| S9_k141_111769 | 1014 | No | NA | 0 | 1 | Not-determined | Genome-fragment | NA | | 0 |
| S9_k141_111947 | 1718 | No | NA | 0 | 2 | Not-determined | Genome-fragment | NA | | 0 |
| S1-final-viral-combined_k141_13762\|\|lt2gene | 1073 | No | NA | 1 | 0 | Low-quality | Genome-fragment | | 1.51 | 0 |
| S9_k141_112192 | 1043 | No | NA | 0 | 0 | Not-determined | Genome-fragment | NA | | 0 |
| S9_k141_112196 | 1631 | No | NA | 0 | 1 | Not-determined | Genome-fragment | NA | | 0 |
| S9_k141_112201 | 4184 | No | NA | 0 | 4 | Not-determined | Genome-fragment | NA | | 0 |
| S1_k141_133010 | 1073 | No | NA | 2 | 0 | Low-quality | Genome-fragment | | 9.98 | 0 |
| S11-final-viral-combined_k141_190241\|\|full | 1349 | No | NA | 0 | 0 | Not-determined | Genome-fragment | NA | | 0 |
| S1_k141_122264 | 1073 | No | NA | 0 | 0 | Low-quality | Genome-fragment | | 0.61 | 0 |
| S17-final-viral-combined_k141_193187\|\|full | 1073 | No | NA | 0 | 1 | Low-quality | Genome-fragment | | 0.64 | 0 |
| S11-final-viral-combined_k141_179156\|\|full | 1688 | No | NA | 0 | 2 | Not-determined | Genome-fragment | NA | | 0 |
| S11-final-viral-combined_k141_55135\|\|full | 1888 | No | NA | 0 | 0 | Not-determined | Genome-fragment | NA | | 0 |
| S11-final-viral-combined_k141_96474\|\|full | 2250 | No | NA | 0 | 0 | Not-determined | Genome-fragment | NA | | 0 |
| S11-final-viral-combined_k141_137838\|\|full | 1703 | No | NA | 0 | 0 | Not-determined | Genome-fragment | NA | | 0 |
| S17-final-viral-combined_k141_71414\|\|full | 1073 | No | NA | 0 | 1 | Low-quality | Genome-fragment | | 1.46 | 0 |
| S11-final-viral-combined_k141_110257\|\|full | 1844 | No | NA | 0 | 2 | Not-determined | Genome-fragment | NA | | 0 |
| S11-final-viral-combined_k141_27578\|\|full | 1291 | No | NA | 0 | 0 | Not-determined | Genome-fragment | NA | | 0 |
| S17-k141_88347 | 1073 | No | NA | 0 | 0 | Low-quality | Genome-fragment | | 1.27 | 0 |
| S11-final-viral-combined_k141_206749\|\|full | 1071 | No | NA | 0 | 0 | Not-determined | Genome-fragment | NA | | 0 |
| S4-final-viral-combined_k141_117215\|\|full | 1073 | No | NA | 0 | 0 | Low-quality | Genome-fragment | | 2.47 | 0 |
| S11-final-viral-combined_k141_137877\|\|full | 1139 | No | NA | 0 | 0 | Not-determined | Genome-fragment | NA | | 0 |
| S6-final-viral-combined_k141_19288\|\|full | 1073 | No | NA | 0 | 0 | Low-quality | Genome-fragment | | 1.79 | 0 |
| S11-final-viral-combined_k141_13874\|\|full | 2756 | No | NA | 0 | 2 | Not-determined | Genome-fragment | NA | | 0 |
| S11-final-viral-combined_k141_317024\|\|full | 2208 | No | NA | 0 | 2 | Not-determined | Genome-fragment | NA | | 0 |
| S11-final-viral-combined_k141_137899\|\|full | 4922 | No | NA | 0 | 5 | Not-determined | Genome-fragment | NA | | 0 |
| S11-final-viral-combined_k141_206772\|\|full | 4054 | No | NA | 0 | 3 | Not-determined | Genome-fragment | NA | | 0 |
| S11-final-viral-combined_k141_303240\|\|full | 7401 | No | NA | 0 | 1 | Not-determined | Genome-fragment | NA | | 0 |
| S11-final-viral-combined_k141_151648\|\|full | 2560 | No | NA | 0 | 0 | Not-determined | Genome-fragment | NA | | 0 |
| S11-final-viral-combined_k141_248132\|\|full | 2705 | No | NA | 0 | 1 | Not-determined | Genome-fragment | NA | | 0 |
| S11-final-viral-combined_k141_317033\|\|full | 9395 | No | NA | 0 | 7 | Not-determined | Genome-fragment | NA | | 0 |
| S6_k141_110998 | 1073 | No | NA | 0 | 1 | Low-quality | Genome-fragment | | 0.84 | 0 |
| S1-final-viral-combined_k141_14767\|\|full | 1074 | No | NA | 0 | 0 | Low-quality | Genome-fragment | | 1.69 | 0 |
| S1-final-viral-combined_k141_277753\|\|lt2gene | 1074 | No | NA | 2 | 0 | Low-quality | Genome-fragment | | 2.3 | 0 |
| S11-final-viral-combined_k141_41367\|\|full | 39181 | No | NA | 12 | 2 | Medium-quality | Genome-fragment | | 60.35 | 0 |
| S11-final-viral-combined_k141_317054\|\|full | 2873 | No | NA | 0 | 1 | Not-determined | Genome-fragment | NA | | 0 |
| S1_k141_209565 | 1074 | No | NA | 0 | 1 | Low-quality | Genome-fragment | | 1.8 | 0 |
| S7_k141_228500 | 1074 | No | NA | 0 | 0 | Low-quality | Genome-fragment | | 2.17 | 0 |
| S12-final-viral-combined_k141_389889\|\|full | 1074 | No | NA | 0 | 0 | Low-quality | Genome-fragment | | 2.06 | 0 |
| S11-final-viral-combined_k141_192971\|\|full | 2196 | No | NA | 0 | 4 | Not-determined | Genome-fragment | NA | | 0 |
| S11-final-viral-combined_k141_82799\|\|full | 1401 | No | NA | 0 | 0 | Not-determined | Genome-fragment | NA | | 0 |
| S12-final-viral-combined_k141_205760\|\|full | 1074 | No | NA | 0 | 2 | Low-quality | Genome-fragment | | 0.53 | 0 |
| S11-final-viral-combined_k141_110327\|\|full | 5631 | No | NA | 0 | 7 | Not-determined | Genome-fragment | NA | | 0 |
| S11-final-viral-combined_k141_192994\|\|full | 1494 | No | NA | 0 | 1 | Not-determined | Genome-fragment | NA | | 0 |
| S11-final-viral-combined_k141_206830\|\|full | 1223 | No | NA | 0 | 0 | Not-determined | Genome-fragment | NA | | 0 |
| S12-final-viral-combined_k141_104751\|\|lt2gene | 1074 | No | NA | 1 | 0 | Low-quality | Genome-fragment | | 1.69 | 0 |
| S11-final-viral-combined_k141_41400\|\|full | 2528 | No | NA | 0 | 2 | Not-determined | Genome-fragment | NA | | 0 |
| S12_k141_289597 | 1074 | No | NA | 0 | 1 | Low-quality | Genome-fragment | | 0.94 | 0 |
| S12_k141_145908 | 1074 | No | NA | 0 | 0 | Low-quality | Genome-fragment | | 3.18 | 0 |
| S11-final-viral-combined_k141_124198\|\|full | 2965 | No | NA | 0 | 0 | Not-determined | Genome-fragment | NA | | 0 |
| S9-final-viral-combined_k141_112634\|\|full | 1075 | No | NA | 0 | 0 | Low-quality | Genome-fragment | | 2.13 | 0 |
| S11-final-viral-combined_k141_289520\|\|full | 4615 | No | NA | 0 | 3 | Not-determined | Genome-fragment | NA | | 0 |

| | | | | | | | | | | | |
|---|---|---|---|---|---|---|---|---|---|---|---|
| S11-final-viral-combined_k141_289537\|\|full | 1272 | No | NA | | 0 | 1 | Not-determined | Genome-fragment | NA | | 0 |
| S11-final-viral-combined_k141_99776\|\|full | 1075 | No | NA | | 0 | 0 | Low-quality | Genome-fragment | | 1.31 | 0 |
| S11-final-viral-combined_k141_206880\|\|full | 3453 | No | NA | | 0 | 2 | Not-determined | Genome-fragment | NA | | 0 |
| S1-final-viral-combined_k141_357319\|\|full | 1075 | No | NA | | 0 | 0 | Low-quality | Genome-fragment | | 2.22 | 0 |
| S1-final-viral-combined_k141_63314\|\|lt2gene | 1075 | No | NA | | 1 | 0 | Low-quality | Genome-fragment | | 2.27 | 0 |
| S1_k141_290381 | 1075 | No | NA | | 0 | 0 | Low-quality | Genome-fragment | | 0.42 | 0 |
| S11-final-viral-combined_k141_69075\|\|full | 1749 | No | NA | | 0 | 0 | Not-determined | Genome-fragment | NA | | 0 |
| S3_k141_100007 | 1075 | No | NA | | 1 | 0 | Low-quality | Genome-fragment | | 0.49 | 0 |
| S5-final-viral-combined_k141_46073\|\|full | 1075 | No | NA | | 1 | 0 | Low-quality | Genome-fragment | | 2.49 | 0 |
| S17-final-viral-combined_k141_55739\|\|full | 1075 | No | NA | | 0 | 0 | Low-quality | Genome-fragment | | 0.64 | 0 |
| S17-k141_13701 | 1075 | No | NA | | 0 | 0 | Low-quality | Genome-fragment | | 0.81 | 0 |
| S17-k141_58578 | 1075 | No | NA | | 0 | 2 | Low-quality | Genome-fragment | | 0.74 | 0 |
| S11-final-viral-combined_k141_234385\|\|full | 3633 | No | NA | | 0 | 0 | Not-determined | Genome-fragment | NA | | 0 |
| S11-final-viral-combined_k141_165609\|\|full | 2474 | No | NA | | 0 | 0 | Not-determined | Genome-fragment | NA | | 0 |
| S12-final-viral-combined_k141_126693\|\|lt2gene | 1075 | No | NA | | 2 | 0 | Low-quality | Genome-fragment | | 3.11 | 0 |
| S12_k141_381496 | 1075 | No | NA | | 0 | 0 | Low-quality | Genome-fragment | | 2.08 | 0 |
| S2-final-viral-combined_k141_84787\|\|full | 1075 | No | NA | | 0 | 0 | Low-quality | Genome-fragment | | 1.83 | 0 |
| S2-final-viral-combined_k141_107010\|\|full | 1075 | No | NA | | 0 | 0 | Low-quality | Genome-fragment | | 1.58 | 0 |
| S11-final-viral-combined_k141_151854\|\|full | 2466 | No | NA | | 0 | 3 | Not-determined | Genome-fragment | NA | | 0 |
| S11-final-viral-combined_k141_165646\|\|full | 1294 | No | NA | | 0 | 1 | Not-determined | Genome-fragment | NA | | 0 |
| S2-final-viral-combined_k141_89812\|\|lt2gene | 1075 | No | NA | | 1 | 0 | Low-quality | Genome-fragment | | 2.55 | 0 |
| S11-final-viral-combined_k141_193148\|\|full | 39231 | Yes | | 30175 | 5 | 4 | Medium-quality | Genome-fragment | | 67.75 | 23.08 |
| S2_k141_42050 | 1075 | No | NA | | 1 | 0 | Low-quality | Genome-fragment | | 1.61 | 0 |
| S11-final-viral-combined_k141_124324\|\|full | 3304 | No | NA | | 0 | 4 | Not-determined | Genome-fragment | NA | | 0 |
| S8-final-viral-combined_k141_96731\|\|full | 1075 | No | NA | | 0 | 0 | Low-quality | Genome-fragment | | 1.59 | 0 |
| S9_k141_907 | 1076 | No | NA | | 1 | 0 | Low-quality | Genome-fragment | | 11.43 | 0 |
| S11-final-viral-combined_k141_324496\|\|full | 1076 | No | NA | | 0 | 0 | Low-quality | Genome-fragment | | 2.69 | 0 |
| S11-final-viral-combined_k141_234426\|\|full | 13340 | No | NA | | 0 | 9 | Not-determined | Genome-fragment | NA | | 0 |
| S1-final-viral-combined_k141_365998\|\|full | 1076 | No | NA | | 0 | 0 | Low-quality | Genome-fragment | | 0.69 | 0 |
| S11-final-viral-combined_k141_69182\|\|full | 1057 | No | NA | | 0 | 1 | Not-determined | Genome-fragment | NA | | 0 |
| S11-final-viral-combined_k141_124393\|\|full | 2429 | No | NA | | 0 | 0 | Not-determined | Genome-fragment | NA | | 0 |
| S1-final-viral-combined_k141_285671\|\|full | 1076 | No | NA | | 0 | 0 | Low-quality | Genome-fragment | | 0.85 | 0 |
| S11-final-viral-combined_k141_220808\|\|full | 7884 | No | NA | | 0 | 0 | Not-determined | Genome-fragment | NA | | 0 |
| S3-final-viral-combined_k141_116328\|\|full | 1076 | No | NA | | 1 | 0 | Low-quality | Genome-fragment | | 1.78 | 0 |
| S11-final-viral-combined_k141_138099\|\|full | 1288 | No | NA | | 0 | 1 | Not-determined | Genome-fragment | NA | | 0 |
| S5-final-viral-combined_k141_72904\|\|full | 1076 | No | NA | | 3 | 0 | Low-quality | Genome-fragment | | 2.36 | 0 |
| S11-final-viral-combined_k141_14092\|\|full | 2575 | No | NA | | 0 | 0 | Not-determined | Genome-fragment | NA | | 0 |
| S11-final-viral-combined_k141_165709\|\|full | 2871 | No | NA | | 0 | 0 | Not-determined | Genome-fragment | NA | | 0 |
| S7-final-viral-combined_k141_40078\|\|full | 1076 | No | NA | | 1 | 0 | Low-quality | Genome-fragment | | 0.88 | 0 |
| S11-final-viral-combined_k141_207007\|\|full | 52392 | No | NA | | 0 | 23 | Not-determined | Genome-fragment | NA | | 0 |
| S7_k141_143163 | 1076 | No | NA | | 2 | 0 | Low-quality | Genome-fragment | | 2.23 | 0 |
| S11-final-viral-combined_k141_96692\|\|full | 2102 | No | NA | | 0 | 2 | Not-determined | Genome-fragment | NA | | 0 |
| S11-final-viral-combined_k141_262077\|\|full | 1984 | No | NA | | 0 | 0 | Not-determined | Genome-fragment | NA | | 0 |
| S11-final-viral-combined_k141_110485\|\|full | 2196 | No | NA | | 0 | 2 | Not-determined | Genome-fragment | NA | | 0 |
| S11-final-viral-combined_k141_55409\|\|full | 1926 | No | NA | | 0 | 2 | Not-determined | Genome-fragment | NA | | 0 |
| S17-final-viral-combined_k141_104028\|\|full | 1076 | No | NA | | 0 | 2 | Low-quality | Genome-fragment | | 1.42 | 0 |
| S17-final-viral-combined_k141_104099\|\|full | 1076 | No | NA | | 1 | 0 | Low-quality | Genome-fragment | | 1.94 | 0 |
| S11-final-viral-combined_k141_289605\|\|full | 1941 | No | NA | | 0 | 3 | Not-determined | Genome-fragment | NA | | 0 |
| S11-final-viral-combined_k141_41510\|\|full | 6143 | No | NA | | 0 | 6 | Not-determined | Genome-fragment | NA | | 0 |
| S12-final-viral-combined_k141_66048\|\|full | 1076 | No | NA | | 2 | 0 | Low-quality | Genome-fragment | | 1.72 | 0 |
| S12-final-viral-combined_k141_248145\|\|lt2gene | 1076 | No | NA | | 1 | 0 | Low-quality | Genome-fragment | | 1.84 | 0 |
| S11-final-viral-combined_k141_220854\|\|full | 1989 | No | NA | | 0 | 1 | Not-determined | Genome-fragment | NA | | 0 |
| S11-final-viral-combined_k141_69207\|\|full | 8496 | No | NA | | 0 | 6 | Not-determined | Genome-fragment | NA | | 0 |
| S2-final-viral-combined_k141_211951\|\|lt2gene | 1076 | No | NA | | 1 | 0 | Low-quality | Genome-fragment | | 0.58 | 0 |
| S2_k141_64120 | 1076 | No | NA | | 0 | 0 | Low-quality | Genome-fragment | | 1.48 | 0 |
| S11-final-viral-combined_k141_165785\|\|full | 1869 | No | NA | | 0 | 0 | Not-determined | Genome-fragment | NA | | 0 |
| S4-final-viral-combined_k141_111719\|\|full | 1076 | No | NA | | 0 | 0 | Low-quality | Genome-fragment | | 2.05 | 0 |
| S11-final-viral-combined_k141_96737\|\|full | 2752 | No | NA | | 0 | 1 | Not-determined | Genome-fragment | NA | | 0 |
| S11-final-viral-combined_k141_124446\|\|full | 4103 | No | NA | | 0 | 3 | Not-determined | Genome-fragment | NA | | 0 |
| S8-final-viral-combined_k141_258279\|\|full | 1076 | No | NA | | 1 | 0 | Low-quality | Genome-fragment | | 1.65 | 0 |
| S11-final-viral-combined_k141_193271\|\|full | 2104 | No | NA | | 0 | 2 | Not-determined | Genome-fragment | NA | | 0 |
| S11_k141_319123 | 1077 | No | NA | | 0 | 0 | Low-quality | Genome-fragment | | 2.37 | 0 |
| S11_k141_119984 | 1077 | No | NA | | 0 | 1 | Low-quality | Genome-fragment | | 2.13 | 0 |
| S1_k141_64782 | 1077 | No | NA | | 1 | 0 | Low-quality | Genome-fragment | | 0.28 | 0 |
| S17-final-viral-combined_k141_201062\|\|full | 1077 | No | NA | | 0 | 0 | Low-quality | Genome-fragment | | 3.05 | 0 |
| S11-final-viral-combined_k141_303534\|\|full | 2821 | No | NA | | 0 | 3 | Not-determined | Genome-fragment | NA | | 0 |
| S17-k141_200767 | 1077 | No | NA | | 0 | 0 | Low-quality | Genome-fragment | | 1.67 | 0 |
| S11-final-viral-combined_k141_151974\|\|full | 6276 | No | NA | | 0 | 1 | Not-determined | Genome-fragment | NA | | 0 |
| S12_k141_361606 | 1077 | No | NA | | 0 | 1 | Low-quality | Genome-fragment | | 2.47 | 0 |
| S11-final-viral-combined_k141_248401\|\|full | 1839 | No | NA | | 0 | 1 | Not-determined | Genome-fragment | NA | | 0 |
| S2-final-viral-combined_k141_127089\|\|full | 1077 | No | NA | | 0 | 0 | Low-quality | Genome-fragment | | 0.66 | 0 |
| S2-final-viral-combined_k141_99321\|\|full | 1077 | No | NA | | 0 | 2 | Low-quality | Genome-fragment | | 1.44 | 0 |
| S2-final-viral-combined_k141_193804\|\|full | 1077 | No | NA | | 0 | 1 | Low-quality | Genome-fragment | | 2.47 | 0 |
| S11-final-viral-combined_k141_110566\|\|full | 1929 | No | NA | | 0 | 1 | Not-determined | Genome-fragment | NA | | 0 |
| S11-final-viral-combined_k141_138247\|\|full | 2105 | No | NA | | 0 | 2 | Not-determined | Genome-fragment | NA | | 0 |
| S2-final-viral-combined_k141_112723\|\|full | 1077 | No | NA | | 3 | 0 | Low-quality | Genome-fragment | | 1.97 | 0 |
| S11-final-viral-combined_k141_152012\|\|full | 1485 | No | NA | | 0 | 0 | Not-determined | Genome-fragment | NA | | 0 |
| S11-final-viral-combined_k141_83070\|\|full | 3223 | No | NA | | 0 | 1 | Not-determined | Genome-fragment | NA | | 0 |
| S6-final-viral-combined_k141_119045\|\|full | 1077 | No | NA | | 0 | 1 | Low-quality | Genome-fragment | | 0.51 | 0 |
| S6-final-viral-combined_k141_3121\|\|full | 1077 | No | NA | | 0 | 0 | Low-quality | Genome-fragment | | 2.14 | 0 |
| S11-final-viral-combined_k141_124507\|\|full | 5044 | No | NA | | 0 | 1 | Not-determined | Genome-fragment | NA | | 0 |
| S11-final-viral-combined_k141_152036\|\|full | 3174 | No | NA | | 0 | 0 | Not-determined | Genome-fragment | NA | | 0 |
| S11-final-viral-combined_k141_289712\|\|full | 18580 | No | NA | | 0 | 11 | Not-determined | Genome-fragment | NA | | 0 |
| S8-final-viral-combined_k141_187774\|\|full | 1077 | No | NA | | 0 | 0 | Low-quality | Genome-fragment | | 0.66 | 0 |
| S11-final-viral-combined_k141_165943\|\|full | 1150 | No | NA | | 0 | 0 | Not-determined | Genome-fragment | NA | | 0 |
| S8-final-viral-combined_k141_155984\|\|full | 1077 | No | NA | | 0 | 0 | Low-quality | Genome-fragment | | 0.7 | 0 |
| S8_k141_79854 | 1077 | No | NA | | 0 | 0 | Low-quality | Genome-fragment | | 0.86 | 0 |
| S11_k141_319543 | 1078 | No | NA | | 2 | 0 | Low-quality | Genome-fragment | | 2.94 | 0 |
| S11_k141_6006 | 1078 | No | NA | | 1 | 0 | Low-quality | Genome-fragment | | 0.57 | 0 |
| S1-final-viral-combined_k141_142958\|\|full | 1078 | No | NA | | 0 | 0 | Low-quality | Genome-fragment | | 2.35 | 0 |
| S11-final-viral-combined_k141_28013\|\|full | 1332 | No | NA | | 0 | 0 | Not-determined | Genome-fragment | NA | | 0 |
| S11-final-viral-combined_k141_14266\|\|full | 1946 | No | NA | | 0 | 1 | Not-determined | Genome-fragment | NA | | 0 |
| S11-final-viral-combined_k141_289730\|\|full | 1482 | No | NA | | 0 | 0 | Not-determined | Genome-fragment | NA | | 0 |
| S11-final-viral-combined_k141_138299\|\|full | 2429 | No | NA | | 0 | 3 | Not-determined | Genome-fragment | NA | | 0 |
| S11-final-viral-combined_k141_41659\|\|full | 1428 | No | NA | | 0 | 1 | Not-determined | Genome-fragment | NA | | 0 |
| S11-final-viral-combined_k141_289732\|\|full | 1308 | No | NA | | 0 | 1 | Not-determined | Genome-fragment | NA | | 0 |
| S5-final-viral-combined_k141_89346\|\|full | 1078 | No | NA | | 2 | 0 | Low-quality | Genome-fragment | | 2.31 | 0 |
| S5_k141_183108 | 1078 | No | NA | | 0 | 0 | Low-quality | Genome-fragment | | 2.66 | 0 |
| S7-final-viral-combined_k141_15790\|\|full | 1078 | No | NA | | 1 | 0 | Low-quality | Genome-fragment | | 1.83 | 0 |
| S11-final-viral-combined_k141_110636\|\|full | 2419 | No | NA | | 0 | 1 | Not-determined | Genome-fragment | NA | | 0 |
| S11-final-viral-combined_k141_69335\|\|full | 2329 | No | NA | | 0 | 1 | Not-determined | Genome-fragment | NA | | 0 |
| S11-final-viral-combined_k141_83107\|\|full | 1314 | No | NA | | 0 | 0 | Not-determined | Genome-fragment | NA | | 0 |
| S11-final-viral-combined_k141_69337\|\|full | 1968 | No | NA | | 0 | 2 | Not-determined | Genome-fragment | NA | | 0 |
| S7-final-viral-combined_k141_27956\|\|full | 1078 | No | NA | | 0 | 0 | Low-quality | Genome-fragment | | 0.72 | 0 |
| S11-final-viral-combined_k141_14318\|\|full | 1999 | No | NA | | 0 | 1 | Not-determined | Genome-fragment | NA | | 0 |
| S11-final-viral-combined_k141_303682\|\|full | 5729 | No | NA | | 0 | 3 | Not-determined | Genome-fragment | NA | | 0 |
| S11-final-viral-combined_k141_69357\|\|full | 5297 | No | NA | | 0 | 1 | Not-determined | Genome-fragment | NA | | 0 |
| S11-final-viral-combined_k141_220937\|\|full | 2503 | No | NA | | 0 | 1 | Not-determined | Genome-fragment | NA | | 0 |
| S11-final-viral-combined_k141_83135\|\|full | 2540 | No | NA | | 0 | 1 | Not-determined | Genome-fragment | NA | | 0 |
| S11-final-viral-combined_k141_69376\|\|full | 1833 | No | NA | | 0 | 1 | Not-determined | Genome-fragment | NA | | 0 |
| S11-final-viral-combined_k141_248565\|\|full | 5089 | No | NA | | 0 | 3 | Not-determined | Genome-fragment | NA | | 0 |
| S11-final-viral-combined_k141_276066\|\|full | 2238 | No | NA | | 0 | 1 | Not-determined | Genome-fragment | NA | | 0 |
| S11-final-viral-combined_k141_96991\|\|full | 1429 | No | NA | | 0 | 2 | Not-determined | Genome-fragment | NA | | 0 |
| S11-final-viral-combined_k141_179605\|\|full | 43615 | Yes | | 22617 | 12 | 17 | Medium-quality | Genome-fragment | | 54 | 48.14 |
| S7_k141_20563 | 1078 | No | NA | | 0 | 0 | Low-quality | Genome-fragment | | 1.8 | 0 |
| S11-final-viral-combined_k141_14365\|\|full | 2206 | No | NA | | 0 | 2 | Not-determined | Genome-fragment | NA | | 0 |
| S11-final-viral-combined_k141_234757\|\|full | 3517 | No | NA | | 0 | 1 | Not-determined | Genome-fragment | NA | | 0 |
| S17-final-viral-combined_k141_138877\|\|full | 1078 | No | NA | | 2 | 0 | Low-quality | Genome-fragment | | 3.16 | 0 |
| S11-final-viral-combined_k141_234790\|\|full | 1037 | No | NA | | 0 | 1 | Not-determined | Genome-fragment | NA | | 0 |
| S17-final-viral-combined_k141_141231\|\|full | 1078 | No | NA | | 0 | 0 | Low-quality | Genome-fragment | | 2.88 | 0 |
| S11-final-viral-combined_k141_152167\|\|full | 1048 | No | NA | | 0 | 2 | Not-determined | Genome-fragment | NA | | 0 |
| S11-final-viral-combined_k141_28104\|\|full | 1684 | No | NA | | 0 | 2 | Not-determined | Genome-fragment | NA | | 0 |
| S11-final-viral-combined_k141_193408\|\|full | 27118 | No | NA | | 0 | 21 | Not-determined | Genome-fragment | NA | | 0 |
| S11-final-viral-combined_k141_193418\|\|full | 1502 | No | NA | | 0 | 0 | Not-determined | Genome-fragment | NA | | 0 |
| S17-k141_263484 | 1078 | No | NA | | 0 | 0 | Low-quality | Genome-fragment | | 0.86 | 0 |
| S11-final-viral-combined_k141_152188\|\|full | 1580 | No | NA | | 0 | 2 | Not-determined | Genome-fragment | NA | | 0 |
| S11-final-viral-combined_k141_193419\|\|full | 1960 | No | NA | | 0 | 1 | Not-determined | Genome-fragment | NA | | 0 |
| S12-final-viral-combined_k141_219655\|\|full | 1078 | No | NA | | 0 | 2 | Low-quality | Genome-fragment | | 0.34 | 0 |
| S11-final-viral-combined_k141_138346\|\|full | 1540 | No | NA | | 0 | 1 | Not-determined | Genome-fragment | NA | | 0 |
| S12-final-viral-combined_k141_195978\|\|lt2gene | 1078 | No | NA | | 1 | 0 | Low-quality | Genome-fragment | | 2.38 | 0 |
| S4-final-viral-combined_k141_151424\|\|full | 1078 | No | NA | | 2 | 0 | Low-quality | Genome-fragment | | 1.59 | 0 |
| S11-final-viral-combined_k141_97034\|\|full | 2698 | No | NA | | 0 | 2 | Not-determined | Genome-fragment | NA | | 0 |
| S6-final-viral-combined_k141_124395\|\|full | 1078 | No | NA | | 0 | 2 | Low-quality | Genome-fragment | | 0.78 | 0 |
| S11-final-viral-combined_k141_41853\|\|full | 1711 | No | NA | | 0 | 3 | Not-determined | Genome-fragment | NA | | 0 |
| S11-final-viral-combined_k141_248582\|\|full | 14636 | No | NA | | 0 | 3 | Not-determined | Genome-fragment | NA | | 0 |
| S11-final-viral-combined_k141_110756\|\|full | 1467 | No | NA | | 0 | 0 | Not-determined | Genome-fragment | NA | | 0 |
| S11-final-viral-combined_k141_221028\|\|full | 1021 | No | NA | | 0 | 0 | Not-determined | Genome-fragment | NA | | 0 |
| S11-final-viral-combined_k141_289855\|\|full | 4843 | No | NA | | 0 | 6 | Not-determined | Genome-fragment | NA | | 0 |
| S11-final-viral-combined_k141_28164\|\|full | 1133 | No | NA | | 0 | 1 | Not-determined | Genome-fragment | NA | | 0 |
| S11-final-viral-combined_k141_97068\|\|full | 2884 | No | NA | | 0 | 1 | Not-determined | Genome-fragment | NA | | 0 |
| S8_k141_59308 | 1078 | No | NA | | 0 | 0 | Low-quality | Genome-fragment | | 2.77 | 0 |
| S11-final-viral-combined_k141_97086\|\|full | 3110 | No | NA | | 0 | 1 | Not-determined | Genome-fragment | NA | | 0 |
| S11-final-viral-combined_k141_96787\|\|lt2gene | 1079 | No | NA | | 2 | 0 | Low-quality | Genome-fragment | | 2.48 | 0 |
| S11_k141_119503 | 1079 | No | NA | | 1 | 0 | Low-quality | Genome-fragment | | 2.76 | 0 |
| S11-final-viral-combined_k141_166152\|\|full | 1902 | No | NA | | 0 | 1 | Not-determined | Genome-fragment | NA | | 0 |
| S11-final-viral-combined_k141_138475\|\|full | 2004 | No | NA | | 0 | 0 | Not-determined | Genome-fragment | NA | | 0 |
| S11-final-viral-combined_k141_138485\|\|full | 1253 | No | NA | | 0 | 2 | Not-determined | Genome-fragment | NA | | 0 |
| S3_k141_128208 | 1079 | No | NA | | 0 | 1 | Low-quality | Genome-fragment | | 3.24 | 0 |
| S11-final-viral-combined_k141_248664\|\|full | 1516 | No | NA | | 0 | 0 | Not-determined | Genome-fragment | NA | | 0 |
| S11-final-viral-combined_k141_14466\|\|full | 4424 | No | NA | | 0 | 2 | Not-determined | Genome-fragment | NA | | 0 |
| S11-final-viral-combined_k141_289941\|\|full | 2447 | No | NA | | 0 | 1 | Not-determined | Genome-fragment | NA | | 0 |
| S11-final-viral-combined_k141_207291\|\|full | 3391 | No | NA | | 0 | 2 | Not-determined | Genome-fragment | NA | | 0 |
| S5-final-viral-combined_k141_136162\|\|lt2gene | 1079 | No | NA | | 2 | 0 | Low-quality | Genome-fragment | | 1.66 | 0 |
| S11-final-viral-combined_k141_97132\|\|full | 2253 | No | NA | | 0 | 1 | Not-determined | Genome-fragment | NA | | 0 |
| S11-final-viral-combined_k141_289967\|\|full | 2622 | No | NA | | 0 | 2 | Not-determined | Genome-fragment | NA | | 0 |
| S7-final-viral-combined_k141_62448\|\|full | 1079 | No | NA | | 1 | 0 | Low-quality | Genome-fragment | | 1.55 | 0 |
| S11-final-viral-combined_k141_124799\|\|full | 1338 | No | NA | | 0 | 0 | Not-determined | Genome-fragment | NA | | 0 |
| S11-final-viral-combined_k141_83281\|\|full | 4130 | No | NA | | 0 | 3 | Not-determined | Genome-fragment | NA | | 0 |
| S7-final-viral-combined_k141_44478\|\|full | 1079 | No | NA | | 0 | 0 | Low-quality | Genome-fragment | | 3.06 | 0 |
| S11-final-viral-combined_k141_110814\|\|full | 1123 | No | NA | | 0 | 1 | Not-determined | Genome-fragment | NA | | 0 |
| S17-final-viral-combined_k141_111172\|\|full | 1079 | No | NA | | 0 | 0 | Low-quality | Genome-fragment | | 2.13 | 0 |
| S12-final-viral-combined_k141_39316\|\|full | 1079 | No | NA | | 0 | 0 | Low-quality | Genome-fragment | | 3.39 | 0 |
| S12-final-viral-combined_k141_247451\|\|lt2gene | 1079 | No | NA | | 1 | 0 | Low-quality | Genome-fragment | | 2.12 | 0 |
| S11-final-viral-combined_k141_235008\|\|full | 1079 | No | NA | | 0 | 1 | Not-determined | Genome-fragment | NA | | 0 |
| S2_k141_177254 | 1079 | No | NA | | 0 | 0 | Low-quality | Genome-fragment | | 3.7 | 0 |
| S11-final-viral-combined_k141_303892\|\|full | 2867 | No | NA | | 0 | 1 | Not-determined | Genome-fragment | NA | | 0 |
| S11-final-viral-combined_k141_290010\|\|full | 1002 | No | NA | | 0 | 0 | Not-determined | Genome-fragment | NA | | 0 |
| S11-final-viral-combined_k141_221167\|\|full | 1555 | No | NA | | 0 | 0 | Not-determined | Genome-fragment | NA | | 0 |
| S1-final-viral-combined_k141_122213\|\|full | 1080 | No | NA | | 0 | 2 | Low-quality | Genome-fragment | | 0.37 | 0 |
| S11-final-viral-combined_k141_138530\|\|full | 1407 | No | NA | | 0 | 2 | Not-determined | Genome-fragment | NA | | 0 |
| S3-final-viral-combined_k141_10580\|\|full | 1080 | No | NA | | 2 | 0 | Low-quality | Genome-fragment | | 2.6 | 0 |
| S11-final-viral-combined_k141_14547\|\|full | 7471 | No | NA | | 0 | 1 | Not-determined | Genome-fragment | NA | | 0 |
| S11-final-viral-combined_k141_124887\|\|full | 1112 | No | NA | | 0 | 1 | Not-determined | Genome-fragment | NA | | 0 |
| S11-final-viral-combined_k141_290030\|\|full | 1365 | No | NA | | 0 | 1 | Not-determined | Genome-fragment | NA | | 0 |

| | | | | | | | | | |
|---|---|---|---|---|---|---|---|---|---|
| S5-final-viral-combined_k141_3580\|\|full | 1080 | No | NA | 0 | 0 | Low-quality | Genome-fragment | 2.12 | 0 |
| S5-final-viral-combined_k141_85088\|\|full | 1080 | No | NA | 2 | 0 | Low-quality | Genome-fragment | 2.38 | 0 |
| S11-final-viral-combined_k141_152339\|\|full | 7421 | No | NA | 0 | 2 | Not-determined | Genome-fragment | NA | 0 |
| S5-final-viral-combined_k141_21801\|\|full | 1080 | No | NA | 0 | 0 | Low-quality | Genome-fragment | 1.86 | 0 |
| S17-final-viral-combined_k141_47928\|\|full | 1080 | No | NA | 0 | 0 | Low-quality | Genome-fragment | 2.8 | 0 |
| S11-final-viral-combined_k141_124914\|\|full | 2484 | No | NA | 0 | 2 | Not-determined | Genome-fragment | NA | 0 |
| S11-final-viral-combined_k141_28316\|\|full | 2324 | No | NA | 0 | 0 | Not-determined | Genome-fragment | NA | 0 |
| S17-final-viral-combined_k141_265642\|\|lt2gene | 1080 | No | NA | 1 | 0 | Low-quality | Genome-fragment | 1.66 | 0 |
| S11-final-viral-combined_k141_152356\|\|full | 3180 | No | NA | 0 | 0 | Not-determined | Genome-fragment | NA | 0 |
| S2-final-viral-combined_k141_40394\|\|full | 1080 | No | NA | 0 | 0 | Low-quality | Genome-fragment | 2.05 | 0 |
| S11-final-viral-combined_k141_276341\|\|full | 2914 | No | NA | 0 | 0 | Not-determined | Genome-fragment | NA | 0 |
| S11-final-viral-combined_k141_28317\|\|full | 6207 | No | NA | 0 | 4 | Not-determined | Genome-fragment | NA | 0 |
| S11-final-viral-combined_k141_221231\|\|full | 2137 | No | NA | 0 | 0 | Not-determined | Genome-fragment | NA | 0 |
| S11-final-viral-combined_k141_276342\|\|full | 1349 | No | NA | 0 | 1 | Not-determined | Genome-fragment | NA | 0 |
| S11-final-viral-combined_k141_678\|\|full | 7158 | No | NA | 0 | 2 | Not-determined | Genome-fragment | NA | 0 |
| S11-final-viral-combined_k141_193702\|\|full | 3304 | No | NA | 0 | 2 | Not-determined | Genome-fragment | NA | 0 |
| S4-final-viral-combined_k141_10744\|\|full | 1080 | No | NA | 1 | 0 | Low-quality | Genome-fragment | 1.31 | 0 |
| S11-final-viral-combined_k141_303944\|\|full | 1673 | No | NA | 0 | 1 | Not-determined | Genome-fragment | NA | 0 |
| S8-final-viral-combined_k141_266458\|\|full | 1080 | No | NA | 1 | 0 | Low-quality | Genome-fragment | 0.82 | 0 |
| S11-final-viral-combined_k141_97292\|\|full | 2922 | No | NA | 0 | 1 | Not-determined | Genome-fragment | NA | 0 |
| S8-final-viral-combined_k141_245828\|\|full | 1080 | No | NA | 1 | 0 | Low-quality | Genome-fragment | 1.95 | 0 |
| S8_k141_35867 | 1080 | No | NA | 0 | 0 | Low-quality | Genome-fragment | 2.45 | 0 |
| S9-final-viral-combined_k141_8864\|\|lt2gene | 1081 | No | NA | 1 | 0 | Low-quality | Genome-fragment | 2.81 | 0 |
| S11-final-viral-combined_k141_235142\|\|full | 2992 | No | NA | 0 | 2 | Not-determined | Genome-fragment | NA | 0 |
| S11-final-viral-combined_k141_297626\|\|lt2gene | 1081 | No | NA | 1 | 0 | Low-quality | Genome-fragment | 2.47 | 0 |
| S11-final-viral-combined_k141_55982\|\|full | 1896 | No | NA | 0 | 0 | Not-determined | Genome-fragment | NA | 0 |
| S1_k141_169781 | 1081 | No | NA | 0 | 1 | Low-quality | Genome-fragment | 2.77 | 0 |
| S5-final-viral-combined_k141_122425\|\|lt2gene | 1081 | No | NA | 2 | 0 | Low-quality | Genome-fragment | 2.41 | 0 |
| S11-final-viral-combined_k141_235186\|\|full | 1539 | No | NA | 0 | 1 | Not-determined | Genome-fragment | NA | 0 |
| S11-final-viral-combined_k141_166423\|\|full | 1429 | No | NA | 0 | 1 | Not-determined | Genome-fragment | NA | 0 |
| S11-final-viral-combined_k141_110981\|\|full | 1763 | No | NA | 0 | 1 | Not-determined | Genome-fragment | NA | 0 |
| S17-final-viral-combined_k141_206409\|\|full | 1081 | No | NA | 2 | 0 | Low-quality | Genome-fragment | 2.71 | 0 |
| S17-k141_121614 | 1081 | No | NA | 0 | 0 | Low-quality | Genome-fragment | 0.93 | 0 |
| S11-final-viral-combined_k141_207506\|\|full | 1835 | No | NA | 0 | 2 | Not-determined | Genome-fragment | NA | 0 |
| S12-final-viral-combined_k141_152557\|\|full | 1081 | No | NA | 0 | 0 | Low-quality | Genome-fragment | 0.86 | 0 |
| S11-final-viral-combined_k141_28397\|\|full | 1134 | No | NA | 0 | 0 | Not-determined | Genome-fragment | NA | 0 |
| S11-final-viral-combined_k141_221310\|\|full | 32507 | No | NA | 11 | 1 | High-quality | High-quality | 91.28 | 0 |
| S11-final-viral-combined_k141_317802\|\|full | 1124 | No | NA | 0 | 1 | Not-determined | Genome-fragment | NA | 0 |
| S12-final-viral-combined_k141_144712\|\|full | 1081 | No | NA | 0 | 0 | Low-quality | Genome-fragment | 2.27 | 0 |
| S11-final-viral-combined_k141_235212\|\|full | 2812 | No | NA | 0 | 2 | Not-determined | Genome-fragment | NA | 0 |
| S11-final-viral-combined_k141_317806\|\|full | 3456 | No | NA | 0 | 2 | Not-determined | Genome-fragment | NA | 0 |
| S11-final-viral-combined_k141_276481\|\|full | 1948 | No | NA | 0 | 2 | Not-determined | Genome-fragment | NA | 0 |
| S2-final-viral-combined_k141_85560\|\|full | 1081 | No | NA | 0 | 0 | Low-quality | Genome-fragment | 2.6 | 0 |
| S11-final-viral-combined_k141_304041\|\|full | 2567 | No | NA | 0 | 0 | Not-determined | Genome-fragment | NA | 0 |
| S11-final-viral-combined_k141_69727\|\|full | 1233 | No | NA | 0 | 0 | Not-determined | Genome-fragment | NA | 0 |
| S11-final-viral-combined_k141_193830\|\|full | 2039 | No | NA | 0 | 3 | Not-determined | Genome-fragment | NA | 0 |
| S11-final-viral-combined_k141_83551\|\|full | 1533 | No | NA | 0 | 1 | Not-determined | Genome-fragment | NA | 0 |
| S11-final-viral-combined_k141_221357\|\|full | 1644 | No | NA | 0 | 1 | Not-determined | Genome-fragment | NA | 0 |
| S11-final-viral-combined_k141_166477\|\|full | 5569 | No | NA | 0 | 3 | Not-determined | Genome-fragment | NA | 0 |
| S11-final-viral-combined_k141_14636\|\|full | 35938 | No | NA | 20 | 0 | High-quality | High-quality | 92.99 | 0 |
| S11-final-viral-combined_k141_304103\|\|full | 2158 | No | NA | 0 | 1 | Not-determined | Genome-fragment | NA | 0 |
| S2-final-viral-combined_k141_214961\|\|full | 1081 | No | NA | 1 | 0 | Low-quality | Genome-fragment | 2.54 | 0 |
| S4-final-viral-combined_k141_96024\|\|full | 1081 | No | NA | 0 | 0 | Low-quality | Genome-fragment | 0.79 | 0 |
| S6-final-viral-combined_k141_44715\|\|full | 1081 | No | NA | 1 | 0 | Low-quality | Genome-fragment | 2.25 | 0 |
| S11-final-viral-combined_k141_290135\|\|full | 2427 | No | NA | 0 | 1 | Not-determined | Genome-fragment | NA | 0 |
| S6-final-viral-combined_k141_69940\|\|full | 1081 | No | NA | 1 | 0 | Low-quality | Genome-fragment | 0.59 | 0 |
| S6-final-viral-combined_k141_137290\|\|lt2gene | 1081 | No | NA | 1 | 0 | Low-quality | Genome-fragment | 2.62 | 0 |
| S9-final-viral-combined_k141_121678\|\|full | 1082 | No | NA | 0 | 0 | Low-quality | Genome-fragment | 2.82 | 0 |
| S11-final-viral-combined_k141_83571\|\|full | 40621 | Yes | 32207 | 12 | 6 | Medium-quality | Genome-fragment | 82.38 | 20.71 |
| S11-final-viral-combined_k141_14663\|\|full | 1393 | No | NA | 0 | 0 | Not-determined | Genome-fragment | NA | 0 |
| S11-final-viral-combined_k141_166490\|\|full | 1913 | No | NA | 0 | 3 | Not-determined | Genome-fragment | NA | 0 |
| S9-final-viral-combined_k141_89878\|\|full | 1082 | No | NA | 0 | 0 | Low-quality | Genome-fragment | 1.03 | 0 |
| S11-final-viral-combined_k141_125034\|\|full | 2448 | No | NA | 0 | 1 | Not-determined | Genome-fragment | NA | 0 |
| S9-final-viral-combined_k141_64016\|\|full | 1082 | No | NA | 0 | 0 | Low-quality | Genome-fragment | 2.05 | 0 |
| S9_k141_28071 | 1082 | No | NA | 0 | 1 | Low-quality | Genome-fragment | 0.31 | 0 |
| S11-final-viral-combined_k141_83624\|\|full | 4525 | No | NA | 0 | 4 | Not-determined | Genome-fragment | NA | 0 |
| S11-final-viral-combined_k141_189933\|\|lt2gene | 1082 | No | NA | 1 | 0 | Low-quality | Genome-fragment | 0.7 | 0 |
| S11-final-viral-combined_k141_304139\|\|full | 1834 | No | NA | 0 | 2 | Not-determined | Genome-fragment | NA | 0 |
| S1-final-viral-combined_k141_137762\|\|full | 1082 | No | NA | 2 | 0 | Low-quality | Genome-fragment | 2.35 | 0 |
| S1-final-viral-combined_k141_221507\|\|full | 1082 | No | NA | 0 | 0 | Low-quality | Genome-fragment | 0.81 | 0 |
| S11-final-viral-combined_k141_125119\|\|full | 7617 | No | NA | 0 | 5 | Not-determined | Genome-fragment | NA | 0 |
| S11-final-viral-combined_k141_248996\|\|full | 4568 | No | NA | 0 | 3 | Not-determined | Genome-fragment | NA | 0 |
| S11-final-viral-combined_k141_152628\|\|full | 2165 | No | NA | 0 | 0 | Not-determined | Genome-fragment | NA | 0 |
| S11-final-viral-combined_k141_977\|\|full | 3504 | No | NA | 0 | 1 | Not-determined | Genome-fragment | NA | 0 |
| S11-final-viral-combined_k141_276570\|\|full | 17411 | No | NA | 0 | 6 | Not-determined | Genome-fragment | NA | 0 |
| S1-final-viral-combined_k141_106745\|\|full | 1082 | No | NA | 0 | 1 | Low-quality | Genome-fragment | 1.09 | 0 |
| S1_k141_112514 | 1082 | No | NA | 0 | 0 | Low-quality | Genome-fragment | 2.11 | 0 |
| S11-final-viral-combined_k141_83703\|\|full | 1215 | No | NA | 0 | 0 | Not-determined | Genome-fragment | NA | 0 |
| S11-final-viral-combined_k141_28483\|\|full | 6699 | No | NA | 0 | 5 | Not-determined | Genome-fragment | NA | 0 |
| S7_k141_236602 | 1082 | No | NA | 1 | 0 | Low-quality | Genome-fragment | 1.86 | 0 |
| S11-final-viral-combined_k141_166608\|\|full | 1533 | No | NA | 0 | 2 | Not-determined | Genome-fragment | NA | 0 |
| S11-final-viral-combined_k141_207691\|\|full | 2158 | No | NA | 0 | 2 | Not-determined | Genome-fragment | NA | 0 |
| S11-final-viral-combined_k141_180040\|\|full | 1278 | No | NA | 0 | 0 | Not-determined | Genome-fragment | NA | 0 |
| S11-final-viral-combined_k141_304194\|\|full | 3101 | No | NA | 0 | 0 | Not-determined | Genome-fragment | NA | 0 |
| S11-final-viral-combined_k141_152665\|\|full | 2272 | No | NA | 0 | 1 | Not-determined | Genome-fragment | NA | 0 |
| S11-final-viral-combined_k141_152666\|\|full | 4114 | No | NA | 0 | 3 | Not-determined | Genome-fragment | NA | 0 |
| S11-final-viral-combined_k141_14777\|\|full | 3326 | No | NA | 0 | 4 | Not-determined | Genome-fragment | NA | 0 |
| S11-final-viral-combined_k141_83742\|\|full | 1727 | No | NA | 0 | 2 | Not-determined | Genome-fragment | NA | 0 |
| S11-final-viral-combined_k141_97609\|\|full | 2449 | No | NA | 0 | 2 | Not-determined | Genome-fragment | NA | 0 |
| S11-final-viral-combined_k141_180077\|\|full | 2558 | No | NA | 0 | 1 | Not-determined | Genome-fragment | NA | 0 |
| S17-final-viral-combined_k141_178340\|\|full | 1082 | No | NA | 2 | 0 | Low-quality | Genome-fragment | 2.84 | 0 |
| S17-final-viral-combined_k141_179667\|\|full | 1082 | No | NA | 1 | 0 | Low-quality | Genome-fragment | 2.54 | 0 |
| S12-final-viral-combined_k141_116436\|\|full | 1082 | No | NA | 0 | 0 | Low-quality | Genome-fragment | 2.07 | 0 |
| S11-final-viral-combined_k141_221462\|\|full | 3657 | No | NA | 0 | 0 | Not-determined | Genome-fragment | NA | 0 |
| S11-final-viral-combined_k141_290235\|\|full | 3874 | No | NA | 0 | 3 | Not-determined | Genome-fragment | NA | 0 |
| S12-final-viral-combined_k141_285014\|\|lt2gene | 1082 | No | NA | 1 | 0 | Low-quality | Genome-fragment | 3.04 | 0 |
| S2-final-viral-combined_k141_168749\|\|full | 1082 | No | NA | 0 | 0 | Low-quality | Genome-fragment | 2.39 | 0 |
| S8-final-viral-combined_k141_485\|\|full | 1082 | No | NA | 0 | 0 | Low-quality | Genome-fragment | 1.75 | 0 |
| S8-final-viral-combined_k141_228090\|\|full | 1082 | No | NA | 1 | 0 | Low-quality | Genome-fragment | 2.73 | 0 |
| S11-final-viral-combined_k141_180807\|\|full | 1083 | No | NA | 0 | 2 | Low-quality | Genome-fragment | 0.54 | 0 |
| S11-final-viral-combined_k141_42215\|\|full | 2113 | No | NA | 0 | 3 | Not-determined | Genome-fragment | NA | 0 |
| S11-final-viral-combined_k141_276638\|\|full | 1891 | No | NA | 0 | 2 | Not-determined | Genome-fragment | NA | 0 |
| S11-final-viral-combined_k141_167741\|\|full | 1083 | No | NA | 0 | 2 | Low-quality | Genome-fragment | 0.4 | 0 |
| S3-final-viral-combined_k141_63671\|\|full | 1083 | No | NA | 1 | 0 | Low-quality | Genome-fragment | 1.34 | 0 |
| S11-final-viral-combined_k141_125176\|\|full | 67341 | Yes | 22887 | 3 | 25 | Medium-quality | Genome-fragment | 56.45 | 66.01 |
| S11-final-viral-combined_k141_304239\|\|full | 44706 | No | NA | 17 | 0 | High-quality | High-quality | 99.61 | 0 |
| S11-final-viral-combined_k141_83805\|\|full | 1108 | No | NA | 0 | 2 | Not-determined | Genome-fragment | NA | 0 |
| S11-final-viral-combined_k141_56123\|\|full | 1493 | No | NA | 0 | 1 | Not-determined | Genome-fragment | NA | 0 |
| S11-final-viral-combined_k141_152773\|\|full | 4344 | No | NA | 0 | 2 | Not-determined | Genome-fragment | NA | 0 |
| S11-final-viral-combined_k141_290278\|\|full | 1406 | No | NA | 0 | 1 | Not-determined | Genome-fragment | NA | 0 |
| S3_k141_34196 | 1083 | No | NA | 0 | 2 | Low-quality | Genome-fragment | 0.67 | 0 |
| S5-final-viral-combined_k141_153436\|\|full | 1083 | No | NA | 1 | 0 | Low-quality | Genome-fragment | 2.46 | 0 |
| S11-final-viral-combined_k141_304274\|\|full | 4945 | No | NA | 0 | 1 | Not-determined | Genome-fragment | NA | 0 |
| S5_k141_198233 | 1083 | No | NA | 0 | 0 | Low-quality | Genome-fragment | 3.02 | 0 |
| S11-final-viral-combined_k141_207831\|\|full | 2553 | No | NA | 0 | 1 | Not-determined | Genome-fragment | NA | 0 |
| S11-final-viral-combined_k141_249131\|\|full | 1946 | No | NA | 0 | 1 | Not-determined | Genome-fragment | NA | 0 |
| S11-final-viral-combined_k141_193956\|\|full | 3898 | No | NA | 0 | 1 | Not-determined | Genome-fragment | NA | 0 |
| S11-final-viral-combined_k141_235594\|\|full | 4229 | No | NA | 0 | 2 | Not-determined | Genome-fragment | NA | 0 |
| S11-final-viral-combined_k141_152802\|\|full | 1326 | No | NA | 0 | 1 | Not-determined | Genome-fragment | NA | 0 |
| S11-final-viral-combined_k141_14892\|\|full | 1933 | No | NA | 0 | 2 | Not-determined | Genome-fragment | NA | 0 |
| S11-final-viral-combined_k141_221578\|\|full | 1748 | No | NA | 0 | 1 | Not-determined | Genome-fragment | NA | 0 |
| S7-final-viral-combined_k141_34868\|\|full | 1083 | No | NA | 3 | 0 | Low-quality | Genome-fragment | 2.31 | 0 |
| S11-final-viral-combined_k141_28535\|\|full | 1398 | No | NA | 0 | 0 | Not-determined | Genome-fragment | NA | 0 |
| S11-final-viral-combined_k141_193989\|\|full | 10709 | No | NA | 0 | 7 | Not-determined | Genome-fragment | NA | 0 |
| S11-final-viral-combined_k141_14951\|\|full | 1625 | No | NA | 0 | 0 | Not-determined | Genome-fragment | NA | 0 |
| S7_k141_44748 | 1083 | No | NA | 0 | 0 | Low-quality | Genome-fragment | 1.34 | 0 |
| S7_k141_147372 | 1083 | No | NA | 0 | 0 | Low-quality | Genome-fragment | 2.53 | 0 |
| S7_k141_40399 | 1083 | No | NA | 1 | 0 | Low-quality | Genome-fragment | 2.14 | 0 |
| S11-final-viral-combined_k141_28579\|\|full | 1687 | No | NA | 0 | 1 | Not-determined | Genome-fragment | NA | 0 |
| S17-k141_89315 | 1083 | No | NA | 0 | 0 | Low-quality | Genome-fragment | 1.21 | 0 |
| S17-k141_86918 | 1083 | No | NA | 0 | 1 | Low-quality | Genome-fragment | 0.58 | 0 |
| S11-final-viral-combined_k141_262998\|\|full | 1937 | No | NA | 0 | 1 | Not-determined | Genome-fragment | NA | 0 |
| S17-k141_21943 | 1083 | No | NA | 0 | 1 | Low-quality | Genome-fragment | 0.6 | 0 |
| S12-final-viral-combined_k141_268303\|\|full | 1083 | No | NA | 0 | 2 | Low-quality | Genome-fragment | 1 | 0 |
| S2-final-viral-combined_k141_198037\|\|full | 1083 | No | NA | 0 | 1 | Low-quality | Genome-fragment | 0.51 | 0 |
| S11-final-viral-combined_k141_235715\|\|full | 3285 | No | NA | 0 | 4 | Not-determined | Genome-fragment | NA | 0 |
| S11-final-viral-combined_k141_138897\|\|full | 1390 | No | NA | 0 | 1 | Not-determined | Genome-fragment | NA | 0 |
| S11-final-viral-combined_k141_56201\|\|full | 1662 | No | NA | 0 | 0 | Not-determined | Genome-fragment | NA | 0 |
| S6-final-viral-combined_k141_64187\|\|lt2gene | 1083 | No | NA | 2 | 0 | Low-quality | Genome-fragment | 2.45 | 0 |
| S9_k141_69999 | 1084 | No | NA | 0 | 0 | Low-quality | Genome-fragment | 2.08 | 0 |
| S11-final-viral-combined_k141_42398\|\|full | 2063 | No | NA | 0 | 0 | Not-determined | Genome-fragment | NA | 0 |
| S11-final-viral-combined_k141_284655\|\|full | 1084 | No | NA | 0 | 0 | Low-quality | Genome-fragment | 1.3 | 0 |
| S11-final-viral-combined_k141_249267\|\|full | 4115 | No | NA | 0 | 3 | Not-determined | Genome-fragment | NA | 0 |
| S11-final-viral-combined_k141_304378\|\|full | 4477 | No | NA | 0 | 3 | Not-determined | Genome-fragment | NA | 0 |
| S11-final-viral-combined_k141_44971\|\|lt2gene | 1084 | No | NA | 2 | 0 | Low-quality | Genome-fragment | 1.89 | 0 |
| S11-final-viral-combined_k141_125333\|\|full | 1107 | No | NA | 0 | 2 | Not-determined | Genome-fragment | NA | 0 |
| S11-final-viral-combined_k141_208008\|\|full | 2441 | No | NA | 0 | 3 | Not-determined | Genome-fragment | NA | 0 |
| S11-final-viral-combined_k141_276868\|\|full | 1085 | No | NA | 0 | 2 | Not-determined | Genome-fragment | NA | 0 |
| S11-final-viral-combined_k141_180286\|\|full | 1851 | No | NA | 0 | 1 | Not-determined | Genome-fragment | NA | 0 |
| S11-final-viral-combined_k141_208028\|\|full | 2676 | No | NA | 0 | 3 | Not-determined | Genome-fragment | NA | 0 |
| S1-final-viral-combined_k141_180381\|\|full | 1084 | No | NA | 0 | 2 | Low-quality | Genome-fragment | 0.36 | 0 |
| S5-final-viral-combined_k141_130777\|\|lt2gene | 1084 | No | NA | 2 | 0 | Low-quality | Genome-fragment | 1.51 | 0 |
| S11-final-viral-combined_k141_304450\|\|full | 4346 | No | NA | 0 | 2 | Not-determined | Genome-fragment | NA | 0 |
| S5-final-viral-combined_k141_177341\|\|lt2gene | 1084 | No | NA | 2 | 0 | Low-quality | Genome-fragment | 1.34 | 0 |
| S11-final-viral-combined_k141_290474\|\|full | 2726 | No | NA | 0 | 2 | Not-determined | Genome-fragment | NA | 0 |
| S7-final-viral-combined_k141_131833\|\|full | 1084 | No | NA | 0 | 0 | Low-quality | Genome-fragment | 2.16 | 0 |
| S12_k141_271346 | 1084 | No | NA | 0 | 1 | Low-quality | Genome-fragment | 1.08 | 0 |
| S12_k141_325839 | 1084 | No | NA | 0 | 0 | Low-quality | Genome-fragment | 1.65 | 0 |
| S2-final-viral-combined_k141_197679\|\|full | 1084 | No | NA | 1 | 0 | Low-quality | Genome-fragment | 24.52 | 0 |
| S6-final-viral-combined_k141_120952\|\|full | 1084 | No | NA | 2 | 0 | Low-quality | Genome-fragment | 1.01 | 0 |
| S8_k141_262734 | 1084 | No | NA | 0 | 0 | Low-quality | Genome-fragment | 0.81 | 0 |
| S9-final-viral-combined_k141_18395\|\|full | 1085 | No | NA | 3 | 0 | Low-quality | Genome-fragment | 2.44 | 0 |
| S11-final-viral-combined_k141_111363\|\|full | 4188 | No | NA | 0 | 1 | Not-determined | Genome-fragment | NA | 0 |
| S11-final-viral-combined_k141_290506\|\|full | 1120 | No | NA | 0 | 0 | Not-determined | Genome-fragment | NA | 0 |
| S9_k141_49467 | 1085 | No | NA | 0 | 0 | Low-quality | Genome-fragment | 2.18 | 0 |
| S1-final-viral-combined_k141_136303\|\|full | 1085 | No | NA | 0 | 1 | Low-quality | Genome-fragment | 1.74 | 0 |
| S11-final-viral-combined_k141_28648\|\|full | 2878 | No | NA | 0 | 1 | Not-determined | Genome-fragment | NA | 0 |
| S1_k141_286825 | 1085 | No | NA | 0 | 1 | Low-quality | Genome-fragment | 0.68 | 0 |
| S1_k141_207196 | 1085 | No | NA | 0 | 0 | Low-quality | Genome-fragment | 1.01 | 0 |
| S11-final-viral-combined_k141_125424\|\|full | 2776 | No | NA | 0 | 1 | Not-determined | Genome-fragment | NA | 0 |
| S11-final-viral-combined_k141_70034\|\|full | 1178 | No | NA | 0 | 1 | Not-determined | Genome-fragment | NA | 0 |
| S11-final-viral-combined_k141_42544\|\|full | 1389 | No | NA | 0 | 1 | Not-determined | Genome-fragment | NA | 0 |
| S11-final-viral-combined_k141_221651\|\|full | 1919 | No | NA | 0 | 2 | Not-determined | Genome-fragment | NA | 0 |
| S11-final-viral-combined_k141_125431\|\|full | 1526 | No | NA | 0 | 2 | Not-determined | Genome-fragment | NA | 0 |

| | | | | | | | | | |
|---|---|---|---|---|---|---|---|---|---|
| S3-final-viral-combined_k141_3009\|\|full | 1085 | No | NA | 0 | 0 | Low-quality | Genome-fragment | 0.49 | 0 |
| S11-final-viral-combined_k141_304521\|\|full | 1473 | No | NA | 0 | 1 | Not-determined | Genome-fragment | NA | 0 |
| S17-final-viral-combined_k141_248136\|\|full | 1085 | No | NA | 1 | 0 | Low-quality | Genome-fragment | 2.78 | 0 |
| S12-final-viral-combined_k141_311471\|\|full | 1085 | No | NA | 2 | 0 | Low-quality | Genome-fragment | 2.95 | 0 |
| S12-final-viral-combined_k141_95213\|\|full | 1085 | No | NA | 2 | 0 | Low-quality | Genome-fragment | 2.3 | 0 |
| S11-final-viral-combined_k141_98028\|\|full | 1612 | No | NA | 0 | 0 | Not-determined | Genome-fragment | NA | 0 |
| S11-final-viral-combined_k141_98034\|\|full | 2475 | No | NA | 0 | 2 | Not-determined | Genome-fragment | NA | 0 |
| S11-final-viral-combined_k141_208056\|\|full | 3203 | No | NA | 0 | 0 | Not-determined | Genome-fragment | NA | 0 |
| S11-final-viral-combined_k141_98039\|\|full | 2192 | No | NA | 0 | 0 | Not-determined | Genome-fragment | NA | 0 |
| S12-final-viral-combined_k141_113943\|\|full | 1085 | No | NA | 0 | 0 | Low-quality | Genome-fragment | 1.89 | 0 |
| S2-final-viral-combined_k141_233930\|\|full | 1085 | No | NA | 0 | 0 | Low-quality | Genome-fragment | 2.27 | 0 |
| S11-final-viral-combined_k141_15104\|\|full | 1995 | No | NA | 0 | 0 | Not-determined | Genome-fragment | NA | 0 |
| S11-final-viral-combined_k141_180367\|\|full | 2786 | No | NA | 0 | 0 | Not-determined | Genome-fragment | NA | 0 |
| S11-final-viral-combined_k141_263208\|\|full | 3620 | No | NA | 0 | 2 | Not-determined | Genome-fragment | NA | 0 |
| S2-final-viral-combined_k141_202799\|\|full | 1085 | No | NA | 1 | 0 | Low-quality | Genome-fragment | 3.04 | 0 |
| S2_k141_31574 | 1085 | No | NA | 0 | 0 | Low-quality | Genome-fragment | 0.1 | 0 |
| S11-final-viral-combined_k141_318116\|\|full | 2577 | No | NA | 0 | 0 | Not-determined | Genome-fragment | NA | 0 |
| S8-final-viral-combined_k141_147097\|\|full | 1085 | No | NA | 0 | 1 | Low-quality | Genome-fragment | 1.61 | 0 |
| S9-final-viral-combined_k141_114357\|\|full | 1086 | No | NA | 0 | 1 | Low-quality | Genome-fragment | 0.86 | 0 |
| S1-final-viral-combined_k141_283497\|\|full | 1086 | No | NA | 2 | 0 | Low-quality | Genome-fragment | 3.28 | 0 |
| S1_k141_68546 | 1086 | No | NA | 0 | 1 | Low-quality | Genome-fragment | 2.16 | 0 |
| S1_k141_344699 | 1086 | No | NA | 0 | 1 | Low-quality | Genome-fragment | 0.73 | 0 |
| S7-final-viral-combined_k141_216341\|\|full | 1086 | No | NA | 0 | 1 | Low-quality | Genome-fragment | 1.82 | 0 |
| S2-final-viral-combined_k141_175796\|\|full | 1086 | No | NA | 2 | 0 | Low-quality | Genome-fragment | 1.55 | 0 |
| S11-final-viral-combined_k141_194178\|\|full | 2474 | No | NA | 0 | 1 | Not-determined | Genome-fragment | NA | 0 |
| S11-final-viral-combined_k141_194179\|\|full | 1721 | No | NA | 0 | 0 | Not-determined | Genome-fragment | NA | 0 |
| S2_k141_189078 | 1086 | No | NA | 0 | 0 | Low-quality | Genome-fragment | 0.7 | 0 |
| S11-final-viral-combined_k141_70147\|\|full | 1798 | No | NA | 0 | 0 | Not-determined | Genome-fragment | NA | 0 |
| S4-final-viral-combined_k141_149035\|\|lt2gene | 1086 | No | NA | 1 | 0 | Low-quality | Genome-fragment | 2.19 | 0 |
| S8_k141_221822 | 1086 | No | NA | 0 | 1 | Low-quality | Genome-fragment | 0.18 | 0 |
| S11-final-viral-combined_k141_1327\|\|full | 11792 | No | NA | 0 | 6 | Not-determined | Genome-fragment | NA | 0 |
| S11-final-viral-combined_k141_180409\|\|full | 3918 | No | NA | 0 | 3 | Not-determined | Genome-fragment | NA | 0 |
| S8_k141_264579 | 1086 | No | NA | 0 | 0 | Low-quality | Genome-fragment | 2.4 | 0 |
| S9-final-viral-combined_k141_32382\|\|full | 1087 | No | NA | 0 | 1 | Low-quality | Genome-fragment | 2.3 | 0 |
| S11-final-viral-combined_k141_84094\|\|full | 1791 | No | NA | 0 | 1 | Not-determined | Genome-fragment | NA | 0 |
| S9-final-viral-combined_k141_102275\|\|full | 1087 | No | NA | 0 | 0 | Low-quality | Genome-fragment | 1.42 | 0 |
| S11-final-viral-combined_k141_135080\|\|full | 1087 | No | NA | 0 | 1 | Low-quality | Genome-fragment | 0.85 | 0 |
| S11-final-viral-combined_k141_98124\|\|full | 1543 | No | NA | 0 | 2 | Not-determined | Genome-fragment | NA | 0 |
| S11-final-viral-combined_k141_235970\|\|full | 1630 | No | NA | 0 | 0 | Not-determined | Genome-fragment | NA | 0 |
| S11-final-viral-combined_k141_138983\|\|full | 9845 | No | NA | 0 | 5 | Not-determined | Genome-fragment | NA | 0 |
| S11-final-viral-combined_k141_191990\|\|full | 1087 | Yes | 883 | 1 | 1 | Low-quality | Genome-fragment | 1.34 | 18.77 |
| S11-final-viral-combined_k141_138987\|\|full | 7709 | No | NA | 0 | 9 | Not-determined | Genome-fragment | NA | 0 |
| S1-final-viral-combined_k141_105608\|\|full | 1087 | No | NA | 0 | 0 | Low-quality | Genome-fragment | 1.85 | 0 |
| S11-final-viral-combined_k141_194205\|\|full | 35517 | No | NA | 20 | 1 | Medium-quality | Genome-fragment | 57.83 | 0 |
| S11-final-viral-combined_k141_318179\|\|full | 1099 | No | NA | 0 | 1 | Not-determined | Genome-fragment | NA | 0 |
| S3-final-viral-combined_k141_51927\|\|full | 1087 | No | NA | 2 | 0 | Low-quality | Genome-fragment | 2.18 | 0 |
| S11-final-viral-combined_k141_180451\|\|full | 2010 | No | NA | 0 | 2 | Not-determined | Genome-fragment | NA | 0 |
| S11-final-viral-combined_k141_318209\|\|full | 3508 | No | NA | 0 | 2 | Not-determined | Genome-fragment | NA | 0 |
| S11-final-viral-combined_k141_180462\|\|full | 1056 | No | NA | 0 | 0 | Not-determined | Genome-fragment | NA | 0 |
| S11-final-viral-combined_k141_304728\|\|full | 1610 | No | NA | 0 | 1 | Not-determined | Genome-fragment | NA | 0 |
| S11-final-viral-combined_k141_290674\|\|full | 4647 | No | NA | 0 | 3 | Not-determined | Genome-fragment | NA | 0 |
| S7-final-viral-combined_k141_47377\|\|lt2gene | 1087 | No | NA | 1 | 0 | Low-quality | Genome-fragment | 1.02 | 0 |
| S11-final-viral-combined_k141_277120\|\|full | 1646 | No | NA | 0 | 1 | Not-determined | Genome-fragment | NA | 0 |
| S17-final-viral-combined_k141_126659\|\|full | 1087 | No | NA | 1 | 0 | Low-quality | Genome-fragment | 2.53 | 0 |
| S11-final-viral-combined_k141_111441\|\|full | 2336 | No | NA | 0 | 2 | Not-determined | Genome-fragment | NA | 0 |
| S12-final-viral-combined_k141_225744\|\|full | 1087 | No | NA | 0 | 0 | Low-quality | Genome-fragment | 2.32 | 0 |
| S11-final-viral-combined_k141_304737\|\|full | 13474 | No | NA | 0 | 6 | Not-determined | Genome-fragment | NA | 0 |
| S12_k141_188795 | 1087 | No | NA | 0 | 0 | Low-quality | Genome-fragment | 0.69 | 0 |
| S11-final-viral-combined_k141_318242\|\|full | 5155 | No | NA | 0 | 3 | Not-determined | Genome-fragment | NA | 0 |
| S11-final-viral-combined_k141_167054\|\|full | 2831 | No | NA | 0 | 1 | Not-determined | Genome-fragment | NA | 0 |
| S11-final-viral-combined_k141_277171\|\|full | 3497 | No | NA | 0 | 3 | Not-determined | Genome-fragment | NA | 0 |
| S2-final-viral-combined_k141_114733\|\|full | 1087 | No | NA | 0 | 0 | Low-quality | Genome-fragment | 2.55 | 0 |
| S11-final-viral-combined_k141_318255\|\|full | 6149 | No | NA | 0 | 2 | Not-determined | Genome-fragment | NA | 0 |
| S2_k141_176191 | 1087 | No | NA | 0 | 0 | Low-quality | Genome-fragment | 1.68 | 0 |
| S6-final-viral-combined_k141_31686\|\|full | 1087 | No | NA | 0 | 1 | Low-quality | Genome-fragment | 0.77 | 0 |
| S8-final-viral-combined_k141_108470\|\|full | 1087 | No | NA | 0 | 0 | Low-quality | Genome-fragment | 3.26 | 0 |
| S8_k141_217131 | 1087 | No | NA | 0 | 0 | Low-quality | Genome-fragment | 2.28 | 0 |
| S11-final-viral-combined_k141_111189\|\|full | 1088 | No | NA | 0 | 0 | Low-quality | Genome-fragment | 2.5 | 0 |
| S11-final-viral-combined_k141_290741\|\|full | 3672 | No | NA | 0 | 4 | Not-determined | Genome-fragment | NA | 0 |
| S11-final-viral-combined_k141_249558\|\|full | 2159 | No | NA | 0 | 2 | Not-determined | Genome-fragment | NA | 0 |
| S11-final-viral-combined_k141_159708\|\|lt2gene | 1088 | No | NA | 1 | 0 | Low-quality | Genome-fragment | 2.87 | 0 |
| S11_k141_148037 | 1088 | No | NA | 0 | 1 | Low-quality | Genome-fragment | 1.03 | 0 |
| S11-final-viral-combined_k141_221958\|\|full | 5904 | No | NA | 0 | 4 | Not-determined | Genome-fragment | NA | 0 |
| S1-final-viral-combined_k141_47855\|\|full | 1088 | No | NA | 1 | 0 | Low-quality | Genome-fragment | 2.01 | 0 |
| S11-final-viral-combined_k141_208250\|\|full | 7473 | No | NA | 0 | 1 | Not-determined | Genome-fragment | NA | 0 |
| S1-final-viral-combined_k141_182328\|\|lt2gene | 1088 | No | NA | 1 | 0 | Low-quality | Genome-fragment | 1.76 | 0 |
| S1_k141_332685 | 1088 | No | NA | 0 | 0 | Low-quality | Genome-fragment | 1.79 | 0 |
| S11-final-viral-combined_k141_249597\|\|full | 1223 | No | NA | 0 | 0 | Not-determined | Genome-fragment | NA | 0 |
| S11-final-viral-combined_k141_304791\|\|full | 1029 | No | NA | 0 | 1 | Not-determined | Genome-fragment | NA | 0 |
| S3_k141_58021 | 1088 | No | NA | 0 | 0 | Low-quality | Genome-fragment | 3.14 | 0 |
| S11-final-viral-combined_k141_42825\|\|full | 1129 | No | NA | 0 | 0 | Not-determined | Genome-fragment | NA | 0 |
| S11-final-viral-combined_k141_249624\|\|full | 1192 | No | NA | 0 | 1 | Not-determined | Genome-fragment | NA | 0 |
| S11-final-viral-combined_k141_1461\|\|full | 2336 | No | NA | 0 | 0 | Not-determined | Genome-fragment | NA | 0 |
| S11-final-viral-combined_k141_167194\|\|full | 1582 | No | NA | 0 | 0 | Not-determined | Genome-fragment | NA | 0 |
| S11-final-viral-combined_k141_111503\|\|full | 5123 | No | NA | 0 | 5 | Not-determined | Genome-fragment | NA | 0 |
| S11-final-viral-combined_k141_290825\|\|full | 2823 | No | NA | 0 | 2 | Not-determined | Genome-fragment | NA | 0 |
| S11-final-viral-combined_k141_318352\|\|full | 1465 | No | NA | 0 | 1 | Not-determined | Genome-fragment | NA | 0 |
| S11-final-viral-combined_k141_236146\|\|full | 2472 | No | NA | 0 | 0 | Not-determined | Genome-fragment | NA | 0 |
| S11-final-viral-combined_k141_236165\|\|full | 2736 | No | NA | 0 | 2 | Not-determined | Genome-fragment | NA | 0 |
| S11-final-viral-combined_k141_125755\|\|full | 5174 | No | NA | 0 | 4 | Not-determined | Genome-fragment | NA | 0 |
| S11-final-viral-combined_k141_180631\|\|full | 1580 | No | NA | 0 | 0 | Not-determined | Genome-fragment | NA | 0 |
| S11-final-viral-combined_k141_318374\|\|full | 1204 | No | NA | 0 | 0 | Not-determined | Genome-fragment | NA | 0 |
| S11-final-viral-combined_k141_208315\|\|full | 8699 | No | NA | 0 | 3 | Not-determined | Genome-fragment | NA | 0 |
| S11-final-viral-combined_k141_290886\|\|full | 1082 | No | NA | 0 | 1 | Not-determined | Genome-fragment | NA | 0 |
| S11-final-viral-combined_k141_29048\|\|full | 1418 | No | NA | 0 | 1 | Not-determined | Genome-fragment | NA | 0 |
| S11-final-viral-combined_k141_180646\|\|full | 4824 | No | NA | 0 | 2 | Not-determined | Genome-fragment | NA | 0 |
| S3_k141_54716 | 1088 | No | NA | 0 | 0 | Low-quality | Genome-fragment | 2.65 | 0 |
| S11-final-viral-combined_k141_222067\|\|full | 7653 | No | NA | 0 | 4 | Not-determined | Genome-fragment | NA | 0 |
| S11-final-viral-combined_k141_98321\|\|full | 1071 | No | NA | 0 | 3 | Not-determined | Genome-fragment | NA | 0 |
| S7-final-viral-combined_k141_113827\|\|full | 1088 | No | NA | 0 | 0 | Low-quality | Genome-fragment | 1.48 | 0 |
| S11-final-viral-combined_k141_194482\|\|full | 2147 | No | NA | 0 | 1 | Not-determined | Genome-fragment | NA | 0 |
| S11-final-viral-combined_k141_277321\|\|full | 7007 | No | NA | 0 | 0 | Not-determined | Genome-fragment | NA | 0 |
| S11-final-viral-combined_k141_277325\|\|full | 2426 | No | NA | 0 | 1 | Not-determined | Genome-fragment | NA | 0 |
| S11-final-viral-combined_k141_1594\|\|full | 7166 | No | NA | 0 | 3 | Not-determined | Genome-fragment | NA | 0 |
| S11-final-viral-combined_k141_84326\|\|full | 23591 | No | NA | 16 | 0 | Medium-quality | Genome-fragment | 53.16 | 0 |
| S11-final-viral-combined_k141_263485\|\|full | 3947 | No | NA | 0 | 1 | Not-determined | Genome-fragment | NA | 0 |
| S11-final-viral-combined_k141_139196\|\|full | 2052 | No | NA | 0 | 2 | Not-determined | Genome-fragment | NA | 0 |
| S7-final-viral-combined_k141_218744\|\|full | 1088 | No | NA | 0 | 0 | Low-quality | Genome-fragment | 2.55 | 0 |
| S11-final-viral-combined_k141_290937\|\|full | 1379 | No | NA | 0 | 0 | Not-determined | Genome-fragment | NA | 0 |
| S11-final-viral-combined_k141_304863\|\|full | 1960 | No | NA | 0 | 0 | Not-determined | Genome-fragment | NA | 0 |
| S11-final-viral-combined_k141_180685\|\|full | 9701 | No | NA | 0 | 6 | Not-determined | Genome-fragment | NA | 0 |
| S11-final-viral-combined_k141_15480\|\|full | 1784 | No | NA | 0 | 1 | Not-determined | Genome-fragment | NA | 0 |
| S11-final-viral-combined_k141_194525\|\|full | 2249 | No | NA | 0 | 0 | Not-determined | Genome-fragment | NA | 0 |
| S11-final-viral-combined_k141_84363\|\|full | 1336 | No | NA | 0 | 2 | Not-determined | Genome-fragment | NA | 0 |
| S11-final-viral-combined_k141_208394\|\|full | 2383 | No | NA | 0 | 0 | Not-determined | Genome-fragment | NA | 0 |
| S11-final-viral-combined_k141_263516\|\|full | 1363 | No | NA | 0 | 0 | Not-determined | Genome-fragment | NA | 0 |
| S11-final-viral-combined_k141_84378\|\|full | 5350 | No | NA | 0 | 3 | Not-determined | Genome-fragment | NA | 0 |
| S11-final-viral-combined_k141_290980\|\|full | 3348 | No | NA | 0 | 1 | Not-determined | Genome-fragment | NA | 0 |
| S7-final-viral-combined_k141_118922\|\|full | 1088 | No | NA | 1 | 0 | Low-quality | Genome-fragment | 2.46 | 0 |
| S11-final-viral-combined_k141_222198\|\|full | 4449 | No | NA | 0 | 0 | Not-determined | Genome-fragment | NA | 0 |
| S7-final-viral-combined_k141_149122\|\|lt2gene | 1088 | No | NA | 1 | 0 | Low-quality | Genome-fragment | 0.97 | 0 |
| S17-final-viral-combined_k141_27848\|\|full | 1088 | No | NA | 0 | 0 | Low-quality | Genome-fragment | 2.68 | 0 |
| S12-final-viral-combined_k141_252526\|\|full | 1088 | No | NA | 0 | 0 | Low-quality | Genome-fragment | 1.63 | 0 |
| S12-final-viral-combined_k141_262263\|\|full | 1088 | No | NA | 0 | 0 | Low-quality | Genome-fragment | 1.99 | 0 |
| S11-final-viral-combined_k141_236214\|\|full | 93358 | Yes | 32851 | 22 | 50 | Medium-quality | Genome-fragment | 54.08 | 64.81 |
| S11-final-viral-combined_k141_291042\|\|full | 4908 | No | NA | 0 | 2 | Not-determined | Genome-fragment | NA | 0 |
| S12-final-viral-combined_k141_63862\|\|full | 1088 | No | NA | 1 | 0 | Low-quality | Genome-fragment | 1.77 | 0 |
| S12-final-viral-combined_k141_204828\|\|lt2gene | 1088 | No | NA | 1 | 0 | Low-quality | Genome-fragment | 1.99 | 0 |
| S2-final-viral-combined_k141_16642\|\|full | 1088 | No | NA | 0 | 1 | Low-quality | Genome-fragment | 1.18 | 0 |
| S11-final-viral-combined_k141_15586\|\|full | 3064 | No | NA | 0 | 2 | Not-determined | Genome-fragment | NA | 0 |
| S11-final-viral-combined_k141_84433\|\|full | 1136 | No | NA | 0 | 1 | Not-determined | Genome-fragment | NA | 0 |
| S2-final-viral-combined_k141_62857\|\|lt2gene | 1088 | No | NA | 1 | 0 | Low-quality | Genome-fragment | 1.42 | 0 |
| S11-final-viral-combined_k141_42928\|\|full | 2884 | No | NA | 0 | 0 | Not-determined | Genome-fragment | NA | 0 |
| S4-final-viral-combined_k141_107661\|\|full | 1088 | No | NA | 0 | 1 | Low-quality | Genome-fragment | 0.93 | 0 |
| S8-final-viral-combined_k141_83045\|\|full | 1088 | No | NA | 2 | 0 | Low-quality | Genome-fragment | 2.66 | 0 |
| S9-final-viral-combined_k141_96349\|\|full | 1089 | No | NA | 2 | 0 | Low-quality | Genome-fragment | 3.02 | 0 |
| S9-final-viral-combined_k141_118173\|\|lt2gene | 1089 | No | NA | 1 | 0 | Low-quality | Genome-fragment | 2.21 | 0 |
| S11-final-viral-combined_k141_227860\|\|full | 1089 | No | NA | 0 | 0 | Low-quality | Genome-fragment | 1.27 | 0 |
| S11_k141_166508 | 1089 | No | NA | 0 | 2 | Low-quality | Genome-fragment | 2.84 | 0 |
| S1-final-viral-combined_k141_57600\|\|full | 1089 | No | NA | 0 | 1 | Low-quality | Genome-fragment | 0.84 | 0 |
| S11-final-viral-combined_k141_236254\|\|full | 5795 | No | NA | 0 | 0 | Not-determined | Genome-fragment | NA | 0 |
| S11-final-viral-combined_k141_70349\|\|full | 1364 | No | NA | 0 | 2 | Not-determined | Genome-fragment | NA | 0 |
| S11-final-viral-combined_k141_139335\|\|full | 3106 | No | NA | 0 | 3 | Not-determined | Genome-fragment | NA | 0 |
| S11-final-viral-combined_k141_1702\|\|full | 1755 | No | NA | 0 | 0 | Not-determined | Genome-fragment | NA | 0 |
| S11-final-viral-combined_k141_167421\|\|full | 1227 | No | NA | 0 | 0 | Not-determined | Genome-fragment | NA | 0 |
| S7-final-viral-combined_k141_127004\|\|full | 1089 | No | NA | 0 | 0 | Low-quality | Genome-fragment | 1.87 | 0 |
| S7_k141_153329 | 1089 | No | NA | 0 | 1 | Low-quality | Genome-fragment | 2.78 | 0 |
| S17-final-viral-combined_k141_190984\|\|full | 1089 | No | NA | 0 | 0 | Low-quality | Genome-fragment | 1.09 | 0 |
| S17-final-viral-combined_k141_224844\|\|full | 1089 | No | NA | 0 | 1 | Low-quality | Genome-fragment | 0.95 | 0 |
| S11-final-viral-combined_k141_304985\|\|full | 20916 | No | NA | 0 | 12 | Not-determined | Genome-fragment | NA | 0 |
| S17-k141_102882 | 1089 | No | NA | 1 | 0 | Low-quality | Genome-fragment | 1.47 | 0 |
| S11-final-viral-combined_k141_222289\|\|full | 3755 | No | NA | 0 | 4 | Not-determined | Genome-fragment | NA | 0 |
| S4-final-viral-combined_k141_66946\|\|lt2gene | 1089 | No | NA | 0 | 0 | Low-quality | Genome-fragment | 2.44 | 0 |
| S11-final-viral-combined_k141_167448\|\|full | 2472 | No | NA | 0 | 2 | Not-determined | Genome-fragment | NA | 0 |
| S4_k141_140390 | 1089 | No | NA | 0 | 0 | Low-quality | Genome-fragment | 2.76 | 0 |
| S11-final-viral-combined_k141_98435\|\|full | 1658 | No | NA | 0 | 2 | Not-determined | Genome-fragment | NA | 0 |
| S11-final-viral-combined_k141_43002\|\|full | 1570 | No | NA | 0 | 0 | Not-determined | Genome-fragment | NA | 0 |
| S4_k141_127999 | 1089 | No | NA | 0 | 0 | Low-quality | Genome-fragment | 2.29 | 0 |
| S11-final-viral-combined_k141_43020\|\|full | 1574 | No | NA | 0 | 0 | Not-determined | Genome-fragment | NA | 0 |
| S8-final-viral-combined_k141_142404\|\|full | 1089 | No | NA | 0 | 1 | Low-quality | Genome-fragment | 1.64 | 0 |
| S11-final-viral-combined_k141_1715\|\|full | 2274 | No | NA | 0 | 2 | Not-determined | Genome-fragment | NA | 0 |
| S11-final-viral-combined_k141_98447\|\|full | 8569 | No | NA | 0 | 4 | Not-determined | Genome-fragment | NA | 0 |
| S11-final-viral-combined_k141_56859\|\|full | 3570 | No | NA | 0 | 1 | Not-determined | Genome-fragment | NA | 0 |
| S11-final-viral-combined_k141_111715\|\|full | 2297 | No | NA | 0 | 1 | Not-determined | Genome-fragment | NA | 0 |
| S9_k141_47737 | 1090 | No | NA | 0 | 0 | Low-quality | Genome-fragment | 0.85 | 0 |
| S11-final-viral-combined_k141_180865\|\|full | 3308 | No | NA | 0 | 1 | Not-determined | Genome-fragment | NA | 0 |
| S11-final-viral-combined_k141_98465\|\|full | 5033 | No | NA | 0 | 6 | Not-determined | Genome-fragment | NA | 0 |
| S11-final-viral-combined_k141_246626\|\|full | 1090 | No | NA | 0 | 0 | Low-quality | Genome-fragment | 0.98 | 0 |
| S1-final-viral-combined_k141_355791\|\|full | 1090 | No | NA | 1 | 0 | Low-quality | Genome-fragment | 2.02 | 0 |
| S1-final-viral-combined_k141_224840\|\|full | 1090 | No | NA | 1 | 0 | Low-quality | Genome-fragment | 1.25 | 0 |
| S1_k141_247084 | 1090 | No | NA | 0 | 0 | Low-quality | Genome-fragment | 2.33 | 0 |
| S1_k141_361946 | 1090 | No | NA | 0 | 0 | Low-quality | Genome-fragment | 0.85 | 0 |
| S5-final-viral-combined_k141_67957\|\|full | 1090 | No | NA | 0 | 1 | Low-quality | Genome-fragment | 1.33 | 0 |
| S11-final-viral-combined_k141_125887\|\|full | 2383 | No | NA | 0 | 4 | Not-determined | Genome-fragment | NA | 0 |

<table>
<tr><td>S7-final-viral-combined_k141_58301||full</td><td>1090</td><td>No</td><td>NA</td><td></td><td>0</td><td>0</td><td>Low-quality</td><td>Genome-fragment</td><td></td><td>0.42</td><td>0</td></tr>
<tr><td>S11-final-viral-combined_k141_84587||full</td><td>3623</td><td>No</td><td>NA</td><td></td><td>0</td><td>3</td><td>Not-determined</td><td>Genome-fragment</td><td>NA</td><td></td><td>0</td></tr>
<tr><td>S7-final-viral-combined_k141_220265||lt2gene</td><td>1090</td><td>No</td><td>NA</td><td></td><td>1</td><td>0</td><td>Low-quality</td><td>Genome-fragment</td><td></td><td>3.05</td><td>0</td></tr>
<tr><td>S11-final-viral-combined_k141_98540||full</td><td>3233</td><td>No</td><td>NA</td><td></td><td>0</td><td>0</td><td>Not-determined</td><td>Genome-fragment</td><td>NA</td><td></td><td>0</td></tr>
<tr><td>S11-final-viral-combined_k141_305018||full</td><td>3869</td><td>No</td><td>NA</td><td></td><td>0</td><td>4</td><td>Not-determined</td><td>Genome-fragment</td><td>NA</td><td></td><td>0</td></tr>
<tr><td>S7-final-viral-combined_k141_60396||lt2gene</td><td>1090</td><td>No</td><td>NA</td><td></td><td>1</td><td>0</td><td>Low-quality</td><td>Genome-fragment</td><td></td><td>1.43</td><td>0</td></tr>
<tr><td>S17-final-viral-combined_k141_214800||lt2gene</td><td>1090</td><td>No</td><td>NA</td><td></td><td>1</td><td>0</td><td>Low-quality</td><td>Genome-fragment</td><td></td><td>2.44</td><td>0</td></tr>
<tr><td>S11-final-viral-combined_k141_222372||full</td><td>1061</td><td>No</td><td>NA</td><td></td><td>0</td><td>0</td><td>Not-determined</td><td>Genome-fragment</td><td>NA</td><td></td><td>0</td></tr>
<tr><td>S11-final-viral-combined_k141_222378||full</td><td>1909</td><td>No</td><td>NA</td><td></td><td>0</td><td>2</td><td>Not-determined</td><td>Genome-fragment</td><td>NA</td><td></td><td>0</td></tr>
<tr><td>S11-final-viral-combined_k141_29236||full</td><td>3373</td><td>No</td><td>NA</td><td></td><td>0</td><td>2</td><td>Not-determined</td><td>Genome-fragment</td><td>NA</td><td></td><td>0</td></tr>
<tr><td>S11-final-viral-combined_k141_236390||full</td><td>1922</td><td>No</td><td>NA</td><td></td><td>0</td><td>3</td><td>Not-determined</td><td>Genome-fragment</td><td>NA</td><td></td><td>0</td></tr>
<tr><td>S12-final-viral-combined_k141_361210||full</td><td>1090</td><td>No</td><td>NA</td><td></td><td>0</td><td>0</td><td>Low-quality</td><td>Genome-fragment</td><td></td><td>1.99</td><td>0</td></tr>
<tr><td>S11-final-viral-combined_k141_153428||full</td><td>2225</td><td>No</td><td>NA</td><td></td><td>0</td><td>1</td><td>Not-determined</td><td>Genome-fragment</td><td>NA</td><td></td><td>0</td></tr>
<tr><td>S11-final-viral-combined_k141_277598||full</td><td>1242</td><td>No</td><td>NA</td><td></td><td>0</td><td>1</td><td>Not-determined</td><td>Genome-fragment</td><td>NA</td><td></td><td>0</td></tr>
<tr><td>S11-final-viral-combined_k141_208690||full</td><td>3963</td><td>No</td><td>NA</td><td></td><td>0</td><td>3</td><td>Not-determined</td><td>Genome-fragment</td><td>NA</td><td></td><td>0</td></tr>
<tr><td>S11-final-viral-combined_k141_291200||full</td><td>6314</td><td>No</td><td>NA</td><td></td><td>0</td><td>0</td><td>Not-determined</td><td>Genome-fragment</td><td>NA</td><td></td><td>0</td></tr>
<tr><td>S11-final-viral-combined_k141_153451||full</td><td>4906</td><td>No</td><td>NA</td><td></td><td>0</td><td>1</td><td>Not-determined</td><td>Genome-fragment</td><td>NA</td><td></td><td>0</td></tr>
<tr><td>S11-final-viral-combined_k141_139530||full</td><td>3908</td><td>No</td><td>NA</td><td></td><td>0</td><td>2</td><td>Not-determined</td><td>Genome-fragment</td><td>NA</td><td></td><td>0</td></tr>
<tr><td>S12_k141_369377</td><td>1090</td><td>No</td><td>NA</td><td></td><td>0</td><td>1</td><td>Low-quality</td><td>Genome-fragment</td><td></td><td>2.08</td><td>0</td></tr>
<tr><td>S11-final-viral-combined_k141_194736||full</td><td>2452</td><td>No</td><td>NA</td><td></td><td>0</td><td>2</td><td>Not-determined</td><td>Genome-fragment</td><td>NA</td><td></td><td>0</td></tr>
<tr><td>S11-final-viral-combined_k141_98600||full</td><td>1525</td><td>No</td><td>NA</td><td></td><td>0</td><td>0</td><td>Not-determined</td><td>Genome-fragment</td><td>NA</td><td></td><td>0</td></tr>
<tr><td>S2-final-viral-combined_k141_21370||full</td><td>1090</td><td>No</td><td>NA</td><td></td><td>0</td><td>1</td><td>Low-quality</td><td>Genome-fragment</td><td></td><td>2.82</td><td>0</td></tr>
<tr><td>S11-final-viral-combined_k141_15754||full</td><td>1853</td><td>No</td><td>NA</td><td></td><td>0</td><td>3</td><td>Not-determined</td><td>Genome-fragment</td><td>NA</td><td></td><td>0</td></tr>
<tr><td>S6-final-viral-combined_k141_17019||full</td><td>1090</td><td>No</td><td>NA</td><td></td><td>0</td><td>1</td><td>Low-quality</td><td>Genome-fragment</td><td></td><td>1.52</td><td>0</td></tr>
<tr><td>S9-final-viral-combined_k141_84388||full</td><td>1091</td><td>No</td><td>NA</td><td></td><td>1</td><td>0</td><td>Low-quality</td><td>Genome-fragment</td><td></td><td>2.64</td><td>0</td></tr>
<tr><td>S11-final-viral-combined_k141_249860||full</td><td>1702</td><td>No</td><td>NA</td><td></td><td>0</td><td>1</td><td>Not-determined</td><td>Genome-fragment</td><td>NA</td><td></td><td>0</td></tr>
<tr><td>S11-final-viral-combined_k141_111844||full</td><td>5929</td><td>No</td><td>NA</td><td></td><td>0</td><td>4</td><td>Not-determined</td><td>Genome-fragment</td><td>NA</td><td></td><td>0</td></tr>
<tr><td>S11-final-viral-combined_k141_62717||full</td><td>1091</td><td>No</td><td>NA</td><td></td><td>0</td><td>0</td><td>Low-quality</td><td>Genome-fragment</td><td></td><td>2.46</td><td>0</td></tr>
<tr><td>S11-final-viral-combined_k141_43189||full</td><td>1365</td><td>No</td><td>NA</td><td></td><td>0</td><td>1</td><td>Not-determined</td><td>Genome-fragment</td><td>NA</td><td></td><td>0</td></tr>
<tr><td>S11-final-viral-combined_k141_291272||full</td><td>2536</td><td>No</td><td>NA</td><td></td><td>0</td><td>2</td><td>Not-determined</td><td>Genome-fragment</td><td>NA</td><td></td><td>0</td></tr>
<tr><td>S11-final-viral-combined_k141_57082||full</td><td>1033</td><td>No</td><td>NA</td><td></td><td>0</td><td>2</td><td>Not-determined</td><td>Genome-fragment</td><td>NA</td><td></td><td>0</td></tr>
<tr><td>S1-final-viral-combined_k141_329671||full</td><td>1091</td><td>No</td><td>NA</td><td></td><td>1</td><td>0</td><td>Low-quality</td><td>Genome-fragment</td><td></td><td>0.67</td><td>0</td></tr>
<tr><td>S11-final-viral-combined_k141_167608||full</td><td>2858</td><td>No</td><td>NA</td><td></td><td>0</td><td>5</td><td>Not-determined</td><td>Genome-fragment</td><td>NA</td><td></td><td>0</td></tr>
<tr><td>S1-final-viral-combined_k141_53686||full</td><td>1091</td><td>No</td><td>NA</td><td></td><td>2</td><td>0</td><td>Low-quality</td><td>Genome-fragment</td><td></td><td>2.25</td><td>0</td></tr>
<tr><td>S11-final-viral-combined_k141_15775||full</td><td>17898</td><td>No</td><td>NA</td><td></td><td>0</td><td>6</td><td>Not-determined</td><td>Genome-fragment</td><td>NA</td><td></td><td>0</td></tr>
<tr><td>S11-final-viral-combined_k141_277658||full</td><td>2099</td><td>No</td><td>NA</td><td></td><td>0</td><td>2</td><td>Not-determined</td><td>Genome-fragment</td><td>NA</td><td></td><td>0</td></tr>
<tr><td>S5-final-viral-combined_k141_187736||lt2gene</td><td>1091</td><td>No</td><td>NA</td><td></td><td>1</td><td>0</td><td>Low-quality</td><td>Genome-fragment</td><td></td><td>2.67</td><td>0</td></tr>
<tr><td>S17-final-viral-combined_k141_213443||full</td><td>1091</td><td>No</td><td>NA</td><td></td><td>1</td><td>0</td><td>Low-quality</td><td>Genome-fragment</td><td></td><td>2.67</td><td>0</td></tr>
<tr><td>S11-final-viral-combined_k141_111868||full</td><td>2379</td><td>No</td><td>NA</td><td></td><td>0</td><td>0</td><td>Not-determined</td><td>Genome-fragment</td><td>NA</td><td></td><td>0</td></tr>
<tr><td>S17-final-viral-combined_k141_118195||full</td><td>1091</td><td>No</td><td>NA</td><td></td><td>0</td><td>0</td><td>Low-quality</td><td>Genome-fragment</td><td></td><td>1.33</td><td>0</td></tr>
<tr><td>S11-final-viral-combined_k141_181029||full</td><td>2467</td><td>No</td><td>NA</td><td></td><td>0</td><td>1</td><td>Not-determined</td><td>Genome-fragment</td><td>NA</td><td></td><td>0</td></tr>
<tr><td>S11-final-viral-combined_k141_249929||full</td><td>3063</td><td>No</td><td>NA</td><td></td><td>0</td><td>1</td><td>Not-determined</td><td>Genome-fragment</td><td>NA</td><td></td><td>0</td></tr>
<tr><td>S12-final-viral-combined_k141_117327||lt2gene</td><td>1091</td><td>No</td><td>NA</td><td></td><td>1</td><td>0</td><td>Low-quality</td><td>Genome-fragment</td><td></td><td>1.79</td><td>0</td></tr>
<tr><td>S11-final-viral-combined_k141_305209||full</td><td>1496</td><td>No</td><td>NA</td><td></td><td>0</td><td>3</td><td>Not-determined</td><td>Genome-fragment</td><td>NA</td><td></td><td>0</td></tr>
<tr><td>S11-final-viral-combined_k141_291313||full</td><td>10306</td><td>No</td><td>NA</td><td></td><td>0</td><td>1</td><td>Not-determined</td><td>Genome-fragment</td><td>NA</td><td></td><td>0</td></tr>
<tr><td>S11-final-viral-combined_k141_98673||full</td><td>2957</td><td>No</td><td>NA</td><td></td><td>0</td><td>3</td><td>Not-determined</td><td>Genome-fragment</td><td>NA</td><td></td><td>0</td></tr>
<tr><td>S12-final-viral-combined_k141_16617||lt2gene</td><td>1091</td><td>No</td><td>NA</td><td></td><td>2</td><td>0</td><td>Low-quality</td><td>Genome-fragment</td><td></td><td>1.96</td><td>0</td></tr>
<tr><td>S11-final-viral-combined_k141_1906||full</td><td>1647</td><td>No</td><td>NA</td><td></td><td>0</td><td>0</td><td>Not-determined</td><td>Genome-fragment</td><td>NA</td><td></td><td>0</td></tr>
<tr><td>S2-final-viral-combined_k141_244126||full</td><td>1091</td><td>No</td><td>NA</td><td></td><td>0</td><td>0</td><td>Low-quality</td><td>Genome-fragment</td><td></td><td>0.74</td><td>0</td></tr>
<tr><td>S2_k141_129732</td><td>1091</td><td>No</td><td>NA</td><td></td><td>0</td><td>0</td><td>Low-quality</td><td>Genome-fragment</td><td></td><td>1.82</td><td>0</td></tr>
<tr><td>S11-final-viral-combined_k141_318741||full</td><td>1926</td><td>No</td><td>NA</td><td></td><td>0</td><td>0</td><td>Not-determined</td><td>Genome-fragment</td><td>NA</td><td></td><td>0</td></tr>
<tr><td>S4-final-viral-combined_k141_152272||full</td><td>1091</td><td>No</td><td>NA</td><td></td><td>0</td><td>0</td><td>Low-quality</td><td>Genome-fragment</td><td></td><td>2.51</td><td>0</td></tr>
<tr><td>S11-final-viral-combined_k141_236538||full</td><td>1224</td><td>No</td><td>NA</td><td></td><td>0</td><td>0</td><td>Not-determined</td><td>Genome-fragment</td><td>NA</td><td></td><td>0</td></tr>
<tr><td>S11-final-viral-combined_k141_318748||full</td><td>2564</td><td>No</td><td>NA</td><td></td><td>0</td><td>1</td><td>Not-determined</td><td>Genome-fragment</td><td>NA</td><td></td><td>0</td></tr>
<tr><td>S11-final-viral-combined_k141_249989||full</td><td>1055</td><td>No</td><td>NA</td><td></td><td>0</td><td>0</td><td>Not-determined</td><td>Genome-fragment</td><td>NA</td><td></td><td>0</td></tr>
<tr><td>S11-final-viral-combined_k141_318761||full</td><td>2304</td><td>No</td><td>NA</td><td></td><td>0</td><td>1</td><td>Not-determined</td><td>Genome-fragment</td><td>NA</td><td></td><td>0</td></tr>
<tr><td>S11-final-viral-combined_k141_167712||full</td><td>2996</td><td>No</td><td>NA</td><td></td><td>0</td><td>1</td><td>Not-determined</td><td>Genome-fragment</td><td>NA</td><td></td><td>0</td></tr>
<tr><td>S4_k141_49353</td><td>1091</td><td>No</td><td>NA</td><td></td><td>0</td><td>0</td><td>Low-quality</td><td>Genome-fragment</td><td></td><td>0.44</td><td>0</td></tr>
<tr><td>S6_k141_100262</td><td>1091</td><td>No</td><td>NA</td><td></td><td>1</td><td>0</td><td>Low-quality</td><td>Genome-fragment</td><td></td><td>2.25</td><td>0</td></tr>
<tr><td>S11-final-viral-combined_k141_98730||full</td><td>5675</td><td>No</td><td>NA</td><td></td><td>0</td><td>3</td><td>Not-determined</td><td>Genome-fragment</td><td>NA</td><td></td><td>0</td></tr>
<tr><td>S8-final-viral-combined_k141_174057||full</td><td>1091</td><td>No</td><td>NA</td><td></td><td>0</td><td>0</td><td>Low-quality</td><td>Genome-fragment</td><td></td><td>2.06</td><td>0</td></tr>
<tr><td>S11-final-viral-combined_k141_84828||full</td><td>1111</td><td>No</td><td>NA</td><td></td><td>0</td><td>2</td><td>Not-determined</td><td>Genome-fragment</td><td>NA</td><td></td><td>0</td></tr>
<tr><td>S9-final-viral-combined_k141_64693||lt2gene</td><td>1092</td><td>No</td><td>NA</td><td></td><td>1</td><td>0</td><td>Low-quality</td><td>Genome-fragment</td><td></td><td>2.96</td><td>0</td></tr>
<tr><td>S11-final-viral-combined_k141_263865||full</td><td>1537</td><td>No</td><td>NA</td><td></td><td>0</td><td>2</td><td>Not-determined</td><td>Genome-fragment</td><td>NA</td><td></td><td>0</td></tr>
<tr><td>S9_k141_59339</td><td>1092</td><td>No</td><td>NA</td><td></td><td>0</td><td>0</td><td>Low-quality</td><td>Genome-fragment</td><td></td><td>1.82</td><td>0</td></tr>
<tr><td>S11-final-viral-combined_k141_208956||full</td><td>2340</td><td>No</td><td>NA</td><td></td><td>0</td><td>0</td><td>Not-determined</td><td>Genome-fragment</td><td>NA</td><td></td><td>0</td></tr>
<tr><td>S11-final-viral-combined_k141_291410||full</td><td>1489</td><td>No</td><td>NA</td><td></td><td>0</td><td>1</td><td>Not-determined</td><td>Genome-fragment</td><td>NA</td><td></td><td>0</td></tr>
<tr><td>S9_k141_12863</td><td>1092</td><td>No</td><td>NA</td><td></td><td>2</td><td>0</td><td>Low-quality</td><td>Genome-fragment</td><td></td><td>2.66</td><td>0</td></tr>
<tr><td>S11-final-viral-combined_k141_43337||full</td><td>4912</td><td>No</td><td>NA</td><td></td><td>0</td><td>2</td><td>Not-determined</td><td>Genome-fragment</td><td>NA</td><td></td><td>0</td></tr>
<tr><td>S11-final-viral-combined_k141_263889||full</td><td>4397</td><td>No</td><td>NA</td><td></td><td>0</td><td>2</td><td>Not-determined</td><td>Genome-fragment</td><td>NA</td><td></td><td>0</td></tr>
<tr><td>S11-final-viral-combined_k141_160037||lt2gene</td><td>1092</td><td>No</td><td>NA</td><td></td><td>1</td><td>0</td><td>Low-quality</td><td>Genome-fragment</td><td></td><td>2.74</td><td>0</td></tr>
<tr><td>S11-final-viral-combined_k141_98795||full</td><td>2152</td><td>No</td><td>NA</td><td></td><td>0</td><td>0</td><td>Not-determined</td><td>Genome-fragment</td><td>NA</td><td></td><td>0</td></tr>
<tr><td>S11_k141_55103</td><td>1092</td><td>No</td><td>NA</td><td></td><td>0</td><td>0</td><td>Low-quality</td><td>Genome-fragment</td><td></td><td>1.64</td><td>0</td></tr>
<tr><td>S11-final-viral-combined_k141_236617||full</td><td>1236</td><td>No</td><td>NA</td><td></td><td>0</td><td>0</td><td>Not-determined</td><td>Genome-fragment</td><td>NA</td><td></td><td>0</td></tr>
<tr><td>S1-final-viral-combined_k141_154027||lt2gene</td><td>1092</td><td>No</td><td>NA</td><td></td><td>1</td><td>0</td><td>Low-quality</td><td>Genome-fragment</td><td></td><td>2.06</td><td>0</td></tr>
<tr><td>S11-final-viral-combined_k141_305298||full</td><td>1117</td><td>No</td><td>NA</td><td></td><td>0</td><td>0</td><td>Not-determined</td><td>Genome-fragment</td><td>NA</td><td></td><td>0</td></tr>
<tr><td>S11-final-viral-combined_k141_305321||full</td><td>1003</td><td>No</td><td>NA</td><td></td><td>0</td><td>1</td><td>Not-determined</td><td>Genome-fragment</td><td>NA</td><td></td><td>0</td></tr>
<tr><td>S11-final-viral-combined_k141_43400||full</td><td>3299</td><td>No</td><td>NA</td><td></td><td>0</td><td>2</td><td>Not-determined</td><td>Genome-fragment</td><td>NA</td><td></td><td>0</td></tr>
<tr><td>S11-final-viral-combined_k141_70453||full</td><td>1029</td><td>No</td><td>NA</td><td></td><td>0</td><td>0</td><td>Not-determined</td><td>Genome-fragment</td><td>NA</td><td></td><td>0</td></tr>
<tr><td>S11-final-viral-combined_k141_277905||full</td><td>2919</td><td>No</td><td>NA</td><td></td><td>0</td><td>2</td><td>Not-determined</td><td>Genome-fragment</td><td>NA</td><td></td><td>0</td></tr>
<tr><td>S11-final-viral-combined_k141_167763||full</td><td>1135</td><td>No</td><td>NA</td><td></td><td>0</td><td>0</td><td>Not-determined</td><td>Genome-fragment</td><td>NA</td><td></td><td>0</td></tr>
<tr><td>S11-final-viral-combined_k141_291465||full</td><td>1188</td><td>No</td><td>NA</td><td></td><td>0</td><td>1</td><td>Not-determined</td><td>Genome-fragment</td><td>NA</td><td></td><td>0</td></tr>
<tr><td>S1_k141_159061</td><td>1092</td><td>No</td><td>NA</td><td></td><td>0</td><td>0</td><td>Low-quality</td><td>Genome-fragment</td><td></td><td>0.26</td><td>0</td></tr>
<tr><td>S11-final-viral-combined_k141_209025||full</td><td>1289</td><td>No</td><td>NA</td><td></td><td>0</td><td>2</td><td>Not-determined</td><td>Genome-fragment</td><td>NA</td><td></td><td>0</td></tr>
<tr><td>S11-final-viral-combined_k141_43402||full</td><td>12229</td><td>No</td><td>NA</td><td></td><td>0</td><td>4</td><td>Not-determined</td><td>Genome-fragment</td><td>NA</td><td></td><td>0</td></tr>
<tr><td>S11-final-viral-combined_k141_236712||full</td><td>1082</td><td>No</td><td>NA</td><td></td><td>0</td><td>1</td><td>Not-determined</td><td>Genome-fragment</td><td>NA</td><td></td><td>0</td></tr>
<tr><td>S3_k141_45297</td><td>1092</td><td>No</td><td>NA</td><td></td><td>0</td><td>0</td><td>Low-quality</td><td>Genome-fragment</td><td></td><td>0.5</td><td>0</td></tr>
<tr><td>S11-final-viral-combined_k141_236721||full</td><td>2957</td><td>No</td><td>NA</td><td></td><td>0</td><td>1</td><td>Not-determined</td><td>Genome-fragment</td><td>NA</td><td></td><td>0</td></tr>
<tr><td>S11-final-viral-combined_k141_305367||full</td><td>9795</td><td>No</td><td>NA</td><td></td><td>0</td><td>6</td><td>Not-determined</td><td>Genome-fragment</td><td>NA</td><td></td><td>0</td></tr>
<tr><td>S5-final-viral-combined_k141_17875||lt2gene</td><td>1092</td><td>No</td><td>NA</td><td></td><td>1</td><td>0</td><td>Low-quality</td><td>Genome-fragment</td><td></td><td>1.81</td><td>0</td></tr>
<tr><td>S11-final-viral-combined_k141_195011||full</td><td>1524</td><td>No</td><td>NA</td><td></td><td>0</td><td>1</td><td>Not-determined</td><td>Genome-fragment</td><td>NA</td><td></td><td>0</td></tr>
<tr><td>S5-final-viral-combined_k141_189541||lt2gene</td><td>1092</td><td>No</td><td>NA</td><td></td><td>1</td><td>0</td><td>Low-quality</td><td>Genome-fragment</td><td></td><td>0.65</td><td>0</td></tr>
<tr><td>S17-k141_129812</td><td>1092</td><td>No</td><td>NA</td><td></td><td>3</td><td>0</td><td>Low-quality</td><td>Genome-fragment</td><td></td><td>2.55</td><td>0</td></tr>
<tr><td>S11-final-viral-combined_k141_70468||full</td><td>2208</td><td>No</td><td>NA</td><td></td><td>0</td><td>1</td><td>Not-determined</td><td>Genome-fragment</td><td>NA</td><td></td><td>0</td></tr>
<tr><td>S11-final-viral-combined_k141_291493||full</td><td>11081</td><td>No</td><td>NA</td><td></td><td>0</td><td>8</td><td>Not-determined</td><td>Genome-fragment</td><td>NA</td><td></td><td>0</td></tr>
<tr><td>S11-final-viral-combined_k141_139879||full</td><td>6832</td><td>No</td><td>NA</td><td></td><td>0</td><td>3</td><td>Not-determined</td><td>Genome-fragment</td><td>NA</td><td></td><td>0</td></tr>
<tr><td>S11-final-viral-combined_k141_57355||full</td><td>4273</td><td>No</td><td>NA</td><td></td><td>0</td><td>1</td><td>Not-determined</td><td>Genome-fragment</td><td>NA</td><td></td><td>0</td></tr>
<tr><td>S11-final-viral-combined_k141_195033||full</td><td>1242</td><td>No</td><td>NA</td><td></td><td>0</td><td>0</td><td>Not-determined</td><td>Genome-fragment</td><td>NA</td><td></td><td>0</td></tr>
<tr><td>S11-final-viral-combined_k141_84925||full</td><td>2743</td><td>No</td><td>NA</td><td></td><td>0</td><td>0</td><td>Not-determined</td><td>Genome-fragment</td><td>NA</td><td></td><td>0</td></tr>
<tr><td>S11-final-viral-combined_k141_181217||full</td><td>2259</td><td>No</td><td>NA</td><td></td><td>0</td><td>3</td><td>Not-determined</td><td>Genome-fragment</td><td>NA</td><td></td><td>0</td></tr>
<tr><td>S11-final-viral-combined_k141_153678||full</td><td>4348</td><td>No</td><td>NA</td><td></td><td>0</td><td>2</td><td>Not-determined</td><td>Genome-fragment</td><td>NA</td><td></td><td>0</td></tr>
<tr><td>S11-final-viral-combined_k141_16103||full</td><td>2391</td><td>No</td><td>NA</td><td></td><td>0</td><td>1</td><td>Not-determined</td><td>Genome-fragment</td><td>NA</td><td></td><td>0</td></tr>
<tr><td>S2-final-viral-combined_k141_101970||full</td><td>1092</td><td>No</td><td>NA</td><td></td><td>0</td><td>0</td><td>Low-quality</td><td>Genome-fragment</td><td></td><td>0.94</td><td>0</td></tr>
<tr><td>S11-final-viral-combined_k141_84956||full</td><td>1375</td><td>No</td><td>NA</td><td></td><td>0</td><td>2</td><td>Not-determined</td><td>Genome-fragment</td><td>NA</td><td></td><td>0</td></tr>
<tr><td>S2-final-viral-combined_k141_194839||full</td><td>1092</td><td>No</td><td>NA</td><td></td><td>0</td><td>1</td><td>Low-quality</td><td>Genome-fragment</td><td></td><td>1.36</td><td>0</td></tr>
<tr><td>S11-final-viral-combined_k141_112100||full</td><td>1866</td><td>No</td><td>NA</td><td></td><td>0</td><td>2</td><td>Not-determined</td><td>Genome-fragment</td><td>NA</td><td></td><td>0</td></tr>
<tr><td>S11-final-viral-combined_k141_222729||full</td><td>1271</td><td>No</td><td>NA</td><td></td><td>0</td><td>1</td><td>Not-determined</td><td>Genome-fragment</td><td>NA</td><td></td><td>0</td></tr>
<tr><td>S11-final-viral-combined_k141_167828||full</td><td>1709</td><td>No</td><td>NA</td><td></td><td>0</td><td>1</td><td>Not-determined</td><td>Genome-fragment</td><td>NA</td><td></td><td>0</td></tr>
<tr><td>S2-final-viral-combined_k141_111400||full</td><td>1092</td><td>No</td><td>NA</td><td></td><td>0</td><td>0</td><td>Low-quality</td><td>Genome-fragment</td><td></td><td>2.67</td><td>0</td></tr>
<tr><td>S11-final-viral-combined_k141_278092||full</td><td>1577</td><td>No</td><td>NA</td><td></td><td>0</td><td>0</td><td>Not-determined</td><td>Genome-fragment</td><td>NA</td><td></td><td>0</td></tr>
<tr><td>S2_k141_238357</td><td>1092</td><td>No</td><td>NA</td><td></td><td>1</td><td>0</td><td>Low-quality</td><td>Genome-fragment</td><td></td><td>2.81</td><td>0</td></tr>
<tr><td>S11-final-viral-combined_k141_57407||full</td><td>7371</td><td>No</td><td>NA</td><td></td><td>0</td><td>5</td><td>Not-determined</td><td>Genome-fragment</td><td>NA</td><td></td><td>0</td></tr>
<tr><td>S11-final-viral-combined_k141_139912||full</td><td>1417</td><td>No</td><td>NA</td><td></td><td>0</td><td>0</td><td>Not-determined</td><td>Genome-fragment</td><td>NA</td><td></td><td>0</td></tr>
<tr><td>S11-final-viral-combined_k141_319018||full</td><td>3487</td><td>No</td><td>NA</td><td></td><td>0</td><td>0</td><td>Not-determined</td><td>Genome-fragment</td><td>NA</td><td></td><td>0</td></tr>
<tr><td>S11-final-viral-combined_k141_57412||full</td><td>1996</td><td>No</td><td>NA</td><td></td><td>0</td><td>2</td><td>Not-determined</td><td>Genome-fragment</td><td>NA</td><td></td><td>0</td></tr>
<tr><td>S11-final-viral-combined_k141_70570||full</td><td>1184</td><td>No</td><td>NA</td><td></td><td>0</td><td>0</td><td>Not-determined</td><td>Genome-fragment</td><td>NA</td><td></td><td>0</td></tr>
<tr><td>S11-final-viral-combined_k141_236884||full</td><td>1821</td><td>No</td><td>NA</td><td></td><td>0</td><td>2</td><td>Not-determined</td><td>Genome-fragment</td><td>NA</td><td></td><td>0</td></tr>
<tr><td>S11-final-viral-combined_k141_236894||full</td><td>2215</td><td>No</td><td>NA</td><td></td><td>0</td><td>1</td><td>Not-determined</td><td>Genome-fragment</td><td>NA</td><td></td><td>0</td></tr>
<tr><td>S11-final-viral-combined_k141_264050||full</td><td>97251</td><td>Yes</td><td></td><td>49308</td><td>22</td><td>32</td><td>Complete</td><td>High-quality</td><td></td><td>100</td><td>49.3</td></tr>
<tr><td>S4-final-viral-combined_k141_120179||full</td><td>1092</td><td>No</td><td>NA</td><td></td><td>0</td><td>2</td><td>Low-quality</td><td>Genome-fragment</td><td></td><td>1.48</td><td>0</td></tr>
<tr><td>S11-final-viral-combined_k141_139935||full</td><td>10462</td><td>No</td><td>NA</td><td></td><td>0</td><td>3</td><td>Not-determined</td><td>Genome-fragment</td><td>NA</td><td></td><td>0</td></tr>
<tr><td>S11-final-viral-combined_k141_98951||full</td><td>1074</td><td>No</td><td>NA</td><td></td><td>0</td><td>1</td><td>Not-determined</td><td>Genome-fragment</td><td>NA</td><td></td><td>0</td></tr>
<tr><td>S8_k141_255645</td><td>1092</td><td>No</td><td>NA</td><td></td><td>0</td><td>1</td><td>Low-quality</td><td>Genome-fragment</td><td></td><td>0.39</td><td>0</td></tr>
<tr><td>S9-final-viral-combined_k141_98497||full</td><td>1093</td><td>No</td><td>NA</td><td></td><td>1</td><td>0</td><td>Low-quality</td><td>Genome-fragment</td><td></td><td>2.9</td><td>0</td></tr>
<tr><td>S11-final-viral-combined_k141_156475||full</td><td>1093</td><td>No</td><td>NA</td><td></td><td>1</td><td>0</td><td>Low-quality</td><td>Genome-fragment</td><td></td><td>3.04</td><td>0</td></tr>
<tr><td>S1-final-viral-combined_k141_133892||full</td><td>1093</td><td>No</td><td>NA</td><td></td><td>0</td><td>0</td><td>Low-quality</td><td>Genome-fragment</td><td></td><td>2.94</td><td>0</td></tr>
<tr><td>S11-final-viral-combined_k141_2197||full</td><td>1493</td><td>No</td><td>NA</td><td></td><td>0</td><td>0</td><td>Not-determined</td><td>Genome-fragment</td><td>NA</td><td></td><td>0</td></tr>
<tr><td>S1-final-viral-combined_k141_257785||full</td><td>1093</td><td>Yes</td><td></td><td>880</td><td>1</td><td>1</td><td>Low-quality</td><td>Genome-fragment</td><td></td><td>2.66</td><td>19.49</td></tr>
<tr><td>S11-final-viral-combined_k141_139941||full</td><td>20245</td><td>No</td><td>NA</td><td></td><td>0</td><td>11</td><td>Not-determined</td><td>Genome-fragment</td><td>NA</td><td></td><td>0</td></tr>
<tr><td>S1-final-viral-combined_k141_94176||full</td><td>1093</td><td>No</td><td>NA</td><td></td><td>0</td><td>1</td><td>Low-quality</td><td>Genome-fragment</td><td></td><td>1.37</td><td>0</td></tr>
<tr><td>S3_k141_117633</td><td>1093</td><td>No</td><td>NA</td><td></td><td>0</td><td>0</td><td>Low-quality</td><td>Genome-fragment</td><td></td><td>2.22</td><td>0</td></tr>
<tr><td>S11-final-viral-combined_k141_209287||full</td><td>1199</td><td>No</td><td>NA</td><td></td><td>0</td><td>1</td><td>Not-determined</td><td>Genome-fragment</td><td>NA</td><td></td><td>0</td></tr>
<tr><td>S3_k141_78672</td><td>1093</td><td>No</td><td>NA</td><td></td><td>0</td><td>0</td><td>Low-quality</td><td>Genome-fragment</td><td></td><td>1.83</td><td>0</td></tr>
<tr><td>S11-final-viral-combined_k141_209295||full</td><td>3044</td><td>No</td><td>NA</td><td></td><td>0</td><td>2</td><td>Not-determined</td><td>Genome-fragment</td><td>NA</td><td></td><td>0</td></tr>
<tr><td>S11-final-viral-combined_k141_126308||full</td><td>3276</td><td>No</td><td>NA</td><td></td><td>0</td><td>2</td><td>Not-determined</td><td>Genome-fragment</td><td>NA</td><td></td><td>0</td></tr>
<tr><td>S12-final-viral-combined_k141_151925||full</td><td>1093</td><td>No</td><td>NA</td><td></td><td>0</td><td>0</td><td>Low-quality</td><td>Genome-fragment</td><td></td><td>2.39</td><td>0</td></tr>
<tr><td>S12-final-viral-combined_k141_221613||full</td><td>1093</td><td>No</td><td>NA</td><td></td><td>0</td><td>0</td><td>Low-quality</td><td>Genome-fragment</td><td></td><td>2.2</td><td>0</td></tr>
<tr><td>S11-final-viral-combined_k141_112147||full</td><td>19697</td><td>No</td><td>NA</td><td></td><td>3</td><td>0</td><td>Medium-quality</td><td>Genome-fragment</td><td></td><td>50.22</td><td>0</td></tr>
<tr><td>S2-final-viral-combined_k141_198978||full</td><td>1093</td><td>No</td><td>NA</td><td></td><td>0</td><td>1</td><td>Low-quality</td><td>Genome-fragment</td><td></td><td>0.36</td><td>0</td></tr>
<tr><td>S11-final-viral-combined_k141_70697||full</td><td>2112</td><td>No</td><td>NA</td><td></td><td>0</td><td>2</td><td>Not-determined</td><td>Genome-fragment</td><td>NA</td><td></td><td>0</td></tr>
<tr><td>S11-final-viral-combined_k141_16155||full</td><td>3812</td><td>No</td><td>NA</td><td></td><td>0</td><td>3</td><td>Not-determined</td><td>Genome-fragment</td><td>NA</td><td></td><td>0</td></tr>
<tr><td>S2-final-viral-combined_k141_56830||full</td><td>1093</td><td>No</td><td>NA</td><td></td><td>0</td><td>0</td><td>Low-quality</td><td>Genome-fragment</td><td></td><td>1.93</td><td>0</td></tr>
<tr><td>S8-final-viral-combined_k141_243307||full</td><td>1093</td><td>No</td><td>NA</td><td></td><td>2</td><td>0</td><td>Low-quality</td><td>Genome-fragment</td><td></td><td>2.12</td><td>0</td></tr>
<tr><td>S11-final-viral-combined_k141_126341||full</td><td>2990</td><td>No</td><td>NA</td><td></td><td>0</td><td>2</td><td>Not-determined</td><td>Genome-fragment</td><td>NA</td><td></td><td>0</td></tr>
<tr><td>S11-final-viral-combined_k141_126352||full</td><td>1957</td><td>No</td><td>NA</td><td></td><td>0</td><td>1</td><td>Not-determined</td><td>Genome-fragment</td><td>NA</td><td></td><td>0</td></tr>
<tr><td>S11-final-viral-combined_k141_319045||full</td><td>16235</td><td>No</td><td>NA</td><td></td><td>0</td><td>10</td><td>Not-determined</td><td>Genome-fragment</td><td>NA</td><td></td><td>0</td></tr>
<tr><td>S11-final-viral-combined_k141_2297||full</td><td>2622</td><td>No</td><td>NA</td><td></td><td>0</td><td>2</td><td>Not-determined</td><td>Genome-fragment</td><td>NA</td><td></td><td>0</td></tr>
<tr><td>S11-final-viral-combined_k141_264168||full</td><td>5957</td><td>No</td><td>NA</td><td></td><td>0</td><td>4</td><td>Not-determined</td><td>Genome-fragment</td><td>NA</td><td></td><td>0</td></tr>
<tr><td>S11-final-viral-combined_k141_305611||full</td><td>1626</td><td>No</td><td>NA</td><td></td><td>0</td><td>0</td><td>Not-determined</td><td>Genome-fragment</td><td>NA</td><td></td><td>0</td></tr>
<tr><td>S11-final-viral-combined_k141_29788||full</td><td>3329</td><td>No</td><td>NA</td><td></td><td>0</td><td>2</td><td>Not-determined</td><td>Genome-fragment</td><td>NA</td><td></td><td>0</td></tr>
<tr><td>S8-final-viral-combined_k141_67710||lt2gene</td><td>1093</td><td>No</td><td>NA</td><td></td><td>1</td><td>0</td><td>Low-quality</td><td>Genome-fragment</td><td></td><td>2.92</td><td>0</td></tr>
<tr><td>S11-final-viral-combined_k141_140009||full</td><td>54887</td><td>Yes</td><td></td><td>33397</td><td>22</td><td>14</td><td>High-quality</td><td>High-quality</td><td></td><td>96.44</td><td>39.15</td></tr>
<tr><td>S11-final-viral-combined_k141_2336||full</td><td>2731</td><td>No</td><td>NA</td><td></td><td>0</td><td>2</td><td>Not-determined</td><td>Genome-fragment</td><td>NA</td><td></td><td>0</td></tr>
<tr><td>S11-final-viral-combined_k141_4744||full</td><td>1094</td><td>No</td><td>NA</td><td></td><td>0</td><td>0</td><td>Low-quality</td><td>Genome-fragment</td><td></td><td>0.7</td><td>0</td></tr>
<tr><td>S11-final-viral-combined_k141_29819||full</td><td>3968</td><td>No</td><td>NA</td><td></td><td>0</td><td>0</td><td>Not-determined</td><td>Genome-fragment</td><td>NA</td><td></td><td>0</td></tr>
<tr><td>S11-final-viral-combined_k141_209357||full</td><td>1440</td><td>No</td><td>NA</td><td></td><td>0</td><td>2</td><td>Not-determined</td><td>Genome-fragment</td><td>NA</td><td></td><td>0</td></tr>
<tr><td>S11-final-viral-combined_k141_140040||full</td><td>6389</td><td>No</td><td>NA</td><td></td><td>0</td><td>3</td><td>Not-determined</td><td>Genome-fragment</td><td>NA</td><td></td><td>0</td></tr>
<tr><td>S11-final-viral-combined_k141_222766||full</td><td>1186</td><td>No</td><td>NA</td><td></td><td>0</td><td>1</td><td>Not-determined</td><td>Genome-fragment</td><td>NA</td><td></td><td>0</td></tr>
<tr><td>S11_k141_282279</td><td>1094</td><td>No</td><td>NA</td><td></td><td>0</td><td>0</td><td>Low-quality</td><td>Genome-fragment</td><td></td><td>2.71</td><td>0</td></tr>
<tr><td>S1-final-viral-combined_k141_268607||full</td><td>1094</td><td>No</td><td>NA</td><td></td><td>0</td><td>0</td><td>Low-quality</td><td>Genome-fragment</td><td></td><td>2.13</td><td>0</td></tr>
<tr><td>S1_k141_166642</td><td>1094</td><td>No</td><td>NA</td><td></td><td>0</td><td>0</td><td>Low-quality</td><td>Genome-fragment</td><td></td><td>1.16</td><td>0</td></tr>
<tr><td>S11-final-viral-combined_k141_209410||full</td><td>2029</td><td>No</td><td>NA</td><td></td><td>0</td><td>1</td><td>Not-determined</td><td>Genome-fragment</td><td>NA</td><td></td><td>0</td></tr>
<tr><td>S3-final-viral-combined_k141_72673||full</td><td>1094</td><td>No</td><td>NA</td><td></td><td>0</td><td>0</td><td>Low-quality</td><td>Genome-fragment</td><td></td><td>1.87</td><td>0</td></tr>
<tr><td>S3-final-viral-combined_k141_67912||full</td><td>1094</td><td>No</td><td>NA</td><td></td><td>0</td><td>0</td><td>Low-quality</td><td>Genome-fragment</td><td></td><td>1.88</td><td>0</td></tr>
<tr><td>S5-final-viral-combined_k141_11939||lt2gene</td><td>1094</td><td>No</td><td>NA</td><td></td><td>1</td><td>0</td><td>Low-quality</td><td>Genome-fragment</td><td></td><td>1.62</td><td>0</td></tr>
<tr><td>S7_k141_78925</td><td>1094</td><td>No</td><td>NA</td><td></td><td>0</td><td>0</td><td>Low-quality</td><td>Genome-fragment</td><td></td><td>1.84</td><td>0</td></tr>
<tr><td>S12-final-viral-combined_k141_120319||full</td><td>1094</td><td>No</td><td>NA</td><td></td><td>1</td><td>0</td><td>Low-quality</td><td>Genome-fragment</td><td></td><td>0.77</td><td>0</td></tr>
<tr><td>S12_k141_380517</td><td>1094</td><td>No</td><td>NA</td><td></td><td>0</td><td>0</td><td>Low-quality</td><td>Genome-fragment</td><td></td><td>3.14</td><td>0</td></tr>
<tr><td>S12_k141_28743</td><td>1094</td><td>No</td><td>NA</td><td></td><td>0</td><td>0</td><td>Low-quality</td><td>Genome-fragment</td><td></td><td>2</td><td>0</td></tr>
<tr><td>S11-final-viral-combined_k141_2432||full</td><td>2553</td><td>No</td><td>NA</td><td></td><td>0</td><td>2</td><td>Not-determined</td><td>Genome-fragment</td><td>NA</td><td></td><td>0</td></tr>
<tr><td>S11-final-viral-combined_k141_29856||full</td><td>1175</td><td>No</td><td>NA</td><td></td><td>0</td><td>2</td><td>Not-determined</td><td>Genome-fragment</td><td>NA</td><td></td><td>0</td></tr>
<tr><td>S11-final-viral-combined_k141_43692||full</td><td>1612</td><td>No</td><td>NA</td><td></td><td>0</td><td>2</td><td>Not-determined</td><td>Genome-fragment</td><td>NA</td><td></td><td>0</td></tr>
<tr><td>S4_k141_161777</td><td>1094</td><td>No</td><td>NA</td><td></td><td>0</td><td>0</td><td>Low-quality</td><td>Genome-fragment</td><td></td><td>0.88</td><td>0</td></tr>
<tr><td>S8-final-viral-combined_k141_108354||full</td><td>1094</td><td>No</td><td>NA</td><td></td><td>1</td><td>0</td><td>Low-quality</td><td>Genome-fragment</td><td></td><td>0.47</td><td>0</td></tr>
</table>

| | | | | | | | | | |
|---|---|---|---|---|---|---|---|---|---|
| S11-final-viral-combined_k141_70845\|\|full | 2785 | No | NA | 0 | 0 | Not-determined | Genome-fragment | NA | 0 |
| S8-final-viral-combined_k141_49344\|\|lt2gene | 1094 | No | NA | 0 | 1 | Low-quality | Genome-fragment | 1.74 | 0 |
| S11-final-viral-combined_k141_305809\|\|full | 2121 | No | NA | 0 | 0 | Not-determined | Genome-fragment | NA | 0 |
| S11-final-viral-combined_k141_85099\|\|full | 1802 | No | NA | 0 | 0 | Not-determined | Genome-fragment | NA | 0 |
| S11-final-viral-combined_k141_278316\|\|full | 1475 | No | NA | 0 | 1 | Not-determined | Genome-fragment | NA | 0 |
| S11-final-viral-combined_k141_250516\|\|full | 1573 | No | NA | 0 | 1 | Not-determined | Genome-fragment | NA | 0 |
| S11-final-viral-combined_k141_209521\|\|full | 1256 | No | NA | 0 | 1 | Not-determined | Genome-fragment | NA | 0 |
| S9-final-viral-combined_k141_22349\|\|full | 1095 | No | NA | 1 | 0 | Low-quality | Genome-fragment | 2.37 | 0 |
| S11-final-viral-combined_k141_209524\|\|full | 4955 | No | NA | 0 | 2 | Not-determined | Genome-fragment | NA | 0 |
| S11-final-viral-combined_k141_126580\|\|full | 1776 | No | NA | 0 | 2 | Not-determined | Genome-fragment | NA | 0 |
| S11-final-viral-combined_k141_168048\|\|full | 52585 | Yes | 29594 | 20 | 10 | Complete | High-quality | 100 | 43.72 |
| S11-final-viral-combined_k141_278337\|\|full | 3373 | No | NA | 0 | 0 | Not-determined | Genome-fragment | NA | 0 |
| S11-final-viral-combined_k141_321266\|\|full | 1095 | No | NA | 0 | 0 | Low-quality | Genome-fragment | 1.32 | 0 |
| S1-final-viral-combined_k141_234097\|\|full | 1095 | No | NA | 0 | 3 | Low-quality | Genome-fragment | 0.32 | 0 |
| S1-final-viral-combined_k141_123886\|\|full | 1095 | No | NA | 0 | 0 | Low-quality | Genome-fragment | 2.59 | 0 |
| S11-final-viral-combined_k141_222957\|\|full | 4457 | No | NA | 0 | 6 | Not-determined | Genome-fragment | NA | 0 |
| S5-final-viral-combined_k141_167942\|\|full | 1095 | No | NA | 0 | 0 | Low-quality | Genome-fragment | 2.25 | 0 |
| S11-final-viral-combined_k141_168091\|\|full | 2826 | No | NA | 0 | 2 | Not-determined | Genome-fragment | NA | 0 |
| S11-final-viral-combined_k141_140237\|\|full | 1643 | No | NA | 0 | 2 | Not-determined | Genome-fragment | NA | 0 |
| S7-final-viral-combined_k141_239555\|\|full | 1095 | No | NA | 1 | 0 | Low-quality | Genome-fragment | 0.77 | 0 |
| S11-final-viral-combined_k141_57802\|\|full | 1624 | No | NA | 0 | 1 | Not-determined | Genome-fragment | NA | 0 |
| S11-final-viral-combined_k141_140250\|\|full | 6783 | No | NA | 0 | 3 | Not-determined | Genome-fragment | NA | 0 |
| S7_k141_28532 | 1095 | No | NA | 0 | 0 | Low-quality | Genome-fragment | 2.63 | 0 |
| S12-final-viral-combined_k141_188308\|\|lt2gene | 1095 | No | NA | 1 | 0 | Low-quality | Genome-fragment | 1.2 | 0 |
| S11-final-viral-combined_k141_2587\|\|full | 1570 | No | NA | 0 | 2 | Not-determined | Genome-fragment | NA | 0 |
| S11-final-viral-combined_k141_181414\|\|full | 18242 | No | NA | 0 | 14 | Not-determined | Genome-fragment | NA | 0 |
| S11-final-viral-combined_k141_140272\|\|full | 1717 | No | NA | 0 | 2 | Not-determined | Genome-fragment | NA | 0 |
| S11-final-viral-combined_k141_209597\|\|full | 1279 | No | NA | 0 | 1 | Not-determined | Genome-fragment | NA | 0 |
| S12-final-viral-combined_k141_71082\|\|lt2gene | 1095 | No | NA | 1 | 0 | Low-quality | Genome-fragment | 1.97 | 0 |
| S11-final-viral-combined_k141_43818\|\|full | 3280 | No | NA | 0 | 2 | Not-determined | Genome-fragment | NA | 0 |
| S11-final-viral-combined_k141_154076\|\|full | 1004 | No | NA | 0 | 0 | Not-determined | Genome-fragment | NA | 0 |
| S11-final-viral-combined_k141_30034\|\|full | 1935 | No | NA | 0 | 2 | Not-determined | Genome-fragment | NA | 0 |
| S11-final-viral-combined_k141_99216\|\|full | 1503 | No | NA | 0 | 1 | Not-determined | Genome-fragment | NA | 0 |
| S11-final-viral-combined_k141_237088\|\|full | 1058 | No | NA | 0 | 0 | Not-determined | Genome-fragment | NA | 0 |
| S11-final-viral-combined_k141_57868\|\|full | 6395 | No | NA | 0 | 4 | Not-determined | Genome-fragment | NA | 0 |
| S11-final-viral-combined_k141_2619\|\|full | 1832 | No | NA | 0 | 0 | Not-determined | Genome-fragment | NA | 0 |
| S12-final-viral-combined_k141_356955\|\|lt2gene | 1095 | No | NA | 1 | 0 | Low-quality | Genome-fragment | 1.48 | 0 |
| S11-final-viral-combined_k141_168179\|\|full | 1797 | No | NA | 0 | 1 | Not-determined | Genome-fragment | NA | 0 |
| S11-final-viral-combined_k141_71008\|\|full | 4359 | No | NA | 0 | 4 | Not-determined | Genome-fragment | NA | 0 |
| S12_k141_137048 | 1095 | No | NA | 1 | 0 | Low-quality | Genome-fragment | 2.13 | 0 |
| S12_k141_304895 | 1095 | No | NA | 0 | 0 | Low-quality | Genome-fragment | 2.3 | 0 |
| S2-final-viral-combined_k141_130159\|\|full | 1095 | No | NA | 2 | 0 | Low-quality | Genome-fragment | 1.47 | 0 |
| S11-final-viral-combined_k141_195395\|\|full | 1126 | No | NA | 0 | 1 | Not-determined | Genome-fragment | NA | 0 |
| S11-final-viral-combined_k141_112367\|\|full | 1498 | No | NA | 0 | 0 | Not-determined | Genome-fragment | NA | 0 |
| S11-final-viral-combined_k141_16242\|\|full | 4543 | No | NA | 0 | 3 | Not-determined | Genome-fragment | NA | 0 |
| S2-final-viral-combined_k141_61690\|\|full | 1095 | No | NA | 0 | 0 | Low-quality | Genome-fragment | 2.11 | 0 |
| S2-final-viral-combined_k141_174231\|\|lt2gene | 1095 | No | NA | 1 | 0 | Low-quality | Genome-fragment | 1.23 | 0 |
| S11-final-viral-combined_k141_305945\|\|full | 16105 | No | NA | 0 | 4 | Not-determined | Genome-fragment | NA | 0 |
| S11-final-viral-combined_k141_264325\|\|full | 1355 | No | NA | 0 | 2 | Not-determined | Genome-fragment | NA | 0 |
| S11-final-viral-combined_k141_278498\|\|full | 1730 | No | NA | 0 | 0 | Not-determined | Genome-fragment | NA | 0 |
| S11-final-viral-combined_k141_278504\|\|full | 1838 | No | NA | 0 | 0 | Not-determined | Genome-fragment | NA | 0 |
| S11-final-viral-combined_k141_43904\|\|full | 1967 | No | NA | 0 | 1 | Not-determined | Genome-fragment | NA | 0 |
| S11-final-viral-combined_k141_126619\|\|full | 2005 | No | NA | 0 | 0 | Not-determined | Genome-fragment | NA | 0 |
| S11-final-viral-combined_k141_126622\|\|full | 1951 | No | NA | 0 | 2 | Not-determined | Genome-fragment | NA | 0 |
| S2_k141_232206 | 1095 | No | NA | 0 | 1 | Low-quality | Genome-fragment | 0.29 | 0 |
| S4-final-viral-combined_k141_85774\|\|full | 1095 | No | NA | 0 | 0 | Low-quality | Genome-fragment | 2.15 | 0 |
| S4-final-viral-combined_k141_60163\|\|full | 1095 | No | NA | 0 | 0 | Low-quality | Genome-fragment | 2.35 | 0 |
| S11-final-viral-combined_k141_305980\|\|full | 2416 | No | NA | 0 | 3 | Not-determined | Genome-fragment | NA | 0 |
| S4_k141_126461 | 1095 | No | NA | 0 | 0 | Low-quality | Genome-fragment | 1.15 | 0 |
| S11-final-viral-combined_k141_264382\|\|full | 1876 | No | NA | 0 | 1 | Not-determined | Genome-fragment | NA | 0 |
| S9-final-viral-combined_k141_88662\|\|full | 1096 | No | NA | 0 | 0 | Low-quality | Genome-fragment | 2.7 | 0 |
| S11-final-viral-combined_k141_81233\|\|full | 1096 | No | NA | 1 | 0 | Low-quality | Genome-fragment | 2.03 | 0 |
| S1-final-viral-combined_k141_280478\|\|full | 1096 | No | NA | 3 | 0 | Low-quality | Genome-fragment | 3.13 | 0 |
| S1_k141_393818 | 1096 | No | NA | 0 | 0 | Low-quality | Genome-fragment | 0.69 | 0 |
| S11-final-viral-combined_k141_140338\|\|full | 2324 | No | NA | 0 | 3 | Not-determined | Genome-fragment | NA | 0 |
| S11-final-viral-combined_k141_112434\|\|full | 2033 | No | NA | 0 | 2 | Not-determined | Genome-fragment | NA | 0 |
| S3-final-viral-combined_k141_6930\|\|full | 1096 | No | NA | 0 | 0 | Low-quality | Genome-fragment | 1.76 | 0 |
| S11-final-viral-combined_k141_181538\|\|full | 4750 | No | NA | 0 | 2 | Not-determined | Genome-fragment | NA | 0 |
| S11-final-viral-combined_k141_2743\|\|full | 4672 | No | NA | 0 | 0 | Not-determined | Genome-fragment | NA | 0 |
| S5-final-viral-combined_k141_135712\|\|full | 1096 | No | NA | 0 | 0 | Low-quality | Genome-fragment | 1.33 | 0 |
| S11-final-viral-combined_k141_305996\|\|full | 1018 | No | NA | 0 | 1 | Not-determined | Genome-fragment | NA | 0 |
| S11-final-viral-combined_k141_112475\|\|full | 1090 | No | NA | 0 | 1 | Not-determined | Genome-fragment | NA | 0 |
| S5-final-viral-combined_k141_64376\|\|full | 1096 | No | NA | 1 | 0 | Low-quality | Genome-fragment | 2.38 | 0 |
| S11-final-viral-combined_k141_195425\|\|full | 29097 | No | NA | 6 | 2 | Medium-quality | Genome-fragment | 67.93 | 0 |
| S5-final-viral-combined_k141_209623\|\|full | 1096 | No | NA | 1 | 0 | Low-quality | Genome-fragment | 1.88 | 0 |
| S11-final-viral-combined_k141_209769\|\|full | 2583 | No | NA | 0 | 0 | Not-determined | Genome-fragment | NA | 0 |
| S5-final-viral-combined_k141_58369\|\|full | 1096 | No | NA | 0 | 0 | Low-quality | Genome-fragment | 2.58 | 0 |
| S11-final-viral-combined_k141_223216\|\|full | 1246 | No | NA | 0 | 0 | Not-determined | Genome-fragment | NA | 0 |
| S5-final-viral-combined_k141_181491\|\|lt2gene | 1096 | No | NA | 2 | 0 | Low-quality | Genome-fragment | 1.35 | 0 |
| S7-final-viral-combined_k141_170368\|\|full | 1096 | No | NA | 0 | 0 | Low-quality | Genome-fragment | 0.57 | 0 |
| S7-final-viral-combined_k141_51532\|\|lt2gene | 1096 | No | NA | 1 | 0 | Low-quality | Genome-fragment | 0.76 | 0 |
| S11-final-viral-combined_k141_30178\|\|full | 1697 | No | NA | 0 | 0 | Not-determined | Genome-fragment | NA | 0 |
| S17-final-viral-combined_k141_85241\|\|full | 1096 | No | NA | 2 | 0 | Low-quality | Genome-fragment | 2.92 | 0 |
| S11-final-viral-combined_k141_319432\|\|full | 1999 | No | NA | 0 | 1 | Not-determined | Genome-fragment | NA | 0 |
| S17-final-viral-combined_k141_263280\|\|lt2gene | 1096 | No | NA | 1 | 0 | Low-quality | Genome-fragment | 2.33 | 0 |
| S12-final-viral-combined_k141_35770\|\|full | 1096 | No | NA | 2 | 0 | Low-quality | Genome-fragment | 2.17 | 0 |
| S11-final-viral-combined_k141_112535\|\|full | 12484 | No | NA | 0 | 9 | Not-determined | Genome-fragment | NA | 0 |
| S11-final-viral-combined_k141_58063\|\|full | 5498 | No | NA | 0 | 6 | Not-determined | Genome-fragment | NA | 0 |
| S11-final-viral-combined_k141_209823\|\|full | 2018 | No | NA | 0 | 2 | Not-determined | Genome-fragment | NA | 0 |
| S12-final-viral-combined_k141_216548\|\|full | 1096 | No | NA | 0 | 1 | Low-quality | Genome-fragment | 0.82 | 0 |
| S11-final-viral-combined_k141_278616\|\|full | 6260 | No | NA | 0 | 3 | Not-determined | Genome-fragment | NA | 0 |
| S11-final-viral-combined_k141_278622\|\|full | 2558 | No | NA | 0 | 3 | Not-determined | Genome-fragment | NA | 0 |
| S12-final-viral-combined_k141_310809\|\|lt2gene | 1096 | No | NA | 2 | 0 | Low-quality | Genome-fragment | 1.35 | 0 |
| S11-final-viral-combined_k141_85317\|\|full | 4878 | No | NA | 0 | 3 | Not-determined | Genome-fragment | NA | 0 |
| S11-final-viral-combined_k141_168339\|\|full | 5631 | No | NA | 0 | 2 | Not-determined | Genome-fragment | NA | 0 |
| S12_k141_59398 | 1096 | No | NA | 0 | 1 | Low-quality | Genome-fragment | 1.21 | 0 |
| S11-final-viral-combined_k141_112546\|\|full | 3378 | No | NA | 0 | 3 | Not-determined | Genome-fragment | NA | 0 |
| S11-final-viral-combined_k141_154333\|\|full | 3327 | No | NA | 0 | 2 | Not-determined | Genome-fragment | NA | 0 |
| S4-final-viral-combined_k141_27992\|\|full | 1096 | No | NA | 0 | 0 | Low-quality | Genome-fragment | 3.17 | 0 |
| S11-final-viral-combined_k141_250800\|\|full | 4425 | No | NA | 0 | 3 | Not-determined | Genome-fragment | NA | 0 |
| S4-final-viral-combined_k141_144693\|\|full | 1096 | No | NA | 2 | 0 | Low-quality | Genome-fragment | 2.77 | 0 |
| S6_k141_66036 | 1096 | No | NA | 0 | 2 | Low-quality | Genome-fragment | 1.39 | 0 |
| S8-final-viral-combined_k141_232863\|\|full | 1096 | No | NA | 0 | 1 | Low-quality | Genome-fragment | 1.43 | 0 |
| S8-final-viral-combined_k141_168306\|\|full | 1096 | No | NA | 0 | 3 | Low-quality | Genome-fragment | 1.34 | 0 |
| S1-final-viral-combined_k141_8013\|\|full | 1097 | No | NA | 0 | 1 | Low-quality | Genome-fragment | 0.8 | 0 |
| S11-final-viral-combined_k141_126689\|\|full | 1068 | No | NA | 0 | 0 | Not-determined | Genome-fragment | NA | 0 |
| S11-final-viral-combined_k141_44034\|\|full | 1979 | No | NA | 0 | 0 | Not-determined | Genome-fragment | NA | 0 |
| S3_k141_43350 | 1097 | No | NA | 0 | 1 | Low-quality | Genome-fragment | 0.98 | 0 |
| S11-final-viral-combined_k141_168389\|\|full | 1864 | No | NA | 0 | 1 | Not-determined | Genome-fragment | NA | 0 |
| S11-final-viral-combined_k141_99466\|\|full | 1287 | No | NA | 0 | 1 | Not-determined | Genome-fragment | NA | 0 |
| S11-final-viral-combined_k141_264551\|\|full | 2864 | No | NA | 0 | 0 | Not-determined | Genome-fragment | NA | 0 |
| S5_k141_149634 | 1097 | No | NA | 2 | 0 | Low-quality | Genome-fragment | 2.04 | 0 |
| S11-final-viral-combined_k141_44071\|\|full | 1884 | No | NA | 0 | 2 | Not-determined | Genome-fragment | NA | 0 |
| S7-final-viral-combined_k141_163862\|\|lt2gene | 1097 | No | NA | 0 | 0 | Low-quality | Genome-fragment | 2.65 | 0 |
| S17-final-viral-combined_k141_58415\|\|lt2gene | 1097 | No | NA | 1 | 0 | Low-quality | Genome-fragment | 2.82 | 0 |
| S12_k141_236282 | 1097 | No | NA | 0 | 1 | Low-quality | Genome-fragment | 1.89 | 0 |
| S11-final-viral-combined_k141_44125\|\|full | 3365 | No | NA | 0 | 2 | Not-determined | Genome-fragment | NA | 0 |
| S11-final-viral-combined_k141_126781\|\|full | 2606 | No | NA | 0 | 2 | Not-determined | Genome-fragment | NA | 0 |
| S2-final-viral-combined_k141_46532\|\|lt2gene | 1097 | No | NA | 1 | 0 | Low-quality | Genome-fragment | 2.23 | 0 |
| S2-final-viral-combined_k141_216099\|\|lt2gene | 1097 | No | NA | 1 | 0 | Low-quality | Genome-fragment | 2.73 | 0 |
| S11-final-viral-combined_k141_154394\|\|full | 1445 | No | NA | 0 | 2 | Not-determined | Genome-fragment | NA | 0 |
| S8-final-viral-combined_k141_200502\|\|lt2gene | 1097 | No | NA | 1 | 0 | Low-quality | Genome-fragment | 2.67 | 0 |
| S11-final-viral-combined_k141_223391\|\|full | 1480 | No | NA | 0 | 0 | Not-determined | Genome-fragment | NA | 0 |
| S11-final-viral-combined_k141_168515\|\|full | 1873 | No | NA | 0 | 0 | Not-determined | Genome-fragment | NA | 0 |
| S11-final-viral-combined_k141_16494\|\|full | 6970 | No | NA | 0 | 6 | Not-determined | Genome-fragment | NA | 0 |
| S11-final-viral-combined_k141_292043\|\|full | 2728 | No | NA | 0 | 3 | Not-determined | Genome-fragment | NA | 0 |
| S11-final-viral-combined_k141_58228\|\|full | 1536 | No | NA | 0 | 1 | Not-determined | Genome-fragment | NA | 0 |
| S8_k141_201130 | 1097 | No | NA | 0 | 0 | Low-quality | Genome-fragment | 0.78 | 0 |
| S11-final-viral-combined_k141_98309\|\|full | 1098 | No | NA | 0 | 0 | Low-quality | Genome-fragment | 2.48 | 0 |
| S11-final-viral-combined_k141_122375\|\|full | 1098 | No | NA | 0 | 0 | Low-quality | Genome-fragment | 2.3 | 0 |
| S11-final-viral-combined_k141_85484\|\|full | 4792 | No | NA | 0 | 4 | Not-determined | Genome-fragment | NA | 0 |
| S11_k141_138549 | 1098 | No | NA | 1 | 0 | Low-quality | Genome-fragment | 1.75 | 0 |
| S11-final-viral-combined_k141_223435\|\|full | 3352 | No | NA | 0 | 4 | Not-determined | Genome-fragment | NA | 0 |
| S11-final-viral-combined_k141_306288\|\|full | 4708 | No | NA | 0 | 3 | Not-determined | Genome-fragment | NA | 0 |
| S11-final-viral-combined_k141_126840\|\|full | 4587 | No | NA | 0 | 4 | Not-determined | Genome-fragment | NA | 0 |
| S11-final-viral-combined_k141_181717\|\|full | 1917 | No | NA | 0 | 1 | Not-determined | Genome-fragment | NA | 0 |
| S1-final-viral-combined_k141_11249\|\|full | 1098 | No | NA | 1 | 0 | Low-quality | Genome-fragment | 2.61 | 0 |
| S5-final-viral-combined_k141_11926\|\|full | 1098 | No | NA | 0 | 0 | Low-quality | Genome-fragment | 2.84 | 0 |
| S11-final-viral-combined_k141_237557\|\|full | 8994 | No | NA | 0 | 6 | Not-determined | Genome-fragment | NA | 0 |
| S11-final-viral-combined_k141_181745\|\|full | 1799 | No | NA | 0 | 0 | Not-determined | Genome-fragment | NA | 0 |
| S11-final-viral-combined_k141_126850\|\|full | 2725 | No | NA | 0 | 1 | Not-determined | Genome-fragment | NA | 0 |
| S7_k141_219939 | 1098 | No | NA | 0 | 0 | Low-quality | Genome-fragment | 0.82 | 0 |
| S11-final-viral-combined_k141_181770\|\|full | 1638 | No | NA | 0 | 0 | Not-determined | Genome-fragment | NA | 0 |
| S17-k141_74001 | 1098 | No | NA | 0 | 3 | Low-quality | Genome-fragment | 3.1 | 0 |
| S11-final-viral-combined_k141_58323\|\|full | 2916 | No | NA | 0 | 3 | Not-determined | Genome-fragment | NA | 0 |
| S11-final-viral-combined_k141_126864\|\|full | 2595 | No | NA | 0 | 2 | Not-determined | Genome-fragment | NA | 0 |
| S11-final-viral-combined_k141_30396\|\|full | 2842 | No | NA | 0 | 1 | Not-determined | Genome-fragment | NA | 0 |
| S11-final-viral-combined_k141_112733\|\|full | 34643 | No | NA | 22 | 1 | High-quality | High-quality | 95.1 | 0 |
| S12-final-viral-combined_k141_383132\|\|lt2gene | 1098 | No | NA | 1 | 0 | Low-quality | Genome-fragment | 2.55 | 0 |
| S12_k141_59332 | 1098 | No | NA | 0 | 1 | Low-quality | Genome-fragment | 0.37 | 0 |
| S11-final-viral-combined_k141_210043\|\|full | 1013 | No | NA | 0 | 2 | Not-determined | Genome-fragment | NA | 0 |
| S4-final-viral-combined_k141_107094\|\|lt2gene | 1098 | No | NA | 1 | 0 | Low-quality | Genome-fragment | 6.43 | 0 |
| S11-final-viral-combined_k141_168654\|\|full | 2833 | No | NA | 0 | 2 | Not-determined | Genome-fragment | NA | 0 |
| S9_k141_105499 | 1099 | No | NA | 0 | 0 | Low-quality | Genome-fragment | 2.56 | 0 |
| S11-final-viral-combined_k141_3041\|\|full | 1292 | No | NA | 0 | 1 | Not-determined | Genome-fragment | NA | 0 |
| S11-final-viral-combined_k141_238404\|\|full | 1099 | No | NA | 0 | 0 | Low-quality | Genome-fragment | 2.73 | 0 |
| S11-final-viral-combined_k141_238790\|\|full | 1099 | No | NA | 0 | 1 | Low-quality | Genome-fragment | 0.82 | 0 |
| S11-final-viral-combined_k141_140706\|\|full | 3472 | No | NA | 0 | 3 | Not-determined | Genome-fragment | NA | 0 |
| S11_k141_265804 | 1099 | No | NA | 1 | 0 | Low-quality | Genome-fragment | 2.99 | 0 |
| S11-final-viral-combined_k141_58388\|\|full | 6454 | No | NA | 0 | 4 | Not-determined | Genome-fragment | NA | 0 |
| S11_k141_173161 | 1099 | No | NA | 0 | 0 | Low-quality | Genome-fragment | 0.62 | 0 |
| S11_k141_173719 | 1099 | No | NA | 1 | 0 | Low-quality | Genome-fragment | 1.83 | 0 |
| S1-final-viral-combined_k141_363124\|\|full | 1099 | No | NA | 1 | 0 | Low-quality | Genome-fragment | 2.43 | 0 |
| S11-final-viral-combined_k141_250998\|\|full | 1961 | No | NA | 0 | 0 | Not-determined | Genome-fragment | NA | 0 |
| S11-final-viral-combined_k141_210116\|\|full | 2869 | No | NA | 0 | 3 | Not-determined | Genome-fragment | NA | 0 |
| S11-final-viral-combined_k141_223530\|\|full | 5243 | No | NA | 0 | 4 | Not-determined | Genome-fragment | NA | 0 |
| S1-final-viral-combined_k141_275741\|\|full | 1099 | No | NA | 2 | 0 | Low-quality | Genome-fragment | 2.51 | 0 |
| S11-final-viral-combined_k141_126984\|\|full | 5222 | No | NA | 0 | 4 | Not-determined | Genome-fragment | NA | 0 |
| S3-final-viral-combined_k141_43716\|\|full | 1099 | No | NA | 0 | 0 | Low-quality | Genome-fragment | 1.95 | 0 |
| S5_k141_125357 | 1099 | No | NA | 1 | 0 | Low-quality | Genome-fragment | 2.57 | 0 |
| S11-final-viral-combined_k141_140743\|\|full | 2718 | No | NA | 0 | 0 | Not-determined | Genome-fragment | NA | 0 |
| S11-final-viral-combined_k141_112847\|\|full | 3397 | No | NA | 0 | 3 | Not-determined | Genome-fragment | NA | 0 |
| S11-final-viral-combined_k141_168697\|\|full | 3683 | No | NA | 0 | 2 | Not-determined | Genome-fragment | NA | 0 |
| S7_k141_96765 | 1099 | No | NA | 0 | 0 | Low-quality | Genome-fragment | 1.13 | 0 |

| | | | | | | | | | |
|---|---|---|---|---|---|---|---|---|---|
| S12-final-viral-combined_k141_323559\|\|lt2gene | 1099 | No | NA | 1 | 0 | Low-quality | Genome-fragment | 2.41 | 0 |
| S2-final-viral-combined_k141_4517\|\|full | 1099 | No | NA | 1 | 0 | Low-quality | Genome-fragment | 2.74 | 0 |
| S11-final-viral-combined_k141_306508\|\|full | 3742 | No | NA | 0 | 2 | Not-determined | Genome-fragment | NA | 0 |
| S11-final-viral-combined_k141_30534\|\|full | 1796 | No | NA | 0 | 0 | Not-determined | Genome-fragment | NA | 0 |
| S11-final-viral-combined_k141_30538\|\|full | 1441 | No | NA | 0 | 0 | Not-determined | Genome-fragment | NA | 0 |
| S2_k141_230241 | 1099 | No | NA | 0 | 0 | Low-quality | Genome-fragment | 1.57 | 0 |
| S2_k141_79670 | 1099 | No | NA | 0 | 0 | Low-quality | Genome-fragment | 0.52 | 0 |
| S11-final-viral-combined_k141_237709\|\|full | 1256 | No | NA | 0 | 0 | Not-determined | Genome-fragment | NA | 0 |
| S4-final-viral-combined_k141_21354\|\|lt2gene | 1099 | No | NA | 1 | 0 | Low-quality | Genome-fragment | 1.48 | 0 |
| S11-final-viral-combined_k141_99757\|\|full | 15586 | No | NA | 0 | 13 | Not-determined | Genome-fragment | NA | 0 |
| S11-final-viral-combined_k141_99758\|\|full | 1109 | No | NA | 0 | 1 | Not-determined | Genome-fragment | NA | 0 |
| S6-final-viral-combined_k141_66711\|\|full | 1099 | No | NA | 1 | 0 | Low-quality | Genome-fragment | 1.98 | 0 |
| S8-final-viral-combined_k141_25523\|\|lt2gene | 1099 | No | NA | 0 | 0 | Low-quality | Genome-fragment | 1.55 | 0 |
| S11-final-viral-combined_k141_30567\|\|full | 1946 | No | NA | 0 | 2 | Not-determined | Genome-fragment | NA | 0 |
| S8-final-viral-combined_k141_132765\|\|lt2gene | 1099 | No | NA | 1 | 0 | Low-quality | Genome-fragment | 1.23 | 0 |
| S11-final-viral-combined_k141_140835\|\|full | 4220 | No | NA | 0 | 4 | Not-determined | Genome-fragment | NA | 0 |
| S11-final-viral-combined_k141_99763\|\|full | 3416 | No | NA | 0 | 0 | Not-determined | Genome-fragment | NA | 0 |
| S3_k141_65342 | 1100 | No | NA | 0 | 0 | Low-quality | Genome-fragment | 2.37 | 0 |
| S11-final-viral-combined_k141_210173\|\|full | 2708 | No | NA | 0 | 2 | Not-determined | Genome-fragment | NA | 0 |
| S11-final-viral-combined_k141_210183\|\|full | 1568 | No | NA | 0 | 1 | Not-determined | Genome-fragment | NA | 0 |
| S5-final-viral-combined_k141_206150\|\|full | 1100 | No | NA | 0 | 1 | Low-quality | Genome-fragment | 1.57 | 0 |
| S5-final-viral-combined_k141_38691\|\|full | 1100 | No | NA | 0 | 0 | Low-quality | Genome-fragment | 2.44 | 0 |
| S5_k141_106412 | 1100 | No | NA | 1 | 0 | Low-quality | Genome-fragment | 0.74 | 0 |
| S11-final-viral-combined_k141_237759\|\|full | 3709 | No | NA | 0 | 3 | Not-determined | Genome-fragment | NA | 0 |
| S7-final-viral-combined_k141_120762\|\|full | 1100 | No | NA | 0 | 0 | Low-quality | Genome-fragment | 2.35 | 0 |
| S17-final-viral-combined_k141_81699\|\|full | 1100 | No | NA | 0 | 1 | Low-quality | Genome-fragment | 1.83 | 0 |
| S11-final-viral-combined_k141_292215\|\|full | 1036 | No | NA | 0 | 1 | Not-determined | Genome-fragment | NA | 0 |
| S11-final-viral-combined_k141_168794\|\|full | 2853 | No | NA | 0 | 2 | Not-determined | Genome-fragment | NA | 0 |
| S11-final-viral-combined_k141_154801\|\|full | 2012 | No | NA | 0 | 0 | Not-determined | Genome-fragment | NA | 0 |
| S11-final-viral-combined_k141_58538\|\|full | 35663 | No | NA | 19 | 1 | Medium-quality | Genome-fragment | 68.08 | 0 |
| S17-final-viral-combined_k141_140852\|\|full | 1100 | No | NA | 0 | 2 | Low-quality | Genome-fragment | 1.4 | 0 |
| S17-final-viral-combined_k141_211305\|\|lt2gene | 1100 | No | NA | 1 | 0 | Low-quality | Genome-fragment | 1.77 | 0 |
| S11-final-viral-combined_k141_251133\|\|full | 1404 | No | NA | 0 | 2 | Not-determined | Genome-fragment | NA | 0 |
| S11-final-viral-combined_k141_279015\|\|full | 1557 | No | NA | 0 | 2 | Not-determined | Genome-fragment | NA | 0 |
| S17-k141_141849 | 1100 | No | NA | 0 | 0 | Low-quality | Genome-fragment | 2.17 | 0 |
| S11-final-viral-combined_k141_264960\|\|full | 1323 | No | NA | 0 | 2 | Not-determined | Genome-fragment | NA | 0 |
| S12-final-viral-combined_k141_18825\|\|full | 1100 | No | NA | 1 | 0 | Low-quality | Genome-fragment | 2.58 | 0 |
| S11-final-viral-combined_k141_3171\|\|full | 1639 | No | NA | 0 | 2 | Not-determined | Genome-fragment | NA | 0 |
| S12-final-viral-combined_k141_270434\|\|full | 1100 | No | NA | 0 | 1 | Low-quality | Genome-fragment | 1.31 | 0 |
| S12-final-viral-combined_k141_213960\|\|full | 1100 | No | NA | 0 | 0 | Low-quality | Genome-fragment | 0.64 | 0 |
| S11-final-viral-combined_k141_71617\|\|full | 1489 | No | NA | 0 | 1 | Not-determined | Genome-fragment | NA | 0 |
| S11-final-viral-combined_k141_16852\|\|full | 2856 | No | NA | 0 | 0 | Not-determined | Genome-fragment | NA | 0 |
| S11-final-viral-combined_k141_195895\|\|full | 3898 | No | NA | 0 | 0 | Not-determined | Genome-fragment | NA | 0 |
| S11-final-viral-combined_k141_127152\|\|full | 1173 | No | NA | 0 | 0 | Not-determined | Genome-fragment | NA | 0 |
| S4-final-viral-combined_k141_118932\|\|full | 1100 | No | NA | 0 | 1 | Low-quality | Genome-fragment | 0.66 | 0 |
| S4-final-viral-combined_k141_59354\|\|lt2gene | 1100 | No | NA | 2 | 0 | Low-quality | Genome-fragment | 3.68 | 0 |
| S11-final-viral-combined_k141_319717\|\|full | 1902 | No | NA | 0 | 1 | Not-determined | Genome-fragment | NA | 0 |
| S11-final-viral-combined_k141_168886\|\|full | 3414 | No | NA | 0 | 2 | Not-determined | Genome-fragment | NA | 0 |
| S4_k141_137683 | 1100 | No | NA | 1 | 0 | Low-quality | Genome-fragment | 0.42 | 0 |
| S6-final-viral-combined_k141_123476\|\|full | 1100 | No | NA | 0 | 0 | Low-quality | Genome-fragment | 1.28 | 0 |
| S8_k141_273351 | 1100 | No | NA | 0 | 0 | Low-quality | Genome-fragment | 1.17 | 0 |
| S11-final-viral-combined_k141_168894\|\|full | 1469 | No | NA | 0 | 1 | Not-determined | Genome-fragment | NA | 0 |
| S1-final-viral-combined_k141_191475\|\|full | 1101 | No | NA | 1 | 0 | Low-quality | Genome-fragment | 2.24 | 0 |
| S11-final-viral-combined_k141_195935\|\|full | 2459 | No | NA | 0 | 0 | Not-determined | Genome-fragment | NA | 0 |
| S1-final-viral-combined_k141_198022\|\|lt2gene | 1101 | No | NA | 1 | 0 | Low-quality | Genome-fragment | 2.52 | 0 |
| S1_k141_275097 | 1101 | No | NA | 0 | 0 | Low-quality | Genome-fragment | 2.8 | 0 |
| S7-final-viral-combined_k141_185762\|\|lt2gene | 1101 | No | NA | 1 | 0 | Low-quality | Genome-fragment | 2.72 | 0 |
| S11-final-viral-combined_k141_319774\|\|full | 1705 | No | NA | 0 | 2 | Not-determined | Genome-fragment | NA | 0 |
| S17-final-viral-combined_k141_151021\|\|full | 1101 | No | NA | 0 | 2 | Low-quality | Genome-fragment | 2.51 | 0 |
| S11-final-viral-combined_k141_99916\|\|full | 3504 | No | NA | 0 | 0 | Not-determined | Genome-fragment | NA | 0 |
| S12-final-viral-combined_k141_116057\|\|full | 1101 | No | NA | 0 | 2 | Low-quality | Genome-fragment | 2.31 | 0 |
| S2-final-viral-combined_k141_101010\|\|full | 1101 | No | NA | 0 | 0 | Low-quality | Genome-fragment | 2.24 | 0 |
| S6-final-viral-combined_k141_84259\|\|full | 1101 | No | NA | 0 | 1 | Low-quality | Genome-fragment | 1.59 | 0 |
| S6-final-viral-combined_k141_92039\|\|full | 1101 | No | NA | 0 | 1 | Low-quality | Genome-fragment | 1.27 | 0 |
| S11-final-viral-combined_k141_58607\|\|full | 1644 | No | NA | 0 | 1 | Not-determined | Genome-fragment | NA | 0 |
| S11-final-viral-combined_k141_279160\|\|full | 3143 | No | NA | 0 | 1 | Not-determined | Genome-fragment | NA | 0 |
| S6-final-viral-combined_k141_121166\|\|full | 1101 | No | NA | 0 | 0 | Low-quality | Genome-fragment | 2.68 | 0 |
| S11-final-viral-combined_k141_127241\|\|full | 3269 | No | NA | 0 | 1 | Not-determined | Genome-fragment | NA | 0 |
| S8-final-viral-combined_k141_224221\|\|lt2gene | 1101 | No | NA | 1 | 0 | Low-quality | Genome-fragment | 1.03 | 0 |
| S11-final-viral-combined_k141_155656\|\|lt2gene | 1102 | No | NA | 0 | 1 | Low-quality | Genome-fragment | 1.2 | 0 |
| S11-final-viral-combined_k141_210192\|\|full | 1368 | No | NA | 0 | 1 | Not-determined | Genome-fragment | NA | 0 |
| S11-final-viral-combined_k141_237896\|\|full | 7225 | No | NA | 0 | 1 | Not-determined | Genome-fragment | NA | 0 |
| S11-final-viral-combined_k141_306746\|\|full | 1397 | No | NA | 0 | 1 | Not-determined | Genome-fragment | NA | 0 |
| S11-final-viral-combined_k141_196003\|\|full | 25024 | No | NA | 0 | 16 | Not-determined | Genome-fragment | NA | 0 |
| S11-final-viral-combined_k141_237902\|\|full | 2731 | No | NA | 0 | 2 | Not-determined | Genome-fragment | NA | 0 |
| S11-final-viral-combined_k141_30808\|\|full | 2259 | No | NA | 0 | 0 | Not-determined | Genome-fragment | NA | 0 |
| S11-final-viral-combined_k141_265060\|\|full | 1768 | No | NA | 0 | 2 | Not-determined | Genome-fragment | NA | 0 |
| S11-final-viral-combined_k141_58622\|\|full | 2061 | No | NA | 0 | 1 | Not-determined | Genome-fragment | NA | 0 |
| S1-final-viral-combined_k141_168174\|\|full | 1102 | No | NA | 0 | 2 | Low-quality | Genome-fragment | 0.53 | 0 |
| S11-final-viral-combined_k141_154845\|\|full | 25037 | No | NA | 22 | 0 | Medium-quality | Genome-fragment | 84.36 | 0 |
| S3_k141_89452 | 1102 | No | NA | 0 | 0 | Low-quality | Genome-fragment | 0.93 | 0 |
| S7-final-viral-combined_k141_138383\|\|full | 1102 | No | NA | 1 | 0 | Low-quality | Genome-fragment | 2.9 | 0 |
| S11-final-viral-combined_k141_279215\|\|full | 7111 | No | NA | 0 | 5 | Not-determined | Genome-fragment | NA | 0 |
| S11-final-viral-combined_k141_237940\|\|full | 3000 | No | NA | 0 | 0 | Not-determined | Genome-fragment | NA | 0 |
| S11-final-viral-combined_k141_154875\|\|full | 2638 | No | NA | 0 | 0 | Not-determined | Genome-fragment | NA | 0 |
| S7-final-viral-combined_k141_109828\|\|full | 1102 | No | NA | 0 | 0 | Low-quality | Genome-fragment | 2.46 | 0 |
| S11-final-viral-combined_k141_306804\|\|full | 3154 | No | NA | 0 | 0 | Not-determined | Genome-fragment | NA | 0 |
| S11-final-viral-combined_k141_100032\|\|full | 1272 | No | NA | 0 | 0 | Not-determined | Genome-fragment | NA | 0 |
| S11-final-viral-combined_k141_85931\|\|full | 2164 | No | NA | 0 | 2 | Not-determined | Genome-fragment | NA | 0 |
| S11-final-viral-combined_k141_100035\|\|full | 1369 | No | NA | 0 | 1 | Not-determined | Genome-fragment | NA | 0 |
| S7_k141_213637 | 1102 | No | NA | 0 | 1 | Low-quality | Genome-fragment | 2.21 | 0 |
| S17-final-viral-combined_k141_5177\|\|full | 1102 | No | NA | 0 | 0 | Low-quality | Genome-fragment | 2.18 | 0 |
| S17-final-viral-combined_k141_252406\|\|full | 1102 | No | NA | 0 | 1 | Low-quality | Genome-fragment | 1.55 | 0 |
| S11-final-viral-combined_k141_279251\|\|full | 2228 | No | NA | 0 | 2 | Not-determined | Genome-fragment | NA | 0 |
| S11-final-viral-combined_k141_154907\|\|full | 1422 | No | NA | 0 | 0 | Not-determined | Genome-fragment | NA | 0 |
| S12_k141_189259 | 1102 | No | NA | 0 | 0 | Low-quality | Genome-fragment | 2.11 | 0 |
| S11-final-viral-combined_k141_279272\|\|full | 8112 | No | NA | 0 | 4 | Not-determined | Genome-fragment | NA | 0 |
| S11-final-viral-combined_k141_319895\|\|full | 2995 | No | NA | 0 | 1 | Not-determined | Genome-fragment | NA | 0 |
| S12_k141_284395 | 1102 | No | NA | 0 | 0 | Low-quality | Genome-fragment | 16 | 0 |
| S11-final-viral-combined_k141_100079\|\|full | 7099 | No | NA | 0 | 5 | Not-determined | Genome-fragment | NA | 0 |
| S2_k141_115476 | 1102 | No | NA | 0 | 0 | Low-quality | Genome-fragment | 3.34 | 0 |
| S11-final-viral-combined_k141_265141\|\|full | 2626 | No | NA | 0 | 1 | Not-determined | Genome-fragment | NA | 0 |
| S6-final-viral-combined_k141_91275\|\|full | 1102 | No | NA | 0 | 0 | Low-quality | Genome-fragment | 1.74 | 0 |
| S11-final-viral-combined_k141_100091\|\|full | 5189 | No | NA | 0 | 4 | Not-determined | Genome-fragment | NA | 0 |
| S8-final-viral-combined_k141_219217\|\|full | 1102 | No | NA | 0 | 1 | Low-quality | Genome-fragment | 1.96 | 0 |
| S8-final-viral-combined_k141_225844\|\|lt2gene | 1102 | No | NA | 1 | 0 | Low-quality | Genome-fragment | 2.53 | 0 |
| S9-final-viral-combined_k141_51973\|\|full | 1103 | No | NA | 1 | 0 | Low-quality | Genome-fragment | 1.36 | 0 |
| S11-final-viral-combined_k141_198523\|\|full | 1103 | No | NA | 0 | 0 | Low-quality | Genome-fragment | 0.73 | 0 |
| S11-final-viral-combined_k141_141082\|\|full | 1695 | No | NA | 0 | 1 | Not-determined | Genome-fragment | NA | 0 |
| S11-final-viral-combined_k141_30888\|\|full | 2978 | No | NA | 0 | 3 | Not-determined | Genome-fragment | NA | 0 |
| S11-final-viral-combined_k141_154976\|\|full | 1699 | No | NA | 0 | 2 | Not-determined | Genome-fragment | NA | 0 |
| S11-final-viral-combined_k141_100124\|\|full | 2476 | No | NA | 0 | 0 | Not-determined | Genome-fragment | NA | 0 |
| S1-final-viral-combined_k141_7191\|\|full | 1103 | No | NA | 0 | 3 | Low-quality | Genome-fragment | 0.84 | 0 |
| S11-final-viral-combined_k141_3381\|\|full | 6826 | No | NA | 0 | 3 | Not-determined | Genome-fragment | NA | 0 |
| S11-final-viral-combined_k141_17029\|\|full | 30219 | No | NA | 5 | 1 | Medium-quality | Genome-fragment | 54.28 | 0 |
| S1_k141_234796 | 1103 | No | NA | 0 | 0 | Low-quality | Genome-fragment | 2.58 | 0 |
| S5-final-viral-combined_k141_169196\|\|full | 1103 | No | NA | 1 | 0 | Low-quality | Genome-fragment | 0.52 | 0 |
| S5-final-viral-combined_k141_20205\|\|lt2gene | 1103 | No | NA | 2 | 0 | Low-quality | Genome-fragment | 2.17 | 0 |
| S11-final-viral-combined_k141_127327\|\|full | 2000 | No | NA | 0 | 2 | Not-determined | Genome-fragment | NA | 0 |
| S7-final-viral-combined_k141_80103\|\|lt2gene | 1103 | No | NA | 1 | 0 | Low-quality | Genome-fragment | 1.86 | 0 |
| S11-final-viral-combined_k141_292437\|\|full | 1860 | No | NA | 0 | 1 | Not-determined | Genome-fragment | NA | 0 |
| S11-final-viral-combined_k141_320010\|\|full | 1810 | No | NA | 0 | 0 | Not-determined | Genome-fragment | NA | 0 |
| S11-final-viral-combined_k141_44721\|\|full | 2058 | No | NA | 0 | 2 | Not-determined | Genome-fragment | NA | 0 |
| S11-final-viral-combined_k141_210369\|\|full | 6782 | No | NA | 0 | 3 | Not-determined | Genome-fragment | NA | 0 |
| S11-final-viral-combined_k141_100171\|\|full | 5209 | No | NA | 0 | 5 | Not-determined | Genome-fragment | NA | 0 |
| S7_k141_20925 | 1103 | Yes | 617 | 1 | 1 | Low-quality | Genome-fragment | 1.52 | 44.06 |
| S11-final-viral-combined_k141_306971\|\|full | 2249 | No | NA | 0 | 1 | Not-determined | Genome-fragment | NA | 0 |
| S12_k141_167148 | 1103 | No | NA | 1 | 0 | Low-quality | Genome-fragment | 1.9 | 0 |
| S3-final-viral-combined_k141_23875\|\|full | 1104 | No | NA | 1 | 0 | Low-quality | Genome-fragment | 1.82 | 0 |
| S11-final-viral-combined_k141_210383\|\|full | 3325 | No | NA | 0 | 2 | Not-determined | Genome-fragment | NA | 0 |
| S3-final-viral-combined_k141_56749\|\|full | 1104 | No | NA | 0 | 1 | Low-quality | Genome-fragment | 1.25 | 0 |
| S11-final-viral-combined_k141_44751\|\|full | 19980 | No | NA | 0 | 16 | Not-determined | Genome-fragment | NA | 0 |
| S11-final-viral-combined_k141_169244\|\|full | 1716 | No | NA | 0 | 0 | Not-determined | Genome-fragment | NA | 0 |
| S11-final-viral-combined_k141_169247\|\|full | 2445 | No | NA | 0 | 3 | Not-determined | Genome-fragment | NA | 0 |
| S5-final-viral-combined_k141_125167\|\|full | 1104 | No | NA | 1 | 0 | Low-quality | Genome-fragment | 1.22 | 0 |
| S11-final-viral-combined_k141_292514\|\|full | 3758 | No | NA | 0 | 0 | Not-determined | Genome-fragment | NA | 0 |
| S11-final-viral-combined_k141_113239\|\|full | 2196 | No | NA | 0 | 1 | Not-determined | Genome-fragment | NA | 0 |
| S5-final-viral-combined_k141_135395\|\|full | 1104 | No | NA | 0 | 2 | Low-quality | Genome-fragment | 0.32 | 0 |
| S7-final-viral-combined_k141_60979\|\|lt2gene | 1104 | No | NA | 1 | 0 | Low-quality | Genome-fragment | 4.41 | 0 |
| S17-final-viral-combined_k141_72412\|\|full | 1104 | No | NA | 0 | 0 | Low-quality | Genome-fragment | 2.43 | 0 |
| S17-k141_164075 | 1104 | No | NA | 0 | 1 | Low-quality | Genome-fragment | 2.26 | 0 |
| S11-final-viral-combined_k141_141197\|\|full | 1868 | No | NA | 0 | 2 | Not-determined | Genome-fragment | NA | 0 |
| S11-final-viral-combined_k141_238131\|\|full | 1505 | No | NA | 0 | 2 | Not-determined | Genome-fragment | NA | 0 |
| S12-final-viral-combined_k141_50847\|\|full | 1104 | No | NA | 1 | 0 | Low-quality | Genome-fragment | 1.01 | 0 |
| S12-final-viral-combined_k141_230375\|\|lt2gene | 1104 | No | NA | 1 | 0 | Low-quality | Genome-fragment | 0.76 | 0 |
| S12_k141_322180 | 1104 | No | NA | 0 | 0 | Low-quality | Genome-fragment | 0.62 | 0 |
| S11-final-viral-combined_k141_169317\|\|full | 5954 | No | NA | 0 | 3 | Not-determined | Genome-fragment | NA | 0 |
| S11-final-viral-combined_k141_31018\|\|full | 2045 | No | NA | 0 | 1 | Not-determined | Genome-fragment | NA | 0 |
| S11-final-viral-combined_k141_71915\|\|full | 9424 | No | NA | 0 | 1 | Not-determined | Genome-fragment | NA | 0 |
| S2-final-viral-combined_k141_20766\|\|full | 1104 | No | NA | 1 | 0 | Low-quality | Genome-fragment | 3.76 | 0 |
| S2-final-viral-combined_k141_114710\|\|full | 1104 | No | NA | 0 | 0 | Low-quality | Genome-fragment | 0.81 | 0 |
| S11-final-viral-combined_k141_292605\|\|full | 1474 | No | NA | 0 | 0 | Not-determined | Genome-fragment | NA | 0 |
| S11-final-viral-combined_k141_182617\|\|full | 4298 | No | NA | 0 | 0 | Not-determined | Genome-fragment | NA | 0 |
| S2_k141_212493 | 1104 | No | NA | 0 | 0 | Low-quality | Genome-fragment | 1.75 | 0 |
| S11-final-viral-combined_k141_100323\|\|full | 1922 | No | NA | 0 | 1 | Not-determined | Genome-fragment | NA | 0 |
| S11-final-viral-combined_k141_155163\|\|full | 1164 | No | NA | 0 | 1 | Not-determined | Genome-fragment | NA | 0 |
| S11-final-viral-combined_k141_279469\|\|full | 3712 | No | NA | 0 | 0 | Not-determined | Genome-fragment | NA | 0 |
| S11-final-viral-combined_k141_113315\|\|full | 5021 | No | NA | 0 | 3 | Not-determined | Genome-fragment | NA | 0 |
| S11-final-viral-combined_k141_3507\|\|full | 82880 | Yes | 41645 | 18 | 35 | Complete | High-quality | 100 | 49.75 |
| S11-final-viral-combined_k141_71977\|\|full | 3178 | No | NA | 0 | 2 | Not-determined | Genome-fragment | NA | 0 |
| S11-final-viral-combined_k141_224183\|\|full | 1045 | No | NA | 0 | 0 | Not-determined | Genome-fragment | NA | 0 |
| S2_k141_26004 | 1104 | No | NA | 0 | 1 | Low-quality | Genome-fragment | 0.26 | 0 |
| S6-final-viral-combined_k141_41071\|\|full | 1104 | No | NA | 2 | 0 | Low-quality | Genome-fragment | 2 | 0 |
| S11-final-viral-combined_k141_3516\|\|full | 1204 | No | NA | 0 | 1 | Not-determined | Genome-fragment | NA | 0 |
| S11-final-viral-combined_k141_251437\|\|full | 10600 | No | NA | 0 | 9 | Not-determined | Genome-fragment | NA | 0 |
| S11-final-viral-combined_k141_44934\|\|full | 2353 | No | NA | 0 | 0 | Not-determined | Genome-fragment | NA | 0 |
| S11-final-viral-combined_k141_320191\|\|full | 4334 | No | NA | 0 | 2 | Not-determined | Genome-fragment | NA | 0 |
| S6_k141_60055 | 1104 | Yes | 527 | 1 | 1 | Low-quality | Genome-fragment | 0.15 | 52.26 |
| S11-final-viral-combined_k141_44944\|\|full | 2892 | No | NA | 0 | 2 | Not-determined | Genome-fragment | NA | 0 |
| S8_k141_156309 | 1104 | No | NA | 1 | 0 | Low-quality | Genome-fragment | 1.88 | 0 |
| S1-final-viral-combined_k141_102578\|\|full | 1105 | No | NA | 0 | 0 | Low-quality | Genome-fragment | 1.32 | 0 |
| S1-final-viral-combined_k141_131229\|\|lt2gene | 1105 | No | NA | 1 | 0 | Low-quality | Genome-fragment | 1.82 | 0 |
| S11-final-viral-combined_k141_210623\|\|full | 1395 | No | NA | 0 | 0 | Not-determined | Genome-fragment | NA | 0 |
| S11-final-viral-combined_k141_31075\|\|full | 1227 | No | NA | 0 | 1 | Not-determined | Genome-fragment | NA | 0 |

| | | | | | | | | | | | |
|---|---|---|---|---|---|---|---|---|---|---|---|
| S1-final-viral-combined_k141_147009||lt2gene | 1105 | No | NA | | 1 | 0 | Low-quality | Genome-fragment | | 3.02 | 0 |
| S11-final-viral-combined_k141_224240||full | 1252 | No | NA | | 0 | 0 | Not-determined | Genome-fragment | NA | | 0 |
| S11-final-viral-combined_k141_196187||full | 2285 | No | NA | | 0 | 2 | Not-determined | Genome-fragment | NA | | 0 |
| S1_k141_314062 | 1105 | No | NA | | 0 | 2 | Low-quality | Genome-fragment | | 0.35 | 0 |
| S1_k141_134301 | 1105 | No | NA | | 0 | 1 | Low-quality | Genome-fragment | | 2.47 | 0 |
| S11-final-viral-combined_k141_44977||full | 14535 | No | NA | | 0 | 6 | Not-determined | Genome-fragment | NA | | 0 |
| S11-final-viral-combined_k141_86440||full | 1117 | No | NA | | 0 | 1 | Not-determined | Genome-fragment | NA | | 0 |
| S11-final-viral-combined_k141_279539||full | 4149 | No | NA | | 0 | 3 | Not-determined | Genome-fragment | NA | | 0 |
| S11-final-viral-combined_k141_210655||full | 1658 | No | NA | | 0 | 2 | Not-determined | Genome-fragment | NA | | 0 |
| S11-final-viral-combined_k141_141351||full | 4306 | No | NA | | 0 | 6 | Not-determined | Genome-fragment | NA | | 0 |
| S3-final-viral-combined_k141_55832||full | 1105 | No | NA | | 0 | 0 | Low-quality | Genome-fragment | | 2.03 | 0 |
| S11-final-viral-combined_k141_320266||full | 1154 | No | NA | | 0 | 0 | Not-determined | Genome-fragment | NA | | 0 |
| S5-final-viral-combined_k141_205106||lt2gene | 1105 | No | NA | | 2 | 0 | Low-quality | Genome-fragment | | 2.38 | 0 |
| S5-final-viral-combined_k141_173360||lt2gene | 1105 | No | NA | | 2 | 0 | Low-quality | Genome-fragment | | 1.34 | 0 |
| S12-final-viral-combined_k141_80405||full | 1105 | No | NA | | 1 | 0 | Low-quality | Genome-fragment | | 1.68 | 0 |
| S11-final-viral-combined_k141_210668||full | 2311 | No | NA | | 0 | 3 | Not-determined | Genome-fragment | NA | | 0 |
| S12-final-viral-combined_k141_50052||full | 1105 | No | NA | | 0 | 0 | Low-quality | Genome-fragment | | 1.49 | 0 |
| S12-final-viral-combined_k141_158420||lt2gene | 1105 | No | NA | | 1 | 0 | Low-quality | Genome-fragment | | 1.97 | 0 |
| S11-final-viral-combined_k141_100495||full | 1573 | No | NA | | 0 | 0 | Not-determined | Genome-fragment | NA | | 0 |
| S2-final-viral-combined_k141_235562||full | 1105 | No | NA | | 1 | 0 | Low-quality | Genome-fragment | | 1.71 | 0 |
| S6-final-viral-combined_k141_111708||full | 1105 | No | NA | | 0 | 0 | Low-quality | Genome-fragment | | 0.64 | 0 |
| S11-final-viral-combined_k141_196225||full | 3954 | No | NA | | 0 | 1 | Not-determined | Genome-fragment | NA | | 0 |
| S8-final-viral-combined_k141_118713||full | 1105 | No | NA | | 0 | 0 | Low-quality | Genome-fragment | | 0.99 | 0 |
| S11-final-viral-combined_k141_182785||full | 4590 | No | NA | | 0 | 1 | Not-determined | Genome-fragment | NA | | 0 |
| S8-final-viral-combined_k141_82407||lt2gene | 1105 | No | NA | | 1 | 0 | Low-quality | Genome-fragment | | 1.27 | 0 |
| S11-final-viral-combined_k141_101819||lt2gene | 1106 | No | NA | | 1 | 0 | Low-quality | Genome-fragment | | 1.02 | 0 |
| S11-final-viral-combined_k141_279617||full | 2506 | No | NA | | 0 | 2 | Not-determined | Genome-fragment | NA | | 0 |
| S11-final-viral-combined_k141_141366||full | 1487 | No | NA | | 0 | 0 | Not-determined | Genome-fragment | NA | | 0 |
| S11-final-viral-combined_k141_141367||full | 1212 | No | NA | | 0 | 0 | Not-determined | Genome-fragment | NA | | 0 |
| S11-final-viral-combined_k141_127635||full | 2045 | No | NA | | 0 | 2 | Not-determined | Genome-fragment | NA | | 0 |
| S11-final-viral-combined_k141_3589||full | 25278 | No | NA | | 0 | 15 | Not-determined | Genome-fragment | NA | | 0 |
| S11-final-viral-combined_k141_123914||lt2gene | 1106 | No | NA | | 1 | 0 | Low-quality | Genome-fragment | | 3.11 | 0 |
| S11-final-viral-combined_k141_210725||full | 4802 | No | NA | | 0 | 2 | Not-determined | Genome-fragment | NA | | 0 |
| S1_k141_37854 | 1106 | No | NA | | 0 | 0 | Low-quality | Genome-fragment | | 0.63 | 0 |
| S11-final-viral-combined_k141_141402||full | 2618 | No | NA | | 0 | 1 | Not-determined | Genome-fragment | NA | | 0 |
| S11-final-viral-combined_k141_292874||full | 10593 | No | NA | | 0 | 5 | Not-determined | Genome-fragment | NA | | 0 |
| S11-final-viral-combined_k141_141417||full | 1948 | No | NA | | 0 | 2 | Not-determined | Genome-fragment | NA | | 0 |
| S11-final-viral-combined_k141_279651||full | 2028 | No | NA | | 0 | 1 | Not-determined | Genome-fragment | NA | | 0 |
| S1_k141_338709 | 1106 | No | NA | | 0 | 1 | Low-quality | Genome-fragment | | 2.42 | 0 |
| S3-final-viral-combined_k141_134633||lt2gene | 1106 | No | NA | | 1 | 0 | Low-quality | Genome-fragment | | 1.78 | 0 |
| S7_k141_189616 | 1106 | No | NA | | 0 | 0 | Low-quality | Genome-fragment | | 1.05 | 0 |
| S11-final-viral-combined_k141_113444||full | 4636 | No | NA | | 0 | 3 | Not-determined | Genome-fragment | NA | | 0 |
| S12-final-viral-combined_k141_65313||full | 1106 | No | NA | | 2 | 0 | Low-quality | Genome-fragment | | 2.73 | 0 |
| S11-final-viral-combined_k141_113448||full | 6419 | No | NA | | 0 | 1 | Not-determined | Genome-fragment | NA | | 0 |
| S11-final-viral-combined_k141_86587||full | 5982 | No | NA | | 0 | 3 | Not-determined | Genome-fragment | NA | | 0 |
| S12-final-viral-combined_k141_378751||lt2gene | 1106 | No | NA | | 1 | 0 | Low-quality | Genome-fragment | | 2.45 | 0 |
| S11-final-viral-combined_k141_155350||full | 1383 | No | NA | | 0 | 0 | Not-determined | Genome-fragment | NA | | 0 |
| S12_k141_271250 | 1106 | No | NA | | 0 | 0 | Low-quality | Genome-fragment | | 1.05 | 0 |
| S11-final-viral-combined_k141_127706||full | 1152 | No | NA | | 0 | 1 | Not-determined | Genome-fragment | NA | | 0 |
| S11-final-viral-combined_k141_265453||full | 4483 | No | NA | | 1 | 0 | High-quality | High-quality | | 100 | 0 |
| S11-final-viral-combined_k141_320409||full | 2418 | No | NA | | 0 | 1 | Not-determined | Genome-fragment | NA | | 0 |
| S11-final-viral-combined_k141_141474||full | 3338 | No | NA | | 0 | 0 | Not-determined | Genome-fragment | NA | | 0 |
| S12_k141_125715 | 1106 | No | NA | | 0 | 0 | Low-quality | Genome-fragment | | 1.8 | 0 |
| S11-final-viral-combined_k141_155379||full | 1244 | No | NA | | 0 | 1 | Not-determined | Genome-fragment | NA | | 0 |
| S2-final-viral-combined_k141_171994||full | 1106 | No | NA | | 0 | 1 | Low-quality | Genome-fragment | | 1.47 | 0 |
| S6_k141_124363 | 1106 | No | NA | | 0 | 1 | Low-quality | Genome-fragment | | 0.41 | 0 |
| S8-final-viral-combined_k141_235206||full | 1106 | No | NA | | 1 | 0 | Low-quality | Genome-fragment | | 2.8 | 0 |
| S8-final-viral-combined_k141_205250||full | 1106 | No | NA | | 0 | 1 | Low-quality | Genome-fragment | | 0.69 | 0 |
| S11-final-viral-combined_k141_265511||full | 1870 | No | NA | | 0 | 1 | Not-determined | Genome-fragment | NA | | 0 |
| S11-final-viral-combined_k141_155429||full | 2755 | No | NA | | 0 | 4 | Not-determined | Genome-fragment | NA | | 0 |
| S11-final-viral-combined_k141_3692||full | 7314 | No | NA | | 0 | 4 | Not-determined | Genome-fragment | NA | | 0 |
| S9-final-viral-combined_k141_103096||full | 1107 | No | NA | | 0 | 0 | Low-quality | Genome-fragment | | 3.02 | 0 |
| S11-final-viral-combined_k141_31984||full | 1107 | No | NA | | 2 | 0 | Low-quality | Genome-fragment | | 1.97 | 0 |
| S1_k141_269605 | 1107 | No | NA | | 0 | 1 | Low-quality | Genome-fragment | | 0.31 | 0 |
| S11-final-viral-combined_k141_86699||full | 1544 | No | NA | | 0 | 1 | Not-determined | Genome-fragment | NA | | 0 |
| S11-final-viral-combined_k141_141550||full | 1770 | No | NA | | 0 | 1 | Not-determined | Genome-fragment | NA | | 0 |
| S3_k141_100439 | 1107 | Yes | | 517 | 1 | 1 | Low-quality | Genome-fragment | | 0.24 | 53.3 |
| S7-final-viral-combined_k141_127462||lt2gene | 1107 | No | NA | | 2 | 0 | Low-quality | Genome-fragment | | 2.33 | 0 |
| S7_k141_102034 | 1107 | No | NA | | 0 | 0 | Low-quality | Genome-fragment | | 1.04 | 0 |
| S11-final-viral-combined_k141_141573||full | 1900 | No | NA | | 0 | 3 | Not-determined | Genome-fragment | NA | | 0 |
| S17-final-viral-combined_k141_160582||full | 1107 | No | NA | | 0 | 0 | Low-quality | Genome-fragment | | 2.04 | 0 |
| S12-final-viral-combined_k141_122837||full | 1107 | No | NA | | 0 | 0 | Low-quality | Genome-fragment | | 3.91 | 0 |
| S12-final-viral-combined_k141_224893||full | 1107 | No | NA | | 0 | 0 | Low-quality | Genome-fragment | | 0.49 | 0 |
| S12-final-viral-combined_k141_208239||lt2gene | 1107 | No | NA | | 1 | 0 | Low-quality | Genome-fragment | | 3.23 | 0 |
| S12_k141_371686 | 1107 | No | NA | | 0 | 0 | Low-quality | Genome-fragment | | 0.61 | 0 |
| S2-final-viral-combined_k141_244626||full | 1107 | No | NA | | 1 | 0 | Low-quality | Genome-fragment | | 2.38 | 0 |
| S11-final-viral-combined_k141_86725||full | 3391 | No | NA | | 0 | 1 | Not-determined | Genome-fragment | NA | | 0 |
| S4-final-viral-combined_k141_121387||full | 1107 | No | NA | | 0 | 1 | Low-quality | Genome-fragment | | 0.6 | 0 |
| S9-final-viral-combined_k141_71606||full | 1108 | No | NA | | 0 | 0 | Low-quality | Genome-fragment | | 1.36 | 0 |
| S11-final-viral-combined_k141_293050||full | 1160 | No | NA | | 0 | 3 | Not-determined | Genome-fragment | NA | | 0 |
| S11-final-viral-combined_k141_279848||full | 1012 | No | NA | | 0 | 1 | Not-determined | Genome-fragment | NA | | 0 |
| S11-final-viral-combined_k141_155521||full | 3228 | No | NA | | 0 | 0 | Not-determined | Genome-fragment | NA | | 0 |
| S11-final-viral-combined_k141_128336||full | 1108 | No | NA | | 0 | 0 | Low-quality | Genome-fragment | | 2.22 | 0 |
| S11-final-viral-combined_k141_31377||full | 2221 | No | NA | | 0 | 3 | Not-determined | Genome-fragment | NA | | 0 |
| S11-final-viral-combined_k141_279870||full | 1116 | No | NA | | 0 | 1 | Not-determined | Genome-fragment | NA | | 0 |
| S11-final-viral-combined_k141_155555||full | 1946 | No | NA | | 0 | 1 | Not-determined | Genome-fragment | NA | | 0 |
| S11-final-viral-combined_k141_224464||full | 2882 | No | NA | | 0 | 3 | Not-determined | Genome-fragment | NA | | 0 |
| S11-final-viral-combined_k141_59289||full | 2632 | No | NA | | 0 | 1 | Not-determined | Genome-fragment | NA | | 0 |
| S11-final-viral-combined_k141_261821||full | 1108 | No | NA | | 0 | 1 | Low-quality | Genome-fragment | | 1.65 | 0 |
| S11-final-viral-combined_k141_17428||full | 1254 | No | NA | | 0 | 0 | Not-determined | Genome-fragment | NA | | 0 |
| S11-final-viral-combined_k141_196476||full | 1552 | No | NA | | 0 | 2 | Not-determined | Genome-fragment | NA | | 0 |
| S11-final-viral-combined_k141_293133||full | 3673 | No | NA | | 0 | 2 | Not-determined | Genome-fragment | NA | | 0 |
| S11-final-viral-combined_k141_59315||full | 1446 | No | NA | | 0 | 1 | Not-determined | Genome-fragment | NA | | 0 |
| S1-final-viral-combined_k141_159363||full | 1108 | No | NA | | 2 | 0 | Low-quality | Genome-fragment | | 2.44 | 0 |
| S11-final-viral-combined_k141_100734||full | 2012 | No | NA | | 0 | 1 | Not-determined | Genome-fragment | NA | | 0 |
| S11-final-viral-combined_k141_238424||full | 1120 | No | NA | | 0 | 2 | Not-determined | Genome-fragment | NA | | 0 |
| S11-final-viral-combined_k141_320569||full | 4590 | No | NA | | 0 | 1 | Not-determined | Genome-fragment | NA | | 0 |
| S11-final-viral-combined_k141_72318||full | 1930 | No | NA | | 0 | 2 | Not-determined | Genome-fragment | NA | | 0 |
| S11-final-viral-combined_k141_17449||full | 1319 | No | NA | | 0 | 0 | Not-determined | Genome-fragment | NA | | 0 |
| S5-final-viral-combined_k141_94235||full | 1108 | No | NA | | 1 | 0 | Low-quality | Genome-fragment | | 2.83 | 0 |
| S5-final-viral-combined_k141_39700||lt2gene | 1108 | No | NA | | 1 | 0 | Low-quality | Genome-fragment | | 2.55 | 0 |
| S11-final-viral-combined_k141_113569||full | 1309 | No | NA | | 0 | 0 | Not-determined | Genome-fragment | NA | | 0 |
| S11-final-viral-combined_k141_31399||full | 1307 | No | NA | | 0 | 2 | Not-determined | Genome-fragment | NA | | 0 |
| S11-final-viral-combined_k141_86834||full | 3422 | No | NA | | 0 | 2 | Not-determined | Genome-fragment | NA | | 0 |
| S11-final-viral-combined_k141_293157||full | 8579 | No | NA | | 0 | 1 | Not-determined | Genome-fragment | NA | | 0 |
| S5_k141_113537 | 1108 | No | NA | | 0 | 0 | Low-quality | Genome-fragment | | 2.11 | 0 |
| S11-final-viral-combined_k141_127870||full | 3563 | No | NA | | 0 | 5 | Not-determined | Genome-fragment | NA | | 0 |
| S11-final-viral-combined_k141_100749||full | 2523 | No | NA | | 0 | 2 | Not-determined | Genome-fragment | NA | | 0 |
| S11-final-viral-combined_k141_45309||full | 1551 | No | NA | | 0 | 0 | Not-determined | Genome-fragment | NA | | 0 |
| S11-final-viral-combined_k141_251870||full | 5498 | No | NA | | 0 | 0 | Not-determined | Genome-fragment | NA | | 0 |
| S11-final-viral-combined_k141_320601||full | 5306 | No | NA | | 0 | 1 | Not-determined | Genome-fragment | NA | | 0 |
| S7-final-viral-combined_k141_63944||full | 1108 | No | NA | | 0 | 0 | Low-quality | Genome-fragment | | 2.82 | 0 |
| S11-final-viral-combined_k141_251886||full | 4078 | No | NA | | 0 | 3 | Not-determined | Genome-fragment | NA | | 0 |
| S7-final-viral-combined_k141_55363||full | 1108 | No | NA | | 1 | 0 | Low-quality | Genome-fragment | | 0.69 | 0 |
| S11-final-viral-combined_k141_224496||full | 2155 | No | NA | | 0 | 1 | Not-determined | Genome-fragment | NA | | 0 |
| S17-final-viral-combined_k141_247066||full | 1108 | No | NA | | 0 | 0 | Low-quality | Genome-fragment | | 5.23 | 0 |
| S17-final-viral-combined_k141_237991||full | 1108 | No | NA | | 0 | 1 | Low-quality | Genome-fragment | | 0.85 | 0 |
| S11-final-viral-combined_k141_3889||full | 1371 | No | NA | | 0 | 1 | Not-determined | Genome-fragment | NA | | 0 |
| S11-final-viral-combined_k141_113605||full | 1691 | No | NA | | 0 | 0 | Not-determined | Genome-fragment | NA | | 0 |
| S12_k141_302691 | 1108 | No | NA | | 0 | 1 | Low-quality | Genome-fragment | | 0.63 | 0 |
| S11-final-viral-combined_k141_279980||full | 7494 | No | NA | | 0 | 6 | Not-determined | Genome-fragment | NA | | 0 |
| S11-final-viral-combined_k141_72366||full | 8725 | No | NA | | 0 | 8 | Not-determined | Genome-fragment | NA | | 0 |
| S4-final-viral-combined_k141_88055||lt2gene | 1108 | No | NA | | 0 | 0 | Low-quality | Genome-fragment | | 2.56 | 0 |
| S11-final-viral-combined_k141_113627||full | 5037 | No | NA | | 0 | 2 | Not-determined | Genome-fragment | NA | | 0 |
| S11-final-viral-combined_k141_224538||full | 5670 | No | NA | | 0 | 1 | Not-determined | Genome-fragment | NA | | 0 |
| S6-final-viral-combined_k141_140197||full | 1108 | No | NA | | 0 | 0 | Low-quality | Genome-fragment | | 0.71 | 0 |
| S11-final-viral-combined_k141_100782||full | 3276 | No | NA | | 0 | 2 | Not-determined | Genome-fragment | NA | | 0 |
| S11-final-viral-combined_k141_280054||full | 4466 | No | NA | | 0 | 3 | Not-determined | Genome-fragment | NA | | 0 |
| S11-final-viral-combined_k141_300273||full | 1109 | No | NA | | 1 | 0 | Low-quality | Genome-fragment | | 2.94 | 0 |
| S11-final-viral-combined_k141_265795||full | 1457 | No | NA | | 0 | 0 | Not-determined | Genome-fragment | NA | | 0 |
| S11-final-viral-combined_k141_72418||full | 6660 | No | NA | | 0 | 7 | Not-determined | Genome-fragment | NA | | 0 |
| S11-final-viral-combined_k141_84132||lt2gene | 1109 | No | NA | | 2 | 0 | Low-quality | Genome-fragment | | 3.27 | 0 |
| S11-final-viral-combined_k141_169829||full | 6475 | No | NA | | 0 | 4 | Not-determined | Genome-fragment | NA | | 0 |
| S1-final-viral-combined_k141_362249||full | 1109 | No | NA | | 0 | 0 | Low-quality | Genome-fragment | | 0.83 | 0 |
| S11-final-viral-combined_k141_45457||full | 1515 | No | NA | | 0 | 1 | Not-determined | Genome-fragment | NA | | 0 |
| S11-final-viral-combined_k141_141868||full | 2893 | No | NA | | 0 | 2 | Not-determined | Genome-fragment | NA | | 0 |
| S11-final-viral-combined_k141_211311||full | 2345 | No | NA | | 0 | 3 | Not-determined | Genome-fragment | NA | | 0 |
| S11-final-viral-combined_k141_141880||full | 4066 | No | NA | | 0 | 5 | Not-determined | Genome-fragment | NA | | 0 |
| S11-final-viral-combined_k141_196625||full | 2544 | No | NA | | 0 | 3 | Not-determined | Genome-fragment | NA | | 0 |
| S11-final-viral-combined_k141_211342||full | 1681 | No | NA | | 0 | 0 | Not-determined | Genome-fragment | NA | | 0 |
| S1-final-viral-combined_k141_15988||full | 1109 | No | NA | | 0 | 0 | Low-quality | Genome-fragment | | 2.46 | 0 |
| S11-final-viral-combined_k141_141901||full | 2500 | No | NA | | 0 | 2 | Not-determined | Genome-fragment | NA | | 0 |
| S11-final-viral-combined_k141_238736||full | 3794 | No | NA | | 0 | 4 | Not-determined | Genome-fragment | NA | | 0 |
| S11-final-viral-combined_k141_252044||full | 1278 | No | NA | | 0 | 0 | Not-determined | Genome-fragment | NA | | 0 |
| S11-final-viral-combined_k141_307549||full | 1042 | No | NA | | 0 | 1 | Not-determined | Genome-fragment | NA | | 0 |
| S11-final-viral-combined_k141_265892||full | 3137 | No | NA | | 0 | 3 | Not-determined | Genome-fragment | NA | | 0 |
| S11-final-viral-combined_k141_293302||full | 3103 | No | NA | | 0 | 1 | Not-determined | Genome-fragment | NA | | 0 |
| S11-final-viral-combined_k141_59424||full | 3312 | No | NA | | 0 | 2 | Not-determined | Genome-fragment | NA | | 0 |
| S11-final-viral-combined_k141_72444||full | 2445 | No | NA | | 0 | 0 | Not-determined | Genome-fragment | NA | | 0 |
| S11-final-viral-combined_k141_293323||full | 1882 | No | NA | | 0 | 1 | Not-determined | Genome-fragment | NA | | 0 |
| S3-final-viral-combined_k141_104631||lt2gene | 1109 | No | NA | | 1 | 0 | Low-quality | Genome-fragment | | 4.08 | 0 |
| S11-final-viral-combined_k141_224695||full | 1757 | No | NA | | 0 | 0 | Not-determined | Genome-fragment | NA | | 0 |
| S11-final-viral-combined_k141_224703||full | 1602 | No | NA | | 0 | 1 | Not-determined | Genome-fragment | NA | | 0 |
| S5-final-viral-combined_k141_125717||full | 1109 | No | NA | | 0 | 1 | Low-quality | Genome-fragment | | 0.86 | 0 |
| S5-final-viral-combined_k141_136352||full | 1109 | No | NA | | 1 | 0 | Low-quality | Genome-fragment | | 3.19 | 0 |
| S17-final-viral-combined_k141_249961||full | 1109 | No | NA | | 0 | 0 | Low-quality | Genome-fragment | | 1.31 | 0 |
| S17-final-viral-combined_k141_55532||full | 1109 | No | NA | | 0 | 2 | Low-quality | Genome-fragment | | 0.42 | 0 |
| S17-final-viral-combined_k141_69863||lt2gene | 1109 | No | NA | | 1 | 0 | Low-quality | Genome-fragment | | 3.24 | 0 |
| S11-final-viral-combined_k141_252077||full | 2818 | No | NA | | 0 | 1 | Not-determined | Genome-fragment | NA | | 0 |
| S17-k141_247035 | 1109 | No | NA | | 0 | 0 | Low-quality | Genome-fragment | | 2.35 | 0 |
| S8-final-viral-combined_k141_243785||full | 1109 | No | NA | | 0 | 1 | Low-quality | Genome-fragment | | 0.95 | 0 |
| S11-final-viral-combined_k141_4024||full | 3783 | No | NA | | 0 | 3 | Not-determined | Genome-fragment | NA | | 0 |
| S11-final-viral-combined_k141_211449||full | 1155 | No | NA | | 0 | 1 | Not-determined | Genome-fragment | NA | | 0 |
| S11-final-viral-combined_k141_141980||full | 5595 | No | NA | | 0 | 2 | Not-determined | Genome-fragment | NA | | 0 |
| S8-final-viral-combined_k141_180108||lt2gene | 1109 | No | NA | | 1 | 0 | Low-quality | Genome-fragment | | 2.17 | 0 |
| S11-final-viral-combined_k141_238840||full | 1170 | No | NA | | 0 | 1 | Not-determined | Genome-fragment | NA | | 0 |
| S9-final-viral-combined_k141_22147||full | 1110 | No | NA | | 2 | 0 | Low-quality | Genome-fragment | | 1.82 | 0 |
| S11-final-viral-combined_k141_196723||full | 4995 | No | NA | | 0 | 2 | Not-determined | Genome-fragment | NA | | 0 |
| S11_k141_3965 | 1110 | No | NA | | 1 | 0 | Low-quality | Genome-fragment | | 1.37 | 0 |
| S11-final-viral-combined_k141_155744||full | 4127 | No | NA | | 0 | 3 | Not-determined | Genome-fragment | NA | | 0 |
| S11-final-viral-combined_k141_142011||full | 3762 | No | NA | | 0 | 2 | Not-determined | Genome-fragment | NA | | 0 |
| S5-final-viral-combined_k141_136852||full | 1110 | No | NA | | 0 | 3 | Low-quality | Genome-fragment | | 1.51 | 0 |
| S11-final-viral-combined_k141_142024||full | 1402 | No | NA | | 0 | 0 | Not-determined | Genome-fragment | NA | | 0 |
| S11-final-viral-combined_k141_128089||full | 4590 | No | NA | | 0 | 2 | Not-determined | Genome-fragment | NA | | 0 |

<table>
<tr><th></th><th></th><th></th><th></th><th></th><th></th><th></th><th></th><th></th><th></th><th></th><th></th></tr>
<tr><td>S11-final-viral-combined_k141_128090||full</td><td>5380</td><td>No</td><td>NA</td><td></td><td>0</td><td>1</td><td>Not-determined</td><td>Genome-fragment</td><td>NA</td><td></td><td>0</td></tr>
<tr><td>S11-final-viral-combined_k141_320710||full</td><td>44314</td><td>No</td><td>NA</td><td></td><td>7</td><td>0</td><td>High-quality</td><td>High-quality</td><td></td><td>99.26</td><td>0</td></tr>
<tr><td>S7-final-viral-combined_k141_142477||full</td><td>1110</td><td>No</td><td>NA</td><td></td><td>0</td><td>0</td><td>Low-quality</td><td>Genome-fragment</td><td></td><td>2.4</td><td>0</td></tr>
<tr><td>S17-final-viral-combined_k141_240018||full</td><td>1110</td><td>No</td><td>NA</td><td></td><td>0</td><td>0</td><td>Low-quality</td><td>Genome-fragment</td><td></td><td>2.06</td><td>0</td></tr>
<tr><td>S11-final-viral-combined_k141_59465||full</td><td>1441</td><td>No</td><td>NA</td><td></td><td>0</td><td>0</td><td>Not-determined</td><td>Genome-fragment</td><td>NA</td><td></td><td>0</td></tr>
<tr><td>S11-final-viral-combined_k141_87174||full</td><td>1997</td><td>No</td><td>NA</td><td></td><td>0</td><td>1</td><td>Not-determined</td><td>Genome-fragment</td><td>NA</td><td></td><td>0</td></tr>
<tr><td>S17-final-viral-combined_k141_41741||lt2gene</td><td>1110</td><td>No</td><td>NA</td><td></td><td>0</td><td>0</td><td>Low-quality</td><td>Genome-fragment</td><td></td><td>2.05</td><td>0</td></tr>
<tr><td>S11-final-viral-combined_k141_59468||full</td><td>4626</td><td>No</td><td>NA</td><td></td><td>0</td><td>4</td><td>Not-determined</td><td>Genome-fragment</td><td>NA</td><td></td><td>0</td></tr>
<tr><td>S11-final-viral-combined_k141_293463||full</td><td>3246</td><td>No</td><td>NA</td><td></td><td>0</td><td>3</td><td>Not-determined</td><td>Genome-fragment</td><td>NA</td><td></td><td>0</td></tr>
<tr><td>S11-final-viral-combined_k141_224781||full</td><td>1613</td><td>No</td><td>NA</td><td></td><td>0</td><td>1</td><td>Not-determined</td><td>Genome-fragment</td><td>NA</td><td></td><td>0</td></tr>
<tr><td>S11-final-viral-combined_k141_196765||full</td><td>2632</td><td>No</td><td>NA</td><td></td><td>0</td><td>1</td><td>Not-determined</td><td>Genome-fragment</td><td>NA</td><td></td><td>0</td></tr>
<tr><td>S11-final-viral-combined_k141_4071||full</td><td>26339</td><td>No</td><td>NA</td><td></td><td>0</td><td>18</td><td>Not-determined</td><td>Genome-fragment</td><td>NA</td><td></td><td>0</td></tr>
<tr><td>S8-final-viral-combined_k141_166079||full</td><td>1110</td><td>No</td><td>NA</td><td></td><td>1</td><td>0</td><td>Low-quality</td><td>Genome-fragment</td><td></td><td>2.98</td><td>0</td></tr>
<tr><td>S8-final-viral-combined_k141_75485||full</td><td>1110</td><td>No</td><td>NA</td><td></td><td>0</td><td>0</td><td>Low-quality</td><td>Genome-fragment</td><td></td><td>0.58</td><td>0</td></tr>
<tr><td>S11-final-viral-combined_k141_87209||full</td><td>1872</td><td>No</td><td>NA</td><td></td><td>0</td><td>0</td><td>Not-determined</td><td>Genome-fragment</td><td>NA</td><td></td><td>0</td></tr>
<tr><td>S11-final-viral-combined_k141_183386||full</td><td>2873</td><td>No</td><td>NA</td><td></td><td>0</td><td>2</td><td>Not-determined</td><td>Genome-fragment</td><td>NA</td><td></td><td>0</td></tr>
<tr><td>S8-final-viral-combined_k141_200966||full</td><td>1110</td><td>No</td><td>NA</td><td></td><td>0</td><td>0</td><td>Low-quality</td><td>Genome-fragment</td><td></td><td>1.78</td><td>0</td></tr>
<tr><td>S9-final-viral-combined_k141_70257||full</td><td>1111</td><td>No</td><td>NA</td><td></td><td>0</td><td>2</td><td>Low-quality</td><td>Genome-fragment</td><td></td><td>0.34</td><td>0</td></tr>
<tr><td>S9-final-viral-combined_k141_91066||lt2gene</td><td>1111</td><td>No</td><td>NA</td><td></td><td>1</td><td>0</td><td>Low-quality</td><td>Genome-fragment</td><td></td><td>1.65</td><td>0</td></tr>
<tr><td>S11-final-viral-combined_k141_59520||full</td><td>1269</td><td>No</td><td>NA</td><td></td><td>0</td><td>3</td><td>Not-determined</td><td>Genome-fragment</td><td>NA</td><td></td><td>0</td></tr>
<tr><td>S9_k141_7934</td><td>1111</td><td>No</td><td>NA</td><td></td><td>0</td><td>0</td><td>Low-quality</td><td>Genome-fragment</td><td></td><td>2.73</td><td>0</td></tr>
<tr><td>S17-final-viral-combined_k141_196092||lt2gene</td><td>1111</td><td>No</td><td>NA</td><td></td><td>0</td><td>0</td><td>Low-quality</td><td>Genome-fragment</td><td></td><td>2.64</td><td>0</td></tr>
<tr><td>S11-final-viral-combined_k141_224831||full</td><td>1431</td><td>No</td><td>NA</td><td></td><td>0</td><td>1</td><td>Not-determined</td><td>Genome-fragment</td><td>NA</td><td></td><td>0</td></tr>
<tr><td>S12-final-viral-combined_k141_84515||full</td><td>1111</td><td>No</td><td>NA</td><td></td><td>2</td><td>0</td><td>Low-quality</td><td>Genome-fragment</td><td></td><td>1.18</td><td>0</td></tr>
<tr><td>S11-final-viral-combined_k141_211569||full</td><td>2831</td><td>No</td><td>NA</td><td></td><td>0</td><td>0</td><td>Not-determined</td><td>Genome-fragment</td><td>NA</td><td></td><td>0</td></tr>
<tr><td>S11-final-viral-combined_k141_211570||full</td><td>1226</td><td>No</td><td>NA</td><td></td><td>0</td><td>1</td><td>Not-determined</td><td>Genome-fragment</td><td>NA</td><td></td><td>0</td></tr>
<tr><td>S11-final-viral-combined_k141_238964||full</td><td>3568</td><td>No</td><td>NA</td><td></td><td>0</td><td>0</td><td>Not-determined</td><td>Genome-fragment</td><td>NA</td><td></td><td>0</td></tr>
<tr><td>S12-final-viral-combined_k141_213761||full</td><td>1111</td><td>No</td><td>NA</td><td></td><td>2</td><td>0</td><td>Low-quality</td><td>Genome-fragment</td><td></td><td>1.38</td><td>0</td></tr>
<tr><td>S11-final-viral-combined_k141_87242||full</td><td>1783</td><td>No</td><td>NA</td><td></td><td>0</td><td>0</td><td>Not-determined</td><td>Genome-fragment</td><td>NA</td><td></td><td>0</td></tr>
<tr><td>S4_k141_118996</td><td>1111</td><td>No</td><td>NA</td><td></td><td>0</td><td>1</td><td>Low-quality</td><td>Genome-fragment</td><td></td><td>2.5</td><td>0</td></tr>
<tr><td>S11-final-viral-combined_k141_45766||full</td><td>1375</td><td>No</td><td>NA</td><td></td><td>0</td><td>1</td><td>Not-determined</td><td>Genome-fragment</td><td>NA</td><td></td><td>0</td></tr>
<tr><td>S11-final-viral-combined_k141_31735||full</td><td>1804</td><td>No</td><td>NA</td><td></td><td>0</td><td>1</td><td>Not-determined</td><td>Genome-fragment</td><td>NA</td><td></td><td>0</td></tr>
<tr><td>S8_k141_220634</td><td>1111</td><td>No</td><td>NA</td><td></td><td>1</td><td>0</td><td>Low-quality</td><td>Genome-fragment</td><td></td><td>1.12</td><td>0</td></tr>
<tr><td>S11-final-viral-combined_k141_19002||full</td><td>1112</td><td>No</td><td>NA</td><td></td><td>0</td><td>1</td><td>Low-quality</td><td>Genome-fragment</td><td></td><td>1.71</td><td>0</td></tr>
<tr><td>S11-final-viral-combined_k141_196849||full</td><td>1255</td><td>No</td><td>NA</td><td></td><td>0</td><td>1</td><td>Not-determined</td><td>Genome-fragment</td><td>NA</td><td></td><td>0</td></tr>
<tr><td>S11_k141_9081</td><td>1112</td><td>No</td><td>NA</td><td></td><td>0</td><td>0</td><td>Low-quality</td><td>Genome-fragment</td><td></td><td>1.73</td><td>0</td></tr>
<tr><td>S1-final-viral-combined_k141_1801||full</td><td>1112</td><td>No</td><td>NA</td><td></td><td>0</td><td>0</td><td>Low-quality</td><td>Genome-fragment</td><td></td><td>1.56</td><td>0</td></tr>
<tr><td>S5-final-viral-combined_k141_157644||full</td><td>1112</td><td>No</td><td>NA</td><td></td><td>2</td><td>0</td><td>Low-quality</td><td>Genome-fragment</td><td></td><td>2.3</td><td>0</td></tr>
<tr><td>S5_k141_24110</td><td>1112</td><td>No</td><td>NA</td><td></td><td>2</td><td>0</td><td>Low-quality</td><td>Genome-fragment</td><td></td><td>3.41</td><td>0</td></tr>
<tr><td>S17-final-viral-combined_k141_138583||full</td><td>1112</td><td>No</td><td>NA</td><td></td><td>0</td><td>0</td><td>Low-quality</td><td>Genome-fragment</td><td></td><td>1.62</td><td>0</td></tr>
<tr><td>S17-k141_72106</td><td>1112</td><td>No</td><td>NA</td><td></td><td>0</td><td>0</td><td>Low-quality</td><td>Genome-fragment</td><td></td><td>0.94</td><td>0</td></tr>
<tr><td>S11-final-viral-combined_k141_128238||full</td><td>1578</td><td>No</td><td>NA</td><td></td><td>0</td><td>0</td><td>Not-determined</td><td>Genome-fragment</td><td>NA</td><td></td><td>0</td></tr>
<tr><td>S11-final-viral-combined_k141_224892||full</td><td>3009</td><td>No</td><td>NA</td><td></td><td>0</td><td>2</td><td>Not-determined</td><td>Genome-fragment</td><td>NA</td><td></td><td>0</td></tr>
<tr><td>S11-final-viral-combined_k141_101124||full</td><td>2664</td><td>No</td><td>NA</td><td></td><td>0</td><td>2</td><td>Not-determined</td><td>Genome-fragment</td><td>NA</td><td></td><td>0</td></tr>
<tr><td>S11-final-viral-combined_k141_280409||full</td><td>5485</td><td>No</td><td>NA</td><td></td><td>0</td><td>5</td><td>Not-determined</td><td>Genome-fragment</td><td>NA</td><td></td><td>0</td></tr>
<tr><td>S11-final-viral-combined_k141_252337||full</td><td>1729</td><td>No</td><td>NA</td><td></td><td>0</td><td>1</td><td>Not-determined</td><td>Genome-fragment</td><td>NA</td><td></td><td>0</td></tr>
<tr><td>S11-final-viral-combined_k141_4199||full</td><td>2023</td><td>No</td><td>NA</td><td></td><td>0</td><td>0</td><td>Not-determined</td><td>Genome-fragment</td><td>NA</td><td></td><td>0</td></tr>
<tr><td>S12-final-viral-combined_k141_357122||full</td><td>1112</td><td>No</td><td>NA</td><td></td><td>0</td><td>0</td><td>Low-quality</td><td>Genome-fragment</td><td></td><td>2.23</td><td>0</td></tr>
<tr><td>S11-final-viral-combined_k141_293637||full</td><td>1292</td><td>No</td><td>NA</td><td></td><td>0</td><td>0</td><td>Not-determined</td><td>Genome-fragment</td><td>NA</td><td></td><td>0</td></tr>
<tr><td>S11-final-viral-combined_k141_183471||full</td><td>3734</td><td>No</td><td>NA</td><td></td><td>0</td><td>2</td><td>Not-determined</td><td>Genome-fragment</td><td>NA</td><td></td><td>0</td></tr>
<tr><td>S12-final-viral-combined_k141_66758||full</td><td>1112</td><td>No</td><td>NA</td><td></td><td>0</td><td>0</td><td>Low-quality</td><td>Genome-fragment</td><td></td><td>0.85</td><td>0</td></tr>
<tr><td>S11-final-viral-combined_k141_239067||full</td><td>1660</td><td>No</td><td>NA</td><td></td><td>0</td><td>2</td><td>Not-determined</td><td>Genome-fragment</td><td>NA</td><td></td><td>0</td></tr>
<tr><td>S11-final-viral-combined_k141_59649||full</td><td>3961</td><td>No</td><td>NA</td><td></td><td>0</td><td>2</td><td>Not-determined</td><td>Genome-fragment</td><td>NA</td><td></td><td>0</td></tr>
<tr><td>S12_k141_320047</td><td>1112</td><td>No</td><td>NA</td><td></td><td>0</td><td>0</td><td>Low-quality</td><td>Genome-fragment</td><td></td><td>0.98</td><td>0</td></tr>
<tr><td>S4-final-viral-combined_k141_108662||full</td><td>1112</td><td>No</td><td>NA</td><td></td><td>0</td><td>1</td><td>Low-quality</td><td>Genome-fragment</td><td></td><td>0.83</td><td>0</td></tr>
<tr><td>S4-final-viral-combined_k141_18445||full</td><td>1112</td><td>No</td><td>NA</td><td></td><td>0</td><td>0</td><td>Low-quality</td><td>Genome-fragment</td><td></td><td>1.09</td><td>0</td></tr>
<tr><td>S6-final-viral-combined_k141_74577||full</td><td>1112</td><td>No</td><td>NA</td><td></td><td>1</td><td>0</td><td>Low-quality</td><td>Genome-fragment</td><td></td><td>1.03</td><td>0</td></tr>
<tr><td>S11-final-viral-combined_k141_224945||full</td><td>3849</td><td>No</td><td>NA</td><td></td><td>0</td><td>4</td><td>Not-determined</td><td>Genome-fragment</td><td>NA</td><td></td><td>0</td></tr>
<tr><td>S11-final-viral-combined_k141_266239||full</td><td>1191</td><td>No</td><td>NA</td><td></td><td>0</td><td>0</td><td>Not-determined</td><td>Genome-fragment</td><td>NA</td><td></td><td>0</td></tr>
<tr><td>S8-final-viral-combined_k141_41875||full</td><td>1112</td><td>No</td><td>NA</td><td></td><td>0</td><td>0</td><td>Low-quality</td><td>Genome-fragment</td><td></td><td>1.36</td><td>0</td></tr>
<tr><td>S11-final-viral-combined_k141_239117||full</td><td>3082</td><td>No</td><td>NA</td><td></td><td>0</td><td>3</td><td>Not-determined</td><td>Genome-fragment</td><td>NA</td><td></td><td>0</td></tr>
<tr><td>S8-final-viral-combined_k141_275718||lt2gene</td><td>1112</td><td>No</td><td>NA</td><td></td><td>1</td><td>0</td><td>Low-quality</td><td>Genome-fragment</td><td></td><td>0.47</td><td>0</td></tr>
<tr><td>S11-final-viral-combined_k141_266244||full</td><td>1275</td><td>No</td><td>NA</td><td></td><td>0</td><td>1</td><td>Not-determined</td><td>Genome-fragment</td><td>NA</td><td></td><td>0</td></tr>
<tr><td>S11-final-viral-combined_k141_183534||full</td><td>2312</td><td>No</td><td>NA</td><td></td><td>0</td><td>0</td><td>Not-determined</td><td>Genome-fragment</td><td>NA</td><td></td><td>0</td></tr>
<tr><td>S11-final-viral-combined_k141_155959||full</td><td>2869</td><td>No</td><td>NA</td><td></td><td>0</td><td>2</td><td>Not-determined</td><td>Genome-fragment</td><td>NA</td><td></td><td>0</td></tr>
<tr><td>S11-final-viral-combined_k141_4269||full</td><td>1274</td><td>No</td><td>NA</td><td></td><td>0</td><td>1</td><td>Not-determined</td><td>Genome-fragment</td><td>NA</td><td></td><td>0</td></tr>
<tr><td>S9_k141_30345</td><td>1113</td><td>No</td><td>NA</td><td></td><td>1</td><td>0</td><td>Low-quality</td><td>Genome-fragment</td><td></td><td>3.54</td><td>0</td></tr>
<tr><td>S11-final-viral-combined_k141_72737||full</td><td>5612</td><td>No</td><td>NA</td><td></td><td>0</td><td>5</td><td>Not-determined</td><td>Genome-fragment</td><td>NA</td><td></td><td>0</td></tr>
<tr><td>S11-final-viral-combined_k141_4274||full</td><td>3639</td><td>No</td><td>NA</td><td></td><td>0</td><td>1</td><td>Not-determined</td><td>Genome-fragment</td><td>NA</td><td></td><td>0</td></tr>
<tr><td>S11-final-viral-combined_k141_17703||full</td><td>1967</td><td>No</td><td>NA</td><td></td><td>0</td><td>2</td><td>Not-determined</td><td>Genome-fragment</td><td>NA</td><td></td><td>0</td></tr>
<tr><td>S11-final-viral-combined_k141_307896||full</td><td>1123</td><td>No</td><td>NA</td><td></td><td>0</td><td>0</td><td>Not-determined</td><td>Genome-fragment</td><td>NA</td><td></td><td>0</td></tr>
<tr><td>S11_k141_31639</td><td>1113</td><td>No</td><td>NA</td><td></td><td>1</td><td>0</td><td>Low-quality</td><td>Genome-fragment</td><td></td><td>2.48</td><td>0</td></tr>
<tr><td>S1-final-viral-combined_k141_320066||full</td><td>1113</td><td>No</td><td>NA</td><td></td><td>1</td><td>0</td><td>Low-quality</td><td>Genome-fragment</td><td></td><td>2.36</td><td>0</td></tr>
<tr><td>S5_k141_169765</td><td>1113</td><td>No</td><td>NA</td><td></td><td>2</td><td>0</td><td>Low-quality</td><td>Genome-fragment</td><td></td><td>2.72</td><td>0</td></tr>
<tr><td>S7-final-viral-combined_k141_222901||full</td><td>1113</td><td>No</td><td>NA</td><td></td><td>2</td><td>0</td><td>Low-quality</td><td>Genome-fragment</td><td></td><td>2.65</td><td>0</td></tr>
<tr><td>S11-final-viral-combined_k141_196991||full</td><td>11885</td><td>No</td><td>NA</td><td></td><td>0</td><td>6</td><td>Not-determined</td><td>Genome-fragment</td><td>NA</td><td></td><td>0</td></tr>
<tr><td>S11-final-viral-combined_k141_128342||full</td><td>8797</td><td>No</td><td>NA</td><td></td><td>0</td><td>5</td><td>Not-determined</td><td>Genome-fragment</td><td>NA</td><td></td><td>0</td></tr>
<tr><td>S17-final-viral-combined_k141_95250||full</td><td>1113</td><td>No</td><td>NA</td><td></td><td>0</td><td>0</td><td>Low-quality</td><td>Genome-fragment</td><td></td><td>1.36</td><td>0</td></tr>
<tr><td>S17-final-viral-combined_k141_30386||full</td><td>1113</td><td>No</td><td>NA</td><td></td><td>0</td><td>1</td><td>Low-quality</td><td>Genome-fragment</td><td></td><td>2.18</td><td>0</td></tr>
<tr><td>S11-final-viral-combined_k141_101322||full</td><td>1262</td><td>No</td><td>NA</td><td></td><td>0</td><td>1</td><td>Not-determined</td><td>Genome-fragment</td><td>NA</td><td></td><td>0</td></tr>
<tr><td>S11-final-viral-combined_k141_320937||full</td><td>3301</td><td>No</td><td>NA</td><td></td><td>0</td><td>0</td><td>Not-determined</td><td>Genome-fragment</td><td>NA</td><td></td><td>0</td></tr>
<tr><td>S17-final-viral-combined_k141_210921||full</td><td>1113</td><td>No</td><td>NA</td><td></td><td>1</td><td>0</td><td>Low-quality</td><td>Genome-fragment</td><td></td><td>3.27</td><td>0</td></tr>
<tr><td>S17-k141_171356</td><td>1113</td><td>No</td><td>NA</td><td></td><td>2</td><td>0</td><td>Low-quality</td><td>Genome-fragment</td><td></td><td>2.75</td><td>0</td></tr>
<tr><td>S11-final-viral-combined_k141_128374||full</td><td>1526</td><td>No</td><td>NA</td><td></td><td>0</td><td>1</td><td>Not-determined</td><td>Genome-fragment</td><td>NA</td><td></td><td>0</td></tr>
<tr><td>S11-final-viral-combined_k141_239191||full</td><td>1094</td><td>No</td><td>NA</td><td></td><td>0</td><td>1</td><td>Not-determined</td><td>Genome-fragment</td><td>NA</td><td></td><td>0</td></tr>
<tr><td>S11-final-viral-combined_k141_239194||full</td><td>3607</td><td>No</td><td>NA</td><td></td><td>0</td><td>0</td><td>Not-determined</td><td>Genome-fragment</td><td>NA</td><td></td><td>0</td></tr>
<tr><td>S11-final-viral-combined_k141_45984||full</td><td>1285</td><td>No</td><td>NA</td><td></td><td>0</td><td>1</td><td>Not-determined</td><td>Genome-fragment</td><td>NA</td><td></td><td>0</td></tr>
<tr><td>S11-final-viral-combined_k141_156020||full</td><td>6290</td><td>No</td><td>NA</td><td></td><td>0</td><td>3</td><td>Not-determined</td><td>Genome-fragment</td><td>NA</td><td></td><td>0</td></tr>
<tr><td>S11-final-viral-combined_k141_31957||full</td><td>1173</td><td>No</td><td>NA</td><td></td><td>0</td><td>1</td><td>Not-determined</td><td>Genome-fragment</td><td>NA</td><td></td><td>0</td></tr>
<tr><td>S17-k141_243312</td><td>1113</td><td>No</td><td>NA</td><td></td><td>0</td><td>0</td><td>Low-quality</td><td>Genome-fragment</td><td></td><td>1.71</td><td>0</td></tr>
<tr><td>S11-final-viral-combined_k141_128396||full</td><td>2171</td><td>No</td><td>NA</td><td></td><td>0</td><td>0</td><td>Not-determined</td><td>Genome-fragment</td><td>NA</td><td></td><td>0</td></tr>
<tr><td>S11-final-viral-combined_k141_72833||full</td><td>2651</td><td>No</td><td>NA</td><td></td><td>0</td><td>1</td><td>Not-determined</td><td>Genome-fragment</td><td>NA</td><td></td><td>0</td></tr>
<tr><td>S11-final-viral-combined_k141_72837||full</td><td>5810</td><td>No</td><td>NA</td><td></td><td>0</td><td>3</td><td>Not-determined</td><td>Genome-fragment</td><td>NA</td><td></td><td>0</td></tr>
<tr><td>S12-final-viral-combined_k141_23492||full</td><td>1113</td><td>No</td><td>NA</td><td></td><td>0</td><td>0</td><td>Low-quality</td><td>Genome-fragment</td><td></td><td>1.54</td><td>0</td></tr>
<tr><td>S12-final-viral-combined_k141_11517||lt2gene</td><td>1113</td><td>No</td><td>NA</td><td></td><td>1</td><td>0</td><td>Low-quality</td><td>Genome-fragment</td><td></td><td>2.44</td><td>0</td></tr>
<tr><td>S12_k141_365228</td><td>1113</td><td>No</td><td>NA</td><td></td><td>0</td><td>2</td><td>Low-quality</td><td>Genome-fragment</td><td></td><td>0.41</td><td>0</td></tr>
<tr><td>S11-final-viral-combined_k141_280643||full</td><td>4488</td><td>No</td><td>NA</td><td></td><td>0</td><td>7</td><td>Not-determined</td><td>Genome-fragment</td><td>NA</td><td></td><td>0</td></tr>
<tr><td>S2-final-viral-combined_k141_214238||lt2gene</td><td>1113</td><td>No</td><td>NA</td><td></td><td>1</td><td>0</td><td>Low-quality</td><td>Genome-fragment</td><td></td><td>3.18</td><td>0</td></tr>
<tr><td>S4-final-viral-combined_k141_98497||full</td><td>1113</td><td>No</td><td>NA</td><td></td><td>0</td><td>0</td><td>Low-quality</td><td>Genome-fragment</td><td></td><td>5.13</td><td>0</td></tr>
<tr><td>S6_k141_100515</td><td>1113</td><td>No</td><td>NA</td><td></td><td>0</td><td>0</td><td>Low-quality</td><td>Genome-fragment</td><td></td><td>3.29</td><td>0</td></tr>
<tr><td>S11-final-viral-combined_k141_17823||full</td><td>1120</td><td>No</td><td>NA</td><td></td><td>0</td><td>1</td><td>Not-determined</td><td>Genome-fragment</td><td>NA</td><td></td><td>0</td></tr>
<tr><td>S11-final-viral-combined_k141_72881||full</td><td>3767</td><td>No</td><td>NA</td><td></td><td>0</td><td>1</td><td>Not-determined</td><td>Genome-fragment</td><td>NA</td><td></td><td>0</td></tr>
<tr><td>S9_k141_34500</td><td>1114</td><td>No</td><td>NA</td><td></td><td>0</td><td>1</td><td>Low-quality</td><td>Genome-fragment</td><td></td><td>0.79</td><td>0</td></tr>
<tr><td>S11-final-viral-combined_k141_101337||full</td><td>78390</td><td>Yes</td><td></td><td>25287</td><td>13</td><td>28</td><td>Medium-quality</td><td>Genome-fragment</td><td></td><td>66.28</td><td>67.74</td></tr>
<tr><td>S5-final-viral-combined_k141_197499||full</td><td>1114</td><td>No</td><td>NA</td><td></td><td>0</td><td>1</td><td>Low-quality</td><td>Genome-fragment</td><td></td><td>2.35</td><td>0</td></tr>
<tr><td>S5-final-viral-combined_k141_140133||full</td><td>1114</td><td>No</td><td>NA</td><td></td><td>0</td><td>1</td><td>Low-quality</td><td>Genome-fragment</td><td></td><td>3.56</td><td>0</td></tr>
<tr><td>S11-final-viral-combined_k141_59771||full</td><td>2047</td><td>No</td><td>NA</td><td></td><td>0</td><td>0</td><td>Not-determined</td><td>Genome-fragment</td><td>NA</td><td></td><td>0</td></tr>
<tr><td>S17-k141_61519</td><td>1114</td><td>No</td><td>NA</td><td></td><td>0</td><td>0</td><td>Low-quality</td><td>Genome-fragment</td><td></td><td>2.23</td><td>0</td></tr>
<tr><td>S11-final-viral-combined_k141_211748||full</td><td>1271</td><td>No</td><td>NA</td><td></td><td>0</td><td>0</td><td>Not-determined</td><td>Genome-fragment</td><td>NA</td><td></td><td>0</td></tr>
<tr><td>S12-final-viral-combined_k141_120861||full</td><td>1114</td><td>No</td><td>NA</td><td></td><td>0</td><td>0</td><td>Low-quality</td><td>Genome-fragment</td><td></td><td>2.7</td><td>0</td></tr>
<tr><td>S11-final-viral-combined_k141_239251||full</td><td>2923</td><td>No</td><td>NA</td><td></td><td>0</td><td>2</td><td>Not-determined</td><td>Genome-fragment</td><td>NA</td><td></td><td>0</td></tr>
<tr><td>S11-final-viral-combined_k141_225160||full</td><td>2855</td><td>No</td><td>NA</td><td></td><td>0</td><td>2</td><td>Not-determined</td><td>Genome-fragment</td><td>NA</td><td></td><td>0</td></tr>
<tr><td>S11-final-viral-combined_k141_142382||full</td><td>2907</td><td>No</td><td>NA</td><td></td><td>0</td><td>3</td><td>Not-determined</td><td>Genome-fragment</td><td>NA</td><td></td><td>0</td></tr>
<tr><td>S11-final-viral-combined_k141_17859||full</td><td>1461</td><td>No</td><td>NA</td><td></td><td>0</td><td>1</td><td>Not-determined</td><td>Genome-fragment</td><td>NA</td><td></td><td>0</td></tr>
<tr><td>S11-final-viral-combined_k141_321043||full</td><td>3471</td><td>No</td><td>NA</td><td></td><td>0</td><td>2</td><td>Not-determined</td><td>Genome-fragment</td><td>NA</td><td></td><td>0</td></tr>
<tr><td>S11-final-viral-combined_k141_72904||full</td><td>3507</td><td>No</td><td>NA</td><td></td><td>0</td><td>0</td><td>Not-determined</td><td>Genome-fragment</td><td>NA</td><td></td><td>0</td></tr>
<tr><td>S12-final-viral-combined_k141_372428||lt2gene</td><td>1114</td><td>No</td><td>NA</td><td></td><td>1</td><td>0</td><td>Low-quality</td><td>Genome-fragment</td><td></td><td>26.77</td><td>0</td></tr>
<tr><td>S11-final-viral-combined_k141_170361||full</td><td>20669</td><td>No</td><td>NA</td><td></td><td>0</td><td>13</td><td>Not-determined</td><td>Genome-fragment</td><td>NA</td><td></td><td>0</td></tr>
<tr><td>S12-final-viral-combined_k141_5533||lt2gene</td><td>1114</td><td>No</td><td>NA</td><td></td><td>1</td><td>0</td><td>Low-quality</td><td>Genome-fragment</td><td></td><td>2.62</td><td>0</td></tr>
<tr><td>S11-final-viral-combined_k141_114169||full</td><td>3584</td><td>No</td><td>NA</td><td></td><td>0</td><td>0</td><td>Not-determined</td><td>Genome-fragment</td><td>NA</td><td></td><td>0</td></tr>
<tr><td>S11-final-viral-combined_k141_252598||full</td><td>1660</td><td>No</td><td>NA</td><td></td><td>0</td><td>0</td><td>Not-determined</td><td>Genome-fragment</td><td>NA</td><td></td><td>0</td></tr>
<tr><td>S11-final-viral-combined_k141_101386||full</td><td>5797</td><td>No</td><td>NA</td><td></td><td>0</td><td>4</td><td>Not-determined</td><td>Genome-fragment</td><td>NA</td><td></td><td>0</td></tr>
<tr><td>S12_k141_332850</td><td>1114</td><td>No</td><td>NA</td><td></td><td>0</td><td>0</td><td>Low-quality</td><td>Genome-fragment</td><td></td><td>2.71</td><td>0</td></tr>
<tr><td>S11-final-viral-combined_k141_114181||full</td><td>2508</td><td>No</td><td>NA</td><td></td><td>0</td><td>3</td><td>Not-determined</td><td>Genome-fragment</td><td>NA</td><td></td><td>0</td></tr>
<tr><td>S11-final-viral-combined_k141_142436||full</td><td>1232</td><td>No</td><td>NA</td><td></td><td>0</td><td>0</td><td>Not-determined</td><td>Genome-fragment</td><td>NA</td><td></td><td>0</td></tr>
<tr><td>S11-final-viral-combined_k141_87425||full</td><td>1359</td><td>No</td><td>NA</td><td></td><td>0</td><td>1</td><td>Not-determined</td><td>Genome-fragment</td><td>NA</td><td></td><td>0</td></tr>
<tr><td>S11-final-viral-combined_k141_128480||full</td><td>1608</td><td>No</td><td>NA</td><td></td><td>0</td><td>0</td><td>Not-determined</td><td>Genome-fragment</td><td>NA</td><td></td><td>0</td></tr>
<tr><td>S2-final-viral-combined_k141_204760||lt2gene</td><td>1114</td><td>No</td><td>NA</td><td></td><td>1</td><td>0</td><td>Low-quality</td><td>Genome-fragment</td><td></td><td>2.35</td><td>0</td></tr>
<tr><td>S11-final-viral-combined_k141_183782||full</td><td>5951</td><td>No</td><td>NA</td><td></td><td>0</td><td>3</td><td>Not-determined</td><td>Genome-fragment</td><td>NA</td><td></td><td>0</td></tr>
<tr><td>S11-final-viral-combined_k141_197126||full</td><td>1541</td><td>No</td><td>NA</td><td></td><td>0</td><td>1</td><td>Not-determined</td><td>Genome-fragment</td><td>NA</td><td></td><td>0</td></tr>
<tr><td>S2_k141_35286</td><td>1114</td><td>No</td><td>NA</td><td></td><td>1</td><td>0</td><td>Low-quality</td><td>Genome-fragment</td><td></td><td>2.45</td><td>0</td></tr>
<tr><td>S11-final-viral-combined_k141_321069||full</td><td>1554</td><td>No</td><td>NA</td><td></td><td>0</td><td>0</td><td>Not-determined</td><td>Genome-fragment</td><td>NA</td><td></td><td>0</td></tr>
<tr><td>S11-final-viral-combined_k141_32064||full</td><td>9622</td><td>No</td><td>NA</td><td></td><td>0</td><td>5</td><td>Not-determined</td><td>Genome-fragment</td><td>NA</td><td></td><td>0</td></tr>
<tr><td>S2_k141_57237</td><td>1114</td><td>No</td><td>NA</td><td></td><td>0</td><td>0</td><td>Low-quality</td><td>Genome-fragment</td><td></td><td>2.04</td><td>0</td></tr>
<tr><td>S11-final-viral-combined_k141_239278||full</td><td>1369</td><td>No</td><td>NA</td><td></td><td>0</td><td>1</td><td>Not-determined</td><td>Genome-fragment</td><td>NA</td><td></td><td>0</td></tr>
<tr><td>S11-final-viral-combined_k141_59819||full</td><td>2970</td><td>No</td><td>NA</td><td></td><td>0</td><td>3</td><td>Not-determined</td><td>Genome-fragment</td><td>NA</td><td></td><td>0</td></tr>
<tr><td>S11-final-viral-combined_k141_225258||full</td><td>1838</td><td>No</td><td>NA</td><td></td><td>0</td><td>1</td><td>Not-determined</td><td>Genome-fragment</td><td>NA</td><td></td><td>0</td></tr>
<tr><td>S11-final-viral-combined_k141_239286||full</td><td>8211</td><td>No</td><td>NA</td><td></td><td>0</td><td>5</td><td>Not-determined</td><td>Genome-fragment</td><td>NA</td><td></td><td>0</td></tr>
<tr><td>S11-final-viral-combined_k141_225273||full</td><td>3414</td><td>No</td><td>NA</td><td></td><td>0</td><td>0</td><td>Not-determined</td><td>Genome-fragment</td><td>NA</td><td></td><td>0</td></tr>
<tr><td>S8-final-viral-combined_k141_190417||full</td><td>1114</td><td>No</td><td>NA</td><td></td><td>0</td><td>2</td><td>Low-quality</td><td>Genome-fragment</td><td></td><td>0.92</td><td>0</td></tr>
<tr><td>S1-final-viral-combined_k141_334434||full</td><td>1115</td><td>No</td><td>NA</td><td></td><td>1</td><td>0</td><td>Low-quality</td><td>Genome-fragment</td><td></td><td>2.56</td><td>0</td></tr>
<tr><td>S1-final-viral-combined_k141_306287||full</td><td>1115</td><td>No</td><td>NA</td><td></td><td>2</td><td>0</td><td>Low-quality</td><td>Genome-fragment</td><td></td><td>8.96</td><td>0</td></tr>
<tr><td>S1-final-viral-combined_k141_372179||full</td><td>1115</td><td>No</td><td>NA</td><td></td><td>0</td><td>0</td><td>Low-quality</td><td>Genome-fragment</td><td></td><td>2.14</td><td>0</td></tr>
<tr><td>S1-final-viral-combined_k141_123028||lt2gene</td><td>1115</td><td>No</td><td>NA</td><td></td><td>1</td><td>0</td><td>Low-quality</td><td>Genome-fragment</td><td></td><td>1.63</td><td>0</td></tr>
<tr><td>S11-final-viral-combined_k141_128542||full</td><td>1966</td><td>No</td><td>NA</td><td></td><td>0</td><td>0</td><td>Not-determined</td><td>Genome-fragment</td><td>NA</td><td></td><td>0</td></tr>
<tr><td>S11-final-viral-combined_k141_59850||full</td><td>1708</td><td>No</td><td>NA</td><td></td><td>0</td><td>3</td><td>Not-determined</td><td>Genome-fragment</td><td>NA</td><td></td><td>0</td></tr>
<tr><td>S1_k141_67728</td><td>1115</td><td>No</td><td>NA</td><td></td><td>0</td><td>1</td><td>Low-quality</td><td>Genome-fragment</td><td></td><td>0.63</td><td>0</td></tr>
<tr><td>S11-final-viral-combined_k141_308080||full</td><td>4058</td><td>No</td><td>NA</td><td></td><td>0</td><td>3</td><td>Not-determined</td><td>Genome-fragment</td><td>NA</td><td></td><td>0</td></tr>
<tr><td>S3-final-viral-combined_k141_29177||lt2gene</td><td>1115</td><td>No</td><td>NA</td><td></td><td>0</td><td>0</td><td>Low-quality</td><td>Genome-fragment</td><td></td><td>0.73</td><td>0</td></tr>
<tr><td>S11-final-viral-combined_k141_32126||full</td><td>3174</td><td>No</td><td>NA</td><td></td><td>0</td><td>2</td><td>Not-determined</td><td>Genome-fragment</td><td>NA</td><td></td><td>0</td></tr>
<tr><td>S11-final-viral-combined_k141_17913||full</td><td>1858</td><td>No</td><td>NA</td><td></td><td>0</td><td>2</td><td>Not-determined</td><td>Genome-fragment</td><td>NA</td><td></td><td>0</td></tr>
<tr><td>S11-final-viral-combined_k141_197192||full</td><td>2678</td><td>No</td><td>NA</td><td></td><td>0</td><td>2</td><td>Not-determined</td><td>Genome-fragment</td><td>NA</td><td></td><td>0</td></tr>
<tr><td>S11-final-viral-combined_k141_239323||full</td><td>1434</td><td>No</td><td>NA</td><td></td><td>0</td><td>2</td><td>Not-determined</td><td>Genome-fragment</td><td>NA</td><td></td><td>0</td></tr>
<tr><td>S11-final-viral-combined_k141_128548||full</td><td>7927</td><td>No</td><td>NA</td><td></td><td>0</td><td>4</td><td>Not-determined</td><td>Genome-fragment</td><td>NA</td><td></td><td>0</td></tr>
<tr><td>S11-final-viral-combined_k141_170434||full</td><td>1582</td><td>No</td><td>NA</td><td></td><td>0</td><td>3</td><td>Not-determined</td><td>Genome-fragment</td><td>NA</td><td></td><td>0</td></tr>
<tr><td>S3_k141_79065</td><td>1115</td><td>No</td><td>NA</td><td></td><td>0</td><td>0</td><td>Low-quality</td><td>Genome-fragment</td><td></td><td>1.65</td><td>0</td></tr>
<tr><td>S3_k141_16261</td><td>1115</td><td>No</td><td>NA</td><td></td><td>0</td><td>0</td><td>Low-quality</td><td>Genome-fragment</td><td></td><td>1.88</td><td>0</td></tr>
<tr><td>S11-final-viral-combined_k141_321139||full</td><td>1354</td><td>No</td><td>NA</td><td></td><td>0</td><td>3</td><td>Not-determined</td><td>Genome-fragment</td><td>NA</td><td></td><td>0</td></tr>
<tr><td>S11-final-viral-combined_k141_142548||full</td><td>2196</td><td>No</td><td>NA</td><td></td><td>0</td><td>2</td><td>Not-determined</td><td>Genome-fragment</td><td>NA</td><td></td><td>0</td></tr>
<tr><td>S5-final-viral-combined_k141_109930||full</td><td>1115</td><td>No</td><td>NA</td><td></td><td>0</td><td>0</td><td>Low-quality</td><td>Genome-fragment</td><td></td><td>1.6</td><td>0</td></tr>
<tr><td>S7-final-viral-combined_k141_70850||full</td><td>1115</td><td>No</td><td>NA</td><td></td><td>0</td><td>0</td><td>Low-quality</td><td>Genome-fragment</td><td></td><td>2.92</td><td>0</td></tr>
<tr><td>S11-final-viral-combined_k141_225377||full</td><td>2149</td><td>No</td><td>NA</td><td></td><td>0</td><td>2</td><td>Not-determined</td><td>Genome-fragment</td><td>NA</td><td></td><td>0</td></tr>
<tr><td>S7_k141_201950</td><td>1115</td><td>No</td><td>NA</td><td></td><td>0</td><td>0</td><td>Low-quality</td><td>Genome-fragment</td><td></td><td>0.84</td><td>0</td></tr>
<tr><td>S11-final-viral-combined_k141_17943||full</td><td>12845</td><td>No</td><td>NA</td><td></td><td>0</td><td>2</td><td>Not-determined</td><td>Genome-fragment</td><td>NA</td><td></td><td>0</td></tr>
<tr><td>S17-final-viral-combined_k141_215753||full</td><td>1115</td><td>No</td><td>NA</td><td></td><td>0</td><td>1</td><td>Low-quality</td><td>Genome-fragment</td><td></td><td>3.16</td><td>0</td></tr>
<tr><td>S17-final-viral-combined_k141_92726||full</td><td>1115</td><td>No</td><td>NA</td><td></td><td>0</td><td>0</td><td>Low-quality</td><td>Genome-fragment</td><td></td><td>3.08</td><td>0</td></tr>
<tr><td>S11-final-viral-combined_k141_266567||full</td><td>2760</td><td>No</td><td>NA</td><td></td><td>0</td><td>3</td><td>Not-determined</td><td>Genome-fragment</td><td>NA</td><td></td><td>0</td></tr>
<tr><td>S17-k141_187981</td><td>1115</td><td>No</td><td>NA</td><td></td><td>0</td><td>1</td><td>Low-quality</td><td>Genome-fragment</td><td></td><td>2.15</td><td>0</td></tr>
<tr><td>S12-final-viral-combined_k141_156144||lt2gene</td><td>1115</td><td>No</td><td>NA</td><td></td><td>1</td><td>0</td><td>Low-quality</td><td>Genome-fragment</td><td></td><td>2.08</td><td>0</td></tr>
<tr><td>S11-final-viral-combined_k141_225408||full</td><td>1306</td><td>No</td><td>NA</td><td></td><td>0</td><td>2</td><td>Not-determined</td><td>Genome-fragment</td><td>NA</td><td></td><td>0</td></tr>
<tr><td>S11-final-viral-combined_k141_266569||full</td><td>2128</td><td>No</td><td>NA</td><td></td><td>0</td><td>0</td><td>Not-determined</td><td>Genome-fragment</td><td>NA</td><td></td><td>0</td></tr>
<tr><td>S11-final-viral-combined_k141_252701||full</td><td>3423</td><td>No</td><td>NA</td><td></td><td>0</td><td>1</td><td>Not-determined</td><td>Genome-fragment</td><td>NA</td><td></td><td>0</td></tr>
<tr><td>S4-final-viral-combined_k141_20921||full</td><td>1115</td><td>No</td><td>NA</td><td></td><td>0</td><td>0</td><td>Low-quality</td><td>Genome-fragment</td><td></td><td>2.38</td><td>0</td></tr>
<tr><td>S4-final-viral-combined_k141_152897||full</td><td>1115</td><td>No</td><td>NA</td><td></td><td>0</td><td>0</td><td>Low-quality</td><td>Genome-fragment</td><td></td><td>0.66</td><td>0</td></tr>
</table>

| | | | | | | | | | |
|---|---|---|---|---|---|---|---|---|---|
| S11-final-viral-combined_k141_321203\|\|full | 11275 | No | NA | 0 | 7 | Not-determined | Genome-fragment | NA | 0 |
| S8-final-viral-combined_k141_76406\|\|lt2gene | 1115 | No | NA | 1 | 0 | Low-quality | Genome-fragment | 2.26 | 0 |
| S11-final-viral-combined_k141_293937\|\|full | 1658 | No | NA | 0 | 1 | Not-determined | Genome-fragment | NA | 0 |
| S11-final-viral-combined_k141_32211\|\|full | 1310 | No | NA | 0 | 0 | Not-determined | Genome-fragment | NA | 0 |
| S11-final-viral-combined_k141_280838\|\|full | 3539 | No | NA | 0 | 2 | Not-determined | Genome-fragment | NA | 0 |
| S9-final-viral-combined_k141_86507\|\|lt2gene | 1116 | No | NA | 1 | 0 | Low-quality | Genome-fragment | 2.44 | 0 |
| S11-final-viral-combined_k141_117161\|\|full | 1116 | No | NA | 0 | 0 | Low-quality | Genome-fragment | 3.03 | 0 |
| S11-final-viral-combined_k141_293955\|\|full | 1458 | No | NA | 0 | 1 | Not-determined | Genome-fragment | NA | 0 |
| S11-final-viral-combined_k141_280851\|\|full | 2343 | No | NA | 0 | 2 | Not-determined | Genome-fragment | NA | 0 |
| S1-final-viral-combined_k141_388509\|\|full | 1116 | No | NA | 0 | 0 | Low-quality | Genome-fragment | 2.87 | 0 |
| S11-final-viral-combined_k141_59983\|\|full | 8832 | No | NA | 0 | 2 | Not-determined | Genome-fragment | NA | 0 |
| S1_k141_121468 | 1116 | No | NA | 0 | 1 | Low-quality | Genome-fragment | 2.18 | 0 |
| S5_k141_165069 | 1116 | No | NA | 0 | 3 | Low-quality | Genome-fragment | 3.27 | 0 |
| S11-final-viral-combined_k141_18011\|\|full | 2225 | No | NA | 0 | 1 | Not-determined | Genome-fragment | NA | 0 |
| S7-final-viral-combined_k141_72885\|\|full | 1116 | No | NA | 1 | 0 | Low-quality | Genome-fragment | 2.87 | 0 |
| S17-final-viral-combined_k141_254372\|\|full | 1116 | No | NA | 1 | 0 | Low-quality | Genome-fragment | 3.43 | 0 |
| S11-final-viral-combined_k141_46317\|\|full | 1098 | No | NA | 0 | 1 | Not-determined | Genome-fragment | NA | 0 |
| S11-final-viral-combined_k141_73113\|\|full | 2972 | No | NA | 0 | 2 | Not-determined | Genome-fragment | NA | 0 |
| S11-final-viral-combined_k141_252803\|\|full | 1158 | No | NA | 0 | 1 | Not-determined | Genome-fragment | NA | 0 |
| S11-final-viral-combined_k141_18042\|\|full | 3092 | No | NA | 0 | 1 | Not-determined | Genome-fragment | NA | 0 |
| S11-final-viral-combined_k141_46321\|\|full | 1617 | No | NA | 0 | 1 | Not-determined | Genome-fragment | NA | 0 |
| S12-final-viral-combined_k141_18685\|\|full | 1116 | No | NA | 0 | 2 | Low-quality | Genome-fragment | 0.35 | 0 |
| S12_k141_260654 | 1116 | No | NA | 0 | 1 | Low-quality | Genome-fragment | 0.54 | 0 |
| S11-final-viral-combined_k141_197365\|\|full | 1134 | No | NA | 0 | 1 | Not-determined | Genome-fragment | NA | 0 |
| S11-final-viral-combined_k141_156384\|\|full | 2394 | No | NA | 0 | 0 | Not-determined | Genome-fragment | NA | 0 |
| S11-final-viral-combined_k141_321320\|\|full | 8480 | No | NA | 0 | 2 | Not-determined | Genome-fragment | NA | 0 |
| S11-final-viral-combined_k141_18057\|\|full | 3008 | No | NA | 0 | 2 | Not-determined | Genome-fragment | NA | 0 |
| S2-final-viral-combined_k141_113103\|\|full | 1116 | No | NA | 0 | 0 | Low-quality | Genome-fragment | 2.63 | 0 |
| S11-final-viral-combined_k141_114373\|\|full | 1697 | No | NA | 0 | 0 | Not-determined | Genome-fragment | NA | 0 |
| S2-final-viral-combined_k141_136713\|\|lt2gene | 1116 | No | NA | 1 | 0 | Low-quality | Genome-fragment | 3.6 | 0 |
| S2-final-viral-combined_k141_91452\|\|lt2gene | 1116 | No | NA | 1 | 0 | Low-quality | Genome-fragment | 2.41 | 0 |
| S2_k141_105983 | 1116 | No | NA | 0 | 1 | Low-quality | Genome-fragment | 0.73 | 0 |
| S11-final-viral-combined_k141_73170\|\|full | 2674 | No | NA | 0 | 1 | Not-determined | Genome-fragment | NA | 0 |
| S11-final-viral-combined_k141_32294\|\|full | 1992 | No | NA | 0 | 0 | Not-determined | Genome-fragment | NA | 0 |
| S11-final-viral-combined_k141_170679\|\|full | 1548 | No | NA | 0 | 1 | Not-determined | Genome-fragment | NA | 0 |
| S9-final-viral-combined_k141_121607\|\|full | 1117 | No | NA | 1 | 0 | Low-quality | Genome-fragment | 2.4 | 0 |
| S9-final-viral-combined_k141_120499\|\|lt2gene | 1117 | No | NA | 1 | 0 | Low-quality | Genome-fragment | 3.18 | 0 |
| S11-final-viral-combined_k141_225613\|\|full | 2008 | No | NA | 0 | 2 | Not-determined | Genome-fragment | NA | 0 |
| S11-final-viral-combined_k141_101738\|\|full | 2146 | No | NA | 0 | 2 | Not-determined | Genome-fragment | NA | 0 |
| S9-final-viral-combined_k141_14919\|\|lt2gene | 1117 | No | NA | 2 | 0 | Low-quality | Genome-fragment | 1.62 | 0 |
| S11-final-viral-combined_k141_219267\|\|full | 1117 | No | NA | 0 | 0 | Low-quality | Genome-fragment | 0.8 | 0 |
| S11-final-viral-combined_k141_4781\|\|full | 7037 | No | NA | 0 | 6 | Not-determined | Genome-fragment | NA | 0 |
| S11-final-viral-combined_k141_290097\|\|lt2gene | 1117 | No | NA | 1 | 0 | Low-quality | Genome-fragment | 3.18 | 0 |
| S11-final-viral-combined_k141_184590\|\|lt2gene | 1117 | No | NA | 2 | 0 | Low-quality | Genome-fragment | 1.36 | 0 |
| S11-final-viral-combined_k141_118549\|\|lt2gene | 1117 | No | NA | 1 | 0 | Low-quality | Genome-fragment | 0.43 | 0 |
| S11-final-viral-combined_k141_73204\|\|full | 68219 | No | NA | 3 | 13 | Medium-quality | Genome-fragment | 61.42 | 0 |
| S1-final-viral-combined_k141_229217\|\|full | 1117 | No | NA | 0 | 1 | Low-quality | Genome-fragment | 0.66 | 0 |
| S1-final-viral-combined_k141_109906\|\|lt2gene | 1117 | No | NA | 2 | 0 | Low-quality | Genome-fragment | 3.41 | 0 |
| S11-final-viral-combined_k141_128850\|\|full | 1278 | No | NA | 0 | 1 | Not-determined | Genome-fragment | NA | 0 |
| S11-final-viral-combined_k141_87734\|\|full | 1461 | No | NA | 0 | 0 | Not-determined | Genome-fragment | NA | 0 |
| S1_k141_177163 | 1117 | No | NA | 0 | 1 | Low-quality | Genome-fragment | 0.83 | 0 |
| S3-final-viral-combined_k141_12098\|\|full | 1117 | No | NA | 0 | 0 | Low-quality | Genome-fragment | 0.84 | 0 |
| S3_k141_92974 | 1117 | No | NA | 1 | 0 | Low-quality | Genome-fragment | 1.86 | 0 |
| S11-final-viral-combined_k141_281156\|\|full | 2820 | No | NA | 0 | 2 | Not-determined | Genome-fragment | NA | 0 |
| S11-final-viral-combined_k141_60101\|\|full | 1853 | No | NA | 0 | 1 | Not-determined | Genome-fragment | NA | 0 |
| S5-final-viral-combined_k141_71676\|\|full | 1117 | No | NA | 0 | 0 | Low-quality | Genome-fragment | 0.58 | 0 |
| S11-final-viral-combined_k141_308351\|\|full | 7416 | No | NA | 0 | 0 | Not-determined | Genome-fragment | NA | 0 |
| S7-final-viral-combined_k141_197113\|\|full | 1117 | No | NA | 0 | 0 | Low-quality | Genome-fragment | 1.37 | 0 |
| S7_k141_220450 | 1117 | No | NA | 0 | 0 | Low-quality | Genome-fragment | 2.43 | 0 |
| S17-final-viral-combined_k141_189489\|\|full | 1117 | No | NA | 0 | 0 | Low-quality | Genome-fragment | 2.58 | 0 |
| S11-final-viral-combined_k141_142849\|\|full | 1321 | No | NA | 0 | 1 | Not-determined | Genome-fragment | NA | 0 |
| S11-final-viral-combined_k141_114605\|\|full | 2373 | No | NA | 0 | 2 | Not-determined | Genome-fragment | NA | 0 |
| S11-final-viral-combined_k141_60166\|\|full | 1586 | No | NA | 0 | 1 | Not-determined | Genome-fragment | NA | 0 |
| S11-final-viral-combined_k141_239689\|\|full | 1577 | No | NA | 0 | 1 | Not-determined | Genome-fragment | NA | 0 |
| S17-final-viral-combined_k141_34137\|\|full | 1117 | No | NA | 0 | 0 | Low-quality | Genome-fragment | 1.48 | 0 |
| S12-final-viral-combined_k141_206145\|\|lt2gene | 1117 | No | NA | 1 | 0 | Low-quality | Genome-fragment | 2.04 | 0 |
| S11-final-viral-combined_k141_184106\|\|full | 2715 | No | NA | 0 | 0 | Not-determined | Genome-fragment | NA | 0 |
| S2-final-viral-combined_k141_99034\|\|full | 1117 | No | NA | 1 | 0 | Low-quality | Genome-fragment | 3.2 | 0 |
| S11-final-viral-combined_k141_60207\|\|full | 1289 | No | NA | 0 | 1 | Not-determined | Genome-fragment | NA | 0 |
| S11-final-viral-combined_k141_308412\|\|full | 1855 | No | NA | 0 | 1 | Not-determined | Genome-fragment | NA | 0 |
| S11-final-viral-combined_k141_73299\|\|full | 3029 | No | NA | 0 | 2 | Not-determined | Genome-fragment | NA | 0 |
| S11-final-viral-combined_k141_239757\|\|full | 1506 | No | NA | 0 | 0 | Not-determined | Genome-fragment | NA | 0 |
| S11-final-viral-combined_k141_101935\|\|full | 1397 | No | NA | 0 | 0 | Not-determined | Genome-fragment | NA | 0 |
| S11-final-viral-combined_k141_128971\|\|full | 6508 | No | NA | 0 | 2 | Not-determined | Genome-fragment | NA | 0 |
| S4-final-viral-combined_k141_149870\|\|full | 1117 | No | NA | 0 | 0 | Low-quality | Genome-fragment | 2.88 | 0 |
| S11-final-viral-combined_k141_212302\|\|full | 4453 | No | NA | 0 | 3 | Not-determined | Genome-fragment | NA | 0 |
| S11-final-viral-combined_k141_18264\|\|full | 1985 | No | NA | 0 | 2 | Not-determined | Genome-fragment | NA | 0 |
| S11-final-viral-combined_k141_294209\|\|full | 1300 | No | NA | 0 | 1 | Not-determined | Genome-fragment | NA | 0 |
| S11-final-viral-combined_k141_128982\|\|full | 1716 | No | NA | 0 | 1 | Not-determined | Genome-fragment | NA | 0 |
| S11-final-viral-combined_k141_101945\|\|full | 1171 | No | NA | 0 | 1 | Not-determined | Genome-fragment | NA | 0 |
| S4-final-viral-combined_k141_73918\|\|full | 1117 | No | NA | 0 | 1 | Low-quality | Genome-fragment | 1.63 | 0 |
| S4_k141_75828 | 1117 | No | NA | 0 | 0 | Low-quality | Genome-fragment | 2.32 | 0 |
| S11-final-viral-combined_k141_270124\|\|lt2gene | 1118 | No | NA | 1 | 0 | Low-quality | Genome-fragment | 2.44 | 0 |
| S11-final-viral-combined_k141_170863\|\|full | 2819 | No | NA | 0 | 3 | Not-determined | Genome-fragment | NA | 0 |
| S5_k141_152380 | 1118 | No | NA | 0 | 0 | Low-quality | Genome-fragment | 1.49 | 0 |
| S11-final-viral-combined_k141_46519\|\|full | 4119 | No | NA | 0 | 1 | Not-determined | Genome-fragment | NA | 0 |
| S11-final-viral-combined_k141_308486\|\|full | 2291 | No | NA | 0 | 0 | Not-determined | Genome-fragment | NA | 0 |
| S11-final-viral-combined_k141_101985\|\|full | 1526 | No | NA | 0 | 1 | Not-determined | Genome-fragment | NA | 0 |
| S5_k141_140802 | 1118 | No | NA | 0 | 0 | Low-quality | Genome-fragment | 1.88 | 0 |
| S11-final-viral-combined_k141_321507\|\|full | 5593 | No | NA | 0 | 4 | Not-determined | Genome-fragment | NA | 0 |
| S7-final-viral-combined_k141_235223\|\|full | 1118 | No | NA | 0 | 1 | Low-quality | Genome-fragment | 3.57 | 0 |
| S7-final-viral-combined_k141_108001\|\|full | 1118 | No | NA | 0 | 0 | Low-quality | Genome-fragment | 2.75 | 0 |
| S11-final-viral-combined_k141_32620\|\|full | 1682 | No | NA | 0 | 0 | Not-determined | Genome-fragment | NA | 0 |
| S7-final-viral-combined_k141_99458\|\|full | 1118 | No | NA | 1 | 0 | Low-quality | Genome-fragment | 2.51 | 0 |
| S7_k141_162243 | 1118 | No | NA | 0 | 0 | Low-quality | Genome-fragment | 0.33 | 0 |
| S17-final-viral-combined_k141_152278\|\|full | 1118 | No | NA | 1 | 0 | Low-quality | Genome-fragment | 1.96 | 0 |
| S11-final-viral-combined_k141_321533\|\|full | 1280 | No | NA | 0 | 2 | Not-determined | Genome-fragment | NA | 0 |
| S12-final-viral-combined_k141_235099\|\|full | 1118 | No | NA | 0 | 0 | Low-quality | Genome-fragment | 2.15 | 0 |
| S6-final-viral-combined_k141_34400\|\|full | 1118 | No | NA | 0 | 0 | Low-quality | Genome-fragment | 1.33 | 0 |
| S6-final-viral-combined_k141_102260\|\|lt2gene | 1118 | No | NA | 1 | 0 | Low-quality | Genome-fragment | 3.47 | 0 |
| S8-final-viral-combined_k141_260314\|\|full | 1118 | No | NA | 2 | 0 | Low-quality | Genome-fragment | 2.72 | 0 |
| S11-final-viral-combined_k141_143006\|\|full | 1860 | No | NA | 0 | 1 | Not-determined | Genome-fragment | NA | 0 |
| S8_k141_90445 | 1118 | No | NA | 0 | 0 | Low-quality | Genome-fragment | 2.58 | 0 |
| S11_k141_18771 | 1119 | No | NA | 0 | 1 | Low-quality | Genome-fragment | 0.52 | 0 |
| S11-final-viral-combined_k141_225789\|\|full | 3044 | No | NA | 0 | 2 | Not-determined | Genome-fragment | NA | 0 |
| S11_k141_40108 | 1119 | No | NA | 0 | 1 | Low-quality | Genome-fragment | 1.34 | 0 |
| S11-final-viral-combined_k141_281391\|\|full | 2907 | No | NA | 0 | 2 | Not-determined | Genome-fragment | NA | 0 |
| S3_k141_91325 | 1119 | No | NA | 0 | 0 | Low-quality | Genome-fragment | 1.07 | 0 |
| S5-final-viral-combined_k141_184785\|\|lt2gene | 1119 | No | NA | 2 | 0 | Low-quality | Genome-fragment | 2.54 | 0 |
| S11-final-viral-combined_k141_239905\|\|full | 3719 | No | NA | 0 | 1 | Not-determined | Genome-fragment | NA | 0 |
| S11-final-viral-combined_k141_281396\|\|full | 2329 | No | NA | 0 | 0 | Not-determined | Genome-fragment | NA | 0 |
| S11-final-viral-combined_k141_281397\|\|full | 2075 | No | NA | 0 | 2 | Not-determined | Genome-fragment | NA | 0 |
| S11-final-viral-combined_k141_266936\|\|full | 3232 | No | NA | 0 | 0 | Not-determined | Genome-fragment | NA | 0 |
| S5_k141_113752 | 1119 | Yes | 503 | 1 | 1 | Low-quality | Genome-fragment | 1.12 | 55.05 |
| S11-final-viral-combined_k141_253181\|\|full | 1885 | No | NA | 0 | 0 | Not-determined | Genome-fragment | NA | 0 |
| S11-final-viral-combined_k141_87960\|\|full | 2559 | No | NA | 0 | 2 | Not-determined | Genome-fragment | NA | 0 |
| S11-final-viral-combined_k141_4989\|\|full | 1456 | No | NA | 0 | 1 | Not-determined | Genome-fragment | NA | 0 |
| S7-final-viral-combined_k141_37013\|\|full | 1119 | No | NA | 0 | 0 | Low-quality | Genome-fragment | 0.77 | 0 |
| S11-final-viral-combined_k141_170949\|\|full | 3632 | No | NA | 0 | 3 | Not-determined | Genome-fragment | NA | 0 |
| S12-final-viral-combined_k141_129767\|\|lt2gene | 1119 | No | NA | 1 | 0 | Low-quality | Genome-fragment | 1 | 0 |
| S2_k141_97706 | 1119 | No | NA | 0 | 0 | Low-quality | Genome-fragment | 2.47 | 0 |
| S4-final-viral-combined_k141_49501\|\|full | 1119 | No | NA | 0 | 0 | Low-quality | Genome-fragment | 2.61 | 0 |
| S11-final-viral-combined_k141_212400\|\|full | 2151 | No | NA | 0 | 0 | Not-determined | Genome-fragment | NA | 0 |
| S11-final-viral-combined_k141_102128\|\|full | 1781 | No | NA | 0 | 1 | Not-determined | Genome-fragment | NA | 0 |
| S11-final-viral-combined_k141_88009\|\|full | 1624 | No | NA | 0 | 0 | Not-determined | Genome-fragment | NA | 0 |
| S11-final-viral-combined_k141_143062\|\|full | 1460 | No | NA | 0 | 2 | Not-determined | Genome-fragment | NA | 0 |
| S8_k141_113601 | 1119 | No | NA | 0 | 1 | Low-quality | Genome-fragment | 1.72 | 0 |
| S9-final-viral-combined_k141_90448\|\|lt2gene | 1120 | No | NA | 1 | 0 | Low-quality | Genome-fragment | 2.43 | 0 |
| S9_k141_72399 | 1120 | No | NA | 0 | 0 | Low-quality | Genome-fragment | 1.87 | 0 |
| S11-final-viral-combined_k141_184209\|\|full | 1461 | No | NA | 0 | 1 | Not-determined | Genome-fragment | NA | 0 |
| S11-final-viral-combined_k141_5089\|\|full | 2305 | No | NA | 0 | 3 | Not-determined | Genome-fragment | NA | 0 |
| S11-final-viral-combined_k141_267016\|\|full | 8364 | No | NA | 0 | 0 | Not-determined | Genome-fragment | NA | 0 |
| S11-final-viral-combined_k141_51087\|\|full | 1120 | No | NA | 1 | 0 | Low-quality | Genome-fragment | 0.68 | 0 |
| S11_k141_86251 | 1120 | No | NA | 1 | 0 | Low-quality | Genome-fragment | 2.61 | 0 |
| S1_k141_141084 | 1120 | No | NA | 0 | 1 | Low-quality | Genome-fragment | 0.52 | 0 |
| S11-final-viral-combined_k141_212474\|\|full | 2949 | No | NA | 0 | 2 | Not-determined | Genome-fragment | NA | 0 |
| S11-final-viral-combined_k141_156734\|\|full | 1820 | No | NA | 0 | 1 | Not-determined | Genome-fragment | NA | 0 |
| S1_k141_79330 | 1120 | No | NA | 0 | 0 | Low-quality | Genome-fragment | 2.05 | 0 |
| S3-final-viral-combined_k141_33599\|\|full | 1120 | No | NA | 1 | 0 | Low-quality | Genome-fragment | 2.91 | 0 |
| S11-final-viral-combined_k141_156746\|\|full | 2407 | No | NA | 0 | 3 | Not-determined | Genome-fragment | NA | 0 |
| S3_k141_98656 | 1120 | No | NA | 0 | 0 | Low-quality | Genome-fragment | 2.88 | 0 |
| S5-final-viral-combined_k141_71291\|\|full | 1120 | No | NA | 0 | 0 | Low-quality | Genome-fragment | 2.71 | 0 |
| S17-final-viral-combined_k141_173708\|\|full | 1120 | No | NA | 0 | 0 | Low-quality | Genome-fragment | 2.48 | 0 |
| S17-final-viral-combined_k141_232695\|\|full | 1120 | No | NA | 0 | 0 | Low-quality | Genome-fragment | 2.08 | 0 |
| S11-final-viral-combined_k141_114879\|\|full | 1871 | No | NA | 0 | 2 | Not-determined | Genome-fragment | NA | 0 |
| S11-final-viral-combined_k141_267118\|\|full | 3038 | No | NA | 0 | 2 | Not-determined | Genome-fragment | NA | 0 |
| S11-final-viral-combined_k141_294458\|\|full | 1620 | No | NA | 0 | 0 | Not-determined | Genome-fragment | NA | 0 |
| S11-final-viral-combined_k141_60490\|\|full | 6613 | No | NA | 0 | 3 | Not-determined | Genome-fragment | NA | 0 |
| S11-final-viral-combined_k141_267120\|\|full | 1600 | No | NA | 0 | 1 | Not-determined | Genome-fragment | NA | 0 |
| S11-final-viral-combined_k141_143164\|\|full | 1040 | No | NA | 0 | 0 | Not-determined | Genome-fragment | NA | 0 |
| S11-final-viral-combined_k141_156752\|\|full | 2539 | No | NA | 0 | 0 | Not-determined | Genome-fragment | NA | 0 |
| S11-final-viral-combined_k141_294469\|\|full | 2621 | No | NA | 0 | 1 | Not-determined | Genome-fragment | NA | 0 |
| S11-final-viral-combined_k141_281599\|\|full | 1175 | No | NA | 0 | 0 | Not-determined | Genome-fragment | NA | 0 |
| S11-final-viral-combined_k141_184317\|\|full | 1218 | No | NA | 0 | 2 | Not-determined | Genome-fragment | NA | 0 |
| S12_k141_280272 | 1120 | No | NA | 0 | 0 | Low-quality | Genome-fragment | 3.04 | 0 |
| S11-final-viral-combined_k141_32725\|\|full | 2628 | No | NA | 0 | 2 | Not-determined | Genome-fragment | NA | 0 |
| S11-final-viral-combined_k141_308741\|\|full | 1062 | No | NA | 0 | 0 | Not-determined | Genome-fragment | NA | 0 |
| S11-final-viral-combined_k141_143193\|\|full | 3726 | No | NA | 0 | 1 | Not-determined | Genome-fragment | NA | 0 |
| S2-final-viral-combined_k141_170669\|\|full | 1120 | No | NA | 0 | 0 | Low-quality | Genome-fragment | 2.17 | 0 |
| S11-final-viral-combined_k141_102257\|\|full | 1366 | No | NA | 0 | 1 | Not-determined | Genome-fragment | NA | 0 |
| S2-final-viral-combined_k141_245134\|\|full | 1120 | No | NA | 2 | 0 | Low-quality | Genome-fragment | 2.42 | 0 |
| S11-final-viral-combined_k141_18533\|\|full | 2368 | No | NA | 0 | 2 | Not-determined | Genome-fragment | NA | 0 |
| S11-final-viral-combined_k141_281637\|\|full | 3665 | No | NA | 0 | 4 | Not-determined | Genome-fragment | NA | 0 |
| S11-final-viral-combined_k141_143204\|\|full | 6095 | No | NA | 0 | 2 | Not-determined | Genome-fragment | NA | 0 |
| S2_k141_170009 | 1120 | No | NA | 0 | 1 | Low-quality | Genome-fragment | 2.5 | 0 |
| S11-final-viral-combined_k141_102266\|\|full | 9586 | No | NA | 0 | 6 | Not-determined | Genome-fragment | NA | 0 |
| S11-final-viral-combined_k141_114954\|\|full | 1192 | No | NA | 0 | 3 | Not-determined | Genome-fragment | NA | 0 |
| S11-final-viral-combined_k141_253255\|\|full | 1743 | No | NA | 0 | 3 | Not-determined | Genome-fragment | NA | 0 |
| S11-final-viral-combined_k141_281649\|\|full | 2587 | No | NA | 0 | 1 | Not-determined | Genome-fragment | NA | 0 |
| S11-final-viral-combined_k141_197938\|\|full | 10593 | No | NA | 0 | 2 | Not-determined | Genome-fragment | NA | 0 |
| S4_k141_161422 | 1120 | No | NA | 0 | 0 | Low-quality | Genome-fragment | 1.87 | 0 |
| S8-final-viral-combined_k141_272835\|\|lt2gene | 1120 | No | NA | 1 | 0 | Low-quality | Genome-fragment | 1.64 | 0 |
| S3_k141_1207 | 1121 | No | NA | 0 | 0 | Low-quality | Genome-fragment | 0.52 | 0 |
| S5-final-viral-combined_k141_50475\|\|full | 1121 | No | NA | 1 | 0 | Low-quality | Genome-fragment | 0.77 | 0 |

| | | | | | | | | | | | |
|---|---|---|---|---|---|---|---|---|---|---|---|
| S11-final-viral-combined_k141_18562\|\|full | 2755 | No | NA | | 0 | 2 | Not-determined | Genome-fragment | NA | | 0 |
| S11-final-viral-combined_k141_5246\|\|full | 1235 | No | NA | | 0 | 1 | Not-determined | Genome-fragment | NA | | 0 |
| S5_k141_83478 | 1121 | No | NA | | 0 | 1 | Low-quality | Genome-fragment | | 3.58 | 0 |
| S7-final-viral-combined_k141_12371\|\|full | 1121 | No | NA | | 1 | 0 | Low-quality | Genome-fragment | | 1.61 | 0 |
| S11-final-viral-combined_k141_184382\|\|full | 1577 | No | NA | | 0 | 1 | Not-determined | Genome-fragment | NA | | 0 |
| S11-final-viral-combined_k141_240139\|\|full | 7259 | No | NA | | 0 | 2 | Not-determined | Genome-fragment | NA | | 0 |
| S7-final-viral-combined_k141_198265\|\|full | 1121 | No | NA | | 1 | 0 | Low-quality | Genome-fragment | | 2.75 | 0 |
| S7_k141_126320 | 1121 | No | NA | | 0 | 1 | Low-quality | Genome-fragment | | 0.37 | 0 |
| S11-final-viral-combined_k141_32810\|\|full | 5526 | No | NA | | 0 | 5 | Not-determined | Genome-fragment | NA | | 0 |
| S11-final-viral-combined_k141_156843\|\|full | 2314 | No | NA | | 0 | 1 | Not-determined | Genome-fragment | NA | | 0 |
| S11-final-viral-combined_k141_321706\|\|full | 2631 | No | NA | | 0 | 2 | Not-determined | Genome-fragment | NA | | 0 |
| S17-final-viral-combined_k141_171932\|\|full | 1121 | No | NA | | 0 | 0 | Low-quality | Genome-fragment | | 3.04 | 0 |
| S12-final-viral-combined_k141_43283\|\|full | 1121 | No | NA | | 1 | 0 | Low-quality | Genome-fragment | | 2.23 | 0 |
| S11-final-viral-combined_k141_212683\|\|full | 2859 | No | NA | | 0 | 0 | Not-determined | Genome-fragment | NA | | 0 |
| S11-final-viral-combined_k141_129337\|\|full | 1715 | No | NA | | 0 | 1 | Not-determined | Genome-fragment | NA | | 0 |
| S11-final-viral-combined_k141_267154\|\|full | 1019 | No | NA | | 0 | 2 | Not-determined | Genome-fragment | NA | | 0 |
| S11-final-viral-combined_k141_184420\|\|full | 2304 | No | NA | | 0 | 1 | Not-determined | Genome-fragment | NA | | 0 |
| S12-final-viral-combined_k141_9685\|\|full | 1121 | No | NA | | 0 | 0 | Low-quality | Genome-fragment | | 2.14 | 0 |
| S12-final-viral-combined_k141_49973\|\|full | 1121 | No | NA | | 0 | 1 | Low-quality | Genome-fragment | | 0.58 | 0 |
| S11-final-viral-combined_k141_73611\|\|full | 2491 | No | NA | | 0 | 0 | Not-determined | Genome-fragment | NA | | 0 |
| S12-final-viral-combined_k141_290242\|\|lt2gene | 1121 | No | NA | | 1 | 0 | Low-quality | Genome-fragment | | 1.47 | 0 |
| S12_k141_284464 | 1121 | No | NA | | 0 | 0 | Low-quality | Genome-fragment | | 0.52 | 0 |
| S11-final-viral-combined_k141_294568\|\|full | 1201 | No | NA | | 0 | 1 | Not-determined | Genome-fragment | NA | | 0 |
| S2-final-viral-combined_k141_104771\|\|full | 1121 | No | NA | | 2 | 0 | Low-quality | Genome-fragment | | 2.69 | 0 |
| S2-final-viral-combined_k141_171337\|\|full | 1121 | No | NA | | 0 | 0 | Low-quality | Genome-fragment | | 0.62 | 0 |
| S11-final-viral-combined_k141_46816\|\|full | 29694 | No | NA | | 8 | 0 | Medium-quality | Genome-fragment | | 62.99 | 0 |
| S11-final-viral-combined_k141_171066\|\|full | 3031 | No | NA | | 0 | 1 | Not-determined | Genome-fragment | NA | | 0 |
| S11-final-viral-combined_k141_32902\|\|full | 5899 | No | NA | | 0 | 3 | Not-determined | Genome-fragment | NA | | 0 |
| S2-final-viral-combined_k141_90845\|\|full | 1121 | No | NA | | 0 | 0 | Low-quality | Genome-fragment | | 3.6 | 0 |
| S4_k141_163236 | 1121 | No | NA | | 0 | 0 | Low-quality | Genome-fragment | | 0.93 | 0 |
| S11-final-viral-combined_k141_73663\|\|full | 1350 | No | NA | | 0 | 0 | Not-determined | Genome-fragment | NA | | 0 |
| S11-final-viral-combined_k141_46838\|\|full | 3952 | No | NA | | 0 | 2 | Not-determined | Genome-fragment | NA | | 0 |
| S11-final-viral-combined_k141_115152\|\|full | 1453 | No | NA | | 0 | 0 | Not-determined | Genome-fragment | NA | | 0 |
| S11-final-viral-combined_k141_171084\|\|full | 3657 | No | NA | | 0 | 3 | Not-determined | Genome-fragment | NA | | 0 |
| S11-final-viral-combined_k141_156954\|\|full | 5358 | No | NA | | 0 | 2 | Not-determined | Genome-fragment | NA | | 0 |
| S11-final-viral-combined_k141_294612\|\|full | 1328 | No | NA | | 0 | 0 | Not-determined | Genome-fragment | NA | | 0 |
| S6_k141_139142 | 1121 | No | NA | | 0 | 0 | Low-quality | Genome-fragment | | 2.23 | 0 |
| S8-final-viral-combined_k141_176008\|\|full | 1121 | No | NA | | 0 | 1 | Low-quality | Genome-fragment | | 2.92 | 0 |
| S11-final-viral-combined_k141_46844\|\|full | 7709 | No | NA | | 0 | 7 | Not-determined | Genome-fragment | NA | | 0 |
| S11-final-viral-combined_k141_143285\|\|full | 1249 | No | NA | | 0 | 1 | Not-determined | Genome-fragment | NA | | 0 |
| S11-final-viral-combined_k141_156974\|\|full | 1185 | No | NA | | 0 | 0 | Not-determined | Genome-fragment | NA | | 0 |
| S11-final-viral-combined_k141_115172\|\|full | 3313 | No | NA | | 0 | 3 | Not-determined | Genome-fragment | NA | | 0 |
| S11-final-viral-combined_k141_294627\|\|full | 3425 | No | NA | | 0 | 3 | Not-determined | Genome-fragment | NA | | 0 |
| S11-final-viral-combined_k141_5402\|\|full | 1572 | No | NA | | 0 | 1 | Not-determined | Genome-fragment | NA | | 0 |
| S8-final-viral-combined_k141_4556\|\|lt2gene | 1121 | No | NA | | 1 | 0 | Low-quality | Genome-fragment | | 2.31 | 0 |
| S8_k141_35475 | 1121 | No | NA | | 0 | 1 | Low-quality | Genome-fragment | | 0.4 | 0 |
| S11-final-viral-combined_k141_198080\|\|full | 21682 | No | NA | | 0 | 13 | Not-determined | Genome-fragment | NA | | 0 |
| S11-final-viral-combined_k141_294666\|\|full | 1076 | No | NA | | 0 | 0 | Not-determined | Genome-fragment | NA | | 0 |
| S11-final-viral-combined_k141_156986\|\|full | 2721 | No | NA | | 0 | 3 | Not-determined | Genome-fragment | NA | | 0 |
| S11-final-viral-combined_k141_294673\|\|full | 1440 | No | NA | | 0 | 1 | Not-determined | Genome-fragment | NA | | 0 |
| S11-final-viral-combined_k141_245733\|\|full | 1122 | No | NA | | 2 | 0 | Low-quality | Genome-fragment | | 3.02 | 0 |
| S11_k141_223054 | 1122 | No | NA | | 1 | 0 | Low-quality | Genome-fragment | | 0.4 | 0 |
| S11_k141_50654 | 1122 | No | NA | | 0 | 1 | Low-quality | Genome-fragment | | 1.88 | 0 |
| S5-final-viral-combined_k141_105411\|\|full | 1122 | No | NA | | 1 | 0 | Low-quality | Genome-fragment | | 2.49 | 0 |
| S7-final-viral-combined_k141_49363\|\|full | 1122 | No | NA | | 1 | 0 | Low-quality | Genome-fragment | | 1.68 | 0 |
| S11-final-viral-combined_k141_294692\|\|full | 1634 | No | NA | | 0 | 0 | Not-determined | Genome-fragment | NA | | 0 |
| S7-final-viral-combined_k141_146858\|\|lt2gene | 1122 | No | NA | | 0 | 0 | Low-quality | Genome-fragment | | 2.67 | 0 |
| S11-final-viral-combined_k141_294704\|\|full | 2735 | No | NA | | 0 | 2 | Not-determined | Genome-fragment | NA | | 0 |
| S11-final-viral-combined_k141_88295\|\|full | 1400 | No | NA | | 0 | 2 | Not-determined | Genome-fragment | NA | | 0 |
| S17-final-viral-combined_k141_188794\|\|lt2gene | 1122 | No | NA | | 1 | 0 | Low-quality | Genome-fragment | | 3.62 | 0 |
| S17-k141_192648 | 1122 | No | NA | | 0 | 1 | Low-quality | Genome-fragment | | 0.49 | 0 |
| S17-k141_260762 | 1122 | No | NA | | 0 | 0 | Low-quality | Genome-fragment | | 3.11 | 0 |
| S17-k141_52707 | 1122 | No | NA | | 0 | 1 | Low-quality | Genome-fragment | | 0.79 | 0 |
| S11-final-viral-combined_k141_240405\|\|full | 4886 | No | NA | | 0 | 1 | Not-determined | Genome-fragment | NA | | 0 |
| S11-final-viral-combined_k141_171175\|\|full | 2002 | No | NA | | 0 | 0 | Not-determined | Genome-fragment | NA | | 0 |
| S11-final-viral-combined_k141_321834\|\|full | 2398 | No | NA | | 0 | 2 | Not-determined | Genome-fragment | NA | | 0 |
| S12_k141_132836 | 1122 | No | NA | | 0 | 1 | Low-quality | Genome-fragment | | 0.38 | 0 |
| S11-final-viral-combined_k141_88332\|\|full | 6184 | No | NA | | 0 | 1 | Not-determined | Genome-fragment | NA | | 0 |
| S4-final-viral-combined_k141_158481\|\|lt2gene | 1122 | No | NA | | 1 | 0 | Low-quality | Genome-fragment | | 3.7 | 0 |
| S6_k141_79827 | 1122 | No | NA | | 0 | 1 | Low-quality | Genome-fragment | | 0.73 | 0 |
| S11-final-viral-combined_k141_71774\|\|lt2gene | 1123 | No | NA | | 1 | 0 | Low-quality | Genome-fragment | | 2.85 | 0 |
| S11_k141_63829 | 1123 | No | NA | | 0 | 1 | Low-quality | Genome-fragment | | 1.42 | 0 |
| S1-final-viral-combined_k141_40606\|\|full | 1123 | No | NA | | 1 | 0 | Low-quality | Genome-fragment | | 2.78 | 0 |
| S11-final-viral-combined_k141_253582\|\|full | 1998 | No | NA | | 0 | 1 | Not-determined | Genome-fragment | NA | | 0 |
| S1-final-viral-combined_k141_10739\|\|lt2gene | 1123 | No | NA | | 1 | 0 | Low-quality | Genome-fragment | | 2.62 | 0 |
| S1-final-viral-combined_k141_262888\|\|lt2gene | 1123 | No | NA | | 1 | 0 | Low-quality | Genome-fragment | | 1.83 | 0 |
| S11-final-viral-combined_k141_226135\|\|full | 3347 | No | NA | | 0 | 2 | Not-determined | Genome-fragment | NA | | 0 |
| S11-final-viral-combined_k141_184668\|\|full | 4157 | No | NA | | 0 | 1 | Not-determined | Genome-fragment | NA | | 0 |
| S11-final-viral-combined_k141_294784\|\|full | 1267 | No | NA | | 0 | 1 | Not-determined | Genome-fragment | NA | | 0 |
| S11-final-viral-combined_k141_267318\|\|full | 1848 | No | NA | | 0 | 2 | Not-determined | Genome-fragment | NA | | 0 |
| S11-final-viral-combined_k141_321860\|\|full | 2792 | No | NA | | 0 | 1 | Not-determined | Genome-fragment | NA | | 0 |
| S5-final-viral-combined_k141_7351\|\|full | 1123 | No | NA | | 1 | 0 | Low-quality | Genome-fragment | | 2.49 | 0 |
| S11-final-viral-combined_k141_115309\|\|full | 1433 | No | NA | | 0 | 1 | Not-determined | Genome-fragment | NA | | 0 |
| S11-final-viral-combined_k141_129506\|\|full | 1692 | No | NA | | 0 | 1 | Not-determined | Genome-fragment | NA | | 0 |
| S5-final-viral-combined_k141_16226\|\|full | 1123 | No | NA | | 1 | 0 | Low-quality | Genome-fragment | | 0.77 | 0 |
| S5-final-viral-combined_k141_170243\|\|lt2gene | 1123 | No | NA | | 1 | 0 | Low-quality | Genome-fragment | | 0.61 | 0 |
| S11-final-viral-combined_k141_129543\|\|full | 2241 | No | NA | | 0 | 2 | Not-determined | Genome-fragment | NA | | 0 |
| S5-final-viral-combined_k141_91722\|\|lt2gene | 1123 | No | NA | | 1 | 0 | Low-quality | Genome-fragment | | 2.84 | 0 |
| S11-final-viral-combined_k141_33110\|\|full | 4127 | No | NA | | 0 | 1 | Not-determined | Genome-fragment | NA | | 0 |
| S17-final-viral-combined_k141_185565\|\|full | 1123 | No | NA | | 1 | 0 | Low-quality | Genome-fragment | | 2.06 | 0 |
| S11-final-viral-combined_k141_47099\|\|full | 2004 | No | NA | | 0 | 3 | Not-determined | Genome-fragment | NA | | 0 |
| S11-final-viral-combined_k141_226165\|\|full | 33207 | No | NA | | 8 | 3 | Medium-quality | Genome-fragment | | 78.45 | 0 |
| S11-final-viral-combined_k141_33113\|\|full | 6894 | No | NA | | 0 | 7 | Not-determined | Genome-fragment | NA | | 0 |
| S12-final-viral-combined_k141_67946\|\|full | 1123 | No | NA | | 1 | 0 | Low-quality | Genome-fragment | | 0.71 | 0 |
| S11-final-viral-combined_k141_5563\|\|full | 4271 | No | NA | | 0 | 3 | Not-determined | Genome-fragment | NA | | 0 |
| S12-final-viral-combined_k141_371844\|\|full | 1123 | No | NA | | 0 | 0 | Low-quality | Genome-fragment | | 1.93 | 0 |
| S11-final-viral-combined_k141_47141\|\|full | 2093 | No | NA | | 0 | 1 | Not-determined | Genome-fragment | NA | | 0 |
| S12-final-viral-combined_k141_379252\|\|full | 1123 | No | NA | | 0 | 1 | Low-quality | Genome-fragment | | 1.84 | 0 |
| S12-final-viral-combined_k141_300508\|\|full | 1123 | No | NA | | 0 | 0 | Low-quality | Genome-fragment | | 0.76 | 0 |
| S11-final-viral-combined_k141_267363\|\|full | 2317 | No | NA | | 0 | 0 | Not-determined | Genome-fragment | NA | | 0 |
| S12-final-viral-combined_k141_387549\|\|lt2gene | 1123 | No | NA | | 1 | 0 | Low-quality | Genome-fragment | | 2.75 | 0 |
| S11-final-viral-combined_k141_129593\|\|full | 1972 | No | NA | | 0 | 1 | Not-determined | Genome-fragment | NA | | 0 |
| S11-final-viral-combined_k141_171340\|\|full | 1388 | No | NA | | 0 | 0 | Not-determined | Genome-fragment | NA | | 0 |
| S12_k141_180810 | 1123 | No | NA | | 0 | 0 | Low-quality | Genome-fragment | | 0.6 | 0 |
| S11-final-viral-combined_k141_184770\|\|full | 3195 | No | NA | | 0 | 1 | Not-determined | Genome-fragment | NA | | 0 |
| S2-final-viral-combined_k141_152660\|\|full | 1123 | No | NA | | 0 | 0 | Low-quality | Genome-fragment | | 2.23 | 0 |
| S2_k141_4420 | 1123 | No | NA | | 0 | 0 | Low-quality | Genome-fragment | | 2.71 | 0 |
| S11-final-viral-combined_k141_240616\|\|full | 1668 | No | NA | | 0 | 1 | Not-determined | Genome-fragment | NA | | 0 |
| S11-final-viral-combined_k141_184791\|\|full | 8204 | No | NA | | 0 | 6 | Not-determined | Genome-fragment | NA | | 0 |
| S8_k141_97849 | 1123 | No | NA | | 0 | 0 | Low-quality | Genome-fragment | | 1.12 | 0 |
| S9-final-viral-combined_k141_123687\|\|lt2gene | 1124 | No | NA | | 1 | 0 | Low-quality | Genome-fragment | | 2.83 | 0 |
| S11-final-viral-combined_k141_130410\|\|full | 1124 | No | NA | | 0 | 0 | Low-quality | Genome-fragment | | 2.32 | 0 |
| S11-final-viral-combined_k141_309270\|\|full | 4510 | No | NA | | 0 | 3 | Not-determined | Genome-fragment | NA | | 0 |
| S11-final-viral-combined_k141_321938\|\|full | 42506 | Yes | | 39787 | 7 | 7 | High-quality | High-quality | | 92.89 | 6.4 |
| S11-final-viral-combined_k141_242641\|\|full | 1124 | No | NA | | 0 | 0 | Low-quality | Genome-fragment | | 2.73 | 0 |
| S11-final-viral-combined_k141_226236\|\|full | 3272 | No | NA | | 0 | 4 | Not-determined | Genome-fragment | NA | | 0 |
| S11-final-viral-combined_k141_283895\|\|full | 1124 | No | NA | | 0 | 0 | Low-quality | Genome-fragment | | 1.46 | 0 |
| S11_k141_119680 | 1124 | No | NA | | 0 | 0 | Low-quality | Genome-fragment | | 2.98 | 0 |
| S11-final-viral-combined_k141_33212\|\|full | 1762 | No | NA | | 0 | 1 | Not-determined | Genome-fragment | NA | | 0 |
| S1-final-viral-combined_k141_152916\|\|full | 1124 | No | NA | | 0 | 0 | Low-quality | Genome-fragment | | 1.31 | 0 |
| S11-final-viral-combined_k141_129642\|\|full | 3035 | No | NA | | 0 | 2 | Not-determined | Genome-fragment | NA | | 0 |
| S11-final-viral-combined_k141_267475\|\|full | 5970 | No | NA | | 0 | 2 | Not-determined | Genome-fragment | NA | | 0 |
| S11-final-viral-combined_k141_226248\|\|full | 2138 | No | NA | | 0 | 0 | Not-determined | Genome-fragment | NA | | 0 |
| S11-final-viral-combined_k141_33239\|\|full | 3753 | No | NA | | 0 | 0 | Not-determined | Genome-fragment | NA | | 0 |
| S1-final-viral-combined_k141_5534\|\|full | 1124 | No | NA | | 0 | 0 | Low-quality | Genome-fragment | | 1.89 | 0 |
| S11-final-viral-combined_k141_226260\|\|full | 1096 | No | NA | | 0 | 3 | Not-determined | Genome-fragment | NA | | 0 |
| S1-final-viral-combined_k141_253685\|\|full | 1124 | No | NA | | 1 | 0 | Low-quality | Genome-fragment | | 1.47 | 0 |
| S1-final-viral-combined_k141_347276\|\|lt2gene | 1124 | No | NA | | 1 | 0 | Low-quality | Genome-fragment | | 3.15 | 0 |
| S1_k141_165134 | 1124 | No | NA | | 0 | 0 | Low-quality | Genome-fragment | | 1.87 | 0 |
| S3-final-viral-combined_k141_38501\|\|full | 1124 | No | NA | | 0 | 1 | Low-quality | Genome-fragment | | 0.69 | 0 |
| S11-final-viral-combined_k141_102820\|\|full | 3130 | No | NA | | 0 | 3 | Not-determined | Genome-fragment | NA | | 0 |
| S11-final-viral-combined_k141_143622\|\|full | 4110 | No | NA | | 0 | 2 | Not-determined | Genome-fragment | NA | | 0 |
| S5-final-viral-combined_k141_108527\|\|lt2gene | 1124 | No | NA | | 0 | 0 | Low-quality | Genome-fragment | | 2.81 | 0 |
| S17-final-viral-combined_k141_99861\|\|full | 1124 | No | NA | | 0 | 1 | Low-quality | Genome-fragment | | 0.55 | 0 |
| S12-final-viral-combined_k141_222173\|\|lt2gene | 1124 | No | NA | | 1 | 0 | Low-quality | Genome-fragment | | 1.42 | 0 |
| S12-final-viral-combined_k141_191767\|\|lt2gene | 1124 | No | NA | | 0 | 0 | Low-quality | Genome-fragment | | 2 | 0 |
| S11-final-viral-combined_k141_253869\|\|full | 1539 | No | NA | | 0 | 0 | Not-determined | Genome-fragment | NA | | 0 |
| S11-final-viral-combined_k141_295020\|\|full | 1278 | No | NA | | 0 | 2 | Not-determined | Genome-fragment | NA | | 0 |
| S11-final-viral-combined_k141_213079\|\|full | 1387 | No | NA | | 0 | 2 | Not-determined | Genome-fragment | NA | | 0 |
| S12-final-viral-combined_k141_145140\|\|lt2gene | 1124 | No | NA | | 1 | 0 | Low-quality | Genome-fragment | | 2.91 | 0 |
| S12-final-viral-combined_k141_216401\|\|lt2gene | 1124 | No | NA | | 1 | 0 | Low-quality | Genome-fragment | | 0.73 | 0 |
| S11-final-viral-combined_k141_240699\|\|full | 2872 | No | NA | | 0 | 2 | Not-determined | Genome-fragment | NA | | 0 |
| S11-final-viral-combined_k141_213099\|\|full | 1737 | No | NA | | 0 | 2 | Not-determined | Genome-fragment | NA | | 0 |
| S11-final-viral-combined_k141_309356\|\|full | 3029 | No | NA | | 0 | 1 | Not-determined | Genome-fragment | NA | | 0 |
| S11-final-viral-combined_k141_5677\|\|full | 27110 | No | NA | | 0 | 13 | Not-determined | Genome-fragment | NA | | 0 |
| S12_k141_306609 | 1124 | No | NA | | 2 | 0 | Low-quality | Genome-fragment | | 2.45 | 0 |
| S2-final-viral-combined_k141_140001\|\|full | 1124 | No | NA | | 0 | 1 | Low-quality | Genome-fragment | | 0.98 | 0 |
| S4-final-viral-combined_k141_123459\|\|full | 1124 | No | NA | | 0 | 0 | Low-quality | Genome-fragment | | 2.29 | 0 |
| S11-final-viral-combined_k141_5688\|\|full | 5923 | No | NA | | 0 | 2 | Not-determined | Genome-fragment | NA | | 0 |
| S4-final-viral-combined_k141_124102\|\|full | 1124 | No | NA | | 1 | 0 | Low-quality | Genome-fragment | | 0.99 | 0 |
| S11-final-viral-combined_k141_88560\|\|full | 1958 | No | NA | | 0 | 2 | Not-determined | Genome-fragment | NA | | 0 |
| S11-final-viral-combined_k141_309384\|\|full | 2203 | No | NA | | 0 | 0 | Not-determined | Genome-fragment | NA | | 0 |
| S11-final-viral-combined_k141_5699\|\|full | 1302 | No | NA | | 0 | 2 | Not-determined | Genome-fragment | NA | | 0 |
| S11-final-viral-combined_k141_282052\|\|full | 3434 | No | NA | | 0 | 2 | Not-determined | Genome-fragment | NA | | 0 |
| S11-final-viral-combined_k141_226380\|\|full | 2118 | No | NA | | 0 | 3 | Not-determined | Genome-fragment | NA | | 0 |
| S8-final-viral-combined_k141_197780\|\|full | 1124 | No | NA | | 0 | 0 | Low-quality | Genome-fragment | | 1.19 | 0 |
| S11-final-viral-combined_k141_253934\|\|full | 6029 | No | NA | | 0 | 1 | Not-determined | Genome-fragment | NA | | 0 |
| S11-final-viral-combined_k141_322062\|\|full | 1482 | No | NA | | 0 | 2 | Not-determined | Genome-fragment | NA | | 0 |
| S11-final-viral-combined_k141_253948\|\|full | 1824 | No | NA | | 0 | 1 | Not-determined | Genome-fragment | NA | | 0 |
| S11-final-viral-combined_k141_61033\|\|full | 2335 | No | NA | | 0 | 2 | Not-determined | Genome-fragment | NA | | 0 |
| S11-final-viral-combined_k141_213161\|\|full | 3307 | No | NA | | 0 | 0 | Not-determined | Genome-fragment | NA | | 0 |
| S11-final-viral-combined_k141_253144\|\|full | 1125 | No | NA | | 2 | 0 | Low-quality | Genome-fragment | | 1.73 | 0 |
| S11-final-viral-combined_k141_129751\|\|full | 1367 | No | NA | | 0 | 1 | Not-determined | Genome-fragment | NA | | 0 |
| S11-final-viral-combined_k141_240783\|\|full | 1770 | No | NA | | 0 | 0 | Not-determined | Genome-fragment | NA | | 0 |
| S11-final-viral-combined_k141_47377\|\|full | 15742 | No | NA | | 0 | 11 | Not-determined | Genome-fragment | NA | | 0 |
| S11-final-viral-combined_k141_7949\|\|full | 1125 | No | NA | | 0 | 0 | Low-quality | Genome-fragment | | 2.89 | 0 |
| S11-final-viral-combined_k141_18915\|\|full | 3029 | No | NA | | 0 | 1 | Not-determined | Genome-fragment | NA | | 0 |
| S11-final-viral-combined_k141_187736\|\|full | 1125 | No | NA | | 0 | 0 | Low-quality | Genome-fragment | | 2.45 | 0 |
| S11-final-viral-combined_k141_240798\|\|full | 3716 | No | NA | | 0 | 1 | Not-determined | Genome-fragment | NA | | 0 |
| S11-final-viral-combined_k141_115425\|\|full | 1700 | No | NA | | 0 | 1 | Not-determined | Genome-fragment | NA | | 0 |
| S1-final-viral-combined_k141_46294\|\|full | 1125 | No | NA | | 0 | 0 | Low-quality | Genome-fragment | | 1.24 | 0 |
| S1-final-viral-combined_k141_328239\|\|full | 1125 | No | NA | | 0 | 0 | Low-quality | Genome-fragment | | 2.17 | 0 |
| S11-final-viral-combined_k141_171499\|\|full | 2649 | No | NA | | 0 | 3 | Not-determined | Genome-fragment | NA | | 0 |
| S1_k141_300389 | 1125 | No | NA | | 0 | 0 | Low-quality | Genome-fragment | | 2.06 | 0 |

| | | | | | | | | | |
|---|---|---|---|---|---|---|---|---|---|
| S11-final-viral-combined_k141_143768\|\|full | 2984 | No | NA | 0 | 2 | Not-determined | Genome-fragment | NA | 0 |
| S11-final-viral-combined_k141_198512\|\|full | 41546 | Yes | 36129 | 9 | 3 | Medium-quality | Genome-fragment | 66.2 | 13.04 |
| S11-final-viral-combined_k141_171514\|\|full | 3260 | No | NA | 0 | 2 | Not-determined | Genome-fragment | NA | 0 |
| S1_k141_45831 | 1125 | No | NA | 0 | 1 | Low-quality | Genome-fragment | 3.72 | 0 |
| S5-final-viral-combined_k141_108199\|\|lt2gene | 1125 | No | NA | 1 | 0 | Low-quality | Genome-fragment | 3.33 | 0 |
| S11-final-viral-combined_k141_115461\|\|full | 1087 | No | NA | 0 | 0 | Not-determined | Genome-fragment | NA | 0 |
| S7-final-viral-combined_k141_26649\|\|full | 1125 | No | NA | 0 | 2 | Low-quality | Genome-fragment | 2.59 | 0 |
| S11-final-viral-combined_k141_226492\|\|full | 1329 | No | NA | 0 | 0 | Not-determined | Genome-fragment | NA | 0 |
| S11-final-viral-combined_k141_282155\|\|full | 2555 | No | NA | 0 | 1 | Not-determined | Genome-fragment | NA | 0 |
| S11-final-viral-combined_k141_171529\|\|full | 2066 | No | NA | 0 | 3 | Not-determined | Genome-fragment | NA | 0 |
| S11-final-viral-combined_k141_184917\|\|full | 1710 | No | NA | 0 | 1 | Not-determined | Genome-fragment | NA | 0 |
| S11-final-viral-combined_k141_322136\|\|full | 1337 | No | NA | 0 | 0 | Not-determined | Genome-fragment | NA | 0 |
| S11-final-viral-combined_k141_322149\|\|full | 2473 | No | NA | 0 | 2 | Not-determined | Genome-fragment | NA | 0 |
| S11-final-viral-combined_k141_157537\|\|full | 1219 | No | NA | 0 | 1 | Not-determined | Genome-fragment | NA | 0 |
| S17-final-viral-combined_k141_232319\|\|lt2gene | 1125 | No | NA | 1 | 0 | Low-quality | Genome-fragment | 2.54 | 0 |
| S11-final-viral-combined_k141_198559\|\|full | 8454 | No | NA | 0 | 7 | Not-determined | Genome-fragment | NA | 0 |
| S11-final-viral-combined_k141_226519\|\|full | 1349 | No | NA | 0 | 0 | Not-determined | Genome-fragment | NA | 0 |
| S12-final-viral-combined_k141_184339\|\|full | 1125 | No | NA | 0 | 0 | Low-quality | Genome-fragment | 0.82 | 0 |
| S11-final-viral-combined_k141_267814\|\|full | 2597 | No | NA | 0 | 2 | Not-determined | Genome-fragment | NA | 0 |
| S12-final-viral-combined_k141_259427\|\|full | 1125 | No | NA | 0 | 0 | Low-quality | Genome-fragment | 0.87 | 0 |
| S11-final-viral-combined_k141_157590\|\|full | 4156 | No | NA | 0 | 1 | Not-determined | Genome-fragment | NA | 0 |
| S12-final-viral-combined_k141_210363\|\|full | 1125 | No | NA | 1 | 0 | Low-quality | Genome-fragment | 1.81 | 0 |
| S11-final-viral-combined_k141_322218\|\|full | 1688 | No | NA | 0 | 1 | Not-determined | Genome-fragment | NA | 0 |
| S11-final-viral-combined_k141_19057\|\|full | 1050 | No | NA | 0 | 1 | Not-determined | Genome-fragment | NA | 0 |
| S4-final-viral-combined_k141_122922\|\|lt2gene | 1125 | No | NA | 1 | 0 | Low-quality | Genome-fragment | 2.89 | 0 |
| S11-final-viral-combined_k141_184984\|\|full | 2428 | No | NA | 0 | 2 | Not-determined | Genome-fragment | NA | 0 |
| S11-final-viral-combined_k141_240890\|\|full | 24596 | No | NA | 0 | 18 | Not-determined | Genome-fragment | NA | 0 |
| S11-final-viral-combined_k141_282227\|\|full | 27969 | No | NA | 16 | 3 | Medium-quality | Genome-fragment | 59.35 | 0 |
| S11-final-viral-combined_k141_103072\|\|full | 2025 | No | NA | 0 | 1 | Not-determined | Genome-fragment | NA | 0 |
| S9-final-viral-combined_k141_67816\|\|full | 1126 | No | NA | 0 | 0 | Low-quality | Genome-fragment | 0.63 | 0 |
| S11-final-viral-combined_k141_213368\|\|full | 4328 | No | NA | 0 | 3 | Not-determined | Genome-fragment | NA | 0 |
| S11-final-viral-combined_k141_322286\|\|full | 6907 | No | NA | 0 | 3 | Not-determined | Genome-fragment | NA | 0 |
| S11-final-viral-combined_k141_306546\|\|full | 1126 | No | NA | 0 | 0 | Low-quality | Genome-fragment | 2.63 | 0 |
| S11-final-viral-combined_k141_157704\|\|full | 1671 | No | NA | 0 | 1 | Not-determined | Genome-fragment | NA | 0 |
| S1-final-viral-combined_k141_369485\|\|lt2gene | 1126 | No | NA | 1 | 0 | Low-quality | Genome-fragment | 18.95 | 0 |
| S1-final-viral-combined_k141_206538\|\|lt2gene | 1126 | No | NA | 2 | 0 | Low-quality | Genome-fragment | 2.44 | 0 |
| S1_k141_270609 | 1126 | No | NA | 0 | 0 | Low-quality | Genome-fragment | 0.91 | 0 |
| S11-final-viral-combined_k141_61241\|\|full | 6106 | No | NA | 0 | 4 | Not-determined | Genome-fragment | NA | 0 |
| S11-final-viral-combined_k141_295252\|\|full | 1729 | No | NA | 0 | 2 | Not-determined | Genome-fragment | NA | 0 |
| S11-final-viral-combined_k141_19156\|\|full | 1372 | No | NA | 0 | 0 | Not-determined | Genome-fragment | NA | 0 |
| S11-final-viral-combined_k141_282320\|\|full | 1145 | No | NA | 0 | 0 | Not-determined | Genome-fragment | NA | 0 |
| S11-final-viral-combined_k141_226624\|\|full | 1965 | No | NA | 0 | 2 | Not-determined | Genome-fragment | NA | 0 |
| S11-final-viral-combined_k141_198700\|\|full | 1241 | No | NA | 0 | 1 | Not-determined | Genome-fragment | NA | 0 |
| S3-final-viral-combined_k141_43122\|\|full | 1126 | No | NA | 0 | 0 | Low-quality | Genome-fragment | 2.25 | 0 |
| S11-final-viral-combined_k141_322338\|\|full | 3842 | No | NA | 0 | 2 | Not-determined | Genome-fragment | NA | 0 |
| S11-final-viral-combined_k141_254282\|\|full | 4445 | No | NA | 0 | 2 | Not-determined | Genome-fragment | NA | 0 |
| S11-final-viral-combined_k141_322354\|\|full | 1799 | No | NA | 0 | 0 | Not-determined | Genome-fragment | NA | 0 |
| S5-final-viral-combined_k141_681\|\|full | 1126 | Yes | 723 | 1 | 1 | Low-quality | Genome-fragment | 1.8 | 35.79 |
| S5-final-viral-combined_k141_58271\|\|full | 1126 | No | NA | 0 | 1 | Low-quality | Genome-fragment | 0.47 | 0 |
| S11-final-viral-combined_k141_33645\|\|full | 1113 | No | NA | 0 | 1 | Not-determined | Genome-fragment | NA | 0 |
| S7-final-viral-combined_k141_122794\|\|full | 1126 | No | NA | 1 | 0 | Low-quality | Genome-fragment | 3.06 | 0 |
| S7-final-viral-combined_k141_53857\|\|full | 1126 | No | NA | 0 | 1 | Low-quality | Genome-fragment | 5.91 | 0 |
| S11-final-viral-combined_k141_254333\|\|full | 1996 | No | NA | 0 | 1 | Not-determined | Genome-fragment | NA | 0 |
| S11-final-viral-combined_k141_61339\|\|full | 2617 | No | NA | 0 | 1 | Not-determined | Genome-fragment | NA | 0 |
| S7-final-viral-combined_k141_9110\|\|full | 1126 | No | NA | 1 | 0 | Low-quality | Genome-fragment | 0.69 | 0 |
| S11-final-viral-combined_k141_47668\|\|full | 1338 | No | NA | 0 | 1 | Not-determined | Genome-fragment | NA | 0 |
| S7_k141_44491 | 1126 | No | NA | 1 | 0 | Low-quality | Genome-fragment | 1.4 | 0 |
| S17-final-viral-combined_k141_109193\|\|full | 1126 | No | NA | 1 | 0 | Low-quality | Genome-fragment | 2.06 | 0 |
| S11-final-viral-combined_k141_295357\|\|full | 3206 | No | NA | 0 | 2 | Not-determined | Genome-fragment | NA | 0 |
| S17-k141_127114 | 1126 | No | NA | 5 | 0 | Low-quality | Genome-fragment | 3.71 | 0 |
| S11-final-viral-combined_k141_322466\|\|full | 1661 | No | NA | 0 | 0 | Not-determined | Genome-fragment | NA | 0 |
| S11-final-viral-combined_k141_144014\|\|full | 4971 | No | NA | 0 | 2 | Not-determined | Genome-fragment | NA | 0 |
| S17-k141_128814 | 1126 | No | NA | 0 | 1 | Low-quality | Genome-fragment | 0.69 | 0 |
| S11-final-viral-combined_k141_213459\|\|full | 2362 | No | NA | 0 | 2 | Not-determined | Genome-fragment | NA | 0 |
| S11-final-viral-combined_k141_213474\|\|full | 2424 | No | NA | 0 | 1 | Not-determined | Genome-fragment | NA | 0 |
| S11-final-viral-combined_k141_185140\|\|full | 3038 | No | NA | 0 | 2 | Not-determined | Genome-fragment | NA | 0 |
| S11-final-viral-combined_k141_33758\|\|full | 1141 | No | NA | 0 | 0 | Not-determined | Genome-fragment | NA | 0 |
| S12-final-viral-combined_k141_132699\|\|full | 1126 | No | NA | 1 | 0 | Low-quality | Genome-fragment | 2.21 | 0 |
| S2-final-viral-combined_k141_168373\|\|full | 1126 | No | NA | 1 | 0 | Low-quality | Genome-fragment | 2.73 | 0 |
| S2-final-viral-combined_k141_244329\|\|full | 1126 | No | NA | 0 | 1 | Low-quality | Genome-fragment | 1.57 | 0 |
| S11-final-viral-combined_k141_144079\|\|full | 1180 | No | NA | 0 | 0 | Not-determined | Genome-fragment | NA | 0 |
| S4-final-viral-combined_k141_57995\|\|lt2gene | 1126 | No | NA | 1 | 0 | Low-quality | Genome-fragment | 2.93 | 0 |
| S6-final-viral-combined_k141_53903\|\|full | 1126 | No | NA | 0 | 0 | Low-quality | Genome-fragment | 1.34 | 0 |
| S8-final-viral-combined_k141_250707\|\|lt2gene | 1126 | No | NA | 0 | 0 | Low-quality | Genome-fragment | 2.44 | 0 |
| S11-final-viral-combined_k141_267993\|\|full | 74748 | Yes | 64139 | 26 | 5 | High-quality | High-quality | 100 | 14.19 |
| S9-final-viral-combined_k141_33673\|\|full | 1127 | No | NA | 2 | 0 | Low-quality | Genome-fragment | 2.5 | 0 |
| S11_k141_297500 | 1127 | No | NA | 1 | 0 | Low-quality | Genome-fragment | 1.78 | 0 |
| S11-final-viral-combined_k141_198863\|\|full | 2613 | No | NA | 0 | 2 | Not-determined | Genome-fragment | NA | 0 |
| S11-final-viral-combined_k141_241192\|\|full | 1646 | No | NA | 0 | 1 | Not-determined | Genome-fragment | NA | 0 |
| S11-final-viral-combined_k141_157977\|\|full | 5705 | No | NA | 0 | 4 | Not-determined | Genome-fragment | NA | 0 |
| S11-final-viral-combined_k141_88966\|\|full | 1506 | No | NA | 0 | 1 | Not-determined | Genome-fragment | NA | 0 |
| S1-final-viral-combined_k141_362117\|\|full | 1127 | No | NA | 1 | 0 | Low-quality | Genome-fragment | 2.2 | 0 |
| S11-final-viral-combined_k141_295490\|\|full | 1685 | No | NA | 0 | 0 | Not-determined | Genome-fragment | NA | 0 |
| S1-final-viral-combined_k141_350441\|\|full | 1127 | No | NA | 0 | 0 | Low-quality | Genome-fragment | 0.9 | 0 |
| S5-final-viral-combined_k141_112842\|\|full | 1127 | No | NA | 0 | 2 | Low-quality | Genome-fragment | 0.78 | 0 |
| S11-final-viral-combined_k141_144137\|\|full | 3229 | No | NA | 0 | 1 | Not-determined | Genome-fragment | NA | 0 |
| S7-final-viral-combined_k141_187752\|\|full | 1127 | No | NA | 0 | 0 | Low-quality | Genome-fragment | 1.91 | 0 |
| S7-final-viral-combined_k141_171838\|\|full | 1127 | No | NA | 0 | 0 | Low-quality | Genome-fragment | 2.75 | 0 |
| S11-final-viral-combined_k141_103309\|\|full | 1662 | No | NA | 0 | 1 | Not-determined | Genome-fragment | NA | 0 |
| S11-final-viral-combined_k141_254464\|\|full | 5746 | No | NA | 0 | 1 | Not-determined | Genome-fragment | NA | 0 |
| S12_k141_191382 | 1127 | No | NA | 0 | 1 | Low-quality | Genome-fragment | 2.46 | 0 |
| S11-final-viral-combined_k141_171863\|\|full | 6406 | No | NA | 0 | 2 | Not-determined | Genome-fragment | NA | 0 |
| S11-final-viral-combined_k141_158015\|\|full | 1731 | No | NA | 0 | 2 | Not-determined | Genome-fragment | NA | 0 |
| S11-final-viral-combined_k141_309920\|\|full | 2898 | No | NA | 0 | 0 | Not-determined | Genome-fragment | NA | 0 |
| S11-final-viral-combined_k141_171865\|\|full | 2401 | No | NA | 0 | 3 | Not-determined | Genome-fragment | NA | 0 |
| S2-final-viral-combined_k141_173907\|\|full | 1127 | No | NA | 0 | 1 | Low-quality | Genome-fragment | 0.52 | 0 |
| S11-final-viral-combined_k141_268061\|\|full | 2823 | No | NA | 0 | 2 | Not-determined | Genome-fragment | NA | 0 |
| S11-final-viral-combined_k141_282615\|\|full | 1661 | No | NA | 0 | 0 | Not-determined | Genome-fragment | NA | 0 |
| S2-final-viral-combined_k141_196382\|\|full | 1127 | No | NA | 0 | 0 | Low-quality | Genome-fragment | 1.95 | 0 |
| S2-final-viral-combined_k141_82409\|\|full | 1127 | No | NA | 0 | 0 | Low-quality | Genome-fragment | 1.76 | 0 |
| S11-final-viral-combined_k141_116018\|\|full | 1531 | No | NA | 0 | 1 | Not-determined | Genome-fragment | NA | 0 |
| S11-final-viral-combined_k141_268086\|\|full | 1783 | No | NA | 0 | 0 | Not-determined | Genome-fragment | NA | 0 |
| S11-final-viral-combined_k141_282630\|\|full | 6359 | No | NA | 0 | 3 | Not-determined | Genome-fragment | NA | 0 |
| S11-final-viral-combined_k141_19371\|\|full | 4078 | No | NA | 0 | 2 | Not-determined | Genome-fragment | NA | 0 |
| S2-final-viral-combined_k141_180386\|\|lt2gene | 1127 | No | NA | 1 | 0 | Low-quality | Genome-fragment | 2.58 | 0 |
| S6-final-viral-combined_k141_64605\|\|full | 1127 | No | NA | 1 | 0 | Low-quality | Genome-fragment | 2.23 | 0 |
| S8-final-viral-combined_k141_146599\|\|full | 1127 | No | NA | 0 | 0 | Low-quality | Genome-fragment | 3.54 | 0 |
| S8_k141_17927 | 1127 | No | NA | 0 | 0 | Low-quality | Genome-fragment | 1.97 | 0 |
| S11-final-viral-combined_k141_47739\|\|full | 1820 | No | NA | 0 | 1 | Not-determined | Genome-fragment | NA | 0 |
| S11-final-viral-combined_k141_6271\|\|full | 1154 | No | NA | 0 | 1 | Not-determined | Genome-fragment | NA | 0 |
| S11-final-viral-combined_k141_74670\|\|full | 1141 | No | NA | 0 | 2 | Not-determined | Genome-fragment | NA | 0 |
| S9-final-viral-combined_k141_78073\|\|lt2gene | 1128 | No | NA | 1 | 0 | Low-quality | Genome-fragment | 1.04 | 0 |
| S1-final-viral-combined_k141_214642\|\|lt2gene | 1128 | No | NA | 1 | 0 | Low-quality | Genome-fragment | 2.32 | 0 |
| S11-final-viral-combined_k141_309974\|\|full | 7093 | No | NA | 0 | 9 | Not-determined | Genome-fragment | NA | 0 |
| S1-final-viral-combined_k141_2632\|\|lt2gene | 1128 | No | NA | 1 | 0 | Low-quality | Genome-fragment | 0.4 | 0 |
| S11-final-viral-combined_k141_171951\|\|full | 1896 | No | NA | 0 | 3 | Not-determined | Genome-fragment | NA | 0 |
| S11-final-viral-combined_k141_74678\|\|full | 2952 | No | NA | 0 | 2 | Not-determined | Genome-fragment | NA | 0 |
| S11-final-viral-combined_k141_61692\|\|full | 2279 | No | NA | 0 | 1 | Not-determined | Genome-fragment | NA | 0 |
| S1_k141_222745 | 1128 | No | NA | 1 | 0 | Low-quality | Genome-fragment | 2.41 | 0 |
| S11-final-viral-combined_k141_310011\|\|full | 1398 | No | NA | 0 | 0 | Not-determined | Genome-fragment | NA | 0 |
| S11-final-viral-combined_k141_61709\|\|full | 1025 | No | NA | 0 | 0 | Not-determined | Genome-fragment | NA | 0 |
| S11-final-viral-combined_k141_241434\|\|full | 1269 | No | NA | 0 | 1 | Not-determined | Genome-fragment | NA | 0 |
| S3-final-viral-combined_k141_70592\|\|full | 1128 | No | NA | 0 | 0 | Low-quality | Genome-fragment | 1.37 | 0 |
| S11-final-viral-combined_k141_213710\|\|full | 5127 | No | NA | 0 | 1 | Not-determined | Genome-fragment | NA | 0 |
| S11-final-viral-combined_k141_310045\|\|full | 2762 | No | NA | 0 | 1 | Not-determined | Genome-fragment | NA | 0 |
| S11-final-viral-combined_k141_227013\|\|full | 10273 | No | NA | 0 | 4 | Not-determined | Genome-fragment | NA | 0 |
| S11-final-viral-combined_k141_322787\|\|full | 1470 | No | NA | 0 | 2 | Not-determined | Genome-fragment | NA | 0 |
| S11-final-viral-combined_k141_89079\|\|full | 1945 | No | NA | 0 | 2 | Not-determined | Genome-fragment | NA | 0 |
| S11-final-viral-combined_k141_198995\|\|full | 6653 | No | NA | 0 | 1 | Not-determined | Genome-fragment | NA | 0 |
| S11-final-viral-combined_k141_19470\|\|full | 2769 | No | NA | 0 | 1 | Not-determined | Genome-fragment | NA | 0 |
| S5-final-viral-combined_k141_101636\|\|lt2gene | 1128 | No | NA | 2 | 0 | Low-quality | Genome-fragment | 2.52 | 0 |
| S11-final-viral-combined_k141_241481\|\|full | 1992 | No | NA | 0 | 1 | Not-determined | Genome-fragment | NA | 0 |
| S7-final-viral-combined_k141_138248\|\|full | 1128 | No | NA | 0 | 1 | Low-quality | Genome-fragment | 0.82 | 0 |
| S11-final-viral-combined_k141_6394\|\|full | 1941 | No | NA | 0 | 1 | Not-determined | Genome-fragment | NA | 0 |
| S2-final-viral-combined_k141_172619\|\|lt2gene | 1128 | No | NA | 1 | 0 | Low-quality | Genome-fragment | 1.41 | 0 |
| S11-final-viral-combined_k141_213736\|\|full | 1360 | No | NA | 0 | 2 | Not-determined | Genome-fragment | NA | 0 |
| S4_k141_121786 | 1128 | No | NA | 0 | 2 | Low-quality | Genome-fragment | 0.65 | 0 |
| S11-final-viral-combined_k141_322809\|\|full | 1438 | No | NA | 0 | 0 | Not-determined | Genome-fragment | NA | 0 |
| S11-final-viral-combined_k141_130247\|\|full | 2196 | No | NA | 0 | 0 | Not-determined | Genome-fragment | NA | 0 |
| S9-final-viral-combined_k141_67856\|\|full | 1129 | No | NA | 0 | 0 | Low-quality | Genome-fragment | 2.15 | 0 |
| S11-final-viral-combined_k141_268286\|\|full | 2820 | No | NA | 0 | 0 | Not-determined | Genome-fragment | NA | 0 |
| S11-final-viral-combined_k141_227046\|\|full | 3420 | No | NA | 0 | 0 | Not-determined | Genome-fragment | NA | 0 |
| S11-final-viral-combined_k141_137956\|\|full | 1129 | No | NA | 0 | 0 | Low-quality | Genome-fragment | 2.81 | 0 |
| S11-final-viral-combined_k141_154096\|\|lt2gene | 1129 | No | NA | 1 | 0 | Low-quality | Genome-fragment | 2.48 | 0 |
| S11-final-viral-combined_k141_103483\|\|full | 1169 | No | NA | 0 | 1 | Not-determined | Genome-fragment | NA | 0 |
| S11_k141_260207 | 1129 | No | NA | 0 | 1 | Low-quality | Genome-fragment | 1.53 | 0 |
| S1-final-viral-combined_k141_273282\|\|full | 1129 | No | NA | 0 | 1 | Low-quality | Genome-fragment | 2.37 | 0 |
| S1-final-viral-combined_k141_12549\|\|full | 1129 | No | NA | 0 | 1 | Low-quality | Genome-fragment | 0.88 | 0 |
| S11-final-viral-combined_k141_34002\|\|full | 3042 | No | NA | 0 | 2 | Not-determined | Genome-fragment | NA | 0 |
| S1_k141_264555 | 1129 | No | NA | 0 | 0 | Low-quality | Genome-fragment | 1.94 | 0 |
| S3-final-viral-combined_k141_65566\|\|full | 1129 | No | NA | 0 | 0 | Low-quality | Genome-fragment | 1.88 | 0 |
| S11-final-viral-combined_k141_322857\|\|full | 1115 | No | NA | 0 | 0 | Not-determined | Genome-fragment | NA | 0 |
| S3-final-viral-combined_k141_101540\|\|lt2gene | 1129 | No | NA | 1 | 0 | Low-quality | Genome-fragment | 2.47 | 0 |
| S7-final-viral-combined_k141_51463\|\|full | 1129 | No | NA | 0 | 1 | Low-quality | Genome-fragment | 2.11 | 0 |
| S11-final-viral-combined_k141_103559\|\|full | 2430 | No | NA | 0 | 2 | Not-determined | Genome-fragment | NA | 0 |
| S7_k141_188268 | 1129 | No | NA | 1 | 0 | Low-quality | Genome-fragment | 1.72 | 0 |
| S11-final-viral-combined_k141_158306\|\|full | 3402 | No | NA | 0 | 3 | Not-determined | Genome-fragment | NA | 0 |
| S11-final-viral-combined_k141_61885\|\|full | 2853 | No | NA | 0 | 1 | Not-determined | Genome-fragment | NA | 0 |
| S17-k141_179063 | 1129 | No | NA | 0 | 0 | Low-quality | Genome-fragment | 0.58 | 0 |
| S12-final-viral-combined_k141_190870\|\|full | 1129 | No | NA | 0 | 0 | Low-quality | Genome-fragment | 0.54 | 0 |
| S11-final-viral-combined_k141_254765\|\|full | 4084 | No | NA | 0 | 3 | Not-determined | Genome-fragment | NA | 0 |
| S12-final-viral-combined_k141_371092\|\|lt2gene | 1129 | No | NA | 1 | 0 | Low-quality | Genome-fragment | 2.54 | 0 |
| S11-final-viral-combined_k141_74851\|\|full | 2387 | No | NA | 0 | 2 | Not-determined | Genome-fragment | NA | 0 |
| S12-final-viral-combined_k141_140149\|\|lt2gene | 1129 | No | NA | 1 | 0 | Low-quality | Genome-fragment | 1.85 | 0 |
| S8-final-viral-combined_k141_34114\|\|full | 1129 | No | NA | 0 | 0 | Low-quality | Genome-fragment | 1.16 | 0 |
| S11-final-viral-combined_k141_61897\|\|full | 4819 | No | NA | 0 | 3 | Not-determined | Genome-fragment | NA | 0 |
| S11_k141_127515 | 1130 | No | NA | 0 | 1 | Low-quality | Genome-fragment | 0.42 | 0 |
| S11-final-viral-combined_k141_254779\|\|full | 4367 | No | NA | 0 | 3 | Not-determined | Genome-fragment | NA | 0 |
| S11-final-viral-combined_k141_213863\|\|full | 2154 | No | NA | 0 | 1 | Not-determined | Genome-fragment | NA | 0 |
| S11-final-viral-combined_k141_172205\|\|full | 4086 | No | NA | 0 | 3 | Not-determined | Genome-fragment | NA | 0 |
| S11-final-viral-combined_k141_310229\|\|full | 4643 | No | NA | 0 | 3 | Not-determined | Genome-fragment | NA | 0 |
| S1-final-viral-combined_k141_202534\|\|full | 1130 | No | NA | 1 | 0 | Low-quality | Genome-fragment | 2.24 | 0 |
| S11-final-viral-combined_k141_103605\|\|full | 1753 | No | NA | 0 | 2 | Not-determined | Genome-fragment | NA | 0 |
| S11-final-viral-combined_k141_158379\|\|full | 1782 | No | NA | 0 | 3 | Not-determined | Genome-fragment | NA | 0 |

| | | | | | | | | | | | |
|---|---|---|---|---|---|---|---|---|---|---|---|
| S1-final-viral-combined_k141_30840\|\|full | 1130 | No | NA | | 2 | 0 | Low-quality | Genome-fragment | | 2.5 | 0 |
| S7-final-viral-combined_k141_57550\|\|full | 1130 | No | NA | | 0 | 0 | Low-quality | Genome-fragment | | 3.25 | 0 |
| S17-final-viral-combined_k141_11208\|\|full | 1130 | No | NA | | 0 | 0 | Low-quality | Genome-fragment | | 2.61 | 0 |
| S11-final-viral-combined_k141_268379\|\|full | 1564 | No | NA | | 0 | 1 | Not-determined | Genome-fragment | NA | | 0 |
| S2-final-viral-combined_k141_110588\|\|full | 1130 | No | NA | | 0 | 0 | Low-quality | Genome-fragment | | 2.14 | 0 |
| S6-final-viral-combined_k141_58691\|\|full | 1130 | No | NA | | 0 | 0 | Low-quality | Genome-fragment | | 2.74 | 0 |
| S8-final-viral-combined_k141_140201\|\|full | 1130 | No | NA | | 0 | 1 | Low-quality | Genome-fragment | | 2.19 | 0 |
| S11-final-viral-combined_k141_310317\|\|full | 1729 | No | NA | | 0 | 2 | Not-determined | Genome-fragment | NA | | 0 |
| S8-final-viral-combined_k141_33846\|\|lt2gene | 1130 | No | NA | | 1 | 0 | Low-quality | Genome-fragment | | 2.4 | 0 |
| S9-final-viral-combined_k141_67159\|\|full | 1131 | No | NA | | 0 | 0 | Low-quality | Genome-fragment | | 2.25 | 0 |
| S11-final-viral-combined_k141_74947\|\|full | 1510 | No | NA | | 0 | 0 | Not-determined | Genome-fragment | NA | | 0 |
| S11-final-viral-combined_k141_310323\|\|full | 3595 | No | NA | | 0 | 4 | Not-determined | Genome-fragment | NA | | 0 |
| S11-final-viral-combined_k141_32838\|\|full | 1131 | No | NA | | 0 | 1 | Low-quality | Genome-fragment | | 1.16 | 0 |
| S11-final-viral-combined_k141_20009\|\|full | 1131 | No | NA | | 0 | 1 | Low-quality | Genome-fragment | | 0.76 | 0 |
| S11-final-viral-combined_k141_89287\|\|full | 1039 | No | NA | | 0 | 1 | Not-determined | Genome-fragment | NA | | 0 |
| S1-final-viral-combined_k141_188668\|\|full | 1131 | No | NA | | 1 | 0 | Low-quality | Genome-fragment | | 2.07 | 0 |
| S11-final-viral-combined_k141_241674\|\|full | 1996 | No | NA | | 0 | 1 | Not-determined | Genome-fragment | NA | | 0 |
| S11-final-viral-combined_k141_19660\|\|full | 2202 | No | NA | | 0 | 2 | Not-determined | Genome-fragment | NA | | 0 |
| S11-final-viral-combined_k141_61989\|\|full | 2112 | No | NA | | 0 | 2 | Not-determined | Genome-fragment | NA | | 0 |
| S11-final-viral-combined_k141_116251\|\|full | 13998 | No | NA | | 0 | 12 | Not-determined | Genome-fragment | NA | | 0 |
| S1-final-viral-combined_k141_28615\|\|full | 1131 | No | NA | | 0 | 0 | Low-quality | Genome-fragment | | 2.74 | 0 |
| S1-final-viral-combined_k141_72490\|\|lt2gene | 1131 | No | NA | | 2 | 0 | Low-quality | Genome-fragment | | 1.79 | 0 |
| S11-final-viral-combined_k141_172346\|\|full | 1674 | No | NA | | 0 | 0 | Not-determined | Genome-fragment | NA | | 0 |
| S5_k141_21009 | 1131 | No | NA | | 0 | 0 | Low-quality | Genome-fragment | | 2.01 | 0 |
| S5_k141_104942 | 1131 | No | NA | | 0 | 1 | Low-quality | Genome-fragment | | 2.37 | 0 |
| S11-final-viral-combined_k141_282865\|\|full | 2412 | No | NA | | 0 | 2 | Not-determined | Genome-fragment | NA | | 0 |
| S11-final-viral-combined_k141_47974\|\|full | 2931 | No | NA | | 0 | 3 | Not-determined | Genome-fragment | NA | | 0 |
| S11-final-viral-combined_k141_6644\|\|full | 8228 | No | NA | | 0 | 2 | Not-determined | Genome-fragment | NA | | 0 |
| S7-final-viral-combined_k141_227077\|\|full | 1131 | No | NA | | 0 | 0 | Low-quality | Genome-fragment | | 0.9 | 0 |
| S11-final-viral-combined_k141_47980\|\|full | 2776 | No | NA | | 0 | 2 | Not-determined | Genome-fragment | NA | | 0 |
| S11-final-viral-combined_k141_254913\|\|full | 1401 | No | NA | | 0 | 1 | Not-determined | Genome-fragment | NA | | 0 |
| S11-final-viral-combined_k141_62015\|\|full | 1794 | No | NA | | 0 | 0 | Not-determined | Genome-fragment | NA | | 0 |
| S11-final-viral-combined_k141_74983\|\|full | 49793 | No | NA | | 20 | 0 | High-quality | High-quality | | 99.25 | 0 |
| S11-final-viral-combined_k141_34202\|\|full | 11240 | No | NA | | 0 | 0 | Not-determined | Genome-fragment | NA | | 0 |
| S12-final-viral-combined_k141_382454\|\|full | 1131 | No | NA | | 0 | 1 | Low-quality | Genome-fragment | | 1.6 | 0 |
| S11-final-viral-combined_k141_103695\|\|full | 6662 | No | NA | | 0 | 0 | Not-determined | Genome-fragment | NA | | 0 |
| S11-final-viral-combined_k141_254940\|\|full | 4438 | No | NA | | 0 | 5 | Not-determined | Genome-fragment | NA | | 0 |
| S11-final-viral-combined_k141_144563\|\|full | 3065 | No | NA | | 0 | 3 | Not-determined | Genome-fragment | NA | | 0 |
| S12-final-viral-combined_k141_267302\|\|full | 1131 | No | NA | | 0 | 0 | Low-quality | Genome-fragment | | 2.69 | 0 |
| S11-final-viral-combined_k141_282903\|\|full | 9631 | No | NA | | 0 | 3 | Not-determined | Genome-fragment | NA | | 0 |
| S11-final-viral-combined_k141_116333\|\|full | 2336 | No | NA | | 0 | 2 | Not-determined | Genome-fragment | NA | | 0 |
| S11-final-viral-combined_k141_310363\|\|full | 9043 | No | NA | | 0 | 2 | Not-determined | Genome-fragment | NA | | 0 |
| S11-final-viral-combined_k141_241759\|\|full | 2958 | No | NA | | 0 | 2 | Not-determined | Genome-fragment | NA | | 0 |
| S12_k141_260067 | 1131 | No | NA | | 0 | 0 | Low-quality | Genome-fragment | | 2.22 | 0 |
| S2-final-viral-combined_k141_101145\|\|lt2gene | 1131 | No | NA | | 1 | 0 | Low-quality | Genome-fragment | | 1.64 | 0 |
| S4-final-viral-combined_k141_150867\|\|full | 1131 | No | NA | | 0 | 0 | Low-quality | Genome-fragment | | 0.99 | 0 |
| S8-final-viral-combined_k141_66369\|\|full | 1131 | No | NA | | 1 | 0 | Low-quality | Genome-fragment | | 3.43 | 0 |
| S11-final-viral-combined_k141_227368\|\|full | 1659 | No | NA | | 0 | 1 | Not-determined | Genome-fragment | NA | | 0 |
| S8_k141_116247 | 1131 | No | NA | | 0 | 0 | Low-quality | Genome-fragment | | 1.69 | 0 |
| S11-final-viral-combined_k141_89382\|\|full | 1922 | No | NA | | 0 | 3 | Not-determined | Genome-fragment | NA | | 0 |
| S11-final-viral-combined_k141_212064\|\|full | 1132 | No | NA | | 0 | 0 | Low-quality | Genome-fragment | | 1.99 | 0 |
| S11-final-viral-combined_k141_6725\|\|full | 2162 | No | NA | | 0 | 1 | Not-determined | Genome-fragment | NA | | 0 |
| S11-final-viral-combined_k141_213977\|\|full | 5453 | No | NA | | 0 | 0 | Not-determined | Genome-fragment | NA | | 0 |
| S1-final-viral-combined_k141_134118\|\|full | 1132 | No | NA | | 1 | 0 | Low-quality | Genome-fragment | | 2.86 | 0 |
| S1-final-viral-combined_k141_315802\|\|full | 1132 | No | NA | | 0 | 0 | Low-quality | Genome-fragment | | 2.64 | 0 |
| S11-final-viral-combined_k141_199259\|\|full | 4112 | No | NA | | 0 | 5 | Not-determined | Genome-fragment | NA | | 0 |
| S5-final-viral-combined_k141_99962\|\|full | 1132 | No | NA | | 0 | 0 | Low-quality | Genome-fragment | | 3.25 | 0 |
| S5-final-viral-combined_k141_194184\|\|full | 1132 | No | NA | | 0 | 0 | Low-quality | Genome-fragment | | 1.72 | 0 |
| S11-final-viral-combined_k141_34236\|\|full | 1809 | No | NA | | 0 | 1 | Not-determined | Genome-fragment | NA | | 0 |
| S7-final-viral-combined_k141_135524\|\|full | 1132 | No | NA | | 0 | 1 | Low-quality | Genome-fragment | | 2.14 | 0 |
| S11-final-viral-combined_k141_144596\|\|full | 2080 | No | NA | | 0 | 1 | Not-determined | Genome-fragment | NA | | 0 |
| S11-final-viral-combined_k141_227391\|\|full | 4881 | No | NA | | 0 | 2 | Not-determined | Genome-fragment | NA | | 0 |
| S7-final-viral-combined_k141_145732\|\|full | 1132 | No | NA | | 0 | 0 | Low-quality | Genome-fragment | | 2.38 | 0 |
| S7_k141_158372 | 1132 | No | NA | | 0 | 0 | Low-quality | Genome-fragment | | 0.74 | 0 |
| S12_k141_138998 | 1132 | No | NA | | 2 | 0 | Low-quality | Genome-fragment | | 2.51 | 0 |
| S4-final-viral-combined_k141_123827\|\|lt2gene | 1132 | No | NA | | 0 | 0 | Low-quality | Genome-fragment | | 2.23 | 0 |
| S11-final-viral-combined_k141_322973\|\|full | 2030 | No | NA | | 0 | 1 | Not-determined | Genome-fragment | NA | | 0 |
| S11-final-viral-combined_k141_62134\|\|full | 2457 | No | NA | | 0 | 1 | Not-determined | Genome-fragment | NA | | 0 |
| S6-final-viral-combined_k141_38998\|\|full | 1132 | No | NA | | 0 | 0 | Low-quality | Genome-fragment | | 3.43 | 0 |
| S8-final-viral-combined_k141_142329\|\|full | 1132 | No | NA | | 0 | 0 | Low-quality | Genome-fragment | | 2.51 | 0 |
| S11-final-viral-combined_k141_62139\|\|full | 1475 | No | NA | | 0 | 0 | Not-determined | Genome-fragment | NA | | 0 |
| S11-final-viral-combined_k141_322981\|\|full | 3603 | No | NA | | 0 | 1 | Not-determined | Genome-fragment | NA | | 0 |
| S11-final-viral-combined_k141_99648\|\|full | 1133 | No | NA | | 1 | 0 | Low-quality | Genome-fragment | | 1.62 | 0 |
| S11-final-viral-combined_k141_307155\|\|full | 1133 | No | NA | | 0 | 2 | Low-quality | Genome-fragment | | 0.82 | 0 |
| S11-final-viral-combined_k141_295868\|\|full | 1924 | No | NA | | 0 | 2 | Not-determined | Genome-fragment | NA | | 0 |
| S11-final-viral-combined_k141_33655\|\|lt2gene | 1133 | No | NA | | 1 | 0 | Low-quality | Genome-fragment | | 0.69 | 0 |
| S11-final-viral-combined_k141_34302\|\|full | 1129 | No | NA | | 0 | 2 | Not-determined | Genome-fragment | NA | | 0 |
| S1-final-viral-combined_k141_314547\|\|full | 1133 | No | NA | | 1 | 0 | Low-quality | Genome-fragment | | 2.02 | 0 |
| S11-final-viral-combined_k141_34306\|\|full | 2000 | No | NA | | 0 | 1 | Not-determined | Genome-fragment | NA | | 0 |
| S11-final-viral-combined_k141_255061\|\|full | 3198 | No | NA | | 0 | 0 | Not-determined | Genome-fragment | NA | | 0 |
| S11-final-viral-combined_k141_268581\|\|full | 1291 | No | NA | | 0 | 1 | Not-determined | Genome-fragment | NA | | 0 |
| S1-final-viral-combined_k141_373640\|\|full | 1133 | No | NA | | 0 | 0 | Low-quality | Genome-fragment | | 1 | 0 |
| S11-final-viral-combined_k141_6801\|\|full | 2415 | No | NA | | 0 | 1 | Not-determined | Genome-fragment | NA | | 0 |
| S1-final-viral-combined_k141_277046\|\|full | 1133 | No | NA | | 0 | 0 | Low-quality | Genome-fragment | | 2.35 | 0 |
| S11-final-viral-combined_k141_89478\|\|full | 2183 | No | NA | | 0 | 0 | Not-determined | Genome-fragment | NA | | 0 |
| S11-final-viral-combined_k141_89482\|\|full | 3493 | No | NA | | 0 | 2 | Not-determined | Genome-fragment | NA | | 0 |
| S1_k141_199478 | 1133 | No | NA | | 0 | 0 | Low-quality | Genome-fragment | | 2.07 | 0 |
| S17-final-viral-combined_k141_78042\|\|full | 1133 | No | NA | | 0 | 2 | Low-quality | Genome-fragment | | 2.53 | 0 |
| S11-final-viral-combined_k141_323003\|\|full | 6416 | No | NA | | 0 | 1 | Not-determined | Genome-fragment | NA | | 0 |
| S11-final-viral-combined_k141_310499\|\|full | 2198 | No | NA | | 0 | 1 | Not-determined | Genome-fragment | NA | | 0 |
| S17-final-viral-combined_k141_171657\|\|full | 1133 | No | NA | | 0 | 0 | Low-quality | Genome-fragment | | 2.11 | 0 |
| S11-final-viral-combined_k141_310510\|\|full | 1018 | No | NA | | 0 | 1 | Not-determined | Genome-fragment | NA | | 0 |
| S17-final-viral-combined_k141_205645\|\|full | 1133 | No | NA | | 0 | 0 | Low-quality | Genome-fragment | | 0.66 | 0 |
| S11-final-viral-combined_k141_310515\|\|full | 1088 | No | NA | | 0 | 0 | Not-determined | Genome-fragment | NA | | 0 |
| S17-k141_53661 | 1133 | No | NA | | 0 | 0 | Low-quality | Genome-fragment | | 0.72 | 0 |
| S11-final-viral-combined_k141_158709\|\|full | 2265 | No | NA | | 0 | 2 | Not-determined | Genome-fragment | NA | | 0 |
| S11-final-viral-combined_k141_172444\|\|full | 1961 | No | NA | | 0 | 2 | Not-determined | Genome-fragment | NA | | 0 |
| S11-final-viral-combined_k141_158713\|\|full | 1627 | No | NA | | 0 | 2 | Not-determined | Genome-fragment | NA | | 0 |
| S11-final-viral-combined_k141_116484\|\|full | 1937 | No | NA | | 0 | 1 | Not-determined | Genome-fragment | NA | | 0 |
| S11-final-viral-combined_k141_75185\|\|full | 1237 | No | NA | | 0 | 1 | Not-determined | Genome-fragment | NA | | 0 |
| S12-final-viral-combined_k141_174841\|\|lt2gene | 1133 | No | NA | | 1 | 0 | Low-quality | Genome-fragment | | 1.59 | 0 |
| S11-final-viral-combined_k141_116489\|\|full | 1715 | No | NA | | 0 | 1 | Not-determined | Genome-fragment | NA | | 0 |
| S12-final-viral-combined_k141_116452\|\|lt2gene | 1133 | No | NA | | 1 | 0 | Low-quality | Genome-fragment | | 2.43 | 0 |
| S11-final-viral-combined_k141_268653\|\|full | 1356 | No | NA | | 0 | 0 | Not-determined | Genome-fragment | NA | | 0 |
| S11-final-viral-combined_k141_48135\|\|full | 2578 | No | NA | | 0 | 2 | Not-determined | Genome-fragment | NA | | 0 |
| S11-final-viral-combined_k141_323046\|\|full | 2840 | No | NA | | 0 | 2 | Not-determined | Genome-fragment | NA | | 0 |
| S11-final-viral-combined_k141_144734\|\|full | 2510 | No | NA | | 0 | 2 | Not-determined | Genome-fragment | NA | | 0 |
| S12_k141_160604 | 1133 | No | NA | | 2 | 0 | Low-quality | Genome-fragment | | 2.6 | 0 |
| S11-final-viral-combined_k141_323051\|\|full | 4670 | No | NA | | 0 | 3 | Not-determined | Genome-fragment | NA | | 0 |
| S11-final-viral-combined_k141_310594\|\|full | 2109 | No | NA | | 0 | 3 | Not-determined | Genome-fragment | NA | | 0 |
| S2-final-viral-combined_k141_14270\|\|full | 1133 | No | NA | | 0 | 1 | Low-quality | Genome-fragment | | 2.3 | 0 |
| S11-final-viral-combined_k141_214134\|\|full | 6240 | No | NA | | 0 | 3 | Not-determined | Genome-fragment | NA | | 0 |
| S9-final-viral-combined_k141_15894\|\|full | 1134 | No | NA | | 0 | 1 | Low-quality | Genome-fragment | | 0.68 | 0 |
| S11-final-viral-combined_k141_19943\|\|full | 8416 | No | NA | | 0 | 2 | Not-determined | Genome-fragment | NA | | 0 |
| S11-final-viral-combined_k141_130522\|\|full | 5428 | No | NA | | 0 | 3 | Not-determined | Genome-fragment | NA | | 0 |
| S9-final-viral-combined_k141_76899\|\|lt2gene | 1134 | No | NA | | 2 | 0 | Low-quality | Genome-fragment | | 0.67 | 0 |
| S11-final-viral-combined_k141_199387\|\|full | 1709 | No | NA | | 0 | 2 | Not-determined | Genome-fragment | NA | | 0 |
| S11-final-viral-combined_k141_6909\|\|full | 4035 | No | NA | | 0 | 3 | Not-determined | Genome-fragment | NA | | 0 |
| S11-final-viral-combined_k141_185731\|\|full | 2307 | No | NA | | 0 | 1 | Not-determined | Genome-fragment | NA | | 0 |
| S11-final-viral-combined_k141_130546\|\|full | 1132 | No | NA | | 0 | 0 | Not-determined | Genome-fragment | NA | | 0 |
| S11-final-viral-combined_k141_135167\|\|lt2gene | 1134 | No | NA | | 1 | 0 | Low-quality | Genome-fragment | | 2.63 | 0 |
| S11-final-viral-combined_k141_130565\|\|full | 1658 | No | NA | | 0 | 0 | Not-determined | Genome-fragment | NA | | 0 |
| S1-final-viral-combined_k141_330154\|\|lt2gene | 1134 | No | NA | | 1 | 0 | Low-quality | Genome-fragment | | 2.84 | 0 |
| S11-final-viral-combined_k141_323081\|\|full | 1340 | No | NA | | 0 | 0 | Not-determined | Genome-fragment | NA | | 0 |
| S12-final-viral-combined_k141_393326\|\|full | 1134 | No | NA | | 0 | 0 | Low-quality | Genome-fragment | | 1.35 | 0 |
| S11-final-viral-combined_k141_89595\|\|full | 1540 | No | NA | | 0 | 0 | Not-determined | Genome-fragment | NA | | 0 |
| S11-final-viral-combined_k141_62349\|\|full | 2023 | No | NA | | 0 | 2 | Not-determined | Genome-fragment | NA | | 0 |
| S11-final-viral-combined_k141_241917\|\|full | 6489 | No | NA | | 0 | 3 | Not-determined | Genome-fragment | NA | | 0 |
| S4-final-viral-combined_k141_154972\|\|full | 1134 | No | NA | | 0 | 1 | Low-quality | Genome-fragment | | 0.56 | 0 |
| S8-final-viral-combined_k141_149766\|\|full | 1134 | No | NA | | 0 | 0 | Low-quality | Genome-fragment | | 2.62 | 0 |
| S11-final-viral-combined_k141_6937\|\|full | 4593 | No | NA | | 0 | 0 | Not-determined | Genome-fragment | NA | | 0 |
| S11-final-viral-combined_k141_20013\|\|full | 2995 | No | NA | | 0 | 2 | Not-determined | Genome-fragment | NA | | 0 |
| S9-final-viral-combined_k141_122830\|\|lt2gene | 1135 | No | NA | | 1 | 0 | Low-quality | Genome-fragment | | 2.9 | 0 |
| S11-final-viral-combined_k141_89616\|\|full | 2105 | No | NA | | 0 | 2 | Not-determined | Genome-fragment | NA | | 0 |
| S11-final-viral-combined_k141_310684\|\|full | 4421 | No | NA | | 0 | 2 | Not-determined | Genome-fragment | NA | | 0 |
| S11-final-viral-combined_k141_227630\|\|full | 1118 | No | NA | | 0 | 0 | Not-determined | Genome-fragment | NA | | 0 |
| S9_k141_28102 | 1135 | No | NA | | 0 | 0 | Low-quality | Genome-fragment | | 2.89 | 0 |
| S11-final-viral-combined_k141_241944\|\|full | 3301 | No | NA | | 0 | 2 | Not-determined | Genome-fragment | NA | | 0 |
| S11-final-viral-combined_k141_20038\|\|full | 1622 | No | NA | | 0 | 0 | Not-determined | Genome-fragment | NA | | 0 |
| S11-final-viral-combined_k141_172559\|\|full | 1362 | No | NA | | 0 | 1 | Not-determined | Genome-fragment | NA | | 0 |
| S11-final-viral-combined_k141_20045\|\|full | 2709 | No | NA | | 0 | 0 | Not-determined | Genome-fragment | NA | | 0 |
| S11-final-viral-combined_k141_310738\|\|full | 3034 | No | NA | | 0 | 0 | Not-determined | Genome-fragment | NA | | 0 |
| S11-final-viral-combined_k141_116589\|\|full | 25615 | No | NA | | 1 | 11 | Medium-quality | Genome-fragment | | 70.25 | 0 |
| S11-final-viral-combined_k141_289690\|\|full | 1135 | No | NA | | 2 | 0 | Low-quality | Genome-fragment | | 1.76 | 0 |
| S11-final-viral-combined_k141_20063\|\|full | 2862 | No | NA | | 0 | 3 | Not-determined | Genome-fragment | NA | | 0 |
| S1-final-viral-combined_k141_91357\|\|lt2gene | 1135 | No | NA | | 2 | 0 | Low-quality | Genome-fragment | | 2.73 | 0 |
| S11-final-viral-combined_k141_268767\|\|full | 2437 | No | NA | | 0 | 2 | Not-determined | Genome-fragment | NA | | 0 |
| S5-final-viral-combined_k141_84835\|\|full | 1135 | No | NA | | 1 | 0 | Low-quality | Genome-fragment | | 2.83 | 0 |
| S5-final-viral-combined_k141_165525\|\|full | 1135 | No | NA | | 2 | 0 | Low-quality | Genome-fragment | | 2.75 | 0 |
| S7-final-viral-combined_k141_77260\|\|full | 1135 | No | NA | | 1 | 0 | Low-quality | Genome-fragment | | 2.29 | 0 |
| S2-final-viral-combined_k141_211975\|\|full | 1135 | No | NA | | 0 | 1 | Low-quality | Genome-fragment | | 1.65 | 0 |
| S11-final-viral-combined_k141_255255\|\|full | 1784 | No | NA | | 0 | 1 | Not-determined | Genome-fragment | NA | | 0 |
| S2-final-viral-combined_k141_194282\|\|lt2gene | 1135 | No | NA | | 2 | 0 | Low-quality | Genome-fragment | | 1.8 | 0 |
| S11-final-viral-combined_k141_296048\|\|full | 2381 | No | NA | | 0 | 1 | Not-determined | Genome-fragment | NA | | 0 |
| S11-final-viral-combined_k141_214256\|\|full | 2163 | No | NA | | 0 | 1 | Not-determined | Genome-fragment | NA | | 0 |
| S11-final-viral-combined_k141_130609\|\|full | 2879 | No | NA | | 0 | 0 | Not-determined | Genome-fragment | NA | | 0 |
| S11-final-viral-combined_k141_172648\|\|full | 1087 | No | NA | | 0 | 1 | Not-determined | Genome-fragment | NA | | 0 |
| S4-final-viral-combined_k141_75661\|\|full | 1135 | No | NA | | 1 | 0 | Low-quality | Genome-fragment | | 2.42 | 0 |
| S8-final-viral-combined_k141_254566\|\|full | 1135 | No | NA | | 0 | 0 | Low-quality | Genome-fragment | | 1.23 | 0 |
| S11-final-viral-combined_k141_34581\|\|full | 2812 | No | NA | | 0 | 2 | Not-determined | Genome-fragment | NA | | 0 |
| S11-final-viral-combined_k141_283148\|\|full | 2380 | No | NA | | 0 | 0 | Not-determined | Genome-fragment | NA | | 0 |
| S8-final-viral-combined_k141_32326\|\|full | 1135 | No | NA | | 2 | 0 | Low-quality | Genome-fragment | | 2.82 | 0 |
| S9-final-viral-combined_k141_37250\|\|full | 1136 | No | NA | | 0 | 0 | Low-quality | Genome-fragment | | 2.03 | 0 |
| S11-final-viral-combined_k141_62468\|\|full | 2054 | No | NA | | 0 | 2 | Not-determined | Genome-fragment | NA | | 0 |
| S11-final-viral-combined_k141_145751\|\|full | 1136 | No | NA | | 1 | 0 | Low-quality | Genome-fragment | | 1.38 | 0 |
| S11-final-viral-combined_k141_54951\|\|full | 1136 | No | NA | | 1 | 0 | Low-quality | Genome-fragment | | 2.5 | 0 |
| S1-final-viral-combined_k141_39174\|\|full | 1136 | No | NA | | 0 | 0 | Low-quality | Genome-fragment | | 2.92 | 0 |
| S11-final-viral-combined_k141_7054\|\|full | 4526 | No | NA | | 0 | 2 | Not-determined | Genome-fragment | NA | | 0 |
| S11-final-viral-combined_k141_283178\|\|full | 1004 | No | NA | | 0 | 0 | Not-determined | Genome-fragment | NA | | 0 |
| S11-final-viral-combined_k141_62481\|\|full | 2617 | No | NA | | 0 | 1 | Not-determined | Genome-fragment | NA | | 0 |
| S11-final-viral-combined_k141_75358\|\|full | 1428 | No | NA | | 0 | 0 | Not-determined | Genome-fragment | NA | | 0 |
| S1-final-viral-combined_k141_262209\|\|lt2gene | 1136 | No | NA | | 1 | 0 | Low-quality | Genome-fragment | | 2.9 | 0 |
| S11-final-viral-combined_k141_310841\|\|full | 3506 | No | NA | | 0 | 0 | Not-determined | Genome-fragment | NA | | 0 |
| S11-final-viral-combined_k141_48246\|\|full | 48975 | Yes | | 41339 | 9 | 5 | Medium-quality | Genome-fragment | | 71 | 15.59 |

| | | | | | | | | | | |
|---|---|---|---|---|---|---|---|---|---|---|
| S3_k141_126467 | 1136 | No | NA | 1 | 0 | Low-quality | Genome-fragment | | 0.88 | 0 |
| S5-final-viral-combined_k141_207864\|\|full | 1136 | No | NA | 0 | 0 | Low-quality | Genome-fragment | | 2.61 | 0 |
| S11-final-viral-combined_k141_283206\|\|full | 4754 | No | NA | 0 | 2 | Not-determined | Genome-fragment | NA | | 0 |
| S11-final-viral-combined_k141_296138\|\|full | 2463 | No | NA | 0 | 0 | Not-determined | Genome-fragment | NA | | 0 |
| S11-final-viral-combined_k141_255314\|\|full | 2981 | No | NA | 0 | 2 | Not-determined | Genome-fragment | NA | | 0 |
| S11-final-viral-combined_k141_75384\|\|full | 1197 | No | NA | 0 | 1 | Not-determined | Genome-fragment | NA | | 0 |
| S11-final-viral-combined_k141_48303\|\|full | 1118 | No | NA | 0 | 1 | Not-determined | Genome-fragment | NA | | 0 |
| S11-final-viral-combined_k141_199584\|\|full | 2017 | No | NA | 0 | 1 | Not-determined | Genome-fragment | NA | | 0 |
| S11-final-viral-combined_k141_89746\|\|full | 2645 | No | NA | 0 | 2 | Not-determined | Genome-fragment | NA | | 0 |
| S5-final-viral-combined_k141_146271\|\|full | 1136 | No | NA | 2 | 0 | Low-quality | Genome-fragment | | 2.96 | 0 |
| S17-final-viral-combined_k141_193410\|\|full | 1136 | No | NA | 1 | 0 | Low-quality | Genome-fragment | | 2.03 | 0 |
| S17-final-viral-combined_k141_94826\|\|full | 1136 | No | NA | 0 | 0 | Low-quality | Genome-fragment | | 2.24 | 0 |
| S17-final-viral-combined_k141_117455\|\|lt2gene | 1136 | No | NA | 1 | 0 | Low-quality | Genome-fragment | | 1.68 | 0 |
| S11-final-viral-combined_k141_283220\|\|full | 34186 | No | NA | 16 | 1 | High-quality | High-quality | | 95.51 | 0 |
| S17-k141_194942 | 1136 | No | NA | 0 | 1 | Low-quality | Genome-fragment | | 0.89 | 0 |
| S11-final-viral-combined_k141_7143\|\|full | 2117 | No | NA | 0 | 1 | Not-determined | Genome-fragment | NA | | 0 |
| S12-final-viral-combined_k141_353053\|\|full | 1136 | No | NA | 0 | 0 | Low-quality | Genome-fragment | | 1.67 | 0 |
| S4-final-viral-combined_k141_159776\|\|full | 1136 | No | NA | 0 | 0 | Low-quality | Genome-fragment | | 2.94 | 0 |
| S11-final-viral-combined_k141_214365\|\|full | 1426 | No | NA | 0 | 0 | Not-determined | Genome-fragment | NA | | 0 |
| S11-final-viral-combined_k141_62588\|\|full | 1396 | No | NA | 0 | 1 | Not-determined | Genome-fragment | NA | | 0 |
| S6-final-viral-combined_k141_82804\|\|lt2gene | 1136 | No | NA | 1 | 0 | Low-quality | Genome-fragment | | 1.31 | 0 |
| S11-final-viral-combined_k141_34753\|\|full | 1418 | No | NA | 0 | 0 | Not-determined | Genome-fragment | NA | | 0 |
| S11-final-viral-combined_k141_214378\|\|full | 1514 | No | NA | 0 | 2 | Not-determined | Genome-fragment | NA | | 0 |
| S11-final-viral-combined_k141_104191\|\|full | 11212 | No | NA | 0 | 5 | Not-determined | Genome-fragment | NA | | 0 |
| S11-final-viral-combined_k141_75425\|\|full | 2411 | No | NA | 0 | 0 | Not-determined | Genome-fragment | NA | | 0 |
| S11-final-viral-combined_k141_242159\|\|full | 1392 | No | NA | 0 | 1 | Not-determined | Genome-fragment | NA | | 0 |
| S11-final-viral-combined_k141_212074\|\|full | 1137 | No | NA | 0 | 0 | Low-quality | Genome-fragment | | 2.47 | 0 |
| S1-final-viral-combined_k141_88030\|\|full | 1137 | No | NA | 0 | 0 | Low-quality | Genome-fragment | | 2.76 | 0 |
| S11-final-viral-combined_k141_7201\|\|full | 1488 | No | NA | 0 | 1 | Not-determined | Genome-fragment | NA | | 0 |
| S1-final-viral-combined_k141_192250\|\|full | 1137 | No | NA | 0 | 1 | Low-quality | Genome-fragment | | 0.95 | 0 |
| S11-final-viral-combined_k141_296193\|\|full | 1027 | No | NA | 0 | 0 | Not-determined | Genome-fragment | NA | | 0 |
| S1_k141_158589 | 1137 | No | NA | 2 | 0 | Low-quality | Genome-fragment | | 2.09 | 0 |
| S11-final-viral-combined_k141_62642\|\|full | 1283 | No | NA | 0 | 0 | Not-determined | Genome-fragment | NA | | 0 |
| S5_k141_150561 | 1137 | No | NA | 0 | 1 | Low-quality | Genome-fragment | | 0.76 | 0 |
| S11-final-viral-combined_k141_242200\|\|full | 7074 | No | NA | 0 | 7 | Not-determined | Genome-fragment | NA | | 0 |
| S11-final-viral-combined_k141_116729\|\|full | 1169 | No | NA | 0 | 2 | Not-determined | Genome-fragment | NA | | 0 |
| S11-final-viral-combined_k141_34814\|\|full | 1159 | No | NA | 0 | 1 | Not-determined | Genome-fragment | NA | | 0 |
| S11-final-viral-combined_k141_145114\|\|full | 2950 | No | NA | 0 | 3 | Not-determined | Genome-fragment | NA | | 0 |
| S11-final-viral-combined_k141_283286\|\|full | 1313 | No | NA | 0 | 2 | Not-determined | Genome-fragment | NA | | 0 |
| S7-final-viral-combined_k141_100936\|\|lt2gene | 1137 | No | NA | 2 | 0 | Low-quality | Genome-fragment | | 2.56 | 0 |
| S11-final-viral-combined_k141_172889\|\|full | 29228 | No | NA | 9 | 0 | Medium-quality | Genome-fragment | | 55.57 | 0 |
| S17-final-viral-combined_k141_243866\|\|full | 1137 | No | NA | 0 | 0 | Low-quality | Genome-fragment | | 0.63 | 0 |
| S11-final-viral-combined_k141_214450\|\|full | 1334 | No | NA | 0 | 1 | Not-determined | Genome-fragment | NA | | 0 |
| S17-final-viral-combined_k141_99992\|\|full | 1137 | No | NA | 0 | 3 | Low-quality | Genome-fragment | | 0.33 | 0 |
| S17-final-viral-combined_k141_197082\|\|lt2gene | 1137 | No | NA | 1 | 0 | Low-quality | Genome-fragment | | 3.12 | 0 |
| S11-final-viral-combined_k141_130829\|\|full | 3411 | No | NA | 0 | 0 | Not-determined | Genome-fragment | NA | | 0 |
| S12-final-viral-combined_k141_18143\|\|full | 1137 | No | NA | 0 | 1 | Low-quality | Genome-fragment | | 2.04 | 0 |
| S11-final-viral-combined_k141_62713\|\|full | 2241 | No | NA | 0 | 3 | Not-determined | Genome-fragment | NA | | 0 |
| S2-final-viral-combined_k141_137135\|\|full | 1137 | No | NA | 0 | 0 | Low-quality | Genome-fragment | | 2.81 | 0 |
| S6-final-viral-combined_k141_109618\|\|full | 1137 | No | NA | 1 | 0 | Low-quality | Genome-fragment | | 4 | 0 |
| S11-final-viral-combined_k141_20306\|\|full | 2660 | No | NA | 0 | 1 | Not-determined | Genome-fragment | NA | | 0 |
| S11-final-viral-combined_k141_255547\|\|full | 1815 | No | NA | 0 | 2 | Not-determined | Genome-fragment | NA | | 0 |
| S11-final-viral-combined_k141_130848\|\|full | 6171 | No | NA | 0 | 5 | Not-determined | Genome-fragment | NA | | 0 |
| S11-final-viral-combined_k141_158946\|\|full | 3921 | No | NA | 0 | 2 | Not-determined | Genome-fragment | NA | | 0 |
| S11-final-viral-combined_k141_89888\|\|full | 1621 | No | NA | 0 | 2 | Not-determined | Genome-fragment | NA | | 0 |
| S11-final-viral-combined_k141_296287\|\|full | 1707 | No | NA | 0 | 1 | Not-determined | Genome-fragment | NA | | 0 |
| S11-final-viral-combined_k141_145188\|\|full | 1882 | No | NA | 0 | 2 | Not-determined | Genome-fragment | NA | | 0 |
| S11-final-viral-combined_k141_145195\|\|full | 1171 | No | NA | 0 | 2 | Not-determined | Genome-fragment | NA | | 0 |
| S11-final-viral-combined_k141_62740\|\|full | 3443 | No | NA | 0 | 2 | Not-determined | Genome-fragment | NA | | 0 |
| S11-final-viral-combined_k141_172953\|\|full | 3342 | No | NA | 0 | 2 | Not-determined | Genome-fragment | NA | | 0 |
| S11-final-viral-combined_k141_268967\|\|full | 17804 | No | NA | 11 | 0 | Medium-quality | Genome-fragment | | 50.48 | 0 |
| S11-final-viral-combined_k141_114309\|\|full | 1138 | No | NA | 0 | 1 | Low-quality | Genome-fragment | | 2.37 | 0 |
| S1-final-viral-combined_k141_95384\|\|lt2gene | 1138 | No | NA | 1 | 0 | Low-quality | Genome-fragment | | 2.62 | 0 |
| S11-final-viral-combined_k141_199715\|\|full | 1469 | No | NA | 0 | 1 | Not-determined | Genome-fragment | NA | | 0 |
| S11-final-viral-combined_k141_130858\|\|full | 4004 | No | NA | 0 | 1 | Not-determined | Genome-fragment | NA | | 0 |
| S11-final-viral-combined_k141_34911\|\|full | 2471 | No | NA | 0 | 1 | Not-determined | Genome-fragment | NA | | 0 |
| S11-final-viral-combined_k141_172968\|\|full | 4215 | No | NA | 0 | 2 | Not-determined | Genome-fragment | NA | | 0 |
| S11-final-viral-combined_k141_75613\|\|full | 7842 | No | NA | 0 | 5 | Not-determined | Genome-fragment | NA | | 0 |
| S11-final-viral-combined_k141_62752\|\|full | 1426 | No | NA | 0 | 0 | Not-determined | Genome-fragment | NA | | 0 |
| S7_k141_129436 | 1138 | No | NA | 0 | 0 | Low-quality | Genome-fragment | | 8.43 | 0 |
| S11-final-viral-combined_k141_116860\|\|full | 4752 | No | NA | 0 | 2 | Not-determined | Genome-fragment | NA | | 0 |
| S11-final-viral-combined_k141_255630\|\|full | 1254 | No | NA | 0 | 0 | Not-determined | Genome-fragment | NA | | 0 |
| S17-final-viral-combined_k141_143518\|\|full | 1138 | No | NA | 1 | 0 | Low-quality | Genome-fragment | | 3.18 | 0 |
| S12-final-viral-combined_k141_224009\|\|full | 1138 | No | NA | 0 | 0 | Low-quality | Genome-fragment | | 2.08 | 0 |
| S12-final-viral-combined_k141_192011\|\|lt2gene | 1138 | No | NA | 1 | 0 | Low-quality | Genome-fragment | | 2.39 | 0 |
| S11-final-viral-combined_k141_173006\|\|full | 1185 | No | NA | 0 | 1 | Not-determined | Genome-fragment | NA | | 0 |
| S11-final-viral-combined_k141_255672\|\|full | 1737 | No | NA | 0 | 1 | Not-determined | Genome-fragment | NA | | 0 |
| S11-final-viral-combined_k141_199781\|\|full | 3534 | No | NA | 0 | 2 | Not-determined | Genome-fragment | NA | | 0 |
| S11-final-viral-combined_k141_296376\|\|full | 2750 | No | NA | 0 | 2 | Not-determined | Genome-fragment | NA | | 0 |
| S11-final-viral-combined_k141_48581\|\|full | 2864 | No | NA | 0 | 0 | Not-determined | Genome-fragment | NA | | 0 |
| S8_k141_243122 | 1138 | No | NA | 0 | 0 | Low-quality | Genome-fragment | | 2.38 | 0 |
| S11-final-viral-combined_k141_130913\|\|full | 1689 | No | NA | 0 | 0 | Not-determined | Genome-fragment | NA | | 0 |
| S11-final-viral-combined_k141_186079\|\|full | 1778 | No | NA | 0 | 2 | Not-determined | Genome-fragment | NA | | 0 |
| S11-final-viral-combined_k141_104323\|\|full | 10685 | No | NA | 0 | 6 | Not-determined | Genome-fragment | NA | | 0 |
| S9-final-viral-combined_k141_125289\|\|full | 1139 | No | NA | 1 | 0 | Low-quality | Genome-fragment | | 0.78 | 0 |
| S11-final-viral-combined_k141_35001\|\|full | 5316 | No | NA | 0 | 0 | Not-determined | Genome-fragment | NA | | 0 |
| S11-final-viral-combined_k141_255685\|\|full | 22563 | No | NA | 0 | 18 | Not-determined | Genome-fragment | NA | | 0 |
| S11-final-viral-combined_k141_104339\|\|full | 3068 | No | NA | 0 | 2 | Not-determined | Genome-fragment | NA | | 0 |
| S11-final-viral-combined_k141_283452\|\|full | 1630 | No | NA | 0 | 0 | Not-determined | Genome-fragment | NA | | 0 |
| S11-final-viral-combined_k141_201510\|\|full | 1139 | No | NA | 0 | 0 | Low-quality | Genome-fragment | | 1.31 | 0 |
| S11-final-viral-combined_k141_242389\|\|full | 3488 | No | NA | 0 | 2 | Not-determined | Genome-fragment | NA | | 0 |
| S11-final-viral-combined_k141_246599\|\|full | 1139 | No | NA | 0 | 0 | Low-quality | Genome-fragment | | 2.47 | 0 |
| S11-final-viral-combined_k141_75697\|\|full | 1284 | No | NA | 0 | 1 | Not-determined | Genome-fragment | NA | | 0 |
| S11-final-viral-combined_k141_116954\|\|full | 1970 | No | NA | 0 | 0 | Not-determined | Genome-fragment | NA | | 0 |
| S11-final-viral-combined_k141_199834\|\|full | 1121 | No | NA | 0 | 0 | Not-determined | Genome-fragment | NA | | 0 |
| S11-final-viral-combined_k141_186127\|\|full | 1205 | No | NA | 0 | 1 | Not-determined | Genome-fragment | NA | | 0 |
| S11-final-viral-combined_k141_255749\|\|full | 2763 | No | NA | 0 | 1 | Not-determined | Genome-fragment | NA | | 0 |
| S11-final-viral-combined_k141_104371\|\|full | 2909 | No | NA | 0 | 0 | Not-determined | Genome-fragment | NA | | 0 |
| S11_k141_244342 | 1139 | No | NA | 0 | 0 | Low-quality | Genome-fragment | | 3.8 | 0 |
| S11-final-viral-combined_k141_131016\|\|full | 2256 | No | NA | 0 | 1 | Not-determined | Genome-fragment | NA | | 0 |
| S5_k141_186479 | 1139 | No | NA | 2 | 0 | Low-quality | Genome-fragment | | 1.89 | 0 |
| S11-final-viral-combined_k141_323536\|\|full | 5205 | No | NA | 0 | 3 | Not-determined | Genome-fragment | NA | | 0 |
| S17-final-viral-combined_k141_201413\|\|full | 1139 | No | NA | 2 | 0 | Low-quality | Genome-fragment | | 2.6 | 0 |
| S17-k141_158073 | 1139 | No | NA | 2 | 0 | Low-quality | Genome-fragment | | 2.23 | 0 |
| S11-final-viral-combined_k141_7443\|\|full | 1460 | No | NA | 0 | 2 | Not-determined | Genome-fragment | NA | | 0 |
| S11-final-viral-combined_k141_311182\|\|full | 2012 | No | NA | 0 | 0 | Not-determined | Genome-fragment | NA | | 0 |
| S12-final-viral-combined_k141_21269\|\|lt2gene | 1139 | No | NA | 1 | 0 | Low-quality | Genome-fragment | | 1.98 | 0 |
| S11-final-viral-combined_k141_35124\|\|full | 3379 | No | NA | 0 | 1 | Not-determined | Genome-fragment | NA | | 0 |
| S12_k141_352897 | 1139 | No | NA | 0 | 0 | Low-quality | Genome-fragment | | 4.1 | 0 |
| S12_k141_36578 | 1139 | No | NA | 1 | 0 | Low-quality | Genome-fragment | | 15.26 | 0 |
| S11-final-viral-combined_k141_35138\|\|full | 1928 | No | NA | 0 | 1 | Not-determined | Genome-fragment | NA | | 0 |
| S11-final-viral-combined_k141_296536\|\|full | 2632 | No | NA | 0 | 1 | Not-determined | Genome-fragment | NA | | 0 |
| S11-final-viral-combined_k141_75797\|\|full | 2701 | No | NA | 0 | 2 | Not-determined | Genome-fragment | NA | | 0 |
| S2-final-viral-combined_k141_189136\|\|lt2gene | 1139 | No | NA | 1 | 0 | Low-quality | Genome-fragment | | 1.66 | 0 |
| S2_k141_218123 | 1139 | No | NA | 0 | 0 | Low-quality | Genome-fragment | | 2.22 | 0 |
| S11-final-viral-combined_k141_214759\|\|full | 1140 | No | NA | 0 | 0 | Low-quality | Genome-fragment | | 2.52 | 0 |
| S11_k141_313893 | 1140 | No | NA | 0 | 1 | Low-quality | Genome-fragment | | 1.1 | 0 |
| S1_k141_143386 | 1140 | No | NA | 0 | 0 | Low-quality | Genome-fragment | | 3.01 | 0 |
| S11-final-viral-combined_k141_35175\|\|full | 1963 | No | NA | 0 | 1 | Not-determined | Genome-fragment | NA | | 0 |
| S5-final-viral-combined_k141_138505\|\|full | 1140 | No | NA | 0 | 0 | Low-quality | Genome-fragment | | 0.72 | 0 |
| S7-final-viral-combined_k141_194127\|\|full | 1140 | No | NA | 1 | 0 | Low-quality | Genome-fragment | | 1.72 | 0 |
| S11-final-viral-combined_k141_173219\|\|full | 2964 | No | NA | 0 | 3 | Not-determined | Genome-fragment | NA | | 0 |
| S11-final-viral-combined_k141_323612\|\|full | 5764 | No | NA | 0 | 3 | Not-determined | Genome-fragment | NA | | 0 |
| S11-final-viral-combined_k141_173230\|\|full | 1664 | No | NA | 0 | 2 | Not-determined | Genome-fragment | NA | | 0 |
| S7-final-viral-combined_k141_76010\|\|full | 1140 | No | NA | 0 | 0 | Low-quality | Genome-fragment | | 1.75 | 0 |
| S7-final-viral-combined_k141_234223\|\|lt2gene | 1140 | No | NA | 1 | 0 | Low-quality | Genome-fragment | | 3.34 | 0 |
| S11-final-viral-combined_k141_117121\|\|full | 4319 | No | NA | 0 | 2 | Not-determined | Genome-fragment | NA | | 0 |
| S17-final-viral-combined_k141_152468\|\|full | 1140 | No | NA | 0 | 0 | Low-quality | Genome-fragment | | 1.43 | 0 |
| S11-final-viral-combined_k141_159198\|\|full | 11160 | No | NA | 0 | 9 | Not-determined | Genome-fragment | NA | | 0 |
| S11-final-viral-combined_k141_214692\|\|full | 5415 | No | NA | 0 | 2 | Not-determined | Genome-fragment | NA | | 0 |
| S17-final-viral-combined_k141_188853\|\|full | 1140 | No | NA | 0 | 1 | Low-quality | Genome-fragment | | 1.46 | 0 |
| S17-k141_133285 | 1140 | No | NA | 0 | 1 | Low-quality | Genome-fragment | | 0.37 | 0 |
| S11-final-viral-combined_k141_35275\|\|full | 1198 | No | NA | 0 | 2 | Not-determined | Genome-fragment | NA | | 0 |
| S11-final-viral-combined_k141_242624\|\|full | 6531 | No | NA | 0 | 4 | Not-determined | Genome-fragment | NA | | 0 |
| S12-final-viral-combined_k141_324669\|\|full | 1140 | No | NA | 0 | 0 | Low-quality | Genome-fragment | | 1.94 | 0 |
| S12-final-viral-combined_k141_299995\|\|full | 1140 | No | NA | 0 | 1 | Low-quality | Genome-fragment | | 0.34 | 0 |
| S11-final-viral-combined_k141_228262\|\|full | 1537 | No | NA | 0 | 1 | Not-determined | Genome-fragment | NA | | 0 |
| S11-final-viral-combined_k141_311294\|\|full | 1112 | No | NA | 0 | 0 | Not-determined | Genome-fragment | NA | | 0 |
| S2-final-viral-combined_k141_234088\|\|full | 1140 | No | NA | 0 | 2 | Low-quality | Genome-fragment | | 0.64 | 0 |
| S4-final-viral-combined_k141_62016\|\|full | 1140 | No | NA | 0 | 0 | Low-quality | Genome-fragment | | 3.04 | 0 |
| S11-final-viral-combined_k141_173341\|\|full | 5297 | No | NA | 0 | 3 | Not-determined | Genome-fragment | NA | | 0 |
| S4_k141_155938 | 1140 | No | NA | 1 | 0 | Low-quality | Genome-fragment | | 1.38 | 0 |
| S8-final-viral-combined_k141_184476\|\|full | 1140 | No | NA | 0 | 1 | Low-quality | Genome-fragment | | 1.67 | 0 |
| S8_k141_40114 | 1140 | No | NA | 0 | 1 | Low-quality | Genome-fragment | | 0.7 | 0 |
| S11-final-viral-combined_k141_159271\|\|full | 2979 | No | NA | 0 | 4 | Not-determined | Genome-fragment | NA | | 0 |
| S11-final-viral-combined_k141_200084\|\|full | 38510 | No | NA | 15 | 1 | Medium-quality | Genome-fragment | | 63.01 | 0 |
| S8_k141_110884 | 1140 | No | NA | 0 | 1 | Low-quality | Genome-fragment | | 3.64 | 0 |
| S9-final-viral-combined_k141_37848\|\|full | 1141 | No | NA | 0 | 2 | Low-quality | Genome-fragment | | 0.84 | 0 |
| S9-final-viral-combined_k141_87806\|\|full | 1141 | No | NA | 0 | 1 | Low-quality | Genome-fragment | | 1.91 | 0 |
| S11_k141_92491 | 1141 | No | NA | 0 | 0 | Low-quality | Genome-fragment | | 1.91 | 0 |
| S1-final-viral-combined_k141_86126\|\|full | 1141 | No | NA | 0 | 1 | Low-quality | Genome-fragment | | 2.37 | 0 |
| S11-final-viral-combined_k141_311331\|\|full | 1470 | No | NA | 0 | 0 | Not-determined | Genome-fragment | NA | | 0 |
| S7-final-viral-combined_k141_179007\|\|lt2gene | 1141 | No | NA | 0 | 0 | Low-quality | Genome-fragment | | 2.68 | 0 |
| S17-k141_46904 | 1141 | No | NA | 0 | 2 | Low-quality | Genome-fragment | | 0.22 | 0 |
| S12-final-viral-combined_k141_14199\|\|full | 1141 | No | NA | 0 | 0 | Low-quality | Genome-fragment | | 2.37 | 0 |
| S11-final-viral-combined_k141_90352\|\|full | 6629 | No | NA | 0 | 2 | Not-determined | Genome-fragment | NA | | 0 |
| S11-final-viral-combined_k141_159321\|\|full | 9146 | No | NA | 0 | 3 | Not-determined | Genome-fragment | NA | | 0 |
| S12-final-viral-combined_k141_122243\|\|lt2gene | 1141 | No | NA | 2 | 0 | Low-quality | Genome-fragment | | 3.02 | 0 |
| S11-final-viral-combined_k141_186413\|\|full | 3351 | No | NA | 0 | 3 | Not-determined | Genome-fragment | NA | | 0 |
| S2-final-viral-combined_k141_215443\|\|full | 1141 | No | NA | 0 | 0 | Low-quality | Genome-fragment | | 2.58 | 0 |
| S11-final-viral-combined_k141_63110\|\|full | 2038 | No | NA | 0 | 1 | Not-determined | Genome-fragment | NA | | 0 |
| S11-final-viral-combined_k141_173471\|\|full | 3445 | No | NA | 0 | 2 | Not-determined | Genome-fragment | NA | | 0 |
| S6_k141_126190 | 1141 | No | NA | 0 | 1 | Low-quality | Genome-fragment | | 0.39 | 0 |
| S8-final-viral-combined_k141_119403\|\|full | 1141 | No | NA | 0 | 0 | Low-quality | Genome-fragment | | 2.01 | 0 |
| S8-final-viral-combined_k141_247991\|\|full | 1141 | No | NA | 0 | 0 | Low-quality | Genome-fragment | | 1.79 | 0 |
| S11-final-viral-combined_k141_296760\|\|full | 1091 | No | NA | 0 | 0 | Not-determined | Genome-fragment | NA | | 0 |
| S11-final-viral-combined_k141_7641\|\|full | 4477 | No | NA | 0 | 2 | Not-determined | Genome-fragment | NA | | 0 |
| S11-final-viral-combined_k141_256055\|\|full | 1980 | No | NA | 0 | 1 | Not-determined | Genome-fragment | NA | | 0 |
| S11-final-viral-combined_k141_269433\|\|full | 2748 | No | NA | 0 | 1 | Not-determined | Genome-fragment | NA | | 0 |
| S8_k141_90868 | 1141 | No | NA | 0 | 0 | Low-quality | Genome-fragment | | 1.27 | 0 |
| S11-final-viral-combined_k141_214847\|\|full | 1586 | No | NA | 0 | 1 | Not-determined | Genome-fragment | NA | | 0 |
| S11-final-viral-combined_k141_145490\|\|full | 1342 | No | NA | 0 | 1 | Not-determined | Genome-fragment | NA | | 0 |
| S11-final-viral-combined_k141_228403\|\|full | 5657 | No | NA | 0 | 3 | Not-determined | Genome-fragment | NA | | 0 |
| S11-final-viral-combined_k141_35492\|\|full | 2701 | No | NA | 0 | 2 | Not-determined | Genome-fragment | NA | | 0 |

| | | | | | | | | | | | |
|---|---|---|---|---|---|---|---|---|---|---|---|
| S11-final-viral-combined_k141_173511\|\|full | 1852 | No | NA | | 0 | 2 | Not-determined | Genome-fragment | NA | | 0 |
| S11-final-viral-combined_k141_7686\|\|full | 1358 | No | NA | | 0 | 1 | Not-determined | Genome-fragment | NA | | 0 |
| S11-final-viral-combined_k141_214891\|\|full | 1593 | No | NA | | 0 | 1 | Not-determined | Genome-fragment | NA | | 0 |
| S9-final-viral-combined_k141_91439\|\|full | 1142 | Yes | | 553 | 2 | 1 | Low-quality | Genome-fragment | | 1.46 | 51.58 |
| S11-final-viral-combined_k141_63199\|\|full | 1240 | No | NA | | 0 | 0 | Not-determined | Genome-fragment | NA | | 0 |
| S11-final-viral-combined_k141_231969\|\|full | 1142 | No | NA | | 0 | 1 | Low-quality | Genome-fragment | | 1.95 | 0 |
| S11-final-viral-combined_k141_104672\|\|full | 1662 | No | NA | | 0 | 1 | Not-determined | Genome-fragment | NA | | 0 |
| S11-final-viral-combined_k141_228417\|\|full | 3415 | No | NA | | 0 | 0 | Not-determined | Genome-fragment | NA | | 0 |
| S11-final-viral-combined_k141_283660\|\|full | 1688 | No | NA | | 0 | 1 | Not-determined | Genome-fragment | NA | | 0 |
| S1_k141_335417 | 1142 | No | NA | | 0 | 1 | Low-quality | Genome-fragment | | 0.45 | 0 |
| S11-final-viral-combined_k141_35521\|\|full | 1395 | No | NA | | 0 | 1 | Not-determined | Genome-fragment | NA | | 0 |
| S11-final-viral-combined_k141_104730\|\|full | 2241 | No | NA | | 0 | 3 | Not-determined | Genome-fragment | NA | | 0 |
| S11-final-viral-combined_k141_296844\|\|full | 2650 | No | NA | | 0 | 1 | Not-determined | Genome-fragment | NA | | 0 |
| S11-final-viral-combined_k141_35532\|\|full | 2050 | No | NA | | 0 | 1 | Not-determined | Genome-fragment | NA | | 0 |
| S11-final-viral-combined_k141_117370\|\|full | 2550 | No | NA | | 0 | 1 | Not-determined | Genome-fragment | NA | | 0 |
| S11-final-viral-combined_k141_159502\|\|full | 1705 | No | NA | | 0 | 2 | Not-determined | Genome-fragment | NA | | 0 |
| S5-final-viral-combined_k141_142196\|\|full | 1142 | No | NA | | 0 | 1 | Low-quality | Genome-fragment | | 0.75 | 0 |
| S11-final-viral-combined_k141_173590\|\|full | 17118 | No | NA | | 0 | 5 | Not-determined | Genome-fragment | NA | | 0 |
| S11-final-viral-combined_k141_117394\|\|full | 2322 | No | NA | | 0 | 2 | Not-determined | Genome-fragment | NA | | 0 |
| S11-final-viral-combined_k141_228485\|\|full | 2400 | No | NA | | 0 | 2 | Not-determined | Genome-fragment | NA | | 0 |
| S11-final-viral-combined_k141_145644\|\|full | 3018 | No | NA | | 0 | 2 | Not-determined | Genome-fragment | NA | | 0 |
| S7-final-viral-combined_k141_47799\|\|full | 1142 | No | NA | | 0 | 0 | Low-quality | Genome-fragment | | 2.24 | 0 |
| S11-final-viral-combined_k141_296893\|\|full | 9369 | No | NA | | 0 | 0 | Not-determined | Genome-fragment | NA | | 0 |
| S11-final-viral-combined_k141_173605\|\|full | 1313 | No | NA | | 0 | 0 | Not-determined | Genome-fragment | NA | | 0 |
| S11-final-viral-combined_k141_63289\|\|full | 2413 | No | NA | | 0 | 2 | Not-determined | Genome-fragment | NA | | 0 |
| S7_k141_215898 | 1142 | No | NA | | 0 | 0 | Low-quality | Genome-fragment | | 2.69 | 0 |
| S12-final-viral-combined_k141_260760\|\|full | 1142 | No | NA | | 0 | 0 | Low-quality | Genome-fragment | | 2.28 | 0 |
| S2-final-viral-combined_k141_146634\|\|full | 1142 | No | NA | | 1 | 0 | Low-quality | Genome-fragment | | 1.92 | 0 |
| S11-final-viral-combined_k141_7913\|\|full | 1362 | No | NA | | 0 | 2 | Not-determined | Genome-fragment | NA | | 0 |
| S2-final-viral-combined_k141_236670\|\|full | 1142 | No | NA | | 0 | 2 | Low-quality | Genome-fragment | | 0.35 | 0 |
| S11-final-viral-combined_k141_323901\|\|full | 1548 | No | NA | | 0 | 3 | Not-determined | Genome-fragment | NA | | 0 |
| S11-final-viral-combined_k141_311546\|\|full | 4490 | No | NA | | 0 | 5 | Not-determined | Genome-fragment | NA | | 0 |
| S2_k141_199357 | 1142 | No | NA | | 0 | 1 | Low-quality | Genome-fragment | | 1.95 | 0 |
| S6-final-viral-combined_k141_133134\|\|full | 1142 | No | NA | | 0 | 0 | Low-quality | Genome-fragment | | 0.62 | 0 |
| S11-final-viral-combined_k141_90574\|\|full | 5545 | No | NA | | 0 | 3 | Not-determined | Genome-fragment | NA | | 0 |
| S6-final-viral-combined_k141_108236\|\|lt2gene | 1142 | No | NA | | 1 | 0 | Low-quality | Genome-fragment | | 2.51 | 0 |
| S11-final-viral-combined_k141_7955\|\|full | 7709 | No | NA | | 0 | 4 | Not-determined | Genome-fragment | NA | | 0 |
| S11-final-viral-combined_k141_159604\|\|full | 3133 | No | NA | | 0 | 0 | Not-determined | Genome-fragment | NA | | 0 |
| S6_k141_2392 | 1142 | No | NA | | 0 | 0 | Low-quality | Genome-fragment | | 0.21 | 0 |
| S11-final-viral-combined_k141_296954\|\|full | 1157 | No | NA | | 0 | 1 | Not-determined | Genome-fragment | NA | | 0 |
| S11-final-viral-combined_k141_269656\|\|full | 3411 | No | NA | | 0 | 2 | Not-determined | Genome-fragment | NA | | 0 |
| S1-final-viral-combined_k141_246824\|\|full | 1143 | No | NA | | 1 | 0 | Low-quality | Genome-fragment | | 1.05 | 0 |
| S11-final-viral-combined_k141_242964\|\|full | 5761 | No | NA | | 0 | 0 | Not-determined | Genome-fragment | NA | | 0 |
| S1-final-viral-combined_k141_371734\|\|full | 1143 | No | NA | | 0 | 0 | Low-quality | Genome-fragment | | 2.53 | 0 |
| S1-final-viral-combined_k141_158852\|\|full | 1143 | No | NA | | 1 | 0 | Low-quality | Genome-fragment | | 2.51 | 0 |
| S11-final-viral-combined_k141_269679\|\|full | 2686 | No | NA | | 0 | 1 | Not-determined | Genome-fragment | NA | | 0 |
| S11-final-viral-combined_k141_269680\|\|full | 1653 | No | NA | | 0 | 0 | Not-determined | Genome-fragment | NA | | 0 |
| S11-final-viral-combined_k141_90610\|\|full | 1682 | No | NA | | 0 | 2 | Not-determined | Genome-fragment | NA | | 0 |
| S11-final-viral-combined_k141_131398\|\|full | 1828 | No | NA | | 0 | 1 | Not-determined | Genome-fragment | NA | | 0 |
| S5_k141_122453 | 1143 | No | NA | | 1 | 0 | Low-quality | Genome-fragment | | 2.65 | 0 |
| S11-final-viral-combined_k141_63359\|\|full | 1301 | No | NA | | 0 | 0 | Not-determined | Genome-fragment | NA | | 0 |
| S11-final-viral-combined_k141_117535\|\|full | 1414 | No | NA | | 0 | 2 | Not-determined | Genome-fragment | NA | | 0 |
| S11-final-viral-combined_k141_242986\|\|full | 2632 | No | NA | | 0 | 3 | Not-determined | Genome-fragment | NA | | 0 |
| S12-final-viral-combined_k141_141857\|\|full | 1143 | No | NA | | 0 | 0 | Low-quality | Genome-fragment | | 2.86 | 0 |
| S11-final-viral-combined_k141_49206\|\|full | 1033 | No | NA | | 0 | 0 | Not-determined | Genome-fragment | NA | | 0 |
| S11-final-viral-combined_k141_131414\|\|full | 2757 | No | NA | | 0 | 1 | Not-determined | Genome-fragment | NA | | 0 |
| S11-final-viral-combined_k141_20818\|\|full | 2660 | No | NA | | 0 | 2 | Not-determined | Genome-fragment | NA | | 0 |
| S12_k141_357386 | 1143 | No | NA | | 0 | 0 | Low-quality | Genome-fragment | | 0.61 | 0 |
| S8_k141_155572 | 1143 | No | NA | | 1 | 0 | Low-quality | Genome-fragment | | 1.79 | 0 |
| S9-final-viral-combined_k141_123414\|\|full | 1144 | No | NA | | 1 | 0 | Low-quality | Genome-fragment | | 1.73 | 0 |
| S11-final-viral-combined_k141_296992\|\|full | 1065 | No | NA | | 0 | 1 | Not-determined | Genome-fragment | NA | | 0 |
| S5-final-viral-combined_k141_71809\|\|full | 1144 | No | NA | | 0 | 2 | Low-quality | Genome-fragment | | 0.45 | 0 |
| S5-final-viral-combined_k141_24756\|\|full | 1144 | No | NA | | 0 | 0 | Low-quality | Genome-fragment | | 0.98 | 0 |
| S5_k141_90386 | 1144 | No | NA | | 2 | 0 | Low-quality | Genome-fragment | | 3.46 | 0 |
| S11-final-viral-combined_k141_215046\|\|full | 1458 | No | NA | | 0 | 1 | Not-determined | Genome-fragment | NA | | 0 |
| S11-final-viral-combined_k141_145757\|\|full | 2232 | No | NA | | 0 | 2 | Not-determined | Genome-fragment | NA | | 0 |
| S12-final-viral-combined_k141_343544\|\|full | 1144 | No | NA | | 0 | 0 | Low-quality | Genome-fragment | | 2.52 | 0 |
| S11-final-viral-combined_k141_131470\|\|full | 3303 | No | NA | | 0 | 1 | Not-determined | Genome-fragment | NA | | 0 |
| S11-final-viral-combined_k141_200511\|\|full | 1472 | No | NA | | 0 | 2 | Not-determined | Genome-fragment | NA | | 0 |
| S2-final-viral-combined_k141_21377\|\|full | 1144 | No | NA | | 0 | 0 | Low-quality | Genome-fragment | | 2.19 | 0 |
| S11-final-viral-combined_k141_8117\|\|full | 2044 | No | NA | | 0 | 0 | Not-determined | Genome-fragment | NA | | 0 |
| S11-final-viral-combined_k141_117637\|\|full | 2773 | No | NA | | 0 | 1 | Not-determined | Genome-fragment | NA | | 0 |
| S11-final-viral-combined_k141_104971\|\|full | 1890 | No | NA | | 0 | 1 | Not-determined | Genome-fragment | NA | | 0 |
| S11-final-viral-combined_k141_200553\|\|full | 3028 | No | NA | | 0 | 2 | Not-determined | Genome-fragment | NA | | 0 |
| S11-final-viral-combined_k141_90741\|\|full | 1156 | No | NA | | 0 | 0 | Not-determined | Genome-fragment | NA | | 0 |
| S11-final-viral-combined_k141_215112\|\|full | 1617 | No | NA | | 0 | 0 | Not-determined | Genome-fragment | NA | | 0 |
| S11-final-viral-combined_k141_20887\|\|full | 12109 | No | NA | | 0 | 6 | Not-determined | Genome-fragment | NA | | 0 |
| S4-final-viral-combined_k141_127595\|\|full | 1144 | No | NA | | 0 | 0 | Low-quality | Genome-fragment | | 2.62 | 0 |
| S11-final-viral-combined_k141_8815\|\|full | 1145 | No | NA | | 2 | 0 | Low-quality | Genome-fragment | | 1.9 | 0 |
| S11-final-viral-combined_k141_90765\|\|full | 3339 | No | NA | | 0 | 1 | Not-determined | Genome-fragment | NA | | 0 |
| S11-final-viral-combined_k141_200580\|\|full | 5103 | No | NA | | 0 | 0 | Not-determined | Genome-fragment | NA | | 0 |
| S11-final-viral-combined_k141_228746\|\|full | 2133 | No | NA | | 0 | 2 | Not-determined | Genome-fragment | NA | | 0 |
| S11-final-viral-combined_k141_32024\|\|lt2gene | 1145 | No | NA | | 2 | 0 | Low-quality | Genome-fragment | | 3.28 | 0 |
| S11-final-viral-combined_k141_131561\|\|full | 1244 | No | NA | | 0 | 1 | Not-determined | Genome-fragment | NA | | 0 |
| S1_k141_29737 | 1145 | No | NA | | 0 | 0 | Low-quality | Genome-fragment | | 1.44 | 0 |
| S11-final-viral-combined_k141_145831\|\|full | 2401 | No | NA | | 0 | 1 | Not-determined | Genome-fragment | NA | | 0 |
| S3-final-viral-combined_k141_64919\|\|full | 1145 | No | NA | | 0 | 0 | Low-quality | Genome-fragment | | 2.73 | 0 |
| S3-final-viral-combined_k141_59557\|\|full | 1145 | No | NA | | 0 | 1 | Low-quality | Genome-fragment | | 0.6 | 0 |
| S5_k141_190309 | 1145 | No | NA | | 0 | 1 | Low-quality | Genome-fragment | | 0.76 | 0 |
| S11-final-viral-combined_k141_76369\|\|full | 2922 | No | NA | | 0 | 3 | Not-determined | Genome-fragment | NA | | 0 |
| S11-final-viral-combined_k141_215154\|\|full | 1025 | No | NA | | 0 | 1 | Not-determined | Genome-fragment | NA | | 0 |
| S11-final-viral-combined_k141_145880\|\|full | 3320 | No | NA | | 0 | 2 | Not-determined | Genome-fragment | NA | | 0 |
| S11-final-viral-combined_k141_186860\|\|full | 2860 | No | NA | | 0 | 1 | Not-determined | Genome-fragment | NA | | 0 |
| S7_k141_113281 | 1145 | No | NA | | 0 | 0 | Low-quality | Genome-fragment | | 0.81 | 0 |
| S11-final-viral-combined_k141_159741\|\|full | 6106 | No | NA | | 0 | 4 | Not-determined | Genome-fragment | NA | | 0 |
| S11-final-viral-combined_k141_35854\|\|full | 2952 | No | NA | | 0 | 0 | Not-determined | Genome-fragment | NA | | 0 |
| S11-final-viral-combined_k141_76407\|\|full | 5055 | No | NA | | 0 | 2 | Not-determined | Genome-fragment | NA | | 0 |
| S17-final-viral-combined_k141_128478\|\|full | 1145 | No | NA | | 1 | 0 | Low-quality | Genome-fragment | | 2.69 | 0 |
| S17-final-viral-combined_k141_166560\|\|full | 1145 | No | NA | | 1 | 0 | Low-quality | Genome-fragment | | 2.37 | 0 |
| S11-final-viral-combined_k141_76408\|\|full | 10091 | No | NA | | 0 | 6 | Not-determined | Genome-fragment | NA | | 0 |
| S11-final-viral-combined_k141_269903\|\|full | 19350 | No | NA | | 0 | 7 | Not-determined | Genome-fragment | NA | | 0 |
| S12-final-viral-combined_k141_307092\|\|full | 1145 | No | NA | | 0 | 1 | Low-quality | Genome-fragment | | 3.12 | 0 |
| S12-final-viral-combined_k141_78936\|\|full | 1145 | No | NA | | 0 | 0 | Low-quality | Genome-fragment | | 2.5 | 0 |
| S11-final-viral-combined_k141_76437\|\|full | 1252 | No | NA | | 0 | 0 | Not-determined | Genome-fragment | NA | | 0 |
| S12-final-viral-combined_k141_104045\|\|lt2gene | 1145 | No | NA | | 1 | 0 | Low-quality | Genome-fragment | | 2.45 | 0 |
| S11-final-viral-combined_k141_186907\|\|full | 1125 | No | NA | | 0 | 1 | Not-determined | Genome-fragment | NA | | 0 |
| S11-final-viral-combined_k141_145957\|\|full | 1857 | No | NA | | 0 | 0 | Not-determined | Genome-fragment | NA | | 0 |
| S11-final-viral-combined_k141_311888\|\|full | 1080 | No | NA | | 0 | 0 | Not-determined | Genome-fragment | NA | | 0 |
| S2-final-viral-combined_k141_64739\|\|full | 1145 | No | NA | | 0 | 0 | Low-quality | Genome-fragment | | 1.62 | 0 |
| S2_k141_55892 | 1145 | No | NA | | 0 | 0 | Low-quality | Genome-fragment | | 3.1 | 0 |
| S11-final-viral-combined_k141_269955\|\|full | 1333 | No | NA | | 0 | 2 | Not-determined | Genome-fragment | NA | | 0 |
| S11-final-viral-combined_k141_35947\|\|full | 1070 | No | NA | | 0 | 1 | Not-determined | Genome-fragment | NA | | 0 |
| S4_k141_164042 | 1145 | No | NA | | 0 | 1 | Low-quality | Genome-fragment | | 0.3 | 0 |
| S8-final-viral-combined_k141_109159\|\|full | 1145 | No | NA | | 0 | 0 | Low-quality | Genome-fragment | | 2.43 | 0 |
| S8_k141_118792 | 1145 | No | NA | | 0 | 1 | Low-quality | Genome-fragment | | 0.43 | 0 |
| S9-final-viral-combined_k141_118774\|\|full | 1146 | No | NA | | 1 | 0 | Low-quality | Genome-fragment | | 2.49 | 0 |
| S11-final-viral-combined_k141_117756\|\|full | 25717 | No | NA | | 10 | 0 | Medium-quality | Genome-fragment | | 61.58 | 0 |
| S11-final-viral-combined_k141_263112\|\|full | 1146 | No | NA | | 0 | 1 | Low-quality | Genome-fragment | | 0.84 | 0 |
| S11-final-viral-combined_k141_256513\|\|full | 2282 | No | NA | | 0 | 0 | Not-determined | Genome-fragment | NA | | 0 |
| S11-final-viral-combined_k141_234153\|\|full | 1146 | No | NA | | 1 | 0 | Low-quality | Genome-fragment | | 2.39 | 0 |
| S11-final-viral-combined_k141_90905\|\|full | 1663 | No | NA | | 0 | 3 | Not-determined | Genome-fragment | NA | | 0 |
| S11-final-viral-combined_k141_159829\|\|full | 1760 | No | NA | | 0 | 0 | Not-determined | Genome-fragment | NA | | 0 |
| S11-final-viral-combined_k141_146039\|\|full | 2227 | No | NA | | 0 | 1 | Not-determined | Genome-fragment | NA | | 0 |
| S11-final-viral-combined_k141_284113\|\|full | 2574 | No | NA | | 0 | 1 | Not-determined | Genome-fragment | NA | | 0 |
| S11-final-viral-combined_k141_146044\|\|full | 2736 | No | NA | | 0 | 0 | Not-determined | Genome-fragment | NA | | 0 |
| S11-final-viral-combined_k141_324180\|\|full | 4953 | No | NA | | 0 | 2 | Not-determined | Genome-fragment | NA | | 0 |
| S1-final-viral-combined_k141_387585\|\|full | 1146 | No | NA | | 0 | 1 | Low-quality | Genome-fragment | | 1.16 | 0 |
| S11-final-viral-combined_k141_284142\|\|full | 4145 | No | NA | | 0 | 4 | Not-determined | Genome-fragment | NA | | 0 |
| S1-final-viral-combined_k141_13078\|\|full | 1146 | No | NA | | 0 | 0 | Low-quality | Genome-fragment | | 0.88 | 0 |
| S11-final-viral-combined_k141_146066\|\|full | 2075 | No | NA | | 0 | 0 | Not-determined | Genome-fragment | NA | | 0 |
| S3_k141_75038 | 1146 | No | NA | | 0 | 2 | Low-quality | Genome-fragment | | 3.66 | 0 |
| S11-final-viral-combined_k141_284157\|\|full | 1160 | No | NA | | 0 | 1 | Not-determined | Genome-fragment | NA | | 0 |
| S11-final-viral-combined_k141_117825\|\|full | 1515 | No | NA | | 0 | 1 | Not-determined | Genome-fragment | NA | | 0 |
| S7-final-viral-combined_k141_123292\|\|lt2gene | 1146 | No | NA | | 1 | 0 | Low-quality | Genome-fragment | | 2.43 | 0 |
| S11-final-viral-combined_k141_146086\|\|full | 1911 | No | NA | | 0 | 1 | Not-determined | Genome-fragment | NA | | 0 |
| S17-final-viral-combined_k141_181115\|\|full | 1146 | No | NA | | 0 | 1 | Low-quality | Genome-fragment | | 1.8 | 0 |
| S11-final-viral-combined_k141_256556\|\|full | 2030 | No | NA | | 0 | 2 | Not-determined | Genome-fragment | NA | | 0 |
| S11-final-viral-combined_k141_297261\|\|full | 1957 | No | NA | | 0 | 1 | Not-determined | Genome-fragment | NA | | 0 |
| S17-final-viral-combined_k141_178578\|\|lt2gene | 1146 | No | NA | | 1 | 0 | Low-quality | Genome-fragment | | 25.69 | 0 |
| S17-final-viral-combined_k141_93059\|\|lt2gene | 1146 | No | NA | | 1 | 0 | Low-quality | Genome-fragment | | 2.7 | 0 |
| S11-final-viral-combined_k141_90974\|\|full | 1446 | No | NA | | 0 | 1 | Not-determined | Genome-fragment | NA | | 0 |
| S17-k141_119324 | 1146 | No | NA | | 0 | 1 | Low-quality | Genome-fragment | | 0.75 | 0 |
| S11-final-viral-combined_k141_8478\|\|full | 6155 | No | NA | | 0 | 3 | Not-determined | Genome-fragment | NA | | 0 |
| S11-final-viral-combined_k141_63652\|\|full | 2044 | No | NA | | 0 | 2 | Not-determined | Genome-fragment | NA | | 0 |
| S11-final-viral-combined_k141_200776\|\|full | 1462 | No | NA | | 0 | 0 | Not-determined | Genome-fragment | NA | | 0 |
| S12-final-viral-combined_k141_57435\|\|full | 1146 | No | NA | | 2 | 0 | Low-quality | Genome-fragment | | 2.29 | 0 |
| S11-final-viral-combined_k141_159936\|\|full | 1252 | No | NA | | 0 | 0 | Not-determined | Genome-fragment | NA | | 0 |
| S11-final-viral-combined_k141_187046\|\|full | 3591 | No | NA | | 0 | 2 | Not-determined | Genome-fragment | NA | | 0 |
| S11-final-viral-combined_k141_200797\|\|full | 1152 | No | NA | | 0 | 1 | Not-determined | Genome-fragment | NA | | 0 |
| S11-final-viral-combined_k141_311910\|\|full | 1509 | No | NA | | 0 | 1 | Not-determined | Genome-fragment | NA | | 0 |
| S12-final-viral-combined_k141_63982\|\|full | 1146 | No | NA | | 0 | 0 | Low-quality | Genome-fragment | | 1.2 | 0 |
| S8_k141_81409 | 1146 | No | NA | | 1 | 0 | Low-quality | Genome-fragment | | 2.96 | 0 |
| S9-final-viral-combined_k141_69805\|\|full | 1147 | No | NA | | 0 | 0 | Low-quality | Genome-fragment | | 2.39 | 0 |
| S9-final-viral-combined_k141_3339\|\|full | 1147 | No | NA | | 2 | 0 | Low-quality | Genome-fragment | | 2.87 | 0 |
| S11-final-viral-combined_k141_324239\|\|full | 1774 | No | NA | | 0 | 0 | Not-determined | Genome-fragment | NA | | 0 |
| S11-final-viral-combined_k141_9190\|\|full | 1147 | No | NA | | 0 | 0 | Low-quality | Genome-fragment | | 0.8 | 0 |
| S11-final-viral-combined_k141_21145\|\|full | 1189 | No | NA | | 0 | 1 | Not-determined | Genome-fragment | NA | | 0 |
| S11-final-viral-combined_k141_117860\|\|lt2gene | 1147 | No | NA | | 1 | 0 | Low-quality | Genome-fragment | | 0.69 | 0 |
| S11-final-viral-combined_k141_8530\|\|full | 1659 | No | NA | | 0 | 2 | Not-determined | Genome-fragment | NA | | 0 |
| S5-final-viral-combined_k141_111573\|\|full | 1147 | No | NA | | 1 | 0 | Low-quality | Genome-fragment | | 3.08 | 0 |
| S11-final-viral-combined_k141_63698\|\|full | 2049 | No | NA | | 0 | 2 | Not-determined | Genome-fragment | NA | | 0 |
| S11-final-viral-combined_k141_105307\|\|full | 12937 | No | NA | | 0 | 5 | Not-determined | Genome-fragment | NA | | 0 |
| S5-final-viral-combined_k141_115629\|\|full | 1147 | No | NA | | 0 | 0 | Low-quality | Genome-fragment | | 2.26 | 0 |
| S5_k141_161117 | 1147 | No | NA | | 1 | 0 | Low-quality | Genome-fragment | | 8.56 | 0 |
| S7-final-viral-combined_k141_182843\|\|full | 1147 | No | NA | | 0 | 0 | Low-quality | Genome-fragment | | 0.86 | 0 |
| S17-final-viral-combined_k141_76751\|\|full | 1147 | No | NA | | 0 | 0 | Low-quality | Genome-fragment | | 1.93 | 0 |
| S11-final-viral-combined_k141_311954\|\|full | 2211 | No | NA | | 0 | 2 | Not-determined | Genome-fragment | NA | | 0 |
| S11-final-viral-combined_k141_117959\|\|full | 4204 | No | NA | | 0 | 4 | Not-determined | Genome-fragment | NA | | 0 |
| S11-final-viral-combined_k141_311965\|\|full | 1674 | No | NA | | 0 | 1 | Not-determined | Genome-fragment | NA | | 0 |
| S11-final-viral-combined_k141_160032\|\|full | 1666 | No | NA | | 0 | 0 | Not-determined | Genome-fragment | NA | | 0 |
| S11-final-viral-combined_k141_243372\|\|full | 5748 | No | NA | | 0 | 3 | Not-determined | Genome-fragment | NA | | 0 |
| S11-final-viral-combined_k141_8606\|\|full | 1869 | No | NA | | 0 | 0 | Not-determined | Genome-fragment | NA | | 0 |
| S11-final-viral-combined_k141_229003\|\|full | 1401 | No | NA | | 0 | 0 | Not-determined | Genome-fragment | NA | | 0 |
| S11-final-viral-combined_k141_243397\|\|full | 1029 | No | NA | | 0 | 1 | Not-determined | Genome-fragment | NA | | 0 |

| | | | | | | | | | |
|---|---|---|---|---|---|---|---|---|---|
| S17-final-viral-combined_k141_226504\|\|lt2gene | 1147 | No | NA | 1 | 0 | Low-quality | Genome-fragment | 2.37 | 0 |
| S12-final-viral-combined_k141_74394\|\|full | 1147 | No | NA | 0 | 1 | Low-quality | Genome-fragment | 1.56 | 0 |
| S2-final-viral-combined_k141_189141\|\|full | 1147 | No | NA | 0 | 1 | Low-quality | Genome-fragment | 0.98 | 0 |
| S2-final-viral-combined_k141_202360\|\|full | 1147 | No | NA | 0 | 1 | Low-quality | Genome-fragment | 0.6 | 0 |
| S2-final-viral-combined_k141_122371\|\|full | 1147 | No | NA | 0 | 0 | Low-quality | Genome-fragment | 2.34 | 0 |
| S11-final-viral-combined_k141_8639\|\|full | 2051 | No | NA | 0 | 3 | Not-determined | Genome-fragment | NA | 0 |
| S4-final-viral-combined_k141_106803\|\|lt2gene | 1147 | No | NA | 1 | 0 | Low-quality | Genome-fragment | 2.96 | 0 |
| S8-final-viral-combined_k141_172201\|\|full | 1147 | No | NA | 1 | 0 | Low-quality | Genome-fragment | 2.31 | 0 |
| S11-final-viral-combined_k141_131845\|\|full | 1743 | No | NA | 0 | 1 | Not-determined | Genome-fragment | NA | 0 |
| S11-final-viral-combined_k141_49632\|\|full | 21007 | No | NA | 12 | 0 | Medium-quality | Genome-fragment | 56.04 | 0 |
| S11_k141_172266 | 1148 | No | NA | 0 | 0 | Low-quality | Genome-fragment | 1.52 | 0 |
| S11-final-viral-combined_k141_131846\|\|full | 5253 | No | NA | 0 | 4 | Not-determined | Genome-fragment | NA | 0 |
| S11-final-viral-combined_k141_324336\|\|full | 1148 | No | NA | 0 | 2 | Not-determined | Genome-fragment | NA | 0 |
| S11-final-viral-combined_k141_229055\|\|full | 4955 | No | NA | 0 | 5 | Not-determined | Genome-fragment | NA | 0 |
| S11-final-viral-combined_k141_174092\|\|full | 2213 | No | NA | 0 | 1 | Not-determined | Genome-fragment | NA | 0 |
| S11-final-viral-combined_k141_21287\|\|full | 6609 | No | NA | 0 | 6 | Not-determined | Genome-fragment | NA | 0 |
| S1-final-viral-combined_k141_126742\|\|lt2gene | 1148 | No | NA | 1 | 0 | Low-quality | Genome-fragment | 1.39 | 0 |
| S11-final-viral-combined_k141_243426\|\|full | 2585 | No | NA | 0 | 2 | Not-determined | Genome-fragment | NA | 0 |
| S11-final-viral-combined_k141_63770\|\|full | 1180 | No | NA | 0 | 0 | Not-determined | Genome-fragment | NA | 0 |
| S5-final-viral-combined_k141_134834\|\|full | 1148 | No | NA | 1 | 0 | Low-quality | Genome-fragment | 2.39 | 0 |
| S5-final-viral-combined_k141_130724\|\|lt2gene | 1148 | No | NA | 1 | 0 | Low-quality | Genome-fragment | 1.61 | 0 |
| S11-final-viral-combined_k141_200950\|\|full | 4449 | No | NA | 0 | 1 | Not-determined | Genome-fragment | NA | 0 |
| S11-final-viral-combined_k141_21302\|\|full | 3908 | No | NA | 0 | 1 | Not-determined | Genome-fragment | NA | 0 |
| S5_k141_129685 | 1148 | No | NA | 1 | 0 | Low-quality | Genome-fragment | 0.44 | 0 |
| S11-final-viral-combined_k141_76759\|\|full | 1582 | No | NA | 0 | 1 | Not-determined | Genome-fragment | NA | 0 |
| S5_k141_41786 | 1148 | No | NA | 0 | 1 | Low-quality | Genome-fragment | 1.41 | 0 |
| S11-final-viral-combined_k141_324364\|\|full | 2334 | No | NA | 0 | 1 | Not-determined | Genome-fragment | NA | 0 |
| S11-final-viral-combined_k141_284436\|\|full | 3035 | No | NA | 0 | 1 | Not-determined | Genome-fragment | NA | 0 |
| S11-final-viral-combined_k141_76789\|\|full | 2375 | No | NA | 0 | 1 | Not-determined | Genome-fragment | NA | 0 |
| S7_k141_219311 | 1148 | Yes | 927 | 1 | 1 | Low-quality | Genome-fragment | 0.64 | 19.25 |
| S7_k141_192318 | 1148 | No | NA | 0 | 0 | Low-quality | Genome-fragment | 2.2 | 0 |
| S17-final-viral-combined_k141_188410\|\|full | 1148 | No | NA | 0 | 1 | Low-quality | Genome-fragment | 2.24 | 0 |
| S17-k141_192595 | 1148 | No | NA | 0 | 0 | Low-quality | Genome-fragment | 2.74 | 0 |
| S11-final-viral-combined_k141_201025\|\|full | 6857 | No | NA | 0 | 2 | Not-determined | Genome-fragment | NA | 0 |
| S11-final-viral-combined_k141_91146\|\|full | 1652 | No | NA | 0 | 1 | Not-determined | Genome-fragment | NA | 0 |
| S11-final-viral-combined_k141_91150\|\|full | 1421 | No | NA | 0 | 0 | Not-determined | Genome-fragment | NA | 0 |
| S11-final-viral-combined_k141_118119\|\|full | 2202 | No | NA | 0 | 1 | Not-determined | Genome-fragment | NA | 0 |
| S11-final-viral-combined_k141_284491\|\|full | 1500 | No | NA | 0 | 1 | Not-determined | Genome-fragment | NA | 0 |
| S17-k141_131326 | 1148 | No | NA | 0 | 0 | Low-quality | Genome-fragment | 1.79 | 0 |
| S11-final-viral-combined_k141_187263\|\|full | 1381 | No | NA | 0 | 0 | Not-determined | Genome-fragment | NA | 0 |
| S12-final-viral-combined_k141_12158\|\|lt2gene | 1148 | No | NA | 1 | 0 | Low-quality | Genome-fragment | 2.72 | 0 |
| S11-final-viral-combined_k141_174223\|\|full | 3102 | No | NA | 0 | 1 | Not-determined | Genome-fragment | NA | 0 |
| S2-final-viral-combined_k141_28991\|\|lt2gene | 1148 | No | NA | 1 | 0 | Low-quality | Genome-fragment | 2.63 | 0 |
| S11-final-viral-combined_k141_118142\|\|full | 3527 | No | NA | 0 | 1 | Not-determined | Genome-fragment | NA | 0 |
| S11-final-viral-combined_k141_21382\|\|full | 5263 | No | NA | 0 | 3 | Not-determined | Genome-fragment | NA | 0 |
| S8-final-viral-combined_k141_256045\|\|full | 1148 | No | NA | 0 | 0 | Low-quality | Genome-fragment | 2.39 | 0 |
| S8-final-viral-combined_k141_22912\|\|lt2gene | 1148 | No | NA | 1 | 0 | Low-quality | Genome-fragment | 2.78 | 0 |
| S9-final-viral-combined_k141_41476\|\|full | 1149 | No | NA | 1 | 0 | Low-quality | Genome-fragment | 1.29 | 0 |
| S11-final-viral-combined_k141_105531\|\|full | 3936 | No | NA | 0 | 1 | Not-determined | Genome-fragment | NA | 0 |
| S9_k141_123911 | 1149 | No | NA | 0 | 0 | Low-quality | Genome-fragment | 1.47 | 0 |
| S11-final-viral-combined_k141_51467\|\|lt2gene | 1149 | No | NA | 2 | 0 | Low-quality | Genome-fragment | 3.42 | 0 |
| S1-final-viral-combined_k141_54236\|\|full | 1149 | No | NA | 2 | 0 | Low-quality | Genome-fragment | 2.68 | 0 |
| S11-final-viral-combined_k141_76887\|\|full | 12398 | No | NA | 0 | 3 | Not-determined | Genome-fragment | NA | 0 |
| S1-final-viral-combined_k141_59781\|\|full | 1149 | No | NA | 1 | 0 | Low-quality | Genome-fragment | 2.42 | 0 |
| S1-final-viral-combined_k141_137142\|\|lt2gene | 1149 | No | NA | 1 | 0 | Low-quality | Genome-fragment | 2.07 | 0 |
| S5-final-viral-combined_k141_153749\|\|full | 1149 | No | NA | 1 | 0 | Low-quality | Genome-fragment | 2.76 | 0 |
| S11-final-viral-combined_k141_76897\|\|full | 10453 | No | NA | 0 | 6 | Not-determined | Genome-fragment | NA | 0 |
| S7-final-viral-combined_k141_235968\|\|full | 1149 | No | NA | 1 | 0 | Low-quality | Genome-fragment | 3 | 0 |
| S11-final-viral-combined_k141_63818\|\|full | 100466 | Yes | 52817 | 11 | 31 | Complete | High-quality | 100 | 47.43 |
| S7-final-viral-combined_k141_88624\|\|full | 1149 | No | NA | 0 | 0 | Low-quality | Genome-fragment | 2.08 | 0 |
| S11-final-viral-combined_k141_105559\|\|full | 1898 | No | NA | 0 | 1 | Not-determined | Genome-fragment | NA | 0 |
| S7-final-viral-combined_k141_141389\|\|lt2gene | 1149 | No | NA | 1 | 0 | Low-quality | Genome-fragment | 3.28 | 0 |
| S11-final-viral-combined_k141_201090\|\|full | 2746 | No | NA | 0 | 2 | Not-determined | Genome-fragment | NA | 0 |
| S7_k141_56990 | 1149 | No | NA | 0 | 0 | Low-quality | Genome-fragment | 2.67 | 0 |
| S7_k141_172470 | 1149 | No | NA | 0 | 1 | Low-quality | Genome-fragment | 0.62 | 0 |
| S17-final-viral-combined_k141_12473\|\|full | 1149 | No | NA | 0 | 2 | Low-quality | Genome-fragment | 0.38 | 0 |
| S12_k141_191658 | 1149 | No | NA | 1 | 0 | Low-quality | Genome-fragment | 3.05 | 0 |
| S2_k141_64136 | 1149 | No | NA | 0 | 1 | Low-quality | Genome-fragment | 1.87 | 0 |
| S11-final-viral-combined_k141_118185\|\|full | 1106 | No | NA | 0 | 2 | Not-determined | Genome-fragment | NA | 0 |
| S11-final-viral-combined_k141_160311\|\|full | 1628 | No | NA | 0 | 0 | Not-determined | Genome-fragment | NA | 0 |
| S6-final-viral-combined_k141_45959\|\|full | 1149 | No | NA | 1 | 0 | Low-quality | Genome-fragment | 1.4 | 0 |
| S11-final-viral-combined_k141_63854\|\|full | 5196 | No | NA | 0 | 1 | Not-determined | Genome-fragment | NA | 0 |
| S11-final-viral-combined_k141_270194\|\|full | 11651 | No | NA | 0 | 5 | Not-determined | Genome-fragment | NA | 0 |
| S9_k141_67177 | 1150 | No | NA | 1 | 0 | Low-quality | Genome-fragment | 2.66 | 0 |
| S11-final-viral-combined_k141_243594\|\|full | 2264 | No | NA | 0 | 0 | Not-determined | Genome-fragment | NA | 0 |
| S11-final-viral-combined_k141_146463\|\|full | 1519 | No | NA | 0 | 2 | Not-determined | Genome-fragment | NA | 0 |
| S1-final-viral-combined_k141_316511\|\|full | 1150 | Yes | 730 | 1 | 1 | Low-quality | Genome-fragment | 1.64 | 36.52 |
| S1-final-viral-combined_k141_15850\|\|full | 1150 | No | NA | 0 | 1 | Low-quality | Genome-fragment | 1.93 | 0 |
| S11-final-viral-combined_k141_257023\|\|full | 1408 | No | NA | 0 | 1 | Not-determined | Genome-fragment | NA | 0 |
| S11-final-viral-combined_k141_146470\|\|full | 1736 | No | NA | 0 | 2 | Not-determined | Genome-fragment | NA | 0 |
| S5-final-viral-combined_k141_72654\|\|lt2gene | 1150 | No | NA | 1 | 0 | Low-quality | Genome-fragment | 1.68 | 0 |
| S5-final-viral-combined_k141_203314\|\|lt2gene | 1150 | No | NA | 1 | 0 | Low-quality | Genome-fragment | 2.91 | 0 |
| S11-final-viral-combined_k141_8931\|\|full | 4325 | No | NA | 0 | 6 | Not-determined | Genome-fragment | NA | 0 |
| S7-final-viral-combined_k141_46181\|\|lt2gene | 1150 | No | NA | 0 | 2 | Low-quality | Genome-fragment | 1.05 | 0 |
| S17-final-viral-combined_k141_776\|\|lt2gene | 1150 | No | NA | 1 | 0 | Low-quality | Genome-fragment | 2.15 | 0 |
| S17-k141_77302 | 1150 | No | NA | 1 | 0 | Low-quality | Genome-fragment | 1.68 | 0 |
| S12_k141_83915 | 1150 | No | NA | 0 | 0 | Low-quality | Genome-fragment | 0.12 | 0 |
| S11-final-viral-combined_k141_312215\|\|full | 2213 | No | NA | 0 | 3 | Not-determined | Genome-fragment | NA | 0 |
| S2_k141_243167 | 1150 | No | NA | 1 | 0 | Low-quality | Genome-fragment | 1.78 | 0 |
| S4_k141_145082 | 1150 | No | NA | 0 | 1 | Low-quality | Genome-fragment | 2.12 | 0 |
| S6-final-viral-combined_k141_58884\|\|full | 1150 | No | NA | 1 | 0 | Low-quality | Genome-fragment | 1.83 | 0 |
| S9-final-viral-combined_k141_38999\|\|lt2gene | 1151 | No | NA | 1 | 0 | Low-quality | Genome-fragment | 2.29 | 0 |
| S9_k141_80562 | 1151 | No | NA | 0 | 1 | Low-quality | Genome-fragment | 0.93 | 0 |
| S1-final-viral-combined_k141_168287\|\|full | 1151 | No | NA | 0 | 0 | Low-quality | Genome-fragment | 2.71 | 0 |
| S11-final-viral-combined_k141_187372\|\|full | 4317 | No | NA | 0 | 3 | Not-determined | Genome-fragment | NA | 0 |
| S11-final-viral-combined_k141_284552\|\|full | 2861 | No | NA | 0 | 3 | Not-determined | Genome-fragment | NA | 0 |
| S1-final-viral-combined_k141_93706\|\|full | 1151 | No | NA | 0 | 1 | Low-quality | Genome-fragment | 0.52 | 0 |
| S1-final-viral-combined_k141_261365\|\|lt2gene | 1151 | No | NA | 1 | 0 | Low-quality | Genome-fragment | 3.27 | 0 |
| S11-final-viral-combined_k141_9043\|\|full | 3187 | No | NA | 0 | 2 | Not-determined | Genome-fragment | NA | 0 |
| S11-final-viral-combined_k141_105710\|\|full | 1432 | No | NA | 0 | 0 | Not-determined | Genome-fragment | NA | 0 |
| S11-final-viral-combined_k141_9046\|\|full | 6583 | No | NA | 0 | 8 | Not-determined | Genome-fragment | NA | 0 |
| S5-final-viral-combined_k141_189996\|\|full | 1151 | No | NA | 0 | 1 | Low-quality | Genome-fragment | 0.59 | 0 |
| S11-final-viral-combined_k141_36434\|\|full | 1250 | No | NA | 0 | 0 | Not-determined | Genome-fragment | NA | 0 |
| S11-final-viral-combined_k141_215723\|\|full | 64981 | Yes | 42332 | 6 | 16 | High-quality | High-quality | 96.66 | 34.85 |
| S11-final-viral-combined_k141_297660\|\|full | 4939 | No | NA | 0 | 5 | Not-determined | Genome-fragment | NA | 0 |
| S5-final-viral-combined_k141_96375\|\|full | 1151 | No | NA | 0 | 1 | Low-quality | Genome-fragment | 3.59 | 0 |
| S11-final-viral-combined_k141_284599\|\|full | 2223 | No | NA | 0 | 0 | Not-determined | Genome-fragment | NA | 0 |
| S7-final-viral-combined_k141_20971\|\|full | 1151 | No | NA | 3 | 0 | Low-quality | Genome-fragment | 1.9 | 0 |
| S11-final-viral-combined_k141_270331\|\|full | 4076 | No | NA | 0 | 1 | Not-determined | Genome-fragment | NA | 0 |
| S7-final-viral-combined_k141_167675\|\|full | 1151 | No | NA | 0 | 0 | Low-quality | Genome-fragment | 2.66 | 0 |
| S11-final-viral-combined_k141_160426\|\|full | 4055 | No | NA | 0 | 1 | Not-determined | Genome-fragment | NA | 0 |
| S11-final-viral-combined_k141_64040\|\|full | 1089 | No | NA | 0 | 1 | Not-determined | Genome-fragment | NA | 0 |
| S12-final-viral-combined_k141_68083\|\|full | 1151 | No | NA | 0 | 1 | Low-quality | Genome-fragment | 1.56 | 0 |
| S12-final-viral-combined_k141_38424\|\|lt2gene | 1151 | No | NA | 1 | 0 | Low-quality | Genome-fragment | 1.68 | 0 |
| S11-final-viral-combined_k141_36438\|\|full | 1845 | No | NA | 0 | 1 | Not-determined | Genome-fragment | NA | 0 |
| S11-final-viral-combined_k141_270355\|\|full | 5619 | No | NA | 0 | 4 | Not-determined | Genome-fragment | NA | 0 |
| S11-final-viral-combined_k141_105769\|\|full | 11230 | No | NA | 0 | 10 | Not-determined | Genome-fragment | NA | 0 |
| S11-final-viral-combined_k141_297698\|\|full | 2428 | No | NA | 0 | 1 | Not-determined | Genome-fragment | NA | 0 |
| S11-final-viral-combined_k141_50117\|\|full | 3237 | No | NA | 0 | 2 | Not-determined | Genome-fragment | NA | 0 |
| S11-final-viral-combined_k141_160470\|\|full | 8912 | No | NA | 0 | 4 | Not-determined | Genome-fragment | NA | 0 |
| S12-final-viral-combined_k141_242853\|\|lt2gene | 1151 | No | NA | 1 | 0 | Low-quality | Genome-fragment | 1 | 0 |
| S11-final-viral-combined_k141_215781\|\|full | 1165 | No | NA | 0 | 1 | Not-determined | Genome-fragment | NA | 0 |
| S11-final-viral-combined_k141_201355\|\|full | 1234 | No | NA | 0 | 0 | Not-determined | Genome-fragment | NA | 0 |
| S11-final-viral-combined_k141_284666\|\|full | 1224 | No | NA | 0 | 0 | Not-determined | Genome-fragment | NA | 0 |
| S2_k141_155384 | 1151 | No | NA | 0 | 1 | Low-quality | Genome-fragment | 0.42 | 0 |
| S2_k141_239314 | 1151 | No | NA | 2 | 0 | Low-quality | Genome-fragment | 3.19 | 0 |
| S11-final-viral-combined_k141_50186\|\|full | 1644 | No | NA | 0 | 2 | Not-determined | Genome-fragment | NA | 0 |
| S11-final-viral-combined_k141_146564\|\|full | 2062 | No | NA | 0 | 0 | Not-determined | Genome-fragment | NA | 0 |
| S2_k141_12262 | 1151 | No | NA | 0 | 1 | Low-quality | Genome-fragment | 0.59 | 0 |
| S2_k141_7501 | 1151 | No | NA | 0 | 1 | Low-quality | Genome-fragment | 0.42 | 0 |
| S4-final-viral-combined_k141_154643\|\|full | 1151 | No | NA | 0 | 1 | Low-quality | Genome-fragment | 2.24 | 0 |
| S4-final-viral-combined_k141_132402\|\|lt2gene | 1151 | No | NA | 1 | 0 | Low-quality | Genome-fragment | 2.63 | 0 |
| S6_k141_78117 | 1151 | No | NA | 0 | 0 | Low-quality | Genome-fragment | 1.17 | 0 |
| S11-final-viral-combined_k141_50209\|\|full | 6574 | No | NA | 0 | 1 | Not-determined | Genome-fragment | NA | 0 |
| S11-final-viral-combined_k141_257180\|\|full | 1715 | No | NA | 0 | 0 | Not-determined | Genome-fragment | NA | 0 |
| S8-final-viral-combined_k141_206813\|\|full | 1151 | No | NA | 0 | 0 | Low-quality | Genome-fragment | 2.69 | 0 |
| S11-final-viral-combined_k141_118403\|\|full | 1514 | No | NA | 0 | 0 | Not-determined | Genome-fragment | NA | 0 |
| S11-final-viral-combined_k141_304333\|\|lt2gene | 1152 | No | NA | 1 | 0 | Low-quality | Genome-fragment | 0.8 | 0 |
| S11-final-viral-combined_k141_297775\|\|full | 1320 | No | NA | 0 | 1 | Not-determined | Genome-fragment | NA | 0 |
| S1-final-viral-combined_k141_160675\|\|full | 1152 | No | NA | 3 | 0 | Low-quality | Genome-fragment | 2.39 | 0 |
| S7-final-viral-combined_k141_217112\|\|full | 1152 | No | NA | 0 | 1 | Low-quality | Genome-fragment | 2.45 | 0 |
| S11-final-viral-combined_k141_187552\|\|full | 2542 | No | NA | 0 | 0 | Not-determined | Genome-fragment | NA | 0 |
| S11-final-viral-combined_k141_257238\|\|full | 1206 | No | NA | 0 | 1 | Not-determined | Genome-fragment | NA | 0 |
| S7-final-viral-combined_k141_137834\|\|lt2gene | 1152 | No | NA | 1 | 0 | Low-quality | Genome-fragment | 3.52 | 0 |
| S17-final-viral-combined_k141_118327\|\|full | 1152 | No | NA | 0 | 2 | Low-quality | Genome-fragment | 0.89 | 0 |
| S4-final-viral-combined_k141_127363\|\|full | 1152 | No | NA | 0 | 0 | Low-quality | Genome-fragment | 2.92 | 0 |
| S11-final-viral-combined_k141_118442\|\|full | 1115 | No | NA | 0 | 2 | Not-determined | Genome-fragment | NA | 0 |
| S4-final-viral-combined_k141_19125\|\|full | 1152 | No | NA | 0 | 0 | Low-quality | Genome-fragment | 2.92 | 0 |
| S8-final-viral-combined_k141_109797\|\|full | 1152 | No | NA | 0 | 0 | Low-quality | Genome-fragment | 2.26 | 0 |
| S11-final-viral-combined_k141_64174\|\|full | 1803 | No | NA | 0 | 1 | Not-determined | Genome-fragment | NA | 0 |
| S11-final-viral-combined_k141_50335\|\|full | 1677 | No | NA | 0 | 2 | Not-determined | Genome-fragment | NA | 0 |
| S11-final-viral-combined_k141_50336\|\|full | 2122 | No | NA | 0 | 1 | Not-determined | Genome-fragment | NA | 0 |
| S9-final-viral-combined_k141_10451\|\|full | 1153 | No | NA | 0 | 0 | Low-quality | Genome-fragment | 2.59 | 0 |
| S1_k141_192725 | 1153 | No | NA | 0 | 1 | Low-quality | Genome-fragment | 2.94 | 0 |
| S3-final-viral-combined_k141_111019\|\|full | 1153 | No | NA | 1 | 0 | Low-quality | Genome-fragment | 3.03 | 0 |
| S11-final-viral-combined_k141_105933\|\|full | 1685 | No | NA | 0 | 2 | Not-determined | Genome-fragment | NA | 0 |
| S3_k141_5143 | 1153 | No | NA | 0 | 1 | Low-quality | Genome-fragment | 1.14 | 0 |
| S11-final-viral-combined_k141_64190\|\|full | 4665 | No | NA | 0 | 1 | Not-determined | Genome-fragment | NA | 0 |
| S11-final-viral-combined_k141_229592\|\|full | 2381 | No | NA | 0 | 2 | Not-determined | Genome-fragment | NA | 0 |
| S11-final-viral-combined_k141_270514\|\|full | 1320 | No | NA | 0 | 2 | Not-determined | Genome-fragment | NA | 0 |
| S7-final-viral-combined_k141_133332\|\|full | 1153 | No | NA | 0 | 0 | Low-quality | Genome-fragment | 2.29 | 0 |
| S11-final-viral-combined_k141_174588\|\|full | 4409 | No | NA | 0 | 0 | Not-determined | Genome-fragment | NA | 0 |
| S11-final-viral-combined_k141_284817\|\|full | 1569 | No | NA | 0 | 2 | Not-determined | Genome-fragment | NA | 0 |
| S7-final-viral-combined_k141_236689\|\|full | 1153 | No | NA | 0 | 0 | Low-quality | Genome-fragment | 0.75 | 0 |
| S11-final-viral-combined_k141_64222\|\|full | 2228 | No | NA | 0 | 2 | Not-determined | Genome-fragment | NA | 0 |
| S17-final-viral-combined_k141_5677\|\|full | 1153 | No | NA | 0 | 0 | Low-quality | Genome-fragment | 0.69 | 0 |
| S11-final-viral-combined_k141_257354\|\|full | 1217 | No | NA | 0 | 0 | Not-determined | Genome-fragment | NA | 0 |
| S11-final-viral-combined_k141_160694\|\|full | 2631 | No | NA | 0 | 2 | Not-determined | Genome-fragment | NA | 0 |
| S11-final-viral-combined_k141_146694\|\|full | 8601 | No | NA | 0 | 6 | Not-determined | Genome-fragment | NA | 0 |
| S11-final-viral-combined_k141_105993\|\|full | 8014 | No | NA | 0 | 6 | Not-determined | Genome-fragment | NA | 0 |
| S17-final-viral-combined_k141_254250\|\|full | 1153 | No | NA | 0 | 0 | Low-quality | Genome-fragment | 0.84 | 0 |
| S12-final-viral-combined_k141_72615\|\|full | 1153 | No | NA | 1 | 0 | Low-quality | Genome-fragment | 2.49 | 0 |
| S11-final-viral-combined_k141_118596\|\|full | 3332 | No | NA | 0 | 2 | Not-determined | Genome-fragment | NA | 0 |
| S11-final-viral-combined_k141_257374\|\|full | 2596 | No | NA | 0 | 2 | Not-determined | Genome-fragment | NA | 0 |

| | | | | | | | | | |
|---|---|---|---|---|---|---|---|---|---|
| S11-final-viral-combined_k141_284919\|\|full | 1464 | No | NA | 0 | 1 | Not-determined | Genome-fragment | NA | 0 |
| S11-final-viral-combined_k141_132055\|\|full | 15504 | No | NA | 0 | 14 | Not-determined | Genome-fragment | NA | 0 |
| S2-final-viral-combined_k141_67473\|\|full | 1153 | No | NA | 0 | 0 | Low-quality | Genome-fragment | 3.63 | 0 |
| S8-final-viral-combined_k141_134290\|\|full | 1153 | No | NA | 0 | 0 | Low-quality | Genome-fragment | 2.14 | 0 |
| S11-final-viral-combined_k141_284937\|\|full | 2437 | No | NA | 0 | 1 | Not-determined | Genome-fragment | NA | 0 |
| S9_k141_112081 | 1154 | No | NA | 1 | 0 | Low-quality | Genome-fragment | 3.14 | 0 |
| S11-final-viral-combined_k141_243951\|\|full | 2171 | No | NA | 0 | 1 | Not-determined | Genome-fragment | NA | 0 |
| S11-final-viral-combined_k141_9436\|\|lt2gene | 1154 | No | NA | 1 | 0 | Low-quality | Genome-fragment | 2.43 | 0 |
| S1-final-viral-combined_k141_144834\|\|full | 1154 | No | NA | 1 | 0 | Low-quality | Genome-fragment | 1.91 | 0 |
| S1_k141_103405 | 1154 | No | NA | 2 | 0 | Low-quality | Genome-fragment | 3.01 | 0 |
| S1_k141_356689 | 1154 | No | NA | 0 | 1 | Low-quality | Genome-fragment | 0.56 | 0 |
| S3-final-viral-combined_k141_35406\|\|full | 1154 | No | NA | 0 | 1 | Low-quality | Genome-fragment | 1.94 | 0 |
| S7-final-viral-combined_k141_193156\|\|full | 1154 | No | NA | 0 | 0 | Low-quality | Genome-fragment | 2.84 | 0 |
| S7_k141_122108 | 1154 | No | NA | 0 | 0 | Low-quality | Genome-fragment | 1.86 | 0 |
| S11-final-viral-combined_k141_146837\|\|full | 1220 | No | NA | 0 | 0 | Not-determined | Genome-fragment | NA | 0 |
| S11-final-viral-combined_k141_187827\|\|full | 2027 | No | NA | 0 | 1 | Not-determined | Genome-fragment | NA | 0 |
| S11-final-viral-combined_k141_324950\|\|full | 1366 | No | NA | 0 | 0 | Not-determined | Genome-fragment | NA | 0 |
| S11-final-viral-combined_k141_132150\|\|full | 1435 | No | NA | 0 | 1 | Not-determined | Genome-fragment | NA | 0 |
| S17-final-viral-combined_k141_13639\|\|full | 1154 | No | NA | 1 | 0 | Low-quality | Genome-fragment | 2.38 | 0 |
| S17-k141_107905 | 1154 | No | NA | 0 | 0 | Low-quality | Genome-fragment | 2.32 | 0 |
| S11-final-viral-combined_k141_146851\|\|full | 1422 | No | NA | 0 | 1 | Not-determined | Genome-fragment | NA | 0 |
| S11-final-viral-combined_k141_187855\|\|full | 1524 | No | NA | 0 | 1 | Not-determined | Genome-fragment | NA | 0 |
| S2-final-viral-combined_k141_105177\|\|full | 1154 | No | NA | 0 | 0 | Low-quality | Genome-fragment | 2.12 | 0 |
| S11-final-viral-combined_k141_118722\|\|full | 1937 | No | NA | 0 | 2 | Not-determined | Genome-fragment | NA | 0 |
| S8_k141_218954 | 1154 | No | NA | 0 | 1 | Low-quality | Genome-fragment | 3.38 | 0 |
| S11-final-viral-combined_k141_270681\|\|full | 10387 | No | NA | 0 | 3 | Not-determined | Genome-fragment | NA | 0 |
| S11-final-viral-combined_k141_187891\|\|full | 2214 | No | NA | 0 | 0 | Not-determined | Genome-fragment | NA | 0 |
| S11-final-viral-combined_k141_106222\|\|full | 2302 | No | NA | 0 | 2 | Not-determined | Genome-fragment | NA | 0 |
| S11-final-viral-combined_k141_105597\|\|full | 1155 | No | NA | 0 | 0 | Low-quality | Genome-fragment | 2.13 | 0 |
| S11-final-viral-combined_k141_160832\|\|full | 1431 | No | NA | 0 | 1 | Not-determined | Genome-fragment | NA | 0 |
| S11-final-viral-combined_k141_91798\|\|full | 1474 | No | NA | 0 | 2 | Not-determined | Genome-fragment | NA | 0 |
| S11-final-viral-combined_k141_229852\|\|full | 1903 | No | NA | 0 | 0 | Not-determined | Genome-fragment | NA | 0 |
| S11-final-viral-combined_k141_146928\|\|full | 4729 | No | NA | 0 | 1 | Not-determined | Genome-fragment | NA | 0 |
| S11-final-viral-combined_k141_187926\|\|full | 2421 | No | NA | 0 | 0 | Not-determined | Genome-fragment | NA | 0 |
| S11-final-viral-combined_k141_215987\|\|full | 5326 | No | NA | 0 | 4 | Not-determined | Genome-fragment | NA | 0 |
| S11-final-viral-combined_k141_312698\|\|full | 1253 | No | NA | 0 | 2 | Not-determined | Genome-fragment | NA | 0 |
| S11-final-viral-combined_k141_257559\|\|full | 3372 | No | NA | 0 | 2 | Not-determined | Genome-fragment | NA | 0 |
| S11-final-viral-combined_k141_298037\|\|full | 1335 | No | NA | 0 | 0 | Not-determined | Genome-fragment | NA | 0 |
| S1-final-viral-combined_k141_102533\|\|full | 1155 | No | NA | 1 | 0 | Low-quality | Genome-fragment | 7.15 | 0 |
| S5_k141_213459 | 1155 | No | NA | 0 | 0 | Low-quality | Genome-fragment | 3.38 | 0 |
| S11-final-viral-combined_k141_187966\|\|full | 1162 | No | NA | 0 | 1 | Not-determined | Genome-fragment | NA | 0 |
| S11-final-viral-combined_k141_216014\|\|full | 3309 | No | NA | 0 | 2 | Not-determined | Genome-fragment | NA | 0 |
| S11-final-viral-combined_k141_132236\|\|full | 4274 | No | NA | 0 | 4 | Not-determined | Genome-fragment | NA | 0 |
| S12-final-viral-combined_k141_324172\|\|full | 1155 | No | NA | 2 | 0 | Low-quality | Genome-fragment | 1.97 | 0 |
| S11-final-viral-combined_k141_285099\|\|full | 5957 | No | NA | 0 | 1 | Not-determined | Genome-fragment | NA | 0 |
| S12_k141_220389 | 1155 | No | NA | 1 | 0 | Low-quality | Genome-fragment | 0.73 | 0 |
| S12_k141_45481 | 1155 | No | NA | 1 | 0 | Low-quality | Genome-fragment | 0.81 | 0 |
| S11-final-viral-combined_k141_325095\|\|full | 3168 | No | NA | 0 | 1 | Not-determined | Genome-fragment | NA | 0 |
| S2-final-viral-combined_k141_12618\|\|lt2gene | 1155 | No | NA | 1 | 0 | Low-quality | Genome-fragment | 2.39 | 0 |
| S11-final-viral-combined_k141_187998\|\|full | 1639 | No | NA | 0 | 0 | Not-determined | Genome-fragment | NA | 0 |
| S11-final-viral-combined_k141_325131\|\|full | 1030 | No | NA | 0 | 0 | Not-determined | Genome-fragment | NA | 0 |
| S11-final-viral-combined_k141_174806\|\|full | 1428 | No | NA | 0 | 1 | Not-determined | Genome-fragment | NA | 0 |
| S11-final-viral-combined_k141_216094\|\|full | 4020 | No | NA | 0 | 5 | Not-determined | Genome-fragment | NA | 0 |
| S11-final-viral-combined_k141_312775\|\|full | 2477 | No | NA | 0 | 1 | Not-determined | Genome-fragment | NA | 0 |
| S6-final-viral-combined_k141_106465\|\|lt2gene | 1155 | No | NA | 1 | 0 | Low-quality | Genome-fragment | 1.89 | 0 |
| S11-final-viral-combined_k141_321695\|\|full | 1156 | No | NA | 0 | 0 | Low-quality | Genome-fragment | 1.96 | 0 |
| S11-final-viral-combined_k141_257621\|\|full | 1611 | No | NA | 0 | 1 | Not-determined | Genome-fragment | NA | 0 |
| S5-final-viral-combined_k141_156005\|\|full | 1156 | No | NA | 3 | 0 | Low-quality | Genome-fragment | 3.54 | 0 |
| S11-final-viral-combined_k141_188033\|\|full | 1140 | No | NA | 0 | 1 | Not-determined | Genome-fragment | NA | 0 |
| S11-final-viral-combined_k141_147034\|\|full | 1570 | No | NA | 0 | 2 | Not-determined | Genome-fragment | NA | 0 |
| S7-final-viral-combined_k141_14247\|\|full | 1156 | No | NA | 0 | 0 | Low-quality | Genome-fragment | 2.49 | 0 |
| S7-final-viral-combined_k141_163345\|\|full | 1156 | No | NA | 0 | 0 | Low-quality | Genome-fragment | 2.81 | 0 |
| S11-final-viral-combined_k141_285215\|\|full | 4845 | No | NA | 0 | 2 | Not-determined | Genome-fragment | NA | 0 |
| S11-final-viral-combined_k141_64392\|\|full | 42456 | No | NA | 9 | 3 | High-quality | High-quality | 99.12 | 0 |
| S11-final-viral-combined_k141_161005\|\|full | 4739 | No | NA | 0 | 1 | Not-determined | Genome-fragment | NA | 0 |
| S7-final-viral-combined_k141_129787\|\|lt2gene | 1156 | No | NA | 1 | 0 | Low-quality | Genome-fragment | 2.73 | 0 |
| S17-final-viral-combined_k141_23886\|\|full | 1156 | No | NA | 0 | 1 | Low-quality | Genome-fragment | 1.75 | 0 |
| S11-final-viral-combined_k141_77553\|\|full | 2160 | No | NA | 0 | 1 | Not-determined | Genome-fragment | NA | 0 |
| S8-final-viral-combined_k141_66537\|\|full | 1156 | No | NA | 1 | 0 | Low-quality | Genome-fragment | 2.62 | 0 |
| S8_k141_23147 | 1156 | No | NA | 0 | 0 | Low-quality | Genome-fragment | 1.41 | 0 |
| S11-final-viral-combined_k141_312811\|\|full | 1626 | No | NA | 0 | 0 | Not-determined | Genome-fragment | NA | 0 |
| S9_k141_118315 | 1157 | No | NA | 0 | 2 | Low-quality | Genome-fragment | 0.64 | 0 |
| S11-final-viral-combined_k141_244239\|\|full | 1662 | No | NA | 0 | 1 | Not-determined | Genome-fragment | NA | 0 |
| S11-final-viral-combined_k141_125399\|\|lt2gene | 1157 | No | NA | 0 | 0 | Low-quality | Genome-fragment | 2.15 | 0 |
| S11-final-viral-combined_k141_9732\|\|full | 1217 | No | NA | 0 | 2 | Not-determined | Genome-fragment | NA | 0 |
| S1-final-viral-combined_k141_348384\|\|full | 1157 | No | NA | 0 | 0 | Low-quality | Genome-fragment | 2.42 | 0 |
| S11-final-viral-combined_k141_50768\|\|full | 1810 | No | NA | 0 | 0 | Not-determined | Genome-fragment | NA | 0 |
| S11-final-viral-combined_k141_64448\|\|full | 4342 | No | NA | 0 | 3 | Not-determined | Genome-fragment | NA | 0 |
| S11-final-viral-combined_k141_270847\|\|full | 1979 | No | NA | 0 | 0 | Not-determined | Genome-fragment | NA | 0 |
| S11-final-viral-combined_k141_132290\|\|full | 1537 | No | NA | 0 | 0 | Not-determined | Genome-fragment | NA | 0 |
| S1-final-viral-combined_k141_184627\|\|full | 1157 | No | NA | 0 | 1 | Low-quality | Genome-fragment | 0.68 | 0 |
| S11-final-viral-combined_k141_91959\|\|full | 1493 | No | NA | 0 | 2 | Not-determined | Genome-fragment | NA | 0 |
| S11-final-viral-combined_k141_244273\|\|full | 2926 | No | NA | 0 | 2 | Not-determined | Genome-fragment | NA | 0 |
| S11-final-viral-combined_k141_216193\|\|full | 1197 | No | NA | 0 | 2 | Not-determined | Genome-fragment | NA | 0 |
| S11-final-viral-combined_k141_50800\|\|full | 9429 | No | NA | 0 | 6 | Not-determined | Genome-fragment | NA | 0 |
| S1-final-viral-combined_k141_111446\|\|full | 1157 | No | NA | 1 | 0 | Low-quality | Genome-fragment | 0.73 | 0 |
| S5-final-viral-combined_k141_6831\|\|full | 1157 | No | NA | 1 | 0 | Low-quality | Genome-fragment | 2.47 | 0 |
| S5-final-viral-combined_k141_109528\|\|lt2gene | 1157 | No | NA | 2 | 0 | Low-quality | Genome-fragment | 3.11 | 0 |
| S5_k141_109261 | 1157 | No | NA | 0 | 0 | Low-quality | Genome-fragment | 9.48 | 0 |
| S11-final-viral-combined_k141_106428\|\|full | 8622 | No | NA | 0 | 5 | Not-determined | Genome-fragment | NA | 0 |
| S11-final-viral-combined_k141_9778\|\|full | 1495 | No | NA | 0 | 3 | Not-determined | Genome-fragment | NA | 0 |
| S11-final-viral-combined_k141_229981\|\|full | 1457 | No | NA | 0 | 0 | Not-determined | Genome-fragment | NA | 0 |
| S12-final-viral-combined_k141_77285\|\|full | 1157 | No | NA | 0 | 0 | Low-quality | Genome-fragment | 0.75 | 0 |
| S11-final-viral-combined_k141_298241\|\|full | 1233 | No | NA | 0 | 1 | Not-determined | Genome-fragment | NA | 0 |
| S11-final-viral-combined_k141_325227\|\|full | 1584 | No | NA | 0 | 0 | Not-determined | Genome-fragment | NA | 0 |
| S11-final-viral-combined_k141_92019\|\|full | 4681 | No | NA | 0 | 3 | Not-determined | Genome-fragment | NA | 0 |
| S11-final-viral-combined_k141_174976\|\|full | 1004 | No | NA | 0 | 1 | Not-determined | Genome-fragment | NA | 0 |
| S11-final-viral-combined_k141_50841\|\|full | 3452 | No | NA | 0 | 3 | Not-determined | Genome-fragment | NA | 0 |
| S11-final-viral-combined_k141_161111\|\|full | 4925 | No | NA | 0 | 2 | Not-determined | Genome-fragment | NA | 0 |
| S11-final-viral-combined_k141_77639\|\|full | 1039 | No | NA | 0 | 1 | Not-determined | Genome-fragment | NA | 0 |
| S11-final-viral-combined_k141_106448\|\|full | 1676 | No | NA | 0 | 2 | Not-determined | Genome-fragment | NA | 0 |
| S11-final-viral-combined_k141_132383\|\|full | 2414 | No | NA | 0 | 2 | Not-determined | Genome-fragment | NA | 0 |
| S11-final-viral-combined_k141_77680\|\|full | 1123 | No | NA | 0 | 2 | Not-determined | Genome-fragment | NA | 0 |
| S11-final-viral-combined_k141_64569\|\|full | 3171 | No | NA | 0 | 0 | Not-determined | Genome-fragment | NA | 0 |
| S2_k141_180733 | 1157 | No | NA | 2 | 0 | Low-quality | Genome-fragment | 3.03 | 0 |
| S8-final-viral-combined_k141_185608\|\|full | 1157 | No | NA | 0 | 0 | Low-quality | Genome-fragment | 3.49 | 0 |
| S11-final-viral-combined_k141_77717\|\|full | 3687 | No | NA | 0 | 2 | Not-determined | Genome-fragment | NA | 0 |
| S11-final-viral-combined_k141_77720\|\|full | 1903 | No | NA | 0 | 2 | Not-determined | Genome-fragment | NA | 0 |
| S11-final-viral-combined_k141_257839\|\|full | 1393 | No | NA | 0 | 1 | Not-determined | Genome-fragment | NA | 0 |
| S11-final-viral-combined_k141_244352\|\|full | 2472 | No | NA | 0 | 1 | Not-determined | Genome-fragment | NA | 0 |
| S5_k141_74 | 1158 | No | NA | 0 | 1 | Low-quality | Genome-fragment | 0.75 | 0 |
| S11-final-viral-combined_k141_92058\|\|full | 1062 | No | NA | 0 | 1 | Not-determined | Genome-fragment | NA | 0 |
| S7-final-viral-combined_k141_202583\|\|lt2gene | 1158 | No | NA | 1 | 0 | Low-quality | Genome-fragment | 2.64 | 0 |
| S11-final-viral-combined_k141_37075\|\|full | 1347 | No | NA | 0 | 1 | Not-determined | Genome-fragment | NA | 0 |
| S11-final-viral-combined_k141_21950\|\|full | 4432 | No | NA | 0 | 6 | Not-determined | Genome-fragment | NA | 0 |
| S17-final-viral-combined_k141_22030\|\|full | 1158 | No | NA | 0 | 0 | Low-quality | Genome-fragment | 2.58 | 0 |
| S11-final-viral-combined_k141_325295\|\|full | 2238 | No | NA | 0 | 2 | Not-determined | Genome-fragment | NA | 0 |
| S11-final-viral-combined_k141_298381\|\|full | 1573 | No | NA | 0 | 0 | Not-determined | Genome-fragment | NA | 0 |
| S12-final-viral-combined_k141_298635\|\|full | 1158 | No | NA | 4 | 0 | Low-quality | Genome-fragment | 3.35 | 0 |
| S11-final-viral-combined_k141_147287\|\|full | 1219 | No | NA | 0 | 0 | Not-determined | Genome-fragment | NA | 0 |
| S11-final-viral-combined_k141_202201\|\|full | 1257 | No | NA | 0 | 2 | Not-determined | Genome-fragment | NA | 0 |
| S12_k141_154407 | 1158 | No | NA | 1 | 0 | Low-quality | Genome-fragment | 1.89 | 0 |
| S11-final-viral-combined_k141_202207\|\|full | 7291 | No | NA | 0 | 4 | Not-determined | Genome-fragment | NA | 0 |
| S11-final-viral-combined_k141_9897\|\|full | 1536 | No | NA | 0 | 2 | Not-determined | Genome-fragment | NA | 0 |
| S11-final-viral-combined_k141_257911\|\|full | 3067 | No | NA | 0 | 4 | Not-determined | Genome-fragment | NA | 0 |
| S2-final-viral-combined_k141_3888\|\|full | 1158 | No | NA | 1 | 0 | Low-quality | Genome-fragment | 2.49 | 0 |
| S11-final-viral-combined_k141_270995\|\|full | 2308 | No | NA | 0 | 0 | Not-determined | Genome-fragment | NA | 0 |
| S11-final-viral-combined_k141_257931\|\|full | 2201 | No | NA | 0 | 2 | Not-determined | Genome-fragment | NA | 0 |
| S2-final-viral-combined_k141_26346\|\|full | 1158 | No | NA | 1 | 0 | Low-quality | Genome-fragment | 1.72 | 0 |
| S2-final-viral-combined_k141_184830\|\|lt2gene | 1158 | No | NA | 1 | 0 | Low-quality | Genome-fragment | 2.91 | 0 |
| S4_k141_550 | 1158 | No | NA | 0 | 0 | Low-quality | Genome-fragment | 2.97 | 0 |
| S11-final-viral-combined_k141_119150\|\|full | 3434 | No | NA | 0 | 1 | Not-determined | Genome-fragment | NA | 0 |
| S4_k141_101770 | 1158 | No | NA | 0 | 1 | Low-quality | Genome-fragment | 3.14 | 0 |
| S11-final-viral-combined_k141_51036\|\|full | 1884 | No | NA | 0 | 2 | Not-determined | Genome-fragment | NA | 0 |
| S11-final-viral-combined_k141_51040\|\|full | 2345 | No | NA | 0 | 3 | Not-determined | Genome-fragment | NA | 0 |
| S11-final-viral-combined_k141_92114\|\|full | 48959 | Yes | 26924 | 7 | 13 | Medium-quality | Genome-fragment | 58.85 | 45.01 |
| S11-final-viral-combined_k141_313604\|\|full | 1159 | No | NA | 0 | 0 | Low-quality | Genome-fragment | 1.98 | 0 |
| S11-final-viral-combined_k141_202257\|\|full | 4943 | No | NA | 0 | 4 | Not-determined | Genome-fragment | NA | 0 |
| S11-final-viral-combined_k141_191509\|\|lt2gene | 1159 | No | NA | 1 | 0 | Low-quality | Genome-fragment | 1.93 | 0 |
| S1-final-viral-combined_k141_151228\|\|full | 1159 | No | NA | 0 | 0 | Low-quality | Genome-fragment | 2.18 | 0 |
| S1-final-viral-combined_k141_88160\|\|full | 1159 | No | NA | 0 | 0 | Low-quality | Genome-fragment | 2.49 | 0 |
| S1-final-viral-combined_k141_241218\|\|full | 1159 | No | NA | 1 | 0 | Low-quality | Genome-fragment | 2.98 | 0 |
| S1-final-viral-combined_k141_161477\|\|full | 1159 | No | NA | 0 | 1 | Low-quality | Genome-fragment | 3.19 | 0 |
| S1-final-viral-combined_k141_223079\|\|lt2gene | 1159 | No | NA | 1 | 0 | Low-quality | Genome-fragment | 1.06 | 0 |
| S3_k141_16872 | 1159 | No | NA | 0 | 1 | Low-quality | Genome-fragment | 1.57 | 0 |
| S5-final-viral-combined_k141_96000\|\|full | 1159 | No | NA | 1 | 0 | Low-quality | Genome-fragment | 0.75 | 0 |
| S11-final-viral-combined_k141_77799\|\|full | 2061 | No | NA | 0 | 2 | Not-determined | Genome-fragment | NA | 0 |
| S11-final-viral-combined_k141_92172\|\|full | 2155 | No | NA | 0 | 2 | Not-determined | Genome-fragment | NA | 0 |
| S11-final-viral-combined_k141_258048\|\|full | 1724 | No | NA | 0 | 0 | Not-determined | Genome-fragment | NA | 0 |
| S11-final-viral-combined_k141_119215\|\|full | 27769 | No | NA | 14 | 2 | Medium-quality | Genome-fragment | 73.83 | 0 |
| S11-final-viral-combined_k141_106590\|\|full | 1708 | No | NA | 0 | 2 | Not-determined | Genome-fragment | NA | 0 |
| S11-final-viral-combined_k141_119233\|\|full | 2005 | No | NA | 0 | 0 | Not-determined | Genome-fragment | NA | 0 |
| S11-final-viral-combined_k141_188358\|\|full | 4547 | No | NA | 0 | 3 | Not-determined | Genome-fragment | NA | 0 |
| S11-final-viral-combined_k141_216453\|\|full | 5389 | No | NA | 0 | 4 | Not-determined | Genome-fragment | NA | 0 |
| S7-final-viral-combined_k141_119201\|\|full | 1159 | No | NA | 0 | 0 | Low-quality | Genome-fragment | 1.22 | 0 |
| S11-final-viral-combined_k141_106610\|\|full | 1242 | No | NA | 0 | 0 | Not-determined | Genome-fragment | NA | 0 |
| S11-final-viral-combined_k141_230211\|\|full | 1068 | No | NA | 0 | 1 | Not-determined | Genome-fragment | NA | 0 |
| S11-final-viral-combined_k141_147471\|\|full | 1109 | No | NA | 0 | 1 | Not-determined | Genome-fragment | NA | 0 |
| S7-final-viral-combined_k141_9628\|\|lt2gene | 1159 | No | NA | 1 | 0 | Low-quality | Genome-fragment | 0.18 | 0 |
| S11-final-viral-combined_k141_298516\|\|full | 1726 | No | NA | 0 | 3 | Not-determined | Genome-fragment | NA | 0 |
| S11-final-viral-combined_k141_175235\|\|full | 3864 | No | NA | 0 | 1 | Not-determined | Genome-fragment | NA | 0 |
| S11-final-viral-combined_k141_92234\|\|full | 2810 | No | NA | 0 | 2 | Not-determined | Genome-fragment | NA | 0 |
| S7_k141_136557 | 1159 | No | NA | 0 | 1 | Low-quality | Genome-fragment | 0.59 | 0 |
| S7_k141_159273 | 1159 | No | NA | 0 | 0 | Low-quality | Genome-fragment | 0.48 | 0 |
| S11-final-viral-combined_k141_119250\|\|full | 3278 | No | NA | 0 | 2 | Not-determined | Genome-fragment | NA | 0 |
| S17-final-viral-combined_k141_90127\|\|full | 1159 | No | NA | 2 | 0 | Low-quality | Genome-fragment | 2.76 | 0 |
| S11-final-viral-combined_k141_202349\|\|full | 2955 | No | NA | 0 | 2 | Not-determined | Genome-fragment | NA | 0 |
| S11-final-viral-combined_k141_132591\|\|full | 5418 | No | NA | 0 | 1 | Not-determined | Genome-fragment | NA | 0 |
| S11-final-viral-combined_k141_230239\|\|full | 3186 | No | NA | 0 | 2 | Not-determined | Genome-fragment | NA | 0 |
| S17-final-viral-combined_k141_42955\|\|full | 1159 | No | NA | 0 | 0 | Low-quality | Genome-fragment | 2.31 | 0 |
| S11-final-viral-combined_k141_132604\|\|full | 3501 | No | NA | 0 | 0 | Not-determined | Genome-fragment | NA | 0 |
| S11-final-viral-combined_k141_325514\|\|full | 3886 | No | NA | 0 | 0 | Not-determined | Genome-fragment | NA | 0 |
| S17-final-viral-combined_k141_159988\|\|lt2gene | 1159 | No | NA | 2 | 0 | Low-quality | Genome-fragment | 3.83 | 0 |
| S11-final-viral-combined_k141_202383\|\|full | 5218 | No | NA | 0 | 4 | Not-determined | Genome-fragment | NA | 0 |

| | | | | | | | | | | |
|---|---|---|---|---|---|---|---|---|---|---|
| S12-final-viral-combined_k141_239852\|\|lt2gene | 1159 | No | NA | 1 | 0 | Low-quality | Genome-fragment | | 2.39 | 0 |
| S11-final-viral-combined_k141_271085\|\|full | 2928 | No | NA | 0 | 1 | Not-determined | Genome-fragment | NA | | 0 |
| S11-final-viral-combined_k141_51234\|\|full | 1728 | No | NA | 0 | 2 | Not-determined | Genome-fragment | NA | | 0 |
| S11-final-viral-combined_k141_271088\|\|full | 1958 | No | NA | 0 | 0 | Not-determined | Genome-fragment | NA | | 0 |
| S11-final-viral-combined_k141_92282\|\|full | 2399 | No | NA | 0 | 1 | Not-determined | Genome-fragment | NA | | 0 |
| S11-final-viral-combined_k141_202396\|\|full | 1371 | No | NA | 0 | 1 | Not-determined | Genome-fragment | NA | | 0 |
| S2_k141_47323 | 1159 | No | NA | 0 | 1 | Low-quality | Genome-fragment | | 0.55 | 0 |
| S8-final-viral-combined_k141_15694\|\|full | 1159 | No | NA | 0 | 0 | Low-quality | Genome-fragment | | 1.94 | 0 |
| S11-final-viral-combined_k141_202401\|\|full | 2760 | No | NA | 0 | 2 | Not-determined | Genome-fragment | NA | | 0 |
| S9_k141_63565 | 1160 | No | NA | 0 | 0 | Low-quality | Genome-fragment | | 2.87 | 0 |
| S11-final-viral-combined_k141_271097\|\|full | 1154 | No | NA | 0 | 1 | Not-determined | Genome-fragment | NA | | 0 |
| S11-final-viral-combined_k141_92308\|\|full | 1669 | No | NA | 0 | 2 | Not-determined | Genome-fragment | NA | | 0 |
| S11-final-viral-combined_k141_204555\|\|full | 1160 | No | NA | 0 | 0 | Low-quality | Genome-fragment | | 2.78 | 0 |
| S11-final-viral-combined_k141_64820\|\|full | 1679 | No | NA | 0 | 3 | Not-determined | Genome-fragment | NA | | 0 |
| S11-final-viral-combined_k141_313267\|\|full | 1000 | No | NA | 0 | 0 | Not-determined | Genome-fragment | NA | | 0 |
| S11-final-viral-combined_k141_291012\|\|lt2gene | 1160 | No | NA | 1 | 0 | Low-quality | Genome-fragment | | 1.52 | 0 |
| S11-final-viral-combined_k141_51270\|\|full | 3471 | No | NA | 0 | 1 | Not-determined | Genome-fragment | NA | | 0 |
| S11-final-viral-combined_k141_64832\|\|full | 1886 | No | NA | 0 | 2 | Not-determined | Genome-fragment | NA | | 0 |
| S11-final-viral-combined_k141_285651\|\|full | 3006 | No | NA | 0 | 1 | Not-determined | Genome-fragment | NA | | 0 |
| S11-final-viral-combined_k141_77906\|\|full | 2251 | No | NA | 0 | 2 | Not-determined | Genome-fragment | NA | | 0 |
| S11-final-viral-combined_k141_64878\|\|full | 4049 | No | NA | 0 | 5 | Not-determined | Genome-fragment | NA | | 0 |
| S1-final-viral-combined_k141_32380\|\|lt2gene | 1160 | No | NA | 1 | 0 | Low-quality | Genome-fragment | | 3.07 | 0 |
| S1_k141_213359 | 1160 | No | NA | 0 | 2 | Low-quality | Genome-fragment | | 0.88 | 0 |
| S3-final-viral-combined_k141_30952\|\|lt2gene | 1160 | No | NA | 1 | 0 | Low-quality | Genome-fragment | | 3 | 0 |
| S11-final-viral-combined_k141_161396\|\|full | 2317 | No | NA | 0 | 1 | Not-determined | Genome-fragment | NA | | 0 |
| S11-final-viral-combined_k141_258190\|\|full | 7507 | No | NA | 0 | 6 | Not-determined | Genome-fragment | NA | | 0 |
| S5_k141_190372 | 1160 | No | NA | 0 | 1 | Low-quality | Genome-fragment | | 1.03 | 0 |
| S11-final-viral-combined_k141_147637\|\|full | 4936 | No | NA | 0 | 2 | Not-determined | Genome-fragment | NA | | 0 |
| S11-final-viral-combined_k141_132714\|\|full | 3398 | No | NA | 0 | 1 | Not-determined | Genome-fragment | NA | | 0 |
| S17-final-viral-combined_k141_233749\|\|lt2gene | 1160 | No | NA | 1 | 0 | Low-quality | Genome-fragment | | 2.86 | 0 |
| S11-final-viral-combined_k141_147642\|\|full | 1426 | No | NA | 0 | 0 | Not-determined | Genome-fragment | NA | | 0 |
| S12-final-viral-combined_k141_380417\|\|full | 1160 | No | NA | 0 | 0 | Low-quality | Genome-fragment | | 1.75 | 0 |
| S8-final-viral-combined_k141_52757\|\|full | 1160 | No | NA | 0 | 0 | Low-quality | Genome-fragment | | 5.46 | 0 |
| S11-final-viral-combined_k141_161427\|\|full | 8073 | No | NA | 0 | 3 | Not-determined | Genome-fragment | NA | | 0 |
| S8-final-viral-combined_k141_78336\|\|full | 1160 | No | NA | 1 | 0 | Low-quality | Genome-fragment | | 2.14 | 0 |
| S9_k141_9380 | 1161 | No | NA | 0 | 2 | Low-quality | Genome-fragment | | 2.43 | 0 |
| S11_k141_311053 | 1161 | No | NA | 2 | 0 | Low-quality | Genome-fragment | | 2.47 | 0 |
| S11_k141_133361 | 1161 | No | NA | 0 | 0 | Low-quality | Genome-fragment | | 2.95 | 0 |
| S11-final-viral-combined_k141_132765\|\|full | 1155 | No | NA | 0 | 1 | Not-determined | Genome-fragment | NA | | 0 |
| S11-final-viral-combined_k141_64962\|\|full | 2617 | No | NA | 0 | 3 | Not-determined | Genome-fragment | NA | | 0 |
| S1-final-viral-combined_k141_24380\|\|full | 1161 | No | NA | 0 | 1 | Low-quality | Genome-fragment | | 2.73 | 0 |
| S11-final-viral-combined_k141_298764\|\|full | 7041 | No | NA | 0 | 4 | Not-determined | Genome-fragment | NA | | 0 |
| S11-final-viral-combined_k141_175474\|\|full | 2364 | No | NA | 0 | 2 | Not-determined | Genome-fragment | NA | | 0 |
| S11-final-viral-combined_k141_258291\|\|full | 1361 | No | NA | 0 | 0 | Not-determined | Genome-fragment | NA | | 0 |
| S11-final-viral-combined_k141_106846\|\|full | 2976 | No | NA | 0 | 2 | Not-determined | Genome-fragment | NA | | 0 |
| S1_k141_291706 | 1161 | No | NA | 0 | 0 | Low-quality | Genome-fragment | | 1.39 | 0 |
| S5-final-viral-combined_k141_2297\|\|full | 1161 | No | NA | 0 | 2 | Low-quality | Genome-fragment | | 0.65 | 0 |
| S7-final-viral-combined_k141_50240\|\|full | 1161 | No | NA | 0 | 0 | Low-quality | Genome-fragment | | 1.79 | 0 |
| S11-final-viral-combined_k141_78056\|\|full | 4060 | No | NA | 0 | 1 | Not-determined | Genome-fragment | NA | | 0 |
| S11-final-viral-combined_k141_64994\|\|full | 2752 | No | NA | 0 | 2 | Not-determined | Genome-fragment | NA | | 0 |
| S11-final-viral-combined_k141_161520\|\|full | 4379 | No | NA | 0 | 3 | Not-determined | Genome-fragment | NA | | 0 |
| S11-final-viral-combined_k141_202544\|\|full | 2510 | No | NA | 0 | 2 | Not-determined | Genome-fragment | NA | | 0 |
| S11-final-viral-combined_k141_65004\|\|full | 6394 | No | NA | 0 | 4 | Not-determined | Genome-fragment | NA | | 0 |
| S7-final-viral-combined_k141_160921\|\|lt2gene | 1161 | No | NA | 1 | 0 | Low-quality | Genome-fragment | | 1.7 | 0 |
| S11-final-viral-combined_k141_132826\|\|full | 1711 | No | NA | 0 | 2 | Not-determined | Genome-fragment | NA | | 0 |
| S17-final-viral-combined_k141_70231\|\|full | 1161 | No | NA | 0 | 1 | Low-quality | Genome-fragment | | 2.52 | 0 |
| S11-final-viral-combined_k141_78101\|\|full | 1491 | No | NA | 0 | 2 | Not-determined | Genome-fragment | NA | | 0 |
| S11-final-viral-combined_k141_22350\|\|full | 1184 | No | NA | 0 | 0 | Not-determined | Genome-fragment | NA | | 0 |
| S11-final-viral-combined_k141_119495\|\|full | 1748 | No | NA | 0 | 1 | Not-determined | Genome-fragment | NA | | 0 |
| S11-final-viral-combined_k141_298795\|\|full | 1504 | No | NA | 0 | 2 | Not-determined | Genome-fragment | NA | | 0 |
| S17-final-viral-combined_k141_73393\|\|full | 1161 | No | NA | 1 | 0 | Low-quality | Genome-fragment | | 2.42 | 0 |
| S11-final-viral-combined_k141_132854\|\|full | 1045 | No | NA | 0 | 1 | Not-determined | Genome-fragment | NA | | 0 |
| S11-final-viral-combined_k141_216811\|\|full | 2117 | No | NA | 0 | 3 | Not-determined | Genome-fragment | NA | | 0 |
| S11-final-viral-combined_k141_37534\|\|full | 5111 | No | NA | 0 | 1 | Not-determined | Genome-fragment | NA | | 0 |
| S12-final-viral-combined_k141_265119\|\|full | 1161 | No | NA | 0 | 0 | Low-quality | Genome-fragment | | 2.49 | 0 |
| S11-final-viral-combined_k141_37546\|\|full | 1492 | No | NA | 0 | 1 | Not-determined | Genome-fragment | NA | | 0 |
| S11-final-viral-combined_k141_313437\|\|full | 1116 | No | NA | 0 | 2 | Not-determined | Genome-fragment | NA | | 0 |
| S12-final-viral-combined_k141_13613\|\|lt2gene | 1161 | No | NA | 1 | 0 | Low-quality | Genome-fragment | | 2.61 | 0 |
| S2-final-viral-combined_k141_229361\|\|full | 1161 | No | NA | 0 | 0 | Low-quality | Genome-fragment | | 0.64 | 0 |
| S2-final-viral-combined_k141_189009\|\|full | 1161 | No | NA | 0 | 1 | Low-quality | Genome-fragment | | 0.71 | 0 |
| S11-final-viral-combined_k141_132895\|\|full | 10610 | No | NA | 0 | 7 | Not-determined | Genome-fragment | NA | | 0 |
| S8-final-viral-combined_k141_109685\|\|full | 1161 | No | NA | 0 | 0 | Low-quality | Genome-fragment | | 2.8 | 0 |
| S8_k141_258310 | 1161 | No | NA | 0 | 0 | Low-quality | Genome-fragment | | 2.63 | 0 |
| S9-final-viral-combined_k141_56932\|\|full | 1162 | No | NA | 0 | 0 | Low-quality | Genome-fragment | | 0.81 | 0 |
| S3-final-viral-combined_k141_37267\|\|full | 1162 | No | NA | 0 | 1 | Low-quality | Genome-fragment | | 0.42 | 0 |
| S5-final-viral-combined_k141_65518\|\|full | 1162 | No | NA | 0 | 0 | Low-quality | Genome-fragment | | 1.93 | 0 |
| S11-final-viral-combined_k141_78177\|\|full | 10409 | No | NA | 0 | 5 | Not-determined | Genome-fragment | NA | | 0 |
| S11-final-viral-combined_k141_92581\|\|full | 1269 | No | NA | 0 | 1 | Not-determined | Genome-fragment | NA | | 0 |
| S11-final-viral-combined_k141_22448\|\|full | 1406 | No | NA | 0 | 2 | Not-determined | Genome-fragment | NA | | 0 |
| S11-final-viral-combined_k141_244878\|\|full | 1148 | No | NA | 0 | 1 | Not-determined | Genome-fragment | NA | | 0 |
| S11-final-viral-combined_k141_325689\|\|full | 1791 | No | NA | 0 | 1 | Not-determined | Genome-fragment | NA | | 0 |
| S17-final-viral-combined_k141_6437\|\|full | 1162 | No | NA | 0 | 1 | Low-quality | Genome-fragment | | 0.93 | 0 |
| S11-final-viral-combined_k141_244885\|\|full | 2818 | No | NA | 0 | 2 | Not-determined | Genome-fragment | NA | | 0 |
| S11-final-viral-combined_k141_78203\|\|full | 12085 | No | NA | 0 | 10 | Not-determined | Genome-fragment | NA | | 0 |
| S12-final-viral-combined_k141_82658\|\|full | 1162 | No | NA | 0 | 1 | Low-quality | Genome-fragment | | 0.54 | 0 |
| S11-final-viral-combined_k141_92612\|\|full | 2229 | No | NA | 0 | 0 | Not-determined | Genome-fragment | NA | | 0 |
| S11-final-viral-combined_k141_106993\|\|full | 3344 | No | NA | 0 | 0 | Not-determined | Genome-fragment | NA | | 0 |
| S2_k141_192361 | 1162 | No | NA | 0 | 0 | Low-quality | Genome-fragment | | 2.12 | 0 |
| S11-final-viral-combined_k141_271415\|\|full | 1428 | No | NA | 0 | 2 | Not-determined | Genome-fragment | NA | | 0 |
| S4_k141_107021 | 1162 | No | NA | 0 | 1 | Low-quality | Genome-fragment | | 1.71 | 0 |
| S11-final-viral-combined_k141_298862\|\|full | 2146 | No | NA | 0 | 1 | Not-determined | Genome-fragment | NA | | 0 |
| S11-final-viral-combined_k141_313520\|\|full | 3493 | No | NA | 0 | 2 | Not-determined | Genome-fragment | NA | | 0 |
| S11-final-viral-combined_k141_188652\|\|full | 1744 | No | NA | 0 | 1 | Not-determined | Genome-fragment | NA | | 0 |
| S11-final-viral-combined_k141_10225\|\|full | 2503 | No | NA | 0 | 0 | Not-determined | Genome-fragment | NA | | 0 |
| S8-final-viral-combined_k141_55024\|\|full | 1162 | No | NA | 0 | 0 | Low-quality | Genome-fragment | | 0.74 | 0 |
| S8_k141_85761 | 1162 | No | NA | 0 | 0 | Low-quality | Genome-fragment | | 1.56 | 0 |
| S1-final-viral-combined_k141_104591\|\|lt2gene | 1163 | No | NA | 0 | 1 | Low-quality | Genome-fragment | | 6.93 | 0 |
| S5-final-viral-combined_k141_175117\|\|full | 1163 | No | NA | 2 | 0 | Low-quality | Genome-fragment | | 2.39 | 0 |
| S5_k141_33909 | 1163 | No | NA | 0 | 0 | Low-quality | Genome-fragment | | 0.47 | 0 |
| S7-final-viral-combined_k141_72393\|\|full | 1163 | No | NA | 1 | 0 | Low-quality | Genome-fragment | | 3.09 | 0 |
| S11-final-viral-combined_k141_202748\|\|full | 2379 | No | NA | 0 | 1 | Not-determined | Genome-fragment | NA | | 0 |
| S12-final-viral-combined_k141_309396\|\|full | 1163 | No | NA | 0 | 0 | Low-quality | Genome-fragment | | 2.48 | 0 |
| S6-final-viral-combined_k141_132470\|\|full | 1163 | No | NA | 0 | 1 | Low-quality | Genome-fragment | | 0.54 | 0 |
| S11-final-viral-combined_k141_313553\|\|full | 4095 | No | NA | 0 | 3 | Not-determined | Genome-fragment | NA | | 0 |
| S11-final-viral-combined_k141_216898\|\|full | 1209 | No | NA | 0 | 0 | Not-determined | Genome-fragment | NA | | 0 |
| S6_k141_19090 | 1163 | No | NA | 1 | 0 | Low-quality | Genome-fragment | | 3.56 | 0 |
| S11-final-viral-combined_k141_161719\|\|full | 2818 | No | NA | 0 | 2 | Not-determined | Genome-fragment | NA | | 0 |
| S6_k141_21461 | 1163 | No | NA | 0 | 1 | Low-quality | Genome-fragment | | 3.86 | 0 |
| S9-final-viral-combined_k141_57841\|\|full | 1164 | No | NA | 1 | 0 | Low-quality | Genome-fragment | | 2.08 | 0 |
| S11-final-viral-combined_k141_170146\|\|full | 1164 | No | NA | 0 | 0 | Low-quality | Genome-fragment | | 1.78 | 0 |
| S1-final-viral-combined_k141_102501\|\|full | 1164 | No | NA | 0 | 1 | Low-quality | Genome-fragment | | 1.42 | 0 |
| S11-final-viral-combined_k141_202796\|\|full | 2395 | No | NA | 0 | 1 | Not-determined | Genome-fragment | NA | | 0 |
| S1-final-viral-combined_k141_27965\|\|full | 1164 | No | NA | 0 | 0 | Low-quality | Genome-fragment | | 0.8 | 0 |
| S11-final-viral-combined_k141_107078\|\|full | 3355 | No | NA | 0 | 1 | Not-determined | Genome-fragment | NA | | 0 |
| S11-final-viral-combined_k141_147914\|\|full | 4338 | No | NA | 0 | 2 | Not-determined | Genome-fragment | NA | | 0 |
| S11-final-viral-combined_k141_65264\|\|full | 2830 | No | NA | 0 | 1 | Not-determined | Genome-fragment | NA | | 0 |
| S1_k141_292778 | 1164 | No | NA | 0 | 0 | Low-quality | Genome-fragment | | 2.43 | 0 |
| S11-final-viral-combined_k141_230669\|\|full | 2712 | No | NA | 0 | 2 | Not-determined | Genome-fragment | NA | | 0 |
| S5-final-viral-combined_k141_35864\|\|lt2gene | 1164 | No | NA | 1 | 0 | Low-quality | Genome-fragment | | 2.61 | 0 |
| S5_k141_110042 | 1164 | No | NA | 0 | 1 | Low-quality | Genome-fragment | | 0.74 | 0 |
| S6-final-viral-combined_k141_66055\|\|full | 1164 | No | NA | 2 | 0 | Low-quality | Genome-fragment | | 1.95 | 0 |
| S11-final-viral-combined_k141_328530\|\|full | 1165 | No | NA | 2 | 0 | Low-quality | Genome-fragment | | 3.39 | 0 |
| S11-final-viral-combined_k141_78354\|\|full | 2297 | No | NA | 0 | 2 | Not-determined | Genome-fragment | NA | | 0 |
| S11-final-viral-combined_k141_258156\|\|lt2gene | 1165 | No | NA | 1 | 0 | Low-quality | Genome-fragment | | 2.53 | 0 |
| S11-final-viral-combined_k141_325817\|\|full | 2866 | No | NA | 0 | 0 | Not-determined | Genome-fragment | NA | | 0 |
| S1-final-viral-combined_k141_162643\|\|full | 1165 | No | NA | 0 | 0 | Low-quality | Genome-fragment | | 2.49 | 0 |
| S11-final-viral-combined_k141_51768\|\|full | 7033 | No | NA | 0 | 3 | Not-determined | Genome-fragment | NA | | 0 |
| S11-final-viral-combined_k141_325821\|\|full | 3108 | No | NA | 0 | 2 | Not-determined | Genome-fragment | NA | | 0 |
| S11-final-viral-combined_k141_78380\|\|full | 1014 | No | NA | 0 | 0 | Not-determined | Genome-fragment | NA | | 0 |
| S11-final-viral-combined_k141_202859\|\|full | 2400 | No | NA | 0 | 0 | Not-determined | Genome-fragment | NA | | 0 |
| S5-final-viral-combined_k141_93876\|\|full | 1165 | No | NA | 1 | 0 | Low-quality | Genome-fragment | | 2.1 | 0 |
| S11-final-viral-combined_k141_188809\|\|full | 4400 | No | NA | 0 | 1 | Not-determined | Genome-fragment | NA | | 0 |
| S11-final-viral-combined_k141_147972\|\|full | 1574 | No | NA | 0 | 1 | Not-determined | Genome-fragment | NA | | 0 |
| S11-final-viral-combined_k141_51791\|\|full | 2345 | No | NA | 0 | 0 | Not-determined | Genome-fragment | NA | | 0 |
| S11-final-viral-combined_k141_299014\|\|full | 2029 | No | NA | 0 | 1 | Not-determined | Genome-fragment | NA | | 0 |
| S7_k141_32133 | 1165 | No | NA | 0 | 0 | Low-quality | Genome-fragment | | 2.58 | 0 |
| S17-final-viral-combined_k141_248504\|\|full | 1165 | No | NA | 0 | 2 | Low-quality | Genome-fragment | | 0.48 | 0 |
| S11-final-viral-combined_k141_299036\|\|full | 2012 | No | NA | 0 | 3 | Not-determined | Genome-fragment | NA | | 0 |
| S11-final-viral-combined_k141_37809\|\|full | 1084 | No | NA | 0 | 0 | Not-determined | Genome-fragment | NA | | 0 |
| S17-final-viral-combined_k141_180515\|\|lt2gene | 1165 | No | NA | 1 | 0 | Low-quality | Genome-fragment | | 2.45 | 0 |
| S12_k141_5436 | 1165 | No | NA | 0 | 0 | Low-quality | Genome-fragment | | 1.24 | 0 |
| S2-final-viral-combined_k141_219676\|\|full | 1165 | No | NA | 1 | 0 | Low-quality | Genome-fragment | | 2.81 | 0 |
| S11-final-viral-combined_k141_78453\|\|full | 2591 | No | NA | 0 | 0 | Not-determined | Genome-fragment | NA | | 0 |
| S11-final-viral-combined_k141_325889\|\|full | 6911 | No | NA | 0 | 3 | Not-determined | Genome-fragment | NA | | 0 |
| S11-final-viral-combined_k141_202958\|\|full | 2142 | No | NA | 0 | 2 | Not-determined | Genome-fragment | NA | | 0 |
| S2-final-viral-combined_k141_107128\|\|full | 1165 | No | NA | 0 | 0 | Low-quality | Genome-fragment | | 0.69 | 0 |
| S2_k141_122135 | 1165 | No | NA | 0 | 1 | Low-quality | Genome-fragment | | 2.41 | 0 |
| S11-final-viral-combined_k141_161836\|\|full | 2527 | No | NA | 0 | 2 | Not-determined | Genome-fragment | NA | | 0 |
| S6-final-viral-combined_k141_92592\|\|full | 1165 | No | NA | 0 | 0 | Low-quality | Genome-fragment | | 1.47 | 0 |
| S8-final-viral-combined_k141_222829\|\|full | 1165 | No | NA | 2 | 0 | Low-quality | Genome-fragment | | 1.25 | 0 |
| S11-final-viral-combined_k141_107223\|\|full | 2402 | No | NA | 0 | 1 | Not-determined | Genome-fragment | NA | | 0 |
| S9-final-viral-combined_k141_63461\|\|full | 1166 | No | NA | 1 | 0 | Low-quality | Genome-fragment | | 2.13 | 0 |
| S11-final-viral-combined_k141_313750\|\|full | 2903 | No | NA | 0 | 2 | Not-determined | Genome-fragment | NA | | 0 |
| S9-final-viral-combined_k141_55045\|\|lt2gene | 1166 | No | NA | 1 | 0 | Low-quality | Genome-fragment | | 2.87 | 0 |
| S11-final-viral-combined_k141_51877\|\|full | 5668 | No | NA | 0 | 4 | Not-determined | Genome-fragment | NA | | 0 |
| S11-final-viral-combined_k141_114811\|\|full | 1166 | No | NA | 0 | 1 | Low-quality | Genome-fragment | | 3.3 | 0 |
| S11-final-viral-combined_k141_51893\|\|full | 1132 | No | NA | 0 | 0 | Not-determined | Genome-fragment | NA | | 0 |
| S1-final-viral-combined_k141_132131\|\|full | 1166 | No | NA | 2 | 0 | Low-quality | Genome-fragment | | 2.77 | 0 |
| S11-final-viral-combined_k141_107248\|\|full | 2085 | No | NA | 0 | 1 | Not-determined | Genome-fragment | NA | | 0 |
| S11-final-viral-combined_k141_230836\|\|full | 1440 | No | NA | 0 | 0 | Not-determined | Genome-fragment | NA | | 0 |
| S1-final-viral-combined_k141_227209\|\|full | 1166 | No | NA | 0 | 2 | Low-quality | Genome-fragment | | 0.37 | 0 |
| S1-final-viral-combined_k141_98472\|\|full | 1166 | No | NA | 0 | 0 | Low-quality | Genome-fragment | | 0.69 | 0 |
| S5-final-viral-combined_k141_8651\|\|full | 1166 | No | NA | 1 | 0 | Low-quality | Genome-fragment | | 1.86 | 0 |
| S5_k141_83186 | 1166 | No | NA | 0 | 0 | Low-quality | Genome-fragment | | 2.37 | 0 |
| S9-final-viral-combined_k141_82483\|\|full | 1167 | No | NA | 0 | 1 | Low-quality | Genome-fragment | | 0.65 | 0 |
| S11_k141_151010 | 1167 | No | NA | 0 | 0 | Low-quality | Genome-fragment | | 2.15 | 0 |
| S1-final-viral-combined_k141_61896\|\|full | 1167 | No | NA | 0 | 0 | Low-quality | Genome-fragment | | 1.9 | 0 |
| S1_k141_70609 | 1167 | No | NA | 1 | 0 | Low-quality | Genome-fragment | | 1.92 | 0 |
| S11-final-viral-combined_k141_107280\|\|full | 1312 | No | NA | 0 | 0 | Not-determined | Genome-fragment | NA | | 0 |
| S5-final-viral-combined_k141_80306\|\|full | 1167 | No | NA | 1 | 0 | Low-quality | Genome-fragment | | 1.67 | 0 |
| S11-final-viral-combined_k141_133201\|\|full | 3030 | No | NA | 0 | 2 | Not-determined | Genome-fragment | NA | | 0 |
| S11-final-viral-combined_k141_161935\|\|full | 1045 | No | NA | 0 | 0 | Not-determined | Genome-fragment | NA | | 0 |
| S17-final-viral-combined_k141_206937\|\|lt2gene | 1167 | No | NA | 1 | 0 | Low-quality | Genome-fragment | | 3.48 | 0 |
| S11-final-viral-combined_k141_51946\|\|full | 9480 | No | NA | 0 | 4 | Not-determined | Genome-fragment | NA | | 0 |

| | | | | | | | | | | | |
|---|---|---|---|---|---|---|---|---|---|---|---|
| S11-final-viral-combined_k141_188983\|\|full | 1518 | No | NA | | 0 | 0 | Not-determined | Genome-fragment | NA | | 0 |
| S12-final-viral-combined_k141_88246\|\|full | 1167 | No | NA | | 0 | 2 | Low-quality | Genome-fragment | | 0.68 | 0 |
| S11-final-viral-combined_k141_313814\|\|full | 1343 | No | NA | | 0 | 0 | Not-determined | Genome-fragment | NA | | 0 |
| S12-final-viral-combined_k141_343602\|\|full | 1167 | No | NA | | 1 | 0 | Low-quality | Genome-fragment | | 1.53 | 0 |
| S11-final-viral-combined_k141_92934\|\|full | 1815 | No | NA | | 0 | 0 | Not-determined | Genome-fragment | NA | | 0 |
| S11-final-viral-combined_k141_299255\|\|full | 1297 | No | NA | | 0 | 2 | Not-determined | Genome-fragment | NA | | 0 |
| S12-final-viral-combined_k141_345117\|\|full | 1167 | No | NA | | 0 | 2 | Low-quality | Genome-fragment | | 0.06 | 0 |
| S8-final-viral-combined_k141_210\|\|full | 1167 | No | NA | | 0 | 0 | Low-quality | Genome-fragment | | 2.45 | 0 |
| S9-final-viral-combined_k141_33556\|\|lt2gene | 1168 | No | NA | | 1 | 0 | Low-quality | Genome-fragment | | 0.75 | 0 |
| S11-final-viral-combined_k141_319394\|\|full | 1168 | No | NA | | 1 | 0 | Low-quality | Genome-fragment | | 2.57 | 0 |
| S11-final-viral-combined_k141_299292\|\|full | 1978 | No | NA | | 0 | 1 | Not-determined | Genome-fragment | NA | | 0 |
| S11-final-viral-combined_k141_119944\|\|full | 3663 | No | NA | | 0 | 2 | Not-determined | Genome-fragment | NA | | 0 |
| S11-final-viral-combined_k141_258785\|\|full | 2268 | No | NA | | 0 | 1 | Not-determined | Genome-fragment | NA | | 0 |
| S11-final-viral-combined_k141_90103\|\|full | 1168 | No | NA | | 0 | 0 | Low-quality | Genome-fragment | | 2.01 | 0 |
| S11-final-viral-combined_k141_286138\|\|full | 2337 | No | NA | | 0 | 1 | Not-determined | Genome-fragment | NA | | 0 |
| S11-final-viral-combined_k141_299304\|\|full | 2123 | No | NA | | 0 | 0 | Not-determined | Genome-fragment | NA | | 0 |
| S1-final-viral-combined_k141_111992\|\|full | 1168 | No | NA | | 0 | 1 | Low-quality | Genome-fragment | | 0.71 | 0 |
| S1_k141_376664 | 1168 | No | NA | | 1 | 0 | Low-quality | Genome-fragment | | 0.59 | 0 |
| S5-final-viral-combined_k141_206022\|\|full | 1168 | No | NA | | 1 | 0 | Low-quality | Genome-fragment | | 2 | 0 |
| S5-final-viral-combined_k141_7867\|\|full | 1168 | No | NA | | 1 | 0 | Low-quality | Genome-fragment | | 2.45 | 0 |
| S11-final-viral-combined_k141_22874\|\|full | 4029 | No | NA | | 0 | 2 | Not-determined | Genome-fragment | NA | | 0 |
| S5-final-viral-combined_k141_94860\|\|full | 1168 | No | NA | | 0 | 1 | Low-quality | Genome-fragment | | 1.59 | 0 |
| S5-final-viral-combined_k141_137060\|\|lt2gene | 1168 | No | NA | | 2 | 0 | Low-quality | Genome-fragment | | 2.77 | 0 |
| S17-final-viral-combined_k141_78491\|\|full | 1168 | Yes | | 769 | 2 | 1 | Low-quality | Genome-fragment | | 1.59 | 34.16 |
| S17-final-viral-combined_k141_51250\|\|full | 1168 | No | NA | | 1 | 0 | Low-quality | Genome-fragment | | 2.83 | 0 |
| S11-final-viral-combined_k141_271772\|\|full | 4643 | No | NA | | 0 | 4 | Not-determined | Genome-fragment | NA | | 0 |
| S11-final-viral-combined_k141_22908\|\|full | 1571 | No | NA | | 0 | 2 | Not-determined | Genome-fragment | NA | | 0 |
| S11-final-viral-combined_k141_78633\|\|full | 2775 | No | NA | | 0 | 3 | Not-determined | Genome-fragment | NA | | 0 |
| S11-final-viral-combined_k141_258844\|\|full | 5543 | No | NA | | 0 | 3 | Not-determined | Genome-fragment | NA | | 0 |
| S11-final-viral-combined_k141_107400\|\|full | 3653 | No | NA | | 0 | 2 | Not-determined | Genome-fragment | NA | | 0 |
| S11-final-viral-combined_k141_245309\|\|full | 2514 | No | NA | | 0 | 2 | Not-determined | Genome-fragment | NA | | 0 |
| S11-final-viral-combined_k141_148191\|\|full | 6996 | No | NA | | 0 | 3 | Not-determined | Genome-fragment | NA | | 0 |
| S11-final-viral-combined_k141_120030\|\|full | 2932 | No | NA | | 0 | 0 | Not-determined | Genome-fragment | NA | | 0 |
| S11-final-viral-combined_k141_38161\|\|full | 3140 | No | NA | | 0 | 2 | Not-determined | Genome-fragment | NA | | 0 |
| S11-final-viral-combined_k141_286234\|\|full | 5886 | No | NA | | 0 | 4 | Not-determined | Genome-fragment | NA | | 0 |
| S11-final-viral-combined_k141_107461\|\|full | 2948 | No | NA | | 0 | 4 | Not-determined | Genome-fragment | NA | | 0 |
| S11-final-viral-combined_k141_52134\|\|full | 2643 | No | NA | | 0 | 0 | Not-determined | Genome-fragment | NA | | 0 |
| S11-final-viral-combined_k141_10605\|\|full | 1344 | No | NA | | 0 | 1 | Not-determined | Genome-fragment | NA | | 0 |
| S17-k141_222528 | 1168 | No | NA | | 0 | 0 | Low-quality | Genome-fragment | | 0.72 | 0 |
| S12-final-viral-combined_k141_331766\|\|full | 1168 | No | NA | | 0 | 1 | Low-quality | Genome-fragment | | 0.37 | 0 |
| S11-final-viral-combined_k141_22952\|\|full | 1505 | No | NA | | 0 | 1 | Not-determined | Genome-fragment | NA | | 0 |
| S2-final-viral-combined_k141_40290\|\|full | 1168 | No | NA | | 0 | 0 | Low-quality | Genome-fragment | | 2.54 | 0 |
| S11-final-viral-combined_k141_133385\|\|full | 1385 | No | NA | | 0 | 1 | Not-determined | Genome-fragment | NA | | 0 |
| S11-final-viral-combined_k141_231071\|\|full | 1529 | No | NA | | 0 | 0 | Not-determined | Genome-fragment | NA | | 0 |
| S11-final-viral-combined_k141_52162\|\|full | 1155 | No | NA | | 0 | 1 | Not-determined | Genome-fragment | NA | | 0 |
| S11-final-viral-combined_k141_133410\|\|full | 3031 | No | NA | | 0 | 1 | Not-determined | Genome-fragment | NA | | 0 |
| S11-final-viral-combined_k141_38224\|\|full | 2374 | No | NA | | 0 | 3 | Not-determined | Genome-fragment | NA | | 0 |
| S2_k141_148825 | 1168 | No | NA | | 0 | 0 | Low-quality | Genome-fragment | | 2.73 | 0 |
| S11-final-viral-combined_k141_217300\|\|full | 2111 | No | NA | | 0 | 2 | Not-determined | Genome-fragment | NA | | 0 |
| S11-final-viral-combined_k141_52209\|\|full | 2261 | No | NA | | 0 | 2 | Not-determined | Genome-fragment | NA | | 0 |
| S4_k141_166750 | 1168 | No | NA | | 2 | 0 | Low-quality | Genome-fragment | | 0.67 | 0 |
| S11-final-viral-combined_k141_258961\|\|full | 1665 | No | NA | | 0 | 1 | Not-determined | Genome-fragment | NA | | 0 |
| S11-final-viral-combined_k141_23003\|\|full | 2179 | No | NA | | 0 | 1 | Not-determined | Genome-fragment | NA | | 0 |
| S8-final-viral-combined_k141_184719\|\|lt2gene | 1168 | No | NA | | 1 | 0 | Low-quality | Genome-fragment | | 2.14 | 0 |
| S11-final-viral-combined_k141_299519\|\|full | 1423 | No | NA | | 0 | 2 | Not-determined | Genome-fragment | NA | | 0 |
| S11-final-viral-combined_k141_258998\|\|full | 2696 | No | NA | | 0 | 2 | Not-determined | Genome-fragment | NA | | 0 |
| S11-final-viral-combined_k141_52256\|\|full | 1178 | No | NA | | 0 | 0 | Not-determined | Genome-fragment | NA | | 0 |
| S11-final-viral-combined_k141_52257\|\|full | 4068 | No | NA | | 0 | 2 | Not-determined | Genome-fragment | NA | | 0 |
| S11-final-viral-combined_k141_65801\|\|full | 1230 | No | NA | | 0 | 0 | Not-determined | Genome-fragment | NA | | 0 |
| S8-final-viral-combined_k141_241257\|\|lt2gene | 1168 | No | NA | | 1 | 0 | Low-quality | Genome-fragment | | 1.1 | 0 |
| S11-final-viral-combined_k141_23019\|\|full | 1345 | No | NA | | 0 | 0 | Not-determined | Genome-fragment | NA | | 0 |
| S11-final-viral-combined_k141_93196\|\|full | 1640 | No | NA | | 0 | 1 | Not-determined | Genome-fragment | NA | | 0 |
| S9-final-viral-combined_k141_104791\|\|full | 1169 | No | NA | | 1 | 0 | Low-quality | Genome-fragment | | 2.79 | 0 |
| S11-final-viral-combined_k141_65829\|\|full | 10638 | No | NA | | 0 | 8 | Not-determined | Genome-fragment | NA | | 0 |
| S11-final-viral-combined_k141_326164\|\|full | 2250 | No | NA | | 0 | 2 | Not-determined | Genome-fragment | NA | | 0 |
| S9-final-viral-combined_k141_21289\|\|full | 1169 | No | NA | | 1 | 0 | Low-quality | Genome-fragment | | 2.54 | 0 |
| S11-final-viral-combined_k141_38308\|\|full | 1488 | No | NA | | 0 | 0 | Not-determined | Genome-fragment | NA | | 0 |
| S11-final-viral-combined_k141_133539\|\|full | 1058 | No | NA | | 0 | 1 | Not-determined | Genome-fragment | NA | | 0 |
| S11_k141_24683 | 1169 | No | NA | | 2 | 0 | Low-quality | Genome-fragment | | 2.06 | 0 |
| S1-final-viral-combined_k141_205908\|\|full | 1169 | No | NA | | 3 | 0 | Low-quality | Genome-fragment | | 2.49 | 0 |
| S1-final-viral-combined_k141_371924\|\|full | 1169 | No | NA | | 0 | 0 | Low-quality | Genome-fragment | | 1.58 | 0 |
| S11-final-viral-combined_k141_286378\|\|full | 2079 | No | NA | | 0 | 2 | Not-determined | Genome-fragment | NA | | 0 |
| S11-final-viral-combined_k141_148345\|\|full | 3428 | No | NA | | 0 | 2 | Not-determined | Genome-fragment | NA | | 0 |
| S11-final-viral-combined_k141_326184\|\|full | 1763 | No | NA | | 0 | 1 | Not-determined | Genome-fragment | NA | | 0 |
| S11-final-viral-combined_k141_78790\|\|full | 1032 | No | NA | | 0 | 1 | Not-determined | Genome-fragment | NA | | 0 |
| S11-final-viral-combined_k141_93262\|\|full | 2691 | No | NA | | 0 | 2 | Not-determined | Genome-fragment | NA | | 0 |
| S11-final-viral-combined_k141_148351\|\|full | 1415 | No | NA | | 0 | 1 | Not-determined | Genome-fragment | NA | | 0 |
| S1-final-viral-combined_k141_78730\|\|lt2gene | 1169 | No | NA | | 1 | 0 | Low-quality | Genome-fragment | | 2.05 | 0 |
| S11-final-viral-combined_k141_176210\|\|full | 12254 | No | NA | | 0 | 10 | Not-determined | Genome-fragment | NA | | 0 |
| S11-final-viral-combined_k141_162328\|\|full | 2803 | No | NA | | 0 | 2 | Not-determined | Genome-fragment | NA | | 0 |
| S5-final-viral-combined_k141_189616\|\|full | 1169 | No | NA | | 0 | 2 | Low-quality | Genome-fragment | | 0.53 | 0 |
| S7-final-viral-combined_k141_226015\|\|full | 1169 | No | NA | | 0 | 0 | Low-quality | Genome-fragment | | 2.97 | 0 |
| S11-final-viral-combined_k141_93325\|\|full | 3002 | No | NA | | 0 | 4 | Not-determined | Genome-fragment | NA | | 0 |
| S11-final-viral-combined_k141_148412\|\|full | 3186 | No | NA | | 0 | 2 | Not-determined | Genome-fragment | NA | | 0 |
| S11-final-viral-combined_k141_245608\|\|full | 3718 | No | NA | | 0 | 3 | Not-determined | Genome-fragment | NA | | 0 |
| S11-final-viral-combined_k141_326255\|\|full | 2117 | No | NA | | 0 | 0 | Not-determined | Genome-fragment | NA | | 0 |
| S17-final-viral-combined_k141_185368\|\|full | 1169 | No | NA | | 0 | 1 | Low-quality | Genome-fragment | | 2.55 | 0 |
| S11-final-viral-combined_k141_299603\|\|full | 1820 | No | NA | | 0 | 0 | Not-determined | Genome-fragment | NA | | 0 |
| S2-final-viral-combined_k141_88013\|\|full | 1169 | No | NA | | 0 | 1 | Low-quality | Genome-fragment | | 0.6 | 0 |
| S2-final-viral-combined_k141_195413\|\|lt2gene | 1169 | No | NA | | 1 | 0 | Low-quality | Genome-fragment | | 2.58 | 0 |
| S2_k141_125372 | 1169 | No | NA | | 0 | 3 | Low-quality | Genome-fragment | | 3.05 | 0 |
| S2_k141_234573 | 1169 | No | NA | | 0 | 3 | Low-quality | Genome-fragment | | 1 | 0 |
| S11-final-viral-combined_k141_314054\|\|full | 1078 | No | NA | | 0 | 1 | Not-determined | Genome-fragment | NA | | 0 |
| S4_k141_70074 | 1169 | No | NA | | 0 | 0 | Low-quality | Genome-fragment | | 1.64 | 0 |
| S11-final-viral-combined_k141_65983\|\|full | 1258 | No | NA | | 0 | 1 | Not-determined | Genome-fragment | NA | | 0 |
| S11-final-viral-combined_k141_299635\|\|full | 9977 | No | NA | | 0 | 2 | Not-determined | Genome-fragment | NA | | 0 |
| S11-final-viral-combined_k141_314066\|\|full | 3355 | No | NA | | 0 | 1 | Not-determined | Genome-fragment | NA | | 0 |
| S8_k141_147792 | 1169 | No | NA | | 0 | 0 | Low-quality | Genome-fragment | | 1.88 | 0 |
| S11-final-viral-combined_k141_93388\|\|full | 4997 | No | NA | | 0 | 3 | Not-determined | Genome-fragment | NA | | 0 |
| S8_k141_57259 | 1169 | No | NA | | 0 | 0 | Low-quality | Genome-fragment | | 2.25 | 0 |
| S9-final-viral-combined_k141_25771\|\|full | 1170 | No | NA | | 0 | 2 | Low-quality | Genome-fragment | | 1.96 | 0 |
| S9-final-viral-combined_k141_46702\|\|full | 1170 | No | NA | | 0 | 0 | Low-quality | Genome-fragment | | 0.8 | 0 |
| S9_k141_12982 | 1170 | No | NA | | 1 | 0 | Low-quality | Genome-fragment | | 2.56 | 0 |
| S11-final-viral-combined_k141_217499\|\|full | 5368 | No | NA | | 0 | 2 | Not-determined | Genome-fragment | NA | | 0 |
| S11-final-viral-combined_k141_176324\|\|full | 5945 | No | NA | | 0 | 5 | Not-determined | Genome-fragment | NA | | 0 |
| S11-final-viral-combined_k141_184028\|\|lt2gene | 1170 | No | NA | | 1 | 0 | Low-quality | Genome-fragment | | 2.54 | 0 |
| S11-final-viral-combined_k141_10780\|\|full | 2101 | No | NA | | 0 | 2 | Not-determined | Genome-fragment | NA | | 0 |
| S11-final-viral-combined_k141_25850\|\|lt2gene | 1170 | No | NA | | 0 | 0 | Low-quality | Genome-fragment | | 3.07 | 0 |
| S11_k141_245645 | 1170 | No | NA | | 0 | 0 | Low-quality | Genome-fragment | | 1.75 | 0 |
| S11-final-viral-combined_k141_286527\|\|full | 3901 | No | NA | | 0 | 2 | Not-determined | Genome-fragment | NA | | 0 |
| S1-final-viral-combined_k141_159835\|\|full | 1170 | No | NA | | 0 | 0 | Low-quality | Genome-fragment | | 2.75 | 0 |
| S11-final-viral-combined_k141_162474\|\|full | 2666 | No | NA | | 0 | 0 | Not-determined | Genome-fragment | NA | | 0 |
| S11-final-viral-combined_k141_66052\|\|full | 7901 | No | NA | | 0 | 6 | Not-determined | Genome-fragment | NA | | 0 |
| S1_k141_152318 | 1170 | No | NA | | 0 | 0 | Low-quality | Genome-fragment | | 2.09 | 0 |
| S1_k141_236392 | 1170 | No | NA | | 0 | 0 | Low-quality | Genome-fragment | | 2.23 | 0 |
| S11-final-viral-combined_k141_217555\|\|full | 1013 | No | NA | | 0 | 0 | Not-determined | Genome-fragment | NA | | 0 |
| S11-final-viral-combined_k141_217557\|\|full | 2425 | No | NA | | 0 | 0 | Not-determined | Genome-fragment | NA | | 0 |
| S5-final-viral-combined_k141_166393\|\|full | 1170 | No | NA | | 2 | 0 | Low-quality | Genome-fragment | | 2.69 | 0 |
| S11-final-viral-combined_k141_217568\|\|full | 1066 | No | NA | | 0 | 0 | Not-determined | Genome-fragment | NA | | 0 |
| S11-final-viral-combined_k141_259209\|\|full | 1274 | No | NA | | 0 | 0 | Not-determined | Genome-fragment | NA | | 0 |
| S11-final-viral-combined_k141_286549\|\|full | 3433 | No | NA | | 0 | 3 | Not-determined | Genome-fragment | NA | | 0 |
| S11-final-viral-combined_k141_176392\|\|full | 3156 | No | NA | | 0 | 2 | Not-determined | Genome-fragment | NA | | 0 |
| S5-final-viral-combined_k141_789\|\|lt2gene | 1170 | No | NA | | 1 | 0 | Low-quality | Genome-fragment | | 0.84 | 0 |
| S7-final-viral-combined_k141_130309\|\|full | 1170 | No | NA | | 0 | 0 | Low-quality | Genome-fragment | | 2.9 | 0 |
| S11-final-viral-combined_k141_314161\|\|full | 1610 | No | NA | | 0 | 1 | Not-determined | Genome-fragment | NA | | 0 |
| S11-final-viral-combined_k141_245701\|\|full | 1938 | No | NA | | 0 | 2 | Not-determined | Genome-fragment | NA | | 0 |
| S17-final-viral-combined_k141_20297\|\|full | 1170 | No | NA | | 1 | 0 | Low-quality | Genome-fragment | | 2.31 | 0 |
| S17-final-viral-combined_k141_197638\|\|lt2gene | 1170 | No | NA | | 1 | 0 | Low-quality | Genome-fragment | | 4.01 | 0 |
| S11-final-viral-combined_k141_148553\|\|full | 3622 | No | NA | | 0 | 3 | Not-determined | Genome-fragment | NA | | 0 |
| S2_k141_228768 | 1170 | No | NA | | 1 | 0 | Low-quality | Genome-fragment | | 1.15 | 0 |
| S11-final-viral-combined_k141_52476\|\|full | 2382 | No | NA | | 0 | 1 | Not-determined | Genome-fragment | NA | | 0 |
| S2_k141_90985 | 1170 | No | NA | | 0 | 0 | Low-quality | Genome-fragment | | 1 | 0 |
| S4-final-viral-combined_k141_16991\|\|full | 1170 | No | NA | | 0 | 0 | Low-quality | Genome-fragment | | 3.53 | 0 |
| S8-final-viral-combined_k141_156366\|\|full | 1170 | No | NA | | 0 | 0 | Low-quality | Genome-fragment | | 1.7 | 0 |
| S11-final-viral-combined_k141_326367\|\|full | 1495 | No | NA | | 0 | 2 | Not-determined | Genome-fragment | NA | | 0 |
| S9-final-viral-combined_k141_120263\|\|lt2gene | 1171 | No | NA | | 1 | 0 | Low-quality | Genome-fragment | | 1.53 | 0 |
| S11-final-viral-combined_k141_185615\|\|lt2gene | 1171 | No | NA | | 1 | 0 | Low-quality | Genome-fragment | | 2.7 | 0 |
| S11-final-viral-combined_k141_10923\|\|full | 1563 | No | NA | | 0 | 0 | Not-determined | Genome-fragment | NA | | 0 |
| S11-final-viral-combined_k141_217663\|\|lt2gene | 1171 | No | NA | | 2 | 0 | Low-quality | Genome-fragment | | 2.63 | 0 |
| S11-final-viral-combined_k141_107873\|\|full | 4457 | No | NA | | 0 | 2 | Not-determined | Genome-fragment | NA | | 0 |
| S3-final-viral-combined_k141_106458\|\|full | 1171 | No | NA | | 1 | 0 | Low-quality | Genome-fragment | | 2.64 | 0 |
| S5-final-viral-combined_k141_202083\|\|full | 1171 | No | NA | | 0 | 0 | Low-quality | Genome-fragment | | 2.89 | 0 |
| S17-final-viral-combined_k141_185396\|\|full | 1171 | No | NA | | 1 | 0 | Low-quality | Genome-fragment | | 1.66 | 0 |
| S12-final-viral-combined_k141_187898\|\|full | 1171 | No | NA | | 0 | 1 | Low-quality | Genome-fragment | | 2.51 | 0 |
| S2-final-viral-combined_k141_12552\|\|full | 1171 | No | NA | | 1 | 0 | Low-quality | Genome-fragment | | 2.66 | 0 |
| S11-final-viral-combined_k141_299828\|\|full | 2445 | No | NA | | 0 | 1 | Not-determined | Genome-fragment | NA | | 0 |
| S11-final-viral-combined_k141_259323\|\|full | 1130 | No | NA | | 0 | 2 | Not-determined | Genome-fragment | NA | | 0 |
| S11-final-viral-combined_k141_148618\|\|full | 2983 | No | NA | | 0 | 1 | Not-determined | Genome-fragment | NA | | 0 |
| S11-final-viral-combined_k141_38556\|\|full | 5955 | No | NA | | 0 | 4 | Not-determined | Genome-fragment | NA | | 0 |
| S2-final-viral-combined_k141_241261\|\|lt2gene | 1171 | No | NA | | 1 | 0 | Low-quality | Genome-fragment | | 2.47 | 0 |
| S11-final-viral-combined_k141_79002\|\|full | 4180 | No | NA | | 0 | 1 | Not-determined | Genome-fragment | NA | | 0 |
| S6-final-viral-combined_k141_126628\|\|lt2gene | 1171 | No | NA | | 0 | 0 | Low-quality | Genome-fragment | | 2.91 | 0 |
| S11-final-viral-combined_k141_120242\|\|full | 1342 | No | NA | | 0 | 1 | Not-determined | Genome-fragment | NA | | 0 |
| S11-final-viral-combined_k141_52586\|\|full | 1432 | No | NA | | 0 | 1 | Not-determined | Genome-fragment | NA | | 0 |
| S6-final-viral-combined_k141_121394\|\|lt2gene | 1171 | No | NA | | 1 | 0 | Low-quality | Genome-fragment | | 3.14 | 0 |
| S11-final-viral-combined_k141_286692\|\|full | 1772 | No | NA | | 0 | 1 | Not-determined | Genome-fragment | NA | | 0 |
| S11-final-viral-combined_k141_299858\|\|full | 2725 | No | NA | | 0 | 2 | Not-determined | Genome-fragment | NA | | 0 |
| S11-final-viral-combined_k141_231430\|\|full | 4783 | No | NA | | 0 | 1 | Not-determined | Genome-fragment | NA | | 0 |
| S11-final-viral-combined_k141_326483\|\|full | 2401 | No | NA | | 0 | 2 | Not-determined | Genome-fragment | NA | | 0 |
| S11-final-viral-combined_k141_314220\|\|full | 1026 | No | NA | | 0 | 2 | Not-determined | Genome-fragment | NA | | 0 |
| S8-final-viral-combined_k141_263253\|\|lt2gene | 1171 | No | NA | | 1 | 0 | Low-quality | Genome-fragment | | 0.76 | 0 |
| S11-final-viral-combined_k141_87047\|\|full | 1172 | No | NA | | 3 | 0 | Low-quality | Genome-fragment | | 2.47 | 0 |
| S1-final-viral-combined_k141_2758\|\|full | 1172 | No | NA | | 0 | 0 | Low-quality | Genome-fragment | | 0.65 | 0 |
| S11-final-viral-combined_k141_38625\|\|full | 4074 | No | NA | | 0 | 0 | Not-determined | Genome-fragment | NA | | 0 |
| S11-final-viral-combined_k141_217681\|\|full | 1108 | No | NA | | 0 | 2 | Not-determined | Genome-fragment | NA | | 0 |
| S11-final-viral-combined_k141_120257\|\|full | 35507 | Yes | | 27209 | 14 | 12 | Medium-quality | Genome-fragment | | 57.27 | 23.37 |
| S11-final-viral-combined_k141_272238\|\|full | 5660 | No | NA | | 0 | 1 | Not-determined | Genome-fragment | NA | | 0 |
| S1-final-viral-combined_k141_261273\|\|full | 1172 | No | NA | | 2 | 0 | Low-quality | Genome-fragment | | 1.6 | 0 |
| S1-final-viral-combined_k141_97527\|\|full | 1172 | No | NA | | 0 | 0 | Low-quality | Genome-fragment | | 2.83 | 0 |
| S11-final-viral-combined_k141_79075\|\|full | 2988 | No | NA | | 0 | 3 | Not-determined | Genome-fragment | NA | | 0 |
| S17-final-viral-combined_k141_89137\|\|full | 1172 | No | NA | | 0 | 0 | Low-quality | Genome-fragment | | 3.14 | 0 |
| S11-final-viral-combined_k141_314244\|\|full | 1838 | No | NA | | 0 | 2 | Not-determined | Genome-fragment | NA | | 0 |
| S11-final-viral-combined_k141_52713\|\|full | 1895 | No | NA | | 0 | 2 | Not-determined | Genome-fragment | NA | | 0 |
| S2-final-viral-combined_k141_79365\|\|full | 1172 | No | NA | | 1 | 0 | Low-quality | Genome-fragment | | 3.32 | 0 |

| | | | | | | | | | | | |
|---|---|---|---|---|---|---|---|---|---|---|---|
| S2-final-viral-combined_k141_175769\|\|full | 1172 | No | NA | | 0 | 1 | Low-quality | Genome-fragment | | 0.89 | 0 |
| S11-final-viral-combined_k141_52722\|\|full | 1438 | No | NA | | 0 | 1 | Not-determined | Genome-fragment | NA | | 0 |
| S4-final-viral-combined_k141_32145\|\|lt2gene | 1172 | No | NA | | 0 | 1 | Low-quality | Genome-fragment | | 1.27 | 0 |
| S8-final-viral-combined_k141_234012\|\|full | 1172 | No | NA | | 0 | 0 | Low-quality | Genome-fragment | | 2.52 | 0 |
| S11-final-viral-combined_k141_11116\|\|full | 2205 | No | NA | | 0 | 2 | Not-determined | Genome-fragment | NA | | 0 |
| S11-final-viral-combined_k141_299954\|\|full | 26312 | No | NA | | 7 | 0 | Medium-quality | Genome-fragment | | 70.68 | 0 |
| S11-final-viral-combined_k141_314271\|\|full | 1493 | No | NA | | 0 | 0 | Not-determined | Genome-fragment | NA | | 0 |
| S11-final-viral-combined_k141_286765\|\|full | 2758 | No | NA | | 0 | 2 | Not-determined | Genome-fragment | NA | | 0 |
| S11-final-viral-combined_k141_176572\|\|full | 4084 | No | NA | | 0 | 2 | Not-determined | Genome-fragment | NA | | 0 |
| S11-final-viral-combined_k141_176586\|\|full | 2459 | No | NA | | 0 | 0 | Not-determined | Genome-fragment | NA | | 0 |
| S11-final-viral-combined_k141_162800\|\|full | 1576 | No | NA | | 0 | 0 | Not-determined | Genome-fragment | NA | | 0 |
| S11-final-viral-combined_k141_314292\|\|full | 2791 | No | NA | | 0 | 1 | Not-determined | Genome-fragment | NA | | 0 |
| S8-final-viral-combined_k141_216775\|\|lt2gene | 1172 | No | NA | | 1 | 0 | Low-quality | Genome-fragment | | 2.54 | 0 |
| S11-final-viral-combined_k141_271676\|\|full | 1173 | No | NA | | 3 | 0 | Low-quality | Genome-fragment | | 2.53 | 0 |
| S1_k141_268405 | 1173 | No | NA | | 0 | 1 | Low-quality | Genome-fragment | | 0.47 | 0 |
| S1_k141_141330 | 1173 | No | NA | | 0 | 1 | Low-quality | Genome-fragment | | 3.31 | 0 |
| S11-final-viral-combined_k141_162850\|\|full | 2655 | No | NA | | 0 | 4 | Not-determined | Genome-fragment | NA | | 0 |
| S3_k141_58161 | 1173 | No | NA | | 0 | 0 | Low-quality | Genome-fragment | | 1.9 | 0 |
| S11-final-viral-combined_k141_176636\|\|full | 4554 | No | NA | | 0 | 1 | Not-determined | Genome-fragment | NA | | 0 |
| S5-final-viral-combined_k141_61727\|\|full | 1173 | No | NA | | 0 | 0 | Low-quality | Genome-fragment | | 2.99 | 0 |
| S5_k141_141932 | 1173 | No | NA | | 0 | 1 | Low-quality | Genome-fragment | | 0.32 | 0 |
| S11-final-viral-combined_k141_23496\|\|full | 1005 | No | NA | | 0 | 2 | Not-determined | Genome-fragment | NA | | 0 |
| S12-final-viral-combined_k141_251464\|\|full | 1173 | No | NA | | 1 | 0 | Low-quality | Genome-fragment | | 2.54 | 0 |
| S11-final-viral-combined_k141_120395\|\|full | 3673 | No | NA | | 0 | 3 | Not-determined | Genome-fragment | NA | | 0 |
| S11-final-viral-combined_k141_108088\|\|full | 3610 | No | NA | | 0 | 3 | Not-determined | Genome-fragment | NA | | 0 |
| S12-final-viral-combined_k141_3454\|\|full | 1173 | No | NA | | 0 | 0 | Low-quality | Genome-fragment | | 2.59 | 0 |
| S11-final-viral-combined_k141_52861\|\|full | 2944 | No | NA | | 0 | 1 | Not-determined | Genome-fragment | NA | | 0 |
| S12-final-viral-combined_k141_206205\|\|full | 1173 | No | NA | | 0 | 1 | Low-quality | Genome-fragment | | 1.14 | 0 |
| S12_k141_145657 | 1173 | No | NA | | 0 | 1 | Low-quality | Genome-fragment | | 0.77 | 0 |
| S11-final-viral-combined_k141_203779\|\|full | 1368 | No | NA | | 0 | 0 | Not-determined | Genome-fragment | NA | | 0 |
| S2_k141_151046 | 1173 | No | NA | | 0 | 0 | Low-quality | Genome-fragment | | 3.55 | 0 |
| S11-final-viral-combined_k141_259526\|\|full | 1555 | No | NA | | 0 | 2 | Not-determined | Genome-fragment | NA | | 0 |
| S9-final-viral-combined_k141_11877\|\|full | 1174 | No | NA | | 0 | 1 | Low-quality | Genome-fragment | | 0.92 | 0 |
| S9_k141_15183 | 1174 | No | NA | | 0 | 2 | Low-quality | Genome-fragment | | 0.63 | 0 |
| S5-final-viral-combined_k141_169526\|\|full | 1174 | No | NA | | 0 | 1 | Low-quality | Genome-fragment | | 3.1 | 0 |
| S11-final-viral-combined_k141_326648\|\|full | 1569 | No | NA | | 0 | 2 | Not-determined | Genome-fragment | NA | | 0 |
| S5_k141_53117 | 1174 | No | NA | | 0 | 1 | Low-quality | Genome-fragment | | 3 | 0 |
| S7-final-viral-combined_k141_39488\|\|lt2gene | 1174 | No | NA | | 1 | 0 | Low-quality | Genome-fragment | | 0.37 | 0 |
| S12-final-viral-combined_k141_2844\|\|full | 1174 | No | NA | | 0 | 2 | Low-quality | Genome-fragment | | 1.81 | 0 |
| S11-final-viral-combined_k141_108155\|\|full | 2332 | No | NA | | 0 | 1 | Not-determined | Genome-fragment | NA | | 0 |
| S12-final-viral-combined_k141_365411\|\|lt2gene | 1174 | No | NA | | 1 | 0 | Low-quality | Genome-fragment | | 1.51 | 0 |
| S2-final-viral-combined_k141_147301\|\|full | 1174 | No | NA | | 1 | 0 | Low-quality | Genome-fragment | | 2.56 | 0 |
| S11-final-viral-combined_k141_79302\|\|full | 2189 | No | NA | | 0 | 1 | Not-determined | Genome-fragment | NA | | 0 |
| S11-final-viral-combined_k141_108180\|\|full | 1168 | No | NA | | 0 | 0 | Not-determined | Genome-fragment | NA | | 0 |
| S11-final-viral-combined_k141_217790\|\|full | 54204 | Yes | | 40062 | 5 | 6 | Medium-quality | Genome-fragment | | 66.2 | 26.09 |
| S2-final-viral-combined_k141_128817\|\|full | 1174 | No | NA | | 2 | 0 | Low-quality | Genome-fragment | | 1.94 | 0 |
| S2-final-viral-combined_k141_245271\|\|full | 1174 | No | NA | | 1 | 0 | Low-quality | Genome-fragment | | 2.75 | 0 |
| S4-final-viral-combined_k141_122412\|\|full | 1174 | No | NA | | 1 | 0 | Low-quality | Genome-fragment | | 2.31 | 0 |
| S4_k141_107795 | 1174 | No | NA | | 0 | 1 | Low-quality | Genome-fragment | | 0.9 | 0 |
| S4_k141_117836 | 1174 | No | NA | | 0 | 1 | Low-quality | Genome-fragment | | 0.71 | 0 |
| S11-final-viral-combined_k141_148887\|\|full | 13818 | No | NA | | 0 | 6 | Not-determined | Genome-fragment | NA | | 0 |
| S11-final-viral-combined_k141_300173\|\|full | 3838 | No | NA | | 0 | 2 | Not-determined | Genome-fragment | NA | | 0 |
| S6-final-viral-combined_k141_3811\|\|full | 1174 | No | NA | | 1 | 0 | Low-quality | Genome-fragment | | 1.68 | 0 |
| S8-final-viral-combined_k141_163927\|\|full | 1174 | No | NA | | 0 | 0 | Low-quality | Genome-fragment | | 2.44 | 0 |
| S11-final-viral-combined_k141_38926\|\|full | 2361 | No | NA | | 0 | 0 | Not-determined | Genome-fragment | NA | | 0 |
| S8-final-viral-combined_k141_192886\|\|full | 1174 | No | NA | | 0 | 0 | Low-quality | Genome-fragment | | 2.05 | 0 |
| S11-final-viral-combined_k141_66410\|\|full | 2340 | No | NA | | 0 | 2 | Not-determined | Genome-fragment | NA | | 0 |
| S11-final-viral-combined_k141_300220\|\|full | 5841 | No | NA | | 0 | 1 | Not-determined | Genome-fragment | NA | | 0 |
| S11-final-viral-combined_k141_93844\|\|full | 2080 | No | NA | | 0 | 1 | Not-determined | Genome-fragment | NA | | 0 |
| S8_k141_84575 | 1174 | No | NA | | 1 | 0 | Low-quality | Genome-fragment | | 2.17 | 0 |
| S11-final-viral-combined_k141_231847\|\|full | 3277 | No | NA | | 0 | 3 | Not-determined | Genome-fragment | NA | | 0 |
| S11-final-viral-combined_k141_52991\|\|full | 2605 | No | NA | | 0 | 2 | Not-determined | Genome-fragment | NA | | 0 |
| S9-final-viral-combined_k141_43179\|\|full | 1175 | No | NA | | 0 | 0 | Low-quality | Genome-fragment | | 2.02 | 0 |
| S11-final-viral-combined_k141_246160\|\|full | 3172 | No | NA | | 0 | 3 | Not-determined | Genome-fragment | NA | | 0 |
| S11-final-viral-combined_k141_234411\|\|full | 1175 | No | NA | | 1 | 0 | Low-quality | Genome-fragment | | 2.1 | 0 |
| S11-final-viral-combined_k141_66488\|\|full | 1922 | No | NA | | 0 | 0 | Not-determined | Genome-fragment | NA | | 0 |
| S11-final-viral-combined_k141_163036\|\|full | 1810 | No | NA | | 0 | 1 | Not-determined | Genome-fragment | NA | | 0 |
| S11-final-viral-combined_k141_93898\|\|full | 2959 | No | NA | | 0 | 2 | Not-determined | Genome-fragment | NA | | 0 |
| S11-final-viral-combined_k141_189723\|\|full | 7594 | No | NA | | 0 | 7 | Not-determined | Genome-fragment | NA | | 0 |
| S11-final-viral-combined_k141_66558\|\|full | 2522 | No | NA | | 0 | 3 | Not-determined | Genome-fragment | NA | | 0 |
| S11-final-viral-combined_k141_23676\|\|full | 2425 | No | NA | | 0 | 3 | Not-determined | Genome-fragment | NA | | 0 |
| S11-final-viral-combined_k141_300270\|\|full | 1877 | No | NA | | 0 | 2 | Not-determined | Genome-fragment | NA | | 0 |
| S11-final-viral-combined_k141_120632\|\|full | 1499 | No | NA | | 0 | 0 | Not-determined | Genome-fragment | NA | | 0 |
| S11-final-viral-combined_k141_302505\|\|full | 1175 | No | NA | | 0 | 0 | Low-quality | Genome-fragment | | 0.92 | 0 |
| S1-final-viral-combined_k141_38241\|\|full | 1175 | No | NA | | 1 | 0 | Low-quality | Genome-fragment | | 0.47 | 0 |
| S1_k141_366987 | 1175 | No | NA | | 0 | 0 | Low-quality | Genome-fragment | | 1.76 | 0 |
| S11-final-viral-combined_k141_259765\|\|full | 1340 | No | NA | | 0 | 1 | Not-determined | Genome-fragment | NA | | 0 |
| S11-final-viral-combined_k141_38992\|\|full | 1122 | No | NA | | 0 | 1 | Not-determined | Genome-fragment | NA | | 0 |
| S1_k141_246642 | 1175 | No | NA | | 0 | 1 | Low-quality | Genome-fragment | | 2.68 | 0 |
| S11-final-viral-combined_k141_39009\|\|full | 2521 | No | NA | | 0 | 2 | Not-determined | Genome-fragment | NA | | 0 |
| S11-final-viral-combined_k141_93928\|\|full | 5712 | No | NA | | 0 | 6 | Not-determined | Genome-fragment | NA | | 0 |
| S3-final-viral-combined_k141_51419\|\|full | 1175 | No | NA | | 0 | 0 | Low-quality | Genome-fragment | | 2.7 | 0 |
| S3-final-viral-combined_k141_17879\|\|lt2gene | 1175 | No | NA | | 0 | 0 | Low-quality | Genome-fragment | | 3.22 | 0 |
| S11-final-viral-combined_k141_189779\|\|full | 20977 | No | NA | | 0 | 13 | Not-determined | Genome-fragment | NA | | 0 |
| S5-final-viral-combined_k141_50421\|\|lt2gene | 1175 | No | NA | | 1 | 0 | Low-quality | Genome-fragment | | 2.84 | 0 |
| S11-final-viral-combined_k141_272702\|\|full | 1010 | No | NA | | 0 | 1 | Not-determined | Genome-fragment | NA | | 0 |
| S11-final-viral-combined_k141_53200\|\|full | 7248 | No | NA | | 0 | 1 | Not-determined | Genome-fragment | NA | | 0 |
| S11-final-viral-combined_k141_120734\|\|full | 1859 | No | NA | | 0 | 1 | Not-determined | Genome-fragment | NA | | 0 |
| S11-final-viral-combined_k141_149038\|\|full | 1432 | No | NA | | 0 | 1 | Not-determined | Genome-fragment | NA | | 0 |
| S7-final-viral-combined_k141_128569\|\|full | 1175 | No | NA | | 0 | 0 | Low-quality | Genome-fragment | | 1.31 | 0 |
| S11-final-viral-combined_k141_39068\|\|full | 5045 | No | NA | | 0 | 2 | Not-determined | Genome-fragment | NA | | 0 |
| S7-final-viral-combined_k141_222100\|\|full | 1175 | No | NA | | 1 | 0 | Low-quality | Genome-fragment | | 2.76 | 0 |
| S7_k141_219413 | 1175 | No | NA | | 1 | 0 | Low-quality | Genome-fragment | | 0.74 | 0 |
| S11-final-viral-combined_k141_11421\|\|full | 1180 | No | NA | | 0 | 0 | Not-determined | Genome-fragment | NA | | 0 |
| S11-final-viral-combined_k141_11424\|\|full | 5971 | No | NA | | 0 | 5 | Not-determined | Genome-fragment | NA | | 0 |
| S11-final-viral-combined_k141_189844\|\|full | 10912 | No | NA | | 0 | 6 | Not-determined | Genome-fragment | NA | | 0 |
| S11-final-viral-combined_k141_326909\|\|full | 5257 | No | NA | | 0 | 3 | Not-determined | Genome-fragment | NA | | 0 |
| S17-final-viral-combined_k141_24759\|\|lt2gene | 1175 | No | NA | | 2 | 0 | Low-quality | Genome-fragment | | 3.13 | 0 |
| S17-k141_115246 | 1175 | No | NA | | 0 | 0 | Low-quality | Genome-fragment | | 0.19 | 0 |
| S11-final-viral-combined_k141_287220\|\|full | 1270 | No | NA | | 0 | 0 | Not-determined | Genome-fragment | NA | | 0 |
| S11-final-viral-combined_k141_134462\|\|full | 1721 | No | NA | | 0 | 2 | Not-determined | Genome-fragment | NA | | 0 |
| S11-final-viral-combined_k141_300466\|\|full | 3280 | No | NA | | 0 | 3 | Not-determined | Genome-fragment | NA | | 0 |
| S12-final-viral-combined_k141_376910\|\|full | 1175 | No | NA | | 0 | 0 | Low-quality | Genome-fragment | | 2.15 | 0 |
| S11-final-viral-combined_k141_176995\|\|full | 2799 | No | NA | | 0 | 1 | Not-determined | Genome-fragment | NA | | 0 |
| S11-final-viral-combined_k141_66735\|\|full | 3356 | No | NA | | 0 | 3 | Not-determined | Genome-fragment | NA | | 0 |
| S11-final-viral-combined_k141_189869\|\|full | 1058 | No | NA | | 0 | 1 | Not-determined | Genome-fragment | NA | | 0 |
| S11-final-viral-combined_k141_232051\|\|full | 3535 | No | NA | | 0 | 2 | Not-determined | Genome-fragment | NA | | 0 |
| S11-final-viral-combined_k141_108513\|\|full | 2955 | No | NA | | 0 | 2 | Not-determined | Genome-fragment | NA | | 0 |
| S11-final-viral-combined_k141_120785\|\|full | 1363 | No | NA | | 0 | 0 | Not-determined | Genome-fragment | NA | | 0 |
| S11-final-viral-combined_k141_108514\|\|full | 4966 | No | NA | | 0 | 2 | Not-determined | Genome-fragment | NA | | 0 |
| S11-final-viral-combined_k141_177022\|\|full | 1248 | No | NA | | 0 | 1 | Not-determined | Genome-fragment | NA | | 0 |
| S12-final-viral-combined_k141_399617\|\|full | 1175 | No | NA | | 0 | 0 | Low-quality | Genome-fragment | | 2.68 | 0 |
| S2_k141_104226 | 1175 | No | NA | | 0 | 0 | Low-quality | Genome-fragment | | 3.26 | 0 |
| S4_k141_75956 | 1175 | No | NA | | 1 | 0 | Low-quality | Genome-fragment | | 1.19 | 0 |
| S8-final-viral-combined_k141_186354\|\|full | 1175 | No | NA | | 2 | 0 | Low-quality | Genome-fragment | | 0.86 | 0 |
| S11-final-viral-combined_k141_149186\|\|full | 1141 | No | NA | | 0 | 1 | Not-determined | Genome-fragment | NA | | 0 |
| S9-final-viral-combined_k141_98560\|\|full | 1176 | No | NA | | 0 | 0 | Low-quality | Genome-fragment | | 0.44 | 0 |
| S9-final-viral-combined_k141_40981\|\|full | 1176 | No | NA | | 2 | 0 | Low-quality | Genome-fragment | | 3.85 | 0 |
| S9-final-viral-combined_k141_113251\|\|lt2gene | 1176 | No | NA | | 1 | 0 | Low-quality | Genome-fragment | | 2.49 | 0 |
| S11-final-viral-combined_k141_301502\|\|full | 1176 | No | NA | | 3 | 0 | Low-quality | Genome-fragment | | 1.87 | 0 |
| S1-final-viral-combined_k141_11153\|\|full | 1176 | No | NA | | 0 | 0 | Low-quality | Genome-fragment | | 1.54 | 0 |
| S3-final-viral-combined_k141_126242\|\|lt2gene | 1176 | No | NA | | 1 | 0 | Low-quality | Genome-fragment | | 1.65 | 0 |
| S7-final-viral-combined_k141_237370\|\|full | 1176 | No | NA | | 0 | 0 | Low-quality | Genome-fragment | | 1 | 0 |
| S7-final-viral-combined_k141_187254\|\|full | 1176 | No | NA | | 0 | 0 | Low-quality | Genome-fragment | | 1.14 | 0 |
| S12-final-viral-combined_k141_18122\|\|lt2gene | 1176 | No | NA | | 2 | 0 | Low-quality | Genome-fragment | | 3.14 | 0 |
| S11-final-viral-combined_k141_11572\|\|full | 2683 | No | NA | | 0 | 3 | Not-determined | Genome-fragment | NA | | 0 |
| S11-final-viral-combined_k141_149267\|\|full | 2734 | No | NA | | 0 | 3 | Not-determined | Genome-fragment | NA | | 0 |
| S12_k141_78446 | 1176 | No | NA | | 0 | 1 | Low-quality | Genome-fragment | | 0.77 | 0 |
| S11-final-viral-combined_k141_163336\|\|full | 1607 | No | NA | | 0 | 1 | Not-determined | Genome-fragment | NA | | 0 |
| S2-final-viral-combined_k141_177677\|\|lt2gene | 1176 | No | NA | | 1 | 0 | Low-quality | Genome-fragment | | 1.32 | 0 |
| S11-final-viral-combined_k141_23942\|\|full | 2155 | No | NA | | 0 | 2 | Not-determined | Genome-fragment | NA | | 0 |
| S11-final-viral-combined_k141_177123\|\|full | 1972 | No | NA | | 0 | 0 | Not-determined | Genome-fragment | NA | | 0 |
| S8-final-viral-combined_k141_90048\|\|full | 1176 | No | NA | | 0 | 1 | Low-quality | Genome-fragment | | 2.03 | 0 |
| S11-final-viral-combined_k141_23949\|\|full | 2660 | No | NA | | 0 | 2 | Not-determined | Genome-fragment | NA | | 0 |
| S11-final-viral-combined_k141_39279\|\|full | 2899 | No | NA | | 0 | 1 | Not-determined | Genome-fragment | NA | | 0 |
| S8-final-viral-combined_k141_119208\|\|lt2gene | 1176 | No | NA | | 1 | 0 | Low-quality | Genome-fragment | | 2.13 | 0 |
| S11-final-viral-combined_k141_53292\|\|full | 3475 | No | NA | | 0 | 3 | Not-determined | Genome-fragment | NA | | 0 |
| S11-final-viral-combined_k141_79765\|\|full | 1297 | No | NA | | 0 | 0 | Not-determined | Genome-fragment | NA | | 0 |
| S11-final-viral-combined_k141_120946\|\|full | 1240 | No | NA | | 0 | 0 | Not-determined | Genome-fragment | NA | | 0 |
| S11-final-viral-combined_k141_246608\|\|full | 1079 | No | NA | | 0 | 1 | Not-determined | Genome-fragment | NA | | 0 |
| S11-final-viral-combined_k141_39310\|\|full | 2144 | No | NA | | 0 | 2 | Not-determined | Genome-fragment | NA | | 0 |
| S8_k141_269704 | 1176 | No | NA | | 0 | 0 | Low-quality | Genome-fragment | | 0.69 | 0 |
| S11-final-viral-combined_k141_79794\|\|full | 1192 | No | NA | | 0 | 1 | Not-determined | Genome-fragment | NA | | 0 |
| S9-final-viral-combined_k141_74455\|\|full | 1177 | No | NA | | 0 | 0 | Low-quality | Genome-fragment | | 1.8 | 0 |
| S11-final-viral-combined_k141_200093\|\|full | 1177 | No | NA | | 0 | 0 | Low-quality | Genome-fragment | | 2.65 | 0 |
| S1-final-viral-combined_k141_49114\|\|full | 1177 | No | NA | | 3 | 0 | Low-quality | Genome-fragment | | 3.05 | 0 |
| S11-final-viral-combined_k141_189966\|\|full | 1248 | No | NA | | 0 | 1 | Not-determined | Genome-fragment | NA | | 0 |
| S1-final-viral-combined_k141_336052\|\|lt2gene | 1177 | No | NA | | 1 | 0 | Low-quality | Genome-fragment | | 2.61 | 0 |
| S11-final-viral-combined_k141_218090\|\|full | 1508 | No | NA | | 0 | 0 | Not-determined | Genome-fragment | NA | | 0 |
| S11-final-viral-combined_k141_314613\|\|full | 1103 | No | NA | | 0 | 0 | Not-determined | Genome-fragment | NA | | 0 |
| S11-final-viral-combined_k141_190000\|\|full | 3884 | No | NA | | 0 | 0 | Not-determined | Genome-fragment | NA | | 0 |
| S11-final-viral-combined_k141_314635\|\|full | 5451 | No | NA | | 0 | 1 | Not-determined | Genome-fragment | NA | | 0 |
| S11-final-viral-combined_k141_108744\|\|full | 7120 | No | NA | | 0 | 1 | Not-determined | Genome-fragment | NA | | 0 |
| S11-final-viral-combined_k141_287393\|\|full | 1190 | No | NA | | 0 | 2 | Not-determined | Genome-fragment | NA | | 0 |
| S1_k141_346805 | 1177 | No | NA | | 0 | 0 | Low-quality | Genome-fragment | | 2.29 | 0 |
| S11-final-viral-combined_k141_24088\|\|full | 2916 | No | NA | | 0 | 2 | Not-determined | Genome-fragment | NA | | 0 |
| S3_k141_80913 | 1177 | No | NA | | 0 | 1 | Low-quality | Genome-fragment | | 0.76 | 0 |
| S11-final-viral-combined_k141_177232\|\|full | 2724 | No | NA | | 0 | 3 | Not-determined | Genome-fragment | NA | | 0 |
| S7_k141_32103 | 1177 | No | NA | | 0 | 0 | Low-quality | Genome-fragment | | 1.92 | 0 |
| S11-final-viral-combined_k141_163470\|\|full | 5623 | No | NA | | 0 | 4 | Not-determined | Genome-fragment | NA | | 0 |
| S7_k141_197928 | 1177 | No | NA | | 0 | 0 | Low-quality | Genome-fragment | | 0.96 | 0 |
| S11-final-viral-combined_k141_190052\|\|full | 1597 | No | NA | | 0 | 0 | Not-determined | Genome-fragment | NA | | 0 |
| S17-final-viral-combined_k141_56950\|\|full | 1177 | No | NA | | 0 | 0 | Low-quality | Genome-fragment | | 2.79 | 0 |
| S17-final-viral-combined_k141_115570\|\|full | 1177 | No | NA | | 0 | 1 | Low-quality | Genome-fragment | | 0.98 | 0 |
| S12-final-viral-combined_k141_322764\|\|full | 1177 | No | NA | | 0 | 2 | Low-quality | Genome-fragment | | 2.22 | 0 |
| S2_k141_219310 | 1177 | No | NA | | 0 | 0 | Low-quality | Genome-fragment | | 3.55 | 0 |
| S11-final-viral-combined_k141_149463\|\|full | 3628 | No | NA | | 0 | 3 | Not-determined | Genome-fragment | NA | | 0 |
| S11-final-viral-combined_k141_300632\|\|full | 1971 | No | NA | | 0 | 2 | Not-determined | Genome-fragment | NA | | 0 |
| S11-final-viral-combined_k141_163520\|\|full | 2002 | No | NA | | 0 | 0 | Not-determined | Genome-fragment | NA | | 0 |
| S11-final-viral-combined_k141_134772\|\|full | 1489 | No | NA | | 0 | 1 | Not-determined | Genome-fragment | NA | | 0 |
| S11-final-viral-combined_k141_24156\|\|full | 1089 | No | NA | | 0 | 0 | Not-determined | Genome-fragment | NA | | 0 |
| S8-final-viral-combined_k141_97281\|\|full | 1177 | No | NA | | 1 | 0 | Low-quality | Genome-fragment | | 2.02 | 0 |
| S8_k141_37330 | 1177 | No | NA | | 1 | 0 | Low-quality | Genome-fragment | | 1.91 | 0 |
| S9-final-viral-combined_k141_33112\|\|full | 1178 | No | NA | | 1 | 0 | Low-quality | Genome-fragment | | 2.62 | 0 |

| | | | | | | | | | | |
|---|---|---|---|---|---|---|---|---|---|---|
| S11-final-viral-combined_k141_94135\|\|full | 1889 | No | NA | 0 | 1 | Not-determined | Genome-fragment | NA | | 0 |
| S9-final-viral-combined_k141_33313\|\|full | 1178 | No | NA | 0 | 0 | Low-quality | Genome-fragment | | 2.85 | 0 |
| S11-final-viral-combined_k141_287511\|\|full | 1662 | No | NA | 0 | 1 | Not-determined | Genome-fragment | NA | | 0 |
| S11-final-viral-combined_k141_207475\|\|full | 1178 | No | NA | 0 | 1 | Low-quality | Genome-fragment | | 0.73 | 0 |
| S11-final-viral-combined_k141_10419\|\|full | 1178 | No | NA | 0 | 0 | Low-quality | Genome-fragment | | 2.12 | 0 |
| S1-final-viral-combined_k141_327445\|\|full | 1178 | No | NA | 0 | 2 | Low-quality | Genome-fragment | | 2.16 | 0 |
| S11-final-viral-combined_k141_149549\|\|full | 1604 | No | NA | 0 | 1 | Not-determined | Genome-fragment | NA | | 0 |
| S5-final-viral-combined_k141_9588\|\|full | 1178 | No | NA | 1 | 0 | Low-quality | Genome-fragment | | 2.15 | 0 |
| S11-final-viral-combined_k141_327235\|\|full | 1106 | No | NA | 0 | 1 | Not-determined | Genome-fragment | NA | | 0 |
| S11-final-viral-combined_k141_67020\|\|full | 1961 | No | NA | 0 | 1 | Not-determined | Genome-fragment | NA | | 0 |
| S11-final-viral-combined_k141_218247\|\|full | 1220 | No | NA | 0 | 0 | Not-determined | Genome-fragment | NA | | 0 |
| S11-final-viral-combined_k141_287555\|\|full | 4921 | No | NA | 0 | 0 | Not-determined | Genome-fragment | NA | | 0 |
| S5-final-viral-combined_k141_110414\|\|full | 1178 | No | NA | 1 | 0 | Low-quality | Genome-fragment | | 1.85 | 0 |
| S11-final-viral-combined_k141_149563\|\|full | 9238 | No | NA | 0 | 5 | Not-determined | Genome-fragment | NA | | 0 |
| S5-final-viral-combined_k141_117603\|\|lt2gene | 1178 | No | NA | 1 | 0 | Low-quality | Genome-fragment | | 2.23 | 0 |
| S11-final-viral-combined_k141_11859\|\|full | 1098 | No | NA | 0 | 0 | Not-determined | Genome-fragment | NA | | 0 |
| S11-final-viral-combined_k141_218283\|\|full | 1329 | No | NA | 0 | 0 | Not-determined | Genome-fragment | NA | | 0 |
| S11-final-viral-combined_k141_149569\|\|full | 5724 | No | NA | 0 | 3 | Not-determined | Genome-fragment | NA | | 0 |
| S11-final-viral-combined_k141_300761\|\|full | 3392 | No | NA | 0 | 3 | Not-determined | Genome-fragment | NA | | 0 |
| S11-final-viral-combined_k141_232358\|\|full | 1286 | No | NA | 0 | 1 | Not-determined | Genome-fragment | NA | | 0 |
| S11-final-viral-combined_k141_300762\|\|full | 4623 | No | NA | 0 | 5 | Not-determined | Genome-fragment | NA | | 0 |
| S5-final-viral-combined_k141_129991\|\|lt2gene | 1178 | No | NA | 2 | 0 | Low-quality | Genome-fragment | | 2.01 | 0 |
| S11-final-viral-combined_k141_149573\|\|full | 1785 | No | NA | 0 | 1 | Not-determined | Genome-fragment | NA | | 0 |
| S11-final-viral-combined_k141_94206\|\|full | 1225 | No | NA | 0 | 0 | Not-determined | Genome-fragment | NA | | 0 |
| S11-final-viral-combined_k141_218294\|\|full | 1241 | No | NA | 0 | 0 | Not-determined | Genome-fragment | NA | | 0 |
| S11-final-viral-combined_k141_300777\|\|full | 2895 | No | NA | 0 | 3 | Not-determined | Genome-fragment | NA | | 0 |
| S7-final-viral-combined_k141_221855\|\|full | 1178 | No | NA | 0 | 0 | Low-quality | Genome-fragment | | 1.24 | 0 |
| S7-final-viral-combined_k141_243737\|\|full | 1178 | No | NA | 0 | 1 | Low-quality | Genome-fragment | | 2.52 | 0 |
| S7_k141_5955 | 1178 | No | NA | 0 | 0 | Low-quality | Genome-fragment | | 2.87 | 0 |
| S11-final-viral-combined_k141_232381\|\|full | 2659 | No | NA | 0 | 1 | Not-determined | Genome-fragment | NA | | 0 |
| S17-final-viral-combined_k141_225934\|\|lt2gene | 1178 | No | NA | 1 | 0 | Low-quality | Genome-fragment | | 3.88 | 0 |
| S11-final-viral-combined_k141_39616\|\|full | 3186 | No | NA | 0 | 2 | Not-determined | Genome-fragment | NA | | 0 |
| S11-final-viral-combined_k141_300795\|\|full | 1967 | No | NA | 0 | 0 | Not-determined | Genome-fragment | NA | | 0 |
| S11-final-viral-combined_k141_218314\|\|full | 3111 | No | NA | 0 | 2 | Not-determined | Genome-fragment | NA | | 0 |
| S11-final-viral-combined_k141_67111\|\|full | 3318 | No | NA | 0 | 3 | Not-determined | Genome-fragment | NA | | 0 |
| S11-final-viral-combined_k141_246860\|\|full | 2451 | No | NA | 0 | 1 | Not-determined | Genome-fragment | NA | | 0 |
| S11-final-viral-combined_k141_11918\|\|full | 2298 | No | NA | 0 | 3 | Not-determined | Genome-fragment | NA | | 0 |
| S2_k141_48539 | 1178 | No | NA | 0 | 0 | Low-quality | Genome-fragment | | 3.22 | 0 |
| S2_k141_89081 | 1178 | No | NA | 0 | 1 | Low-quality | Genome-fragment | | 3.96 | 0 |
| S4_k141_36994 | 1178 | No | NA | 0 | 0 | Low-quality | Genome-fragment | | 3.2 | 0 |
| S6-final-viral-combined_k141_113300\|\|lt2gene | 1178 | No | NA | 1 | 0 | Low-quality | Genome-fragment | | 2.69 | 0 |
| S11-final-viral-combined_k141_149626\|\|full | 1818 | No | NA | 0 | 2 | Not-determined | Genome-fragment | NA | | 0 |
| S11-final-viral-combined_k141_273141\|\|full | 1454 | No | NA | 0 | 2 | Not-determined | Genome-fragment | NA | | 0 |
| S11-final-viral-combined_k141_314841\|\|full | 3042 | No | NA | 0 | 3 | Not-determined | Genome-fragment | NA | | 0 |
| S8-final-viral-combined_k141_37209\|\|full | 1178 | No | NA | 0 | 0 | Low-quality | Genome-fragment | | 2.89 | 0 |
| S9-final-viral-combined_k141_14380\|\|full | 1179 | No | NA | 0 | 0 | Low-quality | Genome-fragment | | 2.52 | 0 |
| S9_k141_102230 | 1179 | No | NA | 0 | 0 | Low-quality | Genome-fragment | | 1.97 | 0 |
| S11-final-viral-combined_k141_53659\|\|full | 2212 | No | NA | 0 | 2 | Not-determined | Genome-fragment | NA | | 0 |
| S1-final-viral-combined_k141_18177\|\|full | 1179 | No | NA | 0 | 1 | Low-quality | Genome-fragment | | 0.59 | 0 |
| S3-final-viral-combined_k141_32553\|\|full | 1179 | No | NA | 0 | 0 | Low-quality | Genome-fragment | | 2.49 | 0 |
| S11-final-viral-combined_k141_163688\|\|full | 1239 | No | NA | 0 | 1 | Not-determined | Genome-fragment | NA | | 0 |
| S11-final-viral-combined_k141_53678\|\|full | 1079 | No | NA | 0 | 1 | Not-determined | Genome-fragment | NA | | 0 |
| S11-final-viral-combined_k141_327401\|\|full | 2976 | No | NA | 0 | 3 | Not-determined | Genome-fragment | NA | | 0 |
| S11-final-viral-combined_k141_177536\|\|full | 1356 | No | NA | 0 | 0 | Not-determined | Genome-fragment | NA | | 0 |
| S3-final-viral-combined_k141_45698\|\|full | 1179 | No | NA | 0 | 0 | Low-quality | Genome-fragment | | 0.84 | 0 |
| S7-final-viral-combined_k141_3097\|\|full | 1179 | No | NA | 1 | 0 | Low-quality | Genome-fragment | | 2.92 | 0 |
| S7-final-viral-combined_k141_209842\|\|full | 1179 | No | NA | 0 | 0 | Low-quality | Genome-fragment | | 2.22 | 0 |
| S2-final-viral-combined_k141_142120\|\|full | 1179 | No | NA | 0 | 0 | Low-quality | Genome-fragment | | 2.08 | 0 |
| S11-final-viral-combined_k141_67193\|\|full | 1489 | No | NA | 0 | 2 | Not-determined | Genome-fragment | NA | | 0 |
| S11-final-viral-combined_k141_67204\|\|full | 1531 | No | NA | 0 | 3 | Not-determined | Genome-fragment | NA | | 0 |
| S4_k141_116818 | 1179 | No | NA | 0 | 0 | Low-quality | Genome-fragment | | 2.43 | 0 |
| S8-final-viral-combined_k141_62412\|\|full | 1179 | No | NA | 0 | 0 | Low-quality | Genome-fragment | | 1.38 | 0 |
| S1-final-viral-combined_k141_239630\|\|full | 1180 | No | NA | 0 | 1 | Low-quality | Genome-fragment | | 0.54 | 0 |
| S11-final-viral-combined_k141_135052\|\|full | 1804 | No | NA | 0 | 2 | Not-determined | Genome-fragment | NA | | 0 |
| S1-final-viral-combined_k141_98282\|\|full | 1180 | No | NA | 0 | 0 | Low-quality | Genome-fragment | | 0.79 | 0 |
| S3-final-viral-combined_k141_86000\|\|lt2gene | 1180 | No | NA | 2 | 0 | Low-quality | Genome-fragment | | 2.54 | 0 |
| S11-final-viral-combined_k141_135076\|\|full | 2620 | No | NA | 0 | 1 | Not-determined | Genome-fragment | NA | | 0 |
| S17-final-viral-combined_k141_71068\|\|lt2gene | 1180 | No | NA | 2 | 0 | Low-quality | Genome-fragment | | 3.05 | 0 |
| S17-k141_204970 | 1180 | No | NA | 0 | 1 | Low-quality | Genome-fragment | | 1.11 | 0 |
| S11-final-viral-combined_k141_287787\|\|full | 3604 | No | NA | 0 | 0 | Not-determined | Genome-fragment | NA | | 0 |
| S12-final-viral-combined_k141_189237\|\|full | 1180 | No | NA | 0 | 0 | Low-quality | Genome-fragment | | 2.03 | 0 |
| S11-final-viral-combined_k141_327506\|\|full | 1833 | No | NA | 0 | 0 | Not-determined | Genome-fragment | NA | | 0 |
| S12-final-viral-combined_k141_379109\|\|full | 1180 | No | NA | 1 | 0 | Low-quality | Genome-fragment | | 2.67 | 0 |
| S11-final-viral-combined_k141_327515\|\|full | 5378 | No | NA | 0 | 2 | Not-determined | Genome-fragment | NA | | 0 |
| S11-final-viral-combined_k141_135121\|\|full | 1836 | No | NA | 0 | 1 | Not-determined | Genome-fragment | NA | | 0 |
| S11-final-viral-combined_k141_300884\|\|full | 1226 | No | NA | 0 | 1 | Not-determined | Genome-fragment | NA | | 0 |
| S2-final-viral-combined_k141_55686\|\|full | 1180 | No | NA | 0 | 0 | Low-quality | Genome-fragment | | 2.31 | 0 |
| S11-final-viral-combined_k141_177619\|\|full | 5825 | No | NA | 0 | 1 | Not-determined | Genome-fragment | NA | | 0 |
| S11-final-viral-combined_k141_247052\|\|full | 1889 | No | NA | 0 | 1 | Not-determined | Genome-fragment | NA | | 0 |
| S4-final-viral-combined_k141_71645\|\|full | 1180 | No | NA | 0 | 0 | Low-quality | Genome-fragment | | 2.76 | 0 |
| S11_k141_145671 | 1181 | No | NA | 0 | 0 | Low-quality | Genome-fragment | | 2.5 | 0 |
| S11-final-viral-combined_k141_109071\|\|full | 3147 | No | NA | 0 | 1 | Not-determined | Genome-fragment | NA | | 0 |
| S1-final-viral-combined_k141_360582\|\|full | 1181 | No | NA | 0 | 2 | Low-quality | Genome-fragment | | 0.75 | 0 |
| S11-final-viral-combined_k141_53770\|\|full | 1760 | No | NA | 0 | 1 | Not-determined | Genome-fragment | NA | | 0 |
| S11-final-viral-combined_k141_190420\|\|full | 1243 | No | NA | 0 | 0 | Not-determined | Genome-fragment | NA | | 0 |
| S11-final-viral-combined_k141_327565\|\|full | 1983 | No | NA | 0 | 0 | Not-determined | Genome-fragment | NA | | 0 |
| S1_k141_51933 | 1181 | No | NA | 0 | 0 | Low-quality | Genome-fragment | | 1.75 | 0 |
| S11-final-viral-combined_k141_190424\|\|full | 5442 | No | NA | 0 | 1 | Not-determined | Genome-fragment | NA | | 0 |
| S11-final-viral-combined_k141_163916\|\|full | 4063 | No | NA | 0 | 3 | Not-determined | Genome-fragment | NA | | 0 |
| S1_k141_193370 | 1181 | No | NA | 1 | 0 | Low-quality | Genome-fragment | | 1.43 | 0 |
| S3-final-viral-combined_k141_105525\|\|full | 1181 | No | NA | 0 | 0 | Low-quality | Genome-fragment | | 3.21 | 0 |
| S5-final-viral-combined_k141_77674\|\|full | 1181 | No | NA | 3 | 0 | Low-quality | Genome-fragment | | 3.21 | 0 |
| S11-final-viral-combined_k141_39912\|\|full | 1553 | No | NA | 0 | 0 | Not-determined | Genome-fragment | NA | | 0 |
| S5-final-viral-combined_k141_160659\|\|lt2gene | 1181 | No | NA | 1 | 0 | Low-quality | Genome-fragment | | 1.77 | 0 |
| S11-final-viral-combined_k141_39918\|\|full | 2254 | No | NA | 0 | 1 | Not-determined | Genome-fragment | NA | | 0 |
| S7-final-viral-combined_k141_234938\|\|full | 1181 | No | NA | 0 | 0 | Low-quality | Genome-fragment | | 2.46 | 0 |
| S11-final-viral-combined_k141_315046\|\|full | 1471 | No | NA | 0 | 0 | Not-determined | Genome-fragment | NA | | 0 |
| S17-final-viral-combined_k141_33740\|\|full | 1181 | No | NA | 0 | 0 | Low-quality | Genome-fragment | | 1.89 | 0 |
| S11-final-viral-combined_k141_39930\|\|full | 1404 | No | NA | 0 | 0 | Not-determined | Genome-fragment | NA | | 0 |
| S11-final-viral-combined_k141_94498\|\|full | 3503 | No | NA | 0 | 4 | Not-determined | Genome-fragment | NA | | 0 |
| S11-final-viral-combined_k141_204522\|\|full | 30685 | No | NA | 12 | 0 | Medium-quality | Genome-fragment | | 66.54 | 0 |
| S11-final-viral-combined_k141_67279\|\|full | 3404 | No | NA | 0 | 1 | Not-determined | Genome-fragment | NA | | 0 |
| S12-final-viral-combined_k141_121545\|\|full | 1181 | No | NA | 1 | 0 | Low-quality | Genome-fragment | | 1.91 | 0 |
| S12-final-viral-combined_k141_361985\|\|full | 1181 | No | NA | 0 | 0 | Low-quality | Genome-fragment | | 1.88 | 0 |
| S12-final-viral-combined_k141_249522\|\|full | 1181 | No | NA | 0 | 1 | Low-quality | Genome-fragment | | 0.36 | 0 |
| S12_k141_232515 | 1181 | No | NA | 0 | 0 | Low-quality | Genome-fragment | | 3.02 | 0 |
| S12_k141_114490 | 1181 | No | NA | 0 | 2 | Low-quality | Genome-fragment | | 0.49 | 0 |
| S11-final-viral-combined_k141_164006\|\|full | 1523 | No | NA | 0 | 0 | Not-determined | Genome-fragment | NA | | 0 |
| S4-final-viral-combined_k141_134635\|\|full | 1181 | No | NA | 0 | 1 | Low-quality | Genome-fragment | | 1.63 | 0 |
| S11-final-viral-combined_k141_232649\|\|full | 1890 | No | NA | 0 | 0 | Not-determined | Genome-fragment | NA | | 0 |
| S11-final-viral-combined_k141_190522\|\|full | 1894 | No | NA | 0 | 1 | Not-determined | Genome-fragment | NA | | 0 |
| S11-final-viral-combined_k141_247182\|\|full | 2407 | No | NA | 0 | 1 | Not-determined | Genome-fragment | NA | | 0 |
| S11-final-viral-combined_k141_190526\|\|full | 1183 | No | NA | 0 | 1 | Not-determined | Genome-fragment | NA | | 0 |
| S11-final-viral-combined_k141_288028\|\|full | 1221 | No | NA | 0 | 0 | Not-determined | Genome-fragment | NA | | 0 |
| S4-final-viral-combined_k141_13785\|\|full | 1181 | No | NA | 0 | 1 | Low-quality | Genome-fragment | | 1.28 | 0 |
| S11-final-viral-combined_k141_80324\|\|full | 1372 | No | NA | 0 | 0 | Not-determined | Genome-fragment | NA | | 0 |
| S6-final-viral-combined_k141_76479\|\|full | 1181 | No | NA | 0 | 0 | Low-quality | Genome-fragment | | 0.78 | 0 |
| S11-final-viral-combined_k141_288046\|\|full | 1549 | No | NA | 0 | 2 | Not-determined | Genome-fragment | NA | | 0 |
| S11-final-viral-combined_k141_135223\|\|full | 1464 | No | NA | 0 | 0 | Not-determined | Genome-fragment | NA | | 0 |
| S11-final-viral-combined_k141_53880\|\|full | 1701 | No | NA | 0 | 2 | Not-determined | Genome-fragment | NA | | 0 |
| S11-final-viral-combined_k141_94598\|\|full | 2479 | No | NA | 0 | 3 | Not-determined | Genome-fragment | NA | | 0 |
| S11-final-viral-combined_k141_164043\|\|full | 2099 | No | NA | 0 | 0 | Not-determined | Genome-fragment | NA | | 0 |
| S11-final-viral-combined_k141_247220\|\|full | 1640 | No | NA | 0 | 1 | Not-determined | Genome-fragment | NA | | 0 |
| S11-final-viral-combined_k141_149928\|\|full | 1639 | No | NA | 0 | 0 | Not-determined | Genome-fragment | NA | | 0 |
| S11-final-viral-combined_k141_121616\|\|full | 1471 | No | NA | 0 | 1 | Not-determined | Genome-fragment | NA | | 0 |
| S8-final-viral-combined_k141_176625\|\|full | 1181 | No | NA | 1 | 0 | Low-quality | Genome-fragment | | 0.71 | 0 |
| S8-final-viral-combined_k141_96488\|\|full | 1181 | No | NA | 0 | 0 | Low-quality | Genome-fragment | | 2.63 | 0 |
| S8-final-viral-combined_k141_146960\|\|full | 1181 | No | NA | 1 | 0 | Low-quality | Genome-fragment | | 2.6 | 0 |
| S11-final-viral-combined_k141_190594\|\|full | 2720 | No | NA | 0 | 0 | Not-determined | Genome-fragment | NA | | 0 |
| S11-final-viral-combined_k141_149961\|\|full | 1388 | No | NA | 0 | 1 | Not-determined | Genome-fragment | NA | | 0 |
| S8_k141_12422 | 1181 | No | NA | 0 | 0 | Low-quality | Genome-fragment | | 0.44 | 0 |
| S11-final-viral-combined_k141_173658\|\|full | 1182 | No | NA | 0 | 1 | Low-quality | Genome-fragment | | 0.54 | 0 |
| S11-final-viral-combined_k141_176502\|\|full | 1182 | No | NA | 1 | 0 | Low-quality | Genome-fragment | | 2.01 | 0 |
| S1-final-viral-combined_k141_35082\|\|full | 1182 | No | NA | 0 | 0 | Low-quality | Genome-fragment | | 1.44 | 0 |
| S1_k141_100511 | 1182 | No | NA | 0 | 1 | Low-quality | Genome-fragment | | 0.79 | 0 |
| S11-final-viral-combined_k141_190624\|\|full | 2130 | No | NA | 0 | 1 | Not-determined | Genome-fragment | NA | | 0 |
| S11-final-viral-combined_k141_80385\|\|full | 1952 | No | NA | 0 | 2 | Not-determined | Genome-fragment | NA | | 0 |
| S5-final-viral-combined_k141_23788\|\|full | 1182 | No | NA | 2 | 0 | Low-quality | Genome-fragment | | 2.21 | 0 |
| S11-final-viral-combined_k141_232774\|\|full | 2128 | No | NA | 0 | 1 | Not-determined | Genome-fragment | NA | | 0 |
| S5-final-viral-combined_k141_77934\|\|lt2gene | 1182 | No | NA | 2 | 0 | Low-quality | Genome-fragment | | 2.63 | 0 |
| S11-final-viral-combined_k141_12240\|\|full | 1372 | No | NA | 0 | 0 | Not-determined | Genome-fragment | NA | | 0 |
| S11-final-viral-combined_k141_177894\|\|full | 2476 | No | NA | 0 | 1 | Not-determined | Genome-fragment | NA | | 0 |
| S11-final-viral-combined_k141_315198\|\|full | 1285 | No | NA | 0 | 0 | Not-determined | Genome-fragment | NA | | 0 |
| S5_k141_20540 | 1182 | No | NA | 0 | 0 | Low-quality | Genome-fragment | | 2.39 | 0 |
| S7-final-viral-combined_k141_209631\|\|full | 1182 | No | NA | 2 | 0 | Low-quality | Genome-fragment | | 1.91 | 0 |
| S11-final-viral-combined_k141_109231\|\|full | 4063 | No | NA | 0 | 2 | Not-determined | Genome-fragment | NA | | 0 |
| S17-k141_258606 | 1182 | No | NA | 0 | 0 | Low-quality | Genome-fragment | | 1.66 | 0 |
| S11-final-viral-combined_k141_260603\|\|full | 2783 | No | NA | 0 | 1 | Not-determined | Genome-fragment | NA | | 0 |
| S2-final-viral-combined_k141_133608\|\|full | 1182 | No | NA | 0 | 2 | Low-quality | Genome-fragment | | 1.65 | 0 |
| S11-final-viral-combined_k141_247323\|\|full | 5327 | No | NA | 0 | 4 | Not-determined | Genome-fragment | NA | | 0 |
| S2_k141_128440 | 1182 | No | NA | 1 | 0 | Low-quality | Genome-fragment | | 2.4 | 0 |
| S11-final-viral-combined_k141_327765\|\|full | 1003 | No | NA | 0 | 0 | Not-determined | Genome-fragment | NA | | 0 |
| S11-final-viral-combined_k141_247333\|\|full | 2049 | No | NA | 0 | 1 | Not-determined | Genome-fragment | NA | | 0 |
| S4_k141_99229 | 1182 | No | NA | 0 | 1 | Low-quality | Genome-fragment | | 2.57 | 0 |
| S11-final-viral-combined_k141_273513\|\|full | 1644 | No | NA | 0 | 2 | Not-determined | Genome-fragment | NA | | 0 |
| S6_k141_124448 | 1182 | No | NA | 1 | 0 | Low-quality | Genome-fragment | | 0.75 | 0 |
| S11-final-viral-combined_k141_273529\|\|full | 3632 | No | NA | 0 | 2 | Not-determined | Genome-fragment | NA | | 0 |
| S11-final-viral-combined_k141_218752\|\|full | 2069 | No | NA | 0 | 0 | Not-determined | Genome-fragment | NA | | 0 |
| S11-final-viral-combined_k141_218769\|\|full | 1351 | No | NA | 0 | 0 | Not-determined | Genome-fragment | NA | | 0 |
| S11-final-viral-combined_k141_12389\|\|full | 4400 | No | NA | 0 | 2 | Not-determined | Genome-fragment | NA | | 0 |
| S11-final-viral-combined_k141_24779\|\|full | 2881 | No | NA | 0 | 1 | Not-determined | Genome-fragment | NA | | 0 |
| S11-final-viral-combined_k141_301122\|\|full | 1125 | No | NA | 0 | 1 | Not-determined | Genome-fragment | NA | | 0 |
| S9-final-viral-combined_k141_43712\|\|full | 1183 | No | NA | 0 | 0 | Low-quality | Genome-fragment | | 2.9 | 0 |
| S11-final-viral-combined_k141_178041\|\|full | 1342 | No | NA | 0 | 1 | Not-determined | Genome-fragment | NA | | 0 |
| S9_k141_111764 | 1183 | No | NA | 0 | 0 | Low-quality | Genome-fragment | | 1.59 | 0 |
| S11-final-viral-combined_k141_190789\|\|full | 2101 | No | NA | 0 | 1 | Not-determined | Genome-fragment | NA | | 0 |
| S11-final-viral-combined_k141_40252\|\|full | 1105 | No | NA | 0 | 1 | Not-determined | Genome-fragment | NA | | 0 |
| S11-final-viral-combined_k141_199640\|\|full | 1183 | No | NA | 1 | 0 | Low-quality | Genome-fragment | | 1.89 | 0 |
| S11-final-viral-combined_k141_40256\|\|full | 2396 | No | NA | 0 | 1 | Not-determined | Genome-fragment | NA | | 0 |
| S11-final-viral-combined_k141_269969\|\|full | 1183 | No | NA | 1 | 0 | Low-quality | Genome-fragment | | 0.75 | 0 |
| S11-final-viral-combined_k141_40274\|\|full | 1992 | No | NA | 0 | 1 | Not-determined | Genome-fragment | NA | | 0 |
| S11-final-viral-combined_k141_54027\|\|full | 6540 | No | NA | 0 | 6 | Not-determined | Genome-fragment | NA | | 0 |
| S11-final-viral-combined_k141_259984\|\|lt2gene | 1183 | No | NA | 2 | 0 | Low-quality | Genome-fragment | | 3.19 | 0 |
| S11-final-viral-combined_k141_273619\|\|full | 17297 | No | NA | 0 | 9 | Not-determined | Genome-fragment | NA | | 0 |

| | | | | | | | | | | | |
|---|---|---|---|---|---|---|---|---|---|---|---|
| S11-final-viral-combined_k141_40306\|\|full | 1937 | No | NA | | 0 | 2 | Not-determined | Genome-fragment | NA | | 0 |
| S1_k141_193675 | 1183 | No | NA | | 0 | 0 | Low-quality | Genome-fragment | | 0.77 | 0 |
| S11-final-viral-combined_k141_247376\|\|full | 1327 | No | NA | | 0 | 0 | Not-determined | Genome-fragment | NA | | 0 |
| S11-final-viral-combined_k141_150192\|\|full | 4416 | No | NA | | 0 | 0 | Not-determined | Genome-fragment | NA | | 0 |
| S11-final-viral-combined_k141_273646\|\|full | 3588 | No | NA | | 0 | 1 | Not-determined | Genome-fragment | NA | | 0 |
| S17-final-viral-combined_k141_258258\|\|full | 1183 | No | NA | | 1 | 0 | Low-quality | Genome-fragment | | 2.87 | 0 |
| S11-final-viral-combined_k141_150210\|\|full | 1758 | No | NA | | 0 | 2 | Not-determined | Genome-fragment | NA | | 0 |
| S11-final-viral-combined_k141_67643\|\|full | 7699 | No | NA | | 0 | 2 | Not-determined | Genome-fragment | NA | | 0 |
| S17-k141_115366 | 1183 | No | NA | | 0 | 0 | Low-quality | Genome-fragment | | 0.82 | 0 |
| S12_k141_70007 | 1183 | No | NA | | 0 | 0 | Low-quality | Genome-fragment | | 0.67 | 0 |
| S11-final-viral-combined_k141_260837\|\|full | 9683 | No | NA | | 0 | 4 | Not-determined | Genome-fragment | NA | | 0 |
| S2-final-viral-combined_k141_214549\|\|full | 1183 | No | NA | | 0 | 0 | Low-quality | Genome-fragment | | 2.19 | 0 |
| S2-final-viral-combined_k141_185872\|\|lt2gene | 1183 | No | NA | | 0 | 0 | Low-quality | Genome-fragment | | 2.76 | 0 |
| S2_k141_63579 | 1183 | No | NA | | 0 | 1 | Low-quality | Genome-fragment | | 0.31 | 0 |
| S11-final-viral-combined_k141_301161\|\|full | 3371 | No | NA | | 0 | 2 | Not-determined | Genome-fragment | NA | | 0 |
| S11-final-viral-combined_k141_121821\|\|full | 1965 | No | NA | | 0 | 0 | Not-determined | Genome-fragment | NA | | 0 |
| S2_k141_131801 | 1183 | No | NA | | 0 | 0 | Low-quality | Genome-fragment | | 1.23 | 0 |
| S6-final-viral-combined_k141_103191\|\|full | 1183 | No | NA | | 0 | 0 | Low-quality | Genome-fragment | | 0.91 | 0 |
| S8_k141_102680 | 1183 | No | NA | | 0 | 0 | Low-quality | Genome-fragment | | 7.61 | 0 |
| S11-final-viral-combined_k141_178107\|\|full | 1125 | No | NA | | 0 | 1 | Not-determined | Genome-fragment | NA | | 0 |
| S11-final-viral-combined_k141_40383\|\|full | 1214 | No | NA | | 0 | 0 | Not-determined | Genome-fragment | NA | | 0 |
| S11-final-viral-combined_k141_200302\|\|full | 1184 | No | NA | | 0 | 0 | Low-quality | Genome-fragment | | 2.07 | 0 |
| S11-final-viral-combined_k141_12564\|\|full | 2530 | No | NA | | 0 | 2 | Not-determined | Genome-fragment | NA | | 0 |
| S11-final-viral-combined_k141_135631\|\|full | 2304 | No | NA | | 0 | 2 | Not-determined | Genome-fragment | NA | | 0 |
| S11-final-viral-combined_k141_327900\|\|full | 3173 | No | NA | | 0 | 2 | Not-determined | Genome-fragment | NA | | 0 |
| S11-final-viral-combined_k141_327903\|\|full | 1782 | No | NA | | 0 | 2 | Not-determined | Genome-fragment | NA | | 0 |
| S1-final-viral-combined_k141_250855\|\|full | 1184 | No | NA | | 3 | 0 | Low-quality | Genome-fragment | | 2.95 | 0 |
| S11-final-viral-combined_k141_288277\|\|full | 5935 | No | NA | | 0 | 4 | Not-determined | Genome-fragment | NA | | 0 |
| S1-final-viral-combined_k141_303952\|\|full | 1184 | No | NA | | 0 | 0 | Low-quality | Genome-fragment | | 2.89 | 0 |
| S11-final-viral-combined_k141_301257\|\|full | 2657 | No | NA | | 0 | 2 | Not-determined | Genome-fragment | NA | | 0 |
| S1-final-viral-combined_k141_109605\|\|full | 1184 | No | NA | | 0 | 0 | Low-quality | Genome-fragment | | 1.81 | 0 |
| S11-final-viral-combined_k141_135685\|\|full | 1881 | No | NA | | 0 | 3 | Not-determined | Genome-fragment | NA | | 0 |
| S11-final-viral-combined_k141_190972\|\|full | 1391 | No | NA | | 0 | 2 | Not-determined | Genome-fragment | NA | | 0 |
| S11-final-viral-combined_k141_327931\|\|full | 1145 | No | NA | | 0 | 2 | Not-determined | Genome-fragment | NA | | 0 |
| S11-final-viral-combined_k141_190975\|\|full | 1973 | No | NA | | 0 | 1 | Not-determined | Genome-fragment | NA | | 0 |
| S1-final-viral-combined_k141_91380\|\|lt2gene | 1184 | No | NA | | 0 | 0 | Low-quality | Genome-fragment | | 2.46 | 0 |
| S1-final-viral-combined_k141_390483\|\|lt2gene | 1184 | No | NA | | 1 | 0 | Low-quality | Genome-fragment | | 2.79 | 0 |
| S1_k141_170410 | 1184 | No | NA | | 0 | 1 | Low-quality | Genome-fragment | | 0.44 | 0 |
| S11-final-viral-combined_k141_109637\|\|full | 1292 | No | NA | | 0 | 2 | Not-determined | Genome-fragment | NA | | 0 |
| S11-final-viral-combined_k141_150384\|\|full | 1727 | No | NA | | 0 | 0 | Not-determined | Genome-fragment | NA | | 0 |
| S11-final-viral-combined_k141_150388\|\|full | 1844 | No | NA | | 0 | 1 | Not-determined | Genome-fragment | NA | | 0 |
| S7_k141_75336 | 1184 | No | NA | | 0 | 2 | Low-quality | Genome-fragment | | 0.77 | 0 |
| S17-k141_193920 | 1184 | No | NA | | 0 | 0 | Low-quality | Genome-fragment | | 1.61 | 0 |
| S11-final-viral-combined_k141_164284\|\|full | 1228 | No | NA | | 0 | 3 | Not-determined | Genome-fragment | NA | | 0 |
| S12-final-viral-combined_k141_81888\|\|full | 1184 | No | NA | | 3 | 0 | Low-quality | Genome-fragment | | 3.17 | 0 |
| S11-final-viral-combined_k141_12637\|\|full | 1567 | No | NA | | 0 | 3 | Not-determined | Genome-fragment | NA | | 0 |
| S11-final-viral-combined_k141_67845\|\|full | 1609 | No | NA | | 0 | 1 | Not-determined | Genome-fragment | NA | | 0 |
| S12-final-viral-combined_k141_350420\|\|full | 1184 | No | NA | | 1 | 0 | Low-quality | Genome-fragment | | 2.52 | 0 |
| S11-final-viral-combined_k141_80600\|\|full | 1465 | No | NA | | 0 | 1 | Not-determined | Genome-fragment | NA | | 0 |
| S11-final-viral-combined_k141_191059\|\|full | 1791 | No | NA | | 0 | 1 | Not-determined | Genome-fragment | NA | | 0 |
| S11-final-viral-combined_k141_273868\|\|full | 1985 | No | NA | | 0 | 2 | Not-determined | Genome-fragment | NA | | 0 |
| S12_k141_342329 | 1184 | No | NA | | 0 | 2 | Low-quality | Genome-fragment | | 1.96 | 0 |
| S11-final-viral-combined_k141_204970\|\|full | 2173 | No | NA | | 0 | 1 | Not-determined | Genome-fragment | NA | | 0 |
| S2-final-viral-combined_k141_219177\|\|full | 1184 | No | NA | | 1 | 0 | Low-quality | Genome-fragment | | 1.73 | 0 |
| S11-final-viral-combined_k141_247594\|\|full | 6155 | No | NA | | 0 | 3 | Not-determined | Genome-fragment | NA | | 0 |
| S8-final-viral-combined_k141_108233\|\|full | 1184 | No | NA | | 0 | 0 | Low-quality | Genome-fragment | | 0.68 | 0 |
| S11-final-viral-combined_k141_109719\|\|full | 1728 | No | NA | | 0 | 1 | Not-determined | Genome-fragment | NA | | 0 |
| S11-final-viral-combined_k141_40545\|\|full | 1185 | No | NA | | 0 | 1 | Low-quality | Genome-fragment | | 1.97 | 0 |
| S11-final-viral-combined_k141_122010\|\|full | 7761 | No | NA | | 0 | 8 | Not-determined | Genome-fragment | NA | | 0 |
| S11-final-viral-combined_k141_54280\|\|full | 3638 | No | NA | | 0 | 0 | Not-determined | Genome-fragment | NA | | 0 |
| S11-final-viral-combined_k141_328025\|\|full | 5836 | No | NA | | 0 | 4 | Not-determined | Genome-fragment | NA | | 0 |
| S11-final-viral-combined_k141_25022\|\|full | 2420 | No | NA | | 0 | 0 | Not-determined | Genome-fragment | NA | | 0 |
| S11_k141_30556 | 1185 | No | NA | | 1 | 0 | Low-quality | Genome-fragment | | 2.04 | 0 |
| S11-final-viral-combined_k141_288422\|\|full | 2361 | No | NA | | 0 | 1 | Not-determined | Genome-fragment | NA | | 0 |
| S11-final-viral-combined_k141_67915\|\|full | 1158 | No | NA | | 0 | 0 | Not-determined | Genome-fragment | NA | | 0 |
| S11-final-viral-combined_k141_80708\|\|full | 1024 | No | NA | | 0 | 2 | Not-determined | Genome-fragment | NA | | 0 |
| S11-final-viral-combined_k141_315601\|\|full | 2054 | No | NA | | 0 | 1 | Not-determined | Genome-fragment | NA | | 0 |
| S1-final-viral-combined_k141_348217\|\|full | 1185 | No | NA | | 0 | 0 | Low-quality | Genome-fragment | | 1.02 | 0 |
| S11-final-viral-combined_k141_219078\|\|full | 2330 | No | NA | | 0 | 2 | Not-determined | Genome-fragment | NA | | 0 |
| S11-final-viral-combined_k141_135832\|\|full | 1541 | No | NA | | 0 | 1 | Not-determined | Genome-fragment | NA | | 0 |
| S11-final-viral-combined_k141_301389\|\|full | 3150 | No | NA | | 0 | 2 | Not-determined | Genome-fragment | NA | | 0 |
| S11-final-viral-combined_k141_274039\|\|full | 3273 | No | NA | | 0 | 3 | Not-determined | Genome-fragment | NA | | 0 |
| S1-final-viral-combined_k141_372440\|\|full | 1185 | No | NA | | 0 | 1 | Low-quality | Genome-fragment | | 9.49 | 0 |
| S11-final-viral-combined_k141_122075\|\|full | 1078 | No | NA | | 0 | 0 | Not-determined | Genome-fragment | NA | | 0 |
| S11-final-viral-combined_k141_315651\|\|full | 2619 | No | NA | | 0 | 3 | Not-determined | Genome-fragment | NA | | 0 |
| S5-final-viral-combined_k141_82132\|\|full | 1185 | No | NA | | 1 | 0 | Low-quality | Genome-fragment | | 0.78 | 0 |
| S11-final-viral-combined_k141_12804\|\|full | 3022 | No | NA | | 0 | 1 | Not-determined | Genome-fragment | NA | | 0 |
| S11-final-viral-combined_k141_233322\|\|full | 3682 | No | NA | | 0 | 6 | Not-determined | Genome-fragment | NA | | 0 |
| S5-final-viral-combined_k141_158247\|\|full | 1185 | No | NA | | 0 | 0 | Low-quality | Genome-fragment | | 0.68 | 0 |
| S11-final-viral-combined_k141_315674\|\|full | 1778 | No | NA | | 0 | 1 | Not-determined | Genome-fragment | NA | | 0 |
| S11-final-viral-combined_k141_301444\|\|full | 2584 | No | NA | | 0 | 3 | Not-determined | Genome-fragment | NA | | 0 |
| S11-final-viral-combined_k141_54454\|\|full | 1702 | No | NA | | 0 | 2 | Not-determined | Genome-fragment | NA | | 0 |
| S5_k141_126809 | 1185 | No | NA | | 0 | 0 | Low-quality | Genome-fragment | | 2.86 | 0 |
| S11-final-viral-combined_k141_135934\|\|full | 5264 | No | NA | | 0 | 4 | Not-determined | Genome-fragment | NA | | 0 |
| S11-final-viral-combined_k141_288610\|\|full | 2083 | No | NA | | 0 | 1 | Not-determined | Genome-fragment | NA | | 0 |
| S7_k141_150040 | 1185 | No | NA | | 0 | 0 | Low-quality | Genome-fragment | | 2.17 | 0 |
| S11-final-viral-combined_k141_233370\|\|full | 2004 | No | NA | | 0 | 2 | Not-determined | Genome-fragment | NA | | 0 |
| S11-final-viral-combined_k141_247835\|\|full | 2222 | No | NA | | 0 | 2 | Not-determined | Genome-fragment | NA | | 0 |
| S11-final-viral-combined_k141_135946\|\|full | 2674 | No | NA | | 0 | 2 | Not-determined | Genome-fragment | NA | | 0 |
| S11-final-viral-combined_k141_301482\|\|full | 3005 | No | NA | | 0 | 1 | Not-determined | Genome-fragment | NA | | 0 |
| S11-final-viral-combined_k141_219172\|\|full | 3655 | No | NA | | 0 | 3 | Not-determined | Genome-fragment | NA | | 0 |
| S11-final-viral-combined_k141_315728\|\|full | 3274 | No | NA | | 0 | 3 | Not-determined | Genome-fragment | NA | | 0 |
| S11-final-viral-combined_k141_261182\|\|full | 7049 | No | NA | | 0 | 4 | Not-determined | Genome-fragment | NA | | 0 |
| S11-final-viral-combined_k141_219179\|\|full | 2822 | No | NA | | 0 | 1 | Not-determined | Genome-fragment | NA | | 0 |
| S12-final-viral-combined_k141_69674\|\|full | 1185 | No | NA | | 1 | 0 | Low-quality | Genome-fragment | | 2.17 | 0 |
| S11-final-viral-combined_k141_205155\|\|full | 1801 | No | NA | | 0 | 0 | Not-determined | Genome-fragment | NA | | 0 |
| S12-final-viral-combined_k141_311441\|\|full | 1185 | No | NA | | 0 | 0 | Low-quality | Genome-fragment | | 1.1 | 0 |
| S8-final-viral-combined_k141_137774\|\|full | 1185 | No | NA | | 0 | 0 | Low-quality | Genome-fragment | | 0.95 | 0 |
| S11-final-viral-combined_k141_40799\|\|full | 1318 | No | NA | | 0 | 1 | Not-determined | Genome-fragment | NA | | 0 |
| S11-final-viral-combined_k141_25222\|\|full | 1720 | No | NA | | 0 | 2 | Not-determined | Genome-fragment | NA | | 0 |
| S11-final-viral-combined_k141_219200\|\|full | 1149 | No | NA | | 0 | 1 | Not-determined | Genome-fragment | NA | | 0 |
| S11-final-viral-combined_k141_274122\|\|full | 1238 | No | NA | | 0 | 1 | Not-determined | Genome-fragment | NA | | 0 |
| S8-final-viral-combined_k141_186398\|\|lt2gene | 1185 | No | NA | | 2 | 0 | Low-quality | Genome-fragment | | 2.19 | 0 |
| S11-final-viral-combined_k141_68126\|\|full | 1152 | No | NA | | 0 | 1 | Not-determined | Genome-fragment | NA | | 0 |
| S11-final-viral-combined_k141_219208\|\|full | 1622 | No | NA | | 0 | 1 | Not-determined | Genome-fragment | NA | | 0 |
| S9-final-viral-combined_k141_94703\|\|full | 1186 | No | NA | | 0 | 0 | Low-quality | Genome-fragment | | 2.99 | 0 |
| S7-final-viral-combined_k141_125265\|\|lt2gene | 1186 | No | NA | | 1 | 0 | Low-quality | Genome-fragment | | 0.68 | 0 |
| S17-k141_171229 | 1186 | No | NA | | 2 | 0 | Low-quality | Genome-fragment | | 3.16 | 0 |
| S11-final-viral-combined_k141_95220\|\|full | 1475 | No | NA | | 0 | 0 | Not-determined | Genome-fragment | NA | | 0 |
| S12-final-viral-combined_k141_163170\|\|full | 1186 | No | NA | | 2 | 0 | Low-quality | Genome-fragment | | 2.92 | 0 |
| S4-final-viral-combined_k141_136223\|\|full | 1186 | No | NA | | 0 | 1 | Low-quality | Genome-fragment | | 0.69 | 0 |
| S8-final-viral-combined_k141_77581\|\|full | 1186 | No | NA | | 0 | 1 | Low-quality | Genome-fragment | | 7.56 | 0 |
| S11-final-viral-combined_k141_95225\|\|full | 8766 | No | NA | | 0 | 2 | Not-determined | Genome-fragment | NA | | 0 |
| S11-final-viral-combined_k141_25252\|\|full | 2644 | No | NA | | 0 | 2 | Not-determined | Genome-fragment | NA | | 0 |
| S11-final-viral-combined_k141_68174\|\|full | 3936 | No | NA | | 0 | 2 | Not-determined | Genome-fragment | NA | | 0 |
| S11-final-viral-combined_k141_191337\|\|full | 3240 | No | NA | | 0 | 0 | Not-determined | Genome-fragment | NA | | 0 |
| S11-final-viral-combined_k141_150758\|\|full | 1550 | No | NA | | 0 | 2 | Not-determined | Genome-fragment | NA | | 0 |
| S8-final-viral-combined_k141_204506\|\|full | 1186 | No | NA | | 0 | 0 | Low-quality | Genome-fragment | | 2.34 | 0 |
| S11-final-viral-combined_k141_294583\|\|full | 1187 | No | NA | | 1 | 0 | Low-quality | Genome-fragment | | 2.99 | 0 |
| S11-final-viral-combined_k141_219258\|\|full | 1344 | No | NA | | 0 | 1 | Not-determined | Genome-fragment | NA | | 0 |
| S11-final-viral-combined_k141_315820\|\|full | 1640 | No | NA | | 0 | 2 | Not-determined | Genome-fragment | NA | | 0 |
| S11-final-viral-combined_k141_193435\|\|lt2gene | 1187 | No | NA | | 1 | 0 | Low-quality | Genome-fragment | | 1.07 | 0 |
| S11-final-viral-combined_k141_136036\|\|full | 2158 | No | NA | | 0 | 0 | Not-determined | Genome-fragment | NA | | 0 |
| S11-final-viral-combined_k141_95263\|\|full | 1209 | No | NA | | 0 | 1 | Not-determined | Genome-fragment | NA | | 0 |
| S11_k141_276873 | 1187 | No | NA | | 0 | 1 | Low-quality | Genome-fragment | | 0.52 | 0 |
| S11-final-viral-combined_k141_219271\|\|full | 1316 | No | NA | | 0 | 0 | Not-determined | Genome-fragment | NA | | 0 |
| S11-final-viral-combined_k141_191365\|\|full | 1753 | No | NA | | 0 | 0 | Not-determined | Genome-fragment | NA | | 0 |
| S11-final-viral-combined_k141_164615\|\|full | 1202 | No | NA | | 0 | 0 | Not-determined | Genome-fragment | NA | | 0 |
| S11-final-viral-combined_k141_261270\|\|full | 2406 | No | NA | | 0 | 1 | Not-determined | Genome-fragment | NA | | 0 |
| S11-final-viral-combined_k141_150816\|\|full | 1012 | No | NA | | 0 | 1 | Not-determined | Genome-fragment | NA | | 0 |
| S11-final-viral-combined_k141_288693\|\|full | 2536 | No | NA | | 0 | 1 | Not-determined | Genome-fragment | NA | | 0 |
| S11-final-viral-combined_k141_328271\|\|full | 3002 | No | NA | | 0 | 2 | Not-determined | Genome-fragment | NA | | 0 |
| S1-final-viral-combined_k141_199166\|\|lt2gene | 1187 | No | NA | | 1 | 0 | Low-quality | Genome-fragment | | 2.56 | 0 |
| S11-final-viral-combined_k141_261302\|\|full | 4488 | No | NA | | 0 | 2 | Not-determined | Genome-fragment | NA | | 0 |
| S11-final-viral-combined_k141_13067\|\|full | 2890 | No | NA | | 0 | 0 | Not-determined | Genome-fragment | NA | | 0 |
| S1-final-viral-combined_k141_51000\|\|lt2gene | 1187 | No | NA | | 1 | 0 | Low-quality | Genome-fragment | | 2.7 | 0 |
| S11-final-viral-combined_k141_95372\|\|full | 1515 | No | NA | | 0 | 1 | Not-determined | Genome-fragment | NA | | 0 |
| S1_k141_296370 | 1187 | No | NA | | 0 | 1 | Low-quality | Genome-fragment | | 0.66 | 0 |
| S5_k141_47642 | 1187 | No | NA | | 0 | 0 | Low-quality | Genome-fragment | | 0.65 | 0 |
| S7-final-viral-combined_k141_14181\|\|full | 1187 | No | NA | | 0 | 1 | Low-quality | Genome-fragment | | 0.54 | 0 |
| S7-final-viral-combined_k141_58578\|\|full | 1187 | No | NA | | 0 | 0 | Low-quality | Genome-fragment | | 2.72 | 0 |
| S11-final-viral-combined_k141_248034\|\|full | 5985 | No | NA | | 0 | 3 | Not-determined | Genome-fragment | NA | | 0 |
| S12_k141_191473 | 1187 | No | NA | | 0 | 1 | Low-quality | Genome-fragment | | 1.33 | 0 |
| S11-final-viral-combined_k141_110185\|\|full | 1587 | No | NA | | 0 | 0 | Not-determined | Genome-fragment | NA | | 0 |
| S11-final-viral-combined_k141_301630\|\|full | 5034 | No | NA | | 0 | 4 | Not-determined | Genome-fragment | NA | | 0 |
| S11-final-viral-combined_k141_261397\|\|full | 1434 | No | NA | | 0 | 0 | Not-determined | Genome-fragment | NA | | 0 |
| S2-final-viral-combined_k141_482\|\|full | 1187 | No | NA | | 1 | 0 | Low-quality | Genome-fragment | | 1.9 | 0 |
| S2-final-viral-combined_k141_137315\|\|full | 1187 | No | NA | | 0 | 0 | Low-quality | Genome-fragment | | 1.75 | 0 |
| S11-final-viral-combined_k141_191463\|\|full | 1691 | No | NA | | 0 | 1 | Not-determined | Genome-fragment | NA | | 0 |
| S2-final-viral-combined_k141_142357\|\|full | 1187 | No | NA | | 0 | 0 | Low-quality | Genome-fragment | | 2.75 | 0 |
| S4-final-viral-combined_k141_162395\|\|full | 1187 | No | NA | | 0 | 1 | Low-quality | Genome-fragment | | 0.57 | 0 |
| S9-final-viral-combined_k141_82726\|\|full | 1188 | No | NA | | 0 | 0 | Low-quality | Genome-fragment | | 1.8 | 0 |
| S11-final-viral-combined_k141_191472\|\|full | 1153 | No | NA | | 0 | 1 | Not-determined | Genome-fragment | NA | | 0 |
| S11-final-viral-combined_k141_25450\|\|full | 1119 | No | NA | | 0 | 1 | Not-determined | Genome-fragment | NA | | 0 |
| S11-final-viral-combined_k141_95439\|\|full | 4285 | No | NA | | 0 | 3 | Not-determined | Genome-fragment | NA | | 0 |
| S9_k141_73694 | 1188 | No | NA | | 0 | 0 | Low-quality | Genome-fragment | | 0.46 | 0 |
| S1-final-viral-combined_k141_160413\|\|lt2gene | 1188 | No | NA | | 1 | 0 | Low-quality | Genome-fragment | | 2.87 | 0 |
| S5_k141_47667 | 1188 | No | NA | | 0 | 1 | Low-quality | Genome-fragment | | 1.27 | 0 |
| S17-final-viral-combined_k141_2901\|\|full | 1188 | Yes | | 492 | 1 | 1 | Low-quality | Genome-fragment | | 0.6 | 58.59 |
| S17-k141_236914 | 1188 | No | NA | | 0 | 1 | Low-quality | Genome-fragment | | 0.31 | 0 |
| S11-final-viral-combined_k141_274349\|\|full | 4427 | No | NA | | 0 | 3 | Not-determined | Genome-fragment | NA | | 0 |
| S12-final-viral-combined_k141_231325\|\|full | 1188 | No | NA | | 1 | 0 | Low-quality | Genome-fragment | | 2.27 | 0 |
| S12_k141_321567 | 1188 | No | NA | | 0 | 0 | Low-quality | Genome-fragment | | 2.54 | 0 |
| S11-final-viral-combined_k141_136252\|\|full | 1241 | No | NA | | 0 | 0 | Not-determined | Genome-fragment | NA | | 0 |
| S4_k141_112641 | 1188 | No | NA | | 1 | 0 | Low-quality | Genome-fragment | | 1.17 | 0 |
| S8-final-viral-combined_k141_77506\|\|full | 1188 | No | NA | | 2 | 0 | Low-quality | Genome-fragment | | 3.28 | 0 |
| S11-final-viral-combined_k141_41093\|\|full | 2508 | No | NA | | 0 | 2 | Not-determined | Genome-fragment | NA | | 0 |
| S9-final-viral-combined_k141_98596\|\|full | 1189 | No | NA | | 0 | 1 | Low-quality | Genome-fragment | | 0.99 | 0 |
| S11-final-viral-combined_k141_117912\|\|full | 1189 | No | NA | | 0 | 2 | Low-quality | Genome-fragment | | 0.9 | 0 |
| S1-final-viral-combined_k141_297322\|\|lt2gene | 1189 | No | NA | | 1 | 0 | Low-quality | Genome-fragment | | 2.81 | 0 |
| S11-final-viral-combined_k141_41114\|\|full | 4969 | No | NA | | 0 | 2 | Not-determined | Genome-fragment | NA | | 0 |
| S11-final-viral-combined_k141_178794\|\|full | 1489 | No | NA | | 0 | 1 | Not-determined | Genome-fragment | NA | | 0 |
| S7-final-viral-combined_k141_45526\|\|full | 1189 | No | NA | | 0 | 0 | Low-quality | Genome-fragment | | 0.75 | 0 |
| S11-final-viral-combined_k141_219584\|\|full | 1755 | No | NA | | 0 | 1 | Not-determined | Genome-fragment | NA | | 0 |

| | | | | | | | | | | | |
|---|---|---|---|---|---|---|---|---|---|---|---|
| S4-final-viral-combined_k141_95728\|\|lt2gene | 1189 | No | NA | | 1 | 0 | Low-quality | Genome-fragment | | 1.64 | 0 |
| S4-final-viral-combined_k141_62003\|\|lt2gene | 1189 | No | NA | | 1 | 0 | Low-quality | Genome-fragment | | 2.5 | 0 |
| S11-final-viral-combined_k141_233842\|\|full | 2020 | No | NA | | 0 | 2 | Not-determined | Genome-fragment | NA | | 0 |
| S6_k141_110392 | 1189 | No | NA | | 0 | 0 | Low-quality | Genome-fragment | | 1.36 | 0 |
| S11-final-viral-combined_k141_301929\|\|full | 1271 | No | NA | | 0 | 0 | Not-determined | Genome-fragment | NA | | 0 |
| S11-final-viral-combined_k141_32453\|\|full | 1190 | No | NA | | 0 | 0 | Low-quality | Genome-fragment | | 2.33 | 0 |
| S11-final-viral-combined_k141_301938\|\|full | 1386 | No | NA | | 0 | 0 | Not-determined | Genome-fragment | NA | | 0 |
| S11-final-viral-combined_k141_316033\|\|full | 2732 | No | NA | | 0 | 0 | Not-determined | Genome-fragment | NA | | 0 |
| S11_k141_142811 | 1190 | No | NA | | 0 | 1 | Low-quality | Genome-fragment | | 1.14 | 0 |
| S11-final-viral-combined_k141_191723\|\|full | 3240 | No | NA | | 0 | 3 | Not-determined | Genome-fragment | NA | | 0 |
| S1-final-viral-combined_k141_287671\|\|full | 1190 | No | NA | | 0 | 2 | Low-quality | Genome-fragment | | 0.64 | 0 |
| S1-final-viral-combined_k141_276653\|\|full | 1190 | No | NA | | 0 | 1 | Low-quality | Genome-fragment | | 0.67 | 0 |
| S11-final-viral-combined_k141_328443\|\|full | 1925 | No | NA | | 0 | 1 | Not-determined | Genome-fragment | NA | | 0 |
| S11-final-viral-combined_k141_233923\|\|full | 1017 | No | NA | | 0 | 1 | Not-determined | Genome-fragment | NA | | 0 |
| S5-final-viral-combined_k141_78977\|\|lt2gene | 1190 | No | NA | | 1 | 0 | Low-quality | Genome-fragment | | 2.85 | 0 |
| S7-final-viral-combined_k141_220653\|\|full | 1190 | No | NA | | 0 | 1 | Low-quality | Genome-fragment | | 0.7 | 0 |
| S11-final-viral-combined_k141_233930\|\|full | 1526 | No | NA | | 0 | 0 | Not-determined | Genome-fragment | NA | | 0 |
| S12-final-viral-combined_k141_374465\|\|lt2gene | 1190 | No | NA | | 1 | 0 | Low-quality | Genome-fragment | | 0.46 | 0 |
| S2-final-viral-combined_k141_228742\|\|full | 1190 | No | NA | | 1 | 0 | Low-quality | Genome-fragment | | 2.96 | 0 |
| S11-final-viral-combined_k141_68502\|\|full | 2346 | No | NA | | 0 | 0 | Not-determined | Genome-fragment | NA | | 0 |
| S11-final-viral-combined_k141_219686\|\|full | 3860 | No | NA | | 0 | 3 | Not-determined | Genome-fragment | NA | | 0 |
| S11-final-viral-combined_k141_289006\|\|full | 1139 | No | NA | | 0 | 0 | Not-determined | Genome-fragment | NA | | 0 |
| S11-final-viral-combined_k141_191787\|\|full | 4495 | No | NA | | 0 | 1 | Not-determined | Genome-fragment | NA | | 0 |
| S11-final-viral-combined_k141_81271\|\|full | 2545 | No | NA | | 0 | 2 | Not-determined | Genome-fragment | NA | | 0 |
| S11-final-viral-combined_k141_289023\|\|full | 1281 | No | NA | | 0 | 1 | Not-determined | Genome-fragment | NA | | 0 |
| S11-final-viral-combined_k141_122693\|\|full | 2094 | No | NA | | 0 | 2 | Not-determined | Genome-fragment | NA | | 0 |
| S11-final-viral-combined_k141_136444\|\|full | 16632 | No | NA | | 0 | 3 | Not-determined | Genome-fragment | NA | | 0 |
| S2-final-viral-combined_k141_182956\|\|full | 1190 | No | NA | | 0 | 0 | Low-quality | Genome-fragment | | 2.22 | 0 |
| S11-final-viral-combined_k141_328498\|\|full | 3237 | No | NA | | 0 | 1 | Not-determined | Genome-fragment | NA | | 0 |
| S11-final-viral-combined_k141_302027\|\|full | 2079 | No | NA | | 0 | 3 | Not-determined | Genome-fragment | NA | | 0 |
| S11-final-viral-combined_k141_219733\|\|full | 1323 | No | NA | | 0 | 2 | Not-determined | Genome-fragment | NA | | 0 |
| S11-final-viral-combined_k141_55003\|\|full | 1996 | No | NA | | 0 | 3 | Not-determined | Genome-fragment | NA | | 0 |
| S2-final-viral-combined_k141_123516\|\|full | 1190 | No | NA | | 1 | 0 | Low-quality | Genome-fragment | | 2.09 | 0 |
| S2-final-viral-combined_k141_162342\|\|lt2gene | 1190 | No | NA | | 2 | 0 | Low-quality | Genome-fragment | | 2.47 | 0 |
| S2_k141_208006 | 1190 | No | NA | | 0 | 2 | Low-quality | Genome-fragment | | 2.36 | 0 |
| S11-final-viral-combined_k141_68527\|\|full | 2390 | No | NA | | 0 | 3 | Not-determined | Genome-fragment | NA | | 0 |
| S8_k141_186645 | 1190 | No | NA | | 0 | 0 | Low-quality | Genome-fragment | | 0.95 | 0 |
| S8_k141_211098 | 1190 | No | NA | | 0 | 0 | Low-quality | Genome-fragment | | 1.72 | 0 |
| S9_k141_106062 | 1191 | No | NA | | 0 | 1 | Low-quality | Genome-fragment | | 0.8 | 0 |
| S11-final-viral-combined_k141_194006\|\|full | 1191 | No | NA | | 1 | 0 | Low-quality | Genome-fragment | | 1.33 | 0 |
| S11-final-viral-combined_k141_20181\|\|full | 1191 | No | NA | | 0 | 1 | Low-quality | Genome-fragment | | 0.71 | 0 |
| S11-final-viral-combined_k141_261762\|\|full | 1472 | No | NA | | 0 | 2 | Not-determined | Genome-fragment | NA | | 0 |
| S11-final-viral-combined_k141_151165\|\|full | 9167 | No | NA | | 0 | 6 | Not-determined | Genome-fragment | NA | | 0 |
| S11-final-viral-combined_k141_274574\|\|full | 1220 | No | NA | | 0 | 0 | Not-determined | Genome-fragment | NA | | 0 |
| S1-final-viral-combined_k141_200911\|\|lt2gene | 1191 | No | NA | | 1 | 0 | Low-quality | Genome-fragment | | 2.54 | 0 |
| S11-final-viral-combined_k141_328565\|\|full | 4168 | No | NA | | 0 | 1 | Not-determined | Genome-fragment | NA | | 0 |
| S11-final-viral-combined_k141_55077\|\|full | 1706 | No | NA | | 0 | 0 | Not-determined | Genome-fragment | NA | | 0 |
| S11-final-viral-combined_k141_274602\|\|full | 1562 | No | NA | | 0 | 1 | Not-determined | Genome-fragment | NA | | 0 |
| S11-final-viral-combined_k141_274604\|\|full | 1978 | No | NA | | 0 | 0 | Not-determined | Genome-fragment | NA | | 0 |
| S1_k141_301193 | 1191 | No | NA | | 0 | 1 | Low-quality | Genome-fragment | | 0.29 | 0 |
| S11-final-viral-combined_k141_178953\|\|full | 6371 | No | NA | | 0 | 2 | Not-determined | Genome-fragment | NA | | 0 |
| S11-final-viral-combined_k141_219811\|\|full | 2467 | No | NA | | 0 | 0 | Not-determined | Genome-fragment | NA | | 0 |
| S11-final-viral-combined_k141_136561\|\|full | 3583 | No | NA | | 0 | 2 | Not-determined | Genome-fragment | NA | | 0 |
| S11-final-viral-combined_k141_205702\|\|full | 1178 | No | NA | | 0 | 0 | Not-determined | Genome-fragment | NA | | 0 |
| S3_k141_124910 | 1191 | No | NA | | 0 | 0 | Low-quality | Genome-fragment | | 2.22 | 0 |
| S5-final-viral-combined_k141_146839\|\|full | 1191 | No | NA | | 0 | 0 | Low-quality | Genome-fragment | | 3.47 | 0 |
| S5_k141_88083 | 1191 | No | NA | | 0 | 0 | Low-quality | Genome-fragment | | 2.48 | 0 |
| S11-final-viral-combined_k141_25886\|\|full | 2033 | No | NA | | 0 | 0 | Not-determined | Genome-fragment | NA | | 0 |
| S11-final-viral-combined_k141_151242\|\|full | 3679 | No | NA | | 0 | 4 | Not-determined | Genome-fragment | NA | | 0 |
| S11-final-viral-combined_k141_95855\|\|full | 2712 | No | NA | | 0 | 0 | Not-determined | Genome-fragment | NA | | 0 |
| S12_k141_4486 | 1191 | No | NA | | 1 | 0 | Low-quality | Genome-fragment | | 3.29 | 0 |
| S4-final-viral-combined_k141_78182\|\|full | 1191 | No | NA | | 0 | 0 | Low-quality | Genome-fragment | | 1.45 | 0 |
| S4_k141_36250 | 1191 | No | NA | | 0 | 0 | Low-quality | Genome-fragment | | 1.19 | 0 |
| S6-final-viral-combined_k141_139316\|\|lt2gene | 1191 | No | NA | | 1 | 0 | Low-quality | Genome-fragment | | 2.16 | 0 |
| S11-final-viral-combined_k141_136596\|\|full | 5533 | No | NA | | 0 | 1 | Not-determined | Genome-fragment | NA | | 0 |
| S8-final-viral-combined_k141_131304\|\|full | 1191 | No | NA | | 0 | 1 | Low-quality | Genome-fragment | | 0.64 | 0 |
| S11-final-viral-combined_k141_289191\|\|full | 5275 | No | NA | | 0 | 3 | Not-determined | Genome-fragment | NA | | 0 |
| S8-final-viral-combined_k141_145326\|\|full | 1191 | No | NA | | 0 | 0 | Low-quality | Genome-fragment | | 1.6 | 0 |
| S9-final-viral-combined_k141_74074\|\|lt2gene | 1192 | No | NA | | 1 | 0 | Low-quality | Genome-fragment | | 3.3 | 0 |
| S11-final-viral-combined_k141_165226\|\|full | 2058 | No | NA | | 0 | 2 | Not-determined | Genome-fragment | NA | | 0 |
| S9_k141_58903 | 1192 | No | NA | | 0 | 0 | Low-quality | Genome-fragment | | 1.22 | 0 |
| S11-final-viral-combined_k141_25941\|\|full | 3404 | No | NA | | 0 | 1 | Not-determined | Genome-fragment | NA | | 0 |
| S11-final-viral-combined_k141_98316\|\|lt2gene | 1192 | No | NA | | 1 | 0 | Low-quality | Genome-fragment | | 3.1 | 0 |
| S11-final-viral-combined_k141_136615\|\|full | 5494 | No | NA | | 0 | 4 | Not-determined | Genome-fragment | NA | | 0 |
| S11-final-viral-combined_k141_136618\|\|full | 1900 | No | NA | | 0 | 3 | Not-determined | Genome-fragment | NA | | 0 |
| S11-final-viral-combined_k141_328682\|\|full | 6458 | No | NA | | 0 | 5 | Not-determined | Genome-fragment | NA | | 0 |
| S1-final-viral-combined_k141_141716\|\|full | 1192 | Yes | | 384 | 1 | 1 | Low-quality | Genome-fragment | | 0.71 | 67.79 |
| S11-final-viral-combined_k141_328693\|\|full | 1128 | No | NA | | 0 | 1 | Not-determined | Genome-fragment | NA | | 0 |
| S5-final-viral-combined_k141_125162\|\|full | 1192 | No | NA | | 3 | 0 | Low-quality | Genome-fragment | | 2.56 | 0 |
| S11-final-viral-combined_k141_179090\|\|full | 2107 | No | NA | | 0 | 2 | Not-determined | Genome-fragment | NA | | 0 |
| S12-final-viral-combined_k141_103187\|\|lt2gene | 1192 | No | NA | | 1 | 0 | Low-quality | Genome-fragment | | 0.77 | 0 |
| S6-final-viral-combined_k141_47267\|\|full | 1192 | No | NA | | 2 | 0 | Low-quality | Genome-fragment | | 2.47 | 0 |
| S9-final-viral-combined_k141_102627\|\|full | 1193 | No | NA | | 2 | 0 | Low-quality | Genome-fragment | | 1.93 | 0 |
| S11-final-viral-combined_k141_294678\|\|lt2gene | 1193 | No | NA | | 1 | 0 | Low-quality | Genome-fragment | | 3.34 | 0 |
| S1-final-viral-combined_k141_65482\|\|full | 1193 | No | NA | | 0 | 0 | Low-quality | Genome-fragment | | 1.8 | 0 |
| S7-final-viral-combined_k141_175465\|\|full | 1193 | No | NA | | 0 | 0 | Low-quality | Genome-fragment | | 2.35 | 0 |
| S11-final-viral-combined_k141_26014\|\|full | 1423 | No | NA | | 0 | 0 | Not-determined | Genome-fragment | NA | | 0 |
| S11-final-viral-combined_k141_191955\|\|full | 2430 | No | NA | | 0 | 2 | Not-determined | Genome-fragment | NA | | 0 |
| S11-final-viral-combined_k141_81598\|\|full | 1993 | No | NA | | 0 | 2 | Not-determined | Genome-fragment | NA | | 0 |
| S7-final-viral-combined_k141_198945\|\|full | 1193 | No | NA | | 0 | 0 | Low-quality | Genome-fragment | | 2.06 | 0 |
| S7_k141_51282 | 1193 | No | NA | | 0 | 1 | Low-quality | Genome-fragment | | 0.52 | 0 |
| S12-final-viral-combined_k141_258059\|\|full | 1193 | No | NA | | 1 | 0 | Low-quality | Genome-fragment | | 2.81 | 0 |
| S12-final-viral-combined_k141_275929\|\|full | 1193 | No | NA | | 0 | 0 | Low-quality | Genome-fragment | | 0.86 | 0 |
| S12-final-viral-combined_k141_336782\|\|lt2gene | 1193 | No | NA | | 1 | 0 | Low-quality | Genome-fragment | | 1.19 | 0 |
| S11-final-viral-combined_k141_136744\|\|full | 3205 | No | NA | | 0 | 2 | Not-determined | Genome-fragment | NA | | 0 |
| S8-final-viral-combined_k141_217599\|\|full | 1193 | No | NA | | 0 | 1 | Low-quality | Genome-fragment | | 2.02 | 0 |
| S9-final-viral-combined_k141_101861\|\|full | 1194 | No | NA | | 0 | 0 | Low-quality | Genome-fragment | | 2.86 | 0 |
| S11-final-viral-combined_k141_26097\|\|full | 1608 | No | NA | | 0 | 2 | Not-determined | Genome-fragment | NA | | 0 |
| S11-final-viral-combined_k141_13761\|\|full | 2478 | No | NA | | 0 | 1 | Not-determined | Genome-fragment | NA | | 0 |
| S9_k141_82087 | 1194 | No | NA | | 0 | 0 | Low-quality | Genome-fragment | | 2.88 | 0 |
| S11-final-viral-combined_k141_124687\|\|full | 1194 | No | NA | | 0 | 0 | Low-quality | Genome-fragment | | 0.32 | 0 |
| S11-final-viral-combined_k141_81646\|\|full | 2258 | No | NA | | 0 | 2 | Not-determined | Genome-fragment | NA | | 0 |
| S1-final-viral-combined_k141_136345\|\|full | 1194 | No | NA | | 0 | 1 | Low-quality | Genome-fragment | | 0.85 | 0 |
| S11-final-viral-combined_k141_136796\|\|full | 2167 | No | NA | | 0 | 0 | Not-determined | Genome-fragment | NA | | 0 |
| S1-final-viral-combined_k141_276092\|\|full | 1194 | No | NA | | 1 | 0 | Low-quality | Genome-fragment | | 0.77 | 0 |
| S1-final-viral-combined_k141_20116\|\|lt2gene | 1194 | No | NA | | 2 | 0 | Low-quality | Genome-fragment | | 2.57 | 0 |
| S11-final-viral-combined_k141_302484\|\|full | 1306 | No | NA | | 0 | 0 | Not-determined | Genome-fragment | NA | | 0 |
| S1_k141_58778 | 1194 | No | NA | | 0 | 1 | Low-quality | Genome-fragment | | 2.44 | 0 |
| S5-final-viral-combined_k141_106260\|\|full | 1194 | No | NA | | 1 | 0 | Low-quality | Genome-fragment | | 1.89 | 0 |
| S11-final-viral-combined_k141_328798\|\|full | 2610 | No | NA | | 0 | 0 | Not-determined | Genome-fragment | NA | | 0 |
| S17-final-viral-combined_k141_78993\|\|lt2gene | 1194 | No | NA | | 1 | 0 | Low-quality | Genome-fragment | | 0.74 | 0 |
| S12_k141_300859 | 1194 | No | NA | | 0 | 1 | Low-quality | Genome-fragment | | 3.37 | 0 |
| S2-final-viral-combined_k141_244388\|\|full | 1194 | No | NA | | 0 | 1 | Low-quality | Genome-fragment | | 2.85 | 0 |
| S2-final-viral-combined_k141_7392\|\|full | 1194 | No | NA | | 1 | 0 | Low-quality | Genome-fragment | | 2.57 | 0 |
| S11-final-viral-combined_k141_328840\|\|full | 4511 | No | NA | | 0 | 3 | Not-determined | Genome-fragment | NA | | 0 |
| S4_k141_13000 | 1194 | No | NA | | 2 | 0 | Low-quality | Genome-fragment | | 1.91 | 0 |
| S11-final-viral-combined_k141_151446\|\|full | 2083 | No | NA | | 0 | 3 | Not-determined | Genome-fragment | NA | | 0 |
| S11-final-viral-combined_k141_302584\|\|full | 1106 | No | NA | | 0 | 0 | Not-determined | Genome-fragment | NA | | 0 |
| S8_k141_50788 | 1194 | No | NA | | 0 | 0 | Low-quality | Genome-fragment | | 1.55 | 0 |
| S11-final-viral-combined_k141_151488\|\|full | 1018 | No | NA | | 0 | 1 | Not-determined | Genome-fragment | NA | | 0 |
| S11-final-viral-combined_k141_136951\|\|full | 1692 | No | NA | | 0 | 2 | Not-determined | Genome-fragment | NA | | 0 |
| S9_k141_123755 | 1195 | No | NA | | 0 | 2 | Low-quality | Genome-fragment | | 0.37 | 0 |
| S11-final-viral-combined_k141_275068\|\|full | 2749 | No | NA | | 0 | 3 | Not-determined | Genome-fragment | NA | | 0 |
| S9_k141_119413 | 1195 | No | NA | | 0 | 0 | Low-quality | Genome-fragment | | 2.4 | 0 |
| S11-final-viral-combined_k141_207825\|\|full | 1195 | No | NA | | 1 | 0 | Low-quality | Genome-fragment | | 2.26 | 0 |
| S11_k141_253537 | 1195 | No | NA | | 0 | 0 | Low-quality | Genome-fragment | | 1.81 | 0 |
| S11-final-viral-combined_k141_81877\|\|full | 4097 | No | NA | | 0 | 3 | Not-determined | Genome-fragment | NA | | 0 |
| S11-final-viral-combined_k141_68874\|\|full | 2190 | No | NA | | 0 | 1 | Not-determined | Genome-fragment | NA | | 0 |
| S11-final-viral-combined_k141_123287\|\|full | 2795 | No | NA | | 0 | 0 | Not-determined | Genome-fragment | NA | | 0 |
| S11-final-viral-combined_k141_137033\|\|full | 6213 | No | NA | | 0 | 7 | Not-determined | Genome-fragment | NA | | 0 |
| S1_k141_364305 | 1195 | No | NA | | 0 | 1 | Low-quality | Genome-fragment | | 0.95 | 0 |
| S11-final-viral-combined_k141_137044\|\|full | 1053 | No | NA | | 0 | 0 | Not-determined | Genome-fragment | NA | | 0 |
| S11-final-viral-combined_k141_81916\|\|full | 2654 | No | NA | | 0 | 2 | Not-determined | Genome-fragment | NA | | 0 |
| S5-final-viral-combined_k141_5385\|\|lt2gene | 1195 | No | NA | | 0 | 0 | Low-quality | Genome-fragment | | 2.47 | 0 |
| S7-final-viral-combined_k141_13842\|\|full | 1195 | No | NA | | 0 | 1 | Low-quality | Genome-fragment | | 1.27 | 0 |
| S11-final-viral-combined_k141_137073\|\|full | 2055 | No | NA | | 0 | 0 | Not-determined | Genome-fragment | NA | | 0 |
| S11-final-viral-combined_k141_26402\|\|full | 2251 | No | NA | | 0 | 1 | Not-determined | Genome-fragment | NA | | 0 |
| S7_k141_107846 | 1195 | No | NA | | 0 | 0 | Low-quality | Genome-fragment | | 2.2 | 0 |
| S11-final-viral-combined_k141_96357\|\|full | 2237 | No | NA | | 0 | 1 | Not-determined | Genome-fragment | NA | | 0 |
| S17-final-viral-combined_k141_257058\|\|full | 1195 | No | NA | | 1 | 0 | Low-quality | Genome-fragment | | 2.43 | 0 |
| S17-k141_8364 | 1195 | No | NA | | 0 | 0 | Low-quality | Genome-fragment | | 2.6 | 0 |
| S11-final-viral-combined_k141_192361\|\|full | 2335 | No | NA | | 0 | 1 | Not-determined | Genome-fragment | NA | | 0 |
| S11-final-viral-combined_k141_192363\|\|full | 1790 | No | NA | | 0 | 2 | Not-determined | Genome-fragment | NA | | 0 |
| S11-final-viral-combined_k141_123409\|\|full | 2848 | No | NA | | 0 | 1 | Not-determined | Genome-fragment | NA | | 0 |
| S12-final-viral-combined_k141_104024\|\|lt2gene | 1195 | No | NA | | 1 | 0 | Low-quality | Genome-fragment | | 2.56 | 0 |
| S11-final-viral-combined_k141_316608\|\|full | 1399 | No | NA | | 0 | 2 | Not-determined | Genome-fragment | NA | | 0 |
| S12_k141_246229 | 1195 | No | NA | | 0 | 0 | Low-quality | Genome-fragment | | 1.97 | 0 |
| S2-final-viral-combined_k141_65502\|\|full | 1195 | Yes | | 335 | 1 | 1 | Low-quality | Genome-fragment | | 0.17 | 71.97 |
| S2-final-viral-combined_k141_3663\|\|lt2gene | 1195 | No | NA | | 1 | 0 | Low-quality | Genome-fragment | | 2.41 | 0 |
| S11-final-viral-combined_k141_26585\|\|full | 10287 | No | NA | | 0 | 8 | Not-determined | Genome-fragment | NA | | 0 |
| S2-final-viral-combined_k141_27746\|\|lt2gene | 1195 | No | NA | | 1 | 0 | Low-quality | Genome-fragment | | 2.97 | 0 |
| S11-final-viral-combined_k141_329005\|\|full | 1086 | No | NA | | 0 | 2 | Not-determined | Genome-fragment | NA | | 0 |
| S11-final-viral-combined_k141_329008\|\|full | 1770 | No | NA | | 0 | 1 | Not-determined | Genome-fragment | NA | | 0 |
| S11-final-viral-combined_k141_192453\|\|full | 2940 | No | NA | | 0 | 1 | Not-determined | Genome-fragment | NA | | 0 |
| S4_k141_21422 | 1195 | No | NA | | 0 | 0 | Low-quality | Genome-fragment | | 1.99 | 0 |
| S11-final-viral-combined_k141_275323\|\|full | 11311 | No | NA | | 0 | 9 | Not-determined | Genome-fragment | NA | | 0 |
| S11-final-viral-combined_k141_26628\|\|full | 2788 | No | NA | | 0 | 1 | Not-determined | Genome-fragment | NA | | 0 |
| S8_k141_125646 | 1195 | No | NA | | 1 | 0 | Low-quality | Genome-fragment | | 1.19 | 0 |
| S8_k141_92110 | 1195 | No | NA | | 2 | 0 | Low-quality | Genome-fragment | | 3.71 | 0 |
| S1-final-viral-combined_k141_151648\|\|full | 1196 | No | NA | | 0 | 0 | Low-quality | Genome-fragment | | 3.39 | 0 |
| S11-final-viral-combined_k141_26670\|\|full | 1103 | No | NA | | 0 | 1 | Not-determined | Genome-fragment | NA | | 0 |
| S11-final-viral-combined_k141_275376\|\|full | 1837 | No | NA | | 0 | 1 | Not-determined | Genome-fragment | NA | | 0 |
| S1-final-viral-combined_k141_218381\|\|full | 1196 | No | NA | | 0 | 2 | Low-quality | Genome-fragment | | 1.75 | 0 |
| S11-final-viral-combined_k141_82137\|\|full | 2940 | No | NA | | 0 | 3 | Not-determined | Genome-fragment | NA | | 0 |
| S1-final-viral-combined_k141_329009\|\|full | 1196 | No | NA | | 0 | 0 | Low-quality | Genome-fragment | | 2.51 | 0 |
| S11-final-viral-combined_k141_123643\|\|full | 2085 | No | NA | | 0 | 2 | Not-determined | Genome-fragment | NA | | 0 |
| S11-final-viral-combined_k141_220472\|\|full | 1306 | No | NA | | 0 | 1 | Not-determined | Genome-fragment | NA | | 0 |
| S11-final-viral-combined_k141_82177\|\|full | 1015 | No | NA | | 0 | 2 | Not-determined | Genome-fragment | NA | | 0 |
| S11-final-viral-combined_k141_26706\|\|full | 1622 | No | NA | | 0 | 1 | Not-determined | Genome-fragment | NA | | 0 |
| S11-final-viral-combined_k141_123658\|\|full | 1860 | No | NA | | 0 | 1 | Not-determined | Genome-fragment | NA | | 0 |
| S5_k141_125564 | 1196 | No | NA | | 0 | 1 | Low-quality | Genome-fragment | | 0.79 | 0 |
| S17-final-viral-combined_k141_51629\|\|full | 1196 | No | NA | | 0 | 0 | Low-quality | Genome-fragment | | 0.79 | 0 |
| S11-final-viral-combined_k141_137427\|\|full | 3130 | No | NA | | 0 | 3 | Not-determined | Genome-fragment | NA | | 0 |
| S12_k141_5446 | 1196 | No | NA | | 1 | 0 | Low-quality | Genome-fragment | | 1.37 | 0 |

| | | | | | | | | | | |
|---|---|---|---|---|---|---|---|---|---|---|
| S11-final-viral-combined_k141_303066\|\|full | 2255 | No | NA | 0 | 3 | Not-determined | Genome-fragment | NA | | 0 |
| S12_k141_362016 | 1196 | No | NA | 1 | 0 | Low-quality | Genome-fragment | | 2.97 | 0 |
| S2-final-viral-combined_k141_57049\|\|full | 1196 | No | NA | 0 | 1 | Low-quality | Genome-fragment | | 2.45 | 0 |
| S11-final-viral-combined_k141_137498\|\|full | 4177 | No | NA | 0 | 1 | Not-determined | Genome-fragment | NA | | 0 |
| S2-final-viral-combined_k141_196488\|\|full | 1196 | No | NA | 0 | 0 | Low-quality | Genome-fragment | | 1.46 | 0 |
| S11-final-viral-combined_k141_137509\|\|full | 2420 | No | NA | 0 | 1 | Not-determined | Genome-fragment | NA | | 0 |
| S11-final-viral-combined_k141_192708\|\|full | 1096 | No | NA | 0 | 1 | Not-determined | Genome-fragment | NA | | 0 |
| S11-final-viral-combined_k141_137547\|\|full | 1126 | No | NA | 0 | 1 | Not-determined | Genome-fragment | NA | | 0 |
| S2-final-viral-combined_k141_127322\|\|lt2gene | 1196 | No | NA | 1 | 0 | Low-quality | Genome-fragment | | 2.83 | 0 |
| S11-final-viral-combined_k141_329193\|\|full | 2169 | No | NA | 0 | 1 | Not-determined | Genome-fragment | NA | | 0 |
| S11-final-viral-combined_k141_82361\|\|full | 14273 | No | NA | 0 | 12 | Not-determined | Genome-fragment | NA | | 0 |
| S11-final-viral-combined_k141_316880\|\|full | 2304 | No | NA | 0 | 2 | Not-determined | Genome-fragment | NA | | 0 |
| S11-final-viral-combined_k141_192773\|\|full | 5206 | No | NA | 0 | 2 | Not-determined | Genome-fragment | NA | | 0 |
| S8-final-viral-combined_k141_115793\|\|full | 1196 | No | NA | 0 | 0 | Low-quality | Genome-fragment | | 1 | 0 |
| S11-final-viral-combined_k141_206350\|\|full | 12767 | No | NA | 0 | 8 | Not-determined | Genome-fragment | NA | | 0 |
| S11-final-viral-combined_k141_275525\|\|full | 11803 | No | NA | 0 | 9 | Not-determined | Genome-fragment | NA | | 0 |
| S11-final-viral-combined_k141_329260\|\|full | 9328 | No | NA | 0 | 3 | Not-determined | Genome-fragment | NA | | 0 |
| S8-final-viral-combined_k141_87995\|\|full | 1196 | No | NA | 0 | 1 | Low-quality | Genome-fragment | | 2.27 | 0 |
| S11-final-viral-combined_k141_26933\|\|full | 1043 | No | NA | 0 | 0 | Not-determined | Genome-fragment | NA | | 0 |
| S11-final-viral-combined_k141_26956\|\|full | 1366 | No | NA | 0 | 0 | Not-determined | Genome-fragment | NA | | 0 |
| S8-final-viral-combined_k141_67175\|\|full | 1196 | No | NA | 0 | 2 | Low-quality | Genome-fragment | | 2.75 | 0 |
| S11-final-viral-combined_k141_206462\|\|full | 2926 | No | NA | 0 | 2 | Not-determined | Genome-fragment | NA | | 0 |
| S11-final-viral-combined_k141_329343\|\|full | 1303 | No | NA | 0 | 1 | Not-determined | Genome-fragment | NA | | 0 |
| S9-final-viral-combined_k141_100194\|\|full | 1197 | No | NA | 1 | 0 | Low-quality | Genome-fragment | | 2.69 | 0 |
| S11-final-viral-combined_k141_329421\|\|full | 2051 | No | NA | 0 | 3 | Not-determined | Genome-fragment | NA | | 0 |
| S9-final-viral-combined_k141_28565\|\|lt2gene | 1197 | No | NA | 1 | 0 | Low-quality | Genome-fragment | | 1.79 | 0 |
| S11_k141_295013 | 1197 | No | NA | 0 | 2 | Low-quality | Genome-fragment | | 0.71 | 0 |
| S11-final-viral-combined_k141_27115\|\|full | 1681 | No | NA | 0 | 1 | Not-determined | Genome-fragment | NA | | 0 |
| S11-final-viral-combined_k141_206655\|\|full | 2551 | No | NA | 0 | 0 | Not-determined | Genome-fragment | NA | | 0 |
| S11-final-viral-combined_k141_27141\|\|full | 3494 | No | NA | 0 | 4 | Not-determined | Genome-fragment | NA | | 0 |
| S11-final-viral-combined_k141_329493\|\|full | 51811 | No | NA | 1 | 29 | High-quality | High-quality | | 100 | 0 |
| S1_k141_107624 | 1197 | No | NA | 0 | 0 | Low-quality | Genome-fragment | | 3.07 | 0 |
| S11-final-viral-combined_k141_329522\|\|full | 7380 | No | NA | 0 | 6 | Not-determined | Genome-fragment | NA | | 0 |
| S11-final-viral-combined_k141_27202\|\|full | 1973 | No | NA | 0 | 2 | Not-determined | Genome-fragment | NA | | 0 |
| S11-final-viral-combined_k141_27219\|\|full | 3045 | No | NA | 0 | 1 | Not-determined | Genome-fragment | NA | | 0 |
| S11-final-viral-combined_k141_27282\|\|full | 2612 | No | NA | 0 | 0 | Not-determined | Genome-fragment | NA | | 0 |
| S1_k141_193419 | 1197 | No | NA | 0 | 1 | Low-quality | Genome-fragment | | 0.67 | 0 |
| S11-final-viral-combined_k141_329809\|\|full | 1281 | No | NA | 0 | 0 | Not-determined | Genome-fragment | NA | | 0 |
| S11-final-viral-combined_k141_329864\|\|full | 3938 | No | NA | 0 | 2 | Not-determined | Genome-fragment | NA | | 0 |
| S3-final-viral-combined_k141_22812\|\|lt2gene | 1197 | No | NA | 0 | 0 | Low-quality | Genome-fragment | | 2.11 | 0 |
| S11-final-viral-combined_k141_330012\|\|full | 1157 | No | NA | 0 | 1 | Not-determined | Genome-fragment | NA | | 0 |
| S11-final-viral-combined_k141_330119\|\|full | 1613 | No | NA | 0 | 1 | Not-determined | Genome-fragment | NA | | 0 |
| S11-final-viral-combined_k141_330252\|\|full | 6534 | No | NA | 0 | 0 | Not-determined | Genome-fragment | NA | | 0 |
| S11-final-viral-combined_k141_330345\|\|full | 1780 | No | NA | 0 | 2 | Not-determined | Genome-fragment | NA | | 0 |
| S11-final-viral-combined_k141_330368\|\|full | 4243 | No | NA | 0 | 1 | Not-determined | Genome-fragment | NA | | 0 |
| S3_k141_130305 | 1197 | No | NA | 0 | 2 | Low-quality | Genome-fragment | | 1.57 | 0 |
| S11-final-viral-combined_k141_124528\|\|0_partial | 12304 | No | NA | 0 | 4 | Not-determined | Genome-fragment | NA | | 0 |
| S11-final-viral-combined_k141_96978\|\|0_partial | 16088 | No | NA | 0 | 12 | Not-determined | Genome-fragment | NA | | 0 |
| S3_k141_124419 | 1197 | No | NA | 0 | 0 | Low-quality | Genome-fragment | | 0.55 | 0 |
| S11-final-viral-combined_k141_248668\|\|0_partial | 10925 | No | NA | 0 | 6 | Not-determined | Genome-fragment | NA | | 0 |
| S5-final-viral-combined_k141_21394\|\|full | 1197 | No | NA | 0 | 1 | Low-quality | Genome-fragment | | 2.11 | 0 |
| S11-final-viral-combined_k141_236046\|\|0_partial | 22654 | No | NA | 0 | 16 | Not-determined | Genome-fragment | NA | | 0 |
| S17-final-viral-combined_k141_167568\|\|full | 1197 | No | NA | 0 | 2 | Low-quality | Genome-fragment | | 0.46 | 0 |
| S11-final-viral-combined_k141_153467\|\|0_partial | 25494 | No | NA | 0 | 15 | Not-determined | Genome-fragment | NA | | 0 |
| S17-final-viral-combined_k141_108127\|\|full | 1197 | No | NA | 0 | 0 | Low-quality | Genome-fragment | | 0.78 | 0 |
| S12-final-viral-combined_k141_271871\|\|full | 1197 | No | NA | 0 | 0 | Low-quality | Genome-fragment | | 2.72 | 0 |
| S12_k141_87408 | 1197 | No | NA | 0 | 0 | Low-quality | Genome-fragment | | 0.65 | 0 |
| S12_k141_144612 | 1197 | No | NA | 0 | 0 | Low-quality | Genome-fragment | | 2.05 | 0 |
| S2-final-viral-combined_k141_104938\|\|lt2gene | 1197 | No | NA | 1 | 0 | Low-quality | Genome-fragment | | 1.57 | 0 |
| S6-final-viral-combined_k141_78058\|\|full | 1197 | No | NA | 0 | 0 | Low-quality | Genome-fragment | | 2.77 | 0 |
| S8-final-viral-combined_k141_231507\|\|full | 1197 | No | NA | 0 | 1 | Low-quality | Genome-fragment | | 0.78 | 0 |
| S8-final-viral-combined_k141_212425\|\|full | 1197 | No | NA | 0 | 0 | Low-quality | Genome-fragment | | 0.78 | 0 |
| S8_k141_87954 | 1197 | No | NA | 1 | 0 | Low-quality | Genome-fragment | | 1.91 | 0 |
| S9-final-viral-combined_k141_76842\|\|full | 1198 | No | NA | 0 | 0 | Low-quality | Genome-fragment | | 2.8 | 0 |
| S11-final-viral-combined_k141_18338\|\|0_partial | 13664 | No | NA | 0 | 8 | Not-determined | Genome-fragment | NA | | 0 |
| S9-final-viral-combined_k141_89724\|\|full | 1198 | No | NA | 0 | 0 | Low-quality | Genome-fragment | | 0.35 | 0 |
| S11-final-viral-combined_k141_36901\|\|full | 1198 | No | NA | 0 | 0 | Low-quality | Genome-fragment | | 1.8 | 0 |
| S11_k141_191183 | 1198 | No | NA | 0 | 0 | Low-quality | Genome-fragment | | 2.12 | 0 |
| S11-final-viral-combined_k141_129668\|\|0_partial | 15245 | No | NA | 0 | 11 | Not-determined | Genome-fragment | NA | | 0 |
| S1-final-viral-combined_k141_197635\|\|full | 1198 | No | NA | 0 | 1 | Low-quality | Genome-fragment | | 1.64 | 0 |
| S1-final-viral-combined_k141_369279\|\|full | 1198 | No | NA | 0 | 0 | Low-quality | Genome-fragment | | 2.05 | 0 |
| S11-final-viral-combined_k141_47427\|\|0_partial | 30930 | No | NA | 0 | 16 | Not-determined | Genome-fragment | NA | | 0 |
| S11-final-viral-combined_k141_199102\|\|0_partial | 21178 | No | NA | 0 | 17 | Not-determined | Genome-fragment | NA | | 0 |
| S1-final-viral-combined_k141_289473\|\|full | 1198 | No | NA | 0 | 1 | Low-quality | Genome-fragment | | 0.7 | 0 |
| S11-final-viral-combined_k141_283224\|\|0_partial | 11396 | No | NA | 0 | 7 | Not-determined | Genome-fragment | NA | | 0 |
| S12-final-viral-combined_k141_325590\|\|full | 1198 | No | NA | 0 | 1 | Low-quality | Genome-fragment | | 0.47 | 0 |
| S11-final-viral-combined_k141_200131\|\|0_partial | 13297 | No | NA | 0 | 8 | Not-determined | Genome-fragment | NA | | 0 |
| S11-final-viral-combined_k141_242954\|\|0_partial | 13557 | No | NA | 0 | 9 | Not-determined | Genome-fragment | NA | | 0 |
| S12_k141_137877 | 1198 | No | NA | 0 | 0 | Low-quality | Genome-fragment | | 2.76 | 0 |
| S11-final-viral-combined_k141_146499\|\|0_partial | 10736 | No | NA | 0 | 3 | Not-determined | Genome-fragment | NA | | 0 |
| S2-final-viral-combined_k141_151538\|\|full | 1198 | No | NA | 0 | 1 | Low-quality | Genome-fragment | | 1.63 | 0 |
| S6_k141_118051 | 1198 | No | NA | 0 | 1 | Low-quality | Genome-fragment | | 4 | 0 |
| S8-final-viral-combined_k141_179335\|\|full | 1198 | No | NA | 1 | 0 | Low-quality | Genome-fragment | | 2.71 | 0 |
| S11-final-viral-combined_k141_4559\|\|full | 1199 | No | NA | 0 | 0 | Low-quality | Genome-fragment | | 2.36 | 0 |
| S11-final-viral-combined_k141_313109\|\|full | 1199 | No | NA | 0 | 1 | Low-quality | Genome-fragment | | 2.2 | 0 |
| S11-final-viral-combined_k141_161582\|\|full | 1199 | No | NA | 0 | 0 | Low-quality | Genome-fragment | | 2.12 | 0 |
| S11_k141_22994 | 1199 | No | NA | 0 | 0 | Low-quality | Genome-fragment | | 0.9 | 0 |
| S1-final-viral-combined_k141_134486\|\|full | 1199 | No | NA | 0 | 0 | Low-quality | Genome-fragment | | 2.66 | 0 |
| S11-final-viral-combined_k141_275441\|\|0_partial | 11597 | No | NA | 0 | 6 | Not-determined | Genome-fragment | NA | | 0 |
| S1-final-viral-combined_k141_173800\|\|full | 1199 | No | NA | 3 | 0 | Low-quality | Genome-fragment | | 3.2 | 0 |
| S1_k141_15332 | 1199 | No | NA | 2 | 0 | Low-quality | Genome-fragment | | 1.44 | 0 |
| S11-final-viral-combined_k141_234401\|\|lt2gene | 1243 | No | NA | 0 | 0 | Not-determined | Genome-fragment | NA | | 0 |
| S11-final-viral-combined_k141_55359\|\|lt2gene | 1106 | No | NA | 0 | 0 | Not-determined | Genome-fragment | NA | | 0 |
| S17-final-viral-combined_k141_206889\|\|lt2gene | 1199 | No | NA | 1 | 0 | Low-quality | Genome-fragment | | 31.1 | 0 |
| S17-k141_11545 | 1199 | No | NA | 0 | 1 | Low-quality | Genome-fragment | | 0.45 | 0 |
| S2-final-viral-combined_k141_91251\|\|full | 1199 | No | NA | 0 | 0 | Low-quality | Genome-fragment | | 2.59 | 0 |
| S11-final-viral-combined_k141_317354\|\|lt2gene | 1102 | No | NA | 0 | 0 | Not-determined | Genome-fragment | NA | | 0 |
| S11-final-viral-combined_k141_152129\|\|lt2gene | 1111 | No | NA | 0 | 0 | Not-determined | Genome-fragment | NA | | 0 |
| S4-final-viral-combined_k141_72548\|\|full | 1199 | No | NA | 0 | 0 | Low-quality | Genome-fragment | | 2.79 | 0 |
| S8_k141_210104 | 1199 | No | NA | 0 | 2 | Low-quality | Genome-fragment | | 0.6 | 0 |
| S9-final-viral-combined_k141_32219\|\|full | 1200 | No | NA | 3 | 0 | Low-quality | Genome-fragment | | 2.6 | 0 |
| S11-final-viral-combined_k141_78924\|\|full | 1200 | No | NA | 0 | 2 | Low-quality | Genome-fragment | | 0.75 | 0 |
| S1-final-viral-combined_k141_162482\|\|full | 1200 | No | NA | 1 | 0 | Low-quality | Genome-fragment | | 2.2 | 0 |
| S11-final-viral-combined_k141_110829\|\|lt2gene | 1730 | No | NA | 0 | 0 | Not-determined | Genome-fragment | NA | | 0 |
| S11-final-viral-combined_k141_166256\|\|lt2gene | 1091 | No | NA | 0 | 0 | Not-determined | Genome-fragment | NA | | 0 |
| S5-final-viral-combined_k141_49840\|\|full | 1200 | No | NA | 1 | 0 | Low-quality | Genome-fragment | | 2.44 | 0 |
| S11-final-viral-combined_k141_166357\|\|lt2gene | 1285 | No | NA | 0 | 0 | Not-determined | Genome-fragment | NA | | 0 |
| S11-final-viral-combined_k141_179840\|\|lt2gene | 1264 | No | NA | 0 | 0 | Not-determined | Genome-fragment | NA | | 0 |
| S7_k141_121767 | 1200 | No | NA | 0 | 0 | Low-quality | Genome-fragment | | 0.97 | 0 |
| S11-final-viral-combined_k141_262751\|\|lt2gene | 1425 | No | NA | 0 | 0 | Not-determined | Genome-fragment | NA | | 0 |
| S11-final-viral-combined_k141_42167\|\|lt2gene | 1013 | No | NA | 0 | 1 | Not-determined | Genome-fragment | NA | | 0 |
| S11-final-viral-combined_k141_262805\|\|lt2gene | 1482 | No | NA | 0 | 0 | Not-determined | Genome-fragment | NA | | 0 |
| S17-final-viral-combined_k141_121124\|\|full | 1200 | No | NA | 1 | 0 | Low-quality | Genome-fragment | | 2.78 | 0 |
| S17-k141_255435 | 1200 | No | NA | 1 | 0 | Low-quality | Genome-fragment | | 2.23 | 0 |
| S8-final-viral-combined_k141_252531\|\|full | 1200 | No | NA | 0 | 0 | Low-quality | Genome-fragment | | 2.34 | 0 |
| S9-final-viral-combined_k141_104994\|\|full | 1201 | No | NA | 0 | 0 | Low-quality | Genome-fragment | | 3.02 | 0 |
| S11-final-viral-combined_k141_30592\|\|full | 1201 | No | NA | 0 | 1 | Low-quality | Genome-fragment | | 2.24 | 0 |
| S11-final-viral-combined_k141_30348\|\|lt2gene | 1201 | No | NA | 1 | 0 | Low-quality | Genome-fragment | | 0.69 | 0 |
| S11-final-viral-combined_k141_70145\|\|lt2gene | 1122 | No | NA | 0 | 0 | Not-determined | Genome-fragment | NA | | 0 |
| S1-final-viral-combined_k141_323914\|\|lt2gene | 1201 | No | NA | 2 | 0 | Low-quality | Genome-fragment | | 2.14 | 0 |
| S1-final-viral-combined_k141_30235\|\|lt2gene | 1201 | No | NA | 1 | 0 | Low-quality | Genome-fragment | | 3.74 | 0 |
| S3-final-viral-combined_k141_51708\|\|full | 1201 | No | NA | 0 | 0 | Low-quality | Genome-fragment | | 1.08 | 0 |
| S11-final-viral-combined_k141_263698\|\|lt2gene | 1086 | No | NA | 0 | 0 | Not-determined | Genome-fragment | NA | | 0 |
| S5-final-viral-combined_k141_17230\|\|full | 1201 | No | NA | 0 | 0 | Low-quality | Genome-fragment | | 2.14 | 0 |
| S11-final-viral-combined_k141_208973\|\|lt2gene | 2022 | No | NA | 0 | 0 | Not-determined | Genome-fragment | NA | | 0 |
| S11-final-viral-combined_k141_209406\|\|lt2gene | 1223 | No | NA | 0 | 0 | Not-determined | Genome-fragment | NA | | 0 |
| S7_k141_76279 | 1201 | No | NA | 0 | 0 | Low-quality | Genome-fragment | | 2.79 | 0 |
| S11-final-viral-combined_k141_264263\|\|lt2gene | 3639 | No | NA | 0 | 0 | Not-determined | Genome-fragment | NA | | 0 |
| S12-final-viral-combined_k141_175188\|\|full | 1201 | No | NA | 0 | 0 | Low-quality | Genome-fragment | | 1.28 | 0 |
| S4-final-viral-combined_k141_4296\|\|full | 1201 | No | NA | 0 | 1 | Low-quality | Genome-fragment | | 0.8 | 0 |
| S4-final-viral-combined_k141_27134\|\|full | 1201 | No | NA | 0 | 2 | Low-quality | Genome-fragment | | 0.75 | 0 |
| S8-final-viral-combined_k141_172713\|\|lt2gene | 1201 | No | NA | 1 | 0 | Low-quality | Genome-fragment | | 1.87 | 0 |
| S8_k141_184903 | 1201 | No | NA | 0 | 1 | Low-quality | Genome-fragment | | 0.52 | 0 |
| S9-final-viral-combined_k141_28563\|\|full | 1202 | No | NA | 0 | 0 | Low-quality | Genome-fragment | | 2.44 | 0 |
| S11-final-viral-combined_k141_154342\|\|lt2gene | 1435 | No | NA | 0 | 1 | Not-determined | Genome-fragment | NA | | 0 |
| S11-final-viral-combined_k141_223315\|\|lt2gene | 1141 | No | NA | 0 | 1 | Not-determined | Genome-fragment | NA | | 0 |
| S9-final-viral-combined_k141_124894\|\|full | 1202 | No | NA | 0 | 1 | Low-quality | Genome-fragment | | 0.95 | 0 |
| S11-final-viral-combined_k141_182608\|\|lt2gene | 1202 | No | NA | 1 | 0 | Low-quality | Genome-fragment | | 2.63 | 0 |
| S11_k141_180688 | 1202 | No | NA | 0 | 1 | Low-quality | Genome-fragment | | 0.38 | 0 |
| S1_k141_3150 | 1202 | No | NA | 0 | 2 | Low-quality | Genome-fragment | | 1.83 | 0 |
| S3-final-viral-combined_k141_130553\|\|full | 1202 | No | NA | 0 | 0 | Low-quality | Genome-fragment | | 2.62 | 0 |
| S11-final-viral-combined_k141_168705\|\|lt2gene | 1904 | No | NA | 0 | 1 | Not-determined | Genome-fragment | NA | | 0 |
| S5-final-viral-combined_k141_134950\|\|full | 1202 | No | NA | 1 | 0 | Low-quality | Genome-fragment | | 2.71 | 0 |
| S5-final-viral-combined_k141_79767\|\|lt2gene | 1202 | No | NA | 2 | 0 | Low-quality | Genome-fragment | | 2.57 | 0 |
| S5_k141_110240 | 1202 | No | NA | 3 | 0 | Low-quality | Genome-fragment | | 3.4 | 0 |
| S7-final-viral-combined_k141_104701\|\|full | 1202 | No | NA | 0 | 0 | Low-quality | Genome-fragment | | 1.66 | 0 |
| S17-final-viral-combined_k141_181424\|\|full | 1202 | No | NA | 1 | 0 | Low-quality | Genome-fragment | | 3.03 | 0 |
| S17-final-viral-combined_k141_188106\|\|full | 1202 | No | NA | 0 | 0 | Low-quality | Genome-fragment | | 2.06 | 0 |
| S17-final-viral-combined_k141_19169\|\|full | 1202 | No | NA | 0 | 0 | Low-quality | Genome-fragment | | 0.97 | 0 |
| S12-final-viral-combined_k141_131712\|\|full | 1202 | No | NA | 0 | 0 | Low-quality | Genome-fragment | | 3.52 | 0 |
| S2_k141_132694 | 1202 | No | NA | 0 | 0 | Low-quality | Genome-fragment | | 2.08 | 0 |
| S6-final-viral-combined_k141_99955\|\|full | 1202 | No | NA | 0 | 0 | Low-quality | Genome-fragment | | 1.56 | 0 |
| S6-final-viral-combined_k141_39108\|\|full | 1202 | No | NA | 0 | 0 | Low-quality | Genome-fragment | | 1.66 | 0 |
| S8-final-viral-combined_k141_74933\|\|full | 1202 | No | NA | 1 | 0 | Low-quality | Genome-fragment | | 2.41 | 0 |
| S9-final-viral-combined_k141_49619\|\|lt2gene | 1203 | No | NA | 1 | 0 | Low-quality | Genome-fragment | | 2.64 | 0 |
| S11-final-viral-combined_k141_72347\|\|lt2gene | 1576 | No | NA | 0 | 2 | Not-determined | Genome-fragment | NA | | 0 |
| S11-final-viral-combined_k141_228326\|\|full | 1203 | No | NA | 0 | 0 | Low-quality | Genome-fragment | | 0.49 | 0 |
| S3-final-viral-combined_k141_98328\|\|full | 1203 | No | NA | 0 | 0 | Low-quality | Genome-fragment | | 2.67 | 0 |
| S3-final-viral-combined_k141_51666\|\|lt2gene | 1203 | No | NA | 1 | 0 | Low-quality | Genome-fragment | | 1.93 | 0 |
| S5-final-viral-combined_k141_197232\|\|full | 1203 | No | NA | 0 | 0 | Low-quality | Genome-fragment | | 2.67 | 0 |
| S7-final-viral-combined_k141_88778\|\|full | 1203 | No | NA | 0 | 2 | Low-quality | Genome-fragment | | 0.41 | 0 |
| S12-final-viral-combined_k141_173332\|\|full | 1203 | No | NA | 0 | 0 | Low-quality | Genome-fragment | | 3.41 | 0 |
| S11-final-viral-combined_k141_252296\|\|lt2gene | 1107 | No | NA | 0 | 0 | Not-determined | Genome-fragment | NA | | 0 |
| S2-final-viral-combined_k141_24291\|\|lt2gene | 1203 | No | NA | 1 | 0 | Low-quality | Genome-fragment | | 2.7 | 0 |
| S2_k141_128097 | 1203 | No | NA | 0 | 1 | Low-quality | Genome-fragment | | 4.03 | 0 |
| S11-final-viral-combined_k141_183775\|\|lt2gene | 1116 | No | NA | 0 | 1 | Not-determined | Genome-fragment | NA | | 0 |
| S8_k141_248379 | 1203 | No | NA | 0 | 0 | Low-quality | Genome-fragment | | 1.72 | 0 |
| S11-final-viral-combined_k141_114239\|\|lt2gene | 1021 | No | NA | 0 | 0 | Not-determined | Genome-fragment | NA | | 0 |
| S9-final-viral-combined_k141_4206\|\|full | 1204 | No | NA | 0 | 0 | Low-quality | Genome-fragment | | 1.77 | 0 |
| S11-final-viral-combined_k141_146157\|\|full | 1204 | No | NA | 1 | 0 | Low-quality | Genome-fragment | | 1.97 | 0 |
| S11-final-viral-combined_k141_212001\|\|lt2gene | 1415 | No | NA | 0 | 0 | Not-determined | Genome-fragment | NA | | 0 |
| S11-final-viral-combined_k141_294012\|\|lt2gene | 1252 | No | NA | 0 | 0 | Not-determined | Genome-fragment | NA | | 0 |
| S11-final-viral-combined_k141_174757\|\|full | 1204 | No | NA | 0 | 0 | Low-quality | Genome-fragment | | 1.71 | 0 |
| S11-final-viral-combined_k141_212232\|\|lt2gene | 1077 | No | NA | 0 | 0 | Not-determined | Genome-fragment | NA | | 0 |

| | | | | | | | | | | | |
|---|---|---|---|---|---|---|---|---|---|---|---|
| S1-final-viral-combined_k141_343573\|\|full | 1204 | No | NA | | 0 | 1 | Low-quality | Genome-fragment | | 1.64 | 0 |
| S1_k141_153504 | 1204 | No | NA | | 0 | 2 | Low-quality | Genome-fragment | | 3.01 | 0 |
| S5-final-viral-combined_k141_60746\|\|lt2gene | 1204 | No | NA | | 1 | 0 | Low-quality | Genome-fragment | | 1.96 | 0 |
| S11-final-viral-combined_k141_267134\|\|lt2gene | 2447 | No | NA | | 0 | 1 | Not-determined | Genome-fragment | NA | | 0 |
| S11-final-viral-combined_k141_253375\|\|lt2gene | 2268 | No | NA | | 0 | 0 | Not-determined | Genome-fragment | NA | | 0 |
| S5_k141_134041 | 1204 | No | NA | | 0 | 0 | Low-quality | Genome-fragment | | 2.8 | 0 |
| S5_k141_32244 | 1204 | No | NA | | 0 | 1 | Low-quality | Genome-fragment | | 2.02 | 0 |
| S5_k141_42187 | 1204 | No | NA | | 0 | 2 | Low-quality | Genome-fragment | | 2.16 | 0 |
| S7-final-viral-combined_k141_170268\|\|full | 1204 | No | NA | | 1 | 0 | Low-quality | Genome-fragment | | 3.6 | 0 |
| S7_k141_211687 | 1204 | No | NA | | 0 | 1 | Low-quality | Genome-fragment | | 0.32 | 0 |
| S11-final-viral-combined_k141_102591\|\|lt2gene | 1241 | No | NA | | 0 | 0 | Not-determined | Genome-fragment | NA | | 0 |
| S17-final-viral-combined_k141_245967\|\|lt2gene | 1204 | No | NA | | 1 | 0 | Low-quality | Genome-fragment | | 2.21 | 0 |
| S17-k141_10510 | 1204 | No | NA | | 0 | 0 | Low-quality | Genome-fragment | | 1.16 | 0 |
| S11-final-viral-combined_k141_74132\|\|lt2gene | 1171 | No | NA | | 0 | 1 | Not-determined | Genome-fragment | NA | | 0 |
| S11-final-viral-combined_k141_157344\|\|lt2gene | 1022 | No | NA | | 0 | 0 | Not-determined | Genome-fragment | NA | | 0 |
| S12-final-viral-combined_k141_50442\|\|full | 1204 | No | NA | | 0 | 0 | Low-quality | Genome-fragment | | 2.06 | 0 |
| S12-final-viral-combined_k141_314578\|\|full | 1204 | No | NA | | 0 | 0 | Low-quality | Genome-fragment | | 2.76 | 0 |
| S12_k141_238449 | 1204 | No | NA | | 0 | 0 | Low-quality | Genome-fragment | | 1.69 | 0 |
| S11-final-viral-combined_k141_309559\|\|lt2gene | 2216 | No | NA | | 0 | 0 | Not-determined | Genome-fragment | NA | | 0 |
| S2_k141_40416 | 1204 | No | NA | | 0 | 1 | Low-quality | Genome-fragment | | 0.65 | 0 |
| S11-final-viral-combined_k141_74444\|\|lt2gene | 1628 | No | NA | | 0 | 0 | Not-determined | Genome-fragment | NA | | 0 |
| S8-final-viral-combined_k141_70213\|\|full | 1204 | No | NA | | 0 | 0 | Low-quality | Genome-fragment | | 0.9 | 0 |
| S11-final-viral-combined_k141_295345\|\|lt2gene | 1385 | No | NA | | 0 | 1 | Not-determined | Genome-fragment | NA | | 0 |
| S11-final-viral-combined_k141_309868\|\|lt2gene | 1267 | No | NA | | 0 | 1 | Not-determined | Genome-fragment | NA | | 0 |
| S11-final-viral-combined_k141_236973\|\|full | 1205 | No | NA | | 1 | 0 | Low-quality | Genome-fragment | | 3.2 | 0 |
| S11-final-viral-combined_k141_100255\|\|full | 1205 | No | NA | | 1 | 0 | Low-quality | Genome-fragment | | 1.86 | 0 |
| S11-final-viral-combined_k141_198511\|\|full | 1205 | No | NA | | 0 | 2 | Low-quality | Genome-fragment | | 0.36 | 0 |
| S11-final-viral-combined_k141_326167\|\|full | 1205 | No | NA | | 1 | 0 | Low-quality | Genome-fragment | | 2.55 | 0 |
| S11_k141_321186 | 1205 | No | NA | | 0 | 1 | Low-quality | Genome-fragment | | 1.46 | 0 |
| S1-final-viral-combined_k141_120667\|\|full | 1205 | No | NA | | 1 | 0 | Low-quality | Genome-fragment | | 0.48 | 0 |
| S11-final-viral-combined_k141_172134\|\|lt2gene | 1981 | No | NA | | 0 | 0 | Not-determined | Genome-fragment | NA | | 0 |
| S11-final-viral-combined_k141_172285\|\|lt2gene | 1141 | No | NA | | 0 | 1 | Not-determined | Genome-fragment | NA | | 0 |
| S11-final-viral-combined_k141_48007\|\|lt2gene | 1708 | No | NA | | 0 | 0 | Not-determined | Genome-fragment | NA | | 0 |
| S1-final-viral-combined_k141_230061\|\|full | 1205 | No | NA | | 0 | 2 | Low-quality | Genome-fragment | | 2.1 | 0 |
| S1-final-viral-combined_k141_32504\|\|full | 1205 | No | NA | | 0 | 0 | Low-quality | Genome-fragment | | 2.78 | 0 |
| S5-final-viral-combined_k141_142545\|\|full | 1205 | No | NA | | 0 | 0 | Low-quality | Genome-fragment | | 0.75 | 0 |
| S11-final-viral-combined_k141_199423\|\|lt2gene | 2659 | No | NA | | 0 | 1 | Not-determined | Genome-fragment | NA | | 0 |
| S7-final-viral-combined_k141_48740\|\|full | 1205 | No | NA | | 2 | 0 | Low-quality | Genome-fragment | | 3.35 | 0 |
| S7-final-viral-combined_k141_185308\|\|lt2gene | 1205 | No | NA | | 2 | 0 | Low-quality | Genome-fragment | | 3.49 | 0 |
| S7-final-viral-combined_k141_49041\|\|lt2gene | 1205 | No | NA | | 2 | 0 | Low-quality | Genome-fragment | | 2.43 | 0 |
| S17-final-viral-combined_k141_219670\|\|full | 1205 | No | NA | | 0 | 0 | Low-quality | Genome-fragment | | 1.87 | 0 |
| S12_k141_151420 | 1205 | No | NA | | 1 | 0 | Low-quality | Genome-fragment | | 3.14 | 0 |
| S11-final-viral-combined_k141_283307\|\|lt2gene | 1079 | No | NA | | 0 | 1 | Not-determined | Genome-fragment | NA | | 0 |
| S12_k141_97556 | 1205 | No | NA | | 0 | 0 | Low-quality | Genome-fragment | | 1.22 | 0 |
| S11-final-viral-combined_k141_214660\|\|lt2gene | 1179 | No | NA | | 0 | 1 | Not-determined | Genome-fragment | NA | | 0 |
| S8-final-viral-combined_k141_195815\|\|full | 1205 | No | NA | | 0 | 0 | Low-quality | Genome-fragment | | 2.9 | 0 |
| S9-final-viral-combined_k141_17156\|\|full | 1206 | No | NA | | 0 | 0 | Low-quality | Genome-fragment | | 2.78 | 0 |
| S9-final-viral-combined_k141_89485\|\|lt2gene | 1206 | No | NA | | 0 | 1 | Low-quality | Genome-fragment | | 2.16 | 0 |
| S9-final-viral-combined_k141_9730\|\|lt2gene | 1206 | No | NA | | 1 | 0 | Low-quality | Genome-fragment | | 2.93 | 0 |
| S9_k141_99754 | 1206 | No | NA | | 0 | 0 | Low-quality | Genome-fragment | | 0.13 | 0 |
| S11_k141_221243 | 1206 | No | NA | | 2 | 0 | Low-quality | Genome-fragment | | 2.47 | 0 |
| S5-final-viral-combined_k141_46648\|\|full | 1206 | No | NA | | 0 | 0 | Low-quality | Genome-fragment | | 0.61 | 0 |
| S5-final-viral-combined_k141_56475\|\|full | 1206 | No | NA | | 0 | 4 | Low-quality | Genome-fragment | | 0.35 | 0 |
| S5_k141_154735 | 1206 | No | NA | | 0 | 0 | Low-quality | Genome-fragment | | 2.01 | 0 |
| S5_k141_164351 | 1206 | No | NA | | 0 | 1 | Low-quality | Genome-fragment | | 1.83 | 0 |
| S17-final-viral-combined_k141_119274\|\|full | 1206 | No | NA | | 1 | 0 | Low-quality | Genome-fragment | | 2.39 | 0 |
| S2-final-viral-combined_k141_236495\|\|full | 1206 | No | NA | | 0 | 1 | Low-quality | Genome-fragment | | 1.97 | 0 |
| S2-final-viral-combined_k141_61183\|\|full | 1206 | No | NA | | 0 | 2 | Low-quality | Genome-fragment | | 3.07 | 0 |
| S2-final-viral-combined_k141_52815\|\|lt2gene | 1206 | No | NA | | 1 | 0 | Low-quality | Genome-fragment | | 2.23 | 0 |
| S2_k141_67303 | 1206 | No | NA | | 0 | 2 | Low-quality | Genome-fragment | | 1.15 | 0 |
| S9-final-viral-combined_k141_104303\|\|full | 1207 | No | NA | | 0 | 1 | Low-quality | Genome-fragment | | 1 | 0 |
| S11-final-viral-combined_k141_49820\|\|lt2gene | 3119 | No | NA | | 0 | 0 | Not-determined | Genome-fragment | NA | | 0 |
| S11-final-viral-combined_k141_246168\|\|full | 1207 | No | NA | | 1 | 0 | Low-quality | Genome-fragment | | 0.63 | 0 |
| S11-final-viral-combined_k141_3364\|\|lt2gene | 1207 | No | NA | | 1 | 0 | Low-quality | Genome-fragment | | 3.27 | 0 |
| S11-final-viral-combined_k141_215778\|\|lt2gene | 1695 | No | NA | | 0 | 0 | Not-determined | Genome-fragment | NA | | 0 |
| S11-final-viral-combined_k141_297882\|\|lt2gene | 1474 | No | NA | | 0 | 0 | Not-determined | Genome-fragment | NA | | 0 |
| S1-final-viral-combined_k141_379162\|\|lt2gene | 1207 | No | NA | | 1 | 0 | Low-quality | Genome-fragment | | 3.11 | 0 |
| S1-final-viral-combined_k141_28659\|\|lt2gene | 1207 | No | NA | | 2 | 0 | Low-quality | Genome-fragment | | 2.7 | 0 |
| S5-final-viral-combined_k141_60396\|\|full | 1207 | No | NA | | 0 | 0 | Low-quality | Genome-fragment | | 0.86 | 0 |
| S11-final-viral-combined_k141_187742\|\|lt2gene | 1650 | No | NA | | 0 | 1 | Not-determined | Genome-fragment | NA | | 0 |
| S17-final-viral-combined_k141_55864\|\|full | 1207 | No | NA | | 0 | 0 | Low-quality | Genome-fragment | | 2.84 | 0 |
| S17-final-viral-combined_k141_117005\|\|lt2gene | 1207 | No | NA | | 1 | 0 | Low-quality | Genome-fragment | | 2.99 | 0 |
| S12-final-viral-combined_k141_137414\|\|full | 1207 | No | NA | | 2 | 0 | Low-quality | Genome-fragment | | 1.21 | 0 |
| S2_k141_54522 | 1207 | No | NA | | 0 | 1 | Low-quality | Genome-fragment | | 0.53 | 0 |
| S11-final-viral-combined_k141_92255\|\|lt2gene | 1012 | No | NA | | 0 | 0 | Not-determined | Genome-fragment | NA | | 0 |
| S6_k141_81841 | 1207 | No | NA | | 1 | 0 | Low-quality | Genome-fragment | | 2.24 | 0 |
| S8-final-viral-combined_k141_238854\|\|full | 1207 | No | NA | | 0 | 0 | Low-quality | Genome-fragment | | 1.77 | 0 |
| S11-final-viral-combined_k141_92505\|\|lt2gene | 1884 | No | NA | | 0 | 1 | Not-determined | Genome-fragment | NA | | 0 |
| S8-final-viral-combined_k141_68664\|\|full | 1207 | No | NA | | 0 | 0 | Low-quality | Genome-fragment | | 2.64 | 0 |
| S9-final-viral-combined_k141_106750\|\|full | 1208 | No | NA | | 0 | 1 | Low-quality | Genome-fragment | | 0.59 | 0 |
| S11_k141_95751 | 1208 | No | NA | | 0 | 0 | Low-quality | Genome-fragment | | 2.41 | 0 |
| S1-final-viral-combined_k141_319764\|\|full | 1208 | No | NA | | 0 | 0 | Low-quality | Genome-fragment | | 3.12 | 0 |
| S1_k141_221333 | 1208 | No | NA | | 0 | 0 | Low-quality | Genome-fragment | | 6.92 | 0 |
| S5-final-viral-combined_k141_58107\|\|full | 1208 | No | NA | | 1 | 0 | Low-quality | Genome-fragment | | 0.83 | 0 |
| S5_k141_36810 | 1208 | No | NA | | 0 | 0 | Low-quality | Genome-fragment | | 1.85 | 0 |
| S5_k141_5150 | 1208 | No | NA | | 0 | 1 | Low-quality | Genome-fragment | | 2.59 | 0 |
| S17-k141_223433 | 1208 | No | NA | | 1 | 0 | Low-quality | Genome-fragment | | 1.19 | 0 |
| S12-final-viral-combined_k141_52293\|\|lt2gene | 1208 | No | NA | | 1 | 0 | Low-quality | Genome-fragment | | 2.43 | 0 |
| S11-final-viral-combined_k141_133362\|\|lt2gene | 1466 | No | NA | | 0 | 1 | Not-determined | Genome-fragment | NA | | 0 |
| S11-final-viral-combined_k141_133368\|\|lt2gene | 1322 | No | NA | | 0 | 0 | Not-determined | Genome-fragment | NA | | 0 |
| S11-final-viral-combined_k141_299486\|\|lt2gene | 1534 | No | NA | | 0 | 0 | Not-determined | Genome-fragment | NA | | 0 |
| S2-final-viral-combined_k141_238444\|\|full | 1208 | No | NA | | 0 | 1 | Low-quality | Genome-fragment | | 8.93 | 0 |
| S11-final-viral-combined_k141_245491\|\|lt2gene | 1159 | No | NA | | 0 | 0 | Not-determined | Genome-fragment | NA | | 0 |
| S2_k141_3467 | 1208 | No | NA | | 0 | 0 | Low-quality | Genome-fragment | | 1.57 | 0 |
| S4-final-viral-combined_k141_92344\|\|lt2gene | 1208 | No | NA | | 1 | 0 | Low-quality | Genome-fragment | | 1.95 | 0 |
| S6-final-viral-combined_k141_87499\|\|full | 1208 | No | NA | | 0 | 1 | Low-quality | Genome-fragment | | 0.77 | 0 |
| S11-final-viral-combined_k141_52520\|\|lt2gene | 1289 | No | NA | | 0 | 0 | Not-determined | Genome-fragment | NA | | 0 |
| S9-final-viral-combined_k141_105401\|\|lt2gene | 1209 | No | NA | | 1 | 0 | Low-quality | Genome-fragment | | 1.41 | 0 |
| S11-final-viral-combined_k141_323955\|\|full | 1209 | No | NA | | 1 | 0 | Low-quality | Genome-fragment | | 2.73 | 0 |
| S1-final-viral-combined_k141_151522\|\|full | 1209 | No | NA | | 0 | 1 | Low-quality | Genome-fragment | | 0.7 | 0 |
| S5-final-viral-combined_k141_31755\|\|full | 1209 | No | NA | | 0 | 0 | Low-quality | Genome-fragment | | 2.53 | 0 |
| S11-final-viral-combined_k141_203764\|\|lt2gene | 3361 | No | NA | | 0 | 1 | Not-determined | Genome-fragment | NA | | 0 |
| S5-final-viral-combined_k141_127001\|\|lt2gene | 1209 | No | NA | | 1 | 0 | Low-quality | Genome-fragment | | 2.74 | 0 |
| S7-final-viral-combined_k141_105038\|\|full | 1209 | No | NA | | 0 | 0 | Low-quality | Genome-fragment | | 2.54 | 0 |
| S7_k141_188626 | 1209 | No | NA | | 0 | 0 | Low-quality | Genome-fragment | | 2.75 | 0 |
| S12-final-viral-combined_k141_61143\|\|full | 1209 | No | NA | | 0 | 0 | Low-quality | Genome-fragment | | 1.57 | 0 |
| S12-final-viral-combined_k141_94434\|\|full | 1209 | No | NA | | 0 | 0 | Low-quality | Genome-fragment | | 3.56 | 0 |
| S12-final-viral-combined_k141_356060\|\|lt2gene | 1209 | No | NA | | 1 | 0 | Low-quality | Genome-fragment | | 1.32 | 0 |
| S11-final-viral-combined_k141_259887\|\|lt2gene | 1084 | No | NA | | 0 | 0 | Not-determined | Genome-fragment | NA | | 0 |
| S11-final-viral-combined_k141_326970\|\|lt2gene | 2305 | No | NA | | 0 | 1 | Not-determined | Genome-fragment | NA | | 0 |
| S12-final-viral-combined_k141_14071\|\|lt2gene | 1209 | No | NA | | 1 | 0 | Low-quality | Genome-fragment | | 3.71 | 0 |
| S8-final-viral-combined_k141_174862\|\|full | 1209 | No | NA | | 0 | 0 | Low-quality | Genome-fragment | | 8.37 | 0 |
| S11-final-viral-combined_k141_314649\|\|lt2gene | 1725 | No | NA | | 0 | 1 | Not-determined | Genome-fragment | NA | | 0 |
| S11-final-viral-combined_k141_94169\|\|lt2gene | 1261 | No | NA | | 0 | 0 | Not-determined | Genome-fragment | NA | | 0 |
| S8-final-viral-combined_k141_245753\|\|full | 1209 | No | NA | | 1 | 0 | Low-quality | Genome-fragment | | 2.48 | 0 |
| S11-final-viral-combined_k141_287719\|\|lt2gene | 1740 | No | NA | | 0 | 0 | Not-determined | Genome-fragment | NA | | 0 |
| S8-final-viral-combined_k141_120292\|\|lt2gene | 1209 | No | NA | | 1 | 0 | Low-quality | Genome-fragment | | 1.29 | 0 |
| S11-final-viral-combined_k141_224003\|\|full | 1210 | No | NA | | 1 | 0 | Low-quality | Genome-fragment | | 2.75 | 0 |
| S11-final-viral-combined_k141_174266\|\|full | 1210 | No | NA | | 0 | 0 | Low-quality | Genome-fragment | | 2.64 | 0 |
| S5-final-viral-combined_k141_109141\|\|full | 1210 | No | NA | | 0 | 0 | Low-quality | Genome-fragment | | 1.9 | 0 |
| S7-final-viral-combined_k141_147086\|\|full | 1210 | No | NA | | 0 | 0 | Low-quality | Genome-fragment | | 2.17 | 0 |
| S12-final-viral-combined_k141_342425\|\|lt2gene | 1210 | No | NA | | 1 | 0 | Low-quality | Genome-fragment | | 2.04 | 0 |
| S12_k141_372566 | 1210 | No | NA | | 1 | 0 | Low-quality | Genome-fragment | | 0.65 | 0 |
| S2-final-viral-combined_k141_209302\|\|lt2gene | 1210 | No | NA | | 1 | 0 | Low-quality | Genome-fragment | | 0.78 | 0 |
| S2_k141_115807 | 1210 | No | NA | | 0 | 0 | Low-quality | Genome-fragment | | 1.72 | 0 |
| S4_k141_40472 | 1210 | No | NA | | 0 | 0 | Low-quality | Genome-fragment | | 1.96 | 0 |
| S11-final-viral-combined_k141_315339\|\|lt2gene | 1276 | No | NA | | 0 | 0 | Not-determined | Genome-fragment | NA | | 0 |
| S11-final-viral-combined_k141_109764\|\|lt2gene | 1329 | No | NA | | 0 | 0 | Not-determined | Genome-fragment | NA | | 0 |
| S11-final-viral-combined_k141_247683\|\|lt2gene | 1036 | No | NA | | 0 | 0 | Not-determined | Genome-fragment | NA | | 0 |
| S11-final-viral-combined_k141_54378\|\|lt2gene | 1003 | No | NA | | 0 | 0 | Not-determined | Genome-fragment | NA | | 0 |
| S11-final-viral-combined_k141_247740\|\|lt2gene | 1382 | No | NA | | 0 | 0 | Not-determined | Genome-fragment | NA | | 0 |
| S11-final-viral-combined_k141_301420\|\|lt2gene | 2040 | No | NA | | 0 | 1 | Not-determined | Genome-fragment | NA | | 0 |
| S11-final-viral-combined_k141_71632\|\|full | 1211 | No | NA | | 1 | 0 | Low-quality | Genome-fragment | | 3.04 | 0 |
| S3-final-viral-combined_k141_35845\|\|full | 1211 | No | NA | | 0 | 1 | Low-quality | Genome-fragment | | 1.65 | 0 |
| S11-final-viral-combined_k141_219235\|\|lt2gene | 1365 | No | NA | | 0 | 0 | Not-determined | Genome-fragment | NA | | 0 |
| S11-final-viral-combined_k141_95305\|\|lt2gene | 1332 | No | NA | | 0 | 0 | Not-determined | Genome-fragment | NA | | 0 |
| S3_k141_125143 | 1211 | No | NA | | 0 | 0 | Low-quality | Genome-fragment | | 2.43 | 0 |
| S5-final-viral-combined_k141_119200\|\|full | 1211 | No | NA | | 1 | 0 | Low-quality | Genome-fragment | | 1.74 | 0 |
| S11-final-viral-combined_k141_178905\|\|lt2gene | 3008 | No | NA | | 0 | 1 | Not-determined | Genome-fragment | NA | | 0 |
| S7-final-viral-combined_k141_39329\|\|lt2gene | 1211 | No | NA | | 1 | 0 | Low-quality | Genome-fragment | | 3.01 | 0 |
| S17-final-viral-combined_k141_217916\|\|full | 1211 | No | NA | | 1 | 0 | Low-quality | Genome-fragment | | 2.72 | 0 |
| S17-final-viral-combined_k141_30210\|\|full | 1211 | No | NA | | 0 | 0 | Low-quality | Genome-fragment | | 2.17 | 0 |
| S12-final-viral-combined_k141_139857\|\|full | 1211 | No | NA | | 0 | 0 | Low-quality | Genome-fragment | | 2.02 | 0 |
| S2-final-viral-combined_k141_130015\|\|full | 1211 | No | NA | | 0 | 0 | Low-quality | Genome-fragment | | 0.89 | 0 |
| S4-final-viral-combined_k141_42772\|\|full | 1211 | Yes | | 497 | 1 | 1 | Low-quality | Genome-fragment | | 1.25 | 58.96 |
| S4-final-viral-combined_k141_156790\|\|lt2gene | 1211 | No | NA | | 1 | 0 | Low-quality | Genome-fragment | | 3.22 | 0 |
| S6-final-viral-combined_k141_57653\|\|full | 1211 | No | NA | | 0 | 0 | Low-quality | Genome-fragment | | 3.01 | 0 |
| S6_k141_47697 | 1211 | No | NA | | 0 | 1 | Low-quality | Genome-fragment | | 0.52 | 0 |
| S8-final-viral-combined_k141_190664\|\|full | 1211 | No | NA | | 0 | 0 | Low-quality | Genome-fragment | | 1.16 | 0 |
| S8-final-viral-combined_k141_195067\|\|full | 1211 | No | NA | | 0 | 0 | Low-quality | Genome-fragment | | 3.04 | 0 |
| S11-final-viral-combined_k141_27367\|\|lt2gene | 1325 | No | NA | | 0 | 1 | Not-determined | Genome-fragment | NA | | 0 |
| S11_k141_13825 | 1783 | No | NA | | 0 | 0 | Not-determined | Genome-fragment | NA | | 0 |
| S11_k141_275662 | 3899 | No | NA | | 0 | 3 | Not-determined | Genome-fragment | NA | | 0 |
| S8_k141_223889 | 1211 | No | NA | | 0 | 0 | Low-quality | Genome-fragment | | 1.88 | 0 |
| S11_k141_96490 | 1396 | No | NA | | 0 | 2 | Not-determined | Genome-fragment | NA | | 0 |
| S11_k141_55146 | 1286 | No | NA | | 0 | 0 | Not-determined | Genome-fragment | NA | | 0 |
| S8_k141_112434 | 1211 | No | NA | | 1 | 0 | Low-quality | Genome-fragment | | 3.75 | 0 |
| S11_k141_289478 | 1132 | No | NA | | 0 | 1 | Not-determined | Genome-fragment | NA | | 0 |
| S11_k141_248134 | 1243 | No | NA | | 0 | 1 | Not-determined | Genome-fragment | NA | | 0 |
| S9_k141_110372 | 1212 | No | NA | | 0 | 0 | Low-quality | Genome-fragment | | 2.06 | 0 |
| S11_k141_137920 | 1224 | No | NA | | 0 | 3 | Not-determined | Genome-fragment | NA | | 0 |
| S11_k141_151683 | 1402 | No | NA | | 0 | 1 | Not-determined | Genome-fragment | NA | | 0 |
| S11_k141_110332 | 1156 | No | NA | | 0 | 0 | Not-determined | Genome-fragment | NA | | 0 |
| S11_k141_192995 | 1334 | No | NA | | 0 | 0 | Not-determined | Genome-fragment | NA | | 0 |
| S11_k141_220598 | 4952 | No | NA | | 0 | 5 | Not-determined | Genome-fragment | NA | | 0 |
| S11-final-viral-combined_k141_209317\|\|full | 1212 | No | NA | | 0 | 0 | Low-quality | Genome-fragment | | 3.16 | 0 |
| S11_k141_289518 | 1491 | No | NA | | 0 | 1 | Not-determined | Genome-fragment | NA | | 0 |
| S11_k141_137951 | 1147 | No | NA | | 0 | 0 | Not-determined | Genome-fragment | NA | | 0 |
| S11_k141_220623 | 4368 | No | NA | | 0 | 3 | Not-determined | Genome-fragment | NA | | 0 |
| S1-final-viral-combined_k141_109767\|\|full | 1212 | No | NA | | 0 | 1 | Low-quality | Genome-fragment | | 0.82 | 0 |
| S1_k141_100081 | 1212 | No | NA | | 0 | 1 | Low-quality | Genome-fragment | | 2.08 | 0 |
| S11_k141_27600 | 1926 | No | NA | | 0 | 1 | Not-determined | Genome-fragment | NA | | 0 |
| S11_k141_220641 | 1986 | No | NA | | 0 | 1 | Not-determined | Genome-fragment | NA | | 0 |
| S5-final-viral-combined_k141_95856\|\|full | 1212 | No | NA | | 0 | 0 | Low-quality | Genome-fragment | | 2.02 | 0 |

| | | | | | | | | | |
|---|---|---|---|---|---|---|---|---|---|
| S12-final-viral-combined_k141_74596\|\|full | 1212 | No | NA | 1 | 0 | Low-quality | Genome-fragment | 2.45 | 0 |
| S11_k141_69070 | 3140 | No | NA | 0 | 2 | Not-determined | Genome-fragment | NA | 0 |
| S11_k141_317161 | 1391 | No | NA | 0 | 1 | Not-determined | Genome-fragment | NA | 0 |
| S2-final-viral-combined_k141_233876\|\|full | 1212 | No | NA | 1 | 0 | Low-quality | Genome-fragment | 2.73 | 0 |
| S11_k141_234402 | 1150 | No | NA | 0 | 1 | Not-determined | Genome-fragment | NA | 0 |
| S11_k141_124294 | 1285 | No | NA | 0 | 0 | Not-determined | Genome-fragment | NA | 0 |
| S11_k141_69147 | 2861 | No | NA | 0 | 1 | Not-determined | Genome-fragment | NA | 0 |
| S11_k141_55340 | 1176 | No | NA | 0 | 1 | Not-determined | Genome-fragment | NA | 0 |
| S11_k141_303398 | 25741 | No | NA | 4 | 0 | Medium-quality | Genome-fragment | 59.61 | 0 |
| S11_k141_317187 | 6935 | No | NA | 0 | 5 | Not-determined | Genome-fragment | NA | 0 |
| S11_k141_317193 | 1152 | No | NA | 0 | 0 | Not-determined | Genome-fragment | NA | 0 |
| S11_k141_193176 | 5286 | No | NA | 0 | 4 | Not-determined | Genome-fragment | NA | 0 |
| S11_k141_289586 | 1460 | No | NA | 0 | 1 | Not-determined | Genome-fragment | NA | 0 |
| S11_k141_69159 | 2835 | No | NA | 0 | 0 | Not-determined | Genome-fragment | NA | 0 |
| S2_k141_41821 | 1212 | No | NA | 0 | 1 | Low-quality | Genome-fragment | 0.53 | 0 |
| S4_k141_68919 | 1212 | No | NA | 0 | 0 | Low-quality | Genome-fragment | 1.39 | 0 |
| S8-final-viral-combined_k141_95571\|\|full | 1212 | No | NA | 0 | 2 | Low-quality | Genome-fragment | 1.22 | 0 |
| S11_k141_151897 | 1009 | No | NA | 0 | 1 | Not-determined | Genome-fragment | NA | 0 |
| S11_k141_234460 | 1275 | No | NA | 0 | 0 | Not-determined | Genome-fragment | NA | 0 |
| S9-final-viral-combined_k141_79144\|\|full | 1213 | No | NA | 0 | 0 | Low-quality | Genome-fragment | 3.12 | 0 |
| S11_k141_179422 | 1007 | No | NA | 0 | 0 | Not-determined | Genome-fragment | NA | 0 |
| S11_k141_110470 | 1558 | No | NA | 0 | 0 | Not-determined | Genome-fragment | NA | 0 |
| S1_k141_18532 | 1213 | No | NA | 1 | 0 | Low-quality | Genome-fragment | 3.6 | 0 |
| S11_k141_165734 | 1329 | No | NA | 0 | 1 | Not-determined | Genome-fragment | NA | 0 |
| S17-final-viral-combined_k141_22629\|\|lt2gene | 1213 | No | NA | 1 | 0 | Low-quality | Genome-fragment | 2.95 | 0 |
| S11_k141_193248 | 1488 | No | NA | 0 | 2 | Not-determined | Genome-fragment | NA | 0 |
| S11_k141_165752 | 1250 | No | NA | 0 | 3 | Not-determined | Genome-fragment | NA | 0 |
| S11_k141_220873 | 1120 | No | NA | 0 | 1 | Not-determined | Genome-fragment | NA | 0 |
| S11_k141_27835 | 1258 | No | NA | 0 | 1 | Not-determined | Genome-fragment | NA | 0 |
| S11_k141_124458 | 4269 | No | NA | 0 | 3 | Not-determined | Genome-fragment | NA | 0 |
| S17-k141_34166 | 1213 | No | NA | 0 | 0 | Low-quality | Genome-fragment | 2.84 | 0 |
| S11_k141_165812 | 5881 | No | NA | 0 | 5 | Not-determined | Genome-fragment | NA | 0 |
| S17-k141_239172 | 1213 | No | NA | 0 | 1 | Low-quality | Genome-fragment | 0.83 | 0 |
| S11_k141_207078 | 1128 | No | NA | 0 | 1 | Not-determined | Genome-fragment | NA | 0 |
| S11_k141_165831 | 1826 | No | NA | 0 | 1 | Not-determined | Genome-fragment | NA | 0 |
| S11_k141_193295 | 1164 | No | NA | 0 | 1 | Not-determined | Genome-fragment | NA | 0 |
| S11_k141_248378 | 1318 | No | NA | 0 | 0 | Not-determined | Genome-fragment | NA | 0 |
| S17-k141_175284 | 1213 | No | NA | 0 | 0 | Low-quality | Genome-fragment | 2.79 | 0 |
| S11_k141_110548 | 1585 | No | NA | 0 | 0 | Not-determined | Genome-fragment | NA | 0 |
| S12-final-viral-combined_k141_228179\|\|full | 1213 | No | NA | 1 | 0 | Low-quality | Genome-fragment | 3.04 | 0 |
| S11_k141_96843 | 1112 | No | NA | 0 | 1 | Not-determined | Genome-fragment | NA | 0 |
| S11_k141_152004 | 1191 | No | NA | 0 | 0 | Not-determined | Genome-fragment | NA | 0 |
| S11_k141_165906 | 9212 | No | NA | 0 | 3 | Not-determined | Genome-fragment | NA | 0 |
| S11_k141_193357 | 4053 | No | NA | 0 | 1 | Not-determined | Genome-fragment | NA | 0 |
| S11_k141_41607 | 9671 | No | NA | 0 | 6 | Not-determined | Genome-fragment | NA | 0 |
| S2-final-viral-combined_k141_210184\|\|full | 1213 | No | NA | 2 | 0 | Low-quality | Genome-fragment | 2.59 | 0 |
| S11_k141_152031 | 1170 | No | NA | 0 | 0 | Not-determined | Genome-fragment | NA | 0 |
| S11_k141_124508 | 1684 | No | NA | 0 | 0 | Not-determined | Genome-fragment | NA | 0 |
| S11_k141_28007 | 1114 | No | NA | 0 | 0 | Not-determined | Genome-fragment | NA | 0 |
| S11_k141_110599 | 2991 | No | NA | 0 | 2 | Not-determined | Genome-fragment | NA | 0 |
| S2-final-viral-combined_k141_78542\|\|full | 1213 | No | NA | 1 | 0 | Low-quality | Genome-fragment | 2.68 | 0 |
| S11_k141_124518 | 6073 | No | NA | 0 | 2 | Not-determined | Genome-fragment | NA | 0 |
| S11_k141_275977 | 1071 | No | NA | 0 | 0 | Not-determined | Genome-fragment | NA | 0 |
| S9-final-viral-combined_k141_35052\|\|full | 1214 | No | NA | 1 | 0 | Low-quality | Genome-fragment | 2.7 | 0 |
| S11_k141_165970 | 1327 | No | NA | 0 | 0 | Not-determined | Genome-fragment | NA | 0 |
| S11_k141_152076 | 8724 | No | NA | 0 | 5 | Not-determined | Genome-fragment | NA | 0 |
| S11_k141_276013 | 1247 | No | NA | 0 | 0 | Not-determined | Genome-fragment | NA | 0 |
| S11_k141_220939 | 1624 | No | NA | 0 | 1 | Not-determined | Genome-fragment | NA | 0 |
| S11_k141_276045 | 2511 | No | NA | 0 | 1 | Not-determined | Genome-fragment | NA | 0 |
| S11_k141_41710 | 5452 | No | NA | 0 | 0 | Not-determined | Genome-fragment | NA | 0 |
| S11_k141_124601 | 6510 | No | NA | 0 | 2 | Not-determined | Genome-fragment | NA | 0 |
| S9_k141_4083 | 1214 | No | NA | 0 | 0 | Low-quality | Genome-fragment | 2.41 | 0 |
| S11_k141_193395 | 1600 | No | NA | 0 | 0 | Not-determined | Genome-fragment | NA | 0 |
| S11_k141_474 | 6135 | No | NA | 0 | 0 | Not-determined | Genome-fragment | NA | 0 |
| S11_k141_152143 | 1816 | No | NA | 0 | 1 | Not-determined | Genome-fragment | NA | 0 |
| S11_k141_317508 | 2225 | No | NA | 0 | 1 | Not-determined | Genome-fragment | NA | 0 |
| S11-final-viral-combined_k141_323594\|\|full | 1214 | No | NA | 0 | 0 | Low-quality | Genome-fragment | 2.06 | 0 |
| S11_k141_152145 | 5554 | No | NA | 0 | 2 | Not-determined | Genome-fragment | NA | 0 |
| S11_k141_55610 | 58459 | No | NA | 27 | 2 | Medium-quality | Genome-fragment | 78.18 | 0 |
| S11_k141_28096 | 5032 | No | NA | 0 | 4 | Not-determined | Genome-fragment | NA | 0 |
| S11-final-viral-combined_k141_132632\|\|full | 1214 | No | NA | 3 | 0 | Low-quality | Genome-fragment | 2.68 | 0 |
| S11-final-viral-combined_k141_303499\|\|lt2gene | 1214 | No | NA | 2 | 0 | Low-quality | Genome-fragment | 1.73 | 0 |
| S11_k141_289837 | 2224 | No | NA | 0 | 0 | Not-determined | Genome-fragment | NA | 0 |
| S11_k141_234833 | 7240 | No | NA | 0 | 4 | Not-determined | Genome-fragment | NA | 0 |
| S11_k141_14390 | 52675 | No | NA | 16 | 2 | Complete | High-quality | 100 | 0 |
| S11_k141_289849 | 4677 | No | NA | 0 | 1 | Not-determined | Genome-fragment | NA | 0 |
| S11_k141_193489 | 5847 | No | NA | 0 | 3 | Not-determined | Genome-fragment | NA | 0 |
| S1-final-viral-combined_k141_377767\|\|full | 1214 | No | NA | 1 | 0 | Low-quality | Genome-fragment | 2.86 | 0 |
| S11_k141_234871 | 1358 | No | NA | 0 | 0 | Not-determined | Genome-fragment | NA | 0 |
| S1-final-viral-combined_k141_161820\|\|lt2gene | 1214 | No | NA | 2 | 0 | Low-quality | Genome-fragment | 2.99 | 0 |
| S3_k141_78616 | 1214 | No | NA | 1 | 0 | Low-quality | Genome-fragment | 3.15 | 0 |
| S7-final-viral-combined_k141_93029\|\|full | 1214 | No | NA | 0 | 0 | Low-quality | Genome-fragment | 1.87 | 0 |
| S11_k141_193551 | 1387 | No | NA | 0 | 0 | Not-determined | Genome-fragment | NA | 0 |
| S11_k141_276200 | 1533 | No | NA | 0 | 1 | Not-determined | Genome-fragment | NA | 0 |
| S11_k141_193566 | 3730 | No | NA | 0 | 1 | Not-determined | Genome-fragment | NA | 0 |
| S7-final-viral-combined_k141_141821\|\|full | 1214 | No | NA | 0 | 2 | Low-quality | Genome-fragment | 0.39 | 0 |
| S11_k141_152284 | 1756 | No | NA | 0 | 0 | Not-determined | Genome-fragment | NA | 0 |
| S11_k141_124809 | 1603 | No | NA | 0 | 2 | Not-determined | Genome-fragment | NA | 0 |
| S11_k141_625 | 1073 | No | NA | 0 | 1 | Not-determined | Genome-fragment | NA | 0 |
| S17-final-viral-combined_k141_191529\|\|full | 1214 | No | NA | 1 | 0 | Low-quality | Genome-fragment | 1.7 | 0 |
| S11_k141_289996 | 1033 | No | NA | 0 | 1 | Not-determined | Genome-fragment | NA | 0 |
| S11_k141_97176 | 1176 | No | NA | 0 | 1 | Not-determined | Genome-fragment | NA | 0 |
| S17-final-viral-combined_k141_98303\|\|full | 1214 | No | NA | 1 | 0 | Low-quality | Genome-fragment | 2.47 | 0 |
| S11_k141_83308 | 4753 | No | NA | 0 | 1 | Not-determined | Genome-fragment | NA | 0 |
| S11_k141_69569 | 2665 | No | NA | 0 | 2 | Not-determined | Genome-fragment | NA | 0 |
| S11_k141_193635 | 1856 | No | NA | 0 | 2 | Not-determined | Genome-fragment | NA | 0 |
| S11_k141_193655 | 1045 | No | NA | 0 | 1 | Not-determined | Genome-fragment | NA | 0 |
| S11_k141_97223 | 1191 | No | NA | 0 | 1 | Not-determined | Genome-fragment | NA | 0 |
| S11_k141_276339 | 2177 | No | NA | 0 | 1 | Not-determined | Genome-fragment | NA | 0 |
| S11_k141_110886 | 1342 | No | NA | 0 | 1 | Not-determined | Genome-fragment | NA | 0 |
| S12_k141_98019 | 1214 | No | NA | 0 | 0 | Low-quality | Genome-fragment | 2.61 | 0 |
| S11_k141_207380 | 1321 | No | NA | 0 | 1 | Not-determined | Genome-fragment | NA | 0 |
| S2-final-viral-combined_k141_85209\|\|full | 1214 | No | NA | 0 | 0 | Low-quality | Genome-fragment | 2.93 | 0 |
| S11_k141_290078 | 1232 | No | NA | 0 | 1 | Not-determined | Genome-fragment | NA | 0 |
| S11_k141_69651 | 7232 | No | NA | 0 | 6 | Not-determined | Genome-fragment | NA | 0 |
| S11_k141_193716 | 1942 | No | NA | 0 | 2 | Not-determined | Genome-fragment | NA | 0 |
| S11_k141_138544 | 1039 | No | NA | 0 | 2 | Not-determined | Genome-fragment | NA | 0 |
| S2-final-viral-combined_k141_8661\|\|full | 1214 | No | NA | 0 | 0 | Low-quality | Genome-fragment | 2.15 | 0 |
| S11_k141_152423 | 1036 | No | NA | 0 | 1 | Not-determined | Genome-fragment | NA | 0 |
| S11_k141_303977 | 4802 | No | NA | 0 | 2 | Not-determined | Genome-fragment | NA | 0 |
| S11_k141_28333 | 4189 | No | NA | 0 | 4 | Not-determined | Genome-fragment | NA | 0 |
| S11_k141_758 | 4660 | No | NA | 0 | 1 | Not-determined | Genome-fragment | NA | 0 |
| S11_k141_97342 | 1323 | No | NA | 0 | 1 | Not-determined | Genome-fragment | NA | 0 |
| S11_k141_790 | 1099 | No | NA | 0 | 0 | Not-determined | Genome-fragment | NA | 0 |
| S11_k141_262664 | 1305 | No | NA | 0 | 1 | Not-determined | Genome-fragment | NA | 0 |
| S11_k141_820 | 3390 | No | NA | 0 | 2 | Not-determined | Genome-fragment | NA | 0 |
| S11_k141_276471 | 4483 | No | NA | 0 | 0 | Not-determined | Genome-fragment | NA | 0 |
| S11_k141_138641 | 1219 | No | NA | 0 | 1 | Not-determined | Genome-fragment | NA | 0 |
| S4-final-viral-combined_k141_23531\|\|full | 1214 | No | NA | 0 | 0 | Low-quality | Genome-fragment | 1.57 | 0 |
| S11_k141_28409 | 4185 | No | NA | 0 | 3 | Not-determined | Genome-fragment | NA | 0 |
| S4_k141_128382 | 1214 | No | NA | 0 | 1 | Low-quality | Genome-fragment | 0.43 | 0 |
| S11_k141_179873 | 2831 | No | NA | 0 | 2 | Not-determined | Genome-fragment | NA | 0 |
| S11_k141_28421 | 1068 | No | NA | 0 | 1 | Not-determined | Genome-fragment | NA | 0 |
| S6-final-viral-combined_k141_44845\|\|full | 1214 | No | NA | 0 | 0 | Low-quality | Genome-fragment | 3.05 | 0 |
| S11_k141_235285 | 1394 | No | NA | 0 | 1 | Not-determined | Genome-fragment | NA | 0 |
| S6-final-viral-combined_k141_134369\|\|full | 1214 | No | NA | 0 | 1 | Low-quality | Genome-fragment | 0.44 | 0 |
| S11_k141_235313 | 2204 | No | NA | 0 | 2 | Not-determined | Genome-fragment | NA | 0 |
| S11_k141_207625 | 4441 | No | NA | 0 | 3 | Not-determined | Genome-fragment | NA | 0 |
| S11_k141_111136 | 2575 | No | NA | 0 | 0 | Not-determined | Genome-fragment | NA | 0 |
| S11_k141_179994 | 2102 | No | NA | 0 | 1 | Not-determined | Genome-fragment | NA | 0 |
| S11_k141_249009 | 1842 | No | NA | 0 | 1 | Not-determined | Genome-fragment | NA | 0 |
| S11_k141_97574 | 1111 | No | NA | 0 | 0 | Not-determined | Genome-fragment | NA | 0 |
| S11_k141_166613 | 2755 | No | NA | 0 | 3 | Not-determined | Genome-fragment | NA | 0 |
| S11_k141_304203 | 3181 | No | NA | 0 | 2 | Not-determined | Genome-fragment | NA | 0 |
| S11_k141_42194 | 4668 | No | NA | 0 | 2 | Not-determined | Genome-fragment | NA | 0 |
| S11_k141_249062 | 1408 | No | NA | 0 | 1 | Not-determined | Genome-fragment | NA | 0 |
| S11_k141_166679 | 1160 | No | NA | 0 | 1 | Not-determined | Genome-fragment | NA | 0 |
| S11_k141_1030 | 2785 | No | NA | 0 | 0 | Not-determined | Genome-fragment | NA | 0 |
| S11_k141_152723 | 1070 | No | NA | 0 | 0 | Not-determined | Genome-fragment | NA | 0 |
| S11_k141_152748 | 3936 | No | NA | 0 | 2 | Not-determined | Genome-fragment | NA | 0 |
| S11_k141_14851 | 1738 | No | NA | 0 | 1 | Not-determined | Genome-fragment | NA | 0 |
| S11_k141_262907 | 1019 | No | NA | 0 | 0 | Not-determined | Genome-fragment | NA | 0 |
| S11_k141_221549 | 1436 | No | NA | 0 | 1 | Not-determined | Genome-fragment | NA | 0 |
| S11_k141_249127 | 1611 | No | NA | 0 | 1 | Not-determined | Genome-fragment | NA | 0 |
| S6-final-viral-combined_k141_134838\|\|full | 1214 | No | NA | 0 | 0 | Low-quality | Genome-fragment | 1.45 | 0 |
| S11_k141_180181 | 14561 | No | NA | 0 | 3 | Not-determined | Genome-fragment | NA | 0 |
| S11_k141_28549 | 1207 | No | NA | 0 | 0 | Not-determined | Genome-fragment | NA | 0 |
| S8-final-viral-combined_k141_115210\|\|full | 1214 | No | NA | 0 | 0 | Low-quality | Genome-fragment | 2.02 | 0 |
| S11_k141_262965 | 1248 | No | NA | 0 | 0 | Not-determined | Genome-fragment | NA | 0 |
| S11_k141_42346 | 1255 | No | NA | 0 | 2 | Not-determined | Genome-fragment | NA | 0 |
| S8-final-viral-combined_k141_78306\|\|full | 1214 | No | NA | 1 | 0 | Low-quality | Genome-fragment | 2.5 | 0 |
| S11_k141_111273 | 1804 | No | NA | 0 | 2 | Not-determined | Genome-fragment | NA | 0 |
| S11_k141_1167 | 3161 | No | NA | 0 | 0 | Not-determined | Genome-fragment | NA | 0 |
| S11_k141_194015 | 1776 | No | NA | 0 | 0 | Not-determined | Genome-fragment | NA | 0 |
| S11_k141_207945 | 1782 | No | NA | 0 | 1 | Not-determined | Genome-fragment | NA | 0 |
| S11-final-viral-combined_k141_180782\|\|full | 1215 | No | NA | 0 | 0 | Low-quality | Genome-fragment | 2.99 | 0 |
| S11_k141_249236 | 1047 | No | NA | 0 | 1 | Not-determined | Genome-fragment | NA | 0 |
| S11_k141_56214 | 2090 | No | NA | 0 | 0 | Not-determined | Genome-fragment | NA | 0 |
| S11_k141_207992 | 1144 | No | NA | 0 | 1 | Not-determined | Genome-fragment | NA | 0 |
| S11_k141_180264 | 1799 | No | NA | 0 | 2 | Not-determined | Genome-fragment | NA | 0 |
| S11_k141_263076 | 3498 | No | NA | 0 | 5 | Not-determined | Genome-fragment | NA | 0 |
| S11_k141_235809 | 1325 | No | NA | 0 | 1 | Not-determined | Genome-fragment | NA | 0 |
| S11-final-viral-combined_k141_24371\|\|full | 1215 | No | NA | 0 | 0 | Low-quality | Genome-fragment | 3.53 | 0 |
| S11_k141_166890 | 1119 | No | NA | 0 | 1 | Not-determined | Genome-fragment | NA | 0 |
| S11_k141_125365 | 1798 | No | NA | 0 | 1 | Not-determined | Genome-fragment | NA | 0 |
| S1_k141_265845 | 1215 | No | NA | 1 | 0 | Low-quality | Genome-fragment | 0.52 | 0 |
| S11_k141_15040 | 2347 | No | NA | 0 | 1 | Not-determined | Genome-fragment | NA | 0 |
| S11_k141_249332 | 1170 | No | NA | 0 | 1 | Not-determined | Genome-fragment | NA | 0 |
| S3_k141_104129 | 1215 | No | NA | 3 | 0 | Low-quality | Genome-fragment | 3.21 | 0 |
| S5-final-viral-combined_k141_206875\|\|full | 1215 | Yes | 807 | 1 | 1 | Low-quality | Genome-fragment | 2.06 | 33.58 |
| S5-final-viral-combined_k141_94215\|\|lt2gene | 1215 | No | NA | 1 | 0 | Low-quality | Genome-fragment | 2.95 | 0 |
| S11_k141_70036 | 3240 | No | NA | 0 | 0 | Not-determined | Genome-fragment | NA | 0 |
| S11_k141_221652 | 3998 | No | NA | 0 | 3 | Not-determined | Genome-fragment | NA | 0 |
| S7-final-viral-combined_k141_99942\|\|full | 1215 | No | NA | 1 | 0 | Low-quality | Genome-fragment | 2.62 | 0 |

| | | | | | | | | | | | |
|---|---|---|---|---|---|---|---|---|---|---|---|
| S11_k141_70078 | 2142 | No | NA | | 0 | 1 | Not-determined | Genome-fragment | NA | | 0 |
| S11_k141_28735 | 1724 | No | NA | | 0 | 1 | Not-determined | Genome-fragment | NA | | 0 |
| S7-final-viral-combined_k141_4571\|\|lt2gene | 1215 | No | NA | | 1 | 0 | Low-quality | Genome-fragment | | 2.59 | 0 |
| S11_k141_318103 | 2153 | No | NA | | 0 | 2 | Not-determined | Genome-fragment | NA | | 0 |
| S11_k141_208089 | 1048 | No | NA | | 0 | 1 | Not-determined | Genome-fragment | NA | | 0 |
| S11_k141_263223 | 2307 | No | NA | | 0 | 1 | Not-determined | Genome-fragment | NA | | 0 |
| S11_k141_152933 | 2432 | No | NA | | 0 | 2 | Not-determined | Genome-fragment | NA | | 0 |
| S11_k141_290586 | 1327 | No | NA | | 0 | 2 | Not-determined | Genome-fragment | NA | | 0 |
| S17-final-viral-combined_k141_15164\|\|full | 1215 | No | NA | | 1 | 0 | Low-quality | Genome-fragment | | 0.43 | 0 |
| S11_k141_180387 | 1046 | No | NA | | 0 | 0 | Not-determined | Genome-fragment | NA | | 0 |
| S11_k141_70149 | 1258 | No | NA | | 0 | 0 | Not-determined | Genome-fragment | NA | | 0 |
| S11_k141_290598 | 1650 | No | NA | | 0 | 0 | Not-determined | Genome-fragment | NA | | 0 |
| S11_k141_180423 | 4813 | No | NA | | 0 | 4 | Not-determined | Genome-fragment | NA | | 0 |
| S11_k141_15162 | 3300 | No | NA | | 0 | 2 | Not-determined | Genome-fragment | NA | | 0 |
| S11_k141_166981 | 1136 | No | NA | | 0 | 1 | Not-determined | Genome-fragment | NA | | 0 |
| S11_k141_249504 | 4957 | No | NA | | 0 | 2 | Not-determined | Genome-fragment | NA | | 0 |
| S11_k141_15196 | 1887 | No | NA | | 0 | 2 | Not-determined | Genome-fragment | NA | | 0 |
| S11_k141_249511 | 1029 | No | NA | | 0 | 2 | Not-determined | Genome-fragment | NA | | 0 |
| S11_k141_318192 | 1129 | No | NA | | 0 | 1 | Not-determined | Genome-fragment | NA | | 0 |
| S17-final-viral-combined_k141_163542\|\|full | 1215 | Yes | | 471 | 1 | 1 | Low-quality | Genome-fragment | | 0.98 | 61.23 |
| S12-final-viral-combined_k141_124016\|\|full | 1215 | No | NA | | 1 | 0 | Low-quality | Genome-fragment | | 2.23 | 0 |
| S12-final-viral-combined_k141_95069\|\|lt2gene | 1215 | No | NA | | 1 | 0 | Low-quality | Genome-fragment | | 3.45 | 0 |
| S11_k141_15269 | 1035 | No | NA | | 0 | 1 | Not-determined | Genome-fragment | NA | | 0 |
| S11_k141_139058 | 1122 | No | NA | | 0 | 2 | Not-determined | Genome-fragment | NA | | 0 |
| S11_k141_194326 | 15603 | No | NA | | 0 | 7 | Not-determined | Genome-fragment | NA | | 0 |
| S11_k141_42731 | 5776 | No | NA | | 0 | 3 | Not-determined | Genome-fragment | NA | | 0 |
| S11_k141_249555 | 1852 | No | NA | | 0 | 0 | Not-determined | Genome-fragment | NA | | 0 |
| S11_k141_1421 | 1335 | No | NA | | 0 | 0 | Not-determined | Genome-fragment | NA | | 0 |
| S11_k141_180532 | 1028 | No | NA | | 0 | 1 | Not-determined | Genome-fragment | NA | | 0 |
| S2-final-viral-combined_k141_75390\|\|full | 1215 | No | NA | | 1 | 0 | Low-quality | Genome-fragment | | 2.48 | 0 |
| S11_k141_1432 | 12745 | No | NA | | 0 | 5 | Not-determined | Genome-fragment | NA | | 0 |
| S11_k141_221981 | 1096 | No | NA | | 0 | 0 | Not-determined | Genome-fragment | NA | | 0 |
| S11_k141_263351 | 1698 | No | NA | | 0 | 3 | Not-determined | Genome-fragment | NA | | 0 |
| S11_k141_139113 | 3497 | No | NA | | 0 | 3 | Not-determined | Genome-fragment | NA | | 0 |
| S11_k141_167160 | 2555 | No | NA | | 0 | 0 | Not-determined | Genome-fragment | NA | | 0 |
| S11_k141_153073 | 3250 | No | NA | | 0 | 0 | Not-determined | Genome-fragment | NA | | 0 |
| S11_k141_111498 | 1369 | No | NA | | 0 | 0 | Not-determined | Genome-fragment | NA | | 0 |
| S11_k141_208306 | 4111 | No | NA | | 0 | 1 | Not-determined | Genome-fragment | NA | | 0 |
| S11_k141_153097 | 2029 | No | NA | | 0 | 2 | Not-determined | Genome-fragment | NA | | 0 |
| S2_k141_23464 | 1215 | No | NA | | 1 | 0 | Low-quality | Genome-fragment | | 1.37 | 0 |
| S11_k141_84301 | 3462 | No | NA | | 0 | 1 | Not-determined | Genome-fragment | NA | | 0 |
| S8-final-viral-combined_k141_274295\|\|full | 1215 | No | NA | | 0 | 0 | Low-quality | Genome-fragment | | 2.83 | 0 |
| S11_k141_111551 | 3702 | No | NA | | 0 | 1 | Not-determined | Genome-fragment | NA | | 0 |
| S11_k141_56661 | 3355 | No | NA | | 0 | 2 | Not-determined | Genome-fragment | NA | | 0 |
| S8-final-viral-combined_k141_218676\|\|full | 1215 | No | NA | | 0 | 0 | Low-quality | Genome-fragment | | 1.93 | 0 |
| S9-final-viral-combined_k141_85819\|\|full | 1216 | No | NA | | 0 | 0 | Low-quality | Genome-fragment | | 2.48 | 0 |
| S11_k141_139232 | 1195 | No | NA | | 0 | 1 | Not-determined | Genome-fragment | NA | | 0 |
| S11-final-viral-combined_k141_43924\|\|full | 1216 | No | NA | | 0 | 2 | Low-quality | Genome-fragment | | 4.01 | 0 |
| S11_k141_318440 | 1621 | No | NA | | 0 | 0 | Not-determined | Genome-fragment | NA | | 0 |
| S11-final-viral-combined_k141_256929\|\|full | 1216 | No | NA | | 1 | 0 | Low-quality | Genome-fragment | | 0.79 | 0 |
| S11_k141_153247 | 3195 | No | NA | | 0 | 1 | Not-determined | Genome-fragment | NA | | 0 |
| S11_k141_84404 | 1152 | No | NA | | 0 | 1 | Not-determined | Genome-fragment | NA | | 0 |
| S11-final-viral-combined_k141_149692\|\|full | 1216 | No | NA | | 0 | 0 | Low-quality | Genome-fragment | | 0.79 | 0 |
| S11_k141_208431 | 1138 | No | NA | | 0 | 1 | Not-determined | Genome-fragment | NA | | 0 |
| S11_k141_111626 | 1262 | No | NA | | 0 | 1 | Not-determined | Genome-fragment | NA | | 0 |
| S11_k141_236242 | 2678 | No | NA | | 0 | 2 | Not-determined | Genome-fragment | NA | | 0 |
| S11_k141_180781 | 1460 | No | NA | | 0 | 0 | Not-determined | Genome-fragment | NA | | 0 |
| S11_k141_56762 | 7865 | No | NA | | 0 | 3 | Not-determined | Genome-fragment | NA | | 0 |
| S11_k141_222262 | 1124 | No | NA | | 0 | 0 | Not-determined | Genome-fragment | NA | | 0 |
| S11_k141_194597 | 1175 | No | NA | | 0 | 0 | Not-determined | Genome-fragment | NA | | 0 |
| S11_k141_56786 | 1630 | No | NA | | 0 | 0 | Not-determined | Genome-fragment | NA | | 0 |
| S11_k141_277464 | 1101 | No | NA | | 0 | 0 | Not-determined | Genome-fragment | NA | | 0 |
| S11_k141_208515 | 5999 | No | NA | | 0 | 2 | Not-determined | Genome-fragment | NA | | 0 |
| S1-final-viral-combined_k141_202036\|\|full | 1216 | No | NA | | 0 | 0 | Low-quality | Genome-fragment | | 2.61 | 0 |
| S11_k141_249794 | 3833 | No | NA | | 0 | 1 | Not-determined | Genome-fragment | NA | | 0 |
| S1-final-viral-combined_k141_344835\|\|full | 1216 | No | NA | | 0 | 1 | Low-quality | Genome-fragment | | 2.87 | 0 |
| S1-final-viral-combined_k141_122176\|\|lt2gene | 1216 | No | NA | | 1 | 0 | Low-quality | Genome-fragment | | 2.26 | 0 |
| S11_k141_98487 | 1102 | No | NA | | 0 | 1 | Not-determined | Genome-fragment | NA | | 0 |
| S11_k141_111752 | 2358 | No | NA | | 0 | 1 | Not-determined | Genome-fragment | NA | | 0 |
| S11_k141_125896 | 1377 | No | NA | | 0 | 0 | Not-determined | Genome-fragment | NA | | 0 |
| S11_k141_153395 | 1021 | No | NA | | 0 | 1 | Not-determined | Genome-fragment | NA | | 0 |
| S1_k141_174599 | 1216 | No | NA | | 0 | 0 | Low-quality | Genome-fragment | | 2.03 | 0 |
| S11_k141_222396 | 1108 | No | NA | | 0 | 0 | Not-determined | Genome-fragment | NA | | 0 |
| S3_k141_41591 | 1216 | No | NA | | 0 | 0 | Low-quality | Genome-fragment | | 7.33 | 0 |
| S11_k141_84637 | 1546 | No | NA | | 0 | 3 | Not-determined | Genome-fragment | NA | | 0 |
| S7-final-viral-combined_k141_71384\|\|full | 1216 | No | NA | | 1 | 0 | Low-quality | Genome-fragment | | 3.22 | 0 |
| S11_k141_194754 | 1109 | No | NA | | 0 | 0 | Not-determined | Genome-fragment | NA | | 0 |
| S11_k141_249838 | 1064 | No | NA | | 0 | 0 | Not-determined | Genome-fragment | NA | | 0 |
| S17-final-viral-combined_k141_142573\|\|full | 1216 | No | NA | | 1 | 0 | Low-quality | Genome-fragment | | 2.08 | 0 |
| S11_k141_167569 | 5025 | No | NA | | 0 | 3 | Not-determined | Genome-fragment | NA | | 0 |
| S11_k141_125994 | 1989 | No | NA | | 0 | 0 | Not-determined | Genome-fragment | NA | | 0 |
| S11_k141_249883 | 1147 | No | NA | | 0 | 0 | Not-determined | Genome-fragment | NA | | 0 |
| S11_k141_236471 | 2019 | No | NA | | 0 | 0 | Not-determined | Genome-fragment | NA | | 0 |
| S11_k141_98648 | 1078 | No | NA | | 0 | 2 | Not-determined | Genome-fragment | NA | | 0 |
| S11_k141_208787 | 1058 | No | NA | | 0 | 1 | Not-determined | Genome-fragment | NA | | 0 |
| S11_k141_98658 | 1159 | No | NA | | 0 | 1 | Not-determined | Genome-fragment | NA | | 0 |
| S6-final-viral-combined_k141_110879\|\|full | 1216 | No | NA | | 0 | 2 | Low-quality | Genome-fragment | | 0.54 | 0 |
| S11_k141_84775 | 1568 | No | NA | | 0 | 0 | Not-determined | Genome-fragment | NA | | 0 |
| S11_k141_277740 | 1610 | No | NA | | 0 | 0 | Not-determined | Genome-fragment | NA | | 0 |
| S11_k141_1921 | 1631 | No | NA | | 0 | 1 | Not-determined | Genome-fragment | NA | | 0 |
| S8-final-viral-combined_k141_215\|\|full | 1216 | No | NA | | 2 | 0 | Low-quality | Genome-fragment | | 2.2 | 0 |
| S11_k141_15863 | 1657 | No | NA | | 0 | 2 | Not-determined | Genome-fragment | NA | | 0 |
| S11_k141_318755 | 1234 | No | NA | | 0 | 1 | Not-determined | Genome-fragment | NA | | 0 |
| S11-final-viral-combined_k141_46076\|\|full | 1217 | No | NA | | 0 | 0 | Low-quality | Genome-fragment | | 2.96 | 0 |
| S11_k141_181062 | 1027 | No | NA | | 0 | 0 | Not-determined | Genome-fragment | NA | | 0 |
| S11_k141_98728 | 1152 | No | NA | | 0 | 1 | Not-determined | Genome-fragment | NA | | 0 |
| S11_k141_263848 | 2028 | No | NA | | 0 | 1 | Not-determined | Genome-fragment | NA | | 0 |
| S11_k141_263867 | 1551 | No | NA | | 0 | 1 | Not-determined | Genome-fragment | NA | | 0 |
| S11_k141_117873 | 1217 | No | NA | | 0 | 0 | Low-quality | Genome-fragment | | 3.05 | 0 |
| S11_k141_277834 | 2450 | No | NA | | 0 | 0 | Not-determined | Genome-fragment | NA | | 0 |
| S11_k141_318838 | 1024 | No | NA | | 0 | 1 | Not-determined | Genome-fragment | NA | | 0 |
| S11_k141_181129 | 2531 | No | NA | | 0 | 2 | Not-determined | Genome-fragment | NA | | 0 |
| S1-final-viral-combined_k141_92728\|\|full | 1217 | No | NA | | 2 | 0 | Low-quality | Genome-fragment | | 2.21 | 0 |
| S11_k141_208972 | 1128 | No | NA | | 0 | 2 | Not-determined | Genome-fragment | NA | | 0 |
| S11_k141_139747 | 1202 | No | NA | | 0 | 2 | Not-determined | Genome-fragment | NA | | 0 |
| S11_k141_277865 | 1136 | No | NA | | 0 | 1 | Not-determined | Genome-fragment | NA | | 0 |
| S11_k141_126172 | 1030 | No | NA | | 0 | 2 | Not-determined | Genome-fragment | NA | | 0 |
| S11_k141_2055 | 1538 | No | NA | | 0 | 1 | Not-determined | Genome-fragment | NA | | 0 |
| S11_k141_153579 | 6515 | No | NA | | 0 | 0 | Not-determined | Genome-fragment | NA | | 0 |
| S11_k141_318865 | 1134 | No | NA | | 0 | 1 | Not-determined | Genome-fragment | NA | | 0 |
| S11_k141_209016 | 3466 | No | NA | | 0 | 2 | Not-determined | Genome-fragment | NA | | 0 |
| S11_k141_305338 | 1326 | No | NA | | 0 | 1 | Not-determined | Genome-fragment | NA | | 0 |
| S1-final-viral-combined_k141_301569\|\|lt2gene | 1217 | No | NA | | 1 | 0 | Low-quality | Genome-fragment | | 2.03 | 0 |
| S11_k141_236685 | 1542 | No | NA | | 0 | 2 | Not-determined | Genome-fragment | NA | | 0 |
| S11_k141_139811 | 1232 | No | NA | | 0 | 0 | Not-determined | Genome-fragment | NA | | 0 |
| S11_k141_250126 | 1331 | No | NA | | 0 | 1 | Not-determined | Genome-fragment | NA | | 0 |
| S3-final-viral-combined_k141_54798\|\|full | 1217 | No | NA | | 0 | 0 | Low-quality | Genome-fragment | | 0.79 | 0 |
| S11_k141_277988 | 2095 | No | NA | | 0 | 2 | Not-determined | Genome-fragment | NA | | 0 |
| S11_k141_57339 | 1130 | No | NA | | 0 | 1 | Not-determined | Genome-fragment | NA | | 0 |
| S11_k141_318965 | 3063 | No | NA | | 0 | 0 | Not-determined | Genome-fragment | NA | | 0 |
| S11_k141_16088 | 1157 | No | NA | | 0 | 2 | Not-determined | Genome-fragment | NA | | 0 |
| S11_k141_98841 | 1075 | No | NA | | 0 | 1 | Not-determined | Genome-fragment | NA | | 0 |
| S11_k141_222692 | 3870 | No | NA | | 0 | 3 | Not-determined | Genome-fragment | NA | | 0 |
| S11_k141_70520 | 1679 | No | NA | | 0 | 1 | Not-determined | Genome-fragment | NA | | 0 |
| S11_k141_209154 | 1679 | No | NA | | 0 | 1 | Not-determined | Genome-fragment | NA | | 0 |
| S11_k141_70565 | 1331 | No | NA | | 0 | 1 | Not-determined | Genome-fragment | NA | | 0 |
| S3_k141_61697 | 1217 | No | NA | | 0 | 0 | Low-quality | Genome-fragment | | 0.94 | 0 |
| S11_k141_209181 | 4757 | No | NA | | 0 | 1 | Not-determined | Genome-fragment | NA | | 0 |
| S11_k141_43504 | 1737 | No | NA | | 0 | 0 | Not-determined | Genome-fragment | NA | | 0 |
| S5-final-viral-combined_k141_154951\|\|lt2gene | 1217 | No | NA | | 2 | 0 | Low-quality | Genome-fragment | | 2.97 | 0 |
| S11_k141_43509 | 1699 | No | NA | | 0 | 1 | Not-determined | Genome-fragment | NA | | 0 |
| S11_k141_57493 | 1800 | No | NA | | 0 | 0 | Not-determined | Genome-fragment | NA | | 0 |
| S11_k141_305507 | 3402 | No | NA | | 0 | 2 | Not-determined | Genome-fragment | NA | | 0 |
| S11_k141_85034 | 1039 | No | NA | | 0 | 2 | Not-determined | Genome-fragment | NA | | 0 |
| S11_k141_209262 | 3321 | No | NA | | 0 | 1 | Not-determined | Genome-fragment | NA | | 0 |
| S7-final-viral-combined_k141_228197\|\|full | 1217 | No | NA | | 0 | 1 | Low-quality | Genome-fragment | | 0.72 | 0 |
| S11_k141_278183 | 3744 | No | NA | | 0 | 4 | Not-determined | Genome-fragment | NA | | 0 |
| S11_k141_126326 | 1038 | No | NA | | 0 | 1 | Not-determined | Genome-fragment | NA | | 0 |
| S11_k141_195214 | 1499 | No | NA | | 0 | 1 | Not-determined | Genome-fragment | NA | | 0 |
| S11_k141_278198 | 1449 | No | NA | | 0 | 0 | Not-determined | Genome-fragment | NA | | 0 |
| S11_k141_264171 | 1365 | No | NA | | 0 | 0 | Not-determined | Genome-fragment | NA | | 0 |
| S11_k141_57568 | 1008 | No | NA | | 0 | 1 | Not-determined | Genome-fragment | NA | | 0 |
| S7-final-viral-combined_k141_50814\|\|full | 1217 | No | NA | | 1 | 0 | Low-quality | Genome-fragment | | 3.19 | 0 |
| S11_k141_153822 | 3123 | No | NA | | 0 | 0 | Not-determined | Genome-fragment | NA | | 0 |
| S11_k141_29815 | 4198 | No | NA | | 0 | 3 | Not-determined | Genome-fragment | NA | | 0 |
| S11_k141_250423 | 1169 | No | NA | | 0 | 0 | Not-determined | Genome-fragment | NA | | 0 |
| S11_k141_112187 | 1205 | No | NA | | 0 | 1 | Not-determined | Genome-fragment | NA | | 0 |
| S7_k141_191781 | 1217 | No | NA | | 2 | 0 | Low-quality | Genome-fragment | | 3.35 | 0 |
| S11_k141_70787 | 1309 | No | NA | | 0 | 0 | Not-determined | Genome-fragment | NA | | 0 |
| S12-final-viral-combined_k141_236477\|\|full | 1217 | No | NA | | 0 | 0 | Low-quality | Genome-fragment | | 1.87 | 0 |
| S11_k141_236984 | 2322 | No | NA | | 0 | 1 | Not-determined | Genome-fragment | NA | | 0 |
| S12-final-viral-combined_k141_80236\|\|full | 1217 | No | NA | | 2 | 0 | Low-quality | Genome-fragment | | 3.08 | 0 |
| S12-final-viral-combined_k141_367305\|\|full | 1217 | No | NA | | 1 | 0 | Low-quality | Genome-fragment | | 1.49 | 0 |
| S12_k141_380729 | 1217 | No | NA | | 0 | 1 | Low-quality | Genome-fragment | | 0.74 | 0 |
| S11_k141_2406 | 1163 | No | NA | | 0 | 1 | Not-determined | Genome-fragment | NA | | 0 |
| S11_k141_305745 | 1357 | No | NA | | 0 | 1 | Not-determined | Genome-fragment | NA | | 0 |
| S11_k141_126480 | 3315 | No | NA | | 0 | 1 | Not-determined | Genome-fragment | NA | | 0 |
| S11_k141_43679 | 1075 | No | NA | | 0 | 0 | Not-determined | Genome-fragment | NA | | 0 |
| S11_k141_305773 | 3434 | No | NA | | 0 | 1 | Not-determined | Genome-fragment | NA | | 0 |
| S11_k141_112253 | 1109 | No | NA | | 0 | 2 | Not-determined | Genome-fragment | NA | | 0 |
| S11_k141_126517 | 1084 | No | NA | | 0 | 1 | Not-determined | Genome-fragment | NA | | 0 |
| S2-final-viral-combined_k141_199793\|\|full | 1217 | No | NA | | 0 | 1 | Low-quality | Genome-fragment | | 1.56 | 0 |
| S11_k141_2515 | 1425 | No | NA | | 0 | 1 | Not-determined | Genome-fragment | NA | | 0 |
| S4-final-viral-combined_k141_102863\|\|full | 1217 | No | NA | | 0 | 1 | Low-quality | Genome-fragment | | 1.99 | 0 |
| S4-final-viral-combined_k141_161639\|\|lt2gene | 1217 | No | NA | | 1 | 0 | Low-quality | Genome-fragment | | 0.75 | 0 |
| S11_k141_70903 | 1426 | No | NA | | 0 | 1 | Not-determined | Genome-fragment | NA | | 0 |
| S11_k141_319255 | 1683 | No | NA | | 0 | 2 | Not-determined | Genome-fragment | NA | | 0 |
| S11_k141_99161 | 1534 | No | NA | | 0 | 0 | Not-determined | Genome-fragment | NA | | 0 |
| S11_k141_209564 | 1061 | No | NA | | 0 | 0 | Not-determined | Genome-fragment | NA | | 0 |
| S11_k141_29974 | 1833 | No | NA | | 0 | 1 | Not-determined | Genome-fragment | NA | | 0 |
| S11_k141_209579 | 1917 | No | NA | | 0 | 1 | Not-determined | Genome-fragment | NA | | 0 |
| S11_k141_250572 | 9488 | No | NA | | 0 | 5 | Not-determined | Genome-fragment | NA | | 0 |
| S11_k141_57846 | 1905 | No | NA | | 0 | 1 | Not-determined | Genome-fragment | NA | | 0 |
| S11_k141_278441 | 1905 | No | NA | | 0 | 0 | Not-determined | Genome-fragment | NA | | 0 |

| | | | | | | | | | |
|---|---|---|---|---|---|---|---|---|---|
| S11_k141_319308 | 3376 | No | NA | 0 | 1 | Not-determined | Genome-fragment | NA | 0 |
| S11_k141_154099 | 1173 | No | NA | 0 | 1 | Not-determined | Genome-fragment | NA | 0 |
| S8-final-viral-combined_k141_19941\|\|full | 1217 | No | NA | 2 | 0 | Low-quality | Genome-fragment | 3.53 | 0 |
| S8_k141_195681 | 1217 | No | NA | 0 | 0 | Low-quality | Genome-fragment | 2.1 | 0 |
| S11_k141_30058 | 1017 | No | NA | 0 | 0 | Not-determined | Genome-fragment | NA | 0 |
| S11_k141_16241 | 1120 | No | NA | 0 | 1 | Not-determined | Genome-fragment | NA | 0 |
| S11-final-viral-combined_k141_300241\|\|full | 1218 | No | NA | 0 | 0 | Low-quality | Genome-fragment | 3.92 | 0 |
| S11_k141_209642 | 1295 | No | NA | 0 | 1 | Not-determined | Genome-fragment | NA | 0 |
| S11_k141_223124 | 4336 | No | NA | 0 | 1 | Not-determined | Genome-fragment | NA | 0 |
| S11_k141_237173 | 1059 | No | NA | 0 | 0 | Not-determined | Genome-fragment | NA | 0 |
| S11_k141_43929 | 1632 | No | NA | 0 | 1 | Not-determined | Genome-fragment | NA | 0 |
| S11_k141_223143 | 1132 | No | NA | 0 | 1 | Not-determined | Genome-fragment | NA | 0 |
| S11_k141_57964 | 1503 | No | NA | 0 | 1 | Not-determined | Genome-fragment | NA | 0 |
| S11_k141_264378 | 1757 | No | NA | 0 | 0 | Not-determined | Genome-fragment | NA | 0 |
| S11_k141_278558 | 3324 | No | NA | 0 | 0 | Not-determined | Genome-fragment | NA | 0 |
| S11-final-viral-combined_k141_41053\|\|full | 1218 | No | NA | 0 | 1 | Low-quality | Genome-fragment | 1.6 | 0 |
| S11_k141_319376 | 1456 | No | NA | 0 | 2 | Not-determined | Genome-fragment | NA | 0 |
| S1-final-viral-combined_k141_193472\|\|full | 1218 | No | NA | 1 | 0 | Low-quality | Genome-fragment | 3.7 | 0 |
| S11_k141_16327 | 1769 | No | NA | 0 | 0 | Not-determined | Genome-fragment | NA | 0 |
| S11_k141_99393 | 5795 | No | NA | 0 | 4 | Not-determined | Genome-fragment | NA | 0 |
| S11_k141_2787 | 1274 | No | NA | 0 | 1 | Not-determined | Genome-fragment | NA | 0 |
| S11_k141_181594 | 2639 | No | NA | 0 | 2 | Not-determined | Genome-fragment | NA | 0 |
| S11_k141_154329 | 1922 | No | NA | 0 | 2 | Not-determined | Genome-fragment | NA | 0 |
| S11_k141_154330 | 1094 | No | NA | 0 | 1 | Not-determined | Genome-fragment | NA | 0 |
| S11_k141_85351 | 1962 | No | NA | 0 | 1 | Not-determined | Genome-fragment | NA | 0 |
| S11_k141_140485 | 1457 | No | NA | 0 | 0 | Not-determined | Genome-fragment | NA | 0 |
| S11_k141_223290 | 1377 | No | NA | 0 | 1 | Not-determined | Genome-fragment | NA | 0 |
| S1_k141_346203 | 1218 | No | NA | 0 | 0 | Low-quality | Genome-fragment | 2.9 | 0 |
| S11_k141_264539 | 3271 | No | NA | 0 | 1 | Not-determined | Genome-fragment | NA | 0 |
| S11_k141_58172 | 1304 | No | NA | 0 | 1 | Not-determined | Genome-fragment | NA | 0 |
| S11_k141_44081 | 1038 | No | NA | 0 | 1 | Not-determined | Genome-fragment | NA | 0 |
| S11_k141_44095 | 2471 | No | NA | 0 | 1 | Not-determined | Genome-fragment | NA | 0 |
| S11_k141_264583 | 1023 | No | NA | 0 | 2 | Not-determined | Genome-fragment | NA | 0 |
| S7-final-viral-combined_k141_2982\|\|lt2gene | 1218 | No | NA | 0 | 1 | Low-quality | Genome-fragment | 0.29 | 0 |
| S17-final-viral-combined_k141_179024\|\|full | 1218 | No | NA | 0 | 0 | Low-quality | Genome-fragment | 2.23 | 0 |
| S11_k141_58212 | 3061 | No | NA | 0 | 1 | Not-determined | Genome-fragment | NA | 0 |
| S11_k141_44116 | 1354 | No | NA | 0 | 1 | Not-determined | Genome-fragment | NA | 0 |
| S11_k141_168454 | 2691 | No | NA | 0 | 0 | Not-determined | Genome-fragment | NA | 0 |
| S11_k141_291961 | 6857 | No | NA | 0 | 6 | Not-determined | Genome-fragment | NA | 0 |
| S11_k141_223347 | 5407 | No | NA | 0 | 3 | Not-determined | Genome-fragment | NA | 0 |
| S17-final-viral-combined_k141_227022\|\|full | 1218 | No | NA | 3 | 0 | Low-quality | Genome-fragment | 1.71 | 0 |
| S17-final-viral-combined_k141_231452\|\|full | 1218 | No | NA | 0 | 0 | Low-quality | Genome-fragment | 15.2 | 0 |
| S11_k141_71311 | 1317 | No | NA | 0 | 0 | Not-determined | Genome-fragment | NA | 0 |
| S11_k141_154373 | 2068 | No | NA | 0 | 1 | Not-determined | Genome-fragment | NA | 0 |
| S11_k141_2947 | 1071 | No | NA | 0 | 0 | Not-determined | Genome-fragment | NA | 0 |
| S11_k141_306219 | 2009 | No | NA | 0 | 2 | Not-determined | Genome-fragment | NA | 0 |
| S11_k141_292003 | 2158 | No | NA | 0 | 1 | Not-determined | Genome-fragment | NA | 0 |
| S17-final-viral-combined_k141_254342\|\|full | 1218 | No | NA | 0 | 1 | Low-quality | Genome-fragment | 2.66 | 0 |
| S11_k141_237505 | 1582 | No | NA | 0 | 0 | Not-determined | Genome-fragment | NA | 0 |
| S11_k141_16505 | 1233 | No | NA | 0 | 0 | Not-determined | Genome-fragment | NA | 0 |
| S2-final-viral-combined_k141_62558\|\|lt2gene | 1218 | No | NA | 1 | 0 | Low-quality | Genome-fragment | 3.93 | 0 |
| S11_k141_181710 | 1646 | No | NA | 0 | 0 | Not-determined | Genome-fragment | NA | 0 |
| S11_k141_112724 | 3824 | No | NA | 0 | 2 | Not-determined | Genome-fragment | NA | 0 |
| S11_k141_44247 | 1681 | No | NA | 0 | 1 | Not-determined | Genome-fragment | NA | 0 |
| S11_k141_85518 | 1329 | No | NA | 0 | 1 | Not-determined | Genome-fragment | NA | 0 |
| S11_k141_30366 | 1567 | No | NA | 0 | 1 | Not-determined | Genome-fragment | NA | 0 |
| S11_k141_223450 | 2771 | No | NA | 0 | 1 | Not-determined | Genome-fragment | NA | 0 |
| S11_k141_168612 | 1111 | No | NA | 0 | 1 | Not-determined | Genome-fragment | NA | 0 |
| S11_k141_140666 | 1089 | No | NA | 0 | 1 | Not-determined | Genome-fragment | NA | 0 |
| S11_k141_3034 | 3115 | No | NA | 0 | 0 | Not-determined | Genome-fragment | NA | 0 |
| S11_k141_319535 | 3696 | No | NA | 0 | 1 | Not-determined | Genome-fragment | NA | 0 |
| S4-final-viral-combined_k141_160754\|\|full | 1218 | No | NA | 0 | 1 | Low-quality | Genome-fragment | 1 | 0 |
| S11_k141_168651 | 1122 | No | NA | 0 | 2 | Not-determined | Genome-fragment | NA | 0 |
| S11_k141_210060 | 3461 | No | NA | 0 | 1 | Not-determined | Genome-fragment | NA | 0 |
| S8-final-viral-combined_k141_198167\|\|full | 1218 | No | NA | 0 | 1 | Low-quality | Genome-fragment | 1.19 | 0 |
| S11_k141_195720 | 1373 | No | NA | 0 | 0 | Not-determined | Genome-fragment | NA | 0 |
| S11_k141_154513 | 1003 | No | NA | 0 | 0 | Not-determined | Genome-fragment | NA | 0 |
| S8-final-viral-combined_k141_233020\|\|full | 1218 | No | NA | 0 | 0 | Low-quality | Genome-fragment | 2.77 | 0 |
| S8-final-viral-combined_k141_201046\|\|full | 1218 | No | NA | 0 | 2 | Low-quality | Genome-fragment | 1.06 | 0 |
| S11_k141_112808 | 15664 | No | NA | 0 | 10 | Not-determined | Genome-fragment | NA | 0 |
| S8-final-viral-combined_k141_223139\|\|lt2gene | 1218 | Yes | 617 | 1 | 1 | Low-quality | Genome-fragment | 0.97 | 49.34 |
| S11_k141_306479 | 1338 | No | NA | 0 | 0 | Not-determined | Genome-fragment | NA | 0 |
| S11_k141_168694 | 1533 | No | NA | 0 | 0 | Not-determined | Genome-fragment | NA | 0 |
| S11_k141_278950 | 5061 | No | NA | 0 | 1 | Not-determined | Genome-fragment | NA | 0 |
| S11_k141_58482 | 1290 | No | NA | 0 | 2 | Not-determined | Genome-fragment | NA | 0 |
| S9-final-viral-combined_k141_11266\|\|full | 1219 | No | NA | 1 | 0 | Low-quality | Genome-fragment | 3.09 | 0 |
| S11_k141_112976 | 1013 | No | NA | 0 | 2 | Not-determined | Genome-fragment | NA | 0 |
| S11_k141_154748 | 6306 | No | NA | 0 | 12 | Not-determined | Genome-fragment | NA | 0 |
| S11_k141_195808 | 4951 | No | NA | 0 | 2 | Not-determined | Genome-fragment | NA | 0 |
| S11_k141_210187 | 3650 | No | NA | 0 | 1 | Not-determined | Genome-fragment | NA | 0 |
| S11_k141_3116 | 38537 | Yes | 31746 | 12 | 4 | Medium-quality | Genome-fragment | 88.7 | 17.62 |
| S11_k141_30609 | 1078 | No | NA | 0 | 0 | Not-determined | Genome-fragment | NA | 0 |
| S9_k141_91796 | 1219 | No | NA | 0 | 0 | Low-quality | Genome-fragment | 1.63 | 0 |
| S11_k141_251106 | 1058 | No | NA | 0 | 1 | Not-determined | Genome-fragment | NA | 0 |
| S11_k141_182093 | 8205 | No | NA | 0 | 0 | Not-determined | Genome-fragment | NA | 0 |
| S11_k141_44351 | 2276 | No | NA | 0 | 2 | Not-determined | Genome-fragment | NA | 0 |
| S11_k141_251140 | 1083 | No | NA | 0 | 1 | Not-determined | Genome-fragment | NA | 0 |
| S11_k141_30653 | 3047 | No | NA | 0 | 2 | Not-determined | Genome-fragment | NA | 0 |
| S11_k141_195898 | 1057 | No | NA | 0 | 1 | Not-determined | Genome-fragment | NA | 0 |
| S11_k141_71644 | 1257 | No | NA | 0 | 0 | Not-determined | Genome-fragment | NA | 0 |
| S11_k141_127188 | 1269 | No | NA | 0 | 1 | Not-determined | Genome-fragment | NA | 0 |
| S11_k141_279117 | 5563 | No | NA | 0 | 1 | Not-determined | Genome-fragment | NA | 0 |
| S11_k141_3241 | 1081 | No | NA | 0 | 0 | Not-determined | Genome-fragment | NA | 0 |
| S11_k141_58605 | 1034 | No | NA | 0 | 0 | Not-determined | Genome-fragment | NA | 0 |
| S11_k141_279202 | 1741 | No | NA | 0 | 0 | Not-determined | Genome-fragment | NA | 0 |
| S11-final-viral-combined_k141_80519\|\|full | 1219 | No | NA | 0 | 0 | Low-quality | Genome-fragment | 0.92 | 0 |
| S11_k141_210218 | 3456 | No | NA | 0 | 1 | Not-determined | Genome-fragment | NA | 0 |
| S11_k141_251212 | 1117 | No | NA | 0 | 1 | Not-determined | Genome-fragment | NA | 0 |
| S11_k141_58639 | 1731 | No | NA | 0 | 1 | Not-determined | Genome-fragment | NA | 0 |
| S11_k141_85898 | 4738 | No | NA | 0 | 0 | Not-determined | Genome-fragment | NA | 0 |
| S11_k141_265092 | 1390 | No | NA | 0 | 2 | Not-determined | Genome-fragment | NA | 0 |
| S11_k141_210222 | 3775 | No | NA | 0 | 2 | Not-determined | Genome-fragment | NA | 0 |
| S11_k141_265104 | 6150 | No | NA | 0 | 0 | Not-determined | Genome-fragment | NA | 0 |
| S11_k141_182252 | 1829 | No | NA | 0 | 2 | Not-determined | Genome-fragment | NA | 0 |
| S11_k141_85929 | 1347 | No | NA | 0 | 1 | Not-determined | Genome-fragment | NA | 0 |
| S11_k141_154887 | 1187 | No | NA | 0 | 0 | Not-determined | Genome-fragment | NA | 0 |
| S11_k141_223884 | 3636 | No | NA | 0 | 3 | Not-determined | Genome-fragment | NA | 0 |
| S11_k141_16953 | 2819 | No | NA | 0 | 0 | Not-determined | Genome-fragment | NA | 0 |
| S11-final-viral-combined_k141_173797\|\|lt2gene | 1219 | No | NA | 1 | 0 | Low-quality | Genome-fragment | 3.3 | 0 |
| S11_k141_154938 | 2514 | No | NA | 0 | 0 | Not-determined | Genome-fragment | NA | 0 |
| S11_k141_265167 | 3571 | No | NA | 0 | 2 | Not-determined | Genome-fragment | NA | 0 |
| S11_k141_319945 | 1791 | No | NA | 0 | 0 | Not-determined | Genome-fragment | NA | 0 |
| S11_k141_141104 | 1304 | No | NA | 0 | 1 | Not-determined | Genome-fragment | NA | 0 |
| S11_k141_231620 | 1219 | No | NA | 0 | 0 | Low-quality | Genome-fragment | 2.04 | 0 |
| S11_k141_279354 | 1093 | No | NA | 0 | 1 | Not-determined | Genome-fragment | NA | 0 |
| S11_k141_113146 | 5316 | No | NA | 0 | 0 | Not-determined | Genome-fragment | NA | 0 |
| S1_k141_186486 | 1219 | No | NA | 0 | 2 | Low-quality | Genome-fragment | 0.36 | 0 |
| S11_k141_319985 | 1267 | No | NA | 0 | 1 | Not-determined | Genome-fragment | NA | 0 |
| S11_k141_319990 | 2451 | No | NA | 0 | 1 | Not-determined | Genome-fragment | NA | 0 |
| S11_k141_292421 | 6384 | No | NA | 0 | 1 | Not-determined | Genome-fragment | NA | 0 |
| S5-final-viral-combined_k141_202004\|\|full | 1219 | No | NA | 0 | 2 | Low-quality | Genome-fragment | 0.81 | 0 |
| S11_k141_210374 | 1060 | No | NA | 0 | 1 | Not-determined | Genome-fragment | NA | 0 |
| S5-final-viral-combined_k141_102114\|\|lt2gene | 1219 | No | NA | 1 | 0 | Low-quality | Genome-fragment | 3.06 | 0 |
| S11_k141_58743 | 1063 | No | NA | 0 | 0 | Not-determined | Genome-fragment | NA | 0 |
| S11_k141_86123 | 1750 | No | NA | 0 | 1 | Not-determined | Genome-fragment | NA | 0 |
| S5-final-viral-combined_k141_129123\|\|lt2gene | 1219 | No | NA | 1 | 0 | Low-quality | Genome-fragment | 2.78 | 0 |
| S12-final-viral-combined_k141_352593\|\|full | 1219 | No | NA | 0 | 1 | Low-quality | Genome-fragment | 1.42 | 0 |
| S11_k141_292520 | 3131 | No | NA | 0 | 0 | Not-determined | Genome-fragment | NA | 0 |
| S11_k141_30972 | 1261 | No | NA | 0 | 1 | Not-determined | Genome-fragment | NA | 0 |
| S12-final-viral-combined_k141_173829\|\|lt2gene | 1219 | No | NA | 1 | 0 | Low-quality | Genome-fragment | 2.9 | 0 |
| S11_k141_86198 | 1026 | No | NA | 0 | 0 | Not-determined | Genome-fragment | NA | 0 |
| S2_k141_247298 | 1219 | No | NA | 0 | 0 | Low-quality | Genome-fragment | 2.94 | 0 |
| S11_k141_155154 | 1071 | No | NA | 0 | 0 | Not-determined | Genome-fragment | NA | 0 |
| S11_k141_279460 | 1069 | No | NA | 0 | 3 | Not-determined | Genome-fragment | NA | 0 |
| S11_k141_86310 | 2719 | No | NA | 0 | 2 | Not-determined | Genome-fragment | NA | 0 |
| S6_k141_23701 | 1219 | No | NA | 0 | 0 | Low-quality | Genome-fragment | 3.6 | 0 |
| S11_k141_113322 | 4061 | No | NA | 0 | 0 | Not-determined | Genome-fragment | NA | 0 |
| S11_k141_196153 | 1310 | No | NA | 0 | 0 | Not-determined | Genome-fragment | NA | 0 |
| S11_k141_141303 | 1307 | No | NA | 0 | 1 | Not-determined | Genome-fragment | NA | 0 |
| S11_k141_251462 | 7019 | No | NA | 0 | 7 | Not-determined | Genome-fragment | NA | 0 |
| S8-final-viral-combined_k141_201964\|\|full | 1219 | No | NA | 0 | 1 | Low-quality | Genome-fragment | 0.83 | 0 |
| S11_k141_31076 | 1162 | No | NA | 0 | 2 | Not-determined | Genome-fragment | NA | 0 |
| S11_k141_58900 | 3738 | No | NA | 0 | 1 | Not-determined | Genome-fragment | NA | 0 |
| S11_k141_320258 | 3012 | No | NA | 0 | 1 | Not-determined | Genome-fragment | NA | 0 |
| S11_k141_292829 | 2807 | No | NA | 0 | 1 | Not-determined | Genome-fragment | NA | 0 |
| S11_k141_265304 | 2729 | No | NA | 0 | 0 | Not-determined | Genome-fragment | NA | 0 |
| S11_k141_100489 | 1449 | No | NA | 0 | 0 | Not-determined | Genome-fragment | NA | 0 |
| S9_k141_92492 | 1220 | No | NA | 0 | 1 | Low-quality | Genome-fragment | 0.81 | 0 |
| S11-final-viral-combined_k141_217260\|\|lt2gene | 1220 | No | NA | 0 | 0 | Low-quality | Genome-fragment | 2.61 | 0 |
| S11_k141_196245 | 8940 | No | NA | 0 | 4 | Not-determined | Genome-fragment | NA | 0 |
| S11_k141_86545 | 1444 | No | NA | 0 | 1 | Not-determined | Genome-fragment | NA | 0 |
| S1_k141_113604 | 1220 | No | NA | 1 | 0 | Low-quality | Genome-fragment | 2.56 | 0 |
| S11_k141_251681 | 1225 | No | NA | 0 | 1 | Not-determined | Genome-fragment | NA | 0 |
| S11_k141_72149 | 1562 | No | NA | 0 | 1 | Not-determined | Genome-fragment | NA | 0 |
| S11_k141_224395 | 3132 | No | NA | 0 | 1 | Not-determined | Genome-fragment | NA | 0 |
| S11_k141_31250 | 1488 | No | NA | 0 | 1 | Not-determined | Genome-fragment | NA | 0 |
| S3-final-viral-combined_k141_49729\|\|full | 1220 | No | NA | 1 | 0 | Low-quality | Genome-fragment | 3.2 | 0 |
| S11_k141_279802 | 1083 | No | NA | 0 | 2 | Not-determined | Genome-fragment | NA | 0 |
| S11_k141_113483 | 3983 | No | NA | 0 | 3 | Not-determined | Genome-fragment | NA | 0 |
| S11_k141_141553 | 4341 | No | NA | 0 | 0 | Not-determined | Genome-fragment | NA | 0 |
| S11_k141_210963 | 1071 | No | NA | 0 | 1 | Not-determined | Genome-fragment | NA | 0 |
| S3-final-viral-combined_k141_44024\|\|lt2gene | 1220 | No | NA | 1 | 0 | Low-quality | Genome-fragment | 3.17 | 0 |
| S11_k141_3723 | 1039 | No | NA | 0 | 1 | Not-determined | Genome-fragment | NA | 0 |
| S11_k141_265545 | 8265 | No | NA | 0 | 3 | Not-determined | Genome-fragment | NA | 0 |
| S3_k141_54194 | 1220 | No | NA | 0 | 0 | Low-quality | Genome-fragment | 2.29 | 0 |
| S7-final-viral-combined_k141_132857\|\|full | 1220 | No | NA | 0 | 0 | Low-quality | Genome-fragment | 3.21 | 0 |
| S11_k141_224458 | 1187 | No | NA | 0 | 2 | Not-determined | Genome-fragment | NA | 0 |
| S11_k141_224460 | 1244 | No | NA | 0 | 1 | Not-determined | Genome-fragment | NA | 0 |
| S7-final-viral-combined_k141_70210\|\|full | 1220 | No | NA | 1 | 0 | Low-quality | Genome-fragment | 19.08 | 0 |
| S11_k141_320521 | 4453 | No | NA | 0 | 1 | Not-determined | Genome-fragment | NA | 0 |
| S7-final-viral-combined_k141_41988\|\|lt2gene | 1220 | No | NA | 0 | 0 | Low-quality | Genome-fragment | 2.61 | 0 |
| S17-k141_82930 | 1220 | No | NA | 0 | 0 | Low-quality | Genome-fragment | 0.97 | 0 |
| S11_k141_86807 | 1144 | No | NA | 0 | 2 | Not-determined | Genome-fragment | NA | 0 |
| S4_k141_43994 | 1220 | No | NA | 0 | 0 | Low-quality | Genome-fragment | 1.23 | 0 |

| | | | | | | | | | | | |
|---|---|---|---|---|---|---|---|---|---|---|---|
| S11_k141_320555 | 31066 | No | NA | | 11 | 1 | Medium-quality | Genome-fragment | | 60.9 | 0 |
| S11_k141_211099 | 4665 | No | NA | | 0 | 5 | Not-determined | Genome-fragment | NA | | 0 |
| S11_k141_265652 | 3153 | No | NA | | 0 | 2 | Not-determined | Genome-fragment | NA | | 0 |
| S11_k141_31428 | 1027 | No | NA | | 0 | 0 | Not-determined | Genome-fragment | NA | | 0 |
| S8-final-viral-combined_k141_93421\|\|full | 1220 | No | NA | | 0 | 0 | Low-quality | Genome-fragment | | 2.34 | 0 |
| S11_k141_155622 | 1835 | No | NA | | 0 | 4 | Not-determined | Genome-fragment | NA | | 0 |
| S9-final-viral-combined_k141_83714\|\|full | 1221 | No | NA | | 0 | 0 | Low-quality | Genome-fragment | | 3.07 | 0 |
| S11_k141_127920 | 3011 | No | NA | | 0 | 1 | Not-determined | Genome-fragment | NA | | 0 |
| S11-final-viral-combined_k141_21893\|\|full | 1221 | No | NA | | 0 | 0 | Low-quality | Genome-fragment | | 3.15 | 0 |
| S11_k141_3906 | 1309 | No | NA | | 0 | 1 | Not-determined | Genome-fragment | NA | | 0 |
| S11_k141_141799 | 2136 | No | NA | | 0 | 1 | Not-determined | Genome-fragment | NA | | 0 |
| S11_k141_155670 | 1614 | No | NA | | 0 | 1 | Not-determined | Genome-fragment | NA | | 0 |
| S11_k141_72396 | 3201 | No | NA | | 0 | 2 | Not-determined | Genome-fragment | NA | | 0 |
| S11-final-viral-combined_k141_313619\|\|lt2gene | 1221 | No | NA | | 1 | 0 | Low-quality | Genome-fragment | | 0.84 | 0 |
| S11_k141_265796 | 4641 | No | NA | | 0 | 6 | Not-determined | Genome-fragment | NA | | 0 |
| S11_k141_178787 | 1221 | No | NA | | 1 | 0 | Low-quality | Genome-fragment | | 3.22 | 0 |
| S1-final-viral-combined_k141_39209\|\|full | 1221 | No | NA | | 1 | 0 | Low-quality | Genome-fragment | | 2.87 | 0 |
| S1-final-viral-combined_k141_174326\|\|full | 1221 | No | NA | | 0 | 0 | Low-quality | Genome-fragment | | 4.86 | 0 |
| S1-final-viral-combined_k141_201392\|\|lt2gene | 1221 | No | NA | | 0 | 1 | Low-quality | Genome-fragment | | 1.67 | 0 |
| S11_k141_87006 | 2723 | No | NA | | 0 | 2 | Not-determined | Genome-fragment | NA | | 0 |
| S1_k141_300402 | 1221 | No | NA | | 2 | 0 | Low-quality | Genome-fragment | | 0.92 | 0 |
| S11_k141_141895 | 1029 | No | NA | | 0 | 1 | Not-determined | Genome-fragment | NA | | 0 |
| S11_k141_252028 | 1718 | No | NA | | 0 | 1 | Not-determined | Genome-fragment | NA | | 0 |
| S11_k141_265877 | 1001 | No | NA | | 0 | 1 | Not-determined | Genome-fragment | NA | | 0 |
| S11_k141_211356 | 2508 | No | NA | | 0 | 0 | Not-determined | Genome-fragment | NA | | 0 |
| S11_k141_224654 | 1991 | No | NA | | 0 | 1 | Not-determined | Genome-fragment | NA | | 0 |
| S11_k141_211370 | 1906 | No | NA | | 0 | 1 | Not-determined | Genome-fragment | NA | | 0 |
| S11_k141_169920 | 2932 | No | NA | | 0 | 2 | Not-determined | Genome-fragment | NA | | 0 |
| S11_k141_238785 | 1590 | No | NA | | 0 | 1 | Not-determined | Genome-fragment | NA | | 0 |
| S11_k141_183224 | 1460 | No | NA | | 0 | 1 | Not-determined | Genome-fragment | NA | | 0 |
| S7-final-viral-combined_k141_2625\|\|full | 1221 | No | NA | | 1 | 0 | Low-quality | Genome-fragment | | 3 | 0 |
| S11_k141_4026 | 1113 | No | NA | | 0 | 0 | Not-determined | Genome-fragment | NA | | 0 |
| S7-final-viral-combined_k141_157950\|\|full | 1221 | No | NA | | 0 | 0 | Low-quality | Genome-fragment | | 0.98 | 0 |
| S11_k141_100981 | 1049 | No | NA | | 0 | 1 | Not-determined | Genome-fragment | NA | | 0 |
| S7_k141_188805 | 1221 | No | NA | | 0 | 0 | Low-quality | Genome-fragment | | 2.5 | 0 |
| S11_k141_155745 | 1688 | No | NA | | 0 | 2 | Not-determined | Genome-fragment | NA | | 0 |
| S12-final-viral-combined_k141_200672\|\|lt2gene | 1221 | No | NA | | 1 | 0 | Low-quality | Genome-fragment | | 2.62 | 0 |
| S11_k141_307602 | 3067 | No | NA | | 0 | 2 | Not-determined | Genome-fragment | NA | | 0 |
| S11_k141_169996 | 2388 | No | NA | | 0 | 1 | Not-determined | Genome-fragment | NA | | 0 |
| S11_k141_252146 | 3010 | No | NA | | 0 | 0 | Not-determined | Genome-fragment | NA | | 0 |
| S11_k141_252148 | 5297 | No | NA | | 0 | 0 | Not-determined | Genome-fragment | NA | | 0 |
| S12_k141_253233 | 1221 | No | NA | | 0 | 0 | Low-quality | Genome-fragment | | 1.24 | 0 |
| S11_k141_31644 | 1019 | No | NA | | 0 | 0 | Not-determined | Genome-fragment | NA | | 0 |
| S11_k141_320738 | 1430 | No | NA | | 0 | 1 | Not-determined | Genome-fragment | NA | | 0 |
| S11_k141_155790 | 1856 | No | NA | | 0 | 2 | Not-determined | Genome-fragment | NA | | 0 |
| S4_k141_135533 | 1221 | No | NA | | 0 | 0 | Low-quality | Genome-fragment | | 1.87 | 0 |
| S11_k141_238874 | 1573 | No | NA | | 0 | 2 | Not-determined | Genome-fragment | NA | | 0 |
| S9-final-viral-combined_k141_32868\|\|lt2gene | 1222 | No | NA | | 1 | 0 | Low-quality | Genome-fragment | | 2.7 | 0 |
| S11_k141_252222 | 4432 | No | NA | | 0 | 2 | Not-determined | Genome-fragment | NA | | 0 |
| S11_k141_142080 | 1832 | No | NA | | 0 | 0 | Not-determined | Genome-fragment | NA | | 0 |
| S11-final-viral-combined_k141_302966\|\|full | 1222 | Yes | | 727 | 1 | 1 | Low-quality | Genome-fragment | | 1.15 | 40.51 |
| S11-final-viral-combined_k141_46804\|\|lt2gene | 1222 | No | NA | | 1 | 0 | Low-quality | Genome-fragment | | 0.74 | 0 |
| S11_k141_252272 | 4954 | No | NA | | 0 | 5 | Not-determined | Genome-fragment | NA | | 0 |
| S11_k141_101057 | 1713 | No | NA | | 0 | 1 | Not-determined | Genome-fragment | NA | | 0 |
| S11_k141_87265 | 1142 | No | NA | | 0 | 1 | Not-determined | Genome-fragment | NA | | 0 |
| S11_k141_266138 | 1289 | No | NA | | 0 | 0 | Not-determined | Genome-fragment | NA | | 0 |
| S11-final-viral-combined_k141_200031\|\|lt2gene | 1222 | No | NA | | 1 | 0 | Low-quality | Genome-fragment | | 2.57 | 0 |
| S1-final-viral-combined_k141_246721\|\|full | 1222 | No | NA | | 0 | 1 | Low-quality | Genome-fragment | | 1.12 | 0 |
| S11_k141_101116 | 2250 | No | NA | | 0 | 1 | Not-determined | Genome-fragment | NA | | 0 |
| S11_k141_72605 | 1190 | No | NA | | 0 | 0 | Not-determined | Genome-fragment | NA | | 0 |
| S11_k141_87293 | 37508 | No | NA | | 10 | 0 | Complete | High-quality | | 100 | 0 |
| S11_k141_59660 | 3319 | No | NA | | 0 | 3 | Not-determined | Genome-fragment | NA | | 0 |
| S11_k141_280452 | 1134 | No | NA | | 0 | 1 | Not-determined | Genome-fragment | NA | | 0 |
| S11_k141_87300 | 1186 | No | NA | | 0 | 0 | Not-determined | Genome-fragment | NA | | 0 |
| S11_k141_266247 | 1079 | No | NA | | 0 | 2 | Not-determined | Genome-fragment | NA | | 0 |
| S3_k141_9753 | 1222 | No | NA | | 0 | 0 | Low-quality | Genome-fragment | | 1.96 | 0 |
| S11_k141_266315 | 2193 | No | NA | | 0 | 1 | Not-determined | Genome-fragment | NA | | 0 |
| S11_k141_211662 | 1182 | No | NA | | 0 | 0 | Not-determined | Genome-fragment | NA | | 0 |
| S11_k141_31865 | 3709 | No | NA | | 0 | 0 | Not-determined | Genome-fragment | NA | | 0 |
| S11_k141_266343 | 1497 | No | NA | | 0 | 2 | Not-determined | Genome-fragment | NA | | 0 |
| S12-final-viral-combined_k141_326325\|\|full | 1222 | No | NA | | 0 | 0 | Low-quality | Genome-fragment | | 3.8 | 0 |
| S11_k141_266396 | 3009 | No | NA | | 0 | 0 | Not-determined | Genome-fragment | NA | | 0 |
| S11_k141_45976 | 1036 | No | NA | | 0 | 1 | Not-determined | Genome-fragment | NA | | 0 |
| S11_k141_320969 | 1452 | No | NA | | 0 | 1 | Not-determined | Genome-fragment | NA | | 0 |
| S11_k141_72821 | 1086 | No | NA | | 0 | 1 | Not-determined | Genome-fragment | NA | | 0 |
| S12-final-viral-combined_k141_82962\|\|full | 1222 | No | NA | | 0 | 0 | Low-quality | Genome-fragment | | 3.73 | 0 |
| S11_k141_183640 | 1671 | No | NA | | 0 | 2 | Not-determined | Genome-fragment | NA | | 0 |
| S12_k141_56236 | 1222 | No | NA | | 0 | 0 | Low-quality | Genome-fragment | | 1.88 | 0 |
| S11_k141_183699 | 2495 | No | NA | | 0 | 0 | Not-determined | Genome-fragment | NA | | 0 |
| S11_k141_87374 | 1206 | No | NA | | 0 | 1 | Not-determined | Genome-fragment | NA | | 0 |
| S11_k141_183717 | 1219 | No | NA | | 0 | 2 | Not-determined | Genome-fragment | NA | | 0 |
| S4-final-viral-combined_k141_133668\|\|lt2gene | 1222 | No | NA | | 1 | 0 | Low-quality | Genome-fragment | | 1.64 | 0 |
| S11_k141_46106 | 2136 | No | NA | | 0 | 4 | Not-determined | Genome-fragment | NA | | 0 |
| S11_k141_4475 | 1968 | No | NA | | 0 | 2 | Not-determined | Genome-fragment | NA | | 0 |
| S11_k141_128477 | 3377 | No | NA | | 0 | 3 | Not-determined | Genome-fragment | NA | | 0 |
| S11_k141_225246 | 1037 | No | NA | | 0 | 0 | Not-determined | Genome-fragment | NA | | 0 |
| S11_k141_4510 | 1051 | No | NA | | 0 | 1 | Not-determined | Genome-fragment | NA | | 0 |
| S11_k141_4545 | 1280 | No | NA | | 0 | 1 | Not-determined | Genome-fragment | NA | | 0 |
| S4_k141_37182 | 1222 | No | NA | | 1 | 0 | Low-quality | Genome-fragment | | 2.14 | 0 |
| S11_k141_156190 | 1146 | No | NA | | 0 | 0 | Not-determined | Genome-fragment | NA | | 0 |
| S11_k141_183803 | 3487 | No | NA | | 0 | 3 | Not-determined | Genome-fragment | NA | | 0 |
| S11_k141_32131 | 1085 | No | NA | | 0 | 0 | Not-determined | Genome-fragment | NA | | 0 |
| S11_k141_170443 | 4149 | No | NA | | 0 | 2 | Not-determined | Genome-fragment | NA | | 0 |
| S8-final-viral-combined_k141_54260\|\|full | 1222 | No | NA | | 0 | 0 | Low-quality | Genome-fragment | | 0.72 | 0 |
| S11_k141_321171 | 1031 | No | NA | | 0 | 2 | Not-determined | Genome-fragment | NA | | 0 |
| S11_k141_46222 | 1051 | No | NA | | 0 | 0 | Not-determined | Genome-fragment | NA | | 0 |
| S11_k141_225393 | 1104 | No | NA | | 0 | 1 | Not-determined | Genome-fragment | NA | | 0 |
| S11_k141_183845 | 4229 | No | NA | | 0 | 0 | Not-determined | Genome-fragment | NA | | 0 |
| S8_k141_166767 | 1222 | No | NA | | 0 | 1 | Low-quality | Genome-fragment | | 2.2 | 0 |
| S11_k141_239363 | 3153 | No | NA | | 0 | 3 | Not-determined | Genome-fragment | NA | | 0 |
| S11-final-viral-combined_k141_305022\|\|full | 1223 | No | NA | | 0 | 0 | Low-quality | Genome-fragment | | 2.09 | 0 |
| S11-final-viral-combined_k141_23352\|\|lt2gene | 1223 | No | NA | | 0 | 0 | Low-quality | Genome-fragment | | 0.8 | 0 |
| S11_k141_170502 | 1590 | No | NA | | 0 | 1 | Not-determined | Genome-fragment | NA | | 0 |
| S11_k141_128609 | 1260 | No | NA | | 0 | 1 | Not-determined | Genome-fragment | NA | | 0 |
| S11_k141_168491 | 1223 | No | NA | | 0 | 0 | Low-quality | Genome-fragment | | 2.66 | 0 |
| S11_k141_114260 | 3455 | No | NA | | 0 | 2 | Not-determined | Genome-fragment | NA | | 0 |
| S11_k141_308167 | 3271 | No | NA | | 0 | 3 | Not-determined | Genome-fragment | NA | | 0 |
| S11_k141_321198 | 1508 | No | NA | | 0 | 1 | Not-determined | Genome-fragment | NA | | 0 |
| S11_k141_114275 | 6262 | No | NA | | 0 | 1 | Not-determined | Genome-fragment | NA | | 0 |
| S5-final-viral-combined_k141_207620\|\|full | 1223 | No | NA | | 0 | 0 | Low-quality | Genome-fragment | | 1.42 | 0 |
| S11_k141_225481 | 3302 | No | NA | | 0 | 3 | Not-determined | Genome-fragment | NA | | 0 |
| S11_k141_183913 | 1565 | No | NA | | 0 | 1 | Not-determined | Genome-fragment | NA | | 0 |
| S11_k141_142640 | 3076 | No | NA | | 0 | 2 | Not-determined | Genome-fragment | NA | | 0 |
| S11_k141_4688 | 1683 | No | NA | | 0 | 0 | Not-determined | Genome-fragment | NA | | 0 |
| S11_k141_252777 | 1200 | No | NA | | 0 | 2 | Not-determined | Genome-fragment | NA | | 0 |
| S11_k141_142680 | 1844 | No | NA | | 0 | 0 | Not-determined | Genome-fragment | NA | | 0 |
| S12-final-viral-combined_k141_184160\|\|full | 1223 | No | NA | | 0 | 0 | Low-quality | Genome-fragment | | 2.34 | 0 |
| S11_k141_142681 | 3539 | No | NA | | 0 | 0 | Not-determined | Genome-fragment | NA | | 0 |
| S12-final-viral-combined_k141_294318\|\|full | 1223 | No | NA | | 1 | 0 | Low-quality | Genome-fragment | | 2.52 | 0 |
| S11_k141_239475 | 1145 | No | NA | | 0 | 0 | Not-determined | Genome-fragment | NA | | 0 |
| S11_k141_18045 | 13513 | No | NA | | 0 | 0 | Not-determined | Genome-fragment | NA | | 0 |
| S11_k141_142705 | 1219 | No | NA | | 0 | 2 | Not-determined | Genome-fragment | NA | | 0 |
| S11_k141_321349 | 1090 | No | NA | | 0 | 1 | Not-determined | Genome-fragment | NA | | 0 |
| S11_k141_294099 | 1453 | No | NA | | 0 | 1 | Not-determined | Genome-fragment | NA | | 0 |
| S12-final-viral-combined_k141_77365\|\|full | 1223 | No | NA | | 1 | 0 | Low-quality | Genome-fragment | | 1.88 | 0 |
| S2-final-viral-combined_k141_211108\|\|full | 1223 | No | NA | | 0 | 0 | Low-quality | Genome-fragment | | 2.81 | 0 |
| S11_k141_197417 | 1107 | No | NA | | 0 | 0 | Not-determined | Genome-fragment | NA | | 0 |
| S11_k141_252896 | 1101 | No | NA | | 0 | 0 | Not-determined | Genome-fragment | NA | | 0 |
| S11_k141_225596 | 1673 | No | NA | | 0 | 0 | Not-determined | Genome-fragment | NA | | 0 |
| S11_k141_321378 | 1395 | No | NA | | 0 | 1 | Not-determined | Genome-fragment | NA | | 0 |
| S11_k141_294125 | 1420 | No | NA | | 0 | 1 | Not-determined | Genome-fragment | NA | | 0 |
| S2-final-viral-combined_k141_179439\|\|lt2gene | 1223 | No | NA | | 2 | 0 | Low-quality | Genome-fragment | | 3.59 | 0 |
| S11_k141_156492 | 1498 | No | NA | | 0 | 1 | Not-determined | Genome-fragment | NA | | 0 |
| S11_k141_183997 | 1568 | No | NA | | 0 | 1 | Not-determined | Genome-fragment | NA | | 0 |
| S4_k141_153718 | 1223 | No | NA | | 1 | 0 | Low-quality | Genome-fragment | | 0.7 | 0 |
| S11_k141_114533 | 2678 | No | NA | | 0 | 0 | Not-determined | Genome-fragment | NA | | 0 |
| S11_k141_18130 | 1055 | No | NA | | 0 | 1 | Not-determined | Genome-fragment | NA | | 0 |
| S11_k141_32418 | 2610 | No | NA | | 0 | 0 | Not-determined | Genome-fragment | NA | | 0 |
| S11_k141_197501 | 1104 | No | NA | | 0 | 1 | Not-determined | Genome-fragment | NA | | 0 |
| S11_k141_142842 | 4890 | No | NA | | 0 | 2 | Not-determined | Genome-fragment | NA | | 0 |
| S11_k141_73232 | 5174 | No | NA | | 0 | 2 | Not-determined | Genome-fragment | NA | | 0 |
| S11_k141_32479 | 10399 | No | NA | | 0 | 4 | Not-determined | Genome-fragment | NA | | 0 |
| S11_k141_142867 | 1304 | No | NA | | 0 | 1 | Not-determined | Genome-fragment | NA | | 0 |
| S11_k141_101907 | 3524 | No | NA | | 0 | 3 | Not-determined | Genome-fragment | NA | | 0 |
| S6_k141_190 | 1223 | No | NA | | 0 | 0 | Low-quality | Genome-fragment | | 2.98 | 0 |
| S11-final-viral-combined_k141_156536\|\|full | 1224 | No | NA | | 1 | 0 | Low-quality | Genome-fragment | | 3.38 | 0 |
| S11-final-viral-combined_k141_324622\|\|full | 1224 | No | NA | | 1 | 0 | Low-quality | Genome-fragment | | 3.43 | 0 |
| S1-final-viral-combined_k141_329369\|\|full | 1224 | No | NA | | 0 | 0 | Low-quality | Genome-fragment | | 1.91 | 0 |
| S11_k141_156566 | 1236 | No | NA | | 0 | 1 | Not-determined | Genome-fragment | NA | | 0 |
| S11_k141_142919 | 1248 | No | NA | | 0 | 1 | Not-determined | Genome-fragment | NA | | 0 |
| S1-final-viral-combined_k141_124331\|\|full | 1224 | No | NA | | 0 | 1 | Low-quality | Genome-fragment | | 1.88 | 0 |
| S11_k141_266915 | 2339 | No | NA | | 0 | 0 | Not-determined | Genome-fragment | NA | | 0 |
| S11_k141_321484 | 1295 | No | NA | | 0 | 0 | Not-determined | Genome-fragment | NA | | 0 |
| S11_k141_142963 | 1321 | No | NA | | 0 | 1 | Not-determined | Genome-fragment | NA | | 0 |
| S3-final-viral-combined_k141_96267\|\|full | 1224 | No | NA | | 1 | 0 | Low-quality | Genome-fragment | | 2.62 | 0 |
| S7-final-viral-combined_k141_188352\|\|full | 1224 | No | NA | | 0 | 0 | Low-quality | Genome-fragment | | 9.54 | 0 |
| S11_k141_281348 | 3528 | No | NA | | 0 | 2 | Not-determined | Genome-fragment | NA | | 0 |
| S11_k141_143005 | 1216 | No | NA | | 0 | 0 | Not-determined | Genome-fragment | NA | | 0 |
| S17-k141_173260 | 1224 | No | NA | | 0 | 0 | Low-quality | Genome-fragment | | 4.23 | 0 |
| S11_k141_4976 | 3308 | No | NA | | 0 | 3 | Not-determined | Genome-fragment | NA | | 0 |
| S11_k141_87956 | 1195 | No | NA | | 0 | 0 | Not-determined | Genome-fragment | NA | | 0 |
| S11_k141_266944 | 1653 | No | NA | | 0 | 0 | Not-determined | Genome-fragment | NA | | 0 |
| S11_k141_321582 | 3158 | No | NA | | 0 | 2 | Not-determined | Genome-fragment | NA | | 0 |
| S11_k141_294337 | 2458 | No | NA | | 0 | 0 | Not-determined | Genome-fragment | NA | | 0 |
| S12-final-viral-combined_k141_51258\|\|full | 1224 | No | NA | | 1 | 0 | Low-quality | Genome-fragment | | 2.67 | 0 |
| S11_k141_281447 | 1492 | No | NA | | 0 | 1 | Not-determined | Genome-fragment | NA | | 0 |
| S11_k141_294349 | 1651 | No | NA | | 0 | 0 | Not-determined | Genome-fragment | NA | | 0 |
| S11_k141_294353 | 1954 | No | NA | | 0 | 0 | Not-determined | Genome-fragment | NA | | 0 |
| S11_k141_239961 | 2373 | No | NA | | 0 | 1 | Not-determined | Genome-fragment | NA | | 0 |
| S11_k141_102155 | 1310 | No | NA | | 0 | 1 | Not-determined | Genome-fragment | NA | | 0 |
| S12-final-viral-combined_k141_219819\|\|full | 1224 | No | NA | | 2 | 0 | Low-quality | Genome-fragment | | 2.01 | 0 |
| S12_k141_378331 | 1224 | No | NA | | 0 | 2 | Low-quality | Genome-fragment | | 1.58 | 0 |
| S12_k141_244439 | 1224 | No | NA | | 0 | 0 | Low-quality | Genome-fragment | | 2.7 | 0 |
| S11_k141_88048 | 3569 | No | NA | | 0 | 2 | Not-determined | Genome-fragment | NA | | 0 |

| | | | | | | | | | | |
|---|---|---|---|---|---|---|---|---|---|---|
| S11_k141_294430 | 1953 | No | NA | 0 | 1 | Not-determined | Genome-fragment | NA | | 0 |
| S11_k141_308659 | 3793 | No | NA | 0 | 1 | Not-determined | Genome-fragment | NA | | 0 |
| S11_k141_156721 | 1036 | No | NA | 0 | 1 | Not-determined | Genome-fragment | NA | | 0 |
| S11_k141_197822 | 1035 | No | NA | 0 | 0 | Not-determined | Genome-fragment | NA | | 0 |
| S11_k141_308703 | 2105 | No | NA | 0 | 0 | Not-determined | Genome-fragment | NA | | 0 |
| S11_k141_32691 | 155831 | No | NA | 1 | 11 | Medium-quality | Genome-fragment | | 70.25 | 0 |
| S11_k141_308724 | 1366 | No | NA | 0 | 1 | Not-determined | Genome-fragment | NA | | 0 |
| S2-final-viral-combined_k141_140603\|\|full | 1224 | No | NA | 4 | 0 | Low-quality | Genome-fragment | | 2.28 | 0 |
| S11_k141_32727 | 1431 | No | NA | 0 | 1 | Not-determined | Genome-fragment | NA | | 0 |
| S11_k141_281617 | 1419 | No | NA | 0 | 1 | Not-determined | Genome-fragment | NA | | 0 |
| S11_k141_129201 | 1070 | No | NA | 0 | 1 | Not-determined | Genome-fragment | NA | | 0 |
| S6_k141_57252 | 1224 | No | NA | 0 | 0 | Low-quality | Genome-fragment | | 1.45 | 0 |
| S11_k141_212577 | 3105 | No | NA | 0 | 2 | Not-determined | Genome-fragment | NA | | 0 |
| S11_k141_18554 | 1010 | No | NA | 0 | 2 | Not-determined | Genome-fragment | NA | | 0 |
| S11_k141_88119 | 1496 | No | NA | 0 | 1 | Not-determined | Genome-fragment | NA | | 0 |
| S11_k141_5222 | 5412 | No | NA | 0 | 4 | Not-determined | Genome-fragment | NA | | 0 |
| S11_k141_32788 | 1488 | No | NA | 0 | 2 | Not-determined | Genome-fragment | NA | | 0 |
| S11_k141_184366 | 1541 | No | NA | 0 | 1 | Not-determined | Genome-fragment | NA | | 0 |
| S11_k141_240138 | 8458 | No | NA | 0 | 2 | Not-determined | Genome-fragment | NA | | 0 |
| S11_k141_60538 | 1124 | No | NA | 0 | 0 | Not-determined | Genome-fragment | NA | | 0 |
| S8_k141_83765 | 1224 | No | NA | 0 | 0 | Low-quality | Genome-fragment | | 0.87 | 0 |
| S11_k141_156845 | 1662 | No | NA | 0 | 0 | Not-determined | Genome-fragment | NA | | 0 |
| S11_k141_143274 | 1338 | No | NA | 0 | 1 | Not-determined | Genome-fragment | NA | | 0 |
| S11-final-viral-combined_k141_176248\|\|full | 1225 | No | NA | 0 | 0 | Low-quality | Genome-fragment | | 0.95 | 0 |
| S11_k141_102357 | 7962 | No | NA | 0 | 1 | Not-determined | Genome-fragment | NA | | 0 |
| S3_k141_97369 | 1225 | No | NA | 0 | 0 | Low-quality | Genome-fragment | | 2.13 | 0 |
| S11_k141_226077 | 2750 | No | NA | 0 | 0 | Not-determined | Genome-fragment | NA | | 0 |
| S11_k141_198020 | 1369 | No | NA | 0 | 1 | Not-determined | Genome-fragment | NA | | 0 |
| S12_k141_246534 | 1225 | No | NA | 0 | 0 | Low-quality | Genome-fragment | | 3.04 | 0 |
| S4_k141_29360 | 1225 | No | NA | 0 | 1 | Low-quality | Genome-fragment | | 0.66 | 0 |
| S11_k141_102416 | 2974 | No | NA | 0 | 0 | Not-determined | Genome-fragment | NA | | 0 |
| S11_k141_321756 | 1083 | No | NA | 0 | 1 | Not-determined | Genome-fragment | NA | | 0 |
| S6-final-viral-combined_k141_32751\|\|full | 1225 | No | NA | 4 | 0 | Low-quality | Genome-fragment | | 2.67 | 0 |
| S11_k141_102437 | 3217 | No | NA | 0 | 3 | Not-determined | Genome-fragment | NA | | 0 |
| S11_k141_253455 | 1372 | No | NA | 0 | 2 | Not-determined | Genome-fragment | NA | | 0 |
| S6-final-viral-combined_k141_128555\|\|full | 1225 | No | NA | 0 | 0 | Low-quality | Genome-fragment | | 2.59 | 0 |
| S11_k141_32975 | 1165 | No | NA | 0 | 1 | Not-determined | Genome-fragment | NA | | 0 |
| S11_k141_171112 | 4208 | No | NA | 0 | 2 | Not-determined | Genome-fragment | NA | | 0 |
| S11_k141_32994 | 1305 | No | NA | 0 | 0 | Not-determined | Genome-fragment | NA | | 0 |
| S11_k141_171119 | 1642 | No | NA | 0 | 1 | Not-determined | Genome-fragment | NA | | 0 |
| S11_k141_171121 | 2648 | No | NA | 0 | 1 | Not-determined | Genome-fragment | NA | | 0 |
| S11_k141_308958 | 3355 | No | NA | 0 | 2 | Not-determined | Genome-fragment | NA | | 0 |
| S11_k141_294719 | 1092 | No | NA | 0 | 0 | Not-determined | Genome-fragment | NA | | 0 |
| S1-final-viral-combined_k141_149079\|\|full | 1226 | No | NA | 2 | 0 | Low-quality | Genome-fragment | | 2.75 | 0 |
| S11_k141_157024 | 1568 | No | NA | 0 | 1 | Not-determined | Genome-fragment | NA | | 0 |
| S11_k141_309009 | 1066 | No | NA | 0 | 1 | Not-determined | Genome-fragment | NA | | 0 |
| S1-final-viral-combined_k141_142473\|\|full | 1226 | No | NA | 0 | 0 | Low-quality | Genome-fragment | | 1.69 | 0 |
| S11_k141_129476 | 2116 | No | NA | 0 | 0 | Not-determined | Genome-fragment | NA | | 0 |
| S5-final-viral-combined_k141_213093\|\|full | 1226 | No | NA | 1 | 0 | Low-quality | Genome-fragment | | 2.54 | 0 |
| S11_k141_157076 | 5853 | No | NA | 0 | 1 | Not-determined | Genome-fragment | NA | | 0 |
| S5_k141_65740 | 1226 | No | NA | 0 | 0 | Low-quality | Genome-fragment | | 19.96 | 0 |
| S7-final-viral-combined_k141_244769\|\|lt2gene | 1226 | No | NA | 1 | 0 | Low-quality | Genome-fragment | | 1.79 | 0 |
| S11_k141_226153 | 1271 | No | NA | 0 | 1 | Not-determined | Genome-fragment | NA | | 0 |
| S11_k141_143496 | 4427 | No | NA | 0 | 3 | Not-determined | Genome-fragment | NA | | 0 |
| S17-final-viral-combined_k141_13609\|\|full | 1226 | No | NA | 1 | 0 | Low-quality | Genome-fragment | | 0.74 | 0 |
| S17-final-viral-combined_k141_105096\|\|full | 1226 | No | NA | 0 | 1 | Low-quality | Genome-fragment | | 2.15 | 0 |
| S11_k141_171300 | 9777 | No | NA | 0 | 4 | Not-determined | Genome-fragment | NA | | 0 |
| S11_k141_226170 | 3873 | No | NA | 0 | 3 | Not-determined | Genome-fragment | NA | | 0 |
| S11_k141_33141 | 1292 | No | NA | 0 | 2 | Not-determined | Genome-fragment | NA | | 0 |
| S11_k141_171317 | 3793 | No | NA | 0 | 2 | Not-determined | Genome-fragment | NA | | 0 |
| S11_k141_294900 | 3329 | No | NA | 0 | 1 | Not-determined | Genome-fragment | NA | | 0 |
| S11_k141_88493 | 1353 | No | NA | 0 | 1 | Not-determined | Genome-fragment | NA | | 0 |
| S17-final-viral-combined_k141_186042\|\|full | 1226 | No | NA | 1 | 0 | Low-quality | Genome-fragment | | 2.51 | 0 |
| S17-final-viral-combined_k141_232422\|\|full | 1226 | No | NA | 1 | 0 | Low-quality | Genome-fragment | | 3.35 | 0 |
| S11_k141_74031 | 3426 | No | NA | 0 | 3 | Not-determined | Genome-fragment | NA | | 0 |
| S11_k141_253793 | 1077 | No | NA | 0 | 1 | Not-determined | Genome-fragment | NA | | 0 |
| S11_k141_88512 | 1633 | No | NA | 0 | 1 | Not-determined | Genome-fragment | NA | | 0 |
| S11_k141_281933 | 1815 | No | NA | 0 | 1 | Not-determined | Genome-fragment | NA | | 0 |
| S11_k141_33254 | 1392 | No | NA | 0 | 0 | Not-determined | Genome-fragment | NA | | 0 |
| S12-final-viral-combined_k141_248892\|\|full | 1226 | No | NA | 0 | 0 | Low-quality | Genome-fragment | | 2.39 | 0 |
| S11_k141_213055 | 2472 | No | NA | 0 | 2 | Not-determined | Genome-fragment | NA | | 0 |
| S6-final-viral-combined_k141_76992\|\|full | 1226 | No | NA | 0 | 1 | Low-quality | Genome-fragment | | 2.09 | 0 |
| S11_k141_157290 | 1284 | No | NA | 0 | 1 | Not-determined | Genome-fragment | NA | | 0 |
| S11_k141_240687 | 1091 | No | NA | 0 | 0 | Not-determined | Genome-fragment | NA | | 0 |
| S11_k141_213082 | 1201 | No | NA | 0 | 1 | Not-determined | Genome-fragment | NA | | 0 |
| S11_k141_198458 | 1077 | No | NA | 0 | 1 | Not-determined | Genome-fragment | NA | | 0 |
| S11_k141_47298 | 17525 | No | NA | 0 | 7 | Not-determined | Genome-fragment | NA | | 0 |
| S11_k141_157328 | 3563 | No | NA | 0 | 3 | Not-determined | Genome-fragment | NA | | 0 |
| S11_k141_102849 | 3427 | No | NA | 0 | 1 | Not-determined | Genome-fragment | NA | | 0 |
| S11_k141_129680 | 1620 | No | NA | 0 | 1 | Not-determined | Genome-fragment | NA | | 0 |
| S11_k141_47307 | 1283 | No | NA | 0 | 1 | Not-determined | Genome-fragment | NA | | 0 |
| S5-final-viral-combined_k141_194785\|\|lt2gene | 1227 | No | NA | 1 | 0 | Low-quality | Genome-fragment | | 0.94 | 0 |
| S7-final-viral-combined_k141_193534\|\|full | 1227 | No | NA | 0 | 1 | Low-quality | Genome-fragment | | 1.63 | 0 |
| S11_k141_5726 | 2291 | No | NA | 0 | 1 | Not-determined | Genome-fragment | NA | | 0 |
| S11_k141_184834 | 1088 | No | NA | 0 | 0 | Not-determined | Genome-fragment | NA | | 0 |
| S17-k141_243019 | 1227 | No | NA | 0 | 0 | Low-quality | Genome-fragment | | 2.33 | 0 |
| S11_k141_171487 | 6578 | No | NA | 0 | 5 | Not-determined | Genome-fragment | NA | | 0 |
| S11_k141_129813 | 1093 | No | NA | 0 | 0 | Not-determined | Genome-fragment | NA | | 0 |
| S11_k141_129827 | 1694 | No | NA | 0 | 0 | Not-determined | Genome-fragment | NA | | 0 |
| S11_k141_171503 | 2184 | No | NA | 0 | 0 | Not-determined | Genome-fragment | NA | | 0 |
| S11_k141_33446 | 1072 | No | NA | 0 | 1 | Not-determined | Genome-fragment | NA | | 0 |
| S11_k141_47398 | 3222 | No | NA | 0 | 2 | Not-determined | Genome-fragment | NA | | 0 |
| S11_k141_254067 | 1672 | No | NA | 0 | 1 | Not-determined | Genome-fragment | NA | | 0 |
| S11_k141_184893 | 1886 | No | NA | 0 | 1 | Not-determined | Genome-fragment | NA | | 0 |
| S12-final-viral-combined_k141_126541\|\|full | 1227 | No | NA | 2 | 0 | Low-quality | Genome-fragment | | 2.92 | 0 |
| S11_k141_184924 | 2102 | No | NA | 0 | 0 | Not-determined | Genome-fragment | NA | | 0 |
| S11_k141_74301 | 3031 | No | NA | 0 | 1 | Not-determined | Genome-fragment | NA | | 0 |
| S11_k141_115492 | 2418 | No | NA | 0 | 2 | Not-determined | Genome-fragment | NA | | 0 |
| S11_k141_254133 | 3537 | No | NA | 0 | 3 | Not-determined | Genome-fragment | NA | | 0 |
| S11_k141_5827 | 1001 | No | NA | 0 | 1 | Not-determined | Genome-fragment | NA | | 0 |
| S11_k141_322217 | 2517 | No | NA | 0 | 2 | Not-determined | Genome-fragment | NA | | 0 |
| S11_k141_322234 | 1020 | No | NA | 0 | 0 | Not-determined | Genome-fragment | NA | | 0 |
| S11_k141_143870 | 1004 | No | NA | 0 | 1 | Not-determined | Genome-fragment | NA | | 0 |
| S12-final-viral-combined_k141_250065\|\|full | 1227 | No | NA | 0 | 0 | Low-quality | Genome-fragment | | 2.3 | 0 |
| S12-final-viral-combined_k141_73995\|\|lt2gene | 1227 | No | NA | 1 | 0 | Low-quality | Genome-fragment | | 0.79 | 0 |
| S11_k141_226583 | 1276 | No | NA | 0 | 1 | Not-determined | Genome-fragment | NA | | 0 |
| S2-final-viral-combined_k141_107872\|\|full | 1227 | No | NA | 0 | 1 | Low-quality | Genome-fragment | | 2.68 | 0 |
| S11_k141_267863 | 1329 | No | NA | 0 | 1 | Not-determined | Genome-fragment | NA | | 0 |
| S11_k141_309645 | 3952 | No | NA | 0 | 2 | Not-determined | Genome-fragment | NA | | 0 |
| S11_k141_47569 | 5513 | No | NA | 0 | 4 | Not-determined | Genome-fragment | NA | | 0 |
| S11_k141_198693 | 1056 | No | NA | 0 | 0 | Not-determined | Genome-fragment | NA | | 0 |
| S11_k141_5945 | 1774 | No | NA | 0 | 1 | Not-determined | Genome-fragment | NA | | 0 |
| S11_k141_171685 | 3820 | No | NA | 0 | 3 | Not-determined | Genome-fragment | NA | | 0 |
| S11_k141_185049 | 3118 | No | NA | 0 | 3 | Not-determined | Genome-fragment | NA | | 0 |
| S11_k141_295305 | 12142 | No | NA | 0 | 7 | Not-determined | Genome-fragment | NA | | 0 |
| S11_k141_33647 | 1992 | No | NA | 0 | 2 | Not-determined | Genome-fragment | NA | | 0 |
| S11_k141_88781 | 3666 | No | NA | 0 | 2 | Not-determined | Genome-fragment | NA | | 0 |
| S4_k141_16729 | 1227 | No | NA | 0 | 0 | Low-quality | Genome-fragment | | 2.15 | 0 |
| S8-final-viral-combined_k141_135273\|\|full | 1227 | No | NA | 0 | 0 | Low-quality | Genome-fragment | | 2.28 | 0 |
| S11_k141_226672 | 2197 | No | NA | 0 | 2 | Not-determined | Genome-fragment | NA | | 0 |
| S11_k141_6021 | 1027 | No | NA | 0 | 1 | Not-determined | Genome-fragment | NA | | 0 |
| S11_k141_33670 | 1537 | No | NA | 0 | 2 | Not-determined | Genome-fragment | NA | | 0 |
| S8-final-viral-combined_k141_148082\|\|full | 1227 | No | NA | 0 | 0 | Low-quality | Genome-fragment | | 1.62 | 0 |
| S11_k141_19215 | 8203 | No | NA | 0 | 3 | Not-determined | Genome-fragment | NA | | 0 |
| S11_k141_267972 | 3581 | No | NA | 0 | 3 | Not-determined | Genome-fragment | NA | | 0 |
| S11_k141_309756 | 1094 | No | NA | 0 | 0 | Not-determined | Genome-fragment | NA | | 0 |
| S11_k141_267973 | 1517 | No | NA | 0 | 1 | Not-determined | Genome-fragment | NA | | 0 |
| S11_k141_115749 | 1228 | No | NA | 0 | 1 | Not-determined | Genome-fragment | NA | | 0 |
| S11_k141_33688 | 6066 | No | NA | 0 | 2 | Not-determined | Genome-fragment | NA | | 0 |
| S11_k141_267987 | 2295 | No | NA | 0 | 0 | Not-determined | Genome-fragment | NA | | 0 |
| S11_k141_295349 | 6316 | No | NA | 0 | 2 | Not-determined | Genome-fragment | NA | | 0 |
| S8-final-viral-combined_k141_183418\|\|lt2gene | 1227 | No | NA | 0 | 0 | Low-quality | Genome-fragment | | 2.47 | 0 |
| S11_k141_115796 | 1477 | No | NA | 0 | 1 | Not-determined | Genome-fragment | NA | | 0 |
| S11_k141_144040 | 1234 | No | NA | 0 | 1 | Not-determined | Genome-fragment | NA | | 0 |
| S11_k141_144059 | 1198 | No | NA | 0 | 2 | Not-determined | Genome-fragment | NA | | 0 |
| S11_k141_198852 | 5054 | No | NA | 0 | 2 | Not-determined | Genome-fragment | NA | | 0 |
| S11_k141_115886 | 9283 | No | NA | 0 | 4 | Not-determined | Genome-fragment | NA | | 0 |
| S11_k141_322586 | 1154 | No | NA | 0 | 1 | Not-determined | Genome-fragment | NA | | 0 |
| S11_k141_171838 | 1158 | No | NA | 0 | 0 | Not-determined | Genome-fragment | NA | | 0 |
| S11_k141_241187 | 1379 | No | NA | 0 | 1 | Not-determined | Genome-fragment | NA | | 0 |
| S11_k141_144145 | 1250 | No | NA | 0 | 1 | Not-determined | Genome-fragment | NA | | 0 |
| S11_k141_144163 | 1126 | No | NA | 0 | 0 | Not-determined | Genome-fragment | NA | | 0 |
| S11_k141_61574 | 1296 | No | NA | 0 | 1 | Not-determined | Genome-fragment | NA | | 0 |
| S11_k141_6203 | 45102 | No | NA | 1 | 9 | Medium-quality | Genome-fragment | | 50.63 | 0 |
| S11_k141_158032 | 2830 | No | NA | 0 | 1 | Not-determined | Genome-fragment | NA | | 0 |
| S9-final-viral-combined_k141_106820\|\|full | 1228 | No | NA | 0 | 0 | Low-quality | Genome-fragment | | 0.69 | 0 |
| S11_k141_74646 | 2112 | No | NA | 0 | 1 | Not-determined | Genome-fragment | NA | | 0 |
| S11_k141_309932 | 3747 | No | NA | 0 | 2 | Not-determined | Genome-fragment | NA | | 0 |
| S11_k141_61636 | 1114 | No | NA | 0 | 1 | Not-determined | Genome-fragment | NA | | 0 |
| S11_k141_19395 | 2167 | No | NA | 0 | 2 | Not-determined | Genome-fragment | NA | | 0 |
| S11_k141_47732 | 3945 | No | NA | 0 | 0 | Not-determined | Genome-fragment | NA | | 0 |
| S11_k141_171937 | 1192 | No | NA | 0 | 1 | Not-determined | Genome-fragment | NA | | 0 |
| S11_k141_130151 | 1414 | No | NA | 0 | 2 | Not-determined | Genome-fragment | NA | | 0 |
| S11-final-viral-combined_k141_187000\|\|full | 1228 | No | NA | 0 | 0 | Low-quality | Genome-fragment | | 1.27 | 0 |
| S11_k141_185280 | 3636 | No | NA | 0 | 2 | Not-determined | Genome-fragment | NA | | 0 |
| S11_k141_198945 | 3317 | No | NA | 0 | 2 | Not-determined | Genome-fragment | NA | | 0 |
| S11-final-viral-combined_k141_312468\|\|full | 1228 | No | NA | 0 | 1 | Low-quality | Genome-fragment | | 2.96 | 0 |
| S11_k141_268174 | 1195 | No | NA | 0 | 0 | Not-determined | Genome-fragment | NA | | 0 |
| S11_k141_144291 | 2479 | No | NA | 0 | 0 | Not-determined | Genome-fragment | NA | | 0 |
| S11_k141_185318 | 1741 | No | NA | 0 | 1 | Not-determined | Genome-fragment | NA | | 0 |
| S11_k141_310018 | 1254 | No | NA | 0 | 1 | Not-determined | Genome-fragment | NA | | 0 |
| S11_k141_6375 | 1093 | No | NA | 0 | 1 | Not-determined | Genome-fragment | NA | | 0 |
| S11_k141_241483 | 1285 | No | NA | 0 | 2 | Not-determined | Genome-fragment | NA | | 0 |
| S11_k141_268261 | 1051 | No | NA | 0 | 1 | Not-determined | Genome-fragment | NA | | 0 |
| S11_k141_310089 | 1408 | No | NA | 0 | 1 | Not-determined | Genome-fragment | NA | | 0 |
| S11_k141_322807 | 1090 | No | NA | 0 | 1 | Not-determined | Genome-fragment | NA | | 0 |
| S11-final-viral-combined_k141_137806\|\|full | 1228 | No | NA | 0 | 0 | Low-quality | Genome-fragment | | 2.48 | 0 |
| S11_k141_19499 | 6399 | No | NA | 0 | 3 | Not-determined | Genome-fragment | NA | | 0 |
| S11_k141_310100 | 1113 | No | NA | 0 | 1 | Not-determined | Genome-fragment | NA | | 0 |
| S11_k141_172103 | 1235 | No | NA | 0 | 2 | Not-determined | Genome-fragment | NA | | 0 |
| S11_k141_89113 | 1322 | No | NA | 0 | 0 | Not-determined | Genome-fragment | NA | | 0 |
| S11_k141_310110 | 1484 | No | NA | 0 | 1 | Not-determined | Genome-fragment | NA | | 0 |
| S1-final-viral-combined_k141_285189\|\|full | 1228 | No | NA | 0 | 0 | Low-quality | Genome-fragment | | 0.83 | 0 |
| S11_k141_213759 | 2562 | No | NA | 0 | 1 | Not-determined | Genome-fragment | NA | | 0 |

| | | | | | | | | | | |
|---|---|---|---|---|---|---|---|---|---|---|
| S11_k141_61811 | 4298 | No | NA | 0 | 3 | Not-determined | Genome-fragment | NA | | 0 |
| S1-final-viral-combined_k141_194928\|\|full | 1228 | No | NA | 0 | 0 | Low-quality | Genome-fragment | | 3.28 | 0 |
| S11_k141_295614 | 4760 | No | NA | 0 | 0 | Not-determined | Genome-fragment | NA | | 0 |
| S11_k141_295622 | 1096 | No | NA | 0 | 1 | Not-determined | Genome-fragment | NA | | 0 |
| S11_k141_227122 | 1106 | No | NA | 0 | 1 | Not-determined | Genome-fragment | NA | | 0 |
| S11_k141_74823 | 1186 | No | NA | 0 | 3 | Not-determined | Genome-fragment | NA | | 0 |
| S11_k141_213817 | 1116 | No | NA | 0 | 0 | Not-determined | Genome-fragment | NA | | 0 |
| S11_k141_295627 | 3074 | No | NA | 0 | 2 | Not-determined | Genome-fragment | NA | | 0 |
| S1_k141_346514 | 1228 | No | NA | 0 | 0 | Low-quality | Genome-fragment | | 22.21 | 0 |
| S11_k141_116154 | 2118 | No | NA | 0 | 1 | Not-determined | Genome-fragment | NA | | 0 |
| S11_k141_241558 | 1612 | No | NA | 0 | 2 | Not-determined | Genome-fragment | NA | | 0 |
| S7-final-viral-combined_k141_49830\|\|full | 1228 | No | NA | 0 | 0 | Low-quality | Genome-fragment | | 5.83 | 0 |
| S17-final-viral-combined_k141_8181\|\|full | 1228 | No | NA | 0 | 0 | Low-quality | Genome-fragment | | 2.76 | 0 |
| S11_k141_310268 | 1331 | No | NA | 0 | 1 | Not-determined | Genome-fragment | NA | | 0 |
| S11_k141_268382 | 1524 | No | NA | 0 | 1 | Not-determined | Genome-fragment | NA | | 0 |
| S11_k141_213877 | 1050 | No | NA | 0 | 2 | Not-determined | Genome-fragment | NA | | 0 |
| S11_k141_34127 | 1028 | No | NA | 0 | 2 | Not-determined | Genome-fragment | NA | | 0 |
| S11_k141_158427 | 3104 | No | NA | 0 | 0 | Not-determined | Genome-fragment | NA | | 0 |
| S2-final-viral-combined_k141_28343\|\|full | 1228 | No | NA | 0 | 0 | Low-quality | Genome-fragment | | 2.13 | 0 |
| S4-final-viral-combined_k141_40149\|\|full | 1228 | No | NA | 0 | 1 | Low-quality | Genome-fragment | | 0.71 | 0 |
| S4_k141_146941 | 1228 | No | NA | 0 | 0 | Low-quality | Genome-fragment | | 0.58 | 0 |
| S11_k141_172281 | 1007 | No | NA | 0 | 1 | Not-determined | Genome-fragment | NA | | 0 |
| S11_k141_89268 | 1684 | No | NA | 0 | 0 | Not-determined | Genome-fragment | NA | | 0 |
| S11_k141_268393 | 1846 | No | NA | 0 | 0 | Not-determined | Genome-fragment | NA | | 0 |
| S11_k141_74957 | 1393 | No | NA | 0 | 0 | Not-determined | Genome-fragment | NA | | 0 |
| S4_k141_18733 | 1228 | No | NA | 0 | 1 | Low-quality | Genome-fragment | | 0.86 | 0 |
| S11_k141_322932 | 1304 | No | NA | 0 | 1 | Not-determined | Genome-fragment | NA | | 0 |
| S11_k141_144480 | 2122 | No | NA | 0 | 3 | Not-determined | Genome-fragment | NA | | 0 |
| S11_k141_103668 | 1533 | No | NA | 0 | 0 | Not-determined | Genome-fragment | NA | | 0 |
| S6_k141_66074 | 1228 | No | NA | 0 | 1 | Low-quality | Genome-fragment | | 2.38 | 0 |
| S11_k141_213922 | 1078 | No | NA | 0 | 1 | Not-determined | Genome-fragment | NA | | 0 |
| S8-final-viral-combined_k141_31823\|\|full | 1228 | No | NA | 2 | 0 | Low-quality | Genome-fragment | | 1.86 | 0 |
| S11_k141_241744 | 1013 | No | NA | 0 | 0 | Not-determined | Genome-fragment | NA | | 0 |
| S9-final-viral-combined_k141_35731\|\|full | 1229 | No | NA | 0 | 0 | Low-quality | Genome-fragment | | 1.1 | 0 |
| S11_k141_75002 | 2947 | No | NA | 0 | 1 | Not-determined | Genome-fragment | NA | | 0 |
| S9-final-viral-combined_k141_97417\|\|lt2gene | 1229 | No | NA | 1 | 0 | Low-quality | Genome-fragment | | 1.61 | 0 |
| S11_k141_116361 | 1005 | No | NA | 0 | 1 | Not-determined | Genome-fragment | NA | | 0 |
| S11_k141_116381 | 3407 | No | NA | 0 | 0 | Not-determined | Genome-fragment | NA | | 0 |
| S11_k141_199273 | 1006 | No | NA | 0 | 1 | Not-determined | Genome-fragment | NA | | 0 |
| S11_k141_19801 | 1479 | No | NA | 0 | 2 | Not-determined | Genome-fragment | NA | | 0 |
| S11_k141_268564 | 3320 | No | NA | 0 | 0 | Not-determined | Genome-fragment | NA | | 0 |
| S11_k141_268578 | 9152 | No | NA | 0 | 3 | Not-determined | Genome-fragment | NA | | 0 |
| S11_k141_185659 | 1157 | No | NA | 0 | 1 | Not-determined | Genome-fragment | NA | | 0 |
| S11_k141_75075 | 1615 | No | NA | 0 | 0 | Not-determined | Genome-fragment | NA | | 0 |
| S1-final-viral-combined_k141_200958\|\|full | 1229 | No | NA | 1 | 0 | Low-quality | Genome-fragment | | 4.28 | 0 |
| S11_k141_227508 | 3797 | No | NA | 0 | 1 | Not-determined | Genome-fragment | NA | | 0 |
| S11_k141_214083 | 6181 | No | NA | 0 | 4 | Not-determined | Genome-fragment | NA | | 0 |
| S11_k141_34410 | 1059 | No | NA | 0 | 0 | Not-determined | Genome-fragment | NA | | 0 |
| S1-final-viral-combined_k141_270021\|\|lt2gene | 1229 | No | NA | 1 | 0 | Low-quality | Genome-fragment | | 3.52 | 0 |
| S11_k141_48130 | 1605 | No | NA | 0 | 2 | Not-determined | Genome-fragment | NA | | 0 |
| S5_k141_64176 | 1229 | No | NA | 1 | 0 | Low-quality | Genome-fragment | | 2.45 | 0 |
| S11_k141_19927 | 1620 | No | NA | 0 | 1 | Not-determined | Genome-fragment | NA | | 0 |
| S11_k141_116530 | 1983 | No | NA | 0 | 1 | Not-determined | Genome-fragment | NA | | 0 |
| S11_k141_130559 | 1620 | No | NA | 0 | 2 | Not-determined | Genome-fragment | NA | | 0 |
| S11_k141_172510 | 1397 | No | NA | 0 | 1 | Not-determined | Genome-fragment | NA | | 0 |
| S11_k141_310651 | 2998 | No | NA | 0 | 1 | Not-determined | Genome-fragment | NA | | 0 |
| S11_k141_214185 | 2246 | No | NA | 0 | 0 | Not-determined | Genome-fragment | NA | | 0 |
| S11_k141_295963 | 1103 | No | NA | 0 | 0 | Not-determined | Genome-fragment | NA | | 0 |
| S11_k141_144801 | 1123 | No | NA | 0 | 0 | Not-determined | Genome-fragment | NA | | 0 |
| S11_k141_62383 | 1062 | No | NA | 0 | 1 | Not-determined | Genome-fragment | NA | | 0 |
| S11_k141_323109 | 1261 | No | NA | 0 | 1 | Not-determined | Genome-fragment | NA | | 0 |
| S17-k141_106903 | 1229 | No | NA | 0 | 1 | Low-quality | Genome-fragment | | 0.33 | 0 |
| S4_k141_31795 | 1229 | No | NA | 0 | 0 | Low-quality | Genome-fragment | | 1.3 | 0 |
| S11_k141_283072 | 1101 | No | NA | 0 | 1 | Not-determined | Genome-fragment | NA | | 0 |
| S8-final-viral-combined_k141_268542\|\|full | 1229 | No | NA | 0 | 1 | Low-quality | Genome-fragment | | 0.86 | 0 |
| S11_k141_323163 | 1514 | No | NA | 0 | 1 | Not-determined | Genome-fragment | NA | | 0 |
| S8-final-viral-combined_k141_273811\|\|full | 1229 | No | NA | 0 | 0 | Low-quality | Genome-fragment | | 0.8 | 0 |
| S11_k141_283116 | 1342 | No | NA | 0 | 0 | Not-determined | Genome-fragment | NA | | 0 |
| S11_k141_89678 | 1087 | No | NA | 0 | 0 | Not-determined | Genome-fragment | NA | | 0 |
| S11_k141_34576 | 1117 | No | NA | 0 | 0 | Not-determined | Genome-fragment | NA | | 0 |
| S8-final-viral-combined_k141_265227\|\|lt2gene | 1229 | No | NA | 1 | 0 | Low-quality | Genome-fragment | | 0.69 | 0 |
| S9-final-viral-combined_k141_40092\|\|full | 1230 | No | NA | 0 | 1 | Low-quality | Genome-fragment | | 1.52 | 0 |
| S11_k141_20129 | 3620 | No | NA | 0 | 3 | Not-determined | Genome-fragment | NA | | 0 |
| S11_k141_62504 | 1660 | No | NA | 0 | 0 | Not-determined | Genome-fragment | NA | | 0 |
| S11-final-viral-combined_k141_983\|\|full | 1230 | No | NA | 0 | 0 | Low-quality | Genome-fragment | | 2.42 | 0 |
| S11_k141_255327 | 1003 | No | NA | 0 | 0 | Not-determined | Genome-fragment | NA | | 0 |
| S11_k141_214335 | 4386 | No | NA | 0 | 4 | Not-determined | Genome-fragment | NA | | 0 |
| S11_k141_227747 | 3210 | No | NA | 0 | 1 | Not-determined | Genome-fragment | NA | | 0 |
| S11-final-viral-combined_k141_211973\|\|lt2gene | 1230 | No | NA | 1 | 0 | Low-quality | Genome-fragment | | 0.48 | 0 |
| S11_k141_145003 | 1991 | No | NA | 0 | 0 | Not-determined | Genome-fragment | NA | | 0 |
| S11_k141_268890 | 1413 | No | NA | 0 | 1 | Not-determined | Genome-fragment | NA | | 0 |
| S11_k141_62576 | 4067 | No | NA | 0 | 0 | Not-determined | Genome-fragment | NA | | 0 |
| S11_k141_48366 | 3355 | No | NA | 0 | 2 | Not-determined | Genome-fragment | NA | | 0 |
| S11_k141_20234 | 2744 | No | NA | 0 | 2 | Not-determined | Genome-fragment | NA | | 0 |
| S11_k141_242148 | 3701 | No | NA | 0 | 1 | Not-determined | Genome-fragment | NA | | 0 |
| S11_k141_62593 | 1264 | No | NA | 0 | 3 | Not-determined | Genome-fragment | NA | | 0 |
| S11_k141_80131 | 1230 | No | NA | 1 | 0 | Low-quality | Genome-fragment | | 2.66 | 0 |
| S5-final-viral-combined_k141_37717\|\|full | 1230 | No | NA | 1 | 0 | Low-quality | Genome-fragment | | 2.76 | 0 |
| S11_k141_20253 | 1247 | No | NA | 0 | 1 | Not-determined | Genome-fragment | NA | | 0 |
| S11_k141_268920 | 1702 | No | NA | 0 | 1 | Not-determined | Genome-fragment | NA | | 0 |
| S11_k141_75440 | 3710 | No | NA | 0 | 3 | Not-determined | Genome-fragment | NA | | 0 |
| S11_k141_130764 | 1683 | No | NA | 0 | 2 | Not-determined | Genome-fragment | NA | | 0 |
| S11_k141_75470 | 1179 | No | NA | 0 | 1 | Not-determined | Genome-fragment | NA | | 0 |
| S5-final-viral-combined_k141_22282\|\|full | 1230 | No | NA | 0 | 0 | Low-quality | Genome-fragment | | 1.67 | 0 |
| S11_k141_323330 | 3156 | No | NA | 0 | 2 | Not-determined | Genome-fragment | NA | | 0 |
| S11_k141_130808 | 1808 | No | NA | 0 | 0 | Not-determined | Genome-fragment | NA | | 0 |
| S11_k141_323355 | 1833 | No | NA | 0 | 2 | Not-determined | Genome-fragment | NA | | 0 |
| S11_k141_255519 | 1452 | No | NA | 0 | 1 | Not-determined | Genome-fragment | NA | | 0 |
| S11_k141_242259 | 1192 | No | NA | 0 | 1 | Not-determined | Genome-fragment | NA | | 0 |
| S11_k141_172904 | 1178 | No | NA | 0 | 0 | Not-determined | Genome-fragment | NA | | 0 |
| S5_k141_17740 | 1230 | No | NA | 1 | 0 | Low-quality | Genome-fragment | | 3.6 | 0 |
| S11_k141_130846 | 3405 | No | NA | 0 | 0 | Not-determined | Genome-fragment | NA | | 0 |
| S7-final-viral-combined_k141_127117\|\|full | 1230 | No | NA | 0 | 0 | Low-quality | Genome-fragment | | 20.86 | 0 |
| S11_k141_89875 | 6523 | No | NA | 0 | 2 | Not-determined | Genome-fragment | NA | | 0 |
| S11_k141_104253 | 1145 | No | NA | 0 | 0 | Not-determined | Genome-fragment | NA | | 0 |
| S12_k141_318936 | 1230 | No | NA | 0 | 1 | Low-quality | Genome-fragment | | 0.8 | 0 |
| S11_k141_34921 | 3306 | No | NA | 0 | 1 | Not-determined | Genome-fragment | NA | | 0 |
| S11_k141_62773 | 1091 | No | NA | 0 | 0 | Not-determined | Genome-fragment | NA | | 0 |
| S11_k141_228018 | 1115 | No | NA | 0 | 0 | Not-determined | Genome-fragment | NA | | 0 |
| S11_k141_228021 | 1191 | No | NA | 0 | 0 | Not-determined | Genome-fragment | NA | | 0 |
| S11_k141_62802 | 1105 | No | NA | 0 | 1 | Not-determined | Genome-fragment | NA | | 0 |
| S8_k141_218140 | 1230 | No | NA | 0 | 0 | Low-quality | Genome-fragment | | 0.75 | 0 |
| S11_k141_116893 | 1458 | No | NA | 0 | 0 | Not-determined | Genome-fragment | NA | | 0 |
| S11_k141_228072 | 1606 | No | NA | 0 | 1 | Not-determined | Genome-fragment | NA | | 0 |
| S9-final-viral-combined_k141_86625\|\|full | 1231 | No | NA | 0 | 0 | Low-quality | Genome-fragment | | 3.15 | 0 |
| S11_k141_130959 | 4611 | No | NA | 0 | 2 | Not-determined | Genome-fragment | NA | | 0 |
| S11_k141_323507 | 3255 | No | NA | 0 | 1 | Not-determined | Genome-fragment | NA | | 0 |
| S11_k141_255720 | 4258 | No | NA | 0 | 3 | Not-determined | Genome-fragment | NA | | 0 |
| S11_k141_311103 | 1207 | No | NA | 0 | 1 | Not-determined | Genome-fragment | NA | | 0 |
| S11_k141_323522 | 1631 | No | NA | 0 | 1 | Not-determined | Genome-fragment | NA | | 0 |
| S11-final-viral-combined_k141_259525\|\|full | 1231 | No | NA | 0 | 1 | Low-quality | Genome-fragment | | 2.19 | 0 |
| S11-final-viral-combined_k141_126464\|\|lt2gene | 1231 | No | NA | 1 | 0 | Low-quality | Genome-fragment | | 2.77 | 0 |
| S11_k141_48693 | 1304 | No | NA | 0 | 1 | Not-determined | Genome-fragment | NA | | 0 |
| S3-final-viral-combined_k141_61607\|\|full | 1231 | No | NA | 0 | 0 | Low-quality | Genome-fragment | | 1.28 | 0 |
| S11_k141_90106 | 1449 | No | NA | 0 | 1 | Not-determined | Genome-fragment | NA | | 0 |
| S11_k141_104393 | 2290 | No | NA | 0 | 1 | Not-determined | Genome-fragment | NA | | 0 |
| S5-final-viral-combined_k141_90994\|\|full | 1231 | No | NA | 1 | 0 | Low-quality | Genome-fragment | | 2.94 | 0 |
| S11_k141_48732 | 1525 | No | NA | 0 | 2 | Not-determined | Genome-fragment | NA | | 0 |
| S5-final-viral-combined_k141_42096\|\|full | 1231 | No | NA | 0 | 0 | Low-quality | Genome-fragment | | 1.19 | 0 |
| S11_k141_62950 | 3859 | No | NA | 0 | 2 | Not-determined | Genome-fragment | NA | | 0 |
| S5-final-viral-combined_k141_87428\|\|full | 1231 | No | NA | 0 | 0 | Low-quality | Genome-fragment | | 2.68 | 0 |
| S11_k141_242547 | 1964 | No | NA | 0 | 1 | Not-determined | Genome-fragment | NA | | 0 |
| S11_k141_35219 | 1206 | No | NA | 0 | 0 | Not-determined | Genome-fragment | NA | | 0 |
| S11_k141_186225 | 3100 | No | NA | 0 | 2 | Not-determined | Genome-fragment | NA | | 0 |
| S7-final-viral-combined_k141_227967\|\|full | 1231 | No | NA | 0 | 0 | Low-quality | Genome-fragment | | 2.33 | 0 |
| S11_k141_228205 | 1058 | No | NA | 0 | 1 | Not-determined | Genome-fragment | NA | | 0 |
| S11_k141_200073 | 1173 | No | NA | 0 | 1 | Not-determined | Genome-fragment | NA | | 0 |
| S11_k141_311320 | 1820 | No | NA | 0 | 0 | Not-determined | Genome-fragment | NA | | 0 |
| S11_k141_159258 | 2012 | No | NA | 0 | 0 | Not-determined | Genome-fragment | NA | | 0 |
| S11_k141_104432 | 3538 | No | NA | 0 | 2 | Not-determined | Genome-fragment | NA | | 0 |
| S11_k141_145452 | 1035 | No | NA | 0 | 0 | Not-determined | Genome-fragment | NA | | 0 |
| S11_k141_131220 | 1287 | No | NA | 0 | 2 | Not-determined | Genome-fragment | NA | | 0 |
| S12-final-viral-combined_k141_146895\|\|full | 1231 | No | NA | 0 | 0 | Low-quality | Genome-fragment | | 2.64 | 0 |
| S11_k141_283597 | 1811 | No | NA | 0 | 0 | Not-determined | Genome-fragment | NA | | 0 |
| S12-final-viral-combined_k141_309233\|\|lt2gene | 1231 | No | NA | 2 | 0 | Low-quality | Genome-fragment | | 2.5 | 0 |
| S11_k141_214826 | 1014 | No | NA | 0 | 1 | Not-determined | Genome-fragment | NA | | 0 |
| S11_k141_269403 | 1395 | No | NA | 0 | 1 | Not-determined | Genome-fragment | NA | | 0 |
| S11_k141_145471 | 1393 | No | NA | 0 | 2 | Not-determined | Genome-fragment | NA | | 0 |
| S11_k141_186473 | 3066 | No | NA | 0 | 1 | Not-determined | Genome-fragment | NA | | 0 |
| S11_k141_159380 | 1196 | No | NA | 0 | 2 | Not-determined | Genome-fragment | NA | | 0 |
| S2-final-viral-combined_k141_75160\|\|full | 1231 | No | NA | 0 | 0 | Low-quality | Genome-fragment | | 3.39 | 0 |
| S6-final-viral-combined_k141_80841\|\|full | 1231 | No | NA | 0 | 0 | Low-quality | Genome-fragment | | 3.93 | 0 |
| S11_k141_173510 | 1720 | No | NA | 0 | 2 | Not-determined | Genome-fragment | NA | | 0 |
| S11_k141_159404 | 1255 | No | NA | 0 | 0 | Not-determined | Genome-fragment | NA | | 0 |
| S11-final-viral-combined_k141_86451\|\|full | 1232 | No | NA | 0 | 0 | Low-quality | Genome-fragment | | 2.62 | 0 |
| S11_k141_296800 | 1296 | No | NA | 0 | 1 | Not-determined | Genome-fragment | NA | | 0 |
| S11_k141_214893 | 1021 | No | NA | 0 | 1 | Not-determined | Genome-fragment | NA | | 0 |
| S11_k141_20701 | 1138 | No | NA | 0 | 1 | Not-determined | Genome-fragment | NA | | 0 |
| S11_k141_200254 | 1164 | No | NA | 0 | 1 | Not-determined | Genome-fragment | NA | | 0 |
| S11_k141_173540 | 1347 | No | NA | 0 | 1 | Not-determined | Genome-fragment | NA | | 0 |
| S11-final-viral-combined_k141_214944\|\|full | 1232 | No | NA | 1 | 0 | Low-quality | Genome-fragment | | 2.93 | 0 |
| S11_k141_269510 | 1018 | No | NA | 0 | 1 | Not-determined | Genome-fragment | NA | | 0 |
| S11_k141_35522 | 1412 | No | NA | 0 | 0 | Not-determined | Genome-fragment | NA | | 0 |
| S11_k141_269523 | 1214 | No | NA | 0 | 1 | Not-determined | Genome-fragment | NA | | 0 |
| S11_k141_242882 | 2548 | No | NA | 0 | 2 | Not-determined | Genome-fragment | NA | | 0 |
| S11_k141_7859 | 1043 | No | NA | 0 | 0 | Not-determined | Genome-fragment | NA | | 0 |
| S1-final-viral-combined_k141_352017\|\|full | 1232 | No | NA | 0 | 0 | Low-quality | Genome-fragment | | 3.05 | 0 |
| S11_k141_269614 | 1018 | No | NA | 0 | 1 | Not-determined | Genome-fragment | NA | | 0 |
| S11_k141_104841 | 4064 | No | NA | 0 | 3 | Not-determined | Genome-fragment | NA | | 0 |
| S11_k141_117411 | 1039 | No | NA | 0 | 0 | Not-determined | Genome-fragment | NA | | 0 |
| S11_k141_311538 | 4706 | No | NA | 0 | 3 | Not-determined | Genome-fragment | NA | | 0 |
| S11_k141_76117 | 4320 | No | NA | 0 | 3 | Not-determined | Genome-fragment | NA | | 0 |
| S11_k141_7952 | 2240 | No | NA | 0 | 0 | Not-determined | Genome-fragment | NA | | 0 |
| S11_k141_256225 | 1127 | No | NA | 0 | 1 | Not-determined | Genome-fragment | NA | | 0 |

| | | | | | | | | | | |
|---|---|---|---|---|---|---|---|---|---|---|
| S11_k141_200413 | 1606 | No | NA | 0 | 0 | Not-determined | Genome-fragment | NA | | 0 |
| S11_k141_49195 | 1120 | No | NA | 0 | 0 | Not-determined | Genome-fragment | NA | | 0 |
| S11_k141_228642 | 1021 | No | NA | 0 | 0 | Not-determined | Genome-fragment | NA | | 0 |
| S11_k141_323959 | 1157 | No | NA | 0 | 1 | Not-determined | Genome-fragment | NA | | 0 |
| S7_k141_184430 | 1232 | No | NA | 0 | 0 | Low-quality | Genome-fragment | | 1.2 | 0 |
| S11_k141_49202 | 1449 | No | NA | 0 | 2 | Not-determined | Genome-fragment | NA | | 0 |
| S17-final-viral-combined_k141_109987\|\|full | 1232 | No | NA | 0 | 1 | Low-quality | Genome-fragment | | 1.67 | 0 |
| S12-final-viral-combined_k141_305606\|\|full | 1232 | No | NA | 0 | 0 | Low-quality | Genome-fragment | | 1.97 | 0 |
| S11_k141_117607 | 1281 | No | NA | 0 | 0 | Not-determined | Genome-fragment | NA | | 0 |
| S11_k141_63390 | 2892 | No | NA | 0 | 0 | Not-determined | Genome-fragment | NA | | 0 |
| S12_k141_101223 | 1232 | No | NA | 2 | 0 | Low-quality | Genome-fragment | | 2.93 | 0 |
| S11_k141_269752 | 1811 | No | NA | 0 | 1 | Not-determined | Genome-fragment | NA | | 0 |
| S4-final-viral-combined_k141_166272\|\|full | 1232 | No | NA | 0 | 0 | Low-quality | Genome-fragment | | 3.31 | 0 |
| S11_k141_131501 | 1074 | No | NA | 0 | 0 | Not-determined | Genome-fragment | NA | | 0 |
| S11_k141_311662 | 2152 | No | NA | 0 | 2 | Not-determined | Genome-fragment | NA | | 0 |
| S4-final-viral-combined_k141_12297\|\|lt2gene | 1232 | No | NA | 1 | 0 | Low-quality | Genome-fragment | | 1.62 | 0 |
| S11_k141_104981 | 2075 | No | NA | 0 | 1 | Not-determined | Genome-fragment | NA | | 0 |
| S4_k141_158992 | 1232 | No | NA | 1 | 0 | Low-quality | Genome-fragment | | 0.71 | 0 |
| S4_k141_27810 | 1232 | No | NA | 1 | 0 | Low-quality | Genome-fragment | | 0.4 | 0 |
| S11_k141_173730 | 1104 | No | NA | 0 | 1 | Not-determined | Genome-fragment | NA | | 0 |
| S11_k141_311717 | 5215 | No | NA | 0 | 1 | Not-determined | Genome-fragment | NA | | 0 |
| S11_k141_311737 | 3593 | No | NA | 0 | 2 | Not-determined | Genome-fragment | NA | | 0 |
| S11-final-viral-combined_k141_279270\|\|full | 1233 | No | NA | 0 | 0 | Low-quality | Genome-fragment | | 3.85 | 0 |
| S11_k141_131558 | 1191 | No | NA | 0 | 1 | Not-determined | Genome-fragment | NA | | 0 |
| S11_k141_269860 | 1127 | No | NA | 0 | 0 | Not-determined | Genome-fragment | NA | | 0 |
| S11_k141_8187 | 1026 | No | NA | 0 | 0 | Not-determined | Genome-fragment | NA | | 0 |
| S11_k141_49279 | 3264 | No | NA | 0 | 1 | Not-determined | Genome-fragment | NA | | 0 |
| S11_k141_311789 | 1247 | No | NA | 0 | 1 | Not-determined | Genome-fragment | NA | | 0 |
| S11_k141_269885 | 1289 | No | NA | 0 | 0 | Not-determined | Genome-fragment | NA | | 0 |
| S11_k141_49322 | 4555 | No | NA | 0 | 1 | Not-determined | Genome-fragment | NA | | 0 |
| S11_k141_311801 | 1723 | No | NA | 0 | 2 | Not-determined | Genome-fragment | NA | | 0 |
| S11-final-viral-combined_k141_197395\|\|lt2gene | 1233 | No | NA | 1 | 0 | Low-quality | Genome-fragment | | 1.13 | 0 |
| S11_k141_284004 | 1349 | No | NA | 0 | 1 | Not-determined | Genome-fragment | NA | | 0 |
| S11_k141_76406 | 1123 | No | NA | 0 | 1 | Not-determined | Genome-fragment | NA | | 0 |
| S1-final-viral-combined_k141_192956\|\|full | 1233 | No | NA | 0 | 0 | Low-quality | Genome-fragment | | 0.76 | 0 |
| S11_k141_269922 | 1066 | No | NA | 0 | 0 | Not-determined | Genome-fragment | NA | | 0 |
| S11_k141_215201 | 2009 | No | NA | 0 | 2 | Not-determined | Genome-fragment | NA | | 0 |
| S11_k141_105099 | 5115 | No | NA | 0 | 2 | Not-determined | Genome-fragment | NA | | 0 |
| S11_k141_243219 | 4802 | No | NA | 0 | 2 | Not-determined | Genome-fragment | NA | | 0 |
| S5-final-viral-combined_k141_198660\|\|lt2gene | 1233 | No | NA | 1 | 0 | Low-quality | Genome-fragment | | 0.76 | 0 |
| S11_k141_173885 | 4421 | No | NA | 0 | 3 | Not-determined | Genome-fragment | NA | | 0 |
| S11_k141_284136 | 1600 | No | NA | 0 | 0 | Not-determined | Genome-fragment | NA | | 0 |
| S7-final-viral-combined_k141_11020\|\|full | 1233 | No | NA | 0 | 2 | Low-quality | Genome-fragment | | 2.72 | 0 |
| S11_k141_243270 | 2788 | No | NA | 0 | 0 | Not-determined | Genome-fragment | NA | | 0 |
| S11_k141_90940 | 4050 | No | NA | 0 | 2 | Not-determined | Genome-fragment | NA | | 0 |
| S11_k141_49483 | 2669 | No | NA | 0 | 4 | Not-determined | Genome-fragment | NA | | 0 |
| S11_k141_187007 | 1190 | No | NA | 0 | 0 | Not-determined | Genome-fragment | NA | | 0 |
| S11_k141_105237 | 3589 | No | NA | 0 | 1 | Not-determined | Genome-fragment | NA | | 0 |
| S17-final-viral-combined_k141_167710\|\|full | 1233 | No | NA | 0 | 0 | Low-quality | Genome-fragment | | 1.93 | 0 |
| S17-final-viral-combined_k141_17736\|\|full | 1233 | No | NA | 0 | 0 | Low-quality | Genome-fragment | | 3.08 | 0 |
| S11_k141_215367 | 4872 | No | NA | 0 | 3 | Not-determined | Genome-fragment | NA | | 0 |
| S11_k141_187075 | 1151 | No | NA | 0 | 0 | Not-determined | Genome-fragment | NA | | 0 |
| S11_k141_297373 | 1431 | No | NA | 0 | 0 | Not-determined | Genome-fragment | NA | | 0 |
| S11_k141_36067 | 5147 | No | NA | 0 | 4 | Not-determined | Genome-fragment | NA | | 0 |
| S17-final-viral-combined_k141_182284\|\|lt2gene | 1233 | No | NA | 1 | 0 | Low-quality | Genome-fragment | | 3.08 | 0 |
| S11_k141_117946 | 1182 | No | NA | 0 | 1 | Not-determined | Genome-fragment | NA | | 0 |
| S11_k141_284305 | 4821 | No | NA | 0 | 3 | Not-determined | Genome-fragment | NA | | 0 |
| S11_k141_228983 | 1778 | No | NA | 0 | 1 | Not-determined | Genome-fragment | NA | | 0 |
| S17-k141_152454 | 1233 | No | NA | 0 | 0 | Low-quality | Genome-fragment | | 2.06 | 0 |
| S11_k141_63750 | 1094 | No | NA | 0 | 1 | Not-determined | Genome-fragment | NA | | 0 |
| S11_k141_131817 | 2154 | No | NA | 0 | 0 | Not-determined | Genome-fragment | NA | | 0 |
| S11_k141_187160 | 1357 | No | NA | 0 | 1 | Not-determined | Genome-fragment | NA | | 0 |
| S11_k141_297407 | 8265 | No | NA | 0 | 4 | Not-determined | Genome-fragment | NA | | 0 |
| S11_k141_256724 | 1692 | No | NA | 0 | 1 | Not-determined | Genome-fragment | NA | | 0 |
| S11_k141_131829 | 2112 | No | NA | 0 | 2 | Not-determined | Genome-fragment | NA | | 0 |
| S11_k141_284360 | 1068 | No | NA | 0 | 0 | Not-determined | Genome-fragment | NA | | 0 |
| S11_k141_146200 | 1380 | No | NA | 0 | 1 | Not-determined | Genome-fragment | NA | | 0 |
| S12-final-viral-combined_k141_218539\|\|full | 1233 | No | NA | 0 | 0 | Low-quality | Genome-fragment | | 1.7 | 0 |
| S11_k141_311999 | 3547 | No | NA | 0 | 3 | Not-determined | Genome-fragment | NA | | 0 |
| S11_k141_297437 | 1958 | No | NA | 0 | 1 | Not-determined | Genome-fragment | NA | | 0 |
| S11_k141_200935 | 6390 | No | NA | 0 | 2 | Not-determined | Genome-fragment | NA | | 0 |
| S11_k141_324354 | 1024 | No | NA | 0 | 4 | Not-determined | Genome-fragment | NA | | 0 |
| S11_k141_63767 | 4145 | No | NA | 0 | 2 | Not-determined | Genome-fragment | NA | | 0 |
| S11_k141_118037 | 1133 | No | NA | 0 | 2 | Not-determined | Genome-fragment | NA | | 0 |
| S11_k141_200951 | 1149 | No | NA | 0 | 1 | Not-determined | Genome-fragment | NA | | 0 |
| S11_k141_200999 | 4704 | No | NA | 0 | 2 | Not-determined | Genome-fragment | NA | | 0 |
| S11_k141_201004 | 1767 | No | NA | 0 | 2 | Not-determined | Genome-fragment | NA | | 0 |
| S11_k141_76788 | 1046 | No | NA | 0 | 1 | Not-determined | Genome-fragment | NA | | 0 |
| S11_k141_229105 | 1193 | No | NA | 0 | 1 | Not-determined | Genome-fragment | NA | | 0 |
| S11_k141_256886 | 1529 | No | NA | 0 | 1 | Not-determined | Genome-fragment | NA | | 0 |
| S11_k141_229129 | 1190 | No | NA | 0 | 1 | Not-determined | Genome-fragment | NA | | 0 |
| S12-final-viral-combined_k141_231558\|\|full | 1233 | No | NA | 0 | 0 | Low-quality | Genome-fragment | | 1.02 | 0 |
| S11_k141_324401 | 4511 | No | NA | 0 | 1 | Not-determined | Genome-fragment | NA | | 0 |
| S11_k141_76846 | 1173 | No | NA | 0 | 0 | Not-determined | Genome-fragment | NA | | 0 |
| S11_k141_21408 | 1377 | No | NA | 0 | 1 | Not-determined | Genome-fragment | NA | | 0 |
| S11_k141_146378 | 1090 | No | NA | 0 | 0 | Not-determined | Genome-fragment | NA | | 0 |
| S11_k141_91215 | 1489 | No | NA | 0 | 1 | Not-determined | Genome-fragment | NA | | 0 |
| S12-final-viral-combined_k141_231530\|\|lt2gene | 1233 | No | NA | 1 | 0 | Low-quality | Genome-fragment | | 0.47 | 0 |
| S4-final-viral-combined_k141_148690\|\|full | 1233 | No | NA | 0 | 0 | Low-quality | Genome-fragment | | 2.13 | 0 |
| S4_k141_117760 | 1233 | No | NA | 0 | 0 | Low-quality | Genome-fragment | | 0.53 | 0 |
| S11_k141_324474 | 3179 | No | NA | 0 | 4 | Not-determined | Genome-fragment | NA | | 0 |
| S11_k141_49875 | 1025 | No | NA | 0 | 1 | Not-determined | Genome-fragment | NA | | 0 |
| S11_k141_8849 | 3857 | No | NA | 0 | 1 | Not-determined | Genome-fragment | NA | | 0 |
| S8-final-viral-combined_k141_271227\|\|full | 1233 | No | NA | 0 | 0 | Low-quality | Genome-fragment | | 1.79 | 0 |
| S9-final-viral-combined_k141_82235\|\|full | 1234 | No | NA | 1 | 0 | Low-quality | Genome-fragment | | 1.99 | 0 |
| S17-final-viral-combined_k141_116998\|\|full | 1234 | No | NA | 0 | 0 | Low-quality | Genome-fragment | | 2 | 0 |
| S4-final-viral-combined_k141_160636\|\|full | 1234 | No | NA | 0 | 2 | Low-quality | Genome-fragment | | 0.87 | 0 |
| S11_k141_63849 | 1999 | No | NA | 0 | 1 | Not-determined | Genome-fragment | NA | | 0 |
| S11_k141_8895 | 3624 | No | NA | 0 | 3 | Not-determined | Genome-fragment | NA | | 0 |
| S11_k141_118189 | 1502 | No | NA | 0 | 4 | Not-determined | Genome-fragment | NA | | 0 |
| S11_k141_160324 | 1117 | No | NA | 0 | 1 | Not-determined | Genome-fragment | NA | | 0 |
| S11_k141_91268 | 7536 | No | NA | 0 | 2 | Not-determined | Genome-fragment | NA | | 0 |
| S8-final-viral-combined_k141_172064\|\|full | 1234 | No | NA | 0 | 1 | Low-quality | Genome-fragment | | 2.32 | 0 |
| S11_k141_243628 | 1214 | No | NA | 0 | 2 | Not-determined | Genome-fragment | NA | | 0 |
| S11_k141_243649 | 3228 | No | NA | 0 | 1 | Not-determined | Genome-fragment | NA | | 0 |
| S11_k141_105653 | 1479 | No | NA | 0 | 1 | Not-determined | Genome-fragment | NA | | 0 |
| S9-final-viral-combined_k141_79303\|\|full | 1235 | No | NA | 0 | 0 | Low-quality | Genome-fragment | | 2.79 | 0 |
| S11_k141_257056 | 1053 | No | NA | 0 | 1 | Not-determined | Genome-fragment | NA | | 0 |
| S11-final-viral-combined_k141_399\|\|full | 1235 | No | NA | 1 | 0 | Low-quality | Genome-fragment | | 3.39 | 0 |
| S11_k141_284533 | 1000 | No | NA | 0 | 0 | Not-determined | Genome-fragment | NA | | 0 |
| S11_k141_270247 | 1198 | No | NA | 0 | 1 | Not-determined | Genome-fragment | NA | | 0 |
| S11_k141_49994 | 3046 | No | NA | 0 | 1 | Not-determined | Genome-fragment | NA | | 0 |
| S11_k141_312264 | 1312 | No | NA | 0 | 0 | Not-determined | Genome-fragment | NA | | 0 |
| S11_k141_324527 | 1002 | No | NA | 0 | 1 | Not-determined | Genome-fragment | NA | | 0 |
| S11_k141_201265 | 1629 | No | NA | 0 | 0 | Not-determined | Genome-fragment | NA | | 0 |
| S1-final-viral-combined_k141_54451\|\|full | 1235 | No | NA | 0 | 2 | Low-quality | Genome-fragment | | 0.39 | 0 |
| S11_k141_187442 | 1720 | No | NA | 0 | 2 | Not-determined | Genome-fragment | NA | | 0 |
| S1-final-viral-combined_k141_386392\|\|full | 1235 | No | NA | 1 | 0 | Low-quality | Genome-fragment | | 1.86 | 0 |
| S11_k141_312324 | 1047 | No | NA | 0 | 0 | Not-determined | Genome-fragment | NA | | 0 |
| S11_k141_105758 | 4371 | No | NA | 0 | 0 | Not-determined | Genome-fragment | NA | | 0 |
| S11_k141_105766 | 1141 | No | NA | 0 | 1 | Not-determined | Genome-fragment | NA | | 0 |
| S11_k141_21535 | 1015 | No | NA | 0 | 1 | Not-determined | Genome-fragment | NA | | 0 |
| S1_k141_177159 | 1235 | No | NA | 2 | 0 | Low-quality | Genome-fragment | | 1.99 | 0 |
| S11_k141_201344 | 6828 | No | NA | 0 | 3 | Not-determined | Genome-fragment | NA | | 0 |
| S11_k141_21572 | 3393 | No | NA | 0 | 4 | Not-determined | Genome-fragment | NA | | 0 |
| S5-final-viral-combined_k141_22059\|\|full | 1235 | No | NA | 1 | 0 | Low-quality | Genome-fragment | | 3.52 | 0 |
| S11_k141_21622 | 5846 | No | NA | 0 | 1 | Not-determined | Genome-fragment | NA | | 0 |
| S11_k141_50239 | 1176 | No | NA | 0 | 0 | Not-determined | Genome-fragment | NA | | 0 |
| S11_k141_297793 | 1658 | No | NA | 0 | 1 | Not-determined | Genome-fragment | NA | | 0 |
| S11_k141_201429 | 1129 | No | NA | 0 | 1 | Not-determined | Genome-fragment | NA | | 0 |
| S5-final-viral-combined_k141_131787\|\|full | 1235 | No | NA | 3 | 0 | Low-quality | Genome-fragment | | 2.89 | 0 |
| S11_k141_297808 | 3713 | No | NA | 0 | 3 | Not-determined | Genome-fragment | NA | | 0 |
| S11_k141_270427 | 1605 | No | NA | 0 | 0 | Not-determined | Genome-fragment | NA | | 0 |
| S11_k141_297837 | 2502 | No | NA | 0 | 1 | Not-determined | Genome-fragment | NA | | 0 |
| S11_k141_36536 | 1022 | No | NA | 0 | 1 | Not-determined | Genome-fragment | NA | | 0 |
| S11_k141_284762 | 2754 | No | NA | 0 | 0 | Not-determined | Genome-fragment | NA | | 0 |
| S11_k141_270475 | 1157 | No | NA | 0 | 0 | Not-determined | Genome-fragment | NA | | 0 |
| S17-final-viral-combined_k141_112910\|\|full | 1235 | No | NA | 0 | 0 | Low-quality | Genome-fragment | | 2.22 | 0 |
| S11_k141_229574 | 1466 | No | NA | 0 | 1 | Not-determined | Genome-fragment | NA | | 0 |
| S11_k141_91546 | 1237 | No | NA | 0 | 1 | Not-determined | Genome-fragment | NA | | 0 |
| S17-final-viral-combined_k141_195822\|\|full | 1235 | No | NA | 0 | 0 | Low-quality | Genome-fragment | | 3.16 | 0 |
| S11_k141_324811 | 1613 | No | NA | 0 | 1 | Not-determined | Genome-fragment | NA | | 0 |
| S2-final-viral-combined_k141_101394\|\|full | 1235 | No | NA | 0 | 0 | Low-quality | Genome-fragment | | 2.29 | 0 |
| S11_k141_284840 | 1018 | No | NA | 0 | 0 | Not-determined | Genome-fragment | NA | | 0 |
| S11_k141_229649 | 1359 | No | NA | 0 | 1 | Not-determined | Genome-fragment | NA | | 0 |
| S11_k141_118583 | 3069 | No | NA | 0 | 2 | Not-determined | Genome-fragment | NA | | 0 |
| S11_k141_229654 | 1511 | No | NA | 0 | 1 | Not-determined | Genome-fragment | NA | | 0 |
| S6_k141_127704 | 1235 | No | NA | 0 | 1 | Low-quality | Genome-fragment | | 1.56 | 0 |
| S11_k141_64247 | 1008 | No | NA | 0 | 1 | Not-determined | Genome-fragment | NA | | 0 |
| S11_k141_270561 | 1015 | No | NA | 0 | 1 | Not-determined | Genome-fragment | NA | | 0 |
| S11_k141_160723 | 3362 | No | NA | 0 | 3 | Not-determined | Genome-fragment | NA | | 0 |
| S11_k141_77228 | 3519 | No | NA | 0 | 3 | Not-determined | Genome-fragment | NA | | 0 |
| S8-final-viral-combined_k141_172723\|\|full | 1235 | No | NA | 0 | 0 | Low-quality | Genome-fragment | | 3.15 | 0 |
| S11_k141_174658 | 1257 | No | NA | 0 | 1 | Not-determined | Genome-fragment | NA | | 0 |
| S11_k141_324915 | 6418 | No | NA | 0 | 1 | Not-determined | Genome-fragment | NA | | 0 |
| S8-final-viral-combined_k141_11041\|\|full | 1235 | No | NA | 0 | 1 | Low-quality | Genome-fragment | | 0.86 | 0 |
| S11_k141_243985 | 4033 | No | NA | 0 | 0 | Not-determined | Genome-fragment | NA | | 0 |
| S11_k141_257478 | 3505 | No | NA | 0 | 0 | Not-determined | Genome-fragment | NA | | 0 |
| S11_k141_201669 | 1005 | No | NA | 0 | 0 | Not-determined | Genome-fragment | NA | | 0 |
| S11_k141_118665 | 1907 | No | NA | 0 | 2 | Not-determined | Genome-fragment | NA | | 0 |
| S9-final-viral-combined_k141_79124\|\|lt2gene | 1236 | No | NA | 1 | 0 | Low-quality | Genome-fragment | | 3.07 | 0 |
| S5-final-viral-combined_k141_16579\|\|full | 1236 | No | NA | 1 | 0 | Low-quality | Genome-fragment | | 2.34 | 0 |
| S11_k141_257502 | 3124 | No | NA | 0 | 1 | Not-determined | Genome-fragment | NA | | 0 |
| S11_k141_257508 | 6092 | No | NA | 0 | 3 | Not-determined | Genome-fragment | NA | | 0 |
| S7-final-viral-combined_k141_27129\|\|full | 1236 | No | NA | 1 | 0 | Low-quality | Genome-fragment | | 2.83 | 0 |
| S11_k141_201716 | 1211 | No | NA | 0 | 1 | Not-determined | Genome-fragment | NA | | 0 |
| S7_k141_54765 | 1236 | No | NA | 1 | 0 | Low-quality | Genome-fragment | | 2.24 | 0 |
| S11_k141_174743 | 1008 | No | NA | 0 | 1 | Not-determined | Genome-fragment | NA | | 0 |
| S11_k141_312656 | 1020 | No | NA | 0 | 1 | Not-determined | Genome-fragment | NA | | 0 |
| S11_k141_36785 | 1176 | No | NA | 0 | 1 | Not-determined | Genome-fragment | NA | | 0 |
| S2-final-viral-combined_k141_67502\|\|full | 1236 | No | NA | 0 | 2 | Low-quality | Genome-fragment | | 0.5 | 0 |
| S11_k141_216016 | 1025 | No | NA | 0 | 0 | Not-determined | Genome-fragment | NA | | 0 |
| S11_k141_146961 | 1042 | No | NA | 0 | 2 | Not-determined | Genome-fragment | NA | | 0 |
| S11_k141_21847 | 1521 | No | NA | 0 | 1 | Not-determined | Genome-fragment | NA | | 0 |

| | | | | | | | | | | |
|---|---|---|---|---|---|---|---|---|---|---|
| S11_k141_270774 | 3079 | No | NA | 0 | 0 | Not-determined | Genome-fragment | NA | | 0 |
| S11_k141_36840 | 1042 | No | NA | 0 | 0 | Not-determined | Genome-fragment | NA | | 0 |
| S11_k141_118792 | 6573 | No | NA | 0 | 5 | Not-determined | Genome-fragment | NA | | 0 |
| S8_k141_27172 | 1236 | No | NA | 0 | 0 | Low-quality | Genome-fragment | | 2 | 0 |
| S11_k141_147008 | 1005 | No | NA | 0 | 1 | Not-determined | Genome-fragment | NA | | 0 |
| S11-final-viral-combined_k141_231733\|\|full | 1237 | No | NA | 1 | 0 | Low-quality | Genome-fragment | | 0.75 | 0 |
| S11_k141_312781 | 1087 | No | NA | 0 | 1 | Not-determined | Genome-fragment | NA | | 0 |
| S1-final-viral-combined_k141_29523\|\|full | 1237 | No | NA | 0 | 0 | Low-quality | Genome-fragment | | 2.49 | 0 |
| S11_k141_201880 | 13760 | No | NA | 0 | 12 | Not-determined | Genome-fragment | NA | | 0 |
| S11_k141_201884 | 1002 | No | NA | 0 | 1 | Not-determined | Genome-fragment | NA | | 0 |
| S5-final-viral-combined_k141_121263\|\|full | 1237 | No | NA | 1 | 0 | Low-quality | Genome-fragment | | 2.24 | 0 |
| S11_k141_64400 | 1114 | No | NA | 0 | 0 | Not-determined | Genome-fragment | NA | | 0 |
| S11_k141_64410 | 1660 | No | NA | 0 | 1 | Not-determined | Genome-fragment | NA | | 0 |
| S11_k141_161025 | 1355 | No | NA | 0 | 1 | Not-determined | Genome-fragment | NA | | 0 |
| S11_k141_312812 | 3782 | No | NA | 0 | 0 | Not-determined | Genome-fragment | NA | | 0 |
| S11_k141_201984 | 3899 | No | NA | 0 | 2 | Not-determined | Genome-fragment | NA | | 0 |
| S11_k141_106378 | 1378 | No | NA | 0 | 1 | Not-determined | Genome-fragment | NA | | 0 |
| S11_k141_201993 | 1111 | No | NA | 0 | 1 | Not-determined | Genome-fragment | NA | | 0 |
| S11_k141_132332 | 1319 | No | NA | 0 | 0 | Not-determined | Genome-fragment | NA | | 0 |
| S11_k141_325187 | 6715 | No | NA | 0 | 3 | Not-determined | Genome-fragment | NA | | 0 |
| S5-final-viral-combined_k141_68743\|\|lt2gene | 1237 | No | NA | 1 | 0 | Low-quality | Genome-fragment | | 2.15 | 0 |
| S11_k141_270881 | 1093 | No | NA | 0 | 1 | Not-determined | Genome-fragment | NA | | 0 |
| S11_k141_312906 | 1055 | No | NA | 0 | 1 | Not-determined | Genome-fragment | NA | | 0 |
| S11_k141_64506 | 1322 | No | NA | 0 | 0 | Not-determined | Genome-fragment | NA | | 0 |
| S5_k141_98771 | 1237 | No | NA | 0 | 0 | Low-quality | Genome-fragment | | 2.53 | 0 |
| S11_k141_188172 | 1027 | No | NA | 0 | 2 | Not-determined | Genome-fragment | NA | | 0 |
| S11_k141_230016 | 4361 | No | NA | 0 | 2 | Not-determined | Genome-fragment | NA | | 0 |
| S11_k141_106474 | 8121 | No | NA | 0 | 5 | Not-determined | Genome-fragment | NA | | 0 |
| S11_k141_106491 | 1286 | No | NA | 0 | 0 | Not-determined | Genome-fragment | NA | | 0 |
| S11_k141_257827 | 1074 | No | NA | 0 | 1 | Not-determined | Genome-fragment | NA | | 0 |
| S2-final-viral-combined_k141_33422\|\|full | 1237 | No | NA | 0 | 1 | Low-quality | Genome-fragment | | 1.69 | 0 |
| S11_k141_270907 | 1173 | No | NA | 0 | 1 | Not-determined | Genome-fragment | NA | | 0 |
| S11_k141_147244 | 1608 | No | NA | 0 | 0 | Not-determined | Genome-fragment | NA | | 0 |
| S11_k141_202183 | 1826 | No | NA | 0 | 1 | Not-determined | Genome-fragment | NA | | 0 |
| S11_k141_325317 | 1668 | No | NA | 0 | 2 | Not-determined | Genome-fragment | NA | | 0 |
| S9_k141_9494 | 1238 | No | NA | 0 | 0 | Low-quality | Genome-fragment | | 3.78 | 0 |
| S11_k141_285473 | 3504 | No | NA | 0 | 1 | Not-determined | Genome-fragment | NA | | 0 |
| S11-final-viral-combined_k141_39438\|\|full | 1238 | No | NA | 0 | 2 | Low-quality | Genome-fragment | | 1.95 | 0 |
| S11_k141_257935 | 1556 | No | NA | 0 | 1 | Not-determined | Genome-fragment | NA | | 0 |
| S11_k141_244498 | 1498 | No | NA | 0 | 0 | Not-determined | Genome-fragment | NA | | 0 |
| S11_k141_175166 | 1098 | No | NA | 0 | 1 | Not-determined | Genome-fragment | NA | | 0 |
| S11_k141_302054 | 1238 | No | NA | 0 | 0 | Low-quality | Genome-fragment | | 2.27 | 0 |
| S11_k141_161264 | 1090 | No | NA | 0 | 1 | Not-determined | Genome-fragment | NA | | 0 |
| S11_k141_325406 | 1087 | No | NA | 0 | 1 | Not-determined | Genome-fragment | NA | | 0 |
| S11_k141_92143 | 1224 | No | NA | 0 | 0 | Not-determined | Genome-fragment | NA | | 0 |
| S1-final-viral-combined_k141_329408\|\|full | 1238 | No | NA | 0 | 0 | Low-quality | Genome-fragment | | 2.85 | 0 |
| S11_k141_132554 | 3759 | No | NA | 0 | 3 | Not-determined | Genome-fragment | NA | | 0 |
| S11_k141_77822 | 1217 | No | NA | 0 | 1 | Not-determined | Genome-fragment | NA | | 0 |
| S3_k141_93549 | 1238 | No | NA | 0 | 1 | Low-quality | Genome-fragment | | 0.33 | 0 |
| S5-final-viral-combined_k141_187618\|\|full | 1238 | No | NA | 1 | 0 | Low-quality | Genome-fragment | | 2.85 | 0 |
| S7-final-viral-combined_k141_46216\|\|full | 1238 | No | NA | 0 | 1 | Low-quality | Genome-fragment | | 1.11 | 0 |
| S11_k141_175239 | 1008 | No | NA | 0 | 1 | Not-determined | Genome-fragment | NA | | 0 |
| S11_k141_64765 | 2631 | No | NA | 0 | 0 | Not-determined | Genome-fragment | NA | | 0 |
| S11_k141_51214 | 7051 | No | NA | 0 | 6 | Not-determined | Genome-fragment | NA | | 0 |
| S11_k141_51219 | 5074 | No | NA | 0 | 0 | Not-determined | Genome-fragment | NA | | 0 |
| S11_k141_244593 | 1142 | No | NA | 0 | 0 | Not-determined | Genome-fragment | NA | | 0 |
| S11_k141_64777 | 1798 | No | NA | 0 | 0 | Not-determined | Genome-fragment | NA | | 0 |
| S11_k141_298585 | 1125 | No | NA | 0 | 0 | Not-determined | Genome-fragment | NA | | 0 |
| S11_k141_175294 | 1003 | No | NA | 0 | 1 | Not-determined | Genome-fragment | NA | | 0 |
| S11_k141_106732 | 3295 | No | NA | 0 | 2 | Not-determined | Genome-fragment | NA | | 0 |
| S11_k141_10024 | 2283 | No | NA | 0 | 2 | Not-determined | Genome-fragment | NA | | 0 |
| S11_k141_92388 | 1471 | No | NA | 0 | 1 | Not-determined | Genome-fragment | NA | | 0 |
| S11_k141_119381 | 1046 | No | NA | 0 | 1 | Not-determined | Genome-fragment | NA | | 0 |
| S11_k141_188491 | 1075 | No | NA | 0 | 1 | Not-determined | Genome-fragment | NA | | 0 |
| S11_k141_325535 | 2195 | No | NA | 0 | 1 | Not-determined | Genome-fragment | NA | | 0 |
| S11_k141_77928 | 1448 | No | NA | 0 | 1 | Not-determined | Genome-fragment | NA | | 0 |
| S11_k141_106772 | 7204 | No | NA | 0 | 0 | Not-determined | Genome-fragment | NA | | 0 |
| S11_k141_37405 | 1282 | No | NA | 0 | 0 | Not-determined | Genome-fragment | NA | | 0 |
| S11_k141_22205 | 3540 | No | NA | 0 | 4 | Not-determined | Genome-fragment | NA | | 0 |
| S7_k141_243336 | 1238 | No | NA | 0 | 3 | Low-quality | Genome-fragment | | 3.23 | 0 |
| S11_k141_106812 | 2519 | No | NA | 0 | 1 | Not-determined | Genome-fragment | NA | | 0 |
| S11_k141_175439 | 1012 | No | NA | 0 | 1 | Not-determined | Genome-fragment | NA | | 0 |
| S17-final-viral-combined_k141_125357\|\|full | 1238 | No | NA | 0 | 2 | Low-quality | Genome-fragment | | 0.9 | 0 |
| S11_k141_106832 | 3963 | No | NA | 0 | 1 | Not-determined | Genome-fragment | NA | | 0 |
| S17-final-viral-combined_k141_83549\|\|full | 1238 | No | NA | 0 | 0 | Low-quality | Genome-fragment | | 1.35 | 0 |
| S11_k141_51400 | 5370 | No | NA | 0 | 3 | Not-determined | Genome-fragment | NA | | 0 |
| S17-final-viral-combined_k141_60519\|\|full | 1238 | No | NA | 1 | 0 | Low-quality | Genome-fragment | | 2.55 | 0 |
| S11_k141_313382 | 2284 | No | NA | 0 | 2 | Not-determined | Genome-fragment | NA | | 0 |
| S12-final-viral-combined_k141_331875\|\|lt2gene | 1238 | No | NA | 2 | 0 | Low-quality | Genome-fragment | | 9.62 | 0 |
| S11_k141_298787 | 1911 | No | NA | 0 | 2 | Not-determined | Genome-fragment | NA | | 0 |
| S11_k141_22345 | 1135 | No | NA | 0 | 2 | Not-determined | Genome-fragment | NA | | 0 |
| S4-final-viral-combined_k141_34844\|\|full | 1238 | No | NA | 2 | 0 | Low-quality | Genome-fragment | | 3.47 | 0 |
| S11_k141_313402 | 2209 | No | NA | 0 | 2 | Not-determined | Genome-fragment | NA | | 0 |
| S11_k141_10123 | 1229 | No | NA | 0 | 1 | Not-determined | Genome-fragment | NA | | 0 |
| S11_k141_230488 | 1161 | No | NA | 0 | 0 | Not-determined | Genome-fragment | NA | | 0 |
| S11_k141_10144 | 1180 | No | NA | 0 | 1 | Not-determined | Genome-fragment | NA | | 0 |
| S11_k141_119535 | 4371 | No | NA | 0 | 2 | Not-determined | Genome-fragment | NA | | 0 |
| S11_k141_313467 | 1041 | No | NA | 0 | 0 | Not-determined | Genome-fragment | NA | | 0 |
| S11_k141_258391 | 1027 | No | NA | 0 | 1 | Not-determined | Genome-fragment | NA | | 0 |
| S11_k141_271385 | 1935 | No | NA | 0 | 0 | Not-determined | Genome-fragment | NA | | 0 |
| S11_k141_22475 | 1231 | No | NA | 0 | 2 | Not-determined | Genome-fragment | NA | | 0 |
| S11_k141_78266 | 1817 | No | NA | 0 | 0 | Not-determined | Genome-fragment | NA | | 0 |
| S11_k141_271461 | 1320 | No | NA | 0 | 1 | Not-determined | Genome-fragment | NA | | 0 |
| S4-final-viral-combined_k141_147053\|\|full | 1238 | No | NA | 0 | 2 | Low-quality | Genome-fragment | | 1.16 | 0 |
| S8-final-viral-combined_k141_98942\|\|full | 1238 | No | NA | 1 | 0 | Low-quality | Genome-fragment | | 2.97 | 0 |
| S11_k141_188691 | 1448 | No | NA | 0 | 1 | Not-determined | Genome-fragment | NA | | 0 |
| S11_k141_188697 | 1069 | No | NA | 0 | 1 | Not-determined | Genome-fragment | NA | | 0 |
| S11_k141_325756 | 1462 | No | NA | 0 | 1 | Not-determined | Genome-fragment | NA | | 0 |
| S11_k141_175671 | 1024 | No | NA | 0 | 0 | Not-determined | Genome-fragment | NA | | 0 |
| S11_k141_258513 | 1008 | No | NA | 0 | 0 | Not-determined | Genome-fragment | NA | | 0 |
| S8-final-viral-combined_k141_113379\|\|full | 1238 | No | NA | 0 | 2 | Low-quality | Genome-fragment | | 0.66 | 0 |
| S11_k141_51724 | 1547 | No | NA | 0 | 1 | Not-determined | Genome-fragment | NA | | 0 |
| S11_k141_78315 | 6288 | No | NA | 0 | 3 | Not-determined | Genome-fragment | NA | | 0 |
| S11_k141_188763 | 1305 | No | NA | 0 | 1 | Not-determined | Genome-fragment | NA | | 0 |
| S11_k141_271551 | 1033 | No | NA | 0 | 1 | Not-determined | Genome-fragment | NA | | 0 |
| S9-final-viral-combined_k141_55092\|\|full | 1239 | No | NA | 0 | 0 | Low-quality | Genome-fragment | | 1.91 | 0 |
| S11_k141_230715 | 1042 | No | NA | 0 | 0 | Not-determined | Genome-fragment | NA | | 0 |
| S11_k141_133089 | 2354 | No | NA | 0 | 0 | Not-determined | Genome-fragment | NA | | 0 |
| S11_k141_299016 | 2991 | No | NA | 0 | 1 | Not-determined | Genome-fragment | NA | | 0 |
| S11_k141_217004 | 5728 | No | NA | 0 | 3 | Not-determined | Genome-fragment | NA | | 0 |
| S11-final-viral-combined_k141_95848\|\|full | 1239 | No | NA | 0 | 0 | Low-quality | Genome-fragment | | 2.31 | 0 |
| S11_k141_107189 | 3327 | No | NA | 0 | 1 | Not-determined | Genome-fragment | NA | | 0 |
| S11_k141_175792 | 2178 | No | NA | 0 | 1 | Not-determined | Genome-fragment | NA | | 0 |
| S17-final-viral-combined_k141_257896\|\|full | 1239 | No | NA | 0 | 1 | Low-quality | Genome-fragment | | 0.59 | 0 |
| S11_k141_37858 | 1140 | No | NA | 0 | 1 | Not-determined | Genome-fragment | NA | | 0 |
| S11_k141_202984 | 1905 | No | NA | 0 | 1 | Not-determined | Genome-fragment | NA | | 0 |
| S17-final-viral-combined_k141_163119\|\|full | 1239 | No | NA | 0 | 0 | Low-quality | Genome-fragment | | 0.89 | 0 |
| S11_k141_286045 | 1177 | No | NA | 0 | 0 | Not-determined | Genome-fragment | NA | | 0 |
| S11_k141_22797 | 1122 | No | NA | 0 | 1 | Not-determined | Genome-fragment | NA | | 0 |
| S11_k141_286065 | 1066 | No | NA | 0 | 0 | Not-determined | Genome-fragment | NA | | 0 |
| S11_k141_188942 | 2904 | No | NA | 0 | 0 | Not-determined | Genome-fragment | NA | | 0 |
| S11_k141_203053 | 1141 | No | NA | 0 | 1 | Not-determined | Genome-fragment | NA | | 0 |
| S11_k141_188974 | 1130 | No | NA | 0 | 1 | Not-determined | Genome-fragment | NA | | 0 |
| S11_k141_325956 | 2321 | No | NA | 0 | 0 | Not-determined | Genome-fragment | NA | | 0 |
| S12-final-viral-combined_k141_20393\|\|full | 1239 | No | NA | 0 | 1 | Low-quality | Genome-fragment | | 0.87 | 0 |
| S11_k141_325968 | 13756 | No | NA | 0 | 4 | Not-determined | Genome-fragment | NA | | 0 |
| S11_k141_217188 | 1483 | No | NA | 0 | 1 | Not-determined | Genome-fragment | NA | | 0 |
| S11_k141_258765 | 1086 | No | NA | 0 | 0 | Not-determined | Genome-fragment | NA | | 0 |
| S11_k141_299273 | 1141 | No | NA | 0 | 1 | Not-determined | Genome-fragment | NA | | 0 |
| S11_k141_245235 | 1687 | No | NA | 0 | 3 | Not-determined | Genome-fragment | NA | | 0 |
| S11_k141_325998 | 1041 | No | NA | 0 | 2 | Not-determined | Genome-fragment | NA | | 0 |
| S11_k141_119974 | 1245 | No | NA | 0 | 1 | Not-determined | Genome-fragment | NA | | 0 |
| S12_k141_287867 | 1239 | No | NA | 0 | 0 | Low-quality | Genome-fragment | | 2.2 | 0 |
| S2-final-viral-combined_k141_17602\|\|lt2gene | 1239 | No | NA | 2 | 0 | Low-quality | Genome-fragment | | 4.51 | 0 |
| S11_k141_189035 | 1041 | No | NA | 0 | 0 | Not-determined | Genome-fragment | NA | | 0 |
| S11_k141_22882 | 3084 | No | NA | 0 | 3 | Not-determined | Genome-fragment | NA | | 0 |
| S11_k141_10588 | 1409 | No | NA | 0 | 1 | Not-determined | Genome-fragment | NA | | 0 |
| S11_k141_78616 | 1378 | No | NA | 0 | 0 | Not-determined | Genome-fragment | NA | | 0 |
| S2_k141_157116 | 1239 | No | NA | 1 | 0 | Low-quality | Genome-fragment | | 4.2 | 0 |
| S11_k141_93056 | 1790 | No | NA | 0 | 0 | Not-determined | Genome-fragment | NA | | 0 |
| S4-final-viral-combined_k141_29409\|\|full | 1239 | No | NA | 1 | 0 | Low-quality | Genome-fragment | | 3.56 | 0 |
| S11_k141_52143 | 1047 | No | NA | 0 | 0 | Not-determined | Genome-fragment | NA | | 0 |
| S4_k141_149820 | 1239 | No | NA | 0 | 2 | Low-quality | Genome-fragment | | 2.08 | 0 |
| S8_k141_26796 | 1239 | No | NA | 0 | 0 | Low-quality | Genome-fragment | | 1.16 | 0 |
| S8_k141_10245 | 1239 | No | NA | 0 | 0 | Low-quality | Genome-fragment | | 1.26 | 0 |
| S11_k141_176121 | 1338 | No | NA | 0 | 2 | Not-determined | Genome-fragment | NA | | 0 |
| S11-final-viral-combined_k141_153026\|\|full | 1240 | No | NA | 0 | 0 | Low-quality | Genome-fragment | | 2.71 | 0 |
| S11_k141_162197 | 1150 | No | NA | 0 | 1 | Not-determined | Genome-fragment | NA | | 0 |
| S11_k141_286285 | 1267 | No | NA | 0 | 0 | Not-determined | Genome-fragment | NA | | 0 |
| S11-final-viral-combined_k141_268505\|\|full | 1240 | No | NA | 0 | 1 | Low-quality | Genome-fragment | | 0.73 | 0 |
| S11_k141_271886 | 1529 | No | NA | 0 | 1 | Not-determined | Genome-fragment | NA | | 0 |
| S11-final-viral-combined_k141_160226\|\|full | 1240 | No | NA | 1 | 0 | Low-quality | Genome-fragment | | 1.61 | 0 |
| S11_k141_299501 | 3074 | No | NA | 0 | 3 | Not-determined | Genome-fragment | NA | | 0 |
| S1-final-viral-combined_k141_33912\|\|full | 1240 | No | NA | 0 | 0 | Low-quality | Genome-fragment | | 0.98 | 0 |
| S3_k141_99966 | 1240 | No | NA | 0 | 0 | Low-quality | Genome-fragment | | 0.81 | 0 |
| S3_k141_8814 | 1240 | No | NA | 0 | 1 | Low-quality | Genome-fragment | | 0.87 | 0 |
| S5-final-viral-combined_k141_168657\|\|full | 1240 | No | NA | 4 | 0 | Low-quality | Genome-fragment | | 3.07 | 0 |
| S11_k141_107618 | 11448 | No | NA | 0 | 10 | Not-determined | Genome-fragment | NA | | 0 |
| S5-final-viral-combined_k141_204359\|\|full | 1240 | No | NA | 1 | 0 | Low-quality | Genome-fragment | | 2.87 | 0 |
| S11_k141_133535 | 3719 | No | NA | 0 | 1 | Not-determined | Genome-fragment | NA | | 0 |
| S11_k141_107672 | 1446 | No | NA | 0 | 1 | Not-determined | Genome-fragment | NA | | 0 |
| S11_k141_245533 | 1072 | No | NA | 0 | 1 | Not-determined | Genome-fragment | NA | | 0 |
| S11_k141_231187 | 1353 | No | NA | 0 | 2 | Not-determined | Genome-fragment | NA | | 0 |
| S5-final-viral-combined_k141_136011\|\|lt2gene | 1240 | No | NA | 2 | 0 | Low-quality | Genome-fragment | | 2.96 | 0 |
| S12-final-viral-combined_k141_153804\|\|full | 1240 | No | NA | 0 | 1 | Low-quality | Genome-fragment | | 2.84 | 0 |
| S11_k141_245620 | 2657 | No | NA | 0 | 2 | Not-determined | Genome-fragment | NA | | 0 |
| S11_k141_162416 | 1047 | No | NA | 0 | 0 | Not-determined | Genome-fragment | NA | | 0 |
| S11_k141_286497 | 5594 | No | NA | 0 | 3 | Not-determined | Genome-fragment | NA | | 0 |
| S12-final-viral-combined_k141_276585\|\|full | 1240 | No | NA | 0 | 2 | Low-quality | Genome-fragment | | 1.65 | 0 |
| S2-final-viral-combined_k141_148107\|\|full | 1240 | No | NA | 2 | 0 | Low-quality | Genome-fragment | | 3.07 | 0 |
| S11_k141_65988 | 1066 | No | NA | 0 | 0 | Not-determined | Genome-fragment | NA | | 0 |
| S11_k141_259105 | 1206 | No | NA | 0 | 2 | Not-determined | Genome-fragment | NA | | 0 |
| S2-final-viral-combined_k141_81580\|\|full | 1240 | No | NA | 0 | 1 | Low-quality | Genome-fragment | | 1.57 | 0 |
| S4-final-viral-combined_k141_159098\|\|full | 1240 | No | NA | 1 | 0 | Low-quality | Genome-fragment | | 2.96 | 0 |

| | | | | | | | | | | |
|---|---|---|---|---|---|---|---|---|---|---|
| S11_k141_176327 | 1857 | No | NA | 0 | 1 | Not-determined | Genome-fragment | NA | | 0 |
| S11_k141_272042 | 7974 | No | NA | 0 | 3 | Not-determined | Genome-fragment | NA | | 0 |
| S4_k141_35287 | 1240 | No | NA | 0 | 0 | Low-quality | Genome-fragment | | 1.24 | 0 |
| S8-final-viral-combined_k141_245197\|\|full | 1240 | No | NA | 0 | 0 | Low-quality | Genome-fragment | | 3.41 | 0 |
| S11_k141_38455 | 1202 | No | NA | 0 | 0 | Not-determined | Genome-fragment | NA | | 0 |
| S11_k141_217562 | 1315 | No | NA | 0 | 2 | Not-determined | Genome-fragment | NA | | 0 |
| S11_k141_78886 | 1145 | No | NA | 0 | 1 | Not-determined | Genome-fragment | NA | | 0 |
| S1-final-viral-combined_k141_321653\|\|full | 1241 | No | NA | 0 | 0 | Low-quality | Genome-fragment | | 3.35 | 0 |
| S11_k141_217596 | 1542 | No | NA | 0 | 0 | Not-determined | Genome-fragment | NA | | 0 |
| S1-final-viral-combined_k141_8498\|\|full | 1241 | No | NA | 0 | 0 | Low-quality | Genome-fragment | | 2.2 | 0 |
| S11_k141_272105 | 16657 | No | NA | 0 | 12 | Not-determined | Genome-fragment | NA | | 0 |
| S11_k141_93539 | 1030 | No | NA | 0 | 1 | Not-determined | Genome-fragment | NA | | 0 |
| S11_k141_10911 | 1074 | No | NA | 0 | 1 | Not-determined | Genome-fragment | NA | | 0 |
| S11_k141_189371 | 3670 | No | NA | 0 | 1 | Not-determined | Genome-fragment | NA | | 0 |
| S11_k141_38531 | 1353 | No | NA | 0 | 2 | Not-determined | Genome-fragment | NA | | 0 |
| S11_k141_299810 | 1469 | No | NA | 0 | 0 | Not-determined | Genome-fragment | NA | | 0 |
| S11_k141_38554 | 1443 | No | NA | 0 | 4 | Not-determined | Genome-fragment | NA | | 0 |
| S11_k141_162643 | 1000 | No | NA | 0 | 2 | Not-determined | Genome-fragment | NA | | 0 |
| S11_k141_79032 | 1006 | No | NA | 0 | 1 | Not-determined | Genome-fragment | NA | | 0 |
| S11_k141_299864 | 2172 | No | NA | 0 | 1 | Not-determined | Genome-fragment | NA | | 0 |
| S11_k141_259360 | 1401 | No | NA | 0 | 4 | Not-determined | Genome-fragment | NA | | 0 |
| S11_k141_176490 | 1571 | No | NA | 0 | 1 | Not-determined | Genome-fragment | NA | | 0 |
| S11_k141_120259 | 2207 | No | NA | 0 | 0 | Not-determined | Genome-fragment | NA | | 0 |
| S11_k141_133991 | 1098 | No | NA | 0 | 1 | Not-determined | Genome-fragment | NA | | 0 |
| S17-final-viral-combined_k141_119017\|\|full | 1241 | No | NA | 0 | 0 | Low-quality | Genome-fragment | | 0.82 | 0 |
| S11_k141_134004 | 1710 | No | NA | 0 | 1 | Not-determined | Genome-fragment | NA | | 0 |
| S17-final-viral-combined_k141_143004\|\|full | 1241 | No | NA | 1 | 0 | Low-quality | Genome-fragment | | 3.49 | 0 |
| S11_k141_286748 | 2022 | No | NA | 0 | 2 | Not-determined | Genome-fragment | NA | | 0 |
| S17-final-viral-combined_k141_109414\|\|full | 1241 | No | NA | 1 | 0 | Low-quality | Genome-fragment | | 2.63 | 0 |
| S11_k141_217700 | 1149 | No | NA | 0 | 0 | Not-determined | Genome-fragment | NA | | 0 |
| S11_k141_52760 | 1009 | No | NA | 0 | 1 | Not-determined | Genome-fragment | NA | | 0 |
| S11_k141_23477 | 2050 | No | NA | 0 | 1 | Not-determined | Genome-fragment | NA | | 0 |
| S12-final-viral-combined_k141_61405\|\|full | 1241 | No | NA | 2 | 0 | Low-quality | Genome-fragment | | 2.66 | 0 |
| S11_k141_79182 | 1244 | No | NA | 0 | 0 | Not-determined | Genome-fragment | NA | | 0 |
| S11_k141_176640 | 1149 | No | NA | 0 | 2 | Not-determined | Genome-fragment | NA | | 0 |
| S12-final-viral-combined_k141_212896\|\|full | 1241 | No | NA | 0 | 0 | Low-quality | Genome-fragment | | 3.07 | 0 |
| S11_k141_108102 | 3027 | No | NA | 0 | 1 | Not-determined | Genome-fragment | NA | | 0 |
| S12_k141_323563 | 1241 | No | NA | 0 | 1 | Low-quality | Genome-fragment | | 0.8 | 0 |
| S11_k141_11210 | 1254 | No | NA | 0 | 0 | Not-determined | Genome-fragment | NA | | 0 |
| S11_k141_134180 | 1829 | No | NA | 0 | 0 | Not-determined | Genome-fragment | NA | | 0 |
| S11_k141_203817 | 1266 | No | NA | 0 | 1 | Not-determined | Genome-fragment | NA | | 0 |
| S11_k141_326644 | 1001 | No | NA | 0 | 0 | Not-determined | Genome-fragment | NA | | 0 |
| S11_k141_326661 | 3827 | No | NA | 0 | 4 | Not-determined | Genome-fragment | NA | | 0 |
| S11_k141_120505 | 4952 | No | NA | 0 | 2 | Not-determined | Genome-fragment | NA | | 0 |
| S2_k141_99960 | 1241 | No | NA | 0 | 0 | Low-quality | Genome-fragment | | 2.99 | 0 |
| S11_k141_79316 | 1050 | No | NA | 0 | 0 | Not-determined | Genome-fragment | NA | | 0 |
| S11_k141_300179 | 1398 | No | NA | 0 | 1 | Not-determined | Genome-fragment | NA | | 0 |
| S11_k141_286979 | 5397 | No | NA | 0 | 2 | Not-determined | Genome-fragment | NA | | 0 |
| S11_k141_286984 | 1043 | No | NA | 0 | 0 | Not-determined | Genome-fragment | NA | | 0 |
| S11_k141_203924 | 1313 | No | NA | 0 | 0 | Not-determined | Genome-fragment | NA | | 0 |
| S11_k141_38940 | 1192 | No | NA | 0 | 0 | Not-determined | Genome-fragment | NA | | 0 |
| S11_k141_148902 | 1362 | No | NA | 0 | 1 | Not-determined | Genome-fragment | NA | | 0 |
| S11_k141_108236 | 2090 | No | NA | 0 | 0 | Not-determined | Genome-fragment | NA | | 0 |
| S11_k141_23607 | 1377 | No | NA | 0 | 1 | Not-determined | Genome-fragment | NA | | 0 |
| S11_k141_53001 | 1750 | No | NA | 0 | 1 | Not-determined | Genome-fragment | NA | | 0 |
| S11_k141_246159 | 1143 | No | NA | 0 | 1 | Not-determined | Genome-fragment | NA | | 0 |
| S11_k141_66478 | 1039 | No | NA | 0 | 0 | Not-determined | Genome-fragment | NA | | 0 |
| S11_k141_148956 | 1051 | No | NA | 0 | 0 | Not-determined | Genome-fragment | NA | | 0 |
| S2_k141_196633 | 1241 | No | NA | 0 | 0 | Low-quality | Genome-fragment | | 0.8 | 0 |
| S4-final-viral-combined_k141_143599\|\|full | 1241 | No | NA | 1 | 0 | Low-quality | Genome-fragment | | 2.94 | 0 |
| S11_k141_246210 | 1212 | No | NA | 0 | 0 | Not-determined | Genome-fragment | NA | | 0 |
| S11_k141_326784 | 4573 | No | NA | 0 | 2 | Not-determined | Genome-fragment | NA | | 0 |
| S8-final-viral-combined_k141_78024\|\|lt2gene | 1241 | No | NA | 2 | 0 | Low-quality | Genome-fragment | | 2.05 | 0 |
| S11_k141_319127 | 1242 | No | NA | 0 | 0 | Low-quality | Genome-fragment | | 2.13 | 0 |
| S1-final-viral-combined_k141_100712\|\|full | 1242 | No | NA | 0 | 0 | Low-quality | Genome-fragment | | 2.51 | 0 |
| S11_k141_53137 | 3481 | No | NA | 0 | 2 | Not-determined | Genome-fragment | NA | | 0 |
| S1-final-viral-combined_k141_102843\|\|full | 1242 | No | NA | 0 | 0 | Low-quality | Genome-fragment | | 5 | 0 |
| S3-final-viral-combined_k141_85250\|\|full | 1242 | No | NA | 0 | 0 | Low-quality | Genome-fragment | | 0.99 | 0 |
| S7-final-viral-combined_k141_120748\|\|full | 1242 | No | NA | 0 | 3 | Low-quality | Genome-fragment | | 0.98 | 0 |
| S11_k141_287146 | 2322 | No | NA | 0 | 2 | Not-determined | Genome-fragment | NA | | 0 |
| S7_k141_99173 | 1242 | No | NA | 0 | 1 | Low-quality | Genome-fragment | | 0.33 | 0 |
| S11_k141_108393 | 1420 | No | NA | 0 | 3 | Not-determined | Genome-fragment | NA | | 0 |
| S11_k141_120710 | 3682 | No | NA | 0 | 0 | Not-determined | Genome-fragment | NA | | 0 |
| S11_k141_23784 | 4481 | No | NA | 0 | 3 | Not-determined | Genome-fragment | NA | | 0 |
| S17-final-viral-combined_k141_202776\|\|full | 1242 | No | NA | 0 | 2 | Low-quality | Genome-fragment | | 0.86 | 0 |
| S11_k141_300384 | 2412 | No | NA | 0 | 1 | Not-determined | Genome-fragment | NA | | 0 |
| S11_k141_272738 | 1302 | No | NA | 0 | 1 | Not-determined | Genome-fragment | NA | | 0 |
| S11_k141_246377 | 1124 | No | NA | 0 | 0 | Not-determined | Genome-fragment | NA | | 0 |
| S11_k141_176949 | 5860 | No | NA | 0 | 3 | Not-determined | Genome-fragment | NA | | 0 |
| S17-final-viral-combined_k141_5417\|\|full | 1242 | No | NA | 0 | 0 | Low-quality | Genome-fragment | | 1.89 | 0 |
| S17-final-viral-combined_k141_66351\|\|lt2gene | 1242 | No | NA | 1 | 0 | Low-quality | Genome-fragment | | 2 | 0 |
| S11_k141_300440 | 1305 | No | NA | 0 | 1 | Not-determined | Genome-fragment | NA | | 0 |
| S11_k141_314514 | 1025 | No | NA | 0 | 0 | Not-determined | Genome-fragment | NA | | 0 |
| S11_k141_108469 | 1040 | No | NA | 0 | 1 | Not-determined | Genome-fragment | NA | | 0 |
| S11_k141_39149 | 2533 | No | NA | 0 | 0 | Not-determined | Genome-fragment | NA | | 0 |
| S11_k141_246471 | 1127 | No | NA | 0 | 1 | Not-determined | Genome-fragment | NA | | 0 |
| S11_k141_287287 | 1266 | No | NA | 0 | 1 | Not-determined | Genome-fragment | NA | | 0 |
| S11_k141_79665 | 1159 | No | NA | 0 | 1 | Not-determined | Genome-fragment | NA | | 0 |
| S11_k141_66793 | 1224 | No | NA | 0 | 1 | Not-determined | Genome-fragment | NA | | 0 |
| S11_k141_11514 | 2155 | No | NA | 0 | 1 | Not-determined | Genome-fragment | NA | | 0 |
| S17-k141_247546 | 1242 | No | NA | 0 | 0 | Low-quality | Genome-fragment | | 2.92 | 0 |
| S11_k141_79679 | 4049 | No | NA | 0 | 0 | Not-determined | Genome-fragment | NA | | 0 |
| S11_k141_11529 | 1012 | No | NA | 0 | 2 | Not-determined | Genome-fragment | NA | | 0 |
| S11_k141_177089 | 3450 | No | NA | 0 | 3 | Not-determined | Genome-fragment | NA | | 0 |
| S11_k141_246544 | 1906 | No | NA | 0 | 2 | Not-determined | Genome-fragment | NA | | 0 |
| S11_k141_79732 | 1279 | No | NA | 0 | 0 | Not-determined | Genome-fragment | NA | | 0 |
| S11_k141_108644 | 1397 | No | NA | 0 | 1 | Not-determined | Genome-fragment | NA | | 0 |
| S11_k141_53283 | 4331 | No | NA | 0 | 3 | Not-determined | Genome-fragment | NA | | 0 |
| S11_k141_120934 | 1735 | No | NA | 0 | 0 | Not-determined | Genome-fragment | NA | | 0 |
| S11_k141_246602 | 1147 | No | NA | 0 | 1 | Not-determined | Genome-fragment | NA | | 0 |
| S2-final-viral-combined_k141_192277\|\|full | 1242 | No | NA | 1 | 0 | Low-quality | Genome-fragment | | 2.53 | 0 |
| S4-final-viral-combined_k141_164207\|\|full | 1242 | No | NA | 0 | 0 | Low-quality | Genome-fragment | | 3.13 | 0 |
| S11_k141_218069 | 1480 | No | NA | 0 | 1 | Not-determined | Genome-fragment | NA | | 0 |
| S6-final-viral-combined_k141_129259\|\|full | 1242 | No | NA | 0 | 0 | Low-quality | Genome-fragment | | 0.53 | 0 |
| S11_k141_177145 | 1188 | No | NA | 0 | 2 | Not-determined | Genome-fragment | NA | | 0 |
| S11_k141_23973 | 1101 | No | NA | 0 | 1 | Not-determined | Genome-fragment | NA | | 0 |
| S11_k141_121000 | 2073 | No | NA | 0 | 3 | Not-determined | Genome-fragment | NA | | 0 |
| S8_k141_54615 | 1242 | No | NA | 1 | 0 | Low-quality | Genome-fragment | | 2.22 | 0 |
| S11_k141_246639 | 3850 | No | NA | 0 | 0 | Not-determined | Genome-fragment | NA | | 0 |
| S11_k141_24017 | 3174 | No | NA | 0 | 1 | Not-determined | Genome-fragment | NA | | 0 |
| S11_k141_177192 | 1158 | No | NA | 0 | 1 | Not-determined | Genome-fragment | NA | | 0 |
| S11_k141_260030 | 1184 | No | NA | 0 | 1 | Not-determined | Genome-fragment | NA | | 0 |
| S11_k141_246666 | 1248 | No | NA | 0 | 0 | Not-determined | Genome-fragment | NA | | 0 |
| S11_k141_121021 | 1060 | No | NA | 0 | 1 | Not-determined | Genome-fragment | NA | | 0 |
| S11_k141_121026 | 1159 | No | NA | 0 | 0 | Not-determined | Genome-fragment | NA | | 0 |
| S11_k141_177243 | 1947 | No | NA | 0 | 1 | Not-determined | Genome-fragment | NA | | 0 |
| S11_k141_108786 | 3065 | No | NA | 0 | 3 | Not-determined | Genome-fragment | NA | | 0 |
| S11_k141_190050 | 13936 | No | NA | 0 | 4 | Not-determined | Genome-fragment | NA | | 0 |
| S11_k141_314693 | 1348 | No | NA | 0 | 0 | Not-determined | Genome-fragment | NA | | 0 |
| S11_k141_11780 | 1830 | No | NA | 0 | 0 | Not-determined | Genome-fragment | NA | | 0 |
| S11_k141_11789 | 1537 | No | NA | 0 | 0 | Not-determined | Genome-fragment | NA | | 0 |
| S9-final-viral-combined_k141_26848\|\|full | 1243 | No | NA | 0 | 0 | Low-quality | Genome-fragment | | 1.75 | 0 |
| S11_k141_108832 | 2630 | No | NA | 0 | 3 | Not-determined | Genome-fragment | NA | | 0 |
| S9_k141_12534 | 1243 | No | NA | 0 | 1 | Low-quality | Genome-fragment | | 0.3 | 0 |
| S11-final-viral-combined_k141_84223\|\|full | 1243 | No | NA | 0 | 0 | Low-quality | Genome-fragment | | 2.59 | 0 |
| S11_k141_94148 | 1394 | No | NA | 0 | 1 | Not-determined | Genome-fragment | NA | | 0 |
| S11_k141_134846 | 1074 | No | NA | 0 | 1 | Not-determined | Genome-fragment | NA | | 0 |
| S11_k141_163577 | 1006 | No | NA | 0 | 1 | Not-determined | Genome-fragment | NA | | 0 |
| S11_k141_79996 | 2385 | No | NA | 0 | 1 | Not-determined | Genome-fragment | NA | | 0 |
| S11_k141_24255 | 4156 | No | NA | 0 | 1 | Not-determined | Genome-fragment | NA | | 0 |
| S11_k141_134883 | 3690 | No | NA | 0 | 1 | Not-determined | Genome-fragment | NA | | 0 |
| S11-final-viral-combined_k141_274560\|\|full | 1243 | No | NA | 2 | 0 | Low-quality | Genome-fragment | | 2.81 | 0 |
| S11_k141_306871 | 1243 | No | NA | 0 | 1 | Low-quality | Genome-fragment | | 0.59 | 0 |
| S11_k141_260222 | 1331 | No | NA | 0 | 1 | Not-determined | Genome-fragment | NA | | 0 |
| S11_k141_80060 | 1126 | No | NA | 0 | 0 | Not-determined | Genome-fragment | NA | | 0 |
| S11_k141_287630 | 11510 | No | NA | 0 | 2 | Not-determined | Genome-fragment | NA | | 0 |
| S11_k141_39648 | 1580 | No | NA | 0 | 1 | Not-determined | Genome-fragment | NA | | 0 |
| S1_k141_313080 | 1243 | No | NA | 0 | 0 | Low-quality | Genome-fragment | | 1.19 | 0 |
| S11_k141_53654 | 3340 | No | NA | 0 | 0 | Not-determined | Genome-fragment | NA | | 0 |
| S5-final-viral-combined_k141_56285\|\|full | 1243 | No | NA | 0 | 0 | Low-quality | Genome-fragment | | 1.86 | 0 |
| S11_k141_134977 | 5032 | No | NA | 0 | 1 | Not-determined | Genome-fragment | NA | | 0 |
| S11_k141_163684 | 9784 | No | NA | 0 | 3 | Not-determined | Genome-fragment | NA | | 0 |
| S7-final-viral-combined_k141_243892\|\|full | 1243 | No | NA | 0 | 0 | Low-quality | Genome-fragment | | 1.69 | 0 |
| S11_k141_135007 | 4722 | No | NA | 0 | 3 | Not-determined | Genome-fragment | NA | | 0 |
| S11_k141_135014 | 1216 | No | NA | 0 | 0 | Not-determined | Genome-fragment | NA | | 0 |
| S11_k141_327436 | 1029 | No | NA | 0 | 0 | Not-determined | Genome-fragment | NA | | 0 |
| S11_k141_260322 | 1221 | No | NA | 0 | 1 | Not-determined | Genome-fragment | NA | | 0 |
| S11_k141_260356 | 1331 | No | NA | 0 | 2 | Not-determined | Genome-fragment | NA | | 0 |
| S11_k141_24458 | 3620 | No | NA | 0 | 2 | Not-determined | Genome-fragment | NA | | 0 |
| S11_k141_39777 | 1056 | No | NA | 0 | 1 | Not-determined | Genome-fragment | NA | | 0 |
| S11_k141_314959 | 1097 | No | NA | 0 | 0 | Not-determined | Genome-fragment | NA | | 0 |
| S11_k141_39828 | 1090 | No | NA | 0 | 0 | Not-determined | Genome-fragment | NA | | 0 |
| S11_k141_327547 | 1045 | No | NA | 0 | 1 | Not-determined | Genome-fragment | NA | | 0 |
| S11_k141_327564 | 1469 | No | NA | 0 | 0 | Not-determined | Genome-fragment | NA | | 0 |
| S7_k141_57380 | 1243 | No | NA | 0 | 1 | Low-quality | Genome-fragment | | 2.28 | 0 |
| S17-final-viral-combined_k141_248207\|\|full | 1243 | No | NA | 0 | 1 | Low-quality | Genome-fragment | | 1.83 | 0 |
| S11_k141_190467 | 1585 | No | NA | 0 | 1 | Not-determined | Genome-fragment | NA | | 0 |
| S17-final-viral-combined_k141_195811\|\|full | 1243 | No | NA | 0 | 0 | Low-quality | Genome-fragment | | 0.74 | 0 |
| S11_k141_232619 | 1689 | No | NA | 0 | 2 | Not-determined | Genome-fragment | NA | | 0 |
| S11_k141_301005 | 1225 | No | NA | 0 | 0 | Not-determined | Genome-fragment | NA | | 0 |
| S17-final-viral-combined_k141_85517\|\|full | 1243 | No | NA | 0 | 0 | Low-quality | Genome-fragment | | 1.74 | 0 |
| S11_k141_121560 | 1625 | No | NA | 0 | 2 | Not-determined | Genome-fragment | NA | | 0 |
| S11_k141_24622 | 1336 | No | NA | 0 | 2 | Not-determined | Genome-fragment | NA | | 0 |
| S11_k141_53862 | 1191 | No | NA | 0 | 0 | Not-determined | Genome-fragment | NA | | 0 |
| S12-final-viral-combined_k141_126075\|\|lt2gene | 1243 | No | NA | 1 | 0 | Low-quality | Genome-fragment | | 2.45 | 0 |
| S11_k141_149896 | 7229 | No | NA | 0 | 2 | Not-determined | Genome-fragment | NA | | 0 |
| S11_k141_204594 | 4009 | No | NA | 0 | 2 | Not-determined | Genome-fragment | NA | | 0 |
| S11_k141_164040 | 4136 | No | NA | 0 | 7 | Not-determined | Genome-fragment | NA | | 0 |
| S2_k141_146210 | 1243 | No | NA | 0 | 0 | Low-quality | Genome-fragment | | 2.11 | 0 |
| S8-final-viral-combined_k141_173973\|\|full | 1243 | No | NA | 0 | 0 | Low-quality | Genome-fragment | | 0.81 | 0 |
| S9-final-viral-combined_k141_36470\|\|full | 1244 | No | NA | 0 | 0 | Low-quality | Genome-fragment | | 0.8 | 0 |
| S11_k141_190581 | 2082 | No | NA | 0 | 1 | Not-determined | Genome-fragment | NA | | 0 |
| S11_k141_260516 | 1175 | No | NA | 0 | 0 | Not-determined | Genome-fragment | NA | | 0 |
| S11-final-viral-combined_k141_136643\|\|full | 1244 | No | NA | 0 | 2 | Low-quality | Genome-fragment | | 0.91 | 0 |

| | | | | | | | | | | |
|---|---|---|---|---|---|---|---|---|---|---|
| S1-final-viral-combined_k141_304828\|\|full | 1244 | No | NA | 0 | 0 | Low-quality | Genome-fragment | | 2.15 | 0 |
| S11_k141_327708 | 3343 | No | NA | 0 | 2 | Not-determined | Genome-fragment | NA | | 0 |
| S1-final-viral-combined_k141_178208\|\|full | 1244 | No | NA | 0 | 0 | Low-quality | Genome-fragment | | 2.98 | 0 |
| S11_k141_12231 | 1540 | No | NA | 0 | 1 | Not-determined | Genome-fragment | NA | | 0 |
| S11_k141_121674 | 1372 | No | NA | 0 | 1 | Not-determined | Genome-fragment | NA | | 0 |
| S1_k141_363294 | 1244 | No | NA | 0 | 0 | Low-quality | Genome-fragment | | 1.42 | 0 |
| S1_k141_209013 | 1244 | No | NA | 0 | 0 | Low-quality | Genome-fragment | | 3.37 | 0 |
| S11_k141_40114 | 3697 | No | NA | 0 | 2 | Not-determined | Genome-fragment | NA | | 0 |
| S11_k141_327748 | 1510 | No | NA | 0 | 0 | Not-determined | Genome-fragment | NA | | 0 |
| S11_k141_260610 | 1142 | No | NA | 0 | 0 | Not-determined | Genome-fragment | NA | | 0 |
| S11_k141_24709 | 2224 | No | NA | 0 | 1 | Not-determined | Genome-fragment | NA | | 0 |
| S11_k141_24733 | 1574 | No | NA | 0 | 2 | Not-determined | Genome-fragment | NA | | 0 |
| S11_k141_67487 | 2666 | No | NA | 0 | 2 | Not-determined | Genome-fragment | NA | | 0 |
| S11_k141_327800 | 2100 | No | NA | 0 | 1 | Not-determined | Genome-fragment | NA | | 0 |
| S11_k141_218783 | 4240 | No | NA | 0 | 3 | Not-determined | Genome-fragment | NA | | 0 |
| S11_k141_260706 | 1194 | No | NA | 0 | 0 | Not-determined | Genome-fragment | NA | | 0 |
| S11_k141_24801 | 1167 | No | NA | 0 | 2 | Not-determined | Genome-fragment | NA | | 0 |
| S11_k141_260725 | 1408 | No | NA | 0 | 1 | Not-determined | Genome-fragment | NA | | 0 |
| S11_k141_67558 | 1462 | No | NA | 0 | 1 | Not-determined | Genome-fragment | NA | | 0 |
| S11_k141_12432 | 1530 | No | NA | 0 | 0 | Not-determined | Genome-fragment | NA | | 0 |
| S11_k141_53993 | 1065 | No | NA | 0 | 2 | Not-determined | Genome-fragment | NA | | 0 |
| S11_k141_260756 | 1044 | No | NA | 0 | 0 | Not-determined | Genome-fragment | NA | | 0 |
| S11_k141_247363 | 1125 | No | NA | 0 | 1 | Not-determined | Genome-fragment | NA | | 0 |
| S5-final-viral-combined_k141_94576\|\|full | 1244 | No | NA | 1 | 0 | Low-quality | Genome-fragment | | 0.85 | 0 |
| S11_k141_54105 | 1209 | No | NA | 0 | 0 | Not-determined | Genome-fragment | NA | | 0 |
| S7-final-viral-combined_k141_189787\|\|full | 1244 | No | NA | 0 | 0 | Low-quality | Genome-fragment | | 2.44 | 0 |
| S11_k141_109518 | 2607 | No | NA | 0 | 1 | Not-determined | Genome-fragment | NA | | 0 |
| S11_k141_54123 | 1616 | No | NA | 0 | 0 | Not-determined | Genome-fragment | NA | | 0 |
| S11_k141_327862 | 1456 | No | NA | 0 | 1 | Not-determined | Genome-fragment | NA | | 0 |
| S11_k141_301185 | 1244 | No | NA | 0 | 2 | Not-determined | Genome-fragment | NA | | 0 |
| S11_k141_178105 | 1791 | No | NA | 0 | 2 | Not-determined | Genome-fragment | NA | | 0 |
| S11_k141_150292 | 1531 | No | NA | 0 | 0 | Not-determined | Genome-fragment | NA | | 0 |
| S11_k141_273733 | 1161 | No | NA | 0 | 0 | Not-determined | Genome-fragment | NA | | 0 |
| S7_k141_166795 | 1244 | No | NA | 0 | 0 | Low-quality | Genome-fragment | | 1.55 | 0 |
| S17-final-viral-combined_k141_253647\|\|full | 1244 | No | NA | 1 | 0 | Low-quality | Genome-fragment | | 2.45 | 0 |
| S11_k141_24911 | 1272 | No | NA | 0 | 0 | Not-determined | Genome-fragment | NA | | 0 |
| S11_k141_12603 | 1988 | No | NA | 0 | 2 | Not-determined | Genome-fragment | NA | | 0 |
| S11_k141_233179 | 12033 | No | NA | 0 | 4 | Not-determined | Genome-fragment | NA | | 0 |
| S17-k141_254861 | 1244 | No | NA | 0 | 0 | Low-quality | Genome-fragment | | 1.74 | 0 |
| S12-final-viral-combined_k141_387081\|\|full | 1244 | No | NA | 0 | 0 | Low-quality | Genome-fragment | | 2.34 | 0 |
| S11_k141_288352 | 1180 | No | NA | 0 | 2 | Not-determined | Genome-fragment | NA | | 0 |
| S11_k141_191030 | 1830 | No | NA | 0 | 2 | Not-determined | Genome-fragment | NA | | 0 |
| S11_k141_288369 | 1710 | No | NA | 0 | 1 | Not-determined | Genome-fragment | NA | | 0 |
| S11_k141_12639 | 1211 | No | NA | 0 | 1 | Not-determined | Genome-fragment | NA | | 0 |
| S12-final-viral-combined_k141_243985\|\|full | 1244 | No | NA | 0 | 0 | Low-quality | Genome-fragment | | 0.89 | 0 |
| S11_k141_109691 | 1486 | No | NA | 0 | 1 | Not-determined | Genome-fragment | NA | | 0 |
| S11_k141_164316 | 1740 | No | NA | 0 | 0 | Not-determined | Genome-fragment | NA | | 0 |
| S11_k141_261021 | 2870 | No | NA | 0 | 1 | Not-determined | Genome-fragment | NA | | 0 |
| S11_k141_12683 | 3017 | No | NA | 0 | 2 | Not-determined | Genome-fragment | NA | | 0 |
| S11_k141_67891 | 3072 | No | NA | 0 | 3 | Not-determined | Genome-fragment | NA | | 0 |
| S11_k141_219045 | 4213 | No | NA | 0 | 2 | Not-determined | Genome-fragment | NA | | 0 |
| S11_k141_67909 | 1198 | No | NA | 0 | 1 | Not-determined | Genome-fragment | NA | | 0 |
| S11_k141_233239 | 1442 | No | NA | 0 | 0 | Not-determined | Genome-fragment | NA | | 0 |
| S11_k141_328028 | 3809 | No | NA | 0 | 0 | Not-determined | Genome-fragment | NA | | 0 |
| S12-final-viral-combined_k141_69093\|\|lt2gene | 1244 | No | NA | 1 | 0 | Low-quality | Genome-fragment | | 2.68 | 0 |
| S11_k141_164413 | 1272 | No | NA | 0 | 3 | Not-determined | Genome-fragment | NA | | 0 |
| S11_k141_40617 | 1109 | No | NA | 0 | 1 | Not-determined | Genome-fragment | NA | | 0 |
| S11_k141_301394 | 2043 | No | NA | 0 | 2 | Not-determined | Genome-fragment | NA | | 0 |
| S11_k141_205109 | 1162 | No | NA | 0 | 2 | Not-determined | Genome-fragment | NA | | 0 |
| S11_k141_191150 | 1255 | No | NA | 0 | 0 | Not-determined | Genome-fragment | NA | | 0 |
| S12_k141_90111 | 1244 | No | NA | 1 | 0 | Low-quality | Genome-fragment | | 3.22 | 0 |
| S11_k141_150562 | 2144 | No | NA | 0 | 1 | Not-determined | Genome-fragment | NA | | 0 |
| S11_k141_178374 | 1812 | No | NA | 0 | 0 | Not-determined | Genome-fragment | NA | | 0 |
| S11_k141_233331 | 9311 | No | NA | 0 | 18 | Not-determined | Genome-fragment | NA | | 0 |
| S2-final-viral-combined_k141_7737\|\|full | 1244 | No | NA | 2 | 0 | Low-quality | Genome-fragment | | 2.8 | 0 |
| S11_k141_164441 | 1094 | No | NA | 0 | 1 | Not-determined | Genome-fragment | NA | | 0 |
| S4_k141_29431 | 1244 | No | NA | 1 | 0 | Low-quality | Genome-fragment | | 3.57 | 0 |
| S11_k141_135922 | 1593 | No | NA | 0 | 0 | Not-determined | Genome-fragment | NA | | 0 |
| S6-final-viral-combined_k141_85567\|\|full | 1244 | No | NA | 0 | 1 | Low-quality | Genome-fragment | | 2.49 | 0 |
| S11_k141_95138 | 3834 | No | NA | 0 | 2 | Not-determined | Genome-fragment | NA | | 0 |
| S11_k141_178425 | 1872 | No | NA | 0 | 0 | Not-determined | Genome-fragment | NA | | 0 |
| S8-final-viral-combined_k141_13154\|\|full | 1244 | No | NA | 0 | 0 | Low-quality | Genome-fragment | | 1.76 | 0 |
| S11_k141_68095 | 1512 | No | NA | 0 | 0 | Not-determined | Genome-fragment | NA | | 0 |
| S8-final-viral-combined_k141_108645\|\|full | 1244 | No | NA | 0 | 0 | Low-quality | Genome-fragment | | 0.78 | 0 |
| S9_k141_23515 | 1245 | No | NA | 0 | 0 | Low-quality | Genome-fragment | | 2.98 | 0 |
| S11-final-viral-combined_k141_278457\|\|full | 1245 | No | NA | 0 | 1 | Low-quality | Genome-fragment | | 1.54 | 0 |
| S1-final-viral-combined_k141_194042\|\|full | 1245 | No | NA | 0 | 1 | Low-quality | Genome-fragment | | 7.48 | 0 |
| S11_k141_288655 | 1776 | No | NA | 0 | 1 | Not-determined | Genome-fragment | NA | | 0 |
| S11_k141_68181 | 1268 | No | NA | 0 | 1 | Not-determined | Genome-fragment | NA | | 0 |
| S11_k141_150759 | 2653 | No | NA | 0 | 1 | Not-determined | Genome-fragment | NA | | 0 |
| S11_k141_219251 | 1074 | No | NA | 0 | 1 | Not-determined | Genome-fragment | NA | | 0 |
| S11_k141_261243 | 3031 | No | NA | 0 | 0 | Not-determined | Genome-fragment | NA | | 0 |
| S11_k141_164606 | 1393 | No | NA | 0 | 1 | Not-determined | Genome-fragment | NA | | 0 |
| S11_k141_40904 | 1913 | No | NA | 0 | 1 | Not-determined | Genome-fragment | NA | | 0 |
| S1-final-viral-combined_k141_376156\|\|full | 1245 | No | NA | 0 | 0 | Low-quality | Genome-fragment | | 0.86 | 0 |
| S11_k141_274188 | 1892 | No | NA | 0 | 1 | Not-determined | Genome-fragment | NA | | 0 |
| S1-final-viral-combined_k141_15883\|\|lt2gene | 1245 | No | NA | 2 | 0 | Low-quality | Genome-fragment | | 3.16 | 0 |
| S11_k141_315827 | 1368 | No | NA | 0 | 1 | Not-determined | Genome-fragment | NA | | 0 |
| S1_k141_306582 | 1245 | No | NA | 0 | 2 | Low-quality | Genome-fragment | | 0.58 | 0 |
| S11_k141_233587 | 1308 | No | NA | 0 | 0 | Not-determined | Genome-fragment | NA | | 0 |
| S17-final-viral-combined_k141_203812\|\|full | 1245 | No | NA | 0 | 0 | Low-quality | Genome-fragment | | 2.08 | 0 |
| S17-final-viral-combined_k141_38866\|\|lt2gene | 1245 | No | NA | 1 | 0 | Low-quality | Genome-fragment | | 3.66 | 0 |
| S17-k141_237720 | 1245 | No | NA | 0 | 0 | Low-quality | Genome-fragment | | 1.59 | 0 |
| S11_k141_80997 | 15746 | No | NA | 0 | 6 | Not-determined | Genome-fragment | NA | | 0 |
| S11_k141_54715 | 1880 | No | NA | 0 | 1 | Not-determined | Genome-fragment | NA | | 0 |
| S17-k141_108394 | 1245 | No | NA | 0 | 0 | Low-quality | Genome-fragment | | 1.87 | 0 |
| S11_k141_54756 | 1589 | No | NA | 0 | 1 | Not-determined | Genome-fragment | NA | | 0 |
| S11_k141_288772 | 1642 | No | NA | 0 | 0 | Not-determined | Genome-fragment | NA | | 0 |
| S11_k141_122402 | 7036 | No | NA | 0 | 2 | Not-determined | Genome-fragment | NA | | 0 |
| S12-final-viral-combined_k141_335813\|\|full | 1245 | No | NA | 0 | 0 | Low-quality | Genome-fragment | | 1.71 | 0 |
| S11_k141_150932 | 2684 | No | NA | 0 | 1 | Not-determined | Genome-fragment | NA | | 0 |
| S12-final-viral-combined_k141_379982\|\|full | 1245 | No | NA | 0 | 0 | Low-quality | Genome-fragment | | 2.48 | 0 |
| S11_k141_191526 | 1052 | No | NA | 0 | 1 | Not-determined | Genome-fragment | NA | | 0 |
| S11_k141_95486 | 1338 | No | NA | 0 | 1 | Not-determined | Genome-fragment | NA | | 0 |
| S12_k141_316090 | 1245 | No | NA | 1 | 0 | Low-quality | Genome-fragment | | 2.96 | 0 |
| S11_k141_13259 | 1908 | No | NA | 0 | 1 | Not-determined | Genome-fragment | NA | | 0 |
| S11_k141_95499 | 1502 | No | NA | 0 | 0 | Not-determined | Genome-fragment | NA | | 0 |
| S11_k141_164833 | 4605 | No | NA | 0 | 4 | Not-determined | Genome-fragment | NA | | 0 |
| S11_k141_191560 | 3972 | No | NA | 0 | 2 | Not-determined | Genome-fragment | NA | | 0 |
| S11_k141_191569 | 1207 | No | NA | 0 | 0 | Not-determined | Genome-fragment | NA | | 0 |
| S2_k141_240119 | 1245 | No | NA | 0 | 0 | Low-quality | Genome-fragment | | 2.44 | 0 |
| S6-final-viral-combined_k141_3558\|\|full | 1245 | No | NA | 0 | 2 | Low-quality | Genome-fragment | | 1.52 | 0 |
| S11_k141_136316 | 1132 | No | NA | 0 | 2 | Not-determined | Genome-fragment | NA | | 0 |
| S11_k141_122517 | 1872 | No | NA | 0 | 2 | Not-determined | Genome-fragment | NA | | 0 |
| S9-final-viral-combined_k141_70474\|\|full | 1246 | No | NA | 0 | 0 | Low-quality | Genome-fragment | | 2.08 | 0 |
| S11_k141_54883 | 1018 | No | NA | 0 | 0 | Not-determined | Genome-fragment | NA | | 0 |
| S11_k141_205476 | 1159 | No | NA | 0 | 0 | Not-determined | Genome-fragment | NA | | 0 |
| S11-final-viral-combined_k141_97491\|\|full | 1246 | No | NA | 0 | 0 | Low-quality | Genome-fragment | | 2.57 | 0 |
| S11_k141_54900 | 3683 | No | NA | 0 | 3 | Not-determined | Genome-fragment | NA | | 0 |
| S11_k141_81187 | 1003 | No | NA | 0 | 0 | Not-determined | Genome-fragment | NA | | 0 |
| S11_k141_191712 | 1724 | No | NA | 0 | 0 | Not-determined | Genome-fragment | NA | | 0 |
| S11_k141_288977 | 22884 | No | NA | 0 | 4 | Not-determined | Genome-fragment | NA | | 0 |
| S1-final-viral-combined_k141_193460\|\|full | 1246 | No | NA | 2 | 0 | Low-quality | Genome-fragment | | 2.92 | 0 |
| S11_k141_41200 | 1700 | No | NA | 0 | 0 | Not-determined | Genome-fragment | NA | | 0 |
| S11_k141_191816 | 1261 | No | NA | 0 | 1 | Not-determined | Genome-fragment | NA | | 0 |
| S1-final-viral-combined_k141_150924\|\|lt2gene | 1246 | No | NA | 1 | 0 | Low-quality | Genome-fragment | | 2.86 | 0 |
| S1_k141_131115 | 1246 | No | NA | 0 | 0 | Low-quality | Genome-fragment | | 1.01 | 0 |
| S11_k141_178902 | 3006 | No | NA | 0 | 2 | Not-determined | Genome-fragment | NA | | 0 |
| S3-final-viral-combined_k141_39415\|\|lt2gene | 1246 | No | NA | 1 | 0 | Low-quality | Genome-fragment | | 2.52 | 0 |
| S11_k141_178908 | 1006 | No | NA | 0 | 0 | Not-determined | Genome-fragment | NA | | 0 |
| S3_k141_127769 | 1246 | No | NA | 0 | 1 | Low-quality | Genome-fragment | | 0.62 | 0 |
| S11_k141_234077 | 2675 | No | NA | 0 | 1 | Not-determined | Genome-fragment | NA | | 0 |
| S11_k141_41317 | 1232 | No | NA | 0 | 1 | Not-determined | Genome-fragment | NA | | 0 |
| S11_k141_136549 | 1032 | No | NA | 0 | 1 | Not-determined | Genome-fragment | NA | | 0 |
| S11_k141_328624 | 3795 | No | NA | 0 | 5 | Not-determined | Genome-fragment | NA | | 0 |
| S11_k141_316247 | 1046 | No | NA | 0 | 1 | Not-determined | Genome-fragment | NA | | 0 |
| S5-final-viral-combined_k141_4633\|\|full | 1246 | No | NA | 3 | 0 | Low-quality | Genome-fragment | | 3.11 | 0 |
| S12-final-viral-combined_k141_351680\|\|full | 1246 | No | NA | 0 | 1 | Low-quality | Genome-fragment | | 0.57 | 0 |
| S11_k141_13630 | 1089 | No | NA | 0 | 0 | Not-determined | Genome-fragment | NA | | 0 |
| S12-final-viral-combined_k141_303671\|\|full | 1246 | No | NA | 0 | 0 | Low-quality | Genome-fragment | | 2.72 | 0 |
| S8-final-viral-combined_k141_68815\|\|full | 1246 | No | NA | 1 | 0 | Low-quality | Genome-fragment | | 2.18 | 0 |
| S11_k141_165277 | 1492 | No | NA | 0 | 2 | Not-determined | Genome-fragment | NA | | 0 |
| S9-final-viral-combined_k141_35256\|\|full | 1247 | No | NA | 0 | 1 | Low-quality | Genome-fragment | | 1.69 | 0 |
| S11-final-viral-combined_k141_250804\|\|full | 1247 | No | NA | 0 | 0 | Low-quality | Genome-fragment | | 1.92 | 0 |
| S11_k141_316317 | 1122 | No | NA | 0 | 1 | Not-determined | Genome-fragment | NA | | 0 |
| S11_k141_165319 | 1119 | No | NA | 0 | 2 | Not-determined | Genome-fragment | NA | | 0 |
| S11_k141_151398 | 1119 | No | NA | 0 | 0 | Not-determined | Genome-fragment | NA | | 0 |
| S11_k141_26046 | 1232 | No | NA | 0 | 1 | Not-determined | Genome-fragment | NA | | 0 |
| S11_k141_316359 | 7547 | No | NA | 0 | 4 | Not-determined | Genome-fragment | NA | | 0 |
| S11_k141_289343 | 3212 | No | NA | 0 | 1 | Not-determined | Genome-fragment | NA | | 0 |
| S11_k141_289388 | 1515 | No | NA | 0 | 0 | Not-determined | Genome-fragment | NA | | 0 |
| S11_k141_274853 | 2575 | No | NA | 0 | 0 | Not-determined | Genome-fragment | NA | | 0 |
| S11_k141_96102 | 5004 | No | NA | 0 | 0 | Not-determined | Genome-fragment | NA | | 0 |
| S11-final-viral-combined_k141_38922\|\|full | 1247 | No | NA | 0 | 0 | Low-quality | Genome-fragment | | 3.02 | 0 |
| S11_k141_68780 | 5674 | No | NA | 0 | 0 | Not-determined | Genome-fragment | NA | | 0 |
| S11_k141_26243 | 1265 | No | NA | 0 | 0 | Not-determined | Genome-fragment | NA | | 0 |
| S11_k141_302544 | 3691 | No | NA | 0 | 2 | Not-determined | Genome-fragment | NA | | 0 |
| S11_k141_81762 | 1104 | No | NA | 0 | 0 | Not-determined | Genome-fragment | NA | | 0 |
| S11_k141_192129 | 2969 | No | NA | 0 | 1 | Not-determined | Genome-fragment | NA | | 0 |
| S11_k141_136900 | 1266 | No | NA | 0 | 1 | Not-determined | Genome-fragment | NA | | 0 |
| S11_k141_81783 | 1101 | No | NA | 0 | 0 | Not-determined | Genome-fragment | NA | | 0 |
| S11_k141_220170 | 1674 | No | NA | 0 | 2 | Not-determined | Genome-fragment | NA | | 0 |
| S11_k141_206083 | 1216 | No | NA | 0 | 1 | Not-determined | Genome-fragment | NA | | 0 |
| S1_k141_309870 | 1247 | No | NA | 0 | 1 | Low-quality | Genome-fragment | | 0.7 | 0 |
| S11_k141_26312 | 1051 | No | NA | 0 | 3 | Not-determined | Genome-fragment | NA | | 0 |
| S11_k141_26327 | 1035 | No | NA | 0 | 0 | Not-determined | Genome-fragment | NA | | 0 |
| S11_k141_328875 | 3328 | No | NA | 0 | 1 | Not-determined | Genome-fragment | NA | | 0 |
| S11_k141_151552 | 1034 | No | NA | 0 | 1 | Not-determined | Genome-fragment | NA | | 0 |
| S11_k141_192272 | 3356 | No | NA | 0 | 1 | Not-determined | Genome-fragment | NA | | 0 |
| S11_k141_328921 | 1236 | No | NA | 0 | 0 | Not-determined | Genome-fragment | NA | | 0 |
| S3_k141_66996 | 1247 | No | NA | 1 | 0 | Low-quality | Genome-fragment | | 3.43 | 0 |
| S11_k141_137054 | 1409 | No | NA | 0 | 1 | Not-determined | Genome-fragment | NA | | 0 |
| S11_k141_206131 | 1349 | No | NA | 0 | 0 | Not-determined | Genome-fragment | NA | | 0 |
| S11_k141_302808 | 6197 | No | NA | 0 | 4 | Not-determined | Genome-fragment | NA | | 0 |

| | | | | | | | | | | |
|---|---|---|---|---|---|---|---|---|---|---|
| S11_k141_137136 | 8446 | No | NA | 0 | 4 | Not-determined | Genome-fragment | NA | | 0 |
| S11_k141_123428 | 1129 | No | NA | 0 | 0 | Not-determined | Genome-fragment | NA | | 0 |
| S5-final-viral-combined_k141_136927\|\|full | 1247 | No | NA | 0 | 1 | Low-quality | Genome-fragment | | 1.5 | 0 |
| S5-final-viral-combined_k141_67965\|\|full | 1247 | No | NA | 1 | 0 | Low-quality | Genome-fragment | | 2.33 | 0 |
| S11_k141_316632 | 3745 | No | NA | 0 | 3 | Not-determined | Genome-fragment | NA | | 0 |
| S11_k141_328990 | 1019 | No | NA | 0 | 0 | Not-determined | Genome-fragment | NA | | 0 |
| S11_k141_192429 | 1355 | No | NA | 0 | 2 | Not-determined | Genome-fragment | NA | | 0 |
| S11_k141_68900 | 1105 | No | NA | 0 | 0 | Not-determined | Genome-fragment | NA | | 0 |
| S11_k141_26594 | 4478 | No | NA | 0 | 2 | Not-determined | Genome-fragment | NA | | 0 |
| S11_k141_137281 | 1273 | No | NA | 0 | 0 | Not-determined | Genome-fragment | NA | | 0 |
| S11_k141_302950 | 1000 | No | NA | 0 | 0 | Not-determined | Genome-fragment | NA | | 0 |
| S11_k141_220436 | 2012 | No | NA | 0 | 1 | Not-determined | Genome-fragment | NA | | 0 |
| S5-final-viral-combined_k141_185175\|\|full | 1247 | No | NA | 0 | 2 | Low-quality | Genome-fragment | | 2.79 | 0 |
| S11_k141_137441 | 1570 | No | NA | 0 | 0 | Not-determined | Genome-fragment | NA | | 0 |
| S11_k141_275463 | 1521 | No | NA | 0 | 2 | Not-determined | Genome-fragment | NA | | 0 |
| S11_k141_316875 | 1199 | No | NA | 0 | 1 | Not-determined | Genome-fragment | NA | | 0 |
| S7-final-viral-combined_k141_236659\|\|lt2gene | 1247 | No | NA | 2 | 0 | Low-quality | Genome-fragment | | 2.51 | 0 |
| S7-final-viral-combined_k141_55397\|\|lt2gene | 1247 | No | NA | 0 | 0 | Low-quality | Genome-fragment | | 3.64 | 0 |
| S11_k141_26900 | 5247 | No | NA | 0 | 2 | Not-determined | Genome-fragment | NA | | 0 |
| S7-final-viral-combined_k141_120003\|\|lt2gene | 1247 | No | NA | 2 | 0 | Low-quality | Genome-fragment | | 2.01 | 0 |
| S11_k141_275614 | 2814 | No | NA | 0 | 0 | Not-determined | Genome-fragment | NA | | 0 |
| S17-final-viral-combined_k141_6034\|\|full | 1247 | No | NA | 0 | 0 | Low-quality | Genome-fragment | | 0.79 | 0 |
| S11_k141_124028 | 1592 | No | NA | 0 | 2 | Not-determined | Genome-fragment | NA | | 0 |
| S11_k141_137801 | 1195 | No | NA | 0 | 0 | Not-determined | Genome-fragment | NA | | 0 |
| S11_k141_27139 | 1111 | No | NA | 0 | 1 | Not-determined | Genome-fragment | NA | | 0 |
| S11_k141_330074 | 1008 | No | NA | 0 | 1 | Not-determined | Genome-fragment | NA | | 0 |
| S4-final-viral-combined_k141_121544\|\|full | 1247 | No | NA | 2 | 0 | Low-quality | Genome-fragment | | 1.9 | 0 |
| S11_k141_330477 | 5123 | No | NA | 0 | 0 | Not-determined | Genome-fragment | NA | | 0 |
| S11_k141_330479 | 36558 | No | NA | 8 | 0 | Complete | High-quality | | 100 | 0 |
| S11_k141_330484 | 4823 | No | NA | 0 | 0 | Not-determined | Genome-fragment | NA | | 0 |
| S8-final-viral-combined_k141_79910\|\|full | 1247 | No | NA | 0 | 0 | Low-quality | Genome-fragment | | 1.91 | 0 |
| S11_k141_330585 | 1029 | No | NA | 0 | 0 | Not-determined | Genome-fragment | NA | | 0 |
| S8-final-viral-combined_k141_241728\|\|full | 1247 | No | NA | 0 | 0 | Low-quality | Genome-fragment | | 0.81 | 0 |
| S1-final-viral-combined_k141_70298\|\|full | 2245 | No | NA | 0 | 0 | Not-determined | Genome-fragment | NA | | 0 |
| S1-final-viral-combined_k141_324053\|\|full | 41421 | No | NA | 23 | 0 | High-quality | High-quality | | 96.35 | 0 |
| S1-final-viral-combined_k141_359778\|\|full | 6875 | No | NA | 0 | 0 | Not-determined | Genome-fragment | NA | | 0 |
| S8_k141_113905 | 1247 | No | NA | 0 | 0 | Low-quality | Genome-fragment | | 2.79 | 0 |
| S1-final-viral-combined_k141_394572\|\|full | 4760 | No | NA | 0 | 0 | Not-determined | Genome-fragment | NA | | 0 |
| S1-final-viral-combined_k141_394574\|\|full | 46833 | No | NA | 15 | 1 | High-quality | High-quality | | 100 | 0 |
| S11-final-viral-combined_k141_79427\|\|lt2gene | 1248 | No | NA | 2 | 0 | Low-quality | Genome-fragment | | 3.15 | 0 |
| S1-final-viral-combined_k141_394628\|\|full | 10807 | No | NA | 0 | 1 | Medium-quality | Genome-fragment | | 73.96 | 0 |
| S11-final-viral-combined_k141_150592\|\|lt2gene | 1248 | No | NA | 1 | 0 | Low-quality | Genome-fragment | | 3.07 | 0 |
| S1-final-viral-combined_k141_180985\|\|full | 9945 | No | NA | 0 | 6 | Not-determined | Genome-fragment | NA | | 0 |
| S1-final-viral-combined_k141_206076\|\|full | 1248 | No | NA | 0 | 0 | Low-quality | Genome-fragment | | 2.57 | 0 |
| S1-final-viral-combined_k141_106935\|\|full | 1248 | No | NA | 0 | 0 | Low-quality | Genome-fragment | | 2.69 | 0 |
| S1-final-viral-combined_k141_164556\|\|full | 4037 | No | NA | 0 | 1 | Not-determined | Genome-fragment | NA | | 0 |
| S1-final-viral-combined_k141_279715\|\|full | 9462 | No | NA | 0 | 7 | Not-determined | Genome-fragment | NA | | 0 |
| S1-final-viral-combined_k141_131658\|\|full | 1645 | No | NA | 0 | 0 | Not-determined | Genome-fragment | NA | | 0 |
| S1-final-viral-combined_k141_49387\|\|full | 3092 | No | NA | 0 | 1 | Not-determined | Genome-fragment | NA | | 0 |
| S1-final-viral-combined_k141_93906\|\|full | 1248 | No | NA | 0 | 0 | Low-quality | Genome-fragment | | 2.6 | 0 |
| S1-final-viral-combined_k141_345549\|\|full | 1238 | No | NA | 0 | 1 | Not-determined | Genome-fragment | NA | | 0 |
| S1-final-viral-combined_k141_361999\|\|full | 1012 | No | NA | 0 | 1 | Not-determined | Genome-fragment | NA | | 0 |
| S1-final-viral-combined_k141_179474\|\|full | 1248 | No | NA | 0 | 0 | Low-quality | Genome-fragment | | 0.92 | 0 |
| S1-final-viral-combined_k141_164566\|\|full | 2328 | No | NA | 0 | 1 | Not-determined | Genome-fragment | NA | | 0 |
| S1-final-viral-combined_k141_263274\|\|full | 1900 | No | NA | 0 | 0 | Not-determined | Genome-fragment | NA | | 0 |
| S1-final-viral-combined_k141_16472\|\|full | 3693 | No | NA | 0 | 2 | Not-determined | Genome-fragment | NA | | 0 |
| S1-final-viral-combined_k141_31527\|\|full | 1248 | No | NA | 0 | 0 | Low-quality | Genome-fragment | | 1.31 | 0 |
| S1-final-viral-combined_k141_49429\|\|full | 6245 | No | NA | 0 | 0 | Not-determined | Genome-fragment | NA | | 0 |
| S1-final-viral-combined_k141_32969\|\|full | 2815 | No | NA | 0 | 1 | Not-determined | Genome-fragment | NA | | 0 |
| S1-final-viral-combined_k141_148133\|\|full | 4223 | No | NA | 0 | 5 | Not-determined | Genome-fragment | NA | | 0 |
| S1-final-viral-combined_k141_82325\|\|full | 1223 | No | NA | 0 | 2 | Not-determined | Genome-fragment | NA | | 0 |
| S7-final-viral-combined_k141_220032\|\|full | 1248 | No | NA | 1 | 0 | Low-quality | Genome-fragment | | 3.08 | 0 |
| S7-final-viral-combined_k141_141731\|\|full | 1248 | No | NA | 1 | 0 | Low-quality | Genome-fragment | | 3.16 | 0 |
| S7-final-viral-combined_k141_204679\|\|lt2gene | 1248 | No | NA | 1 | 0 | Low-quality | Genome-fragment | | 2.08 | 0 |
| S7_k141_60452 | 1248 | No | NA | 1 | 0 | Low-quality | Genome-fragment | | 3.44 | 0 |
| S2-final-viral-combined_k141_157508\|\|full | 1248 | No | NA | 0 | 0 | Low-quality | Genome-fragment | | 3.05 | 0 |
| S1-final-viral-combined_k141_345591\|\|full | 6096 | No | NA | 0 | 4 | Not-determined | Genome-fragment | NA | | 0 |
| S1-final-viral-combined_k141_100\|\|full | 1612 | No | NA | 0 | 3 | Not-determined | Genome-fragment | NA | | 0 |
| S1-final-viral-combined_k141_329133\|\|full | 1097 | No | NA | 0 | 0 | Not-determined | Genome-fragment | NA | | 0 |
| S1-final-viral-combined_k141_263299\|\|full | 1643 | No | NA | 0 | 2 | Not-determined | Genome-fragment | NA | | 0 |
| S1-final-viral-combined_k141_131740\|\|full | 1336 | No | NA | 0 | 1 | Not-determined | Genome-fragment | NA | | 0 |
| S2-final-viral-combined_k141_86410\|\|full | 1248 | No | NA | 0 | 1 | Low-quality | Genome-fragment | | 0.95 | 0 |
| S2_k141_137370 | 1248 | No | NA | 1 | 0 | Low-quality | Genome-fragment | | 2.46 | 0 |
| S1-final-viral-combined_k141_123\|\|full | 4501 | No | NA | 0 | 0 | Not-determined | Genome-fragment | NA | | 0 |
| S4_k141_81945 | 1248 | No | NA | 0 | 1 | Low-quality | Genome-fragment | | 0.42 | 0 |
| S4_k141_54047 | 1248 | No | NA | 1 | 0 | Low-quality | Genome-fragment | | 0.7 | 0 |
| S1-final-viral-combined_k141_362058\|\|full | 2733 | No | NA | 0 | 1 | Not-determined | Genome-fragment | NA | | 0 |
| S1-final-viral-combined_k141_115273\|\|full | 1534 | No | NA | 0 | 1 | Not-determined | Genome-fragment | NA | | 0 |
| S1-final-viral-combined_k141_246845\|\|full | 1418 | No | NA | 0 | 2 | Not-determined | Genome-fragment | NA | | 0 |
| S1-final-viral-combined_k141_33039\|\|full | 1619 | No | NA | 0 | 1 | Not-determined | Genome-fragment | NA | | 0 |
| S11-final-viral-combined_k141_125856\|\|full | 1249 | No | NA | 2 | 0 | Low-quality | Genome-fragment | | 2.48 | 0 |
| S11-final-viral-combined_k141_142090\|\|full | 1249 | No | NA | 0 | 0 | Low-quality | Genome-fragment | | 2.49 | 0 |
| S1-final-viral-combined_k141_230440\|\|full | 4314 | No | NA | 0 | 2 | Not-determined | Genome-fragment | NA | | 0 |
| S1-final-viral-combined_k141_246847\|\|full | 8531 | No | NA | 0 | 0 | Not-determined | Genome-fragment | NA | | 0 |
| S1-final-viral-combined_k141_39539\|\|lt2gene | 1249 | No | NA | 0 | 0 | Low-quality | Genome-fragment | | 2.29 | 0 |
| S1_k141_178479 | 1249 | No | NA | 0 | 0 | Low-quality | Genome-fragment | | 0.21 | 0 |
| S1-final-viral-combined_k141_115301\|\|full | 1168 | No | NA | 0 | 0 | Not-determined | Genome-fragment | NA | | 0 |
| S1-final-viral-combined_k141_115308\|\|full | 1495 | No | NA | 0 | 1 | Not-determined | Genome-fragment | NA | | 0 |
| S1-final-viral-combined_k141_213971\|\|full | 1146 | No | NA | 0 | 0 | Not-determined | Genome-fragment | NA | | 0 |
| S1-final-viral-combined_k141_164618\|\|full | 2006 | No | NA | 0 | 0 | Not-determined | Genome-fragment | NA | | 0 |
| S7-final-viral-combined_k141_236250\|\|full | 1249 | No | NA | 0 | 0 | Low-quality | Genome-fragment | | 1.56 | 0 |
| S12-final-viral-combined_k141_325856\|\|full | 1249 | No | NA | 0 | 0 | Low-quality | Genome-fragment | | 2.78 | 0 |
| S1-final-viral-combined_k141_345645\|\|full | 7264 | No | NA | 0 | 4 | Not-determined | Genome-fragment | NA | | 0 |
| S1-final-viral-combined_k141_345650\|\|full | 4889 | No | NA | 0 | 3 | Not-determined | Genome-fragment | NA | | 0 |
| S12_k141_331028 | 1249 | No | NA | 1 | 0 | Low-quality | Genome-fragment | | 2.28 | 0 |
| S1-final-viral-combined_k141_164641\|\|full | 1522 | No | NA | 0 | 2 | Not-determined | Genome-fragment | NA | | 0 |
| S1-final-viral-combined_k141_49525\|\|full | 6238 | No | NA | 0 | 3 | Not-determined | Genome-fragment | NA | | 0 |
| S2-final-viral-combined_k141_115957\|\|lt2gene | 1249 | No | NA | 2 | 0 | Low-quality | Genome-fragment | | 2.73 | 0 |
| S8-final-viral-combined_k141_232367\|\|full | 1249 | No | NA | 0 | 1 | Low-quality | Genome-fragment | | 1.98 | 0 |
| S8-final-viral-combined_k141_186988\|\|lt2gene | 1249 | No | NA | 2 | 0 | Low-quality | Genome-fragment | | 24.05 | 0 |
| S1-final-viral-combined_k141_279838\|\|full | 1865 | No | NA | 0 | 0 | Not-determined | Genome-fragment | NA | | 0 |
| S1-final-viral-combined_k141_296311\|\|full | 2788 | No | NA | 0 | 1 | Not-determined | Genome-fragment | NA | | 0 |
| S1-final-viral-combined_k141_312828\|\|full | 4702 | No | NA | 0 | 1 | Not-determined | Genome-fragment | NA | | 0 |
| S8_k141_63002 | 1249 | No | NA | 1 | 0 | Low-quality | Genome-fragment | | 3.65 | 0 |
| S1-final-viral-combined_k141_98846\|\|full | 2623 | No | NA | 0 | 2 | Not-determined | Genome-fragment | NA | | 0 |
| S1-final-viral-combined_k141_312847\|\|full | 4048 | No | NA | 0 | 2 | Not-determined | Genome-fragment | NA | | 0 |
| S9_k141_127222 | 1250 | No | NA | 1 | 0 | Low-quality | Genome-fragment | | 3.02 | 0 |
| S1-final-viral-combined_k141_329213\|\|full | 1017 | No | NA | 0 | 0 | Not-determined | Genome-fragment | NA | | 0 |
| S1-final-viral-combined_k141_131842\|\|full | 1897 | No | NA | 0 | 2 | Not-determined | Genome-fragment | NA | | 0 |
| S1-final-viral-combined_k141_197551\|\|full | 1738 | No | NA | 0 | 1 | Not-determined | Genome-fragment | NA | | 0 |
| S1-final-viral-combined_k141_246918\|\|full | 1139 | No | NA | 0 | 0 | Not-determined | Genome-fragment | NA | | 0 |
| S1-final-viral-combined_k141_16599\|\|full | 6002 | No | NA | 0 | 2 | Not-determined | Genome-fragment | NA | | 0 |
| S1-final-viral-combined_k141_230510\|\|full | 1180 | No | NA | 0 | 1 | Not-determined | Genome-fragment | NA | | 0 |
| S1-final-viral-combined_k141_131856\|\|full | 1634 | No | NA | 0 | 2 | Not-determined | Genome-fragment | NA | | 0 |
| S11-final-viral-combined_k141_299497\|\|full | 1250 | No | NA | 0 | 1 | Low-quality | Genome-fragment | | 1.85 | 0 |
| S11-final-viral-combined_k141_163914\|\|full | 1250 | No | NA | 0 | 2 | Low-quality | Genome-fragment | | 2.35 | 0 |
| S1-final-viral-combined_k141_197561\|\|full | 2858 | No | NA | 0 | 0 | Not-determined | Genome-fragment | NA | | 0 |
| S1-final-viral-combined_k141_65855\|\|full | 5397 | No | NA | 0 | 2 | Not-determined | Genome-fragment | NA | | 0 |
| S11-final-viral-combined_k141_32982\|\|lt2gene | 1250 | No | NA | 2 | 0 | Low-quality | Genome-fragment | | 2.92 | 0 |
| S1-final-viral-combined_k141_312862\|\|full | 1523 | No | NA | 0 | 2 | Not-determined | Genome-fragment | NA | | 0 |
| S1-final-viral-combined_k141_362174\|\|full | 4671 | No | NA | 0 | 2 | Not-determined | Genome-fragment | NA | | 0 |
| S1-final-viral-combined_k141_65862\|\|full | 2109 | No | NA | 0 | 2 | Not-determined | Genome-fragment | NA | | 0 |
| S1-final-viral-combined_k141_16621\|\|full | 2421 | No | NA | 0 | 2 | Not-determined | Genome-fragment | NA | | 0 |
| S1-final-viral-combined_k141_246923\|\|full | 30540 | No | NA | 9 | 0 | Medium-quality | Genome-fragment | | 58.68 | 0 |
| S1-final-viral-combined_k141_164693\|\|full | 31466 | No | NA | 0 | 17 | Not-determined | Genome-fragment | NA | | 0 |
| S1-final-viral-combined_k141_362181\|\|full | 1359 | No | NA | 0 | 1 | Not-determined | Genome-fragment | NA | | 0 |
| S1-final-viral-combined_k141_296349\|\|full | 1975 | No | NA | 0 | 2 | Not-determined | Genome-fragment | NA | | 0 |
| S1-final-viral-combined_k141_104145\|\|full | 1250 | No | NA | 0 | 1 | Low-quality | Genome-fragment | | 3.76 | 0 |
| S1-final-viral-combined_k141_6228\|\|full | 1250 | No | NA | 4 | 0 | Low-quality | Genome-fragment | | 2.84 | 0 |
| S1-final-viral-combined_k141_131904\|\|full | 1036 | No | NA | 0 | 0 | Not-determined | Genome-fragment | NA | | 0 |
| S1-final-viral-combined_k141_205779\|\|full | 1250 | No | NA | 2 | 0 | Low-quality | Genome-fragment | | 0.92 | 0 |
| S1_k141_182070 | 1250 | No | NA | 0 | 0 | Low-quality | Genome-fragment | | 2.99 | 0 |
| S3-final-viral-combined_k141_58480\|\|full | 1250 | No | NA | 0 | 0 | Low-quality | Genome-fragment | | 2.46 | 0 |
| S1-final-viral-combined_k141_197622\|\|full | 3109 | No | NA | 0 | 2 | Not-determined | Genome-fragment | NA | | 0 |
| S3-final-viral-combined_k141_36652\|\|lt2gene | 1250 | No | NA | 2 | 0 | Low-quality | Genome-fragment | | 2.76 | 0 |
| S5-final-viral-combined_k141_157670\|\|lt2gene | 1250 | No | NA | 1 | 0 | Low-quality | Genome-fragment | | 1.97 | 0 |
| S1-final-viral-combined_k141_296379\|\|full | 3834 | No | NA | 0 | 2 | Not-determined | Genome-fragment | NA | | 0 |
| S1-final-viral-combined_k141_263400\|\|full | 1731 | No | NA | 0 | 0 | Not-determined | Genome-fragment | NA | | 0 |
| S7-final-viral-combined_k141_185228\|\|full | 1250 | No | NA | 0 | 0 | Low-quality | Genome-fragment | | 3 | 0 |
| S1-final-viral-combined_k141_279969\|\|full | 1850 | No | NA | 0 | 0 | Not-determined | Genome-fragment | NA | | 0 |
| S1-final-viral-combined_k141_296413\|\|full | 2249 | No | NA | 0 | 0 | Not-determined | Genome-fragment | NA | | 0 |
| S1-final-viral-combined_k141_345719\|\|full | 1304 | No | NA | 0 | 0 | Not-determined | Genome-fragment | NA | | 0 |
| S7-final-viral-combined_k141_219151\|\|full | 1250 | No | NA | 1 | 0 | Low-quality | Genome-fragment | | 2.92 | 0 |
| S1-final-viral-combined_k141_115431\|\|full | 5502 | No | NA | 0 | 3 | Not-determined | Genome-fragment | NA | | 0 |
| S1-final-viral-combined_k141_345728\|\|full | 1759 | No | NA | 0 | 3 | Not-determined | Genome-fragment | NA | | 0 |
| S17-final-viral-combined_k141_120805\|\|full | 1250 | No | NA | 0 | 0 | Low-quality | Genome-fragment | | 2.11 | 0 |
| S12_k141_39131 | 1250 | No | NA | 0 | 0 | Low-quality | Genome-fragment | | 2.77 | 0 |
| S8-final-viral-combined_k141_180127\|\|lt2gene | 1250 | No | NA | 1 | 0 | Low-quality | Genome-fragment | | 2.84 | 0 |
| S1-final-viral-combined_k141_164775\|\|full | 2449 | No | NA | 0 | 0 | Not-determined | Genome-fragment | NA | | 0 |
| S1-final-viral-combined_k141_131979\|\|full | 1309 | No | NA | 0 | 0 | Not-determined | Genome-fragment | NA | | 0 |
| S1-final-viral-combined_k141_98968\|\|full | 2423 | No | NA | 0 | 1 | Not-determined | Genome-fragment | NA | | 0 |
| S1-final-viral-combined_k141_131989\|\|full | 2979 | No | NA | 0 | 2 | Not-determined | Genome-fragment | NA | | 0 |
| S11-final-viral-combined_k141_321053\|\|lt2gene | 1251 | No | NA | 1 | 0 | Low-quality | Genome-fragment | | 0.81 | 0 |
| S1-final-viral-combined_k141_59467\|\|full | 1251 | No | NA | 1 | 0 | Low-quality | Genome-fragment | | 2.58 | 0 |
| S1-final-viral-combined_k141_258321\|\|full | 1251 | No | NA | 0 | 0 | Low-quality | Genome-fragment | | 2.29 | 0 |
| S1-final-viral-combined_k141_49597\|\|full | 2649 | No | NA | 0 | 3 | Not-determined | Genome-fragment | NA | | 0 |
| S1-final-viral-combined_k141_230689\|\|full | 1097 | No | NA | 0 | 0 | Not-determined | Genome-fragment | NA | | 0 |
| S7-final-viral-combined_k141_16549\|\|full | 1251 | No | NA | 0 | 0 | Low-quality | Genome-fragment | | 2.93 | 0 |
| S1-final-viral-combined_k141_16783\|\|full | 3449 | No | NA | 0 | 1 | Not-determined | Genome-fragment | NA | | 0 |
| S1-final-viral-combined_k141_164818\|\|full | 2083 | No | NA | 0 | 2 | Not-determined | Genome-fragment | NA | | 0 |
| S17-k141_76634 | 1251 | No | NA | 0 | 1 | Low-quality | Genome-fragment | | 0.33 | 0 |
| S12-final-viral-combined_k141_98847\|\|full | 1251 | No | NA | 0 | 1 | Low-quality | Genome-fragment | | 0.51 | 0 |
| S1-final-viral-combined_k141_181277\|\|full | 6877 | No | NA | 0 | 1 | Not-determined | Genome-fragment | NA | | 0 |
| S1-final-viral-combined_k141_115473\|\|full | 1841 | No | NA | 0 | 2 | Not-determined | Genome-fragment | NA | | 0 |
| S1-final-viral-combined_k141_329318\|\|full | 2509 | No | NA | 0 | 1 | Not-determined | Genome-fragment | NA | | 0 |
| S1-final-viral-combined_k141_520\|\|full | 5155 | No | NA | 0 | 3 | Not-determined | Genome-fragment | NA | | 0 |
| S1-final-viral-combined_k141_49641\|\|full | 5667 | No | NA | 0 | 2 | Not-determined | Genome-fragment | NA | | 0 |
| S1-final-viral-combined_k141_329334\|\|full | 1549 | No | NA | 0 | 1 | Not-determined | Genome-fragment | NA | | 0 |
| S1-final-viral-combined_k141_148362\|\|full | 4458 | No | NA | 0 | 5 | Not-determined | Genome-fragment | NA | | 0 |
| S12-final-viral-combined_k141_384594\|\|full | 1251 | No | NA | 1 | 0 | Low-quality | Genome-fragment | | 1.96 | 0 |
| S1-final-viral-combined_k141_378728\|\|full | 3309 | No | NA | 0 | 1 | Not-determined | Genome-fragment | NA | | 0 |
| S1-final-viral-combined_k141_132065\|\|full | 1111 | No | NA | 0 | 1 | Not-determined | Genome-fragment | NA | | 0 |
| S1-final-viral-combined_k141_230768\|\|full | 7742 | No | NA | 0 | 1 | Not-determined | Genome-fragment | NA | | 0 |
| S1-final-viral-combined_k141_345795\|\|full | 1614 | No | NA | 0 | 2 | Not-determined | Genome-fragment | NA | | 0 |

| | | | | | | | | | | | |
|---|---|---|---|---|---|---|---|---|---|---|---|
| S1-final-viral-combined_k141_181306\|\|full | 1755 | No | NA | | 0 | 2 | Not-determined | Genome-fragment | NA | | 0 |
| S2-final-viral-combined_k141_172083\|\|full | 1251 | No | NA | | 0 | 0 | Low-quality | Genome-fragment | | 2.58 | 0 |
| S4-final-viral-combined_k141_9637\|\|full | 1251 | No | NA | | 0 | 0 | Low-quality | Genome-fragment | | 3.26 | 0 |
| S1-final-viral-combined_k141_296563\|\|full | 1392 | No | NA | | 0 | 0 | Not-determined | Genome-fragment | NA | | 0 |
| S8-final-viral-combined_k141_40783\|\|full | 1251 | No | NA | | 0 | 0 | Low-quality | Genome-fragment | | 2.38 | 0 |
| S8_k141_1032 | 1251 | No | NA | | 0 | 0 | Low-quality | Genome-fragment | | 0.9 | 0 |
| S1-final-viral-combined_k141_569\|\|full | 3426 | No | NA | | 0 | 2 | Not-determined | Genome-fragment | NA | | 0 |
| S1-final-viral-combined_k141_362379\|\|full | 5941 | No | NA | | 0 | 4 | Not-determined | Genome-fragment | NA | | 0 |
| S9-final-viral-combined_k141_14472\|\|full | 1252 | No | NA | | 0 | 1 | Low-quality | Genome-fragment | | 0.55 | 0 |
| S9_k141_8396 | 1252 | No | NA | | 0 | 0 | Low-quality | Genome-fragment | | 1.51 | 0 |
| S1-final-viral-combined_k141_313044\|\|full | 2141 | No | NA | | 0 | 3 | Not-determined | Genome-fragment | NA | | 0 |
| S1-final-viral-combined_k141_362385\|\|full | 4873 | No | NA | | 0 | 3 | Not-determined | Genome-fragment | NA | | 0 |
| S11-final-viral-combined_k141_3767\|\|lt2gene | 1252 | No | NA | | 0 | 0 | Low-quality | Genome-fragment | | 2.83 | 0 |
| S1_k141_341663 | 1252 | No | NA | | 0 | 0 | Low-quality | Genome-fragment | | 2.02 | 0 |
| S1-final-viral-combined_k141_263562\|\|full | 29679 | No | NA | | 4 | 0 | Medium-quality | Genome-fragment | | 64.61 | 0 |
| S1-final-viral-combined_k141_115571\|\|full | 1874 | No | NA | | 0 | 3 | Not-determined | Genome-fragment | NA | | 0 |
| S1-final-viral-combined_k141_33145\|\|full | 4351 | No | NA | | 0 | 2 | Not-determined | Genome-fragment | NA | | 0 |
| S1-final-viral-combined_k141_49700\|\|full | 2906 | No | NA | | 0 | 0 | Not-determined | Genome-fragment | NA | | 0 |
| S1-final-viral-combined_k141_362395\|\|full | 5235 | No | NA | | 0 | 3 | Not-determined | Genome-fragment | NA | | 0 |
| S1-final-viral-combined_k141_230822\|\|full | 3064 | No | NA | | 0 | 1 | Not-determined | Genome-fragment | NA | | 0 |
| S1-final-viral-combined_k141_132120\|\|full | 1370 | No | NA | | 0 | 2 | Not-determined | Genome-fragment | NA | | 0 |
| S1-final-viral-combined_k141_197846\|\|full | 1026 | No | NA | | 0 | 2 | Not-determined | Genome-fragment | NA | | 0 |
| S1-final-viral-combined_k141_99095\|\|full | 1314 | No | NA | | 0 | 2 | Not-determined | Genome-fragment | NA | | 0 |
| S1-final-viral-combined_k141_164894\|\|full | 29496 | No | NA | | 21 | 0 | Medium-quality | Genome-fragment | | 85.65 | 0 |
| S1-final-viral-combined_k141_280157\|\|full | 2889 | No | NA | | 0 | 1 | Not-determined | Genome-fragment | NA | | 0 |
| S1-final-viral-combined_k141_280172\|\|full | 3616 | No | NA | | 0 | 1 | Not-determined | Genome-fragment | NA | | 0 |
| S7-final-viral-combined_k141_22946\|\|full | 1252 | No | NA | | 1 | 0 | Low-quality | Genome-fragment | | 5.94 | 0 |
| S17-final-viral-combined_k141_55103\|\|full | 1252 | No | NA | | 0 | 1 | Low-quality | Genome-fragment | | 2.17 | 0 |
| S1-final-viral-combined_k141_33184\|\|full | 1076 | No | NA | | 0 | 1 | Not-determined | Genome-fragment | NA | | 0 |
| S12-final-viral-combined_k141_156170\|\|full | 1252 | No | NA | | 1 | 0 | Low-quality | Genome-fragment | | 1.72 | 0 |
| S12-final-viral-combined_k141_332767\|\|lt2gene | 1252 | No | NA | | 1 | 0 | Low-quality | Genome-fragment | | 2.64 | 0 |
| S12_k141_76718 | 1252 | No | NA | | 0 | 0 | Low-quality | Genome-fragment | | 2.41 | 0 |
| S12_k141_365527 | 1252 | No | NA | | 0 | 0 | Low-quality | Genome-fragment | | 2.31 | 0 |
| S1-final-viral-combined_k141_263600\|\|full | 1973 | No | NA | | 0 | 1 | Not-determined | Genome-fragment | NA | | 0 |
| S1-final-viral-combined_k141_66039\|\|full | 1937 | No | NA | | 0 | 1 | Not-determined | Genome-fragment | NA | | 0 |
| S2-final-viral-combined_k141_29076\|\|full | 1252 | No | NA | | 2 | 0 | Low-quality | Genome-fragment | | 2 | 0 |
| S1-final-viral-combined_k141_132134\|\|full | 9922 | No | NA | | 0 | 9 | Not-determined | Genome-fragment | NA | | 0 |
| S1-final-viral-combined_k141_33199\|\|full | 1519 | No | NA | | 0 | 1 | Not-determined | Genome-fragment | NA | | 0 |
| S1-final-viral-combined_k141_181436\|\|full | 1352 | No | NA | | 0 | 1 | Not-determined | Genome-fragment | NA | | 0 |
| S1-final-viral-combined_k141_49766\|\|full | 1294 | No | NA | | 0 | 0 | Not-determined | Genome-fragment | NA | | 0 |
| S8-final-viral-combined_k141_241935\|\|full | 1252 | No | NA | | 0 | 0 | Low-quality | Genome-fragment | | 2.75 | 0 |
| S1-final-viral-combined_k141_329445\|\|full | 1792 | No | NA | | 0 | 0 | Not-determined | Genome-fragment | NA | | 0 |
| S1-final-viral-combined_k141_280210\|\|full | 1105 | No | NA | | 0 | 1 | Not-determined | Genome-fragment | NA | | 0 |
| S1-final-viral-combined_k141_181457\|\|full | 1798 | No | NA | | 0 | 1 | Not-determined | Genome-fragment | NA | | 0 |
| S1-final-viral-combined_k141_280217\|\|full | 1290 | No | NA | | 0 | 0 | Not-determined | Genome-fragment | NA | | 0 |
| S1-final-viral-combined_k141_230849\|\|full | 1348 | No | NA | | 0 | 0 | Not-determined | Genome-fragment | NA | | 0 |
| S1-final-viral-combined_k141_230856\|\|full | 1528 | No | NA | | 0 | 1 | Not-determined | Genome-fragment | NA | | 0 |
| S1-final-viral-combined_k141_115656\|\|full | 2196 | No | NA | | 0 | 1 | Not-determined | Genome-fragment | NA | | 0 |
| S11_k141_135261 | 1253 | No | NA | | 0 | 0 | Low-quality | Genome-fragment | | 2.08 | 0 |
| S1-final-viral-combined_k141_296624\|\|full | 1938 | No | NA | | 0 | 1 | Not-determined | Genome-fragment | NA | | 0 |
| S1_k141_83431 | 1253 | No | NA | | 0 | 1 | Low-quality | Genome-fragment | | 0.22 | 0 |
| S1-final-viral-combined_k141_313129\|\|full | 2009 | No | NA | | 0 | 1 | Not-determined | Genome-fragment | NA | | 0 |
| S1-final-viral-combined_k141_378876\|\|full | 2433 | No | NA | | 0 | 2 | Not-determined | Genome-fragment | NA | | 0 |
| S1-final-viral-combined_k141_378882\|\|full | 1069 | No | NA | | 0 | 0 | Not-determined | Genome-fragment | NA | | 0 |
| S1-final-viral-combined_k141_148488\|\|full | 14737 | No | NA | | 0 | 11 | Not-determined | Genome-fragment | NA | | 0 |
| S1-final-viral-combined_k141_230872\|\|full | 1919 | No | NA | | 0 | 2 | Not-determined | Genome-fragment | NA | | 0 |
| S2_k141_185164 | 1253 | No | NA | | 0 | 1 | Low-quality | Genome-fragment | | 1.08 | 0 |
| S1-final-viral-combined_k141_99204\|\|full | 8186 | No | NA | | 0 | 5 | Not-determined | Genome-fragment | NA | | 0 |
| S1-final-viral-combined_k141_115697\|\|full | 4304 | No | NA | | 0 | 1 | Not-determined | Genome-fragment | NA | | 0 |
| S1-final-viral-combined_k141_16922\|\|full | 1604 | No | NA | | 0 | 0 | Not-determined | Genome-fragment | NA | | 0 |
| S1-final-viral-combined_k141_132190\|\|full | 2921 | No | NA | | 0 | 0 | Not-determined | Genome-fragment | NA | | 0 |
| S4-final-viral-combined_k141_40084\|\|full | 1253 | No | NA | | 0 | 0 | Low-quality | Genome-fragment | | 0.78 | 0 |
| S1-final-viral-combined_k141_115702\|\|full | 8565 | No | NA | | 0 | 6 | Not-determined | Genome-fragment | NA | | 0 |
| S1-final-viral-combined_k141_329493\|\|full | 3415 | No | NA | | 0 | 1 | Not-determined | Genome-fragment | NA | | 0 |
| S1-final-viral-combined_k141_82836\|\|full | 3954 | No | NA | | 0 | 4 | Not-determined | Genome-fragment | NA | | 0 |
| S1-final-viral-combined_k141_99231\|\|full | 1200 | No | NA | | 0 | 1 | Not-determined | Genome-fragment | NA | | 0 |
| S1-final-viral-combined_k141_132226\|\|full | 1685 | No | NA | | 0 | 2 | Not-determined | Genome-fragment | NA | | 0 |
| S1-final-viral-combined_k141_66078\|\|full | 7206 | No | NA | | 0 | 4 | Not-determined | Genome-fragment | NA | | 0 |
| S1-final-viral-combined_k141_296707\|\|full | 1491 | No | NA | | 0 | 0 | Not-determined | Genome-fragment | NA | | 0 |
| S8-final-viral-combined_k141_94913\|\|full | 1253 | No | NA | | 0 | 0 | Low-quality | Genome-fragment | | 3 | 0 |
| S1-final-viral-combined_k141_181565\|\|full | 1189 | No | NA | | 0 | 1 | Not-determined | Genome-fragment | NA | | 0 |
| S11-final-viral-combined_k141_267883\|\|full | 1254 | No | NA | | 0 | 1 | Low-quality | Genome-fragment | | 2.76 | 0 |
| S1_k141_213612 | 1254 | No | NA | | 0 | 0 | Low-quality | Genome-fragment | | 3.8 | 0 |
| S1_k141_148001 | 1254 | No | NA | | 0 | 1 | Low-quality | Genome-fragment | | 4 | 0 |
| S17-final-viral-combined_k141_37822\|\|lt2gene | 1254 | No | NA | | 0 | 2 | Low-quality | Genome-fragment | | 0.95 | 0 |
| S12-final-viral-combined_k141_3232\|\|full | 1254 | No | NA | | 0 | 2 | Low-quality | Genome-fragment | | 1.72 | 0 |
| S1-final-viral-combined_k141_66136\|\|full | 1579 | No | NA | | 0 | 0 | Not-determined | Genome-fragment | NA | | 0 |
| S2-final-viral-combined_k141_99010\|\|full | 1254 | No | NA | | 0 | 0 | Low-quality | Genome-fragment | | 2.69 | 0 |
| S1-final-viral-combined_k141_296744\|\|full | 1077 | No | NA | | 0 | 0 | Not-determined | Genome-fragment | NA | | 0 |
| S1-final-viral-combined_k141_66159\|\|full | 1712 | No | NA | | 0 | 0 | Not-determined | Genome-fragment | NA | | 0 |
| S1-final-viral-combined_k141_181626\|\|full | 4731 | No | NA | | 0 | 3 | Not-determined | Genome-fragment | NA | | 0 |
| S1-final-viral-combined_k141_346049\|\|full | 7612 | No | NA | | 0 | 7 | Not-determined | Genome-fragment | NA | | 0 |
| S1-final-viral-combined_k141_115820\|\|full | 1216 | No | NA | | 0 | 0 | Not-determined | Genome-fragment | NA | | 0 |
| S1-final-viral-combined_k141_66176\|\|full | 8491 | No | NA | | 0 | 2 | Not-determined | Genome-fragment | NA | | 0 |
| S1-final-viral-combined_k141_296777\|\|full | 1489 | No | NA | | 0 | 0 | Not-determined | Genome-fragment | NA | | 0 |
| S1-final-viral-combined_k141_132334\|\|full | 3908 | No | NA | | 0 | 1 | Not-determined | Genome-fragment | NA | | 0 |
| S2-final-viral-combined_k141_184686\|\|full | 1254 | No | NA | | 1 | 0 | Low-quality | Genome-fragment | | 0.83 | 0 |
| S1-final-viral-combined_k141_148617\|\|full | 2261 | No | NA | | 0 | 2 | Not-determined | Genome-fragment | NA | | 0 |
| S6_k141_66521 | 1254 | No | NA | | 0 | 0 | Low-quality | Genome-fragment | | 2.45 | 0 |
| S1-final-viral-combined_k141_198030\|\|full | 1150 | No | NA | | 0 | 2 | Not-determined | Genome-fragment | NA | | 0 |
| S8-final-viral-combined_k141_8549\|\|lt2gene | 1254 | No | NA | | 2 | 0 | Low-quality | Genome-fragment | | 1.96 | 0 |
| S9_k141_125496 | 1255 | No | NA | | 0 | 1 | Low-quality | Genome-fragment | | 3.54 | 0 |
| S11-final-viral-combined_k141_111032\|\|full | 1255 | No | NA | | 1 | 0 | Low-quality | Genome-fragment | | 2.63 | 0 |
| S1-final-viral-combined_k141_115877\|\|full | 1096 | No | NA | | 0 | 2 | Not-determined | Genome-fragment | NA | | 0 |
| S1-final-viral-combined_k141_82984\|\|full | 1845 | No | NA | | 0 | 1 | Not-determined | Genome-fragment | NA | | 0 |
| S1-final-viral-combined_k141_17057\|\|full | 2661 | No | NA | | 0 | 0 | Not-determined | Genome-fragment | NA | | 0 |
| S11_k141_208527 | 1255 | No | NA | | 0 | 0 | Low-quality | Genome-fragment | | 2.76 | 0 |
| S1-final-viral-combined_k141_198055\|\|full | 2480 | No | NA | | 0 | 3 | Not-determined | Genome-fragment | NA | | 0 |
| S1-final-viral-combined_k141_17065\|\|full | 1807 | No | NA | | 0 | 0 | Not-determined | Genome-fragment | NA | | 0 |
| S1_k141_271227 | 1255 | No | NA | | 1 | 0 | Low-quality | Genome-fragment | | 2.67 | 0 |
| S3-final-viral-combined_k141_60057\|\|full | 1255 | No | NA | | 1 | 0 | Low-quality | Genome-fragment | | 2.28 | 0 |
| S1-final-viral-combined_k141_263835\|\|full | 6120 | No | NA | | 0 | 3 | Not-determined | Genome-fragment | NA | | 0 |
| S5-final-viral-combined_k141_194600\|\|lt2gene | 1255 | No | NA | | 1 | 0 | Low-quality | Genome-fragment | | 0.73 | 0 |
| S1-final-viral-combined_k141_362573\|\|full | 1045 | No | NA | | 0 | 2 | Not-determined | Genome-fragment | NA | | 0 |
| S7-final-viral-combined_k141_150578\|\|full | 1255 | No | NA | | 0 | 0 | Low-quality | Genome-fragment | | 2.82 | 0 |
| S1-final-viral-combined_k141_247416\|\|full | 3573 | No | NA | | 0 | 1 | Not-determined | Genome-fragment | NA | | 0 |
| S2-final-viral-combined_k141_82342\|\|lt2gene | 1255 | No | NA | | 1 | 0 | Low-quality | Genome-fragment | | 2.42 | 0 |
| S4-final-viral-combined_k141_84318\|\|full | 1255 | No | NA | | 1 | 0 | Low-quality | Genome-fragment | | 2.95 | 0 |
| S6-final-viral-combined_k141_132593\|\|full | 1255 | No | NA | | 0 | 1 | Low-quality | Genome-fragment | | 2.1 | 0 |
| S8-final-viral-combined_k141_75795\|\|full | 1255 | No | NA | | 1 | 0 | Low-quality | Genome-fragment | | 2.08 | 0 |
| S1-final-viral-combined_k141_115950\|\|full | 3853 | No | NA | | 0 | 0 | Not-determined | Genome-fragment | NA | | 0 |
| S1-final-viral-combined_k141_165159\|\|full | 4317 | No | NA | | 0 | 1 | Not-determined | Genome-fragment | NA | | 0 |
| S1-final-viral-combined_k141_346214\|\|full | 1734 | No | NA | | 0 | 2 | Not-determined | Genome-fragment | NA | | 0 |
| S1-final-viral-combined_k141_137654\|\|full | 1256 | No | NA | | 0 | 0 | Low-quality | Genome-fragment | | 1.5 | 0 |
| S1_k141_212297 | 1256 | No | NA | | 0 | 1 | Low-quality | Genome-fragment | | 0.47 | 0 |
| S17-k141_24160 | 1256 | No | NA | | 0 | 2 | Low-quality | Genome-fragment | | 0.36 | 0 |
| S12-final-viral-combined_k141_393318\|\|full | 1256 | No | NA | | 1 | 0 | Low-quality | Genome-fragment | | 2.11 | 0 |
| S1-final-viral-combined_k141_346236\|\|full | 7326 | No | NA | | 0 | 5 | Not-determined | Genome-fragment | NA | | 0 |
| S1-final-viral-combined_k141_329694\|\|full | 1051 | No | NA | | 0 | 0 | Not-determined | Genome-fragment | NA | | 0 |
| S2-final-viral-combined_k141_92149\|\|full | 1256 | No | NA | | 0 | 0 | Low-quality | Genome-fragment | | 3.38 | 0 |
| S1-final-viral-combined_k141_346243\|\|full | 1951 | No | NA | | 0 | 0 | Not-determined | Genome-fragment | NA | | 0 |
| S4-final-viral-combined_k141_72799\|\|full | 1256 | No | NA | | 0 | 0 | Low-quality | Genome-fragment | | 1.94 | 0 |
| S6-final-viral-combined_k141_133667\|\|full | 1256 | No | NA | | 1 | 0 | Low-quality | Genome-fragment | | 3.45 | 0 |
| S9_k141_117264 | 1257 | No | NA | | 0 | 0 | Low-quality | Genome-fragment | | 2.03 | 0 |
| S11_k141_162534 | 1257 | No | NA | | 0 | 0 | Low-quality | Genome-fragment | | 1.95 | 0 |
| S1-final-viral-combined_k141_165204\|\|full | 19280 | No | NA | | 0 | 13 | Not-determined | Genome-fragment | NA | | 0 |
| S1-final-viral-combined_k141_296801\|\|full | 1488 | No | NA | | 0 | 0 | Not-determined | Genome-fragment | NA | | 0 |
| S1-final-viral-combined_k141_292365\|\|full | 1257 | No | NA | | 1 | 0 | Low-quality | Genome-fragment | | 3.35 | 0 |
| S1_k141_92191 | 1257 | No | NA | | 2 | 0 | Low-quality | Genome-fragment | | 3.5 | 0 |
| S1-final-viral-combined_k141_148726\|\|full | 2511 | No | NA | | 0 | 2 | Not-determined | Genome-fragment | NA | | 0 |
| S1-final-viral-combined_k141_66238\|\|full | 4280 | No | NA | | 0 | 4 | Not-determined | Genome-fragment | NA | | 0 |
| S1-final-viral-combined_k141_379037\|\|full | 3595 | No | NA | | 0 | 1 | Not-determined | Genome-fragment | NA | | 0 |
| S1_k141_78736 | 1257 | No | NA | | 0 | 1 | Low-quality | Genome-fragment | | 0.42 | 0 |
| S1-final-viral-combined_k141_362683\|\|full | 1893 | No | NA | | 0 | 0 | Not-determined | Genome-fragment | NA | | 0 |
| S5-final-viral-combined_k141_6870\|\|full | 1257 | No | NA | | 0 | 0 | Low-quality | Genome-fragment | | 3.16 | 0 |
| S5-final-viral-combined_k141_158416\|\|lt2gene | 1257 | No | NA | | 1 | 0 | Low-quality | Genome-fragment | | 0.69 | 0 |
| S1-final-viral-combined_k141_231001\|\|full | 5638 | No | NA | | 0 | 3 | Not-determined | Genome-fragment | NA | | 0 |
| S1-final-viral-combined_k141_927\|\|full | 1954 | No | NA | | 0 | 2 | Not-determined | Genome-fragment | NA | | 0 |
| S7-final-viral-combined_k141_203239\|\|full | 1257 | No | NA | | 2 | 0 | Low-quality | Genome-fragment | | 3.39 | 0 |
| S1-final-viral-combined_k141_17181\|\|full | 1423 | No | NA | | 0 | 0 | Not-determined | Genome-fragment | NA | | 0 |
| S1-final-viral-combined_k141_83124\|\|full | 1993 | No | NA | | 0 | 0 | Not-determined | Genome-fragment | NA | | 0 |
| S12-final-viral-combined_k141_22510\|\|full | 1257 | No | NA | | 1 | 0 | Low-quality | Genome-fragment | | 3.67 | 0 |
| S12-final-viral-combined_k141_162794\|\|full | 1257 | No | NA | | 0 | 0 | Low-quality | Genome-fragment | | 1.84 | 0 |
| S12-final-viral-combined_k141_197981\|\|full | 1257 | No | NA | | 2 | 0 | Low-quality | Genome-fragment | | 2.49 | 0 |
| S12_k141_77351 | 1257 | No | NA | | 0 | 1 | Low-quality | Genome-fragment | | 0.43 | 0 |
| S1-final-viral-combined_k141_116093\|\|full | 2600 | No | NA | | 0 | 2 | Not-determined | Genome-fragment | NA | | 0 |
| S1-final-viral-combined_k141_198227\|\|full | 2396 | No | NA | | 0 | 2 | Not-determined | Genome-fragment | NA | | 0 |
| S1-final-viral-combined_k141_247557\|\|full | 3920 | No | NA | | 0 | 1 | Not-determined | Genome-fragment | NA | | 0 |
| S1-final-viral-combined_k141_313279\|\|full | 2341 | No | NA | | 0 | 1 | Not-determined | Genome-fragment | NA | | 0 |
| S1-final-viral-combined_k141_280547\|\|full | 3342 | No | NA | | 0 | 2 | Not-determined | Genome-fragment | NA | | 0 |
| S1-final-viral-combined_k141_264022\|\|full | 1672 | No | NA | | 0 | 3 | Not-determined | Genome-fragment | NA | | 0 |
| S2-final-viral-combined_k141_54558\|\|lt2gene | 1257 | No | NA | | 2 | 0 | Low-quality | Genome-fragment | | 3.29 | 0 |
| S1-final-viral-combined_k141_247577\|\|full | 3867 | No | NA | | 0 | 3 | Not-determined | Genome-fragment | NA | | 0 |
| S1-final-viral-combined_k141_247578\|\|full | 4106 | No | NA | | 0 | 3 | Not-determined | Genome-fragment | NA | | 0 |
| S1-final-viral-combined_k141_346382\|\|full | 9721 | No | NA | | 0 | 5 | Not-determined | Genome-fragment | NA | | 0 |
| S2_k141_71516 | 1257 | No | NA | | 0 | 1 | Low-quality | Genome-fragment | | 0.78 | 0 |
| S1-final-viral-combined_k141_346392\|\|full | 1568 | No | NA | | 0 | 1 | Not-determined | Genome-fragment | NA | | 0 |
| S1-final-viral-combined_k141_1014\|\|full | 2051 | No | NA | | 0 | 2 | Not-determined | Genome-fragment | NA | | 0 |
| S1-final-viral-combined_k141_313366\|\|full | 1089 | No | NA | | 0 | 0 | Not-determined | Genome-fragment | NA | | 0 |
| S1-final-viral-combined_k141_313369\|\|full | 2881 | No | NA | | 0 | 3 | Not-determined | Genome-fragment | NA | | 0 |
| S1-final-viral-combined_k141_296921\|\|full | 3878 | No | NA | | 0 | 3 | Not-determined | Genome-fragment | NA | | 0 |
| S1-final-viral-combined_k141_231084\|\|full | 2193 | No | NA | | 0 | 2 | Not-determined | Genome-fragment | NA | | 0 |
| S1-final-viral-combined_k141_148824\|\|full | 3095 | No | NA | | 0 | 2 | Not-determined | Genome-fragment | NA | | 0 |
| S4-final-viral-combined_k141_74858\|\|full | 1257 | No | NA | | 0 | 2 | Low-quality | Genome-fragment | | 2.09 | 0 |
| S9-final-viral-combined_k141_27549\|\|full | 1258 | Yes | | 773 | 2 | 1 | Low-quality | Genome-fragment | | 1.75 | 38.55 |
| S9-final-viral-combined_k141_92090\|\|full | 1258 | No | NA | | 0 | 0 | Low-quality | Genome-fragment | | 2.19 | 0 |
| S1-final-viral-combined_k141_214898\|\|full | 2240 | No | NA | | 0 | 0 | Not-determined | Genome-fragment | NA | | 0 |
| S1-final-viral-combined_k141_329783\|\|full | 2111 | No | NA | | 0 | 1 | Not-determined | Genome-fragment | NA | | 0 |
| S1-final-viral-combined_k141_64309\|\|full | 1258 | No | NA | | 1 | 0 | Low-quality | Genome-fragment | | 3.57 | 0 |
| S1-final-viral-combined_k141_1080\|\|full | 4002 | No | NA | | 0 | 1 | Not-determined | Genome-fragment | NA | | 0 |
| S3-final-viral-combined_k141_1896\|\|full | 1258 | No | NA | | 1 | 0 | Low-quality | Genome-fragment | | 2.2 | 0 |
| S5-final-viral-combined_k141_170758\|\|full | 1258 | No | NA | | 1 | 0 | Low-quality | Genome-fragment | | 2.12 | 0 |
| S1-final-viral-combined_k141_231131\|\|full | 4792 | No | NA | | 0 | 1 | Not-determined | Genome-fragment | NA | | 0 |
| S1-final-viral-combined_k141_296985\|\|full | 3839 | No | NA | | 0 | 0 | Not-determined | Genome-fragment | NA | | 0 |

| | | | | | | | | | | | |
|---|---|---|---|---|---|---|---|---|---|---|---|
| S5-final-viral-combined_k141_129244\|\|full | 1258 | No | NA | | 1 | 0 | Low-quality | Genome-fragment | | 8.68 | 0 |
| S1-final-viral-combined_k141_1114\|\|full | 5544 | No | NA | | 0 | 3 | Not-determined | Genome-fragment | NA | | 0 |
| S12-final-viral-combined_k141_169520\|\|full | 1258 | No | NA | | 0 | 0 | Low-quality | Genome-fragment | | 3.44 | 0 |
| S1-final-viral-combined_k141_346507\|\|full | 1520 | No | NA | | 0 | 0 | Not-determined | Genome-fragment | NA | | 0 |
| S1-final-viral-combined_k141_379264\|\|full | 1178 | No | NA | | 0 | 0 | Not-determined | Genome-fragment | NA | | 0 |
| S12-final-viral-combined_k141_182199\|\|full | 1258 | No | NA | | 2 | 0 | Low-quality | Genome-fragment | | 2.79 | 0 |
| S2-final-viral-combined_k141_57188\|\|full | 1258 | No | NA | | 2 | 0 | Low-quality | Genome-fragment | | 2.92 | 0 |
| S2-final-viral-combined_k141_238112\|\|full | 1258 | No | NA | | 0 | 2 | Low-quality | Genome-fragment | | 2.85 | 0 |
| S4-final-viral-combined_k141_21756\|\|full | 1258 | No | NA | | 0 | 0 | Low-quality | Genome-fragment | | 0.79 | 0 |
| S1-final-viral-combined_k141_132662\|\|full | 8323 | No | NA | | 0 | 10 | Not-determined | Genome-fragment | NA | | 0 |
| S6-final-viral-combined_k141_136267\|\|full | 1258 | No | NA | | 1 | 0 | Low-quality | Genome-fragment | | 1.46 | 0 |
| S6-final-viral-combined_k141_97993\|\|full | 1258 | No | NA | | 1 | 0 | Low-quality | Genome-fragment | | 2.76 | 0 |
| S8-final-viral-combined_k141_218896\|\|full | 1258 | No | NA | | 0 | 1 | Low-quality | Genome-fragment | | 1.62 | 0 |
| S1-final-viral-combined_k141_231210\|\|full | 1596 | No | NA | | 0 | 0 | Not-determined | Genome-fragment | NA | | 0 |
| S1-final-viral-combined_k141_379289\|\|full | 6739 | No | NA | | 0 | 6 | Not-determined | Genome-fragment | NA | | 0 |
| S8-final-viral-combined_k141_128089\|\|full | 1258 | No | NA | | 0 | 1 | Low-quality | Genome-fragment | | 2.97 | 0 |
| S11-final-viral-combined_k141_314082\|\|full | 1259 | No | NA | | 0 | 0 | Low-quality | Genome-fragment | | 2.03 | 0 |
| S1-final-viral-combined_k141_147340\|\|full | 1259 | No | NA | | 0 | 0 | Low-quality | Genome-fragment | | 2.93 | 0 |
| S1-final-viral-combined_k141_362955\|\|full | 1349 | No | NA | | 0 | 0 | Not-determined | Genome-fragment | NA | | 0 |
| S1-final-viral-combined_k141_1173\|\|full | 1774 | No | NA | | 0 | 2 | Not-determined | Genome-fragment | NA | | 0 |
| S1-final-viral-combined_k141_329842\|\|full | 2244 | No | NA | | 0 | 1 | Not-determined | Genome-fragment | NA | | 0 |
| S1-final-viral-combined_k141_215037\|\|full | 2623 | No | NA | | 0 | 2 | Not-determined | Genome-fragment | NA | | 0 |
| S1-final-viral-combined_k141_171238\|\|lt2gene | 1259 | No | NA | | 2 | 0 | Low-quality | Genome-fragment | | 2.25 | 0 |
| S1-final-viral-combined_k141_66406\|\|full | 7675 | No | NA | | 0 | 8 | Not-determined | Genome-fragment | NA | | 0 |
| S1_k141_174759 | 1259 | No | NA | | 1 | 0 | Low-quality | Genome-fragment | | 3.57 | 0 |
| S3_k141_111915 | 1259 | No | NA | | 0 | 1 | Low-quality | Genome-fragment | | 0.47 | 0 |
| S5-final-viral-combined_k141_24518\|\|full | 1259 | No | NA | | 0 | 0 | Low-quality | Genome-fragment | | 0.78 | 0 |
| S1-final-viral-combined_k141_231234\|\|full | 26780 | No | NA | | 0 | 0 | Medium-quality | Genome-fragment | | 65.27 | 0 |
| S17-k141_238843 | 1259 | No | NA | | 1 | 0 | Low-quality | Genome-fragment | | 2.67 | 0 |
| S1-final-viral-combined_k141_66458\|\|full | 4385 | No | NA | | 0 | 4 | Not-determined | Genome-fragment | NA | | 0 |
| S1-final-viral-combined_k141_66459\|\|full | 3202 | No | NA | | 0 | 1 | Not-determined | Genome-fragment | NA | | 0 |
| S1-final-viral-combined_k141_363002\|\|full | 3731 | No | NA | | 0 | 2 | Not-determined | Genome-fragment | NA | | 0 |
| S12-final-viral-combined_k141_318265\|\|full | 1259 | No | NA | | 1 | 0 | Low-quality | Genome-fragment | | 1.43 | 0 |
| S12_k141_186392 | 1259 | No | NA | | 0 | 1 | Low-quality | Genome-fragment | | 1.74 | 0 |
| S1-final-viral-combined_k141_198425\|\|full | 1762 | No | NA | | 0 | 1 | Not-determined | Genome-fragment | NA | | 0 |
| S1-final-viral-combined_k141_99599\|\|full | 1235 | No | NA | | 0 | 1 | Not-determined | Genome-fragment | NA | | 0 |
| S12_k141_198242 | 1259 | No | NA | | 0 | 0 | Low-quality | Genome-fragment | | 1.92 | 0 |
| S2-final-viral-combined_k141_53599\|\|full | 1259 | No | NA | | 0 | 0 | Low-quality | Genome-fragment | | 2.93 | 0 |
| S11-final-viral-combined_k141_209477\|\|full | 1260 | No | NA | | 2 | 0 | Low-quality | Genome-fragment | | 2.61 | 0 |
| S1-final-viral-combined_k141_66476\|\|full | 2057 | No | NA | | 0 | 0 | Not-determined | Genome-fragment | NA | | 0 |
| S1-final-viral-combined_k141_66480\|\|full | 1278 | No | NA | | 0 | 1 | Not-determined | Genome-fragment | NA | | 0 |
| S11-final-viral-combined_k141_255447\|\|full | 1260 | No | NA | | 0 | 0 | Low-quality | Genome-fragment | | 1.7 | 0 |
| S1-final-viral-combined_k141_33611\|\|full | 1255 | No | NA | | 0 | 0 | Not-determined | Genome-fragment | NA | | 0 |
| S1-final-viral-combined_k141_17366\|\|full | 1716 | No | NA | | 0 | 0 | Not-determined | Genome-fragment | NA | | 0 |
| S1-final-viral-combined_k141_346613\|\|full | 2854 | No | NA | | 0 | 2 | Not-determined | Genome-fragment | NA | | 0 |
| S1-final-viral-combined_k141_17380\|\|full | 2636 | No | NA | | 0 | 1 | Not-determined | Genome-fragment | NA | | 0 |
| S3-final-viral-combined_k141_8866\|\|lt2gene | 1260 | No | NA | | 2 | 0 | Low-quality | Genome-fragment | | 2.36 | 0 |
| S1-final-viral-combined_k141_33639\|\|full | 1070 | No | NA | | 0 | 0 | Not-determined | Genome-fragment | NA | | 0 |
| S1-final-viral-combined_k141_17397\|\|full | 1952 | No | NA | | 0 | 2 | Not-determined | Genome-fragment | NA | | 0 |
| S5-final-viral-combined_k141_54800\|\|full | 1260 | No | NA | | 0 | 0 | Low-quality | Genome-fragment | | 1.96 | 0 |
| S1-final-viral-combined_k141_1271\|\|full | 2498 | No | NA | | 0 | 0 | Not-determined | Genome-fragment | NA | | 0 |
| S5_k141_158512 | 1260 | No | NA | | 0 | 0 | Low-quality | Genome-fragment | | 2.43 | 0 |
| S7-final-viral-combined_k141_129716\|\|full | 1260 | No | NA | | 0 | 0 | Low-quality | Genome-fragment | | 1.68 | 0 |
| S1-final-viral-combined_k141_83362\|\|full | 4290 | No | NA | | 0 | 2 | Not-determined | Genome-fragment | NA | | 0 |
| S1-final-viral-combined_k141_165469\|\|full | 2502 | No | NA | | 0 | 0 | Not-determined | Genome-fragment | NA | | 0 |
| S1-final-viral-combined_k141_264264\|\|full | 4517 | No | NA | | 0 | 0 | Not-determined | Genome-fragment | NA | | 0 |
| S1-final-viral-combined_k141_49936\|\|full | 3706 | No | NA | | 0 | 3 | Not-determined | Genome-fragment | NA | | 0 |
| S1-final-viral-combined_k141_329891\|\|full | 2841 | No | NA | | 0 | 1 | Not-determined | Genome-fragment | NA | | 0 |
| S1-final-viral-combined_k141_280807\|\|full | 9154 | No | NA | | 0 | 0 | Not-determined | Genome-fragment | NA | | 0 |
| S17-final-viral-combined_k141_51532\|\|full | 1260 | No | NA | | 1 | 0 | Low-quality | Genome-fragment | | 2.59 | 0 |
| S1-final-viral-combined_k141_280811\|\|full | 1108 | No | NA | | 0 | 0 | Not-determined | Genome-fragment | NA | | 0 |
| S1-final-viral-combined_k141_346665\|\|full | 6147 | No | NA | | 0 | 1 | Not-determined | Genome-fragment | NA | | 0 |
| S2_k141_229975 | 1260 | No | NA | | 0 | 1 | Low-quality | Genome-fragment | | 2.1 | 0 |
| S1-final-viral-combined_k141_1318\|\|full | 2439 | No | NA | | 0 | 1 | Not-determined | Genome-fragment | NA | | 0 |
| S8-final-viral-combined_k141_92304\|\|lt2gene | 1260 | No | NA | | 1 | 0 | Low-quality | Genome-fragment | | 2.89 | 0 |
| S1-final-viral-combined_k141_231354\|\|full | 3544 | No | NA | | 0 | 0 | Not-determined | Genome-fragment | NA | | 0 |
| S1_k141_69059 | 1261 | No | NA | | 0 | 0 | Low-quality | Genome-fragment | | 0.12 | 0 |
| S3_k141_47123 | 1261 | No | NA | | 0 | 0 | Low-quality | Genome-fragment | | 3.34 | 0 |
| S1-final-viral-combined_k141_231366\|\|full | 1547 | No | NA | | 0 | 1 | Not-determined | Genome-fragment | NA | | 0 |
| S1-final-viral-combined_k141_1349\|\|full | 2020 | No | NA | | 0 | 1 | Not-determined | Genome-fragment | NA | | 0 |
| S1-final-viral-combined_k141_83405\|\|full | 1705 | No | NA | | 0 | 1 | Not-determined | Genome-fragment | NA | | 0 |
| S1-final-viral-combined_k141_264291\|\|full | 22782 | No | NA | | 0 | 3 | Not-determined | Genome-fragment | NA | | 0 |
| S5-final-viral-combined_k141_151518\|\|full | 1261 | No | NA | | 2 | 0 | Low-quality | Genome-fragment | | 3.11 | 0 |
| S1-final-viral-combined_k141_297288\|\|full | 1838 | No | NA | | 0 | 1 | Not-determined | Genome-fragment | NA | | 0 |
| S1-final-viral-combined_k141_198541\|\|full | 6626 | No | NA | | 0 | 3 | Not-determined | Genome-fragment | NA | | 0 |
| S1-final-viral-combined_k141_17449\|\|full | 4689 | No | NA | | 0 | 3 | Not-determined | Genome-fragment | NA | | 0 |
| S17-final-viral-combined_k141_38687\|\|full | 1261 | No | NA | | 0 | 2 | Low-quality | Genome-fragment | | 0.6 | 0 |
| S1-final-viral-combined_k141_280825\|\|full | 4670 | No | NA | | 0 | 4 | Not-determined | Genome-fragment | NA | | 0 |
| S17-final-viral-combined_k141_263398\|\|full | 1261 | No | NA | | 0 | 2 | Low-quality | Genome-fragment | | 0.62 | 0 |
| S17-k141_75494 | 1261 | No | NA | | 0 | 1 | Low-quality | Genome-fragment | | 1.12 | 0 |
| S1-final-viral-combined_k141_247921\|\|full | 2429 | No | NA | | 0 | 1 | Not-determined | Genome-fragment | NA | | 0 |
| S12-final-viral-combined_k141_89539\|\|full | 1261 | No | NA | | 0 | 0 | Low-quality | Genome-fragment | | 0.75 | 0 |
| S12-final-viral-combined_k141_142756\|\|full | 1261 | No | NA | | 0 | 0 | Low-quality | Genome-fragment | | 0.63 | 0 |
| S12_k141_75666 | 1261 | No | NA | | 0 | 1 | Low-quality | Genome-fragment | | 0.65 | 0 |
| S12_k141_11294 | 1261 | No | NA | | 0 | 1 | Low-quality | Genome-fragment | | 0.7 | 0 |
| S1-final-viral-combined_k141_1415\|\|full | 2973 | No | NA | | 0 | 1 | Not-determined | Genome-fragment | NA | | 0 |
| S1-final-viral-combined_k141_280879\|\|full | 4978 | No | NA | | 0 | 2 | Not-determined | Genome-fragment | NA | | 0 |
| S1-final-viral-combined_k141_231411\|\|full | 12380 | No | NA | | 1 | 0 | Medium-quality | Genome-fragment | | 83.34 | 0 |
| S4-final-viral-combined_k141_8479\|\|full | 1261 | No | NA | | 0 | 0 | Low-quality | Genome-fragment | | 1.34 | 0 |
| S4-final-viral-combined_k141_94351\|\|full | 1261 | No | NA | | 0 | 0 | Low-quality | Genome-fragment | | 2.08 | 0 |
| S1-final-viral-combined_k141_198600\|\|full | 6249 | No | NA | | 0 | 1 | Not-determined | Genome-fragment | NA | | 0 |
| S1-final-viral-combined_k141_280887\|\|full | 1568 | No | NA | | 0 | 0 | Not-determined | Genome-fragment | NA | | 0 |
| S1-final-viral-combined_k141_99749\|\|full | 1461 | No | NA | | 0 | 2 | Not-determined | Genome-fragment | NA | | 0 |
| S1-final-viral-combined_k141_132934\|\|full | 51171 | Yes | | 43190 | 16 | 7 | Complete | High-quality | | 100 | 15.6 |
| S1-final-viral-combined_k141_99759\|\|full | 2867 | No | NA | | 0 | 0 | Not-determined | Genome-fragment | NA | | 0 |
| S1-final-viral-combined_k141_66554\|\|full | 2727 | No | NA | | 0 | 2 | Not-determined | Genome-fragment | NA | | 0 |
| S1-final-viral-combined_k141_198626\|\|full | 1157 | No | NA | | 0 | 1 | Not-determined | Genome-fragment | NA | | 0 |
| S4-final-viral-combined_k141_74761\|\|full | 1261 | No | NA | | 0 | 1 | Low-quality | Genome-fragment | | 1.2 | 0 |
| S1-final-viral-combined_k141_248007\|\|full | 3599 | No | NA | | 0 | 2 | Not-determined | Genome-fragment | NA | | 0 |
| S1-final-viral-combined_k141_33813\|\|full | 16823 | No | NA | | 0 | 12 | Not-determined | Genome-fragment | NA | | 0 |
| S9-final-viral-combined_k141_105521\|\|full | 1262 | No | NA | | 0 | 0 | Low-quality | Genome-fragment | | 2.51 | 0 |
| S9-final-viral-combined_k141_92151\|\|lt2gene | 1262 | No | NA | | 1 | 0 | Low-quality | Genome-fragment | | 2.12 | 0 |
| S1-final-viral-combined_k141_132983\|\|full | 4415 | No | NA | | 0 | 4 | Not-determined | Genome-fragment | NA | | 0 |
| S7-final-viral-combined_k141_9721\|\|full | 1262 | No | NA | | 0 | 0 | Low-quality | Genome-fragment | | 2.13 | 0 |
| S1-final-viral-combined_k141_215337\|\|full | 6484 | No | NA | | 0 | 5 | Not-determined | Genome-fragment | NA | | 0 |
| S17-final-viral-combined_k141_102433\|\|full | 1262 | No | NA | | 1 | 0 | Low-quality | Genome-fragment | | 1.2 | 0 |
| S1-final-viral-combined_k141_182154\|\|full | 1442 | No | NA | | 0 | 1 | Not-determined | Genome-fragment | NA | | 0 |
| S1-final-viral-combined_k141_50047\|\|full | 4594 | No | NA | | 0 | 4 | Not-determined | Genome-fragment | NA | | 0 |
| S17-final-viral-combined_k141_248665\|\|full | 1262 | No | NA | | 1 | 0 | Low-quality | Genome-fragment | | 0.81 | 0 |
| S1-final-viral-combined_k141_215349\|\|full | 8512 | No | NA | | 0 | 5 | Not-determined | Genome-fragment | NA | | 0 |
| S12-final-viral-combined_k141_138509\|\|full | 1262 | No | NA | | 0 | 0 | Low-quality | Genome-fragment | | 2.36 | 0 |
| S12-final-viral-combined_k141_86548\|\|lt2gene | 1262 | No | NA | | 1 | 0 | Low-quality | Genome-fragment | | 3.02 | 0 |
| S2-final-viral-combined_k141_87233\|\|full | 1262 | No | NA | | 0 | 0 | Low-quality | Genome-fragment | | 2.44 | 0 |
| S2-final-viral-combined_k141_110072\|\|full | 1262 | No | NA | | 1 | 0 | Low-quality | Genome-fragment | | 2.85 | 0 |
| S1-final-viral-combined_k141_248087\|\|full | 2419 | No | NA | | 0 | 1 | Not-determined | Genome-fragment | NA | | 0 |
| S4_k141_142401 | 1262 | No | NA | | 2 | 0 | Low-quality | Genome-fragment | | 0.73 | 0 |
| S1-final-viral-combined_k141_182241\|\|full | 1177 | No | NA | | 0 | 1 | Not-determined | Genome-fragment | NA | | 0 |
| S1-final-viral-combined_k141_33908\|\|full | 1454 | No | NA | | 0 | 1 | Not-determined | Genome-fragment | NA | | 0 |
| S1-final-viral-combined_k141_313824\|\|full | 4085 | No | NA | | 0 | 4 | Not-determined | Genome-fragment | NA | | 0 |
| S6_k141_111742 | 1262 | No | NA | | 0 | 0 | Low-quality | Genome-fragment | | 1.32 | 0 |
| S1-final-viral-combined_k141_281012\|\|full | 3269 | No | NA | | 0 | 1 | Not-determined | Genome-fragment | NA | | 0 |
| S1-final-viral-combined_k141_165561\|\|full | 2062 | No | NA | | 0 | 0 | Not-determined | Genome-fragment | NA | | 0 |
| S1-final-viral-combined_k141_281018\|\|full | 5269 | No | NA | | 0 | 4 | Not-determined | Genome-fragment | NA | | 0 |
| S1-final-viral-combined_k141_346881\|\|full | 2845 | No | NA | | 0 | 0 | Not-determined | Genome-fragment | NA | | 0 |
| S9-final-viral-combined_k141_16039\|\|full | 1263 | No | NA | | 1 | 0 | Low-quality | Genome-fragment | | 1.77 | 0 |
| S1-final-viral-combined_k141_281033\|\|full | 1082 | No | NA | | 0 | 1 | Not-determined | Genome-fragment | NA | | 0 |
| S1-final-viral-combined_k141_165607\|\|full | 2641 | No | NA | | 0 | 0 | Not-determined | Genome-fragment | NA | | 0 |
| S1-final-viral-combined_k141_165608\|\|full | 1401 | No | NA | | 0 | 0 | Not-determined | Genome-fragment | NA | | 0 |
| S11-final-viral-combined_k141_187844\|\|full | 1263 | No | NA | | 1 | 0 | Low-quality | Genome-fragment | | 2.8 | 0 |
| S1-final-viral-combined_k141_116554\|\|full | 1799 | No | NA | | 0 | 0 | Not-determined | Genome-fragment | NA | | 0 |
| S1-final-viral-combined_k141_116556\|\|full | 1108 | No | NA | | 0 | 0 | Not-determined | Genome-fragment | NA | | 0 |
| S1-final-viral-combined_k141_72777\|\|full | 1263 | No | NA | | 0 | 0 | Low-quality | Genome-fragment | | 1.91 | 0 |
| S1-final-viral-combined_k141_328207\|\|lt2gene | 1263 | No | NA | | 1 | 0 | Low-quality | Genome-fragment | | 1.18 | 0 |
| S1_k141_218752 | 1263 | No | NA | | 0 | 0 | Low-quality | Genome-fragment | | 2.72 | 0 |
| S1-final-viral-combined_k141_231492\|\|full | 24462 | No | NA | | 0 | 13 | Not-determined | Genome-fragment | NA | | 0 |
| S1_k141_73160 | 1263 | No | NA | | 0 | 0 | Low-quality | Genome-fragment | | 3.28 | 0 |
| S1-final-viral-combined_k141_281062\|\|full | 4248 | No | NA | | 0 | 1 | Not-determined | Genome-fragment | NA | | 0 |
| S1-final-viral-combined_k141_17651\|\|full | 7507 | No | NA | | 1 | 0 | High-quality | High-quality | | 100 | 0 |
| S1-final-viral-combined_k141_165641\|\|full | 3142 | No | NA | | 0 | 2 | Not-determined | Genome-fragment | NA | | 0 |
| S1-final-viral-combined_k141_297485\|\|full | 9631 | No | NA | | 0 | 6 | Not-determined | Genome-fragment | NA | | 0 |
| S3_k141_118011 | 1263 | No | NA | | 0 | 0 | Low-quality | Genome-fragment | | 0.95 | 0 |
| S1-final-viral-combined_k141_313851\|\|full | 13334 | No | NA | | 5 | 0 | High-quality | High-quality | | 96 | 0 |
| S7-final-viral-combined_k141_70245\|\|lt2gene | 1263 | No | NA | | 1 | 0 | Low-quality | Genome-fragment | | 0.87 | 0 |
| S17-final-viral-combined_k141_228696\|\|lt2gene | 1263 | No | NA | | 2 | 0 | Low-quality | Genome-fragment | | 2.65 | 0 |
| S17-k141_228362 | 1263 | No | NA | | 0 | 0 | Low-quality | Genome-fragment | | 3.1 | 0 |
| S1-final-viral-combined_k141_313865\|\|full | 1924 | No | NA | | 0 | 2 | Not-determined | Genome-fragment | NA | | 0 |
| S1-final-viral-combined_k141_1583\|\|full | 2544 | No | NA | | 0 | 1 | Not-determined | Genome-fragment | NA | | 0 |
| S1-final-viral-combined_k141_17683\|\|full | 3754 | No | NA | | 0 | 4 | Not-determined | Genome-fragment | NA | | 0 |
| S1-final-viral-combined_k141_99918\|\|full | 1945 | No | NA | | 0 | 0 | Not-determined | Genome-fragment | NA | | 0 |
| S1-final-viral-combined_k141_17689\|\|full | 1087 | No | NA | | 0 | 1 | Not-determined | Genome-fragment | NA | | 0 |
| S1-final-viral-combined_k141_50160\|\|full | 3125 | No | NA | | 0 | 0 | Not-determined | Genome-fragment | NA | | 0 |
| S12-final-viral-combined_k141_88161\|\|full | 1263 | No | NA | | 1 | 0 | Low-quality | Genome-fragment | | 3.08 | 0 |
| S12-final-viral-combined_k141_360711\|\|lt2gene | 1263 | No | NA | | 2 | 0 | Low-quality | Genome-fragment | | 2.06 | 0 |
| S1-final-viral-combined_k141_231562\|\|full | 1779 | No | NA | | 0 | 0 | Not-determined | Genome-fragment | NA | | 0 |
| S1-final-viral-combined_k141_116633\|\|full | 1623 | No | NA | | 0 | 2 | Not-determined | Genome-fragment | NA | | 0 |
| S1-final-viral-combined_k141_297511\|\|full | 1326 | No | NA | | 0 | 2 | Not-determined | Genome-fragment | NA | | 0 |
| S1-final-viral-combined_k141_1602\|\|full | 2568 | No | NA | | 0 | 1 | Not-determined | Genome-fragment | NA | | 0 |
| S1-final-viral-combined_k141_133144\|\|full | 7309 | No | NA | | 0 | 1 | Not-determined | Genome-fragment | NA | | 0 |
| S1-final-viral-combined_k141_182370\|\|full | 40907 | No | NA | | 0 | 18 | Not-determined | Genome-fragment | NA | | 0 |
| S1-final-viral-combined_k141_182372\|\|full | 1768 | No | NA | | 0 | 1 | Not-determined | Genome-fragment | NA | | 0 |
| S8-final-viral-combined_k141_151700\|\|full | 1263 | No | NA | | 0 | 0 | Low-quality | Genome-fragment | | 2.09 | 0 |
| S8-final-viral-combined_k141_77966\|\|full | 1263 | No | NA | | 0 | 1 | Low-quality | Genome-fragment | | 1.36 | 0 |
| S8_k141_126740 | 1263 | No | NA | | 0 | 2 | Low-quality | Genome-fragment | | 2.61 | 0 |
| S1-final-viral-combined_k141_149272\|\|full | 7892 | No | NA | | 0 | 3 | Not-determined | Genome-fragment | NA | | 0 |
| S11-final-viral-combined_k141_329672\|\|full | 1264 | No | NA | | 0 | 0 | Low-quality | Genome-fragment | | 2.89 | 0 |
| S1-final-viral-combined_k141_50193\|\|full | 14977 | No | NA | | 0 | 10 | Not-determined | Genome-fragment | NA | | 0 |
| S1-final-viral-combined_k141_133193\|\|full | 2543 | No | NA | | 0 | 2 | Not-determined | Genome-fragment | NA | | 0 |
| S1-final-viral-combined_k141_379564\|\|full | 1660 | No | NA | | 0 | 2 | Not-determined | Genome-fragment | NA | | 0 |
| S11_k141_213778 | 1264 | No | NA | | 1 | 0 | Low-quality | Genome-fragment | | 2.93 | 0 |
| S1-final-viral-combined_k141_83571\|\|full | 1142 | No | NA | | 0 | 0 | Not-determined | Genome-fragment | NA | | 0 |
| S1-final-viral-combined_k141_242032\|\|lt2gene | 1264 | No | NA | | 1 | 0 | Low-quality | Genome-fragment | | 2.21 | 0 |
| S5-final-viral-combined_k141_66500\|\|full | 1264 | No | NA | | 1 | 0 | Low-quality | Genome-fragment | | 2.41 | 0 |
| S1-final-viral-combined_k141_83585\|\|full | 1408 | No | NA | | 0 | 1 | Not-determined | Genome-fragment | NA | | 0 |
| S1-final-viral-combined_k141_198931\|\|full | 1623 | No | NA | | 0 | 0 | Not-determined | Genome-fragment | NA | | 0 |

| | | | | | | | | | | | |
|---|---|---|---|---|---|---|---|---|---|---|---|
| S1-final-viral-combined_k141_379598\|\|full | 3489 | No | NA | | 0 | 3 | Not-determined | Genome-fragment | NA | | 0 |
| S1-final-viral-combined_k141_116691\|\|full | 2948 | No | NA | | 0 | 2 | Not-determined | Genome-fragment | NA | | 0 |
| S5_k141_64805 | 1264 | No | NA | | 1 | 0 | Low-quality | Genome-fragment | | 1.85 | 0 |
| S2-final-viral-combined_k141_6993\|\|full | 1264 | No | NA | | 0 | 0 | Low-quality | Genome-fragment | | 2.2 | 0 |
| S1-final-viral-combined_k141_231572\|\|full | 4555 | No | NA | | 0 | 1 | Not-determined | Genome-fragment | NA | | 0 |
| S2_k141_183774 | 1264 | No | NA | | 0 | 0 | Low-quality | Genome-fragment | | 0.78 | 0 |
| S1-final-viral-combined_k141_314006\|\|full | 1934 | No | NA | | 0 | 2 | Not-determined | Genome-fragment | NA | | 0 |
| S1-final-viral-combined_k141_17781\|\|full | 2233 | No | NA | | 0 | 1 | Not-determined | Genome-fragment | NA | | 0 |
| S1-final-viral-combined_k141_149340\|\|full | 5690 | No | NA | | 0 | 4 | Not-determined | Genome-fragment | NA | | 0 |
| S8-final-viral-combined_k141_49187\|\|full | 1264 | No | NA | | 0 | 0 | Low-quality | Genome-fragment | | 2.71 | 0 |
| S9_k141_90026 | 1265 | No | NA | | 2 | 0 | Low-quality | Genome-fragment | | 3.15 | 0 |
| S11-final-viral-combined_k141_175283\|\|full | 1265 | No | NA | | 2 | 0 | Low-quality | Genome-fragment | | 1.1 | 0 |
| S1-final-viral-combined_k141_34176\|\|full | 1578 | No | NA | | 0 | 2 | Not-determined | Genome-fragment | NA | | 0 |
| S1-final-viral-combined_k141_347139\|\|full | 5145 | No | NA | | 0 | 4 | Not-determined | Genome-fragment | NA | | 0 |
| S1-final-viral-combined_k141_21789\|\|full | 1265 | No | NA | | 1 | 0 | Low-quality | Genome-fragment | | 3.57 | 0 |
| S1-final-viral-combined_k141_50280\|\|full | 2116 | No | NA | | 0 | 3 | Not-determined | Genome-fragment | NA | | 0 |
| S1-final-viral-combined_k141_330329\|\|full | 33155 | No | NA | | 7 | 4 | Medium-quality | Genome-fragment | | 77.41 | 0 |
| S1-final-viral-combined_k141_141459\|\|full | 1265 | No | NA | | 0 | 1 | Low-quality | Genome-fragment | | 1.64 | 0 |
| S5-final-viral-combined_k141_178108\|\|full | 1265 | No | NA | | 1 | 0 | Low-quality | Genome-fragment | | 1.42 | 0 |
| S1-final-viral-combined_k141_182446\|\|full | 1108 | No | NA | | 0 | 0 | Not-determined | Genome-fragment | NA | | 0 |
| S1-final-viral-combined_k141_50297\|\|full | 1910 | No | NA | | 0 | 0 | Not-determined | Genome-fragment | NA | | 0 |
| S1-final-viral-combined_k141_50304\|\|full | 1883 | No | NA | | 0 | 2 | Not-determined | Genome-fragment | NA | | 0 |
| S7-final-viral-combined_k141_218510\|\|full | 1265 | No | NA | | 0 | 1 | Low-quality | Genome-fragment | | 0.99 | 0 |
| S1-final-viral-combined_k141_50306\|\|full | 2559 | No | NA | | 0 | 0 | Not-determined | Genome-fragment | NA | | 0 |
| S1-final-viral-combined_k141_215482\|\|full | 3289 | No | NA | | 0 | 2 | Not-determined | Genome-fragment | NA | | 0 |
| S1-final-viral-combined_k141_133285\|\|full | 1286 | No | NA | | 0 | 1 | Not-determined | Genome-fragment | NA | | 0 |
| S1-final-viral-combined_k141_149378\|\|full | 2141 | No | NA | | 0 | 2 | Not-determined | Genome-fragment | NA | | 0 |
| S7-final-viral-combined_k141_180068\|\|full | 1265 | No | NA | | 0 | 1 | Low-quality | Genome-fragment | | 2.75 | 0 |
| S7-final-viral-combined_k141_243108\|\|full | 1265 | No | NA | | 0 | 1 | Low-quality | Genome-fragment | | 1.95 | 0 |
| S1-final-viral-combined_k141_297659\|\|full | 13147 | No | NA | | 0 | 7 | Not-determined | Genome-fragment | NA | | 0 |
| S1-final-viral-combined_k141_215511\|\|full | 3293 | No | NA | | 0 | 2 | Not-determined | Genome-fragment | NA | | 0 |
| S1-final-viral-combined_k141_34278\|\|full | 1536 | No | NA | | 0 | 2 | Not-determined | Genome-fragment | NA | | 0 |
| S17-final-viral-combined_k141_189598\|\|lt2gene | 1265 | No | NA | | 1 | 0 | Low-quality | Genome-fragment | | 1.19 | 0 |
| S1-final-viral-combined_k141_83726\|\|full | 3759 | No | NA | | 0 | 3 | Not-determined | Genome-fragment | NA | | 0 |
| S12-final-viral-combined_k141_179970\|\|full | 1265 | No | NA | | 0 | 1 | Low-quality | Genome-fragment | | 1.5 | 0 |
| S1-final-viral-combined_k141_149420\|\|full | 3087 | No | NA | | 0 | 2 | Not-determined | Genome-fragment | NA | | 0 |
| S12-final-viral-combined_k141_32847\|\|full | 1265 | No | NA | | 1 | 0 | Low-quality | Genome-fragment | | 2.04 | 0 |
| S2-final-viral-combined_k141_227638\|\|lt2gene | 1265 | No | NA | | 2 | 0 | Low-quality | Genome-fragment | | 4.44 | 0 |
| S1-final-viral-combined_k141_215575\|\|full | 2424 | No | NA | | 0 | 1 | Not-determined | Genome-fragment | NA | | 0 |
| S6-final-viral-combined_k141_35870\|\|full | 1265 | No | NA | | 0 | 0 | Low-quality | Genome-fragment | | 0.9 | 0 |
| S9-final-viral-combined_k141_63495\|\|full | 1266 | No | NA | | 0 | 1 | Low-quality | Genome-fragment | | 0.91 | 0 |
| S1-final-viral-combined_k141_248221\|\|full | 3655 | No | NA | | 0 | 2 | Not-determined | Genome-fragment | NA | | 0 |
| S1-final-viral-combined_k141_83783\|\|full | 4480 | No | NA | | 0 | 0 | Not-determined | Genome-fragment | NA | | 0 |
| S1-final-viral-combined_k141_165771\|\|full | 2697 | No | NA | | 0 | 0 | Not-determined | Genome-fragment | NA | | 0 |
| S1-final-viral-combined_k141_50386\|\|full | 1062 | No | NA | | 0 | 0 | Not-determined | Genome-fragment | NA | | 0 |
| S1-final-viral-combined_k141_231792\|\|full | 3453 | No | NA | | 0 | 2 | Not-determined | Genome-fragment | NA | | 0 |
| S1-final-viral-combined_k141_297754\|\|full | 2068 | No | NA | | 0 | 0 | Not-determined | Genome-fragment | NA | | 0 |
| S1-final-viral-combined_k141_231798\|\|full | 6162 | No | NA | | 0 | 5 | Not-determined | Genome-fragment | NA | | 0 |
| S11-final-viral-combined_k141_96324\|\|full | 1266 | No | NA | | 0 | 1 | Low-quality | Genome-fragment | | 1.08 | 0 |
| S1-final-viral-combined_k141_50405\|\|full | 4526 | No | NA | | 0 | 4 | Not-determined | Genome-fragment | NA | | 0 |
| S1-final-viral-combined_k141_215650\|\|full | 3054 | No | NA | | 0 | 3 | Not-determined | Genome-fragment | NA | | 0 |
| S7_k141_224000 | 1266 | No | NA | | 0 | 0 | Low-quality | Genome-fragment | | 2.17 | 0 |
| S1-final-viral-combined_k141_248293\|\|full | 1693 | No | NA | | 0 | 2 | Not-determined | Genome-fragment | NA | | 0 |
| S1-final-viral-combined_k141_149472\|\|full | 23957 | No | NA | | 0 | 12 | Not-determined | Genome-fragment | NA | | 0 |
| S1-final-viral-combined_k141_281370\|\|full | 3032 | No | NA | | 0 | 3 | Not-determined | Genome-fragment | NA | | 0 |
| S1-final-viral-combined_k141_314247\|\|full | 1717 | No | NA | | 0 | 0 | Not-determined | Genome-fragment | NA | | 0 |
| S1-final-viral-combined_k141_248315\|\|full | 3898 | No | NA | | 0 | 2 | Not-determined | Genome-fragment | NA | | 0 |
| S1-final-viral-combined_k141_330509\|\|full | 3074 | No | NA | | 0 | 1 | Not-determined | Genome-fragment | NA | | 0 |
| S1-final-viral-combined_k141_149498\|\|full | 1593 | No | NA | | 0 | 0 | Not-determined | Genome-fragment | NA | | 0 |
| S1-final-viral-combined_k141_330512\|\|full | 1601 | No | NA | | 0 | 1 | Not-determined | Genome-fragment | NA | | 0 |
| S17-final-viral-combined_k141_258108\|\|full | 1266 | No | NA | | 0 | 0 | Low-quality | Genome-fragment | | 0.79 | 0 |
| S1-final-viral-combined_k141_379919\|\|full | 5614 | No | NA | | 0 | 1 | Not-determined | Genome-fragment | NA | | 0 |
| S17-final-viral-combined_k141_94140\|\|full | 1266 | No | NA | | 0 | 1 | Low-quality | Genome-fragment | | 1.8 | 0 |
| S1-final-viral-combined_k141_149506\|\|full | 4629 | No | NA | | 0 | 3 | Not-determined | Genome-fragment | NA | | 0 |
| S17-final-viral-combined_k141_63298\|\|lt2gene | 1266 | No | NA | | 2 | 0 | Low-quality | Genome-fragment | | 2.74 | 0 |
| S1-final-viral-combined_k141_149508\|\|full | 1199 | No | NA | | 0 | 1 | Not-determined | Genome-fragment | NA | | 0 |
| S1-final-viral-combined_k141_379959\|\|full | 2161 | No | NA | | 0 | 2 | Not-determined | Genome-fragment | NA | | 0 |
| S1-final-viral-combined_k141_50436\|\|full | 5139 | No | NA | | 0 | 1 | Not-determined | Genome-fragment | NA | | 0 |
| S1-final-viral-combined_k141_34429\|\|full | 2830 | No | NA | | 0 | 3 | Not-determined | Genome-fragment | NA | | 0 |
| S1-final-viral-combined_k141_281425\|\|full | 1255 | No | NA | | 0 | 0 | Not-determined | Genome-fragment | NA | | 0 |
| S1-final-viral-combined_k141_182634\|\|full | 1867 | No | NA | | 0 | 1 | Not-determined | Genome-fragment | NA | | 0 |
| S1-final-viral-combined_k141_248392\|\|full | 10209 | No | NA | | 0 | 3 | Not-determined | Genome-fragment | NA | | 0 |
| S1-final-viral-combined_k141_248394\|\|full | 2444 | No | NA | | 0 | 0 | Not-determined | Genome-fragment | NA | | 0 |
| S12-final-viral-combined_k141_340915\|\|full | 1266 | No | NA | | 1 | 0 | Low-quality | Genome-fragment | | 1.3 | 0 |
| S1-final-viral-combined_k141_2019\|\|full | 2850 | No | NA | | 0 | 0 | Not-determined | Genome-fragment | NA | | 0 |
| S12-final-viral-combined_k141_359373\|\|full | 1266 | No | NA | | 3 | 0 | Low-quality | Genome-fragment | | 2.72 | 0 |
| S1-final-viral-combined_k141_133538\|\|full | 2158 | No | NA | | 0 | 1 | Not-determined | Genome-fragment | NA | | 0 |
| S1-final-viral-combined_k141_2030\|\|full | 7214 | No | NA | | 0 | 3 | Not-determined | Genome-fragment | NA | | 0 |
| S1-final-viral-combined_k141_182676\|\|full | 2046 | No | NA | | 0 | 1 | Not-determined | Genome-fragment | NA | | 0 |
| S1-final-viral-combined_k141_199239\|\|full | 1356 | No | NA | | 0 | 2 | Not-determined | Genome-fragment | NA | | 0 |
| S1-final-viral-combined_k141_18089\|\|full | 3427 | No | NA | | 0 | 2 | Not-determined | Genome-fragment | NA | | 0 |
| S1-final-viral-combined_k141_133562\|\|full | 1398 | No | NA | | 0 | 0 | Not-determined | Genome-fragment | NA | | 0 |
| S1-final-viral-combined_k141_330606\|\|full | 2498 | No | NA | | 0 | 2 | Not-determined | Genome-fragment | NA | | 0 |
| S1-final-viral-combined_k141_50483\|\|full | 1952 | No | NA | | 0 | 1 | Not-determined | Genome-fragment | NA | | 0 |
| S1-final-viral-combined_k141_83918\|\|full | 2606 | No | NA | | 0 | 3 | Not-determined | Genome-fragment | NA | | 0 |
| S1-final-viral-combined_k141_116855\|\|full | 1907 | No | NA | | 0 | 1 | Not-determined | Genome-fragment | NA | | 0 |
| S1-final-viral-combined_k141_231924\|\|full | 7654 | No | NA | | 0 | 4 | Not-determined | Genome-fragment | NA | | 0 |
| S1-final-viral-combined_k141_231927\|\|full | 1181 | No | NA | | 0 | 1 | Not-determined | Genome-fragment | NA | | 0 |
| S2-final-viral-combined_k141_206725\|\|full | 1266 | No | NA | | 0 | 0 | Low-quality | Genome-fragment | | 1.87 | 0 |
| S4-final-viral-combined_k141_119859\|\|full | 1266 | No | NA | | 2 | 0 | Low-quality | Genome-fragment | | 2.86 | 0 |
| S1-final-viral-combined_k141_231935\|\|full | 1137 | No | NA | | 0 | 0 | Not-determined | Genome-fragment | NA | | 0 |
| S1-final-viral-combined_k141_116870\|\|full | 1422 | No | NA | | 0 | 1 | Not-determined | Genome-fragment | NA | | 0 |
| S1-final-viral-combined_k141_18104\|\|full | 4658 | No | NA | | 0 | 0 | Not-determined | Genome-fragment | NA | | 0 |
| S1-final-viral-combined_k141_199268\|\|full | 1580 | No | NA | | 0 | 1 | Not-determined | Genome-fragment | NA | | 0 |
| S1-final-viral-combined_k141_50508\|\|full | 4230 | No | NA | | 0 | 0 | Not-determined | Genome-fragment | NA | | 0 |
| S1-final-viral-combined_k141_100196\|\|full | 1288 | No | NA | | 0 | 1 | Not-determined | Genome-fragment | NA | | 0 |
| S1-final-viral-combined_k141_133606\|\|full | 2147 | No | NA | | 0 | 2 | Not-determined | Genome-fragment | NA | | 0 |
| S1-final-viral-combined_k141_149656\|\|full | 1023 | No | NA | | 0 | 1 | Not-determined | Genome-fragment | NA | | 0 |
| S4-final-viral-combined_k141_59766\|\|full | 1266 | No | NA | | 0 | 1 | Low-quality | Genome-fragment | | 1.97 | 0 |
| S11-final-viral-combined_k141_295353\|\|full | 1267 | No | NA | | 1 | 0 | Low-quality | Genome-fragment | | 2.61 | 0 |
| S1-final-viral-combined_k141_363666\|\|full | 3581 | No | NA | | 0 | 2 | Not-determined | Genome-fragment | NA | | 0 |
| S1-final-viral-combined_k141_49448\|\|full | 1267 | No | NA | | 0 | 0 | Low-quality | Genome-fragment | | 1.91 | 0 |
| S1-final-viral-combined_k141_1861\|\|full | 1267 | No | NA | | 2 | 0 | Low-quality | Genome-fragment | | 3.37 | 0 |
| S1-final-viral-combined_k141_167084\|\|full | 1267 | No | NA | | 2 | 0 | Low-quality | Genome-fragment | | 2.59 | 0 |
| S1-final-viral-combined_k141_373692\|\|full | 1267 | No | NA | | 1 | 0 | Low-quality | Genome-fragment | | 2.68 | 0 |
| S1-final-viral-combined_k141_165809\|\|full | 1287 | No | NA | | 0 | 1 | Not-determined | Genome-fragment | NA | | 0 |
| S1-final-viral-combined_k141_116927\|\|full | 2484 | No | NA | | 0 | 3 | Not-determined | Genome-fragment | NA | | 0 |
| S1-final-viral-combined_k141_215796\|\|full | 2222 | No | NA | | 0 | 2 | Not-determined | Genome-fragment | NA | | 0 |
| S1-final-viral-combined_k141_363693\|\|full | 1100 | No | NA | | 0 | 1 | Not-determined | Genome-fragment | NA | | 0 |
| S1-final-viral-combined_k141_100258\|\|full | 1129 | No | NA | | 0 | 1 | Not-determined | Genome-fragment | NA | | 0 |
| S5-final-viral-combined_k141_209650\|\|full | 1267 | No | NA | | 1 | 0 | Low-quality | Genome-fragment | | 0.92 | 0 |
| S1-final-viral-combined_k141_231995\|\|full | 1557 | No | NA | | 0 | 2 | Not-determined | Genome-fragment | NA | | 0 |
| S17-final-viral-combined_k141_123339\|\|full | 1267 | No | NA | | 2 | 0 | Low-quality | Genome-fragment | | 3.48 | 0 |
| S1-final-viral-combined_k141_215806\|\|full | 12770 | No | NA | | 0 | 10 | Not-determined | Genome-fragment | NA | | 0 |
| S1-final-viral-combined_k141_330694\|\|full | 4167 | No | NA | | 0 | 2 | Not-determined | Genome-fragment | NA | | 0 |
| S12_k141_116012 | 1267 | No | NA | | 0 | 0 | Low-quality | Genome-fragment | | 0.76 | 0 |
| S1-final-viral-combined_k141_165834\|\|full | 3191 | No | NA | | 0 | 2 | Not-determined | Genome-fragment | NA | | 0 |
| S1-final-viral-combined_k141_100287\|\|full | 1261 | No | NA | | 0 | 1 | Not-determined | Genome-fragment | NA | | 0 |
| S1-final-viral-combined_k141_215846\|\|full | 3825 | No | NA | | 0 | 1 | Not-determined | Genome-fragment | NA | | 0 |
| S8-final-viral-combined_k141_50868\|\|lt2gene | 1267 | No | NA | | 2 | 0 | Low-quality | Genome-fragment | | 1.98 | 0 |
| S11-final-viral-combined_k141_293636\|\|full | 1268 | No | NA | | 0 | 0 | Low-quality | Genome-fragment | | 2.12 | 0 |
| S11-final-viral-combined_k141_189106\|\|lt2gene | 1268 | No | NA | | 2 | 0 | Low-quality | Genome-fragment | | 3.71 | 0 |
| S1-final-viral-combined_k141_284382\|\|full | 1268 | No | NA | | 0 | 1 | Low-quality | Genome-fragment | | 1.5 | 0 |
| S1-final-viral-combined_k141_116938\|\|full | 24124 | No | NA | | 18 | 1 | Medium-quality | Genome-fragment | | 68.29 | 0 |
| S1-final-viral-combined_k141_363720\|\|full | 3342 | No | NA | | 0 | 0 | Not-determined | Genome-fragment | NA | | 0 |
| S1-final-viral-combined_k141_100316\|\|full | 1494 | No | NA | | 0 | 1 | Not-determined | Genome-fragment | NA | | 0 |
| S1-final-viral-combined_k141_281674\|\|full | 1491 | No | NA | | 0 | 1 | Not-determined | Genome-fragment | NA | | 0 |
| S1-final-viral-combined_k141_2186\|\|full | 1001 | No | NA | | 0 | 0 | Not-determined | Genome-fragment | NA | | 0 |
| S1-final-viral-combined_k141_281685\|\|full | 2332 | No | NA | | 0 | 0 | Not-determined | Genome-fragment | NA | | 0 |
| S1-final-viral-combined_k141_215880\|\|full | 5433 | No | NA | | 0 | 5 | Not-determined | Genome-fragment | NA | | 0 |
| S1-final-viral-combined_k141_182835\|\|full | 1166 | No | NA | | 0 | 0 | Not-determined | Genome-fragment | NA | | 0 |
| S1-final-viral-combined_k141_330737\|\|full | 4652 | No | NA | | 0 | 3 | Not-determined | Genome-fragment | NA | | 0 |
| S1-final-viral-combined_k141_2222\|\|full | 1255 | No | NA | | 0 | 1 | Not-determined | Genome-fragment | NA | | 0 |
| S1-final-viral-combined_k141_330745\|\|full | 4079 | No | NA | | 0 | 0 | Not-determined | Genome-fragment | NA | | 0 |
| S1-final-viral-combined_k141_232732\|\|lt2gene | 1268 | No | NA | | 0 | 0 | Low-quality | Genome-fragment | | 9.37 | 0 |
| S1-final-viral-combined_k141_264709\|\|full | 1460 | No | NA | | 0 | 1 | Not-determined | Genome-fragment | NA | | 0 |
| S1-final-viral-combined_k141_248622\|\|full | 22409 | No | NA | | 0 | 3 | Medium-quality | Genome-fragment | | 64.62 | 0 |
| S1-final-viral-combined_k141_100347\|\|full | 1286 | No | NA | | 0 | 1 | Not-determined | Genome-fragment | NA | | 0 |
| S1-final-viral-combined_k141_199398\|\|full | 2876 | No | NA | | 0 | 0 | Not-determined | Genome-fragment | NA | | 0 |
| S1_k141_345241 | 1268 | No | NA | | 0 | 1 | Low-quality | Genome-fragment | | 1.99 | 0 |
| S1-final-viral-combined_k141_66977\|\|full | 2000 | No | NA | | 0 | 2 | Not-determined | Genome-fragment | NA | | 0 |
| S5_k141_86962 | 1268 | No | NA | | 0 | 0 | Low-quality | Genome-fragment | | 0.75 | 0 |
| S1-final-viral-combined_k141_116993\|\|full | 2178 | No | NA | | 0 | 2 | Not-determined | Genome-fragment | NA | | 0 |
| S1-final-viral-combined_k141_182900\|\|full | 1624 | No | NA | | 0 | 2 | Not-determined | Genome-fragment | NA | | 0 |
| S7-final-viral-combined_k141_143503\|\|full | 1268 | Yes | | 902 | 1 | 1 | Low-quality | Genome-fragment | | 0.07 | 28.86 |
| S1-final-viral-combined_k141_165889\|\|full | 1234 | No | NA | | 0 | 0 | Not-determined | Genome-fragment | NA | | 0 |
| S1-final-viral-combined_k141_50640\|\|full | 2374 | No | NA | | 0 | 2 | Not-determined | Genome-fragment | NA | | 0 |
| S1-final-viral-combined_k141_347563\|\|full | 2261 | No | NA | | 0 | 1 | Not-determined | Genome-fragment | NA | | 0 |
| S1-final-viral-combined_k141_314438\|\|full | 12494 | No | NA | | 0 | 7 | Not-determined | Genome-fragment | NA | | 0 |
| S7-final-viral-combined_k141_5570\|\|full | 1268 | No | NA | | 0 | 0 | Low-quality | Genome-fragment | | 2.37 | 0 |
| S1-final-viral-combined_k141_18312\|\|full | 1560 | No | NA | | 0 | 1 | Not-determined | Genome-fragment | NA | | 0 |
| S12-final-viral-combined_k141_112953\|\|lt2gene | 1268 | No | NA | | 1 | 0 | Low-quality | Genome-fragment | | 0.38 | 0 |
| S1-final-viral-combined_k141_67035\|\|full | 2328 | No | NA | | 0 | 0 | Not-determined | Genome-fragment | NA | | 0 |
| S1-final-viral-combined_k141_133794\|\|full | 1244 | No | NA | | 0 | 0 | Not-determined | Genome-fragment | NA | | 0 |
| S1-final-viral-combined_k141_133795\|\|full | 1381 | No | NA | | 0 | 2 | Not-determined | Genome-fragment | NA | | 0 |
| S1-final-viral-combined_k141_380184\|\|full | 6487 | No | NA | | 0 | 3 | Not-determined | Genome-fragment | NA | | 0 |
| S1-final-viral-combined_k141_67049\|\|full | 4664 | No | NA | | 0 | 3 | Not-determined | Genome-fragment | NA | | 0 |
| S1-final-viral-combined_k141_50688\|\|full | 6550 | No | NA | | 0 | 5 | Not-determined | Genome-fragment | NA | | 0 |
| S1-final-viral-combined_k141_133811\|\|full | 2234 | No | NA | | 0 | 1 | Not-determined | Genome-fragment | NA | | 0 |
| S1-final-viral-combined_k141_380204\|\|full | 1244 | No | NA | | 0 | 0 | Not-determined | Genome-fragment | NA | | 0 |
| S6-final-viral-combined_k141_6951\|\|full | 1268 | No | NA | | 0 | 0 | Low-quality | Genome-fragment | | 2.67 | 0 |
| S8-final-viral-combined_k141_107694\|\|full | 1268 | No | NA | | 1 | 0 | Low-quality | Genome-fragment | | 3.19 | 0 |
| S11_k141_156038 | 1269 | No | NA | | 0 | 0 | Low-quality | Genome-fragment | | 0.89 | 0 |
| S1-final-viral-combined_k141_304890\|\|full | 1269 | No | NA | | 0 | 0 | Low-quality | Genome-fragment | | 2.28 | 0 |
| S1-final-viral-combined_k141_387981\|\|lt2gene | 1269 | No | NA | | 1 | 0 | Low-quality | Genome-fragment | | 3.37 | 0 |
| S1-final-viral-combined_k141_100430\|\|full | 33842 | No | NA | | 19 | 1 | Medium-quality | Genome-fragment | | 71.89 | 0 |
| S1-final-viral-combined_k141_84201\|\|full | 2307 | No | NA | | 0 | 2 | Not-determined | Genome-fragment | NA | | 0 |
| S5-final-viral-combined_k141_144533\|\|lt2gene | 1269 | No | NA | | 1 | 0 | Low-quality | Genome-fragment | | 3.64 | 0 |
| S7-final-viral-combined_k141_5746\|\|full | 1269 | No | NA | | 0 | 1 | Low-quality | Genome-fragment | | 3.18 | 0 |
| S1-final-viral-combined_k141_182958\|\|full | 2801 | No | NA | | 0 | 2 | Not-determined | Genome-fragment | NA | | 0 |
| S17-final-viral-combined_k141_115186\|\|full | 1269 | No | NA | | 0 | 0 | Low-quality | Genome-fragment | | 2.54 | 0 |
| S1-final-viral-combined_k141_363851\|\|full | 4337 | No | NA | | 0 | 3 | Not-determined | Genome-fragment | NA | | 0 |
| S17-k141_17326 | 1269 | No | NA | | 0 | 1 | Low-quality | Genome-fragment | | 3.82 | 0 |
| S1-final-viral-combined_k141_18367\|\|full | 6352 | No | NA | | 0 | 2 | Not-determined | Genome-fragment | NA | | 0 |

| | | | | | | | | | | |
|---|---|---|---|---|---|---|---|---|---|---|
| S1-final-viral-combined_k141_298237\|\|full | 2076 | No | NA | 0 | 2 | Not-determined | Genome-fragment | NA | | 0 |
| S1-final-viral-combined_k141_232159\|\|full | 6812 | No | NA | 0 | 6 | Not-determined | Genome-fragment | NA | | 0 |
| S1-final-viral-combined_k141_347641\|\|full | 2238 | No | NA | 0 | 1 | Not-determined | Genome-fragment | NA | | 0 |
| S12-final-viral-combined_k141_363815\|\|lt2gene | 1269 | No | NA | 1 | 0 | Low-quality | Genome-fragment | | 3.15 | 0 |
| S1-final-viral-combined_k141_149844\|\|full | 1817 | No | NA | 0 | 1 | Not-determined | Genome-fragment | NA | | 0 |
| S1-final-viral-combined_k141_84250\|\|full | 1770 | No | NA | 0 | 0 | Not-determined | Genome-fragment | NA | | 0 |
| S2_k141_23623 | 1269 | No | NA | 0 | 0 | Low-quality | Genome-fragment | | 1.3 | 0 |
| S1-final-viral-combined_k141_363895\|\|full | 1868 | No | NA | 0 | 2 | Not-determined | Genome-fragment | NA | | 0 |
| S1-final-viral-combined_k141_50804\|\|full | 1474 | No | NA | 0 | 1 | Not-determined | Genome-fragment | NA | | 0 |
| S1-final-viral-combined_k141_84278\|\|full | 2303 | No | NA | 0 | 1 | Not-determined | Genome-fragment | NA | | 0 |
| S4-final-viral-combined_k141_112866\|\|full | 1269 | No | NA | 1 | 0 | Low-quality | Genome-fragment | | 9.92 | 0 |
| S6-final-viral-combined_k141_2095\|\|full | 1269 | No | NA | 0 | 0 | Low-quality | Genome-fragment | | 0.81 | 0 |
| S6_k141_33901 | 1269 | No | NA | 0 | 1 | Low-quality | Genome-fragment | | 0.61 | 0 |
| S1-final-viral-combined_k141_166012\|\|full | 1602 | No | NA | 0 | 1 | Not-determined | Genome-fragment | NA | | 0 |
| S9-final-viral-combined_k141_47314\|\|full | 1270 | No | NA | 0 | 0 | Low-quality | Genome-fragment | | 3.2 | 0 |
| S1-final-viral-combined_k141_330799\|\|full | 1587 | No | NA | 0 | 1 | Not-determined | Genome-fragment | NA | | 0 |
| S11-final-viral-combined_k141_72132\|\|full | 1270 | No | NA | 1 | 0 | Low-quality | Genome-fragment | | 2.67 | 0 |
| S11-final-viral-combined_k141_241991\|\|full | 1270 | No | NA | 0 | 0 | Low-quality | Genome-fragment | | 2.58 | 0 |
| S11_k141_196667 | 1270 | No | NA | 3 | 0 | Low-quality | Genome-fragment | | 3.05 | 0 |
| S1-final-viral-combined_k141_84323\|\|full | 2179 | No | NA | 0 | 1 | Not-determined | Genome-fragment | NA | | 0 |
| S1-final-viral-combined_k141_133924\|\|full | 2043 | No | NA | 0 | 2 | Not-determined | Genome-fragment | NA | | 0 |
| S7-final-viral-combined_k141_143953\|\|full | 1270 | No | NA | 0 | 0 | Low-quality | Genome-fragment | | 2 | 0 |
| S1-final-viral-combined_k141_183059\|\|full | 1145 | No | NA | 0 | 0 | Not-determined | Genome-fragment | NA | | 0 |
| S7_k141_237871 | 1270 | No | NA | 0 | 1 | Low-quality | Genome-fragment | | 0.3 | 0 |
| S12-final-viral-combined_k141_68224\|\|full | 1270 | No | NA | 1 | 0 | Low-quality | Genome-fragment | | 2.03 | 0 |
| S12-final-viral-combined_k141_1557\|\|full | 1270 | No | NA | 0 | 2 | Low-quality | Genome-fragment | | 0.57 | 0 |
| S1-final-viral-combined_k141_84331\|\|full | 1456 | No | NA | 0 | 1 | Not-determined | Genome-fragment | NA | | 0 |
| S1-final-viral-combined_k141_248802\|\|full | 2049 | No | NA | 0 | 2 | Not-determined | Genome-fragment | NA | | 0 |
| S1-final-viral-combined_k141_347705\|\|full | 1947 | No | NA | 0 | 2 | Not-determined | Genome-fragment | NA | | 0 |
| S1-final-viral-combined_k141_298364\|\|full | 8315 | No | NA | 0 | 6 | Not-determined | Genome-fragment | NA | | 0 |
| S2-final-viral-combined_k141_149185\|\|full | 1270 | No | NA | 0 | 2 | Low-quality | Genome-fragment | | 3.04 | 0 |
| S2-final-viral-combined_k141_77122\|\|full | 1270 | No | NA | 0 | 0 | Low-quality | Genome-fragment | | 0.66 | 0 |
| S2-final-viral-combined_k141_144077\|\|full | 1270 | No | NA | 0 | 0 | Low-quality | Genome-fragment | | 2.36 | 0 |
| S1-final-viral-combined_k141_363959\|\|full | 1139 | No | NA | 0 | 1 | Not-determined | Genome-fragment | NA | | 0 |
| S1-final-viral-combined_k141_298369\|\|full | 4282 | No | NA | 0 | 4 | Not-determined | Genome-fragment | NA | | 0 |
| S2_k141_217002 | 1270 | No | NA | 0 | 1 | Low-quality | Genome-fragment | | 0.45 | 0 |
| S1-final-viral-combined_k141_298371\|\|full | 1669 | No | NA | 0 | 1 | Not-determined | Genome-fragment | NA | | 0 |
| S6-final-viral-combined_k141_74650\|\|full | 1270 | No | NA | 0 | 0 | Low-quality | Genome-fragment | | 0.28 | 0 |
| S11-final-viral-combined_k141_146178\|\|full | 1271 | No | NA | 0 | 0 | Low-quality | Genome-fragment | | 0.84 | 0 |
| S1-final-viral-combined_k141_50899\|\|full | 2221 | No | NA | 0 | 1 | Not-determined | Genome-fragment | NA | | 0 |
| S1-final-viral-combined_k141_264932\|\|full | 1841 | No | NA | 0 | 1 | Not-determined | Genome-fragment | NA | | 0 |
| S1-final-viral-combined_k141_149946\|\|full | 1764 | No | NA | 0 | 1 | Not-determined | Genome-fragment | NA | | 0 |
| S1-final-viral-combined_k141_134002\|\|full | 2384 | No | NA | 0 | 2 | Not-determined | Genome-fragment | NA | | 0 |
| S11_k141_54609 | 1271 | No | NA | 0 | 0 | Low-quality | Genome-fragment | | 1.1 | 0 |
| S1-final-viral-combined_k141_100575\|\|full | 2589 | No | NA | 0 | 0 | Not-determined | Genome-fragment | NA | | 0 |
| S1-final-viral-combined_k141_216142\|\|full | 1587 | No | NA | 0 | 1 | Not-determined | Genome-fragment | NA | | 0 |
| S1-final-viral-combined_k141_218209\|\|lt2gene | 1271 | No | NA | 0 | 0 | Low-quality | Genome-fragment | | 2.74 | 0 |
| S1_k141_383660 | 1271 | No | NA | 0 | 0 | Low-quality | Genome-fragment | | 5.56 | 0 |
| S1-final-viral-combined_k141_248922\|\|full | 1475 | No | NA | 0 | 0 | Not-determined | Genome-fragment | NA | | 0 |
| S1-final-viral-combined_k141_232342\|\|full | 1878 | No | NA | 0 | 1 | Not-determined | Genome-fragment | NA | | 0 |
| S1-final-viral-combined_k141_380353\|\|full | 3713 | No | NA | 0 | 5 | Not-determined | Genome-fragment | NA | | 0 |
| S1-final-viral-combined_k141_150026\|\|full | 2384 | No | NA | 0 | 1 | Not-determined | Genome-fragment | NA | | 0 |
| S17-final-viral-combined_k141_136747\|\|full | 1271 | No | NA | 1 | 0 | Low-quality | Genome-fragment | | 0.82 | 0 |
| S17-final-viral-combined_k141_84807\|\|lt2gene | 1271 | No | NA | 1 | 0 | Low-quality | Genome-fragment | | 2.86 | 0 |
| S1-final-viral-combined_k141_232363\|\|full | 1320 | No | NA | 0 | 0 | Not-determined | Genome-fragment | NA | | 0 |
| S17-final-viral-combined_k141_230229\|\|lt2gene | 1271 | No | NA | 2 | 0 | Low-quality | Genome-fragment | | 2.29 | 0 |
| S12-final-viral-combined_k141_202568\|\|full | 1271 | No | NA | 1 | 0 | Low-quality | Genome-fragment | | 2.27 | 0 |
| S1-final-viral-combined_k141_248961\|\|full | 1870 | No | NA | 0 | 0 | Not-determined | Genome-fragment | NA | | 0 |
| S4_k141_52887 | 1271 | No | NA | 0 | 0 | Low-quality | Genome-fragment | | 2.34 | 0 |
| S1-final-viral-combined_k141_248980\|\|full | 3016 | No | NA | 0 | 0 | Not-determined | Genome-fragment | NA | | 0 |
| S8-final-viral-combined_k141_202732\|\|full | 1271 | No | NA | 0 | 0 | Low-quality | Genome-fragment | | 3.09 | 0 |
| S9-final-viral-combined_k141_117119\|\|full | 1272 | No | NA | 1 | 0 | Low-quality | Genome-fragment | | 2.6 | 0 |
| S1-final-viral-combined_k141_364030\|\|full | 1950 | No | NA | 0 | 1 | Not-determined | Genome-fragment | NA | | 0 |
| S1-final-viral-combined_k141_183180\|\|full | 2674 | No | NA | 0 | 1 | Not-determined | Genome-fragment | NA | | 0 |
| S11-final-viral-combined_k141_276370\|\|full | 1272 | No | NA | 2 | 0 | Low-quality | Genome-fragment | | 3.54 | 0 |
| S1-final-viral-combined_k141_314820\|\|full | 7045 | No | NA | 0 | 3 | Not-determined | Genome-fragment | NA | | 0 |
| S1-final-viral-combined_k141_216260\|\|full | 10510 | No | NA | 0 | 2 | Not-determined | Genome-fragment | NA | | 0 |
| S11_k141_310567 | 1272 | No | NA | 0 | 0 | Low-quality | Genome-fragment | | 1.82 | 0 |
| S1-final-viral-combined_k141_6053\|\|full | 1272 | No | NA | 0 | 0 | Low-quality | Genome-fragment | | 3.41 | 0 |
| S1-final-viral-combined_k141_134149\|\|full | 1014 | No | NA | 0 | 1 | Not-determined | Genome-fragment | NA | | 0 |
| S1-final-viral-combined_k141_51109\|\|full | 13768 | No | NA | 0 | 11 | Not-determined | Genome-fragment | NA | | 0 |
| S1-final-viral-combined_k141_330923\|\|full | 1360 | No | NA | 0 | 1 | Not-determined | Genome-fragment | NA | | 0 |
| S1-final-viral-combined_k141_78447\|\|full | 1272 | No | NA | 0 | 1 | Low-quality | Genome-fragment | | 1.08 | 0 |
| S1-final-viral-combined_k141_2529\|\|full | 3354 | No | NA | 0 | 1 | Not-determined | Genome-fragment | NA | | 0 |
| S1-final-viral-combined_k141_298421\|\|full | 1191 | No | NA | 0 | 2 | Not-determined | Genome-fragment | NA | | 0 |
| S1-final-viral-combined_k141_282012\|\|full | 2092 | No | NA | 0 | 1 | Not-determined | Genome-fragment | NA | | 0 |
| S1-final-viral-combined_k141_84504\|\|full | 2605 | No | NA | 0 | 1 | Not-determined | Genome-fragment | NA | | 0 |
| S1-final-viral-combined_k141_249068\|\|full | 2202 | No | NA | 0 | 1 | Not-determined | Genome-fragment | NA | | 0 |
| S1-final-viral-combined_k141_216301\|\|full | 3221 | No | NA | 0 | 1 | Not-determined | Genome-fragment | NA | | 0 |
| S1-final-viral-combined_k141_183239\|\|full | 2621 | No | NA | 0 | 3 | Not-determined | Genome-fragment | NA | | 0 |
| S1-final-viral-combined_k141_364086\|\|full | 2317 | No | NA | 0 | 0 | Not-determined | Genome-fragment | NA | | 0 |
| S1-final-viral-combined_k141_199714\|\|full | 20970 | No | NA | 0 | 10 | Not-determined | Genome-fragment | NA | | 0 |
| S1-final-viral-combined_k141_364091\|\|full | 1930 | No | NA | 0 | 0 | Not-determined | Genome-fragment | NA | | 0 |
| S3-final-viral-combined_k141_47567\|\|full | 1272 | No | NA | 0 | 0 | Low-quality | Genome-fragment | | 1.14 | 0 |
| S1-final-viral-combined_k141_265058\|\|full | 1656 | No | NA | 0 | 2 | Not-determined | Genome-fragment | NA | | 0 |
| S3-final-viral-combined_k141_100208\|\|full | 1272 | No | NA | 0 | 0 | Low-quality | Genome-fragment | | 0.82 | 0 |
| S12-final-viral-combined_k141_303071\|\|full | 1272 | No | NA | 1 | 0 | Low-quality | Genome-fragment | | 2.98 | 0 |
| S1-final-viral-combined_k141_216341\|\|full | 1234 | No | NA | 0 | 2 | Not-determined | Genome-fragment | NA | | 0 |
| S1-final-viral-combined_k141_298484\|\|full | 6273 | No | NA | 0 | 4 | Not-determined | Genome-fragment | NA | | 0 |
| S12-final-viral-combined_k141_230220\|\|full | 1272 | No | NA | 1 | 0 | Low-quality | Genome-fragment | | 0.78 | 0 |
| S1-final-viral-combined_k141_298499\|\|full | 1444 | No | NA | 0 | 0 | Not-determined | Genome-fragment | NA | | 0 |
| S1-final-viral-combined_k141_100703\|\|full | 1037 | No | NA | 0 | 2 | Not-determined | Genome-fragment | NA | | 0 |
| S1-final-viral-combined_k141_100709\|\|full | 1086 | No | NA | 0 | 1 | Not-determined | Genome-fragment | NA | | 0 |
| S2-final-viral-combined_k141_101933\|\|lt2gene | 1272 | No | NA | 1 | 0 | Low-quality | Genome-fragment | | 0.82 | 0 |
| S8-final-viral-combined_k141_197749\|\|full | 1272 | No | NA | 0 | 0 | Low-quality | Genome-fragment | | 0.97 | 0 |
| S9-final-viral-combined_k141_24029\|\|full | 1273 | No | NA | 0 | 2 | Low-quality | Genome-fragment | | 0.4 | 0 |
| S1_k141_331007 | 1273 | No | NA | 0 | 0 | Low-quality | Genome-fragment | | 1.94 | 0 |
| S3-final-viral-combined_k141_117359\|\|full | 1273 | No | NA | 0 | 0 | Low-quality | Genome-fragment | | 1.29 | 0 |
| S3_k141_33476 | 1273 | No | NA | 0 | 0 | Low-quality | Genome-fragment | | 2.74 | 0 |
| S1-final-viral-combined_k141_298524\|\|full | 1858 | No | NA | 0 | 2 | Not-determined | Genome-fragment | NA | | 0 |
| S7-final-viral-combined_k141_4826\|\|lt2gene | 1273 | No | NA | 1 | 0 | Low-quality | Genome-fragment | | 3.06 | 0 |
| S12_k141_340996 | 1273 | No | NA | 3 | 0 | Low-quality | Genome-fragment | | 2.72 | 0 |
| S1-final-viral-combined_k141_18536\|\|full | 2350 | No | NA | 0 | 1 | Not-determined | Genome-fragment | NA | | 0 |
| S1-final-viral-combined_k141_314909\|\|full | 2072 | No | NA | 0 | 2 | Not-determined | Genome-fragment | NA | | 0 |
| S1-final-viral-combined_k141_232545\|\|full | 2315 | No | NA | 0 | 3 | Not-determined | Genome-fragment | NA | | 0 |
| S1-final-viral-combined_k141_166301\|\|full | 4165 | No | NA | 0 | 4 | Not-determined | Genome-fragment | NA | | 0 |
| S2-final-viral-combined_k141_218351\|\|full | 1273 | No | NA | 1 | 0 | Low-quality | Genome-fragment | | 2.19 | 0 |
| S2-final-viral-combined_k141_75321\|\|full | 1273 | No | NA | 1 | 0 | Low-quality | Genome-fragment | | 3.05 | 0 |
| S1-final-viral-combined_k141_51246\|\|full | 8429 | No | NA | 0 | 2 | Not-determined | Genome-fragment | NA | | 0 |
| S1-final-viral-combined_k141_51259\|\|full | 2502 | No | NA | 0 | 1 | Not-determined | Genome-fragment | NA | | 0 |
| S2-final-viral-combined_k141_159714\|\|full | 1273 | No | NA | 1 | 0 | Low-quality | Genome-fragment | | 3.64 | 0 |
| S1-final-viral-combined_k141_314950\|\|full | 7784 | No | NA | 0 | 7 | Not-determined | Genome-fragment | NA | | 0 |
| S1-final-viral-combined_k141_93536\|\|full | 1274 | No | NA | 0 | 0 | Low-quality | Genome-fragment | | 1.8 | 0 |
| S1-final-viral-combined_k141_314958\|\|full | 1718 | No | NA | 0 | 0 | Not-determined | Genome-fragment | NA | | 0 |
| S5_k141_3420 | 1274 | No | NA | 0 | 0 | Low-quality | Genome-fragment | | 2.01 | 0 |
| S1-final-viral-combined_k141_380551\|\|full | 8050 | No | NA | 0 | 5 | Not-determined | Genome-fragment | NA | | 0 |
| S7-final-viral-combined_k141_34292\|\|full | 1274 | No | NA | 0 | 0 | Low-quality | Genome-fragment | | 3.21 | 0 |
| S1-final-viral-combined_k141_18586\|\|full | 7915 | No | NA | 0 | 8 | Not-determined | Genome-fragment | NA | | 0 |
| S1-final-viral-combined_k141_216457\|\|full | 3126 | No | NA | 0 | 3 | Not-determined | Genome-fragment | NA | | 0 |
| S7_k141_191709 | 1274 | No | NA | 0 | 1 | Low-quality | Genome-fragment | | 2.88 | 0 |
| S17-final-viral-combined_k141_166828\|\|lt2gene | 1274 | No | NA | 1 | 0 | Low-quality | Genome-fragment | | 1.86 | 0 |
| S1-final-viral-combined_k141_117457\|\|full | 5007 | No | NA | 0 | 3 | Not-determined | Genome-fragment | NA | | 0 |
| S1-final-viral-combined_k141_18591\|\|full | 3694 | No | NA | 0 | 1 | Not-determined | Genome-fragment | NA | | 0 |
| S1-final-viral-combined_k141_380562\|\|full | 2154 | No | NA | 0 | 3 | Not-determined | Genome-fragment | NA | | 0 |
| S1-final-viral-combined_k141_183359\|\|full | 1487 | No | NA | 0 | 0 | Not-determined | Genome-fragment | NA | | 0 |
| S1-final-viral-combined_k141_117464\|\|full | 3600 | No | NA | 0 | 3 | Not-determined | Genome-fragment | NA | | 0 |
| S17-final-viral-combined_k141_227178\|\|lt2gene | 1274 | No | NA | 1 | 0 | Low-quality | Genome-fragment | | 3.12 | 0 |
| S1-final-viral-combined_k141_35165\|\|full | 1984 | No | NA | 0 | 0 | Not-determined | Genome-fragment | NA | | 0 |
| S1-final-viral-combined_k141_67547\|\|full | 8953 | No | NA | 0 | 5 | Not-determined | Genome-fragment | NA | | 0 |
| S12-final-viral-combined_k141_93953\|\|full | 1274 | No | NA | 0 | 0 | Low-quality | Genome-fragment | | 3.37 | 0 |
| S12-final-viral-combined_k141_143423\|\|lt2gene | 1274 | No | NA | 1 | 0 | Low-quality | Genome-fragment | | 2.57 | 0 |
| S12_k141_391441 | 1274 | No | NA | 0 | 0 | Low-quality | Genome-fragment | | 1.92 | 0 |
| S1-final-viral-combined_k141_265210\|\|full | 5624 | No | NA | 0 | 3 | Not-determined | Genome-fragment | NA | | 0 |
| S1-final-viral-combined_k141_51345\|\|full | 3565 | No | NA | 0 | 3 | Not-determined | Genome-fragment | NA | | 0 |
| S1-final-viral-combined_k141_18646\|\|full | 1326 | No | NA | 0 | 2 | Not-determined | Genome-fragment | NA | | 0 |
| S2-final-viral-combined_k141_100377\|\|lt2gene | 1274 | No | NA | 1 | 0 | Low-quality | Genome-fragment | | 3.59 | 0 |
| S1-final-viral-combined_k141_216511\|\|full | 1899 | No | NA | 0 | 1 | Not-determined | Genome-fragment | NA | | 0 |
| S1-final-viral-combined_k141_380613\|\|full | 1442 | No | NA | 0 | 1 | Not-determined | Genome-fragment | NA | | 0 |
| S1-final-viral-combined_k141_364260\|\|full | 2646 | No | NA | 0 | 3 | Not-determined | Genome-fragment | NA | | 0 |
| S1-final-viral-combined_k141_166375\|\|full | 13085 | No | NA | 0 | 5 | Not-determined | Genome-fragment | NA | | 0 |
| S1-final-viral-combined_k141_232661\|\|full | 1185 | No | NA | 0 | 2 | Not-determined | Genome-fragment | NA | | 0 |
| S2_k141_53791 | 1274 | No | NA | 1 | 0 | Low-quality | Genome-fragment | | 2.74 | 0 |
| S4-final-viral-combined_k141_57793\|\|full | 1274 | No | NA | 0 | 1 | Low-quality | Genome-fragment | | 0.74 | 0 |
| S1-final-viral-combined_k141_18667\|\|full | 3195 | No | NA | 0 | 3 | Not-determined | Genome-fragment | NA | | 0 |
| S4_k141_65180 | 1274 | No | NA | 0 | 0 | Low-quality | Genome-fragment | | 1.39 | 0 |
| S1-final-viral-combined_k141_35203\|\|full | 1571 | No | NA | 0 | 1 | Not-determined | Genome-fragment | NA | | 0 |
| S8-final-viral-combined_k141_219661\|\|full | 1274 | No | NA | 0 | 1 | Low-quality | Genome-fragment | | 2.25 | 0 |
| S1-final-viral-combined_k141_265224\|\|full | 13415 | No | NA | 0 | 4 | Not-determined | Genome-fragment | NA | | 0 |
| S1-final-viral-combined_k141_249288\|\|full | 1911 | No | NA | 0 | 1 | Not-determined | Genome-fragment | NA | | 0 |
| S1-final-viral-combined_k141_67591\|\|full | 1622 | No | NA | 0 | 0 | Not-determined | Genome-fragment | NA | | 0 |
| S1-final-viral-combined_k141_298661\|\|full | 1890 | No | NA | 0 | 0 | Not-determined | Genome-fragment | NA | | 0 |
| S1-final-viral-combined_k141_249299\|\|full | 7775 | No | NA | 0 | 0 | Not-determined | Genome-fragment | NA | | 0 |
| S1-final-viral-combined_k141_166392\|\|full | 19346 | No | NA | 10 | 0 | Medium-quality | Genome-fragment | | 50.16 | 0 |
| S8-final-viral-combined_k141_30683\|\|full | 1274 | No | NA | 0 | 0 | Low-quality | Genome-fragment | | 2.27 | 0 |
| S1-final-viral-combined_k141_67598\|\|full | 1226 | No | NA | 0 | 0 | Not-determined | Genome-fragment | NA | | 0 |
| S1-final-viral-combined_k141_2710\|\|full | 2471 | No | NA | 0 | 0 | Not-determined | Genome-fragment | NA | | 0 |
| S8-final-viral-combined_k141_68778\|\|full | 1274 | No | NA | 0 | 0 | Low-quality | Genome-fragment | | 3.42 | 0 |
| S1-final-viral-combined_k141_51405\|\|full | 2195 | No | NA | 0 | 1 | Not-determined | Genome-fragment | NA | | 0 |
| S1-final-viral-combined_k141_249364\|\|full | 1332 | No | NA | 0 | 0 | Not-determined | Genome-fragment | NA | | 0 |
| S11-final-viral-combined_k141_58996\|\|full | 1275 | No | NA | 0 | 0 | Low-quality | Genome-fragment | | 1.41 | 0 |
| S17-final-viral-combined_k141_173968\|\|full | 1275 | No | NA | 0 | 1 | Low-quality | Genome-fragment | | 2.23 | 0 |
| S17-k141_215519 | 1275 | No | NA | 1 | 0 | Low-quality | Genome-fragment | | 2.36 | 0 |
| S12-final-viral-combined_k141_84953\|\|full | 1275 | No | NA | 0 | 0 | Low-quality | Genome-fragment | | 2.25 | 0 |
| S1-final-viral-combined_k141_315090\|\|full | 3547 | No | NA | 0 | 0 | Not-determined | Genome-fragment | NA | | 0 |
| S12-final-viral-combined_k141_44620\|\|full | 1275 | No | NA | 3 | 0 | Low-quality | Genome-fragment | | 3.73 | 0 |
| S1-final-viral-combined_k141_232722\|\|full | 1797 | No | NA | 0 | 1 | Not-determined | Genome-fragment | NA | | 0 |
| S2_k141_122549 | 1275 | No | NA | 0 | 0 | Low-quality | Genome-fragment | | 1.65 | 0 |
| S4_k141_147136 | 1275 | No | NA | 0 | 0 | Low-quality | Genome-fragment | | 0.84 | 0 |
| S1-final-viral-combined_k141_84824\|\|full | 7870 | No | NA | 0 | 5 | Not-determined | Genome-fragment | NA | | 0 |
| S8_k141_214746 | 1275 | No | NA | 0 | 1 | Low-quality | Genome-fragment | | 4.07 | 0 |
| S1-final-viral-combined_k141_2792\|\|full | 3573 | No | NA | 0 | 1 | Not-determined | Genome-fragment | NA | | 0 |
| S9-final-viral-combined_k141_89872\|\|full | 1276 | No | NA | 0 | 0 | Low-quality | Genome-fragment | | 1.55 | 0 |
| S1-final-viral-combined_k141_331161\|\|full | 7521 | No | NA | 0 | 1 | Not-determined | Genome-fragment | NA | | 0 |
| S1-final-viral-combined_k141_364387\|\|full | 2514 | No | NA | 0 | 0 | Not-determined | Genome-fragment | NA | | 0 |

| | | | | | | | | | | |
|---|---|---|---|---|---|---|---|---|---|---|
| S9-final-viral-combined_k141_13135\|\|lt2gene | 1276 | No | NA | 1 | 0 | Low-quality | Genome-fragment | | 4.04 | 0 |
| S1-final-viral-combined_k141_2801\|\|full | 2086 | No | NA | 0 | 3 | Not-determined | Genome-fragment | NA | | 0 |
| S1-final-viral-combined_k141_380734\|\|full | 1827 | No | NA | 0 | 0 | Not-determined | Genome-fragment | NA | | 0 |
| S9_k141_97622 | 1276 | No | NA | 0 | 1 | Low-quality | Genome-fragment | | 3.14 | 0 |
| S1-final-viral-combined_k141_298784\|\|full | 1141 | No | NA | 0 | 0 | Not-determined | Genome-fragment | NA | | 0 |
| S1-final-viral-combined_k141_232747\|\|full | 3481 | No | NA | 0 | 3 | Not-determined | Genome-fragment | NA | | 0 |
| S1-final-viral-combined_k141_265302\|\|full | 4589 | No | NA | 0 | 2 | Not-determined | Genome-fragment | NA | | 0 |
| S1-final-viral-combined_k141_150348\|\|full | 2404 | No | NA | 0 | 1 | Not-determined | Genome-fragment | NA | | 0 |
| S11_k141_144273 | 1276 | No | NA | 1 | 0 | Low-quality | Genome-fragment | | 2.28 | 0 |
| S1-final-viral-combined_k141_298822\|\|full | 4036 | No | NA | 0 | 3 | Not-determined | Genome-fragment | NA | | 0 |
| S7-final-viral-combined_k141_73133\|\|full | 1276 | No | NA | 1 | 0 | Low-quality | Genome-fragment | | 2.69 | 0 |
| S12-final-viral-combined_k141_228962\|\|full | 1276 | No | NA | 0 | 0 | Low-quality | Genome-fragment | | 1.13 | 0 |
| S4_k141_161294 | 1276 | No | NA | 0 | 1 | Low-quality | Genome-fragment | | 0.21 | 0 |
| S1-final-viral-combined_k141_166494\|\|full | 2066 | No | NA | 0 | 1 | Not-determined | Genome-fragment | NA | | 0 |
| S1-final-viral-combined_k141_331225\|\|full | 2170 | No | NA | 0 | 1 | Not-determined | Genome-fragment | NA | | 0 |
| S6-final-viral-combined_k141_132581\|\|full | 1276 | No | NA | 0 | 0 | Low-quality | Genome-fragment | | 0.81 | 0 |
| S1-final-viral-combined_k141_100856\|\|full | 3924 | No | NA | 0 | 4 | Not-determined | Genome-fragment | NA | | 0 |
| S8-final-viral-combined_k141_199135\|\|full | 1276 | No | NA | 3 | 0 | Low-quality | Genome-fragment | | 2.61 | 0 |
| S1-final-viral-combined_k141_15910\|\|full | 1277 | No | NA | 0 | 0 | Low-quality | Genome-fragment | | 2.43 | 0 |
| S1-final-viral-combined_k141_282300\|\|full | 2805 | No | NA | 0 | 3 | Not-determined | Genome-fragment | NA | | 0 |
| S5-final-viral-combined_k141_155443\|\|full | 1277 | No | NA | 0 | 0 | Low-quality | Genome-fragment | | 2 | 0 |
| S7-final-viral-combined_k141_207165\|\|full | 1277 | No | NA | 1 | 0 | Low-quality | Genome-fragment | | 3.42 | 0 |
| S1-final-viral-combined_k141_298862\|\|full | 1153 | No | NA | 0 | 1 | Not-determined | Genome-fragment | NA | | 0 |
| S7-final-viral-combined_k141_40177\|\|full | 1277 | No | NA | 1 | 0 | Low-quality | Genome-fragment | | 3.87 | 0 |
| S1-final-viral-combined_k141_282312\|\|full | 1880 | No | NA | 0 | 0 | Not-determined | Genome-fragment | NA | | 0 |
| S1-final-viral-combined_k141_166512\|\|full | 2342 | No | NA | 0 | 3 | Not-determined | Genome-fragment | NA | | 0 |
| S7-final-viral-combined_k141_79832\|\|full | 1277 | No | NA | 0 | 1 | Low-quality | Genome-fragment | | 1.85 | 0 |
| S1-final-viral-combined_k141_232865\|\|full | 1514 | No | NA | 0 | 2 | Not-determined | Genome-fragment | NA | | 0 |
| S1-final-viral-combined_k141_134565\|\|full | 2563 | No | NA | 0 | 2 | Not-determined | Genome-fragment | NA | | 0 |
| S12-final-viral-combined_k141_282931\|\|full | 1277 | No | NA | 0 | 1 | Low-quality | Genome-fragment | | 0.75 | 0 |
| S8-final-viral-combined_k141_164626\|\|full | 1277 | No | NA | 0 | 0 | Low-quality | Genome-fragment | | 2.78 | 0 |
| S1-final-viral-combined_k141_282352\|\|full | 2157 | No | NA | 0 | 0 | Not-determined | Genome-fragment | NA | | 0 |
| S9-final-viral-combined_k141_30932\|\|full | 1278 | No | NA | 0 | 1 | Low-quality | Genome-fragment | | 2.56 | 0 |
| S9_k141_97946 | 1278 | No | NA | 2 | 0 | Low-quality | Genome-fragment | | 3.75 | 0 |
| S9_k141_28440 | 1278 | No | NA | 0 | 0 | Low-quality | Genome-fragment | | 1.37 | 0 |
| S1-final-viral-combined_k141_315214\|\|full | 1904 | No | NA | 0 | 2 | Not-determined | Genome-fragment | NA | | 0 |
| S1-final-viral-combined_k141_134610\|\|full | 2055 | No | NA | 0 | 1 | Not-determined | Genome-fragment | NA | | 0 |
| S1-final-viral-combined_k141_100929\|\|full | 8310 | No | NA | 0 | 5 | Not-determined | Genome-fragment | NA | | 0 |
| S1-final-viral-combined_k141_200146\|\|full | 1553 | No | NA | 0 | 0 | Not-determined | Genome-fragment | NA | | 0 |
| S1-final-viral-combined_k141_100934\|\|full | 1982 | No | NA | 0 | 0 | Not-determined | Genome-fragment | NA | | 0 |
| S1-final-viral-combined_k141_35429\|\|full | 10905 | No | NA | 0 | 8 | Not-determined | Genome-fragment | NA | | 0 |
| S1-final-viral-combined_k141_35438\|\|full | 1072 | No | NA | 0 | 0 | Not-determined | Genome-fragment | NA | | 0 |
| S1-final-viral-combined_k141_315245\|\|full | 1040 | No | NA | 0 | 1 | Not-determined | Genome-fragment | NA | | 0 |
| S11-final-viral-combined_k141_277392\|\|full | 1278 | No | NA | 2 | 0 | Low-quality | Genome-fragment | | 2.52 | 0 |
| S1-final-viral-combined_k141_18900\|\|full | 2155 | No | NA | 0 | 1 | Not-determined | Genome-fragment | NA | | 0 |
| S11-final-viral-combined_k141_215820\|\|full | 1278 | No | NA | 1 | 0 | Low-quality | Genome-fragment | | 2.47 | 0 |
| S1-final-viral-combined_k141_85004\|\|full | 9532 | No | NA | 0 | 8 | Not-determined | Genome-fragment | NA | | 0 |
| S11_k141_34522 | 1278 | No | NA | 2 | 0 | Low-quality | Genome-fragment | | 1.55 | 0 |
| S1-final-viral-combined_k141_117799\|\|full | 1339 | No | NA | 0 | 0 | Not-determined | Genome-fragment | NA | | 0 |
| S1-final-viral-combined_k141_163768\|\|full | 1278 | No | NA | 0 | 1 | Low-quality | Genome-fragment | | 1.74 | 0 |
| S1-final-viral-combined_k141_117811\|\|full | 2672 | No | NA | 0 | 2 | Not-determined | Genome-fragment | NA | | 0 |
| S1-final-viral-combined_k141_18934\|\|full | 3151 | No | NA | 0 | 2 | Not-determined | Genome-fragment | NA | | 0 |
| S1-final-viral-combined_k141_323733\|\|lt2gene | 1278 | No | NA | 1 | 0 | Low-quality | Genome-fragment | | 2.01 | 0 |
| S1-final-viral-combined_k141_265385\|\|full | 7166 | No | NA | 0 | 2 | Not-determined | Genome-fragment | NA | | 0 |
| S1-final-viral-combined_k141_348182\|\|full | 1133 | No | NA | 0 | 2 | Not-determined | Genome-fragment | NA | | 0 |
| S3-final-viral-combined_k141_40112\|\|full | 1278 | No | NA | 0 | 0 | Low-quality | Genome-fragment | | 1.52 | 0 |
| S3-final-viral-combined_k141_130695\|\|lt2gene | 1278 | No | NA | 1 | 0 | Low-quality | Genome-fragment | | 2.89 | 0 |
| S1-final-viral-combined_k141_282406\|\|full | 1849 | No | NA | 0 | 2 | Not-determined | Genome-fragment | NA | | 0 |
| S5-final-viral-combined_k141_154669\|\|full | 1278 | No | NA | 1 | 0 | Low-quality | Genome-fragment | | 0.73 | 0 |
| S1-final-viral-combined_k141_331403\|\|full | 1776 | No | NA | 0 | 2 | Not-determined | Genome-fragment | NA | | 0 |
| S5_k141_148029 | 1278 | No | NA | 0 | 1 | Low-quality | Genome-fragment | | 3.08 | 0 |
| S1-final-viral-combined_k141_67893\|\|full | 1732 | No | NA | 0 | 0 | Not-determined | Genome-fragment | NA | | 0 |
| S17-k141_126021 | 1278 | No | NA | 0 | 0 | Low-quality | Genome-fragment | | 2.24 | 0 |
| S12-final-viral-combined_k141_236201\|\|full | 1278 | No | NA | 0 | 0 | Low-quality | Genome-fragment | | 2.87 | 0 |
| S12_k141_116336 | 1278 | No | NA | 0 | 0 | Low-quality | Genome-fragment | | 2.36 | 0 |
| S1-final-viral-combined_k141_364641\|\|full | 12703 | No | NA | 0 | 5 | Not-determined | Genome-fragment | NA | | 0 |
| S1-final-viral-combined_k141_298963\|\|full | 3330 | No | NA | 0 | 3 | Not-determined | Genome-fragment | NA | | 0 |
| S2-final-viral-combined_k141_94967\|\|full | 1278 | No | NA | 0 | 0 | Low-quality | Genome-fragment | | 1.1 | 0 |
| S1-final-viral-combined_k141_216905\|\|full | 2094 | No | NA | 0 | 0 | Not-determined | Genome-fragment | NA | | 0 |
| S2-final-viral-combined_k141_7633\|\|full | 1278 | No | NA | 0 | 0 | Low-quality | Genome-fragment | | 1.89 | 0 |
| S1-final-viral-combined_k141_249603\|\|full | 2015 | No | NA | 0 | 0 | Not-determined | Genome-fragment | NA | | 0 |
| S4-final-viral-combined_k141_61823\|\|full | 1278 | No | NA | 0 | 2 | Low-quality | Genome-fragment | | 0.93 | 0 |
| S4_k141_69956 | 1278 | No | NA | 0 | 0 | Low-quality | Genome-fragment | | 2.31 | 0 |
| S9-final-viral-combined_k141_110446\|\|full | 1279 | No | NA | 0 | 0 | Low-quality | Genome-fragment | | 2.56 | 0 |
| S1-final-viral-combined_k141_7635\|\|full | 1279 | No | NA | 1 | 0 | Low-quality | Genome-fragment | | 0.83 | 0 |
| S1-final-viral-combined_k141_19017\|\|full | 9596 | No | NA | 0 | 6 | Not-determined | Genome-fragment | NA | | 0 |
| S1-final-viral-combined_k141_35544\|\|full | 2790 | No | NA | 0 | 1 | Not-determined | Genome-fragment | NA | | 0 |
| S1-final-viral-combined_k141_282465\|\|full | 16050 | No | NA | 0 | 13 | Not-determined | Genome-fragment | NA | | 0 |
| S1-final-viral-combined_k141_364710\|\|full | 5270 | No | NA | 0 | 4 | Not-determined | Genome-fragment | NA | | 0 |
| S1-final-viral-combined_k141_19031\|\|full | 32923 | No | NA | 24 | 0 | High-quality | High-quality | | 90.89 | 0 |
| S1-final-viral-combined_k141_35585\|\|full | 1758 | No | NA | 0 | 0 | Not-determined | Genome-fragment | NA | | 0 |
| S1-final-viral-combined_k141_134769\|\|full | 2160 | No | NA | 0 | 2 | Not-determined | Genome-fragment | NA | | 0 |
| S1-final-viral-combined_k141_361745\|\|full | 1279 | No | NA | 0 | 1 | Low-quality | Genome-fragment | | 2.27 | 0 |
| S1-final-viral-combined_k141_101131\|\|full | 2438 | No | NA | 0 | 0 | Not-determined | Genome-fragment | NA | | 0 |
| S1_k141_197480 | 1279 | No | NA | 0 | 1 | Low-quality | Genome-fragment | | 4.46 | 0 |
| S3-final-viral-combined_k141_118590\|\|lt2gene | 1279 | No | NA | 1 | 0 | Low-quality | Genome-fragment | | 3.87 | 0 |
| S1-final-viral-combined_k141_117936\|\|full | 3822 | No | NA | 0 | 3 | Not-determined | Genome-fragment | NA | | 0 |
| S1-final-viral-combined_k141_150685\|\|full | 1465 | No | NA | 0 | 2 | Not-determined | Genome-fragment | NA | | 0 |
| S5-final-viral-combined_k141_193833\|\|full | 1279 | No | NA | 0 | 0 | Low-quality | Genome-fragment | | 1.13 | 0 |
| S1-final-viral-combined_k141_282546\|\|full | 1444 | No | NA | 0 | 1 | Not-determined | Genome-fragment | NA | | 0 |
| S5-final-viral-combined_k141_105197\|\|lt2gene | 1279 | No | NA | 1 | 0 | Low-quality | Genome-fragment | | 1.8 | 0 |
| S1-final-viral-combined_k141_117951\|\|full | 1724 | No | NA | 0 | 0 | Not-determined | Genome-fragment | NA | | 0 |
| S1-final-viral-combined_k141_85124\|\|full | 1783 | No | NA | 0 | 1 | Not-determined | Genome-fragment | NA | | 0 |
| S17-final-viral-combined_k141_249103\|\|full | 1279 | No | NA | 0 | 1 | Low-quality | Genome-fragment | | 0.78 | 0 |
| S1-final-viral-combined_k141_67972\|\|full | 1606 | No | NA | 0 | 1 | Not-determined | Genome-fragment | NA | | 0 |
| S1-final-viral-combined_k141_233047\|\|full | 7150 | No | NA | 0 | 0 | Not-determined | Genome-fragment | NA | | 0 |
| S1-final-viral-combined_k141_348379\|\|full | 3411 | No | NA | 0 | 1 | Not-determined | Genome-fragment | NA | | 0 |
| S17-final-viral-combined_k141_114672\|\|lt2gene | 1279 | No | NA | 1 | 0 | Low-quality | Genome-fragment | | 2.13 | 0 |
| S2-final-viral-combined_k141_38518\|\|lt2gene | 1279 | No | NA | 1 | 0 | Low-quality | Genome-fragment | | 3.78 | 0 |
| S11-final-viral-combined_k141_200768\|\|full | 1280 | No | NA | 0 | 1 | Low-quality | Genome-fragment | | 1.04 | 0 |
| S11-final-viral-combined_k141_165338\|\|lt2gene | 1280 | No | NA | 1 | 0 | Low-quality | Genome-fragment | | 2.33 | 0 |
| S1-final-viral-combined_k141_299172\|\|full | 1093 | No | NA | 0 | 2 | Not-determined | Genome-fragment | NA | | 0 |
| S1-final-viral-combined_k141_192703\|\|full | 1280 | No | NA | 0 | 1 | Low-quality | Genome-fragment | | 2.28 | 0 |
| S1-final-viral-combined_k141_166678\|\|full | 1838 | No | NA | 0 | 0 | Not-determined | Genome-fragment | NA | | 0 |
| S1-final-viral-combined_k141_51737\|\|full | 1002 | No | NA | 0 | 1 | Not-determined | Genome-fragment | NA | | 0 |
| S1-final-viral-combined_k141_249800\|\|full | 1242 | No | NA | 0 | 1 | Not-determined | Genome-fragment | NA | | 0 |
| S5-final-viral-combined_k141_117598\|\|full | 1280 | No | NA | 0 | 0 | Low-quality | Genome-fragment | | 2.24 | 0 |
| S1-final-viral-combined_k141_35647\|\|full | 2276 | No | NA | 0 | 3 | Not-determined | Genome-fragment | NA | | 0 |
| S1-final-viral-combined_k141_364786\|\|full | 1786 | No | NA | 0 | 0 | Not-determined | Genome-fragment | NA | | 0 |
| S1-final-viral-combined_k141_348455\|\|full | 2650 | No | NA | 0 | 1 | Not-determined | Genome-fragment | NA | | 0 |
| S1-final-viral-combined_k141_35652\|\|full | 1658 | No | NA | 0 | 2 | Not-determined | Genome-fragment | NA | | 0 |
| S1-final-viral-combined_k141_315474\|\|full | 3917 | No | NA | 0 | 2 | Not-determined | Genome-fragment | NA | | 0 |
| S17-final-viral-combined_k141_5963\|\|full | 1280 | No | NA | 1 | 0 | Low-quality | Genome-fragment | | 1.83 | 0 |
| S1-final-viral-combined_k141_200283\|\|full | 2335 | No | NA | 0 | 3 | Not-determined | Genome-fragment | NA | | 0 |
| S1-final-viral-combined_k141_166713\|\|full | 7384 | No | NA | 0 | 1 | Not-determined | Genome-fragment | NA | | 0 |
| S1-final-viral-combined_k141_183965\|\|full | 1463 | No | NA | 0 | 1 | Not-determined | Genome-fragment | NA | | 0 |
| S1-final-viral-combined_k141_200289\|\|full | 5690 | No | NA | 0 | 1 | Not-determined | Genome-fragment | NA | | 0 |
| S12-final-viral-combined_k141_237871\|\|full | 1280 | No | NA | 0 | 1 | Low-quality | Genome-fragment | | 2.41 | 0 |
| S12-final-viral-combined_k141_129958\|\|full | 1280 | No | NA | 0 | 0 | Low-quality | Genome-fragment | | 1.82 | 0 |
| S1-final-viral-combined_k141_315507\|\|full | 1661 | No | NA | 0 | 1 | Not-determined | Genome-fragment | NA | | 0 |
| S1-final-viral-combined_k141_183975\|\|full | 1105 | No | NA | 0 | 1 | Not-determined | Genome-fragment | NA | | 0 |
| S1-final-viral-combined_k141_364829\|\|full | 2216 | No | NA | 0 | 2 | Not-determined | Genome-fragment | NA | | 0 |
| S1-final-viral-combined_k141_35686\|\|full | 4294 | No | NA | 0 | 1 | Not-determined | Genome-fragment | NA | | 0 |
| S1-final-viral-combined_k141_315517\|\|full | 2443 | No | NA | 0 | 1 | Not-determined | Genome-fragment | NA | | 0 |
| S1-final-viral-combined_k141_282686\|\|full | 1498 | No | NA | 0 | 3 | Not-determined | Genome-fragment | NA | | 0 |
| S1-final-viral-combined_k141_35710\|\|full | 1666 | No | NA | 0 | 0 | Not-determined | Genome-fragment | NA | | 0 |
| S1-final-viral-combined_k141_348530\|\|full | 1998 | No | NA | 0 | 2 | Not-determined | Genome-fragment | NA | | 0 |
| S1-final-viral-combined_k141_134917\|\|full | 18137 | No | NA | 0 | 8 | Not-determined | Genome-fragment | NA | | 0 |
| S2-final-viral-combined_k141_222099\|\|lt2gene | 1280 | No | NA | 2 | 0 | Low-quality | Genome-fragment | | 4.66 | 0 |
| S2_k141_111917 | 1280 | No | NA | 0 | 0 | Low-quality | Genome-fragment | | 1.75 | 0 |
| S4-final-viral-combined_k141_76405\|\|full | 1280 | No | NA | 3 | 0 | Low-quality | Genome-fragment | | 2.99 | 0 |
| S1-final-viral-combined_k141_150879\|\|full | 1424 | No | NA | 0 | 0 | Not-determined | Genome-fragment | NA | | 0 |
| S1-final-viral-combined_k141_118064\|\|full | 5054 | No | NA | 0 | 1 | Not-determined | Genome-fragment | NA | | 0 |
| S1-final-viral-combined_k141_315593\|\|full | 1263 | No | NA | 0 | 3 | Not-determined | Genome-fragment | NA | | 0 |
| S6_k141_7765 | 1280 | No | NA | 0 | 3 | Low-quality | Genome-fragment | | 0.71 | 0 |
| S8-final-viral-combined_k141_221263\|\|full | 1280 | No | NA | 0 | 0 | Low-quality | Genome-fragment | | 2.81 | 0 |
| S9-final-viral-combined_k141_70282\|\|full | 1281 | No | NA | 0 | 0 | Low-quality | Genome-fragment | | 3.63 | 0 |
| S1-final-viral-combined_k141_200349\|\|full | 3169 | No | NA | 0 | 3 | Not-determined | Genome-fragment | NA | | 0 |
| S11_k141_24207 | 1281 | No | NA | 0 | 0 | Low-quality | Genome-fragment | | 2.82 | 0 |
| S1-final-viral-combined_k141_239601\|\|lt2gene | 1281 | No | NA | 0 | 0 | Low-quality | Genome-fragment | | 8.28 | 0 |
| S7-final-viral-combined_k141_238797\|\|full | 1281 | No | NA | 0 | 0 | Low-quality | Genome-fragment | | 2.01 | 0 |
| S7-final-viral-combined_k141_232581\|\|full | 1281 | No | NA | 0 | 0 | Low-quality | Genome-fragment | | 3.28 | 0 |
| S1-final-viral-combined_k141_249928\|\|full | 2678 | No | NA | 0 | 1 | Not-determined | Genome-fragment | NA | | 0 |
| S1-final-viral-combined_k141_51810\|\|full | 32421 | No | NA | 0 | 14 | Not-determined | Genome-fragment | NA | | 0 |
| S1-final-viral-combined_k141_166759\|\|full | 7186 | No | NA | 0 | 2 | Not-determined | Genome-fragment | NA | | 0 |
| S1-final-viral-combined_k141_299404\|\|full | 6504 | No | NA | 0 | 5 | Not-determined | Genome-fragment | NA | | 0 |
| S1-final-viral-combined_k141_299412\|\|full | 2256 | No | NA | 0 | 0 | Not-determined | Genome-fragment | NA | | 0 |
| S1-final-viral-combined_k141_150933\|\|full | 2101 | No | NA | 0 | 1 | Not-determined | Genome-fragment | NA | | 0 |
| S12_k141_369283 | 1281 | No | NA | 0 | 0 | Low-quality | Genome-fragment | | 1.33 | 0 |
| S12_k141_392111 | 1281 | No | NA | 2 | 0 | Low-quality | Genome-fragment | | 1.98 | 0 |
| S1-final-viral-combined_k141_35798\|\|full | 1779 | No | NA | 0 | 0 | Not-determined | Genome-fragment | NA | | 0 |
| S2_k141_43093 | 1281 | No | NA | 0 | 0 | Low-quality | Genome-fragment | | 2.14 | 0 |
| S9-final-viral-combined_k141_38572\|\|full | 1282 | No | NA | 1 | 0 | Low-quality | Genome-fragment | | 2.27 | 0 |
| S9-final-viral-combined_k141_6880\|\|full | 1282 | No | NA | 0 | 0 | Low-quality | Genome-fragment | | 3.58 | 0 |
| S11-final-viral-combined_k141_329364\|\|lt2gene | 1282 | No | NA | 0 | 0 | Low-quality | Genome-fragment | | 3.51 | 0 |
| S1-final-viral-combined_k141_51856\|\|full | 4104 | No | NA | 0 | 3 | Not-determined | Genome-fragment | NA | | 0 |
| S11_k141_268446 | 1282 | No | NA | 0 | 1 | Low-quality | Genome-fragment | | 2.86 | 0 |
| S1-final-viral-combined_k141_202535\|\|full | 1282 | No | NA | 0 | 0 | Low-quality | Genome-fragment | | 2.29 | 0 |
| S1-final-viral-combined_k141_101293\|\|full | 4123 | No | NA | 0 | 0 | Not-determined | Genome-fragment | NA | | 0 |
| S1-final-viral-combined_k141_217105\|\|full | 1748 | No | NA | 0 | 0 | Not-determined | Genome-fragment | NA | | 0 |
| S1-final-viral-combined_k141_315670\|\|full | 1413 | No | NA | 0 | 0 | Not-determined | Genome-fragment | NA | | 0 |
| S1-final-viral-combined_k141_3084\|\|full | 2491 | No | NA | 0 | 3 | Not-determined | Genome-fragment | NA | | 0 |
| S1-final-viral-combined_k141_101299\|\|full | 3051 | No | NA | 0 | 2 | Not-determined | Genome-fragment | NA | | 0 |
| S1-final-viral-combined_k141_188400\|\|full | 1282 | No | NA | 0 | 0 | Low-quality | Genome-fragment | | 3.12 | 0 |
| S2-final-viral-combined_k141_52921\|\|full | 1282 | No | NA | 0 | 1 | Low-quality | Genome-fragment | | 2.21 | 0 |
| S2-final-viral-combined_k141_51420\|\|full | 1282 | No | NA | 3 | 0 | Low-quality | Genome-fragment | | 2.67 | 0 |
| S1-final-viral-combined_k141_265741\|\|full | 4039 | No | NA | 0 | 0 | Not-determined | Genome-fragment | NA | | 0 |
| S2-final-viral-combined_k141_245322\|\|full | 1282 | No | NA | 0 | 0 | Low-quality | Genome-fragment | | 3.74 | 0 |
| S1-final-viral-combined_k141_19316\|\|full | 2977 | No | NA | 0 | 2 | Not-determined | Genome-fragment | NA | | 0 |
| S4-final-viral-combined_k141_17605\|\|full | 1282 | No | NA | 0 | 4 | Low-quality | Genome-fragment | | 0.37 | 0 |
| S4-final-viral-combined_k141_97251\|\|full | 1282 | No | NA | 0 | 2 | Low-quality | Genome-fragment | | 2.26 | 0 |
| S6-final-viral-combined_k141_71264\|\|full | 1282 | No | NA | 0 | 1 | Low-quality | Genome-fragment | | 0.94 | 0 |
| S1-final-viral-combined_k141_348641\|\|full | 3050 | No | NA | 0 | 1 | Not-determined | Genome-fragment | NA | | 0 |
| S1-final-viral-combined_k141_51908\|\|full | 1454 | No | NA | 0 | 1 | Not-determined | Genome-fragment | NA | | 0 |

| | | | | | | | | | |
|---|---|---|---|---|---|---|---|---|---|
| S1-final-viral-combined_k141_118160\|\|full | 2847 | No | NA | 0 | 4 | Not-determined | Genome-fragment | NA | 0 |
| S9-final-viral-combined_k141_94893\|\|full | 1283 | No | NA | 1 | 0 | Low-quality | Genome-fragment | 3.33 | 0 |
| S1-final-viral-combined_k141_184217\|\|full | 2419 | No | NA | 0 | 1 | Not-determined | Genome-fragment | NA | 0 |
| S9-final-viral-combined_k141_419\|\|lt2gene | 1283 | No | NA | 1 | 0 | Low-quality | Genome-fragment | 2.91 | 0 |
| S1-final-viral-combined_k141_258647\|\|full | 1283 | No | NA | 2 | 0 | Low-quality | Genome-fragment | 2.15 | 0 |
| S1-final-viral-combined_k141_98280\|\|full | 1283 | No | NA | 0 | 0 | Low-quality | Genome-fragment | 3 | 0 |
| S1-final-viral-combined_k141_184239\|\|full | 1975 | No | NA | 0 | 0 | Not-determined | Genome-fragment | NA | 0 |
| S1-final-viral-combined_k141_44716\|\|lt2gene | 1283 | No | NA | 1 | 0 | Low-quality | Genome-fragment | 2.81 | 0 |
| S1-final-viral-combined_k141_315698\|\|full | 2708 | No | NA | 0 | 3 | Not-determined | Genome-fragment | NA | 0 |
| S1_k141_101906 | 1283 | No | NA | 1 | 0 | Low-quality | Genome-fragment | 2.97 | 0 |
| S1_k141_40501 | 1283 | No | NA | 0 | 0 | Low-quality | Genome-fragment | 2.88 | 0 |
| S1-final-viral-combined_k141_282791\|\|full | 2242 | No | NA | 0 | 2 | Not-determined | Genome-fragment | NA | 0 |
| S1-final-viral-combined_k141_3151\|\|full | 1035 | No | NA | 0 | 0 | Not-determined | Genome-fragment | NA | 0 |
| S7_k141_197203 | 1283 | No | NA | 0 | 0 | Low-quality | Genome-fragment | 3.09 | 0 |
| S8-final-viral-combined_k141_69892\|\|full | 1283 | No | NA | 0 | 0 | Low-quality | Genome-fragment | 2.09 | 0 |
| S1-final-viral-combined_k141_151032\|\|full | 1537 | No | NA | 0 | 1 | Not-determined | Genome-fragment | NA | 0 |
| S1-final-viral-combined_k141_381237\|\|full | 5758 | No | NA | 0 | 2 | Not-determined | Genome-fragment | NA | 0 |
| S1-final-viral-combined_k141_118167\|\|full | 50249 | No | NA | 19 | 1 | Medium-quality | Genome-fragment | 82.74 | 0 |
| S1-final-viral-combined_k141_348744\|\|full | 6467 | No | NA | 0 | 3 | Not-determined | Genome-fragment | NA | 0 |
| S1-final-viral-combined_k141_118179\|\|full | 1306 | No | NA | 0 | 2 | Not-determined | Genome-fragment | NA | 0 |
| S1-final-viral-combined_k141_365071\|\|full | 2683 | No | NA | 0 | 1 | Not-determined | Genome-fragment | NA | 0 |
| S1-final-viral-combined_k141_299558\|\|full | 4412 | No | NA | 0 | 0 | Not-determined | Genome-fragment | NA | 0 |
| S1-final-viral-combined_k141_166838\|\|full | 2515 | No | NA | 0 | 0 | Not-determined | Genome-fragment | NA | 0 |
| S1-final-viral-combined_k141_331775\|\|full | 1468 | No | NA | 0 | 0 | Not-determined | Genome-fragment | NA | 0 |
| S1-final-viral-combined_k141_68288\|\|full | 2902 | No | NA | 0 | 1 | Not-determined | Genome-fragment | NA | 0 |
| S1-final-viral-combined_k141_166846\|\|full | 4543 | No | NA | 0 | 7 | Not-determined | Genome-fragment | NA | 0 |
| S8-final-viral-combined_k141_188585\|\|full | 1283 | No | NA | 1 | 0 | Low-quality | Genome-fragment | 2.19 | 0 |
| S1-final-viral-combined_k141_19376\|\|full | 2693 | No | NA | 0 | 1 | Not-determined | Genome-fragment | NA | 0 |
| S1-final-viral-combined_k141_299574\|\|full | 7245 | No | NA | 0 | 7 | Not-determined | Genome-fragment | NA | 0 |
| S9-final-viral-combined_k141_16105\|\|full | 1284 | No | NA | 0 | 0 | Low-quality | Genome-fragment | 0.91 | 0 |
| S11-final-viral-combined_k141_153401\|\|full | 1284 | No | NA | 2 | 0 | Low-quality | Genome-fragment | 2.12 | 0 |
| S1-final-viral-combined_k141_382148\|\|full | 1284 | No | NA | 1 | 0 | Low-quality | Genome-fragment | 2.36 | 0 |
| S1-final-viral-combined_k141_101441\|\|full | 7714 | No | NA | 0 | 7 | Not-determined | Genome-fragment | NA | 0 |
| S1-final-viral-combined_k141_184317\|\|full | 2425 | No | NA | 0 | 1 | Not-determined | Genome-fragment | NA | 0 |
| S1-final-viral-combined_k141_166892\|\|full | 1159 | No | NA | 0 | 1 | Not-determined | Genome-fragment | NA | 0 |
| S1-final-viral-combined_k141_52059\|\|full | 1919 | No | NA | 0 | 3 | Not-determined | Genome-fragment | NA | 0 |
| S1-final-viral-combined_k141_183843\|\|lt2gene | 1284 | No | NA | 1 | 0 | Low-quality | Genome-fragment | 0.69 | 0 |
| S5_k141_27573 | 1284 | No | NA | 0 | 0 | Low-quality | Genome-fragment | 3.25 | 0 |
| S1-final-viral-combined_k141_151062\|\|full | 2658 | No | NA | 0 | 2 | Not-determined | Genome-fragment | NA | 0 |
| S1-final-viral-combined_k141_135108\|\|full | 3830 | No | NA | 0 | 4 | Not-determined | Genome-fragment | NA | 0 |
| S1-final-viral-combined_k141_135117\|\|full | 2467 | No | NA | 0 | 2 | Not-determined | Genome-fragment | NA | 0 |
| S7-final-viral-combined_k141_57547\|\|full | 1284 | No | NA | 0 | 0 | Low-quality | Genome-fragment | 1.17 | 0 |
| S1-final-viral-combined_k141_331801\|\|full | 1086 | No | NA | 0 | 1 | Not-determined | Genome-fragment | NA | 0 |
| S17-final-viral-combined_k141_117860\|\|full | 1284 | No | NA | 0 | 0 | Low-quality | Genome-fragment | 10.2 | 0 |
| S17-k141_256949 | 1284 | No | NA | 0 | 0 | Low-quality | Genome-fragment | 2.71 | 0 |
| S1-final-viral-combined_k141_331826\|\|full | 1787 | No | NA | 0 | 1 | Not-determined | Genome-fragment | NA | 0 |
| S17-k141_1719 | 1284 | No | NA | 3 | 0 | Low-quality | Genome-fragment | 2.88 | 0 |
| S1-final-viral-combined_k141_250104\|\|full | 3834 | No | NA | 0 | 0 | Not-determined | Genome-fragment | NA | 0 |
| S1-final-viral-combined_k141_166940\|\|full | 2541 | No | NA | 0 | 2 | Not-determined | Genome-fragment | NA | 0 |
| S1-final-viral-combined_k141_315820\|\|full | 3107 | No | NA | 0 | 1 | Not-determined | Genome-fragment | NA | 0 |
| S1-final-viral-combined_k141_348843\|\|full | 1999 | No | NA | 0 | 2 | Not-determined | Genome-fragment | NA | 0 |
| S12-final-viral-combined_k141_304077\|\|full | 1284 | No | NA | 0 | 1 | Low-quality | Genome-fragment | 0.78 | 0 |
| S1-final-viral-combined_k141_166959\|\|full | 6692 | No | NA | 0 | 5 | Not-determined | Genome-fragment | NA | 0 |
| S12-final-viral-combined_k141_80969\|\|full | 1284 | No | NA | 1 | 0 | Low-quality | Genome-fragment | 0.59 | 0 |
| S12_k141_362757 | 1284 | No | NA | 0 | 0 | Low-quality | Genome-fragment | 0.7 | 0 |
| S1-final-viral-combined_k141_118306\|\|full | 1556 | No | NA | 0 | 1 | Not-determined | Genome-fragment | NA | 0 |
| S1-final-viral-combined_k141_118308\|\|full | 4195 | No | NA | 0 | 5 | Not-determined | Genome-fragment | NA | 0 |
| S2_k141_80903 | 1284 | No | NA | 1 | 0 | Low-quality | Genome-fragment | 3.04 | 0 |
| S1-final-viral-combined_k141_283014\|\|full | 2199 | No | NA | 0 | 2 | Not-determined | Genome-fragment | NA | 0 |
| S1-final-viral-combined_k141_381368\|\|full | 3482 | No | NA | 0 | 2 | Not-determined | Genome-fragment | NA | 0 |
| S1-final-viral-combined_k141_283024\|\|full | 2296 | No | NA | 0 | 2 | Not-determined | Genome-fragment | NA | 0 |
| S1-final-viral-combined_k141_118317\|\|full | 2583 | No | NA | 0 | 0 | Not-determined | Genome-fragment | NA | 0 |
| S1-final-viral-combined_k141_331896\|\|full | 2459 | No | NA | 1 | 0 | Medium-quality | Genome-fragment | 82.78 | 0 |
| S1-final-viral-combined_k141_118338\|\|full | 3406 | No | NA | 0 | 1 | Not-determined | Genome-fragment | NA | 0 |
| S6-final-viral-combined_k141_133644\|\|lt2gene | 1284 | No | NA | 1 | 0 | Low-quality | Genome-fragment | 2.4 | 0 |
| S1-final-viral-combined_k141_184468\|\|full | 1440 | No | NA | 0 | 1 | Not-determined | Genome-fragment | NA | 0 |
| S1-final-viral-combined_k141_52159\|\|full | 1873 | No | NA | 0 | 1 | Not-determined | Genome-fragment | NA | 0 |
| S1-final-viral-combined_k141_151126\|\|full | 9387 | No | NA | 0 | 9 | Not-determined | Genome-fragment | NA | 0 |
| S1-final-viral-combined_k141_19500\|\|full | 8178 | No | NA | 0 | 10 | Not-determined | Genome-fragment | NA | 0 |
| S9-final-viral-combined_k141_10250\|\|full | 1285 | No | NA | 1 | 0 | Low-quality | Genome-fragment | 2.72 | 0 |
| S9-final-viral-combined_k141_108846\|\|lt2gene | 1285 | No | NA | 0 | 0 | Low-quality | Genome-fragment | 3.13 | 0 |
| S9-final-viral-combined_k141_24094\|\|lt2gene | 1285 | No | NA | 1 | 0 | Low-quality | Genome-fragment | 3.46 | 0 |
| S7-final-viral-combined_k141_151312\|\|full | 1285 | No | NA | 0 | 0 | Low-quality | Genome-fragment | 0.88 | 0 |
| S1-final-viral-combined_k141_250168\|\|full | 3547 | No | NA | 0 | 1 | Not-determined | Genome-fragment | NA | 0 |
| S12-final-viral-combined_k141_95238\|\|full | 1285 | No | NA | 0 | 4 | Low-quality | Genome-fragment | 0.42 | 0 |
| S1-final-viral-combined_k141_184510\|\|full | 1893 | No | NA | 0 | 2 | Not-determined | Genome-fragment | NA | 0 |
| S2-final-viral-combined_k141_75818\|\|lt2gene | 1285 | No | NA | 1 | 0 | Low-quality | Genome-fragment | 1.88 | 0 |
| S1-final-viral-combined_k141_299723\|\|full | 2167 | No | NA | 0 | 0 | Not-determined | Genome-fragment | NA | 0 |
| S1-final-viral-combined_k141_167055\|\|full | 5315 | No | NA | 0 | 3 | Not-determined | Genome-fragment | NA | 0 |
| S1-final-viral-combined_k141_3323\|\|full | 2239 | No | NA | 0 | 1 | Not-determined | Genome-fragment | NA | 0 |
| S1-final-viral-combined_k141_200684\|\|full | 3186 | No | NA | 0 | 1 | Not-determined | Genome-fragment | NA | 0 |
| S1-final-viral-combined_k141_118476\|\|full | 1283 | No | NA | 0 | 1 | Not-determined | Genome-fragment | NA | 0 |
| S1-final-viral-combined_k141_381464\|\|full | 6346 | No | NA | 0 | 2 | Not-determined | Genome-fragment | NA | 0 |
| S1-final-viral-combined_k141_200690\|\|full | 5018 | No | NA | 0 | 2 | Not-determined | Genome-fragment | NA | 0 |
| S9_k141_97343 | 1286 | No | NA | 0 | 0 | Low-quality | Genome-fragment | 2.74 | 0 |
| S1-final-viral-combined_k141_19565\|\|full | 2051 | No | NA | 0 | 0 | Not-determined | Genome-fragment | NA | 0 |
| S1-final-viral-combined_k141_36139\|\|full | 3978 | No | NA | 0 | 3 | Not-determined | Genome-fragment | NA | 0 |
| S1-final-viral-combined_k141_283072\|\|full | 1266 | No | NA | 0 | 0 | Not-determined | Genome-fragment | NA | 0 |
| S11-final-viral-combined_k141_201636\|\|full | 1286 | No | NA | 0 | 0 | Low-quality | Genome-fragment | 2.47 | 0 |
| S1-final-viral-combined_k141_19573\|\|full | 3658 | No | NA | 0 | 0 | Not-determined | Genome-fragment | NA | 0 |
| S1-final-viral-combined_k141_331972\|\|full | 3953 | No | NA | 0 | 1 | Not-determined | Genome-fragment | NA | 0 |
| S11-final-viral-combined_k141_135581\|\|full | 1286 | No | NA | 0 | 1 | Low-quality | Genome-fragment | 3.01 | 0 |
| S1-final-viral-combined_k141_318944\|\|full | 1286 | No | NA | 0 | 1 | Low-quality | Genome-fragment | 1.86 | 0 |
| S5-final-viral-combined_k141_71287\|\|full | 1286 | No | NA | 0 | 1 | Low-quality | Genome-fragment | 0.57 | 0 |
| S1-final-viral-combined_k141_217500\|\|full | 2633 | No | NA | 0 | 2 | Not-determined | Genome-fragment | NA | 0 |
| S1-final-viral-combined_k141_331983\|\|full | 4009 | No | NA | 0 | 4 | Not-determined | Genome-fragment | NA | 0 |
| S5-final-viral-combined_k141_87759\|\|full | 1286 | No | NA | 3 | 0 | Low-quality | Genome-fragment | 2.89 | 0 |
| S1-final-viral-combined_k141_184559\|\|full | 6417 | No | NA | 0 | 5 | Not-determined | Genome-fragment | NA | 0 |
| S5-final-viral-combined_k141_34548\|\|full | 1286 | No | NA | 0 | 2 | Low-quality | Genome-fragment | 0.88 | 0 |
| S1-final-viral-combined_k141_167127\|\|full | 1317 | No | NA | 0 | 2 | Not-determined | Genome-fragment | NA | 0 |
| S1-final-viral-combined_k141_200726\|\|full | 7071 | No | NA | 0 | 5 | Not-determined | Genome-fragment | NA | 0 |
| S7-final-viral-combined_k141_1275\|\|full | 1286 | No | NA | 1 | 0 | Low-quality | Genome-fragment | 0.84 | 0 |
| S7-final-viral-combined_k141_29172\|\|full | 1286 | No | NA | 0 | 2 | Low-quality | Genome-fragment | 1.75 | 0 |
| S17-final-viral-combined_k141_217566\|\|full | 1286 | No | NA | 0 | 2 | Low-quality | Genome-fragment | 0.41 | 0 |
| S1-final-viral-combined_k141_184595\|\|full | 1751 | No | NA | 0 | 0 | Not-determined | Genome-fragment | NA | 0 |
| S1-final-viral-combined_k141_349004\|\|full | 9315 | No | NA | 0 | 3 | Not-determined | Genome-fragment | NA | 0 |
| S1-final-viral-combined_k141_3375\|\|full | 2695 | No | NA | 0 | 1 | Not-determined | Genome-fragment | NA | 0 |
| S1-final-viral-combined_k141_365357\|\|full | 1089 | No | NA | 0 | 0 | Not-determined | Genome-fragment | NA | 0 |
| S1-final-viral-combined_k141_250283\|\|full | 7171 | No | NA | 0 | 4 | Not-determined | Genome-fragment | NA | 0 |
| S12-final-viral-combined_k141_274610\|\|full | 1286 | No | NA | 0 | 1 | Low-quality | Genome-fragment | 2.13 | 0 |
| S1-final-viral-combined_k141_381525\|\|full | 1029 | No | NA | 0 | 0 | Not-determined | Genome-fragment | NA | 0 |
| S12-final-viral-combined_k141_293840\|\|full | 1286 | No | NA | 1 | 0 | Low-quality | Genome-fragment | 3.07 | 0 |
| S12-final-viral-combined_k141_110611\|\|lt2gene | 1286 | No | NA | 1 | 0 | Low-quality | Genome-fragment | 2.83 | 0 |
| S1-final-viral-combined_k141_101528\|\|full | 5060 | No | NA | 0 | 0 | Not-determined | Genome-fragment | NA | 0 |
| S1-final-viral-combined_k141_184606\|\|full | 4984 | No | NA | 0 | 2 | Not-determined | Genome-fragment | NA | 0 |
| S2-final-viral-combined_k141_230575\|\|full | 1286 | No | NA | 1 | 0 | Low-quality | Genome-fragment | 2.88 | 0 |
| S1-final-viral-combined_k141_332037\|\|full | 1828 | No | NA | 0 | 0 | Not-determined | Genome-fragment | NA | 0 |
| S1-final-viral-combined_k141_316024\|\|full | 2394 | No | NA | 0 | 1 | Not-determined | Genome-fragment | NA | 0 |
| S1-final-viral-combined_k141_167167\|\|full | 4995 | No | NA | 0 | 5 | Not-determined | Genome-fragment | NA | 0 |
| S1-final-viral-combined_k141_233529\|\|full | 1082 | No | NA | 0 | 0 | Not-determined | Genome-fragment | NA | 0 |
| S2-final-viral-combined_k141_90248\|\|full | 1286 | No | NA | 1 | 0 | Low-quality | Genome-fragment | 2.84 | 0 |
| S4-final-viral-combined_k141_56574\|\|full | 1286 | No | NA | 1 | 0 | Low-quality | Genome-fragment | 2.14 | 0 |
| S1-final-viral-combined_k141_184637\|\|full | 1533 | No | NA | 0 | 0 | Not-determined | Genome-fragment | NA | 0 |
| S1-final-viral-combined_k141_381578\|\|full | 5871 | No | NA | 0 | 3 | Not-determined | Genome-fragment | NA | 0 |
| S1-final-viral-combined_k141_316076\|\|full | 2664 | No | NA | 0 | 1 | Not-determined | Genome-fragment | NA | 0 |
| S1-final-viral-combined_k141_101601\|\|full | 3689 | No | NA | 0 | 3 | Not-determined | Genome-fragment | NA | 0 |
| S1-final-viral-combined_k141_135415\|\|full | 3368 | No | NA | 0 | 1 | Not-determined | Genome-fragment | NA | 0 |
| S1-final-viral-combined_k141_184685\|\|full | 1359 | No | NA | 0 | 0 | Not-determined | Genome-fragment | NA | 0 |
| S1-final-viral-combined_k141_68539\|\|full | 2912 | No | NA | 0 | 1 | Not-determined | Genome-fragment | NA | 0 |
| S1-final-viral-combined_k141_266136\|\|full | 1057 | No | NA | 0 | 1 | Not-determined | Genome-fragment | NA | 0 |
| S1-final-viral-combined_k141_365418\|\|full | 1497 | No | NA | 0 | 0 | Not-determined | Genome-fragment | NA | 0 |
| S1-final-viral-combined_k141_184708\|\|full | 2059 | No | NA | 0 | 2 | Not-determined | Genome-fragment | NA | 0 |
| S4-final-viral-combined_k141_4559\|\|full | 1286 | No | NA | 0 | 0 | Low-quality | Genome-fragment | 2.69 | 0 |
| S1-final-viral-combined_k141_135468\|\|full | 1078 | No | NA | 0 | 2 | Not-determined | Genome-fragment | NA | 0 |
| S1-final-viral-combined_k141_118613\|\|full | 3683 | No | NA | 0 | 5 | Not-determined | Genome-fragment | NA | 0 |
| S4_k141_36257 | 1286 | No | NA | 0 | 0 | Low-quality | Genome-fragment | 1.56 | 0 |
| S1-final-viral-combined_k141_36332\|\|full | 1255 | No | NA | 0 | 3 | Not-determined | Genome-fragment | NA | 0 |
| S6-final-viral-combined_k141_104109\|\|lt2gene | 1286 | No | NA | 2 | 0 | Low-quality | Genome-fragment | 1.56 | 0 |
| S1-final-viral-combined_k141_135497\|\|full | 2828 | No | NA | 0 | 0 | Not-determined | Genome-fragment | NA | 0 |
| S1-final-viral-combined_k141_283237\|\|full | 7063 | No | NA | 0 | 6 | Not-determined | Genome-fragment | NA | 0 |
| S8-final-viral-combined_k141_163460\|\|full | 1286 | No | NA | 1 | 0 | Low-quality | Genome-fragment | 2.46 | 0 |
| S8-final-viral-combined_k141_94779\|\|lt2gene | 1286 | No | NA | 1 | 0 | Low-quality | Genome-fragment | 3.6 | 0 |
| S1-final-viral-combined_k141_85495\|\|full | 1138 | No | NA | 0 | 2 | Not-determined | Genome-fragment | NA | 0 |
| S11-final-viral-combined_k141_185467\|\|full | 1287 | No | NA | 0 | 1 | Low-quality | Genome-fragment | 0.78 | 0 |
| S1-final-viral-combined_k141_316184\|\|full | 1202 | No | NA | 0 | 0 | Not-determined | Genome-fragment | NA | 0 |
| S1-final-viral-combined_k141_388769\|\|full | 1287 | No | NA | 0 | 0 | Low-quality | Genome-fragment | 2.68 | 0 |
| S1-final-viral-combined_k141_151426\|\|full | 1720 | No | NA | 0 | 2 | Not-determined | Genome-fragment | NA | 0 |
| S3-final-viral-combined_k141_54011\|\|full | 1287 | No | NA | 0 | 0 | Low-quality | Genome-fragment | 2.53 | 0 |
| S1-final-viral-combined_k141_85518\|\|full | 5965 | No | NA | 0 | 1 | Not-determined | Genome-fragment | NA | 0 |
| S7-final-viral-combined_k141_160286\|\|full | 1287 | No | NA | 0 | 2 | Low-quality | Genome-fragment | 2.16 | 0 |
| S7-final-viral-combined_k141_143757\|\|lt2gene | 1287 | No | NA | 1 | 0 | Low-quality | Genome-fragment | 2.29 | 0 |
| S2-final-viral-combined_k141_151263\|\|full | 1287 | No | NA | 1 | 0 | Low-quality | Genome-fragment | 2.07 | 0 |
| S1-final-viral-combined_k141_233690\|\|full | 1161 | No | NA | 0 | 0 | Not-determined | Genome-fragment | NA | 0 |
| S1-final-viral-combined_k141_217596\|\|full | 4235 | No | NA | 0 | 2 | Not-determined | Genome-fragment | NA | 0 |
| S2-final-viral-combined_k141_235916\|\|full | 1287 | No | NA | 0 | 0 | Low-quality | Genome-fragment | 0.78 | 0 |
| S6-final-viral-combined_k141_51920\|\|full | 1287 | No | NA | 0 | 0 | Low-quality | Genome-fragment | 2.54 | 0 |
| S1-final-viral-combined_k141_381703\|\|full | 1847 | No | NA | 0 | 2 | Not-determined | Genome-fragment | NA | 0 |
| S1-final-viral-combined_k141_349100\|\|full | 1832 | No | NA | 0 | 0 | Not-determined | Genome-fragment | NA | 0 |
| S8_k141_8339 | 1287 | No | NA | 0 | 3 | Low-quality | Genome-fragment | 0.34 | 0 |
| S9-final-viral-combined_k141_119546\|\|full | 1288 | No | NA | 0 | 0 | Low-quality | Genome-fragment | 3.26 | 0 |
| S1-final-viral-combined_k141_200927\|\|full | 2511 | No | NA | 0 | 0 | Not-determined | Genome-fragment | NA | 0 |
| S9-final-viral-combined_k141_19160\|\|full | 1288 | No | NA | 0 | 1 | Low-quality | Genome-fragment | 2.68 | 0 |
| S9_k141_35112 | 1288 | No | NA | 2 | 0 | Low-quality | Genome-fragment | 3.19 | 0 |
| S11-final-viral-combined_k141_62334\|\|full | 1288 | No | NA | 0 | 0 | Low-quality | Genome-fragment | 1.03 | 0 |
| S1-final-viral-combined_k141_151508\|\|full | 1303 | No | NA | 0 | 1 | Not-determined | Genome-fragment | NA | 0 |
| S1-final-viral-combined_k141_249955\|\|full | 1288 | No | NA | 0 | 1 | Low-quality | Genome-fragment | 0.65 | 0 |
| S1-final-viral-combined_k141_277548\|\|full | 1288 | No | NA | 0 | 2 | Low-quality | Genome-fragment | 0.41 | 0 |
| S5-final-viral-combined_k141_192569\|\|full | 1288 | No | NA | 1 | 0 | Low-quality | Genome-fragment | 2.6 | 0 |
| S7-final-viral-combined_k141_62068\|\|full | 1288 | No | NA | 0 | 0 | Low-quality | Genome-fragment | 2.9 | 0 |
| S1-final-viral-combined_k141_19809\|\|full | 1036 | No | NA | 0 | 2 | Not-determined | Genome-fragment | NA | 0 |
| S1-final-viral-combined_k141_151536\|\|full | 1055 | No | NA | 0 | 1 | Not-determined | Genome-fragment | NA | 0 |
| S1-final-viral-combined_k141_250503\|\|full | 1618 | No | NA | 0 | 1 | Not-determined | Genome-fragment | NA | 0 |
| S1-final-viral-combined_k141_19821\|\|full | 1381 | No | NA | 0 | 1 | Not-determined | Genome-fragment | NA | 0 |
| S1-final-viral-combined_k141_266361\|\|full | 1647 | No | NA | 0 | 2 | Not-determined | Genome-fragment | NA | 0 |

| | | | | | | | | | |
|---|---|---|---|---|---|---|---|---|---|
| S1-final-viral-combined_k141_19846\|\|full | 1540 | No | NA | 0 | 0 | Not-determined | Genome-fragment | NA | 0 |
| S1-final-viral-combined_k141_19850\|\|full | 1845 | No | NA | 0 | 1 | Not-determined | Genome-fragment | NA | 0 |
| S1-final-viral-combined_k141_250537\|\|full | 1509 | No | NA | 0 | 0 | Not-determined | Genome-fragment | NA | 0 |
| S12-final-viral-combined_k141_360730\|\|full | 1288 | No | NA | 0 | 0 | Low-quality | Genome-fragment | 2 | 0 |
| S12_k141_292167 | 1288 | No | NA | 0 | 0 | Low-quality | Genome-fragment | 2.79 | 0 |
| S4-final-viral-combined_k141_40748\|\|lt2gene | 1288 | No | NA | 0 | 0 | Low-quality | Genome-fragment | 3.59 | 0 |
| S1-final-viral-combined_k141_151589\|\|full | 2845 | No | NA | 0 | 2 | Not-determined | Genome-fragment | NA | 0 |
| S1-final-viral-combined_k141_118763\|\|full | 1787 | No | NA | 0 | 1 | Not-determined | Genome-fragment | NA | 0 |
| S4_k141_43729 | 1288 | No | NA | 1 | 0 | Low-quality | Genome-fragment | 2.33 | 0 |
| S8-final-viral-combined_k141_228522\|\|full | 1288 | No | NA | 0 | 0 | Low-quality | Genome-fragment | 1.03 | 0 |
| S1-final-viral-combined_k141_118766\|\|full | 1036 | No | NA | 0 | 0 | Not-determined | Genome-fragment | NA | 0 |
| S1-final-viral-combined_k141_332236\|\|full | 30000 | No | NA | 18 | 0 | Medium-quality | Genome-fragment | 55.15 | 0 |
| S9-final-viral-combined_k141_66392\|\|full | 1289 | No | NA | 0 | 0 | Low-quality | Genome-fragment | 3.29 | 0 |
| S1-final-viral-combined_k141_118771\|\|full | 1154 | No | NA | 0 | 2 | Not-determined | Genome-fragment | NA | 0 |
| S1-final-viral-combined_k141_151600\|\|full | 1631 | No | NA | 0 | 0 | Not-determined | Genome-fragment | NA | 0 |
| S1-final-viral-combined_k141_332245\|\|full | 1598 | No | NA | 0 | 1 | Not-determined | Genome-fragment | NA | 0 |
| S11-final-viral-combined_k141_78557\|\|full | 1289 | No | NA | 0 | 0 | Low-quality | Genome-fragment | 1.27 | 0 |
| S11_k141_68054 | 1289 | No | NA | 0 | 0 | Low-quality | Genome-fragment | 1.32 | 0 |
| S1-final-viral-combined_k141_151634\|\|full | 1626 | No | NA | 0 | 4 | Not-determined | Genome-fragment | NA | 0 |
| S1-final-viral-combined_k141_52510\|\|full | 1697 | No | NA | 0 | 1 | Not-determined | Genome-fragment | NA | 0 |
| S5-final-viral-combined_k141_190993\|\|full | 1289 | No | NA | 0 | 0 | Low-quality | Genome-fragment | 3.08 | 0 |
| S1-final-viral-combined_k141_283401\|\|full | 1409 | No | NA | 0 | 0 | Not-determined | Genome-fragment | NA | 0 |
| S5-final-viral-combined_k141_23745\|\|full | 1289 | No | NA | 1 | 0 | Low-quality | Genome-fragment | 1.42 | 0 |
| S1-final-viral-combined_k141_381793\|\|full | 1424 | No | NA | 0 | 0 | Not-determined | Genome-fragment | NA | 0 |
| S1-final-viral-combined_k141_52552\|\|full | 1194 | No | NA | 0 | 0 | Not-determined | Genome-fragment | NA | 0 |
| S5_k141_74201 | 1289 | No | NA | 0 | 0 | Low-quality | Genome-fragment | 2.58 | 0 |
| S1-final-viral-combined_k141_250666\|\|full | 1888 | No | NA | 0 | 0 | Not-determined | Genome-fragment | NA | 0 |
| S7_k141_124388 | 1289 | No | NA | 0 | 0 | Low-quality | Genome-fragment | 2.61 | 0 |
| S1-final-viral-combined_k141_19954\|\|full | 2345 | No | NA | 0 | 2 | Not-determined | Genome-fragment | NA | 0 |
| S1-final-viral-combined_k141_365658\|\|full | 1132 | No | NA | 0 | 2 | Not-determined | Genome-fragment | NA | 0 |
| S1-final-viral-combined_k141_365659\|\|full | 1509 | No | NA | 0 | 2 | Not-determined | Genome-fragment | NA | 0 |
| S1-final-viral-combined_k141_381811\|\|full | 2194 | No | NA | 0 | 2 | Not-determined | Genome-fragment | NA | 0 |
| S1-final-viral-combined_k141_151691\|\|full | 1324 | No | NA | 0 | 0 | Not-determined | Genome-fragment | NA | 0 |
| S17-final-viral-combined_k141_64203\|\|full | 1289 | No | NA | 2 | 0 | Low-quality | Genome-fragment | 3.3 | 0 |
| S12_k141_313414 | 1289 | No | NA | 0 | 0 | Low-quality | Genome-fragment | 0.77 | 0 |
| S1-final-viral-combined_k141_52592\|\|full | 2694 | No | NA | 0 | 2 | Not-determined | Genome-fragment | NA | 0 |
| S2-final-viral-combined_k141_53326\|\|lt2gene | 1289 | No | NA | 0 | 0 | Low-quality | Genome-fragment | 3.26 | 0 |
| S4-final-viral-combined_k141_97248\|\|full | 1289 | No | NA | 0 | 0 | Low-quality | Genome-fragment | 0.79 | 0 |
| S1-final-viral-combined_k141_184924\|\|full | 2907 | No | NA | 0 | 0 | Not-determined | Genome-fragment | NA | 0 |
| S1-final-viral-combined_k141_3702\|\|full | 2646 | No | NA | 0 | 1 | Not-determined | Genome-fragment | NA | 0 |
| S1-final-viral-combined_k141_250698\|\|full | 1687 | No | NA | 0 | 2 | Not-determined | Genome-fragment | NA | 0 |
| S1-final-viral-combined_k141_250700\|\|full | 1273 | No | NA | 0 | 2 | Not-determined | Genome-fragment | NA | 0 |
| S1-final-viral-combined_k141_68649\|\|full | 7904 | No | NA | 0 | 3 | Not-determined | Genome-fragment | NA | 0 |
| S8_k141_255566 | 1289 | No | NA | 0 | 1 | Low-quality | Genome-fragment | 2.03 | 0 |
| S1-final-viral-combined_k141_316446\|\|full | 6007 | No | NA | 0 | 4 | Not-determined | Genome-fragment | NA | 0 |
| S8_k141_101791 | 1289 | No | NA | 1 | 0 | Low-quality | Genome-fragment | 1.72 | 0 |
| S9-final-viral-combined_k141_85456\|\|full | 1290 | No | NA | 1 | 0 | Low-quality | Genome-fragment | 2.44 | 0 |
| S1-final-viral-combined_k141_283463\|\|full | 1831 | No | NA | 0 | 1 | Not-determined | Genome-fragment | NA | 0 |
| S11-final-viral-combined_k141_76403\|\|full | 1290 | No | NA | 1 | 0 | Low-quality | Genome-fragment | 1.44 | 0 |
| S1-final-viral-combined_k141_135673\|\|full | 4556 | No | NA | 0 | 3 | Not-determined | Genome-fragment | NA | 0 |
| S1-final-viral-combined_k141_300212\|\|full | 5209 | No | NA | 0 | 0 | Not-determined | Genome-fragment | NA | 0 |
| S1-final-viral-combined_k141_233933\|\|full | 1148 | No | NA | 0 | 1 | Not-determined | Genome-fragment | NA | 0 |
| S11-final-viral-combined_k141_189068\|\|lt2gene | 1290 | No | NA | 1 | 0 | Low-quality | Genome-fragment | 2.95 | 0 |
| S11_k141_174589 | 1290 | No | NA | 0 | 2 | Low-quality | Genome-fragment | 0.21 | 0 |
| S1-final-viral-combined_k141_233954\|\|full | 2405 | No | NA | 0 | 1 | Not-determined | Genome-fragment | NA | 0 |
| S1-final-viral-combined_k141_184976\|\|full | 3945 | No | NA | 0 | 2 | Not-determined | Genome-fragment | NA | 0 |
| S1-final-viral-combined_k141_151708\|\|full | 13557 | No | NA | 2 | 1 | Medium-quality | Genome-fragment | 87.49 | 0 |
| S1-final-viral-combined_k141_33837\|\|full | 1290 | No | NA | 0 | 0 | Low-quality | Genome-fragment | 0.82 | 0 |
| S1-final-viral-combined_k141_168672\|\|full | 1290 | No | NA | 0 | 1 | Low-quality | Genome-fragment | 3.33 | 0 |
| S7-final-viral-combined_k141_78892\|\|full | 1290 | No | NA | 0 | 0 | Low-quality | Genome-fragment | 2.14 | 0 |
| S1-final-viral-combined_k141_233991\|\|full | 5685 | No | NA | 0 | 4 | Not-determined | Genome-fragment | NA | 0 |
| S12-final-viral-combined_k141_117211\|\|full | 1290 | No | NA | 0 | 0 | Low-quality | Genome-fragment | 2.59 | 0 |
| S1-final-viral-combined_k141_283520\|\|full | 2752 | No | NA | 0 | 1 | Not-determined | Genome-fragment | NA | 0 |
| S1-final-viral-combined_k141_52643\|\|full | 23536 | No | NA | 8 | 0 | Medium-quality | Genome-fragment | 56.07 | 0 |
| S4_k141_19346 | 1290 | No | NA | 0 | 1 | Low-quality | Genome-fragment | 1.42 | 0 |
| S6-final-viral-combined_k141_48187\|\|full | 1290 | No | NA | 0 | 0 | Low-quality | Genome-fragment | 1.8 | 0 |
| S1-final-viral-combined_k141_349308\|\|full | 2168 | No | NA | 0 | 1 | Not-determined | Genome-fragment | NA | 0 |
| S1-final-viral-combined_k141_217640\|\|full | 2069 | No | NA | 0 | 1 | Not-determined | Genome-fragment | NA | 0 |
| S1-final-viral-combined_k141_101951\|\|full | 3580 | No | NA | 0 | 2 | Not-determined | Genome-fragment | NA | 0 |
| S8-final-viral-combined_k141_12110\|\|lt2gene | 1290 | No | NA | 1 | 0 | Low-quality | Genome-fragment | 3.42 | 0 |
| S9-final-viral-combined_k141_62825\|\|lt2gene | 1291 | No | NA | 2 | 0 | Low-quality | Genome-fragment | 3.14 | 0 |
| S1-final-viral-combined_k141_101958\|\|full | 2110 | No | NA | 0 | 2 | Not-determined | Genome-fragment | NA | 0 |
| S1-final-viral-combined_k141_118921\|\|full | 1021 | No | NA | 0 | 0 | Not-determined | Genome-fragment | NA | 0 |
| S1-final-viral-combined_k141_118928\|\|full | 3159 | No | NA | 0 | 0 | Not-determined | Genome-fragment | NA | 0 |
| S1-final-viral-combined_k141_283600\|\|full | 14499 | No | NA | 0 | 12 | Not-determined | Genome-fragment | NA | 0 |
| S1-final-viral-combined_k141_332485\|\|full | 2344 | No | NA | 0 | 0 | Not-determined | Genome-fragment | NA | 0 |
| S1-final-viral-combined_k141_266623\|\|full | 1848 | No | NA | 0 | 2 | Not-determined | Genome-fragment | NA | 0 |
| S1-final-viral-combined_k141_201170\|\|full | 3655 | No | NA | 0 | 4 | Not-determined | Genome-fragment | NA | 0 |
| S1-final-viral-combined_k141_52701\|\|full | 3376 | No | NA | 0 | 3 | Not-determined | Genome-fragment | NA | 0 |
| S1-final-viral-combined_k141_363910\|\|full | 1291 | No | NA | 0 | 0 | Low-quality | Genome-fragment | 0.51 | 0 |
| S1-final-viral-combined_k141_152645\|\|full | 1291 | No | NA | 0 | 0 | Low-quality | Genome-fragment | 0.89 | 0 |
| S3-final-viral-combined_k141_127278\|\|full | 1291 | No | NA | 0 | 0 | Low-quality | Genome-fragment | 2.14 | 0 |
| S1-final-viral-combined_k141_266673\|\|full | 12403 | No | NA | 0 | 3 | Not-determined | Genome-fragment | NA | 0 |
| S1-final-viral-combined_k141_167554\|\|full | 7832 | No | NA | 0 | 6 | Not-determined | Genome-fragment | NA | 0 |
| S1-final-viral-combined_k141_201201\|\|full | 4084 | No | NA | 0 | 3 | Not-determined | Genome-fragment | NA | 0 |
| S1-final-viral-combined_k141_118971\|\|full | 1803 | No | NA | 0 | 2 | Not-determined | Genome-fragment | NA | 0 |
| S1-final-viral-combined_k141_52708\|\|full | 51903 | No | NA | 3 | 34 | High-quality | High-quality | 100 | 0 |
| S1-final-viral-combined_k141_217721\|\|full | 3624 | No | NA | 0 | 2 | Not-determined | Genome-fragment | NA | 0 |
| S1-final-viral-combined_k141_185075\|\|full | 3523 | No | NA | 0 | 0 | Not-determined | Genome-fragment | NA | 0 |
| S1-final-viral-combined_k141_52728\|\|full | 2957 | No | NA | 0 | 1 | Not-determined | Genome-fragment | NA | 0 |
| S3-final-viral-combined_k141_60798\|\|full | 1291 | No | NA | 0 | 0 | Low-quality | Genome-fragment | 0.97 | 0 |
| S1-final-viral-combined_k141_250889\|\|full | 1037 | No | NA | 0 | 0 | Not-determined | Genome-fragment | NA | 0 |
| S1-final-viral-combined_k141_167587\|\|full | 3081 | No | NA | 0 | 1 | Not-determined | Genome-fragment | NA | 0 |
| S1-final-viral-combined_k141_234102\|\|full | 1077 | No | NA | 0 | 1 | Not-determined | Genome-fragment | NA | 0 |
| S7-final-viral-combined_k141_188081\|\|lt2gene | 1291 | No | NA | 1 | 0 | Low-quality | Genome-fragment | 3.36 | 0 |
| S1-final-viral-combined_k141_201261\|\|full | 2229 | No | NA | 0 | 3 | Not-determined | Genome-fragment | NA | 0 |
| S1-final-viral-combined_k141_266706\|\|full | 1698 | No | NA | 0 | 2 | Not-determined | Genome-fragment | NA | 0 |
| S17-final-viral-combined_k141_169200\|\|full | 1291 | No | NA | 0 | 0 | Low-quality | Genome-fragment | 2.08 | 0 |
| S1-final-viral-combined_k141_266731\|\|full | 3347 | No | NA | 0 | 1 | Not-determined | Genome-fragment | NA | 0 |
| S17-k141_177889 | 1291 | No | NA | 0 | 0 | Low-quality | Genome-fragment | 2.5 | 0 |
| S1-final-viral-combined_k141_382081\|\|full | 3345 | No | NA | 0 | 3 | Not-determined | Genome-fragment | NA | 0 |
| S17-k141_253373 | 1291 | No | NA | 0 | 3 | Low-quality | Genome-fragment | 0.12 | 0 |
| S4-final-viral-combined_k141_21661\|\|full | 1291 | No | NA | 0 | 0 | Low-quality | Genome-fragment | 2.61 | 0 |
| S9-final-viral-combined_k141_34456\|\|full | 1292 | No | NA | 1 | 0 | Low-quality | Genome-fragment | 3.08 | 0 |
| S11-final-viral-combined_k141_130518\|\|full | 1292 | No | NA | 1 | 0 | Low-quality | Genome-fragment | 3.55 | 0 |
| S1-final-viral-combined_k141_266749\|\|full | 2576 | No | NA | 0 | 2 | Not-determined | Genome-fragment | NA | 0 |
| S11-final-viral-combined_k141_75755\|\|full | 1292 | No | NA | 0 | 0 | Low-quality | Genome-fragment | 2.82 | 0 |
| S1-final-viral-combined_k141_300462\|\|full | 1427 | No | NA | 0 | 1 | Not-determined | Genome-fragment | NA | 0 |
| S1-final-viral-combined_k141_52817\|\|full | 1182 | No | NA | 0 | 1 | Not-determined | Genome-fragment | NA | 0 |
| S1-final-viral-combined_k141_332633\|\|full | 2498 | No | NA | 0 | 1 | Not-determined | Genome-fragment | NA | 0 |
| S11-final-viral-combined_k141_261318\|\|full | 1292 | No | NA | 1 | 0 | Low-quality | Genome-fragment | 2.88 | 0 |
| S1-final-viral-combined_k141_250985\|\|full | 2030 | No | NA | 0 | 1 | Not-determined | Genome-fragment | NA | 0 |
| S11_k141_199458 | 1292 | No | NA | 0 | 1 | Low-quality | Genome-fragment | 0.29 | 0 |
| S1-final-viral-combined_k141_316835\|\|full | 2680 | No | NA | 0 | 2 | Not-determined | Genome-fragment | NA | 0 |
| S1-final-viral-combined_k141_316836\|\|full | 2063 | No | NA | 0 | 1 | Not-determined | Genome-fragment | NA | 0 |
| S1-final-viral-combined_k141_382136\|\|full | 4480 | No | NA | 0 | 2 | Not-determined | Genome-fragment | NA | 0 |
| S1-final-viral-combined_k141_102063\|\|full | 2332 | No | NA | 0 | 2 | Not-determined | Genome-fragment | NA | 0 |
| S1-final-viral-combined_k141_297503\|\|full | 1292 | No | NA | 0 | 0 | Low-quality | Genome-fragment | 1.88 | 0 |
| S1-final-viral-combined_k141_167618\|\|full | 1077 | No | NA | 0 | 0 | Not-determined | Genome-fragment | NA | 0 |
| S1_k141_115751 | 1292 | No | NA | 0 | 1 | Low-quality | Genome-fragment | 2.72 | 0 |
| S1-final-viral-combined_k141_349500\|\|full | 11922 | No | NA | 0 | 0 | Not-determined | Genome-fragment | NA | 0 |
| S5_k141_81734 | 1292 | No | NA | 0 | 0 | Low-quality | Genome-fragment | 3.19 | 0 |
| S1-final-viral-combined_k141_283694\|\|full | 1836 | No | NA | 0 | 1 | Not-determined | Genome-fragment | NA | 0 |
| S7-final-viral-combined_k141_130918\|\|full | 1292 | No | NA | 0 | 1 | Low-quality | Genome-fragment | 2.89 | 0 |
| S7_k141_115274 | 1292 | No | NA | 0 | 0 | Low-quality | Genome-fragment | 1.57 | 0 |
| S1-final-viral-combined_k141_300514\|\|full | 1042 | No | NA | 0 | 0 | Not-determined | Genome-fragment | NA | 0 |
| S1-final-viral-combined_k141_85909\|\|full | 5249 | No | NA | 0 | 3 | Not-determined | Genome-fragment | NA | 0 |
| S1-final-viral-combined_k141_85916\|\|full | 1918 | No | NA | 0 | 1 | Not-determined | Genome-fragment | NA | 0 |
| S12-final-viral-combined_k141_91899\|\|full | 1292 | No | NA | 0 | 1 | Low-quality | Genome-fragment | 1.52 | 0 |
| S1-final-viral-combined_k141_20231\|\|full | 1920 | No | NA | 0 | 0 | Not-determined | Genome-fragment | NA | 0 |
| S1-final-viral-combined_k141_167648\|\|full | 1583 | No | NA | 0 | 2 | Not-determined | Genome-fragment | NA | 0 |
| S2_k141_238330 | 1292 | No | NA | 0 | 1 | Low-quality | Genome-fragment | 0.84 | 0 |
| S1-final-viral-combined_k141_217776\|\|full | 1079 | No | NA | 0 | 1 | Not-determined | Genome-fragment | NA | 0 |
| S4-final-viral-combined_k141_36688\|\|full | 1292 | No | NA | 0 | 0 | Low-quality | Genome-fragment | 2.79 | 0 |
| S4-final-viral-combined_k141_33604\|\|full | 1292 | No | NA | 0 | 1 | Low-quality | Genome-fragment | 2.01 | 0 |
| S1-final-viral-combined_k141_217787\|\|full | 1681 | No | NA | 0 | 0 | Not-determined | Genome-fragment | NA | 0 |
| S1-final-viral-combined_k141_167672\|\|full | 2954 | No | NA | 0 | 2 | Not-determined | Genome-fragment | NA | 0 |
| S4-final-viral-combined_k141_90355\|\|full | 1292 | No | NA | 0 | 0 | Low-quality | Genome-fragment | 2.09 | 0 |
| S4-final-viral-combined_k141_48978\|\|lt2gene | 1292 | No | NA | 1 | 0 | Low-quality | Genome-fragment | 2.07 | 0 |
| S4_k141_121282 | 1292 | No | NA | 0 | 0 | Low-quality | Genome-fragment | 1.21 | 0 |
| S6-final-viral-combined_k141_21890\|\|full | 1292 | No | NA | 2 | 0 | Low-quality | Genome-fragment | 3.76 | 0 |
| S1-final-viral-combined_k141_185282\|\|full | 1501 | No | NA | 0 | 0 | Not-determined | Genome-fragment | NA | 0 |
| S1-final-viral-combined_k141_251126\|\|full | 1753 | No | NA | 0 | 2 | Not-determined | Genome-fragment | NA | 0 |
| S1-final-viral-combined_k141_251139\|\|full | 2517 | No | NA | 0 | 3 | Not-determined | Genome-fragment | NA | 0 |
| S1-final-viral-combined_k141_167694\|\|full | 1355 | No | NA | 0 | 0 | Not-determined | Genome-fragment | NA | 0 |
| S1-final-viral-combined_k141_167711\|\|full | 3534 | No | NA | 0 | 2 | Not-determined | Genome-fragment | NA | 0 |
| S1-final-viral-combined_k141_36590\|\|full | 4185 | No | NA | 0 | 1 | Not-determined | Genome-fragment | NA | 0 |
| S1-final-viral-combined_k141_52983\|\|full | 1319 | No | NA | 0 | 1 | Not-determined | Genome-fragment | NA | 0 |
| S1-final-viral-combined_k141_266948\|\|full | 1055 | No | NA | 0 | 1 | Not-determined | Genome-fragment | NA | 0 |
| S1-final-viral-combined_k141_3928\|\|full | 2805 | No | NA | 0 | 1 | Not-determined | Genome-fragment | NA | 0 |
| S1-final-viral-combined_k141_185336\|\|full | 2431 | No | NA | 1 | 0 | Medium-quality | Genome-fragment | 56.15 | 0 |
| S11-final-viral-combined_k141_96823\|\|full | 1293 | No | NA | 1 | 0 | Low-quality | Genome-fragment | 3.31 | 0 |
| S11-final-viral-combined_k141_177925\|\|full | 1293 | No | NA | 1 | 0 | Low-quality | Genome-fragment | 1.44 | 0 |
| S1-final-viral-combined_k141_281660\|\|full | 1293 | No | NA | 2 | 0 | Low-quality | Genome-fragment | 2.95 | 0 |
| S1-final-viral-combined_k141_5850\|\|full | 1293 | No | NA | 1 | 0 | Low-quality | Genome-fragment | 0.49 | 0 |
| S1-final-viral-combined_k141_102192\|\|full | 4919 | No | NA | 0 | 2 | Not-determined | Genome-fragment | NA | 0 |
| S1-final-viral-combined_k141_86042\|\|full | 14024 | No | NA | 0 | 2 | Not-determined | Genome-fragment | NA | 0 |
| S1_k141_284281 | 1293 | No | NA | 0 | 2 | Low-quality | Genome-fragment | 2.16 | 0 |
| S1_k141_322530 | 1293 | No | NA | 0 | 2 | Low-quality | Genome-fragment | 2.49 | 0 |
| S3-final-viral-combined_k141_125594\|\|lt2gene | 1293 | No | NA | 1 | 0 | Low-quality | Genome-fragment | 3.13 | 0 |
| S1-final-viral-combined_k141_167726\|\|full | 4106 | No | NA | 0 | 1 | Not-determined | Genome-fragment | NA | 0 |
| S1-final-viral-combined_k141_300668\|\|full | 1232 | No | NA | 0 | 0 | Not-determined | Genome-fragment | NA | 0 |
| S1-final-viral-combined_k141_102222\|\|full | 1021 | No | NA | 0 | 0 | Not-determined | Genome-fragment | NA | 0 |
| S7-final-viral-combined_k141_159424\|\|full | 1293 | No | NA | 0 | 0 | Low-quality | Genome-fragment | 0.8 | 0 |
| S7_k141_30825 | 1293 | No | NA | 0 | 0 | Low-quality | Genome-fragment | 0.21 | 0 |
| S1-final-viral-combined_k141_300679\|\|full | 1068 | No | NA | 0 | 1 | Not-determined | Genome-fragment | NA | 0 |
| S17-final-viral-combined_k141_137284\|\|full | 1293 | No | NA | 1 | 0 | Low-quality | Genome-fragment | 2.3 | 0 |
| S17-k141_258331 | 1293 | No | NA | 0 | 1 | Low-quality | Genome-fragment | 1.09 | 0 |
| S1-final-viral-combined_k141_349643\|\|full | 2253 | No | NA | 0 | 1 | Not-determined | Genome-fragment | NA | 0 |
| S4_k141_37345 | 1293 | No | NA | 0 | 0 | Low-quality | Genome-fragment | 2.74 | 0 |
| S8-final-viral-combined_k141_210025\|\|full | 1293 | No | NA | 3 | 0 | Low-quality | Genome-fragment | 2.11 | 0 |
| S1-final-viral-combined_k141_152130\|\|full | 1340 | No | NA | 0 | 1 | Not-determined | Genome-fragment | NA | 0 |
| S1-final-viral-combined_k141_86098\|\|full | 1049 | No | NA | 0 | 0 | Not-determined | Genome-fragment | NA | 0 |
| S8_k141_50437 | 1293 | No | NA | 0 | 0 | Low-quality | Genome-fragment | 1.98 | 0 |

| | | | | | | | | | | | |
|---|---|---|---|---|---|---|---|---|---|---|---|
| S11-final-viral-combined_k141_138238\|\|full | 1294 | No | NA | | 0 | 1 | Low-quality | Genome-fragment | | 1.94 | 0 |
| S1-final-viral-combined_k141_119322\|\|full | 13601 | No | NA | | 0 | 8 | Not-determined | Genome-fragment | NA | | 0 |
| S1-final-viral-combined_k141_36707\|\|full | 6204 | No | NA | | 0 | 3 | Not-determined | Genome-fragment | NA | | 0 |
| S1-final-viral-combined_k141_362288\|\|full | 1294 | No | NA | | 2 | 0 | Low-quality | Genome-fragment | | 3.55 | 0 |
| S1-final-viral-combined_k141_349701\|\|full | 1041 | No | NA | | 0 | 0 | Not-determined | Genome-fragment | NA | | 0 |
| S1-final-viral-combined_k141_217981\|\|full | 2649 | No | NA | | 0 | 0 | Not-determined | Genome-fragment | NA | | 0 |
| S1-final-viral-combined_k141_332863\|\|full | 3847 | No | NA | | 0 | 0 | Not-determined | Genome-fragment | NA | | 0 |
| S1-final-viral-combined_k141_294232\|\|full | 1294 | No | NA | | 0 | 0 | Low-quality | Genome-fragment | | 3.18 | 0 |
| S1-final-viral-combined_k141_185476\|\|full | 2040 | No | NA | | 0 | 1 | Not-determined | Genome-fragment | NA | | 0 |
| S1_k141_394414 | 1294 | No | NA | | 0 | 2 | Low-quality | Genome-fragment | | 1.91 | 0 |
| S1-final-viral-combined_k141_365922\|\|full | 4433 | No | NA | | 0 | 3 | Not-determined | Genome-fragment | NA | | 0 |
| S1-final-viral-combined_k141_349753\|\|full | 4712 | No | NA | | 0 | 2 | Not-determined | Genome-fragment | NA | | 0 |
| S1-final-viral-combined_k141_234464\|\|full | 3147 | No | NA | | 0 | 3 | Not-determined | Genome-fragment | NA | | 0 |
| S1-final-viral-combined_k141_68998\|\|full | 56973 | Yes | | 55700 | 22 | 2 | High-quality | High-quality | | 100 | 2.23 |
| S7-final-viral-combined_k141_73265\|\|lt2gene | 1294 | No | NA | | 1 | 0 | Low-quality | Genome-fragment | | 3.17 | 0 |
| S1-final-viral-combined_k141_217999\|\|full | 3033 | No | NA | | 0 | 2 | Not-determined | Genome-fragment | NA | | 0 |
| S2-final-viral-combined_k141_177997\|\|full | 1294 | No | NA | | 0 | 0 | Low-quality | Genome-fragment | | 3.13 | 0 |
| S11-final-viral-combined_k141_96575\|\|full | 1295 | No | NA | | 0 | 0 | Low-quality | Genome-fragment | | 1.53 | 0 |
| S11_k141_58805 | 1295 | No | NA | | 0 | 1 | Low-quality | Genome-fragment | | 0.95 | 0 |
| S1_k141_353480 | 1295 | No | NA | | 0 | 0 | Low-quality | Genome-fragment | | 0.99 | 0 |
| S1-final-viral-combined_k141_251366\|\|full | 6845 | No | NA | | 0 | 4 | Not-determined | Genome-fragment | NA | | 0 |
| S1-final-viral-combined_k141_36762\|\|full | 1412 | No | NA | | 0 | 0 | Not-determined | Genome-fragment | NA | | 0 |
| S1-final-viral-combined_k141_317138\|\|full | 19449 | No | NA | | 0 | 12 | Not-determined | Genome-fragment | NA | | 0 |
| S1-final-viral-combined_k141_167848\|\|full | 2286 | No | NA | | 0 | 2 | Not-determined | Genome-fragment | NA | | 0 |
| S1-final-viral-combined_k141_152133\|\|full | 1595 | No | NA | | 0 | 2 | Not-determined | Genome-fragment | NA | | 0 |
| S1-final-viral-combined_k141_349820\|\|full | 3028 | No | NA | | 0 | 3 | Not-determined | Genome-fragment | NA | | 0 |
| S1-final-viral-combined_k141_201696\|\|full | 2966 | No | NA | | 0 | 0 | Not-determined | Genome-fragment | NA | | 0 |
| S1-final-viral-combined_k141_69079\|\|full | 1407 | No | NA | | 0 | 1 | Not-determined | Genome-fragment | NA | | 0 |
| S1-final-viral-combined_k141_251378\|\|full | 1333 | No | NA | | 0 | 1 | Not-determined | Genome-fragment | NA | | 0 |
| S1_k141_338639 | 1295 | No | NA | | 0 | 1 | Low-quality | Genome-fragment | | 0.72 | 0 |
| S3-final-viral-combined_k141_133260\|\|full | 1295 | No | NA | | 1 | 0 | Low-quality | Genome-fragment | | 2.21 | 0 |
| S1-final-viral-combined_k141_251387\|\|full | 3571 | No | NA | | 0 | 3 | Not-determined | Genome-fragment | NA | | 0 |
| S1-final-viral-combined_k141_283999\|\|full | 1838 | No | NA | | 0 | 1 | Not-determined | Genome-fragment | NA | | 0 |
| S1-final-viral-combined_k141_349869\|\|full | 2049 | No | NA | | 0 | 1 | Not-determined | Genome-fragment | NA | | 0 |
| S1-final-viral-combined_k141_300776\|\|full | 2146 | No | NA | | 0 | 0 | Not-determined | Genome-fragment | NA | | 0 |
| S5-final-viral-combined_k141_110719\|\|full | 1295 | No | NA | | 0 | 1 | Low-quality | Genome-fragment | | 1.69 | 0 |
| S1-final-viral-combined_k141_267198\|\|full | 4386 | No | NA | | 0 | 2 | Not-determined | Genome-fragment | NA | | 0 |
| S7-final-viral-combined_k141_51555\|\|full | 1295 | No | NA | | 0 | 2 | Low-quality | Genome-fragment | | 0.82 | 0 |
| S7-final-viral-combined_k141_120959\|\|lt2gene | 1295 | No | NA | | 2 | 0 | Low-quality | Genome-fragment | | 2.85 | 0 |
| S12-final-viral-combined_k141_372250\|\|full | 1295 | Yes | | 504 | 1 | 1 | Low-quality | Genome-fragment | | 0.62 | 61.08 |
| S1-final-viral-combined_k141_4054\|\|full | 6441 | No | NA | | 0 | 1 | Not-determined | Genome-fragment | NA | | 0 |
| S1-final-viral-combined_k141_349888\|\|full | 2505 | No | NA | | 0 | 2 | Not-determined | Genome-fragment | NA | | 0 |
| S12-final-viral-combined_k141_92224\|\|full | 1295 | No | NA | | 0 | 0 | Low-quality | Genome-fragment | | 3.9 | 0 |
| S1-final-viral-combined_k141_167919\|\|full | 1848 | No | NA | | 0 | 1 | Not-determined | Genome-fragment | NA | | 0 |
| S1-final-viral-combined_k141_36852\|\|full | 3191 | No | NA | | 0 | 2 | Not-determined | Genome-fragment | NA | | 0 |
| S1-final-viral-combined_k141_382380\|\|full | 1780 | No | NA | | 0 | 0 | Not-determined | Genome-fragment | NA | | 0 |
| S1-final-viral-combined_k141_201769\|\|full | 2819 | No | NA | | 0 | 2 | Not-determined | Genome-fragment | NA | | 0 |
| S12-final-viral-combined_k141_367716\|\|full | 1295 | No | NA | | 0 | 2 | Low-quality | Genome-fragment | | 0.64 | 0 |
| S1-final-viral-combined_k141_185613\|\|full | 1174 | No | NA | | 0 | 1 | Not-determined | Genome-fragment | NA | | 0 |
| S2-final-viral-combined_k141_16746\|\|full | 1295 | No | NA | | 1 | 0 | Low-quality | Genome-fragment | | 2.21 | 0 |
| S1-final-viral-combined_k141_300893\|\|full | 5554 | No | NA | | 0 | 0 | Not-determined | Genome-fragment | NA | | 0 |
| S1-final-viral-combined_k141_284081\|\|full | 6143 | No | NA | | 0 | 4 | Not-determined | Genome-fragment | NA | | 0 |
| S1-final-viral-combined_k141_152265\|\|full | 1631 | No | NA | | 0 | 0 | Not-determined | Genome-fragment | NA | | 0 |
| S1-final-viral-combined_k141_251587\|\|full | 1388 | No | NA | | 0 | 1 | Not-determined | Genome-fragment | NA | | 0 |
| S6-final-viral-combined_k141_59075\|\|full | 1295 | No | NA | | 1 | 0 | Low-quality | Genome-fragment | | 2.54 | 0 |
| S1-final-viral-combined_k141_366151\|\|full | 1085 | No | NA | | 0 | 1 | Not-determined | Genome-fragment | NA | | 0 |
| S6-final-viral-combined_k141_50310\|\|full | 1295 | No | NA | | 0 | 2 | Low-quality | Genome-fragment | | 0.65 | 0 |
| S11-final-viral-combined_k141_91228\|\|full | 1296 | No | NA | | 1 | 0 | Low-quality | Genome-fragment | | 3.7 | 0 |
| S1-final-viral-combined_k141_251599\|\|full | 7549 | No | NA | | 0 | 4 | Not-determined | Genome-fragment | NA | | 0 |
| S11-final-viral-combined_k141_68813\|\|lt2gene | 1296 | No | NA | | 1 | 0 | Low-quality | Genome-fragment | | 2.91 | 0 |
| S1-final-viral-combined_k141_317268\|\|full | 2956 | No | NA | | 0 | 2 | Not-determined | Genome-fragment | NA | | 0 |
| S1-final-viral-combined_k141_382442\|\|full | 2054 | No | NA | | 0 | 1 | Not-determined | Genome-fragment | NA | | 0 |
| S11_k141_218652 | 1296 | No | NA | | 0 | 0 | Low-quality | Genome-fragment | | 2.2 | 0 |
| S1-final-viral-combined_k141_333127\|\|full | 1808 | No | NA | | 0 | 2 | Not-determined | Genome-fragment | NA | | 0 |
| S3_k141_51247 | 1296 | No | NA | | 0 | 0 | Low-quality | Genome-fragment | | 2.99 | 0 |
| S1-final-viral-combined_k141_284134\|\|full | 1722 | No | NA | | 0 | 1 | Not-determined | Genome-fragment | NA | | 0 |
| S1-final-viral-combined_k141_350110\|\|full | 1209 | No | NA | | 0 | 0 | Not-determined | Genome-fragment | NA | | 0 |
| S1-final-viral-combined_k141_333140\|\|full | 4169 | No | NA | | 0 | 2 | Not-determined | Genome-fragment | NA | | 0 |
| S1-final-viral-combined_k141_119692\|\|full | 4446 | No | NA | | 0 | 0 | Not-determined | Genome-fragment | NA | | 0 |
| S17-final-viral-combined_k141_100062\|\|full | 1296 | No | NA | | 0 | 0 | Low-quality | Genome-fragment | | 0.63 | 0 |
| S1-final-viral-combined_k141_350141\|\|full | 2972 | No | NA | | 0 | 1 | Not-determined | Genome-fragment | NA | | 0 |
| S17-final-viral-combined_k141_102652\|\|full | 1296 | No | NA | | 0 | 0 | Low-quality | Genome-fragment | | 1.85 | 0 |
| S17-final-viral-combined_k141_172669\|\|full | 1296 | No | NA | | 0 | 2 | Low-quality | Genome-fragment | | 0.61 | 0 |
| S1-final-viral-combined_k141_102476\|\|full | 1613 | No | NA | | 0 | 0 | Not-determined | Genome-fragment | NA | | 0 |
| S1-final-viral-combined_k141_382517\|\|full | 1815 | No | NA | | 0 | 3 | Not-determined | Genome-fragment | NA | | 0 |
| S1-final-viral-combined_k141_185766\|\|full | 1701 | No | NA | | 0 | 1 | Not-determined | Genome-fragment | NA | | 0 |
| S12-final-viral-combined_k141_366846\|\|full | 1296 | No | NA | | 0 | 1 | Low-quality | Genome-fragment | | 0.66 | 0 |
| S2-final-viral-combined_k141_241957\|\|full | 1296 | No | NA | | 0 | 0 | Low-quality | Genome-fragment | | 1.63 | 0 |
| S1-final-viral-combined_k141_251694\|\|full | 2833 | No | NA | | 0 | 0 | Not-determined | Genome-fragment | NA | | 0 |
| S1-final-viral-combined_k141_152349\|\|full | 1809 | No | NA | | 0 | 1 | Not-determined | Genome-fragment | NA | | 0 |
| S1-final-viral-combined_k141_317361\|\|full | 1597 | No | NA | | 0 | 1 | Not-determined | Genome-fragment | NA | | 0 |
| S4_k141_23528 | 1296 | No | NA | | 0 | 0 | Low-quality | Genome-fragment | | 2.59 | 0 |
| S1-final-viral-combined_k141_284189\|\|full | 4651 | No | NA | | 0 | 0 | Not-determined | Genome-fragment | NA | | 0 |
| S1-final-viral-combined_k141_37083\|\|full | 2826 | No | NA | | 0 | 0 | Not-determined | Genome-fragment | NA | | 0 |
| S1-final-viral-combined_k141_152380\|\|full | 6320 | No | NA | | 0 | 1 | Not-determined | Genome-fragment | NA | | 0 |
| S8-final-viral-combined_k141_74832\|\|full | 1296 | No | NA | | 0 | 1 | Low-quality | Genome-fragment | | 0.85 | 0 |
| S1-final-viral-combined_k141_20801\|\|full | 1892 | No | NA | | 0 | 1 | Not-determined | Genome-fragment | NA | | 0 |
| S1-final-viral-combined_k141_323250\|\|full | 1297 | No | NA | | 0 | 0 | Low-quality | Genome-fragment | | 2.92 | 0 |
| S1-final-viral-combined_k141_382567\|\|full | 1862 | No | NA | | 0 | 1 | Not-determined | Genome-fragment | NA | | 0 |
| S1-final-viral-combined_k141_317391\|\|full | 7534 | No | NA | | 0 | 0 | Not-determined | Genome-fragment | NA | | 0 |
| S1_k141_233371 | 1297 | No | NA | | 0 | 0 | Low-quality | Genome-fragment | | 3.41 | 0 |
| S1-final-viral-combined_k141_102499\|\|full | 2667 | No | NA | | 0 | 3 | Not-determined | Genome-fragment | NA | | 0 |
| S3_k141_109237 | 1297 | No | NA | | 0 | 0 | Low-quality | Genome-fragment | | 3.09 | 0 |
| S7-final-viral-combined_k141_506\|\|full | 1297 | No | NA | | 0 | 0 | Low-quality | Genome-fragment | | 1.64 | 0 |
| S1-final-viral-combined_k141_284219\|\|full | 3774 | No | NA | | 0 | 4 | Not-determined | Genome-fragment | NA | | 0 |
| S1-final-viral-combined_k141_382586\|\|full | 2080 | No | NA | | 0 | 2 | Not-determined | Genome-fragment | NA | | 0 |
| S7-final-viral-combined_k141_64158\|\|full | 1297 | No | NA | | 0 | 0 | Low-quality | Genome-fragment | | 2.2 | 0 |
| S7_k141_204446 | 1297 | No | NA | | 0 | 0 | Low-quality | Genome-fragment | | 1.22 | 0 |
| S1-final-viral-combined_k141_152405\|\|full | 1345 | No | NA | | 0 | 2 | Not-determined | Genome-fragment | NA | | 0 |
| S1-final-viral-combined_k141_37145\|\|full | 1967 | No | NA | | 0 | 0 | Not-determined | Genome-fragment | NA | | 0 |
| S1-final-viral-combined_k141_119827\|\|full | 6494 | No | NA | | 0 | 4 | Not-determined | Genome-fragment | NA | | 0 |
| S17-final-viral-combined_k141_59498\|\|full | 1297 | No | NA | | 0 | 2 | Low-quality | Genome-fragment | | 1 | 0 |
| S17-final-viral-combined_k141_199368\|\|full | 1297 | No | NA | | 0 | 0 | Low-quality | Genome-fragment | | 3.24 | 0 |
| S4-final-viral-combined_k141_166683\|\|full | 1297 | No | NA | | 0 | 2 | Low-quality | Genome-fragment | | 2.39 | 0 |
| S1-final-viral-combined_k141_382618\|\|full | 1159 | No | NA | | 0 | 2 | Not-determined | Genome-fragment | NA | | 0 |
| S1-final-viral-combined_k141_53438\|\|full | 1362 | No | NA | | 0 | 3 | Not-determined | Genome-fragment | NA | | 0 |
| S1-final-viral-combined_k141_69226\|\|full | 4163 | No | NA | | 0 | 3 | Not-determined | Genome-fragment | NA | | 0 |
| S6-final-viral-combined_k141_100526\|\|lt2gene | 1297 | No | NA | | 2 | 0 | Low-quality | Genome-fragment | | 4.03 | 0 |
| S1-final-viral-combined_k141_69229\|\|full | 1348 | No | NA | | 0 | 0 | Not-determined | Genome-fragment | NA | | 0 |
| S1-final-viral-combined_k141_37155\|\|full | 2040 | No | NA | | 0 | 1 | Not-determined | Genome-fragment | NA | | 0 |
| S1-final-viral-combined_k141_202031\|\|full | 2428 | No | NA | | 0 | 3 | Not-determined | Genome-fragment | NA | | 0 |
| S6_k141_130921 | 1297 | No | NA | | 0 | 0 | Low-quality | Genome-fragment | | 0.77 | 0 |
| S1-final-viral-combined_k141_267514\|\|full | 1609 | No | NA | | 0 | 2 | Not-determined | Genome-fragment | NA | | 0 |
| S1-final-viral-combined_k141_53455\|\|full | 2567 | No | NA | | 0 | 0 | Not-determined | Genome-fragment | NA | | 0 |
| S1-final-viral-combined_k141_136479\|\|full | 2497 | No | NA | | 0 | 2 | Not-determined | Genome-fragment | NA | | 0 |
| S11-final-viral-combined_k141_108122\|\|full | 1298 | No | NA | | 0 | 0 | Low-quality | Genome-fragment | | 2.67 | 0 |
| S11_k141_240901 | 1298 | No | NA | | 0 | 0 | Low-quality | Genome-fragment | | 1.39 | 0 |
| S1-final-viral-combined_k141_202043\|\|full | 6612 | No | NA | | 0 | 2 | Not-determined | Genome-fragment | NA | | 0 |
| S1-final-viral-combined_k141_168253\|\|full | 1466 | No | NA | | 0 | 1 | Not-determined | Genome-fragment | NA | | 0 |
| S1-final-viral-combined_k141_318313\|\|full | 1298 | No | NA | | 1 | 0 | Low-quality | Genome-fragment | | 0.79 | 0 |
| S1-final-viral-combined_k141_69258\|\|full | 2359 | No | NA | | 0 | 2 | Not-determined | Genome-fragment | NA | | 0 |
| S1-final-viral-combined_k141_333282\|\|full | 3042 | No | NA | | 0 | 2 | Not-determined | Genome-fragment | NA | | 0 |
| S1-final-viral-combined_k141_69264\|\|full | 2270 | No | NA | | 0 | 1 | Not-determined | Genome-fragment | NA | | 0 |
| S1-final-viral-combined_k141_284284\|\|full | 6439 | No | NA | | 0 | 2 | Not-determined | Genome-fragment | NA | | 0 |
| S1-final-viral-combined_k141_119899\|\|full | 1647 | No | NA | | 0 | 1 | Not-determined | Genome-fragment | NA | | 0 |
| S5_k141_71222 | 1298 | No | NA | | 0 | 1 | Low-quality | Genome-fragment | | 2.84 | 0 |
| S7-final-viral-combined_k141_118310\|\|full | 1298 | No | NA | | 1 | 0 | Low-quality | Genome-fragment | | 3.32 | 0 |
| S1-final-viral-combined_k141_53493\|\|full | 1764 | No | NA | | 0 | 2 | Not-determined | Genome-fragment | NA | | 0 |
| S7-final-viral-combined_k141_97333\|\|full | 1298 | No | NA | | 1 | 0 | Low-quality | Genome-fragment | | 1.69 | 0 |
| S1-final-viral-combined_k141_86424\|\|full | 3212 | No | NA | | 0 | 2 | Not-determined | Genome-fragment | NA | | 0 |
| S1-final-viral-combined_k141_168280\|\|full | 2887 | No | NA | | 0 | 2 | Not-determined | Genome-fragment | NA | | 0 |
| S7-final-viral-combined_k141_100964\|\|full | 1298 | No | NA | | 0 | 2 | Low-quality | Genome-fragment | | 0.78 | 0 |
| S17-k141_103544 | 1298 | No | NA | | 0 | 0 | Low-quality | Genome-fragment | | 5.16 | 0 |
| S1-final-viral-combined_k141_53509\|\|full | 1206 | No | NA | | 0 | 3 | Not-determined | Genome-fragment | NA | | 0 |
| S1-final-viral-combined_k141_69280\|\|full | 7371 | No | NA | | 0 | 5 | Not-determined | Genome-fragment | NA | | 0 |
| S1-final-viral-combined_k141_4229\|\|full | 1382 | No | NA | | 0 | 0 | Not-determined | Genome-fragment | NA | | 0 |
| S1-final-viral-combined_k141_53519\|\|full | 2423 | No | NA | | 0 | 0 | Not-determined | Genome-fragment | NA | | 0 |
| S1-final-viral-combined_k141_333331\|\|full | 1443 | No | NA | | 0 | 1 | Not-determined | Genome-fragment | NA | | 0 |
| S2-final-viral-combined_k141_117106\|\|lt2gene | 1298 | No | NA | | 2 | 0 | Low-quality | Genome-fragment | | 2.16 | 0 |
| S1-final-viral-combined_k141_168302\|\|full | 5547 | No | NA | | 0 | 0 | Not-determined | Genome-fragment | NA | | 0 |
| S6_k141_130749 | 1298 | No | NA | | 1 | 0 | Low-quality | Genome-fragment | | 1.62 | 0 |
| S8-final-viral-combined_k141_197826\|\|full | 1298 | No | NA | | 0 | 0 | Low-quality | Genome-fragment | | 2.04 | 0 |
| S1-final-viral-combined_k141_317531\|\|full | 4932 | No | NA | | 0 | 3 | Not-determined | Genome-fragment | NA | | 0 |
| S8-final-viral-combined_k141_152396\|\|full | 1298 | No | NA | | 0 | 1 | Low-quality | Genome-fragment | | 0.82 | 0 |
| S1-final-viral-combined_k141_53562\|\|full | 2021 | No | NA | | 0 | 0 | Not-determined | Genome-fragment | NA | | 0 |
| S9-final-viral-combined_k141_14862\|\|lt2gene | 1299 | No | NA | | 1 | 0 | Low-quality | Genome-fragment | | 2.99 | 0 |
| S11-final-viral-combined_k141_162382\|\|full | 1299 | No | NA | | 1 | 0 | Low-quality | Genome-fragment | | 2.14 | 0 |
| S1-final-viral-combined_k141_152507\|\|full | 1688 | No | NA | | 0 | 1 | Not-determined | Genome-fragment | NA | | 0 |
| S1-final-viral-combined_k141_53574\|\|full | 2147 | No | NA | | 0 | 2 | Not-determined | Genome-fragment | NA | | 0 |
| S11-final-viral-combined_k141_93487\|\|lt2gene | 1299 | No | NA | | 1 | 0 | Low-quality | Genome-fragment | | 0.87 | 0 |
| S1-final-viral-combined_k141_382736\|\|full | 1024 | No | NA | | 0 | 0 | Not-determined | Genome-fragment | NA | | 0 |
| S1-final-viral-combined_k141_86472\|\|full | 3924 | No | NA | | 0 | 4 | Not-determined | Genome-fragment | NA | | 0 |
| S1-final-viral-combined_k141_374301\|\|full | 1299 | No | NA | | 2 | 0 | Low-quality | Genome-fragment | | 2.88 | 0 |
| S1-final-viral-combined_k141_301348\|\|full | 2190 | No | NA | | 0 | 1 | Not-determined | Genome-fragment | NA | | 0 |
| S1-final-viral-combined_k141_69297\|\|full | 3193 | No | NA | | 0 | 1 | Not-determined | Genome-fragment | NA | | 0 |
| S1-final-viral-combined_k141_317584\|\|full | 3768 | No | NA | | 0 | 0 | Not-determined | Genome-fragment | NA | | 0 |
| S5-final-viral-combined_k141_90123\|\|full | 1299 | No | NA | | 2 | 0 | Low-quality | Genome-fragment | | 2.99 | 0 |
| S1-final-viral-combined_k141_317585\|\|full | 10279 | No | NA | | 0 | 3 | Not-determined | Genome-fragment | NA | | 0 |
| S1-final-viral-combined_k141_37288\|\|full | 3487 | No | NA | | 0 | 2 | Not-determined | Genome-fragment | NA | | 0 |
| S1-final-viral-combined_k141_136614\|\|full | 23699 | No | NA | | 0 | 17 | Not-determined | Genome-fragment | NA | | 0 |
| S1-final-viral-combined_k141_284391\|\|full | 1829 | No | NA | | 0 | 0 | Not-determined | Genome-fragment | NA | | 0 |
| S1-final-viral-combined_k141_37297\|\|full | 1592 | No | NA | | 0 | 1 | Not-determined | Genome-fragment | NA | | 0 |
| S7-final-viral-combined_k141_42926\|\|lt2gene | 1299 | No | NA | | 1 | 0 | Low-quality | Genome-fragment | | 2.7 | 0 |
| S17-k141_95955 | 1299 | No | NA | | 0 | 0 | Low-quality | Genome-fragment | | 2.17 | 0 |
| S12_k141_208274 | 1299 | No | NA | | 0 | 0 | Low-quality | Genome-fragment | | 1.76 | 0 |
| S12_k141_361979 | 1299 | No | NA | | 1 | 0 | Low-quality | Genome-fragment | | 0.1 | 0 |
| S8-final-viral-combined_k141_240124\|\|full | 1299 | No | NA | | 1 | 0 | Low-quality | Genome-fragment | | 2.14 | 0 |
| S1-final-viral-combined_k141_382784\|\|full | 3661 | No | NA | | 0 | 1 | Not-determined | Genome-fragment | NA | | 0 |
| S1-final-viral-combined_k141_69323\|\|full | 2788 | No | NA | | 0 | 2 | Not-determined | Genome-fragment | NA | | 0 |
| S1-final-viral-combined_k141_218372\|\|full | 1901 | No | NA | | 0 | 1 | Not-determined | Genome-fragment | NA | | 0 |
| S1-final-viral-combined_k141_382799\|\|full | 1658 | No | NA | | 0 | 0 | Not-determined | Genome-fragment | NA | | 0 |
| S1-final-viral-combined_k141_86509\|\|full | 3386 | No | NA | | 0 | 2 | Not-determined | Genome-fragment | NA | | 0 |
| S8-final-viral-combined_k141_57428\|\|full | 1299 | No | NA | | 0 | 1 | Low-quality | Genome-fragment | | 0.6 | 0 |
| S1-final-viral-combined_k141_53652\|\|full | 6252 | No | NA | | 0 | 4 | Not-determined | Genome-fragment | NA | | 0 |

| | | | | | | | | | |
|---|---|---|---|---|---|---|---|---|---|
| S8-final-viral-combined_k141_138770\|\|lt2gene | 1299 | No | NA | 0 | 2 | Low-quality | Genome-fragment | 1.85 | 0 |
| S9-final-viral-combined_k141_97179\|\|full | 1300 | No | NA | 0 | 0 | Low-quality | Genome-fragment | 3.49 | 0 |
| S11-final-viral-combined_k141_153243\|\|full | 1300 | No | NA | 2 | 0 | Low-quality | Genome-fragment | 1.03 | 0 |
| S11-final-viral-combined_k141_95483\|\|full | 1300 | No | NA | 1 | 0 | Low-quality | Genome-fragment | 1.54 | 0 |
| S11_k141_251661 | 1300 | No | NA | 2 | 0 | Low-quality | Genome-fragment | 2.67 | 0 |
| S1-final-viral-combined_k141_238583\|\|full | 1300 | No | NA | 0 | 0 | Low-quality | Genome-fragment | 4.26 | 0 |
| S1-final-viral-combined_k141_382828\|\|full | 3208 | No | NA | 0 | 2 | Not-determined | Genome-fragment | NA | 0 |
| S1-final-viral-combined_k141_53661\|\|full | 1245 | No | NA | 0 | 0 | Not-determined | Genome-fragment | NA | 0 |
| S1-final-viral-combined_k141_366471\|\|full | 3089 | No | NA | 0 | 2 | Not-determined | Genome-fragment | NA | 0 |
| S1-final-viral-combined_k141_382860\|\|full | 5283 | No | NA | 0 | 1 | Not-determined | Genome-fragment | NA | 0 |
| S3-final-viral-combined_k141_10332\|\|full | 1300 | No | NA | 0 | 1 | Low-quality | Genome-fragment | 1.23 | 0 |
| S1-final-viral-combined_k141_366492\|\|full | 1134 | No | NA | 0 | 0 | Not-determined | Genome-fragment | NA | 0 |
| S7_k141_8047 | 1300 | No | NA | 2 | 0 | Low-quality | Genome-fragment | 2.94 | 0 |
| S1-final-viral-combined_k141_366494\|\|full | 3124 | No | NA | 0 | 3 | Not-determined | Genome-fragment | NA | 0 |
| S12-final-viral-combined_k141_154155\|\|lt2gene | 1300 | No | NA | 2 | 0 | Low-quality | Genome-fragment | 2.82 | 0 |
| S1-final-viral-combined_k141_120067\|\|full | 2792 | No | NA | 0 | 2 | Not-determined | Genome-fragment | NA | 0 |
| S2-final-viral-combined_k141_184482\|\|full | 1300 | No | NA | 1 | 0 | Low-quality | Genome-fragment | 2.83 | 0 |
| S2-final-viral-combined_k141_132115\|\|full | 1300 | No | NA | 1 | 0 | Low-quality | Genome-fragment | 3.48 | 0 |
| S4-final-viral-combined_k141_18294\|\|full | 1300 | No | NA | 0 | 0 | Low-quality | Genome-fragment | 0.88 | 0 |
| S6-final-viral-combined_k141_77130\|\|full | 1300 | No | NA | 0 | 0 | Low-quality | Genome-fragment | 1.02 | 0 |
| S1-final-viral-combined_k141_382889\|\|full | 1417 | No | NA | 0 | 0 | Not-determined | Genome-fragment | NA | 0 |
| S1-final-viral-combined_k141_350434\|\|full | 3818 | No | NA | 0 | 2 | Not-determined | Genome-fragment | NA | 0 |
| S1-final-viral-combined_k141_185969\|\|full | 1079 | No | NA | 0 | 0 | Not-determined | Genome-fragment | NA | 0 |
| S6-final-viral-combined_k141_75414\|\|full | 1300 | No | NA | 0 | 0 | Low-quality | Genome-fragment | 1.89 | 0 |
| S6-final-viral-combined_k141_58560\|\|full | 1300 | No | NA | 0 | 0 | Low-quality | Genome-fragment | 0.74 | 0 |
| S1-final-viral-combined_k141_350443\|\|full | 1810 | No | NA | 0 | 2 | Not-determined | Genome-fragment | NA | 0 |
| S1-final-viral-combined_k141_317684\|\|full | 3134 | No | NA | 0 | 3 | Not-determined | Genome-fragment | NA | 0 |
| S8-final-viral-combined_k141_69362\|\|full | 1300 | No | NA | 0 | 1 | Low-quality | Genome-fragment | 0.73 | 0 |
| S8-final-viral-combined_k141_120468\|\|full | 1300 | No | NA | 1 | 0 | Low-quality | Genome-fragment | 2.48 | 0 |
| S1-final-viral-combined_k141_37364\|\|full | 15419 | No | NA | 0 | 9 | Not-determined | Genome-fragment | NA | 0 |
| S1-final-viral-combined_k141_102779\|\|full | 5413 | No | NA | 0 | 4 | Not-determined | Genome-fragment | NA | 0 |
| S1-final-viral-combined_k141_102791\|\|full | 1640 | No | NA | 0 | 2 | Not-determined | Genome-fragment | NA | 0 |
| S1-final-viral-combined_k141_102797\|\|full | 2497 | No | NA | 0 | 5 | Not-determined | Genome-fragment | NA | 0 |
| S1-final-viral-combined_k141_53711\|\|full | 1716 | No | NA | 0 | 2 | Not-determined | Genome-fragment | NA | 0 |
| S8-final-viral-combined_k141_228279\|\|full | 1300 | No | NA | 0 | 0 | Low-quality | Genome-fragment | 1.32 | 0 |
| S11-final-viral-combined_k141_98398\|\|full | 1301 | No | NA | 1 | 0 | Low-quality | Genome-fragment | 2.99 | 0 |
| S1-final-viral-combined_k141_4403\|\|full | 16152 | No | NA | 0 | 10 | Not-determined | Genome-fragment | NA | 0 |
| S11_k141_299058 | 1301 | No | NA | 0 | 2 | Low-quality | Genome-fragment | 1.74 | 0 |
| S1-final-viral-combined_k141_44703\|\|lt2gene | 1301 | No | NA | 1 | 0 | Low-quality | Genome-fragment | 2.88 | 0 |
| S1-final-viral-combined_k141_218466\|\|full | 1369 | No | NA | 0 | 1 | Not-determined | Genome-fragment | NA | 0 |
| S1-final-viral-combined_k141_69467\|\|full | 2541 | No | NA | 0 | 2 | Not-determined | Genome-fragment | NA | 0 |
| S3-final-viral-combined_k141_34956\|\|full | 1301 | No | NA | 1 | 0 | Low-quality | Genome-fragment | 0.96 | 0 |
| S1-final-viral-combined_k141_102801\|\|full | 28850 | No | NA | 0 | 3 | Medium-quality | Genome-fragment | 55.22 | 0 |
| S5-final-viral-combined_k141_8900\|\|full | 1301 | No | NA | 1 | 0 | Low-quality | Genome-fragment | 2.66 | 0 |
| S1-final-viral-combined_k141_234992\|\|full | 1248 | No | NA | 0 | 1 | Not-determined | Genome-fragment | NA | 0 |
| S12-final-viral-combined_k141_217780\|\|full | 1301 | No | NA | 0 | 1 | Low-quality | Genome-fragment | 0.49 | 0 |
| S12-final-viral-combined_k141_235250\|\|full | 1301 | No | NA | 1 | 0 | Low-quality | Genome-fragment | 0.84 | 0 |
| S1-final-viral-combined_k141_136798\|\|full | 1573 | No | NA | 0 | 1 | Not-determined | Genome-fragment | NA | 0 |
| S1-final-viral-combined_k141_120181\|\|full | 1313 | No | NA | 0 | 1 | Not-determined | Genome-fragment | NA | 0 |
| S12-final-viral-combined_k141_288074\|\|full | 1301 | No | NA | 0 | 0 | Low-quality | Genome-fragment | 2.5 | 0 |
| S1-final-viral-combined_k141_350557\|\|full | 1872 | No | NA | 0 | 1 | Not-determined | Genome-fragment | NA | 0 |
| S12-final-viral-combined_k141_262697\|\|full | 1301 | No | NA | 1 | 0 | Low-quality | Genome-fragment | 2.94 | 0 |
| S1-final-viral-combined_k141_218529\|\|full | 2312 | No | NA | 0 | 2 | Not-determined | Genome-fragment | NA | 0 |
| S1-final-viral-combined_k141_69541\|\|full | 1501 | No | NA | 0 | 1 | Not-determined | Genome-fragment | NA | 0 |
| S1-final-viral-combined_k141_37450\|\|full | 2048 | No | NA | 0 | 1 | Not-determined | Genome-fragment | NA | 0 |
| S4-final-viral-combined_k141_73128\|\|full | 1301 | No | NA | 0 | 1 | Low-quality | Genome-fragment | 3.4 | 0 |
| S1-final-viral-combined_k141_37455\|\|full | 1585 | No | NA | 0 | 2 | Not-determined | Genome-fragment | NA | 0 |
| S6-final-viral-combined_k141_42525\|\|lt2gene | 1301 | No | NA | 1 | 0 | Low-quality | Genome-fragment | 4.2 | 0 |
| S1-final-viral-combined_k141_168526\|\|full | 4162 | No | NA | 0 | 0 | Not-determined | Genome-fragment | NA | 0 |
| S8-final-viral-combined_k141_198255\|\|full | 1301 | No | NA | 0 | 1 | Low-quality | Genome-fragment | 2.97 | 0 |
| S1-final-viral-combined_k141_37463\|\|full | 6272 | No | NA | 0 | 4 | Not-determined | Genome-fragment | NA | 0 |
| S1-final-viral-combined_k141_301545\|\|full | 3874 | No | NA | 0 | 2 | Not-determined | Genome-fragment | NA | 0 |
| S1-final-viral-combined_k141_235063\|\|full | 1857 | No | NA | 0 | 1 | Not-determined | Genome-fragment | NA | 0 |
| S1-final-viral-combined_k141_267757\|\|full | 2010 | No | NA | 0 | 0 | Not-determined | Genome-fragment | NA | 0 |
| S1-final-viral-combined_k141_86625\|\|full | 1930 | No | NA | 0 | 0 | Not-determined | Genome-fragment | NA | 0 |
| S1-final-viral-combined_k141_69579\|\|full | 3336 | No | NA | 0 | 3 | Not-determined | Genome-fragment | NA | 0 |
| S1-final-viral-combined_k141_53859\|\|full | 2583 | No | NA | 0 | 2 | Not-determined | Genome-fragment | NA | 0 |
| S1-final-viral-combined_k141_252016\|\|full | 1028 | No | NA | 0 | 1 | Not-determined | Genome-fragment | NA | 0 |
| S1-final-viral-combined_k141_168587\|\|full | 3460 | No | NA | 0 | 1 | Not-determined | Genome-fragment | NA | 0 |
| S1-final-viral-combined_k141_218581\|\|full | 2391 | No | NA | 0 | 0 | Not-determined | Genome-fragment | NA | 0 |
| S9-final-viral-combined_k141_97200\|\|lt2gene | 1302 | No | NA | 1 | 0 | Low-quality | Genome-fragment | 3.3 | 0 |
| S1-final-viral-combined_k141_4564\|\|full | 1165 | No | NA | 0 | 1 | Not-determined | Genome-fragment | NA | 0 |
| S1-final-viral-combined_k141_389538\|\|full | 1302 | No | NA | 0 | 1 | Low-quality | Genome-fragment | 0.63 | 0 |
| S1-final-viral-combined_k141_64679\|\|full | 1302 | No | NA | 0 | 1 | Low-quality | Genome-fragment | 0.89 | 0 |
| S3-final-viral-combined_k141_37123\|\|full | 1302 | No | NA | 0 | 0 | Low-quality | Genome-fragment | 3.61 | 0 |
| S1-final-viral-combined_k141_152756\|\|full | 27818 | No | NA | 0 | 16 | Not-determined | Genome-fragment | NA | 0 |
| S1-final-viral-combined_k141_120296\|\|full | 3574 | No | NA | 0 | 0 | Not-determined | Genome-fragment | NA | 0 |
| S1-final-viral-combined_k141_366632\|\|full | 5534 | No | NA | 0 | 3 | Not-determined | Genome-fragment | NA | 0 |
| S1-final-viral-combined_k141_120300\|\|full | 2008 | No | NA | 0 | 1 | Not-determined | Genome-fragment | NA | 0 |
| S1-final-viral-combined_k141_102920\|\|full | 1296 | No | NA | 0 | 0 | Not-determined | Genome-fragment | NA | 0 |
| S1-final-viral-combined_k141_120311\|\|full | 1672 | No | NA | 0 | 3 | Not-determined | Genome-fragment | NA | 0 |
| S1-final-viral-combined_k141_21333\|\|full | 2458 | No | NA | 0 | 2 | Not-determined | Genome-fragment | NA | 0 |
| S3-final-viral-combined_k141_106407\|\|full | 1302 | No | NA | 0 | 2 | Low-quality | Genome-fragment | 0.96 | 0 |
| S5-final-viral-combined_k141_169014\|\|full | 1302 | No | NA | 1 | 2 | Low-quality | Genome-fragment | 1.39 | 0 |
| S7-final-viral-combined_k141_116722\|\|lt2gene | 1302 | No | NA | 1 | 0 | Low-quality | Genome-fragment | 2.62 | 0 |
| S7_k141_226280 | 1302 | No | NA | 0 | 0 | Low-quality | Genome-fragment | 2.73 | 0 |
| S1-final-viral-combined_k141_53869\|\|full | 1742 | No | NA | 0 | 2 | Not-determined | Genome-fragment | NA | 0 |
| S1-final-viral-combined_k141_317890\|\|full | 11345 | No | NA | 0 | 8 | Not-determined | Genome-fragment | NA | 0 |
| S17-k141_95930 | 1302 | No | NA | 1 | 0 | Low-quality | Genome-fragment | 3.39 | 0 |
| S4-final-viral-combined_k141_138100\|\|full | 1302 | No | NA | 1 | 0 | Low-quality | Genome-fragment | 3.11 | 0 |
| S1-final-viral-combined_k141_235083\|\|full | 3280 | No | NA | 0 | 1 | Not-determined | Genome-fragment | NA | 0 |
| S1-final-viral-combined_k141_120348\|\|full | 2511 | No | NA | 0 | 0 | Not-determined | Genome-fragment | NA | 0 |
| S1-final-viral-combined_k141_383133\|\|full | 1121 | No | NA | 0 | 1 | Not-determined | Genome-fragment | NA | 0 |
| S1-final-viral-combined_k141_152828\|\|full | 5176 | No | NA | 0 | 5 | Not-determined | Genome-fragment | NA | 0 |
| S8-final-viral-combined_k141_266813\|\|full | 1302 | No | NA | 3 | 0 | Low-quality | Genome-fragment | 2.85 | 0 |
| S1-final-viral-combined_k141_267840\|\|full | 1685 | No | NA | 0 | 2 | Not-determined | Genome-fragment | NA | 0 |
| S1-final-viral-combined_k141_301628\|\|full | 8878 | No | NA | 0 | 4 | Not-determined | Genome-fragment | NA | 0 |
| S8_k141_105122 | 1302 | No | NA | 0 | 1 | Low-quality | Genome-fragment | 0.48 | 0 |
| S1-final-viral-combined_k141_202311\|\|full | 2215 | No | NA | 0 | 1 | Not-determined | Genome-fragment | NA | 0 |
| S1-final-viral-combined_k141_284761\|\|full | 1127 | No | NA | 0 | 1 | Not-determined | Genome-fragment | NA | 0 |
| S8_k141_151317 | 1302 | No | NA | 0 | 0 | Low-quality | Genome-fragment | 0.91 | 0 |
| S9-final-viral-combined_k141_93907\|\|full | 1303 | No | NA | 0 | 0 | Low-quality | Genome-fragment | 2.12 | 0 |
| S1-final-viral-combined_k141_350691\|\|full | 1753 | No | NA | 0 | 2 | Not-determined | Genome-fragment | NA | 0 |
| S1-final-viral-combined_k141_383172\|\|full | 1799 | No | NA | 0 | 0 | Not-determined | Genome-fragment | NA | 0 |
| S11-final-viral-combined_k141_122369\|\|full | 1303 | No | NA | 1 | 0 | Low-quality | Genome-fragment | 2.61 | 0 |
| S1-final-viral-combined_k141_366710\|\|full | 13558 | No | NA | 0 | 6 | Not-determined | Genome-fragment | NA | 0 |
| S1-final-viral-combined_k141_218650\|\|full | 15962 | No | NA | 0 | 9 | Not-determined | Genome-fragment | NA | 0 |
| S1-final-viral-combined_k141_202359\|\|full | 2107 | No | NA | 0 | 1 | Not-determined | Genome-fragment | NA | 0 |
| S1-final-viral-combined_k141_37418\|\|full | 1303 | No | NA | 0 | 1 | Low-quality | Genome-fragment | 1.77 | 0 |
| S1-final-viral-combined_k141_225546\|\|full | 1303 | No | NA | 0 | 0 | Low-quality | Genome-fragment | 2.25 | 0 |
| S5-final-viral-combined_k141_34793\|\|full | 1303 | No | NA | 3 | 0 | Low-quality | Genome-fragment | 3.34 | 0 |
| S7-final-viral-combined_k141_95365\|\|full | 1303 | No | NA | 0 | 2 | Low-quality | Genome-fragment | 2.18 | 0 |
| S1-final-viral-combined_k141_366730\|\|full | 2575 | No | NA | 0 | 2 | Not-determined | Genome-fragment | NA | 0 |
| S1-final-viral-combined_k141_86725\|\|full | 2748 | No | NA | 0 | 0 | Not-determined | Genome-fragment | NA | 0 |
| S7-final-viral-combined_k141_224150\|\|full | 1303 | No | NA | 0 | 0 | Low-quality | Genome-fragment | 3.59 | 0 |
| S1-final-viral-combined_k141_4679\|\|full | 8732 | No | NA | 0 | 9 | Not-determined | Genome-fragment | NA | 0 |
| S1-final-viral-combined_k141_120456\|\|full | 5308 | No | NA | 0 | 4 | Not-determined | Genome-fragment | NA | 0 |
| S17-final-viral-combined_k141_256960\|\|lt2gene | 1303 | No | NA | 1 | 0 | Low-quality | Genome-fragment | 2.85 | 0 |
| S12-final-viral-combined_k141_358321\|\|full | 1303 | No | NA | 0 | 1 | Low-quality | Genome-fragment | 3.15 | 0 |
| S12-final-viral-combined_k141_77027\|\|full | 1303 | No | NA | 0 | 0 | Low-quality | Genome-fragment | 2.72 | 0 |
| S1-final-viral-combined_k141_202425\|\|full | 2201 | No | NA | 0 | 1 | Not-determined | Genome-fragment | NA | 0 |
| S1-final-viral-combined_k141_235209\|\|full | 2188 | No | NA | 0 | 1 | Not-determined | Genome-fragment | NA | 0 |
| S12-final-viral-combined_k141_331371\|\|full | 1303 | No | NA | 1 | 0 | Low-quality | Genome-fragment | 0.5 | 0 |
| S1-final-viral-combined_k141_218702\|\|full | 4002 | No | NA | 0 | 2 | Not-determined | Genome-fragment | NA | 0 |
| S12-final-viral-combined_k141_103156\|\|lt2gene | 1303 | No | NA | 0 | 0 | Low-quality | Genome-fragment | 2.56 | 0 |
| S12_k141_323514 | 1303 | No | NA | 0 | 0 | Low-quality | Genome-fragment | 3.07 | 0 |
| S1-final-viral-combined_k141_202439\|\|full | 6438 | No | NA | 0 | 2 | Not-determined | Genome-fragment | NA | 0 |
| S1-final-viral-combined_k141_186188\|\|full | 4922 | No | NA | 0 | 2 | Not-determined | Genome-fragment | NA | 0 |
| S2-final-viral-combined_k141_180810\|\|lt2gene | 1303 | No | NA | 1 | 0 | Low-quality | Genome-fragment | 0.85 | 0 |
| S8-final-viral-combined_k141_219820\|\|full | 1303 | No | NA | 0 | 0 | Low-quality | Genome-fragment | 3.78 | 0 |
| S8-final-viral-combined_k141_11332\|\|lt2gene | 1303 | No | NA | 1 | 0 | Low-quality | Genome-fragment | 2.24 | 0 |
| S1-final-viral-combined_k141_333564\|\|full | 7240 | No | NA | 0 | 5 | Not-determined | Genome-fragment | NA | 0 |
| S1-final-viral-combined_k141_120533\|\|full | 8687 | Yes | 5570 | 1 | 1 | High-quality | High-quality | 100 | 35.88 |
| S1-final-viral-combined_k141_120545\|\|full | 1123 | No | NA | 0 | 0 | Not-determined | Genome-fragment | NA | 0 |
| S1-final-viral-combined_k141_267892\|\|full | 4423 | No | NA | 0 | 6 | Not-determined | Genome-fragment | NA | 0 |
| S11-final-viral-combined_k141_110579\|\|full | 1304 | No | NA | 0 | 0 | Low-quality | Genome-fragment | 2.52 | 0 |
| S1-final-viral-combined_k141_333589\|\|full | 1690 | No | NA | 0 | 2 | Not-determined | Genome-fragment | NA | 0 |
| S1-final-viral-combined_k141_230977\|\|full | 1304 | No | NA | 2 | 0 | Low-quality | Genome-fragment | 1.82 | 0 |
| S1-final-viral-combined_k141_149040\|\|full | 1304 | No | NA | 0 | 0 | Low-quality | Genome-fragment | 1.66 | 0 |
| S1-final-viral-combined_k141_19076\|\|full | 1304 | No | NA | 0 | 0 | Low-quality | Genome-fragment | 3.22 | 0 |
| S1_k141_231223 | 1304 | No | NA | 0 | 1 | Low-quality | Genome-fragment | 0.7 | 0 |
| S1-final-viral-combined_k141_267920\|\|full | 2449 | No | NA | 0 | 2 | Not-determined | Genome-fragment | NA | 0 |
| S7-final-viral-combined_k141_234962\|\|full | 1304 | No | NA | 0 | 0 | Low-quality | Genome-fragment | 3.66 | 0 |
| S1-final-viral-combined_k141_202576\|\|full | 2500 | No | NA | 0 | 2 | Not-determined | Genome-fragment | NA | 0 |
| S1-final-viral-combined_k141_4847\|\|full | 11103 | No | NA | 0 | 10 | Not-determined | Genome-fragment | NA | 0 |
| S1-final-viral-combined_k141_69837\|\|full | 4079 | No | NA | 0 | 0 | Not-determined | Genome-fragment | NA | 0 |
| S12-final-viral-combined_k141_150338\|\|full | 1304 | No | NA | 0 | 2 | Low-quality | Genome-fragment | 2.92 | 0 |
| S1-final-viral-combined_k141_4867\|\|full | 2952 | No | NA | 0 | 1 | Not-determined | Genome-fragment | NA | 0 |
| S1-final-viral-combined_k141_86880\|\|full | 3353 | No | NA | 0 | 2 | Not-determined | Genome-fragment | NA | 0 |
| S4_k141_49987 | 1304 | No | NA | 1 | 0 | Low-quality | Genome-fragment | 0.75 | 0 |
| S11-final-viral-combined_k141_98023\|\|full | 1305 | No | NA | 0 | 0 | Low-quality | Genome-fragment | 3.51 | 0 |
| S1-final-viral-combined_k141_169012\|\|full | 2570 | No | NA | 0 | 0 | Not-determined | Genome-fragment | NA | 0 |
| S1-final-viral-combined_k141_239263\|\|full | 1305 | No | NA | 0 | 0 | Low-quality | Genome-fragment | 1.77 | 0 |
| S1-final-viral-combined_k141_302001\|\|full | 7775 | No | NA | 0 | 3 | Not-determined | Genome-fragment | NA | 0 |
| S1-final-viral-combined_k141_218938\|\|full | 1239 | No | NA | 0 | 0 | Not-determined | Genome-fragment | NA | 0 |
| S1-final-viral-combined_k141_103146\|\|full | 3003 | No | NA | 0 | 3 | Not-determined | Genome-fragment | NA | 0 |
| S1-final-viral-combined_k141_302003\|\|full | 3242 | No | NA | 0 | 0 | Not-determined | Genome-fragment | NA | 0 |
| S1-final-viral-combined_k141_292363\|\|lt2gene | 1305 | No | NA | 1 | 0 | Low-quality | Genome-fragment | 25.65 | 0 |
| S12-final-viral-combined_k141_9231\|\|full | 1305 | No | NA | 3 | 0 | Low-quality | Genome-fragment | 2.78 | 0 |
| S1-final-viral-combined_k141_284782\|\|full | 1165 | No | NA | 0 | 0 | Not-determined | Genome-fragment | NA | 0 |
| S1-final-viral-combined_k141_103157\|\|full | 2664 | No | NA | 0 | 2 | Not-determined | Genome-fragment | NA | 0 |
| S12_k141_358732 | 1305 | No | NA | 1 | 0 | Low-quality | Genome-fragment | 2.93 | 0 |
| S6-final-viral-combined_k141_71736\|\|full | 1305 | No | NA | 0 | 0 | Low-quality | Genome-fragment | 3.69 | 0 |
| S1-final-viral-combined_k141_17010\|\|full | 1306 | No | NA | 0 | 0 | Low-quality | Genome-fragment | 1.01 | 0 |
| S1-final-viral-combined_k141_318268\|\|full | 3755 | No | NA | 0 | 6 | Not-determined | Genome-fragment | NA | 0 |
| S5-final-viral-combined_k141_145209\|\|full | 1306 | No | NA | 2 | 0 | Low-quality | Genome-fragment | 0.69 | 0 |
| S1-final-viral-combined_k141_202678\|\|full | 2525 | No | NA | 0 | 1 | Not-determined | Genome-fragment | NA | 0 |
| S1-final-viral-combined_k141_318278\|\|full | 1072 | No | NA | 0 | 1 | Not-determined | Genome-fragment | NA | 0 |
| S1-final-viral-combined_k141_4934\|\|full | 1453 | No | NA | 0 | 0 | Not-determined | Genome-fragment | NA | 0 |
| S7_k141_124620 | 1306 | No | NA | 0 | 0 | Low-quality | Genome-fragment | 2.85 | 0 |
| S1-final-viral-combined_k141_284811\|\|full | 3450 | No | NA | 0 | 3 | Not-determined | Genome-fragment | NA | 0 |
| S7_k141_207523 | 1306 | No | NA | 0 | 2 | Low-quality | Genome-fragment | 0.39 | 0 |
| S1-final-viral-combined_k141_37914\|\|full | 2070 | No | NA | 0 | 2 | Not-determined | Genome-fragment | NA | 0 |
| S1-final-viral-combined_k141_302048\|\|full | 1244 | No | NA | 0 | 0 | Not-determined | Genome-fragment | NA | 0 |

| | | | | | | | | | |
|---|---|---|---|---|---|---|---|---|---|
| S1-final-viral-combined_k141_367013\|\|full | 2421 | No | NA | 0 | 1 | Not-determined | Genome-fragment | NA | 0 |
| S1-final-viral-combined_k141_302061\|\|full | 1094 | No | NA | 0 | 2 | Not-determined | Genome-fragment | NA | 0 |
| S1-final-viral-combined_k141_54076\|\|full | 2712 | No | NA | 0 | 2 | Not-determined | Genome-fragment | NA | 0 |
| S1-final-viral-combined_k141_202715\|\|full | 1266 | No | NA | 0 | 0 | Not-determined | Genome-fragment | NA | 0 |
| S1-final-viral-combined_k141_218985\|\|full | 1219 | No | NA | 0 | 1 | Not-determined | Genome-fragment | NA | 0 |
| S7_k141_200766 | 1306 | No | NA | 0 | 0 | Low-quality | Genome-fragment | 2 | 0 |
| S1-final-viral-combined_k141_252339\|\|full | 1007 | No | NA | 0 | 1 | Not-determined | Genome-fragment | NA | 0 |
| S12_k141_143456 | 1306 | No | NA | 1 | 0 | Low-quality | Genome-fragment | 2.49 | 0 |
| S2-final-viral-combined_k141_18711\|\|full | 1306 | No | NA | 1 | 0 | Low-quality | Genome-fragment | 2.97 | 0 |
| S8-final-viral-combined_k141_256873\|\|full | 1306 | No | NA | 0 | 1 | Low-quality | Genome-fragment | 0.63 | 0 |
| S1-final-viral-combined_k141_5003\|\|full | 1285 | No | NA | 0 | 1 | Not-determined | Genome-fragment | NA | 0 |
| S8-final-viral-combined_k141_19551\|\|lt2gene | 1306 | No | NA | 0 | 0 | Low-quality | Genome-fragment | 3.78 | 0 |
| S1-final-viral-combined_k141_169086\|\|full | 5509 | No | NA | 0 | 3 | Not-determined | Genome-fragment | NA | 0 |
| S9_k141_65196 | 1307 | No | NA | 0 | 1 | Low-quality | Genome-fragment | 0.85 | 0 |
| S1-final-viral-combined_k141_284864\|\|full | 14383 | No | NA | 0 | 11 | Not-determined | Genome-fragment | NA | 0 |
| S11-final-viral-combined_k141_79340\|\|full | 1307 | No | NA | 0 | 1 | Low-quality | Genome-fragment | 1.41 | 0 |
| S11-final-viral-combined_k141_327383\|\|full | 1307 | No | NA | 0 | 0 | Low-quality | Genome-fragment | 0.85 | 0 |
| S1-final-viral-combined_k141_284875\|\|full | 1890 | No | NA | 0 | 2 | Not-determined | Genome-fragment | NA | 0 |
| S1-final-viral-combined_k141_86969\|\|full | 4824 | No | NA | 0 | 3 | Not-determined | Genome-fragment | NA | 0 |
| S1-final-viral-combined_k141_120757\|\|full | 4324 | No | NA | 0 | 1 | Not-determined | Genome-fragment | NA | 0 |
| S1-final-viral-combined_k141_103281\|\|full | 1915 | No | NA | 0 | 2 | Not-determined | Genome-fragment | NA | 0 |
| S1-final-viral-combined_k141_201878\|\|full | 1307 | No | NA | 0 | 1 | Low-quality | Genome-fragment | 0.67 | 0 |
| S1-final-viral-combined_k141_252397\|\|full | 1303 | No | NA | 0 | 0 | Not-determined | Genome-fragment | NA | 0 |
| S1-final-viral-combined_k141_304753\|\|full | 1307 | No | NA | 0 | 0 | Low-quality | Genome-fragment | 1.89 | 0 |
| S1-final-viral-combined_k141_383579\|\|full | 2222 | No | NA | 0 | 1 | Not-determined | Genome-fragment | NA | 0 |
| S1-final-viral-combined_k141_383581\|\|full | 1218 | No | NA | 0 | 1 | Not-determined | Genome-fragment | NA | 0 |
| S1-final-viral-combined_k141_212742\|\|full | 1307 | No | NA | 0 | 0 | Low-quality | Genome-fragment | 1.92 | 0 |
| S1-final-viral-combined_k141_235508\|\|full | 6577 | No | NA | 0 | 0 | Not-determined | Genome-fragment | NA | 0 |
| S1-final-viral-combined_k141_235519\|\|full | 4101 | No | NA | 0 | 0 | Not-determined | Genome-fragment | NA | 0 |
| S1-final-viral-combined_k141_21475\|\|full | 2875 | No | NA | 0 | 1 | Not-determined | Genome-fragment | NA | 0 |
| S1-final-viral-combined_k141_333758\|\|full | 1687 | No | NA | 0 | 1 | Not-determined | Genome-fragment | NA | 0 |
| S7-final-viral-combined_k141_116417\|\|full | 1307 | No | NA | 0 | 0 | Low-quality | Genome-fragment | 3.08 | 0 |
| S1-final-viral-combined_k141_252439\|\|full | 17383 | No | NA | 11 | 0 | Medium-quality | Genome-fragment | 50.91 | 0 |
| S1-final-viral-combined_k141_268194\|\|full | 2074 | No | NA | 0 | 2 | Not-determined | Genome-fragment | NA | 0 |
| S1-final-viral-combined_k141_54177\|\|full | 3236 | No | NA | 0 | 1 | Not-determined | Genome-fragment | NA | 0 |
| S1-final-viral-combined_k141_202751\|\|full | 2362 | No | NA | 0 | 0 | Not-determined | Genome-fragment | NA | 0 |
| S17-final-viral-combined_k141_249118\|\|full | 1307 | No | NA | 0 | 0 | Low-quality | Genome-fragment | 2.22 | 0 |
| S1-final-viral-combined_k141_318431\|\|full | 4131 | No | NA | 0 | 2 | Not-determined | Genome-fragment | NA | 0 |
| S1-final-viral-combined_k141_302145\|\|full | 16356 | No | NA | 0 | 9 | Not-determined | Genome-fragment | NA | 0 |
| S1-final-viral-combined_k141_351117\|\|full | 1654 | No | NA | 0 | 1 | Not-determined | Genome-fragment | NA | 0 |
| S1-final-viral-combined_k141_383662\|\|full | 1154 | No | NA | 0 | 1 | Not-determined | Genome-fragment | NA | 0 |
| S1-final-viral-combined_k141_153242\|\|full | 1221 | No | NA | 0 | 3 | Not-determined | Genome-fragment | NA | 0 |
| S1-final-viral-combined_k141_219156\|\|full | 2502 | No | NA | 0 | 1 | Not-determined | Genome-fragment | NA | 0 |
| S1-final-viral-combined_k141_383680\|\|full | 1750 | No | NA | 0 | 0 | Not-determined | Genome-fragment | NA | 0 |
| S1-final-viral-combined_k141_21499\|\|full | 2540 | No | NA | 0 | 2 | Not-determined | Genome-fragment | NA | 0 |
| S1-final-viral-combined_k141_318450\|\|full | 18563 | No | NA | 0 | 3 | Not-determined | Genome-fragment | NA | 0 |
| S1-final-viral-combined_k141_186532\|\|full | 2487 | No | NA | 0 | 1 | Not-determined | Genome-fragment | NA | 0 |
| S17-final-viral-combined_k141_185816\|\|full | 1307 | No | NA | 0 | 0 | Low-quality | Genome-fragment | 1.63 | 0 |
| S1-final-viral-combined_k141_186536\|\|full | 2172 | No | NA | 0 | 1 | Not-determined | Genome-fragment | NA | 0 |
| S17-final-viral-combined_k141_121718\|\|full | 1307 | No | NA | 0 | 0 | Low-quality | Genome-fragment | 2.39 | 0 |
| S12-final-viral-combined_k141_305823\|\|full | 1307 | No | NA | 0 | 0 | Low-quality | Genome-fragment | 1.56 | 0 |
| S4-final-viral-combined_k141_50633\|\|full | 1307 | No | NA | 0 | 0 | Low-quality | Genome-fragment | 2.62 | 0 |
| S4-final-viral-combined_k141_163689\|\|full | 1307 | No | NA | 0 | 0 | Low-quality | Genome-fragment | 1.38 | 0 |
| S9-final-viral-combined_k141_105421\|\|full | 1308 | No | NA | 0 | 0 | Low-quality | Genome-fragment | 4.68 | 0 |
| S1-final-viral-combined_k141_5119\|\|full | 1445 | No | NA | 0 | 1 | Not-determined | Genome-fragment | NA | 0 |
| S1-final-viral-combined_k141_299522\|\|full | 1308 | Yes | 804 | 1 | 1 | Low-quality | Genome-fragment | 0.51 | 38.53 |
| S1-final-viral-combined_k141_5124\|\|full | 1270 | No | NA | 0 | 0 | Not-determined | Genome-fragment | NA | 0 |
| S1-final-viral-combined_k141_54278\|\|full | 1407 | No | NA | 0 | 1 | Not-determined | Genome-fragment | NA | 0 |
| S1-final-viral-combined_k141_302166\|\|full | 75458 | Yes | 40046 | 9 | 17 | Medium-quality | Genome-fragment | 74.67 | 46.93 |
| S3-final-viral-combined_k141_2876\|\|full | 1308 | No | NA | 0 | 1 | Low-quality | Genome-fragment | 3.84 | 0 |
| S1-final-viral-combined_k141_333937\|\|full | 1612 | No | NA | 0 | 0 | Not-determined | Genome-fragment | NA | 0 |
| S1-final-viral-combined_k141_351236\|\|full | 3168 | No | NA | 0 | 2 | Not-determined | Genome-fragment | NA | 0 |
| S17-k141_160067 | 1308 | No | NA | 0 | 1 | Low-quality | Genome-fragment | 1.85 | 0 |
| S1-final-viral-combined_k141_235621\|\|full | 1684 | No | NA | 0 | 0 | Not-determined | Genome-fragment | NA | 0 |
| S1-final-viral-combined_k141_70049\|\|full | 9339 | No | NA | 0 | 3 | Not-determined | Genome-fragment | NA | 0 |
| S8-final-viral-combined_k141_33353\|\|full | 1308 | No | NA | 1 | 0 | Low-quality | Genome-fragment | 3.21 | 0 |
| S1-final-viral-combined_k141_38101\|\|full | 1839 | No | NA | 0 | 1 | Not-determined | Genome-fragment | NA | 0 |
| S1-final-viral-combined_k141_87156\|\|full | 1204 | No | NA | 0 | 0 | Not-determined | Genome-fragment | NA | 0 |
| S1-final-viral-combined_k141_153375\|\|full | 1192 | No | NA | 0 | 1 | Not-determined | Genome-fragment | NA | 0 |
| S9-final-viral-combined_k141_103754\|\|lt2gene | 1309 | No | NA | 1 | 0 | Low-quality | Genome-fragment | 0.75 | 0 |
| S1-final-viral-combined_k141_186660\|\|full | 1794 | No | NA | 0 | 1 | Not-determined | Genome-fragment | NA | 0 |
| S11-final-viral-combined_k141_278473\|\|full | 1309 | No | NA | 0 | 0 | Low-quality | Genome-fragment | 1.14 | 0 |
| S1-final-viral-combined_k141_5176\|\|full | 5916 | No | NA | 0 | 3 | Not-determined | Genome-fragment | NA | 0 |
| S11-final-viral-combined_k141_93509\|\|lt2gene | 1309 | No | NA | 2 | 0 | Low-quality | Genome-fragment | 2.99 | 0 |
| S1-final-viral-combined_k141_153389\|\|full | 1173 | No | NA | 0 | 1 | Not-determined | Genome-fragment | NA | 0 |
| S1-final-viral-combined_k141_21596\|\|full | 2560 | No | NA | 0 | 1 | Not-determined | Genome-fragment | NA | 0 |
| S1-final-viral-combined_k141_70078\|\|full | 4001 | No | NA | 0 | 3 | Not-determined | Genome-fragment | NA | 0 |
| S1-final-viral-combined_k141_285012\|\|full | 77510 | Yes | 45999 | 25 | 28 | High-quality | High-quality | 97.02 | 40.65 |
| S1-final-viral-combined_k141_219363\|\|full | 1556 | No | NA | 0 | 2 | Not-determined | Genome-fragment | NA | 0 |
| S5-final-viral-combined_k141_138283\|\|full | 1309 | No | NA | 0 | 2 | Low-quality | Genome-fragment | 1.32 | 0 |
| S1-final-viral-combined_k141_21608\|\|full | 3222 | No | NA | 0 | 0 | Not-determined | Genome-fragment | NA | 0 |
| S1-final-viral-combined_k141_5205\|\|full | 1584 | No | NA | 0 | 0 | Not-determined | Genome-fragment | NA | 0 |
| S1-final-viral-combined_k141_153411\|\|full | 2646 | No | NA | 0 | 3 | Not-determined | Genome-fragment | NA | 0 |
| S1-final-viral-combined_k141_367335\|\|full | 2516 | No | NA | 0 | 1 | Not-determined | Genome-fragment | NA | 0 |
| S7-final-viral-combined_k141_157890\|\|full | 1309 | No | NA | 0 | 0 | Low-quality | Genome-fragment | 2.77 | 0 |
| S17-final-viral-combined_k141_122744\|\|full | 1309 | No | NA | 2 | 0 | Low-quality | Genome-fragment | 2.06 | 0 |
| S2_k141_221845 | 1309 | No | NA | 0 | 1 | Low-quality | Genome-fragment | 1.36 | 0 |
| S4-final-viral-combined_k141_160603\|\|full | 1309 | No | NA | 0 | 0 | Low-quality | Genome-fragment | 3.62 | 0 |
| S1-final-viral-combined_k141_54369\|\|full | 1485 | No | NA | 0 | 2 | Not-determined | Genome-fragment | NA | 0 |
| S1-final-viral-combined_k141_169259\|\|full | 1561 | No | NA | 0 | 1 | Not-determined | Genome-fragment | NA | 0 |
| S4_k141_64029 | 1309 | No | NA | 1 | 0 | Low-quality | Genome-fragment | 1.37 | 0 |
| S1-final-viral-combined_k141_186763\|\|full | 1299 | No | NA | 0 | 0 | Not-determined | Genome-fragment | NA | 0 |
| S1-final-viral-combined_k141_202974\|\|full | 1235 | No | NA | 0 | 0 | Not-determined | Genome-fragment | NA | 0 |
| S1-final-viral-combined_k141_137574\|\|full | 1461 | No | NA | 0 | 2 | Not-determined | Genome-fragment | NA | 0 |
| S1-final-viral-combined_k141_252648\|\|full | 2102 | No | NA | 0 | 0 | Not-determined | Genome-fragment | NA | 0 |
| S8-final-viral-combined_k141_251601\|\|lt2gene | 1309 | No | NA | 1 | 0 | Low-quality | Genome-fragment | 1.78 | 0 |
| S1-final-viral-combined_k141_253446\|\|full | 1310 | No | NA | 0 | 0 | Low-quality | Genome-fragment | 3.35 | 0 |
| S1-final-viral-combined_k141_186802\|\|full | 2008 | No | NA | 0 | 1 | Not-determined | Genome-fragment | NA | 0 |
| S1-final-viral-combined_k141_87251\|\|full | 9550 | No | NA | 0 | 6 | Not-determined | Genome-fragment | NA | 0 |
| S1-final-viral-combined_k141_111151\|\|lt2gene | 1310 | No | NA | 0 | 0 | Low-quality | Genome-fragment | 3.35 | 0 |
| S1-final-viral-combined_k141_203026\|\|full | 1352 | No | NA | 0 | 1 | Not-determined | Genome-fragment | NA | 0 |
| S1-final-viral-combined_k141_137626\|\|full | 2626 | No | NA | 0 | 2 | Not-determined | Genome-fragment | NA | 0 |
| S3_k141_117364 | 1310 | No | NA | 0 | 0 | Low-quality | Genome-fragment | 0.6 | 0 |
| S1-final-viral-combined_k141_153489\|\|full | 1710 | No | NA | 0 | 1 | Not-determined | Genome-fragment | NA | 0 |
| S1-final-viral-combined_k141_137627\|\|full | 13832 | No | NA | 0 | 4 | Not-determined | Genome-fragment | NA | 0 |
| S5-final-viral-combined_k141_69313\|\|full | 1310 | No | NA | 0 | 1 | Low-quality | Genome-fragment | 1.78 | 0 |
| S1-final-viral-combined_k141_302360\|\|full | 2503 | No | NA | 0 | 2 | Not-determined | Genome-fragment | NA | 0 |
| S1-final-viral-combined_k141_351441\|\|full | 1776 | No | NA | 0 | 1 | Not-determined | Genome-fragment | NA | 0 |
| S5-final-viral-combined_k141_50153\|\|lt2gene | 1310 | No | NA | 2 | 0 | Low-quality | Genome-fragment | 3.25 | 0 |
| S1-final-viral-combined_k141_186878\|\|full | 27776 | No | NA | 9 | 1 | Medium-quality | Genome-fragment | 65.78 | 0 |
| S7-final-viral-combined_k141_104856\|\|full | 1310 | No | NA | 0 | 0 | Low-quality | Genome-fragment | 3.2 | 0 |
| S17-final-viral-combined_k141_606\|\|full | 1310 | No | NA | 0 | 0 | Low-quality | Genome-fragment | 1.2 | 0 |
| S1-final-viral-combined_k141_318666\|\|full | 3370 | No | NA | 0 | 2 | Not-determined | Genome-fragment | NA | 0 |
| S17-final-viral-combined_k141_22943\|\|full | 1310 | No | NA | 1 | 0 | Low-quality | Genome-fragment | 2.86 | 0 |
| S1-final-viral-combined_k141_285192\|\|full | 7631 | No | NA | 0 | 3 | Not-determined | Genome-fragment | NA | 0 |
| S17-final-viral-combined_k141_125732\|\|full | 1310 | No | NA | 1 | 0 | Low-quality | Genome-fragment | 1.83 | 0 |
| S1-final-viral-combined_k141_121223\|\|full | 1047 | No | NA | 0 | 2 | Not-determined | Genome-fragment | NA | 0 |
| S17-final-viral-combined_k141_181824\|\|full | 1310 | No | NA | 0 | 0 | Low-quality | Genome-fragment | 0.73 | 0 |
| S1-final-viral-combined_k141_186931\|\|full | 1249 | No | NA | 0 | 1 | Not-determined | Genome-fragment | NA | 0 |
| S1-final-viral-combined_k141_334105\|\|full | 2368 | No | NA | 0 | 2 | Not-determined | Genome-fragment | NA | 0 |
| S1-final-viral-combined_k141_318703\|\|full | 2659 | No | NA | 0 | 0 | Not-determined | Genome-fragment | NA | 0 |
| S12-final-viral-combined_k141_180214\|\|full | 1310 | No | NA | 1 | 0 | Low-quality | Genome-fragment | 2.91 | 0 |
| S12_k141_185509 | 1310 | No | NA | 0 | 0 | Low-quality | Genome-fragment | 2.52 | 0 |
| S1-final-viral-combined_k141_121253\|\|full | 1680 | No | NA | 0 | 2 | Not-determined | Genome-fragment | NA | 0 |
| S2-final-viral-combined_k141_17687\|\|full | 1310 | No | NA | 1 | 0 | Low-quality | Genome-fragment | 2.06 | 0 |
| S6-final-viral-combined_k141_133575\|\|full | 1310 | No | NA | 0 | 1 | Low-quality | Genome-fragment | 1.52 | 0 |
| S1-final-viral-combined_k141_21773\|\|full | 2948 | No | NA | 0 | 2 | Not-determined | Genome-fragment | NA | 0 |
| S1-final-viral-combined_k141_5332\|\|full | 1824 | No | NA | 0 | 1 | Not-determined | Genome-fragment | NA | 0 |
| S6_k141_129639 | 1310 | No | NA | 0 | 0 | Low-quality | Genome-fragment | 4.07 | 0 |
| S11-final-viral-combined_k141_266758\|\|full | 1311 | No | NA | 0 | 1 | Low-quality | Genome-fragment | 2.63 | 0 |
| S1-final-viral-combined_k141_235891\|\|full | 1368 | No | NA | 0 | 0 | Not-determined | Genome-fragment | NA | 0 |
| S11-final-viral-combined_k141_54942\|\|full | 1311 | No | NA | 0 | 0 | Low-quality | Genome-fragment | 2.37 | 0 |
| S11-final-viral-combined_k141_242734\|\|lt2gene | 1311 | No | NA | 2 | 0 | Low-quality | Genome-fragment | 0.91 | 0 |
| S1-final-viral-combined_k141_293303\|\|lt2gene | 1311 | No | NA | 1 | 0 | Low-quality | Genome-fragment | 1.91 | 0 |
| S1-final-viral-combined_k141_235906\|\|full | 1724 | No | NA | 0 | 3 | Not-determined | Genome-fragment | NA | 0 |
| S1-final-viral-combined_k141_252804\|\|full | 10933 | No | NA | 0 | 5 | Not-determined | Genome-fragment | NA | 0 |
| S1-final-viral-combined_k141_169303\|\|full | 1284 | No | NA | 0 | 0 | Not-determined | Genome-fragment | NA | 0 |
| S1-final-viral-combined_k141_302449\|\|full | 1982 | No | NA | 0 | 1 | Not-determined | Genome-fragment | NA | 0 |
| S1_k141_43699 | 1311 | No | NA | 0 | 1 | Low-quality | Genome-fragment | 0.48 | 0 |
| S1-final-viral-combined_k141_203176\|\|full | 25710 | No | NA | 0 | 16 | Not-determined | Genome-fragment | NA | 0 |
| S1_k141_162483 | 1311 | No | NA | 0 | 0 | Low-quality | Genome-fragment | 2.89 | 0 |
| S1-final-viral-combined_k141_252864\|\|full | 3342 | No | NA | 0 | 0 | Not-determined | Genome-fragment | NA | 0 |
| S1-final-viral-combined_k141_70327\|\|full | 2334 | No | NA | 0 | 0 | Not-determined | Genome-fragment | NA | 0 |
| S7_k141_220068 | 1311 | No | NA | 0 | 1 | Low-quality | Genome-fragment | 0.64 | 0 |
| S12-final-viral-combined_k141_41001\|\|lt2gene | 1311 | No | NA | 1 | 0 | Low-quality | Genome-fragment | 0.71 | 0 |
| S1-final-viral-combined_k141_285365\|\|full | 1289 | No | NA | 0 | 0 | Not-determined | Genome-fragment | NA | 0 |
| S1-final-viral-combined_k141_103435\|\|full | 2814 | No | NA | 0 | 1 | Not-determined | Genome-fragment | NA | 0 |
| S1-final-viral-combined_k141_252871\|\|full | 4804 | No | NA | 0 | 3 | Not-determined | Genome-fragment | NA | 0 |
| S2-final-viral-combined_k141_122133\|\|full | 1311 | No | NA | 0 | 2 | Low-quality | Genome-fragment | 1.11 | 0 |
| S1-final-viral-combined_k141_70348\|\|full | 1031 | No | NA | 0 | 0 | Not-determined | Genome-fragment | NA | 0 |
| S4-final-viral-combined_k141_38902\|\|full | 1311 | No | NA | 0 | 0 | Low-quality | Genome-fragment | 2.15 | 0 |
| S9-final-viral-combined_k141_109697\|\|full | 1312 | No | NA | 2 | 0 | Low-quality | Genome-fragment | 2.05 | 0 |
| S9-final-viral-combined_k141_36798\|\|full | 1312 | No | NA | 1 | 0 | Low-quality | Genome-fragment | 2.17 | 0 |
| S1-final-viral-combined_k141_74561\|\|full | 1312 | No | NA | 0 | 0 | Low-quality | Genome-fragment | 2.33 | 0 |
| S1-final-viral-combined_k141_70365\|\|full | 2168 | No | NA | 0 | 3 | Not-determined | Genome-fragment | NA | 0 |
| S1-final-viral-combined_k141_70374\|\|full | 2752 | No | NA | 0 | 0 | Not-determined | Genome-fragment | NA | 0 |
| S5-final-viral-combined_k141_81548\|\|full | 1312 | No | NA | 0 | 0 | Low-quality | Genome-fragment | 1.45 | 0 |
| S1-final-viral-combined_k141_70394\|\|full | 2754 | No | NA | 0 | 1 | Not-determined | Genome-fragment | NA | 0 |
| S17-final-viral-combined_k141_140470\|\|full | 1312 | No | NA | 0 | 2 | Low-quality | Genome-fragment | 0.44 | 0 |
| S1-final-viral-combined_k141_153636\|\|full | 5577 | No | NA | 0 | 6 | Not-determined | Genome-fragment | NA | 0 |
| S1-final-viral-combined_k141_203252\|\|full | 5663 | No | NA | 0 | 0 | Not-determined | Genome-fragment | NA | 0 |
| S1-final-viral-combined_k141_236024\|\|full | 3940 | No | NA | 0 | 1 | Not-determined | Genome-fragment | NA | 0 |
| S1-final-viral-combined_k141_203255\|\|full | 1960 | No | NA | 0 | 1 | Not-determined | Genome-fragment | NA | 0 |
| S1-final-viral-combined_k141_38346\|\|full | 1037 | No | NA | 0 | 2 | Not-determined | Genome-fragment | NA | 0 |
| S12-final-viral-combined_k141_328196\|\|lt2gene | 1312 | No | NA | 2 | 0 | Low-quality | Genome-fragment | 2.24 | 0 |
| S2-final-viral-combined_k141_146195\|\|full | 1312 | No | NA | 0 | 0 | Low-quality | Genome-fragment | 2.36 | 0 |
| S8_k141_35692 | 1312 | No | NA | 0 | 0 | Low-quality | Genome-fragment | 0.85 | 0 |
| S1-final-viral-combined_k141_351619\|\|full | 2102 | No | NA | 0 | 0 | Not-determined | Genome-fragment | NA | 0 |
| S17-final-viral-combined_k141_261343\|\|full | 1313 | No | NA | 0 | 0 | Low-quality | Genome-fragment | 1.62 | 0 |
| S17-k141_242485 | 1313 | No | NA | 0 | 0 | Low-quality | Genome-fragment | 0.74 | 0 |
| S1-final-viral-combined_k141_38380\|\|full | 2375 | No | NA | 0 | 3 | Not-determined | Genome-fragment | NA | 0 |
| S1-final-viral-combined_k141_103497\|\|full | 1746 | No | NA | 0 | 1 | Not-determined | Genome-fragment | NA | 0 |
| S1-final-viral-combined_k141_236067\|\|full | 1366 | No | NA | 0 | 2 | Not-determined | Genome-fragment | NA | 0 |
| S1-final-viral-combined_k141_5453\|\|full | 5301 | No | NA | 0 | 2 | Not-determined | Genome-fragment | NA | 0 |

| | | | | | | | | | | |
|---|---|---|---|---|---|---|---|---|---|---|
| S12-final-viral-combined_k141_44760||full | 1313 | No | NA | 0 | 1 | Low-quality | Genome-fragment | | 2.37 | 0 |
| S1-final-viral-combined_k141_334238||full | 3402 | No | NA | 0 | 2 | Not-determined | Genome-fragment | NA | | 0 |
| S12-final-viral-combined_k141_44771||full | 1313 | No | NA | 0 | 1 | Low-quality | Genome-fragment | | 1.65 | 0 |
| S12-final-viral-combined_k141_348057||full | 1313 | No | NA | 0 | 0 | Low-quality | Genome-fragment | | 1.59 | 0 |
| S12-final-viral-combined_k141_48419||full | 1313 | No | NA | 0 | 0 | Low-quality | Genome-fragment | | 2.09 | 0 |
| S1-final-viral-combined_k141_153670||full | 2781 | No | NA | 1 | 0 | Medium-quality | Genome-fragment | | 50.3 | 0 |
| S1-final-viral-combined_k141_21942||full | 5759 | No | NA | 0 | 3 | Not-determined | Genome-fragment | NA | | 0 |
| S1-final-viral-combined_k141_103520||full | 2663 | No | NA | 0 | 3 | Not-determined | Genome-fragment | NA | | 0 |
| S2-final-viral-combined_k141_1512||full | 1313 | No | NA | 0 | 0 | Low-quality | Genome-fragment | | 3.05 | 0 |
| S1-final-viral-combined_k141_268632||full | 1985 | No | NA | 0 | 0 | Not-determined | Genome-fragment | NA | | 0 |
| S1-final-viral-combined_k141_219707||full | 1308 | No | NA | 0 | 1 | Not-determined | Genome-fragment | NA | | 0 |
| S1-final-viral-combined_k141_70503||full | 1995 | No | NA | 0 | 0 | Not-determined | Genome-fragment | NA | | 0 |
| S1-final-viral-combined_k141_203335||full | 1304 | No | NA | 0 | 0 | Not-determined | Genome-fragment | NA | | 0 |
| S1-final-viral-combined_k141_268648||full | 2559 | No | NA | 0 | 2 | Not-determined | Genome-fragment | NA | | 0 |
| S2-final-viral-combined_k141_93912||lt2gene | 1313 | No | NA | 1 | 0 | Low-quality | Genome-fragment | | 1.92 | 0 |
| S1-final-viral-combined_k141_187235||full | 4769 | No | NA | 0 | 4 | Not-determined | Genome-fragment | NA | | 0 |
| S1-final-viral-combined_k141_252972||full | 1240 | No | NA | 0 | 0 | Not-determined | Genome-fragment | NA | | 0 |
| S1-final-viral-combined_k141_38464||full | 1178 | No | NA | 0 | 1 | Not-determined | Genome-fragment | NA | | 0 |
| S1-final-viral-combined_k141_121339||full | 2537 | No | NA | 0 | 0 | Not-determined | Genome-fragment | NA | | 0 |
| S1-final-viral-combined_k141_5507||full | 4026 | No | NA | 0 | 3 | Not-determined | Genome-fragment | NA | | 0 |
| S1-final-viral-combined_k141_351665||full | 1057 | No | NA | 0 | 2 | Not-determined | Genome-fragment | NA | | 0 |
| S4-final-viral-combined_k141_38466||full | 1313 | No | NA | 0 | 2 | Low-quality | Genome-fragment | | 0.44 | 0 |
| S1-final-viral-combined_k141_169512||full | 1802 | No | NA | 0 | 2 | Not-determined | Genome-fragment | NA | | 0 |
| S4-final-viral-combined_k141_25182||full | 1313 | No | NA | 0 | 1 | Low-quality | Genome-fragment | | 0.38 | 0 |
| S4-final-viral-combined_k141_124174||lt2gene | 1313 | No | NA | 1 | 0 | Low-quality | Genome-fragment | | 3.51 | 0 |
| S1-final-viral-combined_k141_384120||full | 3118 | No | NA | 0 | 3 | Not-determined | Genome-fragment | NA | | 0 |
| S6_k141_100921 | 1313 | No | NA | 0 | 0 | Low-quality | Genome-fragment | | 0.96 | 0 |
| S1-final-viral-combined_k141_5525||full | 2842 | No | NA | 0 | 1 | Not-determined | Genome-fragment | NA | | 0 |
| S9-final-viral-combined_k141_102444||lt2gene | 1314 | No | NA | 2 | 0 | Low-quality | Genome-fragment | | 4.1 | 0 |
| S1-final-viral-combined_k141_285537||full | 2217 | No | NA | 0 | 0 | Not-determined | Genome-fragment | NA | | 0 |
| S1-final-viral-combined_k141_268695||full | 1593 | No | NA | 0 | 1 | Not-determined | Genome-fragment | NA | | 0 |
| S1-final-viral-combined_k141_394078||full | 1314 | No | NA | 0 | 1 | Low-quality | Genome-fragment | | 0.5 | 0 |
| S1-final-viral-combined_k141_285542||full | 4714 | No | NA | 0 | 4 | Not-determined | Genome-fragment | NA | | 0 |
| S1-final-viral-combined_k141_236135||full | 2471 | No | NA | 0 | 1 | Not-determined | Genome-fragment | NA | | 0 |
| S7-final-viral-combined_k141_134799||lt2gene | 1314 | No | NA | 1 | 0 | Low-quality | Genome-fragment | | 2.46 | 0 |
| S1-final-viral-combined_k141_87549||full | 1506 | No | NA | 0 | 1 | Not-determined | Genome-fragment | NA | | 0 |
| S17-k141_194874 | 1314 | No | NA | 0 | 0 | Low-quality | Genome-fragment | | 2.98 | 0 |
| S1-final-viral-combined_k141_367877||full | 2093 | No | NA | 0 | 1 | Not-determined | Genome-fragment | NA | | 0 |
| S12-final-viral-combined_k141_270506||full | 1314 | No | NA | 1 | 0 | Low-quality | Genome-fragment | | 1.92 | 0 |
| S1-final-viral-combined_k141_351734||full | 1582 | No | NA | 0 | 0 | Not-determined | Genome-fragment | NA | | 0 |
| S1-final-viral-combined_k141_169566||full | 1132 | No | NA | 0 | 0 | Not-determined | Genome-fragment | NA | | 0 |
| S6-final-viral-combined_k141_1423||full | 1314 | No | NA | 2 | 0 | Low-quality | Genome-fragment | | 3.61 | 0 |
| S1-final-viral-combined_k141_253045||full | 4336 | No | NA | 0 | 2 | Not-determined | Genome-fragment | NA | | 0 |
| S1-final-viral-combined_k141_5600||full | 5930 | No | NA | 0 | 2 | Not-determined | Genome-fragment | NA | | 0 |
| S1-final-viral-combined_k141_38542||full | 2213 | No | NA | 0 | 0 | Not-determined | Genome-fragment | NA | | 0 |
| S6-final-viral-combined_k141_48055||full | 1314 | No | NA | 1 | 0 | Low-quality | Genome-fragment | | 4.5 | 0 |
| S1-final-viral-combined_k141_236184||full | 2146 | No | NA | 0 | 2 | Not-determined | Genome-fragment | NA | | 0 |
| S8-final-viral-combined_k141_49724||full | 1314 | No | NA | 0 | 0 | Low-quality | Genome-fragment | | 2.33 | 0 |
| S8-final-viral-combined_k141_125660||full | 1314 | No | NA | 0 | 1 | Low-quality | Genome-fragment | | 1.76 | 0 |
| S1-final-viral-combined_k141_268766||full | 1486 | No | NA | 0 | 0 | Not-determined | Genome-fragment | NA | | 0 |
| S1-final-viral-combined_k141_5639||full | 2542 | No | NA | 0 | 1 | Not-determined | Genome-fragment | NA | | 0 |
| S1-final-viral-combined_k141_203505||full | 2110 | No | NA | 0 | 0 | Not-determined | Genome-fragment | NA | | 0 |
| S1-final-viral-combined_k141_87637||full | 12267 | No | NA | 0 | 0 | Not-determined | Genome-fragment | NA | | 0 |
| S1-final-viral-combined_k141_367935||full | 4324 | No | NA | 0 | 3 | Not-determined | Genome-fragment | NA | | 0 |
| S8_k141_274072 | 1314 | No | NA | 0 | 2 | Low-quality | Genome-fragment | | 0.36 | 0 |
| S9-final-viral-combined_k141_95017||full | 1315 | No | NA | 0 | 0 | Low-quality | Genome-fragment | | 3.48 | 0 |
| S1-final-viral-combined_k141_187378||full | 22827 | No | NA | 0 | 10 | Not-determined | Genome-fragment | NA | | 0 |
| S1-final-viral-combined_k141_118882||full | 1315 | No | NA | 0 | 0 | Low-quality | Genome-fragment | | 3.16 | 0 |
| S1-final-viral-combined_k141_91841||full | 1315 | No | NA | 0 | 1 | Low-quality | Genome-fragment | | 5.37 | 0 |
| S1-final-viral-combined_k141_351784||full | 2729 | No | NA | 0 | 1 | Not-determined | Genome-fragment | NA | | 0 |
| S1-final-viral-combined_k141_390523||full | 1315 | No | NA | 0 | 0 | Low-quality | Genome-fragment | | 3.01 | 0 |
| S1_k141_385192 | 1315 | No | NA | 0 | 0 | Low-quality | Genome-fragment | | 3.3 | 0 |
| S1-final-viral-combined_k141_5704||full | 2601 | No | NA | 0 | 2 | Not-determined | Genome-fragment | NA | | 0 |
| S5-final-viral-combined_k141_191946||full | 1315 | No | NA | 0 | 0 | Low-quality | Genome-fragment | | 0.78 | 0 |
| S1-final-viral-combined_k141_203547||full | 1402 | No | NA | 0 | 2 | Not-determined | Genome-fragment | NA | | 0 |
| S1-final-viral-combined_k141_187445||full | 2614 | No | NA | 0 | 1 | Not-determined | Genome-fragment | NA | | 0 |
| S1-final-viral-combined_k141_38627||full | 3864 | No | NA | 0 | 2 | Not-determined | Genome-fragment | NA | | 0 |
| S7-final-viral-combined_k141_165859||full | 1315 | No | NA | 0 | 0 | Low-quality | Genome-fragment | | 1.28 | 0 |
| S7-final-viral-combined_k141_78292||full | 1315 | No | NA | 0 | 0 | Low-quality | Genome-fragment | | 1.18 | 0 |
| S17-final-viral-combined_k141_197067||full | 1315 | No | NA | 0 | 2 | Low-quality | Genome-fragment | | 0.45 | 0 |
| S17-final-viral-combined_k141_158318||lt2gene | 1315 | No | NA | 1 | 0 | Low-quality | Genome-fragment | | 2.51 | 0 |
| S12-final-viral-combined_k141_143363||full | 1315 | No | NA | 0 | 2 | Low-quality | Genome-fragment | | 1.62 | 0 |
| S12-final-viral-combined_k141_133597||full | 1315 | No | NA | 3 | 0 | Low-quality | Genome-fragment | | 3.07 | 0 |
| S12_k141_381962 | 1315 | No | NA | 0 | 1 | Low-quality | Genome-fragment | | 0.89 | 0 |
| S1-final-viral-combined_k141_203572||full | 1600 | No | NA | 0 | 0 | Not-determined | Genome-fragment | NA | | 0 |
| S2-final-viral-combined_k141_198067||lt2gene | 1315 | No | NA | 2 | 0 | Low-quality | Genome-fragment | | 3.35 | 0 |
| S4-final-viral-combined_k141_49645||lt2gene | 1315 | No | NA | 1 | 0 | Low-quality | Genome-fragment | | 2.12 | 0 |
| S6-final-viral-combined_k141_135161||lt2gene | 1315 | No | NA | 1 | 0 | Low-quality | Genome-fragment | | 2.09 | 0 |
| S9-final-viral-combined_k141_88245||full | 1316 | No | NA | 0 | 0 | Low-quality | Genome-fragment | | 2.17 | 0 |
| S1-final-viral-combined_k141_365002||full | 1316 | No | NA | 0 | 0 | Low-quality | Genome-fragment | | 1.27 | 0 |
| S1-final-viral-combined_k141_253159||full | 4554 | No | NA | 0 | 4 | Not-determined | Genome-fragment | NA | | 0 |
| S1-final-viral-combined_k141_371321||full | 1316 | No | NA | 1 | 0 | Low-quality | Genome-fragment | | 2.76 | 0 |
| S1-final-viral-combined_k141_89087||lt2gene | 1316 | No | NA | 1 | 0 | Low-quality | Genome-fragment | | 3.43 | 0 |
| S1-final-viral-combined_k141_153727||full | 2103 | No | NA | 0 | 0 | Not-determined | Genome-fragment | NA | | 0 |
| S5-final-viral-combined_k141_98759||full | 1316 | No | NA | 1 | 0 | Low-quality | Genome-fragment | | 3.73 | 0 |
| S1-final-viral-combined_k141_351889||full | 32026 | No | NA | 12 | 0 | Medium-quality | Genome-fragment | | 77.09 | 0 |
| S1-final-viral-combined_k141_236291||full | 2950 | No | NA | 0 | 0 | Not-determined | Genome-fragment | NA | | 0 |
| S5-final-viral-combined_k141_121774||lt2gene | 1316 | No | NA | 1 | 0 | Low-quality | Genome-fragment | | 2.02 | 0 |
| S12-final-viral-combined_k141_325121||lt2gene | 1316 | No | NA | 1 | 0 | Low-quality | Genome-fragment | | 3.02 | 0 |
| S12-final-viral-combined_k141_60918||lt2gene | 1316 | No | NA | 1 | 0 | Low-quality | Genome-fragment | | 2.92 | 0 |
| S2-final-viral-combined_k141_30630||full | 1316 | No | NA | 0 | 0 | Low-quality | Genome-fragment | | 3.36 | 0 |
| S1-final-viral-combined_k141_153776||full | 1594 | No | NA | 0 | 1 | Not-determined | Genome-fragment | NA | | 0 |
| S1-final-viral-combined_k141_87808||full | 2527 | No | NA | 0 | 0 | Not-determined | Genome-fragment | NA | | 0 |
| S9-final-viral-combined_k141_71354||full | 1317 | No | NA | 0 | 1 | Low-quality | Genome-fragment | | 1.08 | 0 |
| S9-final-viral-combined_k141_51280||full | 1317 | No | NA | 1 | 0 | Low-quality | Genome-fragment | | 3.8 | 0 |
| S1-final-viral-combined_k141_351941||full | 2048 | No | NA | 0 | 1 | Not-determined | Genome-fragment | NA | | 0 |
| S11-final-viral-combined_k141_34700||full | 1317 | No | NA | 0 | 1 | Low-quality | Genome-fragment | | 0.48 | 0 |
| S1-final-viral-combined_k141_169754||full | 1323 | No | NA | 0 | 0 | Not-determined | Genome-fragment | NA | | 0 |
| S11-final-viral-combined_k141_66763||full | 1317 | No | NA | 0 | 0 | Low-quality | Genome-fragment | | 2.33 | 0 |
| S1-final-viral-combined_k141_351948||full | 1820 | No | NA | 0 | 1 | Not-determined | Genome-fragment | NA | | 0 |
| S11-final-viral-combined_k141_91892||lt2gene | 1317 | No | NA | 1 | 0 | Low-quality | Genome-fragment | | 3.09 | 0 |
| S1-final-viral-combined_k141_5832||full | 2233 | No | NA | 0 | 2 | Not-determined | Genome-fragment | NA | | 0 |
| S1-final-viral-combined_k141_169788||full | 4712 | No | NA | 0 | 0 | Medium-quality | Genome-fragment | | 57.96 | 0 |
| S1-final-viral-combined_k141_351965||full | 1532 | No | NA | 0 | 1 | Not-determined | Genome-fragment | NA | | 0 |
| S5-final-viral-combined_k141_66535||full | 1317 | No | NA | 0 | 1 | Low-quality | Genome-fragment | | 0.53 | 0 |
| S1-final-viral-combined_k141_169801||full | 3254 | No | NA | 0 | 2 | Not-determined | Genome-fragment | NA | | 0 |
| S1-final-viral-combined_k141_253248||full | 2245 | No | NA | 0 | 1 | Not-determined | Genome-fragment | NA | | 0 |
| S1-final-viral-combined_k141_253254||full | 4196 | No | NA | 0 | 3 | Not-determined | Genome-fragment | NA | | 0 |
| S7_k141_74041 | 1317 | No | NA | 0 | 3 | Low-quality | Genome-fragment | | 0.43 | 0 |
| S1-final-viral-combined_k141_153900||full | 1446 | No | NA | 0 | 1 | Not-determined | Genome-fragment | NA | | 0 |
| S7_k141_138166 | 1317 | No | NA | 0 | 0 | Low-quality | Genome-fragment | | 1.26 | 0 |
| S17-final-viral-combined_k141_14555||full | 1317 | No | NA | 0 | 2 | Low-quality | Genome-fragment | | 0.98 | 0 |
| S17-final-viral-combined_k141_92388||full | 1317 | No | NA | 0 | 1 | Low-quality | Genome-fragment | | 0.43 | 0 |
| S12-final-viral-combined_k141_210535||full | 1317 | No | NA | 0 | 2 | Low-quality | Genome-fragment | | 1.03 | 0 |
| S1-final-viral-combined_k141_169835||full | 11791 | No | NA | 0 | 5 | Not-determined | Genome-fragment | NA | | 0 |
| S12-final-viral-combined_k141_260738||full | 1317 | No | NA | 0 | 0 | Low-quality | Genome-fragment | | 3.05 | 0 |
| S1-final-viral-combined_k141_236437||full | 1566 | No | NA | 0 | 1 | Not-determined | Genome-fragment | NA | | 0 |
| S1-final-viral-combined_k141_87928||full | 1475 | No | NA | 0 | 1 | Not-determined | Genome-fragment | NA | | 0 |
| S4-final-viral-combined_k141_36983||full | 1317 | No | NA | 0 | 0 | Low-quality | Genome-fragment | | 1.38 | 0 |
| S1-final-viral-combined_k141_87929||full | 2517 | No | NA | 0 | 1 | Not-determined | Genome-fragment | NA | | 0 |
| S1-final-viral-combined_k141_317014||full | 1318 | No | NA | 1 | 0 | Low-quality | Genome-fragment | | 1.08 | 0 |
| S1-final-viral-combined_k141_269061||full | 2738 | No | NA | 0 | 1 | Not-determined | Genome-fragment | NA | | 0 |
| S1-final-viral-combined_k141_87935||full | 1821 | No | NA | 0 | 1 | Not-determined | Genome-fragment | NA | | 0 |
| S1-final-viral-combined_k141_203729||full | 1510 | No | NA | 0 | 3 | Not-determined | Genome-fragment | NA | | 0 |
| S1-final-viral-combined_k141_220022||full | 155946 | No | NA | 4 | 20 | High-quality | High-quality | | 100 | 0 |
| S1-final-viral-combined_k141_253316||full | 3459 | No | NA | 0 | 2 | Not-determined | Genome-fragment | NA | | 0 |
| S1-final-viral-combined_k141_169852||full | 3449 | No | NA | 0 | 2 | Not-determined | Genome-fragment | NA | | 0 |
| S1-final-viral-combined_k141_203744||full | 2729 | No | NA | 0 | 3 | Not-determined | Genome-fragment | NA | | 0 |
| S1-final-viral-combined_k141_253319||full | 2988 | No | NA | 0 | 1 | Not-determined | Genome-fragment | NA | | 0 |
| S1-final-viral-combined_k141_269073||full | 7099 | No | NA | 0 | 2 | Not-determined | Genome-fragment | NA | | 0 |
| S5-final-viral-combined_k141_208913||lt2gene | 1318 | No | NA | 1 | 0 | Low-quality | Genome-fragment | | 0.81 | 0 |
| S5_k141_179658 | 1318 | No | NA | 0 | 0 | Low-quality | Genome-fragment | | 2.2 | 0 |
| S1-final-viral-combined_k141_38861||full | 4142 | No | NA | 0 | 4 | Not-determined | Genome-fragment | NA | | 0 |
| S7-final-viral-combined_k141_110756||full | 1318 | No | NA | 0 | 1 | Low-quality | Genome-fragment | | 0.44 | 0 |
| S1-final-viral-combined_k141_38889||full | 8266 | No | NA | 0 | 6 | Not-determined | Genome-fragment | NA | | 0 |
| S4-final-viral-combined_k141_4318||full | 1318 | No | NA | 0 | 2 | Low-quality | Genome-fragment | | 0.42 | 0 |
| S1-final-viral-combined_k141_169879||full | 32857 | No | NA | 17 | 0 | Medium-quality | Genome-fragment | | 51.42 | 0 |
| S1-final-viral-combined_k141_220063||full | 3879 | No | NA | 0 | 1 | Not-determined | Genome-fragment | NA | | 0 |
| S1-final-viral-combined_k141_187538||full | 1882 | No | NA | 0 | 1 | Not-determined | Genome-fragment | NA | | 0 |
| S1-final-viral-combined_k141_269146||full | 1854 | No | NA | 0 | 0 | Not-determined | Genome-fragment | NA | | 0 |
| S1-final-viral-combined_k141_104034||full | 3008 | No | NA | 0 | 3 | Not-determined | Genome-fragment | NA | | 0 |
| S1-final-viral-combined_k141_121817||full | 1603 | No | NA | 0 | 0 | Not-determined | Genome-fragment | NA | | 0 |
| S1-final-viral-combined_k141_285847||full | 3006 | No | NA | 0 | 0 | Not-determined | Genome-fragment | NA | | 0 |
| S1-final-viral-combined_k141_269160||full | 1653 | No | NA | 0 | 2 | Not-determined | Genome-fragment | NA | | 0 |
| S4_k141_131788 | 1318 | No | NA | 0 | 2 | Low-quality | Genome-fragment | | 0.48 | 0 |
| S1-final-viral-combined_k141_220103||full | 3239 | No | NA | 0 | 3 | Not-determined | Genome-fragment | NA | | 0 |
| S1-final-viral-combined_k141_285855||full | 6341 | No | NA | 0 | 4 | Not-determined | Genome-fragment | NA | | 0 |
| S1-final-viral-combined_k141_334609||full | 2774 | No | NA | 0 | 3 | Not-determined | Genome-fragment | NA | | 0 |
| S8-final-viral-combined_k141_211647||full | 1318 | No | NA | 0 | 1 | Low-quality | Genome-fragment | | 1.05 | 0 |
| S1-final-viral-combined_k141_55191||full | 3078 | No | NA | 0 | 3 | Not-determined | Genome-fragment | NA | | 0 |
| S1-final-viral-combined_k141_22266||full | 1677 | No | NA | 0 | 3 | Not-determined | Genome-fragment | NA | | 0 |
| S1-final-viral-combined_k141_285876||full | 1519 | No | NA | 0 | 1 | Not-determined | Genome-fragment | NA | | 0 |
| S8_k141_226956 | 1318 | No | NA | 1 | 0 | Low-quality | Genome-fragment | | 1.34 | 0 |
| S1-final-viral-combined_k141_302982||full | 1338 | No | NA | 0 | 0 | Not-determined | Genome-fragment | NA | | 0 |
| S9-final-viral-combined_k141_26643||full | 1319 | No | NA | 0 | 2 | Low-quality | Genome-fragment | | 0.75 | 0 |
| S1-final-viral-combined_k141_380623||full | 1319 | No | NA | 0 | 0 | Low-quality | Genome-fragment | | 2.8 | 0 |
| S1-final-viral-combined_k141_55231||full | 1510 | No | NA | 0 | 1 | Not-determined | Genome-fragment | NA | | 0 |
| S1-final-viral-combined_k141_227494||full | 1319 | No | NA | 0 | 0 | Low-quality | Genome-fragment | | 2.55 | 0 |
| S1-final-viral-combined_k141_303005||full | 1804 | No | NA | 0 | 3 | Not-determined | Genome-fragment | NA | | 0 |
| S1-final-viral-combined_k141_310886||full | 1319 | No | NA | 0 | 0 | Low-quality | Genome-fragment | | 1.82 | 0 |
| S5-final-viral-combined_k141_179838||full | 1319 | No | NA | 0 | 0 | Low-quality | Genome-fragment | | 1.6 | 0 |
| S1-final-viral-combined_k141_121860||full | 2148 | No | NA | 0 | 1 | Not-determined | Genome-fragment | NA | | 0 |
| S1-final-viral-combined_k141_319432||full | 1583 | No | NA | 0 | 1 | Not-determined | Genome-fragment | NA | | 0 |
| S1-final-viral-combined_k141_104104||full | 1058 | No | NA | 0 | 1 | Not-determined | Genome-fragment | NA | | 0 |
| S12-final-viral-combined_k141_83790||full | 1319 | No | NA | 0 | 0 | Low-quality | Genome-fragment | | 2.57 | 0 |
| S1-final-viral-combined_k141_71089||full | 3325 | No | NA | 0 | 3 | Not-determined | Genome-fragment | NA | | 0 |
| S1-final-viral-combined_k141_319448||full | 1594 | No | NA | 0 | 0 | Not-determined | Genome-fragment | NA | | 0 |
| S1-final-viral-combined_k141_352221||full | 1852 | No | NA | 0 | 2 | Not-determined | Genome-fragment | NA | | 0 |
| S12-final-viral-combined_k141_45300||full | 1319 | No | NA | 1 | 0 | Low-quality | Genome-fragment | | 2.59 | 0 |
| S1-final-viral-combined_k141_170003||full | 1178 | No | NA | 0 | 0 | Not-determined | Genome-fragment | NA | | 0 |
| S1-final-viral-combined_k141_22336||full | 1526 | No | NA | 0 | 0 | Not-determined | Genome-fragment | NA | | 0 |
| S12-final-viral-combined_k141_296597||full | 1319 | No | NA | 2 | 0 | Low-quality | Genome-fragment | | 2.15 | 0 |
| S1-final-viral-combined_k141_170016||full | 1693 | No | NA | 0 | 2 | Not-determined | Genome-fragment | NA | | 0 |
| S12-final-viral-combined_k141_214850||full | 1319 | No | NA | 0 | 0 | Low-quality | Genome-fragment | | 2.91 | 0 |

| | | | | | | | | | | | |
|---|---|---|---|---|---|---|---|---|---|---|---|
| S12-final-viral-combined_k141_137041\|\|lt2gene | 1319 | No | NA | | 1 | 0 | Low-quality | Genome-fragment | | 2.76 | 0 |
| S12-final-viral-combined_k141_275829\|\|lt2gene | 1319 | No | NA | | 1 | 0 | Low-quality | Genome-fragment | | 2.98 | 0 |
| S1-final-viral-combined_k141_88053\|\|full | 2156 | No | NA | | 0 | 2 | Not-determined | Genome-fragment | NA | | 0 |
| S4-final-viral-combined_k141_114974\|\|full | 1319 | No | NA | | 2 | 0 | Low-quality | Genome-fragment | | 2.2 | 0 |
| S6_k141_94898 | 1319 | No | NA | | 0 | 0 | Low-quality | Genome-fragment | | 0.93 | 0 |
| S1-final-viral-combined_k141_71163\|\|full | 1368 | No | NA | | 0 | 1 | Not-determined | Genome-fragment | NA | | 0 |
| S8_k141_107882 | 1319 | No | NA | | 0 | 0 | Low-quality | Genome-fragment | | 2.17 | 0 |
| S1-final-viral-combined_k141_269281\|\|full | 1014 | No | NA | | 0 | 1 | Not-determined | Genome-fragment | NA | | 0 |
| S9-final-viral-combined_k141_46890\|\|full | 1320 | No | NA | | 0 | 1 | Low-quality | Genome-fragment | | 1.56 | 0 |
| S1-final-viral-combined_k141_104184\|\|full | 3411 | No | NA | | 0 | 1 | Not-determined | Genome-fragment | NA | | 0 |
| S1-final-viral-combined_k141_334743\|\|full | 2502 | No | NA | | 0 | 0 | Not-determined | Genome-fragment | NA | | 0 |
| S11-final-viral-combined_k141_133912\|\|lt2gene | 1320 | No | NA | | 1 | 0 | Low-quality | Genome-fragment | | 1.96 | 0 |
| S1-final-viral-combined_k141_154040\|\|full | 4254 | No | NA | | 0 | 1 | Not-determined | Genome-fragment | NA | | 0 |
| S1-final-viral-combined_k141_22445\|\|full | 3909 | No | NA | | 0 | 1 | Not-determined | Genome-fragment | NA | | 0 |
| S1-final-viral-combined_k141_253629\|\|full | 2093 | No | NA | | 0 | 1 | Not-determined | Genome-fragment | NA | | 0 |
| S1-final-viral-combined_k141_286070\|\|full | 1951 | No | NA | | 0 | 2 | Not-determined | Genome-fragment | NA | | 0 |
| S1-final-viral-combined_k141_253642\|\|full | 1489 | No | NA | | 0 | 2 | Not-determined | Genome-fragment | NA | | 0 |
| S1_k141_291681 | 1320 | No | NA | | 1 | 0 | Low-quality | Genome-fragment | | 2.26 | 0 |
| S3-final-viral-combined_k141_59265\|\|lt2gene | 1320 | No | NA | | 1 | 0 | Low-quality | Genome-fragment | | 2.6 | 0 |
| S1-final-viral-combined_k141_352329\|\|full | 1448 | No | NA | | 0 | 1 | Not-determined | Genome-fragment | NA | | 0 |
| S7-final-viral-combined_k141_134296\|\|full | 1320 | No | NA | | 0 | 0 | Low-quality | Genome-fragment | | 3.15 | 0 |
| S1-final-viral-combined_k141_55415\|\|full | 2894 | No | NA | | 0 | 2 | Not-determined | Genome-fragment | NA | | 0 |
| S7-final-viral-combined_k141_155553\|\|lt2gene | 1320 | No | NA | | 1 | 0 | Low-quality | Genome-fragment | | 2.78 | 0 |
| S17-final-viral-combined_k141_222413\|\|lt2gene | 1320 | No | NA | | 1 | 0 | Low-quality | Genome-fragment | | 2.92 | 0 |
| S12-final-viral-combined_k141_138323\|\|full | 1320 | No | NA | | 0 | 0 | Low-quality | Genome-fragment | | 2.03 | 0 |
| S1-final-viral-combined_k141_6158\|\|full | 1751 | No | NA | | 0 | 2 | Not-determined | Genome-fragment | NA | | 0 |
| S1-final-viral-combined_k141_154109\|\|full | 2476 | No | NA | | 0 | 0 | Not-determined | Genome-fragment | NA | | 0 |
| S1-final-viral-combined_k141_352371\|\|full | 2414 | No | NA | | 0 | 2 | Not-determined | Genome-fragment | NA | | 0 |
| S8-final-viral-combined_k141_105590\|\|full | 1320 | No | NA | | 0 | 0 | Low-quality | Genome-fragment | | 0.45 | 0 |
| S1-final-viral-combined_k141_204097\|\|full | 1027 | No | NA | | 0 | 0 | Not-determined | Genome-fragment | NA | | 0 |
| S1-final-viral-combined_k141_154138\|\|full | 6770 | No | NA | | 0 | 4 | Not-determined | Genome-fragment | NA | | 0 |
| S1-final-viral-combined_k141_88224\|\|full | 2599 | No | NA | | 0 | 0 | Not-determined | Genome-fragment | NA | | 0 |
| S11-final-viral-combined_k141_171872\|\|full | 1321 | No | NA | | 0 | 1 | Low-quality | Genome-fragment | | 1.55 | 0 |
| S1-final-viral-combined_k141_170125\|\|full | 2362 | No | NA | | 0 | 3 | Not-determined | Genome-fragment | NA | | 0 |
| S11_k141_153219 | 1321 | No | NA | | 0 | 1 | Low-quality | Genome-fragment | | 0.36 | 0 |
| S1-final-viral-combined_k141_347072\|\|full | 1321 | No | NA | | 0 | 0 | Low-quality | Genome-fragment | | 3.06 | 0 |
| S1-final-viral-combined_k141_187867\|\|full | 1373 | No | NA | | 0 | 2 | Not-determined | Genome-fragment | NA | | 0 |
| S1-final-viral-combined_k141_334900\|\|full | 1360 | No | NA | | 0 | 1 | Not-determined | Genome-fragment | NA | | 0 |
| S1-final-viral-combined_k141_71304\|\|full | 3044 | No | NA | | 0 | 1 | Not-determined | Genome-fragment | NA | | 0 |
| S5-final-viral-combined_k141_56100\|\|full | 1321 | No | NA | | 1 | 0 | Low-quality | Genome-fragment | | 3.42 | 0 |
| S12-final-viral-combined_k141_293130\|\|full | 1321 | No | NA | | 0 | 1 | Low-quality | Genome-fragment | | 2.2 | 0 |
| S12_k141_366528 | 1321 | No | NA | | 0 | 1 | Low-quality | Genome-fragment | | 0.25 | 0 |
| S1-final-viral-combined_k141_236738\|\|full | 1035 | No | NA | | 0 | 0 | Not-determined | Genome-fragment | NA | | 0 |
| S2_k141_1430 | 1321 | No | NA | | 1 | 0 | Low-quality | Genome-fragment | | 3.19 | 0 |
| S1-final-viral-combined_k141_71342\|\|full | 1080 | No | NA | | 0 | 0 | Not-determined | Genome-fragment | NA | | 0 |
| S4_k141_62876 | 1321 | No | NA | | 0 | 0 | Low-quality | Genome-fragment | | 2.58 | 0 |
| S1-final-viral-combined_k141_269442\|\|full | 2346 | No | NA | | 0 | 2 | Not-determined | Genome-fragment | NA | | 0 |
| S1-final-viral-combined_k141_6241\|\|full | 1168 | No | NA | | 0 | 1 | Not-determined | Genome-fragment | NA | | 0 |
| S1-final-viral-combined_k141_71349\|\|full | 6290 | No | NA | | 0 | 5 | Not-determined | Genome-fragment | NA | | 0 |
| S6-final-viral-combined_k141_37217\|\|full | 1321 | No | NA | | 1 | 0 | Low-quality | Genome-fragment | | 4.44 | 0 |
| S11-final-viral-combined_k141_71780\|\|full | 1322 | No | NA | | 0 | 1 | Low-quality | Genome-fragment | | 1.96 | 0 |
| S1-final-viral-combined_k141_334958\|\|full | 3247 | No | NA | | 0 | 2 | Not-determined | Genome-fragment | NA | | 0 |
| S1-final-viral-combined_k141_6287\|\|full | 2265 | No | NA | | 0 | 2 | Not-determined | Genome-fragment | NA | | 0 |
| S11-final-viral-combined_k141_161250\|\|full | 1322 | No | NA | | 0 | 0 | Low-quality | Genome-fragment | | 1.92 | 0 |
| S1-final-viral-combined_k141_368164\|\|full | 5465 | No | NA | | 0 | 1 | Not-determined | Genome-fragment | NA | | 0 |
| S11-final-viral-combined_k141_320817\|\|lt2gene | 1322 | No | NA | | 2 | 0 | Low-quality | Genome-fragment | | 1.88 | 0 |
| S1-final-viral-combined_k141_138381\|\|full | 1960 | No | NA | | 0 | 2 | Not-determined | Genome-fragment | NA | | 0 |
| S11_k141_249518 | 1322 | No | NA | | 0 | 0 | Low-quality | Genome-fragment | | 2.64 | 0 |
| S1-final-viral-combined_k141_241671\|\|lt2gene | 1322 | No | NA | | 1 | 0 | Low-quality | Genome-fragment | | 2.16 | 0 |
| S1-final-viral-combined_k141_88337\|\|full | 2282 | No | NA | | 0 | 0 | Not-determined | Genome-fragment | NA | | 0 |
| S1-final-viral-combined_k141_138393\|\|full | 3534 | No | NA | | 0 | 0 | Not-determined | Genome-fragment | NA | | 0 |
| S7-final-viral-combined_k141_205879\|\|full | 1322 | No | NA | | 0 | 2 | Low-quality | Genome-fragment | | 0.78 | 0 |
| S1-final-viral-combined_k141_88380\|\|full | 1579 | No | NA | | 0 | 1 | Not-determined | Genome-fragment | NA | | 0 |
| S17-final-viral-combined_k141_239966\|\|full | 1322 | No | NA | | 0 | 0 | Low-quality | Genome-fragment | | 2.37 | 0 |
| S1-final-viral-combined_k141_204228\|\|full | 3133 | No | NA | | 0 | 2 | Not-determined | Genome-fragment | NA | | 0 |
| S4-final-viral-combined_k141_80025\|\|full | 1322 | No | NA | | 2 | 0 | Low-quality | Genome-fragment | | 2.41 | 0 |
| S1-final-viral-combined_k141_71457\|\|full | 2191 | No | NA | | 0 | 2 | Not-determined | Genome-fragment | NA | | 0 |
| S1-final-viral-combined_k141_71467\|\|full | 1335 | No | NA | | 0 | 1 | Not-determined | Genome-fragment | NA | | 0 |
| S1-final-viral-combined_k141_335033\|\|full | 3609 | No | NA | | 0 | 3 | Not-determined | Genome-fragment | NA | | 0 |
| S6-final-viral-combined_k141_27244\|\|full | 1322 | No | NA | | 0 | 0 | Low-quality | Genome-fragment | | 2.81 | 0 |
| S9_k141_77050 | 1323 | No | NA | | 0 | 0 | Low-quality | Genome-fragment | | 2.21 | 0 |
| S1-final-viral-combined_k141_188004\|\|full | 2333 | No | NA | | 0 | 1 | Not-determined | Genome-fragment | NA | | 0 |
| S1-final-viral-combined_k141_269559\|\|full | 3015 | No | NA | | 0 | 1 | Not-determined | Genome-fragment | NA | | 0 |
| S11-final-viral-combined_k141_195284\|\|full | 1323 | No | NA | | 1 | 0 | Low-quality | Genome-fragment | | 2.09 | 0 |
| S5-final-viral-combined_k141_173411\|\|full | 1323 | No | NA | | 0 | 0 | Low-quality | Genome-fragment | | 1.18 | 0 |
| S1-final-viral-combined_k141_319772\|\|full | 2371 | No | NA | | 0 | 3 | Not-determined | Genome-fragment | NA | | 0 |
| S1-final-viral-combined_k141_385001\|\|full | 3902 | No | NA | | 0 | 0 | Not-determined | Genome-fragment | NA | | 0 |
| S1-final-viral-combined_k141_253948\|\|full | 1905 | No | NA | | 0 | 1 | Not-determined | Genome-fragment | NA | | 0 |
| S5-final-viral-combined_k141_200211\|\|full | 1323 | No | NA | | 0 | 0 | Low-quality | Genome-fragment | | 2.97 | 0 |
| S1-final-viral-combined_k141_236949\|\|full | 2730 | No | NA | | 0 | 0 | Not-determined | Genome-fragment | NA | | 0 |
| S7-final-viral-combined_k141_164830\|\|full | 1323 | No | NA | | 0 | 1 | Low-quality | Genome-fragment | | 2.27 | 0 |
| S12-final-viral-combined_k141_314404\|\|lt2gene | 1323 | No | NA | | 1 | 0 | Low-quality | Genome-fragment | | 0.72 | 0 |
| S1-final-viral-combined_k141_236957\|\|full | 3898 | No | NA | | 0 | 3 | Not-determined | Genome-fragment | NA | | 0 |
| S12-final-viral-combined_k141_33182\|\|lt2gene | 1323 | No | NA | | 1 | 0 | Low-quality | Genome-fragment | | 0.91 | 0 |
| S1-final-viral-combined_k141_204356\|\|full | 1105 | No | NA | | 0 | 2 | Not-determined | Genome-fragment | NA | | 0 |
| S1-final-viral-combined_k141_236972\|\|full | 1777 | No | NA | | 0 | 0 | Not-determined | Genome-fragment | NA | | 0 |
| S17-final-viral-combined_k141_30320\|\|full | 1324 | No | NA | | 0 | 0 | Low-quality | Genome-fragment | | 2.73 | 0 |
| S1-final-viral-combined_k141_236997\|\|full | 3461 | No | NA | | 0 | 1 | Not-determined | Genome-fragment | NA | | 0 |
| S1-final-viral-combined_k141_269581\|\|full | 3561 | No | NA | | 0 | 2 | Not-determined | Genome-fragment | NA | | 0 |
| S12-final-viral-combined_k141_203407\|\|full | 1324 | No | NA | | 0 | 0 | Low-quality | Genome-fragment | | 1.25 | 0 |
| S1-final-viral-combined_k141_39344\|\|full | 1041 | No | NA | | 0 | 0 | Not-determined | Genome-fragment | NA | | 0 |
| S1-final-viral-combined_k141_368329\|\|full | 1188 | No | NA | | 0 | 1 | Not-determined | Genome-fragment | NA | | 0 |
| S1-final-viral-combined_k141_286280\|\|full | 2694 | No | NA | | 0 | 0 | Not-determined | Genome-fragment | NA | | 0 |
| S1-final-viral-combined_k141_22802\|\|full | 1482 | No | NA | | 0 | 1 | Not-determined | Genome-fragment | NA | | 0 |
| S1-final-viral-combined_k141_122415\|\|full | 3573 | No | NA | | 0 | 2 | Not-determined | Genome-fragment | NA | | 0 |
| S12_k141_398855 | 1324 | No | NA | | 0 | 2 | Low-quality | Genome-fragment | | 0.9 | 0 |
| S1-final-viral-combined_k141_55716\|\|full | 2494 | No | NA | | 0 | 0 | Not-determined | Genome-fragment | NA | | 0 |
| S1-final-viral-combined_k141_269609\|\|full | 1403 | No | NA | | 0 | 2 | Not-determined | Genome-fragment | NA | | 0 |
| S1-final-viral-combined_k141_220465\|\|full | 1161 | No | NA | | 0 | 0 | Not-determined | Genome-fragment | NA | | 0 |
| S2-final-viral-combined_k141_42847\|\|full | 1324 | No | NA | | 0 | 0 | Low-quality | Genome-fragment | | 3.7 | 0 |
| S1-final-viral-combined_k141_220467\|\|full | 1491 | No | NA | | 0 | 0 | Not-determined | Genome-fragment | NA | | 0 |
| S1-final-viral-combined_k141_269614\|\|full | 3384 | No | NA | | 0 | 0 | Not-determined | Genome-fragment | NA | | 0 |
| S2_k141_55909 | 1324 | No | NA | | 0 | 0 | Low-quality | Genome-fragment | | 2.13 | 0 |
| S1-final-viral-combined_k141_204444\|\|full | 3789 | No | NA | | 0 | 3 | Not-determined | Genome-fragment | NA | | 0 |
| S1-final-viral-combined_k141_6516\|\|full | 3905 | No | NA | | 0 | 1 | Not-determined | Genome-fragment | NA | | 0 |
| S1-final-viral-combined_k141_237077\|\|full | 3517 | No | NA | | 0 | 2 | Not-determined | Genome-fragment | NA | | 0 |
| S4-final-viral-combined_k141_93550\|\|full | 1324 | No | NA | | 0 | 1 | Low-quality | Genome-fragment | | 2.5 | 0 |
| S11-final-viral-combined_k141_216036\|\|full | 1325 | No | NA | | 3 | 0 | Low-quality | Genome-fragment | | 6.25 | 0 |
| S1-final-viral-combined_k141_55767\|\|full | 1984 | No | NA | | 0 | 0 | Not-determined | Genome-fragment | NA | | 0 |
| S1-final-viral-combined_k141_4446\|\|full | 1325 | No | NA | | 0 | 0 | Low-quality | Genome-fragment | | 0.62 | 0 |
| S1-final-viral-combined_k141_154436\|\|full | 1325 | No | NA | | 1 | 0 | Low-quality | Genome-fragment | | 2.37 | 0 |
| S1_k141_321268 | 1325 | No | NA | | 0 | 0 | Low-quality | Genome-fragment | | 2.92 | 0 |
| S1-final-viral-combined_k141_6542\|\|full | 1314 | No | NA | | 0 | 0 | Not-determined | Genome-fragment | NA | | 0 |
| S1-final-viral-combined_k141_319959\|\|full | 1412 | No | NA | | 0 | 1 | Not-determined | Genome-fragment | NA | | 0 |
| S3_k141_92733 | 1325 | No | NA | | 1 | 0 | Low-quality | Genome-fragment | | 0.61 | 0 |
| S1-final-viral-combined_k141_55824\|\|full | 1234 | No | NA | | 0 | 1 | Not-determined | Genome-fragment | NA | | 0 |
| S1-final-viral-combined_k141_71645\|\|full | 1476 | No | NA | | 0 | 0 | Not-determined | Genome-fragment | NA | | 0 |
| S5-final-viral-combined_k141_129521\|\|full | 1325 | No | NA | | 0 | 0 | Low-quality | Genome-fragment | | 1.73 | 0 |
| S17-k141_164207 | 1325 | No | NA | | 1 | 0 | Low-quality | Genome-fragment | | 1.95 | 0 |
| S4-final-viral-combined_k141_69730\|\|full | 1325 | No | NA | | 0 | 0 | Low-quality | Genome-fragment | | 0.77 | 0 |
| S1-final-viral-combined_k141_319988\|\|full | 2929 | No | NA | | 0 | 2 | Not-determined | Genome-fragment | NA | | 0 |
| S1-final-viral-combined_k141_39461\|\|full | 2406 | No | NA | | 0 | 2 | Not-determined | Genome-fragment | NA | | 0 |
| S1-final-viral-combined_k141_237175\|\|full | 3330 | No | NA | | 0 | 4 | Not-determined | Genome-fragment | NA | | 0 |
| S4-final-viral-combined_k141_55369\|\|full | 1325 | Yes | | 1053 | 1 | 1 | Low-quality | Genome-fragment | | 0.42 | 20.53 |
| S1-final-viral-combined_k141_279200\|\|lt2gene | 1326 | No | NA | | 0 | 0 | Low-quality | Genome-fragment | | 2.29 | 0 |
| S1-final-viral-combined_k141_154423\|\|full | 3502 | No | NA | | 0 | 0 | Not-determined | Genome-fragment | NA | | 0 |
| S12-final-viral-combined_k141_173211\|\|full | 1326 | No | NA | | 0 | 0 | Low-quality | Genome-fragment | | 2.81 | 0 |
| S1-final-viral-combined_k141_385327\|\|full | 1122 | No | NA | | 0 | 0 | Not-determined | Genome-fragment | NA | | 0 |
| S12-final-viral-combined_k141_6284\|\|full | 1326 | No | NA | | 0 | 0 | Low-quality | Genome-fragment | | 1.61 | 0 |
| S1-final-viral-combined_k141_335205\|\|full | 1369 | No | NA | | 0 | 0 | Not-determined | Genome-fragment | NA | | 0 |
| S1-final-viral-combined_k141_71697\|\|full | 2815 | No | NA | | 0 | 1 | Not-determined | Genome-fragment | NA | | 0 |
| S12-final-viral-combined_k141_195791\|\|lt2gene | 1326 | No | NA | | 1 | 0 | Low-quality | Genome-fragment | | 2.85 | 0 |
| S6-final-viral-combined_k141_119352\|\|full | 1326 | No | NA | | 0 | 0 | Low-quality | Genome-fragment | | 1.39 | 0 |
| S8-final-viral-combined_k141_125689\|\|full | 1326 | No | NA | | 0 | 0 | Low-quality | Genome-fragment | | 3.08 | 0 |
| S1-final-viral-combined_k141_204572\|\|full | 2335 | No | NA | | 0 | 1 | Not-determined | Genome-fragment | NA | | 0 |
| S1-final-viral-combined_k141_71711\|\|full | 2555 | No | NA | | 0 | 1 | Not-determined | Genome-fragment | NA | | 0 |
| S1-final-viral-combined_k141_385364\|\|full | 3816 | No | NA | | 0 | 1 | Not-determined | Genome-fragment | NA | | 0 |
| S1-final-viral-combined_k141_154463\|\|full | 1601 | No | NA | | 0 | 0 | Not-determined | Genome-fragment | NA | | 0 |
| S1-final-viral-combined_k141_335225\|\|full | 2051 | No | NA | | 0 | 3 | Not-determined | Genome-fragment | NA | | 0 |
| S1-final-viral-combined_k141_352880\|\|full | 3316 | No | NA | | 0 | 3 | Not-determined | Genome-fragment | NA | | 0 |
| S1-final-viral-combined_k141_320039\|\|full | 11688 | No | NA | | 0 | 1 | Not-determined | Genome-fragment | NA | | 0 |
| S9-final-viral-combined_k141_83619\|\|full | 1327 | No | NA | | 0 | 0 | Low-quality | Genome-fragment | | 3.3 | 0 |
| S1-final-viral-combined_k141_303562\|\|full | 1469 | No | NA | | 0 | 0 | Not-determined | Genome-fragment | NA | | 0 |
| S1-final-viral-combined_k141_138779\|\|full | 2099 | No | NA | | 0 | 1 | Not-determined | Genome-fragment | NA | | 0 |
| S1-final-viral-combined_k141_55885\|\|full | 1331 | No | NA | | 0 | 2 | Not-determined | Genome-fragment | NA | | 0 |
| S1-final-viral-combined_k141_237252\|\|full | 2423 | No | NA | | 0 | 2 | Not-determined | Genome-fragment | NA | | 0 |
| S9_k141_109611 | 1327 | No | NA | | 0 | 1 | Low-quality | Genome-fragment | | 2.22 | 0 |
| S1-final-viral-combined_k141_320070\|\|full | 2528 | No | NA | | 0 | 3 | Not-determined | Genome-fragment | NA | | 0 |
| S1-final-viral-combined_k141_88754\|\|full | 1340 | No | NA | | 0 | 1 | Not-determined | Genome-fragment | NA | | 0 |
| S11-final-viral-combined_k141_292739\|\|full | 1327 | No | NA | | 0 | 0 | Low-quality | Genome-fragment | | 3.27 | 0 |
| S11-final-viral-combined_k141_41873\|\|lt2gene | 1327 | No | NA | | 1 | 0 | Low-quality | Genome-fragment | | 2.75 | 0 |
| S3-final-viral-combined_k141_28006\|\|full | 1327 | No | NA | | 0 | 1 | Low-quality | Genome-fragment | | 1.22 | 0 |
| S1-final-viral-combined_k141_71754\|\|full | 8817 | No | NA | | 0 | 0 | Not-determined | Genome-fragment | NA | | 0 |
| S1-final-viral-combined_k141_368528\|\|full | 3359 | No | NA | | 0 | 0 | Not-determined | Genome-fragment | NA | | 0 |
| S5-final-viral-combined_k141_100193\|\|full | 1327 | No | NA | | 0 | 0 | Low-quality | Genome-fragment | | 2.19 | 0 |
| S1-final-viral-combined_k141_104641\|\|full | 5201 | No | NA | | 0 | 4 | Not-determined | Genome-fragment | NA | | 0 |
| S17-final-viral-combined_k141_113062\|\|full | 1327 | No | NA | | 0 | 0 | Low-quality | Genome-fragment | | 3.89 | 0 |
| S1-final-viral-combined_k141_204651\|\|full | 1418 | No | NA | | 0 | 0 | Not-determined | Genome-fragment | NA | | 0 |
| S1-final-viral-combined_k141_254239\|\|full | 1270 | No | NA | | 0 | 1 | Not-determined | Genome-fragment | NA | | 0 |
| S1-final-viral-combined_k141_55914\|\|full | 1016 | No | NA | | 0 | 1 | Not-determined | Genome-fragment | NA | | 0 |
| S12-final-viral-combined_k141_254145\|\|full | 1327 | No | NA | | 0 | 0 | Low-quality | Genome-fragment | | 1.89 | 0 |
| S12_k141_316447 | 1327 | No | NA | | 0 | 0 | Low-quality | Genome-fragment | | 0.71 | 0 |
| S1-final-viral-combined_k141_88783\|\|full | 4582 | No | NA | | 0 | 3 | Not-determined | Genome-fragment | NA | | 0 |
| S1-final-viral-combined_k141_138826\|\|full | 12830 | No | NA | | 0 | 11 | Not-determined | Genome-fragment | NA | | 0 |
| S1-final-viral-combined_k141_6714\|\|full | 1729 | No | NA | | 0 | 1 | Not-determined | Genome-fragment | NA | | 0 |
| S1-final-viral-combined_k141_269868\|\|full | 1295 | No | NA | | 0 | 1 | Not-determined | Genome-fragment | NA | | 0 |
| S1-final-viral-combined_k141_122680\|\|full | 1316 | No | NA | | 0 | 0 | Not-determined | Genome-fragment | NA | | 0 |
| S1-final-viral-combined_k141_23049\|\|full | 2383 | No | NA | | 0 | 2 | Not-determined | Genome-fragment | NA | | 0 |
| S1-final-viral-combined_k141_23065\|\|full | 1347 | No | NA | | 0 | 0 | Not-determined | Genome-fragment | NA | | 0 |
| S4_k141_128961 | 1327 | No | NA | | 1 | 0 | Low-quality | Genome-fragment | | 1.11 | 0 |
| S1-final-viral-combined_k141_104689\|\|full | 2863 | No | NA | | 0 | 0 | Not-determined | Genome-fragment | NA | | 0 |
| S1-final-viral-combined_k141_320142\|\|full | 1054 | No | NA | | 0 | 1 | Not-determined | Genome-fragment | NA | | 0 |
| S1-final-viral-combined_k141_320146\|\|full | 2087 | No | NA | | 0 | 1 | Not-determined | Genome-fragment | NA | | 0 |
| S1-final-viral-combined_k141_286411\|\|full | 1822 | No | NA | | 0 | 3 | Not-determined | Genome-fragment | NA | | 0 |

<table>
<tr><td></td><td></td><td></td><td></td><td></td><td></td><td></td><td></td><td></td><td></td></tr>
<tr><td>S8_k141_271</td><td>1327</td><td>No</td><td>NA</td><td>0</td><td>0</td><td>Low-quality</td><td>Genome-fragment</td><td>2.15</td><td>0</td></tr>
<tr><td>S1-final-viral-combined_k141_170521||full</td><td>2425</td><td>No</td><td>NA</td><td>0</td><td>3</td><td>Not-determined</td><td>Genome-fragment</td><td>NA</td><td>0</td></tr>
<tr><td>S9-final-viral-combined_k141_18028||full</td><td>1328</td><td>No</td><td>NA</td><td>1</td><td>0</td><td>Low-quality</td><td>Genome-fragment</td><td>2.89</td><td>0</td></tr>
<tr><td>S1-final-viral-combined_k141_170529||full</td><td>6376</td><td>No</td><td>NA</td><td>0</td><td>4</td><td>Not-determined</td><td>Genome-fragment</td><td>NA</td><td>0</td></tr>
<tr><td>S1-final-viral-combined_k141_220777||full</td><td>1798</td><td>No</td><td>NA</td><td>0</td><td>1</td><td>Not-determined</td><td>Genome-fragment</td><td>NA</td><td>0</td></tr>
<tr><td>S1-final-viral-combined_k141_104716||full</td><td>2363</td><td>No</td><td>NA</td><td>0</td><td>2</td><td>Not-determined</td><td>Genome-fragment</td><td>NA</td><td>0</td></tr>
<tr><td>S11-final-viral-combined_k141_172357||full</td><td>1328</td><td>No</td><td>NA</td><td>0</td><td>0</td><td>Low-quality</td><td>Genome-fragment</td><td>1.77</td><td>0</td></tr>
<tr><td>S1-final-viral-combined_k141_170552||full</td><td>7229</td><td>No</td><td>NA</td><td>0</td><td>0</td><td>Not-determined</td><td>Genome-fragment</td><td>NA</td><td>0</td></tr>
<tr><td>S11-final-viral-combined_k141_254981||full</td><td>1328</td><td>No</td><td>NA</td><td>0</td><td>0</td><td>Low-quality</td><td>Genome-fragment</td><td>1.17</td><td>0</td></tr>
<tr><td>S1-final-viral-combined_k141_368653||full</td><td>1596</td><td>No</td><td>NA</td><td>0</td><td>0</td><td>Not-determined</td><td>Genome-fragment</td><td>NA</td><td>0</td></tr>
<tr><td>S11_k141_256167</td><td>1328</td><td>No</td><td>NA</td><td>1</td><td>0</td><td>Low-quality</td><td>Genome-fragment</td><td>1.53</td><td>0</td></tr>
<tr><td>S1-final-viral-combined_k141_56006||full</td><td>1810</td><td>No</td><td>NA</td><td>0</td><td>1</td><td>Not-determined</td><td>Genome-fragment</td><td>NA</td><td>0</td></tr>
<tr><td>S1-final-viral-combined_k141_56014||full</td><td>1565</td><td>No</td><td>NA</td><td>0</td><td>2</td><td>Not-determined</td><td>Genome-fragment</td><td>NA</td><td>0</td></tr>
<tr><td>S3-final-viral-combined_k141_134054||full</td><td>1328</td><td>No</td><td>NA</td><td>0</td><td>0</td><td>Low-quality</td><td>Genome-fragment</td><td>3.43</td><td>0</td></tr>
<tr><td>S1-final-viral-combined_k141_122761||full</td><td>9263</td><td>No</td><td>NA</td><td>0</td><td>5</td><td>Not-determined</td><td>Genome-fragment</td><td>NA</td><td>0</td></tr>
<tr><td>S1-final-viral-combined_k141_254315||full</td><td>1057</td><td>No</td><td>NA</td><td>0</td><td>1</td><td>Not-determined</td><td>Genome-fragment</td><td>NA</td><td>0</td></tr>
<tr><td>S7-final-viral-combined_k141_77223||full</td><td>1328</td><td>No</td><td>NA</td><td>0</td><td>1</td><td>Low-quality</td><td>Genome-fragment</td><td>0.73</td><td>0</td></tr>
<tr><td>S12-final-viral-combined_k141_40353||full</td><td>1328</td><td>No</td><td>NA</td><td>1</td><td>0</td><td>Low-quality</td><td>Genome-fragment</td><td>3.84</td><td>0</td></tr>
<tr><td>S1-final-viral-combined_k141_56069||full</td><td>2100</td><td>No</td><td>NA</td><td>0</td><td>1</td><td>Not-determined</td><td>Genome-fragment</td><td>NA</td><td>0</td></tr>
<tr><td>S12-final-viral-combined_k141_394747||full</td><td>1328</td><td>No</td><td>NA</td><td>0</td><td>0</td><td>Low-quality</td><td>Genome-fragment</td><td>2.57</td><td>0</td></tr>
<tr><td>S1-final-viral-combined_k141_88935||full</td><td>1795</td><td>No</td><td>NA</td><td>0</td><td>0</td><td>Not-determined</td><td>Genome-fragment</td><td>NA</td><td>0</td></tr>
<tr><td>S2-final-viral-combined_k141_132507||full</td><td>1328</td><td>No</td><td>NA</td><td>0</td><td>1</td><td>Low-quality</td><td>Genome-fragment</td><td>1.09</td><td>0</td></tr>
<tr><td>S11-final-viral-combined_k141_269216||full</td><td>1329</td><td>No</td><td>NA</td><td>0</td><td>1</td><td>Low-quality</td><td>Genome-fragment</td><td>2.62</td><td>0</td></tr>
<tr><td>S1-final-viral-combined_k141_220828||full</td><td>1551</td><td>No</td><td>NA</td><td>0</td><td>1</td><td>Not-determined</td><td>Genome-fragment</td><td>NA</td><td>0</td></tr>
<tr><td>S11_k141_225105</td><td>1329</td><td>No</td><td>NA</td><td>0</td><td>1</td><td>Low-quality</td><td>Genome-fragment</td><td>2.17</td><td>0</td></tr>
<tr><td>S1-final-viral-combined_k141_39616||full</td><td>1892</td><td>No</td><td>NA</td><td>0</td><td>1</td><td>Not-determined</td><td>Genome-fragment</td><td>NA</td><td>0</td></tr>
<tr><td>S1-final-viral-combined_k141_320324||full</td><td>2336</td><td>No</td><td>NA</td><td>0</td><td>3</td><td>Not-determined</td><td>Genome-fragment</td><td>NA</td><td>0</td></tr>
<tr><td>S1-final-viral-combined_k141_181133||full</td><td>1329</td><td>No</td><td>NA</td><td>0</td><td>2</td><td>Low-quality</td><td>Genome-fragment</td><td>1</td><td>0</td></tr>
<tr><td>S1-final-viral-combined_k141_353113||full</td><td>2736</td><td>No</td><td>NA</td><td>0</td><td>1</td><td>Not-determined</td><td>Genome-fragment</td><td>NA</td><td>0</td></tr>
<tr><td>S1-final-viral-combined_k141_320339||full</td><td>1584</td><td>No</td><td>NA</td><td>0</td><td>1</td><td>Not-determined</td><td>Genome-fragment</td><td>NA</td><td>0</td></tr>
<tr><td>S1-final-viral-combined_k141_39641||full</td><td>4304</td><td>No</td><td>NA</td><td>0</td><td>3</td><td>Not-determined</td><td>Genome-fragment</td><td>NA</td><td>0</td></tr>
<tr><td>S1-final-viral-combined_k141_353591||lt2gene</td><td>1329</td><td>No</td><td>NA</td><td>1</td><td>0</td><td>Low-quality</td><td>Genome-fragment</td><td>3.08</td><td>0</td></tr>
<tr><td>S1-final-viral-combined_k141_220843||full</td><td>2294</td><td>No</td><td>NA</td><td>0</td><td>0</td><td>Not-determined</td><td>Genome-fragment</td><td>NA</td><td>0</td></tr>
<tr><td>S3_k141_3140</td><td>1329</td><td>No</td><td>NA</td><td>0</td><td>0</td><td>Low-quality</td><td>Genome-fragment</td><td>3.28</td><td>0</td></tr>
<tr><td>S5-final-viral-combined_k141_85091||full</td><td>1329</td><td>No</td><td>NA</td><td>0</td><td>0</td><td>Low-quality</td><td>Genome-fragment</td><td>1.13</td><td>0</td></tr>
<tr><td>S5-final-viral-combined_k141_43874||full</td><td>1329</td><td>No</td><td>NA</td><td>0</td><td>2</td><td>Low-quality</td><td>Genome-fragment</td><td>3.95</td><td>0</td></tr>
<tr><td>S1-final-viral-combined_k141_56158||full</td><td>3130</td><td>No</td><td>NA</td><td>0</td><td>1</td><td>Not-determined</td><td>Genome-fragment</td><td>NA</td><td>0</td></tr>
<tr><td>S1-final-viral-combined_k141_138938||full</td><td>3370</td><td>No</td><td>NA</td><td>0</td><td>1</td><td>Not-determined</td><td>Genome-fragment</td><td>NA</td><td>0</td></tr>
<tr><td>S1-final-viral-combined_k141_6880||full</td><td>2517</td><td>No</td><td>NA</td><td>0</td><td>2</td><td>Not-determined</td><td>Genome-fragment</td><td>NA</td><td>0</td></tr>
<tr><td>S1-final-viral-combined_k141_368751||full</td><td>4795</td><td>No</td><td>NA</td><td>0</td><td>1</td><td>Not-determined</td><td>Genome-fragment</td><td>NA</td><td>0</td></tr>
<tr><td>S2_k141_134693</td><td>1329</td><td>No</td><td>NA</td><td>0</td><td>1</td><td>Low-quality</td><td>Genome-fragment</td><td>3.52</td><td>0</td></tr>
<tr><td>S1-final-viral-combined_k141_335455||full</td><td>4119</td><td>No</td><td>NA</td><td>0</td><td>3</td><td>Not-determined</td><td>Genome-fragment</td><td>NA</td><td>0</td></tr>
<tr><td>S8_k141_127274</td><td>1329</td><td>No</td><td>NA</td><td>0</td><td>1</td><td>Low-quality</td><td>Genome-fragment</td><td>2.6</td><td>0</td></tr>
<tr><td>S7-final-viral-combined_k141_140089||full</td><td>1330</td><td>No</td><td>NA</td><td>1</td><td>0</td><td>Low-quality</td><td>Genome-fragment</td><td>2.58</td><td>0</td></tr>
<tr><td>S7_k141_197583</td><td>1330</td><td>No</td><td>NA</td><td>0</td><td>2</td><td>Low-quality</td><td>Genome-fragment</td><td>0.3</td><td>0</td></tr>
<tr><td>S1-final-viral-combined_k141_353148||full</td><td>8373</td><td>No</td><td>NA</td><td>0</td><td>4</td><td>Not-determined</td><td>Genome-fragment</td><td>NA</td><td>0</td></tr>
<tr><td>S1-final-viral-combined_k141_188439||full</td><td>1975</td><td>No</td><td>NA</td><td>0</td><td>1</td><td>Not-determined</td><td>Genome-fragment</td><td>NA</td><td>0</td></tr>
<tr><td>S1-final-viral-combined_k141_303886||full</td><td>1647</td><td>No</td><td>NA</td><td>0</td><td>2</td><td>Not-determined</td><td>Genome-fragment</td><td>NA</td><td>0</td></tr>
<tr><td>S1-final-viral-combined_k141_154710||full</td><td>1716</td><td>No</td><td>NA</td><td>0</td><td>2</td><td>Not-determined</td><td>Genome-fragment</td><td>NA</td><td>0</td></tr>
<tr><td>S1-final-viral-combined_k141_88984||full</td><td>2221</td><td>No</td><td>NA</td><td>0</td><td>2</td><td>Not-determined</td><td>Genome-fragment</td><td>NA</td><td>0</td></tr>
<tr><td>S7_k141_122529</td><td>1330</td><td>No</td><td>NA</td><td>0</td><td>1</td><td>Low-quality</td><td>Genome-fragment</td><td>2.64</td><td>0</td></tr>
<tr><td>S1-final-viral-combined_k141_188475||full</td><td>2154</td><td>No</td><td>NA</td><td>0</td><td>1</td><td>Not-determined</td><td>Genome-fragment</td><td>NA</td><td>0</td></tr>
<tr><td>S1-final-viral-combined_k141_286614||full</td><td>3265</td><td>No</td><td>NA</td><td>0</td><td>0</td><td>Not-determined</td><td>Genome-fragment</td><td>NA</td><td>0</td></tr>
<tr><td>S17-final-viral-combined_k141_114252||lt2gene</td><td>1330</td><td>No</td><td>NA</td><td>1</td><td>0</td><td>Low-quality</td><td>Genome-fragment</td><td>3.88</td><td>0</td></tr>
<tr><td>S12-final-viral-combined_k141_51789||full</td><td>1330</td><td>No</td><td>NA</td><td>0</td><td>0</td><td>Low-quality</td><td>Genome-fragment</td><td>2.56</td><td>0</td></tr>
<tr><td>S2-final-viral-combined_k141_110530||full</td><td>1330</td><td>No</td><td>NA</td><td>0</td><td>0</td><td>Low-quality</td><td>Genome-fragment</td><td>3.63</td><td>0</td></tr>
<tr><td>S6_k141_52926</td><td>1330</td><td>No</td><td>NA</td><td>1</td><td>0</td><td>Low-quality</td><td>Genome-fragment</td><td>1.06</td><td>0</td></tr>
<tr><td>S1-final-viral-combined_k141_23248||full</td><td>83257</td><td>Yes</td><td>29089</td><td>23</td><td>35</td><td>Complete</td><td>High-quality</td><td>100</td><td>65.06</td></tr>
<tr><td>S1-final-viral-combined_k141_139011||full</td><td>1462</td><td>No</td><td>NA</td><td>0</td><td>1</td><td>Not-determined</td><td>Genome-fragment</td><td>NA</td><td>0</td></tr>
<tr><td>S1-final-viral-combined_k141_204943||full</td><td>1817</td><td>No</td><td>NA</td><td>0</td><td>1</td><td>Not-determined</td><td>Genome-fragment</td><td>NA</td><td>0</td></tr>
<tr><td>S1-final-viral-combined_k141_188509||full</td><td>3297</td><td>No</td><td>NA</td><td>0</td><td>2</td><td>Not-determined</td><td>Genome-fragment</td><td>NA</td><td>0</td></tr>
<tr><td>S6_k141_20299</td><td>1330</td><td>No</td><td>NA</td><td>0</td><td>0</td><td>Low-quality</td><td>Genome-fragment</td><td>1.5</td><td>0</td></tr>
<tr><td>S1-final-viral-combined_k141_102768||full</td><td>1331</td><td>No</td><td>NA</td><td>0</td><td>0</td><td>Low-quality</td><td>Genome-fragment</td><td>2.18</td><td>0</td></tr>
<tr><td>S1-final-viral-combined_k141_335515||full</td><td>1905</td><td>No</td><td>NA</td><td>0</td><td>0</td><td>Not-determined</td><td>Genome-fragment</td><td>NA</td><td>0</td></tr>
<tr><td>S1-final-viral-combined_k141_303928||full</td><td>3706</td><td>No</td><td>NA</td><td>0</td><td>1</td><td>Not-determined</td><td>Genome-fragment</td><td>NA</td><td>0</td></tr>
<tr><td>S1-final-viral-combined_k141_324904||full</td><td>1331</td><td>No</td><td>NA</td><td>0</td><td>0</td><td>Low-quality</td><td>Genome-fragment</td><td>4.04</td><td>0</td></tr>
<tr><td>S1-final-viral-combined_k141_62540||full</td><td>1331</td><td>No</td><td>NA</td><td>1</td><td>0</td><td>Low-quality</td><td>Genome-fragment</td><td>3.04</td><td>0</td></tr>
<tr><td>S1-final-viral-combined_k141_286676||full</td><td>1902</td><td>No</td><td>NA</td><td>0</td><td>0</td><td>Not-determined</td><td>Genome-fragment</td><td>NA</td><td>0</td></tr>
<tr><td>S1-final-viral-combined_k141_154779||full</td><td>2656</td><td>No</td><td>NA</td><td>0</td><td>1</td><td>Not-determined</td><td>Genome-fragment</td><td>NA</td><td>0</td></tr>
<tr><td>S3-final-viral-combined_k141_121393||full</td><td>1331</td><td>No</td><td>NA</td><td>0</td><td>0</td><td>Low-quality</td><td>Genome-fragment</td><td>1.87</td><td>0</td></tr>
<tr><td>S7-final-viral-combined_k141_196516||full</td><td>1331</td><td>No</td><td>NA</td><td>0</td><td>0</td><td>Low-quality</td><td>Genome-fragment</td><td>0.82</td><td>0</td></tr>
<tr><td>S17-final-viral-combined_k141_111542||full</td><td>1331</td><td>No</td><td>NA</td><td>1</td><td>0</td><td>Low-quality</td><td>Genome-fragment</td><td>2.7</td><td>0</td></tr>
<tr><td>S1-final-viral-combined_k141_188545||full</td><td>10480</td><td>No</td><td>NA</td><td>0</td><td>7</td><td>Not-determined</td><td>Genome-fragment</td><td>NA</td><td>0</td></tr>
<tr><td>S1-final-viral-combined_k141_353246||full</td><td>1788</td><td>No</td><td>NA</td><td>0</td><td>1</td><td>Not-determined</td><td>Genome-fragment</td><td>NA</td><td>0</td></tr>
<tr><td>S1-final-viral-combined_k141_7081||full</td><td>3028</td><td>No</td><td>NA</td><td>0</td><td>2</td><td>Not-determined</td><td>Genome-fragment</td><td>NA</td><td>0</td></tr>
<tr><td>S1-final-viral-combined_k141_286747||full</td><td>1768</td><td>No</td><td>NA</td><td>0</td><td>1</td><td>Not-determined</td><td>Genome-fragment</td><td>NA</td><td>0</td></tr>
<tr><td>S1-final-viral-combined_k141_237642||full</td><td>15115</td><td>No</td><td>NA</td><td>0</td><td>11</td><td>Not-determined</td><td>Genome-fragment</td><td>NA</td><td>0</td></tr>
<tr><td>S1-final-viral-combined_k141_335579||full</td><td>2820</td><td>No</td><td>NA</td><td>0</td><td>2</td><td>Not-determined</td><td>Genome-fragment</td><td>NA</td><td>0</td></tr>
<tr><td>S1-final-viral-combined_k141_188615||full</td><td>1636</td><td>No</td><td>NA</td><td>0</td><td>0</td><td>Not-determined</td><td>Genome-fragment</td><td>NA</td><td>0</td></tr>
<tr><td>S1-final-viral-combined_k141_286810||full</td><td>1208</td><td>No</td><td>NA</td><td>0</td><td>0</td><td>Not-determined</td><td>Genome-fragment</td><td>NA</td><td>0</td></tr>
<tr><td>S1-final-viral-combined_k141_353292||full</td><td>1044</td><td>No</td><td>NA</td><td>0</td><td>0</td><td>Not-determined</td><td>Genome-fragment</td><td>NA</td><td>0</td></tr>
<tr><td>S1-final-viral-combined_k141_237668||full</td><td>1040</td><td>No</td><td>NA</td><td>0</td><td>0</td><td>Not-determined</td><td>Genome-fragment</td><td>NA</td><td>0</td></tr>
<tr><td>S1-final-viral-combined_k141_385639||full</td><td>4015</td><td>No</td><td>NA</td><td>0</td><td>3</td><td>Not-determined</td><td>Genome-fragment</td><td>NA</td><td>0</td></tr>
<tr><td>S17-final-viral-combined_k141_249778||full</td><td>1331</td><td>No</td><td>NA</td><td>0</td><td>0</td><td>Low-quality</td><td>Genome-fragment</td><td>2.27</td><td>0</td></tr>
<tr><td>S1-final-viral-combined_k141_72128||full</td><td>1570</td><td>No</td><td>NA</td><td>0</td><td>1</td><td>Not-determined</td><td>Genome-fragment</td><td>NA</td><td>0</td></tr>
<tr><td>S1-final-viral-combined_k141_254645||full</td><td>2462</td><td>No</td><td>NA</td><td>0</td><td>3</td><td>Not-determined</td><td>Genome-fragment</td><td>NA</td><td>0</td></tr>
<tr><td>S8_k141_249435</td><td>1331</td><td>No</td><td>NA</td><td>1</td><td>0</td><td>Low-quality</td><td>Genome-fragment</td><td>1.59</td><td>0</td></tr>
<tr><td>S1-final-viral-combined_k141_254654||full</td><td>3063</td><td>No</td><td>NA</td><td>0</td><td>0</td><td>Not-determined</td><td>Genome-fragment</td><td>NA</td><td>0</td></tr>
<tr><td>S1-final-viral-combined_k141_188651||full</td><td>3225</td><td>No</td><td>NA</td><td>0</td><td>0</td><td>Not-determined</td><td>Genome-fragment</td><td>NA</td><td>0</td></tr>
<tr><td>S1-final-viral-combined_k141_254665||full</td><td>8648</td><td>No</td><td>NA</td><td>0</td><td>7</td><td>Not-determined</td><td>Genome-fragment</td><td>NA</td><td>0</td></tr>
<tr><td>S1-final-viral-combined_k141_72143||full</td><td>2209</td><td>No</td><td>NA</td><td>0</td><td>2</td><td>Not-determined</td><td>Genome-fragment</td><td>NA</td><td>0</td></tr>
<tr><td>S1-final-viral-combined_k141_72148||full</td><td>1751</td><td>No</td><td>NA</td><td>0</td><td>1</td><td>Not-determined</td><td>Genome-fragment</td><td>NA</td><td>0</td></tr>
<tr><td>S1-final-viral-combined_k141_237340||full</td><td>1332</td><td>Yes</td><td>675</td><td>1</td><td>1</td><td>Low-quality</td><td>Genome-fragment</td><td>1.12</td><td>49.32</td></tr>
<tr><td>S1-final-viral-combined_k141_353336||full</td><td>1297</td><td>No</td><td>NA</td><td>0</td><td>0</td><td>Not-determined</td><td>Genome-fragment</td><td>NA</td><td>0</td></tr>
<tr><td>S1-final-viral-combined_k141_205068||full</td><td>1739</td><td>No</td><td>NA</td><td>0</td><td>0</td><td>Not-determined</td><td>Genome-fragment</td><td>NA</td><td>0</td></tr>
<tr><td>S1-final-viral-combined_k141_312413||full</td><td>1332</td><td>No</td><td>NA</td><td>0</td><td>0</td><td>Low-quality</td><td>Genome-fragment</td><td>2.49</td><td>0</td></tr>
<tr><td>S1-final-viral-combined_k141_188697||full</td><td>14428</td><td>No</td><td>NA</td><td>0</td><td>9</td><td>Not-determined</td><td>Genome-fragment</td><td>NA</td><td>0</td></tr>
<tr><td>S1-final-viral-combined_k141_72167||full</td><td>34592</td><td>No</td><td>NA</td><td>13</td><td>1</td><td>High-quality</td><td>High-quality</td><td>100</td><td>0</td></tr>
<tr><td>S1_k141_188975</td><td>1332</td><td>No</td><td>NA</td><td>0</td><td>1</td><td>Low-quality</td><td>Genome-fragment</td><td>0.96</td><td>0</td></tr>
<tr><td>S1_k141_241860</td><td>1332</td><td>No</td><td>NA</td><td>0</td><td>0</td><td>Low-quality</td><td>Genome-fragment</td><td>0.94</td><td>0</td></tr>
<tr><td>S1-final-viral-combined_k141_104942||full</td><td>7518</td><td>No</td><td>NA</td><td>0</td><td>4</td><td>Not-determined</td><td>Genome-fragment</td><td>NA</td><td>0</td></tr>
<tr><td>S1-final-viral-combined_k141_39805||full</td><td>1251</td><td>No</td><td>NA</td><td>0</td><td>2</td><td>Not-determined</td><td>Genome-fragment</td><td>NA</td><td>0</td></tr>
<tr><td>S1-final-viral-combined_k141_7160||full</td><td>2078</td><td>No</td><td>NA</td><td>0</td><td>2</td><td>Not-determined</td><td>Genome-fragment</td><td>NA</td><td>0</td></tr>
<tr><td>S5-final-viral-combined_k141_91744||full</td><td>1332</td><td>No</td><td>NA</td><td>0</td><td>0</td><td>Low-quality</td><td>Genome-fragment</td><td>1.42</td><td>0</td></tr>
<tr><td>S5-final-viral-combined_k141_75981||full</td><td>1332</td><td>No</td><td>NA</td><td>0</td><td>0</td><td>Low-quality</td><td>Genome-fragment</td><td>2.68</td><td>0</td></tr>
<tr><td>S1-final-viral-combined_k141_56490||full</td><td>1911</td><td>No</td><td>NA</td><td>0</td><td>1</td><td>Not-determined</td><td>Genome-fragment</td><td>NA</td><td>0</td></tr>
<tr><td>S1-final-viral-combined_k141_385740||full</td><td>1176</td><td>No</td><td>NA</td><td>0</td><td>0</td><td>Not-determined</td><td>Genome-fragment</td><td>NA</td><td>0</td></tr>
<tr><td>S5-final-viral-combined_k141_131244||full</td><td>1332</td><td>No</td><td>NA</td><td>1</td><td>0</td><td>Low-quality</td><td>Genome-fragment</td><td>3.19</td><td>0</td></tr>
<tr><td>S1-final-viral-combined_k141_23477||full</td><td>1462</td><td>No</td><td>NA</td><td>0</td><td>2</td><td>Not-determined</td><td>Genome-fragment</td><td>NA</td><td>0</td></tr>
<tr><td>S1-final-viral-combined_k141_23489||full</td><td>1008</td><td>No</td><td>NA</td><td>0</td><td>1</td><td>Not-determined</td><td>Genome-fragment</td><td>NA</td><td>0</td></tr>
<tr><td>S5-final-viral-combined_k141_60096||lt2gene</td><td>1332</td><td>No</td><td>NA</td><td>1</td><td>0</td><td>Low-quality</td><td>Genome-fragment</td><td>1.73</td><td>0</td></tr>
<tr><td>S17-final-viral-combined_k141_16443||full</td><td>1332</td><td>No</td><td>NA</td><td>0</td><td>0</td><td>Low-quality</td><td>Genome-fragment</td><td>0.83</td><td>0</td></tr>
<tr><td>S12-final-viral-combined_k141_66062||full</td><td>1332</td><td>No</td><td>NA</td><td>0</td><td>1</td><td>Low-quality</td><td>Genome-fragment</td><td>3.34</td><td>0</td></tr>
<tr><td>S1-final-viral-combined_k141_39814||full</td><td>4005</td><td>No</td><td>NA</td><td>0</td><td>4</td><td>Not-determined</td><td>Genome-fragment</td><td>NA</td><td>0</td></tr>
<tr><td>S1-final-viral-combined_k141_23497||full</td><td>1630</td><td>No</td><td>NA</td><td>0</td><td>0</td><td>Not-determined</td><td>Genome-fragment</td><td>NA</td><td>0</td></tr>
<tr><td>S1-final-viral-combined_k141_139377||full</td><td>11313</td><td>No</td><td>NA</td><td>0</td><td>9</td><td>Not-determined</td><td>Genome-fragment</td><td>NA</td><td>0</td></tr>
<tr><td>S2-final-viral-combined_k141_74119||full</td><td>1332</td><td>No</td><td>NA</td><td>0</td><td>0</td><td>Low-quality</td><td>Genome-fragment</td><td>1.31</td><td>0</td></tr>
<tr><td>S2_k141_129198</td><td>1332</td><td>No</td><td>NA</td><td>0</td><td>0</td><td>Low-quality</td><td>Genome-fragment</td><td>1.4</td><td>0</td></tr>
<tr><td>S4-final-viral-combined_k141_10555||full</td><td>1332</td><td>No</td><td>NA</td><td>2</td><td>0</td><td>Low-quality</td><td>Genome-fragment</td><td>2.91</td><td>0</td></tr>
<tr><td>S4_k141_127944</td><td>1332</td><td>No</td><td>NA</td><td>0</td><td>1</td><td>Low-quality</td><td>Genome-fragment</td><td>0.76</td><td>0</td></tr>
<tr><td>S1-final-viral-combined_k141_39849||full</td><td>1612</td><td>No</td><td>NA</td><td>0</td><td>2</td><td>Not-determined</td><td>Genome-fragment</td><td>NA</td><td>0</td></tr>
<tr><td>S1-final-viral-combined_k141_205225||full</td><td>5252</td><td>No</td><td>NA</td><td>0</td><td>2</td><td>Not-determined</td><td>Genome-fragment</td><td>NA</td><td>0</td></tr>
<tr><td>S8-final-viral-combined_k141_269324||full</td><td>1332</td><td>No</td><td>NA</td><td>1</td><td>0</td><td>Low-quality</td><td>Genome-fragment</td><td>2.38</td><td>0</td></tr>
<tr><td>S1-final-viral-combined_k141_72339||full</td><td>2731</td><td>No</td><td>NA</td><td>0</td><td>0</td><td>Not-determined</td><td>Genome-fragment</td><td>NA</td><td>0</td></tr>
<tr><td>S9-final-viral-combined_k141_17103||full</td><td>1333</td><td>No</td><td>NA</td><td>0</td><td>1</td><td>Low-quality</td><td>Genome-fragment</td><td>2.28</td><td>0</td></tr>
<tr><td>S1-final-viral-combined_k141_155036||full</td><td>7408</td><td>No</td><td>NA</td><td>0</td><td>5</td><td>Not-determined</td><td>Genome-fragment</td><td>NA</td><td>0</td></tr>
<tr><td>S1-final-viral-combined_k141_23553||full</td><td>1214</td><td>No</td><td>NA</td><td>0</td><td>2</td><td>Not-determined</td><td>Genome-fragment</td><td>NA</td><td>0</td></tr>
<tr><td>S1-final-viral-combined_k141_171103||full</td><td>4032</td><td>No</td><td>NA</td><td>0</td><td>1</td><td>Not-determined</td><td>Genome-fragment</td><td>NA</td><td>0</td></tr>
<tr><td>S1-final-viral-combined_k141_64499||full</td><td>1333</td><td>No</td><td>NA</td><td>0</td><td>0</td><td>Low-quality</td><td>Genome-fragment</td><td>3.14</td><td>0</td></tr>
<tr><td>S7-final-viral-combined_k141_83292||full</td><td>1333</td><td>No</td><td>NA</td><td>0</td><td>0</td><td>Low-quality</td><td>Genome-fragment</td><td>1.07</td><td>0</td></tr>
<tr><td>S17-final-viral-combined_k141_47009||full</td><td>1333</td><td>Yes</td><td>757</td><td>1</td><td>1</td><td>Low-quality</td><td>Genome-fragment</td><td>0.52</td><td>43.21</td></tr>
<tr><td>S1-final-viral-combined_k141_320968||full</td><td>2408</td><td>No</td><td>NA</td><td>0</td><td>0</td><td>Not-determined</td><td>Genome-fragment</td><td>NA</td><td>0</td></tr>
<tr><td>S12-final-viral-combined_k141_14311||full</td><td>1333</td><td>No</td><td>NA</td><td>0</td><td>1</td><td>Low-quality</td><td>Genome-fragment</td><td>2.45</td><td>0</td></tr>
<tr><td>S2-final-viral-combined_k141_231917||lt2gene</td><td>1333</td><td>No</td><td>NA</td><td>1</td><td>0</td><td>Low-quality</td><td>Genome-fragment</td><td>1.75</td><td>0</td></tr>
<tr><td>S1-final-viral-combined_k141_39945||full</td><td>1218</td><td>No</td><td>NA</td><td>0</td><td>0</td><td>Not-determined</td><td>Genome-fragment</td><td>NA</td><td>0</td></tr>
<tr><td>S1-final-viral-combined_k141_335855||full</td><td>1963</td><td>No</td><td>NA</td><td>0</td><td>0</td><td>Not-determined</td><td>Genome-fragment</td><td>NA</td><td>0</td></tr>
<tr><td>S1-final-viral-combined_k141_304191||full</td><td>1531</td><td>No</td><td>NA</td><td>0</td><td>1</td><td>Not-determined</td><td>Genome-fragment</td><td>NA</td><td>0</td></tr>
<tr><td>S2_k141_101837</td><td>1333</td><td>No</td><td>NA</td><td>0</td><td>0</td><td>Low-quality</td><td>Genome-fragment</td><td>2.13</td><td>0</td></tr>
<tr><td>S8-final-viral-combined_k141_204738||full</td><td>1333</td><td>No</td><td>NA</td><td>0</td><td>0</td><td>Low-quality</td><td>Genome-fragment</td><td>2.97</td><td>0</td></tr>
<tr><td>S1-final-viral-combined_k141_254840||full</td><td>1907</td><td>No</td><td>NA</td><td>0</td><td>1</td><td>Not-determined</td><td>Genome-fragment</td><td>NA</td><td>0</td></tr>
<tr><td>S1-final-viral-combined_k141_104412||full</td><td>1334</td><td>No</td><td>NA</td><td>0</td><td>0</td><td>Low-quality</td><td>Genome-fragment</td><td>3.79</td><td>0</td></tr>
<tr><td>S1-final-viral-combined_k141_353510||full</td><td>1792</td><td>No</td><td>NA</td><td>0</td><td>2</td><td>Not-determined</td><td>Genome-fragment</td><td>NA</td><td>0</td></tr>
<tr><td>S1-final-viral-combined_k141_178184||full</td><td>1334</td><td>No</td><td>NA</td><td>1</td><td>0</td><td>Low-quality</td><td>Genome-fragment</td><td>2.86</td><td>0</td></tr>
<tr><td>S1-final-viral-combined_k141_47860||full</td><td>1334</td><td>No</td><td>NA</td><td>3</td><td>0</td><td>Low-quality</td><td>Genome-fragment</td><td>2.71</td><td>0</td></tr>
<tr><td>S1-final-viral-combined_k141_23590||full</td><td>2577</td><td>No</td><td>NA</td><td>0</td><td>1</td><td>Not-determined</td><td>Genome-fragment</td><td>NA</td><td>0</td></tr>
<tr><td>S1-final-viral-combined_k141_304236||full</td><td>3128</td><td>No</td><td>NA</td><td>0</td><td>4</td><td>Not-determined</td><td>Genome-fragment</td><td>NA</td><td>0</td></tr>
<tr><td>S1-final-viral-combined_k141_123387||full</td><td>2810</td><td>No</td><td>NA</td><td>0</td><td>2</td><td>Not-determined</td><td>Genome-fragment</td><td>NA</td><td>0</td></tr>
<tr><td>S1-final-viral-combined_k141_254858||full</td><td>1904</td><td>No</td><td>NA</td><td>0</td><td>0</td><td>Not-determined</td><td>Genome-fragment</td><td>NA</td><td>0</td></tr>
<tr><td>S1-final-viral-combined_k141_221262||full</td><td>1237</td><td>No</td><td>NA</td><td>0</td><td>1</td><td>Not-determined</td><td>Genome-fragment</td><td>NA</td><td>0</td></tr>
<tr><td>S5_k141_101927</td><td>1334</td><td>No</td><td>NA</td><td>0</td><td>1</td><td>Low-quality</td><td>Genome-fragment</td><td>0.39</td><td>0</td></tr>
<tr><td>S7-final-viral-combined_k141_9901||full</td><td>1334</td><td>No</td><td>NA</td><td>1</td><td>0</td><td>Low-quality</td><td>Genome-fragment</td><td>2.47</td><td>0</td></tr>
<tr><td>S1-final-viral-combined_k141_155113||full</td><td>2028</td><td>No</td><td>NA</td><td>0</td><td>0</td><td>Not-determined</td><td>Genome-fragment</td><td>NA</td><td>0</td></tr>
<tr><td>S17-k141_158645</td><td>1334</td><td>No</td><td>NA</td><td>0</td><td>1</td><td>Low-quality</td><td>Genome-fragment</td><td>3.18</td><td>0</td></tr>
<tr><td>S1-final-viral-combined_k141_89478||full</td><td>2734</td><td>No</td><td>NA</td><td>0</td><td>2</td><td>Not-determined</td><td>Genome-fragment</td><td>NA</td><td>0</td></tr>
<tr><td>S12-final-viral-combined_k141_301791||full</td><td>1334</td><td>No</td><td>NA</td><td>1</td><td>0</td><td>Low-quality</td><td>Genome-fragment</td><td>2.18</td><td>0</td></tr>
<tr><td>S12-final-viral-combined_k141_229955||lt2gene</td><td>1334</td><td>No</td><td>NA</td><td>1</td><td>0</td><td>Low-quality</td><td>Genome-fragment</td><td>0.99</td><td>0</td></tr>
<tr><td>S1-final-viral-combined_k141_105166||full</td><td>1366</td><td>No</td><td>NA</td><td>0</td><td>0</td><td>Not-determined</td><td>Genome-fragment</td><td>NA</td><td>0</td></tr>
<tr><td>S1-final-viral-combined_k141_40023||full</td><td>4256</td><td>No</td><td>NA</td><td>0</td><td>4</td><td>Not-determined</td><td>Genome-fragment</td><td>NA</td><td>0</td></tr>
<tr><td>S1-final-viral-combined_k141_40025||full</td><td>1292</td><td>No</td><td>NA</td><td>0</td><td>1</td><td>Not-determined</td><td>Genome-fragment</td><td>NA</td><td>0</td></tr>
<tr><td>S12_k141_227250</td><td>1334</td><td>No</td><td>NA</td><td>0</td><td>1</td><td>Low-quality</td><td>Genome-fragment</td><td>0.88</td><td>0</td></tr>
<tr><td>S1-final-viral-combined_k141_7397||full</td><td>4165</td><td>No</td><td>NA</td><td>0</td><td>3</td><td>Not-determined</td><td>Genome-fragment</td><td>NA</td><td>0</td></tr>
<tr><td>S1-final-viral-combined_k141_254935||full</td><td>1911</td><td>No</td><td>NA</td><td>0</td><td>1</td><td>Not-determined</td><td>Genome-fragment</td><td>NA</td><td>0</td></tr>
<tr><td>S1-final-viral-combined_k141_238091||full</td><td>3362</td><td>No</td><td>NA</td><td>0</td><td>2</td><td>Not-determined</td><td>Genome-fragment</td><td>NA</td><td>0</td></tr>
<tr><td>S1-final-viral-combined_k141_7400||full</td><td>1186</td><td>No</td><td>NA</td><td>0</td><td>0</td><td>Not-determined</td><td>Genome-fragment</td><td>NA</td><td>0</td></tr>
<tr><td>S8-final-viral-combined_k141_149016||full</td><td>1334</td><td>No</td><td>NA</td><td>1</td><td>0</td><td>Low-quality</td><td>Genome-fragment</td><td>0.95</td><td>0</td></tr>
<tr><td>S1-final-viral-combined_k141_171227||full</td><td>1061</td><td>No</td><td>NA</td><td>0</td><td>1</td><td>Not-determined</td><td>Genome-fragment</td><td>NA</td><td>0</td></tr>
<tr><td>S9-final-viral-combined_k141_95758||full</td><td>1335</td><td>No</td><td>NA</td><td>0</td><td>0</td><td>Low-quality</td><td>Genome-fragment</td><td>3.29</td><td>0</td></tr>
<tr><td>S1-final-viral-combined_k141_238125||full</td><td>3581</td><td>No</td><td>NA</td><td>0</td><td>2</td><td>Not-determined</td><td>Genome-fragment</td><td>NA</td><td>0</td></tr>
<tr><td>S1-final-viral-combined_k141_386046||full</td><td>1003</td><td>No</td><td>NA</td><td>0</td><td>0</td><td>Not-determined</td><td>Genome-fragment</td><td>NA</td><td>0</td></tr>
<tr><td>S11-final-viral-combined_k141_170065||full</td><td>1335</td><td>No</td><td>NA</td><td>0</td><td>1</td><td>Low-quality</td><td>Genome-fragment</td><td>1.11</td><td>0</td></tr>
<tr><td>S1-final-viral-combined_k141_335918||full</td><td>1180</td><td>No</td><td>NA</td><td>0</td><td>1</td><td>Not-determined</td><td>Genome-fragment</td><td>NA</td><td>0</td></tr>
<tr><td>S1-final-viral-combined_k141_386079||full</td><td>1614</td><td>No</td><td>NA</td><td>0</td><td>1</td><td>Not-determined</td><td>Genome-fragment</td><td>NA</td><td>0</td></tr>
<tr><td>S1-final-viral-combined_k141_56729||full</td><td>1277</td><td>No</td><td>NA</td><td>0</td><td>1</td><td>Not-determined</td><td>Genome-fragment</td><td>NA</td><td>0</td></tr>
<tr><td>S11-final-viral-combined_k141_32505||full</td><td>1335</td><td>No</td><td>NA</td><td>0</td><td>0</td><td>Low-quality</td><td>Genome-fragment</td><td>2.96</td><td>0</td></tr>
</table>

<table>
<tr><td>S1-final-viral-combined_k141_40115||full</td><td>2384</td><td>No</td><td>NA</td><td>1</td><td>0</td><td>Medium-quality</td><td>Genome-fragment</td><td>66.38</td><td>0</td></tr>
<tr><td>S11-final-viral-combined_k141_325950||full</td><td>1335</td><td>No</td><td>NA</td><td>0</td><td>0</td><td>Low-quality</td><td>Genome-fragment</td><td>2.81</td><td>0</td></tr>
<tr><td>S1-final-viral-combined_k141_315753||lt2gene</td><td>1335</td><td>No</td><td>NA</td><td>2</td><td>0</td><td>Low-quality</td><td>Genome-fragment</td><td>3.16</td><td>0</td></tr>
<tr><td>S1-final-viral-combined_k141_221401||full</td><td>1458</td><td>No</td><td>NA</td><td>0</td><td>2</td><td>Not-determined</td><td>Genome-fragment</td><td>NA</td><td>0</td></tr>
<tr><td>S1-final-viral-combined_k141_304343||full</td><td>1178</td><td>No</td><td>NA</td><td>0</td><td>0</td><td>Not-determined</td><td>Genome-fragment</td><td>NA</td><td>0</td></tr>
<tr><td>S1-final-viral-combined_k141_287147||full</td><td>2003</td><td>No</td><td>NA</td><td>0</td><td>3</td><td>Not-determined</td><td>Genome-fragment</td><td>NA</td><td>0</td></tr>
<tr><td>S1-final-viral-combined_k141_321236||full</td><td>1456</td><td>No</td><td>NA</td><td>0</td><td>0</td><td>Not-determined</td><td>Genome-fragment</td><td>NA</td><td>0</td></tr>
<tr><td>S1-final-viral-combined_k141_23256||lt2gene</td><td>1335</td><td>No</td><td>NA</td><td>0</td><td>0</td><td>Low-quality</td><td>Genome-fragment</td><td>3</td><td>0</td></tr>
<tr><td>S5-final-viral-combined_k141_24621||full</td><td>1335</td><td>Yes</td><td>873</td><td>1</td><td>1</td><td>Low-quality</td><td>Genome-fragment</td><td>0.99</td><td>34.61</td></tr>
<tr><td>S12-final-viral-combined_k141_37631||full</td><td>1335</td><td>No</td><td>NA</td><td>0</td><td>0</td><td>Low-quality</td><td>Genome-fragment</td><td>3.09</td><td>0</td></tr>
<tr><td>S12-final-viral-combined_k141_397033||full</td><td>1335</td><td>No</td><td>NA</td><td>0</td><td>0</td><td>Low-quality</td><td>Genome-fragment</td><td>2.1</td><td>0</td></tr>
<tr><td>S2-final-viral-combined_k141_121658||lt2gene</td><td>1335</td><td>No</td><td>NA</td><td>1</td><td>0</td><td>Low-quality</td><td>Genome-fragment</td><td>3.27</td><td>0</td></tr>
<tr><td>S2_k141_170380</td><td>1335</td><td>No</td><td>NA</td><td>0</td><td>1</td><td>Low-quality</td><td>Genome-fragment</td><td>0.46</td><td>0</td></tr>
<tr><td>S1-final-viral-combined_k141_270684||full</td><td>1747</td><td>No</td><td>NA</td><td>0</td><td>2</td><td>Not-determined</td><td>Genome-fragment</td><td>NA</td><td>0</td></tr>
<tr><td>S1-final-viral-combined_k141_72579||full</td><td>1745</td><td>No</td><td>NA</td><td>0</td><td>3</td><td>Not-determined</td><td>Genome-fragment</td><td>NA</td><td>0</td></tr>
<tr><td>S1-final-viral-combined_k141_205530||full</td><td>32603</td><td>No</td><td>NA</td><td>0</td><td>8</td><td>Not-determined</td><td>Genome-fragment</td><td>NA</td><td>0</td></tr>
<tr><td>S1-final-viral-combined_k141_238241||full</td><td>2301</td><td>No</td><td>NA</td><td>0</td><td>1</td><td>Not-determined</td><td>Genome-fragment</td><td>NA</td><td>0</td></tr>
<tr><td>S1-final-viral-combined_k141_139670||full</td><td>1532</td><td>No</td><td>NA</td><td>0</td><td>1</td><td>Not-determined</td><td>Genome-fragment</td><td>NA</td><td>0</td></tr>
<tr><td>S1-final-viral-combined_k141_105385||full</td><td>1454</td><td>No</td><td>NA</td><td>0</td><td>1</td><td>Not-determined</td><td>Genome-fragment</td><td>NA</td><td>0</td></tr>
<tr><td>S1-final-viral-combined_k141_270711||full</td><td>1321</td><td>No</td><td>NA</td><td>0</td><td>0</td><td>Not-determined</td><td>Genome-fragment</td><td>NA</td><td>0</td></tr>
<tr><td>S4-final-viral-combined_k141_50123||full</td><td>1335</td><td>No</td><td>NA</td><td>0</td><td>0</td><td>Low-quality</td><td>Genome-fragment</td><td>0.56</td><td>0</td></tr>
<tr><td>S8_k141_245075</td><td>1335</td><td>No</td><td>NA</td><td>0</td><td>2</td><td>Low-quality</td><td>Genome-fragment</td><td>0.43</td><td>0</td></tr>
<tr><td>S1-final-viral-combined_k141_40166||full</td><td>2131</td><td>No</td><td>NA</td><td>0</td><td>2</td><td>Not-determined</td><td>Genome-fragment</td><td>NA</td><td>0</td></tr>
<tr><td>S1-final-viral-combined_k141_72605||full</td><td>2239</td><td>No</td><td>NA</td><td>0</td><td>0</td><td>Not-determined</td><td>Genome-fragment</td><td>NA</td><td>0</td></tr>
<tr><td>S1-final-viral-combined_k141_227156||full</td><td>1336</td><td>No</td><td>NA</td><td>0</td><td>0</td><td>Low-quality</td><td>Genome-fragment</td><td>2.41</td><td>0</td></tr>
<tr><td>S1-final-viral-combined_k141_23694||full</td><td>1730</td><td>No</td><td>NA</td><td>0</td><td>2</td><td>Not-determined</td><td>Genome-fragment</td><td>NA</td><td>0</td></tr>
<tr><td>S1_k141_307378</td><td>1336</td><td>No</td><td>NA</td><td>1</td><td>2</td><td>Low-quality</td><td>Genome-fragment</td><td>3.31</td><td>0</td></tr>
<tr><td>S1-final-viral-combined_k141_155280||full</td><td>1423</td><td>No</td><td>NA</td><td>0</td><td>1</td><td>Not-determined</td><td>Genome-fragment</td><td>NA</td><td>0</td></tr>
<tr><td>S1_k141_174636</td><td>1336</td><td>No</td><td>NA</td><td>0</td><td>0</td><td>Low-quality</td><td>Genome-fragment</td><td>2.92</td><td>0</td></tr>
<tr><td>S1-final-viral-combined_k141_369324||full</td><td>2347</td><td>No</td><td>NA</td><td>0</td><td>1</td><td>Not-determined</td><td>Genome-fragment</td><td>NA</td><td>0</td></tr>
<tr><td>S3_k141_69671</td><td>1336</td><td>No</td><td>NA</td><td>0</td><td>1</td><td>Low-quality</td><td>Genome-fragment</td><td>2.23</td><td>0</td></tr>
<tr><td>S5-final-viral-combined_k141_102134||full</td><td>1336</td><td>No</td><td>NA</td><td>0</td><td>0</td><td>Low-quality</td><td>Genome-fragment</td><td>2.79</td><td>0</td></tr>
<tr><td>S7-final-viral-combined_k141_74921||full</td><td>1336</td><td>No</td><td>NA</td><td>0</td><td>0</td><td>Low-quality</td><td>Genome-fragment</td><td>2.47</td><td>0</td></tr>
<tr><td>S1-final-viral-combined_k141_7602||full</td><td>3549</td><td>No</td><td>NA</td><td>0</td><td>0</td><td>Not-determined</td><td>Genome-fragment</td><td>NA</td><td>0</td></tr>
<tr><td>S1-final-viral-combined_k141_40201||full</td><td>1757</td><td>No</td><td>NA</td><td>0</td><td>2</td><td>Not-determined</td><td>Genome-fragment</td><td>NA</td><td>0</td></tr>
<tr><td>S1-final-viral-combined_k141_304377||full</td><td>95231</td><td>Yes</td><td>58512</td><td>31</td><td>29</td><td>Complete</td><td>High-quality</td><td>100</td><td>38.56</td></tr>
<tr><td>S1-final-viral-combined_k141_89651||full</td><td>2227</td><td>No</td><td>NA</td><td>0</td><td>1</td><td>Not-determined</td><td>Genome-fragment</td><td>NA</td><td>0</td></tr>
<tr><td>S1-final-viral-combined_k141_386146||full</td><td>2689</td><td>No</td><td>NA</td><td>0</td><td>2</td><td>Not-determined</td><td>Genome-fragment</td><td>NA</td><td>0</td></tr>
<tr><td>S12-final-viral-combined_k141_329447||full</td><td>1336</td><td>No</td><td>NA</td><td>0</td><td>0</td><td>Low-quality</td><td>Genome-fragment</td><td>3.04</td><td>0</td></tr>
<tr><td>S1-final-viral-combined_k141_321302||full</td><td>1017</td><td>No</td><td>NA</td><td>0</td><td>0</td><td>Not-determined</td><td>Genome-fragment</td><td>NA</td><td>0</td></tr>
<tr><td>S1-final-viral-combined_k141_155374||full</td><td>1284</td><td>No</td><td>NA</td><td>0</td><td>1</td><td>Not-determined</td><td>Genome-fragment</td><td>NA</td><td>0</td></tr>
<tr><td>S1-final-viral-combined_k141_353757||full</td><td>35912</td><td>No</td><td>NA</td><td>11</td><td>2</td><td>High-quality</td><td>High-quality</td><td>98.88</td><td>0</td></tr>
<tr><td>S12-final-viral-combined_k141_47760||lt2gene</td><td>1336</td><td>No</td><td>NA</td><td>1</td><td>0</td><td>Low-quality</td><td>Genome-fragment</td><td>3.76</td><td>0</td></tr>
<tr><td>S1-final-viral-combined_k141_171395||full</td><td>2456</td><td>No</td><td>NA</td><td>0</td><td>3</td><td>Not-determined</td><td>Genome-fragment</td><td>NA</td><td>0</td></tr>
<tr><td>S1-final-viral-combined_k141_221553||full</td><td>3298</td><td>No</td><td>NA</td><td>0</td><td>1</td><td>Not-determined</td><td>Genome-fragment</td><td>NA</td><td>0</td></tr>
<tr><td>S1-final-viral-combined_k141_56913||full</td><td>3428</td><td>No</td><td>NA</td><td>0</td><td>2</td><td>Not-determined</td><td>Genome-fragment</td><td>NA</td><td>0</td></tr>
<tr><td>S2-final-viral-combined_k141_4300||lt2gene</td><td>1336</td><td>No</td><td>NA</td><td>1</td><td>0</td><td>Low-quality</td><td>Genome-fragment</td><td>2.19</td><td>0</td></tr>
<tr><td>S1-final-viral-combined_k141_72675||full</td><td>9589</td><td>No</td><td>NA</td><td>0</td><td>2</td><td>Not-determined</td><td>Genome-fragment</td><td>NA</td><td>0</td></tr>
<tr><td>S4-final-viral-combined_k141_14756||full</td><td>1336</td><td>No</td><td>NA</td><td>0</td><td>0</td><td>Low-quality</td><td>Genome-fragment</td><td>1.07</td><td>0</td></tr>
<tr><td>S1-final-viral-combined_k141_7669||full</td><td>3185</td><td>No</td><td>NA</td><td>0</td><td>1</td><td>Not-determined</td><td>Genome-fragment</td><td>NA</td><td>0</td></tr>
<tr><td>S1-final-viral-combined_k141_386207||full</td><td>1965</td><td>No</td><td>NA</td><td>0</td><td>0</td><td>Not-determined</td><td>Genome-fragment</td><td>NA</td><td>0</td></tr>
<tr><td>S8-final-viral-combined_k141_231568||full</td><td>1336</td><td>No</td><td>NA</td><td>0</td><td>2</td><td>Low-quality</td><td>Genome-fragment</td><td>1</td><td>0</td></tr>
<tr><td>S1-final-viral-combined_k141_321361||full</td><td>3361</td><td>No</td><td>NA</td><td>0</td><td>2</td><td>Not-determined</td><td>Genome-fragment</td><td>NA</td><td>0</td></tr>
<tr><td>S11-final-viral-combined_k141_45197||full</td><td>1337</td><td>No</td><td>NA</td><td>1</td><td>0</td><td>Low-quality</td><td>Genome-fragment</td><td>2.64</td><td>0</td></tr>
<tr><td>S1-final-viral-combined_k141_188182||full</td><td>1337</td><td>No</td><td>NA</td><td>1</td><td>0</td><td>Low-quality</td><td>Genome-fragment</td><td>3</td><td>0</td></tr>
<tr><td>S1-final-viral-combined_k141_353801||full</td><td>2219</td><td>No</td><td>NA</td><td>0</td><td>3</td><td>Not-determined</td><td>Genome-fragment</td><td>NA</td><td>0</td></tr>
<tr><td>S1-final-viral-combined_k141_189195||full</td><td>2729</td><td>No</td><td>NA</td><td>0</td><td>2</td><td>Not-determined</td><td>Genome-fragment</td><td>NA</td><td>0</td></tr>
<tr><td>S1_k141_271463</td><td>1337</td><td>No</td><td>NA</td><td>0</td><td>1</td><td>Low-quality</td><td>Genome-fragment</td><td>1.3</td><td>0</td></tr>
<tr><td>S1-final-viral-combined_k141_23795||full</td><td>1091</td><td>No</td><td>NA</td><td>0</td><td>1</td><td>Not-determined</td><td>Genome-fragment</td><td>NA</td><td>0</td></tr>
<tr><td>S1_k141_57521</td><td>1337</td><td>No</td><td>NA</td><td>0</td><td>0</td><td>Low-quality</td><td>Genome-fragment</td><td>3.83</td><td>0</td></tr>
<tr><td>S5-final-viral-combined_k141_18360||full</td><td>1337</td><td>No</td><td>NA</td><td>2</td><td>0</td><td>Low-quality</td><td>Genome-fragment</td><td>2.86</td><td>0</td></tr>
<tr><td>S1-final-viral-combined_k141_369407||full</td><td>3325</td><td>No</td><td>NA</td><td>0</td><td>2</td><td>Not-determined</td><td>Genome-fragment</td><td>NA</td><td>0</td></tr>
<tr><td>S1-final-viral-combined_k141_287343||full</td><td>1721</td><td>No</td><td>NA</td><td>0</td><td>0</td><td>Not-determined</td><td>Genome-fragment</td><td>NA</td><td>0</td></tr>
<tr><td>S17-final-viral-combined_k141_84512||full</td><td>1337</td><td>No</td><td>NA</td><td>0</td><td>1</td><td>Low-quality</td><td>Genome-fragment</td><td>3.59</td><td>0</td></tr>
<tr><td>S12-final-viral-combined_k141_13815||full</td><td>1337</td><td>No</td><td>NA</td><td>0</td><td>1</td><td>Low-quality</td><td>Genome-fragment</td><td>3.04</td><td>0</td></tr>
<tr><td>S12-final-viral-combined_k141_317442||full</td><td>1337</td><td>No</td><td>NA</td><td>0</td><td>1</td><td>Low-quality</td><td>Genome-fragment</td><td>0.69</td><td>0</td></tr>
<tr><td>S1-final-viral-combined_k141_171472||full</td><td>4811</td><td>No</td><td>NA</td><td>0</td><td>1</td><td>Not-determined</td><td>Genome-fragment</td><td>NA</td><td>0</td></tr>
<tr><td>S1-final-viral-combined_k141_23829||full</td><td>4865</td><td>No</td><td>NA</td><td>0</td><td>3</td><td>Not-determined</td><td>Genome-fragment</td><td>NA</td><td>0</td></tr>
<tr><td>S1-final-viral-combined_k141_89728||full</td><td>1564</td><td>No</td><td>NA</td><td>0</td><td>2</td><td>Not-determined</td><td>Genome-fragment</td><td>NA</td><td>0</td></tr>
<tr><td>S6-final-viral-combined_k141_17839||full</td><td>1337</td><td>No</td><td>NA</td><td>0</td><td>0</td><td>Low-quality</td><td>Genome-fragment</td><td>2.82</td><td>0</td></tr>
<tr><td>S8_k141_3746</td><td>1337</td><td>No</td><td>NA</td><td>2</td><td>0</td><td>Low-quality</td><td>Genome-fragment</td><td>4.16</td><td>0</td></tr>
<tr><td>S1-final-viral-combined_k141_336087||full</td><td>2192</td><td>No</td><td>NA</td><td>0</td><td>2</td><td>Not-determined</td><td>Genome-fragment</td><td>NA</td><td>0</td></tr>
<tr><td>S1-final-viral-combined_k141_221653||full</td><td>2819</td><td>No</td><td>NA</td><td>0</td><td>1</td><td>Not-determined</td><td>Genome-fragment</td><td>NA</td><td>0</td></tr>
<tr><td>S9-final-viral-combined_k141_19115||lt2gene</td><td>1338</td><td>No</td><td>NA</td><td>1</td><td>0</td><td>Low-quality</td><td>Genome-fragment</td><td>3.28</td><td>0</td></tr>
<tr><td>S11-final-viral-combined_k141_58070||full</td><td>1338</td><td>No</td><td>NA</td><td>0</td><td>1</td><td>Low-quality</td><td>Genome-fragment</td><td>1.4</td><td>0</td></tr>
<tr><td>S1-final-viral-combined_k141_123715||full</td><td>1571</td><td>No</td><td>NA</td><td>0</td><td>1</td><td>Not-determined</td><td>Genome-fragment</td><td>NA</td><td>0</td></tr>
<tr><td>S1-final-viral-combined_k141_221664||full</td><td>2598</td><td>No</td><td>NA</td><td>0</td><td>0</td><td>Not-determined</td><td>Genome-fragment</td><td>NA</td><td>0</td></tr>
<tr><td>S1-final-viral-combined_k141_369483||full</td><td>1669</td><td>No</td><td>NA</td><td>0</td><td>0</td><td>Not-determined</td><td>Genome-fragment</td><td>NA</td><td>0</td></tr>
<tr><td>S1-final-viral-combined_k141_114690||full</td><td>1338</td><td>No</td><td>NA</td><td>1</td><td>0</td><td>Low-quality</td><td>Genome-fragment</td><td>1.98</td><td>0</td></tr>
<tr><td>S1-final-viral-combined_k141_287402||full</td><td>1489</td><td>No</td><td>NA</td><td>0</td><td>1</td><td>Not-determined</td><td>Genome-fragment</td><td>NA</td><td>0</td></tr>
<tr><td>S7-final-viral-combined_k141_14010||full</td><td>1338</td><td>No</td><td>NA</td><td>0</td><td>0</td><td>Low-quality</td><td>Genome-fragment</td><td>0.84</td><td>0</td></tr>
<tr><td>S17-final-viral-combined_k141_184913||full</td><td>1338</td><td>No</td><td>NA</td><td>0</td><td>1</td><td>Low-quality</td><td>Genome-fragment</td><td>0.84</td><td>0</td></tr>
<tr><td>S17-final-viral-combined_k141_173328||full</td><td>1338</td><td>No</td><td>NA</td><td>1</td><td>0</td><td>Low-quality</td><td>Genome-fragment</td><td>3.07</td><td>0</td></tr>
<tr><td>S1-final-viral-combined_k141_386329||full</td><td>1899</td><td>No</td><td>NA</td><td>0</td><td>0</td><td>Not-determined</td><td>Genome-fragment</td><td>NA</td><td>0</td></tr>
<tr><td>S1-final-viral-combined_k141_105556||full</td><td>3361</td><td>No</td><td>NA</td><td>0</td><td>3</td><td>Not-determined</td><td>Genome-fragment</td><td>NA</td><td>0</td></tr>
<tr><td>S12-final-viral-combined_k141_177253||full</td><td>1338</td><td>No</td><td>NA</td><td>0</td><td>0</td><td>Low-quality</td><td>Genome-fragment</td><td>4.2</td><td>0</td></tr>
<tr><td>S2-final-viral-combined_k141_1715||full</td><td>1338</td><td>No</td><td>NA</td><td>1</td><td>0</td><td>Low-quality</td><td>Genome-fragment</td><td>3.18</td><td>0</td></tr>
<tr><td>S1-final-viral-combined_k141_139808||full</td><td>1041</td><td>No</td><td>NA</td><td>0</td><td>2</td><td>Not-determined</td><td>Genome-fragment</td><td>NA</td><td>0</td></tr>
<tr><td>S1-final-viral-combined_k141_139812||full</td><td>2300</td><td>No</td><td>NA</td><td>0</td><td>2</td><td>Not-determined</td><td>Genome-fragment</td><td>NA</td><td>0</td></tr>
<tr><td>S2-final-viral-combined_k141_65541||full</td><td>1338</td><td>No</td><td>NA</td><td>0</td><td>0</td><td>Low-quality</td><td>Genome-fragment</td><td>1.74</td><td>0</td></tr>
<tr><td>S1-final-viral-combined_k141_23926||full</td><td>1824</td><td>No</td><td>NA</td><td>0</td><td>3</td><td>Not-determined</td><td>Genome-fragment</td><td>NA</td><td>0</td></tr>
<tr><td>S1-final-viral-combined_k141_369564||full</td><td>2759</td><td>No</td><td>NA</td><td>0</td><td>0</td><td>Not-determined</td><td>Genome-fragment</td><td>NA</td><td>0</td></tr>
<tr><td>S1-final-viral-combined_k141_221729||full</td><td>2369</td><td>No</td><td>NA</td><td>0</td><td>1</td><td>Not-determined</td><td>Genome-fragment</td><td>NA</td><td>0</td></tr>
<tr><td>S11-final-viral-combined_k141_13220||full</td><td>1339</td><td>No</td><td>NA</td><td>0</td><td>0</td><td>Low-quality</td><td>Genome-fragment</td><td>1.99</td><td>0</td></tr>
<tr><td>S11-final-viral-combined_k141_120576||lt2gene</td><td>1339</td><td>No</td><td>NA</td><td>1</td><td>0</td><td>Low-quality</td><td>Genome-fragment</td><td>2.96</td><td>0</td></tr>
<tr><td>S1-final-viral-combined_k141_23944||full</td><td>3188</td><td>No</td><td>NA</td><td>0</td><td>1</td><td>Not-determined</td><td>Genome-fragment</td><td>NA</td><td>0</td></tr>
<tr><td>S1-final-viral-combined_k141_336180||full</td><td>2886</td><td>No</td><td>NA</td><td>0</td><td>2</td><td>Not-determined</td><td>Genome-fragment</td><td>NA</td><td>0</td></tr>
<tr><td>S1-final-viral-combined_k141_353978||full</td><td>7324</td><td>No</td><td>NA</td><td>0</td><td>1</td><td>Not-determined</td><td>Genome-fragment</td><td>NA</td><td>0</td></tr>
<tr><td>S1-final-viral-combined_k141_309564||lt2gene</td><td>1339</td><td>No</td><td>NA</td><td>1</td><td>0</td><td>Low-quality</td><td>Genome-fragment</td><td>3.08</td><td>0</td></tr>
<tr><td>S5_k141_200554</td><td>1339</td><td>No</td><td>NA</td><td>0</td><td>1</td><td>Low-quality</td><td>Genome-fragment</td><td>4.45</td><td>0</td></tr>
<tr><td>S17-final-viral-combined_k141_129508||full</td><td>1339</td><td>No</td><td>NA</td><td>0</td><td>1</td><td>Low-quality</td><td>Genome-fragment</td><td>2.32</td><td>0</td></tr>
<tr><td>S1-final-viral-combined_k141_40359||full</td><td>2256</td><td>No</td><td>NA</td><td>0</td><td>1</td><td>Not-determined</td><td>Genome-fragment</td><td>NA</td><td>0</td></tr>
<tr><td>S17-k141_87581</td><td>1339</td><td>No</td><td>NA</td><td>0</td><td>1</td><td>Low-quality</td><td>Genome-fragment</td><td>1.71</td><td>0</td></tr>
<tr><td>S12-final-viral-combined_k141_34491||full</td><td>1339</td><td>No</td><td>NA</td><td>0</td><td>0</td><td>Low-quality</td><td>Genome-fragment</td><td>2.26</td><td>0</td></tr>
<tr><td>S12-final-viral-combined_k141_365480||full</td><td>1339</td><td>No</td><td>NA</td><td>0</td><td>0</td><td>Low-quality</td><td>Genome-fragment</td><td>1.41</td><td>0</td></tr>
<tr><td>S2-final-viral-combined_k141_160671||full</td><td>1339</td><td>No</td><td>NA</td><td>0</td><td>0</td><td>Low-quality</td><td>Genome-fragment</td><td>3.52</td><td>0</td></tr>
<tr><td>S1-final-viral-combined_k141_270983||full</td><td>5619</td><td>No</td><td>NA</td><td>0</td><td>4</td><td>Not-determined</td><td>Genome-fragment</td><td>NA</td><td>0</td></tr>
<tr><td>S1-final-viral-combined_k141_72817||full</td><td>1386</td><td>No</td><td>NA</td><td>0</td><td>1</td><td>Not-determined</td><td>Genome-fragment</td><td>NA</td><td>0</td></tr>
<tr><td>S1-final-viral-combined_k141_171534||full</td><td>1652</td><td>No</td><td>NA</td><td>0</td><td>1</td><td>Not-determined</td><td>Genome-fragment</td><td>NA</td><td>0</td></tr>
<tr><td>S1-final-viral-combined_k141_238300||full</td><td>1059</td><td>No</td><td>NA</td><td>0</td><td>1</td><td>Not-determined</td><td>Genome-fragment</td><td>NA</td><td>0</td></tr>
<tr><td>S8-final-viral-combined_k141_59194||full</td><td>1339</td><td>No</td><td>NA</td><td>0</td><td>0</td><td>Low-quality</td><td>Genome-fragment</td><td>0.73</td><td>0</td></tr>
<tr><td>S9-final-viral-combined_k141_111265||full</td><td>1340</td><td>No</td><td>NA</td><td>0</td><td>2</td><td>Low-quality</td><td>Genome-fragment</td><td>3.25</td><td>0</td></tr>
<tr><td>S1-final-viral-combined_k141_155544||full</td><td>2159</td><td>No</td><td>NA</td><td>0</td><td>1</td><td>Not-determined</td><td>Genome-fragment</td><td>NA</td><td>0</td></tr>
<tr><td>S1-final-viral-combined_k141_386428||full</td><td>3816</td><td>No</td><td>NA</td><td>0</td><td>4</td><td>Not-determined</td><td>Genome-fragment</td><td>NA</td><td>0</td></tr>
<tr><td>S1-final-viral-combined_k141_155567||full</td><td>2203</td><td>No</td><td>NA</td><td>0</td><td>1</td><td>Not-determined</td><td>Genome-fragment</td><td>NA</td><td>0</td></tr>
<tr><td>S9_k141_81047</td><td>1340</td><td>No</td><td>NA</td><td>1</td><td>0</td><td>Low-quality</td><td>Genome-fragment</td><td>2.85</td><td>0</td></tr>
<tr><td>S11-final-viral-combined_k141_70008||full</td><td>1340</td><td>No</td><td>NA</td><td>0</td><td>0</td><td>Low-quality</td><td>Genome-fragment</td><td>1.64</td><td>0</td></tr>
<tr><td>S1-final-viral-combined_k141_354024||full</td><td>1931</td><td>No</td><td>NA</td><td>0</td><td>1</td><td>Not-determined</td><td>Genome-fragment</td><td>NA</td><td>0</td></tr>
<tr><td>S11-final-viral-combined_k141_130239||full</td><td>1340</td><td>No</td><td>NA</td><td>0</td><td>0</td><td>Low-quality</td><td>Genome-fragment</td><td>2.39</td><td>0</td></tr>
<tr><td>S1-final-viral-combined_k141_155602||full</td><td>1800</td><td>No</td><td>NA</td><td>0</td><td>1</td><td>Not-determined</td><td>Genome-fragment</td><td>NA</td><td>0</td></tr>
<tr><td>S1-final-viral-combined_k141_238357||full</td><td>4145</td><td>No</td><td>NA</td><td>0</td><td>2</td><td>Not-determined</td><td>Genome-fragment</td><td>NA</td><td>0</td></tr>
<tr><td>S11-final-viral-combined_k141_185532||full</td><td>1340</td><td>No</td><td>NA</td><td>0</td><td>2</td><td>Low-quality</td><td>Genome-fragment</td><td>0.84</td><td>0</td></tr>
<tr><td>S1-final-viral-combined_k141_354048||full</td><td>2865</td><td>No</td><td>NA</td><td>0</td><td>3</td><td>Not-determined</td><td>Genome-fragment</td><td>NA</td><td>0</td></tr>
<tr><td>S1-final-viral-combined_k141_189459||full</td><td>4613</td><td>No</td><td>NA</td><td>0</td><td>1</td><td>Not-determined</td><td>Genome-fragment</td><td>NA</td><td>0</td></tr>
<tr><td>S11-final-viral-combined_k141_204859||full</td><td>1340</td><td>No</td><td>NA</td><td>1</td><td>0</td><td>Low-quality</td><td>Genome-fragment</td><td>1.6</td><td>0</td></tr>
<tr><td>S1-final-viral-combined_k141_57147||full</td><td>10578</td><td>No</td><td>NA</td><td>0</td><td>1</td><td>Not-determined</td><td>Genome-fragment</td><td>NA</td><td>0</td></tr>
<tr><td>S1-final-viral-combined_k141_306778||full</td><td>1340</td><td>No</td><td>NA</td><td>1</td><td>0</td><td>Low-quality</td><td>Genome-fragment</td><td>1.22</td><td>0</td></tr>
<tr><td>S1-final-viral-combined_k141_143289||full</td><td>1340</td><td>No</td><td>NA</td><td>0</td><td>0</td><td>Low-quality</td><td>Genome-fragment</td><td>1.17</td><td>0</td></tr>
<tr><td>S1-final-viral-combined_k141_155639||full</td><td>1043</td><td>No</td><td>NA</td><td>0</td><td>0</td><td>Not-determined</td><td>Genome-fragment</td><td>NA</td><td>0</td></tr>
<tr><td>S1-final-viral-combined_k141_62576||full</td><td>1340</td><td>No</td><td>NA</td><td>0</td><td>0</td><td>Low-quality</td><td>Genome-fragment</td><td>3.68</td><td>0</td></tr>
<tr><td>S1-final-viral-combined_k141_89958||full</td><td>6602</td><td>No</td><td>NA</td><td>0</td><td>1</td><td>Not-determined</td><td>Genome-fragment</td><td>NA</td><td>0</td></tr>
<tr><td>S3-final-viral-combined_k141_100425||full</td><td>1340</td><td>No</td><td>NA</td><td>0</td><td>0</td><td>Low-quality</td><td>Genome-fragment</td><td>3.09</td><td>0</td></tr>
<tr><td>S1-final-viral-combined_k141_238388||full</td><td>1217</td><td>No</td><td>NA</td><td>0</td><td>1</td><td>Not-determined</td><td>Genome-fragment</td><td>NA</td><td>0</td></tr>
<tr><td>S1-final-viral-combined_k141_255314||full</td><td>2027</td><td>No</td><td>NA</td><td>0</td><td>1</td><td>Not-determined</td><td>Genome-fragment</td><td>NA</td><td>0</td></tr>
<tr><td>S1-final-viral-combined_k141_271072||full</td><td>1469</td><td>No</td><td>NA</td><td>0</td><td>0</td><td>Not-determined</td><td>Genome-fragment</td><td>NA</td><td>0</td></tr>
<tr><td>S1-final-viral-combined_k141_386520||full</td><td>1654</td><td>No</td><td>NA</td><td>0</td><td>2</td><td>Not-determined</td><td>Genome-fragment</td><td>NA</td><td>0</td></tr>
<tr><td>S5-final-viral-combined_k141_169476||full</td><td>1340</td><td>No</td><td>NA</td><td>0</td><td>0</td><td>Low-quality</td><td>Genome-fragment</td><td>0.69</td><td>0</td></tr>
<tr><td>S1-final-viral-combined_k141_189507||full</td><td>1073</td><td>No</td><td>NA</td><td>0</td><td>1</td><td>Not-determined</td><td>Genome-fragment</td><td>NA</td><td>0</td></tr>
<tr><td>S1-final-viral-combined_k141_89986||full</td><td>1717</td><td>No</td><td>NA</td><td>0</td><td>0</td><td>Not-determined</td><td>Genome-fragment</td><td>NA</td><td>0</td></tr>
<tr><td>S5-final-viral-combined_k141_192161||full</td><td>1340</td><td>No</td><td>NA</td><td>1</td><td>0</td><td>Low-quality</td><td>Genome-fragment</td><td>2.19</td><td>0</td></tr>
<tr><td>S17-k141_45085</td><td>1340</td><td>No</td><td>NA</td><td>0</td><td>2</td><td>Low-quality</td><td>Genome-fragment</td><td>3.42</td><td>0</td></tr>
<tr><td>S12-final-viral-combined_k141_60801||full</td><td>1340</td><td>No</td><td>NA</td><td>0</td><td>0</td><td>Low-quality</td><td>Genome-fragment</td><td>2.74</td><td>0</td></tr>
<tr><td>S1-final-viral-combined_k141_155694||full</td><td>1999</td><td>No</td><td>NA</td><td>0</td><td>1</td><td>Not-determined</td><td>Genome-fragment</td><td>NA</td><td>0</td></tr>
<tr><td>S2-final-viral-combined_k141_35519||full</td><td>1340</td><td>No</td><td>NA</td><td>1</td><td>0</td><td>Low-quality</td><td>Genome-fragment</td><td>2.99</td><td>0</td></tr>
<tr><td>S2-final-viral-combined_k141_224440||full</td><td>1340</td><td>No</td><td>NA</td><td>0</td><td>0</td><td>Low-quality</td><td>Genome-fragment</td><td>0.93</td><td>0</td></tr>
<tr><td>S1-final-viral-combined_k141_238443||full</td><td>1149</td><td>No</td><td>NA</td><td>0</td><td>1</td><td>Not-determined</td><td>Genome-fragment</td><td>NA</td><td>0</td></tr>
<tr><td>S1-final-viral-combined_k141_123964||full</td><td>9518</td><td>No</td><td>NA</td><td>0</td><td>6</td><td>Not-determined</td><td>Genome-fragment</td><td>NA</td><td>0</td></tr>
<tr><td>S1-final-viral-combined_k141_238452||full</td><td>2185</td><td>No</td><td>NA</td><td>0</td><td>2</td><td>Not-determined</td><td>Genome-fragment</td><td>NA</td><td>0</td></tr>
<tr><td>S1-final-viral-combined_k141_386580||full</td><td>3061</td><td>No</td><td>NA</td><td>0</td><td>2</td><td>Not-determined</td><td>Genome-fragment</td><td>NA</td><td>0</td></tr>
<tr><td>S2-final-viral-combined_k141_206395||full</td><td>1340</td><td>No</td><td>NA</td><td>0</td><td>2</td><td>Low-quality</td><td>Genome-fragment</td><td>3.39</td><td>0</td></tr>
<tr><td>S6-final-viral-combined_k141_50563||lt2gene</td><td>1340</td><td>No</td><td>NA</td><td>1</td><td>0</td><td>Low-quality</td><td>Genome-fragment</td><td>0.56</td><td>0</td></tr>
<tr><td>S1-final-viral-combined_k141_123973||full</td><td>2010</td><td>No</td><td>NA</td><td>0</td><td>1</td><td>Not-determined</td><td>Genome-fragment</td><td>NA</td><td>0</td></tr>
<tr><td>S9-final-viral-combined_k141_34957||full</td><td>1341</td><td>No</td><td>NA</td><td>0</td><td>0</td><td>Low-quality</td><td>Genome-fragment</td><td>2.29</td><td>0</td></tr>
<tr><td>S1-final-viral-combined_k141_221900||full</td><td>1132</td><td>No</td><td>NA</td><td>0</td><td>0</td><td>Not-determined</td><td>Genome-fragment</td><td>NA</td><td>0</td></tr>
<tr><td>S1-final-viral-combined_k141_255378||full</td><td>1626</td><td>No</td><td>NA</td><td>0</td><td>0</td><td>Not-determined</td><td>Genome-fragment</td><td>NA</td><td>0</td></tr>
<tr><td>S1-final-viral-combined_k141_336359||full</td><td>1603</td><td>No</td><td>NA</td><td>0</td><td>2</td><td>Not-determined</td><td>Genome-fragment</td><td>NA</td><td>0</td></tr>
<tr><td>S1-final-viral-combined_k141_369702||full</td><td>5629</td><td>No</td><td>NA</td><td>0</td><td>2</td><td>Not-determined</td><td>Genome-fragment</td><td>NA</td><td>0</td></tr>
<tr><td>S11-final-viral-combined_k141_117647||full</td><td>1341</td><td>No</td><td>NA</td><td>0</td><td>0</td><td>Low-quality</td><td>Genome-fragment</td><td>1.22</td><td>0</td></tr>
<tr><td>S1-final-viral-combined_k141_238493||full</td><td>3455</td><td>No</td><td>NA</td><td>0</td><td>3</td><td>Not-determined</td><td>Genome-fragment</td><td>NA</td><td>0</td></tr>
<tr><td>S1-final-viral-combined_k141_321697||full</td><td>2032</td><td>No</td><td>NA</td><td>0</td><td>0</td><td>Not-determined</td><td>Genome-fragment</td><td>NA</td><td>0</td></tr>
<tr><td>S11-final-viral-combined_k141_173766||full</td><td>1341</td><td>No</td><td>NA</td><td>1</td><td>0</td><td>Low-quality</td><td>Genome-fragment</td><td>2.84</td><td>0</td></tr>
<tr><td>S11-final-viral-combined_k141_270678||full</td><td>1341</td><td>No</td><td>NA</td><td>0</td><td>1</td><td>Low-quality</td><td>Genome-fragment</td><td>1.97</td><td>0</td></tr>
<tr><td>S11_k141_69213</td><td>1341</td><td>No</td><td>NA</td><td>0</td><td>0</td><td>Low-quality</td><td>Genome-fragment</td><td>2.32</td><td>0</td></tr>
<tr><td>S1-final-viral-combined_k141_287643||full</td><td>2964</td><td>No</td><td>NA</td><td>0</td><td>1</td><td>Not-determined</td><td>Genome-fragment</td><td>NA</td><td>0</td></tr>
<tr><td>S1-final-viral-combined_k141_189589||full</td><td>1445</td><td>No</td><td>NA</td><td>0</td><td>1</td><td>Not-determined</td><td>Genome-fragment</td><td>NA</td><td>0</td></tr>
<tr><td>S1-final-viral-combined_k141_8058||full</td><td>1483</td><td>No</td><td>NA</td><td>0</td><td>1</td><td>Not-determined</td><td>Genome-fragment</td><td>NA</td><td>0</td></tr>
<tr><td>S1-final-viral-combined_k141_327960||full</td><td>1341</td><td>No</td><td>NA</td><td>0</td><td>0</td><td>Low-quality</td><td>Genome-fragment</td><td>22.83</td><td>0</td></tr>
<tr><td>S1-final-viral-combined_k141_237010||lt2gene</td><td>1341</td><td>No</td><td>NA</td><td>1</td><td>0</td><td>Low-quality</td><td>Genome-fragment</td><td>3.28</td><td>0</td></tr>
<tr><td>S3-final-viral-combined_k141_7521||full</td><td>1341</td><td>No</td><td>NA</td><td>0</td><td>1</td><td>Low-quality</td><td>Genome-fragment</td><td>1.21</td><td>0</td></tr>
<tr><td>S3-final-viral-combined_k141_2234||full</td><td>1341</td><td>No</td><td>NA</td><td>0</td><td>1</td><td>Low-quality</td><td>Genome-fragment</td><td>3.09</td><td>0</td></tr>
<tr><td>S3-final-viral-combined_k141_44100||lt2gene</td><td>1341</td><td>No</td><td>NA</td><td>1</td><td>0</td><td>Low-quality</td><td>Genome-fragment</td><td>3.32</td><td>0</td></tr>
<tr><td>S1-final-viral-combined_k141_206015||full</td><td>1856</td><td>No</td><td>NA</td><td>0</td><td>0</td><td>Not-determined</td><td>Genome-fragment</td><td>NA</td><td>0</td></tr>
<tr><td>S5-final-viral-combined_k141_111783||full</td><td>1341</td><td>No</td><td>NA</td><td>0</td><td>0</td><td>Low-quality</td><td>Genome-fragment</td><td>2.77</td><td>0</td></tr>
<tr><td>S1-final-viral-combined_k141_189619||full</td><td>1050</td><td>No</td><td>NA</td><td>0</td><td>0</td><td>Not-determined</td><td>Genome-fragment</td><td>NA</td><td>0</td></tr>
</table>

| | | | | | | | | | | |
|---|---|---|---|---|---|---|---|---|---|---|
| S5-final-viral-combined_k141_199570\|\|lt2gene | 1341 | No | NA | 1 | 0 | Low-quality | Genome-fragment | | 2.45 | 0 |
| S1-final-viral-combined_k141_369761\|\|full | 1546 | No | NA | 0 | 0 | Not-determined | Genome-fragment | NA | | 0 |
| S1-final-viral-combined_k141_304839\|\|full | 1623 | No | NA | 0 | 0 | Not-determined | Genome-fragment | NA | | 0 |
| S1-final-viral-combined_k141_24198\|\|full | 2006 | No | NA | 0 | 1 | Not-determined | Genome-fragment | NA | | 0 |
| S1-final-viral-combined_k141_73082\|\|full | 1539 | No | NA | 0 | 0 | Not-determined | Genome-fragment | NA | | 0 |
| S7-final-viral-combined_k141_136575\|\|full | 1341 | No | NA | 2 | 0 | Low-quality | Genome-fragment | | 1.61 | 0 |
| S1-final-viral-combined_k141_386667\|\|full | 3839 | No | NA | 0 | 3 | Not-determined | Genome-fragment | NA | | 0 |
| S1-final-viral-combined_k141_336466\|\|full | 4891 | No | NA | 0 | 3 | Not-determined | Genome-fragment | NA | | 0 |
| S7-final-viral-combined_k141_202503\|\|full | 1341 | No | NA | 0 | 0 | Low-quality | Genome-fragment | | 1.46 | 0 |
| S7-final-viral-combined_k141_193415\|\|full | 1341 | No | NA | 1 | 0 | Low-quality | Genome-fragment | | 3.08 | 0 |
| S1-final-viral-combined_k141_369787\|\|full | 2325 | No | NA | 0 | 2 | Not-determined | Genome-fragment | NA | | 0 |
| S12-final-viral-combined_k141_26968\|\|full | 1341 | No | NA | 0 | 0 | Low-quality | Genome-fragment | | 3.25 | 0 |
| S12-final-viral-combined_k141_44914\|\|full | 1341 | No | NA | 0 | 2 | Low-quality | Genome-fragment | | 2.32 | 0 |
| S1-final-viral-combined_k141_189692\|\|full | 12207 | No | NA | 0 | 6 | Not-determined | Genome-fragment | NA | | 0 |
| S12-final-viral-combined_k141_100021\|\|full | 1341 | No | NA | 0 | 0 | Low-quality | Genome-fragment | | 1.8 | 0 |
| S12-final-viral-combined_k141_286806\|\|lt2gene | 1341 | No | NA | 2 | 0 | Low-quality | Genome-fragment | | 1.16 | 0 |
| S1-final-viral-combined_k141_304895\|\|full | 1663 | No | NA | 0 | 2 | Not-determined | Genome-fragment | NA | | 0 |
| S12_k141_291068 | 1341 | No | NA | 0 | 1 | Low-quality | Genome-fragment | | 3.8 | 0 |
| S1-final-viral-combined_k141_321851\|\|full | 5585 | No | NA | 0 | 2 | Not-determined | Genome-fragment | NA | | 0 |
| S2-final-viral-combined_k141_99389\|\|full | 1341 | No | NA | 0 | 1 | Low-quality | Genome-fragment | | 1.05 | 0 |
| S2-final-viral-combined_k141_20537\|\|full | 1341 | No | NA | 0 | 0 | Low-quality | Genome-fragment | | 0.92 | 0 |
| S1-final-viral-combined_k141_336541\|\|full | 2578 | No | NA | 0 | 1 | Not-determined | Genome-fragment | NA | | 0 |
| S8-final-viral-combined_k141_70024\|\|full | 1341 | No | NA | 0 | 0 | Low-quality | Genome-fragment | | 2.1 | 0 |
| S1-final-viral-combined_k141_336555\|\|full | 1012 | No | NA | 0 | 0 | Not-determined | Genome-fragment | NA | | 0 |
| S9-final-viral-combined_k141_6327\|\|full | 1342 | No | NA | 2 | 0 | Low-quality | Genome-fragment | | 2.27 | 0 |
| S1-final-viral-combined_k141_296812\|\|full | 1342 | No | NA | 0 | 0 | Low-quality | Genome-fragment | | 1.92 | 0 |
| S1-final-viral-combined_k141_73131\|\|full | 4383 | No | NA | 0 | 3 | Not-determined | Genome-fragment | NA | | 0 |
| S1-final-viral-combined_k141_8205\|\|full | 1576 | No | NA | 0 | 0 | Not-determined | Genome-fragment | NA | | 0 |
| S1-final-viral-combined_k141_153857\|\|lt2gene | 1342 | No | NA | 1 | 0 | Low-quality | Genome-fragment | | 3.65 | 0 |
| S1-final-viral-combined_k141_238680\|\|full | 1760 | No | NA | 0 | 1 | Not-determined | Genome-fragment | NA | | 0 |
| S5-final-viral-combined_k141_78157\|\|full | 1342 | No | NA | 1 | 0 | Low-quality | Genome-fragment | | 3.03 | 0 |
| S5_k141_68431 | 1342 | No | NA | 0 | 1 | Low-quality | Genome-fragment | | 3.78 | 0 |
| S1-final-viral-combined_k141_354135\|\|full | 3752 | No | NA | 0 | 1 | Not-determined | Genome-fragment | NA | | 0 |
| S1-final-viral-combined_k141_255501\|\|full | 1802 | No | NA | 0 | 0 | Not-determined | Genome-fragment | NA | | 0 |
| S1-final-viral-combined_k141_140027\|\|full | 4093 | No | NA | 0 | 3 | Not-determined | Genome-fragment | NA | | 0 |
| S7-final-viral-combined_k141_215465\|\|full | 1342 | No | NA | 2 | 0 | Low-quality | Genome-fragment | | 3.61 | 0 |
| S1-final-viral-combined_k141_8264\|\|full | 1300 | No | NA | 0 | 0 | Not-determined | Genome-fragment | NA | | 0 |
| S1-final-viral-combined_k141_336640\|\|full | 2548 | No | NA | 0 | 3 | Not-determined | Genome-fragment | NA | | 0 |
| S7_k141_47522 | 1342 | No | NA | 0 | 1 | Low-quality | Genome-fragment | | 0.75 | 0 |
| S12-final-viral-combined_k141_262075\|\|full | 1342 | No | NA | 0 | 2 | Low-quality | Genome-fragment | | 0.4 | 0 |
| S1-final-viral-combined_k141_189784\|\|full | 1827 | No | NA | 0 | 1 | Not-determined | Genome-fragment | NA | | 0 |
| S1-final-viral-combined_k141_255537\|\|full | 2488 | No | NA | 0 | 1 | Not-determined | Genome-fragment | NA | | 0 |
| S1-final-viral-combined_k141_271270\|\|full | 1430 | No | NA | 0 | 1 | Not-determined | Genome-fragment | NA | | 0 |
| S1-final-viral-combined_k141_189791\|\|full | 1663 | No | NA | 0 | 2 | Not-determined | Genome-fragment | NA | | 0 |
| S1-final-viral-combined_k141_287883\|\|full | 2156 | No | NA | 0 | 2 | Not-determined | Genome-fragment | NA | | 0 |
| S1-final-viral-combined_k141_305028\|\|full | 2397 | No | NA | 0 | 1 | Not-determined | Genome-fragment | NA | | 0 |
| S1-final-viral-combined_k141_124184\|\|full | 4174 | No | NA | 0 | 3 | Not-determined | Genome-fragment | NA | | 0 |
| S1-final-viral-combined_k141_386841\|\|full | 1742 | No | NA | 0 | 2 | Not-determined | Genome-fragment | NA | | 0 |
| S1-final-viral-combined_k141_271282\|\|full | 1970 | No | NA | 0 | 2 | Not-determined | Genome-fragment | NA | | 0 |
| S12-final-viral-combined_k141_199921\|\|full | 1342 | No | NA | 0 | 0 | Low-quality | Genome-fragment | | 1.16 | 0 |
| S1-final-viral-combined_k141_287907\|\|full | 1965 | No | NA | 0 | 0 | Not-determined | Genome-fragment | NA | | 0 |
| S1-final-viral-combined_k141_8317\|\|full | 4345 | No | NA | 0 | 4 | Not-determined | Genome-fragment | NA | | 0 |
| S1-final-viral-combined_k141_140052\|\|full | 1950 | No | NA | 0 | 1 | Not-determined | Genome-fragment | NA | | 0 |
| S12-final-viral-combined_k141_231534\|\|lt2gene | 1342 | No | NA | 1 | 0 | Low-quality | Genome-fragment | | 3.17 | 0 |
| S1-final-viral-combined_k141_369976\|\|full | 1070 | No | NA | 0 | 1 | Not-determined | Genome-fragment | NA | | 0 |
| S1-final-viral-combined_k141_171841\|\|full | 2828 | No | NA | 0 | 0 | Not-determined | Genome-fragment | NA | | 0 |
| S12_k141_306697 | 1342 | No | NA | 0 | 0 | Low-quality | Genome-fragment | | 2.38 | 0 |
| S1-final-viral-combined_k141_255611\|\|full | 1549 | No | NA | 0 | 0 | Not-determined | Genome-fragment | NA | | 0 |
| S1-final-viral-combined_k141_189869\|\|full | 3664 | No | NA | 0 | 3 | Not-determined | Genome-fragment | NA | | 0 |
| S12_k141_343857 | 1342 | No | NA | 0 | 0 | Low-quality | Genome-fragment | | 0.74 | 0 |
| S2-final-viral-combined_k141_163580\|\|full | 1342 | No | NA | 0 | 0 | Low-quality | Genome-fragment | | 3.2 | 0 |
| S1-final-viral-combined_k141_354276\|\|full | 1516 | No | NA | 0 | 1 | Not-determined | Genome-fragment | NA | | 0 |
| S2-final-viral-combined_k141_205721\|\|full | 1342 | No | NA | 2 | 0 | Low-quality | Genome-fragment | | 2.36 | 0 |
| S1-final-viral-combined_k141_171862\|\|full | 3750 | No | NA | 0 | 3 | Not-determined | Genome-fragment | NA | | 0 |
| S9-final-viral-combined_k141_19706\|\|full | 1343 | No | NA | 0 | 2 | Low-quality | Genome-fragment | | 1.83 | 0 |
| S1-final-viral-combined_k141_222105\|\|full | 1213 | No | NA | 0 | 2 | Not-determined | Genome-fragment | NA | | 0 |
| S1-final-viral-combined_k141_386903\|\|full | 1297 | No | NA | 0 | 1 | Not-determined | Genome-fragment | NA | | 0 |
| S11-final-viral-combined_k141_229280\|\|full | 1343 | No | NA | 0 | 1 | Low-quality | Genome-fragment | | 2.73 | 0 |
| S11_k141_264131 | 1343 | No | NA | 2 | 0 | Low-quality | Genome-fragment | | 1.01 | 0 |
| S1-final-viral-combined_k141_57370\|\|full | 24795 | Yes | 20871 | 5 | 5 | Medium-quality | Genome-fragment | | 59.24 | 15.83 |
| S1-final-viral-combined_k141_262004\|\|full | 1343 | No | NA | 0 | 0 | Low-quality | Genome-fragment | | 3.76 | 0 |
| S1-final-viral-combined_k141_171909\|\|full | 6593 | No | NA | 0 | 3 | Not-determined | Genome-fragment | NA | | 0 |
| S5-final-viral-combined_k141_171092\|\|full | 1343 | No | NA | 0 | 1 | Low-quality | Genome-fragment | | 1.78 | 0 |
| S7-final-viral-combined_k141_169322\|\|full | 1343 | No | NA | 0 | 0 | Low-quality | Genome-fragment | | 1.67 | 0 |
| S7-final-viral-combined_k141_37947\|\|full | 1343 | No | NA | 1 | 0 | Low-quality | Genome-fragment | | 1.1 | 0 |
| S1-final-viral-combined_k141_370021\|\|full | 2783 | No | NA | 0 | 2 | Not-determined | Genome-fragment | NA | | 0 |
| S1-final-viral-combined_k141_271402\|\|full | 1085 | No | NA | 0 | 2 | Not-determined | Genome-fragment | NA | | 0 |
| S1-final-viral-combined_k141_24416\|\|full | 2159 | No | NA | 0 | 1 | Not-determined | Genome-fragment | NA | | 0 |
| S7_k141_188001 | 1343 | No | NA | 0 | 1 | Low-quality | Genome-fragment | | 2.69 | 0 |
| S17-final-viral-combined_k141_186608\|\|full | 1343 | No | NA | 2 | 0 | Low-quality | Genome-fragment | | 3.58 | 0 |
| S12-final-viral-combined_k141_228650\|\|full | 1343 | No | NA | 0 | 0 | Low-quality | Genome-fragment | | 3.51 | 0 |
| S1-final-viral-combined_k141_354386\|\|full | 2092 | No | NA | 0 | 1 | Not-determined | Genome-fragment | NA | | 0 |
| S1-final-viral-combined_k141_255737\|\|full | 6452 | No | NA | 0 | 2 | Not-determined | Genome-fragment | NA | | 0 |
| S1-final-viral-combined_k141_386975\|\|full | 3555 | No | NA | 0 | 3 | Not-determined | Genome-fragment | NA | | 0 |
| S1-final-viral-combined_k141_206381\|\|full | 2467 | No | NA | 0 | 1 | Not-determined | Genome-fragment | NA | | 0 |
| S2-final-viral-combined_k141_104611\|\|full | 1343 | No | NA | 0 | 0 | Low-quality | Genome-fragment | | 3.7 | 0 |
| S1-final-viral-combined_k141_90307\|\|full | 1128 | No | NA | 0 | 0 | Not-determined | Genome-fragment | NA | | 0 |
| S1-final-viral-combined_k141_90311\|\|full | 1050 | No | NA | 0 | 0 | Not-determined | Genome-fragment | NA | | 0 |
| S1-final-viral-combined_k141_387021\|\|full | 1597 | No | NA | 0 | 1 | Not-determined | Genome-fragment | NA | | 0 |
| S1-final-viral-combined_k141_387026\|\|full | 1245 | No | NA | 0 | 0 | Not-determined | Genome-fragment | NA | | 0 |
| S1-final-viral-combined_k141_90332\|\|full | 2889 | No | NA | 0 | 1 | Not-determined | Genome-fragment | NA | | 0 |
| S2-final-viral-combined_k141_170777\|\|full | 1343 | No | NA | 0 | 1 | Low-quality | Genome-fragment | | 1.52 | 0 |
| S4-final-viral-combined_k141_25684\|\|full | 1343 | No | NA | 0 | 0 | Low-quality | Genome-fragment | | 2.17 | 0 |
| S1-final-viral-combined_k141_24494\|\|full | 4577 | No | NA | 0 | 0 | Not-determined | Genome-fragment | NA | | 0 |
| S9-final-viral-combined_k141_53788\|\|full | 1344 | No | NA | 0 | 0 | Low-quality | Genome-fragment | | 1.5 | 0 |
| S1-final-viral-combined_k141_255823\|\|full | 1713 | No | NA | 0 | 1 | Not-determined | Genome-fragment | NA | | 0 |
| S1-final-viral-combined_k141_238910\|\|full | 1672 | No | NA | 0 | 1 | Not-determined | Genome-fragment | NA | | 0 |
| S1-final-viral-combined_k141_322161\|\|full | 2824 | No | NA | 0 | 2 | Not-determined | Genome-fragment | NA | | 0 |
| S1-final-viral-combined_k141_124452\|\|full | 1636 | No | NA | 0 | 0 | Not-determined | Genome-fragment | NA | | 0 |
| S11-final-viral-combined_k141_242236\|\|full | 1344 | No | NA | 0 | 1 | Low-quality | Genome-fragment | | 0.31 | 0 |
| S1-final-viral-combined_k141_370128\|\|full | 1664 | No | NA | 0 | 2 | Not-determined | Genome-fragment | NA | | 0 |
| S1-final-viral-combined_k141_301690\|\|full | 1344 | No | NA | 0 | 0 | Low-quality | Genome-fragment | | 7.24 | 0 |
| S5_k141_171525 | 1344 | No | NA | 0 | 0 | Low-quality | Genome-fragment | | 1.02 | 0 |
| S7-final-viral-combined_k141_189310\|\|full | 1344 | No | NA | 0 | 3 | Low-quality | Genome-fragment | | 1.19 | 0 |
| S1-final-viral-combined_k141_24515\|\|full | 1255 | No | NA | 0 | 1 | Not-determined | Genome-fragment | NA | | 0 |
| S7-final-viral-combined_k141_179549\|\|full | 1344 | No | NA | 2 | 0 | Low-quality | Genome-fragment | | 1.91 | 0 |
| S1-final-viral-combined_k141_255854\|\|full | 2165 | No | NA | 0 | 1 | Not-determined | Genome-fragment | NA | | 0 |
| S1-final-viral-combined_k141_288059\|\|full | 2307 | No | NA | 0 | 2 | Not-determined | Genome-fragment | NA | | 0 |
| S17-final-viral-combined_k141_56186\|\|full | 1344 | No | NA | 0 | 0 | Low-quality | Genome-fragment | | 2.51 | 0 |
| S17-final-viral-combined_k141_69471\|\|full | 1344 | No | NA | 1 | 0 | Low-quality | Genome-fragment | | 3.79 | 0 |
| S1-final-viral-combined_k141_190157\|\|full | 1215 | No | NA | 0 | 1 | Not-determined | Genome-fragment | NA | | 0 |
| S17-final-viral-combined_k141_265542\|\|full | 1344 | No | NA | 1 | 0 | Low-quality | Genome-fragment | | 3.58 | 0 |
| S12-final-viral-combined_k141_172359\|\|full | 1344 | No | NA | 0 | 0 | Low-quality | Genome-fragment | | 3.28 | 0 |
| S1-final-viral-combined_k141_40822\|\|full | 2397 | No | NA | 0 | 1 | Not-determined | Genome-fragment | NA | | 0 |
| S12-final-viral-combined_k141_383082\|\|lt2gene | 1344 | No | NA | 2 | 0 | Low-quality | Genome-fragment | | 2.92 | 0 |
| S1-final-viral-combined_k141_73338\|\|full | 2748 | No | NA | 0 | 2 | Not-determined | Genome-fragment | NA | | 0 |
| S2-final-viral-combined_k141_173375\|\|full | 1344 | No | NA | 1 | 0 | Low-quality | Genome-fragment | | 3.21 | 0 |
| S4_k141_49111 | 1344 | No | NA | 0 | 0 | Low-quality | Genome-fragment | | 2.33 | 0 |
| S6-final-viral-combined_k141_34480\|\|full | 1344 | No | NA | 0 | 0 | Low-quality | Genome-fragment | | 3.86 | 0 |
| S1-final-viral-combined_k141_40863\|\|full | 1408 | No | NA | 0 | 2 | Not-determined | Genome-fragment | NA | | 0 |
| S8-final-viral-combined_k141_265370\|\|full | 1344 | No | NA | 0 | 0 | Low-quality | Genome-fragment | | 2.27 | 0 |
| S11-final-viral-combined_k141_239790\|\|full | 1345 | No | NA | 0 | 0 | Low-quality | Genome-fragment | | 3.14 | 0 |
| S11-final-viral-combined_k141_62023\|\|full | 1345 | No | NA | 1 | 0 | Low-quality | Genome-fragment | | 3.22 | 0 |
| S1-final-viral-combined_k141_24572\|\|full | 3269 | No | NA | 0 | 1 | Not-determined | Genome-fragment | NA | | 0 |
| S1-final-viral-combined_k141_8502\|\|full | 1679 | No | NA | 0 | 0 | Not-determined | Genome-fragment | NA | | 0 |
| S11-final-viral-combined_k141_227700\|\|full | 1345 | No | NA | 0 | 0 | Low-quality | Genome-fragment | | 2.3 | 0 |
| S5-final-viral-combined_k141_127424\|\|full | 1345 | No | NA | 0 | 1 | Low-quality | Genome-fragment | | 1.34 | 0 |
| S5_k141_111090 | 1345 | No | NA | 0 | 0 | Low-quality | Genome-fragment | | 2.92 | 0 |
| S1-final-viral-combined_k141_124528\|\|full | 4577 | No | NA | 0 | 2 | Not-determined | Genome-fragment | NA | | 0 |
| S7-final-viral-combined_k141_151641\|\|full | 1345 | No | NA | 1 | 0 | Low-quality | Genome-fragment | | 0.86 | 0 |
| S12-final-viral-combined_k141_209557\|\|full | 1345 | No | NA | 0 | 1 | Low-quality | Genome-fragment | | 0.41 | 0 |
| S12-final-viral-combined_k141_65170\|\|full | 1345 | No | NA | 0 | 0 | Low-quality | Genome-fragment | | 3.05 | 0 |
| S1-final-viral-combined_k141_90416\|\|full | 9681 | No | NA | 0 | 7 | Not-determined | Genome-fragment | NA | | 0 |
| S12_k141_47518 | 1345 | No | NA | 0 | 0 | Low-quality | Genome-fragment | | 1.37 | 0 |
| S1-final-viral-combined_k141_155883\|\|full | 2050 | No | NA | 0 | 2 | Not-determined | Genome-fragment | NA | | 0 |
| S2-final-viral-combined_k141_171352\|\|full | 1345 | No | NA | 0 | 1 | Low-quality | Genome-fragment | | 2.27 | 0 |
| S4-final-viral-combined_k141_114901\|\|full | 1345 | No | NA | 1 | 0 | Low-quality | Genome-fragment | | 1.93 | 0 |
| S1-final-viral-combined_k141_305287\|\|full | 2148 | No | NA | 0 | 2 | Not-determined | Genome-fragment | NA | | 0 |
| S1-final-viral-combined_k141_238991\|\|full | 1823 | No | NA | 0 | 1 | Not-determined | Genome-fragment | NA | | 0 |
| S1-final-viral-combined_k141_206505\|\|full | 1442 | No | NA | 0 | 1 | Not-determined | Genome-fragment | NA | | 0 |
| S1-final-viral-combined_k141_190258\|\|full | 4449 | No | NA | 0 | 3 | Not-determined | Genome-fragment | NA | | 0 |
| S1-final-viral-combined_k141_73444\|\|full | 3161 | No | NA | 0 | 2 | Not-determined | Genome-fragment | NA | | 0 |
| S11-final-viral-combined_k141_20498\|\|full | 1346 | No | NA | 0 | 0 | Low-quality | Genome-fragment | | 1.8 | 0 |
| S1-final-viral-combined_k141_255975\|\|full | 3193 | No | NA | 0 | 3 | Not-determined | Genome-fragment | NA | | 0 |
| S11-final-viral-combined_k141_65374\|\|full | 1346 | No | NA | 0 | 0 | Low-quality | Genome-fragment | | 3.35 | 0 |
| S1-final-viral-combined_k141_271693\|\|full | 1234 | No | NA | 0 | 1 | Not-determined | Genome-fragment | NA | | 0 |
| S1-final-viral-combined_k141_336818\|\|full | 3934 | No | NA | 0 | 3 | Not-determined | Genome-fragment | NA | | 0 |
| S1-final-viral-combined_k141_8576\|\|full | 2240 | No | NA | 0 | 0 | Not-determined | Genome-fragment | NA | | 0 |
| S3-final-viral-combined_k141_98273\|\|full | 1346 | No | NA | 0 | 0 | Low-quality | Genome-fragment | | 0.85 | 0 |
| S3-final-viral-combined_k141_6479\|\|full | 1346 | No | NA | 0 | 0 | Low-quality | Genome-fragment | | 0.95 | 0 |
| S3_k141_101694 | 1346 | No | NA | 0 | 0 | Low-quality | Genome-fragment | | 2.25 | 0 |
| S1-final-viral-combined_k141_336847\|\|full | 2490 | No | NA | 0 | 1 | Not-determined | Genome-fragment | NA | | 0 |
| S1-final-viral-combined_k141_90511\|\|full | 1748 | No | NA | 0 | 1 | Not-determined | Genome-fragment | NA | | 0 |
| S7-final-viral-combined_k141_215380\|\|full | 1346 | No | NA | 0 | 2 | Low-quality | Genome-fragment | | 2.53 | 0 |
| S1-final-viral-combined_k141_40968\|\|full | 2165 | No | NA | 0 | 0 | Not-determined | Genome-fragment | NA | | 0 |
| S1-final-viral-combined_k141_124601\|\|full | 1852 | No | NA | 0 | 0 | Not-determined | Genome-fragment | NA | | 0 |
| S7_k141_118027 | 1346 | No | NA | 1 | 0 | Low-quality | Genome-fragment | | 2.5 | 0 |
| S17-final-viral-combined_k141_67500\|\|full | 1346 | No | NA | 0 | 1 | Low-quality | Genome-fragment | | 0.45 | 0 |
| S1-final-viral-combined_k141_73519\|\|full | 2586 | No | NA | 0 | 2 | Not-determined | Genome-fragment | NA | | 0 |
| S12-final-viral-combined_k141_314422\|\|full | 1346 | No | NA | 0 | 0 | Low-quality | Genome-fragment | | 3.82 | 0 |
| S1-final-viral-combined_k141_322336\|\|full | 2309 | No | NA | 0 | 2 | Not-determined | Genome-fragment | NA | | 0 |
| S12-final-viral-combined_k141_182113\|\|full | 1346 | No | NA | 0 | 0 | Low-quality | Genome-fragment | | 1.05 | 0 |
| S12_k141_106959 | 1346 | No | NA | 0 | 1 | Low-quality | Genome-fragment | | 0.51 | 0 |
| S1-final-viral-combined_k141_305400\|\|full | 1369 | No | NA | 0 | 1 | Not-determined | Genome-fragment | NA | | 0 |
| S1-final-viral-combined_k141_57613\|\|full | 6822 | No | NA | 0 | 3 | Not-determined | Genome-fragment | NA | | 0 |
| S1-final-viral-combined_k141_40977\|\|full | 1926 | No | NA | 0 | 2 | Not-determined | Genome-fragment | NA | | 0 |
| S2-final-viral-combined_k141_140664\|\|full | 1346 | No | NA | 0 | 0 | Low-quality | Genome-fragment | | 3.02 | 0 |
| S1-final-viral-combined_k141_40979\|\|full | 3406 | No | NA | 0 | 3 | Not-determined | Genome-fragment | NA | | 0 |
| S6-final-viral-combined_k141_49304\|\|full | 1346 | No | NA | 0 | 0 | Low-quality | Genome-fragment | | 3.28 | 0 |
| S1-final-viral-combined_k141_387285\|\|full | 1396 | No | NA | 0 | 1 | Not-determined | Genome-fragment | NA | | 0 |
| S1-final-viral-combined_k141_8717\|\|full | 1850 | No | NA | 0 | 2 | Not-determined | Genome-fragment | NA | | 0 |
| S1-final-viral-combined_k141_24776\|\|full | 3157 | No | NA | 0 | 2 | Not-determined | Genome-fragment | NA | | 0 |
| S8_k141_148762 | 1346 | No | NA | 0 | 0 | Low-quality | Genome-fragment | | 1.44 | 0 |
| S1-final-viral-combined_k141_155968\|\|full | 2204 | No | NA | 0 | 3 | Not-determined | Genome-fragment | NA | | 0 |

| | | | | | | | | | | |
|---|---|---|---|---|---|---|---|---|---|---|
| S11-final-viral-combined_k141_213524||full | 1347 | No | NA | 1 | 0 | Low-quality | Genome-fragment | | 2.27 | 0 |
| S1_k141_140091 | 1347 | No | NA | 0 | 1 | Low-quality | Genome-fragment | | 0.41 | 0 |
| S1-final-viral-combined_k141_8748||full | 2713 | No | NA | 0 | 1 | Not-determined | Genome-fragment | NA | | 0 |
| S3-final-viral-combined_k141_51948||full | 1347 | No | NA | 0 | 0 | Low-quality | Genome-fragment | | 3.03 | 0 |
| S1-final-viral-combined_k141_222507||full | 1544 | No | NA | 0 | 0 | Not-determined | Genome-fragment | NA | | 0 |
| S1-final-viral-combined_k141_387310||full | 3924 | No | NA | 0 | 2 | Not-determined | Genome-fragment | NA | | 0 |
| S1-final-viral-combined_k141_190441||full | 1510 | No | NA | 0 | 0 | Not-determined | Genome-fragment | NA | | 0 |
| S5-final-viral-combined_k141_31297||lt2gene | 1347 | No | NA | 1 | 0 | Low-quality | Genome-fragment | | 2.9 | 0 |
| S1-final-viral-combined_k141_387324||full | 1596 | No | NA | 0 | 2 | Not-determined | Genome-fragment | NA | | 0 |
| S7-final-viral-combined_k141_30197||full | 1347 | No | NA | 0 | 1 | Low-quality | Genome-fragment | | 2.79 | 0 |
| S1-final-viral-combined_k141_190451||full | 1131 | No | NA | 0 | 1 | Not-determined | Genome-fragment | NA | | 0 |
| S17-k141_29765 | 1347 | No | NA | 0 | 0 | Low-quality | Genome-fragment | | 2.51 | 0 |
| S1-final-viral-combined_k141_288255||full | 13560 | No | NA | 0 | 12 | Not-determined | Genome-fragment | NA | | 0 |
| S1-final-viral-combined_k141_73562||full | 2836 | No | NA | 0 | 1 | Not-determined | Genome-fragment | NA | | 0 |
| S1-final-viral-combined_k141_370410||full | 1298 | No | NA | 0 | 0 | Not-determined | Genome-fragment | NA | | 0 |
| S12-final-viral-combined_k141_72442||full | 1347 | No | NA | 1 | 0 | Low-quality | Genome-fragment | | 0.87 | 0 |
| S12-final-viral-combined_k141_91123||full | 1347 | No | NA | 2 | 0 | Low-quality | Genome-fragment | | 2.79 | 0 |
| S1-final-viral-combined_k141_322413||full | 2955 | No | NA | 0 | 1 | Not-determined | Genome-fragment | NA | | 0 |
| S2-final-viral-combined_k141_145007||full | 1347 | No | NA | 1 | 0 | Low-quality | Genome-fragment | | 1.64 | 0 |
| S1-final-viral-combined_k141_206672||full | 2420 | No | NA | 0 | 0 | Not-determined | Genome-fragment | NA | | 0 |
| S1-final-viral-combined_k141_322416||full | 2455 | No | NA | 0 | 1 | Not-determined | Genome-fragment | NA | | 0 |
| S1-final-viral-combined_k141_256029||full | 4316 | No | NA | 0 | 2 | Not-determined | Genome-fragment | NA | | 0 |
| S1-final-viral-combined_k141_370443||full | 1950 | No | NA | 0 | 1 | Not-determined | Genome-fragment | NA | | 0 |
| S2_k141_116220 | 1347 | No | NA | 0 | 0 | Low-quality | Genome-fragment | | 11.04 | 0 |
| S4-final-viral-combined_k141_121300||full | 1347 | No | NA | 0 | 1 | Low-quality | Genome-fragment | | 2.26 | 0 |
| S1-final-viral-combined_k141_41079||full | 1293 | No | NA | 0 | 1 | Not-determined | Genome-fragment | NA | | 0 |
| S1-final-viral-combined_k141_24864||full | 1915 | No | NA | 0 | 2 | Not-determined | Genome-fragment | NA | | 0 |
| S1-final-viral-combined_k141_8806||full | 1105 | No | NA | 0 | 1 | Not-determined | Genome-fragment | NA | | 0 |
| S8-final-viral-combined_k141_6669||full | 1347 | No | NA | 0 | 0 | Low-quality | Genome-fragment | | 3.43 | 0 |
| S1-final-viral-combined_k141_106396||full | 2043 | No | NA | 0 | 1 | Not-determined | Genome-fragment | NA | | 0 |
| S8_k141_18908 | 1347 | No | NA | 1 | 0 | Low-quality | Genome-fragment | | 0.9 | 0 |
| S1-final-viral-combined_k141_222635||full | 1372 | No | NA | 0 | 0 | Not-determined | Genome-fragment | NA | | 0 |
| S1-final-viral-combined_k141_190531||full | 2639 | No | NA | 0 | 1 | Not-determined | Genome-fragment | NA | | 0 |
| S11-final-viral-combined_k141_207502||full | 1348 | No | NA | 1 | 0 | Low-quality | Genome-fragment | | 3.02 | 0 |
| S11-final-viral-combined_k141_15629||full | 1348 | No | NA | 0 | 0 | Low-quality | Genome-fragment | | 3.03 | 0 |
| S1-final-viral-combined_k141_288322||full | 1119 | No | NA | 0 | 0 | Not-determined | Genome-fragment | NA | | 0 |
| S1-final-viral-combined_k141_90688||full | 1467 | No | NA | 0 | 1 | Not-determined | Genome-fragment | NA | | 0 |
| S11-final-viral-combined_k141_327330||full | 1348 | No | NA | 1 | 0 | Low-quality | Genome-fragment | | 2.47 | 0 |
| S11_k141_230400 | 1348 | No | NA | 0 | 0 | Low-quality | Genome-fragment | | 1.45 | 0 |
| S1-final-viral-combined_k141_354724||full | 2308 | No | NA | 0 | 2 | Not-determined | Genome-fragment | NA | | 0 |
| S1-final-viral-combined_k141_288357||full | 1066 | No | NA | 0 | 2 | Not-determined | Genome-fragment | NA | | 0 |
| S1-final-viral-combined_k141_370543||full | 2383 | No | NA | 0 | 0 | Not-determined | Genome-fragment | NA | | 0 |
| S1-final-viral-combined_k141_124910||full | 1348 | No | NA | 0 | 0 | Low-quality | Genome-fragment | | 0.43 | 0 |
| S1-final-viral-combined_k141_370551||full | 2730 | No | NA | 0 | 1 | Not-determined | Genome-fragment | NA | | 0 |
| S1-final-viral-combined_k141_140372||full | 2207 | No | NA | 0 | 1 | Not-determined | Genome-fragment | NA | | 0 |
| S1-final-viral-combined_k141_258316||full | 1348 | No | NA | 0 | 0 | Low-quality | Genome-fragment | | 2.12 | 0 |
| S1-final-viral-combined_k141_87278||lt2gene | 1348 | No | NA | 2 | 0 | Low-quality | Genome-fragment | | 3.96 | 0 |
| S1-final-viral-combined_k141_222683||full | 1474 | No | NA | 0 | 0 | Not-determined | Genome-fragment | NA | | 0 |
| S1_k141_110103 | 1348 | No | NA | 0 | 2 | Low-quality | Genome-fragment | | 0.35 | 0 |
| S1-final-viral-combined_k141_354739||full | 2778 | No | NA | 0 | 3 | Not-determined | Genome-fragment | NA | | 0 |
| S7-final-viral-combined_k141_41486||full | 1348 | No | NA | 1 | 0 | Low-quality | Genome-fragment | | 1.71 | 0 |
| S7_k141_184709 | 1348 | No | NA | 0 | 0 | Low-quality | Genome-fragment | | 1.42 | 0 |
| S17-k141_6522 | 1348 | No | NA | 0 | 1 | Low-quality | Genome-fragment | | 0.73 | 0 |
| S12-final-viral-combined_k141_23607||full | 1348 | No | NA | 0 | 1 | Low-quality | Genome-fragment | | 0.92 | 0 |
| S2-final-viral-combined_k141_51952||full | 1348 | No | NA | 0 | 0 | Low-quality | Genome-fragment | | 2.27 | 0 |
| S1-final-viral-combined_k141_140412||full | 1274 | No | NA | 0 | 3 | Not-determined | Genome-fragment | NA | | 0 |
| S1-final-viral-combined_k141_288436||full | 1813 | No | NA | 0 | 1 | Not-determined | Genome-fragment | NA | | 0 |
| S2-final-viral-combined_k141_163550||full | 1348 | No | NA | 0 | 0 | Low-quality | Genome-fragment | | 2.67 | 0 |
| S1-final-viral-combined_k141_190626||full | 3578 | No | NA | 0 | 0 | Not-determined | Genome-fragment | NA | | 0 |
| S1-final-viral-combined_k141_190636||full | 2958 | No | NA | 0 | 1 | Not-determined | Genome-fragment | NA | | 0 |
| S1-final-viral-combined_k141_239362||full | 2485 | No | NA | 0 | 3 | Not-determined | Genome-fragment | NA | | 0 |
| S1-final-viral-combined_k141_354782||full | 1387 | No | NA | 0 | 2 | Not-determined | Genome-fragment | NA | | 0 |
| S4_k141_53873 | 1348 | No | NA | 0 | 0 | Low-quality | Genome-fragment | | 1.63 | 0 |
| S9-final-viral-combined_k141_24458||full | 1349 | No | NA | 0 | 0 | Low-quality | Genome-fragment | | 2.25 | 0 |
| S9-final-viral-combined_k141_76805||lt2gene | 1349 | No | NA | 1 | 0 | Low-quality | Genome-fragment | | 3.8 | 0 |
| S1-final-viral-combined_k141_172284||full | 3383 | No | NA | 0 | 1 | Not-determined | Genome-fragment | NA | | 0 |
| S11_k141_242987 | 1349 | No | NA | 1 | 0 | Low-quality | Genome-fragment | | 0.92 | 0 |
| S1-final-viral-combined_k141_41288||full | 3092 | No | NA | 0 | 2 | Not-determined | Genome-fragment | NA | | 0 |
| S1-final-viral-combined_k141_370684||full | 1207 | No | NA | 0 | 1 | Not-determined | Genome-fragment | NA | | 0 |
| S1-final-viral-combined_k141_271958||full | 1890 | No | NA | 0 | 0 | Not-determined | Genome-fragment | NA | | 0 |
| S1-final-viral-combined_k141_352733||full | 1349 | No | NA | 0 | 0 | Low-quality | Genome-fragment | | 2.74 | 0 |
| S1-final-viral-combined_k141_270381||full | 1349 | No | NA | 0 | 0 | Low-quality | Genome-fragment | | 3.52 | 0 |
| S3-final-viral-combined_k141_66341||full | 1349 | No | NA | 0 | 0 | Low-quality | Genome-fragment | | 2.69 | 0 |
| S7-final-viral-combined_k141_8593||lt2gene | 1349 | No | NA | 1 | 0 | Low-quality | Genome-fragment | | 0.93 | 0 |
| S7_k141_49775 | 1349 | No | NA | 0 | 0 | Low-quality | Genome-fragment | | 2.79 | 0 |
| S17-final-viral-combined_k141_44881||full | 1349 | No | NA | 0 | 1 | Low-quality | Genome-fragment | | 0.79 | 0 |
| S12-final-viral-combined_k141_62323||full | 1349 | No | NA | 2 | 0 | Low-quality | Genome-fragment | | 1.36 | 0 |
| S1-final-viral-combined_k141_57839||full | 2059 | No | NA | 0 | 2 | Not-determined | Genome-fragment | NA | | 0 |
| S12-final-viral-combined_k141_216329||full | 1349 | No | NA | 0 | 1 | Low-quality | Genome-fragment | | 1.88 | 0 |
| S1-final-viral-combined_k141_140577||full | 1142 | No | NA | 0 | 0 | Not-determined | Genome-fragment | NA | | 0 |
| S1-final-viral-combined_k141_354899||full | 18395 | No | NA | 16 | 1 | Medium-quality | Genome-fragment | | 72.57 | 0 |
| S1-final-viral-combined_k141_322501||full | 3431 | No | NA | 0 | 3 | Not-determined | Genome-fragment | NA | | 0 |
| S1-final-viral-combined_k141_190702||full | 3774 | No | NA | 0 | 4 | Not-determined | Genome-fragment | NA | | 0 |
| S1-final-viral-combined_k141_25092||full | 2101 | No | NA | 0 | 2 | Not-determined | Genome-fragment | NA | | 0 |
| S1-final-viral-combined_k141_370755||full | 3345 | No | NA | 0 | 2 | Not-determined | Genome-fragment | NA | | 0 |
| S1-final-viral-combined_k141_156205||full | 1018 | No | NA | 0 | 0 | Not-determined | Genome-fragment | NA | | 0 |
| S1-final-viral-combined_k141_354936||full | 2127 | No | NA | 0 | 1 | Not-determined | Genome-fragment | NA | | 0 |
| S1-final-viral-combined_k141_73770||full | 3573 | No | NA | 0 | 2 | Not-determined | Genome-fragment | NA | | 0 |
| S1-final-viral-combined_k141_106563||full | 3362 | No | NA | 0 | 3 | Not-determined | Genome-fragment | NA | | 0 |
| S1-final-viral-combined_k141_156234||full | 6549 | No | NA | 0 | 0 | Not-determined | Genome-fragment | NA | | 0 |
| S1-final-viral-combined_k141_41369||full | 36028 | No | NA | 17 | 1 | Medium-quality | Genome-fragment | | 83.64 | 0 |
| S2-final-viral-combined_k141_15836||full | 1349 | No | NA | 3 | 0 | Low-quality | Genome-fragment | | 2.04 | 0 |
| S2-final-viral-combined_k141_130272||full | 1349 | No | NA | 1 | 0 | Low-quality | Genome-fragment | | 3.79 | 0 |
| S9-final-viral-combined_k141_124536||full | 1350 | No | NA | 1 | 0 | Low-quality | Genome-fragment | | 2.73 | 0 |
| S11-final-viral-combined_k141_18742||lt2gene | 1350 | No | NA | 2 | 0 | Low-quality | Genome-fragment | | 5.94 | 0 |
| S7-final-viral-combined_k141_133769||full | 1350 | No | NA | 0 | 1 | Low-quality | Genome-fragment | | 0.46 | 0 |
| S1-final-viral-combined_k141_370831||full | 1410 | No | NA | 0 | 0 | Not-determined | Genome-fragment | NA | | 0 |
| S17-final-viral-combined_k141_135993||full | 1350 | No | NA | 0 | 0 | Low-quality | Genome-fragment | | 3.68 | 0 |
| S1-final-viral-combined_k141_256361||full | 5195 | No | NA | 0 | 2 | Not-determined | Genome-fragment | NA | | 0 |
| S1-final-viral-combined_k141_125057||full | 1650 | No | NA | 0 | 0 | Not-determined | Genome-fragment | NA | | 0 |
| S2_k141_237059 | 1350 | No | NA | 0 | 0 | Low-quality | Genome-fragment | | 2.66 | 0 |
| S1-final-viral-combined_k141_91001||full | 3962 | No | NA | 0 | 4 | Not-determined | Genome-fragment | NA | | 0 |
| S1-final-viral-combined_k141_140700||full | 4058 | No | NA | 0 | 2 | Not-determined | Genome-fragment | NA | | 0 |
| S8-final-viral-combined_k141_133682||full | 1350 | No | NA | 0 | 0 | Low-quality | Genome-fragment | | 1.44 | 0 |
| S1-final-viral-combined_k141_125075||full | 5175 | No | NA | 0 | 3 | Not-determined | Genome-fragment | NA | | 0 |
| S1-final-viral-combined_k141_41379||full | 10985 | No | NA | 0 | 8 | Not-determined | Genome-fragment | NA | | 0 |
| S1-final-viral-combined_k141_288677||full | 1883 | No | NA | 0 | 2 | Not-determined | Genome-fragment | NA | | 0 |
| S1-final-viral-combined_k141_41387||full | 3852 | No | NA | 0 | 0 | Not-determined | Genome-fragment | NA | | 0 |
| S1-final-viral-combined_k141_156272||full | 1191 | No | NA | 0 | 1 | Not-determined | Genome-fragment | NA | | 0 |
| S11-final-viral-combined_k141_67256||full | 1351 | No | NA | 0 | 1 | Low-quality | Genome-fragment | | 0.45 | 0 |
| S1-final-viral-combined_k141_322615||full | 2495 | No | NA | 0 | 2 | Not-determined | Genome-fragment | NA | | 0 |
| S1-final-viral-combined_k141_9055||full | 6453 | No | NA | 0 | 2 | Not-determined | Genome-fragment | NA | | 0 |
| S1-final-viral-combined_k141_120927||full | 1351 | No | NA | 0 | 0 | Low-quality | Genome-fragment | | 2.35 | 0 |
| S1-final-viral-combined_k141_387706||full | 2088 | No | NA | 0 | 1 | Not-determined | Genome-fragment | NA | | 0 |
| S1-final-viral-combined_k141_73863||full | 1208 | No | NA | 0 | 0 | Not-determined | Genome-fragment | NA | | 0 |
| S1-final-viral-combined_k141_207052||full | 2177 | No | NA | 0 | 3 | Not-determined | Genome-fragment | NA | | 0 |
| S1-final-viral-combined_k141_73865||full | 1058 | No | NA | 0 | 1 | Not-determined | Genome-fragment | NA | | 0 |
| S5-final-viral-combined_k141_180510||full | 1351 | No | NA | 0 | 1 | Low-quality | Genome-fragment | | 1.75 | 0 |
| S1-final-viral-combined_k141_239603||full | 1244 | No | NA | 0 | 1 | Not-determined | Genome-fragment | NA | | 0 |
| S1-final-viral-combined_k141_256430||full | 2323 | No | NA | 0 | 2 | Not-determined | Genome-fragment | NA | | 0 |
| S1-final-viral-combined_k141_9085||full | 1355 | No | NA | 0 | 1 | Not-determined | Genome-fragment | NA | | 0 |
| S1-final-viral-combined_k141_125146||full | 3434 | No | NA | 0 | 2 | Not-determined | Genome-fragment | NA | | 0 |
| S1-final-viral-combined_k141_272193||full | 7269 | No | NA | 0 | 6 | Not-determined | Genome-fragment | NA | | 0 |
| S5-final-viral-combined_k141_19329||lt2gene | 1351 | No | NA | 1 | 0 | Low-quality | Genome-fragment | | 3.02 | 0 |
| S1-final-viral-combined_k141_387752||full | 1907 | No | NA | 0 | 3 | Not-determined | Genome-fragment | NA | | 0 |
| S1-final-viral-combined_k141_305953||full | 1861 | No | NA | 0 | 0 | Not-determined | Genome-fragment | NA | | 0 |
| S1-final-viral-combined_k141_156356||full | 2977 | No | NA | 0 | 1 | Not-determined | Genome-fragment | NA | | 0 |
| S7_k141_125183 | 1351 | No | NA | 0 | 0 | Low-quality | Genome-fragment | | 2.26 | 0 |
| S17-final-viral-combined_k141_184683||full | 1351 | No | NA | 1 | 0 | Low-quality | Genome-fragment | | 26.52 | 0 |
| S17-final-viral-combined_k141_140012||full | 1351 | No | NA | 0 | 0 | Low-quality | Genome-fragment | | 2.1 | 0 |
| S1-final-viral-combined_k141_370973||full | 5800 | No | NA | 0 | 4 | Not-determined | Genome-fragment | NA | | 0 |
| S1-final-viral-combined_k141_25263||full | 3557 | No | NA | 0 | 3 | Not-determined | Genome-fragment | NA | | 0 |
| S1-final-viral-combined_k141_25270||full | 2212 | No | NA | 0 | 0 | Not-determined | Genome-fragment | NA | | 0 |
| S1-final-viral-combined_k141_106706||full | 2003 | No | NA | 0 | 2 | Not-determined | Genome-fragment | NA | | 0 |
| S17-final-viral-combined_k141_86136||full | 1351 | No | NA | 0 | 0 | Low-quality | Genome-fragment | | 3.11 | 0 |
| S1-final-viral-combined_k141_322691||full | 1730 | No | NA | 0 | 0 | Not-determined | Genome-fragment | NA | | 0 |
| S1-final-viral-combined_k141_337370||full | 1371 | No | NA | 0 | 1 | Not-determined | Genome-fragment | NA | | 0 |
| S1-final-viral-combined_k141_322699||full | 1207 | No | NA | 0 | 0 | Not-determined | Genome-fragment | NA | | 0 |
| S1-final-viral-combined_k141_322706||full | 5676 | No | NA | 0 | 3 | Not-determined | Genome-fragment | NA | | 0 |
| S1-final-viral-combined_k141_106730||full | 6847 | No | NA | 0 | 1 | Not-determined | Genome-fragment | NA | | 0 |
| S1-final-viral-combined_k141_9182||full | 1683 | No | NA | 0 | 2 | Not-determined | Genome-fragment | NA | | 0 |
| S1-final-viral-combined_k141_106732||full | 4934 | No | NA | 0 | 3 | Not-determined | Genome-fragment | NA | | 0 |
| S17-final-viral-combined_k141_264920||full | 1351 | No | NA | 0 | 0 | Low-quality | Genome-fragment | | 1.75 | 0 |
| S12-final-viral-combined_k141_68396||full | 1351 | No | NA | 0 | 2 | Low-quality | Genome-fragment | | 0.43 | 0 |
| S1-final-viral-combined_k141_125205||full | 1862 | No | NA | 0 | 1 | Not-determined | Genome-fragment | NA | | 0 |
| S1-final-viral-combined_k141_305987||full | 2649 | No | NA | 0 | 3 | Not-determined | Genome-fragment | NA | | 0 |
| S12_k141_266953 | 1351 | No | NA | 1 | 0 | Low-quality | Genome-fragment | | 2.73 | 0 |
| S1-final-viral-combined_k141_140868||full | 1163 | No | NA | 0 | 1 | Not-determined | Genome-fragment | NA | | 0 |
| S1-final-viral-combined_k141_222988||full | 1539 | No | NA | 0 | 2 | Not-determined | Genome-fragment | NA | | 0 |
| S2-final-viral-combined_k141_78900||full | 1351 | No | NA | 1 | 0 | Low-quality | Genome-fragment | | 2.17 | 0 |
| S4_k141_147421 | 1351 | No | NA | 0 | 1 | Low-quality | Genome-fragment | | 0.75 | 0 |
| S1-final-viral-combined_k141_25376||full | 3237 | No | NA | 0 | 2 | Not-determined | Genome-fragment | NA | | 0 |
| S8-final-viral-combined_k141_85286||full | 1351 | No | NA | 1 | 0 | Low-quality | Genome-fragment | | 2.04 | 0 |
| S1-final-viral-combined_k141_222997||full | 6376 | No | NA | 0 | 4 | Not-determined | Genome-fragment | NA | | 0 |
| S1-final-viral-combined_k141_74001||full | 1009 | No | NA | 0 | 1 | Not-determined | Genome-fragment | NA | | 0 |
| S9_k141_127364 | 1352 | No | NA | 2 | 0 | Low-quality | Genome-fragment | | 3.33 | 0 |
| S11-final-viral-combined_k141_194298||full | 1352 | No | NA | 0 | 0 | Low-quality | Genome-fragment | | 3.09 | 0 |
| S1-final-viral-combined_k141_223024||full | 1994 | No | NA | 0 | 2 | Not-determined | Genome-fragment | NA | | 0 |
| S1-final-viral-combined_k141_288806||full | 4902 | No | NA | 0 | 2 | Not-determined | Genome-fragment | NA | | 0 |
| S1-final-viral-combined_k141_387896||full | 1626 | No | NA | 0 | 1 | Not-determined | Genome-fragment | NA | | 0 |
| S1-final-viral-combined_k141_223052||full | 2683 | No | NA | 0 | 1 | Not-determined | Genome-fragment | NA | | 0 |
| S11-final-viral-combined_k141_174057||full | 1352 | No | NA | 0 | 0 | Low-quality | Genome-fragment | | 2.59 | 0 |
| S1-final-viral-combined_k141_352357||full | 1352 | No | NA | 0 | 2 | Low-quality | Genome-fragment | | 0.46 | 0 |
| S1-final-viral-combined_k141_288835||full | 1899 | No | NA | 0 | 2 | Not-determined | Genome-fragment | NA | | 0 |
| S1-final-viral-combined_k141_89876||full | 1352 | No | NA | 0 | 2 | Low-quality | Genome-fragment | | 2.26 | 0 |
| S1-final-viral-combined_k141_58197||full | 11554 | No | NA | 0 | 7 | Not-determined | Genome-fragment | NA | | 0 |
| S1-final-viral-combined_k141_337458||full | 3360 | No | NA | 0 | 2 | Not-determined | Genome-fragment | NA | | 0 |
| S1-final-viral-combined_k141_106797||full | 1357 | No | NA | 0 | 3 | Not-determined | Genome-fragment | NA | | 0 |
| S1-final-viral-combined_k141_156506||full | 1441 | No | NA | 0 | 2 | Not-determined | Genome-fragment | NA | | 0 |
| S1-final-viral-combined_k141_207263||full | 1834 | No | NA | 0 | 2 | Not-determined | Genome-fragment | NA | | 0 |
| S1-final-viral-combined_k141_106810||full | 1165 | No | NA | 0 | 1 | Not-determined | Genome-fragment | NA | | 0 |
| S1-final-viral-combined_k141_156514||full | 1614 | No | NA | 0 | 0 | Not-determined | Genome-fragment | NA | | 0 |
| S1-final-viral-combined_k141_156523||full | 4747 | No | NA | 0 | 0 | Not-determined | Genome-fragment | NA | | 0 |
| S3-final-viral-combined_k141_56462||full | 1352 | No | NA | 0 | 1 | Low-quality | Genome-fragment | | 0.75 | 0 |

<table>
<tr><td>S1-final-viral-combined_k141_172632||full</td><td>3069</td><td>No</td><td>NA</td><td>0</td><td>1</td><td>Not-determined</td><td>Genome-fragment</td><td>NA</td><td></td><td>0</td></tr>
<tr><td>S1-final-viral-combined_k141_239790||full</td><td>4990</td><td>No</td><td>NA</td><td>1</td><td>0</td><td>Not-determined</td><td>Genome-fragment</td><td>NA</td><td></td><td>0</td></tr>
<tr><td>S5_k141_63420</td><td>1352</td><td>No</td><td>NA</td><td>0</td><td>0</td><td>Low-quality</td><td>Genome-fragment</td><td></td><td>2.81</td><td>0</td></tr>
<tr><td>S1-final-viral-combined_k141_172636||full</td><td>4551</td><td>No</td><td>NA</td><td>0</td><td>3</td><td>Not-determined</td><td>Genome-fragment</td><td>NA</td><td></td><td>0</td></tr>
<tr><td>S7-final-viral-combined_k141_23194||full</td><td>1352</td><td>No</td><td>NA</td><td>1</td><td>0</td><td>Low-quality</td><td>Genome-fragment</td><td></td><td>1.57</td><td>0</td></tr>
<tr><td>S1-final-viral-combined_k141_306142||full</td><td>1797</td><td>No</td><td>NA</td><td>0</td><td>2</td><td>Not-determined</td><td>Genome-fragment</td><td>NA</td><td></td><td>0</td></tr>
<tr><td>S1-final-viral-combined_k141_106867||full</td><td>2344</td><td>No</td><td>NA</td><td>0</td><td>1</td><td>Not-determined</td><td>Genome-fragment</td><td>NA</td><td></td><td>0</td></tr>
<tr><td>S1-final-viral-combined_k141_9350||full</td><td>13160</td><td>No</td><td>NA</td><td>0</td><td>5</td><td>Not-determined</td><td>Genome-fragment</td><td>NA</td><td></td><td>0</td></tr>
<tr><td>S1-final-viral-combined_k141_223081||full</td><td>1704</td><td>No</td><td>NA</td><td>0</td><td>0</td><td>Not-determined</td><td>Genome-fragment</td><td>NA</td><td></td><td>0</td></tr>
<tr><td>S1-final-viral-combined_k141_207327||full</td><td>2608</td><td>No</td><td>NA</td><td>0</td><td>1</td><td>Not-determined</td><td>Genome-fragment</td><td>NA</td><td></td><td>0</td></tr>
<tr><td>S1-final-viral-combined_k141_91342||full</td><td>7354</td><td>No</td><td>NA</td><td>0</td><td>1</td><td>Not-determined</td><td>Genome-fragment</td><td>NA</td><td></td><td>0</td></tr>
<tr><td>S1-final-viral-combined_k141_337549||full</td><td>1411</td><td>No</td><td>NA</td><td>0</td><td>2</td><td>Not-determined</td><td>Genome-fragment</td><td>NA</td><td></td><td>0</td></tr>
<tr><td>S1-final-viral-combined_k141_337559||full</td><td>2228</td><td>No</td><td>NA</td><td>0</td><td>2</td><td>Not-determined</td><td>Genome-fragment</td><td>NA</td><td></td><td>0</td></tr>
<tr><td>S17-final-viral-combined_k141_68903||full</td><td>1352</td><td>No</td><td>NA</td><td>2</td><td>0</td><td>Low-quality</td><td>Genome-fragment</td><td></td><td>3.94</td><td>0</td></tr>
<tr><td>S17-k141_90264</td><td>1352</td><td>No</td><td>NA</td><td>0</td><td>0</td><td>Low-quality</td><td>Genome-fragment</td><td></td><td>0.88</td><td>0</td></tr>
<tr><td>S1-final-viral-combined_k141_156571||full</td><td>2115</td><td>No</td><td>NA</td><td>0</td><td>2</td><td>Not-determined</td><td>Genome-fragment</td><td>NA</td><td></td><td>0</td></tr>
<tr><td>S12_k141_364594</td><td>1352</td><td>No</td><td>NA</td><td>0</td><td>0</td><td>Low-quality</td><td>Genome-fragment</td><td></td><td>2.44</td><td>0</td></tr>
<tr><td>S2-final-viral-combined_k141_69242||full</td><td>1352</td><td>No</td><td>NA</td><td>0</td><td>0</td><td>Low-quality</td><td>Genome-fragment</td><td></td><td>1.93</td><td>0</td></tr>
<tr><td>S1-final-viral-combined_k141_322919||full</td><td>3817</td><td>No</td><td>NA</td><td>0</td><td>3</td><td>Not-determined</td><td>Genome-fragment</td><td>NA</td><td></td><td>0</td></tr>
<tr><td>S11_k141_206084</td><td>1353</td><td>No</td><td>NA</td><td>0</td><td>0</td><td>Low-quality</td><td>Genome-fragment</td><td></td><td>2.21</td><td>0</td></tr>
<tr><td>S1-final-viral-combined_k141_355243||full</td><td>4048</td><td>No</td><td>NA</td><td>0</td><td>4</td><td>Not-determined</td><td>Genome-fragment</td><td>NA</td><td></td><td>0</td></tr>
<tr><td>S1-final-viral-combined_k141_371190||full</td><td>3691</td><td>No</td><td>NA</td><td>0</td><td>2</td><td>Not-determined</td><td>Genome-fragment</td><td>NA</td><td></td><td>0</td></tr>
<tr><td>S1-final-viral-combined_k141_41664||full</td><td>1064</td><td>No</td><td>NA</td><td>0</td><td>0</td><td>Not-determined</td><td>Genome-fragment</td><td>NA</td><td></td><td>0</td></tr>
<tr><td>S1-final-viral-combined_k141_66473||full</td><td>1353</td><td>No</td><td>NA</td><td>0</td><td>0</td><td>Low-quality</td><td>Genome-fragment</td><td></td><td>2.63</td><td>0</td></tr>
<tr><td>S1-final-viral-combined_k141_384458||full</td><td>1353</td><td>No</td><td>NA</td><td>0</td><td>1</td><td>Low-quality</td><td>Genome-fragment</td><td></td><td>2.24</td><td>0</td></tr>
<tr><td>S1-final-viral-combined_k141_306247||full</td><td>7697</td><td>No</td><td>NA</td><td>0</td><td>7</td><td>Not-determined</td><td>Genome-fragment</td><td>NA</td><td></td><td>0</td></tr>
<tr><td>S3-final-viral-combined_k141_76608||full</td><td>1353</td><td>No</td><td>NA</td><td>0</td><td>1</td><td>Low-quality</td><td>Genome-fragment</td><td></td><td>1.41</td><td>0</td></tr>
<tr><td>S5-final-viral-combined_k141_163063||full</td><td>1353</td><td>No</td><td>NA</td><td>0</td><td>1</td><td>Low-quality</td><td>Genome-fragment</td><td></td><td>3.1</td><td>0</td></tr>
<tr><td>S1-final-viral-combined_k141_223210||full</td><td>4751</td><td>No</td><td>NA</td><td>0</td><td>2</td><td>Not-determined</td><td>Genome-fragment</td><td>NA</td><td></td><td>0</td></tr>
<tr><td>S1-final-viral-combined_k141_156637||full</td><td>11186</td><td>No</td><td>NA</td><td>5</td><td>1</td><td>Medium-quality</td><td>Genome-fragment</td><td></td><td>87.8</td><td>0</td></tr>
<tr><td>S1-final-viral-combined_k141_9480||full</td><td>3989</td><td>No</td><td>NA</td><td>0</td><td>2</td><td>Not-determined</td><td>Genome-fragment</td><td>NA</td><td></td><td>0</td></tr>
<tr><td>S1-final-viral-combined_k141_74233||full</td><td>1521</td><td>No</td><td>NA</td><td>0</td><td>0</td><td>Not-determined</td><td>Genome-fragment</td><td>NA</td><td></td><td>0</td></tr>
<tr><td>S1-final-viral-combined_k141_41739||full</td><td>3473</td><td>No</td><td>NA</td><td>0</td><td>1</td><td>Not-determined</td><td>Genome-fragment</td><td>NA</td><td></td><td>0</td></tr>
<tr><td>S1-final-viral-combined_k141_141152||full</td><td>1503</td><td>No</td><td>NA</td><td>0</td><td>2</td><td>Not-determined</td><td>Genome-fragment</td><td>NA</td><td></td><td>0</td></tr>
<tr><td>S5-final-viral-combined_k141_115663||full</td><td>1353</td><td>No</td><td>NA</td><td>0</td><td>0</td><td>Low-quality</td><td>Genome-fragment</td><td></td><td>3.41</td><td>0</td></tr>
<tr><td>S1-final-viral-combined_k141_223253||full</td><td>1149</td><td>No</td><td>NA</td><td>0</td><td>0</td><td>Not-determined</td><td>Genome-fragment</td><td>NA</td><td></td><td>0</td></tr>
<tr><td>S1-final-viral-combined_k141_387998||full</td><td>1351</td><td>No</td><td>NA</td><td>0</td><td>1</td><td>Not-determined</td><td>Genome-fragment</td><td>NA</td><td></td><td>0</td></tr>
<tr><td>S1-final-viral-combined_k141_74265||full</td><td>3061</td><td>No</td><td>NA</td><td>0</td><td>2</td><td>Not-determined</td><td>Genome-fragment</td><td>NA</td><td></td><td>0</td></tr>
<tr><td>S12-final-viral-combined_k141_93654||full</td><td>1353</td><td>No</td><td>NA</td><td>1</td><td>0</td><td>Low-quality</td><td>Genome-fragment</td><td></td><td>2.78</td><td>0</td></tr>
<tr><td>S1-final-viral-combined_k141_322982||full</td><td>1745</td><td>No</td><td>NA</td><td>0</td><td>2</td><td>Not-determined</td><td>Genome-fragment</td><td>NA</td><td></td><td>0</td></tr>
<tr><td>S12-final-viral-combined_k141_92833||lt2gene</td><td>1353</td><td>No</td><td>NA</td><td>2</td><td>0</td><td>Low-quality</td><td>Genome-fragment</td><td></td><td>3.14</td><td>0</td></tr>
<tr><td>S1-final-viral-combined_k141_223296||full</td><td>1717</td><td>No</td><td>NA</td><td>0</td><td>0</td><td>Not-determined</td><td>Genome-fragment</td><td>NA</td><td></td><td>0</td></tr>
<tr><td>S1-final-viral-combined_k141_388049||full</td><td>1066</td><td>No</td><td>NA</td><td>0</td><td>1</td><td>Not-determined</td><td>Genome-fragment</td><td>NA</td><td></td><td>0</td></tr>
<tr><td>S1-final-viral-combined_k141_125392||full</td><td>2693</td><td>No</td><td>NA</td><td>0</td><td>1</td><td>Not-determined</td><td>Genome-fragment</td><td>NA</td><td></td><td>0</td></tr>
<tr><td>S1-final-viral-combined_k141_58377||full</td><td>3266</td><td>No</td><td>NA</td><td>0</td><td>2</td><td>Not-determined</td><td>Genome-fragment</td><td>NA</td><td></td><td>0</td></tr>
<tr><td>S1-final-viral-combined_k141_107040||full</td><td>2210</td><td>No</td><td>NA</td><td>0</td><td>2</td><td>Not-determined</td><td>Genome-fragment</td><td>NA</td><td></td><td>0</td></tr>
<tr><td>S4-final-viral-combined_k141_147200||full</td><td>1353</td><td>No</td><td>NA</td><td>0</td><td>0</td><td>Low-quality</td><td>Genome-fragment</td><td></td><td>3.69</td><td>0</td></tr>
<tr><td>S4-final-viral-combined_k141_94077||full</td><td>1353</td><td>No</td><td>NA</td><td>0</td><td>0</td><td>Low-quality</td><td>Genome-fragment</td><td></td><td>2.72</td><td>0</td></tr>
<tr><td>S1-final-viral-combined_k141_9511||full</td><td>4921</td><td>No</td><td>NA</td><td>0</td><td>1</td><td>Not-determined</td><td>Genome-fragment</td><td>NA</td><td></td><td>0</td></tr>
<tr><td>S4-final-viral-combined_k141_71212||lt2gene</td><td>1353</td><td>No</td><td>NA</td><td>2</td><td>0</td><td>Low-quality</td><td>Genome-fragment</td><td></td><td>2.82</td><td>0</td></tr>
<tr><td>S9_k141_2222</td><td>1354</td><td>No</td><td>NA</td><td>0</td><td>0</td><td>Low-quality</td><td>Genome-fragment</td><td></td><td>4.1</td><td>0</td></tr>
<tr><td>S11-final-viral-combined_k141_210789||full</td><td>1354</td><td>No</td><td>NA</td><td>0</td><td>0</td><td>Low-quality</td><td>Genome-fragment</td><td></td><td>3.84</td><td>0</td></tr>
<tr><td>S1-final-viral-combined_k141_58411||full</td><td>4030</td><td>No</td><td>NA</td><td>0</td><td>3</td><td>Not-determined</td><td>Genome-fragment</td><td>NA</td><td></td><td>0</td></tr>
<tr><td>S1-final-viral-combined_k141_9534||full</td><td>4899</td><td>No</td><td>NA</td><td>0</td><td>3</td><td>Not-determined</td><td>Genome-fragment</td><td>NA</td><td></td><td>0</td></tr>
<tr><td>S1-final-viral-combined_k141_249563||full</td><td>1354</td><td>No</td><td>NA</td><td>0</td><td>0</td><td>Low-quality</td><td>Genome-fragment</td><td></td><td>2.44</td><td>0</td></tr>
<tr><td>S5-final-viral-combined_k141_145589||lt2gene</td><td>1354</td><td>No</td><td>NA</td><td>2</td><td>0</td><td>Low-quality</td><td>Genome-fragment</td><td></td><td>2.66</td><td>0</td></tr>
<tr><td>S17-final-viral-combined_k141_83516||full</td><td>1354</td><td>No</td><td>NA</td><td>0</td><td>0</td><td>Low-quality</td><td>Genome-fragment</td><td></td><td>3.12</td><td>0</td></tr>
<tr><td>S17-k141_129806</td><td>1354</td><td>No</td><td>NA</td><td>0</td><td>0</td><td>Low-quality</td><td>Genome-fragment</td><td></td><td>3.29</td><td>0</td></tr>
<tr><td>S1-final-viral-combined_k141_107073||full</td><td>21709</td><td>No</td><td>NA</td><td>13</td><td>0</td><td>Medium-quality</td><td>Genome-fragment</td><td></td><td>56.42</td><td>0</td></tr>
<tr><td>S6-final-viral-combined_k141_89390||full</td><td>1354</td><td>No</td><td>NA</td><td>0</td><td>0</td><td>Low-quality</td><td>Genome-fragment</td><td></td><td>2.88</td><td>0</td></tr>
<tr><td>S1-final-viral-combined_k141_107102||full</td><td>2689</td><td>No</td><td>NA</td><td>0</td><td>0</td><td>Not-determined</td><td>Genome-fragment</td><td>NA</td><td></td><td>0</td></tr>
<tr><td>S1-final-viral-combined_k141_9568||full</td><td>1437</td><td>No</td><td>NA</td><td>0</td><td>0</td><td>Not-determined</td><td>Genome-fragment</td><td>NA</td><td></td><td>0</td></tr>
<tr><td>S8-final-viral-combined_k141_155484||full</td><td>1354</td><td>No</td><td>NA</td><td>0</td><td>1</td><td>Low-quality</td><td>Genome-fragment</td><td></td><td>1.32</td><td>0</td></tr>
<tr><td>S1-final-viral-combined_k141_74361||full</td><td>4014</td><td>No</td><td>NA</td><td>0</td><td>3</td><td>Not-determined</td><td>Genome-fragment</td><td>NA</td><td></td><td>0</td></tr>
<tr><td>S1-final-viral-combined_k141_388132||full</td><td>5532</td><td>No</td><td>NA</td><td>0</td><td>4</td><td>Not-determined</td><td>Genome-fragment</td><td>NA</td><td></td><td>0</td></tr>
<tr><td>S8_k141_239573</td><td>1354</td><td>No</td><td>NA</td><td>0</td><td>0</td><td>Low-quality</td><td>Genome-fragment</td><td></td><td>0.88</td><td>0</td></tr>
<tr><td>S1-final-viral-combined_k141_191183||full</td><td>1771</td><td>No</td><td>NA</td><td>0</td><td>1</td><td>Not-determined</td><td>Genome-fragment</td><td>NA</td><td></td><td>0</td></tr>
<tr><td>S1-final-viral-combined_k141_236127||full</td><td>1355</td><td>No</td><td>NA</td><td>0</td><td>1</td><td>Low-quality</td><td>Genome-fragment</td><td></td><td>0.72</td><td>0</td></tr>
<tr><td>S5-final-viral-combined_k141_4241||full</td><td>1355</td><td>No</td><td>NA</td><td>0</td><td>0</td><td>Low-quality</td><td>Genome-fragment</td><td></td><td>0.85</td><td>0</td></tr>
<tr><td>S5-final-viral-combined_k141_143873||lt2gene</td><td>1355</td><td>No</td><td>NA</td><td>1</td><td>0</td><td>Low-quality</td><td>Genome-fragment</td><td></td><td>1.52</td><td>0</td></tr>
<tr><td>S1-final-viral-combined_k141_74412||full</td><td>1165</td><td>No</td><td>NA</td><td>0</td><td>1</td><td>Not-determined</td><td>Genome-fragment</td><td>NA</td><td></td><td>0</td></tr>
<tr><td>S7-final-viral-combined_k141_55930||full</td><td>1355</td><td>No</td><td>NA</td><td>1</td><td>0</td><td>Low-quality</td><td>Genome-fragment</td><td></td><td>3.82</td><td>0</td></tr>
<tr><td>S1-final-viral-combined_k141_156796||full</td><td>4204</td><td>No</td><td>NA</td><td>0</td><td>2</td><td>Not-determined</td><td>Genome-fragment</td><td>NA</td><td></td><td>0</td></tr>
<tr><td>S17-k141_155801</td><td>1355</td><td>No</td><td>NA</td><td>0</td><td>2</td><td>Low-quality</td><td>Genome-fragment</td><td></td><td>2.79</td><td>0</td></tr>
<tr><td>S1-final-viral-combined_k141_141285||full</td><td>3915</td><td>No</td><td>NA</td><td>0</td><td>2</td><td>Not-determined</td><td>Genome-fragment</td><td>NA</td><td></td><td>0</td></tr>
<tr><td>S1-final-viral-combined_k141_306336||full</td><td>1582</td><td>No</td><td>NA</td><td>0</td><td>2</td><td>Not-determined</td><td>Genome-fragment</td><td>NA</td><td></td><td>0</td></tr>
<tr><td>S12-final-viral-combined_k141_374418||full</td><td>1355</td><td>No</td><td>NA</td><td>0</td><td>0</td><td>Low-quality</td><td>Genome-fragment</td><td></td><td>2.66</td><td>0</td></tr>
<tr><td>S12-final-viral-combined_k141_160769||full</td><td>1355</td><td>No</td><td>NA</td><td>0</td><td>1</td><td>Low-quality</td><td>Genome-fragment</td><td></td><td>2.13</td><td>0</td></tr>
<tr><td>S1-final-viral-combined_k141_125544||full</td><td>5186</td><td>No</td><td>NA</td><td>0</td><td>4</td><td>Not-determined</td><td>Genome-fragment</td><td>NA</td><td></td><td>0</td></tr>
<tr><td>S1-final-viral-combined_k141_371448||full</td><td>5455</td><td>No</td><td>NA</td><td>0</td><td>3</td><td>Not-determined</td><td>Genome-fragment</td><td>NA</td><td></td><td>0</td></tr>
<tr><td>S1-final-viral-combined_k141_191206||full</td><td>1634</td><td>No</td><td>NA</td><td>0</td><td>2</td><td>Not-determined</td><td>Genome-fragment</td><td>NA</td><td></td><td>0</td></tr>
<tr><td>S1-final-viral-combined_k141_355665||full</td><td>2602</td><td>No</td><td>NA</td><td>0</td><td>3</td><td>Not-determined</td><td>Genome-fragment</td><td>NA</td><td></td><td>0</td></tr>
<tr><td>S1-final-viral-combined_k141_388241||full</td><td>2572</td><td>No</td><td>NA</td><td>0</td><td>3</td><td>Not-determined</td><td>Genome-fragment</td><td>NA</td><td></td><td>0</td></tr>
<tr><td>S12-final-viral-combined_k141_237135||lt2gene</td><td>1355</td><td>No</td><td>NA</td><td>1</td><td>0</td><td>Low-quality</td><td>Genome-fragment</td><td></td><td>0.75</td><td>0</td></tr>
<tr><td>S2-final-viral-combined_k141_35834||full</td><td>1355</td><td>No</td><td>NA</td><td>0</td><td>1</td><td>Low-quality</td><td>Genome-fragment</td><td></td><td>3.17</td><td>0</td></tr>
<tr><td>S1-final-viral-combined_k141_371493||full</td><td>5659</td><td>No</td><td>NA</td><td>0</td><td>7</td><td>Not-determined</td><td>Genome-fragment</td><td>NA</td><td></td><td>0</td></tr>
<tr><td>S8-final-viral-combined_k141_265009||full</td><td>1355</td><td>No</td><td>NA</td><td>0</td><td>0</td><td>Low-quality</td><td>Genome-fragment</td><td></td><td>5.39</td><td>0</td></tr>
<tr><td>S1-final-viral-combined_k141_41971||full</td><td>1643</td><td>No</td><td>NA</td><td>0</td><td>3</td><td>Not-determined</td><td>Genome-fragment</td><td>NA</td><td></td><td>0</td></tr>
<tr><td>S1-final-viral-combined_k141_323197||full</td><td>4581</td><td>No</td><td>NA</td><td>0</td><td>4</td><td>Not-determined</td><td>Genome-fragment</td><td>NA</td><td></td><td>0</td></tr>
<tr><td>S1-final-viral-combined_k141_9760||full</td><td>1316</td><td>No</td><td>NA</td><td>0</td><td>1</td><td>Not-determined</td><td>Genome-fragment</td><td>NA</td><td></td><td>0</td></tr>
<tr><td>S1-final-viral-combined_k141_91800||full</td><td>1833</td><td>No</td><td>NA</td><td>0</td><td>0</td><td>Not-determined</td><td>Genome-fragment</td><td>NA</td><td></td><td>0</td></tr>
<tr><td>S1-final-viral-combined_k141_173115||full</td><td>1511</td><td>No</td><td>NA</td><td>0</td><td>1</td><td>Not-determined</td><td>Genome-fragment</td><td>NA</td><td></td><td>0</td></tr>
<tr><td>S1-final-viral-combined_k141_91809||full</td><td>4005</td><td>No</td><td>NA</td><td>0</td><td>3</td><td>Not-determined</td><td>Genome-fragment</td><td>NA</td><td></td><td>0</td></tr>
<tr><td>S1-final-viral-combined_k141_289223||full</td><td>1207</td><td>No</td><td>NA</td><td>0</td><td>2</td><td>Not-determined</td><td>Genome-fragment</td><td>NA</td><td></td><td>0</td></tr>
<tr><td>S1-final-viral-combined_k141_74545||full</td><td>10676</td><td>No</td><td>NA</td><td>0</td><td>6</td><td>Not-determined</td><td>Genome-fragment</td><td>NA</td><td></td><td>0</td></tr>
<tr><td>S1-final-viral-combined_k141_323240||full</td><td>1798</td><td>No</td><td>NA</td><td>0</td><td>2</td><td>Not-determined</td><td>Genome-fragment</td><td>NA</td><td></td><td>0</td></tr>
<tr><td>S8-final-viral-combined_k141_56067||full</td><td>1355</td><td>No</td><td>NA</td><td>2</td><td>0</td><td>Low-quality</td><td>Genome-fragment</td><td></td><td>2.4</td><td>0</td></tr>
<tr><td>S11-final-viral-combined_k141_20096||full</td><td>1356</td><td>No</td><td>NA</td><td>0</td><td>0</td><td>Low-quality</td><td>Genome-fragment</td><td></td><td>2.41</td><td>0</td></tr>
<tr><td>S1-final-viral-combined_k141_191296||full</td><td>2033</td><td>No</td><td>NA</td><td>0</td><td>2</td><td>Not-determined</td><td>Genome-fragment</td><td>NA</td><td></td><td>0</td></tr>
<tr><td>S1-final-viral-combined_k141_156897||full</td><td>1184</td><td>No</td><td>NA</td><td>0</td><td>2</td><td>Not-determined</td><td>Genome-fragment</td><td>NA</td><td></td><td>0</td></tr>
<tr><td>S1-final-viral-combined_k141_42023||full</td><td>1313</td><td>No</td><td>NA</td><td>0</td><td>1</td><td>Not-determined</td><td>Genome-fragment</td><td>NA</td><td></td><td>0</td></tr>
<tr><td>S1-final-viral-combined_k141_289252||full</td><td>1023</td><td>No</td><td>NA</td><td>0</td><td>1</td><td>Not-determined</td><td>Genome-fragment</td><td>NA</td><td></td><td>0</td></tr>
<tr><td>S1-final-viral-combined_k141_173137||full</td><td>1157</td><td>No</td><td>NA</td><td>0</td><td>0</td><td>Not-determined</td><td>Genome-fragment</td><td>NA</td><td></td><td>0</td></tr>
<tr><td>S1-final-viral-combined_k141_55688||full</td><td>1356</td><td>No</td><td>NA</td><td>0</td><td>0</td><td>Low-quality</td><td>Genome-fragment</td><td></td><td>2.79</td><td>0</td></tr>
<tr><td>S1-final-viral-combined_k141_257156||full</td><td>1449</td><td>No</td><td>NA</td><td>0</td><td>3</td><td>Not-determined</td><td>Genome-fragment</td><td>NA</td><td></td><td>0</td></tr>
<tr><td>S1-final-viral-combined_k141_323295||full</td><td>1010</td><td>No</td><td>NA</td><td>0</td><td>2</td><td>Not-determined</td><td>Genome-fragment</td><td>NA</td><td></td><td>0</td></tr>
<tr><td>S1-final-viral-combined_k141_141431||full</td><td>3026</td><td>No</td><td>NA</td><td>0</td><td>2</td><td>Not-determined</td><td>Genome-fragment</td><td>NA</td><td></td><td>0</td></tr>
<tr><td>S1-final-viral-combined_k141_289291||full</td><td>1221</td><td>No</td><td>NA</td><td>0</td><td>1</td><td>Not-determined</td><td>Genome-fragment</td><td>NA</td><td></td><td>0</td></tr>
<tr><td>S1-final-viral-combined_k141_146617||full</td><td>1356</td><td>No</td><td>NA</td><td>1</td><td>0</td><td>Low-quality</td><td>Genome-fragment</td><td></td><td>2.27</td><td>0</td></tr>
<tr><td>S1-final-viral-combined_k141_306481||full</td><td>1438</td><td>No</td><td>NA</td><td>0</td><td>2</td><td>Not-determined</td><td>Genome-fragment</td><td>NA</td><td></td><td>0</td></tr>
<tr><td>S1-final-viral-combined_k141_25859||full</td><td>5118</td><td>No</td><td>NA</td><td>0</td><td>6</td><td>Not-determined</td><td>Genome-fragment</td><td>NA</td><td></td><td>0</td></tr>
<tr><td>S1-final-viral-combined_k141_289301||full</td><td>1635</td><td>No</td><td>NA</td><td>0</td><td>1</td><td>Not-determined</td><td>Genome-fragment</td><td>NA</td><td></td><td>0</td></tr>
<tr><td>S1-final-viral-combined_k141_16170||lt2gene</td><td>1356</td><td>No</td><td>NA</td><td>1</td><td>0</td><td>Low-quality</td><td>Genome-fragment</td><td></td><td>1.24</td><td>0</td></tr>
<tr><td>S7_k141_90886</td><td>1356</td><td>No</td><td>NA</td><td>0</td><td>0</td><td>Low-quality</td><td>Genome-fragment</td><td></td><td>1.72</td><td>0</td></tr>
<tr><td>S17-final-viral-combined_k141_91780||full</td><td>1356</td><td>No</td><td>NA</td><td>0</td><td>0</td><td>Low-quality</td><td>Genome-fragment</td><td></td><td>2.57</td><td>0</td></tr>
<tr><td>S1-final-viral-combined_k141_257165||full</td><td>1088</td><td>No</td><td>NA</td><td>0</td><td>2</td><td>Not-determined</td><td>Genome-fragment</td><td>NA</td><td></td><td>0</td></tr>
<tr><td>S1-final-viral-combined_k141_141449||full</td><td>6302</td><td>No</td><td>NA</td><td>0</td><td>0</td><td>Not-determined</td><td>Genome-fragment</td><td>NA</td><td></td><td>0</td></tr>
<tr><td>S1-final-viral-combined_k141_306499||full</td><td>2406</td><td>No</td><td>NA</td><td>0</td><td>2</td><td>Not-determined</td><td>Genome-fragment</td><td>NA</td><td></td><td>0</td></tr>
<tr><td>S17-k141_33681</td><td>1356</td><td>No</td><td>NA</td><td>0</td><td>1</td><td>Low-quality</td><td>Genome-fragment</td><td></td><td>0.47</td><td>0</td></tr>
<tr><td>S12-final-viral-combined_k141_211436||lt2gene</td><td>1356</td><td>No</td><td>NA</td><td>1</td><td>0</td><td>Low-quality</td><td>Genome-fragment</td><td></td><td>2.26</td><td>0</td></tr>
<tr><td>S1-final-viral-combined_k141_257176||full</td><td>1276</td><td>No</td><td>NA</td><td>0</td><td>0</td><td>Not-determined</td><td>Genome-fragment</td><td>NA</td><td></td><td>0</td></tr>
<tr><td>S1-final-viral-combined_k141_323361||full</td><td>2268</td><td>No</td><td>NA</td><td>0</td><td>2</td><td>Not-determined</td><td>Genome-fragment</td><td>NA</td><td></td><td>0</td></tr>
<tr><td>S12_k141_127844</td><td>1356</td><td>No</td><td>NA</td><td>0</td><td>0</td><td>Low-quality</td><td>Genome-fragment</td><td></td><td>1.14</td><td>0</td></tr>
<tr><td>S1-final-viral-combined_k141_289369||full</td><td>4427</td><td>No</td><td>NA</td><td>0</td><td>2</td><td>Not-determined</td><td>Genome-fragment</td><td>NA</td><td></td><td>0</td></tr>
<tr><td>S2-final-viral-combined_k141_142957||full</td><td>1356</td><td>No</td><td>NA</td><td>0</td><td>0</td><td>Low-quality</td><td>Genome-fragment</td><td></td><td>2.8</td><td>0</td></tr>
<tr><td>S2_k141_217159</td><td>1356</td><td>No</td><td>NA</td><td>0</td><td>0</td><td>Low-quality</td><td>Genome-fragment</td><td></td><td>1.93</td><td>0</td></tr>
<tr><td>S1-final-viral-combined_k141_306555||full</td><td>5943</td><td>No</td><td>NA</td><td>0</td><td>4</td><td>Not-determined</td><td>Genome-fragment</td><td>NA</td><td></td><td>0</td></tr>
<tr><td>S1-final-viral-combined_k141_74656||full</td><td>1282</td><td>No</td><td>NA</td><td>0</td><td>2</td><td>Not-determined</td><td>Genome-fragment</td><td>NA</td><td></td><td>0</td></tr>
<tr><td>S1-final-viral-combined_k141_240282||full</td><td>2968</td><td>No</td><td>NA</td><td>0</td><td>1</td><td>Not-determined</td><td>Genome-fragment</td><td>NA</td><td></td><td>0</td></tr>
<tr><td>S1-final-viral-combined_k141_337961||full</td><td>1168</td><td>No</td><td>NA</td><td>0</td><td>2</td><td>Not-determined</td><td>Genome-fragment</td><td>NA</td><td></td><td>0</td></tr>
<tr><td>S1-final-viral-combined_k141_337972||full</td><td>2006</td><td>No</td><td>NA</td><td>0</td><td>0</td><td>Not-determined</td><td>Genome-fragment</td><td>NA</td><td></td><td>0</td></tr>
<tr><td>S1-final-viral-combined_k141_173207||full</td><td>1270</td><td>No</td><td>NA</td><td>0</td><td>0</td><td>Not-determined</td><td>Genome-fragment</td><td>NA</td><td></td><td>0</td></tr>
<tr><td>S1-final-viral-combined_k141_191447||full</td><td>1539</td><td>No</td><td>NA</td><td>0</td><td>1</td><td>Not-determined</td><td>Genome-fragment</td><td>NA</td><td></td><td>0</td></tr>
<tr><td>S11-final-viral-combined_k141_170025||lt2gene</td><td>1357</td><td>No</td><td>NA</td><td>0</td><td>0</td><td>Low-quality</td><td>Genome-fragment</td><td></td><td>3.63</td><td>0</td></tr>
<tr><td>S1-final-viral-combined_k141_223426||full</td><td>8237</td><td>No</td><td>NA</td><td>0</td><td>6</td><td>Not-determined</td><td>Genome-fragment</td><td>NA</td><td></td><td>0</td></tr>
<tr><td>S1-final-viral-combined_k141_191473||full</td><td>1902</td><td>No</td><td>NA</td><td>0</td><td>1</td><td>Not-determined</td><td>Genome-fragment</td><td>NA</td><td></td><td>0</td></tr>
<tr><td>S1-final-viral-combined_k141_381385||full</td><td>1357</td><td>No</td><td>NA</td><td>0</td><td>1</td><td>Low-quality</td><td>Genome-fragment</td><td></td><td>1.15</td><td>0</td></tr>
<tr><td>S1-final-viral-combined_k141_223439||full</td><td>1898</td><td>No</td><td>NA</td><td>0</td><td>2</td><td>Not-determined</td><td>Genome-fragment</td><td>NA</td><td></td><td>0</td></tr>
<tr><td>S1-final-viral-combined_k141_23246||full</td><td>1357</td><td>No</td><td>NA</td><td>0</td><td>0</td><td>Low-quality</td><td>Genome-fragment</td><td></td><td>2.28</td><td>0</td></tr>
<tr><td>S1-final-viral-combined_k141_63497||full</td><td>1357</td><td>No</td><td>NA</td><td>0</td><td>0</td><td>Low-quality</td><td>Genome-fragment</td><td></td><td>0.92</td><td>0</td></tr>
<tr><td>S1-final-viral-combined_k141_289478||full</td><td>2737</td><td>No</td><td>NA</td><td>0</td><td>1</td><td>Not-determined</td><td>Genome-fragment</td><td>NA</td><td></td><td>0</td></tr>
<tr><td>S1-final-viral-combined_k141_125700||full</td><td>3378</td><td>No</td><td>NA</td><td>0</td><td>3</td><td>Not-determined</td><td>Genome-fragment</td><td>NA</td><td></td><td>0</td></tr>
<tr><td>S1_k141_305954</td><td>1357</td><td>No</td><td>NA</td><td>3</td><td>0</td><td>Low-quality</td><td>Genome-fragment</td><td></td><td>1.89</td><td>0</td></tr>
<tr><td>S3-final-viral-combined_k141_66423||lt2gene</td><td>1357</td><td>No</td><td>NA</td><td>1</td><td>0</td><td>Low-quality</td><td>Genome-fragment</td><td></td><td>3.68</td><td>0</td></tr>
<tr><td>S5-final-viral-combined_k141_99641||full</td><td>1357</td><td>No</td><td>NA</td><td>0</td><td>2</td><td>Low-quality</td><td>Genome-fragment</td><td></td><td>0.84</td><td>0</td></tr>
<tr><td>S7_k141_113061</td><td>1357</td><td>No</td><td>NA</td><td>0</td><td>0</td><td>Low-quality</td><td>Genome-fragment</td><td></td><td>0.52</td><td>0</td></tr>
<tr><td>S1-final-viral-combined_k141_371637||full</td><td>1592</td><td>No</td><td>NA</td><td>0</td><td>1</td><td>Not-determined</td><td>Genome-fragment</td><td>NA</td><td></td><td>0</td></tr>
<tr><td>S1-final-viral-combined_k141_371653||full</td><td>1413</td><td>No</td><td>NA</td><td>0</td><td>0</td><td>Not-determined</td><td>Genome-fragment</td><td>NA</td><td></td><td>0</td></tr>
<tr><td>S1-final-viral-combined_k141_257326||full</td><td>5277</td><td>No</td><td>NA</td><td>0</td><td>4</td><td>Not-determined</td><td>Genome-fragment</td><td>NA</td><td></td><td>0</td></tr>
<tr><td>S12-final-viral-combined_k141_82537||full</td><td>1357</td><td>No</td><td>NA</td><td>0</td><td>0</td><td>Low-quality</td><td>Genome-fragment</td><td></td><td>2.92</td><td>0</td></tr>
<tr><td>S12-final-viral-combined_k141_334592||full</td><td>1357</td><td>No</td><td>NA</td><td>1</td><td>0</td><td>Low-quality</td><td>Genome-fragment</td><td></td><td>2.44</td><td>0</td></tr>
<tr><td>S1-final-viral-combined_k141_191590||full</td><td>1673</td><td>No</td><td>NA</td><td>0</td><td>0</td><td>Not-determined</td><td>Genome-fragment</td><td>NA</td><td></td><td>0</td></tr>
<tr><td>S1-final-viral-combined_k141_371685||full</td><td>1294</td><td>No</td><td>NA</td><td>0</td><td>0</td><td>Not-determined</td><td>Genome-fragment</td><td>NA</td><td></td><td>0</td></tr>
<tr><td>S12_k141_302033</td><td>1357</td><td>No</td><td>NA</td><td>1</td><td>0</td><td>Low-quality</td><td>Genome-fragment</td><td></td><td>2</td><td>0</td></tr>
<tr><td>S1-final-viral-combined_k141_92003||full</td><td>2440</td><td>No</td><td>NA</td><td>0</td><td>3</td><td>Not-determined</td><td>Genome-fragment</td><td>NA</td><td></td><td>0</td></tr>
<tr><td>S1-final-viral-combined_k141_306743||full</td><td>5882</td><td>No</td><td>NA</td><td>0</td><td>4</td><td>Not-determined</td><td>Genome-fragment</td><td>NA</td><td></td><td>0</td></tr>
<tr><td>S1-final-viral-combined_k141_42225||full</td><td>6330</td><td>No</td><td>NA</td><td>0</td><td>4</td><td>Not-determined</td><td>Genome-fragment</td><td>NA</td><td></td><td>0</td></tr>
<tr><td>S1-final-viral-combined_k141_92013||full</td><td>1035</td><td>No</td><td>NA</td><td>0</td><td>0</td><td>Not-determined</td><td>Genome-fragment</td><td>NA</td><td></td><td>0</td></tr>
<tr><td>S1-final-viral-combined_k141_26125||full</td><td>17878</td><td>No</td><td>NA</td><td>0</td><td>13</td><td>Not-determined</td><td>Genome-fragment</td><td>NA</td><td></td><td>0</td></tr>
<tr><td>S1-final-viral-combined_k141_92035||full</td><td>5893</td><td>No</td><td>NA</td><td>0</td><td>3</td><td>Not-determined</td><td>Genome-fragment</td><td>NA</td><td></td><td>0</td></tr>
<tr><td>S1-final-viral-combined_k141_10093||full</td><td>1982</td><td>No</td><td>NA</td><td>0</td><td>2</td><td>Not-determined</td><td>Genome-fragment</td><td>NA</td><td></td><td>0</td></tr>
<tr><td>S1-final-viral-combined_k141_338175||full</td><td>1683</td><td>No</td><td>NA</td><td>0</td><td>2</td><td>Not-determined</td><td>Genome-fragment</td><td>NA</td><td></td><td>0</td></tr>
<tr><td>S2-final-viral-combined_k141_38194||full</td><td>1357</td><td>No</td><td>NA</td><td>0</td><td>0</td><td>Low-quality</td><td>Genome-fragment</td><td></td><td>2.72</td><td>0</td></tr>
<tr><td>S2_k141_49806</td><td>1357</td><td>No</td><td>NA</td><td>0</td><td>0</td><td>Low-quality</td><td>Genome-fragment</td><td></td><td>1.06</td><td>0</td></tr>
<tr><td>S6_k141_26029</td><td>1357</td><td>No</td><td>NA</td><td>0</td><td>2</td><td>Low-quality</td><td>Genome-fragment</td><td></td><td>1.06</td><td>0</td></tr>
<tr><td>S1-final-viral-combined_k141_107632||full</td><td>1398</td><td>No</td><td>NA</td><td>0</td><td>1</td><td>Not-determined</td><td>Genome-fragment</td><td>NA</td><td></td><td>0</td></tr>
<tr><td>S8-final-viral-combined_k141_65422||full</td><td>1357</td><td>No</td><td>NA</td><td>0</td><td>0</td><td>Low-quality</td><td>Genome-fragment</td><td></td><td>3.45</td><td>0</td></tr>
<tr><td>S1-final-viral-combined_k141_323584||full</td><td>4093</td><td>No</td><td>NA</td><td>0</td><td>3</td><td>Not-determined</td><td>Genome-fragment</td><td>NA</td><td></td><td>0</td></tr>
<tr><td>S1-final-viral-combined_k141_173326||full</td><td>7068</td><td>No</td><td>NA</td><td>0</td><td>5</td><td>Not-determined</td><td>Genome-fragment</td><td>NA</td><td></td><td>0</td></tr>
<tr><td>S1-final-viral-combined_k141_173328||full</td><td>1519</td><td>No</td><td>NA</td><td>0</td><td>0</td><td>Not-determined</td><td>Genome-fragment</td><td>NA</td><td></td><td>0</td></tr>
<tr><td>S1-final-viral-combined_k141_10116||full</td><td>1127</td><td>No</td><td>NA</td><td>0</td><td>0</td><td>Not-determined</td><td>Genome-fragment</td><td>NA</td><td></td><td>0</td></tr>
<tr><td>S1-final-viral-combined_k141_289664||full</td><td>1391</td><td>No</td><td>NA</td><td>0</td><td>2</td><td>Not-determined</td><td>Genome-fragment</td><td>NA</td><td></td><td>0</td></tr>
<tr><td>S11-final-viral-combined_k141_227054||full</td><td>1358</td><td>No</td><td>NA</td><td>0</td><td>0</td><td>Low-quality</td><td>Genome-fragment</td><td></td><td>2.8</td><td>0</td></tr>
<tr><td>S11_k141_113780</td><td>1358</td><td>No</td><td>NA</td><td>0</td><td>1</td><td>Low-quality</td><td>Genome-fragment</td><td></td><td>0.82</td><td>0</td></tr>
</table>

| | | | | | | | | | |
|---|---|---|---|---|---|---|---|---|---|
| S1-final-viral-combined_k141_125837||full | 1053 | No | NA | 0 | 0 | Not-determined | Genome-fragment | NA | 0 |
| S1-final-viral-combined_k141_173350||full | 1381 | No | NA | 0 | 0 | Not-determined | Genome-fragment | NA | 0 |
| S1-final-viral-combined_k141_125841||full | 4904 | No | NA | 0 | 2 | Not-determined | Genome-fragment | NA | 0 |
| S1-final-viral-combined_k141_150632||lt2gene | 1358 | No | NA | 1 | 0 | Low-quality | Genome-fragment | 3.57 | 0 |
| S1-final-viral-combined_k141_338224||full | 1431 | No | NA | 0 | 1 | Not-determined | Genome-fragment | NA | 0 |
| S1-final-viral-combined_k141_371753||full | 2158 | No | NA | 0 | 3 | Not-determined | Genome-fragment | NA | 0 |
| S1-final-viral-combined_k141_173376||full | 6546 | No | NA | 0 | 7 | Not-determined | Genome-fragment | NA | 0 |
| S1-final-viral-combined_k141_191640||full | 9295 | No | NA | 0 | 4 | Not-determined | Genome-fragment | NA | 0 |
| S3-final-viral-combined_k141_89933||full | 1358 | No | NA | 0 | 2 | Low-quality | Genome-fragment | 0.94 | 0 |
| S1-final-viral-combined_k141_157270||full | 3973 | No | NA | 0 | 2 | Not-determined | Genome-fragment | NA | 0 |
| S1-final-viral-combined_k141_356053||full | 1357 | No | NA | 0 | 1 | Not-determined | Genome-fragment | NA | 0 |
| S1-final-viral-combined_k141_289704||full | 2015 | No | NA | 0 | 2 | Not-determined | Genome-fragment | NA | 0 |
| S5-final-viral-combined_k141_181804||lt2gene | 1358 | No | NA | 1 | 0 | Low-quality | Genome-fragment | 1.01 | 0 |
| S7-final-viral-combined_k141_174592||full | 1358 | No | NA | 0 | 0 | Low-quality | Genome-fragment | 2.62 | 0 |
| S1-final-viral-combined_k141_107666||full | 1513 | No | NA | 0 | 1 | Not-determined | Genome-fragment | NA | 0 |
| S17-final-viral-combined_k141_54603||full | 1358 | No | NA | 0 | 1 | Low-quality | Genome-fragment | 0.83 | 0 |
| S2-final-viral-combined_k141_74650||full | 1358 | No | NA | 1 | 0 | Low-quality | Genome-fragment | 3.01 | 0 |
| S9-final-viral-combined_k141_71895||full | 1359 | No | NA | 1 | 0 | Low-quality | Genome-fragment | 4.04 | 0 |
| S1-final-viral-combined_k141_257496||full | 1589 | No | NA | 0 | 1 | Not-determined | Genome-fragment | NA | 0 |
| S1-final-viral-combined_k141_306863||full | 6578 | No | NA | 0 | 3 | Not-determined | Genome-fragment | NA | 0 |
| S11-final-viral-combined_k141_323947||lt2gene | 1359 | No | NA | 1 | 0 | Low-quality | Genome-fragment | 2.19 | 0 |
| S1-final-viral-combined_k141_289714||full | 2537 | No | NA | 0 | 2 | Not-determined | Genome-fragment | NA | 0 |
| S11-final-viral-combined_k141_11921||lt2gene | 1359 | No | NA | 1 | 0 | Low-quality | Genome-fragment | 3.01 | 0 |
| S1-final-viral-combined_k141_356106||full | 1123 | No | NA | 0 | 0 | Not-determined | Genome-fragment | NA | 0 |
| S1-final-viral-combined_k141_240544||full | 2192 | No | NA | 0 | 3 | Not-determined | Genome-fragment | NA | 0 |
| S1-final-viral-combined_k141_371791||full | 9824 | No | NA | 0 | 1 | Not-determined | Genome-fragment | NA | 0 |
| S1-final-viral-combined_k141_257516||full | 3121 | No | NA | 0 | 0 | Not-determined | Genome-fragment | NA | 0 |
| S1_k141_387052 | 1359 | No | NA | 0 | 0 | Low-quality | Genome-fragment | 2.43 | 0 |
| S1-final-viral-combined_k141_208276||full | 3147 | No | NA | 0 | 0 | Not-determined | Genome-fragment | NA | 0 |
| S5-final-viral-combined_k141_34106||full | 1359 | No | NA | 0 | 0 | Low-quality | Genome-fragment | 3.1 | 0 |
| S5-final-viral-combined_k141_97665||full | 1359 | No | NA | 3 | 0 | Low-quality | Genome-fragment | 2.03 | 0 |
| S7-final-viral-combined_k141_89791||full | 1359 | No | NA | 0 | 0 | Low-quality | Genome-fragment | 3.2 | 0 |
| S1-final-viral-combined_k141_42397||full | 2257 | No | NA | 0 | 0 | Not-determined | Genome-fragment | NA | 0 |
| S1-final-viral-combined_k141_74781||full | 1052 | No | NA | 0 | 2 | Not-determined | Genome-fragment | NA | 0 |
| S1-final-viral-combined_k141_74782||full | 1311 | No | NA | 0 | 0 | Not-determined | Genome-fragment | NA | 0 |
| S1-final-viral-combined_k141_42403||full | 1920 | No | NA | 0 | 1 | Not-determined | Genome-fragment | NA | 0 |
| S7_k141_200361 | 1359 | No | NA | 0 | 1 | Low-quality | Genome-fragment | 0.65 | 0 |
| S1-final-viral-combined_k141_141627||full | 2149 | No | NA | 0 | 0 | Not-determined | Genome-fragment | NA | 0 |
| S1-final-viral-combined_k141_191743||full | 1109 | No | NA | 0 | 0 | Not-determined | Genome-fragment | NA | 0 |
| S17-final-viral-combined_k141_113710||full | 1359 | No | NA | 0 | 2 | Low-quality | Genome-fragment | 4.12 | 0 |
| S17-final-viral-combined_k141_96062||full | 1359 | No | NA | 0 | 0 | Low-quality | Genome-fragment | 1.74 | 0 |
| S1-final-viral-combined_k141_289787||full | 2484 | No | NA | 0 | 1 | Not-determined | Genome-fragment | NA | 0 |
| S12-final-viral-combined_k141_72428||full | 1359 | No | NA | 2 | 0 | Low-quality | Genome-fragment | 3.18 | 0 |
| S1-final-viral-combined_k141_273054||full | 1917 | No | NA | 0 | 2 | Not-determined | Genome-fragment | NA | 0 |
| S12-final-viral-combined_k141_275023||full | 1359 | No | NA | 2 | 0 | Low-quality | Genome-fragment | 3.19 | 0 |
| S1-final-viral-combined_k141_74851||full | 3100 | No | NA | 0 | 3 | Not-determined | Genome-fragment | NA | 0 |
| S1-final-viral-combined_k141_356210||full | 4888 | No | NA | 0 | 2 | Not-determined | Genome-fragment | NA | 0 |
| S1-final-viral-combined_k141_257617||full | 9271 | No | NA | 0 | 1 | Not-determined | Genome-fragment | NA | 0 |
| S1-final-viral-combined_k141_173467||full | 3287 | No | NA | 0 | 1 | Not-determined | Genome-fragment | NA | 0 |
| S1-final-viral-combined_k141_338393||full | 3233 | No | NA | 0 | 2 | Not-determined | Genome-fragment | NA | 0 |
| S12-final-viral-combined_k141_232241||lt2gene | 1359 | No | NA | 2 | 0 | Low-quality | Genome-fragment | 3.64 | 0 |
| S4-final-viral-combined_k141_58563||full | 1359 | No | NA | 0 | 1 | Low-quality | Genome-fragment | 1.49 | 0 |
| S1-final-viral-combined_k141_107766||full | 53116 | Yes | 36184 | 10 | 13 | Medium-quality | Genome-fragment | 86.89 | 31.88 |
| S1-final-viral-combined_k141_240655||full | 11704 | No | NA | 0 | 4 | Not-determined | Genome-fragment | NA | 0 |
| S1-final-viral-combined_k141_356255||full | 2072 | No | NA | 0 | 0 | Not-determined | Genome-fragment | NA | 0 |
| S1-final-viral-combined_k141_92329||full | 8493 | No | NA | 0 | 7 | Not-determined | Genome-fragment | NA | 0 |
| S4-final-viral-combined_k141_163862||full | 1359 | No | NA | 1 | 0 | Low-quality | Genome-fragment | 0.86 | 0 |
| S1-final-viral-combined_k141_240679||full | 2128 | No | NA | 0 | 1 | Not-determined | Genome-fragment | NA | 0 |
| S4-final-viral-combined_k141_95825||full | 1359 | No | NA | 0 | 2 | Low-quality | Genome-fragment | 2.25 | 0 |
| S8_k141_10999 | 1359 | No | NA | 0 | 0 | Low-quality | Genome-fragment | 1.72 | 0 |
| S1-final-viral-combined_k141_257700||full | 2642 | No | NA | 0 | 2 | Not-determined | Genome-fragment | NA | 0 |
| S9-final-viral-combined_k141_52301||full | 1360 | No | NA | 1 | 0 | Low-quality | Genome-fragment | 2.98 | 0 |
| S1-final-viral-combined_k141_141883||full | 1360 | No | NA | 2 | 0 | Low-quality | Genome-fragment | 3.25 | 0 |
| S1-final-viral-combined_k141_59018||full | 7793 | No | NA | 0 | 5 | Not-determined | Genome-fragment | NA | 0 |
| S1-final-viral-combined_k141_376631||full | 1360 | No | NA | 0 | 0 | Low-quality | Genome-fragment | 2.74 | 0 |
| S1-final-viral-combined_k141_59021||full | 1806 | No | NA | 0 | 2 | Not-determined | Genome-fragment | NA | 0 |
| S1-final-viral-combined_k141_10264||full | 8107 | No | NA | 0 | 1 | Not-determined | Genome-fragment | NA | 0 |
| S1-final-viral-combined_k141_356345||full | 1377 | No | NA | 0 | 1 | Not-determined | Genome-fragment | NA | 0 |
| S1-final-viral-combined_k141_191846||full | 12676 | No | NA | 0 | 9 | Not-determined | Genome-fragment | NA | 0 |
| S7-final-viral-combined_k141_148260||full | 1360 | No | NA | 0 | 0 | Low-quality | Genome-fragment | 2.36 | 0 |
| S7_k141_130141 | 1360 | No | NA | 0 | 0 | Low-quality | Genome-fragment | 1.08 | 0 |
| S1-final-viral-combined_k141_356352||full | 4104 | No | NA | 0 | 3 | Not-determined | Genome-fragment | NA | 0 |
| S1-final-viral-combined_k141_289954||full | 1967 | No | NA | 0 | 2 | Not-determined | Genome-fragment | NA | 0 |
| S17-final-viral-combined_k141_250269||full | 1360 | No | NA | 1 | 0 | Low-quality | Genome-fragment | 2.42 | 0 |
| S17-k141_256459 | 1360 | No | NA | 0 | 0 | Low-quality | Genome-fragment | 1.89 | 0 |
| S12-final-viral-combined_k141_186985||full | 1360 | No | NA | 1 | 0 | Low-quality | Genome-fragment | 3 | 0 |
| S2-final-viral-combined_k141_231133||full | 1360 | No | NA | 0 | 1 | Low-quality | Genome-fragment | 3.35 | 0 |
| S1-final-viral-combined_k141_141832||full | 2495 | No | NA | 0 | 1 | Not-determined | Genome-fragment | NA | 0 |
| S1-final-viral-combined_k141_289965||full | 2219 | No | NA | 0 | 2 | Not-determined | Genome-fragment | NA | 0 |
| S1-final-viral-combined_k141_173568||full | 1414 | No | NA | 0 | 0 | Not-determined | Genome-fragment | NA | 0 |
| S1-final-viral-combined_k141_26417||full | 14494 | No | NA | 0 | 10 | Not-determined | Genome-fragment | NA | 0 |
| S1-final-viral-combined_k141_126047||full | 3054 | No | NA | 0 | 0 | Not-determined | Genome-fragment | NA | 0 |
| S2-final-viral-combined_k141_60621||full | 1360 | No | NA | 0 | 0 | Low-quality | Genome-fragment | 2.92 | 0 |
| S1-final-viral-combined_k141_126049||full | 2171 | No | NA | 0 | 1 | Not-determined | Genome-fragment | NA | 0 |
| S8-final-viral-combined_k141_124314||lt2gene | 1360 | No | NA | 1 | 0 | Low-quality | Genome-fragment | 2.92 | 0 |
| S1-final-viral-combined_k141_59084||full | 4879 | No | NA | 0 | 0 | Not-determined | Genome-fragment | NA | 0 |
| S1_k141_127924 | 1361 | No | NA | 0 | 1 | Low-quality | Genome-fragment | 2.26 | 0 |
| S1-final-viral-combined_k141_59085||full | 4630 | No | NA | 0 | 1 | Not-determined | Genome-fragment | NA | 0 |
| S7-final-viral-combined_k141_26078||full | 1361 | No | NA | 1 | 0 | Low-quality | Genome-fragment | 1.22 | 0 |
| S17-final-viral-combined_k141_152452||full | 1361 | No | NA | 2 | 0 | Low-quality | Genome-fragment | 2.62 | 0 |
| S1-final-viral-combined_k141_10286||full | 36514 | No | NA | 0 | 20 | Not-determined | Genome-fragment | NA | 0 |
| S17-k141_222839 | 1361 | No | NA | 0 | 1 | Low-quality | Genome-fragment | 4.57 | 0 |
| S1-final-viral-combined_k141_372074||full | 1598 | No | NA | 0 | 0 | Not-determined | Genome-fragment | NA | 0 |
| S17-k141_57873 | 1361 | No | NA | 0 | 0 | Low-quality | Genome-fragment | 2.29 | 0 |
| S1-final-viral-combined_k141_372078||full | 1065 | No | NA | 0 | 0 | Not-determined | Genome-fragment | NA | 0 |
| S12-final-viral-combined_k141_58370||full | 1361 | No | NA | 0 | 0 | Low-quality | Genome-fragment | 2.19 | 0 |
| S1-final-viral-combined_k141_307184||full | 2618 | No | NA | 0 | 2 | Not-determined | Genome-fragment | NA | 0 |
| S1-final-viral-combined_k141_126103||full | 5176 | No | NA | 0 | 3 | Not-determined | Genome-fragment | NA | 0 |
| S1-final-viral-combined_k141_42645||full | 3411 | No | NA | 0 | 3 | Not-determined | Genome-fragment | NA | 0 |
| S1-final-viral-combined_k141_26465||full | 1153 | No | NA | 0 | 0 | Not-determined | Genome-fragment | NA | 0 |
| S1-final-viral-combined_k141_257851||full | 2363 | No | NA | 0 | 1 | Not-determined | Genome-fragment | NA | 0 |
| S12-final-viral-combined_k141_283639||full | 1361 | No | NA | 0 | 0 | Low-quality | Genome-fragment | 2.39 | 0 |
| S6-final-viral-combined_k141_36703||full | 1361 | No | NA | 0 | 0 | Low-quality | Genome-fragment | 1.28 | 0 |
| S1-final-viral-combined_k141_257856||full | 6862 | No | NA | 0 | 4 | Not-determined | Genome-fragment | NA | 0 |
| S1-final-viral-combined_k141_372134||full | 2541 | No | NA | 0 | 0 | Not-determined | Genome-fragment | NA | 0 |
| S6-final-viral-combined_k141_14070||full | 1361 | No | NA | 0 | 0 | Low-quality | Genome-fragment | 3.29 | 0 |
| S1-final-viral-combined_k141_338549||full | 5377 | No | NA | 0 | 5 | Not-determined | Genome-fragment | NA | 0 |
| S1-final-viral-combined_k141_257885||full | 6549 | No | NA | 0 | 4 | Not-determined | Genome-fragment | NA | 0 |
| S1-final-viral-combined_k141_307207||full | 3451 | No | NA | 0 | 2 | Not-determined | Genome-fragment | NA | 0 |
| S8-final-viral-combined_k141_77169||full | 1361 | No | NA | 0 | 0 | Low-quality | Genome-fragment | 0.98 | 0 |
| S1-final-viral-combined_k141_338569||full | 1554 | No | NA | 0 | 0 | Not-determined | Genome-fragment | NA | 0 |
| S11-final-viral-combined_k141_278870||full | 1362 | No | NA | 0 | 0 | Low-quality | Genome-fragment | 0.48 | 0 |
| S11-final-viral-combined_k141_200627||full | 1362 | No | NA | 3 | 0 | Low-quality | Genome-fragment | 4.57 | 0 |
| S1-final-viral-combined_k141_26544||full | 3373 | No | NA | 0 | 2 | Not-determined | Genome-fragment | NA | 0 |
| S5-final-viral-combined_k141_189045||full | 1362 | No | NA | 3 | 0 | Low-quality | Genome-fragment | 1.97 | 0 |
| S1-final-viral-combined_k141_338582||full | 3030 | No | NA | 0 | 0 | Not-determined | Genome-fragment | NA | 0 |
| S1-final-viral-combined_k141_42694||full | 1185 | No | NA | 0 | 2 | Not-determined | Genome-fragment | NA | 0 |
| S7-final-viral-combined_k141_205419||full | 1362 | No | NA | 0 | 1 | Low-quality | Genome-fragment | 1.65 | 0 |
| S1-final-viral-combined_k141_208550||full | 2897 | No | NA | 0 | 2 | Not-determined | Genome-fragment | NA | 0 |
| S1-final-viral-combined_k141_141993||full | 1324 | No | NA | 0 | 0 | Not-determined | Genome-fragment | NA | 0 |
| S1-final-viral-combined_k141_92523||full | 31793 | Yes | 21321 | 10 | 8 | Medium-quality | Genome-fragment | 61.28 | 32.94 |
| S1-final-viral-combined_k141_224136||full | 1047 | No | NA | 0 | 0 | Not-determined | Genome-fragment | NA | 0 |
| S1-final-viral-combined_k141_75145||full | 1030 | No | NA | 0 | 1 | Not-determined | Genome-fragment | NA | 0 |
| S1-final-viral-combined_k141_257962||full | 9387 | No | NA | 0 | 7 | Not-determined | Genome-fragment | NA | 0 |
| S1-final-viral-combined_k141_224160||full | 1863 | No | NA | 0 | 0 | Not-determined | Genome-fragment | NA | 0 |
| S1-final-viral-combined_k141_208588||full | 6162 | No | NA | 0 | 3 | Not-determined | Genome-fragment | NA | 0 |
| S1-final-viral-combined_k141_173782||full | 2331 | No | NA | 0 | 1 | Not-determined | Genome-fragment | NA | 0 |
| S1-final-viral-combined_k141_126302||full | 1213 | No | NA | 0 | 0 | Not-determined | Genome-fragment | NA | 0 |
| S12-final-viral-combined_k141_338824||full | 1362 | No | NA | 0 | 0 | Low-quality | Genome-fragment | 2.67 | 0 |
| S1-final-viral-combined_k141_372268||full | 2254 | No | NA | 0 | 1 | Not-determined | Genome-fragment | NA | 0 |
| S1-final-viral-combined_k141_59189||full | 1792 | No | NA | 0 | 0 | Not-determined | Genome-fragment | NA | 0 |
| S8_k141_201770 | 1362 | No | NA | 0 | 0 | Low-quality | Genome-fragment | 1.75 | 0 |
| S9_k141_125582 | 1363 | No | NA | 1 | 0 | Low-quality | Genome-fragment | 2.5 | 0 |
| S1-final-viral-combined_k141_59204||full | 2603 | No | NA | 0 | 2 | Not-determined | Genome-fragment | NA | 0 |
| S1-final-viral-combined_k141_324115||full | 1649 | No | NA | 0 | 3 | Not-determined | Genome-fragment | NA | 0 |
| S1-final-viral-combined_k141_388583||full | 6837 | No | NA | 0 | 2 | Not-determined | Genome-fragment | NA | 0 |
| S1-final-viral-combined_k141_307299||full | 3198 | No | NA | 0 | 1 | Not-determined | Genome-fragment | NA | 0 |
| S1-final-viral-combined_k141_388584||full | 5613 | No | NA | 0 | 5 | Not-determined | Genome-fragment | NA | 0 |
| S1-final-viral-combined_k141_290203||full | 1976 | No | NA | 0 | 1 | Not-determined | Genome-fragment | NA | 0 |
| S1-final-viral-combined_k141_224189||full | 3708 | No | NA | 0 | 0 | Not-determined | Genome-fragment | NA | 0 |
| S1-final-viral-combined_k141_26615||full | 1487 | No | NA | 0 | 2 | Not-determined | Genome-fragment | NA | 0 |
| S1-final-viral-combined_k141_324141||full | 2377 | No | NA | 0 | 3 | Not-determined | Genome-fragment | NA | 0 |
| S1-final-viral-combined_k141_208661||full | 6595 | No | NA | 0 | 1 | Not-determined | Genome-fragment | NA | 0 |
| S1-final-viral-combined_k141_92636||full | 1470 | No | NA | 0 | 0 | Not-determined | Genome-fragment | NA | 0 |
| S1-final-viral-combined_k141_142067||full | 1200 | No | NA | 0 | 1 | Not-determined | Genome-fragment | NA | 0 |
| S1-final-viral-combined_k141_388606||full | 2761 | No | NA | 0 | 0 | Not-determined | Genome-fragment | NA | 0 |
| S11-final-viral-combined_k141_63822||full | 1363 | No | NA | 0 | 1 | Low-quality | Genome-fragment | 0.3 | 0 |
| S1-final-viral-combined_k141_356572||full | 1989 | No | NA | 0 | 2 | Not-determined | Genome-fragment | NA | 0 |
| S1-final-viral-combined_k141_240987||full | 1298 | No | NA | 0 | 1 | Not-determined | Genome-fragment | NA | 0 |
| S1-final-viral-combined_k141_240990||full | 1070 | No | NA | 0 | 1 | Not-determined | Genome-fragment | NA | 0 |
| S1-final-viral-combined_k141_192004||full | 1033 | No | NA | 0 | 2 | Not-determined | Genome-fragment | NA | 0 |
| S1-final-viral-combined_k141_59261||full | 3074 | No | NA | 0 | 2 | Not-determined | Genome-fragment | NA | 0 |
| S1-final-viral-combined_k141_42351||lt2gene | 1363 | No | NA | 1 | 0 | Low-quality | Genome-fragment | 2.23 | 0 |
| S1-final-viral-combined_k141_208687||full | 5658 | No | NA | 0 | 3 | Not-determined | Genome-fragment | NA | 0 |
| S1-final-viral-combined_k141_338697||full | 1991 | No | NA | 0 | 1 | Not-determined | Genome-fragment | NA | 0 |
| S1-final-viral-combined_k141_208701||full | 1833 | No | NA | 0 | 0 | Not-determined | Genome-fragment | NA | 0 |
| S1-final-viral-combined_k141_208706||full | 1015 | No | NA | 0 | 3 | Not-determined | Genome-fragment | NA | 0 |
| S5-final-viral-combined_k141_110799||full | 1363 | No | NA | 1 | 0 | Low-quality | Genome-fragment | 3.01 | 0 |
| S1-final-viral-combined_k141_258096||full | 2122 | No | NA | 0 | 0 | Not-determined | Genome-fragment | NA | 0 |
| S17-final-viral-combined_k141_31591||full | 1363 | Yes | 378 | 1 | 1 | Low-quality | Genome-fragment | 0.79 | 72.27 |
| S12-final-viral-combined_k141_84908||lt2gene | 1363 | No | NA | 1 | 0 | Low-quality | Genome-fragment | 0.92 | 0 |
| S1-final-viral-combined_k141_208724||full | 1617 | No | NA | 0 | 1 | Not-determined | Genome-fragment | NA | 0 |
| S1-final-viral-combined_k141_192089||full | 1952 | No | NA | 0 | 2 | Not-determined | Genome-fragment | NA | 0 |
| S12_k141_9733 | 1363 | No | NA | 0 | 0 | Low-quality | Genome-fragment | 2.38 | 0 |
| S6-final-viral-combined_k141_85634||full | 1363 | No | NA | 0 | 0 | Low-quality | Genome-fragment | 2.05 | 0 |
| S1-final-viral-combined_k141_59315||full | 3298 | No | NA | 0 | 1 | Not-determined | Genome-fragment | NA | 0 |
| S6-final-viral-combined_k141_40550||lt2gene | 1363 | No | NA | 1 | 0 | Low-quality | Genome-fragment | 4.59 | 0 |
| S8-final-viral-combined_k141_102766||full | 1363 | No | NA | 0 | 0 | Low-quality | Genome-fragment | 2.5 | 0 |
| S1-final-viral-combined_k141_338748||full | 3819 | No | NA | 0 | 1 | Not-determined | Genome-fragment | NA | 0 |
| S1-final-viral-combined_k141_157822||full | 4452 | No | NA | 0 | 2 | Not-determined | Genome-fragment | NA | 0 |
| S9-final-viral-combined_k141_108283||full | 1364 | No | NA | 0 | 1 | Low-quality | Genome-fragment | 1.26 | 0 |
| S1-final-viral-combined_k141_157834||full | 1451 | No | NA | 0 | 0 | Not-determined | Genome-fragment | NA | 0 |
| S1-final-viral-combined_k141_126470||full | 3043 | No | NA | 0 | 3 | Not-determined | Genome-fragment | NA | 0 |
| S1-final-viral-combined_k141_208764||full | 2000 | No | NA | 0 | 1 | Not-determined | Genome-fragment | NA | 0 |

| | | | | | | | | | | |
|---|---|---|---|---|---|---|---|---|---|---|
| S9_k141_84895 | 1364 | No | NA | 0 | 1 | Low-quality | Genome-fragment | | 0.48 | 0 |
| S1-final-viral-combined_k141_208775\|\|full | 4059 | No | NA | 0 | 0 | Not-determined | Genome-fragment | NA | | 0 |
| S1-final-viral-combined_k141_241111\|\|full | 1438 | No | NA | 0 | 1 | Not-determined | Genome-fragment | NA | | 0 |
| S11-final-viral-combined_k141_55419\|\|full | 1364 | No | NA | 1 | 0 | Low-quality | Genome-fragment | | 0.83 | 0 |
| S1-final-viral-combined_k141_338785\|\|full | 1235 | No | NA | 0 | 0 | Not-determined | Genome-fragment | NA | | 0 |
| S1-final-viral-combined_k141_185200\|\|full | 1364 | No | NA | 0 | 0 | Low-quality | Genome-fragment | | 3.74 | 0 |
| S1-final-viral-combined_k141_388731\|\|full | 5268 | No | NA | 0 | 5 | Not-determined | Genome-fragment | NA | | 0 |
| S1-final-viral-combined_k141_258197\|\|full | 2226 | No | NA | 0 | 2 | Not-determined | Genome-fragment | NA | | 0 |
| S7-final-viral-combined_k141_239352\|\|full | 1364 | No | NA | 0 | 0 | Low-quality | Genome-fragment | | 3.04 | 0 |
| S17-k141_149286 | 1364 | No | NA | 2 | 0 | Low-quality | Genome-fragment | | 1.72 | 0 |
| S12-final-viral-combined_k141_376748\|\|full | 1364 | No | NA | 0 | 0 | Low-quality | Genome-fragment | | 2.24 | 0 |
| S12-final-viral-combined_k141_265283\|\|full | 1364 | No | NA | 1 | 0 | Low-quality | Genome-fragment | | 2.84 | 0 |
| S12_k141_124575 | 1364 | No | NA | 0 | 0 | Low-quality | Genome-fragment | | 3.46 | 0 |
| S1-final-viral-combined_k141_338837\|\|full | 6024 | No | NA | 0 | 6 | Not-determined | Genome-fragment | NA | | 0 |
| S2-final-viral-combined_k141_55720\|\|full | 1364 | No | NA | 0 | 0 | Low-quality | Genome-fragment | | 2.4 | 0 |
| S1-final-viral-combined_k141_208837\|\|full | 1137 | No | NA | 0 | 0 | Not-determined | Genome-fragment | NA | | 0 |
| S1-final-viral-combined_k141_290347\|\|full | 2652 | No | NA | 0 | 0 | Not-determined | Genome-fragment | NA | | 0 |
| S1-final-viral-combined_k141_290350\|\|full | 2575 | No | NA | 0 | 0 | Not-determined | Genome-fragment | NA | | 0 |
| S11-final-viral-combined_k141_310043\|\|full | 1365 | No | NA | 0 | 0 | Low-quality | Genome-fragment | | 1.51 | 0 |
| S1-final-viral-combined_k141_165878\|\|full | 1365 | No | NA | 0 | 1 | Low-quality | Genome-fragment | | 1.12 | 0 |
| S1-final-viral-combined_k141_324293\|\|full | 1480 | No | NA | 0 | 1 | Not-determined | Genome-fragment | NA | | 0 |
| S1-final-viral-combined_k141_87489\|\|full | 1365 | No | NA | 0 | 2 | Low-quality | Genome-fragment | | 0.71 | 0 |
| S17-k141_220745 | 1365 | No | NA | 0 | 2 | Low-quality | Genome-fragment | | 0.15 | 0 |
| S1-final-viral-combined_k141_307324\|\|full | 5594 | No | NA | 0 | 1 | Not-determined | Genome-fragment | NA | | 0 |
| S1-final-viral-combined_k141_258249\|\|full | 1265 | No | NA | 0 | 0 | Not-determined | Genome-fragment | NA | | 0 |
| S12-final-viral-combined_k141_86776\|\|full | 1365 | No | NA | 0 | 0 | Low-quality | Genome-fragment | | 3.59 | 0 |
| S12-final-viral-combined_k141_157135\|\|full | 1365 | No | NA | 2 | 0 | Low-quality | Genome-fragment | | 3.44 | 0 |
| S1-final-viral-combined_k141_126617\|\|full | 1255 | No | NA | 0 | 1 | Not-determined | Genome-fragment | NA | | 0 |
| S1-final-viral-combined_k141_126618\|\|full | 1177 | No | NA | 0 | 2 | Not-determined | Genome-fragment | NA | | 0 |
| S1-final-viral-combined_k141_258282\|\|full | 3193 | No | NA | 0 | 0 | Not-determined | Genome-fragment | NA | | 0 |
| S1-final-viral-combined_k141_142368\|\|full | 1347 | No | NA | 0 | 1 | Not-determined | Genome-fragment | NA | | 0 |
| S1-final-viral-combined_k141_142374\|\|full | 4294 | No | NA | 0 | 3 | Not-determined | Genome-fragment | NA | | 0 |
| S12-final-viral-combined_k141_80641\|\|full | 1365 | No | NA | 0 | 0 | Low-quality | Genome-fragment | | 3.1 | 0 |
| S1-final-viral-combined_k141_59551\|\|full | 1063 | No | NA | 0 | 1 | Not-determined | Genome-fragment | NA | | 0 |
| S1-final-viral-combined_k141_10502\|\|full | 7776 | No | NA | 0 | 4 | Not-determined | Genome-fragment | NA | | 0 |
| S2-final-viral-combined_k141_40814\|\|full | 1365 | No | NA | 2 | 0 | Low-quality | Genome-fragment | | 2.97 | 0 |
| S1-final-viral-combined_k141_192321\|\|full | 5826 | No | NA | 0 | 1 | Not-determined | Genome-fragment | NA | | 0 |
| S1-final-viral-combined_k141_290423\|\|full | 3760 | No | NA | 0 | 3 | Not-determined | Genome-fragment | NA | | 0 |
| S1-final-viral-combined_k141_372596\|\|full | 1144 | No | NA | 0 | 1 | Not-determined | Genome-fragment | NA | | 0 |
| S1-final-viral-combined_k141_307388\|\|full | 3367 | No | NA | 0 | 1 | Not-determined | Genome-fragment | NA | | 0 |
| S6-final-viral-combined_k141_22231\|\|full | 1365 | No | NA | 1 | 0 | Low-quality | Genome-fragment | | 1.05 | 0 |
| S1-final-viral-combined_k141_273637\|\|full | 1063 | No | NA | 0 | 0 | Not-determined | Genome-fragment | NA | | 0 |
| S1-final-viral-combined_k141_356823\|\|full | 2818 | No | NA | 0 | 1 | Not-determined | Genome-fragment | NA | | 0 |
| S6_k141_30238 | 1365 | No | NA | 0 | 0 | Low-quality | Genome-fragment | | 3.26 | 0 |
| S8-final-viral-combined_k141_245992\|\|full | 1365 | No | NA | 0 | 0 | Low-quality | Genome-fragment | | 3.52 | 0 |
| S1-final-viral-combined_k141_258317\|\|full | 10332 | No | NA | 0 | 7 | Not-determined | Genome-fragment | NA | | 0 |
| S1-final-viral-combined_k141_75464\|\|full | 3366 | No | NA | 0 | 2 | Not-determined | Genome-fragment | NA | | 0 |
| S8-final-viral-combined_k141_248152\|\|full | 1365 | No | NA | 0 | 0 | Low-quality | Genome-fragment | | 3.43 | 0 |
| S9-final-viral-combined_k141_117660\|\|full | 1366 | No | NA | 0 | 0 | Low-quality | Genome-fragment | | 4.32 | 0 |
| S1-final-viral-combined_k141_26768\|\|full | 4603 | No | NA | 0 | 2 | Not-determined | Genome-fragment | NA | | 0 |
| S1-final-viral-combined_k141_93038\|\|full | 2996 | No | NA | 0 | 0 | Not-determined | Genome-fragment | NA | | 0 |
| S11-final-viral-combined_k141_142\|\|full | 1366 | No | NA | 0 | 0 | Low-quality | Genome-fragment | | 3.09 | 0 |
| S1-final-viral-combined_k141_43167\|\|full | 4573 | No | NA | 0 | 3 | Not-determined | Genome-fragment | NA | | 0 |
| S1_k141_223601 | 1366 | No | NA | 2 | 0 | Low-quality | Genome-fragment | | 2.98 | 0 |
| S1-final-viral-combined_k141_93047\|\|full | 1103 | No | NA | 0 | 0 | Not-determined | Genome-fragment | NA | | 0 |
| S1-final-viral-combined_k141_307405\|\|full | 6449 | No | NA | 0 | 2 | Not-determined | Genome-fragment | NA | | 0 |
| S1-final-viral-combined_k141_142454\|\|full | 2101 | No | NA | 0 | 2 | Not-determined | Genome-fragment | NA | | 0 |
| S1-final-viral-combined_k141_43178\|\|full | 1469 | No | NA | 0 | 1 | Not-determined | Genome-fragment | NA | | 0 |
| S7-final-viral-combined_k141_113697\|\|full | 1366 | No | NA | 2 | 0 | Low-quality | Genome-fragment | | 2.93 | 0 |
| S1-final-viral-combined_k141_174173\|\|full | 1844 | No | NA | 0 | 0 | Not-determined | Genome-fragment | NA | | 0 |
| S1-final-viral-combined_k141_290494\|\|full | 1908 | No | NA | 0 | 1 | Not-determined | Genome-fragment | NA | | 0 |
| S1-final-viral-combined_k141_324368\|\|full | 2937 | No | NA | 0 | 2 | Not-determined | Genome-fragment | NA | | 0 |
| S1-final-viral-combined_k141_174209\|\|full | 1162 | No | NA | 0 | 0 | Not-determined | Genome-fragment | NA | | 0 |
| S1-final-viral-combined_k141_26840\|\|full | 1184 | No | NA | 0 | 0 | Not-determined | Genome-fragment | NA | | 0 |
| S1-final-viral-combined_k141_142461\|\|full | 3111 | No | NA | 0 | 0 | Not-determined | Genome-fragment | NA | | 0 |
| S7-final-viral-combined_k141_4090\|\|full | 1366 | No | NA | 0 | 1 | Low-quality | Genome-fragment | | 2.35 | 0 |
| S1-final-viral-combined_k141_126778\|\|full | 2211 | No | NA | 0 | 0 | Not-determined | Genome-fragment | NA | | 0 |
| S1-final-viral-combined_k141_10609\|\|full | 1942 | No | NA | 0 | 0 | Not-determined | Genome-fragment | NA | | 0 |
| S1-final-viral-combined_k141_388920\|\|full | 2501 | No | NA | 0 | 0 | Not-determined | Genome-fragment | NA | | 0 |
| S7-final-viral-combined_k141_17553\|\|full | 1366 | No | NA | 1 | 0 | Low-quality | Genome-fragment | | 3.68 | 0 |
| S1-final-viral-combined_k141_372636\|\|full | 2537 | No | NA | 0 | 2 | Not-determined | Genome-fragment | NA | | 0 |
| S7_k141_223601 | 1366 | No | NA | 0 | 0 | Low-quality | Genome-fragment | | 0.89 | 0 |
| S1-final-viral-combined_k141_126796\|\|full | 7642 | No | NA | 0 | 4 | Not-determined | Genome-fragment | NA | | 0 |
| S17-final-viral-combined_k141_174723\|\|full | 1366 | No | NA | 0 | 2 | Low-quality | Genome-fragment | | 0.94 | 0 |
| S1-final-viral-combined_k141_356908\|\|full | 2769 | No | NA | 0 | 2 | Not-determined | Genome-fragment | NA | | 0 |
| S1-final-viral-combined_k141_273732\|\|full | 2507 | No | NA | 0 | 0 | Not-determined | Genome-fragment | NA | | 0 |
| S1-final-viral-combined_k141_10650\|\|full | 2964 | No | NA | 0 | 3 | Not-determined | Genome-fragment | NA | | 0 |
| S12-final-viral-combined_k141_385633\|\|full | 1366 | No | NA | 0 | 1 | Low-quality | Genome-fragment | | 1.49 | 0 |
| S12_k141_173641 | 1366 | No | NA | 0 | 0 | Low-quality | Genome-fragment | | 0.63 | 0 |
| S1-final-viral-combined_k141_224578\|\|full | 25656 | No | NA | 10 | 0 | Medium-quality | Genome-fragment | | 59.81 | 0 |
| S1-final-viral-combined_k141_338951\|\|full | 1137 | No | NA | 0 | 0 | Not-determined | Genome-fragment | NA | | 0 |
| S12_k141_31593 | 1366 | No | NA | 0 | 0 | Low-quality | Genome-fragment | | 1.36 | 0 |
| S1-final-viral-combined_k141_307508\|\|full | 1131 | No | NA | 0 | 0 | Not-determined | Genome-fragment | NA | | 0 |
| S1-final-viral-combined_k141_192472\|\|full | 2734 | No | NA | 0 | 3 | Not-determined | Genome-fragment | NA | | 0 |
| S2-final-viral-combined_k141_46120\|\|full | 1366 | No | NA | 0 | 0 | Low-quality | Genome-fragment | | 2.69 | 0 |
| S2-final-viral-combined_k141_80025\|\|full | 1366 | No | NA | 0 | 1 | Low-quality | Genome-fragment | | 0.72 | 0 |
| S1-final-viral-combined_k141_258450\|\|full | 14490 | No | NA | 0 | 11 | Not-determined | Genome-fragment | NA | | 0 |
| S1-final-viral-combined_k141_126855\|\|full | 3339 | No | NA | 0 | 1 | Not-determined | Genome-fragment | NA | | 0 |
| S1-final-viral-combined_k141_200032\|\|full | 1367 | No | NA | 0 | 0 | Low-quality | Genome-fragment | | 0.91 | 0 |
| S1-final-viral-combined_k141_10701\|\|full | 1774 | No | NA | 0 | 1 | Not-determined | Genome-fragment | NA | | 0 |
| S1-final-viral-combined_k141_356923\|\|full | 2853 | No | NA | 0 | 3 | Not-determined | Genome-fragment | NA | | 0 |
| S1-final-viral-combined_k141_319785\|\|full | 1367 | No | NA | 0 | 2 | Low-quality | Genome-fragment | | 2.47 | 0 |
| S1-final-viral-combined_k141_344624\|\|full | 1367 | No | NA | 0 | 1 | Low-quality | Genome-fragment | | 2.55 | 0 |
| S1-final-viral-combined_k141_126927\|\|full | 2900 | No | NA | 0 | 1 | Not-determined | Genome-fragment | NA | | 0 |
| S1-final-viral-combined_k141_307608\|\|full | 2871 | No | NA | 0 | 1 | Not-determined | Genome-fragment | NA | | 0 |
| S1-final-viral-combined_k141_372747\|\|full | 1108 | No | NA | 0 | 2 | Not-determined | Genome-fragment | NA | | 0 |
| S1-final-viral-combined_k141_10770\|\|full | 3126 | No | NA | 0 | 2 | Not-determined | Genome-fragment | NA | | 0 |
| S1-final-viral-combined_k141_75632\|\|full | 2241 | No | NA | 0 | 2 | Not-determined | Genome-fragment | NA | | 0 |
| S3-final-viral-combined_k141_101559\|\|full | 1367 | No | NA | 0 | 1 | Low-quality | Genome-fragment | | 0.72 | 0 |
| S5-final-viral-combined_k141_71535\|\|full | 1367 | No | NA | 0 | 1 | Low-quality | Genome-fragment | | 1.78 | 0 |
| S5-final-viral-combined_k141_80262\|\|lt2gene | 1367 | No | NA | 1 | 0 | Low-quality | Genome-fragment | | 3.49 | 0 |
| S7-final-viral-combined_k141_74437\|\|full | 1367 | No | NA | 1 | 0 | Low-quality | Genome-fragment | | 0.76 | 0 |
| S1-final-viral-combined_k141_290621\|\|full | 2136 | No | NA | 0 | 1 | Not-determined | Genome-fragment | NA | | 0 |
| S1-final-viral-combined_k141_93226\|\|full | 1250 | No | NA | 0 | 2 | Not-determined | Genome-fragment | NA | | 0 |
| S1-final-viral-combined_k141_10801\|\|full | 1596 | No | NA | 0 | 1 | Not-determined | Genome-fragment | NA | | 0 |
| S1-final-viral-combined_k141_126948\|\|full | 2468 | No | NA | 0 | 1 | Not-determined | Genome-fragment | NA | | 0 |
| S17-k141_228107 | 1367 | No | NA | 0 | 0 | Low-quality | Genome-fragment | | 0.78 | 0 |
| S1-final-viral-combined_k141_209205\|\|full | 8161 | No | NA | 0 | 4 | Not-determined | Genome-fragment | NA | | 0 |
| S1-final-viral-combined_k141_290643\|\|full | 2193 | No | NA | 0 | 0 | Not-determined | Genome-fragment | NA | | 0 |
| S12-final-viral-combined_k141_300415\|\|full | 1367 | No | NA | 0 | 0 | Low-quality | Genome-fragment | | 0.86 | 0 |
| S1-final-viral-combined_k141_224718\|\|full | 2108 | No | NA | 0 | 1 | Not-determined | Genome-fragment | NA | | 0 |
| S1-final-viral-combined_k141_158309\|\|full | 4305 | No | NA | 0 | 4 | Not-determined | Genome-fragment | NA | | 0 |
| S12_k141_274374 | 1367 | No | NA | 3 | 0 | Low-quality | Genome-fragment | | 2.64 | 0 |
| S1-final-viral-combined_k141_241508\|\|full | 3962 | No | NA | 0 | 2 | Not-determined | Genome-fragment | NA | | 0 |
| S1-final-viral-combined_k141_43378\|\|full | 1444 | No | NA | 0 | 0 | Not-determined | Genome-fragment | NA | | 0 |
| S1-final-viral-combined_k141_126965\|\|full | 3275 | No | NA | 0 | 3 | Not-determined | Genome-fragment | NA | | 0 |
| S12_k141_96259 | 1367 | No | NA | 0 | 0 | Low-quality | Genome-fragment | | 0.91 | 0 |
| S2-final-viral-combined_k141_44276\|\|full | 1367 | No | NA | 0 | 0 | Low-quality | Genome-fragment | | 1.64 | 0 |
| S2-final-viral-combined_k141_168180\|\|full | 1367 | No | NA | 0 | 1 | Low-quality | Genome-fragment | | 2.31 | 0 |
| S1-final-viral-combined_k141_372867\|\|full | 3087 | No | NA | 0 | 1 | Not-determined | Genome-fragment | NA | | 0 |
| S1-final-viral-combined_k141_27092\|\|full | 1930 | No | NA | 0 | 1 | Not-determined | Genome-fragment | NA | | 0 |
| S2-final-viral-combined_k141_226294\|\|lt2gene | 1367 | No | NA | 1 | 0 | Low-quality | Genome-fragment | | 4.63 | 0 |
| S1-final-viral-combined_k141_59847\|\|full | 2058 | No | NA | 0 | 1 | Not-determined | Genome-fragment | NA | | 0 |
| S1-final-viral-combined_k141_10888\|\|full | 4172 | No | NA | 0 | 2 | Not-determined | Genome-fragment | NA | | 0 |
| S2_k141_142111 | 1367 | No | NA | 0 | 2 | Low-quality | Genome-fragment | | 0.68 | 0 |
| S1-final-viral-combined_k141_324647\|\|full | 2415 | No | NA | 0 | 1 | Not-determined | Genome-fragment | NA | | 0 |
| S1-final-viral-combined_k141_357034\|\|full | 1499 | No | NA | 0 | 0 | Not-determined | Genome-fragment | NA | | 0 |
| S4-final-viral-combined_k141_22531\|\|full | 1367 | No | NA | 0 | 0 | Low-quality | Genome-fragment | | 0.86 | 0 |
| S1-final-viral-combined_k141_93331\|\|full | 1920 | No | NA | 0 | 0 | Not-determined | Genome-fragment | NA | | 0 |
| S1-final-viral-combined_k141_59870\|\|full | 8442 | No | NA | 0 | 7 | Not-determined | Genome-fragment | NA | | 0 |
| S1-final-viral-combined_k141_158350\|\|full | 2154 | No | NA | 0 | 0 | Not-determined | Genome-fragment | NA | | 0 |
| S1-final-viral-combined_k141_389161\|\|full | 3173 | No | NA | 0 | 0 | Not-determined | Genome-fragment | NA | | 0 |
| S8-final-viral-combined_k141_214967\|\|full | 1367 | No | NA | 0 | 0 | Low-quality | Genome-fragment | | 2.04 | 0 |
| S11_k141_311240 | 1368 | No | NA | 0 | 0 | Low-quality | Genome-fragment | | 0.79 | 0 |
| S1-final-viral-combined_k141_372880\|\|full | 2053 | No | NA | 0 | 2 | Not-determined | Genome-fragment | NA | | 0 |
| S1-final-viral-combined_k141_241575\|\|full | 3262 | No | NA | 0 | 2 | Not-determined | Genome-fragment | NA | | 0 |
| S1-final-viral-combined_k141_258671\|\|full | 2040 | No | NA | 0 | 2 | Not-determined | Genome-fragment | NA | | 0 |
| S1-final-viral-combined_k141_357066\|\|full | 1502 | No | NA | 0 | 0 | Not-determined | Genome-fragment | NA | | 0 |
| S1-final-viral-combined_k141_339243\|\|full | 10674 | No | NA | 0 | 5 | Not-determined | Genome-fragment | NA | | 0 |
| S3-final-viral-combined_k141_32075\|\|full | 1368 | No | NA | 2 | 0 | Low-quality | Genome-fragment | | 4.66 | 0 |
| S1-final-viral-combined_k141_273920\|\|full | 2281 | No | NA | 0 | 3 | Not-determined | Genome-fragment | NA | | 0 |
| S1-final-viral-combined_k141_258713\|\|full | 2322 | No | NA | 0 | 1 | Not-determined | Genome-fragment | NA | | 0 |
| S3_k141_132228 | 1368 | No | NA | 0 | 0 | Low-quality | Genome-fragment | | 0.89 | 0 |
| S5_k141_93291 | 1368 | No | NA | 0 | 1 | Low-quality | Genome-fragment | | 0.44 | 0 |
| S1-final-viral-combined_k141_127056\|\|full | 1213 | No | NA | 0 | 1 | Not-determined | Genome-fragment | NA | | 0 |
| S12-final-viral-combined_k141_315485\|\|full | 1368 | No | NA | 0 | 0 | Low-quality | Genome-fragment | | 1.04 | 0 |
| S1-final-viral-combined_k141_241618\|\|full | 1226 | No | NA | 0 | 0 | Not-determined | Genome-fragment | NA | | 0 |
| S1-final-viral-combined_k141_158428\|\|full | 3046 | No | NA | 0 | 3 | Not-determined | Genome-fragment | NA | | 0 |
| S1-final-viral-combined_k141_258750\|\|full | 2296 | No | NA | 0 | 1 | Not-determined | Genome-fragment | NA | | 0 |
| S1-final-viral-combined_k141_11011\|\|full | 2573 | No | NA | 0 | 1 | Not-determined | Genome-fragment | NA | | 0 |
| S1-final-viral-combined_k141_142721\|\|full | 14643 | No | NA | 0 | 6 | Not-determined | Genome-fragment | NA | | 0 |
| S1-final-viral-combined_k141_339289\|\|full | 2443 | No | NA | 0 | 2 | Not-determined | Genome-fragment | NA | | 0 |
| S12-final-viral-combined_k141_156627\|\|lt2gene | 1368 | No | NA | 1 | 0 | Low-quality | Genome-fragment | | 2.15 | 0 |
| S1-final-viral-combined_k141_258764\|\|full | 3699 | No | NA | 0 | 4 | Not-determined | Genome-fragment | NA | | 0 |
| S4-final-viral-combined_k141_49493\|\|full | 1368 | No | NA | 0 | 1 | Low-quality | Genome-fragment | | 1.11 | 0 |
| S1-final-viral-combined_k141_324699\|\|full | 6150 | No | NA | 0 | 0 | Not-determined | Genome-fragment | NA | | 0 |
| S6-final-viral-combined_k141_107922\|\|lt2gene | 1368 | No | NA | 2 | 0 | Low-quality | Genome-fragment | | 4.08 | 0 |
| S8-final-viral-combined_k141_274472\|\|full | 1368 | No | NA | 0 | 0 | Low-quality | Genome-fragment | | 3.01 | 0 |
| S1-final-viral-combined_k141_224870\|\|full | 2416 | No | NA | 0 | 3 | Not-determined | Genome-fragment | NA | | 0 |
| S8_k141_162993 | 1368 | No | NA | 0 | 1 | Low-quality | Genome-fragment | | 0.76 | 0 |
| S11-final-viral-combined_k141_242248\|\|full | 1369 | No | NA | 0 | 0 | Low-quality | Genome-fragment | | 3.07 | 0 |
| S1-final-viral-combined_k141_59925\|\|full | 52971 | No | NA | 0 | 39 | Not-determined | Genome-fragment | NA | | 0 |
| S11-final-viral-combined_k141_11434\|\|full | 1369 | No | NA | 0 | 2 | Low-quality | Genome-fragment | | 0.43 | 0 |
| S1-final-viral-combined_k141_324717\|\|full | 1048 | No | NA | 0 | 2 | Not-determined | Genome-fragment | NA | | 0 |
| S1_k141_295172 | 1369 | No | NA | 0 | 2 | Low-quality | Genome-fragment | | 0.88 | 0 |
| S1-final-viral-combined_k141_209411\|\|full | 1230 | No | NA | 0 | 2 | Not-determined | Genome-fragment | NA | | 0 |
| S1-final-viral-combined_k141_224901\|\|full | 1283 | No | NA | 0 | 1 | Not-determined | Genome-fragment | NA | | 0 |
| S5-final-viral-combined_k141_102894\|\|full | 1369 | No | NA | 0 | 0 | Low-quality | Genome-fragment | | 2.09 | 0 |
| S1-final-viral-combined_k141_75831\|\|full | 1886 | No | NA | 0 | 0 | Not-determined | Genome-fragment | NA | | 0 |
| S1-final-viral-combined_k141_108788\|\|full | 1310 | No | NA | 0 | 1 | Not-determined | Genome-fragment | NA | | 0 |
| S5-final-viral-combined_k141_156960\|\|lt2gene | 1369 | No | NA | 1 | 0 | Low-quality | Genome-fragment | | 5.61 | 0 |
| S1-final-viral-combined_k141_273985\|\|full | 1495 | No | NA | 0 | 2 | Not-determined | Genome-fragment | NA | | 0 |
| S1-final-viral-combined_k141_75865\|\|full | 1677 | No | NA | 0 | 1 | Not-determined | Genome-fragment | NA | | 0 |
| S1-final-viral-combined_k141_93481\|\|full | 2468 | No | NA | 0 | 2 | Not-determined | Genome-fragment | NA | | 0 |

<table>
<tr><th></th><th></th><th></th><th></th><th></th><th></th><th></th><th></th><th></th><th></th></tr>
<tr><td>S1-final-viral-combined_k141_389319||full</td><td>1129</td><td>No</td><td>NA</td><td>0</td><td>0</td><td>Not-determined</td><td>Genome-fragment</td><td>NA</td><td>0</td></tr>
<tr><td>S5_k141_14870</td><td>1369</td><td>No</td><td>NA</td><td>0</td><td>1</td><td>Low-quality</td><td>Genome-fragment</td><td>1.36</td><td>0</td></tr>
<tr><td>S7-final-viral-combined_k141_228383||full</td><td>1369</td><td>No</td><td>NA</td><td>1</td><td>0</td><td>Low-quality</td><td>Genome-fragment</td><td>3.56</td><td>0</td></tr>
<tr><td>S1-final-viral-combined_k141_224943||full</td><td>1659</td><td>No</td><td>NA</td><td>0</td><td>0</td><td>Not-determined</td><td>Genome-fragment</td><td>NA</td><td>0</td></tr>
<tr><td>S12-final-viral-combined_k141_339533||full</td><td>1369</td><td>No</td><td>NA</td><td>1</td><td>0</td><td>Low-quality</td><td>Genome-fragment</td><td>3.05</td><td>0</td></tr>
<tr><td>S12-final-viral-combined_k141_194155||full</td><td>1369</td><td>No</td><td>NA</td><td>0</td><td>0</td><td>Low-quality</td><td>Genome-fragment</td><td>3.56</td><td>0</td></tr>
<tr><td>S1-final-viral-combined_k141_224958||full</td><td>1971</td><td>No</td><td>NA</td><td>0</td><td>3</td><td>Not-determined</td><td>Genome-fragment</td><td>NA</td><td>0</td></tr>
<tr><td>S1-final-viral-combined_k141_27227||full</td><td>2195</td><td>No</td><td>NA</td><td>0</td><td>3</td><td>Not-determined</td><td>Genome-fragment</td><td>NA</td><td>0</td></tr>
<tr><td>S12-final-viral-combined_k141_311372||full</td><td>1369</td><td>No</td><td>NA</td><td>0</td><td>0</td><td>Low-quality</td><td>Genome-fragment</td><td>3.22</td><td>0</td></tr>
<tr><td>S1-final-viral-combined_k141_290967||full</td><td>2186</td><td>No</td><td>NA</td><td>0</td><td>2</td><td>Not-determined</td><td>Genome-fragment</td><td>NA</td><td>0</td></tr>
<tr><td>S1-final-viral-combined_k141_59989||full</td><td>4408</td><td>No</td><td>NA</td><td>0</td><td>2</td><td>Not-determined</td><td>Genome-fragment</td><td>NA</td><td>0</td></tr>
<tr><td>S12_k141_307545</td><td>1369</td><td>No</td><td>NA</td><td>0</td><td>0</td><td>Low-quality</td><td>Genome-fragment</td><td>1.39</td><td>0</td></tr>
<tr><td>S2-final-viral-combined_k141_60484||full</td><td>1369</td><td>No</td><td>NA</td><td>0</td><td>0</td><td>Low-quality</td><td>Genome-fragment</td><td>1.28</td><td>0</td></tr>
<tr><td>S2-final-viral-combined_k141_132864||full</td><td>1369</td><td>No</td><td>NA</td><td>1</td><td>0</td><td>Low-quality</td><td>Genome-fragment</td><td>1.02</td><td>0</td></tr>
<tr><td>S6-final-viral-combined_k141_109738||full</td><td>1369</td><td>No</td><td>NA</td><td>0</td><td>1</td><td>Low-quality</td><td>Genome-fragment</td><td>2.75</td><td>0</td></tr>
<tr><td>S11-final-viral-combined_k141_104404||full</td><td>1370</td><td>No</td><td>NA</td><td>0</td><td>1</td><td>Low-quality</td><td>Genome-fragment</td><td>0.98</td><td>0</td></tr>
<tr><td>S1-final-viral-combined_k141_43587||full</td><td>1040</td><td>No</td><td>NA</td><td>0</td><td>2</td><td>Not-determined</td><td>Genome-fragment</td><td>NA</td><td>0</td></tr>
<tr><td>S1-final-viral-combined_k141_357178||full</td><td>3218</td><td>No</td><td>NA</td><td>0</td><td>1</td><td>Not-determined</td><td>Genome-fragment</td><td>NA</td><td>0</td></tr>
<tr><td>S1-final-viral-combined_k141_339423||full</td><td>1635</td><td>No</td><td>NA</td><td>0</td><td>1</td><td>Not-determined</td><td>Genome-fragment</td><td>NA</td><td>0</td></tr>
<tr><td>S11_k141_310315</td><td>1370</td><td>No</td><td>NA</td><td>0</td><td>0</td><td>Low-quality</td><td>Genome-fragment</td><td>1.12</td><td>0</td></tr>
<tr><td>S1-final-viral-combined_k141_364587||full</td><td>1370</td><td>No</td><td>NA</td><td>0</td><td>1</td><td>Low-quality</td><td>Genome-fragment</td><td>0.98</td><td>0</td></tr>
<tr><td>S1-final-viral-combined_k141_93574||full</td><td>1175</td><td>No</td><td>NA</td><td>0</td><td>1</td><td>Not-determined</td><td>Genome-fragment</td><td>NA</td><td>0</td></tr>
<tr><td>S1-final-viral-combined_k141_366313||full</td><td>1370</td><td>No</td><td>NA</td><td>1</td><td>0</td><td>Low-quality</td><td>Genome-fragment</td><td>2.63</td><td>0</td></tr>
<tr><td>S1-final-viral-combined_k141_43610||full</td><td>1916</td><td>No</td><td>NA</td><td>0</td><td>0</td><td>Not-determined</td><td>Genome-fragment</td><td>NA</td><td>0</td></tr>
<tr><td>S1-final-viral-combined_k141_284338||full</td><td>1370</td><td>No</td><td>NA</td><td>0</td><td>0</td><td>Low-quality</td><td>Genome-fragment</td><td>3.19</td><td>0</td></tr>
<tr><td>S1-final-viral-combined_k141_80577||full</td><td>1370</td><td>No</td><td>NA</td><td>2</td><td>0</td><td>Low-quality</td><td>Genome-fragment</td><td>3.1</td><td>0</td></tr>
<tr><td>S1-final-viral-combined_k141_43614||full</td><td>8848</td><td>No</td><td>NA</td><td>0</td><td>7</td><td>Not-determined</td><td>Genome-fragment</td><td>NA</td><td>0</td></tr>
<tr><td>S1_k141_142451</td><td>1370</td><td>No</td><td>NA</td><td>2</td><td>0</td><td>Low-quality</td><td>Genome-fragment</td><td>3.41</td><td>0</td></tr>
<tr><td>S5-final-viral-combined_k141_152610||full</td><td>1370</td><td>No</td><td>NA</td><td>1</td><td>0</td><td>Low-quality</td><td>Genome-fragment</td><td>3.23</td><td>0</td></tr>
<tr><td>S1-final-viral-combined_k141_225049||full</td><td>2716</td><td>No</td><td>NA</td><td>0</td><td>2</td><td>Not-determined</td><td>Genome-fragment</td><td>NA</td><td>0</td></tr>
<tr><td>S7-final-viral-combined_k141_10772||full</td><td>1370</td><td>No</td><td>NA</td><td>1</td><td>0</td><td>Low-quality</td><td>Genome-fragment</td><td>3.3</td><td>0</td></tr>
<tr><td>S7-final-viral-combined_k141_50217||full</td><td>1370</td><td>No</td><td>NA</td><td>0</td><td>0</td><td>Low-quality</td><td>Genome-fragment</td><td>2.11</td><td>0</td></tr>
<tr><td>S7-final-viral-combined_k141_244328||full</td><td>1370</td><td>No</td><td>NA</td><td>0</td><td>0</td><td>Low-quality</td><td>Genome-fragment</td><td>1.3</td><td>0</td></tr>
<tr><td>S6-final-viral-combined_k141_33383||full</td><td>1370</td><td>No</td><td>NA</td><td>0</td><td>0</td><td>Low-quality</td><td>Genome-fragment</td><td>3.99</td><td>0</td></tr>
<tr><td>S8-final-viral-combined_k141_106898||full</td><td>1370</td><td>No</td><td>NA</td><td>0</td><td>0</td><td>Low-quality</td><td>Genome-fragment</td><td>3.17</td><td>0</td></tr>
<tr><td>S1-final-viral-combined_k141_43654||full</td><td>4173</td><td>No</td><td>NA</td><td>0</td><td>0</td><td>Not-determined</td><td>Genome-fragment</td><td>NA</td><td>0</td></tr>
<tr><td>S8-final-viral-combined_k141_6781||full</td><td>1370</td><td>No</td><td>NA</td><td>0</td><td>1</td><td>Low-quality</td><td>Genome-fragment</td><td>2.62</td><td>0</td></tr>
<tr><td>S1-final-viral-combined_k141_339468||full</td><td>9421</td><td>No</td><td>NA</td><td>0</td><td>7</td><td>Not-determined</td><td>Genome-fragment</td><td>NA</td><td>0</td></tr>
<tr><td>S1-final-viral-combined_k141_11155||full</td><td>1816</td><td>No</td><td>NA</td><td>0</td><td>1</td><td>Not-determined</td><td>Genome-fragment</td><td>NA</td><td>0</td></tr>
<tr><td>S1-final-viral-combined_k141_259053||full</td><td>2814</td><td>No</td><td>NA</td><td>0</td><td>1</td><td>Not-determined</td><td>Genome-fragment</td><td>NA</td><td>0</td></tr>
<tr><td>S3-final-viral-combined_k141_62370||lt2gene</td><td>1371</td><td>No</td><td>NA</td><td>0</td><td>0</td><td>Low-quality</td><td>Genome-fragment</td><td>3.19</td><td>0</td></tr>
<tr><td>S1-final-viral-combined_k141_76038||full</td><td>2678</td><td>No</td><td>NA</td><td>0</td><td>2</td><td>Not-determined</td><td>Genome-fragment</td><td>NA</td><td>0</td></tr>
<tr><td>S1-final-viral-combined_k141_291106||full</td><td>1048</td><td>No</td><td>NA</td><td>0</td><td>2</td><td>Not-determined</td><td>Genome-fragment</td><td>NA</td><td>0</td></tr>
<tr><td>S1-final-viral-combined_k141_339483||full</td><td>1437</td><td>No</td><td>NA</td><td>0</td><td>0</td><td>Not-determined</td><td>Genome-fragment</td><td>NA</td><td>0</td></tr>
<tr><td>S5-final-viral-combined_k141_140824||full</td><td>1371</td><td>No</td><td>NA</td><td>0</td><td>0</td><td>Low-quality</td><td>Genome-fragment</td><td>3.41</td><td>0</td></tr>
<tr><td>S12-final-viral-combined_k141_106240||full</td><td>1371</td><td>No</td><td>NA</td><td>0</td><td>0</td><td>Low-quality</td><td>Genome-fragment</td><td>0.7</td><td>0</td></tr>
<tr><td>S1-final-viral-combined_k141_192842||full</td><td>1530</td><td>No</td><td>NA</td><td>0</td><td>1</td><td>Not-determined</td><td>Genome-fragment</td><td>NA</td><td>0</td></tr>
<tr><td>S8-final-viral-combined_k141_246208||full</td><td>1371</td><td>No</td><td>NA</td><td>0</td><td>0</td><td>Low-quality</td><td>Genome-fragment</td><td>0.74</td><td>0</td></tr>
<tr><td>S1-final-viral-combined_k141_291127||full</td><td>1345</td><td>No</td><td>NA</td><td>0</td><td>2</td><td>Not-determined</td><td>Genome-fragment</td><td>NA</td><td>0</td></tr>
<tr><td>S1-final-viral-combined_k141_241935||full</td><td>2116</td><td>No</td><td>NA</td><td>0</td><td>2</td><td>Not-determined</td><td>Genome-fragment</td><td>NA</td><td>0</td></tr>
<tr><td>S8-final-viral-combined_k141_272042||lt2gene</td><td>1371</td><td>No</td><td>NA</td><td>1</td><td>0</td><td>Low-quality</td><td>Genome-fragment</td><td>2.86</td><td>0</td></tr>
<tr><td>S1-final-viral-combined_k141_142869||full</td><td>3199</td><td>No</td><td>NA</td><td>0</td><td>2</td><td>Not-determined</td><td>Genome-fragment</td><td>NA</td><td>0</td></tr>
<tr><td>S1-final-viral-combined_k141_291153||full</td><td>3375</td><td>No</td><td>NA</td><td>0</td><td>2</td><td>Not-determined</td><td>Genome-fragment</td><td>NA</td><td>0</td></tr>
<tr><td>S11-final-viral-combined_k141_197562||full</td><td>1372</td><td>No</td><td>NA</td><td>0</td><td>0</td><td>Low-quality</td><td>Genome-fragment</td><td>1.09</td><td>0</td></tr>
<tr><td>S11-final-viral-combined_k141_33155||full</td><td>1372</td><td>No</td><td>NA</td><td>0</td><td>0</td><td>Low-quality</td><td>Genome-fragment</td><td>2.44</td><td>0</td></tr>
<tr><td>S1-final-viral-combined_k141_308135||full</td><td>7330</td><td>No</td><td>NA</td><td>0</td><td>5</td><td>Not-determined</td><td>Genome-fragment</td><td>NA</td><td>0</td></tr>
<tr><td>S11-final-viral-combined_k141_50081||full</td><td>1372</td><td>No</td><td>NA</td><td>2</td><td>0</td><td>Low-quality</td><td>Genome-fragment</td><td>0.86</td><td>0</td></tr>
<tr><td>S1-final-viral-combined_k141_324961||full</td><td>1745</td><td>No</td><td>NA</td><td>0</td><td>1</td><td>Not-determined</td><td>Genome-fragment</td><td>NA</td><td>0</td></tr>
<tr><td>S11-final-viral-combined_k141_116114||lt2gene</td><td>1372</td><td>No</td><td>NA</td><td>1</td><td>0</td><td>Low-quality</td><td>Genome-fragment</td><td>2.56</td><td>0</td></tr>
<tr><td>S5-final-viral-combined_k141_206459||full</td><td>1372</td><td>No</td><td>NA</td><td>1</td><td>0</td><td>Low-quality</td><td>Genome-fragment</td><td>2.55</td><td>0</td></tr>
<tr><td>S1-final-viral-combined_k141_209536||full</td><td>1246</td><td>No</td><td>NA</td><td>0</td><td>2</td><td>Not-determined</td><td>Genome-fragment</td><td>NA</td><td>0</td></tr>
<tr><td>S1-final-viral-combined_k141_27327||full</td><td>1287</td><td>No</td><td>NA</td><td>0</td><td>0</td><td>Not-determined</td><td>Genome-fragment</td><td>NA</td><td>0</td></tr>
<tr><td>S1-final-viral-combined_k141_225210||full</td><td>2134</td><td>No</td><td>NA</td><td>0</td><td>3</td><td>Not-determined</td><td>Genome-fragment</td><td>NA</td><td>0</td></tr>
<tr><td>S17-final-viral-combined_k141_172215||lt2gene</td><td>1372</td><td>No</td><td>NA</td><td>1</td><td>0</td><td>Low-quality</td><td>Genome-fragment</td><td>1.22</td><td>0</td></tr>
<tr><td>S17-final-viral-combined_k141_73341||lt2gene</td><td>1372</td><td>No</td><td>NA</td><td>1</td><td>0</td><td>Low-quality</td><td>Genome-fragment</td><td>1.56</td><td>0</td></tr>
<tr><td>S1-final-viral-combined_k141_308160||full</td><td>7772</td><td>No</td><td>NA</td><td>0</td><td>4</td><td>Not-determined</td><td>Genome-fragment</td><td>NA</td><td>0</td></tr>
<tr><td>S17-k141_137054</td><td>1372</td><td>No</td><td>NA</td><td>0</td><td>0</td><td>Low-quality</td><td>Genome-fragment</td><td>3.23</td><td>0</td></tr>
<tr><td>S1-final-viral-combined_k141_43787||full</td><td>1058</td><td>No</td><td>NA</td><td>0</td><td>2</td><td>Not-determined</td><td>Genome-fragment</td><td>NA</td><td>0</td></tr>
<tr><td>S1-final-viral-combined_k141_209557||full</td><td>1260</td><td>No</td><td>NA</td><td>0</td><td>0</td><td>Not-determined</td><td>Genome-fragment</td><td>NA</td><td>0</td></tr>
<tr><td>S12-final-viral-combined_k141_47685||full</td><td>1372</td><td>Yes</td><td>406</td><td>1</td><td>1</td><td>Low-quality</td><td>Genome-fragment</td><td>1.07</td><td>70.41</td></tr>
<tr><td>S1-final-viral-combined_k141_158676||full</td><td>3323</td><td>No</td><td>NA</td><td>0</td><td>1</td><td>Not-determined</td><td>Genome-fragment</td><td>NA</td><td>0</td></tr>
<tr><td>S1-final-viral-combined_k141_357373||full</td><td>2966</td><td>No</td><td>NA</td><td>0</td><td>0</td><td>Medium-quality</td><td>Genome-fragment</td><td>53.65</td><td>0</td></tr>
<tr><td>S6_k141_67651</td><td>1372</td><td>No</td><td>NA</td><td>0</td><td>0</td><td>Low-quality</td><td>Genome-fragment</td><td>1.08</td><td>0</td></tr>
<tr><td>S1-final-viral-combined_k141_357408||full</td><td>2120</td><td>No</td><td>NA</td><td>0</td><td>1</td><td>Not-determined</td><td>Genome-fragment</td><td>NA</td><td>0</td></tr>
<tr><td>S8-final-viral-combined_k141_245718||full</td><td>1372</td><td>No</td><td>NA</td><td>0</td><td>0</td><td>Low-quality</td><td>Genome-fragment</td><td>3.12</td><td>0</td></tr>
<tr><td>S1-final-viral-combined_k141_339560||full</td><td>2117</td><td>No</td><td>NA</td><td>0</td><td>0</td><td>Not-determined</td><td>Genome-fragment</td><td>NA</td><td>0</td></tr>
<tr><td>S1-final-viral-combined_k141_225273||full</td><td>1510</td><td>No</td><td>NA</td><td>0</td><td>1</td><td>Not-determined</td><td>Genome-fragment</td><td>NA</td><td>0</td></tr>
<tr><td>S1-final-viral-combined_k141_373226||full</td><td>1167</td><td>No</td><td>NA</td><td>0</td><td>0</td><td>Not-determined</td><td>Genome-fragment</td><td>NA</td><td>0</td></tr>
<tr><td>S8-final-viral-combined_k141_52337||full</td><td>1372</td><td>No</td><td>NA</td><td>1</td><td>0</td><td>Low-quality</td><td>Genome-fragment</td><td>3.08</td><td>0</td></tr>
<tr><td>S1-final-viral-combined_k141_158702||full</td><td>3677</td><td>No</td><td>NA</td><td>0</td><td>0</td><td>Not-determined</td><td>Genome-fragment</td><td>NA</td><td>0</td></tr>
<tr><td>S1-final-viral-combined_k141_357424||full</td><td>2032</td><td>No</td><td>NA</td><td>0</td><td>0</td><td>Not-determined</td><td>Genome-fragment</td><td>NA</td><td>0</td></tr>
<tr><td>S8_k141_63009</td><td>1372</td><td>No</td><td>NA</td><td>0</td><td>1</td><td>Low-quality</td><td>Genome-fragment</td><td>2.27</td><td>0</td></tr>
<tr><td>S1-final-viral-combined_k141_49799||full</td><td>1373</td><td>No</td><td>NA</td><td>0</td><td>0</td><td>Low-quality</td><td>Genome-fragment</td><td>4.02</td><td>0</td></tr>
<tr><td>S1-final-viral-combined_k141_124868||full</td><td>1373</td><td>No</td><td>NA</td><td>0</td><td>2</td><td>Low-quality</td><td>Genome-fragment</td><td>1.87</td><td>0</td></tr>
<tr><td>S3_k141_119212</td><td>1373</td><td>No</td><td>NA</td><td>0</td><td>1</td><td>Low-quality</td><td>Genome-fragment</td><td>2.96</td><td>0</td></tr>
<tr><td>S1-final-viral-combined_k141_109055||full</td><td>2786</td><td>No</td><td>NA</td><td>0</td><td>3</td><td>Not-determined</td><td>Genome-fragment</td><td>NA</td><td>0</td></tr>
<tr><td>S1-final-viral-combined_k141_373236||full</td><td>1699</td><td>No</td><td>NA</td><td>0</td><td>1</td><td>Not-determined</td><td>Genome-fragment</td><td>NA</td><td>0</td></tr>
<tr><td>S5-final-viral-combined_k141_137903||lt2gene</td><td>1373</td><td>No</td><td>NA</td><td>1</td><td>0</td><td>Low-quality</td><td>Genome-fragment</td><td>3.04</td><td>0</td></tr>
<tr><td>S7-final-viral-combined_k141_219758||full</td><td>1373</td><td>No</td><td>NA</td><td>0</td><td>0</td><td>Low-quality</td><td>Genome-fragment</td><td>2.44</td><td>0</td></tr>
<tr><td>S17-k141_8488</td><td>1373</td><td>No</td><td>NA</td><td>1</td><td>0</td><td>Low-quality</td><td>Genome-fragment</td><td>0.79</td><td>0</td></tr>
<tr><td>S1-final-viral-combined_k141_11293||full</td><td>1122</td><td>No</td><td>NA</td><td>0</td><td>0</td><td>Not-determined</td><td>Genome-fragment</td><td>NA</td><td>0</td></tr>
<tr><td>S1-final-viral-combined_k141_142988||full</td><td>1586</td><td>No</td><td>NA</td><td>0</td><td>0</td><td>Not-determined</td><td>Genome-fragment</td><td>NA</td><td>0</td></tr>
<tr><td>S1-final-viral-combined_k141_142995||full</td><td>1088</td><td>No</td><td>NA</td><td>0</td><td>0</td><td>Not-determined</td><td>Genome-fragment</td><td>NA</td><td>0</td></tr>
<tr><td>S12-final-viral-combined_k141_398105||lt2gene</td><td>1373</td><td>No</td><td>NA</td><td>1</td><td>0</td><td>Low-quality</td><td>Genome-fragment</td><td>2.24</td><td>0</td></tr>
<tr><td>S2-final-viral-combined_k141_15122||full</td><td>1373</td><td>No</td><td>NA</td><td>1</td><td>0</td><td>Low-quality</td><td>Genome-fragment</td><td>0.89</td><td>0</td></tr>
<tr><td>S1-final-viral-combined_k141_27433||full</td><td>1540</td><td>No</td><td>NA</td><td>0</td><td>0</td><td>Not-determined</td><td>Genome-fragment</td><td>NA</td><td>0</td></tr>
<tr><td>S8-final-viral-combined_k141_201866||full</td><td>1373</td><td>No</td><td>NA</td><td>0</td><td>0</td><td>Low-quality</td><td>Genome-fragment</td><td>2.14</td><td>0</td></tr>
<tr><td>S8_k141_40984</td><td>1373</td><td>No</td><td>NA</td><td>0</td><td>1</td><td>Low-quality</td><td>Genome-fragment</td><td>1.3</td><td>0</td></tr>
<tr><td>S9-final-viral-combined_k141_12024||full</td><td>1374</td><td>No</td><td>NA</td><td>1</td><td>0</td><td>Low-quality</td><td>Genome-fragment</td><td>1.58</td><td>0</td></tr>
<tr><td>S1-final-viral-combined_k141_127534||full</td><td>1327</td><td>No</td><td>NA</td><td>0</td><td>0</td><td>Not-determined</td><td>Genome-fragment</td><td>NA</td><td>0</td></tr>
<tr><td>S1-final-viral-combined_k141_389654||full</td><td>1464</td><td>No</td><td>NA</td><td>0</td><td>1</td><td>Not-determined</td><td>Genome-fragment</td><td>NA</td><td>0</td></tr>
<tr><td>S11_k141_17713</td><td>1374</td><td>No</td><td>NA</td><td>0</td><td>1</td><td>Low-quality</td><td>Genome-fragment</td><td>1.42</td><td>0</td></tr>
<tr><td>S1-final-viral-combined_k141_127181||full</td><td>1374</td><td>No</td><td>NA</td><td>0</td><td>1</td><td>Low-quality</td><td>Genome-fragment</td><td>0.35</td><td>0</td></tr>
<tr><td>S1-final-viral-combined_k141_96885||full</td><td>1374</td><td>No</td><td>NA</td><td>1</td><td>0</td><td>Low-quality</td><td>Genome-fragment</td><td>4.24</td><td>0</td></tr>
<tr><td>S1-final-viral-combined_k141_174918||full</td><td>1044</td><td>No</td><td>NA</td><td>0</td><td>1</td><td>Not-determined</td><td>Genome-fragment</td><td>NA</td><td>0</td></tr>
<tr><td>S1-final-viral-combined_k141_76289||full</td><td>1406</td><td>No</td><td>NA</td><td>0</td><td>0</td><td>Not-determined</td><td>Genome-fragment</td><td>NA</td><td>0</td></tr>
<tr><td>S1-final-viral-combined_k141_389674||full</td><td>1513</td><td>No</td><td>NA</td><td>0</td><td>0</td><td>Not-determined</td><td>Genome-fragment</td><td>NA</td><td>0</td></tr>
<tr><td>S1-final-viral-combined_k141_291405||full</td><td>1808</td><td>No</td><td>NA</td><td>0</td><td>2</td><td>Not-determined</td><td>Genome-fragment</td><td>NA</td><td>0</td></tr>
<tr><td>S1_k141_251887</td><td>1374</td><td>No</td><td>NA</td><td>0</td><td>0</td><td>Low-quality</td><td>Genome-fragment</td><td>1.3</td><td>0</td></tr>
<tr><td>S1-final-viral-combined_k141_373308||full</td><td>14841</td><td>No</td><td>NA</td><td>1</td><td>6</td><td>High-quality</td><td>High-quality</td><td>100</td><td>0</td></tr>
<tr><td>S7-final-viral-combined_k141_97086||full</td><td>1374</td><td>No</td><td>NA</td><td>0</td><td>0</td><td>Low-quality</td><td>Genome-fragment</td><td>3.19</td><td>0</td></tr>
<tr><td>S1-final-viral-combined_k141_93949||full</td><td>2152</td><td>No</td><td>NA</td><td>0</td><td>1</td><td>Not-determined</td><td>Genome-fragment</td><td>NA</td><td>0</td></tr>
<tr><td>S1-final-viral-combined_k141_93952||full</td><td>1245</td><td>No</td><td>NA</td><td>0</td><td>3</td><td>Not-determined</td><td>Genome-fragment</td><td>NA</td><td>0</td></tr>
<tr><td>S1-final-viral-combined_k141_325093||full</td><td>3682</td><td>No</td><td>NA</td><td>0</td><td>2</td><td>Not-determined</td><td>Genome-fragment</td><td>NA</td><td>0</td></tr>
<tr><td>S17-final-viral-combined_k141_186018||full</td><td>1374</td><td>No</td><td>NA</td><td>0</td><td>0</td><td>Low-quality</td><td>Genome-fragment</td><td>1.63</td><td>0</td></tr>
<tr><td>S1-final-viral-combined_k141_373316||full</td><td>1320</td><td>No</td><td>NA</td><td>0</td><td>0</td><td>Not-determined</td><td>Genome-fragment</td><td>NA</td><td>0</td></tr>
<tr><td>S12-final-viral-combined_k141_157741||full</td><td>1374</td><td>No</td><td>NA</td><td>0</td><td>0</td><td>Low-quality</td><td>Genome-fragment</td><td>0.4</td><td>0</td></tr>
<tr><td>S1-final-viral-combined_k141_225467||full</td><td>1651</td><td>No</td><td>NA</td><td>0</td><td>1</td><td>Not-determined</td><td>Genome-fragment</td><td>NA</td><td>0</td></tr>
<tr><td>S12-final-viral-combined_k141_313514||lt2gene</td><td>1374</td><td>No</td><td>NA</td><td>1</td><td>0</td><td>Low-quality</td><td>Genome-fragment</td><td>3.42</td><td>0</td></tr>
<tr><td>S1-final-viral-combined_k141_357597||full</td><td>2856</td><td>No</td><td>NA</td><td>0</td><td>0</td><td>Not-determined</td><td>Genome-fragment</td><td>NA</td><td>0</td></tr>
<tr><td>S12_k141_251889</td><td>1374</td><td>No</td><td>NA</td><td>0</td><td>1</td><td>Low-quality</td><td>Genome-fragment</td><td>1.09</td><td>0</td></tr>
<tr><td>S1-final-viral-combined_k141_109189||full</td><td>1068</td><td>No</td><td>NA</td><td>0</td><td>0</td><td>Not-determined</td><td>Genome-fragment</td><td>NA</td><td>0</td></tr>
<tr><td>S1-final-viral-combined_k141_93978||full</td><td>2082</td><td>No</td><td>NA</td><td>0</td><td>1</td><td>Not-determined</td><td>Genome-fragment</td><td>NA</td><td>0</td></tr>
<tr><td>S1-final-viral-combined_k141_308339||full</td><td>1051</td><td>No</td><td>NA</td><td>0</td><td>1</td><td>Not-determined</td><td>Genome-fragment</td><td>NA</td><td>0</td></tr>
<tr><td>S1-final-viral-combined_k141_357608||full</td><td>1136</td><td>No</td><td>NA</td><td>0</td><td>1</td><td>Not-determined</td><td>Genome-fragment</td><td>NA</td><td>0</td></tr>
<tr><td>S1-final-viral-combined_k141_325139||full</td><td>1318</td><td>No</td><td>NA</td><td>0</td><td>0</td><td>Not-determined</td><td>Genome-fragment</td><td>NA</td><td>0</td></tr>
<tr><td>S12_k141_290378</td><td>1374</td><td>No</td><td>NA</td><td>0</td><td>0</td><td>Low-quality</td><td>Genome-fragment</td><td>0.83</td><td>0</td></tr>
<tr><td>S12_k141_263654</td><td>1374</td><td>No</td><td>NA</td><td>0</td><td>0</td><td>Low-quality</td><td>Genome-fragment</td><td>3.09</td><td>0</td></tr>
<tr><td>S1-final-viral-combined_k141_109209||full</td><td>1184</td><td>No</td><td>NA</td><td>0</td><td>0</td><td>Not-determined</td><td>Genome-fragment</td><td>NA</td><td>0</td></tr>
<tr><td>S1-final-viral-combined_k141_242176||full</td><td>1837</td><td>No</td><td>NA</td><td>0</td><td>0</td><td>Not-determined</td><td>Genome-fragment</td><td>NA</td><td>0</td></tr>
<tr><td>S1-final-viral-combined_k141_242177||full</td><td>2512</td><td>No</td><td>NA</td><td>0</td><td>2</td><td>Not-determined</td><td>Genome-fragment</td><td>NA</td><td>0</td></tr>
<tr><td>S2-final-viral-combined_k141_134788||full</td><td>1374</td><td>No</td><td>NA</td><td>0</td><td>0</td><td>Low-quality</td><td>Genome-fragment</td><td>3.3</td><td>0</td></tr>
<tr><td>S1-final-viral-combined_k141_259295||full</td><td>1521</td><td>No</td><td>NA</td><td>0</td><td>1</td><td>Not-determined</td><td>Genome-fragment</td><td>NA</td><td>0</td></tr>
<tr><td>S1-final-viral-combined_k141_389753||full</td><td>6271</td><td>No</td><td>NA</td><td>0</td><td>5</td><td>Not-determined</td><td>Genome-fragment</td><td>NA</td><td>0</td></tr>
<tr><td>S1-final-viral-combined_k141_143184||full</td><td>1064</td><td>No</td><td>NA</td><td>0</td><td>1</td><td>Not-determined</td><td>Genome-fragment</td><td>NA</td><td>0</td></tr>
<tr><td>S1-final-viral-combined_k141_225513||full</td><td>1708</td><td>No</td><td>NA</td><td>0</td><td>0</td><td>Not-determined</td><td>Genome-fragment</td><td>NA</td><td>0</td></tr>
<tr><td>S1-final-viral-combined_k141_94055||full</td><td>3142</td><td>No</td><td>NA</td><td>0</td><td>3</td><td>Not-determined</td><td>Genome-fragment</td><td>NA</td><td>0</td></tr>
<tr><td>S2-final-viral-combined_k141_36066||full</td><td>1374</td><td>No</td><td>NA</td><td>0</td><td>1</td><td>Low-quality</td><td>Genome-fragment</td><td>1.35</td><td>0</td></tr>
<tr><td>S2-final-viral-combined_k141_71630||lt2gene</td><td>1374</td><td>No</td><td>NA</td><td>2</td><td>0</td><td>Low-quality</td><td>Genome-fragment</td><td>3.21</td><td>0</td></tr>
<tr><td>S2_k141_148713</td><td>1374</td><td>No</td><td>NA</td><td>0</td><td>1</td><td>Low-quality</td><td>Genome-fragment</td><td>0.45</td><td>0</td></tr>
<tr><td>S1-final-viral-combined_k141_373408||full</td><td>1444</td><td>No</td><td>NA</td><td>0</td><td>0</td><td>Not-determined</td><td>Genome-fragment</td><td>NA</td><td>0</td></tr>
<tr><td>S1-final-viral-combined_k141_94073||full</td><td>1416</td><td>No</td><td>NA</td><td>0</td><td>0</td><td>Not-determined</td><td>Genome-fragment</td><td>NA</td><td>0</td></tr>
<tr><td>S2_k141_124059</td><td>1374</td><td>No</td><td>NA</td><td>1</td><td>0</td><td>Low-quality</td><td>Genome-fragment</td><td>3.76</td><td>0</td></tr>
<tr><td>S4-final-viral-combined_k141_20824||full</td><td>1374</td><td>No</td><td>NA</td><td>0</td><td>1</td><td>Low-quality</td><td>Genome-fragment</td><td>0.77</td><td>0</td></tr>
<tr><td>S1-final-viral-combined_k141_43992||full</td><td>16717</td><td>No</td><td>NA</td><td>0</td><td>13</td><td>Not-determined</td><td>Genome-fragment</td><td>NA</td><td>0</td></tr>
<tr><td>S1-final-viral-combined_k141_158942||full</td><td>2370</td><td>No</td><td>NA</td><td>0</td><td>0</td><td>Not-determined</td><td>Genome-fragment</td><td>NA</td><td>0</td></tr>
<tr><td>S4-final-viral-combined_k141_22017||lt2gene</td><td>1374</td><td>No</td><td>NA</td><td>1</td><td>0</td><td>Low-quality</td><td>Genome-fragment</td><td>1.29</td><td>0</td></tr>
<tr><td>S1-final-viral-combined_k141_94103||full</td><td>1658</td><td>No</td><td>NA</td><td>0</td><td>1</td><td>Not-determined</td><td>Genome-fragment</td><td>NA</td><td>0</td></tr>
<tr><td>S1-final-viral-combined_k141_158950||full</td><td>3983</td><td>No</td><td>NA</td><td>0</td><td>3</td><td>Not-determined</td><td>Genome-fragment</td><td>NA</td><td>0</td></tr>
<tr><td>S6-final-viral-combined_k141_27266||lt2gene</td><td>1374</td><td>No</td><td>NA</td><td>1</td><td>0</td><td>Low-quality</td><td>Genome-fragment</td><td>2.92</td><td>0</td></tr>
<tr><td>S8_k141_127143</td><td>1374</td><td>No</td><td>NA</td><td>0</td><td>1</td><td>Low-quality</td><td>Genome-fragment</td><td>1.34</td><td>0</td></tr>
<tr><td>S1-final-viral-combined_k141_239248||full</td><td>1375</td><td>No</td><td>NA</td><td>2</td><td>0</td><td>Low-quality</td><td>Genome-fragment</td><td>3.59</td><td>0</td></tr>
<tr><td>S1-final-viral-combined_k141_325276||full</td><td>2940</td><td>No</td><td>NA</td><td>0</td><td>3</td><td>Not-determined</td><td>Genome-fragment</td><td>NA</td><td>0</td></tr>
<tr><td>S1-final-viral-combined_k141_193296||full</td><td>1601</td><td>No</td><td>NA</td><td>0</td><td>2</td><td>Not-determined</td><td>Genome-fragment</td><td>NA</td><td>0</td></tr>
<tr><td>S1-final-viral-combined_k141_78618||full</td><td>1375</td><td>No</td><td>NA</td><td>0</td><td>1</td><td>Low-quality</td><td>Genome-fragment</td><td>3.58</td><td>0</td></tr>
<tr><td>S1-final-viral-combined_k141_37171||lt2gene</td><td>1375</td><td>No</td><td>NA</td><td>1</td><td>0</td><td>Low-quality</td><td>Genome-fragment</td><td>2.6</td><td>0</td></tr>
<tr><td>S1-final-viral-combined_k141_209886||full</td><td>1925</td><td>No</td><td>NA</td><td>0</td><td>1</td><td>Not-determined</td><td>Genome-fragment</td><td>NA</td><td>0</td></tr>
<tr><td>S1-final-viral-combined_k141_193300||full</td><td>3063</td><td>No</td><td>NA</td><td>0</td><td>3</td><td>Not-determined</td><td>Genome-fragment</td><td>NA</td><td>0</td></tr>
<tr><td>S1-final-viral-combined_k141_158978||full</td><td>2188</td><td>No</td><td>NA</td><td>0</td><td>2</td><td>Not-determined</td><td>Genome-fragment</td><td>NA</td><td>0</td></tr>
<tr><td>S1-final-viral-combined_k141_274508||full</td><td>3144</td><td>No</td><td>NA</td><td>0</td><td>2</td><td>Not-determined</td><td>Genome-fragment</td><td>NA</td><td>0</td></tr>
<tr><td>S3_k141_121074</td><td>1375</td><td>No</td><td>NA</td><td>0</td><td>0</td><td>Low-quality</td><td>Genome-fragment</td><td>1.74</td><td>0</td></tr>
<tr><td>S17-final-viral-combined_k141_77050||full</td><td>1375</td><td>No</td><td>NA</td><td>1</td><td>0</td><td>Low-quality</td><td>Genome-fragment</td><td>3.27</td><td>0</td></tr>
<tr><td>S12-final-viral-combined_k141_302956||lt2gene</td><td>1375</td><td>No</td><td>NA</td><td>2</td><td>0</td><td>Low-quality</td><td>Genome-fragment</td><td>4.29</td><td>0</td></tr>
<tr><td>S1-final-viral-combined_k141_60615||full</td><td>1445</td><td>No</td><td>NA</td><td>0</td><td>0</td><td>Not-determined</td><td>Genome-fragment</td><td>NA</td><td>0</td></tr>
<tr><td>S1-final-viral-combined_k141_193328||full</td><td>2848</td><td>No</td><td>NA</td><td>0</td><td>1</td><td>Not-determined</td><td>Genome-fragment</td><td>NA</td><td>0</td></tr>
<tr><td>S12-final-viral-combined_k141_233648||lt2gene</td><td>1375</td><td>No</td><td>NA</td><td>1</td><td>0</td><td>Low-quality</td><td>Genome-fragment</td><td>3.34</td><td>0</td></tr>
<tr><td>S2-final-viral-combined_k141_53778||full</td><td>1375</td><td>No</td><td>NA</td><td>0</td><td>0</td><td>Low-quality</td><td>Genome-fragment</td><td>3.03</td><td>0</td></tr>
<tr><td>S1-final-viral-combined_k141_143322||full</td><td>3643</td><td>No</td><td>NA</td><td>0</td><td>4</td><td>Not-determined</td><td>Genome-fragment</td><td>NA</td><td>0</td></tr>
<tr><td>S1-final-viral-combined_k141_373481||full</td><td>1268</td><td>No</td><td>NA</td><td>0</td><td>1</td><td>Not-determined</td><td>Genome-fragment</td><td>NA</td><td>0</td></tr>
<tr><td>S1-final-viral-combined_k141_357765||full</td><td>2439</td><td>No</td><td>NA</td><td>0</td><td>1</td><td>Not-determined</td><td>Genome-fragment</td><td>NA</td><td>0</td></tr>
<tr><td>S2_k141_101096</td><td>1375</td><td>No</td><td>NA</td><td>1</td><td>0</td><td>Low-quality</td><td>Genome-fragment</td><td>2.34</td><td>0</td></tr>
<tr><td>S2_k141_165353</td><td>1375</td><td>No</td><td>NA</td><td>0</td><td>0</td><td>Low-quality</td><td>Genome-fragment</td><td>1.43</td><td>0</td></tr>
<tr><td>S1-final-viral-combined_k141_159030||full</td><td>3408</td><td>No</td><td>NA</td><td>0</td><td>1</td><td>Not-determined</td><td>Genome-fragment</td><td>NA</td><td>0</td></tr>
<tr><td>S11-final-viral-combined_k141_265617||full</td><td>1376</td><td>No</td><td>NA</td><td>0</td><td>1</td><td>Low-quality</td><td>Genome-fragment</td><td>2.61</td><td>0</td></tr>
</table>

| | | | | | | | | | | | |
|---|---|---|---|---|---|---|---|---|---|---|---|
| S1-final-viral-combined_k141_291710\|\|full | 3103 | No | NA | | 0 | 1 | Not-determined | Genome-fragment | NA | | 0 |
| S1-final-viral-combined_k141_259475\|\|full | 1284 | No | NA | | 0 | 0 | Not-determined | Genome-fragment | NA | | 0 |
| S1-final-viral-combined_k141_175193\|\|full | 1291 | No | NA | | 0 | 1 | Not-determined | Genome-fragment | NA | | 0 |
| S11-final-viral-combined_k141_322564\|\|lt2gene | 1376 | No | NA | | 1 | 0 | Low-quality | Genome-fragment | | 0.95 | 0 |
| S1-final-viral-combined_k141_325353\|\|full | 1454 | No | NA | | 0 | 1 | Not-determined | Genome-fragment | NA | | 0 |
| S3_k141_17429 | 1376 | No | NA | | 0 | 2 | Low-quality | Genome-fragment | | 4.14 | 0 |
| S1-final-viral-combined_k141_193427\|\|full | 1260 | No | NA | | 0 | 0 | Not-determined | Genome-fragment | NA | | 0 |
| S1-final-viral-combined_k141_259508\|\|full | 1693 | No | NA | | 0 | 2 | Not-determined | Genome-fragment | NA | | 0 |
| S5-final-viral-combined_k141_4460\|\|full | 1376 | No | NA | | 0 | 0 | Low-quality | Genome-fragment | | 3.76 | 0 |
| S1-final-viral-combined_k141_94257\|\|full | 6577 | No | NA | | 0 | 5 | Not-determined | Genome-fragment | NA | | 0 |
| S7-final-viral-combined_k141_70393\|\|full | 1376 | Yes | | 1127 | 1 | 1 | Low-quality | Genome-fragment | | 2.26 | 18.1 |
| S1-final-viral-combined_k141_109429\|\|full | 2798 | No | NA | | 0 | 1 | Not-determined | Genome-fragment | NA | | 0 |
| S2-final-viral-combined_k141_134805\|\|full | 1376 | No | NA | | 0 | 0 | Low-quality | Genome-fragment | | 3.26 | 0 |
| S1-final-viral-combined_k141_143382\|\|full | 5793 | No | NA | | 0 | 1 | Not-determined | Genome-fragment | NA | | 0 |
| S1-final-viral-combined_k141_94270\|\|full | 1817 | No | NA | | 0 | 3 | Not-determined | Genome-fragment | NA | | 0 |
| S1-final-viral-combined_k141_308595\|\|full | 2257 | No | NA | | 0 | 3 | Not-determined | Genome-fragment | NA | | 0 |
| S1-final-viral-combined_k141_291803\|\|full | 1933 | No | NA | | 0 | 0 | Not-determined | Genome-fragment | NA | | 0 |
| S1-final-viral-combined_k141_143404\|\|full | 1635 | No | NA | | 0 | 1 | Not-determined | Genome-fragment | NA | | 0 |
| S2-final-viral-combined_k141_93690\|\|full | 1376 | No | NA | | 1 | 0 | Low-quality | Genome-fragment | | 3.03 | 0 |
| S2_k141_134971 | 1376 | No | NA | | 0 | 0 | Low-quality | Genome-fragment | | 2.24 | 0 |
| S1-final-viral-combined_k141_11616\|\|full | 8251 | No | NA | | 0 | 2 | Not-determined | Genome-fragment | NA | | 0 |
| S4-final-viral-combined_k141_70266\|\|full | 1376 | No | NA | | 0 | 0 | Low-quality | Genome-fragment | | 2.02 | 0 |
| S1-final-viral-combined_k141_259602\|\|full | 3074 | No | NA | | 0 | 0 | Not-determined | Genome-fragment | NA | | 0 |
| S8-final-viral-combined_k141_242716\|\|full | 1376 | No | NA | | 0 | 1 | Low-quality | Genome-fragment | | 0.44 | 0 |
| S11-final-viral-combined_k141_91314\|\|full | 1377 | No | NA | | 0 | 0 | Low-quality | Genome-fragment | | 3.22 | 0 |
| S11-final-viral-combined_k141_268295\|\|lt2gene | 1377 | No | NA | | 1 | 0 | Low-quality | Genome-fragment | | 3.83 | 0 |
| S1-final-viral-combined_k141_76546\|\|full | 17519 | No | NA | | 0 | 10 | Not-determined | Genome-fragment | NA | | 0 |
| S1-final-viral-combined_k141_76548\|\|full | 1438 | No | NA | | 0 | 0 | Not-determined | Genome-fragment | NA | | 0 |
| S1-final-viral-combined_k141_133439\|\|lt2gene | 1377 | No | NA | | 1 | 0 | Low-quality | Genome-fragment | | 3.05 | 0 |
| S1-final-viral-combined_k141_255271\|\|lt2gene | 1377 | No | NA | | 0 | 0 | Low-quality | Genome-fragment | | 3.9 | 0 |
| S5-final-viral-combined_k141_90268\|\|full | 1377 | No | NA | | 0 | 0 | Low-quality | Genome-fragment | | 3.13 | 0 |
| S1-final-viral-combined_k141_143459\|\|full | 35960 | No | NA | | 24 | 1 | Medium-quality | Genome-fragment | | 77.24 | 0 |
| S5-final-viral-combined_k141_161464\|\|full | 1377 | No | NA | | 1 | 0 | Low-quality | Genome-fragment | | 1.26 | 0 |
| S7_k141_193569 | 1377 | No | NA | | 0 | 1 | Low-quality | Genome-fragment | | 2.09 | 0 |
| S1-final-viral-combined_k141_193534\|\|full | 2395 | No | NA | | 0 | 2 | Not-determined | Genome-fragment | NA | | 0 |
| S1-final-viral-combined_k141_390031\|\|full | 1120 | No | NA | | 0 | 0 | Not-determined | Genome-fragment | NA | | 0 |
| S17-final-viral-combined_k141_148219\|\|full | 1377 | No | NA | | 0 | 0 | Low-quality | Genome-fragment | | 3.54 | 0 |
| S17-final-viral-combined_k141_194216\|\|full | 1377 | No | NA | | 0 | 1 | Low-quality | Genome-fragment | | 0.86 | 0 |
| S12-final-viral-combined_k141_396379\|\|full | 1377 | No | NA | | 0 | 0 | Low-quality | Genome-fragment | | 2.1 | 0 |
| S1-final-viral-combined_k141_175346\|\|full | 1046 | No | NA | | 0 | 0 | Not-determined | Genome-fragment | NA | | 0 |
| S1-final-viral-combined_k141_11704\|\|full | 1795 | No | NA | | 0 | 0 | Not-determined | Genome-fragment | NA | | 0 |
| S1-final-viral-combined_k141_325510\|\|full | 1748 | No | NA | | 0 | 2 | Not-determined | Genome-fragment | NA | | 0 |
| S1-final-viral-combined_k141_109586\|\|full | 5043 | No | NA | | 0 | 4 | Not-determined | Genome-fragment | NA | | 0 |
| S1-final-viral-combined_k141_259664\|\|full | 5143 | No | NA | | 0 | 0 | Not-determined | Genome-fragment | NA | | 0 |
| S1-final-viral-combined_k141_259667\|\|full | 2235 | No | NA | | 0 | 0 | Not-determined | Genome-fragment | NA | | 0 |
| S1-final-viral-combined_k141_274732\|\|full | 3235 | No | NA | | 0 | 1 | Not-determined | Genome-fragment | NA | | 0 |
| S2-final-viral-combined_k141_137449\|\|full | 1377 | No | NA | | 0 | 0 | Low-quality | Genome-fragment | | 1.24 | 0 |
| S1-final-viral-combined_k141_94446\|\|full | 1551 | No | NA | | 0 | 0 | Not-determined | Genome-fragment | NA | | 0 |
| S1-final-viral-combined_k141_339904\|\|full | 2733 | No | NA | | 0 | 2 | Not-determined | Genome-fragment | NA | | 0 |
| S2-final-viral-combined_k141_75075\|\|full | 1377 | No | NA | | 0 | 1 | Low-quality | Genome-fragment | | 3.35 | 0 |
| S11-final-viral-combined_k141_22425\|\|full | 1378 | No | NA | | 0 | 2 | Low-quality | Genome-fragment | | 0.97 | 0 |
| S1-final-viral-combined_k141_370906\|\|full | 1378 | No | NA | | 0 | 2 | Low-quality | Genome-fragment | | 0.8 | 0 |
| S5-final-viral-combined_k141_129310\|\|full | 1378 | No | NA | | 0 | 1 | Low-quality | Genome-fragment | | 0.71 | 0 |
| S1-final-viral-combined_k141_308752\|\|full | 1352 | No | NA | | 0 | 2 | Not-determined | Genome-fragment | NA | | 0 |
| S1-final-viral-combined_k141_11754\|\|full | 1912 | No | NA | | 0 | 1 | Not-determined | Genome-fragment | NA | | 0 |
| S1-final-viral-combined_k141_94487\|\|full | 1246 | No | NA | | 0 | 1 | Not-determined | Genome-fragment | NA | | 0 |
| S1-final-viral-combined_k141_339949\|\|full | 2358 | No | NA | | 0 | 1 | Not-determined | Genome-fragment | NA | | 0 |
| S7_k141_243494 | 1378 | No | NA | | 0 | 0 | Low-quality | Genome-fragment | | 0.52 | 0 |
| S1-final-viral-combined_k141_76632\|\|full | 1084 | No | NA | | 0 | 1 | Not-determined | Genome-fragment | NA | | 0 |
| S1-final-viral-combined_k141_11768\|\|full | 2216 | No | NA | | 0 | 1 | Not-determined | Genome-fragment | NA | | 0 |
| S1-final-viral-combined_k141_259730\|\|full | 1325 | No | NA | | 0 | 0 | Not-determined | Genome-fragment | NA | | 0 |
| S1-final-viral-combined_k141_325590\|\|full | 2718 | No | NA | | 0 | 0 | Not-determined | Genome-fragment | NA | | 0 |
| S12-final-viral-combined_k141_225055\|\|full | 1378 | No | NA | | 0 | 0 | Low-quality | Genome-fragment | | 1.97 | 0 |
| S1-final-viral-combined_k141_292019\|\|full | 1599 | No | NA | | 0 | 1 | Not-determined | Genome-fragment | NA | | 0 |
| S1-final-viral-combined_k141_339976\|\|full | 4809 | No | NA | | 0 | 4 | Not-determined | Genome-fragment | NA | | 0 |
| S1-final-viral-combined_k141_159242\|\|full | 1339 | No | NA | | 0 | 2 | Not-determined | Genome-fragment | NA | | 0 |
| S1-final-viral-combined_k141_225915\|\|full | 1333 | No | NA | | 0 | 1 | Not-determined | Genome-fragment | NA | | 0 |
| S1-final-viral-combined_k141_325624\|\|full | 2429 | No | NA | | 0 | 1 | Not-determined | Genome-fragment | NA | | 0 |
| S1-final-viral-combined_k141_242525\|\|full | 1657 | No | NA | | 0 | 2 | Not-determined | Genome-fragment | NA | | 0 |
| S1-final-viral-combined_k141_340006\|\|full | 1004 | No | NA | | 0 | 1 | Not-determined | Genome-fragment | NA | | 0 |
| S1-final-viral-combined_k141_109670\|\|full | 1883 | No | NA | | 0 | 0 | Not-determined | Genome-fragment | NA | | 0 |
| S1-final-viral-combined_k141_175395\|\|full | 4979 | No | NA | | 0 | 2 | Not-determined | Genome-fragment | NA | | 0 |
| S1-final-viral-combined_k141_44463\|\|full | 1910 | No | NA | | 0 | 3 | Not-determined | Genome-fragment | NA | | 0 |
| S2-final-viral-combined_k141_10340\|\|lt2gene | 1378 | No | NA | | 1 | 0 | Low-quality | Genome-fragment | | 2.77 | 0 |
| S1-final-viral-combined_k141_28078\|\|full | 1615 | No | NA | | 0 | 0 | Not-determined | Genome-fragment | NA | | 0 |
| S1-final-viral-combined_k141_193687\|\|full | 5738 | No | NA | | 0 | 5 | Not-determined | Genome-fragment | NA | | 0 |
| S6-final-viral-combined_k141_30098\|\|full | 1378 | No | NA | | 1 | 0 | Low-quality | Genome-fragment | | 2.03 | 0 |
| S8_k141_117153 | 1378 | No | NA | | 1 | 0 | Low-quality | Genome-fragment | | 1.89 | 0 |
| S1-final-viral-combined_k141_94540\|\|full | 3204 | No | NA | | 0 | 1 | Not-determined | Genome-fragment | NA | | 0 |
| S1-final-viral-combined_k141_390159\|\|full | 8356 | No | NA | | 0 | 7 | Not-determined | Genome-fragment | NA | | 0 |
| S8_k141_129494 | 1378 | No | NA | | 0 | 0 | Low-quality | Genome-fragment | | 0.73 | 0 |
| S1-final-viral-combined_k141_159314\|\|full | 8804 | No | NA | | 0 | 3 | Not-determined | Genome-fragment | NA | | 0 |
| S1-final-viral-combined_k141_175425\|\|full | 3175 | No | NA | | 0 | 3 | Not-determined | Genome-fragment | NA | | 0 |
| S9-final-viral-combined_k141_74571\|\|full | 1379 | No | NA | | 3 | 0 | Low-quality | Genome-fragment | | 4.33 | 0 |
| S1-final-viral-combined_k141_308848\|\|full | 21928 | No | NA | | 2 | 0 | Medium-quality | Genome-fragment | | 66.34 | 0 |
| S1-final-viral-combined_k141_175439\|\|full | 1526 | No | NA | | 0 | 0 | Not-determined | Genome-fragment | NA | | 0 |
| S11-final-viral-combined_k141_23772\|\|full | 1379 | No | NA | | 0 | 0 | Low-quality | Genome-fragment | | 2.18 | 0 |
| S1-final-viral-combined_k141_188216\|\|full | 1379 | No | NA | | 0 | 0 | Low-quality | Genome-fragment | | 3.27 | 0 |
| S1-final-viral-combined_k141_373837\|\|full | 2562 | No | NA | | 0 | 2 | Not-determined | Genome-fragment | NA | | 0 |
| S7-final-viral-combined_k141_172094\|\|full | 1379 | No | NA | | 0 | 0 | Low-quality | Genome-fragment | | 2.1 | 0 |
| S7_k141_155732 | 1379 | No | NA | | 0 | 0 | Low-quality | Genome-fragment | | 1.3 | 0 |
| S2_k141_98521 | 1379 | No | NA | | 0 | 1 | Low-quality | Genome-fragment | | 1.37 | 0 |
| S6-final-viral-combined_k141_77524\|\|full | 1379 | No | NA | | 0 | 0 | Low-quality | Genome-fragment | | 1.64 | 0 |
| S1-final-viral-combined_k141_308897\|\|full | 1427 | No | NA | | 0 | 2 | Not-determined | Genome-fragment | NA | | 0 |
| S1-final-viral-combined_k141_94600\|\|full | 4172 | No | NA | | 0 | 0 | Not-determined | Genome-fragment | NA | | 0 |
| S9-final-viral-combined_k141_54509\|\|full | 1380 | No | NA | | 0 | 0 | Low-quality | Genome-fragment | | 2.94 | 0 |
| S1-final-viral-combined_k141_292137\|\|full | 2464 | No | NA | | 0 | 1 | Not-determined | Genome-fragment | NA | | 0 |
| S1-final-viral-combined_k141_259876\|\|full | 2698 | No | NA | | 0 | 2 | Not-determined | Genome-fragment | NA | | 0 |
| S1-final-viral-combined_k141_143736\|\|full | 1458 | No | NA | | 0 | 0 | Not-determined | Genome-fragment | NA | | 0 |
| S3-final-viral-combined_k141_39223\|\|lt2gene | 1380 | No | NA | | 1 | 0 | Low-quality | Genome-fragment | | 1.29 | 0 |
| S1-final-viral-combined_k141_76819\|\|full | 3399 | No | NA | | 0 | 1 | Not-determined | Genome-fragment | NA | | 0 |
| S7_k141_134603 | 1380 | No | NA | | 0 | 3 | Low-quality | Genome-fragment | | 3.07 | 0 |
| S1-final-viral-combined_k141_340119\|\|full | 2185 | No | NA | | 0 | 0 | Not-determined | Genome-fragment | NA | | 0 |
| S1-final-viral-combined_k141_358290\|\|full | 7925 | No | NA | | 0 | 2 | Not-determined | Genome-fragment | NA | | 0 |
| S17-final-viral-combined_k141_124786\|\|full | 1380 | No | NA | | 1 | 0 | Low-quality | Genome-fragment | | 1.8 | 0 |
| S1-final-viral-combined_k141_325744\|\|full | 2600 | No | NA | | 0 | 0 | Not-determined | Genome-fragment | NA | | 0 |
| S1-final-viral-combined_k141_292172\|\|full | 3553 | No | NA | | 0 | 0 | Not-determined | Genome-fragment | NA | | 0 |
| S1-final-viral-combined_k141_109782\|\|full | 1092 | No | NA | | 0 | 1 | Not-determined | Genome-fragment | NA | | 0 |
| S17-final-viral-combined_k141_117919\|\|full | 1380 | No | NA | | 2 | 0 | Low-quality | Genome-fragment | | 1.47 | 0 |
| S1-final-viral-combined_k141_94640\|\|full | 1858 | No | NA | | 0 | 2 | Not-determined | Genome-fragment | NA | | 0 |
| S1-final-viral-combined_k141_325770\|\|full | 1456 | No | NA | | 0 | 0 | Not-determined | Genome-fragment | NA | | 0 |
| S1-final-viral-combined_k141_11974\|\|full | 2796 | No | NA | | 0 | 2 | Not-determined | Genome-fragment | NA | | 0 |
| S17-final-viral-combined_k141_132250\|\|full | 1380 | No | NA | | 0 | 0 | Low-quality | Genome-fragment | | 3.38 | 0 |
| S1-final-viral-combined_k141_226060\|\|full | 2384 | No | NA | | 0 | 0 | Not-determined | Genome-fragment | NA | | 0 |
| S1-final-viral-combined_k141_274988\|\|full | 3811 | No | NA | | 0 | 2 | Not-determined | Genome-fragment | NA | | 0 |
| S1-final-viral-combined_k141_76919\|\|full | 1837 | No | NA | | 0 | 0 | Not-determined | Genome-fragment | NA | | 0 |
| S17-k141_144452 | 1380 | No | NA | | 0 | 0 | Low-quality | Genome-fragment | | 3 | 0 |
| S1-final-viral-combined_k141_275003\|\|full | 2284 | No | NA | | 0 | 2 | Not-determined | Genome-fragment | NA | | 0 |
| S1-final-viral-combined_k141_292254\|\|full | 2197 | No | NA | | 0 | 2 | Not-determined | Genome-fragment | NA | | 0 |
| S1-final-viral-combined_k141_109895\|\|full | 2438 | No | NA | | 0 | 2 | Not-determined | Genome-fragment | NA | | 0 |
| S12-final-viral-combined_k141_143272\|\|full | 1380 | No | NA | | 0 | 1 | Low-quality | Genome-fragment | | 1.24 | 0 |
| S1-final-viral-combined_k141_128107\|\|full | 1594 | No | NA | | 0 | 0 | Not-determined | Genome-fragment | NA | | 0 |
| S1-final-viral-combined_k141_340220\|\|full | 1364 | No | NA | | 0 | 1 | Not-determined | Genome-fragment | NA | | 0 |
| S2-final-viral-combined_k141_74355\|\|full | 1380 | No | NA | | 0 | 0 | Low-quality | Genome-fragment | | 1.29 | 0 |
| S1-final-viral-combined_k141_325836\|\|full | 1102 | No | NA | | 0 | 1 | Not-determined | Genome-fragment | NA | | 0 |
| S1-final-viral-combined_k141_374057\|\|full | 4007 | No | NA | | 0 | 2 | Not-determined | Genome-fragment | NA | | 0 |
| S1-final-viral-combined_k141_259967\|\|full | 1129 | No | NA | | 0 | 2 | Not-determined | Genome-fragment | NA | | 0 |
| S1-final-viral-combined_k141_76963\|\|full | 6215 | No | NA | | 0 | 0 | Not-determined | Genome-fragment | NA | | 0 |
| S2-final-viral-combined_k141_55168\|\|full | 1380 | No | NA | | 0 | 2 | Low-quality | Genome-fragment | | 1.88 | 0 |
| S1-final-viral-combined_k141_358456\|\|full | 3028 | No | NA | | 0 | 2 | Not-determined | Genome-fragment | NA | | 0 |
| S1-final-viral-combined_k141_143889\|\|full | 2155 | No | NA | | 0 | 1 | Not-determined | Genome-fragment | NA | | 0 |
| S1-final-viral-combined_k141_175599\|\|full | 3562 | No | NA | | 0 | 3 | Not-determined | Genome-fragment | NA | | 0 |
| S8-final-viral-combined_k141_26546\|\|full | 1380 | No | NA | | 1 | 0 | Low-quality | Genome-fragment | | 3.41 | 0 |
| S1-final-viral-combined_k141_159466\|\|full | 1360 | No | NA | | 0 | 2 | Not-determined | Genome-fragment | NA | | 0 |
| S1-final-viral-combined_k141_44761\|\|full | 3695 | No | NA | | 0 | 0 | Not-determined | Genome-fragment | NA | | 0 |
| S1-final-viral-combined_k141_390370\|\|full | 1380 | No | NA | | 0 | 0 | Not-determined | Genome-fragment | NA | | 0 |
| S1-final-viral-combined_k141_159490\|\|full | 1027 | No | NA | | 0 | 1 | Not-determined | Genome-fragment | NA | | 0 |
| S8_k141_148396 | 1380 | No | NA | | 0 | 0 | Low-quality | Genome-fragment | | 2.18 | 0 |
| S1-final-viral-combined_k141_226179\|\|full | 5210 | No | NA | | 0 | 5 | Not-determined | Genome-fragment | NA | | 0 |
| S11_k141_125990 | 1381 | No | NA | | 0 | 0 | Low-quality | Genome-fragment | | 2.3 | 0 |
| S1-final-viral-combined_k141_340271\|\|full | 2316 | No | NA | | 0 | 1 | Not-determined | Genome-fragment | NA | | 0 |
| S1-final-viral-combined_k141_366264\|\|full | 1381 | No | NA | | 0 | 0 | Low-quality | Genome-fragment | | 3.71 | 0 |
| S1-final-viral-combined_k141_94699\|\|full | 1305 | No | NA | | 0 | 0 | Not-determined | Genome-fragment | NA | | 0 |
| S1-final-viral-combined_k141_374121\|\|full | 2485 | No | NA | | 0 | 2 | Not-determined | Genome-fragment | NA | | 0 |
| S1-final-viral-combined_k141_210573\|\|full | 2846 | No | NA | | 0 | 3 | Not-determined | Genome-fragment | NA | | 0 |
| S1-final-viral-combined_k141_242800\|\|full | 1477 | No | NA | | 0 | 1 | Not-determined | Genome-fragment | NA | | 0 |
| S1-final-viral-combined_k141_16926\|\|lt2gene | 1381 | No | NA | | 1 | 0 | Low-quality | Genome-fragment | | 1.81 | 0 |
| S1-final-viral-combined_k141_12222\|\|full | 1097 | No | NA | | 0 | 0 | Not-determined | Genome-fragment | NA | | 0 |
| S1_k141_149379 | 1381 | No | NA | | 0 | 1 | Low-quality | Genome-fragment | | 0.41 | 0 |
| S1-final-viral-combined_k141_226252\|\|full | 1960 | No | NA | | 0 | 1 | Not-determined | Genome-fragment | NA | | 0 |
| S1-final-viral-combined_k141_159520\|\|full | 1025 | No | NA | | 0 | 0 | Not-determined | Genome-fragment | NA | | 0 |
| S1-final-viral-combined_k141_390446\|\|full | 1343 | No | NA | | 0 | 1 | Not-determined | Genome-fragment | NA | | 0 |
| S1_k141_344850 | 1381 | No | NA | | 0 | 1 | Low-quality | Genome-fragment | | 0.46 | 0 |
| S1-final-viral-combined_k141_77053\|\|full | 5133 | No | NA | | 0 | 2 | Not-determined | Genome-fragment | NA | | 0 |
| S1-final-viral-combined_k141_159544\|\|full | 21136 | No | NA | | 15 | 0 | Medium-quality | Genome-fragment | | 58.23 | 0 |
| S1-final-viral-combined_k141_242858\|\|full | 2447 | No | NA | | 0 | 0 | Not-determined | Genome-fragment | NA | | 0 |
| S5-final-viral-combined_k141_11066\|\|full | 1381 | No | NA | | 0 | 1 | Low-quality | Genome-fragment | | 0.72 | 0 |
| S7-final-viral-combined_k141_164457\|\|full | 1381 | No | NA | | 0 | 0 | Low-quality | Genome-fragment | | 2.46 | 0 |
| S7-final-viral-combined_k141_196228\|\|full | 1381 | No | NA | | 0 | 0 | Low-quality | Genome-fragment | | 0.86 | 0 |
| S1-final-viral-combined_k141_94756\|\|full | 3255 | No | NA | | 0 | 1 | Not-determined | Genome-fragment | NA | | 0 |
| S1-final-viral-combined_k141_309197\|\|full | 1447 | No | NA | | 0 | 0 | Not-determined | Genome-fragment | NA | | 0 |
| S1-final-viral-combined_k141_61490\|\|full | 2132 | No | NA | | 0 | 3 | Not-determined | Genome-fragment | NA | | 0 |
| S1-final-viral-combined_k141_260188\|\|full | 1262 | No | NA | | 0 | 1 | Not-determined | Genome-fragment | NA | | 0 |
| S12-final-viral-combined_k141_115055\|\|full | 1381 | No | NA | | 1 | 0 | Low-quality | Genome-fragment | | 2.29 | 0 |
| S1-final-viral-combined_k141_226314\|\|full | 1444 | No | NA | | 0 | 2 | Not-determined | Genome-fragment | NA | | 0 |
| S1-final-viral-combined_k141_28458\|\|full | 1182 | No | NA | | 0 | 0 | Not-determined | Genome-fragment | NA | | 0 |
| S1-final-viral-combined_k141_77099\|\|full | 8957 | No | NA | | 0 | 6 | Not-determined | Genome-fragment | NA | | 0 |
| S1-final-viral-combined_k141_226324\|\|full | 2543 | No | NA | | 0 | 0 | Not-determined | Genome-fragment | NA | | 0 |
| S1-final-viral-combined_k141_77105\|\|full | 2083 | No | NA | | 0 | 2 | Not-determined | Genome-fragment | NA | | 0 |
| S1-final-viral-combined_k141_326008\|\|full | 1115 | No | NA | | 0 | 0 | Not-determined | Genome-fragment | NA | | 0 |
| S1-final-viral-combined_k141_374219\|\|full | 19640 | No | NA | | 12 | 0 | Medium-quality | Genome-fragment | | 50.09 | 0 |
| S1-final-viral-combined_k141_226358\|\|full | 6207 | No | NA | | 0 | 4 | Not-determined | Genome-fragment | NA | | 0 |
| S8-final-viral-combined_k141_156843\|\|full | 1381 | No | NA | | 1 | 0 | Low-quality | Genome-fragment | | 4.41 | 0 |
| S11-final-viral-combined_k141_267283\|\|full | 1382 | No | NA | | 0 | 1 | Low-quality | Genome-fragment | | 1.3 | 0 |

| | | | | | | | | | | | |
|---|---|---|---|---|---|---|---|---|---|---|---|
| S1-final-viral-combined_k141_292516||full | 4391 | No | NA | | 0 | 3 | Not-determined | Genome-fragment | NA | | 0 |
| S1-final-viral-combined_k141_358683||full | 3643 | No | NA | | 0 | 0 | Not-determined | Genome-fragment | NA | | 0 |
| S1-final-viral-combined_k141_144089||full | 1089 | No | NA | | 0 | 1 | Not-determined | Genome-fragment | NA | | 0 |
| S1-final-viral-combined_k141_242932||full | 1500 | No | NA | | 0 | 0 | Not-determined | Genome-fragment | NA | | 0 |
| S1-final-viral-combined_k141_144092||full | 4427 | No | NA | | 0 | 4 | Not-determined | Genome-fragment | NA | | 0 |
| S1-final-viral-combined_k141_390566||full | 1063 | No | NA | | 0 | 0 | Not-determined | Genome-fragment | NA | | 0 |
| S11-final-viral-combined_k141_288604||full | 1382 | No | NA | | 0 | 1 | Low-quality | Genome-fragment | | 0.99 | 0 |
| S1-final-viral-combined_k141_193884||full | 3087 | No | NA | | 0 | 1 | Not-determined | Genome-fragment | NA | | 0 |
| S1-final-viral-combined_k141_36657||full | 1382 | No | NA | | 2 | 0 | Low-quality | Genome-fragment | | 2.36 | 0 |
| S1-final-viral-combined_k141_340436||full | 1295 | No | NA | | 0 | 0 | Not-determined | Genome-fragment | NA | | 0 |
| S1-final-viral-combined_k141_77188||full | 11572 | No | NA | | 0 | 0 | Not-determined | Genome-fragment | NA | | 0 |
| S1-final-viral-combined_k141_90233||full | 1382 | No | NA | | 0 | 0 | Low-quality | Genome-fragment | | 2.47 | 0 |
| S3_k141_53944 | 1382 | No | NA | | 1 | 0 | Low-quality | Genome-fragment | | 0.8 | 0 |
| S3_k141_96758 | 1382 | No | NA | | 0 | 1 | Low-quality | Genome-fragment | | 0.87 | 0 |
| S3_k141_120095 | 1382 | No | NA | | 0 | 0 | Low-quality | Genome-fragment | | 0.64 | 0 |
| S1-final-viral-combined_k141_61626||full | 7167 | No | NA | | 0 | 7 | Not-determined | Genome-fragment | NA | | 0 |
| S7-final-viral-combined_k141_179588||full | 1382 | No | NA | | 1 | 0 | Low-quality | Genome-fragment | | 1.73 | 0 |
| S1-final-viral-combined_k141_12453||full | 2574 | No | NA | | 0 | 1 | Not-determined | Genome-fragment | NA | | 0 |
| S1-final-viral-combined_k141_128366||full | 1936 | No | NA | | 0 | 0 | Not-determined | Genome-fragment | NA | | 0 |
| S1-final-viral-combined_k141_159670||full | 6533 | No | NA | | 0 | 3 | Not-determined | Genome-fragment | NA | | 0 |
| S7-final-viral-combined_k141_9328||full | 1382 | No | NA | | 0 | 2 | Low-quality | Genome-fragment | | 0.44 | 0 |
| S17-final-viral-combined_k141_29847||full | 1382 | No | NA | | 0 | 2 | Low-quality | Genome-fragment | | 0.49 | 0 |
| S1-final-viral-combined_k141_260367||full | 1150 | No | NA | | 0 | 0 | Not-determined | Genome-fragment | NA | | 0 |
| S1-final-viral-combined_k141_358777||full | 1773 | No | NA | | 0 | 1 | Not-determined | Genome-fragment | NA | | 0 |
| S2-final-viral-combined_k141_128912||lt2gene | 1382 | No | NA | | 0 | 1 | Low-quality | Genome-fragment | | 1.68 | 0 |
| S8-final-viral-combined_k141_201279||full | 1382 | No | NA | | 0 | 0 | Low-quality | Genome-fragment | | 2.69 | 0 |
| S1-final-viral-combined_k141_61657||full | 4586 | No | NA | | 0 | 2 | Not-determined | Genome-fragment | NA | | 0 |
| S1-final-viral-combined_k141_390677||full | 2078 | No | NA | | 0 | 1 | Not-determined | Genome-fragment | NA | | 0 |
| S1-final-viral-combined_k141_144171||full | 3511 | No | NA | | 0 | 4 | Not-determined | Genome-fragment | NA | | 0 |
| S1-final-viral-combined_k141_94851||full | 1587 | No | NA | | 0 | 0 | Not-determined | Genome-fragment | NA | | 0 |
| S11-final-viral-combined_k141_201164||lt2gene | 1383 | No | NA | | 2 | 0 | Low-quality | Genome-fragment | | 4.31 | 0 |
| S1-final-viral-combined_k141_326103||full | 9513 | No | NA | | 0 | 7 | Not-determined | Genome-fragment | NA | | 0 |
| S1-final-viral-combined_k141_358813||full | 1412 | No | NA | | 0 | 0 | Not-determined | Genome-fragment | NA | | 0 |
| S7-final-viral-combined_k141_56638||full | 1383 | No | NA | | 0 | 0 | Low-quality | Genome-fragment | | 2.16 | 0 |
| S1-final-viral-combined_k141_159711||full | 1615 | No | NA | | 0 | 1 | Not-determined | Genome-fragment | NA | | 0 |
| S1-final-viral-combined_k141_45039||full | 1776 | No | NA | | 0 | 1 | Not-determined | Genome-fragment | NA | | 0 |
| S17-final-viral-combined_k141_180519||full | 1383 | No | NA | | 0 | 0 | Low-quality | Genome-fragment | | 3.27 | 0 |
| S1-final-viral-combined_k141_226517||full | 1232 | No | NA | | 0 | 1 | Not-determined | Genome-fragment | NA | | 0 |
| S1-final-viral-combined_k141_110004||full | 2056 | No | NA | | 0 | 2 | Not-determined | Genome-fragment | NA | | 0 |
| S17-final-viral-combined_k141_39093||full | 1383 | No | NA | | 2 | 0 | Low-quality | Genome-fragment | | 3.18 | 0 |
| S17-k141_30728 | 1383 | No | NA | | 1 | 0 | Low-quality | Genome-fragment | | 3.89 | 0 |
| S1-final-viral-combined_k141_374392||full | 1200 | No | NA | | 0 | 1 | Not-determined | Genome-fragment | NA | | 0 |
| S1-final-viral-combined_k141_159733||full | 1456 | No | NA | | 0 | 1 | Not-determined | Genome-fragment | NA | | 0 |
| S2-final-viral-combined_k141_134900||full | 1383 | No | NA | | 0 | 1 | Low-quality | Genome-fragment | | 1.04 | 0 |
| S2-final-viral-combined_k141_231194||full | 1383 | No | NA | | 0 | 0 | Low-quality | Genome-fragment | | 3.54 | 0 |
| S1-final-viral-combined_k141_374410||full | 1754 | No | NA | | 0 | 2 | Not-determined | Genome-fragment | NA | | 0 |
| S1-final-viral-combined_k141_110023||full | 3147 | No | NA | | 0 | 1 | Not-determined | Genome-fragment | NA | | 0 |
| S2_k141_88626 | 1383 | No | NA | | 0 | 1 | Low-quality | Genome-fragment | | 0.82 | 0 |
| S8_k141_201051 | 1383 | No | NA | | 1 | 0 | Low-quality | Genome-fragment | | 0.88 | 0 |
| S1-final-viral-combined_k141_159746||full | 1733 | No | NA | | 0 | 1 | Not-determined | Genome-fragment | NA | | 0 |
| S1-final-viral-combined_k141_45089||full | 2294 | No | NA | | 0 | 0 | Not-determined | Genome-fragment | NA | | 0 |
| S11-final-viral-combined_k141_165966||full | 1384 | No | NA | | 0 | 2 | Low-quality | Genome-fragment | | 0.83 | 0 |
| S1-final-viral-combined_k141_374442||full | 1794 | No | NA | | 0 | 2 | Not-determined | Genome-fragment | NA | | 0 |
| S1-final-viral-combined_k141_110042||full | 3863 | No | NA | | 0 | 2 | Not-determined | Genome-fragment | NA | | 0 |
| S7-final-viral-combined_k141_198085||full | 1384 | No | NA | | 0 | 1 | Low-quality | Genome-fragment | | 1.64 | 0 |
| S1-final-viral-combined_k141_94880||full | 4909 | No | NA | | 0 | 0 | Not-determined | Genome-fragment | NA | | 0 |
| S17-k141_76197 | 1384 | No | NA | | 1 | 0 | Low-quality | Genome-fragment | | 1.26 | 0 |
| S1-final-viral-combined_k141_340546||full | 14534 | No | NA | | 0 | 11 | Not-determined | Genome-fragment | NA | | 0 |
| S1-final-viral-combined_k141_275374||full | 7885 | No | NA | | 0 | 7 | Not-determined | Genome-fragment | NA | | 0 |
| S1-final-viral-combined_k141_326225||full | 2199 | No | NA | | 0 | 2 | Not-determined | Genome-fragment | NA | | 0 |
| S1-final-viral-combined_k141_243144||full | 8426 | No | NA | | 0 | 3 | Not-determined | Genome-fragment | NA | | 0 |
| S1-final-viral-combined_k141_77320||full | 4371 | No | NA | | 0 | 1 | Not-determined | Genome-fragment | NA | | 0 |
| S1-final-viral-combined_k141_326250||full | 1072 | No | NA | | 0 | 1 | Not-determined | Genome-fragment | NA | | 0 |
| S1-final-viral-combined_k141_340558||full | 27649 | No | NA | | 8 | 0 | Medium-quality | Genome-fragment | | 52.94 | 0 |
| S1-final-viral-combined_k141_194124||full | 14062 | No | NA | | 0 | 3 | Not-determined | Genome-fragment | NA | | 0 |
| S1-final-viral-combined_k141_77327||full | 2946 | No | NA | | 0 | 1 | Not-determined | Genome-fragment | NA | | 0 |
| S1-final-viral-combined_k141_28676||full | 4247 | No | NA | | 0 | 3 | Not-determined | Genome-fragment | NA | | 0 |
| S1-final-viral-combined_k141_358934||full | 1964 | No | NA | | 0 | 0 | Not-determined | Genome-fragment | NA | | 0 |
| S1-final-viral-combined_k141_275444||full | 1161 | No | NA | | 0 | 1 | Not-determined | Genome-fragment | NA | | 0 |
| S1-final-viral-combined_k141_77329||full | 7173 | No | NA | | 0 | 0 | Not-determined | Genome-fragment | NA | | 0 |
| S1-final-viral-combined_k141_292811||full | 3284 | No | NA | | 0 | 3 | Not-determined | Genome-fragment | NA | | 0 |
| S8-final-viral-combined_k141_76422||full | 1384 | No | NA | | 0 | 0 | Low-quality | Genome-fragment | | 3.28 | 0 |
| S1-final-viral-combined_k141_340600||full | 4058 | No | NA | | 0 | 5 | Not-determined | Genome-fragment | NA | | 0 |
| S9_k141_76135 | 1385 | No | NA | | 0 | 2 | Low-quality | Genome-fragment | | 2.17 | 0 |
| S1-final-viral-combined_k141_332161||full | 1385 | No | NA | | 1 | 0 | Low-quality | Genome-fragment | | 1.02 | 0 |
| S1-final-viral-combined_k141_222761||full | 1385 | No | NA | | 0 | 0 | Low-quality | Genome-fragment | | 2.65 | 0 |
| S1-final-viral-combined_k141_77380||full | 3789 | No | NA | | 0 | 2 | Not-determined | Genome-fragment | NA | | 0 |
| S5_k141_192284 | 1385 | No | NA | | 0 | 1 | Low-quality | Genome-fragment | | 0.59 | 0 |
| S1-final-viral-combined_k141_340616||full | 1638 | No | NA | | 0 | 0 | Not-determined | Genome-fragment | NA | | 0 |
| S1-final-viral-combined_k141_77389||full | 1645 | No | NA | | 0 | 0 | Not-determined | Genome-fragment | NA | | 0 |
| S17-final-viral-combined_k141_135615||full | 1385 | No | NA | | 1 | 0 | Low-quality | Genome-fragment | | 3.2 | 0 |
| S12_k141_385376 | 1385 | No | NA | | 1 | 0 | Low-quality | Genome-fragment | | 2.75 | 0 |
| S1-final-viral-combined_k141_12688||full | 2585 | No | NA | | 0 | 3 | Not-determined | Genome-fragment | NA | | 0 |
| S1-final-viral-combined_k141_340631||full | 2990 | No | NA | | 0 | 1 | Not-determined | Genome-fragment | NA | | 0 |
| S12_k141_213592 | 1385 | No | NA | | 0 | 0 | Low-quality | Genome-fragment | | 2.35 | 0 |
| S1-final-viral-combined_k141_194239||full | 3406 | No | NA | | 0 | 0 | Not-determined | Genome-fragment | NA | | 0 |
| S2-final-viral-combined_k141_211250||full | 1385 | No | NA | | 1 | 0 | Low-quality | Genome-fragment | | 2.9 | 0 |
| S2-final-viral-combined_k141_246421||full | 1385 | No | NA | | 0 | 2 | Low-quality | Genome-fragment | | 0.44 | 0 |
| S2_k141_114514 | 1385 | No | NA | | 0 | 0 | Low-quality | Genome-fragment | | 3.28 | 0 |
| S4-final-viral-combined_k141_42788||full | 1385 | No | NA | | 0 | 0 | Low-quality | Genome-fragment | | 2.71 | 0 |
| S4-final-viral-combined_k141_94099||full | 1385 | No | NA | | 1 | 0 | Low-quality | Genome-fragment | | 2.61 | 0 |
| S1-final-viral-combined_k141_374616||full | 1030 | No | NA | | 0 | 1 | Not-determined | Genome-fragment | NA | | 0 |
| S1-final-viral-combined_k141_340654||full | 3865 | No | NA | | 0 | 3 | Not-determined | Genome-fragment | NA | | 0 |
| S1-final-viral-combined_k141_374624||full | 1262 | No | NA | | 0 | 0 | Not-determined | Genome-fragment | NA | | 0 |
| S4-final-viral-combined_k141_161890||lt2gene | 1385 | No | NA | | 1 | 0 | Low-quality | Genome-fragment | | 2.02 | 0 |
| S8-final-viral-combined_k141_257513||full | 1385 | No | NA | | 0 | 0 | Low-quality | Genome-fragment | | 0.56 | 0 |
| S8-final-viral-combined_k141_11209||full | 1385 | No | NA | | 1 | 0 | Low-quality | Genome-fragment | | 3.61 | 0 |
| S1-final-viral-combined_k141_61974||full | 4390 | No | NA | | 0 | 3 | Not-determined | Genome-fragment | NA | | 0 |
| S1-final-viral-combined_k141_77544||full | 1008 | No | NA | | 0 | 0 | Not-determined | Genome-fragment | NA | | 0 |
| S1-final-viral-combined_k141_28797||full | 2571 | No | NA | | 0 | 0 | Not-determined | Genome-fragment | NA | | 0 |
| S1-final-viral-combined_k141_236027||full | 1386 | No | NA | | 1 | 0 | Low-quality | Genome-fragment | | 3.69 | 0 |
| S1-final-viral-combined_k141_77560||full | 2921 | No | NA | | 0 | 2 | Not-determined | Genome-fragment | NA | | 0 |
| S1-final-viral-combined_k141_292934||full | 2316 | No | NA | | 0 | 2 | Not-determined | Genome-fragment | NA | | 0 |
| S1-final-viral-combined_k141_243343||full | 2084 | No | NA | | 0 | 0 | Not-determined | Genome-fragment | NA | | 0 |
| S1-final-viral-combined_k141_28829||full | 2137 | No | NA | | 0 | 0 | Not-determined | Genome-fragment | NA | | 0 |
| S1-final-viral-combined_k141_41148||full | 1386 | No | NA | | 0 | 1 | Low-quality | Genome-fragment | | 4.14 | 0 |
| S1-final-viral-combined_k141_77591||full | 1496 | No | NA | | 0 | 1 | Not-determined | Genome-fragment | NA | | 0 |
| S1_k141_189145 | 1386 | No | NA | | 0 | 0 | Low-quality | Genome-fragment | | 1.31 | 0 |
| S1-final-viral-combined_k141_77598||full | 1349 | No | NA | | 0 | 1 | Not-determined | Genome-fragment | NA | | 0 |
| S5_k141_96058 | 1386 | No | NA | | 0 | 3 | Low-quality | Genome-fragment | | 1.4 | 0 |
| S7-final-viral-combined_k141_95151||full | 1386 | No | NA | | 0 | 0 | Low-quality | Genome-fragment | | 2.9 | 0 |
| S1-final-viral-combined_k141_292971||full | 4485 | No | NA | | 0 | 4 | Not-determined | Genome-fragment | NA | | 0 |
| S12-final-viral-combined_k141_32216||full | 1386 | No | NA | | 0 | 0 | Low-quality | Genome-fragment | | 1.08 | 0 |
| S12_k141_214225 | 1386 | No | NA | | 0 | 2 | Low-quality | Genome-fragment | | 0.71 | 0 |
| S2-final-viral-combined_k141_157549||full | 1386 | No | NA | | 2 | 0 | Low-quality | Genome-fragment | | 0.66 | 0 |
| S8-final-viral-combined_k141_96491||full | 1386 | Yes | | 339 | 1 | 2 | Low-quality | Genome-fragment | | 0.68 | 75.54 |
| S8_k141_68222 | 1386 | No | NA | | 1 | 0 | Low-quality | Genome-fragment | | 1.58 | 0 |
| S9-final-viral-combined_k141_127871||full | 1387 | No | NA | | 2 | 0 | Low-quality | Genome-fragment | | 2.15 | 0 |
| S1-final-viral-combined_k141_391029||full | 1199 | No | NA | | 0 | 2 | Not-determined | Genome-fragment | NA | | 0 |
| S1-final-viral-combined_k141_293030||full | 1006 | No | NA | | 0 | 1 | Not-determined | Genome-fragment | NA | | 0 |
| S11-final-viral-combined_k141_321873||full | 1387 | No | NA | | 0 | 1 | Low-quality | Genome-fragment | | 0.15 | 0 |
| S5-final-viral-combined_k141_126023||lt2gene | 1387 | No | NA | | 2 | 0 | Low-quality | Genome-fragment | | 3.16 | 0 |
| S1-final-viral-combined_k141_293050||full | 2844 | No | NA | | 0 | 3 | Not-determined | Genome-fragment | NA | | 0 |
| S1-final-viral-combined_k141_95099||full | 2114 | No | NA | | 0 | 2 | Not-determined | Genome-fragment | NA | | 0 |
| S1-final-viral-combined_k141_194461||full | 5898 | No | NA | | 0 | 2 | Not-determined | Genome-fragment | NA | | 0 |
| S1-final-viral-combined_k141_243443||full | 1814 | No | NA | | 0 | 0 | Not-determined | Genome-fragment | NA | | 0 |
| S1-final-viral-combined_k141_159970||full | 1530 | No | NA | | 0 | 2 | Not-determined | Genome-fragment | NA | | 0 |
| S1-final-viral-combined_k141_128847||full | 3204 | No | NA | | 0 | 4 | Not-determined | Genome-fragment | NA | | 0 |
| S17-final-viral-combined_k141_199451||full | 1387 | No | NA | | 1 | 0 | Low-quality | Genome-fragment | | 3.06 | 0 |
| S1-final-viral-combined_k141_62134||full | 1775 | No | NA | | 0 | 1 | Not-determined | Genome-fragment | NA | | 0 |
| S1-final-viral-combined_k141_95140||full | 2151 | No | NA | | 0 | 1 | Not-determined | Genome-fragment | NA | | 0 |
| S1-final-viral-combined_k141_211062||full | 1340 | No | NA | | 0 | 2 | Not-determined | Genome-fragment | NA | | 0 |
| S1-final-viral-combined_k141_326505||full | 1334 | No | NA | | 0 | 1 | Not-determined | Genome-fragment | NA | | 0 |
| S12-final-viral-combined_k141_319292||full | 1387 | No | NA | | 0 | 0 | Low-quality | Genome-fragment | | 2.31 | 0 |
| S1-final-viral-combined_k141_128895||full | 1241 | No | NA | | 0 | 1 | Not-determined | Genome-fragment | NA | | 0 |
| S1-final-viral-combined_k141_374730||full | 2357 | No | NA | | 0 | 1 | Not-determined | Genome-fragment | NA | | 0 |
| S1-final-viral-combined_k141_340882||full | 5939 | No | NA | | 0 | 2 | Not-determined | Genome-fragment | NA | | 0 |
| S1-final-viral-combined_k141_194503||full | 2758 | No | NA | | 0 | 2 | Not-determined | Genome-fragment | NA | | 0 |
| S1-final-viral-combined_k141_211079||full | 1567 | No | NA | | 0 | 1 | Not-determined | Genome-fragment | NA | | 0 |
| S1-final-viral-combined_k141_144429||full | 3019 | No | NA | | 0 | 3 | Not-determined | Genome-fragment | NA | | 0 |
| S12-final-viral-combined_k141_227239||full | 1387 | No | NA | | 0 | 1 | Low-quality | Genome-fragment | | 2.14 | 0 |
| S1-final-viral-combined_k141_144432||full | 1108 | No | NA | | 0 | 1 | Not-determined | Genome-fragment | NA | | 0 |
| S1-final-viral-combined_k141_293113||full | 2934 | No | NA | | 0 | 3 | Not-determined | Genome-fragment | NA | | 0 |
| S12-final-viral-combined_k141_50002||full | 1387 | No | NA | | 2 | 0 | Low-quality | Genome-fragment | | 2.92 | 0 |
| S12_k141_47899 | 1387 | No | NA | | 1 | 0 | Low-quality | Genome-fragment | | 2.69 | 0 |
| S2-final-viral-combined_k141_239246||full | 1387 | No | NA | | 0 | 1 | Low-quality | Genome-fragment | | 3.29 | 0 |
| S4-final-viral-combined_k141_54466||full | 1387 | No | NA | | 0 | 0 | Low-quality | Genome-fragment | | 3.71 | 0 |
| S8-final-viral-combined_k141_247928||full | 1387 | No | NA | | 0 | 0 | Low-quality | Genome-fragment | | 2.61 | 0 |
| S1-final-viral-combined_k141_391075||full | 2676 | No | NA | | 0 | 1 | Not-determined | Genome-fragment | NA | | 0 |
| S1-final-viral-combined_k141_226939||full | 2300 | No | NA | | 0 | 1 | Not-determined | Genome-fragment | NA | | 0 |
| S1-final-viral-combined_k141_194589||full | 6538 | No | NA | | 0 | 3 | Not-determined | Genome-fragment | NA | | 0 |
| S9_k141_36117 | 1388 | No | NA | | 0 | 0 | Low-quality | Genome-fragment | | 2.36 | 0 |
| S1-final-viral-combined_k141_95226||full | 1003 | No | NA | | 0 | 1 | Not-determined | Genome-fragment | NA | | 0 |
| S1-final-viral-combined_k141_194599||full | 2503 | No | NA | | 0 | 1 | Not-determined | Genome-fragment | NA | | 0 |
| S1-final-viral-combined_k141_275787||full | 3339 | No | NA | | 0 | 4 | Not-determined | Genome-fragment | NA | | 0 |
| S1-final-viral-combined_k141_77779||full | 8290 | No | NA | | 0 | 1 | Not-determined | Genome-fragment | NA | | 0 |
| S1-final-viral-combined_k141_211188||full | 5708 | No | NA | | 0 | 6 | Not-determined | Genome-fragment | NA | | 0 |
| S11-final-viral-combined_k141_65472||full | 1388 | No | NA | | 0 | 0 | Low-quality | Genome-fragment | | 0.55 | 0 |
| S1-final-viral-combined_k141_243589||full | 2888 | No | NA | | 0 | 2 | Not-determined | Genome-fragment | NA | | 0 |
| S1-final-viral-combined_k141_144502||full | 6069 | No | NA | | 0 | 2 | Not-determined | Genome-fragment | NA | | 0 |
| S1-final-viral-combined_k141_144535||full | 3158 | No | NA | | 0 | 3 | Not-determined | Genome-fragment | NA | | 0 |
| S1-final-viral-combined_k141_160108||full | 3566 | No | NA | | 0 | 2 | Not-determined | Genome-fragment | NA | | 0 |
| S1-final-viral-combined_k141_110220||full | 7891 | No | NA | | 0 | 4 | Not-determined | Genome-fragment | NA | | 0 |
| S1-final-viral-combined_k141_227003||full | 25087 | No | NA | | 9 | 1 | Medium-quality | Genome-fragment | | 56.45 | 0 |
| S1-final-viral-combined_k141_341003||full | 2453 | No | NA | | 0 | 2 | Not-determined | Genome-fragment | NA | | 0 |
| S1-final-viral-combined_k141_374867||full | 1196 | No | NA | | 0 | 1 | Not-determined | Genome-fragment | NA | | 0 |
| S1-final-viral-combined_k141_243633||full | 1652 | No | NA | | 0 | 1 | Not-determined | Genome-fragment | NA | | 0 |
| S11_k141_255229 | 1388 | No | NA | | 0 | 3 | Low-quality | Genome-fragment | | 1.75 | 0 |
| S1-final-viral-combined_k141_260913||full | 2089 | No | NA | | 0 | 2 | Not-determined | Genome-fragment | NA | | 0 |
| S1-final-viral-combined_k141_260914||full | 3318 | No | NA | | 0 | 1 | Not-determined | Genome-fragment | NA | | 0 |
| S1-final-viral-combined_k141_236835||full | 1388 | No | NA | | 0 | 0 | Low-quality | Genome-fragment | | 1.64 | 0 |
| S17-final-viral-combined_k141_11109||full | 1388 | No | NA | | 0 | 0 | Low-quality | Genome-fragment | | 1.87 | 0 |
| S1-final-viral-combined_k141_243666||full | 2772 | No | NA | | 0 | 2 | Not-determined | Genome-fragment | NA | | 0 |
| S1-final-viral-combined_k141_160176||full | 6577 | No | NA | | 0 | 4 | Not-determined | Genome-fragment | NA | | 0 |

| | | | | | | | | | |
|---|---|---|---|---|---|---|---|---|---|
| S1-final-viral-combined_k141_211260\|\|full | 1853 | No | NA | 0 | 2 | Not-determined | Genome-fragment | NA | 0 |
| S1-final-viral-combined_k141_211270\|\|full | 1163 | No | NA | 0 | 2 | Not-determined | Genome-fragment | NA | 0 |
| S1-final-viral-combined_k141_12952\|\|full | 5661 | No | NA | 0 | 4 | Not-determined | Genome-fragment | NA | 0 |
| S1-final-viral-combined_k141_326687\|\|full | 4194 | No | NA | 0 | 0 | Not-determined | Genome-fragment | NA | 0 |
| S12-final-viral-combined_k141_241872\|\|full | 1388 | No | NA | 0 | 0 | Low-quality | Genome-fragment | 1.05 | 0 |
| S1-final-viral-combined_k141_12958\|\|full | 1640 | No | NA | 0 | 2 | Not-determined | Genome-fragment | NA | 0 |
| S12-final-viral-combined_k141_165702\|\|full | 1388 | No | NA | 0 | 1 | Low-quality | Genome-fragment | 1.33 | 0 |
| S1-final-viral-combined_k141_45410\|\|full | 3754 | No | NA | 0 | 5 | Not-determined | Genome-fragment | NA | 0 |
| S12-final-viral-combined_k141_219422\|\|lt2gene | 1388 | No | NA | 1 | 0 | Low-quality | Genome-fragment | 4.51 | 0 |
| S6-final-viral-combined_k141_12825\|\|full | 1388 | No | NA | 0 | 0 | Low-quality | Genome-fragment | 4.01 | 0 |
| S1-final-viral-combined_k141_176160\|\|full | 5334 | No | NA | 0 | 3 | Not-determined | Genome-fragment | NA | 0 |
| S6_k141_18691 | 1388 | No | NA | 0 | 0 | Low-quality | Genome-fragment | 0.74 | 0 |
| S1-final-viral-combined_k141_309869\|\|full | 3454 | No | NA | 0 | 1 | Not-determined | Genome-fragment | NA | 0 |
| S1-final-viral-combined_k141_359429\|\|full | 2840 | No | NA | 0 | 2 | Not-determined | Genome-fragment | NA | 0 |
| S3-final-viral-combined_k141_11545\|\|full | 1389 | No | NA | 0 | 0 | Low-quality | Genome-fragment | 2.93 | 0 |
| S17-final-viral-combined_k141_6027\|\|full | 1389 | No | NA | 0 | 2 | Low-quality | Genome-fragment | 1.13 | 0 |
| S12-final-viral-combined_k141_103812\|\|full | 1389 | No | NA | 1 | 0 | Low-quality | Genome-fragment | 3.02 | 0 |
| S2-final-viral-combined_k141_145329\|\|full | 1389 | No | NA | 0 | 0 | Low-quality | Genome-fragment | 2.82 | 0 |
| S1-final-viral-combined_k141_77881\|\|full | 1455 | No | NA | 0 | 0 | Not-determined | Genome-fragment | NA | 0 |
| S1-final-viral-combined_k141_293333\|\|full | 1512 | No | NA | 0 | 1 | Not-determined | Genome-fragment | NA | 0 |
| S4-final-viral-combined_k141_154077\|\|full | 1389 | No | NA | 0 | 1 | Low-quality | Genome-fragment | 6.61 | 0 |
| S4_k141_102034 | 1389 | No | NA | 1 | 0 | Low-quality | Genome-fragment | 3.48 | 0 |
| S6_k141_139679 | 1389 | No | NA | 2 | 0 | Low-quality | Genome-fragment | 2.16 | 0 |
| S1-final-viral-combined_k141_45489\|\|full | 5207 | No | NA | 0 | 2 | Not-determined | Genome-fragment | NA | 0 |
| S1-final-viral-combined_k141_326800\|\|full | 2165 | No | NA | 0 | 2 | Not-determined | Genome-fragment | NA | 0 |
| S1-final-viral-combined_k141_144713\|\|full | 1031 | No | NA | 0 | 1 | Not-determined | Genome-fragment | NA | 0 |
| S1-final-viral-combined_k141_211406\|\|full | 6409 | No | NA | 0 | 1 | Not-determined | Genome-fragment | NA | 0 |
| S8_k141_142840 | 1389 | No | NA | 0 | 0 | Low-quality | Genome-fragment | 0.98 | 0 |
| S1-final-viral-combined_k141_309943\|\|full | 11578 | No | NA | 0 | 8 | Not-determined | Genome-fragment | NA | 0 |
| S8_k141_271865 | 1389 | No | NA | 0 | 0 | Low-quality | Genome-fragment | 1.68 | 0 |
| S11-final-viral-combined_k141_111656\|\|full | 1390 | No | NA | 0 | 0 | Low-quality | Genome-fragment | 3.2 | 0 |
| S1-final-viral-combined_k141_360449\|\|full | 1390 | No | NA | 0 | 0 | Low-quality | Genome-fragment | 1.91 | 0 |
| S3-final-viral-combined_k141_19147\|\|full | 1390 | No | NA | 0 | 2 | Low-quality | Genome-fragment | 0.5 | 0 |
| S17-final-viral-combined_k141_254900\|\|full | 1390 | No | NA | 0 | 2 | Low-quality | Genome-fragment | 0.87 | 0 |
| S12-final-viral-combined_k141_313859\|\|full | 1390 | No | NA | 0 | 0 | Low-quality | Genome-fragment | 4.22 | 0 |
| S2-final-viral-combined_k141_8038\|\|full | 1390 | No | NA | 0 | 0 | Low-quality | Genome-fragment | 3.89 | 0 |
| S9-final-viral-combined_k141_12352\|\|full | 1391 | No | NA | 1 | 0 | Low-quality | Genome-fragment | 0.96 | 0 |
| S1-final-viral-combined_k141_227203\|\|full | 4267 | No | NA | 0 | 1 | Not-determined | Genome-fragment | NA | 0 |
| S1-final-viral-combined_k141_275961\|\|full | 2403 | No | NA | 0 | 2 | Not-determined | Genome-fragment | NA | 0 |
| S1-final-viral-combined_k141_227206\|\|full | 1566 | No | NA | 0 | 0 | Not-determined | Genome-fragment | NA | 0 |
| S11-final-viral-combined_k141_29300\|\|full | 1391 | No | NA | 1 | 0 | Low-quality | Genome-fragment | 3.05 | 0 |
| S1-final-viral-combined_k141_275978\|\|full | 1039 | No | NA | 0 | 1 | Not-determined | Genome-fragment | NA | 0 |
| S1-final-viral-combined_k141_194848\|\|full | 3888 | No | NA | 0 | 3 | Not-determined | Genome-fragment | NA | 0 |
| S1-final-viral-combined_k141_173515\|\|full | 1391 | No | NA | 2 | 0 | Low-quality | Genome-fragment | 3.33 | 0 |
| S3-final-viral-combined_k141_17845\|\|full | 1391 | No | NA | 0 | 0 | Low-quality | Genome-fragment | 9.49 | 0 |
| S1-final-viral-combined_k141_95486\|\|full | 2101 | No | NA | 0 | 2 | Not-determined | Genome-fragment | NA | 0 |
| S1-final-viral-combined_k141_176320\|\|full | 3528 | No | NA | 0 | 2 | Not-determined | Genome-fragment | NA | 0 |
| S1-final-viral-combined_k141_243919\|\|full | 7021 | No | NA | 0 | 0 | Not-determined | Genome-fragment | NA | 0 |
| S1-final-viral-combined_k141_144772\|\|full | 3714 | No | NA | 0 | 1 | Not-determined | Genome-fragment | NA | 0 |
| S1-final-viral-combined_k141_129288\|\|full | 1697 | No | NA | 0 | 1 | Not-determined | Genome-fragment | NA | 0 |
| S1-final-viral-combined_k141_77993\|\|full | 1768 | No | NA | 0 | 1 | Not-determined | Genome-fragment | NA | 0 |
| S1-final-viral-combined_k141_194879\|\|full | 1682 | No | NA | 0 | 0 | Not-determined | Genome-fragment | NA | 0 |
| S1-final-viral-combined_k141_194880\|\|full | 1197 | No | NA | 0 | 2 | Not-determined | Genome-fragment | NA | 0 |
| S1-final-viral-combined_k141_77995\|\|full | 1663 | No | NA | 0 | 1 | Not-determined | Genome-fragment | NA | 0 |
| S1-final-viral-combined_k141_176340\|\|full | 1658 | No | NA | 0 | 2 | Not-determined | Genome-fragment | NA | 0 |
| S5-final-viral-combined_k141_178336\|\|full | 1391 | No | NA | 0 | 0 | Low-quality | Genome-fragment | 0.69 | 0 |
| S5-final-viral-combined_k141_18560\|\|full | 1391 | No | NA | 0 | 0 | Low-quality | Genome-fragment | 0.84 | 0 |
| S1-final-viral-combined_k141_211506\|\|full | 7339 | No | NA | 0 | 1 | Not-determined | Genome-fragment | NA | 0 |
| S1-final-viral-combined_k141_310044\|\|full | 3514 | No | NA | 0 | 1 | Not-determined | Genome-fragment | NA | 0 |
| S1-final-viral-combined_k141_227265\|\|full | 2707 | No | NA | 0 | 2 | Not-determined | Genome-fragment | NA | 0 |
| S2-final-viral-combined_k141_233342\|\|full | 1391 | No | NA | 1 | 0 | Low-quality | Genome-fragment | 20.82 | 0 |
| S1-final-viral-combined_k141_129338\|\|full | 4388 | No | NA | 0 | 0 | Not-determined | Genome-fragment | NA | 0 |
| S1-final-viral-combined_k141_341227\|\|full | 1670 | No | NA | 0 | 2 | Not-determined | Genome-fragment | NA | 0 |
| S1-final-viral-combined_k141_45594\|\|full | 2018 | No | NA | 0 | 0 | Not-determined | Genome-fragment | NA | 0 |
| S6-final-viral-combined_k141_132374\|\|full | 1391 | No | NA | 0 | 0 | Low-quality | Genome-fragment | 2.78 | 0 |
| S8-final-viral-combined_k141_236227\|\|full | 1391 | No | NA | 1 | 0 | Low-quality | Genome-fragment | 3.18 | 0 |
| S8-final-viral-combined_k141_86842\|\|full | 1391 | No | NA | 0 | 1 | Low-quality | Genome-fragment | 2.39 | 0 |
| S1-final-viral-combined_k141_211536\|\|full | 1260 | No | NA | 0 | 1 | Not-determined | Genome-fragment | NA | 0 |
| S9-final-viral-combined_k141_46556\|\|full | 1392 | No | NA | 0 | 0 | Low-quality | Genome-fragment | 3.35 | 0 |
| S11-final-viral-combined_k141_187869\|\|full | 1392 | No | NA | 0 | 0 | Low-quality | Genome-fragment | 3.49 | 0 |
| S1-final-viral-combined_k141_129384\|\|full | 1395 | No | NA | 0 | 1 | Not-determined | Genome-fragment | NA | 0 |
| S1-final-viral-combined_k141_364145\|\|full | 1392 | No | NA | 1 | 0 | Low-quality | Genome-fragment | 1.91 | 0 |
| S1-final-viral-combined_k141_326953\|\|full | 4893 | No | NA | 0 | 3 | Not-determined | Genome-fragment | NA | 0 |
| S1-final-viral-combined_k141_13200\|\|full | 2387 | No | NA | 0 | 2 | Not-determined | Genome-fragment | NA | 0 |
| S1-final-viral-combined_k141_261287\|\|full | 6326 | No | NA | 0 | 3 | Not-determined | Genome-fragment | NA | 0 |
| S1-final-viral-combined_k141_13210\|\|full | 2614 | No | NA | 0 | 1 | Not-determined | Genome-fragment | NA | 0 |
| S1-final-viral-combined_k141_326978\|\|full | 1762 | No | NA | 0 | 1 | Not-determined | Genome-fragment | NA | 0 |
| S1-final-viral-combined_k141_375225\|\|full | 2161 | No | NA | 0 | 3 | Not-determined | Genome-fragment | NA | 0 |
| S1-final-viral-combined_k141_95553\|\|full | 2461 | No | NA | 0 | 0 | Not-determined | Genome-fragment | NA | 0 |
| S1-final-viral-combined_k141_45647\|\|full | 2024 | No | NA | 0 | 1 | Not-determined | Genome-fragment | NA | 0 |
| S1-final-viral-combined_k141_375241\|\|full | 2027 | No | NA | 0 | 2 | Not-determined | Genome-fragment | NA | 0 |
| S1-final-viral-combined_k141_78024\|\|full | 1440 | No | NA | 0 | 0 | Not-determined | Genome-fragment | NA | 0 |
| S3-final-viral-combined_k141_112493\|\|full | 1392 | No | NA | 0 | 0 | Low-quality | Genome-fragment | 2.86 | 0 |
| S1-final-viral-combined_k141_13242\|\|full | 3388 | No | NA | 0 | 1 | Not-determined | Genome-fragment | NA | 0 |
| S1-final-viral-combined_k141_310152\|\|full | 1567 | No | NA | 0 | 1 | Not-determined | Genome-fragment | NA | 0 |
| S1-final-viral-combined_k141_95582\|\|full | 1138 | No | NA | 0 | 0 | Not-determined | Genome-fragment | NA | 0 |
| S1-final-viral-combined_k141_144933\|\|full | 2901 | No | NA | 0 | 4 | Not-determined | Genome-fragment | NA | 0 |
| S7-final-viral-combined_k141_211524\|\|full | 1392 | No | NA | 0 | 0 | Low-quality | Genome-fragment | 2.73 | 0 |
| S1-final-viral-combined_k141_195010\|\|full | 1349 | No | NA | 0 | 1 | Not-determined | Genome-fragment | NA | 0 |
| S1-final-viral-combined_k141_176469\|\|full | 7374 | No | NA | 0 | 5 | Not-determined | Genome-fragment | NA | 0 |
| S1-final-viral-combined_k141_211726\|\|full | 1197 | No | NA | 0 | 2 | Not-determined | Genome-fragment | NA | 0 |
| S1-final-viral-combined_k141_29201\|\|full | 3024 | No | NA | 0 | 0 | Not-determined | Genome-fragment | NA | 0 |
| S7-final-viral-combined_k141_113437\|\|lt2gene | 1392 | No | NA | 1 | 0 | Low-quality | Genome-fragment | 0.96 | 0 |
| S1-final-viral-combined_k141_211738\|\|full | 1098 | No | NA | 0 | 1 | Not-determined | Genome-fragment | NA | 0 |
| S17-final-viral-combined_k141_57682\|\|full | 1392 | No | NA | 1 | 0 | Low-quality | Genome-fragment | 2.52 | 0 |
| S17-final-viral-combined_k141_120268\|\|full | 1392 | No | NA | 0 | 0 | Low-quality | Genome-fragment | 2.65 | 0 |
| S17-final-viral-combined_k141_152774\|\|full | 1392 | No | NA | 0 | 1 | Low-quality | Genome-fragment | 0.94 | 0 |
| S12_k141_35626 | 1392 | No | NA | 0 | 1 | Low-quality | Genome-fragment | 0.68 | 0 |
| S1-final-viral-combined_k141_276250\|\|full | 1538 | No | NA | 0 | 0 | Not-determined | Genome-fragment | NA | 0 |
| S1-final-viral-combined_k141_160638\|\|full | 4579 | No | NA | 0 | 1 | Not-determined | Genome-fragment | NA | 0 |
| S1-final-viral-combined_k141_145002\|\|full | 3910 | No | NA | 0 | 0 | Not-determined | Genome-fragment | NA | 0 |
| S2_k141_25223 | 1392 | No | NA | 1 | 0 | Low-quality | Genome-fragment | 3.05 | 0 |
| S1-final-viral-combined_k141_359658\|\|full | 1125 | No | NA | 0 | 1 | Not-determined | Genome-fragment | NA | 0 |
| S4-final-viral-combined_k141_124979\|\|full | 1392 | No | NA | 1 | 0 | Low-quality | Genome-fragment | 2.75 | 0 |
| S4-final-viral-combined_k141_62557\|\|full | 1392 | No | NA | 0 | 0 | Low-quality | Genome-fragment | 3.75 | 0 |
| S1-final-viral-combined_k141_375331\|\|full | 2857 | No | NA | 0 | 0 | Not-determined | Genome-fragment | NA | 0 |
| S9-final-viral-combined_k141_10887\|\|full | 1393 | No | NA | 1 | 0 | Low-quality | Genome-fragment | 1.72 | 0 |
| S9-final-viral-combined_k141_108965\|\|full | 1393 | No | NA | 0 | 3 | Low-quality | Genome-fragment | 0.35 | 0 |
| S11-final-viral-combined_k141_110701\|\|full | 1393 | No | NA | 0 | 0 | Low-quality | Genome-fragment | 3.65 | 0 |
| S1-final-viral-combined_k141_244188\|\|full | 2690 | No | NA | 0 | 2 | Not-determined | Genome-fragment | NA | 0 |
| S1-final-viral-combined_k141_341429\|\|full | 1603 | No | NA | 0 | 1 | Not-determined | Genome-fragment | NA | 0 |
| S1-final-viral-combined_k141_391471\|\|full | 1393 | No | NA | 0 | 1 | Low-quality | Genome-fragment | 2.42 | 0 |
| S1-final-viral-combined_k141_238969\|\|lt2gene | 1393 | No | NA | 1 | 0 | Low-quality | Genome-fragment | 3.31 | 0 |
| S5-final-viral-combined_k141_64358\|\|full | 1393 | No | NA | 3 | 0 | Low-quality | Genome-fragment | 2.26 | 0 |
| S1-final-viral-combined_k141_176552\|\|full | 1403 | No | NA | 0 | 0 | Not-determined | Genome-fragment | NA | 0 |
| S1-final-viral-combined_k141_160702\|\|full | 1434 | No | NA | 0 | 2 | Not-determined | Genome-fragment | NA | 0 |
| S1-final-viral-combined_k141_227480\|\|full | 1630 | No | NA | 0 | 2 | Not-determined | Genome-fragment | NA | 0 |
| S7-final-viral-combined_k141_190937\|\|full | 1393 | No | NA | 0 | 0 | Low-quality | Genome-fragment | 3.06 | 0 |
| S7-final-viral-combined_k141_24719\|\|lt2gene | 1393 | No | NA | 1 | 0 | Low-quality | Genome-fragment | 3.62 | 0 |
| S1-final-viral-combined_k141_244241\|\|full | 6631 | No | NA | 0 | 5 | Not-determined | Genome-fragment | NA | 0 |
| S12-final-viral-combined_k141_26460\|\|full | 1393 | No | NA | 1 | 0 | Low-quality | Genome-fragment | 1.94 | 0 |
| S1-final-viral-combined_k141_45768\|\|full | 1400 | No | NA | 0 | 1 | Not-determined | Genome-fragment | NA | 0 |
| S1-final-viral-combined_k141_261473\|\|full | 3267 | No | NA | 0 | 0 | Not-determined | Genome-fragment | NA | 0 |
| S1-final-viral-combined_k141_78110\|\|full | 2783 | No | NA | 0 | 1 | Not-determined | Genome-fragment | NA | 0 |
| S1-final-viral-combined_k141_276375\|\|full | 4618 | No | NA | 0 | 3 | Not-determined | Genome-fragment | NA | 0 |
| S2-final-viral-combined_k141_228507\|\|full | 1393 | No | NA | 0 | 1 | Low-quality | Genome-fragment | 2.33 | 0 |
| S1-final-viral-combined_k141_195174\|\|full | 2046 | No | NA | 0 | 1 | Not-determined | Genome-fragment | NA | 0 |
| S1-final-viral-combined_k141_341508\|\|full | 5068 | No | NA | 0 | 0 | Not-determined | Genome-fragment | NA | 0 |
| S2-final-viral-combined_k141_22016\|\|full | 1393 | No | NA | 0 | 0 | Low-quality | Genome-fragment | 1.79 | 0 |
| S1-final-viral-combined_k141_211897\|\|full | 1033 | No | NA | 0 | 1 | Not-determined | Genome-fragment | NA | 0 |
| S2-final-viral-combined_k141_57801\|\|lt2gene | 1393 | No | NA | 1 | 0 | Low-quality | Genome-fragment | 23.47 | 0 |
| S8-final-viral-combined_k141_71481\|\|full | 1393 | No | NA | 1 | 0 | Low-quality | Genome-fragment | 3.26 | 0 |
| S11-final-viral-combined_k141_251369\|\|full | 1394 | No | NA | 0 | 0 | Low-quality | Genome-fragment | 2.41 | 0 |
| S1-final-viral-combined_k141_129690\|\|full | 9792 | No | NA | 0 | 6 | Not-determined | Genome-fragment | NA | 0 |
| S11-final-viral-combined_k141_68864\|\|full | 1394 | No | NA | 0 | 2 | Low-quality | Genome-fragment | 0.51 | 0 |
| S1-final-viral-combined_k141_145154\|\|full | 3138 | No | NA | 0 | 2 | Not-determined | Genome-fragment | NA | 0 |
| S1-final-viral-combined_k141_110752\|\|full | 2287 | No | NA | 0 | 1 | Not-determined | Genome-fragment | NA | 0 |
| S1-final-viral-combined_k141_276419\|\|full | 7039 | No | NA | 0 | 3 | Not-determined | Genome-fragment | NA | 0 |
| S1-final-viral-combined_k141_145157\|\|full | 1885 | No | NA | 0 | 1 | Not-determined | Genome-fragment | NA | 0 |
| S11-final-viral-combined_k141_73465\|\|lt2gene | 1394 | No | NA | 1 | 0 | Low-quality | Genome-fragment | 0.53 | 0 |
| S1-final-viral-combined_k141_327085\|\|full | 2197 | No | NA | 0 | 0 | Not-determined | Genome-fragment | NA | 0 |
| S1-final-viral-combined_k141_176656\|\|full | 1495 | No | NA | 0 | 2 | Not-determined | Genome-fragment | NA | 0 |
| S1-final-viral-combined_k141_375389\|\|full | 1066 | No | NA | 0 | 0 | Not-determined | Genome-fragment | NA | 0 |
| S1-final-viral-combined_k141_176664\|\|full | 1637 | No | NA | 0 | 0 | Not-determined | Genome-fragment | NA | 0 |
| S1-final-viral-combined_k141_293800\|\|full | 1516 | No | NA | 0 | 1 | Not-determined | Genome-fragment | NA | 0 |
| S1-final-viral-combined_k141_45826\|\|full | 1894 | No | NA | 0 | 1 | Not-determined | Genome-fragment | NA | 0 |
| S1-final-viral-combined_k141_375397\|\|full | 3445 | No | NA | 0 | 3 | Not-determined | Genome-fragment | NA | 0 |
| S1-final-viral-combined_k141_280149\|\|full | 1394 | No | NA | 1 | 0 | Low-quality | Genome-fragment | 3.31 | 0 |
| S1-final-viral-combined_k141_17061\|\|full | 1394 | No | NA | 0 | 1 | Low-quality | Genome-fragment | 3.09 | 0 |
| S1-final-viral-combined_k141_62680\|\|full | 8004 | No | NA | 0 | 1 | Not-determined | Genome-fragment | NA | 0 |
| S1-final-viral-combined_k141_62703\|\|full | 2980 | No | NA | 0 | 2 | Not-determined | Genome-fragment | NA | 0 |
| S1-final-viral-combined_k141_244380\|\|full | 2220 | No | NA | 0 | 0 | Not-determined | Genome-fragment | NA | 0 |
| S1-final-viral-combined_k141_195247\|\|full | 1993 | No | NA | 0 | 3 | Not-determined | Genome-fragment | NA | 0 |
| S1_k141_18432 | 1394 | No | NA | 1 | 0 | Low-quality | Genome-fragment | 3.09 | 0 |
| S1-final-viral-combined_k141_293843\|\|full | 1283 | No | NA | 0 | 1 | Not-determined | Genome-fragment | NA | 0 |
| S1_k141_108487 | 1394 | No | NA | 1 | 0 | Low-quality | Genome-fragment | 2.27 | 0 |
| S5-final-viral-combined_k141_58671\|\|full | 1394 | No | NA | 0 | 0 | Low-quality | Genome-fragment | 3.09 | 0 |
| S1-final-viral-combined_k141_293849\|\|full | 2971 | No | NA | 0 | 3 | Not-determined | Genome-fragment | NA | 0 |
| S1-final-viral-combined_k141_95908\|\|full | 1328 | No | NA | 0 | 1 | Not-determined | Genome-fragment | NA | 0 |
| S1-final-viral-combined_k141_391684\|\|full | 1456 | No | NA | 0 | 0 | Not-determined | Genome-fragment | NA | 0 |
| S1-final-viral-combined_k141_293865\|\|full | 1028 | No | NA | 0 | 1 | Not-determined | Genome-fragment | NA | 0 |
| S17-final-viral-combined_k141_174083\|\|full | 1394 | No | NA | 0 | 2 | Low-quality | Genome-fragment | 2.62 | 0 |
| S8-final-viral-combined_k141_13623\|\|full | 1394 | Yes | 1139 | 1 | 1 | Low-quality | Genome-fragment | 2.37 | 18.29 |
| S1-final-viral-combined_k141_62759\|\|full | 1023 | No | NA | 0 | 1 | Not-determined | Genome-fragment | NA | 0 |
| S1-final-viral-combined_k141_244453\|\|full | 1230 | No | NA | 0 | 0 | Not-determined | Genome-fragment | NA | 0 |
| S1-final-viral-combined_k141_129804\|\|full | 3564 | No | NA | 0 | 3 | Not-determined | Genome-fragment | NA | 0 |
| S1-final-viral-combined_k141_176753\|\|full | 2490 | No | NA | 0 | 1 | Not-determined | Genome-fragment | NA | 0 |
| S8-final-viral-combined_k141_268108\|\|lt2gene | 1394 | No | NA | 1 | 0 | Low-quality | Genome-fragment | 3.47 | 0 |
| S5-final-viral-combined_k141_32112\|\|full | 1395 | No | NA | 0 | 0 | Low-quality | Genome-fragment | 0.82 | 0 |
| S1-final-viral-combined_k141_95965\|\|full | 1171 | No | NA | 0 | 0 | Not-determined | Genome-fragment | NA | 0 |
| S1-final-viral-combined_k141_227663\|\|full | 1474 | No | NA | 0 | 0 | Not-determined | Genome-fragment | NA | 0 |
| S1-final-viral-combined_k141_29364\|\|full | 1510 | No | NA | 0 | 1 | Not-determined | Genome-fragment | NA | 0 |
| S1-final-viral-combined_k141_13617\|\|full | 1620 | No | NA | 0 | 1 | Not-determined | Genome-fragment | NA | 0 |
| S1-final-viral-combined_k141_244499\|\|full | 2123 | No | NA | 0 | 1 | Not-determined | Genome-fragment | NA | 0 |

| | | | | | | | | | | | |
|---|---|---|---|---|---|---|---|---|---|---|---|
| S1-final-viral-combined_k141_78357\|\|full | 2025 | No | NA | | 0 | 1 | Not-determined | Genome-fragment | NA | | 0 |
| S1-final-viral-combined_k141_110909\|\|full | 1558 | No | NA | | 0 | 0 | Not-determined | Genome-fragment | NA | | 0 |
| S1-final-viral-combined_k141_78362\|\|full | 3283 | No | NA | | 0 | 0 | Not-determined | Genome-fragment | NA | | 0 |
| S1-final-viral-combined_k141_96007\|\|full | 1398 | No | NA | | 0 | 0 | Not-determined | Genome-fragment | NA | | 0 |
| S1-final-viral-combined_k141_244507\|\|full | 5859 | No | NA | | 0 | 4 | Not-determined | Genome-fragment | NA | | 0 |
| S17-final-viral-combined_k141_73315\|\|full | 1395 | No | NA | | 0 | 0 | Low-quality | Genome-fragment | | 2.21 | 0 |
| S1-final-viral-combined_k141_310323\|\|full | 1010 | No | NA | | 0 | 1 | Not-determined | Genome-fragment | NA | | 0 |
| S1-final-viral-combined_k141_13643\|\|full | 1999 | No | NA | | 0 | 2 | Not-determined | Genome-fragment | NA | | 0 |
| S1-final-viral-combined_k141_212110\|\|full | 3464 | No | NA | | 0 | 3 | Not-determined | Genome-fragment | NA | | 0 |
| S1-final-viral-combined_k141_160964\|\|full | 16754 | No | NA | | 0 | 9 | Not-determined | Genome-fragment | NA | | 0 |
| S12-final-viral-combined_k141_148867\|\|full | 1395 | Yes | | 996 | 1 | 1 | Low-quality | Genome-fragment | | 1.78 | 28.6 |
| S1-final-viral-combined_k141_359901\|\|full | 2255 | No | NA | | 0 | 2 | Not-determined | Genome-fragment | NA | | 0 |
| S1-final-viral-combined_k141_78379\|\|full | 3396 | No | NA | | 0 | 0 | Not-determined | Genome-fragment | NA | | 0 |
| S1-final-viral-combined_k141_78382\|\|full | 1435 | No | NA | | 0 | 2 | Not-determined | Genome-fragment | NA | | 0 |
| S1-final-viral-combined_k141_129871\|\|full | 1876 | No | NA | | 0 | 0 | Not-determined | Genome-fragment | NA | | 0 |
| S2-final-viral-combined_k141_113474\|\|full | 1395 | No | NA | | 0 | 1 | Low-quality | Genome-fragment | | 0.72 | 0 |
| S1-final-viral-combined_k141_46048\|\|full | 1880 | No | NA | | 0 | 2 | Not-determined | Genome-fragment | NA | | 0 |
| S4-final-viral-combined_k141_30438\|\|lt2gene | 1395 | No | NA | | 2 | 0 | Low-quality | Genome-fragment | | 2.09 | 0 |
| S1-final-viral-combined_k141_46059\|\|full | 2879 | No | NA | | 0 | 2 | Not-determined | Genome-fragment | NA | | 0 |
| S1-final-viral-combined_k141_227745\|\|full | 3662 | No | NA | | 0 | 2 | Not-determined | Genome-fragment | NA | | 0 |
| S8-final-viral-combined_k141_207258\|\|full | 1395 | No | NA | | 0 | 2 | Low-quality | Genome-fragment | | 0.75 | 0 |
| S1-final-viral-combined_k141_261620\|\|full | 2151 | No | NA | | 0 | 2 | Not-determined | Genome-fragment | NA | | 0 |
| S11-final-viral-combined_k141_124189\|\|full | 1396 | No | NA | | 0 | 1 | Low-quality | Genome-fragment | | 0.79 | 0 |
| S1-final-viral-combined_k141_195464\|\|full | 7560 | No | NA | | 0 | 6 | Not-determined | Genome-fragment | NA | | 0 |
| S1-final-viral-combined_k141_110985\|\|full | 20869 | No | NA | | 0 | 15 | Not-determined | Genome-fragment | NA | | 0 |
| S1-final-viral-combined_k141_227795\|\|full | 5097 | No | NA | | 0 | 2 | Not-determined | Genome-fragment | NA | | 0 |
| S11-final-viral-combined_k141_96640\|\|full | 1396 | No | NA | | 0 | 0 | Low-quality | Genome-fragment | | 3.29 | 0 |
| S11-final-viral-combined_k141_58764\|\|full | 1396 | No | NA | | 1 | 0 | Low-quality | Genome-fragment | | 2.97 | 0 |
| S1-final-viral-combined_k141_244629\|\|full | 1202 | No | NA | | 0 | 0 | Not-determined | Genome-fragment | NA | | 0 |
| S11_k141_227042 | 1396 | No | NA | | 0 | 0 | Low-quality | Genome-fragment | | 2.41 | 0 |
| S1-final-viral-combined_k141_244654\|\|full | 2958 | No | NA | | 0 | 3 | Not-determined | Genome-fragment | NA | | 0 |
| S1-final-viral-combined_k141_293998\|\|full | 1456 | No | NA | | 0 | 0 | Not-determined | Genome-fragment | NA | | 0 |
| S3_k141_36125 | 1396 | No | NA | | 0 | 0 | Low-quality | Genome-fragment | | 2.33 | 0 |
| S1-final-viral-combined_k141_261681\|\|full | 1244 | No | NA | | 0 | 2 | Not-determined | Genome-fragment | NA | | 0 |
| S1-final-viral-combined_k141_62943\|\|full | 1380 | No | NA | | 0 | 0 | Not-determined | Genome-fragment | NA | | 0 |
| S1-final-viral-combined_k141_111063\|\|full | 3028 | No | NA | | 0 | 0 | Not-determined | Genome-fragment | NA | | 0 |
| S5-final-viral-combined_k141_194871\|\|full | 1396 | No | NA | | 0 | 2 | Low-quality | Genome-fragment | | 0.85 | 0 |
| S5_k141_11531 | 1396 | No | NA | | 1 | 0 | Low-quality | Genome-fragment | | 0.9 | 0 |
| S17-final-viral-combined_k141_226139\|\|full | 1396 | No | NA | | 0 | 1 | Low-quality | Genome-fragment | | 0.44 | 0 |
| S1-final-viral-combined_k141_62976\|\|full | 1676 | No | NA | | 0 | 1 | Not-determined | Genome-fragment | NA | | 0 |
| S1-final-viral-combined_k141_195557\|\|full | 1598 | No | NA | | 0 | 1 | Not-determined | Genome-fragment | NA | | 0 |
| S1-final-viral-combined_k141_391816\|\|full | 2178 | No | NA | | 0 | 1 | Not-determined | Genome-fragment | NA | | 0 |
| S1-final-viral-combined_k141_391822\|\|full | 1460 | No | NA | | 0 | 2 | Not-determined | Genome-fragment | NA | | 0 |
| S1-final-viral-combined_k141_195570\|\|full | 3113 | No | NA | | 0 | 2 | Not-determined | Genome-fragment | NA | | 0 |
| S1-final-viral-combined_k141_63027\|\|full | 1695 | No | NA | | 0 | 2 | Not-determined | Genome-fragment | NA | | 0 |
| S1-final-viral-combined_k141_130071\|\|full | 6617 | No | NA | | 0 | 3 | Not-determined | Genome-fragment | NA | | 0 |
| S1-final-viral-combined_k141_244754\|\|full | 2296 | No | NA | | 0 | 0 | Not-determined | Genome-fragment | NA | | 0 |
| S17-final-viral-combined_k141_216990\|\|full | 1396 | No | NA | | 1 | 0 | Low-quality | Genome-fragment | | 3.49 | 0 |
| S1-final-viral-combined_k141_195608\|\|full | 2408 | No | NA | | 0 | 3 | Not-determined | Genome-fragment | NA | | 0 |
| S1-final-viral-combined_k141_375745\|\|full | 2017 | No | NA | | 0 | 2 | Not-determined | Genome-fragment | NA | | 0 |
| S12-final-viral-combined_k141_385080\|\|full | 1396 | No | NA | | 0 | 2 | Low-quality | Genome-fragment | | 1.86 | 0 |
| S12_k141_391482 | 1396 | No | NA | | 0 | 0 | Low-quality | Genome-fragment | | 3.57 | 0 |
| S1-final-viral-combined_k141_375782\|\|full | 1375 | No | NA | | 0 | 1 | Not-determined | Genome-fragment | NA | | 0 |
| S1-final-viral-combined_k141_111169\|\|full | 2231 | No | NA | | 0 | 0 | Not-determined | Genome-fragment | NA | | 0 |
| S1-final-viral-combined_k141_130175\|\|full | 1428 | No | NA | | 0 | 1 | Not-determined | Genome-fragment | NA | | 0 |
| S1-final-viral-combined_k141_341944\|\|full | 2525 | No | NA | | 0 | 2 | Not-determined | Genome-fragment | NA | | 0 |
| S2-final-viral-combined_k141_52343\|\|full | 1396 | No | NA | | 1 | 0 | Low-quality | Genome-fragment | | 3.32 | 0 |
| S1-final-viral-combined_k141_111214\|\|full | 1093 | No | NA | | 0 | 0 | Not-determined | Genome-fragment | NA | | 0 |
| S2-final-viral-combined_k141_146419\|\|full | 1396 | No | NA | | 0 | 0 | Low-quality | Genome-fragment | | 3.04 | 0 |
| S1-final-viral-combined_k141_391910\|\|full | 7107 | No | NA | | 0 | 5 | Not-determined | Genome-fragment | NA | | 0 |
| S1-final-viral-combined_k141_63151\|\|full | 2518 | No | NA | | 0 | 0 | Not-determined | Genome-fragment | NA | | 0 |
| S2_k141_63752 | 1396 | No | NA | | 0 | 0 | Low-quality | Genome-fragment | | 2.33 | 0 |
| S1-final-viral-combined_k141_391913\|\|full | 1051 | No | NA | | 0 | 2 | Not-determined | Genome-fragment | NA | | 0 |
| S4-final-viral-combined_k141_114998\|\|full | 1396 | No | NA | | 0 | 0 | Low-quality | Genome-fragment | | 2.69 | 0 |
| S1-final-viral-combined_k141_212378\|\|full | 1772 | No | NA | | 0 | 1 | Not-determined | Genome-fragment | NA | | 0 |
| S1-final-viral-combined_k141_276816\|\|full | 1225 | No | NA | | 0 | 0 | Not-determined | Genome-fragment | NA | | 0 |
| S1-final-viral-combined_k141_276817\|\|full | 1535 | No | NA | | 0 | 0 | Not-determined | Genome-fragment | NA | | 0 |
| S1-final-viral-combined_k141_96143\|\|full | 1130 | No | NA | | 0 | 0 | Not-determined | Genome-fragment | NA | | 0 |
| S1-final-viral-combined_k141_227917\|\|full | 18499 | No | NA | | 7 | 1 | Medium-quality | Genome-fragment | | 50.48 | 0 |
| S1-final-viral-combined_k141_227929\|\|full | 2381 | No | NA | | 0 | 1 | Not-determined | Genome-fragment | NA | | 0 |
| S1-final-viral-combined_k141_244857\|\|full | 6672 | No | NA | | 0 | 5 | Not-determined | Genome-fragment | NA | | 0 |
| S4-final-viral-combined_k141_160390\|\|lt2gene | 1396 | No | NA | | 1 | 0 | Low-quality | Genome-fragment | | 2.25 | 0 |
| S8-final-viral-combined_k141_184391\|\|full | 1396 | No | NA | | 1 | 0 | Low-quality | Genome-fragment | | 3.4 | 0 |
| S1-final-viral-combined_k141_360189\|\|full | 2983 | No | NA | | 0 | 3 | Not-determined | Genome-fragment | NA | | 0 |
| S1-final-viral-combined_k141_244871\|\|full | 1869 | No | NA | | 0 | 0 | Not-determined | Genome-fragment | NA | | 0 |
| S8-final-viral-combined_k141_257723\|\|full | 1396 | No | NA | | 0 | 0 | Low-quality | Genome-fragment | | 1.25 | 0 |
| S1-final-viral-combined_k141_347093\|\|full | 1397 | No | NA | | 0 | 0 | Low-quality | Genome-fragment | | 2.96 | 0 |
| S5_k141_54507 | 1397 | No | NA | | 1 | 0 | Low-quality | Genome-fragment | | 3.25 | 0 |
| S1-final-viral-combined_k141_161386\|\|full | 3092 | No | NA | | 0 | 2 | Not-determined | Genome-fragment | NA | | 0 |
| S17-final-viral-combined_k141_52862\|\|lt2gene | 1397 | No | NA | | 1 | 0 | Low-quality | Genome-fragment | | 3.46 | 0 |
| S1-final-viral-combined_k141_29625\|\|full | 4934 | No | NA | | 0 | 0 | Not-determined | Genome-fragment | NA | | 0 |
| S1-final-viral-combined_k141_391970\|\|full | 1650 | No | NA | | 0 | 0 | Not-determined | Genome-fragment | NA | | 0 |
| S1-final-viral-combined_k141_342044\|\|full | 1582 | No | NA | | 0 | 0 | Not-determined | Genome-fragment | NA | | 0 |
| S1-final-viral-combined_k141_111297\|\|full | 1918 | No | NA | | 0 | 2 | Not-determined | Genome-fragment | NA | | 0 |
| S12-final-viral-combined_k141_205905\|\|full | 1397 | No | NA | | 1 | 0 | Low-quality | Genome-fragment | | 3.3 | 0 |
| S1-final-viral-combined_k141_391974\|\|full | 4486 | No | NA | | 0 | 0 | Not-determined | Genome-fragment | NA | | 0 |
| S1-final-viral-combined_k141_212493\|\|full | 1886 | No | NA | | 0 | 0 | Not-determined | Genome-fragment | NA | | 0 |
| S12-final-viral-combined_k141_56856\|\|full | 1397 | No | NA | | 1 | 0 | Low-quality | Genome-fragment | | 2.6 | 0 |
| S6-final-viral-combined_k141_36578\|\|full | 1397 | No | NA | | 0 | 2 | Low-quality | Genome-fragment | | 1.79 | 0 |
| S1-final-viral-combined_k141_262040\|\|full | 2262 | No | NA | | 0 | 1 | Not-determined | Genome-fragment | NA | | 0 |
| S1-final-viral-combined_k141_276911\|\|full | 7487 | No | NA | | 0 | 1 | Not-determined | Genome-fragment | NA | | 0 |
| S1-final-viral-combined_k141_212516\|\|full | 1184 | No | NA | | 0 | 1 | Not-determined | Genome-fragment | NA | | 0 |
| S8-final-viral-combined_k141_71446\|\|full | 1397 | No | NA | | 0 | 0 | Low-quality | Genome-fragment | | 2.88 | 0 |
| S1-final-viral-combined_k141_63216\|\|full | 5240 | No | NA | | 0 | 4 | Not-determined | Genome-fragment | NA | | 0 |
| S1-final-viral-combined_k141_14015\|\|full | 5176 | No | NA | | 0 | 1 | Not-determined | Genome-fragment | NA | | 0 |
| S1-final-viral-combined_k141_195867\|\|full | 1579 | No | NA | | 0 | 0 | Not-determined | Genome-fragment | NA | | 0 |
| S9-final-viral-combined_k141_85306\|\|full | 1398 | No | NA | | 0 | 0 | Low-quality | Genome-fragment | | 3.32 | 0 |
| S11-final-viral-combined_k141_11005\|\|full | 1398 | No | NA | | 1 | 0 | Low-quality | Genome-fragment | | 3.15 | 0 |
| S1-final-viral-combined_k141_111366\|\|full | 1731 | No | NA | | 0 | 0 | Not-determined | Genome-fragment | NA | | 0 |
| S11-final-viral-combined_k141_54592\|\|full | 1398 | No | NA | | 0 | 0 | Low-quality | Genome-fragment | | 2.94 | 0 |
| S1-final-viral-combined_k141_161472\|\|full | 1471 | No | NA | | 0 | 0 | Not-determined | Genome-fragment | NA | | 0 |
| S1-final-viral-combined_k141_244986\|\|full | 1754 | No | NA | | 0 | 0 | Not-determined | Genome-fragment | NA | | 0 |
| S3-final-viral-combined_k141_55328\|\|full | 1398 | No | NA | | 0 | 1 | Low-quality | Genome-fragment | | 0.66 | 0 |
| S1-final-viral-combined_k141_195909\|\|full | 1267 | No | NA | | 0 | 2 | Not-determined | Genome-fragment | NA | | 0 |
| S5-final-viral-combined_k141_47566\|\|full | 1398 | No | NA | | 0 | 0 | Low-quality | Genome-fragment | | 1.99 | 0 |
| S1-final-viral-combined_k141_327240\|\|full | 3339 | No | NA | | 0 | 0 | Not-determined | Genome-fragment | NA | | 0 |
| S1-final-viral-combined_k141_360285\|\|full | 40364 | No | NA | | 17 | 4 | Medium-quality | Genome-fragment | | 81.89 | 0 |
| S1-final-viral-combined_k141_111382\|\|full | 2261 | No | NA | | 0 | 2 | Not-determined | Genome-fragment | NA | | 0 |
| S1-final-viral-combined_k141_262091\|\|full | 12847 | No | NA | | 0 | 7 | Not-determined | Genome-fragment | NA | | 0 |
| S5-final-viral-combined_k141_32405\|\|full | 1398 | No | NA | | 2 | 0 | Low-quality | Genome-fragment | | 2.25 | 0 |
| S1-final-viral-combined_k141_130297\|\|full | 1936 | No | NA | | 0 | 0 | Not-determined | Genome-fragment | NA | | 0 |
| S5-final-viral-combined_k141_194497\|\|full | 1398 | No | NA | | 4 | 0 | Low-quality | Genome-fragment | | 3.49 | 0 |
| S1-final-viral-combined_k141_29703\|\|full | 1147 | No | NA | | 0 | 0 | Not-determined | Genome-fragment | NA | | 0 |
| S1-final-viral-combined_k141_78621\|\|full | 1694 | No | NA | | 0 | 2 | Not-determined | Genome-fragment | NA | | 0 |
| S17-final-viral-combined_k141_179235\|\|full | 1398 | No | NA | | 0 | 0 | Low-quality | Genome-fragment | | 3.48 | 0 |
| S12-final-viral-combined_k141_262316\|\|full | 1398 | No | NA | | 0 | 1 | Low-quality | Genome-fragment | | 2.03 | 0 |
| S12-final-viral-combined_k141_66411\|\|lt2gene | 1398 | No | NA | | 1 | 0 | Low-quality | Genome-fragment | | 2.56 | 0 |
| S2-final-viral-combined_k141_138318\|\|full | 1398 | No | NA | | 0 | 0 | Low-quality | Genome-fragment | | 2.4 | 0 |
| S6-final-viral-combined_k141_70158\|\|full | 1398 | No | NA | | 0 | 1 | Low-quality | Genome-fragment | | 1.66 | 0 |
| S11-final-viral-combined_k141_304497\|\|full | 1399 | No | NA | | 0 | 0 | Low-quality | Genome-fragment | | 1.64 | 0 |
| S11-final-viral-combined_k141_239327\|\|full | 1399 | No | NA | | 1 | 0 | Low-quality | Genome-fragment | | 2.53 | 0 |
| S1-final-viral-combined_k141_145813\|\|full | 2205 | No | NA | | 0 | 1 | Not-determined | Genome-fragment | NA | | 0 |
| S1-final-viral-combined_k141_104718\|\|full | 1399 | No | NA | | 2 | 0 | Low-quality | Genome-fragment | | 2.29 | 0 |
| S1-final-viral-combined_k141_14061\|\|full | 2093 | No | NA | | 0 | 2 | Not-determined | Genome-fragment | NA | | 0 |
| S1-final-viral-combined_k141_376024\|\|full | 4398 | No | NA | | 0 | 5 | Not-determined | Genome-fragment | NA | | 0 |
| S3_k141_71076 | 1399 | No | NA | | 0 | 0 | Low-quality | Genome-fragment | | 2.84 | 0 |
| S1-final-viral-combined_k141_310716\|\|full | 2560 | No | NA | | 0 | 1 | Not-determined | Genome-fragment | NA | | 0 |
| S1-final-viral-combined_k141_294277\|\|full | 1729 | No | NA | | 0 | 1 | Not-determined | Genome-fragment | NA | | 0 |
| S1-final-viral-combined_k141_29754\|\|full | 2482 | No | NA | | 0 | 3 | Not-determined | Genome-fragment | NA | | 0 |
| S1-final-viral-combined_k141_212638\|\|full | 2887 | No | NA | | 0 | 3 | Not-determined | Genome-fragment | NA | | 0 |
| S1-final-viral-combined_k141_310739\|\|full | 1218 | No | NA | | 0 | 1 | Not-determined | Genome-fragment | NA | | 0 |
| S5-final-viral-combined_k141_81182\|\|full | 1399 | No | NA | | 1 | 0 | Low-quality | Genome-fragment | | 2.85 | 0 |
| S1-final-viral-combined_k141_195996\|\|full | 1841 | No | NA | | 0 | 1 | Not-determined | Genome-fragment | NA | | 0 |
| S1-final-viral-combined_k141_262173\|\|full | 2923 | No | NA | | 0 | 3 | Not-determined | Genome-fragment | NA | | 0 |
| S1-final-viral-combined_k141_294318\|\|full | 4880 | No | NA | | 0 | 3 | Not-determined | Genome-fragment | NA | | 0 |
| S1-final-viral-combined_k141_310784\|\|full | 2162 | No | NA | | 0 | 0 | Not-determined | Genome-fragment | NA | | 0 |
| S7-final-viral-combined_k141_55126\|\|full | 1399 | No | NA | | 0 | 2 | Low-quality | Genome-fragment | | 3.59 | 0 |
| S1-final-viral-combined_k141_228215\|\|full | 1126 | No | NA | | 0 | 2 | Not-determined | Genome-fragment | NA | | 0 |
| S17-final-viral-combined_k141_156640\|\|full | 1399 | No | NA | | 0 | 0 | Low-quality | Genome-fragment | | 2.43 | 0 |
| S1-final-viral-combined_k141_96322\|\|full | 7654 | No | NA | | 0 | 5 | Not-determined | Genome-fragment | NA | | 0 |
| S17-final-viral-combined_k141_115031\|\|full | 1399 | Yes | | 633 | 1 | 1 | Low-quality | Genome-fragment | | 1.77 | 54.75 |
| S1-final-viral-combined_k141_130382\|\|full | 1803 | No | NA | | 0 | 0 | Not-determined | Genome-fragment | NA | | 0 |
| S1-final-viral-combined_k141_14169\|\|full | 3598 | No | NA | | 0 | 2 | Not-determined | Genome-fragment | NA | | 0 |
| S1-final-viral-combined_k141_46389\|\|full | 5208 | No | NA | | 0 | 7 | Not-determined | Genome-fragment | NA | | 0 |
| S1-final-viral-combined_k141_14173\|\|full | 1397 | No | NA | | 0 | 1 | Not-determined | Genome-fragment | NA | | 0 |
| S17-final-viral-combined_k141_148647\|\|full | 1399 | No | NA | | 0 | 1 | Low-quality | Genome-fragment | | 0.47 | 0 |
| S1-final-viral-combined_k141_111476\|\|full | 2056 | No | NA | | 0 | 1 | Not-determined | Genome-fragment | NA | | 0 |
| S1-final-viral-combined_k141_294390\|\|full | 2646 | No | NA | | 0 | 2 | Not-determined | Genome-fragment | NA | | 0 |
| S17-final-viral-combined_k141_197240\|\|full | 1399 | No | NA | | 0 | 0 | Low-quality | Genome-fragment | | 3.06 | 0 |
| S12-final-viral-combined_k141_38355\|\|full | 1399 | No | NA | | 0 | 0 | Low-quality | Genome-fragment | | 1.74 | 0 |
| S1-final-viral-combined_k141_310870\|\|full | 2227 | No | NA | | 0 | 0 | Not-determined | Genome-fragment | NA | | 0 |
| S2-final-viral-combined_k141_117584\|\|full | 1399 | No | NA | | 0 | 0 | Low-quality | Genome-fragment | | 2.56 | 0 |
| S2-final-viral-combined_k141_30471\|\|lt2gene | 1399 | No | NA | | 1 | 0 | Low-quality | Genome-fragment | | 4.51 | 0 |
| S4-final-viral-combined_k141_121007\|\|full | 1399 | No | NA | | 0 | 0 | Low-quality | Genome-fragment | | 1.9 | 0 |
| S9-final-viral-combined_k141_81461\|\|full | 1400 | No | NA | | 1 | 0 | Low-quality | Genome-fragment | | 1.76 | 0 |
| S1-final-viral-combined_k141_78848\|\|full | 1825 | No | NA | | 0 | 2 | Not-determined | Genome-fragment | NA | | 0 |
| S9-final-viral-combined_k141_58435\|\|full | 1400 | No | NA | | 0 | 0 | Low-quality | Genome-fragment | | 3.13 | 0 |
| S5-final-viral-combined_k141_194557\|\|full | 1400 | No | NA | | 1 | 0 | Low-quality | Genome-fragment | | 2.35 | 0 |
| S1-final-viral-combined_k141_196149\|\|full | 1759 | No | NA | | 0 | 2 | Not-determined | Genome-fragment | NA | | 0 |
| S5-final-viral-combined_k141_123129\|\|full | 1400 | No | NA | | 0 | 0 | Low-quality | Genome-fragment | | 1.75 | 0 |
| S1-final-viral-combined_k141_327482\|\|full | 3683 | No | NA | | 0 | 0 | Not-determined | Genome-fragment | NA | | 0 |
| S1-final-viral-combined_k141_63515\|\|full | 4684 | No | NA | | 0 | 2 | Not-determined | Genome-fragment | NA | | 0 |
| S17-final-viral-combined_k141_222894\|\|full | 1400 | No | NA | | 0 | 0 | Low-quality | Genome-fragment | | 3.89 | 0 |
| S17-final-viral-combined_k141_71269\|\|full | 1400 | No | NA | | 1 | 0 | Low-quality | Genome-fragment | | 3.22 | 0 |
| S1-final-viral-combined_k141_360494\|\|full | 5356 | No | NA | | 0 | 6 | Not-determined | Genome-fragment | NA | | 0 |
| S1-final-viral-combined_k141_46509\|\|full | 1197 | No | NA | | 0 | 1 | Not-determined | Genome-fragment | NA | | 0 |
| S12-final-viral-combined_k141_264015\|\|full | 1400 | No | NA | | 2 | 0 | Low-quality | Genome-fragment | | 3.12 | 0 |
| S1-final-viral-combined_k141_63549\|\|full | 2599 | No | NA | | 0 | 2 | Not-determined | Genome-fragment | NA | | 0 |
| S12-final-viral-combined_k141_84111\|\|lt2gene | 1400 | No | NA | | 1 | 0 | Low-quality | Genome-fragment | | 2.74 | 0 |
| S12-final-viral-combined_k141_93445\|\|lt2gene | 1400 | No | NA | | 1 | 0 | Low-quality | Genome-fragment | | 3.59 | 0 |
| S1-final-viral-combined_k141_294545\|\|full | 2722 | No | NA | | 0 | 1 | Not-determined | Genome-fragment | NA | | 0 |
| S1-final-viral-combined_k141_245194\|\|full | 2435 | No | NA | | 0 | 1 | Not-determined | Genome-fragment | NA | | 0 |
| S2-final-viral-combined_k141_236801\|\|full | 1400 | No | NA | | 0 | 0 | Low-quality | Genome-fragment | | 2.33 | 0 |
| S6-final-viral-combined_k141_44423\|\|full | 1400 | No | NA | | 0 | 3 | Low-quality | Genome-fragment | | 0.41 | 0 |

| | | | | | | | | | |
|---|---|---|---|---|---|---|---|---|---|
| S9-final-viral-combined_k141_54866\|\|full | 1401 | No | NA | 1 | 0 | Low-quality | Genome-fragment | 3.52 | 0 |
| S1-final-viral-combined_k141_342349\|\|full | 9185 | No | NA | 0 | 5 | Not-determined | Genome-fragment | NA | 0 |
| S11-final-viral-combined_k141_181809\|\|full | 1401 | No | NA | 0 | 0 | Low-quality | Genome-fragment | 2.22 | 0 |
| S1-final-viral-combined_k141_262365\|\|full | 1712 | No | NA | 0 | 2 | Not-determined | Genome-fragment | NA | 0 |
| S1-final-viral-combined_k141_31434\|\|full | 1401 | No | NA | 0 | 1 | Low-quality | Genome-fragment | 1.08 | 0 |
| S1-final-viral-combined_k141_392237\|\|full | 2003 | No | NA | 0 | 1 | Not-determined | Genome-fragment | NA | 0 |
| S1-final-viral-combined_k141_196245\|\|full | 1752 | No | NA | 0 | 2 | Not-determined | Genome-fragment | NA | 0 |
| S3-final-viral-combined_k141_79537\|\|lt2gene | 1401 | No | NA | 1 | 0 | Low-quality | Genome-fragment | 3.25 | 0 |
| S1-final-viral-combined_k141_177679\|\|full | 1057 | No | NA | 0 | 2 | Not-determined | Genome-fragment | NA | 0 |
| S1-final-viral-combined_k141_130517\|\|full | 1803 | No | NA | 0 | 2 | Not-determined | Genome-fragment | NA | 0 |
| S7-final-viral-combined_k141_228835\|\|full | 1401 | No | NA | 2 | 0 | Low-quality | Genome-fragment | 2.5 | 0 |
| S1-final-viral-combined_k141_161787\|\|full | 1046 | No | NA | 0 | 1 | Not-determined | Genome-fragment | NA | 0 |
| S1-final-viral-combined_k141_294609\|\|full | 1940 | No | NA | 0 | 0 | Not-determined | Genome-fragment | NA | 0 |
| S1-final-viral-combined_k141_342407\|\|full | 6208 | No | NA | 0 | 2 | Not-determined | Genome-fragment | NA | 0 |
| S1-final-viral-combined_k141_161810\|\|full | 2700 | No | NA | 0 | 1 | Not-determined | Genome-fragment | NA | 0 |
| S17-k141_143001 | 1401 | No | NA | 0 | 2 | Low-quality | Genome-fragment | 2.77 | 0 |
| S1-final-viral-combined_k141_130541\|\|full | 2838 | No | NA | 0 | 1 | Not-determined | Genome-fragment | NA | 0 |
| S1-final-viral-combined_k141_342429\|\|full | 5111 | No | NA | 0 | 3 | Not-determined | Genome-fragment | NA | 0 |
| S1-final-viral-combined_k141_63621\|\|full | 9431 | No | NA | 0 | 4 | Not-determined | Genome-fragment | NA | 0 |
| S1-final-viral-combined_k141_96569\|\|full | 2603 | No | NA | 0 | 0 | Not-determined | Genome-fragment | NA | 0 |
| S12-final-viral-combined_k141_335881\|\|full | 1401 | No | NA | 1 | 0 | Low-quality | Genome-fragment | 2.37 | 0 |
| S1-final-viral-combined_k141_392304\|\|full | 3564 | No | NA | 0 | 3 | Not-determined | Genome-fragment | NA | 0 |
| S1-final-viral-combined_k141_30095\|\|full | 1682 | No | NA | 0 | 0 | Not-determined | Genome-fragment | NA | 0 |
| S1-final-viral-combined_k141_212892\|\|full | 22088 | No | NA | 9 | 0 | Medium-quality | Genome-fragment | 56.33 | 0 |
| S2-final-viral-combined_k141_20217\|\|full | 1401 | No | NA | 1 | 0 | Low-quality | Genome-fragment | 2.24 | 0 |
| S1-final-viral-combined_k141_342497\|\|full | 1736 | No | NA | 0 | 2 | Not-determined | Genome-fragment | NA | 0 |
| S1-final-viral-combined_k141_294694\|\|full | 2242 | No | NA | 0 | 1 | Not-determined | Genome-fragment | NA | 0 |
| S11-final-viral-combined_k141_235220\|\|full | 1402 | No | NA | 1 | 0 | Low-quality | Genome-fragment | 2.17 | 0 |
| S11-final-viral-combined_k141_214665\|\|full | 1402 | No | NA | 0 | 0 | Low-quality | Genome-fragment | 3.81 | 0 |
| S1-final-viral-combined_k141_196382\|\|full | 19410 | No | NA | 0 | 5 | Not-determined | Genome-fragment | NA | 0 |
| S11_k141_253637 | 1402 | No | NA | 0 | 1 | Low-quality | Genome-fragment | 3.95 | 0 |
| S1-final-viral-combined_k141_342553\|\|full | 2033 | No | NA | 0 | 2 | Not-determined | Genome-fragment | NA | 0 |
| S1-final-viral-combined_k141_294729\|\|full | 6787 | No | NA | 0 | 6 | Not-determined | Genome-fragment | NA | 0 |
| S1-final-viral-combined_k141_30137\|\|full | 1901 | No | NA | 0 | 2 | Not-determined | Genome-fragment | NA | 0 |
| S1-final-viral-combined_k141_262512\|\|full | 8540 | No | NA | 0 | 5 | Not-determined | Genome-fragment | NA | 0 |
| S1-final-viral-combined_k141_96704\|\|full | 1770 | No | NA | 0 | 1 | Not-determined | Genome-fragment | NA | 0 |
| S5-final-viral-combined_k141_163326\|\|full | 1402 | No | NA | 1 | 0 | Low-quality | Genome-fragment | 1.57 | 0 |
| S1-final-viral-combined_k141_96708\|\|full | 4235 | No | NA | 0 | 3 | Not-determined | Genome-fragment | NA | 0 |
| S5-final-viral-combined_k141_120206\|\|full | 1402 | No | NA | 0 | 1 | Low-quality | Genome-fragment | 2.87 | 0 |
| S7-final-viral-combined_k141_154160\|\|full | 1402 | No | NA | 0 | 0 | Low-quality | Genome-fragment | 2.73 | 0 |
| S17-final-viral-combined_k141_146853\|\|full | 1402 | No | NA | 0 | 2 | Low-quality | Genome-fragment | 3.27 | 0 |
| S12-final-viral-combined_k141_262365\|\|full | 1402 | No | NA | 1 | 0 | Low-quality | Genome-fragment | 4.38 | 0 |
| S1-final-viral-combined_k141_30190\|\|full | 2281 | No | NA | 0 | 1 | Not-determined | Genome-fragment | NA | 0 |
| S4_k141_25661 | 1402 | No | NA | 0 | 1 | Low-quality | Genome-fragment | 2.39 | 0 |
| S1-final-viral-combined_k141_96744\|\|full | 1305 | No | NA | 0 | 3 | Not-determined | Genome-fragment | NA | 0 |
| S1-final-viral-combined_k141_14442\|\|full | 2168 | No | NA | 0 | 2 | Not-determined | Genome-fragment | NA | 0 |
| S8-final-viral-combined_k141_93827\|\|full | 1402 | No | NA | 0 | 2 | Low-quality | Genome-fragment | 0.48 | 0 |
| S1-final-viral-combined_k141_177882\|\|full | 2511 | No | NA | 0 | 0 | Not-determined | Genome-fragment | NA | 0 |
| S1-final-viral-combined_k141_130673\|\|full | 1372 | No | NA | 0 | 1 | Not-determined | Genome-fragment | NA | 0 |
| S1-final-viral-combined_k141_327763\|\|full | 1665 | No | NA | 0 | 1 | Not-determined | Genome-fragment | NA | 0 |
| S9_k141_71579 | 1403 | No | NA | 1 | 0 | Low-quality | Genome-fragment | 3.9 | 0 |
| S1-final-viral-combined_k141_30268\|\|full | 1399 | No | NA | 0 | 1 | Not-determined | Genome-fragment | NA | 0 |
| S11_k141_256064 | 1403 | No | NA | 1 | 0 | Low-quality | Genome-fragment | 1.49 | 0 |
| S1-final-viral-combined_k141_85652\|\|full | 1403 | No | NA | 0 | 2 | Low-quality | Genome-fragment | 0.9 | 0 |
| S5-final-viral-combined_k141_123314\|\|full | 1403 | No | NA | 1 | 0 | Low-quality | Genome-fragment | 0.87 | 0 |
| S1-final-viral-combined_k141_96848\|\|full | 1534 | No | NA | 0 | 1 | Not-determined | Genome-fragment | NA | 0 |
| S1-final-viral-combined_k141_14519\|\|full | 3505 | No | NA | 0 | 0 | Not-determined | Genome-fragment | NA | 0 |
| S1-final-viral-combined_k141_63789\|\|full | 7809 | No | NA | 0 | 2 | Not-determined | Genome-fragment | NA | 0 |
| S5-final-viral-combined_k141_9241\|\|lt2gene | 1403 | No | NA | 1 | 0 | Low-quality | Genome-fragment | 0.91 | 0 |
| S1-final-viral-combined_k141_277430\|\|full | 9829 | No | NA | 0 | 5 | Not-determined | Genome-fragment | NA | 0 |
| S7_k141_235448 | 1403 | No | NA | 0 | 1 | Low-quality | Genome-fragment | 0.37 | 0 |
| S1-final-viral-combined_k141_14545\|\|full | 1915 | No | NA | 0 | 1 | Not-determined | Genome-fragment | NA | 0 |
| S17-final-viral-combined_k141_175998\|\|full | 1403 | No | NA | 1 | 0 | Low-quality | Genome-fragment | 1.03 | 0 |
| S1-final-viral-combined_k141_162071\|\|full | 7200 | No | NA | 0 | 4 | Not-determined | Genome-fragment | NA | 0 |
| S17-k141_187443 | 1403 | No | NA | 0 | 0 | Low-quality | Genome-fragment | 3.14 | 0 |
| S1-final-viral-combined_k141_245656\|\|full | 2212 | No | NA | 0 | 1 | Not-determined | Genome-fragment | NA | 0 |
| S1-final-viral-combined_k141_111757\|\|full | 2907 | No | NA | 0 | 2 | Not-determined | Genome-fragment | NA | 0 |
| S1-final-viral-combined_k141_245665\|\|full | 2715 | No | NA | 0 | 2 | Not-determined | Genome-fragment | NA | 0 |
| S1-final-viral-combined_k141_14568\|\|full | 2291 | No | NA | 0 | 0 | Not-determined | Genome-fragment | NA | 0 |
| S12_k141_167245 | 1403 | No | NA | 0 | 1 | Low-quality | Genome-fragment | 0.87 | 0 |
| S1-final-viral-combined_k141_360771\|\|full | 15514 | No | NA | 0 | 12 | Not-determined | Genome-fragment | NA | 0 |
| S1-final-viral-combined_k141_262668\|\|full | 7816 | No | NA | 0 | 3 | Not-determined | Genome-fragment | NA | 0 |
| S2-final-viral-combined_k141_159159\|\|full | 1403 | No | NA | 0 | 0 | Low-quality | Genome-fragment | 3.27 | 0 |
| S2-final-viral-combined_k141_68212\|\|full | 1403 | No | NA | 0 | 1 | Low-quality | Genome-fragment | 2.52 | 0 |
| S1-final-viral-combined_k141_360796\|\|full | 1400 | No | NA | 0 | 0 | Not-determined | Genome-fragment | NA | 0 |
| S1-final-viral-combined_k141_277494\|\|full | 2251 | No | NA | 0 | 0 | Not-determined | Genome-fragment | NA | 0 |
| S4-final-viral-combined_k141_109734\|\|full | 1403 | No | NA | 0 | 0 | Low-quality | Genome-fragment | 2.83 | 0 |
| S1-final-viral-combined_k141_162158\|\|full | 4450 | No | NA | 0 | 3 | Not-determined | Genome-fragment | NA | 0 |
| S1-final-viral-combined_k141_311423\|\|full | 10389 | No | NA | 0 | 11 | Not-determined | Genome-fragment | NA | 0 |
| S6-final-viral-combined_k141_92261\|\|lt2gene | 1403 | No | NA | 1 | 0 | Low-quality | Genome-fragment | 2.82 | 0 |
| S1-final-viral-combined_k141_178042\|\|full | 2484 | No | NA | 0 | 1 | Not-determined | Genome-fragment | NA | 0 |
| S8-final-viral-combined_k141_162590\|\|full | 1403 | No | NA | 0 | 0 | Low-quality | Genome-fragment | 1.9 | 0 |
| S8-final-viral-combined_k141_197065\|\|full | 1403 | No | NA | 0 | 0 | Low-quality | Genome-fragment | 2.8 | 0 |
| S1-final-viral-combined_k141_392563\|\|full | 1935 | No | NA | 0 | 2 | Not-determined | Genome-fragment | NA | 0 |
| S8-final-viral-combined_k141_250362\|\|full | 1403 | No | NA | 0 | 1 | Low-quality | Genome-fragment | 2.56 | 0 |
| S1-final-viral-combined_k141_96933\|\|full | 2481 | No | NA | 0 | 2 | Not-determined | Genome-fragment | NA | 0 |
| S11-final-viral-combined_k141_138104\|\|full | 1404 | No | NA | 0 | 0 | Low-quality | Genome-fragment | 1.08 | 0 |
| S1-final-viral-combined_k141_14620\|\|full | 2688 | No | NA | 0 | 3 | Not-determined | Genome-fragment | NA | 0 |
| S1-final-viral-combined_k141_277574\|\|full | 1274 | No | NA | 0 | 1 | Not-determined | Genome-fragment | NA | 0 |
| S1-final-viral-combined_k141_342839\|\|full | 6548 | No | NA | 0 | 3 | Not-determined | Genome-fragment | NA | 0 |
| S1-final-viral-combined_k141_241449\|\|full | 1404 | No | NA | 0 | 1 | Low-quality | Genome-fragment | 2.57 | 0 |
| S17-final-viral-combined_k141_32825\|\|full | 1404 | No | NA | 0 | 1 | Low-quality | Genome-fragment | 0.68 | 0 |
| S12-final-viral-combined_k141_256488\|\|full | 1404 | No | NA | 2 | 0 | Low-quality | Genome-fragment | 2.78 | 0 |
| S12_k141_286254 | 1404 | No | NA | 0 | 0 | Low-quality | Genome-fragment | 1.39 | 0 |
| S4_k141_153089 | 1404 | No | NA | 0 | 0 | Low-quality | Genome-fragment | 1.41 | 0 |
| S9_k141_64718 | 1405 | No | NA | 1 | 0 | Low-quality | Genome-fragment | 2.79 | 0 |
| S1-final-viral-combined_k141_299371\|\|full | 1405 | No | NA | 0 | 0 | Low-quality | Genome-fragment | 3.65 | 0 |
| S5-final-viral-combined_k141_52073\|\|full | 1405 | No | NA | 0 | 0 | Low-quality | Genome-fragment | 2.45 | 0 |
| S1-final-viral-combined_k141_30471\|\|full | 3012 | No | NA | 0 | 2 | Not-determined | Genome-fragment | NA | 0 |
| S1-final-viral-combined_k141_96973\|\|full | 2784 | No | NA | 0 | 2 | Not-determined | Genome-fragment | NA | 0 |
| S1-final-viral-combined_k141_360908\|\|full | 2331 | No | NA | 0 | 1 | Not-determined | Genome-fragment | NA | 0 |
| S1-final-viral-combined_k141_130899\|\|full | 1532 | No | NA | 0 | 0 | Not-determined | Genome-fragment | NA | 0 |
| S7_k141_32386 | 1405 | No | NA | 0 | 0 | Low-quality | Genome-fragment | 1.33 | 0 |
| S1-final-viral-combined_k141_96982\|\|full | 1183 | No | NA | 0 | 1 | Not-determined | Genome-fragment | NA | 0 |
| S1-final-viral-combined_k141_262781\|\|full | 2045 | No | NA | 0 | 2 | Not-determined | Genome-fragment | NA | 0 |
| S17-final-viral-combined_k141_67205\|\|lt2gene | 1405 | No | NA | 1 | 0 | Low-quality | Genome-fragment | 3.83 | 0 |
| S17-final-viral-combined_k141_204368\|\|lt2gene | 1405 | No | NA | 1 | 0 | Low-quality | Genome-fragment | 2.19 | 0 |
| S12_k141_198456 | 1405 | No | NA | 0 | 0 | Low-quality | Genome-fragment | 0.77 | 0 |
| S2-final-viral-combined_k141_176384\|\|full | 1405 | No | NA | 0 | 0 | Low-quality | Genome-fragment | 2.87 | 0 |
| S1-final-viral-combined_k141_111905\|\|full | 8392 | No | NA | 0 | 6 | Not-determined | Genome-fragment | NA | 0 |
| S2-final-viral-combined_k141_75930\|\|full | 1405 | Yes | 346 | 1 | 1 | Low-quality | Genome-fragment | 0.14 | 75.37 |
| S2-final-viral-combined_k141_246569\|\|full | 1405 | No | NA | 0 | 2 | Low-quality | Genome-fragment | 0.45 | 0 |
| S1-final-viral-combined_k141_342937\|\|full | 1493 | No | NA | 0 | 0 | Not-determined | Genome-fragment | NA | 0 |
| S4-final-viral-combined_k141_130968\|\|full | 1405 | No | NA | 0 | 0 | Low-quality | Genome-fragment | 3.67 | 0 |
| S1-final-viral-combined_k141_392674\|\|full | 8872 | No | NA | 0 | 6 | Not-determined | Genome-fragment | NA | 0 |
| S4-final-viral-combined_k141_34100\|\|full | 1405 | No | NA | 3 | 0 | Low-quality | Genome-fragment | 2.33 | 0 |
| S8-final-viral-combined_k141_196854\|\|full | 1405 | No | NA | 0 | 2 | Low-quality | Genome-fragment | 1.01 | 0 |
| S8_k141_63621 | 1405 | No | NA | 0 | 0 | Low-quality | Genome-fragment | 0.91 | 0 |
| S1-final-viral-combined_k141_111943\|\|full | 3930 | No | NA | 0 | 4 | Not-determined | Genome-fragment | NA | 0 |
| S9-final-viral-combined_k141_73568\|\|full | 1406 | No | NA | 0 | 2 | Low-quality | Genome-fragment | 0.36 | 0 |
| S3-final-viral-combined_k141_70155\|\|full | 1406 | No | NA | 0 | 2 | Low-quality | Genome-fragment | 1.23 | 0 |
| S7-final-viral-combined_k141_233893\|\|full | 1406 | No | NA | 0 | 0 | Low-quality | Genome-fragment | 3.25 | 0 |
| S12-final-viral-combined_k141_152710\|\|full | 1406 | No | NA | 2 | 0 | Low-quality | Genome-fragment | 2.51 | 0 |
| S12-final-viral-combined_k141_106609\|\|full | 1406 | No | NA | 0 | 1 | Low-quality | Genome-fragment | 1.03 | 0 |
| S1-final-viral-combined_k141_196594\|\|full | 18806 | No | NA | 0 | 10 | Not-determined | Genome-fragment | NA | 0 |
| S1-final-viral-combined_k141_14799\|\|full | 1184 | No | NA | 0 | 2 | Not-determined | Genome-fragment | NA | 0 |
| S12-final-viral-combined_k141_326650\|\|full | 1406 | No | NA | 2 | 0 | Low-quality | Genome-fragment | 2.96 | 0 |
| S12_k141_249948 | 1406 | No | NA | 0 | 1 | Low-quality | Genome-fragment | 0.42 | 0 |
| S1-final-viral-combined_k141_196600\|\|full | 2384 | No | NA | 0 | 1 | Not-determined | Genome-fragment | NA | 0 |
| S2-final-viral-combined_k141_124498\|\|full | 1406 | No | NA | 0 | 0 | Low-quality | Genome-fragment | 1.33 | 0 |
| S1-final-viral-combined_k141_213047\|\|full | 5460 | No | NA | 0 | 1 | Not-determined | Genome-fragment | NA | 0 |
| S4-final-viral-combined_k141_146310\|\|full | 1406 | No | NA | 0 | 0 | Low-quality | Genome-fragment | 2.41 | 0 |
| S4-final-viral-combined_k141_7350\|\|full | 1406 | No | NA | 0 | 0 | Low-quality | Genome-fragment | 0.9 | 0 |
| S4-final-viral-combined_k141_58287\|\|full | 1406 | No | NA | 1 | 0 | Low-quality | Genome-fragment | 1.81 | 0 |
| S1-final-viral-combined_k141_376712\|\|full | 3755 | No | NA | 0 | 0 | Not-determined | Genome-fragment | NA | 0 |
| S1-final-viral-combined_k141_146421\|\|full | 3926 | No | NA | 0 | 3 | Not-determined | Genome-fragment | NA | 0 |
| S4-final-viral-combined_k141_149485\|\|lt2gene | 1406 | No | NA | 2 | 0 | Low-quality | Genome-fragment | 2.85 | 0 |
| S1-final-viral-combined_k141_178244\|\|full | 13070 | No | NA | 0 | 9 | Not-determined | Genome-fragment | NA | 0 |
| S1-final-viral-combined_k141_162364\|\|full | 1666 | No | NA | 0 | 0 | Not-determined | Genome-fragment | NA | 0 |
| S1-final-viral-combined_k141_282570\|\|full | 1407 | No | NA | 1 | 0 | Low-quality | Genome-fragment | 2.54 | 0 |
| S1-final-viral-combined_k141_208248\|\|full | 1407 | No | NA | 1 | 0 | Low-quality | Genome-fragment | 1.48 | 0 |
| S1-final-viral-combined_k141_228729\|\|full | 3059 | No | NA | 0 | 1 | Not-determined | Genome-fragment | NA | 0 |
| S1-final-viral-combined_k141_97138\|\|full | 1304 | No | NA | 0 | 1 | Not-determined | Genome-fragment | NA | 0 |
| S1-final-viral-combined_k141_361004\|\|full | 2927 | No | NA | 0 | 0 | Not-determined | Genome-fragment | NA | 0 |
| S1-final-viral-combined_k141_162420\|\|full | 1768 | No | NA | 0 | 1 | Not-determined | Genome-fragment | NA | 0 |
| S1-final-viral-combined_k141_311738\|\|full | 5704 | No | NA | 0 | 3 | Not-determined | Genome-fragment | NA | 0 |
| S1-final-viral-combined_k141_392782\|\|full | 1378 | No | NA | 0 | 1 | Not-determined | Genome-fragment | NA | 0 |
| S7-final-viral-combined_k141_43126\|\|lt2gene | 1407 | No | NA | 1 | 0 | Low-quality | Genome-fragment | 3.59 | 0 |
| S1-final-viral-combined_k141_262826\|\|full | 4131 | No | NA | 0 | 2 | Not-determined | Genome-fragment | NA | 0 |
| S1-final-viral-combined_k141_262833\|\|full | 2349 | No | NA | 0 | 3 | Not-determined | Genome-fragment | NA | 0 |
| S1-final-viral-combined_k141_262837\|\|full | 2044 | No | NA | 0 | 2 | Not-determined | Genome-fragment | NA | 0 |
| S1-final-viral-combined_k141_361045\|\|full | 1143 | No | NA | 0 | 0 | Not-determined | Genome-fragment | NA | 0 |
| S6-final-viral-combined_k141_8089\|\|full | 1407 | No | NA | 0 | 0 | Low-quality | Genome-fragment | 0.54 | 0 |
| S6_k141_71719 | 1407 | No | NA | 0 | 0 | Low-quality | Genome-fragment | 4.53 | 0 |
| S8_k141_236701 | 1407 | No | NA | 1 | 0 | Low-quality | Genome-fragment | 1.1 | 0 |
| S1-final-viral-combined_k141_131088\|\|full | 2101 | No | NA | 0 | 1 | Not-determined | Genome-fragment | NA | 0 |
| S1-final-viral-combined_k141_293308\|\|full | 1408 | No | NA | 0 | 2 | Low-quality | Genome-fragment | 0.79 | 0 |
| S1-final-viral-combined_k141_229196\|\|full | 1408 | No | NA | 1 | 0 | Low-quality | Genome-fragment | 3.32 | 0 |
| S1-final-viral-combined_k141_213093\|\|full | 2618 | No | NA | 0 | 2 | Not-determined | Genome-fragment | NA | 0 |
| S1-final-viral-combined_k141_14924\|\|full | 1701 | No | NA | 0 | 2 | Not-determined | Genome-fragment | NA | 0 |
| S7-final-viral-combined_k141_153946\|\|full | 1408 | No | NA | 0 | 0 | Low-quality | Genome-fragment | 1.32 | 0 |
| S1-final-viral-combined_k141_311832\|\|full | 1962 | No | NA | 0 | 0 | Not-determined | Genome-fragment | NA | 0 |
| S1-final-viral-combined_k141_131134\|\|full | 2251 | No | NA | 0 | 1 | Not-determined | Genome-fragment | NA | 0 |
| S1-final-viral-combined_k141_146542\|\|full | 1289 | No | NA | 0 | 1 | Not-determined | Genome-fragment | NA | 0 |
| S7-final-viral-combined_k141_189269\|\|full | 1408 | No | NA | 1 | 0 | Low-quality | Genome-fragment | 3.85 | 0 |
| S17-final-viral-combined_k141_39513\|\|full | 1408 | No | NA | 0 | 0 | Low-quality | Genome-fragment | 2.82 | 0 |
| S17-final-viral-combined_k141_63315\|\|lt2gene | 1408 | No | NA | 1 | 0 | Low-quality | Genome-fragment | 2.06 | 0 |
| S1-final-viral-combined_k141_277867\|\|full | 5089 | No | NA | 0 | 1 | Not-determined | Genome-fragment | NA | 0 |
| S1-final-viral-combined_k141_277869\|\|full | 1217 | No | NA | 0 | 0 | Not-determined | Genome-fragment | NA | 0 |
| S17-k141_98910 | 1408 | No | NA | 0 | 1 | Low-quality | Genome-fragment | 1.8 | 0 |
| S2-final-viral-combined_k141_5247\|\|full | 1408 | No | NA | 0 | 2 | Low-quality | Genome-fragment | 2.16 | 0 |
| S1-final-viral-combined_k141_178504\|\|full | 1005 | No | NA | 0 | 0 | Not-determined | Genome-fragment | NA | 0 |
| S2_k141_230770 | 1408 | No | NA | 0 | 0 | Low-quality | Genome-fragment | 3.02 | 0 |
| S9-final-viral-combined_k141_125114\|\|full | 1409 | No | NA | 2 | 0 | Low-quality | Genome-fragment | 2.77 | 0 |

| | | | | | | | | | | | |
|---|---|---|---|---|---|---|---|---|---|---|---|
| S1-final-viral-combined_k141_198889\|\|full | 1409 | No | NA | | 1 | 0 | Low-quality | Genome-fragment | | 3.73 | 0 |
| S1-final-viral-combined_k141_95686\|\|full | 1409 | No | NA | | 1 | 0 | Low-quality | Genome-fragment | | 0.91 | 0 |
| S3-final-viral-combined_k141_117599\|\|full | 1409 | No | NA | | 0 | 0 | Low-quality | Genome-fragment | | 30.22 | 0 |
| S1-final-viral-combined_k141_15006\|\|full | 2288 | No | NA | | 0 | 1 | Not-determined | Genome-fragment | NA | | 0 |
| S1-final-viral-combined_k141_311924\|\|full | 1070 | No | NA | | 0 | 2 | Not-determined | Genome-fragment | NA | | 0 |
| S1-final-viral-combined_k141_277956\|\|full | 1334 | No | NA | | 0 | 1 | Not-determined | Genome-fragment | NA | | 0 |
| S1-final-viral-combined_k141_30797\|\|full | 2608 | No | NA | | 0 | 2 | Not-determined | Genome-fragment | NA | | 0 |
| S5-final-viral-combined_k141_41053\|\|full | 1409 | No | NA | | 0 | 1 | Low-quality | Genome-fragment | | 3.41 | 0 |
| S1-final-viral-combined_k141_246071\|\|full | 1277 | No | NA | | 0 | 2 | Not-determined | Genome-fragment | NA | | 0 |
| S1-final-viral-combined_k141_377005\|\|full | 5271 | No | NA | | 0 | 5 | Not-determined | Genome-fragment | NA | | 0 |
| S1-final-viral-combined_k141_162599\|\|full | 1421 | No | NA | | 0 | 2 | Not-determined | Genome-fragment | NA | | 0 |
| S7-final-viral-combined_k141_234312\|\|full | 1409 | No | NA | | 0 | 0 | Low-quality | Genome-fragment | | 2.92 | 0 |
| S17-final-viral-combined_k141_48340\|\|full | 1409 | No | NA | | 1 | 0 | Low-quality | Genome-fragment | | 3.07 | 0 |
| S12-final-viral-combined_k141_323151\|\|full | 1409 | No | NA | | 1 | 0 | Low-quality | Genome-fragment | | 1.8 | 0 |
| S12-final-viral-combined_k141_246838\|\|full | 1409 | No | NA | | 0 | 1 | Low-quality | Genome-fragment | | 0.89 | 0 |
| S1-final-viral-combined_k141_196823\|\|full | 2800 | No | NA | | 0 | 3 | Not-determined | Genome-fragment | NA | | 0 |
| S1-final-viral-combined_k141_246087\|\|full | 1274 | No | NA | | 0 | 2 | Not-determined | Genome-fragment | NA | | 0 |
| S1-final-viral-combined_k141_196828\|\|full | 7360 | No | NA | | 0 | 4 | Not-determined | Genome-fragment | NA | | 0 |
| S1-final-viral-combined_k141_343254\|\|full | 5265 | No | NA | | 0 | 3 | Not-determined | Genome-fragment | NA | | 0 |
| S1-final-viral-combined_k141_263032\|\|full | 4804 | No | NA | | 0 | 0 | Not-determined | Genome-fragment | NA | | 0 |
| S12-final-viral-combined_k141_50133\|\|full | 1409 | No | NA | | 0 | 2 | Low-quality | Genome-fragment | | 0.45 | 0 |
| S1-final-viral-combined_k141_46780\|\|full | 1133 | No | NA | | 0 | 1 | Not-determined | Genome-fragment | NA | | 0 |
| S1-final-viral-combined_k141_377044\|\|full | 2306 | No | NA | | 0 | 2 | Not-determined | Genome-fragment | NA | | 0 |
| S4-final-viral-combined_k141_17882\|\|lt2gene | 1409 | No | NA | | 1 | 0 | Low-quality | Genome-fragment | | 3.28 | 0 |
| S1-final-viral-combined_k141_343285\|\|full | 1188 | No | NA | | 0 | 0 | Not-determined | Genome-fragment | NA | | 0 |
| S1-final-viral-combined_k141_79745\|\|full | 3058 | No | NA | | 0 | 0 | Not-determined | Genome-fragment | NA | | 0 |
| S6-final-viral-combined_k141_21410\|\|full | 1409 | No | NA | | 1 | 0 | Low-quality | Genome-fragment | | 2.37 | 0 |
| S8-final-viral-combined_k141_206698\|\|full | 1409 | No | NA | | 0 | 0 | Low-quality | Genome-fragment | | 2.93 | 0 |
| S8-final-viral-combined_k141_184955\|\|lt2gene | 1409 | No | NA | | 2 | 0 | Low-quality | Genome-fragment | | 2.36 | 0 |
| S8_k141_169428 | 1409 | No | NA | | 0 | 0 | Low-quality | Genome-fragment | | 2.27 | 0 |
| S1-final-viral-combined_k141_295384\|\|full | 3321 | No | NA | | 0 | 3 | Not-determined | Genome-fragment | NA | | 0 |
| S1-final-viral-combined_k141_392866\|\|full | 2404 | No | NA | | 0 | 1 | Not-determined | Genome-fragment | NA | | 0 |
| S9_k141_30140 | 1410 | No | NA | | 1 | 0 | Low-quality | Genome-fragment | | 2.87 | 0 |
| S1-final-viral-combined_k141_97358\|\|full | 4144 | No | NA | | 0 | 1 | Not-determined | Genome-fragment | NA | | 0 |
| S5-final-viral-combined_k141_205934\|\|full | 1410 | No | NA | | 3 | 0 | Low-quality | Genome-fragment | | 4.4 | 0 |
| S1-final-viral-combined_k141_343320\|\|full | 1332 | No | NA | | 0 | 1 | Not-determined | Genome-fragment | NA | | 0 |
| S1-final-viral-combined_k141_131270\|\|full | 38955 | No | NA | | 18 | 0 | High-quality | High-quality | | 92.32 | 0 |
| S1-final-viral-combined_k141_278071\|\|full | 2025 | No | NA | | 0 | 1 | Not-determined | Genome-fragment | NA | | 0 |
| S1-final-viral-combined_k141_15111\|\|full | 11982 | No | NA | | 0 | 9 | Not-determined | Genome-fragment | NA | | 0 |
| S17-final-viral-combined_k141_39129\|\|full | 1410 | No | NA | | 0 | 0 | Low-quality | Genome-fragment | | 4.46 | 0 |
| S17-k141_188948 | 1410 | No | NA | | 1 | 0 | Low-quality | Genome-fragment | | 4.34 | 0 |
| S1-final-viral-combined_k141_328479\|\|full | 6665 | No | NA | | 0 | 2 | Not-determined | Genome-fragment | NA | | 0 |
| S1-final-viral-combined_k141_263102\|\|full | 1171 | No | NA | | 0 | 2 | Not-determined | Genome-fragment | NA | | 0 |
| S1-final-viral-combined_k141_30961\|\|full | 8961 | No | NA | | 0 | 5 | Not-determined | Genome-fragment | NA | | 0 |
| S1-final-viral-combined_k141_97406\|\|full | 6420 | No | NA | | 0 | 3 | Not-determined | Genome-fragment | NA | | 0 |
| S17-k141_41652 | 1410 | No | NA | | 0 | 2 | Low-quality | Genome-fragment | | 0.79 | 0 |
| S12-final-viral-combined_k141_138893\|\|full | 1410 | No | NA | | 0 | 0 | Low-quality | Genome-fragment | | 3.59 | 0 |
| S1-final-viral-combined_k141_361307\|\|full | 2051 | No | NA | | 0 | 0 | Not-determined | Genome-fragment | NA | | 0 |
| S4-final-viral-combined_k141_119987\|\|full | 1410 | No | NA | | 0 | 0 | Low-quality | Genome-fragment | | 2.04 | 0 |
| S1-final-viral-combined_k141_328503\|\|full | 7085 | No | NA | | 0 | 3 | Not-determined | Genome-fragment | NA | | 0 |
| S4-final-viral-combined_k141_30509\|\|full | 1410 | No | NA | | 0 | 0 | Low-quality | Genome-fragment | | 2.72 | 0 |
| S1-final-viral-combined_k141_97446\|\|full | 1581 | No | NA | | 0 | 1 | Not-determined | Genome-fragment | NA | | 0 |
| S1-final-viral-combined_k141_162832\|\|full | 3707 | No | NA | | 0 | 2 | Not-determined | Genome-fragment | NA | | 0 |
| S4_k141_58524 | 1410 | No | NA | | 0 | 0 | Low-quality | Genome-fragment | | 1.37 | 0 |
| S8-final-viral-combined_k141_247623\|\|full | 1410 | No | NA | | 0 | 2 | Low-quality | Genome-fragment | | 2.36 | 0 |
| S1-final-viral-combined_k141_295527\|\|full | 1996 | No | NA | | 0 | 0 | Not-determined | Genome-fragment | NA | | 0 |
| S1-final-viral-combined_k141_79885\|\|full | 2466 | No | NA | | 0 | 1 | Not-determined | Genome-fragment | NA | | 0 |
| S11-final-viral-combined_k141_2552\|\|full | 1411 | No | NA | | 0 | 0 | Low-quality | Genome-fragment | | 1.75 | 0 |
| S1-final-viral-combined_k141_278178\|\|full | 2993 | No | NA | | 0 | 2 | Not-determined | Genome-fragment | NA | | 0 |
| S1-final-viral-combined_k141_213333\|\|full | 21039 | No | NA | | 0 | 7 | Not-determined | Genome-fragment | NA | | 0 |
| S11-final-viral-combined_k141_99632\|\|full | 1411 | No | NA | | 0 | 0 | Low-quality | Genome-fragment | | 1.86 | 0 |
| S1-final-viral-combined_k141_146820\|\|full | 1665 | No | NA | | 0 | 1 | Not-determined | Genome-fragment | NA | | 0 |
| S1-final-viral-combined_k141_219971\|\|full | 1411 | No | NA | | 0 | 1 | Low-quality | Genome-fragment | | 2.55 | 0 |
| S17-final-viral-combined_k141_11887\|\|full | 1411 | No | NA | | 0 | 0 | Low-quality | Genome-fragment | | 2 | 0 |
| S1-final-viral-combined_k141_343426\|\|full | 2842 | No | NA | | 0 | 3 | Not-determined | Genome-fragment | NA | | 0 |
| S1-final-viral-combined_k141_295578\|\|full | 1682 | No | NA | | 0 | 1 | Not-determined | Genome-fragment | NA | | 0 |
| S12-final-viral-combined_k141_151482\|\|full | 1411 | No | NA | | 0 | 0 | Low-quality | Genome-fragment | | 3.9 | 0 |
| S12-final-viral-combined_k141_220976\|\|full | 1411 | No | NA | | 0 | 0 | Low-quality | Genome-fragment | | 2.9 | 0 |
| S12-final-viral-combined_k141_21678\|\|full | 1411 | No | NA | | 0 | 2 | Low-quality | Genome-fragment | | 0.83 | 0 |
| S1-final-viral-combined_k141_131364\|\|full | 3594 | No | NA | | 0 | 2 | Not-determined | Genome-fragment | NA | | 0 |
| S12-final-viral-combined_k141_276744\|\|full | 1411 | No | NA | | 0 | 0 | Low-quality | Genome-fragment | | 2.59 | 0 |
| S1-final-viral-combined_k141_229228\|\|full | 1698 | No | NA | | 0 | 1 | Not-determined | Genome-fragment | NA | | 0 |
| S1-final-viral-combined_k141_328545\|\|full | 1454 | No | NA | | 0 | 1 | Not-determined | Genome-fragment | NA | | 0 |
| S1-final-viral-combined_k141_64440\|\|full | 2187 | No | NA | | 0 | 3 | Not-determined | Genome-fragment | NA | | 0 |
| S1-final-viral-combined_k141_178932\|\|full | 4991 | No | NA | | 0 | 4 | Not-determined | Genome-fragment | NA | | 0 |
| S12-final-viral-combined_k141_46389\|\|full | 1411 | No | NA | | 0 | 0 | Low-quality | Genome-fragment | | 1.71 | 0 |
| S1-final-viral-combined_k141_213445\|\|full | 1736 | No | NA | | 0 | 1 | Not-determined | Genome-fragment | NA | | 0 |
| S1-final-viral-combined_k141_15256\|\|full | 1758 | No | NA | | 0 | 2 | Not-determined | Genome-fragment | NA | | 0 |
| S1-final-viral-combined_k141_377333\|\|full | 2265 | No | NA | | 0 | 2 | Not-determined | Genome-fragment | NA | | 0 |
| S12_k141_396602 | 1411 | No | NA | | 0 | 1 | Low-quality | Genome-fragment | | 2.4 | 0 |
| S1-final-viral-combined_k141_112235\|\|full | 1577 | No | NA | | 0 | 1 | Not-determined | Genome-fragment | NA | | 0 |
| S2-final-viral-combined_k141_121736\|\|full | 1411 | No | NA | | 0 | 0 | Low-quality | Genome-fragment | | 1.65 | 0 |
| S1-final-viral-combined_k141_361494\|\|full | 3565 | No | NA | | 0 | 3 | Not-determined | Genome-fragment | NA | | 0 |
| S2-final-viral-combined_k141_9760\|\|full | 1411 | No | NA | | 0 | 1 | Low-quality | Genome-fragment | | 1.84 | 0 |
| S1-final-viral-combined_k141_229306\|\|full | 2127 | No | NA | | 0 | 0 | Not-determined | Genome-fragment | NA | | 0 |
| S1-final-viral-combined_k141_328594\|\|full | 1584 | No | NA | | 0 | 1 | Not-determined | Genome-fragment | NA | | 0 |
| S1-final-viral-combined_k141_246266\|\|full | 1756 | No | NA | | 0 | 2 | Not-determined | Genome-fragment | NA | | 0 |
| S1-final-viral-combined_k141_178979\|\|full | 3064 | No | NA | | 0 | 2 | Not-determined | Genome-fragment | NA | | 0 |
| S1-final-viral-combined_k141_263231\|\|full | 9264 | No | NA | | 0 | 3 | Not-determined | Genome-fragment | NA | | 0 |
| S1-final-viral-combined_k141_97684\|\|full | 1982 | No | NA | | 0 | 1 | Not-determined | Genome-fragment | NA | | 0 |
| S1-final-viral-combined_k141_393095\|\|full | 2552 | No | NA | | 0 | 1 | Not-determined | Genome-fragment | NA | | 0 |
| S1-final-viral-combined_k141_213472\|\|full | 1728 | No | NA | | 0 | 1 | Not-determined | Genome-fragment | NA | | 0 |
| S1-final-viral-combined_k141_31166\|\|full | 1633 | No | NA | | 0 | 0 | Not-determined | Genome-fragment | NA | | 0 |
| S2-final-viral-combined_k141_33223\|\|lt2gene | 1411 | No | NA | | 1 | 0 | Low-quality | Genome-fragment | | 4.78 | 0 |
| S4_k141_157726 | 1411 | No | NA | | 0 | 0 | Low-quality | Genome-fragment | | 2.17 | 0 |
| S1-final-viral-combined_k141_31177\|\|full | 1327 | No | NA | | 0 | 1 | Not-determined | Genome-fragment | NA | | 0 |
| S1-final-viral-combined_k141_229344\|\|full | 5530 | No | NA | | 0 | 2 | Not-determined | Genome-fragment | NA | | 0 |
| S9-final-viral-combined_k141_45440\|\|full | 1412 | No | NA | | 0 | 1 | Low-quality | Genome-fragment | | 2.93 | 0 |
| S11-final-viral-combined_k141_263877\|\|full | 1412 | No | NA | | 0 | 1 | Low-quality | Genome-fragment | | 2.37 | 0 |
| S11-final-viral-combined_k141_313222\|\|full | 1412 | No | NA | | 0 | 1 | Low-quality | Genome-fragment | | 1.92 | 0 |
| S11-final-viral-combined_k141_247574\|\|full | 1412 | No | NA | | 0 | 0 | Low-quality | Genome-fragment | | 2.19 | 0 |
| S1-final-viral-combined_k141_163070\|\|full | 6143 | No | NA | | 0 | 0 | Not-determined | Genome-fragment | NA | | 0 |
| S11-final-viral-combined_k141_48295\|\|lt2gene | 1412 | No | NA | | 1 | 0 | Low-quality | Genome-fragment | | 2.47 | 0 |
| S1-final-viral-combined_k141_112310\|\|full | 1788 | No | NA | | 0 | 2 | Not-determined | Genome-fragment | NA | | 0 |
| S1-final-viral-combined_k141_64598\|\|full | 4833 | No | NA | | 0 | 1 | Not-determined | Genome-fragment | NA | | 0 |
| S5-final-viral-combined_k141_10916\|\|full | 1412 | No | NA | | 2 | 0 | Low-quality | Genome-fragment | | 3.86 | 0 |
| S7-final-viral-combined_k141_5962\|\|lt2gene | 1412 | No | NA | | 1 | 0 | Low-quality | Genome-fragment | | 0.81 | 0 |
| S17-final-viral-combined_k141_263632\|\|lt2gene | 1412 | No | NA | | 1 | 0 | Low-quality | Genome-fragment | | 0.91 | 0 |
| S1-final-viral-combined_k141_64632\|\|full | 1708 | No | NA | | 0 | 0 | Not-determined | Genome-fragment | NA | | 0 |
| S12-final-viral-combined_k141_228435\|\|full | 1412 | No | NA | | 2 | 0 | Low-quality | Genome-fragment | | 2.26 | 0 |
| S12-final-viral-combined_k141_283526\|\|full | 1412 | No | NA | | 4 | 0 | Low-quality | Genome-fragment | | 3.97 | 0 |
| S1-final-viral-combined_k141_64646\|\|full | 2442 | No | NA | | 0 | 2 | Not-determined | Genome-fragment | NA | | 0 |
| S4-final-viral-combined_k141_121890\|\|lt2gene | 1412 | No | NA | | 1 | 0 | Low-quality | Genome-fragment | | 3.11 | 0 |
| S1-final-viral-combined_k141_312273\|\|full | 4589 | No | NA | | 0 | 3 | Not-determined | Genome-fragment | NA | | 0 |
| S1-final-viral-combined_k141_47220\|\|full | 3629 | No | NA | | 0 | 2 | Not-determined | Genome-fragment | NA | | 0 |
| S1-final-viral-combined_k141_179105\|\|full | 2751 | No | NA | | 0 | 1 | Not-determined | Genome-fragment | NA | | 0 |
| S1-final-viral-combined_k141_377516\|\|full | 4950 | No | NA | | 0 | 4 | Not-determined | Genome-fragment | NA | | 0 |
| S4_k141_116765 | 1412 | No | NA | | 0 | 0 | Low-quality | Genome-fragment | | 1.93 | 0 |
| S9-final-viral-combined_k141_607\|\|full | 1413 | No | NA | | 1 | 0 | Low-quality | Genome-fragment | | 0.84 | 0 |
| S11-final-viral-combined_k141_126391\|\|full | 1413 | No | NA | | 1 | 0 | Low-quality | Genome-fragment | | 1.93 | 0 |
| S11-final-viral-combined_k141_45066\|\|full | 1413 | No | NA | | 0 | 0 | Low-quality | Genome-fragment | | 2.99 | 0 |
| S11-final-viral-combined_k141_87218\|\|full | 1413 | No | NA | | 0 | 0 | Low-quality | Genome-fragment | | 1.13 | 0 |
| S1-final-viral-combined_k141_393186\|\|full | 1084 | No | NA | | 0 | 0 | Not-determined | Genome-fragment | NA | | 0 |
| S11-final-viral-combined_k141_10579\|\|full | 1413 | No | NA | | 0 | 0 | Low-quality | Genome-fragment | | 0.99 | 0 |
| S11_k141_217532 | 1413 | No | NA | | 1 | 0 | Low-quality | Genome-fragment | | 33.95 | 0 |
| S1-final-viral-combined_k141_47266\|\|full | 1802 | No | NA | | 0 | 0 | Not-determined | Genome-fragment | NA | | 0 |
| S1-final-viral-combined_k141_264703\|\|full | 1413 | No | NA | | 0 | 0 | Low-quality | Genome-fragment | | 3.93 | 0 |
| S1-final-viral-combined_k141_213652\|\|full | 1610 | No | NA | | 0 | 2 | Not-determined | Genome-fragment | NA | | 0 |
| S1-final-viral-combined_k141_156495\|\|full | 1413 | No | NA | | 0 | 0 | Low-quality | Genome-fragment | | 1.04 | 0 |
| S1-final-viral-combined_k141_97901\|\|full | 3768 | No | NA | | 0 | 2 | Not-determined | Genome-fragment | NA | | 0 |
| S5-final-viral-combined_k141_43177\|\|full | 1413 | No | NA | | 1 | 0 | Low-quality | Genome-fragment | | 3.2 | 0 |
| S1-final-viral-combined_k141_328849\|\|full | 5550 | No | NA | | 0 | 3 | Not-determined | Genome-fragment | NA | | 0 |
| S7_k141_33379 | 1413 | No | NA | | 1 | 0 | Low-quality | Genome-fragment | | 3.36 | 0 |
| S1-final-viral-combined_k141_163172\|\|full | 3059 | No | NA | | 0 | 1 | Not-determined | Genome-fragment | NA | | 0 |
| S17-final-viral-combined_k141_165682\|\|full | 1413 | No | NA | | 0 | 2 | Low-quality | Genome-fragment | | 0.93 | 0 |
| S12_k141_159583 | 1413 | No | NA | | 0 | 0 | Low-quality | Genome-fragment | | 0.65 | 0 |
| S1-final-viral-combined_k141_295836\|\|full | 9231 | No | NA | | 0 | 7 | Not-determined | Genome-fragment | NA | | 0 |
| S12_k141_384040 | 1413 | No | NA | | 0 | 1 | Low-quality | Genome-fragment | | 2.49 | 0 |
| S1-final-viral-combined_k141_15568\|\|full | 5924 | No | NA | | 0 | 4 | Not-determined | Genome-fragment | NA | | 0 |
| S1-final-viral-combined_k141_147093\|\|full | 5411 | No | NA | | 0 | 1 | Not-determined | Genome-fragment | NA | | 0 |
| S2-final-viral-combined_k141_190714\|\|full | 1413 | Yes | | 861 | 1 | 1 | Low-quality | Genome-fragment | | 0.35 | 39.07 |
| S1-final-viral-combined_k141_343723\|\|full | 3737 | No | NA | | 0 | 3 | Not-determined | Genome-fragment | NA | | 0 |
| S1-final-viral-combined_k141_179302\|\|full | 1280 | No | NA | | 0 | 1 | Not-determined | Genome-fragment | NA | | 0 |
| S1-final-viral-combined_k141_131545\|\|full | 3674 | No | NA | | 0 | 4 | Not-determined | Genome-fragment | NA | | 0 |
| S1-final-viral-combined_k141_15606\|\|full | 3910 | No | NA | | 0 | 2 | Not-determined | Genome-fragment | NA | | 0 |
| S4-final-viral-combined_k141_45389\|\|full | 1413 | No | NA | | 2 | 0 | Low-quality | Genome-fragment | | 2.64 | 0 |
| S1-final-viral-combined_k141_278337\|\|full | 1813 | No | NA | | 0 | 1 | Not-determined | Genome-fragment | NA | | 0 |
| S4-final-viral-combined_k141_40460\|\|full | 1413 | No | NA | | 0 | 0 | Low-quality | Genome-fragment | | 3.46 | 0 |
| S11-final-viral-combined_k141_223232\|\|full | 1414 | No | NA | | 1 | 0 | Low-quality | Genome-fragment | | 2.24 | 0 |
| S1-final-viral-combined_k141_163265\|\|full | 3138 | No | NA | | 0 | 3 | Not-determined | Genome-fragment | NA | | 0 |
| S1-final-viral-combined_k141_98058\|\|full | 4343 | No | NA | | 0 | 5 | Not-determined | Genome-fragment | NA | | 0 |
| S11-final-viral-combined_k141_294429\|\|full | 1414 | No | NA | | 0 | 1 | Low-quality | Genome-fragment | | 2.44 | 0 |
| S1-final-viral-combined_k141_64844\|\|full | 1754 | No | NA | | 0 | 2 | Not-determined | Genome-fragment | NA | | 0 |
| S11_k141_263031 | 1414 | No | NA | | 1 | 0 | Low-quality | Genome-fragment | | 2.52 | 0 |
| S1-final-viral-combined_k141_39170\|\|full | 1414 | No | NA | | 0 | 1 | Low-quality | Genome-fragment | | 2.57 | 0 |
| S1-final-viral-combined_k141_80424\|\|full | 1240 | No | NA | | 0 | 1 | Not-determined | Genome-fragment | NA | | 0 |
| S1-final-viral-combined_k141_278405\|\|full | 14224 | No | NA | | 0 | 11 | Not-determined | Genome-fragment | NA | | 0 |
| S1-final-viral-combined_k141_312504\|\|full | 7191 | No | NA | | 0 | 0 | Not-determined | Genome-fragment | NA | | 0 |
| S5-final-viral-combined_k141_113617\|\|full | 1414 | No | NA | | 2 | 0 | Low-quality | Genome-fragment | | 3.55 | 0 |
| S17-final-viral-combined_k141_141200\|\|full | 1414 | No | NA | | 0 | 0 | Low-quality | Genome-fragment | | 0.78 | 0 |
| S17-final-viral-combined_k141_84342\|\|lt2gene | 1414 | No | NA | | 1 | 0 | Low-quality | Genome-fragment | | 0.87 | 0 |
| S1-final-viral-combined_k141_312515\|\|full | 1665 | No | NA | | 0 | 2 | Not-determined | Genome-fragment | NA | | 0 |
| S1-final-viral-combined_k141_197351\|\|full | 1492 | No | NA | | 0 | 1 | Not-determined | Genome-fragment | NA | | 0 |
| S1-final-viral-combined_k141_361735\|\|full | 2477 | No | NA | | 0 | 0 | Not-determined | Genome-fragment | NA | | 0 |
| S17-k141_222205 | 1414 | No | NA | | 0 | 1 | Low-quality | Genome-fragment | | 2.47 | 0 |
| S2_k141_64847 | 1414 | No | NA | | 0 | 0 | Low-quality | Genome-fragment | | 0.76 | 0 |
| S4-final-viral-combined_k141_151552\|\|full | 1414 | No | NA | | 1 | 0 | Low-quality | Genome-fragment | | 2.13 | 0 |
| S1-final-viral-combined_k141_31355\|\|full | 2282 | No | NA | | 0 | 1 | Not-determined | Genome-fragment | NA | | 0 |
| S1-final-viral-combined_k141_361752\|\|full | 6111 | No | NA | | 0 | 0 | Not-determined | Genome-fragment | NA | | 0 |
| S8-final-viral-combined_k141_211075\|\|full | 1414 | No | NA | | 0 | 2 | Low-quality | Genome-fragment | | 2.17 | 0 |
| S11_k141_124461 | 1415 | No | NA | | 0 | 0 | Low-quality | Genome-fragment | | 1.5 | 0 |
| S1-final-viral-combined_k141_64942\|\|full | 2878 | No | NA | | 0 | 3 | Not-determined | Genome-fragment | NA | | 0 |
| S1-final-viral-combined_k141_229531\|\|full | 1339 | No | NA | | 0 | 1 | Not-determined | Genome-fragment | NA | | 0 |
| S1-final-viral-combined_k141_197382\|\|full | 2266 | No | NA | | 0 | 0 | Not-determined | Genome-fragment | NA | | 0 |

| | | | | | | | | | |
|---|---|---|---|---|---|---|---|---|---|
| S1-final-viral-combined_k141_31407\|\|full | 1428 | No | NA | 0 | 1 | Not-determined | Genome-fragment | NA | 0 |
| S1-final-viral-combined_k141_15714\|\|full | 1355 | No | NA | 0 | 3 | Not-determined | Genome-fragment | NA | 0 |
| S1-final-viral-combined_k141_181844\|\|lt2gene | 1415 | No | NA | 0 | 0 | Low-quality | Genome-fragment | 2.08 | 0 |
| S1-final-viral-combined_k141_64969\|\|full | 6637 | No | NA | 0 | 3 | Not-determined | Genome-fragment | NA | 0 |
| S5_k141_79694 | 1415 | No | NA | 0 | 0 | Low-quality | Genome-fragment | 5.63 | 0 |
| S1-final-viral-combined_k141_343831\|\|full | 10144 | No | NA | 0 | 6 | Not-determined | Genome-fragment | NA | 0 |
| S7-final-viral-combined_k141_155719\|\|full | 1415 | No | NA | 1 | 0 | Low-quality | Genome-fragment | 3.43 | 0 |
| S17-final-viral-combined_k141_188874\|\|lt2gene | 1415 | No | NA | 0 | 0 | Low-quality | Genome-fragment | 3.45 | 0 |
| S1-final-viral-combined_k141_377814\|\|full | 1699 | No | NA | 0 | 0 | Not-determined | Genome-fragment | NA | 0 |
| S1-final-viral-combined_k141_163363\|\|full | 1845 | No | NA | 0 | 0 | Not-determined | Genome-fragment | NA | 0 |
| S12-final-viral-combined_k141_51066\|\|full | 1415 | No | NA | 1 | 0 | Low-quality | Genome-fragment | 4.16 | 0 |
| S1-final-viral-combined_k141_197401\|\|full | 53457 | No | NA | 4 | 4 | Medium-quality | Genome-fragment | 76.98 | 0 |
| S12-final-viral-combined_k141_34755\|\|full | 1415 | No | NA | 0 | 2 | Low-quality | Genome-fragment | 1.33 | 0 |
| S2-final-viral-combined_k141_197665\|\|full | 1415 | No | NA | 0 | 0 | Low-quality | Genome-fragment | 2.44 | 0 |
| S1-final-viral-combined_k141_296047\|\|full | 2583 | No | NA | 0 | 0 | Not-determined | Genome-fragment | NA | 0 |
| S2-final-viral-combined_k141_127633\|\|full | 1415 | No | NA | 2 | 0 | Low-quality | Genome-fragment | 3.23 | 0 |
| S1-final-viral-combined_k141_179395\|\|full | 1544 | No | NA | 0 | 1 | Not-determined | Genome-fragment | NA | 0 |
| S4_k141_130909 | 1415 | No | NA | 0 | 0 | Low-quality | Genome-fragment | 3.59 | 0 |
| S1-final-viral-combined_k141_163388\|\|full | 2196 | No | NA | 0 | 2 | Not-determined | Genome-fragment | NA | 0 |
| S1-final-viral-combined_k141_361829\|\|full | 6103 | No | NA | 0 | 0 | Not-determined | Genome-fragment | NA | 0 |
| S9-final-viral-combined_k141_18144\|\|full | 1416 | No | NA | 0 | 0 | Low-quality | Genome-fragment | 2.77 | 0 |
| S11-final-viral-combined_k141_321652\|\|full | 1416 | No | NA | 0 | 1 | Low-quality | Genome-fragment | 1.65 | 0 |
| S11-final-viral-combined_k141_227464\|\|full | 1416 | No | NA | 1 | 0 | Low-quality | Genome-fragment | 2.38 | 0 |
| S1-final-viral-combined_k141_163434\|\|full | 1415 | No | NA | 0 | 1 | Not-determined | Genome-fragment | NA | 0 |
| S1-final-viral-combined_k141_147332\|\|full | 1690 | No | NA | 0 | 1 | Not-determined | Genome-fragment | NA | 0 |
| S1_k141_197509 | 1416 | No | NA | 0 | 1 | Low-quality | Genome-fragment | 2.39 | 0 |
| S3-final-viral-combined_k141_59119\|\|full | 1416 | No | NA | 0 | 0 | Low-quality | Genome-fragment | 1.13 | 0 |
| S1-final-viral-combined_k141_15827\|\|full | 2315 | No | NA | 0 | 1 | Not-determined | Genome-fragment | NA | 0 |
| S1-final-viral-combined_k141_377904\|\|full | 1493 | No | NA | 0 | 1 | Not-determined | Genome-fragment | NA | 0 |
| S1-final-viral-combined_k141_47549\|\|full | 1457 | No | NA | 0 | 0 | Not-determined | Genome-fragment | NA | 0 |
| S12-final-viral-combined_k141_136425\|\|full | 1416 | No | NA | 0 | 1 | Low-quality | Genome-fragment | 1.22 | 0 |
| S4_k141_99721 | 1416 | No | NA | 0 | 2 | Low-quality | Genome-fragment | 0.34 | 0 |
| S6-final-viral-combined_k141_136388\|\|full | 1416 | No | NA | 0 | 1 | Low-quality | Genome-fragment | 3.02 | 0 |
| S1-final-viral-combined_k141_278676\|\|full | 4967 | No | NA | 0 | 0 | Not-determined | Genome-fragment | NA | 0 |
| S8_k141_228984 | 1416 | No | NA | 0 | 0 | Low-quality | Genome-fragment | 2.69 | 0 |
| S1-final-viral-combined_k141_361884\|\|full | 5842 | No | NA | 0 | 2 | Not-determined | Genome-fragment | NA | 0 |
| S1-final-viral-combined_k141_31461\|\|full | 6984 | No | NA | 0 | 5 | Not-determined | Genome-fragment | NA | 0 |
| S11-final-viral-combined_k141_240644\|\|lt2gene | 1417 | No | NA | 2 | 0 | Low-quality | Genome-fragment | 2.2 | 0 |
| S11_k141_273849 | 1417 | No | NA | 0 | 0 | Low-quality | Genome-fragment | 0.92 | 0 |
| S1-final-viral-combined_k141_19104\|\|full | 1417 | No | NA | 0 | 0 | Low-quality | Genome-fragment | 2.98 | 0 |
| S1-final-viral-combined_k141_19507\|\|full | 1417 | No | NA | 0 | 1 | Low-quality | Genome-fragment | 4.04 | 0 |
| S1-final-viral-combined_k141_112660\|\|full | 2902 | No | NA | 0 | 3 | Not-determined | Genome-fragment | NA | 0 |
| S1-final-viral-combined_k141_163526\|\|full | 2018 | No | NA | 0 | 0 | Not-determined | Genome-fragment | NA | 0 |
| S5-final-viral-combined_k141_155366\|\|full | 1417 | No | NA | 0 | 2 | Low-quality | Genome-fragment | 2.87 | 0 |
| S5-final-viral-combined_k141_24199\|\|full | 1417 | No | NA | 0 | 2 | Low-quality | Genome-fragment | 1.16 | 0 |
| S1-final-viral-combined_k141_31487\|\|full | 2413 | No | NA | 0 | 2 | Not-determined | Genome-fragment | NA | 0 |
| S17-final-viral-combined_k141_230847\|\|full | 1417 | No | NA | 1 | 0 | Low-quality | Genome-fragment | 2.1 | 0 |
| S2-final-viral-combined_k141_115757\|\|full | 1417 | No | NA | 0 | 1 | Low-quality | Genome-fragment | 3.09 | 0 |
| S11-final-viral-combined_k141_138732\|\|lt2gene | 1418 | No | NA | 0 | 0 | Low-quality | Genome-fragment | 3 | 0 |
| S1-final-viral-combined_k141_15947\|\|full | 1432 | No | NA | 0 | 0 | Not-determined | Genome-fragment | NA | 0 |
| S11_k141_257500 | 1418 | No | NA | 0 | 1 | Low-quality | Genome-fragment | 0.4 | 0 |
| S1-final-viral-combined_k141_62595\|\|full | 1418 | No | NA | 0 | 0 | Low-quality | Genome-fragment | 3.26 | 0 |
| S1-final-viral-combined_k141_278812\|\|full | 5625 | No | NA | 0 | 2 | Not-determined | Genome-fragment | NA | 0 |
| S1-final-viral-combined_k141_378018\|\|full | 2935 | No | NA | 0 | 3 | Not-determined | Genome-fragment | NA | 0 |
| S1-final-viral-combined_k141_246683\|\|full | 3237 | No | NA | 0 | 0 | Not-determined | Genome-fragment | NA | 0 |
| S3-final-viral-combined_k141_100413\|\|lt2gene | 1418 | No | NA | 1 | 0 | Low-quality | Genome-fragment | 3.21 | 0 |
| S1-final-viral-combined_k141_278831\|\|full | 2926 | No | NA | 0 | 1 | Not-determined | Genome-fragment | NA | 0 |
| S17-final-viral-combined_k141_201383\|\|full | 1418 | No | NA | 0 | 1 | Low-quality | Genome-fragment | 0.54 | 0 |
| S1-final-viral-combined_k141_246694\|\|full | 10495 | No | NA | 0 | 5 | Not-determined | Genome-fragment | NA | 0 |
| S1-final-viral-combined_k141_163625\|\|full | 1814 | No | NA | 0 | 1 | Not-determined | Genome-fragment | NA | 0 |
| S1-final-viral-combined_k141_393651\|\|full | 3295 | No | NA | 0 | 2 | Not-determined | Genome-fragment | NA | 0 |
| S1-final-viral-combined_k141_47745\|\|full | 1241 | No | NA | 0 | 2 | Not-determined | Genome-fragment | NA | 0 |
| S1-final-viral-combined_k141_112803\|\|full | 2216 | No | NA | 0 | 0 | Not-determined | Genome-fragment | NA | 0 |
| S1-final-viral-combined_k141_147553\|\|full | 2070 | No | NA | 0 | 2 | Not-determined | Genome-fragment | NA | 0 |
| S17-final-viral-combined_k141_179834\|\|full | 1418 | No | NA | 0 | 0 | Low-quality | Genome-fragment | 1.4 | 0 |
| S1-final-viral-combined_k141_147557\|\|full | 1515 | No | NA | 0 | 1 | Not-determined | Genome-fragment | NA | 0 |
| S1-final-viral-combined_k141_344130\|\|full | 4503 | No | NA | 0 | 2 | Not-determined | Genome-fragment | NA | 0 |
| S17-k141_21015 | 1418 | No | NA | 0 | 0 | Low-quality | Genome-fragment | 2.54 | 0 |
| S1-final-viral-combined_k141_112833\|\|full | 3460 | No | NA | 0 | 5 | Not-determined | Genome-fragment | NA | 0 |
| S1-final-viral-combined_k141_278877\|\|full | 1488 | No | NA | 0 | 0 | Not-determined | Genome-fragment | NA | 0 |
| S1-final-viral-combined_k141_163677\|\|full | 1944 | No | NA | 0 | 1 | Not-determined | Genome-fragment | NA | 0 |
| S1-final-viral-combined_k141_229765\|\|full | 1745 | No | NA | 0 | 2 | Not-determined | Genome-fragment | NA | 0 |
| S12-final-viral-combined_k141_355952\|\|full | 1418 | No | NA | 0 | 1 | Low-quality | Genome-fragment | 0.72 | 0 |
| S1-final-viral-combined_k141_393680\|\|full | 2520 | No | NA | 0 | 3 | Not-determined | Genome-fragment | NA | 0 |
| S12-final-viral-combined_k141_164783\|\|full | 1418 | No | NA | 0 | 2 | Low-quality | Genome-fragment | 0.77 | 0 |
| S1-final-viral-combined_k141_393733\|\|full | 1566 | No | NA | 0 | 1 | Not-determined | Genome-fragment | NA | 0 |
| S12_k141_153770 | 1418 | No | NA | 0 | 1 | Low-quality | Genome-fragment | 1.01 | 0 |
| S2_k141_150337 | 1418 | No | NA | 0 | 2 | Low-quality | Genome-fragment | 2.8 | 0 |
| S1-final-viral-combined_k141_112909\|\|full | 8288 | No | NA | 0 | 1 | Not-determined | Genome-fragment | NA | 0 |
| S4_k141_134795 | 1418 | No | NA | 0 | 0 | Low-quality | Genome-fragment | 3.9 | 0 |
| S4_k141_3573 | 1418 | No | NA | 0 | 0 | Low-quality | Genome-fragment | 3.66 | 0 |
| S4_k141_66216 | 1418 | No | NA | 0 | 1 | Low-quality | Genome-fragment | 1.72 | 0 |
| S1-final-viral-combined_k141_65340\|\|full | 2116 | No | NA | 0 | 2 | Not-determined | Genome-fragment | NA | 0 |
| S1-final-viral-combined_k141_112929\|\|full | 1558 | No | NA | 0 | 1 | Not-determined | Genome-fragment | NA | 0 |
| S1-final-viral-combined_k141_229859\|\|full | 2572 | No | NA | 0 | 2 | Not-determined | Genome-fragment | NA | 0 |
| S9-final-viral-combined_k141_116329\|\|full | 1419 | No | NA | 0 | 1 | Low-quality | Genome-fragment | 2.26 | 0 |
| S9_k141_106044 | 1419 | No | NA | 0 | 1 | Low-quality | Genome-fragment | 3.28 | 0 |
| S11-final-viral-combined_k141_65485\|\|full | 1419 | No | NA | 0 | 1 | Low-quality | Genome-fragment | 1.08 | 0 |
| S1-final-viral-combined_k141_16114\|\|full | 1347 | No | NA | 0 | 1 | Not-determined | Genome-fragment | NA | 0 |
| S1-final-viral-combined_k141_344339\|\|full | 1223 | No | NA | 0 | 0 | Not-determined | Genome-fragment | NA | 0 |
| S1-final-viral-combined_k141_112967\|\|full | 2238 | No | NA | 0 | 4 | Not-determined | Genome-fragment | NA | 0 |
| S5-final-viral-combined_k141_29255\|\|full | 1419 | No | NA | 2 | 0 | Low-quality | Genome-fragment | 2.34 | 0 |
| S1-final-viral-combined_k141_47967\|\|full | 1727 | No | NA | 0 | 0 | Not-determined | Genome-fragment | NA | 0 |
| S1-final-viral-combined_k141_16175\|\|full | 3217 | No | NA | 0 | 2 | Not-determined | Genome-fragment | NA | 0 |
| S17-final-viral-combined_k141_27209\|\|full | 1419 | No | NA | 0 | 0 | Low-quality | Genome-fragment | 0.69 | 0 |
| S17-k141_258737 | 1419 | No | NA | 1 | 0 | Low-quality | Genome-fragment | 1.87 | 0 |
| S1-final-viral-combined_k141_393855\|\|full | 5875 | No | NA | 0 | 2 | Not-determined | Genome-fragment | NA | 0 |
| S6-final-viral-combined_k141_65946\|\|full | 1419 | No | NA | 0 | 0 | Low-quality | Genome-fragment | 2.79 | 0 |
| S6-final-viral-combined_k141_104907\|\|full | 1419 | No | NA | 2 | 0 | Low-quality | Genome-fragment | 3.11 | 0 |
| S1-final-viral-combined_k141_81266\|\|full | 3600 | No | NA | 0 | 2 | Not-determined | Genome-fragment | NA | 0 |
| S1-final-viral-combined_k141_393889\|\|full | 3128 | No | NA | 0 | 2 | Not-determined | Genome-fragment | NA | 0 |
| S9-final-viral-combined_k141_121686\|\|full | 1420 | No | NA | 2 | 0 | Low-quality | Genome-fragment | 3.02 | 0 |
| S1-final-viral-combined_k141_230006\|\|full | 2257 | No | NA | 0 | 1 | Not-determined | Genome-fragment | NA | 0 |
| S11-final-viral-combined_k141_83043\|\|full | 1420 | No | NA | 2 | 0 | Low-quality | Genome-fragment | 3.06 | 0 |
| S1-final-viral-combined_k141_393911\|\|full | 3184 | No | NA | 0 | 2 | Not-determined | Genome-fragment | NA | 0 |
| S11-final-viral-combined_k141_79313\|\|full | 1420 | No | NA | 0 | 0 | Low-quality | Genome-fragment | 1.26 | 0 |
| S1-final-viral-combined_k141_300391\|\|full | 1420 | No | NA | 1 | 0 | Low-quality | Genome-fragment | 2.93 | 0 |
| S1-final-viral-combined_k141_98643\|\|full | 5861 | No | NA | 0 | 1 | Not-determined | Genome-fragment | NA | 0 |
| S1-final-viral-combined_k141_356848\|\|full | 1420 | No | NA | 0 | 1 | Low-quality | Genome-fragment | 2.4 | 0 |
| S1-final-viral-combined_k141_53379\|\|lt2gene | 1420 | No | NA | 2 | 0 | Low-quality | Genome-fragment | 3.76 | 0 |
| S7-final-viral-combined_k141_177496\|\|full | 1420 | No | NA | 1 | 0 | Low-quality | Genome-fragment | 2.19 | 0 |
| S1-final-viral-combined_k141_344487\|\|full | 2801 | No | NA | 0 | 1 | Not-determined | Genome-fragment | NA | 0 |
| S1-final-viral-combined_k141_98669\|\|full | 1048 | No | NA | 0 | 0 | Not-determined | Genome-fragment | NA | 0 |
| S7_k141_59063 | 1420 | No | NA | 0 | 0 | Low-quality | Genome-fragment | 3.45 | 0 |
| S17-final-viral-combined_k141_38196\|\|full | 1420 | No | NA | 0 | 0 | Low-quality | Genome-fragment | 4.01 | 0 |
| S1-final-viral-combined_k141_344538\|\|full | 5812 | No | NA | 0 | 2 | Not-determined | Genome-fragment | NA | 0 |
| S1-final-viral-combined_k141_65577\|\|full | 2304 | No | NA | 0 | 2 | Not-determined | Genome-fragment | NA | 0 |
| S1-final-viral-combined_k141_230112\|\|full | 2050 | No | NA | 0 | 1 | Not-determined | Genome-fragment | NA | 0 |
| S1-final-viral-combined_k141_98682\|\|full | 1334 | No | NA | 0 | 0 | Not-determined | Genome-fragment | NA | 0 |
| S1-final-viral-combined_k141_344559\|\|full | 10060 | No | NA | 0 | 1 | Not-determined | Genome-fragment | NA | 0 |
| S1-final-viral-combined_k141_394044\|\|full | 1624 | No | NA | 0 | 1 | Not-determined | Genome-fragment | NA | 0 |
| S2_k141_89273 | 1420 | No | NA | 0 | 0 | Low-quality | Genome-fragment | 2.83 | 0 |
| S1-final-viral-combined_k141_147807\|\|full | 6130 | No | NA | 0 | 5 | Not-determined | Genome-fragment | NA | 0 |
| S1-final-viral-combined_k141_16338\|\|full | 5343 | No | NA | 0 | 3 | Not-determined | Genome-fragment | NA | 0 |
| S1-final-viral-combined_k141_16340\|\|full | 1439 | No | NA | 0 | 0 | Not-determined | Genome-fragment | NA | 0 |
| S1-final-viral-combined_k141_65587\|\|full | 1537 | No | NA | 0 | 1 | Not-determined | Genome-fragment | NA | 0 |
| S8-final-viral-combined_k141_250821\|\|full | 1420 | No | NA | 0 | 1 | Low-quality | Genome-fragment | 3.98 | 0 |
| S1-final-viral-combined_k141_31984\|\|full | 1592 | No | NA | 0 | 1 | Not-determined | Genome-fragment | NA | 0 |
| S1-final-viral-combined_k141_16347\|\|full | 2347 | No | NA | 0 | 0 | Not-determined | Genome-fragment | NA | 0 |
| S11-final-viral-combined_k141_117611\|\|full | 1421 | No | NA | 1 | 0 | Low-quality | Genome-fragment | 3.1 | 0 |
| S1-final-viral-combined_k141_230199\|\|full | 4661 | No | NA | 0 | 4 | Not-determined | Genome-fragment | NA | 0 |
| S1-final-viral-combined_k141_279260\|\|full | 1230 | No | NA | 0 | 0 | Not-determined | Genome-fragment | NA | 0 |
| S1-final-viral-combined_k141_16388\|\|full | 9905 | No | NA | 0 | 4 | Not-determined | Genome-fragment | NA | 0 |
| S1-final-viral-combined_k141_180198\|\|full | 1850 | No | NA | 0 | 1 | Not-determined | Genome-fragment | NA | 0 |
| S1-final-viral-combined_k141_279287\|\|full | 1062 | No | NA | 0 | 0 | Not-determined | Genome-fragment | NA | 0 |
| S11-final-viral-combined_k141_160367\|\|full | 1421 | No | NA | 1 | 0 | Low-quality | Genome-fragment | 3.97 | 0 |
| S1-final-viral-combined_k141_164133\|\|full | 1232 | No | NA | 0 | 0 | Not-determined | Genome-fragment | NA | 0 |
| S1-final-viral-combined_k141_81501\|\|full | 1355 | No | NA | 0 | 0 | Not-determined | Genome-fragment | NA | 0 |
| S1-final-viral-combined_k141_180265\|\|full | 6112 | No | NA | 0 | 5 | Not-determined | Genome-fragment | NA | 0 |
| S1-final-viral-combined_k141_81544\|\|full | 3598 | No | NA | 0 | 1 | Not-determined | Genome-fragment | NA | 0 |
| S11-final-viral-combined_k141_151217\|\|full | 1421 | No | NA | 0 | 2 | Low-quality | Genome-fragment | 4.43 | 0 |
| S1-final-viral-combined_k141_150725\|\|full | 1421 | No | NA | 2 | 0 | Low-quality | Genome-fragment | 2.53 | 0 |
| S1-final-viral-combined_k141_32084\|\|full | 1598 | No | NA | 0 | 2 | Not-determined | Genome-fragment | NA | 0 |
| S1-final-viral-combined_k141_232700\|\|lt2gene | 1421 | No | NA | 1 | 0 | Low-quality | Genome-fragment | 3.38 | 0 |
| S1-final-viral-combined_k141_164218\|\|full | 4350 | No | NA | 0 | 5 | Not-determined | Genome-fragment | NA | 0 |
| S5-final-viral-combined_k141_32249\|\|lt2gene | 1421 | No | NA | 1 | 0 | Low-quality | Genome-fragment | 0.76 | 0 |
| S5-final-viral-combined_k141_211676\|\|lt2gene | 1421 | Yes | 767 | 1 | 1 | Low-quality | Genome-fragment | 1.97 | 46.02 |
| S1-final-viral-combined_k141_32158\|\|full | 2006 | No | NA | 0 | 0 | Not-determined | Genome-fragment | NA | 0 |
| S7-final-viral-combined_k141_239085\|\|full | 1421 | No | NA | 2 | 0 | Low-quality | Genome-fragment | 3.32 | 0 |
| S17-final-viral-combined_k141_214846\|\|full | 1421 | No | NA | 0 | 0 | Low-quality | Genome-fragment | 0.9 | 0 |
| S1-final-viral-combined_k141_32191\|\|full | 4281 | No | NA | 0 | 3 | Not-determined | Genome-fragment | NA | 0 |
| S17-final-viral-combined_k141_228804\|\|full | 1421 | No | NA | 0 | 2 | Low-quality | Genome-fragment | 1.14 | 0 |
| S2-final-viral-combined_k141_83679\|\|full | 1421 | No | NA | 0 | 1 | Low-quality | Genome-fragment | 1.93 | 0 |
| S1-final-viral-combined_k141_180399\|\|full | 2259 | No | NA | 0 | 0 | Not-determined | Genome-fragment | NA | 0 |
| S6-final-viral-combined_k141_17667\|\|full | 1421 | No | NA | 2 | 0 | Low-quality | Genome-fragment | 2.88 | 0 |
| S1-final-viral-combined_k141_180418\|\|full | 3326 | No | NA | 0 | 1 | Not-determined | Genome-fragment | NA | 0 |
| S8_k141_15634 | 1421 | No | NA | 0 | 0 | Low-quality | Genome-fragment | 1.41 | 0 |
| S1-final-viral-combined_k141_394327\|\|full | 2698 | No | NA | 0 | 1 | Not-determined | Genome-fragment | NA | 0 |
| S1-final-viral-combined_k141_344889\|\|full | 1003 | No | NA | 0 | 0 | Not-determined | Genome-fragment | NA | 0 |
| S1-final-viral-combined_k141_394332\|\|full | 3514 | No | NA | 0 | 2 | Not-determined | Genome-fragment | NA | 0 |
| S1-final-viral-combined_k141_81739\|\|full | 2093 | No | NA | 0 | 2 | Not-determined | Genome-fragment | NA | 0 |
| S1-final-viral-combined_k141_164401\|\|full | 3099 | No | NA | 0 | 3 | Not-determined | Genome-fragment | NA | 0 |
| S1-final-viral-combined_k141_180468\|\|full | 2632 | No | NA | 0 | 1 | Not-determined | Genome-fragment | NA | 0 |
| S9-final-viral-combined_k141_16364\|\|full | 1422 | No | NA | 0 | 0 | Low-quality | Genome-fragment | 1.38 | 0 |
| S1-final-viral-combined_k141_113633\|\|full | 1532 | No | NA | 0 | 0 | Not-determined | Genome-fragment | NA | 0 |
| S1-final-viral-combined_k141_48389\|\|full | 3839 | No | NA | 0 | 3 | Not-determined | Genome-fragment | NA | 0 |
| S1-final-viral-combined_k141_388921\|\|full | 1422 | No | NA | 1 | 0 | Low-quality | Genome-fragment | 0.96 | 0 |
| S3-final-viral-combined_k141_95249\|\|full | 1422 | No | NA | 1 | 0 | Low-quality | Genome-fragment | 3.5 | 0 |
| S1-final-viral-combined_k141_394391\|\|full | 2595 | No | NA | 0 | 2 | Not-determined | Genome-fragment | NA | 0 |
| S1-final-viral-combined_k141_394396\|\|full | 2809 | No | NA | 0 | 3 | Not-determined | Genome-fragment | NA | 0 |
| S1-final-viral-combined_k141_279492\|\|full | 29085 | No | NA | 15 | 1 | Medium-quality | Genome-fragment | 81.84 | 0 |
| S1-final-viral-combined_k141_81854\|\|full | 1747 | No | NA | 0 | 1 | Not-determined | Genome-fragment | NA | 0 |
| S2-final-viral-combined_k141_57371\|\|full | 1422 | No | NA | 1 | 0 | Low-quality | Genome-fragment | 0.23 | 0 |
| S1-final-viral-combined_k141_345059\|\|full | 8482 | No | NA | 0 | 1 | Not-determined | Genome-fragment | NA | 0 |

| | | | | | | | | | |
|---|---|---|---|---|---|---|---|---|---|
| S1-final-viral-combined_k141_180603\|\|full | 2608 | No | NA | 0 | 0 | Not-determined | Genome-fragment | NA | 0 |
| S2-final-viral-combined_k141_71970\|\|full | 1422 | No | NA | 0 | 1 | Low-quality | Genome-fragment | 1.89 | 0 |
| S1-final-viral-combined_k141_32497\|\|full | 2082 | No | NA | 0 | 1 | Not-determined | Genome-fragment | NA | 0 |
| S1-final-viral-combined_k141_48530\|\|full | 1964 | No | NA | 0 | 0 | Not-determined | Genome-fragment | NA | 0 |
| S4-final-viral-combined_k141_15020\|\|full | 1422 | Yes | 750 | 1 | 1 | Low-quality | Genome-fragment | 1.06 | 47.26 |
| S9_k141_32953 | 1423 | No | NA | 0 | 0 | Low-quality | Genome-fragment | 2.38 | 0 |
| S5-final-viral-combined_k141_129375\|\|full | 1423 | No | NA | 0 | 2 | Low-quality | Genome-fragment | 0.4 | 0 |
| S5-final-viral-combined_k141_104587\|\|full | 1423 | No | NA | 0 | 0 | Low-quality | Genome-fragment | 2.05 | 0 |
| S7_k141_145339 | 1423 | No | NA | 0 | 0 | Low-quality | Genome-fragment | 3.1 | 0 |
| S1-final-viral-combined_k141_32547\|\|full | 1576 | No | NA | 0 | 0 | Not-determined | Genome-fragment | NA | 0 |
| S1-final-viral-combined_k141_345112\|\|full | 1311 | No | NA | 0 | 0 | Not-determined | Genome-fragment | NA | 0 |
| S1-final-viral-combined_k141_164436\|\|full | 5584 | No | NA | 0 | 3 | Not-determined | Genome-fragment | NA | 0 |
| S1-final-viral-combined_k141_164439\|\|full | 2106 | No | NA | 0 | 1 | Not-determined | Genome-fragment | NA | 0 |
| S17-final-viral-combined_k141_84431\|\|full | 1423 | No | NA | 2 | 0 | Low-quality | Genome-fragment | 3.52 | 0 |
| S1-final-viral-combined_k141_164485\|\|full | 13714 | No | NA | 1 | 2 | Medium-quality | Genome-fragment | 89.89 | 0 |
| S1-final-viral-combined_k141_48676\|\|full | 1195 | No | NA | 0 | 1 | Not-determined | Genome-fragment | NA | 0 |
| S17-final-viral-combined_k141_230826\|\|full | 1423 | No | NA | 1 | 0 | Low-quality | Genome-fragment | 2.18 | 0 |
| S12-final-viral-combined_k141_140064\|\|full | 1423 | No | NA | 0 | 0 | Low-quality | Genome-fragment | 2.17 | 0 |
| S1-final-viral-combined_k141_279611\|\|full | 1716 | No | NA | 0 | 1 | Not-determined | Genome-fragment | NA | 0 |
| S1-final-viral-combined_k141_180779\|\|full | 1388 | No | NA | 0 | 0 | Not-determined | Genome-fragment | NA | 0 |
| S1-final-viral-combined_k141_180781\|\|full | 1442 | No | NA | 0 | 0 | Not-determined | Genome-fragment | NA | 0 |
| S2-final-viral-combined_k141_230261\|\|full | 1423 | No | NA | 0 | 0 | Low-quality | Genome-fragment | 3.71 | 0 |
| S1-final-viral-combined_k141_345168\|\|full | 1677 | No | NA | 0 | 1 | Not-determined | Genome-fragment | NA | 0 |
| S1-final-viral-combined_k141_32738\|\|full | 3179 | No | NA | 0 | 3 | Not-determined | Genome-fragment | NA | 0 |
| S1-final-viral-combined_k141_180806\|\|full | 2018 | No | NA | 0 | 1 | Not-determined | Genome-fragment | NA | 0 |
| S1-final-viral-combined_k141_180807\|\|full | 2337 | No | NA | 0 | 0 | Not-determined | Genome-fragment | NA | 0 |
| S1-final-viral-combined_k141_345238\|\|full | 7040 | No | NA | 0 | 2 | Not-determined | Genome-fragment | NA | 0 |
| S2-final-viral-combined_k141_122264\|\|full | 1423 | No | NA | 0 | 0 | Low-quality | Genome-fragment | 0.94 | 0 |
| S1-final-viral-combined_k141_345249\|\|full | 2666 | No | NA | 0 | 1 | Not-determined | Genome-fragment | NA | 0 |
| S1-final-viral-combined_k141_82151\|\|full | 2117 | No | NA | 0 | 2 | Not-determined | Genome-fragment | NA | 0 |
| S1-final-viral-combined_k141_48809\|\|full | 2078 | No | NA | 0 | 3 | Not-determined | Genome-fragment | NA | 0 |
| S2_k141_23862 | 1423 | No | NA | 0 | 0 | Low-quality | Genome-fragment | 3.67 | 0 |
| S1-final-viral-combined_k141_114145\|\|full | 2105 | No | NA | 0 | 2 | Not-determined | Genome-fragment | NA | 0 |
| S1-final-viral-combined_k141_82187\|\|full | 2508 | No | NA | 0 | 0 | Not-determined | Genome-fragment | NA | 0 |
| S2_k141_143844 | 1423 | No | NA | 0 | 1 | Low-quality | Genome-fragment | 0.86 | 0 |
| S1-final-viral-combined_k141_345325\|\|full | 2041 | No | NA | 0 | 0 | Not-determined | Genome-fragment | NA | 0 |
| S1-final-viral-combined_k141_82205\|\|full | 1118 | No | NA | 0 | 1 | Not-determined | Genome-fragment | NA | 0 |
| S1-final-viral-combined_k141_48883\|\|full | 9453 | No | NA | 0 | 7 | Not-determined | Genome-fragment | NA | 0 |
| S1-final-viral-combined_k141_345351\|\|full | 3352 | No | NA | 0 | 2 | Not-determined | Genome-fragment | NA | 0 |
| S1-final-viral-combined_k141_345383\|\|full | 1542 | No | NA | 0 | 1 | Not-determined | Genome-fragment | NA | 0 |
| S4-final-viral-combined_k141_10245\|\|full | 1423 | No | NA | 0 | 0 | Low-quality | Genome-fragment | 1.87 | 0 |
| S1-final-viral-combined_k141_32863\|\|full | 15902 | No | NA | 0 | 10 | Medium-quality | Genome-fragment | 50.78 | 0 |
| S1-final-viral-combined_k141_48966\|\|full | 3073 | No | NA | 0 | 1 | Not-determined | Genome-fragment | NA | 0 |
| S1-final-viral-combined_k141_32868\|\|full | 6744 | No | NA | 0 | 5 | Not-determined | Genome-fragment | NA | 0 |
| S1-final-viral-combined_k141_114282\|\|full | 2133 | No | NA | 0 | 2 | Not-determined | Genome-fragment | NA | 0 |
| S1-final-viral-combined_k141_345404\|\|full | 1314 | No | NA | 0 | 0 | Not-determined | Genome-fragment | NA | 0 |
| S1-final-viral-combined_k141_345429\|\|full | 1091 | No | NA | 0 | 0 | Not-determined | Genome-fragment | NA | 0 |
| S8-final-viral-combined_k141_246658\|\|full | 1423 | No | NA | 0 | 1 | Low-quality | Genome-fragment | 2.18 | 0 |
| S1-final-viral-combined_k141_32883\|\|full | 3720 | No | NA | 0 | 0 | Not-determined | Genome-fragment | NA | 0 |
| S1-final-viral-combined_k141_114382\|\|full | 1539 | No | NA | 0 | 1 | Not-determined | Genome-fragment | NA | 0 |
| S1-final-viral-combined_k141_114474\|\|full | 2678 | No | NA | 0 | 2 | Not-determined | Genome-fragment | NA | 0 |
| S9-final-viral-combined_k141_75458\|\|full | 1424 | No | NA | 1 | 0 | Low-quality | Genome-fragment | 0.54 | 0 |
| S9-final-viral-combined_k141_56527\|\|full | 1424 | No | NA | 1 | 0 | Low-quality | Genome-fragment | 3.16 | 0 |
| S9_k141_32613 | 1424 | No | NA | 0 | 0 | Low-quality | Genome-fragment | 2.38 | 0 |
| S9_k141_21954 | 1424 | No | NA | 0 | 1 | Low-quality | Genome-fragment | 3.35 | 0 |
| S1-final-viral-combined_k141_49249\|\|full | 2946 | No | NA | 0 | 3 | Not-determined | Genome-fragment | NA | 0 |
| S11-final-viral-combined_k141_195610\|\|full | 1424 | No | NA | 1 | 0 | Low-quality | Genome-fragment | 1.03 | 0 |
| S1-final-viral-combined_k141_49284\|\|full | 2005 | No | NA | 0 | 1 | Not-determined | Genome-fragment | NA | 0 |
| S1-final-viral-combined_k141_284667\|\|full | 1424 | No | NA | 0 | 1 | Low-quality | Genome-fragment | 2.21 | 0 |
| S5_k141_85022 | 1424 | No | NA | 1 | 0 | Low-quality | Genome-fragment | 3.26 | 0 |
| S17-final-viral-combined_k141_47580\|\|full | 1424 | No | NA | 0 | 1 | Low-quality | Genome-fragment | 0.71 | 0 |
| S12-final-viral-combined_k141_188638\|\|full | 1424 | No | NA | 1 | 0 | Low-quality | Genome-fragment | 3.19 | 0 |
| S1-final-viral-combined_k141_114978\|\|full | 2619 | No | NA | 0 | 3 | Not-determined | Genome-fragment | NA | 0 |
| S1-final-viral-combined_k141_115088\|\|full | 3609 | No | NA | 0 | 1 | Not-determined | Genome-fragment | NA | 0 |
| S1-final-viral-combined_k141_115104\|\|full | 1284 | No | NA | 0 | 2 | Not-determined | Genome-fragment | NA | 0 |
| S2-final-viral-combined_k141_238421\|\|full | 1424 | No | NA | 0 | 2 | Low-quality | Genome-fragment | 0.75 | 0 |
| S9-final-viral-combined_k141_89585\|\|full | 1425 | No | NA | 0 | 0 | Low-quality | Genome-fragment | 0.8 | 0 |
| S1-final-viral-combined_k141_34689\|\|full | 1425 | No | NA | 0 | 0 | Low-quality | Genome-fragment | 3.78 | 0 |
| S1-final-viral-combined_k141_125241\|\|full | 1425 | No | NA | 0 | 0 | Low-quality | Genome-fragment | 2.22 | 0 |
| S1-final-viral-combined_k141_2551\|\|0_partial | 17087 | No | NA | 0 | 10 | Not-determined | Genome-fragment | NA | 0 |
| S3-final-viral-combined_k141_85139\|\|lt2gene | 1425 | No | NA | 1 | 0 | Low-quality | Genome-fragment | 2.08 | 0 |
| S7-final-viral-combined_k141_128011\|\|lt2gene | 1425 | No | NA | 1 | 0 | Low-quality | Genome-fragment | 1.34 | 0 |
| S7_k141_17556 | 1425 | No | NA | 0 | 0 | Low-quality | Genome-fragment | 1.76 | 0 |
| S1-final-viral-combined_k141_331544\|\|0_partial | 26230 | Yes | 14540 | 7 | 8 | Complete | High-quality | 100 | 44.57 |
| S7_k141_60291 | 1425 | No | NA | 0 | 0 | Low-quality | Genome-fragment | 3.23 | 0 |
| S1-final-viral-combined_k141_68332\|\|0_partial | 12047 | No | NA | 0 | 7 | Not-determined | Genome-fragment | NA | 0 |
| S1-final-viral-combined_k141_265860\|\|0_partial | 23566 | No | NA | 0 | 13 | Not-determined | Genome-fragment | NA | 0 |
| S1-final-viral-combined_k141_85225\|\|0_partial | 67554 | Yes | 41415 | 21 | 20 | High-quality | High-quality | 100 | 38.69 |
| S17-final-viral-combined_k141_218694\|\|full | 1425 | No | NA | 2 | 0 | Low-quality | Genome-fragment | 2.54 | 0 |
| S1-final-viral-combined_k141_36114\|\|0_partial | 10668 | No | NA | 0 | 9 | Not-determined | Genome-fragment | NA | 0 |
| S12-final-viral-combined_k141_243470\|\|full | 1425 | No | NA | 0 | 0 | Low-quality | Genome-fragment | 3.44 | 0 |
| S2-final-viral-combined_k141_47288\|\|full | 1425 | No | NA | 0 | 0 | Low-quality | Genome-fragment | 23.23 | 0 |
| S1-final-viral-combined_k141_136097\|\|0_partial | 19804 | No | NA | 1 | 6 | Medium-quality | Genome-fragment | 50.57 | 0 |
| S1-final-viral-combined_k141_168463\|\|0_partial | 23512 | No | NA | 0 | 7 | Not-determined | Genome-fragment | NA | 0 |
| S1-final-viral-combined_k141_284778\|\|0_partial | 14860 | No | NA | 0 | 8 | Not-determined | Genome-fragment | NA | 0 |
| S2-final-viral-combined_k141_144466\|\|lt2gene | 1425 | No | NA | 0 | 0 | Low-quality | Genome-fragment | 2.98 | 0 |
| S1-final-viral-combined_k141_169272\|\|0_partial | 12125 | No | NA | 0 | 12 | Not-determined | Genome-fragment | NA | 0 |
| S1-final-viral-combined_k141_121278\|\|0_partial | 11574 | No | NA | 0 | 9 | Not-determined | Genome-fragment | NA | 0 |
| S4-final-viral-combined_k141_104762\|\|full | 1425 | No | NA | 0 | 0 | Low-quality | Genome-fragment | 1.13 | 0 |
| S8-final-viral-combined_k141_109996\|\|full | 1425 | No | NA | 0 | 0 | Low-quality | Genome-fragment | 2.6 | 0 |
| S9-final-viral-combined_k141_91942\|\|lt2gene | 1426 | No | NA | 1 | 0 | Low-quality | Genome-fragment | 3.34 | 0 |
| S11-final-viral-combined_k141_255992\|\|full | 1426 | No | NA | 2 | 0 | Low-quality | Genome-fragment | 3.34 | 0 |
| S1-final-viral-combined_k141_138958\|\|0_partial | 13426 | No | NA | 0 | 10 | Not-determined | Genome-fragment | NA | 0 |
| S1-final-viral-combined_k141_255289\|\|1_partial | 11584 | No | NA | 0 | 5 | Not-determined | Genome-fragment | NA | 0 |
| S11_k141_165217 | 1426 | No | NA | 0 | 1 | Low-quality | Genome-fragment | 2.86 | 0 |
| S1-final-viral-combined_k141_155743\|\|0_partial | 15180 | No | NA | 0 | 11 | Not-determined | Genome-fragment | NA | 0 |
| S7-final-viral-combined_k141_86098\|\|full | 1426 | No | NA | 0 | 0 | Low-quality | Genome-fragment | 2.45 | 0 |
| S1-final-viral-combined_k141_273012\|\|0_partial | 28053 | No | NA | 0 | 18 | Not-determined | Genome-fragment | NA | 0 |
| S1-final-viral-combined_k141_307323\|\|0_partial | 70097 | Yes | 33763 | 16 | 19 | Medium-quality | Genome-fragment | 88.54 | 51.83 |
| S17-final-viral-combined_k141_70831\|\|full | 1426 | No | NA | 0 | 0 | Low-quality | Genome-fragment | 3.05 | 0 |
| S17-k141_49920 | 1426 | No | NA | 0 | 1 | Low-quality | Genome-fragment | 4.02 | 0 |
| S1-final-viral-combined_k141_326034\|\|0_partial | 18298 | No | NA | 0 | 9 | Not-determined | Genome-fragment | NA | 0 |
| S1-final-viral-combined_k141_94988\|\|0_partial | 39556 | Yes | 28256 | 7 | 19 | Medium-quality | Genome-fragment | 68.23 | 28.57 |
| S12-final-viral-combined_k141_201923\|\|full | 1426 | No | NA | 0 | 0 | Low-quality | Genome-fragment | 2.83 | 0 |
| S1-final-viral-combined_k141_375477\|\|0_partial | 12579 | No | NA | 0 | 5 | Not-determined | Genome-fragment | NA | 0 |
| S12_k141_122617 | 1426 | No | NA | 1 | 0 | Low-quality | Genome-fragment | 0.78 | 0 |
| S1-final-viral-combined_k141_294159\|\|0_partial | 18405 | No | NA | 0 | 9 | Not-determined | Genome-fragment | NA | 0 |
| S1-final-viral-combined_k141_294159\|\|1_partial | 13349 | No | NA | 0 | 6 | Not-determined | Genome-fragment | NA | 0 |
| S2-final-viral-combined_k141_114024\|\|full | 1426 | No | NA | 1 | 0 | Low-quality | Genome-fragment | 1.67 | 0 |
| S2-final-viral-combined_k141_126674\|\|full | 1426 | No | NA | 0 | 0 | Low-quality | Genome-fragment | 2.6 | 0 |
| S1-final-viral-combined_k141_115303\|\|lt2gene | 1159 | No | NA | 0 | 0 | Not-determined | Genome-fragment | NA | 0 |
| S11-final-viral-combined_k141_281579\|\|full | 1427 | No | NA | 0 | 0 | Low-quality | Genome-fragment | 3.42 | 0 |
| S1-final-viral-combined_k141_164803\|\|full | 1427 | No | NA | 0 | 0 | Low-quality | Genome-fragment | 1.46 | 0 |
| S1-final-viral-combined_k141_296380\|\|lt2gene | 1223 | No | NA | 0 | 2 | Not-determined | Genome-fragment | NA | 0 |
| S5-final-viral-combined_k141_70563\|\|lt2gene | 1427 | No | NA | 1 | 0 | Low-quality | Genome-fragment | 3.13 | 0 |
| S7_k141_244184 | 1427 | No | NA | 0 | 0 | Low-quality | Genome-fragment | 2.79 | 0 |
| S17-k141_143253 | 1427 | No | NA | 0 | 1 | Low-quality | Genome-fragment | 0.53 | 0 |
| S4-final-viral-combined_k141_29280\|\|full | 1427 | No | NA | 0 | 0 | Low-quality | Genome-fragment | 2.17 | 0 |
| S4-final-viral-combined_k141_166564\|\|full | 1427 | No | NA | 1 | 0 | Low-quality | Genome-fragment | 2.85 | 0 |
| S8-final-viral-combined_k141_187829\|\|full | 1427 | No | NA | 0 | 0 | Low-quality | Genome-fragment | 0.92 | 0 |
| S8_k141_51616 | 1427 | No | NA | 1 | 0 | Low-quality | Genome-fragment | 4.04 | 0 |
| S11-final-viral-combined_k141_80281\|\|full | 1428 | No | NA | 1 | 0 | Low-quality | Genome-fragment | 3.7 | 0 |
| S3-final-viral-combined_k141_15094\|\|lt2gene | 1428 | No | NA | 2 | 0 | Low-quality | Genome-fragment | 4.08 | 0 |
| S3_k141_25468 | 1428 | No | NA | 0 | 1 | Low-quality | Genome-fragment | 3.02 | 0 |
| S1-final-viral-combined_k141_296926\|\|lt2gene | 1405 | No | NA | 0 | 2 | Not-determined | Genome-fragment | NA | 0 |
| S1-final-viral-combined_k141_264200\|\|lt2gene | 1778 | No | NA | 0 | 0 | Not-determined | Genome-fragment | NA | 0 |
| S1-final-viral-combined_k141_329884\|\|lt2gene | 1175 | No | NA | 0 | 0 | Not-determined | Genome-fragment | NA | 0 |
| S5-final-viral-combined_k141_184449\|\|full | 1428 | No | NA | 3 | 0 | Low-quality | Genome-fragment | 3.56 | 0 |
| S5-final-viral-combined_k141_20609\|\|lt2gene | 1428 | No | NA | 1 | 0 | Low-quality | Genome-fragment | 2.48 | 0 |
| S7-final-viral-combined_k141_84874\|\|full | 1428 | No | NA | 0 | 0 | Low-quality | Genome-fragment | 3.28 | 0 |
| S17-final-viral-combined_k141_28222\|\|full | 1428 | No | NA | 3 | 0 | Low-quality | Genome-fragment | 3.06 | 0 |
| S1-final-viral-combined_k141_165741\|\|lt2gene | 1364 | No | NA | 0 | 0 | Not-determined | Genome-fragment | NA | 0 |
| S1-final-viral-combined_k141_17936\|\|lt2gene | 1082 | No | NA | 0 | 0 | Not-determined | Genome-fragment | NA | 0 |
| S12-final-viral-combined_k141_268597\|\|full | 1428 | No | NA | 0 | 0 | Low-quality | Genome-fragment | 3.48 | 0 |
| S12-final-viral-combined_k141_228184\|\|full | 1428 | No | NA | 0 | 0 | Low-quality | Genome-fragment | 0.96 | 0 |
| S6-final-viral-combined_k141_113492\|\|lt2gene | 1428 | No | NA | 1 | 0 | Low-quality | Genome-fragment | 4.33 | 0 |
| S1-final-viral-combined_k141_264563\|\|lt2gene | 1433 | No | NA | 0 | 0 | Not-determined | Genome-fragment | NA | 0 |
| S1-final-viral-combined_k141_314281\|\|lt2gene | 1802 | No | NA | 0 | 0 | Not-determined | Genome-fragment | NA | 0 |
| S1-final-viral-combined_k141_100187\|\|lt2gene | 1441 | No | NA | 0 | 2 | Not-determined | Genome-fragment | NA | 0 |
| S6_k141_33179 | 1428 | No | NA | 0 | 1 | Low-quality | Genome-fragment | 4.03 | 0 |
| S1-final-viral-combined_k141_2463\|\|lt2gene | 1055 | No | NA | 0 | 0 | Not-determined | Genome-fragment | NA | 0 |
| S8-final-viral-combined_k141_17333\|\|full | 1428 | No | NA | 0 | 0 | Low-quality | Genome-fragment | 3.83 | 0 |
| S9_k141_62998 | 1429 | No | NA | 1 | 0 | Low-quality | Genome-fragment | 2.47 | 0 |
| S11-final-viral-combined_k141_17850\|\|full | 1429 | No | NA | 0 | 3 | Low-quality | Genome-fragment | 0.84 | 0 |
| S1-final-viral-combined_k141_315185\|\|lt2gene | 1705 | No | NA | 0 | 1 | Not-determined | Genome-fragment | NA | 0 |
| S1-final-viral-combined_k141_364578\|\|lt2gene | 1903 | No | NA | 0 | 0 | Not-determined | Genome-fragment | NA | 0 |
| S1-final-viral-combined_k141_331427\|\|lt2gene | 1451 | No | NA | 0 | 1 | Not-determined | Genome-fragment | NA | 0 |
| S1-final-viral-combined_k141_119081\|\|full | 1429 | No | NA | 0 | 2 | Low-quality | Genome-fragment | 1.94 | 0 |
| S3-final-viral-combined_k141_85640\|\|full | 1429 | No | NA | 0 | 0 | Low-quality | Genome-fragment | 3.08 | 0 |
| S5-final-viral-combined_k141_141693\|\|full | 1429 | No | NA | 0 | 0 | Low-quality | Genome-fragment | 0.8 | 0 |
| S7-final-viral-combined_k141_223893\|\|full | 1429 | No | NA | 0 | 0 | Low-quality | Genome-fragment | 4.17 | 0 |
| S12-final-viral-combined_k141_88600\|\|full | 1429 | No | NA | 0 | 0 | Low-quality | Genome-fragment | 1.05 | 0 |
| S12_k141_141966 | 1429 | No | NA | 0 | 0 | Low-quality | Genome-fragment | 2.35 | 0 |
| S4_k141_103831 | 1429 | No | NA | 0 | 0 | Low-quality | Genome-fragment | 1.08 | 0 |
| S1-final-viral-combined_k141_200516\|\|lt2gene | 1187 | No | NA | 0 | 0 | Not-determined | Genome-fragment | NA | 0 |
| S6-final-viral-combined_k141_107985\|\|full | 1429 | No | NA | 0 | 0 | Low-quality | Genome-fragment | 2.67 | 0 |
| S9-final-viral-combined_k141_2500\|\|full | 1430 | No | NA | 0 | 0 | Low-quality | Genome-fragment | 1.2 | 0 |
| S9-final-viral-combined_k141_12642\|\|full | 1430 | No | NA | 2 | 0 | Low-quality | Genome-fragment | 3.72 | 0 |
| S11-final-viral-combined_k141_114741\|\|full | 1430 | No | NA | 0 | 0 | Low-quality | Genome-fragment | 0.16 | 0 |
| S1-final-viral-combined_k141_151873\|\|full | 1430 | No | NA | 0 | 2 | Low-quality | Genome-fragment | 1.74 | 0 |
| S1-final-viral-combined_k141_167241\|\|lt2gene | 1092 | No | NA | 0 | 0 | Not-determined | Genome-fragment | NA | 0 |
| S1-final-viral-combined_k141_381630\|\|lt2gene | 1422 | No | NA | 0 | 0 | Not-determined | Genome-fragment | NA | 0 |
| S1-final-viral-combined_k141_250422\|\|lt2gene | 1072 | No | NA | 0 | 0 | Not-determined | Genome-fragment | NA | 0 |
| S1-final-viral-combined_k141_193282\|\|full | 1430 | No | NA | 0 | 2 | Low-quality | Genome-fragment | 0.8 | 0 |
| S1-final-viral-combined_k141_167259\|\|lt2gene | 1084 | No | NA | 0 | 0 | Not-determined | Genome-fragment | NA | 0 |
| S1_k141_374469 | 1430 | No | NA | 0 | 1 | Low-quality | Genome-fragment | 4.64 | 0 |
| S1-final-viral-combined_k141_349201\|\|lt2gene | 1349 | No | NA | 0 | 0 | Not-determined | Genome-fragment | NA | 0 |
| S7-final-viral-combined_k141_113747\|\|full | 1430 | No | NA | 0 | 1 | Low-quality | Genome-fragment | 0.14 | 0 |
| S12_k141_158026 | 1430 | No | NA | 0 | 1 | Low-quality | Genome-fragment | 3.14 | 0 |
| S12_k141_213044 | 1430 | No | NA | 0 | 1 | Low-quality | Genome-fragment | 1.62 | 0 |
| S4-final-viral-combined_k141_50697\|\|full | 1430 | No | NA | 0 | 0 | Low-quality | Genome-fragment | 3.56 | 0 |
| S4-final-viral-combined_k141_73867\|\|full | 1430 | No | NA | 1 | 0 | Low-quality | Genome-fragment | 2.46 | 0 |
| S4_k141_92764 | 1430 | No | NA | 0 | 0 | Low-quality | Genome-fragment | 0.24 | 0 |
| S6-final-viral-combined_k141_9720\|\|lt2gene | 1430 | No | NA | 0 | 0 | Low-quality | Genome-fragment | 2.61 | 0 |

| | | | | | | | | | | |
|---|---|---|---|---|---|---|---|---|---|---|
| S8-final-viral-combined_k141_9687\|\|full | 1430 | No | NA | 0 | 0 | Low-quality | Genome-fragment | | 3.75 | 0 |
| S11-final-viral-combined_k141_140585\|\|full | 1431 | No | NA | 0 | 0 | Low-quality | Genome-fragment | | 2.86 | 0 |
| S1-final-viral-combined_k141_3923\|\|lt2gene | 1091 | No | NA | 0 | 0 | Not-determined | Genome-fragment | NA | | 0 |
| S1-final-viral-combined_k141_168077\|\|lt2gene | 1585 | No | NA | 0 | 1 | Not-determined | Genome-fragment | NA | | 0 |
| S1-final-viral-combined_k141_185804\|\|lt2gene | 2118 | No | NA | 0 | 0 | Not-determined | Genome-fragment | NA | | 0 |
| S1-final-viral-combined_k141_284006\|\|full | 1431 | No | NA | 0 | 1 | Low-quality | Genome-fragment | | 0.45 | 0 |
| S5-final-viral-combined_k141_45645\|\|full | 1431 | No | NA | 0 | 1 | Low-quality | Genome-fragment | | 2.6 | 0 |
| S12-final-viral-combined_k141_232380\|\|full | 1431 | No | NA | 0 | 1 | Low-quality | Genome-fragment | | 1.2 | 0 |
| S12-final-viral-combined_k141_364929\|\|full | 1431 | No | NA | 1 | 0 | Low-quality | Genome-fragment | | 3.19 | 0 |
| S6-final-viral-combined_k141_140622\|\|full | 1431 | No | NA | 3 | 0 | Low-quality | Genome-fragment | | 2.37 | 0 |
| S9-final-viral-combined_k141_104199\|\|full | 1432 | No | NA | 1 | 0 | Low-quality | Genome-fragment | | 3.51 | 0 |
| S9-final-viral-combined_k141_127682\|\|full | 1432 | No | NA | 1 | 0 | Low-quality | Genome-fragment | | 2.41 | 0 |
| S11-final-viral-combined_k141_146760\|\|lt2gene | 1432 | No | NA | 1 | 0 | Low-quality | Genome-fragment | | 3.26 | 0 |
| S1-final-viral-combined_k141_21210\|\|lt2gene | 1432 | No | NA | 1 | 0 | Low-quality | Genome-fragment | | 2.24 | 0 |
| S1-final-viral-combined_k141_168797\|\|lt2gene | 1142 | No | NA | 0 | 0 | Not-determined | Genome-fragment | NA | | 0 |
| S7-final-viral-combined_k141_126180\|\|full | 1432 | No | NA | 0 | 0 | Low-quality | Genome-fragment | | 0.83 | 0 |
| S1-final-viral-combined_k141_4853\|\|lt2gene | 7370 | No | NA | 0 | 1 | Not-determined | Genome-fragment | NA | | 0 |
| S12-final-viral-combined_k141_251840\|\|full | 1432 | No | NA | 0 | 1 | Low-quality | Genome-fragment | | 2.5 | 0 |
| S1-final-viral-combined_k141_5016\|\|lt2gene | 1588 | No | NA | 0 | 1 | Not-determined | Genome-fragment | NA | | 0 |
| S12-final-viral-combined_k141_331259\|\|full | 1432 | No | NA | 0 | 2 | Low-quality | Genome-fragment | | 2.37 | 0 |
| S1-final-viral-combined_k141_302302\|\|lt2gene | 1208 | No | NA | 0 | 0 | Not-determined | Genome-fragment | NA | | 0 |
| S2-final-viral-combined_k141_174596\|\|full | 1432 | No | NA | 0 | 0 | Low-quality | Genome-fragment | | 1.08 | 0 |
| S9_k141_69702 | 1433 | No | NA | 0 | 0 | Low-quality | Genome-fragment | | 2.39 | 0 |
| S1-final-viral-combined_k141_169564\|\|lt2gene | 1161 | No | NA | 0 | 1 | Not-determined | Genome-fragment | NA | | 0 |
| S1-final-viral-combined_k141_152652\|\|full | 1433 | No | NA | 1 | 0 | Low-quality | Genome-fragment | | 2.96 | 0 |
| S1-final-viral-combined_k141_219842\|\|lt2gene | 1013 | No | NA | 0 | 0 | Not-determined | Genome-fragment | NA | | 0 |
| S1-final-viral-combined_k141_137939\|\|lt2gene | 1401 | No | NA | 0 | 0 | Not-determined | Genome-fragment | NA | | 0 |
| S7-final-viral-combined_k141_119825\|\|full | 1433 | No | NA | 0 | 0 | Low-quality | Genome-fragment | | 2.35 | 0 |
| S1-final-viral-combined_k141_319129\|\|lt2gene | 3578 | No | NA | 0 | 0 | Not-determined | Genome-fragment | NA | | 0 |
| S12-final-viral-combined_k141_178275\|\|lt2gene | 1433 | No | NA | 1 | 0 | Low-quality | Genome-fragment | | 3.63 | 0 |
| S1-final-viral-combined_k141_121748\|\|lt2gene | 1116 | No | NA | 0 | 0 | Not-determined | Genome-fragment | NA | | 0 |
| S1-final-viral-combined_k141_384707\|\|lt2gene | 1387 | No | NA | 0 | 2 | Not-determined | Genome-fragment | NA | | 0 |
| S2_k141_159607 | 1433 | No | NA | 0 | 1 | Low-quality | Genome-fragment | | 0.45 | 0 |
| S4_k141_59304 | 1433 | No | NA | 0 | 0 | Low-quality | Genome-fragment | | 2.08 | 0 |
| S8-final-viral-combined_k141_245074\|\|full | 1433 | No | NA | 1 | 0 | Low-quality | Genome-fragment | | 4.38 | 0 |
| S9-final-viral-combined_k141_7398\|\|full | 1434 | No | NA | 0 | 0 | Low-quality | Genome-fragment | | 3.12 | 0 |
| S1-final-viral-combined_k141_301253\|\|full | 1434 | No | NA | 0 | 0 | Low-quality | Genome-fragment | | 3.93 | 0 |
| S1-final-viral-combined_k141_55595\|\|full | 1434 | No | NA | 2 | 0 | Low-quality | Genome-fragment | | 2.83 | 0 |
| S5-final-viral-combined_k141_84977\|\|full | 1434 | No | NA | 0 | 1 | Low-quality | Genome-fragment | | 0.64 | 0 |
| S17-final-viral-combined_k141_190807\|\|full | 1434 | No | NA | 2 | 0 | Low-quality | Genome-fragment | | 3.66 | 0 |
| S17-final-viral-combined_k141_59779\|\|full | 1434 | No | NA | 0 | 0 | Low-quality | Genome-fragment | | 3.66 | 0 |
| S1-final-viral-combined_k141_254354\|\|lt2gene | 1030 | No | NA | 0 | 0 | Not-determined | Genome-fragment | NA | | 0 |
| S1-final-viral-combined_k141_71935\|\|lt2gene | 1665 | No | NA | 0 | 0 | Not-determined | Genome-fragment | NA | | 0 |
| S2-final-viral-combined_k141_150199\|\|full | 1434 | No | NA | 0 | 2 | Low-quality | Genome-fragment | | 0.86 | 0 |
| S8-final-viral-combined_k141_247523\|\|full | 1434 | No | NA | 0 | 0 | Low-quality | Genome-fragment | | 1.22 | 0 |
| S11-final-viral-combined_k141_54116\|\|full | 1435 | No | NA | 0 | 0 | Low-quality | Genome-fragment | | 3.49 | 0 |
| S1-final-viral-combined_k141_67577\|\|full | 1435 | No | NA | 0 | 1 | Low-quality | Genome-fragment | | 0.52 | 0 |
| S1-final-viral-combined_k141_70953\|\|full | 1435 | No | NA | 0 | 0 | Low-quality | Genome-fragment | | 3.36 | 0 |
| S1-final-viral-combined_k141_222638\|\|full | 1435 | No | NA | 1 | 0 | Low-quality | Genome-fragment | | 2.38 | 0 |
| S17-final-viral-combined_k141_43956\|\|full | 1435 | No | NA | 0 | 0 | Low-quality | Genome-fragment | | 2.65 | 0 |
| S1-final-viral-combined_k141_89267\|\|lt2gene | 3957 | No | NA | 0 | 1 | Not-determined | Genome-fragment | NA | | 0 |
| S1-final-viral-combined_k141_221209\|\|lt2gene | 1753 | No | NA | 0 | 0 | Not-determined | Genome-fragment | NA | | 0 |
| S1-final-viral-combined_k141_188898\|\|lt2gene | 3077 | No | NA | 0 | 0 | Not-determined | Genome-fragment | NA | | 0 |
| S12-final-viral-combined_k141_175032\|\|full | 1435 | No | NA | 3 | 0 | Low-quality | Genome-fragment | | 2.81 | 0 |
| S2-final-viral-combined_k141_125071\|\|full | 1435 | No | NA | 3 | 0 | Low-quality | Genome-fragment | | 4.01 | 0 |
| S4_k141_164089 | 1435 | No | NA | 0 | 0 | Low-quality | Genome-fragment | | 2.4 | 0 |
| S6_k141_36596 | 1435 | No | NA | 0 | 0 | Low-quality | Genome-fragment | | 1.7 | 0 |
| S8-final-viral-combined_k141_178292\|\|full | 1435 | No | NA | 0 | 0 | Low-quality | Genome-fragment | | 2.96 | 0 |
| S11_k141_260199 | 1436 | No | NA | 0 | 0 | Low-quality | Genome-fragment | | 1.58 | 0 |
| S1_k141_96876 | 1436 | No | NA | 0 | 0 | Low-quality | Genome-fragment | | 2.87 | 0 |
| S5-final-viral-combined_k141_188391\|\|full | 1436 | No | NA | 0 | 0 | Low-quality | Genome-fragment | | 1.2 | 0 |
| S1-final-viral-combined_k141_369669\|\|lt2gene | 2403 | No | NA | 0 | 0 | Not-determined | Genome-fragment | NA | | 0 |
| S1-final-viral-combined_k141_369676\|\|lt2gene | 2031 | No | NA | 0 | 0 | Not-determined | Genome-fragment | NA | | 0 |
| S7-final-viral-combined_k141_200930\|\|full | 1436 | No | NA | 1 | 0 | Low-quality | Genome-fragment | | 2.29 | 0 |
| S1-final-viral-combined_k141_336489\|\|lt2gene | 1108 | No | NA | 0 | 2 | Not-determined | Genome-fragment | NA | | 0 |
| S1-final-viral-combined_k141_386715\|\|lt2gene | 2317 | No | NA | 0 | 1 | Not-determined | Genome-fragment | NA | | 0 |
| S12_k141_93753 | 1436 | No | NA | 0 | 0 | Low-quality | Genome-fragment | | 1.43 | 0 |
| S2_k141_79681 | 1436 | No | NA | 1 | 0 | Low-quality | Genome-fragment | | 2.68 | 0 |
| S8-final-viral-combined_k141_208289\|\|full | 1436 | No | NA | 0 | 0 | Low-quality | Genome-fragment | | 3.39 | 0 |
| S9-final-viral-combined_k141_85194\|\|full | 1437 | No | NA | 0 | 0 | Low-quality | Genome-fragment | | 1.74 | 0 |
| S1-final-viral-combined_k141_354598\|\|lt2gene | 1508 | No | NA | 0 | 0 | Not-determined | Genome-fragment | NA | | 0 |
| S11-final-viral-combined_k141_196784\|\|full | 1437 | No | NA | 0 | 2 | Low-quality | Genome-fragment | | 0.96 | 0 |
| S11-final-viral-combined_k141_59852\|\|full | 1437 | No | NA | 0 | 0 | Low-quality | Genome-fragment | | 2.63 | 0 |
| S11-final-viral-combined_k141_326912\|\|lt2gene | 1437 | No | NA | 1 | 0 | Low-quality | Genome-fragment | | 3.68 | 0 |
| S1_k141_377661 | 1437 | No | NA | 0 | 0 | Low-quality | Genome-fragment | | 1.92 | 0 |
| S5-final-viral-combined_k141_190554\|\|full | 1437 | No | NA | 1 | 0 | Low-quality | Genome-fragment | | 3.11 | 0 |
| S1-final-viral-combined_k141_337301\|\|lt2gene | 5748 | No | NA | 0 | 1 | Not-determined | Genome-fragment | NA | | 0 |
| S5-final-viral-combined_k141_14430\|\|full | 1437 | No | NA | 2 | 0 | Low-quality | Genome-fragment | | 2.88 | 0 |
| S1-final-viral-combined_k141_322652\|\|lt2gene | 1580 | No | NA | 0 | 0 | Not-determined | Genome-fragment | NA | | 0 |
| S17-final-viral-combined_k141_166993\|\|full | 1437 | No | NA | 1 | 0 | Low-quality | Genome-fragment | | 3.36 | 0 |
| S1-final-viral-combined_k141_355110\|\|lt2gene | 1189 | No | NA | 0 | 0 | Not-determined | Genome-fragment | NA | | 0 |
| S17-final-viral-combined_k141_81451\|\|full | 1437 | No | NA | 0 | 0 | Low-quality | Genome-fragment | | 1 | 0 |
| S6-final-viral-combined_k141_138698\|\|full | 1437 | No | NA | 0 | 1 | Low-quality | Genome-fragment | | 2.11 | 0 |
| S8-final-viral-combined_k141_74322\|\|full | 1437 | No | NA | 0 | 0 | Low-quality | Genome-fragment | | 3.07 | 0 |
| S9-final-viral-combined_k141_56382\|\|full | 1438 | No | NA | 0 | 0 | Low-quality | Genome-fragment | | 5.05 | 0 |
| S9-final-viral-combined_k141_4589\|\|full | 1438 | No | NA | 1 | 0 | Low-quality | Genome-fragment | | 2.04 | 0 |
| S9_k141_76277 | 1438 | No | NA | 0 | 0 | Low-quality | Genome-fragment | | 0.58 | 0 |
| S1-final-viral-combined_k141_289087\|\|lt2gene | 1056 | No | NA | 0 | 0 | Not-determined | Genome-fragment | NA | | 0 |
| S11_k141_142257 | 1438 | No | NA | 0 | 1 | Low-quality | Genome-fragment | | 2.81 | 0 |
| S1-final-viral-combined_k141_377976\|\|full | 1438 | No | NA | 0 | 0 | Low-quality | Genome-fragment | | 3.04 | 0 |
| S1-final-viral-combined_k141_49157\|\|full | 1438 | No | NA | 0 | 1 | Low-quality | Genome-fragment | | 0.45 | 0 |
| S3-final-viral-combined_k141_6823\|\|full | 1438 | No | NA | 0 | 0 | Low-quality | Genome-fragment | | 2.24 | 0 |
| S5-final-viral-combined_k141_71431\|\|full | 1438 | No | NA | 1 | 0 | Low-quality | Genome-fragment | | 2.15 | 0 |
| S1-final-viral-combined_k141_58869\|\|lt2gene | 1039 | No | NA | 0 | 0 | Not-determined | Genome-fragment | NA | | 0 |
| S1-final-viral-combined_k141_157389\|\|lt2gene | 2225 | No | NA | 0 | 0 | Not-determined | Genome-fragment | NA | | 0 |
| S5_k141_202695 | 1438 | No | NA | 0 | 1 | Low-quality | Genome-fragment | | 4.68 | 0 |
| S7-final-viral-combined_k141_167275\|\|full | 1438 | No | NA | 0 | 0 | Low-quality | Genome-fragment | | 2.07 | 0 |
| S1-final-viral-combined_k141_191955\|\|lt2gene | 1952 | No | NA | 0 | 2 | Not-determined | Genome-fragment | NA | | 0 |
| S17-final-viral-combined_k141_67371\|\|full | 1438 | No | NA | 1 | 0 | Low-quality | Genome-fragment | | 0.93 | 0 |
| S1-final-viral-combined_k141_173913\|\|lt2gene | 2100 | No | NA | 0 | 0 | Not-determined | Genome-fragment | NA | | 0 |
| S1-final-viral-combined_k141_142217\|\|lt2gene | 1077 | No | NA | 0 | 1 | Not-determined | Genome-fragment | NA | | 0 |
| S1-final-viral-combined_k141_224355\|\|lt2gene | 1142 | No | NA | 0 | 1 | Not-determined | Genome-fragment | NA | | 0 |
| S17-final-viral-combined_k141_163596\|\|full | 1438 | No | NA | 0 | 0 | Low-quality | Genome-fragment | | 3.09 | 0 |
| S12-final-viral-combined_k141_224226\|\|full | 1438 | No | NA | 0 | 1 | Low-quality | Genome-fragment | | 0.41 | 0 |
| S4_k141_115441 | 1438 | No | NA | 0 | 0 | Low-quality | Genome-fragment | | 3.35 | 0 |
| S6_k141_98240 | 1438 | No | NA | 0 | 2 | Low-quality | Genome-fragment | | 0.36 | 0 |
| S1-final-viral-combined_k141_273937\|\|lt2gene | 1060 | No | NA | 0 | 1 | Not-determined | Genome-fragment | NA | | 0 |
| S1-final-viral-combined_k141_192678\|\|lt2gene | 1017 | No | NA | 0 | 1 | Not-determined | Genome-fragment | NA | | 0 |
| S9-final-viral-combined_k141_12886\|\|full | 1439 | No | NA | 0 | 0 | Low-quality | Genome-fragment | | 1.77 | 0 |
| S11-final-viral-combined_k141_223596\|\|full | 1439 | No | NA | 1 | 0 | Low-quality | Genome-fragment | | 2.94 | 0 |
| S11-final-viral-combined_k141_319754\|\|full | 1439 | No | NA | 0 | 0 | Low-quality | Genome-fragment | | 2.06 | 0 |
| S7-final-viral-combined_k141_224211\|\|full | 1439 | No | NA | 0 | 2 | Low-quality | Genome-fragment | | 0.45 | 0 |
| S17-final-viral-combined_k141_189176\|\|lt2gene | 1439 | No | NA | 0 | 0 | Low-quality | Genome-fragment | | 2.01 | 0 |
| S1-final-viral-combined_k141_325182\|\|lt2gene | 2435 | No | NA | 0 | 2 | Not-determined | Genome-fragment | NA | | 0 |
| S12-final-viral-combined_k141_19548\|\|full | 1439 | No | NA | 0 | 2 | Low-quality | Genome-fragment | | 1 | 0 |
| S2-final-viral-combined_k141_137432\|\|full | 1439 | No | NA | 0 | 1 | Low-quality | Genome-fragment | | 0.77 | 0 |
| S8-final-viral-combined_k141_157432\|\|lt2gene | 1439 | No | NA | 1 | 0 | Low-quality | Genome-fragment | | 3.14 | 0 |
| S1-final-viral-combined_k141_340043\|\|lt2gene | 1042 | No | NA | 0 | 1 | Not-determined | Genome-fragment | NA | | 0 |
| S1-final-viral-combined_k141_283217\|\|lt2gene | 1440 | No | NA | 1 | 0 | Low-quality | Genome-fragment | | 3.17 | 0 |
| S7_k141_242461 | 1440 | No | NA | 0 | 0 | Low-quality | Genome-fragment | | 4.06 | 0 |
| S17-final-viral-combined_k141_216559\|\|lt2gene | 1440 | No | NA | 1 | 0 | Low-quality | Genome-fragment | | 3.62 | 0 |
| S12-final-viral-combined_k141_76724\|\|lt2gene | 1440 | No | NA | 1 | 0 | Low-quality | Genome-fragment | | 1.28 | 0 |
| S12-final-viral-combined_k141_109395\|\|lt2gene | 1440 | No | NA | 1 | 0 | Low-quality | Genome-fragment | | 3.6 | 0 |
| S12_k141_266257 | 1440 | No | NA | 1 | 0 | Low-quality | Genome-fragment | | 1.33 | 0 |
| S2-final-viral-combined_k141_137082\|\|full | 1440 | No | NA | 0 | 1 | Low-quality | Genome-fragment | | 1.34 | 0 |
| S2_k141_1397 | 1440 | No | NA | 0 | 0 | Low-quality | Genome-fragment | | 2.87 | 0 |
| S1-final-viral-combined_k141_94751\|\|lt2gene | 2734 | No | NA | 0 | 0 | Not-determined | Genome-fragment | NA | | 0 |
| S2_k141_155308 | 1440 | No | NA | 0 | 0 | Low-quality | Genome-fragment | | 3.28 | 0 |
| S9-final-viral-combined_k141_81970\|\|full | 1441 | No | NA | 3 | 0 | Low-quality | Genome-fragment | | 3.09 | 0 |
| S1-final-viral-combined_k141_374489\|\|lt2gene | 1432 | No | NA | 0 | 1 | Not-determined | Genome-fragment | NA | | 0 |
| S1-final-viral-combined_k141_210819\|\|lt2gene | 1117 | No | NA | 0 | 0 | Not-determined | Genome-fragment | NA | | 0 |
| S9-final-viral-combined_k141_60427\|\|lt2gene | 1441 | No | NA | 2 | 0 | Low-quality | Genome-fragment | | 3.36 | 0 |
| S11_k141_81558 | 1441 | No | NA | 1 | 0 | Low-quality | Genome-fragment | | 3.38 | 0 |
| S1-final-viral-combined_k141_217253\|\|full | 1441 | No | NA | 0 | 0 | Low-quality | Genome-fragment | | 1.75 | 0 |
| S1-final-viral-combined_k141_392369\|\|full | 1441 | No | NA | 0 | 0 | Low-quality | Genome-fragment | | 1.29 | 0 |
| S1-final-viral-combined_k141_164517\|\|full | 1441 | No | NA | 0 | 0 | Low-quality | Genome-fragment | | 1.45 | 0 |
| S1-final-viral-combined_k141_283192\|\|lt2gene | 1441 | No | NA | 1 | 0 | Low-quality | Genome-fragment | | 3.16 | 0 |
| S1-final-viral-combined_k141_160504\|\|lt2gene | 2007 | No | NA | 0 | 2 | Not-determined | Genome-fragment | NA | | 0 |
| S3-final-viral-combined_k141_120133\|\|full | 1441 | No | NA | 0 | 0 | Low-quality | Genome-fragment | | 3.7 | 0 |
| S3-final-viral-combined_k141_30993\|\|lt2gene | 1441 | No | NA | 0 | 0 | Low-quality | Genome-fragment | | 9.31 | 0 |
| S5_k141_18681 | 1441 | No | NA | 1 | 0 | Low-quality | Genome-fragment | | 1.63 | 0 |
| S7-final-viral-combined_k141_197354\|\|full | 1441 | No | NA | 0 | 2 | Low-quality | Genome-fragment | | 1.04 | 0 |
| S7-final-viral-combined_k141_169261\|\|full | 1441 | No | NA | 0 | 0 | Low-quality | Genome-fragment | | 2.54 | 0 |
| S17-final-viral-combined_k141_37689\|\|full | 1441 | No | NA | 1 | 0 | Low-quality | Genome-fragment | | 11.27 | 0 |
| S12-final-viral-combined_k141_84541\|\|full | 1441 | No | NA | 0 | 0 | Low-quality | Genome-fragment | | 5.06 | 0 |
| S12-final-viral-combined_k141_386907\|\|full | 1441 | No | NA | 2 | 0 | Low-quality | Genome-fragment | | 4.16 | 0 |
| S1-final-viral-combined_k141_46442\|\|lt2gene | 1282 | No | NA | 0 | 0 | Not-determined | Genome-fragment | NA | | 0 |
| S12-final-viral-combined_k141_342475\|\|lt2gene | 1441 | No | NA | 1 | 0 | Low-quality | Genome-fragment | | 2.98 | 0 |
| S2-final-viral-combined_k141_40053\|\|full | 1441 | No | NA | 1 | 0 | Low-quality | Genome-fragment | | 3.16 | 0 |
| S1-final-viral-combined_k141_212895\|\|lt2gene | 1124 | No | NA | 0 | 0 | Not-determined | Genome-fragment | NA | | 0 |
| S2-final-viral-combined_k141_56373\|\|lt2gene | 1441 | No | NA | 0 | 0 | Low-quality | Genome-fragment | | 3.29 | 0 |
| S4_k141_108597 | 1441 | No | NA | 0 | 0 | Low-quality | Genome-fragment | | 3.24 | 0 |
| S1-final-viral-combined_k141_30341\|\|lt2gene | 3423 | No | NA | 0 | 0 | Not-determined | Genome-fragment | NA | | 0 |
| S6-final-viral-combined_k141_65897\|\|full | 1441 | No | NA | 0 | 0 | Low-quality | Genome-fragment | | 2.19 | 0 |
| S1-final-viral-combined_k141_160972\|\|full | 1442 | No | NA | 0 | 0 | Low-quality | Genome-fragment | | 2.11 | 0 |
| S1-final-viral-combined_k141_296762\|\|lt2gene | 1442 | No | NA | 1 | 0 | Low-quality | Genome-fragment | | 3.09 | 0 |
| S1-final-viral-combined_k141_146608\|\|lt2gene | 1697 | No | NA | 0 | 0 | Not-determined | Genome-fragment | NA | | 0 |
| S1_k141_304077 | 1442 | No | NA | 0 | 0 | Low-quality | Genome-fragment | | 2.76 | 0 |
| S1-final-viral-combined_k141_178609\|\|lt2gene | 1602 | No | NA | 0 | 0 | Not-determined | Genome-fragment | NA | | 0 |
| S1-final-viral-combined_k141_278030\|\|lt2gene | 1042 | No | NA | 0 | 0 | Not-determined | Genome-fragment | NA | | 0 |
| S1-final-viral-combined_k141_97776\|\|lt2gene | 1148 | No | NA | 0 | 0 | Not-determined | Genome-fragment | NA | | 0 |
| S3-final-viral-combined_k141_43729\|\|full | 1442 | No | NA | 0 | 0 | Low-quality | Genome-fragment | | 2.71 | 0 |
| S3-final-viral-combined_k141_21607\|\|full | 1442 | No | NA | 0 | 0 | Low-quality | Genome-fragment | | 3.18 | 0 |
| S1-final-viral-combined_k141_278470\|\|lt2gene | 3557 | No | NA | 0 | 0 | Not-determined | Genome-fragment | NA | | 0 |
| S5-final-viral-combined_k141_132615\|\|full | 1442 | No | NA | 0 | 1 | Low-quality | Genome-fragment | | 0.31 | 0 |
| S5-final-viral-combined_k141_150349\|\|full | 1442 | No | NA | 0 | 0 | Low-quality | Genome-fragment | | 2.65 | 0 |
| S7-final-viral-combined_k141_139907\|\|full | 1442 | No | NA | 1 | 0 | Low-quality | Genome-fragment | | 3.43 | 0 |
| S17-final-viral-combined_k141_229164\|\|full | 1442 | No | NA | 0 | 0 | Low-quality | Genome-fragment | | 2.85 | 0 |
| S12_k141_346626 | 1442 | No | NA | 0 | 0 | Low-quality | Genome-fragment | | 3.91 | 0 |
| S1-final-viral-combined_k141_31879\|\|lt2gene | 1084 | No | NA | 0 | 0 | Not-determined | Genome-fragment | NA | | 0 |
| S9-final-viral-combined_k141_26784\|\|full | 1443 | No | NA | 0 | 2 | Low-quality | Genome-fragment | | 0.86 | 0 |
| S11-final-viral-combined_k141_37556\|\|full | 1443 | No | NA | 0 | 0 | Low-quality | Genome-fragment | | 1.75 | 0 |
| S1-final-viral-combined_k141_33113\|\|full | 1443 | No | NA | 0 | 2 | Low-quality | Genome-fragment | | 0.66 | 0 |
| S1-final-viral-combined_k141_83735\|\|full | 1443 | No | NA | 0 | 0 | Low-quality | Genome-fragment | | 1.05 | 0 |

| | | | | | | | | | |
|---|---|---|---|---|---|---|---|---|---|
| S1-final-viral-combined_k141_114999\|\|lt2gene | 2803 | No | NA | 0 | 0 | Not-determined | Genome-fragment | NA | 0 |
| S1_k141_49376 | 3307 | No | NA | 0 | 0 | Not-determined | Genome-fragment | NA | 0 |
| S1_k141_378447 | 1365 | No | NA | 0 | 2 | Not-determined | Genome-fragment | NA | 0 |
| S1_k141_329087 | 1020 | No | NA | 0 | 1 | Not-determined | Genome-fragment | NA | 0 |
| S1_k141_312637 | 1403 | No | NA | 0 | 0 | Not-determined | Genome-fragment | NA | 0 |
| S1_k141_345535 | 1064 | No | NA | 0 | 1 | Not-determined | Genome-fragment | NA | 0 |
| S1_k141_16464 | 3900 | No | NA | 0 | 2 | Not-determined | Genome-fragment | NA | 0 |
| S5-final-viral-combined_k141_67929\|\|full | 1443 | No | NA | 0 | 2 | Low-quality | Genome-fragment | 3.41 | 0 |
| S1_k141_32948 | 1152 | No | NA | 0 | 1 | Not-determined | Genome-fragment | NA | 0 |
| S1_k141_312671 | 1025 | No | NA | 0 | 1 | Not-determined | Genome-fragment | NA | 0 |
| S1_k141_197462 | 3644 | No | NA | 0 | 8 | Not-determined | Genome-fragment | NA | 0 |
| S17-final-viral-combined_k141_201716\|\|full | 1443 | No | NA | 1 | 0 | Low-quality | Genome-fragment | 2.13 | 0 |
| S1_k141_246805 | 1324 | No | NA | 0 | 1 | Not-determined | Genome-fragment | NA | 0 |
| S1_k141_378494 | 2025 | No | NA | 0 | 0 | Not-determined | Genome-fragment | NA | 0 |
| S1_k141_329114 | 2306 | No | NA | 0 | 2 | Not-determined | Genome-fragment | NA | 0 |
| S1_k141_33005 | 1003 | No | NA | 0 | 2 | Not-determined | Genome-fragment | NA | 0 |
| S12-final-viral-combined_k141_329084\|\|full | 1443 | No | NA | 2 | 0 | Low-quality | Genome-fragment | 2.65 | 0 |
| S1_k141_263297 | 1310 | No | NA | 0 | 1 | Not-determined | Genome-fragment | NA | 0 |
| S1_k141_148157 | 2635 | No | NA | 0 | 0 | Not-determined | Genome-fragment | NA | 0 |
| S12-final-viral-combined_k141_45789\|\|full | 1443 | No | NA | 0 | 0 | Low-quality | Genome-fragment | 2.18 | 0 |
| S1_k141_181066 | 1039 | No | NA | 0 | 0 | Not-determined | Genome-fragment | NA | 0 |
| S1_k141_378514 | 1203 | No | NA | 0 | 2 | Not-determined | Genome-fragment | NA | 0 |
| S1_k141_329152 | 1221 | No | NA | 0 | 1 | Not-determined | Genome-fragment | NA | 0 |
| S1_k141_329161 | 1527 | No | NA | 0 | 0 | Not-determined | Genome-fragment | NA | 0 |
| S1_k141_152 | 1493 | No | NA | 0 | 0 | Not-determined | Genome-fragment | NA | 0 |
| S1_k141_296285 | 3781 | No | NA | 0 | 1 | Not-determined | Genome-fragment | NA | 0 |
| S1_k141_345622 | 1420 | No | NA | 0 | 0 | Not-determined | Genome-fragment | NA | 0 |
| S1_k141_296300 | 1409 | No | NA | 0 | 1 | Not-determined | Genome-fragment | NA | 0 |
| S1_k141_115317 | 1086 | No | NA | 0 | 1 | Not-determined | Genome-fragment | NA | 0 |
| S1_k141_164646 | 1545 | No | NA | 0 | 0 | Not-determined | Genome-fragment | NA | 0 |
| S12_k141_316218 | 1443 | No | NA | 0 | 2 | Low-quality | Genome-fragment | 0.72 | 0 |
| S1_k141_98823 | 1712 | No | NA | 0 | 0 | Not-determined | Genome-fragment | NA | 0 |
| S1_k141_164656 | 1051 | No | NA | 0 | 0 | Not-determined | Genome-fragment | NA | 0 |
| S1_k141_16574 | 1056 | No | NA | 0 | 1 | Not-determined | Genome-fragment | NA | 0 |
| S1_k141_263323 | 2916 | No | NA | 0 | 0 | Not-determined | Genome-fragment | NA | 0 |
| S1_k141_148259 | 1581 | No | NA | 0 | 0 | Not-determined | Genome-fragment | NA | 0 |
| S1_k141_197558 | 2032 | No | NA | 0 | 0 | Not-determined | Genome-fragment | NA | 0 |
| S4-final-viral-combined_k141_16737\|\|full | 1443 | No | NA | 1 | 0 | Low-quality | Genome-fragment | 2.78 | 0 |
| S1_k141_263333 | 1395 | No | NA | 0 | 1 | Not-determined | Genome-fragment | NA | 0 |
| S1_k141_362171 | 1136 | No | NA | 0 | 0 | Not-determined | Genome-fragment | NA | 0 |
| S1_k141_181157 | 7315 | No | NA | 0 | 0 | Not-determined | Genome-fragment | NA | 0 |
| S1_k141_181161 | 1161 | No | NA | 0 | 1 | Not-determined | Genome-fragment | NA | 0 |
| S1_k141_148277 | 1204 | No | NA | 0 | 2 | Not-determined | Genome-fragment | NA | 0 |
| S1_k141_131885 | 2046 | No | NA | 0 | 1 | Not-determined | Genome-fragment | NA | 0 |
| S6-final-viral-combined_k141_44970\|\|full | 1443 | No | NA | 0 | 2 | Low-quality | Genome-fragment | 0.6 | 0 |
| S1_k141_148294 | 1041 | No | NA | 0 | 2 | Not-determined | Genome-fragment | NA | 0 |
| S1_k141_263360 | 1054 | No | NA | 0 | 0 | Not-determined | Genome-fragment | NA | 0 |
| S1_k141_197596 | 1059 | No | NA | 0 | 0 | Not-determined | Genome-fragment | NA | 0 |
| S1_k141_279900 | 4048 | No | NA | 0 | 0 | Not-determined | Genome-fragment | NA | 0 |
| S1_k141_181180 | 1088 | No | NA | 0 | 0 | Not-determined | Genome-fragment | NA | 0 |
| S1_k141_230587 | 1208 | No | NA | 0 | 1 | Not-determined | Genome-fragment | NA | 0 |
| S8-final-viral-combined_k141_271824\|\|full | 1443 | No | NA | 0 | 0 | Low-quality | Genome-fragment | 1.02 | 0 |
| S8_k141_267963 | 1443 | No | NA | 0 | 1 | Low-quality | Genome-fragment | 4.07 | 0 |
| S1_k141_181198 | 3673 | No | NA | 0 | 8 | Not-determined | Genome-fragment | NA | 0 |
| S1_k141_329277 | 1169 | No | NA | 0 | 1 | Not-determined | Genome-fragment | NA | 0 |
| S1_k141_353 | 1393 | No | NA | 0 | 2 | Not-determined | Genome-fragment | NA | 0 |
| S1_k141_230595 | 1170 | No | NA | 0 | 0 | Not-determined | Genome-fragment | NA | 0 |
| S1_k141_16701 | 4312 | No | NA | 0 | 3 | Not-determined | Genome-fragment | NA | 0 |
| S11-final-viral-combined_k141_27757\|\|full | 1444 | No | NA | 0 | 1 | Low-quality | Genome-fragment | 2.04 | 0 |
| S1_k141_362207 | 2010 | No | NA | 0 | 3 | Not-determined | Genome-fragment | NA | 0 |
| S11-final-viral-combined_k141_149277\|\|full | 1444 | No | NA | 0 | 0 | Low-quality | Genome-fragment | 0.57 | 0 |
| S1_k141_230630 | 3470 | No | NA | 0 | 1 | Not-determined | Genome-fragment | NA | 0 |
| S1_k141_410 | 2008 | No | NA | 0 | 2 | Not-determined | Genome-fragment | NA | 0 |
| S1_k141_280017 | 1118 | No | NA | 0 | 1 | Not-determined | Genome-fragment | NA | 0 |
| S1_k141_65939 | 1198 | No | NA | 0 | 1 | Not-determined | Genome-fragment | NA | 0 |
| S1_k141_65940 | 1273 | No | NA | 0 | 1 | Not-determined | Genome-fragment | NA | 0 |
| S1_k141_230702 | 4605 | No | NA | 0 | 1 | Not-determined | Genome-fragment | NA | 0 |
| S1_k141_312980 | 1292 | No | NA | 0 | 0 | Not-determined | Genome-fragment | NA | 0 |
| S1-final-viral-combined_k141_386694\|\|full | 1444 | No | NA | 0 | 0 | Low-quality | Genome-fragment | 1.26 | 0 |
| S1_k141_115468 | 1089 | No | NA | 0 | 0 | Not-determined | Genome-fragment | NA | 0 |
| S1-final-viral-combined_k141_236275\|\|lt2gene | 1444 | No | NA | 2 | 0 | Low-quality | Genome-fragment | 3.1 | 0 |
| S1_k141_362303 | 1103 | No | NA | 0 | 1 | Not-determined | Genome-fragment | NA | 0 |
| S1_k141_230767 | 2393 | No | NA | 0 | 1 | Not-determined | Genome-fragment | NA | 0 |
| S1_k141_132060 | 1269 | No | NA | 0 | 1 | Not-determined | Genome-fragment | NA | 0 |
| S3-final-viral-combined_k141_2170\|\|full | 1444 | No | NA | 1 | 0 | Low-quality | Genome-fragment | 2.69 | 0 |
| S1_k141_148367 | 2326 | No | NA | 0 | 3 | Not-determined | Genome-fragment | NA | 0 |
| S1_k141_296543 | 1032 | No | NA | 0 | 1 | Not-determined | Genome-fragment | NA | 0 |
| S1_k141_296545 | 2004 | No | NA | 0 | 1 | Not-determined | Genome-fragment | NA | 0 |
| S7-final-viral-combined_k141_18334\|\|full | 1444 | No | NA | 0 | 0 | Low-quality | Genome-fragment | 1.81 | 0 |
| S1_k141_362352 | 1801 | No | NA | 0 | 1 | Not-determined | Genome-fragment | NA | 0 |
| S1_k141_148425 | 1073 | No | NA | 0 | 1 | Not-determined | Genome-fragment | NA | 0 |
| S1_k141_82755 | 3302 | No | NA | 0 | 3 | Not-determined | Genome-fragment | NA | 0 |
| S7-final-viral-combined_k141_193996\|\|full | 1444 | No | NA | 0 | 1 | Low-quality | Genome-fragment | 1.4 | 0 |
| S1_k141_247158 | 1102 | No | NA | 0 | 1 | Not-determined | Genome-fragment | NA | 0 |
| S1_k141_49689 | 1359 | No | NA | 0 | 1 | Not-determined | Genome-fragment | NA | 0 |
| S1_k141_33138 | 4323 | No | NA | 0 | 6 | Not-determined | Genome-fragment | NA | 0 |
| S1_k141_33140 | 3356 | No | NA | 0 | 0 | Not-determined | Genome-fragment | NA | 0 |
| S17-k141_165843 | 1444 | No | NA | 0 | 1 | Low-quality | Genome-fragment | 3.27 | 0 |
| S1_k141_247167 | 2280 | No | NA | 0 | 1 | Not-determined | Genome-fragment | NA | 0 |
| S1_k141_345875 | 3968 | No | NA | 0 | 3 | Not-determined | Genome-fragment | NA | 0 |
| S1_k141_263569 | 4738 | No | NA | 0 | 0 | Not-determined | Genome-fragment | NA | 0 |
| S1_k141_263570 | 2641 | No | NA | 0 | 6 | Not-determined | Genome-fragment | NA | 0 |
| S1_k141_230826 | 1106 | No | NA | 0 | 1 | Not-determined | Genome-fragment | NA | 0 |
| S1_k141_99097 | 2270 | No | NA | 0 | 1 | Not-determined | Genome-fragment | NA | 0 |
| S2-final-viral-combined_k141_140621\|\|full | 1444 | No | NA | 0 | 1 | Low-quality | Genome-fragment | 4.34 | 0 |
| S1_k141_115609 | 2774 | No | NA | 0 | 1 | Not-determined | Genome-fragment | NA | 0 |
| S1_k141_49730 | 3742 | No | NA | 0 | 0 | Not-determined | Genome-fragment | NA | 0 |
| S4-final-viral-combined_k141_46786\|\|full | 1444 | No | NA | 0 | 0 | Low-quality | Genome-fragment | 0.95 | 0 |
| S1_k141_378796 | 1733 | No | NA | 0 | 0 | Not-determined | Genome-fragment | NA | 0 |
| S1_k141_16854 | 1404 | No | NA | 0 | 1 | Not-determined | Genome-fragment | NA | 0 |
| S1_k141_197883 | 1816 | No | NA | 0 | 0 | Not-determined | Genome-fragment | NA | 0 |
| S1_k141_99134 | 1481 | No | NA | 0 | 2 | Not-determined | Genome-fragment | NA | 0 |
| S1_k141_313098 | 1046 | No | NA | 0 | 0 | Not-determined | Genome-fragment | NA | 0 |
| S1_k141_230843 | 1922 | No | NA | 0 | 1 | Not-determined | Genome-fragment | NA | 0 |
| S1_k141_214325 | 2067 | No | NA | 0 | 4 | Not-determined | Genome-fragment | NA | 0 |
| S1_k141_329453 | 7011 | No | NA | 0 | 4 | Not-determined | Genome-fragment | NA | 0 |
| S11-final-viral-combined_k141_42755\|\|full | 1445 | No | NA | 1 | 0 | Low-quality | Genome-fragment | 3.61 | 0 |
| S1_k141_378877 | 1294 | No | NA | 0 | 2 | Not-determined | Genome-fragment | NA | 0 |
| S1_k141_230864 | 5119 | No | NA | 0 | 2 | Not-determined | Genome-fragment | NA | 0 |
| S1_k141_164963 | 1307 | No | NA | 0 | 1 | Not-determined | Genome-fragment | NA | 0 |
| S1_k141_378891 | 3188 | No | NA | 0 | 1 | Not-determined | Genome-fragment | NA | 0 |
| S1_k141_197951 | 1278 | No | NA | 0 | 1 | Not-determined | Genome-fragment | NA | 0 |
| S1_k141_296663 | 1428 | No | NA | 0 | 0 | Not-determined | Genome-fragment | NA | 0 |
| S11-final-viral-combined_k141_50886\|\|full | 1445 | No | NA | 0 | 1 | Low-quality | Genome-fragment | 1.37 | 0 |
| S1-final-viral-combined_k141_24921\|\|full | 1445 | No | NA | 0 | 0 | Low-quality | Genome-fragment | 2.83 | 0 |
| S1_k141_345988 | 1304 | No | NA | 0 | 0 | Not-determined | Genome-fragment | NA | 0 |
| S1_k141_378918 | 3846 | No | NA | 0 | 2 | Not-determined | Genome-fragment | NA | 0 |
| S1_k141_378927 | 1126 | No | NA | 0 | 0 | Not-determined | Genome-fragment | NA | 0 |
| S1_k141_115723 | 3860 | No | NA | 0 | 0 | Not-determined | Genome-fragment | NA | 0 |
| S1_k141_214385 | 1203 | No | NA | 0 | 0 | Not-determined | Genome-fragment | NA | 0 |
| S1_k141_66082 | 1347 | No | NA | 0 | 1 | Not-determined | Genome-fragment | NA | 0 |
| S1-final-viral-combined_k141_156970\|\|lt2gene | 1445 | No | NA | 1 | 0 | Low-quality | Genome-fragment | 0.78 | 0 |
| S3_k141_83786 | 1445 | No | NA | 1 | 0 | Low-quality | Genome-fragment | 2.03 | 0 |
| S1_k141_263698 | 1284 | No | NA | 0 | 0 | Not-determined | Genome-fragment | NA | 0 |
| S5-final-viral-combined_k141_1305\|\|lt2gene | 1445 | No | NA | 1 | 0 | Low-quality | Genome-fragment | 3.73 | 0 |
| S1_k141_230917 | 5964 | No | NA | 0 | 1 | Not-determined | Genome-fragment | NA | 0 |
| S5_k141_16453 | 1445 | No | NA | 0 | 0 | Low-quality | Genome-fragment | 2.35 | 0 |
| S1_k141_247360 | 4461 | No | NA | 0 | 0 | Not-determined | Genome-fragment | NA | 0 |
| S1_k141_263779 | 3050 | No | NA | 0 | 2 | Not-determined | Genome-fragment | NA | 0 |
| S1_k141_115810 | 1082 | No | NA | 0 | 1 | Not-determined | Genome-fragment | NA | 0 |
| S12-final-viral-combined_k141_74326\|\|full | 1445 | No | NA | 0 | 0 | Low-quality | Genome-fragment | 2.49 | 0 |
| S1_k141_214521 | 3187 | No | NA | 0 | 2 | Not-determined | Genome-fragment | NA | 0 |
| S1_k141_115861 | 1105 | No | NA | 0 | 1 | Not-determined | Genome-fragment | NA | 0 |
| S4-final-viral-combined_k141_98590\|\|full | 1445 | Yes | 731 | 1 | 1 | Low-quality | Genome-fragment | 1.67 | 49.41 |
| S1_k141_176510 | 1446 | No | NA | 0 | 1 | Low-quality | Genome-fragment | 2.47 | 0 |
| S1_k141_66204 | 4540 | No | NA | 0 | 1 | Not-determined | Genome-fragment | NA | 0 |
| S1_k141_247400 | 1008 | No | NA | 0 | 1 | Not-determined | Genome-fragment | NA | 0 |
| S5_k141_63606 | 1446 | No | NA | 0 | 2 | Low-quality | Genome-fragment | 2.86 | 0 |
| S1_k141_148650 | 1375 | No | NA | 0 | 2 | Not-determined | Genome-fragment | NA | 0 |
| S1_k141_198088 | 7010 | No | NA | 0 | 2 | Not-determined | Genome-fragment | NA | 0 |
| S2-final-viral-combined_k141_243742\|\|full | 1446 | Yes | 410 | 1 | 1 | Low-quality | Genome-fragment | 1.02 | 71.65 |
| S1_k141_214691 | 1015 | No | NA | 0 | 1 | Not-determined | Genome-fragment | NA | 0 |
| S1_k141_296810 | 1210 | No | NA | 0 | 2 | Not-determined | Genome-fragment | NA | 0 |
| S1_k141_165231 | 1007 | No | NA | 0 | 1 | Not-determined | Genome-fragment | NA | 0 |
| S1_k141_132457 | 1007 | No | NA | 0 | 0 | Not-determined | Genome-fragment | NA | 0 |
| S1_k141_247527 | 1374 | No | NA | 0 | 1 | Not-determined | Genome-fragment | NA | 0 |
| S1_k141_280492 | 1064 | No | NA | 0 | 0 | Not-determined | Genome-fragment | NA | 0 |
| S1_k141_296850 | 49921 | No | NA | 19 | 1 | High-quality | High-quality | 94.65 | 0 |
| S1_k141_132530 | 1342 | No | NA | 0 | 1 | Not-determined | Genome-fragment | NA | 0 |
| S1_k141_148775 | 6970 | No | NA | 0 | 1 | Not-determined | Genome-fragment | NA | 0 |
| S1_k141_181903 | 1102 | No | NA | 0 | 1 | Not-determined | Genome-fragment | NA | 0 |
| S2-final-viral-combined_k141_224531\|\|full | 1446 | No | NA | 0 | 1 | Low-quality | Genome-fragment | 1.39 | 0 |
| S1_k141_313388 | 11884 | No | NA | 0 | 6 | Not-determined | Genome-fragment | NA | 0 |
| S1_k141_198298 | 4491 | No | NA | 0 | 1 | Not-determined | Genome-fragment | NA | 0 |
| S8-final-viral-combined_k141_90330\|\|full | 1446 | No | NA | 2 | 0 | Low-quality | Genome-fragment | 4.35 | 0 |
| S1_k141_1081 | 1191 | No | NA | 0 | 0 | Not-determined | Genome-fragment | NA | 0 |
| S8-final-viral-combined_k141_275979\|\|full | 1446 | No | NA | 0 | 1 | Low-quality | Genome-fragment | 0.61 | 0 |
| S1_k141_214936 | 1425 | No | NA | 0 | 0 | Not-determined | Genome-fragment | NA | 0 |
| S1_k141_198315 | 7127 | No | NA | 0 | 5 | Not-determined | Genome-fragment | NA | 0 |
| S1_k141_198318 | 1562 | No | NA | 0 | 0 | Not-determined | Genome-fragment | NA | 0 |
| S1_k141_280653 | 1651 | No | NA | 0 | 0 | Not-determined | Genome-fragment | NA | 0 |
| S8-final-viral-combined_k141_130050\|\|lt2gene | 1446 | No | NA | 1 | 0 | Low-quality | Genome-fragment | 1.36 | 0 |
| S1_k141_379272 | 11296 | No | NA | 0 | 6 | Not-determined | Genome-fragment | NA | 0 |
| S3-final-viral-combined_k141_77674\|\|full | 1447 | No | NA | 1 | 0 | Low-quality | Genome-fragment | 3.12 | 0 |
| S1_k141_280703 | 1322 | No | NA | 0 | 1 | Not-determined | Genome-fragment | NA | 0 |
| S3_k141_66527 | 1447 | No | NA | 0 | 0 | Low-quality | Genome-fragment | 1.32 | 0 |
| S1_k141_116337 | 3592 | No | NA | 0 | 3 | Not-determined | Genome-fragment | NA | 0 |
| S1_k141_17283 | 1087 | No | NA | 0 | 2 | Not-determined | Genome-fragment | NA | 0 |
| S5-final-viral-combined_k141_56605\|\|full | 1447 | No | NA | 0 | 1 | Low-quality | Genome-fragment | 0.85 | 0 |
| S1_k141_198387 | 1039 | No | NA | 0 | 0 | Not-determined | Genome-fragment | NA | 0 |
| S17-final-viral-combined_k141_34419\|\|full | 1447 | No | NA | 0 | 0 | Low-quality | Genome-fragment | 2.2 | 0 |
| S1_k141_1192 | 1094 | No | NA | 0 | 1 | Not-determined | Genome-fragment | NA | 0 |
| S4-final-viral-combined_k141_112823\|\|full | 1447 | No | NA | 0 | 1 | Low-quality | Genome-fragment | 11.25 | 0 |
| S8_k141_86261 | 1447 | No | NA | 0 | 0 | Low-quality | Genome-fragment | 3.65 | 0 |

| | | | | | | | | | | | |
|---|---|---|---|---|---|---|---|---|---|---|---|
| S11-final-viral-combined_k141_212727\|\|full | 1448 | No | NA | | 0 | 1 | Low-quality | Genome-fragment | | 0.75 | 0 |
| S1_k141_362985 | 1255 | No | NA | | 0 | 0 | Not-determined | Genome-fragment | NA | | 0 |
| S11-final-viral-combined_k141_77871\|\|full | 1448 | No | NA | | 0 | 2 | Low-quality | Genome-fragment | | 3.68 | 0 |
| S1_k141_297144 | 1091 | No | NA | | 0 | 2 | Not-determined | Genome-fragment | NA | | 0 |
| S11-final-viral-combined_k141_115652\|\|lt2gene | 1448 | No | NA | | 2 | 0 | Low-quality | Genome-fragment | | 3.8 | 0 |
| S1_k141_247808 | 1842 | No | NA | | 0 | 1 | Not-determined | Genome-fragment | NA | | 0 |
| S1_k141_264193 | 1124 | No | NA | | 0 | 2 | Not-determined | Genome-fragment | NA | | 0 |
| S1_k141_379343 | 4168 | No | NA | | 0 | 0 | Not-determined | Genome-fragment | NA | | 0 |
| S1_k141_264205 | 1160 | No | NA | | 0 | 1 | Not-determined | Genome-fragment | NA | | 0 |
| S1_k141_280758 | 1839 | No | NA | | 0 | 1 | Not-determined | Genome-fragment | NA | | 0 |
| S1_k141_1244 | 1507 | No | NA | | 0 | 1 | Not-determined | Genome-fragment | NA | | 0 |
| S1_k141_215101 | 1069 | No | NA | | 0 | 1 | Not-determined | Genome-fragment | NA | | 0 |
| S1_k141_379361 | 1117 | No | NA | | 0 | 0 | Not-determined | Genome-fragment | NA | | 0 |
| S1-final-viral-combined_k141_353806\|\|full | 1448 | No | NA | | 0 | 0 | Low-quality | Genome-fragment | | 1.35 | 0 |
| S1_k141_280780 | 1268 | No | NA | | 0 | 1 | Not-determined | Genome-fragment | NA | | 0 |
| S1_k141_313558 | 7695 | No | NA | | 0 | 4 | Not-determined | Genome-fragment | NA | | 0 |
| S1_k141_280793 | 1330 | No | NA | | 0 | 0 | Not-determined | Genome-fragment | NA | | 0 |
| S1_k141_1282 | 1346 | No | NA | | 0 | 1 | Not-determined | Genome-fragment | NA | | 0 |
| S1_k141_379373 | 2984 | No | NA | | 0 | 2 | Not-determined | Genome-fragment | NA | | 0 |
| S1-final-viral-combined_k141_324199\|\|full | 1448 | No | NA | | 0 | 0 | Low-quality | Genome-fragment | | 2.38 | 0 |
| S1-final-viral-combined_k141_393222\|\|full | 1448 | No | NA | | 0 | 0 | Low-quality | Genome-fragment | | 2.37 | 0 |
| S1_k141_17423 | 1478 | No | NA | | 0 | 0 | Not-determined | Genome-fragment | NA | | 0 |
| S1_k141_215157 | 1302 | No | NA | | 0 | 1 | Not-determined | Genome-fragment | NA | | 0 |
| S1_k141_379405 | 8039 | No | NA | | 0 | 3 | Not-determined | Genome-fragment | NA | | 0 |
| S1_k141_136803 | 1448 | No | NA | | 1 | 0 | Low-quality | Genome-fragment | | 1.02 | 0 |
| S1_k141_33707 | 1571 | No | NA | | 0 | 2 | Not-determined | Genome-fragment | NA | | 0 |
| S1_k141_1353 | 1104 | No | NA | | 0 | 1 | Not-determined | Genome-fragment | NA | | 0 |
| S1_k141_264294 | 1280 | No | NA | | 0 | 0 | Not-determined | Genome-fragment | NA | | 0 |
| S7-final-viral-combined_k141_127306\|\|full | 1448 | No | NA | | 0 | 1 | Low-quality | Genome-fragment | | 3.95 | 0 |
| S1_k141_329973 | 1314 | No | NA | | 0 | 0 | Not-determined | Genome-fragment | NA | | 0 |
| S7_k141_207728 | 1448 | No | NA | | 1 | 0 | Low-quality | Genome-fragment | | 1.13 | 0 |
| S1_k141_247964 | 1056 | No | NA | | 0 | 1 | Not-determined | Genome-fragment | NA | | 0 |
| S17-final-viral-combined_k141_39597\|\|full | 1448 | No | NA | | 2 | 0 | Low-quality | Genome-fragment | | 2.94 | 0 |
| S1_k141_363087 | 2027 | No | NA | | 0 | 2 | Not-determined | Genome-fragment | NA | | 0 |
| S17-k141_125546 | 1448 | No | NA | | 1 | 0 | Low-quality | Genome-fragment | | 1.5 | 0 |
| S1_k141_149099 | 1287 | No | NA | | 0 | 0 | Not-determined | Genome-fragment | NA | | 0 |
| S12-final-viral-combined_k141_242906\|\|full | 1448 | No | NA | | 1 | 0 | Low-quality | Genome-fragment | | 0.79 | 0 |
| S12_k141_375002 | 1448 | No | NA | | 1 | 0 | Low-quality | Genome-fragment | | 0.78 | 0 |
| S1_k141_33806 | 3408 | No | NA | | 0 | 1 | Not-determined | Genome-fragment | NA | | 0 |
| S1_k141_363106 | 7523 | No | NA | | 0 | 1 | Not-determined | Genome-fragment | NA | | 0 |
| S2-final-viral-combined_k141_196986\|\|full | 1448 | No | NA | | 0 | 0 | Low-quality | Genome-fragment | | 2.96 | 0 |
| S4_k141_111661 | 1448 | No | NA | | 1 | 0 | Low-quality | Genome-fragment | | 1.95 | 0 |
| S1_k141_379465 | 1196 | No | NA | | 0 | 1 | Not-determined | Genome-fragment | NA | | 0 |
| S1_k141_313751 | 1654 | No | NA | | 0 | 0 | Not-determined | Genome-fragment | NA | | 0 |
| S1_k141_198678 | 3495 | No | NA | | 0 | 0 | Not-determined | Genome-fragment | NA | | 0 |
| S1_k141_66572 | 3948 | No | NA | | 0 | 2 | Not-determined | Genome-fragment | NA | | 0 |
| S1_k141_248067 | 1604 | No | NA | | 0 | 1 | Not-determined | Genome-fragment | NA | | 0 |
| S1_k141_379498 | 5113 | No | NA | | 0 | 2 | Not-determined | Genome-fragment | NA | | 0 |
| S8-final-viral-combined_k141_10336\|\|full | 1448 | No | NA | | 1 | 0 | Low-quality | Genome-fragment | | 2.87 | 0 |
| S11-final-viral-combined_k141_319881\|\|full | 1449 | No | NA | | 0 | 0 | Low-quality | Genome-fragment | | 1.44 | 0 |
| S1_k141_346852 | 1005 | No | NA | | 0 | 0 | Not-determined | Genome-fragment | NA | | 0 |
| S1_k141_17614 | 1633 | No | NA | | 0 | 1 | Not-determined | Genome-fragment | NA | | 0 |
| S1_k141_363193 | 5948 | No | NA | | 0 | 2 | Not-determined | Genome-fragment | NA | | 0 |
| S11-final-viral-combined_k141_160341\|\|full | 1449 | Yes | | 1249 | 1 | 1 | Low-quality | Genome-fragment | | 1.92 | 13.8 |
| S1-final-viral-combined_k141_162165\|\|full | 1449 | No | NA | | 0 | 2 | Low-quality | Genome-fragment | | 1.03 | 0 |
| S1_k141_17628 | 1589 | No | NA | | 0 | 0 | Not-determined | Genome-fragment | NA | | 0 |
| S1_k141_116555 | 2359 | No | NA | | 0 | 0 | Not-determined | Genome-fragment | NA | | 0 |
| S1_k141_248108 | 1682 | No | NA | | 0 | 1 | Not-determined | Genome-fragment | NA | | 0 |
| S1_k141_50131 | 3336 | No | NA | | 0 | 2 | Not-determined | Genome-fragment | NA | | 0 |
| S1_k141_363254 | 1098 | No | NA | | 0 | 1 | Not-determined | Genome-fragment | NA | | 0 |
| S1_k141_50140 | 1057 | No | NA | | 0 | 0 | Not-determined | Genome-fragment | NA | | 0 |
| S1_k141_281104 | 1023 | No | NA | | 0 | 1 | Not-determined | Genome-fragment | NA | | 0 |
| S1_k141_363266 | 3186 | No | NA | | 0 | 1 | Not-determined | Genome-fragment | NA | | 0 |
| S1_k141_165673 | 1031 | No | NA | | 0 | 1 | Not-determined | Genome-fragment | NA | | 0 |
| S1_k141_17719 | 3571 | No | NA | | 0 | 1 | Not-determined | Genome-fragment | NA | | 0 |
| S1_k141_281128 | 1510 | No | NA | | 0 | 0 | Not-determined | Genome-fragment | NA | | 0 |
| S1_k141_34021 | 1456 | No | NA | | 0 | 2 | Not-determined | Genome-fragment | NA | | 0 |
| S1-final-viral-combined_k141_187745\|\|lt2gene | 1449 | No | NA | | 1 | 0 | Low-quality | Genome-fragment | | 2.12 | 0 |
| S1_k141_354483 | 1449 | No | NA | | 0 | 1 | Low-quality | Genome-fragment | | 1.29 | 0 |
| S1_k141_379562 | 4587 | No | NA | | 0 | 2 | Not-determined | Genome-fragment | NA | | 0 |
| S1_k141_264373 | 1102 | No | NA | | 0 | 0 | Not-determined | Genome-fragment | NA | | 0 |
| S1_k141_330273 | 1585 | No | NA | | 0 | 1 | Not-determined | Genome-fragment | NA | | 0 |
| S7_k141_141066 | 1449 | No | NA | | 0 | 0 | Low-quality | Genome-fragment | | 1.65 | 0 |
| S1_k141_83582 | 1238 | No | NA | | 0 | 1 | Not-determined | Genome-fragment | NA | | 0 |
| S1_k141_198939 | 1399 | No | NA | | 0 | 2 | Not-determined | Genome-fragment | NA | | 0 |
| S1_k141_165709 | 1337 | No | NA | | 0 | 0 | Not-determined | Genome-fragment | NA | | 0 |
| S1_k141_264419 | 11105 | No | NA | | 0 | 7 | Not-determined | Genome-fragment | NA | | 0 |
| S1_k141_313991 | 11007 | No | NA | | 4 | 0 | Medium-quality | Genome-fragment | | 80.73 | 0 |
| S17-final-viral-combined_k141_112600\|\|full | 1449 | No | NA | | 0 | 0 | Low-quality | Genome-fragment | | 3.81 | 0 |
| S1_k141_264426 | 1298 | No | NA | | 0 | 2 | Not-determined | Genome-fragment | NA | | 0 |
| S17-final-viral-combined_k141_114548\|\|full | 1449 | No | NA | | 1 | 0 | Low-quality | Genome-fragment | | 3.37 | 0 |
| S1_k141_34159 | 1654 | No | NA | | 0 | 1 | Not-determined | Genome-fragment | NA | | 0 |
| S1_k141_379651 | 1044 | No | NA | | 0 | 0 | Not-determined | Genome-fragment | NA | | 0 |
| S12-final-viral-combined_k141_308675\|\|full | 1449 | No | NA | | 2 | 0 | Low-quality | Genome-fragment | | 3.11 | 0 |
| S12_k141_140012 | 1449 | No | NA | | 0 | 1 | Low-quality | Genome-fragment | | 0.88 | 0 |
| S1_k141_330348 | 1189 | No | NA | | 0 | 0 | Not-determined | Genome-fragment | NA | | 0 |
| S1_k141_379734 | 1233 | No | NA | | 0 | 1 | Not-determined | Genome-fragment | NA | | 0 |
| S2_k141_133130 | 1449 | No | NA | | 2 | 0 | Low-quality | Genome-fragment | | 2.45 | 0 |
| S6-final-viral-combined_k141_71302\|\|lt2gene | 1449 | No | NA | | 1 | 0 | Low-quality | Genome-fragment | | 1.9 | 0 |
| S1_k141_1775 | 4505 | No | NA | | 0 | 7 | Not-determined | Genome-fragment | NA | | 0 |
| S1_k141_34276 | 1056 | No | NA | | 0 | 1 | Not-determined | Genome-fragment | NA | | 0 |
| S1_k141_297677 | 1359 | No | NA | | 0 | 2 | Not-determined | Genome-fragment | NA | | 0 |
| S1_k141_182490 | 2817 | No | NA | | 0 | 1 | Not-determined | Genome-fragment | NA | | 0 |
| S1_k141_363350 | 1471 | No | NA | | 0 | 0 | Not-determined | Genome-fragment | NA | | 0 |
| S1_k141_363359 | 1604 | No | NA | | 0 | 0 | Not-determined | Genome-fragment | NA | | 0 |
| S1_k141_66750 | 1139 | No | NA | | 0 | 0 | Not-determined | Genome-fragment | NA | | 0 |
| S1_k141_83750 | 1180 | No | NA | | 0 | 0 | Not-determined | Genome-fragment | NA | | 0 |
| S1_k141_182522 | 1819 | No | NA | | 0 | 0 | Not-determined | Genome-fragment | NA | | 0 |
| S1_k141_116736 | 2340 | No | NA | | 0 | 1 | Not-determined | Genome-fragment | NA | | 0 |
| S1_k141_314147 | 4185 | No | NA | | 0 | 1 | Not-determined | Genome-fragment | NA | | 0 |
| S1_k141_116753 | 1052 | No | NA | | 0 | 2 | Not-determined | Genome-fragment | NA | | 0 |
| S1_k141_231737 | 2532 | No | NA | | 0 | 0 | Not-determined | Genome-fragment | NA | | 0 |
| S1_k141_165760 | 1475 | No | NA | | 0 | 1 | Not-determined | Genome-fragment | NA | | 0 |
| S1_k141_363413 | 1105 | No | NA | | 0 | 1 | Not-determined | Genome-fragment | NA | | 0 |
| S1_k141_248227 | 6517 | No | NA | | 0 | 1 | Not-determined | Genome-fragment | NA | | 0 |
| S1_k141_314183 | 1977 | No | NA | | 0 | 1 | Not-determined | Genome-fragment | NA | | 0 |
| S11-final-viral-combined_k141_321116\|\|full | 1450 | No | NA | | 0 | 1 | Low-quality | Genome-fragment | | 1.84 | 0 |
| S1_k141_314187 | 5510 | No | NA | | 0 | 0 | Not-determined | Genome-fragment | NA | | 0 |
| S1-final-viral-combined_k141_223663\|\|full | 1450 | No | NA | | 0 | 1 | Low-quality | Genome-fragment | | 1.76 | 0 |
| S1_k141_379883 | 1020 | No | NA | | 0 | 1 | Not-determined | Genome-fragment | NA | | 0 |
| S1_k141_149474 | 1246 | No | NA | | 0 | 1 | Not-determined | Genome-fragment | NA | | 0 |
| S1_k141_215661 | 1388 | No | NA | | 0 | 1 | Not-determined | Genome-fragment | NA | | 0 |
| S1_k141_17967 | 2605 | No | NA | | 0 | 0 | Not-determined | Genome-fragment | NA | | 0 |
| S17-final-viral-combined_k141_247219\|\|full | 1450 | No | NA | | 0 | 0 | Low-quality | Genome-fragment | | 4.35 | 0 |
| S1_k141_100010 | 45981 | No | NA | | 10 | 0 | High-quality | High-quality | | 100 | 0 |
| S1_k141_379917 | 1231 | No | NA | | 0 | 0 | Not-determined | Genome-fragment | NA | | 0 |
| S2-final-viral-combined_k141_13826\|\|full | 1450 | No | NA | | 0 | 1 | Low-quality | Genome-fragment | | 3.49 | 0 |
| S1_k141_116805 | 3376 | No | NA | | 0 | 1 | Not-determined | Genome-fragment | NA | | 0 |
| S1_k141_116808 | 2143 | No | NA | | 0 | 2 | Not-determined | Genome-fragment | NA | | 0 |
| S1_k141_379925 | 1724 | No | NA | | 0 | 1 | Not-determined | Genome-fragment | NA | | 0 |
| S1_k141_281408 | 1181 | No | NA | | 0 | 1 | Not-determined | Genome-fragment | NA | | 0 |
| S1_k141_100052 | 1039 | No | NA | | 0 | 2 | Not-determined | Genome-fragment | NA | | 0 |
| S1_k141_264541 | 2294 | No | NA | | 0 | 0 | Not-determined | Genome-fragment | NA | | 0 |
| S1_k141_133501 | 3270 | No | NA | | 0 | 1 | Not-determined | Genome-fragment | NA | | 0 |
| S2_k141_247491 | 1450 | No | NA | | 0 | 0 | Low-quality | Genome-fragment | | 8.03 | 0 |
| S4-final-viral-combined_k141_86285\|\|full | 1450 | No | NA | | 0 | 0 | Low-quality | Genome-fragment | | 3.07 | 0 |
| S1_k141_1974 | 4146 | No | NA | | 0 | 2 | Not-determined | Genome-fragment | NA | | 0 |
| S1_k141_281443 | 3039 | No | NA | | 0 | 1 | Not-determined | Genome-fragment | NA | | 0 |
| S1_k141_281452 | 1093 | No | NA | | 0 | 0 | Not-determined | Genome-fragment | NA | | 0 |
| S4-final-viral-combined_k141_72035\|\|lt2gene | 1450 | No | NA | | 0 | 1 | Low-quality | Genome-fragment | | 1.68 | 0 |
| S4_k141_36792 | 1450 | No | NA | | 0 | 0 | Low-quality | Genome-fragment | | 1.46 | 0 |
| S1_k141_314308 | 1681 | No | NA | | 0 | 0 | Not-determined | Genome-fragment | NA | | 0 |
| S1_k141_18075 | 1751 | No | NA | | 0 | 1 | Not-determined | Genome-fragment | NA | | 0 |
| S1_k141_330591 | 1236 | No | NA | | 0 | 0 | Not-determined | Genome-fragment | NA | | 0 |
| S1_k141_330599 | 1520 | No | NA | | 0 | 1 | Not-determined | Genome-fragment | NA | | 0 |
| S1_k141_231917 | 2123 | No | NA | | 0 | 0 | Not-determined | Genome-fragment | NA | | 0 |
| S1_k141_100137 | 1501 | No | NA | | 0 | 2 | Not-determined | Genome-fragment | NA | | 0 |
| S1_k141_149619 | 1075 | No | NA | | 0 | 0 | Not-determined | Genome-fragment | NA | | 0 |
| S1_k141_363612 | 1526 | No | NA | | 0 | 2 | Not-determined | Genome-fragment | NA | | 0 |
| S1_k141_330613 | 5616 | No | NA | | 0 | 1 | Not-determined | Genome-fragment | NA | | 0 |
| S1_k141_248457 | 1683 | No | NA | | 0 | 1 | Not-determined | Genome-fragment | NA | | 0 |
| S1_k141_2081 | 11897 | No | NA | | 0 | 8 | Not-determined | Genome-fragment | NA | | 0 |
| S1_k141_297985 | 1036 | No | NA | | 0 | 1 | Not-determined | Genome-fragment | NA | | 0 |
| S1_k141_330669 | 5062 | No | NA | | 0 | 2 | Not-determined | Genome-fragment | NA | | 0 |
| S1_k141_100205 | 3115 | No | NA | | 0 | 1 | Not-determined | Genome-fragment | NA | | 0 |
| S1_k141_380049 | 1070 | No | NA | | 0 | 2 | Not-determined | Genome-fragment | NA | | 0 |
| S1_k141_133629 | 1691 | No | NA | | 0 | 1 | Not-determined | Genome-fragment | NA | | 0 |
| S1_k141_330680 | 3474 | No | NA | | 0 | 4 | Not-determined | Genome-fragment | NA | | 0 |
| S1_k141_215765 | 1055 | No | NA | | 0 | 0 | Not-determined | Genome-fragment | NA | | 0 |
| S1_k141_380063 | 5052 | No | NA | | 0 | 3 | Not-determined | Genome-fragment | NA | | 0 |
| S1_k141_2118 | 1417 | No | NA | | 0 | 1 | Not-determined | Genome-fragment | NA | | 0 |
| S1_k141_199294 | 3462 | No | NA | | 0 | 1 | Not-determined | Genome-fragment | NA | | 0 |
| S1_k141_50538 | 1205 | No | NA | | 0 | 1 | Not-determined | Genome-fragment | NA | | 0 |
| S1_k141_149674 | 1630 | No | NA | | 0 | 0 | Not-determined | Genome-fragment | NA | | 0 |
| S1_k141_149683 | 1668 | No | NA | | 0 | 0 | Not-determined | Genome-fragment | NA | | 0 |
| S1_k141_199324 | 1226 | No | NA | | 0 | 1 | Not-determined | Genome-fragment | NA | | 0 |
| S1_k141_264627 | 1460 | No | NA | | 0 | 0 | Not-determined | Genome-fragment | NA | | 0 |
| S1_k141_182788 | 2143 | No | NA | | 0 | 1 | Not-determined | Genome-fragment | NA | | 0 |
| S6-final-viral-combined_k141_95829\|\|full | 1450 | No | NA | | 0 | 2 | Low-quality | Genome-fragment | | 0.64 | 0 |
| S1_k141_66889 | 1139 | No | NA | | 0 | 0 | Not-determined | Genome-fragment | NA | | 0 |
| S1_k141_380136 | 1056 | No | NA | | 0 | 2 | Not-determined | Genome-fragment | NA | | 0 |
| S1_k141_165873 | 3046 | No | NA | | 0 | 2 | Not-determined | Genome-fragment | NA | | 0 |
| S1_k141_264655 | 1154 | No | NA | | 0 | 0 | Not-determined | Genome-fragment | NA | | 0 |
| S1_k141_100319 | 1465 | No | NA | | 0 | 1 | Not-determined | Genome-fragment | NA | | 0 |
| S1_k141_2194 | 1120 | No | NA | | 0 | 0 | Not-determined | Genome-fragment | NA | | 0 |
| S6-final-viral-combined_k141_62300\|\|full | 1450 | No | NA | | 0 | 0 | Low-quality | Genome-fragment | | 2.28 | 0 |
| S1_k141_182830 | 1456 | No | NA | | 0 | 2 | Not-determined | Genome-fragment | NA | | 0 |
| S8-final-viral-combined_k141_224303\|\|lt2gene | 1450 | No | NA | | 1 | 0 | Low-quality | Genome-fragment | | 2.53 | 0 |
| S8-final-viral-combined_k141_181655\|\|lt2gene | 1450 | No | NA | | 1 | 0 | Low-quality | Genome-fragment | | 2.45 | 0 |
| S1_k141_34669 | 1371 | No | NA | | 0 | 1 | Not-determined | Genome-fragment | NA | | 0 |
| S1_k141_182855 | 4604 | No | NA | | 0 | 1 | Not-determined | Genome-fragment | NA | | 0 |
| S9-final-viral-combined_k141_57824\|\|full | 1451 | No | NA | | 0 | 1 | Low-quality | Genome-fragment | | 1.71 | 0 |
| S1_k141_149761 | 1864 | No | NA | | 0 | 1 | Not-determined | Genome-fragment | NA | | 0 |
| S1_k141_149782 | 2412 | No | NA | | 0 | 2 | Not-determined | Genome-fragment | NA | | 0 |

| | | | | | | | | | | | |
|---|---|---|---|---|---|---|---|---|---|---|---|
| S1_k141_314455 | 1823 | No | NA | | 0 | 0 | Not-determined | Genome-fragment | NA | | 0 |
| S9-final-viral-combined_k141_68467\|\|full | 1451 | No | NA | | 1 | 0 | Low-quality | Genome-fragment | | 2.39 | 0 |
| S1_k141_100412 | 3309 | No | NA | | 0 | 2 | Not-determined | Genome-fragment | NA | | 0 |
| S1_k141_67027 | 2823 | No | NA | | 0 | 2 | Not-determined | Genome-fragment | NA | | 0 |
| S1_k141_314467 | 1283 | No | NA | | 0 | 1 | Not-determined | Genome-fragment | NA | | 0 |
| S1_k141_264767 | 1195 | No | NA | | 0 | 1 | Not-determined | Genome-fragment | NA | | 0 |
| S1_k141_363799 | 8029 | No | NA | | 0 | 3 | Not-determined | Genome-fragment | NA | | 0 |
| S1_k141_18342 | 1545 | No | NA | | 0 | 1 | Not-determined | Genome-fragment | NA | | 0 |
| S1_k141_165946 | 3053 | No | NA | | 0 | 1 | Not-determined | Genome-fragment | NA | | 0 |
| S1_k141_149824 | 1801 | No | NA | | 0 | 1 | Not-determined | Genome-fragment | NA | | 0 |
| S11-final-viral-combined_k141_318046\|\|full | 1451 | No | NA | | 0 | 1 | Low-quality | Genome-fragment | | 3.09 | 0 |
| S1_k141_50723 | 1426 | No | NA | | 0 | 0 | Not-determined | Genome-fragment | NA | | 0 |
| S1_k141_18358 | 1973 | No | NA | | 0 | 0 | Not-determined | Genome-fragment | NA | | 0 |
| S11-final-viral-combined_k141_162604\|\|full | 1451 | No | NA | | 1 | 0 | Low-quality | Genome-fragment | | 4.05 | 0 |
| S1_k141_84213 | 3808 | No | NA | | 0 | 0 | Not-determined | Genome-fragment | NA | | 0 |
| S1_k141_18362 | 3772 | No | NA | | 0 | 4 | Not-determined | Genome-fragment | NA | | 0 |
| S1_k141_182975 | 2938 | No | NA | | 0 | 1 | Not-determined | Genome-fragment | NA | | 0 |
| S1_k141_84236 | 1069 | No | NA | | 0 | 1 | Not-determined | Genome-fragment | NA | | 0 |
| S1_k141_307242 | 1451 | No | NA | | 0 | 0 | Low-quality | Genome-fragment | | 1.54 | 0 |
| S1_k141_182981 | 1496 | No | NA | | 0 | 1 | Not-determined | Genome-fragment | NA | | 0 |
| S1_k141_363880 | 1131 | No | NA | | 0 | 1 | Not-determined | Genome-fragment | NA | | 0 |
| S1_k141_281789 | 3119 | No | NA | | 0 | 1 | Not-determined | Genome-fragment | NA | | 0 |
| S3-final-viral-combined_k141_46198\|\|full | 1451 | No | NA | | 0 | 1 | Low-quality | Genome-fragment | | 0.84 | 0 |
| S1_k141_216057 | 1034 | No | NA | | 0 | 0 | Not-determined | Genome-fragment | NA | | 0 |
| S1_k141_347671 | 1609 | No | NA | | 0 | 0 | Not-determined | Genome-fragment | NA | | 0 |
| S5-final-viral-combined_k141_112040\|\|full | 1451 | No | NA | | 3 | 0 | Low-quality | Genome-fragment | | 3.79 | 0 |
| S1_k141_50867 | 1028 | No | NA | | 0 | 1 | Not-determined | Genome-fragment | NA | | 0 |
| S5_k141_102696 | 1451 | No | NA | | 1 | 0 | Low-quality | Genome-fragment | | 2.66 | 0 |
| S1_k141_347723 | 3193 | No | NA | | 0 | 3 | Not-determined | Genome-fragment | NA | | 0 |
| S1_k141_100554 | 2956 | No | NA | | 0 | 0 | Not-determined | Genome-fragment | NA | | 0 |
| S1_k141_264900 | 2156 | No | NA | | 0 | 2 | Not-determined | Genome-fragment | NA | | 0 |
| S1_k141_232280 | 1220 | No | NA | | 0 | 1 | Not-determined | Genome-fragment | NA | | 0 |
| S1_k141_232309 | 1601 | No | NA | | 0 | 1 | Not-determined | Genome-fragment | NA | | 0 |
| S17-final-viral-combined_k141_176353\|\|full | 1451 | No | NA | | 0 | 0 | Low-quality | Genome-fragment | | 1.14 | 0 |
| S12_k141_17531 | 1451 | Yes | | 1014 | 1 | 1 | Low-quality | Genome-fragment | | 0.61 | 30.12 |
| S2-final-viral-combined_k141_219962\|\|lt2gene | 1451 | No | NA | | 1 | 0 | Low-quality | Genome-fragment | | 2.42 | 0 |
| S1_k141_34881 | 3329 | No | NA | | 0 | 0 | Not-determined | Genome-fragment | NA | | 0 |
| S1_k141_380339 | 1409 | No | NA | | 0 | 2 | Not-determined | Genome-fragment | NA | | 0 |
| S4-final-viral-combined_k141_154216\|\|full | 1451 | No | NA | | 0 | 0 | Low-quality | Genome-fragment | | 1.95 | 0 |
| S1_k141_232385 | 1126 | No | NA | | 0 | 0 | Not-determined | Genome-fragment | NA | | 0 |
| S1_k141_134063 | 1977 | No | NA | | 0 | 1 | Not-determined | Genome-fragment | NA | | 0 |
| S1_k141_51057 | 1541 | No | NA | | 0 | 0 | Not-determined | Genome-fragment | NA | | 0 |
| S1_k141_380378 | 3583 | No | NA | | 0 | 3 | Not-determined | Genome-fragment | NA | | 0 |
| S1_k141_67296 | 1154 | No | NA | | 0 | 0 | Not-determined | Genome-fragment | NA | | 0 |
| S1_k141_183171 | 1052 | No | NA | | 0 | 0 | Not-determined | Genome-fragment | NA | | 0 |
| S1_k141_281927 | 4114 | No | NA | | 0 | 6 | Not-determined | Genome-fragment | NA | | 0 |
| S4-final-viral-combined_k141_87860\|\|full | 1451 | No | NA | | 0 | 1 | Low-quality | Genome-fragment | | 0.9 | 0 |
| S4-final-viral-combined_k141_23385\|\|lt2gene | 1451 | No | NA | | 0 | 0 | Low-quality | Genome-fragment | | 1.44 | 0 |
| S1_k141_298418 | 4244 | No | NA | | 0 | 3 | Not-determined | Genome-fragment | NA | | 0 |
| S17-final-viral-combined_k141_226377\|\|full | 1452 | No | NA | | 1 | 0 | Low-quality | Genome-fragment | | 3.31 | 0 |
| S1_k141_34981 | 1307 | No | NA | | 0 | 0 | Not-determined | Genome-fragment | NA | | 0 |
| S1_k141_265012 | 2578 | No | NA | | 0 | 1 | Not-determined | Genome-fragment | NA | | 0 |
| S1_k141_298457 | 1694 | No | NA | | 0 | 1 | Not-determined | Genome-fragment | NA | | 0 |
| S1_k141_282043 | 4788 | No | NA | | 0 | 2 | Not-determined | Genome-fragment | NA | | 0 |
| S1_k141_199721 | 1655 | No | NA | | 0 | 0 | Not-determined | Genome-fragment | NA | | 0 |
| S1_k141_249091 | 1176 | No | NA | | 0 | 0 | Not-determined | Genome-fragment | NA | | 0 |
| S1_k141_265070 | 1264 | No | NA | | 0 | 1 | Not-determined | Genome-fragment | NA | | 0 |
| S17-k141_88476 | 1452 | No | NA | | 1 | 0 | Low-quality | Genome-fragment | | 3.47 | 0 |
| S1_k141_84536 | 1293 | No | NA | | 0 | 1 | Not-determined | Genome-fragment | NA | | 0 |
| S1_k141_282073 | 1003 | No | NA | | 0 | 1 | Not-determined | Genome-fragment | NA | | 0 |
| S1_k141_347874 | 1830 | No | NA | | 0 | 3 | Not-determined | Genome-fragment | NA | | 0 |
| S1_k141_298525 | 1033 | No | NA | | 0 | 0 | Not-determined | Genome-fragment | NA | | 0 |
| S12-final-viral-combined_k141_137896\|\|full | 1452 | No | NA | | 0 | 0 | Low-quality | Genome-fragment | | 3.39 | 0 |
| S1_k141_166290 | 1550 | No | NA | | 0 | 0 | Not-determined | Genome-fragment | NA | | 0 |
| S2-final-viral-combined_k141_30449\|\|full | 1452 | No | NA | | 1 | 0 | Low-quality | Genome-fragment | | 3.85 | 0 |
| S1_k141_347910 | 2793 | No | NA | | 0 | 0 | Not-determined | Genome-fragment | NA | | 0 |
| S1_k141_380529 | 1130 | No | NA | | 0 | 0 | Not-determined | Genome-fragment | NA | | 0 |
| S1_k141_364203 | 2099 | No | NA | | 0 | 0 | Not-determined | Genome-fragment | NA | | 0 |
| S1_k141_84655 | 1220 | No | NA | | 0 | 2 | Not-determined | Genome-fragment | NA | | 0 |
| S1_k141_183343 | 1102 | No | NA | | 0 | 0 | Not-determined | Genome-fragment | NA | | 0 |
| S1_k141_331074 | 1144 | No | NA | | 0 | 0 | Not-determined | Genome-fragment | NA | | 0 |
| S1_k141_282083 | 2576 | No | NA | | 0 | 0 | Not-determined | Genome-fragment | NA | | 0 |
| S1_k141_232626 | 1424 | No | NA | | 0 | 1 | Not-determined | Genome-fragment | NA | | 0 |
| S4-final-viral-combined_k141_113593\|\|full | 1452 | No | NA | | 0 | 0 | Low-quality | Genome-fragment | | 0.82 | 0 |
| S1_k141_117474 | 7870 | No | NA | | 0 | 1 | Not-determined | Genome-fragment | NA | | 0 |
| S1_k141_249224 | 1893 | No | NA | | 0 | 2 | Not-determined | Genome-fragment | NA | | 0 |
| S1_k141_331086 | 1517 | No | NA | | 0 | 0 | Not-determined | Genome-fragment | NA | | 0 |
| S1_k141_298619 | 4821 | No | NA | | 0 | 4 | Not-determined | Genome-fragment | NA | | 0 |
| S1_k141_51322 | 1056 | No | NA | | 0 | 0 | Not-determined | Genome-fragment | NA | | 0 |
| S1_k141_282133 | 2254 | No | NA | | 0 | 2 | Not-determined | Genome-fragment | NA | | 0 |
| S1_k141_216487 | 4121 | No | NA | | 0 | 1 | Not-determined | Genome-fragment | NA | | 0 |
| S4-final-viral-combined_k141_166529\|\|full | 1452 | No | NA | | 0 | 2 | Low-quality | Genome-fragment | | 0.86 | 0 |
| S1_k141_150207 | 3054 | No | NA | | 0 | 3 | Not-determined | Genome-fragment | NA | | 0 |
| S1_k141_2669 | 2193 | No | NA | | 0 | 2 | Not-determined | Genome-fragment | NA | | 0 |
| S1_k141_18704 | 1024 | No | NA | | 0 | 2 | Not-determined | Genome-fragment | NA | | 0 |
| S6_k141_66944 | 1452 | No | NA | | 1 | 0 | Low-quality | Genome-fragment | | 0.78 | 0 |
| S1_k141_2699 | 4456 | No | NA | | 0 | 0 | Not-determined | Genome-fragment | NA | | 0 |
| S1_k141_364312 | 1342 | No | NA | | 0 | 2 | Not-determined | Genome-fragment | NA | | 0 |
| S6_k141_19955 | 1452 | No | NA | | 0 | 1 | Low-quality | Genome-fragment | | 0.37 | 0 |
| S1_k141_18714 | 1477 | No | NA | | 0 | 1 | Not-determined | Genome-fragment | NA | | 0 |
| S1_k141_265274 | 1099 | No | NA | | 0 | 1 | Not-determined | Genome-fragment | NA | | 0 |
| S1_k141_84776 | 1413 | No | NA | | 0 | 3 | Not-determined | Genome-fragment | NA | | 0 |
| S8-final-viral-combined_k141_209529\|\|full | 1452 | No | NA | | 0 | 0 | Low-quality | Genome-fragment | | 3.23 | 0 |
| S8_k141_103828 | 1452 | No | NA | | 0 | 0 | Low-quality | Genome-fragment | | 0.68 | 0 |
| S1_k141_100773 | 12895 | No | NA | | 0 | 6 | Not-determined | Genome-fragment | NA | | 0 |
| S1_k141_216642 | 7322 | No | NA | | 0 | 2 | Not-determined | Genome-fragment | NA | | 0 |
| S9-final-viral-combined_k141_79931\|\|full | 1453 | No | NA | | 0 | 0 | Low-quality | Genome-fragment | | 2.27 | 0 |
| S1_k141_134452 | 1759 | No | NA | | 0 | 1 | Not-determined | Genome-fragment | NA | | 0 |
| S1_k141_265285 | 39134 | No | NA | | 24 | 1 | High-quality | High-quality | | 100 | 0 |
| S1_k141_380711 | 3410 | No | NA | | 0 | 4 | Not-determined | Genome-fragment | NA | | 0 |
| S1_k141_84826 | 3675 | No | NA | | 0 | 1 | Not-determined | Genome-fragment | NA | | 0 |
| S1_k141_331162 | 1379 | No | NA | | 0 | 1 | Not-determined | Genome-fragment | NA | | 0 |
| S1_k141_282238 | 1263 | No | NA | | 0 | 0 | Not-determined | Genome-fragment | NA | | 0 |
| S1_k141_166470 | 3136 | No | NA | | 0 | 3 | Not-determined | Genome-fragment | NA | | 0 |
| S1_k141_298799 | 1111 | No | NA | | 0 | 0 | Not-determined | Genome-fragment | NA | | 0 |
| S1_k141_331173 | 3080 | No | NA | | 0 | 1 | Not-determined | Genome-fragment | NA | | 0 |
| S1_k141_265308 | 1106 | No | NA | | 0 | 0 | Not-determined | Genome-fragment | NA | | 0 |
| S1_k141_166490 | 1680 | No | NA | | 0 | 1 | Not-determined | Genome-fragment | NA | | 0 |
| S1_k141_150361 | 1375 | No | NA | | 0 | 0 | Not-determined | Genome-fragment | NA | | 0 |
| S1_k141_84890 | 2597 | No | NA | | 0 | 2 | Not-determined | Genome-fragment | NA | | 0 |
| S1_k141_2845 | 1130 | No | NA | | 0 | 1 | Not-determined | Genome-fragment | NA | | 0 |
| S9_k141_21474 | 1453 | No | NA | | 0 | 0 | Low-quality | Genome-fragment | | 2.43 | 0 |
| S1_k141_200044 | 1216 | No | NA | | 0 | 0 | Not-determined | Genome-fragment | NA | | 0 |
| S1_k141_84931 | 1377 | No | NA | | 0 | 2 | Not-determined | Genome-fragment | NA | | 0 |
| S11-final-viral-combined_k141_35338\|\|full | 1453 | No | NA | | 0 | 1 | Low-quality | Genome-fragment | | 1.9 | 0 |
| S11_k141_2200 | 1453 | No | NA | | 0 | 1 | Low-quality | Genome-fragment | | 2.74 | 0 |
| S1_k141_18826 | 3555 | No | NA | | 0 | 3 | Not-determined | Genome-fragment | NA | | 0 |
| S1-final-viral-combined_k141_244617\|\|full | 1453 | No | NA | | 0 | 0 | Low-quality | Genome-fragment | | 3.48 | 0 |
| S1_k141_216774 | 1944 | No | NA | | 0 | 1 | Not-determined | Genome-fragment | NA | | 0 |
| S1_k141_380817 | 1484 | No | NA | | 0 | 1 | Not-determined | Genome-fragment | NA | | 0 |
| S1-final-viral-combined_k141_377526\|\|full | 1453 | No | NA | | 0 | 0 | Low-quality | Genome-fragment | | 4.57 | 0 |
| S1-final-viral-combined_k141_387434\|\|lt2gene | 1453 | No | NA | | 2 | 0 | Low-quality | Genome-fragment | | 3.2 | 0 |
| S1_k141_100919 | 1292 | No | NA | | 0 | 2 | Not-determined | Genome-fragment | NA | | 0 |
| S1_k141_51467 | 1735 | No | NA | | 0 | 0 | Not-determined | Genome-fragment | NA | | 0 |
| S1_k141_150484 | 1261 | No | NA | | 0 | 0 | Not-determined | Genome-fragment | NA | | 0 |
| S1_k141_364542 | 1934 | No | NA | | 0 | 1 | Not-determined | Genome-fragment | NA | | 0 |
| S1_k141_18884 | 1272 | No | NA | | 0 | 0 | Not-determined | Genome-fragment | NA | | 0 |
| S1_k141_183626 | 1044 | No | NA | | 0 | 1 | Not-determined | Genome-fragment | NA | | 0 |
| S1_k141_216827 | 3124 | No | NA | | 0 | 3 | Not-determined | Genome-fragment | NA | | 0 |
| S17-final-viral-combined_k141_213547\|\|full | 1453 | No | NA | | 0 | 0 | Low-quality | Genome-fragment | | 11.46 | 0 |
| S1_k141_51531 | 3667 | No | NA | | 0 | 1 | Not-determined | Genome-fragment | NA | | 0 |
| S1_k141_265362 | 1283 | No | NA | | 0 | 0 | Not-determined | Genome-fragment | NA | | 0 |
| S1_k141_134643 | 1059 | No | NA | | 0 | 1 | Not-determined | Genome-fragment | NA | | 0 |
| S1_k141_67874 | 1450 | No | NA | | 0 | 2 | Not-determined | Genome-fragment | NA | | 0 |
| S1_k141_51565 | 5463 | No | NA | | 0 | 3 | Not-determined | Genome-fragment | NA | | 0 |
| S17-final-viral-combined_k141_216708\|\|lt2gene | 1453 | No | NA | | 1 | 0 | Low-quality | Genome-fragment | | 3.86 | 0 |
| S1_k141_183694 | 1187 | No | NA | | 0 | 4 | Not-determined | Genome-fragment | NA | | 0 |
| S1_k141_380881 | 3026 | No | NA | | 0 | 2 | Not-determined | Genome-fragment | NA | | 0 |
| S12-final-viral-combined_k141_133547\|\|full | 1453 | No | NA | | 0 | 1 | Low-quality | Genome-fragment | | 2.68 | 0 |
| S1_k141_232991 | 3397 | No | NA | | 0 | 1 | Not-determined | Genome-fragment | NA | | 0 |
| S1_k141_2995 | 1685 | No | NA | | 0 | 3 | Not-determined | Genome-fragment | NA | | 0 |
| S1_k141_249638 | 1983 | No | NA | | 0 | 0 | Not-determined | Genome-fragment | NA | | 0 |
| S1_k141_331483 | 7120 | No | NA | | 0 | 16 | Not-determined | Genome-fragment | NA | | 0 |
| S1_k141_249663 | 1888 | No | NA | | 0 | 1 | Not-determined | Genome-fragment | NA | | 0 |
| S1_k141_265501 | 1296 | No | NA | | 0 | 3 | Not-determined | Genome-fragment | NA | | 0 |
| S1_k141_101108 | 4047 | No | NA | | 0 | 1 | Not-determined | Genome-fragment | NA | | 0 |
| S12_k141_80099 | 1453 | No | NA | | 1 | 0 | Low-quality | Genome-fragment | | 2.62 | 0 |
| S1_k141_134772 | 1062 | No | NA | | 0 | 0 | Not-determined | Genome-fragment | NA | | 0 |
| S4-final-viral-combined_k141_56055\|\|full | 1453 | No | NA | | 0 | 0 | Low-quality | Genome-fragment | | 0.87 | 0 |
| S1_k141_19046 | 3956 | No | NA | | 0 | 1 | Not-determined | Genome-fragment | NA | | 0 |
| S1_k141_249732 | 2104 | No | NA | | 0 | 0 | Not-determined | Genome-fragment | NA | | 0 |
| S1_k141_85108 | 1484 | No | NA | | 0 | 2 | Not-determined | Genome-fragment | NA | | 0 |
| S1_k141_101164 | 1673 | No | NA | | 0 | 1 | Not-determined | Genome-fragment | NA | | 0 |
| S1_k141_85132 | 1070 | No | NA | | 0 | 1 | Not-determined | Genome-fragment | NA | | 0 |
| S8-final-viral-combined_k141_127658\|\|lt2gene | 1453 | No | NA | | 1 | 0 | Low-quality | Genome-fragment | | 1 | 0 |
| S1_k141_67976 | 2578 | No | NA | | 0 | 1 | Not-determined | Genome-fragment | NA | | 0 |
| S8_k141_276011 | 1453 | No | NA | | 0 | 3 | Low-quality | Genome-fragment | | 0.49 | 0 |
| S1_k141_249771 | 1022 | No | NA | | 0 | 0 | Not-determined | Genome-fragment | NA | | 0 |
| S1_k141_117975 | 11716 | No | NA | | 0 | 4 | Not-determined | Genome-fragment | NA | | 0 |
| S1_k141_249825 | 1220 | No | NA | | 0 | 0 | Not-determined | Genome-fragment | NA | | 0 |
| S1_k141_150790 | 4814 | No | NA | | 0 | 3 | Not-determined | Genome-fragment | NA | | 0 |
| S1_k141_381023 | 1777 | No | NA | | 0 | 1 | Not-determined | Genome-fragment | NA | | 0 |
| S11_k141_125010 | 1454 | No | NA | | 0 | 0 | Low-quality | Genome-fragment | | 3.78 | 0 |
| S1-final-viral-combined_k141_306652\|\|full | 1454 | No | NA | | 0 | 0 | Low-quality | Genome-fragment | | 3.34 | 0 |
| S1_k141_299239 | 5622 | No | NA | | 0 | 3 | Not-determined | Genome-fragment | NA | | 0 |
| S1_k141_249863 | 1134 | No | NA | | 0 | 1 | Not-determined | Genome-fragment | NA | | 0 |
| S1_k141_348487 | 5167 | No | NA | | 0 | 5 | Not-determined | Genome-fragment | NA | | 0 |
| S1_k141_249880 | 1425 | No | NA | | 0 | 2 | Not-determined | Genome-fragment | NA | | 0 |
| S1_k141_118005 | 3785 | No | NA | | 0 | 1 | Not-determined | Genome-fragment | NA | | 0 |
| S12-final-viral-combined_k141_366114\|\|full | 1454 | No | NA | | 1 | 0 | Low-quality | Genome-fragment | | 3.27 | 0 |
| S1_k141_200310 | 2167 | No | NA | | 0 | 1 | Not-determined | Genome-fragment | NA | | 0 |
| S1_k141_265588 | 1441 | No | NA | | 0 | 1 | Not-determined | Genome-fragment | NA | | 0 |
| S1_k141_233106 | 3485 | No | NA | | 0 | 0 | Not-determined | Genome-fragment | NA | | 0 |
| S2_k141_216316 | 1454 | No | NA | | 0 | 0 | Low-quality | Genome-fragment | | 2.83 | 0 |

| | | | | | | | | | |
|---|---|---|---|---|---|---|---|---|---|
| S1_k141_315574 | 3599 | No | NA | 0 | 2 | Not-determined | Genome-fragment | NA | 0 |
| S1_k141_315578 | 1104 | No | NA | 0 | 1 | Not-determined | Genome-fragment | NA | 0 |
| S4-final-viral-combined_k141_138662\|\|full | 1454 | No | NA | 0 | 0 | Low-quality | Genome-fragment | 0.9 | 0 |
| S1_k141_282743 | 1737 | No | NA | 0 | 1 | Not-determined | Genome-fragment | NA | 0 |
| S6-final-viral-combined_k141_41527\|\|full | 1454 | No | NA | 0 | 1 | Low-quality | Genome-fragment | 0.94 | 0 |
| S1_k141_35744 | 1602 | No | NA | 0 | 2 | Not-determined | Genome-fragment | NA | 0 |
| S1_k141_331580 | 4461 | No | NA | 0 | 4 | Not-determined | Genome-fragment | NA | 0 |
| S8-final-viral-combined_k141_135637\|\|lt2gene | 1454 | Yes | 1260 | 1 | 1 | Low-quality | Genome-fragment | 1.22 | 13.34 |
| S1_k141_299419 | 1497 | No | NA | 0 | 1 | Not-determined | Genome-fragment | NA | 0 |
| S1_k141_200403 | 1175 | No | NA | 0 | 1 | Not-determined | Genome-fragment | NA | 0 |
| S1_k141_101280 | 11549 | No | NA | 0 | 1 | Not-determined | Genome-fragment | NA | 0 |
| S1_k141_381142 | 3254 | No | NA | 0 | 2 | Not-determined | Genome-fragment | NA | 0 |
| S1_k141_134954 | 1763 | No | NA | 0 | 1 | Not-determined | Genome-fragment | NA | 0 |
| S1_k141_184173 | 1403 | No | NA | 0 | 1 | Not-determined | Genome-fragment | NA | 0 |
| S9-final-viral-combined_k141_55427\|\|full | 1455 | No | NA | 0 | 1 | Low-quality | Genome-fragment | 3.76 | 0 |
| S1_k141_299455 | 1358 | No | NA | 0 | 1 | Not-determined | Genome-fragment | NA | 0 |
| S9-final-viral-combined_k141_50996\|\|full | 1455 | No | NA | 0 | 0 | Low-quality | Genome-fragment | 24.07 | 0 |
| S3-final-viral-combined_k141_72261\|\|full | 1455 | No | NA | 0 | 1 | Low-quality | Genome-fragment | 1.02 | 0 |
| S1_k141_233209 | 2430 | No | NA | 0 | 0 | Not-determined | Genome-fragment | NA | 0 |
| S1_k141_184212 | 1590 | No | NA | 0 | 0 | Not-determined | Genome-fragment | NA | 0 |
| S1_k141_3120 | 2323 | No | NA | 0 | 2 | Not-determined | Genome-fragment | NA | 0 |
| S1_k141_118162 | 1399 | No | NA | 0 | 1 | Not-determined | Genome-fragment | NA | 0 |
| S1_k141_299482 | 6573 | No | NA | 0 | 4 | Not-determined | Genome-fragment | NA | 0 |
| S1_k141_365034 | 1025 | No | NA | 0 | 0 | Not-determined | Genome-fragment | NA | 0 |
| S1_k141_35852 | 4374 | No | NA | 0 | 1 | Not-determined | Genome-fragment | NA | 0 |
| S7-final-viral-combined_k141_146633\|\|full | 1455 | No | NA | 0 | 0 | Low-quality | Genome-fragment | 4.16 | 0 |
| S1_k141_184235 | 1102 | No | NA | 0 | 0 | Not-determined | Genome-fragment | NA | 0 |
| S1_k141_184236 | 1315 | No | NA | 0 | 0 | Not-determined | Genome-fragment | NA | 0 |
| S1_k141_365063 | 2173 | No | NA | 0 | 2 | Not-determined | Genome-fragment | NA | 0 |
| S7-final-viral-combined_k141_105982\|\|full | 1455 | No | NA | 0 | 2 | Low-quality | Genome-fragment | 0.76 | 0 |
| S1_k141_315699 | 1212 | No | NA | 0 | 0 | Not-determined | Genome-fragment | NA | 0 |
| S7_k141_25010 | 1455 | No | NA | 0 | 1 | Low-quality | Genome-fragment | 0.4 | 0 |
| S17-k141_189390 | 1455 | No | NA | 0 | 0 | Low-quality | Genome-fragment | 2.86 | 0 |
| S1_k141_184249 | 1600 | No | NA | 0 | 1 | Not-determined | Genome-fragment | NA | 0 |
| S17-k141_216899 | 1455 | No | NA | 0 | 0 | Low-quality | Genome-fragment | 1.78 | 0 |
| S1_k141_200489 | 7746 | No | NA | 0 | 0 | Not-determined | Genome-fragment | NA | 0 |
| S2-final-viral-combined_k141_219098\|\|full | 1455 | No | NA | 0 | 0 | Low-quality | Genome-fragment | 2.7 | 0 |
| S1_k141_282869 | 1406 | No | NA | 0 | 0 | Not-determined | Genome-fragment | NA | 0 |
| S1_k141_348764 | 4484 | No | NA | 0 | 4 | Not-determined | Genome-fragment | NA | 0 |
| S1_k141_166857 | 2060 | No | NA | 0 | 1 | Not-determined | Genome-fragment | NA | 0 |
| S1_k141_217265 | 6420 | No | NA | 0 | 4 | Not-determined | Genome-fragment | NA | 0 |
| S1_k141_52061 | 3024 | No | NA | 0 | 2 | Not-determined | Genome-fragment | NA | 0 |
| S2-final-viral-combined_k141_163891\|\|full | 1455 | No | NA | 0 | 0 | Low-quality | Genome-fragment | 3.01 | 0 |
| S8-final-viral-combined_k141_245448\|\|full | 1455 | No | NA | 0 | 0 | Low-quality | Genome-fragment | 3.34 | 0 |
| S1_k141_265870 | 4535 | No | NA | 0 | 4 | Not-determined | Genome-fragment | NA | 0 |
| S11-final-viral-combined_k141_69208\|\|full | 1456 | No | NA | 0 | 1 | Low-quality | Genome-fragment | 4.65 | 0 |
| S11-final-viral-combined_k141_108853\|\|full | 1456 | No | NA | 1 | 0 | Low-quality | Genome-fragment | 0.9 | 0 |
| S1_k141_233385 | 3681 | No | NA | 0 | 2 | Not-determined | Genome-fragment | NA | 0 |
| S1_k141_118297 | 1107 | No | NA | 0 | 2 | Not-determined | Genome-fragment | NA | 0 |
| S1_k141_331873 | 3627 | No | NA | 0 | 2 | Not-determined | Genome-fragment | NA | 0 |
| S1_k141_265905 | 1094 | No | NA | 0 | 1 | Not-determined | Genome-fragment | NA | 0 |
| S1_k141_200564 | 1440 | No | NA | 0 | 2 | Not-determined | Genome-fragment | NA | 0 |
| S1-final-viral-combined_k141_121334\|\|full | 1456 | No | NA | 0 | 0 | Low-quality | Genome-fragment | 3.02 | 0 |
| S1_k141_135202 | 1905 | No | NA | 0 | 1 | Not-determined | Genome-fragment | NA | 0 |
| S3-final-viral-combined_k141_52734\|\|full | 1456 | No | NA | 1 | 0 | Low-quality | Genome-fragment | 3.09 | 0 |
| S1_k141_118344 | 1346 | No | NA | 0 | 0 | Not-determined | Genome-fragment | NA | 0 |
| S1_k141_151125 | 1530 | No | NA | 0 | 2 | Not-determined | Genome-fragment | NA | 0 |
| S1_k141_52154 | 1298 | No | NA | 0 | 0 | Not-determined | Genome-fragment | NA | 0 |
| S1_k141_315901 | 2168 | No | NA | 0 | 1 | Not-determined | Genome-fragment | NA | 0 |
| S1_k141_118391 | 1275 | No | NA | 0 | 1 | Not-determined | Genome-fragment | NA | 0 |
| S1_k141_381436 | 1173 | No | NA | 0 | 0 | Not-determined | Genome-fragment | NA | 0 |
| S1_k141_331935 | 3644 | No | NA | 0 | 0 | Not-determined | Genome-fragment | NA | 0 |
| S12_k141_184196 | 1456 | No | NA | 0 | 1 | Low-quality | Genome-fragment | 0.74 | 0 |
| S1_k141_217470 | 7997 | No | NA | 0 | 1 | Not-determined | Genome-fragment | NA | 0 |
| S1_k141_85381 | 1267 | No | NA | 0 | 1 | Not-determined | Genome-fragment | NA | 0 |
| S1_k141_52234 | 1618 | No | NA | 0 | 1 | Not-determined | Genome-fragment | NA | 0 |
| S1_k141_283070 | 2049 | No | NA | 0 | 0 | Not-determined | Genome-fragment | NA | 0 |
| S1_k141_250266 | 1047 | No | NA | 0 | 1 | Not-determined | Genome-fragment | NA | 0 |
| S1_k141_52265 | 1314 | No | NA | 0 | 2 | Not-determined | Genome-fragment | NA | 0 |
| S2-final-viral-combined_k141_242108\|\|full | 1456 | No | NA | 0 | 0 | Low-quality | Genome-fragment | 1.14 | 0 |
| S1_k141_52314 | 2197 | No | NA | 0 | 3 | Not-determined | Genome-fragment | NA | 0 |
| S1_k141_266028 | 3587 | No | NA | 0 | 2 | Not-determined | Genome-fragment | NA | 0 |
| S1_k141_381523 | 1531 | No | NA | 0 | 1 | Not-determined | Genome-fragment | NA | 0 |
| S1_k141_118552 | 2074 | No | NA | 0 | 5 | Not-determined | Genome-fragment | NA | 0 |
| S1_k141_167168 | 1159 | No | NA | 0 | 1 | Not-determined | Genome-fragment | NA | 0 |
| S1_k141_283139 | 4712 | No | NA | 0 | 0 | Not-determined | Genome-fragment | NA | 0 |
| S1_k141_151295 | 1012 | No | NA | 0 | 1 | Not-determined | Genome-fragment | NA | 0 |
| S1_k141_299787 | 1337 | No | NA | 0 | 1 | Not-determined | Genome-fragment | NA | 0 |
| S1_k141_184636 | 1432 | No | NA | 0 | 1 | Not-determined | Genome-fragment | NA | 0 |
| S1_k141_36257 | 1275 | No | NA | 0 | 0 | Not-determined | Genome-fragment | NA | 0 |
| S1_k141_266051 | 3974 | No | NA | 0 | 0 | Not-determined | Genome-fragment | NA | 0 |
| S1_k141_250318 | 1594 | No | NA | 0 | 0 | Not-determined | Genome-fragment | NA | 0 |
| S1_k141_283179 | 1175 | No | NA | 0 | 1 | Not-determined | Genome-fragment | NA | 0 |
| S6-final-viral-combined_k141_57036\|\|full | 1456 | No | NA | 0 | 0 | Low-quality | Genome-fragment | 3.41 | 0 |
| S1_k141_68505 | 6935 | No | NA | 0 | 5 | Not-determined | Genome-fragment | NA | 0 |
| S11-final-viral-combined_k141_29467\|\|full | 1457 | No | NA | 0 | 0 | Low-quality | Genome-fragment | 3.72 | 0 |
| S1_k141_68526 | 1251 | No | NA | 0 | 2 | Not-determined | Genome-fragment | NA | 0 |
| S1_k141_101632 | 1330 | No | NA | 0 | 1 | Not-determined | Genome-fragment | NA | 0 |
| S1-final-viral-combined_k141_99541\|\|full | 1457 | No | NA | 0 | 0 | Low-quality | Genome-fragment | 1.05 | 0 |
| S1_k141_101635 | 1033 | No | NA | 0 | 0 | Not-determined | Genome-fragment | NA | 0 |
| S1_k141_151389 | 1789 | No | NA | 0 | 4 | Not-determined | Genome-fragment | NA | 0 |
| S1-final-viral-combined_k141_323644\|\|full | 1457 | No | NA | 4 | 0 | Low-quality | Genome-fragment | 3.43 | 0 |
| S1_k141_266226 | 1489 | No | NA | 0 | 0 | Not-determined | Genome-fragment | NA | 0 |
| S1_k141_200857 | 5149 | No | NA | 0 | 5 | Not-determined | Genome-fragment | NA | 0 |
| S1_k141_200890 | 1329 | No | NA | 0 | 0 | Not-determined | Genome-fragment | NA | 0 |
| S7-final-viral-combined_k141_71380\|\|full | 1457 | No | NA | 0 | 2 | Low-quality | Genome-fragment | 1.57 | 0 |
| S12-final-viral-combined_k141_102677\|\|full | 1457 | No | NA | 0 | 0 | Low-quality | Genome-fragment | 1.16 | 0 |
| S1_k141_266278 | 1268 | No | NA | 0 | 2 | Not-determined | Genome-fragment | NA | 0 |
| S1_k141_200923 | 1910 | No | NA | 0 | 1 | Not-determined | Genome-fragment | NA | 0 |
| S1_k141_266289 | 1192 | No | NA | 0 | 0 | Not-determined | Genome-fragment | NA | 0 |
| S1_k141_381734 | 1338 | No | NA | 0 | 1 | Not-determined | Genome-fragment | NA | 0 |
| S1_k141_68607 | 1697 | No | NA | 0 | 0 | Not-determined | Genome-fragment | NA | 0 |
| S12-final-viral-combined_k141_220303\|\|full | 1457 | No | NA | 0 | 1 | Low-quality | Genome-fragment | 0.63 | 0 |
| S1_k141_85577 | 1591 | No | NA | 0 | 1 | Not-determined | Genome-fragment | NA | 0 |
| S1_k141_349160 | 1225 | No | NA | 0 | 2 | Not-determined | Genome-fragment | NA | 0 |
| S12_k141_192967 | 1457 | No | NA | 1 | 0 | Low-quality | Genome-fragment | 0.79 | 0 |
| S9-final-viral-combined_k141_26573\|\|full | 1458 | No | NA | 4 | 0 | Low-quality | Genome-fragment | 3.5 | 0 |
| S1_k141_167311 | 9702 | No | NA | 0 | 6 | Not-determined | Genome-fragment | NA | 0 |
| S1_k141_365621 | 1092 | No | NA | 0 | 1 | Not-determined | Genome-fragment | NA | 0 |
| S1_k141_184868 | 2362 | No | NA | 0 | 0 | Not-determined | Genome-fragment | NA | 0 |
| S9_k141_34362 | 1458 | No | NA | 2 | 0 | Low-quality | Genome-fragment | 2.61 | 0 |
| S11-final-viral-combined_k141_227607\|\|full | 1458 | No | NA | 1 | 0 | Low-quality | Genome-fragment | 2.63 | 0 |
| S1_k141_135652 | 1160 | No | NA | 0 | 1 | Not-determined | Genome-fragment | NA | 0 |
| S1_k141_151630 | 2805 | No | NA | 0 | 0 | Not-determined | Genome-fragment | NA | 0 |
| S1-final-viral-combined_k141_313743\|\|full | 1458 | No | NA | 1 | 0 | Low-quality | Genome-fragment | 3.6 | 0 |
| S1_k141_52521 | 1338 | No | NA | 0 | 0 | Not-determined | Genome-fragment | NA | 0 |
| S1_k141_167371 | 1025 | No | NA | 0 | 1 | Not-determined | Genome-fragment | NA | 0 |
| S1_k141_365640 | 1620 | No | NA | 0 | 3 | Not-determined | Genome-fragment | NA | 0 |
| S1_k141_52538 | 1012 | No | NA | 0 | 0 | Not-determined | Genome-fragment | NA | 0 |
| S1-final-viral-combined_k141_188532\|\|full | 1458 | No | NA | 0 | 1 | Low-quality | Genome-fragment | 3.52 | 0 |
| S1_k141_283403 | 4574 | No | NA | 0 | 1 | Not-determined | Genome-fragment | NA | 0 |
| S1_k141_201009 | 1109 | No | NA | 0 | 1 | Not-determined | Genome-fragment | NA | 0 |
| S1_k141_19946 | 1206 | No | NA | 0 | 1 | Not-determined | Genome-fragment | NA | 0 |
| S1_k141_184905 | 3195 | No | NA | 0 | 2 | Not-determined | Genome-fragment | NA | 0 |
| S1_k141_316417 | 1145 | No | NA | 0 | 2 | Not-determined | Genome-fragment | NA | 0 |
| S1-final-viral-combined_k141_44035\|\|lt2gene | 1458 | No | NA | 1 | 0 | Low-quality | Genome-fragment | 3.82 | 0 |
| S1_k141_36486 | 1103 | No | NA | 0 | 0 | Not-determined | Genome-fragment | NA | 0 |
| S17-final-viral-combined_k141_188786\|\|full | 1458 | No | NA | 0 | 1 | Low-quality | Genome-fragment | 1.17 | 0 |
| S1_k141_266500 | 4818 | No | NA | 0 | 5 | Not-determined | Genome-fragment | NA | 0 |
| S1_k141_266510 | 1026 | No | NA | 0 | 1 | Not-determined | Genome-fragment | NA | 0 |
| S1_k141_85698 | 7226 | No | NA | 0 | 4 | Not-determined | Genome-fragment | NA | 0 |
| S1_k141_283468 | 1266 | No | NA | 0 | 0 | Not-determined | Genome-fragment | NA | 0 |
| S1_k141_52608 | 3408 | No | NA | 0 | 3 | Not-determined | Genome-fragment | NA | 0 |
| S1_k141_85717 | 1653 | No | NA | 0 | 0 | Not-determined | Genome-fragment | NA | 0 |
| S1_k141_135692 | 6092 | No | NA | 0 | 7 | Not-determined | Genome-fragment | NA | 0 |
| S1_k141_85727 | 1106 | No | NA | 0 | 2 | Not-determined | Genome-fragment | NA | 0 |
| S1_k141_381907 | 1011 | No | NA | 0 | 0 | Not-determined | Genome-fragment | NA | 0 |
| S1_k141_349276 | 3120 | No | NA | 0 | 1 | Not-determined | Genome-fragment | NA | 0 |
| S1_k141_185014 | 1915 | No | NA | 0 | 0 | Not-determined | Genome-fragment | NA | 0 |
| S12_k141_189780 | 1458 | No | NA | 1 | 0 | Low-quality | Genome-fragment | 1.54 | 0 |
| S2-final-viral-combined_k141_178421\|\|full | 1458 | No | NA | 0 | 0 | Low-quality | Genome-fragment | 2.7 | 0 |
| S1_k141_233994 | 4499 | No | NA | 0 | 2 | Not-determined | Genome-fragment | NA | 0 |
| S1_k141_332409 | 3011 | No | NA | 0 | 1 | Not-determined | Genome-fragment | NA | 0 |
| S1_k141_101929 | 1176 | No | NA | 0 | 1 | Not-determined | Genome-fragment | NA | 0 |
| S1_k141_234010 | 1587 | No | NA | 0 | 2 | Not-determined | Genome-fragment | NA | 0 |
| S1_k141_332422 | 2836 | No | NA | 0 | 3 | Not-determined | Genome-fragment | NA | 0 |
| S1_k141_151740 | 5898 | No | NA | 0 | 3 | Not-determined | Genome-fragment | NA | 0 |
| S1_k141_332451 | 3877 | No | NA | 0 | 0 | Not-determined | Genome-fragment | NA | 0 |
| S1_k141_316607 | 3410 | No | NA | 0 | 4 | Not-determined | Genome-fragment | NA | 0 |
| S1_k141_85804 | 1081 | No | NA | 0 | 1 | Not-determined | Genome-fragment | NA | 0 |
| S1_k141_381997 | 2151 | No | NA | 0 | 2 | Not-determined | Genome-fragment | NA | 0 |
| S1_k141_135733 | 1274 | No | NA | 0 | 2 | Not-determined | Genome-fragment | NA | 0 |
| S1_k141_266619 | 1677 | No | NA | 0 | 0 | Not-determined | Genome-fragment | NA | 0 |
| S1_k141_185046 | 29667 | No | NA | 6 | 1 | Medium-quality | Genome-fragment | 70.38 | 0 |
| S2-final-viral-combined_k141_232280\|\|full | 1458 | No | NA | 1 | 0 | Low-quality | Genome-fragment | 3.46 | 0 |
| S9-final-viral-combined_k141_31489\|\|full | 1459 | No | NA | 0 | 1 | Low-quality | Genome-fragment | 0.72 | 0 |
| S11-final-viral-combined_k141_153743\|\|full | 1459 | No | NA | 0 | 1 | Low-quality | Genome-fragment | 1.87 | 0 |
| S1-final-viral-combined_k141_335374\|\|full | 1459 | No | NA | 0 | 0 | Low-quality | Genome-fragment | 3.38 | 0 |
| S1_k141_300411 | 1206 | No | NA | 0 | 0 | Not-determined | Genome-fragment | NA | 0 |
| S1_k141_332502 | 1561 | No | NA | 0 | 1 | Not-determined | Genome-fragment | NA | 0 |
| S5_k141_51165 | 1459 | No | NA | 0 | 0 | Low-quality | Genome-fragment | 3.07 | 0 |
| S4-final-viral-combined_k141_127807\|\|full | 1459 | No | NA | 0 | 0 | Low-quality | Genome-fragment | 3.52 | 0 |
| S1_k141_349415 | 2564 | No | NA | 0 | 0 | Not-determined | Genome-fragment | NA | 0 |
| S1_k141_185095 | 3431 | No | NA | 0 | 3 | Not-determined | Genome-fragment | NA | 0 |
| S1_k141_119026 | 1844 | No | NA | 0 | 2 | Not-determined | Genome-fragment | NA | 0 |
| S1_k141_167612 | 1012 | No | NA | 0 | 0 | Not-determined | Genome-fragment | NA | 0 |
| S1_k141_119047 | 1643 | No | NA | 0 | 1 | Not-determined | Genome-fragment | NA | 0 |
| S1_k141_300450 | 1202 | No | NA | 0 | 1 | Not-determined | Genome-fragment | NA | 0 |
| S1_k141_316800 | 1003 | No | NA | 0 | 1 | Not-determined | Genome-fragment | NA | 0 |
| S1_k141_151897 | 1222 | No | NA | 0 | 0 | Not-determined | Genome-fragment | NA | 0 |
| S1_k141_234158 | 1411 | No | NA | 0 | 1 | Not-determined | Genome-fragment | NA | 0 |
| S1_k141_52845 | 1217 | No | NA | 0 | 1 | Not-determined | Genome-fragment | NA | 0 |
| S1_k141_300485 | 3963 | No | NA | 0 | 1 | Not-determined | Genome-fragment | NA | 0 |
| S1_k141_36560 | 9361 | No | NA | 0 | 3 | Not-determined | Genome-fragment | NA | 0 |

| | | | | | | | | | |
|---|---|---|---|---|---|---|---|---|---|
| S1_k141_85890 | 1117 | No | NA | 0 | 1 | Not-determined | Genome-fragment | NA | 0 |
| S1_k141_300504 | 1187 | No | NA | 0 | 2 | Not-determined | Genome-fragment | NA | 0 |
| S6_k141_104818 | 1459 | No | NA | 0 | 0 | Low-quality | Genome-fragment | 2.13 | 0 |
| S1_k141_310340 | 1460 | No | NA | 0 | 1 | Low-quality | Genome-fragment | 2.87 | 0 |
| S5-final-viral-combined_k141_19352\|\|full | 1460 | No | NA | 0 | 0 | Low-quality | Genome-fragment | 2.93 | 0 |
| S1_k141_217774 | 2073 | No | NA | 0 | 0 | Not-determined | Genome-fragment | NA | 0 |
| S1_k141_52922 | 1223 | No | NA | 0 | 1 | Not-determined | Genome-fragment | NA | 0 |
| S1_k141_135974 | 1048 | No | NA | 0 | 1 | Not-determined | Genome-fragment | NA | 0 |
| S5-final-viral-combined_k141_63937\|\|full | 1460 | No | NA | 0 | 0 | Low-quality | Genome-fragment | 2.64 | 0 |
| S1_k141_217819 | 1389 | No | NA | 0 | 0 | Not-determined | Genome-fragment | NA | 0 |
| S1_k141_185265 | 11712 | No | NA | 0 | 4 | Not-determined | Genome-fragment | NA | 0 |
| S1_k141_102124 | 1261 | No | NA | 0 | 1 | Not-determined | Genome-fragment | NA | 0 |
| S1_k141_85998 | 3338 | No | NA | 0 | 2 | Not-determined | Genome-fragment | NA | 0 |
| S1_k141_349540 | 1080 | No | NA | 0 | 1 | Not-determined | Genome-fragment | NA | 0 |
| S1_k141_152030 | 1380 | No | NA | 0 | 1 | Not-determined | Genome-fragment | NA | 0 |
| S5-final-viral-combined_k141_111255\|\|full | 1460 | No | NA | 0 | 0 | Low-quality | Genome-fragment | 1.64 | 0 |
| S1_k141_349544 | 1126 | No | NA | 0 | 0 | Not-determined | Genome-fragment | NA | 0 |
| S1_k141_365799 | 1590 | No | NA | 0 | 1 | Not-determined | Genome-fragment | NA | 0 |
| S7-final-viral-combined_k141_180484\|\|full | 1460 | No | NA | 0 | 1 | Low-quality | Genome-fragment | 1.56 | 0 |
| S1_k141_217850 | 1054 | No | NA | 0 | 1 | Not-determined | Genome-fragment | NA | 0 |
| S1_k141_36582 | 1525 | No | NA | 0 | 0 | Not-determined | Genome-fragment | NA | 0 |
| S1_k141_365806 | 1487 | No | NA | 0 | 1 | Not-determined | Genome-fragment | NA | 0 |
| S12-final-viral-combined_k141_382642\|\|full | 1460 | No | NA | 0 | 0 | Low-quality | Genome-fragment | 3.43 | 0 |
| S12-final-viral-combined_k141_148869\|\|full | 1460 | No | NA | 0 | 1 | Low-quality | Genome-fragment | 0.74 | 0 |
| S1_k141_53006 | 4438 | No | NA | 0 | 11 | Not-determined | Genome-fragment | NA | 0 |
| S1_k141_36621 | 3463 | No | NA | 0 | 0 | Not-determined | Genome-fragment | NA | 0 |
| S12-final-viral-combined_k141_34333\|\|lt2gene | 1460 | No | NA | 1 | 0 | Low-quality | Genome-fragment | 2.46 | 0 |
| S2-final-viral-combined_k141_157787\|\|full | 1460 | No | NA | 3 | 0 | Low-quality | Genome-fragment | 4.18 | 0 |
| S1_k141_68926 | 1168 | No | NA | 0 | 1 | Not-determined | Genome-fragment | NA | 0 |
| S1_k141_201544 | 2005 | No | NA | 0 | 0 | Not-determined | Genome-fragment | NA | 0 |
| S1_k141_185395 | 3594 | No | NA | 0 | 4 | Not-determined | Genome-fragment | NA | 0 |
| S1_k141_251246 | 1024 | No | NA | 0 | 2 | Not-determined | Genome-fragment | NA | 0 |
| S1_k141_86082 | 1161 | No | NA | 0 | 1 | Not-determined | Genome-fragment | NA | 0 |
| S1_k141_68980 | 2234 | No | NA | 0 | 1 | Not-determined | Genome-fragment | NA | 0 |
| S2-final-viral-combined_k141_216555\|\|full | 1460 | No | NA | 0 | 0 | Low-quality | Genome-fragment | 0.99 | 0 |
| S2-final-viral-combined_k141_59696\|\|lt2gene | 1460 | No | NA | 1 | 0 | Low-quality | Genome-fragment | 3.02 | 0 |
| S8-final-viral-combined_k141_82017\|\|full | 1460 | No | NA | 0 | 0 | Low-quality | Genome-fragment | 2.57 | 0 |
| S11-final-viral-combined_k141_15646\|\|full | 1461 | No | NA | 1 | 0 | Low-quality | Genome-fragment | 2.64 | 0 |
| S1_k141_349728 | 1024 | No | NA | 0 | 0 | Not-determined | Genome-fragment | NA | 0 |
| S1_k141_20467 | 3988 | No | NA | 0 | 0 | Not-determined | Genome-fragment | NA | 0 |
| S1_k141_283882 | 1223 | No | NA | 0 | 0 | Not-determined | Genome-fragment | NA | 0 |
| S1_k141_185489 | 4074 | No | NA | 0 | 1 | Not-determined | Genome-fragment | NA | 0 |
| S1_k141_201641 | 3597 | No | NA | 0 | 1 | Not-determined | Genome-fragment | NA | 0 |
| S1_k141_317129 | 3268 | No | NA | 0 | 3 | Not-determined | Genome-fragment | NA | 0 |
| S1_k141_234465 | 1037 | No | NA | 0 | 1 | Not-determined | Genome-fragment | NA | 0 |
| S1_k141_317134 | 1507 | No | NA | 0 | 1 | Not-determined | Genome-fragment | NA | 0 |
| S1_k141_283925 | 3005 | No | NA | 0 | 2 | Not-determined | Genome-fragment | NA | 0 |
| S1-final-viral-combined_k141_159306\|\|full | 1461 | No | NA | 0 | 1 | Low-quality | Genome-fragment | 2.53 | 0 |
| S1_k141_300716 | 1379 | No | NA | 0 | 1 | Not-determined | Genome-fragment | NA | 0 |
| S5_k141_165774 | 1461 | No | NA | 0 | 1 | Low-quality | Genome-fragment | 0.84 | 0 |
| S7-final-viral-combined_k141_86304\|\|full | 1461 | No | NA | 0 | 1 | Low-quality | Genome-fragment | 1.21 | 0 |
| S1_k141_36778 | 2536 | No | NA | 0 | 1 | Not-determined | Genome-fragment | NA | 0 |
| S1_k141_365974 | 1639 | No | NA | 0 | 1 | Not-determined | Genome-fragment | NA | 0 |
| S1_k141_382330 | 1081 | No | NA | 0 | 1 | Not-determined | Genome-fragment | NA | 0 |
| S1_k141_20539 | 1028 | No | NA | 0 | 2 | Not-determined | Genome-fragment | NA | 0 |
| S1_k141_267188 | 1466 | No | NA | 0 | 1 | Not-determined | Genome-fragment | NA | 0 |
| S17-final-viral-combined_k141_161285\|\|full | 1461 | No | NA | 1 | 0 | Low-quality | Genome-fragment | 2.52 | 0 |
| S1_k141_332917 | 1129 | No | NA | 0 | 0 | Not-determined | Genome-fragment | NA | 0 |
| S1_k141_185562 | 1016 | No | NA | 0 | 1 | Not-determined | Genome-fragment | NA | 0 |
| S1_k141_152143 | 1153 | No | NA | 0 | 0 | Not-determined | Genome-fragment | NA | 0 |
| S1_k141_152153 | 1375 | No | NA | 0 | 2 | Not-determined | Genome-fragment | NA | 0 |
| S1_k141_349910 | 1144 | No | NA | 0 | 0 | Not-determined | Genome-fragment | NA | 0 |
| S1_k141_152170 | 3313 | No | NA | 0 | 1 | Not-determined | Genome-fragment | NA | 0 |
| S2_k141_115581 | 1461 | No | NA | 0 | 0 | Low-quality | Genome-fragment | 0.25 | 0 |
| S1_k141_152179 | 1498 | No | NA | 0 | 1 | Not-determined | Genome-fragment | NA | 0 |
| S1_k141_300845 | 1138 | No | NA | 0 | 1 | Not-determined | Genome-fragment | NA | 0 |
| S9-final-viral-combined_k141_36940\|\|full | 1462 | No | NA | 0 | 0 | Low-quality | Genome-fragment | 1.77 | 0 |
| S1_k141_251477 | 1520 | No | NA | 0 | 0 | Not-determined | Genome-fragment | NA | 0 |
| S1_k141_332973 | 1406 | No | NA | 0 | 0 | Not-determined | Genome-fragment | NA | 0 |
| S1_k141_332982 | 3498 | No | NA | 0 | 1 | Not-determined | Genome-fragment | NA | 0 |
| S1_k141_349956 | 1180 | No | NA | 0 | 1 | Not-determined | Genome-fragment | NA | 0 |
| S1_k141_218112 | 2429 | No | NA | 0 | 2 | Not-determined | Genome-fragment | NA | 0 |
| S1_k141_119545 | 2926 | No | NA | 0 | 2 | Not-determined | Genome-fragment | NA | 0 |
| S1_k141_167983 | 4055 | No | NA | 0 | 3 | Not-determined | Genome-fragment | NA | 0 |
| S1_k141_102342 | 79954 | No | NA | 20 | 4 | Medium-quality | Genome-fragment | 58.02 | 0 |
| S1_k141_218148 | 1738 | No | NA | 0 | 0 | Not-determined | Genome-fragment | NA | 0 |
| S1_k141_36928 | 1067 | No | NA | 0 | 2 | Not-determined | Genome-fragment | NA | 0 |
| S1_k141_366116 | 4705 | No | NA | 0 | 2 | Not-determined | Genome-fragment | NA | 0 |
| S1_k141_36932 | 1006 | No | NA | 0 | 0 | Not-determined | Genome-fragment | NA | 0 |
| S1_k141_86210 | 1807 | No | NA | 0 | 0 | Not-determined | Genome-fragment | NA | 0 |
| S1_k141_350011 | 1045 | No | NA | 0 | 0 | Not-determined | Genome-fragment | NA | 0 |
| S11-final-viral-combined_k141_229040\|\|full | 1462 | No | NA | 0 | 1 | Low-quality | Genome-fragment | 1.9 | 0 |
| S11-final-viral-combined_k141_215961\|\|full | 1462 | No | NA | 0 | 0 | Low-quality | Genome-fragment | 3.5 | 0 |
| S1_k141_119607 | 1160 | No | NA | 0 | 2 | Not-determined | Genome-fragment | NA | 0 |
| S11-final-viral-combined_k141_325553\|\|lt2gene | 1462 | No | NA | 0 | 0 | Low-quality | Genome-fragment | 0.95 | 0 |
| S1_k141_36959 | 1002 | No | NA | 0 | 2 | Not-determined | Genome-fragment | NA | 0 |
| S1_k141_152303 | 1761 | No | NA | 0 | 0 | Not-determined | Genome-fragment | NA | 0 |
| S1_k141_185686 | 1007 | No | NA | 0 | 0 | Not-determined | Genome-fragment | NA | 0 |
| S1_k141_301017 | 1060 | No | NA | 0 | 1 | Not-determined | Genome-fragment | NA | 0 |
| S1-final-viral-combined_k141_89367\|\|full | 1462 | No | NA | 0 | 0 | Low-quality | Genome-fragment | 0.5 | 0 |
| S1_k141_267383 | 1115 | No | NA | 0 | 2 | Not-determined | Genome-fragment | NA | 0 |
| S7-final-viral-combined_k141_30799\|\|full | 1462 | No | NA | 2 | 0 | Low-quality | Genome-fragment | 3.33 | 0 |
| S1_k141_119658 | 5080 | No | NA | 0 | 3 | Not-determined | Genome-fragment | NA | 0 |
| S1_k141_20724 | 1470 | No | NA | 0 | 1 | Not-determined | Genome-fragment | NA | 0 |
| S1_k141_102432 | 1731 | No | NA | 0 | 2 | Not-determined | Genome-fragment | NA | 0 |
| S1_k141_136283 | 1663 | No | NA | 0 | 2 | Not-determined | Genome-fragment | NA | 0 |
| S1_k141_317295 | 1849 | No | NA | 0 | 2 | Not-determined | Genome-fragment | NA | 0 |
| S1_k141_366216 | 1352 | No | NA | 0 | 1 | Not-determined | Genome-fragment | NA | 0 |
| S1_k141_317309 | 6320 | No | NA | 0 | 5 | Not-determined | Genome-fragment | NA | 0 |
| S1_k141_234637 | 1797 | No | NA | 0 | 3 | Not-determined | Genome-fragment | NA | 0 |
| S1_k141_333166 | 1168 | No | NA | 0 | 0 | Not-determined | Genome-fragment | NA | 0 |
| S1_k141_37050 | 1337 | No | NA | 0 | 1 | Not-determined | Genome-fragment | NA | 0 |
| S1_k141_152347 | 2026 | No | NA | 0 | 0 | Not-determined | Genome-fragment | NA | 0 |
| S1_k141_102483 | 9764 | No | NA | 0 | 2 | Not-determined | Genome-fragment | NA | 0 |
| S1_k141_69195 | 4476 | No | NA | 0 | 0 | Not-determined | Genome-fragment | NA | 0 |
| S7_k141_209053 | 1462 | No | NA | 0 | 0 | Low-quality | Genome-fragment | 2.41 | 0 |
| S1_k141_382563 | 1750 | No | NA | 0 | 1 | Not-determined | Genome-fragment | NA | 0 |
| S1_k141_69203 | 7419 | No | NA | 0 | 3 | Not-determined | Genome-fragment | NA | 0 |
| S1_k141_4131 | 1686 | No | NA | 0 | 1 | Not-determined | Genome-fragment | NA | 0 |
| S1_k141_136383 | 4193 | No | NA | 0 | 0 | Not-determined | Genome-fragment | NA | 0 |
| S1_k141_382582 | 1948 | No | NA | 0 | 2 | Not-determined | Genome-fragment | NA | 0 |
| S12-final-viral-combined_k141_8598\|\|full | 1462 | No | NA | 0 | 1 | Low-quality | Genome-fragment | 1.9 | 0 |
| S4_k141_14074 | 1462 | No | NA | 0 | 0 | Low-quality | Genome-fragment | 0.99 | 0 |
| S1_k141_37125 | 1303 | No | NA | 0 | 1 | Not-determined | Genome-fragment | NA | 0 |
| S6-final-viral-combined_k141_113942\|\|full | 1462 | No | NA | 0 | 1 | Low-quality | Genome-fragment | 1.04 | 0 |
| S1_k141_350231 | 1921 | No | NA | 0 | 2 | Not-determined | Genome-fragment | NA | 0 |
| S1_k141_168183 | 1025 | No | NA | 0 | 1 | Not-determined | Genome-fragment | NA | 0 |
| S1_k141_201990 | 3869 | No | NA | 0 | 2 | Not-determined | Genome-fragment | NA | 0 |
| S1_k141_382610 | 1299 | No | NA | 0 | 1 | Not-determined | Genome-fragment | NA | 0 |
| S6_k141_75354 | 1462 | No | NA | 0 | 0 | Low-quality | Genome-fragment | 0.96 | 0 |
| S1_k141_53441 | 3677 | No | NA | 0 | 2 | Not-determined | Genome-fragment | NA | 0 |
| S1_k141_317471 | 1112 | No | NA | 0 | 1 | Not-determined | Genome-fragment | NA | 0 |
| S1_k141_382624 | 1036 | No | NA | 0 | 1 | Not-determined | Genome-fragment | NA | 0 |
| S1_k141_251749 | 4007 | No | NA | 0 | 0 | Not-determined | Genome-fragment | NA | 0 |
| S1_k141_317475 | 1669 | No | NA | 0 | 1 | Not-determined | Genome-fragment | NA | 0 |
| S1_k141_37184 | 1326 | No | NA | 0 | 0 | Not-determined | Genome-fragment | NA | 0 |
| S1_k141_53474 | 1580 | No | NA | 0 | 1 | Not-determined | Genome-fragment | NA | 0 |
| S1_k141_119887 | 1085 | No | NA | 0 | 0 | Not-determined | Genome-fragment | NA | 0 |
| S5-final-viral-combined_k141_143301\|\|full | 1463 | No | NA | 0 | 0 | Low-quality | Genome-fragment | 2.28 | 0 |
| S1_k141_69266 | 3067 | No | NA | 0 | 0 | Not-determined | Genome-fragment | NA | 0 |
| S1_k141_168270 | 1355 | No | NA | 0 | 1 | Not-determined | Genome-fragment | NA | 0 |
| S1_k141_301289 | 5492 | No | NA | 0 | 1 | Not-determined | Genome-fragment | NA | 0 |
| S7-final-viral-combined_k141_218195\|\|full | 1463 | No | NA | 0 | 1 | Low-quality | Genome-fragment | 2.45 | 0 |
| S1_k141_366348 | 1502 | No | NA | 0 | 0 | Not-determined | Genome-fragment | NA | 0 |
| S1_k141_333340 | 1086 | No | NA | 0 | 1 | Not-determined | Genome-fragment | NA | 0 |
| S1_k141_136572 | 1262 | No | NA | 0 | 1 | Not-determined | Genome-fragment | NA | 0 |
| S1_k141_333353 | 3304 | No | NA | 0 | 1 | Not-determined | Genome-fragment | NA | 0 |
| S1_k141_218289 | 1692 | No | NA | 0 | 1 | Not-determined | Genome-fragment | NA | 0 |
| S1_k141_4253 | 4416 | No | NA | 0 | 0 | Not-determined | Genome-fragment | NA | 0 |
| S1_k141_53581 | 1055 | No | NA | 0 | 1 | Not-determined | Genome-fragment | NA | 0 |
| S1_k141_317580 | 1210 | No | NA | 0 | 1 | Not-determined | Genome-fragment | NA | 0 |
| S1_k141_267614 | 1414 | No | NA | 0 | 1 | Not-determined | Genome-fragment | NA | 0 |
| S1_k141_152540 | 1101 | No | NA | 0 | 3 | Not-determined | Genome-fragment | NA | 0 |
| S1_k141_350367 | 1264 | No | NA | 0 | 1 | Not-determined | Genome-fragment | NA | 0 |
| S1_k141_317590 | 1144 | No | NA | 0 | 0 | Not-determined | Genome-fragment | NA | 0 |
| S1_k141_152559 | 1189 | No | NA | 0 | 1 | Not-determined | Genome-fragment | NA | 0 |
| S7-final-viral-combined_k141_71005\|\|full | 1463 | No | NA | 0 | 2 | Low-quality | Genome-fragment | 1.48 | 0 |
| S12-final-viral-combined_k141_183815\|\|full | 1463 | No | NA | 0 | 0 | Low-quality | Genome-fragment | 1.09 | 0 |
| S1_k141_4277 | 31752 | Yes | 29534 | 14 | 3 | Medium-quality | Genome-fragment | 82.52 | 6.99 |
| S1_k141_317638 | 1310 | No | NA | 0 | 1 | Not-determined | Genome-fragment | NA | 0 |
| S1_k141_120017 | 1056 | No | NA | 0 | 0 | Not-determined | Genome-fragment | NA | 0 |
| S12-final-viral-combined_k141_149701\|\|full | 1463 | No | NA | 1 | 0 | Low-quality | Genome-fragment | 2.51 | 0 |
| S1_k141_350405 | 3848 | No | NA | 0 | 4 | Not-determined | Genome-fragment | NA | 0 |
| S1_k141_53670 | 1358 | No | NA | 0 | 2 | Not-determined | Genome-fragment | NA | 0 |
| S1_k141_37344 | 1372 | No | NA | 0 | 0 | Not-determined | Genome-fragment | NA | 0 |
| S1_k141_120062 | 1396 | No | NA | 0 | 1 | Not-determined | Genome-fragment | NA | 0 |
| S1_k141_102729 | 1011 | No | NA | 0 | 1 | Not-determined | Genome-fragment | NA | 0 |
| S1_k141_234869 | 5796 | No | NA | 0 | 1 | Not-determined | Genome-fragment | NA | 0 |
| S1_k141_185953 | 2473 | No | NA | 0 | 3 | Not-determined | Genome-fragment | NA | 0 |
| S1_k141_382901 | 4073 | No | NA | 0 | 1 | Not-determined | Genome-fragment | NA | 0 |
| S1_k141_234894 | 1498 | No | NA | 0 | 2 | Not-determined | Genome-fragment | NA | 0 |
| S1_k141_234896 | 1376 | No | NA | 0 | 0 | Not-determined | Genome-fragment | NA | 0 |
| S2-final-viral-combined_k141_95065\|\|full | 1463 | No | NA | 1 | 0 | Low-quality | Genome-fragment | 2.17 | 0 |
| S2-final-viral-combined_k141_214462\|\|full | 1463 | No | NA | 0 | 2 | Low-quality | Genome-fragment | 3.35 | 0 |
| S2_k141_166881 | 1463 | No | NA | 1 | 0 | Low-quality | Genome-fragment | 5.22 | 0 |
| S1_k141_350466 | 1028 | No | NA | 0 | 0 | Not-determined | Genome-fragment | NA | 0 |
| S1_k141_234917 | 1249 | No | NA | 0 | 0 | Not-determined | Genome-fragment | NA | 0 |
| S8-final-viral-combined_k141_263569\|\|full | 1463 | No | NA | 1 | 0 | Low-quality | Genome-fragment | 1.31 | 0 |
| S1_k141_202160 | 1208 | No | NA | 0 | 0 | Not-determined | Genome-fragment | NA | 0 |
| S1_k141_37385 | 1092 | No | NA | 0 | 0 | Not-determined | Genome-fragment | NA | 0 |
| S1_k141_234979 | 1303 | No | NA | 0 | 1 | Not-determined | Genome-fragment | NA | 0 |
| S1_k141_234983 | 1133 | No | NA | 0 | 0 | Not-determined | Genome-fragment | NA | 0 |
| S8-final-viral-combined_k141_121668\|\|lt2gene | 1463 | No | NA | 1 | 0 | Low-quality | Genome-fragment | 4.55 | 0 |
| S1_k141_350520 | 3045 | No | NA | 0 | 0 | Not-determined | Genome-fragment | NA | 0 |

| | | | | | | | | | | | |
|---|---|---|---|---|---|---|---|---|---|---|---|
| S8_k141_106522 | 1463 | No | NA | | 0 | 0 | Low-quality | Genome-fragment | | 1.11 | 0 |
| S1_k141_317798 | 1127 | No | NA | | 0 | 0 | Not-determined | Genome-fragment | NA | | 0 |
| S1_k141_4458 | 4426 | No | NA | | 0 | 0 | Not-determined | Genome-fragment | NA | | 0 |
| S1_k141_366607 | 6406 | No | NA | | 0 | 1 | Not-determined | Genome-fragment | NA | | 0 |
| S9-final-viral-combined_k141_53253\|\|full | 1464 | No | NA | | 1 | 0 | Low-quality | Genome-fragment | | 3.53 | 0 |
| S1_k141_37442 | 1050 | No | NA | | 0 | 2 | Not-determined | Genome-fragment | NA | | 0 |
| S1_k141_284621 | 1137 | No | NA | | 0 | 1 | Not-determined | Genome-fragment | NA | | 0 |
| S1-final-viral-combined_k141_216456\|\|full | 1464 | No | NA | | 1 | 0 | Low-quality | Genome-fragment | | 3.58 | 0 |
| S1_k141_136857 | 1059 | No | NA | | 0 | 2 | Not-determined | Genome-fragment | NA | | 0 |
| S1_k141_383005 | 3024 | No | NA | | 0 | 1 | Not-determined | Genome-fragment | NA | | 0 |
| S1_k141_251999 | 1594 | No | NA | | 0 | 2 | Not-determined | Genome-fragment | NA | | 0 |
| S1_k141_366619 | 1515 | No | NA | | 0 | 0 | Not-determined | Genome-fragment | NA | | 0 |
| S3-final-viral-combined_k141_127910\|\|full | 1464 | No | NA | | 0 | 0 | Low-quality | Genome-fragment | | 1.78 | 0 |
| S5-final-viral-combined_k141_53073\|\|full | 1464 | No | NA | | 0 | 0 | Low-quality | Genome-fragment | | 1.96 | 0 |
| S5-final-viral-combined_k141_39548\|\|lt2gene | 1464 | No | NA | | 1 | 0 | Low-quality | Genome-fragment | | 1.42 | 0 |
| S1_k141_102898 | 4006 | No | NA | | 0 | 0 | Not-determined | Genome-fragment | NA | | 0 |
| S7-final-viral-combined_k141_86208\|\|full | 1464 | No | NA | | 1 | 0 | Low-quality | Genome-fragment | | 0.93 | 0 |
| S1_k141_21315 | 3898 | No | NA | | 0 | 3 | Not-determined | Genome-fragment | NA | | 0 |
| S1_k141_152782 | 1116 | No | NA | | 0 | 1 | Not-determined | Genome-fragment | NA | | 0 |
| S1_k141_202297 | 1071 | No | NA | | 0 | 0 | Not-determined | Genome-fragment | NA | | 0 |
| S1_k141_267845 | 3644 | No | NA | | 0 | 0 | Not-determined | Genome-fragment | NA | | 0 |
| S17-final-viral-combined_k141_39939\|\|full | 1464 | No | NA | | 2 | 0 | Low-quality | Genome-fragment | | 4.04 | 0 |
| S1_k141_136913 | 3043 | No | NA | | 0 | 1 | Not-determined | Genome-fragment | NA | | 0 |
| S1_k141_86692 | 3662 | No | NA | | 0 | 0 | Not-determined | Genome-fragment | NA | | 0 |
| S1_k141_168735 | 2187 | No | NA | | 0 | 0 | Not-determined | Genome-fragment | NA | | 0 |
| S17-final-viral-combined_k141_177319\|\|full | 1464 | No | NA | | 0 | 2 | Low-quality | Genome-fragment | | 0.43 | 0 |
| S1_k141_136963 | 4921 | No | NA | | 0 | 2 | Not-determined | Genome-fragment | NA | | 0 |
| S17-k141_702 | 1464 | No | NA | | 0 | 0 | Low-quality | Genome-fragment | | 2.98 | 0 |
| S1_k141_383227 | 1103 | No | NA | | 0 | 2 | Not-determined | Genome-fragment | NA | | 0 |
| S1_k141_69659 | 5005 | No | NA | | 0 | 4 | Not-determined | Genome-fragment | NA | | 0 |
| S1_k141_318017 | 9706 | No | NA | | 0 | 4 | Not-determined | Genome-fragment | NA | | 0 |
| S1_k141_383266 | 1432 | No | NA | | 0 | 1 | Not-determined | Genome-fragment | NA | | 0 |
| S1_k141_218736 | 1644 | No | NA | | 0 | 0 | Not-determined | Genome-fragment | NA | | 0 |
| S1_k141_186227 | 1143 | No | NA | | 0 | 1 | Not-determined | Genome-fragment | NA | | 0 |
| S12-final-viral-combined_k141_122696\|\|full | 1464 | No | NA | | 3 | 0 | Low-quality | Genome-fragment | | 3.04 | 0 |
| S1_k141_252229 | 2720 | No | NA | | 0 | 2 | Not-determined | Genome-fragment | NA | | 0 |
| S1_k141_235294 | 4527 | No | NA | | 0 | 0 | Not-determined | Genome-fragment | NA | | 0 |
| S1_k141_366807 | 3664 | No | NA | | 0 | 1 | Not-determined | Genome-fragment | NA | | 0 |
| S1_k141_69779 | 10151 | No | NA | | 0 | 10 | Not-determined | Genome-fragment | NA | | 0 |
| S1_k141_153002 | 2227 | No | NA | | 0 | 0 | Not-determined | Genome-fragment | NA | | 0 |
| S1_k141_318132 | 1000 | No | NA | | 0 | 1 | Not-determined | Genome-fragment | NA | | 0 |
| S1_k141_366827 | 1661 | No | NA | | 0 | 0 | Not-determined | Genome-fragment | NA | | 0 |
| S1_k141_69795 | 4133 | No | NA | | 0 | 0 | Not-determined | Genome-fragment | NA | | 0 |
| S1_k141_186314 | 1174 | No | NA | | 0 | 1 | Not-determined | Genome-fragment | NA | | 0 |
| S1_k141_252304 | 1046 | No | NA | | 0 | 1 | Not-determined | Genome-fragment | NA | | 0 |
| S8-final-viral-combined_k141_112653\|\|full | 1464 | No | NA | | 1 | 0 | Low-quality | Genome-fragment | | 2.22 | 0 |
| S1_k141_202556 | 3310 | No | NA | | 0 | 0 | Not-determined | Genome-fragment | NA | | 0 |
| S1_k141_318181 | 1108 | No | NA | | 0 | 0 | Not-determined | Genome-fragment | NA | | 0 |
| S1_k141_120575 | 1446 | No | NA | | 0 | 2 | Not-determined | Genome-fragment | NA | | 0 |
| S1_k141_86836 | 1037 | No | NA | | 0 | 1 | Not-determined | Genome-fragment | NA | | 0 |
| S1_k141_168966 | 3115 | No | NA | | 0 | 3 | Not-determined | Genome-fragment | NA | | 0 |
| S1_k141_235363 | 1051 | No | NA | | 0 | 1 | Not-determined | Genome-fragment | NA | | 0 |
| S1_k141_4874 | 1839 | No | NA | | 0 | 1 | Not-determined | Genome-fragment | NA | | 0 |
| S8_k141_60784 | 1464 | No | NA | | 0 | 1 | Low-quality | Genome-fragment | | 1.15 | 0 |
| S1_k141_153095 | 2947 | No | NA | | 0 | 2 | Not-determined | Genome-fragment | NA | | 0 |
| S1_k141_37873 | 1037 | No | NA | | 0 | 1 | Not-determined | Genome-fragment | NA | | 0 |
| S1_k141_137199 | 1014 | No | NA | | 0 | 0 | Not-determined | Genome-fragment | NA | | 0 |
| S12-final-viral-combined_k141_399875\|\|lt2gene | 1465 | No | NA | | 1 | 0 | Low-quality | Genome-fragment | | 3.58 | 0 |
| S1_k141_235413 | 1079 | No | NA | | 0 | 1 | Not-determined | Genome-fragment | NA | | 0 |
| S1_k141_54049 | 1627 | No | NA | | 0 | 1 | Not-determined | Genome-fragment | NA | | 0 |
| S1_k141_302058 | 1010 | No | NA | | 0 | 0 | Not-determined | Genome-fragment | NA | | 0 |
| S1_k141_333652 | 1769 | No | NA | | 0 | 1 | Not-determined | Genome-fragment | NA | | 0 |
| S1_k141_367048 | 1316 | No | NA | | 0 | 1 | Not-determined | Genome-fragment | NA | | 0 |
| S1_k141_120729 | 4968 | No | NA | | 0 | 1 | Not-determined | Genome-fragment | NA | | 0 |
| S1_k141_268097 | 11452 | No | NA | | 0 | 6 | Not-determined | Genome-fragment | NA | | 0 |
| S1_k141_302105 | 2033 | No | NA | | 0 | 0 | Not-determined | Genome-fragment | NA | | 0 |
| S1_k141_284894 | 1136 | No | NA | | 0 | 0 | Not-determined | Genome-fragment | NA | | 0 |
| S1_k141_103317 | 1087 | No | NA | | 0 | 1 | Not-determined | Genome-fragment | NA | | 0 |
| S1_k141_383572 | 2032 | No | NA | | 0 | 1 | Not-determined | Genome-fragment | NA | | 0 |
| S1_k141_367114 | 8354 | No | NA | | 0 | 1 | Not-determined | Genome-fragment | NA | | 0 |
| S1_k141_120791 | 1339 | No | NA | | 0 | 0 | Not-determined | Genome-fragment | NA | | 0 |
| S1_k141_219071 | 2798 | No | NA | | 0 | 1 | Not-determined | Genome-fragment | NA | | 0 |
| S1_k141_252429 | 2258 | No | NA | | 0 | 1 | Not-determined | Genome-fragment | NA | | 0 |
| S1_k141_268158 | 1080 | No | NA | | 0 | 1 | Not-determined | Genome-fragment | NA | | 0 |
| S1_k141_235525 | 2985 | No | NA | | 0 | 0 | Not-determined | Genome-fragment | NA | | 0 |
| S1_k141_120821 | 7741 | No | NA | | 0 | 3 | Not-determined | Genome-fragment | NA | | 0 |
| S1_k141_268174 | 1042 | No | NA | | 0 | 0 | Not-determined | Genome-fragment | NA | | 0 |
| S1_k141_87035 | 3281 | No | NA | | 0 | 2 | Not-determined | Genome-fragment | NA | | 0 |
| S12_k141_368088 | 1465 | No | NA | | 0 | 0 | Low-quality | Genome-fragment | | 3.73 | 0 |
| S1_k141_333767 | 4247 | No | NA | | 0 | 1 | Not-determined | Genome-fragment | NA | | 0 |
| S1_k141_284942 | 1096 | No | NA | | 0 | 1 | Not-determined | Genome-fragment | NA | | 0 |
| S1_k141_284952 | 1184 | No | NA | | 0 | 1 | Not-determined | Genome-fragment | NA | | 0 |
| S6-final-viral-combined_k141_6156\|\|full | 1465 | No | NA | | 0 | 1 | Low-quality | Genome-fragment | | 0.76 | 0 |
| S6-final-viral-combined_k141_74076\|\|full | 1465 | No | NA | | 0 | 0 | Low-quality | Genome-fragment | | 3.71 | 0 |
| S1_k141_302150 | 1058 | No | NA | | 0 | 0 | Not-determined | Genome-fragment | NA | | 0 |
| S1_k141_54188 | 3572 | No | NA | | 0 | 2 | Not-determined | Genome-fragment | NA | | 0 |
| S1_k141_153229 | 1471 | No | NA | | 0 | 1 | Not-determined | Genome-fragment | NA | | 0 |
| S1_k141_137383 | 1014 | No | NA | | 0 | 1 | Not-determined | Genome-fragment | NA | | 0 |
| S9-final-viral-combined_k141_32442\|\|full | 1466 | No | NA | | 0 | 0 | Low-quality | Genome-fragment | | 3.36 | 0 |
| S1_k141_137402 | 3583 | No | NA | | 0 | 1 | Not-determined | Genome-fragment | NA | | 0 |
| S11_k141_52237 | 1466 | No | NA | | 1 | 0 | Low-quality | Genome-fragment | | 0.72 | 0 |
| S1_k141_137425 | 3567 | No | NA | | 0 | 0 | Not-determined | Genome-fragment | NA | | 0 |
| S1_k141_21516 | 1068 | No | NA | | 0 | 0 | Not-determined | Genome-fragment | NA | | 0 |
| S1_k141_120934 | 1323 | No | NA | | 0 | 0 | Not-determined | Genome-fragment | NA | | 0 |
| S1_k141_268283 | 3444 | No | NA | | 0 | 3 | Not-determined | Genome-fragment | NA | | 0 |
| S1_k141_252532 | 3347 | No | NA | | 0 | 1 | Not-determined | Genome-fragment | NA | | 0 |
| S1_k141_284997 | 1458 | No | NA | | 0 | 1 | Not-determined | Genome-fragment | NA | | 0 |
| S1_k141_54271 | 2269 | No | NA | | 0 | 2 | Not-determined | Genome-fragment | NA | | 0 |
| S1_k141_252540 | 7494 | No | NA | | 0 | 6 | Not-determined | Genome-fragment | NA | | 0 |
| S1_k141_54291 | 1279 | No | NA | | 0 | 1 | Not-determined | Genome-fragment | NA | | 0 |
| S1_k141_54303 | 5149 | No | NA | | 0 | 5 | Not-determined | Genome-fragment | NA | | 0 |
| S3-final-viral-combined_k141_43945\|\|full | 1466 | No | NA | | 1 | 0 | Low-quality | Genome-fragment | | 3.51 | 0 |
| S1_k141_351239 | 1451 | No | NA | | 0 | 2 | Not-determined | Genome-fragment | NA | | 0 |
| S1_k141_318549 | 7120 | No | NA | | 0 | 1 | Not-determined | Genome-fragment | NA | | 0 |
| S1_k141_351273 | 1315 | No | NA | | 0 | 0 | Not-determined | Genome-fragment | NA | | 0 |
| S1_k141_169175 | 1321 | No | NA | | 0 | 1 | Not-determined | Genome-fragment | NA | | 0 |
| S1_k141_202895 | 1645 | No | NA | | 0 | 1 | Not-determined | Genome-fragment | NA | | 0 |
| S3-final-viral-combined_k141_29758\|\|lt2gene | 1466 | No | NA | | 1 | 0 | Low-quality | Genome-fragment | | 0.8 | 0 |
| S5-final-viral-combined_k141_28559\|\|full | 1466 | No | NA | | 1 | 0 | Low-quality | Genome-fragment | | 2.36 | 0 |
| S1_k141_318558 | 3368 | No | NA | | 0 | 0 | Not-determined | Genome-fragment | NA | | 0 |
| S5-final-viral-combined_k141_28805\|\|lt2gene | 1466 | No | NA | | 1 | 0 | Low-quality | Genome-fragment | | 2.14 | 0 |
| S1_k141_351286 | 3791 | No | NA | | 0 | 1 | Not-determined | Genome-fragment | NA | | 0 |
| S7-final-viral-combined_k141_92065\|\|full | 1466 | Yes | | 615 | 1 | 1 | Low-quality | Genome-fragment | | 1.45 | 58.05 |
| S7_k141_103936 | 1466 | No | NA | | 0 | 0 | Low-quality | Genome-fragment | | 0.25 | 0 |
| S1_k141_252615 | 5087 | No | NA | | 0 | 2 | Not-determined | Genome-fragment | NA | | 0 |
| S1_k141_186739 | 1635 | No | NA | | 0 | 1 | Not-determined | Genome-fragment | NA | | 0 |
| S1_k141_351336 | 1552 | No | NA | | 0 | 1 | Not-determined | Genome-fragment | NA | | 0 |
| S12-final-viral-combined_k141_180247\|\|lt2gene | 1466 | No | NA | | 1 | 0 | Low-quality | Genome-fragment | | 2.22 | 0 |
| S4-final-viral-combined_k141_117416\|\|full | 1466 | No | NA | | 0 | 1 | Low-quality | Genome-fragment | | 2.07 | 0 |
| S1_k141_351360 | 1248 | No | NA | | 0 | 0 | Not-determined | Genome-fragment | NA | | 0 |
| S1-final-viral-combined_k141_182581\|\|full | 1467 | No | NA | | 0 | 0 | Low-quality | Genome-fragment | | 2.48 | 0 |
| S1-final-viral-combined_k141_62116\|\|lt2gene | 1467 | No | NA | | 1 | 0 | Low-quality | Genome-fragment | | 1.01 | 0 |
| S1_k141_252633 | 1010 | No | NA | | 0 | 1 | Not-determined | Genome-fragment | NA | | 0 |
| S1_k141_285081 | 2769 | No | NA | | 0 | 0 | Not-determined | Genome-fragment | NA | | 0 |
| S1_k141_268402 | 1950 | No | NA | | 0 | 0 | Not-determined | Genome-fragment | NA | | 0 |
| S3_k141_37487 | 1467 | No | NA | | 1 | 0 | Low-quality | Genome-fragment | | 0.95 | 0 |
| S1_k141_235774 | 1109 | No | NA | | 0 | 2 | Not-determined | Genome-fragment | NA | | 0 |
| S1_k141_351382 | 1035 | No | NA | | 0 | 1 | Not-determined | Genome-fragment | NA | | 0 |
| S1_k141_252699 | 2352 | No | NA | | 0 | 2 | Not-determined | Genome-fragment | NA | | 0 |
| S1_k141_87272 | 1196 | No | NA | | 0 | 2 | Not-determined | Genome-fragment | NA | | 0 |
| S1_k141_235809 | 1084 | No | NA | | 0 | 1 | Not-determined | Genome-fragment | NA | | 0 |
| S1_k141_121177 | 1088 | No | NA | | 0 | 0 | Not-determined | Genome-fragment | NA | | 0 |
| S5-final-viral-combined_k141_39181\|\|full | 1467 | No | NA | | 0 | 0 | Low-quality | Genome-fragment | | 3.57 | 0 |
| S7-final-viral-combined_k141_6007\|\|full | 1467 | No | NA | | 0 | 0 | Low-quality | Genome-fragment | | 0.58 | 0 |
| S1_k141_54432 | 1065 | No | NA | | 0 | 3 | Not-determined | Genome-fragment | NA | | 0 |
| S1_k141_367456 | 1437 | No | NA | | 0 | 1 | Not-determined | Genome-fragment | NA | | 0 |
| S1_k141_121207 | 1678 | No | NA | | 0 | 1 | Not-determined | Genome-fragment | NA | | 0 |
| S7-final-viral-combined_k141_7091\|\|full | 1467 | No | NA | | 0 | 0 | Low-quality | Genome-fragment | | 1.72 | 0 |
| S1_k141_5310 | 3206 | No | NA | | 0 | 2 | Not-determined | Genome-fragment | NA | | 0 |
| S1_k141_285235 | 2423 | No | NA | | 0 | 1 | Not-determined | Genome-fragment | NA | | 0 |
| S1_k141_169279 | 3212 | No | NA | | 0 | 1 | Not-determined | Genome-fragment | NA | | 0 |
| S1_k141_54503 | 1142 | No | NA | | 0 | 1 | Not-determined | Genome-fragment | NA | | 0 |
| S1_k141_268484 | 4310 | No | NA | | 0 | 1 | Not-determined | Genome-fragment | NA | | 0 |
| S1_k141_367503 | 1069 | No | NA | | 0 | 1 | Not-determined | Genome-fragment | NA | | 0 |
| S7-final-viral-combined_k141_109227\|\|full | 1467 | No | NA | | 1 | 0 | Low-quality | Genome-fragment | | 3.68 | 0 |
| S1_k141_351537 | 1075 | No | NA | | 0 | 1 | Not-determined | Genome-fragment | NA | | 0 |
| S1_k141_70248 | 2192 | No | NA | | 0 | 3 | Not-determined | Genome-fragment | NA | | 0 |
| S1_k141_87368 | 1008 | No | NA | | 0 | 0 | Not-determined | Genome-fragment | NA | | 0 |
| S1_k141_103383 | 1121 | No | NA | | 0 | 1 | Not-determined | Genome-fragment | NA | | 0 |
| S1_k141_121267 | 1096 | No | NA | | 0 | 1 | Not-determined | Genome-fragment | NA | | 0 |
| S1_k141_38248 | 1872 | No | NA | | 0 | 2 | Not-determined | Genome-fragment | NA | | 0 |
| S7-final-viral-combined_k141_227626\|\|lt2gene | 1467 | No | NA | | 1 | 0 | Low-quality | Genome-fragment | | 3.71 | 0 |
| S1_k141_87387 | 1056 | No | NA | | 0 | 0 | Not-determined | Genome-fragment | NA | | 0 |
| S1_k141_137751 | 12244 | No | NA | | 0 | 3 | Not-determined | Genome-fragment | NA | | 0 |
| S1_k141_54523 | 1393 | No | NA | | 0 | 2 | Not-determined | Genome-fragment | NA | | 0 |
| S1_k141_103432 | 3256 | No | NA | | 0 | 1 | Not-determined | Genome-fragment | NA | | 0 |
| S1_k141_235966 | 1610 | No | NA | | 0 | 1 | Not-determined | Genome-fragment | NA | | 0 |
| S1_k141_268563 | 3972 | No | NA | | 0 | 3 | Not-determined | Genome-fragment | NA | | 0 |
| S1_k141_383973 | 1955 | No | NA | | 0 | 1 | Not-determined | Genome-fragment | NA | | 0 |
| S1_k141_153615 | 2783 | No | NA | | 1 | 0 | Medium-quality | Genome-fragment | | 50.34 | 0 |
| S12-final-viral-combined_k141_280661\|\|full | 1467 | No | NA | | 0 | 0 | Low-quality | Genome-fragment | | 2.46 | 0 |
| S1_k141_137796 | 1550 | No | NA | | 0 | 2 | Not-determined | Genome-fragment | NA | | 0 |
| S1_k141_169394 | 1008 | No | NA | | 0 | 1 | Not-determined | Genome-fragment | NA | | 0 |
| S1_k141_5403 | 1869 | No | NA | | 0 | 2 | Not-determined | Genome-fragment | NA | | 0 |
| S1_k141_87450 | 2148 | No | NA | | 0 | 3 | Not-determined | Genome-fragment | NA | | 0 |
| S1_k141_38364 | 4443 | No | NA | | 0 | 0 | Not-determined | Genome-fragment | NA | | 0 |
| S6-final-viral-combined_k141_51819\|\|full | 1467 | No | NA | | 0 | 0 | Low-quality | Genome-fragment | | 1.32 | 0 |
| S1_k141_5448 | 1353 | No | NA | | 0 | 1 | Not-determined | Genome-fragment | NA | | 0 |
| S1_k141_137804 | 1096 | No | NA | | 0 | 1 | Not-determined | Genome-fragment | NA | | 0 |
| S1_k141_187188 | 1134 | No | NA | | 0 | 0 | Not-determined | Genome-fragment | NA | | 0 |
| S1_k141_334270 | 1116 | No | NA | | 0 | 0 | Not-determined | Genome-fragment | NA | | 0 |
| S1_k141_203320 | 2221 | No | NA | | 0 | 1 | Not-determined | Genome-fragment | NA | | 0 |
| S1_k141_367781 | 1720 | No | NA | | 0 | 2 | Not-determined | Genome-fragment | NA | | 0 |
| S1_k141_103524 | 1434 | No | NA | | 0 | 0 | Not-determined | Genome-fragment | NA | | 0 |

| | | | | | | | | | |
|---|---|---|---|---|---|---|---|---|---|
| S1_k141_384095 | 1004 | No | NA | 0 | 3 | Not-determined | Genome-fragment | NA | 0 |
| S1_k141_121337 | 1031 | No | NA | 0 | 1 | Not-determined | Genome-fragment | NA | 0 |
| S1_k141_169516 | 3753 | No | NA | 0 | 0 | Not-determined | Genome-fragment | NA | 0 |
| S1_k141_236105 | 22362 | No | NA | 13 | 0 | Medium-quality | Genome-fragment | 50.89 | 0 |
| S11-final-viral-combined_k141_168293\|\|full | 1468 | No | NA | 0 | 0 | Low-quality | Genome-fragment | 3.69 | 0 |
| S1_k141_367840 | 2361 | No | NA | 0 | 1 | Not-determined | Genome-fragment | NA | 0 |
| S1_k141_103570 | 1097 | No | NA | 0 | 1 | Not-determined | Genome-fragment | NA | 0 |
| S1_k141_187282 | 1120 | No | NA | 0 | 0 | Not-determined | Genome-fragment | NA | 0 |
| S1_k141_121370 | 1038 | No | NA | 0 | 1 | Not-determined | Genome-fragment | NA | 0 |
| S1_k141_103608 | 2956 | No | NA | 0 | 1 | Not-determined | Genome-fragment | NA | 0 |
| S1_k141_318883 | 1191 | No | NA | 0 | 0 | Not-determined | Genome-fragment | NA | 0 |
| S1_k141_351743 | 1523 | No | NA | 0 | 1 | Not-determined | Genome-fragment | NA | 0 |
| S1_k141_384200 | 1364 | No | NA | 0 | 4 | Not-determined | Genome-fragment | NA | 0 |
| S11_k141_75278 | 1468 | No | NA | 0 | 0 | Low-quality | Genome-fragment | 3.19 | 0 |
| S1_k141_302752 | 1678 | No | NA | 0 | 1 | Not-determined | Genome-fragment | NA | 0 |
| S1_k141_203470 | 1217 | No | NA | 0 | 1 | Not-determined | Genome-fragment | NA | 0 |
| S1_k141_38547 | 1898 | No | NA | 0 | 0 | Not-determined | Genome-fragment | NA | 0 |
| S1_k141_302776 | 1115 | No | NA | 0 | 1 | Not-determined | Genome-fragment | NA | 0 |
| S1_k141_367916 | 1750 | No | NA | 0 | 0 | Not-determined | Genome-fragment | NA | 0 |
| S1_k141_219826 | 1172 | No | NA | 0 | 2 | Not-determined | Genome-fragment | NA | 0 |
| S1_k141_103649 | 2515 | No | NA | 0 | 0 | Not-determined | Genome-fragment | NA | 0 |
| S11_k141_299006 | 1468 | No | NA | 1 | 0 | Low-quality | Genome-fragment | 2.81 | 0 |
| S1_k141_236216 | 1369 | No | NA | 0 | 1 | Not-determined | Genome-fragment | NA | 0 |
| S1_k141_54804 | 1104 | No | NA | 0 | 1 | Not-determined | Genome-fragment | NA | 0 |
| S1_k141_70674 | 1438 | No | NA | 0 | 1 | Not-determined | Genome-fragment | NA | 0 |
| S1_k141_384300 | 1225 | No | NA | 0 | 1 | Not-determined | Genome-fragment | NA | 0 |
| S5-final-viral-combined_k141_85210\|\|full | 1468 | No | NA | 0 | 0 | Low-quality | Genome-fragment | 1.19 | 0 |
| S5-final-viral-combined_k141_123419\|\|lt2gene | 1468 | No | NA | 1 | 0 | Low-quality | Genome-fragment | 23.76 | 0 |
| S1_k141_54839 | 1252 | No | NA | 0 | 1 | Not-determined | Genome-fragment | NA | 0 |
| S1_k141_54842 | 1793 | No | NA | 0 | 1 | Not-determined | Genome-fragment | NA | 0 |
| S1_k141_54859 | 1680 | No | NA | 0 | 0 | Not-determined | Genome-fragment | NA | 0 |
| S1_k141_334293 | 1073 | No | NA | 0 | 0 | Not-determined | Genome-fragment | NA | 0 |
| S1_k141_268854 | 1704 | No | NA | 0 | 3 | Not-determined | Genome-fragment | NA | 0 |
| S1_k141_38644 | 1710 | No | NA | 0 | 1 | Not-determined | Genome-fragment | NA | 0 |
| S1_k141_351829 | 6288 | No | NA | 0 | 1 | Not-determined | Genome-fragment | NA | 0 |
| S1_k141_121498 | 6289 | No | NA | 0 | 0 | Not-determined | Genome-fragment | NA | 0 |
| S1_k141_351853 | 1108 | No | NA | 0 | 1 | Not-determined | Genome-fragment | NA | 0 |
| S1_k141_70716 | 1422 | No | NA | 0 | 2 | Not-determined | Genome-fragment | NA | 0 |
| S1_k141_137976 | 1587 | No | NA | 0 | 1 | Not-determined | Genome-fragment | NA | 0 |
| S12-final-viral-combined_k141_163046\|\|full | 1468 | No | NA | 2 | 0 | Low-quality | Genome-fragment | 2.51 | 0 |
| S1_k141_319020 | 2421 | No | NA | 0 | 2 | Not-determined | Genome-fragment | NA | 0 |
| S1_k141_368049 | 1041 | No | NA | 0 | 1 | Not-determined | Genome-fragment | NA | 0 |
| S1_k141_169680 | 1236 | No | NA | 0 | 1 | Not-determined | Genome-fragment | NA | 0 |
| S1_k141_236280 | 2813 | No | NA | 0 | 0 | Not-determined | Genome-fragment | NA | 0 |
| S1_k141_169697 | 3932 | No | NA | 0 | 3 | Not-determined | Genome-fragment | NA | 0 |
| S1_k141_153725 | 3139 | No | NA | 0 | 0 | Not-determined | Genome-fragment | NA | 0 |
| S1_k141_220010 | 1873 | No | NA | 0 | 1 | Not-determined | Genome-fragment | NA | 0 |
| S1_k141_253171 | 1240 | No | NA | 0 | 1 | Not-determined | Genome-fragment | NA | 0 |
| S1_k141_87772 | 1466 | No | NA | 0 | 2 | Not-determined | Genome-fragment | NA | 0 |
| S4_k141_51607 | 1468 | No | NA | 2 | 0 | Low-quality | Genome-fragment | 0.86 | 0 |
| S1_k141_70814 | 1328 | No | NA | 0 | 1 | Not-determined | Genome-fragment | NA | 0 |
| S1_k141_268946 | 1272 | No | NA | 0 | 1 | Not-determined | Genome-fragment | NA | 0 |
| S8-final-viral-combined_k141_149008\|\|full | 1468 | No | NA | 1 | 0 | Low-quality | Genome-fragment | 0.93 | 0 |
| S1_k141_54992 | 1089 | No | NA | 0 | 2 | Not-determined | Genome-fragment | NA | 0 |
| S11-final-viral-combined_k141_170229\|\|full | 1469 | No | NA | 1 | 0 | Low-quality | Genome-fragment | 2.49 | 0 |
| S1-final-viral-combined_k141_12158\|\|full | 1469 | No | NA | 0 | 0 | Low-quality | Genome-fragment | 4.3 | 0 |
| S1_k141_121653 | 1392 | No | NA | 0 | 1 | Not-determined | Genome-fragment | NA | 0 |
| S3_k141_99778 | 1469 | No | NA | 1 | 0 | Low-quality | Genome-fragment | 0.68 | 0 |
| S1_k141_334426 | 7546 | No | NA | 0 | 1 | Not-determined | Genome-fragment | NA | 0 |
| S5-final-viral-combined_k141_117469\|\|full | 1469 | No | NA | 2 | 0 | Low-quality | Genome-fragment | 4.18 | 0 |
| S5-final-viral-combined_k141_4725\|\|full | 1469 | No | NA | 1 | 0 | Low-quality | Genome-fragment | 1.87 | 0 |
| S5-final-viral-combined_k141_148888\|\|full | 1469 | No | NA | 0 | 0 | Low-quality | Genome-fragment | 2.89 | 0 |
| S1_k141_351966 | 1049 | No | NA | 0 | 1 | Not-determined | Genome-fragment | NA | 0 |
| S1_k141_203697 | 1001 | No | NA | 0 | 0 | Not-determined | Genome-fragment | NA | 0 |
| S1_k141_103925 | 1040 | No | NA | 0 | 0 | Not-determined | Genome-fragment | NA | 0 |
| S1_k141_169822 | 1517 | No | NA | 0 | 2 | Not-determined | Genome-fragment | NA | 0 |
| S1_k141_236431 | 1154 | No | NA | 0 | 1 | Not-determined | Genome-fragment | NA | 0 |
| S1_k141_253303 | 1804 | No | NA | 0 | 1 | Not-determined | Genome-fragment | NA | 0 |
| S1_k141_187510 | 4562 | No | NA | 0 | 1 | Not-determined | Genome-fragment | NA | 0 |
| S1_k141_38875 | 1095 | No | NA | 0 | 1 | Not-determined | Genome-fragment | NA | 0 |
| S7_k141_217874 | 1469 | No | NA | 1 | 0 | Low-quality | Genome-fragment | 2.51 | 0 |
| S1_k141_103998 | 1098 | No | NA | 0 | 1 | Not-determined | Genome-fragment | NA | 0 |
| S1_k141_22222 | 4563 | No | NA | 0 | 0 | Not-determined | Genome-fragment | NA | 0 |
| S1_k141_38913 | 1719 | No | NA | 0 | 2 | Not-determined | Genome-fragment | NA | 0 |
| S1_k141_187541 | 9571 | No | NA | 0 | 4 | Not-determined | Genome-fragment | NA | 0 |
| S1_k141_384637 | 6675 | No | NA | 0 | 2 | Not-determined | Genome-fragment | NA | 0 |
| S1_k141_334597 | 1044 | No | NA | 0 | 0 | Not-determined | Genome-fragment | NA | 0 |
| S1_k141_285846 | 3371 | No | NA | 0 | 1 | Not-determined | Genome-fragment | NA | 0 |
| S1_k141_187556 | 1349 | No | NA | 0 | 1 | Not-determined | Genome-fragment | NA | 0 |
| S1_k141_384647 | 1486 | No | NA | 0 | 0 | Not-determined | Genome-fragment | NA | 0 |
| S1_k141_121818 | 3012 | No | NA | 0 | 2 | Not-determined | Genome-fragment | NA | 0 |
| S1_k141_319350 | 1072 | No | NA | 0 | 0 | Not-determined | Genome-fragment | NA | 0 |
| S1_k141_253406 | 1067 | No | NA | 0 | 1 | Not-determined | Genome-fragment | NA | 0 |
| S1_k141_236542 | 2105 | No | NA | 0 | 1 | Not-determined | Genome-fragment | NA | 0 |
| S1_k141_22265 | 1059 | No | NA | 0 | 1 | Not-determined | Genome-fragment | NA | 0 |
| S1_k141_5961 | 3223 | No | NA | 0 | 1 | Not-determined | Genome-fragment | NA | 0 |
| S1_k141_87984 | 1458 | No | NA | 0 | 1 | Not-determined | Genome-fragment | NA | 0 |
| S17-final-viral-combined_k141_17213\|\|full | 1469 | No | NA | 0 | 0 | Low-quality | Genome-fragment | 1.09 | 0 |
| S1_k141_5984 | 1055 | No | NA | 0 | 1 | Not-determined | Genome-fragment | NA | 0 |
| S1_k141_319423 | 7338 | No | NA | 0 | 5 | Not-determined | Genome-fragment | NA | 0 |
| S1_k141_352192 | 1187 | No | NA | 0 | 1 | Not-determined | Genome-fragment | NA | 0 |
| S1_k141_6005 | 1297 | No | NA | 0 | 0 | Not-determined | Genome-fragment | NA | 0 |
| S1_k141_121849 | 2063 | No | NA | 0 | 1 | Not-determined | Genome-fragment | NA | 0 |
| S1_k141_38983 | 4772 | No | NA | 0 | 5 | Not-determined | Genome-fragment | NA | 0 |
| S12-final-viral-combined_k141_251216\|\|full | 1469 | No | NA | 0 | 1 | Low-quality | Genome-fragment | 1.09 | 0 |
| S1_k141_203893 | 2759 | No | NA | 0 | 2 | Not-determined | Genome-fragment | NA | 0 |
| S1_k141_121869 | 2243 | No | NA | 0 | 2 | Not-determined | Genome-fragment | NA | 0 |
| S1_k141_352211 | 2015 | No | NA | 0 | 2 | Not-determined | Genome-fragment | NA | 0 |
| S1_k141_22313 | 1046 | No | NA | 0 | 1 | Not-determined | Genome-fragment | NA | 0 |
| S1_k141_121909 | 1347 | No | NA | 0 | 0 | Not-determined | Genome-fragment | NA | 0 |
| S2-final-viral-combined_k141_26756\|\|full | 1469 | No | NA | 1 | 0 | Low-quality | Genome-fragment | 0.96 | 0 |
| S2_k141_115357 | 1469 | No | NA | 0 | 0 | Low-quality | Genome-fragment | 2.45 | 0 |
| S1_k141_121935 | 1585 | No | NA | 0 | 0 | Not-determined | Genome-fragment | NA | 0 |
| S1_k141_187737 | 1290 | No | NA | 0 | 1 | Not-determined | Genome-fragment | NA | 0 |
| S1_k141_253540 | 1182 | No | NA | 0 | 0 | Not-determined | Genome-fragment | NA | 0 |
| S1_k141_269273 | 1259 | No | NA | 0 | 1 | Not-determined | Genome-fragment | NA | 0 |
| S1_k141_71175 | 1379 | No | NA | 0 | 1 | Not-determined | Genome-fragment | NA | 0 |
| S1_k141_138279 | 2062 | No | NA | 0 | 1 | Not-determined | Genome-fragment | NA | 0 |
| S11-final-viral-combined_k141_37059\|\|full | 1470 | No | NA | 0 | 0 | Low-quality | Genome-fragment | 3.22 | 0 |
| S1-final-viral-combined_k141_316121\|\|full | 1470 | No | NA | 0 | 0 | Low-quality | Genome-fragment | 3.02 | 0 |
| S1_k141_154035 | 1397 | No | NA | 0 | 0 | Not-determined | Genome-fragment | NA | 0 |
| S1_k141_352297 | 3322 | No | NA | 0 | 1 | Not-determined | Genome-fragment | NA | 0 |
| S1_k141_6096 | 1293 | No | NA | 0 | 0 | Not-determined | Genome-fragment | NA | 0 |
| S1_k141_71200 | 2519 | No | NA | 0 | 0 | Not-determined | Genome-fragment | NA | 0 |
| S1_k141_88155 | 1347 | No | NA | 0 | 1 | Not-determined | Genome-fragment | NA | 0 |
| S1_k141_6131 | 1059 | No | NA | 0 | 2 | Not-determined | Genome-fragment | NA | 0 |
| S1_k141_204065 | 3505 | No | NA | 0 | 1 | Not-determined | Genome-fragment | NA | 0 |
| S17-final-viral-combined_k141_51751\|\|full | 1470 | Yes | 294 | 1 | 1 | Low-quality | Genome-fragment | 0.15 | 80 |
| S17-final-viral-combined_k141_194185\|\|lt2gene | 1470 | No | NA | 1 | 0 | Low-quality | Genome-fragment | 1.38 | 0 |
| S1_k141_236658 | 1232 | No | NA | 0 | 2 | Not-determined | Genome-fragment | NA | 0 |
| S2-final-viral-combined_k141_182620\|\|full | 1470 | No | NA | 0 | 1 | Low-quality | Genome-fragment | 3.1 | 0 |
| S1_k141_269335 | 1796 | No | NA | 0 | 2 | Not-determined | Genome-fragment | NA | 0 |
| S2-final-viral-combined_k141_37172\|\|lt2gene | 1470 | No | NA | 2 | 0 | Low-quality | Genome-fragment | 3.83 | 0 |
| S1_k141_39114 | 1901 | No | NA | 0 | 0 | Not-determined | Genome-fragment | NA | 0 |
| S1_k141_334791 | 3819 | No | NA | 0 | 4 | Not-determined | Genome-fragment | NA | 0 |
| S1_k141_154140 | 3044 | No | NA | 0 | 3 | Not-determined | Genome-fragment | NA | 0 |
| S1_k141_303172 | 1405 | No | NA | 0 | 0 | Not-determined | Genome-fragment | NA | 0 |
| S1_k141_269372 | 1013 | No | NA | 0 | 1 | Not-determined | Genome-fragment | NA | 0 |
| S1_k141_352408 | 1365 | No | NA | 0 | 0 | Not-determined | Genome-fragment | NA | 0 |
| S1_k141_253771 | 1211 | No | NA | 0 | 0 | Not-determined | Genome-fragment | NA | 0 |
| S1_k141_170115 | 40097 | No | NA | 23 | 1 | High-quality | High-quality | 100 | 0 |
| S1_k141_204136 | 2968 | No | NA | 0 | 2 | Not-determined | Genome-fragment | NA | 0 |
| S1_k141_71292 | 4202 | No | NA | 0 | 0 | Not-determined | Genome-fragment | NA | 0 |
| S1_k141_204142 | 1034 | No | NA | 0 | 2 | Not-determined | Genome-fragment | NA | 0 |
| S1_k141_88280 | 3161 | No | NA | 0 | 2 | Not-determined | Genome-fragment | NA | 0 |
| S1_k141_170161 | 1499 | No | NA | 0 | 0 | Not-determined | Genome-fragment | NA | 0 |
| S8-final-viral-combined_k141_155536\|\|full | 1470 | No | NA | 0 | 0 | Low-quality | Genome-fragment | 1.06 | 0 |
| S11-final-viral-combined_k141_324075\|\|full | 1471 | No | NA | 0 | 0 | Low-quality | Genome-fragment | 3.18 | 0 |
| S1_k141_352438 | 1120 | No | NA | 0 | 0 | Not-determined | Genome-fragment | NA | 0 |
| S3-final-viral-combined_k141_81836\|\|full | 1471 | No | NA | 0 | 0 | Low-quality | Genome-fragment | 1.66 | 0 |
| S5-final-viral-combined_k141_174766\|\|full | 1471 | No | NA | 0 | 0 | Low-quality | Genome-fragment | 2.37 | 0 |
| S7-final-viral-combined_k141_143996\|\|full | 1471 | No | NA | 0 | 0 | Low-quality | Genome-fragment | 2.44 | 0 |
| S1_k141_204192 | 1754 | No | NA | 0 | 1 | Not-determined | Genome-fragment | NA | 0 |
| S1_k141_6291 | 1397 | No | NA | 0 | 1 | Not-determined | Genome-fragment | NA | 0 |
| S7-final-viral-combined_k141_23365\|\|full | 1471 | No | NA | 0 | 1 | Low-quality | Genome-fragment | 2.77 | 0 |
| S1_k141_334971 | 1262 | No | NA | 0 | 2 | Not-determined | Genome-fragment | NA | 0 |
| S1_k141_170214 | 1687 | No | NA | 0 | 0 | Not-determined | Genome-fragment | NA | 0 |
| S1_k141_334981 | 3349 | No | NA | 0 | 2 | Not-determined | Genome-fragment | NA | 0 |
| S7-final-viral-combined_k141_162765\|\|full | 1471 | No | NA | 0 | 1 | Low-quality | Genome-fragment | 0.84 | 0 |
| S1_k141_71398 | 1237 | No | NA | 0 | 0 | Not-determined | Genome-fragment | NA | 0 |
| S1_k141_104388 | 1300 | No | NA | 0 | 2 | Not-determined | Genome-fragment | NA | 0 |
| S12-final-viral-combined_k141_253887\|\|full | 1471 | No | NA | 0 | 0 | Low-quality | Genome-fragment | 3.26 | 0 |
| S1_k141_187939 | 3278 | No | NA | 0 | 1 | Not-determined | Genome-fragment | NA | 0 |
| S12-final-viral-combined_k141_240998\|\|full | 1471 | No | NA | 0 | 0 | Low-quality | Genome-fragment | 1.09 | 0 |
| S1_k141_352525 | 1250 | No | NA | 0 | 1 | Not-determined | Genome-fragment | NA | 0 |
| S1_k141_236890 | 1145 | No | NA | 0 | 0 | Not-determined | Genome-fragment | NA | 0 |
| S4-final-viral-combined_k141_85766\|\|full | 1471 | No | NA | 0 | 0 | Low-quality | Genome-fragment | 1.03 | 0 |
| S1_k141_220391 | 1146 | No | NA | 0 | 0 | Not-determined | Genome-fragment | NA | 0 |
| S8_k141_119825 | 1471 | No | NA | 1 | 0 | Low-quality | Genome-fragment | 0.95 | 0 |
| S1_k141_55663 | 1360 | No | NA | 0 | 0 | Not-determined | Genome-fragment | NA | 0 |
| S1_k141_138520 | 1632 | No | NA | 0 | 0 | Not-determined | Genome-fragment | NA | 0 |
| S1_k141_204318 | 4452 | No | NA | 0 | 4 | Not-determined | Genome-fragment | NA | 0 |
| S1_k141_6409 | 1036 | No | NA | 0 | 2 | Not-determined | Genome-fragment | NA | 0 |
| S11-final-viral-combined_k141_264353\|\|full | 1472 | No | NA | 0 | 2 | Low-quality | Genome-fragment | 1.21 | 0 |
| S1_k141_236970 | 2478 | No | NA | 0 | 0 | Not-determined | Genome-fragment | NA | 0 |
| S1_k141_104465 | 1574 | No | NA | 0 | 2 | Not-determined | Genome-fragment | NA | 0 |
| S1_k141_39334 | 1285 | No | NA | 0 | 1 | Not-determined | Genome-fragment | NA | 0 |
| S1_k141_303335 | 2425 | No | NA | 0 | 1 | Not-determined | Genome-fragment | NA | 0 |
| S1_k141_286268 | 6154 | No | NA | 0 | 3 | Not-determined | Genome-fragment | NA | 0 |
| S1_k141_188124 | 1645 | No | NA | 0 | 0 | Not-determined | Genome-fragment | NA | 0 |
| S1_k141_6504 | 1115 | No | NA | 0 | 0 | Not-determined | Genome-fragment | NA | 0 |
| S11-final-viral-combined_k141_302483\|\|full | 1472 | No | NA | 0 | 0 | Low-quality | Genome-fragment | 2.09 | 0 |
| S1_k141_6512 | 1592 | No | NA | 0 | 2 | Not-determined | Genome-fragment | NA | 0 |
| S1_k141_55729 | 1484 | No | NA | 0 | 1 | Not-determined | Genome-fragment | NA | 0 |

| | | | | | | | | | | |
|---|---|---|---|---|---|---|---|---|---|---|
| S1_k141_335113 | 5677 | No | NA | 0 | 1 | Not-determined | Genome-fragment | NA | | 0 |
| S11-final-viral-combined_k141_42488\|\|lt2gene | 1472 | No | NA | 1 | 0 | Low-quality | Genome-fragment | | 0.91 | 0 |
| S1_k141_138643 | 1635 | No | NA | 0 | 0 | Not-determined | Genome-fragment | NA | | 0 |
| S1_k141_254037 | 1083 | No | NA | 0 | 0 | Not-determined | Genome-fragment | NA | | 0 |
| S1_k141_303394 | 1257 | No | NA | 0 | 3 | Not-determined | Genome-fragment | NA | | 0 |
| S1_k141_204488 | 1015 | No | NA | 0 | 1 | Not-determined | Genome-fragment | NA | | 0 |
| S1_k141_335170 | 1139 | No | NA | 0 | 4 | Not-determined | Genome-fragment | NA | | 0 |
| S1_k141_335171 | 3374 | No | NA | 0 | 1 | Not-determined | Genome-fragment | NA | | 0 |
| S1-final-viral-combined_k141_366109\|\|full | 1472 | No | NA | 0 | 0 | Low-quality | Genome-fragment | | 1.98 | 0 |
| S1_k141_385234 | 1385 | No | NA | 0 | 0 | Not-determined | Genome-fragment | NA | | 0 |
| S1_k141_220544 | 1049 | No | NA | 0 | 1 | Not-determined | Genome-fragment | NA | | 0 |
| S1_k141_104516 | 1394 | No | NA | 0 | 0 | Not-determined | Genome-fragment | NA | | 0 |
| S1_k141_188204 | 1058 | No | NA | 0 | 0 | Not-determined | Genome-fragment | NA | | 0 |
| S1_k141_204519 | 1038 | No | NA | 0 | 1 | Not-determined | Genome-fragment | NA | | 0 |
| S1-final-viral-combined_k141_389726\|\|full | 1472 | No | NA | 1 | 0 | Low-quality | Genome-fragment | | 3.22 | 0 |
| S1_k141_220553 | 1169 | No | NA | 0 | 1 | Not-determined | Genome-fragment | NA | | 0 |
| S3-final-viral-combined_k141_118477\|\|full | 1472 | No | NA | 2 | 0 | Low-quality | Genome-fragment | | 3.75 | 0 |
| S1_k141_122535 | 3589 | No | NA | 0 | 2 | Not-determined | Genome-fragment | NA | | 0 |
| S1_k141_138742 | 1292 | No | NA | 0 | 1 | Not-determined | Genome-fragment | NA | | 0 |
| S1_k141_254106 | 2975 | No | NA | 0 | 2 | Not-determined | Genome-fragment | NA | | 0 |
| S1_k141_88645 | 2135 | No | NA | 0 | 2 | Not-determined | Genome-fragment | NA | | 0 |
| S5_k141_68232 | 1472 | No | NA | 0 | 0 | Low-quality | Genome-fragment | | 2.53 | 0 |
| S1_k141_71673 | 1048 | No | NA | 0 | 1 | Not-determined | Genome-fragment | NA | | 0 |
| S1_k141_254138 | 1933 | No | NA | 0 | 1 | Not-determined | Genome-fragment | NA | | 0 |
| S1_k141_22920 | 4483 | No | NA | 0 | 10 | Not-determined | Genome-fragment | NA | | 0 |
| S1_k141_320024 | 3372 | No | NA | 0 | 1 | Not-determined | Genome-fragment | NA | | 0 |
| S1_k141_220637 | 1174 | No | NA | 0 | 1 | Not-determined | Genome-fragment | NA | | 0 |
| S1_k141_22951 | 4311 | No | NA | 0 | 2 | Not-determined | Genome-fragment | NA | | 0 |
| S1_k141_303546 | 3401 | No | NA | 0 | 2 | Not-determined | Genome-fragment | NA | | 0 |
| S1_k141_104617 | 7989 | No | NA | 0 | 5 | Not-determined | Genome-fragment | NA | | 0 |
| S1_k141_122647 | 1448 | No | NA | 0 | 0 | Not-determined | Genome-fragment | NA | | 0 |
| S1_k141_254254 | 1463 | No | NA | 0 | 1 | Not-determined | Genome-fragment | NA | | 0 |
| S7-final-viral-combined_k141_189969\|\|full | 1472 | No | NA | 1 | 0 | Low-quality | Genome-fragment | | 1.7 | 0 |
| S17-final-viral-combined_k141_205240\|\|full | 1472 | No | NA | 0 | 0 | Low-quality | Genome-fragment | | 2.63 | 0 |
| S1_k141_55933 | 3084 | No | NA | 0 | 1 | Not-determined | Genome-fragment | NA | | 0 |
| S1_k141_104665 | 1323 | No | NA | 0 | 1 | Not-determined | Genome-fragment | NA | | 0 |
| S1_k141_368586 | 1162 | No | NA | 0 | 0 | Not-determined | Genome-fragment | NA | | 0 |
| S1_k141_254300 | 1583 | No | NA | 0 | 0 | Not-determined | Genome-fragment | NA | | 0 |
| S1_k141_104710 | 2023 | No | NA | 0 | 1 | Not-determined | Genome-fragment | NA | | 0 |
| S1_k141_353013 | 1053 | No | NA | 0 | 2 | Not-determined | Genome-fragment | NA | | 0 |
| S1_k141_335317 | 6501 | No | NA | 0 | 6 | Not-determined | Genome-fragment | NA | | 0 |
| S1_k141_368616 | 1000 | No | NA | 0 | 1 | Not-determined | Genome-fragment | NA | | 0 |
| S1_k141_71855 | 2036 | No | NA | 0 | 1 | Not-determined | Genome-fragment | NA | | 0 |
| S1_k141_286456 | 1590 | No | NA | 0 | 0 | Not-determined | Genome-fragment | NA | | 0 |
| S1_k141_303680 | 1103 | No | NA | 0 | 1 | Not-determined | Genome-fragment | NA | | 0 |
| S1_k141_6742 | 1276 | No | NA | 0 | 3 | Not-determined | Genome-fragment | NA | | 0 |
| S1_k141_353026 | 5866 | No | NA | 0 | 0 | Not-determined | Genome-fragment | NA | | 0 |
| S12-final-viral-combined_k141_47381\|\|full | 1472 | No | NA | 0 | 1 | Low-quality | Genome-fragment | | 1.69 | 0 |
| S2-final-viral-combined_k141_229970\|\|full | 1472 | No | NA | 0 | 0 | Low-quality | Genome-fragment | | 2.61 | 0 |
| S4-final-viral-combined_k141_153121\|\|full | 1472 | No | NA | 1 | 0 | Low-quality | Genome-fragment | | 3.25 | 0 |
| S1_k141_122789 | 3353 | No | NA | 0 | 2 | Not-determined | Genome-fragment | NA | | 0 |
| S9_k141_127904 | 1473 | No | NA | 0 | 1 | Low-quality | Genome-fragment | | 0.25 | 0 |
| S1_k141_170614 | 4193 | No | NA | 0 | 4 | Not-determined | Genome-fragment | NA | | 0 |
| S1_k141_23148 | 1013 | No | NA | 0 | 1 | Not-determined | Genome-fragment | NA | | 0 |
| S1_k141_385473 | 1072 | No | NA | 0 | 0 | Not-determined | Genome-fragment | NA | | 0 |
| S1_k141_254362 | 1249 | No | NA | 0 | 2 | Not-determined | Genome-fragment | NA | | 0 |
| S11-final-viral-combined_k141_270484\|\|full | 1473 | No | NA | 2 | 0 | Low-quality | Genome-fragment | | 1.78 | 0 |
| S1_k141_56099 | 3969 | No | NA | 0 | 1 | Not-determined | Genome-fragment | NA | | 0 |
| S1_k141_23185 | 1105 | No | NA | 0 | 0 | Not-determined | Genome-fragment | NA | | 0 |
| S1_k141_353103 | 1665 | No | NA | 0 | 0 | Not-determined | Genome-fragment | NA | | 0 |
| S11_k141_44174 | 1473 | No | NA | 0 | 0 | Low-quality | Genome-fragment | | 1.2 | 0 |
| S1_k141_39650 | 1173 | No | NA | 0 | 1 | Not-determined | Genome-fragment | NA | | 0 |
| S1_k141_154632 | 1875 | No | NA | 0 | 1 | Not-determined | Genome-fragment | NA | | 0 |
| S1_k141_188426 | 1168 | No | NA | 0 | 1 | Not-determined | Genome-fragment | NA | | 0 |
| S1_k141_204866 | 4425 | No | NA | 0 | 5 | Not-determined | Genome-fragment | NA | | 0 |
| S1-final-viral-combined_k141_108867\|\|full | 1473 | No | NA | 0 | 0 | Low-quality | Genome-fragment | | 2.36 | 0 |
| S1_k141_320365 | 4138 | No | NA | 0 | 1 | Not-determined | Genome-fragment | NA | | 0 |
| S17-final-viral-combined_k141_195469\|\|full | 1473 | No | NA | 1 | 0 | Low-quality | Genome-fragment | | 3.93 | 0 |
| S1_k141_170734 | 1139 | No | NA | 0 | 0 | Not-determined | Genome-fragment | NA | | 0 |
| S1_k141_385550 | 1196 | No | NA | 0 | 0 | Not-determined | Genome-fragment | NA | | 0 |
| S1_k141_122980 | 1481 | No | NA | 0 | 1 | Not-determined | Genome-fragment | NA | | 0 |
| S1_k141_23261 | 2711 | No | NA | 0 | 1 | Not-determined | Genome-fragment | NA | | 0 |
| S1_k141_237594 | 3659 | No | NA | 0 | 0 | Not-determined | Genome-fragment | NA | | 0 |
| S1_k141_335507 | 7287 | No | NA | 0 | 5 | Not-determined | Genome-fragment | NA | | 0 |
| S1_k141_170828 | 1367 | No | NA | 0 | 0 | Not-determined | Genome-fragment | NA | | 0 |
| S1_k141_270060 | 2270 | No | NA | 0 | 2 | Not-determined | Genome-fragment | NA | | 0 |
| S1_k141_7044 | 4460 | No | NA | 0 | 2 | Not-determined | Genome-fragment | NA | | 0 |
| S1_k141_353255 | 1275 | No | NA | 0 | 1 | Not-determined | Genome-fragment | NA | | 0 |
| S1_k141_188585 | 1009 | No | NA | 0 | 1 | Not-determined | Genome-fragment | NA | | 0 |
| S1_k141_123088 | 1726 | No | NA | 0 | 1 | Not-determined | Genome-fragment | NA | | 0 |
| S1_k141_188613 | 4496 | No | NA | 0 | 2 | Not-determined | Genome-fragment | NA | | 0 |
| S1_k141_353282 | 1246 | No | NA | 0 | 1 | Not-determined | Genome-fragment | NA | | 0 |
| S1_k141_170908 | 1176 | No | NA | 0 | 1 | Not-determined | Genome-fragment | NA | | 0 |
| S2-final-viral-combined_k141_208625\|\|full | 1473 | No | NA | 0 | 0 | Low-quality | Genome-fragment | | 3.69 | 0 |
| S1_k141_254633 | 2237 | No | NA | 0 | 1 | Not-determined | Genome-fragment | NA | | 0 |
| S2-final-viral-combined_k141_95005\|\|full | 1473 | No | NA | 2 | 0 | Low-quality | Genome-fragment | | 3.31 | 0 |
| S1_k141_320615 | 1044 | No | NA | 0 | 1 | Not-determined | Genome-fragment | NA | | 0 |
| S1_k141_170958 | 1742 | No | NA | 0 | 1 | Not-determined | Genome-fragment | NA | | 0 |
| S1_k141_139213 | 2406 | No | NA | 0 | 1 | Not-determined | Genome-fragment | NA | | 0 |
| S1_k141_270220 | 1557 | No | NA | 0 | 1 | Not-determined | Genome-fragment | NA | | 0 |
| S8-final-viral-combined_k141_119505\|\|lt2gene | 1473 | No | NA | 1 | 0 | Low-quality | Genome-fragment | | 2.15 | 0 |
| S11-final-viral-combined_k141_279556\|\|full | 1474 | No | NA | 0 | 1 | Low-quality | Genome-fragment | | 2.43 | 0 |
| S1_k141_56428 | 1034 | No | NA | 0 | 0 | Not-determined | Genome-fragment | NA | | 0 |
| S1_k141_89218 | 1268 | No | NA | 0 | 0 | Not-determined | Genome-fragment | NA | | 0 |
| S1_k141_205108 | 1152 | No | NA | 0 | 1 | Not-determined | Genome-fragment | NA | | 0 |
| S1_k141_56462 | 2102 | No | NA | 0 | 0 | Not-determined | Genome-fragment | NA | | 0 |
| S1_k141_353371 | 1974 | No | NA | 0 | 1 | Not-determined | Genome-fragment | NA | | 0 |
| S1_k141_89220 | 1116 | No | NA | 0 | 1 | Not-determined | Genome-fragment | NA | | 0 |
| S11-final-viral-combined_k141_81374\|\|lt2gene | 1474 | No | NA | 1 | 0 | Low-quality | Genome-fragment | | 4 | 0 |
| S3-final-viral-combined_k141_20806\|\|full | 1474 | No | NA | 0 | 0 | Low-quality | Genome-fragment | | 4.12 | 0 |
| S1_k141_353413 | 2106 | No | NA | 0 | 1 | Not-determined | Genome-fragment | NA | | 0 |
| S1_k141_353416 | 1221 | No | NA | 0 | 0 | Not-determined | Genome-fragment | NA | | 0 |
| S1_k141_154959 | 1198 | No | NA | 0 | 0 | Not-determined | Genome-fragment | NA | | 0 |
| S1_k141_123240 | 2041 | No | NA | 0 | 0 | Not-determined | Genome-fragment | NA | | 0 |
| S1_k141_154968 | 1054 | No | NA | 0 | 2 | Not-determined | Genome-fragment | NA | | 0 |
| S1_k141_320856 | 8935 | No | NA | 0 | 8 | Not-determined | Genome-fragment | NA | | 0 |
| S1_k141_368991 | 4794 | No | NA | 0 | 5 | Not-determined | Genome-fragment | NA | | 0 |
| S1_k141_72322 | 3078 | No | NA | 0 | 1 | Not-determined | Genome-fragment | NA | | 0 |
| S1_k141_89329 | 1487 | No | NA | 0 | 1 | Not-determined | Genome-fragment | NA | | 0 |
| S1_k141_139416 | 2867 | No | NA | 0 | 0 | Not-determined | Genome-fragment | NA | | 0 |
| S1_k141_320873 | 7594 | No | NA | 0 | 2 | Not-determined | Genome-fragment | NA | | 0 |
| S3-final-viral-combined_k141_62630\|\|full | 1474 | No | NA | 0 | 0 | Low-quality | Genome-fragment | | 2.92 | 0 |
| S1_k141_89349 | 1061 | No | NA | 0 | 1 | Not-determined | Genome-fragment | NA | | 0 |
| S1_k141_39883 | 1545 | No | NA | 0 | 1 | Not-determined | Genome-fragment | NA | | 0 |
| S1_k141_72360 | 1015 | No | NA | 0 | 0 | Not-determined | Genome-fragment | NA | | 0 |
| S1_k141_7235 | 1445 | No | NA | 0 | 0 | Not-determined | Genome-fragment | NA | | 0 |
| S7-final-viral-combined_k141_156394\|\|full | 1474 | No | NA | 0 | 1 | Low-quality | Genome-fragment | | 3.02 | 0 |
| S1_k141_304196 | 1101 | No | NA | 0 | 0 | Not-determined | Genome-fragment | NA | | 0 |
| S17-final-viral-combined_k141_218427\|\|full | 1474 | No | NA | 2 | 0 | Low-quality | Genome-fragment | | 2.85 | 0 |
| S17-k141_193249 | 1474 | No | NA | 2 | 0 | Low-quality | Genome-fragment | | 3.44 | 0 |
| S1_k141_369054 | 1209 | No | NA | 0 | 1 | Not-determined | Genome-fragment | NA | | 0 |
| S1_k141_123357 | 1371 | No | NA | 0 | 1 | Not-determined | Genome-fragment | NA | | 0 |
| S12-final-viral-combined_k141_53177\|\|lt2gene | 1474 | No | NA | 0 | 0 | Low-quality | Genome-fragment | | 1.82 | 0 |
| S1_k141_188885 | 1497 | No | NA | 0 | 1 | Not-determined | Genome-fragment | NA | | 0 |
| S1_k141_221240 | 1014 | No | NA | 0 | 0 | Not-determined | Genome-fragment | NA | | 0 |
| S1_k141_123406 | 4346 | No | NA | 0 | 0 | Not-determined | Genome-fragment | NA | | 0 |
| S1_k141_39984 | 4909 | No | NA | 0 | 1 | Not-determined | Genome-fragment | NA | | 0 |
| S1_k141_139473 | 2852 | No | NA | 0 | 4 | Not-determined | Genome-fragment | NA | | 0 |
| S1_k141_23609 | 1076 | No | NA | 0 | 2 | Not-determined | Genome-fragment | NA | | 0 |
| S1_k141_56603 | 1077 | No | NA | 0 | 0 | Not-determined | Genome-fragment | NA | | 0 |
| S1_k141_369111 | 3402 | No | NA | 0 | 2 | Not-determined | Genome-fragment | NA | | 0 |
| S1_k141_254890 | 3240 | No | NA | 0 | 3 | Not-determined | Genome-fragment | NA | | 0 |
| S1_k141_321061 | 1013 | No | NA | 0 | 0 | Not-determined | Genome-fragment | NA | | 0 |
| S1_k141_353564 | 1375 | No | NA | 0 | 0 | Not-determined | Genome-fragment | NA | | 0 |
| S1_k141_40044 | 9105 | No | NA | 0 | 3 | Not-determined | Genome-fragment | NA | | 0 |
| S1_k141_321108 | 1013 | No | NA | 0 | 1 | Not-determined | Genome-fragment | NA | | 0 |
| S1_k141_56640 | 2535 | No | NA | 0 | 2 | Not-determined | Genome-fragment | NA | | 0 |
| S1_k141_221330 | 1389 | No | NA | 0 | 0 | Not-determined | Genome-fragment | NA | | 0 |
| S12_k141_77159 | 1474 | No | NA | 0 | 1 | Low-quality | Genome-fragment | | 0.6 | 0 |
| S1_k141_254959 | 3919 | No | NA | 0 | 10 | Not-determined | Genome-fragment | NA | | 0 |
| S1_k141_238113 | 1010 | No | NA | 0 | 0 | Not-determined | Genome-fragment | NA | | 0 |
| S1_k141_7437 | 5808 | No | NA | 0 | 4 | Not-determined | Genome-fragment | NA | | 0 |
| S2-final-viral-combined_k141_204279\|\|lt2gene | 1474 | No | NA | 2 | 0 | Low-quality | Genome-fragment | | 3.12 | 0 |
| S4-final-viral-combined_k141_17812\|\|full | 1474 | No | NA | 0 | 2 | Low-quality | Genome-fragment | | 0.4 | 0 |
| S1_k141_72528 | 1990 | No | NA | 0 | 4 | Not-determined | Genome-fragment | NA | | 0 |
| S8-final-viral-combined_k141_220108\|\|full | 1474 | No | NA | 1 | 0 | Low-quality | Genome-fragment | | 0.99 | 0 |
| S11-final-viral-combined_k141_72266\|\|full | 1475 | No | NA | 1 | 0 | Low-quality | Genome-fragment | | 2.86 | 0 |
| S1_k141_270631 | 2072 | No | NA | 0 | 2 | Not-determined | Genome-fragment | NA | | 0 |
| S11_k141_83002 | 1475 | No | NA | 0 | 2 | Low-quality | Genome-fragment | | 0.48 | 0 |
| S1_k141_238176 | 1817 | No | NA | 0 | 1 | Not-determined | Genome-fragment | NA | | 0 |
| S1-final-viral-combined_k141_341574\|\|full | 1475 | No | NA | 0 | 0 | Low-quality | Genome-fragment | | 2.47 | 0 |
| S1_k141_105330 | 1313 | No | NA | 0 | 0 | Not-determined | Genome-fragment | NA | | 0 |
| S1_k141_238213 | 8233 | No | NA | 0 | 8 | Not-determined | Genome-fragment | NA | | 0 |
| S1_k141_304374 | 1380 | No | NA | 0 | 2 | Not-determined | Genome-fragment | NA | | 0 |
| S1_k141_367376 | 1475 | No | NA | 0 | 0 | Low-quality | Genome-fragment | | 3.96 | 0 |
| S1_k141_221425 | 1480 | No | NA | 0 | 1 | Not-determined | Genome-fragment | NA | | 0 |
| S1_k141_171323 | 1025 | No | NA | 0 | 1 | Not-determined | Genome-fragment | NA | | 0 |
| S7-final-viral-combined_k141_69851\|\|full | 1475 | No | NA | 0 | 3 | Low-quality | Genome-fragment | | 0.45 | 0 |
| S1_k141_188985 | 1558 | No | NA | 0 | 1 | Not-determined | Genome-fragment | NA | | 0 |
| S1_k141_369263 | 1324 | No | NA | 0 | 1 | Not-determined | Genome-fragment | NA | | 0 |
| S1_k141_238218 | 6530 | No | NA | 0 | 1 | Not-determined | Genome-fragment | NA | | 0 |
| S1_k141_155219 | 1115 | No | NA | 0 | 0 | Not-determined | Genome-fragment | NA | | 0 |
| S17-k141_263437 | 1475 | No | NA | 0 | 0 | Low-quality | Genome-fragment | | 0.35 | 0 |
| S1_k141_72575 | 1114 | No | NA | 0 | 1 | Not-determined | Genome-fragment | NA | | 0 |
| S1_k141_7546 | 1528 | No | NA | 0 | 1 | Not-determined | Genome-fragment | NA | | 0 |
| S1_k141_56843 | 1451 | No | NA | 0 | 1 | Not-determined | Genome-fragment | NA | | 0 |
| S1_k141_255099 | 2000 | No | NA | 0 | 1 | Not-determined | Genome-fragment | NA | | 0 |
| S1_k141_155250 | 1073 | No | NA | 0 | 2 | Not-determined | Genome-fragment | NA | | 0 |
| S1_k141_270713 | 1398 | No | NA | 0 | 0 | Not-determined | Genome-fragment | NA | | 0 |
| S2-final-viral-combined_k141_242102\|\|full | 1475 | No | NA | 0 | 1 | Low-quality | Genome-fragment | | 0.84 | 0 |
| S8-final-viral-combined_k141_223006\|\|full | 1475 | No | NA | 0 | 0 | Low-quality | Genome-fragment | | 3.42 | 0 |
| S1_k141_287245 | 1474 | No | NA | 0 | 1 | Not-determined | Genome-fragment | NA | | 0 |
| S1_k141_123596 | 5818 | No | NA | 0 | 5 | Not-determined | Genome-fragment | NA | | 0 |
| S1_k141_221519 | 1690 | No | NA | 0 | 1 | Not-determined | Genome-fragment | NA | | 0 |

| | | | | | | | | | |
|---|---|---|---|---|---|---|---|---|---|
| S1_k141_321309 | 1261 | No | NA | 0 | 1 | Not-determined | Genome-fragment | NA | 0 |
| S1_k141_123616 | 1501 | No | NA | 0 | 1 | Not-determined | Genome-fragment | NA | 0 |
| S1_k141_155378 | 6534 | No | NA | 0 | 3 | Not-determined | Genome-fragment | NA | 0 |
| S1_k141_89666 | 1515 | No | NA | 0 | 2 | Not-determined | Genome-fragment | NA | 0 |
| S8-final-viral-combined_k141_216696\|\|full | 1475 | No | NA | 1 | 0 | Low-quality | Genome-fragment | 2.84 | 0 |
| S1_k141_56921 | 1050 | No | NA | 0 | 1 | Not-determined | Genome-fragment | NA | 0 |
| S1_k141_221595 | 1925 | No | NA | 0 | 1 | Not-determined | Genome-fragment | NA | 0 |
| S1_k141_304463 | 3168 | No | NA | 0 | 1 | Not-determined | Genome-fragment | NA | 0 |
| S1_k141_56941 | 1481 | No | NA | 0 | 0 | Not-determined | Genome-fragment | NA | 0 |
| S1_k141_189199 | 1222 | No | NA | 0 | 0 | Not-determined | Genome-fragment | NA | 0 |
| S1_k141_386234 | 1573 | No | NA | 0 | 1 | Not-determined | Genome-fragment | NA | 0 |
| S1_k141_321392 | 4265 | No | NA | 0 | 3 | Not-determined | Genome-fragment | NA | 0 |
| S1_k141_304489 | 1726 | No | NA | 0 | 1 | Not-determined | Genome-fragment | NA | 0 |
| S1_k141_369418 | 1538 | No | NA | 0 | 0 | Not-determined | Genome-fragment | NA | 0 |
| S1_k141_270869 | 1922 | No | NA | 0 | 0 | Not-determined | Genome-fragment | NA | 0 |
| S1_k141_221626 | 10940 | No | NA | 0 | 3 | Not-determined | Genome-fragment | NA | 0 |
| S1_k141_72730 | 1064 | No | NA | 0 | 0 | Not-determined | Genome-fragment | NA | 0 |
| S1_k141_7700 | 30471 | No | NA | 0 | 8 | Not-determined | Genome-fragment | NA | 0 |
| S1_k141_189234 | 13335 | No | NA | 0 | 7 | Not-determined | Genome-fragment | NA | 0 |
| S8_k141_64681 | 1475 | No | NA | 2 | 0 | Low-quality | Genome-fragment | 3.08 | 0 |
| S1_k141_353862 | 1174 | No | NA | 0 | 1 | Not-determined | Genome-fragment | NA | 0 |
| S1_k141_155467 | 1363 | No | NA | 0 | 1 | Not-determined | Genome-fragment | NA | 0 |
| S1_k141_57068 | 1137 | No | NA | 0 | 0 | Not-determined | Genome-fragment | NA | 0 |
| S1_k141_189258 | 2813 | No | NA | 0 | 2 | Not-determined | Genome-fragment | NA | 0 |
| S1_k141_205794 | 1427 | No | NA | 0 | 1 | Not-determined | Genome-fragment | NA | 0 |
| S1_k141_89770 | 1280 | No | NA | 0 | 1 | Not-determined | Genome-fragment | NA | 0 |
| S1_k141_386360 | 3950 | No | NA | 0 | 0 | Not-determined | Genome-fragment | NA | 0 |
| S1_k141_40330 | 1724 | No | NA | 0 | 0 | Not-determined | Genome-fragment | NA | 0 |
| S1_k141_353950 | 1158 | No | NA | 0 | 2 | Not-determined | Genome-fragment | NA | 0 |
| S1_k141_221731 | 1042 | No | NA | 0 | 2 | Not-determined | Genome-fragment | NA | 0 |
| S1_k141_386385 | 1834 | No | NA | 0 | 0 | Not-determined | Genome-fragment | NA | 0 |
| S1_k141_336177 | 1278 | No | NA | 0 | 0 | Not-determined | Genome-fragment | NA | 0 |
| S1_k141_72760 | 2876 | No | NA | 0 | 1 | Not-determined | Genome-fragment | NA | 0 |
| S1_k141_221757 | 2496 | No | NA | 0 | 0 | Not-determined | Genome-fragment | NA | 0 |
| S1_k141_270973 | 3317 | No | NA | 0 | 2 | Not-determined | Genome-fragment | NA | 0 |
| S1_k141_105675 | 1308 | No | NA | 0 | 1 | Not-determined | Genome-fragment | NA | 0 |
| S1_k141_238292 | 4146 | No | NA | 0 | 0 | Not-determined | Genome-fragment | NA | 0 |
| S1_k141_105702 | 3054 | No | NA | 0 | 2 | Not-determined | Genome-fragment | NA | 0 |
| S9-final-viral-combined_k141_29136\|\|full | 1476 | No | NA | 0 | 0 | Low-quality | Genome-fragment | 3.57 | 0 |
| S1_k141_354014 | 5130 | No | NA | 0 | 3 | Not-determined | Genome-fragment | NA | 0 |
| S1_k141_189419 | 1382 | No | NA | 0 | 1 | Not-determined | Genome-fragment | NA | 0 |
| S1_k141_139920 | 1803 | No | NA | 0 | 1 | Not-determined | Genome-fragment | NA | 0 |
| S1_k141_139931 | 1534 | No | NA | 0 | 0 | Not-determined | Genome-fragment | NA | 0 |
| S1_k141_40429 | 4226 | No | NA | 0 | 2 | Not-determined | Genome-fragment | NA | 0 |
| S1_k141_221834 | 1953 | No | NA | 0 | 0 | Not-determined | Genome-fragment | NA | 0 |
| S9-final-viral-combined_k141_110343\|\|full | 1476 | No | NA | 2 | 0 | Low-quality | Genome-fragment | 3.21 | 0 |
| S1-final-viral-combined_k141_294977\|\|full | 1476 | No | NA | 0 | 0 | Low-quality | Genome-fragment | 4.22 | 0 |
| S1_k141_155604 | 2156 | No | NA | 0 | 0 | Not-determined | Genome-fragment | NA | 0 |
| S1_k141_24041 | 1062 | No | NA | 0 | 0 | Not-determined | Genome-fragment | NA | 0 |
| S1_k141_51945 | 1476 | No | NA | 0 | 0 | Low-quality | Genome-fragment | 3.3 | 0 |
| S1_k141_7963 | 2989 | No | NA | 0 | 1 | Not-determined | Genome-fragment | NA | 0 |
| S1_k141_369649 | 1309 | No | NA | 0 | 0 | Not-determined | Genome-fragment | NA | 0 |
| S1_k141_155672 | 1050 | No | NA | 0 | 1 | Not-determined | Genome-fragment | NA | 0 |
| S1_k141_7979 | 3491 | No | NA | 0 | 3 | Not-determined | Genome-fragment | NA | 0 |
| S1_k141_171611 | 1586 | No | NA | 0 | 0 | Not-determined | Genome-fragment | NA | 0 |
| S1_k141_221879 | 1256 | No | NA | 0 | 1 | Not-determined | Genome-fragment | NA | 0 |
| S5-final-viral-combined_k141_141931\|\|full | 1476 | No | NA | 2 | 0 | Low-quality | Genome-fragment | 2.44 | 0 |
| S1_k141_155712 | 3508 | No | NA | 0 | 1 | Not-determined | Genome-fragment | NA | 0 |
| S1_k141_369684 | 4047 | No | NA | 0 | 8 | Not-determined | Genome-fragment | NA | 0 |
| S1_k141_238468 | 1431 | No | NA | 0 | 1 | Not-determined | Genome-fragment | NA | 0 |
| S1_k141_105829 | 1451 | No | NA | 0 | 0 | Not-determined | Genome-fragment | NA | 0 |
| S1_k141_336387 | 2133 | No | NA | 0 | 2 | Not-determined | Genome-fragment | NA | 0 |
| S1_k141_287648 | 1845 | No | NA | 0 | 2 | Not-determined | Genome-fragment | NA | 0 |
| S7_k141_104547 | 1476 | No | NA | 0 | 0 | Low-quality | Genome-fragment | 2.14 | 0 |
| S1_k141_271238 | 1160 | No | NA | 0 | 0 | Not-determined | Genome-fragment | NA | 0 |
| S17-final-viral-combined_k141_222385\|\|full | 1476 | No | NA | 0 | 0 | Low-quality | Genome-fragment | 3.63 | 0 |
| S1_k141_255433 | 1262 | No | NA | 0 | 1 | Not-determined | Genome-fragment | NA | 0 |
| S1_k141_171695 | 1323 | No | NA | 0 | 0 | Not-determined | Genome-fragment | NA | 0 |
| S1_k141_57248 | 1259 | No | NA | 0 | 0 | Not-determined | Genome-fragment | NA | 0 |
| S17-k141_31485 | 1476 | No | NA | 2 | 0 | Low-quality | Genome-fragment | 5.38 | 0 |
| S1-final-viral-combined_k141_289920\|\|full | 1477 | No | NA | 0 | 0 | Low-quality | Genome-fragment | 3.79 | 0 |
| S1_k141_321801 | 1111 | No | NA | 0 | 1 | Not-determined | Genome-fragment | NA | 0 |
| S1_k141_304861 | 9729 | No | NA | 0 | 1 | Not-determined | Genome-fragment | NA | 0 |
| S1_k141_171726 | 1286 | No | NA | 0 | 1 | Not-determined | Genome-fragment | NA | 0 |
| S1_k141_369797 | 1034 | No | NA | 0 | 0 | Not-determined | Genome-fragment | NA | 0 |
| S1_k141_321835 | 2189 | No | NA | 0 | 2 | Not-determined | Genome-fragment | NA | 0 |
| S5-final-viral-combined_k141_207005\|\|full | 1477 | No | NA | 0 | 0 | Low-quality | Genome-fragment | 2.82 | 0 |
| S1_k141_386714 | 1266 | No | NA | 0 | 1 | Not-determined | Genome-fragment | NA | 0 |
| S1_k141_105940 | 3570 | No | NA | 0 | 4 | Not-determined | Genome-fragment | NA | 0 |
| S1_k141_8164 | 1583 | No | NA | 0 | 1 | Not-determined | Genome-fragment | NA | 0 |
| S1_k141_206094 | 1523 | No | NA | 0 | 1 | Not-determined | Genome-fragment | NA | 0 |
| S1_k141_304925 | 1308 | No | NA | 0 | 1 | Not-determined | Genome-fragment | NA | 0 |
| S1_k141_90126 | 3432 | No | NA | 0 | 1 | Not-determined | Genome-fragment | NA | 0 |
| S1_k141_90161 | 1145 | No | NA | 0 | 2 | Not-determined | Genome-fragment | NA | 0 |
| S1_k141_304960 | 3045 | No | NA | 0 | 2 | Not-determined | Genome-fragment | NA | 0 |
| S7-final-viral-combined_k141_243164\|\|full | 1477 | No | NA | 0 | 0 | Low-quality | Genome-fragment | 3.43 | 0 |
| S1_k141_287830 | 1014 | No | NA | 0 | 1 | Not-determined | Genome-fragment | NA | 0 |
| S1_k141_171785 | 1032 | No | NA | 0 | 1 | Not-determined | Genome-fragment | NA | 0 |
| S17-final-viral-combined_k141_156193\|\|full | 1477 | No | NA | 1 | 0 | Low-quality | Genome-fragment | 2.1 | 0 |
| S17-final-viral-combined_k141_227357\|\|full | 1477 | No | NA | 0 | 1 | Low-quality | Genome-fragment | 0.62 | 0 |
| S1_k141_287842 | 1428 | No | NA | 0 | 1 | Not-determined | Genome-fragment | NA | 0 |
| S1_k141_369862 | 1205 | No | NA | 0 | 1 | Not-determined | Genome-fragment | NA | 0 |
| S17-final-viral-combined_k141_210942\|\|full | 1477 | No | NA | 0 | 0 | Low-quality | Genome-fragment | 2.44 | 0 |
| S17-final-viral-combined_k141_97288\|\|full | 1477 | No | NA | 0 | 0 | Low-quality | Genome-fragment | 2.75 | 0 |
| S1_k141_73166 | 9961 | No | NA | 0 | 7 | Not-determined | Genome-fragment | NA | 0 |
| S1_k141_222008 | 1023 | No | NA | 0 | 1 | Not-determined | Genome-fragment | NA | 0 |
| S1_k141_305016 | 1232 | No | NA | 0 | 0 | Not-determined | Genome-fragment | NA | 0 |
| S1_k141_354176 | 1105 | No | NA | 0 | 1 | Not-determined | Genome-fragment | NA | 0 |
| S1_k141_189831 | 1775 | No | NA | 0 | 0 | Not-determined | Genome-fragment | NA | 0 |
| S1_k141_73204 | 1151 | No | NA | 0 | 0 | Not-determined | Genome-fragment | NA | 0 |
| S1_k141_189847 | 1138 | No | NA | 0 | 1 | Not-determined | Genome-fragment | NA | 0 |
| S1_k141_140080 | 1216 | No | NA | 0 | 0 | Not-determined | Genome-fragment | NA | 0 |
| S12-final-viral-combined_k141_361968\|\|full | 1477 | No | NA | 0 | 0 | Low-quality | Genome-fragment | 2.24 | 0 |
| S1_k141_171835 | 1028 | No | NA | 0 | 0 | Not-determined | Genome-fragment | NA | 0 |
| S1_k141_321969 | 7531 | No | NA | 0 | 8 | Not-determined | Genome-fragment | NA | 0 |
| S1_k141_124253 | 1171 | No | NA | 0 | 1 | Not-determined | Genome-fragment | NA | 0 |
| S1_k141_354275 | 1128 | No | NA | 0 | 1 | Not-determined | Genome-fragment | NA | 0 |
| S1_k141_222104 | 1372 | No | NA | 0 | 1 | Not-determined | Genome-fragment | NA | 0 |
| S1_k141_124278 | 2302 | No | NA | 0 | 2 | Not-determined | Genome-fragment | NA | 0 |
| S1_k141_386908 | 1769 | No | NA | 0 | 1 | Not-determined | Genome-fragment | NA | 0 |
| S8-final-viral-combined_k141_57029\|\|full | 1477 | No | NA | 0 | 1 | Low-quality | Genome-fragment | 0.72 | 0 |
| S8-final-viral-combined_k141_47399\|\|lt2gene | 1477 | No | NA | 1 | 0 | Low-quality | Genome-fragment | 3.26 | 0 |
| S11-final-viral-combined_k141_89401\|\|full | 1478 | No | NA | 0 | 2 | Low-quality | Genome-fragment | 1.39 | 0 |
| S1_k141_238887 | 1168 | No | NA | 0 | 1 | Not-determined | Genome-fragment | NA | 0 |
| S1_k141_124340 | 1051 | No | NA | 0 | 0 | Not-determined | Genome-fragment | NA | 0 |
| S1_k141_73288 | 2363 | No | NA | 0 | 1 | Not-determined | Genome-fragment | NA | 0 |
| S1_k141_90282 | 3097 | No | NA | 0 | 3 | Not-determined | Genome-fragment | NA | 0 |
| S1_k141_322070 | 1038 | No | NA | 0 | 1 | Not-determined | Genome-fragment | NA | 0 |
| S1_k141_106115 | 26905 | No | NA | 7 | 0 | Medium-quality | Genome-fragment | 78.86 | 0 |
| S1-final-viral-combined_k141_299305\|\|lt2gene | 1478 | No | NA | 1 | 0 | Low-quality | Genome-fragment | 1.02 | 0 |
| S1_k141_255757 | 1163 | No | NA | 0 | 0 | Not-determined | Genome-fragment | NA | 0 |
| S1_k141_57430 | 6268 | No | NA | 0 | 2 | Not-determined | Genome-fragment | NA | 0 |
| S1_k141_305123 | 1108 | No | NA | 0 | 3 | Not-determined | Genome-fragment | NA | 0 |
| S1_k141_222240 | 1580 | No | NA | 0 | 1 | Not-determined | Genome-fragment | NA | 0 |
| S1_k141_206400 | 1266 | No | NA | 0 | 1 | Not-determined | Genome-fragment | NA | 0 |
| S1_k141_171938 | 6123 | No | NA | 0 | 1 | Not-determined | Genome-fragment | NA | 0 |
| S5_k141_62303 | 1478 | No | NA | 0 | 0 | Low-quality | Genome-fragment | 0.97 | 0 |
| S1_k141_40761 | 3297 | No | NA | 0 | 1 | Not-determined | Genome-fragment | NA | 0 |
| S7-final-viral-combined_k141_158064\|\|full | 1478 | No | NA | 0 | 0 | Low-quality | Genome-fragment | 2.67 | 0 |
| S7-final-viral-combined_k141_46309\|\|full | 1478 | No | NA | 0 | 0 | Low-quality | Genome-fragment | 3.06 | 0 |
| S7_k141_235807 | 1478 | No | NA | 0 | 1 | Low-quality | Genome-fragment | 2.6 | 0 |
| S1_k141_73305 | 2220 | No | NA | 0 | 2 | Not-determined | Genome-fragment | NA | 0 |
| S17-final-viral-combined_k141_111596\|\|full | 1478 | No | NA | 0 | 0 | Low-quality | Genome-fragment | 3.64 | 0 |
| S17-final-viral-combined_k141_135192\|\|full | 1478 | No | NA | 0 | 2 | Low-quality | Genome-fragment | 3.73 | 0 |
| S1_k141_387063 | 3415 | No | NA | 0 | 0 | Not-determined | Genome-fragment | NA | 0 |
| S1_k141_387064 | 1347 | No | NA | 0 | 0 | Not-determined | Genome-fragment | NA | 0 |
| S1_k141_90345 | 1291 | No | NA | 0 | 2 | Not-determined | Genome-fragment | NA | 0 |
| S17-final-viral-combined_k141_16176\|\|full | 1478 | No | NA | 0 | 0 | Low-quality | Genome-fragment | 3.62 | 0 |
| S1_k141_106193 | 2243 | No | NA | 0 | 0 | Not-determined | Genome-fragment | NA | 0 |
| S1_k141_106201 | 1657 | No | NA | 0 | 1 | Not-determined | Genome-fragment | NA | 0 |
| S1_k141_354498 | 1648 | No | NA | 0 | 2 | Not-determined | Genome-fragment | NA | 0 |
| S1_k141_24530 | 4667 | No | NA | 0 | 1 | Not-determined | Genome-fragment | NA | 0 |
| S1_k141_57495 | 3248 | No | NA | 0 | 1 | Not-determined | Genome-fragment | NA | 0 |
| S1_k141_106233 | 1014 | No | NA | 0 | 0 | Not-determined | Genome-fragment | NA | 0 |
| S1_k141_90382 | 2958 | No | NA | 0 | 2 | Not-determined | Genome-fragment | NA | 0 |
| S1_k141_40850 | 1027 | No | NA | 0 | 1 | Not-determined | Genome-fragment | NA | 0 |
| S1_k141_155841 | 5048 | No | NA | 0 | 1 | Not-determined | Genome-fragment | NA | 0 |
| S17-final-viral-combined_k141_63314\|\|full | 1478 | No | NA | 1 | 0 | Low-quality | Genome-fragment | 3 | 0 |
| S2_k141_220206 | 1478 | No | NA | 0 | 0 | Low-quality | Genome-fragment | 0.85 | 0 |
| S1_k141_172005 | 3574 | No | NA | 0 | 2 | Not-determined | Genome-fragment | NA | 0 |
| S1_k141_24580 | 6468 | No | NA | 0 | 1 | Not-determined | Genome-fragment | NA | 0 |
| S4-final-viral-combined_k141_49781\|\|full | 1478 | No | NA | 1 | 0 | Low-quality | Genome-fragment | 0.95 | 0 |
| S4-final-viral-combined_k141_120456\|\|full | 1478 | Yes | 1115 | 1 | 1 | Low-quality | Genome-fragment | 2.25 | 24.56 |
| S1_k141_370220 | 1862 | No | NA | 0 | 2 | Not-determined | Genome-fragment | NA | 0 |
| S1_k141_354553 | 1391 | No | NA | 0 | 0 | Not-determined | Genome-fragment | NA | 0 |
| S1_k141_255961 | 1098 | No | NA | 0 | 0 | Not-determined | Genome-fragment | NA | 0 |
| S1_k141_155909 | 3318 | No | NA | 0 | 1 | Not-determined | Genome-fragment | NA | 0 |
| S1_k141_239043 | 1172 | No | NA | 0 | 0 | Not-determined | Genome-fragment | NA | 0 |
| S1_k141_40943 | 4073 | No | NA | 0 | 2 | Not-determined | Genome-fragment | NA | 0 |
| S8_k141_70646 | 1478 | No | NA | 0 | 0 | Low-quality | Genome-fragment | 2.06 | 0 |
| S1_k141_24717 | 1071 | No | NA | 0 | 0 | Not-determined | Genome-fragment | NA | 0 |
| S1_k141_82882 | 1479 | No | NA | 0 | 0 | Low-quality | Genome-fragment | 3.14 | 0 |
| S1_k141_305376 | 3881 | No | NA | 0 | 1 | Not-determined | Genome-fragment | NA | 0 |
| S17-final-viral-combined_k141_122518\|\|full | 1479 | No | NA | 0 | 0 | Low-quality | Genome-fragment | 3.32 | 0 |
| S17-final-viral-combined_k141_36242\|\|full | 1479 | No | NA | 2 | 0 | Low-quality | Genome-fragment | 3.91 | 0 |
| S12-final-viral-combined_k141_109682\|\|full | 1479 | No | NA | 0 | 0 | Low-quality | Genome-fragment | 1.64 | 0 |
| S1_k141_90597 | 2892 | No | NA | 0 | 0 | Not-determined | Genome-fragment | NA | 0 |
| S1_k141_8763 | 1173 | No | NA | 0 | 0 | Not-determined | Genome-fragment | NA | 0 |
| S1_k141_24816 | 2092 | No | NA | 0 | 1 | Not-determined | Genome-fragment | NA | 0 |
| S1_k141_73561 | 1903 | No | NA | 0 | 2 | Not-determined | Genome-fragment | NA | 0 |
| S1_k141_41041 | 3984 | No | NA | 0 | 3 | Not-determined | Genome-fragment | NA | 0 |
| S1_k141_222550 | 1228 | No | NA | 0 | 2 | Not-determined | Genome-fragment | NA | 0 |
| S1_k141_41052 | 1397 | No | NA | 0 | 0 | Not-determined | Genome-fragment | NA | 0 |
| S1_k141_337072 | 1202 | No | NA | 0 | 2 | Not-determined | Genome-fragment | NA | 0 |

| | | | | | | | | | | | |
|---|---|---|---|---|---|---|---|---|---|---|---|
| S1_k141_24860 | 1129 | No | NA | | 0 | 0 | Not-determined | Genome-fragment | NA | | 0 |
| S1_k141_239228 | 1082 | No | NA | | 0 | 1 | Not-determined | Genome-fragment | NA | | 0 |
| S1_k141_206693 | 3467 | No | NA | | 0 | 2 | Not-determined | Genome-fragment | NA | | 0 |
| S12-final-viral-combined_k141_366666\|\|full | 1479 | No | NA | | 2 | 0 | Low-quality | Genome-fragment | | 3.41 | 0 |
| S1_k141_206720 | 1809 | No | NA | | 0 | 0 | Not-determined | Genome-fragment | NA | | 0 |
| S1_k141_370496 | 1243 | No | NA | | 0 | 2 | Not-determined | Genome-fragment | NA | | 0 |
| S1_k141_305535 | 1399 | No | NA | | 0 | 0 | Not-determined | Genome-fragment | NA | | 0 |
| S2-final-viral-combined_k141_171239\|\|full | 1479 | No | NA | | 1 | 0 | Low-quality | Genome-fragment | | 4.64 | 0 |
| S1_k141_24885 | 3173 | No | NA | | 0 | 2 | Not-determined | Genome-fragment | NA | | 0 |
| S4-final-viral-combined_k141_105080\|\|full | 1479 | No | NA | | 0 | 1 | Low-quality | Genome-fragment | | 1.1 | 0 |
| S3-final-viral-combined_k141_57879\|\|full | 1480 | No | NA | | 0 | 1 | Low-quality | Genome-fragment | | 0.91 | 0 |
| S1_k141_90701 | 2632 | No | NA | | 0 | 0 | Not-determined | Genome-fragment | NA | | 0 |
| S1_k141_156050 | 6820 | No | NA | | 0 | 0 | Not-determined | Genome-fragment | NA | | 0 |
| S1_k141_354719 | 1305 | No | NA | | 0 | 0 | Not-determined | Genome-fragment | NA | | 0 |
| S1_k141_305564 | 1780 | No | NA | | 0 | 1 | Not-determined | Genome-fragment | NA | | 0 |
| S1_k141_370527 | 4004 | No | NA | | 0 | 2 | Not-determined | Genome-fragment | NA | | 0 |
| S1_k141_24915 | 1049 | No | NA | | 0 | 1 | Not-determined | Genome-fragment | NA | | 0 |
| S1_k141_124821 | 1716 | No | NA | | 0 | 0 | Not-determined | Genome-fragment | NA | | 0 |
| S1_k141_206805 | 1070 | No | NA | | 0 | 0 | Not-determined | Genome-fragment | NA | | 0 |
| S1_k141_271859 | 1267 | No | NA | | 0 | 1 | Not-determined | Genome-fragment | NA | | 0 |
| S1_k141_57719 | 1953 | No | NA | | 0 | 1 | Not-determined | Genome-fragment | NA | | 0 |
| S1_k141_206811 | 4634 | No | NA | | 0 | 2 | Not-determined | Genome-fragment | NA | | 0 |
| S5-final-viral-combined_k141_135667\|\|full | 1480 | No | NA | | 0 | 0 | Low-quality | Genome-fragment | | 1.64 | 0 |
| S7-final-viral-combined_k141_131406\|\|full | 1480 | No | NA | | 0 | 0 | Low-quality | Genome-fragment | | 3.7 | 0 |
| S1_k141_206864 | 10080 | No | NA | | 0 | 6 | Not-determined | Genome-fragment | NA | | 0 |
| S1_k141_57781 | 2569 | No | NA | | 0 | 1 | Not-determined | Genome-fragment | NA | | 0 |
| S17-final-viral-combined_k141_174031\|\|full | 1480 | No | NA | | 0 | 1 | Low-quality | Genome-fragment | | 0.88 | 0 |
| S1_k141_41293 | 1145 | No | NA | | 0 | 2 | Not-determined | Genome-fragment | NA | | 0 |
| S1_k141_239426 | 1256 | No | NA | | 0 | 0 | Not-determined | Genome-fragment | NA | | 0 |
| S1_k141_73727 | 1608 | No | NA | | 0 | 1 | Not-determined | Genome-fragment | NA | | 0 |
| S1_k141_222733 | 1095 | No | NA | | 0 | 1 | Not-determined | Genome-fragment | NA | | 0 |
| S1_k141_239438 | 1152 | No | NA | | 0 | 1 | Not-determined | Genome-fragment | NA | | 0 |
| S12-final-viral-combined_k141_175444\|\|full | 1480 | No | NA | | 0 | 2 | Low-quality | Genome-fragment | | 0.81 | 0 |
| S1_k141_288518 | 1875 | No | NA | | 0 | 1 | Not-determined | Genome-fragment | NA | | 0 |
| S4-final-viral-combined_k141_82837\|\|full | 1480 | No | NA | | 0 | 0 | Low-quality | Genome-fragment | | 3.57 | 0 |
| S1_k141_354894 | 1252 | No | NA | | 0 | 1 | Not-determined | Genome-fragment | NA | | 0 |
| S1_k141_140569 | 1074 | No | NA | | 0 | 1 | Not-determined | Genome-fragment | NA | | 0 |
| S1_k141_124961 | 1158 | No | NA | | 0 | 0 | Not-determined | Genome-fragment | NA | | 0 |
| S1_k141_124968 | 4343 | No | NA | | 0 | 0 | Not-determined | Genome-fragment | NA | | 0 |
| S1_k141_8955 | 3790 | No | NA | | 0 | 1 | Not-determined | Genome-fragment | NA | | 0 |
| S1_k141_370747 | 1115 | No | NA | | 0 | 0 | Not-determined | Genome-fragment | NA | | 0 |
| S4_k141_95147 | 1480 | No | NA | | 0 | 1 | Low-quality | Genome-fragment | | 0.71 | 0 |
| S8-final-viral-combined_k141_231764\|\|full | 1480 | No | NA | | 0 | 0 | Low-quality | Genome-fragment | | 3.41 | 0 |
| S1_k141_206968 | 5324 | No | NA | | 0 | 1 | Not-determined | Genome-fragment | NA | | 0 |
| S1_k141_73747 | 1874 | No | NA | | 0 | 2 | Not-determined | Genome-fragment | NA | | 0 |
| S1_k141_8998 | 3058 | No | NA | | 0 | 3 | Not-determined | Genome-fragment | NA | | 0 |
| S1_k141_305822 | 2640 | No | NA | | 0 | 1 | Not-determined | Genome-fragment | NA | | 0 |
| S1_k141_125031 | 1116 | No | NA | | 0 | 1 | Not-determined | Genome-fragment | NA | | 0 |
| S1_k141_57924 | 1015 | No | NA | | 0 | 2 | Not-determined | Genome-fragment | NA | | 0 |
| S1_k141_90974 | 1031 | No | NA | | 0 | 0 | Not-determined | Genome-fragment | NA | | 0 |
| S1_k141_106579 | 4621 | No | NA | | 0 | 0 | Not-determined | Genome-fragment | NA | | 0 |
| S1_k141_239545 | 1200 | No | NA | | 0 | 0 | Not-determined | Genome-fragment | NA | | 0 |
| S1_k141_288652 | 1040 | No | NA | | 0 | 0 | Not-determined | Genome-fragment | NA | | 0 |
| S8-final-viral-combined_k141_60347\|\|full | 1480 | No | NA | | 0 | 1 | Low-quality | Genome-fragment | | 2.75 | 0 |
| S1_k141_73834 | 1219 | No | NA | | 0 | 1 | Not-determined | Genome-fragment | NA | | 0 |
| S1_k141_25184 | 1012 | No | NA | | 0 | 1 | Not-determined | Genome-fragment | NA | | 0 |
| S1_k141_222845 | 1820 | No | NA | | 0 | 4 | Not-determined | Genome-fragment | NA | | 0 |
| S1_k141_125103 | 1170 | No | NA | | 0 | 0 | Not-determined | Genome-fragment | NA | | 0 |
| S1_k141_322616 | 1395 | No | NA | | 0 | 0 | Not-determined | Genome-fragment | NA | | 0 |
| S8-final-viral-combined_k141_264487\|\|full | 1480 | No | NA | | 0 | 0 | Low-quality | Genome-fragment | | 2.22 | 0 |
| S1_k141_305911 | 1149 | No | NA | | 0 | 1 | Not-determined | Genome-fragment | NA | | 0 |
| S1_k141_288699 | 1488 | No | NA | | 0 | 1 | Not-determined | Genome-fragment | NA | | 0 |
| S1_k141_207046 | 1076 | No | NA | | 0 | 1 | Not-determined | Genome-fragment | NA | | 0 |
| S1_k141_73859 | 1341 | No | NA | | 0 | 0 | Not-determined | Genome-fragment | NA | | 0 |
| S1_k141_370915 | 1157 | No | NA | | 0 | 0 | Not-determined | Genome-fragment | NA | | 0 |
| S1_k141_125128 | 1464 | No | NA | | 0 | 1 | Not-determined | Genome-fragment | NA | | 0 |
| S1_k141_222898 | 1212 | No | NA | | 0 | 0 | Not-determined | Genome-fragment | NA | | 0 |
| S8_k141_4615 | 1480 | No | NA | | 1 | 0 | Low-quality | Genome-fragment | | 2.37 | 0 |
| S1_k141_272176 | 1431 | No | NA | | 0 | 1 | Not-determined | Genome-fragment | NA | | 0 |
| S1_k141_58013 | 1182 | No | NA | | 0 | 1 | Not-determined | Genome-fragment | NA | | 0 |
| S1_k141_25217 | 1505 | No | NA | | 0 | 1 | Not-determined | Genome-fragment | NA | | 0 |
| S1_k141_256451 | 1253 | No | NA | | 0 | 0 | Not-determined | Genome-fragment | NA | | 0 |
| S7-final-viral-combined_k141_13781\|\|full | 1481 | No | NA | | 1 | 0 | Low-quality | Genome-fragment | | 2.76 | 0 |
| S17-final-viral-combined_k141_113623\|\|full | 1481 | No | NA | | 1 | 0 | Low-quality | Genome-fragment | | 2.52 | 0 |
| S1_k141_156369 | 1034 | No | NA | | 0 | 0 | Not-determined | Genome-fragment | NA | | 0 |
| S17-final-viral-combined_k141_251966\|\|full | 1481 | No | NA | | 1 | 0 | Low-quality | Genome-fragment | | 3.22 | 0 |
| S1_k141_125195 | 1521 | No | NA | | 0 | 1 | Not-determined | Genome-fragment | NA | | 0 |
| S1_k141_106726 | 1315 | No | NA | | 0 | 1 | Not-determined | Genome-fragment | NA | | 0 |
| S1_k141_387799 | 3676 | No | NA | | 0 | 1 | Not-determined | Genome-fragment | NA | | 0 |
| S1_k141_239674 | 1384 | No | NA | | 0 | 0 | Not-determined | Genome-fragment | NA | | 0 |
| S1_k141_207110 | 1326 | No | NA | | 0 | 0 | Not-determined | Genome-fragment | NA | | 0 |
| S1_k141_190812 | 1412 | No | NA | | 0 | 0 | Not-determined | Genome-fragment | NA | | 0 |
| S1_k141_256554 | 1692 | No | NA | | 0 | 2 | Not-determined | Genome-fragment | NA | | 0 |
| S2_k141_127978 | 1481 | No | NA | | 0 | 1 | Low-quality | Genome-fragment | | 0.96 | 0 |
| S1_k141_322729 | 1140 | No | NA | | 0 | 0 | Not-determined | Genome-fragment | NA | | 0 |
| S1_k141_73961 | 1159 | No | NA | | 0 | 0 | Not-determined | Genome-fragment | NA | | 0 |
| S1_k141_172518 | 1077 | No | NA | | 0 | 1 | Not-determined | Genome-fragment | NA | | 0 |
| S1_k141_172528 | 3362 | No | NA | | 0 | 3 | Not-determined | Genome-fragment | NA | | 0 |
| S1_k141_272270 | 1858 | No | NA | | 0 | 1 | Not-determined | Genome-fragment | NA | | 0 |
| S1_k141_272272 | 1330 | No | NA | | 0 | 0 | Not-determined | Genome-fragment | NA | | 0 |
| S1_k141_73986 | 2810 | No | NA | | 0 | 1 | Not-determined | Genome-fragment | NA | | 0 |
| S1_k141_305996 | 1569 | No | NA | | 0 | 1 | Not-determined | Genome-fragment | NA | | 0 |
| S1_k141_222958 | 1222 | No | NA | | 0 | 0 | Not-determined | Genome-fragment | NA | | 0 |
| S8-final-viral-combined_k141_153736\|\|full | 1481 | No | NA | | 1 | 0 | Low-quality | Genome-fragment | | 2.39 | 0 |
| S1_k141_106752 | 1307 | No | NA | | 0 | 1 | Not-determined | Genome-fragment | NA | | 0 |
| S8-final-viral-combined_k141_28866\|\|full | 1481 | No | NA | | 0 | 0 | Low-quality | Genome-fragment | | 2.67 | 0 |
| S11-final-viral-combined_k141_146725\|\|lt2gene | 1482 | No | NA | | 1 | 0 | Low-quality | Genome-fragment | | 1.94 | 0 |
| S1_k141_41534 | 1029 | No | NA | | 0 | 1 | Not-determined | Genome-fragment | NA | | 0 |
| S1_k141_272325 | 1576 | No | NA | | 0 | 0 | Not-determined | Genome-fragment | NA | | 0 |
| S1-final-viral-combined_k141_4299\|\|full | 1482 | No | NA | | 0 | 0 | Low-quality | Genome-fragment | | 1.21 | 0 |
| S1_k141_172591 | 1078 | No | NA | | 0 | 1 | Not-determined | Genome-fragment | NA | | 0 |
| S1_k141_337422 | 1094 | No | NA | | 0 | 1 | Not-determined | Genome-fragment | NA | | 0 |
| S1_k141_91234 | 1051 | No | NA | | 0 | 2 | Not-determined | Genome-fragment | NA | | 0 |
| S1_k141_223034 | 1063 | No | NA | | 0 | 0 | Not-determined | Genome-fragment | NA | | 0 |
| S1_k141_91241 | 1257 | No | NA | | 0 | 1 | Not-determined | Genome-fragment | NA | | 0 |
| S1_k141_74036 | 3340 | No | NA | | 0 | 1 | Not-determined | Genome-fragment | NA | | 0 |
| S1_k141_306084 | 1918 | No | NA | | 0 | 1 | Not-determined | Genome-fragment | NA | | 0 |
| S1_k141_207244 | 1136 | No | NA | | 0 | 1 | Not-determined | Genome-fragment | NA | | 0 |
| S1_k141_387910 | 7215 | No | NA | | 0 | 3 | Not-determined | Genome-fragment | NA | | 0 |
| S1-final-viral-combined_k141_340191\|\|full | 1482 | No | NA | | 0 | 0 | Low-quality | Genome-fragment | | 3.13 | 0 |
| S1_k141_9327 | 1239 | No | NA | | 0 | 1 | Not-determined | Genome-fragment | NA | | 0 |
| S1-final-viral-combined_k141_122299\|\|lt2gene | 1482 | No | NA | | 1 | 0 | Low-quality | Genome-fragment | | 3.29 | 0 |
| S1_k141_371121 | 4139 | No | NA | | 0 | 1 | Not-determined | Genome-fragment | NA | | 0 |
| S1_k141_272400 | 1401 | No | NA | | 0 | 0 | Not-determined | Genome-fragment | NA | | 0 |
| S1_k141_280898 | 1482 | No | NA | | 0 | 0 | Low-quality | Genome-fragment | | 3.6 | 0 |
| S1_k141_190948 | 1069 | No | NA | | 0 | 1 | Not-determined | Genome-fragment | NA | | 0 |
| S1_k141_25488 | 1501 | No | NA | | 0 | 0 | Not-determined | Genome-fragment | NA | | 0 |
| S1_k141_140975 | 1380 | No | NA | | 0 | 2 | Not-determined | Genome-fragment | NA | | 0 |
| S3_k141_134249 | 1482 | No | NA | | 0 | 0 | Low-quality | Genome-fragment | | 1.88 | 0 |
| S5-final-viral-combined_k141_77911\|\|full | 1482 | No | NA | | 0 | 0 | Low-quality | Genome-fragment | | 2.87 | 0 |
| S1_k141_239821 | 1011 | No | NA | | 0 | 1 | Not-determined | Genome-fragment | NA | | 0 |
| S1_k141_207358 | 3856 | No | NA | | 0 | 0 | Not-determined | Genome-fragment | NA | | 0 |
| S1_k141_106929 | 1192 | No | NA | | 0 | 0 | Not-determined | Genome-fragment | NA | | 0 |
| S5_k141_208592 | 1482 | No | NA | | 0 | 1 | Low-quality | Genome-fragment | | 0.71 | 0 |
| S1_k141_125355 | 1115 | No | NA | | 0 | 1 | Not-determined | Genome-fragment | NA | | 0 |
| S7-final-viral-combined_k141_40206\|\|full | 1482 | No | NA | | 0 | 0 | Low-quality | Genome-fragment | | 2.08 | 0 |
| S1_k141_355256 | 1116 | No | NA | | 0 | 0 | Not-determined | Genome-fragment | NA | | 0 |
| S17-final-viral-combined_k141_55769\|\|full | 1482 | No | NA | | 1 | 0 | Low-quality | Genome-fragment | | 1.19 | 0 |
| S1_k141_191014 | 1157 | No | NA | | 0 | 1 | Not-determined | Genome-fragment | NA | | 0 |
| S1_k141_337616 | 2548 | No | NA | | 0 | 1 | Not-determined | Genome-fragment | NA | | 0 |
| S1_k141_91409 | 1218 | No | NA | | 0 | 1 | Not-determined | Genome-fragment | NA | | 0 |
| S1_k141_172792 | 2121 | No | NA | | 0 | 1 | Not-determined | Genome-fragment | NA | | 0 |
| S1_k141_288954 | 1548 | No | NA | | 0 | 0 | Not-determined | Genome-fragment | NA | | 0 |
| S1_k141_239871 | 1039 | No | NA | | 0 | 1 | Not-determined | Genome-fragment | NA | | 0 |
| S1_k141_207480 | 1039 | No | NA | | 0 | 0 | Not-determined | Genome-fragment | NA | | 0 |
| S1_k141_371256 | 1039 | No | NA | | 0 | 0 | Not-determined | Genome-fragment | NA | | 0 |
| S1_k141_141160 | 1684 | No | NA | | 0 | 1 | Not-determined | Genome-fragment | NA | | 0 |
| S12-final-viral-combined_k141_343635\|\|full | 1482 | No | NA | | 1 | 0 | Low-quality | Genome-fragment | | 3.7 | 0 |
| S2-final-viral-combined_k141_60674\|\|full | 1482 | No | NA | | 0 | 1 | Low-quality | Genome-fragment | | 3.22 | 0 |
| S11-final-viral-combined_k141_195273\|\|full | 1483 | No | NA | | 0 | 0 | Low-quality | Genome-fragment | | 2.09 | 0 |
| S1_k141_371273 | 1051 | No | NA | | 0 | 1 | Not-determined | Genome-fragment | NA | | 0 |
| S11-final-viral-combined_k141_155774\|\|full | 1483 | No | NA | | 0 | 0 | Low-quality | Genome-fragment | | 2.62 | 0 |
| S1_k141_58342 | 1183 | No | NA | | 0 | 0 | Not-determined | Genome-fragment | NA | | 0 |
| S1_k141_207507 | 2216 | No | NA | | 0 | 2 | Not-determined | Genome-fragment | NA | | 0 |
| S1_k141_74275 | 2242 | No | NA | | 0 | 2 | Not-determined | Genome-fragment | NA | | 0 |
| S1_k141_322996 | 2117 | No | NA | | 0 | 2 | Not-determined | Genome-fragment | NA | | 0 |
| S1_k141_156695 | 1629 | No | NA | | 0 | 1 | Not-determined | Genome-fragment | NA | | 0 |
| S1_k141_107027 | 2324 | No | NA | | 0 | 0 | Not-determined | Genome-fragment | NA | | 0 |
| S1_k141_355457 | 1013 | No | NA | | 0 | 1 | Not-determined | Genome-fragment | NA | | 0 |
| S1_k141_91595 | 1214 | No | NA | | 0 | 1 | Not-determined | Genome-fragment | NA | | 0 |
| S11-final-viral-combined_k141_231498\|\|full | 1483 | No | NA | | 0 | 1 | Low-quality | Genome-fragment | | 0.95 | 0 |
| S1_k141_74329 | 1086 | No | NA | | 0 | 1 | Not-determined | Genome-fragment | NA | | 0 |
| S1_k141_207582 | 2502 | No | NA | | 0 | 2 | Not-determined | Genome-fragment | NA | | 0 |
| S1_k141_355494 | 3355 | No | NA | | 0 | 1 | Not-determined | Genome-fragment | NA | | 0 |
| S1_k141_172935 | 1499 | No | NA | | 0 | 1 | Not-determined | Genome-fragment | NA | | 0 |
| S11-final-viral-combined_k141_232305\|\|full | 1483 | No | NA | | 0 | 2 | Low-quality | Genome-fragment | | 0.4 | 0 |
| S1_k141_337651 | 1148 | No | NA | | 0 | 1 | Not-determined | Genome-fragment | NA | | 0 |
| S1_k141_9547 | 1559 | No | NA | | 0 | 1 | Not-determined | Genome-fragment | NA | | 0 |
| S1_k141_9575 | 1678 | No | NA | | 0 | 1 | Not-determined | Genome-fragment | NA | | 0 |
| S1_k141_156772 | 1450 | No | NA | | 0 | 1 | Not-determined | Genome-fragment | NA | | 0 |
| S1_k141_107118 | 2950 | No | NA | | 0 | 2 | Not-determined | Genome-fragment | NA | | 0 |
| S1_k141_256991 | 1428 | No | NA | | 0 | 1 | Not-determined | Genome-fragment | NA | | 0 |
| S1_k141_371383 | 1692 | No | NA | | 0 | 1 | Not-determined | Genome-fragment | NA | | 0 |
| S1_k141_9594 | 1573 | No | NA | | 0 | 0 | Not-determined | Genome-fragment | NA | | 0 |
| S1_k141_388137 | 1998 | No | NA | | 0 | 2 | Not-determined | Genome-fragment | NA | | 0 |
| S1_k141_41848 | 1003 | No | NA | | 0 | 1 | Not-determined | Genome-fragment | NA | | 0 |
| S1_k141_91637 | 3450 | No | NA | | 0 | 1 | Not-determined | Genome-fragment | NA | | 0 |
| S1_k141_323078 | 1383 | No | NA | | 0 | 1 | Not-determined | Genome-fragment | NA | | 0 |
| S1-final-viral-combined_k141_5235\|\|full | 1483 | Yes | | 1217 | 1 | 1 | Low-quality | Genome-fragment | | 1.38 | 17.94 |
| S1_k141_323082 | 3149 | No | NA | | 0 | 2 | Not-determined | Genome-fragment | NA | | 0 |
| S5-final-viral-combined_k141_7180\|\|full | 1483 | No | NA | | 1 | 0 | Low-quality | Genome-fragment | | 3.8 | 0 |
| S1_k141_25702 | 1293 | No | NA | | 0 | 1 | Not-determined | Genome-fragment | NA | | 0 |
| S1_k141_257035 | 1320 | No | NA | | 0 | 1 | Not-determined | Genome-fragment | NA | | 0 |
| S1_k141_9663 | 8777 | No | NA | | 0 | 5 | Not-determined | Genome-fragment | NA | | 0 |

| | | | | | | | | | | |
|---|---|---|---|---|---|---|---|---|---|---|
| S1_k141_388205 | 1048 | No | NA | 0 | 0 | Not-determined | Genome-fragment | NA | | 0 |
| S1_k141_289195 | 1933 | No | NA | 0 | 0 | Not-determined | Genome-fragment | NA | | 0 |
| S1_k141_289196 | 1142 | No | NA | 0 | 1 | Not-determined | Genome-fragment | NA | | 0 |
| S1_k141_41918 | 4137 | No | NA | 0 | 2 | Not-determined | Genome-fragment | NA | | 0 |
| S5-final-viral-combined_k141_130470\|\|full | 1483 | No | NA | 0 | 0 | Low-quality | Genome-fragment | | 3.19 | 0 |
| S5_k141_74763 | 1483 | No | NA | 0 | 0 | Low-quality | Genome-fragment | | 2 | 0 |
| S1_k141_355675 | 2083 | No | NA | 0 | 2 | Not-determined | Genome-fragment | NA | | 0 |
| S17-final-viral-combined_k141_259285\|\|full | 1483 | No | NA | 0 | 0 | Low-quality | Genome-fragment | | 2.39 | 0 |
| S1_k141_91732 | 1035 | No | NA | 0 | 0 | Not-determined | Genome-fragment | NA | | 0 |
| S12-final-viral-combined_k141_114366\|\|full | 1483 | No | NA | 0 | 0 | Low-quality | Genome-fragment | | 1.01 | 0 |
| S2-final-viral-combined_k141_125971\|\|full | 1483 | No | NA | 0 | 1 | Low-quality | Genome-fragment | | 1.96 | 0 |
| S1_k141_25780 | 3684 | No | NA | 0 | 1 | Not-determined | Genome-fragment | NA | | 0 |
| S1_k141_207672 | 3005 | No | NA | 0 | 1 | Not-determined | Genome-fragment | NA | | 0 |
| S1_k141_388292 | 3129 | No | NA | 0 | 0 | Medium-quality | Genome-fragment | | 51.19 | 0 |
| S1_k141_74530 | 1051 | No | NA | 0 | 1 | Not-determined | Genome-fragment | NA | | 0 |
| S1_k141_289208 | 3276 | No | NA | 0 | 3 | Not-determined | Genome-fragment | NA | | 0 |
| S2-final-viral-combined_k141_181770\|\|full | 1483 | No | NA | 0 | 0 | Low-quality | Genome-fragment | | 2.62 | 0 |
| S1_k141_257095 | 1027 | No | NA | 0 | 0 | Not-determined | Genome-fragment | NA | | 0 |
| S1_k141_306402 | 1113 | No | NA | 0 | 1 | Not-determined | Genome-fragment | NA | | 0 |
| S1_k141_58476 | 5638 | No | NA | 0 | 4 | Not-determined | Genome-fragment | NA | | 0 |
| S1_k141_306403 | 1850 | No | NA | 0 | 0 | Not-determined | Genome-fragment | NA | | 0 |
| S1_k141_289249 | 3509 | No | NA | 0 | 4 | Not-determined | Genome-fragment | NA | | 0 |
| S1_k141_257134 | 3141 | No | NA | 0 | 0 | Not-determined | Genome-fragment | NA | | 0 |
| S1_k141_257143 | 1005 | No | NA | 0 | 1 | Not-determined | Genome-fragment | NA | | 0 |
| S1_k141_173160 | 1067 | No | NA | 0 | 0 | Not-determined | Genome-fragment | NA | | 0 |
| S1_k141_141458 | 1087 | No | NA | 0 | 0 | Not-determined | Genome-fragment | NA | | 0 |
| S1_k141_191366 | 3662 | No | NA | 0 | 4 | Not-determined | Genome-fragment | NA | | 0 |
| S1_k141_25921 | 1753 | No | NA | 0 | 1 | Not-determined | Genome-fragment | NA | | 0 |
| S1_k141_9923 | 3088 | No | NA | 0 | 2 | Not-determined | Genome-fragment | NA | | 0 |
| S1_k141_25953 | 3215 | No | NA | 0 | 2 | Not-determined | Genome-fragment | NA | | 0 |
| S1_k141_141492 | 3183 | No | NA | 0 | 1 | Not-determined | Genome-fragment | NA | | 0 |
| S1_k141_306561 | 1332 | No | NA | 0 | 0 | Not-determined | Genome-fragment | NA | | 0 |
| S1_k141_58596 | 1275 | No | NA | 0 | 1 | Not-determined | Genome-fragment | NA | | 0 |
| S1_k141_223414 | 3342 | No | NA | 0 | 0 | Not-determined | Genome-fragment | NA | | 0 |
| S1_k141_42112 | 1018 | No | NA | 0 | 1 | Not-determined | Genome-fragment | NA | | 0 |
| S1_k141_125683 | 1132 | No | NA | 0 | 1 | Not-determined | Genome-fragment | NA | | 0 |
| S6-final-viral-combined_k141_64198\|\|full | 1483 | No | NA | 1 | 0 | Low-quality | Genome-fragment | | 1.94 | 0 |
| S6_k141_77104 | 1483 | No | NA | 0 | 0 | Low-quality | Genome-fragment | | 1.37 | 0 |
| S1_k141_306588 | 1071 | No | NA | 0 | 1 | Not-determined | Genome-fragment | NA | | 0 |
| S1_k141_9977 | 1154 | No | NA | 0 | 0 | Not-determined | Genome-fragment | NA | | 0 |
| S1_k141_223487 | 3234 | No | NA | 0 | 2 | Not-determined | Genome-fragment | NA | | 0 |
| S1_k141_157028 | 1208 | No | NA | 0 | 2 | Not-determined | Genome-fragment | NA | | 0 |
| S1_k141_107506 | 1128 | No | NA | 0 | 0 | Not-determined | Genome-fragment | NA | | 0 |
| S1-final-viral-combined_k141_18251\|\|full | 1484 | No | NA | 1 | 0 | Low-quality | Genome-fragment | | 42.99 | 0 |
| S1_k141_240361 | 1128 | No | NA | 0 | 2 | Not-determined | Genome-fragment | NA | | 0 |
| S1_k141_91966 | 1395 | No | NA | 0 | 1 | Not-determined | Genome-fragment | NA | | 0 |
| S1_k141_240383 | 1109 | No | NA | 0 | 1 | Not-determined | Genome-fragment | NA | | 0 |
| S1_k141_134637 | 1484 | No | NA | 0 | 0 | Low-quality | Genome-fragment | | 3.22 | 0 |
| S1_k141_323487 | 1775 | No | NA | 0 | 3 | Not-determined | Genome-fragment | NA | | 0 |
| S1_k141_208064 | 5094 | No | NA | 0 | 3 | Not-determined | Genome-fragment | NA | | 0 |
| S7-final-viral-combined_k141_167095\|\|full | 1484 | No | NA | 0 | 0 | Low-quality | Genome-fragment | | 2.91 | 0 |
| S1_k141_157105 | 1255 | No | NA | 0 | 0 | Not-determined | Genome-fragment | NA | | 0 |
| S1_k141_289593 | 1319 | No | NA | 0 | 0 | Not-determined | Genome-fragment | NA | | 0 |
| S1_k141_191613 | 1190 | No | NA | 0 | 1 | Not-determined | Genome-fragment | NA | | 0 |
| S7-final-viral-combined_k141_240531\|\|lt2gene | 1484 | No | NA | 1 | 0 | Low-quality | Genome-fragment | | 1.39 | 0 |
| S1_k141_223605 | 1323 | No | NA | 0 | 1 | Not-determined | Genome-fragment | NA | | 0 |
| S1_k141_157173 | 1172 | No | NA | 0 | 1 | Not-determined | Genome-fragment | NA | | 0 |
| S12-final-viral-combined_k141_109501\|\|full | 1484 | No | NA | 0 | 0 | Low-quality | Genome-fragment | | 0.86 | 0 |
| S1_k141_208136 | 1001 | No | NA | 0 | 1 | Not-determined | Genome-fragment | NA | | 0 |
| S2-final-viral-combined_k141_111915\|\|full | 1484 | No | NA | 1 | 0 | Low-quality | Genome-fragment | | 2.61 | 0 |
| S1_k141_42248 | 1028 | No | NA | 0 | 0 | Not-determined | Genome-fragment | NA | | 0 |
| S1_k141_257394 | 1276 | No | NA | 0 | 1 | Not-determined | Genome-fragment | NA | | 0 |
| S1_k141_173357 | 1633 | No | NA | 0 | 0 | Not-determined | Genome-fragment | NA | | 0 |
| S1_k141_42290 | 4600 | No | NA | 0 | 1 | Not-determined | Genome-fragment | NA | | 0 |
| S1_k141_223620 | 31814 | No | NA | 15 | 0 | High-quality | High-quality | | 96.36 | 0 |
| S1_k141_223643 | 3175 | No | NA | 0 | 1 | Not-determined | Genome-fragment | NA | | 0 |
| S1_k141_92117 | 2526 | No | NA | 0 | 2 | Not-determined | Genome-fragment | NA | | 0 |
| S1_k141_208193 | 1187 | No | NA | 0 | 1 | Not-determined | Genome-fragment | NA | | 0 |
| S1_k141_58830 | 3914 | No | NA | 0 | 0 | Not-determined | Genome-fragment | NA | | 0 |
| S1-final-viral-combined_k141_116860\|\|full | 1485 | No | NA | 0 | 0 | Low-quality | Genome-fragment | | 3.62 | 0 |
| S1_k141_157271 | 1818 | No | NA | 0 | 1 | Not-determined | Genome-fragment | NA | | 0 |
| S1_k141_26266 | 1552 | No | NA | 0 | 1 | Not-determined | Genome-fragment | NA | | 0 |
| S1_k141_323641 | 1534 | No | NA | 0 | 0 | Not-determined | Genome-fragment | NA | | 0 |
| S1_k141_240529 | 1497 | No | NA | 0 | 1 | Not-determined | Genome-fragment | NA | | 0 |
| S1_k141_107682 | 1457 | No | NA | 0 | 0 | Not-determined | Genome-fragment | NA | | 0 |
| S1-final-viral-combined_k141_333605\|\|full | 1485 | No | NA | 0 | 2 | Low-quality | Genome-fragment | | 2.16 | 0 |
| S1_k141_356094 | 1260 | No | NA | 0 | 1 | Not-determined | Genome-fragment | NA | | 0 |
| S1_k141_306879 | 1017 | No | NA | 0 | 1 | Not-determined | Genome-fragment | NA | | 0 |
| S1_k141_157322 | 1479 | No | NA | 0 | 2 | Not-determined | Genome-fragment | NA | | 0 |
| S1_k141_248018 | 1485 | No | NA | 1 | 0 | Low-quality | Genome-fragment | | 5.09 | 0 |
| S1_k141_223682 | 10659 | No | NA | 0 | 5 | Not-determined | Genome-fragment | NA | | 0 |
| S1_k141_289730 | 1715 | No | NA | 0 | 0 | Not-determined | Genome-fragment | NA | | 0 |
| S1_k141_107726 | 3387 | No | NA | 0 | 1 | Not-determined | Genome-fragment | NA | | 0 |
| S1_k141_42385 | 1483 | No | NA | 0 | 1 | Not-determined | Genome-fragment | NA | | 0 |
| S5-final-viral-combined_k141_108018\|\|full | 1485 | No | NA | 0 | 1 | Low-quality | Genome-fragment | | 3.3 | 0 |
| S1_k141_26335 | 3465 | No | NA | 0 | 3 | Not-determined | Genome-fragment | NA | | 0 |
| S1_k141_26350 | 1108 | No | NA | 0 | 0 | Not-determined | Genome-fragment | NA | | 0 |
| S1_k141_208307 | 3094 | No | NA | 0 | 0 | Not-determined | Genome-fragment | NA | | 0 |
| S1_k141_74789 | 1266 | No | NA | 0 | 0 | Not-determined | Genome-fragment | NA | | 0 |
| S1_k141_289757 | 1236 | No | NA | 0 | 1 | Not-determined | Genome-fragment | NA | | 0 |
| S5-final-viral-combined_k141_150713\|\|full | 1485 | No | NA | 0 | 0 | Low-quality | Genome-fragment | | 3.54 | 0 |
| S1_k141_42425 | 1182 | No | NA | 0 | 1 | Not-determined | Genome-fragment | NA | | 0 |
| S1_k141_141629 | 1631 | No | NA | 0 | 1 | Not-determined | Genome-fragment | NA | | 0 |
| S1_k141_371835 | 1330 | No | NA | 0 | 1 | Not-determined | Genome-fragment | NA | | 0 |
| S17-final-viral-combined_k141_224926\|\|full | 1485 | No | NA | 0 | 0 | Low-quality | Genome-fragment | | 3.6 | 0 |
| S1_k141_240622 | 1683 | No | NA | 0 | 0 | Not-determined | Genome-fragment | NA | | 0 |
| S1_k141_273080 | 1752 | No | NA | 0 | 2 | Not-determined | Genome-fragment | NA | | 0 |
| S1_k141_208338 | 1023 | No | NA | 0 | 1 | Not-determined | Genome-fragment | NA | | 0 |
| S1_k141_157417 | 1595 | No | NA | 0 | 1 | Not-determined | Genome-fragment | NA | | 0 |
| S12-final-viral-combined_k141_400839\|\|full | 1485 | No | NA | 0 | 0 | Low-quality | Genome-fragment | | 3.08 | 0 |
| S1_k141_208343 | 3119 | No | NA | 0 | 1 | Not-determined | Genome-fragment | NA | | 0 |
| S1_k141_306977 | 1129 | No | NA | 0 | 1 | Not-determined | Genome-fragment | NA | | 0 |
| S1_k141_58992 | 1046 | No | NA | 0 | 1 | Not-determined | Genome-fragment | NA | | 0 |
| S1_k141_42475 | 5283 | No | NA | 0 | 1 | Not-determined | Genome-fragment | NA | | 0 |
| S2_k141_115289 | 1485 | No | NA | 0 | 1 | Low-quality | Genome-fragment | | 0.89 | 0 |
| S1_k141_42482 | 8193 | No | NA | 0 | 0 | Not-determined | Genome-fragment | NA | | 0 |
| S1_k141_157462 | 1148 | No | NA | 0 | 1 | Not-determined | Genome-fragment | NA | | 0 |
| S1_k141_141757 | 3622 | No | NA | 0 | 2 | Not-determined | Genome-fragment | NA | | 0 |
| S1_k141_223868 | 1290 | No | NA | 0 | 1 | Not-determined | Genome-fragment | NA | | 0 |
| S1_k141_356304 | 1154 | No | NA | 0 | 0 | Not-determined | Genome-fragment | NA | | 0 |
| S1_k141_371966 | 1090 | No | NA | 0 | 0 | Not-determined | Genome-fragment | NA | | 0 |
| S1_k141_26400 | 1066 | No | NA | 0 | 0 | Not-determined | Genome-fragment | NA | | 0 |
| S1_k141_157496 | 3001 | No | NA | 0 | 1 | Not-determined | Genome-fragment | NA | | 0 |
| S9-final-viral-combined_k141_71998\|\|full | 1486 | No | NA | 0 | 2 | Low-quality | Genome-fragment | | 1.2 | 0 |
| S1_k141_338427 | 1135 | No | NA | 0 | 1 | Not-determined | Genome-fragment | NA | | 0 |
| S1_k141_323844 | 2260 | No | NA | 0 | 1 | Not-determined | Genome-fragment | NA | | 0 |
| S9_k141_105694 | 1486 | No | NA | 0 | 1 | Low-quality | Genome-fragment | | 0.95 | 0 |
| S1_k141_323856 | 1321 | No | NA | 0 | 0 | Not-determined | Genome-fragment | NA | | 0 |
| S1_k141_257772 | 1107 | No | NA | 0 | 1 | Not-determined | Genome-fragment | NA | | 0 |
| S1_k141_372014 | 1848 | No | NA | 0 | 1 | Not-determined | Genome-fragment | NA | | 0 |
| S1_k141_173555 | 1307 | No | NA | 0 | 2 | Not-determined | Genome-fragment | NA | | 0 |
| S1_k141_289967 | 3525 | No | NA | 0 | 1 | Not-determined | Genome-fragment | NA | | 0 |
| S1_k141_240790 | 2960 | No | NA | 0 | 1 | Not-determined | Genome-fragment | NA | | 0 |
| S1_k141_157576 | 1046 | No | NA | 0 | 0 | Not-determined | Genome-fragment | NA | | 0 |
| S1_k141_356368 | 4225 | No | NA | 0 | 5 | Not-determined | Genome-fragment | NA | | 0 |
| S1-final-viral-combined_k141_264947\|\|full | 1486 | No | NA | 1 | 0 | Low-quality | Genome-fragment | | 2.75 | 0 |
| S1_k141_92459 | 6325 | No | NA | 0 | 1 | Not-determined | Genome-fragment | NA | | 0 |
| S1-final-viral-combined_k141_328153\|\|full | 1486 | No | NA | 1 | 0 | Low-quality | Genome-fragment | | 11.52 | 0 |
| S1-final-viral-combined_k141_81669\|\|full | 1486 | No | NA | 1 | 0 | Low-quality | Genome-fragment | | 3.77 | 0 |
| S12-final-viral-combined_k141_3675\|\|full | 1486 | No | NA | 0 | 0 | Low-quality | Genome-fragment | | 0.93 | 0 |
| S1_k141_323949 | 1176 | No | NA | 0 | 2 | Not-determined | Genome-fragment | NA | | 0 |
| S1_k141_26472 | 1024 | No | NA | 0 | 0 | Not-determined | Genome-fragment | NA | | 0 |
| S2-final-viral-combined_k141_176727\|\|full | 1486 | No | NA | 2 | 0 | Low-quality | Genome-fragment | | 3.3 | 0 |
| S1_k141_26481 | 1001 | No | NA | 0 | 3 | Not-determined | Genome-fragment | NA | | 0 |
| S1_k141_75051 | 1656 | No | NA | 0 | 0 | Not-determined | Genome-fragment | NA | | 0 |
| S1_k141_290068 | 1210 | No | NA | 0 | 0 | Not-determined | Genome-fragment | NA | | 0 |
| S1_k141_10353 | 1124 | No | NA | 0 | 1 | Not-determined | Genome-fragment | NA | | 0 |
| S1_k141_141935 | 1159 | No | NA | 0 | 1 | Not-determined | Genome-fragment | NA | | 0 |
| S1_k141_307219 | 1142 | No | NA | 0 | 0 | Not-determined | Genome-fragment | NA | | 0 |
| S1_k141_356427 | 1202 | No | NA | 0 | 0 | Not-determined | Genome-fragment | NA | | 0 |
| S1_k141_26531 | 1069 | No | NA | 0 | 1 | Not-determined | Genome-fragment | NA | | 0 |
| S1_k141_173711 | 1203 | No | NA | 0 | 1 | Not-determined | Genome-fragment | NA | | 0 |
| S1_k141_157678 | 1484 | No | NA | 0 | 1 | Not-determined | Genome-fragment | NA | | 0 |
| S1_k141_141947 | 1925 | No | NA | 0 | 1 | Not-determined | Genome-fragment | NA | | 0 |
| S1_k141_224118 | 1121 | No | NA | 0 | 0 | Not-determined | Genome-fragment | NA | | 0 |
| S1_k141_75122 | 3142 | No | NA | 0 | 2 | Not-determined | Genome-fragment | NA | | 0 |
| S1_k141_356460 | 1037 | No | NA | 0 | 0 | Not-determined | Genome-fragment | NA | | 0 |
| S2_k141_72586 | 1486 | No | NA | 1 | 0 | Low-quality | Genome-fragment | | 2.13 | 0 |
| S1_k141_324088 | 1397 | No | NA | 0 | 0 | Not-determined | Genome-fragment | NA | | 0 |
| S8-final-viral-combined_k141_81964\|\|full | 1486 | No | NA | 0 | 0 | Low-quality | Genome-fragment | | 2.81 | 0 |
| S8-final-viral-combined_k141_2539\|\|full | 1486 | No | NA | 1 | 0 | Low-quality | Genome-fragment | | 1.13 | 0 |
| S8-final-viral-combined_k141_200646\|\|full | 1486 | No | NA | 0 | 1 | Low-quality | Genome-fragment | | 3.63 | 0 |
| S1_k141_307304 | 1272 | No | NA | 0 | 2 | Not-determined | Genome-fragment | NA | | 0 |
| S1_k141_356557 | 1299 | No | NA | 0 | 1 | Not-determined | Genome-fragment | NA | | 0 |
| S1_k141_75214 | 2137 | No | NA | 0 | 0 | Not-determined | Genome-fragment | NA | | 0 |
| S1_k141_59226 | 1260 | No | NA | 0 | 0 | Not-determined | Genome-fragment | NA | | 0 |
| S9-final-viral-combined_k141_43150\|\|lt2gene | 1487 | No | NA | 1 | 0 | Low-quality | Genome-fragment | | 3.86 | 0 |
| S1_k141_142071 | 1000 | No | NA | 0 | 1 | Not-determined | Genome-fragment | NA | | 0 |
| S1_k141_92656 | 1193 | No | NA | 0 | 1 | Not-determined | Genome-fragment | NA | | 0 |
| S1_k141_224204 | 1811 | No | NA | 0 | 1 | Not-determined | Genome-fragment | NA | | 0 |
| S1_k141_92661 | 1109 | No | NA | 0 | 0 | Not-determined | Genome-fragment | NA | | 0 |
| S1_k141_108097 | 3945 | No | NA | 0 | 0 | Not-determined | Genome-fragment | NA | | 0 |
| S5-final-viral-combined_k141_136357\|\|full | 1487 | No | NA | 0 | 0 | Low-quality | Genome-fragment | | 2.06 | 0 |
| S1_k141_75230 | 1122 | No | NA | 0 | 1 | Not-determined | Genome-fragment | NA | | 0 |
| S7_k141_210657 | 1487 | No | NA | 0 | 0 | Low-quality | Genome-fragment | | 2.93 | 0 |
| S1_k141_192053 | 3034 | No | NA | 0 | 1 | Not-determined | Genome-fragment | NA | | 0 |
| S7_k141_190897 | 1487 | No | NA | 0 | 0 | Low-quality | Genome-fragment | | 1.45 | 0 |
| S1_k141_324203 | 1187 | No | NA | 0 | 1 | Not-determined | Genome-fragment | NA | | 0 |
| S1_k141_75247 | 1329 | No | NA | 0 | 2 | Not-determined | Genome-fragment | NA | | 0 |
| S1_k141_42837 | 1104 | No | NA | 0 | 1 | Not-determined | Genome-fragment | NA | | 0 |
| S1_k141_92721 | 1174 | No | NA | 0 | 1 | Not-determined | Genome-fragment | NA | | 0 |
| S1_k141_273434 | 1770 | No | NA | 0 | 1 | Not-determined | Genome-fragment | NA | | 0 |
| S1_k141_356613 | 1038 | No | NA | 0 | 0 | Not-determined | Genome-fragment | NA | | 0 |
| S1_k141_142140 | 1964 | No | NA | 0 | 2 | Not-determined | Genome-fragment | NA | | 0 |

| | | | | | | | | | |
|---|---|---|---|---|---|---|---|---|---|
| S1_k141_324248 | 1018 | No | NA | 0 | 1 | Not-determined | Genome-fragment | NA | 0 |
| S1_k141_192117 | 4494 | No | NA | 0 | 1 | Not-determined | Genome-fragment | NA | 0 |
| S1_k141_126468 | 6594 | No | NA | 0 | 5 | Not-determined | Genome-fragment | NA | 0 |
| S1_k141_42911 | 1071 | No | NA | 0 | 1 | Not-determined | Genome-fragment | NA | 0 |
| S17-final-viral-combined_k141_35700\|\|full | 1487 | No | NA | 0 | 2 | Low-quality | Genome-fragment | 1.7 | 0 |
| S1_k141_241091 | 1085 | No | NA | 0 | 2 | Not-determined | Genome-fragment | NA | 0 |
| S17-final-viral-combined_k141_48656\|\|full | 1487 | No | NA | 0 | 0 | Low-quality | Genome-fragment | 3.15 | 0 |
| S1_k141_192164 | 1020 | No | NA | 0 | 2 | Not-determined | Genome-fragment | NA | 0 |
| S17-final-viral-combined_k141_7838\|\|full | 1487 | Yes | 423 | 1 | 1 | Low-quality | Genome-fragment | 0.2 | 71.55 |
| S1_k141_241131 | 3465 | No | NA | 0 | 3 | Not-determined | Genome-fragment | NA | 0 |
| S1_k141_92809 | 10998 | No | NA | 0 | 8 | Not-determined | Genome-fragment | NA | 0 |
| S1_k141_142238 | 1605 | No | NA | 0 | 1 | Not-determined | Genome-fragment | NA | 0 |
| S17-final-viral-combined_k141_252727\|\|full | 1487 | No | NA | 2 | 0 | Low-quality | Genome-fragment | 3.18 | 0 |
| S1_k141_92852 | 1155 | No | NA | 0 | 1 | Not-determined | Genome-fragment | NA | 0 |
| S12-final-viral-combined_k141_49238\|\|full | 1487 | No | NA | 0 | 2 | Low-quality | Genome-fragment | 2.49 | 0 |
| S1_k141_388771 | 2977 | No | NA | 0 | 0 | Not-determined | Genome-fragment | NA | 0 |
| S1_k141_388774 | 1210 | No | NA | 0 | 0 | Not-determined | Genome-fragment | NA | 0 |
| S1_k141_356733 | 4811 | No | NA | 0 | 2 | Not-determined | Genome-fragment | NA | 0 |
| S1_k141_208811 | 1114 | No | NA | 0 | 1 | Not-determined | Genome-fragment | NA | 0 |
| S1_k141_208814 | 1729 | No | NA | 0 | 0 | Not-determined | Genome-fragment | NA | 0 |
| S1_k141_273527 | 2210 | No | NA | 0 | 0 | Not-determined | Genome-fragment | NA | 0 |
| S1_k141_92901 | 3381 | No | NA | 0 | 4 | Not-determined | Genome-fragment | NA | 0 |
| S1_k141_10426 | 2939 | No | NA | 0 | 2 | Not-determined | Genome-fragment | NA | 0 |
| S1_k141_208863 | 1044 | No | NA | 0 | 1 | Not-determined | Genome-fragment | NA | 0 |
| S1_k141_157984 | 3441 | No | NA | 0 | 1 | Not-determined | Genome-fragment | NA | 0 |
| S2_k141_213031 | 1487 | No | NA | 0 | 1 | Low-quality | Genome-fragment | 1.76 | 0 |
| S1_k141_241248 | 2422 | No | NA | 0 | 2 | Not-determined | Genome-fragment | NA | 0 |
| S1_k141_241263 | 2132 | No | NA | 0 | 1 | Not-determined | Genome-fragment | NA | 0 |
| S1_k141_241267 | 1336 | No | NA | 0 | 1 | Not-determined | Genome-fragment | NA | 0 |
| S1_k141_75381 | 1077 | No | NA | 0 | 1 | Not-determined | Genome-fragment | NA | 0 |
| S1_k141_208875 | 18901 | No | NA | 3 | 0 | Medium-quality | Genome-fragment | 50.43 | 0 |
| S1_k141_290405 | 1022 | No | NA | 0 | 1 | Not-determined | Genome-fragment | NA | 0 |
| S1_k141_43071 | 1237 | No | NA | 0 | 1 | Not-determined | Genome-fragment | NA | 0 |
| S1_k141_43079 | 3362 | No | NA | 0 | 3 | Not-determined | Genome-fragment | NA | 0 |
| S6_k141_122835 | 1487 | No | NA | 0 | 0 | Low-quality | Genome-fragment | 1.05 | 0 |
| S8-final-viral-combined_k141_195921\|\|full | 1487 | No | NA | 0 | 1 | Low-quality | Genome-fragment | 1.67 | 0 |
| S1_k141_224464 | 1013 | No | NA | 0 | 2 | Not-determined | Genome-fragment | NA | 0 |
| S1_k141_388864 | 1084 | No | NA | 0 | 2 | Not-determined | Genome-fragment | NA | 0 |
| S1_k141_273594 | 1873 | No | NA | 0 | 0 | Not-determined | Genome-fragment | NA | 0 |
| S1_k141_158041 | 1064 | No | NA | 0 | 1 | Not-determined | Genome-fragment | NA | 0 |
| S1_k141_241333 | 1411 | No | NA | 0 | 1 | Not-determined | Genome-fragment | NA | 0 |
| S1_k141_59568 | 4455 | No | NA | 0 | 3 | Not-determined | Genome-fragment | NA | 0 |
| S1_k141_93004 | 1013 | No | NA | 0 | 1 | Not-determined | Genome-fragment | NA | 0 |
| S1_k141_356819 | 1005 | No | NA | 0 | 0 | Not-determined | Genome-fragment | NA | 0 |
| S1_k141_290442 | 1285 | No | NA | 0 | 0 | Not-determined | Genome-fragment | NA | 0 |
| S1_k141_174152 | 4004 | No | NA | 0 | 2 | Not-determined | Genome-fragment | NA | 0 |
| S1_k141_290464 | 1282 | No | NA | 0 | 0 | Not-determined | Genome-fragment | NA | 0 |
| S8-final-viral-combined_k141_236609\|\|full | 1487 | No | NA | 0 | 0 | Low-quality | Genome-fragment | 0.97 | 0 |
| S1_k141_356859 | 3153 | No | NA | 0 | 0 | Not-determined | Genome-fragment | NA | 0 |
| S8_k141_253395 | 1487 | No | NA | 0 | 1 | Low-quality | Genome-fragment | 0.52 | 0 |
| S1_k141_126735 | 3038 | No | NA | 0 | 1 | Not-determined | Genome-fragment | NA | 0 |
| S9-final-viral-combined_k141_10324\|\|full | 1488 | No | NA | 2 | 0 | Low-quality | Genome-fragment | 3.35 | 0 |
| S1_k141_43186 | 3920 | No | NA | 0 | 0 | Not-determined | Genome-fragment | NA | 0 |
| S1_k141_75498 | 1208 | No | NA | 0 | 0 | Not-determined | Genome-fragment | NA | 0 |
| S1_k141_192409 | 1024 | No | NA | 0 | 1 | Not-determined | Genome-fragment | NA | 0 |
| S1_k141_43187 | 10589 | No | NA | 0 | 10 | Not-determined | Genome-fragment | NA | 0 |
| S1_k141_174210 | 1444 | No | NA | 0 | 5 | Not-determined | Genome-fragment | NA | 0 |
| S1-final-viral-combined_k141_87181\|\|full | 1488 | No | NA | 1 | 0 | Low-quality | Genome-fragment | 4.38 | 0 |
| S1_k141_43205 | 1390 | No | NA | 0 | 0 | Not-determined | Genome-fragment | NA | 0 |
| S1_k141_26850 | 5057 | No | NA | 0 | 1 | Not-determined | Genome-fragment | NA | 0 |
| S1_k141_10635 | 1461 | No | NA | 0 | 0 | Not-determined | Genome-fragment | NA | 0 |
| S1_k141_388936 | 1248 | No | NA | 0 | 0 | Not-determined | Genome-fragment | NA | 0 |
| S3_k141_92635 | 1488 | No | NA | 0 | 1 | Low-quality | Genome-fragment | 0.97 | 0 |
| S1_k141_372652 | 1626 | No | NA | 0 | 0 | Not-determined | Genome-fragment | NA | 0 |
| S1_k141_126824 | 1267 | No | NA | 0 | 0 | Not-determined | Genome-fragment | NA | 0 |
| S1_k141_388959 | 2065 | No | NA | 0 | 0 | Not-determined | Genome-fragment | NA | 0 |
| S1_k141_224579 | 11026 | No | NA | 0 | 7 | Not-determined | Genome-fragment | NA | 0 |
| S1_k141_93131 | 13737 | No | NA | 0 | 3 | Not-determined | Genome-fragment | NA | 0 |
| S5_k141_39897 | 1488 | No | NA | 0 | 0 | Low-quality | Genome-fragment | 2.76 | 0 |
| S1_k141_209086 | 1433 | No | NA | 0 | 1 | Not-determined | Genome-fragment | NA | 0 |
| S12-final-viral-combined_k141_301624\|\|full | 1488 | No | NA | 0 | 0 | Low-quality | Genome-fragment | 1.61 | 0 |
| S1_k141_241380 | 3616 | No | NA | 0 | 2 | Not-determined | Genome-fragment | NA | 0 |
| S1_k141_26965 | 4474 | No | NA | 0 | 0 | Not-determined | Genome-fragment | NA | 0 |
| S1_k141_224641 | 1292 | No | NA | 0 | 2 | Not-determined | Genome-fragment | NA | 0 |
| S1_k141_142569 | 3543 | No | NA | 0 | 1 | Not-determined | Genome-fragment | NA | 0 |
| S1_k141_43321 | 1582 | No | NA | 0 | 1 | Not-determined | Genome-fragment | NA | 0 |
| S1_k141_126934 | 1221 | No | NA | 0 | 2 | Not-determined | Genome-fragment | NA | 0 |
| S1_k141_192547 | 3269 | No | NA | 0 | 2 | Not-determined | Genome-fragment | NA | 0 |
| S1_k141_126943 | 10456 | No | NA | 0 | 6 | Not-determined | Genome-fragment | NA | 0 |
| S1_k141_27013 | 1113 | No | NA | 0 | 0 | Not-determined | Genome-fragment | NA | 0 |
| S1_k141_324588 | 1594 | No | NA | 0 | 1 | Not-determined | Genome-fragment | NA | 0 |
| S1_k141_372816 | 1177 | No | NA | 0 | 2 | Not-determined | Genome-fragment | NA | 0 |
| S1_k141_258556 | 1487 | No | NA | 0 | 0 | Not-determined | Genome-fragment | NA | 0 |
| S1_k141_258560 | 2554 | No | NA | 0 | 2 | Not-determined | Genome-fragment | NA | 0 |
| S1_k141_241501 | 4128 | No | NA | 0 | 1 | Not-determined | Genome-fragment | NA | 0 |
| S12-final-viral-combined_k141_101135\|\|full | 1488 | No | NA | 1 | 0 | Low-quality | Genome-fragment | 0.68 | 0 |
| S1_k141_241509 | 1147 | No | NA | 0 | 0 | Not-determined | Genome-fragment | NA | 0 |
| S1_k141_290680 | 1789 | No | NA | 0 | 2 | Not-determined | Genome-fragment | NA | 0 |
| S12-final-viral-combined_k141_106113\|\|full | 1488 | No | NA | 0 | 0 | Low-quality | Genome-fragment | 3.16 | 0 |
| S1_k141_59810 | 1327 | No | NA | 0 | 1 | Not-determined | Genome-fragment | NA | 0 |
| S1_k141_389132 | 2625 | No | NA | 0 | 1 | Not-determined | Genome-fragment | NA | 0 |
| S1_k141_126977 | 1553 | No | NA | 0 | 2 | Not-determined | Genome-fragment | NA | 0 |
| S1_k141_27101 | 1328 | No | NA | 0 | 0 | Not-determined | Genome-fragment | NA | 0 |
| S1_k141_290704 | 1137 | No | NA | 0 | 2 | Not-determined | Genome-fragment | NA | 0 |
| S1_k141_389143 | 1781 | No | NA | 0 | 1 | Not-determined | Genome-fragment | NA | 0 |
| S12-final-viral-combined_k141_130126\|\|lt2gene | 1488 | No | NA | 1 | 0 | Low-quality | Genome-fragment | 3.61 | 0 |
| S12_k141_73745 | 1488 | No | NA | 0 | 1 | Low-quality | Genome-fragment | 1.5 | 0 |
| S1_k141_209311 | 3120 | No | NA | 0 | 1 | Not-determined | Genome-fragment | NA | 0 |
| S1_k141_192631 | 1243 | No | NA | 0 | 1 | Not-determined | Genome-fragment | NA | 0 |
| S11-final-viral-combined_k141_181773\|\|lt2gene | 1489 | No | NA | 1 | 0 | Low-quality | Genome-fragment | 2.35 | 0 |
| S1_k141_389196 | 1383 | No | NA | 0 | 0 | Not-determined | Genome-fragment | NA | 0 |
| S1_k141_10975 | 1706 | No | NA | 0 | 0 | Not-determined | Genome-fragment | NA | 0 |
| S1-final-viral-combined_k141_253917\|\|full | 1489 | No | NA | 0 | 0 | Low-quality | Genome-fragment | 1.58 | 0 |
| S1_k141_224821 | 8426 | No | NA | 0 | 8 | Not-determined | Genome-fragment | NA | 0 |
| S1_k141_174403 | 1607 | No | NA | 0 | 1 | Not-determined | Genome-fragment | NA | 0 |
| S1_k141_258722 | 1264 | No | NA | 0 | 1 | Not-determined | Genome-fragment | NA | 0 |
| S1_k141_75727 | 1164 | No | NA | 0 | 0 | Not-determined | Genome-fragment | NA | 0 |
| S1_k141_258741 | 1074 | No | NA | 0 | 0 | Not-determined | Genome-fragment | NA | 0 |
| S1_k141_142731 | 1035 | No | NA | 0 | 1 | Not-determined | Genome-fragment | NA | 0 |
| S1_k141_389237 | 1125 | No | NA | 0 | 1 | Not-determined | Genome-fragment | NA | 0 |
| S1_k141_62638 | 1489 | No | NA | 0 | 0 | Low-quality | Genome-fragment | 3.05 | 0 |
| S1_k141_75779 | 5450 | No | NA | 0 | 0 | Not-determined | Genome-fragment | NA | 0 |
| S1_k141_258805 | 1052 | No | NA | 0 | 2 | Not-determined | Genome-fragment | NA | 0 |
| S1_k141_307855 | 4218 | No | NA | 0 | 1 | Not-determined | Genome-fragment | NA | 0 |
| S1_k141_142757 | 1062 | No | NA | 0 | 0 | Not-determined | Genome-fragment | NA | 0 |
| S1_k141_373012 | 1485 | No | NA | 0 | 1 | Not-determined | Genome-fragment | NA | 0 |
| S1_k141_389270 | 1455 | No | NA | 0 | 0 | Not-determined | Genome-fragment | NA | 0 |
| S1_k141_209421 | 1782 | No | NA | 0 | 1 | Not-determined | Genome-fragment | NA | 0 |
| S1_k141_373022 | 1086 | No | NA | 0 | 0 | Not-determined | Genome-fragment | NA | 0 |
| S1_k141_174477 | 3046 | No | NA | 0 | 2 | Not-determined | Genome-fragment | NA | 0 |
| S5-final-viral-combined_k141_185792\|\|full | 1489 | No | NA | 0 | 0 | Low-quality | Genome-fragment | 3.65 | 0 |
| S1_k141_108805 | 1857 | No | NA | 0 | 1 | Not-determined | Genome-fragment | NA | 0 |
| S1_k141_290928 | 1114 | No | NA | 0 | 1 | Not-determined | Genome-fragment | NA | 0 |
| S1_k141_209461 | 1222 | No | NA | 0 | 0 | Not-determined | Genome-fragment | NA | 0 |
| S7-final-viral-combined_k141_143106\|\|full | 1489 | No | NA | 0 | 0 | Low-quality | Genome-fragment | 0.94 | 0 |
| S1_k141_43552 | 1046 | No | NA | 0 | 0 | Not-determined | Genome-fragment | NA | 0 |
| S1_k141_75911 | 3407 | No | NA | 0 | 4 | Not-determined | Genome-fragment | NA | 0 |
| S1_k141_307980 | 3178 | No | NA | 0 | 1 | Not-determined | Genome-fragment | NA | 0 |
| S1_k141_373087 | 2051 | No | NA | 0 | 0 | Not-determined | Genome-fragment | NA | 0 |
| S1_k141_357148 | 3556 | No | NA | 0 | 1 | Not-determined | Genome-fragment | NA | 0 |
| S12-final-viral-combined_k141_354878\|\|full | 1489 | No | NA | 0 | 0 | Low-quality | Genome-fragment | 2.25 | 0 |
| S12-final-viral-combined_k141_254701\|\|full | 1489 | No | NA | 0 | 0 | Low-quality | Genome-fragment | 2.94 | 0 |
| S2-final-viral-combined_k141_122368\|\|full | 1489 | No | NA | 0 | 0 | Low-quality | Genome-fragment | 2.97 | 0 |
| S1_k141_307994 | 1152 | No | NA | 0 | 1 | Not-determined | Genome-fragment | NA | 0 |
| S1_k141_324816 | 1651 | No | NA | 0 | 1 | Not-determined | Genome-fragment | NA | 0 |
| S1_k141_373101 | 1168 | No | NA | 0 | 0 | Not-determined | Genome-fragment | NA | 0 |
| S1_k141_127210 | 1043 | No | NA | 0 | 0 | Not-determined | Genome-fragment | NA | 0 |
| S1_k141_60032 | 3075 | No | NA | 0 | 4 | Not-determined | Genome-fragment | NA | 0 |
| S1_k141_241813 | 2133 | No | NA | 0 | 1 | Not-determined | Genome-fragment | NA | 0 |
| S1_k141_225015 | 1336 | No | NA | 0 | 1 | Not-determined | Genome-fragment | NA | 0 |
| S1_k141_389394 | 1091 | No | NA | 0 | 1 | Not-determined | Genome-fragment | NA | 0 |
| S1_k141_75950 | 7752 | No | NA | 0 | 1 | Not-determined | Genome-fragment | NA | 0 |
| S4-final-viral-combined_k141_53315\|\|full | 1489 | No | NA | 0 | 0 | Low-quality | Genome-fragment | 3.03 | 0 |
| S8-final-viral-combined_k141_198670\|\|full | 1489 | No | NA | 0 | 0 | Low-quality | Genome-fragment | 3.1 | 0 |
| S1_k141_339440 | 1967 | No | NA | 0 | 0 | Not-determined | Genome-fragment | NA | 0 |
| S1_k141_274045 | 5612 | No | NA | 0 | 1 | Not-determined | Genome-fragment | NA | 0 |
| S11-final-viral-combined_k141_238357\|\|full | 1490 | No | NA | 1 | 0 | Low-quality | Genome-fragment | 2.65 | 0 |
| S1_k141_192769 | 1486 | No | NA | 0 | 2 | Not-determined | Genome-fragment | NA | 0 |
| S11-final-viral-combined_k141_55037\|\|full | 1490 | No | NA | 1 | 0 | Low-quality | Genome-fragment | 3.65 | 0 |
| S11-final-viral-combined_k141_276620\|\|lt2gene | 1490 | No | NA | 1 | 0 | Low-quality | Genome-fragment | 3.12 | 0 |
| S1_k141_274084 | 1034 | No | NA | 0 | 0 | Not-determined | Genome-fragment | NA | 0 |
| S11-final-viral-combined_k141_276648\|\|lt2gene | 1490 | No | NA | 1 | 0 | Low-quality | Genome-fragment | 3.12 | 0 |
| S1_k141_60093 | 3884 | No | NA | 0 | 1 | Not-determined | Genome-fragment | NA | 0 |
| S1-final-viral-combined_k141_44771\|\|lt2gene | 1490 | No | NA | 0 | 0 | Low-quality | Genome-fragment | 2.81 | 0 |
| S1_k141_324892 | 1101 | No | NA | 0 | 1 | Not-determined | Genome-fragment | NA | 0 |
| S1_k141_191225 | 1490 | No | NA | 0 | 0 | Low-quality | Genome-fragment | 2.55 | 0 |
| S17-final-viral-combined_k141_33676\|\|full | 1490 | No | NA | 0 | 0 | Low-quality | Genome-fragment | 2.62 | 0 |
| S1_k141_43695 | 1056 | No | NA | 0 | 1 | Not-determined | Genome-fragment | NA | 0 |
| S17-final-viral-combined_k141_240555\|\|full | 1490 | No | NA | 1 | 0 | Low-quality | Genome-fragment | 1.71 | 0 |
| S1_k141_43719 | 4945 | No | NA | 0 | 8 | Not-determined | Genome-fragment | NA | 0 |
| S1_k141_373176 | 1241 | No | NA | 0 | 0 | Not-determined | Genome-fragment | NA | 0 |
| S17-final-viral-combined_k141_231437\|\|full | 1490 | No | NA | 0 | 0 | Low-quality | Genome-fragment | 2.2 | 0 |
| S1_k141_108998 | 1316 | No | NA | 0 | 2 | Not-determined | Genome-fragment | NA | 0 |
| S17-final-viral-combined_k141_77406\|\|full | 1490 | No | NA | 2 | 0 | Low-quality | Genome-fragment | 3.76 | 0 |
| S17-k141_92619 | 1490 | Yes | 308 | 1 | 1 | Low-quality | Genome-fragment | 0.2 | 79.33 |
| S12-final-viral-combined_k141_244433\|\|full | 1490 | No | NA | 0 | 0 | Low-quality | Genome-fragment | 2.73 | 0 |
| S8-final-viral-combined_k141_206785\|\|full | 1490 | No | NA | 0 | 1 | Low-quality | Genome-fragment | 0.88 | 0 |
| S1_k141_27329 | 3239 | No | NA | 0 | 2 | Not-determined | Genome-fragment | NA | 0 |
| S1_k141_43776 | 2402 | No | NA | 0 | 0 | Not-determined | Genome-fragment | NA | 0 |
| S1_k141_27338 | 1740 | No | NA | 0 | 1 | Not-determined | Genome-fragment | NA | 0 |
| S9-final-viral-combined_k141_48062\|\|full | 1491 | No | NA | 1 | 0 | Low-quality | Genome-fragment | 3.99 | 0 |
| S1-final-viral-combined_k141_288174\|\|full | 1491 | No | NA | 0 | 0 | Low-quality | Genome-fragment | 3.49 | 0 |
| S1_k141_291267 | 1076 | No | NA | 0 | 1 | Not-determined | Genome-fragment | NA | 0 |
| S1_k141_274252 | 8363 | No | NA | 0 | 1 | Not-determined | Genome-fragment | NA | 0 |

| | | | | | | | | | | | |
|---|---|---|---|---|---|---|---|---|---|---|---|
| S1-final-viral-combined_k141_126361\|\|lt2gene | 1491 | No | NA | | 1 | 0 | Low-quality | Genome-fragment | | 5.05 | 0 |
| S1_k141_109053 | 3785 | No | NA | | 0 | 1 | Not-determined | Genome-fragment | NA | | 0 |
| S1_k141_93802 | 2098 | No | NA | | 0 | 2 | Not-determined | Genome-fragment | NA | | 0 |
| S1_k141_357447 | 1250 | No | NA | | 0 | 0 | Not-determined | Genome-fragment | NA | | 0 |
| S1_k141_76213 | 1515 | No | NA | | 0 | 1 | Not-determined | Genome-fragment | NA | | 0 |
| S1_k141_209643 | 1680 | No | NA | | 0 | 1 | Not-determined | Genome-fragment | NA | | 0 |
| S1_k141_225303 | 2504 | No | NA | | 0 | 0 | Not-determined | Genome-fragment | NA | | 0 |
| S1_k141_158711 | 1310 | No | NA | | 0 | 1 | Not-determined | Genome-fragment | NA | | 0 |
| S1_k141_76234 | 1318 | No | NA | | 0 | 0 | Not-determined | Genome-fragment | NA | | 0 |
| S1_k141_259199 | 1309 | No | NA | | 0 | 1 | Not-determined | Genome-fragment | NA | | 0 |
| S1_k141_60337 | 1915 | No | NA | | 0 | 4 | Not-determined | Genome-fragment | NA | | 0 |
| S1_k141_259204 | 6960 | No | NA | | 0 | 1 | Not-determined | Genome-fragment | NA | | 0 |
| S7-final-viral-combined_k141_10277\|\|full | 1491 | Yes | | 666 | 1 | 1 | Low-quality | Genome-fragment | | 1.75 | 55.33 |
| S1_k141_274333 | 1299 | No | NA | | 0 | 1 | Not-determined | Genome-fragment | NA | | 0 |
| S1_k141_339597 | 4674 | No | NA | | 0 | 3 | Not-determined | Genome-fragment | NA | | 0 |
| S7-final-viral-combined_k141_176302\|\|full | 1491 | No | NA | | 0 | 2 | Low-quality | Genome-fragment | | 2.06 | 0 |
| S1_k141_339606 | 6414 | No | NA | | 0 | 4 | Not-determined | Genome-fragment | NA | | 0 |
| S1_k141_76266 | 1245 | No | NA | | 0 | 1 | Not-determined | Genome-fragment | NA | | 0 |
| S1_k141_193029 | 1522 | No | NA | | 0 | 0 | Not-determined | Genome-fragment | NA | | 0 |
| S1_k141_109140 | 1224 | No | NA | | 0 | 0 | Not-determined | Genome-fragment | NA | | 0 |
| S1_k141_109149 | 1443 | No | NA | | 0 | 1 | Not-determined | Genome-fragment | NA | | 0 |
| S17-final-viral-combined_k141_231082\|\|full | 1491 | No | NA | | 2 | 0 | Low-quality | Genome-fragment | | 3.49 | 0 |
| S1_k141_93935 | 1010 | No | NA | | 0 | 1 | Not-determined | Genome-fragment | NA | | 0 |
| S1_k141_11367 | 1197 | No | NA | | 0 | 1 | Not-determined | Genome-fragment | NA | | 0 |
| S2-final-viral-combined_k141_190946\|\|full | 1491 | No | NA | | 0 | 0 | Low-quality | Genome-fragment | | 1.01 | 0 |
| S1_k141_291415 | 1201 | No | NA | | 0 | 1 | Not-determined | Genome-fragment | NA | | 0 |
| S1_k141_274374 | 1045 | No | NA | | 0 | 1 | Not-determined | Genome-fragment | NA | | 0 |
| S8_k141_53529 | 1491 | No | NA | | 1 | 0 | Low-quality | Genome-fragment | | 0.97 | 0 |
| S1_k141_174969 | 5579 | No | NA | | 0 | 4 | Not-determined | Genome-fragment | NA | | 0 |
| S11-final-viral-combined_k141_23872\|\|full | 1492 | No | NA | | 0 | 3 | Low-quality | Genome-fragment | | 0.88 | 0 |
| S1_k141_308340 | 1015 | No | NA | | 0 | 1 | Not-determined | Genome-fragment | NA | | 0 |
| S11_k141_230203 | 1492 | No | NA | | 0 | 0 | Low-quality | Genome-fragment | | 3.49 | 0 |
| S1_k141_175006 | 1165 | No | NA | | 0 | 0 | Not-determined | Genome-fragment | NA | | 0 |
| S1_k141_308384 | 2552 | No | NA | | 0 | 3 | Not-determined | Genome-fragment | NA | | 0 |
| S1-final-viral-combined_k141_114663\|\|full | 1492 | No | NA | | 1 | 0 | Low-quality | Genome-fragment | | 2.21 | 0 |
| S1_k141_193195 | 2063 | No | NA | | 0 | 0 | Not-determined | Genome-fragment | NA | | 0 |
| S3-final-viral-combined_k141_135079\|\|full | 1492 | No | NA | | 1 | 0 | Low-quality | Genome-fragment | | 3.13 | 0 |
| S1_k141_259302 | 1637 | No | NA | | 0 | 0 | Not-determined | Genome-fragment | NA | | 0 |
| S1_k141_291539 | 2220 | No | NA | | 0 | 3 | Not-determined | Genome-fragment | NA | | 0 |
| S1_k141_143226 | 1125 | No | NA | | 0 | 0 | Not-determined | Genome-fragment | NA | | 0 |
| S1_k141_325228 | 1177 | No | NA | | 0 | 1 | Not-determined | Genome-fragment | NA | | 0 |
| S1_k141_158951 | 4704 | No | NA | | 0 | 3 | Not-determined | Genome-fragment | NA | | 0 |
| S1_k141_109303 | 1555 | No | NA | | 0 | 1 | Not-determined | Genome-fragment | NA | | 0 |
| S1_k141_143258 | 1034 | No | NA | | 0 | 0 | Not-determined | Genome-fragment | NA | | 0 |
| S1_k141_158977 | 1846 | No | NA | | 0 | 2 | Not-determined | Genome-fragment | NA | | 0 |
| S5_k141_163756 | 1492 | No | NA | | 0 | 1 | Low-quality | Genome-fragment | | 3.47 | 0 |
| S1_k141_76412 | 2109 | No | NA | | 0 | 0 | Not-determined | Genome-fragment | NA | | 0 |
| S1_k141_109335 | 1050 | No | NA | | 0 | 0 | Not-determined | Genome-fragment | NA | | 0 |
| S1_k141_143312 | 1103 | No | NA | | 0 | 0 | Not-determined | Genome-fragment | NA | | 0 |
| S12-final-viral-combined_k141_95642\|\|full | 1492 | No | NA | | 0 | 2 | Low-quality | Genome-fragment | | 2.58 | 0 |
| S1_k141_159003 | 1183 | No | NA | | 0 | 0 | Not-determined | Genome-fragment | NA | | 0 |
| S1_k141_159006 | 1341 | No | NA | | 0 | 0 | Not-determined | Genome-fragment | NA | | 0 |
| S1_k141_274533 | 4039 | No | NA | | 0 | 0 | Not-determined | Genome-fragment | NA | | 0 |
| S1_k141_27659 | 1175 | No | NA | | 0 | 0 | Not-determined | Genome-fragment | NA | | 0 |
| S1_k141_127712 | 1722 | No | NA | | 0 | 0 | Not-determined | Genome-fragment | NA | | 0 |
| S12_k141_299931 | 1492 | No | NA | | 0 | 1 | Low-quality | Genome-fragment | | 2.21 | 0 |
| S8_k141_59304 | 1492 | No | NA | | 0 | 0 | Low-quality | Genome-fragment | | 1.12 | 0 |
| S1_k141_209913 | 1493 | No | NA | | 0 | 1 | Not-determined | Genome-fragment | NA | | 0 |
| S1_k141_373509 | 2312 | No | NA | | 0 | 1 | Not-determined | Genome-fragment | NA | | 0 |
| S1_k141_339808 | 6975 | No | NA | | 0 | 1 | Not-determined | Genome-fragment | NA | | 0 |
| S1_k141_159033 | 1078 | No | NA | | 0 | 1 | Not-determined | Genome-fragment | NA | | 0 |
| S8_k141_144085 | 1492 | No | NA | | 0 | 1 | Low-quality | Genome-fragment | | 2.05 | 0 |
| S1_k141_175176 | 1428 | No | NA | | 0 | 1 | Not-determined | Genome-fragment | NA | | 0 |
| S1_k141_109376 | 1514 | No | NA | | 0 | 0 | Not-determined | Genome-fragment | NA | | 0 |
| S1_k141_389841 | 1145 | No | NA | | 0 | 1 | Not-determined | Genome-fragment | NA | | 0 |
| S9-final-viral-combined_k141_48117\|\|full | 1493 | No | NA | | 1 | 0 | Low-quality | Genome-fragment | | 3.61 | 0 |
| S9_k141_122205 | 1493 | No | NA | | 2 | 0 | Low-quality | Genome-fragment | | 3.57 | 0 |
| S1_k141_357842 | 1587 | No | NA | | 0 | 1 | Not-determined | Genome-fragment | NA | | 0 |
| S1_k141_357850 | 5169 | No | NA | | 0 | 3 | Not-determined | Genome-fragment | NA | | 0 |
| S1_k141_159102 | 1186 | No | NA | | 0 | 2 | Not-determined | Genome-fragment | NA | | 0 |
| S7-final-viral-combined_k141_88925\|\|full | 1493 | No | NA | | 0 | 1 | Low-quality | Genome-fragment | | 0.76 | 0 |
| S1_k141_11575 | 1669 | No | NA | | 0 | 0 | Not-determined | Genome-fragment | NA | | 0 |
| S12-final-viral-combined_k141_36040\|\|full | 1493 | No | NA | | 1 | 0 | Low-quality | Genome-fragment | | 2.26 | 0 |
| S1_k141_11592 | 1006 | No | NA | | 0 | 1 | Not-determined | Genome-fragment | NA | | 0 |
| S1_k141_308603 | 2217 | No | NA | | 0 | 2 | Not-determined | Genome-fragment | NA | | 0 |
| S1_k141_291815 | 1177 | No | NA | | 0 | 2 | Not-determined | Genome-fragment | NA | | 0 |
| S12-final-viral-combined_k141_384563\|\|full | 1493 | No | NA | | 0 | 1 | Low-quality | Genome-fragment | | 3.69 | 0 |
| S1_k141_94286 | 1208 | No | NA | | 0 | 1 | Not-determined | Genome-fragment | NA | | 0 |
| S1_k141_291847 | 1608 | No | NA | | 0 | 0 | Not-determined | Genome-fragment | NA | | 0 |
| S1_k141_143448 | 1140 | No | NA | | 0 | 1 | Not-determined | Genome-fragment | NA | | 0 |
| S1_k141_325457 | 1126 | No | NA | | 0 | 0 | Not-determined | Genome-fragment | NA | | 0 |
| S1_k141_109464 | 1066 | No | NA | | 0 | 1 | Not-determined | Genome-fragment | NA | | 0 |
| S1_k141_76561 | 1133 | No | NA | | 0 | 0 | Not-determined | Genome-fragment | NA | | 0 |
| S1_k141_389988 | 1268 | No | NA | | 0 | 2 | Not-determined | Genome-fragment | NA | | 0 |
| S1_k141_225750 | 6459 | No | NA | | 0 | 4 | Not-determined | Genome-fragment | NA | | 0 |
| S1_k141_109499 | 1106 | No | NA | | 0 | 1 | Not-determined | Genome-fragment | NA | | 0 |
| S1_k141_193504 | 1058 | No | NA | | 0 | 1 | Not-determined | Genome-fragment | NA | | 0 |
| S1_k141_225766 | 1356 | No | NA | | 0 | 1 | Not-determined | Genome-fragment | NA | | 0 |
| S2-final-viral-combined_k141_165526\|\|full | 1493 | No | NA | | 1 | 0 | Low-quality | Genome-fragment | | 1 | 0 |
| S1_k141_210083 | 1334 | No | NA | | 0 | 2 | Not-determined | Genome-fragment | NA | | 0 |
| S1_k141_357957 | 1389 | No | NA | | 0 | 1 | Not-determined | Genome-fragment | NA | | 0 |
| S11-final-viral-combined_k141_86223\|\|full | 1494 | No | NA | | 0 | 0 | Low-quality | Genome-fragment | | 1.78 | 0 |
| S1-final-viral-combined_k141_130710\|\|full | 1494 | No | NA | | 0 | 0 | Low-quality | Genome-fragment | | 3.52 | 0 |
| S1_k141_291907 | 1215 | No | NA | | 0 | 0 | Not-determined | Genome-fragment | NA | | 0 |
| S1_k141_308662 | 1031 | No | NA | | 0 | 0 | Not-determined | Genome-fragment | NA | | 0 |
| S5-final-viral-combined_k141_155455\|\|full | 1494 | No | NA | | 1 | 0 | Low-quality | Genome-fragment | | 3.73 | 0 |
| S1_k141_242477 | 1298 | No | NA | | 0 | 1 | Not-determined | Genome-fragment | NA | | 0 |
| S1_k141_339905 | 1326 | No | NA | | 0 | 1 | Not-determined | Genome-fragment | NA | | 0 |
| S1_k141_373728 | 1043 | No | NA | | 0 | 1 | Not-determined | Genome-fragment | NA | | 0 |
| S1_k141_193604 | 3500 | No | NA | | 0 | 2 | Not-determined | Genome-fragment | NA | | 0 |
| S1_k141_358048 | 2235 | No | NA | | 0 | 2 | Not-determined | Genome-fragment | NA | | 0 |
| S1_k141_358056 | 3829 | No | NA | | 0 | 3 | Not-determined | Genome-fragment | NA | | 0 |
| S1_k141_109628 | 3387 | No | NA | | 0 | 2 | Not-determined | Genome-fragment | NA | | 0 |
| S1_k141_60916 | 1612 | No | NA | | 0 | 1 | Not-determined | Genome-fragment | NA | | 0 |
| S5-final-viral-combined_k141_132286\|\|full | 1494 | No | NA | | 1 | 0 | Low-quality | Genome-fragment | | 2.77 | 0 |
| S1_k141_143576 | 1709 | No | NA | | 0 | 2 | Not-determined | Genome-fragment | NA | | 0 |
| S1_k141_292020 | 1399 | No | NA | | 0 | 0 | Not-determined | Genome-fragment | NA | | 0 |
| S1_k141_159243 | 1790 | No | NA | | 0 | 0 | Not-determined | Genome-fragment | NA | | 0 |
| S1_k141_11820 | 1586 | No | NA | | 0 | 1 | Not-determined | Genome-fragment | NA | | 0 |
| S1_k141_390116 | 1125 | No | NA | | 0 | 1 | Not-determined | Genome-fragment | NA | | 0 |
| S1_k141_358139 | 4234 | No | NA | | 0 | 1 | Not-determined | Genome-fragment | NA | | 0 |
| S5-final-viral-combined_k141_175812\|\|lt2gene | 1494 | No | NA | | 1 | 0 | Low-quality | Genome-fragment | | 1.03 | 0 |
| S1_k141_210214 | 2023 | No | NA | | 0 | 1 | Not-determined | Genome-fragment | NA | | 0 |
| S1_k141_143660 | 4048 | No | NA | | 0 | 3 | Not-determined | Genome-fragment | NA | | 0 |
| S1_k141_373808 | 1022 | No | NA | | 0 | 0 | Not-determined | Genome-fragment | NA | | 0 |
| S7-final-viral-combined_k141_175685\|\|full | 1494 | No | NA | | 0 | 0 | Low-quality | Genome-fragment | | 2.27 | 0 |
| S1_k141_109720 | 3350 | No | NA | | 0 | 2 | Not-determined | Genome-fragment | NA | | 0 |
| S7_k141_240983 | 1494 | No | NA | | 0 | 0 | Low-quality | Genome-fragment | | 2.5 | 0 |
| S1_k141_61070 | 1752 | No | NA | | 0 | 2 | Not-determined | Genome-fragment | NA | | 0 |
| S1_k141_44536 | 1150 | No | NA | | 0 | 1 | Not-determined | Genome-fragment | NA | | 0 |
| S1_k141_159358 | 1871 | No | NA | | 0 | 0 | Not-determined | Genome-fragment | NA | | 0 |
| S1_k141_274896 | 3203 | No | NA | | 0 | 3 | Not-determined | Genome-fragment | NA | | 0 |
| S1_k141_242627 | 1405 | No | NA | | 0 | 1 | Not-determined | Genome-fragment | NA | | 0 |
| S1_k141_358305 | 1254 | No | NA | | 0 | 1 | Not-determined | Genome-fragment | NA | | 0 |
| S1_k141_225995 | 47567 | No | NA | | 14 | 1 | High-quality | High-quality | | 100 | 0 |
| S1_k141_274929 | 3591 | No | NA | | 0 | 1 | Not-determined | Genome-fragment | NA | | 0 |
| S1_k141_373919 | 3488 | No | NA | | 0 | 2 | Not-determined | Genome-fragment | NA | | 0 |
| S1_k141_11964 | 2780 | No | NA | | 0 | 0 | Not-determined | Genome-fragment | NA | | 0 |
| S1_k141_76856 | 1075 | No | NA | | 0 | 1 | Not-determined | Genome-fragment | NA | | 0 |
| S1_k141_390214 | 1155 | No | NA | | 0 | 2 | Not-determined | Genome-fragment | NA | | 0 |
| S1_k141_11979 | 3124 | No | NA | | 0 | 1 | Not-determined | Genome-fragment | NA | | 0 |
| S1_k141_226027 | 1542 | No | NA | | 0 | 0 | Not-determined | Genome-fragment | NA | | 0 |
| S1_k141_226043 | 1693 | No | NA | | 0 | 1 | Not-determined | Genome-fragment | NA | | 0 |
| S1_k141_340164 | 1099 | No | NA | | 0 | 0 | Not-determined | Genome-fragment | NA | | 0 |
| S1_k141_292231 | 1264 | No | NA | | 0 | 1 | Not-determined | Genome-fragment | NA | | 0 |
| S1_k141_76896 | 1128 | No | NA | | 0 | 1 | Not-determined | Genome-fragment | NA | | 0 |
| S1_k141_226055 | 1073 | No | NA | | 0 | 0 | Not-determined | Genome-fragment | NA | | 0 |
| S1_k141_242656 | 1016 | No | NA | | 0 | 1 | Not-determined | Genome-fragment | NA | | 0 |
| S1_k141_374008 | 1055 | No | NA | | 0 | 1 | Not-determined | Genome-fragment | NA | | 0 |
| S1_k141_340185 | 1148 | No | NA | | 0 | 0 | Not-determined | Genome-fragment | NA | | 0 |
| S1_k141_44664 | 1754 | No | NA | | 0 | 1 | Not-determined | Genome-fragment | NA | | 0 |
| S1_k141_143848 | 1414 | No | NA | | 0 | 1 | Not-determined | Genome-fragment | NA | | 0 |
| S1_k141_128123 | 1091 | No | NA | | 0 | 0 | Not-determined | Genome-fragment | NA | | 0 |
| S1_k141_358418 | 4069 | No | NA | | 0 | 3 | Not-determined | Genome-fragment | NA | | 0 |
| S1_k141_143858 | 1235 | No | NA | | 0 | 0 | Not-determined | Genome-fragment | NA | | 0 |
| S1_k141_61287 | 1178 | No | NA | | 0 | 1 | Not-determined | Genome-fragment | NA | | 0 |
| S1_k141_175556 | 1425 | No | NA | | 0 | 0 | Not-determined | Genome-fragment | NA | | 0 |
| S1_k141_358450 | 1073 | No | NA | | 0 | 1 | Not-determined | Genome-fragment | NA | | 0 |
| S1_k141_12095 | 2332 | No | NA | | 0 | 1 | Not-determined | Genome-fragment | NA | | 0 |
| S17-final-viral-combined_k141_133974\|\|full | 1494 | No | NA | | 0 | 0 | Low-quality | Genome-fragment | | 2.64 | 0 |
| S1_k141_76992 | 4750 | No | NA | | 0 | 4 | Not-determined | Genome-fragment | NA | | 0 |
| S1_k141_325875 | 1404 | No | NA | | 0 | 1 | Not-determined | Genome-fragment | NA | | 0 |
| S17-final-viral-combined_k141_44119\|\|full | 1494 | No | NA | | 0 | 1 | Low-quality | Genome-fragment | | 3.06 | 0 |
| S1_k141_374113 | 1124 | No | NA | | 0 | 1 | Not-determined | Genome-fragment | NA | | 0 |
| S1_k141_275144 | 1036 | No | NA | | 0 | 1 | Not-determined | Genome-fragment | NA | | 0 |
| S1_k141_374138 | 2357 | No | NA | | 0 | 0 | Not-determined | Genome-fragment | NA | | 0 |
| S1_k141_143961 | 1463 | No | NA | | 0 | 1 | Not-determined | Genome-fragment | NA | | 0 |
| S12-final-viral-combined_k141_176148\|\|full | 1494 | No | NA | | 0 | 0 | Low-quality | Genome-fragment | | 2.03 | 0 |
| S2-final-viral-combined_k141_4224\|\|full | 1494 | No | NA | | 0 | 0 | Low-quality | Genome-fragment | | 2.74 | 0 |
| S1_k141_242821 | 2302 | No | NA | | 0 | 1 | Not-determined | Genome-fragment | NA | | 0 |
| S2-final-viral-combined_k141_58925\|\|full | 1494 | No | NA | | 0 | 0 | Low-quality | Genome-fragment | | 3.54 | 0 |
| S1_k141_94753 | 1635 | No | NA | | 0 | 1 | Not-determined | Genome-fragment | NA | | 0 |
| S1_k141_143985 | 1323 | No | NA | | 0 | 1 | Not-determined | Genome-fragment | NA | | 0 |
| S1_k141_77081 | 1118 | No | NA | | 0 | 1 | Not-determined | Genome-fragment | NA | | 0 |
| S1_k141_309189 | 1051 | No | NA | | 0 | 0 | Not-determined | Genome-fragment | NA | | 0 |
| S9-final-viral-combined_k141_15970\|\|full | 1495 | No | NA | | 0 | 0 | Low-quality | Genome-fragment | | 3.61 | 0 |
| S1_k141_242903 | 1085 | No | NA | | 0 | 1 | Not-determined | Genome-fragment | NA | | 0 |
| S1_k141_28450 | 1144 | No | NA | | 0 | 3 | Not-determined | Genome-fragment | NA | | 0 |
| S9_k141_30596 | 1495 | No | NA | | 0 | 0 | Low-quality | Genome-fragment | | 5.67 | 0 |
| S1-final-viral-combined_k141_367267\|\|full | 1495 | No | NA | | 0 | 1 | Low-quality | Genome-fragment | | 1.18 | 0 |
| S1_k141_44878 | 1658 | No | NA | | 0 | 1 | Not-determined | Genome-fragment | NA | | 0 |
| S1_k141_390541 | 6974 | No | NA | | 0 | 5 | Not-determined | Genome-fragment | NA | | 0 |
| S1_k141_128272 | 5630 | No | NA | | 0 | 5 | Not-determined | Genome-fragment | NA | | 0 |
| S1_k141_326028 | 2368 | No | NA | | 0 | 1 | Not-determined | Genome-fragment | NA | | 0 |

| | | | | | | | | | | | |
|---|---|---|---|---|---|---|---|---|---|---|---|
| S1_k141_309236 | 2370 | No | NA | | 0 | 1 | Not-determined | Genome-fragment | NA | | 0 |
| S1_k141_175711 | 1096 | No | NA | | 0 | 1 | Not-determined | Genome-fragment | NA | | 0 |
| S1_k141_309255 | 1103 | No | NA | | 0 | 0 | Not-determined | Genome-fragment | NA | | 0 |
| S1_k141_193848 | 1106 | No | NA | | 0 | 1 | Not-determined | Genome-fragment | NA | | 0 |
| S1_k141_374250 | 1360 | No | NA | | 0 | 1 | Not-determined | Genome-fragment | NA | | 0 |
| S1-final-viral-combined_k141_205299\|\|full | 1495 | No | NA | | 1 | 0 | Low-quality | Genome-fragment | | 3.52 | 0 |
| S1_k141_358729 | 1022 | No | NA | | 0 | 1 | Not-determined | Genome-fragment | NA | | 0 |
| S1_k141_128334 | 5542 | No | NA | | 0 | 0 | Not-determined | Genome-fragment | NA | | 0 |
| S1_k141_390607 | 1062 | No | NA | | 0 | 1 | Not-determined | Genome-fragment | NA | | 0 |
| S1_k141_374302 | 1075 | No | NA | | 0 | 0 | Not-determined | Genome-fragment | NA | | 0 |
| S1_k141_374305 | 1053 | No | NA | | 0 | 1 | Not-determined | Genome-fragment | NA | | 0 |
| S1_k141_326053 | 1717 | No | NA | | 0 | 1 | Not-determined | Genome-fragment | NA | | 0 |
| S1_k141_275256 | 1181 | No | NA | | 0 | 0 | Not-determined | Genome-fragment | NA | | 0 |
| S1_k141_260361 | 1822 | No | NA | | 0 | 0 | Not-determined | Genome-fragment | NA | | 0 |
| S1_k141_109955 | 1045 | No | NA | | 0 | 0 | Not-determined | Genome-fragment | NA | | 0 |
| S1_k141_292629 | 1277 | No | NA | | 0 | 0 | Not-determined | Genome-fragment | NA | | 0 |
| S1-final-viral-combined_k141_242553\|\|full | 1495 | No | NA | | 0 | 0 | Low-quality | Genome-fragment | | 3.34 | 0 |
| S1_k141_275306 | 1914 | No | NA | | 0 | 0 | Not-determined | Genome-fragment | NA | | 0 |
| S1_k141_128402 | 2058 | No | NA | | 0 | 1 | Not-determined | Genome-fragment | NA | | 0 |
| S1_k141_102204 | 1495 | No | NA | | 0 | 0 | Low-quality | Genome-fragment | | 3.64 | 0 |
| S1_k141_292688 | 1043 | No | NA | | 0 | 0 | Not-determined | Genome-fragment | NA | | 0 |
| S1_k141_109987 | 4035 | No | NA | | 0 | 0 | Not-determined | Genome-fragment | NA | | 0 |
| S1_k141_12510 | 1665 | No | NA | | 0 | 1 | Not-determined | Genome-fragment | NA | | 0 |
| S1_k141_292693 | 1181 | No | NA | | 0 | 1 | Not-determined | Genome-fragment | NA | | 0 |
| S1_k141_159720 | 1073 | No | NA | | 0 | 0 | Not-determined | Genome-fragment | NA | | 0 |
| S1_k141_226516 | 1112 | No | NA | | 0 | 0 | Not-determined | Genome-fragment | NA | | 0 |
| S5-final-viral-combined_k141_141440\|\|full | 1495 | No | NA | | 0 | 1 | Low-quality | Genome-fragment | | 0.6 | 0 |
| S1_k141_309349 | 2714 | No | NA | | 0 | 3 | Not-determined | Genome-fragment | NA | | 0 |
| S1_k141_28651 | 1099 | No | NA | | 0 | 0 | Not-determined | Genome-fragment | NA | | 0 |
| S1_k141_390771 | 3930 | No | NA | | 0 | 2 | Not-determined | Genome-fragment | NA | | 0 |
| S1_k141_275338 | 1760 | No | NA | | 0 | 2 | Not-determined | Genome-fragment | NA | | 0 |
| S1_k141_194079 | 1699 | No | NA | | 0 | 1 | Not-determined | Genome-fragment | NA | | 0 |
| S1_k141_309387 | 1094 | No | NA | | 0 | 0 | Not-determined | Genome-fragment | NA | | 0 |
| S7-final-viral-combined_k141_229060\|\|full | 1495 | No | NA | | 0 | 2 | Low-quality | Genome-fragment | | 0.69 | 0 |
| S7-final-viral-combined_k141_8849\|\|full | 1495 | No | NA | | 1 | 0 | Low-quality | Genome-fragment | | 2.52 | 0 |
| S7_k141_206056 | 1495 | No | NA | | 0 | 0 | Low-quality | Genome-fragment | | 2.46 | 0 |
| S1_k141_243165 | 1095 | No | NA | | 0 | 1 | Not-determined | Genome-fragment | NA | | 0 |
| S1_k141_12618 | 1095 | No | NA | | 0 | 0 | Not-determined | Genome-fragment | NA | | 0 |
| S1_k141_358941 | 1218 | No | NA | | 0 | 1 | Not-determined | Genome-fragment | NA | | 0 |
| S1_k141_374529 | 5331 | No | NA | | 0 | 0 | Not-determined | Genome-fragment | NA | | 0 |
| S1_k141_292821 | 3612 | No | NA | | 0 | 1 | Not-determined | Genome-fragment | NA | | 0 |
| S1_k141_275457 | 1833 | No | NA | | 0 | 0 | Not-determined | Genome-fragment | NA | | 0 |
| S1_k141_61850 | 1529 | No | NA | | 0 | 2 | Not-determined | Genome-fragment | NA | | 0 |
| S1_k141_260486 | 2048 | No | NA | | 0 | 1 | Not-determined | Genome-fragment | NA | | 0 |
| S1_k141_309489 | 15553 | No | NA | | 0 | 29 | Not-determined | Genome-fragment | NA | | 0 |
| S17-final-viral-combined_k141_212373\|\|full | 1495 | No | NA | | 1 | 0 | Low-quality | Genome-fragment | | 2.81 | 0 |
| S1_k141_260562 | 1821 | No | NA | | 0 | 0 | Not-determined | Genome-fragment | NA | | 0 |
| S17-final-viral-combined_k141_42262\|\|lt2gene | 1495 | No | NA | | 0 | 0 | Low-quality | Genome-fragment | | 3.6 | 0 |
| S1_k141_275573 | 3191 | No | NA | | 0 | 1 | Not-determined | Genome-fragment | NA | | 0 |
| S1_k141_374634 | 4127 | No | NA | | 0 | 3 | Not-determined | Genome-fragment | NA | | 0 |
| S1_k141_374645 | 1118 | No | NA | | 0 | 0 | Not-determined | Genome-fragment | NA | | 0 |
| S1_k141_226733 | 1245 | No | NA | | 0 | 1 | Not-determined | Genome-fragment | NA | | 0 |
| S1_k141_390889 | 3201 | No | NA | | 0 | 1 | Not-determined | Genome-fragment | NA | | 0 |
| S1_k141_45228 | 3358 | No | NA | | 0 | 1 | Not-determined | Genome-fragment | NA | | 0 |
| S1_k141_243304 | 3768 | No | NA | | 0 | 0 | Not-determined | Genome-fragment | NA | | 0 |
| S1_k141_210922 | 2912 | No | NA | | 0 | 0 | Not-determined | Genome-fragment | NA | | 0 |
| S1_k141_260609 | 1515 | No | NA | | 0 | 1 | Not-determined | Genome-fragment | NA | | 0 |
| S1_k141_390926 | 1012 | No | NA | | 0 | 0 | Not-determined | Genome-fragment | NA | | 0 |
| S1_k141_62006 | 1017 | No | NA | | 0 | 1 | Not-determined | Genome-fragment | NA | | 0 |
| S1_k141_326413 | 2039 | No | NA | | 0 | 2 | Not-determined | Genome-fragment | NA | | 0 |
| S1_k141_359149 | 1786 | No | NA | | 0 | 1 | Not-determined | Genome-fragment | NA | | 0 |
| S1_k141_28838 | 4081 | No | NA | | 0 | 3 | Not-determined | Genome-fragment | NA | | 0 |
| S1_k141_77616 | 3172 | No | NA | | 0 | 1 | Not-determined | Genome-fragment | NA | | 0 |
| S1_k141_326431 | 2272 | No | NA | | 0 | 1 | Not-determined | Genome-fragment | NA | | 0 |
| S1_k141_175978 | 1363 | No | NA | | 0 | 2 | Not-determined | Genome-fragment | NA | | 0 |
| S1_k141_293004 | 1210 | No | NA | | 0 | 1 | Not-determined | Genome-fragment | NA | | 0 |
| S12-final-viral-combined_k141_112142\|\|full | 1495 | No | NA | | 1 | 0 | Low-quality | Genome-fragment | | 3.83 | 0 |
| S1_k141_293017 | 1059 | No | NA | | 0 | 1 | Not-determined | Genome-fragment | NA | | 0 |
| S1_k141_110068 | 1237 | No | NA | | 0 | 1 | Not-determined | Genome-fragment | NA | | 0 |
| S1_k141_391039 | 1165 | No | NA | | 0 | 1 | Not-determined | Genome-fragment | NA | | 0 |
| S12-final-viral-combined_k141_199378\|\|lt2gene | 1495 | No | NA | | 1 | 0 | Low-quality | Genome-fragment | | 1.03 | 0 |
| S1_k141_309655 | 1303 | No | NA | | 0 | 1 | Not-determined | Genome-fragment | NA | | 0 |
| S4-final-viral-combined_k141_117761\|\|full | 1495 | No | NA | | 1 | 0 | Low-quality | Genome-fragment | | 3.06 | 0 |
| S1_k141_62127 | 2399 | No | NA | | 0 | 2 | Not-determined | Genome-fragment | NA | | 0 |
| S1_k141_293083 | 1890 | No | NA | | 0 | 0 | Not-determined | Genome-fragment | NA | | 0 |
| S8-final-viral-combined_k141_51325\|\|full | 1495 | No | NA | | 0 | 0 | Low-quality | Genome-fragment | | 2.89 | 0 |
| S9-final-viral-combined_k141_16218\|\|full | 1496 | No | NA | | 0 | 0 | Low-quality | Genome-fragment | | 3.28 | 0 |
| S1_k141_28888 | 4476 | No | NA | | 0 | 1 | Not-determined | Genome-fragment | NA | | 0 |
| S1_k141_128911 | 2034 | No | NA | | 0 | 0 | Not-determined | Genome-fragment | NA | | 0 |
| S1_k141_211091 | 4322 | No | NA | | 0 | 2 | Not-determined | Genome-fragment | NA | | 0 |
| S1_k141_62183 | 2386 | No | NA | | 0 | 0 | Not-determined | Genome-fragment | NA | | 0 |
| S1_k141_160035 | 1102 | No | NA | | 0 | 1 | Not-determined | Genome-fragment | NA | | 0 |
| S9_k141_54897 | 1496 | No | NA | | 0 | 1 | Low-quality | Genome-fragment | | 0.36 | 0 |
| S1_k141_95196 | 1089 | No | NA | | 0 | 1 | Not-determined | Genome-fragment | NA | | 0 |
| S11-final-viral-combined_k141_132896\|\|full | 1496 | No | NA | | 1 | 0 | Low-quality | Genome-fragment | | 2.59 | 0 |
| S1_k141_326581 | 1470 | No | NA | | 0 | 0 | Not-determined | Genome-fragment | NA | | 0 |
| S1_k141_309751 | 1370 | No | NA | | 0 | 0 | Not-determined | Genome-fragment | NA | | 0 |
| S1_k141_243544 | 1495 | No | NA | | 0 | 0 | Not-determined | Genome-fragment | NA | | 0 |
| S1_k141_260827 | 6744 | No | NA | | 0 | 5 | Not-determined | Genome-fragment | NA | | 0 |
| S1_k141_326612 | 1445 | No | NA | | 0 | 1 | Not-determined | Genome-fragment | NA | | 0 |
| S1_k141_226951 | 1016 | No | NA | | 0 | 0 | Not-determined | Genome-fragment | NA | | 0 |
| S1_k141_226972 | 1360 | No | NA | | 0 | 0 | Not-determined | Genome-fragment | NA | | 0 |
| S1_k141_128959 | 1014 | No | NA | | 0 | 0 | Not-determined | Genome-fragment | NA | | 0 |
| S1_k141_243593 | 3727 | No | NA | | 0 | 2 | Not-determined | Genome-fragment | NA | | 0 |
| S1_k141_260869 | 1525 | No | NA | | 0 | 1 | Not-determined | Genome-fragment | NA | | 0 |
| S1_k141_340983 | 3949 | No | NA | | 0 | 2 | Not-determined | Genome-fragment | NA | | 0 |
| S1_k141_45377 | 3897 | No | NA | | 0 | 2 | Not-determined | Genome-fragment | NA | | 0 |
| S1_k141_176069 | 1528 | No | NA | | 0 | 1 | Not-determined | Genome-fragment | NA | | 0 |
| S1_k141_95310 | 1184 | No | NA | | 0 | 1 | Not-determined | Genome-fragment | NA | | 0 |
| S1_k141_326640 | 1045 | No | NA | | 0 | 1 | Not-determined | Genome-fragment | NA | | 0 |
| S1_k141_326641 | 2582 | No | NA | | 0 | 2 | Not-determined | Genome-fragment | NA | | 0 |
| S1_k141_326642 | 1006 | No | NA | | 0 | 1 | Not-determined | Genome-fragment | NA | | 0 |
| S7-final-viral-combined_k141_153928\|\|full | 1496 | No | NA | | 0 | 0 | Low-quality | Genome-fragment | | 3.64 | 0 |
| S1_k141_341030 | 1113 | No | NA | | 0 | 1 | Not-determined | Genome-fragment | NA | | 0 |
| S1_k141_275854 | 2130 | No | NA | | 0 | 2 | Not-determined | Genome-fragment | NA | | 0 |
| S1_k141_341036 | 3201 | No | NA | | 0 | 4 | Not-determined | Genome-fragment | NA | | 0 |
| S1_k141_129074 | 1478 | No | NA | | 0 | 2 | Not-determined | Genome-fragment | NA | | 0 |
| S1_k141_12948 | 1213 | No | NA | | 0 | 1 | Not-determined | Genome-fragment | NA | | 0 |
| S1_k141_341047 | 1390 | No | NA | | 0 | 2 | Not-determined | Genome-fragment | NA | | 0 |
| S1_k141_160195 | 1298 | No | NA | | 0 | 2 | Not-determined | Genome-fragment | NA | | 0 |
| S1_k141_374939 | 2552 | No | NA | | 0 | 2 | Not-determined | Genome-fragment | NA | | 0 |
| S1_k141_194742 | 2163 | No | NA | | 0 | 1 | Not-determined | Genome-fragment | NA | | 0 |
| S1_k141_110334 | 1424 | No | NA | | 0 | 1 | Not-determined | Genome-fragment | NA | | 0 |
| S12-final-viral-combined_k141_178542\|\|full | 1496 | No | NA | | 0 | 1 | Low-quality | Genome-fragment | | 0.66 | 0 |
| S1_k141_129129 | 1026 | No | NA | | 0 | 2 | Not-determined | Genome-fragment | NA | | 0 |
| S1_k141_62367 | 3491 | No | NA | | 0 | 3 | Not-determined | Genome-fragment | NA | | 0 |
| S1_k141_211331 | 3503 | No | NA | | 0 | 1 | Not-determined | Genome-fragment | NA | | 0 |
| S1_k141_391178 | 1084 | No | NA | | 0 | 1 | Not-determined | Genome-fragment | NA | | 0 |
| S1_k141_160256 | 5257 | No | NA | | 0 | 2 | Not-determined | Genome-fragment | NA | | 0 |
| S12-final-viral-combined_k141_367003\|\|full | 1496 | No | NA | | 0 | 0 | Low-quality | Genome-fragment | | 14.48 | 0 |
| S1_k141_326769 | 1111 | No | NA | | 0 | 0 | Not-determined | Genome-fragment | NA | | 0 |
| S2-final-viral-combined_k141_106430\|\|full | 1496 | No | NA | | 0 | 0 | Low-quality | Genome-fragment | | 0.94 | 0 |
| S1_k141_261083 | 1243 | No | NA | | 0 | 0 | Not-determined | Genome-fragment | NA | | 0 |
| S1_k141_391222 | 1431 | No | NA | | 0 | 0 | Not-determined | Genome-fragment | NA | | 0 |
| S1_k141_243865 | 1102 | No | NA | | 0 | 0 | Not-determined | Genome-fragment | NA | | 0 |
| S2-final-viral-combined_k141_130373\|\|full | 1496 | No | NA | | 0 | 0 | Low-quality | Genome-fragment | | 1.67 | 0 |
| S1_k141_359534 | 1203 | No | NA | | 0 | 2 | Not-determined | Genome-fragment | NA | | 0 |
| S4_k141_118887 | 1496 | No | NA | | 0 | 0 | Low-quality | Genome-fragment | | 5.13 | 0 |
| S9-final-viral-combined_k141_67190\|\|full | 1497 | No | NA | | 2 | 0 | Low-quality | Genome-fragment | | 4.36 | 0 |
| S1_k141_391284 | 1162 | No | NA | | 0 | 1 | Not-determined | Genome-fragment | NA | | 0 |
| S1_k141_160400 | 1039 | No | NA | | 0 | 0 | Not-determined | Genome-fragment | NA | | 0 |
| S1-final-viral-combined_k141_164947\|\|full | 1497 | No | NA | | 1 | 0 | Low-quality | Genome-fragment | | 3.73 | 0 |
| S1_k141_160417 | 2391 | No | NA | | 0 | 0 | Not-determined | Genome-fragment | NA | | 0 |
| S1_k141_29170 | 1027 | No | NA | | 0 | 1 | Not-determined | Genome-fragment | NA | | 0 |
| S1-final-viral-combined_k141_225225\|\|full | 1497 | No | NA | | 0 | 0 | Low-quality | Genome-fragment | | 3.92 | 0 |
| S1_k141_29173 | 1820 | No | NA | | 0 | 0 | Not-determined | Genome-fragment | NA | | 0 |
| S1_k141_341226 | 1120 | No | NA | | 0 | 1 | Not-determined | Genome-fragment | NA | | 0 |
| S1_k141_375151 | 5140 | No | NA | | 0 | 2 | Not-determined | Genome-fragment | NA | | 0 |
| S1_k141_129364 | 1746 | No | NA | | 0 | 1 | Not-determined | Genome-fragment | NA | | 0 |
| S1_k141_326930 | 1795 | No | NA | | 0 | 1 | Not-determined | Genome-fragment | NA | | 0 |
| S5-final-viral-combined_k141_41153\|\|lt2gene | 1497 | No | NA | | 1 | 0 | Low-quality | Genome-fragment | | 2.82 | 0 |
| S1_k141_176397 | 12993 | No | NA | | 0 | 9 | Not-determined | Genome-fragment | NA | | 0 |
| S1_k141_243978 | 1014 | No | NA | | 0 | 0 | Not-determined | Genome-fragment | NA | | 0 |
| S1_k141_310117 | 3220 | No | NA | | 0 | 2 | Not-determined | Genome-fragment | NA | | 0 |
| S1_k141_95542 | 1038 | No | NA | | 0 | 2 | Not-determined | Genome-fragment | NA | | 0 |
| S1_k141_78008 | 1348 | No | NA | | 0 | 0 | Not-determined | Genome-fragment | NA | | 0 |
| S12-final-viral-combined_k141_86122\|\|full | 1497 | No | NA | | 0 | 1 | Low-quality | Genome-fragment | | 1.99 | 0 |
| S1_k141_13227 | 1078 | No | NA | | 0 | 1 | Not-determined | Genome-fragment | NA | | 0 |
| S1_k141_95552 | 2832 | No | NA | | 0 | 1 | Not-determined | Genome-fragment | NA | | 0 |
| S1_k141_176444 | 1995 | No | NA | | 0 | 0 | Not-determined | Genome-fragment | NA | | 0 |
| S1_k141_327001 | 1232 | No | NA | | 0 | 0 | Not-determined | Genome-fragment | NA | | 0 |
| S1_k141_129464 | 1851 | No | NA | | 0 | 2 | Not-determined | Genome-fragment | NA | | 0 |
| S12-final-viral-combined_k141_358784\|\|full | 1497 | No | NA | | 0 | 2 | Low-quality | Genome-fragment | | 0.45 | 0 |
| S12-final-viral-combined_k141_178475\|\|full | 1497 | No | NA | | 0 | 1 | Low-quality | Genome-fragment | | 1.14 | 0 |
| S12-final-viral-combined_k141_360894\|\|lt2gene | 1497 | No | NA | | 1 | 0 | Low-quality | Genome-fragment | | 0.93 | 0 |
| S2-final-viral-combined_k141_57555\|\|lt2gene | 1497 | Yes | | 826 | 1 | 1 | Low-quality | Genome-fragment | | 2.22 | 44.82 |
| S1_k141_293585 | 1476 | No | NA | | 0 | 1 | Not-determined | Genome-fragment | NA | | 0 |
| S1_k141_341372 | 1013 | No | NA | | 0 | 2 | Not-determined | Genome-fragment | NA | | 0 |
| S1_k141_227380 | 1215 | No | NA | | 0 | 0 | Not-determined | Genome-fragment | NA | | 0 |
| S1_k141_45686 | 2284 | No | NA | | 0 | 0 | Not-determined | Genome-fragment | NA | | 0 |
| S1_k141_129513 | 1566 | No | NA | | 0 | 1 | Not-determined | Genome-fragment | NA | | 0 |
| S1_k141_160613 | 1107 | No | NA | | 0 | 0 | Not-determined | Genome-fragment | NA | | 0 |
| S1_k141_359628 | 1324 | No | NA | | 0 | 1 | Not-determined | Genome-fragment | NA | | 0 |
| S1_k141_45699 | 2063 | No | NA | | 0 | 5 | Not-determined | Genome-fragment | NA | | 0 |
| S4-final-viral-combined_k141_141426\|\|full | 1497 | No | NA | | 0 | 0 | Low-quality | Genome-fragment | | 2.44 | 0 |
| S8-final-viral-combined_k141_243883\|\|full | 1497 | No | NA | | 0 | 0 | Low-quality | Genome-fragment | | 2.21 | 0 |
| S1_k141_375336 | 1872 | No | NA | | 0 | 2 | Not-determined | Genome-fragment | NA | | 0 |
| S9-final-viral-combined_k141_28619\|\|full | 1498 | No | NA | | 0 | 0 | Low-quality | Genome-fragment | | 2.6 | 0 |
| S1_k141_276289 | 1272 | No | NA | | 0 | 1 | Not-determined | Genome-fragment | NA | | 0 |
| S1_k141_195134 | 1280 | No | NA | | 0 | 1 | Not-determined | Genome-fragment | NA | | 0 |
| S1_k141_276317 | 1219 | No | NA | | 0 | 1 | Not-determined | Genome-fragment | NA | | 0 |
| S1-final-viral-combined_k141_49735\|\|full | 1498 | No | NA | | 0 | 2 | Low-quality | Genome-fragment | | 1.2 | 0 |
| S1_k141_327027 | 1317 | No | NA | | 0 | 0 | Not-determined | Genome-fragment | NA | | 0 |
| S1-final-viral-combined_k141_39761\|\|full | 1498 | No | NA | | 1 | 0 | Low-quality | Genome-fragment | | 3.25 | 0 |

| | | | | | | | | | |
|---|---|---|---|---|---|---|---|---|---|
| S1_k141_95750 | 1603 | No | NA | 0 | 0 | Not-determined | Genome-fragment | NA | 0 |
| S1-final-viral-combined_k141_156401\|\|full | 1498 | No | NA | 0 | 1 | Low-quality | Genome-fragment | 0.72 | 0 |
| S1_k141_293684 | 1160 | No | NA | 0 | 1 | Not-determined | Genome-fragment | NA | 0 |
| S1_k141_95764 | 1266 | No | NA | 0 | 1 | Not-determined | Genome-fragment | NA | 0 |
| S1_k141_13398 | 1129 | No | NA | 0 | 1 | Not-determined | Genome-fragment | NA | 0 |
| S1_k141_145114 | 1036 | No | NA | 0 | 1 | Not-determined | Genome-fragment | NA | 0 |
| S1_k141_126124 | 1498 | No | NA | 2 | 0 | Low-quality | Genome-fragment | 2.67 | 0 |
| S5-final-viral-combined_k141_206570\|\|full | 1498 | No | NA | 2 | 0 | Low-quality | Genome-fragment | 2.26 | 0 |
| S1_k141_391556 | 4989 | No | NA | 0 | 0 | Not-determined | Genome-fragment | NA | 0 |
| S1_k141_327075 | 3752 | No | NA | 0 | 3 | Not-determined | Genome-fragment | NA | 0 |
| S1_k141_391561 | 1173 | No | NA | 0 | 2 | Not-determined | Genome-fragment | NA | 0 |
| S17-final-viral-combined_k141_147556\|\|full | 1498 | No | NA | 0 | 0 | Low-quality | Genome-fragment | 3.03 | 0 |
| S17-final-viral-combined_k141_9346\|\|full | 1498 | No | NA | 1 | 0 | Low-quality | Genome-fragment | 2.79 | 0 |
| S1_k141_244325 | 1720 | No | NA | 0 | 0 | Not-determined | Genome-fragment | NA | 0 |
| S12-final-viral-combined_k141_323134\|\|full | 1498 | No | NA | 0 | 1 | Low-quality | Genome-fragment | 1.9 | 0 |
| S1_k141_293772 | 1081 | No | NA | 0 | 0 | Not-determined | Genome-fragment | NA | 0 |
| S12-final-viral-combined_k141_139936\|\|full | 1498 | No | NA | 0 | 2 | Low-quality | Genome-fragment | 1.79 | 0 |
| S1_k141_391603 | 1033 | No | NA | 0 | 0 | Not-determined | Genome-fragment | NA | 0 |
| S2-final-viral-combined_k141_45544\|\|full | 1498 | No | NA | 0 | 0 | Low-quality | Genome-fragment | 2.45 | 0 |
| S1_k141_78232 | 1459 | No | NA | 0 | 1 | Not-determined | Genome-fragment | NA | 0 |
| S1_k141_176690 | 1838 | No | NA | 0 | 1 | Not-determined | Genome-fragment | NA | 0 |
| S1_k141_95887 | 1241 | No | NA | 0 | 1 | Not-determined | Genome-fragment | NA | 0 |
| S1_k141_375450 | 1889 | No | NA | 0 | 0 | Not-determined | Genome-fragment | NA | 0 |
| S1_k141_95889 | 1060 | No | NA | 0 | 0 | Not-determined | Genome-fragment | NA | 0 |
| S1_k141_62722 | 1218 | No | NA | 0 | 0 | Not-determined | Genome-fragment | NA | 0 |
| S2-final-viral-combined_k141_67332\|\|lt2gene | 1498 | No | NA | 1 | 0 | Low-quality | Genome-fragment | 4.01 | 0 |
| S1_k141_212042 | 1251 | No | NA | 0 | 0 | Not-determined | Genome-fragment | NA | 0 |
| S1_k141_29288 | 47233 | No | NA | 17 | 1 | Medium-quality | Genome-fragment | 87 | 0 |
| S4-final-viral-combined_k141_53823\|\|full | 1498 | No | NA | 2 | 0 | Low-quality | Genome-fragment | 2.33 | 0 |
| S8-final-viral-combined_k141_36235\|\|full | 1498 | No | NA | 0 | 0 | Low-quality | Genome-fragment | 1.74 | 0 |
| S1_k141_276545 | 5999 | No | NA | 0 | 3 | Not-determined | Genome-fragment | NA | 0 |
| S9-final-viral-combined_k141_96345\|\|full | 1499 | No | NA | 0 | 0 | Low-quality | Genome-fragment | 17.03 | 0 |
| S1_k141_45965 | 1039 | No | NA | 0 | 1 | Not-determined | Genome-fragment | NA | 0 |
| S1_k141_227687 | 1032 | No | NA | 0 | 0 | Not-determined | Genome-fragment | NA | 0 |
| S1_k141_46004 | 2690 | No | NA | 0 | 1 | Not-determined | Genome-fragment | NA | 0 |
| S1_k141_78367 | 3977 | No | NA | 0 | 1 | Not-determined | Genome-fragment | NA | 0 |
| S11-final-viral-combined_k141_99899\|\|full | 1499 | No | NA | 0 | 2 | Low-quality | Genome-fragment | 1.27 | 0 |
| S1_k141_96021 | 1215 | No | NA | 0 | 0 | Not-determined | Genome-fragment | NA | 0 |
| S1_k141_244547 | 1010 | No | NA | 0 | 0 | Not-determined | Genome-fragment | NA | 0 |
| S1_k141_145419 | 4246 | No | NA | 0 | 2 | Not-determined | Genome-fragment | NA | 0 |
| S11-final-viral-combined_k141_257859\|\|full | 1499 | No | NA | 0 | 0 | Low-quality | Genome-fragment | 1.11 | 0 |
| S1_k141_195463 | 1044 | No | NA | 0 | 0 | Not-determined | Genome-fragment | NA | 0 |
| S1_k141_227782 | 7723 | No | NA | 0 | 5 | Not-determined | Genome-fragment | NA | 0 |
| S1_k141_227794 | 3850 | No | NA | 0 | 3 | Not-determined | Genome-fragment | NA | 0 |
| S7_k141_113762 | 1499 | No | NA | 0 | 0 | Low-quality | Genome-fragment | 2.47 | 0 |
| S1_k141_359974 | 1105 | No | NA | 0 | 0 | Not-determined | Genome-fragment | NA | 0 |
| S17-k141_173680 | 1499 | No | NA | 0 | 0 | Low-quality | Genome-fragment | 2.39 | 0 |
| S12_k141_195817 | 1499 | No | NA | 0 | 0 | Low-quality | Genome-fragment | 0.81 | 0 |
| S1_k141_161085 | 1050 | No | NA | 0 | 1 | Not-determined | Genome-fragment | NA | 0 |
| S1_k141_195503 | 1414 | No | NA | 0 | 1 | Not-determined | Genome-fragment | NA | 0 |
| S1_k141_310407 | 5153 | No | NA | 0 | 1 | Not-determined | Genome-fragment | NA | 0 |
| S1_k141_129998 | 2585 | No | NA | 0 | 1 | Not-determined | Genome-fragment | NA | 0 |
| S12_k141_113847 | 1499 | No | NA | 0 | 0 | Low-quality | Genome-fragment | 1.06 | 0 |
| S2-final-viral-combined_k141_90169\|\|lt2gene | 1499 | No | NA | 0 | 0 | Low-quality | Genome-fragment | 3.87 | 0 |
| S1_k141_161120 | 1849 | No | NA | 0 | 0 | Not-determined | Genome-fragment | NA | 0 |
| S1_k141_261711 | 1668 | No | NA | 0 | 0 | Not-determined | Genome-fragment | NA | 0 |
| S1_k141_391799 | 1328 | No | NA | 0 | 0 | Not-determined | Genome-fragment | NA | 0 |
| S1_k141_176978 | 1219 | No | NA | 0 | 0 | Not-determined | Genome-fragment | NA | 0 |
| S1_k141_13787 | 2373 | No | NA | 0 | 1 | Not-determined | Genome-fragment | NA | 0 |
| S4_k141_2702 | 1499 | No | NA | 0 | 1 | Low-quality | Genome-fragment | 0.42 | 0 |
| S1_k141_176981 | 1819 | No | NA | 0 | 1 | Not-determined | Genome-fragment | NA | 0 |
| S4_k141_108384 | 1499 | No | NA | 1 | 0 | Low-quality | Genome-fragment | 2.39 | 0 |
| S1_k141_294057 | 2444 | No | NA | 0 | 1 | Not-determined | Genome-fragment | NA | 0 |
| S1_k141_195565 | 2408 | No | NA | 0 | 2 | Not-determined | Genome-fragment | NA | 0 |
| S1_k141_375674 | 6554 | No | NA | 0 | 5 | Not-determined | Genome-fragment | NA | 0 |
| S8-final-viral-combined_k141_199834\|\|full | 1499 | No | NA | 0 | 0 | Low-quality | Genome-fragment | 1.75 | 0 |
| S1_k141_227834 | 1284 | No | NA | 0 | 1 | Not-determined | Genome-fragment | NA | 0 |
| S1_k141_294068 | 1230 | No | NA | 0 | 1 | Not-determined | Genome-fragment | NA | 0 |
| S1_k141_78484 | 1123 | No | NA | 0 | 0 | Not-determined | Genome-fragment | NA | 0 |
| S1_k141_261759 | 2456 | No | NA | 0 | 0 | Not-determined | Genome-fragment | NA | 0 |
| S1_k141_227847 | 1023 | No | NA | 0 | 1 | Not-determined | Genome-fragment | NA | 0 |
| S9-final-viral-combined_k141_128110\|\|full | 1500 | No | NA | 1 | 0 | Low-quality | Genome-fragment | 3.78 | 0 |
| S1_k141_176997 | 1277 | No | NA | 0 | 2 | Not-determined | Genome-fragment | NA | 0 |
| S9_k141_99042 | 1500 | No | NA | 0 | 0 | Low-quality | Genome-fragment | 2.86 | 0 |
| S1_k141_360071 | 3382 | No | NA | 0 | 1 | Not-determined | Genome-fragment | NA | 0 |
| S11-final-viral-combined_k141_312160\|\|full | 1500 | No | NA | 0 | 0 | Low-quality | Genome-fragment | 4.1 | 0 |
| S1_k141_46230 | 2004 | No | NA | 0 | 0 | Not-determined | Genome-fragment | NA | 0 |
| S1_k141_145610 | 2271 | No | NA | 0 | 1 | Not-determined | Genome-fragment | NA | 0 |
| S1_k141_195618 | 3248 | No | NA | 0 | 2 | Not-determined | Genome-fragment | NA | 0 |
| S1_k141_111171 | 1398 | No | NA | 0 | 1 | Not-determined | Genome-fragment | NA | 0 |
| S1_k141_130155 | 2432 | No | NA | 0 | 2 | Not-determined | Genome-fragment | NA | 0 |
| S11-final-viral-combined_k141_232933\|\|full | 1500 | No | NA | 1 | 0 | Low-quality | Genome-fragment | 0.95 | 0 |
| S1_k141_294133 | 3582 | No | NA | 0 | 2 | Not-determined | Genome-fragment | NA | 0 |
| S1_k141_261867 | 1067 | No | NA | 0 | 1 | Not-determined | Genome-fragment | NA | 0 |
| S1_k141_78535 | 1800 | No | NA | 0 | 2 | Not-determined | Genome-fragment | NA | 0 |
| S1_k141_244836 | 31397 | No | NA | 9 | 0 | Medium-quality | Genome-fragment | 57.64 | 0 |
| S1-final-viral-combined_k141_149900\|\|full | 1500 | No | NA | 1 | 0 | Low-quality | Genome-fragment | 29.92 | 0 |
| S1-final-viral-combined_k141_283377\|\|full | 1500 | No | NA | 0 | 0 | Low-quality | Genome-fragment | 0.84 | 0 |
| S3-final-viral-combined_k141_80656\|\|full | 1500 | No | NA | 1 | 0 | Low-quality | Genome-fragment | 2.48 | 0 |
| S1_k141_391933 | 1792 | No | NA | 0 | 1 | Not-determined | Genome-fragment | NA | 0 |
| S5-final-viral-combined_k141_195413\|\|full | 1500 | No | NA | 0 | 1 | Low-quality | Genome-fragment | 3.73 | 0 |
| S1_k141_212405 | 1959 | No | NA | 0 | 3 | Not-determined | Genome-fragment | NA | 0 |
| S7-final-viral-combined_k141_204684\|\|full | 1500 | No | NA | 0 | 1 | Low-quality | Genome-fragment | 2.07 | 0 |
| S1_k141_276846 | 1148 | No | NA | 0 | 1 | Not-determined | Genome-fragment | NA | 0 |
| S1_k141_195748 | 1074 | No | NA | 0 | 0 | Not-determined | Genome-fragment | NA | 0 |
| S1_k141_212430 | 1009 | No | NA | 0 | 1 | Not-determined | Genome-fragment | NA | 0 |
| S1_k141_161382 | 18199 | No | NA | 0 | 31 | Not-determined | Genome-fragment | NA | 0 |
| S1_k141_391955 | 4268 | No | NA | 0 | 1 | Not-determined | Genome-fragment | NA | 0 |
| S1_k141_130214 | 40887 | No | NA | 18 | 0 | Complete | High-quality | 100 | 0 |
| S1_k141_261998 | 1025 | No | NA | 0 | 1 | Not-determined | Genome-fragment | NA | 0 |
| S1_k141_294179 | 1674 | No | NA | 0 | 1 | Not-determined | Genome-fragment | NA | 0 |
| S1_k141_227982 | 1192 | No | NA | 0 | 0 | Not-determined | Genome-fragment | NA | 0 |
| S17-final-viral-combined_k141_105422\|\|full | 1500 | No | NA | 2 | 0 | Low-quality | Genome-fragment | 4.02 | 0 |
| S1_k141_46269 | 2494 | No | NA | 0 | 0 | Not-determined | Genome-fragment | NA | 0 |
| S1_k141_145690 | 1067 | No | NA | 0 | 1 | Not-determined | Genome-fragment | NA | 0 |
| S1_k141_360250 | 1158 | No | NA | 0 | 0 | Not-determined | Genome-fragment | NA | 0 |
| S1_k141_111310 | 1092 | No | NA | 0 | 1 | Not-determined | Genome-fragment | NA | 0 |
| S1_k141_375905 | 8836 | No | NA | 0 | 8 | Not-determined | Genome-fragment | NA | 0 |
| S1_k141_276908 | 1174 | No | NA | 0 | 0 | Not-determined | Genome-fragment | NA | 0 |
| S1_k141_96258 | 1165 | No | NA | 0 | 1 | Not-determined | Genome-fragment | NA | 0 |
| S1_k141_310660 | 1750 | No | NA | 0 | 0 | Not-determined | Genome-fragment | NA | 0 |
| S12-final-viral-combined_k141_169768\|\|full | 1500 | No | NA | 0 | 0 | Low-quality | Genome-fragment | 2.17 | 0 |
| S1_k141_228047 | 1332 | No | NA | 0 | 0 | Not-determined | Genome-fragment | NA | 0 |
| S1_k141_276965 | 1004 | No | NA | 0 | 1 | Not-determined | Genome-fragment | NA | 0 |
| S12_k141_341736 | 1500 | No | NA | 0 | 0 | Low-quality | Genome-fragment | 0.79 | 0 |
| S1_k141_130313 | 1031 | No | NA | 0 | 3 | Not-determined | Genome-fragment | NA | 0 |
| S2_k141_11138 | 1500 | No | NA | 0 | 0 | Low-quality | Genome-fragment | 3.08 | 0 |
| S4-final-viral-combined_k141_104933\|\|full | 1500 | No | NA | 0 | 2 | Low-quality | Genome-fragment | 0.93 | 0 |
| S11_k141_326311 | 1501 | No | NA | 0 | 0 | Low-quality | Genome-fragment | 2.72 | 0 |
| S1_k141_277010 | 3006 | No | NA | 0 | 2 | Not-determined | Genome-fragment | NA | 0 |
| S1_k141_228123 | 1023 | No | NA | 0 | 1 | Not-determined | Genome-fragment | NA | 0 |
| S1_k141_195983 | 2023 | No | NA | 0 | 0 | Not-determined | Genome-fragment | NA | 0 |
| S1_k141_78677 | 6683 | No | NA | 0 | 2 | Not-determined | Genome-fragment | NA | 0 |
| S1_k141_111408 | 4537 | No | NA | 0 | 4 | Not-determined | Genome-fragment | NA | 0 |
| S1_k141_63344 | 1850 | No | NA | 0 | 1 | Not-determined | Genome-fragment | NA | 0 |
| S3-final-viral-combined_k141_103080\|\|full | 1501 | No | NA | 0 | 0 | Low-quality | Genome-fragment | 2.29 | 0 |
| S1_k141_245059 | 1039 | No | NA | 0 | 1 | Not-determined | Genome-fragment | NA | 0 |
| S1_k141_327344 | 1012 | No | NA | 0 | 1 | Not-determined | Genome-fragment | NA | 0 |
| S1_k141_46367 | 1021 | No | NA | 0 | 2 | Not-determined | Genome-fragment | NA | 0 |
| S1_k141_294358 | 1383 | No | NA | 0 | 1 | Not-determined | Genome-fragment | NA | 0 |
| S1_k141_376106 | 1512 | No | NA | 0 | 1 | Not-determined | Genome-fragment | NA | 0 |
| S1_k141_177495 | 1135 | No | NA | 0 | 1 | Not-determined | Genome-fragment | NA | 0 |
| S1_k141_145933 | 2124 | No | NA | 0 | 1 | Not-determined | Genome-fragment | NA | 0 |
| S1_k141_14189 | 1082 | No | NA | 0 | 0 | Not-determined | Genome-fragment | NA | 0 |
| S1_k141_392096 | 1198 | No | NA | 0 | 0 | Not-determined | Genome-fragment | NA | 0 |
| S17-final-viral-combined_k141_115834\|\|full | 1501 | No | NA | 0 | 0 | Low-quality | Genome-fragment | 0.77 | 0 |
| S1_k141_96375 | 1200 | No | NA | 0 | 2 | Not-determined | Genome-fragment | NA | 0 |
| S1_k141_46476 | 1208 | No | NA | 0 | 1 | Not-determined | Genome-fragment | NA | 0 |
| S1_k141_177565 | 1122 | No | NA | 0 | 0 | Not-determined | Genome-fragment | NA | 0 |
| S1_k141_392141 | 1320 | No | NA | 0 | 2 | Not-determined | Genome-fragment | NA | 0 |
| S1_k141_294466 | 1169 | No | NA | 0 | 0 | Not-determined | Genome-fragment | NA | 0 |
| S1_k141_342311 | 1755 | No | NA | 0 | 1 | Not-determined | Genome-fragment | NA | 0 |
| S1_k141_262279 | 1716 | No | NA | 0 | 1 | Not-determined | Genome-fragment | NA | 0 |
| S1_k141_245143 | 1568 | No | NA | 0 | 1 | Not-determined | Genome-fragment | NA | 0 |
| S1_k141_376211 | 1044 | No | NA | 0 | 1 | Not-determined | Genome-fragment | NA | 0 |
| S1_k141_161698 | 4421 | No | NA | 0 | 0 | Not-determined | Genome-fragment | NA | 0 |
| S17-final-viral-combined_k141_93626\|\|full | 1501 | No | NA | 0 | 0 | Low-quality | Genome-fragment | 2.65 | 0 |
| S17-k141_170506 | 1501 | No | NA | 5 | 0 | Low-quality | Genome-fragment | 4.96 | 0 |
| S1_k141_14279 | 1231 | No | NA | 0 | 2 | Not-determined | Genome-fragment | NA | 0 |
| S12-final-viral-combined_k141_262051\|\|full | 1501 | No | NA | 0 | 1 | Low-quality | Genome-fragment | 1.27 | 0 |
| S1_k141_196198 | 1095 | No | NA | 0 | 0 | Not-determined | Genome-fragment | NA | 0 |
| S1_k141_277239 | 3374 | No | NA | 0 | 1 | Not-determined | Genome-fragment | NA | 0 |
| S1_k141_262373 | 1086 | No | NA | 0 | 1 | Not-determined | Genome-fragment | NA | 0 |
| S1_k141_177647 | 17690 | No | NA | 0 | 2 | Not-determined | Genome-fragment | NA | 0 |
| S12_k141_269148 | 1501 | No | NA | 0 | 1 | Low-quality | Genome-fragment | 0.39 | 0 |
| S1_k141_196243 | 2796 | No | NA | 0 | 2 | Not-determined | Genome-fragment | NA | 0 |
| S4-final-viral-combined_k141_69810\|\|full | 1501 | Yes | 1348 | 1 | 1 | Low-quality | Genome-fragment | 0.83 | 10.19 |
| S1_k141_311020 | 1411 | No | NA | 0 | 1 | Not-determined | Genome-fragment | NA | 0 |
| S1_k141_327599 | 1680 | No | NA | 0 | 0 | Not-determined | Genome-fragment | NA | 0 |
| S1_k141_262465 | 1127 | No | NA | 0 | 2 | Not-determined | Genome-fragment | NA | 0 |
| S1_k141_63663 | 1434 | No | NA | 0 | 1 | Not-determined | Genome-fragment | NA | 0 |
| S1_k141_14348 | 1790 | No | NA | 0 | 2 | Not-determined | Genome-fragment | NA | 0 |
| S1_k141_96601 | 1236 | No | NA | 0 | 0 | Not-determined | Genome-fragment | NA | 0 |
| S1_k141_130561 | 1251 | No | NA | 0 | 1 | Not-determined | Genome-fragment | NA | 0 |
| S8-final-viral-combined_k141_116701\|\|full | 1501 | No | NA | 0 | 0 | Low-quality | Genome-fragment | 2.52 | 0 |
| S1_k141_245375 | 1119 | No | NA | 0 | 2 | Not-determined | Genome-fragment | NA | 0 |
| S1_k141_262497 | 1172 | No | NA | 0 | 2 | Not-determined | Genome-fragment | NA | 0 |
| S1_k141_342547 | 1450 | No | NA | 0 | 1 | Not-determined | Genome-fragment | NA | 0 |
| S1_k141_130621 | 1353 | No | NA | 0 | 0 | Not-determined | Genome-fragment | NA | 0 |
| S1_k141_262516 | 1063 | No | NA | 0 | 0 | Not-determined | Genome-fragment | NA | 0 |
| S1_k141_196400 | 3263 | No | NA | 0 | 1 | Not-determined | Genome-fragment | NA | 0 |
| S1_k141_14392 | 1641 | No | NA | 0 | 1 | Not-determined | Genome-fragment | NA | 0 |
| S8-final-viral-combined_k141_209772\|\|full | 1501 | No | NA | 1 | 0 | Low-quality | Genome-fragment | 3.28 | 0 |
| S1_k141_96725 | 1282 | No | NA | 0 | 0 | Not-determined | Genome-fragment | NA | 0 |

| | | | | | | | | | | | |
|---|---|---|---|---|---|---|---|---|---|---|---|
| S1_k141_294751 | 1630 | No | NA | | 0 | 1 | Not-determined | Genome-fragment | NA | | 0 |
| S1_k141_327733 | 3107 | No | NA | | 0 | 0 | Not-determined | Genome-fragment | NA | | 0 |
| S1_k141_376420 | 2050 | No | NA | | 0 | 1 | Not-determined | Genome-fragment | NA | | 0 |
| S1_k141_79081 | 5022 | No | NA | | 0 | 2 | Not-determined | Genome-fragment | NA | | 0 |
| S1_k141_14441 | 1258 | No | NA | | 0 | 2 | Not-determined | Genome-fragment | NA | | 0 |
| S9-final-viral-combined_k141_11752\|\|full | 1502 | No | NA | | 0 | 0 | Low-quality | Genome-fragment | | 3.88 | 0 |
| S1_k141_177897 | 1018 | No | NA | | 0 | 1 | Not-determined | Genome-fragment | NA | | 0 |
| S1_k141_130686 | 1309 | No | NA | | 0 | 1 | Not-determined | Genome-fragment | NA | | 0 |
| S1_k141_342628 | 2412 | No | NA | | 0 | 1 | Not-determined | Genome-fragment | NA | | 0 |
| S1_k141_212940 | 59765 | No | NA | | 36 | 1 | High-quality | High-quality | | 99.32 | 0 |
| S1_k141_342631 | 1772 | No | NA | | 0 | 1 | Not-determined | Genome-fragment | NA | | 0 |
| S1_k141_63756 | 7597 | No | NA | | 0 | 2 | Not-determined | Genome-fragment | NA | | 0 |
| S1_k141_294822 | 1089 | No | NA | | 0 | 1 | Not-determined | Genome-fragment | NA | | 0 |
| S11-final-viral-combined_k141_190355\|\|full | 1502 | No | NA | | 0 | 0 | Low-quality | Genome-fragment | | 0.95 | 0 |
| S1_k141_360724 | 1439 | No | NA | | 0 | 1 | Not-determined | Genome-fragment | NA | | 0 |
| S1_k141_392479 | 2312 | No | NA | | 0 | 1 | Not-determined | Genome-fragment | NA | | 0 |
| S1_k141_360751 | 1295 | No | NA | | 0 | 0 | Not-determined | Genome-fragment | NA | | 0 |
| S1_k141_177981 | 1124 | No | NA | | 0 | 1 | Not-determined | Genome-fragment | NA | | 0 |
| S1-final-viral-combined_k141_271638\|\|full | 1502 | No | NA | | 3 | 0 | Low-quality | Genome-fragment | | 1.1 | 0 |
| S1_k141_311393 | 1329 | No | NA | | 0 | 1 | Not-determined | Genome-fragment | NA | | 0 |
| S1_k141_311408 | 2492 | No | NA | | 0 | 0 | Not-determined | Genome-fragment | NA | | 0 |
| S1_k141_327852 | 7740 | No | NA | | 0 | 6 | Not-determined | Genome-fragment | NA | | 0 |
| S3-final-viral-combined_k141_40271\|\|full | 1502 | No | NA | | 0 | 1 | Low-quality | Genome-fragment | | 0.81 | 0 |
| S7_k141_78975 | 1502 | No | NA | | 0 | 0 | Low-quality | Genome-fragment | | 1.99 | 0 |
| S1_k141_342775 | 1033 | No | NA | | 0 | 0 | Not-determined | Genome-fragment | NA | | 0 |
| S1_k141_277497 | 1026 | No | NA | | 0 | 0 | Not-determined | Genome-fragment | NA | | 0 |
| S1_k141_376568 | 1194 | No | NA | | 0 | 0 | Not-determined | Genome-fragment | NA | | 0 |
| S1_k141_327866 | 1332 | No | NA | | 0 | 0 | Not-determined | Genome-fragment | NA | | 0 |
| S1_k141_96922 | 1200 | No | NA | | 0 | 0 | Not-determined | Genome-fragment | NA | | 0 |
| S1_k141_327894 | 3459 | No | NA | | 0 | 1 | Not-determined | Genome-fragment | NA | | 0 |
| S1_k141_178064 | 2554 | No | NA | | 0 | 1 | Not-determined | Genome-fragment | NA | | 0 |
| S1_k141_360859 | 4809 | No | NA | | 0 | 6 | Not-determined | Genome-fragment | NA | | 0 |
| S1_k141_162184 | 1000 | No | NA | | 0 | 0 | Not-determined | Genome-fragment | NA | | 0 |
| S1_k141_360866 | 3023 | No | NA | | 0 | 2 | Not-determined | Genome-fragment | NA | | 0 |
| S1_k141_360872 | 3325 | No | NA | | 0 | 0 | Not-determined | Genome-fragment | NA | | 0 |
| S1_k141_162208 | 1073 | No | NA | | 0 | 1 | Not-determined | Genome-fragment | NA | | 0 |
| S1_k141_228536 | 7871 | No | NA | | 0 | 1 | Not-determined | Genome-fragment | NA | | 0 |
| S1_k141_162216 | 1256 | No | NA | | 0 | 0 | Not-determined | Genome-fragment | NA | | 0 |
| S1_k141_178112 | 1000 | No | NA | | 0 | 1 | Not-determined | Genome-fragment | NA | | 0 |
| S1_k141_327924 | 1011 | No | NA | | 0 | 0 | Not-determined | Genome-fragment | NA | | 0 |
| S1_k141_311510 | 1184 | No | NA | | 0 | 1 | Not-determined | Genome-fragment | NA | | 0 |
| S1_k141_392641 | 1358 | No | NA | | 0 | 1 | Not-determined | Genome-fragment | NA | | 0 |
| S1_k141_146322 | 1602 | No | NA | | 0 | 0 | Not-determined | Genome-fragment | NA | | 0 |
| S1_k141_327945 | 1554 | No | NA | | 0 | 2 | Not-determined | Genome-fragment | NA | | 0 |
| S1_k141_262779 | 6432 | No | NA | | 0 | 5 | Not-determined | Genome-fragment | NA | | 0 |
| S1_k141_376598 | 3395 | No | NA | | 0 | 3 | Not-determined | Genome-fragment | NA | | 0 |
| S1_k141_14739 | 1084 | No | NA | | 0 | 1 | Not-determined | Genome-fragment | NA | | 0 |
| S1_k141_311577 | 2212 | No | NA | | 0 | 2 | Not-determined | Genome-fragment | NA | | 0 |
| S17-final-viral-combined_k141_160420\|\|full | 1502 | No | NA | | 0 | 0 | Low-quality | Genome-fragment | | 2.85 | 0 |
| S12-final-viral-combined_k141_72995\|\|full | 1502 | No | NA | | 2 | 0 | Low-quality | Genome-fragment | | 2.85 | 0 |
| S1_k141_392682 | 4645 | No | NA | | 0 | 3 | Not-determined | Genome-fragment | NA | | 0 |
| S1_k141_130956 | 1307 | No | NA | | 0 | 1 | Not-determined | Genome-fragment | NA | | 0 |
| S1_k141_328014 | 4896 | No | NA | | 0 | 0 | Not-determined | Genome-fragment | NA | | 0 |
| S12-final-viral-combined_k141_360622\|\|full | 1502 | No | NA | | 1 | 0 | Low-quality | Genome-fragment | | 1.01 | 0 |
| S1_k141_162338 | 2051 | No | NA | | 0 | 3 | Not-determined | Genome-fragment | NA | | 0 |
| S1_k141_14800 | 1234 | No | NA | | 0 | 2 | Not-determined | Genome-fragment | NA | | 0 |
| S1_k141_178216 | 3099 | No | NA | | 0 | 1 | Not-determined | Genome-fragment | NA | | 0 |
| S12_k141_251467 | 1502 | No | NA | | 0 | 1 | Low-quality | Genome-fragment | | 0.52 | 0 |
| S1_k141_328051 | 1010 | No | NA | | 0 | 0 | Not-determined | Genome-fragment | NA | | 0 |
| S1_k141_146415 | 1306 | No | NA | | 0 | 0 | Not-determined | Genome-fragment | NA | | 0 |
| S8_k141_206093 | 1502 | No | NA | | 1 | 0 | Low-quality | Genome-fragment | | 1.9 | 0 |
| S1_k141_392732 | 10728 | No | NA | | 0 | 2 | Not-determined | Genome-fragment | NA | | 0 |
| S9-final-viral-combined_k141_7397\|\|full | 1503 | No | NA | | 0 | 0 | Low-quality | Genome-fragment | | 4.32 | 0 |
| S1_k141_328073 | 3330 | No | NA | | 0 | 3 | Not-determined | Genome-fragment | NA | | 0 |
| S1_k141_245951 | 1837 | No | NA | | 0 | 1 | Not-determined | Genome-fragment | NA | | 0 |
| S11_k141_265268 | 1503 | No | NA | | 0 | 0 | Low-quality | Genome-fragment | | 3.89 | 0 |
| S1_k141_79418 | 1438 | No | NA | | 0 | 1 | Not-determined | Genome-fragment | NA | | 0 |
| S1_k141_178281 | 1001 | No | NA | | 0 | 0 | Not-determined | Genome-fragment | NA | | 0 |
| S1_k141_392753 | 1310 | No | NA | | 0 | 1 | Not-determined | Genome-fragment | NA | | 0 |
| S1-final-viral-combined_k141_207568\|\|full | 1503 | No | NA | | 0 | 2 | Low-quality | Genome-fragment | | 3.41 | 0 |
| S1_k141_178317 | 1883 | No | NA | | 0 | 1 | Not-determined | Genome-fragment | NA | | 0 |
| S1_k141_262816 | 1896 | No | NA | | 0 | 2 | Not-determined | Genome-fragment | NA | | 0 |
| S1_k141_361016 | 3758 | No | NA | | 0 | 1 | Not-determined | Genome-fragment | NA | | 0 |
| S1_k141_64130 | 1531 | No | NA | | 0 | 1 | Not-determined | Genome-fragment | NA | | 0 |
| S1_k141_311756 | 1931 | No | NA | | 0 | 0 | Not-determined | Genome-fragment | NA | | 0 |
| S1_k141_311758 | 1054 | No | NA | | 0 | 0 | Not-determined | Genome-fragment | NA | | 0 |
| S3-final-viral-combined_k141_114241\|\|lt2gene | 1503 | No | NA | | 1 | 0 | Low-quality | Genome-fragment | | 2.84 | 0 |
| S1_k141_178353 | 2750 | No | NA | | 0 | 1 | Not-determined | Genome-fragment | NA | | 0 |
| S1_k141_178375 | 1341 | No | NA | | 0 | 0 | Not-determined | Genome-fragment | NA | | 0 |
| S1_k141_262846 | 1710 | No | NA | | 0 | 1 | Not-determined | Genome-fragment | NA | | 0 |
| S17-final-viral-combined_k141_128071\|\|full | 1503 | No | NA | | 1 | 0 | Low-quality | Genome-fragment | | 3.67 | 0 |
| S4-final-viral-combined_k141_34137\|\|full | 1503 | No | NA | | 0 | 0 | Low-quality | Genome-fragment | | 2.45 | 0 |
| S1_k141_213084 | 1865 | No | NA | | 0 | 1 | Not-determined | Genome-fragment | NA | | 0 |
| S1_k141_97218 | 1612 | No | NA | | 0 | 3 | Not-determined | Genome-fragment | NA | | 0 |
| S8-final-viral-combined_k141_29165\|\|full | 1503 | No | NA | | 1 | 0 | Low-quality | Genome-fragment | | 3.7 | 0 |
| S1_k141_112034 | 1077 | No | NA | | 0 | 1 | Not-determined | Genome-fragment | NA | | 0 |
| S1_k141_295232 | 1568 | No | NA | | 0 | 1 | Not-determined | Genome-fragment | NA | | 0 |
| S1_k141_46612 | 1164 | No | NA | | 0 | 1 | Not-determined | Genome-fragment | NA | | 0 |
| S1_k141_295234 | 2726 | No | NA | | 0 | 0 | Not-determined | Genome-fragment | NA | | 0 |
| S1_k141_79574 | 1073 | No | NA | | 0 | 1 | Not-determined | Genome-fragment | NA | | 0 |
| S8-final-viral-combined_k141_77249\|\|full | 1503 | No | NA | | 0 | 0 | Low-quality | Genome-fragment | | 1.44 | 0 |
| S8-final-viral-combined_k141_231538\|\|lt2gene | 1503 | No | NA | | 1 | 0 | Low-quality | Genome-fragment | | 2.45 | 0 |
| S1_k141_376912 | 3720 | No | NA | | 0 | 2 | Not-determined | Genome-fragment | NA | | 0 |
| S11-final-viral-combined_k141_322119\|\|lt2gene | 1504 | No | NA | | 1 | 0 | Low-quality | Genome-fragment | | 1.73 | 0 |
| S1_k141_112075 | 2854 | No | NA | | 0 | 1 | Not-determined | Genome-fragment | NA | | 0 |
| S1_k141_14958 | 1876 | No | NA | | 0 | 2 | Not-determined | Genome-fragment | NA | | 0 |
| S1_k141_79608 | 1258 | No | NA | | 0 | 0 | Not-determined | Genome-fragment | NA | | 0 |
| S1_k141_97302 | 1103 | No | NA | | 0 | 1 | Not-determined | Genome-fragment | NA | | 0 |
| S1-final-viral-combined_k141_82290\|\|full | 1504 | No | NA | | 0 | 0 | Low-quality | Genome-fragment | | 1.1 | 0 |
| S1_k141_277900 | 5152 | No | NA | | 0 | 1 | Not-determined | Genome-fragment | NA | | 0 |
| S1_k141_146593 | 1739 | No | NA | | 0 | 1 | Not-determined | Genome-fragment | NA | | 0 |
| S1_k141_178517 | 3059 | No | NA | | 0 | 3 | Not-determined | Genome-fragment | NA | | 0 |
| S1_k141_14995 | 1147 | No | NA | | 0 | 0 | Not-determined | Genome-fragment | NA | | 0 |
| S1_k141_30791 | 1458 | No | NA | | 0 | 0 | Not-determined | Genome-fragment | NA | | 0 |
| S1_k141_262957 | 1188 | No | NA | | 0 | 1 | Not-determined | Genome-fragment | NA | | 0 |
| S1_k141_376989 | 1075 | No | NA | | 0 | 0 | Not-determined | Genome-fragment | NA | | 0 |
| S1_k141_311941 | 6384 | Yes | | 3223 | 1 | 2 | Not-determined | Genome-fragment | NA | | 49.51 |
| S1_k141_146653 | 1271 | No | NA | | 0 | 0 | Not-determined | Genome-fragment | NA | | 0 |
| S1-final-viral-combined_k141_169393\|\|full | 1504 | No | NA | | 0 | 0 | Low-quality | Genome-fragment | | 2.8 | 0 |
| S17-final-viral-combined_k141_81625\|\|full | 1504 | No | NA | | 0 | 0 | Low-quality | Genome-fragment | | 3.45 | 0 |
| S17-final-viral-combined_k141_91969\|\|full | 1504 | No | NA | | 0 | 2 | Low-quality | Genome-fragment | | 0.82 | 0 |
| S1_k141_15092 | 1581 | No | NA | | 0 | 1 | Not-determined | Genome-fragment | NA | | 0 |
| S1_k141_30944 | 1127 | No | NA | | 0 | 1 | Not-determined | Genome-fragment | NA | | 0 |
| S1_k141_30954 | 1260 | No | NA | | 0 | 0 | Not-determined | Genome-fragment | NA | | 0 |
| S1_k141_162774 | 3834 | No | NA | | 0 | 1 | Not-determined | Genome-fragment | NA | | 0 |
| S1_k141_328489 | 1977 | No | NA | | 0 | 0 | Not-determined | Genome-fragment | NA | | 0 |
| S17-final-viral-combined_k141_198371\|\|full | 1504 | No | NA | | 0 | 0 | Low-quality | Genome-fragment | | 2.07 | 0 |
| S1_k141_343380 | 1446 | No | NA | | 0 | 1 | Not-determined | Genome-fragment | NA | | 0 |
| S1_k141_196965 | 1048 | No | NA | | 0 | 1 | Not-determined | Genome-fragment | NA | | 0 |
| S1_k141_146818 | 1190 | No | NA | | 0 | 0 | Not-determined | Genome-fragment | NA | | 0 |
| S12-final-viral-combined_k141_398640\|\|full | 1504 | No | NA | | 0 | 2 | Low-quality | Genome-fragment | | 2.2 | 0 |
| S12_k141_365515 | 1504 | No | NA | | 0 | 0 | Low-quality | Genome-fragment | | 2.73 | 0 |
| S1_k141_79928 | 3058 | No | NA | | 0 | 1 | Not-determined | Genome-fragment | NA | | 0 |
| S4_k141_159594 | 1504 | No | NA | | 0 | 2 | Low-quality | Genome-fragment | | 0.64 | 0 |
| S1_k141_361406 | 3223 | No | NA | | 0 | 1 | Not-determined | Genome-fragment | NA | | 0 |
| S1_k141_328528 | 2130 | No | NA | | 0 | 2 | Not-determined | Genome-fragment | NA | | 0 |
| S6-final-viral-combined_k141_132416\|\|full | 1504 | No | NA | | 0 | 0 | Low-quality | Genome-fragment | | 1.62 | 0 |
| S1_k141_47001 | 1903 | No | NA | | 0 | 2 | Not-determined | Genome-fragment | NA | | 0 |
| S1_k141_47014 | 2671 | No | NA | | 0 | 0 | Not-determined | Genome-fragment | NA | | 0 |
| S1_k141_377299 | 2436 | No | NA | | 0 | 1 | Not-determined | Genome-fragment | NA | | 0 |
| S9-final-viral-combined_k141_109446\|\|full | 1505 | No | NA | | 1 | 0 | Low-quality | Genome-fragment | | 3.9 | 0 |
| S1_k141_162973 | 2036 | No | NA | | 0 | 1 | Not-determined | Genome-fragment | NA | | 0 |
| S11-final-viral-combined_k141_158405\|\|full | 1505 | No | NA | | 1 | 0 | Low-quality | Genome-fragment | | 4.22 | 0 |
| S1_k141_64476 | 1560 | No | NA | | 0 | 1 | Not-determined | Genome-fragment | NA | | 0 |
| S1_k141_361472 | 3600 | No | NA | | 0 | 1 | Not-determined | Genome-fragment | NA | | 0 |
| S1_k141_244239 | 1505 | No | NA | | 0 | 1 | Low-quality | Genome-fragment | | 0.83 | 0 |
| S1_k141_197081 | 1047 | No | NA | | 0 | 0 | Not-determined | Genome-fragment | NA | | 0 |
| S1_k141_263215 | 7662 | No | NA | | 0 | 3 | Not-determined | Genome-fragment | NA | | 0 |
| S1_k141_246239 | 4053 | No | NA | | 0 | 3 | Not-determined | Genome-fragment | NA | | 0 |
| S7-final-viral-combined_k141_63313\|\|lt2gene | 1505 | No | NA | | 1 | 0 | Low-quality | Genome-fragment | | 3.29 | 0 |
| S1_k141_15274 | 1039 | No | NA | | 0 | 1 | Not-determined | Genome-fragment | NA | | 0 |
| S1_k141_64493 | 1044 | No | NA | | 0 | 1 | Not-determined | Genome-fragment | NA | | 0 |
| S1_k141_377351 | 6497 | No | NA | | 0 | 3 | Not-determined | Genome-fragment | NA | | 0 |
| S1_k141_31155 | 11507 | No | NA | | 0 | 0 | Not-determined | Genome-fragment | NA | | 0 |
| S1_k141_295646 | 1204 | No | NA | | 0 | 2 | Not-determined | Genome-fragment | NA | | 0 |
| S1_k141_328617 | 1517 | No | NA | | 0 | 1 | Not-determined | Genome-fragment | NA | | 0 |
| S1_k141_31170 | 1047 | No | NA | | 0 | 0 | Not-determined | Genome-fragment | NA | | 0 |
| S1_k141_328635 | 1040 | No | NA | | 0 | 1 | Not-determined | Genome-fragment | NA | | 0 |
| S17-k141_50159 | 1505 | No | NA | | 1 | 0 | Low-quality | Genome-fragment | | 0.84 | 0 |
| S12-final-viral-combined_k141_314572\|\|full | 1505 | No | NA | | 0 | 1 | Low-quality | Genome-fragment | | 1.92 | 0 |
| S1_k141_213495 | 1317 | No | NA | | 0 | 0 | Not-determined | Genome-fragment | NA | | 0 |
| S1_k141_80104 | 3442 | No | NA | | 0 | 3 | Not-determined | Genome-fragment | NA | | 0 |
| S1_k141_179017 | 1410 | No | NA | | 0 | 1 | Not-determined | Genome-fragment | NA | | 0 |
| S1_k141_47121 | 1102 | No | NA | | 0 | 1 | Not-determined | Genome-fragment | NA | | 0 |
| S4-final-viral-combined_k141_113649\|\|full | 1505 | No | NA | | 0 | 0 | Low-quality | Genome-fragment | | 1.08 | 0 |
| S1_k141_31242 | 2012 | No | NA | | 0 | 1 | Not-determined | Genome-fragment | NA | | 0 |
| S1_k141_343594 | 1328 | No | NA | | 0 | 2 | Not-determined | Genome-fragment | NA | | 0 |
| S1_k141_377452 | 1231 | No | NA | | 0 | 1 | Not-determined | Genome-fragment | NA | | 0 |
| S1_k141_146985 | 3300 | No | NA | | 0 | 0 | Not-determined | Genome-fragment | NA | | 0 |
| S1_k141_246331 | 1856 | No | NA | | 0 | 1 | Not-determined | Genome-fragment | NA | | 0 |
| S1_k141_197176 | 1270 | No | NA | | 0 | 0 | Not-determined | Genome-fragment | NA | | 0 |
| S1_k141_328736 | 1158 | No | NA | | 0 | 1 | Not-determined | Genome-fragment | NA | | 0 |
| S1_k141_163103 | 1117 | No | NA | | 0 | 1 | Not-determined | Genome-fragment | NA | | 0 |
| S8-final-viral-combined_k141_262298\|\|full | 1505 | No | NA | | 2 | 0 | Low-quality | Genome-fragment | | 3.91 | 0 |
| S1_k141_213570 | 1151 | No | NA | | 0 | 2 | Not-determined | Genome-fragment | NA | | 0 |
| S1_k141_295750 | 1269 | No | NA | | 0 | 3 | Not-determined | Genome-fragment | NA | | 0 |
| S1_k141_229441 | 1353 | No | NA | | 0 | 0 | Not-determined | Genome-fragment | NA | | 0 |
| S1_k141_229443 | 1047 | No | NA | | 0 | 2 | Not-determined | Genome-fragment | NA | | 0 |
| S1_k141_328761 | 3312 | No | NA | | 0 | 3 | Not-determined | Genome-fragment | NA | | 0 |
| S1_k141_31293 | 16897 | No | NA | | 0 | 9 | Not-determined | Genome-fragment | NA | | 0 |
| S9-final-viral-combined_k141_57141\|\|full | 1506 | No | NA | | 3 | 0 | Low-quality | Genome-fragment | | 4.58 | 0 |
| S1_k141_197257 | 3344 | No | NA | | 0 | 2 | Not-determined | Genome-fragment | NA | | 0 |
| S9-final-viral-combined_k141_82348\|\|lt2gene | 1506 | No | NA | | 2 | 0 | Low-quality | Genome-fragment | | 3.71 | 0 |
| S1_k141_246403 | 12655 | No | NA | | 0 | 4 | Not-determined | Genome-fragment | NA | | 0 |

| | | | | | | | | | |
|---|---|---|---|---|---|---|---|---|---|
| S1_k141_179241 | 1949 | No | NA | 0 | 1 | Not-determined | Genome-fragment | NA | 0 |
| S11-final-viral-combined_k141_323943||full | 1506 | No | NA | 0 | 2 | Low-quality | Genome-fragment | 0.93 | 0 |
| S11-final-viral-combined_k141_121633||full | 1506 | No | NA | 0 | 1 | Low-quality | Genome-fragment | 2.01 | 0 |
| S1_k141_112452 | 1630 | No | NA | 0 | 0 | Not-determined | Genome-fragment | NA | 0 |
| S1-final-viral-combined_k141_231276||full | 1506 | No | NA | 0 | 1 | Low-quality | Genome-fragment | 1.02 | 0 |
| S1-final-viral-combined_k141_349273||lt2gene | 1506 | No | NA | 0 | 0 | Low-quality | Genome-fragment | 2.71 | 0 |
| S1_k141_80367 | 1984 | No | NA | 0 | 0 | Not-determined | Genome-fragment | NA | 0 |
| S12-final-viral-combined_k141_211827||full | 1506 | No | NA | 0 | 2 | Low-quality | Genome-fragment | 1.14 | 0 |
| S1_k141_15610 | 1350 | No | NA | 0 | 0 | Not-determined | Genome-fragment | NA | 0 |
| S1_k141_163280 | 2325 | No | NA | 0 | 2 | Not-determined | Genome-fragment | NA | 0 |
| S2_k141_55443 | 1506 | No | NA | 0 | 1 | Low-quality | Genome-fragment | 1.11 | 0 |
| S1_k141_361726 | 1100 | No | NA | 0 | 1 | Not-determined | Genome-fragment | NA | 0 |
| S9-final-viral-combined_k141_59660||full | 1507 | No | NA | 0 | 1 | Low-quality | Genome-fragment | 1.19 | 0 |
| S1_k141_64882 | 4207 | No | NA | 0 | 0 | Not-determined | Genome-fragment | NA | 0 |
| S1_k141_47387 | 1004 | No | NA | 0 | 0 | Not-determined | Genome-fragment | NA | 0 |
| S11-final-viral-combined_k141_225378||full | 1507 | No | NA | 0 | 0 | Low-quality | Genome-fragment | 1.74 | 0 |
| S1_k141_131609 | 1128 | No | NA | 0 | 0 | Not-determined | Genome-fragment | NA | 0 |
| S1_k141_278433 | 1150 | No | NA | 0 | 0 | Not-determined | Genome-fragment | NA | 0 |
| S1_k141_213791 | 3333 | No | NA | 0 | 1 | Not-determined | Genome-fragment | NA | 0 |
| S1_k141_179314 | 1523 | No | NA | 0 | 1 | Not-determined | Genome-fragment | NA | 0 |
| S1_k141_246477 | 1043 | No | NA | 0 | 1 | Not-determined | Genome-fragment | NA | 0 |
| S1_k141_98114 | 1420 | No | NA | 0 | 1 | Not-determined | Genome-fragment | NA | 0 |
| S1_k141_393320 | 1464 | No | NA | 0 | 1 | Not-determined | Genome-fragment | NA | 0 |
| S1_k141_179362 | 1455 | No | NA | 0 | 1 | Not-determined | Genome-fragment | NA | 0 |
| S1_k141_25374 | 1507 | Yes | 237 | 1 | 1 | Low-quality | Genome-fragment | 0.13 | 84.27 |
| S1_k141_47453 | 1335 | No | NA | 0 | 0 | Not-determined | Genome-fragment | NA | 0 |
| S1_k141_393353 | 1293 | No | NA | 0 | 1 | Not-determined | Genome-fragment | NA | 0 |
| S1_k141_98153 | 7386 | No | NA | 0 | 2 | Not-determined | Genome-fragment | NA | 0 |
| S1_k141_377810 | 1103 | No | NA | 0 | 2 | Not-determined | Genome-fragment | NA | 0 |
| S1_k141_246528 | 1121 | No | NA | 0 | 0 | Not-determined | Genome-fragment | NA | 0 |
| S1_k141_278601 | 1621 | No | NA | 0 | 2 | Not-determined | Genome-fragment | NA | 0 |
| S1_k141_293965 | 1507 | No | NA | 0 | 2 | Low-quality | Genome-fragment | 3.17 | 0 |
| S1_k141_296126 | 1461 | No | NA | 0 | 0 | Not-determined | Genome-fragment | NA | 0 |
| S1_k141_147362 | 1032 | No | NA | 0 | 1 | Not-determined | Genome-fragment | NA | 0 |
| S1_k141_278655 | 6103 | No | NA | 0 | 7 | Not-determined | Genome-fragment | NA | 0 |
| S1_k141_15829 | 1316 | No | NA | 0 | 0 | Not-determined | Genome-fragment | NA | 0 |
| S1_k141_15830 | 1360 | No | NA | 0 | 1 | Not-determined | Genome-fragment | NA | 0 |
| S1_k141_179582 | 1481 | No | NA | 0 | 0 | Not-determined | Genome-fragment | NA | 0 |
| S1_k141_147431 | 1965 | No | NA | 0 | 1 | Not-determined | Genome-fragment | NA | 0 |
| S1_k141_278720 | 1253 | No | NA | 0 | 0 | Not-determined | Genome-fragment | NA | 0 |
| S3-final-viral-combined_k141_17987||full | 1507 | No | NA | 2 | 0 | Low-quality | Genome-fragment | 4.05 | 0 |
| S1_k141_246644 | 1704 | No | NA | 0 | 2 | Not-determined | Genome-fragment | NA | 0 |
| S17-final-viral-combined_k141_67376||full | 1507 | No | NA | 0 | 0 | Low-quality | Genome-fragment | 3.41 | 0 |
| S12-final-viral-combined_k141_123716||full | 1507 | No | NA | 1 | 0 | Low-quality | Genome-fragment | 2.29 | 0 |
| S1_k141_361926 | 1219 | No | NA | 0 | 0 | Not-determined | Genome-fragment | NA | 0 |
| S1_k141_344060 | 2604 | No | NA | 0 | 0 | Not-determined | Genome-fragment | NA | 0 |
| S2-final-viral-combined_k141_33248||full | 1507 | No | NA | 1 | 0 | Low-quality | Genome-fragment | 2.55 | 0 |
| S1_k141_65210 | 5587 | No | NA | 0 | 1 | Not-determined | Genome-fragment | NA | 0 |
| S2-final-viral-combined_k141_158809||lt2gene | 1507 | No | NA | 2 | 0 | Low-quality | Genome-fragment | 3.18 | 0 |
| S1_k141_112778 | 2217 | No | NA | 0 | 0 | Not-determined | Genome-fragment | NA | 0 |
| S1_k141_16003 | 3289 | No | NA | 0 | 0 | Not-determined | Genome-fragment | NA | 0 |
| S1_k141_163619 | 3346 | No | NA | 0 | 1 | Not-determined | Genome-fragment | NA | 0 |
| S1_k141_179691 | 1329 | No | NA | 0 | 1 | Not-determined | Genome-fragment | NA | 0 |
| S1_k141_163634 | 1448 | No | NA | 0 | 2 | Not-determined | Genome-fragment | NA | 0 |
| S1_k141_98405 | 1488 | No | NA | 0 | 1 | Not-determined | Genome-fragment | NA | 0 |
| S1_k141_378089 | 2422 | No | NA | 0 | 2 | Not-determined | Genome-fragment | NA | 0 |
| S4-final-viral-combined_k141_57112||full | 1507 | No | NA | 0 | 1 | Low-quality | Genome-fragment | 2.11 | 0 |
| S9-final-viral-combined_k141_39000||full | 1508 | No | NA | 0 | 0 | Low-quality | Genome-fragment | 5.9 | 0 |
| S11-final-viral-combined_k141_127245||full | 1508 | No | NA | 3 | 0 | Low-quality | Genome-fragment | 2.49 | 0 |
| S1_k141_393696 | 4889 | No | NA | 0 | 1 | Not-determined | Genome-fragment | NA | 0 |
| S1_k141_344198 | 1035 | No | NA | 0 | 1 | Not-determined | Genome-fragment | NA | 0 |
| S1_k141_246760 | 1574 | No | NA | 0 | 2 | Not-determined | Genome-fragment | NA | 0 |
| S1-final-viral-combined_k141_47634||full | 1508 | No | NA | 0 | 1 | Low-quality | Genome-fragment | 2.84 | 0 |
| S17-k141_209681 | 1508 | No | NA | 4 | 0 | Low-quality | Genome-fragment | 4.96 | 0 |
| S1_k141_31627 | 1374 | No | NA | 0 | 0 | Not-determined | Genome-fragment | NA | 0 |
| S1_k141_393747 | 1982 | No | NA | 0 | 1 | Not-determined | Genome-fragment | NA | 0 |
| S1_k141_393758 | 1680 | No | NA | 0 | 1 | Not-determined | Genome-fragment | NA | 0 |
| S1_k141_65357 | 1278 | No | NA | 0 | 1 | Not-determined | Genome-fragment | NA | 0 |
| S1_k141_229861 | 3072 | No | NA | 0 | 2 | Not-determined | Genome-fragment | NA | 0 |
| S1_k141_279021 | 1730 | No | NA | 0 | 1 | Not-determined | Genome-fragment | NA | 0 |
| S1_k141_31724 | 4663 | No | NA | 0 | 3 | Not-determined | Genome-fragment | NA | 0 |
| S1_k141_16142 | 1325 | No | NA | 0 | 0 | Not-determined | Genome-fragment | NA | 0 |
| S12-final-viral-combined_k141_257165||full | 1508 | No | NA | 0 | 0 | Low-quality | Genome-fragment | 0.56 | 0 |
| S1_k141_31760 | 2896 | No | NA | 0 | 2 | Not-determined | Genome-fragment | NA | 0 |
| S1_k141_179998 | 1037 | No | NA | 0 | 0 | Not-determined | Genome-fragment | NA | 0 |
| S1_k141_344383 | 1013 | No | NA | 0 | 1 | Not-determined | Genome-fragment | NA | 0 |
| S1_k141_230016 | 3470 | No | NA | 0 | 1 | Not-determined | Genome-fragment | NA | 0 |
| S1_k141_344466 | 1114 | No | NA | 0 | 0 | Not-determined | Genome-fragment | NA | 0 |
| S1_k141_147715 | 1203 | No | NA | 0 | 0 | Not-determined | Genome-fragment | NA | 0 |
| S1_k141_147722 | 1687 | No | NA | 0 | 2 | Not-determined | Genome-fragment | NA | 0 |
| S1_k141_147733 | 3026 | No | NA | 0 | 1 | Not-determined | Genome-fragment | NA | 0 |
| S1_k141_163964 | 4855 | No | NA | 0 | 2 | Not-determined | Genome-fragment | NA | 0 |
| S1_k141_279171 | 1072 | No | NA | 0 | 1 | Not-determined | Genome-fragment | NA | 0 |
| S1_k141_344526 | 3172 | No | NA | 0 | 2 | Not-determined | Genome-fragment | NA | 0 |
| S1_k141_65558 | 1000 | No | NA | 0 | 1 | Not-determined | Genome-fragment | NA | 0 |
| S1_k141_230098 | 1241 | No | NA | 0 | 0 | Not-determined | Genome-fragment | NA | 0 |
| S1_k141_378357 | 1165 | No | NA | 0 | 2 | Not-determined | Genome-fragment | NA | 0 |
| S1_k141_394064 | 2343 | No | NA | 0 | 0 | Not-determined | Genome-fragment | NA | 0 |
| S1_k141_31969 | 1328 | No | NA | 0 | 1 | Not-determined | Genome-fragment | NA | 0 |
| S12_k141_17241 | 1508 | No | NA | 0 | 2 | Low-quality | Genome-fragment | 0.64 | 0 |
| S8_k141_267086 | 1508 | No | NA | 1 | 0 | Low-quality | Genome-fragment | 3.75 | 0 |
| S1_k141_230253 | 1170 | No | NA | 0 | 1 | Not-determined | Genome-fragment | NA | 0 |
| S1_k141_147942 | 1492 | No | NA | 0 | 1 | Not-determined | Genome-fragment | NA | 0 |
| S11-final-viral-combined_k141_319373||full | 1509 | No | NA | 0 | 1 | Low-quality | Genome-fragment | 0.9 | 0 |
| S3_k141_97941 | 1509 | No | NA | 1 | 0 | Low-quality | Genome-fragment | 0.69 | 0 |
| S1_k141_230274 | 1183 | No | NA | 0 | 3 | Not-determined | Genome-fragment | NA | 0 |
| S1_k141_81554 | 1058 | No | NA | 0 | 0 | Not-determined | Genome-fragment | NA | 0 |
| S7-final-viral-combined_k141_167021||full | 1509 | No | NA | 1 | 0 | Low-quality | Genome-fragment | 2.5 | 0 |
| S1_k141_164222 | 1250 | No | NA | 0 | 0 | Not-determined | Genome-fragment | NA | 0 |
| S1_k141_180302 | 2652 | No | NA | 0 | 2 | Not-determined | Genome-fragment | NA | 0 |
| S12-final-viral-combined_k141_26804||full | 1509 | No | NA | 0 | 1 | Low-quality | Genome-fragment | 1.09 | 0 |
| S1_k141_344795 | 1321 | No | NA | 0 | 0 | Not-determined | Genome-fragment | NA | 0 |
| S1_k141_81665 | 1171 | No | NA | 0 | 0 | Not-determined | Genome-fragment | NA | 0 |
| S1_k141_394223 | 1340 | No | NA | 0 | 1 | Not-determined | Genome-fragment | NA | 0 |
| S12_k141_246857 | 1509 | No | NA | 0 | 1 | Low-quality | Genome-fragment | 0.99 | 0 |
| S1_k141_180442 | 1191 | No | NA | 0 | 2 | Not-determined | Genome-fragment | NA | 0 |
| S2_k141_224619 | 1509 | No | NA | 0 | 1 | Low-quality | Genome-fragment | 1 | 0 |
| S1_k141_164358 | 5908 | No | NA | 0 | 3 | Not-determined | Genome-fragment | NA | 0 |
| S1_k141_113579 | 1298 | No | NA | 0 | 0 | Not-determined | Genome-fragment | NA | 0 |
| S1_k141_344906 | 3041 | No | NA | 0 | 3 | Not-determined | Genome-fragment | NA | 0 |
| S1_k141_344921 | 3250 | No | NA | 0 | 2 | Not-determined | Genome-fragment | NA | 0 |
| S1_k141_279430 | 1257 | No | NA | 0 | 0 | Not-determined | Genome-fragment | NA | 0 |
| S5-final-viral-combined_k141_180244||full | 1510 | No | NA | 0 | 0 | Low-quality | Genome-fragment | 2.56 | 0 |
| S7-final-viral-combined_k141_197011||full | 1510 | No | NA | 1 | 0 | Low-quality | Genome-fragment | 3.4 | 0 |
| S1_k141_32362 | 1478 | No | NA | 0 | 1 | Not-determined | Genome-fragment | NA | 0 |
| S1_k141_32371 | 3531 | No | NA | 0 | 0 | Not-determined | Genome-fragment | NA | 0 |
| S1_k141_279488 | 6465 | No | NA | 0 | 1 | Not-determined | Genome-fragment | NA | 0 |
| S1_k141_48434 | 3509 | No | NA | 0 | 0 | Not-determined | Genome-fragment | NA | 0 |
| S7-final-viral-combined_k141_138138||full | 1510 | No | NA | 1 | 0 | Low-quality | Genome-fragment | 3.25 | 0 |
| S1_k141_48448 | 1663 | No | NA | 0 | 0 | Not-determined | Genome-fragment | NA | 0 |
| S1_k141_32403 | 3012 | No | NA | 0 | 2 | Not-determined | Genome-fragment | NA | 0 |
| S1_k141_48455 | 2027 | No | NA | 0 | 2 | Not-determined | Genome-fragment | NA | 0 |
| S12-final-viral-combined_k141_387111||full | 1510 | No | NA | 0 | 2 | Low-quality | Genome-fragment | 1.05 | 0 |
| S1_k141_32424 | 3044 | No | NA | 0 | 1 | Not-determined | Genome-fragment | NA | 0 |
| S1_k141_48488 | 1262 | No | NA | 0 | 1 | Not-determined | Genome-fragment | NA | 0 |
| S1_k141_32459 | 1028 | No | NA | 0 | 1 | Not-determined | Genome-fragment | NA | 0 |
| S1_k141_48513 | 1655 | No | NA | 0 | 0 | Not-determined | Genome-fragment | NA | 0 |
| S1_k141_65753 | 1467 | No | NA | 0 | 0 | Not-determined | Genome-fragment | NA | 0 |
| S1_k141_180663 | 1757 | No | NA | 0 | 1 | Not-determined | Genome-fragment | NA | 0 |
| S1_k141_32584 | 4753 | No | NA | 0 | 3 | Not-determined | Genome-fragment | NA | 0 |
| S1_k141_180699 | 12772 | No | NA | 0 | 5 | Not-determined | Genome-fragment | NA | 0 |
| S1_k141_48681 | 2441 | No | NA | 0 | 5 | Not-determined | Genome-fragment | NA | 0 |
| S8-final-viral-combined_k141_71798||full | 1510 | No | NA | 0 | 0 | Low-quality | Genome-fragment | 2.94 | 0 |
| S1_k141_279603 | 1225 | No | NA | 0 | 0 | Not-determined | Genome-fragment | NA | 0 |
| S1_k141_48724 | 1213 | No | NA | 0 | 0 | Not-determined | Genome-fragment | NA | 0 |
| S1_k141_345166 | 1025 | No | NA | 0 | 0 | Not-determined | Genome-fragment | NA | 0 |
| S1_k141_32725 | 1177 | No | NA | 0 | 2 | Not-determined | Genome-fragment | NA | 0 |
| S1_k141_180798 | 1077 | No | NA | 0 | 0 | Not-determined | Genome-fragment | NA | 0 |
| S1_k141_32750 | 1036 | No | NA | 0 | 0 | Not-determined | Genome-fragment | NA | 0 |
| S8_k141_196658 | 1510 | No | NA | 0 | 1 | Low-quality | Genome-fragment | 5.18 | 0 |
| S1_k141_279652 | 1015 | No | NA | 0 | 1 | Not-determined | Genome-fragment | NA | 0 |
| S1_k141_114025 | 3234 | No | NA | 0 | 0 | Not-determined | Genome-fragment | NA | 0 |
| S1_k141_394571 | 101988 | No | NA | 17 | 3 | Complete | High-quality | 100 | 0 |
| S12-final-viral-combined_k141_193312||full | 1511 | No | NA | 2 | 0 | Low-quality | Genome-fragment | 2.81 | 0 |
| S12-final-viral-combined_k141_181497||full | 1511 | No | NA | 1 | 0 | Low-quality | Genome-fragment | 3.8 | 0 |
| S1_k141_82107 | 1068 | No | NA | 0 | 1 | Not-determined | Genome-fragment | NA | 0 |
| S1_k141_345285 | 1011 | No | NA | 0 | 0 | Not-determined | Genome-fragment | NA | 0 |
| S1_k141_394605 | 1569 | No | NA | 0 | 0 | Not-determined | Genome-fragment | NA | 0 |
| S1_k141_82162 | 7266 | No | NA | 0 | 1 | Not-determined | Genome-fragment | NA | 0 |
| S1_k141_394607 | 3239 | No | NA | 0 | 1 | Not-determined | Genome-fragment | NA | 0 |
| S1_k141_345311 | 12480 | No | NA | 0 | 0 | Not-determined | Genome-fragment | NA | 0 |
| S1_k141_114189 | 1852 | No | NA | 0 | 1 | Not-determined | Genome-fragment | NA | 0 |
| S12_k141_389763 | 1511 | No | NA | 0 | 0 | Low-quality | Genome-fragment | 2.97 | 0 |
| S12_k141_189801 | 1511 | No | NA | 0 | 1 | Low-quality | Genome-fragment | 0.26 | 0 |
| S1_k141_48971 | 1102 | No | NA | 0 | 2 | Not-determined | Genome-fragment | NA | 0 |
| S2-final-viral-combined_k141_216748||lt2gene | 1511 | No | NA | 0 | 0 | Low-quality | Genome-fragment | 2.82 | 0 |
| S1_k141_32878 | 18889 | No | NA | 3 | 0 | Medium-quality | Genome-fragment | 51.68 | 0 |
| S8_k141_66168 | 1511 | No | NA | 0 | 1 | Low-quality | Genome-fragment | 1.45 | 0 |
| S9-final-viral-combined_k141_58804||full | 1512 | No | NA | 0 | 2 | Low-quality | Genome-fragment | 2.19 | 0 |
| S1_k141_49147 | 4323 | No | NA | 0 | 0 | Not-determined | Genome-fragment | NA | 0 |
| S1_k141_114574 | 1257 | No | NA | 0 | 0 | Not-determined | Genome-fragment | NA | 0 |
| S1_k141_114918 | 1797 | No | NA | 0 | 0 | Not-determined | Genome-fragment | NA | 0 |
| S1_k141_114986 | 1157 | No | NA | 0 | 0 | Not-determined | Genome-fragment | NA | 0 |
| S3-final-viral-combined_k141_128066||full | 2531 | No | NA | 0 | 0 | Not-determined | Genome-fragment | NA | 0 |
| S3-final-viral-combined_k141_137874||full | 8924 | No | NA | 0 | 1 | Not-determined | Genome-fragment | NA | 0 |
| S9-final-viral-combined_k141_74790||full | 1512 | No | NA | 0 | 2 | Low-quality | Genome-fragment | 1.06 | 0 |
| S3-final-viral-combined_k141_132259||full | 1787 | No | NA | 0 | 1 | Not-determined | Genome-fragment | NA | 0 |
| S3-final-viral-combined_k141_34515||full | 1965 | No | NA | 0 | 3 | Not-determined | Genome-fragment | NA | 0 |
| S3-final-viral-combined_k141_97763||full | 2003 | No | NA | 0 | 2 | Not-determined | Genome-fragment | NA | 0 |
| S3-final-viral-combined_k141_51767||full | 3838 | No | NA | 0 | 3 | Not-determined | Genome-fragment | NA | 0 |
| S11_k141_196520 | 1512 | No | NA | 0 | 0 | Low-quality | Genome-fragment | 2.21 | 0 |
| S3-final-viral-combined_k141_120780||full | 6704 | No | NA | 0 | 4 | Not-determined | Genome-fragment | NA | 0 |
| S3-final-viral-combined_k141_11526||full | 1892 | No | NA | 0 | 3 | Not-determined | Genome-fragment | NA | 0 |
| S3-final-viral-combined_k141_23051||full | 2559 | No | NA | 0 | 0 | Not-determined | Genome-fragment | NA | 0 |

| | | | | | | | | | | |
|---|---|---|---|---|---|---|---|---|---|---|
| S12-final-viral-combined_k141_358183\|\|full | 1512 | No | NA | 1 | 0 | Low-quality | Genome-fragment | | 4.15 | 0 |
| S3-final-viral-combined_k141_51798\|\|full | 4119 | No | NA | 0 | 2 | Not-determined | Genome-fragment | NA | | 0 |
| S12_k141_300546 | 1512 | No | NA | 0 | 0 | Low-quality | Genome-fragment | | 3.41 | 0 |
| S2-final-viral-combined_k141_157293\|\|full | 1512 | No | NA | 1 | 0 | Low-quality | Genome-fragment | | 5.18 | 0 |
| S3-final-viral-combined_k141_69082\|\|full | 3731 | No | NA | 0 | 2 | Not-determined | Genome-fragment | NA | | 0 |
| S3-final-viral-combined_k141_63347\|\|full | 1040 | No | NA | 0 | 0 | Not-determined | Genome-fragment | NA | | 0 |
| S2-final-viral-combined_k141_134240\|\|full | 1512 | No | NA | 0 | 1 | Low-quality | Genome-fragment | | 2.01 | 0 |
| S3-final-viral-combined_k141_46103\|\|full | 3895 | No | NA | 0 | 3 | Not-determined | Genome-fragment | NA | | 0 |
| S3-final-viral-combined_k141_115104\|\|full | 6170 | No | NA | 0 | 0 | Not-determined | Genome-fragment | NA | | 0 |
| S3-final-viral-combined_k141_34596\|\|full | 1734 | No | NA | 0 | 2 | Not-determined | Genome-fragment | NA | | 0 |
| S3-final-viral-combined_k141_57565\|\|full | 1320 | No | NA | 0 | 0 | Not-determined | Genome-fragment | NA | | 0 |
| S6_k141_85515 | 1512 | No | NA | 0 | 0 | Low-quality | Genome-fragment | | 0.66 | 0 |
| S8-final-viral-combined_k141_47751\|\|lt2gene | 1512 | No | NA | 1 | 0 | Low-quality | Genome-fragment | | 4.44 | 0 |
| S3-final-viral-combined_k141_115138\|\|full | 3507 | No | NA | 0 | 1 | Not-determined | Genome-fragment | NA | | 0 |
| S3-final-viral-combined_k141_34625\|\|full | 5349 | No | NA | 0 | 3 | Not-determined | Genome-fragment | NA | | 0 |
| S3-final-viral-combined_k141_86379\|\|full | 1929 | No | NA | 0 | 3 | Not-determined | Genome-fragment | NA | | 0 |
| S3-final-viral-combined_k141_51849\|\|full | 3389 | No | NA | 0 | 1 | Not-determined | Genome-fragment | NA | | 0 |
| S3-final-viral-combined_k141_5852\|\|full | 1621 | No | NA | 0 | 1 | Not-determined | Genome-fragment | NA | | 0 |
| S8-final-viral-combined_k141_176712\|\|lt2gene | 1512 | No | NA | 0 | 0 | Low-quality | Genome-fragment | | 0.98 | 0 |
| S3-final-viral-combined_k141_74848\|\|full | 2001 | No | NA | 0 | 0 | Not-determined | Genome-fragment | NA | | 0 |
| S9-final-viral-combined_k141_52797\|\|full | 1513 | No | NA | 0 | 1 | Low-quality | Genome-fragment | | 2.01 | 0 |
| S3-final-viral-combined_k141_80585\|\|full | 1598 | No | NA | 0 | 2 | Not-determined | Genome-fragment | NA | | 0 |
| S3-final-viral-combined_k141_103622\|\|full | 2576 | No | NA | 0 | 1 | Not-determined | Genome-fragment | NA | | 0 |
| S3-final-viral-combined_k141_126694\|\|full | 1923 | No | NA | 0 | 2 | Not-determined | Genome-fragment | NA | | 0 |
| S5-final-viral-combined_k141_186746\|\|full | 1513 | No | NA | 0 | 2 | Low-quality | Genome-fragment | | 0.5 | 0 |
| S3-final-viral-combined_k141_40378\|\|full | 2092 | No | NA | 0 | 0 | Not-determined | Genome-fragment | NA | | 0 |
| S3-final-viral-combined_k141_221\|\|full | 1790 | No | NA | 0 | 1 | Not-determined | Genome-fragment | NA | | 0 |
| S3-final-viral-combined_k141_103641\|\|full | 1824 | No | NA | 0 | 1 | Not-determined | Genome-fragment | NA | | 0 |
| S7-final-viral-combined_k141_239305\|\|full | 1513 | No | NA | 0 | 2 | Low-quality | Genome-fragment | | 1.45 | 0 |
| S3-final-viral-combined_k141_132450\|\|full | 3199 | No | NA | 0 | 3 | Not-determined | Genome-fragment | NA | | 0 |
| S3-final-viral-combined_k141_40380\|\|full | 6809 | No | NA | 0 | 4 | Not-determined | Genome-fragment | NA | | 0 |
| S7_k141_12922 | 1513 | No | NA | 0 | 1 | Low-quality | Genome-fragment | | 0.25 | 0 |
| S3-final-viral-combined_k141_115220\|\|full | 7895 | No | NA | 0 | 1 | Not-determined | Genome-fragment | NA | | 0 |
| S3-final-viral-combined_k141_69190\|\|full | 2004 | No | NA | 0 | 1 | Not-determined | Genome-fragment | NA | | 0 |
| S3-final-viral-combined_k141_115230\|\|full | 2650 | No | NA | 0 | 0 | Not-determined | Genome-fragment | NA | | 0 |
| S7_k141_48136 | 1513 | No | NA | 0 | 0 | Low-quality | Genome-fragment | | 2.53 | 0 |
| S3-final-viral-combined_k141_120998\|\|full | 1317 | No | NA | 0 | 1 | Not-determined | Genome-fragment | NA | | 0 |
| S3-final-viral-combined_k141_40410\|\|full | 1015 | No | NA | 0 | 1 | Not-determined | Genome-fragment | NA | | 0 |
| S12_k141_374550 | 1513 | No | NA | 0 | 1 | Low-quality | Genome-fragment | | 1.16 | 0 |
| S3-final-viral-combined_k141_5938\|\|full | 12804 | No | NA | 0 | 9 | Not-determined | Genome-fragment | NA | | 0 |
| S2_k141_98024 | 1513 | No | NA | 0 | 0 | Low-quality | Genome-fragment | | 3.44 | 0 |
| S4-final-viral-combined_k141_132515\|\|full | 1513 | No | NA | 0 | 2 | Low-quality | Genome-fragment | | 0.5 | 0 |
| S3-final-viral-combined_k141_92204\|\|full | 2259 | No | NA | 0 | 1 | Not-determined | Genome-fragment | NA | | 0 |
| S3-final-viral-combined_k141_74975\|\|full | 6971 | No | NA | 0 | 7 | Not-determined | Genome-fragment | NA | | 0 |
| S4-final-viral-combined_k141_36331\|\|lt2gene | 1513 | No | NA | 0 | 0 | Low-quality | Genome-fragment | | 2.68 | 0 |
| S3-final-viral-combined_k141_28937\|\|full | 9541 | No | NA | 0 | 3 | Not-determined | Genome-fragment | NA | | 0 |
| S3-final-viral-combined_k141_57652\|\|full | 6007 | No | NA | 0 | 2 | Not-determined | Genome-fragment | NA | | 0 |
| S3-final-viral-combined_k141_28939\|\|full | 2734 | No | NA | 0 | 2 | Not-determined | Genome-fragment | NA | | 0 |
| S8_k141_110603 | 1513 | No | NA | 0 | 1 | Low-quality | Genome-fragment | | 0.29 | 0 |
| S9-final-viral-combined_k141_108099\|\|full | 1514 | No | NA | 1 | 0 | Low-quality | Genome-fragment | | 3.32 | 0 |
| S3-final-viral-combined_k141_74992\|\|full | 1882 | No | NA | 0 | 0 | Not-determined | Genome-fragment | NA | | 0 |
| S9-final-viral-combined_k141_20349\|\|full | 1514 | No | NA | 0 | 1 | Low-quality | Genome-fragment | | 0.73 | 0 |
| S1-final-viral-combined_k141_2020\|\|full | 1514 | No | NA | 1 | 0 | Low-quality | Genome-fragment | | 1.99 | 0 |
| S1_k141_155192 | 1514 | No | NA | 0 | 1 | Low-quality | Genome-fragment | | 0.57 | 0 |
| S7-final-viral-combined_k141_219822\|\|lt2gene | 1514 | No | NA | 1 | 0 | Low-quality | Genome-fragment | | 2.03 | 0 |
| S17-final-viral-combined_k141_184374\|\|full | 1514 | No | NA | 0 | 0 | Low-quality | Genome-fragment | | 4.84 | 0 |
| S3-final-viral-combined_k141_126809\|\|full | 1897 | No | NA | 0 | 1 | Not-determined | Genome-fragment | NA | | 0 |
| S3-final-viral-combined_k141_126812\|\|full | 2105 | No | NA | 0 | 1 | Not-determined | Genome-fragment | NA | | 0 |
| S3-final-viral-combined_k141_34784\|\|full | 1094 | No | NA | 0 | 2 | Not-determined | Genome-fragment | NA | | 0 |
| S3-final-viral-combined_k141_17459\|\|full | 69116 | No | NA | 0 | 19 | Not-determined | Genome-fragment | NA | | 0 |
| S17-k141_130682 | 1514 | No | NA | 1 | 0 | Low-quality | Genome-fragment | | 3.14 | 0 |
| S12-final-viral-combined_k141_220871\|\|lt2gene | 1514 | No | NA | 1 | 0 | Low-quality | Genome-fragment | | 3.84 | 0 |
| S3-final-viral-combined_k141_11716\|\|full | 1048 | No | NA | 0 | 0 | Not-determined | Genome-fragment | NA | | 0 |
| S3-final-viral-combined_k141_132594\|\|full | 6099 | No | NA | 0 | 0 | Not-determined | Genome-fragment | NA | | 0 |
| S3-final-viral-combined_k141_406\|\|full | 2819 | No | NA | 0 | 2 | Not-determined | Genome-fragment | NA | | 0 |
| S3-final-viral-combined_k141_17478\|\|full | 3953 | No | NA | 0 | 2 | Not-determined | Genome-fragment | NA | | 0 |
| S3-final-viral-combined_k141_46355\|\|full | 1168 | No | NA | 0 | 1 | Not-determined | Genome-fragment | NA | | 0 |
| S3-final-viral-combined_k141_57738\|\|full | 1378 | No | NA | 0 | 1 | Not-determined | Genome-fragment | NA | | 0 |
| S3-final-viral-combined_k141_52088\|\|full | 2109 | No | NA | 0 | 3 | Not-determined | Genome-fragment | NA | | 0 |
| S3-final-viral-combined_k141_40553\|\|full | 5218 | No | NA | 0 | 3 | Not-determined | Genome-fragment | NA | | 0 |
| S3-final-viral-combined_k141_63590\|\|full | 5660 | No | NA | 0 | 2 | Not-determined | Genome-fragment | NA | | 0 |
| S3-final-viral-combined_k141_40561\|\|full | 1591 | No | NA | 0 | 1 | Not-determined | Genome-fragment | NA | | 0 |
| S2_k141_131725 | 1514 | No | NA | 0 | 0 | Low-quality | Genome-fragment | | 2.16 | 0 |
| S3-final-viral-combined_k141_75035\|\|full | 2580 | No | NA | 0 | 2 | Not-determined | Genome-fragment | NA | | 0 |
| S3-final-viral-combined_k141_86723\|\|full | 1170 | No | NA | 0 | 1 | Not-determined | Genome-fragment | NA | | 0 |
| S8-final-viral-combined_k141_141912\|\|full | 1514 | No | NA | 0 | 0 | Low-quality | Genome-fragment | | 1.85 | 0 |
| S3-final-viral-combined_k141_80797\|\|full | 3146 | No | NA | 0 | 2 | Not-determined | Genome-fragment | NA | | 0 |
| S8-final-viral-combined_k141_259871\|\|lt2gene | 1514 | No | NA | 2 | 0 | Low-quality | Genome-fragment | | 3.67 | 0 |
| S3-final-viral-combined_k141_126929\|\|full | 1567 | No | NA | 0 | 1 | Not-determined | Genome-fragment | NA | | 0 |
| S3-final-viral-combined_k141_23437\|\|full | 1026 | No | NA | 0 | 1 | Not-determined | Genome-fragment | NA | | 0 |
| S3-final-viral-combined_k141_69383\|\|full | 1451 | No | NA | 0 | 3 | Not-determined | Genome-fragment | NA | | 0 |
| S11-final-viral-combined_k141_32724\|\|full | 1515 | No | NA | 0 | 0 | Low-quality | Genome-fragment | | 2.45 | 0 |
| S3-final-viral-combined_k141_17572\|\|full | 1412 | No | NA | 0 | 1 | Not-determined | Genome-fragment | NA | | 0 |
| S3-final-viral-combined_k141_98093\|\|full | 2209 | No | NA | 0 | 1 | Not-determined | Genome-fragment | NA | | 0 |
| S3-final-viral-combined_k141_132708\|\|full | 1692 | No | NA | 0 | 1 | Not-determined | Genome-fragment | NA | | 0 |
| S11-final-viral-combined_k141_300551\|\|full | 1515 | No | NA | 2 | 0 | Low-quality | Genome-fragment | | 4.16 | 0 |
| S3-final-viral-combined_k141_481\|\|full | 4745 | No | NA | 0 | 0 | Not-determined | Genome-fragment | NA | | 0 |
| S3-final-viral-combined_k141_75114\|\|full | 2171 | No | NA | 0 | 1 | Not-determined | Genome-fragment | NA | | 0 |
| S3-final-viral-combined_k141_490\|\|full | 1780 | No | NA | 0 | 1 | Not-determined | Genome-fragment | NA | | 0 |
| S3-final-viral-combined_k141_80862\|\|full | 3959 | No | NA | 0 | 2 | Not-determined | Genome-fragment | NA | | 0 |
| S1-final-viral-combined_k141_103787\|\|full | 1515 | No | NA | 0 | 0 | Low-quality | Genome-fragment | | 3.17 | 0 |
| S3-final-viral-combined_k141_75127\|\|full | 1080 | No | NA | 0 | 1 | Not-determined | Genome-fragment | NA | | 0 |
| S3-final-viral-combined_k141_36589\|\|full | 1515 | No | NA | 1 | 0 | Low-quality | Genome-fragment | | 2.84 | 0 |
| S7-final-viral-combined_k141_63872\|\|full | 1515 | No | NA | 1 | 0 | Low-quality | Genome-fragment | | 2.25 | 0 |
| S3-final-viral-combined_k141_6190\|\|full | 1421 | No | NA | 0 | 1 | Not-determined | Genome-fragment | NA | | 0 |
| S3-final-viral-combined_k141_34970\|\|full | 2720 | No | NA | 0 | 1 | Not-determined | Genome-fragment | NA | | 0 |
| S3-final-viral-combined_k141_98117\|\|full | 2071 | No | NA | 0 | 1 | Not-determined | Genome-fragment | NA | | 0 |
| S17-final-viral-combined_k141_93112\|\|full | 1515 | No | NA | 1 | 0 | Low-quality | Genome-fragment | | 2.22 | 0 |
| S12-final-viral-combined_k141_40393\|\|full | 1515 | No | NA | 2 | 0 | Low-quality | Genome-fragment | | 3.83 | 0 |
| S6-final-viral-combined_k141_65092\|\|full | 1515 | No | NA | 0 | 1 | Low-quality | Genome-fragment | | 1.9 | 0 |
| S3-final-viral-combined_k141_40657\|\|full | 2119 | No | NA | 0 | 2 | Not-determined | Genome-fragment | NA | | 0 |
| S3-final-viral-combined_k141_57849\|\|full | 1166 | No | NA | 0 | 2 | Not-determined | Genome-fragment | NA | | 0 |
| S8-final-viral-combined_k141_44070\|\|full | 1515 | No | NA | 1 | 0 | Low-quality | Genome-fragment | | 2.77 | 0 |
| S1-final-viral-combined_k141_350822\|\|lt2gene | 1516 | No | NA | 1 | 0 | Low-quality | Genome-fragment | | 3.41 | 0 |
| S3-final-viral-combined_k141_69470\|\|full | 1671 | No | NA | 0 | 0 | Not-determined | Genome-fragment | NA | | 0 |
| S1-final-viral-combined_k141_289072\|\|lt2gene | 1516 | No | NA | 1 | 0 | Low-quality | Genome-fragment | | 2.22 | 0 |
| S1_k141_381386 | 1516 | No | NA | 0 | 1 | Low-quality | Genome-fragment | | 1.63 | 0 |
| S5-final-viral-combined_k141_68040\|\|full | 1516 | No | NA | 0 | 0 | Low-quality | Genome-fragment | | 2.54 | 0 |
| S3-final-viral-combined_k141_132783\|\|full | 1472 | No | NA | 0 | 2 | Not-determined | Genome-fragment | NA | | 0 |
| S3-final-viral-combined_k141_6239\|\|full | 2244 | No | NA | 0 | 2 | Not-determined | Genome-fragment | NA | | 0 |
| S3-final-viral-combined_k141_92473\|\|full | 2244 | No | NA | 0 | 2 | Not-determined | Genome-fragment | NA | | 0 |
| S3-final-viral-combined_k141_11890\|\|full | 4714 | No | NA | 0 | 2 | Not-determined | Genome-fragment | NA | | 0 |
| S3-final-viral-combined_k141_11897\|\|full | 2622 | No | NA | 0 | 1 | Not-determined | Genome-fragment | NA | | 0 |
| S3-final-viral-combined_k141_11904\|\|full | 1531 | No | NA | 0 | 1 | Not-determined | Genome-fragment | NA | | 0 |
| S3-final-viral-combined_k141_6271\|\|full | 3819 | No | NA | 0 | 2 | Not-determined | Genome-fragment | NA | | 0 |
| S3-final-viral-combined_k141_75205\|\|full | 1219 | No | NA | 0 | 0 | Not-determined | Genome-fragment | NA | | 0 |
| S7-final-viral-combined_k141_199330\|\|full | 1516 | No | NA | 0 | 1 | Low-quality | Genome-fragment | | 0.62 | 0 |
| S3-final-viral-combined_k141_6278\|\|full | 2385 | No | NA | 0 | 3 | Not-determined | Genome-fragment | NA | | 0 |
| S3-final-viral-combined_k141_35059\|\|full | 11579 | No | NA | 0 | 7 | Not-determined | Genome-fragment | NA | | 0 |
| S3-final-viral-combined_k141_86910\|\|full | 3479 | No | NA | 0 | 2 | Not-determined | Genome-fragment | NA | | 0 |
| S7-final-viral-combined_k141_230412\|\|lt2gene | 1516 | No | NA | 0 | 0 | Low-quality | Genome-fragment | | 1.67 | 0 |
| S3-final-viral-combined_k141_86913\|\|full | 3509 | No | NA | 0 | 2 | Not-determined | Genome-fragment | NA | | 0 |
| S3-final-viral-combined_k141_23593\|\|full | 2803 | No | NA | 0 | 3 | Not-determined | Genome-fragment | NA | | 0 |
| S3-final-viral-combined_k141_23594\|\|full | 1950 | No | NA | 0 | 1 | Not-determined | Genome-fragment | NA | | 0 |
| S3-final-viral-combined_k141_98216\|\|full | 7129 | No | NA | 0 | 4 | Not-determined | Genome-fragment | NA | | 0 |
| S3-final-viral-combined_k141_46638\|\|full | 2479 | No | NA | 0 | 1 | Not-determined | Genome-fragment | NA | | 0 |
| S3-final-viral-combined_k141_115597\|\|full | 5103 | No | NA | 0 | 5 | Not-determined | Genome-fragment | NA | | 0 |
| S3-final-viral-combined_k141_6381\|\|full | 3227 | No | NA | 0 | 1 | Not-determined | Genome-fragment | NA | | 0 |
| S3-final-viral-combined_k141_75264\|\|full | 8092 | No | NA | 0 | 1 | Not-determined | Genome-fragment | NA | | 0 |
| S17-final-viral-combined_k141_2849\|\|full | 1516 | No | NA | 1 | 0 | Low-quality | Genome-fragment | | 2.4 | 0 |
| S12-final-viral-combined_k141_154699\|\|full | 1516 | No | NA | 0 | 0 | Low-quality | Genome-fragment | | 1.96 | 0 |
| S3-final-viral-combined_k141_11975\|\|full | 1166 | No | NA | 0 | 2 | Not-determined | Genome-fragment | NA | | 0 |
| S3-final-viral-combined_k141_40838\|\|full | 2080 | No | NA | 0 | 1 | Not-determined | Genome-fragment | NA | | 0 |
| S3-final-viral-combined_k141_35115\|\|full | 3223 | No | NA | 0 | 1 | Not-determined | Genome-fragment | NA | | 0 |
| S3-final-viral-combined_k141_81080\|\|full | 1720 | No | NA | 0 | 1 | Not-determined | Genome-fragment | NA | | 0 |
| S3-final-viral-combined_k141_40857\|\|full | 2797 | No | NA | 0 | 1 | Not-determined | Genome-fragment | NA | | 0 |
| S3-final-viral-combined_k141_132922\|\|full | 2389 | No | NA | 0 | 0 | Not-determined | Genome-fragment | NA | | 0 |
| S3-final-viral-combined_k141_63821\|\|full | 1164 | No | NA | 0 | 0 | Not-determined | Genome-fragment | NA | | 0 |
| S3-final-viral-combined_k141_92586\|\|full | 2032 | No | NA | 0 | 2 | Not-determined | Genome-fragment | NA | | 0 |
| S3-final-viral-combined_k141_98248\|\|full | 3617 | No | NA | 0 | 1 | Not-determined | Genome-fragment | NA | | 0 |
| S3-final-viral-combined_k141_35146\|\|full | 1755 | No | NA | 0 | 1 | Not-determined | Genome-fragment | NA | | 0 |
| S3-final-viral-combined_k141_132926\|\|full | 6650 | No | NA | 0 | 4 | Not-determined | Genome-fragment | NA | | 0 |
| S12-final-viral-combined_k141_175404\|\|full | 1516 | No | NA | 0 | 1 | Low-quality | Genome-fragment | | 5.65 | 0 |
| S3-final-viral-combined_k141_104145\|\|full | 2179 | No | NA | 0 | 2 | Not-determined | Genome-fragment | NA | | 0 |
| S12_k141_203268 | 1516 | No | NA | 0 | 0 | Low-quality | Genome-fragment | | 47.39 | 0 |
| S2_k141_126026 | 1516 | No | NA | 3 | 0 | Low-quality | Genome-fragment | | 4.21 | 0 |
| S3-final-viral-combined_k141_109956\|\|full | 14976 | No | NA | 0 | 1 | Not-determined | Genome-fragment | NA | | 0 |
| S3-final-viral-combined_k141_46700\|\|full | 1731 | No | NA | 0 | 1 | Not-determined | Genome-fragment | NA | | 0 |
| S3-final-viral-combined_k141_40889\|\|full | 3074 | No | NA | 0 | 1 | Not-determined | Genome-fragment | NA | | 0 |
| S3-final-viral-combined_k141_75309\|\|full | 2606 | No | NA | 0 | 2 | Not-determined | Genome-fragment | NA | | 0 |
| S2_k141_193889 | 1516 | No | NA | 0 | 2 | Low-quality | Genome-fragment | | 0.42 | 0 |
| S3-final-viral-combined_k141_127304\|\|full | 1511 | No | NA | 0 | 0 | Not-determined | Genome-fragment | NA | | 0 |
| S6-final-viral-combined_k141_10817\|\|full | 1516 | No | NA | 1 | 0 | Low-quality | Genome-fragment | | 3.91 | 0 |
| S8-final-viral-combined_k141_242270\|\|full | 1516 | No | NA | 0 | 1 | Low-quality | Genome-fragment | | 2.64 | 0 |
| S9-final-viral-combined_k141_26632\|\|full | 1517 | No | NA | 0 | 0 | Low-quality | Genome-fragment | | 3.04 | 0 |
| S9-final-viral-combined_k141_98465\|\|lt2gene | 1517 | No | NA | 1 | 0 | Low-quality | Genome-fragment | | 3.49 | 0 |
| S11-final-viral-combined_k141_225585\|\|full | 1517 | No | NA | 2 | 0 | Low-quality | Genome-fragment | | 3.59 | 0 |
| S11_k141_1635 | 1517 | No | NA | 0 | 0 | Low-quality | Genome-fragment | | 3.99 | 0 |
| S1-final-viral-combined_k141_298528\|\|full | 1517 | No | NA | 2 | 0 | Low-quality | Genome-fragment | | 3.76 | 0 |
| S3-final-viral-combined_k141_12063\|\|full | 2818 | No | NA | 0 | 2 | Not-determined | Genome-fragment | NA | | 0 |
| S7-final-viral-combined_k141_207085\|\|full | 1517 | No | NA | 0 | 0 | Low-quality | Genome-fragment | | 3.45 | 0 |
| S7-final-viral-combined_k141_209743\|\|full | 1517 | No | NA | 1 | 0 | Low-quality | Genome-fragment | | 3.43 | 0 |
| S3-final-viral-combined_k141_6510\|\|full | 1159 | No | NA | 0 | 2 | Not-determined | Genome-fragment | NA | | 0 |
| S3-final-viral-combined_k141_29338\|\|full | 6194 | No | NA | 0 | 4 | Not-determined | Genome-fragment | NA | | 0 |
| S17-final-viral-combined_k141_262930\|\|full | 1517 | No | NA | 0 | 2 | Low-quality | Genome-fragment | | 0.76 | 0 |
| S3-final-viral-combined_k141_121438\|\|full | 1670 | No | NA | 0 | 1 | Not-determined | Genome-fragment | NA | | 0 |
| S17-k141_162884 | 1517 | No | NA | 1 | 0 | Low-quality | Genome-fragment | | 2.59 | 0 |
| S12-final-viral-combined_k141_167405\|\|full | 1517 | No | NA | 0 | 0 | Low-quality | Genome-fragment | | 3.16 | 0 |
| S3-final-viral-combined_k141_104223\|\|full | 2549 | No | NA | 0 | 0 | Not-determined | Genome-fragment | NA | | 0 |
| S12_k141_241359 | 1517 | No | NA | 0 | 1 | Low-quality | Genome-fragment | | 0.39 | 0 |
| S3-final-viral-combined_k141_6582\|\|full | 3084 | No | NA | 0 | 3 | Not-determined | Genome-fragment | NA | | 0 |
| S9-final-viral-combined_k141_32414\|\|lt2gene | 1518 | No | NA | 1 | 0 | Low-quality | Genome-fragment | | 3.87 | 0 |
| S11-final-viral-combined_k141_255129\|\|full | 1518 | No | NA | 0 | 0 | Low-quality | Genome-fragment | | 2.75 | 0 |
| S3-final-viral-combined_k141_29421\|\|full | 2192 | No | NA | 0 | 2 | Not-determined | Genome-fragment | NA | | 0 |

| | | | | | | | | | | | |
|---|---|---|---|---|---|---|---|---|---|---|---|
| S3-final-viral-combined_k141_104247\|\|full | 2091 | No | NA | | 0 | 0 | Not-determined | Genome-fragment | NA | | 0 |
| S11-final-viral-combined_k141_229794\|\|full | 1518 | No | NA | | 0 | 2 | Low-quality | Genome-fragment | | 0.48 | 0 |
| S1-final-viral-combined_k141_169446\|\|lt2gene | 1518 | No | NA | | 1 | 0 | Low-quality | Genome-fragment | | 2.2 | 0 |
| S5-final-viral-combined_k141_63723\|\|full | 1518 | No | NA | | 0 | 1 | Low-quality | Genome-fragment | | 2.77 | 0 |
| S3-final-viral-combined_k141_829\|\|full | 4321 | No | NA | | 0 | 3 | Not-determined | Genome-fragment | NA | | 0 |
| S3-final-viral-combined_k141_29438\|\|full | 3026 | No | NA | | 0 | 1 | Not-determined | Genome-fragment | NA | | 0 |
| S3-final-viral-combined_k141_18003\|\|full | 1527 | No | NA | | 0 | 2 | Not-determined | Genome-fragment | NA | | 0 |
| S7-final-viral-combined_k141_48717\|\|full | 1518 | Yes | | 1287 | 1 | 1 | Low-quality | Genome-fragment | | 2.58 | 15.22 |
| S3-final-viral-combined_k141_127462\|\|full | 2131 | No | NA | | 0 | 1 | Not-determined | Genome-fragment | NA | | 0 |
| S12-final-viral-combined_k141_128269\|\|full | 1518 | No | NA | | 1 | 0 | Low-quality | Genome-fragment | | 2.91 | 0 |
| S3-final-viral-combined_k141_46886\|\|full | 2904 | No | NA | | 0 | 1 | Not-determined | Genome-fragment | NA | | 0 |
| S3-final-viral-combined_k141_64036\|\|full | 1864 | No | NA | | 0 | 3 | Not-determined | Genome-fragment | NA | | 0 |
| S3-final-viral-combined_k141_110095\|\|full | 3544 | No | NA | | 0 | 2 | Not-determined | Genome-fragment | NA | | 0 |
| S3-final-viral-combined_k141_81366\|\|full | 1844 | No | NA | | 0 | 1 | Not-determined | Genome-fragment | NA | | 0 |
| S3-final-viral-combined_k141_898\|\|full | 3872 | No | NA | | 0 | 4 | Not-determined | Genome-fragment | NA | | 0 |
| S3-final-viral-combined_k141_900\|\|full | 4492 | No | NA | | 0 | 3 | Not-determined | Genome-fragment | NA | | 0 |
| S12_k141_89498 | 1518 | No | NA | | 0 | 0 | Low-quality | Genome-fragment | | 1.39 | 0 |
| S3-final-viral-combined_k141_18042\|\|full | 1837 | No | NA | | 0 | 0 | Not-determined | Genome-fragment | NA | | 0 |
| S12_k141_308865 | 1518 | No | NA | | 0 | 1 | Low-quality | Genome-fragment | | 0.61 | 0 |
| S2-final-viral-combined_k141_214434\|\|full | 1518 | No | NA | | 0 | 1 | Low-quality | Genome-fragment | | 2.29 | 0 |
| S2_k141_16560 | 1518 | No | NA | | 2 | 0 | Low-quality | Genome-fragment | | 3.25 | 0 |
| S3-final-viral-combined_k141_133131\|\|full | 2308 | No | NA | | 0 | 2 | Not-determined | Genome-fragment | NA | | 0 |
| S3-final-viral-combined_k141_127507\|\|full | 2223 | No | NA | | 0 | 2 | Not-determined | Genome-fragment | NA | | 0 |
| S11-final-viral-combined_k141_1294\|\|full | 1519 | No | NA | | 0 | 2 | Low-quality | Genome-fragment | | 0.98 | 0 |
| S3-final-viral-combined_k141_81435\|\|full | 1720 | No | NA | | 0 | 2 | Not-determined | Genome-fragment | NA | | 0 |
| S3-final-viral-combined_k141_121555\|\|full | 4283 | No | NA | | 0 | 3 | Not-determined | Genome-fragment | NA | | 0 |
| S3-final-viral-combined_k141_64120\|\|full | 2596 | No | NA | | 0 | 2 | Not-determined | Genome-fragment | NA | | 0 |
| S11-final-viral-combined_k141_169660\|\|full | 1519 | No | NA | | 1 | 0 | Low-quality | Genome-fragment | | 1.71 | 0 |
| S11-final-viral-combined_k141_40503\|\|full | 1519 | No | NA | | 0 | 1 | Low-quality | Genome-fragment | | 1.81 | 0 |
| S3-final-viral-combined_k141_81464\|\|full | 1920 | No | NA | | 0 | 1 | Not-determined | Genome-fragment | NA | | 0 |
| S11-final-viral-combined_k141_71412\|\|lt2gene | 1519 | No | NA | | 1 | 0 | Low-quality | Genome-fragment | | 2.8 | 0 |
| S1_k141_353056 | 1519 | No | NA | | 0 | 0 | Low-quality | Genome-fragment | | 3.17 | 0 |
| S3-final-viral-combined_k141_35405\|\|full | 2080 | No | NA | | 0 | 1 | Not-determined | Genome-fragment | NA | | 0 |
| S3-final-viral-combined_k141_5666\|\|full | 1519 | No | NA | | 0 | 0 | Low-quality | Genome-fragment | | 2.82 | 0 |
| S3-final-viral-combined_k141_58437\|\|full | 5117 | No | NA | | 0 | 3 | Not-determined | Genome-fragment | NA | | 0 |
| S5-final-viral-combined_k141_125352\|\|full | 1519 | No | NA | | 0 | 0 | Low-quality | Genome-fragment | | 0.87 | 0 |
| S3-final-viral-combined_k141_75632\|\|full | 6240 | No | NA | | 0 | 4 | Not-determined | Genome-fragment | NA | | 0 |
| S3-final-viral-combined_k141_69881\|\|full | 3591 | No | NA | | 0 | 2 | Not-determined | Genome-fragment | NA | | 0 |
| S5-final-viral-combined_k141_156565\|\|lt2gene | 1519 | No | NA | | 2 | 0 | Low-quality | Genome-fragment | | 3.71 | 0 |
| S3-final-viral-combined_k141_87399\|\|full | 1348 | No | NA | | 0 | 1 | Not-determined | Genome-fragment | NA | | 0 |
| S17-final-viral-combined_k141_198760\|\|lt2gene | 1519 | No | NA | | 1 | 0 | Low-quality | Genome-fragment | | 3.81 | 0 |
| S3-final-viral-combined_k141_127610\|\|full | 1142 | No | NA | | 0 | 0 | Not-determined | Genome-fragment | NA | | 0 |
| S3-final-viral-combined_k141_69906\|\|full | 2877 | No | NA | | 0 | 3 | Not-determined | Genome-fragment | NA | | 0 |
| S3-final-viral-combined_k141_58458\|\|full | 4006 | No | NA | | 0 | 3 | Not-determined | Genome-fragment | NA | | 0 |
| S3-final-viral-combined_k141_115914\|\|full | 1187 | No | NA | | 0 | 1 | Not-determined | Genome-fragment | NA | | 0 |
| S3-final-viral-combined_k141_18073\|\|full | 3790 | No | NA | | 0 | 2 | Not-determined | Genome-fragment | NA | | 0 |
| S3-final-viral-combined_k141_75687\|\|full | 1165 | No | NA | | 0 | 0 | Not-determined | Genome-fragment | NA | | 0 |
| S3-final-viral-combined_k141_6776\|\|full | 6530 | No | NA | | 0 | 3 | Not-determined | Genome-fragment | NA | | 0 |
| S12-final-viral-combined_k141_185488\|\|full | 1519 | No | NA | | 0 | 3 | Low-quality | Genome-fragment | | 2.21 | 0 |
| S3-final-viral-combined_k141_52765\|\|full | 1601 | No | NA | | 0 | 0 | Not-determined | Genome-fragment | NA | | 0 |
| S4-final-viral-combined_k141_22642\|\|lt2gene | 1519 | No | NA | | 0 | 0 | Low-quality | Genome-fragment | | 1.51 | 0 |
| S3-final-viral-combined_k141_133261\|\|full | 2127 | No | NA | | 0 | 2 | Not-determined | Genome-fragment | NA | | 0 |
| S3-final-viral-combined_k141_127636\|\|full | 1728 | No | NA | | 0 | 0 | Not-determined | Genome-fragment | NA | | 0 |
| S3-final-viral-combined_k141_35483\|\|full | 2174 | No | NA | | 0 | 1 | Not-determined | Genome-fragment | NA | | 0 |
| S3-final-viral-combined_k141_98544\|\|full | 3879 | No | NA | | 0 | 3 | Not-determined | Genome-fragment | NA | | 0 |
| S9-final-viral-combined_k141_5895\|\|full | 1520 | No | NA | | 0 | 1 | Low-quality | Genome-fragment | | 0.71 | 0 |
| S3-final-viral-combined_k141_75741\|\|full | 2810 | No | NA | | 0 | 1 | Not-determined | Genome-fragment | NA | | 0 |
| S1-final-viral-combined_k141_213196\|\|full | 1520 | No | NA | | 0 | 1 | Low-quality | Genome-fragment | | 1.42 | 0 |
| S3-final-viral-combined_k141_64283\|\|full | 3210 | No | NA | | 0 | 1 | Not-determined | Genome-fragment | NA | | 0 |
| S3-final-viral-combined_k141_75749\|\|full | 2690 | No | NA | | 0 | 1 | Not-determined | Genome-fragment | NA | | 0 |
| S5-final-viral-combined_k141_205368\|\|lt2gene | 1520 | No | NA | | 1 | 0 | Low-quality | Genome-fragment | | 2.22 | 0 |
| S3-final-viral-combined_k141_87519\|\|full | 1895 | No | NA | | 0 | 1 | Not-determined | Genome-fragment | NA | | 0 |
| S3-final-viral-combined_k141_98621\|\|full | 2073 | No | NA | | 0 | 1 | Not-determined | Genome-fragment | NA | | 0 |
| S7-final-viral-combined_k141_106347\|\|full | 1520 | No | NA | | 0 | 0 | Low-quality | Genome-fragment | | 1.23 | 0 |
| S3-final-viral-combined_k141_64331\|\|full | 3862 | No | NA | | 0 | 3 | Not-determined | Genome-fragment | NA | | 0 |
| S3-final-viral-combined_k141_6871\|\|full | 2156 | No | NA | | 0 | 2 | Not-determined | Genome-fragment | NA | | 0 |
| S7_k141_131880 | 1520 | No | NA | | 0 | 1 | Low-quality | Genome-fragment | | 1.07 | 0 |
| S3-final-viral-combined_k141_70027\|\|full | 1657 | No | NA | | 0 | 1 | Not-determined | Genome-fragment | NA | | 0 |
| S3-final-viral-combined_k141_18197\|\|full | 1827 | No | NA | | 0 | 2 | Not-determined | Genome-fragment | NA | | 0 |
| S3-final-viral-combined_k141_58583\|\|full | 6487 | No | NA | | 0 | 5 | Not-determined | Genome-fragment | NA | | 0 |
| S12_k141_390299 | 1520 | No | NA | | 1 | 0 | Low-quality | Genome-fragment | | 2.07 | 0 |
| S8-final-viral-combined_k141_219637\|\|full | 1520 | No | NA | | 0 | 0 | Low-quality | Genome-fragment | | 3.59 | 0 |
| S1-final-viral-combined_k141_242169\|\|full | 1521 | No | NA | | 0 | 0 | Low-quality | Genome-fragment | | 2.87 | 0 |
| S3-final-viral-combined_k141_24087\|\|full | 2006 | No | NA | | 0 | 2 | Not-determined | Genome-fragment | NA | | 0 |
| S1-final-viral-combined_k141_76974\|\|full | 1521 | No | NA | | 0 | 1 | Low-quality | Genome-fragment | | 1.58 | 0 |
| S2-final-viral-combined_k141_35871\|\|full | 1521 | No | NA | | 2 | 0 | Low-quality | Genome-fragment | | 4.53 | 0 |
| S3-final-viral-combined_k141_6925\|\|full | 1014 | No | NA | | 0 | 0 | Not-determined | Genome-fragment | NA | | 0 |
| S4-final-viral-combined_k141_142156\|\|full | 1521 | No | NA | | 0 | 0 | Low-quality | Genome-fragment | | 1.16 | 0 |
| S8-final-viral-combined_k141_123556\|\|full | 1521 | No | NA | | 0 | 0 | Low-quality | Genome-fragment | | 3.7 | 0 |
| S3-final-viral-combined_k141_12421\|\|full | 1284 | No | NA | | 0 | 2 | Not-determined | Genome-fragment | NA | | 0 |
| S3-final-viral-combined_k141_133422\|\|full | 1883 | No | NA | | 0 | 1 | Not-determined | Genome-fragment | NA | | 0 |
| S3-final-viral-combined_k141_133428\|\|full | 2244 | No | NA | | 0 | 0 | Not-determined | Genome-fragment | NA | | 0 |
| S3-final-viral-combined_k141_70091\|\|full | 1364 | No | NA | | 0 | 0 | Not-determined | Genome-fragment | NA | | 0 |
| S1_k141_106984 | 1522 | No | NA | | 0 | 3 | Low-quality | Genome-fragment | | 0.44 | 0 |
| S5-final-viral-combined_k141_204451\|\|full | 1522 | No | NA | | 1 | 0 | Low-quality | Genome-fragment | | 2.96 | 0 |
| S3-final-viral-combined_k141_133455\|\|full | 1183 | No | NA | | 0 | 1 | Not-determined | Genome-fragment | NA | | 0 |
| S3-final-viral-combined_k141_12479\|\|full | 2291 | No | NA | | 0 | 0 | Not-determined | Genome-fragment | NA | | 0 |
| S3-final-viral-combined_k141_110452\|\|full | 1349 | No | NA | | 0 | 2 | Not-determined | Genome-fragment | NA | | 0 |
| S3-final-viral-combined_k141_70129\|\|full | 1340 | No | NA | | 0 | 1 | Not-determined | Genome-fragment | NA | | 0 |
| S7-final-viral-combined_k141_35193\|\|full | 1522 | No | NA | | 0 | 0 | Low-quality | Genome-fragment | | 3.97 | 0 |
| S3-final-viral-combined_k141_24191\|\|full | 1793 | No | NA | | 0 | 2 | Not-determined | Genome-fragment | NA | | 0 |
| S3-final-viral-combined_k141_58669\|\|full | 2809 | No | NA | | 0 | 3 | Not-determined | Genome-fragment | NA | | 0 |
| S7_k141_157717 | 1522 | No | NA | | 1 | 0 | Low-quality | Genome-fragment | | 1.04 | 0 |
| S17-final-viral-combined_k141_12785\|\|full | 1522 | No | NA | | 0 | 0 | Low-quality | Genome-fragment | | 3.57 | 0 |
| S17-final-viral-combined_k141_59855\|\|full | 1522 | No | NA | | 1 | 0 | Low-quality | Genome-fragment | | 3.54 | 0 |
| S17-final-viral-combined_k141_249006\|\|full | 1522 | No | NA | | 0 | 0 | Low-quality | Genome-fragment | | 2.63 | 0 |
| S3-final-viral-combined_k141_127894\|\|full | 2040 | No | NA | | 0 | 1 | Not-determined | Genome-fragment | NA | | 0 |
| S2-final-viral-combined_k141_35397\|\|full | 1522 | No | NA | | 0 | 0 | Low-quality | Genome-fragment | | 1.83 | 0 |
| S3-final-viral-combined_k141_41460\|\|full | 1068 | No | NA | | 0 | 1 | Not-determined | Genome-fragment | NA | | 0 |
| S4-final-viral-combined_k141_110128\|\|lt2gene | 1522 | No | NA | | 1 | 0 | Low-quality | Genome-fragment | | 34.04 | 0 |
| S4_k141_59811 | 1522 | No | NA | | 0 | 0 | Low-quality | Genome-fragment | | 2.29 | 0 |
| S3-final-viral-combined_k141_127917\|\|full | 2825 | No | NA | | 0 | 2 | Not-determined | Genome-fragment | NA | | 0 |
| S8_k141_235880 | 1522 | No | NA | | 0 | 0 | Low-quality | Genome-fragment | | 0.64 | 0 |
| S9-final-viral-combined_k141_112112\|\|full | 1523 | No | NA | | 1 | 0 | Low-quality | Genome-fragment | | 4.12 | 0 |
| S1-final-viral-combined_k141_48813\|\|full | 1523 | No | NA | | 3 | 0 | Low-quality | Genome-fragment | | 2.91 | 0 |
| S3-final-viral-combined_k141_93190\|\|full | 1774 | No | NA | | 0 | 0 | Not-determined | Genome-fragment | NA | | 0 |
| S3_k141_116812 | 1523 | No | NA | | 0 | 0 | Low-quality | Genome-fragment | | 2.21 | 0 |
| S3-final-viral-combined_k141_87700\|\|full | 1015 | No | NA | | 0 | 1 | Not-determined | Genome-fragment | NA | | 0 |
| S5-final-viral-combined_k141_21131\|\|full | 1523 | No | NA | | 0 | 3 | Low-quality | Genome-fragment | | 3.07 | 0 |
| S3-final-viral-combined_k141_133564\|\|full | 4133 | No | NA | | 0 | 2 | Not-determined | Genome-fragment | NA | | 0 |
| S5-final-viral-combined_k141_130314\|\|full | 1523 | No | NA | | 0 | 1 | Low-quality | Genome-fragment | | 3.1 | 0 |
| S5-final-viral-combined_k141_38048\|\|lt2gene | 1523 | No | NA | | 1 | 0 | Low-quality | Genome-fragment | | 3.34 | 0 |
| S12-final-viral-combined_k141_168601\|\|full | 1523 | No | NA | | 0 | 0 | Low-quality | Genome-fragment | | 2.45 | 0 |
| S3-final-viral-combined_k141_76025\|\|full | 2750 | No | NA | | 0 | 0 | Not-determined | Genome-fragment | NA | | 0 |
| S3-final-viral-combined_k141_104696\|\|full | 2206 | No | NA | | 0 | 2 | Not-determined | Genome-fragment | NA | | 0 |
| S12-final-viral-combined_k141_315281\|\|full | 1523 | No | NA | | 0 | 0 | Low-quality | Genome-fragment | | 0.98 | 0 |
| S3-final-viral-combined_k141_93232\|\|full | 2177 | No | NA | | 0 | 1 | Not-determined | Genome-fragment | NA | | 0 |
| S12-final-viral-combined_k141_365531\|\|full | 1523 | No | NA | | 0 | 0 | Low-quality | Genome-fragment | | 3.94 | 0 |
| S3-final-viral-combined_k141_58815\|\|full | 1897 | No | NA | | 0 | 0 | Not-determined | Genome-fragment | NA | | 0 |
| S2-final-viral-combined_k141_106609\|\|full | 1523 | No | NA | | 0 | 1 | Low-quality | Genome-fragment | | 2.92 | 0 |
| S2_k141_206297 | 1523 | No | NA | | 0 | 1 | Low-quality | Genome-fragment | | 1.05 | 0 |
| S3-final-viral-combined_k141_76067\|\|full | 2263 | No | NA | | 0 | 1 | Not-determined | Genome-fragment | NA | | 0 |
| S8-final-viral-combined_k141_244965\|\|full | 1523 | No | NA | | 0 | 2 | Low-quality | Genome-fragment | | 0.97 | 0 |
| S3-final-viral-combined_k141_18461\|\|full | 1178 | No | NA | | 0 | 0 | Not-determined | Genome-fragment | NA | | 0 |
| S3-final-viral-combined_k141_35783\|\|full | 4406 | No | NA | | 0 | 3 | Not-determined | Genome-fragment | NA | | 0 |
| S3-final-viral-combined_k141_98872\|\|full | 2993 | No | NA | | 0 | 2 | Not-determined | Genome-fragment | NA | | 0 |
| S3-final-viral-combined_k141_58871\|\|full | 7053 | No | NA | | 0 | 5 | Not-determined | Genome-fragment | NA | | 0 |
| S3-final-viral-combined_k141_98882\|\|full | 1646 | No | NA | | 0 | 1 | Not-determined | Genome-fragment | NA | | 0 |
| S3-final-viral-combined_k141_18468\|\|full | 8276 | No | NA | | 0 | 7 | Not-determined | Genome-fragment | NA | | 0 |
| S3-final-viral-combined_k141_58879\|\|full | 1871 | No | NA | | 0 | 2 | Not-determined | Genome-fragment | NA | | 0 |
| S3-final-viral-combined_k141_128107\|\|full | 1148 | No | NA | | 0 | 0 | Not-determined | Genome-fragment | NA | | 0 |
| S11-final-viral-combined_k141_9724\|\|full | 1524 | No | NA | | 0 | 0 | Low-quality | Genome-fragment | | 4.12 | 0 |
[truncated: 4,633,947 more chars]
