# Supplementary material for: Investigating the Human Intestinal DNA Virome and Predicting Disease-Associated Virus–Host Interactions in Severe Myalgic Encephalomyelitis/Chronic Fatigue Syndrome (ME/CFS)
Source: Int J Mol Sci. 2023 Dec 8;24(24):17267. doi: 10.3390/ijms242417267 (PMC10744171; doi:10.3390/ijms242417267)
Supplement: Supplementary file 1 [file ijms-24-17267-s001.zip › Table S4.pdf]

Table S4. Provirus_and_plasmid_detections

Table S4-1. VLP_provirus_genomad

seq_name	n_genes_with_taxonomy	agreement	taxid	lineage
S17_vs_110666|provirus_6805_70348	58	1	2561	Viruses;Duplodnaviria;Heunggongvirae;Uroviricota;Caudoviricetes
S9_vf_13507|provirus_13823_120230	88	1	2561	Viruses;Duplodnaviria;Heunggongvirae;Uroviricota;Caudoviricetes
S9_vs_46498|provirus_1704_20897	20	1	2561	Viruses;Duplodnaviria;Heunggongvirae;Uroviricota;Caudoviricetes
S9_vs_67792|provirus_23562_67089	41	1	2561	Viruses;Duplodnaviria;Heunggongvirae;Uroviricota;Caudoviricetes
S9_vs_90727|provirus_109214_150749	38	1	2561	Viruses;Duplodnaviria;Heunggongvirae;Uroviricota;Caudoviricetes
S11_vs_45658|provirus_1_50991	48	1	2561	Viruses;Duplodnaviria;Heunggongvirae;Uroviricota;Caudoviricetes
S11_vs_48109|provirus_19503_33025	11	1	2561	Viruses;Duplodnaviria;Heunggongvirae;Uroviricota;Caudoviricetes
S11_vs_48109|provirus_37443_52410	17	1	2561	Viruses;Duplodnaviria;Heunggongvirae;Uroviricota;Caudoviricetes
S11_vs_82331|provirus_1_39593	24	1	2561	Viruses;Duplodnaviria;Heunggongvirae;Uroviricota;Caudoviricetes
S13_vs_9438|provirus_1_26027	15	1	2561	Viruses;Duplodnaviria;Heunggongvirae;Uroviricota;Caudoviricetes
S13_vs_23314|provirus_1_43630	42	1	2561	Viruses;Duplodnaviria;Heunggongvirae;Uroviricota;Caudoviricetes
S13_vs_26756|provirus_1_36490	34	1	2561	Viruses;Duplodnaviria;Heunggongvirae;Uroviricota;Caudoviricetes
S13_vs_30101|provirus_4484_51177	33	0.9912	2561	Viruses;Duplodnaviria;Heunggongvirae;Uroviricota;Caudoviricetes
S13_vs_30167|provirus_1_53839	46	1	2561	Viruses;Duplodnaviria;Heunggongvirae;Uroviricota;Caudoviricetes
S13_vs_33947|provirus_1_26796	14	1	2561	Viruses;Duplodnaviria;Heunggongvirae;Uroviricota;Caudoviricetes
S13_vs_35816|provirus_9638_57224	59	1	2561	Viruses;Duplodnaviria;Heunggongvirae;Uroviricota;Caudoviricetes
S13_vs_45488|provirus_49188_88826	27	1	2561	Viruses;Duplodnaviria;Heunggongvirae;Uroviricota;Caudoviricetes
S13_vs_67429|provirus_51626_91295	36	1	2561	Viruses;Duplodnaviria;Heunggongvirae;Uroviricota;Caudoviricetes
S13_vs_114213|provirus_1_28952	23	1	2561	Viruses;Duplodnaviria;Heunggongvirae;Uroviricota;Caudoviricetes
S14_vs_66139|provirus_119631_178690	40	1	2561	Viruses;Duplodnaviria;Heunggongvirae;Uroviricota;Caudoviricetes
S14_vs_66208|provirus_10049_67174	49	1	2561	Viruses;Duplodnaviria;Heunggongvirae;Uroviricota;Caudoviricetes
S14_vs_96111|provirus_1_24143	26	1	2561	Viruses;Duplodnaviria;Heunggongvirae;Uroviricota;Caudoviricetes
S14_vs_74067|provirus_149062_181135	27	1	2561	Viruses;Duplodnaviria;Heunggongvirae;Uroviricota;Caudoviricetes
S14_vs_74067|provirus_193206_252822	56	1	2561	Viruses;Duplodnaviria;Heunggongvirae;Uroviricota;Caudoviricetes
S14_vs_9162|provirus_77661_102634	28	1	2561	Viruses;Duplodnaviria;Heunggongvirae;Uroviricota;Caudoviricetes
S14_vs_112345|provirus_25548_38263	8	1	2561	Viruses;Duplodnaviria;Heunggongvirae;Uroviricota;Caudoviricetes
S14_vs_39266|provirus_8204_37426	25	1	2561	Viruses;Duplodnaviria;Heunggongvirae;Uroviricota;Caudoviricetes
S14_vs_2870|provirus_1_47896	42	1	2561	Viruses;Duplodnaviria;Heunggongvirae;Uroviricota;Caudoviricetes
S14_vs_32499|provirus_16787_41581	28	1	2561	Viruses;Duplodnaviria;Heunggongvirae;Uroviricota;Caudoviricetes
S14_vs_25852|provirus_1_35257	9	1	2561	Viruses;Duplodnaviria;Heunggongvirae;Uroviricota;Caudoviricetes
S14_vs_25852|provirus_71427_107963	13	1	2561	Viruses;Duplodnaviria;Heunggongvirae;Uroviricota;Caudoviricetes
S14_vs_11510|provirus_1_37329	10	1	2561	Viruses;Duplodnaviria;Heunggongvirae;Uroviricota;Caudoviricetes
S14_vs_78164|provirus_1_33442	35	1	2561	Viruses;Duplodnaviria;Heunggongvirae;Uroviricota;Caudoviricetes
S14_vs_70985|provirus_15261_57392	12	1	2561	Viruses;Duplodnaviria;Heunggongvirae;Uroviricota;Caudoviricetes
S14_vs_70985|provirus_96748_158000	14	1	2561	Viruses;Duplodnaviria;Heunggongvirae;Uroviricota;Caudoviricetes
S1_vs_117317|provirus_1_31247	49	1	2561	Viruses;Duplodnaviria;Heunggongvirae;Uroviricota;Caudoviricetes
S3_vf_106401|provirus_6294_39808	21	1	2561	Viruses;Duplodnaviria;Heunggongvirae;Uroviricota;Caudoviricetes
S3_vs_119605|provirus_10462_56346	45	1	2561	Viruses;Duplodnaviria;Heunggongvirae;Uroviricota;Caudoviricetes
S3_vs_9158|provirus_1_28511	24	0.9779	2561	Viruses;Duplodnaviria;Heunggongvirae;Uroviricota;Caudoviricetes
S3_vs_64424|provirus_1_35095	28	1	2561	Viruses;Duplodnaviria;Heunggongvirae;Uroviricota;Caudoviricetes
S3_vs_17168|provirus_62162_103365	30	1	2561	Viruses;Duplodnaviria;Heunggongvirae;Uroviricota;Caudoviricetes
S3_vs_42569|provirus_1_55470	64	1	2561	Viruses;Duplodnaviria;Heunggongvirae;Uroviricota;Caudoviricetes
S3_vs_18858|provirus_7640_40921	21	1	2561	Viruses;Duplodnaviria;Heunggongvirae;Uroviricota;Caudoviricetes
S3_vs_68031|provirus_1_31195	35	1	2561	Viruses;Duplodnaviria;Heunggongvirae;Uroviricota;Caudoviricetes
S3_vs_6604|provirus_44093_78643	21	1	2561	Viruses;Duplodnaviria;Heunggongvirae;Uroviricota;Caudoviricetes
S3_vs_70508|provirus_10985_57171	30	1	2561	Viruses;Duplodnaviria;Heunggongvirae;Uroviricota;Caudoviricetes
S3_vs_117894|provirus_1_42562	23	1	2561	Viruses;Duplodnaviria;Heunggongvirae;Uroviricota;Caudoviricetes
S5_vs_30490|provirus_19631_56501	24	1	2561	Viruses;Duplodnaviria;Heunggongvirae;Uroviricota;Caudoviricetes
S5_vs_109527|provirus_86293_134147	16	1	2561	Viruses;Duplodnaviria;Heunggongvirae;Uroviricota;Caudoviricetes
S5_vs_123256|provirus_35835_71330	29	1	2561	Viruses;Duplodnaviria;Heunggongvirae;Uroviricota;Caudoviricetes
S5_vs_117319|provirus_37947_108449	65	1	2561	Viruses;Duplodnaviria;Heunggongvirae;Uroviricota;Caudoviricetes
S5_vs_79992|provirus_1_37142	42	1	2561	Viruses;Duplodnaviria;Heunggongvirae;Uroviricota;Caudoviricetes
S5_vs_20500|provirus_26729_71704	33	1	2561	Viruses;Duplodnaviria;Heunggongvirae;Uroviricota;Caudoviricetes
S5_vs_3501|provirus_235_23805	21	1	2561	Viruses;Duplodnaviria;Heunggongvirae;Uroviricota;Caudoviricetes
S5_vs_124677|provirus_1_41828	41	1	2561	Viruses;Duplodnaviria;Heunggongvirae;Uroviricota;Caudoviricetes
S5_vs_130294|provirus_21565_31442	9	1	2561	Viruses;Duplodnaviria;Heunggongvirae;Uroviricota;Caudoviricetes
S5_vs_80496|provirus_58106_119911	51	1	2561	Viruses;Duplodnaviria;Heunggongvirae;Uroviricota;Caudoviricetes
S5_vs_142195|provirus_1_52517	17	1	2561	Viruses;Duplodnaviria;Heunggongvirae;Uroviricota;Caudoviricetes
S5_vs_9747|provirus_14158_64437	20	1	2561	Viruses;Duplodnaviria;Heunggongvirae;Uroviricota;Caudoviricetes
S5_vs_21778|provirus_68731_157098	14	1	2561	Viruses;Duplodnaviria;Heunggongvirae;Uroviricota;Caudoviricetes
S5_vs_131510|provirus_92040_133423	42	1	2561	Viruses;Duplodnaviria;Heunggongvirae;Uroviricota;Caudoviricetes
S5_vs_46394|provirus_33205_42387	9	1	2561	Viruses;Duplodnaviria;Heunggongvirae;Uroviricota;Caudoviricetes
S5_vs_40848|provirus_22909_78371	40	1	2561	Viruses;Duplodnaviria;Heunggongvirae;Uroviricota;Caudoviricetes
S5_vs_72532|provirus_8356_50725	36	1	2561	Viruses;Duplodnaviria;Heunggongvirae;Uroviricota;Caudoviricetes
S5_vs_77593|provirus_49321_99338	54	1	2561	Viruses;Duplodnaviria;Heunggongvirae;Uroviricota;Caudoviricetes
S5_vs_138980|provirus_1_47926	40	1	2561	Viruses;Duplodnaviria;Heunggongvirae;Uroviricota;Caudoviricetes
S5_vs_74199|provirus_14632_30526	9	1	2561	Viruses;Duplodnaviria;Heunggongvirae;Uroviricota;Caudoviricetes
S5_vs_76500|provirus_1_36552	29	1	2561	Viruses;Duplodnaviria;Heunggongvirae;Uroviricota;Caudoviricetes
S7_vf_36627|provirus_69_11085	11	1	2561	Viruses;Duplodnaviria;Heunggongvirae;Uroviricota;Caudoviricetes
S7_vs_11081|provirus_1_46169	41	1	2561	Viruses;Duplodnaviria;Heunggongvirae;Uroviricota;Caudoviricetes
S7_vs_16344|provirus_1_34983	32	1	2561	Viruses;Duplodnaviria;Heunggongvirae;Uroviricota;Caudoviricetes
S7_vs_47050|provirus_1_34528	29	1	2561	Viruses;Duplodnaviria;Heunggongvirae;Uroviricota;Caudoviricetes
S7_vs_49426|provirus_19260_57556	30	1	2561	Viruses;Duplodnaviria;Heunggongvirae;Uroviricota;Caudoviricetes
S7_vs_51332|provirus_1_12316	11	1	2561	Viruses;Duplodnaviria;Heunggongvirae;Uroviricota;Caudoviricetes
S7_vs_97737|provirus_53372_63197	9	1	2561	Viruses;Duplodnaviria;Heunggongvirae;Uroviricota;Caudoviricetes
S7_vs_119679|provirus_14434_51206	39	1	2561	Viruses;Duplodnaviria;Heunggongvirae;Uroviricota;Caudoviricetes
S16_vs_19968|provirus_18399_54417	27	1	2561	Viruses;Duplodnaviria;Heunggongvirae;Uroviricota;Caudoviricetes
S16_vs_32764|provirus_43014_55386	7	1	2561	Viruses;Duplodnaviria;Heunggongvirae;Uroviricota;Caudoviricetes
S16_vs_34021|provirus_1_29929	29	1	2561	Viruses;Duplodnaviria;Heunggongvirae;Uroviricota;Caudoviricetes
S16_vs_48714|provirus_9547_23424	5	0.9272	8016	Viruses;Monodnaviria;Loebvirae;Hofneiviricota;Faserviricetes;Tubulavirales;Inoviridae
S16_vs_82116|provirus_1_40122	45	0.9942	2561	Viruses;Duplodnaviria;Heunggongvirae;Uroviricota;Caudoviricetes
S16_vs_89511|provirus_1_61265	42	1	2561	Viruses;Duplodnaviria;Heunggongvirae;Uroviricota;Caudoviricetes
S16_vs_101072|provirus_6845_31451	27	1	2561	Viruses;Duplodnaviria;Heunggongvirae;Uroviricota;Caudoviricetes
S16_vs_107235|provirus_1_37912	35	1	2561	Viruses;Duplodnaviria;Heunggongvirae;Uroviricota;Caudoviricetes
S16_vs_132979|provirus_1_22166	19	0.9814	2561	Viruses;Duplodnaviria;Heunggongvirae;Uroviricota;Caudoviricetes
S16_vs_140468|provirus_7020_20506	12	1	2561	Viruses;Duplodnaviria;Heunggongvirae;Uroviricota;Caudoviricetes
S16_vs_141218|provirus_1_41081	30	0.9878	2561	Viruses;Duplodnaviria;Heunggongvirae;Uroviricota;Caudoviricetes
S16_vs_142561|provirus_46226_82728	33	1	2561	Viruses;Duplodnaviria;Heunggongvirae;Uroviricota;Caudoviricetes
S10_vs_14557|provirus_24735_75557	31	1	2561	Viruses;Duplodnaviria;Heunggongvirae;Uroviricota;Caudoviricetes
S12_vf_60224|provirus_3687_54526	45	1	2561	Viruses;Duplodnaviria;Heunggongvirae;Uroviricota;Caudoviricetes
S12_vs_71525|provirus_5502_31779	16	1	2561	Viruses;Duplodnaviria;Heunggongvirae;Uroviricota;Caudoviricetes
S12_vs_142625|provirus_29520_70930	33	1	2561	Viruses;Duplodnaviria;Heunggongvirae;Uroviricota;Caudoviricetes
S12_vs_26336|provirus_16576_67092	16	1	2561	Viruses;Duplodnaviria;Heunggongvirae;Uroviricota;Caudoviricetes
S12_vs_121913|provirus_20147_123530	81	1	2561	Viruses;Duplodnaviria;Heunggongvirae;Uroviricota;Caudoviricetes
S12_vs_153780|provirus_11265_61281	31	1	2561	Viruses;Duplodnaviria;Heunggongvirae;Uroviricota;Caudoviricetes
S12_vs_123620|provirus_40480_78363	29	1	2561	Viruses;Duplodnaviria;Heunggongvirae;Uroviricota;Caudoviricetes
S12_vs_133070|provirus_55125_132222	44	1	2561	Viruses;Duplodnaviria;Heunggongvirae;Uroviricota;Caudoviricetes
S12_vs_172379|provirus_92122_132266	25	0.9882	2561	Viruses;Duplodnaviria;Heunggongvirae;Uroviricota;Caudoviricetes
S12_vs_23796|provirus_1_38428	33	1	2561	Viruses;Duplodnaviria;Heunggongvirae;Uroviricota;Caudoviricetes
S15_vs_42043|provirus_134410_193486	49	1	2561	Viruses;Duplodnaviria;Heunggongvirae;Uroviricota;Caudoviricetes
S15_vs_43913|provirus_11358_44889	34	1	2561	Viruses;Duplodnaviria;Heunggongvirae;Uroviricota;Caudoviricetes

S15_vs_71968|provirus_18124_39396	17	1	2561	Viruses;Duplodnaviria;Heunggongvirae;Uroviricota;Caudoviricetes
S2_vs_10289|provirus_7396_46594	37	1	2561	Viruses;Duplodnaviria;Heunggongvirae;Uroviricota;Caudoviricetes
S6_vs_9784|provirus_9084_47596	38	1	2561	Viruses;Duplodnaviria;Heunggongvirae;Uroviricota;Caudoviricetes
S6_vs_54606|provirus_1_21709	30	1	2561	Viruses;Duplodnaviria;Heunggongvirae;Uroviricota;Caudoviricetes
S6_vs_29152|provirus_10612_64693	57	1	2561	Viruses;Duplodnaviria;Heunggongvirae;Uroviricota;Caudoviricetes
S6_vs_10314|provirus_44149_83752	42	1	2561	Viruses;Duplodnaviria;Heunggongvirae;Uroviricota;Caudoviricetes
S6_vs_1146|provirus_1_36227	36	1	2561	Viruses;Duplodnaviria;Heunggongvirae;Uroviricota;Caudoviricetes
S6_vs_46039|provirus_1_38172	12	1	2561	Viruses;Duplodnaviria;Heunggongvirae;Uroviricota;Caudoviricetes
S6_vs_46039|provirus_69634_111911	14	1	2561	Viruses;Duplodnaviria;Heunggongvirae;Uroviricota;Caudoviricetes
S6_vs_34453|provirus_4536_65412	58	1	2561	Viruses;Duplodnaviria;Heunggongvirae;Uroviricota;Caudoviricetes
S6_vs_39025|provirus_18844_51286	38	1	2561	Viruses;Duplodnaviria;Heunggongvirae;Uroviricota;Caudoviricetes
S6_vs_39065|provirus_14912_41773	9	1	2561	Viruses;Duplodnaviria;Heunggongvirae;Uroviricota;Caudoviricetes
S8_vf_28956|provirus_172_25419	22	1	2561	Viruses;Duplodnaviria;Heunggongvirae;Uroviricota;Caudoviricetes
S8_vf_12212|provirus_3_31196	31	1	2561	Viruses;Duplodnaviria;Heunggongvirae;Uroviricota;Caudoviricetes
S8_vf_170878|provirus_6035_59917	54	1	2561	Viruses;Duplodnaviria;Heunggongvirae;Uroviricota;Caudoviricetes
S8_vs_149700|provirus_19406_69356	42	1	2561	Viruses;Duplodnaviria;Heunggongvirae;Uroviricota;Caudoviricetes
S8_vs_115958|provirus_68174_127950	45	1	2561	Viruses;Duplodnaviria;Heunggongvirae;Uroviricota;Caudoviricetes
S8_vs_125717|provirus_34678_52956	23	1	2561	Viruses;Duplodnaviria;Heunggongvirae;Uroviricota;Caudoviricetes
S8_vs_4153|provirus_1_12586	10	1	2561	Viruses;Duplodnaviria;Heunggongvirae;Uroviricota;Caudoviricetes
S8_vs_47265|provirus_19038_69505	46	1	2561	Viruses;Duplodnaviria;Heunggongvirae;Uroviricota;Caudoviricetes
S8_vs_179208|provirus_1_52017	20	1	2561	Viruses;Duplodnaviria;Heunggongvirae;Uroviricota;Caudoviricetes
S8_vs_205257|provirus_1_42712	28	1	2561	Viruses;Duplodnaviria;Heunggongvirae;Uroviricota;Caudoviricetes

Note:

Provirus integrated into plasmid

Table S4-2. WMS_provirus_genomad

seq_name	n_genes_with_taxonomy	agreement	taxid	lineage
S9-wms-combined_64277|provirus_1_50273	40	1	2561	Viruses;Duplodnaviria;Heunggongvirae;Uroviricota;Caudoviricetes
S9-wms-combined_53991|provirus_1_38377	39	1	2561	Viruses;Duplodnaviria;Heunggongvirae;Uroviricota;Caudoviricetes
S9-wms-combined_97755|provirus_1_31801	35	1	2561	Viruses;Duplodnaviria;Heunggongvirae;Uroviricota;Caudoviricetes
S9-wms-combined_33230|provirus_1_29168	30	1	2561	Viruses;Duplodnaviria;Heunggongvirae;Uroviricota;Caudoviricetes
S9-wms-combined_124857|provirus_24439_80893	56	1	2561	Viruses;Duplodnaviria;Heunggongvirae;Uroviricota;Caudoviricetes
S9-wms-combined_28805|provirus_23826_59739	38	1	2561	Viruses;Duplodnaviria;Heunggongvirae;Uroviricota;Caudoviricetes
S9-wms-combined_93232|provirus_5433_50669	48	1	2561	Viruses;Duplodnaviria;Heunggongvirae;Uroviricota;Caudoviricetes
S9-wms-combined_109322|provirus_1_43439	37	1	2561	Viruses;Duplodnaviria;Heunggongvirae;Uroviricota;Caudoviricetes
S9-wms-combined_29611|provirus_307713_413955	93	1	2561	Viruses;Duplodnaviria;Heunggongvirae;Uroviricota;Caudoviricetes
S9-wms-combined_20485|provirus_49228_90295	46	1	2561	Viruses;Duplodnaviria;Heunggongvirae;Uroviricota;Caudoviricetes
S9-wms-combined_9931|provirus_43028_90630	39	1	2561	Viruses;Duplodnaviria;Heunggongvirae;Uroviricota;Caudoviricetes
S9-wms-combined_37587|provirus_1_47703	32	1	2561	Viruses;Duplodnaviria;Heunggongvirae;Uroviricota;Caudoviricetes
S9-wms-combined_5526|provirus_1_38027	32	1	2561	Viruses;Duplodnaviria;Heunggongvirae;Uroviricota;Caudoviricetes
S9-wms-combined_76274|provirus_3258_36781	35	1	2561	Viruses;Duplodnaviria;Heunggongvirae;Uroviricota;Caudoviricetes
S11-wms-combined_193148|provirus_8658_39231	9	1	2561	Viruses;Duplodnaviria;Heunggongvirae;Uroviricota;Caudoviricetes
S11-wms-combined_179605|provirus_18936_43615	24	1	2561	Viruses;Duplodnaviria;Heunggongvirae;Uroviricota;Caudoviricetes
S11-wms-combined_83571|provirus_9777_40621	36	1	2561	Viruses;Duplodnaviria;Heunggongvirae;Uroviricota;Caudoviricetes
S11-wms-combined_125176|provirus_1_23922	22	1	2561	Viruses;Duplodnaviria;Heunggongvirae;Uroviricota;Caudoviricetes
S11-wms-combined_236214|provirus_60975_93358	29	1	2561	Viruses;Duplodnaviria;Heunggongvirae;Uroviricota;Caudoviricetes
S11-wms-combined_264050|provirus_28059_77647	49	1	2561	Viruses;Duplodnaviria;Heunggongvirae;Uroviricota;Caudoviricetes
S11-wms-combined_140009|provirus_19523_54887	37	1	2561	Viruses;Duplodnaviria;Heunggongvirae;Uroviricota;Caudoviricetes
S11-wms-combined_168048|provirus_1_41217	31	1	2561	Viruses;Duplodnaviria;Heunggongvirae;Uroviricota;Caudoviricetes
S11-wms-combined_3507|provirus_29062_70964	52	1	2561	Viruses;Duplodnaviria;Heunggongvirae;Uroviricota;Caudoviricetes
S11-wms-combined_101337|provirus_1_25287	19	1	2561	Viruses;Duplodnaviria;Heunggongvirae;Uroviricota;Caudoviricetes
S11-wms-combined_73204|provirus_23691_55764	27	1	2561	Viruses;Duplodnaviria;Heunggongvirae;Uroviricota;Caudoviricetes
S11-wms-combined_198512|provirus_3772_41546	31	1	2561	Viruses;Duplodnaviria;Heunggongvirae;Uroviricota;Caudoviricetes
S11-wms-combined_267993|provirus_1_65458	54	1	2561	Viruses;Duplodnaviria;Heunggongvirae;Uroviricota;Caudoviricetes
S11-wms-combined_116589|provirus_12509_25615	10	1	2561	Viruses;Duplodnaviria;Heunggongvirae;Uroviricota;Caudoviricetes
S11-wms-combined_48246|provirus_1_44551	43	1	2561	Viruses;Duplodnaviria;Heunggongvirae;Uroviricota;Caudoviricetes
S11-wms-combined_63818|provirus_8325_62965	45	1	2561	Viruses;Duplodnaviria;Heunggongvirae;Uroviricota;Caudoviricetes
S11-wms-combined_215723|provirus_1_42332	30	1	2561	Viruses;Duplodnaviria;Heunggongvirae;Uroviricota;Caudoviricetes
S11-wms-combined_92114|provirus_1_26924	20	1	2561	Viruses;Duplodnaviria;Heunggongvirae;Uroviricota;Caudoviricetes
S11-wms-combined_120257|provirus_8529_35507	33	1	2561	Viruses;Duplodnaviria;Heunggongvirae;Uroviricota;Caudoviricetes
S11-wms-combined_217790|provirus_1_43023	13	1	2561	Viruses;Duplodnaviria;Heunggongvirae;Uroviricota;Caudoviricetes
S11-wms_32691|provirus_3_142121	9	1	2561	Viruses;Duplodnaviria;Heunggongvirae;Uroviricota;Caudoviricetes
S1-wms-combined_52708|provirus_6059_16025	9	1	2561	Viruses;Duplodnaviria;Heunggongvirae;Uroviricota;Caudoviricetes
S1-wms-combined_68998|provirus_3499_56973	63	1	2561	Viruses;Duplodnaviria;Heunggongvirae;Uroviricota;Caudoviricetes
S1-wms-combined_102801|provirus_3620_28850	7	1	2561	Viruses;Duplodnaviria;Heunggongvirae;Uroviricota;Caudoviricetes
S1-wms-combined_302166|provirus_11917_75458	42	1	2561	Viruses;Duplodnaviria;Heunggongvirae;Uroviricota;Caudoviricetes
S1-wms-combined_285012|provirus_1_46216	53	1	2561	Viruses;Duplodnaviria;Heunggongvirae;Uroviricota;Caudoviricetes
S1-wms-combined_220022|provirus_1_85675	4	1	2561	Viruses;Duplodnaviria;Heunggongvirae;Uroviricota;Caudoviricetes
S1-wms-combined_220022|provirus_128734_155946	4	1	2561	Viruses;Duplodnaviria;Heunggongvirae;Uroviricota;Caudoviricetes
S1-wms-combined_23248|provirus_27603_62426	36	1	2561	Viruses;Duplodnaviria;Heunggongvirae;Uroviricota;Caudoviricetes
S1-wms-combined_304377|provirus_1_66513	60	1	2561	Viruses;Duplodnaviria;Heunggongvirae;Uroviricota;Caudoviricetes
S1-wms-combined_57370|provirus_1_21189	19	1	2561	Viruses;Duplodnaviria;Heunggongvirae;Uroviricota;Caudoviricetes
S1-wms-combined_107766|provirus_1_36184	27	1	2561	Viruses;Duplodnaviria;Heunggongvirae;Uroviricota;Caudoviricetes
S1-wms-combined_92523|provirus_13796_31793	13	1	2561	Viruses;Duplodnaviria;Heunggongvirae;Uroviricota;Caudoviricetes
S1-wms-combined_331544|provirus_1_23231	21	1	2561	Viruses;Duplodnaviria;Heunggongvirae;Uroviricota;Caudoviricetes
S1-wms-combined_85225|provirus_1_39579	48	1	2561	Viruses;Duplodnaviria;Heunggongvirae;Uroviricota;Caudoviricetes
S1-wms-combined_136097|provirus_8755_19804	10	1	2561	Viruses;Duplodnaviria;Heunggongvirae;Uroviricota;Caudoviricetes
S1-wms-combined_307323|provirus_1_42035	40	1	2561	Viruses;Duplodnaviria;Heunggongvirae;Uroviricota;Caudoviricetes
S1-wms-combined_94988|provirus_10302_22992	10	1	2561	Viruses;Duplodnaviria;Heunggongvirae;Uroviricota;Caudoviricetes
S5-wms-combined_18356|provirus_1_22757	23	1	2561	Viruses;Duplodnaviria;Heunggongvirae;Uroviricota;Caudoviricetes
S5-wms-combined_161191|provirus_134452_175715	44	1	2561	Viruses;Duplodnaviria;Heunggongvirae;Uroviricota;Caudoviricetes
S5-wms-combined_134381|provirus_32274_74956	57	1	2561	Viruses;Duplodnaviria;Heunggongvirae;Uroviricota;Caudoviricetes
S5-wms-combined_90315|provirus_11740_73154	59	1	2561	Viruses;Duplodnaviria;Heunggongvirae;Uroviricota;Caudoviricetes
S5-wms-combined_134743|provirus_30511_101552	44	0.9919	2561	Viruses;Duplodnaviria;Heunggongvirae;Uroviricota;Caudoviricetes
S5-wms-combined_72410|provirus_71571_129551	66	1	2561	Viruses;Duplodnaviria;Heunggongvirae;Uroviricota;Caudoviricetes
S5-wms-combined_206050|provirus_15780_57403	41	1	2561	Viruses;Duplodnaviria;Heunggongvirae;Uroviricota;Caudoviricetes
S5-wms-combined_108794|provirus_19443_74905	40	1	2561	Viruses;Duplodnaviria;Heunggongvirae;Uroviricota;Caudoviricetes
S5-wms-combined_207291|provirus_13547_57653	31	1	2561	Viruses;Duplodnaviria;Heunggongvirae;Uroviricota;Caudoviricetes
S5-wms-combined_29447|provirus_1_32335	37	1	2561	Viruses;Duplodnaviria;Heunggongvirae;Uroviricota;Caudoviricetes
S5-wms-combined_83370|provirus_1_42289	36	1	2561	Viruses;Duplodnaviria;Heunggongvirae;Uroviricota;Caudoviricetes
S5-wms-combined_66662|provirus_52916_98874	33	1	2561	Viruses;Duplodnaviria;Heunggongvirae;Uroviricota;Caudoviricetes
S5-wms-combined_59064|provirus_35710_77093	42	1	2561	Viruses;Duplodnaviria;Heunggongvirae;Uroviricota;Caudoviricetes
S5-wms-combined_94482|provirus_9030_59881	34	1	2561	Viruses;Duplodnaviria;Heunggongvirae;Uroviricota;Caudoviricetes
S5-wms-combined_67650|provirus_42189_77684	29	1	2561	Viruses;Duplodnaviria;Heunggongvirae;Uroviricota;Caudoviricetes
S5-wms-combined_94786|provirus_4677_40532	25	1	2561	Viruses;Duplodnaviria;Heunggongvirae;Uroviricota;Caudoviricetes
S5-wms-combined_210959|provirus_126240_159070	27	1	2561	Viruses;Duplodnaviria;Heunggongvirae;Uroviricota;Caudoviricetes
S5-wms-combined_78434|provirus_1_90452	15	1	2561	Viruses;Duplodnaviria;Heunggongvirae;Uroviricota;Caudoviricetes
S5-wms-combined_98202|provirus_1_61764	20	0.9818	2561	Viruses;Duplodnaviria;Heunggongvirae;Uroviricota;Caudoviricetes
S5-wms-combined_26971|provirus_4471_34173	35	1	2561	Viruses;Duplodnaviria;Heunggongvirae;Uroviricota;Caudoviricetes
S5-wms-combined_178432|provirus_1_33423	25	1	2561	Viruses;Duplodnaviria;Heunggongvirae;Uroviricota;Caudoviricetes
S5-wms-combined_179241|provirus_5356_19361	9	1	2561	Viruses;Duplodnaviria;Heunggongvirae;Uroviricota;Caudoviricetes
S5-wms-combined_117213|provirus_1_35094	35	1	2561	Viruses;Duplodnaviria;Heunggongvirae;Uroviricota;Caudoviricetes
S5-wms_94965|provirus_4329_41461	34	1	2561	Viruses;Duplodnaviria;Heunggongvirae;Uroviricota;Caudoviricetes
S7-wms-combined_71592|provirus_9021_38751	32	1	2561	Viruses;Duplodnaviria;Heunggongvirae;Uroviricota;Caudoviricetes
S7-wms-combined_20921|provirus_1_139822	92	1	2561	Viruses;Duplodnaviria;Heunggongvirae;Uroviricota;Caudoviricetes
S7-wms-combined_62119|provirus_55121_129634	73	1	2561	Viruses;Duplodnaviria;Heunggongvirae;Uroviricota;Caudoviricetes
S7-wms-combined_185016|provirus_8013_41583	35	1	2561	Viruses;Duplodnaviria;Heunggongvirae;Uroviricota;Caudoviricetes
S7-wms-combined_174935|provirus_1_23182	22	1	2561	Viruses;Duplodnaviria;Heunggongvirae;Uroviricota;Caudoviricetes
S7-wms-combined_134458|provirus_1_46614	49	1	2561	Viruses;Duplodnaviria;Heunggongvirae;Uroviricota;Caudoviricetes
S7-wms-combined_2122|provirus_17514_50765	28	1	2561	Viruses;Duplodnaviria;Heunggongvirae;Uroviricota;Caudoviricetes
S7-wms-combined_135154|provirus_1_28157	17	1	2561	Viruses;Duplodnaviria;Heunggongvirae;Uroviricota;Caudoviricetes
S7-wms-combined_238231|provirus_41225_71249	26	1	2561	Viruses;Duplodnaviria;Heunggongvirae;Uroviricota;Caudoviricetes
S7-wms-combined_43950|provirus_8198_57129	41	1	2561	Viruses;Duplodnaviria;Heunggongvirae;Uroviricota;Caudoviricetes
S7-wms-combined_3697|provirus_66701_124033	46	1	2561	Viruses;Duplodnaviria;Heunggongvirae;Uroviricota;Caudoviricetes
S7-wms-combined_157260|provirus_19304_75227	58	1	2561	Viruses;Duplodnaviria;Heunggongvirae;Uroviricota;Caudoviricetes
S7-wms-combined_74829|provirus_2475_38322	29	1	2561	Viruses;Duplodnaviria;Heunggongvirae;Uroviricota;Caudoviricetes
S7-wms-combined_14267|provirus_25528_51357	23	1	2561	Viruses;Duplodnaviria;Heunggongvirae;Uroviricota;Caudoviricetes
S7-wms-combined_167867|provirus_1_81146	11	1	2561	Viruses;Duplodnaviria;Heunggongvirae;Uroviricota;Caudoviricetes
S7-wms-combined_106253|provirus_1_36567	28	0.9688	2561	Viruses;Duplodnaviria;Heunggongvirae;Uroviricota;Caudoviricetes
S7-wms-combined_14529|provirus_20870_62786	27	1	2561	Viruses;Duplodnaviria;Heunggongvirae;Uroviricota;Caudoviricetes
S7-wms-combined_137858|provirus_28744_38569	9	1	2561	Viruses;Duplodnaviria;Heunggongvirae;Uroviricota;Caudoviricetes
S7-wms-combined_5625|provirus_26522_67320	41	1	2561	Viruses;Duplodnaviria;Heunggongvirae;Uroviricota;Caudoviricetes
S7-wms-combined_190281|provirus_28520_63064	33	1	2561	Viruses;Duplodnaviria;Heunggongvirae;Uroviricota;Caudoviricetes
S7-wms-combined_26217|provirus_37948_88555	44	1	2561	Viruses;Duplodnaviria;Heunggongvirae;Uroviricota;Caudoviricetes
S7-wms-combined_7205|provirus_1_62239	58	1	2561	Viruses;Duplodnaviria;Heunggongvirae;Uroviricota;Caudoviricetes
S7-wms-combined_72070|provirus_3771_55473	32	1	2561	Viruses;Duplodnaviria;Heunggongvirae;Uroviricota;Caudoviricetes
S7-wms-combined_136642|provirus_1_17488	21	1	2561	Viruses;Duplodnaviria;Heunggongvirae;Uroviricota;Caudoviricetes
S7-wms-combined_147158|provirus_1_43742	42	1	2561	Viruses;Duplodnaviria;Heunggongvirae;Uroviricota;Caudoviricetes
S7-wms-combined_218379|provirus_3331_36800	28	1	2561	Viruses;Duplodnaviria;Heunggongvirae;Uroviricota;Caudoviricetes

S7-wms-combined_107679_0|provirus_1_37921	39	1	2561	Viruses;Duplodnaviria;Heunggongvirae;Uroviricota;Caudoviricetes
S7-wms-combined_107679_1|provirus_4391_36183	18	1	2561	Viruses;Duplodnaviria;Heunggongvirae;Uroviricota;Caudoviricetes
S17-wms-combined_233761|provirus_1_38860	25	1	2561	Viruses;Duplodnaviria;Heunggongvirae;Uroviricota;Caudoviricetes
S17-wms-combined_56006|provirus_18810_96019	60	1	2561	Viruses;Duplodnaviria;Heunggongvirae;Uroviricota;Caudoviricetes
S17-wms-combined_56061|provirus_96601_107131	9	1	2561	Viruses;Duplodnaviria;Heunggongvirae;Uroviricota;Caudoviricetes
S17-wms-combined_2234|provirus_16885_70511	61	1	2561	Viruses;Duplodnaviria;Heunggongvirae;Uroviricota;Caudoviricetes
S17-wms-combined_203701|provirus_1_59819	21	1	2561	Viruses;Duplodnaviria;Heunggongvirae;Uroviricota;Caudoviricetes
S17-wms-combined_247494|provirus_1_40245	36	1	2561	Viruses;Duplodnaviria;Heunggongvirae;Uroviricota;Caudoviricetes
S17-wms-combined_126774|provirus_33403_66657	6	1	2561	Viruses;Duplodnaviria;Heunggongvirae;Uroviricota;Caudoviricetes
S17-wms-combined_248321|provirus_1_64257	60	1	2561	Viruses;Duplodnaviria;Heunggongvirae;Uroviricota;Caudoviricetes
S17-wms-combined_248321|provirus_78214_127137	61	1	2561	Viruses;Duplodnaviria;Heunggongvirae;Uroviricota;Caudoviricetes
S17-wms-combined_193958|provirus_41974_101082	50	1	2561	Viruses;Duplodnaviria;Heunggongvirae;Uroviricota;Caudoviricetes
S17-wms-combined_259811|provirus_1_48861	60	1	2561	Viruses;Duplodnaviria;Heunggongvirae;Uroviricota;Caudoviricetes
S17-wms-combined_185239|provirus_13026_23450	4	1	2561	Viruses;Duplodnaviria;Heunggongvirae;Uroviricota;Caudoviricetes
S17-wms-combined_186314|provirus_28354_68895	54	1	2561	Viruses;Duplodnaviria;Heunggongvirae;Uroviricota;Caudoviricetes
S17-wms-combined_162786|provirus_1_130535	29	1	2561	Viruses;Duplodnaviria;Heunggongvirae;Uroviricota;Caudoviricetes
S17-wms-combined_95360|provirus_1_43743	30	1	2561	Viruses;Duplodnaviria;Heunggongvirae;Uroviricota;Caudoviricetes
S17-wms-combined_199746|provirus_311_40236	41	1	2561	Viruses;Duplodnaviria;Heunggongvirae;Uroviricota;Caudoviricetes
S12-wms-combined_334806|provirus_1_39697	21	1	2561	Viruses;Duplodnaviria;Heunggongvirae;Uroviricota;Caudoviricetes
S12-wms-combined_184338|provirus_7702_50077	45	1	2561	Viruses;Duplodnaviria;Heunggongvirae;Uroviricota;Caudoviricetes
S12-wms-combined_368462|provirus_3828_55485	40	1	2561	Viruses;Duplodnaviria;Heunggongvirae;Uroviricota;Caudoviricetes
S12-wms-combined_201125|provirus_169478_224193	46	1	2561	Viruses;Duplodnaviria;Heunggongvirae;Uroviricota;Caudoviricetes
S12-wms-combined_368562|provirus_5352_23237	11	1	2561	Viruses;Duplodnaviria;Heunggongvirae;Uroviricota;Caudoviricetes
S12-wms-combined_117628|provirus_37358_58691	22	1	2561	Viruses;Duplodnaviria;Heunggongvirae;Uroviricota;Caudoviricetes
S12-wms-combined_17617|provirus_14012_54837	45	1	2561	Viruses;Duplodnaviria;Heunggongvirae;Uroviricota;Caudoviricetes
S12-wms-combined_51076|provirus_1_52990	7	1	2561	Viruses;Duplodnaviria;Heunggongvirae;Uroviricota;Caudoviricetes
S12-wms-combined_51076|provirus_63671_110930	11	0.9503	2561	Viruses;Duplodnaviria;Heunggongvirae;Uroviricota;Caudoviricetes
S12-wms-combined_151843|provirus_1_54070	53	1	2561	Viruses;Duplodnaviria;Heunggongvirae;Uroviricota;Caudoviricetes
S12-wms-combined_18287|provirus_31039_78190	48	1	2561	Viruses;Duplodnaviria;Heunggongvirae;Uroviricota;Caudoviricetes
S12-wms-combined_2450|provirus_49617_81938	36	1	2561	Viruses;Duplodnaviria;Heunggongvirae;Uroviricota;Caudoviricetes
S12-wms-combined_320727|provirus_43373_74615	26	1	2561	Viruses;Duplodnaviria;Heunggongvirae;Uroviricota;Caudoviricetes
S12-wms-combined_320727|provirus_86494_158985	72	1	2561	Viruses;Duplodnaviria;Heunggongvirae;Uroviricota;Caudoviricetes
S12-wms-combined_371541|provirus_27093_40221	11	1	2561	Viruses;Duplodnaviria;Heunggongvirae;Uroviricota;Caudoviricetes
S12-wms-combined_120133|provirus_1_28638	29	1	2561	Viruses;Duplodnaviria;Heunggongvirae;Uroviricota;Caudoviricetes
S12-wms-combined_189323|provirus_1_38428	33	1	2561	Viruses;Duplodnaviria;Heunggongvirae;Uroviricota;Caudoviricetes
S12-wms-combined_256299|provirus_6861_53411	38	1	2561	Viruses;Duplodnaviria;Heunggongvirae;Uroviricota;Caudoviricetes
S12-wms-combined_156234|provirus_1_28774	30	1	2561	Viruses;Duplodnaviria;Heunggongvirae;Uroviricota;Caudoviricetes
S12-wms-combined_23517|provirus_1_43179	61	1	2561	Viruses;Duplodnaviria;Heunggongvirae;Uroviricota;Caudoviricetes
S12-wms-combined_375343|provirus_1_29654	26	1	2561	Viruses;Duplodnaviria;Heunggongvirae;Uroviricota;Caudoviricetes
S12-wms-combined_358010|provirus_6040_57083	36	1	2561	Viruses;Duplodnaviria;Heunggongvirae;Uroviricota;Caudoviricetes
S12-wms-combined_358797|provirus_1_26270	22	1	2561	Viruses;Duplodnaviria;Heunggongvirae;Uroviricota;Caudoviricetes
S12-wms-combined_359190|provirus_24482_64793	34	1	2561	Viruses;Duplodnaviria;Heunggongvirae;Uroviricota;Caudoviricetes
S12-wms-combined_328554|provirus_9763_43415	32	1	2561	Viruses;Duplodnaviria;Heunggongvirae;Uroviricota;Caudoviricetes
S12-wms-combined_329242|provirus_1_27761	6	1	2561	Viruses;Duplodnaviria;Heunggongvirae;Uroviricota;Caudoviricetes
S12-wms-combined_313953|provirus_1_52155	31	1	2561	Viruses;Duplodnaviria;Heunggongvirae;Uroviricota;Caudoviricetes
S12-wms-combined_111524|provirus_1_48545	17	1	2561	Viruses;Duplodnaviria;Heunggongvirae;Uroviricota;Caudoviricetes
S12-wms-combined_297545|provirus_1_54974	66	1	2561	Viruses;Duplodnaviria;Heunggongvirae;Uroviricota;Caudoviricetes
S12-wms-combined_129799|provirus_25526_72680	52	1	2561	Viruses;Duplodnaviria;Heunggongvirae;Uroviricota;Caudoviricetes
S12-wms-combined_282594|provirus_1_43487	25	1	2561	Viruses;Duplodnaviria;Heunggongvirae;Uroviricota;Caudoviricetes
S12-wms-combined_147628|provirus_1_66934	47	1	2561	Viruses;Duplodnaviria;Heunggongvirae;Uroviricota;Caudoviricetes
S12-wms-combined_264845|provirus_13087_19738	5	1	2561	Viruses;Duplodnaviria;Heunggongvirae;Uroviricota;Caudoviricetes
S12-wms-combined_299293|provirus_20365_74597	49	1	2561	Viruses;Duplodnaviria;Heunggongvirae;Uroviricota;Caudoviricetes
S12-wms-combined_98046|provirus_43964_89205	38	1	2561	Viruses;Duplodnaviria;Heunggongvirae;Uroviricota;Caudoviricetes
S12-wms-combined_31761|provirus_4908_40932	30	1	2561	Viruses;Duplodnaviria;Heunggongvirae;Uroviricota;Caudoviricetes
S12-wms-combined_368190|provirus_4987_62142	68	1	2561	Viruses;Duplodnaviria;Heunggongvirae;Uroviricota;Caudoviricetes
S12-wms-combined_168259|provirus_1_29820	12	1	2561	Viruses;Duplodnaviria;Heunggongvirae;Uroviricota;Caudoviricetes
S12-wms-combined_270839|provirus_3921_42253	29	1	2561	Viruses;Duplodnaviria;Heunggongvirae;Uroviricota;Caudoviricetes
S12-wms_161239|provirus_5587_25370	24	1	2561	Viruses;Duplodnaviria;Heunggongvirae;Uroviricota;Caudoviricetes
S2-wms-combined_196783|provirus_1_43247	38	1	2561	Viruses;Duplodnaviria;Heunggongvirae;Uroviricota;Caudoviricetes
S2-wms-combined_62519|provirus_6893_41704	41	1	2561	Viruses;Duplodnaviria;Heunggongvirae;Uroviricota;Caudoviricetes
S2-wms-combined_135139|provirus_1_34436	9	1	2561	Viruses;Duplodnaviria;Heunggongvirae;Uroviricota;Caudoviricetes
S2-wms-combined_218002|provirus_41237_76226	35	1	2561	Viruses;Duplodnaviria;Heunggongvirae;Uroviricota;Caudoviricetes
S2-wms-combined_135154|provirus_11137_34738	13	1	2561	Viruses;Duplodnaviria;Heunggongvirae;Uroviricota;Caudoviricetes
S2-wms-combined_93845|provirus_1_41258	39	1	2561	Viruses;Duplodnaviria;Heunggongvirae;Uroviricota;Caudoviricetes
S2-wms-combined_52568|provirus_1_46833	39	1	2561	Viruses;Duplodnaviria;Heunggongvirae;Uroviricota;Caudoviricetes
S2-wms-combined_166665|provirus_1_31971	32	1	2561	Viruses;Duplodnaviria;Heunggongvirae;Uroviricota;Caudoviricetes
S2-wms-combined_229529|provirus_28533_87711	49	1	2561	Viruses;Duplodnaviria;Heunggongvirae;Uroviricota;Caudoviricetes
S2-wms-combined_84608|provirus_18423_96376	30	1	2561	Viruses;Duplodnaviria;Heunggongvirae;Uroviricota;Caudoviricetes
S2-wms-combined_74387|provirus_8639_47478	38	1	2561	Viruses;Duplodnaviria;Heunggongvirae;Uroviricota;Caudoviricetes
S2-wms-combined_147065|provirus_99157_133534	36	1	2561	Viruses;Duplodnaviria;Heunggongvirae;Uroviricota;Caudoviricetes
S2-wms-combined_137119|provirus_23866_96227	57	1	2561	Viruses;Duplodnaviria;Heunggongvirae;Uroviricota;Caudoviricetes
S2-wms-combined_85381|provirus_1_35531	30	1	2561	Viruses;Duplodnaviria;Heunggongvirae;Uroviricota;Caudoviricetes
S2-wms-combined_116221|provirus_11792_43877	36	1	2561	Viruses;Duplodnaviria;Heunggongvirae;Uroviricota;Caudoviricetes
S2-wms-combined_116221|provirus_59302_90374	30	1	2561	Viruses;Duplodnaviria;Heunggongvirae;Uroviricota;Caudoviricetes
S2-wms-combined_157934|provirus_4946_38145	38	1	2561	Viruses;Duplodnaviria;Heunggongvirae;Uroviricota;Caudoviricetes
S2-wms-combined_13246|provirus_142843_195068	54	1	2561	Viruses;Duplodnaviria;Heunggongvirae;Uroviricota;Caudoviricetes
S2-wms-combined_210822|provirus_1_50976	18	1	2561	Viruses;Duplodnaviria;Heunggongvirae;Uroviricota;Caudoviricetes
S2-wms-combined_24549|provirus_1_19009	20	1	2561	Viruses;Duplodnaviria;Heunggongvirae;Uroviricota;Caudoviricetes
S2-wms-combined_24549|provirus_52727_65681	13	1	2561	Viruses;Duplodnaviria;Heunggongvirae;Uroviricota;Caudoviricetes
S2-wms-combined_170845|provirus_1_34623	30	1	2561	Viruses;Duplodnaviria;Heunggongvirae;Uroviricota;Caudoviricetes
S2-wms-combined_159670|provirus_4693_36742	33	1	2561	Viruses;Duplodnaviria;Heunggongvirae;Uroviricota;Caudoviricetes
S2-wms-combined_181391|provirus_93678_123498	27	1	2561	Viruses;Duplodnaviria;Heunggongvirae;Uroviricota;Caudoviricetes
S2-wms-combined_200390|provirus_1_42588	37	1	2561	Viruses;Duplodnaviria;Heunggongvirae;Uroviricota;Caudoviricetes
S2-wms-combined_57293|provirus_12687_28064	9	1	2561	Viruses;Duplodnaviria;Heunggongvirae;Uroviricota;Caudoviricetes
S2-wms-combined_191613|provirus_1_40741	24	1	2561	Viruses;Duplodnaviria;Heunggongvirae;Uroviricota;Caudoviricetes
S2-wms-combined_98888|provirus_46109_107937	81	1	2561	Viruses;Duplodnaviria;Heunggongvirae;Uroviricota;Caudoviricetes
S2-wms-combined_181849|provirus_4199_47914	28	1	2561	Viruses;Duplodnaviria;Heunggongvirae;Uroviricota;Caudoviricetes
S2-wms-combined_99446|provirus_17627_43104	25	1	2561	Viruses;Duplodnaviria;Heunggongvirae;Uroviricota;Caudoviricetes
S2-wms-combined_25727|provirus_4849_37113	22	1	2561	Viruses;Duplodnaviria;Heunggongvirae;Uroviricota;Caudoviricetes
S2-wms-combined_58121|provirus_18687_27276	5	1	2561	Viruses;Duplodnaviria;Heunggongvirae;Uroviricota;Caudoviricetes
S2-wms-combined_140996|provirus_89093_146281	65	1	2561	Viruses;Duplodnaviria;Heunggongvirae;Uroviricota;Caudoviricetes
S2-wms-combined_140996|provirus_204609_222314	14	1	2561	Viruses;Duplodnaviria;Heunggongvirae;Uroviricota;Caudoviricetes
S2-wms-combined_222535|provirus_7864_38886	5	1	2561	Viruses;Duplodnaviria;Heunggongvirae;Uroviricota;Caudoviricetes
S2-wms-combined_58563|provirus_21097_53508	35	1	2561	Viruses;Duplodnaviria;Heunggongvirae;Uroviricota;Caudoviricetes
S2-wms-combined_110215|provirus_1_66879	60	1	2561	Viruses;Duplodnaviria;Heunggongvirae;Uroviricota;Caudoviricetes
S2-wms-combined_5791|provirus_106816_143252	34	1	2561	Viruses;Duplodnaviria;Heunggongvirae;Uroviricota;Caudoviricetes
S2-wms-combined_101826|provirus_12326_53091	34	1	2561	Viruses;Duplodnaviria;Heunggongvirae;Uroviricota;Caudoviricetes
S2-wms-combined_122457|provirus_1_24741	30	1	2561	Viruses;Duplodnaviria;Heunggongvirae;Uroviricota;Caudoviricetes
S2-wms-combined_185494|provirus_1_41920	29	1	2561	Viruses;Duplodnaviria;Heunggongvirae;Uroviricota;Caudoviricetes
S2-wms-combined_225908|provirus_81930_115597	26	1	2561	Viruses;Duplodnaviria;Heunggongvirae;Uroviricota;Caudoviricetes
S2-wms-combined_225908|provirus_180828_195686	7	1	2561	Viruses;Duplodnaviria;Heunggongvirae;Uroviricota;Caudoviricetes
S2-wms-combined_227800|provirus_1_24245	25	1	2561	Viruses;Duplodnaviria;Heunggongvirae;Uroviricota;Caudoviricetes
S2-wms-combined_143737|provirus_1_42150	35	1	2561	Viruses;Duplodnaviria;Heunggongvirae;Uroviricota;Caudoviricetes
S2-wms-combined_7756|provirus_1_48340	17	1	2561	Viruses;Duplodnaviria;Heunggongvirae;Uroviricota;Caudoviricetes

S2-wms_124416|provirus_5437_58906	48	1	2561	Viruses;Duplodnaviria;Heunggongvirae;Uroviricota;Caudoviricetes
S2-wms_156439|provirus_1_21599	19	1	2561	Viruses;Duplodnaviria;Heunggongvirae;Uroviricota;Caudoviricetes
S2-wms_107329|provirus_5393_52664	55	1	2561	Viruses;Duplodnaviria;Heunggongvirae;Uroviricota;Caudoviricetes
S2-wms_130687|provirus_27517_69355	42	0.994	2561	Viruses;Duplodnaviria;Heunggongvirae;Uroviricota;Caudoviricetes
S4-wms-combined_118531|provirus_26963_69321	33	1	2561	Viruses;Duplodnaviria;Heunggongvirae;Uroviricota;Caudoviricetes
S4-wms-combined_22012|provirus_3895_27205	24	1	2561	Viruses;Duplodnaviria;Heunggongvirae;Uroviricota;Caudoviricetes
S4-wms-combined_57123|provirus_1_36340	18	1	2561	Viruses;Duplodnaviria;Heunggongvirae;Uroviricota;Caudoviricetes
S4-wms-combined_64632|provirus_24726_57119	38	1	2561	Viruses;Duplodnaviria;Heunggongvirae;Uroviricota;Caudoviricetes
S4-wms-combined_113721|provirus_1_14776	10	1	2561	Viruses;Duplodnaviria;Heunggongvirae;Uroviricota;Caudoviricetes
S4-wms-combined_57963|provirus_66556_101297	39	1	2561	Viruses;Duplodnaviria;Heunggongvirae;Uroviricota;Caudoviricetes
S4-wms-combined_17284|provirus_1_54754	44	1	2561	Viruses;Duplodnaviria;Heunggongvirae;Uroviricota;Caudoviricetes
S4-wms-combined_121978|provirus_55570_110001	44	1	2561	Viruses;Duplodnaviria;Heunggongvirae;Uroviricota;Caudoviricetes
S4-wms-combined_25201|provirus_1_27684	32	1	2561	Viruses;Duplodnaviria;Heunggongvirae;Uroviricota;Caudoviricetes
S4-wms-combined_41490|provirus_33118_73219	31	1	2561	Viruses;Duplodnaviria;Heunggongvirae;Uroviricota;Caudoviricetes
S4-wms-combined_51079|provirus_1_19968	22	1	2561	Viruses;Duplodnaviria;Heunggongvirae;Uroviricota;Caudoviricetes
S4-wms_50475|provirus_24295_99597	77	0.9974	2561	Viruses;Duplodnaviria;Heunggongvirae;Uroviricota;Caudoviricetes
S6-wms-combined_41246|provirus_15790_40602	5	0.8534	2561	Viruses;Duplodnaviria;Heunggongvirae;Uroviricota;Caudoviricetes
S6-wms-combined_135223|provirus_91822_139676	16	1	2561	Viruses;Duplodnaviria;Heunggongvirae;Uroviricota;Caudoviricetes
S6-wms-combined_88414|provirus_44149_78550	41	1	2561	Viruses;Duplodnaviria;Heunggongvirae;Uroviricota;Caudoviricetes
S6-wms-combined_82597|provirus_164408_202920	38	1	2561	Viruses;Duplodnaviria;Heunggongvirae;Uroviricota;Caudoviricetes
S6-wms-combined_118427|provirus_96034_109457	12	1	2561	Viruses;Duplodnaviria;Heunggongvirae;Uroviricota;Caudoviricetes
S6-wms-combined_118427|provirus_117014_176709	53	1	2561	Viruses;Duplodnaviria;Heunggongvirae;Uroviricota;Caudoviricetes
S6-wms-combined_72228|provirus_24529_52555	15	1	2561	Viruses;Duplodnaviria;Heunggongvirae;Uroviricota;Caudoviricetes
S6-wms-combined_119138|provirus_23040_55482	38	1	2561	Viruses;Duplodnaviria;Heunggongvirae;Uroviricota;Caudoviricetes
S6-wms-combined_8508|provirus_1_42230	49	1	2561	Viruses;Duplodnaviria;Heunggongvirae;Uroviricota;Caudoviricetes
S6-wms-combined_32530|provirus_8460_46060	35	1	2561	Viruses;Duplodnaviria;Heunggongvirae;Uroviricota;Caudoviricetes
S6-wms-combined_126236|provirus_31179_69368	37	1	2561	Viruses;Duplodnaviria;Heunggongvirae;Uroviricota;Caudoviricetes
S6-wms-combined_50721|provirus_71105_115106	43	1	2561	Viruses;Duplodnaviria;Heunggongvirae;Uroviricota;Caudoviricetes
S6-wms-combined_138710|provirus_7149_17026	9	1	2561	Viruses;Duplodnaviria;Heunggongvirae;Uroviricota;Caudoviricetes
S6-wms-combined_56464|provirus_1_23497	25	0.9875	2561	Viruses;Duplodnaviria;Heunggongvirae;Uroviricota;Caudoviricetes
S6-wms-combined_91039|provirus_1_44817	38	1	2561	Viruses;Duplodnaviria;Heunggongvirae;Uroviricota;Caudoviricetes
S8-wms-combined_215102|provirus_23618_66504	26	1	2561	Viruses;Duplodnaviria;Heunggongvirae;Uroviricota;Caudoviricetes
S8-wms-combined_23583|provirus_43975_85815	41	1	2561	Viruses;Duplodnaviria;Heunggongvirae;Uroviricota;Caudoviricetes
S8-wms-combined_48046|provirus_1_29855	22	1	2561	Viruses;Duplodnaviria;Heunggongvirae;Uroviricota;Caudoviricetes
S8-wms-combined_26087|provirus_1_49451	33	1	2561	Viruses;Duplodnaviria;Heunggongvirae;Uroviricota;Caudoviricetes
S8-wms-combined_72911|provirus_1_47687	33	1	2561	Viruses;Duplodnaviria;Heunggongvirae;Uroviricota;Caudoviricetes
S8-wms-combined_49369|provirus_1_39890	24	1	2561	Viruses;Duplodnaviria;Heunggongvirae;Uroviricota;Caudoviricetes
S8-wms-combined_74275|provirus_1_26939	25	1	2561	Viruses;Duplodnaviria;Heunggongvirae;Uroviricota;Caudoviricetes
S8-wms-combined_270362|provirus_10571_69096	55	1	2561	Viruses;Duplodnaviria;Heunggongvirae;Uroviricota;Caudoviricetes
S8-wms-combined_200168|provirus_1_66777	45	1	2561	Viruses;Duplodnaviria;Heunggongvirae;Uroviricota;Caudoviricetes
S8-wms-combined_108917|provirus_9767_70457	48	1	2561	Viruses;Duplodnaviria;Heunggongvirae;Uroviricota;Caudoviricetes
S8-wms-combined_40666|provirus_44493_71582	21	1	2561	Viruses;Duplodnaviria;Heunggongvirae;Uroviricota;Caudoviricetes
S8-wms-combined_169835|provirus_34678_52956	23	1	2561	Viruses;Duplodnaviria;Heunggongvirae;Uroviricota;Caudoviricetes
S8-wms-combined_68780|provirus_22210_73585	54	1	2561	Viruses;Duplodnaviria;Heunggongvirae;Uroviricota;Caudoviricetes
S8-wms-combined_161181|provirus_9043_44282	39	1	2561	Viruses;Duplodnaviria;Heunggongvirae;Uroviricota;Caudoviricetes
S8-wms-combined_246185|provirus_1_35887	11	1	2561	Viruses;Duplodnaviria;Heunggongvirae;Uroviricota;Caudoviricetes
S8-wms_58515|provirus_15402_22507	6	1	2561	Viruses;Duplodnaviria;Heunggongvirae;Uroviricota;Caudoviricetes
S8-wms_48935|provirus_1_52721	54	1	2561	Viruses;Duplodnaviria;Heunggongvirae;Uroviricota;Caudoviricetes

Note:

Provirus integrated into plasmid

Table S4-3. Plasmid_detection_genomad

VLP-HQ-vOTUs:

seq_name	length	topology	n_genes	genetic_code	plasmid_score	fdr	n_hallmarks	marker_enrichment
S16_vs_173141	3774	No terminal repeats	3	11	0.9899	NA	1	0.9293
S5_vs_109527	142384	No terminal repeats	133	11	0.9873	NA	0	9.1116
S17_vs_52890	60210	No terminal repeats	53	11	0.9832	NA	1	1.8946
S6_vs_46039	111911	No terminal repeats	121	11	0.9823	NA	2	0.0396
S14_vs_25852	107963	No terminal repeats	113	11	0.9815	NA	3	1.395
S12_vs_164056	6664	No terminal repeats	9	11	0.9612	NA	0	0.5113

MWS-HQ-vOTUs:

seq_name	length	topology	n_genes	genetic_code	plasmid_score	fdr	n_hallmarks	marker_enrichment
S6-wms-combined_135223	147913	No terminal repeats	138	11	0.9866	NA	0	9.7754
S1-wms-combined_394628	10807	No terminal repeats	13	11	0.9673	NA	0	1.1593
S2-wms-combined_233107	16186	No terminal repeats	16	11	0.9548	NA	1	0.4814
S2-wms-combined_115352	3640	No terminal repeats	6	11	0.9387	NA	0	1.196
S2-wms-combined_132966	4461	No terminal repeats	11	11	0.9346	NA	0	0.8148
S1-wms-combined_169788	4712	No terminal repeats	8	11	0.8482	NA	0	0.876

Note:

Provirus detected

conjugation_genes	amr_genes
NA	NA
NA	NA
NA	NA
MOBV	NF000153
MOBV	NA
NA	NA

conjugation_genes	amr_genes
B_traF	NA
NA	NA
NA	NA
NA	NA
NA	NA
NA	NA
