# Supplementary material for: Investigating the Human Intestinal DNA Virome and Predicting Disease-Associated Virus–Host Interactions in Severe Myalgic Encephalomyelitis/Chronic Fatigue Syndrome (ME/CFS)
Source: Int J Mol Sci. 2023 Dec 8;24(24):17267. doi: 10.3390/ijms242417267 (PMC10744171; doi:10.3390/ijms242417267)
Supplement: Supplementary file 1 [file ijms-24-17267-s001.zip › Table S5.pdf]

Table S5. NC contamination

Table S5-1. NCs_sequences_stats

Sample_IDs	# Raw_reads (PE)	# cleaned_reads (PE)	# Total_raw_contigs	N50 (bp)	Longest_contig_length (bp)
NC1	306630	360240	3597	433	13109
NC2	16623	18592	19	446	910
NC3	72714	111664	2128	495	8397
Sum	395967	490496	5744	1374	22416
Mean	131989.00	163498.67	1914.67	458.00	7472.00
SD	153821.84	176623.79	1798.51	32.70	6151.88

			NC1-3		
	Merged_UViGs	LQ_undetermined_NC-UViGs_checkv	nr-vOTUs_size_filtration_1kb_cut_off	LQ_undetermined_nr-vOTUs_checkv	Notes
# Viral genomes	5953	5953	103	103	All NC viral genomes are low quality and undetermined
# Viral genomes >= 1 kb	255	255	103	103	
# Viral genomes >= 5 kb	13	13	3	3	
# Viral genomes >= 10 kb	4	4	0	0	
N50 (bp)	468	468	NA	NA	
Longest representative (bp)	13109	13109	8080	8080	Not-determined
Shortest representative (bp)	239	239	1040	1040	Not-determined

Table S5-2. NC1_taxa

25.04	45105	45105	U	0	unclassified
74.96	135015	859	R	1	root
74	133297	185	R1	131567	cellular organisms
72.32	130261	475	D	2	Bacteria
58.69	105718	1452	P	1224	Proteobacteria
27.47	49478	53	C	28216	Betaproteobacteria
27.4	49360	1152	O	80840	Burkholderiales
25.49	45914	1107	F	80864	Comamonadaceae
24.53	44185	28928	G	80865	Delftia
6.82	12277	12277	S	558537	Delftia lacustris
1.01	1814	1814	S	180282	Delftia tsuruhatensis
0.47	855	854	S	80866	Delftia acidovorans
0	1	1	S1	398578	Delftia acidovorans SPH-1
0.17	311	67	G1	2613839	unclassified Delftia
0.08	148	148	S	742013	Delftia sp. Cs1-4
0.05	96	96	S	1920191	Delftia sp. HK171
0.07	134	9	G	12916	Acidovorax
0.06	108	0	G1	2684926	unclassified Acidovorax
0.05	82	82	S	358220	Acidovorax sp. KKS102
0.01	11	11	S	232721	Acidovorax sp. JS42
0	7	7	S	2478662	Acidovorax sp. 1608163
0	4	4	S	1842533	Acidovorax sp. RAC01
0	4	4	S	2518343	Acidovorax sp. JMULE5
0.01	14	14	S	553814	Acidovorax carolinensis
0	3	3	S	2743470	Acidovorax antarcticus
0.07	133	0	G	201096	Alicycliphilus
0.07	133	0	S	179636	Alicycliphilus denitrificans
0.07	133	133	S1	596154	Alicycliphilus denitrificans K601
0.07	125	22	G	34072	Variovorax
0.05	95	10	G1	663243	unclassified Variovorax
0.03	63	63	S	1034889	Variovorax sp. HW608
0.01	14	14	S	207745	Variovorax sp. WDL1
0	5	5	S	662548	Variovorax sp. RA8
0	3	3	S	2762322	Variovorax sp. PAMC26660
0	5	5	S	34073	Variovorax paradoxus
0	3	3	S	436515	Variovorax boronicumulans
0.06	105	14	G	283	Comamonas
0.01	22	22	S	160825	Comamonas koreensis
0.01	21	10	S	285	Comamonas testosteroni
0.01	11	11	S1	1392005	Comamonas testosteroni TK102
0.01	20	20	S	1562974	Comamonas piscis
0.01	18	18	S	225991	Comamonas aquatica
0	9	0	G1	2638500	unclassified Comamonas
0	9	9	S	1232667	Comamonas sp. 7D-2
0	1	1	S	1082851	Comamonas serinivorans
0.05	93	0	G	238749	Diaphorobacter
0.04	80	1	G1	2649760	unclassified Diaphorobacter
0.03	46	46	S	2714924	Diaphorobacter sp. HDW4A
0.01	26	26	S	2792224	Diaphorobacter sp. JS3051
0	6	6	S	2735554	Diaphorobacter sp. JS3050
0	1	1	S	2714925	Diaphorobacter sp. HDW4B
0	8	8	S	1288495	Diaphorobacter aerolatus
0	3	3	S	1715720	Diaphorobacter ruginosibacter
0	2	2	S	1546149	Diaphorobacter polyhydroxybutyrativorans
0.01	13	1	G	47420	Hydrogenophaga
0.01	12	3	G1	2610897	unclassified Hydrogenophaga
0	8	8	S	795665	Hydrogenophaga sp. PBC
0	1	1	S	434010	Hydrogenophaga sp. PBL-H3
0	9	0	G	28065	Rhodoferax
0	5	0	G1	2627954	unclassified Rhodoferax
0	5	5	S	2741720	Rhodoferax sp. BAB1
0	4	4	S	1484693	Rhodoferax saidenbachensis
0	6	0	G	665874	Limnohabitans
0	6	0	G1	2626134	unclassified Limnohabitans
0	5	5	S	1678129	Limnohabitans sp. 103DPR2
0	1	1	S	1678128	Limnohabitans sp. 63ED37-2
0	1	0	G	215579	Schlegelella
0	1	1	S	215580	Schlegelella thermodepolymerans
0	1	0	G	364316	Verminephrobacter
0	1	0	S	364317	Verminephrobacter eiseniae
0	1	1	S1	391735	Verminephrobacter eiseniae EF01-2
0	1	0	G	683756	Kinneretia
0	1	0	G1	2632542	unclassified Kinneretia
0	1	1	S	2714952	Kinneretia sp. DAIF2
0	1	0	G	1649468	Melaminivora
0	1	0	G1	2641902	unclassified Melaminivora
0	1	1	S	2109913	Melaminivora sp. SC2-9
0.63	1138	200	F	119060	Burkholderiaceae
0.38	691	327	G	106589	Cupriavidus
0.03	57	57	S	82633	Cupriavidus pauculus
0.03	54	54	S	164546	Cupriavidus taiwanensis
0.03	52	48	S	82541	Cupriavidus gilardii
0	4	4	S1	1267562	Cupriavidus gilardii CR3
0.02	34	34	S	1796606	Cupriavidus nantongensis
0.02	31	30	S	106590	Cupriavidus necator
0	1	1	S1	1042878	Cupriavidus necator N-1
0.02	29	29	S	96344	Cupriavidus oxalaticus
0.01	26	26	S	119219	Cupriavidus metallidurans
0.01	25	25	S	151783	Cupriavidus campinensis
0.01	20	20	S	68895	Cupriavidus basilensis
0.01	13	13	S	1040979	Cupriavidus neocaledonicus
0.01	12	0	G1	2640874	unclassified Cupriavidus
0	6	6	S	876364	Cupriavidus sp. USMAA2-4
0	6	6	S	2771360	Cupriavidus sp. ISTL7
0.01	11	0	S	248026	Cupriavidus pinatubonensis
0.01	11	11	S1	264198	Cupriavidus pinatubonensis JMP134
0.06	104	19	G	48736	Ralstonia
0.03	47	41	S	329	Ralstonia pickettii
0	5	5	S1	428406	Ralstonia pickettii 12D
0	1	1	S1	402626	Ralstonia pickettii 12J
0.01	19	19	S	305	Ralstonia solanacearum
0.01	13	13	S	105219	Ralstonia mannitolilytica
0	6	6	S	190721	Ralstonia insidiosa

0.05	86	3	G	32008	Burkholderia
0.03	59	4	G1	87882	Burkholderia cepacia complex
0.02	35	35	S	95485	Burkholderia stabilis
0.01	14	14	S	95486	Burkholderia cenocepacia
0	4	4	S	488731	Burkholderia seminalis
0	1	0	S	292	Burkholderia cepacia
0	1	1	S1	1395570	Burkholderia cepacia JBK9
0	1	1	S	488732	Burkholderia diffusa
0.01	20	1	G1	2613784	unclassified Burkholderia
0	7	7	S	1097668	Burkholderia sp. YI23
0	5	5	S	1855726	Burkholderia sp. KK1
0	4	4	S	1705310	Burkholderia sp. IDO3
0	2	2	S	1740163	Burkholderia sp. Bp7605
0	1	1	S	758782	Burkholderia sp. THE68
0	2	2	S	28095	Burkholderia gladioli
0	1	1	S	337	Burkholderia glumae
0	1	0	G1	111527	pseudomallei group
0	1	1	S	28450	Burkholderia pseudomallei
0.02	40	7	G	1822464	Paraburkholderia
0	9	0	G1	2615204	unclassified Paraburkholderia
0	7	7	S	1926494	Paraburkholderia sp. SOS3
0	2	2	S	2604047	Paraburkholderia sp. Msb3
0	8	8	S	311230	Paraburkholderia terrae
0	5	5	S	92647	Paraburkholderia tropica
0	4	4	S	311231	Paraburkholderia ginsengisoli
0	3	3	S	2654982	Paraburkholderia atlantica
0	2	0	S	36873	Paraburkholderia xenovorans
0	2	2	S1	266265	Paraburkholderia xenovorans LB400
0	2	0	S	261302	Paraburkholderia phytofirmans
0	2	2	S1	398527	Paraburkholderia phytofirmans PsJN
0.01	14	0	G	93217	Pandoraea
0	6	6	S	93220	Pandoraea pnomenusa
0	5	5	S	1891094	Pandoraea fibrosis
0	1	1	S	445709	Pandoraea thiooxydans
0	1	1	S	656179	Pandoraea faecigallinarum
0	1	0	G1	2624094	unclassified Pandoraea
0	1	1	S	2518599	Pandoraea sp. XY-2
0	2	0	G	1827195	Caballeronia
0	2	0	G1	2646786	unclassified Caballeronia
0	2	2	S	2814653	Caballeronia sp. M1242
0	1	0	G	2571159	Mycetohabitans
0	1	0	S	412963	Mycetohabitans rhizoxinica
0	1	1	S1	882378	Mycetohabitans rhizoxinica HKI 454
0.55	982	114	F	75682	Oxalobacteraceae
0.38	680	115	G	149698	Massilia
0.15	272	40	G1	2609279	unclassified Massilia
0.06	110	110	S	1707785	Massilia sp. WG5
0.04	72	72	S	2769491	Massilia sp. LPB0304
0.01	19	19	S	2765360	Massilia sp. CCM 8941
0.01	15	15	S	1678028	Massilia sp. NR 4-1
0	9	9	S	1593482	Massilia sp. YMA4
0	7	7	S	2709302	Massilia sp. Dwa41.01b
0.1	177	177	S	945844	Massilia oculi
0.02	42	42	S	1141883	Massilia putida
0.01	21	21	S	864828	Massilia umbonata
0.01	20	20	S	2072590	Massilia armeniaca
0.01	19	19	S	871742	Massilia flava
0.01	11	11	S	321984	Massilia plicata
0	3	3	S	2045208	Massilia violaceinigra
0.05	99	0	G	75654	Duganella
0.05	99	1	G1	2636909	unclassified Duganella
0.04	72	72	S	2728020	Duganella sp. GN2-R2
0.01	26	26	S	2728021	Duganella sp. AF9R3
0.03	46	34	G	29580	Janthinobacterium
0	4	0	G1	2610881	unclassified Janthinobacterium
0	2	2	S	1644131	Janthinobacterium sp. 1_2014MBL_MicDiv
0	2	2	S	2497863	Janthinobacterium sp. 17J80-10
0	3	0	S	55508	Janthinobacterium agaricidamnosum
0	3	3	S1	1349767	Janthinobacterium agaricidamnosum NBRC 102515 = DSM 9628
0	3	3	S	368607	Janthinobacterium svalbardensis
0	1	1	S	29581	Janthinobacterium lividum
0	1	1	S	2590869	Janthinobacterium tructae
0.01	16	0	G	963	Herbaspirillum
0.01	11	11	S	863372	Herbaspirillum huttiense
0	3	3	S	964	Herbaspirillum seropedicae
0	1	0	S	80842	Herbaspirillum rubrisubalbicans
0	1	1	S1	1235827	Herbaspirillum rubrisubalbicans Os34
0	1	0	S	341045	Herbaspirillum hiltneri
0	1	1	S1	1262470	Herbaspirillum hiltneri N3
0.01	13	0	G	401469	Undibacterium
0.01	13	5	G1	2630295	unclassified Undibacterium
0	6	6	S	2058625	Undibacterium sp. YM2
0	2	2	S	2058624	Undibacterium sp. KW1
0.01	11	0	G	1344552	Noviherbaspirillum
0.01	11	0	G1	2617509	unclassified Noviherbaspirillum
0.01	11	11	S	2601898	Noviherbaspirillum sp. UKPF54
0	3	0	G	202907	Collimonas
0	2	2	S	279058	Collimonas arenae
0	1	1	S	158899	Collimonas fungivorans
0.05	93	0	F	506	Alcaligenaceae
0.02	39	0	G	507	Alcaligenes
0.02	39	39	S	511	Alcaligenes faecalis
0.01	20	10	G	222	Achromobacter
0	7	6	S	85698	Achromobacter xylosoxidans
0	1	1	S1	762376	Achromobacter xylosoxidans A8
0	1	1	S	217203	Achromobacter spanius
0	1	1	S	1353891	Achromobacter deleyi
0	1	0	G1	2626865	unclassified Achromobacter
0	1	1	S	1758194	Achromobacter sp. AONIH1
0.01	17	0	G	517	Bordetella
0	8	8	S	103855	Bordetella hinzii
0	5	5	S	94624	Bordetella petrii
0	2	2	S	1331258	Bordetella pseudohinzii
0	1	1	S	1416803	Bordetella genomosp. 9

0	1	0	G1	2630031	unclassified Bordetella
0	1	1	S	1746199	Bordetella sp. N
0.01	12	0	G	305976	Pusillimonas
0	7	7	S	2028345	Pusillimonas thiosulfatoxidans
0	5	0	G1	2640016	unclassified Pusillimonas
0	5	5	S	1007105	Pusillimonas sp. T7-7
0	5	0	G	152267	Pigmentiphaga
0	5	0	G1	2626614	unclassified Pigmentiphaga
0	5	5	S	2488560	Pigmentiphaga sp. H8
0.03	51	0	F	995019	Sutterellaceae
0.03	48	0	G	40544	Sutterella
0.02	43	43	S	40545	Sutterella wadsworthensis
0	5	5	S	2494234	Sutterella megalosphaeroides
0	3	0	G	1918598	Turicimonas
0	3	3	S	1796652	Turicimonas muris
0.02	30	0	O1	224471	Burkholderiales genera incertae sedis
0.01	11	0	G	88	Leptothrix
0.01	11	0	S	34029	Leptothrix cholodnii
0.01	11	11	S1	395495	Leptothrix cholodnii SP-6
0	6	3	G	316612	Methylibium
0	3	0	G1	2633235	unclassified Methylibium
0	3	3	S	2082386	Methylibium sp. Pch-M
0	5	0	G	54066	Xylophilus
0	5	5	S	2697032	Xylophilus rhododendri
0	4	0	G	318147	Paucibacter
0	4	0	G1	2642959	unclassified Paucibacter
0	4	4	S	1768242	Paucibacter sp. KCTC 42545
0	3	0	G	212743	Rhizobacter
0	2	2	S	946333	Rhizobacter gummiphilus
0	1	0	G1	2640088	unclassified Rhizobacter
0	1	1	S	2753607	Rhizobacter sp. AJA081-3
0	1	0	G	92793	Aquabacterium
0	1	1	S	1296669	Aquabacterium olei
0.03	50	0	O	206351	Neisseriales
0.02	34	0	F	481	Neisseriaceae
0.01	18	0	G	32257	Kingella
0.01	18	18	S	502	Kingella denitrificans
0.01	15	0	G	482	Neisseria
0	7	7	S	490	Neisseria sicca
0	5	5	S	28449	Neisseria subflava
0	2	2	S	495	Neisseria elongata
0	1	1	S	488	Neisseria mucosa
0	1	0	G	538	Eikenella
0	1	1	S	539	Eikenella corrodens
0.01	16	0	F	1499392	Chromobacteriaceae
0.01	10	0	G	568394	Pseudogulbenkiania
0.01	10	0	G1	2642558	unclassified Pseudogulbenkiania
0.01	10	10	S	748280	Pseudogulbenkiania sp. NH8B
0	5	0	F1	90153	Chromobacterium group
0	5	0	G	535	Chromobacterium
0	2	2	S	536	Chromobacterium violaceum
0	2	2	S	2202141	Chromobacterium phragmitis
0	1	1	S	1108595	Chromobacterium vaccinii
0	1	0	G	407217	Aquitalea
0	1	1	S	1537400	Aquitalea aquatilis
0.01	11	2	O	206389	Rhodocyclales
0	7	0	F	2008794	Zoogloeaceae
0	4	0	G	33057	Thauera
0	2	0	S	59405	Thauera aromatica
0	2	2	S1	44139	Thauera aromatica K172
0	1	1	S	1134435	Thauera humireducens
0	1	1	G1	2609274	unclassified Thauera
0	3	0	G	12960	Azoarcus
0	3	0	G1	2629479	unclassified Azoarcus
0	3	3	S	2016596	Azoarcus sp. M9-3-2
0	2	0	F	75787	Rhodocyclaceae
0	2	1	G	146937	Azospira
0	1	0	S	146939	Azospira oryzae
0	1	1	S1	640081	Azospira oryzae PS
0	4	0	O	32003	Nitrosomonadales
0	2	0	F	2772226	Sulfuricellaceae
0	1	0	G	1778653	Sulfuriferula
0	1	1	S	171865	Sulfuriferula plumbiphila
0	1	0	G	2772198	Sulfurimicrobium
0	1	1	S	2715678	Sulfurimicrobium lacus
0	1	0	F	206379	Nitrosomonadaceae
0	1	0	G	914	Nitrosomonas
0	1	0	S	916	Nitrosomonas eutropha
0	1	1	S1	335283	Nitrosomonas eutropha C91
0	1	0	F	2008790	Thiobacillaceae
0	1	0	G	1938335	Sulfuritortus
0	1	1	S	1914471	Sulfuritortus calidifontis
22.39	40331	353	C	28211	Alphaproteobacteria
19.55	35209	79	O	204457	Sphingomonadales
19.47	35061	1294	F	41297	Sphingomonadaceae
17.77	32003	626	G	13687	Sphingomonas
16.89	30418	30418	S	13689	Sphingomonas paucimobilis
0.15	277	277	S	93064	Sphingomonas koreensis
0.13	236	5	G1	196159	unclassified Sphingomonas
0.03	56	56	S	1961362	Sphingomonas sp. NIC1
0.02	44	44	S	2219696	Sphingomonas sp. FARSPH
0.02	34	34	S	2596913	Sphingomonas sp. NBWT7
0.01	17	17	S	2759526	Sphingomonas sp. DH-S5
0.01	16	16	S	304378	Sphingomonas sp. IC081
0.01	13	13	S	2735134	Sphingomonas sp. AP4-R1
0.01	13	13	S	1523415	Sphingomonas sp. AAP5
0.01	10	10	S	1938607	Sphingomonas sp. LM7
0	8	8	S	1517554	Sphingomonas sp. HMP9
0	7	7	S	2565555	Sphingomonas sp. PAMC26645
0	7	7	S	1390395	Sphingomonas sp. LK11
0	4	4	S	28214	Sphingomonas sp.
0	1	1	S	849864	Sphingomonas sp. SH
0	1	1	S	2653203	Sphingomonas sp. CL5.1
0.12	211	0	S	397260	Sphingomonas sanxanigenens

0.12	211	211	S1	1123269	Sphingomonas sanxanigenens DSM 19645 = NX02
0.09	155	13	S	152682	Sphingomonas melonis
0.08	142	142	S1	621456	Sphingomonas melonis TY
0.02	32	32	S	424800	Sphingomonas insulae
0.01	20	0	S	160791	Sphingomonas wittichii
0.01	20	20	S1	392499	Sphingomonas wittichii RW1
0.01	17	17	S	1609977	Sphingomonas hengshuiensis
0	5	5	S	653931	Sphingomonas alpina
0	4	4	S	1549858	Sphingomonas taxi
0	2	2	S	1813879	Sphingomonas panacisoli
0.69	1248	130	G	165695	Sphingobium
0.17	302	302	S	1673076	Sphingobium hydrophobicum
0.12	222	222	S	13690	Sphingobium yanoikuyae
0.09	169	47	G1	2611147	unclassified Sphingobium
0.02	40	40	S	520734	Sphingobium sp. RSMS
0.02	32	32	S	1315974	Sphingobium sp. TKS
0.02	28	28	S	484429	Sphingobium sp. YBL2
0.01	19	19	S	2082188	Sphingobium sp. YG1
0	2	2	S	2565554	Sphingobium sp. PAMC28499
0	1	1	S	1843368	Sphingobium sp. RAC03
0.08	143	143	S	120107	Sphingobium cloacae
0.07	126	126	S	76947	Sphingobium herbicidovorans
0.05	82	82	S	135719	Sphingobium amiense
0.04	65	65	S	1332080	Sphingobium baderi
0	5	0	S	336203	Sphingobium fuliginis
0	5	5	S1	1208342	Sphingobium fuliginis ATCC 27551
0	2	0	S	332056	Sphingobium japonicum
0	2	2	S1	452662	Sphingobium japonicum UT26S
0	2	2	S	1054037	Sphingobium barthaii
0.11	204	0	G	165696	Novosphingobium
0.1	177	45	G1	2644732	unclassified Novosphingobium
0.05	98	98	S	164608	Novosphingobium sp. KA1
0.01	16	16	S	702113	Novosphingobium sp. PP1Y
0.01	10	10	S	2780074	Novosphingobium sp. ES2-1
0	7	7	S	2571749	Novosphingobium sp. ABRDHK2
0	1	1	S	1609758	Novosphingobium sp. P6W
0.01	22	0	S	205844	Novosphingobium pentaromativorans
0.01	22	22	S1	1088721	Novosphingobium pentaromativorans US6-1
0	5	0	S	48935	Novosphingobium aromaticivorans
0	5	5	S1	279238	Novosphingobium aromaticivorans DSM 12444
0.1	186	16	G	165697	Sphingopyxis
0.07	122	1	G1	2614943	unclassified Sphingopyxis
0.05	89	89	S	292913	Sphingopyxis sp. 113P3
0.01	10	10	S	1914525	Sphingopyxis sp. FD7
0	9	9	S	1874061	Sphingopyxis sp. EG6
0	6	6	S	1357916	Sphingopyxis sp. QXT-31
0	4	4	S	2486273	Sphingopyxis sp. OPL5
0	2	2	S	1866325	Sphingopyxis sp. MG
0	1	1	S	2565556	Sphingopyxis sp. PAMC25046
0.02	32	32	S	33050	Sphingopyxis macrogoltabida
0	9	0	S	117207	Sphingopyxis alaskensis
0	9	9	S1	317655	Sphingopyxis alaskensis RB2256
0	5	5	S	1515612	Sphingopyxis fribergensis
0	2	2	S	267128	Sphingopyxis granuli
0.07	126	0	G	1649486	Rhizorhabdus
0.07	126	126	S	1850238	Rhizorhabdus dicambivorans
0.04	68	4	F	335929	Erythrobacteraceae
0.02	36	0	F1	2800788	Erythrobacter/Porphyrobacter group
0.02	32	0	G	1041	Erythrobacter
0.02	28	0	G1	2633097	unclassified Erythrobacter
0.01	26	26	S	2785907	Erythrobacter sp. A30-3
0	2	2	S	1798193	Erythrobacter sp. HL-111
0	3	0	S	39960	Erythrobacter litoralis
0	3	3	S1	314225	Erythrobacter litoralis HTCC2594
0	1	1	S	1112	Erythrobacter neustonensis
0	4	0	G	1111	Porphyrobacter
0	4	0	G1	2683265	unclassified Porphyrobacter
0	2	2	S	2003315	Porphyrobacter sp. CACIAM 03H1
0	2	2	S	2023229	Porphyrobacter sp. HT-58-2
0.01	13	0	G	1295327	Croceicoccus
0	9	9	S	1348774	Croceicoccus naphthovorans
0	4	0	G1	2629967	unclassified Croceicoccus
0	4	4	S	2798724	Croceicoccus sp. YJ47
0.01	10	0	G	361177	Altererythrobacter
0	9	9	S	1267766	Altererythrobacter atlanticus
0	1	0	G1	2614945	unclassified Altererythrobacter
0	1	1	S	2060312	Altererythrobacter sp. B11
0	5	0	G	2800681	Aurantiacibacter
0	3	3	S	1648404	Aurantiacibacter atlanticus
0	2	2	S	502682	Aurantiacibacter gangjinensis
0	1	0	F	2820280	Sphingosinicellaceae
0	1	0	G	335405	Sphingosinicella
0	1	0	G1	2644549	unclassified Sphingosinicella
0	1	1	S	1892855	Sphingosinicella sp. BN140058
2.25	4051	75	O	356	Hyphomicrobiales
1.87	3371	419	F	119045	Methylobacteriaceae
1.25	2247	176	G	2282523	Methylorubrum
0.85	1523	1433	S	223967	Methylorubrum populi
0.05	90	90	S1	441620	Methylorubrum populi BJ001
0.3	535	55	S	408	Methylorubrum extorquens
0.21	386	386	S1	272630	Methylorubrum extorquens AM1
0.05	83	83	S1	440085	Methylorubrum extorquens CM4
0.01	11	11	S1	419610	Methylorubrum extorquens PA1
0.01	13	13	S	29429	Methylorubrum zatmanii
0.39	697	107	G	407	Methylobacterium
0.27	478	232	G1	2615210	unclassified Methylobacterium
0.06	111	111	S	2067957	Methylobacterium sp. DM1
0.04	67	67	S	925818	Methylobacterium sp. AMS5
0.02	28	28	S	2202828	Methylobacterium sp. 17Sr1-43
0.01	23	23	S	739141	Methylobacterium sp. XJLW
0	9	9	S	426117	Methylobacterium sp. 4-46
0	7	7	S	2603276	Methylobacterium sp. WL1
0	1	1	S	2202826	Methylobacterium sp. 17Sr1-1
0.02	39	39	S	2202825	Methylobacterium durans

0.01	25	25	S	269660	Methylobacterium brachiatum
0.01	18	0	S	39956	Methylobacterium mesophilicum
0.01	18	18	S1	908290	Methylobacterium mesophilicum SR1.6/6
0.01	12	12	S	270351	Methylobacterium aquaticum
0	7	0	S	31998	Methylobacterium radiotolerans
0	7	7	S1	426355	Methylobacterium radiotolerans JCM 2831
0	5	5	S	418223	Methylobacterium phyllosphaerae
0	3	3	S	2051553	Methylobacterium currus
0	2	0	S	114616	Methylobacterium nodulans
0	2	2	S1	460265	Methylobacterium nodulans ORS 2060
0	1	0	S	334852	Methylobacterium oryzae
0	1	1	S1	693986	Methylobacterium oryzae CBMB20
0	8	0	G	186650	Microvirga
0	5	5	S	1882682	Microvirga ossetica
0	3	0	G1	2617746	unclassified Microvirga
0	3	3	S	2807101	Microvirga sp. VF16
0.13	226	3	F	41294	Bradyrhizobiaceae
0.06	117	4	G	85413	Bosea
0.06	106	13	G1	2653178	unclassified Bosea
0.02	45	45	S	1842539	Bosea sp. RAC05
0.01	13	13	S	1867715	Bosea sp. Tri-49
0.01	10	10	S	2020412	Bosea sp. ANAM02
0	9	9	S	2015316	Bosea sp. AS-1
0	8	8	S	1792307	Bosea sp. PAMC 26642
0	8	8	S	2599640	Bosea sp. F3-2
0	7	7	S	1526658	Bosea vaviloviae
0.05	94	39	G	374	Bradyrhizobium
0.02	33	9	G1	2631580	unclassified Bradyrhizobium
0	9	9	S	2057741	Bradyrhizobium sp. SK17
0	6	6	S	2715960	Bradyrhizobium sp. PSBB068
0	3	3	S	1325102	Bradyrhizobium sp. CCBAU 51765
0	3	3	S	2493093	Bradyrhizobium sp. LCT2
0	2	2	S	1325114	Bradyrhizobium sp. CCBAU 53351
0	1	1	S	858422	Bradyrhizobium sp. CCBAU 051011
0	5	5	S	190148	Bradyrhizobium paxllaeri
0	5	5	S	1437360	Bradyrhizobium erythrophlei
0	3	0	S	44255	Bradyrhizobium oligotrophicum
0	3	3	S1	1245469	Bradyrhizobium oligotrophicum S58
0	3	3	S	244734	Bradyrhizobium betae
0	3	3	S	858423	Bradyrhizobium arachidis
0	2	2	S	83637	Bradyrhizobium genosp. L
0	1	1	S	1404864	Bradyrhizobium cosmicum
0	6	0	G	1073	Rhodopseudomonas
0	6	0	S	1076	Rhodopseudomonas palustris
0	6	6	S1	316056	Rhodopseudomonas palustris BisB18
0	3	0	G	1033	Afipia
0	3	3	S	40137	Afipia carboxidovorans
0	3	0	G	1395974	Tardiphaga
0	2	2	S	943830	Tardiphaga robiniae
0	1	1	G1	2631404	unclassified Tardiphaga
0.12	223	23	F	82115	Rhizobiaceae
0.1	172	23	F1	227290	Rhizobium/Agrobacterium group
0.04	80	9	G	357	Agrobacterium
0.03	63	5	G1	1183400	Agrobacterium tumefaciens complex
0.03	58	55	S	358	Agrobacterium tumefaciens
0	3	3	S1	311403	Agrobacterium radiobacter K84
0	5	5	S	359	Agrobacterium rhizogenes
0	3	3	S	160699	Agrobacterium larrymoorei
0.04	68	30	G	379	Rhizobium
0.01	16	10	G1	2613769	unclassified Rhizobium
0	3	3	S	1869170	Rhizobium sp. S41
0	2	2	S	1571470	Rhizobium sp. ACO-34A
0	1	1	S	1981173	Rhizobium sp. NXC14
0	7	7	S	648995	Rhizobium pusense
0	6	6	S	2267833	Rhizobium oryzihabitans
0	4	4	S	384	Rhizobium leguminosarum
0	2	2	S	396	Rhizobium phaseoli
0	2	2	S	1138190	Rhizobium binae
0	1	1	S	56730	Rhizobium gallicum
0	1	0	G	1525371	Neorhizobium
0	1	0	S	399	Neorhizobium galegae
0	1	0	S1	323655	Neorhizobium galegae bv. orientalis
0	1	1	S2	1028800	Neorhizobium galegae bv. orientalis str. HAMBI 540
0.01	16	0	G	323620	Shinella
0.01	16	0	G1	2643062	unclassified Shinella
0.01	12	12	S	2715959	Shinella sp. PSBB067
0	4	4	S	879274	Shinella sp. HZN7
0.01	12	3	F1	227292	Sinorhizobium/Ensifer group
0	8	0	G	28105	Sinorhizobium
0	3	3	S	194963	Sinorhizobium americanum
0	2	2	S	382	Sinorhizobium meliloti
0	2	0	G1	2613772	unclassified Sinorhizobium
0	2	2	S	1449788	Sinorhizobium sp. LM21
0	1	0	S	110321	Sinorhizobium medicae
0	1	1	S1	366394	Sinorhizobium medicae WSM419
0	1	1	G	106591	Ensifer
0.05	88	0	F	69277	Phyllobacteriaceae
0.03	48	0	G	31988	Aminobacter
0.03	48	0	G1	2644704	unclassified Aminobacter
0.03	46	46	S	2774562	Aminobacter sp. SR38
0	2	2	S	374606	Aminobacter sp. MSH1
0.02	39	7	G	68287	Mesorhizobium
0.01	16	5	G1	325217	unclassified Mesorhizobium
0	6	6	S	2744521	Mesorhizobium sp. L-2-11
0	3	3	S	2744518	Mesorhizobium sp. 131-2-1
0	1	1	S	1854057	Mesorhizobium sp. AA22
0	1	1	S	2493673	Mesorhizobium sp. M1B.F.Ca.ET.045.04.1.1
0	5	0	S	39645	Mesorhizobium ciceri
0	5	5	S1	682633	Mesorhizobium ciceri ca181
0	4	4	S	1670800	Mesorhizobium oceanicum
0	3	0	S	381	Mesorhizobium loti
0	3	3	S1	935548	Mesorhizobium loti R88b
0	2	2	S	28104	Mesorhizobium huakuii
0	2	0	S	593909	Mesorhizobium opportunistum

0	2	2	S1	536019	Mesorhizobium opportunistum WSM2075
0	1	0	G	449972	Chelativorans
0	1	0	G1	2643807	unclassified Chelativorans
0	1	1	S	266779	Chelativorans sp. BNC1
0.01	23	0	F	118882	Brucellaceae
0.01	23	9	F1	2826938	Brucella/Ochrobactrum group
0	8	0	G	234	Brucella
0	7	7	S	271865	[Ochrobactrum] quorumnocens
0	1	1	S	529	Brucella anthropi
0	6	0	G	528	Ochrobactrum
0	6	0	G1	239106	unclassified Ochrobactrum
0	5	5	S	1449781	Ochrobactrum sp. LM19
0	1	1	S	2726427	Ochrobactrum sp. MT180101
0.01	14	0	F	255475	Aurantimonadaceae
0.01	12	2	G	293088	Martelella
0.01	10	0	S	293089	Martelella mediterranea
0.01	10	10	S1	1122214	Martelella mediterranea DSM 17316
0	2	0	G	414371	Aureimonas
0	1	1	S	1701758	Aureimonas populi
0	1	0	G1	2615206	unclassified Aureimonas
0	1	1	S	2816454	Aureimonas sp. OT7
0.01	10	5	F	335928	Xanthobacteraceae
0	2	0	G	6	Azorhizobium
0	2	0	S	7	Azorhizobium caulinodans
0	2	2	S1	438753	Azorhizobium caulinodans ORS 571
0	2	0	G	556257	Pseudolabrys
0	2	2	S	331696	Pseudolabrys taiwanensis
0	1	0	G	99	Ancylobacter
0	1	0	G1	2626613	unclassified Ancylobacter
0	1	1	S	1850374	Ancylobacter sp. TS-1
0.01	10	0	F	2821832	Stappiaceae
0	7	0	G	227873	Pannonibacter
0	7	2	S	121719	Pannonibacter phragmitetus
0	5	5	S1	1402210	Pannonibacter phragmitetus BB
0	3	0	G	478070	Labrenzia
0	3	3	G1	2648686	unclassified Labrenzia
0	4	0	F	45401	Hyphomicrobiaceae
0	3	0	G	46913	Devosia
0	3	1	G1	196773	unclassified Devosia
0	2	2	S	2499144	Devosia sp. 1566
0	1	0	G	45402	Aquabacter
0	1	0	G1	2663263	unclassified Aquabacter
0	1	1	S	2820278	Aquabacter sp. L1I39
0	4	0	O1	119042	Rhizobiales incertae sedis
0	4	0	G	1734920	Pseudorhodoplanes
0	4	4	S	1235591	Pseudorhodoplanes sinuspersici
0	2	0	F	45404	Beijerinckiaceae
0	2	0	G	120652	Methylocella
0	2	0	S	199596	Methylocella silvestris
0	2	2	S1	395965	Methylocella silvestris BL2
0	1	0	F	655351	Cohaesibacteraceae
0	1	0	G	1406135	Breoghania
0	1	0	G1	2624130	unclassified Breoghania
0	1	1	S	2304600	Breoghania sp. L-A4
0.24	424	0	O	204458	Caulobacterales
0.24	424	18	F	76892	Caulobacteraceae
0.21	377	112	G	41275	Brevundimonas
0.09	165	38	G1	2622653	unclassified Brevundimonas
0.01	21	21	S	2774190	Brevundimonas sp. LVF2
0.01	21	21	S	2813778	Brevundimonas sp. CS1
0.01	20	20	S	2591463	Brevundimonas sp. M20
0.01	16	16	S	2579977	Brevundimonas sp. SGAir0440
0.01	15	15	S	2752515	Brevundimonas sp. AJA228-03
0.01	12	12	S	2774189	Brevundimonas sp. LVF1
0	7	7	S	2800818	Brevundimonas sp. GRTSA-9
0	6	6	S	1938605	Brevundimonas sp. LM2
0	4	4	S	2561924	Brevundimonas sp. MF30-B
0	4	4	S	2562582	Brevundimonas sp. 'scallop'
0	1	1	S	1827469	Brevundimonas sp. GW460-12-10-14-LB2
0.02	44	44	S	588932	Brevundimonas naejangsanensis
0.02	32	32	S	293	Brevundimonas diminuta
0.01	11	0	S	74313	Brevundimonas subvibrioides
0.01	11	11	S1	633149	Brevundimonas subvibrioides ATCC 15264
0.01	11	11	S	74329	Brevundimonas mediterranea
0	1	1	S	41276	Brevundimonas vesicularis
0	1	1	S	1325724	Brevundimonas vancanneytii
0.01	25	9	G	75	Caulobacter
0	8	8	S	155892	Caulobacter vibrioides
0	4	0	G1	2648921	unclassified Caulobacter
0	4	4	S	69665	Caulobacter sp. FWC26
0	3	3	S	2708539	Caulobacter soli
0	1	1	S	1679497	Caulobacter flavus
0	4	0	G	76890	Asticcacaulis
0	4	1	S	78587	Asticcacaulis excentricus
0	3	3	S1	573065	Asticcacaulis excentricus CB 48
0.11	206	0	O	204455	Rhodobacterales
0.11	206	13	F	31989	Rhodobacteraceae
0.07	125	14	G	265	Paracoccus
0.03	54	0	G1	246570	unclassified Paracoccus
0.03	49	49	S	2500532	Paracoccus sp. Arc7-R13
0	3	3	S	2589076	Paracoccus sp. AK26
0	2	2	S	2812658	Paracoccus sp. H4-D09
0.02	34	34	S	147645	Paracoccus yeei
0	9	9	S	266	Paracoccus denitrificans
0	5	0	S	34003	Paracoccus aminophilus
0	5	5	S1	1367847	Paracoccus aminophilus JCM 7686
0	3	3	S	453842	Paracoccus aestuarii
0	3	3	S	2560053	Paracoccus liaowanqingii
0	1	1	S	82367	Paracoccus pantotrophus
0	1	1	S	1545044	Paracoccus sanguinis
0	1	1	S	1945662	Paracoccus contaminans
0.01	18	0	G	366614	Haematobacter
0.01	18	18	S	195105	Haematobacter massiliensis
0.01	12	0	G	1097466	Defluviimonas

0.01	12	12	S	1335048	Defluviimonas alba
0	9	0	G	92944	Ketogulonicigenium
0	9	9	S	92945	Ketogulonicigenium vulgare
0	5	0	G	2816884	Qingshengfaniella
0	5	5	S	2599296	Qingshengfaniella alkalisoli
0	5	0	G	1060	Rhodobacter
0	5	5	S	1075	Rhodobacter blasticus
0	5	5	G	302485	Phaeobacter
0	3	0	G	991903	Polymorphum
0	3	0	S	991904	Polymorphum gilvum
0	3	3	S1	991905	Polymorphum gilvum SL003B-26A1
0	2	0	G	2613960	Pseudopuniceibacterium
0	2	2	S	2613965	Pseudopuniceibacterium antarcticum
0	2	0	G	1855413	Brevirhabdus
0	2	2	S	1267768	Brevirhabdus pacifica
0	2	0	G	875170	Celeribacter
0	2	2	S	1208324	Celeribacter indicus
0	1	0	G	74030	Roseovarius
0	1	0	G1	2614913	unclassified Roseovarius
0	1	0	S	391613	Roseovarius sp. TM1035
0	1	1	S1	2203213	Roseovarius sp. AK1035
0	1	0	G	74032	Antarctobacter
0	1	1	S	74033	Antarctobacter heliothermus
0	1	0	G	1653176	Cereibacter
0	1	1	S	1063	Cereibacter sphaeroides
0	1	0	G	159345	Roseibacterium
0	1	0	S	159346	Roseibacterium elongatum
0	1	1	S1	1294273	Roseibacterium elongatum DSM 19469
0	1	0	G	335927	Thalassobius
0	1	1	S	53501	Thalassobius gelatinovorus
0.04	75	2	O	204441	Rhodospirillales
0.03	57	0	F	41295	Rhodospirillaceae
0.02	31	0	G	1543704	Niveispirillum
0.02	31	31	S	1612173	Niveispirillum cyanobacteriorum
0	8	0	G	1612157	Pararhodospirillum
0	8	0	S	1084	Pararhodospirillum photometricum
0	8	8	S1	1150469	Pararhodospirillum photometricum DSM 122
0	7	0	G	2705399	Hypericibacter
0	7	7	S	2602015	Hypericibacter terrae
0	6	1	G	191	Azospirillum
0	4	4	S	192	Azospirillum brasilense
0	1	0	G1	2630922	unclassified Azospirillum
0	1	1	S	652764	Azospirillum sp. TSH100
0	4	0	G	2478349	Indioceanicola
0	4	4	S	2220096	Indioceanicola profundi
0	1	0	G	1543705	Nitrospirillum
0	1	0	S	28077	Nitrospirillum amazonense
0	1	1	S1	1441467	Nitrospirillum amazonense CBAmc
0.01	16	3	F	433	Acetobacteraceae
0	4	0	G	2603324	Acidibrevibacterium
0	4	4	S	1969806	Acidibrevibacterium fodinaquatile
0	3	0	G	1434011	Komagataeibacter
0	2	2	S	28448	Komagataeibacter xylinus
0	1	1	S	265960	Komagataeibacter nataicola
0	2	0	G	125216	Roseomonas
0	1	1	S	207340	Roseomonas mucosa
0	1	0	G1	2617492	unclassified Roseomonas
0	1	1	S	2768161	Roseomonas sp. 1318
0	2	0	G	2804525	Lichenicola
0	2	2	S	1484109	Lichenicola cladoniae
0	1	1	G	441	Gluconobacter
0	1	0	G	35812	Roseococcus
0	1	0	G1	2639982	unclassified Roseococcus
0	1	1	S	2771361	Roseococcus sp. NIBR12
0	9	0	C1	82117	Alphaproteobacteria incertae sedis
0	9	0	G	1632780	Phreatobacter
0	9	9	S	1940610	Phreatobacter stygius
0	2	0	O	766	Rickettsiales
0	1	0	F	942	Anaplasmataceae
0	1	0	F1	952	Wolbachieae
0	1	0	G	953	Wolbachia
0	1	0	G1	2640676	unclassified Wolbachia
0	1	1	S	169402	Wolbachia endosymbiont of Folsomia candida
0	1	0	F	1328881	Candidatus Midichloriaceae
0	1	0	G	1802983	Candidatus Fokinia
0	1	1	S	1802984	Candidatus Fokinia solitaria
0	2	0	O	2800059	Maricaulales
0	2	0	F	2800061	Maricaulaceae
0	2	0	G	1649466	Marinicauda
0	2	2	S	2029849	Marinicauda algicola
8	14411	119	C	1236	Gammaproteobacteria
6.02	10837	12	O	135614	Xanthomonadales
6.01	10820	247	F	32033	Xanthomonadaceae
5.83	10504	1358	G	40323	Stenotrophomonas
5	9008	162	G1	995085	Stenotrophomonas maltophilia group
4.9	8832	7952	S	40324	Stenotrophomonas maltophilia
0.44	784	784	S1	391008	Stenotrophomonas maltophilia R551-3
0.05	86	86	S1	522373	Stenotrophomonas maltophilia K279a
0	8	8	S1	868597	Stenotrophomonas maltophilia JV3
0	2	2	S1	1163399	Stenotrophomonas maltophilia D457
0	8	8	S	2072413	Stenotrophomonas sp. SAU14A_NAIMI4_5
0	3	3	S	2072408	Stenotrophomonas sp. YAU14A_MKIMI4_1
0	1	1	S	2072407	Stenotrophomonas sp. YAU14D1_LEIMI4_1
0	1	1	S	2072412	Stenotrophomonas sp. ZAC14A_NAIMI4_1
0	1	1	S	2072414	Stenotrophomonas sp. ESTM1D_MKCIP4_1
0.05	85	4	G1	196198	unclassified Stenotrophomonas
0.01	24	24	S	1904944	Stenotrophomonas sp. LM091
0.01	18	18	S	2005046	Stenotrophomonas sp. WZN-1
0.01	15	15	S	2682487	Stenotrophomonas sp. SXG-1
0	9	9	S	2282124	Stenotrophomonas sp. ASS1
0	7	7	S	2691571	Stenotrophomonas sp. 364
0	6	6	S	2742129	Stenotrophomonas sp. NA06056
0	1	1	S	1827305	Stenotrophomonas sp. MYb57
0	1	1	S	2303750	Stenotrophomonas sp. G4

0.02	37	37	S	216778	Stenotrophomonas rhizophila
0.01	16	16	S	128780	Stenotrophomonas acidaminiphila
0.02	28	10	G	338	Xanthomonas
0	9	0	G1	643453	Xanthomonas citri group
0	9	6	S	346	Xanthomonas citri
0	2	2	S1	473421	Xanthomonas citri pv. glycines
0	1	1	S1	76802	Xanthomonas citri pv. aurantifolii
0	6	6	S	442694	Xanthomonas perforans
0	3	3	S	347	Xanthomonas oryzae
0.01	23	0	G	83618	Pseudoxanthomonas
0.01	23	23	S	128785	Pseudoxanthomonas mexicana
0.01	11	5	G	68	Lysobacter
0	2	2	S	69	Lysobacter enzymogenes
0	2	0	G1	2635362	unclassified Lysobacter
0	2	2	S	2290922	Lysobacter sp. TY2-98
0	1	1	S	2591633	Lysobacter alkalisoli
0	1	1	S	2763317	Lysobacter solisilvae
0	4	0	G	141948	Thermomonas
0	2	2	S	1463158	Thermomonas carbonis
0	2	0	G1	2633315	unclassified Thermomonas
0	2	2	S	2771436	Thermomonas sp. XSG
0	3	0	G	83614	Luteimonas
0	3	0	G1	2629088	unclassified Luteimonas
0	3	3	S	1896164	Luteimonas sp. JM171
0	5	0	F	1775411	Rhodanobacteraceae
0	3	0	G	242605	Luteibacter
0	2	0	S	242606	Luteibacter rhizovicinus
0	2	2	S1	1440763	Luteibacter rhizovicinus DSM 16549
0	1	1	S	2589080	Luteibacter pinisoli
0	2	0	G	231454	Dyella
0	1	1	S	445710	Dyella thiooxydans
0	1	1	S	1849581	Dyella caseinilytica
1.62	2919	4	O	72274	Pseudomonadales
1.09	1962	12	F	468	Moraxellaceae
1.04	1866	665	G	469	Acinetobacter
0.43	779	766	S	40214	Acinetobacter johnsonii
0.01	13	13	S1	1242245	Acinetobacter johnsonii XBB1
0.05	87	87	S	40215	Acinetobacter junii
0.04	76	2	G1	196816	unclassified Acinetobacter
0.01	20	20	S	2743575	Acinetobacter sp. NEB 394
0.01	17	17	S	2004646	Acinetobacter sp. WCHA55
0.01	17	17	S	2004644	Acinetobacter sp. WCHA45
0	8	8	S	2601122	Acinetobacter sp. YH12138
0	5	5	S	1636603	Acinetobacter sp. ACNIH1
0	2	2	S	2545797	Acinetobacter sp. FDAARGOS_724
0	2	2	S	1646498	Acinetobacter sp. TTH0-4
0	1	1	S	1280052	Acinetobacter sp. M131
0	1	1	S	1879049	Acinetobacter sp. WCHAc010034
0	1	1	S	1758189	Acinetobacter sp. ACNIH2
0.03	62	15	G1	909768	Acinetobacter calcoaceticus/baumannii complex
0.02	40	40	S	470	Acinetobacter baumannii
0	4	4	S	1530123	Acinetobacter seifertii
0	3	1	S	48296	Acinetobacter pittii
0	2	2	S1	871585	Acinetobacter pittii PHEA-2
0.02	30	30	S	202956	Acinetobacter towneri
0.01	23	23	S	756892	Acinetobacter indicus
0.01	21	19	S	28090	Acinetobacter lwoffii
0	2	2	S1	1046625	Acinetobacter lwoffii WJ10621
0.01	21	21	S	29430	Acinetobacter haemolyticus
0.01	19	19	S	108980	Acinetobacter ursingii
0.01	16	16	S	108981	Acinetobacter schindleri
0.01	16	16	S	134534	Acinetobacter gyllenbergii
0.01	14	14	S	106648	Acinetobacter bereziniae
0.01	13	13	S	1879050	Acinetobacter wuhouensis
0	9	0	S	52133	Acinetobacter venetianus
0	9	9	S1	1197884	Acinetobacter venetianus VE-C3
0	6	6	S	2715164	Acinetobacter shaoyimingii
0	5	5	S	40216	Acinetobacter radioresistens
0	2	2	S	70348	Acinetobacter dispersus
0	1	1	S	487316	Acinetobacter soli
0	1	1	S	2006115	Acinetobacter piscicola
0.04	69	0	G	475	Moraxella
0.04	69	69	S	34062	Moraxella osloensis
0.01	14	0	G	497	Psychrobacter
0	9	0	G1	196806	unclassified Psychrobacter
0	7	7	S	2733866	Psychrobacter sp. KCTC 72983
0	2	2	S	1699621	Psychrobacter sp. P11F6
0	5	0	S	334543	Psychrobacter arcticus
0	5	5	S1	259536	Psychrobacter arcticus 273-4
0	1	0	G	2824158	Aquirhabdus
0	1	1	S	2283318	Aquirhabdus parva
0.53	953	0	F	135621	Pseudomonadaceae
0.53	953	219	G	286	Pseudomonas
0.22	402	0	G1	136841	Pseudomonas aeruginosa group
0.18	320	320	S	287	Pseudomonas aeruginosa
0.02	40	28	S	300	Pseudomonas mendocina
0.01	11	11	S1	1225174	Pseudomonas mendocina S5.2
0	1	1	S1	399739	Pseudomonas mendocina ymp
0.02	40	0	G2	1232139	Pseudomonas oleovorans/pseudoalcaligenes group
0.02	40	1	S	301	Pseudomonas oleovorans
0.02	39	39	S1	1182590	Pseudomonas pseudoalcaligenes CECT 5344
0	2	0	G2	627141	Pseudomonas nitroreducens/multiresinivorans group
0	2	2	S	46680	Pseudomonas nitroreducens
0.08	152	6	G1	136843	Pseudomonas fluorescens group
0.07	121	120	S	294	Pseudomonas fluorescens
0	1	1	S1	463794	Pseudomonas fluorescens BBc6R8
0	9	9	S	78543	Pseudomonas migulae
0	9	9	S	380021	Pseudomonas protegens
0	7	7	S	29442	Pseudomonas tolaasii
0.03	61	21	G1	196821	unclassified Pseudomonas
0	5	5	S	253237	Pseudomonas sp. phDV1
0	4	4	S	2735906	Pseudomonas sp. B11D7D
0	3	3	S	2774873	Pseudomonas sp. ADPe
0	3	3	S	2762896	Pseudomonas sp. MPDS

0	3	3	S	2731681	Pseudomonas sp. S1-A32-2
0	3	3	S	2614442	Pseudomonas sp. LPB0260
0	3	3	S	2201356	Pseudomonas sp. 31-12
0	3	3	S	1294143	Pseudomonas sp. ATCC 13867
0	2	2	S	2812000	Pseudomonas sp. SDM007
0	2	2	S	2811422	Pseudomonas sp. PDNC002
0	1	1	S	2049589	Pseudomonas sp. HLS-6
0	1	1	S	76885	Pseudomonas sp. K-62
0	1	1	S	2725477	Pseudomonas sp. TUM18999
0	1	1	S	2605424	Pseudomonas sp. J380
0	1	1	S	2587845	Pseudomonas sp. THAF7b
0	1	1	S	2054919	Pseudomonas sp. S09G 359
0	1	1	S	2025658	Pseudomonas sp. NS1(2017)
0	1	1	S	1931241	Pseudomonas sp. S-6-2
0	1	1	S	1898684	Pseudomonas sp. LPH1
0.02	35	0	G1	136845	Pseudomonas putida group
0.02	33	25	S	303	Pseudomonas putida
0	6	6	S1	1211579	Pseudomonas putida NBRC 14164
0	2	2	S1	1384061	Pseudomonas putida S13.1.2
0	2	2	S	47885	Pseudomonas oryzihabitans
0.01	19	0	G1	136846	Pseudomonas stutzeri group
0.01	11	0	G2	578833	Pseudomonas stutzeri subgroup
0.01	11	9	S	316	Pseudomonas stutzeri
0	2	2	S1	1123519	Pseudomonas stutzeri DSM 10701
0	8	8	S	74829	Pseudomonas balearica
0.01	11	0	S	101564	Pseudomonas alcaliphila
0.01	11	11	S1	741155	Pseudomonas alcaliphila JAB1
0.01	10	10	S	359110	Pseudomonas extremaustralis
0	8	8	S	237610	Pseudomonas psychrotolerans
0	6	6	S	65741	Pseudomonas knackmussii
0	6	6	S	216142	Pseudomonas rhizosphaerae
0	4	4	S	104087	Pseudomonas frederiksbergensis
0	3	3	S	2502979	Pseudomonas khazarica
0	3	3	S	1615674	Pseudomonas lactis
0	3	3	S	1499686	Pseudomonas saudiphocaensis
0	3	3	S	237609	Pseudomonas alkylphenolica
0	3	3	S	157782	Pseudomonas parafulva
0	2	2	S	219572	Pseudomonas antarctica
0	1	1	S	1691904	Pseudomonas sediminis
0	1	1	S	395598	Pseudomonas reinekei
0	1	0	G1	136844	Pseudomonas pertucinogena group
0	1	1	S	43306	Pseudomonas denitrificans (nom. rej.)
0.17	302	2	O	91347	Enterobacterales
0.09	168	51	F	543	Enterobacteriaceae
0.04	68	4	G	561	Escherichia
0.03	63	61	S	562	Escherichia coli
0	1	1	S1	2778655	Escherichia coli O167:H26
0	1	1	S1	2763104	Escherichia coli O150:H6
0	1	1	S	208962	Escherichia albertii
0.02	33	22	G	570	Klebsiella
0.01	10	10	S	548	Klebsiella aerogenes
0	1	1	S	571	Klebsiella oxytoca
0	6	0	G	83654	Leclercia
0	5	5	S	83655	Leclercia adecarboxylata
0	1	0	G1	2627398	unclassified Leclercia
0	1	1	S	1920114	Leclercia sp. LSNIH1
0	4	1	G	590	Salmonella
0	3	1	S	28901	Salmonella enterica
0	2	0	S1	59201	Salmonella enterica subsp. enterica
0	1	1	S2	189201	Salmonella enterica subsp. enterica serovar Cubana
0	1	0	S2	598	Salmonella enterica subsp. enterica serovar Rubislaw
0	1	1	S3	938143	Salmonella enterica subsp. enterica serovar Rubislaw str. ATCC 10717
0	3	0	G	544	Citrobacter
0	3	0	G1	1344959	Citrobacter freundii complex
0	3	3	S	546	Citrobacter freundii
0	2	1	G	547	Enterobacter
0	1	1	G1	354276	Enterobacter cloacae complex
0	1	1	G	160674	Raoultella
0.07	124	0	F	1903411	Yersiniaceae
0.07	120	56	G	613	Serratia
0.03	53	50	S	614	Serratia liquefaciens
0	3	3	S1	1346614	Serratia liquefaciens ATCC 27592
0	4	4	S	615	Serratia marcescens
0	4	0	G1	2647522	unclassified Serratia
0	4	4	S	488142	Serratia sp. SCBI
0	3	3	S	2741499	Serratia surfactantfaciens
0	4	0	G	629	Yersinia
0	4	4	S	2607663	Yersinia canariae
0	4	0	F	1903414	Morganellaceae
0	4	0	G	586	Providencia
0	4	4	S	333965	Providencia vermicola
0	2	0	F	1903410	Pectobacteriaceae
0	2	0	G	204037	Dickeya
0	2	2	S	204042	Dickeya zeae
0	1	0	O1	451511	Enterobacterales incertae sedis
0	1	0	G	702	Plesiomonas
0	1	1	S	703	Plesiomonas shigelloides
0	1	0	F	1903409	Erwiniaceae
0	1	0	G	53335	Pantoea
0	1	1	S	66269	Pantoea stewartii
0.04	66	0	O	135624	Aeromonadales
0.04	66	0	F	84642	Aeromonadaceae
0.04	66	30	G	642	Aeromonas
0.01	13	13	S	644	Aeromonas hydrophila
0	8	8	S	651	Aeromonas media
0	7	7	S	648	Aeromonas caviae
0	4	4	S	218936	Aeromonas simiae
0	2	2	S	73010	Aeromonas encheleia
0	1	1	S	654	Aeromonas veronii
0	1	0	G1	257493	unclassified Aeromonas
0	1	1	S	2675707	Aeromonas sp. WP2-W18-CRE-05
0.03	59	0	O	135625	Pasteurellales
0.03	59	0	F	712	Pasteurellaceae
0.03	58	0	G	724	Haemophilus

0.03	48	41	S	729	Haemophilus parainfluenzae
0	7	7	S1	862965	Haemophilus parainfluenzae T3T1
0	7	7	S	726	Haemophilus haemolyticus
0	3	3	S	730	[Haemophilus] ducreyi
0	1	0	G	416916	Aggregatibacter
0	1	1	S	739	Aggregatibacter segnis
0.02	35	0	O	135619	Oceanospirillales
0.01	19	0	F	224372	Alcanivoracaceae
0.01	19	0	G	59753	Alcanivorax
0.01	10	10	S	1094342	Alcanivorax xenomutans
0	6	0	S	285091	Alcanivorax dieselolei
0	6	6	S1	930169	Alcanivorax dieselolei B5
0	3	0	S	1306787	Alcanivorax pacificus
0	3	3	S1	391936	Alcanivorax pacificus W11-5
0.01	16	5	F	28256	Halomonadaceae
0	9	0	G	2745	Halomonas
0	9	5	G1	2609666	unclassified Halomonas
0	3	3	S	2733487	Halomonas sp. MCCC 1A13316
0	1	1	S	2733488	Halomonas sp. MCCC 1A13718
0	1	0	G	42054	Chromohalobacter
0	1	0	S	158080	Chromohalobacter salexigens
0	1	1	S1	290398	Chromohalobacter salexigens DSM 3043
0	1	0	G	204286	Cobetia
0	1	0	G1	2609414	unclassified Cobetia
0	1	1	S	2686360	Cobetia sp. L2A1
0.01	24	0	O	135613	Chromatiales
0.01	13	0	F	1046	Chromatiaceae
0.01	13	0	G	67575	Rheinheimera
0.01	13	0	G1	115860	unclassified Rheinheimera
0.01	10	10	S	2498451	Rheinheimera sp. LHK132
0	3	3	S	1763998	Rheinheimera sp. F8
0	9	0	F	1096778	Thioalkalispiraceae
0	9	0	G	2034504	Sulfurivermis
0	9	9	S	1972068	Sulfurivermis fontis
0	2	0	F	72276	Ectothiorhodospiraceae
0	2	0	G	106633	Thioalkalivibrio
0	2	0	S	1033854	Thioalkalivibrio sulfidiphilus
0	2	2	S1	396588	Thioalkalivibrio sulfidiphilus HL-EbGr7
0.01	17	0	O	135622	Alteromonadales
0	9	0	F	267888	Pseudoalteromonadaceae
0	6	3	G	53246	Pseudoalteromonas
0	2	2	S	166935	Pseudoalteromonas translucida
0	1	1	S	874454	Pseudoalteromonas arabiensis
0	3	0	G	907197	Psychrosphaera
0	3	3	S	1266052	Psychrosphaera aestuarii
0	6	0	F	267890	Shewanellaceae
0	6	0	G	22	Shewanella
0	4	0	G1	196818	unclassified Shewanella
0	4	4	S	94122	Shewanella sp. ANA-3
0	2	0	S	271098	Shewanella halifaxensis
0	2	2	S1	458817	Shewanella halifaxensis HAW-EB4
0	2	0	F	72275	Alteromonadaceae
0	2	0	G	2742	Marinobacter
0	2	2	S	418719	Marinobacter salsuginis
0	9	0	O	72273	Thiotrichales
0	7	0	F	135616	Piscirickettsiaceae
0	7	0	G	28884	Hydrogenovibrio
0	7	7	S	28885	Hydrogenovibrio marinus
0	2	0	F	135617	Thiotrichaceae
0	2	0	G	1030	Thiothrix
0	2	0	G1	2636184	unclassified Thiothrix
0	2	2	S	1032	Thiothrix sp.
0	9	0	O	135615	Cardiobacteriales
0	9	0	F	868	Cardiobacteriaceae
0	9	0	G	2717	Cardiobacterium
0	9	9	S	2718	Cardiobacterium hominis
0	6	0	O	1706369	Cellvibrionales
0	3	0	F	1706371	Cellvibrionaceae
0	3	0	G	10	Cellvibrio
0	3	0	G1	2624793	unclassified Cellvibrio
0	3	3	S	454662	Cellvibrio sp. KY-YJ-3
0	3	0	F	1706373	Microbulbiferaceae
0	3	0	G	48073	Microbulbifer
0	3	0	G1	2619833	unclassified Microbulbifer
0	3	3	S	2745199	Microbulbifer sp. YPW1
0	4	0	O	1934945	Immundisolibacterales
0	4	0	F	1934946	Immundisolibacteraceae
0	4	0	G	1934947	Immundisolibacter
0	4	4	S	1810504	Immundisolibacter cernigliae
0	2	0	O	135618	Methylococcales
0	2	0	F	403	Methylococcaceae
0	2	0	G	416	Methylomonas
0	2	0	S	421	Methylomonas methanica
0	2	2	S1	857087	Methylomonas methanica MC09
0	2	0	O	135623	Vibrionales
0	2	0	F	641	Vibrionaceae
0	2	0	G	662	Vibrio
0	1	1	S	666	Vibrio cholerae
0	1	0	G1	717610	Vibrio harveyi group
0	1	1	S	663	Vibrio alginolyticus
0	1	0	O	1775403	Nevskiales
0	1	0	F	568386	Sinobacteraceae
0	1	0	G	1861863	Sinimarinibacterium
0	1	0	G1	2621506	unclassified Sinimarinibacterium
0	1	1	S	2698684	Sinimarinibacterium sp. NLF-5-8
0.02	28	0	P1	68525	delta/epsilon subdivisions
0.01	15	0	C	28221	Deltaproteobacteria
0.01	14	0	O	29	Myxococcales
0.01	10	0	O1	80812	Sorangiineae
0	8	0	F	1524216	Labilitrichaceae
0	8	0	G	1524217	Labilithrix
0	8	8	S	1391654	Labilithrix luteola
0	2	0	F	49	Polyangiaceae
0	2	0	G	39643	Sorangium

0	2	1	S	56	Sorangium cellulosum
0	1	1	S1	1254432	Sorangium cellulosum So0157-2
0	2	0	O1	80811	Cystobacterineae
0	2	0	F	31	Myxococcaceae
0	2	0	G	32	Myxococcus
0	2	2	S	34	Myxococcus xanthus
0	2	0	O1	224462	Nannocystineae
0	2	0	F	224464	Kofleriaceae
0	2	0	G	162027	Haliangium
0	2	0	S	80816	Haliangium ochraceum
0	2	2	S1	502025	Haliangium ochraceum DSM 14365
0	1	0	O	213115	Desulfovibrionales
0	1	0	F	194924	Desulfovibrionaceae
0	1	0	G	2773294	Desulfolutivibrio
0	1	1	S	2773299	Desulfolutivibrio sulfoxidireducens
0.01	13	0	C	29547	Epsilonproteobacteria
0.01	13	0	O	213849	Campylobacterales
0.01	13	0	F	72294	Campylobacteraceae
0.01	13	2	G	194	Campylobacter
0.01	11	10	S	195	Campylobacter coli
0	1	1	S1	1367491	Campylobacter coli 76339
0.01	11	0	C	2008785	Hydrogenophilalia
0.01	11	0	O	119069	Hydrogenophilales
0.01	11	0	F	206349	Hydrogenophilaceae
0.01	11	0	G	70774	Hydrogenophilus
0.01	11	11	S	297	Hydrogenophilus thermoluteolus
0	7	0	C	1553900	Oligoflexia
0	7	0	O	213481	Bdellovibrionales
0	7	0	F	213483	Bdellovibrionaceae
0	7	0	G	958	Bdellovibrio
0	5	0	S	959	Bdellovibrio bacteriovorus
0	5	5	S1	765869	Bdellovibrio bacteriovorus W
0	2	0	G1	2633795	unclassified Bdellovibrio
0	2	2	S	2231053	Bdellovibrio sp. ZAP7
9.57	17233	29	D1	1783272	Terrabacteria group
7.72	13900	2	P	201174	Actinobacteria
7.69	13850	191	C	1760	Actinomycetia
5.72	10299	5	O	85009	Propionibacteriales
5.69	10254	105	F	31957	Propionibacteriaceae
5.56	10008	72	G	1912216	Cutibacterium
5.44	9805	9781	S	1747	Cutibacterium acnes
0.01	14	1	S1	1734925	Cutibacterium acnes subsp. acnes
0.01	11	11	S2	1114967	Cutibacterium acnes TypeIA2 P.acn17
0	2	2	S2	1114966	Cutibacterium acnes TypeIA2 P.acn33
0	7	7	S1	1134454	Cutibacterium acnes HL096PA1
0	2	2	S1	1234380	Cutibacterium acnes C1
0	1	0	S1	1905725	Cutibacterium acnes subsp. defendens
0	1	1	S2	1091045	Cutibacterium acnes subsp. defendens ATCC 11828
0.07	121	121	S	33011	Cutibacterium granulosum
0.01	10	9	S	33010	Cutibacterium avidum
0	1	1	S1	1170318	Cutibacterium avidum 44067
0.05	96	0	G	1743	Propionibacterium
0.03	49	0	G1	2608905	unclassified Propionibacterium
0.03	49	49	S	671223	Propionibacterium sp. oral taxon 193
0.02	43	17	S	1744	Propionibacterium freudenreichii
0.01	26	26	S1	1752	Propionibacterium freudenreichii subsp. shermanii
0	4	4	S	556499	Propionibacterium acidifaciens
0.01	18	0	G	72763	Tessaracoccus
0.01	14	14	S	399497	Tessaracoccus flavescens
0	3	3	S	1285901	Tessaracoccus defluvii
0	1	1	S	1332264	Tessaracoccus aquimaris
0.01	14	0	G	1912215	Acidipropionibacterium
0.01	13	13	S	1749	Acidipropionibacterium jensenii
0	1	1	S	2057246	Acidipropionibacterium virtanenii
0.01	10	0	G	29404	Microlunatus
0	5	0	S	29405	Microlunatus phosphovorus
0	5	5	S1	1032480	Microlunatus phosphovorus NM-1
0	5	5	S	546874	Microlunatus sagamiharensis
0	3	0	G	1085622	Propioniciclava
0	3	0	G1	2642922	unclassified Propioniciclava
0	3	3	S	2714937	Propioniciclava sp. HDW11
0.02	40	1	F	85015	Nocardioidaceae
0.01	22	7	G	1839	Nocardioides
0	7	0	G1	2615069	unclassified Nocardioides
0	4	4	S	2736757	Nocardioides sp. MC1495
0	1	1	S	2017486	Nocardioides sp. S5
0	1	1	S	2663857	Nocardioides sp. zg-579
0	1	1	S	2760089	Nocardioides sp. S-713
0	4	4	S	200618	Nocardioides aromaticivorans
0	2	2	S	402297	Nocardioides daphniae
0	1	1	S	449461	Nocardioides humi
0	1	1	S	2712223	Nocardioides anomalus
0.01	10	1	G	2040	Aeromicrobium
0	3	0	S	219314	Aeromicrobium marinum
0	3	3	S1	585531	Aeromicrobium marinum DSM 15272
0	3	3	S	1736691	Aeromicrobium choanae
0	2	0	G1	2633570	unclassified Aeromicrobium
0	2	2	S	2663859	Aeromicrobium sp. zg-629
0	1	1	S	2041	Aeromicrobium erythreum
0	7	0	G	116071	Micropruina
0	7	7	S	75385	Micropruina glycogenica
1.33	2389	68	O	85006	Micrococcales
1.1	1989	151	F	85023	Microbacteriaceae
0.85	1540	438	G	33882	Microbacterium
0.24	428	59	G1	2609290	unclassified Microbacterium
0.02	40	40	S	2483401	Microbacterium sp. 10M-3C3
0.02	37	37	S	2014534	Microbacterium sp. PM5
0.01	27	27	S	2567934	Microbacterium sp. 4R-513
0.01	25	25	S	367477	Microbacterium sp. XT11
0.01	25	25	S	2603598	Microbacterium sp. CBA3102
0.01	25	25	S	2782167	Microbacterium sp. A18JL200
0.01	24	24	S	1714373	Microbacterium sp. No. 7
0.01	24	24	S	2268461	Microbacterium sp. ABRD_28
0.01	22	22	S	2782169	Microbacterium sp. NY27

0.01	20	20	S	2782166	Microbacterium sp. A18JL241
0.01	14	14	S	1795053	Microbacterium sp. PAMC 28756
0.01	14	14	S	2763257	Microbacterium sp. YJN-G
0.01	12	12	S	1906742	Microbacterium sp. BH-3-3-3
0.01	11	11	S	2782168	Microbacterium sp. WY121
0.01	10	10	S	2489212	Microbacterium sp. RG1
0	8	8	S	1906274	Microbacterium sp. JZ31
0	7	7	S	2103230	Microbacterium sp. str. 'China'
0	6	6	S	1938334	Microbacterium sp. TPU 3598
0	5	5	S	912630	Microbacterium sp. LKL04
0	5	5	S	2766784	Microbacterium sp. Nx66
0	4	4	S	2048898	Microbacterium sp. Y-01
0	2	2	S	1916917	Microbacterium sp. 1.5R
0	1	1	S	2606451	Microbacterium sp. 1S1
0	1	1	S	2709304	Microbacterium sp. Se63.02b
0.13	240	240	S	36805	Microbacterium aurum
0.08	138	138	S	162426	Microbacterium hominis
0.03	63	63	S	199592	Microbacterium paraoxydans
0.02	42	42	S	1072463	Microbacterium lemovicicum
0.02	33	33	S	104336	Microbacterium foliorum
0.02	30	30	S	273677	Microbacterium oleivorans
0.01	21	21	S	82380	Microbacterium oxydans
0.01	21	21	S	2541726	Microbacterium wangchenii
0.01	18	18	S	84292	Microbacterium chocolatum
0.01	16	16	S	370764	Microbacterium pygmaeum
0.01	11	11	S	2614638	Microbacterium caowuchunii
0.01	10	10	S	904291	Microbacterium sediminis
0	7	7	S	69362	Microbacterium schleiferi
0	7	7	S	743009	Microbacterium oryzae
0	5	5	S	2614639	Microbacterium lushaniae
0	4	4	S	273678	Microbacterium hydrocarbonoxydans
0	3	3	S	2509458	Microbacterium protaetiae
0	2	2	S	57043	Microbacterium esteraromaticum
0	2	2	S	1526412	Microbacterium endophyticum
0	1	0	S	2033	Microbacterium testaceum
0	1	1	S1	979556	Microbacterium testaceum StLB037
0.05	98	2	G	2034	Curtobacterium
0.03	60	0	S	2035	Curtobacterium flaccumfaciens
0.03	60	60	S1	138532	Curtobacterium flaccumfaciens pv. flaccumfaciens
0.02	36	3	G1	257496	unclassified Curtobacterium
0.01	16	16	S	1561023	Curtobacterium sp. MR_MD2014
0.01	10	10	S	2070337	Curtobacterium sp. SGAir0471
0	3	3	S	2495430	Curtobacterium sp. Csp2
0	2	2	S	1905847	Curtobacterium sp. BH-2-1-1
0	1	1	S	2795488	Curtobacterium sp. YC1
0	1	1	S	2804758	Curtobacterium sp. 24E2
0.02	40	0	G	33877	Agromyces
0.02	34	0	G1	2639701	unclassified Agromyces
0.01	16	16	S	2592652	Agromyces sp. KACC 19306
0	9	9	S	2498704	Agromyces sp. LHK192
0	5	5	S	2781962	Agromyces sp. G127AT
0	4	4	S	2585717	Agromyces sp. HY052
0	3	3	S	589382	Agromyces flavus
0	2	2	S	2509455	Agromyces protaetiae
0	1	1	S	2080742	Agromyces badenianii
0.02	30	0	G	33886	Rathayibacter
0.01	21	2	G1	2609250	unclassified Rathayibacter
0.01	16	16	S	2609253	Rathayibacter sp. VKM Ac-2760
0	2	2	S	2609257	Rathayibacter sp. VKM Ac-2804
0	1	1	S	2609254	Rathayibacter sp. VKM Ac-2762
0	8	8	S	59737	Rathayibacter iranicus
0	1	1	S	1671680	Rathayibacter tanaceti
0.01	27	0	G	55968	Leucobacter
0.01	16	16	S	1784719	Leucobacter triazinivorans
0.01	11	11	S	1935379	Leucobacter muris
0.01	20	0	G	110932	Leifsonia
0.01	12	4	G1	2663824	unclassified Leifsonia
0	4	4	S	1798223	Leifsonia sp. 21MFCrub1.1
0	4	4	S	2724914	Leifsonia sp. PS1209
0	8	8	S	150026	Leifsonia shinshuensis
0.01	14	0	G	1573	Clavibacter
0.01	11	0	S	28447	Clavibacter michiganensis
0	6	6	S1	31963	Clavibacter michiganensis subsp. nebraskensis
0	3	3	S1	33013	Clavibacter michiganensis subsp. michiganensis
0	2	2	S1	1874630	Clavibacter michiganensis subsp. capsici
0	3	0	G1	2626594	unclassified Clavibacter
0	3	3	S	2768071	Clavibacter sp. DM1
0.01	12	0	G	1433997	Diaminobutyricimonas
0.01	12	0	G1	2643261	unclassified Diaminobutyricimonas
0.01	12	12	S	2683590	Diaminobutyricimonas sp. LJ205
0	9	0	G	235888	Salinibacterium
0	9	0	G1	2632331	unclassified Salinibacterium
0	4	4	S	2708338	Salinibacterium sp. ZJ450
0	3	3	S	2603292	Salinibacterium sp. dk2585
0	2	2	S	2708084	Salinibacterium sp. ZJ70
0	7	0	G	76634	Mycetocola
0	6	0	G1	2685235	unclassified Mycetocola
0	6	6	S	2116510	Mycetocola sp. JXN-3
0	1	1	S	2079792	Mycetocola zhujimingii
0	7	0	G	190323	Plantibacter
0	6	6	S	150123	Plantibacter flavus
0	1	1	G1	2624265	unclassified Plantibacter
0	6	0	G	1195526	Gryllotalpicola
0	6	6	S	2419771	Gryllotalpicola protaetiae
0	5	0	G	69578	Cryobacterium
0	3	0	G1	2649013	unclassified Cryobacterium
0	3	3	S	1978566	Cryobacterium sp. LW097
0	2	2	S	670052	Cryobacterium arcticum
0	5	0	G	881616	Herbiconiux
0	5	0	G1	2618217	unclassified Herbiconiux
0	5	5	S	2735133	Herbiconiux sp. SALV-R1
0	4	0	G	337004	Microcella
0	4	4	S	279828	Microcella alkaliphila
0	3	0	G	46352	Agrococcus

0	2	2	S	399736	Agrococcus jejuensis
0	1	0	G1	2615065	unclassified Agrococcus
0	1	1	S	2070347	Agrococcus sp. SGAir0287
0	3	0	G	96492	Frigoribacterium
0	3	0	G1	2627005	unclassified Frigoribacterium
0	3	3	S	2596916	Frigoribacterium sp. NBH87
0	3	0	G	447237	Frondihabitans
0	3	0	G1	2626248	unclassified Frondihabitans
0	3	3	S	1795630	Frondihabitans sp. PAMC 28766
0	3	0	G	1331730	Chryseoglobus
0	3	3	S	2750620	Chryseoglobus indicus
0	2	0	G	110934	Agreia
0	2	0	G1	2641148	unclassified Agreia
0	2	2	S	2773266	Agreia sp. COWG
0.09	171	38	F	1268	Micrococcaceae
0.02	44	9	G	1269	Micrococcus
0.02	34	34	S	1270	Micrococcus luteus
0	1	0	G1	2620948	unclassified Micrococcus
0	1	1	S	376418	Micrococcus sp. A7
0.02	28	0	G	32207	Rothia
0.01	15	1	S	2047	Rothia dentocariosa
0.01	14	14	S1	762948	Rothia dentocariosa ATCC 17931
0	8	8	S	37923	Rothia kristinae
0	4	4	S	43675	Rothia mucilaginosa
0	1	1	S	169480	Rothia amarae
0.01	25	1	G	57493	Kocuria
0	9	9	S	71999	Kocuria palustris
0	8	8	S	1272	Kocuria varians
0	7	7	S	72000	Kocuria rhizophila
0.01	12	0	G	1742989	Glutamicibacter
0.01	12	12	S	37929	Glutamicibacter nicotianae
0.01	11	0	G	1663	Arthrobacter
0	9	0	G1	235627	unclassified Arthrobacter
0	6	6	S	1357915	Arthrobacter sp. QXT-31
0	3	3	S	904039	Arthrobacter sp. NEB 688
0	2	2	S	37921	Arthrobacter agilis
0	8	0	G	370735	Zhihengliuella
0	8	0	G1	2626589	unclassified Zhihengliuella
0	8	8	S	2058657	Zhihengliuella sp. ISTPL4
0	4	0	G	1645	Renibacterium
0	4	4	S	1646	Renibacterium salmoninarum
0	1	0	G	1742993	Pseudarthrobacter
0	1	1	S	121292	Pseudarthrobacter sulfonivorans
0.03	58	0	F	85019	Brevibacteriaceae
0.03	58	1	G	1696	Brevibacterium
0.02	30	0	G1	2614124	unclassified Brevibacterium
0.01	12	12	S	1496080	Brevibacterium sp. YB235
0.01	12	12	S	2575923	Brevibacterium sp. CS2
0	6	6	S	1406197	Brevibacterium sp. Ap13
0.01	14	14	S	273384	Brevibacterium aurantiacum
0	8	8	S	1703	Brevibacterium linens
0	5	5	S	629680	Brevibacterium sandarakinum
0.02	38	0	F	85021	Intrasporangiaceae
0.02	37	10	G	53457	Janibacter
0.01	13	13	S	857417	Janibacter indicus
0.01	10	10	S	53458	Janibacter limosus
0	3	3	S	262209	Janibacter melonis
0	1	0	G1	2649294	unclassified Janibacter
0	1	1	S	2761047	Janibacter sp. YB324
0	1	0	G	367298	Phycicoccus
0	1	0	G1	2637926	unclassified Phycicoccus
0	1	1	S	2714941	Phycicoccus sp. HDW14
0.01	21	0	F	85016	Cellulomonadaceae
0.01	20	0	G	1707	Cellulomonas
0	9	0	S	1711	Cellulomonas flavigena
0	9	9	S1	446466	Cellulomonas flavigena DSM 20109
0	7	0	G1	2620175	unclassified Cellulomonas
0	5	5	S	2654191	Cellulomonas sp. JZ18
0	2	2	S	2704467	Cellulomonas sp. H30R-01
0	4	4	S	2729175	Cellulomonas taurus
0	1	0	G	162491	Oerskovia
0	1	0	G1	2619021	unclassified Oerskovia
0	1	1	S	1179673	Oerskovia sp. KBS0722
0.01	17	0	F	2805426	Kytococcaceae
0.01	17	0	G	57499	Kytococcus
0.01	17	17	S	1276	Kytococcus sedentarius
0.01	10	0	F	85017	Promicromonosporaceae
0	7	0	G	157920	Cellulosimicrobium
0	5	5	S	1710	Cellulosimicrobium cellulans
0	2	0	G1	2624466	unclassified Cellulosimicrobium
0	1	1	S	1906273	Cellulosimicrobium sp. JZ28
0	1	1	S	2731680	Cellulosimicrobium sp. 72-3
0	3	0	G	186188	Xylanimonas
0	3	3	S	2509459	Xylanimonas allomyrinae
0	9	0	F	85020	Dermabacteraceae
0	8	0	G	43668	Brachybacterium
0	4	0	S	43669	Brachybacterium faecium
0	4	4	S1	446465	Brachybacterium faecium DSM 4810
0	4	4	S	1331682	Brachybacterium ginsengisoli
0	1	0	G	36739	Dermabacter
0	1	1	S	1630135	Dermabacter vaginalis
0	3	0	F	145357	Dermacoccaceae
0	3	0	G	57495	Dermacoccus
0	3	3	S	1274	Dermacoccus nishinomiyaensis
0	2	0	F	145358	Bogoriellaceae
0	2	0	G	154116	Georgenia
0	1	1	S	2483799	Georgenia faecalis
0	1	0	G1	2626815	unclassified Georgenia
0	1	1	S	2589797	Georgenia sp. Z443
0	2	0	F	145360	Sanguibacteraceae
0	2	0	G	60919	Sanguibacter
0	2	0	S	60920	Sanguibacter keddieii
0	2	2	S1	446469	Sanguibacter keddieii DSM 10542
0	1	0	F	2805590	Ornithinimicrobiaceae

0	1	0	G	265976	Serinicoccus
0	1	1	S	1078471	Serinicoccus profundi
0.43	775	11	O	85007	Corynebacteriales
0.29	517	0	F	1653	Corynebacteriaceae
0.29	517	33	G	1716	Corynebacterium
0.11	194	194	S	1979527	Corynebacterium kefirresidentii
0.06	117	117	S	38304	Corynebacterium tuberculostearicum
0.03	46	46	S	43990	Corynebacterium segmentosum
0.01	24	24	S	161879	Corynebacterium kroppenstedtii
0.01	20	20	S	2594913	Corynebacterium sanguinis
0.01	18	18	S	38290	Corynebacterium macginleyi
0.01	10	0	S	1404244	Corynebacterium glyciniphilum
0.01	10	10	S1	1404245	Corynebacterium glyciniphilum AJ 3170
0.01	10	10	S	401472	Corynebacterium ureicelerivorans
0	6	0	G1	2624378	unclassified Corynebacterium
0	3	3	S	2675216	Corynebacterium sp. 1959
0	2	2	S	2754725	Corynebacterium sp. Marseille-Q3630
0	1	1	S	702967	Corynebacterium sp. NML98-0116
0	6	6	S	169292	Corynebacterium aurimucosum
0	5	5	S	35757	Corynebacterium cystitidis
0	5	5	S	43770	Corynebacterium striatum
0	4	4	S	43768	Corynebacterium matruchotii
0	3	0	S	1230998	Corynebacterium frankenforstense
0	3	3	S1	1437875	Corynebacterium frankenforstense DSM 45800
0	3	0	S	225326	Corynebacterium halotolerans
0	3	3	S1	1121362	Corynebacterium halotolerans YIM 70093 = DSM 44683
0	3	3	S	161899	Corynebacterium singulare
0	2	0	S	575200	Corynebacterium maris
0	2	2	S1	1224163	Corynebacterium maris DSM 45190
0	2	2	S	156976	Corynebacterium riegelii
0	2	2	S	146827	Corynebacterium simulans
0	1	1	S	1471400	Corynebacterium pelargi
0	1	1	S	35755	Corynebacterium kutscheri
0	1	1	S	38289	Corynebacterium jeikeium
0	1	0	S	38305	Corynebacterium vitaeruminis
0	1	1	S1	1224164	Corynebacterium vitaeruminis DSM 20294
0.05	86	0	F	85025	Nocardiaceae
0.05	85	15	G	1827	Rhodococcus
0.03	51	0	G1	192944	unclassified Rhodococcus
0.02	41	41	S	2795031	Rhodococcus sp. P-2
0	5	5	S	2507582	Rhodococcus sp. ABRD24
0	4	4	S	1045808	Rhodococcus sp. YL-1
0	1	1	S	2499145	Rhodococcus sp. X156
0.01	13	13	S	334542	Rhodococcus qingshengii
0	5	5	S	1833	Rhodococcus erythropolis
0	1	1	S	1830	Rhodococcus ruber
0	1	0	G	1817	Nocardia
0	1	1	S	37329	Nocardia farcinica
0.04	80	3	F	1762	Mycobacteriaceae
0.03	52	0	G	1763	Mycobacterium
0.02	36	36	S	1789	Mycobacterium xenopi
0	9	9	S	1389713	Mycobacterium paragordonae
0	5	5	S	1768	Mycobacterium kansasii
0	1	1	S	2492438	Mycobacterium novum
0	1	0	G1	77643	Mycobacterium tuberculosis complex
0	1	1	S	78331	Mycobacterium canettii
0.01	25	2	G	1866885	Mycolicibacterium
0.01	11	11	S	67081	Mycolicibacterium alvei
0	5	5	S	1794	Mycolicibacterium gadium
0	1	1	S	1431246	Mycolicibacterium anyangense
0	1	1	S	1286181	Mycolicibacterium arabiense
0	1	1	S	1286180	Mycolicibacterium sediminis
0	1	1	S	1791	Mycolicibacterium aurum
0	1	1	S	319706	Mycolicibacterium phocaicum
0	1	1	S	53462	Mycolicibacterium mageritense
0	1	0	S	1804	Mycolicibacterium gilvum
0	1	1	S1	350054	Mycolicibacterium gilvum PYR-GCK
0.02	32	0	F	85026	Gordoniaceae
0.02	32	10	G	2053	Gordonia
0.01	12	12	S	2055	Gordonia terrae
0	7	7	S	2054	Gordonia bronchialis
0	2	2	S	36822	Gordonia rubripertincta
0	1	0	S	84595	Gordonia polyisoprenivorans
0	1	1	S1	1112204	Gordonia polyisoprenivorans VH2
0.01	21	0	F	85028	Tsukamurellaceae
0.01	21	1	G	2060	Tsukamurella
0.01	12	12	S	57704	Tsukamurella tyrosinosolvens
0	8	8	S	2061	Tsukamurella paurometabola
0.01	18	0	F	2805586	Lawsonellaceae
0.01	18	0	G	1847725	Lawsonella
0.01	18	18	S	1528099	Lawsonella clevelandensis
0.01	10	0	F	85029	Dietziaceae
0.01	10	1	G	37914	Dietzia
0	5	0	G1	2617939	unclassified Dietzia
0	5	5	S	712270	Dietzia sp. oral taxon 368
0	2	2	S	499555	Dietzia timorensis
0	2	2	S	546160	Dietzia lutea
0.04	65	0	O	85011	Streptomycetales
0.04	65	3	F	2062	Streptomycetaceae
0.03	62	34	G	1883	Streptomyces
0.01	13	2	G1	2593676	unclassified Streptomyces
0	3	3	S	2721173	Streptomyces sp. DSM 40868
0	3	3	S	444103	Streptomyces sp. CNQ-509
0	2	2	S	2203205	Streptomyces sp. WAC 01529
0	1	1	S	2768069	Streptomyces sp. CRXT-Y-14
0	1	1	S	2695266	Streptomyces sp. HM190
0	1	1	S	2059884	Streptomyces sp. CMB-StM0423
0	4	0	S	379067	Streptomyces bingchenggensis
0	4	4	S1	749414	Streptomyces bingchenggensis BCW-1
0	4	4	S	67267	Streptomyces alboflavus
0	3	3	S	51201	Streptomyces griseocarneus
0	2	2	S	80860	Streptomyces cyanogenus
0	1	1	S	40318	Streptomyces nodosus
0	1	0	S	33903	Streptomyces avermitilis

0	1	1	S1	227882	Streptomyces avermitilis MA-4680 = NBRC 14893
0.02	41	0	O	85004	Bifidobacteriales
0.02	41	0	F	31953	Bifidobacteriaceae
0.02	41	6	G	1678	Bifidobacterium
0.01	13	13	S	1681	Bifidobacterium bifidum
0.01	11	11	S	216816	Bifidobacterium longum
0	6	0	S	78448	Bifidobacterium pullorum
0	6	6	S1	78344	Bifidobacterium pullorum subsp. gallinarum
0	3	3	S	1680	Bifidobacterium adolescentis
0	2	2	S	28026	Bifidobacterium pseudocatenulatum
0.02	36	0	O	85010	Pseudonocardiales
0.02	36	1	F	2070	Pseudonocardiaceae
0.01	15	0	G	1835	Saccharopolyspora
0	9	9	S	1836	Saccharopolyspora erythraea
0	4	4	S	2665642	Saccharopolyspora coralli
0	1	1	S	60894	Saccharopolyspora spinosa
0	1	1	S	333966	Saccharopolyspora pogona
0	8	4	G	40566	Actinosynnema
0	4	4	S	42197	Actinosynnema pretiosum
0	3	3	G	1851	Saccharomonospora
0	2	0	G	1813	Amycolatopsis
0	1	1	S	33910	Amycolatopsis mediterranei
0	1	0	G1	2618356	unclassified Amycolatopsis
0	1	1	S	2742131	Amycolatopsis sp. Hca4
0	2	0	G	1847	Pseudonocardia
0	1	0	S	240495	Pseudonocardia dioxanivorans
0	1	1	S1	675635	Pseudonocardia dioxanivorans CB1190
0	1	0	G1	2619320	unclassified Pseudonocardia
0	1	1	S	1641402	Pseudonocardia sp. HH130629-09
0	2	0	G	2071	Saccharothrix
0	1	0	S	103731	Saccharothrix espanaensis
0	1	1	S1	1179773	Saccharothrix espanaensis DSM 44229
0	1	0	G1	2593673	unclassified Saccharothrix
0	1	1	S	2781735	Saccharothrix sp. 6-C
0	2	0	G	1137960	Allokutzneria
0	2	2	S	211114	Allokutzneria albata
0	1	1	G	65496	Actinoalloteichus
0.01	20	0	O	85012	Streptosporangiales
0.01	10	0	F	2004	Streptosporangiaceae
0	6	3	G	2000	Streptosporangium
0	2	0	G1	2632669	unclassified Streptosporangium
0	2	2	S	2202249	Streptosporangium sp. 'caverna'
0	1	0	S	2001	Streptosporangium roseum
0	1	1	S1	479432	Streptosporangium roseum DSM 43021
0	4	2	G	83681	Nonomuraea
0	1	0	G1	2593643	unclassified Nonomuraea
0	1	1	S	1909395	Nonomuraea sp. ATCC 55076
0	1	1	S	2656914	Nonomuraea nitratireducens
0.01	10	0	F	2012	Thermomonosporaceae
0.01	10	0	G	1988	Actinomadura
0.01	10	5	G1	2626254	unclassified Actinomadura
0	3	3	S	2742128	Actinomadura sp. NAK00032
0	1	1	S	1219491	Actinomadura sp. WMMB 499
0	1	1	S	2591108	Actinomadura sp. WMMA1423
0.01	15	0	O	2037	Actinomycetales
0.01	15	1	F	2049	Actinomycetaceae
0.01	12	6	G	1654	Actinomyces
0	4	4	S	1655	Actinomyces naeslundii
0	2	2	S	2057800	Actinomyces qiguomingii
0	1	0	G	1522056	Flaviflexus
0	1	1	S	1282737	Flaviflexus salsibiostraticola
0	1	0	G	2529408	Schaalia
0	1	0	G1	2691889	unclassified Schaalia
0	1	1	S	2758572	Schaalia sp. JY-X169
0.01	10	0	O	85008	Micromonosporales
0.01	10	3	F	28056	Micromonosporaceae
0	4	0	G	1865	Actinoplanes
0	4	0	G1	2626549	unclassified Actinoplanes
0	4	4	S	649831	Actinoplanes sp. N902-109
0	3	0	G	1873	Micromonospora
0	2	2	S	47875	Micromonospora sagamiensis
0	1	1	S	1881	Micromonospora viridifaciens
0	5	0	O	1643682	Geodermatophilales
0	5	0	F	85030	Geodermatophilaceae
0	5	0	G	88138	Modestobacter
0	5	5	S	477641	Modestobacter marinus
0	4	0	O	85013	Frankiales
0	4	0	F	74712	Frankiaceae
0	4	0	G	1854	Frankia
0	3	0	S	1859	Frankia alni
0	3	3	S1	326424	Frankia alni ACN14a
0	1	1	S	106370	Frankia casuarinae
0.02	29	0	C	84995	Rubrobacteria
0.02	29	0	O	84996	Rubrobacterales
0.02	29	0	F	84997	Rubrobacteraceae
0.02	29	0	G	42255	Rubrobacter
0.02	29	14	S	49319	Rubrobacter xylanophilus
0.01	15	15	S1	266117	Rubrobacter xylanophilus DSM 9941
0.01	10	0	C	1497346	Thermoleophilia
0.01	10	0	O	588673	Solirubrobacterales
0.01	10	0	F	320583	Conexibacteraceae
0.01	10	0	G	191494	Conexibacter
0.01	10	0	S	191495	Conexibacter woesei
0.01	10	10	S1	469383	Conexibacter woesei DSM 14684
0	9	0	C	84998	Coriobacteriia
0	6	0	O	84999	Coriobacteriales
0	6	0	F	84107	Coriobacteriaceae
0	6	0	G	102106	Collinsella
0	6	6	S	74426	Collinsella aerofaciens
0	3	0	O	1643822	Eggerthellales
0	3	0	F	1643826	Eggerthellaceae
0	2	0	G	84111	Eggerthella
0	2	2	S	84112	Eggerthella lenta
0	1	1	G	447020	Adlercreutzia

1.79	3219	5	P	1239	Firmicutes
1.4	2522	0	C	91061	Bacilli
0.79	1417	3	O	186826	Lactobacillales
0.68	1221	0	F	33958	Lactobacillaceae
0.66	1195	0	G	1578	Lactobacillus
0.65	1171	282	S	1584	Lactobacillus delbrueckii
0.49	888	888	S1	29397	Lactobacillus delbrueckii subsp. lactis
0	1	0	S1	1537158	Lactobacillus delbrueckii subsp. jakobsenii
0	1	1	S2	1217420	Lactobacillus delbrueckii subsp. jakobsenii ZN7a-9 = DSM 26046
0.01	16	16	S	47770	Lactobacillus crispatus
0	6	6	S	147802	Lactobacillus iners
0	1	0	S	33959	Lactobacillus johnsonii
0	1	1	S1	633699	Lactobacillus johnsonii FI9785
0	1	1	S	109790	Lactobacillus jensenii
0.01	17	0	G	2742598	Limosilactobacillus
0.01	15	15	S	1598	Limosilactobacillus reuteri
0	2	2	S	1633	Limosilactobacillus vaginalis
0	9	1	G	1243	Leuconostoc
0	7	7	S	33964	Leuconostoc citreum
0	1	1	S	1245	Leuconostoc mesenteroides
0.09	165	0	F	1300	Streptococcaceae
0.09	162	38	G	1301	Streptococcus
0.02	41	41	S	28037	Streptococcus mitis
0.01	21	6	G1	2608887	unclassified Streptococcus
0.01	14	14	S	2610896	Streptococcus sp. LPB0220
0	1	1	S	2598453	Streptococcus sp. 116-D4
0.01	16	16	S	1313	Streptococcus pneumoniae
0.01	12	12	S	1308	Streptococcus thermophilus
0.01	11	11	S	1305	Streptococcus sanguinis
0.01	10	10	S	1304	Streptococcus salivarius
0	7	7	S	1302	Streptococcus gordonii
0	4	0	S	1318	Streptococcus parasanguinis
0	2	2	S1	760570	Streptococcus parasanguinis ATCC 15912
0	2	2	S1	1114965	Streptococcus parasanguinis FW213
0	2	2	S	1303	Streptococcus oralis
0	3	0	G	1357	Lactococcus
0	2	2	S	1358	Lactococcus lactis
0	1	1	S	1359	Lactococcus cremoris
0.01	17	0	F	81852	Enterococcaceae
0.01	17	0	G	1350	Enterococcus
0.01	15	15	S	44008	Enterococcus cecorum
0	2	2	S	1351	Enterococcus faecalis
0	6	0	F	186827	Aerococcaceae
0	5	0	G	46123	Abiotrophia
0	5	5	S	46125	Abiotrophia defectiva
0	1	0	G	1375	Aerococcus
0	1	1	S	1377	Aerococcus viridans
0	5	0	F	186828	Carnobacteriaceae
0	5	0	G	29393	Dolosigranulum
0	5	5	S	29394	Dolosigranulum pigrum
0.61	1105	6	O	1385	Bacillales
0.56	1015	3	F	90964	Staphylococcaceae
0.55	991	73	G	1279	Staphylococcus
0.2	366	366	S	29388	Staphylococcus capitis
0.18	332	316	S	1282	Staphylococcus epidermidis
0.01	16	16	S1	1449752	Staphylococcus epidermidis PM221
0.06	113	113	S	33028	Staphylococcus saccharolyticus
0.04	67	67	S	1292	Staphylococcus warneri
0.01	13	13	S	1280	Staphylococcus aureus
0.01	10	10	S	1283	Staphylococcus haemolyticus
0	5	5	S	29385	Staphylococcus saprophyticus
0	5	5	S	1290	Staphylococcus hominis
0	3	3	S	45972	Staphylococcus pasteuri
0	1	1	S	170573	Staphylococcus pettenkoferi
0	1	0	G1	91994	unclassified Staphylococcus
0	1	1	S	2799680	Staphylococcus sp. 11-B-312
0	1	1	S	28035	Staphylococcus lugdunensis
0	1	0	S	1286	Staphylococcus simulans
0	1	1	S1	1287	Staphylococcus simulans bv. staphylolyticus
0.01	13	0	G	2803850	Mammaliicoccus
0.01	13	13	S	42858	Mammaliicoccus lentus
0	8	0	G	227979	Jeotgalicoccus
0	8	8	S	1461582	Jeotgalicoccus saudimassiliensis
0.03	50	6	F	186817	Bacillaceae
0.01	21	0	G	1276290	Caldibacillus
0.01	21	21	S	35841	Caldibacillus thermoamylovorans
0.01	11	0	G	2817139	Weizmannia
0.01	11	11	S	1398	Weizmannia coagulans
0	9	0	G	1386	Bacillus
0	5	5	S	38875	Bacillus oleronius
0	4	0	G1	86661	Bacillus cereus group
0	4	4	S	1392	Bacillus anthracis
0	1	0	G	2675232	Neobacillus
0	1	1	S	1215031	Neobacillus thermocopriae
0	1	0	G	1329200	Fictibacillus
0	1	1	S	255247	Fictibacillus arsenicus
0	1	0	G	150247	Anoxybacillus
0	1	1	S	33934	Anoxybacillus flavithermus
0.01	16	0	O1	539002	Bacillales incertae sedis
0.01	16	0	O2	539738	Bacillales Family XI. Incertae Sedis
0.01	16	3	G	1378	Gemella
0.01	12	12	S	29391	Gemella morbillorum
0	1	1	S	1379	Gemella haemolysans
0	7	1	F	186822	Paenibacillaceae
0	6	2	G	44249	Paenibacillus
0	4	0	G1	185978	unclassified Paenibacillus
0	2	2	S	2660554	Paenibacillus sp. B01
0	2	2	S	2023772	Paenibacillus sp. RUD330
0	7	0	F	186823	Alicyclobacillaceae
0	7	0	G	29330	Alicyclobacillus
0	7	0	G1	2622400	unclassified Alicyclobacillus
0	7	7	S	2665646	Alicyclobacillus sp. SO9
0	4	0	F	186818	Planococcaceae
0	4	0	G	1372	Planococcus

0	4	4	S	1215089	Planococcus halocryophilus
0.23	408	0	C	186801	Clostridia
0.23	408	32	O	186802	Eubacteriales
0.12	209	1	F	216572	Oscillospiraceae
0.09	167	0	G	216851	Faecalibacterium
0.09	167	129	S	853	Faecalibacterium prausnitzii
0.01	26	26	S1	718252	Faecalibacterium prausnitzii L2-6
0.01	12	12	S1	657322	Faecalibacterium prausnitzii SL3/3
0.02	34	0	G	1263	Ruminococcus
0.02	33	33	S	1160721	Ruminococcus bicirculans
0	1	0	S	1264	Ruminococcus albus
0	1	1	S1	697329	Ruminococcus albus 7 = DSM 20455
0	3	0	G	459786	Oscillibacter
0	3	0	G1	2629304	unclassified Oscillibacter
0	2	2	S	2763056	Oscillibacter sp. NSJ-62
0	1	1	S	2109687	Oscillibacter sp. PEA192
0	2	0	G	2591381	Dysosmobacter
0	2	2	S	2093857	Dysosmobacter welbionis
0	1	0	G	1905344	Ruthenibacterium
0	1	1	S	1550024	Ruthenibacterium lactatiformans
0	1	0	G	2304691	Thermoclostridium
0	1	1	S	1510	Thermoclostridium stercorarium
0.07	134	12	F	186803	Lachnospiraceae
0.03	50	0	G	841	Roseburia
0.03	47	19	S	166486	Roseburia intestinalis
0.01	19	19	S1	657315	Roseburia intestinalis M50/1
0	8	8	S1	536231	Roseburia intestinalis L1-82
0	1	1	S1	718255	Roseburia intestinalis XB6B4
0	3	3	S	301301	Roseburia hominis
0.01	17	0	G	572511	Blautia
0.01	13	0	G1	2648079	unclassified Blautia
0.01	13	13	S	2479767	Blautia sp. SC05B48
0	3	0	S	40520	Blautia obeum
0	3	3	S1	657314	Blautia obeum A2-162
0	1	1	S	33035	Blautia producta
0.01	16	0	G	28050	Lachnospira
0.01	16	0	S	39485	Lachnospira eligens
0.01	16	16	S1	515620	[Eubacterium] eligens ATCC 27750
0.01	11	0	G	2316020	Mediterraneibacter
0.01	10	0	S	33039	[Ruminococcus] torques
0.01	10	10	S1	657313	[Ruminococcus] torques L2-14
0	1	1	S	33038	[Ruminococcus] gnavus
0	8	0	G	1506553	Lachnoclostridium
0	5	5	S	29347	[Clostridium] scindens
0	3	3	S	1871021	Lachnoclostridium phocaeense
0	7	0	G	207244	Anaerostipes
0	5	5	S	649756	Anaerostipes hadrus
0	2	2	S	105841	Anaerostipes caccae
0	6	0	G	33042	Coprococcus
0	4	0	G1	2684943	unclassified Coprococcus
0	4	4	S	751585	Coprococcus sp. ART55/1
0	2	2	S	116085	Coprococcus catus
0	3	0	G	830	Butyrivibrio
0	3	3	S	831	Butyrivibrio fibrisolvens
0	2	0	G	2719313	Enterocloster
0	2	2	S	208479	Enterocloster bolteae
0	1	0	G	2569097	Anaerobutyricum
0	1	1	S	39488	Anaerobutyricum hallii
0	1	0	G	2719231	Lacrimispora
0	1	0	S	84030	Lacrimispora saccharolytica
0	1	1	S1	717608	[Clostridium] cf. saccharolyticum K10
0.01	17	0	O1	538999	Eubacteriales incertae sedis
0	8	0	G	2039302	Monoglobus
0	8	8	S	1981510	Monoglobus pectinilyticus
0	7	0	G	1918454	Flintibacter
0	7	0	G1	2610894	unclassified Flintibacter
0	7	7	S	2610895	Flintibacter sp. KGMB00164
0	2	0	G	1392389	Intestinimonas
0	2	2	S	1297617	Intestinimonas butyriciproducens
0.01	11	0	F	31979	Clostridiaceae
0.01	11	4	G	1485	Clostridium
0	3	3	S	1504	Clostridium septicum
0	2	2	S	1491	Clostridium botulinum
0	2	2	S	1502	Clostridium perfringens
0	2	1	F	186804	Peptostreptococcaceae
0	1	0	G	1870884	Clostridioides
0	1	1	S	1496	Clostridioides difficile
0	2	0	F	990719	Christensenellaceae
0	2	0	G	990721	Christensenella
0	2	2	S	626937	Christensenella minuta
0	1	0	F	186806	Eubacteriaceae
0	1	0	G	1730	Eubacterium
0	1	1	S	2041044	Eubacterium maltosivorans
0.14	260	0	C	526524	Erysipelotrichia
0.14	260	0	O	526525	Erysipelotrichales
0.14	258	0	F	2810281	Turicibacteraceae
0.14	258	0	G	191303	Turicibacter
0.09	168	168	S	154288	Turicibacter sanguinis
0.05	90	0	G1	2638206	unclassified Turicibacter
0.05	90	90	S	1712675	Turicibacter sp. H121
0	2	0	F	128827	Erysipelotrichaceae
0	1	0	G	1647	Erysipelothrix
0	1	1	S	1514105	Erysipelothrix larvae
0	1	0	G	1505663	Erysipelatoclostridium
0	1	1	S	1522	[Clostridium] innocuum
0.01	17	0	C	909932	Negativicutes
0.01	10	0	O	1843489	Veillonellales
0.01	10	0	F	31977	Veillonellaceae
0.01	10	0	G	29465	Veillonella
0	5	5	S	39778	Veillonella dispar
0	4	4	S	29466	Veillonella parvula
0	1	1	S	2682456	Veillonella nakazawae
0	5	0	O	909929	Selenomonadales
0	5	0	F	1843491	Selenomonadaceae

0	5	0	G	158846	Megamonas
0	5	5	S	437897	Megamonas funiformis
0	2	0	O	1843488	Acidaminococcales
0	2	0	F	909930	Acidaminococcaceae
0	2	0	G	33024	Phascolarctobacterium
0	2	0	G1	2639160	unclassified Phascolarctobacterium
0	2	2	S	2823317	Phascolarctobacterium sp. Marseille-Q4147
0	7	3	C	1737404	Tissierellia
0	4	0	O	1737405	Tissierellales
0	4	0	F	1570339	Peptoniphilaceae
0	4	0	G	150022	Finegoldia
0	4	4	S	1260	Finegoldia magna
0.03	56	0	P	1297	Deinococcus-Thermus
0.03	56	0	C	188787	Deinococci
0.03	53	0	O	118964	Deinococcales
0.03	53	0	F	183710	Deinococcaceae
0.03	53	12	G	1298	Deinococcus
0.01	11	11	S	1211322	Deinococcus metallilatus
0	8	8	S	1768108	Deinococcus actinosclerus
0	6	5	S	980427	Deinococcus wulumuqiensis
0	1	1	S1	1288484	Deinococcus wulumuqiensis R12
0	5	0	S	309887	Deinococcus maricopensis
0	5	5	S1	709986	Deinococcus maricopensis DSM 21211
0	3	0	S	55148	Deinococcus proteolyticus
0	3	3	S1	693977	Deinococcus proteolyticus MRP
0	2	0	S	310783	Deinococcus deserti
0	2	2	S1	546414	Deinococcus deserti VCD115
0	2	2	S	317577	Deinococcus ficus
0	2	0	S	432329	Deinococcus peraridilitoris
0	2	2	S1	937777	Deinococcus peraridilitoris DSM 19664
0	2	0	G1	2623546	unclassified Deinococcus
0	2	2	S	2652443	Deinococcus sp. AJ005
0	3	0	O	68933	Thermales
0	3	0	F	188786	Thermaceae
0	3	0	G	270	Thermus
0	2	0	S	37636	Thermus scotoductus
0	2	2	S1	743525	Thermus scotoductus SA-01
0	1	0	S	56957	Thermus oshimai
0	1	1	S1	751945	Thermus oshimai JL-2
0.01	26	0	D2	1798711	Cyanobacteria/Melainabacteria group
0.01	26	0	P	1117	Cyanobacteria
0.01	14	0	O	1890424	Synechococcales
0.01	13	0	F	1890426	Synechococcaceae
0.01	13	0	G	1129	Synechococcus
0.01	13	6	G1	2626047	unclassified Synechococcus
0	7	7	S	1916956	Synechococcus sp. SynAce01
0	1	0	F	2303730	Prochlorotrichaceae
0	1	0	G	170610	Halomicronema
0	1	0	S	1209493	Halomicronema hongdechloris
0	1	1	S1	1641165	Halomicronema hongdechloris C2206
0.01	10	0	P1	1301283	Oscillatoriophycideae
0.01	10	0	O	1150	Oscillatoriales
0	9	0	F	1892252	Microcoleaceae
0	9	0	G	44471	Microcoleus
0	9	0	G1	2642155	unclassified Microcoleus
0	9	9	S	1173027	Microcoleus sp. PCC 7113
0	1	0	F	1892254	Oscillatoriaceae
0	1	0	G	1155738	Moorea
0	1	0	S	1155739	Moorea producens
0	1	1	S1	1454205	Moorea producens JHB
0	1	0	O	1161	Nostocales
0	1	0	F	1162	Nostocaceae
0	1	0	G	1177	Nostoc
0	1	0	G1	2593658	unclassified Nostoc
0	1	1	S	1618022	Nostoc sp. 'Lobaria pulmonaria (5183) cyanobiont'
0	1	0	C	307596	Gloeobacteria
0	1	0	O	307595	Gloeobacterales
0	1	0	F	1890422	Gloeobacteraceae
0	1	0	G	33071	Gloeobacter
0	1	0	S	33072	Gloeobacter violaceus
0	1	1	S1	251221	Gloeobacter violaceus PCC 7421
0	2	0	P	200795	Chloroflexi
0	2	0	C	189775	Thermomicrobia
0	2	0	C1	85000	Sphaerobacteridae
0	2	0	O	85001	Sphaerobacterales
0	2	0	O1	255728	Sphaerobacterineae
0	2	0	F	85002	Sphaerobacteraceae
0	2	0	G	2056	Sphaerobacter
0	2	0	S	2057	Sphaerobacter thermophilus
0	2	2	S1	479434	Sphaerobacter thermophilus DSM 20745
0	1	0	P	544448	Tenericutes
0	1	0	C	31969	Mollicutes
0	1	0	O	186329	Acholeplasmatales
0	1	0	F	2146	Acholeplasmataceae
0	1	0	G	2147	Acholeplasma
0	1	1	S	2148	Acholeplasma laidlawii
3.77	6791	0	D1	1783270	FCB group
3.77	6791	0	D2	68336	Bacteroidetes/Chlorobi group
3.77	6791	17	P	976	Bacteroidetes
2.46	4437	0	C	200643	Bacteroidia
2.46	4437	280	O	171549	Bacteroidales
1.88	3381	0	F	815	Bacteroidaceae
1.88	3381	341	G	816	Bacteroides
1.24	2228	2228	S	818	Bacteroides thetaiotaomicron
0.22	401	399	S	817	Bacteroides fragilis
0	1	1	S1	295405	Bacteroides fragilis YCH46
0	1	1	S1	862962	Bacteroides fragilis 638R
0.08	138	138	S	820	Bacteroides uniformis
0.07	127	14	G1	2646097	unclassified Bacteroides
0.02	44	44	S	2785531	Bacteroides sp. HF-162
0.01	26	26	S	2650157	Bacteroides sp. HF-5287
0.01	18	18	S	2763022	Bacteroides sp. M10
0.01	13	13	S	2528203	Bacteroides sp. A1C1
0	8	8	S	2650158	Bacteroides sp. HF-5141

0	4	4	S	2755405	Bacteroides sp. CACC 737
0.04	69	69	S	28116	Bacteroides ovatus
0.02	29	29	S	28111	Bacteroides eggerthii
0.02	29	27	S	371601	Bacteroides xylanisolvens
0	2	2	S1	657309	Bacteroides xylanisolvens XB1A
0.01	15	15	S	246787	Bacteroides cellulosilyticus
0	2	2	S	1796613	Bacteroides caecimuris
0	1	1	S	47678	Bacteroides caccae
0	1	1	S	329854	Bacteroides intestinalis
0.34	619	0	O1	333046	Bacteroidales incertae sedis
0.34	619	71	G	909656	Phocaeicola
0.21	373	373	S	821	Phocaeicola vulgatus
0.09	167	80	S	357276	Phocaeicola dorei
0.05	87	87	S1	997877	Bacteroides dorei CL03T12C01
0	7	7	S	387090	Phocaeicola coprophilus
0	1	0	S	376805	Phocaeicola salanitronis
0	1	1	S1	667015	Phocaeicola salanitronis DSM 18170
0.03	57	0	F	171550	Rikenellaceae
0.03	57	4	G	239759	Alistipes
0.01	25	0	S	328813	Alistipes onderdonkii
0.01	25	25	S1	2585117	Alistipes onderdonkii subsp. vulgaris
0.01	14	14	S	2585118	Alistipes communis
0	8	0	S	214856	Alistipes finegoldii
0	8	8	S1	679935	Alistipes finegoldii DSM 17242
0	2	0	S	328814	Alistipes shahii
0	2	2	S1	717959	Alistipes shahii WAL 8301
0	2	2	S	2364787	Alistipes megaguti
0	1	1	S	626932	Alistipes indistinctus
0	1	1	S	2585119	Alistipes dispar
0.03	57	0	F	1853231	Odoribacteraceae
0.02	34	0	G	283168	Odoribacter
0.02	34	34	S	28118	Odoribacter splanchnicus
0.01	23	9	G	574697	Butyricimonas
0.01	14	14	S	544645	Butyricimonas virosa
0.01	21	0	F	171552	Prevotellaceae
0.01	18	0	G	838	Prevotella
0	7	7	S	165179	Prevotella copri
0	6	6	S	28132	Prevotella melaninogenica
0	4	4	S	28135	Prevotella oris
0	1	0	S	589437	Prevotella scopos
0	1	1	S1	1236518	Prevotella scopos JCM 17725
0	3	0	G	577309	Paraprevotella
0	3	0	S	454155	Paraprevotella xylaniphila
0	3	3	S1	762982	Paraprevotella xylaniphila YIT 11841
0.01	19	0	F	2005525	Tannerellaceae
0.01	19	4	G	375288	Parabacteroides
0.01	14	14	S	823	Parabacteroides distasonis
0	1	1	S	328812	Parabacteroides goldsteinii
0	2	0	F	2005473	Muribaculaceae
0	2	0	G	2518495	Duncaniella
0	2	2	S	2518971	Duncaniella dubosii
0	1	0	F	171551	Porphyromonadaceae
0	1	0	G	836	Porphyromonas
0	1	1	S	837	Porphyromonas gingivalis
0.74	1334	0	C	117743	Flavobacteriia
0.74	1334	7	O	200644	Flavobacteriales
0.72	1295	11	F	2762318	Weeksellaceae
0.38	690	39	F1	2782232	Chryseobacterium group
0.29	529	92	G	59732	Chryseobacterium
0.09	156	156	S	246	Chryseobacterium balustinum
0.08	143	143	S	254	Chryseobacterium indoltheticum
0.04	67	0	G1	2593645	unclassified Chryseobacterium
0.01	11	11	S	2547600	Chryseobacterium sp. NBC 122
0.01	10	10	S	1721091	Chryseobacterium sp. IHB B 17019
0.01	10	10	S	2015076	Chryseobacterium sp. T16E-39
0.01	10	10	S	2478663	Chryseobacterium sp. 3008163
0	8	8	S	2487065	Chryseobacterium sp. G0201
0	7	7	S	2724619	Chryseobacterium sp. NEB161
0	6	6	S	2758037	Chryseobacterium sp. cx-624
0	3	3	S	1932669	Chryseobacterium sp. JV274
0	2	2	S	2039166	Chryseobacterium sp. 6424
0.01	24	24	S	1493872	Chryseobacterium shandongense
0.01	21	21	S	1685010	Chryseobacterium glaciei
0.01	10	10	S	1241982	Chryseobacterium nakagawai
0	7	7	S	2754694	Chryseobacterium manosquense
0	4	4	S	250	Chryseobacterium gleum
0	3	3	S	1324352	Chryseobacterium gallinarum
0	1	1	S	253	Chryseobacterium indologenes
0	1	1	S	1124835	Chryseobacterium carnipullorum
0.05	95	0	G	2782229	Epilithonimonas
0.05	95	95	S	2487072	Epilithonimonas vandammei
0.01	26	0	G	2782231	Kaistella
0.01	23	23	S	421525	Chryseobacterium haifense
0	3	3	S	266748	Kaistella antarctica
0	1	0	G	2782228	Planobacterium
0	1	1	S	536441	Chryseobacterium taklimakanense
0.31	553	39	G	59734	Empedobacter
0.27	480	480	S	343874	Empedobacter falsenii
0.02	28	28	S	1628248	Empedobacter stercoris
0	6	6	S	247	Empedobacter brevis
0.02	29	0	G	501783	Cloacibacterium
0.02	29	29	S	237258	Cloacibacterium normanense
0	6	0	G	308865	Elizabethkingia
0	5	5	S	1756150	Elizabethkingia ursingii
0	1	1	S	1117645	Elizabethkingia anophelis
0	6	0	G	1433995	Cruoricaptor
0	6	6	S	1118202	Cruoricaptor ignavus
0.02	32	0	F	49546	Flavobacteriaceae
0.01	14	1	G	1016	Capnocytophaga
0	7	7	S	2708117	Capnocytophaga endodontalis
0	5	0	G1	2640652	unclassified Capnocytophaga
0	5	5	S	2748316	Capnocytophaga sp. oral taxon 902
0	1	1	S	1019	Capnocytophaga sputigena
0	9	0	G	237	Flavobacterium

0	5	5	S	2175091	Flavobacterium album
0	2	2	S	2172098	Flavobacterium pallidum
0	1	0	G1	196869	unclassified Flavobacterium
0	1	1	S	2294119	Flavobacterium sp. CJ74
0	1	1	S	1306519	Flavobacterium commune
0	4	0	G	363408	Nonlabens
0	4	4	S	331648	Nonlabens spongiae
0	3	0	G	76831	Myroides
0	3	3	S	76832	Myroides odoratimimus
0	2	0	G	104267	Tenacibaculum
0	2	0	G1	2635139	unclassified Tenacibaculum
0	2	2	S	2358479	Tenacibaculum sp. DSM 106434
0.36	643	0	C	768503	Cytophagia
0.36	643	1	O	768507	Cytophagales
0.35	629	2	F	1853232	Hymenobacteraceae
0.35	625	11	G	89966	Hymenobacter
0.33	587	25	G1	2615202	unclassified Hymenobacter
0.28	506	506	S	2584940	Hymenobacter sp. DG01
0.01	19	19	S	2735321	Hymenobacter sp. TS19
0.01	14	14	S	2596915	Hymenobacter sp. NBH84
0	7	7	S	1356852	Hymenobacter sp. APR13
0	5	5	S	1385664	Hymenobacter sp. DG25B
0	4	4	S	2675877	Hymenobacter sp. BRD67
0	4	4	S	2675878	Hymenobacter sp. BRD128
0	1	1	S	1484116	Hymenobacter sp. PAMC 26554
0	1	1	S	1484118	Hymenobacter sp. PAMC 26628
0	1	1	S	2761579	Hymenobacter sp. S2-20-2
0.01	10	10	S	2724192	Hymenobacter russus
0	8	0	S	1446467	Hymenobacter swuensis
0	8	8	S1	1227739	Hymenobacter swuensis DY53
0	6	6	S	2319843	Hymenobacter oligotrophus
0	3	3	S	1385715	Hymenobacter qilianensis
0	2	0	G	323449	Pontibacter
0	2	2	S	2694929	Pontibacter russatus
0	9	0	F	89373	Cytophagaceae
0	9	0	G	107	Spirosoma
0	8	8	S	2666025	Spirosoma endbachense
0	1	0	G1	2621999	unclassified Spirosoma
0	1	1	S	2710596	Spirosoma sp. PL0136
0	4	0	F	563798	Cyclobacteriaceae
0	4	0	G	390846	Echinicola
0	4	0	S	390884	Echinicola vietnamensis
0	4	4	S1	926556	Echinicola vietnamensis DSM 17526
0.19	350	0	C	117747	Sphingobacteriia
0.19	350	0	O	200666	Sphingobacteriales
0.19	350	0	F	84566	Sphingobacteriaceae
0.19	343	13	G	28453	Sphingobacterium
0.11	190	190	S	28454	Sphingobacterium multivorum
0.08	139	13	G1	2609468	unclassified Sphingobacterium
0.06	112	112	S	2795738	Sphingobacterium sp. UDSM-2020
0	7	7	S	2003121	Sphingobacterium sp. G1-14
0	6	6	S	1933220	Sphingobacterium sp. B29
0	1	1	S	1538644	Sphingobacterium sp. ML3W
0	1	1	S	371142	Sphingobacterium daejeonense
0	5	0	G	84567	Pedobacter
0	5	5	S	363852	Pedobacter ginsengisoli
0	2	0	G	1649482	Pseudopedobacter
0	2	0	S	151895	Pseudopedobacter saltans
0	2	2	S1	762903	Pseudopedobacter saltans DSM 12145
0.01	10	0	C	1853228	Chitinophagia
0.01	10	0	O	1853229	Chitinophagales
0.01	10	1	F	563835	Chitinophagaceae
0	6	0	G	79328	Chitinophaga
0	4	4	S	2029983	Chitinophaga caeni
0	2	2	S	2203219	Chitinophaga alhagiae
0	2	0	G	379899	Niabella
0	2	0	S	446683	Niabella soli
0	2	2	S1	929713	Niabella soli DSM 19437
0	1	0	G	1860196	Pseudobacter
0	1	1	S	661488	Pseudobacter ginsenosidimutans
0.01	20	0	D1	1783257	PVC group
0.01	12	0	P	204428	Chlamydiae
0.01	12	1	C	204429	Chlamydiia
0.01	11	0	O	1963360	Parachlamydiales
0.01	11	11	F	92713	Parachlamydiaceae
0	4	0	P	74201	Verrucomicrobia
0	2	0	C	203494	Verrucomicrobiae
0	2	0	O	48461	Verrucomicrobiales
0	2	0	F	1647988	Akkermansiaceae
0	2	0	G	239934	Akkermansia
0	2	2	S	239935	Akkermansia muciniphila
0	1	0	P1	326457	Verrucomicrobia incertae sedis
0	1	0	G	1541670	Methylacidimicrobium
0	1	0	G1	2730358	unclassified Methylacidimicrobium
0	1	1	S	2730359	Methylacidimicrobium sp. AP8
0	1	0	C	414999	Opitutae
0	1	0	O	415000	Opitutales
0	1	0	F	134623	Opitutaceae
0	1	0	G	178440	Opitutus
0	1	0	G1	2649488	unclassified Opitutus
0	1	1	S	1882749	Opitutus sp. GAS368
0	4	0	P	203682	Planctomycetes
0	4	0	C	203683	Planctomycetia
0	4	0	O	2691355	Gemmatales
0	4	0	F	1914233	Gemmataceae
0	4	0	G	113	Gemmata
0	4	4	S	114	Gemmata obscuriglobus
0.01	11	0	P	203691	Spirochaetes
0.01	11	0	C	203692	Spirochaetia
0	8	0	O	1643686	Brachyspirales
0	8	0	F	143786	Brachyspiraceae
0	8	0	G	29521	Brachyspira
0	8	8	S	52584	Brachyspira pilosicoli
0	3	0	O	136	Spirochaetales

0	3	0	F	137	Spirochaetaceae
0	2	2	G	157	Treponema
0	1	0	G	146	Spirochaeta
0	1	0	S	46355	Spirochaeta africana
0	1	1	S1	889378	Spirochaeta africana DSM 8902
0	8	0	P	32066	Fusobacteria
0	8	0	C	203490	Fusobacteriia
0	8	0	O	203491	Fusobacteriales
0	8	0	F	203492	Fusobacteriaceae
0	8	0	G	848	Fusobacterium
0	8	0	S	851	Fusobacterium nucleatum
0	8	8	S1	155615	Fusobacterium nucleatum subsp. vincentii
0	3	0	P	40117	Nitrospirae
0	3	0	C	203693	Nitrospira
0	3	0	O	189778	Nitrospirales
0	3	0	F	189779	Nitrospiraceae
0	3	0	G	1234	Nitrospira
0	3	3	S	42253	Nitrospira moscoviensis
0	2	0	D1	2323	Bacteria incertae sedis
0	2	0	D2	1783234	Bacteria candidate phyla
0	2	0	D3	95901	Candidatus Dependentiae
0	2	0	C	2497643	Candidatus Babeliae
0	2	0	O	2497644	Candidatus Babeliales
0	2	0	F	2497645	Candidatus Babeliaceae
0	2	0	G	1551504	Candidatus Babela
0	2	2	S	673862	Candidatus Babela massiliensis
1.58	2844	21	D	2759	Eukaryota
1.35	2424	0	D1	33154	Opisthokonta
0.86	1553	0	K	4751	Fungi
0.86	1553	0	K1	451864	Dikarya
0.86	1541	0	P	5204	Basidiomycota
0.86	1541	1	P1	452284	Ustilaginomycotina
0.84	1519	0	C	1538075	Malasseziomycetes
0.84	1519	0	O	162474	Malasseziales
0.84	1519	0	F	742845	Malasseziaceae
0.84	1519	0	G	55193	Malassezia
0.84	1519	1519	S	76775	Malassezia restricta
0.01	21	0	C	5257	Ustilaginomycetes
0.01	21	0	O	5267	Ustilaginales
0.01	21	0	F	5268	Ustilaginaceae
0.01	17	0	G	63265	Sporisorium
0.01	17	17	S	280036	Sporisorium graminicola
0	4	0	G	5269	Ustilago
0	4	0	S	5270	Ustilago maydis
0	4	4	S1	237631	Ustilago maydis 521
0.01	12	0	P	4890	Ascomycota
0.01	12	0	P1	716545	saccharomyceta
0	9	0	P2	147537	Saccharomycotina
0	9	0	C	4891	Saccharomycetes
0	9	0	O	4892	Saccharomycetales
0	7	0	F	766764	Debaryomycetaceae
0	7	0	G	4958	Debaryomyces
0	7	0	S	4959	Debaryomyces hansenii
0	7	7	S1	284592	Debaryomyces hansenii CBS767
0	2	0	F	4893	Saccharomycetaceae
0	2	0	G	4930	Saccharomyces
0	2	0	S	4932	Saccharomyces cerevisiae
0	2	2	S1	559292	Saccharomyces cerevisiae S288C
0	3	0	P2	147538	Pezizomycotina
0	3	0	P3	716546	leotiomyceta
0	2	0	P4	715989	sordariomyceta
0	1	0	C	147548	Leotiomycetes
0	1	0	O	5178	Helotiales
0	1	0	F	28983	Sclerotiniaceae
0	1	0	G	33196	Botrytis
0	1	0	S	40559	Botrytis cinerea
0	1	1	S1	332648	Botrytis cinerea B05.10
0	1	0	C	147550	Sordariomycetes
0	1	0	C1	222543	Hypocreomycetidae
0	1	0	O	5125	Hypocreales
0	1	0	F	474942	Ophiocordycipitaceae
0	1	0	G	98402	Drechmeria
0	1	1	S	98403	Drechmeria coniospora
0	1	0	P4	715962	dothideomyceta
0	1	0	C	147541	Dothideomycetes
0	1	0	C1	451867	Dothideomycetidae
0	1	0	O	2726947	Mycosphaerellales
0	1	0	F	93133	Mycosphaerellaceae
0	1	0	G	29002	Cercospora
0	1	1	S	122368	Cercospora beticola
0.48	871	0	K	33208	Metazoa
0.48	871	0	K1	6072	Eumetazoa
0.48	871	0	K2	33213	Bilateria
0.48	871	0	K3	33511	Deuterostomia
0.48	871	0	P	7711	Chordata
0.48	871	0	P1	89593	Craniata
0.48	871	0	P2	7742	Vertebrata
0.48	871	0	P3	7776	Gnathostomata
0.48	871	0	P4	117570	Teleostomi
0.48	871	0	P5	117571	Euteleostomi
0.48	871	0	P6	8287	Sarcopterygii
0.48	871	0	C	1338369	Dipnotetrapodomorpha
0.48	871	0	C1	32523	Tetrapoda
0.48	871	0	C2	32524	Amniota
0.48	871	0	C	40674	Mammalia
0.48	871	0	C1	32525	Theria
0.48	871	0	C2	9347	Eutheria
0.48	871	0	C3	1437010	Boreoeutheria
0.48	871	0	C4	314146	Euarchontoglires
0.48	871	0	O	9443	Primates
0.48	871	0	O1	376913	Haplorrhini
0.48	871	0	O2	314293	Simiiformes
0.48	871	0	O3	9526	Catarrhini
0.48	871	0	O4	314295	Hominoidea

0.48	871	0	F	9604	Hominidae
0.48	871	0	F1	207598	Homininae
0.48	871	0	G	9605	Homo
0.48	871	871	S	9606	Homo sapiens
0.2	359	0	K	33090	Viridiplantae
0.2	359	0	P	35493	Streptophyta
0.2	359	0	P1	131221	Streptophytina
0.2	359	0	P2	3193	Embryophyta
0.2	355	0	P3	58023	Tracheophyta
0.2	355	0	P4	78536	Euphyllophyta
0.2	355	0	P5	58024	Spermatophyta
0.2	355	0	C	3398	Magnoliopsida
0.19	350	1	C1	1437183	Mesangiospermae
0.1	185	0	C2	71240	eudicotyledons
0.1	185	0	C3	91827	Gunneridae
0.1	185	4	C4	1437201	Pentapetalae
0.07	124	0	C5	71275	rosids
0.06	110	3	C6	91835	fabids
0.05	84	0	O	72025	Fabales
0.05	84	0	F	3803	Fabaceae
0.05	84	0	F1	3814	Papilionoideae
0.05	84	0	F2	2231393	50 kb inversion clade
0.04	79	0	F3	2231382	NPAAA clade
0.04	78	0	F4	2233855	indigoferoid/millettioid clade
0.04	78	0	F5	163735	Phaseoleae
0.04	69	0	G	3846	Glycine
0.04	69	0	G1	1462606	Glycine subgen. Soja
0.04	69	69	S	3848	Glycine soja
0	8	0	G	3820	Cajanus
0	8	8	S	3821	Cajanus cajan
0	1	0	G	3883	Phaseolus
0	1	1	S	3885	Phaseolus vulgaris
0	1	0	F4	2233838	Hologalegina
0	1	0	F5	2233839	IRL clade
0	1	0	F6	163742	Trifolieae
0	1	0	G	3877	Medicago
0	1	1	S	3880	Medicago truncatula
0	5	0	F3	2231387	dalbergioids sensu lato
0	5	0	F4	163725	Dalbergieae
0	5	0	F5	2231390	Pterocarpus clade
0	5	5	G	3817	Arachis
0.01	13	0	O	71239	Cucurbitales
0.01	13	3	F	3650	Cucurbitaceae
0	7	0	F1	1003878	Cucurbiteae
0	7	0	G	3660	Cucurbita
0	7	0	S	3663	Cucurbita pepo
0	7	7	S1	3664	Cucurbita pepo subsp. pepo
0	3	0	F1	1003877	Benincaseae
0	3	0	G	102210	Benincasa
0	3	3	S	102211	Benincasa hispida
0	5	0	O	3502	Fagales
0	5	0	F	3503	Fagaceae
0	5	0	G	3511	Quercus
0	5	5	S	97700	Quercus lobata
0	5	0	O	3744	Rosales
0	5	0	F	3745	Rosaceae
0	5	0	F1	171638	Rosoideae
0	5	0	F2	721789	Potentilleae
0	5	0	F3	1184124	Fragariinae
0	5	0	G	3746	Fragaria
0	5	0	S	57918	Fragaria vesca
0	5	5	S1	101020	Fragaria vesca subsp. vesca
0.01	14	0	C6	91836	malvids
0	5	0	O	41937	Sapindales
0	5	0	F	23513	Rutaceae
0	5	0	F1	1728959	Aurantioideae
0	5	0	G	2706	Citrus
0	5	5	S	2711	Citrus sinensis
0	3	0	O	3699	Brassicales
0	3	0	F	3700	Brassicaceae
0	2	0	F1	981071	Brassiceae
0	2	2	G	3705	Brassica
0	1	0	F1	980083	Camelineae
0	1	0	G	71323	Camelina
0	1	1	S	90675	Camelina sativa
0	3	0	O	41938	Malvales
0	3	0	F	3629	Malvaceae
0	2	0	F1	214907	Malvoideae
0	2	1	G	3633	Gossypium
0	1	1	S	29730	Gossypium raimondii
0	1	0	F1	214909	Byttnerioideae
0	1	0	G	3640	Theobroma
0	1	1	S	3641	Theobroma cacao
0	3	0	O	41944	Myrtales
0	2	0	F	3931	Myrtaceae
0	2	0	F1	1699513	Myrtoideae
0	2	0	F2	1699524	Eucalypteae
0	2	0	G	3932	Eucalyptus
0	2	2	S	71139	Eucalyptus grandis
0	1	0	F	3928	Lythraceae
0	1	0	G	22662	Punica
0	1	1	S	22663	Punica granatum
0.03	57	0	C5	71274	asterids
0.03	50	0	C6	91888	lamiids
0.03	50	0	O	4069	Solanales
0.03	50	0	F	4070	Solanaceae
0.03	50	6	F1	424551	Solanoideae
0.02	41	0	F2	424564	Capsiceae
0.02	41	0	G	4071	Capsicum
0.02	41	41	S	4072	Capsicum annuum
0	3	0	F2	424574	Solaneae
0	3	0	G	4107	Solanum
0	3	3	G1	49274	Solanum subgen. Lycopersicon
0	7	0	C6	91882	campanulids

0	4	0	O	4209	Asterales
0	4	0	F	4210	Asteraceae
0	2	0	F1	102804	Asteroideae
0	2	0	F2	911341	Heliantheae alliance
0	2	0	F3	102814	Heliantheae
0	2	0	G	4231	Helianthus
0	2	2	S	4232	Helianthus annuus
0	2	0	F1	219103	Carduoideae
0	2	0	F2	102818	Cardueae
0	2	0	F3	742010	Carduinae
0	2	0	G	4264	Cynara
0	2	0	S	4265	Cynara cardunculus
0	2	0	S1	309979	Cynara cardunculus subsp. cardunculus
0	2	2	S2	59895	Cynara cardunculus var. scolymus
0	3	0	O	4036	Apiales
0	3	0	O1	364270	Apiineae
0	3	0	F	4037	Apiaceae
0	3	0	F1	241778	Apioideae
0	3	0	F2	241789	Scandiceae
0	3	0	F3	241799	Daucinae
0	3	0	G	4038	Daucus
0	3	0	G1	1873447	Daucus sect. Daucus
0	3	0	S	4039	Daucus carota
0	3	3	S1	79200	Daucus carota subsp. sativus
0.07	126	0	C2	4447	Liliopsida
0.07	126	0	C3	1437197	Petrosaviidae
0.07	126	2	C4	4734	commelinids
0.03	57	0	O	4618	Zingiberales
0.03	57	0	F	4637	Musaceae
0.03	57	0	G	4640	Musa
0.03	57	0	S	4641	Musa acuminata
0.03	57	57	S1	214687	Musa acuminata subsp. malaccensis
0.03	51	0	O	38820	Poales
0.03	51	0	F	4479	Poaceae
0.03	47	0	F1	359160	BOP clade
0.02	36	0	F2	147368	Pooideae
0.02	34	0	F3	1648038	Triticodae
0.02	34	0	F4	147389	Triticeae
0.02	34	9	F5	1648030	Triticinae
0.01	25	0	G	4564	Triticum
0.01	25	25	S	85692	Triticum dicoccoides
0	2	0	F3	2822797	Stipodae
0	2	0	F4	147385	Brachypodieae
0	2	0	G	15367	Brachypodium
0	2	2	S	15368	Brachypodium distachyon
0.01	11	0	F2	147367	Oryzoideae
0.01	11	0	F3	147380	Oryzeae
0.01	11	0	F4	1648021	Oryzinae
0.01	11	0	G	4527	Oryza
0.01	11	0	S	4530	Oryza sativa
0.01	11	11	S1	39947	Oryza sativa Japonica Group
0	4	0	F1	147370	PACMAD clade
0	4	0	F2	147369	Panicoideae
0	3	0	F3	1648033	Andropogonodae
0	3	0	F4	147429	Andropogoneae
0	3	0	F5	1648029	Tripsacinae
0	3	0	G	4575	Zea
0	3	3	S	4577	Zea mays
0	1	0	F3	1648036	Panicodae
0	1	0	F4	147428	Paniceae
0	1	0	F5	1293365	Panicinae
0	1	0	G	4539	Panicum
0	1	0	G1	2100772	Panicum sect. Panicum
0	1	1	S	206008	Panicum hallii
0.01	16	0	O	40551	Arecales
0.01	16	0	F	4710	Arecaceae
0.01	16	0	F1	169697	Arecoideae
0.01	16	0	F2	169705	Cocoseae
0.01	16	0	F3	169729	Elaeidinae
0.01	16	0	G	51952	Elaeis
0.01	16	16	S	51953	Elaeis guineensis
0.02	38	0	O	41768	Ranunculales
0.02	38	0	F	3465	Papaveraceae
0.02	38	0	F1	1462614	Papaveroideae
0.02	38	0	G	3468	Papaver
0.02	38	38	S	3469	Papaver somniferum
0	5	0	O	261007	Nymphaeales
0	5	0	F	4410	Nymphaeaceae
0	5	0	G	4418	Nymphaea
0	5	5	S	210225	Nymphaea colorata
0	4	0	P3	3208	Bryophyta
0	4	0	P4	404260	Bryophytina
0	4	0	C	3214	Bryopsida
0	4	0	C1	114656	Funariidae
0	4	0	O	3215	Funariales
0	4	0	F	3216	Funariaceae
0	4	0	G	37414	Physcomitrium
0	4	4	S	3218	Physcomitrium patens
0.02	35	0	D1	2698737	Sar
0.02	35	0	D2	33630	Alveolata
0.02	35	0	P	5794	Apicomplexa
0.02	33	0	C	1280412	Conoidasida
0.02	33	0	C1	5796	Coccidia
0.02	33	0	O	75739	Eucoccidiorida
0.02	33	0	O1	423054	Eimeriorina
0.02	33	0	F	5809	Sarcocystidae
0.02	33	0	G	5810	Toxoplasma
0.02	33	0	S	5811	Toxoplasma gondii
0.02	33	33	S1	508771	Toxoplasma gondii ME49
0	2	0	C	422676	Aconoidasida
0	2	0	O	5863	Piroplasmida
0	2	0	F	32594	Babesiidae
0	2	0	G	5864	Babesia
0	2	2	S	5866	Babesia bigemina

0	4	0	D1	2611352	Discoba
0	4	0	P	33682	Euglenozoa
0	4	0	C	5653	Kinetoplastea
0	4	0	C1	2704647	Metakinetoplastina
0	4	0	O	2704949	Trypanosomatida
0	4	0	F	5654	Trypanosomatidae
0	4	0	F1	1286322	Leishmaniinae
0	4	0	G	5658	Leishmania
0	4	0	G1	38568	Leishmania
0	4	0	G2	38582	Leishmania mexicana species complex
0	4	0	S	5665	Leishmania mexicana
0	4	4	S1	929439	Leishmania mexicana MHOM/GT/2001/U1103
0	1	0	P	2763	Rhodophyta
0	1	0	C	2797	Bangiophyceae
0	1	0	O	265318	Cyanidiales
0	1	0	F	265316	Cyanidiaceae
0	1	0	G	45156	Cyanidioschyzon
0	1	0	S	45157	Cyanidioschyzon merolae
0	1	1	S1	280699	Cyanidioschyzon merolae strain 10D
0	7	0	D	2157	Archaea
0	7	0	P	28890	Euryarchaeota
0	6	0	P1	2290931	Stenosarchaea group
0	3	0	C	183963	Halobacteria
0	3	0	O	1644055	Haloferacales
0	3	0	F	1644056	Haloferacaceae
0	2	0	G	1073986	Halobellus
0	2	2	S	699433	Halobellus limi
0	1	0	G	376170	Haloplanus
0	1	1	S	660522	Haloplanus aerogenes
0	3	0	C	224756	Methanomicrobia
0	3	0	O	94695	Methanosarcinales
0	3	0	F	143067	Methanotrichaceae
0	3	0	G	2222	Methanothrix
0	3	0	S	2223	Methanothrix soehngenii
0	3	3	S1	990316	Methanothrix soehngenii GP6
0	1	0	P1	2283794	Methanomada group
0	1	0	C	183925	Methanobacteria
0	1	0	O	2158	Methanobacteriales
0	1	0	F	2159	Methanobacteriaceae
0	1	0	G	2172	Methanobrevibacter
0	1	1	S	2173	Methanobrevibacter smithii
0.48	859	0	D	10239	Viruses
0.47	854	0	D1	2731341	Duplodnaviria
0.47	854	0	D2	2731360	Heunggongvirae
0.47	854	0	P	2731618	Uroviricota
0.47	854	0	C	2731619	Caudoviricetes
0.47	854	0	O	28883	Caudovirales
0.37	665	0	F	10744	Podoviridae
0.37	665	0	F1	196895	unclassified Podoviridae
0.37	665	0	F2	1978007	crAss-like viruses
0.37	665	0	F3	2315857	environmental samples
0.37	665	665	S	1211417	uncultured crAssphage
0.1	176	0	F	10699	Siphoviridae
0.08	141	20	G	1623305	Skunavirus
0.06	117	56	G1	2050979	unclassified Skunavirus
0	6	6	S	2029658	Lactococcus phage 05601
0	4	4	S	2675246	Lactococcus phage CHPC129
0	4	4	S	2029674	Lactococcus phage 96401
0	4	4	S	2029664	Lactococcus phage 51701
0	3	3	S	2029662	Lactococcus phage 38503
0	3	3	S	1636570	Lactococcus phage 936 group phage Phi5.12
0	3	3	S	1636566	Lactococcus phage 936 group phage Phi4.2
0	2	2	S	2675257	Lactococcus phage CHPC965
0	2	2	S	2029672	Lactococcus phage 79201
0	2	2	S	2029671	Lactococcus phage 66901
0	2	2	S	2029668	Lactococcus phage 62601
0	2	2	S	2029666	Lactococcus phage 56301
0	2	2	S	2029661	Lactococcus phage 37201
0	2	2	S	2029659	Lactococcus phage 16802
0	2	2	S	1874593	Lactococcus phage i0139
0	2	2	S	1636573	Lactococcus phage 936 group phage PhiB1127
0	1	1	S	39838	Lactococcus phage 936
0	1	1	S	2675256	Lactococcus phage CHPC964
0	1	1	S	2675251	Lactococcus phage CHPC52
0	1	1	S	1636560	Lactococcus phage 936 group phage Phi155
0	1	1	S	1636576	Lactococcus phage 936 group phage PhiE1127
0	1	1	S	2675250	Lactococcus phage CHPC362
0	1	1	S	2662298	Lactococcus phage P656
0	1	1	S	2029675	Lactococcus phage 96403
0	1	1	S	213777	Lactococcus lactis phage p272
0	1	1	S	1636583	Lactococcus phage 936 group phage PhiLj
0	1	1	S	213782	Lactococcus phage fd13
0	1	1	S	1262537	Lactococcus phage P680
0	1	1	S	1636724	Lactococcus phage 936 group phage PhiA.16
0	1	1	S	1289467	Lactococcus phage CaseusJM1
0	1	1	S	2029660	Lactococcus phage 30804
0	1	1	S	2027272	Lactococcus phage LP0903
0	2	2	S	287412	Lactococcus virus Sl4
0	2	2	S	665883	Lactococcus virus CB13
0.02	28	22	G	1982251	Pahexavirus
0	3	0	S	1982287	Propionibacterium virus PHL114L00
0	3	3	S1	1235656	Propionibacterium phage PHL114L00
0	2	0	S	1982297	Propionibacterium virus PHL171M01
0	2	2	S1	1500827	Propionibacterium phage PHL171M01
0	1	0	S	1982282	Propionibacterium virus PHL092M00
0	1	1	S1	1500813	Propionibacterium phage PHL092M00
0	7	3	F1	196894	unclassified Siphoviridae
0	3	3	S	1105171	Bacteroides phage B124-14
0	1	1	S	881953	Lactococcus phage 949
0.01	13	0	F	10662	Myoviridae
0.01	12	0	F1	196896	unclassified Myoviridae
0.01	12	12	S	12402	Streptococcus phage EJ-1
0	1	0	G	2733134	Taranisvirus
0	1	0	S	2734146	Faecalibacterium virus Taranis

0	1	1	S1	2070186	Faecalibacterium phage FP_Taranis
0	5	0	D1	2731342	Monodnaviria
0	5	0	K	2732092	Shotokuvirae
0	5	0	P	2732415	Cossaviricota
0	5	0	C	2732421	Papovaviricetes
0	5	0	O	2732532	Sepolyvirales
0	5	0	F	151341	Polyomaviridae
0	5	0	G	1891713	Alphapolyomavirus
0	5	0	S	1891727	Human polyomavirus 8
0	5	5	S1	862909	Trichodysplasia spinulosa-associated polyomavirus

Table S5-3. NC2_taxa

54.1	5029	5029	U	0	unclassified
45.9	4267	99	R	1	root
44.83	4167	5	R1	131567	cellular organisms
42.99	3996	45	D	2	Bacteria
19.95	1855	7	P	1224	Proteobacteria
8.85	823	0	C	28216	Betaproteobacteria
8.84	822	22	O	80840	Burkholderiales
7.99	743	6	F	80864	Comamonadaceae
7.85	730	451	G	80865	Delftia
2.09	194	194	S	558537	Delftia lacustris
0.77	72	72	S	180282	Delftia tsuruhatensis
0.12	11	11	S	80866	Delftia acidovorans
0.02	2	0	G1	2613839	unclassified Delftia
0.01	1	1	S	742013	Delftia sp. Cs1-4
0.01	1	1	S	1920191	Delftia sp. HK171
0.03	3	0	G	201096	Alicycliphilus
0.03	3	0	S	179636	Alicycliphilus denitrificans
0.03	3	3	S1	596154	Alicycliphilus denitrificans K601
0.02	2	0	G	215579	Schlegelella
0.02	2	2	S	215580	Schlegelella thermodepolymerans
0.01	1	0	G	34072	Variovorax
0.01	1	1	S	34073	Variovorax paradoxus
0.01	1	0	G	47420	Hydrogenophaga
0.01	1	1	S	1763535	Hydrogenophaga crassostreae
0.26	24	6	F	75682	Oxalobacteraceae
0.19	18	0	G	149698	Massilia
0.12	11	0	G1	2609279	unclassified Massilia
0.09	8	8	S	2769491	Massilia sp. LPB0304
0.02	2	2	S	2765360	Massilia sp. CCM 8941
0.01	1	1	S	1707785	Massilia sp. WG5
0.05	5	5	S	871742	Massilia flava
0.01	1	1	S	321985	Massilia lutea
0.01	1	1	S	1141883	Massilia putida
0.19	18	11	F	119060	Burkholderiaceae
0.08	7	5	G	106589	Cupriavidus
0.02	2	2	S	82633	Cupriavidus pauculus
0.12	11	0	F	995019	Sutterellaceae
0.12	11	0	G	40544	Sutterella
0.12	11	11	S	40545	Sutterella wadsworthensis
0.03	3	0	F	506	Alcaligenaceae
0.03	3	1	G	517	Bordetella
0.02	2	2	S	123899	Bordetella trematum
0.01	1	0	O1	224471	Burkholderiales genera incertae sedis
0.01	1	1	G	32012	Thiomonas
0.01	1	0	O	206389	Rhodocyclales
0.01	1	0	F	2008794	Zoogloeaceae
0.01	1	0	G	33057	Thauera
0.01	1	1	S	96773	Thauera chlorobenzoica
7.87	732	18	C	28211	Alphaproteobacteria
5.02	467	0	O	204457	Sphingomonadales
4.99	464	15	F	41297	Sphingomonadaceae
4.59	427	19	G	13687	Sphingomonas
4.21	391	391	S	13689	Sphingomonas paucimobilis
0.05	5	0	G1	196159	unclassified Sphingomonas
0.05	5	5	S	2698679	Sphingomonas sp. C33
0.04	4	4	S	93064	Sphingomonas koreensis
0.03	3	3	S	152682	Sphingomonas melonis
0.03	3	0	S	397260	Sphingomonas sanxanigenens
0.03	3	3	S1	1123269	Sphingomonas sanxanigenens DSM 19645 = NX02
0.02	2	2	S	2698828	Sphingomonas lacunae
0.13	12	3	G	165695	Sphingobium
0.06	6	6	S	13690	Sphingobium yanoikuyae
0.03	3	3	S	135719	Sphingobium amiense
0.08	7	3	G	165696	Novosphingobium
0.02	2	2	S	158500	Novosphingobium resinovorum
0.02	2	0	G1	2644732	unclassified Novosphingobium
0.02	2	2	S	164608	Novosphingobium sp. KA1
0.02	2	0	G	1649486	Rhizorhabdus
0.02	2	2	S	1850238	Rhizorhabdus dicambivorans
0.01	1	0	G	165697	Sphingopyxis
0.01	1	1	S	33050	Sphingopyxis macrogoltabida
0.03	3	0	F	335929	Erythrobacteraceae
0.03	3	0	F1	2800788	Erythrobacter/Porphyrobacter group
0.03	3	0	G	1041	Erythrobacter
0.03	3	0	G1	2633097	unclassified Erythrobacter
0.03	3	3	S	2785907	Erythrobacter sp. A30-3
1.91	178	1	O	356	Hyphomicrobiales
1.61	150	27	F	119045	Methylobacteriaceae
1.17	109	14	G	2282523	Methylorubrum
0.55	51	42	S	223967	Methylorubrum populi
0.1	9	9	S1	441620	Methylorubrum populi BJ001
0.47	44	7	S	408	Methylorubrum extorquens
0.29	27	27	S1	272630	Methylorubrum extorquens AM1
0.11	10	10	S1	440085	Methylorubrum extorquens CM4
0.15	14	1	G	407	Methylobacterium
0.11	10	4	G1	2615210	unclassified Methylobacterium
0.06	6	6	S	2067957	Methylobacterium sp. DM1
0.02	2	2	S	270351	Methylobacterium aquaticum
0.01	1	1	S	2202825	Methylobacterium durans
0.11	10	0	F	82115	Rhizobiaceae
0.06	6	0	F1	227292	Sinorhizobium/Ensifer group
0.06	6	0	G	28105	Sinorhizobium
0.06	6	0	G1	663276	Sinorhizobium fredii group
0.06	6	6	S	380	Sinorhizobium fredii
0.04	4	0	F1	227290	Rhizobium/Agrobacterium group
0.02	2	1	G	357	Agrobacterium
0.01	1	1	S	359	Agrobacterium rhizogenes
0.02	2	0	G	1525371	Neorhizobium
0.02	2	0	G1	2629175	unclassified Neorhizobium
0.02	2	2	S	1825976	Neorhizobium sp. NCHU2750
0.06	6	0	F	41294	Bradyrhizobiaceae
0.04	4	2	G	374	Bradyrhizobium
0.02	2	0	G1	2631580	unclassified Bradyrhizobium

0.02	2	2	S	319017	Bradyrhizobium sp. WSM471
0.02	2	0	G	85413	Bosea
0.02	2	0	G1	2653178	unclassified Bosea
0.02	2	2	S	1867715	Bosea sp. Tri-49
0.06	6	0	F	69277	Phyllobacteriaceae
0.06	6	6	G	68287	Mesorhizobium
0.03	3	0	F	118882	Brucellaceae
0.03	3	0	F1	2826938	Brucella/Ochrobactrum group
0.03	3	0	G	234	Brucella
0.03	3	3	S	271865	[Ochrobactrum] quorumnocens
0.01	1	0	F	31993	Methylocystaceae
0.01	1	0	G	133	Methylocystis
0.01	1	1	S	391905	Methylocystis heyeri
0.01	1	0	F	45401	Hyphomicrobiaceae
0.01	1	0	G	1484898	Methyloceanibacter
0.01	1	0	G1	2617503	unclassified Methyloceanibacter
0.01	1	1	S	2170729	Methyloceanibacter sp. wino2
0.48	45	0	O	204455	Rhodobacterales
0.48	45	19	F	31989	Rhodobacteraceae
0.12	11	0	G	1060	Rhodobacter
0.12	11	0	G1	196779	unclassified Rhodobacter
0.12	11	11	S	1850250	Rhodobacter sp. LPB0142
0.05	5	3	G	265	Paracoccus
0.01	1	1	S	135740	Paracoccus kondratievae
0.01	1	0	G1	246570	unclassified Paracoccus
0.01	1	1	S	2500532	Paracoccus sp. Arc7-R13
0.04	4	0	G	366614	Haematobacter
0.04	4	4	S	195105	Haematobacter massiliensis
0.02	2	0	G	1653176	Cereibacter
0.02	2	2	S	1063	Cereibacter sphaeroides
0.02	2	0	G	74030	Roseovarius
0.02	2	2	G1	2614913	unclassified Roseovarius
0.01	1	0	G	53945	Octadecabacter
0.01	1	0	S	53946	Octadecabacter arcticus
0.01	1	1	S1	391616	Octadecabacter arcticus 238
0.01	1	0	G	204456	Gemmobacter
0.01	1	0	G1	2636157	unclassified Gemmobacter
0.01	1	1	S	2169400	Gemmobacter sp. HYN0069
0.24	22	0	O	204458	Caulobacterales
0.24	22	1	F	76892	Caulobacteraceae
0.19	18	1	G	41275	Brevundimonas
0.16	15	0	G1	2622653	unclassified Brevundimonas
0.06	6	6	S	1532555	Brevundimonas sp. DS20
0.05	5	5	S	2752515	Brevundimonas sp. AJA228-03
0.02	2	2	S	2579977	Brevundimonas sp. SGAir0440
0.02	2	2	S	2774189	Brevundimonas sp. LVF1
0.02	2	2	S	293	Brevundimonas diminuta
0.03	3	1	G	75	Caulobacter
0.02	2	0	G1	2648921	unclassified Caulobacter
0.02	2	2	S	69665	Caulobacter sp. FWC26
0.01	1	0	O	204441	Rhodospirillales
0.01	1	0	F	433	Acetobacteraceae
0.01	1	0	G	1654741	Bombella
0.01	1	0	G1	2644098	unclassified Bombella
0.01	1	1	S	2697033	Bombella sp. KACC 21507
0.01	1	0	O	255473	Parvularculales
0.01	1	0	F	255474	Parvularculaceae
0.01	1	0	G	208215	Parvularcula
0.01	1	0	S	208216	Parvularcula bermudensis
0.01	1	1	S1	314260	Parvularcula bermudensis HTCC2503
3	279	0	C	1236	Gammaproteobacteria
1.96	182	2	O	135614	Xanthomonadales
1.94	180	0	F	32033	Xanthomonadaceae
1.93	179	31	G	40323	Stenotrophomonas
1.55	144	0	G1	995085	Stenotrophomonas maltophilia group
1.55	144	137	S	40324	Stenotrophomonas maltophilia
0.08	7	7	S1	391008	Stenotrophomonas maltophilia R551-3
0.03	3	0	G1	196198	unclassified Stenotrophomonas
0.03	3	3	S	2282124	Stenotrophomonas sp. ASS1
0.01	1	1	S	128780	Stenotrophomonas acidaminiphila
0.01	1	0	G	68	Lysobacter
0.01	1	1	S	1324796	Lysobacter lycopersici
0.9	84	0	O	72274	Pseudomonadales
0.55	51	0	F	468	Moraxellaceae
0.55	51	12	G	469	Acinetobacter
0.16	15	15	S	28090	Acinetobacter lwoffii
0.12	11	0	S	52133	Acinetobacter venetianus
0.12	11	11	S1	1197884	Acinetobacter venetianus VE-C3
0.08	7	7	S	40214	Acinetobacter johnsonii
0.04	4	0	G1	196816	unclassified Acinetobacter
0.03	3	3	S	2725684	Acinetobacter sp. NEB149
0.01	1	1	S	2004644	Acinetobacter sp. WCHA45
0.02	2	0	G1	909768	Acinetobacter calcoaceticus/baumannii complex
0.02	2	2	S	470	Acinetobacter baumannii
0.35	33	0	F	135621	Pseudomonadaceae
0.35	33	2	G	286	Pseudomonas
0.22	20	0	G1	136841	Pseudomonas aeruginosa group
0.22	20	20	S	287	Pseudomonas aeruginosa
0.05	5	0	G1	136843	Pseudomonas fluorescens group
0.05	5	5	S	294	Pseudomonas fluorescens
0.03	3	3	S	157782	Pseudomonas parafulva
0.02	2	0	G1	136845	Pseudomonas putida group
0.01	1	1	S	303	Pseudomonas putida
0.01	1	0	S	47880	Pseudomonas fulva
0.01	1	1	S1	743720	Pseudomonas fulva 12-X
0.01	1	0	G1	196821	unclassified Pseudomonas
0.01	1	1	S	2083054	Pseudomonas sp. LG1D9
0.09	8	1	O	91347	Enterobacterales
0.08	7	3	F	543	Enterobacteriaceae
0.04	4	0	G	561	Escherichia
0.04	4	4	S	562	Escherichia coli
0.04	4	0	O	135624	Aeromonadales
0.04	4	0	F	84642	Aeromonadaceae
0.04	4	0	G	642	Aeromonas

0.04	4	0	G1	257493	unclassified Aeromonas
0.04	4	4	S	1636607	Aeromonas sp. ASNIH2
0.01	1	0	O	135625	Pasteurellales
0.01	1	0	F	712	Pasteurellaceae
0.01	1	0	G	724	Haemophilus
0.01	1	1	S	729	Haemophilus parainfluenzae
0.15	14	0	P1	68525	delta/epsilon subdivisions
0.11	10	0	C	28221	Deltaproteobacteria
0.08	7	0	O	29	Myxococcales
0.08	7	0	O1	80811	Cystobacterineae
0.06	6	0	F	31	Myxococcaceae
0.06	6	0	G	83461	Corallococcus
0.06	6	6	S	35	Corallococcus macrosporus
0.01	1	0	F	39	Archangiaceae
0.01	1	0	G	40	Stigmatella
0.01	1	0	S	41	Stigmatella aurantiaca
0.01	1	1	S1	378806	Stigmatella aurantiaca DW4/3-1
0.03	3	0	O	213115	Desulfovibrionales
0.03	3	0	F	194924	Desulfovibrionaceae
0.03	3	0	G	872	Desulfovibrio
0.03	3	0	S	345370	Desulfovibrio carbinoliphilus
0.03	3	3	S1	694327	Desulfovibrio carbinoliphilus subsp. oakridgensis
0.04	4	0	C	29547	Epsilonproteobacteria
0.04	4	0	O	213849	Campylobacterales
0.04	4	0	F	72294	Campylobacteraceae
0.04	4	0	G	194	Campylobacter
0.04	4	4	S	195	Campylobacter coli
14.68	1365	0	D1	1783270	FCB group
14.68	1365	0	D2	68336	Bacteroidetes/Chlorobi group
14.68	1365	1	P	976	Bacteroidetes
14.09	1310	0	C	200643	Bacteroidia
14.09	1310	110	O	171549	Bacteroidales
10.83	1007	0	F	815	Bacteroidaceae
10.83	1007	93	G	816	Bacteroides
6.81	633	633	S	818	Bacteroides thetaiotaomicron
0.75	70	70	S	246787	Bacteroides cellulosilyticus
0.65	60	60	S	820	Bacteroides uniformis
0.43	40	40	S	28111	Bacteroides eggerthii
0.4	37	9	G1	2646097	unclassified Bacteroides
0.11	10	10	S	2650157	Bacteroides sp. HF-5287
0.11	10	10	S	2785531	Bacteroides sp. HF-162
0.05	5	5	S	2528203	Bacteroides sp. A1C1
0.02	2	2	S	2755405	Bacteroides sp. CACC 737
0.01	1	1	S	2763022	Bacteroides sp. M10
0.38	35	35	S	817	Bacteroides fragilis
0.24	22	22	S	28116	Bacteroides ovatus
0.08	7	7	S	329854	Bacteroides intestinalis
0.05	5	5	S	47678	Bacteroides caccae
0.04	4	3	S	371601	Bacteroides xylanisolvens
0.01	1	1	S1	657309	Bacteroides xylanisolvens XB1A
0.01	1	1	S	1796613	Bacteroides caecimuris
1.38	128	0	O1	333046	Bacteroidales incertae sedis
1.38	128	15	G	909656	Phocaeicola
0.73	68	63	S	357276	Phocaeicola dorei
0.05	5	5	S1	997877	Bacteroides dorei CL03T12C01
0.46	43	43	S	821	Phocaeicola vulgatus
0.01	1	0	S	376805	Phocaeicola salanitronis
0.01	1	1	S1	667015	Phocaeicola salanitronis DSM 18170
0.01	1	1	S	387090	Phocaeicola coprophilus
0.47	44	0	F	171550	Rikenellaceae
0.47	44	0	G	239759	Alistipes
0.32	30	0	S	328813	Alistipes onderdonkii
0.32	30	30	S1	2585117	Alistipes onderdonkii subsp. vulgaris
0.06	6	6	S	2585118	Alistipes communis
0.04	4	0	S	214856	Alistipes finegoldii
0.04	4	4	S1	679935	Alistipes finegoldii DSM 17242
0.02	2	0	S	328814	Alistipes shahii
0.02	2	2	S1	717959	Alistipes shahii WAL 8301
0.01	1	1	S	2585119	Alistipes dispar
0.01	1	0	G1	2608932	unclassified Alistipes
0.01	1	1	S	2662363	Alistipes sp. dk3624
0.11	10	0	F	2005525	Tannerellaceae
0.11	10	3	G	375288	Parabacteroides
0.06	6	6	S	823	Parabacteroides distasonis
0.01	1	1	S	328812	Parabacteroides goldsteinii
0.1	9	0	F	1853231	Odoribacteraceae
0.1	9	0	G	574697	Butyricimonas
0.08	7	7	S	544645	Butyricimonas virosa
0.02	2	2	S	2093856	Butyricimonas faecalis
0.01	1	0	F	171552	Prevotellaceae
0.01	1	0	G	838	Prevotella
0.01	1	1	S	1177574	Prevotella jejuni
0.01	1	0	F	2005473	Muribaculaceae
0.01	1	0	G	1918540	Muribaculum
0.01	1	1	S	2530390	Muribaculum gordoncarteri
0.34	32	0	C	768503	Cytophagia
0.34	32	0	O	768507	Cytophagales
0.34	32	0	F	1853232	Hymenobacteraceae
0.34	32	12	G	89966	Hymenobacter
0.15	14	0	G1	2615202	unclassified Hymenobacter
0.12	11	11	S	2675878	Hymenobacter sp. BRD128
0.02	2	2	S	1356852	Hymenobacter sp. APR13
0.01	1	1	S	2584940	Hymenobacter sp. DG01
0.05	5	5	S	2607656	Hymenobacter busanensis
0.01	1	1	S	1385715	Hymenobacter qilianensis
0.24	22	0	C	117743	Flavobacteriia
0.24	22	0	O	200644	Flavobacteriales
0.24	22	0	F	2762318	Weeksellaceae
0.19	18	0	F1	2782232	Chryseobacterium group
0.19	18	1	G	59732	Chryseobacterium
0.08	7	7	S	246	Chryseobacterium balustinum
0.08	7	7	S	558152	Chryseobacterium piperi
0.03	3	0	G1	2593645	unclassified Chryseobacterium
0.03	3	3	S	2487065	Chryseobacterium sp. G0201

0.04	4	4	G	59734	Empedobacter
7.86	731	3	D1	1783272	Terrabacteria group
5.78	537	0	P	201174	Actinobacteria
5.66	526	33	C	1760	Actinomycetia
2.8	260	0	O	85009	Propionibacteriales
2.47	230	4	F	31957	Propionibacteriaceae
2.38	221	0	G	1912216	Cutibacterium
2.37	220	220	S	1747	Cutibacterium acnes
0.01	1	1	S	33011	Cutibacterium granulosum
0.05	5	0	G	1743	Propionibacterium
0.04	4	4	S	1744	Propionibacterium freudenreichii
0.01	1	0	G1	2608905	unclassified Propionibacterium
0.01	1	1	S	671223	Propionibacterium sp. oral taxon 193
0.32	30	3	F	85015	Nocardioidaceae
0.27	25	0	G	1839	Nocardioides
0.12	11	11	S	2518370	Nocardioides euryhalodurans
0.08	7	7	S	433659	Nocardioides mesophilus
0.08	7	0	G1	2615069	unclassified Nocardioides
0.04	4	4	S	2763008	Nocardioides sp. zg-1228
0.02	2	2	S	110319	Nocardioides sp. CF8
0.01	1	1	S	2714939	Nocardioides sp. HDW12B
0.02	2	0	G	86795	Marmoricola
0.02	2	2	S	642780	Marmoricola scoriae
1.42	132	5	O	85006	Micrococcales
0.71	66	0	F	85023	Microbacteriaceae
0.66	61	16	G	33882	Microbacterium
0.17	16	16	S	57043	Microbacterium esteraromaticum
0.11	10	10	S	162426	Microbacterium hominis
0.09	8	0	G1	2609290	unclassified Microbacterium
0.05	5	5	S	2782166	Microbacterium sp. A18JL241
0.02	2	2	S	2048898	Microbacterium sp. Y-01
0.01	1	1	S	2603598	Microbacterium sp. CBA3102
0.05	5	5	S	104336	Microbacterium foliorum
0.03	3	3	S	273677	Microbacterium oleivorans
0.02	2	2	S	69362	Microbacterium schleiferi
0.01	1	1	S	936337	Microbacterium amylolyticum
0.02	2	0	G	110932	Leifsonia
0.02	2	1	G1	2663824	unclassified Leifsonia
0.01	1	1	S	1798223	Leifsonia sp. 21MFCrub1.1
0.01	1	0	G	2034	Curtobacterium
0.01	1	0	S	2035	Curtobacterium flaccumfaciens
0.01	1	1	S1	138532	Curtobacterium flaccumfaciens pv. flaccumfaciens
0.01	1	0	G	33877	Agromyces
0.01	1	0	G1	2639701	unclassified Agromyces
0.01	1	1	S	2498704	Agromyces sp. LHK192
0.01	1	0	G	235888	Salinibacterium
0.01	1	0	G1	2632331	unclassified Salinibacterium
0.01	1	1	S	2708084	Salinibacterium sp. ZJ70
0.3	28	0	F	2805590	Ornithinimicrobiaceae
0.2	19	0	G	265976	Serinicoccus
0.2	19	19	S	1078471	Serinicoccus profundi
0.1	9	0	G	125287	Ornithinimicrobium
0.06	6	6	S	1288636	Ornithinimicrobium flavum
0.03	3	0	G1	2615080	unclassified Ornithinimicrobium
0.02	2	2	S	2594265	Ornithinimicrobium sp. H23M54
0.01	1	1	S	2508882	Ornithinimicrobium sp. HY006
0.28	26	0	F	1268	Micrococcaceae
0.19	18	10	G	1269	Micrococcus
0.09	8	8	S	1270	Micrococcus luteus
0.08	7	0	G	1663	Arthrobacter
0.04	4	4	S	37921	Arthrobacter agilis
0.03	3	0	G1	235627	unclassified Arthrobacter
0.03	3	3	S	2575374	Arthrobacter sp. 24S4-2
0.01	1	0	G	57493	Kocuria
0.01	1	1	S	72000	Kocuria rhizophila
0.04	4	0	F	85019	Brevibacteriaceae
0.04	4	1	G	1696	Brevibacterium
0.03	3	3	S	629680	Brevibacterium sandarakinum
0.03	3	0	F	85021	Intrasporangiaceae
0.03	3	0	G	53457	Janibacter
0.03	3	3	S	53458	Janibacter limosus
0.58	54	0	O	85007	Corynebacteriales
0.33	31	0	F	1653	Corynebacteriaceae
0.33	31	0	G	1716	Corynebacterium
0.13	12	12	S	43990	Corynebacterium segmentosum
0.11	10	0	S	1404244	Corynebacterium glyciniphilum
0.11	10	10	S1	1404245	Corynebacterium glyciniphilum AJ 3170
0.06	6	6	S	1979527	Corynebacterium kefirresidentii
0.03	3	3	S	38304	Corynebacterium tuberculostearicum
0.13	12	0	F	1762	Mycobacteriaceae
0.08	7	5	G	1866885	Mycolicibacterium
0.01	1	1	S	2487344	Mycolicibacterium nivoides
0.01	1	1	S	1794	Mycolicibacterium gadium
0.03	3	3	G	1763	Mycobacterium
0.02	2	0	G	670516	Mycobacteroides
0.02	2	2	S	1774	Mycobacteroides chelonae
0.05	5	0	F	2805586	Lawsonellaceae
0.05	5	0	G	1847725	Lawsonella
0.05	5	5	S	1528099	Lawsonella clevelandensis
0.04	4	0	F	85025	Nocardiaceae
0.04	4	4	G	1827	Rhodococcus
0.02	2	0	F	85029	Dietziaceae
0.02	2	0	G	37914	Dietzia
0.02	2	0	G1	2617939	unclassified Dietzia
0.02	2	2	S	712270	Dietzia sp. oral taxon 368
0.17	16	0	O	85011	Streptomycetales
0.17	16	0	F	2062	Streptomycetaceae
0.17	16	8	G	1883	Streptomyces
0.02	2	0	G1	2593676	unclassified Streptomyces
0.01	1	1	S	2695266	Streptomyces sp. HM190
0.01	1	1	S	646637	Streptomyces sp. M2
0.02	2	2	S	1169025	Streptomyces pratensis
0.02	2	2	S	1927	Streptomyces rimosus
0.01	1	1	S	68270	Streptomyces spectabilis

0.01	1	0	S	1912	Streptomyces hygroscopicus
0.01	1	1	S1	264445	Streptomyces hygroscopicus subsp. limoneus
0.13	12	0	O	85008	Micromonosporales
0.13	12	6	F	28056	Micromonosporaceae
0.06	6	0	G	1873	Micromonospora
0.04	4	0	G1	2617518	unclassified Micromonospora
0.04	4	4	S	2583243	Micromonospora sp. HM134
0.02	2	2	S	261654	Micromonospora auratinigra
0.11	10	0	O	85010	Pseudonocardiales
0.11	10	0	F	2070	Pseudonocardiaceae
0.06	6	0	G	1813	Amycolatopsis
0.06	6	6	S	31958	Amycolatopsis orientalis
0.04	4	0	G	1847	Pseudonocardia
0.04	4	3	G1	2619320	unclassified Pseudonocardia
0.01	1	1	S	2761535	Pseudonocardia sp. CGMCC 4.1532
0.02	2	0	O	2037	Actinomycetales
0.02	2	0	F	2049	Actinomycetaceae
0.02	2	0	G	1654	Actinomyces
0.02	2	2	S	675090	Actinomyces weissii
0.02	2	0	O	85004	Bifidobacteriales
0.02	2	0	F	31953	Bifidobacteriaceae
0.02	2	0	G	1678	Bifidobacterium
0.01	1	1	S	1680	Bifidobacterium adolescentis
0.01	1	1	S	28025	Bifidobacterium animalis
0.02	2	0	O	85013	Frankiales
0.02	2	0	F	74712	Frankiaceae
0.02	2	0	G	1854	Frankia
0.02	2	0	G1	2632575	unclassified Frankia
0.02	2	2	S	710111	Frankia sp. QA3
0.02	2	0	O	1217098	Jiangellales
0.02	2	0	F	1217100	Jiangellaceae
0.02	2	0	G	281472	Jiangella
0.02	2	0	G1	2624397	unclassified Jiangella
0.02	2	2	S	1798224	Jiangella sp. DSM 45060
0.01	1	0	O	85014	Glycomycetales
0.01	1	0	F	85034	Glycomycetaceae
0.01	1	0	G	283810	Stackebrandtia
0.01	1	0	S	283811	Stackebrandtia nassauensis
0.01	1	1	S1	446470	Stackebrandtia nassauensis DSM 44728
0.12	11	0	C	84995	Rubrobacteria
0.12	11	0	O	84996	Rubrobacterales
0.11	10	0	F	84997	Rubrobacteraceae
0.11	10	0	G	42255	Rubrobacter
0.11	10	0	G1	2637691	unclassified Rubrobacter
0.11	10	10	S	2653851	Rubrobacter sp. SCSIO 52909
0.01	1	0	F	2600303	Baekduiaceae
0.01	1	0	G	2600304	Baekduia
0.01	1	1	S	496014	Baekduia soli
1.85	172	3	P	1239	Firmicutes
0.79	73	2	C	91061	Bacilli
0.53	49	0	O	1385	Bacillales
0.53	49	0	F	90964	Staphylococcaceae
0.53	49	8	G	1279	Staphylococcus
0.25	23	23	S	1282	Staphylococcus epidermidis
0.14	13	13	S	29388	Staphylococcus capitis
0.05	5	5	S	1292	Staphylococcus warneri
0.24	22	3	O	186826	Lactobacillales
0.11	10	0	F	1300	Streptococcaceae
0.11	10	3	G	1301	Streptococcus
0.02	2	2	S	28037	Streptococcus mitis
0.02	2	2	S	1308	Streptococcus thermophilus
0.02	2	2	S	1304	Streptococcus salivarius
0.01	1	1	S	1305	Streptococcus sanguinis
0.1	9	0	F	33958	Lactobacillaceae
0.08	7	0	G	1578	Lactobacillus
0.08	7	0	S	1584	Lactobacillus delbrueckii
0.08	7	7	S1	29397	Lactobacillus delbrueckii subsp. lactis
0.01	1	0	G	2767842	Lactiplantibacillus
0.01	1	1	S	1590	Lactiplantibacillus plantarum
0.01	1	0	G	2767885	Latilactobacillus
0.01	1	1	S	1599	Latilactobacillus sakei
0.72	67	0	C	186801	Clostridia
0.72	67	3	O	186802	Eubacteriales
0.37	34	2	F	186803	Lachnospiraceae
0.18	17	1	G	841	Roseburia
0.15	14	7	S	166486	Roseburia intestinalis
0.03	3	3	S1	536231	Roseburia intestinalis L1-82
0.03	3	3	S1	657315	Roseburia intestinalis M50/1
0.01	1	1	S1	718255	Roseburia intestinalis XB6B4
0.02	2	2	S	301301	Roseburia hominis
0.04	4	0	G	28050	Lachnospira
0.04	4	0	S	39485	Lachnospira eligens
0.04	4	4	S1	515620	[Eubacterium] eligens ATCC 27750
0.03	3	0	G	572511	Blautia
0.02	2	0	G1	2648079	unclassified Blautia
0.02	2	2	S	2479767	Blautia sp. SC05B48
0.01	1	0	S	40520	Blautia obeum
0.01	1	1	S1	657314	Blautia obeum A2-162
0.02	2	0	G	2569097	Anaerobutyricum
0.02	2	2	S	39488	Anaerobutyricum hallii
0.02	2	1	G	2719313	Enterocloster
0.01	1	1	S	1531	Enterocloster clostridioformis
0.01	1	0	G	33042	Coprococcus
0.01	1	1	S	410072	Coprococcus comes
0.01	1	1	G	1506553	Lachnoclostridium
0.01	1	0	G	2316020	Mediterraneibacter
0.01	1	0	S	33039	[Ruminococcus] torques
0.01	1	1	S1	657313	[Ruminococcus] torques L2-14
0.01	1	0	G	2719231	Lacrimispora
0.01	1	1	S	84030	Lacrimispora saccharolytica
0.25	23	0	F	216572	Oscillospiraceae
0.18	17	0	G	216851	Faecalibacterium
0.18	17	11	S	853	Faecalibacterium prausnitzii
0.04	4	4	S1	718252	Faecalibacterium prausnitzii L2-6

0.02	2	2	S1	657322	Faecalibacterium prausnitzii SL3/3
0.02	2	0	G	1263	Ruminococcus
0.02	2	2	S	1160721	Ruminococcus bicirculans
0.02	2	0	G	459786	Oscillibacter
0.02	2	0	G1	2629304	unclassified Oscillibacter
0.01	1	1	S	2109687	Oscillibacter sp. PEA192
0.01	1	1	S	2763056	Oscillibacter sp. NSJ-62
0.01	1	0	G	1905344	Ruthenibacterium
0.01	1	1	S	1550024	Ruthenibacterium lactatiformans
0.01	1	0	G	2591381	Dysosmobacter
0.01	1	1	S	2093857	Dysosmobacter welbionis
0.03	3	0	F	186804	Peptostreptococcaceae
0.02	2	0	G	1870884	Clostridioides
0.02	2	2	S	1496	Clostridioides difficile
0.01	1	1	G	1501226	Romboutsia
0.02	2	0	O1	538999	Eubacteriales incertae sedis
0.01	1	0	G	1392389	Intestinimonas
0.01	1	1	S	1297617	Intestinimonas butyriciproducens
0.01	1	0	G	2039302	Monoglobus
0.01	1	1	S	1981510	Monoglobus pectinilyticus
0.01	1	0	F	31979	Clostridiaceae
0.01	1	0	G	1485	Clostridium
0.01	1	1	S	1561	Clostridium baratii
0.01	1	0	F	186806	Eubacteriaceae
0.01	1	0	G	33951	Acetobacterium
0.01	1	0	S	33952	Acetobacterium woodii
0.01	1	1	S1	931626	Acetobacterium woodii DSM 1030
0.16	15	0	C	909932	Negativicutes
0.13	12	0	O	1843489	Veillonellales
0.13	12	0	F	31977	Veillonellaceae
0.13	12	0	G	29465	Veillonella
0.13	12	7	S	29466	Veillonella parvula
0.05	5	5	S1	1316254	Veillonella parvula HSIVP1
0.03	3	0	O	1843488	Acidaminococcales
0.03	3	0	F	909930	Acidaminococcaceae
0.03	3	0	G	33024	Phascolarctobacterium
0.03	3	3	S	33025	Phascolarctobacterium faecium
0.15	14	0	C	526524	Erysipelotrichia
0.15	14	0	O	526525	Erysipelotrichales
0.15	14	0	F	2810281	Turicibacteraceae
0.15	14	0	G	191303	Turicibacter
0.13	12	12	S	154288	Turicibacter sanguinis
0.02	2	0	G1	2638206	unclassified Turicibacter
0.02	2	2	S	1712675	Turicibacter sp. H121
0.2	19	0	D2	1798711	Cyanobacteria/Melainabacteria group
0.2	19	0	P	1117	Cyanobacteria
0.12	11	1	O	1161	Nostocales
0.05	5	0	F	1162	Nostocaceae
0.05	5	5	G	1177	Nostoc
0.05	5	0	F	1182	Scytonemataceae
0.05	5	0	G	1203	Scytonema
0.05	5	0	G1	2618749	unclassified Scytonema
0.05	5	5	S	1137095	Scytonema sp. HK-05
0.08	7	0	O	52604	Pleurocapsales
0.08	7	0	F	1890500	Hyellaceae
0.08	7	0	G	44474	Pleurocapsa
0.08	7	0	S	54308	Pleurocapsa minor
0.08	7	7	S1	118163	Pleurocapsa sp. PCC 7327
0.01	1	0	O	1890424	Synechococcales
0.01	1	0	F	1890438	Leptolyngbyaceae
0.01	1	0	G	47251	Leptolyngbya
0.01	1	0	G1	2650499	unclassified Leptolyngbya
0.01	1	1	S	1752064	Leptolyngbya sp. NIES-3755
1.79	166	0	D	2759	Eukaryota
1.2	112	0	D1	33154	Opisthokonta
1.09	101	0	K	4751	Fungi
1.09	101	0	K1	451864	Dikarya
1.09	101	0	P	5204	Basidiomycota
1.09	101	0	P1	452284	Ustilaginomycotina
1.09	101	0	C	1538075	Malasseziomycetes
1.09	101	0	O	162474	Malasseziales
1.09	101	0	F	742845	Malasseziaceae
1.09	101	0	G	55193	Malassezia
1.09	101	101	S	76775	Malassezia restricta
0.12	11	0	K	33208	Metazoa
0.12	11	0	K1	6072	Eumetazoa
0.12	11	0	K2	33213	Bilateria
0.12	11	0	K3	33511	Deuterostomia
0.12	11	0	P	7711	Chordata
0.12	11	0	P1	89593	Craniata
0.12	11	0	P2	7742	Vertebrata
0.12	11	0	P3	7776	Gnathostomata
0.12	11	0	P4	117570	Teleostomi
0.12	11	0	P5	117571	Euteleostomi
0.12	11	0	P6	8287	Sarcopterygii
0.12	11	0	C	1338369	Dipnotetrapodomorpha
0.12	11	0	C1	32523	Tetrapoda
0.12	11	0	C2	32524	Amniota
0.12	11	0	C	40674	Mammalia
0.12	11	0	C1	32525	Theria
0.12	11	0	C2	9347	Eutheria
0.12	11	0	C3	1437010	Boreoeutheria
0.12	11	0	C4	314146	Euarchontoglires
0.12	11	0	O	9443	Primates
0.12	11	0	O1	376913	Haplorrhini
0.12	11	0	O2	314293	Simiiformes
0.12	11	0	O3	9526	Catarrhini
0.12	11	0	O4	314295	Hominoidea
0.12	11	0	F	9604	Hominidae
0.12	11	0	F1	207598	Homininae
0.12	11	0	G	9605	Homo
0.12	11	11	S	9606	Homo sapiens
0.58	54	0	K	33090	Viridiplantae
0.58	54	0	P	35493	Streptophyta

0.58	54	0	P1	131221	Streptophytina
0.58	54	0	P2	3193	Embryophyta
0.58	54	0	P3	58023	Tracheophyta
0.58	54	0	P4	78536	Euphyllophyta
0.58	54	0	P5	58024	Spermatophyta
0.58	54	0	C	3398	Magnoliopsida
0.58	54	0	C1	1437183	Mesangiospermae
0.42	39	0	C2	4447	Liliopsida
0.42	39	0	C3	1437197	Petrosaviidae
0.42	39	0	C4	4734	commelinids
0.42	39	0	O	38820	Poales
0.42	39	0	F	4479	Poaceae
0.42	39	0	F1	359160	BOP clade
0.42	39	0	F2	147368	Pooideae
0.42	39	0	F3	1648038	Triticodae
0.42	39	0	F4	147389	Triticeae
0.42	39	12	F5	1648030	Triticinae
0.29	27	0	G	4564	Triticum
0.29	27	27	S	85692	Triticum dicoccoides
0.16	15	0	C2	71240	eudicotyledons
0.16	15	0	C3	91827	Gunneridae
0.16	15	0	C4	1437201	Pentapetalae
0.15	14	0	C5	71275	rosids
0.14	13	1	C6	91836	malvids
0.13	12	0	O	41937	Sapindales
0.13	12	0	F	23513	Rutaceae
0.13	12	0	F1	1728959	Aurantioideae
0.13	12	0	G	2706	Citrus
0.13	12	12	S	2711	Citrus sinensis
0.01	1	0	C6	91835	fabids
0.01	1	0	O	72025	Fabales
0.01	1	0	F	3803	Fabaceae
0.01	1	0	F1	3814	Papilionoideae
0.01	1	0	F2	2231393	50 kb inversion clade
0.01	1	0	F3	2231387	dalbergioids sensu lato
0.01	1	0	F4	163725	Dalbergieae
0.01	1	0	F5	2231390	Pterocarpus clade
0.01	1	1	G	3817	Arachis
0.01	1	0	C5	71274	asterids
0.01	1	0	C6	91882	campanulids
0.01	1	0	O	4209	Asterales
0.01	1	0	F	4210	Asteraceae
0.01	1	0	F1	102804	Asteroideae
0.01	1	0	F2	911341	Heliantheae alliance
0.01	1	0	F3	102814	Heliantheae
0.01	1	0	G	4231	Helianthus
0.01	1	1	S	4232	Helianthus annuus
0.01	1	0	D	10239	Viruses
0.01	1	0	D1	2731341	Duplodnaviria
0.01	1	0	D2	2731360	Heunggongvirae
0.01	1	0	P	2731618	Uroviricota
0.01	1	0	C	2731619	Caudoviricetes
0.01	1	0	O	28883	Caudovirales
0.01	1	0	F	10699	Siphoviridae
0.01	1	0	G	1982251	Pahexavirus
0.01	1	0	S	1982277	Propionibacterium virus PHL060L00
0.01	1	1	S1	1235647	Propionibacterium phage PHL060L00

Table S5-4. NC3_taxa

16.8	9381	9381	U	0	unclassified
83.2	46451	0	R	1	root
82.04	45804	13	R1	131567	cellular organisms
75.39	42092	273	D	2	Bacteria
71.67	40013	830	P	1224	Proteobacteria
68.6	38302	8	C	28216	Betaproteobacteria
68.58	38290	1000	O	80840	Burkholderiales
66.65	37212	927	F	80864	Comamonadaceae
64.2	35844	23321	G	80865	Delftia
19.75	11025	11025	S	558537	Delftia lacustris
1.33	742	742	S	80866	Delftia acidovorans
1.01	564	564	S	180282	Delftia tsuruhatensis
0.34	192	25	G1	2613839	unclassified Delftia
0.22	123	123	S	742013	Delftia sp. Cs1-4
0.08	44	44	S	1920191	Delftia sp. HK171
0.28	154	24	G	12916	Acidovorax
0.22	122	5	G1	2684926	unclassified Acidovorax
0.18	102	102	S	358220	Acidovorax sp. KKS102
0.02	9	9	S	232721	Acidovorax sp. JS42
0.01	5	5	S	2518343	Acidovorax sp. JMULE5
0	1	1	S	1842533	Acidovorax sp. RAC01
0.01	8	8	S	553814	Acidovorax carolinensis
0.22	125	0	G	201096	Alicycliphilus
0.22	125	1	S	179636	Alicycliphilus denitrificans
0.22	124	124	S1	596154	Alicycliphilus denitrificans K601
0.09	48	2	G	34072	Variovorax
0.08	46	1	G1	663243	unclassified Variovorax
0.05	27	27	S	1034889	Variovorax sp. HW608
0.02	10	10	S	662548	Variovorax sp. RA8
0.01	8	8	S	207745	Variovorax sp. WDL1
0.09	48	0	G	238749	Diaphorobacter
0.09	48	0	G1	2649760	unclassified Diaphorobacter
0.06	34	34	S	2714924	Diaphorobacter sp. HDW4A
0.03	14	14	S	2792224	Diaphorobacter sp. JS3051
0.08	45	0	G	283	Comamonas
0.05	26	26	S	160825	Comamonas koreensis
0.01	6	0	S	285	Comamonas testosteroni
0.01	6	6	S1	1392005	Comamonas testosteroni TK102
0.01	4	4	S	225992	Comamonas kerstersii
0.01	4	0	G1	2638500	unclassified Comamonas
0.01	4	4	S	1232667	Comamonas sp. 7D-2
0.01	3	3	S	1082851	Comamonas serinivorans
0	1	1	S	225991	Comamonas aquatica
0	1	1	S	363952	Comamonas thiooxydans
0.02	12	0	G	47420	Hydrogenophaga
0.02	12	0	G1	2610897	unclassified Hydrogenophaga
0.02	12	12	S	795665	Hydrogenophaga sp. PBC
0	2	0	G	28065	Rhodoferax
0	1	0	S	192843	Rhodoferax ferrireducens
0	1	1	S1	338969	Rhodoferax ferrireducens T118
0	1	0	G1	2627954	unclassified Rhodoferax
0	1	1	S	2741720	Rhodoferax sp. BAB1
0	2	0	G	52972	Polaromonas
0	2	2	G1	2638319	unclassified Polaromonas
0	2	0	G	215579	Schlegelella
0	2	2	S	215580	Schlegelella thermodepolymerans
0	2	0	G	1649468	Melaminivora
0	2	0	G1	2641902	unclassified Melaminivora
0	2	2	S	2109913	Melaminivora sp. SC2-9
0	1	0	G	2678886	Pulveribacter
0	1	1	S	2116657	Pulveribacter suum
0.09	50	0	F	119060	Burkholderiaceae
0.06	31	0	G	106589	Cupriavidus
0.03	17	17	S	119219	Cupriavidus metallidurans
0.02	13	13	S	1796606	Cupriavidus nantongensis
0	1	1	S	68895	Cupriavidus basilensis
0.02	12	0	G	48736	Ralstonia
0.02	11	11	S	305	Ralstonia solanacearum
0	1	0	S	329	Ralstonia pickettii
0	1	1	S1	428406	Ralstonia pickettii 12D
0.01	5	0	G	32008	Burkholderia
0.01	5	5	G1	87882	Burkholderia cepacia complex
0	2	0	G	93217	Pandoraea
0	1	1	S	93220	Pandoraea pnomenusa
0	1	1	S	573737	Pandoraea oxalativorans
0.02	13	0	F	75682	Oxalobacteraceae
0.02	12	0	G	75654	Duganella
0.02	12	0	G1	2636909	unclassified Duganella
0.02	12	12	S	2728020	Duganella sp. GN2-R2
0	1	0	G	1344552	Noviherbaspirillum
0	1	0	G1	2617509	unclassified Noviherbaspirillum
0	1	1	S	2601898	Noviherbaspirillum sp. UKPF54
0.02	9	0	F	506	Alcaligenaceae
0.01	5	0	G	517	Bordetella
0.01	5	5	S	94624	Bordetella petrii
0	2	1	G	222	Achromobacter
0	1	1	S	85698	Achromobacter xylosoxidans
0	2	0	G	507	Alcaligenes
0	2	2	S	511	Alcaligenes faecalis
0.01	4	0	O1	224471	Burkholderiales genera incertae sedis
0.01	4	0	G	88	Leptothrix
0.01	4	0	S	34029	Leptothrix cholodnii
0.01	4	4	S1	395495	Leptothrix cholodnii SP-6
0	2	0	F	995019	Sutterellaceae
0	2	0	G	40544	Sutterella
0	2	2	S	40545	Sutterella wadsworthensis
0	2	0	O	32003	Nitrosomonadales
0	2	0	F	2008793	Sterolibacteriaceae
0	2	0	G	311181	Denitratisoma
0	2	2	S	311182	Denitratisoma oestradiolicum
0	2	0	O	206389	Rhodocyclales
0	2	0	F	2008794	Zoogloeaceae
0	2	0	G	12960	Azoarcus

0	2	2	G1	2629479	unclassified Azoarcus
1.39	775	16	C	1236	Gammaproteobacteria
0.81	453	0	O	135614	Xanthomonadales
0.81	453	0	F	32033	Xanthomonadaceae
0.8	449	71	G	40323	Stenotrophomonas
0.68	377	0	G1	995085	Stenotrophomonas maltophilia group
0.68	377	374	S	40324	Stenotrophomonas maltophilia
0	2	2	S1	868597	Stenotrophomonas maltophilia JV3
0	1	1	S1	391008	Stenotrophomonas maltophilia R551-3
0	1	0	G1	196198	unclassified Stenotrophomonas
0	1	1	S	2282124	Stenotrophomonas sp. ASS1
0.01	4	0	G	338	Xanthomonas
0.01	3	3	S	339	Xanthomonas campestris
0	1	0	G1	643453	Xanthomonas citri group
0	1	0	S	346	Xanthomonas citri
0	1	1	S1	473421	Xanthomonas citri pv. glycines
0.53	297	0	O	72274	Pseudomonadales
0.52	290	0	F	135621	Pseudomonadaceae
0.52	290	2	G	286	Pseudomonas
0.51	284	0	G1	136841	Pseudomonas aeruginosa group
0.51	283	283	S	287	Pseudomonas aeruginosa
0	1	0	G2	1232139	Pseudomonas oleovorans/pseudoalcaligenes group
0	1	1	S	301	Pseudomonas oleovorans
0	2	0	G1	136842	Pseudomonas chlororaphis group
0	2	2	S	587753	Pseudomonas chlororaphis
0	1	0	G1	136849	Pseudomonas syringae group
0	1	1	G2	251698	Pseudomonas syringae group genomosp. 2
0	1	0	G1	136846	Pseudomonas stutzeri group
0	1	0	G2	578833	Pseudomonas stutzeri subgroup
0	1	1	S	316	Pseudomonas stutzeri
0.01	7	0	F	468	Moraxellaceae
0.01	7	0	G	469	Acinetobacter
0.01	7	7	S	40214	Acinetobacter johnsonii
0.01	5	0	O	91347	Enterobacterales
0.01	5	2	F	543	Enterobacteriaceae
0	1	0	G	547	Enterobacter
0	1	0	G1	354276	Enterobacter cloacae complex
0	1	1	S	208224	Enterobacter kobei
0	1	0	G	561	Escherichia
0	1	1	S	562	Escherichia coli
0	1	1	G	570	Klebsiella
0.01	4	0	O	135625	Pasteurellales
0.01	4	0	F	712	Pasteurellaceae
0.01	4	0	G	2094023	Glaesserella
0.01	4	4	S	738	Glaesserella parasuis
0.15	85	0	C	28211	Alphaproteobacteria
0.13	70	0	O	356	Hyphomicrobiales
0.09	53	0	F	69277	Phyllobacteriaceae
0.08	46	0	G	31988	Aminobacter
0.08	46	0	G1	2644704	unclassified Aminobacter
0.08	46	46	S	2774562	Aminobacter sp. SR38
0.01	7	0	G	28100	Phyllobacterium
0.01	5	5	S	1867719	Phyllobacterium zundukense
0	2	0	G1	2638441	unclassified Phyllobacterium
0	2	2	S	2718938	Phyllobacterium sp. 628
0.02	11	0	F	82115	Rhizobiaceae
0.02	11	0	F1	227290	Rhizobium/Agrobacterium group
0.01	7	0	G	1525371	Neorhizobium
0.01	7	0	S	399	Neorhizobium galegae
0.01	7	0	S1	323655	Neorhizobium galegae bv. orientalis
0.01	7	7	S2	1028800	Neorhizobium galegae bv. orientalis str. HAMBI 540
0.01	4	0	G	357	Agrobacterium
0	2	2	S	28099	Agrobacterium rubi
0	2	0	G1	1183400	Agrobacterium tumefaciens complex
0	2	2	S	358	Agrobacterium tumefaciens
0.01	5	2	F	119045	Methylobacteriaceae
0	2	0	G	2282523	Methylorubrum
0	1	0	S	408	Methylorubrum extorquens
0	1	1	S1	272630	Methylorubrum extorquens AM1
0	1	1	S	223967	Methylorubrum populi
0	1	1	G	407	Methylobacterium
0	1	0	F	41294	Bradyrhizobiaceae
0	1	0	G	374	Bradyrhizobium
0	1	0	G1	2631580	unclassified Bradyrhizobium
0	1	1	S	2493093	Bradyrhizobium sp. LCT2
0.03	14	0	O	204457	Sphingomonadales
0.03	14	0	F	41297	Sphingomonadaceae
0.01	7	0	G	13687	Sphingomonas
0.01	5	5	S	13689	Sphingomonas paucimobilis
0	2	0	G1	196159	unclassified Sphingomonas
0	1	1	S	2698679	Sphingomonas sp. C33
0	1	1	S	304378	Sphingomonas sp. IC081
0.01	4	3	G	165695	Sphingobium
0	1	0	G1	2611147	unclassified Sphingobium
0	1	1	S	2082188	Sphingobium sp. YG1
0.01	3	2	G	165697	Sphingopyxis
0	1	0	S	117207	Sphingopyxis alaskensis
0	1	1	S1	317655	Sphingopyxis alaskensis RB2256
0	1	0	O	204458	Caulobacterales
0	1	0	F	76892	Caulobacteraceae
0	1	1	G	41275	Brevundimonas
0.04	21	0	P1	68525	delta/epsilon subdivisions
0.03	18	0	C	29547	Epsilonproteobacteria
0.03	18	0	O	213849	Campylobacterales
0.03	18	0	F	72294	Campylobacteraceae
0.03	18	7	G	194	Campylobacter
0.02	11	11	S	195	Campylobacter coli
0.01	3	0	C	28221	Deltaproteobacteria
0	2	0	O	69541	Desulfuromonadales
0	2	0	F	2812024	Syntrophotaleaceae
0	2	0	G	2812025	Syntrophotalea
0	2	2	S	1842532	Syntrophotalea acetylenivorans
0	1	0	O	29	Myxococcales
0	1	0	O1	80811	Cystobacterineae

0	1	0	F	31	Myxococcaceae
0	1	0	G	83461	Corallococcus
0	1	1	S	184914	Corallococcus coralloides
2.34	1304	0	D1	1783270	FCB group
2.34	1304	0	D2	68336	Bacteroidetes/Chlorobi group
2.34	1304	3	P	976	Bacteroidetes
2.31	1288	0	C	200643	Bacteroidia
2.31	1288	318	O	171549	Bacteroidales
0.77	429	0	F	815	Bacteroidaceae
0.77	429	80	G	816	Bacteroides
0.19	107	107	S	820	Bacteroides uniformis
0.11	64	20	G1	2646097	unclassified Bacteroides
0.04	24	24	S	2785531	Bacteroides sp. HF-162
0.01	7	7	S	2528203	Bacteroides sp. A1C1
0.01	5	5	S	2650157	Bacteroides sp. HF-5287
0.01	5	5	S	2755405	Bacteroides sp. CACC 737
0	1	1	S	2650158	Bacteroides sp. HF-5141
0	1	1	S	2715212	Bacteroides sp. CBA7301
0	1	1	S	2763022	Bacteroides sp. M10
0.07	40	40	S	28116	Bacteroides ovatus
0.07	38	38	S	28111	Bacteroides eggerthii
0.06	35	35	S	246787	Bacteroides cellulosilyticus
0.05	27	27	S	818	Bacteroides thetaiotaomicron
0.02	10	8	S	371601	Bacteroides xylanisolvens
0	2	2	S1	657309	Bacteroides xylanisolvens XB1A
0.02	9	9	S	47678	Bacteroides caccae
0.01	7	6	S	817	Bacteroides fragilis
0	1	1	S1	295405	Bacteroides fragilis YCH46
0.01	7	7	S	329854	Bacteroides intestinalis
0	2	0	S	290053	Bacteroides helcogenes
0	2	2	S1	693979	Bacteroides helcogenes P 36-108
0	2	2	S	1796613	Bacteroides caecimuris
0	1	1	S	28113	Bacteroides heparinolyticus
0.59	328	0	O1	333046	Bacteroidales incertae sedis
0.59	328	35	G	909656	Phocaeicola
0.28	154	25	S	357276	Phocaeicola dorei
0.23	129	129	S1	997877	Bacteroides dorei CL03T12C01
0.25	139	139	S	821	Phocaeicola vulgatus
0.2	111	1	F	1853231	Odoribacteraceae
0.14	79	2	G	574697	Butyricimonas
0.13	74	74	S	544645	Butyricimonas virosa
0.01	3	3	S	2093856	Butyricimonas faecalis
0.06	31	0	G	283168	Odoribacter
0.06	31	31	S	28118	Odoribacter splanchnicus
0.09	53	0	F	171550	Rikenellaceae
0.09	53	1	G	239759	Alistipes
0.03	17	0	S	328813	Alistipes onderdonkii
0.03	17	17	S1	2585117	Alistipes onderdonkii subsp. vulgaris
0.03	16	0	S	328814	Alistipes shahii
0.03	16	16	S1	717959	Alistipes shahii WAL 8301
0.02	9	0	S	214856	Alistipes finegoldii
0.02	9	9	S1	679935	Alistipes finegoldii DSM 17242
0.01	8	8	S	2585118	Alistipes communis
0	2	2	S	2585119	Alistipes dispar
0.07	39	0	F	171552	Prevotellaceae
0.07	39	0	G	838	Prevotella
0.07	38	38	S	165179	Prevotella copri
0	1	1	S	28132	Prevotella melaninogenica
0.01	7	0	F	2005525	Tannerellaceae
0.01	7	3	G	375288	Parabacteroides
0	2	2	S	823	Parabacteroides distasonis
0	2	2	S	328812	Parabacteroides goldsteinii
0	2	0	F	2005519	Barnesiellaceae
0	2	0	G	397864	Barnesiella
0	2	0	S	397865	Barnesiella viscericola
0	2	2	S1	880074	Barnesiella viscericola DSM 18177
0	1	0	F	171551	Porphyromonadaceae
0	1	0	G	836	Porphyromonas
0	1	1	S	837	Porphyromonas gingivalis
0.01	8	0	C	768503	Cytophagia
0.01	8	5	O	768507	Cytophagales
0.01	3	0	F	1853232	Hymenobacteraceae
0.01	3	0	G	89966	Hymenobacter
0.01	3	0	G1	2615202	unclassified Hymenobacter
0.01	3	3	S	2584940	Hymenobacter sp. DG01
0.01	5	0	C	117743	Flavobacteriia
0.01	5	0	O	200644	Flavobacteriales
0.01	4	0	F	2762318	Weeksellaceae
0.01	4	0	F1	2782232	Chryseobacterium group
0.01	4	0	G	59732	Chryseobacterium
0.01	4	0	G1	2593645	unclassified Chryseobacterium
0.01	4	4	S	2487065	Chryseobacterium sp. G0201
0	1	0	F	49546	Flavobacteriaceae
0	1	1	G	237	Flavobacterium
0.9	502	1	D1	1783272	Terrabacteria group
0.59	329	3	P	1239	Firmicutes
0.29	162	0	C	186801	Clostridia
0.29	161	12	O	186802	Eubacteriales
0.18	101	3	F	216572	Oscillospiraceae
0.16	90	0	G	216851	Faecalibacterium
0.16	90	51	S	853	Faecalibacterium prausnitzii
0.04	21	21	S1	657322	Faecalibacterium prausnitzii SL3/3
0.03	18	18	S1	718252	Faecalibacterium prausnitzii L2-6
0.01	6	1	G	1263	Ruminococcus
0.01	5	5	S	1160721	Ruminococcus bicirculans
0	1	0	G	459786	Oscillibacter
0	1	0	G1	2629304	unclassified Oscillibacter
0	1	1	S	2109687	Oscillibacter sp. PEA192
0	1	0	G	1905344	Ruthenibacterium
0	1	1	S	1550024	Ruthenibacterium lactatiformans
0.04	21	3	F	186803	Lachnospiraceae
0.01	7	0	G	841	Roseburia
0.01	5	5	S	166486	Roseburia intestinalis
0	2	2	S	301301	Roseburia hominis

0	2	0	G	33042	Coprococcus
0	1	1	S	116085	Coprococcus catus
0	1	0	G1	2684943	unclassified Coprococcus
0	1	1	S	751585	Coprococcus sp. ART55/1
0	2	0	G	207244	Anaerostipes
0	2	2	S	649756	Anaerostipes hadrus
0	2	0	G	2719231	Lacrimispora
0	2	0	S	84030	Lacrimispora saccharolytica
0	2	2	S1	717608	[Clostridium] cf. saccharolyticum K10
0	1	0	G	830	Butyrivibrio
0	1	1	S	831	Butyrivibrio fibrisolvens
0	1	0	G	28050	Lachnospira
0	1	0	S	39485	Lachnospira eligens
0	1	1	S1	515620	[Eubacterium] eligens ATCC 27750
0	1	0	G	1506553	Lachnoclostridium
0	1	1	S	29347	[Clostridium] scindens
0	1	0	G	1843210	Anaerocolumna
0	1	1	S	433286	Anaerocolumna cellulosilytica
0	1	0	G	2316020	Mediterraneibacter
0	1	1	S	33038	[Ruminococcus] gnavus
0.03	18	3	F	186804	Peptostreptococcaceae
0.03	14	1	G	1501226	Romboutsia
0.02	12	12	S	1115758	Romboutsia ilealis
0	1	1	S	1507512	Romboutsia hominis
0	1	0	G	2743582	Peptacetobacter
0	1	1	S	89152	Peptacetobacter hiranonis
0.01	5	0	F	31979	Clostridiaceae
0.01	4	1	G	1485	Clostridium
0	1	0	G1	2614128	unclassified Clostridium
0	1	1	S	641107	Clostridium sp. DL-VIII
0	1	1	S	1504	Clostridium septicum
0	1	1	S	1502	Clostridium perfringens
0	1	0	G	1649459	Hungatella
0	1	0	S	154046	Hungatella hathewayi
0	1	1	S1	742737	Hungatella hathewayi WAL-18680
0	2	0	O1	538999	Eubacteriales incertae sedis
0	2	0	G	2039302	Monoglobus
0	2	2	S	1981510	Monoglobus pectinilyticus
0	2	0	F	990719	Christensenellaceae
0	2	0	G	990721	Christensenella
0	1	1	S	626937	Christensenella minuta
0	1	0	G1	2649046	unclassified Christensenella
0	1	1	S	2086585	Christensenella sp. Marseille-P3954
0	1	0	O	485256	Natranaerobiales
0	1	0	F	485255	Natranaerobiaceae
0	1	0	G	375928	Natranaerobius
0	1	0	S	375929	Natranaerobius thermophilus
0	1	1	S1	457570	Natranaerobius thermophilus JW/NM-WN-LF
0.17	95	0	C	526524	Erysipelotrichia
0.17	95	0	O	526525	Erysipelotrichales
0.17	93	0	F	2810281	Turicibacteraceae
0.17	93	0	G	191303	Turicibacter
0.12	65	65	S	154288	Turicibacter sanguinis
0.05	28	0	G1	2638206	unclassified Turicibacter
0.05	28	28	S	1712675	Turicibacter sp. H121
0	2	0	F	128827	Erysipelotrichaceae
0	1	0	G	1918538	Longibaculum
0	1	0	G1	2636055	unclassified Longibaculum
0	1	1	S	2584943	Longibaculum sp. KGMB06250
0	1	0	G	2678885	Faecalibacillus
0	1	1	S	1982626	Faecalibacillus intestinalis
0.11	63	0	C	91061	Bacilli
0.09	53	0	O	1385	Bacillales
0.09	50	0	F	90964	Staphylococcaceae
0.09	50	3	G	1279	Staphylococcus
0.07	40	40	S	1282	Staphylococcus epidermidis
0.01	3	3	S	1290	Staphylococcus hominis
0	2	2	S	29388	Staphylococcus capitis
0	2	2	S	1283	Staphylococcus haemolyticus
0	2	0	F	186817	Bacillaceae
0	2	0	G	1386	Bacillus
0	2	0	G1	86661	Bacillus cereus group
0	2	2	S	64104	Bacillus pseudomycoides
0	1	0	O1	539002	Bacillales incertae sedis
0	1	0	O2	539738	Bacillales Family XI. Incertae Sedis
0	1	0	G	1378	Gemella
0	1	1	S	1379	Gemella haemolysans
0.02	10	1	O	186826	Lactobacillales
0.01	7	0	F	1300	Streptococcaceae
0.01	7	2	G	1301	Streptococcus
0	2	0	G1	2608887	unclassified Streptococcus
0	2	2	S	1759399	Streptococcus sp. A12
0	1	0	S	257758	Streptococcus pseudopneumoniae
0	1	1	S1	1054460	Streptococcus pseudopneumoniae IS7493
0	1	1	S	1304	Streptococcus salivarius
0	1	1	S	1343	Streptococcus vestibularis
0	2	0	F	81852	Enterococcaceae
0	2	0	G	1350	Enterococcus
0	2	2	S	1351	Enterococcus faecalis
0.01	6	0	C	1737404	Tissierellia
0.01	6	0	O	1737405	Tissierellales
0.01	6	0	F	1570339	Peptoniphilaceae
0.01	6	0	G	165779	Anaerococcus
0.01	6	6	S	1870984	Anaerococcus mediterraneensis
0.31	171	0	P	201174	Actinobacteria
0.29	164	0	C	1760	Actinomycetia
0.24	136	0	O	85009	Propionibacteriales
0.24	133	3	F	31957	Propionibacteriaceae
0.23	130	3	G	1912216	Cutibacterium
0.22	121	121	S	1747	Cutibacterium acnes
0.01	5	5	S	33011	Cutibacterium granulosum
0	1	1	S	33010	Cutibacterium avidum
0.01	3	0	F	85015	Nocardioidaceae
0.01	3	0	G	1839	Nocardioides

0.01	3	0	G1	2615069	unclassified Nocardioides
0	2	2	S	2763008	Nocardioides sp. zg-1228
0	1	1	S	2017486	Nocardioides sp. S5
0.03	14	0	O	85006	Micrococcales
0.01	6	0	F	1268	Micrococcaceae
0.01	6	0	G	32207	Rothia
0.01	5	5	S	172042	Rothia aeria
0	1	1	S	2047	Rothia dentocariosa
0.01	5	0	F	85020	Dermabacteraceae
0.01	5	0	G	36739	Dermabacter
0.01	5	5	S	1630135	Dermabacter vaginalis
0.01	3	2	F	85023	Microbacteriaceae
0	1	0	G	33882	Microbacterium
0	1	1	S	104336	Microbacterium foliorum
0.02	9	0	O	85007	Corynebacteriales
0.02	9	0	F	1653	Corynebacteriaceae
0.02	9	0	G	1716	Corynebacterium
0.01	5	5	S	38304	Corynebacterium tuberculostearicum
0	2	2	S	161879	Corynebacterium kroppenstedtii
0	1	0	G1	2624378	unclassified Corynebacterium
0	1	1	S	2778078	Corynebacterium sp. FDAARGOS 1242
0	1	1	S	161899	Corynebacterium singulare
0.01	5	0	O	85004	Bifidobacteriales
0.01	5	0	F	31953	Bifidobacteriaceae
0.01	4	0	G	1678	Bifidobacterium
0	2	2	S	1681	Bifidobacterium bifidum
0	1	1	S	1680	Bifidobacterium adolescentis
0	1	1	S	1685	Bifidobacterium breve
0	1	0	G	2701	Gardnerella
0	1	1	S	2702	Gardnerella vaginalis
0.01	7	0	C	84998	Coriobacteriia
0.01	6	0	O	84999	Coriobacteriales
0.01	6	0	F	84107	Coriobacteriaceae
0.01	6	0	G	102106	Collinsella
0.01	6	6	S	74426	Collinsella aerofaciens
0	1	0	O	1643822	Eggerthellales
0	1	0	F	1643826	Eggerthellaceae
0	1	0	G	644652	Gordonibacter
0	1	0	S	471189	Gordonibacter pamelaeae
0	1	1	S1	657308	Gordonibacter pamelaeae 7-10-1-b
0	1	0	P	544448	Tenericutes
0	1	0	C	31969	Mollicutes
0	1	0	O	186328	Entomoplasmatales
0	1	0	F	2131	Spiroplasmataceae
0	1	1	G	2132	Spiroplasma
6.63	3699	2	D	2759	Eukaryota
6.59	3680	0	D1	33154	Opisthokonta
6.55	3655	0	K	4751	Fungi
6.55	3655	0	K1	451864	Dikarya
6.54	3654	0	P	5204	Basidiomycota
6.54	3654	0	P1	452284	Ustilaginomycotina
6.54	3654	0	C	1538075	Malasseziomycetes
6.54	3654	0	O	162474	Malasseziales
6.54	3654	0	F	742845	Malasseziaceae
6.54	3654	0	G	55193	Malassezia
6.54	3654	3654	S	76775	Malassezia restricta
0	1	0	P	4890	Ascomycota
0	1	0	P1	716545	saccharomyceta
0	1	0	P2	147538	Pezizomycotina
0	1	0	P3	716546	leotiomyceta
0	1	0	P4	715962	dothideomyceta
0	1	0	C	147541	Dothideomycetes
0	1	0	C1	451867	Dothideomycetidae
0	1	0	O	2726947	Mycosphaerellales
0	1	0	F	93133	Mycosphaerellaceae
0	1	0	G	1047167	Zymoseptoria
0	1	0	S	1047171	Zymoseptoria tritici
0	1	1	S1	336722	Zymoseptoria tritici IPO323
0.04	25	0	K	33208	Metazoa
0.04	25	0	K1	6072	Eumetazoa
0.04	25	0	K2	33213	Bilateria
0.04	25	0	K3	33511	Deuterostomia
0.04	25	0	P	7711	Chordata
0.04	25	0	P1	89593	Craniata
0.04	25	0	P2	7742	Vertebrata
0.04	25	0	P3	7776	Gnathostomata
0.04	25	0	P4	117570	Teleostomi
0.04	25	0	P5	117571	Euteleostomi
0.04	25	0	P6	8287	Sarcopterygii
0.04	25	0	C	1338369	Dipnotetrapodomorpha
0.04	25	0	C1	32523	Tetrapoda
0.04	25	0	C2	32524	Amniota
0.04	25	0	C	40674	Mammalia
0.04	25	0	C1	32525	Theria
0.04	25	0	C2	9347	Eutheria
0.04	25	0	C3	1437010	Boreoeutheria
0.04	25	0	C4	314146	Euarchontoglires
0.04	25	0	O	9443	Primates
0.04	25	0	O1	376913	Haplorrhini
0.04	25	0	O2	314293	Simiiformes
0.04	25	0	O3	9526	Catarrhini
0.04	25	0	O4	314295	Hominoidea
0.04	25	0	F	9604	Hominidae
0.04	25	0	F1	207598	Homininae
0.04	25	0	G	9605	Homo
0.04	25	25	S	9606	Homo sapiens
0.03	17	0	K	33090	Viridiplantae
0.03	17	0	P	35493	Streptophyta
0.03	17	0	P1	131221	Streptophytina
0.03	17	0	P2	3193	Embryophyta
0.03	17	0	P3	58023	Tracheophyta
0.03	17	0	P4	78536	Euphyllophyta
0.03	17	0	P5	58024	Spermatophyta
0.03	17	0	C	3398	Magnoliopsida

0.03	17	0	C1	1437183	Mesangiospermae
0.01	8	0	C2	71240	eudicotyledons
0.01	8	0	C3	91827	Gunneridae
0.01	8	0	C4	1437201	Pentapetalae
0.01	5	0	C5	71275	rosids
0.01	4	0	C6	91835	fabids
0	1	0	O	3502	Fagales
0	1	0	F	3503	Fagaceae
0	1	0	G	3511	Quercus
0	1	1	S	97700	Quercus lobata
0	1	0	O	3744	Rosales
0	1	0	F	3745	Rosaceae
0	1	0	F1	171638	Rosoideae
0	1	0	F2	1176516	Rosoideae incertae sedis
0	1	0	G	3764	Rosa
0	1	1	S	74649	Rosa chinensis
0	1	0	O	72025	Fabales
0	1	0	F	3803	Fabaceae
0	1	0	F1	3814	Papilionoideae
0	1	0	F2	2231393	50 kb inversion clade
0	1	0	F3	2231382	NPAAA clade
0	1	0	F4	2233855	indigoferoid/millettioid clade
0	1	0	F5	163735	Phaseoleae
0	1	0	G	3820	Cajanus
0	1	1	S	3821	Cajanus cajan
0	1	0	O	233875	Celastrales
0	1	0	F	4305	Celastraceae
0	1	0	G	123484	Tripterygium
0	1	1	S	458696	Tripterygium wilfordii
0	1	0	C6	91834	rosids incertae sedis
0	1	0	O	403667	Vitales
0	1	0	F	3602	Vitaceae
0	1	0	F1	2304100	Viteae
0	1	1	G	3603	Vitis
0.01	3	0	C5	71274	asterids
0.01	3	0	C6	91888	lamiids
0.01	3	0	O	4069	Solanales
0.01	3	0	F	4070	Solanaceae
0.01	3	0	F1	424551	Solanoideae
0.01	3	0	F2	424564	Capsiceae
0.01	3	0	G	4071	Capsicum
0.01	3	3	S	4072	Capsicum annuum
0.01	5	0	C2	4447	Liliopsida
0.01	5	0	C3	1437197	Petrosaviidae
0.01	5	0	C4	4734	commelinids
0.01	4	0	O	38820	Poales
0.01	4	0	F	4479	Poaceae
0.01	3	0	F1	359160	BOP clade
0.01	3	0	F2	147368	Pooideae
0.01	3	0	F3	1648038	Triticodae
0.01	3	0	F4	147389	Triticeae
0.01	3	0	F5	1648030	Triticinae
0	2	0	G	4564	Triticum
0	2	2	S	85692	Triticum dicoccoides
0	1	0	G	4480	Aegilops
0	1	0	S	37682	Aegilops tauschii
0	1	1	S1	200361	Aegilops tauschii subsp. strangulata
0	1	0	F1	147370	PACMAD clade
0	1	0	F2	147369	Panicoideae
0	1	0	F3	1648033	Andropogonodae
0	1	0	F4	147429	Andropogoneae
0	1	0	F5	1648029	Tripsacinae
0	1	0	G	4575	Zea
0	1	1	S	4577	Zea mays
0	1	0	O	4618	Zingiberales
0	1	0	F	4637	Musaceae
0	1	0	G	4640	Musa
0	1	0	S	4641	Musa acuminata
0	1	1	S1	214687	Musa acuminata subsp. malaccensis
0.01	4	0	O	41768	Ranunculales
0.01	4	0	F	3465	Papaveraceae
0.01	4	0	F1	1462614	Papaveroideae
0.01	4	0	G	3468	Papaver
0.01	4	4	S	3469	Papaver somniferum
1.16	647	0	D	10239	Viruses
1.16	647	0	D1	2731341	Duplodnaviria
1.16	647	0	D2	2731360	Heunggongvirae
1.16	647	0	P	2731618	Uroviricota
1.16	647	0	C	2731619	Caudoviricetes
1.16	647	0	O	28883	Caudovirales
1.12	626	0	F	10744	Podoviridae
1.12	626	0	F1	196895	unclassified Podoviridae
1.12	626	0	F2	1978007	crAss-like viruses
1.12	626	0	F3	2315857	environmental samples
1.12	626	626	S	1211417	uncultured crAssphage
0.04	21	0	F	10699	Siphoviridae
0.02	10	1	G	186532	Ceduovirus
0.01	7	4	G1	329156	unclassified Ceduovirus
0	2	2	S	1862960	Lactococcus phage M5938
0	1	1	S	1862959	Lactococcus phage D4412
0	2	2	S	36343	Lactococcus virus bIL67
0.02	9	2	G	1623305	Skunavirus
0.01	7	3	G1	2050979	unclassified Skunavirus
0	2	2	S	2029660	Lactococcus phage 30804
0	1	1	S	39838	Lactococcus phage 936
0	1	1	S	2029671	Lactococcus phage 66901
0	1	1	G	1982251	Pahexavirus
0	1	1	G	1623304	Moineauvirus
