# Supplementary material for: Investigating the Human Intestinal DNA Virome and Predicting Disease-Associated Virus–Host Interactions in Severe Myalgic Encephalomyelitis/Chronic Fatigue Syndrome (ME/CFS)
Source: Int J Mol Sci. 2023 Dec 8;24(24):17267. doi: 10.3390/ijms242417267 (PMC10744171; doi:10.3390/ijms242417267)
Supplement: Supplementary file 1 [file ijms-24-17267-s001.zip › Table S6.pdf]

Table S6. Viral taxonomic annotations

Table S6-1. VLP_HQ_vOTUs_taxonomies

Scaffold	Closer	Accession	Status	VC	Level	Weight	Host	BaltimoreGroup	Realm	Kingdom	Phylum	Class	Order	Family	Subfamily	Genus
S10_vf_18671	Parabacteroides phage PDS1	MN929097	Clustered	VC_855_0	C1	169	Parabacteroides	Group I	Duplodnaviria	Heunggongvirae	Uroviricota	Caudoviricetes	n.a.	Unclassified	Unclassified	Unclassified
S10_vf_24791	Faecalibacterium phage FP_Epona	MG711462	Clustered	VC_723_0	C2	95	Faecalibacterium	Group I	Duplodnaviria	Heunggongvirae	Uroviricota	Caudoviricetes	n.a.	Unclassified	Unclassified	Eponavirus
S10_vf_25241	n.a.	n.a.	Clustered	VC_1558_0	F	n.a.	n.a.	n.a.	n.a.	n.a.	n.a.	n.a.	n.a.	n.a.	n.a.	n.a.
S10_vf_3644	uncultured phage cr109_1	MT774399	Clustered	VC_519_7	N2	277	Unspecified	Group I	Duplodnaviria	Heunggongvirae	Uroviricota	Caudoviricetes	Crassvirales	Suoliviridae	O	O
S10_vf_36880	uncultured phage cr127_1	MT774393	Clustered	VC_693_0	C1	105	Unspecified	Group I	Duplodnaviria	Heunggongvirae	Uroviricota	Caudoviricetes	Crassvirales	Crevaviridae	Doltivirinae	Kahucivirus
S10_vf_64371	n.a.	n.a.	Clustered	VC_1564_0	F	n.a.	n.a.	n.a.	n.a.	n.a.	n.a.	n.a.	n.a.	n.a.	n.a.	n.a.
S10_vf_80712	n.a.	n.a.	Clustered	VC_219_0	A	n.a.	n.a.	n.a.	n.a.	n.a.	n.a.	n.a.	n.a.	n.a.	n.a.	n.a.
S10_vs_120489	n.a.	n.a.	Clustered	VC_1566_0	A	n.a.	n.a.	n.a.	n.a.	n.a.	n.a.	n.a.	n.a.	n.a.	n.a.	n.a.
S10_vs_120541	n.a.	n.a.	Clustered	VC_1567_0	A	n.a.	n.a.	n.a.	n.a.	n.a.	n.a.	n.a.	n.a.	n.a.	n.a.	n.a.
S10_vs_14557	Flavobacterium phage vB_FspS_laban6-1	MN812211	Clustered	VC_1062_0	N5	6	Flavobacterium	Group I	Duplodnaviria	Heunggongvirae	Uroviricota	Caudoviricetes	n.a.	Duneviridae	O	O
S10_vs_18901	n.a.	n.a.	Clustered	VC_1291_0	A	n.a.	n.a.	n.a.	n.a.	n.a.	n.a.	n.a.	n.a.	n.a.	n.a.	n.a.
S10_vs_2538	Lactobacillus phage c5	EU340421	Clustered	VC_658_0	N10	6	Lactobacillus	Group I	Duplodnaviria	Heunggongvirae	Uroviricota	Caudoviricetes	n.a.	Unclassified	O	O
S10_vs_26365	Faecalibacterium phage FP_oengus	MG711463	Clustered	VC_1085_1	N20	67	Faecalibacterium	Group I	Duplodnaviria	Heunggongvirae	Uroviricota	Caudoviricetes	n.a.	Unclassified	O	O
S10_vs_35854	Bacteroides phage LoVEphage	MW660583	Clustered	VC_1527_0	N4	24	Bacteroides	Group I	Duplodnaviria	Heunggongvirae	Uroviricota	Caudoviricetes	n.a.	Unclassified	O	O
S10_vs_36407	n.a.	n.a.	Clustered	VC_1578_0	A	n.a.	n.a.	n.a.	n.a.	n.a.	n.a.	n.a.	n.a.	n.a.	n.a.	n.a.
S10_vs_36598	n.a.	n.a.	Clustered	VC_1579_0	A	n.a.	n.a.	n.a.	n.a.	n.a.	n.a.	n.a.	n.a.	n.a.	n.a.	n.a.
S10_vs_43473	Microviridae sp.	MG945488	Clustered	VC_1334_0	C1	19	Unspecified	Group II	Monodnaviria	Sangervirae	Phixviricota	Malgrandaviricetes	Petitvirales	Microviridae	Unclassified	Unclassified
S10_vs_58014	Bacteroides phage p00	BK010646	Clustered	VC_695_0	C2	93	Bacteroides	Group I	Duplodnaviria	Heunggongvirae	Uroviricota	Caudoviricetes	n.a.	Unclassified	Unclassified	Unclassified
S10_vs_67471	n.a.	n.a.	Clustered	VC_950_0	A	n.a.	n.a.	n.a.	n.a.	n.a.	n.a.	n.a.	n.a.	n.a.	n.a.	n.a.
S10_vs_71900	n.a.	n.a.	Clustered	VC_1576_0	A	n.a.	n.a.	n.a.	n.a.	n.a.	n.a.	n.a.	n.a.	n.a.	n.a.	n.a.
S11_vf_104035	uncultured phage cr53_1	MT774396	Clustered	VC_519_0	N2	233	Unspecified	Group I	Duplodnaviria	Heunggongvirae	Uroviricota	Caudoviricetes	Crassvirales	Suoliviridae	O	O
S11_vf_104044	uncultured phage cr85_1	MT774390	Clustered	VC_1054_0	C1	300	Unspecified	Group I	Duplodnaviria	Heunggongvirae	Uroviricota	Caudoviricetes	Crassvirales	Steigviridae	Asinivirinae	Kahnovirus
S11_vf_104048	Akkermansia phage DTMo-2021a	CP084202	Clustered	VC_750_0	N2	1	Akkermansia	Group I	Duplodnaviria	Heunggongvirae	Uroviricota	Caudoviricetes	n.a.	Unclassified	O	O
S11_vf_104052	Bacillus phage vB_BtS_BMBtp15	KX190835	Clustered	VC_759_0	N6	9	Bacillus	Group I	Duplodnaviria	Heunggongvirae	Uroviricota	Caudoviricetes	n.a.	Unclassified	O	O
S11_vf_104059	uncultured phage cr110_1	MT774386	Clustered	VC_691_0	C1	193	Unspecified	Group I	Duplodnaviria	Heunggongvirae	Uroviricota	Caudoviricetes	Crassvirales	Intestiviridae	Crudevirinae	Delmidovirus
S11_vf_104070	Clostridium phage phiMMP01	LN681541	Clustered	VC_150_0	N17	13	Clostridium	Group I	Duplodnaviria	Heunggongvirae	Uroviricota	Caudoviricetes	n.a.	Unclassified	O	O
S11_vf_104071	Croceibacter phage P2559Y	KC688701	Clustered	VC_1045_0	N2	16	Croceibacter	Group I	Duplodnaviria	Heunggongvirae	Uroviricota	Caudoviricetes	n.a.	Unclassified	O	O
S11_vf_10907	Staphylococcus phage PI-Sepi-HH2	MT880871	Clustered	VC_759_0	N11	3	Staphylococcus	Group I	Duplodnaviria	Heunggongvirae	Uroviricota	Caudoviricetes	n.a.	Unclassified	O	O
S11_vf_13033	Faecalibacterium phage FP_oengus	MG711463	Clustered	VC_1085_1	N20	65	Faecalibacterium	Group I	Duplodnaviria	Heunggongvirae	Uroviricota	Caudoviricetes	n.a.	Unclassified	O	O
S11_vf_27845	n.a.	n.a.	Clustered	VC_1564_0	F	n.a.	n.a.	n.a.	n.a.	n.a.	n.a.	n.a.	n.a.	n.a.	n.a.	n.a.
S11_vf_28515	Bacillus phage BUCT082	MZ969646	Clustered	VC_219_0	N9	5	Bacillus	Group I	Duplodnaviria	Heunggongvirae	Uroviricota	Caudoviricetes	n.a.	Unclassified	O	O
S11_vf_28645	n.a.	n.a.	Clustered	VC_1558_0	F	n.a.	n.a.	n.a.	n.a.	n.a.	n.a.	n.a.	n.a.	n.a.	n.a.	n.a.
S11_vf_34224	Clostridium phage phiMMP01	LN681541	Clustered	VC_150_0	N17	13	Clostridium	Group I	Duplodnaviria	Heunggongvirae	Uroviricota	Caudoviricetes	n.a.	Unclassified	O	O
S11_vf_43997	CrAssphage sp. O-152	MK069403	Clustered	VC_691_0	C1	131	Unspecified	Group I	Duplodnaviria	Heunggongvirae	Uroviricota	Caudoviricetes	Crassvirales	Unclassified	Unclassified	Unclassified
S11_vf_47632	Butyrivibrio phage Idris	MN882554	Clustered	VC_1289_0	N7	31	Butyrivibrio	Group I	Duplodnaviria	Heunggongvirae	Uroviricota	Caudoviricetes	n.a.	Unclassified	O	O
S11_vf_66160	Roseburia phage Shimadzu	LR596903	Clustered	VC_1256_0	N5	4	Roseburia	Group I	Duplodnaviria	Heunggongvirae	Uroviricota	Caudoviricetes	n.a.	Unclassified	O	O
S11_vf_66522	Actinomyces phage LC001	ON920540	Clustered	VC_1435_0	C1	131	Actinomyces	Group I	Duplodnaviria	Heunggongvirae	Uroviricota	Caudoviricetes	n.a.	Unclassified	Unclassified	Unclassified
S11_vf_68693	n.a.	n.a.	Clustered	VC_1559_0	A	n.a.	n.a.	n.a.	n.a.	n.a.	n.a.	n.a.	n.a.	n.a.	n.a.	n.a.
S11_vf_84303	n.a.	n.a.	Clustered	VC_1552_0	A	n.a.	n.a.	n.a.	n.a.	n.a.	n.a.	n.a.	n.a.	n.a.	n.a.	n.a.
S11_vf_92391	n.a.	n.a.	Clustered	VC_1585_0	A	n.a.	n.a.	n.a.	n.a.	n.a.	n.a.	n.a.	n.a.	n.a.	n.a.	n.a.
S11_vf_95067	n.a.	n.a.	Clustered	VC_1552_0	A	n.a.	n.a.	n.a.	n.a.	n.a.	n.a.	n.a.	n.a.	n.a.	n.a.	n.a.
S11_vs_10172	Faecalibacterium phage FP_Epona	MG711462	Clustered	VC_723_0	C13	55	Faecalibacterium	Group I	Duplodnaviria	Heunggongvirae	Uroviricota	Caudoviricetes	n.a.	Unclassified	Unclassified	Eponavirus
S11_vs_104032	Bdellovibrio phage phi1422	JQ177062	Clustered	VC_940_0	C1	26	Bdellovibrio	Group I	Duplodnaviria	Heunggongvirae	Uroviricota	Caudoviricetes	n.a.	Unclassified	Unclassified	Unclassified
S11_vs_104038	Bacillus phage vB_BtS_BMBtp13	KX190832	Clustered	VC_667_0	C4	10	Bacillus	Group I	Duplodnaviria	Heunggongvirae	Uroviricota	Caudoviricetes	n.a.	Unclassified	Unclassified	Unclassified
S11_vs_104041	Clostridioides phage CD1801	MW512570	Clustered	VC_669_0	N4	5	Clostridioides	Group I	Duplodnaviria	Heunggongvirae	Uroviricota	Caudoviricetes	n.a.	Unclassified	O	O
S11_vs_104047	Streptococcus phage D4446	MN938931	Clustered	VC_643_0	N8	9	Streptococcus	Group I	Duplodnaviria	Heunggongvirae	Uroviricota	Caudoviricetes	n.a.	Unclassified	O	O
S11_vs_104053	Paenibacillus phage PG1	HQ332138	Clustered	VC_946_0	N10	2	Paenibacillus	Group I	Duplodnaviria	Heunggongvirae	Uroviricota	Caudoviricetes	n.a.	Unclassified	O	O
S11_vs_104054	n.a.	n.a.	Clustered	VC_1567_0	A	n.a.	n.a.	n.a.	n.a.	n.a.	n.a.	n.a.	n.a.	n.a.	n.a.	n.a.
S11_vs_104064	Faecalibacterium phage FP_oengus	MG711463	Clustered	VC_1085_1	N19	69	Faecalibacterium	Group I	Duplodnaviria	Heunggongvirae	Uroviricota	Caudoviricetes	n.a.	Unclassified	O	O
S11_vs_11267	Geobacillus phage GBSV1	DQ340064	Clustered	VC_778_0	N24	8	Geobacillus	Group I	Duplodnaviria	Heunggongvirae	Uroviricota	Caudoviricetes	n.a.	Unclassified	O	O
S11_vs_11994	Faecalibacterium phage FP_Taranis	MG711467	Clustered	VC_732_0	N3	96	Faecalibacterium	Group I	Duplodnaviria	Heunggongvirae	Uroviricota	Caudoviricetes	n.a.	Unclassified	O	O
S11_vs_14882	n.a.	n.a.	Clustered	VC_1570_0	A	n.a.	n.a.	n.a.	n.a.	n.a.	n.a.	n.a.	n.a.	n.a.	n.a.	n.a.
S11_vs_1937	Phage vB_RanS_PJN03	ON245412	Clustered	VC_951_0	N13	6	Unspecified	Group I	Duplodnaviria	Heunggongvirae	Uroviricota	Caudoviricetes	n.a.	Unclassified	O	O
S11_vs_19892	Faecalibacterium phage FP_Epona	MG711462	Clustered	VC_723_0	C13	50	Faecalibacterium	Group I	Duplodnaviria	Heunggongvirae	Uroviricota	Caudoviricetes	n.a.	Unclassified	Unclassified	Eponavirus
S11_vs_23188	n.a.	n.a.	Clustered	VC_1559_0	A	n.a.	n.a.	n.a.	n.a.	n.a.	n.a.	n.a.	n.a.	n.a.	n.a.	n.a.
S11_vs_23357	Faecalibacterium phage FP_Epona	MG711462	Clustered	VC_723_0	C13	52	Faecalibacterium	Group I	Duplodnaviria	Heunggongvirae	Uroviricota	Caudoviricetes	n.a.	Unclassified	Unclassified	Eponavirus
S11_vs_26767	Streptococcus phage MismyG	OL774867	Clustered	VC_152_0	N13	9	Streptococcus	Group I	Duplodnaviria	Heunggongvirae	Uroviricota	Caudoviricetes	n.a.	Unclassified	O	O
S11_vs_30739	Faecalibacterium phage FP_Lagaffe	MG711461	Clustered	VC_1289_0	N24	3	Faecalibacterium	Group I	Duplodnaviria	Heunggongvirae	Uroviricota	Caudoviricetes	n.a.	Unclassified	O	O
S11_vs_3277	Bacteroides phage p00	BK010646	Clustered	VC_695_0	C4	43	Bacteroides	Group I	Duplodnaviria	Heunggongvirae	Uroviricota	Caudoviricetes	n.a.	Unclassified	Unclassified	Unclassified
S11_vs_34137	Geobacillus phage GBSV1	DQ340064	Clustered	VC_779_0	N25	6	Geobacillus	Group I	Duplodnaviria	Heunggongvirae	Uroviricota	Caudoviricetes	n.a.	Unclassified	O	O
S11_vs_37666	Clostridium phage HM2	LT600745	Clustered	VC_1258_0	N48	4	Clostridium	Group I	Duplodnaviria	Heunggongvirae	Uroviricota	Caudoviricetes	n.a.	Salasmaviridae	O	O
S11_vs_40945	Faecalibacterium phage FP_Epona	MG711462	Clustered	VC_723_0	C13	55	Faecalibacterium	Group I	Duplodnaviria	Heunggongvirae	Uroviricota	Caudoviricetes	n.a.	Unclassified	Unclassified	Eponavirus
S11_vs_41107	Clostridium phage phiMMP01	LN681541	Clustered	VC_151_0	N20	16	Clostridium	Group I	Duplodnaviria	Heunggongvirae	Uroviricota	Caudoviricetes	n.a.	Unclassified	O	O
S11_vs_42927	Streptococcus phage Javan630	MK448997	Clustered	VC_649_1	C1	104	Streptococcus	Group I	Duplodnaviria	Heunggongvirae	Uroviricota	Caudoviricetes	n.a.	Unclassified	Unclassified	Unclassified
S11_vs_5077	Escherichia phage ESSI2_ev015	LR597649	Clustered	VC_5_0	C1	115	Escherichia	Group I	Duplodnaviria	Heunggongvirae	Uroviricota	Caudoviricetes	n.a.	Peduoviridae	Unclassified	Quadragintavirus
S11_vs_52577	Faecalibacterium phage FP_Mushu	MG711460	Clustered	VC_1288_0	C1	145	Faecalibacterium	Group I	Duplodnaviria	Heunggongvirae	Uroviricota	Caudoviricetes	n.a.	Unclassified	Unclassified	Mushuvirus
S11_vs_58702	Clostridium phage phiMMP01	LN681541	Clustered	VC_151_0	N17	32	Clostridium	Group I	Duplodnaviria	Heunggongvirae	Uroviricota	Caudoviricetes	n.a.	Unclassified	O	O
S11_vs_58930	Bacteroides phage p00	BK010646	Clustered	VC_695_0	C3	46	Bacteroides	Group I	Duplodnaviria	Heunggongvirae	Uroviricota	Caudoviricetes	n.a.	Unclassified	Unclassified	Unclassified
S11_vs_62573	Roseburia phage Shimadzu	LR596903	Clustered	VC_1256_0	N5	2	Roseburia	Group I	Duplodnaviria	Heunggongvirae	Uroviricota	Caudoviricetes	n.a.	Unclassified	O	O
S11_vs_6458	n.a.	n.a.	Clustered	VC_1595_0	A	n.a.	n.a.	n.a.	n.a.	n.a.	n.a.	n.a.	n.a.	n.a.	n.a.	n.a.
S11_vs_68151	Faecalibacterium phage FP_oengus	MG711463	Clustered	VC_1085_1	N19	65	Faecalibacterium	Group I	Duplodnaviria	Heunggongvirae	Uroviricota	Caudoviricetes	n.a.	Unclassified	O	O
S11_vs_76784	Streptococcus phage Javan580	MK448989	Clustered	VC_558_0	N12	8	Streptococcus	Group I	Duplodnaviria	Heunggongvirae	Uroviricota	Caudoviricetes	n.a.	Unclassified	O	O
S11_vs_79275	n.a.	n.a.	Clustered	VC_1570_0	A	n.a.	n.a.	n.a.	n.a.	n.a.	n.a.	n.a.	n.a.	n.a.	n.a.	n.a.
S11_vs_81775	Bacillus phage vB_BtS_BMBtp13	KX190832	Clustered	VC_667_0	C4	10	Bacillus	Group I	Duplodnaviria	Heunggongvirae	Uroviricota	Caudoviricetes	n.a.	Unclassified	Unclassified	Unclassified
S11_vs_8456	Caldibacillus phage CBP1	MF595878	Clustered	VC_349_0	C2	5	Caldibacillus	Group I	Duplodnaviria	Heunggongvirae	Uroviricota	Caudoviricetes	n.a.	Unclassified	Unclassified	Unclassified
S11_vs_84627	n.a.	n.a.	Clustered	VC_1596_0	A	n.a.	n.a.	n.a.	n.a.	n.a.	n.a.	n.a.	n.a.	n.a.	n.a.	n.a.
S11_vs_84848	Bacteroides phage p00	BK010646	Clustered	VC_695_0	C1	73	Bacteroides	Group I	Duplodnaviria	Heunggongvirae	Uroviricota	Caudoviricetes	n.a.	Unclassified	Unclassified	Unclassified
S11_vs_93101	Bdellovibrio phage phi1402	JF344709	Clustered	VC_967_0	C4	37	Bdellovibrio	Group I	Duplodnaviria	Heunggongvirae	Uroviricota	Caudoviricetes	n.a.	Unclassified	Unclassified	Unclassified

S11_vs_98193	Bacteroides phage p00	BK010646	Clustered	VC_695_0	C6	22	Bacteroides	Group I	Duplodnaviria	Heunggongvirae	Uroviricota	Caudoviricetes	n.a.	Unclassified	Unclassified	Unclassified
S12_vf_10887	n.a.	n.a.	Clustered	VC_1597_0	F	n.a.	n.a.	n.a.	n.a.	n.a.	n.a.	n.a.	n.a.	n.a.	n.a.	n.a.
S12_vf_128794	n.a.	n.a.	Clustered	VC_1552_0	A	n.a.	n.a.	n.a.	n.a.	n.a.	n.a.	n.a.	n.a.	n.a.	n.a.	n.a.
S12_vf_169796	Bacillus phage vB_BcoS-136	MH884508	Clustered	VC_491_0	N22	2	Bacillus	Group I	Duplodnaviria	Heunggongvirae	Uroviricota	Caudoviricetes	n.a.	Unclassified	O	O
S12_vf_173208	n.a.	n.a.	Clustered	VC_1274_0	A	n.a.	n.a.	n.a.	n.a.	n.a.	n.a.	n.a.	n.a.	n.a.	n.a.	n.a.
S12_vf_174374	n.a.	n.a.	Clustered	VC_1576_0	A	n.a.	n.a.	n.a.	n.a.	n.a.	n.a.	n.a.	n.a.	n.a.	n.a.	n.a.
S12_vf_176437	Clostridium phage HM2	LT600745	Clustered	VC_1261_0	N43	4	Clostridium	Group I	Duplodnaviria	Heunggongvirae	Uroviricota	Caudoviricetes	n.a.	Salasmaviridae	O	O
S12_vf_181024	Bacillus phage BUCT082	MZ969646	Clustered	VC_1040_0	N19	1	Bacillus	Group I	Duplodnaviria	Heunggongvirae	Uroviricota	Caudoviricetes	n.a.	Unclassified	O	O
S12_vf_188822	n.a.	n.a.	Clustered	VC_1597_0	F	n.a.	n.a.	n.a.	n.a.	n.a.	n.a.	n.a.	n.a.	n.a.	n.a.	n.a.
S12_vf_188824	Phage DP SC_6_H4_2017	MT121964	Clustered	VC_1490_0	N7	121	Unspecified	Group I	Duplodnaviria	Heunggongvirae	Uroviricota	Caudoviricetes	n.a.	Unclassified	O	O
S12_vf_188830	n.a.	n.a.	Clustered	VC_1598_0	F	n.a.	n.a.	n.a.	n.a.	n.a.	n.a.	n.a.	n.a.	n.a.	n.a.	n.a.
S12_vf_188838	Inovirus sp.	MT135293	Clustered	VC_1495_0	C1	12	Unspecified	Group II	Monodnaviria	Loebvirae	Hofneiviricota	Faserviricetes	Tubulavirales	Inoviridae	Unclassified	Inovirus
S12_vf_48278	Myoviridae sp.	MW202455	Clustered	VC_439_0	N3	3	Unspecified	Group I	Duplodnaviria	Heunggongvirae	Uroviricota	Caudoviricetes	n.a.	Unclassified	O	O
S12_vf_55175	Faecalibacterium phage FP_Lagaffe	MG711461	Clustered	VC_1290_0	N30	3	Faecalibacterium	Group I	Duplodnaviria	Heunggongvirae	Uroviricota	Caudoviricetes	n.a.	Unclassified	O	O
S12_vf_60224	Phage vB_RanS_PJN03	ON245412	Clustered	VC_950_0	N14	7	Unspecified	Group I	Duplodnaviria	Heunggongvirae	Uroviricota	Caudoviricetes	n.a.	Unclassified	O	O
S12_vf_69136	Phage vB_RanS_PJN03	ON245412	Clustered	VC_950_0	N14	4	Unspecified	Group I	Duplodnaviria	Heunggongvirae	Uroviricota	Caudoviricetes	n.a.	Unclassified	O	O
S12_vf_79666	n.a.	n.a.	Clustered	VC_1552_0	A	n.a.	n.a.	n.a.	n.a.	n.a.	n.a.	n.a.	n.a.	n.a.	n.a.	n.a.
S12_vs_106329	Geobacillus phage GBSV1	DQ340064	Clustered	VC_779_0	N44	1	Geobacillus	Group I	Duplodnaviria	Heunggongvirae	Uroviricota	Caudoviricetes	n.a.	Unclassified	O	O
S12_vs_111815	n.a.	n.a.	Clustered	VC_1553_0	A	n.a.	n.a.	n.a.	n.a.	n.a.	n.a.	n.a.	n.a.	n.a.	n.a.	n.a.
S12_vs_121913	n.a.	n.a.	Clustered	VC_1582_0	A	n.a.	n.a.	n.a.	n.a.	n.a.	n.a.	n.a.	n.a.	n.a.	n.a.	n.a.
S12_vs_137069	n.a.	n.a.	Clustered	VC_1601_0	F	n.a.	n.a.	n.a.	n.a.	n.a.	n.a.	n.a.	n.a.	n.a.	n.a.	n.a.
S12_vs_138261	n.a.	n.a.	Clustered	VC_1583_0	A	n.a.	n.a.	n.a.	n.a.	n.a.	n.a.	n.a.	n.a.	n.a.	n.a.	n.a.
S12_vs_141337	Bacteroides phage p00	BK010646	Clustered	VC_695_0	C1	127	Bacteroides	Group I	Duplodnaviria	Heunggongvirae	Uroviricota	Caudoviricetes	n.a.	Unclassified	Unclassified	Unclassified
S12_vs_14927	uncultured phage cr3_1	MT774381	Clustered	VC_691_0	C1	165	Unspecified	Group I	Duplodnaviria	Heunggongvirae	Uroviricota	Caudoviricetes	Crassvirales	Intestiviridae	Crudevirinae	Diorhovirus
S12_vs_150812	Clostridium phage phiMMP01	LN681541	Clustered	VC_151_0	N18	25	Clostridium	Group I	Duplodnaviria	Heunggongvirae	Uroviricota	Caudoviricetes	n.a.	Unclassified	O	O
S12_vs_151292	Bacteroides phage LoVEphage	MW660583	Clustered	VC_1526_0	C2	199	Bacteroides	Group I	Duplodnaviria	Heunggongvirae	Uroviricota	Caudoviricetes	n.a.	Unclassified	Unclassified	Unclassified
S12_vs_163990	Faecalibacterium phage FP_oengus	MG711463	Clustered	VC_1085_1	N18	66	Faecalibacterium	Group I	Duplodnaviria	Heunggongvirae	Uroviricota	Caudoviricetes	n.a.	Unclassified	O	O
S12_vs_183752	Clostridium phage HM2	LT600745	Clustered	VC_1261_0	N43	4	Clostridium	Group I	Duplodnaviria	Heunggongvirae	Uroviricota	Caudoviricetes	n.a.	Salasmaviridae	O	O
S12_vs_183891	n.a.	n.a.	Clustered	VC_1603_0	F	n.a.	n.a.	n.a.	n.a.	n.a.	n.a.	n.a.	n.a.	n.a.	n.a.	n.a.
S12_vs_188820	n.a.	n.a.	Clustered	VC_1553_0	A	n.a.	n.a.	n.a.	n.a.	n.a.	n.a.	n.a.	n.a.	n.a.	n.a.	n.a.
S12_vs_188821	Faecalibacterium phage FP_oengus	MG711463	Clustered	VC_1085_1	N15	79	Faecalibacterium	Group I	Duplodnaviria	Heunggongvirae	Uroviricota	Caudoviricetes	n.a.	Unclassified	O	O
S12_vs_188823	n.a.	n.a.	Clustered	VC_1566_0	A	n.a.	n.a.	n.a.	n.a.	n.a.	n.a.	n.a.	n.a.	n.a.	n.a.	n.a.
S12_vs_188828	n.a.	n.a.	Clustered	VC_1568_0	A	n.a.	n.a.	n.a.	n.a.	n.a.	n.a.	n.a.	n.a.	n.a.	n.a.	n.a.
S12_vs_188832	Tortoise microvirus 9	MK765559	Clustered	VC_1161_0	C1	9	Unspecified	Group II	Monodnaviria	Sangervirae	Phixviricota	Malgrandaviricetes	Petitvirales	Microviridae	Unclassified	Unclassified
S12_vs_188833	n.a.	n.a.	Clustered	VC_1274_0	A	n.a.	n.a.	n.a.	n.a.	n.a.	n.a.	n.a.	n.a.	n.a.	n.a.	n.a.
S12_vs_23796	Bacteroides phage p00	BK010646	Clustered	VC_695_0	C3	48	Bacteroides	Group I	Duplodnaviria	Heunggongvirae	Uroviricota	Caudoviricetes	n.a.	Unclassified	Unclassified	Unclassified
S12_vs_26336	n.a.	n.a.	Clustered	VC_1605_0	A	n.a.	n.a.	n.a.	n.a.	n.a.	n.a.	n.a.	n.a.	n.a.	n.a.	n.a.
S12_vs_48045	Exiguobacterium phage vB_EauM-23	MH844558	Clustered	VC_145_0	C2	24	Exiguobacterium	Group I	Duplodnaviria	Heunggongvirae	Uroviricota	Caudoviricetes	n.a.	Unclassified	Unclassified	Unclassified
S12_vs_58560	Faecalibacterium phage FP_oengus	MG711463	Clustered	VC_1085_1	N20	40	Faecalibacterium	Group I	Duplodnaviria	Heunggongvirae	Uroviricota	Caudoviricetes	n.a.	Unclassified	O	O
S12_vs_5857	Bacillus phage vB_BtS_BMBtp13	KX190832	Clustered	VC_1230_0	N12	5	Bacillus	Group I	Duplodnaviria	Heunggongvirae	Uroviricota	Caudoviricetes	n.a.	Unclassified	O	O
S12_vs_6836	Clostridium phage phiMMP01	LN681541	Clustered	VC_151_0	N10	26	Clostridium	Group I	Duplodnaviria	Heunggongvirae	Uroviricota	Caudoviricetes	n.a.	Unclassified	O	O
S12_vs_71525	n.a.	n.a.	Clustered	VC_1606_0	A	n.a.	n.a.	n.a.	n.a.	n.a.	n.a.	n.a.	n.a.	n.a.	n.a.	n.a.
S12_vs_79785	Clostridium phage phiMMP01	LN681541	Clustered	VC_151_0	N21	23	Clostridium	Group I	Duplodnaviria	Heunggongvirae	Uroviricota	Caudoviricetes	n.a.	Unclassified	O	O
S12_vs_86928	Akkermansia phage DTMo-2021a	CP084202	Clustered	VC_752_0	N2	7	Akkermansia	Group I	Duplodnaviria	Heunggongvirae	Uroviricota	Caudoviricetes	n.a.	Unclassified	O	O
S12_vs_92868	Faecalibacterium phage FP_oengus	MG711463	Clustered	VC_1085_0	N18	72	Faecalibacterium	Group I	Duplodnaviria	Heunggongvirae	Uroviricota	Caudoviricetes	n.a.	Unclassified	O	O
S12_vs_93357	Acetobacter phage phiAO1	LC644971	Clustered	VC_383_0	N4	18	Acetobacter	Group I	Duplodnaviria	Heunggongvirae	Uroviricota	Caudoviricetes	n.a.	Unclassified	O	O
S12_vs_99092	Geobacillus phage GBSV1	DQ340064	Clustered	VC_778_0	N35	6	Geobacillus	Group I	Duplodnaviria	Heunggongvirae	Uroviricota	Caudoviricetes	n.a.	Unclassified	O	O
S13_vf_109235	n.a.	n.a.	Clustered	VC_1552_0	A	n.a.	n.a.	n.a.	n.a.	n.a.	n.a.	n.a.	n.a.	n.a.	n.a.	n.a.
S13_vf_11246	uncultured phage cr53_1	MT774396	Clustered	VC_519_0	N2	225	Unspecified	Group I	Duplodnaviria	Heunggongvirae	Uroviricota	Caudoviricetes	Crassvirales	Suoliviridae	O	O
S13_vf_131208	Clostridium phage HM2	LT600745	Clustered	VC_1261_0	N40	4	Clostridium	Group I	Duplodnaviria	Heunggongvirae	Uroviricota	Caudoviricetes	n.a.	Salasmaviridae	O	O
S13_vf_143422	uncultured phage cr10_1	MT774382	Clustered	VC_519_11	C3	112	Unspecified	Group I	Duplodnaviria	Heunggongvirae	Uroviricota	Caudoviricetes	Crassvirales	Suoliviridae	Boorivirinae	Canhaevirus
S13_vf_150407	Inovirus sp.	MT135292	Clustered	VC_1495_0	C1	23	Unspecified	Group II	Monodnaviria	Loebvirae	Hofneiviricota	Faserviricetes	Tubulavirales	Inoviridae	Unclassified	Inovirus
S13_vf_151045	Phage DP SC_6_H4_2017	MT121964	Clustered	VC_1490_0	N6	122	Unspecified	Group I	Duplodnaviria	Heunggongvirae	Uroviricota	Caudoviricetes	n.a.	Unclassified	O	O
S13_vf_151755	Delftia phage IME-DE1	KR153873	Clustered	VC_86_0	N3	6	Delftia	Group I	Duplodnaviria	Heunggongvirae	Uroviricota	Caudoviricetes	n.a.	Autographiviridae	O	O
S13_vf_22175	Exiguobacterium phage vB_EauS-123	KU160495	Clustered	VC_779_0	N38	3	Exiguobacterium	Group I	Duplodnaviria	Heunggongvirae	Uroviricota	Caudoviricetes	n.a.	Unclassified	O	O
S13_vf_28309	Clostridium phage HM2	LT600745	Clustered	VC_1258_0	N39	4	Clostridium	Group I	Duplodnaviria	Heunggongvirae	Uroviricota	Caudoviricetes	n.a.	Salasmaviridae	O	O
S13_vf_56074	Phage DP SC_6_H4_2017	MT121964	Clustered	VC_1490_1	C3	269	Unspecified	Group I	Duplodnaviria	Heunggongvirae	Uroviricota	Caudoviricetes	n.a.	Unclassified	Unclassified	Unclassified
S13_vf_7670	n.a.	n.a.	Clustered	VC_1598_0	F	n.a.	n.a.	n.a.	n.a.	n.a.	n.a.	n.a.	n.a.	n.a.	n.a.	n.a.
S13_vf_87873	Parabacteroides phage PDS1	MN929097	Clustered	VC_855_0	C4	115	Parabacteroides	Group I	Duplodnaviria	Heunggongvirae	Uroviricota	Caudoviricetes	n.a.	Unclassified	Unclassified	Unclassified
S13_vs_113593	Bifidobacterium phage BitterVaud1	MT006236	Clustered	VC_826_0	C1	52	Bifidobacterium	Group I	Duplodnaviria	Heunggongvirae	Uroviricota	Caudoviricetes	n.a.	Unclassified	Unclassified	Unclassified
S13_vs_121307	n.a.	n.a.	Clustered	VC_905_0	A	n.a.	n.a.	n.a.	n.a.	n.a.	n.a.	n.a.	n.a.	n.a.	n.a.	n.a.
S13_vs_12957	Bacteriophage sp.	OP549880	Clustered	VC_1549_0	N5	13	Unspecified	Unclassified	Unclassified	n.a.	n.a.	n.a.	n.a.	Unclassified	O	O
S13_vs_148294	Streptococcus phage Javan336	MK448910	Clustered	VC_152_0	N13	7	Streptococcus	Group I	Duplodnaviria	Heunggongvirae	Uroviricota	Caudoviricetes	n.a.	Unclassified	O	O
S13_vs_151510	uncultured phage cr128_1	MT774392	Clustered	VC_1054_0	C1	175	Unspecified	Group I	Duplodnaviria	Heunggongvirae	Uroviricota	Caudoviricetes	Crassvirales	Steigviridae	Asinivirinae	Mahlunavirus
S13_vs_152223	Bifidobacterium phage BitterVaud1	MT006236	Clustered	VC_826_0	C1	30	Bifidobacterium	Group I	Duplodnaviria	Heunggongvirae	Uroviricota	Caudoviricetes	n.a.	Unclassified	Unclassified	Unclassified
S13_vs_15645	Cellulophaga phage phi17:2	KC821609	Clustered	VC_1055_0	N8	6	Cellulophaga	Group I	Duplodnaviria	Heunggongvirae	Uroviricota	Caudoviricetes	n.a.	Unclassified	O	O
S13_vs_23314	Streptococcus phage Javan191	MK448700	Clustered	VC_649_1	C2	72	Streptococcus	Group I	Duplodnaviria	Heunggongvirae	Uroviricota	Caudoviricetes	n.a.	Unclassified	Unclassified	Unclassified
S13_vs_30101	Streptococcus phage Javan630	MK448997	Clustered	VC_649_3	N6	37	Streptococcus	Group I	Duplodnaviria	Heunggongvirae	Uroviricota	Caudoviricetes	n.a.	Unclassified	O	O
S13_vs_30167	Streptococcus phage Javan630	MK448997	Clustered	VC_649_2	N3	71	Streptococcus	Group I	Duplodnaviria	Heunggongvirae	Uroviricota	Caudoviricetes	n.a.	Unclassified	O	O
S13_vs_33947	n.a.	n.a.	Clustered	VC_1291_0	A	n.a.	n.a.	n.a.	n.a.	n.a.	n.a.	n.a.	n.a.	n.a.	n.a.	n.a.
S13_vs_35816	Faecalibacterium phage FP_Lagaffe	MG711461	Clustered	VC_727_0	C4	107	Faecalibacterium	Group I	Duplodnaviria	Heunggongvirae	Uroviricota	Caudoviricetes	n.a.	Unclassified	Unclassified	Lagaffevirus
S13_vs_67429	n.a.	n.a.	Clustered	VC_153_0	A	n.a.	n.a.	n.a.	n.a.	n.a.	n.a.	n.a.	n.a.	n.a.	n.a.	n.a.
S13_vs_68102	Bacillus phage vB_BsuP-Goe1	KU831549	Clustered	VC_847_0	N5	7	Bacillus	Group I	Duplodnaviria	Heunggongvirae	Uroviricota	Caudoviricetes	n.a.	Salasmaviridae	O	O
S13_vs_83447	n.a.	n.a.	Clustered	VC_1565_0	A	n.a.	n.a.	n.a.	n.a.	n.a.	n.a.	n.a.	n.a.	n.a.	n.a.	n.a.
S13_vs_87108	n.a.	n.a.	Clustered	VC_1607_0	F	n.a.	n.a.	n.a.	n.a.	n.a.	n.a.	n.a.	n.a.	n.a.	n.a.	n.a.
S13_vs_90967	Tortoise microvirus 19	MK765569	Clustered	VC_1300_0	N4	5	Unspecified	Group II	Monodnaviria	Sangervirae	Phixviricota	Malgrandaviricetes	Petitvirales	Microviridae	O	O
S13_vs_9438	n.a.	n.a.	Clustered	VC_1596_0	A	n.a.	n.a.	n.a.	n.a.	n.a.	n.a.	n.a.	n.a.	n.a.	n.a.	n.a.
S13_vs_98591	Myoviridae sp.	MW202455	Clustered	VC_439_0	N3	5	Unspecified	Group I	Duplodnaviria	Heunggongvirae	Uroviricota	Caudoviricetes	n.a.	Unclassified	O	O
S14_vf_106156	Lactobacillus phage JNU_P11	MN830257	Clustered	VC_275_0	C1	88	Lactobacillus	Group I	Duplodnaviria	Heunggongvirae	Uroviricota	Caudoviricetes	n.a.	Unclassified	Unclassified	Unclassified
S14_vf_109545	n.a.	n.a.	Clustered	VC_1421_0	A	n.a.	n.a.	n.a.	n.a.	n.a.	n.a.	n.a.	n.a.	n.a.	n.a.	n.a.
S14_vf_114491	n.a.	n.a.	Clustered	VC_1258_0	A	n.a.	n.a.	n.a.	n.a.	n.a.	n.a.	n.a.	n.a.	n.a.	n.a.	n.a.
S14_vf_117150	Clostridium phage phiCp-B	MW478290	Clustered	VC_21_0	N4	11	Clostridium	Group I	Duplodnaviria	Heunggongvirae	Uroviricota	Caudoviricetes	n.a.	Unclassified	O	O

S14_vf_117157	n.a.	n.a.	Clustered	VC_1564_0	F	n.a.	n.a.	n.a.	n.a.	n.a.	n.a.	n.a.	n.a.	n.a.	n.a.	n.a.
S14_vf_117164	n.a.	n.a.	Clustered	VC_946_0	A	n.a.	n.a.	n.a.	n.a.	n.a.	n.a.	n.a.	n.a.	n.a.	n.a.	n.a.
S14_vf_37500	Microviridae sp.	MG945488	Clustered	VC_1334_0	C1	19	Unspecified	Group II	Monodnaviria	Sangervirae	Phixviricota	Malgrandaviricetes	Petitvirales	Microviridae	Unclassified	Unclassified
S14_vf_44452	n.a.	n.a.	Clustered	VC_1511_0	A	n.a.	n.a.	n.a.	n.a.	n.a.	n.a.	n.a.	n.a.	n.a.	n.a.	n.a.
S14_vf_48775	n.a.	n.a.	Clustered	VC_1597_0	F	n.a.	n.a.	n.a.	n.a.	n.a.	n.a.	n.a.	n.a.	n.a.	n.a.	n.a.
S14_vf_56130	Clostridium phage phiMMP01	LN681541	Clustered	VC_151_0	N22	20	Clostridium	Group I	Duplodnaviria	Heunggongvirae	Uroviricota	Caudoviricetes	n.a.	Unclassified	O	O
S14_vf_58919	Faecalibacterium phage FP_Mushu	MG711460	Clustered	VC_1288_0	C1	76	Faecalibacterium	Group I	Duplodnaviria	Heunggongvirae	Uroviricota	Caudoviricetes	n.a.	Unclassified	Unclassified	Mushuvirus
S14_vf_72335	Butyrivibrio virus Arian	MN882551	Clustered	VC_720_0	N6	14	Butyrivibrio	Group I	Duplodnaviria	Heunggongvirae	Uroviricota	Caudoviricetes	n.a.	Unclassified	O	O
S14_vf_72953	Lactobacillus phage phiEF-1.1	MF041990	Clustered	VC_315_0	C1	23	Lactobacillus	Group I	Duplodnaviria	Heunggongvirae	Uroviricota	Caudoviricetes	n.a.	Unclassified	Unclassified	Unclassified
S14_vf_78139	Lactobacillus phage JCL1032	EU409559	Clustered	VC_390_0	C1	155	Lactobacillus	Group I	Duplodnaviria	Heunggongvirae	Uroviricota	Caudoviricetes	n.a.	Unclassified	Unclassified	Unclassified
S14_vf_92240	Butyrivibrio phage Idris	MN882554	Clustered	VC_1289_0	N4	48	Butyrivibrio	Group I	Duplodnaviria	Heunggongvirae	Uroviricota	Caudoviricetes	n.a.	Unclassified	O	O
S14_vf_99344	n.a.	n.a.	Clustered	VC_1258_0	A	n.a.	n.a.	n.a.	n.a.	n.a.	n.a.	n.a.	n.a.	n.a.	n.a.	n.a.
S14_vs_102698	Bacillus phage 250	GU229986	Clustered	VC_782_0	N8	3	Bacillus	Group I	Duplodnaviria	Heunggongvirae	Uroviricota	Caudoviricetes	n.a.	Unclassified	O	O
S14_vs_110290	n.a.	n.a.	Clustered	VC_782_0	A	n.a.	n.a.	n.a.	n.a.	n.a.	n.a.	n.a.	n.a.	n.a.	n.a.	n.a.
S14_vs_11510	n.a.	n.a.	Clustered	VC_1612_1	A	n.a.	n.a.	n.a.	n.a.	n.a.	n.a.	n.a.	n.a.	n.a.	n.a.	n.a.
S14_vs_117158	Streptococcus phage MismyG	OL774867	Clustered	VC_152_0	N11	7	Streptococcus	Group I	Duplodnaviria	Heunggongvirae	Uroviricota	Caudoviricetes	n.a.	Unclassified	O	O
S14_vs_117160	n.a.	n.a.	Clustered	VC_658_0	A	n.a.	n.a.	n.a.	n.a.	n.a.	n.a.	n.a.	n.a.	n.a.	n.a.	n.a.
S14_vs_117161	Butyrivibrio phage Idris	MN882554	Clustered	VC_1289_0	N8	34	Butyrivibrio	Group I	Duplodnaviria	Heunggongvirae	Uroviricota	Caudoviricetes	n.a.	Unclassified	O	O
S14_vs_117165	Butyrivibrio virus Ceridwen	MN882553	Clustered	VC_1479_0	N39	2	Butyrivibrio	Group I	Duplodnaviria	Heunggongvirae	Uroviricota	Caudoviricetes	n.a.	Unclassified	O	O
S14_vs_117166	n.a.	n.a.	Clustered	VC_779_0	A	n.a.	n.a.	n.a.	n.a.	n.a.	n.a.	n.a.	n.a.	n.a.	n.a.	n.a.
S14_vs_14737	Faecalibacterium phage FP_Lugh	MG711464	Clustered	VC_923_0	N4	12	Faecalibacterium	Group I	Duplodnaviria	Heunggongvirae	Uroviricota	Caudoviricetes	n.a.	Unclassified	O	O
S14_vs_15228	Cellulophaga phage phi17:2	KC821609	Clustered	VC_1055_1	N8	6	Cellulophaga	Group I	Duplodnaviria	Heunggongvirae	Uroviricota	Caudoviricetes	n.a.	Unclassified	O	O
S14_vs_25852	n.a.	n.a.	Clustered	VC_1612_0	A	n.a.	n.a.	n.a.	n.a.	n.a.	n.a.	n.a.	n.a.	n.a.	n.a.	n.a.
S14_vs_2870	Acetobacter phage phiAO1	LC644971	Clustered	VC_383_0	N4	16	Acetobacter	Group I	Duplodnaviria	Heunggongvirae	Uroviricota	Caudoviricetes	n.a.	Unclassified	O	O
S14_vs_32499	Bacteroides phage F2	MT806187	Clustered	VC_1505_0	N11	5	Bacteroides	Group I	Duplodnaviria	Heunggongvirae	Uroviricota	Caudoviricetes	n.a.	Unclassified	O	O
S14_vs_3251	n.a.	n.a.	Clustered	VC_1294_0	A	n.a.	n.a.	n.a.	n.a.	n.a.	n.a.	n.a.	n.a.	n.a.	n.a.	n.a.
S14_vs_39797	Clostridium phage phiCD111	LN681535	Clustered	VC_923_0	N5	11	Clostridium	Group I	Duplodnaviria	Heunggongvirae	Uroviricota	Caudoviricetes	n.a.	Unclassified	O	O
S14_vs_59347	Butyrivibrio virus Arian	MN882551	Clustered	VC_720_0	N5	17	Butyrivibrio	Group I	Duplodnaviria	Heunggongvirae	Uroviricota	Caudoviricetes	n.a.	Unclassified	O	O
S14_vs_61530	n.a.	n.a.	Clustered	VC_779_0	A	n.a.	n.a.	n.a.	n.a.	n.a.	n.a.	n.a.	n.a.	n.a.	n.a.	n.a.
S14_vs_62178	Geobacillus phage GBSV1	DQ340064	Clustered	VC_778_0	N43	6	Geobacillus	Group I	Duplodnaviria	Heunggongvirae	Uroviricota	Caudoviricetes	n.a.	Unclassified	O	O
S14_vs_632	Clostridium phage phiMMP01	LN681541	Clustered	VC_151_0	N18	24	Clostridium	Group I	Duplodnaviria	Heunggongvirae	Uroviricota	Caudoviricetes	n.a.	Unclassified	O	O
S14_vs_66139	Streptococcus phage Javan630	MK448997	Clustered	VC_649_0	N6	52	Streptococcus	Group I	Duplodnaviria	Heunggongvirae	Uroviricota	Caudoviricetes	n.a.	Unclassified	O	O
S14_vs_66208	Phage vB_RanS_PJN03	ON245412	Clustered	VC_950_0	N13	7	Unspecified	Group I	Duplodnaviria	Heunggongvirae	Uroviricota	Caudoviricetes	n.a.	Unclassified	O	O
S14_vs_66841	Erysipelothrix phage SE-1	KR816341	Clustered	VC_1149_0	N3	15	Erysipelothrix	Group I	Duplodnaviria	Heunggongvirae	Uroviricota	Caudoviricetes	n.a.	Unclassified	O	O
S14_vs_67094	n.a.	n.a.	Clustered	VC_1614_0	F	n.a.	n.a.	n.a.	n.a.	n.a.	n.a.	n.a.	n.a.	n.a.	n.a.	n.a.
S14_vs_73301	Clostridium phage phiCTP1	HM159959	Clustered	VC_920_0	N5	7	Clostridium	Group I	Duplodnaviria	Heunggongvirae	Uroviricota	Caudoviricetes	n.a.	Unclassified	O	O
S14_vs_78164	Winogradskyella phage Peternella_1	MT732475	Clustered	VC_694_0	N5	17	Winogradskyella	Group I	Duplodnaviria	Heunggongvirae	Uroviricota	Caudoviricetes	n.a.	Winoviridae	O	O
S14_vs_88666	Clostridium phage phiCp-A	MW478289	Clustered	VC_1035_0	N2	14	Clostridium	Group I	Duplodnaviria	Heunggongvirae	Uroviricota	Caudoviricetes	n.a.	Unclassified	O	O
S14_vs_96111	Streptococcus phage Javan105	MK448667	Clustered	VC_649_1	C1	58	Streptococcus	Group I	Duplodnaviria	Heunggongvirae	Uroviricota	Caudoviricetes	n.a.	Unclassified	Unclassified	Unclassified
S14_vs_9676	Faecalibacterium phage FP_Lagaffe	MG711461	Clustered	VC_1291_0	N43	3	Faecalibacterium	Group I	Duplodnaviria	Heunggongvirae	Uroviricota	Caudoviricetes	n.a.	Unclassified	O	O
S15_vf_2032	Bacteroides phage LoVEphage	MW660583	Clustered	VC_1527_0	N3	36	Bacteroides	Group I	Duplodnaviria	Heunggongvirae	Uroviricota	Caudoviricetes	n.a.	Unclassified	O	O
S15_vf_20644	Phage DP SC_6_H4_2017	MT121964	Clustered	VC_1490_1	C3	268	Unspecified	Group I	Duplodnaviria	Heunggongvirae	Uroviricota	Caudoviricetes	n.a.	Unclassified	Unclassified	Unclassified
S15_vf_2513	n.a.	n.a.	Clustered	VC_1254_2	A	n.a.	n.a.	n.a.	n.a.	n.a.	n.a.	n.a.	n.a.	n.a.	n.a.	n.a.
S15_vf_55639	n.a.	n.a.	Clustered	VC_946_0	A	n.a.	n.a.	n.a.	n.a.	n.a.	n.a.	n.a.	n.a.	n.a.	n.a.	n.a.
S15_vf_74586	Phage DP SC_6_H4_2017	MT121964	Clustered	VC_1490_0	N8	118	Unspecified	Group I	Duplodnaviria	Heunggongvirae	Uroviricota	Caudoviricetes	n.a.	Unclassified	O	O
S15_vs_1407	n.a.	n.a.	Clustered	VC_946_0	A	n.a.	n.a.	n.a.	n.a.	n.a.	n.a.	n.a.	n.a.	n.a.	n.a.	n.a.
S15_vs_18274	n.a.	n.a.	Clustered	VC_1602_0	A	n.a.	n.a.	n.a.	n.a.	n.a.	n.a.	n.a.	n.a.	n.a.	n.a.	n.a.
S15_vs_27510	n.a.	n.a.	Clustered	VC_1559_1	A	n.a.	n.a.	n.a.	n.a.	n.a.	n.a.	n.a.	n.a.	n.a.	n.a.	n.a.
S15_vs_35445	n.a.	n.a.	Clustered	VC_1599_0	A	n.a.	n.a.	n.a.	n.a.	n.a.	n.a.	n.a.	n.a.	n.a.	n.a.	n.a.
S15_vs_7103	Eggerthella phage PMBT5	MH626557	Clustered	VC_1421_0	N35	3	Eggerthella	Group I	Duplodnaviria	Heunggongvirae	Uroviricota	Caudoviricetes	n.a.	Unclassified	O	O
S15_vs_71968	Pasteurella phage AFS-2018a	MH238466	Clustered	VC_630_0	N4	6	Pasteurella	Group I	Duplodnaviria	Heunggongvirae	Uroviricota	Caudoviricetes	n.a.	Unclassified	O	O
S15_vs_76390	Bdellovibrio phage phi1402	JF344709	Clustered	VC_967_0	C4	30	Bdellovibrio	Group I	Duplodnaviria	Heunggongvirae	Uroviricota	Caudoviricetes	n.a.	Unclassified	Unclassified	Unclassified
S16_vf_100235	n.a.	n.a.	Clustered	VC_1258_0	A	n.a.	n.a.	n.a.	n.a.	n.a.	n.a.	n.a.	n.a.	n.a.	n.a.	n.a.
S16_vf_100378	Bifidobacterium phage BitterVaud1	MT006236	Clustered	VC_826_0	C3	22	Bifidobacterium	Group I	Duplodnaviria	Heunggongvirae	Uroviricota	Caudoviricetes	n.a.	Unclassified	Unclassified	Unclassified
S16_vf_1037	n.a.	n.a.	Clustered	VC_1254_1	A	n.a.	n.a.	n.a.	n.a.	n.a.	n.a.	n.a.	n.a.	n.a.	n.a.	n.a.
S16_vf_119834	Clostridium phage HM2	LT600745	Clustered	VC_1261_0	N34	4	Clostridium	Group I	Duplodnaviria	Heunggongvirae	Uroviricota	Caudoviricetes	n.a.	Salasmaviridae	O	O
S16_vf_151450	uncultured phage cr127_1	MT774393	Clustered	VC_693_0	C2	64	Unspecified	Group I	Duplodnaviria	Heunggongvirae	Uroviricota	Caudoviricetes	Crassvirales	Crevaviridae	Doltivirinae	Kahucivirus
S16_vf_164913	n.a.	n.a.	Clustered	VC_1607_0	F	n.a.	n.a.	n.a.	n.a.	n.a.	n.a.	n.a.	n.a.	n.a.	n.a.	n.a.
S16_vf_169895	Clostridium phage HM2	LT600745	Clustered	VC_1261_0	N41	3	Clostridium	Group I	Duplodnaviria	Heunggongvirae	Uroviricota	Caudoviricetes	n.a.	Salasmaviridae	O	O
S16_vf_169985	Phage DP SC_6_H4_2017	MT121964	Clustered	VC_1490_0	N8	118	Unspecified	Group I	Duplodnaviria	Heunggongvirae	Uroviricota	Caudoviricetes	n.a.	Unclassified	O	O
S16_vf_173109	Bifidobacterium phage BlindBasel1	MT006235	Clustered	VC_1485_0	C1	48	Bifidobacterium	Group I	Duplodnaviria	Heunggongvirae	Uroviricota	Caudoviricetes	n.a.	Unclassified	Unclassified	Unclassified
S16_vf_36146	Clostridium phage phiMMP01	LN681541	Clustered	VC_151_0	N18	18	Clostridium	Group I	Duplodnaviria	Heunggongvirae	Uroviricota	Caudoviricetes	n.a.	Unclassified	O	O
S16_vf_5270	Faecalibacterium phage FP_oengus	MG711463	Clustered	VC_1085_1	N19	46	Faecalibacterium	Group I	Duplodnaviria	Heunggongvirae	Uroviricota	Caudoviricetes	n.a.	Unclassified	O	O
S16_vf_6102	Faecalibacterium phage FP_Toutatis	MG711466	Clustered	VC_728_0	C4	65	Faecalibacterium	Group I	Duplodnaviria	Heunggongvirae	Uroviricota	Caudoviricetes	n.a.	Unclassified	Unclassified	Toutatisvirus
S16_vf_87190	Cellulophaga phage phi13:1	KC821625	Clustered	VC_963_0	N5	17	Cellulophaga	Group I	Duplodnaviria	Heunggongvirae	Uroviricota	Caudoviricetes	n.a.	Unclassified	O	O
S16_vf_90328	Butyrivibrio phage Idris	MN882554	Clustered	VC_1477_0	N16	1	Butyrivibrio	Group I	Duplodnaviria	Heunggongvirae	Uroviricota	Caudoviricetes	n.a.	Unclassified	O	O
S16_vf_97279	Faecalibacterium phage FP_Epona	MG711462	Clustered	VC_723_0	C1	113	Faecalibacterium	Group I	Duplodnaviria	Heunggongvirae	Uroviricota	Caudoviricetes	n.a.	Unclassified	Unclassified	Eponavirus
S16_vs_103726	n.a.	n.a.	Clustered	VC_779_0	A	n.a.	n.a.	n.a.	n.a.	n.a.	n.a.	n.a.	n.a.	n.a.	n.a.	n.a.
S16_vs_116956	n.a.	n.a.	Clustered	VC_442_0	A	n.a.	n.a.	n.a.	n.a.	n.a.	n.a.	n.a.	n.a.	n.a.	n.a.	n.a.
S16_vs_120622	Acetobacter phage phiAO1	LC644971	Clustered	VC_383_0	N4	14	Acetobacter	Group I	Duplodnaviria	Heunggongvirae	Uroviricota	Caudoviricetes	n.a.	Unclassified	O	O
S16_vs_124703	Bacillus phage vB_BtS_BMBtp13	KX190832	Clustered	VC_667_0	C9	8	Bacillus	Group I	Duplodnaviria	Heunggongvirae	Uroviricota	Caudoviricetes	n.a.	Unclassified	Unclassified	Unclassified
S16_vs_127669	Bacteroides phage LoVEphage	MW660583	Clustered	VC_1526_0	C1	297	Bacteroides	Group I	Duplodnaviria	Heunggongvirae	Uroviricota	Caudoviricetes	n.a.	Unclassified	Unclassified	Unclassified
S16_vs_127905	n.a.	n.a.	Clustered	VC_1559_1	A	n.a.	n.a.	n.a.	n.a.	n.a.	n.a.	n.a.	n.a.	n.a.	n.a.	n.a.
S16_vs_136935	n.a.	n.a.	Clustered	VC_1063_0	A	n.a.	n.a.	n.a.	n.a.	n.a.	n.a.	n.a.	n.a.	n.a.	n.a.	n.a.
S16_vs_142561	Pasteurella phage AFS-2018a	MH238466	Clustered	VC_630_0	N5	4	Pasteurella	Group I	Duplodnaviria	Heunggongvirae	Uroviricota	Caudoviricetes	n.a.	Unclassified	O	O
S16_vs_173079	Faecalibacterium phage FP_Brigit	MG711465	Clustered	VC_442_0	N4	34	Faecalibacterium	Group I	Duplodnaviria	Heunggongvirae	Uroviricota	Caudoviricetes	n.a.	Unclassified	O	O
S16_vs_173096	Akkermansia phage DTMo-2021a	CP084202	Clustered	VC_751_0	C1	90	Akkermansia	Group I	Duplodnaviria	Heunggongvirae	Uroviricota	Caudoviricetes	n.a.	Unclassified	Unclassified	Unclassified
S16_vs_173102	Parabacteroides phage PDS1	MN929097	Clustered	VC_855_0	C4	114	Parabacteroides	Group I	Duplodnaviria	Heunggongvirae	Uroviricota	Caudoviricetes	n.a.	Unclassified	Unclassified	Unclassified
S16_vs_18440	Faecalibacterium phage FP_Toutatis	MG711466	Clustered	VC_728_0	C1	121	Faecalibacterium	Group I	Duplodnaviria	Heunggongvirae	Uroviricota	Caudoviricetes	n.a.	Unclassified	Unclassified	Toutatisvirus
S16_vs_19320	Bacillus phage vB_BtS_BMBtp13	KX190832	Clustered	VC_1230_0	N9	5	Bacillus	Group I	Duplodnaviria	Heunggongvirae	Uroviricota	Caudoviricetes	n.a.	Unclassified	O	O
S16_vs_19968	n.a.	n.a.	Clustered	VC_1591_0	A	n.a.	n.a.	n.a.	n.a.	n.a.	n.a.	n.a.	n.a.	n.a.	n.a.	n.a.
S16_vs_26078	Bacillus phage 1	DQ840344	Clustered	VC_778_0	N28	8	Bacillus	Group I	Duplodnaviria	Heunggongvirae	Uroviricota	Caudoviricetes	n.a.	Unclassified	O	O

S16_vs_32764	n.a.	n.a.	Clustered	VC_1599_0	A	n.a.	n.a.	n.a.	n.a.	n.a.	n.a.	n.a.	n.a.	n.a.	n.a.	n.a.
S16_vs_33281	n.a.	n.a.	Clustered	VC_1591_0	A	n.a.	n.a.	n.a.	n.a.	n.a.	n.a.	n.a.	n.a.	n.a.	n.a.	n.a.
S16_vs_4662	Bacillus phage vB_BtS_BMBtp13	KX190832	Clustered	VC_667_0	C7	5	Bacillus	Group I	Duplodnaviria	Heunggongvirae	Uroviricota	Caudoviricetes	n.a.	Unclassified	Unclassified	Unclassified
S16_vs_48714	n.a.	n.a.	Clustered	VC_1616_0	F	n.a.	n.a.	n.a.	n.a.	n.a.	n.a.	n.a.	n.a.	n.a.	n.a.	n.a.
S16_vs_51901	n.a.	n.a.	Clustered	VC_1602_0	A	n.a.	n.a.	n.a.	n.a.	n.a.	n.a.	n.a.	n.a.	n.a.	n.a.	n.a.
S16_vs_64584	n.a.	n.a.	Clustered	VC_1611_0	A	n.a.	n.a.	n.a.	n.a.	n.a.	n.a.	n.a.	n.a.	n.a.	n.a.	n.a.
S16_vs_75108	n.a.	n.a.	Clustered	VC_1290_0	A	n.a.	n.a.	n.a.	n.a.	n.a.	n.a.	n.a.	n.a.	n.a.	n.a.	n.a.
S16_vs_78361	Bifidobacterium phage PMBT6	MH444512	Clustered	VC_878_0	N2	52	Bifidobacterium	Group I	Duplodnaviria	Heunggongvirae	Uroviricota	Caudoviricetes	n.a.	Unclassified	O	O
S16_vs_80244	n.a.	n.a.	Clustered	VC_1579_0	A	n.a.	n.a.	n.a.	n.a.	n.a.	n.a.	n.a.	n.a.	n.a.	n.a.	n.a.
S16_vs_86116	Faecalibacterium phage FP_Lagaffe	MG711461	Clustered	VC_727_0	C1	169	Faecalibacterium	Group I	Duplodnaviria	Heunggongvirae	Uroviricota	Caudoviricetes	n.a.	Unclassified	Unclassified	Lagaffevirus
S16_vs_89511	Clostridioides phage CD1801	MW512570	Clustered	VC_670_0	N12	5	Clostridioides	Group I	Duplodnaviria	Heunggongvirae	Uroviricota	Caudoviricetes	n.a.	Unclassified	O	O
S16_vs_92150	Faecalibacterium phage FP_Lugh	MG711464	Clustered	VC_779_0	N27	7	Faecalibacterium	Group I	Duplodnaviria	Heunggongvirae	Uroviricota	Caudoviricetes	n.a.	Unclassified	O	O
S17_vf_148303	n.a.	n.a.	Clustered	VC_1258_0	A	n.a.	n.a.	n.a.	n.a.	n.a.	n.a.	n.a.	n.a.	n.a.	n.a.	n.a.
S17_vf_172132	n.a.	n.a.	Clustered	VC_1607_0	F	n.a.	n.a.	n.a.	n.a.	n.a.	n.a.	n.a.	n.a.	n.a.	n.a.	n.a.
S17_vf_174033	Bacillus phage SPR	OM236515	Clustered	VC_1040_0	N10	3	Bacillus	Group I	Duplodnaviria	Heunggongvirae	Uroviricota	Caudoviricetes	n.a.	Unclassified	O	O
S17_vf_174203	Clostridium phage HM2	LT600745	Clustered	VC_1258_0	N48	4	Clostridium	Group I	Duplodnaviria	Heunggongvirae	Uroviricota	Caudoviricetes	n.a.	Salasmaviridae	O	O
S17_vf_177197	n.a.	n.a.	Clustered	VC_1617_0	F	n.a.	n.a.	n.a.	n.a.	n.a.	n.a.	n.a.	n.a.	n.a.	n.a.	n.a.
S17_vf_177199	Enterobacter phage phiEap-2	KT287080	Clustered	VC_560_0	C1	93	Enterobacter	Group I	Duplodnaviria	Heunggongvirae	Uroviricota	Caudoviricetes	n.a.	Unclassified	Guernseyvirinae	Cornellvirus
S17_vf_35138	Phage vB_RanS_PJN03	ON245412	Clustered	VC_949_0	N12	2	Unspecified	Group I	Duplodnaviria	Heunggongvirae	Uroviricota	Caudoviricetes	n.a.	Unclassified	O	O
S17_vf_67042	Clostridium phage HM2	LT600745	Clustered	VC_1261_0	N27	8	Clostridium	Group I	Duplodnaviria	Heunggongvirae	Uroviricota	Caudoviricetes	n.a.	Salasmaviridae	O	O
S17_vf_77057	n.a.	n.a.	Clustered	VC_1585_0	A	n.a.	n.a.	n.a.	n.a.	n.a.	n.a.	n.a.	n.a.	n.a.	n.a.	n.a.
S17_vf_79151	n.a.	n.a.	Clustered	VC_1258_0	A	n.a.	n.a.	n.a.	n.a.	n.a.	n.a.	n.a.	n.a.	n.a.	n.a.	n.a.
S17_vf_81232	uncultured phage cr10_1	MT774382	Clustered	VC_519_11	C1	292	Unspecified	Group I	Duplodnaviria	Heunggongvirae	Uroviricota	Caudoviricetes	Crassvirales	Suoliviridae	Boorivirinae	Canhaevirus
S17_vf_97671	Faecalibacterium phage FP_Mushu	MG711460	Clustered	VC_1288_0	C3	78	Faecalibacterium	Group I	Duplodnaviria	Heunggongvirae	Uroviricota	Caudoviricetes	n.a.	Unclassified	Unclassified	Mushuvirus
S17_vs_103009	n.a.	n.a.	Clustered	VC_1291_0	A	n.a.	n.a.	n.a.	n.a.	n.a.	n.a.	n.a.	n.a.	n.a.	n.a.	n.a.
S17_vs_116640	Bacteroides phage F4	MT806185	Clustered	VC_1505_0	N12	2	Bacteroides	Group I	Duplodnaviria	Heunggongvirae	Uroviricota	Caudoviricetes	n.a.	Unclassified	O	O
S17_vs_121041	Geobacillus phage GBSV1	DQ340064	Clustered	VC_778_0	N52	6	Geobacillus	Group I	Duplodnaviria	Heunggongvirae	Uroviricota	Caudoviricetes	n.a.	Unclassified	O	O
S17_vs_127519	n.a.	n.a.	Clustered	VC_1290_0	A	n.a.	n.a.	n.a.	n.a.	n.a.	n.a.	n.a.	n.a.	n.a.	n.a.	n.a.
S17_vs_138036	Bdellovibrio phage phi1402	JF344709	Clustered	VC_967_0	C4	31	Bdellovibrio	Group I	Duplodnaviria	Heunggongvirae	Uroviricota	Caudoviricetes	n.a.	Unclassified	Unclassified	Unclassified
S17_vs_160502	uncultured phage cr116_1	MT774389	Clustered	VC_948_0	N13	4	Unspecified	Group I	Duplodnaviria	Heunggongvirae	Uroviricota	Caudoviricetes	Crassvirales	Steigviridae	O	O
S17_vs_177196	n.a.	n.a.	Clustered	VC_1602_0	A	n.a.	n.a.	n.a.	n.a.	n.a.	n.a.	n.a.	n.a.	n.a.	n.a.	n.a.
S17_vs_2079	Bacteroides phage p00	BK010646	Clustered	VC_695_0	C1	38	Bacteroides	Group I	Duplodnaviria	Heunggongvirae	Uroviricota	Caudoviricetes	n.a.	Unclassified	Unclassified	Unclassified
S17_vs_39515	uncultured phage cr271_1	MT774394	Clustered	VC_948_0	N12	2	Unspecified	Group I	Duplodnaviria	Heunggongvirae	Uroviricota	Caudoviricetes	Crassvirales	Intestiviridae	O	O
S17_vs_41845	Ruminococcus phage phiRM10	MT980841	Clustered	VC_779_0	N55	3	Ruminococcus	Group I	Duplodnaviria	Heunggongvirae	Uroviricota	Caudoviricetes	n.a.	Unclassified	O	O
S17_vs_43307	n.a.	n.a.	Clustered	VC_1583_0	A	n.a.	n.a.	n.a.	n.a.	n.a.	n.a.	n.a.	n.a.	n.a.	n.a.	n.a.
S17_vs_58025	n.a.	n.a.	Clustered	VC_1607_0	F	n.a.	n.a.	n.a.	n.a.	n.a.	n.a.	n.a.	n.a.	n.a.	n.a.	n.a.
S17_vs_73830	n.a.	n.a.	Clustered	VC_779_0	A	n.a.	n.a.	n.a.	n.a.	n.a.	n.a.	n.a.	n.a.	n.a.	n.a.	n.a.
S1_vf_125650	n.a.	n.a.	Clustered	VC_1607_0	F	n.a.	n.a.	n.a.	n.a.	n.a.	n.a.	n.a.	n.a.	n.a.	n.a.	n.a.
S1_vf_158961	Faecalibacterium phage FP_Lagaffe	MG711461	Clustered	VC_1256_0	N4	8	Faecalibacterium	Group I	Duplodnaviria	Heunggongvirae	Uroviricota	Caudoviricetes	n.a.	Unclassified	O	O
S1_vf_159951	Bacteroides phage SJC03	MT074146	Clustered	VC_1489_0	N6	15	Bacteroides	Group I	Duplodnaviria	Heunggongvirae	Uroviricota	Caudoviricetes	n.a.	Unclassified	O	O
S1_vf_22149	n.a.	n.a.	Clustered	VC_1552_0	A	n.a.	n.a.	n.a.	n.a.	n.a.	n.a.	n.a.	n.a.	n.a.	n.a.	n.a.
S1_vf_50591	Streptococcus phage Javan261	MK448720	Clustered	VC_905_0	N4	10	Streptococcus	Group I	Duplodnaviria	Heunggongvirae	Uroviricota	Caudoviricetes	n.a.	Unclassified	O	O
S1_vs_100019	Tortoise microvirus 19	MK765569	Clustered	VC_1300_0	N4	4	Unspecified	Group II	Monodnaviria	Sangervirae	Phixviricota	Malgrandaviricetes	Petitvirales	Microviridae	O	O
S1_vs_143929	n.a.	n.a.	Clustered	VC_1554_0	A	n.a.	n.a.	n.a.	n.a.	n.a.	n.a.	n.a.	n.a.	n.a.	n.a.	n.a.
S1_vs_3435	Microviridae sp.	MG945598	Clustered	VC_1385_0	C1	4	Unspecified	Group II	Monodnaviria	Sangervirae	Phixviricota	Malgrandaviricetes	Petitvirales	Microviridae	Unclassified	Unclassified
S1_vs_62409	Geobacillus phage GBSV1	DQ340064	Clustered	VC_778_0	N20	9	Geobacillus	Group I	Duplodnaviria	Heunggongvirae	Uroviricota	Caudoviricetes	n.a.	Unclassified	O	O
S1_vs_91270	n.a.	n.a.	Clustered	VC_782_0	A	n.a.	n.a.	n.a.	n.a.	n.a.	n.a.	n.a.	n.a.	n.a.	n.a.	n.a.
S2_vf_20910	n.a.	n.a.	Clustered	VC_1585_0	A	n.a.	n.a.	n.a.	n.a.	n.a.	n.a.	n.a.	n.a.	n.a.	n.a.	n.a.
S2_vf_21511	Faecalibacterium phage FP_oengus	MG711463	Clustered	VC_1085_1	N21	70	Faecalibacterium	Group I	Duplodnaviria	Heunggongvirae	Uroviricota	Caudoviricetes	n.a.	Unclassified	O	O
S2_vf_21630	Clostridium phage phiCTP1	HM159959	Clustered	VC_921_0	N5	6	Clostridium	Group I	Duplodnaviria	Heunggongvirae	Uroviricota	Caudoviricetes	n.a.	Unclassified	O	O
S2_vf_3356	n.a.	n.a.	Clustered	VC_1607_0	F	n.a.	n.a.	n.a.	n.a.	n.a.	n.a.	n.a.	n.a.	n.a.	n.a.	n.a.
S2_vf_4910	Clostridium phage HM2	LT600745	Clustered	VC_1261_0	N27	7	Clostridium	Group I	Duplodnaviria	Heunggongvirae	Uroviricota	Caudoviricetes	n.a.	Salasmaviridae	O	O
S2_vs_10289	Bacteroides phage p00	BK010646	Clustered	VC_695_0	C1	47	Bacteroides	Group I	Duplodnaviria	Heunggongvirae	Uroviricota	Caudoviricetes	n.a.	Unclassified	Unclassified	Unclassified
S2_vs_7306	Parabacteroides phage PDS1	MN929097	Clustered	VC_855_0	C2	160	Parabacteroides	Group I	Duplodnaviria	Heunggongvirae	Uroviricota	Caudoviricetes	n.a.	Unclassified	Unclassified	Unclassified
S3_vf_100440	Clostridium phage phiMMP01	LN681541	Clustered	VC_151_0	N20	17	Clostridium	Group I	Duplodnaviria	Heunggongvirae	Uroviricota	Caudoviricetes	n.a.	Unclassified	O	O
S3_vf_106401	Clostridium phage phiCD6356	GU949551	Clustered	VC_430_0	N3	17	Clostridium	Group I	Duplodnaviria	Heunggongvirae	Uroviricota	Caudoviricetes	n.a.	Unclassified	O	O
S3_vf_109277	n.a.	n.a.	Clustered	VC_1558_0	F	n.a.	n.a.	n.a.	n.a.	n.a.	n.a.	n.a.	n.a.	n.a.	n.a.	n.a.
S3_vf_111776	Clostridium phage HM2	LT600745	Clustered	VC_1261_0	N36	4	Clostridium	Group I	Duplodnaviria	Heunggongvirae	Uroviricota	Caudoviricetes	n.a.	Salasmaviridae	O	O
S3_vf_115304	uncultured phage cr125_1	MT774407	Clustered	VC_519_2	C1	239	Unspecified	Group I	Duplodnaviria	Heunggongvirae	Uroviricota	Caudoviricetes	Crassvirales	Suoliviridae	Uncouvirinae	Aurodevirus
S3_vf_127168	n.a.	n.a.	Clustered	VC_1607_0	F	n.a.	n.a.	n.a.	n.a.	n.a.	n.a.	n.a.	n.a.	n.a.	n.a.	n.a.
S3_vf_127399	Bacteroides phage SJC03	MT074146	Clustered	VC_1489_0	N6	13	Bacteroides	Group I	Duplodnaviria	Heunggongvirae	Uroviricota	Caudoviricetes	n.a.	Unclassified	O	O
S3_vf_127405	Clostridium phage phiSM101	CP000315	Clustered	VC_21_0	N4	11	Clostridium	Group I	Duplodnaviria	Heunggongvirae	Uroviricota	Caudoviricetes	n.a.	Unclassified	O	O
S3_vf_127406	Phage DP SC_6_H4_2017	MT121964	Clustered	VC_1490_1	C3	269	Unspecified	Group I	Duplodnaviria	Heunggongvirae	Uroviricota	Caudoviricetes	n.a.	Unclassified	Unclassified	Unclassified
S3_vf_127409	Clostridium phage phiMMP01	LN681541	Clustered	VC_151_0	N19	21	Clostridium	Group I	Duplodnaviria	Heunggongvirae	Uroviricota	Caudoviricetes	n.a.	Unclassified	O	O
S3_vf_127423	n.a.	n.a.	Clustered	VC_1618_0	F	n.a.	n.a.	n.a.	n.a.	n.a.	n.a.	n.a.	n.a.	n.a.	n.a.	n.a.
S3_vf_127426	Gokushovirus WZ-2015a	KT264756	Clustered	VC_1145_0	C1	14	Unspecified	Group II	Monodnaviria	Sangervirae	Phixviricota	Malgrandaviricetes	Petitvirales	Microviridae	Gokushovirinae	Gokushovirus
S3_vf_127431	Inovirus sp.	MT135308	Clustered	VC_1492_0	C1	36	Unspecified	Group II	Monodnaviria	Loebvirae	Hofneiviricota	Faserviricetes	Tubulavirales	Inoviridae	Unclassified	Inovirus
S3_vf_127447	uncultured phage cr126_1	MT774391	Clustered	VC_1054_1	C1	233	Unspecified	Group I	Duplodnaviria	Heunggongvirae	Uroviricota	Caudoviricetes	Crassvirales	Steigviridae	Asinivirinae	Kolpuevirus
S3_vf_17748	Carjivirus communis	BK010471	Clustered	VC_691_0	C1	284	Unspecified	Group I	Duplodnaviria	Heunggongvirae	Uroviricota	Caudoviricetes	Crassvirales	Intestiviridae	Crudevirinae	Carjivirus
S3_vf_18080	n.a.	n.a.	Clustered	VC_1576_0	A	n.a.	n.a.	n.a.	n.a.	n.a.	n.a.	n.a.	n.a.	n.a.	n.a.	n.a.
S3_vf_18829	Phage DP SC_6_H4_2017	MT121964	Clustered	VC_1490_0	N6	121	Unspecified	Group I	Duplodnaviria	Heunggongvirae	Uroviricota	Caudoviricetes	n.a.	Unclassified	O	O
S3_vf_25163	uncultured phage cr109_1	MT774399	Clustered	VC_519_7	N1	300	Unspecified	Group I	Duplodnaviria	Heunggongvirae	Uroviricota	Caudoviricetes	Crassvirales	Suoliviridae	O	O
S3_vf_35073	Faecalibacterium phage FP_oengus	MG711463	Clustered	VC_1085_1	N20	53	Faecalibacterium	Group I	Duplodnaviria	Heunggongvirae	Uroviricota	Caudoviricetes	n.a.	Unclassified	O	O
S3_vf_44405	Siphovirus 29632	KY053532	Clustered	VC_826_0	C2	16	Unspecified	Group I	Duplodnaviria	Heunggongvirae	Uroviricota	Caudoviricetes	n.a.	Unclassified	Unclassified	Unclassified
S3_vf_54353	Clavibacter phage CMP1	GQ241246	Clustered	VC_880_0	C2	14	Clavibacter	Group I	Duplodnaviria	Heunggongvirae	Uroviricota	Caudoviricetes	n.a.	Unclassified	Unclassified	Cimpunavirus
S3_vf_54498	Microviridae sp.	MG945511	Clustered	VC_1299_0	C1	17	Unspecified	Group II	Monodnaviria	Sangervirae	Phixviricota	Malgrandaviricetes	Petitvirales	Microviridae	Unclassified	Unclassified
S3_vf_57422	n.a.	n.a.	Clustered	VC_1258_0	A	n.a.	n.a.	n.a.	n.a.	n.a.	n.a.	n.a.	n.a.	n.a.	n.a.	n.a.
S3_vf_67333	Roseburia phage Jekyll	LR596902	Clustered	VC_1254_0	N20	6	Roseburia	Group I	Duplodnaviria	Heunggongvirae	Uroviricota	Caudoviricetes	n.a.	Unclassified	O	O
S3_vf_67828	Tortoise microvirus 13	MK765563	Clustered	VC_1299_0	C1	21	Unspecified	Group II	Monodnaviria	Sangervirae	Phixviricota	Malgrandaviricetes	Petitvirales	Microviridae	Unclassified	Unclassified
S3_vf_70746	Bifidobacterium phage BigBern1	MT006237	Clustered	VC_879_0	C1	17	Bifidobacterium	Group I	Duplodnaviria	Heunggongvirae	Uroviricota	Caudoviricetes	n.a.	Unclassified	Unclassified	Unclassified
S3_vf_73597	Phage vB_RanS_PJN03	ON245412	Clustered	VC_948_0	N21	2	Unspecified	Group I	Duplodnaviria	Heunggongvirae	Uroviricota	Caudoviricetes	n.a.	Unclassified	O	O
S3_vf_80287	Clostridium phage phiCT453A	KM983327	Clustered	VC_151_0	N23	22	Clostridium	Group I	Duplodnaviria	Heunggongvirae	Uroviricota	Caudoviricetes	n.a.	Unclassified	O	O

S3_vf_8058	Faecalibacterium phage FP_Epona	MG711462	Clustered	VC_723_0	C5	91	Faecalibacterium	Group I	Duplodnaviria	Heunggongvirae	Uroviricota	Caudoviricetes	n.a.	Unclassified	Unclassified	Eponavirus
S3_vf_82571	Caldibacillus phage CBP1	MF595878	Clustered	VC_1274_0	N18	4	Caldibacillus	Group I	Duplodnaviria	Heunggongvirae	Uroviricota	Caudoviricetes	n.a.	Unclassified	O	O
S3_vf_83536	n.a.	n.a.	Clustered	VC_1258_0	A	n.a.	n.a.	n.a.	n.a.	n.a.	n.a.	n.a.	n.a.	n.a.	n.a.	n.a.
S3_vf_84832	n.a.	n.a.	Clustered	VC_1258_0	A	n.a.	n.a.	n.a.	n.a.	n.a.	n.a.	n.a.	n.a.	n.a.	n.a.	n.a.
S3_vf_96222	Lactococcus phage CaseusJM1	KC522412	Clustered	VC_210_0	C1	46	Lactococcus	Group I	Duplodnaviria	Heunggongvirae	Uroviricota	Caudoviricetes	n.a.	Unclassified	Unclassified	Skunavirus
S3_vf_99734	Faecalibacterium phage FP_Lagaffe	MG711461	Clustered	VC_727_0	C5	112	Faecalibacterium	Group I	Duplodnaviria	Heunggongvirae	Uroviricota	Caudoviricetes	n.a.	Unclassified	Unclassified	Lagaffevirus
S3_vs_100785	Bacillus phage vB_BtS_BMBtp13	KX190832	Clustered	VC_905_0	N21	4	Bacillus	Group I	Duplodnaviria	Heunggongvirae	Uroviricota	Caudoviricetes	n.a.	Unclassified	O	O
S3_vs_108076	Streptococcus phage MismyG	OL774867	Clustered	VC_152_0	N12	5	Streptococcus	Group I	Duplodnaviria	Heunggongvirae	Uroviricota	Caudoviricetes	n.a.	Unclassified	O	O
S3_vs_114271	n.a.	n.a.	Clustered	VC_1274_0	A	n.a.	n.a.	n.a.	n.a.	n.a.	n.a.	n.a.	n.a.	n.a.	n.a.	n.a.
S3_vs_1143	Faecalibacterium phage FP_Epona	MG711462	Clustered	VC_723_0	C5	92	Faecalibacterium	Group I	Duplodnaviria	Heunggongvirae	Uroviricota	Caudoviricetes	n.a.	Unclassified	Unclassified	Eponavirus
S3_vs_117894	n.a.	n.a.	Clustered	VC_1606_0	A	n.a.	n.a.	n.a.	n.a.	n.a.	n.a.	n.a.	n.a.	n.a.	n.a.	n.a.
S3_vs_119605	Streptococcus phage Javan630	MK448997	Clustered	VC_649_2	N10	41	Streptococcus	Group I	Duplodnaviria	Heunggongvirae	Uroviricota	Caudoviricetes	n.a.	Unclassified	O	O
S3_vs_119660	Bacteroides phage p00	BK010646	Clustered	VC_695_0	C3	62	Bacteroides	Group I	Duplodnaviria	Heunggongvirae	Uroviricota	Caudoviricetes	n.a.	Unclassified	Unclassified	Unclassified
S3_vs_123834	Bifidobacterium phage PMBT6	MH444512	Clustered	VC_878_0	N2	52	Bifidobacterium	Group I	Duplodnaviria	Heunggongvirae	Uroviricota	Caudoviricetes	n.a.	Unclassified	O	O
S3_vs_127387	Butyrivibrio virus Arian	MN882551	Clustered	VC_720_0	N6	16	Butyrivibrio	Group I	Duplodnaviria	Heunggongvirae	Uroviricota	Caudoviricetes	n.a.	Unclassified	O	O
S3_vs_127391	n.a.	n.a.	Clustered	VC_641_0	A	n.a.	n.a.	n.a.	n.a.	n.a.	n.a.	n.a.	n.a.	n.a.	n.a.	n.a.
S3_vs_127404	Parabacteroides phage PDS1	MN929097	Clustered	VC_855_0	C1	84	Parabacteroides	Group I	Duplodnaviria	Heunggongvirae	Uroviricota	Caudoviricetes	n.a.	Unclassified	Unclassified	Unclassified
S3_vs_127413	Croceibacter phage P2559Y	KC688701	Clustered	VC_1045_0	N2	13	Croceibacter	Group I	Duplodnaviria	Heunggongvirae	Uroviricota	Caudoviricetes	n.a.	Unclassified	O	O
S3_vs_127442	Poophage MBI-2016a	LT986678	Clustered	VC_1267_0	C1	24	Unspecified	Group II	Monodnaviria	Sangervirae	Phixviricota	Malgrandaviricetes	Petitvirales	Microviridae	Unclassified	Unclassified
S3_vs_127445	Phage DP SC_6_H4_2017	MT121964	Clustered	VC_1490_1	C2	172	Unspecified	Group I	Duplodnaviria	Heunggongvirae	Uroviricota	Caudoviricetes	n.a.	Unclassified	Unclassified	Unclassified
S3_vs_12973	n.a.	n.a.	Clustered	VC_1611_0	A	n.a.	n.a.	n.a.	n.a.	n.a.	n.a.	n.a.	n.a.	n.a.	n.a.	n.a.
S3_vs_12985	Butyrivibrio phage Idris	MN882554	Clustered	VC_1289_0	N5	30	Butyrivibrio	Group I	Duplodnaviria	Heunggongvirae	Uroviricota	Caudoviricetes	n.a.	Unclassified	O	O
S3_vs_17168	Pasteurella phage AFS-2018a	MH238466	Clustered	VC_630_0	N4	4	Pasteurella	Group I	Duplodnaviria	Heunggongvirae	Uroviricota	Caudoviricetes	n.a.	Unclassified	O	O
S3_vs_18858	n.a.	n.a.	Clustered	VC_1291_0	A	n.a.	n.a.	n.a.	n.a.	n.a.	n.a.	n.a.	n.a.	n.a.	n.a.	n.a.
S3_vs_28402	n.a.	n.a.	Clustered	VC_779_0	A	n.a.	n.a.	n.a.	n.a.	n.a.	n.a.	n.a.	n.a.	n.a.	n.a.	n.a.
S3_vs_29077	Streptococcus phage Javan90	MK449010	Clustered	VC_643_0	N5	15	Streptococcus	Group I	Duplodnaviria	Heunggongvirae	Uroviricota	Caudoviricetes	n.a.	Unclassified	O	O
S3_vs_42569	Faecalibacterium phage FP_Toutatis	MG711466	Clustered	VC_728_0	C1	146	Faecalibacterium	Group I	Duplodnaviria	Heunggongvirae	Uroviricota	Caudoviricetes	n.a.	Unclassified	Unclassified	Toutatisvirus
S3_vs_45147	n.a.	n.a.	Clustered	VC_1560_0	A	n.a.	n.a.	n.a.	n.a.	n.a.	n.a.	n.a.	n.a.	n.a.	n.a.	n.a.
S3_vs_49835	n.a.	n.a.	Clustered	VC_1291_0	A	n.a.	n.a.	n.a.	n.a.	n.a.	n.a.	n.a.	n.a.	n.a.	n.a.	n.a.
S3_vs_50924	n.a.	n.a.	Clustered	VC_1613_0	A	n.a.	n.a.	n.a.	n.a.	n.a.	n.a.	n.a.	n.a.	n.a.	n.a.	n.a.
S3_vs_54303	Faecalibacterium phage FP_Epona	MG711462	Clustered	VC_723_0	C13	56	Faecalibacterium	Group I	Duplodnaviria	Heunggongvirae	Uroviricota	Caudoviricetes	n.a.	Unclassified	Unclassified	Eponavirus
S3_vs_64424	n.a.	n.a.	Clustered	VC_1063_0	A	n.a.	n.a.	n.a.	n.a.	n.a.	n.a.	n.a.	n.a.	n.a.	n.a.	n.a.
S3_vs_70508	n.a.	n.a.	Clustered	VC_1572_0	A	n.a.	n.a.	n.a.	n.a.	n.a.	n.a.	n.a.	n.a.	n.a.	n.a.	n.a.
S3_vs_72118	n.a.	n.a.	Clustered	VC_1570_0	A	n.a.	n.a.	n.a.	n.a.	n.a.	n.a.	n.a.	n.a.	n.a.	n.a.	n.a.
S3_vs_73361	n.a.	n.a.	Clustered	VC_1231_0	A	n.a.	n.a.	n.a.	n.a.	n.a.	n.a.	n.a.	n.a.	n.a.	n.a.	n.a.
S3_vs_77156	Microviridae sp.	MG945455	Clustered	VC_1267_0	C1	23	Unspecified	Group II	Monodnaviria	Sangervirae	Phixviricota	Malgrandaviricetes	Petitvirales	Microviridae	Unclassified	Unclassified
S3_vs_80151	Cellulophaga phage phi17:2	KC821609	Clustered	VC_1056_0	N8	4	Cellulophaga	Group I	Duplodnaviria	Heunggongvirae	Uroviricota	Caudoviricetes	n.a.	Unclassified	O	O
S3_vs_82895	Cellulophaga phage phi17:2	KC821609	Clustered	VC_1055_1	N8	6	Cellulophaga	Group I	Duplodnaviria	Heunggongvirae	Uroviricota	Caudoviricetes	n.a.	Unclassified	O	O
S3_vs_89898	Parabacteroides phage PDS1	MN929097	Clustered	VC_855_0	C5	106	Parabacteroides	Group I	Duplodnaviria	Heunggongvirae	Uroviricota	Caudoviricetes	n.a.	Unclassified	Unclassified	Unclassified
S3_vs_90341	n.a.	n.a.	Clustered	VC_1620_0	F	n.a.	n.a.	n.a.	n.a.	n.a.	n.a.	n.a.	n.a.	n.a.	n.a.	n.a.
S3_vs_95574	n.a.	n.a.	Clustered	VC_1291_0	A	n.a.	n.a.	n.a.	n.a.	n.a.	n.a.	n.a.	n.a.	n.a.	n.a.	n.a.
S3_vs_98675	Clostridium phage CWou-2020a	CP063964	Clustered	VC_747_0	N2	5	Clostridium	Group I	Duplodnaviria	Heunggongvirae	Uroviricota	Caudoviricetes	n.a.	Unclassified	O	O
S4_vf_10506	n.a.	n.a.	Clustered	VC_1617_0	F	n.a.	n.a.	n.a.	n.a.	n.a.	n.a.	n.a.	n.a.	n.a.	n.a.	n.a.
S4_vf_12614	n.a.	n.a.	Clustered	VC_949_0	A	n.a.	n.a.	n.a.	n.a.	n.a.	n.a.	n.a.	n.a.	n.a.	n.a.	n.a.
S4_vf_12624	Curtobacterium phage Penoan	MZ333133	Clustered	VC_1195_0	N3	4	Curtobacterium	Group I	Duplodnaviria	Heunggongvirae	Uroviricota	Caudoviricetes	n.a.	Unclassified	O	O
S4_vf_13627	n.a.	n.a.	Clustered	VC_1258_0	A	n.a.	n.a.	n.a.	n.a.	n.a.	n.a.	n.a.	n.a.	n.a.	n.a.	n.a.
S4_vf_13759	Bacillus phage vB_BtS_BMBtp13	KX190832	Clustered	VC_667_0	C1	13	Bacillus	Group I	Duplodnaviria	Heunggongvirae	Uroviricota	Caudoviricetes	n.a.	Unclassified	Unclassified	Unclassified
S4_vf_18803	Bacteroides phage F4	MT806185	Clustered	VC_1044_0	C2	59	Bacteroides	Group I	Duplodnaviria	Heunggongvirae	Uroviricota	Caudoviricetes	n.a.	Unclassified	Unclassified	Unclassified
S4_vf_22219	Bacillus phage BCD7	JN712910	Clustered	VC_995_0	C1	49	Bacillus	Group I	Duplodnaviria	Heunggongvirae	Uroviricota	Caudoviricetes	n.a.	Unclassified	Unclassified	Becedseptimavirus
S4_vf_2524	n.a.	n.a.	Clustered	VC_946_0	A	n.a.	n.a.	n.a.	n.a.	n.a.	n.a.	n.a.	n.a.	n.a.	n.a.	n.a.
S4_vf_26417	uncultured phage cr127_1	MT774393	Clustered	VC_693_0	C1	264	Unspecified	Group I	Duplodnaviria	Heunggongvirae	Uroviricota	Caudoviricetes	Crassvirales	Crevaviridae	Doltivirinae	Kahucivirus
S4_vf_27861	Phage DP SC_6_H4_2017	MT121964	Clustered	VC_1490_0	N7	120	Unspecified	Group I	Duplodnaviria	Heunggongvirae	Uroviricota	Caudoviricetes	n.a.	Unclassified	O	O
S4_vf_28601	Bifidobacterium phage BigBern1	MT006237	Clustered	VC_879_0	C4	6	Bifidobacterium	Group I	Duplodnaviria	Heunggongvirae	Uroviricota	Caudoviricetes	n.a.	Unclassified	Unclassified	Unclassified
S4_vf_31978	n.a.	n.a.	Clustered	VC_1254_1	A	n.a.	n.a.	n.a.	n.a.	n.a.	n.a.	n.a.	n.a.	n.a.	n.a.	n.a.
S4_vf_31981	uncultured phage cr118_1	MT774379	Clustered	VC_519_10	C1	201	Unspecified	Group I	Duplodnaviria	Heunggongvirae	Uroviricota	Caudoviricetes	Crassvirales	Suoliviridae	Uncouvirinae	Besingivirus
S4_vf_4838	uncultured phage cr50_1	MT774375	Clustered	VC_519_11	C1	272	Unspecified	Group I	Duplodnaviria	Heunggongvirae	Uroviricota	Caudoviricetes	Crassvirales	Suoliviridae	Boorivirinae	Cohcovirus
S4_vf_4891	n.a.	n.a.	Clustered	VC_1561_0	A	n.a.	n.a.	n.a.	n.a.	n.a.	n.a.	n.a.	n.a.	n.a.	n.a.	n.a.
S4_vf_5021	Clostridium phage HM2	LT600745	Clustered	VC_1258_0	N38	3	Clostridium	Group I	Duplodnaviria	Heunggongvirae	Uroviricota	Caudoviricetes	n.a.	Salasmaviridae	O	O
S4_vs_VIRSorter_1135-cat_2	Clostridium phage phiMMP03	LN681542	Clustered	VC_151_0	N19	22	Clostridium	Group I	Duplodnaviria	Heunggongvirae	Uroviricota	Caudoviricetes	n.a.	Unclassified	O	O
S4_vs_VIRSorter_12831-circular-cat_2	Ruminococcus phage phiRgPS_6	MT980839	Clustered	VC_779_0	N31	5	Ruminococcus	Group I	Duplodnaviria	Heunggongvirae	Uroviricota	Caudoviricetes	n.a.	Unclassified	O	O
S4_vs_VIRSorter_13190-cat_2	Cellulophaga phage phi17:2	KC821609	Clustered	VC_1056_0	N8	3	Cellulophaga	Group I	Duplodnaviria	Heunggongvirae	Uroviricota	Caudoviricetes	n.a.	Unclassified	O	O
S4_vs_VIRSorter_13488-cat_2	n.a.	n.a.	Clustered	VC_1611_0	A	n.a.	n.a.	n.a.	n.a.	n.a.	n.a.	n.a.	n.a.	n.a.	n.a.	n.a.
S4_vs_VIRSorter_15112-cat_2	n.a.	n.a.	Clustered	VC_1604_0	A	n.a.	n.a.	n.a.	n.a.	n.a.	n.a.	n.a.	n.a.	n.a.	n.a.	n.a.
S4_vs_VIRSorter_15837-cat_2	Clostridium phage phiCT453A	KM983327	Clustered	VC_152_0	N19	8	Clostridium	Group I	Duplodnaviria	Heunggongvirae	Uroviricota	Caudoviricetes	n.a.	Unclassified	O	O
S4_vs_VIRSorter_16493-cat_2	Bifidobacterium phage Bbif-1	GQ141189	Clustered	VC_876_0	C1	150	Bifidobacterium	Group I	Duplodnaviria	Heunggongvirae	Uroviricota	Caudoviricetes	n.a.	Unclassified	Unclassified	Unclassified
S4_vs_VIRSorter_19324-cat_2	Erysipelothrix phage SE-1	KR816341	Clustered	VC_1149_0	N3	18	Erysipelothrix	Group I	Duplodnaviria	Heunggongvirae	Uroviricota	Caudoviricetes	n.a.	Unclassified	O	O
S4_vs_VIRSorter_3006-cat_1	Faecalibacterium phage FP_oengus	MG711463	Clustered	VC_1085_1	N21	64	Faecalibacterium	Group I	Duplodnaviria	Heunggongvirae	Uroviricota	Caudoviricetes	n.a.	Unclassified	O	O
S4_vs_VIRSorter_30222-cat_2	Faecalibacterium phage FP_oengus	MG711463	Clustered	VC_1085_0	N17	71	Faecalibacterium	Group I	Duplodnaviria	Heunggongvirae	Uroviricota	Caudoviricetes	n.a.	Unclassified	O	O
S4_vs_VIRSorter_31171-circular-cat_2	Faecalibacterium phage FP_Lugh	MG711464	Clustered	VC_923_0	N5	13	Faecalibacterium	Group I	Duplodnaviria	Heunggongvirae	Uroviricota	Caudoviricetes	n.a.	Unclassified	O	O
S4_vs_VIRSorter_31196-circular-cat_2	n.a.	n.a.	Clustered	VC_1481_0	A	n.a.	n.a.	n.a.	n.a.	n.a.	n.a.	n.a.	n.a.	n.a.	n.a.	n.a.
S4_vs_VIRSorter_31560-circular-cat_2	Butyrivibrio virus Ceridwen	MN882553	Clustered	VC_1481_1	N49	1	Butyrivibrio	Group I	Duplodnaviria	Heunggongvirae	Uroviricota	Caudoviricetes	n.a.	Unclassified	O	O
S4_vs_VIRSorter_31976-circular-cat_2	n.a.	n.a.	Clustered	VC_641_0	A	n.a.	n.a.	n.a.	n.a.	n.a.	n.a.	n.a.	n.a.	n.a.	n.a.	n.a.
S4_vs_VIRSorter_31977-circular-cat_2	Faecalibacterium phage FP_Lugh	MG711464	Clustered	VC_923_0	N23	3	Faecalibacterium	Group I	Duplodnaviria	Heunggongvirae	Uroviricota	Caudoviricetes	n.a.	Unclassified	O	O
S4_vs_VIRSorter_31980-circular-cat_2	Phage WC36-2	OL791266	Clustered	VC_249_1	C1	37	Unspecified	Group I	Duplodnaviria	Heunggongvirae	Uroviricota	Caudoviricetes	n.a.	Unclassified	Unclassified	Unclassified
S4_vs_VIRSorter_4746-cat_2	Clostridium phage HM2	LT600745	Clustered	VC_1265_0	N19	4	Clostridium	Group I	Duplodnaviria	Heunggongvirae	Uroviricota	Caudoviricetes	n.a.	Salasmaviridae	O	O
S4_vs_VIRSorter_4839-cat_2	Faecalibacterium phage FP_oengus	MG711463	Clustered	VC_1085_1	N20	65	Faecalibacterium	Group I	Duplodnaviria	Heunggongvirae	Uroviricota	Caudoviricetes	n.a.	Unclassified	O	O
S4_vs_VIRSorter_4959-cat_2	Clostridioides phage CD1801	MW512570	Clustered	VC_670_0	N7	13	Clostridioides	Group I	Duplodnaviria	Heunggongvirae	Uroviricota	Caudoviricetes	n.a.	Unclassified	O	O
S4_vs_VIRSorter_6022-circular-cat_1	Parabacteroides phage PDS1	MN929097	Clustered	VC_855_0	C3	147	Parabacteroides	Group I	Duplodnaviria	Heunggongvirae	Uroviricota	Caudoviricetes	n.a.	Unclassified	Unclassified	Unclassified
S4_vs_VIRSorter_7386-circular-cat_2	Faecalibacterium phage FP_Epona	MG711462	Clustered	VC_723_0	C14	48	Faecalibacterium	Group I	Duplodnaviria	Heunggongvirae	Uroviricota	Caudoviricetes	n.a.	Unclassified	Unclassified	Eponavirus
S4_vs_VIRSorter_8622-cat_2	Faecalibacterium phage FP_Lagaffe	MG711461	Clustered	VC_727_0	C5	99	Faecalibacterium	Group I	Duplodnaviria	Heunggongvirae	Uroviricota	Caudoviricetes	n.a.	Unclassified	Unclassified	Lagaffevirus
S4_vs_VIRSorter_9913-cat_1	Parabacteroides phage PDS1	MN929097	Clustered	VC_855_0	C5	83	Parabacteroides	Group I	Duplodnaviria	Heunggongvirae	Uroviricota	Caudoviricetes	n.a.	Unclassified	Unclassified	Unclassified
S5_vf_110171	n.a.	n.a.	Clustered	VC_1552_0	A	n.a.	n.a.	n.a.	n.a.	n.a.	n.a.	n.a.	n.a.	n.a.	n.a.	n.a.

S5_vf_11450	Geobacillus phage GBSV1	DQ340064	Clustered	VC_778_0	N37	6	Geobacillus	Group I	Duplodnaviria	Heunggongvirae	Uroviricota	Caudoviricetes	n.a.	Unclassified	O	O
S5_vf_122568	Faecalibacterium phage FP_Taranis	MG711467	Clustered	VC_732_0	N3	98	Faecalibacterium	Group I	Duplodnaviria	Heunggongvirae	Uroviricota	Caudoviricetes	n.a.	Unclassified	O	O
S5_vf_123990	n.a.	n.a.	Clustered	VC_1258_0	A	n.a.	n.a.	n.a.	n.a.	n.a.	n.a.	n.a.	n.a.	n.a.	n.a.	n.a.
S5_vf_134040	Faecalibacterium phage FP_Mushu	MG711460	Clustered	VC_1288_0	C3	100	Faecalibacterium	Group I	Duplodnaviria	Heunggongvirae	Uroviricota	Caudoviricetes	n.a.	Unclassified	Unclassified	Mushuvirus
S5_vf_136076	n.a.	n.a.	Clustered	VC_1552_0	A	n.a.	n.a.	n.a.	n.a.	n.a.	n.a.	n.a.	n.a.	n.a.	n.a.	n.a.
S5_vf_137260	Bacteriophage sp.	OP549880	Clustered	VC_1549_0	N5	16	Unspecified	Unclassified	Unclassified	n.a.	n.a.	n.a.	n.a.	Unclassified	O	O
S5_vf_145585	Bacillus phage vB_BpsS-140	MH884512	Clustered	VC_638_0	C1	6	Bacillus	Group I	Duplodnaviria	Heunggongvirae	Uroviricota	Caudoviricetes	n.a.	Unclassified	Unclassified	Unclassified
S5_vf_145588	Clostridium phage phiCTP1	HM159959	Clustered	VC_921_0	N4	8	Clostridium	Group I	Duplodnaviria	Heunggongvirae	Uroviricota	Caudoviricetes	n.a.	Unclassified	O	O
S5_vf_18461	Phage vB_RanS_PJN03	ON245412	Clustered	VC_951_1	N15	4	Unspecified	Group I	Duplodnaviria	Heunggongvirae	Uroviricota	Caudoviricetes	n.a.	Unclassified	O	O
S5_vf_25423	Lactococcus phage 936 group phage Phi4.2	KP793123	Clustered	VC_210_0	C1	76	Lactococcus	Group I	Duplodnaviria	Heunggongvirae	Uroviricota	Caudoviricetes	n.a.	Unclassified	Unclassified	Skunavirus
S5_vf_28061	n.a.	n.a.	Clustered	VC_430_0	A	n.a.	n.a.	n.a.	n.a.	n.a.	n.a.	n.a.	n.a.	n.a.	n.a.	n.a.
S5_vf_55415	Bacteroides phage crAss001	MH675552	Clustered	VC_1054_1	C1	261	Bacteroides	Group I	Duplodnaviria	Heunggongvirae	Uroviricota	Caudoviricetes	Crassvirales	Steigviridae	Asinivirinae	Kehishuvirus
S5_vf_6032	n.a.	n.a.	Clustered	VC_1511_0	A	n.a.	n.a.	n.a.	n.a.	n.a.	n.a.	n.a.	n.a.	n.a.	n.a.	n.a.
S5_vf_6506	Clostridium phage vB_CpeS-1181	MW478292	Clustered	VC_923_0	N1	28	Clostridium	Group I	Duplodnaviria	Heunggongvirae	Uroviricota	Caudoviricetes	n.a.	Unclassified	O	O
S5_vf_74661	Escherichia phage TL-2011b	JQ011317	Clustered	VC_492_0	C1	112	Escherichia	Group I	Duplodnaviria	Heunggongvirae	Uroviricota	Caudoviricetes	n.a.	Unclassified	Unclassified	Unclassified
S5_vf_77701	Bifidobacterium phage BitterVaud1	MT006236	Clustered	VC_826_0	C2	11	Bifidobacterium	Group I	Duplodnaviria	Heunggongvirae	Uroviricota	Caudoviricetes	n.a.	Unclassified	Unclassified	Unclassified
S5_vf_8965	Bacteroides phage F4	MT806185	Clustered	VC_1505_0	N9	11	Bacteroides	Group I	Duplodnaviria	Heunggongvirae	Uroviricota	Caudoviricetes	n.a.	Unclassified	O	O
S5_vs_107105	Butyrivibrio phage Idris	MN882554	Clustered	VC_724_0	N15	4	Butyrivibrio	Group I	Duplodnaviria	Heunggongvirae	Uroviricota	Caudoviricetes	n.a.	Unclassified	O	O
S5_vs_116235	Streptococcus phage Javan261	MK448720	Clustered	VC_905_0	N10	7	Streptococcus	Group I	Duplodnaviria	Heunggongvirae	Uroviricota	Caudoviricetes	n.a.	Unclassified	O	O
S5_vs_120079	Roseburia phage Jekyll	LR596902	Clustered	VC_1252_0	C1	68	Roseburia	Group I	Duplodnaviria	Heunggongvirae	Uroviricota	Caudoviricetes	n.a.	Unclassified	Unclassified	Unclassified
S5_vs_121419	n.a.	n.a.	Clustered	VC_1570_0	A	n.a.	n.a.	n.a.	n.a.	n.a.	n.a.	n.a.	n.a.	n.a.	n.a.	n.a.
S5_vs_123256	Bacteroides phage p00	BK010646	Clustered	VC_695_0	C5	15	Bacteroides	Group I	Duplodnaviria	Heunggongvirae	Uroviricota	Caudoviricetes	n.a.	Unclassified	Unclassified	Unclassified
S5_vs_124035	n.a.	n.a.	Clustered	VC_1614_0	F	n.a.	n.a.	n.a.	n.a.	n.a.	n.a.	n.a.	n.a.	n.a.	n.a.	n.a.
S5_vs_129970	n.a.	n.a.	Clustered	VC_1572_0	A	n.a.	n.a.	n.a.	n.a.	n.a.	n.a.	n.a.	n.a.	n.a.	n.a.	n.a.
S5_vs_13090	n.a.	n.a.	Clustered	VC_1587_0	A	n.a.	n.a.	n.a.	n.a.	n.a.	n.a.	n.a.	n.a.	n.a.	n.a.	n.a.
S5_vs_130958	Faecalibacterium phage FP_Taranis	MG711467	Clustered	VC_732_0	N3	75	Faecalibacterium	Group I	Duplodnaviria	Heunggongvirae	Uroviricota	Caudoviricetes	n.a.	Unclassified	O	O
S5_vs_1338	n.a.	n.a.	Clustered	VC_1582_0	A	n.a.	n.a.	n.a.	n.a.	n.a.	n.a.	n.a.	n.a.	n.a.	n.a.	n.a.
S5_vs_136679	n.a.	n.a.	Clustered	VC_1291_0	A	n.a.	n.a.	n.a.	n.a.	n.a.	n.a.	n.a.	n.a.	n.a.	n.a.	n.a.
S5_vs_137154	Clostridium phage PhiS63	JQ660954	Clustered	VC_747_0	N2	5	Clostridium	Group I	Duplodnaviria	Heunggongvirae	Uroviricota	Caudoviricetes	n.a.	Unclassified	O	O
S5_vs_138980	Clostridium phage phiCT19406B	KM983331	Clustered	VC_660_0	N9	9	Clostridium	Group I	Duplodnaviria	Heunggongvirae	Uroviricota	Caudoviricetes	n.a.	Unclassified	O	O
S5_vs_139401	n.a.	n.a.	Clustered	VC_1587_0	A	n.a.	n.a.	n.a.	n.a.	n.a.	n.a.	n.a.	n.a.	n.a.	n.a.	n.a.
S5_vs_140946	n.a.	n.a.	Clustered	VC_1613_0	A	n.a.	n.a.	n.a.	n.a.	n.a.	n.a.	n.a.	n.a.	n.a.	n.a.	n.a.
S5_vs_142195	n.a.	n.a.	Clustered	VC_1605_0	A	n.a.	n.a.	n.a.	n.a.	n.a.	n.a.	n.a.	n.a.	n.a.	n.a.	n.a.
S5_vs_142450	Clostridium phage HM2	LT600745	Clustered	VC_1261_0	N36	4	Clostridium	Group I	Duplodnaviria	Heunggongvirae	Uroviricota	Caudoviricetes	n.a.	Salasmaviridae	O	O
S5_vs_145026	n.a.	n.a.	Clustered	VC_1291_0	A	n.a.	n.a.	n.a.	n.a.	n.a.	n.a.	n.a.	n.a.	n.a.	n.a.	n.a.
S5_vs_145581	n.a.	n.a.	Clustered	VC_658_0	A	n.a.	n.a.	n.a.	n.a.	n.a.	n.a.	n.a.	n.a.	n.a.	n.a.	n.a.
S5_vs_145590	Enterococcus phage EFC-1	KJ608188	Clustered	VC_278_0	N12	14	Enterococcus	Group I	Duplodnaviria	Heunggongvirae	Uroviricota	Caudoviricetes	n.a.	Unclassified	O	O
S5_vs_145592	n.a.	n.a.	Clustered	VC_1554_0	A	n.a.	n.a.	n.a.	n.a.	n.a.	n.a.	n.a.	n.a.	n.a.	n.a.	n.a.
S5_vs_145593	n.a.	n.a.	Clustered	VC_1615_0	F	n.a.	n.a.	n.a.	n.a.	n.a.	n.a.	n.a.	n.a.	n.a.	n.a.	n.a.
S5_vs_145597	Thermoanaerobacterium phage THSA-485A	CP003186	Clustered	VC_724_0	N12	3	Thermoanaerobacterium	Group I	Duplodnaviria	Heunggongvirae	Uroviricota	Caudoviricetes	n.a.	Unclassified	O	O
S5_vs_145598	Clostridium phage CDMH1	HG531805	Clustered	VC_151_0	N11	25	Clostridium	Group I	Duplodnaviria	Heunggongvirae	Uroviricota	Caudoviricetes	n.a.	Unclassified	O	O
S5_vs_145599	Bacteriophage sp.	LT996078	Clustered	VC_278_0	N26	4	Unspecified	Unclassified	Unclassified	n.a.	n.a.	n.a.	n.a.	Unclassified	O	O
S5_vs_145601	Streptococcus phage MismyG	OL774867	Clustered	VC_152_0	N11	7	Streptococcus	Group I	Duplodnaviria	Heunggongvirae	Uroviricota	Caudoviricetes	n.a.	Unclassified	O	O
S5_vs_145602	Enterococcus phage EFC-1	KJ608188	Clustered	VC_278_0	N11	13	Enterococcus	Group I	Duplodnaviria	Heunggongvirae	Uroviricota	Caudoviricetes	n.a.	Unclassified	O	O
S5_vs_145605	Bacillus phage vB_BanS_Athena	OK500002	Clustered	VC_946_0	N8	2	Bacillus	Group I	Duplodnaviria	Heunggongvirae	Uroviricota	Caudoviricetes	n.a.	Unclassified	O	O
S5_vs_1572	Parabacteroides phage PDS1	MN929097	Clustered	VC_855_0	C4	120	Parabacteroides	Group I	Duplodnaviria	Heunggongvirae	Uroviricota	Caudoviricetes	n.a.	Unclassified	Unclassified	Unclassified
S5_vs_17520	n.a.	n.a.	Clustered	VC_1587_0	A	n.a.	n.a.	n.a.	n.a.	n.a.	n.a.	n.a.	n.a.	n.a.	n.a.	n.a.
S5_vs_20268	Paenibacillus phage PG1	HQ332138	Clustered	VC_349_0	C1	11	Paenibacillus	Group I	Duplodnaviria	Heunggongvirae	Uroviricota	Caudoviricetes	n.a.	Unclassified	Unclassified	Unclassified
S5_vs_20500	Pasteurella phage AFS-2018a	MH238466	Clustered	VC_630_0	N4	5	Pasteurella	Group I	Duplodnaviria	Heunggongvirae	Uroviricota	Caudoviricetes	n.a.	Unclassified	O	O
S5_vs_28263	n.a.	n.a.	Clustered	VC_1587_0	A	n.a.	n.a.	n.a.	n.a.	n.a.	n.a.	n.a.	n.a.	n.a.	n.a.	n.a.
S5_vs_2876	Lactococcus phage CAP	MZ308445	Clustered	VC_278_0	N13	5	Lactococcus	Group I	Duplodnaviria	Heunggongvirae	Uroviricota	Caudoviricetes	n.a.	Unclassified	O	O
S5_vs_3183	Bacillus phage vB_BpsS-140	MH884512	Clustered	VC_782_0	N11	1	Bacillus	Group I	Duplodnaviria	Heunggongvirae	Uroviricota	Caudoviricetes	n.a.	Unclassified	O	O
S5_vs_3501	Clostridium phage vB_CpeS-CP51	KC237729	Clustered	VC_1035_0	N2	11	Clostridium	Group I	Duplodnaviria	Heunggongvirae	Uroviricota	Caudoviricetes	n.a.	Unclassified	O	O
S5_vs_38949	Faecalibacterium phage FP_Epona	MG711462	Clustered	VC_723_0	C13	52	Faecalibacterium	Group I	Duplodnaviria	Heunggongvirae	Uroviricota	Caudoviricetes	n.a.	Unclassified	Unclassified	Eponavirus
S5_vs_40848	Streptococcus phage Javan630	MK448997	Clustered	VC_649_0	N6	56	Streptococcus	Group I	Duplodnaviria	Heunggongvirae	Uroviricota	Caudoviricetes	n.a.	Unclassified	O	O
S5_vs_49480	Geobacillus phage GBSV1	DQ340064	Clustered	VC_778_0	N37	6	Geobacillus	Group I	Duplodnaviria	Heunggongvirae	Uroviricota	Caudoviricetes	n.a.	Unclassified	O	O
S5_vs_56083	n.a.	n.a.	Clustered	VC_1274_0	A	n.a.	n.a.	n.a.	n.a.	n.a.	n.a.	n.a.	n.a.	n.a.	n.a.	n.a.
S5_vs_58766	Clostridium phage phiCD38-2	HM568888	Clustered	VC_923_0	N14	8	Clostridium	Group I	Duplodnaviria	Heunggongvirae	Uroviricota	Caudoviricetes	n.a.	Unclassified	O	O
S5_vs_58859	Geobacillus phage GBSV1	DQ340064	Clustered	VC_779_0	N41	2	Geobacillus	Group I	Duplodnaviria	Heunggongvirae	Uroviricota	Caudoviricetes	n.a.	Unclassified	O	O
S5_vs_63924	Bifidobacterium phage BigBern1	MT006237	Clustered	VC_879_0	C2	32	Bifidobacterium	Group I	Duplodnaviria	Heunggongvirae	Uroviricota	Caudoviricetes	n.a.	Unclassified	Unclassified	Unclassified
S5_vs_67958	n.a.	n.a.	Clustered	VC_1554_0	A	n.a.	n.a.	n.a.	n.a.	n.a.	n.a.	n.a.	n.a.	n.a.	n.a.	n.a.
S5_vs_69083	Winogradskyella phage Peternella_1	MT732475	Clustered	VC_696_0	N3	27	Winogradskyella	Group I	Duplodnaviria	Heunggongvirae	Uroviricota	Caudoviricetes	n.a.	Winoviridae	O	O
S5_vs_74199	n.a.	n.a.	Clustered	VC_1291_0	A	n.a.	n.a.	n.a.	n.a.	n.a.	n.a.	n.a.	n.a.	n.a.	n.a.	n.a.
S5_vs_7444	n.a.	n.a.	Clustered	VC_1614_0	F	n.a.	n.a.	n.a.	n.a.	n.a.	n.a.	n.a.	n.a.	n.a.	n.a.	n.a.
S5_vs_76500	Bacteroides phage p00	BK010646	Clustered	VC_695_0	C6	20	Bacteroides	Group I	Duplodnaviria	Heunggongvirae	Uroviricota	Caudoviricetes	n.a.	Unclassified	Unclassified	Unclassified
S5_vs_79992	Winogradskyella phage Peternella_1	MT732475	Clustered	VC_696_0	N3	24	Winogradskyella	Group I	Duplodnaviria	Heunggongvirae	Uroviricota	Caudoviricetes	n.a.	Winoviridae	O	O
S5_vs_80498	Lactococcus phage Tuc2009	AF109874	Clustered	VC_278_0	N9	11	Lactococcus	Group I	Duplodnaviria	Heunggongvirae	Uroviricota	Caudoviricetes	n.a.	Unclassified	O	O
S5_vs_83886	n.a.	n.a.	Clustered	VC_920_0	A	n.a.	n.a.	n.a.	n.a.	n.a.	n.a.	n.a.	n.a.	n.a.	n.a.	n.a.
S5_vs_84946	Streptococcus phage Javan290	MK448900	Clustered	VC_332_0	N27	5	Streptococcus	Group I	Duplodnaviria	Heunggongvirae	Uroviricota	Caudoviricetes	n.a.	Unclassified	O	O
S5_vs_85065	Faecalibacterium phage FP_oengus	MG711463	Clustered	VC_1085_1	N17	70	Faecalibacterium	Group I	Duplodnaviria	Heunggongvirae	Uroviricota	Caudoviricetes	n.a.	Unclassified	O	O
S5_vs_86562	Akkermansia phage DTMo-2021a	CP084202	Clustered	VC_752_0	N2	15	Akkermansia	Group I	Duplodnaviria	Heunggongvirae	Uroviricota	Caudoviricetes	n.a.	Unclassified	O	O
S5_vs_8847	Streptococcus phage Javan191	MK448700	Clustered	VC_649_1	C2	63	Streptococcus	Group I	Duplodnaviria	Heunggongvirae	Uroviricota	Caudoviricetes	n.a.	Unclassified	Unclassified	Unclassified
S5_vs_93839	Flavobacterium phage fF4	MN850656	Clustered	VC_694_0	N2	23	Flavobacterium	Group I	Duplodnaviria	Heunggongvirae	Uroviricota	Caudoviricetes	n.a.	Unclassified	O	O
S5_vs_9747	n.a.	n.a.	Clustered	VC_1595_0	A	n.a.	n.a.	n.a.	n.a.	n.a.	n.a.	n.a.	n.a.	n.a.	n.a.	n.a.
S6_vf_25710	n.a.	n.a.	Clustered	VC_750_0	A	n.a.	n.a.	n.a.	n.a.	n.a.	n.a.	n.a.	n.a.	n.a.	n.a.	n.a.
S6_vf_3435	Lactococcus phage 62403	MF443120	Clustered	VC_706_0	C1	75	Lactococcus	Group I	Duplodnaviria	Heunggongvirae	Uroviricota	Caudoviricetes	n.a.	Unclassified	Unclassified	Ceduovirus
S6_vf_45228	Clostridium phage HM2	LT600745	Clustered	VC_1261_0	N33	4	Clostridium	Group I	Duplodnaviria	Heunggongvirae	Uroviricota	Caudoviricetes	n.a.	Salasmaviridae	O	O
S6_vf_45618	Eggerthella phage PMBT5	MH626557	Clustered	VC_1421_0	N42	2	Eggerthella	Group I	Duplodnaviria	Heunggongvirae	Uroviricota	Caudoviricetes	n.a.	Unclassified	O	O
S6_vf_56486	Bacteroides phage SJC03	MT074146	Clustered	VC_963_0	N5	49	Bacteroides	Group I	Duplodnaviria	Heunggongvirae	Uroviricota	Caudoviricetes	n.a.	Unclassified	O	O
S6_vf_6314	n.a.	n.a.	Clustered	VC_1607_0	F	n.a.	n.a.	n.a.	n.a.	n.a.	n.a.	n.a.	n.a.	n.a.	n.a.	n.a.
S6_vs_10314	Butyrivibrio phage Idris	MN882554	Clustered	VC_1289_0	N4	38	Butyrivibrio	Group I	Duplodnaviria	Heunggongvirae	Uroviricota	Caudoviricetes	n.a.	Unclassified	O	O
S6_vs_12945	Clostridioides phage CD1801	MW512570	Clustered	VC_670_0	N10	7	Clostridioides	Group I	Duplodnaviria	Heunggongvirae	Uroviricota	Caudoviricetes	n.a.	Unclassified	O	O

S6_vs_15813	n.a.	n.a.	Clustered	VC_1481_0	A	n.a.	n.a.	n.a.	n.a.	n.a.	n.a.	n.a.	n.a.	n.a.	n.a.	n.a.
S6_vs_32886	n.a.	n.a.	Clustered	VC_1590_0	A	n.a.	n.a.	n.a.	n.a.	n.a.	n.a.	n.a.	n.a.	n.a.	n.a.	n.a.
S6_vs_33711	Bacillus phage vB_BtS_BMBtp13	KX190832	Clustered	VC_667_0	C7	7	Bacillus	Group I	Duplodnaviria	Heunggongvirae	Uroviricota	Caudoviricetes	n.a.	Unclassified	Unclassified	Unclassified
S6_vs_38250	Bacillus phage vB_BtS_BMBtp13	KX190832	Clustered	VC_667_0	C8	6	Bacillus	Group I	Duplodnaviria	Heunggongvirae	Uroviricota	Caudoviricetes	n.a.	Unclassified	Unclassified	Unclassified
S6_vs_39025	Clostridioides phage CD1801	MW512570	Clustered	VC_670_0	N11	6	Clostridioides	Group I	Duplodnaviria	Heunggongvirae	Uroviricota	Caudoviricetes	n.a.	Unclassified	O	O
S6_vs_46039	n.a.	n.a.	Clustered	VC_1612_0	A	n.a.	n.a.	n.a.	n.a.	n.a.	n.a.	n.a.	n.a.	n.a.	n.a.	n.a.
S6_vs_54606	Butyrivibrio phage Idris	MN882554	Clustered	VC_1289_0	N4	33	Butyrivibrio	Group I	Duplodnaviria	Heunggongvirae	Uroviricota	Caudoviricetes	n.a.	Unclassified	O	O
S6_vs_56490	Geobacillus phage GBSV1	DQ340064	Clustered	VC_778_0	N39	6	Geobacillus	Group I	Duplodnaviria	Heunggongvirae	Uroviricota	Caudoviricetes	n.a.	Unclassified	O	O
S6_vs_56491	Lactococcus phage CAP	MZ308445	Clustered	VC_278_0	N22	2	Lactococcus	Group I	Duplodnaviria	Heunggongvirae	Uroviricota	Caudoviricetes	n.a.	Unclassified	O	O
S6_vs_56493	n.a.	n.a.	Clustered	VC_1601_0	F	n.a.	n.a.	n.a.	n.a.	n.a.	n.a.	n.a.	n.a.	n.a.	n.a.	n.a.
S6_vs_56496	Faecalibacterium phage FP_Epona	MG711462	Clustered	VC_723_0	C2	104	Faecalibacterium	Group I	Duplodnaviria	Heunggongvirae	Uroviricota	Caudoviricetes	n.a.	Unclassified	Unclassified	Eponavirus
S6_vs_56498	Butyrivibrio phage Idris	MN882554	Clustered	VC_1289_0	N5	41	Butyrivibrio	Group I	Duplodnaviria	Heunggongvirae	Uroviricota	Caudoviricetes	n.a.	Unclassified	O	O
S6_vs_9784	n.a.	n.a.	Clustered	VC_1505_0	A	n.a.	n.a.	n.a.	n.a.	n.a.	n.a.	n.a.	n.a.	n.a.	n.a.	n.a.
S7_vf_114476	Butyrivibrio virus Arian	MN882551	Clustered	VC_720_0	N6	14	Butyrivibrio	Group I	Duplodnaviria	Heunggongvirae	Uroviricota	Caudoviricetes	n.a.	Unclassified	O	O
S7_vf_117697	n.a.	n.a.	Clustered	VC_1576_0	A	n.a.	n.a.	n.a.	n.a.	n.a.	n.a.	n.a.	n.a.	n.a.	n.a.	n.a.
S7_vf_134023	n.a.	n.a.	Clustered	VC_1590_0	A	n.a.	n.a.	n.a.	n.a.	n.a.	n.a.	n.a.	n.a.	n.a.	n.a.	n.a.
S7_vf_135778	Bacillus phage vB_BanS_Athena	OK500002	Clustered	VC_946_0	N8	2	Bacillus	Group I	Duplodnaviria	Heunggongvirae	Uroviricota	Caudoviricetes	n.a.	Unclassified	O	O
S7_vf_138019	n.a.	n.a.	Clustered	VC_1615_0	F	n.a.	n.a.	n.a.	n.a.	n.a.	n.a.	n.a.	n.a.	n.a.	n.a.	n.a.
S7_vf_138069	n.a.	n.a.	Clustered	VC_1564_0	F	n.a.	n.a.	n.a.	n.a.	n.a.	n.a.	n.a.	n.a.	n.a.	n.a.	n.a.
S7_vf_19866	Clostridium phage HM2	LT600745	Clustered	VC_1258_0	N44	3	Clostridium	Group I	Duplodnaviria	Heunggongvirae	Uroviricota	Caudoviricetes	n.a.	Salasmaviridae	O	O
S7_vf_20129	Bacteriophage sp.	OP549880	Clustered	VC_1549_0	N5	16	Unspecified	Unclassified	Unclassified	n.a.	n.a.	n.a.	n.a.	Unclassified	O	O
S7_vf_27437	n.a.	n.a.	Clustered	VC_1607_0	F	n.a.	n.a.	n.a.	n.a.	n.a.	n.a.	n.a.	n.a.	n.a.	n.a.	n.a.
S7_vf_28429	Cellulophaga phage phi17:2	KC821609	Clustered	VC_1055_0	N8	4	Cellulophaga	Group I	Duplodnaviria	Heunggongvirae	Uroviricota	Caudoviricetes	n.a.	Unclassified	O	O
S7_vf_29196	n.a.	n.a.	Clustered	VC_1554_0	A	n.a.	n.a.	n.a.	n.a.	n.a.	n.a.	n.a.	n.a.	n.a.	n.a.	n.a.
S7_vf_32559	n.a.	n.a.	Clustered	VC_1258_0	A	n.a.	n.a.	n.a.	n.a.	n.a.	n.a.	n.a.	n.a.	n.a.	n.a.	n.a.
S7_vf_36627	n.a.	n.a.	Clustered	VC_1258_0	A	n.a.	n.a.	n.a.	n.a.	n.a.	n.a.	n.a.	n.a.	n.a.	n.a.	n.a.
S7_vf_38697	Faecalibacterium phage FP_oengus	MG711463	Clustered	VC_1085_1	N18	59	Faecalibacterium	Group I	Duplodnaviria	Heunggongvirae	Uroviricota	Caudoviricetes	n.a.	Unclassified	O	O
S7_vf_39724	Faecalibacterium phage FP_Mushu	MG711460	Clustered	VC_1288_0	C1	171	Faecalibacterium	Group I	Duplodnaviria	Heunggongvirae	Uroviricota	Caudoviricetes	n.a.	Unclassified	Unclassified	Mushuvirus
S7_vf_5439	Faecalibacterium phage FP_Epona	MG711462	Clustered	VC_723_0	C1	118	Faecalibacterium	Group I	Duplodnaviria	Heunggongvirae	Uroviricota	Caudoviricetes	n.a.	Unclassified	Unclassified	Eponavirus
S7_vf_5843	Curtobacterium phage Penoan	MZ333133	Clustered	VC_1195_0	N3	4	Curtobacterium	Group I	Duplodnaviria	Heunggongvirae	Uroviricota	Caudoviricetes	n.a.	Unclassified	O	O
S7_vf_60968	Ruminococcus phage phiRgPS_6	MT980839	Clustered	VC_278_0	N1	47	Ruminococcus	Group I	Duplodnaviria	Heunggongvirae	Uroviricota	Caudoviricetes	n.a.	Unclassified	O	O
S7_vf_63894	Leuconostoc phage 1-A4	GQ451696	Clustered	VC_889_0	C2	116	Leuconostoc	Group I	Duplodnaviria	Heunggongvirae	Uroviricota	Caudoviricetes	n.a.	Unclassified	Mccleskeyvirinae	Unaquatrovirus
S7_vf_68546	Clostridium phage phiCp-D	MW478291	Clustered	VC_921_0	N4	12	Clostridium	Group I	Duplodnaviria	Heunggongvirae	Uroviricota	Caudoviricetes	n.a.	Unclassified	O	O
S7_vf_69348	n.a.	n.a.	Clustered	VC_1258_0	A	n.a.	n.a.	n.a.	n.a.	n.a.	n.a.	n.a.	n.a.	n.a.	n.a.	n.a.
S7_vf_71527	n.a.	n.a.	Clustered	VC_1616_0	F	n.a.	n.a.	n.a.	n.a.	n.a.	n.a.	n.a.	n.a.	n.a.	n.a.	n.a.
S7_vf_76382	Clostridium phage HM2	LT600745	Clustered	VC_1261_0	N20	7	Clostridium	Group I	Duplodnaviria	Heunggongvirae	Uroviricota	Caudoviricetes	n.a.	Salasmaviridae	O	O
S7_vf_76463	n.a.	n.a.	Clustered	VC_1552_0	A	n.a.	n.a.	n.a.	n.a.	n.a.	n.a.	n.a.	n.a.	n.a.	n.a.	n.a.
S7_vf_84974	Clostridioides phage ES-S-0173-01	CP067352	Clustered	VC_748_0	N12	1	Clostridioides	Group I	Duplodnaviria	Heunggongvirae	Uroviricota	Caudoviricetes	n.a.	Unclassified	O	O
S7_vf_89197	Lactococcus phage vB_LLc_bIBB14	MH779519	Clustered	VC_706_0	C1	113	Lactococcus	Group I	Duplodnaviria	Heunggongvirae	Uroviricota	Caudoviricetes	n.a.	Unclassified	Unclassified	Ceduovirus
S7_vs_100640	n.a.	n.a.	Clustered	VC_1587_0	A	n.a.	n.a.	n.a.	n.a.	n.a.	n.a.	n.a.	n.a.	n.a.	n.a.	n.a.
S7_vs_11081	Bacteroides phage p00	BK010646	Clustered	VC_695_0	C5	43	Bacteroides	Group I	Duplodnaviria	Heunggongvirae	Uroviricota	Caudoviricetes	n.a.	Unclassified	Unclassified	Unclassified
S7_vs_111831	Streptococcus phage Javan580	MK448989	Clustered	VC_558_0	N10	8	Streptococcus	Group I	Duplodnaviria	Heunggongvirae	Uroviricota	Caudoviricetes	n.a.	Unclassified	O	O
S7_vs_118295	n.a.	n.a.	Clustered	VC_1481_0	A	n.a.	n.a.	n.a.	n.a.	n.a.	n.a.	n.a.	n.a.	n.a.	n.a.	n.a.
S7_vs_119492	Clostridium phage HM2	LT600745	Clustered	VC_1261_0	N36	4	Clostridium	Group I	Duplodnaviria	Heunggongvirae	Uroviricota	Caudoviricetes	n.a.	Salasmaviridae	O	O
S7_vs_119679	Winogradskyella phage Peternella_1	MT732475	Clustered	VC_696_0	N4	22	Winogradskyella	Group I	Duplodnaviria	Heunggongvirae	Uroviricota	Caudoviricetes	n.a.	Winoviridae	O	O
S7_vs_122151	n.a.	n.a.	Clustered	VC_779_0	A	n.a.	n.a.	n.a.	n.a.	n.a.	n.a.	n.a.	n.a.	n.a.	n.a.	n.a.
S7_vs_129271	n.a.	n.a.	Clustered	VC_1578_0	A	n.a.	n.a.	n.a.	n.a.	n.a.	n.a.	n.a.	n.a.	n.a.	n.a.	n.a.
S7_vs_129858	n.a.	n.a.	Clustered	VC_1620_0	F	n.a.	n.a.	n.a.	n.a.	n.a.	n.a.	n.a.	n.a.	n.a.	n.a.	n.a.
S7_vs_13041	Butyrivibrio virus Arian	MN882551	Clustered	VC_720_0	N6	14	Butyrivibrio	Group I	Duplodnaviria	Heunggongvirae	Uroviricota	Caudoviricetes	n.a.	Unclassified	O	O
S7_vs_134391	n.a.	n.a.	Clustered	VC_1568_0	A	n.a.	n.a.	n.a.	n.a.	n.a.	n.a.	n.a.	n.a.	n.a.	n.a.	n.a.
S7_vs_134583	Faecalibacterium phage FP_Toutatis	MG711466	Clustered	VC_728_0	C4	56	Faecalibacterium	Group I	Duplodnaviria	Heunggongvirae	Uroviricota	Caudoviricetes	n.a.	Unclassified	Unclassified	Toutatisvirus
S7_vs_135828	Microviridae sp.	MG945563	Clustered	VC_1335_0	C1	9	Unspecified	Group II	Monodnaviria	Sangervirae	Phixviricota	Malgrandaviricetes	Petitvirales	Microviridae	Unclassified	Unclassified
S7_vs_136164	n.a.	n.a.	Clustered	VC_1570_0	A	n.a.	n.a.	n.a.	n.a.	n.a.	n.a.	n.a.	n.a.	n.a.	n.a.	n.a.
S7_vs_137915	Bacteroides phage DAC16	MT074137	Clustered	VC_963_0	N5	42	Bacteroides	Group I	Duplodnaviria	Heunggongvirae	Uroviricota	Caudoviricetes	n.a.	Unclassified	O	O
S7_vs_13799	Roseburia phage Jekyll	LR596902	Clustered	VC_278_0	N14	5	Roseburia	Group I	Duplodnaviria	Heunggongvirae	Uroviricota	Caudoviricetes	n.a.	Unclassified	O	O
S7_vs_138096	n.a.	n.a.	Clustered	VC_1570_0	A	n.a.	n.a.	n.a.	n.a.	n.a.	n.a.	n.a.	n.a.	n.a.	n.a.	n.a.
S7_vs_138109	Lactococcus phage CAP	MZ308445	Clustered	VC_278_0	N13	8	Lactococcus	Group I	Duplodnaviria	Heunggongvirae	Uroviricota	Caudoviricetes	n.a.	Unclassified	O	O
S7_vs_138173	Phage vB_RanS_PJN03	ON245412	Clustered	VC_950_0	N13	5	Unspecified	Group I	Duplodnaviria	Heunggongvirae	Uroviricota	Caudoviricetes	n.a.	Unclassified	O	O
S7_vs_20200	n.a.	n.a.	Clustered	VC_1291_0	A	n.a.	n.a.	n.a.	n.a.	n.a.	n.a.	n.a.	n.a.	n.a.	n.a.	n.a.
S7_vs_2599	n.a.	n.a.	Clustered	VC_1572_0	A	n.a.	n.a.	n.a.	n.a.	n.a.	n.a.	n.a.	n.a.	n.a.	n.a.	n.a.
S7_vs_27530	n.a.	n.a.	Clustered	VC_1567_0	A	n.a.	n.a.	n.a.	n.a.	n.a.	n.a.	n.a.	n.a.	n.a.	n.a.	n.a.
S7_vs_28733	Faecalibacterium phage FP_Lugh	MG711464	Clustered	VC_923_0	N19	4	Faecalibacterium	Group I	Duplodnaviria	Heunggongvirae	Uroviricota	Caudoviricetes	n.a.	Unclassified	O	O
S7_vs_38113	Geobacillus phage GBSV1	DQ340064	Clustered	VC_779_0	N42	2	Geobacillus	Group I	Duplodnaviria	Heunggongvirae	Uroviricota	Caudoviricetes	n.a.	Unclassified	O	O
S7_vs_38834	Exiguobacterium phage vB_EauS-123	KU160495	Clustered	VC_779_0	N30	5	Exiguobacterium	Group I	Duplodnaviria	Heunggongvirae	Uroviricota	Caudoviricetes	n.a.	Unclassified	O	O
S7_vs_41098	Faecalibacterium phage FP_oengus	MG711463	Clustered	VC_1085_1	N20	65	Faecalibacterium	Group I	Duplodnaviria	Heunggongvirae	Uroviricota	Caudoviricetes	n.a.	Unclassified	O	O
S7_vs_41534	Clostridium phage HM2	LT600745	Clustered	VC_1265_0	N19	4	Clostridium	Group I	Duplodnaviria	Heunggongvirae	Uroviricota	Caudoviricetes	n.a.	Salasmaviridae	O	O
S7_vs_42563	n.a.	n.a.	Clustered	VC_1291_0	A	n.a.	n.a.	n.a.	n.a.	n.a.	n.a.	n.a.	n.a.	n.a.	n.a.	n.a.
S7_vs_47050	Butyrivibrio virus Arian	MN882551	Clustered	VC_720_0	N1	32	Butyrivibrio	Group I	Duplodnaviria	Heunggongvirae	Uroviricota	Caudoviricetes	n.a.	Unclassified	O	O
S7_vs_48523	Streptococcus phage MismyG	OL774867	Clustered	VC_152_0	N34	2	Streptococcus	Group I	Duplodnaviria	Heunggongvirae	Uroviricota	Caudoviricetes	n.a.	Unclassified	O	O
S7_vs_48708	Clostridium phage Clo-PEP-1	KY206887	Clustered	VC_442_0	N3	25	Clostridium	Group I	Duplodnaviria	Heunggongvirae	Uroviricota	Caudoviricetes	n.a.	Unclassified	O	O
S7_vs_48832	Bacteriophage sp.	LT996078	Clustered	VC_278_0	N14	5	Unspecified	Unclassified	Unclassified	n.a.	n.a.	n.a.	n.a.	Unclassified	O	O
S7_vs_49426	Erysipelothrix phage SE-1	KR816341	Clustered	VC_1149_0	N3	11	Erysipelothrix	Group I	Duplodnaviria	Heunggongvirae	Uroviricota	Caudoviricetes	n.a.	Unclassified	O	O
S7_vs_4956	Faecalibacterium phage FP_Epona	MG711462	Clustered	VC_723_0	C13	52	Faecalibacterium	Group I	Duplodnaviria	Heunggongvirae	Uroviricota	Caudoviricetes	n.a.	Unclassified	Unclassified	Eponavirus
S7_vs_50194	n.a.	n.a.	Clustered	VC_1602_0	A	n.a.	n.a.	n.a.	n.a.	n.a.	n.a.	n.a.	n.a.	n.a.	n.a.	n.a.
S7_vs_5236	Butyrivibrio phage Idris	MN882554	Clustered	VC_1289_0	N7	31	Butyrivibrio	Group I	Duplodnaviria	Heunggongvirae	Uroviricota	Caudoviricetes	n.a.	Unclassified	O	O
S7_vs_55409	Bacillus phage 1	DQ840344	Clustered	VC_778_0	N28	7	Bacillus	Group I	Duplodnaviria	Heunggongvirae	Uroviricota	Caudoviricetes	n.a.	Unclassified	O	O
S7_vs_557	Bacteroides phage F2	MT806187	Clustered	VC_1505_0	N12	9	Bacteroides	Group I	Duplodnaviria	Heunggongvirae	Uroviricota	Caudoviricetes	n.a.	Unclassified	O	O
S7_vs_56529	Bacillus phage vB_BtS_BMBtp13	KX190832	Clustered	VC_667_0	C9	5	Bacillus	Group I	Duplodnaviria	Heunggongvirae	Uroviricota	Caudoviricetes	n.a.	Unclassified	Unclassified	Unclassified
S7_vs_59465	Clostridium phage phiMMP01	LN681541	Clustered	VC_151_0	N16	27	Clostridium	Group I	Duplodnaviria	Heunggongvirae	Uroviricota	Caudoviricetes	n.a.	Unclassified	O	O
S7_vs_6626	Faecalibacterium phage FP_Epona	MG711462	Clustered	VC_723_0	C13	49	Faecalibacterium	Group I	Duplodnaviria	Heunggongvirae	Uroviricota	Caudoviricetes	n.a.	Unclassified	Unclassified	Eponavirus
S7_vs_69630	Clostridium phage phiCT19406B	KM983331	Clustered	VC_660_0	N8	10	Clostridium	Group I	Duplodnaviria	Heunggongvirae	Uroviricota	Caudoviricetes	n.a.	Unclassified	O	O
S7_vs_73280	Microbacterium phage Min1	EF579802	Clustered	VC_642_0	C1	13	Microbacterium	Group I	Duplodnaviria	Heunggongvirae	Uroviricota	Caudoviricetes	n.a.	Unclassified	Unclassified	Minunavirus

S7_vs_82140	Geobacillus phage GBSV1	DQ340064	Clustered	VC_779_0	N41	2	Geobacillus	Group I	Duplodnaviria	Heunggongvirae	Uroviricota	Caudoviricetes	n.a.	Unclassified	O	O
S7_vs_83100	Streptococcus phage MismyG	OL774867	Clustered	VC_152_0	N17	5	Streptococcus	Group I	Duplodnaviria	Heunggongvirae	Uroviricota	Caudoviricetes	n.a.	Unclassified	O	O
S7_vs_86377	Streptococcus phage Javan290	MK448900	Clustered	VC_332_0	N27	2	Streptococcus	Group I	Duplodnaviria	Heunggongvirae	Uroviricota	Caudoviricetes	n.a.	Unclassified	O	O
S7_vs_87439	n.a.	n.a.	Clustered	VC_1479_0	A	n.a.	n.a.	n.a.	n.a.	n.a.	n.a.	n.a.	n.a.	n.a.	n.a.	n.a.
S7_vs_90915	Streptococcus phage MismyG	OL774867	Clustered	VC_152_0	N15	6	Streptococcus	Group I	Duplodnaviria	Heunggongvirae	Uroviricota	Caudoviricetes	n.a.	Unclassified	O	O
S7_vs_944	n.a.	n.a.	Clustered	VC_1505_0	A	n.a.	n.a.	n.a.	n.a.	n.a.	n.a.	n.a.	n.a.	n.a.	n.a.	n.a.
S8_vf_101283	Faecalibacterium phage FP_Toutatis	MG711466	Clustered	VC_728_0	C1	101	Faecalibacterium	Group I	Duplodnaviria	Heunggongvirae	Uroviricota	Caudoviricetes	n.a.	Unclassified	Unclassified	Toutatisvirus
S8_vf_106946	Ralstonia phage RPSC1	MF893341	Clustered	VC_86_0	N3	7	Ralstonia	Group I	Duplodnaviria	Heunggongvirae	Uroviricota	Caudoviricetes	n.a.	Autographiviridae	O	O
S8_vf_111790	Eggerthella phage PMBT5	MH626557	Clustered	VC_1421_0	N20	4	Eggerthella	Group I	Duplodnaviria	Heunggongvirae	Uroviricota	Caudoviricetes	n.a.	Unclassified	O	O
S8_vf_117796	Bacteroides phage p00	BK010646	Clustered	VC_695_0	C5	43	Bacteroides	Group I	Duplodnaviria	Heunggongvirae	Uroviricota	Caudoviricetes	n.a.	Unclassified	Unclassified	Unclassified
S8_vf_12212	n.a.	n.a.	Clustered	VC_153_0	A	n.a.	n.a.	n.a.	n.a.	n.a.	n.a.	n.a.	n.a.	n.a.	n.a.	n.a.
S8_vf_129423	Leuconostoc phage 1-A4	GQ451696	Clustered	VC_889_0	C1	127	Leuconostoc	Group I	Duplodnaviria	Heunggongvirae	Uroviricota	Caudoviricetes	n.a.	Unclassified	Mccleskeyvirinae	Unaquatrovirus
S8_vf_131009	Roseburia phage Jekyll	LR596902	Clustered	VC_1254_2	N26	1	Roseburia	Group I	Duplodnaviria	Heunggongvirae	Uroviricota	Caudoviricetes	n.a.	Unclassified	O	O
S8_vf_131802	uncultured phage cr56_1	MT774397	Clustered	VC_519_5	C1	300	Unspecified	Group I	Duplodnaviria	Heunggongvirae	Uroviricota	Caudoviricetes	Crassvirales	Suoliviridae	Loutivirinae	Buchavirus
S8_vf_132697	Roseburia phage Jekyll	LR596902	Clustered	VC_1254_0	N19	10	Roseburia	Group I	Duplodnaviria	Heunggongvirae	Uroviricota	Caudoviricetes	n.a.	Unclassified	O	O
S8_vf_134943	n.a.	n.a.	Clustered	VC_1564_0	F	n.a.	n.a.	n.a.	n.a.	n.a.	n.a.	n.a.	n.a.	n.a.	n.a.	n.a.
S8_vf_136235	Bacteriophage sp.	OP549880	Clustered	VC_1549_0	N5	16	Unspecified	Unclassified	Unclassified	n.a.	n.a.	n.a.	n.a.	Unclassified	O	O
S8_vf_13758	Butyrivibrio phage Idris	MN882554	Clustered	VC_1477_0	N13	2	Butyrivibrio	Group I	Duplodnaviria	Heunggongvirae	Uroviricota	Caudoviricetes	n.a.	Unclassified	O	O
S8_vf_142700	Bacteriophage sp.	OP549880	Clustered	VC_1549_0	N5	17	Unspecified	Unclassified	Unclassified	n.a.	n.a.	n.a.	n.a.	Unclassified	O	O
S8_vf_147128	Phage DP SC_6_H4_2017	MT121964	Clustered	VC_1490_1	C2	174	Unspecified	Group I	Duplodnaviria	Heunggongvirae	Uroviricota	Caudoviricetes	n.a.	Unclassified	Unclassified	Unclassified
S8_vf_151384	Faecalibacterium phage FP_Epona	MG711462	Clustered	VC_723_0	C2	83	Faecalibacterium	Group I	Duplodnaviria	Heunggongvirae	Uroviricota	Caudoviricetes	n.a.	Unclassified	Unclassified	Eponavirus
S8_vf_158225	uncultured phage cr3_1	MT774381	Clustered	VC_691_0	C1	148	Unspecified	Group I	Duplodnaviria	Heunggongvirae	Uroviricota	Caudoviricetes	Crassvirales	Intestiviridae	Crudevirinae	Diorhovirus
S8_vf_16172	n.a.	n.a.	Clustered	VC_1583_0	A	n.a.	n.a.	n.a.	n.a.	n.a.	n.a.	n.a.	n.a.	n.a.	n.a.	n.a.
S8_vf_163760	Faecalibacterium phage FP_oengus	MG711463	Clustered	VC_1085_1	N15	73	Faecalibacterium	Group I	Duplodnaviria	Heunggongvirae	Uroviricota	Caudoviricetes	n.a.	Unclassified	O	O
S8_vf_166403	n.a.	n.a.	Clustered	VC_1294_0	A	n.a.	n.a.	n.a.	n.a.	n.a.	n.a.	n.a.	n.a.	n.a.	n.a.	n.a.
S8_vf_166677	uncultured phage cr8_1	MT774384	Clustered	VC_691_1	C1	191	Unspecified	Group I	Duplodnaviria	Heunggongvirae	Uroviricota	Caudoviricetes	Crassvirales	Intestiviridae	Obtuvirinae	Fohxhuevirus
S8_vf_170430	Clavibacter phage CMP1	GQ241246	Clustered	VC_880_0	C2	14	Clavibacter	Group I	Duplodnaviria	Heunggongvirae	Uroviricota	Caudoviricetes	n.a.	Unclassified	Unclassified	Cimpunavirus
S8_vf_170878	Faecalibacterium phage FP_Toutatis	MG711466	Clustered	VC_728_0	C4	72	Faecalibacterium	Group I	Duplodnaviria	Heunggongvirae	Uroviricota	Caudoviricetes	n.a.	Unclassified	Unclassified	Toutatisvirus
S8_vf_174559	Clostridium phage HM2	LT600745	Clustered	VC_1261_0	N32	4	Clostridium	Group I	Duplodnaviria	Heunggongvirae	Uroviricota	Caudoviricetes	n.a.	Salasmaviridae	O	O
S8_vf_183045	Bacteroides phage F4	MT806185	Clustered	VC_1044_0	C1	94	Bacteroides	Group I	Duplodnaviria	Heunggongvirae	Uroviricota	Caudoviricetes	n.a.	Unclassified	Unclassified	Unclassified
S8_vf_187742	Geobacillus virus E3	KP144388	Clustered	VC_748_0	N17	2	Geobacillus	Group I	Duplodnaviria	Heunggongvirae	Uroviricota	Caudoviricetes	n.a.	Unclassified	O	O
S8_vf_196670	n.a.	n.a.	Clustered	VC_1258_0	A	n.a.	n.a.	n.a.	n.a.	n.a.	n.a.	n.a.	n.a.	n.a.	n.a.	n.a.
S8_vf_197061	Bifidobacterium phage BigBern1	MT006237	Clustered	VC_879_0	C2	34	Bifidobacterium	Group I	Duplodnaviria	Heunggongvirae	Uroviricota	Caudoviricetes	n.a.	Unclassified	Unclassified	Unclassified
S8_vf_203044	Bacillus phage vB_BsuP-Goe1	KU831549	Clustered	VC_847_0	N20	3	Bacillus	Group I	Duplodnaviria	Heunggongvirae	Uroviricota	Caudoviricetes	n.a.	Salasmaviridae	O	O
S8_vf_208102	n.a.	n.a.	Clustered	VC_1619_0	F	n.a.	n.a.	n.a.	n.a.	n.a.	n.a.	n.a.	n.a.	n.a.	n.a.	n.a.
S8_vf_208107	n.a.	n.a.	Clustered	VC_1619_0	F	n.a.	n.a.	n.a.	n.a.	n.a.	n.a.	n.a.	n.a.	n.a.	n.a.	n.a.
S8_vf_208110	Bacillus phage vB_BcoS-136	MH884508	Clustered	VC_491_0	N19	3	Bacillus	Group I	Duplodnaviria	Heunggongvirae	Uroviricota	Caudoviricetes	n.a.	Unclassified	O	O
S8_vf_208119	Gokushovirus WZ-2015a	KT264793	Clustered	VC_1170_0	C1	27	Unspecified	Group II	Monodnaviria	Sangervirae	Phixviricota	Malgrandaviricetes	Petitvirales	Microviridae	Gokushovirinae	Gokushovirus
S8_vf_208120	Phage vB_RanS_PJN03	ON245412	Clustered	VC_951_1	N14	12	Unspecified	Group I	Duplodnaviria	Heunggongvirae	Uroviricota	Caudoviricetes	n.a.	Unclassified	O	O
S8_vf_208126	Clostridium phage phiMMP01	LN681541	Clustered	VC_151_0	N19	27	Clostridium	Group I	Duplodnaviria	Heunggongvirae	Uroviricota	Caudoviricetes	n.a.	Unclassified	O	O
S8_vf_208146	Human gut gokushovirus	KX513867	Clustered	VC_1184_0	C1	28	Unspecified	Group II	Monodnaviria	Sangervirae	Phixviricota	Malgrandaviricetes	Petitvirales	Microviridae	Gokushovirinae	Unclassified
S8_vf_41376	Faecalibacterium phage FP_oengus	MG711463	Clustered	VC_1085_1	N21	59	Faecalibacterium	Group I	Duplodnaviria	Heunggongvirae	Uroviricota	Caudoviricetes	n.a.	Unclassified	O	O
S8_vf_43483	n.a.	n.a.	Clustered	VC_1607_0	F	n.a.	n.a.	n.a.	n.a.	n.a.	n.a.	n.a.	n.a.	n.a.	n.a.	n.a.
S8_vf_45776	Lactococcus phage 62605	MF448565	Clustered	VC_210_0	C1	141	Lactococcus	Group I	Duplodnaviria	Heunggongvirae	Uroviricota	Caudoviricetes	n.a.	Unclassified	Unclassified	Skunavirus
S8_vf_52050	Clostridium phage HM2	LT600745	Clustered	VC_1258_0	N38	4	Clostridium	Group I	Duplodnaviria	Heunggongvirae	Uroviricota	Caudoviricetes	n.a.	Salasmaviridae	O	O
S8_vf_55476	n.a.	n.a.	Clustered	VC_1481_1	A	n.a.	n.a.	n.a.	n.a.	n.a.	n.a.	n.a.	n.a.	n.a.	n.a.	n.a.
S8_vf_71214	Arthrobacter phage Orcanus	MT952853	Clustered	VC_1511_0	N3	1	Arthrobacter	Group I	Duplodnaviria	Heunggongvirae	Uroviricota	Caudoviricetes	n.a.	Unclassified	O	O
S8_vf_72773	Faecalibacterium phage FP_Lagaffe	MG711461	Clustered	VC_1291_0	N40	3	Faecalibacterium	Group I	Duplodnaviria	Heunggongvirae	Uroviricota	Caudoviricetes	n.a.	Unclassified	O	O
S8_vf_79769	n.a.	n.a.	Clustered	VC_1561_0	A	n.a.	n.a.	n.a.	n.a.	n.a.	n.a.	n.a.	n.a.	n.a.	n.a.	n.a.
S8_vf_87755	CrAssphage sp. C0521BD4	MW067000	Clustered	VC_691_0	C2	168	Unspecified	Group I	Duplodnaviria	Heunggongvirae	Uroviricota	Caudoviricetes	Crassvirales	Unclassified	Unclassified	Unclassified
S8_vf_93976	n.a.	n.a.	Clustered	VC_1570_0	A	n.a.	n.a.	n.a.	n.a.	n.a.	n.a.	n.a.	n.a.	n.a.	n.a.	n.a.
S8_vf_94361	Microviridae sp.	MG945227	Clustered	VC_1145_0	C1	20	Unspecified	Group II	Monodnaviria	Sangervirae	Phixviricota	Malgrandaviricetes	Petitvirales	Microviridae	Unclassified	Unclassified
S8_vs_125717	n.a.	n.a.	Clustered	VC_1274_0	A	n.a.	n.a.	n.a.	n.a.	n.a.	n.a.	n.a.	n.a.	n.a.	n.a.	n.a.
S8_vs_132359	n.a.	n.a.	Clustered	VC_1618_0	F	n.a.	n.a.	n.a.	n.a.	n.a.	n.a.	n.a.	n.a.	n.a.	n.a.	n.a.
S8_vs_134805	Faecalibacterium phage FP_Mushu	MG711460	Clustered	VC_1288_0	C1	147	Faecalibacterium	Group I	Duplodnaviria	Heunggongvirae	Uroviricota	Caudoviricetes	n.a.	Unclassified	Unclassified	Mushuvirus
S8_vs_136922	Clostridium phage phiCTP1	HM159959	Clustered	VC_921_0	N7	4	Clostridium	Group I	Duplodnaviria	Heunggongvirae	Uroviricota	Caudoviricetes	n.a.	Unclassified	O	O
S8_vs_142097	n.a.	n.a.	Clustered	VC_1258_0	A	n.a.	n.a.	n.a.	n.a.	n.a.	n.a.	n.a.	n.a.	n.a.	n.a.	n.a.
S8_vs_143608	Streptococcus phage Javan261	MK448720	Clustered	VC_778_0	N20	6	Streptococcus	Group I	Duplodnaviria	Heunggongvirae	Uroviricota	Caudoviricetes	n.a.	Unclassified	O	O
S8_vs_145173	Faecalibacterium phage FP_Taranis	MG711467	Clustered	VC_1290_0	N36	2	Faecalibacterium	Group I	Duplodnaviria	Heunggongvirae	Uroviricota	Caudoviricetes	n.a.	Unclassified	O	O
S8_vs_148389	Bacteroides phage F2	MT806186	Clustered	VC_1507_0	N4	4	Bacteroides	Group I	Duplodnaviria	Heunggongvirae	Uroviricota	Caudoviricetes	n.a.	Unclassified	O	O
S8_vs_149700	Streptococcus phage Javan630	MK448997	Clustered	VC_649_3	N2	71	Streptococcus	Group I	Duplodnaviria	Heunggongvirae	Uroviricota	Caudoviricetes	n.a.	Unclassified	O	O
S8_vs_154231	n.a.	n.a.	Clustered	VC_779_0	A	n.a.	n.a.	n.a.	n.a.	n.a.	n.a.	n.a.	n.a.	n.a.	n.a.	n.a.
S8_vs_162053	Clostridium phage HM2	LT600745	Clustered	VC_1258_0	N38	4	Clostridium	Group I	Duplodnaviria	Heunggongvirae	Uroviricota	Caudoviricetes	n.a.	Salasmaviridae	O	O
S8_vs_175235	Bacillus phage vB_BtS_BMBtp13	KX190832	Clustered	VC_667_0	C10	7	Bacillus	Group I	Duplodnaviria	Heunggongvirae	Uroviricota	Caudoviricetes	n.a.	Unclassified	Unclassified	Unclassified
S8_vs_176604	n.a.	n.a.	Clustered	VC_1231_0	A	n.a.	n.a.	n.a.	n.a.	n.a.	n.a.	n.a.	n.a.	n.a.	n.a.	n.a.
S8_vs_176720	Bacillus phage vB_BtS_BMBtp13	KX190832	Clustered	VC_667_0	C7	9	Bacillus	Group I	Duplodnaviria	Heunggongvirae	Uroviricota	Caudoviricetes	n.a.	Unclassified	Unclassified	Unclassified
S8_vs_179208	n.a.	n.a.	Clustered	VC_1612_1	A	n.a.	n.a.	n.a.	n.a.	n.a.	n.a.	n.a.	n.a.	n.a.	n.a.	n.a.
S8_vs_182288	Cellulophaga phage phi17:2	KC821609	Clustered	VC_1055_1	N8	4	Cellulophaga	Group I	Duplodnaviria	Heunggongvirae	Uroviricota	Caudoviricetes	n.a.	Unclassified	O	O
S8_vs_182331	Clostridium phage phiMMP04	JX145342	Clustered	VC_669_0	N2	4	Clostridium	Group I	Duplodnaviria	Heunggongvirae	Uroviricota	Caudoviricetes	n.a.	Unclassified	O	O
S8_vs_193843	n.a.	n.a.	Clustered	VC_1566_0	A	n.a.	n.a.	n.a.	n.a.	n.a.	n.a.	n.a.	n.a.	n.a.	n.a.	n.a.
S8_vs_204260	n.a.	n.a.	Clustered	VC_1291_0	A	n.a.	n.a.	n.a.	n.a.	n.a.	n.a.	n.a.	n.a.	n.a.	n.a.	n.a.
S8_vs_205257	n.a.	n.a.	Clustered	VC_1291_0	A	n.a.	n.a.	n.a.	n.a.	n.a.	n.a.	n.a.	n.a.	n.a.	n.a.	n.a.
S8_vs_206526	Roseburia phage Shimadzu	LR596903	Clustered	VC_727_0	C1	214	Roseburia	Group I	Duplodnaviria	Heunggongvirae	Uroviricota	Caudoviricetes	n.a.	Unclassified	Unclassified	Unclassified
S8_vs_208103	Bacteroides phage SJC03	MT074146	Clustered	VC_963_0	N5	35	Bacteroides	Group I	Duplodnaviria	Heunggongvirae	Uroviricota	Caudoviricetes	n.a.	Unclassified	O	O
S8_vs_208113	n.a.	n.a.	Clustered	VC_1291_0	A	n.a.	n.a.	n.a.	n.a.	n.a.	n.a.	n.a.	n.a.	n.a.	n.a.	n.a.
S8_vs_208114	Psychrobacter phage pOW20-A	JF974295	Clustered	VC_439_0	N3	2	Psychrobacter	Group I	Duplodnaviria	Heunggongvirae	Uroviricota	Caudoviricetes	n.a.	Unclassified	O	O
S8_vs_208118	Streptococcus phage MismyG	OL774867	Clustered	VC_152_0	N15	4	Streptococcus	Group I	Duplodnaviria	Heunggongvirae	Uroviricota	Caudoviricetes	n.a.	Unclassified	O	O
S8_vs_208129	Bdellovibrio phage phi1402	JF344709	Clustered	VC_967_0	C4	37	Bdellovibrio	Group I	Duplodnaviria	Heunggongvirae	Uroviricota	Caudoviricetes	n.a.	Unclassified	Unclassified	Unclassified
S8_vs_28311	Flavobacterium phage vB_FspS_laban6-1	MN812211	Clustered	VC_1062_0	N5	8	Flavobacterium	Group I	Duplodnaviria	Heunggongvirae	Uroviricota	Caudoviricetes	n.a.	Duneviridae	O	O
S8_vs_4153	n.a.	n.a.	Clustered	VC_1507_0	A	n.a.	n.a.	n.a.	n.a.	n.a.	n.a.	n.a.	n.a.	n.a.	n.a.	n.a.
S8_vs_47265	Faecalibacterium phage FP_Epona	MG711462	Clustered	VC_723_0	C5	81	Faecalibacterium	Group I	Duplodnaviria	Heunggongvirae	Uroviricota	Caudoviricetes	n.a.	Unclassified	Unclassified	Eponavirus
S8_vs_4883	n.a.	n.a.	Clustered	VC_1554_0	A	n.a.	n.a.	n.a.	n.a.	n.a.	n.a.	n.a.	n.a.	n.a.	n.a.	n.a.

S8_vs_50431	n.a.	n.a.	Clustered	VC_1567_0	A	n.a.	n.a.	n.a.	n.a.	n.a.	n.a.	n.a.	n.a.	n.a.	n.a.	n.a.
S8_vs_5211	n.a.	n.a.	Clustered	VC_1611_0	A	n.a.	n.a.	n.a.	n.a.	n.a.	n.a.	n.a.	n.a.	n.a.	n.a.	n.a.
S8_vs_71212	n.a.	n.a.	Clustered	VC_1479_0	A	n.a.	n.a.	n.a.	n.a.	n.a.	n.a.	n.a.	n.a.	n.a.	n.a.	n.a.
S8_vs_76852	Streptococcus phage Javan261	MK448720	Clustered	VC_724_0	N30	2	Streptococcus	Group I	Duplodnaviria	Heunggongvirae	Uroviricota	Caudoviricetes	n.a.	Unclassified	O	O
S8_vs_9114	n.a.	n.a.	Clustered	VC_1576_0	A	n.a.	n.a.	n.a.	n.a.	n.a.	n.a.	n.a.	n.a.	n.a.	n.a.	n.a.
S8_vs_99362	Bacteroides phage p00	BK010646	Clustered	VC_695_0	C5	45	Bacteroides	Group I	Duplodnaviria	Heunggongvirae	Uroviricota	Caudoviricetes	n.a.	Unclassified	Unclassified	Unclassified
S8_vs_9987	Phage vB_RanS_PJN03	ON245412	Clustered	VC_951_0	N14	7	Unspecified	Group I	Duplodnaviria	Heunggongvirae	Uroviricota	Caudoviricetes	n.a.	Unclassified	O	O
S9_vf_15012	Streptococcus phage Javan372	MK448920	Clustered	VC_344_0	C1	124	Streptococcus	Group I	Duplodnaviria	Heunggongvirae	Uroviricota	Caudoviricetes	n.a.	Unclassified	Unclassified	Unclassified
S9_vf_23901	Tortoise microvirus 13	MK765563	Clustered	VC_1299_0	C1	26	Unspecified	Group II	Monodnaviria	Sangervirae	Phixviricota	Malgrandaviricetes	Petitvirales	Microviridae	Unclassified	Unclassified
S9_vf_26805	Microviridae sp.	MG945488	Clustered	VC_1334_0	C1	20	Unspecified	Group II	Monodnaviria	Sangervirae	Phixviricota	Malgrandaviricetes	Petitvirales	Microviridae	Unclassified	Unclassified
S9_vf_34736	Clostridium phage HM2	LT600745	Clustered	VC_1261_0	N28	8	Clostridium	Group I	Duplodnaviria	Heunggongvirae	Uroviricota	Caudoviricetes	n.a.	Salasmaviridae	O	O
S9_vf_4115	Streptococcus phage Javan318	MK448905	Clustered	VC_425_0	C1	98	Streptococcus	Group I	Duplodnaviria	Heunggongvirae	Uroviricota	Caudoviricetes	n.a.	Unclassified	Unclassified	Unclassified
S9_vf_51656	uncultured phage cr8_1	MT774384	Clustered	VC_691_1	C1	246	Unspecified	Group I	Duplodnaviria	Heunggongvirae	Uroviricota	Caudoviricetes	Crassvirales	Intestiviridae	Obtuvirinae	Fohxhuevirus
S9_vf_64844	Faecalibacterium phage FP_Lagaffe	MG711461	Clustered	VC_727_0	C4	104	Faecalibacterium	Group I	Duplodnaviria	Heunggongvirae	Uroviricota	Caudoviricetes	n.a.	Unclassified	Unclassified	Lagaffevirus
S9_vf_70236	n.a.	n.a.	Clustered	VC_1258_0	A	n.a.	n.a.	n.a.	n.a.	n.a.	n.a.	n.a.	n.a.	n.a.	n.a.	n.a.
S9_vf_73311	Exiguobacterium phage vB_EauM-23	MH844558	Clustered	VC_145_0	C2	22	Exiguobacterium	Group I	Duplodnaviria	Heunggongvirae	Uroviricota	Caudoviricetes	n.a.	Unclassified	Unclassified	Unclassified
S9_vf_75363	n.a.	n.a.	Clustered	VC_1258_0	A	n.a.	n.a.	n.a.	n.a.	n.a.	n.a.	n.a.	n.a.	n.a.	n.a.	n.a.
S9_vf_76769	uncultured phage cr52_1	MT774395	Clustered	VC_519_6	C1	199	Unspecified	Group I	Duplodnaviria	Heunggongvirae	Uroviricota	Caudoviricetes	Crassvirales	Suoliviridae	Loutivirinae	Buchavirus
S9_vf_79919	n.a.	n.a.	Clustered	VC_779_0	A	n.a.	n.a.	n.a.	n.a.	n.a.	n.a.	n.a.	n.a.	n.a.	n.a.	n.a.
S9_vf_90852	Inovirus sp.	MT135289	Clustered	VC_1492_0	C1	31	Unspecified	Group II	Monodnaviria	Loebvirae	Hofneiviricota	Faserviricetes	Tubulavirales	Inoviridae	Unclassified	Inovirus
S9_vf_90866	Streptococcus phage phiZJ20091101-2	KX077893	Clustered	VC_1229_0	C1	16	Streptococcus	Unclassified	Unclassified	n.a.	n.a.	n.a.	n.a.	Unclassified	Unclassified	Unclassified
S9_vf_90871	Clostridium phage phiMMP01	LN681541	Clustered	VC_150_0	N16	10	Clostridium	Group I	Duplodnaviria	Heunggongvirae	Uroviricota	Caudoviricetes	n.a.	Unclassified	O	O
S9_vs_1966	Streptococcus phage Javan290	MK448900	Clustered	VC_332_0	N19	3	Streptococcus	Group I	Duplodnaviria	Heunggongvirae	Uroviricota	Caudoviricetes	n.a.	Unclassified	O	O
S9_vs_21850	Bacteroides phage p00	BK010646	Clustered	VC_695_0	C4	42	Bacteroides	Group I	Duplodnaviria	Heunggongvirae	Uroviricota	Caudoviricetes	n.a.	Unclassified	Unclassified	Unclassified
S9_vs_26179	n.a.	n.a.	Clustered	VC_782_0	A	n.a.	n.a.	n.a.	n.a.	n.a.	n.a.	n.a.	n.a.	n.a.	n.a.	n.a.
S9_vs_27813	Faecalibacterium phage FP_Epona	MG711462	Clustered	VC_723_0	C13	52	Faecalibacterium	Group I	Duplodnaviria	Heunggongvirae	Uroviricota	Caudoviricetes	n.a.	Unclassified	Unclassified	Eponavirus
S9_vs_28197	Bacteroides phage F4	MT806185	Clustered	VC_1505_0	N7	7	Bacteroides	Group I	Duplodnaviria	Heunggongvirae	Uroviricota	Caudoviricetes	n.a.	Unclassified	O	O
S9_vs_32588	Faecalibacterium phage FP_oengus	MG711463	Clustered	VC_1085_1	N16	70	Faecalibacterium	Group I	Duplodnaviria	Heunggongvirae	Uroviricota	Caudoviricetes	n.a.	Unclassified	O	O
S9_vs_34898	Lactococcus phage CAP	MZ308445	Clustered	VC_278_0	N12	9	Lactococcus	Group I	Duplodnaviria	Heunggongvirae	Uroviricota	Caudoviricetes	n.a.	Unclassified	O	O
S9_vs_4343	n.a.	n.a.	Clustered	VC_1291_0	A	n.a.	n.a.	n.a.	n.a.	n.a.	n.a.	n.a.	n.a.	n.a.	n.a.	n.a.
S9_vs_4558	Faecalibacterium phage FP_oengus	MG711463	Clustered	VC_1085_1	N18	68	Faecalibacterium	Group I	Duplodnaviria	Heunggongvirae	Uroviricota	Caudoviricetes	n.a.	Unclassified	O	O
S9_vs_45812	Bacillus phage vB_BtS_BMBtp13	KX190832	Clustered	VC_667_0	C7	7	Bacillus	Group I	Duplodnaviria	Heunggongvirae	Uroviricota	Caudoviricetes	n.a.	Unclassified	Unclassified	Unclassified
S9_vs_46498	Geobacillus phage GBSV1	DQ340064	Clustered	VC_779_0	N29	4	Geobacillus	Group I	Duplodnaviria	Heunggongvirae	Uroviricota	Caudoviricetes	n.a.	Unclassified	O	O
S9_vs_48424	n.a.	n.a.	Clustered	VC_1565_0	A	n.a.	n.a.	n.a.	n.a.	n.a.	n.a.	n.a.	n.a.	n.a.	n.a.	n.a.
S9_vs_56354	Faecalibacterium phage FP_Epona	MG711462	Clustered	VC_723_0	C13	56	Faecalibacterium	Group I	Duplodnaviria	Heunggongvirae	Uroviricota	Caudoviricetes	n.a.	Unclassified	Unclassified	Eponavirus
S9_vs_5676	Streptococcus phage Javan261	MK448720	Clustered	VC_724_0	N45	1	Streptococcus	Group I	Duplodnaviria	Heunggongvirae	Uroviricota	Caudoviricetes	n.a.	Unclassified	O	O
S9_vs_58402	n.a.	n.a.	Clustered	VC_1613_0	A	n.a.	n.a.	n.a.	n.a.	n.a.	n.a.	n.a.	n.a.	n.a.	n.a.	n.a.
S9_vs_63485	n.a.	n.a.	Clustered	VC_1604_0	A	n.a.	n.a.	n.a.	n.a.	n.a.	n.a.	n.a.	n.a.	n.a.	n.a.	n.a.
S9_vs_73764	Geobacillus phage GBSV1	DQ340064	Clustered	VC_778_0	N36	7	Geobacillus	Group I	Duplodnaviria	Heunggongvirae	Uroviricota	Caudoviricetes	n.a.	Unclassified	O	O
S9_vs_75050	Geobacillus phage GBSV1	DQ340064	Clustered	VC_778_0	N20	9	Geobacillus	Group I	Duplodnaviria	Heunggongvirae	Uroviricota	Caudoviricetes	n.a.	Unclassified	O	O
S9_vs_75290	n.a.	n.a.	Clustered	VC_1607_0	F	n.a.	n.a.	n.a.	n.a.	n.a.	n.a.	n.a.	n.a.	n.a.	n.a.	n.a.
S9_vs_7881	Clostridium phage phiMMP01	LN681541	Clustered	VC_151_0	N12	27	Clostridium	Group I	Duplodnaviria	Heunggongvirae	Uroviricota	Caudoviricetes	n.a.	Unclassified	O	O
S9_vs_83108	n.a.	n.a.	Clustered	VC_1560_0	A	n.a.	n.a.	n.a.	n.a.	n.a.	n.a.	n.a.	n.a.	n.a.	n.a.	n.a.
S9_vs_84046	Butyrivibrio phage Idris	MN882554	Clustered	VC_724_0	N27	4	Butyrivibrio	Group I	Duplodnaviria	Heunggongvirae	Uroviricota	Caudoviricetes	n.a.	Unclassified	O	O
S9_vs_90849	n.a.	n.a.	Clustered	VC_1291_0	A	n.a.	n.a.	n.a.	n.a.	n.a.	n.a.	n.a.	n.a.	n.a.	n.a.	n.a.
S9_vs_90851	Clostridium phage phiMMP01	LN681541	Clustered	VC_151_0	N21	23	Clostridium	Group I	Duplodnaviria	Heunggongvirae	Uroviricota	Caudoviricetes	n.a.	Unclassified	O	O
S9_vs_90857	n.a.	n.a.	Clustered	VC_1479_0	A	n.a.	n.a.	n.a.	n.a.	n.a.	n.a.	n.a.	n.a.	n.a.	n.a.	n.a.
S9_vs_90860	Butyrivibrio phage Idris	MN882554	Clustered	VC_724_0	N36	4	Butyrivibrio	Group I	Duplodnaviria	Heunggongvirae	Uroviricota	Caudoviricetes	n.a.	Unclassified	O	O
S9_vs_90863	n.a.	n.a.	Clustered	VC_1603_0	F	n.a.	n.a.	n.a.	n.a.	n.a.	n.a.	n.a.	n.a.	n.a.	n.a.	n.a.
S10_vf_120738	n.a.	n.a.	Clustered/Singleton	VC_491_3	A	n.a.	n.a.	n.a.	n.a.	n.a.	n.a.	n.a.	n.a.	n.a.	n.a.	n.a.
S10_vf_43964	Bacteroides phage LoVEphage	MW660583	Clustered/Singleton	VC_1525_2	N5	14	Bacteroides	Group I	Duplodnaviria	Heunggongvirae	Uroviricota	Caudoviricetes	n.a.	Unclassified	O	O
S10_vs_37379	n.a.	n.a.	Clustered/Singleton	VC_1055_2	A	n.a.	n.a.	n.a.	n.a.	n.a.	n.a.	n.a.	n.a.	n.a.	n.a.	n.a.
S10_vs_62776	Bacteroides phage LoVEphage	MW660583	Clustered/Singleton	VC_1525_0	N2	32	Bacteroides	Group I	Duplodnaviria	Heunggongvirae	Uroviricota	Caudoviricetes	n.a.	Unclassified	O	O
S10_vs_77004	n.a.	n.a.	Clustered/Singleton	VC_922_0	A	n.a.	n.a.	n.a.	n.a.	n.a.	n.a.	n.a.	n.a.	n.a.	n.a.	n.a.
S11_vf_104033	Bacteroides phage SJC03	MT074146	Clustered/Singleton	VC_963_1	N4	51	Bacteroides	Group I	Duplodnaviria	Heunggongvirae	Uroviricota	Caudoviricetes	n.a.	Unclassified	O	O
S11_vf_3790	Geobacillus virus E3	KP144388	Clustered/Singleton	VC_490_0	N9	1	Geobacillus	Group I	Duplodnaviria	Heunggongvirae	Uroviricota	Caudoviricetes	n.a.	Unclassified	O	O
S11_vf_70015	Phage vB_RanS_PJN03	ON245412	Clustered/Singleton	VC_951_2	N20	7	Unspecified	Group I	Duplodnaviria	Heunggongvirae	Uroviricota	Caudoviricetes	n.a.	Unclassified	O	O
S11_vs_75554	n.a.	n.a.	Clustered/Singleton	VC_1264_0	A	n.a.	n.a.	n.a.	n.a.	n.a.	n.a.	n.a.	n.a.	n.a.	n.a.	n.a.
S12_vf_188819	Clostridium phage c-st	AP008983	Clustered/Singleton	VC_490_3	N7	5	Clostridium	Group I	Duplodnaviria	Heunggongvirae	Uroviricota	Caudoviricetes	n.a.	Unclassified	O	O
S12_vf_67909	Enterococcus phage EF1	MF001358	Clustered/Singleton	VC_221_0	N2	11	Enterococcus	Group I	Duplodnaviria	Heunggongvirae	Uroviricota	Caudoviricetes	n.a.	Unclassified	O	O
S12_vs_123620	n.a.	n.a.	Clustered/Singleton	VC_1295_0	A	n.a.	n.a.	n.a.	n.a.	n.a.	n.a.	n.a.	n.a.	n.a.	n.a.	n.a.
S12_vs_172379	n.a.	n.a.	Clustered/Singleton	VC_1477_1	A	n.a.	n.a.	n.a.	n.a.	n.a.	n.a.	n.a.	n.a.	n.a.	n.a.	n.a.
S13_vf_38708	n.a.	n.a.	Clustered/Singleton	VC_1292_0	A	n.a.	n.a.	n.a.	n.a.	n.a.	n.a.	n.a.	n.a.	n.a.	n.a.	n.a.
S13_vf_4901	Bacillus phage vB_BcoS-136	MH884508	Clustered/Singleton	VC_491_1	N10	2	Bacillus	Group I	Duplodnaviria	Heunggongvirae	Uroviricota	Caudoviricetes	n.a.	Unclassified	O	O
S13_vs_26756	n.a.	n.a.	Clustered/Singleton	VC_1295_1	A	n.a.	n.a.	n.a.	n.a.	n.a.	n.a.	n.a.	n.a.	n.a.	n.a.	n.a.
S14_vs_70985	n.a.	n.a.	Clustered/Singleton	VC_1612_2	A	n.a.	n.a.	n.a.	n.a.	n.a.	n.a.	n.a.	n.a.	n.a.	n.a.	n.a.
S14_vs_74067	n.a.	n.a.	Clustered/Singleton	VC_1582_2	A	n.a.	n.a.	n.a.	n.a.	n.a.	n.a.	n.a.	n.a.	n.a.	n.a.	n.a.
S15_vf_75924	uncultured phage cr116_1	MT774389	Clustered/Singleton	VC_1079_0	N6	6	Unspecified	Group I	Duplodnaviria	Heunggongvirae	Uroviricota	Caudoviricetes	Crassvirales	Steigviridae	O	O
S15_vs_42043	n.a.	n.a.	Clustered/Singleton	VC_1559_2	A	n.a.	n.a.	n.a.	n.a.	n.a.	n.a.	n.a.	n.a.	n.a.	n.a.	n.a.
S16_vf_108554	n.a.	n.a.	Clustered/Singleton	VC_1554_1	A	n.a.	n.a.	n.a.	n.a.	n.a.	n.a.	n.a.	n.a.	n.a.	n.a.	n.a.
S16_vf_173099	n.a.	n.a.	Clustered/Singleton	VC_1586_0	A	n.a.	n.a.	n.a.	n.a.	n.a.	n.a.	n.a.	n.a.	n.a.	n.a.	n.a.
S16_vf_96483	n.a.	n.a.	Clustered/Singleton	VC_1557_0	A	n.a.	n.a.	n.a.	n.a.	n.a.	n.a.	n.a.	n.a.	n.a.	n.a.	n.a.
S16_vs_15093	Butyrivibrio virus Ceridwen	MN882553	Clustered/Singleton	VC_1434_0	N12	8	Butyrivibrio	Group I	Duplodnaviria	Heunggongvirae	Uroviricota	Caudoviricetes	n.a.	Unclassified	O	O
S16_vs_34751	n.a.	n.a.	Clustered/Singleton	VC_780_0	A	n.a.	n.a.	n.a.	n.a.	n.a.	n.a.	n.a.	n.a.	n.a.	n.a.	n.a.
S3_vf_108959	n.a.	n.a.	Clustered/Singleton	VC_1254_3	A	n.a.	n.a.	n.a.	n.a.	n.a.	n.a.	n.a.	n.a.	n.a.	n.a.	n.a.
S3_vf_61860	Clostridium phage vB_CpeS-1181	MW478292	Clustered/Singleton	VC_923_1	N2	21	Clostridium	Group I	Duplodnaviria	Heunggongvirae	Uroviricota	Caudoviricetes	n.a.	Unclassified	O	O
S3_vs_16825	Roseburia phage Jekyll	LR596902	Clustered/Singleton	VC_1254_4	N19	10	Roseburia	Group I	Duplodnaviria	Heunggongvirae	Uroviricota	Caudoviricetes	n.a.	Unclassified	O	O
S3_vs_39774	n.a.	n.a.	Clustered/Singleton	VC_1582_1	A	n.a.	n.a.	n.a.	n.a.	n.a.	n.a.	n.a.	n.a.	n.a.	n.a.	n.a.
S3_vs_68031	Faecalibacterium phage FP_oengus	MG711463	Clustered/Singleton	VC_1242_0	N2	101	Faecalibacterium	Group I	Duplodnaviria	Heunggongvirae	Uroviricota	Caudoviricetes	n.a.	Unclassified	O	O
S3_vs_81793	n.a.	n.a.	Clustered/Singleton	VC_1586_1	A	n.a.	n.a.	n.a.	n.a.	n.a.	n.a.	n.a.	n.a.	n.a.	n.a.	n.a.
S4_vf_31973	n.a.	n.a.	Clustered/Singleton	VC_490_2	A	n.a.	n.a.	n.a.	n.a.	n.a.	n.a.	n.a.	n.a.	n.a.	n.a.	n.a.
S4_vs_VIRSorter_31975-circular-cat_1	Phage vB_RanS_PJN03	ON245412	Clustered/Singleton	VC_948_1	N24	3	Unspecified	Group I	Duplodnaviria	Heunggongvirae	Uroviricota	Caudoviricetes	n.a.	Unclassified	O	O

S5_vf_145579	uncultured phage cr85_1	MT774390	Clustered/Singleton	VC_1079_2	N6	12	Unspecified	Group I	Duplodnaviria	Heunggongvirae	Uroviricota	Caudoviricetes	Crassvirales	Steigviridae	O	O
S5_vf_67622	IAS virus	KJ003983	Clustered/Singleton	VC_1079_1	N6	11	Unspecified	Group I	Duplodnaviria	Heunggongvirae	Uroviricota	Caudoviricetes	Crassvirales	Steigviridae	O	O
S5_vs_109527	n.a.	n.a.	Clustered/Singleton	VC_1612_3	A	n.a.	n.a.	n.a.	n.a.	n.a.	n.a.	n.a.	n.a.	n.a.	n.a.	n.a.
S5_vs_122344	Roseburia phage Jekyll	LR596902	Clustered/Singleton	VC_1252_1	C2	7	Roseburia	Group I	Duplodnaviria	Heunggongvirae	Uroviricota	Caudoviricetes	n.a.	Unclassified	Unclassified	O
S5_vs_124677	Faecalibacterium phage FP_Toutatis	MG711466	Clustered/Singleton	VC_728_1	C3	63	Faecalibacterium	Group I	Duplodnaviria	Heunggongvirae	Uroviricota	Caudoviricetes	n.a.	Unclassified	Unclassified	O
S5_vs_131510	Bacteroides phage p00	BK010646	Clustered/Singleton	VC_695_1	C1	71	Bacteroides	Group I	Duplodnaviria	Heunggongvirae	Uroviricota	Caudoviricetes	n.a.	Unclassified	Unclassified	O
S5_vs_77593	Clostridium phage phiMMP01	LN681541	Clustered/Singleton	VC_151_1	N22	16	Clostridium	Group I	Duplodnaviria	Heunggongvirae	Uroviricota	Caudoviricetes	n.a.	Unclassified	O	O
S5_vs_97574	Clostridium phage c-st	AP008983	Clustered/Singleton	VC_219_1	N8	13	Clostridium	Group I	Duplodnaviria	Heunggongvirae	Uroviricota	Caudoviricetes	n.a.	Unclassified	O	O
S6_vs_29152	Faecalibacterium phage FP_Brigit	MG711465	Clustered/Singleton	VC_923_2	N16	5	Faecalibacterium	Group I	Duplodnaviria	Heunggongvirae	Uroviricota	Caudoviricetes	n.a.	Unclassified	O	O
S7_vf_37279	Bacteroides phage LoVEphage	MW660583	Clustered/Singleton	VC_1525_1	N6	15	Bacteroides	Group I	Duplodnaviria	Heunggongvirae	Uroviricota	Caudoviricetes	n.a.	Unclassified	O	O
S7_vs_103530	n.a.	n.a.	Clustered/Singleton	VC_1554_2	A	n.a.	n.a.	n.a.	n.a.	n.a.	n.a.	n.a.	n.a.	n.a.	n.a.	n.a.
S7_vs_135679	n.a.	n.a.	Clustered/Singleton	VC_1594_0	A	n.a.	n.a.	n.a.	n.a.	n.a.	n.a.	n.a.	n.a.	n.a.	n.a.	n.a.
S7_vs_137611	Brevibacillus phage Sundance	KT151959	Clustered/Singleton	VC_219_2	N20	1	Brevibacillus	Group I	Duplodnaviria	Heunggongvirae	Uroviricota	Caudoviricetes	n.a.	Unclassified	O	O
S7_vs_54322	n.a.	n.a.	Clustered/Singleton	VC_1565_1	A	n.a.	n.a.	n.a.	n.a.	n.a.	n.a.	n.a.	n.a.	n.a.	n.a.	n.a.
S7_vs_78507	n.a.	n.a.	Clustered/Singleton	VC_1481_2	A	n.a.	n.a.	n.a.	n.a.	n.a.	n.a.	n.a.	n.a.	n.a.	n.a.	n.a.
S8_vf_11794	Bacillus phage vB_BcoS-136	MH884508	Clustered/Singleton	VC_491_2	N17	3	Bacillus	Group I	Duplodnaviria	Heunggongvirae	Uroviricota	Caudoviricetes	n.a.	Unclassified	O	O
S8_vf_161703	Bacillus phage vB_BsuS_PJN02	OM634653	Clustered/Singleton	VC_1040_1	N9	8	Bacillus	Group I	Duplodnaviria	Heunggongvirae	Uroviricota	Caudoviricetes	n.a.	Unclassified	O	O
S8_vf_173225	Geobacillus virus E3	KP144388	Clustered/Singleton	VC_490_1	N10	3	Geobacillus	Group I	Duplodnaviria	Heunggongvirae	Uroviricota	Caudoviricetes	n.a.	Unclassified	O	O
S8_vf_23955	Faecalibacterium phage FP_oengus	MG711463	Clustered/Singleton	VC_1085_2	N21	60	Faecalibacterium	Group I	Duplodnaviria	Heunggongvirae	Uroviricota	Caudoviricetes	n.a.	Unclassified	O	O
S8_vf_54514	Butyrivibrio virus Ceridwen	MN882553	Clustered/Singleton	VC_1554_5	N61	5	Butyrivibrio	Group I	Duplodnaviria	Heunggongvirae	Uroviricota	Caudoviricetes	n.a.	Unclassified	O	O
S8_vs_115958	Streptococcus phage Javan630	MK448997	Clustered/Singleton	VC_649_4	C9	43	Streptococcus	Group I	Duplodnaviria	Heunggongvirae	Uroviricota	Caudoviricetes	n.a.	Unclassified	Unclassified	O
S8_vs_59736	Clostridium phage c-st	AP008983	Clustered/Singleton	VC_219_3	N7	16	Clostridium	Group I	Duplodnaviria	Heunggongvirae	Uroviricota	Caudoviricetes	n.a.	Unclassified	O	O
S9_vf_13507	n.a.	n.a.	Clustered/Singleton	VC_1582_3	A	n.a.	n.a.	n.a.	n.a.	n.a.	n.a.	n.a.	n.a.	n.a.	n.a.	n.a.
S9_vf_57277	n.a.	n.a.	Clustered/Singleton	VC_1554_4	A	n.a.	n.a.	n.a.	n.a.	n.a.	n.a.	n.a.	n.a.	n.a.	n.a.	n.a.
S9_vf_90847	Enterococcus phage EF1	MF001358	Clustered/Singleton	VC_221_1	N2	25	Enterococcus	Group I	Duplodnaviria	Heunggongvirae	Uroviricota	Caudoviricetes	n.a.	Unclassified	O	O
S9_vs_10424	n.a.	n.a.	Clustered/Singleton	VC_1421_1	A	n.a.	n.a.	n.a.	n.a.	n.a.	n.a.	n.a.	n.a.	n.a.	n.a.	n.a.
S9_vs_50439	n.a.	n.a.	Clustered/Singleton	VC_1554_3	A	n.a.	n.a.	n.a.	n.a.	n.a.	n.a.	n.a.	n.a.	n.a.	n.a.	n.a.
S9_vs_90727	Streptococcus phage Javan630	MK448997	Clustered/Singleton	VC_649_5	C5	55	Streptococcus	Group I	Duplodnaviria	Heunggongvirae	Uroviricota	Caudoviricetes	n.a.	Unclassified	Unclassified	O
S9_vs_90846	Faecalibacterium phage FP_Lugh	MG711464	Clustered/Singleton	VC_640_0	N5	19	Faecalibacterium	Group I	Duplodnaviria	Heunggongvirae	Uroviricota	Caudoviricetes	n.a.	Unclassified	O	O
S9_vs_90859	Bacteriophage sp.	LT996078	Clustered/Singleton	VC_278_1	N17	4	Unspecified	Unclassified	Unclassified	n.a.	n.a.	n.a.	n.a.	Unclassified	O	O
S10_vf_47170	n.a.	n.a.	Outlier	n.a.	F	n.a.	n.a.	n.a.	n.a.	n.a.	n.a.	n.a.	n.a.	n.a.	n.a.	n.a.
S10_vf_62230	Streptococcus phage Javan576	MK448987	Outlier	n.a.	N1	17	Streptococcus	Group I	Duplodnaviria	Heunggongvirae	Uroviricota	Caudoviricetes	n.a.	Unclassified	O	O
S10_vs_106834	Riemerella phage vB_RanS_PT03	MZ995504	Outlier	n.a.	N4	11	Riemerella	Group I	Duplodnaviria	Heunggongvirae	Uroviricota	Caudoviricetes	n.a.	Unclassified	O	O
S10_vs_37694	Cellulophaga phage Ingeline_1	MT732435	Outlier	n.a.	N4	7	Cellulophaga	Group I	Duplodnaviria	Heunggongvirae	Uroviricota	Caudoviricetes	n.a.	Duneviridae	O	O
S11_vf_33433	Tenacibaculum phage Gundel_1	MT732474	Outlier	n.a.	N1	11	Tenacibaculum	Group I	Duplodnaviria	Heunggongvirae	Uroviricota	Caudoviricetes	n.a.	Pachyviridae	O	O
S11_vf_99751	n.a.	n.a.	Outlier	n.a.	A	n.a.	n.a.	n.a.	n.a.	n.a.	n.a.	n.a.	n.a.	n.a.	n.a.	n.a.
S11_vs_20225	Faecalibacterium phage FP_Lugh	MG711464	Outlier	n.a.	N2	8	Faecalibacterium	Group I	Duplodnaviria	Heunggongvirae	Uroviricota	Caudoviricetes	n.a.	Unclassified	O	O
S11_vs_48109	Streptococcus phage Javan630	MK448997	Outlier	n.a.	N9	26	Streptococcus	Group I	Duplodnaviria	Heunggongvirae	Uroviricota	Caudoviricetes	n.a.	Unclassified	O	O
S11_vs_53675	n.a.	n.a.	Outlier	n.a.	A	n.a.	n.a.	n.a.	n.a.	n.a.	n.a.	n.a.	n.a.	n.a.	n.a.	n.a.
S11_vs_55144	n.a.	n.a.	Outlier	n.a.	A	n.a.	n.a.	n.a.	n.a.	n.a.	n.a.	n.a.	n.a.	n.a.	n.a.	n.a.
S11_vs_56762	n.a.	n.a.	Outlier	n.a.	A	n.a.	n.a.	n.a.	n.a.	n.a.	n.a.	n.a.	n.a.	n.a.	n.a.	n.a.
S11_vs_67573	n.a.	n.a.	Outlier	n.a.	A	n.a.	n.a.	n.a.	n.a.	n.a.	n.a.	n.a.	n.a.	n.a.	n.a.	n.a.
S11_vs_68348	Streptococcus phage IPP10	KY065452	Outlier	n.a.	N3	19	Streptococcus	Group I	Duplodnaviria	Heunggongvirae	Uroviricota	Caudoviricetes	n.a.	Unclassified	O	O
S11_vs_6878	Clostridium phage phiCT9441A	KM983329	Outlier	n.a.	N3	14	Clostridium	Group I	Duplodnaviria	Heunggongvirae	Uroviricota	Caudoviricetes	n.a.	Unclassified	O	O
S11_vs_79831	n.a.	n.a.	Outlier	n.a.	A	n.a.	n.a.	n.a.	n.a.	n.a.	n.a.	n.a.	n.a.	n.a.	n.a.	n.a.
S11_vs_82331	Klebsiella phage ST11-OXA245phi3.2	MK416010	Outlier	n.a.	N1	18	Klebsiella	Group I	Duplodnaviria	Heunggongvirae	Uroviricota	Caudoviricetes	n.a.	Unclassified	O	O
S11_vs_89911	n.a.	n.a.	Outlier	n.a.	A	n.a.	n.a.	n.a.	n.a.	n.a.	n.a.	n.a.	n.a.	n.a.	n.a.	n.a.
S12_vf_105542	Streptococcus phage SF39	OK173051	Outlier	n.a.	N3	3	Streptococcus	Group I	Duplodnaviria	Heunggongvirae	Uroviricota	Caudoviricetes	n.a.	Unclassified	O	O
S12_vf_18902	Bacillus phage PK-3	ON881243	Outlier	n.a.	N14	11	Bacillus	Group I	Duplodnaviria	Heunggongvirae	Uroviricota	Caudoviricetes	n.a.	Unclassified	O	O
S12_vs_118154	Butyrivibrio phage Idris	MN882554	Outlier	n.a.	N20	4	Butyrivibrio	Group I	Duplodnaviria	Heunggongvirae	Uroviricota	Caudoviricetes	n.a.	Unclassified	O	O
S12_vs_133070	Bacillus phage BUCT082	MZ969646	Outlier	n.a.	N4	8	Bacillus	Group I	Duplodnaviria	Heunggongvirae	Uroviricota	Caudoviricetes	n.a.	Unclassified	O	O
S12_vs_142625	Elizabethkingia phage TCUEAP2	OK632025	Outlier	n.a.	N2	17	Elizabethkingia	Group I	Duplodnaviria	Heunggongvirae	Uroviricota	Caudoviricetes	n.a.	Unclassified	O	O
S12_vs_14467	n.a.	n.a.	Outlier	n.a.	A	n.a.	n.a.	n.a.	n.a.	n.a.	n.a.	n.a.	n.a.	n.a.	n.a.	n.a.
S12_vs_153780	Acidithiobacillus phage AcaML1	JX507079	Outlier	n.a.	N1	25	Acidithiobacillus	Group I	Duplodnaviria	Heunggongvirae	Uroviricota	Caudoviricetes	n.a.	Unclassified	O	O
S12_vs_188825	n.a.	n.a.	Outlier	n.a.	A	n.a.	n.a.	n.a.	n.a.	n.a.	n.a.	n.a.	n.a.	n.a.	n.a.	n.a.
S12_vs_1980	Elizabethkingia phage TCUEAP2	OK632025	Outlier	n.a.	N7	25	Elizabethkingia	Group I	Duplodnaviria	Heunggongvirae	Uroviricota	Caudoviricetes	n.a.	Unclassified	O	O
S12_vs_22853	Streptococcus phage SM1	AY007505	Outlier	n.a.	N2	24	Streptococcus	Group I	Duplodnaviria	Heunggongvirae	Uroviricota	Caudoviricetes	n.a.	Unclassified	O	O
S12_vs_28661	n.a.	n.a.	Outlier	n.a.	A	n.a.	n.a.	n.a.	n.a.	n.a.	n.a.	n.a.	n.a.	n.a.	n.a.	n.a.
S12_vs_32669	n.a.	n.a.	Outlier	n.a.	A	n.a.	n.a.	n.a.	n.a.	n.a.	n.a.	n.a.	n.a.	n.a.	n.a.	n.a.
S12_vs_74280	n.a.	n.a.	Outlier	n.a.	A	n.a.	n.a.	n.a.	n.a.	n.a.	n.a.	n.a.	n.a.	n.a.	n.a.	n.a.
S12_vs_75098	Lactobacillus phage c5	EU340421	Outlier	n.a.	N5	6	Lactobacillus	Group I	Duplodnaviria	Heunggongvirae	Uroviricota	Caudoviricetes	n.a.	Unclassified	O	O
S12_vs_81729	Bacillus phage vB_BtS_BMBtp13	KX190832	Outlier	n.a.	N9	4	Bacillus	Group I	Duplodnaviria	Heunggongvirae	Uroviricota	Caudoviricetes	n.a.	Unclassified	O	O
S12_vs_8204	Bacillus phage G	JN638751	Outlier	n.a.	N15	4	Bacillus	Group I	Duplodnaviria	Heunggongvirae	Uroviricota	Caudoviricetes	n.a.	Unclassified	O	O
S12_vs_87088	Faecalibacterium phage FP_Lugh	MG711464	Outlier	n.a.	N1	32	Faecalibacterium	Group I	Duplodnaviria	Heunggongvirae	Uroviricota	Caudoviricetes	n.a.	Unclassified	O	O
S12_vs_94042	n.a.	n.a.	Outlier	n.a.	A	n.a.	n.a.	n.a.	n.a.	n.a.	n.a.	n.a.	n.a.	n.a.	n.a.	n.a.
S12_vs_99219	n.a.	n.a.	Outlier	n.a.	A	n.a.	n.a.	n.a.	n.a.	n.a.	n.a.	n.a.	n.a.	n.a.	n.a.	n.a.
S13_vf_147396	n.a.	n.a.	Outlier	n.a.	A	n.a.	n.a.	n.a.	n.a.	n.a.	n.a.	n.a.	n.a.	n.a.	n.a.	n.a.
S13_vf_1770	Pectobacterium phage DU_PP_III	MF979562	Outlier	n.a.	N2	13	Pectobacterium	Group I	Duplodnaviria	Heunggongvirae	Uroviricota	Caudoviricetes	n.a.	Unclassified	O	O
S13_vf_54201	Faecalibacterium phage FP_Brigit	MG711465	Outlier	n.a.	N1	18	Faecalibacterium	Group I	Duplodnaviria	Heunggongvirae	Uroviricota	Caudoviricetes	n.a.	Unclassified	O	O
S13_vf_66508	Bacteroides phage ARB14	MT074134	Outlier	n.a.	N1	51	Bacteroides	Group I	Duplodnaviria	Heunggongvirae	Uroviricota	Caudoviricetes	n.a.	Unclassified	O	O
S13_vs_114213	Faecalibacterium phage FP_Mushu	MG711460	Outlier	n.a.	N4	4	Faecalibacterium	Group I	Duplodnaviria	Heunggongvirae	Uroviricota	Caudoviricetes	n.a.	Unclassified	O	O
S13_vs_13129	Bacillus phage vB_BtS_BMBtp13	KX190832	Outlier	n.a.	N22	2	Bacillus	Group I	Duplodnaviria	Heunggongvirae	Uroviricota	Caudoviricetes	n.a.	Unclassified	O	O
S13_vs_14794	Aeromonas phage P5	OM519319	Outlier	n.a.	N1	2	Aeromonas	Group I	Duplodnaviria	Heunggongvirae	Uroviricota	Caudoviricetes	n.a.	Unclassified	O	O
S13_vs_27462	Streptococcus phage Dp-1	HQ268735	Outlier	n.a.	N1	35	Streptococcus	Group I	Duplodnaviria	Heunggongvirae	Uroviricota	Caudoviricetes	n.a.	Unclassified	O	O
S13_vs_45488	Curvibacter phage TJ1	MH766655	Outlier	n.a.	N1	13	Curvibacter	Group I	Duplodnaviria	Heunggongvirae	Uroviricota	Caudoviricetes	n.a.	Unclassified	O	O
S13_vs_57267	Prevotella phage Lak-C1	MK250029	Outlier	n.a.	N1	4	Prevotella	Group I	Duplodnaviria	Heunggongvirae	Uroviricota	Caudoviricetes	n.a.	Unclassified	O	O
S14_vf_117147	Bacillus phage B13	OP066531	Outlier	n.a.	N15	6	Bacillus	Group I	Duplodnaviria	Heunggongvirae	Uroviricota	Caudoviricetes	n.a.	Unclassified	O	O
S14_vf_51134	Streptococcus phage OlisA2	OL774869	Outlier	n.a.	N5	22	Streptococcus	Group I	Duplodnaviria	Heunggongvirae	Uroviricota	Caudoviricetes	n.a.	Unclassified	O	O
S14_vf_57689	Microbacterium phage Rasputia	MT639646	Outlier	n.a.	N1	17	Microbacterium	Group I	Duplodnaviria	Heunggongvirae	Uroviricota	Caudoviricetes	n.a.	Unclassified	O	O
S14_vs_100227	Streptococcus phage phiARI0746	KT337365	Outlier	n.a.	N8	12	Streptococcus	Group I	Duplodnaviria	Heunggongvirae	Uroviricota	Caudoviricetes	n.a.	Unclassified	O	O
S14_vs_101482	n.a.	n.a.	Outlier	n.a.	A	n.a.	n.a.	n.a.	n.a.	n.a.	n.a.	n.a.	n.a.	n.a.	n.a.	n.a.
S14_vs_108376	Clostridium phage phiCT453A	KM983327	Outlier	n.a.	N5	10	Clostridium	Group I	Duplodnaviria	Heunggongvirae	Uroviricota	Caudoviricetes	n.a.	Unclassified	O	O

S14_vs_112345	n.a.	n.a.	Outlier	n.a.	A	n.a.	n.a.	n.a.	n.a.	n.a.	n.a.	n.a.	n.a.	n.a.	n.a.	n.a.
S14_vs_115377	Roseburia phage Jekyll	LR596902	Outlier	n.a.	N13	9	Roseburia	Group I	Duplodnaviria	Heunggongvirae	Uroviricota	Caudoviricetes	n.a.	Unclassified	O	O
S14_vs_115455	Clostridium phage phiSM101	CP000315	Outlier	n.a.	N2	16	Clostridium	Group I	Duplodnaviria	Heunggongvirae	Uroviricota	Caudoviricetes	n.a.	Unclassified	O	O
S14_vs_20193	Faecalibacterium phage FP_Lagaffe	MG711461	Outlier	n.a.	N2	50	Faecalibacterium	Group I	Duplodnaviria	Heunggongvirae	Uroviricota	Caudoviricetes	n.a.	Unclassified	O	O
S14_vs_26320	Geobacillus virus E3	KP144388	Outlier	n.a.	N16	4	Geobacillus	Group I	Duplodnaviria	Heunggongvirae	Uroviricota	Caudoviricetes	n.a.	Unclassified	O	O
S14_vs_30108	n.a.	n.a.	Outlier	n.a.	A	n.a.	n.a.	n.a.	n.a.	n.a.	n.a.	n.a.	n.a.	n.a.	n.a.	n.a.
S14_vs_30566	Streptococcus phage phiARI0746	KT337365	Outlier	n.a.	N3	11	Streptococcus	Group I	Duplodnaviria	Heunggongvirae	Uroviricota	Caudoviricetes	n.a.	Unclassified	O	O
S14_vs_79505	n.a.	n.a.	Outlier	n.a.	A	n.a.	n.a.	n.a.	n.a.	n.a.	n.a.	n.a.	n.a.	n.a.	n.a.	n.a.
S14_vs_9162	Streptococcus phage Javan242	MK448882	Outlier	n.a.	N11	14	Streptococcus	Group I	Duplodnaviria	Heunggongvirae	Uroviricota	Caudoviricetes	n.a.	Unclassified	O	O
S14_vs_93565	Streptococcus phage Javan422	MK448934	Outlier	n.a.	N7	6	Streptococcus	Group I	Duplodnaviria	Heunggongvirae	Uroviricota	Caudoviricetes	n.a.	Unclassified	O	O
S14_vs_96286	Streptococcus phage Javan583	MK448814	Outlier	n.a.	N6	21	Streptococcus	Group I	Duplodnaviria	Heunggongvirae	Uroviricota	Caudoviricetes	n.a.	Unclassified	O	O
S15_vf_58467	Enterococcus phage 9183	MT939241	Outlier	n.a.	N1	11	Enterococcus	Group I	Duplodnaviria	Heunggongvirae	Uroviricota	Caudoviricetes	n.a.	Unclassified	O	O
S16_vf_172897	Bifidobacterium phage BigBern1	MT006237	Outlier	n.a.	N6	9	Bifidobacterium	Group I	Duplodnaviria	Heunggongvirae	Uroviricota	Caudoviricetes	n.a.	Unclassified	O	O
S16_vf_32359	n.a.	n.a.	Outlier	n.a.	A	n.a.	n.a.	n.a.	n.a.	n.a.	n.a.	n.a.	n.a.	n.a.	n.a.	n.a.
S16_vf_33218	Lactococcus phage CHPC965	MN689525	Outlier	n.a.	N1	62	Lactococcus	Group I	Duplodnaviria	Heunggongvirae	Uroviricota	Caudoviricetes	n.a.	Unclassified	O	O
S16_vf_9270	n.a.	n.a.	Outlier	n.a.	A	n.a.	n.a.	n.a.	n.a.	n.a.	n.a.	n.a.	n.a.	n.a.	n.a.	n.a.
S16_vs_10622	n.a.	n.a.	Outlier	n.a.	A	n.a.	n.a.	n.a.	n.a.	n.a.	n.a.	n.a.	n.a.	n.a.	n.a.	n.a.
S16_vs_120149	n.a.	n.a.	Outlier	n.a.	A	n.a.	n.a.	n.a.	n.a.	n.a.	n.a.	n.a.	n.a.	n.a.	n.a.	n.a.
S16_vs_137222	Faecalibacterium phage FP_Lagaffe	MG711461	Outlier	n.a.	N3	48	Faecalibacterium	Group I	Duplodnaviria	Heunggongvirae	Uroviricota	Caudoviricetes	n.a.	Unclassified	O	O
S16_vs_141218	n.a.	n.a.	Outlier	n.a.	A	n.a.	n.a.	n.a.	n.a.	n.a.	n.a.	n.a.	n.a.	n.a.	n.a.	n.a.
S16_vs_165662	Clostridium phage HM T	KU517658	Outlier	n.a.	N8	2	Clostridium	Group I	Duplodnaviria	Heunggongvirae	Uroviricota	Caudoviricetes	n.a.	Unclassified	O	O
S16_vs_18293	Streptococcus phage Javan261	MK448720	Outlier	n.a.	N4	12	Streptococcus	Group I	Duplodnaviria	Heunggongvirae	Uroviricota	Caudoviricetes	n.a.	Unclassified	O	O
S16_vs_34021	n.a.	n.a.	Outlier	n.a.	A	n.a.	n.a.	n.a.	n.a.	n.a.	n.a.	n.a.	n.a.	n.a.	n.a.	n.a.
S16_vs_35031	n.a.	n.a.	Outlier	n.a.	A	n.a.	n.a.	n.a.	n.a.	n.a.	n.a.	n.a.	n.a.	n.a.	n.a.	n.a.
S16_vs_38863	Clostridium phage phiMMP01	LN681541	Outlier	n.a.	N9	19	Clostridium	Group I	Duplodnaviria	Heunggongvirae	Uroviricota	Caudoviricetes	n.a.	Unclassified	O	O
S16_vs_54250	n.a.	n.a.	Outlier	n.a.	A	n.a.	n.a.	n.a.	n.a.	n.a.	n.a.	n.a.	n.a.	n.a.	n.a.	n.a.
S16_vs_81805	Faecalibacterium phage FP_Lugh	MG711464	Outlier	n.a.	N3	19	Faecalibacterium	Group I	Duplodnaviria	Heunggongvirae	Uroviricota	Caudoviricetes	n.a.	Unclassified	O	O
S17_vf_111931	Clostridium phage DCp1	OP256049	Outlier	n.a.	N1	10	Clostridium	Group I	Duplodnaviria	Heunggongvirae	Uroviricota	Caudoviricetes	n.a.	Guelinviridae	O	O
S17_vf_46388	Ruminococcus phage phiRg507T2_3	MT980837	Outlier	n.a.	N6	4	Ruminococcus	Group I	Duplodnaviria	Heunggongvirae	Uroviricota	Caudoviricetes	n.a.	Unclassified	O	O
S17_vs_110666	Faecalibacterium phage FP_Brigit	MG711465	Outlier	n.a.	N2	29	Faecalibacterium	Group I	Duplodnaviria	Heunggongvirae	Uroviricota	Caudoviricetes	n.a.	Unclassified	O	O
S17_vs_117507	n.a.	n.a.	Outlier	n.a.	A	n.a.	n.a.	n.a.	n.a.	n.a.	n.a.	n.a.	n.a.	n.a.	n.a.	n.a.
S17_vs_177202	Faecalibacterium phage FP_Lugh	MG711464	Outlier	n.a.	N15	9	Faecalibacterium	Group I	Duplodnaviria	Heunggongvirae	Uroviricota	Caudoviricetes	n.a.	Unclassified	O	O
S17_vs_177203	Acetobacter phage phiAO1	LC644971	Outlier	n.a.	N1	26	Acetobacter	Group I	Duplodnaviria	Heunggongvirae	Uroviricota	Caudoviricetes	n.a.	Unclassified	O	O
S17_vs_31654	Streptococcus phage Javan74	MK449005	Outlier	n.a.	N2	26	Streptococcus	Group I	Duplodnaviria	Heunggongvirae	Uroviricota	Caudoviricetes	n.a.	Unclassified	O	O
S17_vs_52890	n.a.	n.a.	Outlier	n.a.	A	n.a.	n.a.	n.a.	n.a.	n.a.	n.a.	n.a.	n.a.	n.a.	n.a.	n.a.
S1_vf_138157	n.a.	n.a.	Outlier	n.a.	A	n.a.	n.a.	n.a.	n.a.	n.a.	n.a.	n.a.	n.a.	n.a.	n.a.	n.a.
S1_vf_140259	Microviridae sp.	MG945534	Outlier	n.a.	N1	6	Unspecified	Group II	Monodnaviria	Sangervirae	Phixviricota	Malgrandaviricetes	Petitvirales	Microviridae	O	O
S1_vf_28398	n.a.	n.a.	Outlier	n.a.	A	n.a.	n.a.	n.a.	n.a.	n.a.	n.a.	n.a.	n.a.	n.a.	n.a.	n.a.
S1_vs_133577	Faecalibacterium phage FP_Lugh	MG711464	Outlier	n.a.	N4	19	Faecalibacterium	Group I	Duplodnaviria	Heunggongvirae	Uroviricota	Caudoviricetes	n.a.	Unclassified	O	O
S1_vs_23682	Butyrivibrio virus Ceridwen	MN882553	Outlier	n.a.	N9	17	Butyrivibrio	Group I	Duplodnaviria	Heunggongvirae	Uroviricota	Caudoviricetes	n.a.	Unclassified	O	O
S2_vs_4368	Butyrivibrio phage Idris	MN882554	Outlier	n.a.	N9	1	Butyrivibrio	Group I	Duplodnaviria	Heunggongvirae	Uroviricota	Caudoviricetes	n.a.	Unclassified	O	O
S3_vf_101591	Gokushovirus WZ-2015a	KT264789	Outlier	n.a.	N1	8	Unspecified	Group II	Monodnaviria	Sangervirae	Phixviricota	Malgrandaviricetes	Petitvirales	Microviridae	O	O
S3_vf_101611	Streptococcus phage Javan74	MK449005	Outlier	n.a.	N3	14	Streptococcus	Group I	Duplodnaviria	Heunggongvirae	Uroviricota	Caudoviricetes	n.a.	Unclassified	O	O
S3_vf_111893	Streptococcus phage Javan583	MK448814	Outlier	n.a.	N4	23	Streptococcus	Group I	Duplodnaviria	Heunggongvirae	Uroviricota	Caudoviricetes	n.a.	Unclassified	O	O
S3_vf_127392	Roseburia phage Jekyll	LR596902	Outlier	n.a.	N28	7	Roseburia	Group I	Duplodnaviria	Heunggongvirae	Uroviricota	Caudoviricetes	n.a.	Unclassified	O	O
S3_vf_127408	Bifidobacterium phage BlindBasel1	MT006235	Outlier	n.a.	N1	28	Bifidobacterium	Group I	Duplodnaviria	Heunggongvirae	Uroviricota	Caudoviricetes	n.a.	Unclassified	O	O
S3_vf_2186	n.a.	n.a.	Outlier	n.a.	F	n.a.	n.a.	n.a.	n.a.	n.a.	n.a.	n.a.	n.a.	n.a.	n.a.	n.a.
S3_vf_23042	Lactococcus phage P656	MN552149	Outlier	n.a.	N1	50	Lactococcus	Group I	Duplodnaviria	Heunggongvirae	Uroviricota	Caudoviricetes	n.a.	Unclassified	O	O
S3_vf_5525	n.a.	n.a.	Outlier	n.a.	A	n.a.	n.a.	n.a.	n.a.	n.a.	n.a.	n.a.	n.a.	n.a.	n.a.	n.a.
S3_vf_56363	Clostridium phage phiCDKH01	MN718463	Outlier	n.a.	N1	26	Clostridium	Group I	Duplodnaviria	Heunggongvirae	Uroviricota	Caudoviricetes	n.a.	Unclassified	O	O
S3_vf_99626	Propionibacterium phage B3	KX620749	Outlier	n.a.	N2	11	Propionibacterium	Group I	Duplodnaviria	Heunggongvirae	Uroviricota	Caudoviricetes	n.a.	Unclassified	O	O
S3_vs_1031	n.a.	n.a.	Outlier	n.a.	A	n.a.	n.a.	n.a.	n.a.	n.a.	n.a.	n.a.	n.a.	n.a.	n.a.	n.a.
S3_vs_119771	Faecalibacterium phage FP_Lugh	MG711464	Outlier	n.a.	N2	17	Faecalibacterium	Group I	Duplodnaviria	Heunggongvirae	Uroviricota	Caudoviricetes	n.a.	Unclassified	O	O
S3_vs_122706	Streptococcus phage phiARI0746	KT337365	Outlier	n.a.	N6	2	Streptococcus	Group I	Duplodnaviria	Heunggongvirae	Uroviricota	Caudoviricetes	n.a.	Unclassified	O	O
S3_vs_127395	n.a.	n.a.	Outlier	n.a.	A	n.a.	n.a.	n.a.	n.a.	n.a.	n.a.	n.a.	n.a.	n.a.	n.a.	n.a.
S3_vs_127397	Streptococcus phage Javan278	MK448896	Outlier	n.a.	N12	6	Streptococcus	Group I	Duplodnaviria	Heunggongvirae	Uroviricota	Caudoviricetes	n.a.	Unclassified	O	O
S3_vs_127401	Streptococcus phage phiARI0746	KT337365	Outlier	n.a.	N5	12	Streptococcus	Group I	Duplodnaviria	Heunggongvirae	Uroviricota	Caudoviricetes	n.a.	Unclassified	O	O
S3_vs_127410	Clostridium phage phiCD38-2	HM568888	Outlier	n.a.	N15	3	Clostridium	Group I	Duplodnaviria	Heunggongvirae	Uroviricota	Caudoviricetes	n.a.	Unclassified	O	O
S3_vs_15450	Clostridium phage phiCT453A	KM983327	Outlier	n.a.	N16	5	Clostridium	Group I	Duplodnaviria	Heunggongvirae	Uroviricota	Caudoviricetes	n.a.	Unclassified	O	O
S3_vs_23194	Cellulophaga phage Ingeline_1	MT732435	Outlier	n.a.	N2	7	Cellulophaga	Group I	Duplodnaviria	Heunggongvirae	Uroviricota	Caudoviricetes	n.a.	Duneviridae	O	O
S3_vs_40939	n.a.	n.a.	Outlier	n.a.	A	n.a.	n.a.	n.a.	n.a.	n.a.	n.a.	n.a.	n.a.	n.a.	n.a.	n.a.
S3_vs_56292	Elizabethkingia phage TCUEAP3	OK632026	Outlier	n.a.	N12	6	Elizabethkingia	Group I	Duplodnaviria	Heunggongvirae	Uroviricota	Caudoviricetes	n.a.	Unclassified	O	O
S3_vs_62410	n.a.	n.a.	Outlier	n.a.	A	n.a.	n.a.	n.a.	n.a.	n.a.	n.a.	n.a.	n.a.	n.a.	n.a.	n.a.
S3_vs_6604	Flavobacterium phage vB_FspS_laban6-1	MN812211	Outlier	n.a.	N4	12	Flavobacterium	Group I	Duplodnaviria	Heunggongvirae	Uroviricota	Caudoviricetes	n.a.	Duneviridae	O	O
S3_vs_70093	n.a.	n.a.	Outlier	n.a.	A	n.a.	n.a.	n.a.	n.a.	n.a.	n.a.	n.a.	n.a.	n.a.	n.a.	n.a.
S3_vs_94577	Lactococcus phage C41431	KX160219	Outlier	n.a.	N2	26	Lactococcus	Group I	Duplodnaviria	Heunggongvirae	Uroviricota	Caudoviricetes	n.a.	Unclassified	O	O
S3_vs_97827	n.a.	n.a.	Outlier	n.a.	A	n.a.	n.a.	n.a.	n.a.	n.a.	n.a.	n.a.	n.a.	n.a.	n.a.	n.a.
S4_vf_13831	n.a.	n.a.	Outlier	n.a.	A	n.a.	n.a.	n.a.	n.a.	n.a.	n.a.	n.a.	n.a.	n.a.	n.a.	n.a.
S4_vf_20440	Riemerella phage RAP44	HQ396194	Outlier	n.a.	N11	5	Riemerella	Group I	Duplodnaviria	Heunggongvirae	Uroviricota	Caudoviricetes	n.a.	Unclassified	O	O
S4_vf_31982	Lactobacillus phage Ld3	KJ564038	Outlier	n.a.	N1	41	Lactobacillus	Group I	Duplodnaviria	Heunggongvirae	Uroviricota	Caudoviricetes	n.a.	Unclassified	O	O
S4_vs_VIRSorter_2744-cat_3	Bifidobacterium phage Bbif-1	GQ141189	Outlier	n.a.	N5	9	Bifidobacterium	Group I	Duplodnaviria	Heunggongvirae	Uroviricota	Caudoviricetes	n.a.	Unclassified	O	O
S4_vs_VIRSorter_31972-circular-cat_2	Faecalibacterium phage FP_Lugh	MG711464	Outlier	n.a.	N18	4	Faecalibacterium	Group I	Duplodnaviria	Heunggongvirae	Uroviricota	Caudoviricetes	n.a.	Unclassified	O	O
S4_vs_VIRSorter_5698-cat_2	Bifidobacterium phage Bbif-1	GQ141189	Outlier	n.a.	N3	17	Bifidobacterium	Group I	Duplodnaviria	Heunggongvirae	Uroviricota	Caudoviricetes	n.a.	Unclassified	O	O
S5_vf_103206	n.a.	n.a.	Outlier	n.a.	F	n.a.	n.a.	n.a.	n.a.	n.a.	n.a.	n.a.	n.a.	n.a.	n.a.	n.a.
S5_vf_116300	n.a.	n.a.	Outlier	n.a.	A	n.a.	n.a.	n.a.	n.a.	n.a.	n.a.	n.a.	n.a.	n.a.	n.a.	n.a.
S5_vf_55687	Lactococcus phage M5938	KX373687	Outlier	n.a.	N1	58	Lactococcus	Group I	Duplodnaviria	Heunggongvirae	Uroviricota	Caudoviricetes	n.a.	Unclassified	O	O
S5_vf_8808	Lactobacillus phage c5	EU340421	Outlier	n.a.	N5	8	Lactobacillus	Group I	Duplodnaviria	Heunggongvirae	Uroviricota	Caudoviricetes	n.a.	Unclassified	O	O
S5_vs_10428	Faecalibacterium phage FP_Taranis	MG711467	Outlier	n.a.	N3	17	Faecalibacterium	Group I	Duplodnaviria	Heunggongvirae	Uroviricota	Caudoviricetes	n.a.	Unclassified	O	O
S5_vs_112452	Clostridium phage phiCT453A	KM983327	Outlier	n.a.	N7	16	Clostridium	Group I	Duplodnaviria	Heunggongvirae	Uroviricota	Caudoviricetes	n.a.	Unclassified	O	O
S5_vs_113386	n.a.	n.a.	Outlier	n.a.	A	n.a.	n.a.	n.a.	n.a.	n.a.	n.a.	n.a.	n.a.	n.a.	n.a.	n.a.
S5_vs_118168	n.a.	n.a.	Outlier	n.a.	A	n.a.	n.a.	n.a.	n.a.	n.a.	n.a.	n.a.	n.a.	n.a.	n.a.	n.a.
S5_vs_120584	Streptococcus phage Dp-1	HQ268735	Outlier	n.a.	N2	13	Streptococcus	Group I	Duplodnaviria	Heunggongvirae	Uroviricota	Caudoviricetes	n.a.	Unclassified	O	O

S5_vs_121411	n.a.	n.a.	Outlier	n.a.	A	n.a.	n.a.	n.a.	n.a.	n.a.	n.a.	n.a.	n.a.	n.a.	n.a.	n.a.
S5_vs_122618	n.a.	n.a.	Outlier	n.a.	A	n.a.	n.a.	n.a.	n.a.	n.a.	n.a.	n.a.	n.a.	n.a.	n.a.	n.a.
S5_vs_128398	Faecalibacterium phage FP_Epona	MG711462	Outlier	n.a.	N10	30	Faecalibacterium	Group I	Duplodnaviria	Heunggongvirae	Uroviricota	Caudoviricetes	n.a.	Unclassified	O	O
S5_vs_128881	n.a.	n.a.	Outlier	n.a.	A	n.a.	n.a.	n.a.	n.a.	n.a.	n.a.	n.a.	n.a.	n.a.	n.a.	n.a.
S5_vs_130032	Escherichia phage 2H10	LR595862	Outlier	n.a.	N1	65	Escherichia	Group I	Duplodnaviria	Heunggongvirae	Uroviricota	Caudoviricetes	n.a.	Unclassified	O	O
S5_vs_130476	n.a.	n.a.	Outlier	n.a.	A	n.a.	n.a.	n.a.	n.a.	n.a.	n.a.	n.a.	n.a.	n.a.	n.a.	n.a.
S5_vs_141621	Clostridium phage phiCT19406B	KM983331	Outlier	n.a.	N4	10	Clostridium	Group I	Duplodnaviria	Heunggongvirae	Uroviricota	Caudoviricetes	n.a.	Unclassified	O	O
S5_vs_145582	Phage WC36-2	OL791266	Outlier	n.a.	N4	23	Unspecified	Group I	Duplodnaviria	Heunggongvirae	Uroviricota	Caudoviricetes	n.a.	Unclassified	O	O
S5_vs_145611	Clostridium phage phiCT453A	KM983327	Outlier	n.a.	N7	13	Clostridium	Group I	Duplodnaviria	Heunggongvirae	Uroviricota	Caudoviricetes	n.a.	Unclassified	O	O
S5_vs_32885	Faecalibacterium phage FP_Lagaffe	MG711461	Outlier	n.a.	N3	13	Faecalibacterium	Group I	Duplodnaviria	Heunggongvirae	Uroviricota	Caudoviricetes	n.a.	Unclassified	O	O
S5_vs_37659	n.a.	n.a.	Outlier	n.a.	A	n.a.	n.a.	n.a.	n.a.	n.a.	n.a.	n.a.	n.a.	n.a.	n.a.	n.a.
S5_vs_43207	Faecalibacterium phage FP_Lugh	MG711464	Outlier	n.a.	N15	1	Faecalibacterium	Group I	Duplodnaviria	Heunggongvirae	Uroviricota	Caudoviricetes	n.a.	Unclassified	O	O
S5_vs_46394	n.a.	n.a.	Outlier	n.a.	A	n.a.	n.a.	n.a.	n.a.	n.a.	n.a.	n.a.	n.a.	n.a.	n.a.	n.a.
S5_vs_4737	n.a.	n.a.	Outlier	n.a.	A	n.a.	n.a.	n.a.	n.a.	n.a.	n.a.	n.a.	n.a.	n.a.	n.a.	n.a.
S5_vs_51416	Faecalibacterium phage FP_Lugh	MG711464	Outlier	n.a.	N26	1	Faecalibacterium	Group I	Duplodnaviria	Heunggongvirae	Uroviricota	Caudoviricetes	n.a.	Unclassified	O	O
S5_vs_59798	Lactobacillus phage c5	EU340421	Outlier	n.a.	N10	6	Lactobacillus	Group I	Duplodnaviria	Heunggongvirae	Uroviricota	Caudoviricetes	n.a.	Unclassified	O	O
S5_vs_72532	Bacteroides phage p00	BK010646	Outlier	n.a.	N2	30	Bacteroides	Group I	Duplodnaviria	Heunggongvirae	Uroviricota	Caudoviricetes	n.a.	Unclassified	O	O
S5_vs_80911	Clostridium phage phiSM101	CP000315	Outlier	n.a.	N4	3	Clostridium	Group I	Duplodnaviria	Heunggongvirae	Uroviricota	Caudoviricetes	n.a.	Unclassified	O	O
S5_vs_8125	Clostridium phage phiSM101	CP000315	Outlier	n.a.	N6	8	Clostridium	Group I	Duplodnaviria	Heunggongvirae	Uroviricota	Caudoviricetes	n.a.	Unclassified	O	O
S5_vs_850	n.a.	n.a.	Outlier	n.a.	A	n.a.	n.a.	n.a.	n.a.	n.a.	n.a.	n.a.	n.a.	n.a.	n.a.	n.a.
S5_vs_89540	Lactobacillus phage CL2	KR905067	Outlier	n.a.	N1	19	Lactobacillus	Group I	Duplodnaviria	Heunggongvirae	Uroviricota	Caudoviricetes	n.a.	Unclassified	O	O
S6_vf_47238	Pectobacterium phage DU_PP_III	MF979562	Outlier	n.a.	N2	13	Pectobacterium	Group I	Duplodnaviria	Heunggongvirae	Uroviricota	Caudoviricetes	n.a.	Unclassified	O	O
S6_vf_56484	Faecalibacterium phage FP_Lugh	MG711464	Outlier	n.a.	N9	9	Faecalibacterium	Group I	Duplodnaviria	Heunggongvirae	Uroviricota	Caudoviricetes	n.a.	Unclassified	O	O
S6_vs_37812	Rhodobacter phage RcOceanus	MW677520	Outlier	n.a.	N1	9	Rhodobacter	Group I	Duplodnaviria	Heunggongvirae	Uroviricota	Caudoviricetes	n.a.	Unclassified	O	O
S6_vs_39065	n.a.	n.a.	Outlier	n.a.	A	n.a.	n.a.	n.a.	n.a.	n.a.	n.a.	n.a.	n.a.	n.a.	n.a.	n.a.
S6_vs_44070	Bacteriophage sp.	MW202489	Outlier	n.a.	N1	11	Unspecified	Unclassified	Unclassified	n.a.	n.a.	n.a.	n.a.	Unclassified	O	O
S7_vf_13614	Streptococcus phage Javan583	MK448814	Outlier	n.a.	N3	23	Streptococcus	Group I	Duplodnaviria	Heunggongvirae	Uroviricota	Caudoviricetes	n.a.	Unclassified	O	O
S7_vf_138225	Ralstonia phage RPSC1	MF893341	Outlier	n.a.	N3	4	Ralstonia	Group I	Duplodnaviria	Heunggongvirae	Uroviricota	Caudoviricetes	n.a.	Autographiviridae	O	O
S7_vf_29941	Lactococcus phage P680	KC182551	Outlier	n.a.	N1	60	Lactococcus	Group I	Duplodnaviria	Heunggongvirae	Uroviricota	Caudoviricetes	n.a.	Unclassified	O	O
S7_vf_5801	Riemerella phage RAP44	HQ396194	Outlier	n.a.	N1	20	Riemerella	Group I	Duplodnaviria	Heunggongvirae	Uroviricota	Caudoviricetes	n.a.	Unclassified	O	O
S7_vf_68239	Lactococcus phage 62601	MF448564	Outlier	n.a.	N1	46	Lactococcus	Group I	Duplodnaviria	Heunggongvirae	Uroviricota	Caudoviricetes	n.a.	Unclassified	O	O
S7_vf_83624	Clostridium phage HM2	LT600745	Outlier	n.a.	N28	4	Clostridium	Group I	Duplodnaviria	Heunggongvirae	Uroviricota	Caudoviricetes	n.a.	Salasmaviridae	O	O
S7_vf_9334	Podoviridae sp.	MW202898	Outlier	n.a.	N1	9	Unspecified	Group I	Duplodnaviria	Heunggongvirae	Uroviricota	Caudoviricetes	n.a.	Unclassified	O	O
S7_vs_134784	Faecalibacterium phage FP_Lugh	MG711464	Outlier	n.a.	N4	7	Faecalibacterium	Group I	Duplodnaviria	Heunggongvirae	Uroviricota	Caudoviricetes	n.a.	Unclassified	O	O
S7_vs_138140	n.a.	n.a.	Outlier	n.a.	A	n.a.	n.a.	n.a.	n.a.	n.a.	n.a.	n.a.	n.a.	n.a.	n.a.	n.a.
S7_vs_14275	n.a.	n.a.	Outlier	n.a.	A	n.a.	n.a.	n.a.	n.a.	n.a.	n.a.	n.a.	n.a.	n.a.	n.a.	n.a.
S7_vs_16344	Campylobacter phage PC10	MZ047271	Outlier	n.a.	N1	15	Campylobacter	Group I	Duplodnaviria	Heunggongvirae	Uroviricota	Caudoviricetes	n.a.	Unclassified	O	O
S7_vs_21521	n.a.	n.a.	Outlier	n.a.	A	n.a.	n.a.	n.a.	n.a.	n.a.	n.a.	n.a.	n.a.	n.a.	n.a.	n.a.
S7_vs_26235	n.a.	n.a.	Outlier	n.a.	A	n.a.	n.a.	n.a.	n.a.	n.a.	n.a.	n.a.	n.a.	n.a.	n.a.	n.a.
S7_vs_29595	Pseudomonas phage BHU-1	OK638201	Outlier	n.a.	N1	9	Pseudomonas	Group I	Duplodnaviria	Heunggongvirae	Uroviricota	Caudoviricetes	n.a.	Unclassified	O	O
S7_vs_36656	Clostridium phage phiCT453A	KM983327	Outlier	n.a.	N1	16	Clostridium	Group I	Duplodnaviria	Heunggongvirae	Uroviricota	Caudoviricetes	n.a.	Unclassified	O	O
S7_vs_40259	Clostridium phage phiCDHM19	LK985322	Outlier	n.a.	N7	9	Clostridium	Group I	Duplodnaviria	Heunggongvirae	Uroviricota	Caudoviricetes	n.a.	Unclassified	O	O
S7_vs_51332	n.a.	n.a.	Outlier	n.a.	A	n.a.	n.a.	n.a.	n.a.	n.a.	n.a.	n.a.	n.a.	n.a.	n.a.	n.a.
S7_vs_55031	n.a.	n.a.	Outlier	n.a.	A	n.a.	n.a.	n.a.	n.a.	n.a.	n.a.	n.a.	n.a.	n.a.	n.a.	n.a.
S7_vs_70265	Clostridium phage HM2	LT600745	Outlier	n.a.	N32	4	Clostridium	Group I	Duplodnaviria	Heunggongvirae	Uroviricota	Caudoviricetes	n.a.	Salasmaviridae	O	O
S7_vs_72737	n.a.	n.a.	Outlier	n.a.	A	n.a.	n.a.	n.a.	n.a.	n.a.	n.a.	n.a.	n.a.	n.a.	n.a.	n.a.
S8_vf_122206	Bacteroides phage LoVEphage	MW660583	Outlier	n.a.	N8	13	Bacteroides	Group I	Duplodnaviria	Heunggongvirae	Uroviricota	Caudoviricetes	n.a.	Unclassified	O	O
S8_vf_13709	Faecalibacterium phage FP_Lugh	MG711464	Outlier	n.a.	N8	6	Faecalibacterium	Group I	Duplodnaviria	Heunggongvirae	Uroviricota	Caudoviricetes	n.a.	Unclassified	O	O
S8_vf_147338	Faecalibacterium phage FP_oengus	MG711463	Outlier	n.a.	N21	32	Faecalibacterium	Group I	Duplodnaviria	Heunggongvirae	Uroviricota	Caudoviricetes	n.a.	Unclassified	O	O
S8_vf_192724	Clostridium phage HM2	LT600745	Outlier	n.a.	N2	4	Clostridium	Group I	Duplodnaviria	Heunggongvirae	Uroviricota	Caudoviricetes	n.a.	Salasmaviridae	O	O
S8_vf_65522	n.a.	n.a.	Outlier	n.a.	A	n.a.	n.a.	n.a.	n.a.	n.a.	n.a.	n.a.	n.a.	n.a.	n.a.	n.a.
S8_vs_110692	Streptococcus phage Javan278	MK448896	Outlier	n.a.	N10	8	Streptococcus	Group I	Duplodnaviria	Heunggongvirae	Uroviricota	Caudoviricetes	n.a.	Unclassified	O	O
S8_vs_113059	Gokushovirus WZ-2015a	KT264759	Outlier	n.a.	N1	14	Unspecified	Group II	Monodnaviria	Sangervirae	Phixviricota	Malgrandaviricetes	Petitvirales	Microviridae	O	O
S8_vs_126582	Phage DP SC_6_H4_2017	MT121964	Outlier	n.a.	N1	97	Unspecified	Group I	Duplodnaviria	Heunggongvirae	Uroviricota	Caudoviricetes	n.a.	Unclassified	O	O
S8_vs_132472	n.a.	n.a.	Outlier	n.a.	A	n.a.	n.a.	n.a.	n.a.	n.a.	n.a.	n.a.	n.a.	n.a.	n.a.	n.a.
S8_vs_147670	Clostridioides phage CD1801	MW512570	Outlier	n.a.	N1	9	Clostridioides	Group I	Duplodnaviria	Heunggongvirae	Uroviricota	Caudoviricetes	n.a.	Unclassified	O	O
S8_vs_147697	n.a.	n.a.	Outlier	n.a.	A	n.a.	n.a.	n.a.	n.a.	n.a.	n.a.	n.a.	n.a.	n.a.	n.a.	n.a.
S8_vs_185041	Gardnerella phage vB_Gva_AB1	MW387018	Outlier	n.a.	N4	38	Gardnerella	Group I	Duplodnaviria	Heunggongvirae	Uroviricota	Caudoviricetes	n.a.	Unclassified	O	O
S8_vs_3224	n.a.	n.a.	Outlier	n.a.	A	n.a.	n.a.	n.a.	n.a.	n.a.	n.a.	n.a.	n.a.	n.a.	n.a.	n.a.
S8_vs_63277	Faecalibacterium phage FP_Lugh	MG711464	Outlier	n.a.	N1	37	Faecalibacterium	Group I	Duplodnaviria	Heunggongvirae	Uroviricota	Caudoviricetes	n.a.	Unclassified	O	O
S8_vs_72117	n.a.	n.a.	Outlier	n.a.	A	n.a.	n.a.	n.a.	n.a.	n.a.	n.a.	n.a.	n.a.	n.a.	n.a.	n.a.
S9_vf_19284	n.a.	n.a.	Outlier	n.a.	A	n.a.	n.a.	n.a.	n.a.	n.a.	n.a.	n.a.	n.a.	n.a.	n.a.	n.a.
S9_vf_934	Curtobacterium phage Penoan	MZ333133	Outlier	n.a.	N1	13	Curtobacterium	Group I	Duplodnaviria	Heunggongvirae	Uroviricota	Caudoviricetes	n.a.	Unclassified	O	O
S9_vs_14235	n.a.	n.a.	Outlier	n.a.	A	n.a.	n.a.	n.a.	n.a.	n.a.	n.a.	n.a.	n.a.	n.a.	n.a.	n.a.
S9_vs_19689	n.a.	n.a.	Outlier	n.a.	A	n.a.	n.a.	n.a.	n.a.	n.a.	n.a.	n.a.	n.a.	n.a.	n.a.	n.a.
S9_vs_2799	n.a.	n.a.	Outlier	n.a.	A	n.a.	n.a.	n.a.	n.a.	n.a.	n.a.	n.a.	n.a.	n.a.	n.a.	n.a.
S9_vs_41371	Psychrobacillus phage PVJ1	MZ983385	Outlier	n.a.	N3	14	Psychrobacillus	Group I	Duplodnaviria	Heunggongvirae	Uroviricota	Caudoviricetes	n.a.	Unclassified	O	O
S9_vs_44331	Ruminococcus phage phiRg519T2	MT980838	Outlier	n.a.	N7	9	Ruminococcus	Group I	Duplodnaviria	Heunggongvirae	Uroviricota	Caudoviricetes	n.a.	Unclassified	O	O
S9_vs_55738	Streptococcus phage Javan355	MK448738	Outlier	n.a.	N6	8	Streptococcus	Group I	Duplodnaviria	Heunggongvirae	Uroviricota	Caudoviricetes	n.a.	Unclassified	O	O
S9_vs_58086	Streptococcus phage Javan290	MK448900	Outlier	n.a.	N15	5	Streptococcus	Group I	Duplodnaviria	Heunggongvirae	Uroviricota	Caudoviricetes	n.a.	Unclassified	O	O
S9_vs_62803	Faecalibacterium phage FP_Lugh	MG711464	Outlier	n.a.	N3	22	Faecalibacterium	Group I	Duplodnaviria	Heunggongvirae	Uroviricota	Caudoviricetes	n.a.	Unclassified	O	O
S9_vs_66877	Faecalibacterium phage FP_Lugh	MG711464	Outlier	n.a.	N3	16	Faecalibacterium	Group I	Duplodnaviria	Heunggongvirae	Uroviricota	Caudoviricetes	n.a.	Unclassified	O	O
S9_vs_67792	Phage WC36-2	OL791266	Outlier	n.a.	N2	23	Unspecified	Group I	Duplodnaviria	Heunggongvirae	Uroviricota	Caudoviricetes	n.a.	Unclassified	O	O
S14_vf_117162	Bacteroides phage EMB2	ON721385	Overlap (VC_1044/VC_1047)	n.a.	C7	57	Bacteroides	Group I	Duplodnaviria	Heunggongvirae	Uroviricota	Caudoviricetes	n.a.	Unclassified	Unclassified	O
S17_vf_177200	Bacteroides phage EMB2	ON721385	Overlap (VC_1044/VC_1047)	n.a.	C7	57	Bacteroides	Group I	Duplodnaviria	Heunggongvirae	Uroviricota	Caudoviricetes	n.a.	Unclassified	Unclassified	O
S11_vf_104062	Bacteroides phage EMB2	ON721385	Overlap (VC_1044/VC_1047/VC_1488)	n.a.	C4	143	Bacteroides	Group I	Duplodnaviria	Heunggongvirae	Uroviricota	Caudoviricetes	n.a.	Unclassified	Unclassified	O
S7_vf_27309	Bacteroides phage EMB2	ON721385	Overlap (VC_1044/VC_1047/VC_1488)	n.a.	C1	150	Bacteroides	Group I	Duplodnaviria	Heunggongvirae	Uroviricota	Caudoviricetes	n.a.	Unclassified	Unclassified	O
S12_vf_145044	n.a.	n.a.	Overlap (VC_1080/VC_1510)	n.a.	A	n.a.	n.a.	n.a.	n.a.	n.a.	n.a.	n.a.	n.a.	n.a.	n.a.	n.a.
S12_vf_32615	n.a.	n.a.	Overlap (VC_1080/VC_1510)	n.a.	A	n.a.	n.a.	n.a.	n.a.	n.a.	n.a.	n.a.	n.a.	n.a.	n.a.	n.a.
S15_vf_33505	uncultured phage cr53_1	MT774396	Overlap (VC_1080/VC_1510)	n.a.	N6	2	Unspecified	Group I	Duplodnaviria	Heunggongvirae	Uroviricota	Caudoviricetes	Crassvirales	Suoliviridae	O	O
S5_vs_117319	Faecalibacterium phage FP_oengus	MG711463	Overlap (VC_1086/VC_1243)	n.a.	C1	149	Faecalibacterium	Group I	Duplodnaviria	Heunggongvirae	Uroviricota	Caudoviricetes	n.a.	Unclassified	Unclassified	O
S6_vs_34453	Faecalibacterium phage FP_oengus	MG711463	Overlap (VC_1086/VC_1243)	n.a.	C1	143	Faecalibacterium	Group I	Duplodnaviria	Heunggongvirae	Uroviricota	Caudoviricetes	n.a.	Unclassified	Unclassified	O

S7_vs_87279	Faecalibacterium phage FP_oengus	MG711463	Overlap (VC_1086/VC_1243)	n.a.	C1	178	Faecalibacterium	Group I	Duplodnaviria	Heunggongvirae	Uroviricota	Caudoviricetes	n.a.	Unclassified	Unclassified	O
S3_vs_51931	Gokushovirus WZ-2015a	KT264829	Overlap (VC_1098/VC_1100)	n.a.	C1	28	Unspecified	Group II	Monodnaviria	Sangervirae	Phixviricota	Malgrandaviricetes	Petitvirales	Microviridae	Gokushovirinae	O
S16_vs_101671	Microviridae sp.	MG945704	Overlap (VC_1160/VC_1164)	n.a.	C1	23	Unspecified	Group II	Monodnaviria	Sangervirae	Phixviricota	Malgrandaviricetes	Petitvirales	Microviridae	Unclassified	O
S17_vs_177210	Microviridae sp.	MG945704	Overlap (VC_1160/VC_1164)	n.a.	C1	17	Unspecified	Group II	Monodnaviria	Sangervirae	Phixviricota	Malgrandaviricetes	Petitvirales	Microviridae	Unclassified	O
S1_vs_3267	Microviridae sp.	MG945704	Overlap (VC_1160/VC_1164)	n.a.	C1	22	Unspecified	Group II	Monodnaviria	Sangervirae	Phixviricota	Malgrandaviricetes	Petitvirales	Microviridae	Unclassified	O
S3_vs_51005	Microviridae sp.	MG945704	Overlap (VC_1160/VC_1164)	n.a.	C1	19	Unspecified	Group II	Monodnaviria	Sangervirae	Phixviricota	Malgrandaviricetes	Petitvirales	Microviridae	Unclassified	O
S16_vs_118029	Roseburia phage Jekyll	LR596902	Overlap (VC_1254/VC_1595)	n.a.	N16	5	Roseburia	Group I	Duplodnaviria	Heunggongvirae	Uroviricota	Caudoviricetes	n.a.	Unclassified	O	O
S7_vs_30640	Faecalibacterium phage FP_Lugh	MG711464	Overlap (VC_1254/VC_1595)	n.a.	N20	1	Faecalibacterium	Group I	Duplodnaviria	Heunggongvirae	Uroviricota	Caudoviricetes	n.a.	Unclassified	O	O
S11_vs_49071	n.a.	n.a.	Overlap (VC_1255/VC_1558)	n.a.	A	n.a.	n.a.	n.a.	n.a.	n.a.	n.a.	n.a.	n.a.	n.a.	n.a.	n.a.
S7_vs_101751	n.a.	n.a.	Overlap (VC_1255/VC_1558)	n.a.	A	n.a.	n.a.	n.a.	n.a.	n.a.	n.a.	n.a.	n.a.	n.a.	n.a.	n.a.
S7_vs_137998	n.a.	n.a.	Overlap (VC_1255/VC_1558)	n.a.	A	n.a.	n.a.	n.a.	n.a.	n.a.	n.a.	n.a.	n.a.	n.a.	n.a.	n.a.
S12_vs_26847	n.a.	n.a.	Overlap (VC_1255/VC_1593)	n.a.	A	n.a.	n.a.	n.a.	n.a.	n.a.	n.a.	n.a.	n.a.	n.a.	n.a.	n.a.
S16_vf_5809	n.a.	n.a.	Overlap (VC_1255/VC_1593)	n.a.	A	n.a.	n.a.	n.a.	n.a.	n.a.	n.a.	n.a.	n.a.	n.a.	n.a.	n.a.
S16_vf_84462	n.a.	n.a.	Overlap (VC_1255/VC_1594)	n.a.	A	n.a.	n.a.	n.a.	n.a.	n.a.	n.a.	n.a.	n.a.	n.a.	n.a.	n.a.
S5_vf_60597	n.a.	n.a.	Overlap (VC_1255/VC_1594)	n.a.	A	n.a.	n.a.	n.a.	n.a.	n.a.	n.a.	n.a.	n.a.	n.a.	n.a.	n.a.
S8_vf_201695	n.a.	n.a.	Overlap (VC_1255/VC_1594)	n.a.	A	n.a.	n.a.	n.a.	n.a.	n.a.	n.a.	n.a.	n.a.	n.a.	n.a.	n.a.
S3_vs_127421	Roseburia phage Jekyll	LR596902	Overlap (VC_1256/VC_1564)	n.a.	N2	26	Roseburia	Group I	Duplodnaviria	Heunggongvirae	Uroviricota	Caudoviricetes	n.a.	Unclassified	O	O
S8_vs_173961	Roseburia phage Jekyll	LR596902	Overlap (VC_1256/VC_1564)	n.a.	N2	24	Roseburia	Group I	Duplodnaviria	Heunggongvirae	Uroviricota	Caudoviricetes	n.a.	Unclassified	O	O
S11_vf_58587	n.a.	n.a.	Overlap (VC_1259/VC_1261)	n.a.	A	n.a.	n.a.	n.a.	n.a.	n.a.	n.a.	n.a.	n.a.	n.a.	n.a.	n.a.
S12_vf_170614	Clostridium phage HM2	LT600745	Overlap (VC_1259/VC_1261)	n.a.	N34	4	Clostridium	Group I	Duplodnaviria	Heunggongvirae	Uroviricota	Caudoviricetes	n.a.	Salasmaviridae	O	O
S16_vf_100473	n.a.	n.a.	Overlap (VC_1259/VC_1261)	n.a.	A	n.a.	n.a.	n.a.	n.a.	n.a.	n.a.	n.a.	n.a.	n.a.	n.a.	n.a.
S16_vf_94866	n.a.	n.a.	Overlap (VC_1259/VC_1261)	n.a.	A	n.a.	n.a.	n.a.	n.a.	n.a.	n.a.	n.a.	n.a.	n.a.	n.a.	n.a.
S5_vf_38011	n.a.	n.a.	Overlap (VC_1259/VC_1261)	n.a.	A	n.a.	n.a.	n.a.	n.a.	n.a.	n.a.	n.a.	n.a.	n.a.	n.a.	n.a.
S6_vf_24553	n.a.	n.a.	Overlap (VC_1259/VC_1261)	n.a.	A	n.a.	n.a.	n.a.	n.a.	n.a.	n.a.	n.a.	n.a.	n.a.	n.a.	n.a.
S7_vf_32396	n.a.	n.a.	Overlap (VC_1259/VC_1261)	n.a.	A	n.a.	n.a.	n.a.	n.a.	n.a.	n.a.	n.a.	n.a.	n.a.	n.a.	n.a.
S7_vf_89668	n.a.	n.a.	Overlap (VC_1259/VC_1261)	n.a.	A	n.a.	n.a.	n.a.	n.a.	n.a.	n.a.	n.a.	n.a.	n.a.	n.a.	n.a.
S8_vf_153696	n.a.	n.a.	Overlap (VC_1259/VC_1261)	n.a.	A	n.a.	n.a.	n.a.	n.a.	n.a.	n.a.	n.a.	n.a.	n.a.	n.a.	n.a.
S10_vf_57705	n.a.	n.a.	Overlap (VC_1259/VC_1563)	n.a.	A	n.a.	n.a.	n.a.	n.a.	n.a.	n.a.	n.a.	n.a.	n.a.	n.a.	n.a.
S12_vf_8173	n.a.	n.a.	Overlap (VC_1259/VC_1563)	n.a.	A	n.a.	n.a.	n.a.	n.a.	n.a.	n.a.	n.a.	n.a.	n.a.	n.a.	n.a.
S15_vf_71616	n.a.	n.a.	Overlap (VC_1259/VC_1563)	n.a.	A	n.a.	n.a.	n.a.	n.a.	n.a.	n.a.	n.a.	n.a.	n.a.	n.a.	n.a.
S7_vs_107876	Clostridium phage HM2	LT600745	Overlap (VC_1260/VC_1267)	n.a.	N11	7	Clostridium	Group I	Duplodnaviria	Heunggongvirae	Uroviricota	Caudoviricetes	n.a.	Salasmaviridae	O	O
S8_vf_3410	Clostridium phage HM2	LT600745	Overlap (VC_1260/VC_1267)	n.a.	N11	7	Clostridium	Group I	Duplodnaviria	Heunggongvirae	Uroviricota	Caudoviricetes	n.a.	Salasmaviridae	O	O
S12_vf_61299	Clostridium phage HM2	LT600745	Overlap (VC_1262/VC_1263)	n.a.	N30	4	Clostridium	Group I	Duplodnaviria	Heunggongvirae	Uroviricota	Caudoviricetes	n.a.	Salasmaviridae	O	O
S14_vf_93134	Clostridium phage HM2	LT600745	Overlap (VC_1262/VC_1263)	n.a.	N30	4	Clostridium	Group I	Duplodnaviria	Heunggongvirae	Uroviricota	Caudoviricetes	n.a.	Salasmaviridae	O	O
S17_vf_36452	Clostridium phage HM2	LT600745	Overlap (VC_1262/VC_1263)	n.a.	N30	4	Clostridium	Group I	Duplodnaviria	Heunggongvirae	Uroviricota	Caudoviricetes	n.a.	Salasmaviridae	O	O
S2_vf_17850	Clostridium phage HM2	LT600745	Overlap (VC_1262/VC_1263)	n.a.	N30	4	Clostridium	Group I	Duplodnaviria	Heunggongvirae	Uroviricota	Caudoviricetes	n.a.	Salasmaviridae	O	O
S16_vf_147326	Clostridium phage HM2	LT600745	Overlap (VC_1262/VC_1265)	n.a.	N18	8	Clostridium	Group I	Duplodnaviria	Heunggongvirae	Uroviricota	Caudoviricetes	n.a.	Salasmaviridae	O	O
S8_vf_159173	Clostridium phage HM2	LT600745	Overlap (VC_1262/VC_1267)	n.a.	N30	4	Clostridium	Group I	Duplodnaviria	Heunggongvirae	Uroviricota	Caudoviricetes	n.a.	Salasmaviridae	O	O
S13_vf_97702	Clostridium phage HM2	LT600745	Overlap (VC_1264/VC_1267)	n.a.	N6	7	Clostridium	Group I	Duplodnaviria	Heunggongvirae	Uroviricota	Caudoviricetes	n.a.	Salasmaviridae	O	O
S7_vs_13903	Clostridium phage HM2	LT600745	Overlap (VC_1264/VC_1267)	n.a.	N6	7	Clostridium	Group I	Duplodnaviria	Heunggongvirae	Uroviricota	Caudoviricetes	n.a.	Salasmaviridae	O	O
S1_vs_145792	n.a.	n.a.	Overlap (VC_1291/VC_1578)	n.a.	A	n.a.	n.a.	n.a.	n.a.	n.a.	n.a.	n.a.	n.a.	n.a.	n.a.	n.a.
S3_vs_25896	n.a.	n.a.	Overlap (VC_1291/VC_1578)	n.a.	A	n.a.	n.a.	n.a.	n.a.	n.a.	n.a.	n.a.	n.a.	n.a.	n.a.	n.a.
S5_vs_145591	n.a.	n.a.	Overlap (VC_1291/VC_1578)	n.a.	A	n.a.	n.a.	n.a.	n.a.	n.a.	n.a.	n.a.	n.a.	n.a.	n.a.	n.a.
S5_vs_27918	n.a.	n.a.	Overlap (VC_1291/VC_1578)	n.a.	A	n.a.	n.a.	n.a.	n.a.	n.a.	n.a.	n.a.	n.a.	n.a.	n.a.	n.a.
S7_vf_72493	n.a.	n.a.	Overlap (VC_1291/VC_1578)	n.a.	A	n.a.	n.a.	n.a.	n.a.	n.a.	n.a.	n.a.	n.a.	n.a.	n.a.	n.a.
S9_vs_69322	n.a.	n.a.	Overlap (VC_1291/VC_1578)	n.a.	A	n.a.	n.a.	n.a.	n.a.	n.a.	n.a.	n.a.	n.a.	n.a.	n.a.	n.a.
S9_vs_88286	n.a.	n.a.	Overlap (VC_1291/VC_1578)	n.a.	A	n.a.	n.a.	n.a.	n.a.	n.a.	n.a.	n.a.	n.a.	n.a.	n.a.	n.a.
S13_vs_49889	n.a.	n.a.	Overlap (VC_1292/VC_1574)	n.a.	A	n.a.	n.a.	n.a.	n.a.	n.a.	n.a.	n.a.	n.a.	n.a.	n.a.	n.a.
S16_vs_59697	n.a.	n.a.	Overlap (VC_1292/VC_1574)	n.a.	A	n.a.	n.a.	n.a.	n.a.	n.a.	n.a.	n.a.	n.a.	n.a.	n.a.	n.a.
S8_vs_152553	n.a.	n.a.	Overlap (VC_1292/VC_1574)	n.a.	A	n.a.	n.a.	n.a.	n.a.	n.a.	n.a.	n.a.	n.a.	n.a.	n.a.	n.a.
S14_vs_4887	n.a.	n.a.	Overlap (VC_1292/VC_1575)	n.a.	A	n.a.	n.a.	n.a.	n.a.	n.a.	n.a.	n.a.	n.a.	n.a.	n.a.	n.a.
S7_vs_95276	n.a.	n.a.	Overlap (VC_1292/VC_1575)	n.a.	A	n.a.	n.a.	n.a.	n.a.	n.a.	n.a.	n.a.	n.a.	n.a.	n.a.	n.a.
S14_vs_90934	n.a.	n.a.	Overlap (VC_1292/VC_1610)	n.a.	A	n.a.	n.a.	n.a.	n.a.	n.a.	n.a.	n.a.	n.a.	n.a.	n.a.	n.a.
S5_vs_30490	n.a.	n.a.	Overlap (VC_1292/VC_1610)	n.a.	A	n.a.	n.a.	n.a.	n.a.	n.a.	n.a.	n.a.	n.a.	n.a.	n.a.	n.a.
S5_vs_36605	Faecalibacterium phage FP_Lugh	MG711464	Overlap (VC_1294/VC_1575)	n.a.	N14	3	Faecalibacterium	Group I	Duplodnaviria	Heunggongvirae	Uroviricota	Caudoviricetes	n.a.	Unclassified	O	O
S6_vs_22637	n.a.	n.a.	Overlap (VC_1294/VC_1575)	n.a.	A	n.a.	n.a.	n.a.	n.a.	n.a.	n.a.	n.a.	n.a.	n.a.	n.a.	n.a.
S12_vf_188846	Tortoise microvirus 19	MK765569	Overlap (VC_1300/VC_1301)	n.a.	N4	9	Unspecified	Group II	Monodnaviria	Sangervirae	Phixviricota	Malgrandaviricetes	Petitvirales	Microviridae	O	O
S8_vf_208115	Tortoise microvirus 19	MK765569	Overlap (VC_1300/VC_1301)	n.a.	N3	9	Unspecified	Group II	Monodnaviria	Sangervirae	Phixviricota	Malgrandaviricetes	Petitvirales	Microviridae	O	O
S14_vf_43340	Tortoise microvirus 14	MK765564	Overlap (VC_1300/VC_1302)	n.a.	N2	4	Unspecified	Group II	Monodnaviria	Sangervirae	Phixviricota	Malgrandaviricetes	Petitvirales	Microviridae	O	O
S3_vf_59078	Tortoise microvirus 14	MK765564	Overlap (VC_1300/VC_1302)	n.a.	N2	4	Unspecified	Group II	Monodnaviria	Sangervirae	Phixviricota	Malgrandaviricetes	Petitvirales	Microviridae	O	O
S17_vf_128147	Microvirus mar63	MZ089809	Overlap (VC_1335/VC_1337)	n.a.	C1	17	Unspecified	Group II	Monodnaviria	Sangervirae	Phixviricota	Malgrandaviricetes	Petitvirales	Microviridae	Unclassified	O
S14_vf_115736	Butyrivibrio virus Ceridwen	MN882553	Overlap (VC_1481/VC_1482)	n.a.	C31	6	Butyrivibrio	Group I	Duplodnaviria	Heunggongvirae	Uroviricota	Caudoviricetes	n.a.	Unclassified	Unclassified	O
S16_vs_20060	Butyrivibrio virus Ceridwen	MN882553	Overlap (VC_1481/VC_1482)	n.a.	C35	2	Butyrivibrio	Group I	Duplodnaviria	Heunggongvirae	Uroviricota	Caudoviricetes	n.a.	Unclassified	Unclassified	O
S1_vf_136526	Butyrivibrio virus Ceridwen	MN882553	Overlap (VC_1481/VC_1482)	n.a.	C35	3	Butyrivibrio	Group I	Duplodnaviria	Heunggongvirae	Uroviricota	Caudoviricetes	n.a.	Unclassified	Unclassified	O
S1_vf_6272	Butyrivibrio virus Ceridwen	MN882553	Overlap (VC_1481/VC_1482)	n.a.	C36	6	Butyrivibrio	Group I	Duplodnaviria	Heunggongvirae	Uroviricota	Caudoviricetes	n.a.	Unclassified	Unclassified	O
S1_vs_21439	Butyrivibrio virus Ceridwen	MN882553	Overlap (VC_1481/VC_1482)	n.a.	C32	2	Butyrivibrio	Group I	Duplodnaviria	Heunggongvirae	Uroviricota	Caudoviricetes	n.a.	Unclassified	Unclassified	O
S3_vf_127400	Butyrivibrio virus Ceridwen	MN882553	Overlap (VC_1481/VC_1482)	n.a.	C36	2	Butyrivibrio	Group I	Duplodnaviria	Heunggongvirae	Uroviricota	Caudoviricetes	n.a.	Unclassified	Unclassified	O
S8_vs_88013	Butyrivibrio virus Ceridwen	MN882553	Overlap (VC_1481/VC_1482)	n.a.	C30	7	Butyrivibrio	Group I	Duplodnaviria	Heunggongvirae	Uroviricota	Caudoviricetes	n.a.	Unclassified	Unclassified	O
S14_vs_112068	n.a.	n.a.	Overlap (VC_1482/VC_1570)	n.a.	A	n.a.	n.a.	n.a.	n.a.	n.a.	n.a.	n.a.	n.a.	n.a.	n.a.	n.a.
S6_vs_56492	n.a.	n.a.	Overlap (VC_1482/VC_1570)	n.a.	A	n.a.	n.a.	n.a.	n.a.	n.a.	n.a.	n.a.	n.a.	n.a.	n.a.	n.a.
S7_vs_43529	n.a.	n.a.	Overlap (VC_1482/VC_1570)	n.a.	A	n.a.	n.a.	n.a.	n.a.	n.a.	n.a.	n.a.	n.a.	n.a.	n.a.	n.a.
S17_vf_140653	Bacteroides phage F4	MT806185	Overlap (VC_1506/VC_1507)	n.a.	N12	1	Bacteroides	Group I	Duplodnaviria	Heunggongvirae	Uroviricota	Caudoviricetes	n.a.	Unclassified	O	O
S3_vs_9158	n.a.	n.a.	Overlap (VC_1506/VC_1507)	n.a.	A	n.a.	n.a.	n.a.	n.a.	n.a.	n.a.	n.a.	n.a.	n.a.	n.a.	n.a.
S8_vf_28956	n.a.	n.a.	Overlap (VC_1506/VC_1507)	n.a.	A	n.a.	n.a.	n.a.	n.a.	n.a.	n.a.	n.a.	n.a.	n.a.	n.a.	n.a.
S11_vs_45658	Campylobacter phage PC10	MZ047271	Overlap (VC_1530/VC_1560)	n.a.	N8	6	Campylobacter	Group I	Duplodnaviria	Heunggongvirae	Uroviricota	Caudoviricetes	n.a.	Unclassified	O	O
S12_vs_111286	n.a.	n.a.	Overlap (VC_1530/VC_1560)	n.a.	A	n.a.	n.a.	n.a.	n.a.	n.a.	n.a.	n.a.	n.a.	n.a.	n.a.	n.a.
S5_vs_80496	n.a.	n.a.	Overlap (VC_1530/VC_1560)	n.a.	A	n.a.	n.a.	n.a.	n.a.	n.a.	n.a.	n.a.	n.a.	n.a.	n.a.	n.a.
S5_vs_117417	n.a.	n.a.	Overlap (VC_155/VC_1589)	n.a.	A	n.a.	n.a.	n.a.	n.a.	n.a.	n.a.	n.a.	n.a.	n.a.	n.a.	n.a.
S7_vs_28150	n.a.	n.a.	Overlap (VC_155/VC_1589)	n.a.	A	n.a.	n.a.	n.a.	n.a.	n.a.	n.a.	n.a.	n.a.	n.a.	n.a.	n.a.
S11_vs_104049	Clostridium phage phiMMP04	JX145342	Overlap (VC_155/VC_661)	n.a.	N7	9	Clostridium	Group I	Duplodnaviria	Heunggongvirae	Uroviricota	Caudoviricetes	n.a.	Unclassified	O	O
S14_vs_56410	Clostridium phage phiMMP04	JX145342	Overlap (VC_155/VC_661)	n.a.	N5	8	Clostridium	Group I	Duplodnaviria	Heunggongvirae	Uroviricota	Caudoviricetes	n.a.	Unclassified	O	O
S5_vs_69980	Clostridium phage phiCT453B	KM983328	Overlap (VC_155/VC_661)	n.a.	N5	10	Clostridium	Group I	Duplodnaviria	Heunggongvirae	Uroviricota	Caudoviricetes	n.a.	Unclassified	O	O

S3_vf_7137	Bacteriophage sp.	OP549880	Overlap (VC_1550/VC_1551)	n.a.	C3	24	Unspecified	Unclassified	Unclassified	n.a.	n.a.	n.a.	n.a.	Unclassified	Unclassified	O
S4_vf_30103	Bacteriophage sp.	OP549880	Overlap (VC_1550/VC_1551)	n.a.	C3	25	Unspecified	Unclassified	Unclassified	n.a.	n.a.	n.a.	n.a.	Unclassified	Unclassified	O
S10_vf_113489	n.a.	n.a.	Overlap (VC_1553/VC_1555)	n.a.	A	n.a.	n.a.	n.a.	n.a.	n.a.	n.a.	n.a.	n.a.	n.a.	n.a.	n.a.
S16_vf_52513	n.a.	n.a.	Overlap (VC_1555/VC_1556)	n.a.	A	n.a.	n.a.	n.a.	n.a.	n.a.	n.a.	n.a.	n.a.	n.a.	n.a.	n.a.
S8_vs_51368	n.a.	n.a.	Overlap (VC_1555/VC_1556)	n.a.	A	n.a.	n.a.	n.a.	n.a.	n.a.	n.a.	n.a.	n.a.	n.a.	n.a.	n.a.
S16_vs_76823	n.a.	n.a.	Overlap (VC_1555/VC_1557)	n.a.	A	n.a.	n.a.	n.a.	n.a.	n.a.	n.a.	n.a.	n.a.	n.a.	n.a.	n.a.
S8_vs_102608	n.a.	n.a.	Overlap (VC_1555/VC_1557)	n.a.	A	n.a.	n.a.	n.a.	n.a.	n.a.	n.a.	n.a.	n.a.	n.a.	n.a.	n.a.
S11_vs_36748	n.a.	n.a.	Overlap (VC_1566/VC_1581)	n.a.	A	n.a.	n.a.	n.a.	n.a.	n.a.	n.a.	n.a.	n.a.	n.a.	n.a.	n.a.
S17_vs_157209	n.a.	n.a.	Overlap (VC_1566/VC_1581)	n.a.	A	n.a.	n.a.	n.a.	n.a.	n.a.	n.a.	n.a.	n.a.	n.a.	n.a.	n.a.
S3_vs_89485	n.a.	n.a.	Overlap (VC_1566/VC_1581/VC_1585)	n.a.	A	n.a.	n.a.	n.a.	n.a.	n.a.	n.a.	n.a.	n.a.	n.a.	n.a.	n.a.
S8_vs_40014	n.a.	n.a.	Overlap (VC_1566/VC_1581/VC_1585)	n.a.	A	n.a.	n.a.	n.a.	n.a.	n.a.	n.a.	n.a.	n.a.	n.a.	n.a.	n.a.
S5_vs_84574	n.a.	n.a.	Overlap (VC_1571/VC_1590)	n.a.	A	n.a.	n.a.	n.a.	n.a.	n.a.	n.a.	n.a.	n.a.	n.a.	n.a.	n.a.
S9_vs_88847	n.a.	n.a.	Overlap (VC_1571/VC_1590)	n.a.	A	n.a.	n.a.	n.a.	n.a.	n.a.	n.a.	n.a.	n.a.	n.a.	n.a.	n.a.
S13_vf_147589	n.a.	n.a.	Overlap (VC_1601/VC_1616)	n.a.	F	n.a.	n.a.	n.a.	n.a.	n.a.	n.a.	n.a.	n.a.	n.a.	n.a.	n.a.
S16_vf_173133	n.a.	n.a.	Overlap (VC_1601/VC_1616)	n.a.	F	n.a.	n.a.	n.a.	n.a.	n.a.	n.a.	n.a.	n.a.	n.a.	n.a.	n.a.
S5_vs_145633	n.a.	n.a.	Overlap (VC_1601/VC_1616)	n.a.	F	n.a.	n.a.	n.a.	n.a.	n.a.	n.a.	n.a.	n.a.	n.a.	n.a.	n.a.
S13_vf_91189	n.a.	n.a.	Overlap (VC_1608/VC_1609)	n.a.	F	n.a.	n.a.	n.a.	n.a.	n.a.	n.a.	n.a.	n.a.	n.a.	n.a.	n.a.
S4_vf_16485	n.a.	n.a.	Overlap (VC_1608/VC_1609)	n.a.	F	n.a.	n.a.	n.a.	n.a.	n.a.	n.a.	n.a.	n.a.	n.a.	n.a.	n.a.
S5_vs_130294	n.a.	n.a.	Overlap (VC_1610/VC_1611)	n.a.	A	n.a.	n.a.	n.a.	n.a.	n.a.	n.a.	n.a.	n.a.	n.a.	n.a.	n.a.
S7_vs_97737	n.a.	n.a.	Overlap (VC_1610/VC_1611)	n.a.	A	n.a.	n.a.	n.a.	n.a.	n.a.	n.a.	n.a.	n.a.	n.a.	n.a.	n.a.
S9_vf_90856	Streptococcus phage Javan535	MK448799	Overlap (VC_262/VC_427)	n.a.	C1	73	Streptococcus	Group I	Duplodnaviria	Heunggongvirae	Uroviricota	Caudoviricetes	n.a.	Unclassified	Unclassified	O
S5_vs_60507	Streptococcus phage Javan573	MK448810	Overlap (VC_265/VC_330)	n.a.	C1	43	Streptococcus	Group I	Duplodnaviria	Heunggongvirae	Uroviricota	Caudoviricetes	n.a.	Unclassified	Unclassified	O
S5_vs_6478	Lactobacillus phage phiadh	AJ131519	Overlap (VC_271/VC_315)	n.a.	C1	86	Lactobacillus	Group I	Duplodnaviria	Heunggongvirae	Uroviricota	Caudoviricetes	n.a.	Unclassified	Unclassified	O
S11_vs_13652	Streptococcus phage Javan407	MK448753	Overlap (VC_326/VC_644)	n.a.	C7	15	Streptococcus	Group I	Duplodnaviria	Heunggongvirae	Uroviricota	Caudoviricetes	n.a.	Unclassified	Unclassified	O
S11_vs_3006	Streptococcus phage Javan407	MK448753	Overlap (VC_326/VC_644)	n.a.	C7	15	Streptococcus	Group I	Duplodnaviria	Heunggongvirae	Uroviricota	Caudoviricetes	n.a.	Unclassified	Unclassified	O
S5_vs_109085	Streptococcus phage Javan407	MK448753	Overlap (VC_326/VC_644)	n.a.	C7	11	Streptococcus	Group I	Duplodnaviria	Heunggongvirae	Uroviricota	Caudoviricetes	n.a.	Unclassified	Unclassified	O
S7_vs_23770	Streptococcus phage Javan407	MK448753	Overlap (VC_326/VC_644)	n.a.	C7	16	Streptococcus	Group I	Duplodnaviria	Heunggongvirae	Uroviricota	Caudoviricetes	n.a.	Unclassified	Unclassified	O
S11_vs_38860	n.a.	n.a.	Overlap (VC_333/VC_645)	n.a.	A	n.a.	n.a.	n.a.	n.a.	n.a.	n.a.	n.a.	n.a.	n.a.	n.a.	n.a.
S12_vs_98175	Clostridium phage phiCD38-2	HM568888	Overlap (VC_333/VC_645)	n.a.	N24	2	Clostridium	Group I	Duplodnaviria	Heunggongvirae	Uroviricota	Caudoviricetes	n.a.	Unclassified	O	O
S14_vs_117153	Roseburia phage Jekyll	LR596902	Overlap (VC_333/VC_645)	n.a.	N37	3	Roseburia	Group I	Duplodnaviria	Heunggongvirae	Uroviricota	Caudoviricetes	n.a.	Unclassified	O	O
S16_vs_140709	n.a.	n.a.	Overlap (VC_333/VC_645)	n.a.	A	n.a.	n.a.	n.a.	n.a.	n.a.	n.a.	n.a.	n.a.	n.a.	n.a.	n.a.
S16_vs_172749	Clostridium phage phiCD38-2	HM568888	Overlap (VC_333/VC_645)	n.a.	N34	4	Clostridium	Group I	Duplodnaviria	Heunggongvirae	Uroviricota	Caudoviricetes	n.a.	Unclassified	O	O
S4_vf_31682	Bacillus phage PBC1	JQ619704	Overlap (VC_333/VC_645)	n.a.	N32	3	Bacillus	Group I	Duplodnaviria	Heunggongvirae	Uroviricota	Caudoviricetes	n.a.	Unclassified	O	O
S4_vs_VIRSorter_31916-circular-cat_1	Clostridium phage phiCD38-2	HM568888	Overlap (VC_333/VC_645)	n.a.	N15	6	Clostridium	Group I	Duplodnaviria	Heunggongvirae	Uroviricota	Caudoviricetes	n.a.	Unclassified	O	O
S5_vs_145578	Faecalibacterium phage FP_Lugh	MG711464	Overlap (VC_333/VC_645)	n.a.	N9	15	Faecalibacterium	Group I	Duplodnaviria	Heunggongvirae	Uroviricota	Caudoviricetes	n.a.	Unclassified	O	O
S7_vs_60775	Clostridium phage phiCD38-2	HM568888	Overlap (VC_333/VC_645)	n.a.	N33	3	Clostridium	Group I	Duplodnaviria	Heunggongvirae	Uroviricota	Caudoviricetes	n.a.	Unclassified	O	O
S10_vs_120570	n.a.	n.a.	Overlap (VC_334/VC_1572)	n.a.	A	n.a.	n.a.	n.a.	n.a.	n.a.	n.a.	n.a.	n.a.	n.a.	n.a.	n.a.
S16_vs_107235	n.a.	n.a.	Overlap (VC_334/VC_1572)	n.a.	A	n.a.	n.a.	n.a.	n.a.	n.a.	n.a.	n.a.	n.a.	n.a.	n.a.	n.a.
S5_vs_95307	Solobacterium phage SMO_1P	MZ503613	Overlap (VC_334/VC_556)	n.a.	C3	26	Solobacterium	Group I	Duplodnaviria	Heunggongvirae	Uroviricota	Caudoviricetes	n.a.	Unclassified	Unclassified	O
S9_vs_48240	Solobacterium phage SMO_1P	MZ503613	Overlap (VC_334/VC_556)	n.a.	C3	26	Solobacterium	Group I	Duplodnaviria	Heunggongvirae	Uroviricota	Caudoviricetes	n.a.	Unclassified	Unclassified	O
S16_vs_82116	Solobacterium phage SMO_1P	MZ503613	Overlap (VC_334/VC_556/VC_1037)	n.a.	C6	13	Solobacterium	Group I	Duplodnaviria	Heunggongvirae	Uroviricota	Caudoviricetes	n.a.	Unclassified	Unclassified	O
S4_vf_11308	Solobacterium phage SMO_1P	MZ503613	Overlap (VC_334/VC_556/VC_1037)	n.a.	C8	15	Solobacterium	Group I	Duplodnaviria	Heunggongvirae	Uroviricota	Caudoviricetes	n.a.	Unclassified	Unclassified	O
S16_vs_132979	Faecalibacterium phage FP_Lugh	MG711464	Overlap (VC_335/VC_558/VC_559)	n.a.	N7	4	Faecalibacterium	Group I	Duplodnaviria	Heunggongvirae	Uroviricota	Caudoviricetes	n.a.	Unclassified	O	O
S6_vs_53966	Faecalibacterium phage FP_Lugh	MG711464	Overlap (VC_335/VC_558/VC_559)	n.a.	N5	10	Faecalibacterium	Group I	Duplodnaviria	Heunggongvirae	Uroviricota	Caudoviricetes	n.a.	Unclassified	O	O
S12_vs_58148	Geobacillus phage GBSV1	DQ340064	Overlap (VC_346/VC_779)	n.a.	C3	11	Geobacillus	Group I	Duplodnaviria	Heunggongvirae	Uroviricota	Caudoviricetes	n.a.	Unclassified	Unclassified	O
S16_vs_54616	Geobacillus phage GBSV1	DQ340064	Overlap (VC_346/VC_779)	n.a.	C3	15	Geobacillus	Group I	Duplodnaviria	Heunggongvirae	Uroviricota	Caudoviricetes	n.a.	Unclassified	Unclassified	O
S11_vs_104069	Vibrio phage vB_VspS_VS-ABTNL-3	MF497422	Overlap (VC_354/VC_1271)	n.a.	C1	11	Vibrio	Group I	Duplodnaviria	Heunggongvirae	Uroviricota	Caudoviricetes	n.a.	Unclassified	Unclassified	O
S15_vs_39804	Bacillus phage vB_BtS_BMBtp13	KX190832	Overlap (VC_433/VC_668)	n.a.	N2	18	Bacillus	Group I	Duplodnaviria	Heunggongvirae	Uroviricota	Caudoviricetes	n.a.	Unclassified	O	O
S3_vs_20315	Bacillus phage vB_BtS_BMBtp13	KX190832	Overlap (VC_433/VC_668)	n.a.	N2	21	Bacillus	Group I	Duplodnaviria	Heunggongvirae	Uroviricota	Caudoviricetes	n.a.	Unclassified	O	O
S2_vf_4130	uncultured phage cr6_1	MT774401	Overlap (VC_520/VC_522)	n.a.	C3	282	Unspecified	Group I	Duplodnaviria	Heunggongvirae	Uroviricota	Caudoviricetes	Crassvirales	Suoliviridae	Bearivirinae	O
S4_vf_31825	uncultured phage cr6_1	MT774401	Overlap (VC_520/VC_522)	n.a.	C5	216	Unspecified	Group I	Duplodnaviria	Heunggongvirae	Uroviricota	Caudoviricetes	Crassvirales	Suoliviridae	Bearivirinae	O
S8_vf_49282	uncultured phage cr6_1	MT774401	Overlap (VC_520/VC_522)	n.a.	C5	188	Unspecified	Group I	Duplodnaviria	Heunggongvirae	Uroviricota	Caudoviricetes	Crassvirales	Suoliviridae	Bearivirinae	O
S10_vs_46819	Faecalibacterium phage FP_Lugh	MG711464	Overlap (VC_558/VC_559)	n.a.	N12	10	Faecalibacterium	Group I	Duplodnaviria	Heunggongvirae	Uroviricota	Caudoviricetes	n.a.	Unclassified	O	O
S4_vs_VIRSorter_27316-cat_2	Faecalibacterium phage FP_Lugh	MG711464	Overlap (VC_558/VC_559)	n.a.	N2	46	Faecalibacterium	Group I	Duplodnaviria	Heunggongvirae	Uroviricota	Caudoviricetes	n.a.	Unclassified	O	O
S6_vs_1146	Streptococcus phage Javan240	MK448881	Overlap (VC_558/VC_559)	n.a.	N13	5	Streptococcus	Group I	Duplodnaviria	Heunggongvirae	Uroviricota	Caudoviricetes	n.a.	Unclassified	O	O
S14_vf_85122	Clostridium phage vB_CpeS-1181	MW478292	Overlap (VC_563/VC_842/VC_924)	n.a.	C4	25	Clostridium	Group I	Duplodnaviria	Heunggongvirae	Uroviricota	Caudoviricetes	n.a.	Unclassified	Unclassified	O
S5_vf_145583	Clostridium phage vB_CpeS-1181	MW478292	Overlap (VC_563/VC_842/VC_924)	n.a.	C4	7	Clostridium	Group I	Duplodnaviria	Heunggongvirae	Uroviricota	Caudoviricetes	n.a.	Unclassified	Unclassified	O
S10_vf_46328	Roseburia phage Jekyll	LR596902	Overlap (VC_627/VC_1564)	n.a.	N19	7	Roseburia	Group I	Duplodnaviria	Heunggongvirae	Uroviricota	Caudoviricetes	n.a.	Unclassified	O	O
S11_vf_34008	Roseburia phage Jekyll	LR596902	Overlap (VC_627/VC_1564)	n.a.	N18	3	Roseburia	Group I	Duplodnaviria	Heunggongvirae	Uroviricota	Caudoviricetes	n.a.	Unclassified	O	O
S11_vs_82368	Roseburia phage Jekyll	LR596902	Overlap (VC_627/VC_1564)	n.a.	N15	10	Roseburia	Group I	Duplodnaviria	Heunggongvirae	Uroviricota	Caudoviricetes	n.a.	Unclassified	O	O
S3_vf_11428	Roseburia phage Jekyll	LR596902	Overlap (VC_627/VC_1564)	n.a.	N13	17	Roseburia	Group I	Duplodnaviria	Heunggongvirae	Uroviricota	Caudoviricetes	n.a.	Unclassified	O	O
S4_vf_13353	Roseburia phage Jekyll	LR596902	Overlap (VC_627/VC_1564)	n.a.	N12	23	Roseburia	Group I	Duplodnaviria	Heunggongvirae	Uroviricota	Caudoviricetes	n.a.	Unclassified	O	O
S5_vf_105728	Roseburia phage Jekyll	LR596902	Overlap (VC_627/VC_1564)	n.a.	N16	7	Roseburia	Group I	Duplodnaviria	Heunggongvirae	Uroviricota	Caudoviricetes	n.a.	Unclassified	O	O
S5_vf_145604	Roseburia phage Jekyll	LR596902	Overlap (VC_627/VC_1564)	n.a.	N14	18	Roseburia	Group I	Duplodnaviria	Heunggongvirae	Uroviricota	Caudoviricetes	n.a.	Unclassified	O	O
S5_vs_68254	Roseburia phage Jekyll	LR596902	Overlap (VC_627/VC_1564)	n.a.	N18	7	Roseburia	Group I	Duplodnaviria	Heunggongvirae	Uroviricota	Caudoviricetes	n.a.	Unclassified	O	O
S7_vf_49485	Roseburia phage Jekyll	LR596902	Overlap (VC_627/VC_1564)	n.a.	N13	7	Roseburia	Group I	Duplodnaviria	Heunggongvirae	Uroviricota	Caudoviricetes	n.a.	Unclassified	O	O
S7_vs_65719	Roseburia phage Jekyll	LR596902	Overlap (VC_627/VC_1564)	n.a.	N27	2	Roseburia	Group I	Duplodnaviria	Heunggongvirae	Uroviricota	Caudoviricetes	n.a.	Unclassified	O	O
S9_vs_90848	Roseburia phage Jekyll	LR596902	Overlap (VC_627/VC_1564)	n.a.	N16	9	Roseburia	Group I	Duplodnaviria	Heunggongvirae	Uroviricota	Caudoviricetes	n.a.	Unclassified	O	O
S11_vs_83666	Faecalibacterium phage FP_Lugh	MG711464	Overlap (VC_641/VC_1595)	n.a.	N5	20	Faecalibacterium	Group I	Duplodnaviria	Heunggongvirae	Uroviricota	Caudoviricetes	n.a.	Unclassified	O	O
S14_vs_56251	Clostridium phage phiCD38-2	HM568888	Overlap (VC_641/VC_1595)	n.a.	N13	4	Clostridium	Group I	Duplodnaviria	Heunggongvirae	Uroviricota	Caudoviricetes	n.a.	Unclassified	O	O
S4_vs_VIRSorter_21643-cat_2	Faecalibacterium phage FP_Lugh	MG711464	Overlap (VC_641/VC_1595)	n.a.	N3	21	Faecalibacterium	Group I	Duplodnaviria	Heunggongvirae	Uroviricota	Caudoviricetes	n.a.	Unclassified	O	O
S6_vs_56485	Faecalibacterium phage FP_Lugh	MG711464	Overlap (VC_641/VC_925)	n.a.	N18	3	Faecalibacterium	Group I	Duplodnaviria	Heunggongvirae	Uroviricota	Caudoviricetes	n.a.	Unclassified	O	O
S9_vs_37437	Clostridium phage phiCD111	LN681535	Overlap (VC_641/VC_925)	n.a.	N12	5	Clostridium	Group I	Duplodnaviria	Heunggongvirae	Uroviricota	Caudoviricetes	n.a.	Unclassified	O	O
S14_vs_112252	n.a.	n.a.	Overlap (VC_660/VC_734)	n.a.	A	n.a.	n.a.	n.a.	n.a.	n.a.	n.a.	n.a.	n.a.	n.a.	n.a.	n.a.
S5_vs_49011	Clostridium phage phiCT19406B	KM983331	Overlap (VC_660/VC_734)	n.a.	N9	5	Clostridium	Group I	Duplodnaviria	Heunggongvirae	Uroviricota	Caudoviricetes	n.a.	Unclassified	O	O
S3_vf_8164	Bacillus phage vB_BtS_BMBtp13	KX190832	Overlap (VC_668/VC_907)	n.a.	N12	7	Bacillus	Group I	Duplodnaviria	Heunggongvirae	Uroviricota	Caudoviricetes	n.a.	Unclassified	O	O
S7_vf_8332	Bacillus phage vB_BtS_BMBtp13	KX190832	Overlap (VC_668/VC_907)	n.a.	N9	8	Bacillus	Group I	Duplodnaviria	Heunggongvirae	Uroviricota	Caudoviricetes	n.a.	Unclassified	O	O
S13_vf_3728	Bacteroides phage crAss002	MN917146	Overlap (VC_692/VC_693)	n.a.	C1	233	Bacteroides	Group I	Duplodnaviria	Heunggongvirae	Uroviricota	Caudoviricetes	Crassvirales	Intestiviridae	Churivirinae	O
S16_vf_173095	Bacteroides phage crAss002	MN917146	Overlap (VC_692/VC_693)	n.a.	C1	184	Bacteroides	Group I	Duplodnaviria	Heunggongvirae	Uroviricota	Caudoviricetes	Crassvirales	Intestiviridae	Churivirinae	O
S12_vf_173666	n.a.	n.a.	Overlap (VC_725/VC_1221)	n.a.	A	n.a.	n.a.	n.a.	n.a.	n.a.	n.a.	n.a.	n.a.	n.a.	n.a.	n.a.
S7_vf_67910	n.a.	n.a.	Overlap (VC_725/VC_1221)	n.a.	A	n.a.	n.a.	n.a.	n.a.	n.a.	n.a.	n.a.	n.a.	n.a.	n.a.	n.a.
S8_vf_195032	Roseburia phage Shimadzu	LR596903	Overlap (VC_725/VC_1221)	n.a.	N4	1	Roseburia	Group I	Duplodnaviria	Heunggongvirae	Uroviricota	Caudoviricetes	n.a.	Unclassified	O	O

S11_vf_33117	n.a.	n.a.	Overlap (VC_730/VC_1293)	n.a.	A	n.a.	n.a.	n.a.	n.a.	n.a.	n.a.	n.a.	n.a.	n.a.	n.a.	n.a.	n.a.
S8_vs_208127	Faecalibacterium phage FP_Lagaffe	MG711461	Overlap (VC_730/VC_1293)	n.a.	N16	2	Faecalibacterium	Group I	Duplodnaviria	Heunggongvirae	Uroviricota	Caudoviricetes	n.a.	Unclassified	O	O
S7_vs_135322	Faecalibacterium phage FP_Taranis	MG711467	Overlap (VC_730/VC_733)	n.a.	C1	222	Faecalibacterium	Group I	Duplodnaviria	Heunggongvirae	Uroviricota	Caudoviricetes	n.a.	Unclassified	Unclassified	O
S3_vs_90331	Faecalibacterium phage FP_Taranis	MG711467	Overlap (VC_730/VC_733/VC_1293)	n.a.	C4	84	Faecalibacterium	Group I	Duplodnaviria	Heunggongvirae	Uroviricota	Caudoviricetes	n.a.	Unclassified	Unclassified	O
S8_vs_179529	Faecalibacterium phage FP_Taranis	MG711467	Overlap (VC_730/VC_733/VC_1293)	n.a.	C6	73	Faecalibacterium	Group I	Duplodnaviria	Heunggongvirae	Uroviricota	Caudoviricetes	n.a.	Unclassified	Unclassified	O
S3_vs_30318	n.a.	n.a.	Overlap (VC_734/VC_1576)	n.a.	A	n.a.	n.a.	n.a.	n.a.	n.a.	n.a.	n.a.	n.a.	n.a.	n.a.	n.a.	n.a.
S5_vf_145586	n.a.	n.a.	Overlap (VC_734/VC_1576)	n.a.	A	n.a.	n.a.	n.a.	n.a.	n.a.	n.a.	n.a.	n.a.	n.a.	n.a.	n.a.	n.a.
S11_vs_104042	n.a.	n.a.	Overlap (VC_760/VC_888)	n.a.	A	n.a.	n.a.	n.a.	n.a.	n.a.	n.a.	n.a.	n.a.	n.a.	n.a.	n.a.	n.a.
S16_vs_101072	Stenotrophomonas phage vB_SmeS_BUCT702	OM735687	Overlap (VC_760/VC_888)	n.a.	N5	4	Stenotrophomonas	Group I	Duplodnaviria	Heunggongvirae	Uroviricota	Caudoviricetes	n.a.	Unclassified	O	O
S9_vs_90850	Stenotrophomonas phage vB_SmeS_BUCT702	OM735687	Overlap (VC_760/VC_888)	n.a.	N7	3	Stenotrophomonas	Group I	Duplodnaviria	Heunggongvirae	Uroviricota	Caudoviricetes	n.a.	Unclassified	O	O
S13_vs_149860	Bifidobacterium phage BadAztec1	MT006233	Overlap (VC_762/VC_763)	n.a.	C1	26	Bifidobacterium	Group I	Duplodnaviria	Heunggongvirae	Uroviricota	Caudoviricetes	n.a.	Unclassified	Unclassified	O
S2_vf_16829	Actinomyces phage Av-1	DQ123818	Overlap (VC_762/VC_763)	n.a.	C5	15	Actinomyces	Group I	Duplodnaviria	Heunggongvirae	Uroviricota	Caudoviricetes	n.a.	Unclassified	Unclassified	O
S3_vf_33389	Actinomyces phage Av-1	DQ123818	Overlap (VC_762/VC_763)	n.a.	C5	15	Actinomyces	Group I	Duplodnaviria	Heunggongvirae	Uroviricota	Caudoviricetes	n.a.	Unclassified	Unclassified	O
S8_vf_91033	Actinomyces phage Av-1	DQ123818	Overlap (VC_762/VC_763)	n.a.	C5	15	Actinomyces	Group I	Duplodnaviria	Heunggongvirae	Uroviricota	Caudoviricetes	n.a.	Unclassified	Unclassified	O
S14_vs_30439	Geobacillus phage GBSV1	DQ340064	Overlap (VC_779/VC_781)	n.a.	C18	6	Geobacillus	Group I	Duplodnaviria	Heunggongvirae	Uroviricota	Caudoviricetes	n.a.	Unclassified	Unclassified	O
S4_vs_VIRSorter_16803-cat_2	Geobacillus phage GBSV1	DQ340064	Overlap (VC_779/VC_781)	n.a.	C11	6	Geobacillus	Group I	Duplodnaviria	Heunggongvirae	Uroviricota	Caudoviricetes	n.a.	Unclassified	Unclassified	O
S6_vs_46091	Geobacillus phage GBSV1	DQ340064	Overlap (VC_779/VC_782)	n.a.	C7	9	Geobacillus	Group I	Duplodnaviria	Heunggongvirae	Uroviricota	Caudoviricetes	n.a.	Unclassified	Unclassified	O
S9_vs_48347	Geobacillus phage GBSV1	DQ340064	Overlap (VC_779/VC_782)	n.a.	C13	6	Geobacillus	Group I	Duplodnaviria	Heunggongvirae	Uroviricota	Caudoviricetes	n.a.	Unclassified	Unclassified	O
S14_vf_117155	Bifidobacterium phage BitterVaud1	MT006236	Overlap (VC_827/VC_828)	n.a.	N2	23	Bifidobacterium	Group I	Duplodnaviria	Heunggongvirae	Uroviricota	Caudoviricetes	n.a.	Unclassified	O	O
S16_vf_16182	Bifidobacterium phage BitterVaud1	MT006236	Overlap (VC_827/VC_828)	n.a.	N4	15	Bifidobacterium	Group I	Duplodnaviria	Heunggongvirae	Uroviricota	Caudoviricetes	n.a.	Unclassified	O	O
S7_vf_37293	Bifidobacterium phage BitterVaud1	MT006236	Overlap (VC_827/VC_829)	n.a.	N3	18	Bifidobacterium	Group I	Duplodnaviria	Heunggongvirae	Uroviricota	Caudoviricetes	n.a.	Unclassified	O	O
S7_vf_49007	Bifidobacterium phage BitterVaud1	MT006236	Overlap (VC_827/VC_829)	n.a.	N11	8	Bifidobacterium	Group I	Duplodnaviria	Heunggongvirae	Uroviricota	Caudoviricetes	n.a.	Unclassified	O	O
S12_vs_167155	Butyrivibrio virus Ceridwen	MN882553	Overlap (VC_841/VC_1435/VC_1481)	n.a.	C22	17	Butyrivibrio	Group I	Duplodnaviria	Heunggongvirae	Uroviricota	Caudoviricetes	n.a.	Unclassified	Unclassified	O
S4_vf_31979	Butyrivibrio virus Ceridwen	MN882553	Overlap (VC_841/VC_1435/VC_1481)	n.a.	C12	20	Butyrivibrio	Group I	Duplodnaviria	Heunggongvirae	Uroviricota	Caudoviricetes	n.a.	Unclassified	Unclassified	O
S10_vs_20400	Butyrivibrio virus Ceridwen	MN882553	Overlap (VC_841/VC_1481)	n.a.	C23	18	Butyrivibrio	Group I	Duplodnaviria	Heunggongvirae	Uroviricota	Caudoviricetes	n.a.	Unclassified	Unclassified	O
S11_vf_104034	Butyrivibrio virus Ceridwen	MN882553	Overlap (VC_841/VC_1481)	n.a.	C20	34	Butyrivibrio	Group I	Duplodnaviria	Heunggongvirae	Uroviricota	Caudoviricetes	n.a.	Unclassified	Unclassified	O
S16_vf_12551	Butyrivibrio virus Ceridwen	MN882553	Overlap (VC_841/VC_1481)	n.a.	C30	29	Butyrivibrio	Group I	Duplodnaviria	Heunggongvirae	Uroviricota	Caudoviricetes	n.a.	Unclassified	Unclassified	O
S16_vs_78429	Butyrivibrio virus Ceridwen	MN882553	Overlap (VC_841/VC_1481)	n.a.	C20	38	Butyrivibrio	Group I	Duplodnaviria	Heunggongvirae	Uroviricota	Caudoviricetes	n.a.	Unclassified	Unclassified	O
S1_vs_117317	Butyrivibrio virus Ceridwen	MN882553	Overlap (VC_841/VC_1481)	n.a.	C25	34	Butyrivibrio	Group I	Duplodnaviria	Heunggongvirae	Uroviricota	Caudoviricetes	n.a.	Unclassified	Unclassified	O
S1_vs_155183	Butyrivibrio virus Ceridwen	MN882553	Overlap (VC_841/VC_1481)	n.a.	C24	31	Butyrivibrio	Group I	Duplodnaviria	Heunggongvirae	Uroviricota	Caudoviricetes	n.a.	Unclassified	Unclassified	O
S4_vs_VIRSorter_29332-cat_2	Butyrivibrio virus Ceridwen	MN882553	Overlap (VC_841/VC_1481)	n.a.	C27	19	Butyrivibrio	Group I	Duplodnaviria	Heunggongvirae	Uroviricota	Caudoviricetes	n.a.	Unclassified	Unclassified	O
S5_vf_21869	Butyrivibrio virus Ceridwen	MN882553	Overlap (VC_841/VC_1481)	n.a.	C26	28	Butyrivibrio	Group I	Duplodnaviria	Heunggongvirae	Uroviricota	Caudoviricetes	n.a.	Unclassified	Unclassified	O
S7_vs_4114	Butyrivibrio virus Ceridwen	MN882553	Overlap (VC_841/VC_1481)	n.a.	C28	14	Butyrivibrio	Group I	Duplodnaviria	Heunggongvirae	Uroviricota	Caudoviricetes	n.a.	Unclassified	Unclassified	O
S7_vs_83474	Butyrivibrio virus Ceridwen	MN882553	Overlap (VC_841/VC_1481)	n.a.	C26	21	Butyrivibrio	Group I	Duplodnaviria	Heunggongvirae	Uroviricota	Caudoviricetes	n.a.	Unclassified	Unclassified	O
S7_vs_94738	Butyrivibrio virus Ceridwen	MN882553	Overlap (VC_841/VC_1481)	n.a.	C28	31	Butyrivibrio	Group I	Duplodnaviria	Heunggongvirae	Uroviricota	Caudoviricetes	n.a.	Unclassified	Unclassified	O
S8_vf_72329	Butyrivibrio virus Ceridwen	MN882553	Overlap (VC_841/VC_1481)	n.a.	C26	25	Butyrivibrio	Group I	Duplodnaviria	Heunggongvirae	Uroviricota	Caudoviricetes	n.a.	Unclassified	Unclassified	O
S8_vs_139391	Butyrivibrio virus Ceridwen	MN882553	Overlap (VC_841/VC_1481)	n.a.	C25	32	Butyrivibrio	Group I	Duplodnaviria	Heunggongvirae	Uroviricota	Caudoviricetes	n.a.	Unclassified	Unclassified	O
S8_vs_35308	Butyrivibrio virus Ceridwen	MN882553	Overlap (VC_841/VC_1481)	n.a.	C23	37	Butyrivibrio	Group I	Duplodnaviria	Heunggongvirae	Uroviricota	Caudoviricetes	n.a.	Unclassified	Unclassified	O
S8_vs_54183	Butyrivibrio virus Ceridwen	MN882553	Overlap (VC_841/VC_1481)	n.a.	C27	28	Butyrivibrio	Group I	Duplodnaviria	Heunggongvirae	Uroviricota	Caudoviricetes	n.a.	Unclassified	Unclassified	O
S10_vs_119298	Croceibacter phage P2559Y	KC688701	Overlap (VC_856/VC_857)	n.a.	N4	46	Croceibacter	Group I	Duplodnaviria	Heunggongvirae	Uroviricota	Caudoviricetes	n.a.	Unclassified	O	O
S11_vs_104036	Croceibacter phage P2559Y	KC688701	Overlap (VC_856/VC_857)	n.a.	N4	46	Croceibacter	Group I	Duplodnaviria	Heunggongvirae	Uroviricota	Caudoviricetes	n.a.	Unclassified	O	O
S16_vs_11649	Croceibacter phage P2559Y	KC688701	Overlap (VC_856/VC_857)	n.a.	N4	44	Croceibacter	Group I	Duplodnaviria	Heunggongvirae	Uroviricota	Caudoviricetes	n.a.	Unclassified	O	O
S6_vs_15390	Croceibacter phage P2559Y	KC688701	Overlap (VC_856/VC_857)	n.a.	N4	41	Croceibacter	Group I	Duplodnaviria	Heunggongvirae	Uroviricota	Caudoviricetes	n.a.	Unclassified	O	O
S3_vs_50557	Bifidobacterium phage BigBern1	MT006237	Overlap (VC_880/VC_1487)	n.a.	N5	5	Bifidobacterium	Group I	Duplodnaviria	Heunggongvirae	Uroviricota	Caudoviricetes	n.a.	Unclassified	O	O
S5_vf_29332	Bifidobacterium phage BigBern1	MT006237	Overlap (VC_880/VC_1487)	n.a.	N4	8	Bifidobacterium	Group I	Duplodnaviria	Heunggongvirae	Uroviricota	Caudoviricetes	n.a.	Unclassified	O	O
S7_vs_55526	n.a.	n.a.	Overlap (VC_880/VC_1487)	n.a.	A	n.a.	n.a.	n.a.	n.a.	n.a.	n.a.	n.a.	n.a.	n.a.	n.a.	n.a.	n.a.
S16_vs_17493	n.a.	n.a.	Overlap (VC_921/VC_1292)	n.a.	A	n.a.	n.a.	n.a.	n.a.	n.a.	n.a.	n.a.	n.a.	n.a.	n.a.	n.a.	n.a.
S14_vs_38905	n.a.	n.a.	Overlap (VC_923/VC_1582)	n.a.	A	n.a.	n.a.	n.a.	n.a.	n.a.	n.a.	n.a.	n.a.	n.a.	n.a.	n.a.	n.a.
S5_vs_13254	n.a.	n.a.	Overlap (VC_923/VC_1582)	n.a.	A	n.a.	n.a.	n.a.	n.a.	n.a.	n.a.	n.a.	n.a.	n.a.	n.a.	n.a.	n.a.
S14_vs_39266	Clostridium phage vB_CpeS-1181	MW478292	Overlap (VC_923/VC_924)	n.a.	C13	2	Clostridium	Group I	Duplodnaviria	Heunggongvirae	Uroviricota	Caudoviricetes	n.a.	Unclassified	Unclassified	O
S12_vf_16374	Phage vB_RanS_PJN03	ON245412	Overlap (VC_952/VC_1529)	n.a.	N15	7	Unspecified	Group I	Duplodnaviria	Heunggongvirae	Uroviricota	Caudoviricetes	n.a.	Unclassified	O	O
S15_vs_3868	Phage vB_RanS_PJN03	ON245412	Overlap (VC_952/VC_1529)	n.a.	N17	4	Unspecified	Group I	Duplodnaviria	Heunggongvirae	Uroviricota	Caudoviricetes	n.a.	Unclassified	O	O
S15_vs_43913	Phage vB_RanS_PJN03	ON245412	Overlap (VC_952/VC_1529)	n.a.	N14	3	Unspecified	Group I	Duplodnaviria	Heunggongvirae	Uroviricota	Caudoviricetes	n.a.	Unclassified	O	O
S11_vf_104046	n.a.	n.a.	Singleton	n.a.	G	n.a.	n.a.	n.a.	n.a.	n.a.	n.a.	n.a.	n.a.	n.a.	n.a.	n.a.	n.a.
S11_vf_89309	n.a.	n.a.	Singleton	n.a.	G	n.a.	n.a.	n.a.	n.a.	n.a.	n.a.	n.a.	n.a.	n.a.	n.a.	n.a.	n.a.
S11_vs_104037	n.a.	n.a.	Singleton	n.a.	G	n.a.	n.a.	n.a.	n.a.	n.a.	n.a.	n.a.	n.a.	n.a.	n.a.	n.a.	n.a.
S12_vf_187941	n.a.	n.a.	Singleton	n.a.	G	n.a.	n.a.	n.a.	n.a.	n.a.	n.a.	n.a.	n.a.	n.a.	n.a.	n.a.	n.a.
S12_vf_188827	n.a.	n.a.	Singleton	n.a.	G	n.a.	n.a.	n.a.	n.a.	n.a.	n.a.	n.a.	n.a.	n.a.	n.a.	n.a.	n.a.
S12_vs_164056	n.a.	n.a.	Singleton	n.a.	G	n.a.	n.a.	n.a.	n.a.	n.a.	n.a.	n.a.	n.a.	n.a.	n.a.	n.a.	n.a.
S12_vs_70241	n.a.	n.a.	Singleton	n.a.	G	n.a.	n.a.	n.a.	n.a.	n.a.	n.a.	n.a.	n.a.	n.a.	n.a.	n.a.	n.a.
S14_vs_97835	n.a.	n.a.	Singleton	n.a.	G	n.a.	n.a.	n.a.	n.a.	n.a.	n.a.	n.a.	n.a.	n.a.	n.a.	n.a.	n.a.
S16_vs_120605	n.a.	n.a.	Singleton	n.a.	G	n.a.	n.a.	n.a.	n.a.	n.a.	n.a.	n.a.	n.a.	n.a.	n.a.	n.a.	n.a.
S16_vs_140468	n.a.	n.a.	Singleton	n.a.	G	n.a.	n.a.	n.a.	n.a.	n.a.	n.a.	n.a.	n.a.	n.a.	n.a.	n.a.	n.a.
S16_vs_173141	n.a.	n.a.	Singleton	n.a.	G	n.a.	n.a.	n.a.	n.a.	n.a.	n.a.	n.a.	n.a.	n.a.	n.a.	n.a.	n.a.
S17_vf_21718	n.a.	n.a.	Singleton	n.a.	G	n.a.	n.a.	n.a.	n.a.	n.a.	n.a.	n.a.	n.a.	n.a.	n.a.	n.a.	n.a.
S17_vs_133231	n.a.	n.a.	Singleton	n.a.	G	n.a.	n.a.	n.a.	n.a.	n.a.	n.a.	n.a.	n.a.	n.a.	n.a.	n.a.	n.a.
S17_vs_8690	n.a.	n.a.	Singleton	n.a.	G	n.a.	n.a.	n.a.	n.a.	n.a.	n.a.	n.a.	n.a.	n.a.	n.a.	n.a.	n.a.
S3_vf_127450	n.a.	n.a.	Singleton	n.a.	G	n.a.	n.a.	n.a.	n.a.	n.a.	n.a.	n.a.	n.a.	n.a.	n.a.	n.a.	n.a.
S3_vf_22968	n.a.	n.a.	Singleton	n.a.	G	n.a.	n.a.	n.a.	n.a.	n.a.	n.a.	n.a.	n.a.	n.a.	n.a.	n.a.	n.a.
S3_vf_87896	n.a.	n.a.	Singleton	n.a.	G	n.a.	n.a.	n.a.	n.a.	n.a.	n.a.	n.a.	n.a.	n.a.	n.a.	n.a.	n.a.
S3_vs_127411	n.a.	n.a.	Singleton	n.a.	G	n.a.	n.a.	n.a.	n.a.	n.a.	n.a.	n.a.	n.a.	n.a.	n.a.	n.a.	n.a.
S3_vs_39940	n.a.	n.a.	Singleton	n.a.	G	n.a.	n.a.	n.a.	n.a.	n.a.	n.a.	n.a.	n.a.	n.a.	n.a.	n.a.	n.a.
S3_vs_87728	n.a.	n.a.	Singleton	n.a.	G	n.a.	n.a.	n.a.	n.a.	n.a.	n.a.	n.a.	n.a.	n.a.	n.a.	n.a.	n.a.
S4_vf_17948	n.a.	n.a.	Singleton	n.a.	G	n.a.	n.a.	n.a.	n.a.	n.a.	n.a.	n.a.	n.a.	n.a.	n.a.	n.a.	n.a.
S5_vs_21778	n.a.	n.a.	Singleton	n.a.	G	n.a.	n.a.	n.a.	n.a.	n.a.	n.a.	n.a.	n.a.	n.a.	n.a.	n.a.	n.a.
S5_vs_41663	n.a.	n.a.	Singleton	n.a.	G	n.a.	n.a.	n.a.	n.a.	n.a.	n.a.	n.a.	n.a.	n.a.	n.a.	n.a.	n.a.
S5_vs_65490	n.a.	n.a.	Singleton	n.a.	G	n.a.	n.a.	n.a.	n.a.	n.a.	n.a.	n.a.	n.a.	n.a.	n.a.	n.a.	n.a.
S7_vf_11744	n.a.	n.a.	Singleton	n.a.	G	n.a.	n.a.	n.a.	n.a.	n.a.	n.a.	n.a.	n.a.	n.a.	n.a.	n.a.	n.a.
S7_vs_13370	n.a.	n.a.	Singleton	n.a.	G	n.a.	n.a.	n.a.	n.a.	n.a.	n.a.	n.a.	n.a.	n.a.	n.a.	n.a.	n.a.
S7_vs_138116	n.a.	n.a.	Singleton	n.a.	G	n.a.	n.a.	n.a.	n.a.	n.a.	n.a.	n.a.	n.a.	n.a.	n.a.	n.a.	n.a.
S7_vs_26757	n.a.	n.a.	Singleton	n.a.	G	n.a.	n.a.	n.a.	n.a.	n.a.	n.a.	n.a.	n.a.	n.a.	n.a.	n.a.	n.a.
S7_vs_30665	n.a.	n.a.	Singleton	n.a.	G	n.a.	n.a.	n.a.	n.a.	n.a.	n.a.	n.a.	n.a.	n.a.	n.a.	n.a.	n.a.

S8_vf_102508	n.a.	n.a.	Singleton	n.a.	G	n.a.	n.a.	n.a.	n.a.	n.a.	n.a.	n.a.	n.a.	n.a.	n.a.	n.a.
S8_vf_208111	n.a.	n.a.	Singleton	n.a.	G	n.a.	n.a.	n.a.	n.a.	n.a.	n.a.	n.a.	n.a.	n.a.	n.a.	n.a.
S8_vf_7243	n.a.	n.a.	Singleton	n.a.	G	n.a.	n.a.	n.a.	n.a.	n.a.	n.a.	n.a.	n.a.	n.a.	n.a.	n.a.
S8_vs_196247	n.a.	n.a.	Singleton	n.a.	G	n.a.	n.a.	n.a.	n.a.	n.a.	n.a.	n.a.	n.a.	n.a.	n.a.	n.a.
S9_vs_42609	n.a.	n.a.	Singleton	n.a.	G	n.a.	n.a.	n.a.	n.a.	n.a.	n.a.	n.a.	n.a.	n.a.	n.a.	n.a.

Table S6-2. WMS_HQ_vOTUs_taxonomies

Scaffold	Closer	Accession	Status	VC	Level	Weight	Host	BaltimoreGroup	Realm	Kingdom	Phylum	Class	Order	Family	Subfamily	Genus
S1-wms-combined_107073	Burkholderia phage BcepMu	AY539836	Clustered	VC_610_0	N4	5	Burkholderia	Group I	Duplodnaviria	Heunggongvirae	Uroviricota	Caudoviricetes	n.a.	Unclassified	O	O
S1-wms-combined_118167	Clostridium phage phiMMP02	JX145341	Clustered	VC_825_0	N11	2	Clostridium	Group I	Duplodnaviria	Heunggongvirae	Uroviricota	Caudoviricetes	n.a.	Unclassified	O	O
S1-wms-combined_120533	n.a.	n.a.	Clustered	VC_1481_0	F	n.a.	n.a.	n.a.	n.a.	n.a.	n.a.	n.a.	n.a.	n.a.	n.a.	n.a.
S1-wms-combined_132934	Faecalibacterium phage FP_Epona	MG711462	Clustered	VC_707_0	N10	46	Faecalibacterium	Group I	Duplodnaviria	Heunggongvirae	Uroviricota	Caudoviricetes	n.a.	Unclassified	O	O
S1-wms-combined_151708	n.a.	n.a.	Clustered	VC_1484_0	A	n.a.	n.a.	n.a.	n.a.	n.a.	n.a.	n.a.	n.a.	n.a.	n.a.	n.a.
S1-wms-combined_156637	n.a.	n.a.	Clustered	VC_1487_0	F	n.a.	n.a.	n.a.	n.a.	n.a.	n.a.	n.a.	n.a.	n.a.	n.a.	n.a.
S1-wms-combined_159544	Pseudomonas phage phi3	KT887559	Clustered	VC_5_0	C2	32	Pseudomonas	Group I	Duplodnaviria	Heunggongvirae	Uroviricota	Caudoviricetes	n.a.	Peduoviridae	Unclassified	Phitrevirus
S1-wms-combined_164485	n.a.	n.a.	Clustered	VC_1484_0	A	n.a.	n.a.	n.a.	n.a.	n.a.	n.a.	n.a.	n.a.	n.a.	n.a.	n.a.
S1-wms-combined_169879	Bacillus phage BalMu-1	KP063902	Clustered	VC_825_0	N9	12	Bacillus	Group I	Duplodnaviria	Heunggongvirae	Uroviricota	Caudoviricetes	n.a.	Unclassified	O	O
S1-wms-combined_185336	n.a.	n.a.	Clustered	VC_1491_0	F	n.a.	n.a.	n.a.	n.a.	n.a.	n.a.	n.a.	n.a.	n.a.	n.a.	n.a.
S1-wms-combined_19031	Mannheimia phage vB_MhM_3927AP2	KP137440	Clustered	VC_181_0	C1	48	Mannheimia	Group I	Duplodnaviria	Heunggongvirae	Uroviricota	Caudoviricetes	n.a.	Unclassified	Unclassified	Unclassified
S1-wms-combined_197401	n.a.	n.a.	Clustered	VC_1408_0	A	n.a.	n.a.	n.a.	n.a.	n.a.	n.a.	n.a.	n.a.	n.a.	n.a.	n.a.
S1-wms-combined_224578	Roseburia phage Jekyll	LR596902	Clustered	VC_418_0	C5	9	Roseburia	Group I	Duplodnaviria	Heunggongvirae	Uroviricota	Caudoviricetes	n.a.	Unclassified	Unclassified	Unclassified
S1-wms-combined_23248	Faecalibacterium phage FP_Lugh	MG711464	Clustered	VC_621_0	C2	71	Faecalibacterium	Group I	Duplodnaviria	Heunggongvirae	Uroviricota	Caudoviricetes	n.a.	Unclassified	Unclassified	Lughvirus
S1-wms-combined_246923	Flavobacterium phage fF4	MN850656	Clustered	VC_679_0	N12	13	Flavobacterium	Group I	Duplodnaviria	Heunggongvirae	Uroviricota	Caudoviricetes	n.a.	Unclassified	O	O
S1-wms-combined_248622	n.a.	n.a.	Clustered	VC_1494_0	F	n.a.	n.a.	n.a.	n.a.	n.a.	n.a.	n.a.	n.a.	n.a.	n.a.	n.a.
S1-wms-combined_263562	n.a.	n.a.	Clustered	VC_1437_0	A	n.a.	n.a.	n.a.	n.a.	n.a.	n.a.	n.a.	n.a.	n.a.	n.a.	n.a.
S1-wms-combined_302166	Faecalibacterium phage FP_Lagaffe	MG711461	Clustered	VC_715_0	N8	11	Faecalibacterium	Group I	Duplodnaviria	Heunggongvirae	Uroviricota	Caudoviricetes	n.a.	Unclassified	O	O
S1-wms-combined_304377	Faecalibacterium phage FP_oengus	MG711463	Clustered	VC_804_0	C1	134	Faecalibacterium	Group I	Duplodnaviria	Heunggongvirae	Uroviricota	Caudoviricetes	n.a.	Unclassified	Unclassified	Oengusvirus
S1-wms-combined_307323	Bifidobacterium phage PMBT6	MH444512	Clustered	VC_798_0	N3	41	Bifidobacterium	Group I	Duplodnaviria	Heunggongvirae	Uroviricota	Caudoviricetes	n.a.	Unclassified	O	O
S1-wms-combined_313851	n.a.	n.a.	Clustered	VC_1496_0	F	n.a.	n.a.	n.a.	n.a.	n.a.	n.a.	n.a.	n.a.	n.a.	n.a.	n.a.
S1-wms-combined_324053	Myoviridae sp.	MW202455	Clustered	VC_427_0	C1	7	Unspecified	Group I	Duplodnaviria	Heunggongvirae	Uroviricota	Caudoviricetes	n.a.	Unclassified	Unclassified	Unclassified
S1-wms-combined_331544	n.a.	n.a.	Clustered	VC_1497_0	F	n.a.	n.a.	n.a.	n.a.	n.a.	n.a.	n.a.	n.a.	n.a.	n.a.	n.a.
S1-wms-combined_331896	n.a.	n.a.	Clustered	VC_1481_0	F	n.a.	n.a.	n.a.	n.a.	n.a.	n.a.	n.a.	n.a.	n.a.	n.a.	n.a.
S1-wms-combined_332236	Streptococcus phage Javan630	MK448997	Clustered	VC_626_0	C5	55	Streptococcus	Group I	Duplodnaviria	Heunggongvirae	Uroviricota	Caudoviricetes	n.a.	Unclassified	Unclassified	Unclassified
S1-wms-combined_340558	Flavobacterium phage fF4	MN850656	Clustered	VC_679_0	N18	4	Flavobacterium	Group I	Duplodnaviria	Heunggongvirae	Uroviricota	Caudoviricetes	n.a.	Unclassified	O	O
S1-wms-combined_353757	Clostridium phage phiCT19406B	KM983331	Clustered	VC_650_0	N16	7	Clostridium	Group I	Duplodnaviria	Heunggongvirae	Uroviricota	Caudoviricetes	n.a.	Unclassified	O	O
S1-wms-combined_354899	n.a.	n.a.	Clustered	VC_1498_0	F	n.a.	n.a.	n.a.	n.a.	n.a.	n.a.	n.a.	n.a.	n.a.	n.a.	n.a.
S1-wms-combined_357373	Tortoise microvirus 19	MK765569	Clustered	VC_1243_0	C1	9	Unspecified	Group II	Monodnaviria	Sangervirae	Phixviricota	Malgrandaviricetes	Petitvirales	Microviridae	Unclassified	Unclassified
S1-wms-combined_360285	Exiguobacterium phage vB_EalM-137	MH884510	Clustered	VC_716_0	N2	13	Exiguobacterium	Group I	Duplodnaviria	Heunggongvirae	Uroviricota	Caudoviricetes	n.a.	Unclassified	O	O
S1-wms-combined_394574	Parabacteroides phage PDS1	MN929097	Clustered	VC_831_0	C1	164	Parabacteroides	Group I	Duplodnaviria	Heunggongvirae	Uroviricota	Caudoviricetes	n.a.	Unclassified	Unclassified	Unclassified
S1-wms-combined_394628	n.a.	n.a.	Clustered	VC_1500_0	F	n.a.	n.a.	n.a.	n.a.	n.a.	n.a.	n.a.	n.a.	n.a.	n.a.	n.a.
S1-wms-combined_40115	n.a.	n.a.	Clustered	VC_1491_0	F	n.a.	n.a.	n.a.	n.a.	n.a.	n.a.	n.a.	n.a.	n.a.	n.a.	n.a.
S1-wms-combined_41369	Roseburia phage Jekyll	LR596902	Clustered	VC_1209_0	N12	5	Roseburia	Group I	Duplodnaviria	Heunggongvirae	Uroviricota	Caudoviricetes	n.a.	Unclassified	O	O
S1-wms-combined_52643	Bacteroides phage p00	BK010646	Clustered	VC_679_0	N6	14	Bacteroides	Group I	Duplodnaviria	Heunggongvirae	Uroviricota	Caudoviricetes	n.a.	Unclassified	O	O
S1-wms-combined_68998	Faecalibacterium phage FP_Taranis	MG711467	Clustered	VC_711_0	C1	223	Faecalibacterium	Group I	Duplodnaviria	Heunggongvirae	Uroviricota	Caudoviricetes	n.a.	Unclassified	Unclassified	Taranisvirus
S1-wms-combined_72167	Listeria phage LWP01	CP011103	Clustered	VC_420_0	N10	3	Listeria	Group I	Duplodnaviria	Heunggongvirae	Uroviricota	Caudoviricetes	n.a.	Unclassified	O	O
S1-wms_100010	n.a.	n.a.	Clustered	VC_1492_0	A	n.a.	n.a.	n.a.	n.a.	n.a.	n.a.	n.a.	n.a.	n.a.	n.a.	n.a.
S1-wms_130214	Butyrivibrio virus Ceridwen	MN882553	Clustered	VC_1209_0	N11	6	Butyrivibrio	Group I	Duplodnaviria	Heunggongvirae	Uroviricota	Caudoviricetes	n.a.	Unclassified	O	O
S1-wms_170115	Clostridium phage phiCD38-2	HM568888	Clustered	VC_627_0	N9	6	Clostridium	Group I	Duplodnaviria	Heunggongvirae	Uroviricota	Caudoviricetes	n.a.	Unclassified	O	O
S1-wms_185046	n.a.	n.a.	Clustered	VC_1239_0	A	n.a.	n.a.	n.a.	n.a.	n.a.	n.a.	n.a.	n.a.	n.a.	n.a.	n.a.
S1-wms_212940	Faecalibacterium phage FP_oengus	MG711463	Clustered	VC_804_2	N10	46	Faecalibacterium	Group I	Duplodnaviria	Heunggongvirae	Uroviricota	Caudoviricetes	n.a.	Unclassified	O	O
S1-wms_225995	n.a.	n.a.	Clustered	VC_1492_0	A	n.a.	n.a.	n.a.	n.a.	n.a.	n.a.	n.a.	n.a.	n.a.	n.a.	n.a.
S1-wms_244836	n.a.	n.a.	Clustered	VC_1473_0	A	n.a.	n.a.	n.a.	n.a.	n.a.	n.a.	n.a.	n.a.	n.a.	n.a.	n.a.
S1-wms_296850	Faecalibacterium phage FP_Toutatis	MG711466	Clustered	VC_710_0	C6	68	Faecalibacterium	Group I	Duplodnaviria	Heunggongvirae	Uroviricota	Caudoviricetes	n.a.	Unclassified	Unclassified	Toutatisvirus
S1-wms_313991	n.a.	n.a.	Clustered	VC_1496_0	F	n.a.	n.a.	n.a.	n.a.	n.a.	n.a.	n.a.	n.a.	n.a.	n.a.	n.a.
S1-wms_4277	Faecalibacterium phage FP_Mushu	MG711460	Clustered	VC_1235_0	C5	126	Faecalibacterium	Group I	Duplodnaviria	Heunggongvirae	Uroviricota	Caudoviricetes	n.a.	Unclassified	Unclassified	Mushuvirus
S11-wms-combined_119215	Clostridium phage phiCT453B	KM983328	Clustered	VC_650_0	N16	3	Clostridium	Group I	Duplodnaviria	Heunggongvirae	Uroviricota	Caudoviricetes	n.a.	Unclassified	O	O
S11-wms-combined_125176	Campylobacter phage PC10	MZ047271	Clustered	VC_1456_0	N7	6	Campylobacter	Group I	Duplodnaviria	Heunggongvirae	Uroviricota	Caudoviricetes	n.a.	Unclassified	O	O
S11-wms-combined_140009	Faecalibacterium phage FP_Lugh	MG711464	Clustered	VC_621_0	C1	94	Faecalibacterium	Group I	Duplodnaviria	Heunggongvirae	Uroviricota	Caudoviricetes	n.a.	Unclassified	Unclassified	Lughvirus
S11-wms-combined_154845	Escherichia phage ESSI2_ev015	LR597649	Clustered	VC_5_0	C1	105	Escherichia	Group I	Duplodnaviria	Heunggongvirae	Uroviricota	Caudoviricetes	n.a.	Peduoviridae	Unclassified	Quadragintavirus
S11-wms-combined_17029	n.a.	n.a.	Clustered	VC_1502_0	A	n.a.	n.a.	n.a.	n.a.	n.a.	n.a.	n.a.	n.a.	n.a.	n.a.	n.a.
S11-wms-combined_172889	Flavobacterium phage fF4	MN850656	Clustered	VC_679_0	N17	7	Flavobacterium	Group I	Duplodnaviria	Heunggongvirae	Uroviricota	Caudoviricetes	n.a.	Unclassified	O	O
S11-wms-combined_193148	n.a.	n.a.	Clustered	VC_616_0	A	n.a.	n.a.	n.a.	n.a.	n.a.	n.a.	n.a.	n.a.	n.a.	n.a.	n.a.
S11-wms-combined_194205	Streptococcus phage Javan630	MK448997	Clustered	VC_626_0	C3	66	Streptococcus	Group I	Duplodnaviria	Heunggongvirae	Uroviricota	Caudoviricetes	n.a.	Unclassified	Unclassified	Unclassified
S11-wms-combined_195425	Bacteroides phage p00	BK010646	Clustered	VC_677_0	C2	65	Bacteroides	Group I	Duplodnaviria	Heunggongvirae	Uroviricota	Caudoviricetes	n.a.	Unclassified	Unclassified	Unclassified
S11-wms-combined_198512	Roseburia phage Shimadzu	LR596903	Clustered	VC_715_0	N3	5	Roseburia	Group I	Duplodnaviria	Heunggongvirae	Uroviricota	Caudoviricetes	n.a.	Unclassified	O	O
S11-wms-combined_200084	Bacillus phage BalMu-1	KP063902	Clustered	VC_825_0	N10	6	Bacillus	Group I	Duplodnaviria	Heunggongvirae	Uroviricota	Caudoviricetes	n.a.	Unclassified	O	O
S11-wms-combined_204522	Burkholderia phage BcepC6B	AY605181	Clustered	VC_616_0	N3	1	Burkholderia	Group I	Duplodnaviria	Heunggongvirae	Uroviricota	Caudoviricetes	n.a.	Unclassified	O	O
S11-wms-combined_217790	n.a.	n.a.	Clustered	VC_1503_0	A	n.a.	n.a.	n.a.	n.a.	n.a.	n.a.	n.a.	n.a.	n.a.	n.a.	n.a.
S11-wms-combined_221310	n.a.	n.a.	Clustered	VC_1504_0	A	n.a.	n.a.	n.a.	n.a.	n.a.	n.a.	n.a.	n.a.	n.a.	n.a.	n.a.
S11-wms-combined_268967	Mannheimia phage vB_MhM_3927AP2	KP137440	Clustered	VC_181_0	C2	23	Mannheimia	Group I	Duplodnaviria	Heunggongvirae	Uroviricota	Caudoviricetes	n.a.	Unclassified	Unclassified	Unclassified
S11-wms-combined_283220	Faecalibacterium phage FP_Mushu	MG711460	Clustered	VC_1235_0	C1	160	Faecalibacterium	Group I	Duplodnaviria	Heunggongvirae	Uroviricota	Caudoviricetes	n.a.	Unclassified	Unclassified	Mushuvirus
S11-wms-combined_299954	n.a.	n.a.	Clustered	VC_650_0	A	n.a.	n.a.	n.a.	n.a.	n.a.	n.a.	n.a.	n.a.	n.a.	n.a.	n.a.
S11-wms-combined_304239	Faecalibacterium phage FP_Epona	MG711462	Clustered	VC_707_0	N10	51	Faecalibacterium	Group I	Duplodnaviria	Heunggongvirae	Uroviricota	Caudoviricetes	n.a.	Unclassified	O	O
S11-wms-combined_41367	n.a.	n.a.	Clustered	VC_1505_0	A	n.a.	n.a.	n.a.	n.a.	n.a.	n.a.	n.a.	n.a.	n.a.	n.a.	n.a.
S11-wms-combined_46816	n.a.	n.a.	Clustered	VC_893_0	A	n.a.	n.a.	n.a.	n.a.	n.a.	n.a.	n.a.	n.a.	n.a.	n.a.	n.a.
S11-wms-combined_48246	Phage vB_RanS_PJN03	ON245412	Clustered	VC_917_0	N10	5	Unspecified	Group I	Duplodnaviria	Heunggongvirae	Uroviricota	Caudoviricetes	n.a.	Unclassified	O	O
S11-wms-combined_49632	Clostridium phage phiCT453B	KM983328	Clustered	VC_650_0	N12	10	Clostridium	Group I	Duplodnaviria	Heunggongvirae	Uroviricota	Caudoviricetes	n.a.	Unclassified	O	O
S11-wms-combined_58538	Deep-sea thermophilic phage D6E	GU568037	Clustered	VC_271_0	N9	2	Unspecified	Group I	Duplodnaviria	Heunggongvirae	Uroviricota	Caudoviricetes	n.a.	Unclassified	O	O
S11-wms-combined_73204	n.a.	n.a.	Clustered	VC_1397_0	A	n.a.	n.a.	n.a.	n.a.	n.a.	n.a.	n.a.	n.a.	n.a.	n.a.	n.a.
S11-wms-combined_74983	Faecalibacterium phage FP_Toutatis	MG711466	Clustered	VC_710_0	C6	71	Faecalibacterium	Group I	Duplodnaviria	Heunggongvirae	Uroviricota	Caudoviricetes	n.a.	Unclassified	Unclassified	Toutatisvirus
S11-wms-combined_83571	Winogradskyella phage Peternella_1	MT732475	Clustered	VC_676_1	N12	19	Winogradskyella	Group I	Duplodnaviria	Heunggongvirae	Uroviricota	Caudoviricetes	n.a.	Winoviridae	O	O
S11-wms-combined_84326	Escherichia phage vB_EcoM-613R2	ON470591	Clustered	VC_716_0	N2	12	Escherichia	Group I	Duplodnaviria	Heunggongvirae	Uroviricota	Caudoviricetes	n.a.	Unclassified	O	O
S11-wms-combined_92114	n.a.	n.a.	Clustered	VC_1014_0	A	n.a.	n.a.	n.a.	n.a.	n.a.	n.a.	n.a.	n.a.	n.a.	n.a.	n.a.
S11-wms_14390	n.a.	n.a.	Clustered	VC_271_0	A	n.a.	n.a.	n.a.	n.a.	n.a.	n.a.	n.a.	n.a.	n.a.	n.a.	n.a.
S11-wms_303398	n.a.	n.a.	Clustered	VC_1239_0	A	n.a.	n.a.	n.a.	n.a.	n.a.	n.a.	n.a.	n.a.	n.a.	n.a.	n.a.
S11-wms_3116	Faecalibacterium phage FP_Mushu	MG711460	Clustered	VC_1235_0	C2	142	Faecalibacterium	Group I	Duplodnaviria	Heunggongvirae	Uroviricota	Caudoviricetes	n.a.	Unclassified	Unclassified	Mushuvirus
S11-wms_320555	Phage vB_RanS_PJN03	ON245412	Clustered	VC_918_0	N5	9	Unspecified	Group I	Duplodnaviria	Heunggongvirae	Uroviricota	Caudoviricetes	n.a.	Unclassified	O	O
S11-wms_330479	Elizabethkingia phage TCUEAP1	MN732896	Clustered	VC_1010_0	C1	8	Elizabethkingia	Unclassified	Unclassified	n.a.	n.a.	n.a.	n.a.	Unclassified	Unclassified	Unclassified
S11-wms_55610	Faecalibacterium phage FP_Brigit	MG711465	Clustered	VC_606_0	C2	149	Faecalibacterium	Group I	Duplodnaviria	Heunggongvirae	Uroviricota	Caudoviricetes	n.a.	Unclassified	Unclassified	Brigitvirus
S12-wms-combined_107942	Bifidobacterium phage BitterVaud1	MT006236	Clustered	VC_804_2	N5	34	Bifidobacterium	Group I	Duplodnaviria	Heunggongvirae	Uroviricota	Caudoviricetes	n.a.	Unclassified	O	O
S12-wms-combined_111524	n.a.	n.a.	Clustered	VC_1503_0	A	n.a.	n.a.	n.a.	n.a.	n.a.	n.a.	n.a.	n.a.	n.a.	n.a.	n.a.
S12-wms-combined_120133	Geobacillus phage GBSV1	DQ340064	Clustered	VC_338_0	N6	6	Geobacillus	Group I	Duplodnaviria	Heunggongvirae	Uroviricota	Caudoviricetes	n.a.	Unclassified	O	O

S12-wms-combined_129554	Faecalibacterium phage FP_Epona	MG711462	Clustered	VC_707_1	C3	50	Faecalibacterium	Group I	Duplodnaviria	Heunggongvirae	Uroviricota	Caudoviricetes	n.a.	Unclassified	Unclassified	Eponavirus
S12-wms-combined_147628	Faecalibacterium phage FP_Taranis	MG711467	Clustered	VC_1238_0	N1	24	Faecalibacterium	Group I	Duplodnaviria	Heunggongvirae	Uroviricota	Caudoviricetes	n.a.	Unclassified	O	O
S12-wms-combined_156234	Faecalibacterium phage FP_Toutatis	MG711466	Clustered	VC_710_0	C8	49	Faecalibacterium	Group I	Duplodnaviria	Heunggongvirae	Uroviricota	Caudoviricetes	n.a.	Unclassified	Unclassified	Toutatisvirus
S12-wms-combined_168259	Akkermansia phage DTMo-2021a	CP084202	Clustered	VC_736_0	N1	13	Akkermansia	Group I	Duplodnaviria	Heunggongvirae	Uroviricota	Caudoviricetes	n.a.	Unclassified	O	O
S12-wms-combined_17617	Faecalibacterium phage FP_Epona	MG711462	Clustered	VC_707_0	N8	53	Faecalibacterium	Group I	Duplodnaviria	Heunggongvirae	Uroviricota	Caudoviricetes	n.a.	Unclassified	O	O
S12-wms-combined_18287	Faecalibacterium phage FP_Epona	MG711462	Clustered	VC_707_0	N10	42	Faecalibacterium	Group I	Duplodnaviria	Heunggongvirae	Uroviricota	Caudoviricetes	n.a.	Unclassified	O	O
S12-wms-combined_191570	Clostridium phage phiCT453A	KM983327	Clustered	VC_732_0	N4	9	Clostridium	Group I	Duplodnaviria	Heunggongvirae	Uroviricota	Caudoviricetes	n.a.	Unclassified	O	O
S12-wms-combined_210942	n.a.	n.a.	Clustered	VC_1487_0	F	n.a.	n.a.	n.a.	n.a.	n.a.	n.a.	n.a.	n.a.	n.a.	n.a.	n.a.
S12-wms-combined_2450	Winogradskyella phage Peternella_1	MT732475	Clustered	VC_676_0	N7	19	Winogradskyella	Group I	Duplodnaviria	Heunggongvirae	Uroviricota	Caudoviricetes	n.a.	Winoviridae	O	O
S12-wms-combined_245755	Butyrivibrio phage Idris	MN882554	Clustered	VC_876_0	N6	38	Butyrivibrio	Group I	Duplodnaviria	Heunggongvirae	Uroviricota	Caudoviricetes	n.a.	Unclassified	O	O
S12-wms-combined_250551	Bacteroides phage p00	BK010646	Clustered	VC_677_0	C1	130	Bacteroides	Group I	Duplodnaviria	Heunggongvirae	Uroviricota	Caudoviricetes	n.a.	Unclassified	Unclassified	Unclassified
S12-wms-combined_256299	n.a.	n.a.	Clustered	VC_1505_0	A	n.a.	n.a.	n.a.	n.a.	n.a.	n.a.	n.a.	n.a.	n.a.	n.a.	n.a.
S12-wms-combined_259783	Roseburia phage Shimadzu	LR596903	Clustered	VC_717_0	C1	228	Roseburia	Group I	Duplodnaviria	Heunggongvirae	Uroviricota	Caudoviricetes	n.a.	Unclassified	Unclassified	Unclassified
S12-wms-combined_260036	uncultured phage cr128_1	MT774392	Clustered	VC_1439_0	N7	3	Unspecified	Group I	Duplodnaviria	Heunggongvirae	Uroviricota	Caudoviricetes	Crassvirales	Steigviridae	O	O
S12-wms-combined_263377	Clostridium phage Clo-PEP-1	KY206887	Clustered	VC_212_0	N5	17	Clostridium	Group I	Duplodnaviria	Heunggongvirae	Uroviricota	Caudoviricetes	n.a.	Unclassified	O	O
S12-wms-combined_264425	n.a.	n.a.	Clustered	VC_1492_0	A	n.a.	n.a.	n.a.	n.a.	n.a.	n.a.	n.a.	n.a.	n.a.	n.a.	n.a.
S12-wms-combined_264845	n.a.	n.a.	Clustered	VC_1507_0	A	n.a.	n.a.	n.a.	n.a.	n.a.	n.a.	n.a.	n.a.	n.a.	n.a.	n.a.
S12-wms-combined_282594	Akkermansia phage DTMo-2021a	CP084202	Clustered	VC_736_0	N3	7	Akkermansia	Group I	Duplodnaviria	Heunggongvirae	Uroviricota	Caudoviricetes	n.a.	Unclassified	O	O
S12-wms-combined_299293	n.a.	n.a.	Clustered	VC_1492_0	A	n.a.	n.a.	n.a.	n.a.	n.a.	n.a.	n.a.	n.a.	n.a.	n.a.	n.a.
S12-wms-combined_299872	Faecalibacterium phage FP_Mushu	MG711460	Clustered	VC_1235_0	C3	71	Faecalibacterium	Group I	Duplodnaviria	Heunggongvirae	Uroviricota	Caudoviricetes	n.a.	Unclassified	Unclassified	Mushuvirus
S12-wms-combined_320727	Flavobacterium phage FPSV-S27	MK764448	Clustered	VC_1397_0	N4	6	Flavobacterium	Group I	Duplodnaviria	Heunggongvirae	Uroviricota	Caudoviricetes	n.a.	Unclassified	O	O
S12-wms-combined_321174	Butyrivibrio phage Idris	MN882554	Clustered	VC_915_0	N8	6	Butyrivibrio	Group I	Duplodnaviria	Heunggongvirae	Uroviricota	Caudoviricetes	n.a.	Unclassified	O	O
S12-wms-combined_329242	n.a.	n.a.	Clustered	VC_1484_0	A	n.a.	n.a.	n.a.	n.a.	n.a.	n.a.	n.a.	n.a.	n.a.	n.a.	n.a.
S12-wms-combined_334806	n.a.	n.a.	Clustered	VC_1239_0	A	n.a.	n.a.	n.a.	n.a.	n.a.	n.a.	n.a.	n.a.	n.a.	n.a.	n.a.
S12-wms-combined_345948	Faecalibacterium phage FP_Lugh	MG711464	Clustered	VC_621_0	C5	43	Faecalibacterium	Group I	Duplodnaviria	Heunggongvirae	Uroviricota	Caudoviricetes	n.a.	Unclassified	Unclassified	Lughvirus
S12-wms-combined_351847	n.a.	n.a.	Clustered	VC_1436_0	A	n.a.	n.a.	n.a.	n.a.	n.a.	n.a.	n.a.	n.a.	n.a.	n.a.	n.a.
S12-wms-combined_358010	Exiguobacterium phage vB_EalM-137	MH884510	Clustered	VC_715_0	N4	9	Exiguobacterium	Group I	Duplodnaviria	Heunggongvirae	Uroviricota	Caudoviricetes	n.a.	Unclassified	O	O
S12-wms-combined_368462	Streptococcus phage Javan630	MK448997	Clustered	VC_626_0	C7	61	Streptococcus	Group I	Duplodnaviria	Heunggongvirae	Uroviricota	Caudoviricetes	n.a.	Unclassified	Unclassified	Unclassified
S12-wms-combined_375343	n.a.	n.a.	Clustered	VC_1502_0	A	n.a.	n.a.	n.a.	n.a.	n.a.	n.a.	n.a.	n.a.	n.a.	n.a.	n.a.
S12-wms-combined_376326	n.a.	n.a.	Clustered	VC_1076_0	A	n.a.	n.a.	n.a.	n.a.	n.a.	n.a.	n.a.	n.a.	n.a.	n.a.	n.a.
S12-wms-combined_393703	n.a.	n.a.	Clustered	VC_1481_0	F	n.a.	n.a.	n.a.	n.a.	n.a.	n.a.	n.a.	n.a.	n.a.	n.a.	n.a.
S12-wms-combined_401379	n.a.	n.a.	Clustered	VC_1500_0	F	n.a.	n.a.	n.a.	n.a.	n.a.	n.a.	n.a.	n.a.	n.a.	n.a.	n.a.
S12-wms-combined_401383	n.a.	n.a.	Clustered	VC_1510_0	F	n.a.	n.a.	n.a.	n.a.	n.a.	n.a.	n.a.	n.a.	n.a.	n.a.	n.a.
S12-wms-combined_41160	n.a.	n.a.	Clustered	VC_1238_0	A	n.a.	n.a.	n.a.	n.a.	n.a.	n.a.	n.a.	n.a.	n.a.	n.a.	n.a.
S12-wms-combined_51076	n.a.	n.a.	Clustered	VC_1479_0	A	n.a.	n.a.	n.a.	n.a.	n.a.	n.a.	n.a.	n.a.	n.a.	n.a.	n.a.
S12-wms-combined_71235	Faecalibacterium phage FP_Lugh	MG711464	Clustered	VC_621_0	C4	57	Faecalibacterium	Group I	Duplodnaviria	Heunggongvirae	Uroviricota	Caudoviricetes	n.a.	Unclassified	Unclassified	Lughvirus
S12-wms-combined_7174	Streptococcus phage Javan630	MK448997	Clustered	VC_626_0	C6	59	Streptococcus	Group I	Duplodnaviria	Heunggongvirae	Uroviricota	Caudoviricetes	n.a.	Unclassified	Unclassified	Unclassified
S12-wms-combined_91231	n.a.	n.a.	Clustered	VC_1439_0	A	n.a.	n.a.	n.a.	n.a.	n.a.	n.a.	n.a.	n.a.	n.a.	n.a.	n.a.
S12-wms-combined_99704	Streptococcus phage Javan105	MK448667	Clustered	VC_626_0	C2	81	Streptococcus	Group I	Duplodnaviria	Heunggongvirae	Uroviricota	Caudoviricetes	n.a.	Unclassified	Unclassified	Unclassified
S12-wms_12658	Phage vB_RanS_PJN03	ON245412	Clustered	VC_918_0	N8	12	Unspecified	Group I	Duplodnaviria	Heunggongvirae	Uroviricota	Caudoviricetes	n.a.	Unclassified	O	O
S12-wms_15700	n.a.	n.a.	Clustered	VC_1511_0	F	n.a.	n.a.	n.a.	n.a.	n.a.	n.a.	n.a.	n.a.	n.a.	n.a.	n.a.
S12-wms_179388	n.a.	n.a.	Clustered	VC_1492_0	A	n.a.	n.a.	n.a.	n.a.	n.a.	n.a.	n.a.	n.a.	n.a.	n.a.	n.a.
S12-wms_193565	Faecalibacterium phage FP_Mushu	MG711460	Clustered	VC_1235_0	C1	150	Faecalibacterium	Group I	Duplodnaviria	Heunggongvirae	Uroviricota	Caudoviricetes	n.a.	Unclassified	Unclassified	Mushuvirus
S12-wms_373775	n.a.	n.a.	Clustered	VC_1507_0	A	n.a.	n.a.	n.a.	n.a.	n.a.	n.a.	n.a.	n.a.	n.a.	n.a.	n.a.
S12-wms_383851	n.a.	n.a.	Clustered	VC_1436_0	A	n.a.	n.a.	n.a.	n.a.	n.a.	n.a.	n.a.	n.a.	n.a.	n.a.	n.a.
S12-wms_386692	n.a.	n.a.	Clustered	VC_1456_0	A	n.a.	n.a.	n.a.	n.a.	n.a.	n.a.	n.a.	n.a.	n.a.	n.a.	n.a.
S12-wms_401366	n.a.	n.a.	Clustered	VC_1492_0	A	n.a.	n.a.	n.a.	n.a.	n.a.	n.a.	n.a.	n.a.	n.a.	n.a.	n.a.
S12-wms_401372	Phage DP SC_6_H4_2017	MT121964	Clustered	VC_1420_0	C2	137	Unspecified	Group I	Duplodnaviria	Heunggongvirae	Uroviricota	Caudoviricetes	n.a.	Unclassified	Unclassified	Unclassified
S12-wms_44817	n.a.	n.a.	Clustered	VC_1492_0	A	n.a.	n.a.	n.a.	n.a.	n.a.	n.a.	n.a.	n.a.	n.a.	n.a.	n.a.
S17-wms-combined_126600	n.a.	n.a.	Clustered	VC_1481_0	F	n.a.	n.a.	n.a.	n.a.	n.a.	n.a.	n.a.	n.a.	n.a.	n.a.	n.a.
S17-wms-combined_126774	n.a.	n.a.	Clustered	VC_1484_0	A	n.a.	n.a.	n.a.	n.a.	n.a.	n.a.	n.a.	n.a.	n.a.	n.a.	n.a.
S17-wms-combined_127688	Faecalibacterium phage FP_Epona	MG711462	Clustered	VC_707_1	C2	106	Faecalibacterium	Group I	Duplodnaviria	Heunggongvirae	Uroviricota	Caudoviricetes	n.a.	Unclassified	Unclassified	Eponavirus
S17-wms-combined_130215	Streptococcus phage Javan261	MK448720	Clustered	VC_338_0	N9	5	Streptococcus	Group I	Duplodnaviria	Heunggongvirae	Uroviricota	Caudoviricetes	n.a.	Unclassified	O	O
S17-wms-combined_136622	n.a.	n.a.	Clustered	VC_650_0	A	n.a.	n.a.	n.a.	n.a.	n.a.	n.a.	n.a.	n.a.	n.a.	n.a.	n.a.
S17-wms-combined_146850	n.a.	n.a.	Clustered	VC_1484_0	A	n.a.	n.a.	n.a.	n.a.	n.a.	n.a.	n.a.	n.a.	n.a.	n.a.	n.a.
S17-wms-combined_157468	n.a.	n.a.	Clustered	VC_1484_0	A	n.a.	n.a.	n.a.	n.a.	n.a.	n.a.	n.a.	n.a.	n.a.	n.a.	n.a.
S17-wms-combined_158354	n.a.	n.a.	Clustered	VC_1505_0	A	n.a.	n.a.	n.a.	n.a.	n.a.	n.a.	n.a.	n.a.	n.a.	n.a.	n.a.
S17-wms-combined_162786	n.a.	n.a.	Clustered	VC_1408_0	A	n.a.	n.a.	n.a.	n.a.	n.a.	n.a.	n.a.	n.a.	n.a.	n.a.	n.a.
S17-wms-combined_16309	Bacteroides phage F4	MT806185	Clustered	VC_1435_0	N11	8	Bacteroides	Group I	Duplodnaviria	Heunggongvirae	Uroviricota	Caudoviricetes	n.a.	Unclassified	O	O
S17-wms-combined_175924	Faecalibacterium phage FP_Mushu	MG711460	Clustered	VC_1235_0	C1	96	Faecalibacterium	Group I	Duplodnaviria	Heunggongvirae	Uroviricota	Caudoviricetes	n.a.	Unclassified	Unclassified	Mushuvirus
S17-wms-combined_179771	n.a.	n.a.	Clustered	VC_1509_0	A	n.a.	n.a.	n.a.	n.a.	n.a.	n.a.	n.a.	n.a.	n.a.	n.a.	n.a.
S17-wms-combined_193958	Faecalibacterium phage FP_Mushu	MG711460	Clustered	VC_1235_0	C3	135	Faecalibacterium	Group I	Duplodnaviria	Heunggongvirae	Uroviricota	Caudoviricetes	n.a.	Unclassified	Unclassified	Mushuvirus
S17-wms-combined_199440	Faecalibacterium phage FP_Epona	MG711462	Clustered	VC_707_0	N10	26	Faecalibacterium	Group I	Duplodnaviria	Heunggongvirae	Uroviricota	Caudoviricetes	n.a.	Unclassified	O	O
S17-wms-combined_199746	Bacteroides phage F4	MT806185	Clustered	VC_1014_0	N10	2	Bacteroides	Group I	Duplodnaviria	Heunggongvirae	Uroviricota	Caudoviricetes	n.a.	Unclassified	O	O
S17-wms-combined_203701	n.a.	n.a.	Clustered	VC_1508_0	A	n.a.	n.a.	n.a.	n.a.	n.a.	n.a.	n.a.	n.a.	n.a.	n.a.	n.a.
S17-wms-combined_223748	n.a.	n.a.	Clustered	VC_1512_0	F	n.a.	n.a.	n.a.	n.a.	n.a.	n.a.	n.a.	n.a.	n.a.	n.a.	n.a.
S17-wms-combined_225356	n.a.	n.a.	Clustered	VC_1513_0	A	n.a.	n.a.	n.a.	n.a.	n.a.	n.a.	n.a.	n.a.	n.a.	n.a.	n.a.
S17-wms-combined_233761	n.a.	n.a.	Clustered	VC_1495_0	A	n.a.	n.a.	n.a.	n.a.	n.a.	n.a.	n.a.	n.a.	n.a.	n.a.	n.a.
S17-wms-combined_247494	Clostridium phage phiMMP04	JX145342	Clustered	VC_650_0	N15	6	Clostridium	Group I	Duplodnaviria	Heunggongvirae	Uroviricota	Caudoviricetes	n.a.	Unclassified	O	O
S17-wms-combined_259811	Faecalibacterium phage FP_oengus	MG711463	Clustered	VC_804_1	N8	29	Faecalibacterium	Group I	Duplodnaviria	Heunggongvirae	Uroviricota	Caudoviricetes	n.a.	Unclassified	O	O
S17-wms-combined_260343	Faecalibacterium phage FP_Toutatis	MG711466	Clustered	VC_710_0	C5	77	Faecalibacterium	Group I	Duplodnaviria	Heunggongvirae	Uroviricota	Caudoviricetes	n.a.	Unclassified	Unclassified	Toutatisvirus
S17-wms-combined_30308	n.a.	n.a.	Clustered	VC_1482_0	F	n.a.	n.a.	n.a.	n.a.	n.a.	n.a.	n.a.	n.a.	n.a.	n.a.	n.a.
S17-wms-combined_47860	Faecalibacterium phage FP_oengus	MG711463	Clustered	VC_804_1	N7	66	Faecalibacterium	Group I	Duplodnaviria	Heunggongvirae	Uroviricota	Caudoviricetes	n.a.	Unclassified	O	O
S17-wms-combined_70983	n.a.	n.a.	Clustered	VC_1494_0	F	n.a.	n.a.	n.a.	n.a.	n.a.	n.a.	n.a.	n.a.	n.a.	n.a.	n.a.
S17-wms-combined_79709	Clostridium phage phiCT453B	KM983328	Clustered	VC_650_0	N14	10	Clostridium	Group I	Duplodnaviria	Heunggongvirae	Uroviricota	Caudoviricetes	n.a.	Unclassified	O	O
S17-wms-combined_90378	Faecalibacterium phage FP_Lugh	MG711464	Clustered	VC_621_0	C2	65	Faecalibacterium	Group I	Duplodnaviria	Heunggongvirae	Uroviricota	Caudoviricetes	n.a.	Unclassified	Unclassified	Lughvirus
S17-wms-combined_9864	Streptococcus phage Javan630	MK448997	Clustered	VC_626_0	C7	56	Streptococcus	Group I	Duplodnaviria	Heunggongvirae	Uroviricota	Caudoviricetes	n.a.	Unclassified	Unclassified	Unclassified
S17-wms_124308	Bacteroides phage F4	MT806185	Clustered	VC_1435_0	N11	4	Bacteroides	Group I	Duplodnaviria	Heunggongvirae	Uroviricota	Caudoviricetes	n.a.	Unclassified	O	O
S17-wms_254872	Microvirus mar62	MZ089808	Clustered	VC_1276_0	C1	18	Unspecified	Group II	Monodnaviria	Sangervirae	Phixviricota	Malgrandaviricetes	Petitvirales	Microviridae	Unclassified	Microvirus
S17-wms_266511	Enterobacter phage phiEap-2	KT287080	Clustered	VC_540_0	C1	92	Enterobacter	Group I	Duplodnaviria	Heunggongvirae	Uroviricota	Caudoviricetes	n.a.	Unclassified	Guernseyvirinae	Cornellvirus
S2-wms-combined_101826	Paenibacillus phage PG1	HQ332138	Clustered	VC_734_0	C2	8	Paenibacillus	Group I	Duplodnaviria	Heunggongvirae	Uroviricota	Caudoviricetes	n.a.	Unclassified	Unclassified	Unclassified
S2-wms-combined_113619	Bacteroides phage LoVEphage	MW660583	Clustered	VC_832_0	N12	3	Bacteroides	Group I	Duplodnaviria	Heunggongvirae	Uroviricota	Caudoviricetes	n.a.	Unclassified	O	O
S2-wms-combined_126134	n.a.	n.a.	Clustered	VC_650_0	A	n.a.	n.a.	n.a.	n.a.	n.a.	n.a.	n.a.	n.a.	n.a.	n.a.	n.a.
S2-wms-combined_132966	Inovirus sp.	MT135284	Clustered	VC_1422_0	C1	28	Unspecified	Group II	Monodnaviria	Loebvirae	Hofneiviricota	Faserviricetes	Tubulavirales	Inoviridae	Unclassified	Inovirus
S2-wms-combined_135139	n.a.	n.a.	Clustered	VC_738_0	A	n.a.	n.a.	n.a.	n.a.	n.a.	n.a.	n.a.	n.a.	n.a.	n.a.	n.a.
S2-wms-combined_135154	n.a.	n.a.	Clustered	VC_947_0	A	n.a.	n.a.	n.a.	n.a.	n.a.	n.a.	n.a.	n.a.	n.a.	n.a.	n.a.

S2-wms-combined_165833	Faecalibacterium phage FP_Mushu	MG711460	Clustered	VC_1235_0	C3	67	Faecalibacterium	Group I	Duplodnaviria	Heunggongvirae	Uroviricota	Caudoviricetes	n.a.	Unclassified	Unclassified	Mushuvirus
S2-wms-combined_166665	Streptococcus phage SM1	AY007505	Clustered	VC_324_0	N6	8	Streptococcus	Group I	Duplodnaviria	Heunggongvirae	Uroviricota	Caudoviricetes	n.a.	Unclassified	O	O
S2-wms-combined_174241	Streptococcus phage Javan630	MK448997	Clustered	VC_626_0	C6	54	Streptococcus	Group I	Duplodnaviria	Heunggongvirae	Uroviricota	Caudoviricetes	n.a.	Unclassified	Unclassified	Unclassified
S2-wms-combined_176376	Clostridioides phage phiCD27	EU719189	Clustered	VC_825_0	N14	2	Clostridioides	Group I	Duplodnaviria	Heunggongvirae	Uroviricota	Caudoviricetes	n.a.	Unclassified	O	O
S2-wms-combined_180640	Faecalibacterium phage FP_Epona	MG711462	Clustered	VC_707_1	C2	54	Faecalibacterium	Group I	Duplodnaviria	Heunggongvirae	Uroviricota	Caudoviricetes	n.a.	Unclassified	Unclassified	Eponavirus
S2-wms-combined_181095	n.a.	n.a.	Clustered	VC_1481_0	F	n.a.	n.a.	n.a.	n.a.	n.a.	n.a.	n.a.	n.a.	n.a.	n.a.	n.a.
S2-wms-combined_196783	Clostridium phage phiCT19406B	KM983331	Clustered	VC_629_0	N10	9	Clostridium	Group I	Duplodnaviria	Heunggongvirae	Uroviricota	Caudoviricetes	n.a.	Unclassified	O	O
S2-wms-combined_197577	Phage DP SC_6_H4_2017	MT121964	Clustered	VC_1420_0	C1	138	Unspecified	Group I	Duplodnaviria	Heunggongvirae	Uroviricota	Caudoviricetes	n.a.	Unclassified	Unclassified	Unclassified
S2-wms-combined_207832	Faecalibacterium phage FP_Lagaffe	MG711461	Clustered	VC_717_0	C3	94	Faecalibacterium	Group I	Duplodnaviria	Heunggongvirae	Uroviricota	Caudoviricetes	n.a.	Unclassified	Unclassified	Lagaffevirus
S2-wms-combined_210822	n.a.	n.a.	Clustered	VC_1495_0	A	n.a.	n.a.	n.a.	n.a.	n.a.	n.a.	n.a.	n.a.	n.a.	n.a.	n.a.
S2-wms-combined_218002	Clostridioides phage CD1801	MW512570	Clustered	VC_148_1	N13	1	Clostridioides	Group I	Duplodnaviria	Heunggongvirae	Uroviricota	Caudoviricetes	n.a.	Unclassified	O	O
S2-wms-combined_227800	Bacillus phage vB_BtS_BMBtp13	KX190832	Clustered	VC_420_0	N7	5	Bacillus	Group I	Duplodnaviria	Heunggongvirae	Uroviricota	Caudoviricetes	n.a.	Unclassified	O	O
S2-wms-combined_229529	Phage vB_RanS_PJN03	ON245412	Clustered	VC_1473_0	N6	2	Unspecified	Group I	Duplodnaviria	Heunggongvirae	Uroviricota	Caudoviricetes	n.a.	Unclassified	O	O
S2-wms-combined_238929	Bacillus phage Carmen17	MG784342	Clustered	VC_732_0	N3	11	Bacillus	Group I	Duplodnaviria	Heunggongvirae	Uroviricota	Caudoviricetes	n.a.	Unclassified	O	O
S2-wms-combined_244564	Streptococcus phage Javan105	MK448667	Clustered	VC_626_0	C2	58	Streptococcus	Group I	Duplodnaviria	Heunggongvirae	Uroviricota	Caudoviricetes	n.a.	Unclassified	Unclassified	Unclassified
S2-wms-combined_246480	Roseburia phage Jekyll	LR596902	Clustered	VC_418_0	C4	14	Roseburia	Group I	Duplodnaviria	Heunggongvirae	Uroviricota	Caudoviricetes	n.a.	Unclassified	Unclassified	Unclassified
S2-wms-combined_248318	n.a.	n.a.	Clustered	VC_1498_0	F	n.a.	n.a.	n.a.	n.a.	n.a.	n.a.	n.a.	n.a.	n.a.	n.a.	n.a.
S2-wms-combined_248329	Butyrivibrio phage Idris	MN882554	Clustered	VC_734_0	N11	3	Butyrivibrio	Group I	Duplodnaviria	Heunggongvirae	Uroviricota	Caudoviricetes	n.a.	Unclassified	O	O
S2-wms-combined_31084	Clostridium phage Clo-PEP-1	KY206887	Clustered	VC_212_1	N4	11	Clostridium	Group I	Duplodnaviria	Heunggongvirae	Uroviricota	Caudoviricetes	n.a.	Unclassified	O	O
S2-wms-combined_4685	n.a.	n.a.	Clustered	VC_1484_0	A	n.a.	n.a.	n.a.	n.a.	n.a.	n.a.	n.a.	n.a.	n.a.	n.a.	n.a.
S2-wms-combined_55495	Geobacillus phage GBSV1	DQ340064	Clustered	VC_338_0	N8	8	Geobacillus	Group I	Duplodnaviria	Heunggongvirae	Uroviricota	Caudoviricetes	n.a.	Unclassified	O	O
S2-wms-combined_58121	n.a.	n.a.	Clustered	VC_1436_0	A	n.a.	n.a.	n.a.	n.a.	n.a.	n.a.	n.a.	n.a.	n.a.	n.a.	n.a.
S2-wms-combined_62377	Bifidobacterium phage BitterVaud1	MT006236	Clustered	VC_804_2	N7	33	Bifidobacterium	Group I	Duplodnaviria	Heunggongvirae	Uroviricota	Caudoviricetes	n.a.	Unclassified	O	O
S2-wms-combined_62519	Clostridioides phage CD1801	MW512570	Clustered	VC_148_0	N11	3	Clostridioides	Group I	Duplodnaviria	Heunggongvirae	Uroviricota	Caudoviricetes	n.a.	Unclassified	O	O
S2-wms-combined_6605	n.a.	n.a.	Clustered	VC_1484_0	A	n.a.	n.a.	n.a.	n.a.	n.a.	n.a.	n.a.	n.a.	n.a.	n.a.	n.a.
S2-wms-combined_85381	Faecalibacterium phage FP_Epona	MG711462	Clustered	VC_707_1	C6	34	Faecalibacterium	Group I	Duplodnaviria	Heunggongvirae	Uroviricota	Caudoviricetes	n.a.	Unclassified	Unclassified	Eponavirus
S2-wms-combined_89742	Burkholderia phage BcepMu	AY539836	Clustered	VC_610_0	N4	4	Burkholderia	Group I	Duplodnaviria	Heunggongvirae	Uroviricota	Caudoviricetes	n.a.	Unclassified	O	O
S2-wms-combined_93845	Faecalibacterium phage FP_Mushu	MG711460	Clustered	VC_271_0	N8	8	Faecalibacterium	Group I	Duplodnaviria	Heunggongvirae	Uroviricota	Caudoviricetes	n.a.	Unclassified	O	O
S2-wms_107329	Faecalibacterium phage FP_Brigit	MG711465	Clustered	VC_606_0	C3	131	Faecalibacterium	Group I	Duplodnaviria	Heunggongvirae	Uroviricota	Caudoviricetes	n.a.	Unclassified	Unclassified	Brigitvirus
S2-wms_110761	n.a.	n.a.	Clustered	VC_1496_0	F	n.a.	n.a.	n.a.	n.a.	n.a.	n.a.	n.a.	n.a.	n.a.	n.a.	n.a.
S2-wms_128831	Phage DP SC_6_H4_2017	MT121964	Clustered	VC_1420_0	C2	137	Unspecified	Group I	Duplodnaviria	Heunggongvirae	Uroviricota	Caudoviricetes	n.a.	Unclassified	Unclassified	Unclassified
S2-wms_128962	uncultured phage cr53_1	MT774396	Clustered	VC_502_0	N2	228	Unspecified	Group I	Duplodnaviria	Heunggongvirae	Uroviricota	Caudoviricetes	Crassvirales	Suoliviridae	O	O
S2-wms_130687	Solobacterium phage SMO_1P	MZ503613	Clustered	VC_417_0	C2	23	Solobacterium	Group I	Duplodnaviria	Heunggongvirae	Uroviricota	Caudoviricetes	n.a.	Unclassified	Unclassified	Unclassified
S2-wms_140933	Roseburia phage Jekyll	LR596902	Clustered	VC_418_0	C4	20	Roseburia	Group I	Duplodnaviria	Heunggongvirae	Uroviricota	Caudoviricetes	n.a.	Unclassified	Unclassified	Unclassified
S2-wms_156439	Bacteroides phage F4	MT806185	Clustered	VC_1435_0	N11	4	Bacteroides	Group I	Duplodnaviria	Heunggongvirae	Uroviricota	Caudoviricetes	n.a.	Unclassified	O	O
S2-wms_23562	n.a.	n.a.	Clustered	VC_1496_0	F	n.a.	n.a.	n.a.	n.a.	n.a.	n.a.	n.a.	n.a.	n.a.	n.a.	n.a.
S2-wms_248315	Faecalibacterium phage FP_oengus	MG711463	Clustered	VC_804_2	N7	45	Faecalibacterium	Group I	Duplodnaviria	Heunggongvirae	Uroviricota	Caudoviricetes	n.a.	Unclassified	O	O
S2-wms_53881	Bacillus phage Chedec 11	ON042750	Clustered	VC_821_0	N2	7	Bacillus	Group I	Duplodnaviria	Heunggongvirae	Uroviricota	Caudoviricetes	n.a.	Salasmaviridae	O	O
S2-wms_79304	Actinomyces phage Av-1	DQ123818	Clustered	VC_744_0	C1	19	Actinomyces	Group I	Duplodnaviria	Heunggongvirae	Uroviricota	Caudoviricetes	n.a.	Unclassified	Unclassified	Dybvigvirus
S2-wms_79944	Faecalibacterium phage FP_Brigit	MG711465	Clustered	VC_825_0	N9	16	Faecalibacterium	Group I	Duplodnaviria	Heunggongvirae	Uroviricota	Caudoviricetes	n.a.	Unclassified	O	O
S3-wms-combined_73262	Bacteroides phage p00	BK010646	Clustered	VC_677_0	C1	108	Bacteroides	Group I	Duplodnaviria	Heunggongvirae	Uroviricota	Caudoviricetes	n.a.	Unclassified	Unclassified	Unclassified
S3-wms_22744	Faecalibacterium phage FP_Lagaffe	MG711461	Clustered	VC_717_0	C3	102	Faecalibacterium	Group I	Duplodnaviria	Heunggongvirae	Uroviricota	Caudoviricetes	n.a.	Unclassified	Unclassified	Lagaffevirus
S3-wms_23958	Caldibacillus phage CBP1	MF595878	Clustered	VC_915_0	N18	5	Caldibacillus	Group I	Duplodnaviria	Heunggongvirae	Uroviricota	Caudoviricetes	n.a.	Unclassified	O	O
S4-wms-combined_113721	n.a.	n.a.	Clustered	VC_893_0	A	n.a.	n.a.	n.a.	n.a.	n.a.	n.a.	n.a.	n.a.	n.a.	n.a.	n.a.
S4-wms-combined_143879	n.a.	n.a.	Clustered	VC_1482_0	F	n.a.	n.a.	n.a.	n.a.	n.a.	n.a.	n.a.	n.a.	n.a.	n.a.	n.a.
S4-wms-combined_14411	Parabacteroides phage PDS1	MN929097	Clustered	VC_831_0	C2	150	Parabacteroides	Group I	Duplodnaviria	Heunggongvirae	Uroviricota	Caudoviricetes	n.a.	Unclassified	Unclassified	Unclassified
S4-wms-combined_17284	Bifidobacterium phage Bbif-1	GQ141189	Clustered	VC_852_0	C1	135	Bifidobacterium	Group I	Duplodnaviria	Heunggongvirae	Uroviricota	Caudoviricetes	n.a.	Unclassified	Unclassified	Unclassified
S4-wms-combined_41490	Burkholderia phage BcepMu	AY539836	Clustered	VC_610_0	N5	2	Burkholderia	Group I	Duplodnaviria	Heunggongvirae	Uroviricota	Caudoviricetes	n.a.	Unclassified	O	O
S4-wms-combined_42647	Faecalibacterium phage FP_Lugh	MG711464	Clustered	VC_621_0	C5	60	Faecalibacterium	Group I	Duplodnaviria	Heunggongvirae	Uroviricota	Caudoviricetes	n.a.	Unclassified	Unclassified	Lughvirus
S4-wms-combined_47565	n.a.	n.a.	Clustered	VC_1495_0	A	n.a.	n.a.	n.a.	n.a.	n.a.	n.a.	n.a.	n.a.	n.a.	n.a.	n.a.
S4-wms-combined_51079	n.a.	n.a.	Clustered	VC_1497_0	F	n.a.	n.a.	n.a.	n.a.	n.a.	n.a.	n.a.	n.a.	n.a.	n.a.	n.a.
S4-wms-combined_57123	n.a.	n.a.	Clustered	VC_1508_0	A	n.a.	n.a.	n.a.	n.a.	n.a.	n.a.	n.a.	n.a.	n.a.	n.a.	n.a.
S4-wms-combined_64632	Roseburia phage Jekyll	LR596902	Clustered	VC_418_0	C5	4	Roseburia	Group I	Duplodnaviria	Heunggongvirae	Uroviricota	Caudoviricetes	n.a.	Unclassified	Unclassified	Unclassified
S4-wms_107182	n.a.	n.a.	Clustered	VC_1513_0	A	n.a.	n.a.	n.a.	n.a.	n.a.	n.a.	n.a.	n.a.	n.a.	n.a.	n.a.
S4-wms_118690	Lactococcus phage phiQ1	AP019527	Clustered	VC_535_0	N2	7	Lactococcus	Group I	Duplodnaviria	Heunggongvirae	Uroviricota	Caudoviricetes	n.a.	Unclassified	O	O
S4-wms_141607	Bacteroides phage F4	MT806185	Clustered	VC_1008_0	C1	77	Bacteroides	Group I	Duplodnaviria	Heunggongvirae	Uroviricota	Caudoviricetes	n.a.	Unclassified	Unclassified	Unclassified
S4-wms_161443	Bacteriophage sp.	OP549880	Clustered	VC_1477_0	C2	25	Unspecified	Unclassified	Unclassified	n.a.	n.a.	n.a.	n.a.	Unclassified	Unclassified	Unclassified
S4-wms_45128	n.a.	n.a.	Clustered	VC_1439_0	A	n.a.	n.a.	n.a.	n.a.	n.a.	n.a.	n.a.	n.a.	n.a.	n.a.	n.a.
S4-wms_62635	Roseburia phage Jekyll	LR596902	Clustered	VC_418_0	C4	29	Roseburia	Group I	Duplodnaviria	Heunggongvirae	Uroviricota	Caudoviricetes	n.a.	Unclassified	Unclassified	Unclassified
S4-wms_73990	uncultured phage cr50_1	MT774375	Clustered	VC_502_1	C1	130	Unspecified	Group I	Duplodnaviria	Heunggongvirae	Uroviricota	Caudoviricetes	Crassvirales	Suoliviridae	Boorivirinae	Cohcovirus
S5-wms-combined_117213	Bifidobacterium phage Bbif-1	GQ141189	Clustered	VC_854_0	N3	19	Bifidobacterium	Group I	Duplodnaviria	Heunggongvirae	Uroviricota	Caudoviricetes	n.a.	Unclassified	O	O
S5-wms-combined_134381	Faecalibacterium phage FP_Lagaffe	MG711461	Clustered	VC_717_0	C3	84	Faecalibacterium	Group I	Duplodnaviria	Heunggongvirae	Uroviricota	Caudoviricetes	n.a.	Unclassified	Unclassified	Lagaffevirus
S5-wms-combined_145067	Clostridium phage phiCT453B	KM983328	Clustered	VC_650_0	N11	8	Clostridium	Group I	Duplodnaviria	Heunggongvirae	Uroviricota	Caudoviricetes	n.a.	Unclassified	O	O
S5-wms-combined_178432	n.a.	n.a.	Clustered	VC_1506_0	A	n.a.	n.a.	n.a.	n.a.	n.a.	n.a.	n.a.	n.a.	n.a.	n.a.	n.a.
S5-wms-combined_18356	Escherichia phage ESSI2_ev239	LR597637	Clustered	VC_5_0	C1	58	Escherichia	Group I	Duplodnaviria	Heunggongvirae	Uroviricota	Caudoviricetes	n.a.	Peduoviridae	Unclassified	Eevirus
S5-wms-combined_206050	Streptococcus phage Javan105	MK448667	Clustered	VC_626_0	C2	92	Streptococcus	Group I	Duplodnaviria	Heunggongvirae	Uroviricota	Caudoviricetes	n.a.	Unclassified	Unclassified	Unclassified
S5-wms-combined_207291	n.a.	n.a.	Clustered	VC_1437_0	A	n.a.	n.a.	n.a.	n.a.	n.a.	n.a.	n.a.	n.a.	n.a.	n.a.	n.a.
S5-wms-combined_26971	Butyrivibrio phage Idris	MN882554	Clustered	VC_876_0	N8	29	Butyrivibrio	Group I	Duplodnaviria	Heunggongvirae	Uroviricota	Caudoviricetes	n.a.	Unclassified	O	O
S5-wms-combined_29272	Streptococcus phage Javan630	MK448997	Clustered	VC_626_0	C7	55	Streptococcus	Group I	Duplodnaviria	Heunggongvirae	Uroviricota	Caudoviricetes	n.a.	Unclassified	Unclassified	Unclassified
S5-wms-combined_29447	Winogradskyella phage Peternella_1	MT732475	Clustered	VC_676_0	N8	17	Winogradskyella	Group I	Duplodnaviria	Heunggongvirae	Uroviricota	Caudoviricetes	n.a.	Winoviridae	O	O
S5-wms-combined_39793	n.a.	n.a.	Clustered	VC_1514_0	F	n.a.	n.a.	n.a.	n.a.	n.a.	n.a.	n.a.	n.a.	n.a.	n.a.	n.a.
S5-wms-combined_52741	Akkermansia phage DTMo-2021a	CP084202	Clustered	VC_736_0	N2	17	Akkermansia	Group I	Duplodnaviria	Heunggongvirae	Uroviricota	Caudoviricetes	n.a.	Unclassified	O	O
S5-wms-combined_59064	Bacteroides phage p00	BK010646	Clustered	VC_677_0	C1	75	Bacteroides	Group I	Duplodnaviria	Heunggongvirae	Uroviricota	Caudoviricetes	n.a.	Unclassified	Unclassified	Unclassified
S5-wms-combined_66017	n.a.	n.a.	Clustered	VC_1499_0	F	n.a.	n.a.	n.a.	n.a.	n.a.	n.a.	n.a.	n.a.	n.a.	n.a.	n.a.
S5-wms-combined_7292	Caldibacillus phage CBP1	MF595878	Clustered	VC_734_0	C2	8	Caldibacillus	Group I	Duplodnaviria	Heunggongvirae	Uroviricota	Caudoviricetes	n.a.	Unclassified	Unclassified	Unclassified
S5-wms-combined_73301	Clostridium phage phiCD38-2	HM568888	Clustered	VC_627_0	N4	8	Clostridium	Group I	Duplodnaviria	Heunggongvirae	Uroviricota	Caudoviricetes	n.a.	Unclassified	O	O
S5-wms-combined_78434	n.a.	n.a.	Clustered	VC_1479_0	A	n.a.	n.a.	n.a.	n.a.	n.a.	n.a.	n.a.	n.a.	n.a.	n.a.	n.a.
S5-wms-combined_83370	Bacteroides phage p00	BK010646	Clustered	VC_680_0	N2	29	Bacteroides	Group I	Duplodnaviria	Heunggongvirae	Uroviricota	Caudoviricetes	n.a.	Unclassified	O	O
S5-wms-combined_83412	Clostridium phage phiSM101	CP000315	Clustered	VC_19_0	C1	15	Clostridium	Group I	Duplodnaviria	Heunggongvirae	Uroviricota	Caudoviricetes	n.a.	Unclassified	Unclassified	Unclassified
S5-wms-combined_98202	n.a.	n.a.	Clustered	VC_1503_0	A	n.a.	n.a.	n.a.	n.a.	n.a.	n.a.	n.a.	n.a.	n.a.	n.a.	n.a.
S5-wms_210460	n.a.	n.a.	Clustered	VC_1499_0	F	n.a.	n.a.	n.a.	n.a.	n.a.	n.a.	n.a.	n.a.	n.a.	n.a.	n.a.
S5-wms_49310	Solobacterium phage SMO_1P	MZ503613	Clustered	VC_417_0	C2	28	Solobacterium	Group I	Duplodnaviria	Heunggongvirae	Uroviricota	Caudoviricetes	n.a.	Unclassified	Unclassified	Unclassified
S5-wms_52566	n.a.	n.a.	Clustered	VC_1481_0	F	n.a.	n.a.	n.a.	n.a.	n.a.	n.a.	n.a.	n.a.	n.a.	n.a.	n.a.
S5-wms_85650	n.a.	n.a.	Clustered	VC_1505_0	A	n.a.	n.a.	n.a.	n.a.	n.a.	n.a.	n.a.	n.a.	n.a.	n.a.	n.a.
S5-wms_94965	Bifidobacterium phage BigBern1	MT006237	Clustered	VC_1198_0	C2	16	Bifidobacterium	Group I	Duplodnaviria	Heunggongvirae	Uroviricota	Caudoviricetes	n.a.	Unclassified	Unclassified	Unclassified
S6-wms-combined_113986	Clostridium phage phiCD38-2	HM568888	Clustered	VC_627_0	N3	9	Clostridium	Group I	Duplodnaviria	Heunggongvirae	Uroviricota	Caudoviricetes	n.a.	Unclassified	O	O

S6-wms-combined_117756	Faecalibacterium phage FP_Epona	MG711462	Clustered	VC_707_1	C3	96	Faecalibacterium	Group I	Duplodnaviria	Heunggongvirae	Uroviricota	Caudoviricetes	n.a.	Unclassified	Unclassified	Eponavirus
S6-wms-combined_118484	Bacteriophage sp.	LT985574	Clustered	VC_338_0	N10	5	Unspecified	Unclassified	Unclassified	n.a.	n.a.	n.a.	n.a.	Unclassified	O	O
S6-wms-combined_119017	Pseudomonas phage phi3	KT887559	Clustered	VC_5_0	C2	39	Pseudomonas	Group I	Duplodnaviria	Heunggongvirae	Uroviricota	Caudoviricetes	n.a.	Peduoviridae	Unclassified	Phitrevirus
S6-wms-combined_119138	Clostridioides phage CD2301	MW512571	Clustered	VC_148_1	N11	2	Clostridioides	Group I	Duplodnaviria	Heunggongvirae	Uroviricota	Caudoviricetes	n.a.	Unclassified	O	O
S6-wms-combined_126236	Faecalibacterium phage FP_Toutatis	MG711466	Clustered	VC_710_0	C2	64	Faecalibacterium	Group I	Duplodnaviria	Heunggongvirae	Uroviricota	Caudoviricetes	n.a.	Unclassified	Unclassified	Toutatisvirus
S6-wms-combined_135223	n.a.	n.a.	Clustered	VC_1508_0	A	n.a.	n.a.	n.a.	n.a.	n.a.	n.a.	n.a.	n.a.	n.a.	n.a.	n.a.
S6-wms-combined_140731	n.a.	n.a.	Clustered	VC_825_0	A	n.a.	n.a.	n.a.	n.a.	n.a.	n.a.	n.a.	n.a.	n.a.	n.a.	n.a.
S6-wms-combined_32530	Clostridioides phage CD1801	MW512570	Clustered	VC_148_1	N10	2	Clostridioides	Group I	Duplodnaviria	Heunggongvirae	Uroviricota	Caudoviricetes	n.a.	Unclassified	O	O
S6-wms-combined_41246	n.a.	n.a.	Clustered	VC_1484_0	A	n.a.	n.a.	n.a.	n.a.	n.a.	n.a.	n.a.	n.a.	n.a.	n.a.	n.a.
S6-wms-combined_49909	Streptococcus phage Javan35	MK448736	Clustered	VC_324_0	N6	8	Streptococcus	Group I	Duplodnaviria	Heunggongvirae	Uroviricota	Caudoviricetes	n.a.	Unclassified	O	O
S6-wms-combined_50721	Winogradskyella phage Peternella_1	MT732475	Clustered	VC_676_1	N11	16	Winogradskyella	Group I	Duplodnaviria	Heunggongvirae	Uroviricota	Caudoviricetes	n.a.	Winoviridae	O	O
S6-wms-combined_72228	n.a.	n.a.	Clustered	VC_1506_0	A	n.a.	n.a.	n.a.	n.a.	n.a.	n.a.	n.a.	n.a.	n.a.	n.a.	n.a.
S6-wms-combined_8508	Faecalibacterium phage FP_Epona	MG711462	Clustered	VC_707_0	N11	42	Faecalibacterium	Group I	Duplodnaviria	Heunggongvirae	Uroviricota	Caudoviricetes	n.a.	Unclassified	O	O
S6-wms-combined_91039	n.a.	n.a.	Clustered	VC_1456_0	A	n.a.	n.a.	n.a.	n.a.	n.a.	n.a.	n.a.	n.a.	n.a.	n.a.	n.a.
S6-wms_132588	Clostridium phage Clo-PEP-1	KY206887	Clustered	VC_212_0	N4	18	Clostridium	Group I	Duplodnaviria	Heunggongvirae	Uroviricota	Caudoviricetes	n.a.	Unclassified	O	O
S6-wms_137591	n.a.	n.a.	Clustered	VC_1496_0	F	n.a.	n.a.	n.a.	n.a.	n.a.	n.a.	n.a.	n.a.	n.a.	n.a.	n.a.
S6-wms_138342	uncultured phage cr53_1	MT774396	Clustered	VC_502_0	N2	233	Unspecified	Group I	Duplodnaviria	Heunggongvirae	Uroviricota	Caudoviricetes	Crassvirales	Suoliviridae	O	O
S6-wms_54578	Parabacteroides phage PDS1	MN929097	Clustered	VC_831_0	C3	113	Parabacteroides	Group I	Duplodnaviria	Heunggongvirae	Uroviricota	Caudoviricetes	n.a.	Unclassified	Unclassified	Unclassified
S7-wms-combined_104872	n.a.	n.a.	Clustered	VC_1437_0	A	n.a.	n.a.	n.a.	n.a.	n.a.	n.a.	n.a.	n.a.	n.a.	n.a.	n.a.
S7-wms-combined_107679_0	Winogradskyella phage Peternella_1	MT732475	Clustered	VC_676_1	N11	17	Winogradskyella	Group I	Duplodnaviria	Heunggongvirae	Uroviricota	Caudoviricetes	n.a.	Winoviridae	O	O
S7-wms-combined_111004	Faecalibacterium phage FP_Taranis	MG711467	Clustered	VC_711_0	C1	200	Faecalibacterium	Group I	Duplodnaviria	Heunggongvirae	Uroviricota	Caudoviricetes	n.a.	Unclassified	Unclassified	Taranisvirus
S7-wms-combined_134458	Faecalibacterium phage FP_Toutatis	MG711466	Clustered	VC_710_0	C4	73	Faecalibacterium	Group I	Duplodnaviria	Heunggongvirae	Uroviricota	Caudoviricetes	n.a.	Unclassified	Unclassified	Toutatisvirus
S7-wms-combined_136642	n.a.	n.a.	Clustered	VC_1497_0	F	n.a.	n.a.	n.a.	n.a.	n.a.	n.a.	n.a.	n.a.	n.a.	n.a.	n.a.
S7-wms-combined_139683	Butyrivibrio phage Idris	MN882554	Clustered	VC_876_0	N6	34	Butyrivibrio	Group I	Duplodnaviria	Heunggongvirae	Uroviricota	Caudoviricetes	n.a.	Unclassified	O	O
S7-wms-combined_14267	n.a.	n.a.	Clustered	VC_1456_0	A	n.a.	n.a.	n.a.	n.a.	n.a.	n.a.	n.a.	n.a.	n.a.	n.a.	n.a.
S7-wms-combined_143218	n.a.	n.a.	Clustered	VC_1514_0	F	n.a.	n.a.	n.a.	n.a.	n.a.	n.a.	n.a.	n.a.	n.a.	n.a.	n.a.
S7-wms-combined_147158	Microbacterium phage Min1	EF579802	Clustered	VC_798_0	N6	14	Microbacterium	Group I	Duplodnaviria	Heunggongvirae	Uroviricota	Caudoviricetes	n.a.	Unclassified	O	O
S7-wms-combined_163980	n.a.	n.a.	Clustered	VC_1014_0	A	n.a.	n.a.	n.a.	n.a.	n.a.	n.a.	n.a.	n.a.	n.a.	n.a.	n.a.
S7-wms-combined_185016	Bacteroides phage F4	MT806185	Clustered	VC_1435_0	N11	3	Bacteroides	Group I	Duplodnaviria	Heunggongvirae	Uroviricota	Caudoviricetes	n.a.	Unclassified	O	O
S7-wms-combined_189250	Bifidobacterium phage BigBern1	MT006237	Clustered	VC_1198_0	C2	5	Bifidobacterium	Group I	Duplodnaviria	Heunggongvirae	Uroviricota	Caudoviricetes	n.a.	Unclassified	Unclassified	Unclassified
S7-wms-combined_225260	Clostridium phage HM2	LT600745	Clustered	VC_822_0	C1	4	Clostridium	Group I	Duplodnaviria	Heunggongvirae	Uroviricota	Caudoviricetes	n.a.	Salasmaviridae	Unclassified	Unclassified
S7-wms-combined_235165	Erysipelothrix phage SE-1	KR816341	Clustered	VC_1109_0	C1	12	Erysipelothrix	Group I	Duplodnaviria	Heunggongvirae	Uroviricota	Caudoviricetes	n.a.	Unclassified	Unclassified	Unclassified
S7-wms-combined_239784	n.a.	n.a.	Clustered	VC_1436_0	A	n.a.	n.a.	n.a.	n.a.	n.a.	n.a.	n.a.	n.a.	n.a.	n.a.	n.a.
S7-wms-combined_28138	Listeria phage LP-030-2	JX120799	Clustered	VC_420_0	N8	6	Listeria	Group I	Duplodnaviria	Heunggongvirae	Uroviricota	Caudoviricetes	n.a.	Unclassified	O	O
S7-wms-combined_31068	n.a.	n.a.	Clustered	VC_650_0	A	n.a.	n.a.	n.a.	n.a.	n.a.	n.a.	n.a.	n.a.	n.a.	n.a.	n.a.
S7-wms-combined_40779	n.a.	n.a.	Clustered	VC_1239_0	A	n.a.	n.a.	n.a.	n.a.	n.a.	n.a.	n.a.	n.a.	n.a.	n.a.	n.a.
S7-wms-combined_43950	Bacteroides phage p00	BK010646	Clustered	VC_677_0	C1	129	Bacteroides	Group I	Duplodnaviria	Heunggongvirae	Uroviricota	Caudoviricetes	n.a.	Unclassified	Unclassified	Unclassified
S7-wms-combined_5625	Bacteroides phage p00	BK010646	Clustered	VC_677_0	C4	35	Bacteroides	Group I	Duplodnaviria	Heunggongvirae	Uroviricota	Caudoviricetes	n.a.	Unclassified	Unclassified	Unclassified
S7-wms-combined_7205	Clostridium phage Clo-PEP-1	KY206887	Clustered	VC_212_1	N5	16	Clostridium	Group I	Duplodnaviria	Heunggongvirae	Uroviricota	Caudoviricetes	n.a.	Unclassified	O	O
S7-wms-combined_72070	n.a.	n.a.	Clustered	VC_1509_0	A	n.a.	n.a.	n.a.	n.a.	n.a.	n.a.	n.a.	n.a.	n.a.	n.a.	n.a.
S7-wms-combined_84688	n.a.	n.a.	Clustered	VC_1484_0	A	n.a.	n.a.	n.a.	n.a.	n.a.	n.a.	n.a.	n.a.	n.a.	n.a.	n.a.
S7-wms-combined_90393	Bacteroides phage F4	MT806185	Clustered	VC_1435_0	N10	8	Bacteroides	Group I	Duplodnaviria	Heunggongvirae	Uroviricota	Caudoviricetes	n.a.	Unclassified	O	O
S7-wms-combined_90728	Bacteroides phage F4	MT806185	Clustered	VC_1435_0	N6	11	Bacteroides	Group I	Duplodnaviria	Heunggongvirae	Uroviricota	Caudoviricetes	n.a.	Unclassified	O	O
S7-wms_113137	Faecalibacterium phage FP_Mushu	MG711460	Clustered	VC_1235_0	C1	170	Faecalibacterium	Group I	Duplodnaviria	Heunggongvirae	Uroviricota	Caudoviricetes	n.a.	Unclassified	Unclassified	Mushuvirus
S7-wms_118607	Faecalibacterium phage FP_Lagaffe	MG711461	Clustered	VC_717_0	C1	230	Faecalibacterium	Group I	Duplodnaviria	Heunggongvirae	Uroviricota	Caudoviricetes	n.a.	Unclassified	Unclassified	Lagaffevirus
S7-wms_123954	n.a.	n.a.	Clustered	VC_1435_0	A	n.a.	n.a.	n.a.	n.a.	n.a.	n.a.	n.a.	n.a.	n.a.	n.a.	n.a.
S7-wms_127801	Clostridium phage phiCD6356	GU949551	Clustered	VC_420_0	N4	6	Clostridium	Group I	Duplodnaviria	Heunggongvirae	Uroviricota	Caudoviricetes	n.a.	Unclassified	O	O
S7-wms_157264	Bacteriophage sp.	OP549880	Clustered	VC_1477_0	C2	24	Unspecified	Unclassified	Unclassified	n.a.	n.a.	n.a.	n.a.	Unclassified	Unclassified	Unclassified
S7-wms_159432	Faecalibacterium phage FP_Epona	MG711462	Clustered	VC_707_1	C4	67	Faecalibacterium	Group I	Duplodnaviria	Heunggongvirae	Uroviricota	Caudoviricetes	n.a.	Unclassified	Unclassified	Eponavirus
S7-wms_200936	Faecalibacterium phage FP_Mushu	MG711460	Clustered	VC_1235_0	C2	142	Faecalibacterium	Group I	Duplodnaviria	Heunggongvirae	Uroviricota	Caudoviricetes	n.a.	Unclassified	Unclassified	Mushuvirus
S7-wms_239833	Bacillus phage vB_BsuP-Goe1	KU831549	Clustered	VC_821_0	N1	10	Bacillus	Group I	Duplodnaviria	Heunggongvirae	Uroviricota	Caudoviricetes	n.a.	Salasmaviridae	O	O
S7-wms_244425	uncultured phage cr127_1	MT774393	Clustered	VC_675_0	C1	70	Unspecified	Group I	Duplodnaviria	Heunggongvirae	Uroviricota	Caudoviricetes	Crassvirales	Crevaviridae	Doltivirinae	Kahucivirus
S7-wms_244866	Bacteroides phage EMB2	ON721385	Clustered	VC_1009_0	C1	149	Bacteroides	Group I	Duplodnaviria	Heunggongvirae	Uroviricota	Caudoviricetes	n.a.	Unclassified	Unclassified	Unclassified
S7-wms_53338	Enterococcus phage Athos	LR990834	Clustered	VC_821_0	N1	9	Enterococcus	Group I	Duplodnaviria	Heunggongvirae	Uroviricota	Caudoviricetes	n.a.	Salasmaviridae	O	O
S7-wms_80255	Streptococcus phage Javan249	MK448716	Clustered	VC_629_0	N2	10	Streptococcus	Group I	Duplodnaviria	Heunggongvirae	Uroviricota	Caudoviricetes	n.a.	Unclassified	O	O
S8-wms-combined_108917	Streptococcus phage Javan630	MK448997	Clustered	VC_626_0	C7	54	Streptococcus	Group I	Duplodnaviria	Heunggongvirae	Uroviricota	Caudoviricetes	n.a.	Unclassified	Unclassified	Unclassified
S8-wms-combined_134098	n.a.	n.a.	Clustered	VC_1239_0	A	n.a.	n.a.	n.a.	n.a.	n.a.	n.a.	n.a.	n.a.	n.a.	n.a.	n.a.
S8-wms-combined_134284	n.a.	n.a.	Clustered	VC_1236_0	A	n.a.	n.a.	n.a.	n.a.	n.a.	n.a.	n.a.	n.a.	n.a.	n.a.	n.a.
S8-wms-combined_140059	n.a.	n.a.	Clustered	VC_420_0	A	n.a.	n.a.	n.a.	n.a.	n.a.	n.a.	n.a.	n.a.	n.a.	n.a.	n.a.
S8-wms-combined_146562	Faecalibacterium phage FP_Mushu	MG711460	Clustered	VC_271_0	N8	13	Faecalibacterium	Group I	Duplodnaviria	Heunggongvirae	Uroviricota	Caudoviricetes	n.a.	Unclassified	O	O
S8-wms-combined_161181	Clostridioides phage CD1801	MW512570	Clustered	VC_148_0	N12	4	Clostridioides	Group I	Duplodnaviria	Heunggongvirae	Uroviricota	Caudoviricetes	n.a.	Unclassified	O	O
S8-wms-combined_169835	n.a.	n.a.	Clustered	VC_915_0	A	n.a.	n.a.	n.a.	n.a.	n.a.	n.a.	n.a.	n.a.	n.a.	n.a.	n.a.
S8-wms-combined_186054	Streptococcus phage Javan630	MK448997	Clustered	VC_626_0	C5	72	Streptococcus	Group I	Duplodnaviria	Heunggongvirae	Uroviricota	Caudoviricetes	n.a.	Unclassified	Unclassified	Unclassified
S8-wms-combined_200168	Faecalibacterium phage FP_Epona	MG711462	Clustered	VC_707_1	C2	42	Faecalibacterium	Group I	Duplodnaviria	Heunggongvirae	Uroviricota	Caudoviricetes	n.a.	Unclassified	Unclassified	Eponavirus
S8-wms-combined_211119	n.a.	n.a.	Clustered	VC_1236_0	A	n.a.	n.a.	n.a.	n.a.	n.a.	n.a.	n.a.	n.a.	n.a.	n.a.	n.a.
S8-wms-combined_215102	n.a.	n.a.	Clustered	VC_1504_0	A	n.a.	n.a.	n.a.	n.a.	n.a.	n.a.	n.a.	n.a.	n.a.	n.a.	n.a.
S8-wms-combined_217866	Faecalibacterium phage FP_Epona	MG711462	Clustered	VC_707_1	C4	84	Faecalibacterium	Group I	Duplodnaviria	Heunggongvirae	Uroviricota	Caudoviricetes	n.a.	Unclassified	Unclassified	Eponavirus
S8-wms-combined_226076	n.a.	n.a.	Clustered	VC_1511_0	F	n.a.	n.a.	n.a.	n.a.	n.a.	n.a.	n.a.	n.a.	n.a.	n.a.	n.a.
S8-wms-combined_234473	n.a.	n.a.	Clustered	VC_1481_0	F	n.a.	n.a.	n.a.	n.a.	n.a.	n.a.	n.a.	n.a.	n.a.	n.a.	n.a.
S8-wms-combined_246185	Akkermansia phage DTMo-2021a	CP084202	Clustered	VC_738_0	N3	1	Akkermansia	Group I	Duplodnaviria	Heunggongvirae	Uroviricota	Caudoviricetes	n.a.	Unclassified	O	O
S8-wms-combined_25607	Faecalibacterium phage FP_Taranis	MG711467	Clustered	VC_1239_0	N20	2	Faecalibacterium	Group I	Duplodnaviria	Heunggongvirae	Uroviricota	Caudoviricetes	n.a.	Unclassified	O	O
S8-wms-combined_26087	Burkholderia phage BcepMu	AY539836	Clustered	VC_610_0	N5	1	Burkholderia	Group I	Duplodnaviria	Heunggongvirae	Uroviricota	Caudoviricetes	n.a.	Unclassified	O	O
S8-wms-combined_264711	n.a.	n.a.	Clustered	VC_1481_0	F	n.a.	n.a.	n.a.	n.a.	n.a.	n.a.	n.a.	n.a.	n.a.	n.a.	n.a.
S8-wms-combined_270362	Phage vB_RanS_PJN03	ON245412	Clustered	VC_917_0	N10	6	Unspecified	Group I	Duplodnaviria	Heunggongvirae	Uroviricota	Caudoviricetes	n.a.	Unclassified	O	O
S8-wms-combined_276204	n.a.	n.a.	Clustered	VC_1501_0	A	n.a.	n.a.	n.a.	n.a.	n.a.	n.a.	n.a.	n.a.	n.a.	n.a.	n.a.
S8-wms-combined_276211	Butyrivibrio virus Ceridwen	MN882553	Clustered	VC_1209_0	N10	11	Butyrivibrio	Group I	Duplodnaviria	Heunggongvirae	Uroviricota	Caudoviricetes	n.a.	Unclassified	O	O
S8-wms-combined_49369	n.a.	n.a.	Clustered	VC_1236_0	A	n.a.	n.a.	n.a.	n.a.	n.a.	n.a.	n.a.	n.a.	n.a.	n.a.	n.a.
S8-wms-combined_68780	n.a.	n.a.	Clustered	VC_271_0	A	n.a.	n.a.	n.a.	n.a.	n.a.	n.a.	n.a.	n.a.	n.a.	n.a.	n.a.
S8-wms-combined_72911	n.a.	n.a.	Clustered	VC_1076_0	A	n.a.	n.a.	n.a.	n.a.	n.a.	n.a.	n.a.	n.a.	n.a.	n.a.	n.a.
S8-wms-combined_97848	Bacteroides phage p00	BK010646	Clustered	VC_677_0	C4	36	Bacteroides	Group I	Duplodnaviria	Heunggongvirae	Uroviricota	Caudoviricetes	n.a.	Unclassified	Unclassified	Unclassified
S8-wms_127191	Faecalibacterium phage FP_oengus	MG711463	Clustered	VC_804_2	N2	66	Faecalibacterium	Group I	Duplodnaviria	Heunggongvirae	Uroviricota	Caudoviricetes	n.a.	Unclassified	O	O
S8-wms_152831	Faecalibacterium phage FP_Toutatis	MG711466	Clustered	VC_710_0	C1	82	Faecalibacterium	Group I	Duplodnaviria	Heunggongvirae	Uroviricota	Caudoviricetes	n.a.	Unclassified	Unclassified	Toutatisvirus
S8-wms_244525	Bacillus phage BeachBum	KY921761	Clustered	VC_821_0	N1	10	Bacillus	Group I	Duplodnaviria	Heunggongvirae	Uroviricota	Caudoviricetes	n.a.	Salasmaviridae	O	O
S8-wms_30498	Faecalibacterium phage FP_Mushu	MG711460	Clustered	VC_1235_0	C4	112	Faecalibacterium	Group I	Duplodnaviria	Heunggongvirae	Uroviricota	Caudoviricetes	n.a.	Unclassified	Unclassified	Mushuvirus
S8-wms_48935	Faecalibacterium phage FP_Toutatis	MG711466	Clustered	VC_710_0	C6	71	Faecalibacterium	Group I	Duplodnaviria	Heunggongvirae	Uroviricota	Caudoviricetes	n.a.	Unclassified	Unclassified	Toutatisvirus
S8-wms_57121	n.a.	n.a.	Clustered	VC_1501_0	A	n.a.	n.a.	n.a.	n.a.	n.a.	n.a.	n.a.	n.a.	n.a.	n.a.	n.a.

S8-wms_82880	Faecalibacterium phage FP_Brigit	MG711465	Clustered	VC_606_0	C3	104	Faecalibacterium	Group I	Duplodnaviria	Heunggongvirae	Uroviricota	Caudoviricetes	n.a.	Unclassified	Unclassified	Brigitvirus
S9-wms-combined_105462	Clostridioides phage phiCD27	EU719189	Clustered	VC_825_0	N9	3	Clostridioides	Group I	Duplodnaviria	Heunggongvirae	Uroviricota	Caudoviricetes	n.a.	Unclassified	O	O
S9-wms-combined_25007	Streptococcus phage Javan422	MK448934	Clustered	VC_626_0	C1	52	Streptococcus	Group I	Duplodnaviria	Heunggongvirae	Uroviricota	Caudoviricetes	n.a.	Unclassified	Unclassified	Unclassified
S9-wms-combined_26410	Faecalibacterium phage FP_oengus	MG711463	Clustered	VC_804_2	N8	48	Faecalibacterium	Group I	Duplodnaviria	Heunggongvirae	Uroviricota	Caudoviricetes	n.a.	Unclassified	O	O
S9-wms-combined_28805	Winogradskyella phage Peternella_1	MT732475	Clustered	VC_676_1	N11	18	Winogradskyella	Group I	Duplodnaviria	Heunggongvirae	Uroviricota	Caudoviricetes	n.a.	Winoviridae	O	O
S9-wms-combined_31456	Bacteroides phage p00	BK010646	Clustered	VC_677_0	C4	33	Bacteroides	Group I	Duplodnaviria	Heunggongvirae	Uroviricota	Caudoviricetes	n.a.	Unclassified	Unclassified	Unclassified
S9-wms-combined_33229	Bacteroides phage F2	MT806187	Clustered	VC_1435_0	N11	7	Bacteroides	Group I	Duplodnaviria	Heunggongvirae	Uroviricota	Caudoviricetes	n.a.	Unclassified	O	O
S9-wms-combined_33230	Bacteroides phage F4	MT806185	Clustered	VC_1435_0	N11	3	Bacteroides	Group I	Duplodnaviria	Heunggongvirae	Uroviricota	Caudoviricetes	n.a.	Unclassified	O	O
S9-wms-combined_36459	Streptococcus phage Javan553	MK448804	Clustered	VC_338_0	N6	6	Streptococcus	Group I	Duplodnaviria	Heunggongvirae	Uroviricota	Caudoviricetes	n.a.	Unclassified	O	O
S9-wms-combined_37587	Phage vB_RanS_PJN03	ON245412	Clustered	VC_1473_0	N5	4	Unspecified	Group I	Duplodnaviria	Heunggongvirae	Uroviricota	Caudoviricetes	n.a.	Unclassified	O	O
S9-wms-combined_3878	n.a.	n.a.	Clustered	VC_1510_0	F	n.a.	n.a.	n.a.	n.a.	n.a.	n.a.	n.a.	n.a.	n.a.	n.a.	n.a.
S9-wms-combined_44402	Bacteroides phage p00	BK010646	Clustered	VC_680_0	N2	31	Bacteroides	Group I	Duplodnaviria	Heunggongvirae	Uroviricota	Caudoviricetes	n.a.	Unclassified	O	O
S9-wms-combined_53991	Phage WC36-2	OL791266	Clustered	VC_947_0	N1	24	Unspecified	Group I	Duplodnaviria	Heunggongvirae	Uroviricota	Caudoviricetes	n.a.	Unclassified	O	O
S9-wms-combined_5526	Bacteroides phage LoVEphage	MW660583	Clustered	VC_832_0	N10	1	Bacteroides	Group I	Duplodnaviria	Heunggongvirae	Uroviricota	Caudoviricetes	n.a.	Unclassified	O	O
S9-wms-combined_56773	n.a.	n.a.	Clustered	VC_1481_0	F	n.a.	n.a.	n.a.	n.a.	n.a.	n.a.	n.a.	n.a.	n.a.	n.a.	n.a.
S9-wms-combined_64277	Burkholderia phage phiE255	CP000622	Clustered	VC_250_0	C1	83	Burkholderia	Group I	Duplodnaviria	Heunggongvirae	Uroviricota	Caudoviricetes	n.a.	Unclassified	Unclassified	Bcepmuvirus
S9-wms-combined_71647	Bacillus phage vB_BtS_BMBtp13	KX190832	Clustered	VC_420_0	N6	5	Bacillus	Group I	Duplodnaviria	Heunggongvirae	Uroviricota	Caudoviricetes	n.a.	Unclassified	O	O
S9-wms-combined_76274	Bifidobacterium phage Bbif-1	GQ141189	Clustered	VC_854_0	N3	19	Bifidobacterium	Group I	Duplodnaviria	Heunggongvirae	Uroviricota	Caudoviricetes	n.a.	Unclassified	O	O
S9-wms-combined_89249	Roseburia phage Jekyll	LR596902	Clustered	VC_947_0	N4	3	Roseburia	Group I	Duplodnaviria	Heunggongvirae	Uroviricota	Caudoviricetes	n.a.	Unclassified	O	O
S9-wms_104169	Inovirus sp.	MT135285	Clustered	VC_1423_0	C1	13	Unspecified	Group II	Monodnaviria	Loebvirae	Hofneiviricota	Faserviricetes	Tubulavirales	Inoviridae	Unclassified	Inovirus
S9-wms_128232	Tortoise microvirus 13	MK765563	Clustered	VC_1243_0	C1	26	Unspecified	Group II	Monodnaviria	Sangervirae	Phixviricota	Malgrandaviricetes	Petitvirales	Microviridae	Unclassified	Unclassified
S9-wms_19025	Lactococcus phage phiQ1	AP019527	Clustered	VC_535_0	N2	9	Lactococcus	Group I	Duplodnaviria	Heunggongvirae	Uroviricota	Caudoviricetes	n.a.	Unclassified	O	O
S9-wms_64472	n.a.	n.a.	Clustered	VC_1512_0	F	n.a.	n.a.	n.a.	n.a.	n.a.	n.a.	n.a.	n.a.	n.a.	n.a.	n.a.
S1-wms-combined_220022	n.a.	n.a.	Clustered/Singleton	VC_1408_1	A	n.a.	n.a.	n.a.	n.a.	n.a.	n.a.	n.a.	n.a.	n.a.	n.a.	n.a.
S1-wms-combined_85225	Propionibacterium phage Anatole	KX620748	Clustered/Singleton	VC_798_1	N4	12	Propionibacterium	Group I	Duplodnaviria	Heunggongvirae	Uroviricota	Caudoviricetes	n.a.	Unclassified	O	O
S1-wms_102342	Bacillus phage vB_BcoS-136	MH884508	Clustered/Singleton	VC_719_0	N2	1	Bacillus	Group I	Duplodnaviria	Heunggongvirae	Uroviricota	Caudoviricetes	n.a.	Unclassified	O	O
S11-wms-combined_264050	Faecalibacterium phage FP_Epona	MG711462	Clustered/Singleton	VC_707_3	C1	64	Faecalibacterium	Group I	Duplodnaviria	Heunggongvirae	Uroviricota	Caudoviricetes	n.a.	Unclassified	Unclassified	O
S11-wms-combined_267993	Bacillus phage BalMu-1	KP063902	Clustered/Singleton	VC_825_1	N11	4	Bacillus	Group I	Duplodnaviria	Heunggongvirae	Uroviricota	Caudoviricetes	n.a.	Unclassified	O	O
S11-wms-combined_3507	Butyrivibrio virus Ceridwen	MN882553	Clustered/Singleton	VC_1209_2	N8	17	Butyrivibrio	Group I	Duplodnaviria	Heunggongvirae	Uroviricota	Caudoviricetes	n.a.	Unclassified	O	O
S12-wms-combined_129799	Faecalibacterium phage FP_Epona	MG711462	Clustered/Singleton	VC_707_2	C1	100	Faecalibacterium	Group I	Duplodnaviria	Heunggongvirae	Uroviricota	Caudoviricetes	n.a.	Unclassified	Unclassified	O
S12-wms-combined_157167	n.a.	n.a.	Clustered/Singleton	VC_1489_0	F	n.a.	n.a.	n.a.	n.a.	n.a.	n.a.	n.a.	n.a.	n.a.	n.a.	n.a.
S12-wms-combined_201125	Phage vB_RanS_PJN03	ON245412	Clustered/Singleton	VC_918_2	N12	10	Unspecified	Group I	Duplodnaviria	Heunggongvirae	Uroviricota	Caudoviricetes	n.a.	Unclassified	O	O
S12-wms-combined_23517	Butyrivibrio virus Ceridwen	MN882553	Clustered/Singleton	VC_1209_1	N11	7	Butyrivibrio	Group I	Duplodnaviria	Heunggongvirae	Uroviricota	Caudoviricetes	n.a.	Unclassified	O	O
S12-wms-combined_323943	Faecalibacterium phage FP_Taranis	MG711467	Clustered/Singleton	VC_711_1	C1	110	Faecalibacterium	Group I	Duplodnaviria	Heunggongvirae	Uroviricota	Caudoviricetes	n.a.	Unclassified	Unclassified	O
S12-wms-combined_358797	Elizabethkingia phage TCUEAP3	OK632026	Clustered/Singleton	VC_678_0	N11	9	Elizabethkingia	Group I	Duplodnaviria	Heunggongvirae	Uroviricota	Caudoviricetes	n.a.	Unclassified	O	O
S12-wms-combined_98046	Streptococcus phage Javan630	MK448997	Clustered/Singleton	VC_626_2	C5	48	Streptococcus	Group I	Duplodnaviria	Heunggongvirae	Uroviricota	Caudoviricetes	n.a.	Unclassified	Unclassified	O
S17-wms-combined_186314	Bifidobacterium phage Bbif-1	GQ141189	Clustered/Singleton	VC_798_2	N5	15	Bifidobacterium	Group I	Duplodnaviria	Heunggongvirae	Uroviricota	Caudoviricetes	n.a.	Unclassified	O	O
S17-wms-combined_2234	Faecalibacterium phage FP_Taranis	MG711467	Clustered/Singleton	VC_711_2	C3	88	Faecalibacterium	Group I	Duplodnaviria	Heunggongvirae	Uroviricota	Caudoviricetes	n.a.	Unclassified	Unclassified	O
S17-wms-combined_248321	Faecalibacterium phage FP_Lagaffe	MG711461	Clustered/Singleton	VC_717_1	C4	90	Faecalibacterium	Group I	Duplodnaviria	Heunggongvirae	Uroviricota	Caudoviricetes	n.a.	Unclassified	Unclassified	O
S17-wms-combined_95360	Clostridium phage phiCT453A	KM983327	Clustered/Singleton	VC_732_1	N4	3	Clostridium	Group I	Duplodnaviria	Heunggongvirae	Uroviricota	Caudoviricetes	n.a.	Unclassified	O	O
S2-wms-combined_116221	Butyrivibrio phage Idris	MN882554	Clustered/Singleton	VC_876_1	N5	33	Butyrivibrio	Group I	Duplodnaviria	Heunggongvirae	Uroviricota	Caudoviricetes	n.a.	Unclassified	O	O
S2-wms-combined_13246	Faecalibacterium phage FP_Toutatis	MG711466	Clustered/Singleton	VC_710_4	C2	69	Faecalibacterium	Group I	Duplodnaviria	Heunggongvirae	Uroviricota	Caudoviricetes	n.a.	Unclassified	Unclassified	O
S2-wms-combined_137119	Faecalibacterium phage FP_Toutatis	MG711466	Clustered/Singleton	VC_710_1	C8	55	Faecalibacterium	Group I	Duplodnaviria	Heunggongvirae	Uroviricota	Caudoviricetes	n.a.	Unclassified	Unclassified	O
S2-wms-combined_140996	Faecalibacterium phage FP_Taranis	MG711467	Clustered/Singleton	VC_711_3	C2	76	Faecalibacterium	Group I	Duplodnaviria	Heunggongvirae	Uroviricota	Caudoviricetes	n.a.	Unclassified	Unclassified	O
S2-wms-combined_157934	Faecalibacterium phage FP_Mushu	MG711460	Clustered/Singleton	VC_1235_1	C4	67	Faecalibacterium	Group I	Duplodnaviria	Heunggongvirae	Uroviricota	Caudoviricetes	n.a.	Unclassified	Unclassified	O
S2-wms-combined_233107	n.a.	n.a.	Clustered/Singleton	VC_1485_0	A	n.a.	n.a.	n.a.	n.a.	n.a.	n.a.	n.a.	n.a.	n.a.	n.a.	n.a.
S2-wms-combined_24549	Clostridioides phage JD033	MT193276	Clustered/Singleton	VC_651_0	N8	10	Clostridioides	Group I	Duplodnaviria	Heunggongvirae	Uroviricota	Caudoviricetes	n.a.	Unclassified	O	O
S2-wms-combined_98888	Faecalibacterium phage FP_oengus	MG711463	Clustered/Singleton	VC_804_3	C9	41	Faecalibacterium	Group I	Duplodnaviria	Heunggongvirae	Uroviricota	Caudoviricetes	n.a.	Unclassified	Unclassified	O
S5-wms-combined_108794	Streptococcus phage Javan422	MK448934	Clustered/Singleton	VC_626_3	C5	55	Streptococcus	Group I	Duplodnaviria	Heunggongvirae	Uroviricota	Caudoviricetes	n.a.	Unclassified	Unclassified	O
S5-wms-combined_134743	n.a.	n.a.	Clustered/Singleton	VC_825_2	A	n.a.	n.a.	n.a.	n.a.	n.a.	n.a.	n.a.	n.a.	n.a.	n.a.	n.a.
S5-wms-combined_66662	Campylobacter phage A11a	MG065691	Clustered/Singleton	VC_1219_1	C1	8	Campylobacter	Unclassified	Unclassified	n.a.	n.a.	n.a.	n.a.	Unclassified	Unclassified	O
S5-wms-combined_72410	Faecalibacterium phage FP_oengus	MG711463	Clustered/Singleton	VC_804_4	C8	41	Faecalibacterium	Group I	Duplodnaviria	Heunggongvirae	Uroviricota	Caudoviricetes	n.a.	Unclassified	Unclassified	O
S5-wms-combined_90315	Phage vB_RanS_PJN03	ON245412	Clustered/Singleton	VC_917_1	N9	10	Unspecified	Group I	Duplodnaviria	Heunggongvirae	Uroviricota	Caudoviricetes	n.a.	Unclassified	O	O
S5-wms_67169	Roseburia phage Shimadzu	LR596903	Clustered/Singleton	VC_914_0	N3	5	Roseburia	Group I	Duplodnaviria	Heunggongvirae	Uroviricota	Caudoviricetes	n.a.	Unclassified	O	O
S6-wms-combined_118427	Faecalibacterium phage FP_Toutatis	MG711466	Clustered/Singleton	VC_710_3	C8	60	Faecalibacterium	Group I	Duplodnaviria	Heunggongvirae	Uroviricota	Caudoviricetes	n.a.	Unclassified	Unclassified	O
S6-wms-combined_82597	n.a.	n.a.	Clustered/Singleton	VC_1435_1	A	n.a.	n.a.	n.a.	n.a.	n.a.	n.a.	n.a.	n.a.	n.a.	n.a.	n.a.
S7-wms-combined_157260	Faecalibacterium phage FP_Toutatis	MG711466	Clustered/Singleton	VC_710_2	C11	24	Faecalibacterium	Group I	Duplodnaviria	Heunggongvirae	Uroviricota	Caudoviricetes	n.a.	Unclassified	Unclassified	O
S7-wms-combined_169375	n.a.	n.a.	Clustered/Singleton	VC_1483_0	A	n.a.	n.a.	n.a.	n.a.	n.a.	n.a.	n.a.	n.a.	n.a.	n.a.	n.a.
S7-wms-combined_20921	Bacillus phage PBC2	KT070867	Clustered/Singleton	VC_719_1	N2	5	Bacillus	Group I	Duplodnaviria	Heunggongvirae	Uroviricota	Caudoviricetes	n.a.	Unclassified	O	O
S7-wms-combined_238231	n.a.	n.a.	Clustered/Singleton	VC_271_2	A	n.a.	n.a.	n.a.	n.a.	n.a.	n.a.	n.a.	n.a.	n.a.	n.a.	n.a.
S7-wms-combined_26217	Phage vB_RanS_PJN03	ON245412	Clustered/Singleton	VC_918_1	N12	9	Unspecified	Group I	Duplodnaviria	Heunggongvirae	Uroviricota	Caudoviricetes	n.a.	Unclassified	O	O
S7-wms-combined_3697	Campylobacter phage A11a	MG065691	Clustered/Singleton	VC_832_1	N13	2	Campylobacter	Unclassified	Unclassified	n.a.	n.a.	n.a.	n.a.	Unclassified	O	O
S7-wms-combined_62119	Bacteroides phage LoVEphage	MW660583	Clustered/Singleton	VC_1221_1	C1	248	Bacteroides	Group I	Duplodnaviria	Heunggongvirae	Uroviricota	Caudoviricetes	n.a.	Unclassified	Unclassified	O
S8-wms-combined_225114	Faecalibacterium phage FP_Taranis	MG711467	Clustered/Singleton	VC_271_1	N1	70	Faecalibacterium	Group I	Duplodnaviria	Heunggongvirae	Uroviricota	Caudoviricetes	n.a.	Unclassified	O	O
S8-wms-combined_40666	n.a.	n.a.	Clustered/Singleton	VC_1483_1	A	n.a.	n.a.	n.a.	n.a.	n.a.	n.a.	n.a.	n.a.	n.a.	n.a.	n.a.
S8-wms_111056	Bacteroides phage crAss002	MN917146	Clustered/Singleton	VC_674_0	N1	134	Bacteroides	Group I	Duplodnaviria	Heunggongvirae	Uroviricota	Caudoviricetes	Crassvirales	Intestiviridae	O	O
S9-wms-combined_20485	Streptococcus phage P0092	KY705252	Clustered/Singleton	VC_255_1	C1	66	Streptococcus	Group I	Duplodnaviria	Heunggongvirae	Uroviricota	Caudoviricetes	n.a.	Unclassified	Unclassified	O
S9-wms-combined_29611	Faecalibacterium phage FP_Brigit	MG711465	Clustered/Singleton	VC_606_1	C3	85	Faecalibacterium	Group I	Duplodnaviria	Heunggongvirae	Uroviricota	Caudoviricetes	n.a.	Unclassified	Unclassified	O
S9-wms-combined_9931	Erysipelothrix phage phi1605	MF172979	Clustered/Singleton	VC_626_1	C6	58	Erysipelothrix	Group I	Duplodnaviria	Heunggongvirae	Uroviricota	Caudoviricetes	n.a.	Unclassified	Unclassified	O
S1-wms-combined_102801	n.a.	n.a.	Outlier	n.a.	A	n.a.	n.a.	n.a.	n.a.	n.a.	n.a.	n.a.	n.a.	n.a.	n.a.	n.a.
S1-wms-combined_116938	Streptococcus phage Javan580	MK448989	Outlier	n.a.	N5	9	Streptococcus	Group I	Duplodnaviria	Heunggongvirae	Uroviricota	Caudoviricetes	n.a.	Unclassified	O	O
S1-wms-combined_131270	Butyrivibrio virus Ceridwen	MN882553	Outlier	n.a.	N2	15	Butyrivibrio	Group I	Duplodnaviria	Heunggongvirae	Uroviricota	Caudoviricetes	n.a.	Unclassified	O	O
S1-wms-combined_136097	n.a.	n.a.	Outlier	n.a.	A	n.a.	n.a.	n.a.	n.a.	n.a.	n.a.	n.a.	n.a.	n.a.	n.a.	n.a.
S1-wms-combined_143459	Pseudomonas phage vB_Pae_CF5a	MK511057	Outlier	n.a.	N1	15	Pseudomonas	Group I	Duplodnaviria	Heunggongvirae	Uroviricota	Caudoviricetes	n.a.	Unclassified	O	O
S1-wms-combined_164894	Faecalibacterium phage FP_Lugh	MG711464	Outlier	n.a.	N4	16	Faecalibacterium	Group I	Duplodnaviria	Heunggongvirae	Uroviricota	Caudoviricetes	n.a.	Unclassified	O	O
S1-wms-combined_186878	n.a.	n.a.	Outlier	n.a.	A	n.a.	n.a.	n.a.	n.a.	n.a.	n.a.	n.a.	n.a.	n.a.	n.a.	n.a.
S1-wms-combined_212892	Butyrivibrio virus Ceridwen	MN882553	Outlier	n.a.	N1	19	Butyrivibrio	Group I	Duplodnaviria	Heunggongvirae	Uroviricota	Caudoviricetes	n.a.	Unclassified	O	O
S1-wms-combined_227003	n.a.	n.a.	Outlier	n.a.	A	n.a.	n.a.	n.a.	n.a.	n.a.	n.a.	n.a.	n.a.	n.a.	n.a.	n.a.
S1-wms-combined_227917	Faecalibacterium phage FP_Lugh	MG711464	Outlier	n.a.	N2	15	Faecalibacterium	Group I	Duplodnaviria	Heunggongvirae	Uroviricota	Caudoviricetes	n.a.	Unclassified	O	O
S1-wms-combined_252439	Bacillus phage vB_BpsS-140	MH884512	Outlier	n.a.	N1	2	Bacillus	Group I	Duplodnaviria	Heunggongvirae	Uroviricota	Caudoviricetes	n.a.	Unclassified	O	O
S1-wms-combined_279492	Streptococcus phage Javan580	MK448989	Outlier	n.a.	N6	8	Streptococcus	Group I	Duplodnaviria	Heunggongvirae	Uroviricota	Caudoviricetes	n.a.	Unclassified	O	O
S1-wms-combined_308848	n.a.	n.a.	Outlier	n.a.	A	n.a.	n.a.	n.a.	n.a.	n.a.	n.a.	n.a.	n.a.	n.a.	n.a.	n.a.
S1-wms-combined_57370	Faecalibacterium phage FP_Lagaffe	MG711461	Outlier	n.a.	N15	3	Faecalibacterium	Group I	Duplodnaviria	Heunggongvirae	Uroviricota	Caudoviricetes	n.a.	Unclassified	O	O
S1-wms-combined_92523	Bacteriophage sp.	MW202489	Outlier	n.a.	N1	6	Unspecified	Unclassified	Unclassified	n.a.	n.a.	n.a.	n.a.	Unclassified	O	O
S1-wms-combined_94988	n.a.	n.a.	Outlier	n.a.	A	n.a.	n.a.	n.a.	n.a.	n.a.	n.a.	n.a.	n.a.	n.a.	n.a.	n.a.
S1-wms_106115	n.a.	n.a.	Outlier	n.a.	A	n.a.	n.a.	n.a.	n.a.	n.a.	n.a.	n.a.	n.a.	n.a.	n.a.	n.a.

S1-wms_208875	Bacteroides phage ARB14	MT074134	Outlier	n.a.	N1	61	Bacteroides	Group I	Duplodnaviria	Heunggongvirae	Uroviricota	Caudoviricetes	n.a.	Unclassified	O	O
S1-wms_236105	Roseburia phage Jekyll	LR596902	Outlier	n.a.	N11	3	Roseburia	Group I	Duplodnaviria	Heunggongvirae	Uroviricota	Caudoviricetes	n.a.	Unclassified	O	O
S1-wms_29288	Faecalibacterium phage FP_Taranis	MG711467	Outlier	n.a.	N2	61	Faecalibacterium	Group I	Duplodnaviria	Heunggongvirae	Uroviricota	Caudoviricetes	n.a.	Unclassified	O	O
S1-wms_394571	Bacteroides phage SJC03	MT074146	Outlier	n.a.	N1	36	Bacteroides	Group I	Duplodnaviria	Heunggongvirae	Uroviricota	Caudoviricetes	n.a.	Unclassified	O	O
S11-wms-combined_101337	Klebsiella phage ST11-OXA245phi3.2	MK416010	Outlier	n.a.	N1	14	Klebsiella	Group I	Duplodnaviria	Heunggongvirae	Uroviricota	Caudoviricetes	n.a.	Unclassified	O	O
S11-wms-combined_112147	n.a.	n.a.	Outlier	n.a.	A	n.a.	n.a.	n.a.	n.a.	n.a.	n.a.	n.a.	n.a.	n.a.	n.a.	n.a.
S11-wms-combined_112733	Alteromonadaceae phage B23	KY939598	Outlier	n.a.	N1	18	Unspecified	Group I	Duplodnaviria	Heunggongvirae	Uroviricota	Caudoviricetes	n.a.	Unclassified	O	O
S11-wms-combined_116589	n.a.	n.a.	Outlier	n.a.	A	n.a.	n.a.	n.a.	n.a.	n.a.	n.a.	n.a.	n.a.	n.a.	n.a.	n.a.
S11-wms-combined_14636	Streptococcus phage Javan68	MK449004	Outlier	n.a.	N3	10	Streptococcus	Group I	Duplodnaviria	Heunggongvirae	Uroviricota	Caudoviricetes	n.a.	Unclassified	O	O
S11-wms-combined_168048	Streptococcus phage Javan1	MK448665	Outlier	n.a.	N2	5	Streptococcus	Group I	Duplodnaviria	Heunggongvirae	Uroviricota	Caudoviricetes	n.a.	Unclassified	O	O
S11-wms-combined_215723	Cellulophaga phage Ingeline_1	MT732435	Outlier	n.a.	N3	8	Cellulophaga	Group I	Duplodnaviria	Heunggongvirae	Uroviricota	Caudoviricetes	n.a.	Duneviridae	O	O
S11-wms-combined_236214	Faecalibacterium phage FP_oengus	MG711463	Outlier	n.a.	N4	32	Faecalibacterium	Group I	Duplodnaviria	Heunggongvirae	Uroviricota	Caudoviricetes	n.a.	Unclassified	O	O
S11-wms-combined_282227	Streptococcus phage Javan422	MK448934	Outlier	n.a.	N4	33	Streptococcus	Group I	Duplodnaviria	Heunggongvirae	Uroviricota	Caudoviricetes	n.a.	Unclassified	O	O
S11-wms-combined_329493	n.a.	n.a.	Outlier	n.a.	A	n.a.	n.a.	n.a.	n.a.	n.a.	n.a.	n.a.	n.a.	n.a.	n.a.	n.a.
S11-wms-combined_63818	Bacteroides phage LoVEphage	MW660583	Outlier	n.a.	N11	8	Bacteroides	Group I	Duplodnaviria	Heunggongvirae	Uroviricota	Caudoviricetes	n.a.	Unclassified	O	O
S11-wms_87293	Yersinia phage YeP4	MK733262	Outlier	n.a.	N1	1	Yersinia	Group I	Duplodnaviria	Heunggongvirae	Uroviricota	Caudoviricetes	n.a.	Unclassified	O	O
S12-wms-combined_151843	Aeribacillus phage AP45	KX965989	Outlier	n.a.	N1	15	Aeribacillus	Group I	Duplodnaviria	Heunggongvirae	Uroviricota	Caudoviricetes	n.a.	Unclassified	O	O
S12-wms-combined_164656	n.a.	n.a.	Outlier	n.a.	A	n.a.	n.a.	n.a.	n.a.	n.a.	n.a.	n.a.	n.a.	n.a.	n.a.	n.a.
S12-wms-combined_190114	Bacteroides phage p00	BK010646	Outlier	n.a.	N10	18	Bacteroides	Group I	Duplodnaviria	Heunggongvirae	Uroviricota	Caudoviricetes	n.a.	Unclassified	O	O
S12-wms-combined_21504	Acetobacter phage phiAO1	LC644971	Outlier	n.a.	N1	10	Acetobacter	Group I	Duplodnaviria	Heunggongvirae	Uroviricota	Caudoviricetes	n.a.	Unclassified	O	O
S12-wms-combined_223043	Faecalibacterium phage FP_Mushu	MG711460	Outlier	n.a.	N6	47	Faecalibacterium	Group I	Duplodnaviria	Heunggongvirae	Uroviricota	Caudoviricetes	n.a.	Unclassified	O	O
S12-wms-combined_264077	Faecalibacterium phage FP_Toutatis	MG711466	Outlier	n.a.	N1	57	Faecalibacterium	Group I	Duplodnaviria	Heunggongvirae	Uroviricota	Caudoviricetes	n.a.	Unclassified	O	O
S12-wms-combined_293354	Clostridium phage phiCP26F	GQ443085	Outlier	n.a.	N4	2	Clostridium	Group I	Duplodnaviria	Heunggongvirae	Uroviricota	Caudoviricetes	n.a.	Unclassified	O	O
S12-wms-combined_302876	Clostridium phage phiCT453B	KM983328	Outlier	n.a.	N1	22	Clostridium	Group I	Duplodnaviria	Heunggongvirae	Uroviricota	Caudoviricetes	n.a.	Unclassified	O	O
S12-wms-combined_313953	Acidithiobacillus phage AcaML1	JX507079	Outlier	n.a.	N1	25	Acidithiobacillus	Group I	Duplodnaviria	Heunggongvirae	Uroviricota	Caudoviricetes	n.a.	Unclassified	O	O
S12-wms-combined_325418	Streptococcus phage Javan422	MK448934	Outlier	n.a.	N9	13	Streptococcus	Group I	Duplodnaviria	Heunggongvirae	Uroviricota	Caudoviricetes	n.a.	Unclassified	O	O
S12-wms-combined_328554	Elizabethkingia phage TCUEAP2	OK632025	Outlier	n.a.	N1	20	Elizabethkingia	Group I	Duplodnaviria	Heunggongvirae	Uroviricota	Caudoviricetes	n.a.	Unclassified	O	O
S12-wms-combined_359190	Streptococcus phage Javan100	MK448838	Outlier	n.a.	N7	26	Streptococcus	Group I	Duplodnaviria	Heunggongvirae	Uroviricota	Caudoviricetes	n.a.	Unclassified	O	O
S12-wms-combined_368562	Bacteroides phage p00	BK010646	Outlier	n.a.	N7	10	Bacteroides	Group I	Duplodnaviria	Heunggongvirae	Uroviricota	Caudoviricetes	n.a.	Unclassified	O	O
S12-wms-combined_371541	n.a.	n.a.	Outlier	n.a.	A	n.a.	n.a.	n.a.	n.a.	n.a.	n.a.	n.a.	n.a.	n.a.	n.a.	n.a.
S12-wms-combined_48648	n.a.	n.a.	Outlier	n.a.	A	n.a.	n.a.	n.a.	n.a.	n.a.	n.a.	n.a.	n.a.	n.a.	n.a.	n.a.
S12-wms-combined_63649	n.a.	n.a.	Outlier	n.a.	A	n.a.	n.a.	n.a.	n.a.	n.a.	n.a.	n.a.	n.a.	n.a.	n.a.	n.a.
S12-wms_135291	Streptococcus phage phi-SgaBSJ31_rum	MN270259	Outlier	n.a.	N2	2	Streptococcus	Group I	Duplodnaviria	Heunggongvirae	Uroviricota	Caudoviricetes	n.a.	Unclassified	O	O
S12-wms_17950	Butyrivibrio virus Arian	MN882551	Outlier	n.a.	N1	2	Butyrivibrio	Group I	Duplodnaviria	Heunggongvirae	Uroviricota	Caudoviricetes	n.a.	Unclassified	O	O
S12-wms_202803	Faecalibacterium phage FP_Lagaffe	MG711461	Outlier	n.a.	N31	3	Faecalibacterium	Group I	Duplodnaviria	Heunggongvirae	Uroviricota	Caudoviricetes	n.a.	Unclassified	O	O
S12-wms_235125	n.a.	n.a.	Outlier	n.a.	A	n.a.	n.a.	n.a.	n.a.	n.a.	n.a.	n.a.	n.a.	n.a.	n.a.	n.a.
S17-wms-combined_106940	Riemerella phage RAP44	HQ396194	Outlier	n.a.	N1	20	Riemerella	Group I	Duplodnaviria	Heunggongvirae	Uroviricota	Caudoviricetes	n.a.	Unclassified	O	O
S17-wms-combined_140517	Streptococcus phage Javan100	MK448838	Outlier	n.a.	N4	35	Streptococcus	Group I	Duplodnaviria	Heunggongvirae	Uroviricota	Caudoviricetes	n.a.	Unclassified	O	O
S17-wms-combined_146050	n.a.	n.a.	Outlier	n.a.	A	n.a.	n.a.	n.a.	n.a.	n.a.	n.a.	n.a.	n.a.	n.a.	n.a.	n.a.
S17-wms-combined_171305	Bacteroides phage LoVEphage	MW660583	Outlier	n.a.	N2	1	Bacteroides	Group I	Duplodnaviria	Heunggongvirae	Uroviricota	Caudoviricetes	n.a.	Unclassified	O	O
S17-wms-combined_185239	n.a.	n.a.	Outlier	n.a.	A	n.a.	n.a.	n.a.	n.a.	n.a.	n.a.	n.a.	n.a.	n.a.	n.a.	n.a.
S17-wms-combined_72837	Bacillus phage B13	OP066531	Outlier	n.a.	N3	8	Bacillus	Group I	Duplodnaviria	Heunggongvirae	Uroviricota	Caudoviricetes	n.a.	Unclassified	O	O
S17-wms_235536	n.a.	n.a.	Outlier	n.a.	F	n.a.	n.a.	n.a.	n.a.	n.a.	n.a.	n.a.	n.a.	n.a.	n.a.	n.a.
S2-wms-combined_110215	Faecalibacterium phage FP_Brigit	MG711465	Outlier	n.a.	N7	23	Faecalibacterium	Group I	Duplodnaviria	Heunggongvirae	Uroviricota	Caudoviricetes	n.a.	Unclassified	O	O
S2-wms-combined_126440	Roseburia phage Jekyll	LR596902	Outlier	n.a.	N2	9	Roseburia	Group I	Duplodnaviria	Heunggongvirae	Uroviricota	Caudoviricetes	n.a.	Unclassified	O	O
S2-wms-combined_127080	n.a.	n.a.	Outlier	n.a.	A	n.a.	n.a.	n.a.	n.a.	n.a.	n.a.	n.a.	n.a.	n.a.	n.a.	n.a.
S2-wms-combined_143737	Cellulophaga phage Ingeline_1	MT732435	Outlier	n.a.	N5	2	Cellulophaga	Group I	Duplodnaviria	Heunggongvirae	Uroviricota	Caudoviricetes	n.a.	Duneviridae	O	O
S2-wms-combined_159670	n.a.	n.a.	Outlier	n.a.	A	n.a.	n.a.	n.a.	n.a.	n.a.	n.a.	n.a.	n.a.	n.a.	n.a.	n.a.
S2-wms-combined_170845	Streptococcus phage Javan261	MK448720	Outlier	n.a.	N8	16	Streptococcus	Group I	Duplodnaviria	Heunggongvirae	Uroviricota	Caudoviricetes	n.a.	Unclassified	O	O
S2-wms-combined_181391	n.a.	n.a.	Outlier	n.a.	A	n.a.	n.a.	n.a.	n.a.	n.a.	n.a.	n.a.	n.a.	n.a.	n.a.	n.a.
S2-wms-combined_185494	Delftia phage IME-DE1	KR153873	Outlier	n.a.	N8	4	Delftia	Group I	Duplodnaviria	Heunggongvirae	Uroviricota	Caudoviricetes	n.a.	Autographiviridae	O	O
S2-wms-combined_190624	n.a.	n.a.	Outlier	n.a.	A	n.a.	n.a.	n.a.	n.a.	n.a.	n.a.	n.a.	n.a.	n.a.	n.a.	n.a.
S2-wms-combined_191613	n.a.	n.a.	Outlier	n.a.	A	n.a.	n.a.	n.a.	n.a.	n.a.	n.a.	n.a.	n.a.	n.a.	n.a.	n.a.
S2-wms-combined_199384	Clostridioides phage phiCD08011	MW512572	Outlier	n.a.	N15	2	Clostridioides	Group I	Duplodnaviria	Heunggongvirae	Uroviricota	Caudoviricetes	n.a.	Unclassified	O	O
S2-wms-combined_200390	Clostridium phage phiCD38-2	HM568888	Outlier	n.a.	N4	4	Clostridium	Group I	Duplodnaviria	Heunggongvirae	Uroviricota	Caudoviricetes	n.a.	Unclassified	O	O
S2-wms-combined_225908	Campylobacter phage A11a	MG065691	Outlier	n.a.	N8	2	Campylobacter	Unclassified	Unclassified	n.a.	n.a.	n.a.	n.a.	Unclassified	O	O
S2-wms-combined_25727	Geobacillus phage GBSV1	DQ340064	Outlier	n.a.	N5	2	Geobacillus	Group I	Duplodnaviria	Heunggongvirae	Uroviricota	Caudoviricetes	n.a.	Unclassified	O	O
S2-wms-combined_51446	n.a.	n.a.	Outlier	n.a.	F	n.a.	n.a.	n.a.	n.a.	n.a.	n.a.	n.a.	n.a.	n.a.	n.a.	n.a.
S2-wms-combined_52568	n.a.	n.a.	Outlier	n.a.	A	n.a.	n.a.	n.a.	n.a.	n.a.	n.a.	n.a.	n.a.	n.a.	n.a.	n.a.
S2-wms-combined_5791	Butyrivibrio phage Idris	MN882554	Outlier	n.a.	N4	15	Butyrivibrio	Group I	Duplodnaviria	Heunggongvirae	Uroviricota	Caudoviricetes	n.a.	Unclassified	O	O
S2-wms-combined_62839	Bacillus phage PK-3	ON881243	Outlier	n.a.	N1	9	Bacillus	Group I	Duplodnaviria	Heunggongvirae	Uroviricota	Caudoviricetes	n.a.	Unclassified	O	O
S2-wms-combined_72725	n.a.	n.a.	Outlier	n.a.	A	n.a.	n.a.	n.a.	n.a.	n.a.	n.a.	n.a.	n.a.	n.a.	n.a.	n.a.
S2-wms-combined_74387	Faecalibacterium phage FP_Lugh	MG711464	Outlier	n.a.	N4	10	Faecalibacterium	Group I	Duplodnaviria	Heunggongvirae	Uroviricota	Caudoviricetes	n.a.	Unclassified	O	O
S2-wms-combined_75144	n.a.	n.a.	Outlier	n.a.	A	n.a.	n.a.	n.a.	n.a.	n.a.	n.a.	n.a.	n.a.	n.a.	n.a.	n.a.
S2-wms-combined_7756	Akkermansia phage DTMo-2021a	CP084202	Outlier	n.a.	N3	11	Akkermansia	Group I	Duplodnaviria	Heunggongvirae	Uroviricota	Caudoviricetes	n.a.	Unclassified	O	O
S2-wms-combined_78670	Bacillus phage vB_BtS_BMBtp15	KX190835	Outlier	n.a.	N3	6	Bacillus	Group I	Duplodnaviria	Heunggongvirae	Uroviricota	Caudoviricetes	n.a.	Unclassified	O	O
S2-wms-combined_84608	Bordetella phage BPP-1	AY029185	Outlier	n.a.	N1	30	Bordetella	Group I	Duplodnaviria	Heunggongvirae	Uroviricota	Caudoviricetes	n.a.	Unclassified	O	O
S2-wms_246296	n.a.	n.a.	Outlier	n.a.	A	n.a.	n.a.	n.a.	n.a.	n.a.	n.a.	n.a.	n.a.	n.a.	n.a.	n.a.
S2-wms_248324	Clostridium phage phiCTP1	HM159959	Outlier	n.a.	N1	6	Clostridium	Group I	Duplodnaviria	Heunggongvirae	Uroviricota	Caudoviricetes	n.a.	Unclassified	O	O
S2-wms_248327	n.a.	n.a.	Outlier	n.a.	F	n.a.	n.a.	n.a.	n.a.	n.a.	n.a.	n.a.	n.a.	n.a.	n.a.	n.a.
S2-wms_6424	Tenacibaculum phage Gundel_1	MT732474	Outlier	n.a.	N1	8	Tenacibaculum	Group I	Duplodnaviria	Heunggongvirae	Uroviricota	Caudoviricetes	n.a.	Pachyviridae	O	O
S3-wms-combined_135428	Propionibacterium phage Anatole	KX620748	Outlier	n.a.	N2	15	Propionibacterium	Group I	Duplodnaviria	Heunggongvirae	Uroviricota	Caudoviricetes	n.a.	Unclassified	O	O
S3-wms-combined_45305	Faecalibacterium phage FP_Lugh	MG711464	Outlier	n.a.	N2	19	Faecalibacterium	Group I	Duplodnaviria	Heunggongvirae	Uroviricota	Caudoviricetes	n.a.	Unclassified	O	O
S3-wms_94494	Bifidobacterium phage PMBT6	MH444512	Outlier	n.a.	N1	29	Bifidobacterium	Group I	Duplodnaviria	Heunggongvirae	Uroviricota	Caudoviricetes	n.a.	Unclassified	O	O
S4-wms-combined_10758	Cellulophaga phage phi17:2	KC821609	Outlier	n.a.	N1	7	Cellulophaga	Group I	Duplodnaviria	Heunggongvirae	Uroviricota	Caudoviricetes	n.a.	Unclassified	O	O
S4-wms-combined_118531	Podoviridae sp.	MW202722	Outlier	n.a.	N1	8	Unspecified	Group I	Duplodnaviria	Heunggongvirae	Uroviricota	Caudoviricetes	n.a.	Unclassified	O	O
S4-wms-combined_121978	Gardnerella phage vB_Gva_AB1	MW387018	Outlier	n.a.	N5	36	Gardnerella	Group I	Duplodnaviria	Heunggongvirae	Uroviricota	Caudoviricetes	n.a.	Unclassified	O	O
S4-wms-combined_1246	n.a.	n.a.	Outlier	n.a.	A	n.a.	n.a.	n.a.	n.a.	n.a.	n.a.	n.a.	n.a.	n.a.	n.a.	n.a.
S4-wms-combined_22012	Propionibacterium phage B3	KX620749	Outlier	n.a.	N3	16	Propionibacterium	Group I	Duplodnaviria	Heunggongvirae	Uroviricota	Caudoviricetes	n.a.	Unclassified	O	O
S4-wms-combined_25201	Streptococcus phage SM1	AY007505	Outlier	n.a.	N7	5	Streptococcus	Group I	Duplodnaviria	Heunggongvirae	Uroviricota	Caudoviricetes	n.a.	Unclassified	O	O
S4-wms-combined_83492	Bacteroides phage p00	BK010646	Outlier	n.a.	N9	18	Bacteroides	Group I	Duplodnaviria	Heunggongvirae	Uroviricota	Caudoviricetes	n.a.	Unclassified	O	O
S4-wms_114120	Pectobacterium phage DU_PP_III	MF979562	Outlier	n.a.	N1	9	Pectobacterium	Group I	Duplodnaviria	Heunggongvirae	Uroviricota	Caudoviricetes	n.a.	Unclassified	O	O
S4-wms_50475	Phage vB_RanS_PJN03	ON245412	Outlier	n.a.	N10	3	Unspecified	Group I	Duplodnaviria	Heunggongvirae	Uroviricota	Caudoviricetes	n.a.	Unclassified	O	O
S5-wms-combined_117095	n.a.	n.a.	Outlier	n.a.	A	n.a.	n.a.	n.a.	n.a.	n.a.	n.a.	n.a.	n.a.	n.a.	n.a.	n.a.
S5-wms-combined_156329	Geobacillus phage GBSV1	DQ340064	Outlier	n.a.	N7	4	Geobacillus	Group I	Duplodnaviria	Heunggongvirae	Uroviricota	Caudoviricetes	n.a.	Unclassified	O	O
S5-wms-combined_161191	n.a.	n.a.	Outlier	n.a.	A	n.a.	n.a.	n.a.	n.a.	n.a.	n.a.	n.a.	n.a.	n.a.	n.a.	n.a.

S5-wms-combined_175145	Streptococcus phage Javan90	MK449010	Outlier	n.a.	N1	14	Streptococcus	Group I	Duplodnaviria	Heunggongvirae	Uroviricota	Caudoviricetes	n.a.	Unclassified	O	O
S5-wms-combined_179241	n.a.	n.a.	Outlier	n.a.	A	n.a.	n.a.	n.a.	n.a.	n.a.	n.a.	n.a.	n.a.	n.a.	n.a.	n.a.
S5-wms-combined_204782	n.a.	n.a.	Outlier	n.a.	A	n.a.	n.a.	n.a.	n.a.	n.a.	n.a.	n.a.	n.a.	n.a.	n.a.	n.a.
S5-wms-combined_3201	Yersinia phage YeP1	MK733259	Outlier	n.a.	N1	26	Yersinia	Group I	Duplodnaviria	Heunggongvirae	Uroviricota	Caudoviricetes	n.a.	Unclassified	O	O
S5-wms-combined_94482	n.a.	n.a.	Outlier	n.a.	A	n.a.	n.a.	n.a.	n.a.	n.a.	n.a.	n.a.	n.a.	n.a.	n.a.	n.a.
S5-wms_164032	Lactococcus phage vB_LacS_15	MN337887	Outlier	n.a.	N1	46	Lactococcus	Group I	Duplodnaviria	Heunggongvirae	Uroviricota	Caudoviricetes	n.a.	Unclassified	O	O
S6-wms-combined_56464	Faecalibacterium phage FP_Lugh	MG711464	Outlier	n.a.	N8	10	Faecalibacterium	Group I	Duplodnaviria	Heunggongvirae	Uroviricota	Caudoviricetes	n.a.	Unclassified	O	O
S6-wms_140743	Bacillus phage vB_BpsS-140	MH884512	Outlier	n.a.	N1	4	Bacillus	Group I	Duplodnaviria	Heunggongvirae	Uroviricota	Caudoviricetes	n.a.	Unclassified	O	O
S7-wms-combined_106253	Listeria phage LWP01	CP011103	Outlier	n.a.	N5	5	Listeria	Group I	Duplodnaviria	Heunggongvirae	Uroviricota	Caudoviricetes	n.a.	Unclassified	O	O
S7-wms-combined_107679_1	Riemerella phage RAP44	HQ396194	Outlier	n.a.	N1	7	Riemerella	Group I	Duplodnaviria	Heunggongvirae	Uroviricota	Caudoviricetes	n.a.	Unclassified	O	O
S7-wms-combined_135154	n.a.	n.a.	Outlier	n.a.	A	n.a.	n.a.	n.a.	n.a.	n.a.	n.a.	n.a.	n.a.	n.a.	n.a.	n.a.
S7-wms-combined_14529	Enterococcus phage phiFL4A	GQ478088	Outlier	n.a.	N1	10	Enterococcus	Group I	Duplodnaviria	Heunggongvirae	Uroviricota	Caudoviricetes	n.a.	Unclassified	O	O
S7-wms-combined_156351	n.a.	n.a.	Outlier	n.a.	A	n.a.	n.a.	n.a.	n.a.	n.a.	n.a.	n.a.	n.a.	n.a.	n.a.	n.a.
S7-wms-combined_174935	Bifidobacterium phage Bbif-1	GQ141189	Outlier	n.a.	N2	8	Bifidobacterium	Group I	Duplodnaviria	Heunggongvirae	Uroviricota	Caudoviricetes	n.a.	Unclassified	O	O
S7-wms-combined_190281	n.a.	n.a.	Outlier	n.a.	A	n.a.	n.a.	n.a.	n.a.	n.a.	n.a.	n.a.	n.a.	n.a.	n.a.	n.a.
S7-wms-combined_2122	Bacteriophage sp.	LT996075	Outlier	n.a.	N1	11	Unspecified	Unclassified	Unclassified	n.a.	n.a.	n.a.	n.a.	Unclassified	O	O
S7-wms-combined_21693	Streptococcus phage Javan100	MK448838	Outlier	n.a.	N5	38	Streptococcus	Group I	Duplodnaviria	Heunggongvirae	Uroviricota	Caudoviricetes	n.a.	Unclassified	O	O
S7-wms-combined_217226	Pseudomonas phage BHU-1	OK638201	Outlier	n.a.	N1	8	Pseudomonas	Group I	Duplodnaviria	Heunggongvirae	Uroviricota	Caudoviricetes	n.a.	Unclassified	O	O
S7-wms-combined_218379	Butyrivibrio virus Arian	MN882551	Outlier	n.a.	N1	24	Butyrivibrio	Group I	Duplodnaviria	Heunggongvirae	Uroviricota	Caudoviricetes	n.a.	Unclassified	O	O
S7-wms-combined_224523	Clostridium phage phiCD38-2	HM568888	Outlier	n.a.	N8	2	Clostridium	Group I	Duplodnaviria	Heunggongvirae	Uroviricota	Caudoviricetes	n.a.	Unclassified	O	O
S7-wms-combined_3669	Microviridae sp.	MG945130	Outlier	n.a.	N1	5	Unspecified	Group II	Monodnaviria	Sangervirae	Phixviricota	Malgrandaviricetes	Petitvirales	Microviridae	O	O
S7-wms-combined_69024	Streptococcus phage OlisA2	OL774869	Outlier	n.a.	N1	26	Streptococcus	Group I	Duplodnaviria	Heunggongvirae	Uroviricota	Caudoviricetes	n.a.	Unclassified	O	O
S7-wms-combined_71592	Streptococcus phage Javan583	MK448814	Outlier	n.a.	N1	26	Streptococcus	Group I	Duplodnaviria	Heunggongvirae	Uroviricota	Caudoviricetes	n.a.	Unclassified	O	O
S7-wms-combined_74829	Streptococcus phage Javan199	MK448702	Outlier	n.a.	N2	9	Streptococcus	Group I	Duplodnaviria	Heunggongvirae	Uroviricota	Caudoviricetes	n.a.	Unclassified	O	O
S7-wms_151112	Butyrivibrio virus Arian	MN882551	Outlier	n.a.	N1	14	Butyrivibrio	Group I	Duplodnaviria	Heunggongvirae	Uroviricota	Caudoviricetes	n.a.	Unclassified	O	O
S7-wms_76437	n.a.	n.a.	Outlier	n.a.	A	n.a.	n.a.	n.a.	n.a.	n.a.	n.a.	n.a.	n.a.	n.a.	n.a.	n.a.
S8-wms-combined_117281	Streptococcus phage Javan105	MK448667	Outlier	n.a.	N12	21	Streptococcus	Group I	Duplodnaviria	Heunggongvirae	Uroviricota	Caudoviricetes	n.a.	Unclassified	O	O
S8-wms-combined_164289	n.a.	n.a.	Outlier	n.a.	A	n.a.	n.a.	n.a.	n.a.	n.a.	n.a.	n.a.	n.a.	n.a.	n.a.	n.a.
S8-wms-combined_180957	Bacteroides phage p00	BK010646	Outlier	n.a.	N9	15	Bacteroides	Group I	Duplodnaviria	Heunggongvirae	Uroviricota	Caudoviricetes	n.a.	Unclassified	O	O
S8-wms-combined_185611	n.a.	n.a.	Outlier	n.a.	A	n.a.	n.a.	n.a.	n.a.	n.a.	n.a.	n.a.	n.a.	n.a.	n.a.	n.a.
S8-wms-combined_23583	n.a.	n.a.	Outlier	n.a.	A	n.a.	n.a.	n.a.	n.a.	n.a.	n.a.	n.a.	n.a.	n.a.	n.a.	n.a.
S8-wms-combined_239791	Olleya phage Harreka_1	MT732457	Outlier	n.a.	N4	3	Olleya	Group I	Duplodnaviria	Heunggongvirae	Uroviricota	Caudoviricetes	n.a.	Aggregaviridae	O	O
S8-wms-combined_48046	n.a.	n.a.	Outlier	n.a.	A	n.a.	n.a.	n.a.	n.a.	n.a.	n.a.	n.a.	n.a.	n.a.	n.a.	n.a.
S8-wms-combined_74275	Bacteroides phage F2	MT806186	Outlier	n.a.	N2	5	Bacteroides	Group I	Duplodnaviria	Heunggongvirae	Uroviricota	Caudoviricetes	n.a.	Unclassified	O	O
S8-wms-combined_81597	Bacteroides phage p00	BK010646	Outlier	n.a.	N9	20	Bacteroides	Group I	Duplodnaviria	Heunggongvirae	Uroviricota	Caudoviricetes	n.a.	Unclassified	O	O
S8-wms_113693	Streptococcus phage Javan553	MK448804	Outlier	n.a.	N3	9	Streptococcus	Group I	Duplodnaviria	Heunggongvirae	Uroviricota	Caudoviricetes	n.a.	Unclassified	O	O
S8-wms_190781	Clostridium phage phiMMP01	LN681541	Outlier	n.a.	N7	11	Clostridium	Group I	Duplodnaviria	Heunggongvirae	Uroviricota	Caudoviricetes	n.a.	Unclassified	O	O
S8-wms_58515	n.a.	n.a.	Outlier	n.a.	A	n.a.	n.a.	n.a.	n.a.	n.a.	n.a.	n.a.	n.a.	n.a.	n.a.	n.a.
S9-wms-combined_109322	Streptococcus phage Javan74	MK449005	Outlier	n.a.	N1	26	Streptococcus	Group I	Duplodnaviria	Heunggongvirae	Uroviricota	Caudoviricetes	n.a.	Unclassified	O	O
S9-wms-combined_122742	n.a.	n.a.	Outlier	n.a.	A	n.a.	n.a.	n.a.	n.a.	n.a.	n.a.	n.a.	n.a.	n.a.	n.a.	n.a.
S9-wms-combined_124857	n.a.	n.a.	Outlier	n.a.	A	n.a.	n.a.	n.a.	n.a.	n.a.	n.a.	n.a.	n.a.	n.a.	n.a.	n.a.
S9-wms-combined_2042	n.a.	n.a.	Outlier	n.a.	A	n.a.	n.a.	n.a.	n.a.	n.a.	n.a.	n.a.	n.a.	n.a.	n.a.	n.a.
S9-wms-combined_3461	n.a.	n.a.	Outlier	n.a.	A	n.a.	n.a.	n.a.	n.a.	n.a.	n.a.	n.a.	n.a.	n.a.	n.a.	n.a.
S9-wms-combined_93232	Klebsiella phage ST101-KPC2phi6.3	MK416017	Outlier	n.a.	N1	72	Klebsiella	Group I	Duplodnaviria	Heunggongvirae	Uroviricota	Caudoviricetes	n.a.	Unclassified	O	O
S9-wms_114971	Clostridium phage phiMMP04	JX145342	Outlier	n.a.	N1	13	Clostridium	Group I	Duplodnaviria	Heunggongvirae	Uroviricota	Caudoviricetes	n.a.	Unclassified	O	O
S12-wms-combined_148741	n.a.	n.a.	Overlap (VC_1103/VC_1240)	n.a.	A	n.a.	n.a.	n.a.	n.a.	n.a.	n.a.	n.a.	n.a.	n.a.	n.a.	n.a.
S12-wms-combined_270839	n.a.	n.a.	Overlap (VC_1103/VC_1240)	n.a.	A	n.a.	n.a.	n.a.	n.a.	n.a.	n.a.	n.a.	n.a.	n.a.	n.a.	n.a.
S1-wms-combined_52708	n.a.	n.a.	Overlap (VC_1221/VC_1237)	n.a.	A	n.a.	n.a.	n.a.	n.a.	n.a.	n.a.	n.a.	n.a.	n.a.	n.a.	n.a.
S17-wms-combined_56061	n.a.	n.a.	Overlap (VC_1221/VC_1237)	n.a.	A	n.a.	n.a.	n.a.	n.a.	n.a.	n.a.	n.a.	n.a.	n.a.	n.a.	n.a.
S2-wms-combined_181849	n.a.	n.a.	Overlap (VC_1221/VC_1237)	n.a.	A	n.a.	n.a.	n.a.	n.a.	n.a.	n.a.	n.a.	n.a.	n.a.	n.a.	n.a.
S2-wms-combined_57293	Campylobacter phage A11a	MG065691	Overlap (VC_1221/VC_1237)	n.a.	N11	5	Campylobacter	Unclassified	Unclassified	n.a.	n.a.	n.a.	n.a.	Unclassified	O	O
S6-wms-combined_110950	n.a.	n.a.	Overlap (VC_1221/VC_1237)	n.a.	A	n.a.	n.a.	n.a.	n.a.	n.a.	n.a.	n.a.	n.a.	n.a.	n.a.	n.a.
S6-wms-combined_138710	n.a.	n.a.	Overlap (VC_1221/VC_1237)	n.a.	A	n.a.	n.a.	n.a.	n.a.	n.a.	n.a.	n.a.	n.a.	n.a.	n.a.	n.a.
S7-wms-combined_137858	n.a.	n.a.	Overlap (VC_1221/VC_1237)	n.a.	A	n.a.	n.a.	n.a.	n.a.	n.a.	n.a.	n.a.	n.a.	n.a.	n.a.	n.a.
S12-wms-combined_75733	Tortoise microvirus 19	MK765569	Overlap (VC_1244/VC_1245)	n.a.	N2	9	Unspecified	Group II	Monodnaviria	Sangervirae	Phixviricota	Malgrandaviricetes	Petitvirales	Microviridae	O	O
S8-wms_270	Tortoise microvirus 19	MK765569	Overlap (VC_1244/VC_1245)	n.a.	N2	9	Unspecified	Group II	Monodnaviria	Sangervirae	Phixviricota	Malgrandaviricetes	Petitvirales	Microviridae	O	O
S1-wms-combined_153670	n.a.	n.a.	Overlap (VC_1245/VC_1487)	n.a.	A	n.a.	n.a.	n.a.	n.a.	n.a.	n.a.	n.a.	n.a.	n.a.	n.a.	n.a.
S1-wms_153615	n.a.	n.a.	Overlap (VC_1245/VC_1487)	n.a.	A	n.a.	n.a.	n.a.	n.a.	n.a.	n.a.	n.a.	n.a.	n.a.	n.a.	n.a.
S12-wms-combined_132907	Clostridium phage phiCT453A	KM983327	Overlap (VC_138/VC_146)	n.a.	C5	31	Clostridium	Group I	Duplodnaviria	Heunggongvirae	Uroviricota	Caudoviricetes	n.a.	Unclassified	Unclassified	O
S12-wms-combined_52101	Thermus phage phi OH2	AB823818	Overlap (VC_138/VC_146)	n.a.	C11	15	Thermus	Group I	Duplodnaviria	Heunggongvirae	Uroviricota	Caudoviricetes	n.a.	Unclassified	Unclassified	O
S17-wms-combined_258087	Thermus phage phi OH2	AB823818	Overlap (VC_138/VC_146)	n.a.	C25	9	Thermus	Group I	Duplodnaviria	Heunggongvirae	Uroviricota	Caudoviricetes	n.a.	Unclassified	Unclassified	O
S17-wms_106671	Clostridium phage phiCT9441A	KM983329	Overlap (VC_138/VC_146)	n.a.	C11	23	Clostridium	Group I	Duplodnaviria	Heunggongvirae	Uroviricota	Caudoviricetes	n.a.	Unclassified	Unclassified	O
S2-wms-combined_135014	Thermus phage phi OH2	AB823818	Overlap (VC_138/VC_146)	n.a.	C29	10	Thermus	Group I	Duplodnaviria	Heunggongvirae	Uroviricota	Caudoviricetes	n.a.	Unclassified	Unclassified	O
S2-wms-combined_207000	Clostridium phage phiCT9441A	KM983329	Overlap (VC_138/VC_146)	n.a.	C11	22	Clostridium	Group I	Duplodnaviria	Heunggongvirae	Uroviricota	Caudoviricetes	n.a.	Unclassified	Unclassified	O
S2-wms-combined_58563	Thermus phage phi OH2	AB823818	Overlap (VC_138/VC_146)	n.a.	C27	13	Thermus	Group I	Duplodnaviria	Heunggongvirae	Uroviricota	Caudoviricetes	n.a.	Unclassified	Unclassified	O
S2-wms_245232	Thermus phage phi OH2	AB823818	Overlap (VC_138/VC_146)	n.a.	C17	19	Thermus	Group I	Duplodnaviria	Heunggongvirae	Uroviricota	Caudoviricetes	n.a.	Unclassified	Unclassified	O
S8-wms-combined_219796	Thermus phage phi OH2	AB823818	Overlap (VC_138/VC_146)	n.a.	C1	19	Thermus	Group I	Duplodnaviria	Heunggongvirae	Uroviricota	Caudoviricetes	n.a.	Unclassified	Unclassified	O
S8-wms_20528	Clostridium phage phiCT453A	KM983327	Overlap (VC_138/VC_146)	n.a.	C15	28	Clostridium	Group I	Duplodnaviria	Heunggongvirae	Uroviricota	Caudoviricetes	n.a.	Unclassified	Unclassified	O
S9-wms-combined_105822	Thermus phage phi OH2	AB823818	Overlap (VC_138/VC_146)	n.a.	C1	25	Thermus	Group I	Duplodnaviria	Heunggongvirae	Uroviricota	Caudoviricetes	n.a.	Unclassified	Unclassified	O
S9-wms-combined_97755	Clostridium phage phiCT9441A	KM983329	Overlap (VC_138/VC_146)	n.a.	C7	30	Clostridium	Group I	Duplodnaviria	Heunggongvirae	Uroviricota	Caudoviricetes	n.a.	Unclassified	Unclassified	O
S1-wms_265285	Thermus phage phi OH2	AB823818	Overlap (VC_138/VC_146/VC_147)	n.a.	C25	16	Thermus	Group I	Duplodnaviria	Heunggongvirae	Uroviricota	Caudoviricetes	n.a.	Unclassified	Unclassified	O
S12-wms_161239	Thermus phage phi OH2	AB823818	Overlap (VC_138/VC_146/VC_147)	n.a.	C21	4	Thermus	Group I	Duplodnaviria	Heunggongvirae	Uroviricota	Caudoviricetes	n.a.	Unclassified	Unclassified	O
S17-wms_116248	Thermus phage phi OH2	AB823818	Overlap (VC_138/VC_146/VC_147)	n.a.	C23	17	Thermus	Group I	Duplodnaviria	Heunggongvirae	Uroviricota	Caudoviricetes	n.a.	Unclassified	Unclassified	O
S17-wms_29082	Thermus phage phi OH2	AB823818	Overlap (VC_138/VC_146/VC_147)	n.a.	C25	15	Thermus	Group I	Duplodnaviria	Heunggongvirae	Uroviricota	Caudoviricetes	n.a.	Unclassified	Unclassified	O
S4-wms-combined_57963	Thermus phage phi OH2	AB823818	Overlap (VC_138/VC_146/VC_147)	n.a.	C25	11	Thermus	Group I	Duplodnaviria	Heunggongvirae	Uroviricota	Caudoviricetes	n.a.	Unclassified	Unclassified	O
S17-wms-combined_66123	Thermus phage phi OH2	AB823818	Overlap (VC_138/VC_146/VC_148)	n.a.	C29	10	Thermus	Group I	Duplodnaviria	Heunggongvirae	Uroviricota	Caudoviricetes	n.a.	Unclassified	Unclassified	O
S17-wms-combined_75892	Psychrobacillus phage PVJ1	MZ983385	Overlap (VC_138/VC_146/VC_148)	n.a.	C27	10	Psychrobacillus	Group I	Duplodnaviria	Heunggongvirae	Uroviricota	Caudoviricetes	n.a.	Unclassified	Unclassified	O
S2-wms-combined_122457	Thermus phage phi OH2	AB823818	Overlap (VC_138/VC_146/VC_148)	n.a.	C33	8	Thermus	Group I	Duplodnaviria	Heunggongvirae	Uroviricota	Caudoviricetes	n.a.	Unclassified	Unclassified	O
S1-wms-combined_373308	n.a.	n.a.	Overlap (VC_1483/VC_1490)	n.a.	F	n.a.	n.a.	n.a.	n.a.	n.a.	n.a.	n.a.	n.a.	n.a.	n.a.	n.a.
S5-wms-combined_163165	n.a.	n.a.	Overlap (VC_1485/VC_1486)	n.a.	A	n.a.	n.a.	n.a.	n.a.	n.a.	n.a.	n.a.	n.a.	n.a.	n.a.	n.a.
S9-wms-combined_10990	n.a.	n.a.	Overlap (VC_1489/VC_1494)	n.a.	A	n.a.	n.a.	n.a.	n.a.	n.a.	n.a.	n.a.	n.a.	n.a.	n.a.	n.a.
S1-wms-combined_17651	n.a.	n.a.	Overlap (VC_1490/VC_1491)	n.a.	F	n.a.	n.a.	n.a.	n.a.	n.a.	n.a.	n.a.	n.a.	n.a.	n.a.	n.a.
S12-wms-combined_133608	n.a.	n.a.	Overlap (VC_1490/VC_1491)	n.a.	F	n.a.	n.a.	n.a.	n.a.	n.a.	n.a.	n.a.	n.a.	n.a.	n.a.	n.a.
S12-wms-combined_117628	Clostridium phage PhiS63	JQ660954	Overlap (VC_20/VC_542)	n.a.	C3	13	Clostridium	Group I	Duplodnaviria	Heunggongvirae	Uroviricota	Caudoviricetes	n.a.	Unclassified	Unclassified	O
S2-wms_161986	Clostridium phage PhiS63	JQ660954	Overlap (VC_20/VC_542)	n.a.	C5	14	Clostridium	Group I	Duplodnaviria	Heunggongvirae	Uroviricota	Caudoviricetes	n.a.	Unclassified	Unclassified	O
S2-wms_192667	Clostridium phage PhiS63	JQ660954	Overlap (VC_20/VC_542)	n.a.	C1	20	Clostridium	Group I	Duplodnaviria	Heunggongvirae	Uroviricota	Caudoviricetes	n.a.	Unclassified	Unclassified	O

S11-wms-combined_320710	Vibrio phage vB_VspS_VS-ABTNL-3	MF497422	Overlap (VC_342/VC_1215)	n.a.	C1	10	Vibrio	Group I	Duplodnaviria	Heunggongvirae	Uroviricota	Caudoviricetes	n.a.	Unclassified	Unclassified	O
S2-wms-combined_158821	Butyrivibrio phage Idris	MN882554	Overlap (VC_422/VC_1413)	n.a.	C5	9	Butyrivibrio	Group I	Duplodnaviria	Heunggongvirae	Uroviricota	Caudoviricetes	n.a.	Unclassified	Unclassified	O
S4-wms-combined_112093	Butyrivibrio phage Idris	MN882554	Overlap (VC_422/VC_1413)	n.a.	C5	7	Butyrivibrio	Group I	Duplodnaviria	Heunggongvirae	Uroviricota	Caudoviricetes	n.a.	Unclassified	Unclassified	O
S17-wms-combined_56006	Faecalibacterium phage FP_Brigit	MG711465	Overlap (VC_607/VC_1238)	n.a.	N5	33	Faecalibacterium	Group I	Duplodnaviria	Heunggongvirae	Uroviricota	Caudoviricetes	n.a.	Unclassified	O	O
S2-wms_124416	Faecalibacterium phage FP_Brigit	MG711465	Overlap (VC_607/VC_1238)	n.a.	N4	17	Faecalibacterium	Group I	Duplodnaviria	Heunggongvirae	Uroviricota	Caudoviricetes	n.a.	Unclassified	O	O
S12-wms-combined_385270	Lactobacillus phage c5	EU340421	Overlap (VC_630/VC_642)	n.a.	N5	6	Lactobacillus	Group I	Duplodnaviria	Heunggongvirae	Uroviricota	Caudoviricetes	n.a.	Unclassified	O	O
S17-wms-combined_179363	Lactobacillus phage c5	EU340421	Overlap (VC_630/VC_642)	n.a.	N4	9	Lactobacillus	Group I	Duplodnaviria	Heunggongvirae	Uroviricota	Caudoviricetes	n.a.	Unclassified	O	O
S2-wms-combined_99446	Clostridium phage phiCT19406B	KM983331	Overlap (VC_630/VC_642)	n.a.	N5	7	Clostridium	Group I	Duplodnaviria	Heunggongvirae	Uroviricota	Caudoviricetes	n.a.	Unclassified	O	O
S5-wms-combined_147993	Lactobacillus phage c5	EU340421	Overlap (VC_630/VC_642)	n.a.	N5	8	Lactobacillus	Group I	Duplodnaviria	Heunggongvirae	Uroviricota	Caudoviricetes	n.a.	Unclassified	O	O
S5-wms-combined_94786	Lactobacillus phage c5	EU340421	Overlap (VC_630/VC_642)	n.a.	N2	7	Lactobacillus	Group I	Duplodnaviria	Heunggongvirae	Uroviricota	Caudoviricetes	n.a.	Unclassified	O	O
S1-wms-combined_374219	n.a.	n.a.	Overlap (VC_651/VC_736)	n.a.	A	n.a.	n.a.	n.a.	n.a.	n.a.	n.a.	n.a.	n.a.	n.a.	n.a.	n.a.
S6-wms-combined_128972	n.a.	n.a.	Overlap (VC_651/VC_736)	n.a.	A	n.a.	n.a.	n.a.	n.a.	n.a.	n.a.	n.a.	n.a.	n.a.	n.a.	n.a.
S1-wms-combined_107766	n.a.	n.a.	Overlap (VC_652/VC_1481)	n.a.	A	n.a.	n.a.	n.a.	n.a.	n.a.	n.a.	n.a.	n.a.	n.a.	n.a.	n.a.
S12-wms-combined_25459	n.a.	n.a.	Overlap (VC_652/VC_1481)	n.a.	A	n.a.	n.a.	n.a.	n.a.	n.a.	n.a.	n.a.	n.a.	n.a.	n.a.	n.a.
S12-wms-combined_320262	n.a.	n.a.	Overlap (VC_652/VC_1481)	n.a.	A	n.a.	n.a.	n.a.	n.a.	n.a.	n.a.	n.a.	n.a.	n.a.	n.a.	n.a.
S2-wms-combined_208559	n.a.	n.a.	Overlap (VC_652/VC_1481)	n.a.	A	n.a.	n.a.	n.a.	n.a.	n.a.	n.a.	n.a.	n.a.	n.a.	n.a.	n.a.
S5-wms-combined_183113	n.a.	n.a.	Overlap (VC_652/VC_1481)	n.a.	A	n.a.	n.a.	n.a.	n.a.	n.a.	n.a.	n.a.	n.a.	n.a.	n.a.	n.a.
S12-wms-combined_184338	Roseburia phage Jekyll	LR596902	Overlap (VC_652/VC_894)	n.a.	N9	4	Roseburia	Group I	Duplodnaviria	Heunggongvirae	Uroviricota	Caudoviricetes	n.a.	Unclassified	O	O
S1-wms-combined_330329	Bacteroides phage p00	BK010646	Overlap (VC_678/VC_679)	n.a.	N6	37	Bacteroides	Group I	Duplodnaviria	Heunggongvirae	Uroviricota	Caudoviricetes	n.a.	Unclassified	O	O
S11-wms-combined_226165	Bacteroides phage p00	BK010646	Overlap (VC_678/VC_679)	n.a.	N10	22	Bacteroides	Group I	Duplodnaviria	Heunggongvirae	Uroviricota	Caudoviricetes	n.a.	Unclassified	O	O
S11-wms-combined_321938	Bacteroides phage p00	BK010646	Overlap (VC_678/VC_679)	n.a.	N4	38	Bacteroides	Group I	Duplodnaviria	Heunggongvirae	Uroviricota	Caudoviricetes	n.a.	Unclassified	O	O
S11-wms-combined_64392	Bacteroides phage p00	BK010646	Overlap (VC_678/VC_679)	n.a.	N2	56	Bacteroides	Group I	Duplodnaviria	Heunggongvirae	Uroviricota	Caudoviricetes	n.a.	Unclassified	O	O
S12-wms-combined_189323	Bacteroides phage p00	BK010646	Overlap (VC_678/VC_679)	n.a.	N2	45	Bacteroides	Group I	Duplodnaviria	Heunggongvirae	Uroviricota	Caudoviricetes	n.a.	Unclassified	O	O
S2-wms-combined_147065	Bacteroides phage p00	BK010646	Overlap (VC_678/VC_679)	n.a.	N7	30	Bacteroides	Group I	Duplodnaviria	Heunggongvirae	Uroviricota	Caudoviricetes	n.a.	Unclassified	O	O
S5-wms-combined_210959	Bacteroides phage p00	BK010646	Overlap (VC_678/VC_679)	n.a.	N10	15	Bacteroides	Group I	Duplodnaviria	Heunggongvirae	Uroviricota	Caudoviricetes	n.a.	Unclassified	O	O
S5-wms-combined_67650	Bacteroides phage p00	BK010646	Overlap (VC_678/VC_679)	n.a.	N11	14	Bacteroides	Group I	Duplodnaviria	Heunggongvirae	Uroviricota	Caudoviricetes	n.a.	Unclassified	O	O
S1-wms-combined_100430	Faecalibacterium phage FP_Taranis	MG711467	Overlap (VC_711/VC_715)	n.a.	C8	25	Faecalibacterium	Group I	Duplodnaviria	Heunggongvirae	Uroviricota	Caudoviricetes	n.a.	Unclassified	Unclassified	O
S1-wms-combined_285012	Faecalibacterium phage FP_Taranis	MG711467	Overlap (VC_711/VC_715)	n.a.	C9	22	Faecalibacterium	Group I	Duplodnaviria	Heunggongvirae	Uroviricota	Caudoviricetes	n.a.	Unclassified	Unclassified	O
S11-wms-combined_120257	Vibrio phage phi 2	KJ545483	Overlap (VC_711/VC_715)	n.a.	C9	8	Vibrio	Group I	Duplodnaviria	Heunggongvirae	Uroviricota	Caudoviricetes	n.a.	Unclassified	Unclassified	O
S17-wms-combined_106262	Faecalibacterium phage FP_Taranis	MG711467	Overlap (VC_711/VC_715)	n.a.	C9	21	Faecalibacterium	Group I	Duplodnaviria	Heunggongvirae	Uroviricota	Caudoviricetes	n.a.	Unclassified	Unclassified	O
S5-wms-combined_199135	n.a.	n.a.	Overlap (VC_733/VC_1494)	n.a.	A	n.a.	n.a.	n.a.	n.a.	n.a.	n.a.	n.a.	n.a.	n.a.	n.a.	n.a.
S17-wms-combined_39687	n.a.	n.a.	Overlap (VC_733/VC_893/VC_1494)	n.a.	A	n.a.	n.a.	n.a.	n.a.	n.a.	n.a.	n.a.	n.a.	n.a.	n.a.	n.a.
S12-wms_184276	Clostridium phage HM T	KU517658	Overlap (VC_735/VC_915)	n.a.	N11	4	Clostridium	Group I	Duplodnaviria	Heunggongvirae	Uroviricota	Caudoviricetes	n.a.	Unclassified	O	O
S5-wms_62679	Paenibacillus phage PG1	HQ332138	Overlap (VC_735/VC_915)	n.a.	N5	6	Paenibacillus	Group I	Duplodnaviria	Heunggongvirae	Uroviricota	Caudoviricetes	n.a.	Unclassified	O	O
S2-wms-combined_201205	Akkermansia phage DTMo-2021a	CP084202	Overlap (VC_737/VC_738)	n.a.	C1	54	Akkermansia	Group I	Duplodnaviria	Heunggongvirae	Uroviricota	Caudoviricetes	n.a.	Unclassified	Unclassified	O
S1-wms-combined_351889	Butyrivibrio virus Ceridwen	MN882553	Overlap (VC_817/VC_1210)	n.a.	C5	40	Butyrivibrio	Group I	Duplodnaviria	Heunggongvirae	Uroviricota	Caudoviricetes	n.a.	Unclassified	Unclassified	O
S2-wms-combined_64692	Butyrivibrio virus Ceridwen	MN882553	Overlap (VC_817/VC_1210)	n.a.	C5	39	Butyrivibrio	Group I	Duplodnaviria	Heunggongvirae	Uroviricota	Caudoviricetes	n.a.	Unclassified	Unclassified	O
S8-wms-combined_246389	Butyrivibrio virus Ceridwen	MN882553	Overlap (VC_817/VC_1210)	n.a.	C5	42	Butyrivibrio	Group I	Duplodnaviria	Heunggongvirae	Uroviricota	Caudoviricetes	n.a.	Unclassified	Unclassified	O
S17-wms-combined_253499	n.a.	n.a.	Overlap (VC_833/VC_1015)	n.a.	A	n.a.	n.a.	n.a.	n.a.	n.a.	n.a.	n.a.	n.a.	n.a.	n.a.	n.a.
S12-wms-combined_31761	Butyrivibrio phage Idris	MN882554	Overlap (VC_877/VC_1413)	n.a.	C1	67	Butyrivibrio	Group I	Duplodnaviria	Heunggongvirae	Uroviricota	Caudoviricetes	n.a.	Unclassified	Unclassified	O
S5-wms-combined_26061	Butyrivibrio phage Idris	MN882554	Overlap (VC_877/VC_1413)	n.a.	C5	58	Butyrivibrio	Group I	Duplodnaviria	Heunggongvirae	Uroviricota	Caudoviricetes	n.a.	Unclassified	Unclassified	O
S6-wms-combined_88414	Butyrivibrio phage Idris	MN882554	Overlap (VC_877/VC_1413)	n.a.	C5	39	Butyrivibrio	Group I	Duplodnaviria	Heunggongvirae	Uroviricota	Caudoviricetes	n.a.	Unclassified	Unclassified	O
S1-wms-combined_166392	Faecalibacterium phage FP_Lugh	MG711464	Overlap (VC_893/VC_894)	n.a.	N9	2	Faecalibacterium	Group I	Duplodnaviria	Heunggongvirae	Uroviricota	Caudoviricetes	n.a.	Unclassified	O	O
S1-wms_223620	Clostridium phage phiCD38-2	HM568888	Overlap (VC_893/VC_894)	n.a.	N10	4	Clostridium	Group I	Duplodnaviria	Heunggongvirae	Uroviricota	Caudoviricetes	n.a.	Unclassified	O	O
S11-wms-combined_179605	n.a.	n.a.	Overlap (VC_893/VC_894/VC_1489/VC_1494)	n.a.	A	n.a.	n.a.	n.a.	n.a.	n.a.	n.a.	n.a.	n.a.	n.a.	n.a.	n.a.
S12-wms-combined_297545	Bacteroides phage LoVEphage	MW660583	Overlap (VC_918/VC_1456)	n.a.	N13	3	Bacteroides	Group I	Duplodnaviria	Heunggongvirae	Uroviricota	Caudoviricetes	n.a.	Unclassified	O	O
S12-wms-combined_368190	Phage vB_RanS_PJN03	ON245412	Overlap (VC_918/VC_1456)	n.a.	N12	7	Unspecified	Group I	Duplodnaviria	Heunggongvirae	Uroviricota	Caudoviricetes	n.a.	Unclassified	O	O
S1-wms-combined_169788	n.a.	n.a.	Singleton	n.a.	G	n.a.	n.a.	n.a.	n.a.	n.a.	n.a.	n.a.	n.a.	n.a.	n.a.	n.a.
S1-wms-combined_231234	n.a.	n.a.	Singleton	n.a.	G	n.a.	n.a.	n.a.	n.a.	n.a.	n.a.	n.a.	n.a.	n.a.	n.a.	n.a.
S1-wms-combined_231411	n.a.	n.a.	Singleton	n.a.	G	n.a.	n.a.	n.a.	n.a.	n.a.	n.a.	n.a.	n.a.	n.a.	n.a.	n.a.
S1-wms-combined_32863	n.a.	n.a.	Singleton	n.a.	G	n.a.	n.a.	n.a.	n.a.	n.a.	n.a.	n.a.	n.a.	n.a.	n.a.	n.a.
S1-wms_32878	n.a.	n.a.	Singleton	n.a.	G	n.a.	n.a.	n.a.	n.a.	n.a.	n.a.	n.a.	n.a.	n.a.	n.a.	n.a.
S1-wms_388292	n.a.	n.a.	Singleton	n.a.	G	n.a.	n.a.	n.a.	n.a.	n.a.	n.a.	n.a.	n.a.	n.a.	n.a.	n.a.
S11-wms-combined_117756	n.a.	n.a.	Singleton	n.a.	G	n.a.	n.a.	n.a.	n.a.	n.a.	n.a.	n.a.	n.a.	n.a.	n.a.	n.a.
S11-wms-combined_265453	n.a.	n.a.	Singleton	n.a.	G	n.a.	n.a.	n.a.	n.a.	n.a.	n.a.	n.a.	n.a.	n.a.	n.a.	n.a.
S11-wms_32691	n.a.	n.a.	Singleton	n.a.	G	n.a.	n.a.	n.a.	n.a.	n.a.	n.a.	n.a.	n.a.	n.a.	n.a.	n.a.
S11-wms_6203	n.a.	n.a.	Singleton	n.a.	G	n.a.	n.a.	n.a.	n.a.	n.a.	n.a.	n.a.	n.a.	n.a.	n.a.	n.a.
S12-wms-combined_117447	n.a.	n.a.	Singleton	n.a.	G	n.a.	n.a.	n.a.	n.a.	n.a.	n.a.	n.a.	n.a.	n.a.	n.a.	n.a.
S12-wms-combined_390985	n.a.	n.a.	Singleton	n.a.	G	n.a.	n.a.	n.a.	n.a.	n.a.	n.a.	n.a.	n.a.	n.a.	n.a.	n.a.
S12-wms-combined_63056	n.a.	n.a.	Singleton	n.a.	G	n.a.	n.a.	n.a.	n.a.	n.a.	n.a.	n.a.	n.a.	n.a.	n.a.	n.a.
S12-wms_223235	n.a.	n.a.	Singleton	n.a.	G	n.a.	n.a.	n.a.	n.a.	n.a.	n.a.	n.a.	n.a.	n.a.	n.a.	n.a.
S12-wms_336780	n.a.	n.a.	Singleton	n.a.	G	n.a.	n.a.	n.a.	n.a.	n.a.	n.a.	n.a.	n.a.	n.a.	n.a.	n.a.
S12-wms_352846	n.a.	n.a.	Singleton	n.a.	G	n.a.	n.a.	n.a.	n.a.	n.a.	n.a.	n.a.	n.a.	n.a.	n.a.	n.a.
S2-wms-combined_115352	n.a.	n.a.	Singleton	n.a.	G	n.a.	n.a.	n.a.	n.a.	n.a.	n.a.	n.a.	n.a.	n.a.	n.a.	n.a.
S2-wms-combined_204225	n.a.	n.a.	Singleton	n.a.	G	n.a.	n.a.	n.a.	n.a.	n.a.	n.a.	n.a.	n.a.	n.a.	n.a.	n.a.
S2-wms-combined_222535	n.a.	n.a.	Singleton	n.a.	G	n.a.	n.a.	n.a.	n.a.	n.a.	n.a.	n.a.	n.a.	n.a.	n.a.	n.a.
S2-wms-combined_230282	n.a.	n.a.	Singleton	n.a.	G	n.a.	n.a.	n.a.	n.a.	n.a.	n.a.	n.a.	n.a.	n.a.	n.a.	n.a.
S2-wms-combined_30750	n.a.	n.a.	Singleton	n.a.	G	n.a.	n.a.	n.a.	n.a.	n.a.	n.a.	n.a.	n.a.	n.a.	n.a.	n.a.
S2-wms-combined_48661	n.a.	n.a.	Singleton	n.a.	G	n.a.	n.a.	n.a.	n.a.	n.a.	n.a.	n.a.	n.a.	n.a.	n.a.	n.a.
S2-wms-combined_52006	n.a.	n.a.	Singleton	n.a.	G	n.a.	n.a.	n.a.	n.a.	n.a.	n.a.	n.a.	n.a.	n.a.	n.a.	n.a.
S2-wms-combined_73327	n.a.	n.a.	Singleton	n.a.	G	n.a.	n.a.	n.a.	n.a.	n.a.	n.a.	n.a.	n.a.	n.a.	n.a.	n.a.
S2-wms-combined_95987	n.a.	n.a.	Singleton	n.a.	G	n.a.	n.a.	n.a.	n.a.	n.a.	n.a.	n.a.	n.a.	n.a.	n.a.	n.a.
S4-wms-combined_24659	n.a.	n.a.	Singleton	n.a.	G	n.a.	n.a.	n.a.	n.a.	n.a.	n.a.	n.a.	n.a.	n.a.	n.a.	n.a.
S5-wms-combined_106597	n.a.	n.a.	Singleton	n.a.	G	n.a.	n.a.	n.a.	n.a.	n.a.	n.a.	n.a.	n.a.	n.a.	n.a.	n.a.
S5-wms-combined_197186	n.a.	n.a.	Singleton	n.a.	G	n.a.	n.a.	n.a.	n.a.	n.a.	n.a.	n.a.	n.a.	n.a.	n.a.	n.a.
S5-wms-combined_205046	n.a.	n.a.	Singleton	n.a.	G	n.a.	n.a.	n.a.	n.a.	n.a.	n.a.	n.a.	n.a.	n.a.	n.a.	n.a.
S5-wms_10783	n.a.	n.a.	Singleton	n.a.	G	n.a.	n.a.	n.a.	n.a.	n.a.	n.a.	n.a.	n.a.	n.a.	n.a.	n.a.
S5-wms_123499	n.a.	n.a.	Singleton	n.a.	G	n.a.	n.a.	n.a.	n.a.	n.a.	n.a.	n.a.	n.a.	n.a.	n.a.	n.a.
S5-wms_212596	n.a.	n.a.	Singleton	n.a.	G	n.a.	n.a.	n.a.	n.a.	n.a.	n.a.	n.a.	n.a.	n.a.	n.a.	n.a.
S6-wms-combined_65225	n.a.	n.a.	Singleton	n.a.	G	n.a.	n.a.	n.a.	n.a.	n.a.	n.a.	n.a.	n.a.	n.a.	n.a.	n.a.
S6-wms-combined_745	n.a.	n.a.	Singleton	n.a.	G	n.a.	n.a.	n.a.	n.a.	n.a.	n.a.	n.a.	n.a.	n.a.	n.a.	n.a.
S7-wms-combined_121062	n.a.	n.a.	Singleton	n.a.	G	n.a.	n.a.	n.a.	n.a.	n.a.	n.a.	n.a.	n.a.	n.a.	n.a.	n.a.
S7-wms-combined_123085	n.a.	n.a.	Singleton	n.a.	G	n.a.	n.a.	n.a.	n.a.	n.a.	n.a.	n.a.	n.a.	n.a.	n.a.	n.a.
S7-wms-combined_134452	n.a.	n.a.	Singleton	n.a.	G	n.a.	n.a.	n.a.	n.a.	n.a.	n.a.	n.a.	n.a.	n.a.	n.a.	n.a.
S7-wms-combined_157434	n.a.	n.a.	Singleton	n.a.	G	n.a.	n.a.	n.a.	n.a.	n.a.	n.a.	n.a.	n.a.	n.a.	n.a.	n.a.
S7-wms-combined_167867	n.a.	n.a.	Singleton	n.a.	G	n.a.	n.a.	n.a.	n.a.	n.a.	n.a.	n.a.	n.a.	n.a.	n.a.	n.a.

S9-wms-combined_27311	n.a.	n.a.	Singleton	n.a.	G	n.a.	n.a.	n.a.	n.a.	n.a.	n.a.	n.a.	n.a.	n.a.	n.a.	n.a.
S9-wms_10480	n.a.	n.a.	Singleton	n.a.	G	n.a.	n.a.	n.a.	n.a.	n.a.	n.a.	n.a.	n.a.	n.a.	n.a.	n.a.

Table S6-3. VC statistiscs	VLP-HQ-vOTUs	WMS-HQ-vOTUs
vOTUs considered as “clustered” or “clustered/singleton”	764	411
vOTUs considered as “singleton”	34	41
vOTUs considered as “outlier”	207	148
vOTUs considered as “overlap” between more than one clusters	226	88
