# Supplementary material for: Investigating the Human Intestinal DNA Virome and Predicting Disease-Associated Virus–Host Interactions in Severe Myalgic Encephalomyelitis/Chronic Fatigue Syndrome (ME/CFS)
Source: Int J Mol Sci. 2023 Dec 8;24(24):17267. doi: 10.3390/ijms242417267 (PMC10744171; doi:10.3390/ijms242417267)
Supplement: Supplementary file 1 [file ijms-24-17267-s001.zip › Table S8.pdf]

TableS8. WMS viral cluster analysis

Table S8-1. WMS_viral_cluster_overview

	VC	Size	Internal Weight	External Weight	Quality	P-value	Min Dist	Max Dist	Total Dist	Below Thres	Taxon Prediction Score	Avg Dist	Genera	Families	Orders	Members
0	VC_0_0	3	77.25303487	0	1	0	1	2	3	3	1	1.57735027	0	0	0	AB002632,KC357596,OP297622
1	VC_1000_0	21	1764.69294	326.8331113	0.843734621	2.47E-08	0	2.236067977	210	210	1	1.45145382	0	0	0	MG945161,MK249131,MK249144,MK249156,MK249159,MK249160,MK249167,MK249179,MK249181,MZ364226,MZ364241,MZ364244,MZ364245,MZ364246,MZ364247,MZ364249,MZ364250,MZ364260,MZ364263,MZ364266,MZ364270
2	VC_1002_0	2	5.394552787	24.43350627	0.180854972	0.279092325	5.567764363	5.567764363	1	1	1	5.56776436	0	0	0	MT152698,MZ334527
3	VC_1003_0	4	613.6260189	90.27156737	0.871754685	0.00016219	5	6.782329983	6	6	1	6.01652811	0	0	0	MN698240,MN698245,MN698246,MN698248
4	VC_1004_0	2	212.1262903	245.2302544	0.463809456	0.057744643	0	0	1	1	1	0	0	0	0	MW202462,MW202544
5	VC_1006_0	8	7918.777591	2772.145143	0.74070104	4.06E-14	0	8.774964387	28	28	1	6.71046686	0	0	0	KC595511,KT070866,MG967616,MG967617,MG967618,MK288022,MW392801,MW392802
6	VC_1007_0	19	26158.92735	4259.906749	0.85995825	5.31E-42	0	4	171	171	1	2.53470896	0	0	0	MT074134,MT074135,MT074143,MT074144,MT074145,MT074147,MT074148,MT074149,MT074150,MT074151,MT074152,MT074153,MT074154,MT074155,MT074156,MT074157,MT074158,MT074159,MT074160
7	VC_1008_0	4	615.7103743	316.9364352	0.660175286	6.46E-05	1.732050808	6	6	6	1	4.45167754	0	0	0	MT635598,MT806185,MT806186,S4-wms_141607
8	VC_1009_0	3	454.8895356	3127.647895	0.126974119	0.001855388	1.732050808	3	3	3	1	2.52015931	0	0	0	ON721384,ON721385,S7-wms_244866
9	VC_100_0	15	18911.93957	10023.80534	0.653583989	3.42E-16	0	8.246211251	105	105	1	5.36854133	0	0	0	KC330679,KC330680,KC330681,KC330682,KC330683,KC330684,KF669648,KF669651,KF669659,KU836751,MG983742,MH884513,MN727882,MT422786,MT514532
10	VC_1010_0	2	7.940981737	31.06519054	0.203582697	0.226627352	5.477225575	5.477225575	1	1	1	5.47722558	0	0	0	MN732896,S11-wms_330479
11	VC_1011_0	9	1542.44428	0	1	0	0	1.732050808	36	36	1	0.96225045	0	0	0	KC821606,KC821623,KC821628,KC821631,MT732445,MT732446,MT732447,MT732448,MT732449
12	VC_1012_0	4	1231.694827	181.6559425	0.871471438	4.61E-05	6.8556546	7.615773106	6	6	1	7.27476053	0	0	0	KC821608,KC821620,KC821622,KC821633
13	VC_1013_0	34	94222.53757	578.6058074	0.993896637	1.78E-58	0	7.141428429	561	561	1	5.0102952	0	0	0	MN812206,MN812207,MN812208,MN812209,MN812210,MN812212,MN812213,MN812214,MN812215,MN812216,MN812217,MN812218,MN812219,MN812220,MN812221,MN812222,MN812223,MN812224,MN812225,MN812226,MN812227,MN812228,MN812229,MN812230,MN812231,MN812232,MN812233,MN812234,MN812235,MN812236,MN812237,MN812238,MN812240,MN812241
14	VC_1014_0	3	50.89806485	200.2682651	0.202646847	0.144087817	7.211102551	9	3	2	0.666666667	8.2904522	0	0	0	S11-wms-combined_92114,S17-wms-combined_199746,S7-wms-combined_163980
15	VC_1015_0	62	553967.0099	2972.02003	0.994663653	1.16E-278	0	3.872983346	1891	1891	1	1.14540492	0	0	0	MT497017,MT497065,MT497066,MT497067,MT497068,MT497069,MT497070,MT497071,MT497072,MT497073,MT497074,MT497075,MT497076,MT497077,MT497078,MT497079,MT497080,MT497081,MT497082,MT497083,MT497084,MT497085,MT497086,MT497087,MT497088,MT497089,MT497090,MT497091,MT497092,MT497093,MT497094,MT497095,MT497096,MT497097,MT497098,MT497099,MT497100,MT497101,MT497102,MT497103,MT497104,MT497105,MT497106,MT497107,MT497108,MT497109,MT497110,MT497111,MT497112,MT497113,MT497114,MT497115,MT497116,MT497117,MT497118,MT497119,MT497120,MT497121,MT497122,MT497123,MT497124,MT497125
16	VC_1016_0	8	5433.763075	297.5763986	0.948079083	9.82E-16	0	0	28	28	1	0	0	0	0	MT732435,MT732436,MT732437,MT732438,MT732439,MT732440,MT732441,MT732442
17	VC_1017_0	5	3000	74.05087486	0.975910979	3.90E-06	0	4.898979486	10	10	1	2.93938769	0	0	0	KC821609,KC821632,KT962245,KT962246,KT962247
18	VC_1018_0	2	188.1416897	2021.96321	0.085127946	0.055980677	5.830951895	5.830951895	1	1	1	5.83095189	0	0	0	KJ003983,MT774389
19	VC_1018_1	10	7017.239745	2316.291925	0.751831139	1.94E-11	2	9.539392014	45	36	0.8	8.18817816	0	0	0	MH675552,MT074136,MT074138,MT774378,MT774385,MT774387,MT774388,MT774390,MT774391,MT774392
20	VC_1019_0	5	3000	464.0184448	0.866046197	1.33E-08	0	2.449489743	10	10	1	1.46969385	0	0	0	KC821612,KC821614,MT732432,MT732433,MT732434
21	VC_101_0	3	497.2311074	876.078347	0.362067781	0.002351157	2.449489743	5.385164807	3	3	1	4.27821818	0	0	0	KY565347,MH884514,OP341625
22	VC_1020_0	5	1846.320447	386.2957948	0.826976178	2.96E-07	0	4.242640687	10	10	1	3.32864269	0	0	0	KC821613,KC821615,KC821617,KC821619,KC821627
23	VC_1021_0	10	4853.66425	614.4769338	0.887625993	1.89E-20	0	6.8556546	45	45	1	4.19134967	0	0	0	MK764442,MK764445,MK764448,MN812203,MN812204,MN812205,MW421581,MW421582,MW421583,MW421584
24	VC_1022_0	4	1476.028711	358.302652	0.804668524	3.91E-05	1	3.605551275	6	6	1	2.46941742	0	0	0	KY206887,MK017819,MN417334,OP381444
25	VC_1023_0	8	4865.42966	809.4334527	0.857365114	4.02E-14	1	5.656854249	28	28	1	3.91833899	0	0	0	KC959568,KU599887,KU599888,KU599889,MK764437,MK764440,MK764450,MW421576
26	VC_1024_0	2	300	0	1	0	0	0	1	1	1	0	0	0	0	KC960489,KF981876
27	VC_1025_0	4	1800	0	1	1	1	3.464101615	6	6	1	2.66527994	0	0	0	KF005317,KF005318,KF005319,KF005320
28	VC_1026_0	2	2.699997311	6.368472666	0.297734603	0.185546685	7.141428429	7.141428429	1	1	1	7.14142843	0	0	0	KX257490,MW805362
29	VC_1027_0	20	17450.45256	7080.649275	0.711360324	9.35E-59	0	7.211102551	190	190	1	5.58509488	0	0	0	KF322026,KF800937,KJ502657,KY290756,KY626176,KY626177,MF481197,MG018929,MG545917,MH113812,MH113813,MH113814,MH113815,MK455769,MK455770,MK820013,MN807240,MT941682,MW451247,MW865291
30	VC_1028_0	8	8400	609.8557848	0.93231237	7.68E-17	1	3.741657387	28	28	1	2.48352807	0	0	0	KU245542,OK638201,OK638202,OK638203,OL343755,OL473897,OL622073,ON857939
31	VC_1029_0	4	1800	198.6255297	0.900618937	4.24E-05	2.449489743	4.582575695	6	6	1	3.64296812	0	0	0	MW677511,MW677527,OP009274,OP009281
32	VC_102_0	2	35.70708253	277.4142268	0.114035939	0.053624467	7.348469228	7.348469228	1	1	1	7.34846923	0	0	0	KJ159566,KT001912
33	VC_1030_0	3	639.8512308	170.5937414	0.789506077	0.004575844	6	7.211102551	3	3	1	6.76072345	0	0	0	MG596799,MK473373,MZ605292
34	VC_1031_0	4	1566.723002	420.5238217	0.788388731	2.38E-05	5.196152423	6.557438524	6	6	1	5.84302362	0	0	0	MN096357,MN096358,MT818426,ON526969
35	VC_1032_0	7	3021.390644	34.59405021	0.988679901	4.96E-06	1.414213562	4.472135955	21	21	1	2.98437575	0	0	0	KF669655,KF669657,KF669660,KM236247,KM236248,KP411017,KT001911
36	VC_1034_0	2	145.6618651	1905.736805	0.071006122	0.046736356	8	8	1	1	1	8	0	0	0	ON645346,OP580516
37	VC_1034_1	1	0	351.2232494	0	0	0	0	1	1	1	0	0	0	0	KF692088
38	VC_1035_0	6	2585.886475	2741.774604	0.485369928	1.23E-10	3.464101615	5.744562647	15	15	1	4.89673425	0	0	0	MH834603,MH834605,MH834611,MH834614,MH834623,ON970561
39	VC_1036_0	11	9323.246069	4889.391776	0.655982807	4.76E-31	2.449489743	9.591663047	55	48	0.872727273	7.82429352	0	0	0	MH834598,MH834612,MH834625,MH910036,MH910037,MH910041,MW712719,ON724017,ON970598,OP434442,OP434463
40	VC_1037_0	6	2456.770905	1156.974224	0.679840669	2.17E-10	3.605551275	6.08276253	15	15	1	4.99225323	0	0	0	MH834621,MH834627,MK392368,MZ150784,MZ274308,ON392166
41	VC_1039_0	5	1699.537165	0	1	0	0	3.605551275	10	10	1	2.6822395	0	0	0	KF751793,KF751794,KF751795,KF751796,KF751797
42	VC_103_0	6	4294.375821	1558.407207	0.733732277	2.22E-10	1	3.464101615	15	15	1	2.59895821	0	0	0	MN176222,MN176223,MN176224,MN176225,MN176226,MN176229
43	VC_1040_0	23	60461.10187	2488.877802	0.960462612	5.32E-41	0	1.732050808	253	253	1	0.20723532	0	0	0	KT876725,KU599880,KU599881,KU599882,KU599883,KU599884,KU599885,MK764436,MK764438,MK764439,MK764443,MK764444,MK764446,MK764447,OK268422,OK268423,OK268424,OK268425,OK268426,OK268427,OK340817,OK340818,OK340819
44	VC_1041_0	9	5541.177777	2930.572458	0.654077094	1.10E-21	0	5.385164807	36	36	1	3.49415694	0	0	0	MG592391,MG592421,MG592436,MG592443,MG592445,MG592449,MG592661,OX241561,OX241569
45	VC_1043_0	2	300	4909.815617	0.057583612	0.043457527	0	0	1	1	1	0	0	0	0	MN183282,MT771347
46	VC_1044_0	3	788.466022	8563.810208	0.084307392	0.001476374	3.31662479	3.872983346	3	3	1	3.64375517	0	0	0	OM913586,ON970564,ON970608
47	VC_1045_0	2	156.6572271	206.1842793	0.431751121	0.05601967	5.916079783	5.916079783	1	1	1	5.91607978	0	0	0	MN988468,MN988516
48	VC_1046_0	7	4220.673924	263.2927084	0.941281296	2.16E-11	0	4.123105626	21	21	1	2.71290761	0	0	0	KJ510413,KM591905,MF155936,MG925349,MH001451,MK494089,MN735432
49	VC_1047_0	5	1430.948215	734.1050901	0.660929785	6.85E-08	0	8.185352772	10	10	1	6.1126874	0	0	0	MW584157,MW584173,MW584189,MW584206,MW584209
50	VC_1048_0	3	280.8724475	0	1	0	1.414213562	1.732050808	3	3	1	1.62610506	0	0	0	KJ617393,LK392619,Z47794
51	VC_1049_0	8	5407.635344	3024.792047	0.641290472	4.95E-18	0	8.888194417	28	28	1	7.27386247	0	0	0	KJ802832,KX987158,KY002061,MH586730,MZ726797,OL604152,ON602746,OP617744
52	VC_104_0	6	2530.405176	521.4160736	0.829145933	4.69E-10	2.645751311	7.810249676	15	15	1	6.10945144	0	0	0	MH752385,MN013088,MN176219,MN176227,MN176228,X97918
53	VC_1050_0	24	80027.74598	71723.91298	0.527359941	1.01E-131	0	6.164414003	276	276	1	1.90522489	0	0	0	MG592411,MG592538,MG592563,MG592564,MG592565,MG592571,MG592632,MG592633,MG592634,MG592635,MG592636,MG592637,MG592638,MG592639,MG592640,MG592642,MG592645,MG592646,MG592648,MG592649,MG592650,MG592651,MG592652,MG592653
54	VC_1050_1	29	68140.94969	72040.74473	0.486090213	2.39E-176	0	9.486832981	406	389	0.958128079	7.89742415	0	0	0	MG592397,MG592406,MG592407,MG592408,MG592416,MG592417,MG592428,MG592463,MG592464,MG592471,MG592475,MG592476,MG592492,MG592493,MG592528,MG592601,MG592602,MG592617,MG592618,MG592655,MH925092,MW824418,MW824430,MW824432,MW824433,MW824434,OX241427,OX241556,OX241563
55	VC_1051_0	2	76.66564858	0	1	0	4.795831523	4.795831523	1	1	1	4.79583152	0	0	0	KM058087,MW900438
56	VC_1052_0	2	300	3558.158445	0.077757304	0.053993533	1	1	1	1	1	1	0	0	0	MT234342,MT234343
57	VC_1052_1	1	0	3801.338718	0	0	0	0	1	1	1	0	0	0	0	KP881232
58	VC_1052_2	1	0	3340.460153	0	0	0	0	1	1	1	0	0	0	0	MF403007
59	VC_1052_3	5	3000	13220.40126	0.184952268	0.013439523	4.582575695	7.141428429	10	10	1	6.23979295	0	0	0	KM199770,MN549360,MN549361,MT778837,MT778839
60	VC_1052_4	6	4500	13966.28309	0.243687372	0.002040746	0	8.660254038	15	15	1	4.7446747	0	0	0	MN988486,MN988509,MN988520,MN988532,MN988534,MN988553
61	VC_1053_0	1	0	62.55824749	0	0	0	0	1	1	1	0	0	0	0	MG945594
62	VC_1054_0	12	366.5388527	913.8579835	0.286269727	0.00253356	0	2.828427125	66	66	1	2.0953888	0	0	0	KM589498,KT264826,KT264827,MG945473,MG945703,MH931003,MK496723,MN379633,MN379651,OM522999,OM523001,OM869624
63	VC_1056_0	4	72.93892166	358.720217	0.168973422	0.158893734	0	2.645751311	6	6	1	1.79428018	0	0	0	KT264807,KT264829,KX513869,MG945560
64	VC_1058_0	2	8.282189768	8.07565711	0.506312954	0.270145687	2.236067977	2.236067977	1	1	1	2.23606798	0	0	0	OM869532,OM869702
65	VC_1059_0	3	18.75727903	28.99904547	0.392770575	0.016005398	1.414213562	2	3	3	1	1.80473785	0	0	0	MG945835,MH992170,MH992193
66	VC_1061_0	2	20.86797703	8.66893268	0.706505089	0.239750061	0	0	1	1	1	0	0	0	0	KM589499,KM589507
67	VC_1062_0	45	6527.953136	178.3586877	0.973404355	0.490501528	0	1.732050808	990	990	1	0.99770742	0	0	0	KM589500,KM589503,KM589504,KM589505,KM589509,KM589510,KM589512,KM589513,KP087948,MG945178,MG945463,MH572367,MH572368,MH572377,MH572378,MH572379,MH572459,MH572460,MH572461,MH572462,MH572463,MH572465,MH992180,MK249133,MK249138,MK249191,MK249199,MK249207,MK249208,MK249228,MK249232,MK765579,MK765582,MK765639,MK765642,MK765643,MK765644,MK765646,MN311488,MN311489,MN311490,MN311491,MZ364242,OL617032,OL617033
68	VC_1066_0	52	16759.48627	5058.433614	0.768152343	1.05E-284	0	2.645751311	1326	1326	1	1.35082908	0	0	0	KM589508,KT264808,KT264809,KT264810,MH992165,MK249127,MK249128,MK249129,MK249139,MK249149,MK249150,MK249153,MK249154,MK249155,MK249158,MK249162,MK249163,MK249166,MK249168,MK249174,MK249176,MK249178,MK249180,MK249188,MK249192,MK249194,MK249195,MK249196,MK249198,MK249204,MK249212,MK249214,MK249219,MN862338,MZ364222,MZ364223,MZ364232,MZ364248,MZ364254,MZ364255,MZ364261,MZ364262,MZ364264,MZ364265,MZ364268,MZ364269,MZ364271,MZ364272,MZ364273,MZ364275,MZ364276,MZ364278
69	VC_1067_0	2	8.583214966	708.0442186	0.011977235	0.141922278	1.414213562	1.414213562	1	1	1	1.41421356	0	0	0	MK249132,MK249205
70	VC_1068_0	5	81.24792706	0	1	0	0	1.414213562	10	10	1	0.88284271	0	0	0	KM589514,MK249222,MZ364231,MZ364280,MZ364284
71	VC_1069_0	4	65.20443085	0	1	0	0	1.414213562	6	6	1	1.1785113	0	0	0	KM589515,KM589516,MT135405,OM869497
72	VC_1070_0	2	300	2008.693666	0.129943615	0.046247325	7.141428429	7.141428429	1	1	1	7.14142843	0	0	0	MH153807,MH153812
73	VC_1070_1	1	0	1264.24364	0	0	0	0	1	1	1	0	0	0	0	KX774321
74	VC_1071_0	20	57000	117435.2819	0.326768756	1.31E-102	2	9.055385138	190	187	0.984210526	6.32864962	0	0	0	MH576964,MH576965,MH576968,MH590597,MH590599,MH727564,MK359332,MK359351,MK801722,MN369750,MN369757,MN428060,MN484599,MW291014,MW507134,ON260819,ON260828,ON970579,ON970590,OP021680
75	VC_1071_1	1	0	12914.8746	0	0	0	0	1	1	1	0	0	0	0	OL829978
76	VC_1071_2	5	3000	53141.80486	0.053436116	2.64E-05	3.464101615	9.38083152	10	9	0.9	7.1747401	0	0	0	MF155946,MT310865,MT684590,MT897905,ON970591
77	VC_1071_3	16	36000	116914.0826	0.235426322	9.11E-57	3.31662479	9.327379053	120	116	0.966666667	7.3676089	0	0	0	KM652554,MF347636,MF347637,MF347638,MF347639,MF358541,MF358542,MK460248,MK494112,MK977711,MN096371,MN369743,MW507136,MZ958750,OK310502,ON108650
78	VC_1071_4	1	0	12034.67914	0	0	0	0	1	1	1	0	0	0	0	MN444876
79	VC_1071_5	1	0	11767.87188	0	0	0	0	1	1	1	0	0	0	0	MT711976
80	VC_1072_0	2	300	11048.06758	0.026436219	0.048825988	3.605551275	3.605551275	1	1	1	3.60555128	0	0	0	MG757153,MK620896
81	VC_1072_1	2	300	11282.93201	0.025900178	0.048719576	4	4	1	1	1	4	0	0	0	MT024865,MT024872
82	VC_1072_2	5	3000	26912.46432	0.100292639	6.85E-06	2.236067977	5.099019514	10	10	1	4.10391909	0	0	0	MH590589,MH651172,MW365952,MZ820095,ON456343
83	VC_1072_3	6	4500	30019.34189	0.130361697	6.48E-08	3.605551275	9.110433579	15	13	0.866666667	6.98070744	0	0	0	MK061412,MT114162,MW435853,MZ820093,MZ820094,ON208830
84	VC_1072_4	4	1800	22659.45077	0.073591186	0.000434408	3.741657387	7.937253933	6	6	1	6.88727558	0	0	0	MH536811,MH536814,MN369754,MT952852
85	VC_1072_5	3	900	18056.33942	0.047477521	0.010026794	6.92820323	8.94427191	3	3	1	8.23474534	0	0	0	MT684598,MW822145,ON645339
86	VC_1073_0	24	56348.25696	10828.27477	0.838808666	3.17E-125	1.414213562	7.141428429	276	276	1	4.35726314	0	0	0	KT365401,KU160651,KU160656,KU160665,KU160668,KU647629,MF189176,MF189177,MF189178,MF324907,MF377441,MF377442,MH825703,MK919479,MN183279,MN183283,MT024868,MT114161,MT310895,MT776811,MT889388,MW862982,OK999980,ON970601
87	VC_1074_0	19	51212.62536	4462.845979	0.919841793	1.25E-88	0	5.477225575	171	171	1	3.06956111	0	0	0	KM879463,KU160660,KX522650,KX670786,KX670787,KX855961,KX855962,MF185718,MF185721,MF185723,MF185724,MF185725,MF668274,MH744419,MK494107,MN204499,MN204503,MN234234,MN703411
88	VC_1075_0	4	1166.060237	6694.97929	0.148334102	1.57E-05	3.872983346	6.633249581	6	6	1	5.16919816	0	0	0	MT498055,MW601223,MZ747522,OL742560
89	VC_1076_0	2	37.25022147	70.38665183	0.346073054	0.361836805	7.874007874	7.874007874	1	1	1	7.87400787	0	0	0	S12-wms-combined_376326,S8-wms-combined_72911
90	VC_1077_0	48	6785.315608	1174.292656	0.852468536	1.82E-44	0	2.236067977	1128	1128	1	1.40732858	0	0	0	KP087938,KP087942,MG883726,MG945129,MG945206,MG945670,MG945836,MG945840,MG945846,MH572362,MH572410,MH572411,MH572412,MH572486,MH572521,MH931004,MH992163,MH992164,MH992192,MH992199,MH992202,MH992206,MH992208,MH992214,MH992215,MH992216,MH992217,MH992219,MH992220,MH992222,MK765645,MN862346,MW202445,MW202609,MW202760,MW202819,MZ089752,MZ364224,MZ364225,MZ364227,MZ364277,MZ364279,MZ364283,MZ364285,MZ364286,MZ364291,OL617030,OL617031
91	VC_1079_0	6	136.6462375	10.27097468	0.930090052	0.109034215	0	2	15	15	1	1.01307683	0	0	0	MH572360,MH572361,MH572409,MH572457,MH572482,MH572483
92	VC_107_0	5	1502.822416	1825.786854	0.45148658	5.28E-08	4	6.92820323	10	10	1	6.06172101	0	0	0	OP341624,OP341626,OP341627,OP341628,OP341630
93	VC_1081_0	1	0	13.04261974	0	0	0	0	1	1	1	0	0	0	0	MN379632
94	VC_1082_0	3	24.02393131	10.66891169	0.69247514	0.380453364	0	1.732050808	3	3	1	1.15470054	0	0	0	MH572366,MW202464,MW202671
95	VC_1084_0	9	557.0840498	292.3370352	0.655839677	1.47E-17	0	1.414213562	36	36	1	0.79125782	0	0	0	MH572287,MH572288,MH572467,MH572468,MH572496,MH572497,MH572498,MH572499,MH572500
96	VC_1085_0	2	25.35975681	17.56982715	0.590729154	0.066807201	0	0	1	1	1	0	0	0	0	KT264812,MG945475
97	VC_1086_0	3	14.03030183	2.639931621	0.841637993	0.185546685	1	1.732050808	3	3	1	1.38208812	0	0	0	KP087943,KP087951,KP087957
98	VC_1087_0	5	24.06238446	5.612535498	0.810866027	0.012404212	1	2.236067977	10	10	1	1.56748611	0	0	0	KP087953,MH572371,MH572373,MH572380,MK249215
99	VC_1088_0	3	16.40130604	4.790390798	0.773949635	0.172889293	0	1	3	3	1	0.66666667	0	0	0	KP087955,MH572364,MH572458
100	VC_1089_0	2	3.175008833	2.639931621	0.54600883	0.5	1	1	1	1	1	1	0	0	0	MH572489,MW202618
101	VC_108_0	33	124113.4748	6664.107101	0.949042435	3.43E-84	0	7.937253933	528	528	1	6.14539779	0	0	0	AB712291,HE962497,JF731128,KF192053,KJ094032,KJ127303,KT932699,KY303907,KY472224,LC547238,MH618488,MH791415,MK570225,MK721189,MK721194,MK800154,MN103542,MN871443,MN939408,MW677132,MZ333457,OL505085,OL870612,ON086985,ON113171,ON113172,ON113173,ON113174,ON113175,ON113177,ON113178,ON113179,ON113180
102	VC_1090_0	4	183.6191782	0	1	0	7.348469228	8.602325267	6	6	1	7.97825176	0	0	0	KP202971,KT001914,MF098558,ON323491
103	VC_1091_0	2	150.9423405	0	1	0	3.741657387	3.741657387	1	1	1	3.74165739	0	0	0	KP836355,KP836356
104	VC_1092_0	12	11676.69435	1452.418658	0.889374198	8.74E-33	0	5.291502622	66	66	1	3.716604	0	0	0	MW314851,MW314853,MW314857,MW584149,MW584151,MW584184,MW584193,MW584194,MW584198,MW584200,MW584205,MW584210
105	VC_1093_0	40	165629.108	23580.81472	0.875372209	2.54E-278	1	7.615773106	780	780	1	5.72824474	0	0	0	KU963250,MF919514,MG812487,MH153809,MH450129,MH479924,MH651166,MH651168,MH976506,MH976507,MH976508,MH976509,MH976510,MH976511,MH976512,MH976513,MH976514,MH976518,MH976519,MK937594,MK937601,MK967396,MK977702,MN329673,MN329677,MT498035,MT639640,MT723934,MT723937,MT723941,MW822146,MW924645,MZ322015,MZ681510,ON081332,ON456336,ON637761,ON724014,OP172877,OP434456
106	VC_1095_0	16	23546.79785	27866.13825	0.457993642	1.40E-70	0	8.831760866	120	120	1	6.18855891	0	0	0	KU963253,MH669000,MK937607,MK937611,MN586026,MN586028,MN586047,MT498036,MW314847,MZ322009,MZ322020,ON392159,ON456337,ON456349,ON755186,OP068342
107	VC_1096_0	5	2079.239624	348.1711688	0.856566853	0.001300234	2.645751311	8.246211251	10	10	1	6.15967245	0	0	0	KR063280,MK279853,MN586022,MZ820089,ON755184
108	VC_1098_0	4	1800	1493.830995	0.546476125	3.54E-05	1	7.810249676	6	6	1	4.67237231	0	0	0	MK814760,MK894436,MN484601,OP297539
109	VC_1098_1	1	0	737.4900434	0	0	0	0	1	1	1	0	0	0	0	KR063281
110	VC_1099_0	2	300	848.430591	0.261226061	0.053268464	2.449489743	2.449489743	1	1	1	2.44948974	0	0	0	MN234206,MN234211
111	VC_1099_1	1	0	670.1181876	0	0	0	0	1	1	1	0	0	0	0	OK040794
112	VC_109_0	3	847.6412643	4121.825521	0.170569862	0.001643767	0	3.16227766	3	3	1	2.10818511	0	0	0	MZ398135,MZ398136,MZ399596
113	VC_10_0	25	32380.36153	61024.34232	0.346667354	5.46E-178	0	9.219544457	300	292	0.973333333	7.3914032	0	0	0	AP011955,AP011956,AY508486,CP062430,CP062436,CP062447,DQ530361,GQ398772,KC595278,KF831354,KJ452292,KJ596420,KT809368,KT809369,KT878766,KY653123,MG029510,MG029512,MH384259,MH401416,MW924889,OP038320,OP136135,OP328423,OP493558
114	VC_1100_0	2	300	2034.126921	0.128527715	0.053268464	3.464101615	3.464101615	1	1	1	3.46410162	0	0	0	MN908685,MZ150783
115	VC_1100_1	2	300	2152.360589	0.122331113	0.053268464	6.08276253	6.08276253	1	1	1	6.08276253	0	0	0	OK040793,ON637770
116	VC_1100_2	1	0	953.1209951	0	0	0	0	1	1	1	0	0	0	0	OK040790
117	VC_1100_3	1	0	1262.046244	0	0	0	0	1	1	1	0	0	0	0	OP068343
118	VC_1101_0	2	255.3834155	5.573705406	0.978641298	0.270145687	2.449489743	2.449489743	1	1	1	2.44948974	0	0	0	KR136259,KR136260
119	VC_1103_0	2	14.75294763	0	1	0	0	0	1	1	1	0	0	0	0	KR704913,KR704915
120	VC_1104_0	154	131803.624	23550.04811	0.848410097	0	0	3.464101615	11781	11781	1	1.80810962	0	0	0	KT264749,KT264750,KT264751,KT264752,KT264753,KT264754,KT264755,KT264756,KT264757,KT264758,KT264759,KT264760,KT264761,KT264762,KT264763,KT264764,KT264765,KT264766,KT264767,KT264768,KT264769,KT264779,KX513865,MG025518,MG641179,MG641180,MG641186,MG883717,MG945153,MG945158,MG945163,MG945190,MG945203,MG945205,MG945207,MG945213,MG945227,MG945237,MG945238,MG945318,MG945325,MG945330,MG945333,MG945346,MG945351,MG945355,MG945361,MG945363,MG945387,MG945393,MG945398,MG945402,MG945407,MG945415,MG945424,MG945429,MG945435,MG945438,MG945444,MG945459,MG945461,MG945467,MG945476,MG945486,MG945499,MG945508,MG945517,MG945526,MG945541,MG945548,MG945559,MG945565,MG945566,MG945570,MG945586,MG945587,MG945589,MG945596,MG945602,MG945646,MG945671,MG945673,MG945674,MG945681,MG945691,MG945699,MG945705,MG945708,MG945710,MG945728,MG945731,MG945744,MG945758,MG945761,MG945765,MG945768,MG945770,MG945786,MG945787,MG945793,MG945796,MG945801,MG945802,MG945820,MG945853,MG945855,MG945862,MG945864,MG945869,MK765620,MT135257,MT135263,MT135264,MT135272,MT135273,MT135278,MT135338,MZ089765,MZ089767,MZ089774,MZ089775,OM869524,OM869525,OM869526,OM869527,OM869528,OM869531,OM869533,OM869534,OM869535,OM869537,OM869538,OM869543,OM869546,OM869547,OM869550,OM869606,OM869635,OM869641,OM869642,OM869643,OM869644,OM869645,OM869664,OM869697,OM869698,OM869700,OM869701,OM869703,OM869704,OP549869,OP549872,OP549875,OP549876
121	VC_1105_0	3	19.55315005	239.4936747	0.075481142	0.005331249	1.732050808	2	3	3	1	1.82136721	0	0	0	MG945597,MK496743,MK496744
122	VC_1108_0	4	98.49456099	1932.484699	0.048496094	9.79E-06	0	2.236067977	6	6	1	1.55840852	0	0	0	KT264775,MG883718,MG945386,MG945775

123	VC_1109_0	2	11.84558033	6.52357444	0.644862569	0.144422183	7.071067812	7.071067812	1	1	1	7.07106781	0	0	0	KR816341,S7-wms-combined_235165
124	VC_110_0	4	786.3198875	6093.327532	0.114296539	1.27E-05	0	6	6	6	1	4.36237312	0	0	0	FR687252,MZ333135,OM513679,OM513680
125	VC_1110_0	8	356.5398977	159.0330362	0.691541146	1.09E-10	0	2.449489743	28	28	1	1.78760465	0	0	0	MG945181,MG945211,MG945232,MG945718,MG945783,MT135261,MT135334,MZ089771
126	VC_1111_0	2	15.53109722	43.42024723	0.2634562	0.101520076	1	1	1	1	1	1	0	0	0	MG945489,MT135282
127	VC_1112_0	2	8.282189768	29.15743477	0.221214552	0.276226452	1.732050808	1.732050808	1	1	1	1.73205081	0	0	0	MK496719,MK496738
128	VC_1113_0	1	0	116.1832688	0	0	0	0	1	1	1	0	0	0	0	MG945591
129	VC_1114_0	2	8.583214966	155.8955653	0.05218433	0.05239548	1.414213562	1.414213562	1	1	1	1.41421356	0	0	0	MG945131,MG945742
130	VC_1116_0	14	989.6359613	258.7226044	0.792749766	5.26E-26	0	2.236067977	91	91	1	1.53975598	0	0	0	KT264776,MG883719,MG945139,MG945339,MG945394,MG945403,MG945542,MG945550,MG945645,MG945736,MG945740,MG945771,MG945819,OP209174
131	VC_1117_0	5	172.0997629	194.9832313	0.468830661	2.61E-07	0	1	10	10	1	0.6	0	0	0	KT264781,MG945481,MG945807,MT135256,MT135266
132	VC_1119_0	20	1151.152751	553.9510847	0.675121788	2.11E-05	0	2.449489743	190	190	1	1.49247807	0	0	0	KT264782,KT264783,KX513866,KX513870,MG945154,MG945472,MG945534,MG945613,MG945826,MK496754,MK496805,MK496812,MK496813,MK765559,MK765603,MK765621,MK765669,MZ089756,MZ089773,OM869503
133	VC_111_0	91	460400.0387	19850.42961	0.958666507	0	0	7.615773106	4095	4095	1	6.03369009	0	0	0	AB716666,GQ413938,KF765493,KM576124,KP708985,KP708986,KT367886,KT964103,KU666550,KX211991,KX237514,KX587949,KX856662,KY000081,KY385423,KY684082,MF612071,MG972768,MH885472,MK135468,MK562503,MK598851,MK693005,MN101217,MN101219,MN101220,MN116494,MN176572,MN176573,MN380459,MN794000,MN794001,MN794002,MN794003,MT090077,MT197175,MT197176,MT360680,MT559526,MT559527,MT682065,MT701589,MT939252,MW590329,MZ208805,MZ424864,MZ571827,MZ602146,MZ634338,MZ634339,MZ634340,MZ634342,MZ634343,OK562429,OK583892,OK583893,OL362278,OL362279,OL474125,OL976437,OL979478,OL979479,OM864357,ON132389,ON146449,ON165415,ON602723,ON602724,ON602725,ON602726,ON602727,ON602728,ON602732,ON602739,ON602740,ON602743,ON602748,ON602752,ON602754,ON602756,ON602757,ON602761,ON602762,ON602767,ON809559,OP114731,OP413829,OU509533,OU509534,OX335379,OX335428
134	VC_1120_0	7	139.7627318	380.2096518	0.268788759	0.004813204	1	2	21	21	1	1.55863437	0	0	0	MG945704,MZ089755,MZ089759,MZ089761,MZ089764,OM869627,OM869629
135	VC_1122_0	10	738.4083418	27.33738998	0.964299651	3.03E-06	0	2.449489743	45	45	1	1.83755917	0	0	0	KT264785,KX513873,MG945186,MG945569,MG945689,MG945797,MG945870,MN379630,MN379640,MT135351
136	VC_1123_0	2	25.35975681	54.22329692	0.31865775	0.067297332	0	0	1	1	1	0	0	0	0	KT264784,KX513864
137	VC_1124_0	10	775.5435605	461.7117706	0.626825798	1.01E-21	0	2.828427125	45	45	1	2.01338599	0	0	0	KT264798,KT264799,KT264800,KT264801,MG945135,MG945136,MG945157,MG945391,MG945867,MT135258
138	VC_1125_0	20	1879.265887	1477.037791	0.559921291	2.38E-35	0	2.645751311	190	190	1	1.77602531	0	0	0	KT264796,KT264797,MG883722,MG945286,MG945319,MG945338,MG945347,MG945385,MG945389,MG945410,MG945416,MG945437,MG945538,MG945635,MG945639,MG945746,MG945767,MG945858,OM869558,OM869663
139	VC_1126_0	8	393.5066845	980.3011821	0.286435021	1.56E-12	0	2.828427125	28	28	1	1.8299748	0	0	0	KT264788,KT264789,KT264790,MG945138,MG945381,MG945382,MG945780,MT135259
140	VC_1127_0	19	3585.755056	1125.35752	0.761127016	1.30E-64	0	2.645751311	171	171	1	1.59146452	0	0	0	KT264791,KT264792,KT264793,KX513868,KX513877,MG945162,MG945233,MG945334,MG945383,MG945388,MG945430,MG945447,MG945514,MG945642,MG945694,MG945753,MG945781,MT135277,MZ821031
141	VC_1128_0	2	13.78291419	138.669696	0.09040786	0.048566868	1.414213562	1.414213562	1	1	1	1.41421356	0	0	0	KT264794,MG945231
142	VC_112_0	8	3390.940014	9975.342762	0.253693571	1.16E-19	3.31662479	6.480740698	28	28	1	4.9008984	0	0	0	KF319020,KM819694,MG575418,MG575419,MK387869,MW358930,OL416096,OM234793
143	VC_1132_0	2	4.09143279	37.74211098	0.097802682	0.168052265	2	2	1	1	1	2	0	0	0	KT264795,MG945738
144	VC_1133_0	4	38.92455113	271.6296645	0.125338988	0.024932995	1.414213562	2.449489743	6	6	1	2.00222711	0	0	0	MG641184,MZ089778,OM869517,OM869640
145	VC_1134_0	5	53.56080877	361.780039	0.128956275	0.236291101	0	2.449489743	10	10	1	1.70951319	0	0	0	MK496714,MK496779,MK496792,MZ089777,OM869545
146	VC_1136_0	2	26.62182152	19.27661572	0.580015859	0.117381831	2	2	1	1	1	2	0	0	0	KT264803,MG945142
147	VC_1137_0	3	63.77440347	5.72793687	0.917586417	0.069320317	1.414213562	1.732050808	3	3	1	1.62610506	0	0	0	KT264802,MG945620,MG945741
148	VC_1138_0	4	137.7798897	8.212978528	0.943743974	0.010350137	1.414213562	2	6	6	1	1.67076326	0	0	0	KT264805,MG945519,MG945693,MG945809
149	VC_1139_0	4	115.3245102	0	1	0	0	2.236067977	6	6	1	1.57868933	0	0	0	KT264806,MG945320,MG945561,MG945721
150	VC_113_0	9	3153.164405	2922.381764	0.518992748	1.08E-22	2	8.717797887	36	36	1	6.87188797	0	0	0	AB720063,AP008979,AY299121,DQ777876,EU717894,MW934263,MZ701794,OK258140,OM913894
151	VC_1140_0	4	42.35797099	277.3736397	0.132479772	0.337928011	1.414213562	2.645751311	6	6	1	2.22279108	0	0	0	KT264820,KT264821,MG945644,MK765602
152	VC_1143_0	9	866.3168129	325.6739771	0.726781465	6.92E-19	0	1.414213562	36	36	1	0.7126904	0	0	0	KT264818,KT264819,KX513867,KX513872,KX513874,MG945573,MG945759,MG945760,MG945811
153	VC_1144_0	7	534.3856341	220.8613688	0.707564058	7.01E-13	0	1.414213562	21	21	1	0.82446231	0	0	0	MG883714,MG945364,MG945525,MG945719,MG945805,MT135337,MT135424
154	VC_1145_0	5	159.9798797	21.66125994	0.88074695	0.000789379	0	0	10	10	1	0	0	0	0	OM869513,OM869516,OM869519,OM869634,OM869637
155	VC_1146_0	3	62.6039311	109.3607466	0.364051106	0.001329228	0	0	3	3	1	0	0	0	0	MG945234,MG945405,MG945827
156	VC_1147_0	3	12.51733192	16.36573266	0.433379633	0.346640909	1.414213562	1.732050808	3	3	1	1.62610506	0	0	0	KT264822,KT264830,MK496745
157	VC_1148_0	16	1653.669296	221.092342	0.882069092	1.68E-24	0	2	120	120	1	1.09933691	0	0	0	KT264823,KT264824,KT264825,MG945179,MG945357,MG945413,MG945506,MG945507,MG945609,MG945676,MG945706,MG945764,MG945817,MT135267,MT135271,MT135281
158	VC_1149_0	5	194.4233581	213.3694372	0.476769968	1.96E-07	0	2	10	10	1	1.21961524	0	0	0	MG945441,MG945487,MG945505,MT135255,MT135275
159	VC_1151_0	9	588.4458773	99.9913509	0.854756038	1.41E-11	0	1.414213562	36	36	1	0.90910895	0	0	0	KT264828,MG945228,MG945426,MG945726,MG945754,MG945816,OP549870,OP549873,OP549877
160	VC_1152_0	4	125.2078622	48.5485187	0.720594326	0.000288354	0	0	6	6	1	0	0	0	0	MG945204,MG945727,MG945785,MG945810
161	VC_1153_0	2	3.644286755	46.24911685	0.073041454	0.194373584	2.236067977	2.236067977	1	1	1	2.23606798	0	0	0	KT264831,MN379641
162	VC_1154_0	5	74.23000791	0	1	0	0	1.414213562	10	10	1	0.84852814	0	0	0	KT264833,MG945418,MG945549,MG945747,OM869677
163	VC_1155_0	2	8.951186952	0	1	0	1.414213562	1.414213562	1	1	1	1.41421356	0	0	0	KT264834,MG945678
164	VC_1156_0	8	6486.058456	1485.393041	0.813660907	1.12E-14	0	6.557438524	28	28	1	4.7186548	0	0	0	KT755656,MH431938,MH454076,MH454080,MH460824,MH460825,MH460826,MH460827
165	VC_1157_0	24	79745.203	0	1	0	0	5	276	276	1	2.88524355	0	0	0	KT876724,KT876726,KU599877,KU599878,KU599879,KU599886,MK764441,MK764449,MW421577,MW421578,MW421579,MW421580,MW442825,MW442826,MW442827,MW442828,MW442829,MW442830,MW448539,MW448540,MW448541,OK340820,OK340821,OK340822
166	VC_1158_0	3	900	1930.902276	0.317919841	0.006644424	0	1.732050808	3	3	1	1.15470054	0	0	0	MH576962,MK620894,MW291021
167	VC_1158_1	2	300	1885.967613	0.137238996	0.101796862	3.464101615	3.464101615	1	1	1	3.46410162	0	0	0	MH744418,MT310898
168	VC_1159_0	3	75.95706824	153.9104374	0.330438476	0.01451641	6.480740698	7.615773106	3	3	1	7.19327619	0	0	0	MF668275,MZ955870,OV696619
169	VC_115_0	2	60.73640616	86.78846027	0.411702838	0.092556008	5.196152423	5.196152423	1	1	1	5.19615242	0	0	0	MG592523,OX241425
170	VC_1160_0	73	350016.3117	5854.845117	0.983547852	0	0	8.366600265	2628	2628	1	5.99761565	0	0	0	KU160640,KU160643,KU160645,KU160648,KU160649,KU160652,KU160653,KU160659,KU160661,KU160662,KU160671,KU160672,KX576640,KX576641,KX621004,KX621005,KX688102,KX688103,MF140400,MF140404,MF140407,MF140424,MF140425,MF668285,MG198779,MG198781,MG198782,MG210949,MH450117,MH450118,MH450121,MH450131,MH744421,MK112526,MK112528,MK112529,MK112531,MK112535,MK112538,MK279844,MK279845,MK279847,MK279851,MK279854,MK279856,MK279860,MK279862,MK279863,MK279867,MK279868,MK279869,MK279872,MK279874,MK279875,MK279876,MK279880,MK279884,MK279886,MK279892,MK279896,MK279901,MK279907,MK660714,MK814749,MK919476,MK919484,MN586061,MN735429,MN813680,MT498045,MT498050,MW314848,OP434444
171	VC_1161_0	2	96.52230796	3163.60741	0.029606892	0.042628099	5.656854249	5.656854249	1	1	1	5.65685425	0	0	0	KX557278,MT771339
172	VC_1162_0	10	8202.982884	6149.279255	0.571546339	7.39E-30	0	5.656854249	45	45	1	3.23890421	0	0	0	MK016495,MK016497,MK737942,MN369765,MT310855,MT498057,MZ150782,MZ747515,ON260821,ON970583
173	VC_1163_0	28	45361.45591	46782.97634	0.492286455	1.05E-220	0	8.306623863	378	378	1	6.11988951	0	0	0	MG839027,MG925343,MG925345,MG925347,MG944216,MG944218,MG944226,MH513982,MH590604,MH590606,MK308637,MK620901,MK620904,MK814762,MK894432,MK937602,MN096367,MN096378,MN284901,MN310543,MT553340,MT657343,MT657344,MZ005670,OK318961,ON526972,ON970571,OP068339
174	VC_1164_0	25	70031.14352	25741.19561	0.731225155	2.60E-139	0	7.141428429	300	300	1	5.34270546	0	0	0	KU160641,KU160646,MF038790,MF038791,MF140401,MF140423,MF140432,MH399789,MH450115,MH450125,MH479926,MH825711,MN234168,MN234194,MN234221,MN284902,MN703412,MW055912,MZ209303,MZ274309,OK310503,ON456335,ON456340,ON645338,OP434462
175	VC_1166_0	20	30763.22744	311.9133725	0.989962608	4.71E-40	1.732050808	7.549834435	190	190	1	5.40681094	0	0	0	KU160644,MF140397,MH834595,MH834606,MH834608,MH834609,MH834616,MH834618,MH834620,MH834624,MN234214,MN484600,MN703409,MT889373,MT952853,MW507131,ON260822,ON970604,OP297550,OP297552
176	VC_1167_0	6	2474.88811	388.9488032	0.86418612	5.45E-07	0	4	15	15	1	2.59761825	0	0	0	KU160650,MF668266,MF668273,MF668279,MH450114,ON337195
177	VC_1168_0	2	39.06987812	355.3833776	0.099048183	0.064498006	4.69041576	4.69041576	1	1	1	4.69041576	0	0	0	MW507126,MW590223
178	VC_1169_0	7	6112.725839	118.016966	0.981058925	4.39E-05	2.236067977	4.472135955	21	21	1	3.65463397	0	0	0	KU160654,KU160664,MF140418,MF140427,MF140434,MG099950,MN585972
179	VC_116_0	13	14237.49555	0	1	0	0	4.795831523	78	78	1	3.10338801	0	0	0	AB720064,LR743523,LR743528,LR743529,LR743531,MW802488,MW822535,MW822536,MW822537,MW822538,MW822539,OK913679,OK913680
180	VC_1170_0	3	900	1847.977088	0.327513648	0.001657868	2.828427125	3.464101615	3	3	1	3.04031862	0	0	0	MN586021,MT889394,OP434452
181	VC_1170_1	3	834.9295532	1876.061447	0.307979463	0.001635534	2.828427125	5	3	3	1	4.27614237	0	0	0	KU160669,KU160673,MW712735
182	VC_1170_2	2	144.4804482	1734.323269	0.076900235	0.045810936	7.549834435	7.549834435	1	1	1	7.54983444	0	0	0	MW862992,OM818331
183	VC_1171_0	21	58214.54746	23487.41835	0.712523216	4.44E-95	0	7.416198487	210	210	1	5.40657882	0	0	0	MH153803,MH153813,MN183284,MT114166,MT639641,MT657333,MW365946,MW365949,MW365950,MW712724,MW712727,MW712728,MW924638,MZ322011,MZ322018,OM203157,OP068331,OP068332,OP068336,OP068337,OP068344
184	VC_1173_0	6	4500	22.25367723	0.995079074	7.04E-06	0	7.071067812	15	15	1	4.99758711	0	0	0	KU230356,MK605242,MK605243,MK605244,MK605245,MK605246
185	VC_1174_0	4	1417.86243	0	1	0	3	4.582575695	6	6	1	3.97998801	0	0	0	KU234532,KU234533,MT426122,MZ078141
186	VC_1175_0	22	41914.35759	49960.57373	0.456211036	1.02E-137	0	8.717797887	231	231	1	4.88055141	0	0	0	KU963246,KU963247,MG099941,MH271295,MK279911,MK279912,MK359313,MK801734,MK875797,MN586055,MT771342,MT818424,MW055908,MW291013,MW507130,MW822147,MZ150788,MZ171371,MZ681509,OM203160,ON645343,OP297544
187	VC_1176_0	41	138099.1816	6392.659702	0.955757642	0	0	9.16515139	820	818	0.997560976	6.92207979	0	0	0	KU963251,KU963254,KU963259,KU998236,KU998248,KX557274,KX557275,MF919521,MH020241,MH153808,MH536818,MH576969,MK501729,MK501730,MK801721,MK801724,MK814757,MK864263,MK878896,MK919470,MK967389,MN010762,MN062704,MN096365,MN234161,MN234179,MN369740,MN585963,MN586031,MT553336,MT723935,MT723936,MT723942,MW291016,MW601225,MZ150785,MZ622164,ON724008,ON724011,ON724012,ON970567
188	VC_1177_0	14	10743.127	2946.929446	0.784739423	3.55E-53	2.449489743	9.273618495	91	90	0.989010989	6.91584715	0	0	0	KU963252,KU998235,KX557279,MH479915,MK061410,MK284521,MK878901,MK937599,MK967377,MN813694,MT498059,MT522006,MT771341,MW055901
189	VC_1178_0	24	67757.39262	3384.346427	0.952428118	1.18E-89	0	7.681145748	276	276	1	5.4285614	0	0	0	KU998234,KX557286,MH479910,MH479917,MH669015,MK305889,MK814761,MK864264,MK864265,MK864266,MK864267,MK937603,MK967381,MN010760,MN284907,MT310872,MT521998,MT639651,MW507125,MZ005680,MZ150791,MZ622177,ON970602,ON970619
190	VC_1179_0	3	900	286.0342172	0.758831395	0.031801285	1.732050808	2.645751311	3	3	1	2.27576395	0	0	0	KU647626,KU647627,MN586053
191	VC_117_0	9	2958.977649	6.703616968	0.997739603	0.010113628	1	6.32455532	36	36	1	4.71599315	0	0	0	AB757800,AB757801,JX880072,KX365747,MH059635,MK574011,MW202665,MW202744,OL959999
192	VC_1180_0	3	528.6241114	495.6882675	0.516077051	0.001821403	2.449489743	4.69041576	3	3	1	3.71330183	0	0	0	MK814759,MZ209302,OM818327
193	VC_1181_0	2	300	6201.586617	0.046142583	0.048735676	7.141428429	7.141428429	1	1	1	7.14142843	0	0	0	KU998245,MK524501
194	VC_1181_1	10	13500	9664.244735	0.582794741	6.04E-22	1.732050808	7.810249676	45	45	1	4.74787631	0	0	0	KU998244,KU998246,KU998247,KX557276,MF919520,MF919537,MK376956,MK376959,MT498054,MZ388552
195	VC_1182_0	4	1800	4526.359409	0.284523829	2.32E-05	6	8.306623863	6	6	1	7.57417286	0	0	0	KX557272,MK977705,MN908691,MT657339
196	VC_1182_1	1	0	1900.171593	0	0	0	0	1	1	1	0	0	0	0	MH590603
197	VC_1183_0	6	4384.377814	21.97870762	0.995012045	3.03E-05	0	6.633249581	15	15	1	3.64029784	0	0	0	MH479913,MH479925,MK919478,MN586014,MN813698,MW601220
198	VC_1184_0	5	1488.123041	769.8319638	0.659057881	1.79E-07	3.872983346	4.795831523	10	10	1	4.37161393	0	0	0	KX557282,MK524505,MK814750,MW862986,ON724009
199	VC_1186_0	4	847.3932277	1001.954357	0.458211985	8.06E-05	3.464101615	5.916079783	6	6	1	5.07847688	0	0	0	KX620750,KX620751,KX620753,KX620754
200	VC_1187_0	3	66.0942646	5.435273925	0.924013575	0.074457337	3.741657387	4.898979486	3	3	1	4.21354562	0	0	0	KX077892,OL774866,OL774876
201	VC_1188_0	3	550.5303161	61.10675933	0.900093108	0.005586199	0	1.414213562	3	3	1	0.94280904	0	0	0	MT344103,MT344104,MT344105
202	VC_1189_0	9	7392.870039	244.540247	0.967981261	1.20E-11	2.236067977	6.244997998	36	36	1	4.69677034	0	0	0	KX455876,KY290947,KY290949,KY290950,KY290952,MH179470,MH179471,MH179479,MT276994
203	VC_118_0	3	457.2346591	185.0008775	0.711942322	0.007885065	1	6.557438524	3	3	1	4.67939307	0	0	0	AB746912,LC545443,LC545444
204	VC_1190_0	2	237.0894915	227.3824808	0.510449512	0.052354615	0	0	1	1	1	0	0	0	0	MG592484,MG592485
205	VC_1191_0	3	23.42188031	142.9237229	0.140802521	0.010230257	1	2.236067977	3	3	1	1.74535599	0	0	0	KX513871,MG945160,MG945185
206	VC_1192_0	3	28.52794827	41.57361211	0.406951687	0.005726732	1.414213562	1.732050808	3	3	1	1.62610506	0	0	0	MT135371,MT135422,MT135432
207	VC_1193_0	3	61.07984183	50.52249268	0.547298962	0.004762206	1	1.414213562	3	3	1	1.13807119	0	0	0	MG945352,MG945735,MG945752
208	VC_1194_0	5	153.4685656	139.6363789	0.523595963	1.01E-06	0	2	10	10	1	1.31209559	0	0	0	MG945210,MG945327,MG945623,MT135440,MT135446
209	VC_1196_0	3	60.70502737	119.0249068	0.337756911	0.00256025	1	1.414213562	3	3	1	1.13807119	0	0	0	MG945748,MG945799,MG945800
210	VC_1197_0	15	536.1783476	60.1606615	0.899116676	0.233916058	0	2	105	105	1	1.56660741	0	0	0	KX513875,MG883712,MG945284,MG945309,MG945433,MG945502,MG945616,MG945794,MK496693,MK496774,MZ089789,OM869564,OM869565,OM869570,OM869709
211	VC_1198_0	3	40.5016314	104.4554578	0.279404282	0.048518588	6.32455532	7.483314774	3	3	1	7.05211311	0	0	0	MT006237,S5-wms_94965,S7-wms-combined_189250
212	VC_1199_0	8	4339.436071	1133.97735	0.792820812	2.37E-17	0	6.708203932	28	28	1	4.79091908	0	0	0	MH727560,MH926061,MK937613,MK977706,MK977710,MK977712,MK977714,MK977715
213	VC_119_0	2	219.3274309	178.2972606	0.551594093	0.081593387	0	0	1	1	1	0	0	0	0	LC545445,LC545446
214	VC_11_0	19	25857.86752	8338.75262	0.756152725	1.59E-90	0	8.366600265	171	171	1	5.93033142	0	0	0	AP019560,AP019561,AP019562,KX827368,KX827369,KX827370,MF428478,MF428479,MF428480,MF428481,MK075003,MK075006,MN028536,MT423823,MT423824,OM373551,OM373552,OM373553,OP168678
215	VC_1200_0	4	1251.179227	188.376379	0.869142687	8.04E-07	0	0	6	6	1	0	0	0	0	MF197383,MH271317,MN234233,OP172878
216	VC_1201_0	2	60.63961284	7.353353373	0.891851264	0.185546685	2	2	1	1	1	2	0	0	0	KY817360,OL656105
217	VC_1202_0	2	24.45666682	0	1	0	1	1	1	1	1	1	0	0	0	LC035386,LC210520
218	VC_1203_0	7	6300	3370.525347	0.65146409	4.57E-11	1.732050808	7	21	21	1	4.51061784	0	0	0	MN988521,MN988522,MN988523,MN988524,MN988525,MN988559,MN988561
219	VC_1203_1	1	0	2200.623707	0	0	0	0	1	1	1	0	0	0	0	MN988560
220	VC_1204_0	2	300	39.73220869	0.883048449	0.080290302	1.414213562	1.414213562	1	1	1	1.41421356	0	0	0	LC644975,LC644976
221	VC_1205_0	3	784.0286798	67.04974997	0.921217895	0.004981863	0	2.828427125	3	3	1	1.88561808	0	0	0	MT740726,MT740743,MT740745
222	VC_1206_0	8	2728.653322	11532.40349	0.191335983	2.29E-18	1	8.888194417	28	28	1	7.51489758	0	0	0	MH271298,MH271308,MH271320,MK880124,MT952845,MW862981,OK040779,ON970568
223	VC_1207_0	29	63820.65503	13768.46972	0.822546397	2.00E-116	0	8.366600265	406	406	1	5.71931372	0	0	0	MH834610,MH834619,MH834629,MK061413,MN703413,MN813676,MN908689,MT024869,MT024871,MT639653,MT889366,MT889375,MT889376,MT897906,MW055910,MZ005682,MZ681506,MZ747516,MZ820087,OK040777,OL455896,OL549188,OL549189,OL549191,OL549192,ON208833,ON528933,ON970616,OP434455
224	VC_1208_0	11	16500	0	1	1	2	6.244997998	55	55	1	4.99526187	0	0	0	LR535901,LR535903,LR535904,LR535905,LR535906,LR535907,LR535908,LR535911,LR535912,LR535913,LR535917
225	VC_1209_0	3	135.7682431	738.2387754	0.155339992	0.008479812	8	8.602325267	3	3	1	8.36253555	0	0	0	S1-wms-combined_41369,S1-wms_130214,S8-wms-combined_276211
226	VC_1209_1	1	0	356.7893026	0	0	0	0	1	1	1	0	0	0	0	S12-wms-combined_23517
227	VC_1209_2	1	0	387.2390441	0	0	0	0	1	1	1	0	0	0	0	S11-wms-combined_3507
228	VC_1210_0	2	300	7881.824057	0.03666664	0.318790317	7.071067812	7.071067812	1	1	1	7.07106781	0	0	0	MK250021,MK250023
229	VC_1210_1	7	6300	17097.63814	0.269257947	0.001258589	3	8.485281374	21	21	1	6.2165533	0	0	0	MK250020,MK250022,MK250024,MK250025,MK250026,MK250027,MK250028
230	VC_1210_2	4	1800	13409.42458	0.118347673	0.079377892	1.414213562	5.099019514	6	6	1	4.04329689	0	0	0	MK250015,MK250016,MK250017,MK250018
231	VC_1210_3	2	300	7901.575323	0.036578339	0.318790317	8.94427191	8.94427191	1	1	1	8.94427191	0	0	0	MK250019,MK250029
232	VC_1211_0	25	3603.464777	0	1	0	0	2.236067977	300	300	1	1.57629033	0	0	0	LT986678,MG945141,MG945453,MG945455,MG945456,MG945498,MG945509,MG945564,MG945664,MG945680,MG945683,MG945713,MG945716,MG945730,MG945784,MG945790,MG945813,MG945845,MK765557,MK765655,MK765661,MK765667,MT135342,OM869561,OM869610
233	VC_1212_0	2	16.30924847	0	1	0	0	0	1	1	1	0	0	0	0	MF101922,MH015251
234	VC_1213_0	2	90.65925481	0	1	0	0	0	1	1	1	0	0	0	0	MF443783,MF443784
235	VC_1215_0	3	451.5757549	0	1	0	0	2.236067977	3	3	1	1.49071198	0	0	0	MF580955,MF580957,MF580958
236	VC_1216_0	3	733.9828396	168.3287969	0.813447162	0.007745576	0	1.732050808	3	3	1	1.15470054	0	0	0	MF580956,MF580959,MF580960
237	VC_1217_0	3	670.0375996	168.3287969	0.799218101	0.007837249	0	2	3	3	1	1.33333333	0	0	0	MF580961,MF580962,MF629150
238	VC_1218_0	4	304.1725247	37.41721771	0.89046153	0.004694091	0	1.732050808	6	6	1	0.8660254	0	0	0	MF979562,MK080312,MT074429,MT074440
239	VC_1219_0	1	0	25.92395319	0	0	0	0	1	1	1	0	0	0	0	MG065691
240	VC_1219_1	1	0	23.61272473	0	0	0	0	1	1	1	0	0	0	0	S5-wms-combined_66662
241	VC_121_0	4	1008.728937	4553.009423	0.181369362	1.47E-05	0	5.744562647	6	6	1	4.71358471	0	0	0	JQ177061,JQ177064,KJ572844,MN379460
242	VC_1221_0	1	0	275.4167061	0	0	0	0	1	1	1	0	0	0	0	MW660583
243	VC_1221_1	1	0	316.0874499	0	0	0	0	1	1	1	0	0	0	0	S7-wms-combined_62119
244	VC_1222_0	3	14.8095565	0	1	1	0	1	3	3	1	0.66666667	0	0	0	MG214764,MW202596,OL617037
245	VC_1223_0	2	3.427695194	0	1	0	5	5	1	1	1	5	0	0	0	MG269973,MW202566
246	VC_1224_0	19	29427.15172	0	1	0	0	6.557438524	171	171	1	4.0781944	0	0	0	MG515223,MG518519,MG518520,MG663582,MG663583,MG663584,MG663585,MG663586,MH155868,MK112550,MN119376,MN284894,MN994275,MT684596,MZ622160,OL455897,ON108647,ON970569,OP021678
247	VC_1225_0	4	411.6172006	16.3108134	0.961884212	0.006251147	1	1.414213562	6	6	1	1.13807119	0	0	0	MG592394,MG592402,MG592427,MG592455
248	VC_1226_0	2	138.4797217	74.67781679	0.64965904	0.131776239	0	0	1	1	1	0	0	0	0	MG592533,MG592560
249	VC_1227_0	6	4247.323365	318.2488974	0.930293755	3.15E-07	0	3.741657387	15	15	1	2.40575792	0	0	0	MG592536,MG592554,MG592555,MG592556,MG592591,MG592625
250	VC_1228_0	2	278.3645715	2.748292682	0.990223526	0.270145687	2.645751311	2.645751311	1	1	1	2.64575131	0	0	0	MK373772,OP136151
251	VC_1229_0	7	4516.020158	34.42698443	0.992434373	1.33E-07	0	7.280109889	21	21	1	4.62133199	0	0	0	MG592596,MW824384,MW824385,MW824386,MW824395,MW824396,MW824397
252	VC_122_0	5	1836.144968	7022.95615	0.207260866	4.49E-08	3.872983346	8.062257748	10	10	1	7.1870679	0	0	0	MG592400,MG592422,MG592425,MG592448,MG592486
253	VC_122_1	1	0	665.783618	0	0	0	0	1	1	1	0	0	0	0	MG592434
254	VC_1230_0	3	10.47501181	11.34108823	0.480150521	0.172889293	2	2	3	3	1	2	0	0	0	MG945523,MG945677,OM869552

255	VC_1231_0	2	13.66481654	19.28092176	0.414767349	0.106686854	1.414213562	1.414213562	1	1	1	1.41421356	0	0	0	MZ089785,OM869555
256	VC_1232_0	2	15.53109722	17.45384775	0.470854119	0.144422183	0	0	1	1	1	0	0	0	0	MG641185,MG945667
257	VC_1233_0	16	1239.958404	142.2362437	0.897093912	8.30E-19	0	2.449489743	120	120	1	1.40364026	0	0	0	MG641188,MG945130,MG945256,MG945457,MG945557,MG945725,MG945757,MN379637,MN379647,MT135339,MT135348,MT135361,MT135384,MT135409,MT135435,OL617023
258	VC_1234_0	44	149904.9295	0	1	0	0	8.602325267	946	946	1	5.8011474	0	0	0	MG670586,MH045566,MH045567,MH045568,MH509447,MH834596,MH834599,MH834602,MH834604,MH834626,MK814753,MK937598,MK937610,MN062714,MN183281,MN329675,MT024860,MT310853,MT310896,MT639649,MT818427,MT889365,MT889372,MT889389,MT889392,MW601221,MW601224,MW712721,MW924640,MW924647,MZ322012,MZ747513,MZ820083,OK318958,OK999979,OK999981,OL742562,OM818329,ON724018,OP172875,OP434439,OP434443,OP434445
259	VC_1235_0	12	6765.183035	1693.982031	0.799745954	2.14E-20	3	8.717797887	66	66	1	5.74834106	0	0	0	MG711460,S1-wms_4277,S11-wms-combined_283220,S11-wms_3116,S12-wms-combined_299872,S12-wms_193565,S17-wms-combined_175924,S17-wms-combined_193958,S2-wms-combined_165833,S7-wms_113137,S7-wms_200936,S8-wms_30498
260	VC_1235_1	1	0	898.6757519	0	0	0	0	1	1	1	0	0	0	0	S2-wms-combined_157934
261	VC_1236_0	3	66.88739326	405.8522353	0.141488865	0.002252261	4.123105626	6.557438524	3	3	1	5.72042828	0	0	0	S8-wms-combined_134284,S8-wms-combined_211119,S8-wms-combined_49369
262	VC_1238_0	2	21.2201661	179.9390334	0.105489414	0.08545176	8.94427191	8.94427191	1	1	1	8.94427191	0	0	0	S12-wms-combined_147628,S12-wms-combined_41160
263	VC_1239_0	6	307.2945309	346.7136276	0.469863452	2.78E-07	5.291502622	8.831760866	15	15	1	6.82157088	0	0	0	S1-wms_185046,S11-wms_303398,S12-wms-combined_334806,S7-wms-combined_40779,S8-wms-combined_134098,S8-wms-combined_25607
264	VC_123_0	23	37012.61764	8289.344677	0.817020185	1.60E-95	4.123105626	8.717797887	253	253	1	6.7634474	0	0	0	JX866719,KJ021043,KX237516,MF663761,MG197810,MG432137,MK521906,MK685667,MK798142,MN689778,MT559528,MT757392,MT894004,MZ374361,MZ398021,MZ398245,MZ504995,OK499978,ON086804,ON602758,ON807605,ON809560,OP114730
265	VC_1240_0	82	413485.3	106963.1009	0.794478952	0	0	8.94427191	3321	3321	1	6.48111885	0	0	0	MG839015,MG839016,MG839017,MG839018,MG839020,MG839021,MG839022,MG839023,MG839026,MG839028,MG839029,MG920061,MG925352,MG944219,MG944222,MG962360,MG962367,MH045554,MH045558,MH045560,MH045562,MH045563,MH045564,MH045565,MH153802,MH153805,MH153806,MH153811,MH513981,MK620895,MK620902,MK620903,MK620905,MK757447,MK919471,MK967388,MK967391,MK967394,MK967400,MK977697,MN062702,MN062709,MN062713,MN234163,MN234177,MN234181,MN234204,MN234222,MN369737,MN617840,MN735430,MN735435,MN807246,MN892483,MT024864,MT310851,MT310856,MT310861,MT310866,MT310873,MT451985,MT498049,MT553339,MT553341,MT553343,MT771350,MT952846,MT986026,MT986027,MW055911,MW291026,MW435859,MZ005684,MZ958749,OK310506,ON260823,ON337194,ON456354,ON970581,ON970605,ON970606,OP021683
266	VC_1243_0	52	6748.865982	645.9660172	0.912646289	0.072916892	0	3.16227766	1326	1326	1	2.07075055	0	0	0	MG883709,MG945260,MG945282,MG945390,MG945396,MG945452,MG945485,MG945511,MG945515,MG945618,MG945634,MG945641,MG945647,MG945743,MH572525,MH572526,MK496694,MK496775,MK765561,MK765563,MK765564,MK765568,MK765569,MK765594,MK765638,MN311493,MT135347,MT135352,MT135355,MT135366,MT135381,MT135382,MT135387,MT135444,MW202703,MW202824,MZ089786,MZ089791,MZ089795,MZ089806,OL617028,OM869549,OM869559,OM869560,OM869562,OM869569,OM869650,OP549868,OP549871,OP549874,S1-wms-combined_357373,S9-wms_128232
267	VC_1245_0	2	14.75294763	0	1	0	0	0	1	1	1	0	0	0	0	MG883710,MG945464
268	VC_1246_0	2	18.57572439	0	1	0	0	0	1	1	1	0	0	0	0	MG883713,MT135394
269	VC_1247_0	7	328.4875003	33.05080297	0.908582845	1.04E-05	0	1.414213562	21	21	1	0.54353398	0	0	0	MG883720,MG945384,MG945482,MG945692,MG945697,MG945698,MG945734
270	VC_124_0	2	93.60729906	144.3398453	0.393395346	0.046629082	6.32455532	6.32455532	1	1	1	6.32455532	0	0	0	KP977490,MW960031
271	VC_1250_0	5	91.09487203	51.60415612	0.638370655	0.000810551	0	1.732050808	10	10	1	1.25850575	0	0	0	MG883724,MG945625,MG945629,MG945632,MG945633
272	VC_1251_0	15	1494.860866	151.1350927	0.908180156	4.90E-21	0	2.645751311	105	105	1	2.13491638	0	0	0	MG945128,MG945159,MG945188,MG945229,MG945255,MG945323,MG945332,MG945401,MG945685,MG945745,MG945774,MG945803,MG945829,MT135336,MT135441
273	VC_1252_0	3	56.32923145	117.6897036	0.32369599	0.002096069	1.414213562	1.414213562	3	3	1	1.41421356	0	0	0	MG945544,MG945723,MT135439
274	VC_1253_0	6	286.7807044	183.5543355	0.60973706	2.35E-09	0	2.236067977	15	15	1	1.52363122	0	0	0	MG945414,MG945445,MG945578,MG945611,MT135367,MT135433
275	VC_1255_0	4	47.73982594	144.7590607	0.24800053	0.001718468	0	2.236067977	6	6	1	1.57868933	0	0	0	MG945460,MG945496,MG945622,MG945830
276	VC_1256_0	4	43.53488751	31.39820017	0.580983499	0.052482568	1	2	6	6	1	1.54875479	0	0	0	MG945241,MG945276,MG945448,MG945643
277	VC_1257_0	6	161.0906594	86.07779142	0.651744423	3.06E-07	0	1	15	15	1	0.33333333	0	0	0	MG945140,MG945257,MG945449,MG945458,MG945662,MG945815
278	VC_1258_0	3	13.11938141	41.05708426	0.242160157	0.140741807	1	1.414213562	3	3	1	1.13807119	0	0	0	MG945580,MG945804,MG945909
279	VC_1259_0	8	604.3146253	130.4088202	0.822506249	3.73E-12	0	1.414213562	28	28	1	0.63672954	0	0	0	MG945166,MG945208,MG945329,MG945365,MG945512,MG945540,MG945687,MT135329
280	VC_125_0	17	13545.03156	1107.121122	0.924439695	2.11E-65	1.732050808	8.246211251	136	136	1	6.4211288	0	0	0	AB775548,GU903191,KM360178,KX231828,KY705409,MH252365,MT012302,MT161381,MT161382,MT161383,MT161384,MT375042,MT613935,MT701586,MT708550,MZ345003,ON853583
281	VC_1260_0	2	9.796278594	0	1	0	1	1	1	1	1	1	0	0	0	MG945165,MT135354
282	VC_1261_0	5	152.6286803	46.88638112	0.764998287	7.00E-05	0	1.414213562	10	10	1	0.96568542	0	0	0	MG945177,MG945349,MG945411,MG945462,MG945495
283	VC_1262_0	2	16.30924847	120.35741	0.119335972	0.043864358	0	0	1	1	1	0	0	0	0	MN311487,MN311492
284	VC_1263_0	2	3.84840074	0	1	0	0	0	1	1	1	0	0	0	0	MG945183,MG945314
285	VC_1264_0	6	52.05710841	21.66126294	0.706161944	0.079442752	0	2	15	15	1	1.21471546	0	0	0	MG945261,MG945420,MG945470,MG945518,MK249148,MK496685
286	VC_1266_0	5	57.93390719	0	1	0	0	2	10	10	1	1.29282032	0	0	0	MG945189,MG945275,MG945408,MG945848,MG945852
287	VC_1267_0	7	233.5670107	0	1	0	0	1.414213562	21	21	1	0.80812204	0	0	0	MG945209,MG945263,MG945279,MG945404,MG945750,MG945776,MG945791
288	VC_1268_0	3	14.50934719	165.3295597	0.08067969	0.313746804	1.414213562	1.414213562	3	3	1	1.41421356	0	0	0	MG945212,MG945582,MT135345
289	VC_126_0	2	81.74986358	410.4355049	0.16609568	0.048184325	5.196152423	5.196152423	1	1	1	5.19615242	0	0	0	EF057797,EU399241
290	VC_1270_0	3	54.79870562	25.69470678	0.680784973	0.013119721	1	1.414213562	3	3	1	1.13807119	0	0	0	MG945214,MT135380,MT135423
291	VC_1271_0	2	24.45666682	0	1	0	0	0	1	1	1	0	0	0	0	MG945230,MT135357
292	VC_1272_0	5	166.2088324	0	1	0	0	1	10	10	1	0.6	0	0	0	MG945235,MG945392,MT135388,MT135443,MT135462
293	VC_1273_0	6	144.1261857	51.03733489	0.738489372	1.96E-05	0	1.414213562	15	15	1	0.81617605	0	0	0	MG945236,MG945822,MT135358,MT135377,MT135426,MT135427
294	VC_1274_0	2	13.23885114	7.492687495	0.638585074	0.270145687	0	0	1	1	1	0	0	0	0	MG945567,MG945631
295	VC_1275_0	4	83.76514101	0	1	0	0	1.732050808	6	6	1	1.21542146	0	0	0	MG945252,MG945584,MN379645,OM869494
296	VC_1276_0	7	240.2427152	36.07126754	0.869455511	0.000175243	0	2.645751311	21	21	1	1.61777442	0	0	0	MG945488,MG945572,MZ089802,MZ089808,MZ089809,OP549867,S17-wms_254872
297	VC_1277_0	14	558.2043093	121.058678	0.821779369	2.24E-09	0	2.236067977	91	91	1	1.53235072	0	0	0	MG945494,MG945563,MG945585,MK765590,MN379638,MN379648,MT135333,MT135379,MT135391,MT135401,MT135437,MT135445,OM869612,OM869668
298	VC_1278_0	3	56.32923145	85.86606972	0.396139893	0.002947141	1	1.414213562	3	3	1	1.13807119	0	0	0	MG945425,MG945789,MT135415
299	VC_1279_0	4	32.68044565	21.26440339	0.605812162	0.230279085	1	2	6	6	1	1.42674632	0	0	0	MG945258,MG945434,MT135434,MT135451
300	VC_127_0	1	0	152.4074347	0	0	0	0	1	1	1	0	0	0	0	MF663786
301	VC_127_1	1	0	131.988923	0	0	0	0	1	1	1	0	0	0	0	OL614104
302	VC_127_2	1	0	165.0256777	0	0	0	0	1	1	1	0	0	0	0	MT711888
303	VC_1280_0	2	9.796278594	131.1654061	0.069496038	0.050553389	0	0	1	1	1	0	0	0	0	MK765572,OM869521
304	VC_1281_0	7	336.1779181	28.75601342	0.921202138	4.71E-05	0	1.414213562	21	21	1	0.90814609	0	0	0	MG945278,MG945285,MG945492,MG945605,MG945751,MG945812,MG945825
305	VC_1282_0	3	14.20750251	0	1	0	0	0	3	3	1	0	0	0	0	MG945280,MK765649,OM869500
306	VC_1283_0	26	6186.976235	5174.279777	0.54456798	9.52E-112	0	3.31662479	325	325	1	2.25786721	0	0	0	MK765552,MK765553,MK765556,MK765560,MK765562,MK765565,MK765591,MK765592,MK765593,MK765596,MK765606,MK765607,MK765609,MK765611,MK765612,MK765613,MK765615,MK765633,MK765648,MK765651,MK765657,MK765658,MK765660,MK765666,MT135276,MT135425
307	VC_1285_0	25	2092.084258	0	1	0	0	2.449489743	300	300	1	1.56008437	0	0	0	MG945316,MG945344,MG945468,MG945524,MG945556,MG945562,MG945579,MG945612,MG945640,MG945668,MG945712,MG945733,MG945806,MG945814,MK765571,MK765574,MK765576,MK765577,MK765580,MT135330,MT135406,MT135431,MT135458,OL617036,OM869623
308	VC_1286_0	1	0	4.936518833	0	0	0	0	1	1	1	0	0	0	0	MG945328
309	VC_1287_0	7	245.4147848	0	1	0	0	1.414213562	21	21	1	0.90814609	0	0	0	MG945331,MG945343,MG945490,MG945501,MG945665,MG945729,MG945782
310	VC_1288_0	3	54.11289743	0	1	0	0	2.236067977	3	3	1	1.49071198	0	0	0	MG945335,MG945778,MT135385
311	VC_1289_0	2	15.53109722	17.79379432	0.466050946	0.144422183	1	1	1	1	1	1	0	0	0	MG945336,MT135365
312	VC_1290_0	2	22.90036782	0	1	0	1	1	1	1	1	1	0	0	0	MG945337,MT135410
313	VC_1291_0	2	3.84840074	0	1	0	1.732050808	1.732050808	1	1	1	1.73205081	0	0	0	MG945345,MG945818
314	VC_1292_0	3	17.37708608	0	1	0	0	1.414213562	3	3	1	0.94280904	0	0	0	MG945348,MG945702,MK496732
315	VC_1293_0	4	77.49339696	21.26440339	0.784681278	0.007107019	1	1.732050808	6	6	1	1.45112372	0	0	0	MG945350,MG945432,MG945755,MT135447
316	VC_1294_0	6	124.4392727	63.36048707	0.662616783	7.28E-06	0	2	15	15	1	1.28448135	0	0	0	MG945354,MG945576,MG945637,MK496765,OM869571,OP209176
317	VC_1295_0	3	76.07927043	0	1	1	0	0	3	3	1	0	0	0	0	MG945360,MG945478,MG945614
318	VC_1296_0	4	33.20055058	5.033427347	0.868351984	0.088483245	0	1	6	6	1	0.5	0	0	0	MG945362,MK496704,MK496729,MK496782
319	VC_1297_0	7	98.07626717	0	1	0	0	1.414213562	21	21	1	0.81290799	0	0	0	MG945395,MG945421,MG945480,MG945539,MG945696,MK496696,MK496711
320	VC_1298_0	3	29.18471806	30.51523872	0.488856603	0.018325274	1	2	3	3	1	1.57735027	0	0	0	MG945397,MT135331,MT135461
321	VC_1299_0	2	19.17778267	0	1	0	0	0	1	1	1	0	0	0	0	MG945409,OM869563
322	VC_129_0	43	122177.171	15725.17723	0.885968749	1.72E-289	0	6.633249581	903	903	1	3.91630731	0	0	0	AF493143,KJ507099,KR091952,KU064779,KY798120,KY798121,KY971611,LC730319,LN889756,LN889995,LN913017,MG250485,MG775258,MN729595,MN729596,MN729597,MN729598,MN729599,MN729600,MT104465,MT104466,MT711889,MT711890,MW812345,MZ501272,MZ598487,MZ826344,MZ826345,MZ826346,MZ826347,MZ826348,MZ826349,MZ826351,MZ826352,MZ826354,OK523998,OK523999,OL581611,OM256450,OM282085,OM471789,OM630590,ON189046
323	VC_12_0	21	20517.63897	69729.17433	0.227350288	2.58E-127	2.828427125	9.219544457	210	201	0.957142857	7.89497415	0	0	0	AP008954,AY954960,AY954961,CP062422,CP062462,CP071049,DQ530362,JQ973847,KJ140076,KJ452291,KP209285,KP893289,KT344895,MK290764,MK618716,MW238767,OM439663,OM439669,OM439672,OM439673,OM439674
324	VC_12_1	2	186.7549706	9526.452074	0.019226911	0.042605046	7.141428429	7.141428429	1	1	1	7.14142843	0	0	0	MG543995,MG564297
325	VC_12_2	19	17763.05043	70266.55697	0.201784956	2.99E-104	0	8.774964387	171	171	1	7.51915864	0	0	0	AB370268,AP001553,AY954962,AY954963,AY954964,AY954965,AY954966,AY954967,AY954968,DQ908929,JX013863,JX094501,KC595279,KP893290,KR709302,KR709303,MK211557,MN904510,ON571632
326	VC_1300_0	2	13.78291419	6.975321055	0.663973311	0.270145687	1	1	1	1	1	1	0	0	0	MG945412,MG945828
327	VC_1301_0	2	29.79354264	0	1	0	0	0	1	1	1	0	0	0	0	MG945417,MG945419
328	VC_1302_0	3	23.13612536	0	1	0	0	1	3	3	1	0.66666667	0	0	0	MG945427,MG945574,MT135411
329	VC_1303_0	6	86.06224956	0	1	0	0	1.732050808	15	15	1	1.26994394	0	0	0	MG945439,MG945628,MK496757,MK765601,MZ089768,OM869696
330	VC_1304_0	7	202.9578452	52.93487507	0.793136456	1.99E-07	0	1.732050808	21	21	1	1.31933397	0	0	0	MZ089804,MZ089807,OM869574,OM869576,OM869577,OM869592,OM869653
331	VC_1305_0	5	64.07936426	19.12586863	0.770136229	0.330149589	0	1.414213562	10	10	1	0.88284271	0	0	0	MG945469,MG945824,MT135353,MT135400,MT135402
332	VC_1306_0	2	15.53109722	0	1	0	0	0	1	1	1	0	0	0	0	MG945483,MT135420
333	VC_1307_0	2	9.796278594	8.969958177	0.522016146	0.270145687	1.732050808	1.732050808	1	1	1	1.73205081	0	0	0	MT135397,MT135407
334	VC_1308_0	12	413.9025686	91.86867639	0.818359234	0.018926896	0	2	66	66	1	1.41851258	0	0	0	MG945500,MG945621,MH572369,MK765583,MT135326,OM869498,OM869502,OM869505,OM869587,OM869604,OM869633,OM869691
335	VC_1309_0	2	4.635491837	10.36788319	0.308963272	0.270145687	0	0	1	1	1	0	0	0	0	MT135328,OM869626
336	VC_130_0	19	21305.78605	13807.89769	0.606765904	7.61E-95	0	5.830951895	171	171	1	4.24465466	0	0	0	AB775549,FR823298,GU583987,JX173487,KP025626,KX431888,KX449361,KX449362,KX449363,MH179472,MH179475,MH179478,MH268168,MK033137,MT094431,MT711887,OK094519,OM131411,ON109291
337	VC_1310_0	2	4.09143279	0	1	0	2.449489743	2.449489743	1	1	1	2.44948974	0	0	0	MG945504,MG945617
338	VC_1311_0	5	157.3924036	31.8757975	0.831583978	0.001209473	0	1	10	10	1	0.6	0	0	0	MG945529,MG945568,MG945717,MG945739,MT135265
339	VC_1312_0	3	71.65227999	0	1	0	1	2	3	3	1	1.57735027	0	0	0	MG945533,MT135343,MT135416
340	VC_1313_0	13	534.5643427	95.76004215	0.848078157	0.125435675	0	2.449489743	78	78	1	1.66468498	0	0	0	MZ089798,MZ089799,MZ089800,MZ089801,MZ089803,MZ089805,OM869557,OM869648,OM869652,OM869654,OM869655,OM869666,OM869684
341	VC_1314_0	4	79.74794015	38.50093659	0.674407591	0.000970101	0	1.414213562	6	6	1	0.70710678	0	0	0	MZ089796,MZ089797,OM869566,OM869568
342	VC_1315_0	4	52.85941249	13.17737036	0.800454083	0.011246719	0	1.414213562	6	6	1	0.97140452	0	0	0	MT135349,MT135363,MT135392,MT135408
343	VC_1319_0	2	8.282189768	0	1	0	0	0	1	1	1	0	0	0	0	MG945554,MG945575
344	VC_131_0	17	40800	111970.7868	0.267066766	4.43E-68	0	7.280109889	136	136	1	3.75175114	0	0	0	HE600015,KJ716335,KM209228,KM209270,KM209320,KY942056,KY942057,MG266157,MH807812,MH807820,MN788369,MN813048,MN813049,MN813050,MN813051,MN813052,MN813053
345	VC_131_1	6	4500	74453.41947	0.056995631	7.64E-09	0	7.348469228	15	15	1	6.03944119	0	0	0	MW650886,OL656110,ON184125,ON184126,ON212266,ON862890
346	VC_131_2	7	6300	85706.09621	0.068473724	8.87E-12	6.8556546	9.16515139	21	19	0.904761905	7.97193823	0	0	0	FJ373894,JX181829,LC373201,MK509462,MT078988,OL615011,OL615013
347	VC_131_3	1	0	14145.51018	0	0	0	0	1	1	1	0	0	0	0	MT857002
348	VC_1321_0	14	920.2479817	193.7988885	0.826040633	7.70E-11	0	2.645751311	91	91	1	1.66339713	0	0	0	MZ089770,MZ089772,MZ089780,OM869514,OM869529,OM869540,OM869542,OM869544,OM869548,OM869553,OM869589,OM869590,OM869638,OM869707
349	VC_1322_0	2	5.033427347	0	1	0	0	0	1	1	1	0	0	0	0	MG945615,MT135372
350	VC_1323_0	6	119.9922707	80.65722269	0.598019306	0.005396109	0	1.732050808	15	15	1	1.18851321	0	0	0	MH572374,MH572375,MH572376,MH572414,MH572485,MH572522
351	VC_1324_0	4	26.18077238	17.39585731	0.600798469	0.455926385	0	1	6	6	1	0.5	0	0	0	MG945619,MH572524,MH992173,MZ364234
352	VC_1325_0	7	496.162381	45.16902334	0.916559389	1.89E-07	0	2.645751311	21	21	1	1.25988158	0	0	0	MK765555,MK765567,MK765600,MK765610,MK765617,MK765659,MK765664
353	VC_1326_0	2	3.644286755	0	1	0	1.414213562	1.414213562	1	1	1	1.41421356	0	0	0	MG945690,MK496692
354	VC_1327_0	4	79.74794015	0	1	0	0	1	6	6	1	0.5	0	0	0	MG945724,MT135383,MT135403,MT135456
355	VC_1328_0	2	3.088008248	0	1	0	2	2	1	1	1	2	0	0	0	MG945766,MG945823
356	VC_1329_0	2	3.644286755	0	1	0	0	0	1	1	1	0	0	0	0	MG945769,MG945792
357	VC_132_0	4	1800	48239.29813	0.035971728	1.22E-05	4.795831523	7.681145748	6	6	1	6.26379699	0	0	0	JX878496,KX452697,MF285619,MH929319
358	VC_132_1	17	40800	193899.6083	0.173839233	1.28E-86	0	9.273618495	136	134	0.985294118	7.34270017	0	0	0	AB797215,KX147096,KY073123,MG428990,MG428991,MG770379,MK373783,MN045230,MN478483,MZ634341,ON202820,ON323462,ON399185,OX335402,OX335403,OX335405,OX335416
359	VC_1330_0	2	15.53109722	0	1	0	0	0	1	1	1	0	0	0	0	MG945788,MG945795
360	VC_1331_0	4	85.81825823	31.05885908	0.734260565	0.001005414	0	1.414213562	6	6	1	0.70710678	0	0	0	MG945798,MK765653,MT135350,MT135457
361	VC_1332_0	2	24.45666682	31.05885908	0.440537425	0.080290302	0	0	1	1	1	0	0	0	0	OM869639,OM869679
362	VC_1333_0	2	19.17778267	0	1	0	0	0	1	1	1	0	0	0	0	MG945808,MG945849
363	VC_1334_0	1	0	13.57530437	0	0	0	0	1	1	1	0	0	0	0	MG945821
364	VC_1335_0	7	252.3056609	50.00905139	0.834579498	1.56E-06	0	1.732050808	21	21	1	1.01731412	0	0	0	MG945831,MT135370,MT135373,MT135418,MT135430,MT135455,MT135459
365	VC_1336_0	3	48.98470788	50.00905139	0.494826222	0.00471016	0	1.414213562	3	3	1	0.94280904	0	0	0	OP549864,OP549865,OP549866
366	VC_1337_0	2	14.98702917	0	1	0	1	1	1	1	1	1	0	0	0	MG945832,MT135421
367	VC_1338_0	4	65.83420152	0	1	0	0	0	6	6	1	0	0	0	0	MG945839,MH572484,MZ364252,MZ364293
368	VC_1339_0	7	211.2939182	18.93990286	0.917736227	0.002276952	0	2	21	21	1	1.36135511	0	0	0	MG945842,MH992159,MH992175,MH992200,MK249218,MK249229,MK249233
369	VC_133_0	2	300	47353.71101	0.006295417	0.059611162	6.32455532	6.32455532	1	1	1	6.32455532	0	0	0	KF669653,KF669654
370	VC_133_1	1	0	30799.98837	0	0	0	0	1	1	1	0	0	0	0	MG428992
371	VC_133_10	10	13500	242843.0135	0.052663811	1.02E-22	1.732050808	7.615773106	45	45	1	5.62342029	0	0	0	MH709121,MN871504,MZ375357,MZ375358,OL964736,OL964737,OL964738,OL964746,OL964747,OL964753
372	VC_133_11	2	300	49793.20862	0.005988836	0.060950463	0	0	1	1	1	0	0	0	0	MW006476,MW006480
373	VC_133_12	2	300	58813.90421	0.005074948	0.132791703	1.732050808	1.732050808	1	1	1	1.73205081	0	0	0	MN871494,MN871495
374	VC_133_13	2	300	58951.9738	0.005063122	0.132052319	7.348469228	7.348469228	1	1	1	7.34846923	0	0	0	JN126049,LR800396
375	VC_133_14	3	900	87520.98781	0.010178579	0.036727444	5.099019514	6.403124237	3	3	1	5.74890213	0	0	0	KX438380,MN953776,OM681507
376	VC_133_15	2	300	58092.73182	0.005137626	0.152135634	4.472135955	4.472135955	1	1	1	4.47213595	0	0	0	KX765865,MW388005
377	VC_133_16	1	0	29932.11568	0	0	0	0	1	1	1	0	0	0	0	KJ174318
378	VC_133_17	1	0	30320.54851	0	0	0	0	1	1	1	0	0	0	0	MH709120
379	VC_133_18	1	0	29829.57656	0	0	0	0	1	1	1	0	0	0	0	KR296692
380	VC_133_19	2	300	58836.74104	0.005072988	0.130335699	6.8556546	6.8556546	1	1	1	6.8556546	0	0	0	MN871500,MT074475
381	VC_133_2	1	0	30925.65194	0	0	0	0	1	1	1	0	0	0	0	MT074472
382	VC_133_20	1	0	29371.99133	0	0	0	0	1	1	1	0	0	0	0	OM367911
383	VC_133_21	1	0	29851.20891	0	0	0	0	1	1	1	0	0	0	0	MK972690
384	VC_133_22	13	23400	267560.268	0.080423352	5.67E-47	1.414213562	10.81665383	78	64	0.820512821	7.867174	0	0	0	FQ312032,JX128257,JX181828,KJ174317,MH427377,MH688040,MH940212,MN066127,MN342150,MW021753,MW021757,OK322699,OP610151
385	VC_133_23	1	0	28414.90037	0	0	0	0	1	1	1	0	0	0	0	MT074470
386	VC_133_24	1	0	29934.31938	0	0	0	0	1	1	1	0	0	0	0	MF001362
387	VC_133_25	1	0	30087.36899	0	0	0	0	1	1	1	0	0	0	0	JN593240
388	VC_133_26	1	0	29602.38895	0	0	0	0	1	1	1	0	0	0	0	JN673056

389	VC_133_27	1	0	30658.75382	0	0	0	0	1	1	1	0	0	0	0	MG383452
390	VC_133_28	3	900	84455.86086	0.010544091	0.023383628	4.795831523	9	3	2	0.666666667	7.26527717	0	0	0	LR811967,LR811969,MW355478
391	VC_133_29	2	300	57614.49581	0.00518005	0.141348401	1	1	1	1	1	1	0	0	0	MN871475,MN871501
392	VC_133_3	3	900	84849.96131	0.010495632	0.00880425	4.358898944	8.124038405	3	3	1	6.67759059	0	0	0	MH370368,MH370371,MT074469
393	VC_133_30	2	300	57389.01091	0.005200297	0.122611286	0	0	1	1	1	0	0	0	0	KX245012,KY000003
394	VC_133_31	6	4500	166641.783	0.026293988	3.06E-06	1.414213562	8.062257748	15	15	1	6.17437227	0	0	0	HQ259103,MH370370,MT074476,MW355450,MW355475,MW355477
395	VC_133_32	1	0	29753.55514	0	0	0	0	1	1	1	0	0	0	0	OM688977
396	VC_133_33	1	0	28471.36416	0	0	0	0	1	1	1	0	0	0	0	MT499896
397	VC_133_34	3	900	87040.36724	0.010234208	0.02630956	7.810249676	9.16515139	3	2	0.666666667	8.30421833	0	0	0	KY630163,KY787213,OK283619
398	VC_133_35	1	0	29910.96093	0	0	0	0	1	1	1	0	0	0	0	MZ156570
399	VC_133_36	2	300	57912.27187	0.005153553	0.135788523	1	1	1	1	1	1	0	0	0	MN871491,MN871492
400	VC_133_37	1	0	29721.8579	0	0	0	0	1	1	1	0	0	0	0	MN871493
401	VC_133_38	1	0	29185.44119	0	0	0	0	1	1	1	0	0	0	0	MN871502
402	VC_133_39	1	0	29958.8431	0	0	0	0	1	1	1	0	0	0	0	MT074478
403	VC_133_4	1	0	30039.16754	0	0	0	0	1	1	1	0	0	0	0	LR811968
404	VC_133_40	1	0	29943.85904	0	0	0	0	1	1	1	0	0	0	0	MH523359
405	VC_133_41	1	0	29493.30729	0	0	0	0	1	1	1	0	0	0	0	MT074474
406	VC_133_42	1	0	28165.3901	0	0	0	0	1	1	1	0	0	0	0	MN445184
407	VC_133_43	4	1800	118001.9253	0.0150248	4.32E-05	1.732050808	2.449489743	6	6	1	2.01585532	0	0	0	MN871496,MN871497,MN871498,MN871499
408	VC_133_44	1	0	29338.26797	0	0	0	0	1	1	1	0	0	0	0	MW021754
409	VC_133_45	2	300	57527.04619	0.005187884	0.051013205	7.549834435	7.549834435	1	1	1	7.54983444	0	0	0	MT074471,MT074480
410	VC_133_46	1	0	29235.47487	0	0	0	0	1	1	1	0	0	0	0	MW021760
411	VC_133_47	1	0	28878.36629	0	0	0	0	1	1	1	0	0	0	0	MT074479
412	VC_133_48	2	300	57909.14032	0.00515383	0.063600477	1.732050808	1.732050808	1	1	1	1.73205081	0	0	0	MK972698,MK972699
413	VC_133_49	1	0	30125.20818	0	0	0	0	1	1	1	0	0	0	0	ON169963
414	VC_133_5	1	0	30484.88589	0	0	0	0	1	1	1	0	0	0	0	MG748547
415	VC_133_50	1	0	30634.82056	0	0	0	0	1	1	1	0	0	0	0	ON169961
416	VC_133_51	1	0	31280.41455	0	0	0	0	1	1	1	0	0	0	0	ON169962
417	VC_133_6	1	0	30595.22212	0	0	0	0	1	1	1	0	0	0	0	MH791405
418	VC_133_7	1	0	30113.50112	0	0	0	0	1	1	1	0	0	0	0	MT974436
419	VC_133_8	1	0	30068.91172	0	0	0	0	1	1	1	0	0	0	0	MT074477
420	VC_133_9	3	900	89951.56384	0.009906269	0.01542891	2.236067977	8.544003745	3	3	1	6.42178437	0	0	0	KP797973,KX171211,MN384979
421	VC_1340_0	5	71.54630972	0	1	0	1.414213562	2	10	10	1	1.58994949	0	0	0	MG945850,MT135335,MT135340,MT135376,MT135393
422	VC_1341_0	2	14.98702917	4.392456787	0.773345031	0.5	1	1	1	1	1	1	0	0	0	MT135269,MT135429
423	VC_1342_0	3	23.2746182	0	1	0	1.414213562	2.236067977	3	3	1	1.79411078	0	0	0	MG945857,MG945859,MG945866
424	VC_1343_0	21	52416.18639	836.4594984	0.984292621	3.56E-52	0	8.246211251	210	210	1	3.55519549	0	0	0	MH271292,MH271301,MK737941,MK801731,MK801732,MN497954,MT522001,MT522003,MT639643,MT684591,MT684592,MT818420,MW055903,MW291025,MZ681513,OK040788,OK318957,ON526977,ON637767,ON755183,OP580517
425	VC_1344_0	78	162099.785	4467.495057	0.973179036	1.99E-73	0	5.477225575	3003	3003	1	3.73403355	0	0	0	MH045557,MH045561,MH371111,MH371118,MH371124,MH399783,MH576963,MH651181,MH744416,MH779512,MH825700,MK524502,MK620897,MK801730,MK878899,MK894434,MN010755,MN010759,MN284892,MN329679,MN369745,MN428062,MN428064,MN585990,MN586008,MN586010,MT310864,MT316458,MT316459,MT316462,MT498038,MT498040,MT498046,MT498047,MT498051,MT498065,MT498067,MT521990,MT639642,MT639647,MT657332,MT776807,MT952850,MW055907,MW055909,MW291020,MW321655,MW507127,MW924642,MZ005676,MZ005678,MZ028628,MZ209304,MZ274307,MZ322006,MZ322008,MZ322014,MZ322027,MZ622180,MZ648031,OK040781,OK040783,OK310504,ON081334,ON108642,ON108648,ON260812,ON260814,ON392161,ON456350,ON645341,ON645345,ON755182,ON755188,ON970573,ON970589,ON970613,OP580518
426	VC_1345_0	2	300	2347.092755	0.113331881	0.044208267	0	0	1	1	1	0	0	0	0	MN703407,MN703414
427	VC_1345_1	2	297.2168991	2812.090542	0.095589421	0.0438062	6.32455532	6.32455532	1	1	1	6.32455532	0	0	0	MH183162,MT639646
428	VC_1346_0	10	8786.703368	2501.558935	0.778392912	1.16E-15	0	3.741657387	45	45	1	2.51186182	0	0	0	MW021755,MW021763,MW021766,OL539438,OL539439,OL539442,OL539455,OL539456,OL539464,OM135608
429	VC_1348_0	5	2158.980254	1793.686937	0.546208459	1.54E-07	1	2.828427125	10	10	1	2.03060005	0	0	0	MH153799,MK737944,MZ456037,MZ622170,ON260825
430	VC_1349_0	7	5184.820658	15593.64902	0.249528514	1.22E-14	2.645751311	8	21	21	1	5.14258405	0	0	0	MH271303,MK937591,MK937606,MK937608,MN813682,MN813684,OK040792
431	VC_134_0	3	900	8514.371509	0.095598522	0.001415056	2	3.741657387	3	3	1	3.06858633	0	0	0	MH363700,MK795384,OK428602
432	VC_1350_0	2	194.2252171	996.3660026	0.16313342	0.046951483	3.16227766	3.16227766	1	1	1	3.16227766	0	0	0	MT889371,ON970592
433	VC_1351_0	2	20.32742815	0	1	0	6.92820323	6.92820323	1	1	1	6.92820323	0	0	0	MH155870,MK240575
434	VC_1352_0	31	118151.4156	21014.00908	0.848999785	2.04E-147	0	7.211102551	465	465	1	4.70966775	0	0	0	MH155873,MH271304,MH271312,MH271321,MH590600,MK524510,MK875798,MK977698,MN062719,MN175603,MN444877,MN585980,MN585986,MN586007,MN586042,MN586059,MT657336,MT818415,MW578837,MW601217,MZ150792,MZ322026,MZ622179,MZ648035,ON081330,ON260815,ON260820,ON970563,ON970612,OP068338,OP297543
435	VC_1353_0	10	11003.33853	0	1	0	0	5.099019514	45	45	1	3.58220563	0	0	0	MH229862,MH229863,MH229864,MH229865,MH536815,MH825706,MN224564,MN224565,ON456338,ON456348
436	VC_1354_0	3	509.5064876	8.832975739	0.982959091	0.040427799	0	4	3	3	1	2.66666667	0	0	0	MH447526,MK064562,MK064563
437	VC_1355_0	3	25.88353688	0	1	0	1.414213562	1.414213562	3	3	1	1.41421356	0	0	0	MH572274,MH572275,MH572276
438	VC_1356_0	8	268.2858067	39.07361524	0.872873215	1.78E-05	0	1.414213562	28	28	1	0.35355339	0	0	0	MH572353,MH572354,MH572355,MH572357,MH572358,MH572407,MH572408,MH572516
439	VC_1357_0	6	141.446599	0	1	0	0	1	15	15	1	0.53333333	0	0	0	MH572490,MH572491,MH572517,MH572518,MH572519,MH572520
440	VC_1358_0	5	2384.922104	0	1	0	0	1	10	10	1	0.4	0	0	0	MH707430,MW354669,MW354676,MW354677,MW354678
441	VC_1359_0	11	4623.84576	0	1	1	0	0	55	55	1	0	0	0	0	MH730557,MZ230992,MZ230993,MZ230994,MZ230995,MZ230996,MZ230997,MZ230998,MZ230999,MZ231000,MZ231001
442	VC_135_0	2	300	9638.212953	0.030186514	0.044425325	0	0	1	1	1	0	0	0	0	MG592609,MG592621
443	VC_135_1	2	300	9735.632693	0.029893481	0.044423634	8.602325267	8.602325267	1	1	1	8.60232527	0	0	0	MW824370,MW824375
444	VC_1360_0	31	58834.4525	7743.30292	0.883695344	7.52E-89	0	7.280109889	465	465	1	5.17055674	0	0	0	MK308638,MK757449,MK878904,MK894435,MK894438,MN310546,MN369747,MT310869,MT310875,MT310887,MT310888,MT310889,MT310890,MT310891,MT498064,MT657334,MT657337,MT684587,MT771344,MW055914,MW291018,MW365947,MW365948,MW507124,MW862991,MZ388554,OM913587,ON081328,ON456346,ON970584,OP297538
445	VC_1361_0	6	2405.982619	7743.30292	0.237059309	6.08E-11	0	4.358898944	15	15	1	2.42354592	0	0	0	MH825698,MN062711,MT024863,ON260817,ON526980,OP068335
446	VC_1362_0	6	663.0981007	0	1	0	1.732050808	4.69041576	15	15	1	3.58062305	0	0	0	MH834607,MH834622,MK757448,MT024867,MT024870,MT889364
447	VC_1363_0	2	188.7261751	37.93253524	0.832644705	0.270145687	0	0	1	1	1	0	0	0	0	MH939157,MK071653
448	VC_1364_0	3	34.64653635	0	1	0	0	1	3	3	1	0.66666667	0	0	0	MH992154,MK249216,MK249217
449	VC_1365_0	3	43.26213071	0	1	0	0	1.732050808	3	3	1	1.15470054	0	0	0	MH992155,MH992196,MH992205
450	VC_1367_0	2	15.53109722	0	1	0	0	0	1	1	1	0	0	0	0	MH992167,MK496682
451	VC_1368_0	2	10.97236665	0	1	0	0	0	1	1	1	0	0	0	0	MH992171,MW202680
452	VC_1369_0	3	377.4785754	7.353353373	0.980892039	0.040427799	1	1.414213562	3	3	1	1.13807119	0	0	0	MK305890,MK620900,OP434453
453	VC_136_0	2	300	6990.138631	0.041151481	0.042587315	3.741657387	3.741657387	1	1	1	3.74165739	0	0	0	MH375644,MT366762
454	VC_1370_0	2	137.3136498	29.85354423	0.821415055	0.087622123	6.32455532	6.32455532	1	1	1	6.32455532	0	0	0	MK411746,MN586027
455	VC_1371_0	5	63.78333871	27.2776659	0.700446245	0.478116184	0	1.732050808	10	10	1	1.17067423	0	0	0	MK496679,MK496680,MK496776,OM869578,OM869711
456	VC_1372_0	2	4.936518833	22.88520911	0.177433941	0.263544628	1.414213562	1.414213562	1	1	1	1.41421356	0	0	0	MK496698,MK496772
457	VC_1373_0	2	29.79354264	16.15131422	0.648463064	0.131776239	0	0	1	1	1	0	0	0	0	MK496681,MK496760
458	VC_1374_0	2	20.86797703	0	1	0	0	0	1	1	1	0	0	0	0	MK496683,MK496761
459	VC_1375_0	2	4.33446634	0	1	0	0	0	1	1	1	0	0	0	0	MK496686,MK496764
460	VC_1376_0	3	36.3442126	0	1	0	0	1.414213562	3	3	1	0.94280904	0	0	0	MK496688,MK496690,MK496766
461	VC_1377_0	3	8.784913575	0	1	1	1	1.732050808	3	3	1	1.38208812	0	0	0	MK496689,MK496700,MK496784
462	VC_1378_0	2	4.635491837	0	1	0	0	0	1	1	1	0	0	0	0	MK496697,MK496780
463	VC_1379_0	2	6.334452844	0	1	0	0	0	1	1	1	0	0	0	0	MK496699,MK496785
464	VC_137_0	3	355.9573481	2795.565648	0.112947724	0.001453003	7.549834435	8.366600265	3	3	1	7.99289748	0	0	0	AJ609634,MK448910,OL774867
465	VC_137_1	1	0	1199.65636	0	0	0	0	1	1	1	0	0	0	0	MK448902
466	VC_1380_0	2	10.97236665	0	1	0	0	0	1	1	1	0	0	0	0	MK496701,MK496702
467	VC_1381_0	3	7.93983353	0	1	0	1	1.414213562	3	3	1	1.13807119	0	0	0	MK496710,MK496767,MK496768
468	VC_1382_0	2	11.67133666	0	1	0	0	0	1	1	1	0	0	0	0	MK496712,MK496787
469	VC_1383_0	2	14.20888126	7.93983353	0.641521704	0.270145687	0	0	1	1	1	0	0	0	0	MK496716,MK496790
470	VC_1384_0	2	9.319160538	0	1	0	0	0	1	1	1	0	0	0	0	MK496717,MK496804
471	VC_1385_0	2	25.35975681	0	1	0	0	0	1	1	1	0	0	0	0	MK496720,MK496796
472	VC_1386_0	3	20.80953851	0	1	1	0	0	3	3	1	0	0	0	0	MK496721,MK496781,MK496786
473	VC_1387_0	3	30.10425588	10.72874778	0.737253035	0.03826125	1	1.414213562	3	3	1	1.13807119	0	0	0	MK496725,MK496746,MK496811
474	VC_1388_0	2	14.75294763	0	1	0	0	0	1	1	1	0	0	0	0	MK496748,MK496815
475	VC_1389_0	2	15.53109722	8.969958177	0.633895029	0.270145687	0	0	1	1	1	0	0	0	0	MK496750,MK496816
476	VC_138_0	2	112.7686339	448.4465939	0.200936518	0.046310926	5.196152423	5.196152423	1	1	1	5.19615242	0	0	0	DQ340064,DQ840344
477	VC_1390_0	2	16.30924847	0	1	0	0	0	1	1	1	0	0	0	0	MK496753,MK496818
478	VC_1391_0	2	9.796278594	0	1	0	1.414213562	1.414213562	1	1	1	1.41421356	0	0	0	MK496756,MK496824
479	VC_1392_0	2	14.75294763	0	1	0	0	0	1	1	1	0	0	0	0	MK496758,MK496808
480	VC_1393_0	2	16.30924847	0	1	0	0	0	1	1	1	0	0	0	0	MK496770,MK496771
481	VC_1394_0	2	3.887317306	0	1	0	0	0	1	1	1	0	0	0	0	MK496820,OM869632
482	VC_1395_0	3	502.6487421	144.9856775	0.776130371	0.036099099	0	2.236067977	3	3	1	1.49071198	0	0	0	MK570053,MK570054,MK570055
483	VC_1396_0	110	1770844.614	0	1	0	0	10.24695077	5995	5843	0.974645538	4.93673592	0	0	0	MK719701,MK719702,MK719703,MK719704,MK719705,MK719706,MK719707,MK719708,MK719709,MK719710,MK719711,MK719712,MK719713,MK719714,MK719715,MK719716,MK719717,MK719718,MK719719,MK719720,MK719721,MK719722,MK719723,MK719724,MK719725,MK719726,MK719727,MK719728,MK719729,MK719730,MK719731,MK719732,MK719733,MK719734,MK719735,MK719736,MK719737,MK719738,MK719739,MK719740,MK719741,MK719742,MK719743,MK719744,MK719745,MK719746,MK719747,MK719748,MK719749,MK719750,MK719751,MK719752,MK719753,MK719754,MT497224,MT497225,MT497226,MT497227,MT497228,MT497229,MT497230,MT497231,MT497232,MT497233,MT497234,MT497235,MT497236,MT497237,MT497238,MT497239,MT497240,MT497241,MT497242,MT497243,MT497244,MT497245,MT497246,MT497247,MT497248,MT497249,MT497250,MT497251,MT497252,MT497253,MT497254,MT497255,MT497256,MT497257,MT497258,MT497259,MT497260,MT497261,MT497262,MT497263,MT497264,MT497265,MT497266,MT497267,MT497268,MT497269,MT497270,MT497271,MT497272,MT497273,MT497274,MT497275,MT497276,MT497277,MT497278,MT497279
484	VC_1397_0	2	165.0302466	73.96689844	0.690511372	0.065366293	8.124038405	8.124038405	1	1	1	8.1240384	0	0	0	S11-wms-combined_73204,S12-wms-combined_320727
485	VC_1398_0	2	20.02287899	0	1	0	0	0	1	1	1	0	0	0	0	MK765551,MK765650
486	VC_1399_0	9	420.1681197	0	1	1	0	0	36	36	1	0	0	0	0	MK765558,MK765581,MK765604,MK765622,MK765640,MK765654,MK765656,MK765663,MK765668
487	VC_139_0	2	186.1419215	2467.976517	0.070133239	0.044898152	6.480740698	6.480740698	1	1	1	6.4807407	0	0	0	AY855346,LK985322
488	VC_139_1	4	1073.274386	4973.384526	0.177498748	1.39E-05	3.605551275	8.426149773	6	6	1	6.97968773	0	0	0	DQ466086,HG531805,LN681541,LN681542
489	VC_1400_0	2	9.319160538	0	1	0	0	0	1	1	1	0	0	0	0	MK765573,MK765662
490	VC_1401_0	2	10.97236665	0	1	0	0	0	1	1	1	0	0	0	0	MK765575,MK765578
491	VC_1402_0	14	412.1442703	2051.859504	0.167266087	0.486096463	0	2.645751311	91	91	1	1.99090085	0	0	0	MK765587,MK765588,MK765598,MK765599,MK765623,MK765625,MK765628,MK765630,MK765631,MK765634,OL617020,OL617024,OL617026,OM782324
492	VC_1403_0	28	8196.936363	2688.272467	0.753034369	2.07E-168	0	2.236067977	378	378	1	0.66608515	0	0	0	MK765589,MN988460,MN988467,MN988469,MN988471,MN988473,MN988474,MN988475,MN988478,MN988479,MN988480,MN988481,MN988489,MN988491,MN988494,MN988498,MN988499,MN988503,MN988504,MN988511,MN988518,MW960029,MW960035,MW980067,OL617021,OL617022,OL617025,OL617027
493	VC_1404_0	1	0	51.76694088	0	0	0	0	1	1	1	0	0	0	0	MK765626
494	VC_1406_0	2	10.27339825	0	1	0	0	0	1	1	1	0	0	0	0	MK765605,MK765641
495	VC_1407_0	2	20.34523526	4.540216016	0.817555408	0.185546685	8.306623863	8.306623863	1	1	1	8.30662386	0	0	0	MK929790,MW202545
496	VC_1408_0	2	10.51240781	51.50282377	0.16951332	0.401293674	8.94427191	8.94427191	1	1	1	8.94427191	0	0	0	S1-wms-combined_197401,S17-wms-combined_162786
497	VC_1408_1	1	0	53.04348343	0	0	0	0	1	1	1	0	0	0	0	S1-wms-combined_220022
498	VC_1409_0	2	13.23885114	0	1	0	0	0	1	1	1	0	0	0	0	MN379629,MN379636
499	VC_140_0	2	217.5902156	311.6650836	0.411125247	0.051752086	4.472135955	4.472135955	1	1	1	4.47213595	0	0	0	DQ003640,MH341453
500	VC_1410_0	2	20.02287899	0	1	0	0	0	1	1	1	0	0	0	0	MN379639,MN379646
501	VC_1411_0	2	14.75294763	0	1	0	0	0	1	1	1	0	0	0	0	MN379643,MN379650
502	VC_1413_0	2	171.3104661	80.74987012	0.679640711	0.105649774	0	0	1	1	1	0	0	0	0	MN882551,MN882552
503	VC_1414_0	4	1800	0	1	1	0	1.732050808	6	6	1	1.09341299	0	0	0	MN988461,MN988462,MN988463,MN988472
504	VC_1415_0	2	15.53109722	0	1	0	0	0	1	1	1	0	0	0	0	MN988485,MN988550
505	VC_1416_0	5	3000	13.62742601	0.995478065	0.000116471	0	6.480740698	10	10	1	4.65916457	0	0	0	MN988515,MN988535,MN988540,MW980063,MW980064
506	VC_1417_0	15	22851.94544	40.83131706	0.99821641	2.09E-10	0	6.403124237	105	105	1	4.04178947	0	0	0	MT732458,MT732459,MT732460,MT732461,MT732462,MT732463,MT732464,MT732465,MT732466,MT732467,MT732468,MT732469,MT732470,MT732471,MT732472
507	VC_1418_0	2	141.7431816	0	1	0	0	0	1	1	1	0	0	0	0	MT006234,MT006235
508	VC_1419_0	6	4500	215.6728999	0.954264661	6.96E-06	0	7.071067812	15	15	1	4.62872666	0	0	0	MT074137,MT074139,MT074140,MT074141,MT074142,MT074146
509	VC_141_0	6	3383.244073	2256.676936	0.599874372	4.94E-11	0	8	15	15	1	5.27587007	0	0	0	KC595514,KC595515,KC595517,KC595518,KT151956,KT151958
510	VC_1420_0	5	1193.292455	0	1	0	2.645751311	8	10	10	1	6.04425093	0	0	0	MT121960,MT121964,S12-wms_401372,S2-wms-combined_197577,S2-wms_128831
511	VC_1421_0	4	59.41518686	0	1	0	0	1.732050808	6	6	1	1.09341299	0	0	0	MT135262,MT135274,MT135389,MT135413
512	VC_1422_0	2	28.13437958	0	1	0	1	1	1	1	1	1	0	0	0	MT135284,S2-wms-combined_132966
513	VC_1423_0	8	524.5818436	60.89151326	0.895996099	0.000160684	0	2.828427125	28	28	1	1.63382316	0	0	0	MT135285,MT135286,MT135289,MT135297,MT135302,MT135308,MT135314,S9-wms_104169
514	VC_1424_0	8	514.7654063	141.6190616	0.784243734	5.40E-13	0	3.605551275	28	28	1	2.56744696	0	0	0	MT135288,MT135290,MT135291,MT135301,MT135303,MT135307,MT135309,MT135311
515	VC_1425_0	7	309.0508475	133.9381279	0.697649072	1.45E-08	0	4	21	21	1	2.66412119	0	0	0	MT135287,MT135294,MT135298,MT135299,MT135304,MT135306,MT135310
516	VC_1426_0	4	103.7350176	0	1	0	0	1.414213562	6	6	1	0.97140452	0	0	0	MT135292,MT135293,MT135296,MT135305
517	VC_1427_0	2	5.334455843	0	1	0	1	1	1	1	1	1	0	0	0	MT135332,MT135436
518	VC_1428_0	2	7.981166171	0	1	0	0	0	1	1	1	0	0	0	0	MT135346,MT135395
519	VC_1429_0	8	279.6760993	8.221783646	0.971442014	0.05482919	1	2.828427125	28	28	1	1.98315598	0	0	0	MT135359,MT135368,MT135374,MT135378,MT135399,MT135414,MT135419,MT135463

520	VC_142_0	2	251.0442322	295.0561832	0.459703427	0.057268646	0	0	1	1	1	0	0	0	0	KM983330,KM983333
521	VC_1430_0	4	36.04712246	0	1	0	1.414213562	2	6	6	1	1.67076326	0	0	0	MT135369,MT135396,MT135454,MT135460
522	VC_1431_0	3	28.18227414	0	1	0	0	1.414213562	3	3	1	0.94280904	0	0	0	MT135375,MT135438,MT135453
523	VC_1433_0	2	300	0	1	0	5.477225575	5.477225575	1	1	1	5.47722558	0	0	0	MT234670,ON014754
524	VC_1434_0	2	273.4640231	39.3646605	0.874165438	0.144422183	1.414213562	1.414213562	1	1	1	1.41421356	0	0	0	ON529863,ON529867
525	VC_1435_0	9	874.6348922	387.3481794	0.693063886	5.34E-08	4.123105626	8	36	36	1	6.48134192	0	0	0	S17-wms-combined_16309,S17-wms_124308,S2-wms_156439,S7-wms-combined_185016,S7-wms-combined_90393,S7-wms-combined_90728,S7-wms_123954,S9-wms-combined_33229,S9-wms-combined_33230
526	VC_1435_1	1	0	183.8748403	0	0	0	0	1	1	1	0	0	0	0	S6-wms-combined_82597
527	VC_1436_0	4	46.74873652	35.12833821	0.570962466	0.03630047	3.872983346	6.480740698	6	6	1	5.70807377	0	0	0	S12-wms-combined_351847,S12-wms_383851,S2-wms-combined_58121,S7-wms-combined_239784
528	VC_1437_0	3	250.8216948	87.23509648	0.741951356	0.008115024	5.291502622	6.32455532	3	3	1	5.92682398	0	0	0	S1-wms-combined_263562,S5-wms-combined_207291,S7-wms-combined_104872
529	VC_1438_0	6	4500	0	1	1	0	3.605551275	15	15	1	1.86005006	0	0	0	MT732450,MT732451,MT732452,MT732453,MT732454,MT732455
530	VC_1439_0	3	38.36357799	47.62584872	0.446142967	0.012635581	5.830951895	7.141428429	3	3	1	6.56019475	0	0	0	S12-wms-combined_260036,S12-wms-combined_91231,S4-wms_45128
531	VC_143_0	2	39.90191747	295.7747569	0.118870093	0.05004329	6.782329983	6.782329983	1	1	1	6.78232998	0	0	0	KT626446,ON699007
532	VC_1440_0	4	663.5497664	0	1	0	0	1	6	6	1	0.66666667	0	0	0	MT952848,MZ150790,OL455892,OP068334
533	VC_1441_0	2	25.35975681	0	1	0	0	0	1	1	1	0	0	0	0	MT975991,OP433453
534	VC_1442_0	2	256.5191023	8.356398473	0.968451599	0.131776239	0	0	1	1	1	0	0	0	0	MW202613,MW202748
535	VC_1443_0	2	194.062967	31.29142199	0.861145718	0.239750061	0	0	1	1	1	0	0	0	0	MW202637,MW202789
536	VC_1444_0	3	89.38062792	0	1	1	0	0	3	3	1	0	0	0	0	MW202486,MW202591,MW202766
537	VC_1445_0	4	1266.227393	14.99889595	0.988293328	0.003842984	1	1.414213562	6	6	1	1.13807119	0	0	0	MW202572,MW202631,MW202645,MW202742
538	VC_1446_0	3	721.8807503	0	1	1	0	0	3	3	1	0	0	0	0	MW202524,MW202718,MW202743
539	VC_1447_0	2	244.0422632	0	1	0	0	0	1	1	1	0	0	0	0	MW202601,MW202604
540	VC_1448_0	5	2085.645793	0	1	0	0	1	10	10	1	0.6	0	0	0	MW202608,MW202725,MW202812,MW202826,MW202842
541	VC_1449_0	2	197.6921866	0	1	0	0	0	1	1	1	0	0	0	0	MW202622,MW202658
542	VC_144_0	2	268.7364357	365.5521083	0.42368168	0.052317582	1	1	1	1	1	1	0	0	0	MN604230,MN604698
543	VC_1450_0	3	23.13612536	0	1	0	0	1	3	3	1	0.66666667	0	0	0	MW202642,MW202843,MW202852
544	VC_1451_0	2	265.2030535	0	1	0	0	0	1	1	1	0	0	0	0	MW202657,MW202864
545	VC_1452_0	2	66.1264962	0	1	0	5	5	1	1	1	5	0	0	0	MW202753,MW202825
546	VC_1453_0	7	1522.810025	147.0001386	0.911965958	0.000486588	0	5.196152423	21	21	1	3.41668908	0	0	0	MW353166,MW353167,MW353168,MW353169,MW353170,MW353171,MW353172
547	VC_1454_0	2	33.93969256	14.98865778	0.693661085	0.144422183	9	9	1	0	0	9	0	0	0	MW462221,MZ047271
548	VC_1456_0	4	551.7060882	349.6011421	0.612117677	0.000122565	5.477225575	8.717797887	6	6	1	7.20663184	0	0	0	S11-wms-combined_125176,S12-wms_386692,S6-wms-combined_91039,S7-wms-combined_14267
549	VC_1457_0	2	11.67133666	0	1	0	0	0	1	1	1	0	0	0	0	MZ089747,MZ089748
550	VC_1458_0	2	4.392456787	0	1	0	1	1	1	1	1	1	0	0	0	MZ089769,OM869588
551	VC_1459_0	2	17.49655	0	1	0	0	0	1	1	1	0	0	0	0	MZ089783,OM869706
552	VC_1460_0	3	41.32207156	11.34108823	0.784648542	0.03826125	0	1.732050808	3	3	1	1.15470054	0	0	0	MZ089787,MZ089788,OM869708
553	VC_1461_0	3	47.37144291	0	1	0	0	0	3	3	1	0	0	0	0	MZ089790,MZ089792,MZ089794
554	VC_1462_0	4	73.07202371	0	1	0	0	1	6	6	1	0.5	0	0	0	MZ089811,OM869593,OM869669,OM869712
555	VC_1463_0	6	126.1913745	0	1	0	0	1	15	15	1	0.33333333	0	0	0	MZ364235,MZ364236,MZ364238,MZ364239,MZ364257,MZ364289
556	VC_1464_0	3	23.42188031	0	1	0	0	2	3	3	1	1.33333333	0	0	0	MZ364251,MZ364282,MZ364294
557	VC_1465_0	2	208.5298712	0	1	0	0	0	1	1	1	0	0	0	0	MZ447863,MZ570427
558	VC_1466_0	2	2.262782824	0	1	0	2.449489743	2.449489743	1	1	1	2.44948974	0	0	0	MZ474488,MZ474489
559	VC_1467_0	3	162.2436747	0	1	0	2.645751311	3.16227766	3	3	1	2.81792676	0	0	0	OL689226,OL689227,OL689230
560	VC_1468_0	2	4.33446634	0	1	0	0	0	1	1	1	0	0	0	0	OM869510,OM869512
561	VC_1469_0	2	20.86797703	0	1	0	0	0	1	1	1	0	0	0	0	OM869572,OM869573
562	VC_1470_0	3	5.276002318	0	1	0	0	0	3	3	1	0	0	0	0	OM869583,OM869672,OM869688
563	VC_1471_0	2	14.20888126	0	1	0	0	0	1	1	1	0	0	0	0	OM869594,OM869713
564	VC_1472_0	2	2.490056751	0	1	0	6.32455532	6.32455532	1	1	1	6.32455532	0	0	0	ON014753,ON014757
565	VC_1473_0	3	222.4261317	84.92048884	0.723697991	0.003868342	7.810249676	9	3	2	0.666666667	8.31142936	0	0	0	S1-wms_244836,S2-wms-combined_229529,S9-wms-combined_37587
566	VC_1474_0	3	900	0	1	1	2	3	3	3	1	2.66666667	0	0	0	ON464759,ON464763,ON464764
567	VC_1475_0	2	300	0	1	0	1.732050808	1.732050808	1	1	1	1.73205081	0	0	0	ON529865,ON529866
568	VC_1476_0	2	16.30924847	0	1	0	0	0	1	1	1	0	0	0	0	OP549878,OP549879
569	VC_1477_0	3	102.2494938	0	1	0	1.732050808	3.16227766	3	3	1	2.63144282	0	0	0	OP549880,S4-wms_161443,S7-wms_157264
570	VC_1478_0	2	29.79354264	0	1	0	0	0	1	1	1	0	0	0	0	PF3COMN,PF3COMNY
571	VC_1479_0	2	205.3385226	214.8549051	0.488676188	0.061538965	7.549834435	7.549834435	1	1	1	7.54983444	0	0	0	S12-wms-combined_51076,S5-wms-combined_78434
572	VC_1481_0	9	152.8826248	9.479170006	0.941616992	0.220826911	1.414213562	3.464101615	36	36	1	2.48166124	0	0	0	S1-wms-combined_120533,S1-wms-combined_331896,S12-wms-combined_393703,S17-wms-combined_126600,S2-wms-combined_181095,S5-wms_52566,S8-wms-combined_234473,S8-wms-combined_264711,S9-wms-combined_56773
573	VC_1482_0	2	9.152822718	16.61800529	0.355162151	0.144422183	1	1	1	1	1	1	0	0	0	S17-wms-combined_30308,S4-wms-combined_143879
574	VC_1483_0	1	0	59.24370107	0	0	0	0	1	1	1	0	0	0	0	S7-wms-combined_169375
575	VC_1483_1	1	0	45.35844511	0	0	0	0	1	1	1	0	0	0	0	S8-wms-combined_40666
576	VC_1484_0	10	1043.57682	464.9838744	0.69176986	2.08E-08	2.236067977	10.58300524	45	40	0.888888889	7.07370741	0	0	0	S1-wms-combined_151708,S1-wms-combined_164485,S12-wms-combined_329242,S17-wms-combined_126774,S17-wms-combined_146850,S17-wms-combined_157468,S2-wms-combined_4685,S2-wms-combined_6605,S6-wms-combined_41246,S7-wms-combined_84688
577	VC_1485_0	1	0	44.10957618	0	0	0	0	1	1	1	0	0	0	0	S2-wms-combined_233107
578	VC_1487_0	2	6.557034017	0	1	0	4.472135955	4.472135955	1	1	1	4.47213595	0	0	0	S1-wms-combined_156637,S12-wms-combined_210942
579	VC_1489_0	1	0	5.515472155	0	0	0	0	1	1	1	0	0	0	0	S12-wms-combined_157167
580	VC_148_0	2	170.0183889	662.0139995	0.20434107	0.046334615	6.244997998	6.244997998	1	1	1	6.244998	0	0	0	S2-wms-combined_62519,S8-wms-combined_161181
581	VC_148_1	3	417.1453056	623.6087881	0.400810632	0.002477882	5.567764363	7.745966692	3	3	1	6.5722851	0	0	0	S2-wms-combined_218002,S6-wms-combined_119138,S6-wms-combined_32530
582	VC_1491_0	2	5.635482839	0	1	0	1	1	1	1	1	1	0	0	0	S1-wms-combined_185336,S1-wms-combined_40115
583	VC_1492_0	7	1518.837228	555.5451018	0.7321877	5.64E-11	3.872983346	9.539392014	21	18	0.857142857	7.74673984	0	0	0	S1-wms_100010,S1-wms_225995,S12-wms-combined_264425,S12-wms-combined_299293,S12-wms_179388,S12-wms_401366,S12-wms_44817
584	VC_1494_0	2	30.24608585	1.463968726	0.953832665	0.5	1.732050808	1.732050808	1	1	1	1.73205081	0	0	0	S1-wms-combined_248622,S17-wms-combined_70983
585	VC_1495_0	3	93.82698037	38.09746008	0.711217573	0.006365682	5	7.810249676	3	3	1	6.76452148	0	0	0	S17-wms-combined_233761,S2-wms-combined_210822,S4-wms-combined_47565
586	VC_1496_0	5	130.1356474	10.89296783	0.922760584	0.011247136	2	3.605551275	10	10	1	2.88307627	0	0	0	S1-wms-combined_313851,S1-wms_313991,S2-wms_110761,S2-wms_23562,S6-wms_137591
587	VC_1497_0	3	210.1504315	0	1	0	3	4.358898944	3	3	1	3.86717988	0	0	0	S1-wms-combined_331544,S4-wms-combined_51079,S7-wms-combined_136642
588	VC_1498_0	2	33.56623516	3.194404025	0.913102598	0.5	4.472135955	4.472135955	1	1	1	4.47213595	0	0	0	S1-wms-combined_354899,S2-wms-combined_248318
589	VC_1499_0	2	1.467479827	3.194404025	0.31478258	0.5	6.782329983	6.782329983	1	1	1	6.78232998	0	0	0	S5-wms-combined_66017,S5-wms_210460
590	VC_149_0	5	3000	103889.1795	0.028066452	1.03E-06	0	1.732050808	10	10	1	0.89746915	0	0	0	MT080592,MT080597,MT080598,MT080599,MT080603
591	VC_149_1	3	900	69214.93003	0.012836068	0.006960472	1	1.414213562	3	3	1	1.13807119	0	0	0	OM850391,OM850392,ON113344
592	VC_149_10	1	0	35853.22012	0	0	0	0	1	1	1	0	0	0	0	KY794641
593	VC_149_11	4	1800	86765.56562	0.020323926	0.000164304	0	8.94427191	6	6	1	4.90603679	0	0	0	MT080582,MT080583,MT080586,MT080594
594	VC_149_12	2	300	67267.03433	0.004440035	0.168432916	7.874007874	7.874007874	1	1	1	7.87400787	0	0	0	MH107769,MN045228
595	VC_149_13	3	900	102060.8519	0.008741186	0.048210974	7.348469228	8.888194417	3	3	1	8.22093781	0	0	0	AB853330,JX878671,LR215719
596	VC_149_14	1	0	35285.51482	0	0	0	0	1	1	1	0	0	0	0	AB903967
597	VC_149_15	16	36000	421023.6356	0.078770543	1.24E-67	0	10.77032961	120	97	0.808333333	7.02767147	0	0	0	JQ686190,KM216423,KY779849,LC574321,LR215718,MF001365,MG557618,MH159197,MK250904,MN304941,MT786458,MW546073,MZ427930,MZ573923,OP235318,OP323059
598	VC_149_16	1	0	33802.20141	0	0	0	0	1	1	1	0	0	0	0	KJ888149
599	VC_149_17	4	1800	136436.969	0.013021119	0.000481854	0	2	6	6	1	1.33333333	0	0	0	MT080585,MT080589,MT080591,MT080601
600	VC_149_18	1	0	34926.54481	0	0	0	0	1	1	1	0	0	0	0	MT554104
601	VC_149_19	1	0	34414.99466	0	0	0	0	1	1	1	0	0	0	0	OP263968
602	VC_149_2	1	0	24781.52512	0	0	0	0	1	1	1	0	0	0	0	LR215720
603	VC_149_20	1	0	33796.75631	0	0	0	0	1	1	1	0	0	0	0	KP027446
604	VC_149_21	1	0	38022.37521	0	0	0	0	1	1	1	0	0	0	0	MG721208
605	VC_149_22	2	300	47850.66213	0.006230444	0.070834384	1	1	1	1	1	1	0	0	0	MT080593,MT080596
606	VC_149_23	1	0	32659.35798	0	0	0	0	1	1	1	0	0	0	0	AB853331
607	VC_149_24	1	0	23304.24333	0	0	0	0	1	1	1	0	0	0	0	MZ417322
608	VC_149_25	14	27300	259934.8427	0.09504418	5.46E-47	2.449489743	10.95445115	91	80	0.879120879	7.00604712	0	0	0	MZ417315,MZ417319,MZ417320,MZ417321,MZ417327,MZ417328,MZ417332,MZ417337,MZ417340,MZ417345,MZ417346,MZ417347,MZ417350,MZ417354
609	VC_149_26	1	0	23636.88817	0	0	0	0	1	1	1	0	0	0	0	MZ417331
610	VC_149_27	1	0	17406.87461	0	0	0	0	1	1	1	0	0	0	0	KX011028
611	VC_149_28	1	0	21591.38333	0	0	0	0	1	1	1	0	0	0	0	MN935200
612	VC_149_29	3	900	73914.6509	0.012029729	0.005514229	6.403124237	8.246211251	3	3	1	7.52886314	0	0	0	KY794642,KY794643,LR215721
613	VC_149_3	1	0	23798.4585	0	0	0	0	1	1	1	0	0	0	0	MT787017
614	VC_149_4	1	0	24237.99408	0	0	0	0	1	1	1	0	0	0	0	OL741057
615	VC_149_5	1	0	25353.63292	0	0	0	0	1	1	1	0	0	0	0	MN091625
616	VC_149_6	1	0	26787.2931	0	0	0	0	1	1	1	0	0	0	0	OP297178
617	VC_149_7	1	0	23354.53964	0	0	0	0	1	1	1	0	0	0	0	LC648442
618	VC_149_8	66	643500	766163.0263	0.456492075	0	0	10.39230485	2145	1755	0.818181818	6.46395765	0	0	0	AY176327,AY954969,EU418428,FR852584,HQ163896,JX080300,JX080301,JX080302,JX080303,JX080304,JX080305,JX875065,KC012913,KF766114,KJ206559,KJ206560,KJ206561,KJ206562,KJ206563,KP687431,KP687432,KR902361,KR908644,KY581279,MF398190,MF405094,MG656408,MH136584,MH844528,MH844529,MK331930,MK331931,MK417514,MK417515,MK417516,MK584893,MK656892,MN047438,MN336261,MN336262,MN336263,MN539736,MN539737,MN539738,MT080600,MT080602,MT226657,MT338525,MW460250,MW528836,MW546064,MW546065,MW546066,MW546067,MW546068,MW546069,MW546070,MW546072,MW546074,OL362280,OL960567,OL960568,OL960569,OL960570,OM030343,OM373557
619	VC_149_9	3	900	106201.1063	0.008403275	0.021454443	7.937253933	8.888194417	3	3	1	8.35721987	0	0	0	AP018375,MT411892,OP263967
620	VC_1500_0	2	43.47911773	1.118016055	0.974930764	0.5	0	0	1	1	1	0	0	0	0	S1-wms-combined_394628,S12-wms-combined_401379
621	VC_1501_0	2	4.619287091	35.46274095	0.115245843	0.280637918	7.874007874	7.874007874	1	1	1	7.87400787	0	0	0	S8-wms-combined_276204,S8-wms_57121
622	VC_1502_0	2	33.367684	50.19727374	0.39930235	0.081867177	7.416198487	7.416198487	1	1	1	7.41619849	0	0	0	S11-wms-combined_17029,S12-wms-combined_375343
623	VC_1503_0	3	354.7435472	34.61511723	0.911097093	0.009451772	5.567764363	7	3	3	1	6.34950169	0	0	0	S11-wms-combined_217790,S12-wms-combined_111524,S5-wms-combined_98202
624	VC_1504_0	2	60.7080931	2.226001104	0.964629647	0.5	6.32455532	6.32455532	1	1	1	6.32455532	0	0	0	S11-wms-combined_221310,S8-wms-combined_215102
625	VC_1505_0	4	365.6148947	19.43590799	0.949523783	0.001207324	5	7.416198487	6	6	1	6.60496004	0	0	0	S11-wms-combined_41367,S12-wms-combined_256299,S17-wms-combined_158354,S5-wms_85650
626	VC_1506_0	2	10.62980693	39.76173325	0.210944275	0.191366544	8.602325267	8.602325267	1	1	1	8.60232527	0	0	0	S5-wms-combined_178432,S6-wms-combined_72228
627	VC_1507_0	2	5.776189	4.860891598	0.543023901	0.270145687	4.69041576	4.69041576	1	1	1	4.69041576	0	0	0	S12-wms-combined_264845,S12-wms_373775
628	VC_1508_0	3	155.4711449	17.71885559	0.897691232	0.040427799	8	9	3	2	0.666666667	8.35408592	0	0	0	S17-wms-combined_203701,S4-wms-combined_57123,S6-wms-combined_135223
629	VC_1509_0	2	164.7898519	111.6200581	0.596179247	0.087622123	3.605551275	3.605551275	1	1	1	3.60555128	0	0	0	S17-wms-combined_179771,S7-wms-combined_72070
630	VC_150_0	13	23400	73432.19069	0.241655175	1.34E-51	0	9	78	77	0.987179487	6.23043855	0	0	0	KF021268,KP027447,MH321490,MH321491,MH542234,MT596501,MT596502,MT596503,MT596504,MT596505,MT596506,MZ417323,MZ417325
631	VC_1510_0	2	10.9800245	0	1	0	0	0	1	1	1	0	0	0	0	S12-wms-combined_401383,S9-wms-combined_3878
632	VC_1511_0	2	7.44029455	0	1	0	5	5	1	1	1	5	0	0	0	S12-wms_15700,S8-wms-combined_226076
633	VC_1512_0	2	2.933115285	0	1	0	0	0	1	1	1	0	0	0	0	S17-wms-combined_223748,S9-wms_64472
634	VC_1513_0	2	15.20842239	1.209466374	0.926332405	0.5	4.898979486	4.898979486	1	1	1	4.89897949	0	0	0	S17-wms-combined_225356,S4-wms_107182
635	VC_1514_0	2	9.826238618	0	1	0	1	1	1	1	1	1	0	0	0	S5-wms-combined_39793,S7-wms-combined_143218
636	VC_151_0	6	4500	56765.36966	0.073450956	1.33E-11	0	8.831760866	15	15	1	6.61829481	0	0	0	KX171212,KX171213,MW430345,MZ417329,MZ417330,MZ417344
637	VC_152_0	1	0	5908.481571	0	0	0	0	1	1	1	0	0	0	0	LC647030
638	VC_152_1	1	0	4169.681694	0	0	0	0	1	1	1	0	0	0	0	MZ417326
639	VC_153_0	25	90000	26444.95332	0.772897386	1.12E-142	0	8.062257748	300	300	1	5.57362611	0	0	0	AP019522,JX194239,JX846612,JX846613,KP881332,KX532239,KY779848,LC492751,LC492752,MT080584,MT080587,MT080588,MT080590,MT080595,MW363798,MW546071,MW546075,MW546076,MW546077,MZ643272,ON814134,ON814135,ON814136,ON866946,OP263969
640	VC_153_1	1	0	8761.775594	0	0	0	0	1	1	1	0	0	0	0	LC576631
641	VC_154_0	4	300.9148492	1402.886472	0.176613814	4.89E-05	4.358898944	6.633249581	6	6	1	5.84551635	0	0	0	AB854109,KY117485,MN844877,OL634959
642	VC_155_0	14	11428.51712	7323.511316	0.60945498	4.62E-55	0	6.782329983	91	91	1	4.2026275	0	0	0	FN594518,MT104468,MT104469,MT104470,MT104471,MT104472,MT104476,MT104477,MT354570,MW627366,MZ868714,MZ868715,MZ868716,MZ868717
643	VC_156_0	12	9950.94663	1029.926012	0.906207271	8.86E-30	1	5.567764363	66	66	1	3.70983969	0	0	0	FR751545,KM247287,MK629528,MN850568,MN850570,MN850583,MN850592,MN850608,MN850617,MN850624,MN850625,MW250275
644	VC_157_0	3	509.7785864	856.0247477	0.373244503	0.00191903	1.414213562	2.828427125	3	3	1	2.23071014	0	0	0	MK962628,MK962638,MK962640
645	VC_159_0	2	160.637858	1040.78759	0.133706056	0.04371377	2.828427125	2.828427125	1	1	1	2.82842712	0	0	0	OM913597,OM913598
646	VC_15_0	3	361.2304471	7148.582097	0.048101127	0.001465399	7	7.280109889	3	3	1	7.16373748	0	0	0	KX827371,MK075002,OM373558
647	VC_15_1	9	2772.329634	12463.93259	0.18195602	7.69E-23	4.472135955	9.486832981	36	32	0.888888889	7.46346854	0	0	0	AY954954,MK075001,MK075004,MK450538,OM373550,OM373554,OM373555,OM373556,OM373561
648	VC_160_0	2	148.853439	151.4831149	0.495622118	0.056361467	3.605551275	3.605551275	1	1	1	3.60555128	0	0	0	MF150911,MN996301
649	VC_161_0	3	487.8368293	356.6612016	0.577664851	0.00195604	1.414213562	3.741657387	3	3	1	2.87332419	0	0	0	AB863625,MT740737,MT740741
650	VC_162_0	18	17769.63491	6234.873331	0.740262401	2.99E-66	1.732050808	9.273618495	153	132	0.862745098	7.09065507	0	0	0	AY349011,FJ937737,JN662425,JX104231,MH638294,MT361768,MT740727,MT740730,MT740731,MT740732,MT740733,MT740736,MT740738,MT740739,MT740740,MT740742,OM283127,OV121131
651	VC_163_0	2	36.54933039	91.01037225	0.286527247	0.058939311	6.708203932	6.708203932	1	1	1	6.70820393	0	0	0	MW202475,MW202867
652	VC_164_0	3	900	29296.33813	0.029804938	0.170162741	2	4	3	3	1	3.15470054	0	0	0	MK268344,MK773491,MT001829
653	VC_164_1	1	0	10350.71338	0	0	0	0	1	1	1	0	0	0	0	MT446416
654	VC_164_10	5	3000	45799.74405	0.061475732	0.035164184	5	8.366600265	10	10	1	6.756909	0	0	0	AB897757,JQ513383,MT708547,OP617745,OU509535
655	VC_164_11	10	13500	76062.51673	0.1507327	1.58E-09	5.744562647	9.746794345	45	41	0.911111111	7.53313291	0	0	0	KC295538,KM507819,LC494302,LT603033,MH383160,MH791411,MK327931,MK817115,MW822008,ON568341
656	VC_164_2	1	0	9649.720417	0	0	0	0	1	1	1	0	0	0	0	MH494197
657	VC_164_3	1	0	10334.39817	0	0	0	0	1	1	1	0	0	0	0	ON286972

658	VC_164_4	1	0	10326.84073	0	0	0	0	1	1	1	0	0	0	0	JN882285
659	VC_164_5	1	0	10243.70835	0	0	0	0	1	1	1	0	0	0	0	KU574722
660	VC_164_6	1	0	10341.51957	0	0	0	0	1	1	1	0	0	0	0	OP292654
661	VC_164_7	7	6300	59614.18713	0.095578817	2.00E-05	3	6.08276253	21	21	1	4.19819628	0	0	0	KY630187,MT939486,MT939487,MT939488,MW879342,MW879343,MW879344
662	VC_164_8	3	900	29243.38875	0.029857293	0.150553512	0	5.656854249	3	3	1	3.77123617	0	0	0	LR596615,LT960551,LT960552
663	VC_164_9	1	0	6339.081617	0	0	0	0	1	1	1	0	0	0	0	MG592671
664	VC_165_0	4	1800	6210.016059	0.224718651	1.67E-05	3	4.69041576	6	6	1	3.87656384	0	0	0	LC727697,LC727698,LR588166,MK797984
665	VC_166_0	26	77776.21373	20382.99439	0.792347608	8.76E-165	0	8.306623863	325	325	1	4.66074837	0	0	0	AM749441,DQ163916,JX131330,KC758116,KT734862,KX198613,KX961385,KY000218,KY000219,KY000220,KY000221,MF490239,MH791403,MH791406,MN871456,MN871463,MN871469,MN871478,MN871482,MN871486,MT118302,MZ089734,MZ089741,OL763419,OM809160,ON165687
666	VC_167_0	3	900	394.5491546	0.695222732	0.00173425	1	2.828427125	3	3	1	2.15805948	0	0	0	AB910392,MK443266,MK803322
667	VC_169_0	20	37837.01925	12415.60697	0.752936157	1.73E-110	1.414213562	7.937253933	190	190	1	5.55601113	0	0	0	JQ067089,JQ067093,KU204984,KX241618,LR743532,MG018224,MT664984,OK275492,OK275493,OL674541,ON005621,ON189045,ON189047,ON331942,ON529854,ON932079,ON932080,ON932084,OP361299,OP380269
668	VC_16_0	12	1426.870011	730.0879617	0.661519617	1.66E-22	0	3.605551275	66	66	1	2.41476988	0	0	0	AB012573,AB012574,AB572858,AF452449,AF453500,AY242528,CP017905,CP017906,D89074,FJ904927,MN536023,MN690600
669	VC_170_0	1	0	12.04923717	0	0	0	0	1	1	1	0	0	0	0	KT001913
670	VC_170_1	1	0	93.05263174	0	0	0	0	1	1	1	0	0	0	0	KY006853
671	VC_171_0	8	4962.696141	3471.388723	0.588409557	8.35E-19	1	7.141428429	28	28	1	4.07582896	0	0	0	KP202969,KP202970,MH746817,MK962627,MK962633,MK962634,MT241605,MT241607
672	VC_172_0	5	1913.78375	3433.349007	0.357908404	3.05E-08	2.236067977	5.830951895	10	10	1	4.58002585	0	0	0	KF626665,KF626668,LR743524,MZ326855,ON189048
673	VC_173_0	2	169.7809307	1953.91116	0.079946114	0.045109088	5.385164807	5.385164807	1	1	1	5.38516481	0	0	0	KT887558,MG707188
674	VC_174_0	3	900	35.19812395	0.962362923	0.006664428	1	1.732050808	3	3	1	1.38208812	0	0	0	AB910393,LC102729,OK094707
675	VC_175_0	4	869.5914639	1391.110991	0.384655425	1.74E-05	3.464101615	7.348469228	6	6	1	5.77007588	0	0	0	MG592578,MG592660,MW865292,OX241572
676	VC_176_0	2	208.9662403	3991.28597	0.049750879	0.043362593	6.92820323	6.92820323	1	1	1	6.92820323	0	0	0	AF165214,HQ711985
677	VC_176_1	2	135.9459687	3613.836762	0.036254359	0.043424325	8.306623863	8.306623863	1	1	1	8.30662386	0	0	0	KT887557,ON857940
678	VC_176_2	1	0	1948.343483	0	0	0	0	1	1	1	0	0	0	0	OM141125
679	VC_176_3	1	0	1502.463013	0	0	0	0	1	1	1	0	0	0	0	KT968831
680	VC_177_0	5	145.4409	49.29896565	0.74684708	0.001143542	1.414213562	3.464101615	10	10	1	2.66576837	0	0	0	AB910602,HG315669,KX181651,KY853667,MN335248
681	VC_178_0	5	227.9381438	66.50805902	0.774124922	1.01E-05	1	2.828427125	10	10	1	2.30117167	0	0	0	MH206183,MH206184,MH218848,MK512531,MN263053
682	VC_179_0	53	217386.3131	20524.01427	0.913732142	0	0	6.708203932	1378	1378	1	4.27507974	0	0	0	AY394005,DQ631426,DQ873690,EU272036,EU272037,HE584812,HM624080,JN808773,JN811560,JQ067085,JQ762257,JX434030,JX434031,JX434032,JX434033,KF475786,KJ477077,KM233689,KT968832,KU199707,KU199708,KU199709,LN610590,LN907801,LT608331,LT999987,MH643777,MH643778,MK144667,MK511017,MK511018,MK511019,MK511020,MK511021,MK511022,MK511023,MK511024,MK511025,MK511026,MK511027,MK511028,MK511029,MK511030,MK511031,MK511032,MK511033,MK511034,MK511035,MK511036,MK511037,MK511038,MW390886,OM541307
683	VC_17_0	9	1323.577384	575.5641335	0.696934574	2.73E-18	1.414213562	2.645751311	36	36	1	1.96619301	0	0	0	AB043678,AB043679,MN719123,MT188662,MT188663,MT188664,MT188665,MT188666,MT193890
684	VC_180_0	4	773.0916907	2986.222222	0.205647017	1.38E-05	0	3.872983346	6	6	1	2.36332418	0	0	0	AB916497,ON170148,ON170170,ON170354
685	VC_181_0	5	457.526721	3459.059229	0.11681774	5.35E-08	4	7.071067812	10	10	1	5.89942223	0	0	0	JF832915,KP137440,MK388859,S1-wms-combined_19031,S11-wms-combined_268967
686	VC_182_0	5	627.5162961	387.2362285	0.618393432	3.26E-07	0	8.366600265	10	10	1	6.39165407	0	0	0	JF974307,JF974308,KT381864,KT381865,KX077179
687	VC_183_0	2	67.83568441	949.0846561	0.06670698	0.045101067	4.69041576	4.69041576	1	1	1	4.69041576	0	0	0	KF302035,MZ568827
688	VC_184_0	2	202.5113226	51.76539008	0.796421035	0.063315229	3.31662479	3.31662479	1	1	1	3.31662479	0	0	0	MH727593,MZ547449
689	VC_185_0	3	16.01522219	19.67897952	0.448678537	0.028519937	6.708203932	7.141428429	3	3	1	6.9259452	0	0	0	LR797314,MF959999,MK088078
690	VC_187_0	4	1800	25432.74038	0.06609691	5.90E-05	6.403124237	7.681145748	6	6	1	7.01248551	0	0	0	KX961630,MN150686,OK499977,OK499983
691	VC_187_1	7	6300	37968.02708	0.142314903	7.64E-14	3.31662479	8.366600265	21	21	1	6.89654448	0	0	0	JN797797,JN797798,KT187252,KT224359,KX987999,MH487649,MN013089
692	VC_187_2	2	300	14036.894	0.020925034	0.069424051	6.782329983	6.782329983	1	1	1	6.78232998	0	0	0	KJ081346,KJ676859
693	VC_187_3	9	10800	44006.57942	0.197056633	2.21E-22	0	9.746794345	36	31	0.861111111	7.93174139	0	0	0	JX094431,KJ451625,KT995480,KU577463,KX495186,MG602477,MH638309,MH638312,OL964058
694	VC_187_4	1	0	7235.550894	0	0	0	0	1	1	1	0	0	0	0	MN364664
695	VC_188_0	13	838.2350435	2.245014199	0.997328891	0.001761978	0	1	78	78	1	0.15384615	0	0	0	AB971354,AF059242,AF059243,EF068134,FJ483840,FJ483841,FJ483842,FJ483843,FJ483844,FJ539132,FJ539133,FJ539134,FJ539135
696	VC_189_0	2	40.28399931	3850.248397	0.010354367	0.060465563	4	4	1	1	1	4	0	0	0	AJ604530,AJ604531
697	VC_18_0	4	732.3688929	72.6237079	0.909783385	0.000176534	1	6.633249581	6	6	1	4.60077735	0	0	0	AB016282,HM072038,OM236514,ON528935
698	VC_190_0	2	300	34290.63765	0.008672867	0.042365267	5.916079783	5.916079783	1	1	1	5.91607978	0	0	0	KP143763,KX130863
699	VC_190_1	1	0	19389.39865	0	0	0	0	1	1	1	0	0	0	0	MK373792
700	VC_190_10	1	0	20350.10251	0	0	0	0	1	1	1	0	0	0	0	KR422353
701	VC_190_11	1	0	15969.89458	0	0	0	0	1	1	1	0	0	0	0	AC171169
702	VC_190_12	1	0	20754.3707	0	0	0	0	1	1	1	0	0	0	0	LR027384
703	VC_190_13	4	1800	81486.62085	0.021612115	2.96E-05	1.414213562	9	6	4	0.666666667	7.43792829	0	0	0	MH370377,MH370378,MN970211,MN994502
704	VC_190_14	3	900	61781.97448	0.014358195	0.003031482	8.717797887	9.16515139	3	2	0.666666667	8.86691572	0	0	0	LR597655,MN022787,MN316588
705	VC_190_15	3	900	60919.38448	0.01455854	0.002405347	8.366600265	9.219544457	3	2	0.666666667	8.71004949	0	0	0	KJ190157,KY677846,MN994496
706	VC_190_16	1	0	21832.32772	0	0	0	0	1	1	1	0	0	0	0	OK272480
707	VC_190_17	1	0	21043.48228	0	0	0	0	1	1	1	0	0	0	0	AP019559
708	VC_190_18	1	0	21629.52886	0	0	0	0	1	1	1	0	0	0	0	MF402939
709	VC_190_19	1	0	20394.03052	0	0	0	0	1	1	1	0	0	0	0	MZ501075
710	VC_190_2	2	300	40921.62771	0.007277733	0.044689377	1.414213562	1.414213562	1	1	1	1.41421356	0	0	0	MK373797,MK373799
711	VC_190_20	4	1800	76642.237	0.022946821	2.11E-05	0	8.544003745	6	6	1	6.79446236	0	0	0	LN887948,MK972715,MK972716,MT843274
712	VC_190_21	1	0	19586.76816	0	0	0	0	1	1	1	0	0	0	0	KY114934
713	VC_190_22	3	900	47611.59315	0.018552266	0.001403304	4	6.92820323	3	3	1	5.95213549	0	0	0	KM979354,KM979355,MH203051
714	VC_190_23	10	13343.52773	176202.3531	0.07039735	1.48E-27	5.477225575	9.695359715	45	38	0.844444444	8.10558088	0	0	0	HQ406778,KY963424,MH102285,MN026741,MT311645,MW149273,MW149275,OK108607,OK254197,OP394141
715	VC_190_24	5	3000	99378.93128	0.029302904	4.03E-07	4	9.38083152	10	7	0.7	8.22481894	0	0	0	HQ665011,LR597659,MZ398246,OK143207,ON185585
716	VC_190_25	1	0	19730.74874	0	0	0	0	1	1	1	0	0	0	0	MN956514
717	VC_190_26	1	0	19443.74958	0	0	0	0	1	1	1	0	0	0	0	MG387042
718	VC_190_27	1	0	19229.34703	0	0	0	0	1	1	1	0	0	0	0	KR233164
719	VC_190_28	5	3000	95735.91224	0.030384081	6.09E-08	1.414213562	4.242640687	10	10	1	3.02850482	0	0	0	OK665835,ON185581,ON185582,ON185583,ON185584
720	VC_190_29	1	0	22183.2875	0	0	0	0	1	1	1	0	0	0	0	MT080103
721	VC_190_3	1	0	20610.01085	0	0	0	0	1	1	1	0	0	0	0	MN994504
722	VC_190_30	1	0	10861.73543	0	0	0	0	1	1	1	0	0	0	0	MF774688
723	VC_190_31	1	0	16233.54405	0	0	0	0	1	1	1	0	0	0	0	MG065692
724	VC_190_32	1	0	21108.90924	0	0	0	0	1	1	1	0	0	0	0	MZ501058
725	VC_190_33	1	0	16783.0568	0	0	0	0	1	1	1	0	0	0	0	MZ501103
726	VC_190_34	1	0	19218.17837	0	0	0	0	1	1	1	0	0	0	0	MN445182
727	VC_190_35	1	0	18558.21395	0	0	0	0	1	1	1	0	0	0	0	OK272477
728	VC_190_36	1	0	20376.27901	0	0	0	0	1	1	1	0	0	0	0	MT653143
729	VC_190_37	1	0	20362.63132	0	0	0	0	1	1	1	0	0	0	0	MT653146
730	VC_190_38	3	900	50631.32546	0.017465105	0.001520484	1.732050808	3.741657387	3	3	1	3.02641982	0	0	0	AY543070,AY587007,AY692264
731	VC_190_39	1	0	19812.33004	0	0	0	0	1	1	1	0	0	0	0	MT233524
732	VC_190_4	1	0	20626.66511	0	0	0	0	1	1	1	0	0	0	0	MT577844
733	VC_190_40	2	300	35807.64594	0.00830849	0.044436046	8.485281374	8.485281374	1	1	1	8.48528137	0	0	0	MG969412,MW006479
734	VC_190_5	1	0	20300.36538	0	0	0	0	1	1	1	0	0	0	0	OM386657
735	VC_190_6	1	0	19253.03664	0	0	0	0	1	1	1	0	0	0	0	MZ501074
736	VC_190_7	7	6300	126108.2022	0.047580134	8.72E-15	0	8.888194417	21	21	1	3.17601008	0	0	0	MF431730,MF431731,MF431732,MF431733,MF431734,MF431735,MT074448
737	VC_190_8	1	0	20290.9754	0	0	0	0	1	1	1	0	0	0	0	OM386660
738	VC_190_9	1	0	19891.84901	0	0	0	0	1	1	1	0	0	0	0	MK373796
739	VC_191_0	2	300	44720.68094	0.006663604	0.042390289	8.124038405	8.124038405	1	1	1	8.1240384	0	0	0	MW006478,MW006481
740	VC_191_1	1	0	27471.69922	0	0	0	0	1	1	1	0	0	0	0	MK370036
741	VC_191_10	1	0	26254.57197	0	0	0	0	1	1	1	0	0	0	0	MN197465
742	VC_191_11	1	0	24943.49915	0	0	0	0	1	1	1	0	0	0	0	MF681663
743	VC_191_12	1	0	26917.93676	0	0	0	0	1	1	1	0	0	0	0	MT074464
744	VC_191_13	5	3000	125656.1973	0.02331796	7.25E-07	0	1.732050808	10	10	1	0.89746915	0	0	0	MK907267,MK907268,MK907269,MK907270,MK907271
745	VC_191_14	1	0	26048.52431	0	0	0	0	1	1	1	0	0	0	0	LR027389
746	VC_191_15	2	300	52544.0857	0.005677078	0.063689909	8.602325267	8.602325267	1	1	1	8.60232527	0	0	0	OL451946,OL502173
747	VC_191_16	12	19800	287909.0451	0.0643465	9.81E-27	1.414213562	9.643650761	66	62	0.939393939	7.00217171	0	0	0	KY787212,MF001363,MH370369,MK393882,MK947458,MK972717,MN218190,MN218191,MN379739,MN994501,MT074450,MT074458
748	VC_191_17	4	1800	106823.2528	0.016571038	0.000699373	6.782329983	9.219544457	6	5	0.833333333	8.00216241	0	0	0	CP000917,MK947459,MZ501105,OL770310
749	VC_191_18	2	300	52167.54735	0.00571782	0.080964108	9	9	1	0	0	9	0	0	0	MT833283,MZ501072
750	VC_191_19	12	19800	282290.8336	0.0655432	2.30E-31	1	9.486832981	66	57	0.863636364	8.01745434	0	0	0	KM236244,KU927497,KX017521,LC659915,MG065642,MH631454,MK728824,MK728825,MN187969,MW357609,OK288022,OP045498
751	VC_191_2	1	0	25778.49115	0	0	0	0	1	1	1	0	0	0	0	MZ501109
752	VC_191_20	1	0	27227.36716	0	0	0	0	1	1	1	0	0	0	0	MK005300
753	VC_191_21	1	0	27051.75957	0	0	0	0	1	1	1	0	0	0	0	MZ501076
754	VC_191_22	1	0	25737.13538	0	0	0	0	1	1	1	0	0	0	0	MW567727
755	VC_191_23	1	0	27061.48273	0	0	0	0	1	1	1	0	0	0	0	MH370379
756	VC_191_24	1	0	26153.08948	0	0	0	0	1	1	1	0	0	0	0	MZ570151
757	VC_191_25	1	0	25347.00592	0	0	0	0	1	1	1	0	0	0	0	MW206381
758	VC_191_26	1	0	21567.70159	0	0	0	0	1	1	1	0	0	0	0	HE956708
759	VC_191_27	1	0	25191.98158	0	0	0	0	1	1	1	0	0	0	0	MK867835
760	VC_191_28	1	0	23661.39424	0	0	0	0	1	1	1	0	0	0	0	MZ501108
761	VC_191_29	1	0	22075.88908	0	0	0	0	1	1	1	0	0	0	0	MH370375
762	VC_191_3	5	3000	126652.1684	0.023138834	1.51E-06	4.358898944	9.797958971	10	8	0.8	7.82941577	0	0	0	MK972704,MK972707,MK972709,MT074453,MT074457
763	VC_191_30	2	300	49258.17705	0.006053491	0.044682493	8.660254038	8.660254038	1	1	1	8.66025404	0	0	0	MT074447,MT074461
764	VC_191_31	2	300	52544.96014	0.005676984	0.075756206	2	2	1	1	1	2	0	0	0	MK770410,MK972708
765	VC_191_32	12	19800	270243.2705	0.068265676	1.33E-36	0	9.746794345	66	50	0.757575758	6.75990426	0	0	0	MF431736,MF431737,MF431738,MF431739,MF431740,MF431741,MK050846,MK809530,MT074449,MT074465,MT653145,ON526840
766	VC_191_33	1	0	23888.23863	0	0	0	0	1	1	1	0	0	0	0	MW423798
767	VC_191_4	2	300	54457.66338	0.005478685	0.110634862	8.602325267	8.602325267	1	1	1	8.60232527	0	0	0	MH370366,MT074455
768	VC_191_5	6	4500	153396.7086	0.028499644	3.93E-08	5.385164807	8.94427191	15	15	1	7.24098642	0	0	0	MH370386,MT004791,MT074452,MT074456,MT074460,MT074463
769	VC_191_6	1	0	26395.09908	0	0	0	0	1	1	1	0	0	0	0	MT074468
770	VC_191_7	1	0	26692.48171	0	0	0	0	1	1	1	0	0	0	0	MT074467
771	VC_191_8	2	300	54163.04677	0.005508322	0.081686479	8.246211251	8.246211251	1	1	1	8.24621125	0	0	0	MH370376,MT074454
772	VC_191_9	5	3000	128725.7283	0.022774594	3.38E-06	1.414213562	9.486832981	10	7	0.7	7.69118478	0	0	0	MH370367,MH370380,MT074459,MT074466,MW411578
773	VC_192_0	15	31500	30622.84972	0.507059804	6.21E-68	2.449489743	5.830951895	105	105	1	4.46625316	0	0	0	KX845404,MG459987,MK521907,MK630230,MN101228,MN163280,MN434094,MN434096,MT380195,MW713578,MZ571828,MZ571830,MZ634344,ON637170,OP171943
774	VC_192_1	1	0	11380.64928	0	0	0	0	1	1	1	0	0	0	0	MZ079855
775	VC_192_2	1	0	7988.346869	0	0	0	0	1	1	1	0	0	0	0	MN478377
776	VC_193_0	2	300	9034.215628	0.032139819	0.04237344	4.795831523	4.795831523	1	1	1	4.79583152	0	0	0	KY363465,MW057857
777	VC_195_0	2	300	2197.117871	0.120138502	0.043199927	6.08276253	6.08276253	1	1	1	6.08276253	0	0	0	MG676224,MG676225
778	VC_195_1	1	0	1388.333863	0	0	0	0	1	1	1	0	0	0	0	MK318083
779	VC_196_0	2	300	13432.15991	0.021846527	0.042373039	4.582575695	4.582575695	1	1	1	4.58257569	0	0	0	MN095770,ON287373
780	VC_197_0	5	42.9951916	281.6890521	0.132421552	0.153015986	0	2.645751311	10	10	1	1.82595123	0	0	0	AE002163,CMU41758,MG641183,MN862345,MW202581
781	VC_198_0	28	2059.615789	6418.464807	0.242934207	0.000288251	0	2.645751311	378	378	1	2.05957055	0	0	0	AJ270057,AJ550635,AY769964,MG883723,MG945324,MG945546,MG945571,MG945669,MH572277,MH572280,MH572282,MH572290,MH572330,MH572334,MH572422,MH572438,MH572444,MH572448,MH572452,MH572454,MH992157,MH992174,MH992182,MH992210,MN862343,MW202732,MW202889,OL617035
782	VC_199_0	12	925.7847476	1140.824961	0.447972708	9.20E-37	0	1.414213562	66	66	1	0.44997704	0	0	0	KC131021,MH572341,MH572342,MH572343,MH572399,MH572400,MH572401,MH572402,MH572403,MH572453,MH572513,MH572514
783	VC_19_0	2	14.94602377	342.7214179	0.041787488	0.079551936	7	7	1	1	1	7	0	0	0	CP000315,S5-wms-combined_83412
784	VC_1_0	54	124307.3014	21467.29167	0.852736398	0	0	8.124038405	1431	1431	1	5.33692535	0	0	0	AB276040,AB981169,GU911303,KP966108,MG747435,MT701591,ON170026,ON170032,ON170053,ON170059,ON170061,ON170067,ON170070,ON170073,ON170076,ON170078,ON170080,ON170081,ON170082,ON170084,ON170128,ON170130,ON170131,ON170135,ON170151,ON170173,ON170210,ON170213,ON170227,ON170230,ON170233,ON170235,ON170237,ON170239,ON170241,ON170243,ON170258,ON170261,ON170263,ON170266,ON170268,ON170270,ON170272,ON170276,ON170304,ON170305,ON170321,ON170325,ON170329,ON170344,ON170347,ON170348,ON170351,ON642070
785	VC_200_0	1	0	38.34816644	0	0	0	0	1	1	1	0	0	0	0	MH992181
786	VC_201_0	3	18.76623677	169.2877527	0.09979175	0.303092391	1.414213562	2	3	3	1	1.80473785	0	0	0	MH572434,MH572475,MZ286294
787	VC_202_0	102	232117.7805	341121.4321	0.404923068	0	0	9.899494937	5151	4057	0.787614056	8.40707448	0	0	0	AF009630,AF011378,DQ054536,DQ227763,DQ227764,EU221285,FJ848881,FJ848882,FJ848883,FJ848884,FJ848885,GQ979703,KC182542,KC182543,KC182544,KC182545,KC182546,KC182547,KC182548,KC182549,KC182550,KC182551,KC182552,KC522412,KF676640,KM091442,KP793104,KP793105,KP793108,KP793109,KP793111,KP793113,KP793115,KP793116,KP793117,KP793118,KP793123,KP793124,KP793129,KP793134,KX346246,KX379667,KX379668,KX379670,KX379671,KY554760,KY554761,MF346933,MF448554,MF448555,MF448556,MF448557,MF448558,MF448559,MF448560,MF448561,MF448562,MF448563,MF448564,MF448565,MF448566,MF448567,MF448568,MF448569,MF448570,MF448571,MF448572,MF775703,MF775704,MF775705,MG253647,MG253648,MG253649,MG253650,MG253651,MG253652,MG253653,MG309717,MG309718,MK301437,MN552148,MN552149,MN552150,MN689514,MN689517,MN689518,MN689519,MN689520,MN689522,MN689523,MN689524,MN689525,MW032477,MW041632,MW041633,MW041634,MW041635,MW041636,MW041637,MW041638,MW041639,MW041640
788	VC_209_0	4	1081.170469	8440.042981	0.113553853	1.22E-05	0	4.123105626	6	6	1	2.06155281	0	0	0	KY888143,KY926698,KY926699,MZ368140
789	VC_20_0	5	479.8840249	613.9025757	0.438736427	6.57E-07	6.32455532	7.615773106	10	10	1	6.90598058	0	0	0	KT372714,KT852578,KX190835,KX534340,MN065183
790	VC_211_0	1	0	455.3646207	0	0	0	0	1	1	1	0	0	0	0	AP008983
791	VC_211_1	1	0	998.0831717	0	0	0	0	1	1	1	0	0	0	0	KP144388

792	VC_211_2	1	0	1373.112111	0	0	0	0	1	1	1	0	0	0	0	KT151959
793	VC_211_3	1	0	654.209181	0	0	0	0	1	1	1	0	0	0	0	MT325768
794	VC_212_0	2	153.6749663	357.7748628	0.300469289	0.053311599	5.477225575	5.477225575	1	1	1	5.47722558	0	0	0	S12-wms-combined_263377,S6-wms_132588
795	VC_212_1	2	86.47065646	440.8725463	0.163974156	0.048000263	7.071067812	7.071067812	1	1	1	7.07106781	0	0	0	S2-wms-combined_31084,S7-wms-combined_7205
796	VC_213_0	2	300	398.8432816	0.429280796	0.046997734	4.358898944	4.358898944	1	1	1	4.35889894	0	0	0	KT429160,MT880872
797	VC_214_0	2	300	1076.689234	0.217914103	0.046411969	1.732050808	1.732050808	1	1	1	1.73205081	0	0	0	ON210834,ON210835
798	VC_214_1	1	0	779.7045849	0	0	0	0	1	1	1	0	0	0	0	OM654379
799	VC_215_0	5	2522.172713	32301.54977	0.072426855	2.54E-08	3.741657387	6.403124237	10	10	1	5.34027508	0	0	0	DQ398043,KF017927,KF416344,MG099947,MN585989
800	VC_215_1	19	42660.29283	59198.23532	0.418819058	3.13E-107	0	6.8556546	171	171	1	3.81392702	0	0	0	AF022214,AP018476,AP018480,KC787102,KC787105,KC787106,KX574455,KX758538,KX897981,LC730318,MH513985,MK494087,MN234169,MT310876,MT310883,MT310884,MT310885,MW924646,OP297553
801	VC_215_2	5	1992.199069	32823.9773	0.057220501	2.57E-08	4.242640687	8.062257748	10	10	1	7.08658422	0	0	0	JF946695,KT381276,MF919536,MH020237,Z18946
802	VC_216_0	4	1618.484435	32067.70813	0.04804593	1.22E-05	2.236067977	4.582575695	6	6	1	3.71206449	0	0	0	MF185727,MF324899,MK801735,MN369748
803	VC_216_1	1	0	9396.898193	0	0	0	0	1	1	1	0	0	0	0	MK814748
804	VC_216_2	2	247.5387595	17613.84865	0.013858876	0.042550033	4.242640687	4.242640687	1	1	1	4.24264069	0	0	0	MN585968,MN586002
805	VC_216_3	8	4913.201601	65160.611	0.070114661	7.36E-20	3.741657387	9.273618495	28	24	0.857142857	7.77084591	0	0	0	GU339467,JN408461,KF024721,KU761558,KU761559,KX641263,MF140403,MH825709
806	VC_216_4	13	19307.44912	97238.69853	0.165663555	5.79E-51	0	8.306623863	78	78	1	4.82818423	0	0	0	KJ959632,KM197169,KM677210,KU297783,KX683875,MF668269,MF919538,MH825701,MK061408,MK284522,MK967395,MZ322019,OM203159
807	VC_216_5	2	195.4757455	20599.53135	0.009400129	0.042521879	7.141428429	7.141428429	1	1	1	7.14142843	0	0	0	MW601216,MZ209305
808	VC_216_6	42	163568.6864	182270.8701	0.472961185	0	0	10	861	719	0.835075494	7.21597085	0	0	0	AP018479,JN408460,JX899358,KF981601,KJ410132,KJ410134,KM463009,KM591906,KT588442,KU985093,KU985095,KX576639,KX619650,KY965065,LC730320,MF472896,MF919490,MF919500,MF919531,MH051252,MH077576,MH338234,MH697591,MH825704,MK524491,MK919472,MN234203,MN585981,MN585983,MN585998,MT310877,MT310886,MT498041,MT498060,MT498066,MT658804,MW924643,MZ355728,MZ622171,MZ622182,MZ820090,OP297537
809	VC_216_7	1	0	8856.177242	0	0	0	0	1	1	1	0	0	0	0	MT310858
810	VC_217_0	5	2289.501023	30890.49691	0.069002446	2.46E-08	3.872983346	6.92820323	10	10	1	5.47677941	0	0	0	EU744250,KU568494,KX758539,MK279857,MN703408
811	VC_217_1	1	0	7912.560291	0	0	0	0	1	1	1	0	0	0	0	MN062708
812	VC_218_0	3	782.2795157	15465.16003	0.048147864	0.001390214	3	5	3	3	1	4	0	0	0	KF861510,MN096376,MT639645
813	VC_219_0	5	1595.157751	18353.03061	0.079965044	2.48E-08	2.828427125	8.485281374	10	10	1	6.38448575	0	0	0	JF957060,KC661275,KP273225,MG793454,MT553337
814	VC_21_0	4	111.4714721	84.21485042	0.569643656	0.127375581	7.681145748	9.110433579	6	4	0.666666667	8.58725488	0	0	0	JF974314,KJ746502,MF614627,MF614628
815	VC_220_0	4	1453.906695	22193.69812	0.061482197	1.20E-05	5.567764363	6.782329983	6	6	1	6.16504923	0	0	0	KR080193,MN310541,MT522004,OM203161
816	VC_221_0	22	67367.22787	23093.52086	0.744712252	2.11E-137	0	5.099019514	231	231	1	3.01920591	0	0	0	KU695581,KY471460,MF140410,MH338236,MH338237,MH536828,MK494130,MK524507,MK524511,MK524513,MK524514,MK524519,MN310547,MN586006,MN703405,MN892487,MW055904,MZ747517,MZ747521,ON108640,OP297532,OP297549
817	VC_222_0	5	1517.821101	25362.36497	0.056466168	2.53E-08	6.244997998	8.426149773	10	10	1	7.63850731	0	0	0	KX657793,MH513984,MK494113,MK524530,OM203158
818	VC_223_0	38	179471.2197	17863.58826	0.909475736	0	0	7.141428429	703	703	1	4.65361014	0	0	0	JF704097,JF937092,JF937094,JN049605,JN698999,JN699019,KF560332,KF560333,KF560334,KM401838,KU695582,KU985096,KX375815,KX576638,KX641261,MF668288,MG099945,MG099952,MH020240,MH051253,MH155876,MH779517,MK359352,MK359360,MK494090,MK894433,MN586039,MN728683,MT024861,MT657338,MW822143,MZ005685,MZ171376,MZ322007,MZ456034,MZ681516,OM913585,OP434447
819	VC_224_0	19	47555.34642	2948.112595	0.941625531	5.60E-92	0	7.745966692	171	171	1	3.91247511	0	0	0	KU998249,KU998250,KU998251,KX557280,KX557283,KX557284,MF668282,MH001454,MH316566,MH513974,MK967382,MN284898,MN369756,MT522007,MZ028625,MZ622176,MZ681508,OM818328,OP021679
820	VC_225_0	32	113319.9526	20305.77267	0.848039944	7.66E-251	0	9.110433579	496	494	0.995967742	5.74579741	0	0	0	JF704110,JN699005,KT285706,KU613353,KU716094,KU716095,KX585252,KX664447,KX685355,MG872835,MG872840,MG872842,MH371122,MK061409,MK468591,MK494108,MK524489,MK559428,MK977713,MN234167,MN234200,MN234209,MN234235,MN310542,MN369741,MN369763,MN369764,MN585974,MN585978,MN585979,MN585994,ON724016
821	VC_227_0	2	1.1225071	2.245014199	0.333333333	1	0	0	1	1	1	0	0	0	0	AF334111,HM066936
822	VC_228_0	60	155914.9098	9598.257091	0.942009103	0	0	7	1770	1770	1	4.92165975	0	0	0	AF063097,AY135486,AY135739,AY251033,B1U32222,HQ110084,KC139521,KC618326,KF322032,KR073660,KR073661,KT630644,KX833211,LR595869,LR595870,LR595873,LR595883,LR595885,LR595887,MF276773,MK416018,MK907236,MK907237,MK907238,MK907239,MK972710,MK972711,MN016939,MT239398,MT374855,MT374857,MT374858,MZ420230,MZ520833,OK490408,OK490461,OK490464,OK490467,OK490470,OK490473,OM046626,OM046627,OM046628,OM140653,OM475426,OM475429,OM475431,OM475433,OM475434,ON146439,ON146440,ON239131,ON470601,ON470606,ON470613,ON782130,OU784549,OU784746,OU784750,OV192280
823	VC_229_0	32	46600.00671	4499.977688	0.911937787	7.67E-112	0	7.483314774	496	496	1	4.47462072	0	0	0	CP025874,CP025901,CP025923,CP052639,KP063541,KY271397,LR595891,MG589387,MK416015,OK490394,OK490396,OK490398,OK490400,OK490402,OK490404,OK490406,OK490412,OK490414,OK490422,OK490424,OK490426,OK490428,OK490432,OK490435,OK490437,OK490440,OK490443,OK490446,OK490449,OK490453,OK490456,OK490459
824	VC_22_0	11	2920.285837	2735.728737	0.516315119	8.71E-27	1	8.062257748	55	55	1	5.49775365	0	0	0	AB036666,HQ906662,HQ906663,HQ906664,KX522565,MK976036,MN180248,MN180249,MN180250,MN180251,MN180252
825	VC_230_0	3	70.61879616	884.6255288	0.07392747	0.006504896	8.185352772	9.327379053	3	2	0.666666667	8.6660044	0	0	0	JQ780327,MK524177,OL964755
826	VC_230_1	1	0	477.3565143	0	0	0	0	1	1	1	0	0	0	0	HM208537
827	VC_231_0	3	157.6153094	510.278316	0.235988642	0.001754965	5.656854249	6.92820323	3	3	1	6.35526606	0	0	0	MT366580,MZ496293,OP121182
828	VC_232_0	2	125.0498273	9.811724398	0.927245948	0.270145687	1.414213562	1.414213562	1	1	1	1.41421356	0	0	0	AF065411,AF301375
829	VC_233_0	2	24.9650949	9.811724398	0.717865964	0.270145687	4.123105626	4.123105626	1	1	1	4.12310563	0	0	0	MH674343,MN737622
830	VC_234_0	21	61521.00418	12109.07517	0.835541734	3.12E-99	0	5.099019514	210	210	1	2.74822547	0	0	0	AP018469,AP018470,AP018471,JN831653,KJ944841,KT361920,KX621007,KY087992,MF140402,MF140435,MH051258,MH926058,MK224497,MK524485,MK524488,MN369761,MN945904,MT310882,MW601218,ON526978,ON645342
831	VC_236_0	72	583676.4075	136578.7826	0.810374456	0	0	8.831760866	2556	2556	1	6.1289474	0	0	0	HM152764,HM152767,JF704105,JF704106,JN185608,JN643714,KJ567045,KM677211,KX585253,KX641264,KX657794,KX657795,KX657796,KY380102,MF140412,MF140428,MF140430,MF377440,MF472892,MF668267,MF919532,MG962364,MG962371,MH001453,MH051246,MH077577,MH371113,MH509444,MH513977,MH536821,MH536822,MH697578,MH727544,MK016503,MK112537,MK460247,MN234182,MN234197,MN234198,MN234205,MN234230,MN234231,MN369742,MN369746,MN908692,MW712722,MW712723,MW712730,MW712733,MW712734,MW862979,MW862984,MW862985,MW862990,MZ005674,MZ355721,MZ648037,OK040778,OK040782,OK040784,OK310505,OM818330,OM913584,ON081329,ON081331,ON260816,ON456339,ON456353,ON526984,ON687735,ON687736,ON724013
832	VC_236_1	5	2221.338035	59790.03023	0.035821465	2.74E-08	2.236067977	8.306623863	10	10	1	6.28854192	0	0	0	MF140416,MN234165,MN234174,MN234201,MN234226
833	VC_237_0	4	1693.871323	37774.13405	0.042917581	1.24E-05	1.732050808	5.830951895	6	6	1	3.97862657	0	0	0	KF713486,MF185722,MK310138,MK494119
834	VC_237_1	13	15105.64098	70088.68106	0.177308072	1.61E-49	0	9.38083152	78	57	0.730769231	6.99515529	0	0	0	JF937104,JX042579,KP027199,KR080206,KX683428,KY087993,MF140398,MF140406,MF324915,MF373841,MN234199,MT723940,MW601222
835	VC_237_2	11	12082.33592	73644.12421	0.140940567	2.39E-36	2	9.055385138	55	54	0.981818182	6.36407234	0	0	0	KY945355,MF324908,MF324909,MF324911,MF324912,MF324913,MF324914,MN234176,MN369762,MN945901,ON456351
836	VC_237_3	8	6807.995757	55536.19069	0.10920017	9.27E-20	2.236067977	8.888194417	28	28	1	5.99764966	0	0	0	MH910038,MH910039,MH910042,MK376955,MK460246,MN284895,MT310859,MT310860
837	VC_238_0	17	33731.46578	21480.73253	0.61094227	3.98E-82	0	7.071067812	136	136	1	5.01230262	0	0	0	JN243855,KJ567042,KM363596,KM923971,KX585251,KX636165,MF185717,MF185719,MF185720,MG962374,MH051255,MH576966,MH651171,MH697592,MW435854,MZ322016,MZ958747
838	VC_23_0	3	519.3022692	686.9090739	0.43052345	0.001626222	3.31662479	4.358898944	3	3	1	3.89184124	0	0	0	AY133112,FN297812,JX556417
839	VC_240_0	4	1290.629844	361.7275595	0.781083948	1.73E-05	0	6.403124237	6	6	1	4.94405078	0	0	0	MW314859,MW584165,MW584169,MW584207
840	VC_241_0	2	49.04344142	632.3689051	0.071973221	0.045664183	7.874007874	7.874007874	1	1	1	7.87400787	0	0	0	MF975720,MT121966
841	VC_242_0	2	212.5664611	2240.2481	0.086662263	0.046207465	2.449489743	2.449489743	1	1	1	2.44948974	0	0	0	KT588074,MK340941
842	VC_242_1	20	11311.49717	5225.186862	0.684024509	1.47E-79	0	10.04987562	190	159	0.836842105	7.84048388	0	0	0	KT630649,MK416007,MK416019,MK422455,MK433579,MK448230,MN098327,MN166823,MT135176,MT701587,MW057855,OK665842,OL791266,OM284015,OM621816,ON470579,ON470582,ON470588,ON470593,OP114732
843	VC_243_0	2	103.5828484	1523.449572	0.063663666	0.043901085	5.830951895	5.830951895	1	1	1	5.83095189	0	0	0	MF695815,MK416014
844	VC_244_0	12	10659.5207	15734.79964	0.403856609	1.30E-41	0	7.211102551	66	66	1	1.63039999	0	0	0	KY271401,MK416017,OK490430,OK490433,OK490438,OK490441,OK490444,OK490447,OK490450,OK490451,OK490454,OK490458
845	VC_245_0	10	2287.093513	10384.39387	0.180491322	1.09E-27	0	9.055385138	45	44	0.977777778	6.40824879	0	0	0	KY653119,LN997803,LR597642,ON470580,ON470583,ON470586,ON470600,ON470607,ON470612,ON470621
846	VC_246_0	3	161.0427249	50.18066681	0.762428458	0.040427799	1.414213562	2.645751311	3	3	1	2.09867762	0	0	0	AY583234,AY583235,AY583236
847	VC_247_0	14	20555.10116	1985.918371	0.91189758	9.30E-25	0	9.486832981	91	88	0.967032967	6.97785304	0	0	0	AF234172,AF234173,AF503408,FO818745,KU760857,MH160767,MH422554,MH445380,MH445381,MZ188976,ON018986,ON470615,ON470617,OP279344
848	VC_247_1	1	0	1916.570517	0	0	0	0	1	1	1	0	0	0	0	MF356679
849	VC_248_0	11	9681.814553	4087.764906	0.703130737	3.27E-31	0	4	55	55	1	1.28942858	0	0	0	HQ316581,MG592412,MG592507,MG592513,MG592524,MG592527,MG592588,MG592589,MG592669,MG592670,MZ568828
850	VC_249_0	5	1074.600487	873.8413154	0.551517877	1.01E-07	0	3.605551275	10	10	1	1.44222051	0	0	0	ON170062,ON170209,ON170231,ON170302,ON170328
851	VC_24_0	3	791.7970641	1499.699553	0.345537086	0.001551749	0	0	3	3	1	0	0	0	0	JX507079,MH142219,MH142220
852	VC_250_0	4	623.7934919	1980.439952	0.239530559	1.76E-05	3.16227766	6.403124237	6	6	1	5.36590709	0	0	0	AY539836,CP000622,ON170188,S9-wms-combined_64277
853	VC_251_0	22	29398.77358	15776.95091	0.65076485	2.43E-130	0	7.615773106	231	231	1	5.1761588	0	0	0	AF232233,JX495041,JX495042,JX495043,LN898172,MH719189,MH719190,MH719191,MH719192,MH719193,MH719194,MH719195,MK511057,MK511058,MK511059,MK511060,MK511061,MK511063,MK511064,MK511065,MK934841,MN536027
854	VC_252_0	121	300525.8772	106891.1821	0.737636951	0	0	9	7260	7259	0.999862259	7.16564614	0	0	0	AF085222,AF115102,AF115103,AF145054,FJ236310,KT717083,KT717084,KT717085,KY705251,KY705256,KY705257,KY705259,KY705260,KY705261,KY705262,KY705263,KY705264,KY705265,KY705266,KY705267,KY705270,KY705271,KY705272,KY705273,KY705274,KY705275,KY705276,KY705282,KY705283,KY705284,KY705287,KY705288,KY705289,KY705290,MF161328,MF580759,MF580760,MF580761,MF580762,MF580763,MF580764,MF580765,MF580766,MF580767,MF580768,MF580769,MF580770,MF580771,MF580772,MF580773,MF580774,MF580775,MG708273,MG708274,MG708275,MH000602,MH000603,MH000604,MH375594,MH375595,MH375596,MH375597,MH375598,MH375599,MH375600,MH892351,MH892352,MH892353,MH892354,MH892356,MH892357,MH892358,MH892359,MH892360,MH892361,MH892375,MH892377,MH892378,MH892379,MH892380,MH892381,MH892382,MH937458,MH937459,MH937461,MH937462,MH937465,MH937466,MH937467,MH937468,MH937469,MH937470,MH937471,MH937472,MH937474,MH937477,MH937480,MH937481,MH937482,MH937483,MH937485,MH937486,MH937487,MH937488,MH937489,MH937490,MH937491,MH937492,MH937493,MH937495,MH937496,MH937497,MH937499,MH937500,MH937501,MH937504,MH937506,MH937508,MH973661,MH973662,MZ090946
855	VC_253_0	1	0	2509.267235	0	0	0	0	1	1	1	0	0	0	0	MK448899
856	VC_254_0	46	82669.64514	77566.04759	0.515925283	0	0	8.485281374	1035	1035	1	6.2363292	0	0	0	AF158600,AY699705,EF529515,FJ226752,HE861935,HG380752,HG424323,KY705258,KY705268,KY705269,KY705277,KY705278,KY705279,KY705280,KY705281,KY705285,KY705286,MH375593,MH892362,MH892363,MH892364,MH892366,MH892376,MH892383,MH892384,MH892385,MH937457,MH937460,MH937463,MH937464,MH937473,MH937475,MH937476,MH937478,MH937479,MH937484,MH937494,MH937498,MH937502,MH937503,MH937505,MH937507,MH937509,MH937510,MH937511,U88974
857	VC_255_0	13	8178.08362	31747.58641	0.204832721	1.70E-51	1.414213562	6.782329983	78	78	1	5.04915111	0	0	0	FJ965538,KX879643,KY705252,KY705253,KY705254,KY705255,MH892355,MH892367,MH892373,MH973663,MK202159,MK202160,MK202161
858	VC_255_1	1	0	1633.64622	0	0	0	0	1	1	1	0	0	0	0	S9-wms-combined_20485
859	VC_256_0	14	11169.0841	35992.45271	0.236826127	2.53E-59	1	6.633249581	91	91	1	3.98042113	0	0	0	KU678389,KU678390,KU678391,KU678392,KX879641,KX879642,MH892350,MH892365,MH892368,MH892369,MH892370,MH892371,MH892372,MH892374
860	VC_259_0	3	715.2446236	5514.662951	0.114808224	0.001413984	0	1.414213562	3	3	1	0.94280904	0	0	0	MK448824,MK449002,MK449003
861	VC_25_0	12	3650.572096	944.3943397	0.794471983	1.57E-31	0	5.385164807	66	66	1	1.9821656	0	0	0	ON170017,ON170042,ON170051,ON170056,ON170060,ON170066,ON170072,ON170166,ON170169,ON170274,ON170278,ON170357
862	VC_260_0	13	5184.965108	8807.861649	0.370544508	5.13E-48	0	8.366600265	78	78	1	6.54712714	0	0	0	AF176025,AF323669,AF489521,AJ245616,LT985568,LT985571,LT993224,MN534316,MN534317,MN689509,MN689516,MN689521,MN689530
863	VC_262_0	5	631.3449179	8090.698635	0.072384977	1.08E-07	5.656854249	8.366600265	10	10	1	7.29115813	0	0	0	KX160204,KX160219,KX160220,KX456211,MN534320
864	VC_263_0	7	1631.360157	5268.286225	0.236441126	3.25E-15	0	7.681145748	21	21	1	5.26347312	0	0	0	AF323668,LT985569,LT985574,LT985578,LT985583,LT993229,LT996082
865	VC_265_0	12	7292.891574	9276.60908	0.44013949	2.46E-41	0	7.211102551	66	66	1	5.33694571	0	0	0	DQ394807,DQ394808,DQ394809,DQ394810,KX456206,KX456208,KX456209,KX456210,KX456213,LT985575,LT985576,LT996081
866	VC_267_0	2	125.4364865	1143.55802	0.098847147	0.044420663	6.164414003	6.164414003	1	1	1	6.164414	0	0	0	GQ452243,KT945994
867	VC_268_0	7	3085.10951	5917.14834	0.342703971	4.14E-15	1.732050808	6.403124237	21	21	1	3.67235089	0	0	0	JX564242,JX567312,KX160206,KX160209,KX160210,KX160211,MN552144
868	VC_269_0	3	56.88816804	235.1701242	0.194783608	0.006918233	5.567764363	7.615773106	3	3	1	6.86657865	0	0	0	KJ608189,KU981050,KY744240
869	VC_26_0	4	67.67918338	23.37704642	0.743268017	0.000638472	0	2.828427125	6	6	1	2.2656847	0	0	0	AY714348,JN848801,MN200777,MN200778
870	VC_271_0	5	529.8493955	823.3568471	0.391551102	1.62E-06	6	9.219544457	10	8	0.8	7.94242244	0	0	0	S11-wms-combined_58538,S11-wms_14390,S2-wms-combined_93845,S8-wms-combined_146562,S8-wms-combined_68780
871	VC_271_1	1	0	523.080296	0	0	0	0	1	1	1	0	0	0	0	S8-wms-combined_225114
872	VC_271_2	1	0	177.8336898	0	0	0	0	1	1	1	0	0	0	0	S7-wms-combined_238231
873	VC_272_0	11	3331.362768	3447.040706	0.491467169	2.32E-30	1.732050808	9.38083152	55	48	0.872727273	7.72100282	0	0	0	AJ251789,AP018361,AY131267,AY605066,EU246945,KC171646,KC171647,MF999224,MH983004,MN830258,MT956952
874	VC_274_0	2	35.63643261	410.4036909	0.079895128	0.046291003	5.656854249	5.656854249	1	1	1	5.65685425	0	0	0	AJ421943,MH220877
875	VC_276_0	2	18.89078523	0	1	0	1	1	1	1	1	1	0	0	0	AF133242,AJ969242
876	VC_277_0	3	129.2226826	0	1	0	1	3.16227766	3	3	1	2.38742589	0	0	0	AF155037,MG966533,MK424903
877	VC_278_0	3	582.1889009	1325.620708	0.305160902	0.001511229	0	0	3	3	1	0	0	0	0	AY029185,AY526908,AY526909
878	VC_279_0	2	54.34164394	233.4106092	0.188848717	0.048753352	7.071067812	7.071067812	1	1	1	7.07106781	0	0	0	AY605181,MH128984
879	VC_27_0	2	99.41867932	0	1	0	2.828427125	2.828427125	1	1	1	2.82842712	0	0	0	AB063393,GQ403789
880	VC_280_0	2	109.4155329	212.4090253	0.339985033	0.048416719	3.16227766	3.16227766	1	1	1	3.16227766	0	0	0	GQ421471,MT084350
881	VC_281_0	3	446.7391569	807.6483375	0.356141271	0.001716806	0	1.414213562	3	3	1	0.94280904	0	0	0	MK733262,MK733263,MK733264
882	VC_283_0	2	300	119789.507	0.002498137	0.050634469	2.236067977	2.236067977	1	1	1	2.23606798	0	0	0	MN508614,MN508618
883	VC_283_1	1	0	57794.13165	0	0	0	0	1	1	1	0	0	0	0	MZ502379
884	VC_283_10	1	0	59310.5022	0	0	0	0	1	1	1	0	0	0	0	AP011113
885	VC_283_100	2	300	111981.7135	0.002671851	0.091082117	1.414213562	1.414213562	1	1	1	1.41421356	0	0	0	MT515752,MT526905
886	VC_283_101	1	0	59790.40236	0	0	0	0	1	1	1	0	0	0	0	LR597660
887	VC_283_102	1	0	35201.49716	0	0	0	0	1	1	1	0	0	0	0	OK570375
888	VC_283_103	1	0	57336.87394	0	0	0	0	1	1	1	0	0	0	0	ON528735
889	VC_283_104	1	0	53313.35613	0	0	0	0	1	1	1	0	0	0	0	OL310488
890	VC_283_105	1	0	56264.59798	0	0	0	0	1	1	1	0	0	0	0	MZ234013
891	VC_283_106	1	0	54679.41562	0	0	0	0	1	1	1	0	0	0	0	MZ753803
892	VC_283_107	1	0	58364.35483	0	0	0	0	1	1	1	0	0	0	0	ON528739
893	VC_283_108	1	0	56752.13998	0	0	0	0	1	1	1	0	0	0	0	OK148441
894	VC_283_109	2	300	112659.7316	0.002655814	0.084642774	1.732050808	1.732050808	1	1	1	1.73205081	0	0	0	KY608967,OK275722
895	VC_283_11	1	0	60377.51078	0	0	0	0	1	1	1	0	0	0	0	ON528741
896	VC_283_110	2	300	111929.1516	0.002673102	0.090895844	1.732050808	1.732050808	1	1	1	1.73205081	0	0	0	MN508616,MN508617
897	VC_283_111	1	0	56430.69191	0	0	0	0	1	1	1	0	0	0	0	KY290975
898	VC_283_112	1	0	58313.26299	0	0	0	0	1	1	1	0	0	0	0	OL870317
899	VC_283_113	1	0	57554.29511	0	0	0	0	1	1	1	0	0	0	0	LR215724
900	VC_283_114	1	0	59367.06972	0	0	0	0	1	1	1	0	0	0	0	KX130861
901	VC_283_115	1	0	58301.33869	0	0	0	0	1	1	1	0	0	0	0	JN986846
902	VC_283_116	1	0	61944.16504	0	0	0	0	1	1	1	0	0	0	0	MZ501089
903	VC_283_117	3	900	169892.4395	0.005269554	0.002479634	1	2.236067977	3	3	1	1.74535599	0	0	0	MZ234031,MZ234035,MZ234038
904	VC_283_118	3	900	166565.7181	0.005374234	0.008892194	3.16227766	6.633249581	3	3	1	5.42542265	0	0	0	MZ501113,OL741432,OL870316
905	VC_283_119	1	0	62238.8327	0	0	0	0	1	1	1	0	0	0	0	MK327939
906	VC_283_12	1	0	60183.16132	0	0	0	0	1	1	1	0	0	0	0	MK373785
907	VC_283_120	1	0	60561.70395	0	0	0	0	1	1	1	0	0	0	0	MK962752
908	VC_283_121	2	300	115568.7289	0.002589137	0.092994048	1.414213562	1.414213562	1	1	1	1.41421356	0	0	0	MK327940,MK327945
909	VC_283_122	1	0	57253.87028	0	0	0	0	1	1	1	0	0	0	0	OK274152
910	VC_283_123	1	0	57651.75063	0	0	0	0	1	1	1	0	0	0	0	OL362041
911	VC_283_124	1	0	58602.44421	0	0	0	0	1	1	1	0	0	0	0	MK373779
912	VC_283_125	1	0	59253.52705	0	0	0	0	1	1	1	0	0	0	0	KX130862
913	VC_283_126	1	0	59527.49259	0	0	0	0	1	1	1	0	0	0	0	OM386666
914	VC_283_127	1	0	55530.80668	0	0	0	0	1	1	1	0	0	0	0	MK327929
915	VC_283_128	1	0	57758.85566	0	0	0	0	1	1	1	0	0	0	0	OK076929
916	VC_283_129	1	0	58587.47647	0	0	0	0	1	1	1	0	0	0	0	MH992510
917	VC_283_13	1	0	55951.3093	0	0	0	0	1	1	1	0	0	0	0	ON528745
918	VC_283_130	1	0	60864.94969	0	0	0	0	1	1	1	0	0	0	0	MN022785

919	VC_283_131	1	0	57161.60568	0	0	0	0	1	1		1	0	0	0	0	MN716856
920	VC_283_132	1	0	57258.18646	0	0	0	0	1	1		1	0	0	0	0	ON528740
921	VC_283_133	1	0	56472.12297	0	0	0	0	1	1		1	0	0	0	0	MK327936
922	VC_283_134	1	0	55392.28672	0	0	0	0	1	1		1	0	0	0	0	MK327944
923	VC_283_135	1	0	58649.98311	0	0	0	0	1	1		1	0	0	0	0	OP168898
924	VC_283_136	2	300	115202.4883	0.002597347	0.058838631	0	0	1	1		1	0	0	0	0	MT515753,MT515755
925	VC_283_137	1	0	58166.83875	0	0	0	0	1	1		1	0	0	0	0	LR597657
926	VC_283_138	1	0	56006.85911	0	0	0	0	1	1		1	0	0	0	0	MG867727
927	VC_283_139	1	0	58494.75773	0	0	0	0	1	1		1	0	0	0	0	KX009778
928	VC_283_14	1	0	59989.80019	0	0	0	0	1	1		1	0	0	0	0	MF001360
929	VC_283_140	2	300	107088.1139	0.002793605	0.061880847	7.416198487	7.416198487	1	1		1	7.41619849	0	0	0	OV876900,OV877085
930	VC_283_141	1	0	54265.91385	0	0	0	0	1	1		1	0	0	0	0	LR746311
931	VC_283_142	2	300	104621.4334	0.002859282	0.057160884	5.916079783	5.916079783	1	1		1	5.91607978	0	0	0	ON528726,ON528744
932	VC_283_143	1	0	53476.04152	0	0	0	0	1	1		1	0	0	0	0	KX452698
933	VC_283_144	1	0	53181.19378	0	0	0	0	1	1		1	0	0	0	0	MW341595
934	VC_283_145	1	0	51542.93427	0	0	0	0	1	1		1	0	0	0	0	KM501444
935	VC_283_146	1	0	52758.63402	0	0	0	0	1	1		1	0	0	0	0	ON528746
936	VC_283_147	1	0	55122.49188	0	0	0	0	1	1		1	0	0	0	0	MZ501096
937	VC_283_148	1	0	52657.83641	0	0	0	0	1	1		1	0	0	0	0	JN202312
938	VC_283_149	1	0	54913.00302	0	0	0	0	1	1		1	0	0	0	0	ON528736
939	VC_283_15	1	0	56866.96279	0	0	0	0	1	1		1	0	0	0	0	LC465543
940	VC_283_150	1	0	55558.58535	0	0	0	0	1	1		1	0	0	0	0	MZ065353
941	VC_283_151	1	0	54893.21566	0	0	0	0	1	1		1	0	0	0	0	MH051915
942	VC_283_152	1	0	57278.48967	0	0	0	0	1	1		1	0	0	0	0	OL960580
943	VC_283_153	1	0	59012.05502	0	0	0	0	1	1		1	0	0	0	0	MW749000
944	VC_283_154	1	0	60428.93842	0	0	0	0	1	1		1	0	0	0	0	HM035025
945	VC_283_155	2	300	110916.1506	0.00269745	0.047142304	1.414213562	1.414213562	1	1		1	1.41421356	0	0	0	MT630408,MZ681930
946	VC_283_156	1	0	55884.30038	0	0	0	0	1	1		1	0	0	0	0	MK327942
947	VC_283_157	1	0	55108.60927	0	0	0	0	1	1		1	0	0	0	0	OL960581
948	VC_283_158	1	0	59233.37871	0	0	0	0	1	1		1	0	0	0	0	MF327007
949	VC_283_159	1	0	54860.41539	0	0	0	0	1	1		1	0	0	0	0	MH837626
950	VC_283_16	1	0	60712.32051	0	0	0	0	1	1		1	0	0	0	0	MK327937
951	VC_283_160	1	0	54251.52797	0	0	0	0	1	1		1	0	0	0	0	ON229909
952	VC_283_161	1	0	53236.83215	0	0	0	0	1	1		1	0	0	0	0	MT179807
953	VC_283_162	1	0	48856.56246	0	0	0	0	1	1		1	0	0	0	0	ON720975
954	VC_283_163	2	300	103200.2982	0.002898542	0.065792539	2.449489743	2.449489743	1	1		1	2.44948974	0	0	0	KF925357,KR233165
955	VC_283_164	2	300	99767.4428	0.002997978	0.065242118	6	6	1	1		1	6	0	0	0	MK886800,MZ502380
956	VC_283_165	1	0	53832.30059	0	0	0	0	1	1		1	0	0	0	0	OK018184
957	VC_283_166	1	0	50780.09139	0	0	0	0	1	1		1	0	0	0	0	MZ189262
958	VC_283_167	1	0	50764.0544	0	0	0	0	1	1		1	0	0	0	0	OM135583
959	VC_283_168	1	0	53099.15931	0	0	0	0	1	1		1	0	0	0	0	MW822007
960	VC_283_169	1	0	61933.86525	0	0	0	0	1	1		1	0	0	0	0	MK962750
961	VC_283_17	1	0	60859.95597	0	0	0	0	1	1		1	0	0	0	0	MK327946
962	VC_283_170	1	0	53059.50458	0	0	0	0	1	1		1	0	0	0	0	MH051916
963	VC_283_171	1	0	53533.24226	0	0	0	0	1	1		1	0	0	0	0	MT682709
964	VC_283_172	1	0	53032.94284	0	0	0	0	1	1		1	0	0	0	0	MW883059
965	VC_283_173	1	0	54677.05552	0	0	0	0	1	1		1	0	0	0	0	MT968995
966	VC_283_174	1	0	56641.60881	0	0	0	0	1	1		1	0	0	0	0	MZ417521
967	VC_283_175	1	0	53799.65906	0	0	0	0	1	1		1	0	0	0	0	MW822011
968	VC_283_176	1	0	52917.87372	0	0	0	0	1	1		1	0	0	0	0	MZ150757
969	VC_283_177	1	0	49687.20753	0	0	0	0	1	1		1	0	0	0	0	OL770073
970	VC_283_178	1	0	51434.2806	0	0	0	0	1	1		1	0	0	0	0	ON645936
971	VC_283_179	1	0	54102.54061	0	0	0	0	1	1		1	0	0	0	0	MN580668
972	VC_283_18	1	0	62863.35367	0	0	0	0	1	1		1	0	0	0	0	MK327928
973	VC_283_180	1	0	60265.38709	0	0	0	0	1	1		1	0	0	0	0	MT515756
974	VC_283_181	1	0	57768.90131	0	0	0	0	1	1		1	0	0	0	0	MH243439
975	VC_283_182	1	0	58640.56435	0	0	0	0	1	1		1	0	0	0	0	LC348379
976	VC_283_183	1	0	55239.42686	0	0	0	0	1	1		1	0	0	0	0	MF001359
977	VC_283_184	2	300	119497.3894	0.002504228	0.064687762	2	2	1	1		1	2	0	0	0	MT884006,MT884007
978	VC_283_185	1	0	59479.61663	0	0	0	0	1	1		1	0	0	0	0	MN994497
979	VC_283_186	1	0	58649.21804	0	0	0	0	1	1		1	0	0	0	0	ON528742
980	VC_283_187	1	0	59458.78497	0	0	0	0	1	1		1	0	0	0	0	AP018932
981	VC_283_188	1	0	62557.4859	0	0	0	0	1	1		1	0	0	0	0	MH992121
982	VC_283_189	1	0	58897.63014	0	0	0	0	1	1		1	0	0	0	0	MF158046
983	VC_283_19	1	0	57093.09284	0	0	0	0	1	1		1	0	0	0	0	MF327008
984	VC_283_190	1	0	58728.9995	0	0	0	0	1	1		1	0	0	0	0	MK977694
985	VC_283_2	2	300	111377.1494	0.002686315	0.061573814	1.732050808	1.732050808	1	1		1	1.73205081	0	0	0	HM997020,KP869105
986	VC_283_20	1	0	57187.35527	0	0	0	0	1	1		1	0	0	0	0	MF327009
987	VC_283_21	1	0	59772.10288	0	0	0	0	1	1		1	0	0	0	0	MN781580
988	VC_283_22	1	0	55714.97966	0	0	0	0	1	1		1	0	0	0	0	OP197928
989	VC_283_23	1	0	56296.8144	0	0	0	0	1	1		1	0	0	0	0	MT828552
990	VC_283_24	1	0	57236.96471	0	0	0	0	1	1		1	0	0	0	0	MG775043
991	VC_283_25	1	0	60232.55887	0	0	0	0	1	1		1	0	0	0	0	KU867876
992	VC_283_26	1	0	59263.77784	0	0	0	0	1	1		1	0	0	0	0	OL615012
993	VC_283_27	1	0	60875.45337	0	0	0	0	1	1		1	0	0	0	0	MT176424
994	VC_283_28	1	0	60868.82152	0	0	0	0	1	1		1	0	0	0	0	MT176426
995	VC_283_29	1	0	60523.66594	0	0	0	0	1	1		1	0	0	0	0	ON528727
996	VC_283_3	3	900	161887.8459	0.005528668	0.003198296	2	3.872983346	3	3		1	3.24865556	0	0	0	FJ839693,KM607004,OK040907
997	VC_283_30	1	0	59203.79146	0	0	0	0	1	1		1	0	0	0	0	ON528728
998	VC_283_31	1	0	59299.02445	0	0	0	0	1	1		1	0	0	0	0	MN895437
999	VC_283_32	1	0	60696.81481	0	0	0	0	1	1		1	0	0	0	0	MZ436830
1000	VC_283_33	4	1800	236990.7064	0.007537982	0.000205293	0	2	6	6		1	1.27614237	0	0	0	LN881726,LN881729,LN881734,LN881737
1001	VC_283_34	4	1800	217882.2524	0.008193652	0.000251904	0	1.732050808	6	6		1	1.09341299	0	0	0	MH809535,MT515751,MT828551,MT828553
1002	VC_283_35	1	0	60693.33042	0	0	0	0	1	1		1	0	0	0	0	MK962755
1003	VC_283_36	1	0	60056.2788	0	0	0	0	1	1		1	0	0	0	0	MZ501065
1004	VC_283_37	1	0	60123.85751	0	0	0	0	1	1		1	0	0	0	0	LN881732
1005	VC_283_38	1	0	58969.3354	0	0	0	0	1	1		1	0	0	0	0	LR990704
1006	VC_283_39	1	0	60195.08236	0	0	0	0	1	1		1	0	0	0	0	MN895434
1007	VC_283_4	5	3000	264553.5642	0.011212708	3.06E-07	0	1	10	10		1	0.4	0	0	0	MZ291552,OP555980,OP555981,OP555982,OP572230
1008	VC_283_40	1	0	59421.36744	0	0	0	0	1	1		1	0	0	0	0	MZ234018
1009	VC_283_41	1	0	59457.10193	0	0	0	0	1	1		1	0	0	0	0	MZ501046
1010	VC_283_42	1	0	57504.08644	0	0	0	0	1	1		1	0	0	0	0	MZ501050
1011	VC_283_43	1	0	60793.60963	0	0	0	0	1	1		1	0	0	0	0	MK373786
1012	VC_283_44	1	0	60470.60139	0	0	0	0	1	1		1	0	0	0	0	MN895438
1013	VC_283_45	1	0	58018.74176	0	0	0	0	1	1		1	0	0	0	0	MH355584
1014	VC_283_46	1	0	60275.03869	0	0	0	0	1	1		1	0	0	0	0	MZ501066
1015	VC_283_47	1	0	61112.49399	0	0	0	0	1	1		1	0	0	0	0	MZ501067
1016	VC_283_48	1	0	61085.71627	0	0	0	0	1	1		1	0	0	0	0	MN895436
1017	VC_283_49	1	0	58118.80707	0	0	0	0	1	1		1	0	0	0	0	MT176425
1018	VC_283_5	1	0	54896.68993	0	0	0	0	1	1		1	0	0	0	0	LC620505
1019	VC_283_50	1	0	59876.25626	0	0	0	0	1	1		1	0	0	0	0	MZ501106
1020	VC_283_51	1	0	58831.11302	0	0	0	0	1	1		1	0	0	0	0	MN895435
1021	VC_283_52	1	0	60956.36154	0	0	0	0	1	1		1	0	0	0	0	MH553563
1022	VC_283_53	1	0	61297.26374	0	0	0	0	1	1		1	0	0	0	0	MK373781
1023	VC_283_54	1	0	61206.44555	0	0	0	0	1	1		1	0	0	0	0	MT682713
1024	VC_283_55	1	0	60110.03395	0	0	0	0	1	1		1	0	0	0	0	ON528743
1025	VC_283_56	1	0	60463.32819	0	0	0	0	1	1		1	0	0	0	0	MZ726795
1026	VC_283_57	1	0	57406.97821	0	0	0	0	1	1		1	0	0	0	0	KT184311
1027	VC_283_58	1	0	60033.79378	0	0	0	0	1	1		1	0	0	0	0	KX130864
1028	VC_283_59	1	0	59742.00578	0	0	0	0	1	1		1	0	0	0	0	OK272473
1029	VC_283_6	1	0	56220.49696	0	0	0	0	1	1		1	0	0	0	0	ON782582
1030	VC_283_60	1	0	58212.43114	0	0	0	0	1	1		1	0	0	0	0	OK272476
1031	VC_283_61	1	0	60550.67538	0	0	0	0	1	1		1	0	0	0	0	MT446387
1032	VC_283_62	1	0	59954.74686	0	0	0	0	1	1		1	0	0	0	0	OK272484
1033	VC_283_63	1	0	57831.52963	0	0	0	0	1	1		1	0	0	0	0	KT184312
1034	VC_283_64	1	0	59467.34018	0	0	0	0	1	1		1	0	0	0	0	MF158045
1035	VC_283_65	1	0	59087.49413	0	0	0	0	1	1		1	0	0	0	0	OL956808
1036	VC_283_66	1	0	57447.13931	0	0	0	0	1	1		1	0	0	0	0	KT184308
1037	VC_283_67	2	300	109440.9693	0.00273371	0.092739974	7.745966692	7.745966692	1	1		1	7.74596669	0	0	0	LR877332,MT515754
1038	VC_283_68	1	0	60660.29529	0	0	0	0	1	1		1	0	0	0	0	MW749001
1039	VC_283_69	1	0	60308.17943	0	0	0	0	1	1		1	0	0	0	0	HE956711
1040	VC_283_7	1	0	58231.65418	0	0	0	0	1	1		1	0	0	0	0	MW286157
1041	VC_283_70	1	0	56516.76489	0	0	0	0	1	1		1	0	0	0	0	MZ501098
1042	VC_283_71	1	0	60240.29782	0	0	0	0	1	1		1	0	0	0	0	LR215723
1043	VC_283_72	1	0	61497.94227	0	0	0	0	1	1		1	0	0	0	0	MT682710
1044	VC_283_73	3	900	173836.8854	0.005150601	0.002546496	0	1	3	3		1	0.66666667	0	0	0	LN881728,LN881733,LN881736
1045	VC_283_74	1	0	58035.56058	0	0	0	0	1	1		1	0	0	0	0	MT682714
1046	VC_283_75	1	0	59072.10958	0	0	0	0	1	1		1	0	0	0	0	MZ170040
1047	VC_283_76	1	0	55588.98912	0	0	0	0	1	1		1	0	0	0	0	MK373787
1048	VC_283_77	2	300	120759.3369	0.002478124	0.045925809	1.732050808	1.732050808	1	1		1	1.73205081	0	0	0	MT682712,MZ170041
1049	VC_283_78	13	23400	645726.1312	0.034970985	7.73E-36	0	11.13552873	78	62		0.794871795	6.93277294	0	0	0	MZ234027,MZ234030,MZ234034,MZ234036,MZ234037,MZ234039,MZ234040,MZ234041,MZ234042,MZ234043,MZ234051,MZ234052,MZ234053
1050	VC_283_79	1	0	55462.94152	0	0	0	0	1	1		1	0	0	0	0	MF001354
1051	VC_283_8	1	0	58502.63356	0	0	0	0	1	1		1	0	0	0	0	KU925172
1052	VC_283_80	1	0	54613.17947	0	0	0	0	1	1		1	0	0	0	0	MT764206
1053	VC_283_81	1	0	55298.89282	0	0	0	0	1	1		1	0	0	0	0	KR269718
1054	VC_283_82	6	4500	342477.0607	0.012969157	2.03E-11	0	2.449489743	15	15		1	1.4946358	0	0	0	KM606994,KM606995,KM606996,KM606997,KM606998,KM606999
1055	VC_283_83	1	0	61796.8049	0	0	0	0	1	1		1	0	0	0	0	KM607000
1056	VC_283_84	1	0	62418.83314	0	0	0	0	1	1		1	0	0	0	0	FJ839692
1057	VC_283_85	1	0	60248.45679	0	0	0	0	1	1		1	0	0	0	0	KX828711
1058	VC_283_86	1	0	57985.64552	0	0	0	0	1	1		1	0	0	0	0	ON528734

1059	VC_283_87	1	0	60161.50195	0	0	0	0	1	1	1	0	0	0	0	ON528738
1060	VC_283_88	2	300	116873.7375	0.002560301	0.044034531	3.464101615	3.464101615	1	1	1	3.46410162	0	0	0	AP018814,MH550421
1061	VC_283_89	2	300	110398.306	0.002710069	0.087352388	2.449489743	2.449489743	1	1	1	2.44948974	0	0	0	MG781190,MG781191
1062	VC_283_9	1	0	57254.40407	0	0	0	0	1	1	1	0	0	0	0	MZ501052
1063	VC_283_90	1	0	58872.61378	0	0	0	0	1	1	1	0	0	0	0	KF208315
1064	VC_283_91	11	16500	562694.5584	0.028487837	2.04E-35	0	7.071067812	55	55	1	3.66904412	0	0	0	AF158101,HM137666,KJ477684,KJ477685,KJ477686,KM607002,KM607003,MT984581,MZ375356,OL964735,OL964745
1065	VC_283_92	4	1800	225479.4939	0.007919764	4.18E-05	1.732050808	3.31662479	6	6	1	2.4902663	0	0	0	AP018813,LC348380,MH751506,OM339528
1066	VC_283_93	1	0	59566.85807	0	0	0	0	1	1	1	0	0	0	0	MT932213
1067	VC_283_94	2	300	113276.3023	0.002641396	0.047062377	0	0	1	1	1	0	0	0	0	DQ904452,KM607001
1068	VC_283_95	1	0	63444.99548	0	0	0	0	1	1	1	0	0	0	0	JX128259
1069	VC_283_96	1	0	62775.39109	0	0	0	0	1	1	1	0	0	0	0	ON506924
1070	VC_283_97	1	0	57778.2252	0	0	0	0	1	1	1	0	0	0	0	LR215722
1071	VC_283_98	1	0	56986.02756	0	0	0	0	1	1	1	0	0	0	0	ON210139
1072	VC_283_99	1	0	59553.26724	0	0	0	0	1	1	1	0	0	0	0	KJ668714
1073	VC_284_0	3	900	17299.91444	0.049450782	0.001390344	6.32455532	7.483314774	3	3	1	7.09706162	0	0	0	KJ025957,MN095771,OM457001
1074	VC_286_0	2	300	40832.54991	0.007293494	0.225370074	7.280109889	7.280109889	1	1	1	7.28010989	0	0	0	MW512264,MZ501047
1075	VC_286_1	21	63000	308533.0289	0.169567697	8.06E-32	0	9.949874371	210	190	0.904761905	7.50975077	0	0	0	KR422352,LR027383,LR027386,LR027387,LR027390,LR027391,MH243438,MK327934,MK327943,MK373778,MK373782,MK373784,MK962754,MK962756,MK962757,MN850579,MN850590,MZ234025,OL960576,OL960577,OM960734
1076	VC_286_10	9	10800	164735.8961	0.061525877	1.22E-06	7.071067812	9.433981132	36	24	0.666666667	8.60363114	0	0	0	AY303349,KF562341,KM407600,KY703222,MH992122,MT446390,MZ501056,OM982647,ON528737
1077	VC_286_11	3	900	60679.26371	0.01461531	0.06539716	6.8556546	8.888194417	3	3	1	8.19186996	0	0	0	MH359124,MK524178,MW882907
1078	VC_286_12	3	900	60572.96841	0.014640581	0.076717347	8.185352772	8.94427191	3	3	1	8.67260637	0	0	0	MG589383,MZ189261,OL770074
1079	VC_286_13	10	13500	179846.1995	0.06982294	7.09E-07	1.414213562	9.110433579	45	43	0.955555556	7.78853948	0	0	0	KF582788,MF044457,MK639187,MT782071,MW630116,MZ311865,MZ560701,MZ868638,OV754623,OV877511
1080	VC_286_14	1	0	21180.8957	0	0	0	0	1	1	1	0	0	0	0	JX536493
1081	VC_286_15	2	300	40797.75938	0.007299668	0.243736918	7.615773106	7.615773106	1	1	1	7.61577311	0	0	0	MK047717,ON528725
1082	VC_286_16	1	0	20764.01845	0	0	0	0	1	1	1	0	0	0	0	KX130865
1083	VC_286_17	8	8400	148648.1791	0.053486771	2.17E-05	0	9.433981132	28	17	0.607142857	7.94377446	0	0	0	KP869101,KP869104,KP869111,KT184309,KT184310,MK047718,MN648445,MN850622
1084	VC_286_2	1	0	20696.92053	0	0	0	0	1	1	1	0	0	0	0	MN850651
1085	VC_286_3	2	300	40730.30271	0.007311669	0.251141957	2	2	1	1	1	2	0	0	0	MK373774,MK373775
1086	VC_286_4	1	0	20834.35497	0	0	0	0	1	1	1	0	0	0	0	MK327933
1087	VC_286_5	1	0	20904.49631	0	0	0	0	1	1	1	0	0	0	0	MT446423
1088	VC_286_6	1	0	21137.69818	0	0	0	0	1	1	1	0	0	0	0	MN864145
1089	VC_286_7	1	0	25804.89913	0	0	0	0	1	1	1	0	0	0	0	OM632671
1090	VC_286_8	1	0	20691.83756	0	0	0	0	1	1	1	0	0	0	0	KU255730
1091	VC_286_9	1	0	22049.33248	0	0	0	0	1	1	1	0	0	0	0	OM386662
1092	VC_287_0	2	300	10443.76437	0.027923174	0.042285388	5.567764363	5.567764363	1	1	1	5.56776436	0	0	0	MT457552,MT457553
1093	VC_287_1	1	0	6203.789024	0	0	0	0	1	1	1	0	0	0	0	KY971610
1094	VC_288_0	3	900	20513.78926	0.042028993	0.001438912	5.656854249	6.244997998	3	3	1	5.8821383	0	0	0	MW358927,MW385300,OK499989
1095	VC_288_1	1	0	6930.472329	0	0	0	0	1	1	1	0	0	0	0	KX078569
1096	VC_288_2	2	300	12829.18591	0.022849855	0.044614412	2.828427125	2.828427125	1	1	1	2.82842712	0	0	0	MG696114,MK295204
1097	VC_288_3	3	900	18997.67191	0.045231422	0.001559747	5.916079783	8.717797887	3	3	1	7.25843537	0	0	0	KP890823,MF001355,MW367898
1098	VC_288_4	1	0	6606.855676	0	0	0	0	1	1	1	0	0	0	0	MW057858
1099	VC_28_0	2	232.4492088	7305.086568	0.030838886	0.042803361	1.732050808	1.732050808	1	1	1	1.73205081	0	0	0	MW722521,MW790496
1100	VC_28_1	53	133920.7211	53018.8005	0.716385278	0	0	9.746794345	1378	1285	0.932510885	7.38035055	0	0	0	AB102868,AB362338,AB426868,AF217253,AF527608,AY052766,BK000583,CP051272,CP051278,CP051279,CP051285,CP051288,DQ003260,EU570103,FJ000341,GQ422450,GQ422451,GU573886,JF900176,JQ806763,KR296686,KR296687,KR296688,KU878967,KU927491,KU927492,KU927494,KU927495,KU927496,KU927498,KX776161,LC730322,LT714109,MF188997,MG407615,MG969415,MH370387,MK770414,MK770415,MK972685,MK972686,MK972687,MK972692,MK972693,MK972694,MN161573,MT774487,MT932212,MW013502,MW013503,MW355480,OP265889,OP377721
1101	VC_290_0	10	13500	124159.6298	0.098067967	3.34E-24	1	8.774964387	45	45	1	7.47403287	0	0	0	MT129652,MT446396,MT446411,MT446412,MT446415,MT446421,MT611523,MW233709,MW250787,MW598459
1102	VC_290_1	8	8400	102585.303	0.075685697	1.77E-15	7.071067812	9.848857802	28	24	0.857142857	8.29522652	0	0	0	GQ981382,KP007360,MT446420,MT446422,MT533174,MW269952,OK254198,OL451225
1103	VC_290_2	2	300	29137.65366	0.01019103	0.076289934	8.124038405	8.124038405	1	1	1	8.1240384	0	0	0	HM563683,KP007361
1104	VC_290_3	2	300	29036.85924	0.010226044	0.077261572	8.888194417	8.888194417	1	1	1	8.88819442	0	0	0	KP007362,MT446392
1105	VC_290_4	2	300	32475.59243	0.009153153	0.096539322	8.366600265	8.366600265	1	1	1	8.36660027	0	0	0	KP007359,OM386655
1106	VC_290_5	14	27300	164639.3613	0.142232421	4.10E-42	5.567764363	9.486832981	91	84	0.923076923	7.82353853	0	0	0	HM071924,HQ829472,KT176190,KU878968,KU878969,MH051917,MK234886,MT150133,MT682711,MW149274,MW679410,MZ561532,OK335775,OL780484
1107	VC_290_6	12	19800	152479.5125	0.11492951	2.07E-30	1	9.899494937	66	54	0.818181818	7.86279076	0	0	0	EF469154,EU863409,MH051913,MH791409,MK310184,MN850609,MN850621,MT129654,OL770107,OP292222,OV876907,OV877765
1108	VC_290_7	1	0	15037.03231	0	0	0	0	1	1	1	0	0	0	0	MK295203
1109	VC_292_0	4	678.1388485	3364.723639	0.167737303	1.33E-05	0	8.660254038	6	6	1	6.62821126	0	0	0	MK448704,MK448707,MK448709,MK448710
1110	VC_293_0	1	0	1271.232262	0	0	0	0	1	1	1	0	0	0	0	MK448750
1111	VC_293_1	1	0	1560.18993	0	0	0	0	1	1	1	0	0	0	0	MK448995
1112	VC_293_2	2	106.5232503	3317.199015	0.031113286	0.042895506	7.211102551	7.211102551	1	1	1	7.21110255	0	0	0	MK448740,MK448917
1113	VC_293_3	19	10734.86464	30128.26795	0.262702929	3.00E-92	1	9.848857802	171	140	0.81871345	8.18874665	0	0	0	MK448675,MK448676,MK448688,MK448690,MK448694,MK448772,MK448778,MK448782,MK448850,MK448859,MK448877,MK448931,MK448955,MK448962,MK448966,MK448973,MK448975,MK449004,OL774870
1114	VC_294_0	2	237.0894915	2597.267606	0.083648419	0.042682242	0	0	1	1	1	0	0	0	0	MK448832,MK449008
1115	VC_295_0	2	53.03792305	1525.75293	0.033594015	0.055357686	8.717797887	8.717797887	1	1	1	8.71779789	0	0	0	MK819239,ON857931
1116	VC_296_0	6	1795.665816	1583.133157	0.531450918	3.90E-11	0	6.08276253	15	15	1	4.38117748	0	0	0	MG592403,MG592404,MG592405,MG592551,MN592897,MW824406
1117	VC_297_0	5	1436.999284	12172.88337	0.105584987	7.48E-08	4.898979486	9.433981132	10	9	0.9	7.68797595	0	0	0	KM389210,KU199710,MW748993,MZ089728,ON778007
1118	VC_297_1	20	27598.51446	21223.32415	0.565290355	3.33E-93	0	9.643650761	190	146	0.768421053	6.30856522	0	0	0	HQ711984,JX403939,KU310943,MK511001,MK511002,MK511003,MK511004,MK511005,MK511006,MK511007,MK511008,MK511009,MK511010,MK511011,MK511012,MK511013,MK511014,MK511015,MK511016,MZ773939
1119	VC_298_0	1	0	368.7083874	0	0	0	0	1	1	1	0	0	0	0	KU708004
1120	VC_298_1	1	0	475.2702627	0	0	0	0	1	1	1	0	0	0	0	MW862109
1121	VC_299_0	24	78018.47597	745.4675623	0.990535421	1.63E-67	0	4.472135955	276	276	1	1.83762828	0	0	0	KX581090,KX581091,KX581092,KX581093,KX581094,KX581095,KX581096,KX581097,KX581098,KX581099,KX581100,KY658673,KY658674,KY658675,KY658676,KY658677,KY658678,KY658679,KY658680,MK672799,MK672800,MK672801,MK672803,MK672804
1122	VC_29_0	10	5406.663895	4973.811783	0.520849339	5.96E-30	0	3	45	45	1	2.23741396	0	0	0	AF157835,EU794049,LR794146,LR794147,LR794148,LR794149,LR794150,LR794151,LR794152,LR797923
1123	VC_2_0	4	765.0153412	5071.903898	0.131064918	1.31E-05	1.414213562	4.358898944	6	6	1	3.48965488	0	0	0	AB008550,MK034952,MK511066,Y13918
1124	VC_300_0	3	123.2750423	888.7675128	0.121808161	0.00158299	6.164414003	9.219544457	3	2	0.666666667	8.05297428	0	0	0	HQ141411,MG788324,OP650937
1125	VC_301_0	2	22.37749083	150.856979	0.129174585	0.050697955	1.732050808	1.732050808	1	1	1	1.73205081	0	0	0	LT985589,LT993247
1126	VC_302_0	2	67.45895315	284.5212238	0.191655546	0.050257969	6.8556546	6.8556546	1	1	1	6.8556546	0	0	0	MH837542,MH837543
1127	VC_303_0	155	170519.0101	357.4815823	0.997907953	4.34E-56	0	2.645751311	11935	11935	1	1.32521393	0	0	0	AF176027,AF176028,AF176029,AF176030,AF176031,AF176032,AF176033,AF176034,AF274751,AF299300,AF299301,AF299302,AF299303,AF299304,AF299305,AF299306,AF299307,AF299308,AF299309,AF299310,AF299311,AF299312,AF299313,AF299314,CP004084,DQ079880,DQ079881,DQ079882,DQ079883,DQ079884,DQ079885,DQ079886,DQ079887,DQ079888,DQ079889,DQ079890,DQ079891,DQ079892,DQ079893,DQ079894,DQ079895,EF380009,EF380010,EF380011,EF380012,EF380013,EF380014,EF380015,EF380016,EF380017,EF380018,EF380019,EF380020,EF380021,EF380022,EF380023,EF380024,EF380025,EF380026,EF380027,EF380028,EF380029,EF380030,EF380031,EF380032,FJ849058,GQ153912,GQ153913,GQ153914,GQ153915,GU385905,HM753658,HM753659,HM753660,HM753661,HM753662,HM753663,HM753664,HM753665,HM753666,HM753667,HM753668,HM753669,HM753670,HM753671,HM753672,HM753673,HM753674,HM753675,HM753676,HM753677,HM753678,HM753679,HM753680,HM753681,HM753682,HM753683,HM753684,HM753685,HM753686,HM753687,HM753688,HM753689,HM753690,HM753691,HM753692,HM753693,HM753694,HM753695,HM753696,HM753697,HM753698,HM753699,HM753700,HM753701,HM753702,HM753703,HM753704,HM753705,HM753706,HM753707,HM753708,HM753709,HM753710,HM753711,HM753712,HM753713,HM753714,HM753715,HM753716,HM753717,HM753718,HM775306,HM775307,HM775308,HM775309,HM775310,HM775311,JF719726,JF719727,JF719728,JQ764986,JQ764987,JQ764988,JQ764989,JQ764990,KJ997912,KY653237,MH378441,MH378442,MH378443,MN385565,MT947439,PX1CG,S13CG
1128	VC_304_0	11	6277.975218	1699.818151	0.786931289	7.13E-25	1	7.348469228	55	55	1	4.61864606	0	0	0	AF189021,FJ867910,FJ867911,FJ867912,FJ867913,FJ867914,JQ809650,MF431615,MF431616,MF431617,MK613346
1129	VC_305_0	12	3948.569345	2924.606861	0.574489759	2.86E-33	4.358898944	9.539392014	66	56	0.848484848	8.14215732	0	0	0	GU557055,MK613343,MK613344,MK613345,MW514246,MW514247,MZ892987,MZ892988,MZ892989,MZ892990,MZ892991,MZ892992
1130	VC_306_0	2	59.68224381	0	1	0	0	0	1	1	1	0	0	0	0	AF191797,MW557853
1131	VC_307_0	2	146.3359871	235.1980221	0.383546377	0.047485936	4.358898944	4.358898944	1	1	1	4.35889894	0	0	0	AJ131519,MW476489
1132	VC_308_0	4	602.3819499	599.1323936	0.501352275	2.58E-05	4.123105626	7.348469228	6	6	1	6.00376476	0	0	0	AF195902,DQ320509,HE956704,KF767351
1133	VC_309_0	3	51.47939396	355.5847171	0.126465077	0.005011038	7.681145748	8.185352772	3	3	1	7.91350213	0	0	0	MF041990,MN830256,X98106
1134	VC_30_0	2	172.3224131	4487.442202	0.036980927	0.042653646	5.477225575	5.477225575	1	1	1	5.47722558	0	0	0	JQ182733,JQ182734
1135	VC_30_1	16	7766.339179	31596.15724	0.197303014	9.00E-73	1.414213562	9.695359715	120	100	0.833333333	8.26512448	0	0	0	AF335538,AF547987,CP000711,JF974339,JN600960,KJ832078,MH717096,MN340231,MT813197,MT833387,MZ043897,OM022251,ON470589,ON470594,ON470605,OV696608
1136	VC_310_0	33	158395.828	22193.20244	0.877106586	9.48E-180	0	6.32455532	528	528	1	4.61339163	0	0	0	AF222060,AY190604,KC292020,KC292022,MN901520,MN901521,MW450817,MZ334492,MZ334493,MZ334494,MZ334495,MZ334496,MZ334497,MZ334498,MZ334499,MZ334500,MZ334501,MZ334502,MZ334503,MZ334504,MZ334505,MZ334506,MZ334507,MZ334508,MZ334509,MZ334510,MZ334511,MZ334512,MZ334513,MZ334514,MZ334515,MZ334516,OK649958
1137	VC_311_0	6	4477.772934	17942.8043	0.199717112	4.85E-11	0	4.582575695	15	15	1	3.55205146	0	0	0	KC117376,KC292021,MZ334517,MZ334518,MZ334519,MZ334520
1138	VC_312_0	2	13.80349458	435.2224777	0.030740971	0.045635733	7.681145748	7.681145748	1	1	1	7.68114575	0	0	0	KC292026,MZ334525
1139	VC_313_0	2	300	1443.237422	0.172093598	0.092556008	2.236067977	2.236067977	1	1	1	2.23606798	0	0	0	KC292029,MZ334524
1140	VC_313_1	3	900	1663.147163	0.351130834	0.001979817	0	6.782329983	3	3	1	4.52155332	0	0	0	KC117377,KC292027,MZ334523
1141	VC_314_0	2	6.936512835	31.43019879	0.180795084	1.68E-07	0	0	1	1	1	0	0	0	0	DQ836063,JX625144
1142	VC_315_0	12	457.8098471	53.88034078	0.894701243	1.12E-26	0	0	66	66	1	0	0	0	0	AF227250,EF108464,EF108465,EF204939,EF204940,FJ483837,FJ483838,FJ483839,JQ966307,LT898437,MK213795,MT830620
1143	VC_316_0	3	681.7498462	199.0749145	0.773990329	0.001795021	1.732050808	2	3	3	1	1.82136721	0	0	0	MZ326866,MZ611865,ON711490
1144	VC_317_0	18	16158.71709	32751.2685	0.330376648	3.34E-95	0	8.306623863	153	153	1	6.22269127	0	0	0	MK448685,MK448763,MK448767,MK448771,MK448773,MK448774,MK448777,MK448786,MK448845,MK448853,MK448855,MK448942,MK448943,MK448963,MK448964,MK448969,MK448972,MK449012
1145	VC_317_1	2	55.07230462	2905.006366	0.018605014	0.043428487	7.549834435	7.549834435	1	1	1	7.54983444	0	0	0	MK448706,MK448721
1146	VC_317_2	20	8886.168695	36092.56177	0.197563795	2.61E-63	1	10.44030651	190	130	0.684210526	8.08112621	0	0	0	AY007505,FR671408,FR671409,KC348600,KC348601,KC348602,KT337360,KX077894,KY065485,KY065503,KY065504,MK448814,MK448881,MK448882,MK448896,MK448909,MK448988,MK448989,OL774869,OL774877
1147	VC_317_3	1	0	1870.892515	0	0	0	0	1	1	1	0	0	0	0	KP208803
1148	VC_317_4	1	0	1849.04137	0	0	0	0	1	1	1	0	0	0	0	MK448900
1149	VC_318_0	3	188.2095086	4360.350714	0.041377821	0.014201475	4.472135955	8.888194417	3	3	1	7.37843159	0	0	0	KY065466,KY065468,MK448982
1150	VC_31_0	9	3330.646199	6365.977827	0.343485134	3.91E-24	0	9.695359715	36	25	0.694444444	7.89867771	0	0	0	KY271395,KY271396,MK448231,MK448233,MK448235,MK714353,OK490410,OK490415,OK490416
1151	VC_31_1	1	0	1126.729457	0	0	0	0	1	1	1	0	0	0	0	OK499971
1152	VC_31_2	1	0	1229.270654	0	0	0	0	1	1	1	0	0	0	0	HQ201308
1153	VC_323_0	2	70.92740272	2449.006464	0.028146533	0.043434336	7.810249676	7.810249676	1	1	1	7.81024968	0	0	0	MK448821,MK448822
1154	VC_323_1	9	3811.405081	17391.77352	0.179756307	5.55E-23	0	9.38083152	36	27	0.75	7.23563587	0	0	0	KY065498,MK448735,MK448757,MK448758,MK448759,MK448893,MK448915,MK448939,OL774872
1155	VC_324_0	2	131.5118847	508.490588	0.205486526	0.044855408	4.242640687	4.242640687	1	1	1	4.24264069	0	0	0	S2-wms-combined_166665,S6-wms-combined_49909
1156	VC_325_0	2	117.4771498	28496.99365	0.004105515	0.044255606	8.831760866	8.831760866	1	1	1	8.83176087	0	0	0	MH513971,ON970586
1157	VC_325_1	2	138.4512245	32281.57147	0.004270547	0.043366516	8.602325267	8.602325267	1	1	1	8.60232527	0	0	0	JN398369,MN617845
1158	VC_325_10	3	588.7649137	48324.42096	0.012036937	0.001408313	4.898979486	8.062257748	3	3	1	6.65374574	0	0	0	MH744414,MK359331,MN119379
1159	VC_325_11	1	0	14416.98793	0	0	0	0	1	1	1	0	0	0	0	MH001458
1160	VC_325_12	1	0	17081.72166	0	0	0	0	1	1	1	0	0	0	0	MN813700
1161	VC_325_13	2	275.2336694	33100.82066	0.008246441	0.042488001	4.242640687	4.242640687	1	1	1	4.24264069	0	0	0	EU744249,EU744252
1162	VC_325_14	3	457.6794697	48240.59259	0.009398269	0.001408411	8.185352772	8.717797887	3	3	1	8.5211349	0	0	0	GU247132,KT818595,MT889384
1163	VC_325_15	1	0	14217.71429	0	0	0	0	1	1	1	0	0	0	0	KJ174156
1164	VC_325_16	2	300	31166.24194	0.009534027	0.042486071	2.449489743	2.449489743	1	1	1	2.44948974	0	0	0	MK310141,MK524523
1165	VC_325_17	1	0	17582.00979	0	0	0	0	1	1	1	0	0	0	0	KC701493
1166	VC_325_18	1	0	15417.33981	0	0	0	0	1	1	1	0	0	0	0	OL549190
1167	VC_325_19	3	492.8467404	45344.75553	0.010752018	0.001407792	6	8.602325267	3	3	1	7.69586888	0	0	0	JN020140,MH338238,MK967387
1168	VC_325_2	1	0	16589.27191	0	0	0	0	1	1	1	0	0	0	0	JF937099
1169	VC_325_20	1	0	15090.15862	0	0	0	0	1	1	1	0	0	0	0	MN585970
1170	VC_325_21	5	1682.031399	78826.66127	0.020892544	2.44E-07	0	9.539392014	10	8	0.8	7.16028134	0	0	0	EU744248,KX522649,KX522943,KX574454,MK524497
1171	VC_325_22	1	0	14933.38509	0	0	0	0	1	1	1	0	0	0	0	MK524499
1172	VC_325_23	17	19144.41287	248067.595	0.071645032	3.39E-75	4.358898944	9.949874371	136	87	0.639705882	8.45332384	0	0	0	JF937103,KC661273,KT259047,MG920060,MG925351,MH020244,MH450113,MH450130,MH576959,MH576971,MH576974,MK359299,MK820638,MT639654,MW507123,MW712732,ON970627
1173	VC_325_24	3	476.5564668	52637.37397	0.008972344	0.001460512	7.681145748	8.94427191	3	3	1	8.35052248	0	0	0	JF957057,MH230876,MN585976
1174	VC_325_25	1	0	16863.18343	0	0	0	0	1	1	1	0	0	0	0	MZ648034
1175	VC_325_26	1	0	16396.86985	0	0	0	0	1	1	1	0	0	0	0	MG944220
1176	VC_325_27	2	136.0071932	30847.581	0.004389653	0.042490455	8.306623863	8.306623863	1	1	1	8.30662386	0	0	0	MH536816,MH632118
1177	VC_325_28	1	0	16171.82291	0	0	0	0	1	1	1	0	0	0	0	KX369584
1178	VC_325_29	3	539.9423373	46303.20516	0.011526602	0.001425585	4.795831523	8.602325267	3	3	1	7.15347151	0	0	0	KP027196,KP027203,MW862983
1179	VC_325_3	1	0	15866.13216	0	0	0	0	1	1	1	0	0	0	0	KF493880
1180	VC_325_30	4	1005.360273	69044.18897	0.014352131	1.38E-05	6.08276253	9.055385138	6	5	0.833333333	7.83237288	0	0	0	KJ409696,KT326768,MK494116,OK040791
1181	VC_325_31	1	0	17686.55402	0	0	0	0	1	1	1	0	0	0	0	MK814754
1182	VC_325_32	1	0	15269.28669	0	0	0	0	1	1	1	0	0	0	0	MW435858
1183	VC_325_33	4	814.7044672	53996.61883	0.014863799	1.44E-05	7.549834435	8.831760866	6	6	1	8.34414842	0	0	0	KF560331,MK112532,MK359316,OM203162
1184	VC_325_34	1	0	17246.96612	0	0	0	0	1	1	1	0	0	0	0	ON677304
1185	VC_325_35	17	20190.06617	255693.2912	0.073183342	2.04E-71	3.16227766	10.63014581	136	105	0.772058824	8.22596587	0	0	0	EU826470,JN572689,JQ698665,JQ809703,KF024724,KF279415,KF279416,KM597531,KR080201,MF668283,MK359312,MK524506,MK524531,MN428054,MN586000,MT657342,MZ322004
1186	VC_325_36	2	147.8237021	34212.86317	0.004302117	0.042488849	8.544003745	8.544003745	1	1	1	8.54400375	0	0	0	MH371110,MN369760
1187	VC_325_37	3	419.9137134	51783.24547	0.008043837	0.003650635	7.937253933	9.055385138	3	2	0.666666667	8.51221427	0	0	0	MK061415,MN585985,MT776812
1188	VC_325_38	6	2478.598114	90495.17242	0.026659111	2.79E-10	2.828427125	9.695359715	15	12	0.8	7.39581648	0	0	0	KX683876,KX702320,MG099951,MH000606,MH651180,MT952847
1189	VC_325_39	2	136.6321074	30523.98669	0.004456274	0.042491111	8.366600265	8.366600265	1	1	1	8.36660027	0	0	0	AF271693,MZ355726
1190	VC_325_4	1	0	15740.99088	0	0	0	0	1	1	1	0	0	0	0	KX369586
1191	VC_325_40	2	126.3905118	31657.62837	0.003976543	0.044256591	8.485281374	8.485281374	1	1	1	8.48528137	0	0	0	KP027205,MH697581

1192	VC_325_41	2	117.7833678	32923.66532	0.003564716	0.059288714	8.774964387	8.774964387	1	1	1	8.77496439	0	0	0	JF937100,MK112530
1193	VC_325_42	4	697.3816868	61478.73303	0.011216231	0.000195584	7.416198487	9.16515139	6	4	0.666666667	8.55261741	0	0	0	JN699016,JX307705,KT363731,MN119377
1194	VC_325_43	1	0	16009.35792	0	0	0	0	1	1	1	0	0	0	0	KX712238
1195	VC_325_44	3	721.8511321	45143.26067	0.015738567	0.00140829	3.741657387	5.916079783	3	3	1	5.01430066	0	0	0	EU744251,JN660814,MH669011
1196	VC_325_45	1	0	16839.78449	0	0	0	0	1	1	1	0	0	0	0	MK524525
1197	VC_325_46	2	135.5831604	33414.17532	0.004041256	0.042483835	8.485281374	8.485281374	1	1	1	8.48528137	0	0	0	MH576975,MK967401
1198	VC_325_47	2	127.1045418	28153.53666	0.004494401	0.042490803	8.366600265	8.366600265	1	1	1	8.36660027	0	0	0	JN699000,MG962370
1199	VC_325_48	2	117.2664193	29691.13608	0.003934005	0.049910593	8.426149773	8.426149773	1	1	1	8.42614977	0	0	0	KY213952,MK112540
1200	VC_325_49	4	886.5199348	62344.19895	0.014020399	4.04E-05	0	8.94427191	6	6	1	7.26307929	0	0	0	AP018477,AP018478,KF416340,MH632117
1201	VC_325_5	11	7704.904691	156370.1075	0.046959647	3.05E-34	6.403124237	9.949874371	55	42	0.763636364	8.50990937	0	0	0	AY500152,JF937108,JF937110,KJ194585,KT626047,MG872833,MG925344,MG962372,MK359354,MN585999,MT310897
1202	VC_325_50	4	1246.485376	67358.66735	0.018168976	1.22E-05	0	8.544003745	6	6	1	5.736322	0	0	0	MG812486,MG812489,MG812495,MG872836
1203	VC_325_51	2	129.7220855	33340.9662	0.003875692	0.043361739	8.426149773	8.426149773	1	1	1	8.42614977	0	0	0	KJ690250,MN585991
1204	VC_325_52	5	1187.163243	79335.26088	0.014743263	9.27E-06	7.211102551	9.327379053	10	7	0.7	8.63969743	0	0	0	JN153085,MH230878,MH697575,MN062710,MN234212
1205	VC_325_53	7	2915.34069	117417.2785	0.024227352	1.13E-12	5.916079783	9.539392014	21	19	0.904761905	8.24863931	0	0	0	KT388014,KX550443,MH020236,MH399782,MH590595,MK359300,MK359304
1206	VC_325_54	1	0	17015.92601	0	0	0	0	1	1	1	0	0	0	0	KY224001
1207	VC_325_55	2	181.3958628	29798.92568	0.006050498	0.042490047	7	7	1	1	1	7	0	0	0	MK305893,MZ355722
1208	VC_325_56	2	116.558312	32060.34917	0.003622421	0.053952016	9	9	1	0	0	9	0	0	0	MF919508,MN585997
1209	VC_325_57	1	0	17230.06883	0	0	0	0	1	1	1	0	0	0	0	ON208829
1210	VC_325_58	1	0	15914.13453	0	0	0	0	1	1	1	0	0	0	0	ON970624
1211	VC_325_59	1	0	15893.16976	0	0	0	0	1	1	1	0	0	0	0	MT771340
1212	VC_325_6	1	0	16105.01703	0	0	0	0	1	1	1	0	0	0	0	MN444869
1213	VC_325_60	2	125.0356719	32679.04259	0.003811589	0.042486995	8.660254038	8.660254038	1	1	1	8.66025404	0	0	0	JN687951,MH338239
1214	VC_325_61	8	4997.55715	121440.9436	0.039525596	1.66E-19	0	9.695359715	28	22	0.785714286	7.44194501	0	0	0	JN153086,JN698998,MG925340,MG925342,MH450133,MH479919,MK310142,MT639644
1215	VC_325_62	3	887.7068008	46311.90201	0.018807503	0.001408061	1.732050808	3.16227766	3	3	1	2.63144282	0	0	0	OL455898,OM105884,OP021677
1216	VC_325_7	3	411.0483533	47825.20079	0.008521565	0.002602184	8	9.219544457	3	2	0.666666667	8.66483628	0	0	0	KR997929,KR997934,MG944221
1217	VC_325_8	2	300	32088.76977	0.00926247	0.042487323	2	2	1	1	1	2	0	0	0	KF024726,KP027204
1218	VC_325_9	2	182.0366701	32716.06605	0.005533349	0.042490663	7.348469228	7.348469228	1	1	1	7.34846923	0	0	0	AY500153,KT438500
1219	VC_326_0	1	0	16.42479477	0	0	0	0	1	1	1	0	0	0	0	MW202895
1220	VC_327_0	12	807.2837481	97.27810752	0.892458313	4.46E-12	0	2	66	66	1	1.23826871	0	0	0	AF306496,MH992158,MH992160,MH992177,MH992178,MH992189,MH992191,MH992194,MH992197,MH992207,MH992218,MW202535
1221	VC_328_0	11	16500	18554.53226	0.470695198	7.57E-37	0	3	55	55	1	2.23376765	0	0	0	EU710883,FQ482083,HQ728263,MG589384,MH426725,MK759884,MK770412,MN604407,OK483342,OM522317,ON706965
1222	VC_329_0	2	263.4920828	51733.14663	0.005067483	0.04535418	8.185352772	8.185352772	1	1	1	8.18535277	0	0	0	MG646672,MH586731
1223	VC_329_1	17	34199.01442	360377.2376	0.086672764	1.42E-57	1.732050808	10.24695077	136	112	0.823529412	7.47264463	0	0	0	AF320576,JF461087,JX181822,KP143762,KY626162,MF370225,MG251391,MG251392,MG969405,MG969406,MK965969,MK965970,MW006474,MW006475,MW006482,MZ327261,MZ327262
1224	VC_329_10	1	0	24312.36389	0	0	0	0	1	1	1	0	0	0	0	MN994498
1225	VC_329_11	15	26960.41785	313734.9721	0.079133498	1.00E-62	0	9.273618495	105	98	0.933333333	7.98197884	0	0	0	KP010413,MG646669,MG646670,MG646671,MN227145,MN481367,MN655999,MW311372,MW321605,MW362867,MZ150758,OL474141,OL800606,OL825705,ON239128
1226	VC_329_12	2	186.965864	45589.79227	0.004084297	0.273945336	8.888194417	8.888194417	1	1	1	8.88819442	0	0	0	MG065664,OK272472
1227	VC_329_13	1	0	25806.9484	0	0	0	0	1	1	1	0	0	0	0	KM657822
1228	VC_329_14	1	0	24594.96613	0	0	0	0	1	1	1	0	0	0	0	MG969404
1229	VC_329_15	1	0	34625.44868	0	0	0	0	1	1	1	0	0	0	0	GU169904
1230	VC_329_16	4	1634.886699	102137.8843	0.015754486	1.93E-05	6.782329983	8.888194417	6	6	1	8.30742248	0	0	0	KF055347,KX405003,MT409176,MW423797
1231	VC_329_17	4	1610.40083	97479.13649	0.016251976	3.75E-05	2	8.888194417	6	6	1	6.82129153	0	0	0	KM092515,MT322327,OK040806,OK040807
1232	VC_329_18	7	5635.440122	166132.2487	0.032808499	4.90E-13	5.830951895	9.486832981	21	17	0.80952381	8.10020853	0	0	0	JX560968,MK524176,MN882542,MT833282,MT887289,MW296865,ON540300
1233	VC_329_19	1	0	25560.90188	0	0	0	0	1	1	1	0	0	0	0	ON868915
1234	VC_329_2	2	255.117896	51899.91047	0.00489153	0.044399149	8.366600265	8.366600265	1	1	1	8.36660027	0	0	0	MN850631,MN850637
1235	VC_329_20	4	1737.835029	98200.81716	0.017389018	1.22E-05	0	7.615773106	6	6	1	4.27929107	0	0	0	LC739530,LC739531,LC739532,ON210138
1236	VC_329_21	1	0	23252.27476	0	0	0	0	1	1	1	0	0	0	0	MH051914
1237	VC_329_22	5	2764.613608	122697.3371	0.022035474	6.49E-08	4.582575695	8.94427191	10	10	1	6.70791858	0	0	0	MZ311863,OM386661,OM386663,OM386664,OM386665
1238	VC_329_3	24	69063.49042	503433.1308	0.12063563	3.77E-140	6.557438524	10.04987562	276	201	0.72826087	8.6040649	0	0	0	KX377933,LR880803,MK962758,MN850564,MN850566,MN850577,MN850580,MN850585,MN850603,MN850605,MN850610,MN850611,MN850614,MN850619,MN850633,MN850635,MN850636,MN850639,MN850644,MN850647,MZ501086,OK272490,OM630585,OX016465
1239	VC_329_4	2	273.6694968	52415.17981	0.005194069	0.042535462	8.366600265	8.366600265	1	1	1	8.36660027	0	0	0	JN225449,KR014248
1240	VC_329_5	1	0	25567.08366	0	0	0	0	1	1	1	0	0	0	0	MN994499
1241	VC_329_6	3	838.9145994	77680.48507	0.01068417	0.001665311	5	8.485281374	3	3	1	7.18251304	0	0	0	LR597658,MZ726791,MZ726792
1242	VC_329_7	18	40984.24225	395811.3452	0.093829341	6.99E-90	1	10.04987562	153	119	0.777777778	7.86997931	0	0	0	EU877232,KP869100,KP869106,KP869109,KP869110,KP869113,KT184313,KT184314,KT184315,KT184316,LC519489,LR699048,MH571750,MK482688,MK482689,MK482690,MT446388,MW481326
1243	VC_329_8	2	242.1912643	49736.22644	0.004845917	0.043459863	8.485281374	8.485281374	1	1	1	8.48528137	0	0	0	LC473039,MN994503
1244	VC_329_9	1	0	25368.62508	0	0	0	0	1	1	1	0	0	0	0	LR597663
1245	VC_32_0	1	0	643.5305745	0	0	0	0	1	1	1	0	0	0	0	MH443100
1246	VC_330_0	27	102495.595	98965.79554	0.508760486	3.75E-212	0	9.591663047	351	304	0.866096866	6.13008013	0	0	0	KM236239,KT001916,KT363872,KT454805,KT454806,KY654690,MF158040,MF158041,MF158043,MF158044,MF327003,MF327004,MF327005,MH791400,MH791413,MH920362,MK562502,MK562504,MK562505,MN342151,MN850587,OK539913,OM953433,ON528729,ON528730,ON528731,ON528732
1247	VC_331_0	4	1800	18562.65746	0.088397107	1.23E-05	0	4.898979486	6	6	1	4.03090892	0	0	0	MK290737,MK290738,MN270891,MN270892
1248	VC_332_0	3	900	21002.73684	0.041090755	0.001412463	3.605551275	4.795831523	3	3	1	4.2146745	0	0	0	MW749004,MW749010,OK283618
1249	VC_333_0	25	29999.05292	34086.20443	0.468111609	1.52E-169	0	8.485281374	300	300	1	6.22138209	0	0	0	FN391954,KT337342,KY065453,KY065454,KY065458,KY065459,KY065460,KY065461,KY065462,KY065463,KY065469,KY065470,KY065471,KY065494,KY065495,KY065499,MK448732,MK448739,MK448901,MK448912,MK448914,MK448918,MK448920,MT900487,OL774868
1250	VC_334_0	1	0	802.8355074	0	0	0	0	1	1	1	0	0	0	0	MK449000
1251	VC_335_0	6	2297.296305	3949.529958	0.367754154	2.58E-11	0	5.656854249	15	15	1	3.14148763	0	0	0	MK448804,MK448805,MK448807,MK448808,MK448815,MK448991
1252	VC_337_0	6	1712.736173	1114.460314	0.605807266	1.15E-10	3.741657387	7.280109889	15	15	1	6.10969157	0	0	0	MT980836,MT980837,MT980838,MT980839,MT980840,MT980841
1253	VC_338_0	5	206.4548077	417.509107	0.330876198	1.31E-07	4.472135955	7.745966692	10	10	1	6.59036485	0	0	0	S12-wms-combined_120133,S17-wms-combined_130215,S2-wms-combined_55495,S6-wms-combined_118484,S9-wms-combined_36459
1254	VC_339_0	9	981.738133	62.08083222	0.940525288	2.29E-06	0	5	36	36	1	3.72198459	0	0	0	AF323671,LT985572,LT985581,LT985584,LT985586,LT985587,LT985593,LT993227,LT996080
1255	VC_33_0	4	369.5376562	5786.734801	0.060026202	5.17E-05	6.633249581	7.483314774	6	6	1	6.99114485	0	0	0	HQ201307,JQ182729,MH178096,MN688132
1256	VC_340_0	31	26290.12297	1240.327757	0.95494706	1.21E-54	0	8.831760866	465	465	1	7.15123817	0	0	0	AF338467,AP013538,AP013539,AY939843,EF372997,GU071093,GU071100,GU071102,GU071104,GU071107,GU943054,HM559717,HQ316584,HQ317388,HQ317389,HQ332137,HQ332140,HQ337022,HQ633062,HQ634152,HQ634188,KC310802,KC310803,KC310804,KC310805,KC310806,MH540083,MK016662,MT424636,MW015080,MZ803112
1257	VC_341_0	3	29.54426776	87.58841794	0.252229065	0.0603454	6.164414003	8.246211251	3	3	1	7.16056436	0	0	0	AF396866,MW202489,OK085710
1258	VC_342_0	2	54.63205894	245.9994971	0.1817243	0.065885926	8	8	1	1	1	8	0	0	0	MN988526,MN988556
1259	VC_344_0	21	63000	52890.80955	0.543615152	5.93E-76	0	7.348469228	210	210	1	4.62905152	0	0	0	AF399011,AP019418,JX233784,KU521356,LC727695,LC727696,LC727699,LC727701,MF805716,MN563784,MN871480,MN871489,MT133560,MT995926,MT995927,MT995928,MW057919,MZ444140,ON631220,OP310983,OP310984
1260	VC_344_1	3	900	24157.56909	0.035917291	0.066511228	2.828427125	3.464101615	3	3	1	3.04031862	0	0	0	MK599315,MN103543,OU343167
1261	VC_344_2	1	0	7196.500975	0	0	0	0	1	1	1	0	0	0	0	MF063068
1262	VC_344_3	1	0	8153.192185	0	0	0	0	1	1	1	0	0	0	0	MK552327
1263	VC_344_4	1	0	8072.992379	0	0	0	0	1	1	1	0	0	0	0	EU197055
1264	VC_344_5	1	0	8311.146891	0	0	0	0	1	1	1	0	0	0	0	MF042360
1265	VC_344_6	1	0	8637.082971	0	0	0	0	1	1	1	0	0	0	0	HQ630627
1266	VC_345_0	2	300	4078.759555	0.068512554	0.043458474	3.605551275	3.605551275	1	1	1	3.60555128	0	0	0	AP017924,AP017925
1267	VC_346_0	4	1800	2107.678745	0.46063152	2.09E-05	0	3.464101615	6	6	1	1.73205081	0	0	0	MK308674,MK308675,MK308676,MK308677
1268	VC_346_1	2	300	2122.465727	0.123840761	0.046465516	2.828427125	2.828427125	1	1	1	2.82842712	0	0	0	MT811961,OM319461
1269	VC_346_2	1	0	1337.595263	0	0	0	0	1	1	1	0	0	0	0	OV032902
1270	VC_346_3	1	0	1409.942802	0	0	0	0	1	1	1	0	0	0	0	MH595538
1271	VC_347_0	3	900	4924.541563	0.154518599	0.001555396	6.403124237	6.782329983	3	3	1	6.58096425	0	0	0	LR881104,MN871444,MN871445
1272	VC_348_0	22	19891.82467	825.1353317	0.960171023	7.82E-21	1	7.810249676	231	231	1	5.56966756	0	0	0	AJ344259,AJ414696,AJ748296,AJ875026,FM164764,KP282675,KX712143,KY744228,KY744229,KY744230,KY744231,KY744232,KY744233,KY744234,KY744235,MK064565,MN876841,MN876842,MN876843,OM401712,OM401713,OM401714
1273	VC_349_0	7	2570.327721	174.7307766	0.936347157	3.52E-08	0	8.660254038	21	21	1	6.02974406	0	0	0	AF440571,AM087120,AM087121,AM087122,AM087123,EU545650,KX467643
1274	VC_34_0	4	1015.147178	1318.866937	0.434936178	1.94E-05	1.732050808	4.358898944	6	6	1	3.20366374	0	0	0	AY374448,CP038007,CP072507,KY271402
1275	VC_350_0	2	78.44058844	163.6877283	0.323962887	0.061345549	3.605551275	3.605551275	1	1	1	3.60555128	0	0	0	AJ854042,MK064564
1276	VC_351_0	3	122.3463943	15.13147339	0.889935204	0.018444213	5.477225575	7.141428429	3	3	1	6.06213946	0	0	0	AM419438,HQ332141,MW344765
1277	VC_352_0	3	672.0697793	247.8105226	0.730605686	0.011327247	1	6.557438524	3	3	1	4.67939307	0	0	0	AF440695,MK002701,MK450543
1278	VC_353_0	6	2137.634998	1784.506164	0.545017354	2.23E-11	0	7.874007874	15	15	1	6.18823565	0	0	0	AF447491,AY453853,CP000625,OK095358,OM892879,OP293079
1279	VC_354_0	4	647.2701708	3053.520662	0.174900501	1.32E-05	7.549834435	9.055385138	6	5	0.833333333	8.23005365	0	0	0	MN512538,MN812722,MT227924,OP125534
1280	VC_355_0	2	14.28598213	264.1878418	0.051300987	0.065816124	7.280109889	7.280109889	1	1	1	7.28010989	0	0	0	MT121967,MW651859
1281	VC_356_0	2	52.50222215	211.042249	0.199215798	0.067889051	6.8556546	6.8556546	1	1	1	6.8556546	0	0	0	HQ634187,MK105819
1282	VC_357_0	53	125880.8187	14320.20516	0.897859482	0	0	6.633249581	1378	1378	1	2.52389239	0	0	0	MT740734,ON170024,ON170027,ON170031,ON170052,ON170057,ON170058,ON170068,ON170071,ON170075,ON170077,ON170079,ON170129,ON170133,ON170134,ON170136,ON170150,ON170172,ON170175,ON170179,ON170181,ON170182,ON170185,ON170187,ON170208,ON170214,ON170228,ON170229,ON170232,ON170234,ON170236,ON170238,ON170240,ON170242,ON170254,ON170257,ON170262,ON170264,ON170265,ON170267,ON170275,ON170279,ON170286,ON170287,ON170303,ON170317,ON170323,ON170324,ON170326,ON170327,ON170343,ON170355,ON170359
1283	VC_358_0	3	146.9906332	138.6304012	0.514635183	0.023427089	0	0	3	3	1	0	0	0	0	ON170149,ON170171,ON170322
1284	VC_359_0	7	2542.15543	4323.533868	0.370269513	4.92E-14	0	8.774964387	21	21	1	6.84867358	0	0	0	JN585957,MF612073,MH426726,MK618715,MT251347,MT251348,MW722080
1285	VC_35_0	6	1238.457604	4255.358782	0.225427557	6.80E-11	5.916079783	9.327379053	15	14	0.933333333	7.84472984	0	0	0	KY271399,MK416008,MK416013,MK416022,MK448234,MK448237
1286	VC_35_1	1	0	780.8240063	0	0	0	0	1	1	1	0	0	0	0	OL617042
1287	VC_360_0	358	933940.8583	37349.42269	0.961546591	0	0	2.449489743	63903	63903	1	1.24209469	0	0	0	AF454431,AY751298,DQ079896,DQ079897,DQ079899,DQ079900,DQ079901,DQ079902,DQ079903,DQ079904,DQ079906,DQ079908,DQ079909,GQ153920,GQ153922,GQ153923,JF719729,JF719730,JF719731,KX266300,KX266301,KX266302,KX266303,KX266304,KX266305,KX266306,KX266307,KX266308,KX266309,KX266310,KX266311,KX266312,KX266313,KX266314,KX266315,KX266316,KX266317,KX266318,KX266319,KX266320,KX266321,KX266322,KX266323,KX266324,KX266325,KX266326,KX266327,KX266328,KX266329,KX266330,KX266331,KX266332,KX266333,KX266334,KX266335,KX266336,KX266337,KX266338,KX266339,KX266340,KX266341,KX266342,KX266343,KX266344,KX266345,KX266346,KX266347,KX266348,KX266349,KX266350,KX266351,KX266352,KX266353,KX266354,KX266355,KX266356,KX266357,KX266358,KX266359,KX266360,KX266361,KX266362,KX266363,KX266364,KX266365,KX266366,KX266367,KX266368,KX266369,KX266370,KX266371,KX266372,KX266373,KX266374,KX266375,KX266376,KX266377,KX266378,KX266379,KX266380,KX266381,KX266382,KX266383,KX266384,KX266385,KX266386,KX266387,KX266388,KX266389,KX266390,KX266391,KX266392,KX266393,KX266394,KX266395,KX266396,KX266397,KX266398,KX266399,KX266400,KX266401,KX266402,KX266403,KX266404,KX266405,KX266406,KX266407,KX266408,KX266409,KX266410,KX266411,KX266412,KX266413,KX266414,KX266415,KX266416,KX266417,KX266418,KX266419,KX266420,KX266421,KX266422,KX266423,KX266424,KX266425,KX266426,KX266427,KX266428,KX266429,KX266430,KX266431,KX266432,KX266433,KX266434,KX266435,KX266436,KX266437,KX266438,KX266439,KX266440,KX266441,KX266442,KX266443,KX266444,KX266445,KX266446,KX266447,KX266448,KX266449,KX266450,KX266451,KX266452,KX266453,KX266454,KX266455,KX266456,KX266457,KX266458,KX266459,KX266460,KX266461,KX266462,KX266463,KX266464,KX266465,KX266466,KX266467,KX266468,KX266469,KX266470,KX266471,KX266472,KX266473,KX266474,KX266475,KX266476,KX266477,KX266478,KX266479,KX266480,KX266481,KX266482,KX266483,KX266484,KX266485,KX266486,KX266487,KX266488,KX266489,KX266490,KX266491,KX266492,KX266493,KX266494,KX266495,KX266496,KX266497,KX266498,KX266499,KX266500,KX266501,KX266502,KX266503,KX266504,KX266505,KX266506,KX266507,KX266508,KX266509,KX266510,KX266511,KX266512,KX266513,KX266514,KX266515,KX266516,KX266517,KX266518,KX266519,KX266520,KX266521,KX266522,KX266523,KX266524,KX266525,KX266526,KX266527,KX266528,KX266529,KX266530,KX266531,KX266532,KX266533,KX266534,KX266535,KX266536,KX266537,KX266538,KX266539,KX266540,KX266541,KX266542,KX266543,KX266544,KX266545,KX266546,KX266547,KX266548,KX266549,KX266550,KX266551,KX266552,KX266553,KX266554,KX266555,KX266556,KX266557,KX266558,KX266559,KX266560,KX266561,KX266562,KX266563,KX266564,KX266565,KX266566,KX266567,KX266568,KX266569,KX266570,KX266571,KX266572,KX266573,KX266574,KX266575,KX266576,KX266577,KX266578,KX266579,KX266580,KX266581,KX266582,KX266583,KX266584,KX266585,KX266586,KX266587,KX266588,KX266589,KX266590,KX266591,KX266592,KX266593,KX266594,KX266595,KX266596,KX266597,KX266598,KX266599,KX266600,KX266601,KX266602,KX266603,KX266604,KX266605,KX266606,KX266607,KX266608,KX266609,KX266610,KX266611,KX266612,KX266613,KX266614,KX266615,KX266616,KX266617,KX266618,KX266619,KX266620,KX266621,KX266622,KX266623,KX266624,KX266625,KX266626,KX266627,KX266628,KX266629,KX266630,KX266631,KX266632,KX266633,KX266634,KX266635,KX266636,KX266637,MG563770
1288	VC_362_0	2	9.319160538	4638.912396	0.002004883	0.166922652	2	2	1	1	1	2	0	0	0	GQ153921,KC237308
1289	VC_363_0	2	28.83930013	7.11248825	0.80216594	0.270145687	0	0	1	1	1	0	0	0	0	OK338687,OM055665
1290	VC_364_0	16	23058.60561	12465.34109	0.649100332	4.60E-59	0	7.810249676	120	120	1	3.92678842	0	0	0	AP008986,KY210139,MG944227,MG944228,MG944229,MG944230,MG944231,MG944232,MG944233,MG944234,MG944235,MG944236,MN461279,OK275494,ON996340,OP067662
1291	VC_369_0	6	2622.442153	1804.123778	0.592432643	1.77E-10	0	8.062257748	15	15	1	5.46937234	0	0	0	JQ177063,KY290953,KY290954,MH179474,MK804891,MK804892
1292	VC_371_0	2	178.4560001	56108.12328	0.003170489	0.052680276	8.717797887	8.717797887	1	1	1	8.71779789	0	0	0	MT897903,ON456342
1293	VC_371_1	11	10733.0163	272398.5923	0.037908224	3.31E-34	0	10.04987562	55	47	0.854545455	7.96321926	0	0	0	KJ595576,KM347890,KR997967,KY006474,MH926060,MK279910,MK524512,MK524526,MN585967,MN586058,MW534377
1294	VC_371_10	1	0	26257.54059	0	0	0	0	1	1	1	0	0	0	0	MH590594
1295	VC_371_11	31	82352.98288	806460.386	0.092654978	3.56E-148	0	10.63014581	465	326	0.701075269	8.52719185	0	0	0	KX592589,MF155947,MF668276,MF919528,MF919539,MG757159,MH230874,MH513980,MH590588,MK112539,MK112546,MK112551,MK279843,MK279865,MK279870,MK279873,MK279878,MK279894,MK279895,MK279897,MN369744,MT310868,MT316460,MT316463,MW534381,MW712720,MW862980,MW862993,MZ648038,ON637769,OP434459
1296	VC_371_12	4	1262.830498	99281.29446	0.012559963	1.51E-05	4.242640687	9.055385138	6	5	0.833333333	7.5280735	0	0	0	MK112544,MT310867,MT952849,ON456332
1297	VC_371_13	33	106710.7158	489555.7132	0.178964823	0	0	10.09950494	528	463	0.876893939	7.68566569	0	0	0	JN192463,KM363597,KR029086,KR029087,KX576645,KX576646,KX576647,KX702319,KY385380,KY385382,KY385383,MF919511,MG757164,MG757165,MG944223,MG962363,MH051251,MH155874,MH316568,MH371107,MH479916,MH479918,MH513973,MH576958,MK279850,MK279858,MK279864,MK279883,MK279890,MK279891,MN585965,MN945903,ON456330
1298	VC_371_14	1	0	26472.93987	0	0	0	0	1	1	1	0	0	0	0	MN369738
1299	VC_371_15	44	166408.6136	1030233.935	0.139062925	0	4.123105626	10.19803903	946	707	0.747357294	8.47829338	0	0	0	GQ303264,GQ303266,JF704103,JF704109,JF937097,JF957056,JN006063,JN638752,JN638753,JN699008,JN699010,KM408320,KP027208,KT599441,KU867907,KX578071,KX670813,KX683292,KX683293,KY676783,MG770212,MG925346,MG925350,MG944225,MH399775,MH399786,MH576970,MH651184,MH825702,MH825705,MK112527,MK112536,MK112555,MK279855,MK279859,MK279861,MK279866,MK279879,MK279881,MK279882,MK279885,MK279887,MK279888,MW712726
1300	VC_371_16	42	154118.0091	994987.1284	0.134120024	1.58E-278	0	10.19803903	861	627	0.728222997	8.4720456	0	0	0	AF547430,DQ398046,FJ174694,GU247133,GU247134,JF704102,JN699009,JX649096,JX649097,JX649100,KJ194580,KJ194583,KX369585,KX620786,MG757158,MG925348,MG962365,MH371114,MH371117,MH371125,MH399773,MH399780,MH450116,MH450122,MH479914,MH479921,MH651175,MH651186,MH727555,MH744417,MH779516,MK112542,MK112553,MK279846,MK279852,MK279871,MK494104,MN945899,MT897909,MW534379,MW534380,MZ622173
1301	VC_371_17	3	668.3153086	87629.97615	0.007568836	0.001883237	0	8.366600265	3	3	1	5.57773351	0	0	0	JF937095,JN698990,JN699017
1302	VC_371_18	10	8923.016739	286616.4264	0.030192304	7.83E-23	0	9.433981132	45	36	0.8	7.68111969	0	0	0	JF704091,JF937109,JN698989,KJ194579,KP027197,KP027209,KR816508,KX576643,KY965066,MT897901
1303	VC_371_19	1	0	28762.5234	0	0	0	0	1	1	1	0	0	0	0	MZ622172
1304	VC_371_2	8	5309.103892	223490.6376	0.023204152	8.79E-17	6.32455532	9.486832981	28	24	0.857142857	8.05241003	0	0	0	KC576784,KJ567044,MG925356,MG962375,MH077582,MK112552,MK279889,MN096366
1305	VC_371_20	1	0	29066.00238	0	0	0	0	1	1	1	0	0	0	0	MN703415
1306	VC_371_21	2	167.8782629	50758.67292	0.003296478	0.042292629	8.888194417	8.888194417	1	1	1	8.88819442	0	0	0	JN006064,KR781349
1307	VC_371_3	22	43298.56312	507088.4314	0.078669306	5.02E-136	5.477225575	9.695359715	231	209	0.904761905	8.01523596	0	0	0	GQ303259,JF704099,JX649098,JX649099,KC661274,KC691258,KF713485,KJ174157,KJ538723,KM236502,MF919503,MF919507,MF919523,MF919530,MG925339,MG962362,MH230875,MH779500,MK279877,MK279908,MN444868,MT897902
1308	VC_371_4	5	1880.778604	137693.1926	0.013475139	2.86E-07	0	8.888194417	10	10	1	7.61835872	0	0	0	KT364588,MH576954,MH576967,MH651177,MK279904
1309	VC_371_5	2	179.2875557	51487.76121	0.003470056	0.042289286	8.602325267	8.602325267	1	1	1	8.60232527	0	0	0	MH051264,MK310139
1310	VC_371_6	1	0	20520.99468	0	0	0	0	1	1	1	0	0	0	0	MF919519
1311	VC_371_7	1	0	25721.24865	0	0	0	0	1	1	1	0	0	0	0	ON970594
1312	VC_371_8	1	0	24054.69344	0	0	0	0	1	1	1	0	0	0	0	MZ648041
1313	VC_371_9	4	1095.139234	99094.75905	0.010930635	2.34E-05	6.244997998	9.38083152	6	4	0.666666667	8.31236511	0	0	0	MT776813,MT889369,MZ681520,ON970577

1314	VC_372_0	24	79908.38324	64356.26351	0.553901354	7.53E-144	0	6.244997998	276	276	1	3.62715277	0	0	0	DQ398044,EU770221,JF704094,JF704104,JN699011,KF279411,KF279412,KF279413,KF493881,KR080194,KR080200,KT270441,KX589269,MH513970,MK524486,MN234171,MN369759,MN428050,MT310870,MT639648,MT818419,MT897910,MW435857,ON970575
1315	VC_372_1	9	9132.603158	37934.01294	0.194035686	3.62E-24	0	8.246211251	36	36	1	5.08013553	0	0	0	JN699007,KF024727,KF416341,KR080199,KY224000,MH727561,MK524490,MN284893,MT889381
1316	VC_372_2	5	1779.442795	22945.72888	0.071968875	3.18E-08	5.656854249	9.055385138	10	9	0.9	7.5712574	0	0	0	MH744423,MK059749,MN617843,MW055906,OL455893
1317	VC_372_3	5	2966.428379	23298.26406	0.11294358	3.30E-08	0	4.472135955	10	10	1	3.7791889	0	0	0	KF024729,KJ433973,KJ433974,KJ433975,KJ433976
1318	VC_372_4	3	523.2325138	15340.53732	0.032982861	0.001467429	6.633249581	8.831760866	3	3	1	7.98343061	0	0	0	MN813693,MZ150786,OP297541
1319	VC_373_0	10	5782.863642	1957.950674	0.747061408	1.69E-21	2	6	45	45	1	4.77180584	0	0	0	AJ006589,AJ550940,JX182372,MK433266,MK450421,MK450426,MK450431,MK450433,MK686068,MK686069
1320	VC_374_0	2	98.89526051	687.0879377	0.125823632	0.044216833	4.123105626	4.123105626	1	1	1	4.12310563	0	0	0	GQ379227,MK937595
1321	VC_375_0	3	331.4591376	1005.620557	0.247897817	0.001927136	0	6.708203932	3	3	1	4.47213595	0	0	0	MG593800,MG593803,MH001457
1322	VC_376_0	3	385.8139242	1228.980825	0.238924436	0.002100806	4	4.358898944	3	3	1	4.16066819	0	0	0	MT701593,MT701595,MT701596
1323	VC_377_0	3	102.0180316	159.1354209	0.390644009	0.002149445	6.08276253	7.416198487	3	3	1	6.85667628	0	0	0	KR905068,KU848187,LC625742
1324	VC_378_0	9	4816.186316	1518.1514	0.760329893	2.30E-17	1.732050808	7.937253933	36	36	1	6.5363143	0	0	0	AJ242593,DQ003637,KJ094022,KP399678,MH341452,MZ361825,MZ361826,MZ361827,ON456151
1325	VC_379_0	5	898.198954	981.3357567	0.477883675	7.73E-08	4.242640687	9	10	9	0.9	7.45893136	0	0	0	AJ312240,DQ003639,JX120799,KJ094023,MT500540
1326	VC_37_0	5	1656.167192	1414.830112	0.539292949	1.31E-07	1.414213562	7.071067812	10	10	1	5.61087099	0	0	0	OM046621,OM046622,OM046623,OM046625,OM046629
1327	VC_37_1	3	144.2631276	1588.359613	0.083262862	0.051536502	8.94427191	8.94427191	3	3	1	8.94427191	0	0	0	KU052037,MN013086,OL396571
1328	VC_380_0	3	237.7284585	1220.057724	0.163074984	0.003249027	7.071067812	7.874007874	3	3	1	7.56368079	0	0	0	DQ003642,KP399677,MH341451
1329	VC_381_0	2	55.31844079	612.2201767	0.082869274	0.046186082	6.92820323	6.92820323	1	1	1	6.92820323	0	0	0	GU323708,HQ141410
1330	VC_385_0	51	161709.5524	23022.52987	0.875373408	0	0	6.164414003	1275	1275	1	4.16327379	0	0	0	AJ251805,AJ318471,EU547803,JX421753,KC960671,KM253764,KP313531,KP791805,KP791806,KP791807,KT008108,KU687347,KU687348,KX130726,KX452696,KX778611,LC730324,MF285616,MF285617,MF285620,MH717709,MK608335,MN013074,MN149904,MT419366,MT813142,MW296877,MW600722,MZ359670,MZ428229,OK149215,OL362281,OL362282,OL362283,OL691920,OL744209,OL744212,OL744217,OL800605,ON528720,ON528721,ON528722,ON528723,ON528724,ON950090,OP617747,OX335419,OX335420,OX335431,OX335434,OX335437
1331	VC_386_0	49	120746.5472	31298.5354	0.794149637	0	0	7	1176	1176	1	5.00025007	0	0	0	AM183667,EU734170,EU734171,FJ194439,GQ468526,HQ333270,JQ965702,KF700371,KM366096,KP090453,KP090454,KY084243,LT960606,LT961836,LT961837,LT961838,LT961840,LT961841,LT961842,LT961843,LT961844,LT961845,LT961846,LT962379,LT962380,LT962475,LT962906,LT962907,MG471392,MH031343,MH809534,MK685668,MK907285,MN080711,MN252582,MT374859,MT515757,MT542512,MT625440,MW147603,MW367418,MW767996,MZ501055,MZ501064,OK663600,OL800603,OL856139,ON754979,ON922920
1332	VC_387_0	3	366.9510029	8293.443365	0.042371165	0.001384107	3.16227766	4.898979486	3	3	1	4.1777977	0	0	0	HQ728265,MN450150,ON715521
1333	VC_388_0	4	596.9021897	16696.71238	0.034515757	1.19E-05	1	7	6	6	1	5.26825212	0	0	0	KT321314,ON602733,OX335378,OX335441
1334	VC_389_0	3	539.8748266	9940.81592	0.051511378	0.001384431	1	1.732050808	3	3	1	1.38208812	0	0	0	MF979561,MW001769,ON995367
1335	VC_38_0	3	900	0	1	1	3.741657387	4.898979486	3	3	1	4.44368421	0	0	0	AB231700,KF356199,OP394178
1336	VC_390_0	4	693.1883555	14133.57017	0.046752522	1.18E-05	3.872983346	5.744562647	6	6	1	4.97314905	0	0	0	EU652770,KX078568,MW358931,OK499982
1337	VC_392_0	126	892534.6732	35195.3221	0.962062968	0	0	7.280109889	7875	7875	1	4.59053969	0	0	0	AY247822,AY264774,AY264775,AY264776,AY264777,AY264778,EU734174,FR667955,GU071091,JQ957925,JQ965700,JQ965701,JQ965703,JX000007,JX872508,KM242061,KU927499,LC730325,LR745706,LR745707,LR745708,LR745709,LR745710,MG488277,MG832642,MG833025,MG966531,MH382198,MH717098,MH717099,MH779619,MK310182,MK542821,MN026740,MN098329,MN481365,MN481366,MT350293,MT543041,MT681669,MW074885,MW883060,MZ220766,MZ318361,MZ318362,MZ318363,MZ375233,MZ375234,MZ375235,MZ375236,MZ375237,MZ375238,MZ375239,MZ375240,MZ375241,MZ375242,MZ375243,MZ375244,MZ375245,MZ375246,MZ375247,MZ375248,MZ375249,MZ375250,MZ375251,MZ375252,MZ375253,MZ375254,MZ375255,MZ375256,MZ375257,MZ375258,MZ375259,MZ375260,MZ375261,MZ375262,MZ375263,MZ375264,MZ375265,MZ375266,MZ375267,MZ375268,MZ375269,MZ375270,MZ375271,MZ375272,MZ375273,MZ375274,MZ375275,MZ375276,MZ375277,MZ375278,MZ375279,MZ375280,MZ375281,MZ375282,MZ375283,MZ375284,MZ375285,MZ375286,MZ375287,MZ375288,MZ375289,MZ375290,MZ375291,MZ375292,MZ375293,MZ375294,MZ501078,MZ501081,MZ833439,OK042080,OL964739,OL964740,OL964741,OL964748,OL964749,OL964750,OM630586,ON528715,ON528716,ON528717,ON528719,ON568193,OP331230,V01146
1338	VC_393_0	7	3186.069379	14766.61339	0.177470377	2.99E-15	1.414213562	4	21	21	1	3.29063102	0	0	0	MH059639,MH460459,MN013082,MN013085,MN812693,MT822288,ON287378
1339	VC_394_0	2	162.6016698	7548.03508	0.021087969	0.042077996	2.236067977	2.236067977	1	1	1	2.23606798	0	0	0	MH370477,MT465573
1340	VC_395_0	5	1818.525602	15988.10076	0.102126341	2.43E-08	1	2.449489743	10	10	1	1.90360089	0	0	0	MN184885,MT822284,MT822285,MT822286,MT822287
1341	VC_396_0	2	111.0109439	1442.856894	0.07144169	0.042406414	4.123105626	4.123105626	1	1	1	4.12310563	0	0	0	MW057854,MW358928
1342	VC_398_0	48	7635.116631	8227.893032	0.481315765	4.49E-81	0	2.449489743	1128	1128	1	1.48586544	0	0	0	KP087939,KP087945,KT264811,MG945164,MG945682,MG945837,MH572269,MH572314,MH572315,MH572316,MH572317,MH572318,MH572319,MH572320,MH572321,MH572325,MH572326,MH572328,MH572329,MH572336,MH572351,MH572394,MH572395,MH572396,MH572397,MH572404,MH572419,MH572431,MH572439,MH572441,MH572443,MH572445,MH572446,MH572451,MH572473,MH572487,MH572492,MH572493,MH572494,MH572503,MH572510,MH572511,MH992195,MH992211,MK249143,MK249151,MK249223,MK249231
1343	VC_39_0	35	145832.3456	190456.2075	0.433652422	0	0	8.774964387	595	595	1	4.20220432	0	0	0	AF125520,AP000363,AP000422,AP004402,AP005153,AP005154,AP012529,AP012531,AP012532,AP012535,EU311208,HM208303,KJ909655,KP682372,KP682373,KP682375,KP682376,KP682377,KP682378,KP682379,KP682380,KP682382,KP682383,KP682384,KP682385,KP682386,KP682387,KP682388,KP682389,KP682390,KP682391,KP682392,KU238068,KU238069,KU238070
1344	VC_3_0	17	13843.20867	10704.70014	0.563926189	1.02E-79	0	6.557438524	136	136	1	4.89830091	0	0	0	CP000623,CP000624,CP008753,DQ087285,GU911304,HM461982,JX681814,LC528882,LC667452,MW072790,MZ375754,OM304382,OM638608,OM638609,OM638610,OM638611,ON135435
1345	VC_400_0	1	0	135.8404689	0	0	0	0	1	1	1	0	0	0	0	MH572340
1346	VC_401_0	1	0	23.24090136	0	0	0	0	1	1	1	0	0	0	0	MH572479
1347	VC_402_0	1	0	26.21969195	0	0	0	0	1	1	1	0	0	0	0	MH572512
1348	VC_403_0	20	3038.785298	1566.935839	0.659784908	3.21E-84	0	2.236067977	190	190	1	1.19888184	0	0	0	KC131023,KC131025,MH572294,MH572295,MH572296,MH572384,MH572385,MH572386,MH572387,MH572388,MH572389,MH572390,MH572391,MH572430,MH572470,MH572471,MH572472,MH572505,MH572506,MH572507
1349	VC_404_0	25	2062.264597	1588.44935	0.564893505	1.16E-22	0	2.449489743	300	300	1	1.79557655	0	0	0	MH572279,MH572300,MH572301,MH572302,MH572303,MH572305,MH572306,MH572307,MH572308,MH572309,MH572310,MH572311,MH572312,MH572313,MH572345,MH572392,MH572393,MH572406,MH572435,MH572436,MH572437,MH572469,MH572480,MH572508,MH572509
1350	VC_406_0	11	9660.292554	563.1494002	0.944915871	1.41E-27	0	6.708203932	55	55	1	5.32343751	0	0	0	KP790011,KU998237,KU998238,KY471268,KY471269,MH651189,MH697580,MN010758,MT553344,MW132713,ON260826
1351	VC_407_0	3	351.7916377	697.822307	0.335162885	0.001752088	3	5.477225575	3	3	1	4.62079679	0	0	0	AJ278322,MG324353,MG324354
1352	VC_408_0	9	6578.977811	1070.851939	0.860016239	1.54E-21	0	0	36	36	1	0	0	0	0	KY566218,KY613597,KY624610,KY624611,KY624612,KY624613,KY624614,KY624615,KY624616
1353	VC_409_0	3	71.91390314	408.9526625	0.149550641	0.017058989	7	8.426149773	3	3	1	7.49907253	0	0	0	KY203335,KY250482,MW582634
1354	VC_40_0	5	1470.851922	15953.28203	0.084414636	5.31E-08	4.123105626	5.477225575	10	10	1	4.96104245	0	0	0	LC567830,LC567838,LC567842,LC616079,ON416862
1355	VC_410_0	7	2464.03842	652.9410414	0.790521224	3.41E-13	2.449489743	7.416198487	21	21	1	6.2683484	0	0	0	MG198783,MG198784,MH271305,MH271318,MT521997,MZ681507,ON456352
1356	VC_411_0	3	75.906291	536.6301669	0.123921262	0.010136594	7	8.717797887	3	3	1	7.75587744	0	0	0	LC644972,LC644974,MT843275
1357	VC_412_0	39	54043.08926	9913.324136	0.844998748	8.06E-302	0	8.94427191	741	741	1	7.18451099	0	0	0	AJ302074,DQ113772,HF563658,JX879087,KC413988,KT337358,KT337364,KT388093,KX550065,KY065455,KY065479,KY065487,KY065490,KY065491,KY065492,KY065493,KY065501,KY065502,KY349816,MK448671,MK448708,MK448714,MK448831,MK448834,MK448861,MK448874,MK448875,MK448886,MK448887,MK448897,MK448898,MK448905,MK448925,MK448927,MK449006,MK449009,MT900488,MW495853,OL774873
1358	VC_413_0	3	189.5358541	4373.925467	0.041533354	0.001582459	7.141428429	8	3	3	1	7.69289412	0	0	0	KY065465,MK448733,MK448904
1359	VC_414_0	37	70652.39027	50121.5382	0.584997037	0	1.414213562	9.591663047	666	631	0.947447447	7.27835871	0	0	0	FR671405,FR671406,FR671407,FR671410,FR671411,HG799490,HG799496,HG799497,KJ417497,KT337339,KT337340,KT337341,KT337352,KT337354,KT337355,KT337356,KT337359,KT337362,KY065450,KY065451,KY065452,KY065456,KY065472,KY065473,KY065474,KY065475,KY065476,KY065477,KY065478,KY065480,KY065486,KY065488,KY065496,KY065497,KY065500,KY065505,OK173051
1360	VC_415_0	1	0	2039.294407	0	0	0	0	1	1	1	0	0	0	0	KY065449
1361	VC_416_0	14	8305.583211	23731.79778	0.259246635	2.19E-58	0	9	91	90	0.989010989	7.18153297	0	0	0	MK448686,MK448762,MK448788,MK448791,MK448793,MK448862,MK448949,MK448951,MK448954,MK448958,MK448959,MK448960,MK448965,MK448977
1362	VC_417_0	5	274.4500849	139.5623016	0.662903077	0.002256602	5.916079783	9.695359715	10	8	0.8	7.59008262	0	0	0	KC237729,MW478289,MZ503613,S2-wms_130687,S5-wms_49310
1363	VC_418_0	6	403.2804723	478.2549863	0.457475044	4.95E-07	6.08276253	9.848857802	15	12	0.8	8.07032403	0	0	0	LR596902,S1-wms-combined_224578,S2-wms-combined_246480,S2-wms_140933,S4-wms-combined_64632,S4-wms_62635
1364	VC_419_0	5	1149.395377	3451.249621	0.249833529	3.43E-08	1.414213562	6.8556546	10	10	1	5.80724635	0	0	0	MK448680,MK448684,MK448698,MK448769,MK448895
1365	VC_41_0	16	22058.91815	86879.96828	0.202488926	1.77E-55	0	11	120	116	0.966666667	4.86693769	0	0	0	AJ556162,JQ347801,LC727891,LC727892,LC727893,LC727894,LC727895,LC727896,LC727897,LC727898,LC727899,LC727900,LC727901,LC727902,ON470597,ON470625
1366	VC_41_1	10	7272.870373	54440.7915	0.117848628	2.75E-30	2.236067977	10	45	37	0.822222222	7.29353099	0	0	0	AP012530,AP012538,AP012539,AP012540,FJ184280,FJ188381,FM180578,LC645408,LC645425,LC645426
1367	VC_420_0	6	92.73700876	313.7146824	0.228162438	0.025379185	6.403124237	8.717797887	15	15	1	7.45694224	0	0	0	S1-wms-combined_72167,S2-wms-combined_227800,S7-wms-combined_28138,S7-wms_127801,S8-wms-combined_140059,S9-wms-combined_71647
1368	VC_422_0	5	1509.058092	4313.102866	0.259192094	2.50E-08	0	3	10	10	1	2.38362334	0	0	0	MF034659,MF166859,MT902335,MZ995503,MZ995506
1369	VC_423_0	45	172111.3091	9361.033478	0.948416197	2.31E-272	0	4.69041576	990	990	1	2.82465251	0	0	0	AJ505558,AM265638,AM910651,EU056923,EU236438,FN263372,JQ307386,JX501340,JX997978,KC969441,KM067278,KR054031,KU743887,KX711710,KY618819,LC727700,LN610574,LN610580,MH107770,MH979674,MN553585,MN553587,MN553588,MN553589,MN602045,MN615698,MN615699,MN692672,MN871455,MN871483,MN901924,MW117144,MW406975,MW406976,MZ553931,MZ687409,OK539824,OL754589,OL802210,ON857926,ON857928,ON857933,ON857935,OP292288,OP556577
1370	VC_424_0	7	1133.815378	119.8008654	0.904435774	0.0001857	0	5	21	21	1	3.30909703	0	0	0	AJ536073,AY257527,AY701338,CP013282,EU408779,MZ089978,MZ089979
1371	VC_426_0	2	157.6968698	640.3204387	0.197610839	0.043560592	4.358898944	4.358898944	1	1	1	4.35889894	0	0	0	AJ560763,GQ866233
1372	VC_426_1	3	113.8338456	874.7302244	0.115150701	0.001953848	7.937253933	8.831760866	3	3	1	8.39838819	0	0	0	CP013974,MG575421,MZ424863
1373	VC_427_0	3	30.36597811	453.6216654	0.062741226	0.031698786	7.681145748	8	3	3	1	7.87279989	0	0	0	JF974295,MW202455,S1-wms-combined_324053
1374	VC_428_0	33	123368.9251	1055.713699	0.991515236	3.33E-84	0	6	528	528	1	3.16629641	0	0	0	KM366099,KX826077,KY608966,MF740800,MH181876,MK568062,MK673511,MK770411,MK770413,MN379740,MT012730,MT074434,MT074435,MT074436,MT074437,MT074438,MT074439,MT074441,MT074442,MT074443,MT074444,MT074445,MT074446,MT663719,MT764207,MT768059,MW413353,MW508890,MW602648,MZ127825,OM313460,OM313461,OP132241
1375	VC_42_0	49	173362.7203	210421.3585	0.451719417	0	0	9.643650761	1176	1011	0.859693878	6.78952839	0	0	0	AP012534,HE664024,HG792102,HG792103,HG792104,HG792105,HG803182,HG803183,HG803184,HG803185,HQ424691,JQ011318,KF971864,KP682371,KP682374,KR781488,KU298437,KY914478,LC645409,LC645410,LC645411,LC645412,LC645413,LC645414,LC645415,LC645416,LC645417,LC645418,LC645419,LC645420,LC645421,LC645422,LC645423,LC645424,LC645427,LC645438,LC645442,LC645443,LC645444,LC645445,LC645446,LC645447,LC645448,LC645449,LC645450,LC645451,LC727903,MG710528,MN067333
1376	VC_431_0	9	2681.014496	13346.99325	0.167270601	9.75E-22	0	8.717797887	36	36	1	6.92677007	0	0	0	MK448692,MK448702,MK448711,MK448716,MK448724,MK448730,MK448744,MK448745,MK448923
1377	VC_432_0	2	300	7992.37339	0.036177821	0.043196036	0	0	1	1	1	0	0	0	0	MN695334,MN695335
1378	VC_432_1	1	0	8278.263398	0	0	0	0	1	1	1	0	0	0	0	MH412654
1379	VC_432_10	2	300	13563.39915	0.021639715	0.042289829	1.732050808	1.732050808	1	1	1	1.73205081	0	0	0	GU071098,HQ317391
1380	VC_432_11	1	0	9722.949975	0	0	0	0	1	1	1	0	0	0	0	HQ633071
1381	VC_432_12	1	0	7041.795082	0	0	0	0	1	1	1	0	0	0	0	MW015081
1382	VC_432_13	1	0	8524.066472	0	0	0	0	1	1	1	0	0	0	0	MK799832
1383	VC_432_14	1	0	8152.101765	0	0	0	0	1	1	1	0	0	0	0	MN018232
1384	VC_432_15	3	900	20632.44506	0.04179739	0.001443179	1.414213562	2.645751311	3	3	1	2.09867762	0	0	0	KU686204,KU686205,KU686206
1385	VC_432_16	1	0	6923.837283	0	0	0	0	1	1	1	0	0	0	0	MH629685
1386	VC_432_2	1	0	10031.8177	0	0	0	0	1	1	1	0	0	0	0	GU071095
1387	VC_432_3	1	0	8850.791003	0	0	0	0	1	1	1	0	0	0	0	MF351863
1388	VC_432_4	1	0	8731.803758	0	0	0	0	1	1	1	0	0	0	0	MT408532
1389	VC_432_5	1	0	10636.28451	0	0	0	0	1	1	1	0	0	0	0	MK867354
1390	VC_432_6	1	0	5226.300768	0	0	0	0	1	1	1	0	0	0	0	MH319749
1391	VC_432_7	1	0	7317.645565	0	0	0	0	1	1	1	0	0	0	0	MH319754
1392	VC_432_8	1	0	6993.109173	0	0	0	0	1	1	1	0	0	0	0	KM359505
1393	VC_432_9	3	900	23316.65955	0.037164498	0.001486876	2.236067977	4.123105626	3	3	1	3.36694366	0	0	0	AY939844,GU071092,HQ632825
1394	VC_433_0	6	4500	52678.17455	0.078701358	2.45E-11	1.414213562	2.449489743	15	15	1	1.93972431	0	0	0	HQ634177,KU686192,KU686193,KU686194,KU686195,KU686196
1395	VC_433_1	9	10800	73079.42889	0.128756241	1.37E-24	1	9.746794345	36	31	0.861111111	6.77186817	0	0	0	KJ019054,KJ019069,KJ019082,KJ019089,KJ019094,KJ019156,KU686200,KU686201,KU686202
1396	VC_433_10	1	0	10400.95313	0	0	0	0	1	1	1	0	0	0	0	KU686211
1397	VC_433_2	1	0	10232.49831	0	0	0	0	1	1	1	0	0	0	0	KF156338
1398	VC_433_3	3	900	29884.90753	0.029235105	0.001871896	1.414213562	2.645751311	3	3	1	2.09867762	0	0	0	KU686197,KU686198,KU686199
1399	VC_433_4	9	10800	73217.09277	0.128545271	9.86E-24	0	6.782329983	36	36	1	4.63153589	0	0	0	GU071105,KU594607,KX349291,KX349292,KX349293,KX349294,KX349295,KX349296,KX349297
1400	VC_433_5	2	300	17008.44074	0.017332584	0.063578424	4.69041576	4.69041576	1	1	1	4.69041576	0	0	0	AJ630128,LN828717
1401	VC_433_6	1	0	11224.04754	0	0	0	0	1	1	1	0	0	0	0	KU594606
1402	VC_433_7	1	0	10852.20659	0	0	0	0	1	1	1	0	0	0	0	MW147367
1403	VC_433_8	1	0	10306.34815	0	0	0	0	1	1	1	0	0	0	0	FM207411
1404	VC_433_9	1	0	10743.62193	0	0	0	0	1	1	1	0	0	0	0	MW117966
1405	VC_435_0	2	300	6789.518525	0.042315991	0.042722815	7.416198487	7.416198487	1	1	1	7.41619849	0	0	0	MT162466,MT162467
1406	VC_435_1	1	0	3805.632018	0	0	0	0	1	1	1	0	0	0	0	MK016664
1407	VC_436_0	4	1800	3624.513577	0.331826988	1.28E-05	0	2.828427125	6	6	1	1.79411078	0	0	0	MZ577095,MZ577096,MZ577097,MZ577098
1408	VC_437_0	4	1800	1460.685381	0.55203118	1.77E-05	2	8.185352772	6	6	1	6.34115588	0	0	0	KF381361,KR052480,KR052481,KR052482
1409	VC_438_0	6	514.4598957	5098.47782	0.091656085	5.73E-05	4.123105626	8.831760866	15	15	1	7.0968541	0	0	0	MH319720,MH319733,MH319742,MH319753,MH319758,MH327487
1410	VC_439_0	2	28.66807063	956.7655289	0.029091834	0.09466152	8.485281374	8.485281374	1	1	1	8.48528137	0	0	0	MH319750,MH319759
1411	VC_43_0	38	82501.86654	57699.31907	0.588453416	0	0	9.797958971	703	679	0.965860597	6.63367767	0	0	0	EU078592,JQ086376,JQ182735,KT232076,LAMCG,LC730321,LR595850,LR595859,LR595860,LR595861,LR595863,LR595864,LR595865,LR595866,LR597635,LR597636,LR597639,LR597643,LR597644,LR597648,LR597650,LR597651,LR597652,LR597653,MF374379,MH547045,OL657226,OL657227,OL657228,OM418625,OM418626,OM418627,OM475427,OM475428,OM475430,OM475432,OM475435,OV696612
1412	VC_440_0	4	181.3255917	0	1	0	1.732050808	3.872983346	6	6	1	2.97497703	0	0	0	AJ635161,AY722806,MN876845,MT047590
1413	VC_442_0	4	1800	19574.82243	0.084211226	3.68E-05	1.732050808	3	6	6	1	2.46111786	0	0	0	MK838116,MN102098,MN131137,MN159079
1414	VC_442_1	3	900	15829.35752	0.053797643	0.005804482	2.645751311	5.196152423	3	3	1	4.31364108	0	0	0	MN642089,ON287370,ON287375
1415	VC_442_2	4	1800	19535.6715	0.084365753	0.000118713	7.416198487	9.486832981	6	5	0.833333333	8.56874713	0	0	0	JN627160,MK689364,MZ348422,MZ501267
1416	VC_442_3	3	900	16112.08532	0.052903567	0.003248195	3	5.656854249	3	3	1	4.61766889	0	0	0	MN032614,MN453779,OL964756
1417	VC_442_4	2	300	11508.22077	0.025406029	0.081405016	6.480740698	6.480740698	1	1	1	6.4807407	0	0	0	MK774614,OM810292
1418	VC_443_0	2	300	10048.63301	0.028989336	0.125903965	5.385164807	5.385164807	1	1	1	5.38516481	0	0	0	MH791414,MW272540
1419	VC_443_1	2	300	10135.57369	0.028747821	0.10652622	3.605551275	3.605551275	1	1	1	3.60555128	0	0	0	GU459069,KY290955
1420	VC_443_2	10	13500	26873.86424	0.334374731	4.86E-23	5	9.949874371	45	42	0.933333333	7.36030341	0	0	0	JX123262,MF448340,MF498773,MF498775,MH791398,MH791404,MN871441,MN871442,MT713136,OM339718
1421	VC_444_0	2	189.2994502	43.3529588	0.813657813	0.270145687	1.732050808	1.732050808	1	1	1	1.73205081	0	0	0	AJ783769,JQ287645
1422	VC_445_0	2	300	454.0852776	0.397832989	0.077364462	2.236067977	2.236067977	1	1	1	2.23606798	0	0	0	MT732443,MT732444
1423	VC_445_1	2	94.59058088	634.4718487	0.129742772	0.069448331	8.717797887	8.717797887	1	1	1	8.71779789	0	0	0	KC821607,KC821618
1424	VC_446_0	4	691.9971506	225.7319334	0.754032059	0.000122324	4.472135955	6	6	6	1	5.16525043	0	0	0	HG322870,KP238129,KP282677,KP282678
1425	VC_447_0	3	585.4749419	122.6626863	0.82678129	0.004900713	0	3.16227766	3	3	1	2.10818511	0	0	0	AJ888457,KX607101,KX607102
1426	VC_448_0	4	1800	4084.747806	0.30587547	0.000211471	0	6.32455532	6	6	1	4.0941984	0	0	0	KU878088,LC680884,MF360957,MW749003
1427	VC_448_1	1	0	1907.899768	0	0	0	0	1	1	1	0	0	0	0	KT895374
1428	VC_449_0	7	6300	4174.678176	0.601450459	1.21E-11	3	8.888194417	21	21	1	7.02623696	0	0	0	LC680885,MN091626,MW218148,MW248466,MW349128,MW349129,MZ779063
1429	VC_450_0	16	36000	1642.758687	0.956359238	1.75E-34	0	2.236067977	120	120	1	0.38569808	0	0	0	AM076770,MN617773,MT366931,MT366932,MT366933,MT366934,MT366935,MT366936,MT366937,MT366938,MT366939,MT366940,MT366941,MT366942,MT366943,MT366944
1430	VC_451_0	80	335903.9773	316.3006065	0.999059246	1.74E-97	0	7.416198487	3160	3160	1	5.57204816	0	0	0	AM084414,DQ111067,EU734172,HG813241,HG818823,HG818824,KC109329,KJ748011,KT990215,KU687349,KU687350,KU687351,KX669227,KX689784,KY295894,KY962008,LT594300,MF630921,MG845865,MH400309,MH464253,MH491167,MK033136,MK301532,MK610268,MK903277,MK903279,MK903281,MN067430,MN218775,MN414250,MN510331,MN518893,MN518894,MN715150,MN812686,MT350292,MT446393,MT446394,MT496969,MT682707,MT808983,MT862763,MW258709,MW345254,MW416012,MW748991,MW876471,MZ223858,MZ234009,MZ234011,MZ234021,MZ234022,MZ234023,MZ234024,MZ234044,MZ234045,MZ234046,MZ234047,MZ311861,MZ311862,MZ398247,MZ648215,MZ726794,MZ923531,OK210074,OL362275,OL362276,OL539729,OL773676,OL773677,OM256482,OM355495,OM457002,OM867676,OM908436,OM937123,ON528733,ON548433,ON637250
1431	VC_452_0	66	229009.5116	18629.77858	0.924770506	3.82E-252	0	8.246211251	2145	2145	1	5.9191448	0	0	0	AM084415,AY288927,AY370673,AY370674,GU595417,JN986844,JQ837901,KM366097,KM370384,KM819695,KM819696,KP890822,KU946962,KX279892,KY295891,KY295892,KY295893,KY295897,KY295899,KY435490,KY744566,LC730323,MG004687,MG251390,MG775042,MH729818,MK373771,MK373773,MK380014,MN384978,MT496971,MT680615,MW394389,MZ031013,MZ234012,MZ234019,MZ234020,MZ234032,MZ234049,MZ234050,MZ398244,MZ573924,MZ747518,MZ997839,MZ997840,MZ997841,OK625527,OL362272,OL362273,OL744215,OL744218,ON148527,ON185580,ON210144,ON210145,ON461912,ON526839,ON602763,ON602764,ON602765,OP018999,OP038547,OP079918,OP413828,OP413830,OX335382
1432	VC_453_0	6	2385.120897	18535.91353	0.114005878	2.24E-11	0	4	15	15	1	2.65757914	0	0	0	EF160123,FQ482084,FQ482086,MG736918,OK570184,OM486945
1433	VC_455_0	18	27764.02161	32622.24104	0.459773803	6.73E-92	0	7.549834435	153	153	1	5.46475294	0	0	0	DQ121662,JF770475,JQ182736,KC333879,KC579452,KF771236,KF771237,KF771238,KF771239,KJ668713,KT825490,MF158039,MF158042,MW286156,OL539447,OL539451,OL539453,OL539467

1434	VC_456_0	62	362422.1017	39104.94971	0.902609427	0	0	8	1891	1891	1	3.83484609	0	0	0	AM156909,JN986845,KX130960,KY398841,MG050172,MG065688,MK372342,MK373793,MK907226,MK907227,MK907228,MK907229,MK907230,MK907231,MK907232,MK907233,MK907234,MK907235,MK907240,MK907241,MK907242,MK907243,MK907244,MK907245,MK907246,MK907247,MK907248,MK907249,MK907250,MK907251,MK907252,MK907253,MK907254,MK907255,MK907256,MK907257,MK907258,MK907259,MK907260,MK907261,MK907262,MK907263,MK907264,MK907265,MK907266,MK907272,MK907273,MK907274,MK907275,MK907276,MN164484,MN840485,MT511058,MT682706,MW057918,MZ501051,MZ501080,MZ501087,OM258170,ON081052,OP087522,OP251154
1435	VC_458_0	5	1650.186607	6863.653045	0.193824018	3.22E-08	0	3.31662479	10	10	1	2.52026691	0	0	0	MH059632,MH807810,MH807813,MH807815,MH807817
1436	VC_459_0	3	230.4568416	39.34927899	0.854157204	0.009451772	4.123105626	5.567764363	3	3	1	5.05603185	0	0	0	KC292023,MG550112,MZ334526
1437	VC_45_0	12	5477.354336	10170.17797	0.350045888	6.45E-40	0	8.774964387	66	66	1	6.99470114	0	0	0	AJ298298,AY055382,KC736978,KC814930,KU927493,MK733259,MK733260,MK733261,MZ501270,ON470584,ON470603,SFU82619
1438	VC_460_0	19	26606.0772	44464.11235	0.374363392	2.87E-105	0	8.544003745	171	171	1	7.15340719	0	0	0	JF974294,JF974312,MK327140,MT682064,MW119258,MW722082,MZ318367,MZ440881,MZ836210,OL362277,OL617041,ON602730,ON602737,ON602738,ON602744,ON602759,ON602760,OP373729,OP481875
1439	VC_461_0	96	663542.7094	49388.65482	0.930724531	0	0	9.327379053	4560	4366	0.95745614	5.93181689	0	0	0	AM491472,KX017520,KX523699,KY860935,MF158038,MH023293,MH321493,MH424446,MH717097,MH791402,MH791410,MH791412,MK112901,MK599416,MK759854,MK947460,MN692673,MN737551,MN871490,MN871503,MT768060,MW427215,MW477799,MZ375232,MZ375295,MZ375296,MZ375297,MZ375298,MZ375299,MZ375300,MZ375301,MZ375302,MZ375303,MZ375304,MZ375305,MZ375306,MZ375307,MZ375308,MZ375309,MZ375310,MZ375311,MZ375312,MZ375313,MZ375314,MZ375315,MZ375316,MZ375317,MZ375318,MZ375319,MZ375320,MZ375321,MZ375322,MZ375323,MZ375324,MZ375325,MZ375326,MZ375327,MZ375328,MZ375329,MZ375330,MZ375331,MZ375332,MZ375333,MZ375334,MZ375335,MZ375336,MZ375337,MZ375338,MZ375339,MZ375340,MZ375341,MZ375342,MZ375343,MZ375344,MZ375345,MZ375346,MZ375347,MZ375348,MZ375349,MZ375350,MZ375351,MZ375352,MZ375353,MZ375354,MZ375355,MZ489634,MZ726796,OL362274,OL964734,OL964742,OL964743,OL964744,OL964751,OL964752,ON239132,ON637251
1440	VC_463_0	4	783.1294826	207.4362324	0.790588116	2.84E-05	1	2.828427125	6	6	1	2.23263402	0	0	0	AM749121,DQ386162,KR153145,MT430910
1441	VC_464_0	8	5833.561973	16351.71835	0.262947409	1.59E-19	1.732050808	8.246211251	28	28	1	5.43831234	0	0	0	JQ067087,KU356689,LN610577,LN610584,LN610585,MT108725,MT108731,MZ926748
1442	VC_465_0	7	5016.018234	10084.03633	0.332185438	2.27E-15	2.236067977	4.123105626	21	21	1	3.17349815	0	0	0	JQ067084,KX129925,KX898399,KX898400,MN504636,MW835180,ON911540
1443	VC_466_0	3	83.54543582	172.4501267	0.326355016	0.002526948	5.744562647	8.366600265	3	3	1	6.78538697	0	0	0	MW091529,MW202777,MW462191
1444	VC_467_0	5	3000	1364.03657	0.687436952	3.79E-08	4	6	10	10	1	4.99669956	0	0	0	MN862068,ON529856,ON529859,ON529862,ON529864
1445	VC_468_0	4	1049.48111	6287.364114	0.143042558	1.52E-05	2.449489743	6.557438524	6	6	1	5.08599019	0	0	0	MN988465,MN988541,MN988554,ON014755
1446	VC_469_0	2	212.6706798	1212.743534	0.149199214	0.043418561	1.732050808	1.732050808	1	1	1	1.73205081	0	0	0	MT536174,OL960029
1447	VC_46_0	2	202.638716	10996.45374	0.018094209	0.042364634	5.477225575	5.477225575	1	1	1	5.47722558	0	0	0	KF766125,KJ603229
1448	VC_470_0	3	286.2659991	229.3067432	0.555238816	0.00176646	5.477225575	7	3	3	1	6.39514317	0	0	0	MN698239,MN698241,MW574966
1449	VC_471_0	2	229.9156141	2630.600977	0.080375557	0.043342814	3.464101615	3.464101615	1	1	1	3.46410162	0	0	0	MW677525,OP009285
1450	VC_472_0	47	214486.1715	23179.59369	0.902469783	5.18E-148	0	7.348469228	1081	1081	1	4.36701973	0	0	0	AM910650,AY078382,HE983844,HF543949,HG518155,JN254801,JX863101,KC294142,KF856712,KP233880,KR054029,LC648443,LN610578,MF768469,MG432151,MG589385,MG589386,MG845684,MH973725,MK837011,MK837012,MN508619,MN553586,MN562749,MN871476,MN871484,MT108723,MT108724,MT118288,MT119362,MT119371,MT119373,MW329986,MW512831,MW526260,MZ089732,MZ089733,MZ089738,MZ089739,MZ089740,MZ089742,OL441337,ON156559,ON376263,ON857927,ON857936,OP342787
1451	VC_473_0	1	0	1085.686134	0	0	0	0	1	1	1	0	0	0	0	CP067352
1452	VC_473_1	1	0	1226.243411	0	0	0	0	1	1	1	0	0	0	0	CP069348
1453	VC_473_2	2	300	2636.857382	0.102150006	0.053977716	0	0	1	1	1	0	0	0	0	CP011970,LN681537
1454	VC_473_3	2	300	2522.140781	0.10630228	0.05796749	5.656854249	5.656854249	1	1	1	5.65685425	0	0	0	MF547662,MF547663
1455	VC_473_4	1	0	1430.683953	0	0	0	0	1	1	1	0	0	0	0	CP067347
1456	VC_474_0	14	27300	4946.05951	0.846615072	4.87E-49	0	8.717797887	91	91	1	5.93881762	0	0	0	HM029250,KF926093,KM677185,KY554768,KY554769,KY554770,KY554771,KY554772,KY554773,KY554774,KY554775,KY554776,KY554777,MN552145
1457	VC_475_0	3	900	10670.67508	0.077782843	0.002016249	5.099019514	7.549834435	3	3	1	6.64298795	0	0	0	OM236516,OM634653,ON133270
1458	VC_475_1	3	900	14572.562	0.058167484	0.011339906	6.480740698	7.745966692	3	3	1	7.00301246	0	0	0	OK499972,OK499987,OK499994
1459	VC_475_10	2	300	10228.17403	0.02849497	0.122788077	8.602325267	8.602325267	1	1	1	8.60232527	0	0	0	OK499992,ON548418
1460	VC_475_2	3	900	14497.59297	0.058450694	0.011169775	7.874007874	8.366600265	3	3	1	8.08020271	0	0	0	KC481682,MT254578,ON548420
1461	VC_475_3	1	0	4268.345023	0	0	0	0	1	1	1	0	0	0	0	ON881243
1462	VC_475_4	1	0	4329.584955	0	0	0	0	1	1	1	0	0	0	0	MH884508
1463	VC_475_5	2	300	9917.889658	0.02936027	0.060126546	7.745966692	7.745966692	1	1	1	7.74596669	0	0	0	ON548417,ON548419
1464	VC_475_6	1	0	5284.575367	0	0	0	0	1	1	1	0	0	0	0	KT070867
1465	VC_475_7	1	0	5343.185285	0	0	0	0	1	1	1	0	0	0	0	MW084976
1466	VC_475_8	2	300	10022.13376	0.029063758	0.098153013	8.94427191	8.94427191	1	1	1	8.94427191	0	0	0	MK288021,OK499991
1467	VC_475_9	1	0	5351.539625	0	0	0	0	1	1	1	0	0	0	0	ON548421
1468	VC_476_0	19	17870.53028	4999.411823	0.781398143	9.35E-88	0	8.185352772	171	171	1	5.57371364	0	0	0	AY150271,DQ126339,JN391180,JQ011317,JQ691610,KC911856,KC911857,KP869107,KP869108,KX231829,MK416010,MK422448,MN187550,MT261384,MT580116,MT580117,MW205203,MW722083,OK490417
1469	VC_477_0	4	1800	30.04838867	0.98358055	0.002510272	0	3.741657387	6	6	1	2.64106598	0	0	0	AP011616,AP011617,DQ997624,MK257744
1470	VC_478_0	2	300	556.0086277	0.350463757	0.052335108	5.744562647	5.744562647	1	1	1	5.74456265	0	0	0	MG592415,OX241562
1471	VC_478_1	1	0	352.5184081	0	0	0	0	1	1	1	0	0	0	0	MW073017
1472	VC_479_0	17	19853.59535	1955.583382	0.910332094	1.98E-55	0	2	136	136	1	1.1373724	0	0	0	OM416763,OM416764,OM416765,OM416766,OM416767,OM416768,OM416769,OM416770,OM416771,OM416772,OM416773,OM416774,OM416775,OM416776,OM416777,OM416778,OM416779
1473	VC_47_0	48	335554.7405	95579.23346	0.778307349	0	0	5.656854249	1128	1128	1	1.06140515	0	0	0	LC616031,LC616032,LC616033,LC616034,LC616035,LC616036,LC616037,LC616038,LC616039,LC616040,LC616041,LC616042,LC616043,LC616044,LC616045,LC616046,LC616047,LC616048,LC616049,LC616050,LC616051,LC616052,LC616053,LC616054,LC616055,LC616056,LC616057,LC616058,LC616059,LC616060,LC616061,LC616062,LC616063,LC616064,LC616065,LC616066,LC616067,LC616068,LC616069,LC616070,LC616071,LC616072,LC616073,LC616074,LC616075,LC616076,LC616077,LC616078
1474	VC_480_0	19	18264.18138	15278.7175	0.544502174	2.23E-94	0	7.348469228	171	171	1	5.68465347	0	0	0	AP013541,AP013542,AP013545,KC465901,MH579717,MH598798,MH598800,MH598802,MH598803,MH598804,MH598805,MH598807,MT375520,MT375521,MT375526,MT375527,MT375528,MT375530,MT375531
1475	VC_481_0	8	1114.725681	7153.139226	0.134826306	3.69E-10	2.828427125	7	28	28	1	6.08748657	0	0	0	AP013544,JX483873,KM199771,KT997847,MT708545,MW023914,MW980070,MZ666938
1476	VC_482_0	4	288.8558882	682.1080933	0.297493927	1.58E-05	4	5.385164807	6	6	1	4.94134621	0	0	0	AP013546,AP013547,AP013548,MW822601
1477	VC_484_0	3	529.0036916	1137.761647	0.317383425	0.001563623	1.414213562	1.732050808	3	3	1	1.62610506	0	0	0	KJ183191,KJ183192,KJ183193
1478	VC_485_0	3	409.0652329	1303.516792	0.238858768	0.001556357	2	8.246211251	3	3	1	6.16414083	0	0	0	MN698242,MN698243,MN698247
1479	VC_486_0	18	19231.23322	2450.376477	0.886983646	1.35E-71	0	8	153	153	1	5.59253698	0	0	0	KC465898,MT375525,MT647605,MW273920,MW273922,MW273923,MW273924,MW273925,MW273926,OP131293,OP131294,OP131295,OP131296,OP131297,OP131299,OP131300,OP131301,OP131302
1480	VC_487_0	2	81.35158442	240.279701	0.252934301	0.050750825	5.099019514	5.099019514	1	1	1	5.09901951	0	0	0	MT375519,MT375529
1481	VC_488_0	8	8400	20194.29086	0.293764935	2.89E-18	0	7.348469228	28	28	1	5.28719818	0	0	0	MN871446,MN871447,MN871448,MN871449,MN871451,MN871452,MN871453,MW394391
1482	VC_488_1	8	8400	20302.90266	0.292653328	3.62E-18	2	6.92820323	28	28	1	4.5257755	0	0	0	MF285618,MK994515,MW021758,OK040170,OL539468,ON287372,ON287376,ON375847
1483	VC_488_2	2	251.6850835	7488.692952	0.032515865	0.044889709	8.717797887	8.717797887	1	1	1	8.71779789	0	0	0	KF623294,MN334766
1484	VC_489_0	3	900	12385.30781	0.067744008	0.017408422	0	8.94427191	3	3	1	5.96284794	0	0	0	MN445183,MW845760,MW879340
1485	VC_489_1	11	16500	19207.23294	0.462091253	1.66E-24	1.732050808	6.708203932	55	55	1	3.30297378	0	0	0	KF806589,KU886222,KU886223,KU886224,KU886225,MG655267,MG655268,MG655269,MG655270,MK514281,MZ501268
1486	VC_489_2	1	0	4738.35674	0	0	0	0	1	1	1	0	0	0	0	MT661596
1487	VC_48_0	10	12285.83385	58308.57339	0.174034096	6.28E-31	0	2.828427125	45	45	1	1.87260851	0	0	0	LC645428,LC645429,LC645430,LC645431,LC645432,LC645433,LC645434,LC645435,LC645436,LC645437
1488	VC_490_0	2	300	29137.2416	0.010191172	0.046551366	4.123105626	4.123105626	1	1	1	4.12310563	0	0	0	AP014714,AP014715
1489	VC_490_1	5	3000	64154.24373	0.044673275	3.84E-08	5.567764363	8.426149773	10	10	1	7.70484516	0	0	0	MN087708,MN882610,MZ348423,OL989991,ON630910
1490	VC_490_2	1	0	14602.08382	0	0	0	0	1	1	1	0	0	0	0	KF835987
1491	VC_490_3	2	300	21981.67049	0.013463982	0.042968507	7.937253933	7.937253933	1	1	1	7.93725393	0	0	0	HE956709,KY593455
1492	VC_490_4	1	0	10553.64871	0	0	0	0	1	1	1	0	0	0	0	KP202158
1493	VC_490_5	1	0	10630.23158	0	0	0	0	1	1	1	0	0	0	0	MH059636
1494	VC_490_6	1	0	10869.36129	0	0	0	0	1	1	1	0	0	0	0	MT920315
1495	VC_491_0	36	189000	165177.5171	0.53363071	0	2.236067977	11.18033989	630	612	0.971428571	5.9839723	0	0	0	CP062992,KR269720,KT239446,KX258185,KY000080,LR877331,MG751100,MH333064,MH729874,MK421971,MN101226,MN101229,MN101230,MN434095,MT580897,MT701588,MW021752,MW239157,MZ560702,MZ602148,MZ890186,OK499980,OK499981,OK499996,OK499997,OK631811,OL539458,OL870318,OL870319,OM337876,OM337877,OM630219,ON550260,OP331213,OP617748,OU509536
1496	VC_491_1	1	0	13554.54626	0	0	0	0	1	1	1	0	0	0	0	MN101225
1497	VC_492_0	2	300	18716.53641	0.015775743	0.3359603	3.16227766	3.16227766	1	1	1	3.16227766	0	0	0	HQ698922,MK240351
1498	VC_492_1	20	57000	79480.64906	0.417641625	3.53E-15	0	5.099019514	190	190	1	4.04707788	0	0	0	CAKLQC020000001,CAKLQD020000001,MH165274,MH460829,MH713599,MN648195,MN662249,MN709128,MN723850,MN732883,MN782535,MT385367,MT409116,MT741943,OL763420,OL770258,OL770259,OL770261,OL770262,OP585103
1499	VC_492_2	8	8400	60408.91322	0.122077208	0.01213426	1	5	28	28	1	3.85204346	0	0	0	MK278860,MN864865,MT949699,MW388001,MW388002,MW388003,MW388004,OL770260
1500	VC_492_3	1	0	9804.541038	0	0	0	0	1	1	1	0	0	0	0	GU911519
1501	VC_492_4	1	0	9696.805758	0	0	0	0	1	1	1	0	0	0	0	HM004124
1502	VC_492_5	1	0	7513.711895	0	0	0	0	1	1	1	0	0	0	0	HM032710
1503	VC_492_6	1	0	7211.166009	0	0	0	0	1	1	1	0	0	0	0	HM114315
1504	VC_493_0	1	0	2878.525935	0	0	0	0	1	1	1	0	0	0	0	KT932418
1505	VC_493_1	1	0	5442.420425	0	0	0	0	1	1	1	0	0	0	0	MG592562
1506	VC_493_10	1	0	4101.868649	0	0	0	0	1	1	1	0	0	0	0	HQ634156
1507	VC_493_11	1	0	2871.129988	0	0	0	0	1	1	1	0	0	0	0	KX507046
1508	VC_493_12	2	300	8856.782758	0.032762599	0.044420764	2.645751311	2.645751311	1	1	1	2.64575131	0	0	0	MW824371,MW824372
1509	VC_493_13	1	0	4797.982562	0	0	0	0	1	1	1	0	0	0	0	MW824376
1510	VC_493_14	1	0	4971.14331	0	0	0	0	1	1	1	0	0	0	0	HQ316579
1511	VC_493_15	1	0	4975.972474	0	0	0	0	1	1	1	0	0	0	0	HQ634195
1512	VC_493_16	1	0	4049.83305	0	0	0	0	1	1	1	0	0	0	0	MG592553
1513	VC_493_2	1	0	4362.879406	0	0	0	0	1	1	1	0	0	0	0	MG592529
1514	VC_493_3	1	0	3738.780113	0	0	0	0	1	1	1	0	0	0	0	OX241437
1515	VC_493_4	1	0	4179.119926	0	0	0	0	1	1	1	0	0	0	0	OX241455
1516	VC_493_5	1	0	2716.689211	0	0	0	0	1	1	1	0	0	0	0	AP014858
1517	VC_493_6	1	0	3317.739637	0	0	0	0	1	1	1	0	0	0	0	MG592495
1518	VC_493_7	1	0	3641.006886	0	0	0	0	1	1	1	0	0	0	0	HQ634157
1519	VC_493_8	1	0	3217.344474	0	0	0	0	1	1	1	0	0	0	0	MG592441
1520	VC_493_9	1	0	3210.396277	0	0	0	0	1	1	1	0	0	0	0	ON922990
1521	VC_495_0	39	222300	499350.4637	0.308043868	2.92E-97	0	7.681145748	741	741	1	3.95220507	0	0	0	KY883638,KY883639,KY883640,MH310933,MH310935,MN419153,MW794150,MW794151,MW794152,MW794153,MW794154,MW794155,MW794156,MW794157,MW794158,MW794159,MW794160,MW794161,MW794162,MW794163,MW794164,MW794165,MW794166,MW794167,MW794168,MW794169,MW794170,MW794171,MW794172,MW794173,MW794176,MW794177,MW794179,MW794180,MW794182,MW794183,MW794188,MW794190,MZ292654
1522	VC_495_1	41	246000	504075.9346	0.327966795	2.55E-115	0	10.44030651	820	747	0.91097561	6.47826863	0	0	0	HQ641347,HQ641348,HQ641349,HQ641350,HQ641351,HQ641352,HQ641353,HQ641354,KY065147,KY883634,KY883635,KY883636,KY883637,MH310934,MH310936,MN402506,MW794140,MW794141,MW794142,MW794143,MW794144,MW794145,MW794146,MW794147,MW794148,MW794149,MW794174,MW794175,MW794178,MW794181,MW794184,MW794185,MW794186,MW794187,MW794189,MW794191,MW794192,MW911612,MW911613,MW911614,MW911615
1523	VC_496_0	1	0	1121.016836	0	0	0	0	1	1	1	0	0	0	0	KF302034
1524	VC_496_1	1	0	794.5361574	0	0	0	0	1	1	1	0	0	0	0	MF370964
1525	VC_496_2	1	0	828.2632615	0	0	0	0	1	1	1	0	0	0	0	KR534323
1526	VC_496_3	1	0	1126.219863	0	0	0	0	1	1	1	0	0	0	0	KX822733
1527	VC_497_0	1	0	1562.597451	0	0	0	0	1	1	1	0	0	0	0	MG592459
1528	VC_497_1	1	0	2869.974651	0	0	0	0	1	1	1	0	0	0	0	MG592473
1529	VC_498_0	8	2780.635543	4193.97987	0.398679408	2.32E-18	1	7.681145748	28	28	1	5.82470113	0	0	0	AP014887,AY328852,AY328853,KF854250,KX196154,KY622015,MG592395,OL658619
1530	VC_499_0	24	30583.14801	5109.085961	0.85685721	1.06E-103	0	9.38083152	276	263	0.952898551	7.68184372	0	0	0	KT160311,MG592392,MG592401,MG592414,MG592451,MG592461,MG592462,MG592467,MG592468,MG592472,MG592499,MG592526,MG592531,MG592541,MG592542,MG592547,MG592561,MG592572,MG592590,MG592592,MG592611,MW824380,MW824381,MW824382
1531	VC_49_0	36	135240.7621	86094.16892	0.611023129	0	0	6.244997998	630	630	1	3.3704152	0	0	0	LC727840,LC727841,LC727842,LC727843,LC727844,LC727845,LC727846,LC727847,LC727848,LC727849,LC727850,LC727851,LC727852,LC727853,LC727854,LC727855,LC727856,LC727857,LC727858,LC727859,LC727860,LC727861,LC727862,LC727863,LC727864,LC727865,LC727866,LC727867,LC727868,LC727869,LC727870,LC727871,LC727872,LC727873,LC727874,LC727875
1532	VC_4_0	7	3658.278678	965.6263913	0.791166476	4.23E-15	0	3.741657387	21	21	1	2.14880429	0	0	0	DQ426904,DQ426905,JN255163,KP137432,KP137434,KP137436,KP137438
1533	VC_500_0	3	548.8343665	114.1463582	0.827828542	0.003082402	0	2.828427125	3	3	1	1.88561808	0	0	0	OM287554,OM732336,OM732337
1534	VC_501_0	16	15471.45467	23.82333559	0.998462542	5.06E-10	0	4.582575695	120	120	1	3.60979912	0	0	0	AP014888,MG592509,MG592546,MG592620,MG592623,OX241426,OX241428,OX241430,OX241431,OX241432,OX241441,OX241448,OX241449,OX241494,OX241567,OX241568
1535	VC_502_0	2	300	3768.636081	0.073734783	0.048286377	5	5	1	1	1	5	0	0	0	S2-wms_128962,S6-wms_138342
1536	VC_502_1	6	1965.585679	6436.022048	0.233953517	1.36E-09	4.123105626	8.888194417	15	15	1	7.28870806	0	0	0	MT774375,MT774376,MT774379,MT774380,MT774382,S4-wms_73990
1537	VC_502_2	1	0	1634.059385	0	0	0	0	1	1	1	0	0	0	0	MT774401
1538	VC_502_3	2	300	3843.04071	0.072410585	0.048286377	4.69041576	4.69041576	1	1	1	4.69041576	0	0	0	MT774399,MT774403
1539	VC_502_4	3	592.2606988	5349.540316	0.099676966	0.002846525	7.348469228	8.602325267	3	3	1	7.94160079	0	0	0	MT774395,MT774397,MT774398
1540	VC_502_5	2	227.3246905	3988.923926	0.053916339	0.064220362	8.888194417	8.888194417	1	1	1	8.88819442	0	0	0	MT774396,MT774405
1541	VC_502_6	2	260.7048296	2981.24832	0.080415977	0.048286377	7.280109889	7.280109889	1	1	1	7.28010989	0	0	0	MT774404,MT774407
1542	VC_502_7	1	0	2103.9841	0	0	0	0	1	1	1	0	0	0	0	MT774400
1543	VC_505_0	3	900	122.4445884	0.880243301	0.031801285	0	1.732050808	3	3	1	1.15470054	0	0	0	MW353175,MW353176,MW353177
1544	VC_506_0	8	8400	2988.501572	0.73758606	3.59E-17	1.414213562	7.141428429	28	28	1	4.73885141	0	0	0	KR560069,MK580972,MN095772,MN098328,MZ042490,MZ326868,MZ398248,OL702939
1545	VC_506_1	1	0	1745.127974	0	0	0	0	1	1	1	0	0	0	0	ON758385
1546	VC_507_0	8	8038.332233	9961.642226	0.446574647	1.48E-19	0	4	28	28	1	2.950966	0	0	0	AP018462,AP018463,KY130461,MH001446,MH001450,MK494114,MK494131,MK494132
1547	VC_508_0	32	148680.6033	15416.97348	0.906049963	2.98E-235	0	3.31662479	496	496	1	1.05395957	0	0	0	AY129334,DQ398048,JN618996,JN698991,JN699004,KF024722,KM101117,KR997932,KT365402,KT880194,KX443696,MG757162,MG812490,MG812491,MH001452,MH051249,MH590591,MH697584,MH727546,MK061411,MK620898,MK814751,MN234225,MN428049,MN586045,MN586046,MN813678,MT639652,MW712731,MZ456036,ON970587,ON970593
1548	VC_509_0	37	199764.5847	6412.154864	0.968899717	2.62E-283	0	4.358898944	666	666	1	2.49468749	0	0	0	DQ398049,EU816589,FJ641182,JF704095,JN698992,JN699003,JN699006,JN699018,KF493879,KJ194581,KJ194584,KP027207,KR080203,KR080205,MG839014,MG920059,MG925338,MG925353,MH051265,MH316565,MH513976,MH576977,MK061414,MK359357,MN096359,MN096360,MN234223,MN444872,MN444873,MT310871,MT310892,MT952851,MW924644,MZ171375,MZ171381,OK318960,OP434441
1549	VC_50_0	9	6174.451903	4988.253745	0.553132197	4.82E-23	1	4.69041576	36	36	1	3.45667293	0	0	0	AF083977,GQ357916,KP010268,LR595867,MT374852,MT374853,MT374854,MT374856,MT591577
1550	VC_510_0	12	10993.69262	16745.87982	0.396318027	1.33E-43	0	9	66	64	0.96969697	7.02803356	0	0	0	MH153810,MK801725,MN234196,MN369758,MN908693,MT657335,OK216879,OM105886,ON081333,ON108644,ON260813,OP434461
1551	VC_511_0	3	598.7489424	4824.004194	0.110414199	0.001413172	6.244997998	7.549834435	3	3	1	6.75852438	0	0	0	JN698995,OP172873,OP172874
1552	VC_513_0	32	106519.9272	48277.93262	0.688122739	1.86E-283	0	7.071067812	496	496	1	4.71123291	0	0	0	AP018485,JF704111,JF957058,KC172839,KJ510415,KM592966,KT326767,KX640831,KX670828,KX683423,KY464936,LC730317,MF373840,MG812492,MH020248,MH047632,MH047633,MH051259,MH316563,MH976517,MK284519,MK494127,MK524496,MN062706,MN062712,MN586063,MT310878,MT310879,MT310880,MT310881,MW712718,ON970623
1553	VC_514_0	75	616547.0641	39213.56701	0.940201401	0	0	8.485281374	2775	2775	1	5.18470342	0	0	0	AY129332,HM755814,JF704098,JF704101,JF704114,KC661272,KC661281,KF279418,KM101119,KM233454,KM233455,KP017310,KP017311,KP027202,KT359365,KT381277,KU255188,KU578077,KU984914,KU985091,KX458237,KX507362,KX523125,KX664448,KX670788,KX808129,KY965063,MF185729,MF185732,MF185733,MG757161,MG925341,MG925355,MH020238,MH077583,MH155864,MH338240,MH371121,MH399776,MH450119,MH450124,MH450126,MH450128,MH513975,MH536813,MH536825,MH576949,MH651179,MH727554,MK112543,MK279903,MK279913,MK284518,MK310140,MK359341,MK433273,MK494096,MK494128,MK875800,MK967399,MN062718,MN096368,MN284900,MN497953,MN585969,MN585984,MN586048,MN813699,MT657331,MT897907,MW507128,MZ681518,MZ747514,OK318959,OK999978
1554	VC_515_0	16	25202.01822	41233.9628	0.379342908	5.71E-78	0	6.8556546	120	120	1	4.47315232	0	0	0	JQ512844,JX307704,JX411619,KC661279,KJ156985,KM101120,MF773750,MH001459,MK494124,MN586038,MN807247,MT522002,MZ322017,MZ355723,MZ622159,ON337196
1555	VC_516_0	3	800.3460439	915.4966441	0.466444884	0.001432024	0	4.795831523	3	3	1	3.19722102	0	0	0	MW314854,MW584159,MW584197
1556	VC_517_0	18	35131.60624	184530.4201	0.159934818	2.46E-95	0	8.485281374	153	153	1	4.57788988	0	0	0	JF704093,JN243857,KX550442,KX579975,KX641260,KY083059,MF668278,MK279898,MK359310,MK359322,MK494115,MK494117,MK494129,MK875799,MN284905,MN813697,MZ322023,ON970614

1557	VC_517_1	108	1122660.617	200717.3553	0.848329533	0	0	9.433981132	5778	5752	0.995500173	6.24169495	0	0	0	GQ303263,HM152766,JF792674,JN243856,JN561150,JN699015,JQ809701,JQ896627,JX307702,JX307703,KC661271,KF024733,KF841475,KF841476,KF954506,KF959563,KJ510414,KP027198,KP057620,KT365397,KT716493,KU055616,KU867906,KU985092,KU985094,KX458236,KX550441,KX557234,KX619651,KX648373,KX808130,KX817174,KX817175,KY006473,KY083058,KY083060,KY083061,KY083062,KY083063,KY204244,KY204245,KY204246,KY204247,KY204248,KY204249,KY204250,KY204251,KY471266,KY549155,MF919496,MF919509,MG099942,MG944217,MG944224,MG962373,MH020249,MH051247,MH077581,MH155875,MH155879,MH230877,MH399777,MH399785,MH479909,MH479911,MH509443,MH536817,MH651170,MH697588,MH727563,MH825710,MK279902,MK310145,MK359301,MK359323,MK359325,MK359340,MK359356,MK494088,MK494093,MK494094,MK494097,MK494101,MK494125,MK494126,MK878895,MK878905,MN728684,MN813696,MT114158,MT490373,MT498056,MT553338,MT553346,MT723939,MT818417,MT889382,MT889386,MT889390,MW291019,MZ005673,MZ171380,MZ322025,MZ355725,MZ747509,ON637771,ON970565,OP297529
1558	VC_518_0	6	2390.572436	16066.30531	0.129522039	1.99E-11	2.236067977	7.615773106	15	15	1	6.25639766	0	0	0	AP018487,FJ168659,HM152765,JN699001,MN234195,MN234236
1559	VC_518_1	2	144.5496243	3695.751541	0.037640179	0.042812394	7.615773106	7.615773106	1	1	1	7.61577311	0	0	0	AY129333,KP027201
1560	VC_519_0	39	161534.1835	36523.81713	0.815590297	0.00E+00	0	8.660254038	741	741	1	4.79325292	0	0	0	JN412591,JN572061,KC691256,KF841477,KP027200,KR080195,KT347313,KU985090,KX641262,MF133446,MF140399,MF281061,MF373843,MF472894,MF919492,MG198785,MK016499,MK494122,MK524508,MK814758,MK820639,MK919480,MK937604,MN010757,MN096356,MN585977,MN807248,MN807249,MN807250,MN892484,MN892486,MN908683,MT024866,MT498061,MW055902,ON637760,ON970574,OP172867,OP434454
1561	VC_51_0	10	2834.000518	13442.39718	0.174117183	1.18E-25	1.414213562	8.544003745	45	45	1	6.62419714	0	0	0	AB285204,JQ182728,LR595868,LR597641,MF807953,MW057859,ON470578,ON470598,ON470618,OV696610
1562	VC_520_0	1	0	11810.96613	0	0	0	0	1	1	1	0	0	0	0	KT309034
1563	VC_520_1	1	0	10197.06664	0	0	0	0	1	1	1	0	0	0	0	MT889374
1564	VC_520_10	1	0	10576.02655	0	0	0	0	1	1	1	0	0	0	0	MN369752
1565	VC_520_11	1	0	11007.27855	0	0	0	0	1	1	1	0	0	0	0	MN585995
1566	VC_520_12	1	0	10563.36717	0	0	0	0	1	1	1	0	0	0	0	MN735431
1567	VC_520_13	1	0	10590.446	0	0	0	0	1	1	1	0	0	0	0	KT281793
1568	VC_520_14	1	0	11296.88435	0	0	0	0	1	1	1	0	0	0	0	MT684595
1569	VC_520_15	2	173.328997	21978.54059	0.007824576	0.042395997	9	9	1	0	0	9	0	0	0	MF668270,MT684593
1570	VC_520_16	1	0	10296.16512	0	0	0	0	1	1	1	0	0	0	0	KX808131
1571	VC_520_17	1	0	8438.314548	0	0	0	0	1	1	1	0	0	0	0	MN369739
1572	VC_520_18	1	0	10684.57847	0	0	0	0	1	1	1	0	0	0	0	MN586001
1573	VC_520_19	2	220.1261144	22286.86624	0.009780343	0.04238448	7.348469228	7.348469228	1	1	1	7.34846923	0	0	0	KM652553,MN369749
1574	VC_520_2	1	0	10487.15943	0	0	0	0	1	1	1	0	0	0	0	KR824843
1575	VC_520_20	1	0	11180.31481	0	0	0	0	1	1	1	0	0	0	0	JN699012
1576	VC_520_21	1	0	10618.41653	0	0	0	0	1	1	1	0	0	0	0	MH669001
1577	VC_520_22	1	0	10108.90708	0	0	0	0	1	1	1	0	0	0	0	OP068340
1578	VC_520_23	2	178.4560001	22956.03437	0.007713851	0.042365924	8.717797887	8.717797887	1	1	1	8.71779789	0	0	0	JN859129,KF024723
1579	VC_520_24	1	0	11039.85058	0	0	0	0	1	1	1	0	0	0	0	EF536069
1580	VC_520_25	1	0	9676.551631	0	0	0	0	1	1	1	0	0	0	0	MH590598
1581	VC_520_26	3	858.7458015	32169.37056	0.026000447	0.001401023	5.099019514	6.244997998	3	3	1	5.69619339	0	0	0	HQ728524,JN698996,JX121091
1582	VC_520_27	2	300	20671.01852	0.014305457	0.042383652	0	0	1	1	1	0	0	0	0	KT281792,KY471267
1583	VC_520_28	2	300	21668.51747	0.013655906	0.042384694	3.605551275	3.605551275	1	1	1	3.60555128	0	0	0	JN020141,MT114167
1584	VC_520_29	1	0	9064.079229	0	0	0	0	1	1	1	0	0	0	0	KM597530
1585	VC_520_3	1	0	9522.278909	0	0	0	0	1	1	1	0	0	0	0	MF668287
1586	VC_520_30	2	161.7085748	22122.93015	0.007256504	0.045550803	8.831760866	8.831760866	1	1	1	8.83176087	0	0	0	MH825707,ON526976
1587	VC_520_31	11	9796.994206	99423.26537	0.089699422	2.18E-36	0	9.848857802	55	33	0.6	8.51832669	0	0	0	FJ174690,GU060500,JF937093,KT281795,MH020235,MH077580,MK016494,MN585973,MW924649,MZ681519,OP297536
1588	VC_520_32	1	0	11923.9228	0	0	0	0	1	1	1	0	0	0	0	MH651178
1589	VC_520_33	2	191.9853909	21888.68256	0.008694728	0.042384286	8.185352772	8.185352772	1	1	1	8.18535277	0	0	0	MH371123,MN369755
1590	VC_520_34	1	0	11816.1091	0	0	0	0	1	1	1	0	0	0	0	MH077578
1591	VC_520_35	3	848.2095059	32936.25638	0.025106494	0.001401434	3	6.480740698	3	3	1	5.07510111	0	0	0	KR997930,KR997931,KR997968
1592	VC_520_36	1	0	9777.770827	0	0	0	0	1	1	1	0	0	0	0	MH744415
1593	VC_520_37	1	0	10379.91041	0	0	0	0	1	1	1	0	0	0	0	JF937102
1594	VC_520_38	6	3172.802843	57905.53997	0.051946446	1.75E-11	3.31662479	10.04987562	15	13	0.866666667	7.79849748	0	0	0	KJ025956,MH155865,MH155871,MH590602,MK524517,MT310893
1595	VC_520_39	1	0	11733.19194	0	0	0	0	1	1	1	0	0	0	0	MH371120
1596	VC_520_4	1	0	10409.715	0	0	0	0	1	1	1	0	0	0	0	MH155866
1597	VC_520_40	4	1188.670512	28795.04166	0.039643874	1.22E-05	5.567764363	7.810249676	6	6	1	7.1365245	0	0	0	KU865302,KY965064,MH371115,MT553345
1598	VC_520_41	2	300	21208.72784	0.013947826	0.042385428	3.741657387	3.741657387	1	1	1	3.74165739	0	0	0	MH651183,MN586011
1599	VC_520_42	1	0	7531.032504	0	0	0	0	1	1	1	0	0	0	0	MH399771
1600	VC_520_43	3	610.5187355	30956.55654	0.019340364	0.001629566	6.92820323	8.717797887	3	3	1	8.08277546	0	0	0	JN020143,ON456356,ON970562
1601	VC_520_44	1	0	11639.30892	0	0	0	0	1	1	1	0	0	0	0	KY348865
1602	VC_520_45	7	4112.764836	60685.73201	0.063470065	5.05E-15	5.744562647	9.110433579	21	17	0.80952381	7.86168387	0	0	0	KT895281,MG872841,MH316564,MH399784,MH651169,MH834617,MK494110
1603	VC_520_46	1	0	9830.676191	0	0	0	0	1	1	1	0	0	0	0	MZ274306
1604	VC_520_47	1	0	10021.24975	0	0	0	0	1	1	1	0	0	0	0	MT684586
1605	VC_520_48	1	0	9422.671119	0	0	0	0	1	1	1	0	0	0	0	MZ171378
1606	VC_520_49	1	0	10598.13752	0	0	0	0	1	1	1	0	0	0	0	KM101122
1607	VC_520_5	1	0	10721.87687	0	0	0	0	1	1	1	0	0	0	0	MH727548
1608	VC_520_50	1	0	10749.61604	0	0	0	0	1	1	1	0	0	0	0	MT897904
1609	VC_520_51	1	0	10465.88992	0	0	0	0	1	1	1	0	0	0	0	MH051257
1610	VC_520_52	1	0	8236.15923	0	0	0	0	1	1	1	0	0	0	0	KC736071
1611	VC_520_53	1	0	9801.354288	0	0	0	0	1	1	1	0	0	0	0	KY385384
1612	VC_520_54	1	0	9383.172625	0	0	0	0	1	1	1	0	0	0	0	OM105885
1613	VC_520_55	1	0	10549.09198	0	0	0	0	1	1	1	0	0	0	0	OP297542
1614	VC_520_56	1	0	8986.963781	0	0	0	0	1	1	1	0	0	0	0	KM279937
1615	VC_520_57	1	0	9563.123029	0	0	0	0	1	1	1	0	0	0	0	MK359343
1616	VC_520_58	3	714.5275273	33340.52854	0.02098154	0.0014007	0	8.185352772	3	3	1	5.45690185	0	0	0	DQ398050,EU816590,ON970595
1617	VC_520_59	5	2704.14804	56150.51157	0.045946201	2.56E-08	1.732050808	7.211102551	10	10	1	5.04516783	0	0	0	JN542517,MH001448,MN585987,MN586005,MZ681512
1618	VC_520_6	3	900	31659.49111	0.02764171	0.001401594	0	1.414213562	3	3	1	0.94280904	0	0	0	MN586004,MT684597,MT684601
1619	VC_520_60	2	227.3489848	24667.77269	0.00913227	0.042387674	6.8556546	6.8556546	1	1	1	6.8556546	0	0	0	DQ398045,KT279576
1620	VC_520_61	1	0	11146.06478	0	0	0	0	1	1	1	0	0	0	0	MW055915
1621	VC_520_62	1	0	10999.16688	0	0	0	0	1	1	1	0	0	0	0	ON970566
1622	VC_520_63	1	0	11617.90847	0	0	0	0	1	1	1	0	0	0	0	MF919501
1623	VC_520_64	1	0	11087.91545	0	0	0	0	1	1	1	0	0	0	0	MG962361
1624	VC_520_65	1	0	11190.68355	0	0	0	0	1	1	1	0	0	0	0	MZ747519
1625	VC_520_66	1	0	10669.52564	0	0	0	0	1	1	1	0	0	0	0	MN586012
1626	VC_520_67	1	0	10218.28713	0	0	0	0	1	1	1	0	0	0	0	ON970610
1627	VC_520_68	1	0	11179.24913	0	0	0	0	1	1	1	0	0	0	0	KM347889
1628	VC_520_69	1	0	11375.71701	0	0	0	0	1	1	1	0	0	0	0	MH669014
1629	VC_520_7	1	0	11016.66876	0	0	0	0	1	1	1	0	0	0	0	MN310548
1630	VC_520_70	1	0	11020.72191	0	0	0	0	1	1	1	0	0	0	0	KC661280
1631	VC_520_71	1	0	10917.0638	0	0	0	0	1	1	1	0	0	0	0	FJ174693
1632	VC_520_72	1	0	11087.27983	0	0	0	0	1	1	1	0	0	0	0	MK494118
1633	VC_520_73	2	251.1239561	23167.32839	0.010723337	0.042372566	6.708203932	6.708203932	1	1	1	6.70820393	0	0	0	KR935214,MH669012
1634	VC_520_74	2	170.2183944	22535.57891	0.007496693	0.04239414	8.888194417	8.888194417	1	1	1	8.88819442	0	0	0	JF704117,MK112556
1635	VC_520_75	1	0	11705.76837	0	0	0	0	1	1	1	0	0	0	0	MN310544
1636	VC_520_76	2	194.3164302	21023.60947	0.009158126	0.042386242	7.745966692	7.745966692	1	1	1	7.74596669	0	0	0	JN398368,MT114164
1637	VC_520_77	3	697.7262384	31307.16208	0.021800615	0.001400887	1.414213562	8.246211251	3	3	1	5.92815441	0	0	0	JQ300538,KX576644,MK016496
1638	VC_520_78	2	186.3957452	23176.20389	0.007978382	0.042388467	8.062257748	8.062257748	1	1	1	8.06225775	0	0	0	MH590596,OP297528
1639	VC_520_79	1	0	10603.49463	0	0	0	0	1	1	1	0	0	0	0	KC661278
1640	VC_520_8	1	0	10591.73324	0	0	0	0	1	1	1	0	0	0	0	MZ958744
1641	VC_520_80	3	900	29587.9148	0.029519894	0.001400963	0	2.645751311	3	3	1	1.76383421	0	0	0	MH651188,MH669003,MK359315
1642	VC_520_81	1	0	11776.88464	0	0	0	0	1	1	1	0	0	0	0	MF919526
1643	VC_520_82	1	0	9603.914151	0	0	0	0	1	1	1	0	0	0	0	MN310545
1644	VC_520_83	1	0	9485.953804	0	0	0	0	1	1	1	0	0	0	0	KX610764
1645	VC_520_84	1	0	11029.18346	0	0	0	0	1	1	1	0	0	0	0	ON970585
1646	VC_520_85	2	300	19927.40518	0.014831364	0.042375953	0	0	1	1	1	0	0	0	0	KT895280,MN369751
1647	VC_520_86	1	0	8826.798885	0	0	0	0	1	1	1	0	0	0	0	MZ747512
1648	VC_520_87	1	0	10766.08325	0	0	0	0	1	1	1	0	0	0	0	MK524503
1649	VC_520_88	1	0	9961.080023	0	0	0	0	1	1	1	0	0	0	0	JN020142
1650	VC_520_89	1	0	10865.89646	0	0	0	0	1	1	1	0	0	0	0	MN585975
1651	VC_520_9	1	0	9166.765787	0	0	0	0	1	1	1	0	0	0	0	KF114874
1652	VC_520_90	1	0	10051.24931	0	0	0	0	1	1	1	0	0	0	0	MK016504
1653	VC_520_91	1	0	11030.35404	0	0	0	0	1	1	1	0	0	0	0	MZ209299
1654	VC_520_92	5	3000	42280.4134	0.066253812	2.55E-08	0	3.16227766	10	10	1	1.51622777	0	0	0	KM402757,MK359307,MN428051,MT889396,MT889397
1655	VC_520_93	2	199.9702461	19105.66095	0.01035813	0.042379207	8.366600265	8.366600265	1	1	1	8.36660027	0	0	0	JF937098,MH632119
1656	VC_520_94	1	0	9545.867104	0	0	0	0	1	1	1	0	0	0	0	MK967397
1657	VC_520_95	1	0	10438.81724	0	0	0	0	1	1	1	0	0	0	0	MK224592
1658	VC_520_96	1	0	10489.48078	0	0	0	0	1	1	1	0	0	0	0	AY129330
1659	VC_520_97	1	0	9270.754592	0	0	0	0	1	1	1	0	0	0	0	MG925354
1660	VC_520_98	1	0	7076.320832	0	0	0	0	1	1	1	0	0	0	0	MG812488
1661	VC_521_0	37	101981.1658	9834.697816	0.912045594	3.31E-267	0	8.306623863	666	666	1	5.61799173	0	0	0	JN256079,JN624851,KC576783,KF986246,KM588359,KU935726,KU935727,KU935728,KU935729,KU935730,KU935731,KX756439,MG099936,MG099948,MH316570,MH399787,MH697576,MH697585,MH697593,MH926055,MK392367,MK524500,MK524509,MK524515,MK524518,MK524520,MK524522,MK524528,MK977708,MT498042,MT498044,MT498048,MT684588,MT723932,MZ622162,OL742561,OP297540
1662	VC_522_0	3	395.6454258	4335.854974	0.083619443	0.001473402	5.744562647	7.416198487	3	3	1	6.79062123	0	0	0	KU963256,MN175604,ON645344
1663	VC_522_1	5	1047.165441	9246.92981	0.101724864	3.27E-08	5.744562647	9.38083152	10	8	0.8	7.78767062	0	0	0	MN428053,MN585966,MT521995,OP297548,OP434457
1664	VC_523_0	1	0	5035.835014	0	0	0	0	1	1	1	0	0	0	0	MK977709
1665	VC_524_0	2	300	2354.064437	0.113034181	0.045224332	1.414213562	1.414213562	1	1	1	1.41421356	0	0	0	MW584176,MW584199
1666	VC_524_1	1	0	1395.526051	0	0	0	0	1	1	1	0	0	0	0	MW584188
1667	VC_525_0	25	55792.22408	65572.42871	0.45970736	3.28E-174	0	8.602325267	300	300	1	5.87900478	0	0	0	KU963255,KU963257,KX557273,KX557287,MH479912,MH479927,MK016492,MK112547,MK801727,MK814755,MK820642,MK875793,MK875796,MK878898,MK919483,MT723938,MT889398,MT952844,MW712717,MW924648,MW924650,MZ171374,MZ274304,OP297546,OP434440
1668	VC_525_1	5	1187.724623	23290.69672	0.048521292	5.28E-07	7.549834435	9	10	9	0.9	8.3861217	0	0	0	KU963249,KU963263,MK919469,MK967384,MT639650
1669	VC_526_0	6	4500	5759.382448	0.438622892	5.13E-10	1.732050808	7.549834435	15	15	1	6.45307399	0	0	0	MG557979,MG765277,MG765279,MH809529,MH809530,MH809531
1670	VC_526_1	1	0	2341.779255	0	0	0	0	1	1	1	0	0	0	0	MF787246
1671	VC_526_2	1	0	1778.168355	0	0	0	0	1	1	1	0	0	0	0	AY682195
1672	VC_526_3	1	0	1359.863636	0	0	0	0	1	1	1	0	0	0	0	MK504444
1673	VC_527_0	3	191.4536294	610.73212	0.23866496	0.001601173	3.872983346	5.099019514	3	3	1	4.51819285	0	0	0	MW202473,MW202478,MW202738
1674	VC_529_0	2	191.413696	180.8907318	0.514132204	0.049743124	1.414213562	1.414213562	1	1	1	1.41421356	0	0	0	MW824391,MW824401
1675	VC_52_0	87	234431.8252	3380.135927	0.985786518	7.49E-138	0	6.08276253	3741	3741	1	1.06138173	0	0	0	AB259123,AB434711,AB937974,AB981170,KX179905,MF716957,ON170020,ON170023,ON170029,ON170030,ON170034,ON170037,ON170041,ON170050,ON170055,ON170087,ON170089,ON170091,ON170093,ON170095,ON170097,ON170101,ON170103,ON170105,ON170107,ON170109,ON170111,ON170113,ON170115,ON170117,ON170119,ON170121,ON170125,ON170127,ON170132,ON170143,ON170145,ON170147,ON170153,ON170155,ON170157,ON170159,ON170161,ON170163,ON170165,ON170168,ON170193,ON170195,ON170197,ON170199,ON170201,ON170203,ON170205,ON170207,ON170212,ON170216,ON170218,ON170220,ON170223,ON170226,ON170245,ON170247,ON170249,ON170251,ON170253,ON170273,ON170277,ON170281,ON170283,ON170285,ON170290,ON170292,ON170294,ON170296,ON170299,ON170301,ON170308,ON170312,ON170314,ON170316,ON170331,ON170333,ON170335,ON170337,ON170339,ON170341,ON170353
1676	VC_530_0	9	6075.930994	1493.315585	0.802712784	7.48E-13	2.645751311	7.071067812	36	36	1	4.52501467	0	0	0	AP019417,KJ936628,MF431726,MG592626,MK483107,MN062185,MW217515,MW451248,MW748992
1677	VC_531_0	9	10800	30734.32405	0.2600259	2.12E-09	2.645751311	6.244997998	36	36	1	4.53935448	0	0	0	KC131129,KP671755,KT919972,KT919973,MK568540,MT612988,MT612989,ON000910,ON190025
1678	VC_531_1	6	4500	25892.57823	0.148062463	0.000213354	2.828427125	8.831760866	15	15	1	5.80804956	0	0	0	AY283928,JN849462,MN794232,MT135026,OK905446,ON190024
1679	VC_531_2	1	0	5809.695788	0	0	0	0	1	1	1	0	0	0	0	HQ317393
1680	VC_531_3	1	0	5807.809061	0	0	0	0	1	1	1	0	0	0	0	MW824377
1681	VC_531_4	1	0	5803.044018	0	0	0	0	1	1	1	0	0	0	0	MG592456
1682	VC_531_5	1	0	8136.748311	0	0	0	0	1	1	1	0	0	0	0	MT135025
1683	VC_531_6	1	0	5779.187796	0	0	0	0	1	1	1	0	0	0	0	MT135024
1684	VC_532_0	2	300	441.4787999	0.404596868	0.105649774	1.414213562	1.414213562	1	1	1	1.41421356	0	0	0	AP019524,AP019525
1685	VC_532_1	1	0	368.5904716	0	0	0	0	1	1	1	0	0	0	0	LC168164
1686	VC_533_0	2	300	806.0109454	0.271245055	0.069143071	0	0	1	1	1	0	0	0	0	LR756501,LR756503
1687	VC_533_1	1	0	877.6125162	0	0	0	0	1	1	1	0	0	0	0	LR745206
1688	VC_533_2	1	0	885.9910368	0	0	0	0	1	1	1	0	0	0	0	LR756502

1689	VC_533_3	1	0	700.3472986	0	0	0	0	1	1	1	0	0	0	0	LR756504
1690	VC_534_0	9	10146.17645	102.1409323	0.990033395	1.19E-09	0	4.242640687	36	36	1	2.75680021	0	0	0	AP019527,FJ429185,KY554762,KY554763,KY554764,KY554765,KY554766,KY554767,MN528768
1691	VC_535_0	2	15.70169036	102.1409323	0.133242879	0.05936654	5.744562647	5.744562647	1	1	1	5.74456265	0	0	0	S4-wms_118690,S9-wms_19025
1692	VC_536_0	3	539.7638945	5604.981907	0.087841534	0.001464102	5.656854249	7.141428429	3	3	1	6.59942756	0	0	0	EF153632,MT708549,MW460246
1693	VC_539_0	3	168.3768118	0	1	0	3	3.741657387	3	3	1	3.35276073	0	0	0	AY048850,BK013337,BK013338
1694	VC_53_0	5	340.1298475	343.2957481	0.4976838	1.92E-06	0	4.123105626	10	10	1	2.47275816	0	0	0	HM064452,ON170025,ON170069,ON170139,ON170320
1695	VC_540_0	15	12482.00302	31065.41727	0.286630136	4.22E-68	1	8.366600265	105	105	1	6.64467241	0	0	0	KC139518,KT287080,MF158036,MF158037,MH424444,MK214385,MK479295,MN070121,MN393079,MT012729,MW725301,MZ004649,MZ501269,ON720976,S17-wms_266511
1696	VC_542_0	2	130.7598395	217.6741788	0.375278626	0.059077917	5.477225575	5.477225575	1	1	1	5.47722558	0	0	0	MW478292,OK247671
1697	VC_543_0	6	3484.410086	548.6388398	0.863964249	5.19E-07	0	3.16227766	15	15	1	1.91304472	0	0	0	MG592399,MG592418,MG592419,MG592510,MG592511,MG592579
1698	VC_544_0	2	244.0422632	449.8887193	0.351680887	0.059409431	0	0	1	1	1	0	0	0	0	MG592447,MG592453
1699	VC_546_0	2	300	21764.79714	0.013596318	0.362514023	8.660254038	8.660254038	1	1	1	8.66025404	0	0	0	JN201525,MH399772
1700	VC_546_1	2	300	21828.05743	0.013557449	0.31594849	7.937253933	7.937253933	1	1	1	7.93725393	0	0	0	MN586029,MW314846
1701	VC_546_10	1	0	11461.18437	0	0	0	0	1	1	1	0	0	0	0	AY129338
1702	VC_546_11	1	0	11323.32455	0	0	0	0	1	1	1	0	0	0	0	JF937101
1703	VC_546_2	3	900	31842.1138	0.027487535	0.147396579	4.69041576	5.656854249	3	3	1	5.14876317	0	0	0	JN698997,KM400683,MF919534
1704	VC_546_3	4	1800	41277.11567	0.041785527	0.078050118	4.358898944	8.485281374	6	6	1	6.59267293	0	0	0	KM925136,MF072690,MF133445,MF668284
1705	VC_546_4	16	36000	107019.4769	0.251713968	1.97E-07	4.472135955	9.591663047	120	113	0.941666667	7.4617397	0	0	0	KF114875,MH077579,MH669017,MH697582,MH697583,MH727551,MK524504,MK524521,MK524527,MK524529,MK967379,MK967402,MN062701,MN183286,MZ622169,MZ958748
1706	VC_546_5	1	0	11219.89964	0	0	0	0	1	1	1	0	0	0	0	MF919504
1707	VC_546_6	2	300	21959.02769	0.013477678	0.218907096	6.32455532	6.32455532	1	1	1	6.32455532	0	0	0	KF006818,MF919512
1708	VC_546_7	4	1800	42115.81183	0.040987515	0.003674896	6.403124237	8.94427191	6	6	1	7.72258529	0	0	0	JF937090,JF957059,KM101123,MK279849
1709	VC_546_8	1	0	11189.44157	0	0	0	0	1	1	1	0	0	0	0	MW132714
1710	VC_546_9	1	0	11190.99746	0	0	0	0	1	1	1	0	0	0	0	MK524516
1711	VC_54_0	6	489.3313593	238.3040791	0.672495227	1.01E-07	0	2.236067977	15	15	1	1.4547417	0	0	0	AB259124,AB828698,AB931172,JQ408219,LC066596,MT495635
1712	VC_550_0	12	18665.12486	4608.706307	0.801979044	2.27E-35	0	8.306623863	66	66	1	5.69683144	0	0	0	MW314855,MW314856,MW314858,MW584152,MW584158,MW584161,MW584164,MW584171,MW584179,MW584180,MW584196,MW584204
1713	VC_551_0	16	34817.81906	345562.9196	0.091534128	1.09E-58	0	10.34408043	120	80	0.666666667	8.30542041	0	0	0	JF937091,JF937106,MG872831,MG872832,MG872843,MH399774,MH399778,MH536820,MK016502,MK359320,MK801726,MK937593,MN096361,MN586013,MT723943,MW291023
1714	VC_551_1	4	1733.230065	107507.4298	0.015866163	0.000316626	6.782329983	9.327379053	6	4	0.666666667	8.39794815	0	0	0	MF919541,MH576956,MN586043,MN586044
1715	VC_551_2	17	40653.29819	377299.1555	0.097267758	2.10E-76	0	9.273618495	136	133	0.977941176	7.37256991	0	0	0	EU816591,JF937096,JN006061,JN006062,KF188414,KR080204,KT222941,KT222942,KU865303,KX817173,KY319168,MH513972,MH513978,MN586032,MN586051,MT114165,MZ274305
1716	VC_551_3	1	0	27822.30359	0	0	0	0	1	1	1	0	0	0	0	KF306380
1717	VC_551_4	2	300	49344.74346	0.006042936	0.043041426	3.741657387	3.741657387	1	1	1	3.74165739	0	0	0	JN412590,KF416343
1718	VC_551_5	2	278.1092754	50903.34479	0.00543379	0.044519377	8.888194417	8.888194417	1	1	1	8.88819442	0	0	0	MK016491,MK016498
1719	VC_551_6	22	67760.06557	490503.9482	0.121376381	4.40E-93	0	10.29563014	231	187	0.80952381	7.78923934	0	0	0	EU816588,KX611831,KX834009,MG757160,MG872837,MH371112,MH513983,MH536829,MH651174,MH669002,MH727557,MH779503,MK559429,MN428059,MN586034,MN586037,MT952854,MZ622157,MZ681505,OL742559,ON970597,ON970600
1720	VC_551_7	28	111344.4161	559083.8721	0.166079532	7.94E-159	0	10.34408043	378	322	0.851851852	7.84633949	0	0	0	AY129331,DQ398041,GQ303265,JN382248,JN391441,KC661277,KC691255,KC748971,KF279417,KT020852,KT373978,KY549152,MF668277,MH000607,MH536827,MH576953,MK359309,MK620893,MK757445,MN586017,MN586019,MN586035,MN586041,MN586050,MZ322024,MZ622158,MZ622163,ON45 6345
1721	VC_551_8	19	50413.44652	434972.1398	0.103862677	5.04E-79	0	9.899494937	171	149	0.871345029	7.47701522	0	0	0	KC748969,KF024732,KF493883,KF562099,KF562100,KF562101,KF562102,MF919506,MF919524,MF919525,MF919529,MF919535,MF919540,MG099953,MH020247,MH590587,MK433262,MN096364,MT522005
1722	VC_554_0	2	77.87004059	261.9314456	0.229163331	0.04806215	6.557438524	6.557438524	1	1	1	6.55743852	0	0	0	JN698994,MPU46938
1723	VC_555_0	13	13808.1958	4563.869163	0.751586489	1.09E-45	0	7.211102551	78	78	1	5.1258068	0	0	0	MW314852,MW584148,MW584150,MW584154,MW584156,MW584162,MW584163,MW584181,MW584182,MW584192,MW584203,MW584208,MW584212
1724	VC_556_0	21	63000	561.4156776	0.991167351	4.11E-67	0	5.099019514	210	210	1	3.64221959	0	0	0	AY129335,JN698993,KF017002,KF024730,KJ829260,KT281796,MG099943,MG872838,MH020246,MH590592,MH926062,MN428052,MN428058,MN585964,MT818418,MT818425,MW314845,MW712737,MZ209306,MZ747511,ON260829
1725	VC_557_0	3	459.9962677	5194.234025	0.081354357	0.00146721	8.717797887	9.16515139	3	2	0.666666667	8.90490338	0	0	0	MK937597,MN096370,OP434458
1726	VC_557_1	11	9997.72515	10220.21677	0.494497669	8.09E-35	2	9.539392014	55	43	0.781818182	8.29724861	0	0	0	MG099939,MH651176,MH651185,MH779504,MK279906,MK376961,MK814752,MZ388553,MZ747510,ON456334,ON526981
1727	VC_557_2	1	0	2254.986348	0	0	0	0	1	1	1	0	0	0	0	MH536812
1728	VC_557_3	1	0	2398.146174	0	0	0	0	1	1	1	0	0	0	0	MW534375
1729	VC_558_0	2	300	92368.60475	0.003237342	0.241415006	8.426149773	8.426149773	1	1	1	8.42614977	0	0	0	JN204348,JN412592
1730	VC_558_1	2	300	92830.90516	0.003221272	0.215949683	9	9	1	0	0	9	0	0	0	GQ303260,MT553335
1731	VC_558_10	1	0	47359.44854	0	0	0	0	1	1	1	0	0	0	0	MK112534
1732	VC_558_11	2	300	92981.86351	0.003216059	0.214714127	4.358898944	4.358898944	1	1	1	4.35889894	0	0	0	KT281790,MT889395
1733	VC_558_12	3	900	138757.9099	0.006444318	0.121457474	7.348469228	7.681145748	3	3	1	7.48193782	0	0	0	KC748970,MH825697,MK359348
1734	VC_558_13	1	0	47215.63528	0	0	0	0	1	1	1	0	0	0	0	MK359321
1735	VC_558_14	8	8400	360021.3364	0.022799982	0.000547538	2.449489743	9.797958971	28	23	0.821428571	7.95553113	0	0	0	MG872839,MH316561,MK359303,MK359311,MK359358,MK450427,ON526982,ON637768
1736	VC_558_15	1	0	47307.97164	0	0	0	0	1	1	1	0	0	0	0	MK359342
1737	VC_558_16	1	0	47349.47565	0	0	0	0	1	1	1	0	0	0	0	ON755189
1738	VC_558_17	1	0	47419.5358	0	0	0	0	1	1	1	0	0	0	0	MK359328
1739	VC_558_18	1	0	47432.14325	0	0	0	0	1	1	1	0	0	0	0	MN183285
1740	VC_558_19	1	0	47438.38156	0	0	0	0	1	1	1	0	0	0	0	ON637764
1741	VC_558_2	2	300	94325.92395	0.003170379	0.331400206	8.366600265	8.366600265	1	1	1	8.36660027	0	0	0	EU826471,MH727543
1742	VC_558_20	17	40800	714683.7422	0.054005133	1.59E-17	0	9.219544457	136	135	0.992647059	5.13099088	0	0	0	JF704096,KR080196,MF919493,MH697586,MH697590,MK359326,MK359335,MK359338,MK359339,MK359347,MK359362,MK450432,MN428066,MZ028626,MZ681511,ON081335,ON260824
1743	VC_558_21	1	0	46878.2198	0	0	0	0	1	1	1	0	0	0	0	MT723945
1744	VC_558_22	4	1800	178073.2196	0.010007048	0.00687181	7.141428429	9.38083152	6	5	0.833333333	8.03161037	0	0	0	JF704092,MH590593,MK450423,MZ958745
1745	VC_558_23	1	0	46933.08472	0	0	0	0	1	1	1	0	0	0	0	KX369583
1746	VC_558_24	1	0	47127.02634	0	0	0	0	1	1	1	0	0	0	0	OP021681
1747	VC_558_25	1	0	47633.32593	0	0	0	0	1	1	1	0	0	0	0	EU826468
1748	VC_558_26	1	0	47564.42932	0	0	0	0	1	1	1	0	0	0	0	EU826469
1749	VC_558_27	2	300	94507.21276	0.003164316	0.358645393	6.708203932	6.708203932	1	1	1	6.70820393	0	0	0	MK359361,MW712729
1750	VC_558_28	1	0	47332.358	0	0	0	0	1	1	1	0	0	0	0	MK359333
1751	VC_558_29	1	0	47620.2144	0	0	0	0	1	1	1	0	0	0	0	MT818416
1752	VC_558_3	1	0	47121.29767	0	0	0	0	1	1	1	0	0	0	0	MK359355
1753	VC_558_30	3	900	140553.3225	0.006362523	0.234259467	6.782329983	8.94427191	3	3	1	7.95021343	0	0	0	EU826467,JN699625,MK359344
1754	VC_558_31	1	0	47664.16351	0	0	0	0	1	1	1	0	0	0	0	DQ398053
1755	VC_558_32	1	0	47617.04415	0	0	0	0	1	1	1	0	0	0	0	MK310143
1756	VC_558_33	1	0	47421.54567	0	0	0	0	1	1	1	0	0	0	0	MH910040
1757	VC_558_34	1	0	47642.99373	0	0	0	0	1	1	1	0	0	0	0	MK112549
1758	VC_558_35	1	0	47493.15754	0	0	0	0	1	1	1	0	0	0	0	MT818423
1759	VC_558_36	4	1800	186052.3017	0.009581996	0.14086148	0	8.602325267	6	6	1	6.72255372	0	0	0	MK359336,MK359363,MK359366,MN234193
1760	VC_558_37	1	0	47370.33283	0	0	0	0	1	1	1	0	0	0	0	KT365400
1761	VC_558_38	2	300	94425.0999	0.003167059	0.356713713	7.615773106	7.615773106	1	1	1	7.61577311	0	0	0	MK878897,MN428065
1762	VC_558_39	3	900	140956.2253	0.006344452	0.261504262	7.810249676	9.327379053	3	2	0.666666667	8.42055571	0	0	0	MK359364,MT723944,OP297534
1763	VC_558_4	1	0	47493.56223	0	0	0	0	1	1	1	0	0	0	0	MK359353
1764	VC_558_40	1	0	47529.22438	0	0	0	0	1	1	1	0	0	0	0	MN276192
1765	VC_558_41	1	0	47691.65039	0	0	0	0	1	1	1	0	0	0	0	MK450424
1766	VC_558_42	1	0	47479.50419	0	0	0	0	1	1	1	0	0	0	0	MZ856343
1767	VC_558_43	1	0	47461.53961	0	0	0	0	1	1	1	0	0	0	0	MK359337
1768	VC_558_44	1	0	46847.27278	0	0	0	0	1	1	1	0	0	0	0	MK359345
1769	VC_558_45	1	0	47381.35474	0	0	0	0	1	1	1	0	0	0	0	MK359327
1770	VC_558_46	1	0	46960.96139	0	0	0	0	1	1	1	0	0	0	0	MK359349
1771	VC_558_47	3	900	140417.0076	0.00636866	0.199560568	6.92820323	8.717797887	3	3	1	7.70977196	0	0	0	MK112541,MK112545,MK112548
1772	VC_558_48	2	300	93781.4661	0.003188726	0.305338744	7.810249676	7.810249676	1	1	1	7.81024968	0	0	0	KF024734,KX721256
1773	VC_558_49	7	6300	318448.6069	0.019399621	0.004896307	6.32455532	9.949874371	21	18	0.857142857	8.26513953	0	0	0	JN624850,KC748968,KX721255,MH779498,MK112554,MK359334,OP068341
1774	VC_558_5	1	0	47488.13148	0	0	0	0	1	1	1	0	0	0	0	KC691257
1775	VC_558_50	15	31500	641172.7558	0.046828119	1.65E-13	4.123105626	9.949874371	105	97	0.923809524	7.41666042	0	0	0	JQ911768,KJ595575,KM881426,KT321476,MF919499,MH051266,MH316567,MH669008,MH825713,MK359314,MK359365,MK450429,MK450434,OK040789,OP021684
1776	VC_558_51	1	0	47204.54291	0	0	0	0	1	1	1	0	0	0	0	MK112533
1777	VC_558_52	1	0	47219.97891	0	0	0	0	1	1	1	0	0	0	0	MH744422
1778	VC_558_53	1	0	47570.91209	0	0	0	0	1	1	1	0	0	0	0	MK359306
1779	VC_558_54	1	0	47180.98025	0	0	0	0	1	1	1	0	0	0	0	MH779502
1780	VC_558_55	1	0	47112.5107	0	0	0	0	1	1	1	0	0	0	0	MF919494
1781	VC_558_56	1	0	47180.19033	0	0	0	0	1	1	1	0	0	0	0	MH727558
1782	VC_558_57	1	0	47038.93777	0	0	0	0	1	1	1	0	0	0	0	MK450425
1783	VC_558_58	1	0	47669.10236	0	0	0	0	1	1	1	0	0	0	0	MZ388551
1784	VC_558_59	1	0	47474.74816	0	0	0	0	1	1	1	0	0	0	0	MK359317
1785	VC_558_6	2	300	93293.9363	0.003205336	0.268507211	8.888194417	8.888194417	1	1	1	8.88819442	0	0	0	JF704116,KT365398
1786	VC_558_60	1	0	47606.57188	0	0	0	0	1	1	1	0	0	0	0	MN183280
1787	VC_558_61	2	300	94444.89654	0.003166397	0.338345214	8.888194417	8.888194417	1	1	1	8.88819442	0	0	0	MH051260,MK359318
1788	VC_558_62	1	0	47432.44668	0	0	0	0	1	1	1	0	0	0	0	MK359305
1789	VC_558_63	1	0	47645.36359	0	0	0	0	1	1	1	0	0	0	0	JN699626
1790	VC_558_64	1	0	47371.4805	0	0	0	0	1	1	1	0	0	0	0	MK359350
1791	VC_558_65	2	300	94787.19385	0.003154999	0.388258183	7.874007874	7.874007874	1	1	1	7.87400787	0	0	0	MK450422,MW291012
1792	VC_558_66	1	0	47654.53093	0	0	0	0	1	1	1	0	0	0	0	MK359330
1793	VC_558_67	1	0	47650.65395	0	0	0	0	1	1	1	0	0	0	0	OP021682
1794	VC_558_68	1	0	47579.9955	0	0	0	0	1	1	1	0	0	0	0	MG812494
1795	VC_558_69	1	0	47454.45477	0	0	0	0	1	1	1	0	0	0	0	MK359329
1796	VC_558_7	1	0	47343.74263	0	0	0	0	1	1	1	0	0	0	0	MH051261
1797	VC_558_70	1	0	47616.04637	0	0	0	0	1	1	1	0	0	0	0	ON456344
1798	VC_558_71	1	0	46911.88748	0	0	0	0	1	1	1	0	0	0	0	MK359346
1799	VC_558_72	1	0	47512.46907	0	0	0	0	1	1	1	0	0	0	0	MG812493
1800	VC_558_73	2	300	91083.29862	0.003282876	0.156251606	6.708203932	6.708203932	1	1	1	6.70820393	0	0	0	MH697579,MK359359
1801	VC_558_74	1	0	46728.57024	0	0	0	0	1	1	1	0	0	0	0	MF919495
1802	VC_558_75	1	0	47470.66446	0	0	0	0	1	1	1	0	0	0	0	ON551388
1803	VC_558_76	1	0	46972.36199	0	0	0	0	1	1	1	0	0	0	0	MW822148
1804	VC_558_77	3	900	137906.3086	0.006483855	0.091220611	7.745966692	9.486832981	3	2	0.666666667	8.36893585	0	0	0	MF919513,MK359308,MN062707
1805	VC_558_78	1	0	47242.75258	0	0	0	0	1	1	1	0	0	0	0	JN699627
1806	VC_558_79	1	0	47305.68387	0	0	0	0	1	1	1	0	0	0	0	MN735434
1807	VC_558_8	2	300	94033.49784	0.003180206	0.319694882	5.744562647	5.744562647	1	1	1	5.74456265	0	0	0	AY129337,JN699013
1808	VC_558_80	1	0	47529.16606	0	0	0	0	1	1	1	0	0	0	0	JN412588
1809	VC_558_81	1	0	46225.18959	0	0	0	0	1	1	1	0	0	0	0	KX752698
1810	VC_558_82	3	900	138687.6647	0.006447561	0.115661263	8	9.433981132	3	2	0.666666667	8.58020166	0	0	0	GQ303262,KX831080,MF919527
1811	VC_558_9	3	900	138960.6583	0.006434976	0.170884714	7.211102551	9.643650761	3	2	0.666666667	8.26400242	0	0	0	KF006817,KX781992,MK359324
1812	VC_559_0	2	300	26550.78442	0.011172858	0.043487187	7.549834435	7.549834435	1	1	1	7.54983444	0	0	0	EU826466,MF668280
1813	VC_559_1	1	0	14785.88997	0	0	0	0	1	1	1	0	0	0	0	MZ820085
1814	VC_55_0	3	412.2044165	1050.709626	0.281769403	0.001498887	1.732050808	2.828427125	3	3	1	2.2655153	0	0	0	CP006891,CP023680,CP023686
1815	VC_563_0	2	300	3827.425423	0.072684536	0.043574004	4.358898944	4.358898944	1	1	1	4.35889894	0	0	0	KJ567043,MK016501
1816	VC_564_0	3	799.5228981	1537.841199	0.342061769	0.001500873	1	2.645751311	3	3	1	2.03174702	0	0	0	MW584155,MW584175,MW584201
1817	VC_565_0	20	50899.66127	9706.93229	0.839837026	1.27E-107	0	5.916079783	190	190	1	2.34081619	0	0	0	DQ398047,DQ398051,EU676000,FJ168660,FJ168661,FJ168662,JF937107,JN699014,KJ194582,MH025888,MH316562,MH399788,MH834615,MK494102,MK524498,MK559427,MN119378,MN234227,MN284908,MT889385
1818	VC_566_0	2	278.0137445	2534.146485	0.098861275	0.043774736	3.16227766	3.16227766	1	1	1	3.16227766	0	0	0	AY129339,MK279909
1819	VC_567_0	8	7341.142214	7534.251051	0.493509118	2.33E-19	2	6.480740698	28	28	1	3.69202683	0	0	0	EU770222,FJ174691,KF986247,KJ567046,MG962376,MH509445,MN010756,MT521996
1820	VC_568_0	8	8286.314136	6156.062465	0.573750039	2.33E-19	1	3.741657387	28	28	1	2.69742317	0	0	0	JF704112,KF416342,KT591076,KY969628,MH001447,MH926059,MK757446,MT684602
1821	VC_569_0	3	900	3397.037133	0.209446642	0.001501332	1.732050808	2.449489743	3	3	1	2.13920284	0	0	0	JN412589,KR997933,ON108649
1822	VC_570_0	9	8792.373367	8827.992161	0.498989272	3.61E-24	0	6.633249581	36	36	1	3.7813655	0	0	0	KX557285,MH025890,MH271313,MH576972,MT639639,MT771345,MT771348,MZ171372,ON970620
1823	VC_571_0	4	1169.929722	4919.904907	0.192111903	1.41E-05	2.828427125	8.246211251	6	6	1	6.39988986	0	0	0	MG757156,MH834613,MN585993,MT776806
1824	VC_572_0	16	31595.28854	11770.00962	0.728584603	2.12E-71	0	6.633249581	120	120	1	4.22128436	0	0	0	MN234162,MN234190,MN234207,MN234220,MN234232,MN586003,MZ005671,OL455887,OL455890,OL455895,ON755185,ON755187,ON970611,OP172868,OP172870,OP172876
1825	VC_573_0	2	300	2427.425616	0.109993834	0.043606824	0	0	1	1	1	0	0	0	0	MN813687,ON108643
1826	VC_574_0	7	3301.506079	8774.589514	0.273391847	8.34E-15	1.732050808	7.681145748	21	21	1	5.26960757	0	0	0	JX901189,KF024728,MK279840,MW460248,MZ152914,OM793623,ON108645

1827	VC_575_0	35	136272.7823	18321.46948	0.881486735	5.26E-211	0	6	595	595	1	4.08958209	0	0	0	AY216660,HM035024,JX912252,KP085586,KU194205,KU194206,KX828710,KY985004,LC516895,MF093736,MF468274,MG049919,MH051911,MH051912,MH285980,MH917278,MK213796,MK335533,MK972831,MN153797,MN296515,MN337573,MN342247,MN393473,MT427400,MT682715,MZ501092,OL770108,OL800706,OL960578,OL960579,OL960582,ON185577,OP094641,OP455115
1828	VC_576_0	4	1163.704331	18025.63302	0.060643279	1.23E-05	2.645751311	8.485281374	6	6	1	6.12265542	0	0	0	MF996376,MH051918,MT023084,MZ501053
1829	VC_577_0	6	1548.022128	122.5337887	0.926650891	7.02E-08	1	8.246211251	15	15	1	5.97461018	0	0	0	AY236756,JN051154,JX486087,MG765275,MG765278,MH809528
1830	VC_578_0	2	22.31017736	108.0381512	0.171158139	0.060263944	7.615773106	7.615773106	1	1	1	7.61577311	0	0	0	MG020111,MK504442
1831	VC_57_0	4	552.7163666	768.5908318	0.418310267	1.33E-05	0	3	6	6	1	1.77614237	0	0	0	ON470596,ON470604,ON470624,ON470627
1832	VC_580_0	2	300	4446.40947	0.063205672	0.042366949	2.645751311	2.645751311	1	1	1	2.64575131	0	0	0	KC954775,OM638103
1833	VC_581_0	1	0	594.9849057	0	0	0	0	1	1	1	0	0	0	0	MW805361
1834	VC_581_1	1	0	692.8442743	0	0	0	0	1	1	1	0	0	0	0	OM982621
1835	VC_582_0	4	1800	28784.84824	0.058852671	3.01E-05	2	8.185352772	6	6	1	5.64411039	0	0	0	MN184887,MN732867,OP480062,OP480063
1836	VC_582_1	3	900	13595.40449	0.062088643	0.001844336	0	1	3	3	1	0.66666667	0	0	0	OK136180,OK181858,OK181861
1837	VC_582_2	1	0	8711.10173	0	0	0	0	1	1	1	0	0	0	0	KM190144
1838	VC_582_3	12	19800	64213.85183	0.235675422	1.08E-32	4.795831523	9.899494937	66	57	0.863636364	7.51381629	0	0	0	KT934943,KX452695,KY652726,MG250486,MK024806,MT178275,MT438396,MT939253,MZ602147,ON602729,ON602736,OP296941
1839	VC_582_4	3	900	24990.57229	0.034761688	0.01826497	7.348469228	8.062257748	3	3	1	7.74032555	0	0	0	JN882284,KF550303,MZ311864
1840	VC_582_5	4	1800	32301.79282	0.052783149	0.000643752	5.477225575	9.486832981	6	5	0.833333333	7.52142506	0	0	0	GU070616,JX181824,MW006477,MZ326168
1841	VC_582_6	1	0	7520.844121	0	0	0	0	1	1	1	0	0	0	0	MG969411
1842	VC_584_0	28	82831.99977	24681.44858	0.770433848	1.19E-184	0	8	378	378	1	5.08482933	0	0	0	AY308796,KM236241,KX015770,KX015771,KY657202,KY694971,LR597656,MG241338,MH102284,MH491968,MH729819,MN994500,MT478992,MW021748,MW021750,MW598258,MZ501059,OK499973,OK499984,OL539443,OL539444,OL539461,OL539462,OL539465,OL539466,ON843697,OP292673,OP292674
1843	VC_585_0	4	1279.394267	22727.52265	0.053292735	1.25E-05	4.242640687	8.124038405	6	6	1	6.34146046	0	0	0	MN019128,MW032452,MZ501077,MZ501085
1844	VC_585_1	3	518.9018679	16078.33367	0.031264355	0.00142332	4.795831523	7.874007874	3	3	1	6.69534596	0	0	0	MK778457,MN090155,MZ501099
1845	VC_585_2	1	0	4166.535327	0	0	0	0	1	1	1	0	0	0	0	MW689258
1846	VC_585_3	1	0	5417.048994	0	0	0	0	1	1	1	0	0	0	0	OM317910
1847	VC_586_0	3	679.2056022	13028.51838	0.049549116	0.001400665	4.472135955	5.385164807	3	3	1	4.88437743	0	0	0	MN101222,OK019720,ON045001
1848	VC_587_0	2	78.10662623	162.1078398	0.325153716	0.088071125	7.681145748	7.681145748	1	1	1	7.68114575	0	0	0	AY320035,KT221034
1849	VC_588_0	2	9.923238075	109.4041428	0.083159774	0.056049052	7.348469228	7.348469228	1	1	1	7.34846923	0	0	0	KU958700,MH744420
1850	VC_589_0	2	36.60716638	0	1	0	2.236067977	2.236067977	1	1	1	2.23606798	0	0	0	AY324828,MN710383
1851	VC_590_0	27	105300	557.7903275	0.994730758	1.48E-107	2.449489743	7.211102551	351	351	1	5.27674722	0	0	0	AY343333,EF437941,EU863408,HE978309,KY683735,MF327006,MH560358,MK327932,MK327935,MK327941,MK327947,MK573636,MK962753,MN655998,MN709127,MN850574,MN850615,MN882349,MN894885,MT478991,MT764208,MW748999,MZ504876,MZ504877,MZ504878,ON286974,OX001802
1852	VC_591_0	14	19127.0017	17769.9264	0.518390085	2.94E-57	0	4.582575695	91	91	1	2.11587904	0	0	0	CP059058,KP063118,MG030346,MN062188,MN604053,MN840486,MW145139,OK499974,OK499975,OK499986,OK499998,OK500000,OL741431,OM962992
1853	VC_592_0	50	261416.1014	95983.34738	0.731439576	0	0	7.416198487	1225	1225	1	4.0712103	0	0	0	JN871591,JQ340774,JX094499,JX296113,KC139512,KC139514,KC139515,KC139519,KC677662,KM366098,KM458633,KR296689,KR296691,KU927500,KX452699,KX452700,KX765862,KX765863,KX765864,KY014601,KY620117,LC553736,LR026998,MG280946,MH631453,MH791395,MH791396,MK249308,MK286578,MN871450,MT499897,MT499898,MT675124,MT675125,MT675126,MW057860,MW149272,MW355449,MW355460,MW355467,MW355470,MW355474,MW355476,MW355479,OL606627,OL699877,OM025231,OP453108,OP535471,OP535472
1854	VC_593_0	5	2754.527615	3222.868399	0.460824012	5.09E-08	0	4.358898944	10	10	1	1.74355958	0	0	0	KM581061,MH015249,MH015252,MH015254,MH015258
1855	VC_594_0	2	207.4114343	2608.698784	0.073651746	0.043141407	3.872983346	3.872983346	1	1	1	3.87298335	0	0	0	KT321316,OK041469
1856	VC_597_0	6	3119.77287	14198.54361	0.180142964	1.89E-11	2	6.782329983	15	15	1	3.92486478	0	0	0	MK838113,MN317029,MT952005,MW978786,OL840901,OL964754
1857	VC_598_0	4	190.1483101	2996.216586	0.059675623	0.006446423	6.403124237	8.544003745	6	6	1	7.9641815	0	0	0	HQ632859,KY249644,MF476925,MW269554
1858	VC_599_0	50	49785.4191	54.95993934	0.998897281	2.66E-10	0	6.92820323	1225	1225	1	4.87863241	0	0	0	AY370762,AY388628,AY423772,EU030938,EU030939,FJ870915,FJ870916,FJ870917,KC618393,KY563228,KY579375,MK054204,MK054205,MK054206,MK054207,MK054208,MK054209,MK054210,MK054211,MK054212,MK054213,MK054214,MK054215,MK054216,MK054217,MK054218,MK054219,MK054220,MK054221,MK054222,MK054223,MK054224,MK054225,MK054226,MK054227,MK054228,MK054229,MK054230,MK054231,MK054232,MK054233,MK054234,MK054235,MK054236,MK054237,MN496305,MN496306,MN496307,MN496308,X07234
1859	VC_5_0	30	23500.21387	859.7231277	0.964707498	3.70E-95	0	7.141428429	435	435	1	5.80011839	0	0	0	AB374228,AF125163,AY027935,BHU24159,DQ114220,DQ674738,HG803181,HQ110083,KF361475,KT887559,KU504502,KX058879,KX774374,KY045851,LR597637,LR597638,LR597640,LR597649,MG592573,MK972712,MK972713,MK972714,MN563793,MT662115,OM095401,OP484843,S1-wms-combined_159544,S11-wms-combined_154845,S5-wms-combined_18356,S6-wms-combined_119017
1860	VC_600_0	3	900	15724.75535	0.054136135	0.028899307	2.449489743	5.830951895	3	3	1	4.6457653	0	0	0	MK577502,MN871507,ON042478
1861	VC_600_1	7	6300	27987.24762	0.183741783	3.05E-08	0	3.16227766	21	21	1	2.5408393	0	0	0	AY375531,AY962392,KY290948,KY290951,KY290956,KY290957,KY290958
1862	VC_600_2	10	13500	30900.04694	0.304053733	2.28E-16	3.872983346	7.280109889	45	45	1	5.62986696	0	0	0	DQ529280,HM452125,JN377894,JN377895,JX306041,MF479730,MH179476,MH179477,MW584871,OL840900
1863	VC_602_0	2	186.7156519	628.600438	0.22901014	0.044394478	1.732050808	1.732050808	1	1	1	1.73205081	0	0	0	JN190960,MW677528
1864	VC_603_0	9	3372.761814	25.6490689	0.992452629	5.86E-08	0	1.414213562	36	36	1	0.31426968	0	0	0	AY441783,AY848684,AY848685,AY848686,AY848687,AY848688,AY848689,OK041468,OM630588
1865	VC_604_0	22	20750.49858	5695.694667	0.784630831	3.31E-82	0	8.124038405	231	231	1	5.77500235	0	0	0	KR093634,KR093635,KR093636,KR093637,KR093639,KR093640,KR093641,KR093642,KR093643,KR093644,KR093645,KR093646,KR093647,KR093648,KR093649,KR093650,KR093651,KR093652,KR093653,KR093654,KR093655,KR093656
1866	VC_606_0	4	872.5909345	1063.605231	0.450672793	4.85E-05	5.830951895	8.602325267	6	6	1	7.726646	0	0	0	MG711465,S11-wms_55610,S2-wms_107329,S8-wms_82880
1867	VC_606_1	1	0	882.4291923	0	0	0	0	1	1	1	0	0	0	0	S9-wms-combined_29611
1868	VC_607_0	16	18489.69643	1654.767957	0.917854954	8.89E-52	0	5.477225575	120	120	1	2.70067024	0	0	0	AY505112,AY510084,JF712866,KM612259,KM612260,KM612261,KM612262,KM612263,KM612264,KM612265,KY883642,KY883647,KY883656,MH105773,MW824387,MW824404
1869	VC_608_0	5	1567.62124	2051.577509	0.433140413	4.03E-08	1.414213562	4.795831523	10	10	1	3.76301008	0	0	0	MG674163,MT992243,MW176032,MW176034,MW590698
1870	VC_609_0	4	169.099382	176.6966874	0.48901476	7.58E-05	6.244997998	7.483314774	6	6	1	6.9621696	0	0	0	MF074189,MW202496,MW202711,MW202879
1871	VC_60_0	5	2441.575006	3321.121314	0.423686217	3.49E-08	0	0	10	10	1	0	0	0	0	OK490460,OK490463,OK490466,OK490469,OK490472
1872	VC_610_0	4	523.2076095	72.77961896	0.877883929	0.000146702	3.605551275	8.774964387	6	6	1	7.16128168	0	0	0	S1-wms-combined_107073,S2-wms-combined_89742,S4-wms-combined_41490,S8-wms-combined_26087
1873	VC_611_0	2	93.91643864	0	1	0	1	1	1	1	1	1	0	0	0	AY569307,GU080336
1874	VC_612_0	9	6923.000841	330.0044463	0.954501006	1.36E-09	0	1.414213562	36	36	1	0.97140452	0	0	0	AY575774,DQ163914,KU884561,KU884562,KU884563,KU884564,KU884565,MK514283,ON857941
1875	VC_613_0	2	12.20284227	330.0044463	0.035659212	0.058056818	4	4	1	1	1	4	0	0	0	JF412296,OM519319
1876	VC_614_0	5	2392.952933	7598.207687	0.239507003	3.05E-08	0	6.164414003	10	10	1	4.58269575	0	0	0	KX198614,MT795651,MW114771,ON457558,ON457559
1877	VC_615_0	2	68.12788864	187.2033649	0.266821581	0.058219991	5.916079783	5.916079783	1	1	1	5.91607978	0	0	0	CAKLQA020000003,MZ326864
1878	VC_616_0	2	11.90255483	4.352786502	0.732224233	0.185546685	5.196152423	5.196152423	1	1	1	5.19615242	0	0	0	S11-wms-combined_193148,S11-wms-combined_204522
1879	VC_618_0	13	21408.53374	12834.19385	0.625199429	1.55E-40	2.828427125	8.185352772	78	78	1	4.43282022	0	0	0	JX128258,MH051335,MH816966,MK373776,MK373777,MK903282,MN038177,MN176217,MT446405,MT446408,OL856099,ON210137,OP352608
1880	VC_61_0	12	1595.563257	494.9021532	0.76325743	6.80E-23	0	1.414213562	66	66	1	0.54913092	0	0	0	AB334720,AB334721,F1CCG,GQ153916,GQ153917,GQ153918,GQ153919,JF719732,JF719733,JF719734,JX412914,PFDCG
1881	VC_620_0	27	91761.93946	12636.70769	0.87895717	4.28E-154	0	6.633249581	351	351	1	2.17482563	0	0	0	KT361654,KT361655,KT361656,KT361657,OP494865,OP503543,OP503968,OP503969,OP503970,OP503971,OP503972,OP503973,OP503974,OP503975,OP503976,OP503977,OP503978,OP503979,OP503980,OP503981,OP503982,OP503983,OP503984,OP503985,OP503986,OP503987,OP503988
1882	VC_621_0	7	1324.371047	965.9188405	0.578254768	7.63E-14	5.567764363	8.485281374	21	21	1	6.90294788	0	0	0	MG711464,S1-wms-combined_23248,S11-wms-combined_140009,S12-wms-combined_345948,S12-wms-combined_71235,S17-wms-combined_90378,S4-wms-combined_42647
1883	VC_622_0	2	15.27436348	286.2217024	0.0506619	0.374060699	6.403124237	6.403124237	1	1	1	6.40312424	0	0	0	MF176161,MH884512
1884	VC_623_0	1	0	723.8746465	0	0	0	0	1	1	1	0	0	0	0	MK448818
1885	VC_625_0	2	31.14175232	1172.949692	0.025863278	0.075194384	8.246211251	8.246211251	1	1	1	8.24621125	0	0	0	MK448890,MK448930
1886	VC_626_0	23	13604.18923	7008.47845	0.659991683	1.21E-89	0	9.899494937	253	212	0.837944664	7.7258118	0	0	0	MF172979,MK448667,MK448700,MK448720,MK448839,MK448841,MK448843,MK448934,MK448997,MW387018,S1-wms-combined_332236,S11-wms-combined_194205,S12-wms-combined_368462,S12-wms-combined_7174,S12-wms-combined_99704,S17-wms-combined_9864,S2-wms-combined_174241,S2-wms-combined_244564,S5-wms-combined_206050,S5-wms-combined_29272,S8-wms-combined_108917,S8-wms-combined_186054,S9-wms-combined_25007
1887	VC_626_1	1	0	1219.139539	0	0	0	0	1	1	1	0	0	0	0	S9-wms-combined_9931
1888	VC_626_2	1	0	830.7329334	0	0	0	0	1	1	1	0	0	0	0	S12-wms-combined_98046
1889	VC_626_3	1	0	1161.335998	0	0	0	0	1	1	1	0	0	0	0	S5-wms-combined_108794
1890	VC_627_0	3	68.07235785	251.9366996	0.212720097	0.00588632	6.480740698	7.615773106	3	3	1	7.05586054	0	0	0	S1-wms_170115,S5-wms-combined_73301,S6-wms-combined_113986
1891	VC_629_0	2	3.36577704	819.335632	0.004091128	0.064504847	7.810249676	7.810249676	1	1	1	7.81024968	0	0	0	S2-wms-combined_196783,S7-wms_80255
1892	VC_630_0	20	37595.95862	6569.501769	0.851252501	1.10E-70	0	6.164414003	190	190	1	3.32817466	0	0	0	AY625898,KC262634,KC900378,LT603684,MF490237,MK511039,MK511040,MK511041,MK511042,MK511043,MK511044,MK511045,MK511046,MK511047,MK511048,MK511049,MK511050,MK511051,MT118301,MZ285879
1893	VC_631_0	3	583.4978387	4225.696033	0.121329656	0.00180801	1.414213562	5.477225575	3	3	1	4.12288824	0	0	0	MT152150,MT491207,MT491208
1894	VC_632_0	37	80454.77271	58027.82614	0.58097388	1.44E-299	0	10.29563014	666	615	0.923423423	6.29049688	0	0	0	FM864213,FN997652,KC348603,KC581799,KT336320,KT336321,KX077896,MK359990,MK448674,MK448741,MK448752,MK448811,MK448819,MK448828,MK448983,MN270258,MN270259,MN270260,MN270261,MN270262,MN270263,MN270264,MN270265,MN270266,MN270267,MN270268,MN270269,MN270270,MN270271,MN270272,MN270273,MN270274,MN270275,MN270276,MN270277,MN270278,MN270279
1895	VC_634_0	3	900	3499.194644	0.2045829	0.001624074	4	5.385164807	3	3	1	4.72699878	0	0	0	KU052488,MG252693,MG765276
1896	VC_634_1	6	3883.235044	5301.34001	0.422799642	4.42E-11	3	8	15	15	1	6.39104702	0	0	0	JX486088,KX223815,KY381600,MG744354,MG765274,ON117106
1897	VC_636_0	9	3588.703747	11229.47162	0.242182567	2.80E-24	0	9.110433579	36	35	0.972222222	7.07986276	0	0	0	KM289195,MK448681,MK448683,MK448783,MK448790,MK448798,MK448833,MK448967,MK449010
1898	VC_637_0	115	515693.5824	189774.6858	0.730994725	0	0	9.643650761	6555	5745	0.876430206	7.89659356	0	0	0	AY730274,EF177456,HE775250,HG934469,HG934470,JQ007353,JX202565,KC139511,KR270151,KT881477,KT962832,KU595434,KU951146,KU951147,KX245013,KX405002,KX649889,KY379853,KY626163,LC507823,MF001356,MF001366,MF431252,MG646668,MG969408,MG969409,MG969410,MH358359,MH370358,MH370359,MH370360,MH370361,MH370362,MH370363,MH370365,MH370372,MH370373,MH370374,MH370381,MH370384,MH370385,MH424443,MH424445,MH992509,MK249126,MK761195,MK761196,MK761197,MK761198,MK761199,MK770409,MK972691,MK972700,MK972701,MK972702,MK972703,MK972705,MK972706,MN336264,MN336265,MN336266,MN393078,MN722429,MN850616,MT074431,MT074432,MT074451,MT074462,MT074473,MT074481,MT074482,MT074483,MT074484,MT588083,MT632017,MT632018,MT632019,MT632020,MT653137,MT653144,MT677934,MT740291,MW201959,MW355463,MW355464,MW355465,MW715619,MW790495,MZ156567,MZ156568,MZ156569,MZ274225,MZ955866,OK108608,OK108609,OK143508,OL436139,OL519843,OL652658,OL770276,OM210019,OM291373,OM339548,ON167532,ON381768,ON409568,ON409569,ON409570,ON454038,ON526838,ON540301,ON720977,ON996339,OP484850,OP494211
1899	VC_637_1	27	54781.84372	157737.5817	0.257773348	6.84E-210	0	9.695359715	351	346	0.985754986	5.51914278	0	0	0	JX297445,KF562864,KF562865,KY176369,MK578530,MK759883,MK931446,MT074430,MT677933,MW355451,MW355452,MW355453,MW355454,MW355455,MW355456,MW355457,MW355458,MW355459,MW355461,MW355462,MW355466,MW355468,MW355469,MW355471,MW355472,MW355473,OK169616
1900	VC_637_2	2	61.84280275	16192.21137	0.003804762	0.071550726	8.717797887	8.717797887	1	1	1	8.71779789	0	0	0	KF148055,KM272358
1901	VC_638_0	45	134780.0923	39334.04894	0.774090441	0	0	8.774964387	990	990	1	7.06682833	0	0	0	GU196277,GU196278,GU196279,GU196280,GU196281,KY295895,KY295896,KY295898,MF153391,MG099933,MG252615,MG852086,MH051334,MK542015,MK737937,MK931438,MK931439,MK931440,MK931441,MN927225,MN927226,MT711523,MT711524,MT711525,MT711526,MT711527,MT711528,MW331437,MW722821,MZ234010,MZ234048,MZ576217,MZ576218,MZ832314,MZ997838,OK539620,OL539727,OM272052,OM632667,OM632668,OM810255,ON001686,ON391668,ON548432,ON556632
1902	VC_639_0	2	146.9309448	379.5334857	0.279089975	0.044730829	5.744562647	5.744562647	1	1	1	5.74456265	0	0	0	MK813940,OM033133
1903	VC_63_0	2	186.1693958	1436.314803	0.114743426	0.043104024	0	0	1	1	1	0	0	0	0	AP009146,DQ785801
1904	VC_640_0	7	3089.679768	333.8623179	0.90248044	2.24E-11	2.449489743	3.872983346	21	21	1	3.31101335	0	0	0	AY739900,EU340421,KF188410,KJ564036,KJ564037,KJ564038,MG913376
1905	VC_642_0	7	2417.598764	13611.55131	0.150825138	4.26E-15	0	9.327379053	21	16	0.761904762	7.93946224	0	0	0	HQ632860,MG592506,MG592545,MG592567,MG592568,MG592600,MG592630
1906	VC_642_1	1	0	2702.709744	0	0	0	0	1	1	1	0	0	0	0	OX241574
1907	VC_642_2	6	1388.437513	6879.206365	0.167936299	3.78E-11	5.744562647	8.124038405	15	15	1	7.21619728	0	0	0	AY772740,JF974313,MG676223,MK097141,ON714422,OP375635
1908	VC_642_3	9	4038.243012	14915.79995	0.21305444	8.04E-19	0	9.327379053	36	25	0.694444444	7.72374278	0	0	0	KX507045,MG592494,MG592501,MG592508,MG592514,MG592583,MG592587,MG592595,MG592644
1909	VC_642_4	2	286.2198086	5230.614639	0.051881167	0.043955187	3.605551275	3.605551275	1	1	1	3.60555128	0	0	0	MW824412,MW824413
1910	VC_642_5	4	842.4429109	8500.710663	0.09016687	3.34E-05	0	8.660254038	6	6	1	6.83296571	0	0	0	MG592544,MG592664,MG592665,OX241573
1911	VC_643_0	2	48.5982064	396.602511	0.109160216	0.049146528	7.681145748	7.681145748	1	1	1	7.68114575	0	0	0	JF974287,MK249872
1912	VC_644_0	5	642.4447683	323.798235	0.664889439	2.49E-07	7.211102551	8.544003745	10	10	1	7.81242481	0	0	0	KC542353,KP994596,KU747973,MK387309,MN313256
1913	VC_645_0	8	3940.101255	8615.808508	0.313804521	1.07E-18	4.472135955	7.348469228	28	28	1	6.58760414	0	0	0	MG592390,MG592420,MG592423,MG592439,MG592444,MG592454,MG592465,MG592466
1914	VC_646_0	29	66273.46258	6306.494515	0.913109696	5.45E-161	0	8.124038405	406	406	1	6.92851206	0	0	0	MG592393,MG592477,MG592478,MG592518,MG592540,MG592552,MG592569,MG592570,MG592608,MG592614,MG592622,MG592627,MG592628,MG592641,MG592647,MG592656,MW824369,MW824373,MW824374,MW824378,MW824379,MW824383,OX241429,OX241438,OX241498,OX241557,OX241558,OX241575,OX241591
1915	VC_647_0	11	7027.924156	4724.127414	0.59801679	3.90E-34	1.732050808	5.567764363	55	55	1	4.2045458	0	0	0	CP069347,HG796225,HG798901,JX145342,LK985321,LN681538,LN681540,MK473382,MT193276,MW512571,MW512572
1916	VC_649_0	3	439.1384791	1011.010578	0.302822994	0.001613846	0	7.874007874	3	3	1	5.24933858	0	0	0	KM983328,KM983331,KM983334
1917	VC_64_0	3	758.319256	619.7639384	0.550271028	0.002143148	2.828427125	3.741657387	3	3	1	3.34472871	0	0	0	JN022534,MK798143,MK798144
1918	VC_650_0	10	1114.899638	1341.973229	0.453788087	2.80E-17	3.872983346	8.426149773	45	45	1	6.8649715	0	0	0	S1-wms-combined_353757,S11-wms-combined_119215,S11-wms-combined_299954,S11-wms-combined_49632,S17-wms-combined_136622,S17-wms-combined_247494,S17-wms-combined_79709,S2-wms-combined_126134,S5-wms-combined_145067,S7-wms-combined_31068
1919	VC_651_0	1	0	311.6378685	0	0	0	0	1	1	1	0	0	0	0	S2-wms-combined_24549
1920	VC_652_0	4	1163.472412	1289.531447	0.47430517	1.92E-05	1	7.141428429	6	6	1	5.58314361	0	0	0	EU874396,GU229986,KX227758,KX227759
1921	VC_654_0	4	816.5700608	2001.800487	0.289731264	1.74E-05	1	7.280109889	6	6	1	5.57740093	0	0	0	KT725776,KX227757,KX227760,MW302330
1922	VC_655_0	2	300	26685.92375	0.011116907	0.042955046	2	2	1	1	1	2	0	0	0	GU071099,MH512890
1923	VC_655_1	1	0	13662.93816	0	0	0	0	1	1	1	0	0	0	0	GU071103
1924	VC_655_2	3	900	38009.82916	0.023130402	0.001392441	2	7.549834435	3	3	1	5.60998144	0	0	0	AY940168,HQ337021,HQ634176
1925	VC_655_3	1	0	14822.53253	0	0	0	0	1	1	1	0	0	0	0	HQ634175
1926	VC_655_4	1	0	14769.21429	0	0	0	0	1	1	1	0	0	0	0	JN371769
1927	VC_655_5	3	900	53412.14728	0.016570879	0.001393701	1	2.449489743	3	3	1	1.89518591	0	0	0	KU686207,KU686208,KU686209
1928	VC_655_6	2	300	38023.22407	0.007828151	0.042248323	2	2	1	1	1	2	0	0	0	GU071106,HQ634190
1929	VC_655_7	3	900	53384.96291	0.016579177	0.001430221	5.385164807	7	3	3	1	5.95413013	0	0	0	HQ634189,MH920639,MH920640
1930	VC_655_8	1	0	18916.10699	0	0	0	0	1	1	1	0	0	0	0	GU071094
1931	VC_655_9	1	0	16998.53129	0	0	0	0	1	1	1	0	0	0	0	GU071097
1932	VC_656_0	2	300	33440.59173	0.008891367	0.077434819	1.414213562	1.414213562	1	1	1	1.41421356	0	0	0	HQ316583,HQ316603
1933	VC_656_1	46	310500	54309.80035	0.85112845	0	0	6.403124237	1035	1035	1	1.32292446	0	0	0	KJ019028,KJ019029,KJ019031,KJ019032,KJ019034,KJ019035,KJ019036,KJ019046,KJ019047,KJ019048,KJ019050,KJ019056,KJ019057,KJ019062,KJ019070,KJ019072,KJ019073,KJ019074,KJ019075,KJ019077,KJ019078,KJ019079,KJ019080,KJ019083,KJ019112,KJ019113,KJ019115,KJ019117,KJ019118,KJ019119,KJ019120,KJ019121,KJ019124,KJ019125,KJ019126,KJ019127,KJ019129,KJ019130,KJ019131,KJ019136,KJ019139,KJ019140,KJ019160,KJ019162,KJ019164,KJ019165
1934	VC_657_0	3	900	40501.8411	0.021738164	0.074718742	3.741657387	5.099019514	3	3	1	4.64656547	0	0	0	KU686212,KU686213,MT586120
1935	VC_657_1	41	246000	82480.51936	0.748902859	8.25E-308	0	7.211102551	820	820	1	4.60136291	0	0	0	KJ019037,KJ019045,KJ019052,KJ019053,KJ019058,KJ019059,KJ019066,KJ019085,KJ019086,KJ019090,KJ019091,KJ019092,KJ019093,KJ019095,KJ019096,KJ019097,KJ019098,KJ019099,KJ019100,KJ019101,KJ019102,KJ019103,KJ019105,KJ019106,KJ019107,KJ019111,KJ019123,KJ019141,KJ019142,KJ019143,KJ019144,KJ019145,KJ019146,KJ019147,KJ019148,KJ019149,KJ019150,KJ019151,KJ019152,KJ019155,KJ019159
1936	VC_659_0	2	5.107437333	196.2328999	0.025367184	0.454010834	5.196152423	5.196152423	1	1	1	5.19615242	0	0	0	MH319737,MH319746
1937	VC_65_0	2	300	1343.601474	0.182525998	0.044411797	6.8556546	6.8556546	1	1	1	6.8556546	0	0	0	MT366760,MT366761
1938	VC_662_0	62	567300	245957.5768	0.697564973	0	2.236067977	7.615773106	1891	1891	1	4.88716728	0	0	0	HQ317290,HQ317291,HQ317292,KX349226,KX349227,KX349228,KX349229,KX349230,KX349231,KX349232,KX349233,KX349234,KX349235,KX349236,KX349237,KX349238,KX349239,KX349240,KX349241,KX349242,KX349243,KX349244,KX349245,KX349246,KX349247,KX349248,KX349249,KX349250,KX349251,KX349252,KX349253,KX349254,KX349255,KX349256,KX349257,KX349258,KX349259,KX349260,KX349261,KX349262,KX349263,KX349264,KX349265,KX349266,KX349267,KX349268,KX349269,KX349270,KX349271,KX349272,KX349273,KX349274,KX349275,KX349276,KX349277,KX349278,KX349279,KX349280,KX349281,KX349282,KX349283,KX349284
1939	VC_664_0	4	1800	5044.937411	0.262968073	1.25E-05	1.732050808	6.92820323	6	6	1	5.17303142	0	0	0	JQ245707,OP053360,OP053361,OP053362
1940	VC_666_0	4	695.0268285	0	1	0	2.449489743	3.741657387	6	6	1	3.32783177	0	0	0	AY950802,JN968479,KC252997,KT809302
1941	VC_667_0	7	6297.013839	9056.773479	0.410127723	6.11E-15	0	7.211102551	21	21	1	5.56469076	0	0	0	KF929199,KJ804259,MT596499,MZ417336,MZ417339,MZ417343,MZ417348
1942	VC_667_1	1	0	2006.743693	0	0	0	0	1	1	1	0	0	0	0	MH356730
1943	VC_667_2	1	0	1615.098383	0	0	0	0	1	1	1	0	0	0	0	MK893987
1944	VC_668_0	25	90000	72484.09913	0.553900354	8.51E-141	3.31662479	9.591663047	300	288	0.96	6.25979871	0	0	0	GU295964,HQ918180,KT001918,KT001919,KT321315,KX130727,LR746310,LT607758,MN101223,MT157285,MW448170,MZ322895,MZ612130,MZ707156,MZ707157,OK631812,OK631813,OM103621,OM971648,OM971649,ON515458,OP045497,OP684128,OX335396,OX335407
1945	VC_668_1	1	0	10389.9482	0	0	0	0	1	1	1	0	0	0	0	OP617746
1946	VC_668_2	1	0	9304.308347	0	0	0	0	1	1	1	0	0	0	0	MN101221
1947	VC_668_3	1	0	6434.059769	0	0	0	0	1	1	1	0	0	0	0	MN101224
1948	VC_669_0	21	63000	110875.7817	0.362327631	2.24E-68	3.741657387	7.937253933	210	210	1	6.05182341	0	0	0	JN882287,KT381880,KX431559,KX431560,KX443552,LC589952,MH823906,MN508621,MN508622,MN508623,MN508624,MT341500,MW021751,MW021756,MW250785,MW629017,MW767161,OL539470,OL539471,OL551674,ON157416

1949	VC_669_1	10	13500	92961.51817	0.126806383	2.05E-12	2.236067977	9.055385138	45	44	0.977777778	7.06680559	0	0	0	AY967407,HE858210,HE981739,HM134276,KC801932,KM236237,KR869820,KX245890,MT334653,MW021749
1950	VC_669_2	1	0	13644.15417	0	0	0	0	1	1	1	0	0	0	0	MN508817
1951	VC_669_3	1	0	13041.52098	0	0	0	0	1	1	1	0	0	0	0	MN038175
1952	VC_669_4	1	0	6577.3216	0	0	0	0	1	1	1	0	0	0	0	OK210075
1953	VC_669_5	1	0	10457.96563	0	0	0	0	1	1	1	0	0	0	0	MT560058
1954	VC_66_0	2	300	1203.753494	0.199500783	0.045125822	3.16227766	3.16227766	1	1	1	3.16227766	0	0	0	MK552140,MK552141
1955	VC_66_1	1	0	886.2692331	0	0	0	0	1	1	1	0	0	0	0	MN094788
1956	VC_66_2	1	0	1071.180453	0	0	0	0	1	1	1	0	0	0	0	MW460245
1957	VC_66_3	1	0	1033.657941	0	0	0	0	1	1	1	0	0	0	0	AB366653
1958	VC_670_0	2	185.2131867	84.17932058	0.687521671	0.270145687	2.645751311	2.645751311	1	1	1	2.64575131	0	0	0	CAKLQA020000002,MW176033
1959	VC_671_0	5	2191.328257	1952.226306	0.528852275	4.43E-08	1.414213562	8.246211251	10	10	1	5.83692164	0	0	0	MN937349,MW393850,MZ326859,MZ398240,MZ398241
1960	VC_672_0	12	1011.273891	0	1	0	0	1.414213562	66	66	1	0.54913092	0	0	0	BCP1,MG883715,MG883725,MG945215,MG945341,MG945520,MG945551,MG945686,MG945756,MT135344,MT135404,MT135412
1961	VC_673_0	15	16448.71563	3527.556499	0.823412673	1.30E-27	0	9.848857802	105	80	0.761904762	6.77141748	0	0	0	BK010471,MH552500,MH616963,MK069403,MK238400,MT006214,MT774381,MT774383,MT774384,MT774386,MT774394,MW067000,MW067001,MW067002,MW067003
1962	VC_674_0	1	0	1009.741299	0	0	0	0	1	1	1	0	0	0	0	S8-wms_111056
1963	VC_675_0	2	70.38024472	798.229082	0.081026352	0.049814183	4.582575695	4.582575695	1	1	1	4.58257569	0	0	0	MT774393,S7-wms_244425
1964	VC_676_0	2	103.9474573	908.9196867	0.102626942	0.048432853	8.185352772	8.185352772	1	1	1	8.18535277	0	0	0	S12-wms-combined_2450,S5-wms-combined_29447
1965	VC_676_1	4	813.3028709	1350.165691	0.37592544	3.07E-05	5.830951895	9.273618495	6	4	0.666666667	7.59263182	0	0	0	S11-wms-combined_83571,S6-wms-combined_50721,S7-wms-combined_107679_0,S9-wms-combined_28805
1966	VC_677_0	9	1998.43589	3278.793801	0.378690337	3.19E-15	3	10.24695077	36	30	0.833333333	6.9847536	0	0	0	BK010646,S11-wms-combined_195425,S12-wms-combined_250551,S3-wms-combined_73262,S5-wms-combined_59064,S7-wms-combined_43950,S7-wms-combined_5625,S8-wms-combined_97848,S9-wms-combined_31456
1967	VC_678_0	1	0	363.4689654	0	0	0	0	1	1	1	0	0	0	0	S12-wms-combined_358797
1968	VC_679_0	4	337.9785062	889.1482352	0.275422656	2.26E-05	2.645751311	6.164414003	6	6	1	5.32643565	0	0	0	S1-wms-combined_246923,S1-wms-combined_340558,S1-wms-combined_52643,S11-wms-combined_172889
1969	VC_67_0	2	300	21184.88311	0.013963306	0.306423895	6.782329983	6.782329983	1	1	1	6.78232998	0	0	0	KY448244,MW366843
1970	VC_67_1	8	8400	70354.72113	0.106660272	0.001067539	0	3.741657387	28	28	1	2.93994676	0	0	0	MZ475891,MZ475892,MZ475893,MZ475894,MZ475895,MZ475896,MZ475897,MZ475898
1971	VC_67_2	2	300	21194.82003	0.013956851	0.306423895	2.645751311	2.645751311	1	1	1	2.64575131	0	0	0	KY984068,MZ443769
1972	VC_67_3	20	57000	103922.2986	0.354208214	2.11E-23	0	8.485281374	190	190	1	6.94207876	0	0	0	MH248138,MH460463,MZ443770,MZ443771,MZ443772,MZ443773,MZ443774,MZ443776,MZ443777,MZ443778,MZ443779,MZ443780,MZ443782,MZ443783,MZ443784,MZ443785,MZ443786,MZ443787,MZ443788,MZ443789
1973	VC_67_4	4	1800	39915.17313	0.043149767	0.090569651	0	4.582575695	6	6	1	3.15953797	0	0	0	MH389777,MH460460,MH460461,MH460462
1974	VC_67_5	1	0	6188.660477	0	0	0	0	1	1	1	0	0	0	0	LT598654
1975	VC_680_0	2	67.76650507	515.0306915	0.116278022	0.050604009	5.830951895	5.830951895	1	1	1	5.83095189	0	0	0	S5-wms-combined_83370,S9-wms-combined_44402
1976	VC_681_0	3	75.85897246	5.549985161	0.931825866	0.185546685	3.741657387	4.472135955	3	3	1	4.2286431	0	0	0	BK013345,BK013346,BK013347
1977	VC_682_0	2	91.75309723	0	1	0	5.744562647	5.744562647	1	1	1	5.74456265	0	0	0	BK059895,OM373202
1978	VC_683_0	5	2481.039374	286.985402	0.896321231	2.57E-07	3.31662479	6.08276253	10	10	1	4.99440478	0	0	0	BK061308,BK061309,BK061310,BK061311,BK061312
1979	VC_684_0	5	2454.127061	3813.759577	0.391539797	5.15E-08	0	5.291502622	10	10	1	3.35337804	0	0	0	MW353181,MW584168,MW584172,MW584178,MW584190
1980	VC_685_0	16	15703.29683	37129.70216	0.297225165	3.64E-76	0	9.433981132	120	110	0.916666667	7.67523254	0	0	0	MG872834,MH513979,MH651167,MK284520,MK433274,MK433275,MK820641,MN062715,MT521999,MW534378,MW601219,MZ322028,MZ622161,MZ622165,ON108651,OP434460
1981	VC_686_0	2	300	1097.01388	0.21474375	0.046080597	2.449489743	2.449489743	1	1	1	2.44948974	0	0	0	MK494099,ON260818
1982	VC_686_1	2	300	1060.930368	0.220437435	0.086720853	2.645751311	2.645751311	1	1	1	2.64575131	0	0	0	KU728633,MK977701
1983	VC_687_0	3	900	632.737921	0.587184533	0.007473349	0	1.414213562	3	3	1	0.94280904	0	0	0	KF114879,KF114880,KX656785
1984	VC_687_1	2	165.531821	576.9118011	0.222955408	0.085815228	4	4	1	1	1	4	0	0	0	KF114876,KF114877
1985	VC_688_0	2	271.5102337	1.476517761	0.994591248	0.5	1.414213562	1.414213562	1	1	1	1.41421356	0	0	0	MF974396,MF974397
1986	VC_689_0	2	68.40404897	86.97364221	0.440243696	0.044175637	4.242640687	4.242640687	1	1	1	4.24264069	0	0	0	DQ490056,MW791312
1987	VC_68_0	1	0	870.9884294	0	0	0	0	1	1	1	0	0	0	0	AP018399
1988	VC_68_1	1	0	891.9527154	0	0	0	0	1	1	1	0	0	0	0	MN047793
1989	VC_690_0	50	146258.3169	2907.587734	0.980507692	1.94E-89	0	3.872983346	1225	1225	1	2.68525706	0	0	0	C2PVCG,KX373685,KX373686,KX373687,KX373688,KX373689,KX373690,KX373691,KX373692,L67CG,MF443119,MF443120,MF443121,MF443122,MF443123,MF443124,MF443125,MF443126,MH779518,MH779519,MH779520,MH779521,MH779522,MH779523,MH779524,MH779525,MH779526,MH779527,MK301442,MK301443,MN337887,MN689503,MN689504,MN689505,MN689506,MN689507,MN689508,MN689510,MN689511,MN689512,MN689513,MN689515,MN689526,MN689527,MN689528,MN689529,MN689531,MN689532,MN689533,MZ955869
1990	VC_691_0	2	300	2550.665151	0.105238597	0.046569618	1	1	1	1	1	1	0	0	0	MT939241,ON506926
1991	VC_691_1	5	3000	3884.292072	0.435774655	6.53E-08	2.828427125	4.242640687	10	10	1	3.51458585	0	0	0	CAJCJZ010000002,LT546029,LT615366,MT119360,MT119361
1992	VC_692_0	2	300	366.1608314	0.450341698	0.046592743	1	1	1	1	1	1	0	0	0	MF001358,MF001361
1993	VC_694_0	1	0	3285.002126	0	0	0	0	1	1	1	0	0	0	0	MW672041
1994	VC_695_0	48	170278.2921	38349.50091	0.816182205	6.73E-251	0	7.874007874	1128	1128	1	5.8264709	0	0	0	CAJDJX010000002,CAJDKF010000002,JX193904,KF728385,KF733017,KJ127304,KR131750,KT932701,KX284704,LC623721,MF678788,MF678789,MF678790,MG264739,MG708276,MH193369,MH203383,MH203384,MH355583,MH375074,MK125140,MK360024,MK721186,MK721187,MK721190,MK721191,MK721199,MK721200,MK982307,MT119359,MT520979,MT627482,MT661597,MT661598,MT829326,MT857001,MZ182246,MZ272341,MZ326865,MZ333461,OL505084,OL539449,OL799256,OL799257,ON113176,ON113334,OP254195,OP559177
1995	VC_696_0	2	269.1717675	1388.432964	0.162385979	0.054263206	2.645751311	2.645751311	1	1	1	2.64575131	0	0	0	KT588075,MZ514874
1996	VC_696_1	8	2948.754805	2460.195169	0.545162151	6.31E-13	2.828427125	9.38083152	28	26	0.928571429	7.53321717	0	0	0	CAKLQA020000001,JX403940,KP861230,KT588073,MH115576,MT361972,MZ675741,ON391949
1997	VC_697_0	5	2170.252717	443.5210452	0.830313912	2.89E-07	2	5.567764363	10	10	1	4.37314319	0	0	0	KJ507100,MT104473,MT104475,MZ348425,MZ868718
1998	VC_698_0	26	56402.45878	3817.346316	0.936609786	1.93E-77	1	8.366600265	325	325	1	6.98170123	0	0	0	HE806280,HM368260,JX976549,KJ817802,KP861229,KP861231,KU510289,KY670595,KY829116,MF346584,MH853786,MH853787,MH853788,MN166083,MN516421,MN516422,MZ593728,MZ618622,MZ712044,OL743187,OM471864,ON036882,ON036883,ON237674,ON480525,OP585104
1999	VC_699_0	71	297286.7845	20605.49821	0.935180879	0	0	6.480740698	2485	2485	1	4.87062132	0	0	0	CAKLQB020000001,CAKLQG020000001,HQ186308,JX658790,KC311669,KR149290,KT339321,KT388102,KT388103,KT804908,KY082667,KY268295,KY268296,KY888680,MF033347,MF033348,MF033349,MF033350,MF033351,MG459218,MG599035,MH042230,MH763831,MH800200,MK089780,MK257719,MK257720,MK257721,MK257722,MK257723,MK278859,MN061582,MN212906,MN294712,MN395291,MN433707,MN604238,MN604239,MN614471,MN651570,MN807295,MT263719,MT633129,MT741944,MW056501,MW056502,MW056503,MW345241,MW366783,MW366784,MW459163,MW926912,MZ099557,MZ593174,MZ868724,MZ868725,MZ868726,MZ936314,MZ936315,MZ936316,MZ967493,OK546191,OL770263,OL964948,OM925528,ON210142,ON513429,OP019135,OP171942,OP291336,OP508218
2000	VC_6_0	44	106631.7623	10800.78664	0.908025614	0	0	6.782329983	946	946	1	4.1988097	0	0	0	AY319521,CP019275,CP025900,HM770079,KT630647,KY271398,MK416009,MK416011,MK416012,MK416020,MK422449,MK422453,MK422454,MK433577,MK433578,MK433580,MK433581,MK433582,OK490395,OK490397,OK490399,OK490401,OK490403,OK490405,OK490407,OK490411,OK490413,OK490418,OK490423,OK490425,OK490427,OK490429,OK490431,OK490434,OK490436,OK490439,OK490442,OK490445,OK490448,OK490452,OK490455,OK490457,ON470620,OV696611
2001	VC_700_0	10	13500	3216.691654	0.807576061	7.57E-25	4.123105626	7.280109889	45	45	1	5.90717957	0	0	0	KJ628499,MH746814,MH800198,MH800199,MK170160,MT188223,MT623546,MW349133,OL960030,OP585105
2002	VC_700_1	1	0	2061.708323	0	0	0	0	1	1	1	0	0	0	0	KJ473422
2003	VC_701_0	2	300	1716.936821	0.148740405	0.047500846	8.602325267	8.602325267	1	1	1	8.60232527	0	0	0	MN276049,MZ477002
2004	VC_701_1	2	300	2000.902268	0.130383634	0.048858364	8.306623863	8.306623863	1	1	1	8.30662386	0	0	0	MH133207,MZ043613
2005	VC_701_2	1	0	1229.569888	0	0	0	0	1	1	1	0	0	0	0	CAKLQE020000001
2006	VC_702_0	5	3000	848.9987429	0.779423481	1.17E-07	5.916079783	7.615773106	10	10	1	6.736914	0	0	0	KM672662,KY000079,MW316731,MW316732,MW316733
2007	VC_704_0	16	16538.32406	6196.970899	0.727429492	7.63E-66	0	8.246211251	120	120	1	4.79801836	0	0	0	DQ150593,DQ222851,DQ222853,DQ222855,DQ289555,DQ289556,JQ062992,KY963369,KY963370,KY963371,MK085976,MT745954,MT745955,MT745956,OK499979,OK499999
2008	VC_705_0	11	6097.60396	784.107456	0.886059236	1.16E-25	2	8.062257748	55	55	1	6.19058444	0	0	0	KF591601,MF614100,MH363708,MK105855,MK125141,MK651787,MN850581,MN850588,MN850623,MW258710,MW258711
2009	VC_706_0	2	152.4024416	0	1	0	2.449489743	2.449489743	1	1	1	2.44948974	0	0	0	CP002495,CP091911
2010	VC_707_0	6	1721.677634	4859.569075	0.26160357	4.64E-11	5.477225575	9.643650761	15	13	0.866666667	7.66282275	0	0	0	S1-wms-combined_132934,S11-wms-combined_304239,S12-wms-combined_17617,S12-wms-combined_18287,S17-wms-combined_199440,S6-wms-combined_8508
2011	VC_707_1	9	2058.584435	5559.310228	0.2702301	4.30E-14	5.099019514	9.643650761	36	32	0.888888889	7.44156844	0	0	0	MG711462,S12-wms-combined_129554,S17-wms-combined_127688,S2-wms-combined_180640,S2-wms-combined_85381,S6-wms-combined_117756,S7-wms_159432,S8-wms-combined_200168,S8-wms-combined_217866
2012	VC_707_2	1	0	1334.336668	0	0	0	0	1	1	1	0	0	0	0	S12-wms-combined_129799
2013	VC_707_3	1	0	1057.448786	0	0	0	0	1	1	1	0	0	0	0	S11-wms-combined_264050
2014	VC_708_0	47	155269.4686	24155.55053	0.865372451	1.07E-270	0	7.810249676	1081	1081	1	5.72726918	0	0	0	KF010834,KP202972,KP296791,KP296793,KP296794,KP296795,KP296796,KT361649,KT361650,KT361652,MG727695,MG727696,MG727698,MG727699,MG727700,MG727701,MG727702,MH431931,MH431932,MH431933,MH431934,MH431935,MH431936,MH431937,MH454077,MH454078,MH454079,MH454082,MH454083,MH454084,MW927511,MW927513,MW927514,MW927515,MW927516,MW927517,MW927519,MW927520,MW927521,MW927522,MW927523,MW927524,MW927525,OP503992,OP503993,OP503994,OP503995
2015	VC_70_0	27	19598.84556	13168.99846	0.598112148	7.79E-104	0	9.110433579	351	347	0.988603989	6.73842252	0	0	0	AB920995,AB983711,GU393987,HG793132,HG796219,HG796220,HG796221,KC853746,KT381879,KX660669,KY316062,KY464836,KY981272,MF621978,MH684921,MN191861,MT740728,MZ326863,OK319016,OK335783,ON170174,ON170178,ON170184,ON170190,ON170318,ON170358,ON529868
2016	VC_710_0	10	2966.817404	9161.037562	0.244628371	2.77E-21	6.244997998	9.899494937	45	37	0.822222222	8.09113901	0	0	0	MG711466,ON470591,S1-wms_296850,S11-wms-combined_74983,S12-wms-combined_156234,S17-wms-combined_260343,S6-wms-combined_126236,S7-wms-combined_134458,S8-wms_152831,S8-wms_48935
2017	VC_710_1	1	0	1507.739953	0	0	0	0	1	1	1	0	0	0	0	S2-wms-combined_137119
2018	VC_710_2	1	0	1051.567598	0	0	0	0	1	1	1	0	0	0	0	S7-wms-combined_157260
2019	VC_710_3	1	0	1573.976143	0	0	0	0	1	1	1	0	0	0	0	S6-wms-combined_118427
2020	VC_710_4	1	0	1459.564153	0	0	0	0	1	1	1	0	0	0	0	S2-wms-combined_13246
2021	VC_711_0	3	591.8326688	1967.749302	0.231222393	0.00157505	5.567764363	7.416198487	3	3	1	6.43617272	0	0	0	MG711467,S1-wms-combined_68998,S7-wms-combined_111004
2022	VC_711_1	1	0	941.7882192	0	0	0	0	1	1	1	0	0	0	0	S12-wms-combined_323943
2023	VC_711_2	1	0	868.7989642	0	0	0	0	1	1	1	0	0	0	0	S17-wms-combined_2234
2024	VC_711_3	1	0	867.1943258	0	0	0	0	1	1	1	0	0	0	0	S2-wms-combined_140996
2025	VC_713_0	3	775.6014264	750.4802458	0.508230615	0.001728838	0	1.732050808	3	3	1	1.15470054	0	0	0	ON464760,ON464761,ON464762
2026	VC_715_0	3	131.093981	307.2034508	0.299098218	0.001865828	7.549834435	8.306623863	3	3	1	7.91015539	0	0	0	S1-wms-combined_302166,S11-wms-combined_198512,S12-wms-combined_358010
2027	VC_716_0	2	46.44857974	406.7762208	0.102484638	0.045370232	5	5	1	1	1	5	0	0	0	S1-wms-combined_360285,S11-wms-combined_84326
2028	VC_717_0	7	2260.479892	1628.032166	0.581322588	6.71E-13	0	9.695359715	21	19	0.904761905	7.32818469	0	0	0	LR596903,MG711461,S12-wms-combined_259783,S2-wms-combined_207832,S3-wms_22744,S5-wms-combined_134381,S7-wms_118607
2029	VC_717_1	1	0	1460.068596	0	0	0	0	1	1	1	0	0	0	0	S17-wms-combined_248321
2030	VC_718_0	4	864.1333326	487.5788638	0.63928796	3.32E-05	3.741657387	9.591663047	6	5	0.833333333	6.91724602	0	0	0	KP137433,KP137435,KP137437,KP137439
2031	VC_719_0	1	0	7.187772826	0	0	0	0	1	1	1	0	0	0	0	S1-wms_102342
2032	VC_719_1	1	0	70.90669296	0	0	0	0	1	1	1	0	0	0	0	S7-wms-combined_20921
2033	VC_720_0	2	300	237.7728731	0.557856328	0.105649774	1	1	1	1	1	1	0	0	0	KT151955,KT151957
2034	VC_720_1	1	0	220.4440851	0	0	0	0	1	1	1	0	0	0	0	CP027117
2035	VC_721_0	9	1114.033729	0	1	0	0	1	36	36	1	0.5	0	0	0	CP045566,MN890136,MN890137,MN890138,MN890139,MN890140,MN890141,MN890142,MN890143
2036	VC_722_0	13	18689.96688	17931.75838	0.510351895	2.49E-47	2.236067977	5.385164807	78	78	1	4.26912753	0	0	0	KJ419279,KP719132,KP719133,KP719134,KY926791,MK165087,MK373794,MK373795,MT682705,MZ501057,MZ501068,OM386652,ON922918
2037	VC_723_0	9	9387.359787	3763.61425	0.713814791	6.13E-20	0	8.717797887	36	36	1	7.24613448	0	0	0	CP053388,CP054387,CP058330,CP101890,CP101896,JQ965645,MK422451,MZ779062,OV696614
2038	VC_724_0	3	900	4773.545586	0.158630963	0.001848425	4.358898944	5.291502622	3	3	1	4.81541103	0	0	0	MT770738,MT778838,MW960030
2039	VC_724_1	6	4500	5776.315294	0.437900149	1.46E-10	0	8.831760866	15	15	1	6.22807669	0	0	0	JX483881,MN988505,MN988506,MN988543,MN988547,MN988548
2040	VC_725_0	4	1800	2417.659507	0.426776983	2.64E-05	2.449489743	7.745966692	6	6	1	6.04416416	0	0	0	MG018927,MT354569,OM140839,OM223115
2041	VC_726_0	3	30.64228132	773.2673444	0.038116575	0.110443923	8.185352772	8.94427191	3	3	1	8.63486302	0	0	0	JN116824,KR053195,MH153804
2042	VC_727_0	11	6153.897136	6022.120403	0.505411323	1.03E-28	0	7.416198487	55	55	1	5.51526529	0	0	0	JX182371,KY092479,KY092480,KY092481,KY092482,KY092483,KY092484,KY676784,MH825699,MK305888,MT711975
2043	VC_728_0	3	896.8620978	1336.013332	0.401662397	0.001497412	2.828427125	3.605551275	3	3	1	3.34650989	0	0	0	MK279841,MT316461,MT723933
2044	VC_72_0	2	155.0281624	1840.655257	0.077681741	0.043835387	3.16227766	3.16227766	1	1	1	3.16227766	0	0	0	JN882298,MG201401
2045	VC_730_0	9	3328.009786	0	1	0	0	5.291502622	36	36	1	3.68257376	0	0	0	CP060690,HQ377372,HQ377373,JF773396,KX879601,KX879602,KY661963,MT899443,MT899444
2046	VC_731_0	2	108.9578026	185.734697	0.369733884	0.050202073	5.830951895	5.830951895	1	1	1	5.83095189	0	0	0	KX190833,MK759918
2047	VC_732_0	2	12.56463272	227.1135663	0.052422927	0.092571862	7.141428429	7.141428429	1	1	1	7.14142843	0	0	0	S12-wms-combined_191570,S2-wms-combined_238929
2048	VC_732_1	1	0	22.88917021	0	0	0	0	1	1	1	0	0	0	0	S17-wms-combined_95360
2049	VC_733_0	5	1751.560852	1675.2474	0.511134771	6.34E-08	0	3.605551275	10	10	1	2.352846	0	0	0	HM568888,KU057941,LN681535,LN681536,LN881738
2050	VC_734_0	6	94.5687546	399.4030455	0.191445655	3.11E-05	6.08276253	8.774964387	15	15	1	7.59908801	0	0	0	HQ332138,KU517658,MF595878,S2-wms-combined_101826,S2-wms-combined_248329,S5-wms-combined_7292
2051	VC_736_0	3	60.19369253	58.85059373	0.505641173	0.048046165	6.164414003	8.124038405	3	3	1	6.97390066	0	0	0	S12-wms-combined_168259,S12-wms-combined_282594,S5-wms-combined_52741
2052	VC_738_0	2	80.52440074	39.50457993	0.670874653	0.144422183	6.32455532	6.32455532	1	1	1	6.32455532	0	0	0	S2-wms-combined_135139,S8-wms-combined_246185
2053	VC_739_0	26	97500	4365.885299	0.95714085	1.15E-120	0	7.681145748	325	325	1	4.32276158	0	0	0	DQ003638,DQ004855,JQ797329,JX126918,KJ094029,KJ094030,KJ094031,KJ094033,KJ535721,KJ535722,KJ586794,KJ586795,KJ591604,KJ591605,KJ668715,KM373208,MK792752,MN128594,MN172529,MN939539,MN939540,MT178766,MT178767,MT438761,MT668623,MT668624
2054	VC_740_0	3	132.766692	524.6203007	0.20196124	0.001881716	3.872983346	6	3	3	1	5.02304526	0	0	0	DQ003641,EU855793,GQ403788
2055	VC_743_0	15	2915.121088	0	1	0	0	2	105	105	1	1.05734224	0	0	0	DQ079870,DQ079871,DQ079872,DQ079873,DQ079874,DQ079875,DQ079876,DQ079878,DQ079879,DQ085810,GQ149088,MG945254,MK629527,X60322,X60323
2056	VC_744_0	10	905.4949876	379.1266269	0.704872919	3.16E-15	1.732050808	5	45	45	1	3.6076412	0	0	0	DQ123818,MK016490,MK016500,MN988551,MZ333134,MZ333136,ON381767,ON724010,OV696617,S2-wms_79304
2057	VC_745_0	4	491.8710019	388.7823469	0.558529645	4.20E-05	0	1.732050808	6	6	1	1.09341299	0	0	0	MT006233,MT006238,MT006239,MT006240
2058	VC_747_0	23	41839.43348	9169.011548	0.820245225	3.54E-98	0	8.185352772	253	253	1	4.27919687	0	0	0	DQ163912,KJ959591,KY707339,MK510962,MK510963,MK510964,MK510965,MK510968,MK510969,MK510970,MK510971,MK510974,MK510975,MK510976,MK510978,MK510984,MK510985,MK510986,MK510987,MK510988,MK510990,MK510992,MK510993
2059	VC_748_0	12	14576.12067	6612.811209	0.687912008	1.44E-38	0	2.449489743	66	66	1	0.52742681	0	0	0	MK510966,MK510967,MK510972,MK510973,MK510977,MK510979,MK510980,MK510981,MK510982,MK510983,MK510989,MK510991
2060	VC_749_0	40	89068.12443	29428.63212	0.751650315	6.72E-208	0	8.717797887	780	780	1	5.69660812	0	0	0	DQ163913,HG962376,JF939047,JQ067092,JQ307387,KM434185,KR537871,KR537872,KR935213,KR935215,KU197014,LC552830,LR743530,MG897800,MK165657,MK165658,MK574078,MN062187,MT118304,MT118305,MW512832,MW677514,MW677526,MZ501266,MZ927745,MZ927746,OK094665,OK330455,OK330456,OK539825,OL802211,OM234791,ON189042,ON189043,ON189044,ON911538,ON932082,ON932085,OP009282,OP422637
2061	VC_74_0	15	12583.74351	11599.27169	0.520354613	3.76E-60	2	7.071067812	105	105	1	4.8986993	0	0	0	KF626667,KU197013,KU595432,KU595433,LR778216,MF375456,MK903278,MN478375,OK258138,OK275495,ON911539,ON932078,ON932081,ON932083,ON960072
2062	VC_750_0	1	0	2714.239436	0	0	0	0	1	1	1	0	0	0	0	MG366115
2063	VC_751_0	4	1284.182478	6000.89537	0.176275739	1.47E-05	1.414213562	2.645751311	6	6	1	1.9463942	0	0	0	KP313532,MK962635,MK962636,MK962637
2064	VC_752_0	3	509.8066057	5959.402942	0.078805085	0.001539802	2	3.872983346	3	3	1	3.15951154	0	0	0	MN734437,MN734438,MN734439
2065	VC_754_0	8	6625.038031	2185.22346	0.751968377	1.29E-18	0	3.605551275	28	28	1	2.35636253	0	0	0	HQ641345,HQ641346,KM224878,KM224879,KY883657,KY883658,MT066160,MT767883
2066	VC_755_0	9	5135.247874	6303.565628	0.448931865	2.54E-23	2.828427125	7.483314774	36	36	1	5.73257279	0	0	0	DQ163915,KU535860,LT986460,MF788075,MG676466,MW929175,MZ089731,MZ727333,ON857932
2067	VC_756_0	2	151.4358819	773.4472642	0.163735151	0.045729859	2.828427125	2.828427125	1	1	1	2.82842712	0	0	0	KC751414,KF302037
2068	VC_757_0	2	219.3274309	2797.325369	0.07270556	0.044726375	0	0	1	1	1	0	0	0	0	MG592599,MG592619
2069	VC_758_0	18	24677.61951	8758.743841	0.738047354	1.21E-80	0	6.708203932	153	153	1	4.34008982	0	0	0	MG592629,MW824407,MW824408,MW824409,MW865297,MW865299,MW865302,MW865307,OL676770,OX241435,OX241436,OX241445,OX241446,OX241447,OX241501,OX241504,OX241559,OX241560
2070	VC_759_0	3	874.4873857	967.5061026	0.47475053	0.001866735	3	5.385164807	3	3	1	4.4280481	0	0	0	MN988507,MN988510,MN988527
2071	VC_75_0	2	163.224133	2735.262044	0.05631358	0.043807214	1.732050808	1.732050808	1	1	1	1.73205081	0	0	0	MZ666965,MZ666966
2072	VC_760_0	3	43.85797604	16.374553	0.728144355	0.014092901	7	7.211102551	3	3	1	7.11751033	0	0	0	MW202785,MW202796,OK172329
2073	VC_761_0	67	462930.5845	54493.33465	0.894683387	0	0	5.477225575	2211	2211	1	2.18578635	0	0	0	DQ398042,EU568876,FJ973624,GQ303261,JN412593,JN699002,KC787103,KC787104,KC787107,KC787108,KC787109,KC787110,KC787111,KC787112,KJ725374,KM923970,KT347314,KT355472,KT355474,KT365399,KX443326,KX534004,KX588251,KX641265,KX641266,KX664455,MF668268,MF668272,MF919497,MG099946,MH001455,MH001456,MH045569,MH077584,MH371116,MH371119,MH450127,MH479920,MH590605,MH779505,MH779507,MH779509,MH779513,MH779514,MH779515,MK305884,MK310146,MK433277,MK433279,MK524493,MK524494,MK919475,MN444875,MT818422,MW119570,MW291028,MZ171377,MZ747508,ON392160,ON392162,ON637759,ON637765,ON637766,ON645337,ON970618,OP172872,OP294982
2074	VC_762_0	9	5276.430779	54317.00831	0.088540465	3.00E-24	3	6.8556546	36	36	1	5.62572368	0	0	0	KJ410133,KR080197,KR080198,KR080202,MF141540,MK494105,MN234183,MN234185,MN234219
2075	VC_763_0	2	300	4620.301278	0.060971876	0.049047866	5.656854249	5.656854249	1	1	1	5.65685425	0	0	0	MW584160,MW584202
2076	VC_763_1	9	10800	18339.30416	0.370633421	1.56E-20	0	6.92820323	36	36	1	3.50469455	0	0	0	JN699628,KC900379,KT591491,MH697587,MK937612,MN586024,MT658803,MW713383,OP297551
2077	VC_763_2	7	6138.695275	17365.66776	0.261172586	1.81E-13	2.645751311	8.717797887	21	21	1	6.23986884	0	0	0	JF937105,KY223999,KY783914,MF319184,MT657341,MT658802,MW578836
2078	VC_764_0	4	1800	3261.795678	0.355605029	2.53E-05	0	3.741657387	6	6	1	2.68732527	0	0	0	DQ398052,KP027195,KY385381,MN585992

2079	VC_765_0	132	640830.6623	0	1	0	0	7.549834435	8646	8646	1	5.80230765	0	0	0	DQ431235,FJ706171,FJ706172,JX262215,JX262216,JX262217,JX262218,JX262219,JX262220,JX262221,JX262222,JX262223,JX262224,JX262225,JX570702,JX570703,JX570704,JX570705,JX570706,JX570707,JX570708,JX570709,JX570710,JX570711,JX570712,JX570713,JX570714,KJ578758,KJ578759,KJ578760,KJ578761,KJ578762,KJ578763,KJ578764,KJ578765,KJ578766,KJ578767,KJ578768,KJ578769,KJ578770,KJ578771,KJ578772,KJ578773,KJ578774,KJ578775,KJ578776,KJ578777,KJ578778,KJ578779,KJ578780,KJ578781,KJ578782,KJ578783,KJ578784,KJ578785,KJ578786,KJ578787,KJ578788,KJ578789,KJ578790,KJ578791,KJ578792,KJ722067,KR337643,KR337644,KR337645,KR337646,KR337647,KR337648,KR337649,KR337650,KR337651,KR337652,KR337653,KR337654,KR347352,KR347353,KR347354,KR347355,KR902978,KR902979,KR902980,KR902981,KR902982,KR902983,KR902984,KR902985,KR902986,KR902987,KT934381,KY926792,KY926793,MF919491,MF919505,MF919515,MF919516,MF919522,MF919533,MG820632,MG820633,MG820634,MG820635,MG820636,MG820637,MG820638,MG820639,MG820640,MG820641,MG820642,MG820643,MG820644,MG820645,MN329678,MN813675,MN813677,MN813679,MN813683,MN813688,MN813689,MN813690,MN813691,MN813692,MT647607,MW161461,MW161462,MW161463,MW161464,MW161465,MW161466,MW161467,MW161468,MW505928
2080	VC_766_0	2	139.3917034	381.9073105	0.267392993	0.050838929	3.605551275	3.605551275	1	1	1	3.60555128	0	0	0	LN681534,MN718463
2081	VC_767_0	34	116922.9948	10538.32175	0.917321411	3.65E-157	0	8.306623863	561	561	1	6.53837321	0	0	0	JF923796,JF923797,KR053201,MF919510,MF919542,MG757157,MG757166,MH536819,MH536824,MH576960,MH669007,MH669013,MH727547,MH727552,MH744424,MH779499,MH779501,MH779508,MH779510,MK376954,MK376960,MK376962,MK433272,MK977699,MN369753,MN369766,MN428063,MT889393,MT986025,MW507129,MZ322010,MZ322022,MZ622178,ON456333
2082	VC_768_0	8	7678.085453	3272.043043	0.70118679	9.30E-19	0	3.741657387	28	28	1	2.76717839	0	0	0	MG198776,MG198777,MG198778,MG198780,MH926056,MH926057,MH976516,MK977700
2083	VC_769_0	2	300	5908.36276	0.048321919	0.044857026	0	0	1	1	1	0	0	0	0	MW452562,MW582531
2084	VC_76_0	37	199800	12675.11806	0.940345401	1.99E-249	0	8.366600265	666	666	1	4.59331518	0	0	0	GU988610,HE983845,HQ832595,KC862297,KC862298,KC862300,KR052143,KT736033,KT804923,KU297675,KU497559,KU761955,KY000083,KY073228,LN610572,LN610575,LN610586,LN610587,MF974178,MH370478,MH725810,MH791399,MH791408,MH791416,MK138526,MK224498,MK340761,MK443264,MN013356,MN563785,MN871470,MN871481,MN871488,MW526259,OK539826,ON169972,ON361135
2085	VC_76_1	1	0	8764.895999	0	0	0	0	1	1	1	0	0	0	0	MK443265
2086	VC_770_0	5	1155.419465	220.9835972	0.839448485	5.95E-06	6.633249581	8.831760866	10	10	1	8.28102619	0	0	0	DQ500118,MN988496,MW960032,OP484857,OP484858
2087	VC_771_0	2	222.8787542	69.62866705	0.761959315	0.083990929	0	0	1	1	1	0	0	0	0	MN988490,MN988502
2088	VC_772_0	6	4500	143183.2802	0.030470612	0.189871701	1	8.485281374	15	15	1	5.12072175	0	0	0	MG065644,MG065662,MG065672,MG065674,MG065677,MG065690
2089	VC_772_1	2	300	50058.43737	0.005957294	0.423176388	8.717797887	8.717797887	1	1	1	8.71779789	0	0	0	MK962749,ON286975
2090	VC_772_10	8	8400	185389.8746	0.043345918	0.091181698	0	9.797958971	28	26	0.928571429	6.61513598	0	0	0	MN850630,MT884008,MT884009,MT884010,MT884011,MZ501082,MZ501083,MZ501102
2091	VC_772_11	6	4500	142956.7099	0.030517431	0.157461973	1.414213562	9.486832981	15	12	0.8	7.8967305	0	0	0	LC739541,LC739542,MH491969,MH752840,MZ501048,MZ501094
2092	VC_772_12	1	0	23826.56746	0	0	0	0	1	1	1	0	0	0	0	MG065659
2093	VC_772_13	1	0	23845.72953	0	0	0	0	1	1	1	0	0	0	0	MG065653
2094	VC_772_14	3	900	74290.53569	0.011969592	0.360204934	1	8.062257748	3	3	1	5.68741925	0	0	0	MG065654,MG065655,MG065666
2095	VC_772_15	5	3000	120477.4574	0.024295933	0.238551388	1.414213562	9.16515139	10	9	0.9	7.61819444	0	0	0	DQ832317,KC690136,KP869102,KP869103,KP869112
2096	VC_772_16	10	13500	226275.5	0.056302666	0.056507198	1.414213562	9.899494937	45	39	0.866666667	7.99138374	0	0	0	KJ190158,LC739538,LC739540,MG065646,MG065647,MG065658,MG065661,MG065682,MG963916,MN850642
2097	VC_772_17	2	300	50118.27029	0.005950224	0.429740878	7.874007874	7.874007874	1	1	1	7.87400787	0	0	0	MW748998,MZ501073
2098	VC_772_18	1	0	25204.93072	0	0	0	0	1	1	1	0	0	0	0	MZ501061
2099	VC_772_19	16	36000	333103.2673	0.09753368	0.002953397	5.477225575	9.591663047	120	97	0.808333333	8.22089808	0	0	0	KT001917,LR699804,MG065635,MK327930,MK373780,MN850576,MN850578,MN850595,MN850597,MN850612,MN850626,MN850627,MZ501054,MZ501062,OK272482,OV101294
2100	VC_772_2	1	0	25318.4133	0	0	0	0	1	1	1	0	0	0	0	MZ501060
2101	VC_772_20	2	300	49946.55334	0.005970559	0.411314129	8.831760866	8.831760866	1	1	1	8.83176087	0	0	0	MN850593,MT682716
2102	VC_772_3	1	0	25289.6848	0	0	0	0	1	1	1	0	0	0	0	MZ501093
2103	VC_772_4	4	1800	97447.29796	0.018136514	0.269816967	5.196152423	8.062257748	6	6	1	6.94872507	0	0	0	JQ031132,MK883717,MK883718,MW883061
2104	VC_772_5	1	0	25323.47938	0	0	0	0	1	1	1	0	0	0	0	MT740314
2105	VC_772_6	3	900	74214.28565	0.011981742	0.360204934	7.874007874	8.185352772	3	3	1	7.99887153	0	0	0	MN850646,MN850649,MZ501111
2106	VC_772_7	2	300	49969.9172	0.005967784	0.411312863	1.414213562	1.414213562	1	1	1	1.41421356	0	0	0	LR694610,LR694611
2107	VC_772_8	2	300	50007.49052	0.005963327	0.417063176	8.426149773	8.426149773	1	1	1	8.42614977	0	0	0	KR698074,ON286973
2108	VC_772_9	7	6300	164419.517	0.036902635	0.122944969	0	8.602325267	21	21	1	7.15404982	0	0	0	KY683736,LN881727,LN881735,LR597647,LR694165,MG022439,MG022440
2109	VC_773_0	23	33128.63817	26951.18396	0.55141039	5.19E-147	0	6.633249581	253	253	1	4.68191574	0	0	0	DQ864624,MK448678,MK448689,MK448693,MK448696,MK448697,MK448699,MK448766,MK448779,MK448784,MK448789,MK448797,MK448852,MK448866,MK448867,MK448868,MK448871,MK448947,MK448952,MK448961,MK448968,MK448974,MK448976
2110	VC_774_0	5	1856.897624	3801.168194	0.328185936	4.45E-08	0	5	10	10	1	3.35761542	0	0	0	GQ478081,GQ478082,GQ478083,GQ478084,GQ478085
2111	VC_775_0	3	244.7177624	6472.95906	0.036428928	0.00149927	8.485281374	8.94427191	3	3	1	8.67729285	0	0	0	MK448854,MK448933,MK448950
2112	VC_775_1	14	7306.152807	18818.92942	0.279660471	1.10E-56	0	10.14889157	91	69	0.758241758	8.12715297	0	0	0	MK448673,MK448765,MK448775,MK448794,MK448795,MK448858,MK448864,MK448941,MK448944,MK448945,MK448946,MK448953,MK448957,MK448970
2113	VC_777_0	3	208.6113942	229.3501718	0.476323519	0.003818798	2.236067977	5	3	3	1	4.04501582	0	0	0	EF537008,KF598865,MT457475
2114	VC_778_0	4	737.3286073	171.2281121	0.811538335	0.000145652	2	4.582575695	6	6	1	3.68696909	0	0	0	DQ875742,OM897575,ON677538,OP056300
2115	VC_779_0	37	118325.4986	89920.22656	0.568201333	0	0	8.544003745	666	666	1	6.24004965	0	0	0	EF056009,HQ259105,JX415535,JX880034,KC206276,KF192075,KF562340,KF620435,KJ135004,LR999871,MG208881,MG770228,MG922974,MH358458,MH669274,MH733496,MH992123,MK931297,MT682708,MW175414,MW630115,MZ358387,MZ394712,MZ501049,OM632664,OM632665,OM632666,OM632669,OM632670,OM632672,OM982646,ON602749,ON602751,ON922919,OP312987,OV049961,OW991345
2116	VC_779_1	11	9792.26289	51899.96447	0.15872766	3.02E-36	0	8.660254038	55	55	1	6.56934382	0	0	0	KF787094,KF787095,KT321317,MG018928,MK962626,MK962629,MK962630,MK962631,MK962632,MK962639,MK962641
2117	VC_779_2	5	2415.600617	25159.05866	0.087602193	3.02E-08	2	7	10	10	1	4.61388751	0	0	0	KF806588,KX098389,KX098390,KX098391,MH160392
2118	VC_779_3	7	2525.695849	30446.6471	0.076600436	2.98E-11	2.645751311	9.695359715	21	15	0.714285714	7.44016366	0	0	0	MG983743,MK863032,MN062186,MZ326856,MZ326857,MZ326861,OM687511
2119	VC_77_0	20	57000	2021.61029	0.965747965	9.64E-94	0	6.708203932	190	190	1	4.2297731	0	0	0	AB472900,KC862295,KC862296,KC862299,KC862301,KM434186,KY994101,LN610573,LN610576,LN610581,LN610582,LN610583,MK799650,MT108728,MT108730,MT118294,MT118295,MT118300,OM421595,ON653033
2120	VC_780_0	8	6297.213526	5796.76405	0.520690028	1.11E-19	3.605551275	7.483314774	28	28	1	5.88829947	0	0	0	KC139517,KC139520,KM236242,MH899585,MN163281,MW544066,OM858838,OM908766
2121	VC_781_0	2	265.8677255	6825.881525	0.037489724	0.043087922	2.236067977	2.236067977	1	1	1	2.23606798	0	0	0	KU356690,KU356691
2122	VC_782_0	3	872.3706223	2074.941541	0.295988539	0.001437147	0	3.464101615	3	3	1	2.30940108	0	0	0	MG592603,MG592604,MG592610
2123	VC_783_0	3	600.9441881	8806.604363	0.063878936	0.001456927	2.236067977	7.745966692	3	3	1	5.88772681	0	0	0	MN228696,MW960036,MW960038
2124	VC_784_0	6	3077.842112	2501.120836	0.55168714	6.34E-11	2.236067977	8.544003745	15	15	1	6.15531127	0	0	0	KC438282,KC438283,KP280062,KY065148,KY612839,OP313112
2125	VC_786_0	3	900	10150.08573	0.081447332	0.001459028	0	0	3	3	1	0	0	0	0	MT234338,MT234339,MT234340
2126	VC_78_0	2	300	9502.196575	0.030605385	0.043826134	6.403124237	6.403124237	1	1	1	6.40312424	0	0	0	MG018930,MZ605293
2127	VC_78_1	6	4500	28536.24847	0.136214014	8.37E-11	0	4.472135955	15	15	1	3.3133152	0	0	0	KU130126,KU130127,KU130128,KU130129,KU130130,KU130131
2128	VC_78_2	1	0	7154.663407	0	0	0	0	1	1	1	0	0	0	0	OK040171
2129	VC_78_3	11	16500	47643.65672	0.257235101	1.76E-14	1.732050808	7.483314774	55	55	1	5.42240434	0	0	0	KJ409772,MN905160,MT670417,MT670418,MT670419,MT670420,MT670421,MT670422,MW175491,MZ348426,MZ868713
2130	VC_78_4	1	0	7281.899923	0	0	0	0	1	1	1	0	0	0	0	MN812694
2131	VC_78_5	6	4500	36884.41982	0.108736573	0.000219177	0	7.348469228	15	15	1	5.27913003	0	0	0	LN887844,MZ826350,MZ826353,OK258139,OK275491,OM982620
2132	VC_78_6	1	0	7501.578133	0	0	0	0	1	1	1	0	0	0	0	MF893340
2133	VC_791_0	3	239.1210589	0	1	0	2.236067977	4.69041576	3	3	1	3.68319645	0	0	0	EF432053,KP282673,KP282674
2134	VC_792_0	35	104343.6366	1823.489572	0.982824348	8.52E-163	0	8.544003745	595	595	1	5.68601442	0	0	0	EF460875,HQ728264,KF534715,KY087898,MF044458,MH191398,MN045229,MN098326,MN117721,MN122072,MN136198,MN812688,MN850594,MT230534,MT446397,MT446398,MT446399,MT446400,MT446402,MT446403,MT446406,MT446409,MT446419,MW147598,MW147599,MW147600,MW147601,MW147602,MW349138,OK073976,OK129343,OK148439,OL741059,OL960574,ON778463
2135	VC_793_0	12	10587.83541	1823.489572	0.853078573	2.49E-39	0	7.071067812	66	66	1	4.73828361	0	0	0	KJ002054,KJ858521,KY709296,LR813619,MT623578,MW380983,MW380984,MW380985,MZ336020,OL770365,OM810291,OP056089
2136	VC_794_0	3	60.36276343	0	1	0	1.414213562	2.236067977	3	3	1	1.79411078	0	0	0	EF506570,SVU28974,X51344
2137	VC_795_0	19	45673.65119	21026.96374	0.684756074	6.72E-94	0	6.403124237	171	171	1	4.52467423	0	0	0	KM236243,KP064094,KX534337,LN881725,LR025195,LR025196,LR025197,LR025198,LT907986,MH230177,MK672798,MZ501071,MZ501091,MZ501104,MZ501110,MZ501112,OL539454,OL960573,OL960575
2138	VC_796_0	5	2913.103985	5777.094967	0.33521718	3.19E-08	0	2	10	10	1	1.4	0	0	0	MK816297,MN029011,MN871471,MN871472,MN871474
2139	VC_798_0	2	81.66170869	367.4662179	0.181822826	0.056539872	8.124038405	8.124038405	1	1	1	8.1240384	0	0	0	S1-wms-combined_307323,S7-wms-combined_147158
2140	VC_798_1	1	0	180.9023059	0	0	0	0	1	1	1	0	0	0	0	S1-wms-combined_85225
2141	VC_798_2	1	0	238.2138971	0	0	0	0	1	1	1	0	0	0	0	S17-wms-combined_186314
2142	VC_799_0	2	300	7884.073077	0.036656564	0.044523346	2.828427125	2.828427125	1	1	1	2.82842712	0	0	0	GU580941,GU580942
2143	VC_799_1	27	80451.01648	12972.86533	0.861139731	6.40E-161	0	7.681145748	351	351	1	5.43141286	0	0	0	MG962368,MG962369,MK376953,MK376957,MN010761,MN204501,MN284896,MN284904,MN585971,MN585982,MN585988,MN585996,MT310850,MT498039,MT498052,MT521994,MW291022,MW862987,MZ005675,MZ005683,MZ171370,MZ209301,MZ388556,MZ622168,ON526971,ON970572,ON970622
2144	VC_79_0	64	340539.6994	473080.0691	0.41854895	0	0	10.19803903	2016	1542	0.764880952	8.23685145	0	0	0	AB560486,AP018815,FM201282,JN254800,KP340287,KP340288,KR869157,KX171208,LC102730,LC105987,LC105988,LC105989,LC105990,LC472883,LC472884,LN610579,LN610588,MF490240,MG897799,MN871458,MN871459,MN871461,MN871462,MN871464,MN871465,MN871466,MN871473,MN871479,MN871485,MN871487,MN871508,MN871509,MT118291,MT118297,MT118298,MT118299,MT119363,MT119365,MT119368,MT119376,MT133561,MT133562,MT349888,MT413450,MT933737,MW512833,MW595221,MZ553930,OL362268,OL362269,OL754588,OM953790,ON324181,ON375838,ON375839,ON375840,ON529291,ON857930,ON857934,ON857942,OP310975,OP310977,OP314870,OP314871
2145	VC_79_1	2	169.6194151	28098.91702	0.00600029	0.042512573	8.124038405	8.124038405	1	1	1	8.1240384	0	0	0	LC472881,MW406974
2146	VC_79_10	1	0	14898.99722	0	0	0	0	1	1	1	0	0	0	0	AP019535
2147	VC_79_11	1	0	14254.58589	0	0	0	0	1	1	1	0	0	0	0	KY000082
2148	VC_79_2	1	0	13697.49644	0	0	0	0	1	1	1	0	0	0	0	FM201281
2149	VC_79_3	1	0	14506.83799	0	0	0	0	1	1	1	0	0	0	0	OP310979
2150	VC_79_4	3	522.9873019	48984.25632	0.010563854	0.003679085	7.615773106	9.219544457	3	2	0.666666667	8.17215444	0	0	0	MF623055,MT118289,MT118292
2151	VC_79_5	2	159.6536676	34526.90695	0.004602753	0.07218034	8.717797887	8.717797887	1	1	1	8.71779789	0	0	0	MK837009,MW595220
2152	VC_79_6	11	9262.439235	166082.4306	0.052824124	4.42E-19	2.236067977	9.539392014	55	41	0.745454545	7.97133203	0	0	0	MK837010,MN871457,MN871460,MN871467,MT108729,MT491204,MT491205,MT491206,MW512830,MW512834,OP310974
2153	VC_79_7	33	94647.75936	343556.7013	0.21598995	5.13E-286	0	10.14889157	528	444	0.840909091	7.87568363	0	0	0	FM887021,FM897211,GU815091,KR054033,KU198331,MF490241,MF768470,MK318076,MN131141,MN131142,MN131143,MN615700,MN615701,MN615702,MT108727,MT118290,MT119366,MT119369,MZ285878,ON857925,ON857929,ON857937,ON857938,OP310970,OP310971,OP310972,OP310973,OP310976,OP310978,OP310980,OP310981,OP310982,OP314872
2154	VC_79_8	1	0	14710.63843	0	0	0	0	1	1	1	0	0	0	0	KM434184
2155	VC_79_9	2	148.7562797	24157.58282	0.006120061	0.051399994	7.416198487	7.416198487	1	1	1	7.41619849	0	0	0	MK340760,MN871468
2156	VC_7_0	28	35372.94817	54946.06413	0.391644542	9.39E-213	0	9.273618495	378	376	0.994708995	7.33007058	0	0	0	AB009866,AB044554,AB243556,AF424783,CP062426,CP062435,CP062445,CP062457,EF462197,EF462199,FJ713816,GQ915271,KC342645,KX232515,KY389063,KY389064,KY653125,KY695153,MH384260,MH401414,MH401415,MW147366,MW192778,MW627293,MW650841,OM439664,OP136133,OP493557
2157	VC_800_0	3	718.9121272	847.0350659	0.459090913	0.001526301	1.414213562	3	3	3	1	2.35332162	0	0	0	EU081845,MK779875,MN552143
2158	VC_801_0	4	1650.228433	1767.891007	0.482788405	1.30E-05	1.414213562	2.236067977	6	6	1	1.71010792	0	0	0	KJ489010,KJ489011,KJ489012,KJ489013
2159	VC_802_0	17	37435.43938	9211.235889	0.802531781	4.85E-78	0	5.291502622	136	136	1	4.31721128	0	0	0	KU160642,KU647628,MF189170,MF189171,MF189172,MF189173,MF189174,MF189175,MF324906,MH450120,MK919473,MK967398,MN284897,MZ209300,MZ209307,MZ747520,OP434451
2160	VC_803_0	3	332.9240126	6162.528472	0.051254938	0.001422636	3.741657387	9.055385138	3	2	0.666666667	7.24710481	0	0	0	MH153800,MH153801,MK814756
2161	VC_804_0	2	134.0553987	2264.409768	0.05589216	0.043092449	8.544003745	8.544003745	1	1	1	8.54400375	0	0	0	MG711463,S1-wms-combined_304377
2162	VC_804_1	2	91.07192788	2113.0944	0.041318083	0.046477822	8.485281374	8.485281374	1	1	1	8.48528137	0	0	0	S17-wms-combined_259811,S17-wms-combined_47860
2163	VC_804_2	6	873.586289	6651.485243	0.116090098	7.83E-10	7.211102551	9.38083152	15	13	0.866666667	8.17427094	0	0	0	S1-wms_212940,S12-wms-combined_107942,S2-wms-combined_62377,S2-wms_248315,S8-wms_127191,S9-wms-combined_26410
2164	VC_804_3	1	0	1436.678512	0	0	0	0	1	1	1	0	0	0	0	S2-wms-combined_98888
2165	VC_804_4	1	0	1261.655568	0	0	0	0	1	1	1	0	0	0	0	S5-wms-combined_72410
2166	VC_805_0	4	1434.451062	145.2511201	0.90805158	0.003773117	2	7.141428429	6	6	1	4.76499877	0	0	0	EU100883,EU100884,KX987127,MH992131
2167	VC_806_0	3	424.7185729	373.5634018	0.532040791	0.00175974	0	4.123105626	3	3	1	2.74873708	0	0	0	KP792623,MW202557,MW202655
2168	VC_807_0	17	14572.45305	1494.023497	0.907009885	6.93E-58	0	6.244997998	136	136	1	3.70243046	0	0	0	EU124666,MG592549,MG592550,MG592581,MG592582,MK796797,MW824388,MW824389,MW824392,MW824393,MW824394,MW824398,MW824399,MW824402,MW824403,MW824405,OL762410
2169	VC_808_0	2	60.17217399	96.02497212	0.385232224	0.05234006	5.656854249	5.656854249	1	1	1	5.65685425	0	0	0	MW202520,MW202674
2170	VC_809_0	2	279.6474462	1274.688656	0.179914399	0.03700094	1.732050808	1.732050808	1	1	1	1.73205081	0	0	0	KJ028219,MG770216
2171	VC_80_0	41	203278.4193	145002.2244	0.583662695	0	0	10.58300524	820	804	0.980487805	5.0654395	0	0	0	DQ163917,EU716414,KF981875,KR054028,KR054030,KT254130,KT254131,KT254132,KT254133,KT372690,KT372691,KT372692,KT372693,KT372694,KT372695,KT372696,KT372697,KT372698,LC472882,LN610589,MH536736,MK317959,MN508620,MN563783,MN564818,MN871454,MT108726,MT118293,MT118303,MT119364,MT119367,MT119370,MT119372,MT119374,MT119375,MT119377,MT119378,MT133563,MW557846,MZ089729,ON361134
2172	VC_810_0	18	44600.26523	4295.853672	0.912143259	4.34E-52	0	2.449489743	153	153	1	0.79758978	0	0	0	EU203571,KJ538722,KT222940,KT246485,KT454972,MF919517,MH697577,MH727549,MK494095,MK919482,MT114159,MT657330,MT684594,MT818421,MW924639,MZ005672,MZ622181,OP297533
2173	VC_812_0	3	900	6767.990741	0.117371034	0.001576207	3.16227766	4.69041576	3	3	1	4.10827646	0	0	0	HM997019,MG873442,MW296032
2174	VC_812_1	4	1800	6144.275433	0.226578247	1.57E-05	3.16227766	4.582575695	6	6	1	3.82889075	0	0	0	LC519601,MT028297,MT664722,MT740244
2175	VC_812_2	2	300	4187.734869	0.066848869	0.044818616	1.732050808	1.732050808	1	1	1	1.73205081	0	0	0	JN882286,MW343794
2176	VC_812_3	1	0	2189.009008	0	0	0	0	1	1	1	0	0	0	0	OL799327
2177	VC_813_0	12	19800	64562.22281	0.234702208	4.84E-40	0	6.32455532	66	66	1	4.7215476	0	0	0	JX415536,LC553734,MK310183,MT446407,MT446413,MT446414,MZ234015,MZ234017,MZ342906,MZ398243,OK148440,OP293233
2178	VC_813_1	24	79583.03124	69161.90049	0.535030204	1.98E-126	3.605551275	8.246211251	276	276	1	6.13883182	0	0	0	EU330206,JX867715,KF981730,KM044272,KP308307,LC553735,MG673519,MG748548,MG976803,MG983840,MK373770,MN045231,MN148435,MN508615,MN715356,MT127620,MT129653,MT129655,MT446386,MT446410,MW741821,MW929182,MZ234028,MZ648214
2179	VC_814_0	3	228.8215809	0	1	0	0	4.242640687	3	3	1	2.82842712	0	0	0	EU350361,KY296500,KY296501
2180	VC_815_0	8	5394.352001	109.2936615	0.980141588	4.67E-12	0	4.898979486	28	28	1	3.69812397	0	0	0	EU588980,GQ443085,JF767208,JF767209,JF767210,MH999280,MZ401006,MZ995505
2181	VC_817_0	14	6440.473296	3714.630564	0.63421048	5.36E-45	0	4.472135955	91	91	1	2.50476061	0	0	0	EU622808,EU771092,KU831549,MF407276,MT459794,MW354668,MW354670,MW477480,MZ570263,OL744111,OM249958,ON042750,PZACG,X99260
2182	VC_818_0	20	18829.97421	782.514746	0.960101201	4.36E-43	0	5.656854249	190	190	1	4.1294355	0	0	0	KC685370,KX349899,KX349900,KX349901,KX961631,KY947509,MF156577,MF156578,MF156580,MH817022,MK084630,MK284526,MK284527,MK284528,MN082624,MT777452,MW012634,MW348917,MZ384014,OL744112
2183	VC_821_0	4	37.37090036	703.7524964	0.050424667	0.340348128	3.16227766	3.872983346	6	6	1	3.52530423	0	0	0	S2-wms_53881,S7-wms_239833,S7-wms_53338,S8-wms_244525
2184	VC_822_0	2	3.698386129	93.65553471	0.037989082	0.105107375	4	4	1	1	1	4	0	0	0	LT600745,S7-wms-combined_225260
2185	VC_823_0	124	592162.9285	31679.87725	0.949218173	0	0	8.485281374	7626	7626	1	6.66394083	0	0	0	EU734173,GQ413937,HG994092,HM480846,KR149291,KT367885,KU183006,KX591654,KX712070,KX712071,KY389315,KY389316,KY450753,KY652723,KY652724,KY652725,LC413193,LC413194,LC413195,LN866626,MF285615,MG835568,MH172261,MH172262,MH172263,MH172264,MH587638,MK134560,MK203841,MK380015,MK380016,MK521905,MK618658,MK795385,MK903728,MN052874,MN101216,MN101218,MN101227,MN149903,MN176574,MN434093,MN585130,MT425185,MT446417,MT446418,MT542697,MT813140,MT813141,MT966872,MT966873,MW042787,MW042788,MW042789,MW042790,MW042791,MW042792,MW042793,MW042794,MW042795,MW042796,MW042797,MW042798,MW042799,MW042800,MW042801,MW042803,MW042804,MW042805,MW042806,MW042807,MW042808,MW042809,MW042810,MW042811,MW042812,MZ424865,MZ571832,OK138556,OL409034,OL799328,OM194188,OM194189,OM194190,ON148528,ON148529,ON571554,ON602742,ON602753,ON602755,ON880501,OP013026,OP045496,OP103917,OP103918,OP103919,OP103920,OP103921,OP103922,OP103923,OP256047,OU342754,OX335359,OX335360,OX335375,OX335376,OX335377,OX335389,OX335390,OX335392,OX335394,OX335398,OX335401,OX335408,OX335409,OX335413,OX335414,OX335418,OX335426,OX335427,OX335433,OX335438,OX335440,OX335443
2186	VC_825_0	7	902.3512775	687.8379329	0.567449	1.58E-10	4.898979486	9.273618495	21	18	0.857142857	7.66483343	0	0	0	S1-wms-combined_118167,S1-wms-combined_169879,S11-wms-combined_200084,S2-wms-combined_176376,S2-wms_79944,S6-wms-combined_140731,S9-wms-combined_105462
2187	VC_825_1	1	0	351.9464578	0	0	0	0	1	1	1	0	0	0	0	S11-wms-combined_267993
2188	VC_825_2	1	0	171.5574266	0	0	0	0	1	1	1	0	0	0	0	S5-wms-combined_134743
2189	VC_826_0	91	648813.6754	4272.64629	0.993457762	1.80E-170	0	4.795831523	4095	4095	1	0.90674291	0	0	0	ON170019,ON170022,ON170028,ON170033,ON170036,ON170038,ON170040,ON170049,ON170054,ON170074,ON170086,ON170088,ON170090,ON170092,ON170094,ON170096,ON170098,ON170100,ON170102,ON170104,ON170106,ON170108,ON170110,ON170112,ON170114,ON170116,ON170118,ON170120,ON170122,ON170124,ON170126,ON170142,ON170144,ON170146,ON170152,ON170154,ON170156,ON170158,ON170160,ON170162,ON170164,ON170167,ON170176,ON170180,ON170186,ON170189,ON170192,ON170194,ON170196,ON170198,ON170200,ON170202,ON170204,ON170206,ON170211,ON170215,ON170217,ON170219,ON170221,ON170222,ON170225,ON170244,ON170246,ON170248,ON170250,ON170252,ON170271,ON170280,ON170282,ON170284,ON170289,ON170291,ON170293,ON170295,ON170297,ON170298,ON170300,ON170306,ON170307,ON170311,ON170313,ON170315,ON170330,ON170332,ON170334,ON170336,ON170338,ON170340,ON170352,ON170356,ON170360
2190	VC_827_0	6	2188.007623	732.1101405	0.74928746	3.97E-10	0	6.403124237	15	15	1	4.83903897	0	0	0	KJ018213,MG592431,MG592432,MG592435,MG592458,MG592666
2191	VC_828_0	3	491.9267258	544.0495258	0.474843632	0.001564446	2	3.741657387	3	3	1	3.06858633	0	0	0	JQ619704,MG784342,MH598512
2192	VC_829_0	5	1661.524261	221.9730378	0.88214847	1.68E-06	1	4.472135955	10	10	1	3.30247059	0	0	0	EU887664,JX887877,KF296717,KX190834,MT136606
2193	VC_82_0	24	47250.58365	17027.60803	0.735095099	1.62E-109	0	3.605551275	276	276	1	1.71232251	0	0	0	DQ029335,FJ409640,HQ641340,HQ641341,HQ641342,HQ641343,HQ641344,JQ780163,KY883641,KY883643,KY883644,KY883645,KY883646,KY883648,KY883649,KY883650,KY883651,KY883652,KY883653,MF574151,MG957431,MT215167,ON464735,ON464736
2194	VC_830_0	6	2978.428392	1578.192926	0.653648435	3.28E-09	2.645751311	4.358898944	15	15	1	3.65314758	0	0	0	FJ008913,HE608841,MN078104,MT630433,MW314138,MW916539
2195	VC_831_0	5	1296.59194	1671.327294	0.436869011	4.80E-07	4.242640687	6.164414003	10	10	1	5.22077311	0	0	0	MN929097,OK632025,S1-wms-combined_394574,S4-wms-combined_14411,S6-wms_54578
2196	VC_832_0	2	88.03814134	287.6297805	0.234350969	0.054738612	7.681145748	7.681145748	1	1	1	7.68114575	0	0	0	S2-wms-combined_113619,S9-wms-combined_5526
2197	VC_832_1	1	0	235.7872272	0	0	0	0	1	1	1	0	0	0	0	S7-wms-combined_3697
2198	VC_833_0	16	21402.09654	1484.576387	0.935133587	1.17E-65	0	7.615773106	120	120	1	6.07199263	0	0	0	FJ591093,FJ591094,FR682616,FR719956,HQ317387,KJ621082,KJ803031,KU885988,KU885989,KU885990,MH015250,MH015253,MH015256,MH015257,MH015259,MZ773648

2199	VC_834_0	7	261.7771128	4.120575994	0.984503152	0.057231067	0	3.16227766	21	21	1	2.38153923	0	0	0	FJ685651,GU321093,JN882264,JN882266,KF056323,MW344764,MW344766
2200	VC_835_0	67	330702.2629	12905.51484	0.962441145	0	0	6.403124237	2211	2211	1	4.99848368	0	0	0	FJ750948,GQ502199,JN984867,JQ086375,JX865427,KM233151,KM896878,KX534335,KX534336,KX534338,KX534339,KX534341,LN881730,LN881731,LR597646,LR990702,LR999870,LT960609,MG065648,MG581355,MK138527,MK373789,MK373790,MK373791,MK728541,MN850575,MN850589,MN850628,MN850629,MN850650,MT331608,MT446389,MT446391,MT478993,MT884012,MT884013,MT884014,MT884015,MT951623,MW749005,MW822006,MW862804,MZ234008,MZ234014,MZ234016,MZ234026,MZ234029,MZ501070,MZ501088,MZ501095,MZ501097,MZ501107,OK272474,OK272488,OK310512,OK310513,OK310514,OK499985,OK499988,OK499993,OL615010,OL770278,OM262540,OM262541,ON184124,ON210136,OP046317
2201	VC_836_0	9	5873.392843	1721.697178	0.773314448	3.12E-17	0	6.164414003	36	36	1	4.39206902	0	0	0	KT367887,MN656993,MT075871,MW419910,OL539440,OM687892,ON602734,ON602747,ON602766
2202	VC_837_0	29	70287.37118	317.8494158	0.995498217	5.79E-49	0	3.741657387	406	406	1	1.76510711	0	0	0	FJ896200,LR794124,MF754111,MF754112,MK474470,MN974282,MT178448,MT501516,MW250641,MW331544,MZ020222,MZ182247,MZ516827,MZ592921,OK349506,OK585158,OK585159,OK585160,OM362522,OM799543,ON080940,ON080941,ON457554,ON457555,ON457556,ON457557,ON864052,ON872379,OP117450
2203	VC_838_0	29	120231.2952	19390.83617	0.861119179	1.36E-130	0	5.291502622	406	406	1	2.26064597	0	0	0	FN422399,HG962375,JX194238,KM411958,KM411959,KM411960,KM974184,KP994390,KR052142,KR054032,KU948710,KX171209,KX171210,MF490236,MF490238,MH356729,MN553583,MN553584,MN906996,MT080102,MZ955867,OK665488,OL519844,OM234792,OM937766,ON815901,ON815902,ON815903,ON857943
2204	VC_839_0	5	3000	15187.25564	0.16495067	5.27E-08	1.414213562	4.123105626	10	10	1	3.16844205	0	0	0	FN422398,LC064302,MT416090,OL519842,OM782452
2205	VC_83_0	6	2461.82057	1363.45482	0.643566886	5.20E-11	1	4.242640687	15	15	1	3.22132324	0	0	0	AB597179,KP343639,OK274245,OK318991,OP313111,OP617560
2206	VC_841_0	10	12061.73826	8051.64278	0.599687255	1.20E-27	0	6.08276253	45	45	1	4.81975698	0	0	0	KY083726,KY514264,KY514265,KY549659,KY769270,MH059638,MN812685,MN812687,MN812691,MZ348421
2207	VC_842_0	2	275.6345664	4652.857405	0.055926756	0.044351492	3.741657387	3.741657387	1	1	1	3.74165739	0	0	0	MG775260,MH179480
2208	VC_843_0	5	3000	7553.916253	0.284254672	5.62E-08	0	7.211102551	10	10	1	5.86727123	0	0	0	FN667788,FN667789,HE815464,MK408758,MT932329
2209	VC_844_0	27	105300	6898.926948	0.938511649	1.26E-123	0	5.744562647	351	351	1	3.55202946	0	0	0	FR823450,GU296433,JN132397,JX569801,KF148616,KX229736,KX236333,KX879627,MH107028,MT863714,MT863715,MT863716,MT863717,MT863718,MT863719,MT863720,MT863721,MT863722,MT863723,MT863724,MT863725,MT863726,MT863727,MT863728,MT863729,MT863730,MT863731
2210	VC_845_0	3	900	1787.95544	0.334826979	0.001685027	4.123105626	6.403124237	3	3	1	5.56354796	0	0	0	MK387337,MW451250,OM249648
2211	VC_846_0	2	300	250.0040779	0.545450501	0.076424876	2.645751311	2.645751311	1	1	1	2.64575131	0	0	0	MK388689,OK570186
2212	VC_847_0	5	3000	45302.50553	0.062108579	0.000780631	0	6.32455532	10	10	1	4.19707798	0	0	0	LC739533,LC739534,LC739535,LC739537,LC739539
2213	VC_847_1	26	97500	56698.39464	0.632302303	1.33E-127	2.645751311	9.16515139	325	319	0.981538462	6.13721713	0	0	0	JX561091,KX552041,KX664695,LR597654,LR746137,MG065650,MH051333,MH160766,MH252123,MK373788,MN850565,MN850571,MN850598,MN850618,MN850648,MT496970,MT944117,MW175890,MZ726793,OL505078,OL739525,OM362897,OM386656,OM386659,OM386667,OP562383
2214	VC_848_0	28	113400	29654.77656	0.792703346	8.02E-191	0	6.92820323	378	378	1	5.01661939	0	0	0	FR775895,KU522583,LC739536,LR865361,MF805809,MG065639,MG065645,MG065656,MG065689,MH675927,MH816848,MN850573,MN850584,MN850601,MN850632,MN850645,MT932211,MZ501063,MZ501084,MZ501100,OK210077,OK272470,OM386653,OM386654,OM386658,OM401812,OP177727,OX090893
2215	VC_848_1	1	0	10018.61617	0	0	0	0	1	1	1	0	0	0	0	OP617743
2216	VC_849_0	3	900	12137.29383	0.069032731	0.001520602	5.830951895	7.681145748	3	3	1	7.02064403	0	0	0	MH533020,ON287369,ON287374
2217	VC_849_1	1	0	6544.136617	0	0	0	0	1	1	1	0	0	0	0	MZ520832
2218	VC_849_2	2	300	11900.64446	0.024588865	0.134862392	8.246211251	8.246211251	1	1	1	8.24621125	0	0	0	JQ015307,KY514263
2219	VC_849_3	2	300	12024.09481	0.024342559	0.148117714	2	2	1	1	1	2	0	0	0	MF979560,MF979563
2220	VC_849_4	2	300	12278.21411	0.023850763	0.135189255	4.242640687	4.242640687	1	1	1	4.24264069	0	0	0	MT552976,MT560059
2221	VC_849_5	3	900	17386.106	0.049217696	0.024252726	4.582575695	6.633249581	3	3	1	5.59452988	0	0	0	JQ691612,KC954774,KT353109
2222	VC_849_6	7	6300	32104.37033	0.16404383	1.40E-08	0	9.110433579	21	20	0.952380952	4.91621841	0	0	0	JQ691611,MN013077,OL744211,OL744214,OL744216,OL744219,OL744220
2223	VC_849_7	1	0	6546.979717	0	0	0	0	1	1	1	0	0	0	0	ON712643
2224	VC_849_8	1	0	6402.655988	0	0	0	0	1	1	1	0	0	0	0	MW367417
2225	VC_849_9	1	0	6604.110858	0	0	0	0	1	1	1	0	0	0	0	MW250786
2226	VC_850_0	3	900	9133.805035	0.08969678	0.001522067	0	1	3	3	1	0.66666667	0	0	0	OK136181,OK181860,OK181862
2227	VC_851_0	2	233.4908123	288.5897858	0.447231353	0.058957805	0	0	1	1	1	0	0	0	0	MH444512,MK932884
2228	VC_852_0	2	134.7280456	196.7969374	0.406388817	0.058101215	5.567764363	5.567764363	1	1	1	5.56776436	0	0	0	GQ141189,S4-wms-combined_17284
2229	VC_853_0	2	2.60778797	7.049965784	0.270020135	0.270145687	7.348469228	7.348469228	1	1	1	7.34846923	0	0	0	MG941013,MW073100
2230	VC_854_0	2	188.4475972	276.2068684	0.405565019	0.055317198	5.567764363	5.567764363	1	1	1	5.56776436	0	0	0	S5-wms-combined_117213,S9-wms-combined_76274
2231	VC_855_0	2	16.90845442	576.0555946	0.028515143	0.106210712	7.211102551	7.211102551	1	1	1	7.21110255	0	0	0	GQ241246,KF669650
2232	VC_856_0	7	6300	11892.15584	0.346303102	9.21E-14	2.449489743	4.123105626	21	21	1	3.44815283	0	0	0	MH371108,MH371109,MH536823,MZ355724,MZ456035,ON260827,ON970603
2233	VC_856_1	13	17124.3608	12155.85791	0.584844019	6.81E-37	0	8.94427191	78	78	1	6.7915944	0	0	0	KJ173786,MH271300,MH271307,MH271310,MH271316,MH399779,MK376963,MK919474,MK937596,MN096374,MN908690,MZ622166,OP068333
2234	VC_857_0	11	13551.06864	401.8774811	0.971197661	4.44E-25	0	4.582575695	55	55	1	2.131513	0	0	0	KU160638,KU160639,KU160647,KU160663,KU160666,MF185731,MN234164,MN234217,MN234224,MT310874,MT986028
2235	VC_859_0	9	9151.351568	556.8340387	0.942642831	3.93E-21	1	6.480740698	36	36	1	4.65190477	0	0	0	MH019215,MH019216,MK305891,MT498037,MT498053,MT897908,MT936332,MZ648033,MZ648036
2236	VC_85_0	3	900	20441.00269	0.042172339	0.001767581	1.414213562	7.745966692	3	3	1	5.59198445	0	0	0	KJ010547,KJ010548,KM051843
2237	VC_85_1	4	1800	33736.21231	0.050652556	3.31E-05	2	6.244997998	6	6	1	5.07471375	0	0	0	MW419084,MW419085,MW419086,MW419087
2238	VC_85_10	1	0	8516.537146	0	0	0	0	1	1	1	0	0	0	0	MN043729
2239	VC_85_11	1	0	8073.157121	0	0	0	0	1	1	1	0	0	0	0	MN940411
2240	VC_85_12	1	0	8893.936149	0	0	0	0	1	1	1	0	0	0	0	MT151604
2241	VC_85_13	1	0	6331.402046	0	0	0	0	1	1	1	0	0	0	0	KU640380
2242	VC_85_2	3	900	26028.53816	0.033421792	0.002697044	6	7.348469228	3	3	1	6.66057294	0	0	0	KC699836,MW749002,MW749007
2243	VC_85_3	19	51300	88428.33059	0.367141007	1.86E-72	0	10.19803903	171	138	0.807017544	7.8270336	0	0	0	AB930182,AP013029,KF669652,LT960608,MF422185,MG000860,MW354667,MZ501260,MZ501261,MZ501262,MZ501263,MZ501265,OM982668,OM982669,OM982670,OM982671,OM982672,OM982673,OM982674
2244	VC_85_4	3	900	29739.90445	0.02937346	0.018747447	4.358898944	6.633249581	3	3	1	5.74571551	0	0	0	KY368640,MN043730,OK349510
2245	VC_85_5	1	0	10667.8394	0	0	0	0	1	1	1	0	0	0	0	MH606185
2246	VC_85_6	1	0	7281.836584	0	0	0	0	1	1	1	0	0	0	0	JX238501
2247	VC_85_7	1	0	7209.823838	0	0	0	0	1	1	1	0	0	0	0	KM236246
2248	VC_85_8	1	0	7158.743652	0	0	0	0	1	1	1	0	0	0	0	KU253712
2249	VC_85_9	1	0	8029.002578	0	0	0	0	1	1	1	0	0	0	0	KM236245
2250	VC_860_0	16	22007.77886	78.94550051	0.996425658	1.56E-34	0	0	120	120	1	0	0	0	0	MN023176,MN023177,MN023178,MN023179,MN023180,MN023181,MN023182,MN023183,MN023184,MN023185,MN023186,MN023187,MN023188,MN023189,MN023190,MN023191
2251	VC_861_0	9	5111.33448	3598.075727	0.586874927	3.76E-19	0	2.449489743	36	36	1	1.77998133	0	0	0	JQ659259,KC013021,KC013022,KC013023,KC013024,KC013025,KX555527,MN552146,OV696621
2252	VC_862_0	11	7454.693584	3598.075727	0.674463872	5.04E-25	0	4.242640687	55	55	1	2.86287286	0	0	0	GQ451696,KC013026,KC013027,KC013028,KC013029,KM262191,KM262192,KX578042,KX578043,KX578044,MN552147
2253	VC_864_0	2	1.001946165	0	1	0	8.660254038	8.660254038	1	1	1	8.66025404	0	0	0	GQ919031,MN234216
2254	VC_865_0	2	300	3616.860453	0.076591955	0.061155602	7.615773106	7.615773106	1	1	1	7.61577311	0	0	0	MK804893,MK813939
2255	VC_865_1	1	0	2218.581146	0	0	0	0	1	1	1	0	0	0	0	MK813938
2256	VC_865_2	1	0	2283.114753	0	0	0	0	1	1	1	0	0	0	0	MK813941
2257	VC_865_3	2	300	3708.914878	0.074833218	0.065309387	2	2	1	1	1	2	0	0	0	OM033134,OM033135
2258	VC_865_4	1	0	2062.491807	0	0	0	0	1	1	1	0	0	0	0	OM033136
2259	VC_866_0	4	1796.549689	20807.86997	0.079477806	1.41E-05	2	3.16227766	6	6	1	2.68284146	0	0	0	JN035618,KR063278,MW534374,ON526973
2260	VC_866_1	20	56219.05479	60569.74447	0.481373686	1.12E-107	0	3.741657387	190	190	1	2.38819705	0	0	0	KU998240,MG099935,MH025889,MK450428,MK450430,MK450435,MK450436,MK494106,MK494109,MK494121,MK878903,MK919477,MK919481,ON724015,ON970582,ON970588,ON970596,ON970607,OP297547,OP434450
2261	VC_866_2	3	857.8383138	17778.32709	0.046030838	0.001495893	0	4.898979486	3	3	1	3.26598632	0	0	0	KR011062,KR011063,KR011064
2262	VC_867_0	6	2391.512762	5076.559507	0.320231604	2.36E-11	1.414213562	8.544003745	15	15	1	6.66807098	0	0	0	KP790008,KP790009,KP790010,KR063279,KX557281,ON526975
2263	VC_868_0	25	87159.07037	63348.60413	0.579100505	1.26E-159	0	6.557438524	300	300	1	4.58591166	0	0	0	KU963245,KU998241,MG099940,MG757152,MG757155,MG770211,MG770214,MK359302,MK433261,MK433263,MK433265,MK433267,MK801728,MK801733,MK967390,MN444874,MN945905,MT114160,MT889368,MT889370,MT889379,MT986029,MW507132,MW507133,ON456331
2264	VC_869_0	9	10652.68933	29946.36197	0.262387642	3.38E-24	0	4.123105626	36	36	1	2.67436644	0	0	0	KU963258,KU963262,KU998242,KU998243,MH576976,MK494098,MN234166,OM203156,OP434448
2265	VC_870_0	5	3000	4074.851555	0.424037166	3.48E-08	0	3.872983346	10	10	1	2.58997055	0	0	0	KU998252,KU998253,KU998254,MH479923,MN096363
2266	VC_872_0	31	92286.42497	20930.86923	0.815126572	1.06E-180	0	9.486832981	465	381	0.819354839	5.5442276	0	0	0	KX670789,KX670790,MF467948,MF467949,MG757154,MH155877,MH155880,MH669006,MK433259,MK433260,MK433268,MK433269,MK967386,MN204492,MN204494,MN204495,MN204496,MN204497,MN204500,MN204504,MN586054,MN586056,MT310852,MT310854,MT657340,MT684589,MT684600,MW055905,MW145539,MW712736,ON108646
2267	VC_873_0	3	171.0361318	16.77012196	0.9107052	0.01623666	4.898979486	6.164414003	3	3	1	5.63144846	0	0	0	GU936715,HM480106,MG271909
2268	VC_874_0	3	510.649276	300.807572	0.629299361	0.001875173	0	1	3	3	1	0.66666667	0	0	0	KR935216,KR935217,KT253150
2269	VC_875_0	10	874.1649441	10.64489206	0.987969288	0.000515666	0	3.16227766	45	45	1	1.949558	0	0	0	GU942563,HQ224500,KJ619459,KP972568,KP972569,KR063267,KR063268,KT728930,KT728931,MF155889
2270	VC_876_0	3	295.8563793	1521.595185	0.16278639	0.002533688	5.291502622	9.055385138	3	2	0.666666667	7.44896259	0	0	0	S12-wms-combined_245755,S5-wms-combined_26971,S7-wms-combined_139683
2271	VC_876_1	1	0	774.6655457	0	0	0	0	1	1	1	0	0	0	0	S2-wms-combined_116221
2272	VC_877_0	2	282.570576	81.45767269	0.776232551	0.131776239	0	0	1	1	1	0	0	0	0	MN830254,MN830255
2273	VC_878_0	32	40879.10866	145.3465115	0.996457076	1.18E-21	0	7.416198487	496	496	1	5.84547735	0	0	0	HE956707,HE956710,JN651747,KC107834,KT184661,KT949345,KX756572,KY318515,LN878149,MF683623,MG878892,MH179473,MH992513,MK053931,MK330684,MK838107,MK838108,MK838109,MK838110,MK838111,MN509793,MT682386,MT682387,MT682388,MT682389,MT682390,MT682391,MW250276,MZ592920,OL362270,OM654404,ON287377
2274	VC_879_0	36	130335.6355	785.5607976	0.994008895	1.45E-62	0	3	630	630	1	1.28263109	0	0	0	HF569088,HF569089,HF569090,HF569091,HF569092,HG428758,JN939331,JN939332,KC556893,KC556894,KC556895,KC556896,KC556897,KC556898,KJ133688,KJ133689,KJ133690,KJ133691,KJ133692,KJ133693,KJ133694,KJ133695,KJ133696,KJ133697,KJ133698,KJ133699,KJ133700,KJ133701,KJ133702,KJ133703,KJ133704,KJ133705,KJ133706,KJ133707,KY056619,MT002975
2275	VC_87_0	8	8400	6749.295291	0.554481237	2.37E-15	3.605551275	6.633249581	28	28	1	5.15779362	0	0	0	FJ230960,KF669649,KY368639,MT601270,MT601271,MW001214,MZ751040,OM236513
2276	VC_87_1	3	900	4601.790606	0.163583107	0.002220758	2.645751311	4.898979486	3	3	1	4.11352077	0	0	0	KC595512,KC595513,KF554508
2277	VC_87_2	1	0	1790.257906	0	0	0	0	1	1	1	0	0	0	0	MH884511
2278	VC_880_0	2	6.494897698	33.74507264	0.161404137	0.165449273	2.828427125	2.828427125	1	1	1	2.82842712	0	0	0	HG007973,HM150760
2279	VC_881_0	2	116.2925148	55.20414358	0.678103678	0.081867177	4.795831523	4.795831523	1	1	1	4.79583152	0	0	0	KP282679,KP282680
2280	VC_882_0	20	56996.48288	477.5613956	0.991690834	3.68E-42	0	5	190	190	1	3.47168261	0	0	0	HG992588,JQ692107,JX556418,MF754113,MF754114,MF754115,MF754116,MG602476,MG893203,MH599087,MH925090,MN215888,MW451246,MW451251,MZ592922,OK483201,OK625546,OL314400,OP547477,OP595601
2281	VC_883_0	10	13500	53499.9303	0.201492747	6.62E-30	2	8.366600265	45	45	1	6.93940589	0	0	0	JF966203,KJ489397,KJ489398,KJ489400,KU737346,MF288921,MF288922,MF498901,MH638310,MK215646
2282	VC_883_1	12	19800	61315.56817	0.244096176	1.58E-29	0	8.888194417	66	66	1	7.23117208	0	0	0	KF208639,KF669647,KJ489402,KT307976,KU737345,KU737347,MF765814,MN038178,MW281503,OK499976,OK499990,OM350011
2283	VC_883_2	2	300	16244.54916	0.01813286	0.119051476	1	1	1	1	1	1	0	0	0	JN790865,JN797796
2284	VC_883_3	2	300	16178.89877	0.0182051	0.120997643	6.08276253	6.08276253	1	1	1	6.08276253	0	0	0	KF669662,KY888882
2285	VC_883_4	1	0	8334.787781	0	0	0	0	1	1	1	0	0	0	0	MN153503
2286	VC_883_5	1	0	8326.763175	0	0	0	0	1	1	1	0	0	0	0	MW787012
2287	VC_883_6	1	0	8412.098617	0	0	0	0	1	1	1	0	0	0	0	OM743306
2288	VC_884_0	28	113400	69177.15993	0.621107263	5.79E-66	0	9.273618495	378	372	0.984126984	5.8583255	0	0	0	KJ489399,KJ489401,KT207918,KU737344,KU737348,KU737349,KU737350,KU737351,KU737352,KX147229,KX156152,KX349902,KX349903,KX961629,KX961632,MF288917,MF288918,MF288919,MF288920,MG763894,MH538193,MH638311,MN038176,MN038179,MT584805,OK500001,OM350012,OM350013
2289	VC_884_1	8	8400	67805.95899	0.1102276	0.001067699	2.236067977	9.797958971	28	21	0.75	6.41675753	0	0	0	HM144387,JN654439,KC430106,KU847400,KX011169,MW392803,MW584228,MW596414
2290	VC_885_0	11	12218.33395	282.7890493	0.977378908	4.89E-26	1.414213562	4.123105626	55	55	1	2.60976373	0	0	0	MW677509,MW677510,MW677512,MW677515,MW677517,MW677520,OP009272,OP009275,OP009278,OP009280,OP009286
2291	VC_886_0	2	254.9815929	1599.035974	0.137529222	0.046476646	5.567764363	5.567764363	1	1	1	5.56776436	0	0	0	MN988459,MN988558
2292	VC_886_1	1	0	947.1266082	0	0	0	0	1	1	1	0	0	0	0	MW677529
2293	VC_887_0	18	45806.16967	79246.03598	0.366296375	1.26E-88	1.732050808	5.830951895	153	153	1	4.11090176	0	0	0	HM152763,JF704108,JF704113,JN600672,MF185726,MH744425,MK279905,MK310144,MK494092,MK494100,MN096382,MN586018,MN586023,MN617841,MT771349,MW435855,MW822149,MZ958746
2294	VC_888_0	28	113369.9368	92859.56387	0.549727059	2.16E-189	0	6.708203932	378	378	1	3.9534891	0	0	0	JF744988,JN680858,KC661276,KC691254,KF024731,KT591489,KU234099,KU997639,KX507361,KX580961,KX580962,MF140422,MF185728,MF185730,MF324910,MH779511,MH834600,MK937600,MN096380,MN234228,MN586030,MN586052,MN703406,MN703410,MT758688,MW578835,MZ171373,OL455899
2295	VC_888_1	3	841.2723336	28264.9645	0.028903507	0.001515524	5.656854249	6.92820323	3	3	1	6.40610235	0	0	0	MH576973,MN234187,MZ005679
2296	VC_889_0	16	35864.16096	84927.65251	0.296908871	2.50E-71	0	6.403124237	120	120	1	4.33119716	0	0	0	KF024725,KT281791,KT281794,KT372003,MF140405,MF140413,MF140414,MF140421,MH727559,MK878900,MN096377,MT889383,MT889391,MW291024,MZ622183,ON755181
2297	VC_88_0	1	0	98.74618067	0	0	0	0	1	1	1	0	0	0	0	KT624200
2298	VC_88_1	1	0	166.3995877	0	0	0	0	1	1	1	0	0	0	0	MF418016
2299	VC_890_0	3	891.4359569	1608.753319	0.356547388	0.001690487	4.472135955	5.744562647	3	3	1	5.13761701	0	0	0	MH651182,MK967378,MT658805
2300	VC_890_1	4	1800	2101.850195	0.461319607	1.54E-05	2	6.164414003	6	6	1	4.34289031	0	0	0	MH976515,MN444870,MT684599,OK040786
2301	VC_891_0	2	195.93564	145.0672348	0.574586476	0.077364462	2.828427125	2.828427125	1	1	1	2.82842712	0	0	0	HQ268735,KY629621
2302	VC_893_0	2	34.39247974	164.4716374	0.172944623	0.061538965	5.656854249	5.656854249	1	1	1	5.65685425	0	0	0	S11-wms-combined_46816,S4-wms-combined_113721
2303	VC_894_0	3	481.1329333	275.5962867	0.63580594	0.003078484	2	5.099019514	3	3	1	3.999333	0	0	0	HM588722,KF302032,KF302033
2304	VC_895_0	2	94.49741148	244.4261373	0.278816305	0.057487462	6.32455532	6.32455532	1	1	1	6.32455532	0	0	0	KF302036,KX365748
2305	VC_896_0	5	809.90674	815.9494359	0.498141688	2.26E-07	4.898979486	7	10	10	1	6.42305023	0	0	0	KR060090,KX912252,KY379511,MF370965,MT002875
2306	VC_897_0	6	890.1520339	1251.605661	0.415617526	3.89E-10	1	3.464101615	15	15	1	2.25807726	0	0	0	HM640230,JN800508,KY996523,MH999279,MW815121,OP022426
2307	VC_898_0	10	3856.088318	1437.859218	0.728395643	3.32E-22	0	3.464101615	45	45	1	2.21477937	0	0	0	JQ729990,JQ729991,JQ729992,MH248069,MW057920,MZ401005,MZ401007,MZ401008,MZ401009,OL547738
2308	VC_899_0	4	349.1158081	965.6244404	0.265539759	2.90E-05	0	4.123105626	6	6	1	2.6970748	0	0	0	MH393889,MK017820,OM638104,OP256049
2309	VC_89_0	2	300	6355.773225	0.045073651	0.071505289	5.656854249	5.656854249	1	1	1	5.65685425	0	0	0	MT601273,MT601274
2310	VC_89_1	2	300	6408.57464	0.044718888	0.053531097	6.633249581	6.633249581	1	1	1	6.63324958	0	0	0	MT601272,OL580764
2311	VC_89_2	5	3000	11756.77214	0.203296491	3.57E-06	1.414213562	7	10	10	1	4.8336581	0	0	0	KY030782,MT366945,MT366946,MT366947,MT366948
2312	VC_89_3	1	0	3619.233123	0	0	0	0	1	1	1	0	0	0	0	AF020713
2313	VC_89_4	1	0	3430.559108	0	0	0	0	1	1	1	0	0	0	0	ON107264
2314	VC_89_5	1	0	3338.672051	0	0	0	0	1	1	1	0	0	0	0	MZ969646
2315	VC_89_6	1	0	2911.32742	0	0	0	0	1	1	1	0	0	0	0	OM236515
2316	VC_8_0	58	213381.62	43508.58969	0.830633523	0	0	9.273618495	1653	1644	0.994555354	6.67411331	0	0	0	AB045978,AF424782,AP009363,AP012341,AY954955,AY954956,AY954957,CP062434,CP062442,CP062444,CP062461,EF462198,EU861005,JQ779023,JQ957932,JX174275,KF598856,KT186243,KY056620,LC541428,MF580410,MG029509,MG029511,MG029513,MG029514,MG029515,MG029516,MG029517,MG029518,MG770897,MH384261,MK348510,MK801680,MK801681,MK940809,MT366568,MT899504,MW924497,OL690335,OL840290,OM304856,OM439665,OM439667,ON856257,ON856258,ON989481,OP038318,OP038319,OP136131,OP136132,OP136134,OP328420,OP328422,OP374154,OP374155,OP374156,OP584903,OP584904
2317	VC_901_0	5	2264.4853	179.5809188	0.92652371	5.03E-06	0	6.92820323	10	10	1	5.20273259	0	0	0	KF623293,MW824400,MW824417,MW824421,MW824431
2318	VC_902_0	3	900	2.027099769	0.997752729	0.124106539	0	7	3	3	1	4.66666667	0	0	0	HQ316582,MG592662,MG592672
2319	VC_903_0	7	3617.529445	1286.438099	0.737674019	7.07E-13	0	5.477225575	21	21	1	3.17546318	0	0	0	MW824414,MW824420,MW824427,MW824428,MW824429,MW865319,OX241434
2320	VC_904_0	6	3965.497854	13054.78931	0.232986542	1.50E-10	0	8.124038405	15	15	1	6.2548271	0	0	0	JF713456,MG592585,MG592586,MG720309,MK559459,MT735629
2321	VC_904_1	4	1800	10316.52946	0.148557391	2.28E-05	0	4.582575695	6	6	1	3.17797618	0	0	0	HQ316604,KX198615,MT451873,ON015836
2322	VC_904_2	7	5573.038396	13932.76392	0.285711826	3.35E-13	0	7.280109889	21	21	1	3.70286489	0	0	0	KY022433,MH925091,MN871506,MW411580,OK483199,OP547478,OV032860
2323	VC_905_0	3	900	1728.865254	0.342353036	0.001762247	3	3.605551275	3	3	1	3.25594298	0	0	0	MG189906,MT110073,MZ326862
2324	VC_907_0	3	661.5756116	334.457848	0.664210228	0.003668864	3	5	3	3	1	4	0	0	0	HQ317384,HQ332142,KM233261
2325	VC_908_0	3	620.8108253	600.4506209	0.508335727	0.002754703	0	6.708203932	3	3	1	4.47213595	0	0	0	MN988533,MN988538,MW980068
2326	VC_909_0	5	1544.119256	540.7655917	0.740625679	1.39E-05	3.872983346	4.795831523	10	10	1	4.32611438	0	0	0	HQ317386,MG592522,MG640035,MT720689,OM837731

2327	VC_90_0	1	0	209.2356595	0	0	0	0	1	1	1	0	0	0	0	LC597490
2328	VC_90_1	1	0	319.5678008	0	0	0	0	1	1	1	0	0	0	0	OM634661
2329	VC_90_2	1	0	115.6778985	0	0	0	0	1	1	1	0	0	0	0	EF583821
2330	VC_910_0	2	92.94972114	1.019761903	0.989147946	0.5	8.306623863	8.306623863	1	1	1	8.30662386	0	0	0	HQ317390,MZ568826
2331	VC_911_0	6	3701.968809	9581.535428	0.278689173	1.51E-11	0	4.358898944	15	15	1	1.82971466	0	0	0	HQ317392,KC821610,KC821616,KC821629,KC821630,KC821634
2332	VC_912_0	76	708837.691	15200.22476	0.979006314	2.10E-297	0	6.08276253	2850	2850	1	3.00676872	0	0	0	KM873719,KY951963,KY951964,KY979235,KY979236,KY979237,KY979238,KY979239,KY979240,KY979241,KY979242,KY979243,KY979244,KY979245,KY979246,KY979247,KY992519,KY992520,MK756083,MK756084,MK756085,MK756086,MK756087,MK756088,MK756089,MK756090,MK756091,MK756092,MK756093,MK756094,MK756095,MK756096,MT431535,MT585273,MT585274,MT585275,MT585276,MT585277,MT585278,MT585279,MT585280,MT585281,MT585282,MT585283,MT585284,MT585285,MT585286,MT585287,MT585288,MT585289,MT585290,MT585291,MT585292,MT585293,MT585294,MT585295,MT585296,MT585297,MT585298,MT585299,MT585300,MT585301,MT585302,MT585303,MT585304,MT585305,MT585306,MT585307,MT585308,MT585309,MT585310,MT585311,MT585312,MT585313,MT585314,MT585315
2333	VC_913_0	2	3.484199298	29.95716172	0.104188322	0.176932247	6.244997998	6.244997998	1	1	1	6.244998	0	0	0	HQ332136,MW452941
2334	VC_914_0	1	0	29.72877948	0	0	0	0	1	1	1	0	0	0	0	S5-wms_67169
2335	VC_915_0	3	134.954144	473.2973843	0.221872265	0.001806511	6.557438524	7.483314774	3	3	1	7.1069544	0	0	0	S12-wms-combined_321174,S3-wms_23958,S8-wms-combined_169835
2336	VC_916_0	4	1477.245847	266.3523491	0.847239834	0.00014426	0	5	6	6	1	2.81649658	0	0	0	HQ396194,MZ995504,ON922917,ON933637
2337	VC_917_0	2	199.4292273	464.5320311	0.300362747	0.05145877	5.744562647	5.744562647	1	1	1	5.74456265	0	0	0	S11-wms-combined_48246,S8-wms-combined_270362
2338	VC_917_1	1	0	261.8601647	0	0	0	0	1	1	1	0	0	0	0	S5-wms-combined_90315
2339	VC_918_0	2	63.55645036	385.7879135	0.141442634	0.071233377	6.633249581	6.633249581	1	1	1	6.63324958	0	0	0	S11-wms_320555,S12-wms_12658
2340	VC_918_1	1	0	363.2801541	0	0	0	0	1	1	1	0	0	0	0	S7-wms-combined_26217
2341	VC_918_2	1	0	322.874506	0	0	0	0	1	1	1	0	0	0	0	S12-wms-combined_201125
2342	VC_919_0	39	100299.6999	10298.62874	0.906882601	7.09E-169	0	8.306623863	741	741	1	6.30544419	0	0	0	HQ403646,KU963260,KU998239,MG770215,MH271302,MH450132,MK279900,MK875791,MK875795,MK967383,MK967385,MN062716,MN234215,MN284906,MN310549,MN444871,MN444878,MN586015,MN586036,MN586049,MN586057,MT776808,MT776809,MT776810,MT889367,MT889378,MW507135,MW862988,MZ150793,MZ322013,MZ622167,OK040785,OL455888,OL829977,ON970576,ON970599,ON970609,ON970621,OP297531
2343	VC_91_0	22	69300	61019.14218	0.531771456	3.97E-111	0	8.426149773	231	231	1	6.25441328	0	0	0	AB609718,AP009390,AP018714,AP018715,CAJDJY010000002,CAJDJZ010000002,KJ801817,KR049063,LC596377,LC596378,LC596379,LR031359,MK268686,MK693030,MK721192,MN854830,MW633168,MZ420150,ON113167,ON113168,ON113169,ON286976
2344	VC_91_1	1	0	7235.329762	0	0	0	0	1	1	1	0	0	0	0	OP559178
2345	VC_920_0	2	300	1495.267577	0.167106009	0.044437036	3	3	1	1	1	3	0	0	0	MN204493,MW822144
2346	VC_921_0	2	204.0635968	11.19622322	0.947987399	0.270145687	0	0	1	1	1	0	0	0	0	HQ632857,HQ632858
2347	VC_922_0	2	36.72054079	50.70014174	0.420044087	0.095215132	6.480740698	6.480740698	1	1	1	6.4807407	0	0	0	MK291441,MK291443
2348	VC_923_0	23	73209.94788	4217.256885	0.945532621	1.58E-92	1	6.403124237	253	253	1	3.99794166	0	0	0	MW677513,MW677516,MW677518,MW677519,MW677521,MW677522,MW677523,MW677524,OP009265,OP009266,OP009267,OP009268,OP009269,OP009270,OP009271,OP009273,OP009276,OP009277,OP009279,OP009283,OP009284,OP009287,OP009288
2349	VC_924_0	34	53696.4037	6355.746279	0.894162885	5.31E-162	0	9.16515139	561	548	0.976827094	7.59217804	0	0	0	MG592442,MG592450,MG592457,MG592460,MG592482,MG592483,MG592488,MG592489,MG592490,MG592502,MG592503,MG592504,MG592505,MG592516,MG592517,MG592525,MG592530,MG592532,MG592534,MG592535,MG592539,MG592543,MG592557,MG592558,MG592559,MG592576,MG592593,MG592594,MG592597,MG592598,MG592605,MG592606,MG592607,MG592631
2350	VC_925_0	1	0	98.13986357	0	0	0	0	1	1	1	0	0	0	0	MN592896
2351	VC_926_0	3	524.6968328	30.84262168	0.944481672	0.005696139	0	5.385164807	3	3	1	3.59010987	0	0	0	MG592520,OX241566,OX241570
2352	VC_927_0	2	1.929920333	20.09416263	0.087627727	0.280637918	8.602325267	8.602325267	1	1	1	8.60232527	0	0	0	HQ698895,KJ410740
2353	VC_928_0	3	700.6199726	192.609007	0.784367714	0.005586199	0	1	3	3	1	0.66666667	0	0	0	JF974286,JF974321,MT490303
2354	VC_929_0	4	1800	327.5743819	0.846033876	0.002777992	0	3.16227766	6	6	1	1.58113883	0	0	0	HQ634192,KC821604,KC821621,KC821625
2355	VC_92_0	22	69300	53583.28562	0.563949765	7.06E-119	2.645751311	8.717797887	231	231	1	6.80538555	0	0	0	CAJDKA010000002,KP339049,KY549443,LR990835,MH791397,MH880817,MN027503,MN241318,MT856905,MT909815,MW004544,MW004545,MZ147816,MZ333462,OL799258,OL799260,OL870611,OM966901,ON113170,ON125307,ON506927,ON506928
2356	VC_930_0	7	5720.530485	5300.767828	0.519043249	8.33E-15	0	6.557438524	21	21	1	3.64779041	0	0	0	KX889068,MG592409,MG592410,MG592470,MG592519,MG592521,MG592668
2357	VC_932_0	2	17.75718474	22.25367723	0.443809102	0.105649774	7.937253933	7.937253933	1	1	1	7.93725393	0	0	0	HQ683709,MG209611
2358	VC_933_0	8	2536.194403	4416.315356	0.364788327	5.85E-18	0	7	28	28	1	5.73451774	0	0	0	KR093625,KR093626,KR093627,KR093628,KR093629,KR093630,KR093631,KR093633
2359	VC_934_0	2	258.2134949	41.57361848	0.861322863	0.075007761	0	0	1	1	1	0	0	0	0	MT119766,MT210154
2360	VC_935_0	2	16.67520639	6.881059614	0.707888355	0.270145687	6.708203932	6.708203932	1	1	1	6.70820393	0	0	0	MN988536,OP028995
2361	VC_936_0	2	300	23.13842396	0.928394699	0.185546685	1.732050808	1.732050808	1	1	1	1.73205081	0	0	0	MN602266,MN602267
2362	VC_937_0	40	141603.9082	66357.10443	0.68091565	0	0	10.19803903	780	730	0.935897436	6.44529351	0	0	0	JN672684,KR269719,KX237515,KY780482,MN013075,MN013076,MN013078,MN013079,MN013080,MN013083,MN013087,MT701592,MW021762,MW021764,MW021765,MW042802,MW417503,MW722081,MZ428221,MZ428224,MZ598515,MZ634348,MZ634350,MZ634351,OL539450,OL539472,OL865411,OM065837,OM065838,OM065839,OM065841,OM328157,OM735690,OP611406,OV877339,OV877387,OV877766,OX335361,OX335395,OX335410
2363	VC_938_0	99	753703.7546	72857.93473	0.911854208	0	0	9.746794345	4851	4816	0.992784993	6.94244397	0	0	0	JF501022,KP658157,KR262148,KT001920,KY575286,LR757892,MF415412,MF612072,MG552615,MG894052,MH445453,MH633484,MH633485,MH633486,MH633487,MH688453,MH844531,MK170446,MK278861,MK521904,MK618657,MK931442,MK931443,MK931444,MK931445,MN379832,MN395285,MN395286,MN689779,MN783016,MT894005,MW655991,MW672037,MZ172460,MZ221764,MZ398242,MZ428222,MZ428223,MZ428225,MZ428226,MZ428227,MZ428228,MZ571831,MZ571833,MZ571834,MZ634345,MZ634346,OK138555,OK655936,OL539448,OL539460,OL674249,OL702938,OL744210,OL744213,OM032871,OM065840,OM065842,OM065843,OM687893,OM938991,OM938992,OM964875,ON489264,ON602735,ON602741,ON602750,ON881905,OP267563,OP413832,OP617742,OU342755,OW251746,OX335369,OX335370,OX335371,OX335380,OX335381,OX335383,OX335384,OX335385,OX335386,OX335387,OX335388,OX335391,OX335393,OX335400,OX335404,OX335411,OX335412,OX335417,OX335421,OX335422,OX335423,OX335429,OX335430,OX335432,OX335435,OX335439
2364	VC_939_0	15	31500	0	1	1	0	4	105	105	1	2.82037923	0	0	0	JF704100,KJ538721,KX159477,MG757167,MH727553,MH727562,MK061407,MK279842,MK305892,MK494123,MN329674,MN329676,MN428056,MN428061,ON637772
2365	VC_93_0	2	300	2522.512373	0.106288285	0.043343284	4.69041576	4.69041576	1	1	1	4.69041576	0	0	0	FJ822135,MT758689
2366	VC_940_0	34	124426.6099	31914.44734	0.795866499	4.34E-174	0	6.708203932	561	561	1	4.80178961	0	0	0	JF704107,JN083852,JN083853,JN408459,JQ684677,JX042578,KF017001,KF493882,KF560330,KM101118,KT004677,KT246486,KT438501,MG099934,MG099937,MG099938,MG099944,MG099949,MH020239,MH051250,MH051254,MH051256,MH338235,MH338241,MK305887,MK494091,MN096372,MN586016,MN586060,MN586062,MN617844,OL455889,ON970625,ON970626
2367	VC_941_0	40	134790.4737	60485.30736	0.690256994	0	0	8.366600265	780	780	1	6.24999193	0	0	0	KC710998,MG065636,MG065640,MG065641,MG065643,MG065649,MG065657,MG065663,MG065665,MG065667,MG065669,MG065670,MG065671,MG065673,MG065675,MG065679,MG065680,MG065681,MG065687,MK373798,MN055691,MN432485,MN850567,MN850569,MN850572,MN850591,MN850600,MN850602,MN850607,MN850640,MT074433,MT127619,MT360681,MZ501079,MZ501090,OK272479,OK272487,OL539459,ON185589,ON528718
2368	VC_942_0	36	149921.4328	41101.33966	0.784835394	0	0	7.810249676	630	630	1	4.77516521	0	0	0	LR027385,LR027388,LR596614,LT841304,LT961732,MF564201,MK962751,MN781674,MN850582,MN850586,MN850596,MN850604,MN850606,MN850613,MN850620,MN850634,MN850638,MN850641,MN850643,MZ234033,MZ501069,MZ501101,OK272471,OK272478,OK272481,OK272483,OK272485,OK272486,OK272489,OL539445,OL539446,ON185586,ON185587,ON185588,ON711395,OU734268
2369	VC_943_0	2	117.8948035	0	1	0	0	0	1	1	1	0	0	0	0	JF917095,JQ010983
2370	VC_944_0	6	2047.827568	450.8353524	0.819569359	3.24E-10	0	8.062257748	15	15	1	6.45356313	0	0	0	MG592398,MG592496,MG592497,MG592584,MG592612,MG592613
2371	VC_946_0	11	14643.69742	5272.956054	0.735248893	5.87E-32	0	5.291502622	55	55	1	3.2186615	0	0	0	JX483876,JX483877,JX483878,MN988482,MN988500,MN988501,MN988508,MN988530,MN988545,MN988549,MW980073
2372	VC_947_0	3	18.34590779	472.5482563	0.03737243	0.154083248	8.062257748	8.94427191	3	3	1	8.47755981	0	0	0	S2-wms-combined_135154,S9-wms-combined_53991,S9-wms-combined_89249
2373	VC_948_0	9	4605.209468	3855.415011	0.544310822	6.34E-23	2.828427125	8.246211251	36	36	1	6.81362184	0	0	0	MG592429,MG592438,MG592487,MG592566,MG592654,MG592657,MG592658,MG592659,MZ369152
2374	VC_949_0	10	1561.773596	1051.267046	0.597684388	3.67E-26	0	5.385164807	45	45	1	4.1036756	0	0	0	JN116822,KR053197,KR053198,KU998255,KU998256,MG812496,MH271296,MK660712,MK801723,MN234213
2375	VC_94_0	2	300	2000.22131	0.130422233	0.042768664	5	5	1	1	1	5	0	0	0	HM242243,OM135992
2376	VC_950_0	38	61189.70439	3610.240361	0.944286367	3.80E-160	0	3.31662479	703	703	1	0.70022435	0	0	0	KT355471,KT355473,KT355475,KT783672,KU160655,KU160657,KU160658,KU160667,KU160670,KU160674,KX443695,KX576642,KX610765,KY434670,MF140409,MF140415,MF140417,MF140419,MF140420,MF140426,MF140429,MF140431,MF140433,MH576948,MH576950,MH576951,MH576952,MH576955,MH576957,MH576961,MH779506,MK494103,MK494111,MK737943,MK820640,MN617842,MN813695,ON970580
2377	VC_951_0	3	170.828764	1457.377717	0.104918366	0.000781363	0	2.645751311	3	3	1	1.76383421	0	0	0	MH834594,MH834597,MH834628
2378	VC_952_0	2	217.1396412	10.08773978	0.955605087	0.113899997	3.31662479	3.31662479	1	1	1	3.31662479	0	0	0	JN116828,MW654182
2379	VC_953_0	2	37.37261764	139.236576	0.21161196	0.051791431	8.246211251	8.246211251	1	1	1	8.24621125	0	0	0	JN116823,MK967393
2380	VC_954_0	13	19765.2031	3137.044472	0.863024602	5.85E-34	0	3.16227766	78	78	1	2.10214822	0	0	0	KU963261,MH399781,MH509446,MK279893,MK305885,MK359319,MK376958,MK967392,MW291015,MW534376,MZ005677,ON637762,ON637763
2381	VC_955_0	3	900	262.0556666	0.774489576	0.008653781	3.31662479	3.741657387	3	3	1	3.45830232	0	0	0	MZ322021,ON970578,OP297535
2382	VC_956_0	39	161105.8903	0	1	0	0	4.898979486	741	741	1	2.34867167	0	0	0	JN116826,JN116827,KT372002,KT375356,KT959213,KT990218,KX550082,KX611788,KX712236,KX712237,KY549153,KY549154,MF189179,MF324898,MF324900,MF324901,MF324902,MF324903,MF324904,MF324905,MF373842,MF537628,MH271291,MH271293,MH271294,MH271297,MH271299,MH271306,MH271309,MH271311,MH271314,MH271315,MH271319,MH316569,MK524487,MK524495,MN945902,MW291027,MZ402608
2383	VC_957_0	1	0	48.27881381	0	0	0	0	1	1	1	0	0	0	0	JN175269
2384	VC_957_1	1	0	239.9820837	0	0	0	0	1	1	1	0	0	0	0	KU862660
2385	VC_958_0	3	472.9777625	2810.343293	0.14405468	0.001519856	4.582575695	5.477225575	3	3	1	5.0853179	0	0	0	MW570842,MW584170,MW584191
2386	VC_959_0	5	3000	57139.48282	0.049884034	7.69E-06	1.732050808	6	10	10	1	4.6732941	0	0	0	JX094500,KX397367,KX397372,MK514282,MW845759
2387	VC_959_1	8	8400	87314.22511	0.08776125	0.015649005	0	5.656854249	28	28	1	3.90289794	0	0	0	KX397368,KX397370,KX397371,MT710307,MW384823,MW812340,MW812342,MW812343
2388	VC_959_2	5	3000	58998.97861	0.048387894	0.106535744	1.414213562	7.549834435	10	10	1	6.37000768	0	0	0	KX397365,KX397366,MW760841,MW812338,MW812341
2389	VC_959_3	3	900	37125.02258	0.023668625	0.240884522	3.31662479	5.916079783	3	3	1	4.96318627	0	0	0	KX397369,MH426724,MW845758
2390	VC_959_4	3	900	37124.50086	0.02366895	0.228611835	3	5.830951895	3	3	1	4.73870557	0	0	0	JX316028,KX397364,KX397373
2391	VC_959_5	19	51300	144057.1605	0.262595954	9.71E-12	0	8.185352772	171	171	1	4.2852566	0	0	0	JN641803,KU726251,LR535902,LR535909,LR535910,LR535914,LR535915,LR535916,LR535918,LR535919,LR535920,LR535921,MH221129,MN445185,MT679221,MT799840,MW812339,OK428535,OM456492
2392	VC_959_6	1	0	12511.73976	0	0	0	0	1	1	1	0	0	0	0	MH221128
2393	VC_95_0	3	900	3402.167684	0.209196867	0.001474233	5	7.810249676	3	3	1	6.80867426	0	0	0	MK504443,MK504446,OM293948
2394	VC_960_0	1	0	10.05535834	0	0	0	0	1	1	1	0	0	0	0	JN712910
2395	VC_960_1	1	0	10.05535834	0	0	0	0	1	1	1	0	0	0	0	KX925554
2396	VC_961_0	3	106.4086542	51.23281036	0.675004222	0.009451772	2.236067977	3.31662479	3	3	1	2.90499014	0	0	0	KX344510,KY264020,MW344767
2397	VC_962_0	2	23.76754747	42.7767567	0.357168773	0.077364462	2	2	1	1	1	2	0	0	0	JN882267,OM621814
2398	VC_963_0	4	228.149705	40.10860974	0.85048512	0.001207324	1	3.741657387	6	6	1	2.46834964	0	0	0	KY965934,MG550110,MG550111,MG550113
2399	VC_965_0	2	217.0759579	684.4014729	0.240800214	0.043100688	2.236067977	2.236067977	1	1	1	2.23606798	0	0	0	MG596800,OM982619
2400	VC_966_0	3	554.985136	1008.96586	0.35486095	0.001596675	2.828427125	3.872983346	3	3	1	3.33934509	0	0	0	MK608336,OK499995,OL539452
2401	VC_967_0	16	10058.69829	1154.346242	0.897053272	2.90E-29	0	3.16227766	120	120	1	2.3244859	0	0	0	JQ309827,KT932700,LT630001,MK721183,MK721184,MK721185,MK721188,MK721193,MK721195,MK721196,MK721197,MK721198,MT747434,MT757399,MW623430,ON352054
2402	VC_968_0	4	485.128389	1154.346242	0.295904786	2.36E-05	0	1.414213562	6	6	1	0.70710678	0	0	0	KT945995,MK360025,OL799259,ON113181
2403	VC_969_0	3	817.6386765	165.7605334	0.831441258	0.002371493	4.69041576	6.244997998	3	3	1	5.44019286	0	0	0	JQ312117,MK637516,MT234341
2404	VC_96_0	30	27741.43008	10893.04648	0.718048555	1.98E-151	0	2.645751311	435	435	1	1.64734655	0	0	0	AB626962,AB626963,AF513032,AF513033,AY954949,EU136189,HF937074,KJ210330,KM366100,KU992911,KY000084,KY000085,LC709172,MG766218,MG766219,MH107118,MH972260,MK764384,MK903033,MK922546,MK922547,MK922548,MK936475,MK936476,MN098325,MN150710,MT926124,MW286254,MW363799,MZ359091
2405	VC_970_0	9	10690.56724	347.8252975	0.968489497	1.75E-17	0	8	36	36	1	5.5057368	0	0	0	MN988470,MN988484,MN988495,MN988531,MN988537,MN988544,MN988546,MN988557,MW980069
2406	VC_971_0	3	900	15520.97344	0.054807957	0.001610862	0	4.69041576	3	3	1	3.12694384	0	0	0	KP280063,KY883654,KY883655
2407	VC_971_1	24	82800	29191.73012	0.739340306	6.62E-124	1	9.486832981	276	246	0.891304348	6.60630262	0	0	0	JQ340389,LC487410,MG649966,MG649967,MK796244,MK895508,MK907780,MN954399,MN958086,MN966730,MN966731,MN966732,MT448616,MT448617,MT459144,MT460514,MT460515,MT460516,MT460517,MT460518,MW344054,MW344055,MW344056,MW398869
2408	VC_971_2	1	0	6336.528846	0	0	0	0	1	1	1	0	0	0	0	KT345706
2409	VC_972_0	6	4500	11062.7367	0.289152229	3.92E-11	1	5.291502622	15	15	1	3.4955718	0	0	0	MH645904,MH925094,MN871505,MN871510,MN871511,MT442039
2410	VC_973_0	8	8400	9394.801359	0.472047978	9.07E-19	0	5.196152423	28	28	1	2.02628859	0	0	0	MW824415,MW824416,MW824419,MW824422,MW824423,MW824424,MW824425,MW824426
2411	VC_974_0	2	187.0839253	0	1	0	2.449489743	2.449489743	1	1	1	2.44948974	0	0	0	JQ823122,JQ823123
2412	VC_975_0	11	11563.78002	373.4405153	0.968716293	4.21E-12	0	6.164414003	55	55	1	3.29509457	0	0	0	KP876465,KP876466,KT989433,KU189325,KU530220,KY365739,MF975638,MH178381,MH178382,MK686071,ON526979
2413	VC_976_0	2	300	9507.483407	0.030588887	0.072506347	4.123105626	4.123105626	1	1	1	4.12310563	0	0	0	ON529850,ON529857
2414	VC_976_1	5	3000	19588.48624	0.132811025	2.92E-07	4.123105626	9	10	9	0.9	7.13503501	0	0	0	ON529851,ON529852,ON529853,ON529855,ON529860
2415	VC_976_2	2	300	10478.7144	0.027832633	0.045892061	5.477225575	5.477225575	1	1	1	5.47722558	0	0	0	JX100810,MH588545
2416	VC_976_3	2	300	11321.42634	0.025814387	0.115971081	5.099019514	5.099019514	1	1	1	5.09901951	0	0	0	JX100814,MH588544
2417	VC_976_4	11	16500	32544.68409	0.336427898	1.46E-28	0	6.32455532	55	55	1	4.14097514	0	0	0	JX100809,JX100811,JX100812,JX100813,JX163858,KY555142,KY555143,KY555144,KY555145,KY555146,KY555147
2418	VC_976_5	1	0	5377.822146	0	0	0	0	1	1	1	0	0	0	0	ON529858
2419	VC_977_0	2	300	3423.122803	0.080577519	0.060796812	2.236067977	2.236067977	1	1	1	2.23606798	0	0	0	KT870145,MH015255
2420	VC_977_1	2	300	3496.813473	0.079013626	0.060228139	5.477225575	5.477225575	1	1	1	5.47722558	0	0	0	MN602268,MT270409
2421	VC_977_2	1	0	2042.548992	0	0	0	0	1	1	1	0	0	0	0	OP186293
2422	VC_978_0	2	150.5403256	2379.365428	0.059504322	0.045886287	8.774964387	8.774964387	1	1	1	8.77496439	0	0	0	KY629563,OL955261
2423	VC_978_1	1	0	1388.167082	0	0	0	0	1	1	1	0	0	0	0	OL963731
2424	VC_97_0	5	111.5900918	1491.210386	0.069621948	0.018861652	2.645751311	4.123105626	10	10	1	3.44567181	0	0	0	AY212251,KJ528544,LC628082,LC644557,ON637248
2425	VC_980_0	10	13500	5.297151533	0.999607772	1.00E-11	0	4.242640687	45	45	1	3.3501184	0	0	0	JX126919,JX126920,JX442241,KJ094020,KJ094021,MH299806,MN114082,MN114083,MN128593,MN904502
2426	VC_981_0	3	715.2495115	12748.06306	0.053125819	0.001463146	1.414213562	4.123105626	3	3	1	3.13676751	0	0	0	JX182369,ON526970,ON526974
2427	VC_981_1	26	61511.05997	26248.4928	0.700904438	2.42E-181	1	8.124038405	325	325	1	6.07667365	0	0	0	JX182370,JX262376,MF766044,MF766045,MF766046,MF766047,MF766048,MG593802,MH001460,MH590601,MH651190,MH669004,MH669016,MK392363,MK392364,MK392365,MK392366,MK460245,MK524524,MN234192,MN234208,MT310863,MT701594,MT701597,MT708548,OK412919
2428	VC_982_0	62	252374.1182	39523.50113	0.86459807	0	0	9.949874371	1891	1405	0.742993125	7.00090512	0	0	0	JX889246,KC700556,KC700557,KC700558,KT124227,KT124228,KT124229,KT152029,KT184390,KT184391,KT186228,KT186229,KX344445,KX507343,KX507344,KX507345,KX815338,MF541403,MF541404,MF541405,MF541406,MF541407,MF541408,MF541409,MF541410,MG298964,MG757163,MH171093,MH171094,MH171095,MH171096,MH171097,MH171098,MH248947,MK433270,MK433271,MK433276,MK433278,MN096373,MN096375,MN096379,MN096381,MN204498,MN284899,MN586009,MT701598,MT711978,MT711979,MW365951,MW365953,MW435856,MZ648032,MZ648039,MZ648040,MZ820088,MZ820091,MZ820092,OL455891,OL455894,OL742563,ON208832,ON526985
2429	VC_984_0	38	103257.469	2561.789282	0.975790897	1.54E-28	0	7.348469228	703	703	1	4.55307746	0	0	0	JX290549,KR604693,KT240186,KX278418,KX278419,KY953156,LC063634,LC375533,MH807811,MH807814,MH807816,MH807818,MH807819,MK095192,MK095193,MK095194,MK095195,MK095196,MK095197,MK095198,MK095199,MK095200,MK095201,MK095202,MK095203,MK095204,MK095205,MK095206,MK095207,MK095208,MK095209,MK095210,MK095211,MN270887,MN270888,MN271656,MN812690,MN812692
2430	VC_987_0	22	21989.08812	2564.710178	0.895547314	6.66E-57	0	8.366600265	231	231	1	6.0284453	0	0	0	JX483874,JX483875,JX483879,JX483880,MF403005,MF403006,MN988466,MN988488,MN988492,MN988493,MN988512,MN988513,MN988514,MN988528,MN988529,MT708544,MT708546,MW960034,MW980062,MW980065,MW980074,MZ462995
2431	VC_988_0	2	161.3318315	855.7179851	0.158627266	0.04731066	2.645751311	2.645751311	1	1	1	2.64575131	0	0	0	MF278336,MW043865
2432	VC_989_0	2	106.3185658	776.1615822	0.120477006	0.047487814	3.741657387	3.741657387	1	1	1	3.74165739	0	0	0	KY065149,MT227925
2433	VC_990_0	3	900	514.4810762	0.636275745	0.001804384	2	6.92820323	3	3	1	5.18715094	0	0	0	KF614509,MN988552,MT778840
2434	VC_991_0	2	90.17782025	149.8211652	0.375742506	0.085815228	4.358898944	4.358898944	1	1	1	4.35889894	0	0	0	KC292024,KC292028
2435	VC_992_0	8	302.3992671	478.3551084	0.387316775	6.08E-16	0	1.732050808	28	28	1	0.93542942	0	0	0	KP087954,MH572347,MH572348,MH572349,MH572405,MH572418,MH572455,MH572515
2436	VC_994_0	1	0	27.21090003	0	0	0	0	1	1	1	0	0	0	0	MK249135
2437	VC_995_0	69	17880.25327	3040.131553	0.854680897	1.19E-10	0	2.645751311	2346	2346	1	1.83381067	0	0	0	KC131022,KM589502,KM589511,KT264813,KT264814,KT264815,MG945202,MG945465,MG945477,MG945663,MG945838,MG945843,MH572283,MH572284,MH572285,MH572286,MH572291,MH572292,MH572293,MH572382,MH572383,MH572423,MH572424,MH572427,MH572428,MH572466,MH572495,MH572504,MH992156,MH992161,MH992184,MH992185,MH992213,MK249141,MK249147,MK249165,MK249185,MK249190,MK249197,MK249201,MK249202,MK249203,MK249211,MK249213,MK249221,MK496825,MN862333,MN862335,MN862341,MW202412,MW202414,MW202466,MW202471,MW202549,MW202587,MW202847,MW202853,MW202883,MZ364228,MZ364233,MZ364240,MZ364243,MZ364256,MZ364258,MZ364259,MZ364267,MZ364274,OL617029,OM523002
2438	VC_996_0	2	4.09143279	92.95456729	0.042159726	0.40961847	2	2	1	1	1	2	0	0	0	MN862339,MN862340
2439	VC_997_0	1	0	266.922191	0	0	0	0	1	1	1	0	0	0	0	MH992169
2440	VC_998_0	2	9.796278594	180.3650543	0.051515618	0.060981907	1.414213562	1.414213562	1	1	1	1.41421356	0	0	0	MH572299,MH572425
2441	VC_999_0	2	4.33446634	154.352742	0.027314529	0.260106748	2	2	1	1	1	2	0	0	0	MH572426,MK249189
2442	VC_99_0	32	45153.49508	5995.79033	0.88277861	5.38E-81	0	4.472135955	496	496	1	3.00063405	0	0	0	AB647160,AB731695,JF734911,JQ617284,KX119174,KX119175,KX119176,KX119177,KX119182,KX119188,KX119189,KX119190,KX119191,KX119192,KX119193,KX119194,KX119195,KX119196,KX119197,KX119198,KX119199,KX119200,KX119201,KX119202,KX119203,KX119204,KX119205,KX119206,MW247144,MW247145,MW247147,OM515228
2443	VC_9_0	38	72396.55326	75510.32145	0.48947389	0	0	8.831760866	703	703	1	7.56657936	0	0	0	AB370205,AF424781,AP008953,AY954951,AY954952,AY954953,CP062427,CP062451,CP062454,DQ517338,DQ530359,DQ530360,EU861004,GU477322,HQ127381,JQ779024,JX274646,JX274647,KC677663,KJ716334,MG557619,MH321492,MK658834,MK801682,MK801683,MT013111,MW554488,OM439662,OM439666,OM439668,OM439670,OM439671,OM439675,ON911714,ON911715,ON911716,ON911717,ON911718

Table S8-2. WMS_genome_by_genome_overview

Genome	Order	Family	Genus	VC	VC Status	Size	VC Subcluster	VC Subcluster Size	Quality	Adj P-value	Topology Confidence Score	Genera in VC	Families in VC	Orders in VC	Genus Confidence Score	Reference
AB002632	Unassigned	Unassigned	Unassigned	0_0	Clustered	3	VC_0_0	3	1	1	1	0	0	0	1	Ref
AB008550	Unassigned	Unassigned	Unassigned	2_0	Clustered	4	VC_2_0	4	0.1311	0.99998692	0.1311	0	0	0	1	Ref
AB009866	Unassigned	Unassigned	Unassigned	7_0	Clustered	28	VC_7_0	28	0.3916	1	0.3916	0	0	0	0.9947	Ref
AB012573	Unassigned	Unassigned	Unassigned	16_0	Clustered	12	VC_16_0	12	0.6615	1	0.6615	0	0	0	1	Ref
AB012574	Unassigned	Unassigned	Unassigned	16_0	Clustered	12	VC_16_0	12	0.6615	1	0.6615	0	0	0	1	Ref
AB016282	Unassigned	Unassigned	Unassigned	18_0	Clustered	4	VC_18_0	4	0.9098	0.99982347	0.9096	0	0	0	1	Ref
AB036666	Unassigned	Unassigned	Unassigned	22_0	Clustered	11	VC_22_0	11	0.5163	1	0.5163	0	0	0	1	Ref
AB043678	Unassigned	Unassigned	Unassigned	17_0	Clustered	9	VC_17_0	9	0.6969	1	0.6969	0	0	0	1	Ref
AB043679	Unassigned	Unassigned	Unassigned	17_0	Clustered	9	VC_17_0	9	0.6969	1	0.6969	0	0	0	1	Ref
AB044554	Unassigned	Unassigned	Unassigned	7_0	Clustered	28	VC_7_0	28	0.3916	1	0.3916	0	0	0	0.9947	Ref
AB045978	Unassigned	Unassigned	Unassigned	8_0	Clustered	58	VC_8_0	58	0.8306	1	0.8306	0	0	0	0.9946	Ref
AB063393	Unassigned	Unassigned	Unassigned	27_0	Clustered	2	VC_27_0	2	1	1	1	0	0	0	1	Ref
AB102868	Unassigned	Unassigned	Unassigned	28_1	Clustered	53	VC_28_1	53	0.7164	1	0.7164	0	0	0	0.9325	Ref
AB231700	Unassigned	Unassigned	Unassigned	38_0	Clustered	3	VC_38_0	3	1	0	0	0	0	0	1	Ref
AB243556	Unassigned	Unassigned	Unassigned	7_0	Clustered	28	VC_7_0	28	0.3916	1	0.3916	0	0	0	0.9947	Ref
AB259123	Unassigned	Unassigned	Unassigned	52_0	Clustered	87	VC_52_0	87	0.9858	1	0.9858	0	0	0	1	Ref
AB259124	Unassigned	Unassigned	Unassigned	54_0	Clustered	6	VC_54_0	6	0.6725	0.9999999	0.6725	0	0	0	1	Ref
AB276040	Unassigned	Unassigned	Unassigned	1_0	Clustered	54	VC_1_0	54	0.8527	1	0.8527	0	0	0	1	Ref
AB285204	Unassigned	Unassigned	Unassigned	51_0	Clustered	10	VC_51_0	10	0.1741	1	0.1741	0	0	0	1	Ref
AB334720	Unassigned	Unassigned	Unassigned	61_0	Clustered	12	VC_61_0	12	0.7633	1	0.7633	0	0	0	1	Ref
AB334721	Unassigned	Unassigned	Unassigned	61_0	Clustered	12	VC_61_0	12	0.7633	1	0.7633	0	0	0	1	Ref
AB362338	Unassigned	Unassigned	Unassigned	28_1	Clustered	53	VC_28_1	53	0.7164	1	0.7164	0	0	0	0.9325	Ref
AB366653	Unassigned	Unassigned	Unassigned	66_3	Clustered/Singleton	1	VC_66_3	1	0	1	0	0	0	0	1	Ref
AB370205	Unassigned	Unassigned	Unassigned	9_0	Clustered	38	VC_9_0	38	0.4895	1	0.4895	0	0	0	1	Ref
AB370268	Unassigned	Unassigned	Unassigned	12_2	Clustered	19	VC_12_2	19	0.2018	1	0.2018	0	0	0	1	Ref
AB374228	Unassigned	Unassigned	Unassigned	5_0	Clustered	30	VC_5_0	30	0.9647	1	0.9647	0	0	0	1	Ref
AB426868	Unassigned	Unassigned	Unassigned	28_1	Clustered	53	VC_28_1	53	0.7164	1	0.7164	0	0	0	0.9325	Ref
AB434711	Unassigned	Unassigned	Unassigned	52_0	Clustered	87	VC_52_0	87	0.9858	1	0.9858	0	0	0	1	Ref
AB472900	Unassigned	Unassigned	Unassigned	77_0	Clustered	20	VC_77_0	20	0.9657	1	0.9657	0	0	0	1	Ref
AB560486	Unassigned	Unassigned	Unassigned	79_0	Clustered	64	VC_79_0	64	0.4185	1	0.4185	0	0	0	0.7649	Ref
AB572858	Unassigned	Unassigned	Unassigned	16_0	Clustered	12	VC_16_0	12	0.6615	1	0.6615	0	0	0	1	Ref
AB597179	Unassigned	Unassigned	Unassigned	83_0	Clustered	6	VC_83_0	6	0.6436	1	0.6436	0	0	0	1	Ref
AB609718	Unassigned	Unassigned	Unassigned	91_0	Clustered	22	VC_91_0	22	0.5318	1	0.5318	0	0	0	1	Ref
AB626962	Unassigned	Unassigned	Unassigned	96_0	Clustered	30	VC_96_0	30	0.718	1	0.718	0	0	0	1	Ref
AB626963	Unassigned	Unassigned	Unassigned	96_0	Clustered	30	VC_96_0	30	0.718	1	0.718	0	0	0	1	Ref
AB647160	Unassigned	Unassigned	Unassigned	99_0	Clustered	32	VC_99_0	32	0.8828	1	0.8828	0	0	0	1	Ref
AB712291	Unassigned	Unassigned	Unassigned	108_0	Clustered	33	VC_108_0	33	0.949	1	0.949	0	0	0	1	Ref
AB716666	Unassigned	Unassigned	Unassigned	111_0	Clustered	91	VC_111_0	91	0.9587	1	0.9587	0	0	0	1	Ref
AB720063	Unassigned	Unassigned	Unassigned	113_0	Clustered	9	VC_113_0	9	0.519	1	0.519	0	0	0	1	Ref
AB720064	Unassigned	Unassigned	Unassigned	116_0	Clustered	13	VC_116_0	13	1	1	1	0	0	0	1	Ref
AB731695	Unassigned	Unassigned	Unassigned	99_0	Clustered	32	VC_99_0	32	0.8828	1	0.8828	0	0	0	1	Ref
AB746912	Unassigned	Unassigned	Unassigned	118_0	Clustered	3	VC_118_0	3	0.7119	0.99211494	0.7063	0	0	0	1	Ref
AB757800	Unassigned	Unassigned	Unassigned	117_0	Clustered	9	VC_117_0	9	0.9977	0.98988637	0.9876	0	0	0	1	Ref
AB757801	Unassigned	Unassigned	Unassigned	117_0	Clustered	9	VC_117_0	9	0.9977	0.98988637	0.9876	0	0	0	1	Ref
AB775548	Unassigned	Unassigned	Unassigned	125_0	Clustered	17	VC_125_0	17	0.9244	1	0.9244	0	0	0	1	Ref
AB775549	Unassigned	Unassigned	Unassigned	130_0	Clustered	19	VC_130_0	19	0.6068	1	0.6068	0	0	0	1	Ref
AB797215	Unassigned	Unassigned	Unassigned	132_1	Clustered	17	VC_132_1	17	0.1738	1	0.1738	0	0	0	0.9853	Ref
AB828698	Unassigned	Unassigned	Unassigned	54_0	Clustered	6	VC_54_0	6	0.6725	0.9999999	0.6725	0	0	0	1	Ref
AB853330	Unassigned	Unassigned	Unassigned	149_13	Clustered	3	VC_149_13	3	0.0087	0.95178903	0.0083	0	0	0	1	Ref
AB853331	Unassigned	Unassigned	Unassigned	149_23	Clustered/Singleton	1	VC_149_23	1	0	1	0	0	0	0	1	Ref
AB854109	Unassigned	Unassigned	Unassigned	154_0	Clustered	4	VC_154_0	4	0.1766	0.99995106	0.1766	0	0	0	1	Ref
AB863625	Unassigned	Unassigned	Unassigned	161_0	Clustered	3	VC_161_0	3	0.5777	0.99804396	0.5765	0	0	0	1	Ref
AB897757	Unassigned	Unassigned	Unassigned	164_10	Clustered	5	VC_164_10	5	0.0615	0.96483582	0.0593	0	0	0	1	Ref
AB903967	Unassigned	Unassigned	Unassigned	149_14	Clustered/Singleton	1	VC_149_14	1	0	1	0	0	0	0	1	Ref
AB910392	Unassigned	Unassigned	Unassigned	167_0	Clustered	3	VC_167_0	3	0.6952	0.99826575	0.694	0	0	0	1	Ref
AB910393	Unassigned	Unassigned	Unassigned	174_0	Clustered	3	VC_174_0	3	0.9624	0.99333557	0.9559	0	0	0	1	Ref
AB910602	Unassigned	Unassigned	Unassigned	177_0	Clustered	5	VC_177_0	5	0.7468	0.99885646	0.746	0	0	0	1	Ref
AB916497	Unassigned	Unassigned	Unassigned	180_0	Clustered	4	VC_180_0	4	0.2056	0.9999862	0.2056	0	0	0	1	Ref
AB920995	Unassigned	Unassigned	Unassigned	70_0	Clustered	27	VC_70_0	27	0.5981	1	0.5981	0	0	0	0.9886	Ref
AB930182	Unassigned	Unassigned	Unassigned	85_3	Clustered	19	VC_85_3	19	0.3671	1	0.3671	0	0	0	0.807	Ref
AB931172	Unassigned	Unassigned	Unassigned	54_0	Clustered	6	VC_54_0	6	0.6725	0.9999999	0.6725	0	0	0	1	Ref
AB937974	Unassigned	Unassigned	Unassigned	52_0	Clustered	87	VC_52_0	87	0.9858	1	0.9858	0	0	0	1	Ref
AB971354	Unassigned	Unassigned	Unassigned	188_0	Clustered	13	VC_188_0	13	0.9973	0.99823802	0.9956	0	0	0	1	Ref
AB981169	Unassigned	Unassigned	Unassigned	1_0	Clustered	54	VC_1_0	54	0.8527	1	0.8527	0	0	0	1	Ref
AB981170	Unassigned	Unassigned	Unassigned	52_0	Clustered	87	VC_52_0	87	0.9858	1	0.9858	0	0	0	1	Ref
AB983711	Unassigned	Unassigned	Unassigned	70_0	Clustered	27	VC_70_0	27	0.5981	1	0.5981	0	0	0	0.9886	Ref
AC171169	Unassigned	Unassigned	Unassigned	190_11	Clustered/Singleton	1	VC_190_11	1	0	1	0	0	0	0	1	Ref
AE002163	Unassigned	Unassigned	Unassigned	197_0	Clustered	5	VC_197_0	5	0.1324	0.84698401	0.1122	0	0	0	1	Ref
AF009630	Unassigned	Unassigned	Unassigned	202_0	Clustered	102	VC_202_0	102	0.4049	1	0.4049	0	0	0	0.7876	Ref
AF011378	Unassigned	Unassigned	Unassigned	202_0	Clustered	102	VC_202_0	102	0.4049	1	0.4049	0	0	0	0.7876	Ref
AF020713	Unassigned	Unassigned	Unassigned	89_3	Clustered/Singleton	1	VC_89_3	1	0	1	0	0	0	0	1	Ref
AF022214	Unassigned	Unassigned	Unassigned	215_1	Clustered	19	VC_215_1	19	0.4188	1	0.4188	0	0	0	1	Ref
AF059242	Unassigned	Unassigned	Unassigned	188_0	Clustered	13	VC_188_0	13	0.9973	0.99823802	0.9956	0	0	0	1	Ref
AF059243	Unassigned	Unassigned	Unassigned	188_0	Clustered	13	VC_188_0	13	0.9973	0.99823802	0.9956	0	0	0	1	Ref
AF063097	Unassigned	Unassigned	Unassigned	228_0	Clustered	60	VC_228_0	60	0.942	1	0.942	0	0	0	1	Ref
AF065411	Unassigned	Unassigned	Unassigned	232_0	Clustered	2	VC_232_0	2	0.9272	0.72985431	0.6768	0	0	0	1	Ref
AF083977	Unassigned	Unassigned	Unassigned	50_0	Clustered	9	VC_50_0	9	0.5531	1	0.5531	0	0	0	1	Ref
AF085222	Unassigned	Unassigned	Unassigned	252_0	Clustered	121	VC_252_0	121	0.7376	1	0.7376	0	0	0	0.9999	Ref

AF115102	Unassigned	Unassigned	Unassigned	252_0	Clustered	121	VC_252_0	121	0.7376	1	0.7376	0	0	0	0.9999	Ref
AF115103	Unassigned	Unassigned	Unassigned	252_0	Clustered	121	VC_252_0	121	0.7376	1	0.7376	0	0	0	0.9999	Ref
AF125163	Unassigned	Unassigned	Unassigned	5_0	Clustered	30	VC_5_0	30	0.9647	1	0.9647	0	0	0	1	Ref
AF125520	Unassigned	Unassigned	Unassigned	39_0	Clustered	35	VC_39_0	35	0.4337	1	0.4337	0	0	0	1	Ref
AF133242	Unassigned	Unassigned	Unassigned	276_0	Clustered	2	VC_276_0	2	1	1	1	0	0	0	1	Ref
AF145054	Unassigned	Unassigned	Unassigned	252_0	Clustered	121	VC_252_0	121	0.7376	1	0.7376	0	0	0	0.9999	Ref
AF155037	Unassigned	Unassigned	Unassigned	277_0	Clustered	3	VC_277_0	3	1	1	1	0	0	0	1	Ref
AF157835	Unassigned	Unassigned	Unassigned	29_0	Clustered	10	VC_29_0	10	0.5208	1	0.5208	0	0	0	1	Ref
AF158101	Unassigned	Unassigned	Unassigned	283_91	Clustered	11	VC_283_91	11	0.0285	1	0.0285	0	0	0	1	Ref
AF158600	Unassigned	Unassigned	Unassigned	254_0	Clustered	46	VC_254_0	46	0.5159	1	0.5159	0	0	0	1	Ref
AF165214	Unassigned	Unassigned	Unassigned	176_0	Clustered	2	VC_176_0	2	0.0498	0.95663741	0.0476	0	0	0	1	Ref
AF176025	Unassigned	Unassigned	Unassigned	260_0	Clustered	13	VC_260_0	13	0.3705	1	0.3705	0	0	0	1	Ref
AF176027	Unassigned	Unassigned	Unassigned	303_0	Clustered	155	VC_303_0	155	0.9979	1	0.9979	0	0	0	1	Ref
AF176028	Unassigned	Unassigned	Unassigned	303_0	Clustered	155	VC_303_0	155	0.9979	1	0.9979	0	0	0	1	Ref
AF176029	Unassigned	Unassigned	Unassigned	303_0	Clustered	155	VC_303_0	155	0.9979	1	0.9979	0	0	0	1	Ref
AF176030	Unassigned	Unassigned	Unassigned	303_0	Clustered	155	VC_303_0	155	0.9979	1	0.9979	0	0	0	1	Ref
AF176031	Unassigned	Unassigned	Unassigned	303_0	Clustered	155	VC_303_0	155	0.9979	1	0.9979	0	0	0	1	Ref
AF176032	Unassigned	Unassigned	Unassigned	303_0	Clustered	155	VC_303_0	155	0.9979	1	0.9979	0	0	0	1	Ref
AF176033	Unassigned	Unassigned	Unassigned	303_0	Clustered	155	VC_303_0	155	0.9979	1	0.9979	0	0	0	1	Ref
AF176034	Unassigned	Unassigned	Unassigned	303_0	Clustered	155	VC_303_0	155	0.9979	1	0.9979	0	0	0	1	Ref
AF189021	Unassigned	Unassigned	Unassigned	304_0	Clustered	11	VC_304_0	11	0.7869	1	0.7869	0	0	0	1	Ref
AF191797	Unassigned	Unassigned	Unassigned	306_0	Clustered	2	VC_306_0	2	1	1	1	0	0	0	1	Ref
AF195902	Unassigned	Unassigned	Unassigned	308_0	Clustered	4	VC_308_0	4	0.5014	0.99997418	0.5013	0	0	0	1	Ref
AF217253	Unassigned	Unassigned	Unassigned	28_1	Clustered	53	VC_28_1	53	0.7164	1	0.7164	0	0	0	0.9325	Ref
AF222060	Unassigned	Unassigned	Unassigned	310_0	Clustered	33	VC_310_0	33	0.8771	1	0.8771	0	0	0	1	Ref
AF227250	Unassigned	Unassigned	Unassigned	315_0	Clustered	12	VC_315_0	12	0.8947	1	0.8947	0	0	0	1	Ref
AF232233	Unassigned	Unassigned	Unassigned	251_0	Clustered	22	VC_251_0	22	0.6508	1	0.6508	0	0	0	1	Ref
AF234172	Unassigned	Unassigned	Unassigned	247_0	Clustered	14	VC_247_0	14	0.9119	1	0.9119	0	0	0	0.967	Ref
AF234173	Unassigned	Unassigned	Unassigned	247_0	Clustered	14	VC_247_0	14	0.9119	1	0.9119	0	0	0	0.967	Ref
AF271693	Unassigned	Unassigned	Unassigned	325_39	Clustered	2	VC_325_39	2	0.0045	0.95750889	0.0043	0	0	0	1	Ref
AF274751	Unassigned	Unassigned	Unassigned	303_0	Clustered	155	VC_303_0	155	0.9979	1	0.9979	0	0	0	1	Ref
AF299300	Unassigned	Unassigned	Unassigned	303_0	Clustered	155	VC_303_0	155	0.9979	1	0.9979	0	0	0	1	Ref
AF299301	Unassigned	Unassigned	Unassigned	303_0	Clustered	155	VC_303_0	155	0.9979	1	0.9979	0	0	0	1	Ref
AF299302	Unassigned	Unassigned	Unassigned	303_0	Clustered	155	VC_303_0	155	0.9979	1	0.9979	0	0	0	1	Ref
AF299303	Unassigned	Unassigned	Unassigned	303_0	Clustered	155	VC_303_0	155	0.9979	1	0.9979	0	0	0	1	Ref
AF299304	Unassigned	Unassigned	Unassigned	303_0	Clustered	155	VC_303_0	155	0.9979	1	0.9979	0	0	0	1	Ref
AF299305	Unassigned	Unassigned	Unassigned	303_0	Clustered	155	VC_303_0	155	0.9979	1	0.9979	0	0	0	1	Ref
AF299306	Unassigned	Unassigned	Unassigned	303_0	Clustered	155	VC_303_0	155	0.9979	1	0.9979	0	0	0	1	Ref
AF299307	Unassigned	Unassigned	Unassigned	303_0	Clustered	155	VC_303_0	155	0.9979	1	0.9979	0	0	0	1	Ref
AF299308	Unassigned	Unassigned	Unassigned	303_0	Clustered	155	VC_303_0	155	0.9979	1	0.9979	0	0	0	1	Ref
AF299309	Unassigned	Unassigned	Unassigned	303_0	Clustered	155	VC_303_0	155	0.9979	1	0.9979	0	0	0	1	Ref
AF299310	Unassigned	Unassigned	Unassigned	303_0	Clustered	155	VC_303_0	155	0.9979	1	0.9979	0	0	0	1	Ref
AF299311	Unassigned	Unassigned	Unassigned	303_0	Clustered	155	VC_303_0	155	0.9979	1	0.9979	0	0	0	1	Ref
AF299312	Unassigned	Unassigned	Unassigned	303_0	Clustered	155	VC_303_0	155	0.9979	1	0.9979	0	0	0	1	Ref
AF299313	Unassigned	Unassigned	Unassigned	303_0	Clustered	155	VC_303_0	155	0.9979	1	0.9979	0	0	0	1	Ref
AF299314	Unassigned	Unassigned	Unassigned	303_0	Clustered	155	VC_303_0	155	0.9979	1	0.9979	0	0	0	1	Ref
AF301375	Unassigned	Unassigned	Unassigned	232_0	Clustered	2	VC_232_0	2	0.9272	0.72985431	0.6768	0	0	0	1	Ref
AF306496	Unassigned	Unassigned	Unassigned	327_0	Clustered	12	VC_327_0	12	0.8925	1	0.8925	0	0	0	1	Ref
AF320576	Unassigned	Unassigned	Unassigned	329_1	Clustered	17	VC_329_1	17	0.0867	1	0.0867	0	0	0	0.8235	Ref
AF323668	Unassigned	Unassigned	Unassigned	263_0	Clustered	7	VC_263_0	7	0.2364	1	0.2364	0	0	0	1	Ref
AF323669	Unassigned	Unassigned	Unassigned	260_0	Clustered	13	VC_260_0	13	0.3705	1	0.3705	0	0	0	1	Ref
AF323671	Unassigned	Unassigned	Unassigned	339_0	Clustered	9	VC_339_0	9	0.9405	0.99999771	0.9405	0	0	0	1	Ref
AF334111	Unassigned	Unassigned	Unassigned	227_0	Clustered	2	VC_227_0	2	0.3333	0	0	0	0	0	1	Ref
AF335538	Unassigned	Unassigned	Unassigned	30_1	Clustered	16	VC_30_1	16	0.1973	1	0.1973	0	0	0	0.8333	Ref
AF338467	Unassigned	Unassigned	Unassigned	340_0	Clustered	31	VC_340_0	31	0.9549	1	0.9549	0	0	0	1	Ref
AF396866	Unassigned	Unassigned	Unassigned	341_0	Clustered	3	VC_341_0	3	0.2522	0.9396546	0.237	0	0	0	1	Ref
AF399011	Unassigned	Unassigned	Unassigned	344_0	Clustered	21	VC_344_0	21	0.5436	1	0.5436	0	0	0	1	Ref
AF424781	Unassigned	Unassigned	Unassigned	9_0	Clustered	38	VC_9_0	38	0.4895	1	0.4895	0	0	0	1	Ref
AF424782	Unassigned	Unassigned	Unassigned	8_0	Clustered	58	VC_8_0	58	0.8306	1	0.8306	0	0	0	0.9946	Ref
AF424783	Unassigned	Unassigned	Unassigned	7_0	Clustered	28	VC_7_0	28	0.3916	1	0.3916	0	0	0	0.9947	Ref
AF440571	Unassigned	Unassigned	Unassigned	349_0	Clustered	7	VC_349_0	7	0.9363	0.99999996	0.9363	0	0	0	1	Ref
AF440695	Unassigned	Unassigned	Unassigned	352_0	Clustered	3	VC_352_0	3	0.7306	0.98867275	0.7223	0	0	0	1	Ref
AF447491	Unassigned	Unassigned	Unassigned	353_0	Clustered	6	VC_353_0	6	0.545	1	0.545	0	0	0	1	Ref
AF452449	Unassigned	Unassigned	Unassigned	16_0	Clustered	12	VC_16_0	12	0.6615	1	0.6615	0	0	0	1	Ref
AF453500	Unassigned	Unassigned	Unassigned	16_0	Clustered	12	VC_16_0	12	0.6615	1	0.6615	0	0	0	1	Ref
AF454431	Unassigned	Unassigned	Unassigned	360_0	Clustered	358	VC_360_0	358	0.9615	1	0.9615	0	0	0	1	Ref
AF489521	Unassigned	Unassigned	Unassigned	260_0	Clustered	13	VC_260_0	13	0.3705	1	0.3705	0	0	0	1	Ref
AF493143	Unassigned	Unassigned	Unassigned	129_0	Clustered	43	VC_129_0	43	0.886	1	0.886	0	0	0	1	Ref
AF503408	Unassigned	Unassigned	Unassigned	247_0	Clustered	14	VC_247_0	14	0.9119	1	0.9119	0	0	0	0.967	Ref
AF513032	Unassigned	Unassigned	Unassigned	96_0	Clustered	30	VC_96_0	30	0.718	1	0.718	0	0	0	1	Ref
AF513033	Unassigned	Unassigned	Unassigned	96_0	Clustered	30	VC_96_0	30	0.718	1	0.718	0	0	0	1	Ref
AF527608	Unassigned	Unassigned	Unassigned	28_1	Clustered	53	VC_28_1	53	0.7164	1	0.7164	0	0	0	0.9325	Ref
AF547430	Unassigned	Unassigned	Unassigned	371_16	Clustered	42	VC_371_16	42	0.1341	1	0.1341	0	0	0	0.7282	Ref
AF547987	Unassigned	Unassigned	Unassigned	30_1	Clustered	16	VC_30_1	16	0.1973	1	0.1973	0	0	0	0.8333	Ref
AJ006589	Unassigned	Unassigned	Unassigned	373_0	Clustered	10	VC_373_0	10	0.7471	1	0.7471	0	0	0	1	Ref
AJ131519	Unassigned	Unassigned	Unassigned	307_0	Clustered	2	VC_307_0	2	0.3835	0.95251406	0.3653	0	0	0	1	Ref
AJ242593	Unassigned	Unassigned	Unassigned	378_0	Clustered	9	VC_378_0	9	0.7603	1	0.7603	0	0	0	1	Ref
AJ245616	Unassigned	Unassigned	Unassigned	260_0	Clustered	13	VC_260_0	13	0.3705	1	0.3705	0	0	0	1	Ref
AJ251789	Unassigned	Unassigned	Unassigned	272_0	Clustered	11	VC_272_0	11	0.4915	1	0.4915	0	0	0	0.8727	Ref
AJ251805	Unassigned	Unassigned	Unassigned	385_0	Clustered	51	VC_385_0	51	0.8754	1	0.8754	0	0	0	1	Ref
AJ270057	Unassigned	Unassigned	Unassigned	198_0	Clustered	28	VC_198_0	28	0.2429	0.99971175	0.2429	0	0	0	1	Ref

AJ278322	Unassigned	Unassigned	Unassigned	407_0	Clustered	3	VC_407_0	3	0.3352	0.99824791	0.3346	0	0	0	1	Ref
AJ298298	Unassigned	Unassigned	Unassigned	45_0	Clustered	12	VC_45_0	12	0.35	1	0.35	0	0	0	1	Ref
AJ302074	Unassigned	Unassigned	Unassigned	412_0	Clustered	39	VC_412_0	39	0.845	1	0.845	0	0	0	1	Ref
AJ312240	Unassigned	Unassigned	Unassigned	379_0	Clustered	5	VC_379_0	5	0.4779	0.99999992	0.4779	0	0	0	0.9	Ref
AJ318471	Unassigned	Unassigned	Unassigned	385_0	Clustered	51	VC_385_0	51	0.8754	1	0.8754	0	0	0	1	Ref
AJ344259	Unassigned	Unassigned	Unassigned	348_0	Clustered	22	VC_348_0	22	0.9602	1	0.9602	0	0	0	1	Ref
AJ414696	Unassigned	Unassigned	Unassigned	348_0	Clustered	22	VC_348_0	22	0.9602	1	0.9602	0	0	0	1	Ref
AJ421943	Unassigned	Unassigned	Unassigned	274_0	Clustered	2	VC_274_0	2	0.0799	0.953709	0.0762	0	0	0	1	Ref
AJ505558	Unassigned	Unassigned	Unassigned	423_0	Clustered	45	VC_423_0	45	0.9484	1	0.9484	0	0	0	1	Ref
AJ536073	Unassigned	Unassigned	Unassigned	424_0	Clustered	7	VC_424_0	7	0.9044	0.9998143	0.9043	0	0	0	1	Ref
AJ550635	Unassigned	Unassigned	Unassigned	198_0	Clustered	28	VC_198_0	28	0.2429	0.99971175	0.2429	0	0	0	1	Ref
AJ550940	Unassigned	Unassigned	Unassigned	373_0	Clustered	10	VC_373_0	10	0.7471	1	0.7471	0	0	0	1	Ref
AJ556162	Unassigned	Unassigned	Unassigned	41_0	Clustered	16	VC_41_0	16	0.2025	1	0.2025	0	0	0	0.9667	Ref
AJ560763	Unassigned	Unassigned	Unassigned	426_0	Clustered	2	VC_426_0	2	0.1976	0.95643941	0.189	0	0	0	1	Ref
AJ604530	Unassigned	Unassigned	Unassigned	189_0	Clustered	2	VC_189_0	2	0.0104	0.93953444	0.0097	0	0	0	1	Ref
AJ604531	Unassigned	Unassigned	Unassigned	189_0	Clustered	2	VC_189_0	2	0.0104	0.93953444	0.0097	0	0	0	1	Ref
AJ609634	Unassigned	Unassigned	Unassigned	137_0	Clustered	3	VC_137_0	3	0.1129	0.998547	0.1128	0	0	0	1	Ref
AJ630128	Unassigned	Unassigned	Unassigned	433_5	Clustered	2	VC_433_5	2	0.0173	0.93642158	0.0162	0	0	0	1	Ref
AJ635161	Unassigned	Unassigned	Unassigned	440_0	Clustered	4	VC_440_0	4	1	1	1	0	0	0	1	Ref
AJ748296	Unassigned	Unassigned	Unassigned	348_0	Clustered	22	VC_348_0	22	0.9602	1	0.9602	0	0	0	1	Ref
AJ783769	Unassigned	Unassigned	Unassigned	444_0	Clustered	2	VC_444_0	2	0.8137	0.72985431	0.5939	0	0	0	1	Ref
AJ854042	Unassigned	Unassigned	Unassigned	350_0	Clustered	2	VC_350_0	2	0.324	0.93865445	0.3041	0	0	0	1	Ref
AJ875026	Unassigned	Unassigned	Unassigned	348_0	Clustered	22	VC_348_0	22	0.9602	1	0.9602	0	0	0	1	Ref
AJ888457	Unassigned	Unassigned	Unassigned	447_0	Clustered	3	VC_447_0	3	0.8268	0.99509929	0.8227	0	0	0	1	Ref
AJ969242	Unassigned	Unassigned	Unassigned	276_0	Clustered	2	VC_276_0	2	1	1	1	0	0	0	1	Ref
AM076770	Unassigned	Unassigned	Unassigned	450_0	Clustered	16	VC_450_0	16	0.9564	1	0.9564	0	0	0	1	Ref
AM084414	Unassigned	Unassigned	Unassigned	451_0	Clustered	80	VC_451_0	80	0.9991	1	0.9991	0	0	0	1	Ref
AM084415	Unassigned	Unassigned	Unassigned	452_0	Clustered	66	VC_452_0	66	0.9248	1	0.9248	0	0	0	1	Ref
AM087120	Unassigned	Unassigned	Unassigned	349_0	Clustered	7	VC_349_0	7	0.9363	0.99999996	0.9363	0	0	0	1	Ref
AM087121	Unassigned	Unassigned	Unassigned	349_0	Clustered	7	VC_349_0	7	0.9363	0.99999996	0.9363	0	0	0	1	Ref
AM087122	Unassigned	Unassigned	Unassigned	349_0	Clustered	7	VC_349_0	7	0.9363	0.99999996	0.9363	0	0	0	1	Ref
AM087123	Unassigned	Unassigned	Unassigned	349_0	Clustered	7	VC_349_0	7	0.9363	0.99999996	0.9363	0	0	0	1	Ref
AM156909	Unassigned	Unassigned	Unassigned	456_0	Clustered	62	VC_456_0	62	0.9026	1	0.9026	0	0	0	1	Ref
AM183667	Unassigned	Unassigned	Unassigned	386_0	Clustered	49	VC_386_0	49	0.7941	1	0.7941	0	0	0	1	Ref
AM265638	Unassigned	Unassigned	Unassigned	423_0	Clustered	45	VC_423_0	45	0.9484	1	0.9484	0	0	0	1	Ref
AM419438	Unassigned	Unassigned	Unassigned	351_0	Clustered	3	VC_351_0	3	0.8899	0.98155579	0.8735	0	0	0	1	Ref
AM491472	Unassigned	Unassigned	Unassigned	461_0	Clustered	96	VC_461_0	96	0.9307	1	0.9307	0	0	0	0.9575	Ref
AM749121	Unassigned	Unassigned	Unassigned	463_0	Clustered	4	VC_463_0	4	0.7906	0.99997156	0.7906	0	0	0	1	Ref
AM749441	Unassigned	Unassigned	Unassigned	166_0	Clustered	26	VC_166_0	26	0.7923	1	0.7923	0	0	0	1	Ref
AM910650	Unassigned	Unassigned	Unassigned	472_0	Clustered	47	VC_472_0	47	0.9025	1	0.9025	0	0	0	1	Ref
AM910651	Unassigned	Unassigned	Unassigned	423_0	Clustered	45	VC_423_0	45	0.9484	1	0.9484	0	0	0	1	Ref
AP000363	Unassigned	Unassigned	Unassigned	39_0	Clustered	35	VC_39_0	35	0.4337	1	0.4337	0	0	0	1	Ref
AP000422	Unassigned	Unassigned	Unassigned	39_0	Clustered	35	VC_39_0	35	0.4337	1	0.4337	0	0	0	1	Ref
AP001553	Unassigned	Unassigned	Unassigned	12_2	Clustered	19	VC_12_2	19	0.2018	1	0.2018	0	0	0	1	Ref
AP004402	Unassigned	Unassigned	Unassigned	39_0	Clustered	35	VC_39_0	35	0.4337	1	0.4337	0	0	0	1	Ref
AP005153	Unassigned	Unassigned	Unassigned	39_0	Clustered	35	VC_39_0	35	0.4337	1	0.4337	0	0	0	1	Ref
AP005154	Unassigned	Unassigned	Unassigned	39_0	Clustered	35	VC_39_0	35	0.4337	1	0.4337	0	0	0	1	Ref
AP008953	Unassigned	Unassigned	Unassigned	9_0	Clustered	38	VC_9_0	38	0.4895	1	0.4895	0	0	0	1	Ref
AP008954	Unassigned	Unassigned	Unassigned	12_0	Clustered	21	VC_12_0	21	0.2274	1	0.2274	0	0	0	0.9571	Ref
AP008979	Unassigned	Unassigned	Unassigned	113_0	Clustered	9	VC_113_0	9	0.519	1	0.519	0	0	0	1	Ref
AP008983	Unassigned	Unassigned	Unassigned	211_0	Clustered/Singleton	1	VC_211_0	1	0	1	0	0	0	0	1	Ref
AP008986	Unassigned	Unassigned	Unassigned	364_0	Clustered	16	VC_364_0	16	0.6491	1	0.6491	0	0	0	1	Ref
AP009146	Unassigned	Unassigned	Unassigned	63_0	Clustered	2	VC_63_0	2	0.1147	0.95689598	0.1098	0	0	0	1	Ref
AP009363	Unassigned	Unassigned	Unassigned	8_0	Clustered	58	VC_8_0	58	0.8306	1	0.8306	0	0	0	0.9946	Ref
AP009390	Unassigned	Unassigned	Unassigned	91_0	Clustered	22	VC_91_0	22	0.5318	1	0.5318	0	0	0	1	Ref
AP011113	Unassigned	Unassigned	Unassigned	283_10	Clustered/Singleton	1	VC_283_10	1	0	1	0	0	0	0	1	Ref
AP011616	Unassigned	Unassigned	Unassigned	477_0	Clustered	4	VC_477_0	4	0.9836	0.99748973	0.9811	0	0	0	1	Ref
AP011617	Unassigned	Unassigned	Unassigned	477_0	Clustered	4	VC_477_0	4	0.9836	0.99748973	0.9811	0	0	0	1	Ref
AP011955	Unassigned	Unassigned	Unassigned	10_0	Clustered	25	VC_10_0	25	0.3467	1	0.3467	0	0	0	0.9733	Ref
AP011956	Unassigned	Unassigned	Unassigned	10_0	Clustered	25	VC_10_0	25	0.3467	1	0.3467	0	0	0	0.9733	Ref
AP012341	Unassigned	Unassigned	Unassigned	8_0	Clustered	58	VC_8_0	58	0.8306	1	0.8306	0	0	0	0.9946	Ref
AP012529	Unassigned	Unassigned	Unassigned	39_0	Clustered	35	VC_39_0	35	0.4337	1	0.4337	0	0	0	1	Ref
AP012530	Unassigned	Unassigned	Unassigned	41_1	Clustered	10	VC_41_1	10	0.1178	1	0.1178	0	0	0	0.8222	Ref
AP012531	Unassigned	Unassigned	Unassigned	39_0	Clustered	35	VC_39_0	35	0.4337	1	0.4337	0	0	0	1	Ref
AP012532	Unassigned	Unassigned	Unassigned	39_0	Clustered	35	VC_39_0	35	0.4337	1	0.4337	0	0	0	1	Ref
AP012534	Unassigned	Unassigned	Unassigned	42_0	Clustered	49	VC_42_0	49	0.4517	1	0.4517	0	0	0	0.8597	Ref
AP012535	Unassigned	Unassigned	Unassigned	39_0	Clustered	35	VC_39_0	35	0.4337	1	0.4337	0	0	0	1	Ref
AP012538	Unassigned	Unassigned	Unassigned	41_1	Clustered	10	VC_41_1	10	0.1178	1	0.1178	0	0	0	0.8222	Ref
AP012539	Unassigned	Unassigned	Unassigned	41_1	Clustered	10	VC_41_1	10	0.1178	1	0.1178	0	0	0	0.8222	Ref
AP012540	Unassigned	Unassigned	Unassigned	41_1	Clustered	10	VC_41_1	10	0.1178	1	0.1178	0	0	0	0.8222	Ref
AP013029	Unassigned	Unassigned	Unassigned	85_3	Clustered	19	VC_85_3	19	0.3671	1	0.3671	0	0	0	0.807	Ref
AP013538	Unassigned	Unassigned	Unassigned	340_0	Clustered	31	VC_340_0	31	0.9549	1	0.9549	0	0	0	1	Ref
AP013539	Unassigned	Unassigned	Unassigned	340_0	Clustered	31	VC_340_0	31	0.9549	1	0.9549	0	0	0	1	Ref
AP013541	Unassigned	Unassigned	Unassigned	480_0	Clustered	19	VC_480_0	19	0.5445	1	0.5445	0	0	0	1	Ref
AP013542	Unassigned	Unassigned	Unassigned	480_0	Clustered	19	VC_480_0	19	0.5445	1	0.5445	0	0	0	1	Ref
AP013544	Unassigned	Unassigned	Unassigned	481_0	Clustered	8	VC_481_0	8	0.1348	1	0.1348	0	0	0	1	Ref
AP013545	Unassigned	Unassigned	Unassigned	480_0	Clustered	19	VC_480_0	19	0.5445	1	0.5445	0	0	0	1	Ref
AP013546	Unassigned	Unassigned	Unassigned	482_0	Clustered	4	VC_482_0	4	0.2975	0.99998416	0.2975	0	0	0	1	Ref
AP013547	Unassigned	Unassigned	Unassigned	482_0	Clustered	4	VC_482_0	4	0.2975	0.99998416	0.2975	0	0	0	1	Ref
AP013548	Unassigned	Unassigned	Unassigned	482_0	Clustered	4	VC_482_0	4	0.2975	0.99998416	0.2975	0	0	0	1	Ref
AP014714	Unassigned	Unassigned	Unassigned	490_0	Clustered	2	VC_490_0	2	0.0102	0.95344863	0.0097	0	0	0	1	Ref

AP014715	Unassigned	Unassigned	Unassigned	490_0	Clustered	2	VC_490_0	2	0.0102	0.95344863	0.0097	0	0	0	1	Ref
AP014858	Unassigned	Unassigned	Unassigned	493_5	Clustered/Singleton	1	VC_493_5	1	0	1	0	0	0	0	1	Ref
AP014887	Unassigned	Unassigned	Unassigned	498_0	Clustered	8	VC_498_0	8	0.3987	1	0.3987	0	0	0	1	Ref
AP014888	Unassigned	Unassigned	Unassigned	501_0	Clustered	16	VC_501_0	16	0.9985	1	0.9985	0	0	0	1	Ref
AP017924	Unassigned	Unassigned	Unassigned	345_0	Clustered	2	VC_345_0	2	0.0685	0.95654153	0.0655	0	0	0	1	Ref
AP017925	Unassigned	Unassigned	Unassigned	345_0	Clustered	2	VC_345_0	2	0.0685	0.95654153	0.0655	0	0	0	1	Ref
AP018361	Unassigned	Unassigned	Unassigned	272_0	Clustered	11	VC_272_0	11	0.4915	1	0.4915	0	0	0	0.8727	Ref
AP018375	Unassigned	Unassigned	Unassigned	149_9	Clustered	3	VC_149_9	3	0.0084	0.97854556	0.0082	0	0	0	1	Ref
AP018399	Unassigned	Unassigned	Unassigned	68_0	Clustered/Singleton	1	VC_68_0	1	0	1	0	0	0	0	1	Ref
AP018462	Unassigned	Unassigned	Unassigned	507_0	Clustered	8	VC_507_0	8	0.4466	1	0.4466	0	0	0	1	Ref
AP018463	Unassigned	Unassigned	Unassigned	507_0	Clustered	8	VC_507_0	8	0.4466	1	0.4466	0	0	0	1	Ref
AP018469	Unassigned	Unassigned	Unassigned	234_0	Clustered	21	VC_234_0	21	0.8355	1	0.8355	0	0	0	1	Ref
AP018470	Unassigned	Unassigned	Unassigned	234_0	Clustered	21	VC_234_0	21	0.8355	1	0.8355	0	0	0	1	Ref
AP018471	Unassigned	Unassigned	Unassigned	234_0	Clustered	21	VC_234_0	21	0.8355	1	0.8355	0	0	0	1	Ref
AP018476	Unassigned	Unassigned	Unassigned	215_1	Clustered	19	VC_215_1	19	0.4188	1	0.4188	0	0	0	1	Ref
AP018477	Unassigned	Unassigned	Unassigned	325_49	Clustered	4	VC_325_49	4	0.014	0.99995965	0.014	0	0	0	1	Ref
AP018478	Unassigned	Unassigned	Unassigned	325_49	Clustered	4	VC_325_49	4	0.014	0.99995965	0.014	0	0	0	1	Ref
AP018479	Unassigned	Unassigned	Unassigned	216_6	Clustered	42	VC_216_6	42	0.473	1	0.473	0	0	0	0.8351	Ref
AP018480	Unassigned	Unassigned	Unassigned	215_1	Clustered	19	VC_215_1	19	0.4188	1	0.4188	0	0	0	1	Ref
AP018485	Unassigned	Unassigned	Unassigned	513_0	Clustered	32	VC_513_0	32	0.6881	1	0.6881	0	0	0	1	Ref
AP018487	Unassigned	Unassigned	Unassigned	518_0	Clustered	6	VC_518_0	6	0.1295	1	0.1295	0	0	0	1	Ref
AP018714	Unassigned	Unassigned	Unassigned	91_0	Clustered	22	VC_91_0	22	0.5318	1	0.5318	0	0	0	1	Ref
AP018715	Unassigned	Unassigned	Unassigned	91_0	Clustered	22	VC_91_0	22	0.5318	1	0.5318	0	0	0	1	Ref
AP018813	Unassigned	Unassigned	Unassigned	283_92	Clustered	4	VC_283_92	4	0.0079	0.9999582	0.0079	0	0	0	1	Ref
AP018814	Unassigned	Unassigned	Unassigned	283_88	Clustered	2	VC_283_88	2	0.0026	0.95596547	0.0024	0	0	0	1	Ref
AP018815	Unassigned	Unassigned	Unassigned	79_0	Clustered	64	VC_79_0	64	0.4185	1	0.4185	0	0	0	0.7649	Ref
AP018932	Unassigned	Unassigned	Unassigned	283_187	Clustered/Singleton	1	VC_283_187	1	0	1	0	0	0	0	1	Ref
AP019417	Unassigned	Unassigned	Unassigned	530_0	Clustered	9	VC_530_0	9	0.8027	1	0.8027	0	0	0	1	Ref
AP019418	Unassigned	Unassigned	Unassigned	344_0	Clustered	21	VC_344_0	21	0.5436	1	0.5436	0	0	0	1	Ref
AP019522	Unassigned	Unassigned	Unassigned	153_0	Clustered	25	VC_153_0	25	0.7729	1	0.7729	0	0	0	1	Ref
AP019524	Unassigned	Unassigned	Unassigned	532_0	Clustered	2	VC_532_0	2	0.4046	0.89435023	0.3619	0	0	0	1	Ref
AP019525	Unassigned	Unassigned	Unassigned	532_0	Clustered	2	VC_532_0	2	0.4046	0.89435023	0.3619	0	0	0	1	Ref
AP019527	Unassigned	Unassigned	Unassigned	534_0	Clustered	9	VC_534_0	9	0.99	1	0.99	0	0	0	1	Ref
AP019535	Unassigned	Unassigned	Unassigned	79_10	Clustered/Singleton	1	VC_79_10	1	0	1	0	0	0	0	1	Ref
AP019559	Unassigned	Unassigned	Unassigned	190_17	Clustered/Singleton	1	VC_190_17	1	0	1	0	0	0	0	1	Ref
AP019560	Unassigned	Unassigned	Unassigned	11_0	Clustered	19	VC_11_0	19	0.7562	1	0.7562	0	0	0	1	Ref
AP019561	Unassigned	Unassigned	Unassigned	11_0	Clustered	19	VC_11_0	19	0.7562	1	0.7562	0	0	0	1	Ref
AP019562	Unassigned	Unassigned	Unassigned	11_0	Clustered	19	VC_11_0	19	0.7562	1	0.7562	0	0	0	1	Ref
AY007505	Unassigned	Unassigned	Unassigned	317_2	Clustered	20	VC_317_2	20	0.1976	1	0.1976	0	0	0	0.6842	Ref
AY027935	Unassigned	Unassigned	Unassigned	5_0	Clustered	30	VC_5_0	30	0.9647	1	0.9647	0	0	0	1	Ref
AY029185	Unassigned	Unassigned	Unassigned	278_0	Clustered	3	VC_278_0	3	0.3052	0.99848877	0.3047	0	0	0	1	Ref
AY048850	Unassigned	Unassigned	Unassigned	539_0	Clustered	3	VC_539_0	3	1	1	1	0	0	0	1	Ref
AY052766	Unassigned	Unassigned	Unassigned	28_1	Clustered	53	VC_28_1	53	0.7164	1	0.7164	0	0	0	0.9325	Ref
AY055382	Unassigned	Unassigned	Unassigned	45_0	Clustered	12	VC_45_0	12	0.35	1	0.35	0	0	0	1	Ref
AY078382	Unassigned	Unassigned	Unassigned	472_0	Clustered	47	VC_472_0	47	0.9025	1	0.9025	0	0	0	1	Ref
AY129330	Unassigned	Unassigned	Unassigned	520_96	Clustered/Singleton	1	VC_520_96	1	0	1	0	0	0	0	1	Ref
AY129331	Unassigned	Unassigned	Unassigned	551_7	Clustered	28	VC_551_7	28	0.1661	1	0.1661	0	0	0	0.8519	Ref
AY129332	Unassigned	Unassigned	Unassigned	514_0	Clustered	75	VC_514_0	75	0.9402	1	0.9402	0	0	0	1	Ref
AY129333	Unassigned	Unassigned	Unassigned	518_1	Clustered	2	VC_518_1	2	0.0376	0.95718761	0.036	0	0	0	1	Ref
AY129334	Unassigned	Unassigned	Unassigned	508_0	Clustered	32	VC_508_0	32	0.906	1	0.906	0	0	0	1	Ref
AY129335	Unassigned	Unassigned	Unassigned	556_0	Clustered	21	VC_556_0	21	0.9912	1	0.9912	0	0	0	1	Ref
AY129337	Unassigned	Unassigned	Unassigned	558_8	Clustered	2	VC_558_8	2	0.0032	0.68030512	0.0022	0	0	0	1	Ref
AY129338	Unassigned	Unassigned	Unassigned	546_10	Clustered/Singleton	1	VC_546_10	1	0	1	0	0	0	0	1	Ref
AY129339	Unassigned	Unassigned	Unassigned	566_0	Clustered	2	VC_566_0	2	0.0989	0.95622526	0.0945	0	0	0	1	Ref
AY131267	Unassigned	Unassigned	Unassigned	272_0	Clustered	11	VC_272_0	11	0.4915	1	0.4915	0	0	0	0.8727	Ref
AY133112	Unassigned	Unassigned	Unassigned	23_0	Clustered	3	VC_23_0	3	0.4305	0.99837378	0.4298	0	0	0	1	Ref
AY135486	Unassigned	Unassigned	Unassigned	228_0	Clustered	60	VC_228_0	60	0.942	1	0.942	0	0	0	1	Ref
AY135739	Unassigned	Unassigned	Unassigned	228_0	Clustered	60	VC_228_0	60	0.942	1	0.942	0	0	0	1	Ref
AY150271	Unassigned	Unassigned	Unassigned	476_0	Clustered	19	VC_476_0	19	0.7814	1	0.7814	0	0	0	1	Ref
AY176327	Unassigned	Unassigned	Unassigned	149_8	Clustered	66	VC_149_8	66	0.4565	1	0.4565	0	0	0	0.8182	Ref
AY190604	Unassigned	Unassigned	Unassigned	310_0	Clustered	33	VC_310_0	33	0.8771	1	0.8771	0	0	0	1	Ref
AY212251	Unassigned	Unassigned	Unassigned	97_0	Clustered	5	VC_97_0	5	0.0696	0.98113835	0.0683	0	0	0	1	Ref
AY216660	Unassigned	Unassigned	Unassigned	575_0	Clustered	35	VC_575_0	35	0.8815	1	0.8815	0	0	0	1	Ref
AY236756	Unassigned	Unassigned	Unassigned	577_0	Clustered	6	VC_577_0	6	0.9267	0.99999993	0.9267	0	0	0	1	Ref
AY242528	Unassigned	Unassigned	Unassigned	16_0	Clustered	12	VC_16_0	12	0.6615	1	0.6615	0	0	0	1	Ref
AY247822	Unassigned	Unassigned	Unassigned	392_0	Clustered	126	VC_392_0	126	0.9621	1	0.9621	0	0	0	1	Ref
AY251033	Unassigned	Unassigned	Unassigned	228_0	Clustered	60	VC_228_0	60	0.942	1	0.942	0	0	0	1	Ref
AY257527	Unassigned	Unassigned	Unassigned	424_0	Clustered	7	VC_424_0	7	0.9044	0.9998143	0.9043	0	0	0	1	Ref
AY264774	Unassigned	Unassigned	Unassigned	392_0	Clustered	126	VC_392_0	126	0.9621	1	0.9621	0	0	0	1	Ref
AY264775	Unassigned	Unassigned	Unassigned	392_0	Clustered	126	VC_392_0	126	0.9621	1	0.9621	0	0	0	1	Ref
AY264776	Unassigned	Unassigned	Unassigned	392_0	Clustered	126	VC_392_0	126	0.9621	1	0.9621	0	0	0	1	Ref
AY264777	Unassigned	Unassigned	Unassigned	392_0	Clustered	126	VC_392_0	126	0.9621	1	0.9621	0	0	0	1	Ref
AY264778	Unassigned	Unassigned	Unassigned	392_0	Clustered	126	VC_392_0	126	0.9621	1	0.9621	0	0	0	1	Ref
AY283928	Unassigned	Unassigned	Unassigned	531_1	Clustered	6	VC_531_1	6	0.1481	0.99978665	0.148	0	0	0	1	Ref
AY288927	Unassigned	Unassigned	Unassigned	452_0	Clustered	66	VC_452_0	66	0.9248	1	0.9248	0	0	0	1	Ref
AY299121	Unassigned	Unassigned	Unassigned	113_0	Clustered	9	VC_113_0	9	0.519	1	0.519	0	0	0	1	Ref
AY303349	Unassigned	Unassigned	Unassigned	286_10	Clustered	9	VC_286_10	9	0.0615	0.99999878	0.0615	0	0	0	0.6667	Ref
AY308796	Unassigned	Unassigned	Unassigned	584_0	Clustered	28	VC_584_0	28	0.7704	1	0.7704	0	0	0	1	Ref
AY319521	Unassigned	Unassigned	Unassigned	6_0	Clustered	44	VC_6_0	44	0.908	1	0.908	0	0	0	1	Ref
AY320035	Unassigned	Unassigned	Unassigned	587_0	Clustered	2	VC_587_0	2	0.3252	0.91192888	0.2965	0	0	0	1	Ref
AY324828	Unassigned	Unassigned	Unassigned	589_0	Clustered	2	VC_589_0	2	1	1	1	0	0	0	1	Ref

AY328852	Unassigned	Unassigned	Unassigned	498_0	Clustered	8	VC_498_0	8	0.3987	1	0.3987	0	0	0	1	Ref
AY328853	Unassigned	Unassigned	Unassigned	498_0	Clustered	8	VC_498_0	8	0.3987	1	0.3987	0	0	0	1	Ref
AY343333	Unassigned	Unassigned	Unassigned	590_0	Clustered	27	VC_590_0	27	0.9947	1	0.9947	0	0	0	1	Ref
AY349011	Unassigned	Unassigned	Unassigned	162_0	Clustered	18	VC_162_0	18	0.7403	1	0.7403	0	0	0	0.8627	Ref
AY370673	Unassigned	Unassigned	Unassigned	452_0	Clustered	66	VC_452_0	66	0.9248	1	0.9248	0	0	0	1	Ref
AY370674	Unassigned	Unassigned	Unassigned	452_0	Clustered	66	VC_452_0	66	0.9248	1	0.9248	0	0	0	1	Ref
AY370762	Unassigned	Unassigned	Unassigned	599_0	Clustered	50	VC_599_0	50	0.9989	1	0.9989	0	0	0	1	Ref
AY374448	Unassigned	Unassigned	Unassigned	34_0	Clustered	4	VC_34_0	4	0.4349	0.99998062	0.4349	0	0	0	1	Ref
AY375531	Unassigned	Unassigned	Unassigned	600_1	Clustered	7	VC_600_1	7	0.1837	0.99999997	0.1837	0	0	0	1	Ref
AY388628	Unassigned	Unassigned	Unassigned	599_0	Clustered	50	VC_599_0	50	0.9989	1	0.9989	0	0	0	1	Ref
AY394005	Unassigned	Unassigned	Unassigned	179_0	Clustered	53	VC_179_0	53	0.9137	1	0.9137	0	0	0	1	Ref
AY423772	Unassigned	Unassigned	Unassigned	599_0	Clustered	50	VC_599_0	50	0.9989	1	0.9989	0	0	0	1	Ref
AY441783	Unassigned	Unassigned	Unassigned	603_0	Clustered	9	VC_603_0	9	0.9925	0.99999994	0.9925	0	0	0	1	Ref
AY453853	Unassigned	Unassigned	Unassigned	353_0	Clustered	6	VC_353_0	6	0.545	1	0.545	0	0	0	1	Ref
AY500152	Unassigned	Unassigned	Unassigned	325_5	Clustered	11	VC_325_5	11	0.047	1	0.047	0	0	0	0.7636	Ref
AY500153	Unassigned	Unassigned	Unassigned	325_9	Clustered	2	VC_325_9	2	0.0055	0.95750934	0.0053	0	0	0	1	Ref
AY505112	Unassigned	Unassigned	Unassigned	607_0	Clustered	16	VC_607_0	16	0.9179	1	0.9179	0	0	0	1	Ref
AY508486	Unassigned	Unassigned	Unassigned	10_0	Clustered	25	VC_10_0	25	0.3467	1	0.3467	0	0	0	0.9733	Ref
AY510084	Unassigned	Unassigned	Unassigned	607_0	Clustered	16	VC_607_0	16	0.9179	1	0.9179	0	0	0	1	Ref
AY526908	Unassigned	Unassigned	Unassigned	278_0	Clustered	3	VC_278_0	3	0.3052	0.99848877	0.3047	0	0	0	1	Ref
AY526909	Unassigned	Unassigned	Unassigned	278_0	Clustered	3	VC_278_0	3	0.3052	0.99848877	0.3047	0	0	0	1	Ref
AY539836	Unassigned	Unassigned	Unassigned	250_0	Clustered	4	VC_250_0	4	0.2395	0.99998241	0.2395	0	0	0	1	Ref
AY543070	Unassigned	Unassigned	Unassigned	190_38	Clustered	3	VC_190_38	3	0.0175	0.99847952	0.0174	0	0	0	1	Ref
AY569307	Unassigned	Unassigned	Unassigned	611_0	Clustered	2	VC_611_0	2	1	1	1	0	0	0	1	Ref
AY575774	Unassigned	Unassigned	Unassigned	612_0	Clustered	9	VC_612_0	9	0.9545	1	0.9545	0	0	0	1	Ref
AY583234	Unassigned	Unassigned	Unassigned	246_0	Clustered	3	VC_246_0	3	0.7624	0.9595722	0.7316	0	0	0	1	Ref
AY583235	Unassigned	Unassigned	Unassigned	246_0	Clustered	3	VC_246_0	3	0.7624	0.9595722	0.7316	0	0	0	1	Ref
AY583236	Unassigned	Unassigned	Unassigned	246_0	Clustered	3	VC_246_0	3	0.7624	0.9595722	0.7316	0	0	0	1	Ref
AY587007	Unassigned	Unassigned	Unassigned	190_38	Clustered	3	VC_190_38	3	0.0175	0.99847952	0.0174	0	0	0	1	Ref
AY605066	Unassigned	Unassigned	Unassigned	272_0	Clustered	11	VC_272_0	11	0.4915	1	0.4915	0	0	0	0.8727	Ref
AY605181	Unassigned	Unassigned	Unassigned	279_0	Clustered	2	VC_279_0	2	0.1888	0.95124665	0.1796	0	0	0	1	Ref
AY625898	Unassigned	Unassigned	Unassigned	630_0	Clustered	20	VC_630_0	20	0.8513	1	0.8513	0	0	0	1	Ref
AY682195	Unassigned	Unassigned	Unassigned	526_2	Clustered/Singleton	1	VC_526_2	1	0	1	0	0	0	0	1	Ref
AY692264	Unassigned	Unassigned	Unassigned	190_38	Clustered	3	VC_190_38	3	0.0175	0.99847952	0.0174	0	0	0	1	Ref
AY699705	Unassigned	Unassigned	Unassigned	254_0	Clustered	46	VC_254_0	46	0.5159	1	0.5159	0	0	0	1	Ref
AY701338	Unassigned	Unassigned	Unassigned	424_0	Clustered	7	VC_424_0	7	0.9044	0.9998143	0.9043	0	0	0	1	Ref
AY714348	Unassigned	Unassigned	Unassigned	26_0	Clustered	4	VC_26_0	4	0.7433	0.99936153	0.7428	0	0	0	1	Ref
AY722806	Unassigned	Unassigned	Unassigned	440_0	Clustered	4	VC_440_0	4	1	1	1	0	0	0	1	Ref
AY730274	Unassigned	Unassigned	Unassigned	637_0	Clustered	115	VC_637_0	115	0.731	1	0.731	0	0	0	0.8764	Ref
AY739900	Unassigned	Unassigned	Unassigned	640_0	Clustered	7	VC_640_0	7	0.9025	1	0.9025	0	0	0	1	Ref
AY751298	Unassigned	Unassigned	Unassigned	360_0	Clustered	358	VC_360_0	358	0.9615	1	0.9615	0	0	0	1	Ref
AY769964	Unassigned	Unassigned	Unassigned	198_0	Clustered	28	VC_198_0	28	0.2429	0.99971175	0.2429	0	0	0	1	Ref
AY772740	Unassigned	Unassigned	Unassigned	642_2	Clustered	6	VC_642_2	6	0.1679	1	0.1679	0	0	0	1	Ref
AY848684	Unassigned	Unassigned	Unassigned	603_0	Clustered	9	VC_603_0	9	0.9925	0.99999994	0.9925	0	0	0	1	Ref
AY848685	Unassigned	Unassigned	Unassigned	603_0	Clustered	9	VC_603_0	9	0.9925	0.99999994	0.9925	0	0	0	1	Ref
AY848686	Unassigned	Unassigned	Unassigned	603_0	Clustered	9	VC_603_0	9	0.9925	0.99999994	0.9925	0	0	0	1	Ref
AY848687	Unassigned	Unassigned	Unassigned	603_0	Clustered	9	VC_603_0	9	0.9925	0.99999994	0.9925	0	0	0	1	Ref
AY848688	Unassigned	Unassigned	Unassigned	603_0	Clustered	9	VC_603_0	9	0.9925	0.99999994	0.9925	0	0	0	1	Ref
AY848689	Unassigned	Unassigned	Unassigned	603_0	Clustered	9	VC_603_0	9	0.9925	0.99999994	0.9925	0	0	0	1	Ref
AY855346	Unassigned	Unassigned	Unassigned	139_0	Clustered	2	VC_139_0	2	0.0701	0.95510185	0.067	0	0	0	1	Ref
AY939843	Unassigned	Unassigned	Unassigned	340_0	Clustered	31	VC_340_0	31	0.9549	1	0.9549	0	0	0	1	Ref
AY939844	Unassigned	Unassigned	Unassigned	432_9	Clustered	3	VC_432_9	3	0.0372	0.99851312	0.0371	0	0	0	1	Ref
AY940168	Unassigned	Unassigned	Unassigned	655_2	Clustered	3	VC_655_2	3	0.0231	0.99860756	0.0231	0	0	0	1	Ref
AY950802	Unassigned	Unassigned	Unassigned	666_0	Clustered	4	VC_666_0	4	1	1	1	0	0	0	1	Ref
AY954949	Unassigned	Unassigned	Unassigned	96_0	Clustered	30	VC_96_0	30	0.718	1	0.718	0	0	0	1	Ref
AY954951	Unassigned	Unassigned	Unassigned	9_0	Clustered	38	VC_9_0	38	0.4895	1	0.4895	0	0	0	1	Ref
AY954952	Unassigned	Unassigned	Unassigned	9_0	Clustered	38	VC_9_0	38	0.4895	1	0.4895	0	0	0	1	Ref
AY954953	Unassigned	Unassigned	Unassigned	9_0	Clustered	38	VC_9_0	38	0.4895	1	0.4895	0	0	0	1	Ref
AY954954	Unassigned	Unassigned	Unassigned	15_1	Clustered	9	VC_15_1	9	0.182	1	0.182	0	0	0	0.8889	Ref
AY954955	Unassigned	Unassigned	Unassigned	8_0	Clustered	58	VC_8_0	58	0.8306	1	0.8306	0	0	0	0.9946	Ref
AY954956	Unassigned	Unassigned	Unassigned	8_0	Clustered	58	VC_8_0	58	0.8306	1	0.8306	0	0	0	0.9946	Ref
AY954957	Unassigned	Unassigned	Unassigned	8_0	Clustered	58	VC_8_0	58	0.8306	1	0.8306	0	0	0	0.9946	Ref
AY954960	Unassigned	Unassigned	Unassigned	12_0	Clustered	21	VC_12_0	21	0.2274	1	0.2274	0	0	0	0.9571	Ref
AY954961	Unassigned	Unassigned	Unassigned	12_0	Clustered	21	VC_12_0	21	0.2274	1	0.2274	0	0	0	0.9571	Ref
AY954962	Unassigned	Unassigned	Unassigned	12_2	Clustered	19	VC_12_2	19	0.2018	1	0.2018	0	0	0	1	Ref
AY954963	Unassigned	Unassigned	Unassigned	12_2	Clustered	19	VC_12_2	19	0.2018	1	0.2018	0	0	0	1	Ref
AY954964	Unassigned	Unassigned	Unassigned	12_2	Clustered	19	VC_12_2	19	0.2018	1	0.2018	0	0	0	1	Ref
AY954965	Unassigned	Unassigned	Unassigned	12_2	Clustered	19	VC_12_2	19	0.2018	1	0.2018	0	0	0	1	Ref
AY954966	Unassigned	Unassigned	Unassigned	12_2	Clustered	19	VC_12_2	19	0.2018	1	0.2018	0	0	0	1	Ref
AY954967	Unassigned	Unassigned	Unassigned	12_2	Clustered	19	VC_12_2	19	0.2018	1	0.2018	0	0	0	1	Ref
AY954968	Unassigned	Unassigned	Unassigned	12_2	Clustered	19	VC_12_2	19	0.2018	1	0.2018	0	0	0	1	Ref
AY954969	Unassigned	Unassigned	Unassigned	149_8	Clustered	66	VC_149_8	66	0.4565	1	0.4565	0	0	0	0.8182	Ref
AY962392	Unassigned	Unassigned	Unassigned	600_1	Clustered	7	VC_600_1	7	0.1837	0.99999997	0.1837	0	0	0	1	Ref
AY967407	Unassigned	Unassigned	Unassigned	669_1	Clustered	10	VC_669_1	10	0.1268	1	0.1268	0	0	0	0.9778	Ref
B1U32222	Unassigned	Unassigned	Unassigned	228_0	Clustered	60	VC_228_0	60	0.942	1	0.942	0	0	0	1	Ref
BCP1	Unassigned	Unassigned	Unassigned	672_0	Clustered	12	VC_672_0	12	1	1	1	0	0	0	1	Ref
BHU24159	Unassigned	Unassigned	Unassigned	5_0	Clustered	30	VC_5_0	30	0.9647	1	0.9647	0	0	0	1	Ref
BK000583	Unassigned	Unassigned	Unassigned	28_1	Clustered	53	VC_28_1	53	0.7164	1	0.7164	0	0	0	0.9325	Ref
BK010471	Unassigned	Unassigned	Unassigned	673_0	Clustered	15	VC_673_0	15	0.8234	1	0.8234	0	0	0	0.7619	Ref
BK010646	Unassigned	Unassigned	Unassigned	677_0	Clustered	9	VC_677_0	9	0.3787	1	0.3787	0	0	0	0.8333	Ref
BK013337	Unassigned	Unassigned	Unassigned	539_0	Clustered	3	VC_539_0	3	1	1	1	0	0	0	1	Ref

BK013338	Unassigned	Unassigned	Unassigned	539_0	Clustered	3	VC_539_0	3	1	1	1	0	0	0	1	Ref
BK013345	Unassigned	Unassigned	Unassigned	681_0	Clustered	3	VC_681_0	3	0.9318	0.81445332	0.7589	0	0	0	1	Ref
BK013346	Unassigned	Unassigned	Unassigned	681_0	Clustered	3	VC_681_0	3	0.9318	0.81445332	0.7589	0	0	0	1	Ref
BK013347	Unassigned	Unassigned	Unassigned	681_0	Clustered	3	VC_681_0	3	0.9318	0.81445332	0.7589	0	0	0	1	Ref
BK059895	Unassigned	Unassigned	Unassigned	682_0	Clustered	2	VC_682_0	2	1	1	1	0	0	0	1	Ref
BK061308	Unassigned	Unassigned	Unassigned	683_0	Clustered	5	VC_683_0	5	0.8963	0.99999974	0.8963	0	0	0	1	Ref
BK061309	Unassigned	Unassigned	Unassigned	683_0	Clustered	5	VC_683_0	5	0.8963	0.99999974	0.8963	0	0	0	1	Ref
BK061310	Unassigned	Unassigned	Unassigned	683_0	Clustered	5	VC_683_0	5	0.8963	0.99999974	0.8963	0	0	0	1	Ref
BK061311	Unassigned	Unassigned	Unassigned	683_0	Clustered	5	VC_683_0	5	0.8963	0.99999974	0.8963	0	0	0	1	Ref
BK061312	Unassigned	Unassigned	Unassigned	683_0	Clustered	5	VC_683_0	5	0.8963	0.99999974	0.8963	0	0	0	1	Ref
C2PVCG	Unassigned	Unassigned	Unassigned	690_0	Clustered	50	VC_690_0	50	0.9805	1	0.9805	0	0	0	1	Ref
CAJCJZ010000002	Unassigned	Unassigned	Unassigned	691_1	Clustered	5	VC_691_1	5	0.4358	0.99999993	0.4358	0	0	0	1	Ref
CAJDJX010000002	Unassigned	Unassigned	Unassigned	695_0	Clustered	48	VC_695_0	48	0.8162	1	0.8162	0	0	0	1	Ref
CAJDJY010000002	Unassigned	Unassigned	Unassigned	91_0	Clustered	22	VC_91_0	22	0.5318	1	0.5318	0	0	0	1	Ref
CAJDJZ010000002	Unassigned	Unassigned	Unassigned	91_0	Clustered	22	VC_91_0	22	0.5318	1	0.5318	0	0	0	1	Ref
CAJDKA010000002	Unassigned	Unassigned	Unassigned	92_0	Clustered	22	VC_92_0	22	0.5639	1	0.5639	0	0	0	1	Ref
CAJDKF010000002	Unassigned	Unassigned	Unassigned	695_0	Clustered	48	VC_695_0	48	0.8162	1	0.8162	0	0	0	1	Ref
CAKLQA020000001	Unassigned	Unassigned	Unassigned	696_1	Clustered	8	VC_696_1	8	0.5452	1	0.5452	0	0	0	0.9286	Ref
CAKLQA020000002	Unassigned	Unassigned	Unassigned	670_0	Clustered	2	VC_670_0	2	0.6875	0.72985431	0.5018	0	0	0	1	Ref
CAKLQA020000003	Unassigned	Unassigned	Unassigned	615_0	Clustered	2	VC_615_0	2	0.2668	0.94178001	0.2513	0	0	0	1	Ref
CAKLQB020000001	Unassigned	Unassigned	Unassigned	699_0	Clustered	71	VC_699_0	71	0.9352	1	0.9352	0	0	0	1	Ref
CAKLQC020000001	Unassigned	Unassigned	Unassigned	492_1	Clustered	20	VC_492_1	20	0.4176	1	0.4176	0	0	0	1	Ref
CAKLQD020000001	Unassigned	Unassigned	Unassigned	492_1	Clustered	20	VC_492_1	20	0.4176	1	0.4176	0	0	0	1	Ref
CAKLQE020000001	Unassigned	Unassigned	Unassigned	701_2	Clustered/Singleton	1	VC_701_2	1	0	1	0	0	0	0	1	Ref
CAKLQG020000001	Unassigned	Unassigned	Unassigned	699_0	Clustered	71	VC_699_0	71	0.9352	1	0.9352	0	0	0	1	Ref
CMU41758	Unassigned	Unassigned	Unassigned	197_0	Clustered	5	VC_197_0	5	0.1324	0.84698401	0.1122	0	0	0	1	Ref
CP000315	Unassigned	Unassigned	Unassigned	19_0	Clustered	2	VC_19_0	2	0.0418	0.92044806	0.0385	0	0	0	1	Ref
CP000622	Unassigned	Unassigned	Unassigned	250_0	Clustered	4	VC_250_0	4	0.2395	0.99998241	0.2395	0	0	0	1	Ref
CP000623	Unassigned	Unassigned	Unassigned	3_0	Clustered	17	VC_3_0	17	0.5639	1	0.5639	0	0	0	1	Ref
CP000624	Unassigned	Unassigned	Unassigned	3_0	Clustered	17	VC_3_0	17	0.5639	1	0.5639	0	0	0	1	Ref
CP000625	Unassigned	Unassigned	Unassigned	353_0	Clustered	6	VC_353_0	6	0.545	1	0.545	0	0	0	1	Ref
CP000711	Unassigned	Unassigned	Unassigned	30_1	Clustered	16	VC_30_1	16	0.1973	1	0.1973	0	0	0	0.8333	Ref
CP000917	Unassigned	Unassigned	Unassigned	191_17	Clustered	4	VC_191_17	4	0.0166	0.99930063	0.0166	0	0	0	0.8333	Ref
CP002495	Unassigned	Unassigned	Unassigned	706_0	Clustered	2	VC_706_0	2	1	1	1	0	0	0	1	Ref
CP004084	Unassigned	Unassigned	Unassigned	303_0	Clustered	155	VC_303_0	155	0.9979	1	0.9979	0	0	0	1	Ref
CP006891	Unassigned	Unassigned	Unassigned	55_0	Clustered	3	VC_55_0	3	0.2818	0.99850111	0.2813	0	0	0	1	Ref
CP008753	Unassigned	Unassigned	Unassigned	3_0	Clustered	17	VC_3_0	17	0.5639	1	0.5639	0	0	0	1	Ref
CP011970	Unassigned	Unassigned	Unassigned	473_2	Clustered	2	VC_473_2	2	0.1022	0.94602228	0.0966	0	0	0	1	Ref
CP013282	Unassigned	Unassigned	Unassigned	424_0	Clustered	7	VC_424_0	7	0.9044	0.9998143	0.9043	0	0	0	1	Ref
CP013974	Unassigned	Unassigned	Unassigned	426_1	Clustered	3	VC_426_1	3	0.1152	0.99804615	0.1149	0	0	0	1	Ref
CP017905	Unassigned	Unassigned	Unassigned	16_0	Clustered	12	VC_16_0	12	0.6615	1	0.6615	0	0	0	1	Ref
CP017906	Unassigned	Unassigned	Unassigned	16_0	Clustered	12	VC_16_0	12	0.6615	1	0.6615	0	0	0	1	Ref
CP019275	Unassigned	Unassigned	Unassigned	6_0	Clustered	44	VC_6_0	44	0.908	1	0.908	0	0	0	1	Ref
CP023680	Unassigned	Unassigned	Unassigned	55_0	Clustered	3	VC_55_0	3	0.2818	0.99850111	0.2813	0	0	0	1	Ref
CP023686	Unassigned	Unassigned	Unassigned	55_0	Clustered	3	VC_55_0	3	0.2818	0.99850111	0.2813	0	0	0	1	Ref
CP025874	Unassigned	Unassigned	Unassigned	229_0	Clustered	32	VC_229_0	32	0.9119	1	0.9119	0	0	0	1	Ref
CP025900	Unassigned	Unassigned	Unassigned	6_0	Clustered	44	VC_6_0	44	0.908	1	0.908	0	0	0	1	Ref
CP025901	Unassigned	Unassigned	Unassigned	229_0	Clustered	32	VC_229_0	32	0.9119	1	0.9119	0	0	0	1	Ref
CP025923	Unassigned	Unassigned	Unassigned	229_0	Clustered	32	VC_229_0	32	0.9119	1	0.9119	0	0	0	1	Ref
CP027117	Unassigned	Unassigned	Unassigned	720_1	Clustered/Singleton	1	VC_720_1	1	0	1	0	0	0	0	1	Ref
CP038007	Unassigned	Unassigned	Unassigned	34_0	Clustered	4	VC_34_0	4	0.4349	0.99998062	0.4349	0	0	0	1	Ref
CP045566	Unassigned	Unassigned	Unassigned	721_0	Clustered	9	VC_721_0	9	1	1	1	0	0	0	1	Ref
CP051272	Unassigned	Unassigned	Unassigned	28_1	Clustered	53	VC_28_1	53	0.7164	1	0.7164	0	0	0	0.9325	Ref
CP051278	Unassigned	Unassigned	Unassigned	28_1	Clustered	53	VC_28_1	53	0.7164	1	0.7164	0	0	0	0.9325	Ref
CP051279	Unassigned	Unassigned	Unassigned	28_1	Clustered	53	VC_28_1	53	0.7164	1	0.7164	0	0	0	0.9325	Ref
CP051285	Unassigned	Unassigned	Unassigned	28_1	Clustered	53	VC_28_1	53	0.7164	1	0.7164	0	0	0	0.9325	Ref
CP051288	Unassigned	Unassigned	Unassigned	28_1	Clustered	53	VC_28_1	53	0.7164	1	0.7164	0	0	0	0.9325	Ref
CP052639	Unassigned	Unassigned	Unassigned	229_0	Clustered	32	VC_229_0	32	0.9119	1	0.9119	0	0	0	1	Ref
CP053388	Unassigned	Unassigned	Unassigned	723_0	Clustered	9	VC_723_0	9	0.7138	1	0.7138	0	0	0	1	Ref
CP054387	Unassigned	Unassigned	Unassigned	723_0	Clustered	9	VC_723_0	9	0.7138	1	0.7138	0	0	0	1	Ref
CP058330	Unassigned	Unassigned	Unassigned	723_0	Clustered	9	VC_723_0	9	0.7138	1	0.7138	0	0	0	1	Ref
CP059058	Unassigned	Unassigned	Unassigned	591_0	Clustered	14	VC_591_0	14	0.5184	1	0.5184	0	0	0	1	Ref
CP060690	Unassigned	Unassigned	Unassigned	730_0	Clustered	9	VC_730_0	9	1	1	1	0	0	0	1	Ref
CP062422	Unassigned	Unassigned	Unassigned	12_0	Clustered	21	VC_12_0	21	0.2274	1	0.2274	0	0	0	0.9571	Ref
CP062426	Unassigned	Unassigned	Unassigned	7_0	Clustered	28	VC_7_0	28	0.3916	1	0.3916	0	0	0	0.9947	Ref
CP062427	Unassigned	Unassigned	Unassigned	9_0	Clustered	38	VC_9_0	38	0.4895	1	0.4895	0	0	0	1	Ref
CP062430	Unassigned	Unassigned	Unassigned	10_0	Clustered	25	VC_10_0	25	0.3467	1	0.3467	0	0	0	0.9733	Ref
CP062434	Unassigned	Unassigned	Unassigned	8_0	Clustered	58	VC_8_0	58	0.8306	1	0.8306	0	0	0	0.9946	Ref
CP062435	Unassigned	Unassigned	Unassigned	7_0	Clustered	28	VC_7_0	28	0.3916	1	0.3916	0	0	0	0.9947	Ref
CP062436	Unassigned	Unassigned	Unassigned	10_0	Clustered	25	VC_10_0	25	0.3467	1	0.3467	0	0	0	0.9733	Ref
CP062442	Unassigned	Unassigned	Unassigned	8_0	Clustered	58	VC_8_0	58	0.8306	1	0.8306	0	0	0	0.9946	Ref
CP062444	Unassigned	Unassigned	Unassigned	8_0	Clustered	58	VC_8_0	58	0.8306	1	0.8306	0	0	0	0.9946	Ref
CP062445	Unassigned	Unassigned	Unassigned	7_0	Clustered	28	VC_7_0	28	0.3916	1	0.3916	0	0	0	0.9947	Ref
CP062447	Unassigned	Unassigned	Unassigned	10_0	Clustered	25	VC_10_0	25	0.3467	1	0.3467	0	0	0	0.9733	Ref
CP062451	Unassigned	Unassigned	Unassigned	9_0	Clustered	38	VC_9_0	38	0.4895	1	0.4895	0	0	0	1	Ref
CP062454	Unassigned	Unassigned	Unassigned	9_0	Clustered	38	VC_9_0	38	0.4895	1	0.4895	0	0	0	1	Ref
CP062457	Unassigned	Unassigned	Unassigned	7_0	Clustered	28	VC_7_0	28	0.3916	1	0.3916	0	0	0	0.9947	Ref
CP062461	Unassigned	Unassigned	Unassigned	8_0	Clustered	58	VC_8_0	58	0.8306	1	0.8306	0	0	0	0.9946	Ref
CP062462	Unassigned	Unassigned	Unassigned	12_0	Clustered	21	VC_12_0	21	0.2274	1	0.2274	0	0	0	0.9571	Ref
CP062992	Unassigned	Unassigned	Unassigned	491_0	Clustered	36	VC_491_0	36	0.5336	1	0.5336	0	0	0	0.9714	Ref
CP067347	Unassigned	Unassigned	Unassigned	473_4	Clustered/Singleton	1	VC_473_4	1	0	1	0	0	0	0	1	Ref

CP067352	Unassigned	Unassigned	Unassigned	473_0	Clustered/Singleton	1	VC_473_0	1	0	1	0	0	0	0	1	Ref
CP069347	Unassigned	Unassigned	Unassigned	647_0	Clustered	11	VC_647_0	11	0.598	1	0.598	0	0	0	1	Ref
CP069348	Unassigned	Unassigned	Unassigned	473_1	Clustered/Singleton	1	VC_473_1	1	0	1	0	0	0	0	1	Ref
CP071049	Unassigned	Unassigned	Unassigned	12_0	Clustered	21	VC_12_0	21	0.2274	1	0.2274	0	0	0	0.9571	Ref
CP072507	Unassigned	Unassigned	Unassigned	34_0	Clustered	4	VC_34_0	4	0.4349	0.99998062	0.4349	0	0	0	1	Ref
CP091911	Unassigned	Unassigned	Unassigned	706_0	Clustered	2	VC_706_0	2	1	1	1	0	0	0	1	Ref
CP101890	Unassigned	Unassigned	Unassigned	723_0	Clustered	9	VC_723_0	9	0.7138	1	0.7138	0	0	0	1	Ref
CP101896	Unassigned	Unassigned	Unassigned	723_0	Clustered	9	VC_723_0	9	0.7138	1	0.7138	0	0	0	1	Ref
D89074	Unassigned	Unassigned	Unassigned	16_0	Clustered	12	VC_16_0	12	0.6615	1	0.6615	0	0	0	1	Ref
DQ003260	Unassigned	Unassigned	Unassigned	28_1	Clustered	53	VC_28_1	53	0.7164	1	0.7164	0	0	0	0.9325	Ref
DQ003637	Unassigned	Unassigned	Unassigned	378_0	Clustered	9	VC_378_0	9	0.7603	1	0.7603	0	0	0	1	Ref
DQ003638	Unassigned	Unassigned	Unassigned	739_0	Clustered	26	VC_739_0	26	0.9571	1	0.9571	0	0	0	1	Ref
DQ003639	Unassigned	Unassigned	Unassigned	379_0	Clustered	5	VC_379_0	5	0.4779	0.99999992	0.4779	0	0	0	0.9	Ref
DQ003640	Unassigned	Unassigned	Unassigned	140_0	Clustered	2	VC_140_0	2	0.4111	0.94824791	0.3898	0	0	0	1	Ref
DQ003641	Unassigned	Unassigned	Unassigned	740_0	Clustered	3	VC_740_0	3	0.202	0.99811828	0.2016	0	0	0	1	Ref
DQ003642	Unassigned	Unassigned	Unassigned	380_0	Clustered	3	VC_380_0	3	0.1631	0.99675097	0.1625	0	0	0	1	Ref
DQ004855	Unassigned	Unassigned	Unassigned	739_0	Clustered	26	VC_739_0	26	0.9571	1	0.9571	0	0	0	1	Ref
DQ029335	Unassigned	Unassigned	Unassigned	82_0	Clustered	24	VC_82_0	24	0.7351	1	0.7351	0	0	0	1	Ref
DQ054536	Unassigned	Unassigned	Unassigned	202_0	Clustered	102	VC_202_0	102	0.4049	1	0.4049	0	0	0	0.7876	Ref
DQ079870	Unassigned	Unassigned	Unassigned	743_0	Clustered	15	VC_743_0	15	1	1	1	0	0	0	1	Ref
DQ079871	Unassigned	Unassigned	Unassigned	743_0	Clustered	15	VC_743_0	15	1	1	1	0	0	0	1	Ref
DQ079872	Unassigned	Unassigned	Unassigned	743_0	Clustered	15	VC_743_0	15	1	1	1	0	0	0	1	Ref
DQ079873	Unassigned	Unassigned	Unassigned	743_0	Clustered	15	VC_743_0	15	1	1	1	0	0	0	1	Ref
DQ079874	Unassigned	Unassigned	Unassigned	743_0	Clustered	15	VC_743_0	15	1	1	1	0	0	0	1	Ref
DQ079875	Unassigned	Unassigned	Unassigned	743_0	Clustered	15	VC_743_0	15	1	1	1	0	0	0	1	Ref
DQ079876	Unassigned	Unassigned	Unassigned	743_0	Clustered	15	VC_743_0	15	1	1	1	0	0	0	1	Ref
DQ079878	Unassigned	Unassigned	Unassigned	743_0	Clustered	15	VC_743_0	15	1	1	1	0	0	0	1	Ref
DQ079879	Unassigned	Unassigned	Unassigned	743_0	Clustered	15	VC_743_0	15	1	1	1	0	0	0	1	Ref
DQ079880	Unassigned	Unassigned	Unassigned	303_0	Clustered	155	VC_303_0	155	0.9979	1	0.9979	0	0	0	1	Ref
DQ079881	Unassigned	Unassigned	Unassigned	303_0	Clustered	155	VC_303_0	155	0.9979	1	0.9979	0	0	0	1	Ref
DQ079882	Unassigned	Unassigned	Unassigned	303_0	Clustered	155	VC_303_0	155	0.9979	1	0.9979	0	0	0	1	Ref
DQ079883	Unassigned	Unassigned	Unassigned	303_0	Clustered	155	VC_303_0	155	0.9979	1	0.9979	0	0	0	1	Ref
DQ079884	Unassigned	Unassigned	Unassigned	303_0	Clustered	155	VC_303_0	155	0.9979	1	0.9979	0	0	0	1	Ref
DQ079885	Unassigned	Unassigned	Unassigned	303_0	Clustered	155	VC_303_0	155	0.9979	1	0.9979	0	0	0	1	Ref
DQ079886	Unassigned	Unassigned	Unassigned	303_0	Clustered	155	VC_303_0	155	0.9979	1	0.9979	0	0	0	1	Ref
DQ079887	Unassigned	Unassigned	Unassigned	303_0	Clustered	155	VC_303_0	155	0.9979	1	0.9979	0	0	0	1	Ref
DQ079888	Unassigned	Unassigned	Unassigned	303_0	Clustered	155	VC_303_0	155	0.9979	1	0.9979	0	0	0	1	Ref
DQ079889	Unassigned	Unassigned	Unassigned	303_0	Clustered	155	VC_303_0	155	0.9979	1	0.9979	0	0	0	1	Ref
DQ079890	Unassigned	Unassigned	Unassigned	303_0	Clustered	155	VC_303_0	155	0.9979	1	0.9979	0	0	0	1	Ref
DQ079891	Unassigned	Unassigned	Unassigned	303_0	Clustered	155	VC_303_0	155	0.9979	1	0.9979	0	0	0	1	Ref
DQ079892	Unassigned	Unassigned	Unassigned	303_0	Clustered	155	VC_303_0	155	0.9979	1	0.9979	0	0	0	1	Ref
DQ079893	Unassigned	Unassigned	Unassigned	303_0	Clustered	155	VC_303_0	155	0.9979	1	0.9979	0	0	0	1	Ref
DQ079894	Unassigned	Unassigned	Unassigned	303_0	Clustered	155	VC_303_0	155	0.9979	1	0.9979	0	0	0	1	Ref
DQ079895	Unassigned	Unassigned	Unassigned	303_0	Clustered	155	VC_303_0	155	0.9979	1	0.9979	0	0	0	1	Ref
DQ079896	Unassigned	Unassigned	Unassigned	360_0	Clustered	358	VC_360_0	358	0.9615	1	0.9615	0	0	0	1	Ref
DQ079897	Unassigned	Unassigned	Unassigned	360_0	Clustered	358	VC_360_0	358	0.9615	1	0.9615	0	0	0	1	Ref
DQ079899	Unassigned	Unassigned	Unassigned	360_0	Clustered	358	VC_360_0	358	0.9615	1	0.9615	0	0	0	1	Ref
DQ079900	Unassigned	Unassigned	Unassigned	360_0	Clustered	358	VC_360_0	358	0.9615	1	0.9615	0	0	0	1	Ref
DQ079901	Unassigned	Unassigned	Unassigned	360_0	Clustered	358	VC_360_0	358	0.9615	1	0.9615	0	0	0	1	Ref
DQ079902	Unassigned	Unassigned	Unassigned	360_0	Clustered	358	VC_360_0	358	0.9615	1	0.9615	0	0	0	1	Ref
DQ079903	Unassigned	Unassigned	Unassigned	360_0	Clustered	358	VC_360_0	358	0.9615	1	0.9615	0	0	0	1	Ref
DQ079904	Unassigned	Unassigned	Unassigned	360_0	Clustered	358	VC_360_0	358	0.9615	1	0.9615	0	0	0	1	Ref
DQ079906	Unassigned	Unassigned	Unassigned	360_0	Clustered	358	VC_360_0	358	0.9615	1	0.9615	0	0	0	1	Ref
DQ079908	Unassigned	Unassigned	Unassigned	360_0	Clustered	358	VC_360_0	358	0.9615	1	0.9615	0	0	0	1	Ref
DQ079909	Unassigned	Unassigned	Unassigned	360_0	Clustered	358	VC_360_0	358	0.9615	1	0.9615	0	0	0	1	Ref
DQ085810	Unassigned	Unassigned	Unassigned	743_0	Clustered	15	VC_743_0	15	1	1	1	0	0	0	1	Ref
DQ087285	Unassigned	Unassigned	Unassigned	3_0	Clustered	17	VC_3_0	17	0.5639	1	0.5639	0	0	0	1	Ref
DQ111067	Unassigned	Unassigned	Unassigned	451_0	Clustered	80	VC_451_0	80	0.9991	1	0.9991	0	0	0	1	Ref
DQ113772	Unassigned	Unassigned	Unassigned	412_0	Clustered	39	VC_412_0	39	0.845	1	0.845	0	0	0	1	Ref
DQ114220	Unassigned	Unassigned	Unassigned	5_0	Clustered	30	VC_5_0	30	0.9647	1	0.9647	0	0	0	1	Ref
DQ121662	Unassigned	Unassigned	Unassigned	455_0	Clustered	18	VC_455_0	18	0.4598	1	0.4598	0	0	0	1	Ref
DQ123818	Unassigned	Unassigned	Unassigned	744_0	Clustered	10	VC_744_0	10	0.7049	1	0.7049	0	0	0	1	Ref
DQ126339	Unassigned	Unassigned	Unassigned	476_0	Clustered	19	VC_476_0	19	0.7814	1	0.7814	0	0	0	1	Ref
DQ150593	Unassigned	Unassigned	Unassigned	704_0	Clustered	16	VC_704_0	16	0.7274	1	0.7274	0	0	0	1	Ref
DQ163912	Unassigned	Unassigned	Unassigned	747_0	Clustered	23	VC_747_0	23	0.8202	1	0.8202	0	0	0	1	Ref
DQ163913	Unassigned	Unassigned	Unassigned	749_0	Clustered	40	VC_749_0	40	0.7517	1	0.7517	0	0	0	1	Ref
DQ163914	Unassigned	Unassigned	Unassigned	612_0	Clustered	9	VC_612_0	9	0.9545	1	0.9545	0	0	0	1	Ref
DQ163915	Unassigned	Unassigned	Unassigned	755_0	Clustered	9	VC_755_0	9	0.4489	1	0.4489	0	0	0	1	Ref
DQ163916	Unassigned	Unassigned	Unassigned	166_0	Clustered	26	VC_166_0	26	0.7923	1	0.7923	0	0	0	1	Ref
DQ163917	Unassigned	Unassigned	Unassigned	80_0	Clustered	41	VC_80_0	41	0.5837	1	0.5837	0	0	0	0.9805	Ref
DQ222851	Unassigned	Unassigned	Unassigned	704_0	Clustered	16	VC_704_0	16	0.7274	1	0.7274	0	0	0	1	Ref
DQ222853	Unassigned	Unassigned	Unassigned	704_0	Clustered	16	VC_704_0	16	0.7274	1	0.7274	0	0	0	1	Ref
DQ222855	Unassigned	Unassigned	Unassigned	704_0	Clustered	16	VC_704_0	16	0.7274	1	0.7274	0	0	0	1	Ref
DQ227763	Unassigned	Unassigned	Unassigned	202_0	Clustered	102	VC_202_0	102	0.4049	1	0.4049	0	0	0	0.7876	Ref
DQ227764	Unassigned	Unassigned	Unassigned	202_0	Clustered	102	VC_202_0	102	0.4049	1	0.4049	0	0	0	0.7876	Ref
DQ289555	Unassigned	Unassigned	Unassigned	704_0	Clustered	16	VC_704_0	16	0.7274	1	0.7274	0	0	0	1	Ref
DQ289556	Unassigned	Unassigned	Unassigned	704_0	Clustered	16	VC_704_0	16	0.7274	1	0.7274	0	0	0	1	Ref
DQ320509	Unassigned	Unassigned	Unassigned	308_0	Clustered	4	VC_308_0	4	0.5014	0.99997418	0.5013	0	0	0	1	Ref
DQ340064	Unassigned	Unassigned	Unassigned	138_0	Clustered	2	VC_138_0	2	0.2009	0.95368907	0.1916	0	0	0	1	Ref
DQ386162	Unassigned	Unassigned	Unassigned	463_0	Clustered	4	VC_463_0	4	0.7906	0.99997156	0.7906	0	0	0	1	Ref
DQ394807	Unassigned	Unassigned	Unassigned	265_0	Clustered	12	VC_265_0	12	0.4401	1	0.4401	0	0	0	1	Ref

DQ394808	Unassigned	Unassigned	Unassigned	265_0	Clustered	12	VC_265_0	12	0.4401	1	0.4401	0	0	0	1	Ref
DQ394809	Unassigned	Unassigned	Unassigned	265_0	Clustered	12	VC_265_0	12	0.4401	1	0.4401	0	0	0	1	Ref
DQ394810	Unassigned	Unassigned	Unassigned	265_0	Clustered	12	VC_265_0	12	0.4401	1	0.4401	0	0	0	1	Ref
DQ398041	Unassigned	Unassigned	Unassigned	551_7	Clustered	28	VC_551_7	28	0.1661	1	0.1661	0	0	0	0.8519	Ref
DQ398042	Unassigned	Unassigned	Unassigned	761_0	Clustered	67	VC_761_0	67	0.8947	1	0.8947	0	0	0	1	Ref
DQ398043	Unassigned	Unassigned	Unassigned	215_0	Clustered	5	VC_215_0	5	0.0724	0.99999997	0.0724	0	0	0	1	Ref
DQ398044	Unassigned	Unassigned	Unassigned	372_0	Clustered	24	VC_372_0	24	0.5539	1	0.5539	0	0	0	1	Ref
DQ398045	Unassigned	Unassigned	Unassigned	520_60	Clustered	2	VC_520_60	2	0.0091	0.95761233	0.0087	0	0	0	1	Ref
DQ398046	Unassigned	Unassigned	Unassigned	371_16	Clustered	42	VC_371_16	42	0.1341	1	0.1341	0	0	0	0.7282	Ref
DQ398047	Unassigned	Unassigned	Unassigned	565_0	Clustered	20	VC_565_0	20	0.8398	1	0.8398	0	0	0	1	Ref
DQ398048	Unassigned	Unassigned	Unassigned	508_0	Clustered	32	VC_508_0	32	0.906	1	0.906	0	0	0	1	Ref
DQ398049	Unassigned	Unassigned	Unassigned	509_0	Clustered	37	VC_509_0	37	0.9689	1	0.9689	0	0	0	1	Ref
DQ398050	Unassigned	Unassigned	Unassigned	520_58	Clustered	3	VC_520_58	3	0.021	0.9985993	0.021	0	0	0	1	Ref
DQ398051	Unassigned	Unassigned	Unassigned	565_0	Clustered	20	VC_565_0	20	0.8398	1	0.8398	0	0	0	1	Ref
DQ398052	Unassigned	Unassigned	Unassigned	764_0	Clustered	4	VC_764_0	4	0.3556	0.99997469	0.3556	0	0	0	1	Ref
DQ398053	Unassigned	Unassigned	Unassigned	558_31	Clustered/Singleton	1	VC_558_31	1	0	1	0	0	0	0	1	Ref
DQ426904	Unassigned	Unassigned	Unassigned	4_0	Clustered	7	VC_4_0	7	0.7912	1	0.7912	0	0	0	1	Ref
DQ426905	Unassigned	Unassigned	Unassigned	4_0	Clustered	7	VC_4_0	7	0.7912	1	0.7912	0	0	0	1	Ref
DQ431235	Unassigned	Unassigned	Unassigned	765_0	Clustered	132	VC_765_0	132	1	1	1	0	0	0	1	Ref
DQ466086	Unassigned	Unassigned	Unassigned	139_1	Clustered	4	VC_139_1	4	0.1775	0.99998609	0.1775	0	0	0	1	Ref
DQ490056	Unassigned	Unassigned	Unassigned	689_0	Clustered	2	VC_689_0	2	0.4402	0.95582436	0.4208	0	0	0	1	Ref
DQ500118	Unassigned	Unassigned	Unassigned	770_0	Clustered	5	VC_770_0	5	0.8394	0.99999405	0.8394	0	0	0	1	Ref
DQ517338	Unassigned	Unassigned	Unassigned	9_0	Clustered	38	VC_9_0	38	0.4895	1	0.4895	0	0	0	1	Ref
DQ529280	Unassigned	Unassigned	Unassigned	600_2	Clustered	10	VC_600_2	10	0.3041	1	0.3041	0	0	0	1	Ref
DQ530359	Unassigned	Unassigned	Unassigned	9_0	Clustered	38	VC_9_0	38	0.4895	1	0.4895	0	0	0	1	Ref
DQ530360	Unassigned	Unassigned	Unassigned	9_0	Clustered	38	VC_9_0	38	0.4895	1	0.4895	0	0	0	1	Ref
DQ530361	Unassigned	Unassigned	Unassigned	10_0	Clustered	25	VC_10_0	25	0.3467	1	0.3467	0	0	0	0.9733	Ref
DQ530362	Unassigned	Unassigned	Unassigned	12_0	Clustered	21	VC_12_0	21	0.2274	1	0.2274	0	0	0	0.9571	Ref
DQ631426	Unassigned	Unassigned	Unassigned	179_0	Clustered	53	VC_179_0	53	0.9137	1	0.9137	0	0	0	1	Ref
DQ674738	Unassigned	Unassigned	Unassigned	5_0	Clustered	30	VC_5_0	30	0.9647	1	0.9647	0	0	0	1	Ref
DQ777876	Unassigned	Unassigned	Unassigned	113_0	Clustered	9	VC_113_0	9	0.519	1	0.519	0	0	0	1	Ref
DQ785801	Unassigned	Unassigned	Unassigned	63_0	Clustered	2	VC_63_0	2	0.1147	0.95689598	0.1098	0	0	0	1	Ref
DQ832317	Unassigned	Unassigned	Unassigned	772_15	Clustered	5	VC_772_15	5	0.0243	0.76144861	0.0185	0	0	0	0.9	Ref
DQ836063	Unassigned	Unassigned	Unassigned	314_0	Clustered	2	VC_314_0	2	0.1808	0.99999983	0.1808	0	0	0	1	Ref
DQ840344	Unassigned	Unassigned	Unassigned	138_0	Clustered	2	VC_138_0	2	0.2009	0.95368907	0.1916	0	0	0	1	Ref
DQ864624	Unassigned	Unassigned	Unassigned	773_0	Clustered	23	VC_773_0	23	0.5514	1	0.5514	0	0	0	1	Ref
DQ873690	Unassigned	Unassigned	Unassigned	179_0	Clustered	53	VC_179_0	53	0.9137	1	0.9137	0	0	0	1	Ref
DQ875742	Unassigned	Unassigned	Unassigned	778_0	Clustered	4	VC_778_0	4	0.8115	0.99985435	0.8114	0	0	0	1	Ref
DQ904452	Unassigned	Unassigned	Unassigned	283_94	Clustered	2	VC_283_94	2	0.0026	0.95293762	0.0025	0	0	0	1	Ref
DQ908929	Unassigned	Unassigned	Unassigned	12_2	Clustered	19	VC_12_2	19	0.2018	1	0.2018	0	0	0	1	Ref
DQ997624	Unassigned	Unassigned	Unassigned	477_0	Clustered	4	VC_477_0	4	0.9836	0.99748973	0.9811	0	0	0	1	Ref
EF056009	Unassigned	Unassigned	Unassigned	779_0	Clustered	37	VC_779_0	37	0.5682	1	0.5682	0	0	0	1	Ref
EF057797	Unassigned	Unassigned	Unassigned	126_0	Clustered	2	VC_126_0	2	0.1661	0.95181568	0.1581	0	0	0	1	Ref
EF068134	Unassigned	Unassigned	Unassigned	188_0	Clustered	13	VC_188_0	13	0.9973	0.99823802	0.9956	0	0	0	1	Ref
EF108464	Unassigned	Unassigned	Unassigned	315_0	Clustered	12	VC_315_0	12	0.8947	1	0.8947	0	0	0	1	Ref
EF108465	Unassigned	Unassigned	Unassigned	315_0	Clustered	12	VC_315_0	12	0.8947	1	0.8947	0	0	0	1	Ref
EF153632	Unassigned	Unassigned	Unassigned	536_0	Clustered	3	VC_536_0	3	0.0878	0.9985359	0.0877	0	0	0	1	Ref
EF160123	Unassigned	Unassigned	Unassigned	453_0	Clustered	6	VC_453_0	6	0.114	1	0.114	0	0	0	1	Ref
EF177456	Unassigned	Unassigned	Unassigned	637_0	Clustered	115	VC_637_0	115	0.731	1	0.731	0	0	0	0.8764	Ref
EF204939	Unassigned	Unassigned	Unassigned	315_0	Clustered	12	VC_315_0	12	0.8947	1	0.8947	0	0	0	1	Ref
EF204940	Unassigned	Unassigned	Unassigned	315_0	Clustered	12	VC_315_0	12	0.8947	1	0.8947	0	0	0	1	Ref
EF372997	Unassigned	Unassigned	Unassigned	340_0	Clustered	31	VC_340_0	31	0.9549	1	0.9549	0	0	0	1	Ref
EF380009	Unassigned	Unassigned	Unassigned	303_0	Clustered	155	VC_303_0	155	0.9979	1	0.9979	0	0	0	1	Ref
EF380010	Unassigned	Unassigned	Unassigned	303_0	Clustered	155	VC_303_0	155	0.9979	1	0.9979	0	0	0	1	Ref
EF380011	Unassigned	Unassigned	Unassigned	303_0	Clustered	155	VC_303_0	155	0.9979	1	0.9979	0	0	0	1	Ref
EF380012	Unassigned	Unassigned	Unassigned	303_0	Clustered	155	VC_303_0	155	0.9979	1	0.9979	0	0	0	1	Ref
EF380013	Unassigned	Unassigned	Unassigned	303_0	Clustered	155	VC_303_0	155	0.9979	1	0.9979	0	0	0	1	Ref
EF380014	Unassigned	Unassigned	Unassigned	303_0	Clustered	155	VC_303_0	155	0.9979	1	0.9979	0	0	0	1	Ref
EF380015	Unassigned	Unassigned	Unassigned	303_0	Clustered	155	VC_303_0	155	0.9979	1	0.9979	0	0	0	1	Ref
EF380016	Unassigned	Unassigned	Unassigned	303_0	Clustered	155	VC_303_0	155	0.9979	1	0.9979	0	0	0	1	Ref
EF380017	Unassigned	Unassigned	Unassigned	303_0	Clustered	155	VC_303_0	155	0.9979	1	0.9979	0	0	0	1	Ref
EF380018	Unassigned	Unassigned	Unassigned	303_0	Clustered	155	VC_303_0	155	0.9979	1	0.9979	0	0	0	1	Ref
EF380019	Unassigned	Unassigned	Unassigned	303_0	Clustered	155	VC_303_0	155	0.9979	1	0.9979	0	0	0	1	Ref
EF380020	Unassigned	Unassigned	Unassigned	303_0	Clustered	155	VC_303_0	155	0.9979	1	0.9979	0	0	0	1	Ref
EF380021	Unassigned	Unassigned	Unassigned	303_0	Clustered	155	VC_303_0	155	0.9979	1	0.9979	0	0	0	1	Ref
EF380022	Unassigned	Unassigned	Unassigned	303_0	Clustered	155	VC_303_0	155	0.9979	1	0.9979	0	0	0	1	Ref
EF380023	Unassigned	Unassigned	Unassigned	303_0	Clustered	155	VC_303_0	155	0.9979	1	0.9979	0	0	0	1	Ref
EF380024	Unassigned	Unassigned	Unassigned	303_0	Clustered	155	VC_303_0	155	0.9979	1	0.9979	0	0	0	1	Ref
EF380025	Unassigned	Unassigned	Unassigned	303_0	Clustered	155	VC_303_0	155	0.9979	1	0.9979	0	0	0	1	Ref
EF380026	Unassigned	Unassigned	Unassigned	303_0	Clustered	155	VC_303_0	155	0.9979	1	0.9979	0	0	0	1	Ref
EF380027	Unassigned	Unassigned	Unassigned	303_0	Clustered	155	VC_303_0	155	0.9979	1	0.9979	0	0	0	1	Ref
EF380028	Unassigned	Unassigned	Unassigned	303_0	Clustered	155	VC_303_0	155	0.9979	1	0.9979	0	0	0	1	Ref
EF380029	Unassigned	Unassigned	Unassigned	303_0	Clustered	155	VC_303_0	155	0.9979	1	0.9979	0	0	0	1	Ref
EF380030	Unassigned	Unassigned	Unassigned	303_0	Clustered	155	VC_303_0	155	0.9979	1	0.9979	0	0	0	1	Ref
EF380031	Unassigned	Unassigned	Unassigned	303_0	Clustered	155	VC_303_0	155	0.9979	1	0.9979	0	0	0	1	Ref
EF380032	Unassigned	Unassigned	Unassigned	303_0	Clustered	155	VC_303_0	155	0.9979	1	0.9979	0	0	0	1	Ref
EF432053	Unassigned	Unassigned	Unassigned	791_0	Clustered	3	VC_791_0	3	1	1	1	0	0	0	1	Ref
EF437941	Unassigned	Unassigned	Unassigned	590_0	Clustered	27	VC_590_0	27	0.9947	1	0.9947	0	0	0	1	Ref
EF460875	Unassigned	Unassigned	Unassigned	792_0	Clustered	35	VC_792_0	35	0.9828	1	0.9828	0	0	0	1	Ref
EF462197	Unassigned	Unassigned	Unassigned	7_0	Clustered	28	VC_7_0	28	0.3916	1	0.3916	0	0	0	0.9947	Ref
EF462198	Unassigned	Unassigned	Unassigned	8_0	Clustered	58	VC_8_0	58	0.8306	1	0.8306	0	0	0	0.9946	Ref

EF462199	Unassigned	Unassigned	Unassigned	7_0	Clustered	28	VC_7_0	28	0.3916	1	0.3916	0	0	0	0.9947	Ref
EF469154	Unassigned	Unassigned	Unassigned	290_6	Clustered	12	VC_290_6	12	0.1149	1	0.1149	0	0	0	0.8182	Ref
EF506570	Unassigned	Unassigned	Unassigned	794_0	Clustered	3	VC_794_0	3	1	1	1	0	0	0	1	Ref
EF529515	Unassigned	Unassigned	Unassigned	254_0	Clustered	46	VC_254_0	46	0.5159	1	0.5159	0	0	0	1	Ref
EF536069	Unassigned	Unassigned	Unassigned	520_24	Clustered/Singleton	1	VC_520_24	1	0	1	0	0	0	0	1	Ref
EF537008	Unassigned	Unassigned	Unassigned	777_0	Clustered	3	VC_777_0	3	0.4763	0.9961812	0.4745	0	0	0	1	Ref
EF583821	Unassigned	Unassigned	Unassigned	90_2	Clustered/Singleton	1	VC_90_2	1	0	1	0	0	0	0	1	Ref
EU030938	Unassigned	Unassigned	Unassigned	599_0	Clustered	50	VC_599_0	50	0.9989	1	0.9989	0	0	0	1	Ref
EU030939	Unassigned	Unassigned	Unassigned	599_0	Clustered	50	VC_599_0	50	0.9989	1	0.9989	0	0	0	1	Ref
EU056923	Unassigned	Unassigned	Unassigned	423_0	Clustered	45	VC_423_0	45	0.9484	1	0.9484	0	0	0	1	Ref
EU078592	Unassigned	Unassigned	Unassigned	43_0	Clustered	38	VC_43_0	38	0.5885	1	0.5885	0	0	0	0.9659	Ref
EU081845	Unassigned	Unassigned	Unassigned	800_0	Clustered	3	VC_800_0	3	0.4591	0.9984737	0.4584	0	0	0	1	Ref
EU100883	Unassigned	Unassigned	Unassigned	805_0	Clustered	4	VC_805_0	4	0.9081	0.99622688	0.9046	0	0	0	1	Ref
EU100884	Unassigned	Unassigned	Unassigned	805_0	Clustered	4	VC_805_0	4	0.9081	0.99622688	0.9046	0	0	0	1	Ref
EU124666	Unassigned	Unassigned	Unassigned	807_0	Clustered	17	VC_807_0	17	0.907	1	0.907	0	0	0	1	Ref
EU136189	Unassigned	Unassigned	Unassigned	96_0	Clustered	30	VC_96_0	30	0.718	1	0.718	0	0	0	1	Ref
EU197055	Unassigned	Unassigned	Unassigned	344_4	Clustered/Singleton	1	VC_344_4	1	0	1	0	0	0	0	1	Ref
EU203571	Unassigned	Unassigned	Unassigned	810_0	Clustered	18	VC_810_0	18	0.9121	1	0.9121	0	0	0	1	Ref
EU221285	Unassigned	Unassigned	Unassigned	202_0	Clustered	102	VC_202_0	102	0.4049	1	0.4049	0	0	0	0.7876	Ref
EU236438	Unassigned	Unassigned	Unassigned	423_0	Clustered	45	VC_423_0	45	0.9484	1	0.9484	0	0	0	1	Ref
EU246945	Unassigned	Unassigned	Unassigned	272_0	Clustered	11	VC_272_0	11	0.4915	1	0.4915	0	0	0	0.8727	Ref
EU272036	Unassigned	Unassigned	Unassigned	179_0	Clustered	53	VC_179_0	53	0.9137	1	0.9137	0	0	0	1	Ref
EU272037	Unassigned	Unassigned	Unassigned	179_0	Clustered	53	VC_179_0	53	0.9137	1	0.9137	0	0	0	1	Ref
EU311208	Unassigned	Unassigned	Unassigned	39_0	Clustered	35	VC_39_0	35	0.4337	1	0.4337	0	0	0	1	Ref
EU330206	Unassigned	Unassigned	Unassigned	813_1	Clustered	24	VC_813_1	24	0.535	1	0.535	0	0	0	1	Ref
EU340421	Unassigned	Unassigned	Unassigned	640_0	Clustered	7	VC_640_0	7	0.9025	1	0.9025	0	0	0	1	Ref
EU350361	Unassigned	Unassigned	Unassigned	814_0	Clustered	3	VC_814_0	3	1	1	1	0	0	0	1	Ref
EU399241	Unassigned	Unassigned	Unassigned	126_0	Clustered	2	VC_126_0	2	0.1661	0.95181568	0.1581	0	0	0	1	Ref
EU408779	Unassigned	Unassigned	Unassigned	424_0	Clustered	7	VC_424_0	7	0.9044	0.9998143	0.9043	0	0	0	1	Ref
EU418428	Unassigned	Unassigned	Unassigned	149_8	Clustered	66	VC_149_8	66	0.4565	1	0.4565	0	0	0	0.8182	Ref
EU545650	Unassigned	Unassigned	Unassigned	349_0	Clustered	7	VC_349_0	7	0.9363	0.99999996	0.9363	0	0	0	1	Ref
EU547803	Unassigned	Unassigned	Unassigned	385_0	Clustered	51	VC_385_0	51	0.8754	1	0.8754	0	0	0	1	Ref
EU568876	Unassigned	Unassigned	Unassigned	761_0	Clustered	67	VC_761_0	67	0.8947	1	0.8947	0	0	0	1	Ref
EU570103	Unassigned	Unassigned	Unassigned	28_1	Clustered	53	VC_28_1	53	0.7164	1	0.7164	0	0	0	0.9325	Ref
EU588980	Unassigned	Unassigned	Unassigned	815_0	Clustered	8	VC_815_0	8	0.9801	1	0.9801	0	0	0	1	Ref
EU622808	Unassigned	Unassigned	Unassigned	817_0	Clustered	14	VC_817_0	14	0.6342	1	0.6342	0	0	0	1	Ref
EU652770	Unassigned	Unassigned	Unassigned	390_0	Clustered	4	VC_390_0	4	0.0468	0.9999882	0.0468	0	0	0	1	Ref
EU676000	Unassigned	Unassigned	Unassigned	565_0	Clustered	20	VC_565_0	20	0.8398	1	0.8398	0	0	0	1	Ref
EU710883	Unassigned	Unassigned	Unassigned	328_0	Clustered	11	VC_328_0	11	0.4707	1	0.4707	0	0	0	1	Ref
EU716414	Unassigned	Unassigned	Unassigned	80_0	Clustered	41	VC_80_0	41	0.5837	1	0.5837	0	0	0	0.9805	Ref
EU717894	Unassigned	Unassigned	Unassigned	113_0	Clustered	9	VC_113_0	9	0.519	1	0.519	0	0	0	1	Ref
EU734170	Unassigned	Unassigned	Unassigned	386_0	Clustered	49	VC_386_0	49	0.7941	1	0.7941	0	0	0	1	Ref
EU734171	Unassigned	Unassigned	Unassigned	386_0	Clustered	49	VC_386_0	49	0.7941	1	0.7941	0	0	0	1	Ref
EU734172	Unassigned	Unassigned	Unassigned	451_0	Clustered	80	VC_451_0	80	0.9991	1	0.9991	0	0	0	1	Ref
EU734173	Unassigned	Unassigned	Unassigned	823_0	Clustered	124	VC_823_0	124	0.9492	1	0.9492	0	0	0	1	Ref
EU734174	Unassigned	Unassigned	Unassigned	392_0	Clustered	126	VC_392_0	126	0.9621	1	0.9621	0	0	0	1	Ref
EU744248	Unassigned	Unassigned	Unassigned	325_21	Clustered	5	VC_325_21	5	0.0209	0.99999976	0.0209	0	0	0	0.8	Ref
EU744249	Unassigned	Unassigned	Unassigned	325_13	Clustered	2	VC_325_13	2	0.0082	0.957512	0.0079	0	0	0	1	Ref
EU744250	Unassigned	Unassigned	Unassigned	217_0	Clustered	5	VC_217_0	5	0.069	0.99999998	0.069	0	0	0	1	Ref
EU744251	Unassigned	Unassigned	Unassigned	325_44	Clustered	3	VC_325_44	3	0.0157	0.99859171	0.0157	0	0	0	1	Ref
EU744252	Unassigned	Unassigned	Unassigned	325_13	Clustered	2	VC_325_13	2	0.0082	0.957512	0.0079	0	0	0	1	Ref
EU770221	Unassigned	Unassigned	Unassigned	372_0	Clustered	24	VC_372_0	24	0.5539	1	0.5539	0	0	0	1	Ref
EU770222	Unassigned	Unassigned	Unassigned	567_0	Clustered	8	VC_567_0	8	0.4935	1	0.4935	0	0	0	1	Ref
EU771092	Unassigned	Unassigned	Unassigned	817_0	Clustered	14	VC_817_0	14	0.6342	1	0.6342	0	0	0	1	Ref
EU794049	Unassigned	Unassigned	Unassigned	29_0	Clustered	10	VC_29_0	10	0.5208	1	0.5208	0	0	0	1	Ref
EU816588	Unassigned	Unassigned	Unassigned	551_6	Clustered	22	VC_551_6	22	0.1214	1	0.1214	0	0	0	0.8095	Ref
EU816589	Unassigned	Unassigned	Unassigned	509_0	Clustered	37	VC_509_0	37	0.9689	1	0.9689	0	0	0	1	Ref
EU816590	Unassigned	Unassigned	Unassigned	520_58	Clustered	3	VC_520_58	3	0.021	0.9985993	0.021	0	0	0	1	Ref
EU816591	Unassigned	Unassigned	Unassigned	551_2	Clustered	17	VC_551_2	17	0.0973	1	0.0973	0	0	0	0.9779	Ref
EU826466	Unassigned	Unassigned	Unassigned	559_0	Clustered	2	VC_559_0	2	0.0112	0.95651281	0.0107	0	0	0	1	Ref
EU826467	Unassigned	Unassigned	Unassigned	558_30	Clustered	3	VC_558_30	3	0.0064	0.76574053	0.0049	0	0	0	1	Ref
EU826468	Unassigned	Unassigned	Unassigned	558_25	Clustered/Singleton	1	VC_558_25	1	0	1	0	0	0	0	1	Ref
EU826469	Unassigned	Unassigned	Unassigned	558_26	Clustered/Singleton	1	VC_558_26	1	0	1	0	0	0	0	1	Ref
EU826470	Unassigned	Unassigned	Unassigned	325_35	Clustered	17	VC_325_35	17	0.0732	1	0.0732	0	0	0	0.7721	Ref
EU826471	Unassigned	Unassigned	Unassigned	558_2	Clustered	2	VC_558_2	2	0.0032	0.66859979	0.0021	0	0	0	1	Ref
EU855793	Unassigned	Unassigned	Unassigned	740_0	Clustered	3	VC_740_0	3	0.202	0.99811828	0.2016	0	0	0	1	Ref
EU861004	Unassigned	Unassigned	Unassigned	9_0	Clustered	38	VC_9_0	38	0.4895	1	0.4895	0	0	0	1	Ref
EU861005	Unassigned	Unassigned	Unassigned	8_0	Clustered	58	VC_8_0	58	0.8306	1	0.8306	0	0	0	0.9946	Ref
EU863408	Unassigned	Unassigned	Unassigned	590_0	Clustered	27	VC_590_0	27	0.9947	1	0.9947	0	0	0	1	Ref
EU863409	Unassigned	Unassigned	Unassigned	290_6	Clustered	12	VC_290_6	12	0.1149	1	0.1149	0	0	0	0.8182	Ref
EU874396	Unassigned	Unassigned	Unassigned	652_0	Clustered	4	VC_652_0	4	0.4743	0.99998076	0.4743	0	0	0	1	Ref
EU877232	Unassigned	Unassigned	Unassigned	329_7	Clustered	18	VC_329_7	18	0.0938	1	0.0938	0	0	0	0.7778	Ref
EU887664	Unassigned	Unassigned	Unassigned	829_0	Clustered	5	VC_829_0	5	0.8821	0.99999832	0.8821	0	0	0	1	Ref
F1CCG	Unassigned	Unassigned	Unassigned	61_0	Clustered	12	VC_61_0	12	0.7633	1	0.7633	0	0	0	1	Ref
FJ000341	Unassigned	Unassigned	Unassigned	28_1	Clustered	53	VC_28_1	53	0.7164	1	0.7164	0	0	0	0.9325	Ref
FJ008913	Unassigned	Unassigned	Unassigned	830_0	Clustered	6	VC_830_0	6	0.6536	1	0.6536	0	0	0	1	Ref
FJ168659	Unassigned	Unassigned	Unassigned	518_0	Clustered	6	VC_518_0	6	0.1295	1	0.1295	0	0	0	1	Ref
FJ168660	Unassigned	Unassigned	Unassigned	565_0	Clustered	20	VC_565_0	20	0.8398	1	0.8398	0	0	0	1	Ref
FJ168661	Unassigned	Unassigned	Unassigned	565_0	Clustered	20	VC_565_0	20	0.8398	1	0.8398	0	0	0	1	Ref
FJ168662	Unassigned	Unassigned	Unassigned	565_0	Clustered	20	VC_565_0	20	0.8398	1	0.8398	0	0	0	1	Ref
FJ174690	Unassigned	Unassigned	Unassigned	520_31	Clustered	11	VC_520_31	11	0.0897	1	0.0897	0	0	0	0.6	Ref

FJ174691	Unassigned	Unassigned	Unassigned	567_0	Clustered	8	VC_567_0	8	0.4935	1	0.4935	0	0	0	1	Ref
FJ174693	Unassigned	Unassigned	Unassigned	520_71	Clustered/Singleton	1	VC_520_71	1	0	1	0	0	0	0	1	Ref
FJ174694	Unassigned	Unassigned	Unassigned	371_16	Clustered	42	VC_371_16	42	0.1341	1	0.1341	0	0	0	0.7282	Ref
FJ184280	Unassigned	Unassigned	Unassigned	41_1	Clustered	10	VC_41_1	10	0.1178	1	0.1178	0	0	0	0.8222	Ref
FJ188381	Unassigned	Unassigned	Unassigned	41_1	Clustered	10	VC_41_1	10	0.1178	1	0.1178	0	0	0	0.8222	Ref
FJ194439	Unassigned	Unassigned	Unassigned	386_0	Clustered	49	VC_386_0	49	0.7941	1	0.7941	0	0	0	1	Ref
FJ226752	Unassigned	Unassigned	Unassigned	254_0	Clustered	46	VC_254_0	46	0.5159	1	0.5159	0	0	0	1	Ref
FJ230960	Unassigned	Unassigned	Unassigned	87_0	Clustered	8	VC_87_0	8	0.5545	1	0.5545	0	0	0	1	Ref
FJ236310	Unassigned	Unassigned	Unassigned	252_0	Clustered	121	VC_252_0	121	0.7376	1	0.7376	0	0	0	0.9999	Ref
FJ373894	Unassigned	Unassigned	Unassigned	131_2	Clustered	7	VC_131_2	7	0.0685	1	0.0685	0	0	0	0.9048	Ref
FJ409640	Unassigned	Unassigned	Unassigned	82_0	Clustered	24	VC_82_0	24	0.7351	1	0.7351	0	0	0	1	Ref
FJ429185	Unassigned	Unassigned	Unassigned	534_0	Clustered	9	VC_534_0	9	0.99	1	0.99	0	0	0	1	Ref
FJ483837	Unassigned	Unassigned	Unassigned	315_0	Clustered	12	VC_315_0	12	0.8947	1	0.8947	0	0	0	1	Ref
FJ483838	Unassigned	Unassigned	Unassigned	315_0	Clustered	12	VC_315_0	12	0.8947	1	0.8947	0	0	0	1	Ref
FJ483839	Unassigned	Unassigned	Unassigned	315_0	Clustered	12	VC_315_0	12	0.8947	1	0.8947	0	0	0	1	Ref
FJ483840	Unassigned	Unassigned	Unassigned	188_0	Clustered	13	VC_188_0	13	0.9973	0.99823802	0.9956	0	0	0	1	Ref
FJ483841	Unassigned	Unassigned	Unassigned	188_0	Clustered	13	VC_188_0	13	0.9973	0.99823802	0.9956	0	0	0	1	Ref
FJ483842	Unassigned	Unassigned	Unassigned	188_0	Clustered	13	VC_188_0	13	0.9973	0.99823802	0.9956	0	0	0	1	Ref
FJ483843	Unassigned	Unassigned	Unassigned	188_0	Clustered	13	VC_188_0	13	0.9973	0.99823802	0.9956	0	0	0	1	Ref
FJ483844	Unassigned	Unassigned	Unassigned	188_0	Clustered	13	VC_188_0	13	0.9973	0.99823802	0.9956	0	0	0	1	Ref
FJ539132	Unassigned	Unassigned	Unassigned	188_0	Clustered	13	VC_188_0	13	0.9973	0.99823802	0.9956	0	0	0	1	Ref
FJ539133	Unassigned	Unassigned	Unassigned	188_0	Clustered	13	VC_188_0	13	0.9973	0.99823802	0.9956	0	0	0	1	Ref
FJ539134	Unassigned	Unassigned	Unassigned	188_0	Clustered	13	VC_188_0	13	0.9973	0.99823802	0.9956	0	0	0	1	Ref
FJ539135	Unassigned	Unassigned	Unassigned	188_0	Clustered	13	VC_188_0	13	0.9973	0.99823802	0.9956	0	0	0	1	Ref
FJ591093	Unassigned	Unassigned	Unassigned	833_0	Clustered	16	VC_833_0	16	0.9351	1	0.9351	0	0	0	1	Ref
FJ591094	Unassigned	Unassigned	Unassigned	833_0	Clustered	16	VC_833_0	16	0.9351	1	0.9351	0	0	0	1	Ref
FJ641182	Unassigned	Unassigned	Unassigned	509_0	Clustered	37	VC_509_0	37	0.9689	1	0.9689	0	0	0	1	Ref
FJ685651	Unassigned	Unassigned	Unassigned	834_0	Clustered	7	VC_834_0	7	0.9845	0.94276893	0.9282	0	0	0	1	Ref
FJ706171	Unassigned	Unassigned	Unassigned	765_0	Clustered	132	VC_765_0	132	1	1	1	0	0	0	1	Ref
FJ706172	Unassigned	Unassigned	Unassigned	765_0	Clustered	132	VC_765_0	132	1	1	1	0	0	0	1	Ref
FJ713816	Unassigned	Unassigned	Unassigned	7_0	Clustered	28	VC_7_0	28	0.3916	1	0.3916	0	0	0	0.9947	Ref
FJ750948	Unassigned	Unassigned	Unassigned	835_0	Clustered	67	VC_835_0	67	0.9624	1	0.9624	0	0	0	1	Ref
FJ822135	Unassigned	Unassigned	Unassigned	93_0	Clustered	2	VC_93_0	2	0.1063	0.95665672	0.1017	0	0	0	1	Ref
FJ839692	Unassigned	Unassigned	Unassigned	283_84	Clustered/Singleton	1	VC_283_84	1	0	1	0	0	0	0	1	Ref
FJ839693	Unassigned	Unassigned	Unassigned	283_3	Clustered	3	VC_283_3	3	0.0055	0.9968017	0.0055	0	0	0	1	Ref
FJ848881	Unassigned	Unassigned	Unassigned	202_0	Clustered	102	VC_202_0	102	0.4049	1	0.4049	0	0	0	0.7876	Ref
FJ848882	Unassigned	Unassigned	Unassigned	202_0	Clustered	102	VC_202_0	102	0.4049	1	0.4049	0	0	0	0.7876	Ref
FJ848883	Unassigned	Unassigned	Unassigned	202_0	Clustered	102	VC_202_0	102	0.4049	1	0.4049	0	0	0	0.7876	Ref
FJ848884	Unassigned	Unassigned	Unassigned	202_0	Clustered	102	VC_202_0	102	0.4049	1	0.4049	0	0	0	0.7876	Ref
FJ848885	Unassigned	Unassigned	Unassigned	202_0	Clustered	102	VC_202_0	102	0.4049	1	0.4049	0	0	0	0.7876	Ref
FJ849058	Unassigned	Unassigned	Unassigned	303_0	Clustered	155	VC_303_0	155	0.9979	1	0.9979	0	0	0	1	Ref
FJ867910	Unassigned	Unassigned	Unassigned	304_0	Clustered	11	VC_304_0	11	0.7869	1	0.7869	0	0	0	1	Ref
FJ867911	Unassigned	Unassigned	Unassigned	304_0	Clustered	11	VC_304_0	11	0.7869	1	0.7869	0	0	0	1	Ref
FJ867912	Unassigned	Unassigned	Unassigned	304_0	Clustered	11	VC_304_0	11	0.7869	1	0.7869	0	0	0	1	Ref
FJ867913	Unassigned	Unassigned	Unassigned	304_0	Clustered	11	VC_304_0	11	0.7869	1	0.7869	0	0	0	1	Ref
FJ867914	Unassigned	Unassigned	Unassigned	304_0	Clustered	11	VC_304_0	11	0.7869	1	0.7869	0	0	0	1	Ref
FJ870915	Unassigned	Unassigned	Unassigned	599_0	Clustered	50	VC_599_0	50	0.9989	1	0.9989	0	0	0	1	Ref
FJ870916	Unassigned	Unassigned	Unassigned	599_0	Clustered	50	VC_599_0	50	0.9989	1	0.9989	0	0	0	1	Ref
FJ870917	Unassigned	Unassigned	Unassigned	599_0	Clustered	50	VC_599_0	50	0.9989	1	0.9989	0	0	0	1	Ref
FJ896200	Unassigned	Unassigned	Unassigned	837_0	Clustered	29	VC_837_0	29	0.9955	1	0.9955	0	0	0	1	Ref
FJ904927	Unassigned	Unassigned	Unassigned	16_0	Clustered	12	VC_16_0	12	0.6615	1	0.6615	0	0	0	1	Ref
FJ937737	Unassigned	Unassigned	Unassigned	162_0	Clustered	18	VC_162_0	18	0.7403	1	0.7403	0	0	0	0.8627	Ref
FJ965538	Unassigned	Unassigned	Unassigned	255_0	Clustered	13	VC_255_0	13	0.2048	1	0.2048	0	0	0	1	Ref
FJ973624	Unassigned	Unassigned	Unassigned	761_0	Clustered	67	VC_761_0	67	0.8947	1	0.8947	0	0	0	1	Ref
FM164764	Unassigned	Unassigned	Unassigned	348_0	Clustered	22	VC_348_0	22	0.9602	1	0.9602	0	0	0	1	Ref
FM180578	Unassigned	Unassigned	Unassigned	41_1	Clustered	10	VC_41_1	10	0.1178	1	0.1178	0	0	0	0.8222	Ref
FM201281	Unassigned	Unassigned	Unassigned	79_2	Clustered/Singleton	1	VC_79_2	1	0	1	0	0	0	0	1	Ref
FM201282	Unassigned	Unassigned	Unassigned	79_0	Clustered	64	VC_79_0	64	0.4185	1	0.4185	0	0	0	0.7649	Ref
FM207411	Unassigned	Unassigned	Unassigned	433_8	Clustered/Singleton	1	VC_433_8	1	0	1	0	0	0	0	1	Ref
FM864213	Unassigned	Unassigned	Unassigned	632_0	Clustered	37	VC_632_0	37	0.581	1	0.581	0	0	0	0.9234	Ref
FM887021	Unassigned	Unassigned	Unassigned	79_7	Clustered	33	VC_79_7	33	0.216	1	0.216	0	0	0	0.8409	Ref
FM897211	Unassigned	Unassigned	Unassigned	79_7	Clustered	33	VC_79_7	33	0.216	1	0.216	0	0	0	0.8409	Ref
FN263372	Unassigned	Unassigned	Unassigned	423_0	Clustered	45	VC_423_0	45	0.9484	1	0.9484	0	0	0	1	Ref
FN297812	Unassigned	Unassigned	Unassigned	23_0	Clustered	3	VC_23_0	3	0.4305	0.99837378	0.4298	0	0	0	1	Ref
FN391954	Unassigned	Unassigned	Unassigned	333_0	Clustered	25	VC_333_0	25	0.4681	1	0.4681	0	0	0	1	Ref
FN422398	Unassigned	Unassigned	Unassigned	839_0	Clustered	5	VC_839_0	5	0.165	0.99999995	0.165	0	0	0	1	Ref
FN422399	Unassigned	Unassigned	Unassigned	838_0	Clustered	29	VC_838_0	29	0.8611	1	0.8611	0	0	0	1	Ref
FN594518	Unassigned	Unassigned	Unassigned	155_0	Clustered	14	VC_155_0	14	0.6095	1	0.6095	0	0	0	1	Ref
FN667788	Unassigned	Unassigned	Unassigned	843_0	Clustered	5	VC_843_0	5	0.2843	0.99999994	0.2843	0	0	0	1	Ref
FN667789	Unassigned	Unassigned	Unassigned	843_0	Clustered	5	VC_843_0	5	0.2843	0.99999994	0.2843	0	0	0	1	Ref
FN997652	Unassigned	Unassigned	Unassigned	632_0	Clustered	37	VC_632_0	37	0.581	1	0.581	0	0	0	0.9234	Ref
FO818745	Unassigned	Unassigned	Unassigned	247_0	Clustered	14	VC_247_0	14	0.9119	1	0.9119	0	0	0	0.967	Ref
FQ312032	Unassigned	Unassigned	Unassigned	133_22	Clustered	13	VC_133_22	13	0.0804	1	0.0804	0	0	0	0.8205	Ref
FQ482083	Unassigned	Unassigned	Unassigned	328_0	Clustered	11	VC_328_0	11	0.4707	1	0.4707	0	0	0	1	Ref
FQ482084	Unassigned	Unassigned	Unassigned	453_0	Clustered	6	VC_453_0	6	0.114	1	0.114	0	0	0	1	Ref
FQ482086	Unassigned	Unassigned	Unassigned	453_0	Clustered	6	VC_453_0	6	0.114	1	0.114	0	0	0	1	Ref
FR667955	Unassigned	Unassigned	Unassigned	392_0	Clustered	126	VC_392_0	126	0.9621	1	0.9621	0	0	0	1	Ref
FR671405	Unassigned	Unassigned	Unassigned	414_0	Clustered	37	VC_414_0	37	0.585	1	0.585	0	0	0	0.9474	Ref
FR671406	Unassigned	Unassigned	Unassigned	414_0	Clustered	37	VC_414_0	37	0.585	1	0.585	0	0	0	0.9474	Ref
FR671407	Unassigned	Unassigned	Unassigned	414_0	Clustered	37	VC_414_0	37	0.585	1	0.585	0	0	0	0.9474	Ref
FR671408	Unassigned	Unassigned	Unassigned	317_2	Clustered	20	VC_317_2	20	0.1976	1	0.1976	0	0	0	0.6842	Ref

FR671409	Unassigned	Unassigned	Unassigned	317_2	Clustered	20	VC_317_2	20	0.1976	1	0.1976	0	0	0	0.6842	Ref
FR671410	Unassigned	Unassigned	Unassigned	414_0	Clustered	37	VC_414_0	37	0.585	1	0.585	0	0	0	0.9474	Ref
FR671411	Unassigned	Unassigned	Unassigned	414_0	Clustered	37	VC_414_0	37	0.585	1	0.585	0	0	0	0.9474	Ref
FR682616	Unassigned	Unassigned	Unassigned	833_0	Clustered	16	VC_833_0	16	0.9351	1	0.9351	0	0	0	1	Ref
FR687252	Unassigned	Unassigned	Unassigned	110_0	Clustered	4	VC_110_0	4	0.1143	0.99998732	0.1143	0	0	0	1	Ref
FR719956	Unassigned	Unassigned	Unassigned	833_0	Clustered	16	VC_833_0	16	0.9351	1	0.9351	0	0	0	1	Ref
FR751545	Unassigned	Unassigned	Unassigned	156_0	Clustered	12	VC_156_0	12	0.9062	1	0.9062	0	0	0	1	Ref
FR775895	Unassigned	Unassigned	Unassigned	848_0	Clustered	28	VC_848_0	28	0.7927	1	0.7927	0	0	0	1	Ref
FR823298	Unassigned	Unassigned	Unassigned	130_0	Clustered	19	VC_130_0	19	0.6068	1	0.6068	0	0	0	1	Ref
FR823450	Unassigned	Unassigned	Unassigned	844_0	Clustered	27	VC_844_0	27	0.9385	1	0.9385	0	0	0	1	Ref
FR852584	Unassigned	Unassigned	Unassigned	149_8	Clustered	66	VC_149_8	66	0.4565	1	0.4565	0	0	0	0.8182	Ref
GQ141189	Unassigned	Unassigned	Unassigned	852_0	Clustered	2	VC_852_0	2	0.4064	0.94189878	0.3828	0	0	0	1	Ref
GQ149088	Unassigned	Unassigned	Unassigned	743_0	Clustered	15	VC_743_0	15	1	1	1	0	0	0	1	Ref
GQ153912	Unassigned	Unassigned	Unassigned	303_0	Clustered	155	VC_303_0	155	0.9979	1	0.9979	0	0	0	1	Ref
GQ153913	Unassigned	Unassigned	Unassigned	303_0	Clustered	155	VC_303_0	155	0.9979	1	0.9979	0	0	0	1	Ref
GQ153914	Unassigned	Unassigned	Unassigned	303_0	Clustered	155	VC_303_0	155	0.9979	1	0.9979	0	0	0	1	Ref
GQ153915	Unassigned	Unassigned	Unassigned	303_0	Clustered	155	VC_303_0	155	0.9979	1	0.9979	0	0	0	1	Ref
GQ153916	Unassigned	Unassigned	Unassigned	61_0	Clustered	12	VC_61_0	12	0.7633	1	0.7633	0	0	0	1	Ref
GQ153917	Unassigned	Unassigned	Unassigned	61_0	Clustered	12	VC_61_0	12	0.7633	1	0.7633	0	0	0	1	Ref
GQ153918	Unassigned	Unassigned	Unassigned	61_0	Clustered	12	VC_61_0	12	0.7633	1	0.7633	0	0	0	1	Ref
GQ153919	Unassigned	Unassigned	Unassigned	61_0	Clustered	12	VC_61_0	12	0.7633	1	0.7633	0	0	0	1	Ref
GQ153920	Unassigned	Unassigned	Unassigned	360_0	Clustered	358	VC_360_0	358	0.9615	1	0.9615	0	0	0	1	Ref
GQ153921	Unassigned	Unassigned	Unassigned	362_0	Clustered	2	VC_362_0	2	0.002	0.83307735	0.0017	0	0	0	1	Ref
GQ153922	Unassigned	Unassigned	Unassigned	360_0	Clustered	358	VC_360_0	358	0.9615	1	0.9615	0	0	0	1	Ref
GQ153923	Unassigned	Unassigned	Unassigned	360_0	Clustered	358	VC_360_0	358	0.9615	1	0.9615	0	0	0	1	Ref
GQ241246	Unassigned	Unassigned	Unassigned	855_0	Clustered	2	VC_855_0	2	0.0285	0.89378929	0.0255	0	0	0	1	Ref
GQ303259	Unassigned	Unassigned	Unassigned	371_3	Clustered	22	VC_371_3	22	0.0787	1	0.0787	0	0	0	0.9048	Ref
GQ303260	Unassigned	Unassigned	Unassigned	558_1	Clustered	2	VC_558_1	2	0.0032	0.78405032	0.0025	0	0	0	0	Ref
GQ303261	Unassigned	Unassigned	Unassigned	761_0	Clustered	67	VC_761_0	67	0.8947	1	0.8947	0	0	0	1	Ref
GQ303262	Unassigned	Unassigned	Unassigned	558_82	Clustered	3	VC_558_82	3	0.0064	0.88433874	0.0057	0	0	0	0.6667	Ref
GQ303263	Unassigned	Unassigned	Unassigned	517_1	Clustered	108	VC_517_1	108	0.8483	1	0.8483	0	0	0	0.9955	Ref
GQ303264	Unassigned	Unassigned	Unassigned	371_15	Clustered	44	VC_371_15	44	0.1391	1	0.1391	0	0	0	0.7474	Ref
GQ303265	Unassigned	Unassigned	Unassigned	551_7	Clustered	28	VC_551_7	28	0.1661	1	0.1661	0	0	0	0.8519	Ref
GQ303266	Unassigned	Unassigned	Unassigned	371_15	Clustered	44	VC_371_15	44	0.1391	1	0.1391	0	0	0	0.7474	Ref
GQ357916	Unassigned	Unassigned	Unassigned	50_0	Clustered	9	VC_50_0	9	0.5531	1	0.5531	0	0	0	1	Ref
GQ379227	Unassigned	Unassigned	Unassigned	374_0	Clustered	2	VC_374_0	2	0.1258	0.95578317	0.1203	0	0	0	1	Ref
GQ398772	Unassigned	Unassigned	Unassigned	10_0	Clustered	25	VC_10_0	25	0.3467	1	0.3467	0	0	0	0.9733	Ref
GQ403788	Unassigned	Unassigned	Unassigned	740_0	Clustered	3	VC_740_0	3	0.202	0.99811828	0.2016	0	0	0	1	Ref
GQ403789	Unassigned	Unassigned	Unassigned	27_0	Clustered	2	VC_27_0	2	1	1	1	0	0	0	1	Ref
GQ413937	Unassigned	Unassigned	Unassigned	823_0	Clustered	124	VC_823_0	124	0.9492	1	0.9492	0	0	0	1	Ref
GQ413938	Unassigned	Unassigned	Unassigned	111_0	Clustered	91	VC_111_0	91	0.9587	1	0.9587	0	0	0	1	Ref
GQ421471	Unassigned	Unassigned	Unassigned	280_0	Clustered	2	VC_280_0	2	0.34	0.95158328	0.3235	0	0	0	1	Ref
GQ422450	Unassigned	Unassigned	Unassigned	28_1	Clustered	53	VC_28_1	53	0.7164	1	0.7164	0	0	0	0.9325	Ref
GQ422451	Unassigned	Unassigned	Unassigned	28_1	Clustered	53	VC_28_1	53	0.7164	1	0.7164	0	0	0	0.9325	Ref
GQ443085	Unassigned	Unassigned	Unassigned	815_0	Clustered	8	VC_815_0	8	0.9801	1	0.9801	0	0	0	1	Ref
GQ451696	Unassigned	Unassigned	Unassigned	862_0	Clustered	11	VC_862_0	11	0.6745	1	0.6745	0	0	0	1	Ref
GQ452243	Unassigned	Unassigned	Unassigned	267_0	Clustered	2	VC_267_0	2	0.0988	0.95557934	0.0945	0	0	0	1	Ref
GQ468526	Unassigned	Unassigned	Unassigned	386_0	Clustered	49	VC_386_0	49	0.7941	1	0.7941	0	0	0	1	Ref
GQ478081	Unassigned	Unassigned	Unassigned	774_0	Clustered	5	VC_774_0	5	0.3282	0.99999996	0.3282	0	0	0	1	Ref
GQ478082	Unassigned	Unassigned	Unassigned	774_0	Clustered	5	VC_774_0	5	0.3282	0.99999996	0.3282	0	0	0	1	Ref
GQ478083	Unassigned	Unassigned	Unassigned	774_0	Clustered	5	VC_774_0	5	0.3282	0.99999996	0.3282	0	0	0	1	Ref
GQ478084	Unassigned	Unassigned	Unassigned	774_0	Clustered	5	VC_774_0	5	0.3282	0.99999996	0.3282	0	0	0	1	Ref
GQ478085	Unassigned	Unassigned	Unassigned	774_0	Clustered	5	VC_774_0	5	0.3282	0.99999996	0.3282	0	0	0	1	Ref
GQ502199	Unassigned	Unassigned	Unassigned	835_0	Clustered	67	VC_835_0	67	0.9624	1	0.9624	0	0	0	1	Ref
GQ866233	Unassigned	Unassigned	Unassigned	426_0	Clustered	2	VC_426_0	2	0.1976	0.95643941	0.189	0	0	0	1	Ref
GQ915271	Unassigned	Unassigned	Unassigned	7_0	Clustered	28	VC_7_0	28	0.3916	1	0.3916	0	0	0	0.9947	Ref
GQ919031	Unassigned	Unassigned	Unassigned	864_0	Clustered	2	VC_864_0	2	1	1	1	0	0	0	1	Ref
GQ979703	Unassigned	Unassigned	Unassigned	202_0	Clustered	102	VC_202_0	102	0.4049	1	0.4049	0	0	0	0.7876	Ref
GQ981382	Unassigned	Unassigned	Unassigned	290_1	Clustered	8	VC_290_1	8	0.0757	1	0.0757	0	0	0	0.8571	Ref
GU060500	Unassigned	Unassigned	Unassigned	520_31	Clustered	11	VC_520_31	11	0.0897	1	0.0897	0	0	0	0.6	Ref
GU070616	Unassigned	Unassigned	Unassigned	582_5	Clustered	4	VC_582_5	4	0.0528	0.99935625	0.0527	0	0	0	0.8333	Ref
GU071091	Unassigned	Unassigned	Unassigned	392_0	Clustered	126	VC_392_0	126	0.9621	1	0.9621	0	0	0	1	Ref
GU071092	Unassigned	Unassigned	Unassigned	432_9	Clustered	3	VC_432_9	3	0.0372	0.99851312	0.0371	0	0	0	1	Ref
GU071093	Unassigned	Unassigned	Unassigned	340_0	Clustered	31	VC_340_0	31	0.9549	1	0.9549	0	0	0	1	Ref
GU071094	Unassigned	Unassigned	Unassigned	655_8	Clustered/Singleton	1	VC_655_8	1	0	1	0	0	0	0	1	Ref
GU071095	Unassigned	Unassigned	Unassigned	432_2	Clustered/Singleton	1	VC_432_2	1	0	1	0	0	0	0	1	Ref
GU071097	Unassigned	Unassigned	Unassigned	655_9	Clustered/Singleton	1	VC_655_9	1	0	1	0	0	0	0	1	Ref
GU071098	Unassigned	Unassigned	Unassigned	432_10	Clustered	2	VC_432_10	2	0.0216	0.95771017	0.0207	0	0	0	1	Ref
GU071099	Unassigned	Unassigned	Unassigned	655_0	Clustered	2	VC_655_0	2	0.0111	0.95704495	0.0106	0	0	0	1	Ref
GU071100	Unassigned	Unassigned	Unassigned	340_0	Clustered	31	VC_340_0	31	0.9549	1	0.9549	0	0	0	1	Ref
GU071102	Unassigned	Unassigned	Unassigned	340_0	Clustered	31	VC_340_0	31	0.9549	1	0.9549	0	0	0	1	Ref
GU071103	Unassigned	Unassigned	Unassigned	655_1	Clustered/Singleton	1	VC_655_1	1	0	1	0	0	0	0	1	Ref
GU071104	Unassigned	Unassigned	Unassigned	340_0	Clustered	31	VC_340_0	31	0.9549	1	0.9549	0	0	0	1	Ref
GU071105	Unassigned	Unassigned	Unassigned	433_4	Clustered	9	VC_433_4	9	0.1285	1	0.1285	0	0	0	1	Ref
GU071106	Unassigned	Unassigned	Unassigned	655_6	Clustered	2	VC_655_6	2	0.0078	0.95775168	0.0075	0	0	0	1	Ref
GU071107	Unassigned	Unassigned	Unassigned	340_0	Clustered	31	VC_340_0	31	0.9549	1	0.9549	0	0	0	1	Ref
GU080336	Unassigned	Unassigned	Unassigned	611_0	Clustered	2	VC_611_0	2	1	1	1	0	0	0	1	Ref
GU169904	Unassigned	Unassigned	Unassigned	329_15	Clustered/Singleton	1	VC_329_15	1	0	1	0	0	0	0	1	Ref
GU196277	Unassigned	Unassigned	Unassigned	638_0	Clustered	45	VC_638_0	45	0.7741	1	0.7741	0	0	0	1	Ref
GU196278	Unassigned	Unassigned	Unassigned	638_0	Clustered	45	VC_638_0	45	0.7741	1	0.7741	0	0	0	1	Ref
GU196279	Unassigned	Unassigned	Unassigned	638_0	Clustered	45	VC_638_0	45	0.7741	1	0.7741	0	0	0	1	Ref

GU196280	Unassigned	Unassigned	Unassigned	638_0	Clustered	45	VC_638_0	45	0.7741	1	0.7741	0	0	0	1	Ref
GU196281	Unassigned	Unassigned	Unassigned	638_0	Clustered	45	VC_638_0	45	0.7741	1	0.7741	0	0	0	1	Ref
GU229986	Unassigned	Unassigned	Unassigned	652_0	Clustered	4	VC_652_0	4	0.4743	0.99998076	0.4743	0	0	0	1	Ref
GU247132	Unassigned	Unassigned	Unassigned	325_14	Clustered	3	VC_325_14	3	0.0094	0.99859159	0.0094	0	0	0	1	Ref
GU247133	Unassigned	Unassigned	Unassigned	371_16	Clustered	42	VC_371_16	42	0.1341	1	0.1341	0	0	0	0.7282	Ref
GU247134	Unassigned	Unassigned	Unassigned	371_16	Clustered	42	VC_371_16	42	0.1341	1	0.1341	0	0	0	0.7282	Ref
GU295964	Unassigned	Unassigned	Unassigned	668_0	Clustered	25	VC_668_0	25	0.5539	1	0.5539	0	0	0	0.96	Ref
GU296433	Unassigned	Unassigned	Unassigned	844_0	Clustered	27	VC_844_0	27	0.9385	1	0.9385	0	0	0	1	Ref
GU321093	Unassigned	Unassigned	Unassigned	834_0	Clustered	7	VC_834_0	7	0.9845	0.94276893	0.9282	0	0	0	1	Ref
GU323708	Unassigned	Unassigned	Unassigned	381_0	Clustered	2	VC_381_0	2	0.0829	0.95381392	0.079	0	0	0	1	Ref
GU339467	Unassigned	Unassigned	Unassigned	216_3	Clustered	8	VC_216_3	8	0.0701	1	0.0701	0	0	0	0.8571	Ref
GU385905	Unassigned	Unassigned	Unassigned	303_0	Clustered	155	VC_303_0	155	0.9979	1	0.9979	0	0	0	1	Ref
GU393987	Unassigned	Unassigned	Unassigned	70_0	Clustered	27	VC_70_0	27	0.5981	1	0.5981	0	0	0	0.9886	Ref
GU459069	Unassigned	Unassigned	Unassigned	443_1	Clustered	2	VC_443_1	2	0.0287	0.89347378	0.0257	0	0	0	1	Ref
GU477322	Unassigned	Unassigned	Unassigned	9_0	Clustered	38	VC_9_0	38	0.4895	1	0.4895	0	0	0	1	Ref
GU557055	Unassigned	Unassigned	Unassigned	305_0	Clustered	12	VC_305_0	12	0.5745	1	0.5745	0	0	0	0.8485	Ref
GU573886	Unassigned	Unassigned	Unassigned	28_1	Clustered	53	VC_28_1	53	0.7164	1	0.7164	0	0	0	0.9325	Ref
GU580941	Unassigned	Unassigned	Unassigned	799_0	Clustered	2	VC_799_0	2	0.0367	0.95547665	0.035	0	0	0	1	Ref
GU580942	Unassigned	Unassigned	Unassigned	799_0	Clustered	2	VC_799_0	2	0.0367	0.95547665	0.035	0	0	0	1	Ref
GU583987	Unassigned	Unassigned	Unassigned	130_0	Clustered	19	VC_130_0	19	0.6068	1	0.6068	0	0	0	1	Ref
GU595417	Unassigned	Unassigned	Unassigned	452_0	Clustered	66	VC_452_0	66	0.9248	1	0.9248	0	0	0	1	Ref
GU815091	Unassigned	Unassigned	Unassigned	79_7	Clustered	33	VC_79_7	33	0.216	1	0.216	0	0	0	0.8409	Ref
GU903191	Unassigned	Unassigned	Unassigned	125_0	Clustered	17	VC_125_0	17	0.9244	1	0.9244	0	0	0	1	Ref
GU911303	Unassigned	Unassigned	Unassigned	1_0	Clustered	54	VC_1_0	54	0.8527	1	0.8527	0	0	0	1	Ref
GU911304	Unassigned	Unassigned	Unassigned	3_0	Clustered	17	VC_3_0	17	0.5639	1	0.5639	0	0	0	1	Ref
GU911519	Unassigned	Unassigned	Unassigned	492_3	Clustered/Singleton	1	VC_492_3	1	0	1	0	0	0	0	1	Ref
GU936715	Unassigned	Unassigned	Unassigned	873_0	Clustered	3	VC_873_0	3	0.9107	0.98376334	0.8959	0	0	0	1	Ref
GU942563	Unassigned	Unassigned	Unassigned	875_0	Clustered	10	VC_875_0	10	0.988	0.99948433	0.9875	0	0	0	1	Ref
GU943054	Unassigned	Unassigned	Unassigned	340_0	Clustered	31	VC_340_0	31	0.9549	1	0.9549	0	0	0	1	Ref
GU988610	Unassigned	Unassigned	Unassigned	76_0	Clustered	37	VC_76_0	37	0.9403	1	0.9403	0	0	0	1	Ref
HE584812	Unassigned	Unassigned	Unassigned	179_0	Clustered	53	VC_179_0	53	0.9137	1	0.9137	0	0	0	1	Ref
HE600015	Unassigned	Unassigned	Unassigned	131_0	Clustered	17	VC_131_0	17	0.2671	1	0.2671	0	0	0	1	Ref
HE608841	Unassigned	Unassigned	Unassigned	830_0	Clustered	6	VC_830_0	6	0.6536	1	0.6536	0	0	0	1	Ref
HE664024	Unassigned	Unassigned	Unassigned	42_0	Clustered	49	VC_42_0	49	0.4517	1	0.4517	0	0	0	0.8597	Ref
HE775250	Unassigned	Unassigned	Unassigned	637_0	Clustered	115	VC_637_0	115	0.731	1	0.731	0	0	0	0.8764	Ref
HE806280	Unassigned	Unassigned	Unassigned	698_0	Clustered	26	VC_698_0	26	0.9366	1	0.9366	0	0	0	1	Ref
HE815464	Unassigned	Unassigned	Unassigned	843_0	Clustered	5	VC_843_0	5	0.2843	0.99999994	0.2843	0	0	0	1	Ref
HE858210	Unassigned	Unassigned	Unassigned	669_1	Clustered	10	VC_669_1	10	0.1268	1	0.1268	0	0	0	0.9778	Ref
HE861935	Unassigned	Unassigned	Unassigned	254_0	Clustered	46	VC_254_0	46	0.5159	1	0.5159	0	0	0	1	Ref
HE956704	Unassigned	Unassigned	Unassigned	308_0	Clustered	4	VC_308_0	4	0.5014	0.99997418	0.5013	0	0	0	1	Ref
HE956707	Unassigned	Unassigned	Unassigned	878_0	Clustered	32	VC_878_0	32	0.9965	1	0.9965	0	0	0	1	Ref
HE956708	Unassigned	Unassigned	Unassigned	191_26	Clustered/Singleton	1	VC_191_26	1	0	1	0	0	0	0	1	Ref
HE956709	Unassigned	Unassigned	Unassigned	490_3	Clustered	2	VC_490_3	2	0.0135	0.95703149	0.0129	0	0	0	1	Ref
HE956710	Unassigned	Unassigned	Unassigned	878_0	Clustered	32	VC_878_0	32	0.9965	1	0.9965	0	0	0	1	Ref
HE956711	Unassigned	Unassigned	Unassigned	283_69	Clustered/Singleton	1	VC_283_69	1	0	1	0	0	0	0	1	Ref
HE962497	Unassigned	Unassigned	Unassigned	108_0	Clustered	33	VC_108_0	33	0.949	1	0.949	0	0	0	1	Ref
HE978309	Unassigned	Unassigned	Unassigned	590_0	Clustered	27	VC_590_0	27	0.9947	1	0.9947	0	0	0	1	Ref
HE981739	Unassigned	Unassigned	Unassigned	669_1	Clustered	10	VC_669_1	10	0.1268	1	0.1268	0	0	0	0.9778	Ref
HE983844	Unassigned	Unassigned	Unassigned	472_0	Clustered	47	VC_472_0	47	0.9025	1	0.9025	0	0	0	1	Ref
HE983845	Unassigned	Unassigned	Unassigned	76_0	Clustered	37	VC_76_0	37	0.9403	1	0.9403	0	0	0	1	Ref
HF543949	Unassigned	Unassigned	Unassigned	472_0	Clustered	47	VC_472_0	47	0.9025	1	0.9025	0	0	0	1	Ref
HF563658	Unassigned	Unassigned	Unassigned	412_0	Clustered	39	VC_412_0	39	0.845	1	0.845	0	0	0	1	Ref
HF569088	Unassigned	Unassigned	Unassigned	879_0	Clustered	36	VC_879_0	36	0.994	1	0.994	0	0	0	1	Ref
HF569089	Unassigned	Unassigned	Unassigned	879_0	Clustered	36	VC_879_0	36	0.994	1	0.994	0	0	0	1	Ref
HF569090	Unassigned	Unassigned	Unassigned	879_0	Clustered	36	VC_879_0	36	0.994	1	0.994	0	0	0	1	Ref
HF569091	Unassigned	Unassigned	Unassigned	879_0	Clustered	36	VC_879_0	36	0.994	1	0.994	0	0	0	1	Ref
HF569092	Unassigned	Unassigned	Unassigned	879_0	Clustered	36	VC_879_0	36	0.994	1	0.994	0	0	0	1	Ref
HF937074	Unassigned	Unassigned	Unassigned	96_0	Clustered	30	VC_96_0	30	0.718	1	0.718	0	0	0	1	Ref
HG007973	Unassigned	Unassigned	Unassigned	880_0	Clustered	2	VC_880_0	2	0.1614	0.83455073	0.1347	0	0	0	1	Ref
HG315669	Unassigned	Unassigned	Unassigned	177_0	Clustered	5	VC_177_0	5	0.7468	0.99885646	0.746	0	0	0	1	Ref
HG322870	Unassigned	Unassigned	Unassigned	446_0	Clustered	4	VC_446_0	4	0.754	0.99987768	0.7539	0	0	0	1	Ref
HG380752	Unassigned	Unassigned	Unassigned	254_0	Clustered	46	VC_254_0	46	0.5159	1	0.5159	0	0	0	1	Ref
HG424323	Unassigned	Unassigned	Unassigned	254_0	Clustered	46	VC_254_0	46	0.5159	1	0.5159	0	0	0	1	Ref
HG428758	Unassigned	Unassigned	Unassigned	879_0	Clustered	36	VC_879_0	36	0.994	1	0.994	0	0	0	1	Ref
HG518155	Unassigned	Unassigned	Unassigned	472_0	Clustered	47	VC_472_0	47	0.9025	1	0.9025	0	0	0	1	Ref
HG531805	Unassigned	Unassigned	Unassigned	139_1	Clustered	4	VC_139_1	4	0.1775	0.99998609	0.1775	0	0	0	1	Ref
HG792102	Unassigned	Unassigned	Unassigned	42_0	Clustered	49	VC_42_0	49	0.4517	1	0.4517	0	0	0	0.8597	Ref
HG792103	Unassigned	Unassigned	Unassigned	42_0	Clustered	49	VC_42_0	49	0.4517	1	0.4517	0	0	0	0.8597	Ref
HG792104	Unassigned	Unassigned	Unassigned	42_0	Clustered	49	VC_42_0	49	0.4517	1	0.4517	0	0	0	0.8597	Ref
HG792105	Unassigned	Unassigned	Unassigned	42_0	Clustered	49	VC_42_0	49	0.4517	1	0.4517	0	0	0	0.8597	Ref
HG793132	Unassigned	Unassigned	Unassigned	70_0	Clustered	27	VC_70_0	27	0.5981	1	0.5981	0	0	0	0.9886	Ref
HG796219	Unassigned	Unassigned	Unassigned	70_0	Clustered	27	VC_70_0	27	0.5981	1	0.5981	0	0	0	0.9886	Ref
HG796220	Unassigned	Unassigned	Unassigned	70_0	Clustered	27	VC_70_0	27	0.5981	1	0.5981	0	0	0	0.9886	Ref
HG796221	Unassigned	Unassigned	Unassigned	70_0	Clustered	27	VC_70_0	27	0.5981	1	0.5981	0	0	0	0.9886	Ref
HG796225	Unassigned	Unassigned	Unassigned	647_0	Clustered	11	VC_647_0	11	0.598	1	0.598	0	0	0	1	Ref
HG798901	Unassigned	Unassigned	Unassigned	647_0	Clustered	11	VC_647_0	11	0.598	1	0.598	0	0	0	1	Ref
HG799490	Unassigned	Unassigned	Unassigned	414_0	Clustered	37	VC_414_0	37	0.585	1	0.585	0	0	0	0.9474	Ref
HG799496	Unassigned	Unassigned	Unassigned	414_0	Clustered	37	VC_414_0	37	0.585	1	0.585	0	0	0	0.9474	Ref
HG799497	Unassigned	Unassigned	Unassigned	414_0	Clustered	37	VC_414_0	37	0.585	1	0.585	0	0	0	0.9474	Ref
HG803181	Unassigned	Unassigned	Unassigned	5_0	Clustered	30	VC_5_0	30	0.9647	1	0.9647	0	0	0	1	Ref
HG803182	Unassigned	Unassigned	Unassigned	42_0	Clustered	49	VC_42_0	49	0.4517	1	0.4517	0	0	0	0.8597	Ref

HG803183	Unassigned	Unassigned	Unassigned	42_0	Clustered	49	VC_42_0	49	0.4517	1	0.4517	0	0	0	0.8597	Ref
HG803184	Unassigned	Unassigned	Unassigned	42_0	Clustered	49	VC_42_0	49	0.4517	1	0.4517	0	0	0	0.8597	Ref
HG803185	Unassigned	Unassigned	Unassigned	42_0	Clustered	49	VC_42_0	49	0.4517	1	0.4517	0	0	0	0.8597	Ref
HG813241	Unassigned	Unassigned	Unassigned	451_0	Clustered	80	VC_451_0	80	0.9991	1	0.9991	0	0	0	1	Ref
HG818823	Unassigned	Unassigned	Unassigned	451_0	Clustered	80	VC_451_0	80	0.9991	1	0.9991	0	0	0	1	Ref
HG818824	Unassigned	Unassigned	Unassigned	451_0	Clustered	80	VC_451_0	80	0.9991	1	0.9991	0	0	0	1	Ref
HG934469	Unassigned	Unassigned	Unassigned	637_0	Clustered	115	VC_637_0	115	0.731	1	0.731	0	0	0	0.8764	Ref
HG934470	Unassigned	Unassigned	Unassigned	637_0	Clustered	115	VC_637_0	115	0.731	1	0.731	0	0	0	0.8764	Ref
HG962375	Unassigned	Unassigned	Unassigned	838_0	Clustered	29	VC_838_0	29	0.8611	1	0.8611	0	0	0	1	Ref
HG962376	Unassigned	Unassigned	Unassigned	749_0	Clustered	40	VC_749_0	40	0.7517	1	0.7517	0	0	0	1	Ref
HG992588	Unassigned	Unassigned	Unassigned	882_0	Clustered	20	VC_882_0	20	0.9917	1	0.9917	0	0	0	1	Ref
HG994092	Unassigned	Unassigned	Unassigned	823_0	Clustered	124	VC_823_0	124	0.9492	1	0.9492	0	0	0	1	Ref
HM004124	Unassigned	Unassigned	Unassigned	492_4	Clustered/Singleton	1	VC_492_4	1	0	1	0	0	0	0	1	Ref
HM029250	Unassigned	Unassigned	Unassigned	474_0	Clustered	14	VC_474_0	14	0.8466	1	0.8466	0	0	0	1	Ref
HM032710	Unassigned	Unassigned	Unassigned	492_5	Clustered/Singleton	1	VC_492_5	1	0	1	0	0	0	0	1	Ref
HM035024	Unassigned	Unassigned	Unassigned	575_0	Clustered	35	VC_575_0	35	0.8815	1	0.8815	0	0	0	1	Ref
HM035025	Unassigned	Unassigned	Unassigned	283_154	Clustered/Singleton	1	VC_283_154	1	0	1	0	0	0	0	1	Ref
HM064452	Unassigned	Unassigned	Unassigned	53_0	Clustered	5	VC_53_0	5	0.4977	0.99999808	0.4977	0	0	0	1	Ref
HM066936	Unassigned	Unassigned	Unassigned	227_0	Clustered	2	VC_227_0	2	0.3333	0	0	0	0	0	1	Ref
HM071924	Unassigned	Unassigned	Unassigned	290_5	Clustered	14	VC_290_5	14	0.1422	1	0.1422	0	0	0	0.9231	Ref
HM072038	Unassigned	Unassigned	Unassigned	18_0	Clustered	4	VC_18_0	4	0.9098	0.99982347	0.9096	0	0	0	1	Ref
HM114315	Unassigned	Unassigned	Unassigned	492_6	Clustered/Singleton	1	VC_492_6	1	0	1	0	0	0	0	1	Ref
HM134276	Unassigned	Unassigned	Unassigned	669_1	Clustered	10	VC_669_1	10	0.1268	1	0.1268	0	0	0	0.9778	Ref
HM137666	Unassigned	Unassigned	Unassigned	283_91	Clustered	11	VC_283_91	11	0.0285	1	0.0285	0	0	0	1	Ref
HM144387	Unassigned	Unassigned	Unassigned	884_1	Clustered	8	VC_884_1	8	0.1102	0.9989323	0.1101	0	0	0	0.75	Ref
HM150760	Unassigned	Unassigned	Unassigned	880_0	Clustered	2	VC_880_0	2	0.1614	0.83455073	0.1347	0	0	0	1	Ref
HM152763	Unassigned	Unassigned	Unassigned	887_0	Clustered	18	VC_887_0	18	0.3663	1	0.3663	0	0	0	1	Ref
HM152764	Unassigned	Unassigned	Unassigned	236_0	Clustered	72	VC_236_0	72	0.8104	1	0.8104	0	0	0	1	Ref
HM152765	Unassigned	Unassigned	Unassigned	518_0	Clustered	6	VC_518_0	6	0.1295	1	0.1295	0	0	0	1	Ref
HM152766	Unassigned	Unassigned	Unassigned	517_1	Clustered	108	VC_517_1	108	0.8483	1	0.8483	0	0	0	0.9955	Ref
HM152767	Unassigned	Unassigned	Unassigned	236_0	Clustered	72	VC_236_0	72	0.8104	1	0.8104	0	0	0	1	Ref
HM208303	Unassigned	Unassigned	Unassigned	39_0	Clustered	35	VC_39_0	35	0.4337	1	0.4337	0	0	0	1	Ref
HM208537	Unassigned	Unassigned	Unassigned	230_1	Clustered/Singleton	1	VC_230_1	1	0	1	0	0	0	0	1	Ref
HM242243	Unassigned	Unassigned	Unassigned	94_0	Clustered	2	VC_94_0	2	0.1304	0.95723134	0.1248	0	0	0	1	Ref
HM368260	Unassigned	Unassigned	Unassigned	698_0	Clustered	26	VC_698_0	26	0.9366	1	0.9366	0	0	0	1	Ref
HM452125	Unassigned	Unassigned	Unassigned	600_2	Clustered	10	VC_600_2	10	0.3041	1	0.3041	0	0	0	1	Ref
HM461982	Unassigned	Unassigned	Unassigned	3_0	Clustered	17	VC_3_0	17	0.5639	1	0.5639	0	0	0	1	Ref
HM480106	Unassigned	Unassigned	Unassigned	873_0	Clustered	3	VC_873_0	3	0.9107	0.98376334	0.8959	0	0	0	1	Ref
HM480846	Unassigned	Unassigned	Unassigned	823_0	Clustered	124	VC_823_0	124	0.9492	1	0.9492	0	0	0	1	Ref
HM559717	Unassigned	Unassigned	Unassigned	340_0	Clustered	31	VC_340_0	31	0.9549	1	0.9549	0	0	0	1	Ref
HM563683	Unassigned	Unassigned	Unassigned	290_2	Clustered	2	VC_290_2	2	0.0102	0.92371007	0.0094	0	0	0	1	Ref
HM568888	Unassigned	Unassigned	Unassigned	733_0	Clustered	5	VC_733_0	5	0.5111	0.99999994	0.5111	0	0	0	1	Ref
HM588722	Unassigned	Unassigned	Unassigned	894_0	Clustered	3	VC_894_0	3	0.6358	0.99692152	0.6338	0	0	0	1	Ref
HM624080	Unassigned	Unassigned	Unassigned	179_0	Clustered	53	VC_179_0	53	0.9137	1	0.9137	0	0	0	1	Ref
HM640230	Unassigned	Unassigned	Unassigned	897_0	Clustered	6	VC_897_0	6	0.4156	1	0.4156	0	0	0	1	Ref
HM753658	Unassigned	Unassigned	Unassigned	303_0	Clustered	155	VC_303_0	155	0.9979	1	0.9979	0	0	0	1	Ref
HM753659	Unassigned	Unassigned	Unassigned	303_0	Clustered	155	VC_303_0	155	0.9979	1	0.9979	0	0	0	1	Ref
HM753660	Unassigned	Unassigned	Unassigned	303_0	Clustered	155	VC_303_0	155	0.9979	1	0.9979	0	0	0	1	Ref
HM753661	Unassigned	Unassigned	Unassigned	303_0	Clustered	155	VC_303_0	155	0.9979	1	0.9979	0	0	0	1	Ref
HM753662	Unassigned	Unassigned	Unassigned	303_0	Clustered	155	VC_303_0	155	0.9979	1	0.9979	0	0	0	1	Ref
HM753663	Unassigned	Unassigned	Unassigned	303_0	Clustered	155	VC_303_0	155	0.9979	1	0.9979	0	0	0	1	Ref
HM753664	Unassigned	Unassigned	Unassigned	303_0	Clustered	155	VC_303_0	155	0.9979	1	0.9979	0	0	0	1	Ref
HM753665	Unassigned	Unassigned	Unassigned	303_0	Clustered	155	VC_303_0	155	0.9979	1	0.9979	0	0	0	1	Ref
HM753666	Unassigned	Unassigned	Unassigned	303_0	Clustered	155	VC_303_0	155	0.9979	1	0.9979	0	0	0	1	Ref
HM753667	Unassigned	Unassigned	Unassigned	303_0	Clustered	155	VC_303_0	155	0.9979	1	0.9979	0	0	0	1	Ref
HM753668	Unassigned	Unassigned	Unassigned	303_0	Clustered	155	VC_303_0	155	0.9979	1	0.9979	0	0	0	1	Ref
HM753669	Unassigned	Unassigned	Unassigned	303_0	Clustered	155	VC_303_0	155	0.9979	1	0.9979	0	0	0	1	Ref
HM753670	Unassigned	Unassigned	Unassigned	303_0	Clustered	155	VC_303_0	155	0.9979	1	0.9979	0	0	0	1	Ref
HM753671	Unassigned	Unassigned	Unassigned	303_0	Clustered	155	VC_303_0	155	0.9979	1	0.9979	0	0	0	1	Ref
HM753672	Unassigned	Unassigned	Unassigned	303_0	Clustered	155	VC_303_0	155	0.9979	1	0.9979	0	0	0	1	Ref
HM753673	Unassigned	Unassigned	Unassigned	303_0	Clustered	155	VC_303_0	155	0.9979	1	0.9979	0	0	0	1	Ref
HM753674	Unassigned	Unassigned	Unassigned	303_0	Clustered	155	VC_303_0	155	0.9979	1	0.9979	0	0	0	1	Ref
HM753675	Unassigned	Unassigned	Unassigned	303_0	Clustered	155	VC_303_0	155	0.9979	1	0.9979	0	0	0	1	Ref
HM753676	Unassigned	Unassigned	Unassigned	303_0	Clustered	155	VC_303_0	155	0.9979	1	0.9979	0	0	0	1	Ref
HM753677	Unassigned	Unassigned	Unassigned	303_0	Clustered	155	VC_303_0	155	0.9979	1	0.9979	0	0	0	1	Ref
HM753678	Unassigned	Unassigned	Unassigned	303_0	Clustered	155	VC_303_0	155	0.9979	1	0.9979	0	0	0	1	Ref
HM753679	Unassigned	Unassigned	Unassigned	303_0	Clustered	155	VC_303_0	155	0.9979	1	0.9979	0	0	0	1	Ref
HM753680	Unassigned	Unassigned	Unassigned	303_0	Clustered	155	VC_303_0	155	0.9979	1	0.9979	0	0	0	1	Ref
HM753681	Unassigned	Unassigned	Unassigned	303_0	Clustered	155	VC_303_0	155	0.9979	1	0.9979	0	0	0	1	Ref
HM753682	Unassigned	Unassigned	Unassigned	303_0	Clustered	155	VC_303_0	155	0.9979	1	0.9979	0	0	0	1	Ref
HM753683	Unassigned	Unassigned	Unassigned	303_0	Clustered	155	VC_303_0	155	0.9979	1	0.9979	0	0	0	1	Ref
HM753684	Unassigned	Unassigned	Unassigned	303_0	Clustered	155	VC_303_0	155	0.9979	1	0.9979	0	0	0	1	Ref
HM753685	Unassigned	Unassigned	Unassigned	303_0	Clustered	155	VC_303_0	155	0.9979	1	0.9979	0	0	0	1	Ref
HM753686	Unassigned	Unassigned	Unassigned	303_0	Clustered	155	VC_303_0	155	0.9979	1	0.9979	0	0	0	1	Ref
HM753687	Unassigned	Unassigned	Unassigned	303_0	Clustered	155	VC_303_0	155	0.9979	1	0.9979	0	0	0	1	Ref
HM753688	Unassigned	Unassigned	Unassigned	303_0	Clustered	155	VC_303_0	155	0.9979	1	0.9979	0	0	0	1	Ref
HM753689	Unassigned	Unassigned	Unassigned	303_0	Clustered	155	VC_303_0	155	0.9979	1	0.9979	0	0	0	1	Ref
HM753690	Unassigned	Unassigned	Unassigned	303_0	Clustered	155	VC_303_0	155	0.9979	1	0.9979	0	0	0	1	Ref
HM753691	Unassigned	Unassigned	Unassigned	303_0	Clustered	155	VC_303_0	155	0.9979	1	0.9979	0	0	0	1	Ref
HM753692	Unassigned	Unassigned	Unassigned	303_0	Clustered	155	VC_303_0	155	0.9979	1	0.9979	0	0	0	1	Ref
HM753693	Unassigned	Unassigned	Unassigned	303_0	Clustered	155	VC_303_0	155	0.9979	1	0.9979	0	0	0	1	Ref

HM753694	Unassigned	Unassigned	Unassigned	303_0	Clustered	155	VC_303_0	155	0.9979	1	0.9979	0	0	0	1	Ref
HM753695	Unassigned	Unassigned	Unassigned	303_0	Clustered	155	VC_303_0	155	0.9979	1	0.9979	0	0	0	1	Ref
HM753696	Unassigned	Unassigned	Unassigned	303_0	Clustered	155	VC_303_0	155	0.9979	1	0.9979	0	0	0	1	Ref
HM753697	Unassigned	Unassigned	Unassigned	303_0	Clustered	155	VC_303_0	155	0.9979	1	0.9979	0	0	0	1	Ref
HM753698	Unassigned	Unassigned	Unassigned	303_0	Clustered	155	VC_303_0	155	0.9979	1	0.9979	0	0	0	1	Ref
HM753699	Unassigned	Unassigned	Unassigned	303_0	Clustered	155	VC_303_0	155	0.9979	1	0.9979	0	0	0	1	Ref
HM753700	Unassigned	Unassigned	Unassigned	303_0	Clustered	155	VC_303_0	155	0.9979	1	0.9979	0	0	0	1	Ref
HM753701	Unassigned	Unassigned	Unassigned	303_0	Clustered	155	VC_303_0	155	0.9979	1	0.9979	0	0	0	1	Ref
HM753702	Unassigned	Unassigned	Unassigned	303_0	Clustered	155	VC_303_0	155	0.9979	1	0.9979	0	0	0	1	Ref
HM753703	Unassigned	Unassigned	Unassigned	303_0	Clustered	155	VC_303_0	155	0.9979	1	0.9979	0	0	0	1	Ref
HM753704	Unassigned	Unassigned	Unassigned	303_0	Clustered	155	VC_303_0	155	0.9979	1	0.9979	0	0	0	1	Ref
HM753705	Unassigned	Unassigned	Unassigned	303_0	Clustered	155	VC_303_0	155	0.9979	1	0.9979	0	0	0	1	Ref
HM753706	Unassigned	Unassigned	Unassigned	303_0	Clustered	155	VC_303_0	155	0.9979	1	0.9979	0	0	0	1	Ref
HM753707	Unassigned	Unassigned	Unassigned	303_0	Clustered	155	VC_303_0	155	0.9979	1	0.9979	0	0	0	1	Ref
HM753708	Unassigned	Unassigned	Unassigned	303_0	Clustered	155	VC_303_0	155	0.9979	1	0.9979	0	0	0	1	Ref
HM753709	Unassigned	Unassigned	Unassigned	303_0	Clustered	155	VC_303_0	155	0.9979	1	0.9979	0	0	0	1	Ref
HM753710	Unassigned	Unassigned	Unassigned	303_0	Clustered	155	VC_303_0	155	0.9979	1	0.9979	0	0	0	1	Ref
HM753711	Unassigned	Unassigned	Unassigned	303_0	Clustered	155	VC_303_0	155	0.9979	1	0.9979	0	0	0	1	Ref
HM753712	Unassigned	Unassigned	Unassigned	303_0	Clustered	155	VC_303_0	155	0.9979	1	0.9979	0	0	0	1	Ref
HM753713	Unassigned	Unassigned	Unassigned	303_0	Clustered	155	VC_303_0	155	0.9979	1	0.9979	0	0	0	1	Ref
HM753714	Unassigned	Unassigned	Unassigned	303_0	Clustered	155	VC_303_0	155	0.9979	1	0.9979	0	0	0	1	Ref
HM753715	Unassigned	Unassigned	Unassigned	303_0	Clustered	155	VC_303_0	155	0.9979	1	0.9979	0	0	0	1	Ref
HM753716	Unassigned	Unassigned	Unassigned	303_0	Clustered	155	VC_303_0	155	0.9979	1	0.9979	0	0	0	1	Ref
HM753717	Unassigned	Unassigned	Unassigned	303_0	Clustered	155	VC_303_0	155	0.9979	1	0.9979	0	0	0	1	Ref
HM753718	Unassigned	Unassigned	Unassigned	303_0	Clustered	155	VC_303_0	155	0.9979	1	0.9979	0	0	0	1	Ref
HM755814	Unassigned	Unassigned	Unassigned	514_0	Clustered	75	VC_514_0	75	0.9402	1	0.9402	0	0	0	1	Ref
HM770079	Unassigned	Unassigned	Unassigned	6_0	Clustered	44	VC_6_0	44	0.908	1	0.908	0	0	0	1	Ref
HM775306	Unassigned	Unassigned	Unassigned	303_0	Clustered	155	VC_303_0	155	0.9979	1	0.9979	0	0	0	1	Ref
HM775307	Unassigned	Unassigned	Unassigned	303_0	Clustered	155	VC_303_0	155	0.9979	1	0.9979	0	0	0	1	Ref
HM775308	Unassigned	Unassigned	Unassigned	303_0	Clustered	155	VC_303_0	155	0.9979	1	0.9979	0	0	0	1	Ref
HM775309	Unassigned	Unassigned	Unassigned	303_0	Clustered	155	VC_303_0	155	0.9979	1	0.9979	0	0	0	1	Ref
HM775310	Unassigned	Unassigned	Unassigned	303_0	Clustered	155	VC_303_0	155	0.9979	1	0.9979	0	0	0	1	Ref
HM775311	Unassigned	Unassigned	Unassigned	303_0	Clustered	155	VC_303_0	155	0.9979	1	0.9979	0	0	0	1	Ref
HM997019	Unassigned	Unassigned	Unassigned	812_0	Clustered	3	VC_812_0	3	0.1174	0.99842379	0.1172	0	0	0	1	Ref
HM997020	Unassigned	Unassigned	Unassigned	283_2	Clustered	2	VC_283_2	2	0.0027	0.93842619	0.0025	0	0	0	1	Ref
HQ110083	Unassigned	Unassigned	Unassigned	5_0	Clustered	30	VC_5_0	30	0.9647	1	0.9647	0	0	0	1	Ref
HQ110084	Unassigned	Unassigned	Unassigned	228_0	Clustered	60	VC_228_0	60	0.942	1	0.942	0	0	0	1	Ref
HQ127381	Unassigned	Unassigned	Unassigned	9_0	Clustered	38	VC_9_0	38	0.4895	1	0.4895	0	0	0	1	Ref
HQ141410	Unassigned	Unassigned	Unassigned	381_0	Clustered	2	VC_381_0	2	0.0829	0.95381392	0.079	0	0	0	1	Ref
HQ141411	Unassigned	Unassigned	Unassigned	300_0	Clustered	3	VC_300_0	3	0.1218	0.99841701	0.1216	0	0	0	0.6667	Ref
HQ163896	Unassigned	Unassigned	Unassigned	149_8	Clustered	66	VC_149_8	66	0.4565	1	0.4565	0	0	0	0.8182	Ref
HQ186308	Unassigned	Unassigned	Unassigned	699_0	Clustered	71	VC_699_0	71	0.9352	1	0.9352	0	0	0	1	Ref
HQ201307	Unassigned	Unassigned	Unassigned	33_0	Clustered	4	VC_33_0	4	0.06	0.99994826	0.06	0	0	0	1	Ref
HQ201308	Unassigned	Unassigned	Unassigned	31_2	Clustered/Singleton	1	VC_31_2	1	0	1	0	0	0	0	1	Ref
HQ224500	Unassigned	Unassigned	Unassigned	875_0	Clustered	10	VC_875_0	10	0.988	0.99948433	0.9875	0	0	0	1	Ref
HQ259103	Unassigned	Unassigned	Unassigned	133_31	Clustered	6	VC_133_31	6	0.0263	0.99999694	0.0263	0	0	0	1	Ref
HQ259105	Unassigned	Unassigned	Unassigned	779_0	Clustered	37	VC_779_0	37	0.5682	1	0.5682	0	0	0	1	Ref
HQ268735	Unassigned	Unassigned	Unassigned	891_0	Clustered	2	VC_891_0	2	0.5746	0.92263554	0.5301	0	0	0	1	Ref
HQ316579	Unassigned	Unassigned	Unassigned	493_14	Clustered/Singleton	1	VC_493_14	1	0	1	0	0	0	0	1	Ref
HQ316581	Unassigned	Unassigned	Unassigned	248_0	Clustered	11	VC_248_0	11	0.7031	1	0.7031	0	0	0	1	Ref
HQ316582	Unassigned	Unassigned	Unassigned	902_0	Clustered	3	VC_902_0	3	0.9978	0.87589346	0.8739	0	0	0	1	Ref
HQ316583	Unassigned	Unassigned	Unassigned	656_0	Clustered	2	VC_656_0	2	0.0089	0.92256518	0.0082	0	0	0	1	Ref
HQ316584	Unassigned	Unassigned	Unassigned	340_0	Clustered	31	VC_340_0	31	0.9549	1	0.9549	0	0	0	1	Ref
HQ316603	Unassigned	Unassigned	Unassigned	656_0	Clustered	2	VC_656_0	2	0.0089	0.92256518	0.0082	0	0	0	1	Ref
HQ316604	Unassigned	Unassigned	Unassigned	904_1	Clustered	4	VC_904_1	4	0.1486	0.99997723	0.1486	0	0	0	1	Ref
HQ317290	Unassigned	Unassigned	Unassigned	662_0	Clustered	62	VC_662_0	62	0.6976	1	0.6976	0	0	0	1	Ref
HQ317291	Unassigned	Unassigned	Unassigned	662_0	Clustered	62	VC_662_0	62	0.6976	1	0.6976	0	0	0	1	Ref
HQ317292	Unassigned	Unassigned	Unassigned	662_0	Clustered	62	VC_662_0	62	0.6976	1	0.6976	0	0	0	1	Ref
HQ317384	Unassigned	Unassigned	Unassigned	907_0	Clustered	3	VC_907_0	3	0.6642	0.99633114	0.6618	0	0	0	1	Ref
HQ317386	Unassigned	Unassigned	Unassigned	909_0	Clustered	5	VC_909_0	5	0.7406	0.9999861	0.7406	0	0	0	1	Ref
HQ317387	Unassigned	Unassigned	Unassigned	833_0	Clustered	16	VC_833_0	16	0.9351	1	0.9351	0	0	0	1	Ref
HQ317388	Unassigned	Unassigned	Unassigned	340_0	Clustered	31	VC_340_0	31	0.9549	1	0.9549	0	0	0	1	Ref
HQ317389	Unassigned	Unassigned	Unassigned	340_0	Clustered	31	VC_340_0	31	0.9549	1	0.9549	0	0	0	1	Ref
HQ317390	Unassigned	Unassigned	Unassigned	910_0	Clustered	2	VC_910_0	2	0.9891	0.5	0.4946	0	0	0	1	Ref
HQ317391	Unassigned	Unassigned	Unassigned	432_10	Clustered	2	VC_432_10	2	0.0216	0.95771017	0.0207	0	0	0	1	Ref
HQ317392	Unassigned	Unassigned	Unassigned	911_0	Clustered	6	VC_911_0	6	0.2787	1	0.2787	0	0	0	1	Ref
HQ317393	Unassigned	Unassigned	Unassigned	531_2	Clustered/Singleton	1	VC_531_2	1	0	1	0	0	0	0	1	Ref
HQ332136	Unassigned	Unassigned	Unassigned	913_0	Clustered	2	VC_913_0	2	0.1042	0.82306775	0.0858	0	0	0	1	Ref
HQ332137	Unassigned	Unassigned	Unassigned	340_0	Clustered	31	VC_340_0	31	0.9549	1	0.9549	0	0	0	1	Ref
HQ332138	Unassigned	Unassigned	Unassigned	734_0	Clustered	6	VC_734_0	6	0.1914	0.99996888	0.1914	0	0	0	1	Ref
HQ332140	Unassigned	Unassigned	Unassigned	340_0	Clustered	31	VC_340_0	31	0.9549	1	0.9549	0	0	0	1	Ref
HQ332141	Unassigned	Unassigned	Unassigned	351_0	Clustered	3	VC_351_0	3	0.8899	0.98155579	0.8735	0	0	0	1	Ref
HQ332142	Unassigned	Unassigned	Unassigned	907_0	Clustered	3	VC_907_0	3	0.6642	0.99633114	0.6618	0	0	0	1	Ref
HQ333270	Unassigned	Unassigned	Unassigned	386_0	Clustered	49	VC_386_0	49	0.7941	1	0.7941	0	0	0	1	Ref
HQ337021	Unassigned	Unassigned	Unassigned	655_2	Clustered	3	VC_655_2	3	0.0231	0.99860756	0.0231	0	0	0	1	Ref
HQ337022	Unassigned	Unassigned	Unassigned	340_0	Clustered	31	VC_340_0	31	0.9549	1	0.9549	0	0	0	1	Ref
HQ377372	Unassigned	Unassigned	Unassigned	730_0	Clustered	9	VC_730_0	9	1	1	1	0	0	0	1	Ref
HQ377373	Unassigned	Unassigned	Unassigned	730_0	Clustered	9	VC_730_0	9	1	1	1	0	0	0	1	Ref
HQ396194	Unassigned	Unassigned	Unassigned	916_0	Clustered	4	VC_916_0	4	0.8472	0.99985574	0.8471	0	0	0	1	Ref
HQ403646	Unassigned	Unassigned	Unassigned	919_0	Clustered	39	VC_919_0	39	0.9069	1	0.9069	0	0	0	1	Ref
HQ406778	Unassigned	Unassigned	Unassigned	190_23	Clustered	10	VC_190_23	10	0.0704	1	0.0704	0	0	0	0.8444	Ref

HQ424691	Unassigned	Unassigned	Unassigned	42_0	Clustered	49	VC_42_0	49	0.4517	1	0.4517	0	0	0	0.8597	Ref
HQ630627	Unassigned	Unassigned	Unassigned	344_6	Clustered/Singleton	1	VC_344_6	1	0	1	0	0	0	0	1	Ref
HQ632825	Unassigned	Unassigned	Unassigned	432_9	Clustered	3	VC_432_9	3	0.0372	0.99851312	0.0371	0	0	0	1	Ref
HQ632857	Unassigned	Unassigned	Unassigned	921_0	Clustered	2	VC_921_0	2	0.948	0.72985431	0.6919	0	0	0	1	Ref
HQ632858	Unassigned	Unassigned	Unassigned	921_0	Clustered	2	VC_921_0	2	0.948	0.72985431	0.6919	0	0	0	1	Ref
HQ632859	Unassigned	Unassigned	Unassigned	598_0	Clustered	4	VC_598_0	4	0.0597	0.99355358	0.0593	0	0	0	1	Ref
HQ632860	Unassigned	Unassigned	Unassigned	642_0	Clustered	7	VC_642_0	7	0.1508	1	0.1508	0	0	0	0.7619	Ref
HQ633062	Unassigned	Unassigned	Unassigned	340_0	Clustered	31	VC_340_0	31	0.9549	1	0.9549	0	0	0	1	Ref
HQ633071	Unassigned	Unassigned	Unassigned	432_11	Clustered/Singleton	1	VC_432_11	1	0	1	0	0	0	0	1	Ref
HQ634152	Unassigned	Unassigned	Unassigned	340_0	Clustered	31	VC_340_0	31	0.9549	1	0.9549	0	0	0	1	Ref
HQ634156	Unassigned	Unassigned	Unassigned	493_10	Clustered/Singleton	1	VC_493_10	1	0	1	0	0	0	0	1	Ref
HQ634157	Unassigned	Unassigned	Unassigned	493_7	Clustered/Singleton	1	VC_493_7	1	0	1	0	0	0	0	1	Ref
HQ634175	Unassigned	Unassigned	Unassigned	655_3	Clustered/Singleton	1	VC_655_3	1	0	1	0	0	0	0	1	Ref
HQ634176	Unassigned	Unassigned	Unassigned	655_2	Clustered	3	VC_655_2	3	0.0231	0.99860756	0.0231	0	0	0	1	Ref
HQ634177	Unassigned	Unassigned	Unassigned	433_0	Clustered	6	VC_433_0	6	0.0787	1	0.0787	0	0	0	1	Ref
HQ634187	Unassigned	Unassigned	Unassigned	356_0	Clustered	2	VC_356_0	2	0.1992	0.93211095	0.1857	0	0	0	1	Ref
HQ634188	Unassigned	Unassigned	Unassigned	340_0	Clustered	31	VC_340_0	31	0.9549	1	0.9549	0	0	0	1	Ref
HQ634189	Unassigned	Unassigned	Unassigned	655_7	Clustered	3	VC_655_7	3	0.0166	0.99856978	0.0166	0	0	0	1	Ref
HQ634190	Unassigned	Unassigned	Unassigned	655_6	Clustered	2	VC_655_6	2	0.0078	0.95775168	0.0075	0	0	0	1	Ref
HQ634192	Unassigned	Unassigned	Unassigned	929_0	Clustered	4	VC_929_0	4	0.846	0.99722201	0.8437	0	0	0	1	Ref
HQ634195	Unassigned	Unassigned	Unassigned	493_15	Clustered/Singleton	1	VC_493_15	1	0	1	0	0	0	0	1	Ref
HQ641340	Unassigned	Unassigned	Unassigned	82_0	Clustered	24	VC_82_0	24	0.7351	1	0.7351	0	0	0	1	Ref
HQ641341	Unassigned	Unassigned	Unassigned	82_0	Clustered	24	VC_82_0	24	0.7351	1	0.7351	0	0	0	1	Ref
HQ641342	Unassigned	Unassigned	Unassigned	82_0	Clustered	24	VC_82_0	24	0.7351	1	0.7351	0	0	0	1	Ref
HQ641343	Unassigned	Unassigned	Unassigned	82_0	Clustered	24	VC_82_0	24	0.7351	1	0.7351	0	0	0	1	Ref
HQ641344	Unassigned	Unassigned	Unassigned	82_0	Clustered	24	VC_82_0	24	0.7351	1	0.7351	0	0	0	1	Ref
HQ641345	Unassigned	Unassigned	Unassigned	754_0	Clustered	8	VC_754_0	8	0.752	1	0.752	0	0	0	1	Ref
HQ641346	Unassigned	Unassigned	Unassigned	754_0	Clustered	8	VC_754_0	8	0.752	1	0.752	0	0	0	1	Ref
HQ641347	Unassigned	Unassigned	Unassigned	495_1	Clustered	41	VC_495_1	41	0.328	1	0.328	0	0	0	0.911	Ref
HQ641348	Unassigned	Unassigned	Unassigned	495_1	Clustered	41	VC_495_1	41	0.328	1	0.328	0	0	0	0.911	Ref
HQ641349	Unassigned	Unassigned	Unassigned	495_1	Clustered	41	VC_495_1	41	0.328	1	0.328	0	0	0	0.911	Ref
HQ641350	Unassigned	Unassigned	Unassigned	495_1	Clustered	41	VC_495_1	41	0.328	1	0.328	0	0	0	0.911	Ref
HQ641351	Unassigned	Unassigned	Unassigned	495_1	Clustered	41	VC_495_1	41	0.328	1	0.328	0	0	0	0.911	Ref
HQ641352	Unassigned	Unassigned	Unassigned	495_1	Clustered	41	VC_495_1	41	0.328	1	0.328	0	0	0	0.911	Ref
HQ641353	Unassigned	Unassigned	Unassigned	495_1	Clustered	41	VC_495_1	41	0.328	1	0.328	0	0	0	0.911	Ref
HQ641354	Unassigned	Unassigned	Unassigned	495_1	Clustered	41	VC_495_1	41	0.328	1	0.328	0	0	0	0.911	Ref
HQ665011	Unassigned	Unassigned	Unassigned	190_24	Clustered	5	VC_190_24	5	0.0293	0.9999996	0.0293	0	0	0	0.7	Ref
HQ683709	Unassigned	Unassigned	Unassigned	932_0	Clustered	2	VC_932_0	2	0.4438	0.89435023	0.3969	0	0	0	1	Ref
HQ698895	Unassigned	Unassigned	Unassigned	927_0	Clustered	2	VC_927_0	2	0.0876	0.71936208	0.063	0	0	0	1	Ref
HQ698922	Unassigned	Unassigned	Unassigned	492_0	Clustered	2	VC_492_0	2	0.0158	0.6640397	0.0105	0	0	0	1	Ref
HQ711984	Unassigned	Unassigned	Unassigned	297_1	Clustered	20	VC_297_1	20	0.5653	1	0.5653	0	0	0	0.7684	Ref
HQ711985	Unassigned	Unassigned	Unassigned	176_0	Clustered	2	VC_176_0	2	0.0498	0.95663741	0.0476	0	0	0	1	Ref
HQ728263	Unassigned	Unassigned	Unassigned	328_0	Clustered	11	VC_328_0	11	0.4707	1	0.4707	0	0	0	1	Ref
HQ728264	Unassigned	Unassigned	Unassigned	792_0	Clustered	35	VC_792_0	35	0.9828	1	0.9828	0	0	0	1	Ref
HQ728265	Unassigned	Unassigned	Unassigned	387_0	Clustered	3	VC_387_0	3	0.0424	0.99861589	0.0423	0	0	0	1	Ref
HQ728524	Unassigned	Unassigned	Unassigned	520_26	Clustered	3	VC_520_26	3	0.026	0.99859898	0.026	0	0	0	1	Ref
HQ829472	Unassigned	Unassigned	Unassigned	290_5	Clustered	14	VC_290_5	14	0.1422	1	0.1422	0	0	0	0.9231	Ref
HQ832595	Unassigned	Unassigned	Unassigned	76_0	Clustered	37	VC_76_0	37	0.9403	1	0.9403	0	0	0	1	Ref
HQ906662	Unassigned	Unassigned	Unassigned	22_0	Clustered	11	VC_22_0	11	0.5163	1	0.5163	0	0	0	1	Ref
HQ906663	Unassigned	Unassigned	Unassigned	22_0	Clustered	11	VC_22_0	11	0.5163	1	0.5163	0	0	0	1	Ref
HQ906664	Unassigned	Unassigned	Unassigned	22_0	Clustered	11	VC_22_0	11	0.5163	1	0.5163	0	0	0	1	Ref
HQ918180	Unassigned	Unassigned	Unassigned	668_0	Clustered	25	VC_668_0	25	0.5539	1	0.5539	0	0	0	0.96	Ref
JF412296	Unassigned	Unassigned	Unassigned	613_0	Clustered	2	VC_613_0	2	0.0357	0.94194318	0.0336	0	0	0	1	Ref
JF461087	Unassigned	Unassigned	Unassigned	329_1	Clustered	17	VC_329_1	17	0.0867	1	0.0867	0	0	0	0.8235	Ref
JF501022	Unassigned	Unassigned	Unassigned	938_0	Clustered	99	VC_938_0	99	0.9119	1	0.9119	0	0	0	0.9928	Ref
JF704091	Unassigned	Unassigned	Unassigned	371_18	Clustered	10	VC_371_18	10	0.0302	1	0.0302	0	0	0	0.8	Ref
JF704092	Unassigned	Unassigned	Unassigned	558_22	Clustered	4	VC_558_22	4	0.01	0.99312819	0.0099	0	0	0	0.8333	Ref
JF704093	Unassigned	Unassigned	Unassigned	517_0	Clustered	18	VC_517_0	18	0.1599	1	0.1599	0	0	0	1	Ref
JF704094	Unassigned	Unassigned	Unassigned	372_0	Clustered	24	VC_372_0	24	0.5539	1	0.5539	0	0	0	1	Ref
JF704095	Unassigned	Unassigned	Unassigned	509_0	Clustered	37	VC_509_0	37	0.9689	1	0.9689	0	0	0	1	Ref
JF704096	Unassigned	Unassigned	Unassigned	558_20	Clustered	17	VC_558_20	17	0.054	1	0.054	0	0	0	0.9926	Ref
JF704097	Unassigned	Unassigned	Unassigned	223_0	Clustered	38	VC_223_0	38	0.9095	1	0.9095	0	0	0	1	Ref
JF704098	Unassigned	Unassigned	Unassigned	514_0	Clustered	75	VC_514_0	75	0.9402	1	0.9402	0	0	0	1	Ref
JF704099	Unassigned	Unassigned	Unassigned	371_3	Clustered	22	VC_371_3	22	0.0787	1	0.0787	0	0	0	0.9048	Ref
JF704100	Unassigned	Unassigned	Unassigned	939_0	Clustered	15	VC_939_0	15	1	0	0	0	0	0	1	Ref
JF704101	Unassigned	Unassigned	Unassigned	514_0	Clustered	75	VC_514_0	75	0.9402	1	0.9402	0	0	0	1	Ref
JF704102	Unassigned	Unassigned	Unassigned	371_16	Clustered	42	VC_371_16	42	0.1341	1	0.1341	0	0	0	0.7282	Ref
JF704103	Unassigned	Unassigned	Unassigned	371_15	Clustered	44	VC_371_15	44	0.1391	1	0.1391	0	0	0	0.7474	Ref
JF704104	Unassigned	Unassigned	Unassigned	372_0	Clustered	24	VC_372_0	24	0.5539	1	0.5539	0	0	0	1	Ref
JF704105	Unassigned	Unassigned	Unassigned	236_0	Clustered	72	VC_236_0	72	0.8104	1	0.8104	0	0	0	1	Ref
JF704106	Unassigned	Unassigned	Unassigned	236_0	Clustered	72	VC_236_0	72	0.8104	1	0.8104	0	0	0	1	Ref
JF704107	Unassigned	Unassigned	Unassigned	940_0	Clustered	34	VC_940_0	34	0.7959	1	0.7959	0	0	0	1	Ref
JF704108	Unassigned	Unassigned	Unassigned	887_0	Clustered	18	VC_887_0	18	0.3663	1	0.3663	0	0	0	1	Ref
JF704109	Unassigned	Unassigned	Unassigned	371_15	Clustered	44	VC_371_15	44	0.1391	1	0.1391	0	0	0	0.7474	Ref
JF704110	Unassigned	Unassigned	Unassigned	225_0	Clustered	32	VC_225_0	32	0.848	1	0.848	0	0	0	0.996	Ref
JF704111	Unassigned	Unassigned	Unassigned	513_0	Clustered	32	VC_513_0	32	0.6881	1	0.6881	0	0	0	1	Ref
JF704112	Unassigned	Unassigned	Unassigned	568_0	Clustered	8	VC_568_0	8	0.5738	1	0.5738	0	0	0	1	Ref
JF704113	Unassigned	Unassigned	Unassigned	887_0	Clustered	18	VC_887_0	18	0.3663	1	0.3663	0	0	0	1	Ref
JF704114	Unassigned	Unassigned	Unassigned	514_0	Clustered	75	VC_514_0	75	0.9402	1	0.9402	0	0	0	1	Ref
JF704116	Unassigned	Unassigned	Unassigned	558_6	Clustered	2	VC_558_6	2	0.0032	0.73149279	0.0023	0	0	0	1	Ref
JF704117	Unassigned	Unassigned	Unassigned	520_74	Clustered	2	VC_520_74	2	0.0075	0.95760586	0.0072	0	0	0	1	Ref

JF712866	Unassigned	Unassigned	Unassigned	607_0	Clustered	16	VC_607_0	16	0.9179	1	0.9179	0	0	0	1	Ref
JF713456	Unassigned	Unassigned	Unassigned	904_0	Clustered	6	VC_904_0	6	0.233	1	0.233	0	0	0	1	Ref
JF719726	Unassigned	Unassigned	Unassigned	303_0	Clustered	155	VC_303_0	155	0.9979	1	0.9979	0	0	0	1	Ref
JF719727	Unassigned	Unassigned	Unassigned	303_0	Clustered	155	VC_303_0	155	0.9979	1	0.9979	0	0	0	1	Ref
JF719728	Unassigned	Unassigned	Unassigned	303_0	Clustered	155	VC_303_0	155	0.9979	1	0.9979	0	0	0	1	Ref
JF719729	Unassigned	Unassigned	Unassigned	360_0	Clustered	358	VC_360_0	358	0.9615	1	0.9615	0	0	0	1	Ref
JF719730	Unassigned	Unassigned	Unassigned	360_0	Clustered	358	VC_360_0	358	0.9615	1	0.9615	0	0	0	1	Ref
JF719731	Unassigned	Unassigned	Unassigned	360_0	Clustered	358	VC_360_0	358	0.9615	1	0.9615	0	0	0	1	Ref
JF719732	Unassigned	Unassigned	Unassigned	61_0	Clustered	12	VC_61_0	12	0.7633	1	0.7633	0	0	0	1	Ref
JF719733	Unassigned	Unassigned	Unassigned	61_0	Clustered	12	VC_61_0	12	0.7633	1	0.7633	0	0	0	1	Ref
JF719734	Unassigned	Unassigned	Unassigned	61_0	Clustered	12	VC_61_0	12	0.7633	1	0.7633	0	0	0	1	Ref
JF731128	Unassigned	Unassigned	Unassigned	108_0	Clustered	33	VC_108_0	33	0.949	1	0.949	0	0	0	1	Ref
JF734911	Unassigned	Unassigned	Unassigned	99_0	Clustered	32	VC_99_0	32	0.8828	1	0.8828	0	0	0	1	Ref
JF744988	Unassigned	Unassigned	Unassigned	888_0	Clustered	28	VC_888_0	28	0.5497	1	0.5497	0	0	0	1	Ref
JF767208	Unassigned	Unassigned	Unassigned	815_0	Clustered	8	VC_815_0	8	0.9801	1	0.9801	0	0	0	1	Ref
JF767209	Unassigned	Unassigned	Unassigned	815_0	Clustered	8	VC_815_0	8	0.9801	1	0.9801	0	0	0	1	Ref
JF767210	Unassigned	Unassigned	Unassigned	815_0	Clustered	8	VC_815_0	8	0.9801	1	0.9801	0	0	0	1	Ref
JF770475	Unassigned	Unassigned	Unassigned	455_0	Clustered	18	VC_455_0	18	0.4598	1	0.4598	0	0	0	1	Ref
JF773396	Unassigned	Unassigned	Unassigned	730_0	Clustered	9	VC_730_0	9	1	1	1	0	0	0	1	Ref
JF792674	Unassigned	Unassigned	Unassigned	517_1	Clustered	108	VC_517_1	108	0.8483	1	0.8483	0	0	0	0.9955	Ref
JF832915	Unassigned	Unassigned	Unassigned	181_0	Clustered	5	VC_181_0	5	0.1168	0.99999995	0.1168	0	0	0	1	Ref
JF900176	Unassigned	Unassigned	Unassigned	28_1	Clustered	53	VC_28_1	53	0.7164	1	0.7164	0	0	0	0.9325	Ref
JF917095	Unassigned	Unassigned	Unassigned	943_0	Clustered	2	VC_943_0	2	1	1	1	0	0	0	1	Ref
JF923796	Unassigned	Unassigned	Unassigned	767_0	Clustered	34	VC_767_0	34	0.9173	1	0.9173	0	0	0	1	Ref
JF923797	Unassigned	Unassigned	Unassigned	767_0	Clustered	34	VC_767_0	34	0.9173	1	0.9173	0	0	0	1	Ref
JF937090	Unassigned	Unassigned	Unassigned	546_7	Clustered	4	VC_546_7	4	0.041	0.9963251	0.0408	0	0	0	1	Ref
JF937091	Unassigned	Unassigned	Unassigned	551_0	Clustered	16	VC_551_0	16	0.0915	1	0.0915	0	0	0	0.6667	Ref
JF937092	Unassigned	Unassigned	Unassigned	223_0	Clustered	38	VC_223_0	38	0.9095	1	0.9095	0	0	0	1	Ref
JF937093	Unassigned	Unassigned	Unassigned	520_31	Clustered	11	VC_520_31	11	0.0897	1	0.0897	0	0	0	0.6	Ref
JF937094	Unassigned	Unassigned	Unassigned	223_0	Clustered	38	VC_223_0	38	0.9095	1	0.9095	0	0	0	1	Ref
JF937095	Unassigned	Unassigned	Unassigned	371_17	Clustered	3	VC_371_17	3	0.0076	0.99811676	0.0076	0	0	0	1	Ref
JF937096	Unassigned	Unassigned	Unassigned	551_2	Clustered	17	VC_551_2	17	0.0973	1	0.0973	0	0	0	0.9779	Ref
JF937097	Unassigned	Unassigned	Unassigned	371_15	Clustered	44	VC_371_15	44	0.1391	1	0.1391	0	0	0	0.7474	Ref
JF937098	Unassigned	Unassigned	Unassigned	520_93	Clustered	2	VC_520_93	2	0.0104	0.95762079	0.0099	0	0	0	1	Ref
JF937099	Unassigned	Unassigned	Unassigned	325_2	Clustered/Singleton	1	VC_325_2	1	0	1	0	0	0	0	1	Ref
JF937100	Unassigned	Unassigned	Unassigned	325_41	Clustered	2	VC_325_41	2	0.0036	0.94071129	0.0034	0	0	0	1	Ref
JF937101	Unassigned	Unassigned	Unassigned	546_11	Clustered/Singleton	1	VC_546_11	1	0	1	0	0	0	0	1	Ref
JF937102	Unassigned	Unassigned	Unassigned	520_37	Clustered/Singleton	1	VC_520_37	1	0	1	0	0	0	0	1	Ref
JF937103	Unassigned	Unassigned	Unassigned	325_23	Clustered	17	VC_325_23	17	0.0716	1	0.0716	0	0	0	0.6397	Ref
JF937104	Unassigned	Unassigned	Unassigned	237_1	Clustered	13	VC_237_1	13	0.1773	1	0.1773	0	0	0	0.7308	Ref
JF937105	Unassigned	Unassigned	Unassigned	763_2	Clustered	7	VC_763_2	7	0.2612	1	0.2612	0	0	0	1	Ref
JF937106	Unassigned	Unassigned	Unassigned	551_0	Clustered	16	VC_551_0	16	0.0915	1	0.0915	0	0	0	0.6667	Ref
JF937107	Unassigned	Unassigned	Unassigned	565_0	Clustered	20	VC_565_0	20	0.8398	1	0.8398	0	0	0	1	Ref
JF937108	Unassigned	Unassigned	Unassigned	325_5	Clustered	11	VC_325_5	11	0.047	1	0.047	0	0	0	0.7636	Ref
JF937109	Unassigned	Unassigned	Unassigned	371_18	Clustered	10	VC_371_18	10	0.0302	1	0.0302	0	0	0	0.8	Ref
JF937110	Unassigned	Unassigned	Unassigned	325_5	Clustered	11	VC_325_5	11	0.047	1	0.047	0	0	0	0.7636	Ref
JF939047	Unassigned	Unassigned	Unassigned	749_0	Clustered	40	VC_749_0	40	0.7517	1	0.7517	0	0	0	1	Ref
JF946695	Unassigned	Unassigned	Unassigned	215_2	Clustered	5	VC_215_2	5	0.0572	0.99999997	0.0572	0	0	0	1	Ref
JF957056	Unassigned	Unassigned	Unassigned	371_15	Clustered	44	VC_371_15	44	0.1391	1	0.1391	0	0	0	0.7474	Ref
JF957057	Unassigned	Unassigned	Unassigned	325_24	Clustered	3	VC_325_24	3	0.009	0.99853949	0.009	0	0	0	1	Ref
JF957058	Unassigned	Unassigned	Unassigned	513_0	Clustered	32	VC_513_0	32	0.6881	1	0.6881	0	0	0	1	Ref
JF957059	Unassigned	Unassigned	Unassigned	546_7	Clustered	4	VC_546_7	4	0.041	0.9963251	0.0408	0	0	0	1	Ref
JF957060	Unassigned	Unassigned	Unassigned	219_0	Clustered	5	VC_219_0	5	0.08	0.99999998	0.08	0	0	0	1	Ref
JF966203	Unassigned	Unassigned	Unassigned	883_0	Clustered	10	VC_883_0	10	0.2015	1	0.2015	0	0	0	1	Ref
JF974286	Unassigned	Unassigned	Unassigned	928_0	Clustered	3	VC_928_0	3	0.7844	0.9944138	0.78	0	0	0	1	Ref
JF974287	Unassigned	Unassigned	Unassigned	643_0	Clustered	2	VC_643_0	2	0.1092	0.95085347	0.1038	0	0	0	1	Ref
JF974294	Unassigned	Unassigned	Unassigned	460_0	Clustered	19	VC_460_0	19	0.3744	1	0.3744	0	0	0	1	Ref
JF974295	Unassigned	Unassigned	Unassigned	427_0	Clustered	3	VC_427_0	3	0.0627	0.96830121	0.0608	0	0	0	1	Ref
JF974307	Unassigned	Unassigned	Unassigned	182_0	Clustered	5	VC_182_0	5	0.6184	0.99999967	0.6184	0	0	0	1	Ref
JF974308	Unassigned	Unassigned	Unassigned	182_0	Clustered	5	VC_182_0	5	0.6184	0.99999967	0.6184	0	0	0	1	Ref
JF974312	Unassigned	Unassigned	Unassigned	460_0	Clustered	19	VC_460_0	19	0.3744	1	0.3744	0	0	0	1	Ref
JF974313	Unassigned	Unassigned	Unassigned	642_2	Clustered	6	VC_642_2	6	0.1679	1	0.1679	0	0	0	1	Ref
JF974314	Unassigned	Unassigned	Unassigned	21_0	Clustered	4	VC_21_0	4	0.5696	0.87262442	0.4971	0	0	0	0.6667	Ref
JF974321	Unassigned	Unassigned	Unassigned	928_0	Clustered	3	VC_928_0	3	0.7844	0.9944138	0.78	0	0	0	1	Ref
JF974339	Unassigned	Unassigned	Unassigned	30_1	Clustered	16	VC_30_1	16	0.1973	1	0.1973	0	0	0	0.8333	Ref
JN006061	Unassigned	Unassigned	Unassigned	551_2	Clustered	17	VC_551_2	17	0.0973	1	0.0973	0	0	0	0.9779	Ref
JN006062	Unassigned	Unassigned	Unassigned	551_2	Clustered	17	VC_551_2	17	0.0973	1	0.0973	0	0	0	0.9779	Ref
JN006063	Unassigned	Unassigned	Unassigned	371_15	Clustered	44	VC_371_15	44	0.1391	1	0.1391	0	0	0	0.7474	Ref
JN006064	Unassigned	Unassigned	Unassigned	371_21	Clustered	2	VC_371_21	2	0.0033	0.95770737	0.0032	0	0	0	1	Ref
JN020140	Unassigned	Unassigned	Unassigned	325_19	Clustered	3	VC_325_19	3	0.0108	0.99859221	0.0107	0	0	0	1	Ref
JN020141	Unassigned	Unassigned	Unassigned	520_28	Clustered	2	VC_520_28	2	0.0137	0.95761531	0.0131	0	0	0	1	Ref
JN020142	Unassigned	Unassigned	Unassigned	520_88	Clustered/Singleton	1	VC_520_88	1	0	1	0	0	0	0	1	Ref
JN020143	Unassigned	Unassigned	Unassigned	520_43	Clustered	3	VC_520_43	3	0.0193	0.99837043	0.0193	0	0	0	1	Ref
JN022534	Unassigned	Unassigned	Unassigned	64_0	Clustered	3	VC_64_0	3	0.5503	0.99785685	0.5491	0	0	0	1	Ref
JN035618	Unassigned	Unassigned	Unassigned	866_0	Clustered	4	VC_866_0	4	0.0795	0.99998595	0.0795	0	0	0	1	Ref
JN049605	Unassigned	Unassigned	Unassigned	223_0	Clustered	38	VC_223_0	38	0.9095	1	0.9095	0	0	0	1	Ref
JN051154	Unassigned	Unassigned	Unassigned	577_0	Clustered	6	VC_577_0	6	0.9267	0.99999993	0.9267	0	0	0	1	Ref
JN083852	Unassigned	Unassigned	Unassigned	940_0	Clustered	34	VC_940_0	34	0.7959	1	0.7959	0	0	0	1	Ref
JN083853	Unassigned	Unassigned	Unassigned	940_0	Clustered	34	VC_940_0	34	0.7959	1	0.7959	0	0	0	1	Ref
JN116822	Unassigned	Unassigned	Unassigned	949_0	Clustered	10	VC_949_0	10	0.5977	1	0.5977	0	0	0	1	Ref
JN116823	Unassigned	Unassigned	Unassigned	953_0	Clustered	2	VC_953_0	2	0.2116	0.94820857	0.2007	0	0	0	1	Ref

JN116824	Unassigned	Unassigned	Unassigned	726_0	Clustered	3	VC_726_0	3	0.0381	0.88955608	0.0339	0	0	0	1	Ref
JN116826	Unassigned	Unassigned	Unassigned	956_0	Clustered	39	VC_956_0	39	1	1	1	0	0	0	1	Ref
JN116827	Unassigned	Unassigned	Unassigned	956_0	Clustered	39	VC_956_0	39	1	1	1	0	0	0	1	Ref
JN116828	Unassigned	Unassigned	Unassigned	952_0	Clustered	2	VC_952_0	2	0.9556	0.8861	0.8468	0	0	0	1	Ref
JN126049	Unassigned	Unassigned	Unassigned	133_13	Clustered	2	VC_133_13	2	0.0051	0.86794768	0.0044	0	0	0	1	Ref
JN132397	Unassigned	Unassigned	Unassigned	844_0	Clustered	27	VC_844_0	27	0.9385	1	0.9385	0	0	0	1	Ref
JN153085	Unassigned	Unassigned	Unassigned	325_52	Clustered	5	VC_325_52	5	0.0147	0.99999073	0.0147	0	0	0	0.7	Ref
JN153086	Unassigned	Unassigned	Unassigned	325_61	Clustered	8	VC_325_61	8	0.0395	1	0.0395	0	0	0	0.7857	Ref
JN175269	Unassigned	Unassigned	Unassigned	957_0	Clustered/Singleton	1	VC_957_0	1	0	1	0	0	0	0	1	Ref
JN185608	Unassigned	Unassigned	Unassigned	236_0	Clustered	72	VC_236_0	72	0.8104	1	0.8104	0	0	0	1	Ref
JN190960	Unassigned	Unassigned	Unassigned	602_0	Clustered	2	VC_602_0	2	0.229	0.95560552	0.2188	0	0	0	1	Ref
JN192463	Unassigned	Unassigned	Unassigned	371_13	Clustered	33	VC_371_13	33	0.179	1	0.179	0	0	0	0.8769	Ref
JN201525	Unassigned	Unassigned	Unassigned	546_0	Clustered	2	VC_546_0	2	0.0136	0.63748598	0.0087	0	0	0	1	Ref
JN202312	Unassigned	Unassigned	Unassigned	283_148	Clustered/Singleton	1	VC_283_148	1	0	1	0	0	0	0	1	Ref
JN204348	Unassigned	Unassigned	Unassigned	558_0	Clustered	2	VC_558_0	2	0.0032	0.75858499	0.0025	0	0	0	1	Ref
JN225449	Unassigned	Unassigned	Unassigned	329_4	Clustered	2	VC_329_4	2	0.0052	0.95746454	0.005	0	0	0	1	Ref
JN243855	Unassigned	Unassigned	Unassigned	238_0	Clustered	17	VC_238_0	17	0.6109	1	0.6109	0	0	0	1	Ref
JN243856	Unassigned	Unassigned	Unassigned	517_1	Clustered	108	VC_517_1	108	0.8483	1	0.8483	0	0	0	0.9955	Ref
JN243857	Unassigned	Unassigned	Unassigned	517_0	Clustered	18	VC_517_0	18	0.1599	1	0.1599	0	0	0	1	Ref
JN254800	Unassigned	Unassigned	Unassigned	79_0	Clustered	64	VC_79_0	64	0.4185	1	0.4185	0	0	0	0.7649	Ref
JN254801	Unassigned	Unassigned	Unassigned	472_0	Clustered	47	VC_472_0	47	0.9025	1	0.9025	0	0	0	1	Ref
JN255163	Unassigned	Unassigned	Unassigned	4_0	Clustered	7	VC_4_0	7	0.7912	1	0.7912	0	0	0	1	Ref
JN256079	Unassigned	Unassigned	Unassigned	521_0	Clustered	37	VC_521_0	37	0.912	1	0.912	0	0	0	1	Ref
JN371769	Unassigned	Unassigned	Unassigned	655_4	Clustered/Singleton	1	VC_655_4	1	0	1	0	0	0	0	1	Ref
JN377894	Unassigned	Unassigned	Unassigned	600_2	Clustered	10	VC_600_2	10	0.3041	1	0.3041	0	0	0	1	Ref
JN377895	Unassigned	Unassigned	Unassigned	600_2	Clustered	10	VC_600_2	10	0.3041	1	0.3041	0	0	0	1	Ref
JN382248	Unassigned	Unassigned	Unassigned	551_7	Clustered	28	VC_551_7	28	0.1661	1	0.1661	0	0	0	0.8519	Ref
JN391180	Unassigned	Unassigned	Unassigned	476_0	Clustered	19	VC_476_0	19	0.7814	1	0.7814	0	0	0	1	Ref
JN391441	Unassigned	Unassigned	Unassigned	551_7	Clustered	28	VC_551_7	28	0.1661	1	0.1661	0	0	0	0.8519	Ref
JN398368	Unassigned	Unassigned	Unassigned	520_76	Clustered	2	VC_520_76	2	0.0092	0.95761376	0.0088	0	0	0	1	Ref
JN398369	Unassigned	Unassigned	Unassigned	325_1	Clustered	2	VC_325_1	2	0.0043	0.95663348	0.0041	0	0	0	1	Ref
JN408459	Unassigned	Unassigned	Unassigned	940_0	Clustered	34	VC_940_0	34	0.7959	1	0.7959	0	0	0	1	Ref
JN408460	Unassigned	Unassigned	Unassigned	216_6	Clustered	42	VC_216_6	42	0.473	1	0.473	0	0	0	0.8351	Ref
JN408461	Unassigned	Unassigned	Unassigned	216_3	Clustered	8	VC_216_3	8	0.0701	1	0.0701	0	0	0	0.8571	Ref
JN412588	Unassigned	Unassigned	Unassigned	558_80	Clustered/Singleton	1	VC_558_80	1	0	1	0	0	0	0	1	Ref
JN412589	Unassigned	Unassigned	Unassigned	569_0	Clustered	3	VC_569_0	3	0.2094	0.99849867	0.2091	0	0	0	1	Ref
JN412590	Unassigned	Unassigned	Unassigned	551_4	Clustered	2	VC_551_4	2	0.006	0.95695857	0.0058	0	0	0	1	Ref
JN412591	Unassigned	Unassigned	Unassigned	519_0	Clustered	39	VC_519_0	39	0.8156	1	0.8156	0	0	0	1	Ref
JN412592	Unassigned	Unassigned	Unassigned	558_0	Clustered	2	VC_558_0	2	0.0032	0.75858499	0.0025	0	0	0	1	Ref
JN412593	Unassigned	Unassigned	Unassigned	761_0	Clustered	67	VC_761_0	67	0.8947	1	0.8947	0	0	0	1	Ref
JN542517	Unassigned	Unassigned	Unassigned	520_59	Clustered	5	VC_520_59	5	0.0459	0.99999997	0.0459	0	0	0	1	Ref
JN561150	Unassigned	Unassigned	Unassigned	517_1	Clustered	108	VC_517_1	108	0.8483	1	0.8483	0	0	0	0.9955	Ref
JN572061	Unassigned	Unassigned	Unassigned	519_0	Clustered	39	VC_519_0	39	0.8156	1	0.8156	0	0	0	1	Ref
JN572689	Unassigned	Unassigned	Unassigned	325_35	Clustered	17	VC_325_35	17	0.0732	1	0.0732	0	0	0	0.7721	Ref
JN585957	Unassigned	Unassigned	Unassigned	359_0	Clustered	7	VC_359_0	7	0.3703	1	0.3703	0	0	0	1	Ref
JN593240	Unassigned	Unassigned	Unassigned	133_25	Clustered/Singleton	1	VC_133_25	1	0	1	0	0	0	0	1	Ref
JN600672	Unassigned	Unassigned	Unassigned	887_0	Clustered	18	VC_887_0	18	0.3663	1	0.3663	0	0	0	1	Ref
JN600960	Unassigned	Unassigned	Unassigned	30_1	Clustered	16	VC_30_1	16	0.1973	1	0.1973	0	0	0	0.8333	Ref
JN618996	Unassigned	Unassigned	Unassigned	508_0	Clustered	32	VC_508_0	32	0.906	1	0.906	0	0	0	1	Ref
JN624850	Unassigned	Unassigned	Unassigned	558_49	Clustered	7	VC_558_49	7	0.0194	0.99510369	0.0193	0	0	0	0.8571	Ref
JN624851	Unassigned	Unassigned	Unassigned	521_0	Clustered	37	VC_521_0	37	0.912	1	0.912	0	0	0	1	Ref
JN627160	Unassigned	Unassigned	Unassigned	442_2	Clustered	4	VC_442_2	4	0.0844	0.99988129	0.0844	0	0	0	0.8333	Ref
JN638752	Unassigned	Unassigned	Unassigned	371_15	Clustered	44	VC_371_15	44	0.1391	1	0.1391	0	0	0	0.7474	Ref
JN638753	Unassigned	Unassigned	Unassigned	371_15	Clustered	44	VC_371_15	44	0.1391	1	0.1391	0	0	0	0.7474	Ref
JN641803	Unassigned	Unassigned	Unassigned	959_5	Clustered	19	VC_959_5	19	0.2626	1	0.2626	0	0	0	1	Ref
JN643714	Unassigned	Unassigned	Unassigned	236_0	Clustered	72	VC_236_0	72	0.8104	1	0.8104	0	0	0	1	Ref
JN651747	Unassigned	Unassigned	Unassigned	878_0	Clustered	32	VC_878_0	32	0.9965	1	0.9965	0	0	0	1	Ref
JN654439	Unassigned	Unassigned	Unassigned	884_1	Clustered	8	VC_884_1	8	0.1102	0.9989323	0.1101	0	0	0	0.75	Ref
JN660814	Unassigned	Unassigned	Unassigned	325_44	Clustered	3	VC_325_44	3	0.0157	0.99859171	0.0157	0	0	0	1	Ref
JN662425	Unassigned	Unassigned	Unassigned	162_0	Clustered	18	VC_162_0	18	0.7403	1	0.7403	0	0	0	0.8627	Ref
JN672684	Unassigned	Unassigned	Unassigned	937_0	Clustered	40	VC_937_0	40	0.6809	1	0.6809	0	0	0	0.9359	Ref
JN673056	Unassigned	Unassigned	Unassigned	133_26	Clustered/Singleton	1	VC_133_26	1	0	1	0	0	0	0	1	Ref
JN680858	Unassigned	Unassigned	Unassigned	888_0	Clustered	28	VC_888_0	28	0.5497	1	0.5497	0	0	0	1	Ref
JN687951	Unassigned	Unassigned	Unassigned	325_60	Clustered	2	VC_325_60	2	0.0038	0.957513	0.0036	0	0	0	1	Ref
JN698989	Unassigned	Unassigned	Unassigned	371_18	Clustered	10	VC_371_18	10	0.0302	1	0.0302	0	0	0	0.8	Ref
JN698990	Unassigned	Unassigned	Unassigned	371_17	Clustered	3	VC_371_17	3	0.0076	0.99811676	0.0076	0	0	0	1	Ref
JN698991	Unassigned	Unassigned	Unassigned	508_0	Clustered	32	VC_508_0	32	0.906	1	0.906	0	0	0	1	Ref
JN698992	Unassigned	Unassigned	Unassigned	509_0	Clustered	37	VC_509_0	37	0.9689	1	0.9689	0	0	0	1	Ref
JN698993	Unassigned	Unassigned	Unassigned	556_0	Clustered	21	VC_556_0	21	0.9912	1	0.9912	0	0	0	1	Ref
JN698994	Unassigned	Unassigned	Unassigned	554_0	Clustered	2	VC_554_0	2	0.2292	0.95193785	0.2181	0	0	0	1	Ref
JN698995	Unassigned	Unassigned	Unassigned	511_0	Clustered	3	VC_511_0	3	0.1104	0.99858683	0.1103	0	0	0	1	Ref
JN698996	Unassigned	Unassigned	Unassigned	520_26	Clustered	3	VC_520_26	3	0.026	0.99859898	0.026	0	0	0	1	Ref
JN698997	Unassigned	Unassigned	Unassigned	546_2	Clustered	3	VC_546_2	3	0.0275	0.85260342	0.0234	0	0	0	1	Ref
JN698998	Unassigned	Unassigned	Unassigned	325_61	Clustered	8	VC_325_61	8	0.0395	1	0.0395	0	0	0	0.7857	Ref
JN698999	Unassigned	Unassigned	Unassigned	223_0	Clustered	38	VC_223_0	38	0.9095	1	0.9095	0	0	0	1	Ref
JN699000	Unassigned	Unassigned	Unassigned	325_47	Clustered	2	VC_325_47	2	0.0045	0.9575092	0.0043	0	0	0	1	Ref
JN699001	Unassigned	Unassigned	Unassigned	518_0	Clustered	6	VC_518_0	6	0.1295	1	0.1295	0	0	0	1	Ref
JN699002	Unassigned	Unassigned	Unassigned	761_0	Clustered	67	VC_761_0	67	0.8947	1	0.8947	0	0	0	1	Ref
JN699003	Unassigned	Unassigned	Unassigned	509_0	Clustered	37	VC_509_0	37	0.9689	1	0.9689	0	0	0	1	Ref
JN699004	Unassigned	Unassigned	Unassigned	508_0	Clustered	32	VC_508_0	32	0.906	1	0.906	0	0	0	1	Ref
JN699005	Unassigned	Unassigned	Unassigned	225_0	Clustered	32	VC_225_0	32	0.848	1	0.848	0	0	0	0.996	Ref

JN699006	Unassigned	Unassigned	Unassigned	509_0	Clustered	37	VC_509_0	37	0.9689	1	0.9689	0	0	0	1	Ref
JN699007	Unassigned	Unassigned	Unassigned	372_1	Clustered	9	VC_372_1	9	0.194	1	0.194	0	0	0	1	Ref
JN699008	Unassigned	Unassigned	Unassigned	371_15	Clustered	44	VC_371_15	44	0.1391	1	0.1391	0	0	0	0.7474	Ref
JN699009	Unassigned	Unassigned	Unassigned	371_16	Clustered	42	VC_371_16	42	0.1341	1	0.1341	0	0	0	0.7282	Ref
JN699010	Unassigned	Unassigned	Unassigned	371_15	Clustered	44	VC_371_15	44	0.1391	1	0.1391	0	0	0	0.7474	Ref
JN699011	Unassigned	Unassigned	Unassigned	372_0	Clustered	24	VC_372_0	24	0.5539	1	0.5539	0	0	0	1	Ref
JN699012	Unassigned	Unassigned	Unassigned	520_20	Clustered/Singleton	1	VC_520_20	1	0	1	0	0	0	0	1	Ref
JN699013	Unassigned	Unassigned	Unassigned	558_8	Clustered	2	VC_558_8	2	0.0032	0.68030512	0.0022	0	0	0	1	Ref
JN699014	Unassigned	Unassigned	Unassigned	565_0	Clustered	20	VC_565_0	20	0.8398	1	0.8398	0	0	0	1	Ref
JN699015	Unassigned	Unassigned	Unassigned	517_1	Clustered	108	VC_517_1	108	0.8483	1	0.8483	0	0	0	0.9955	Ref
JN699016	Unassigned	Unassigned	Unassigned	325_42	Clustered	4	VC_325_42	4	0.0112	0.99980442	0.0112	0	0	0	0.6667	Ref
JN699017	Unassigned	Unassigned	Unassigned	371_17	Clustered	3	VC_371_17	3	0.0076	0.99811676	0.0076	0	0	0	1	Ref
JN699018	Unassigned	Unassigned	Unassigned	509_0	Clustered	37	VC_509_0	37	0.9689	1	0.9689	0	0	0	1	Ref
JN699019	Unassigned	Unassigned	Unassigned	223_0	Clustered	38	VC_223_0	38	0.9095	1	0.9095	0	0	0	1	Ref
JN699625	Unassigned	Unassigned	Unassigned	558_30	Clustered	3	VC_558_30	3	0.0064	0.76574053	0.0049	0	0	0	1	Ref
JN699626	Unassigned	Unassigned	Unassigned	558_63	Clustered/Singleton	1	VC_558_63	1	0	1	0	0	0	0	1	Ref
JN699627	Unassigned	Unassigned	Unassigned	558_78	Clustered/Singleton	1	VC_558_78	1	0	1	0	0	0	0	1	Ref
JN699628	Unassigned	Unassigned	Unassigned	763_1	Clustered	9	VC_763_1	9	0.3706	1	0.3706	0	0	0	1	Ref
JN712910	Unassigned	Unassigned	Unassigned	960_0	Clustered/Singleton	1	VC_960_0	1	0	1	0	0	0	0	1	Ref
JN790865	Unassigned	Unassigned	Unassigned	883_2	Clustered	2	VC_883_2	2	0.0181	0.88094852	0.016	0	0	0	1	Ref
JN797796	Unassigned	Unassigned	Unassigned	883_2	Clustered	2	VC_883_2	2	0.0181	0.88094852	0.016	0	0	0	1	Ref
JN797797	Unassigned	Unassigned	Unassigned	187_1	Clustered	7	VC_187_1	7	0.1423	1	0.1423	0	0	0	1	Ref
JN797798	Unassigned	Unassigned	Unassigned	187_1	Clustered	7	VC_187_1	7	0.1423	1	0.1423	0	0	0	1	Ref
JN800508	Unassigned	Unassigned	Unassigned	897_0	Clustered	6	VC_897_0	6	0.4156	1	0.4156	0	0	0	1	Ref
JN808773	Unassigned	Unassigned	Unassigned	179_0	Clustered	53	VC_179_0	53	0.9137	1	0.9137	0	0	0	1	Ref
JN811560	Unassigned	Unassigned	Unassigned	179_0	Clustered	53	VC_179_0	53	0.9137	1	0.9137	0	0	0	1	Ref
JN831653	Unassigned	Unassigned	Unassigned	234_0	Clustered	21	VC_234_0	21	0.8355	1	0.8355	0	0	0	1	Ref
JN848801	Unassigned	Unassigned	Unassigned	26_0	Clustered	4	VC_26_0	4	0.7433	0.99936153	0.7428	0	0	0	1	Ref
JN849462	Unassigned	Unassigned	Unassigned	531_1	Clustered	6	VC_531_1	6	0.1481	0.99978665	0.148	0	0	0	1	Ref
JN859129	Unassigned	Unassigned	Unassigned	520_23	Clustered	2	VC_520_23	2	0.0077	0.95763408	0.0074	0	0	0	1	Ref
JN871591	Unassigned	Unassigned	Unassigned	592_0	Clustered	50	VC_592_0	50	0.7314	1	0.7314	0	0	0	1	Ref
JN882264	Unassigned	Unassigned	Unassigned	834_0	Clustered	7	VC_834_0	7	0.9845	0.94276893	0.9282	0	0	0	1	Ref
JN882266	Unassigned	Unassigned	Unassigned	834_0	Clustered	7	VC_834_0	7	0.9845	0.94276893	0.9282	0	0	0	1	Ref
JN882267	Unassigned	Unassigned	Unassigned	962_0	Clustered	2	VC_962_0	2	0.3572	0.92263554	0.3295	0	0	0	1	Ref
JN882284	Unassigned	Unassigned	Unassigned	582_4	Clustered	3	VC_582_4	3	0.0348	0.98173503	0.0341	0	0	0	1	Ref
JN882285	Unassigned	Unassigned	Unassigned	164_4	Clustered/Singleton	1	VC_164_4	1	0	1	0	0	0	0	1	Ref
JN882286	Unassigned	Unassigned	Unassigned	812_2	Clustered	2	VC_812_2	2	0.0668	0.95518138	0.0639	0	0	0	1	Ref
JN882287	Unassigned	Unassigned	Unassigned	669_0	Clustered	21	VC_669_0	21	0.3623	1	0.3623	0	0	0	1	Ref
JN882298	Unassigned	Unassigned	Unassigned	72_0	Clustered	2	VC_72_0	2	0.0777	0.95616461	0.0743	0	0	0	1	Ref
JN939331	Unassigned	Unassigned	Unassigned	879_0	Clustered	36	VC_879_0	36	0.994	1	0.994	0	0	0	1	Ref
JN939332	Unassigned	Unassigned	Unassigned	879_0	Clustered	36	VC_879_0	36	0.994	1	0.994	0	0	0	1	Ref
JN968479	Unassigned	Unassigned	Unassigned	666_0	Clustered	4	VC_666_0	4	1	1	1	0	0	0	1	Ref
JN984867	Unassigned	Unassigned	Unassigned	835_0	Clustered	67	VC_835_0	67	0.9624	1	0.9624	0	0	0	1	Ref
JN986844	Unassigned	Unassigned	Unassigned	452_0	Clustered	66	VC_452_0	66	0.9248	1	0.9248	0	0	0	1	Ref
JN986845	Unassigned	Unassigned	Unassigned	456_0	Clustered	62	VC_456_0	62	0.9026	1	0.9026	0	0	0	1	Ref
JN986846	Unassigned	Unassigned	Unassigned	283_115	Clustered/Singleton	1	VC_283_115	1	0	1	0	0	0	0	1	Ref
JQ007353	Unassigned	Unassigned	Unassigned	637_0	Clustered	115	VC_637_0	115	0.731	1	0.731	0	0	0	0.8764	Ref
JQ010983	Unassigned	Unassigned	Unassigned	943_0	Clustered	2	VC_943_0	2	1	1	1	0	0	0	1	Ref
JQ011317	Unassigned	Unassigned	Unassigned	476_0	Clustered	19	VC_476_0	19	0.7814	1	0.7814	0	0	0	1	Ref
JQ011318	Unassigned	Unassigned	Unassigned	42_0	Clustered	49	VC_42_0	49	0.4517	1	0.4517	0	0	0	0.8597	Ref
JQ015307	Unassigned	Unassigned	Unassigned	849_2	Clustered	2	VC_849_2	2	0.0246	0.86513761	0.0213	0	0	0	1	Ref
JQ031132	Unassigned	Unassigned	Unassigned	772_4	Clustered	4	VC_772_4	4	0.0181	0.73018303	0.0132	0	0	0	1	Ref
JQ062992	Unassigned	Unassigned	Unassigned	704_0	Clustered	16	VC_704_0	16	0.7274	1	0.7274	0	0	0	1	Ref
JQ067084	Unassigned	Unassigned	Unassigned	465_0	Clustered	7	VC_465_0	7	0.3322	1	0.3322	0	0	0	1	Ref
JQ067085	Unassigned	Unassigned	Unassigned	179_0	Clustered	53	VC_179_0	53	0.9137	1	0.9137	0	0	0	1	Ref
JQ067087	Unassigned	Unassigned	Unassigned	464_0	Clustered	8	VC_464_0	8	0.2629	1	0.2629	0	0	0	1	Ref
JQ067089	Unassigned	Unassigned	Unassigned	169_0	Clustered	20	VC_169_0	20	0.7529	1	0.7529	0	0	0	1	Ref
JQ067092	Unassigned	Unassigned	Unassigned	749_0	Clustered	40	VC_749_0	40	0.7517	1	0.7517	0	0	0	1	Ref
JQ067093	Unassigned	Unassigned	Unassigned	169_0	Clustered	20	VC_169_0	20	0.7529	1	0.7529	0	0	0	1	Ref
JQ086375	Unassigned	Unassigned	Unassigned	835_0	Clustered	67	VC_835_0	67	0.9624	1	0.9624	0	0	0	1	Ref
JQ086376	Unassigned	Unassigned	Unassigned	43_0	Clustered	38	VC_43_0	38	0.5885	1	0.5885	0	0	0	0.9659	Ref
JQ177061	Unassigned	Unassigned	Unassigned	121_0	Clustered	4	VC_121_0	4	0.1814	0.99998531	0.1814	0	0	0	1	Ref
JQ177063	Unassigned	Unassigned	Unassigned	369_0	Clustered	6	VC_369_0	6	0.5924	1	0.5924	0	0	0	1	Ref
JQ177064	Unassigned	Unassigned	Unassigned	121_0	Clustered	4	VC_121_0	4	0.1814	0.99998531	0.1814	0	0	0	1	Ref
JQ182728	Unassigned	Unassigned	Unassigned	51_0	Clustered	10	VC_51_0	10	0.1741	1	0.1741	0	0	0	1	Ref
JQ182729	Unassigned	Unassigned	Unassigned	33_0	Clustered	4	VC_33_0	4	0.06	0.99994826	0.06	0	0	0	1	Ref
JQ182733	Unassigned	Unassigned	Unassigned	30_0	Clustered	2	VC_30_0	2	0.037	0.95734635	0.0354	0	0	0	1	Ref
JQ182734	Unassigned	Unassigned	Unassigned	30_0	Clustered	2	VC_30_0	2	0.037	0.95734635	0.0354	0	0	0	1	Ref
JQ182735	Unassigned	Unassigned	Unassigned	43_0	Clustered	38	VC_43_0	38	0.5885	1	0.5885	0	0	0	0.9659	Ref
JQ182736	Unassigned	Unassigned	Unassigned	455_0	Clustered	18	VC_455_0	18	0.4598	1	0.4598	0	0	0	1	Ref
JQ245707	Unassigned	Unassigned	Unassigned	664_0	Clustered	4	VC_664_0	4	0.263	0.99998747	0.263	0	0	0	1	Ref
JQ287645	Unassigned	Unassigned	Unassigned	444_0	Clustered	2	VC_444_0	2	0.8137	0.72985431	0.5939	0	0	0	1	Ref
JQ300538	Unassigned	Unassigned	Unassigned	520_77	Clustered	3	VC_520_77	3	0.0218	0.99859911	0.0218	0	0	0	1	Ref
JQ307386	Unassigned	Unassigned	Unassigned	423_0	Clustered	45	VC_423_0	45	0.9484	1	0.9484	0	0	0	1	Ref
JQ307387	Unassigned	Unassigned	Unassigned	749_0	Clustered	40	VC_749_0	40	0.7517	1	0.7517	0	0	0	1	Ref
JQ309827	Unassigned	Unassigned	Unassigned	967_0	Clustered	16	VC_967_0	16	0.8971	1	0.8971	0	0	0	1	Ref
JQ312117	Unassigned	Unassigned	Unassigned	969_0	Clustered	3	VC_969_0	3	0.8314	0.99762851	0.8295	0	0	0	1	Ref
JQ340389	Unassigned	Unassigned	Unassigned	971_1	Clustered	24	VC_971_1	24	0.7393	1	0.7393	0	0	0	0.8913	Ref
JQ340774	Unassigned	Unassigned	Unassigned	592_0	Clustered	50	VC_592_0	50	0.7314	1	0.7314	0	0	0	1	Ref
JQ347801	Unassigned	Unassigned	Unassigned	41_0	Clustered	16	VC_41_0	16	0.2025	1	0.2025	0	0	0	0.9667	Ref
JQ408219	Unassigned	Unassigned	Unassigned	54_0	Clustered	6	VC_54_0	6	0.6725	0.9999999	0.6725	0	0	0	1	Ref

JQ512844	Unassigned	Unassigned	Unassigned	515_0	Clustered	16	VC_515_0	16	0.3793	1	0.3793	0	0	0	1	Ref
JQ513383	Unassigned	Unassigned	Unassigned	164_10	Clustered	5	VC_164_10	5	0.0615	0.96483582	0.0593	0	0	0	1	Ref
JQ617284	Unassigned	Unassigned	Unassigned	99_0	Clustered	32	VC_99_0	32	0.8828	1	0.8828	0	0	0	1	Ref
JQ619704	Unassigned	Unassigned	Unassigned	828_0	Clustered	3	VC_828_0	3	0.4748	0.99843555	0.4741	0	0	0	1	Ref
JQ659259	Unassigned	Unassigned	Unassigned	861_0	Clustered	9	VC_861_0	9	0.5869	1	0.5869	0	0	0	1	Ref
JQ684677	Unassigned	Unassigned	Unassigned	940_0	Clustered	34	VC_940_0	34	0.7959	1	0.7959	0	0	0	1	Ref
JQ686190	Unassigned	Unassigned	Unassigned	149_15	Clustered	16	VC_149_15	16	0.0788	1	0.0788	0	0	0	0.8083	Ref
JQ691610	Unassigned	Unassigned	Unassigned	476_0	Clustered	19	VC_476_0	19	0.7814	1	0.7814	0	0	0	1	Ref
JQ691611	Unassigned	Unassigned	Unassigned	849_6	Clustered	7	VC_849_6	7	0.164	0.99999999	0.164	0	0	0	0.9524	Ref
JQ691612	Unassigned	Unassigned	Unassigned	849_5	Clustered	3	VC_849_5	3	0.0492	0.97574727	0.048	0	0	0	1	Ref
JQ692107	Unassigned	Unassigned	Unassigned	882_0	Clustered	20	VC_882_0	20	0.9917	1	0.9917	0	0	0	1	Ref
JQ698665	Unassigned	Unassigned	Unassigned	325_35	Clustered	17	VC_325_35	17	0.0732	1	0.0732	0	0	0	0.7721	Ref
JQ729990	Unassigned	Unassigned	Unassigned	898_0	Clustered	10	VC_898_0	10	0.7284	1	0.7284	0	0	0	1	Ref
JQ729991	Unassigned	Unassigned	Unassigned	898_0	Clustered	10	VC_898_0	10	0.7284	1	0.7284	0	0	0	1	Ref
JQ729992	Unassigned	Unassigned	Unassigned	898_0	Clustered	10	VC_898_0	10	0.7284	1	0.7284	0	0	0	1	Ref
JQ762257	Unassigned	Unassigned	Unassigned	179_0	Clustered	53	VC_179_0	53	0.9137	1	0.9137	0	0	0	1	Ref
JQ764986	Unassigned	Unassigned	Unassigned	303_0	Clustered	155	VC_303_0	155	0.9979	1	0.9979	0	0	0	1	Ref
JQ764987	Unassigned	Unassigned	Unassigned	303_0	Clustered	155	VC_303_0	155	0.9979	1	0.9979	0	0	0	1	Ref
JQ764988	Unassigned	Unassigned	Unassigned	303_0	Clustered	155	VC_303_0	155	0.9979	1	0.9979	0	0	0	1	Ref
JQ764989	Unassigned	Unassigned	Unassigned	303_0	Clustered	155	VC_303_0	155	0.9979	1	0.9979	0	0	0	1	Ref
JQ764990	Unassigned	Unassigned	Unassigned	303_0	Clustered	155	VC_303_0	155	0.9979	1	0.9979	0	0	0	1	Ref
JQ779023	Unassigned	Unassigned	Unassigned	8_0	Clustered	58	VC_8_0	58	0.8306	1	0.8306	0	0	0	0.9946	Ref
JQ779024	Unassigned	Unassigned	Unassigned	9_0	Clustered	38	VC_9_0	38	0.4895	1	0.4895	0	0	0	1	Ref
JQ780163	Unassigned	Unassigned	Unassigned	82_0	Clustered	24	VC_82_0	24	0.7351	1	0.7351	0	0	0	1	Ref
JQ780327	Unassigned	Unassigned	Unassigned	230_0	Clustered	3	VC_230_0	3	0.0739	0.9934951	0.0734	0	0	0	0.6667	Ref
JQ797329	Unassigned	Unassigned	Unassigned	739_0	Clustered	26	VC_739_0	26	0.9571	1	0.9571	0	0	0	1	Ref
JQ806763	Unassigned	Unassigned	Unassigned	28_1	Clustered	53	VC_28_1	53	0.7164	1	0.7164	0	0	0	0.9325	Ref
JQ809650	Unassigned	Unassigned	Unassigned	304_0	Clustered	11	VC_304_0	11	0.7869	1	0.7869	0	0	0	1	Ref
JQ809701	Unassigned	Unassigned	Unassigned	517_1	Clustered	108	VC_517_1	108	0.8483	1	0.8483	0	0	0	0.9955	Ref
JQ809703	Unassigned	Unassigned	Unassigned	325_35	Clustered	17	VC_325_35	17	0.0732	1	0.0732	0	0	0	0.7721	Ref
JQ823122	Unassigned	Unassigned	Unassigned	974_0	Clustered	2	VC_974_0	2	1	1	1	0	0	0	1	Ref
JQ823123	Unassigned	Unassigned	Unassigned	974_0	Clustered	2	VC_974_0	2	1	1	1	0	0	0	1	Ref
JQ837901	Unassigned	Unassigned	Unassigned	452_0	Clustered	66	VC_452_0	66	0.9248	1	0.9248	0	0	0	1	Ref
JQ896627	Unassigned	Unassigned	Unassigned	517_1	Clustered	108	VC_517_1	108	0.8483	1	0.8483	0	0	0	0.9955	Ref
JQ911768	Unassigned	Unassigned	Unassigned	558_50	Clustered	15	VC_558_50	15	0.0468	1	0.0468	0	0	0	0.9238	Ref
JQ957925	Unassigned	Unassigned	Unassigned	392_0	Clustered	126	VC_392_0	126	0.9621	1	0.9621	0	0	0	1	Ref
JQ957932	Unassigned	Unassigned	Unassigned	8_0	Clustered	58	VC_8_0	58	0.8306	1	0.8306	0	0	0	0.9946	Ref
JQ965645	Unassigned	Unassigned	Unassigned	723_0	Clustered	9	VC_723_0	9	0.7138	1	0.7138	0	0	0	1	Ref
JQ965700	Unassigned	Unassigned	Unassigned	392_0	Clustered	126	VC_392_0	126	0.9621	1	0.9621	0	0	0	1	Ref
JQ965701	Unassigned	Unassigned	Unassigned	392_0	Clustered	126	VC_392_0	126	0.9621	1	0.9621	0	0	0	1	Ref
JQ965702	Unassigned	Unassigned	Unassigned	386_0	Clustered	49	VC_386_0	49	0.7941	1	0.7941	0	0	0	1	Ref
JQ965703	Unassigned	Unassigned	Unassigned	392_0	Clustered	126	VC_392_0	126	0.9621	1	0.9621	0	0	0	1	Ref
JQ966307	Unassigned	Unassigned	Unassigned	315_0	Clustered	12	VC_315_0	12	0.8947	1	0.8947	0	0	0	1	Ref
JQ973847	Unassigned	Unassigned	Unassigned	12_0	Clustered	21	VC_12_0	21	0.2274	1	0.2274	0	0	0	0.9571	Ref
JX000007	Unassigned	Unassigned	Unassigned	392_0	Clustered	126	VC_392_0	126	0.9621	1	0.9621	0	0	0	1	Ref
JX013863	Unassigned	Unassigned	Unassigned	12_2	Clustered	19	VC_12_2	19	0.2018	1	0.2018	0	0	0	1	Ref
JX042578	Unassigned	Unassigned	Unassigned	940_0	Clustered	34	VC_940_0	34	0.7959	1	0.7959	0	0	0	1	Ref
JX042579	Unassigned	Unassigned	Unassigned	237_1	Clustered	13	VC_237_1	13	0.1773	1	0.1773	0	0	0	0.7308	Ref
JX080300	Unassigned	Unassigned	Unassigned	149_8	Clustered	66	VC_149_8	66	0.4565	1	0.4565	0	0	0	0.8182	Ref
JX080301	Unassigned	Unassigned	Unassigned	149_8	Clustered	66	VC_149_8	66	0.4565	1	0.4565	0	0	0	0.8182	Ref
JX080302	Unassigned	Unassigned	Unassigned	149_8	Clustered	66	VC_149_8	66	0.4565	1	0.4565	0	0	0	0.8182	Ref
JX080303	Unassigned	Unassigned	Unassigned	149_8	Clustered	66	VC_149_8	66	0.4565	1	0.4565	0	0	0	0.8182	Ref
JX080304	Unassigned	Unassigned	Unassigned	149_8	Clustered	66	VC_149_8	66	0.4565	1	0.4565	0	0	0	0.8182	Ref
JX080305	Unassigned	Unassigned	Unassigned	149_8	Clustered	66	VC_149_8	66	0.4565	1	0.4565	0	0	0	0.8182	Ref
JX094431	Unassigned	Unassigned	Unassigned	187_3	Clustered	9	VC_187_3	9	0.1971	1	0.1971	0	0	0	0.8611	Ref
JX094499	Unassigned	Unassigned	Unassigned	592_0	Clustered	50	VC_592_0	50	0.7314	1	0.7314	0	0	0	1	Ref
JX094500	Unassigned	Unassigned	Unassigned	959_0	Clustered	5	VC_959_0	5	0.0499	0.99999231	0.0499	0	0	0	1	Ref
JX094501	Unassigned	Unassigned	Unassigned	12_2	Clustered	19	VC_12_2	19	0.2018	1	0.2018	0	0	0	1	Ref
JX100809	Unassigned	Unassigned	Unassigned	976_4	Clustered	11	VC_976_4	11	0.3364	1	0.3364	0	0	0	1	Ref
JX100810	Unassigned	Unassigned	Unassigned	976_2	Clustered	2	VC_976_2	2	0.0278	0.95410794	0.0266	0	0	0	1	Ref
JX100811	Unassigned	Unassigned	Unassigned	976_4	Clustered	11	VC_976_4	11	0.3364	1	0.3364	0	0	0	1	Ref
JX100812	Unassigned	Unassigned	Unassigned	976_4	Clustered	11	VC_976_4	11	0.3364	1	0.3364	0	0	0	1	Ref
JX100813	Unassigned	Unassigned	Unassigned	976_4	Clustered	11	VC_976_4	11	0.3364	1	0.3364	0	0	0	1	Ref
JX100814	Unassigned	Unassigned	Unassigned	976_3	Clustered	2	VC_976_3	2	0.0258	0.88402892	0.0228	0	0	0	1	Ref
JX104231	Unassigned	Unassigned	Unassigned	162_0	Clustered	18	VC_162_0	18	0.7403	1	0.7403	0	0	0	0.8627	Ref
JX120799	Unassigned	Unassigned	Unassigned	379_0	Clustered	5	VC_379_0	5	0.4779	0.99999992	0.4779	0	0	0	0.9	Ref
JX121091	Unassigned	Unassigned	Unassigned	520_26	Clustered	3	VC_520_26	3	0.026	0.99859898	0.026	0	0	0	1	Ref
JX123262	Unassigned	Unassigned	Unassigned	443_2	Clustered	10	VC_443_2	10	0.3344	1	0.3344	0	0	0	0.9333	Ref
JX126918	Unassigned	Unassigned	Unassigned	739_0	Clustered	26	VC_739_0	26	0.9571	1	0.9571	0	0	0	1	Ref
JX126919	Unassigned	Unassigned	Unassigned	980_0	Clustered	10	VC_980_0	10	0.9996	1	0.9996	0	0	0	1	Ref
JX126920	Unassigned	Unassigned	Unassigned	980_0	Clustered	10	VC_980_0	10	0.9996	1	0.9996	0	0	0	1	Ref
JX128257	Unassigned	Unassigned	Unassigned	133_22	Clustered	13	VC_133_22	13	0.0804	1	0.0804	0	0	0	0.8205	Ref
JX128258	Unassigned	Unassigned	Unassigned	618_0	Clustered	13	VC_618_0	13	0.6252	1	0.6252	0	0	0	1	Ref
JX128259	Unassigned	Unassigned	Unassigned	283_95	Clustered/Singleton	1	VC_283_95	1	0	1	0	0	0	0	1	Ref
JX131330	Unassigned	Unassigned	Unassigned	166_0	Clustered	26	VC_166_0	26	0.7923	1	0.7923	0	0	0	1	Ref
JX145342	Unassigned	Unassigned	Unassigned	647_0	Clustered	11	VC_647_0	11	0.598	1	0.598	0	0	0	1	Ref
JX163858	Unassigned	Unassigned	Unassigned	976_4	Clustered	11	VC_976_4	11	0.3364	1	0.3364	0	0	0	1	Ref
JX173487	Unassigned	Unassigned	Unassigned	130_0	Clustered	19	VC_130_0	19	0.6068	1	0.6068	0	0	0	1	Ref
JX174275	Unassigned	Unassigned	Unassigned	8_0	Clustered	58	VC_8_0	58	0.8306	1	0.8306	0	0	0	0.9946	Ref
JX181822	Unassigned	Unassigned	Unassigned	329_1	Clustered	17	VC_329_1	17	0.0867	1	0.0867	0	0	0	0.8235	Ref
JX181824	Unassigned	Unassigned	Unassigned	582_5	Clustered	4	VC_582_5	4	0.0528	0.99935625	0.0527	0	0	0	0.8333	Ref

JX181828	Unassigned	Unassigned	Unassigned	133_22	Clustered	13	VC_133_22	13	0.0804	1	0.0804	0	0	0	0.8205	Ref
JX181829	Unassigned	Unassigned	Unassigned	131_2	Clustered	7	VC_131_2	7	0.0685	1	0.0685	0	0	0	0.9048	Ref
JX182369	Unassigned	Unassigned	Unassigned	981_0	Clustered	3	VC_981_0	3	0.0531	0.99853685	0.053	0	0	0	1	Ref
JX182370	Unassigned	Unassigned	Unassigned	981_1	Clustered	26	VC_981_1	26	0.7009	1	0.7009	0	0	0	1	Ref
JX182371	Unassigned	Unassigned	Unassigned	727_0	Clustered	11	VC_727_0	11	0.5054	1	0.5054	0	0	0	1	Ref
JX182372	Unassigned	Unassigned	Unassigned	373_0	Clustered	10	VC_373_0	10	0.7471	1	0.7471	0	0	0	1	Ref
JX193904	Unassigned	Unassigned	Unassigned	695_0	Clustered	48	VC_695_0	48	0.8162	1	0.8162	0	0	0	1	Ref
JX194238	Unassigned	Unassigned	Unassigned	838_0	Clustered	29	VC_838_0	29	0.8611	1	0.8611	0	0	0	1	Ref
JX194239	Unassigned	Unassigned	Unassigned	153_0	Clustered	25	VC_153_0	25	0.7729	1	0.7729	0	0	0	1	Ref
JX202565	Unassigned	Unassigned	Unassigned	637_0	Clustered	115	VC_637_0	115	0.731	1	0.731	0	0	0	0.8764	Ref
JX233784	Unassigned	Unassigned	Unassigned	344_0	Clustered	21	VC_344_0	21	0.5436	1	0.5436	0	0	0	1	Ref
JX238501	Unassigned	Unassigned	Unassigned	85_6	Clustered/Singleton	1	VC_85_6	1	0	1	0	0	0	0	1	Ref
JX262215	Unassigned	Unassigned	Unassigned	765_0	Clustered	132	VC_765_0	132	1	1	1	0	0	0	1	Ref
JX262216	Unassigned	Unassigned	Unassigned	765_0	Clustered	132	VC_765_0	132	1	1	1	0	0	0	1	Ref
JX262217	Unassigned	Unassigned	Unassigned	765_0	Clustered	132	VC_765_0	132	1	1	1	0	0	0	1	Ref
JX262218	Unassigned	Unassigned	Unassigned	765_0	Clustered	132	VC_765_0	132	1	1	1	0	0	0	1	Ref
JX262219	Unassigned	Unassigned	Unassigned	765_0	Clustered	132	VC_765_0	132	1	1	1	0	0	0	1	Ref
JX262220	Unassigned	Unassigned	Unassigned	765_0	Clustered	132	VC_765_0	132	1	1	1	0	0	0	1	Ref
JX262221	Unassigned	Unassigned	Unassigned	765_0	Clustered	132	VC_765_0	132	1	1	1	0	0	0	1	Ref
JX262222	Unassigned	Unassigned	Unassigned	765_0	Clustered	132	VC_765_0	132	1	1	1	0	0	0	1	Ref
JX262223	Unassigned	Unassigned	Unassigned	765_0	Clustered	132	VC_765_0	132	1	1	1	0	0	0	1	Ref
JX262224	Unassigned	Unassigned	Unassigned	765_0	Clustered	132	VC_765_0	132	1	1	1	0	0	0	1	Ref
JX262225	Unassigned	Unassigned	Unassigned	765_0	Clustered	132	VC_765_0	132	1	1	1	0	0	0	1	Ref
JX262376	Unassigned	Unassigned	Unassigned	981_1	Clustered	26	VC_981_1	26	0.7009	1	0.7009	0	0	0	1	Ref
JX274646	Unassigned	Unassigned	Unassigned	9_0	Clustered	38	VC_9_0	38	0.4895	1	0.4895	0	0	0	1	Ref
JX274647	Unassigned	Unassigned	Unassigned	9_0	Clustered	38	VC_9_0	38	0.4895	1	0.4895	0	0	0	1	Ref
JX290549	Unassigned	Unassigned	Unassigned	984_0	Clustered	38	VC_984_0	38	0.9758	1	0.9758	0	0	0	1	Ref
JX296113	Unassigned	Unassigned	Unassigned	592_0	Clustered	50	VC_592_0	50	0.7314	1	0.7314	0	0	0	1	Ref
JX297445	Unassigned	Unassigned	Unassigned	637_1	Clustered	27	VC_637_1	27	0.2578	1	0.2578	0	0	0	0.9858	Ref
JX306041	Unassigned	Unassigned	Unassigned	600_2	Clustered	10	VC_600_2	10	0.3041	1	0.3041	0	0	0	1	Ref
JX307702	Unassigned	Unassigned	Unassigned	517_1	Clustered	108	VC_517_1	108	0.8483	1	0.8483	0	0	0	0.9955	Ref
JX307703	Unassigned	Unassigned	Unassigned	517_1	Clustered	108	VC_517_1	108	0.8483	1	0.8483	0	0	0	0.9955	Ref
JX307704	Unassigned	Unassigned	Unassigned	515_0	Clustered	16	VC_515_0	16	0.3793	1	0.3793	0	0	0	1	Ref
JX307705	Unassigned	Unassigned	Unassigned	325_42	Clustered	4	VC_325_42	4	0.0112	0.99980442	0.0112	0	0	0	0.6667	Ref
JX316028	Unassigned	Unassigned	Unassigned	959_4	Clustered	3	VC_959_4	3	0.0237	0.77138817	0.0183	0	0	0	1	Ref
JX403939	Unassigned	Unassigned	Unassigned	297_1	Clustered	20	VC_297_1	20	0.5653	1	0.5653	0	0	0	0.7684	Ref
JX403940	Unassigned	Unassigned	Unassigned	696_1	Clustered	8	VC_696_1	8	0.5452	1	0.5452	0	0	0	0.9286	Ref
JX411619	Unassigned	Unassigned	Unassigned	515_0	Clustered	16	VC_515_0	16	0.3793	1	0.3793	0	0	0	1	Ref
JX412914	Unassigned	Unassigned	Unassigned	61_0	Clustered	12	VC_61_0	12	0.7633	1	0.7633	0	0	0	1	Ref
JX415535	Unassigned	Unassigned	Unassigned	779_0	Clustered	37	VC_779_0	37	0.5682	1	0.5682	0	0	0	1	Ref
JX415536	Unassigned	Unassigned	Unassigned	813_0	Clustered	12	VC_813_0	12	0.2347	1	0.2347	0	0	0	1	Ref
JX421753	Unassigned	Unassigned	Unassigned	385_0	Clustered	51	VC_385_0	51	0.8754	1	0.8754	0	0	0	1	Ref
JX434030	Unassigned	Unassigned	Unassigned	179_0	Clustered	53	VC_179_0	53	0.9137	1	0.9137	0	0	0	1	Ref
JX434031	Unassigned	Unassigned	Unassigned	179_0	Clustered	53	VC_179_0	53	0.9137	1	0.9137	0	0	0	1	Ref
JX434032	Unassigned	Unassigned	Unassigned	179_0	Clustered	53	VC_179_0	53	0.9137	1	0.9137	0	0	0	1	Ref
JX434033	Unassigned	Unassigned	Unassigned	179_0	Clustered	53	VC_179_0	53	0.9137	1	0.9137	0	0	0	1	Ref
JX442241	Unassigned	Unassigned	Unassigned	980_0	Clustered	10	VC_980_0	10	0.9996	1	0.9996	0	0	0	1	Ref
JX483873	Unassigned	Unassigned	Unassigned	481_0	Clustered	8	VC_481_0	8	0.1348	1	0.1348	0	0	0	1	Ref
JX483874	Unassigned	Unassigned	Unassigned	987_0	Clustered	22	VC_987_0	22	0.8955	1	0.8955	0	0	0	1	Ref
JX483875	Unassigned	Unassigned	Unassigned	987_0	Clustered	22	VC_987_0	22	0.8955	1	0.8955	0	0	0	1	Ref
JX483876	Unassigned	Unassigned	Unassigned	946_0	Clustered	11	VC_946_0	11	0.7352	1	0.7352	0	0	0	1	Ref
JX483877	Unassigned	Unassigned	Unassigned	946_0	Clustered	11	VC_946_0	11	0.7352	1	0.7352	0	0	0	1	Ref
JX483878	Unassigned	Unassigned	Unassigned	946_0	Clustered	11	VC_946_0	11	0.7352	1	0.7352	0	0	0	1	Ref
JX483879	Unassigned	Unassigned	Unassigned	987_0	Clustered	22	VC_987_0	22	0.8955	1	0.8955	0	0	0	1	Ref
JX483880	Unassigned	Unassigned	Unassigned	987_0	Clustered	22	VC_987_0	22	0.8955	1	0.8955	0	0	0	1	Ref
JX483881	Unassigned	Unassigned	Unassigned	724_1	Clustered	6	VC_724_1	6	0.4379	1	0.4379	0	0	0	1	Ref
JX486087	Unassigned	Unassigned	Unassigned	577_0	Clustered	6	VC_577_0	6	0.9267	0.99999993	0.9267	0	0	0	1	Ref
JX486088	Unassigned	Unassigned	Unassigned	634_1	Clustered	6	VC_634_1	6	0.4228	1	0.4228	0	0	0	1	Ref
JX495041	Unassigned	Unassigned	Unassigned	251_0	Clustered	22	VC_251_0	22	0.6508	1	0.6508	0	0	0	1	Ref
JX495042	Unassigned	Unassigned	Unassigned	251_0	Clustered	22	VC_251_0	22	0.6508	1	0.6508	0	0	0	1	Ref
JX495043	Unassigned	Unassigned	Unassigned	251_0	Clustered	22	VC_251_0	22	0.6508	1	0.6508	0	0	0	1	Ref
JX501340	Unassigned	Unassigned	Unassigned	423_0	Clustered	45	VC_423_0	45	0.9484	1	0.9484	0	0	0	1	Ref
JX507079	Unassigned	Unassigned	Unassigned	24_0	Clustered	3	VC_24_0	3	0.3455	0.99844825	0.345	0	0	0	1	Ref
JX536493	Unassigned	Unassigned	Unassigned	286_14	Clustered/Singleton	1	VC_286_14	1	0	1	0	0	0	0	1	Ref
JX556417	Unassigned	Unassigned	Unassigned	23_0	Clustered	3	VC_23_0	3	0.4305	0.99837378	0.4298	0	0	0	1	Ref
JX556418	Unassigned	Unassigned	Unassigned	882_0	Clustered	20	VC_882_0	20	0.9917	1	0.9917	0	0	0	1	Ref
JX560968	Unassigned	Unassigned	Unassigned	329_18	Clustered	7	VC_329_18	7	0.0328	1	0.0328	0	0	0	0.8095	Ref
JX561091	Unassigned	Unassigned	Unassigned	847_1	Clustered	26	VC_847_1	26	0.6323	1	0.6323	0	0	0	0.9815	Ref
JX564242	Unassigned	Unassigned	Unassigned	268_0	Clustered	7	VC_268_0	7	0.3427	1	0.3427	0	0	0	1	Ref
JX567312	Unassigned	Unassigned	Unassigned	268_0	Clustered	7	VC_268_0	7	0.3427	1	0.3427	0	0	0	1	Ref
JX569801	Unassigned	Unassigned	Unassigned	844_0	Clustered	27	VC_844_0	27	0.9385	1	0.9385	0	0	0	1	Ref
JX570702	Unassigned	Unassigned	Unassigned	765_0	Clustered	132	VC_765_0	132	1	1	1	0	0	0	1	Ref
JX570703	Unassigned	Unassigned	Unassigned	765_0	Clustered	132	VC_765_0	132	1	1	1	0	0	0	1	Ref
JX570704	Unassigned	Unassigned	Unassigned	765_0	Clustered	132	VC_765_0	132	1	1	1	0	0	0	1	Ref
JX570705	Unassigned	Unassigned	Unassigned	765_0	Clustered	132	VC_765_0	132	1	1	1	0	0	0	1	Ref
JX570706	Unassigned	Unassigned	Unassigned	765_0	Clustered	132	VC_765_0	132	1	1	1	0	0	0	1	Ref
JX570707	Unassigned	Unassigned	Unassigned	765_0	Clustered	132	VC_765_0	132	1	1	1	0	0	0	1	Ref
JX570708	Unassigned	Unassigned	Unassigned	765_0	Clustered	132	VC_765_0	132	1	1	1	0	0	0	1	Ref
JX570709	Unassigned	Unassigned	Unassigned	765_0	Clustered	132	VC_765_0	132	1	1	1	0	0	0	1	Ref
JX570710	Unassigned	Unassigned	Unassigned	765_0	Clustered	132	VC_765_0	132	1	1	1	0	0	0	1	Ref
JX570711	Unassigned	Unassigned	Unassigned	765_0	Clustered	132	VC_765_0	132	1	1	1	0	0	0	1	Ref

JX570712	Unassigned	Unassigned	Unassigned	765_0	Clustered	132	VC_765_0	132	1	1	1	0	0	0	1	Ref
JX570713	Unassigned	Unassigned	Unassigned	765_0	Clustered	132	VC_765_0	132	1	1	1	0	0	0	1	Ref
JX570714	Unassigned	Unassigned	Unassigned	765_0	Clustered	132	VC_765_0	132	1	1	1	0	0	0	1	Ref
JX625144	Unassigned	Unassigned	Unassigned	314_0	Clustered	2	VC_314_0	2	0.1808	0.99999983	0.1808	0	0	0	1	Ref
JX649096	Unassigned	Unassigned	Unassigned	371_16	Clustered	42	VC_371_16	42	0.1341	1	0.1341	0	0	0	0.7282	Ref
JX649097	Unassigned	Unassigned	Unassigned	371_16	Clustered	42	VC_371_16	42	0.1341	1	0.1341	0	0	0	0.7282	Ref
JX649098	Unassigned	Unassigned	Unassigned	371_3	Clustered	22	VC_371_3	22	0.0787	1	0.0787	0	0	0	0.9048	Ref
JX649099	Unassigned	Unassigned	Unassigned	371_3	Clustered	22	VC_371_3	22	0.0787	1	0.0787	0	0	0	0.9048	Ref
JX649100	Unassigned	Unassigned	Unassigned	371_16	Clustered	42	VC_371_16	42	0.1341	1	0.1341	0	0	0	0.7282	Ref
JX658790	Unassigned	Unassigned	Unassigned	699_0	Clustered	71	VC_699_0	71	0.9352	1	0.9352	0	0	0	1	Ref
JX681814	Unassigned	Unassigned	Unassigned	3_0	Clustered	17	VC_3_0	17	0.5639	1	0.5639	0	0	0	1	Ref
JX846612	Unassigned	Unassigned	Unassigned	153_0	Clustered	25	VC_153_0	25	0.7729	1	0.7729	0	0	0	1	Ref
JX846613	Unassigned	Unassigned	Unassigned	153_0	Clustered	25	VC_153_0	25	0.7729	1	0.7729	0	0	0	1	Ref
JX863101	Unassigned	Unassigned	Unassigned	472_0	Clustered	47	VC_472_0	47	0.9025	1	0.9025	0	0	0	1	Ref
JX865427	Unassigned	Unassigned	Unassigned	835_0	Clustered	67	VC_835_0	67	0.9624	1	0.9624	0	0	0	1	Ref
JX866719	Unassigned	Unassigned	Unassigned	123_0	Clustered	23	VC_123_0	23	0.817	1	0.817	0	0	0	1	Ref
JX867715	Unassigned	Unassigned	Unassigned	813_1	Clustered	24	VC_813_1	24	0.535	1	0.535	0	0	0	1	Ref
JX872508	Unassigned	Unassigned	Unassigned	392_0	Clustered	126	VC_392_0	126	0.9621	1	0.9621	0	0	0	1	Ref
JX875065	Unassigned	Unassigned	Unassigned	149_8	Clustered	66	VC_149_8	66	0.4565	1	0.4565	0	0	0	0.8182	Ref
JX878496	Unassigned	Unassigned	Unassigned	132_0	Clustered	4	VC_132_0	4	0.036	0.99998777	0.036	0	0	0	1	Ref
JX878671	Unassigned	Unassigned	Unassigned	149_13	Clustered	3	VC_149_13	3	0.0087	0.95178903	0.0083	0	0	0	1	Ref
JX879087	Unassigned	Unassigned	Unassigned	412_0	Clustered	39	VC_412_0	39	0.845	1	0.845	0	0	0	1	Ref
JX880034	Unassigned	Unassigned	Unassigned	779_0	Clustered	37	VC_779_0	37	0.5682	1	0.5682	0	0	0	1	Ref
JX880072	Unassigned	Unassigned	Unassigned	117_0	Clustered	9	VC_117_0	9	0.9977	0.98988637	0.9876	0	0	0	1	Ref
JX887877	Unassigned	Unassigned	Unassigned	829_0	Clustered	5	VC_829_0	5	0.8821	0.99999832	0.8821	0	0	0	1	Ref
JX889246	Unassigned	Unassigned	Unassigned	982_0	Clustered	62	VC_982_0	62	0.8646	1	0.8646	0	0	0	0.743	Ref
JX899358	Unassigned	Unassigned	Unassigned	216_6	Clustered	42	VC_216_6	42	0.473	1	0.473	0	0	0	0.8351	Ref
JX901189	Unassigned	Unassigned	Unassigned	574_0	Clustered	7	VC_574_0	7	0.2734	1	0.2734	0	0	0	1	Ref
JX912252	Unassigned	Unassigned	Unassigned	575_0	Clustered	35	VC_575_0	35	0.8815	1	0.8815	0	0	0	1	Ref
JX976549	Unassigned	Unassigned	Unassigned	698_0	Clustered	26	VC_698_0	26	0.9366	1	0.9366	0	0	0	1	Ref
JX997978	Unassigned	Unassigned	Unassigned	423_0	Clustered	45	VC_423_0	45	0.9484	1	0.9484	0	0	0	1	Ref
KC012913	Unassigned	Unassigned	Unassigned	149_8	Clustered	66	VC_149_8	66	0.4565	1	0.4565	0	0	0	0.8182	Ref
KC013021	Unassigned	Unassigned	Unassigned	861_0	Clustered	9	VC_861_0	9	0.5869	1	0.5869	0	0	0	1	Ref
KC013022	Unassigned	Unassigned	Unassigned	861_0	Clustered	9	VC_861_0	9	0.5869	1	0.5869	0	0	0	1	Ref
KC013023	Unassigned	Unassigned	Unassigned	861_0	Clustered	9	VC_861_0	9	0.5869	1	0.5869	0	0	0	1	Ref
KC013024	Unassigned	Unassigned	Unassigned	861_0	Clustered	9	VC_861_0	9	0.5869	1	0.5869	0	0	0	1	Ref
KC013025	Unassigned	Unassigned	Unassigned	861_0	Clustered	9	VC_861_0	9	0.5869	1	0.5869	0	0	0	1	Ref
KC013026	Unassigned	Unassigned	Unassigned	862_0	Clustered	11	VC_862_0	11	0.6745	1	0.6745	0	0	0	1	Ref
KC013027	Unassigned	Unassigned	Unassigned	862_0	Clustered	11	VC_862_0	11	0.6745	1	0.6745	0	0	0	1	Ref
KC013028	Unassigned	Unassigned	Unassigned	862_0	Clustered	11	VC_862_0	11	0.6745	1	0.6745	0	0	0	1	Ref
KC013029	Unassigned	Unassigned	Unassigned	862_0	Clustered	11	VC_862_0	11	0.6745	1	0.6745	0	0	0	1	Ref
KC107834	Unassigned	Unassigned	Unassigned	878_0	Clustered	32	VC_878_0	32	0.9965	1	0.9965	0	0	0	1	Ref
KC109329	Unassigned	Unassigned	Unassigned	451_0	Clustered	80	VC_451_0	80	0.9991	1	0.9991	0	0	0	1	Ref
KC117376	Unassigned	Unassigned	Unassigned	311_0	Clustered	6	VC_311_0	6	0.1997	1	0.1997	0	0	0	1	Ref
KC117377	Unassigned	Unassigned	Unassigned	313_1	Clustered	3	VC_313_1	3	0.3511	0.99802018	0.3504	0	0	0	1	Ref
KC131021	Unassigned	Unassigned	Unassigned	199_0	Clustered	12	VC_199_0	12	0.448	1	0.448	0	0	0	1	Ref
KC131022	Unassigned	Unassigned	Unassigned	995_0	Clustered	69	VC_995_0	69	0.8547	1	0.8547	0	0	0	1	Ref
KC131023	Unassigned	Unassigned	Unassigned	403_0	Clustered	20	VC_403_0	20	0.6598	1	0.6598	0	0	0	1	Ref
KC131025	Unassigned	Unassigned	Unassigned	403_0	Clustered	20	VC_403_0	20	0.6598	1	0.6598	0	0	0	1	Ref
KC131129	Unassigned	Unassigned	Unassigned	531_0	Clustered	9	VC_531_0	9	0.26	1	0.26	0	0	0	1	Ref
KC139511	Unassigned	Unassigned	Unassigned	637_0	Clustered	115	VC_637_0	115	0.731	1	0.731	0	0	0	0.8764	Ref
KC139512	Unassigned	Unassigned	Unassigned	592_0	Clustered	50	VC_592_0	50	0.7314	1	0.7314	0	0	0	1	Ref
KC139514	Unassigned	Unassigned	Unassigned	592_0	Clustered	50	VC_592_0	50	0.7314	1	0.7314	0	0	0	1	Ref
KC139515	Unassigned	Unassigned	Unassigned	592_0	Clustered	50	VC_592_0	50	0.7314	1	0.7314	0	0	0	1	Ref
KC139517	Unassigned	Unassigned	Unassigned	780_0	Clustered	8	VC_780_0	8	0.5207	1	0.5207	0	0	0	1	Ref
KC139518	Unassigned	Unassigned	Unassigned	540_0	Clustered	15	VC_540_0	15	0.2866	1	0.2866	0	0	0	1	Ref
KC139519	Unassigned	Unassigned	Unassigned	592_0	Clustered	50	VC_592_0	50	0.7314	1	0.7314	0	0	0	1	Ref
KC139520	Unassigned	Unassigned	Unassigned	780_0	Clustered	8	VC_780_0	8	0.5207	1	0.5207	0	0	0	1	Ref
KC139521	Unassigned	Unassigned	Unassigned	228_0	Clustered	60	VC_228_0	60	0.942	1	0.942	0	0	0	1	Ref
KC171646	Unassigned	Unassigned	Unassigned	272_0	Clustered	11	VC_272_0	11	0.4915	1	0.4915	0	0	0	0.8727	Ref
KC171647	Unassigned	Unassigned	Unassigned	272_0	Clustered	11	VC_272_0	11	0.4915	1	0.4915	0	0	0	0.8727	Ref
KC172839	Unassigned	Unassigned	Unassigned	513_0	Clustered	32	VC_513_0	32	0.6881	1	0.6881	0	0	0	1	Ref
KC182542	Unassigned	Unassigned	Unassigned	202_0	Clustered	102	VC_202_0	102	0.4049	1	0.4049	0	0	0	0.7876	Ref
KC182543	Unassigned	Unassigned	Unassigned	202_0	Clustered	102	VC_202_0	102	0.4049	1	0.4049	0	0	0	0.7876	Ref
KC182544	Unassigned	Unassigned	Unassigned	202_0	Clustered	102	VC_202_0	102	0.4049	1	0.4049	0	0	0	0.7876	Ref
KC182545	Unassigned	Unassigned	Unassigned	202_0	Clustered	102	VC_202_0	102	0.4049	1	0.4049	0	0	0	0.7876	Ref
KC182546	Unassigned	Unassigned	Unassigned	202_0	Clustered	102	VC_202_0	102	0.4049	1	0.4049	0	0	0	0.7876	Ref
KC182547	Unassigned	Unassigned	Unassigned	202_0	Clustered	102	VC_202_0	102	0.4049	1	0.4049	0	0	0	0.7876	Ref
KC182548	Unassigned	Unassigned	Unassigned	202_0	Clustered	102	VC_202_0	102	0.4049	1	0.4049	0	0	0	0.7876	Ref
KC182549	Unassigned	Unassigned	Unassigned	202_0	Clustered	102	VC_202_0	102	0.4049	1	0.4049	0	0	0	0.7876	Ref
KC182550	Unassigned	Unassigned	Unassigned	202_0	Clustered	102	VC_202_0	102	0.4049	1	0.4049	0	0	0	0.7876	Ref
KC182551	Unassigned	Unassigned	Unassigned	202_0	Clustered	102	VC_202_0	102	0.4049	1	0.4049	0	0	0	0.7876	Ref
KC182552	Unassigned	Unassigned	Unassigned	202_0	Clustered	102	VC_202_0	102	0.4049	1	0.4049	0	0	0	0.7876	Ref
KC206276	Unassigned	Unassigned	Unassigned	779_0	Clustered	37	VC_779_0	37	0.5682	1	0.5682	0	0	0	1	Ref
KC237308	Unassigned	Unassigned	Unassigned	362_0	Clustered	2	VC_362_0	2	0.002	0.83307735	0.0017	0	0	0	1	Ref
KC237729	Unassigned	Unassigned	Unassigned	417_0	Clustered	5	VC_417_0	5	0.6629	0.9977434	0.6614	0	0	0	0.8	Ref
KC252997	Unassigned	Unassigned	Unassigned	666_0	Clustered	4	VC_666_0	4	1	1	1	0	0	0	1	Ref
KC262634	Unassigned	Unassigned	Unassigned	630_0	Clustered	20	VC_630_0	20	0.8513	1	0.8513	0	0	0	1	Ref
KC292020	Unassigned	Unassigned	Unassigned	310_0	Clustered	33	VC_310_0	33	0.8771	1	0.8771	0	0	0	1	Ref
KC292021	Unassigned	Unassigned	Unassigned	311_0	Clustered	6	VC_311_0	6	0.1997	1	0.1997	0	0	0	1	Ref
KC292022	Unassigned	Unassigned	Unassigned	310_0	Clustered	33	VC_310_0	33	0.8771	1	0.8771	0	0	0	1	Ref

KC292023	Unassigned	Unassigned	Unassigned	459_0	Clustered	3	VC_459_0	3	0.8542	0.99054823	0.8461	0	0	0	1	Ref
KC292024	Unassigned	Unassigned	Unassigned	991_0	Clustered	2	VC_991_0	2	0.3757	0.91418477	0.3435	0	0	0	1	Ref
KC292026	Unassigned	Unassigned	Unassigned	312_0	Clustered	2	VC_312_0	2	0.0307	0.95436427	0.0293	0	0	0	1	Ref
KC292027	Unassigned	Unassigned	Unassigned	313_1	Clustered	3	VC_313_1	3	0.3511	0.99802018	0.3504	0	0	0	1	Ref
KC292028	Unassigned	Unassigned	Unassigned	991_0	Clustered	2	VC_991_0	2	0.3757	0.91418477	0.3435	0	0	0	1	Ref
KC292029	Unassigned	Unassigned	Unassigned	313_0	Clustered	2	VC_313_0	2	0.1721	0.90744399	0.1562	0	0	0	1	Ref
KC294142	Unassigned	Unassigned	Unassigned	472_0	Clustered	47	VC_472_0	47	0.9025	1	0.9025	0	0	0	1	Ref
KC295538	Unassigned	Unassigned	Unassigned	164_11	Clustered	10	VC_164_11	10	0.1507	1	0.1507	0	0	0	0.9111	Ref
KC310802	Unassigned	Unassigned	Unassigned	340_0	Clustered	31	VC_340_0	31	0.9549	1	0.9549	0	0	0	1	Ref
KC310803	Unassigned	Unassigned	Unassigned	340_0	Clustered	31	VC_340_0	31	0.9549	1	0.9549	0	0	0	1	Ref
KC310804	Unassigned	Unassigned	Unassigned	340_0	Clustered	31	VC_340_0	31	0.9549	1	0.9549	0	0	0	1	Ref
KC310805	Unassigned	Unassigned	Unassigned	340_0	Clustered	31	VC_340_0	31	0.9549	1	0.9549	0	0	0	1	Ref
KC310806	Unassigned	Unassigned	Unassigned	340_0	Clustered	31	VC_340_0	31	0.9549	1	0.9549	0	0	0	1	Ref
KC311669	Unassigned	Unassigned	Unassigned	699_0	Clustered	71	VC_699_0	71	0.9352	1	0.9352	0	0	0	1	Ref
KC330679	Unassigned	Unassigned	Unassigned	100_0	Clustered	15	VC_100_0	15	0.6536	1	0.6536	0	0	0	1	Ref
KC330680	Unassigned	Unassigned	Unassigned	100_0	Clustered	15	VC_100_0	15	0.6536	1	0.6536	0	0	0	1	Ref
KC330681	Unassigned	Unassigned	Unassigned	100_0	Clustered	15	VC_100_0	15	0.6536	1	0.6536	0	0	0	1	Ref
KC330682	Unassigned	Unassigned	Unassigned	100_0	Clustered	15	VC_100_0	15	0.6536	1	0.6536	0	0	0	1	Ref
KC330683	Unassigned	Unassigned	Unassigned	100_0	Clustered	15	VC_100_0	15	0.6536	1	0.6536	0	0	0	1	Ref
KC330684	Unassigned	Unassigned	Unassigned	100_0	Clustered	15	VC_100_0	15	0.6536	1	0.6536	0	0	0	1	Ref
KC333879	Unassigned	Unassigned	Unassigned	455_0	Clustered	18	VC_455_0	18	0.4598	1	0.4598	0	0	0	1	Ref
KC342645	Unassigned	Unassigned	Unassigned	7_0	Clustered	28	VC_7_0	28	0.3916	1	0.3916	0	0	0	0.9947	Ref
KC348600	Unassigned	Unassigned	Unassigned	317_2	Clustered	20	VC_317_2	20	0.1976	1	0.1976	0	0	0	0.6842	Ref
KC348601	Unassigned	Unassigned	Unassigned	317_2	Clustered	20	VC_317_2	20	0.1976	1	0.1976	0	0	0	0.6842	Ref
KC348602	Unassigned	Unassigned	Unassigned	317_2	Clustered	20	VC_317_2	20	0.1976	1	0.1976	0	0	0	0.6842	Ref
KC348603	Unassigned	Unassigned	Unassigned	632_0	Clustered	37	VC_632_0	37	0.581	1	0.581	0	0	0	0.9234	Ref
KC357596	Unassigned	Unassigned	Unassigned	0_0	Clustered	3	VC_0_0	3	1	1	1	0	0	0	1	Ref
KC413988	Unassigned	Unassigned	Unassigned	412_0	Clustered	39	VC_412_0	39	0.845	1	0.845	0	0	0	1	Ref
KC430106	Unassigned	Unassigned	Unassigned	884_1	Clustered	8	VC_884_1	8	0.1102	0.9989323	0.1101	0	0	0	0.75	Ref
KC438282	Unassigned	Unassigned	Unassigned	784_0	Clustered	6	VC_784_0	6	0.5517	1	0.5517	0	0	0	1	Ref
KC438283	Unassigned	Unassigned	Unassigned	784_0	Clustered	6	VC_784_0	6	0.5517	1	0.5517	0	0	0	1	Ref
KC465898	Unassigned	Unassigned	Unassigned	486_0	Clustered	18	VC_486_0	18	0.887	1	0.887	0	0	0	1	Ref
KC465901	Unassigned	Unassigned	Unassigned	480_0	Clustered	19	VC_480_0	19	0.5445	1	0.5445	0	0	0	1	Ref
KC481682	Unassigned	Unassigned	Unassigned	475_2	Clustered	3	VC_475_2	3	0.0585	0.98883022	0.0578	0	0	0	1	Ref
KC522412	Unassigned	Unassigned	Unassigned	202_0	Clustered	102	VC_202_0	102	0.4049	1	0.4049	0	0	0	0.7876	Ref
KC542353	Unassigned	Unassigned	Unassigned	644_0	Clustered	5	VC_644_0	5	0.6649	0.99999975	0.6649	0	0	0	1	Ref
KC556893	Unassigned	Unassigned	Unassigned	879_0	Clustered	36	VC_879_0	36	0.994	1	0.994	0	0	0	1	Ref
KC556894	Unassigned	Unassigned	Unassigned	879_0	Clustered	36	VC_879_0	36	0.994	1	0.994	0	0	0	1	Ref
KC556895	Unassigned	Unassigned	Unassigned	879_0	Clustered	36	VC_879_0	36	0.994	1	0.994	0	0	0	1	Ref
KC556896	Unassigned	Unassigned	Unassigned	879_0	Clustered	36	VC_879_0	36	0.994	1	0.994	0	0	0	1	Ref
KC556897	Unassigned	Unassigned	Unassigned	879_0	Clustered	36	VC_879_0	36	0.994	1	0.994	0	0	0	1	Ref
KC556898	Unassigned	Unassigned	Unassigned	879_0	Clustered	36	VC_879_0	36	0.994	1	0.994	0	0	0	1	Ref
KC576783	Unassigned	Unassigned	Unassigned	521_0	Clustered	37	VC_521_0	37	0.912	1	0.912	0	0	0	1	Ref
KC576784	Unassigned	Unassigned	Unassigned	371_2	Clustered	8	VC_371_2	8	0.0232	1	0.0232	0	0	0	0.8571	Ref
KC579452	Unassigned	Unassigned	Unassigned	455_0	Clustered	18	VC_455_0	18	0.4598	1	0.4598	0	0	0	1	Ref
KC581799	Unassigned	Unassigned	Unassigned	632_0	Clustered	37	VC_632_0	37	0.581	1	0.581	0	0	0	0.9234	Ref
KC595278	Unassigned	Unassigned	Unassigned	10_0	Clustered	25	VC_10_0	25	0.3467	1	0.3467	0	0	0	0.9733	Ref
KC595279	Unassigned	Unassigned	Unassigned	12_2	Clustered	19	VC_12_2	19	0.2018	1	0.2018	0	0	0	1	Ref
KC595511	Unassigned	Unassigned	Unassigned	1006_0	Clustered	8	VC_1006_0	8	0.7407	1	0.7407	0	0	0	1	Ref
KC595512	Unassigned	Unassigned	Unassigned	87_1	Clustered	3	VC_87_1	3	0.1636	0.99777924	0.1632	0	0	0	1	Ref
KC595513	Unassigned	Unassigned	Unassigned	87_1	Clustered	3	VC_87_1	3	0.1636	0.99777924	0.1632	0	0	0	1	Ref
KC595514	Unassigned	Unassigned	Unassigned	141_0	Clustered	6	VC_141_0	6	0.5999	1	0.5999	0	0	0	1	Ref
KC595515	Unassigned	Unassigned	Unassigned	141_0	Clustered	6	VC_141_0	6	0.5999	1	0.5999	0	0	0	1	Ref
KC595517	Unassigned	Unassigned	Unassigned	141_0	Clustered	6	VC_141_0	6	0.5999	1	0.5999	0	0	0	1	Ref
KC595518	Unassigned	Unassigned	Unassigned	141_0	Clustered	6	VC_141_0	6	0.5999	1	0.5999	0	0	0	1	Ref
KC618326	Unassigned	Unassigned	Unassigned	228_0	Clustered	60	VC_228_0	60	0.942	1	0.942	0	0	0	1	Ref
KC618393	Unassigned	Unassigned	Unassigned	599_0	Clustered	50	VC_599_0	50	0.9989	1	0.9989	0	0	0	1	Ref
KC661271	Unassigned	Unassigned	Unassigned	517_1	Clustered	108	VC_517_1	108	0.8483	1	0.8483	0	0	0	0.9955	Ref
KC661272	Unassigned	Unassigned	Unassigned	514_0	Clustered	75	VC_514_0	75	0.9402	1	0.9402	0	0	0	1	Ref
KC661273	Unassigned	Unassigned	Unassigned	325_23	Clustered	17	VC_325_23	17	0.0716	1	0.0716	0	0	0	0.6397	Ref
KC661274	Unassigned	Unassigned	Unassigned	371_3	Clustered	22	VC_371_3	22	0.0787	1	0.0787	0	0	0	0.9048	Ref
KC661275	Unassigned	Unassigned	Unassigned	219_0	Clustered	5	VC_219_0	5	0.08	0.99999998	0.08	0	0	0	1	Ref
KC661276	Unassigned	Unassigned	Unassigned	888_0	Clustered	28	VC_888_0	28	0.5497	1	0.5497	0	0	0	1	Ref
KC661277	Unassigned	Unassigned	Unassigned	551_7	Clustered	28	VC_551_7	28	0.1661	1	0.1661	0	0	0	0.8519	Ref
KC661278	Unassigned	Unassigned	Unassigned	520_79	Clustered/Singleton	1	VC_520_79	1	0	1	0	0	0	0	1	Ref
KC661279	Unassigned	Unassigned	Unassigned	515_0	Clustered	16	VC_515_0	16	0.3793	1	0.3793	0	0	0	1	Ref
KC661280	Unassigned	Unassigned	Unassigned	520_70	Clustered/Singleton	1	VC_520_70	1	0	1	0	0	0	0	1	Ref
KC661281	Unassigned	Unassigned	Unassigned	514_0	Clustered	75	VC_514_0	75	0.9402	1	0.9402	0	0	0	1	Ref
KC677662	Unassigned	Unassigned	Unassigned	592_0	Clustered	50	VC_592_0	50	0.7314	1	0.7314	0	0	0	1	Ref
KC677663	Unassigned	Unassigned	Unassigned	9_0	Clustered	38	VC_9_0	38	0.4895	1	0.4895	0	0	0	1	Ref
KC685370	Unassigned	Unassigned	Unassigned	818_0	Clustered	20	VC_818_0	20	0.9601	1	0.9601	0	0	0	1	Ref
KC690136	Unassigned	Unassigned	Unassigned	772_15	Clustered	5	VC_772_15	5	0.0243	0.76144861	0.0185	0	0	0	0.9	Ref
KC691254	Unassigned	Unassigned	Unassigned	888_0	Clustered	28	VC_888_0	28	0.5497	1	0.5497	0	0	0	1	Ref
KC691255	Unassigned	Unassigned	Unassigned	551_7	Clustered	28	VC_551_7	28	0.1661	1	0.1661	0	0	0	0.8519	Ref
KC691256	Unassigned	Unassigned	Unassigned	519_0	Clustered	39	VC_519_0	39	0.8156	1	0.8156	0	0	0	1	Ref
KC691257	Unassigned	Unassigned	Unassigned	558_5	Clustered/Singleton	1	VC_558_5	1	0	1	0	0	0	0	1	Ref
KC691258	Unassigned	Unassigned	Unassigned	371_3	Clustered	22	VC_371_3	22	0.0787	1	0.0787	0	0	0	0.9048	Ref
KC699836	Unassigned	Unassigned	Unassigned	85_2	Clustered	3	VC_85_2	3	0.0334	0.99730296	0.0333	0	0	0	1	Ref
KC700556	Unassigned	Unassigned	Unassigned	982_0	Clustered	62	VC_982_0	62	0.8646	1	0.8646	0	0	0	0.743	Ref
KC700557	Unassigned	Unassigned	Unassigned	982_0	Clustered	62	VC_982_0	62	0.8646	1	0.8646	0	0	0	0.743	Ref
KC700558	Unassigned	Unassigned	Unassigned	982_0	Clustered	62	VC_982_0	62	0.8646	1	0.8646	0	0	0	0.743	Ref

KC701493	Unassigned	Unassigned	Unassigned	325_17	Clustered/Singleton	1	VC_325_17	1	0	1	0	0	0	0	1	Ref
KC710998	Unassigned	Unassigned	Unassigned	941_0	Clustered	40	VC_941_0	40	0.6903	1	0.6903	0	0	0	1	Ref
KC736071	Unassigned	Unassigned	Unassigned	520_52	Clustered/Singleton	1	VC_520_52	1	0	1	0	0	0	0	1	Ref
KC736978	Unassigned	Unassigned	Unassigned	45_0	Clustered	12	VC_45_0	12	0.35	1	0.35	0	0	0	1	Ref
KC748968	Unassigned	Unassigned	Unassigned	558_49	Clustered	7	VC_558_49	7	0.0194	0.99510369	0.0193	0	0	0	0.8571	Ref
KC748969	Unassigned	Unassigned	Unassigned	551_8	Clustered	19	VC_551_8	19	0.1039	1	0.1039	0	0	0	0.8713	Ref
KC748970	Unassigned	Unassigned	Unassigned	558_12	Clustered	3	VC_558_12	3	0.0064	0.87854253	0.0057	0	0	0	1	Ref
KC748971	Unassigned	Unassigned	Unassigned	551_7	Clustered	28	VC_551_7	28	0.1661	1	0.1661	0	0	0	0.8519	Ref
KC751414	Unassigned	Unassigned	Unassigned	756_0	Clustered	2	VC_756_0	2	0.1637	0.95427014	0.1562	0	0	0	1	Ref
KC758116	Unassigned	Unassigned	Unassigned	166_0	Clustered	26	VC_166_0	26	0.7923	1	0.7923	0	0	0	1	Ref
KC787102	Unassigned	Unassigned	Unassigned	215_1	Clustered	19	VC_215_1	19	0.4188	1	0.4188	0	0	0	1	Ref
KC787103	Unassigned	Unassigned	Unassigned	761_0	Clustered	67	VC_761_0	67	0.8947	1	0.8947	0	0	0	1	Ref
KC787104	Unassigned	Unassigned	Unassigned	761_0	Clustered	67	VC_761_0	67	0.8947	1	0.8947	0	0	0	1	Ref
KC787105	Unassigned	Unassigned	Unassigned	215_1	Clustered	19	VC_215_1	19	0.4188	1	0.4188	0	0	0	1	Ref
KC787106	Unassigned	Unassigned	Unassigned	215_1	Clustered	19	VC_215_1	19	0.4188	1	0.4188	0	0	0	1	Ref
KC787107	Unassigned	Unassigned	Unassigned	761_0	Clustered	67	VC_761_0	67	0.8947	1	0.8947	0	0	0	1	Ref
KC787108	Unassigned	Unassigned	Unassigned	761_0	Clustered	67	VC_761_0	67	0.8947	1	0.8947	0	0	0	1	Ref
KC787109	Unassigned	Unassigned	Unassigned	761_0	Clustered	67	VC_761_0	67	0.8947	1	0.8947	0	0	0	1	Ref
KC787110	Unassigned	Unassigned	Unassigned	761_0	Clustered	67	VC_761_0	67	0.8947	1	0.8947	0	0	0	1	Ref
KC787111	Unassigned	Unassigned	Unassigned	761_0	Clustered	67	VC_761_0	67	0.8947	1	0.8947	0	0	0	1	Ref
KC787112	Unassigned	Unassigned	Unassigned	761_0	Clustered	67	VC_761_0	67	0.8947	1	0.8947	0	0	0	1	Ref
KC801932	Unassigned	Unassigned	Unassigned	669_1	Clustered	10	VC_669_1	10	0.1268	1	0.1268	0	0	0	0.9778	Ref
KC814930	Unassigned	Unassigned	Unassigned	45_0	Clustered	12	VC_45_0	12	0.35	1	0.35	0	0	0	1	Ref
KC821604	Unassigned	Unassigned	Unassigned	929_0	Clustered	4	VC_929_0	4	0.846	0.99722201	0.8437	0	0	0	1	Ref
KC821606	Unassigned	Unassigned	Unassigned	1011_0	Clustered	9	VC_1011_0	9	1	1	1	0	0	0	1	Ref
KC821607	Unassigned	Unassigned	Unassigned	445_1	Clustered	2	VC_445_1	2	0.1297	0.93055167	0.1207	0	0	0	1	Ref
KC821608	Unassigned	Unassigned	Unassigned	1012_0	Clustered	4	VC_1012_0	4	0.8715	0.99995392	0.8714	0	0	0	1	Ref
KC821609	Unassigned	Unassigned	Unassigned	1017_0	Clustered	5	VC_1017_0	5	0.9759	0.9999961	0.9759	0	0	0	1	Ref
KC821610	Unassigned	Unassigned	Unassigned	911_0	Clustered	6	VC_911_0	6	0.2787	1	0.2787	0	0	0	1	Ref
KC821612	Unassigned	Unassigned	Unassigned	1019_0	Clustered	5	VC_1019_0	5	0.866	0.99999999	0.866	0	0	0	1	Ref
KC821613	Unassigned	Unassigned	Unassigned	1020_0	Clustered	5	VC_1020_0	5	0.827	0.9999997	0.827	0	0	0	1	Ref
KC821614	Unassigned	Unassigned	Unassigned	1019_0	Clustered	5	VC_1019_0	5	0.866	0.99999999	0.866	0	0	0	1	Ref
KC821615	Unassigned	Unassigned	Unassigned	1020_0	Clustered	5	VC_1020_0	5	0.827	0.9999997	0.827	0	0	0	1	Ref
KC821616	Unassigned	Unassigned	Unassigned	911_0	Clustered	6	VC_911_0	6	0.2787	1	0.2787	0	0	0	1	Ref
KC821617	Unassigned	Unassigned	Unassigned	1020_0	Clustered	5	VC_1020_0	5	0.827	0.9999997	0.827	0	0	0	1	Ref
KC821618	Unassigned	Unassigned	Unassigned	445_1	Clustered	2	VC_445_1	2	0.1297	0.93055167	0.1207	0	0	0	1	Ref
KC821619	Unassigned	Unassigned	Unassigned	1020_0	Clustered	5	VC_1020_0	5	0.827	0.9999997	0.827	0	0	0	1	Ref
KC821620	Unassigned	Unassigned	Unassigned	1012_0	Clustered	4	VC_1012_0	4	0.8715	0.99995392	0.8714	0	0	0	1	Ref
KC821621	Unassigned	Unassigned	Unassigned	929_0	Clustered	4	VC_929_0	4	0.846	0.99722201	0.8437	0	0	0	1	Ref
KC821622	Unassigned	Unassigned	Unassigned	1012_0	Clustered	4	VC_1012_0	4	0.8715	0.99995392	0.8714	0	0	0	1	Ref
KC821623	Unassigned	Unassigned	Unassigned	1011_0	Clustered	9	VC_1011_0	9	1	1	1	0	0	0	1	Ref
KC821625	Unassigned	Unassigned	Unassigned	929_0	Clustered	4	VC_929_0	4	0.846	0.99722201	0.8437	0	0	0	1	Ref
KC821627	Unassigned	Unassigned	Unassigned	1020_0	Clustered	5	VC_1020_0	5	0.827	0.9999997	0.827	0	0	0	1	Ref
KC821628	Unassigned	Unassigned	Unassigned	1011_0	Clustered	9	VC_1011_0	9	1	1	1	0	0	0	1	Ref
KC821629	Unassigned	Unassigned	Unassigned	911_0	Clustered	6	VC_911_0	6	0.2787	1	0.2787	0	0	0	1	Ref
KC821630	Unassigned	Unassigned	Unassigned	911_0	Clustered	6	VC_911_0	6	0.2787	1	0.2787	0	0	0	1	Ref
KC821631	Unassigned	Unassigned	Unassigned	1011_0	Clustered	9	VC_1011_0	9	1	1	1	0	0	0	1	Ref
KC821632	Unassigned	Unassigned	Unassigned	1017_0	Clustered	5	VC_1017_0	5	0.9759	0.9999961	0.9759	0	0	0	1	Ref
KC821633	Unassigned	Unassigned	Unassigned	1012_0	Clustered	4	VC_1012_0	4	0.8715	0.99995392	0.8714	0	0	0	1	Ref
KC821634	Unassigned	Unassigned	Unassigned	911_0	Clustered	6	VC_911_0	6	0.2787	1	0.2787	0	0	0	1	Ref
KC853746	Unassigned	Unassigned	Unassigned	70_0	Clustered	27	VC_70_0	27	0.5981	1	0.5981	0	0	0	0.9886	Ref
KC862295	Unassigned	Unassigned	Unassigned	77_0	Clustered	20	VC_77_0	20	0.9657	1	0.9657	0	0	0	1	Ref
KC862296	Unassigned	Unassigned	Unassigned	77_0	Clustered	20	VC_77_0	20	0.9657	1	0.9657	0	0	0	1	Ref
KC862297	Unassigned	Unassigned	Unassigned	76_0	Clustered	37	VC_76_0	37	0.9403	1	0.9403	0	0	0	1	Ref
KC862298	Unassigned	Unassigned	Unassigned	76_0	Clustered	37	VC_76_0	37	0.9403	1	0.9403	0	0	0	1	Ref
KC862299	Unassigned	Unassigned	Unassigned	77_0	Clustered	20	VC_77_0	20	0.9657	1	0.9657	0	0	0	1	Ref
KC862300	Unassigned	Unassigned	Unassigned	76_0	Clustered	37	VC_76_0	37	0.9403	1	0.9403	0	0	0	1	Ref
KC862301	Unassigned	Unassigned	Unassigned	77_0	Clustered	20	VC_77_0	20	0.9657	1	0.9657	0	0	0	1	Ref
KC900378	Unassigned	Unassigned	Unassigned	630_0	Clustered	20	VC_630_0	20	0.8513	1	0.8513	0	0	0	1	Ref
KC900379	Unassigned	Unassigned	Unassigned	763_1	Clustered	9	VC_763_1	9	0.3706	1	0.3706	0	0	0	1	Ref
KC911856	Unassigned	Unassigned	Unassigned	476_0	Clustered	19	VC_476_0	19	0.7814	1	0.7814	0	0	0	1	Ref
KC911857	Unassigned	Unassigned	Unassigned	476_0	Clustered	19	VC_476_0	19	0.7814	1	0.7814	0	0	0	1	Ref
KC954774	Unassigned	Unassigned	Unassigned	849_5	Clustered	3	VC_849_5	3	0.0492	0.97574727	0.048	0	0	0	1	Ref
KC954775	Unassigned	Unassigned	Unassigned	580_0	Clustered	2	VC_580_0	2	0.0632	0.95763305	0.0605	0	0	0	1	Ref
KC959568	Unassigned	Unassigned	Unassigned	1023_0	Clustered	8	VC_1023_0	8	0.8574	1	0.8574	0	0	0	1	Ref
KC960489	Unassigned	Unassigned	Unassigned	1024_0	Clustered	2	VC_1024_0	2	1	1	1	0	0	0	1	Ref
KC960671	Unassigned	Unassigned	Unassigned	385_0	Clustered	51	VC_385_0	51	0.8754	1	0.8754	0	0	0	1	Ref
KC969441	Unassigned	Unassigned	Unassigned	423_0	Clustered	45	VC_423_0	45	0.9484	1	0.9484	0	0	0	1	Ref
KF005317	Unassigned	Unassigned	Unassigned	1025_0	Clustered	4	VC_1025_0	4	1	0	0	0	0	0	1	Ref
KF005318	Unassigned	Unassigned	Unassigned	1025_0	Clustered	4	VC_1025_0	4	1	0	0	0	0	0	1	Ref
KF005319	Unassigned	Unassigned	Unassigned	1025_0	Clustered	4	VC_1025_0	4	1	0	0	0	0	0	1	Ref
KF005320	Unassigned	Unassigned	Unassigned	1025_0	Clustered	4	VC_1025_0	4	1	0	0	0	0	0	1	Ref
KF006817	Unassigned	Unassigned	Unassigned	558_9	Clustered	3	VC_558_9	3	0.0064	0.82911529	0.0053	0	0	0	0.6667	Ref
KF006818	Unassigned	Unassigned	Unassigned	546_6	Clustered	2	VC_546_6	2	0.0135	0.7810929	0.0105	0	0	0	1	Ref
KF010834	Unassigned	Unassigned	Unassigned	708_0	Clustered	47	VC_708_0	47	0.8654	1	0.8654	0	0	0	1	Ref
KF017001	Unassigned	Unassigned	Unassigned	940_0	Clustered	34	VC_940_0	34	0.7959	1	0.7959	0	0	0	1	Ref
KF017002	Unassigned	Unassigned	Unassigned	556_0	Clustered	21	VC_556_0	21	0.9912	1	0.9912	0	0	0	1	Ref
KF017927	Unassigned	Unassigned	Unassigned	215_0	Clustered	5	VC_215_0	5	0.0724	0.99999997	0.0724	0	0	0	1	Ref
KF021268	Unassigned	Unassigned	Unassigned	150_0	Clustered	13	VC_150_0	13	0.2417	1	0.2417	0	0	0	0.9872	Ref
KF024721	Unassigned	Unassigned	Unassigned	216_3	Clustered	8	VC_216_3	8	0.0701	1	0.0701	0	0	0	0.8571	Ref
KF024722	Unassigned	Unassigned	Unassigned	508_0	Clustered	32	VC_508_0	32	0.906	1	0.906	0	0	0	1	Ref

KF024723	Unassigned	Unassigned	Unassigned	520_23	Clustered	2	VC_520_23	2	0.0077	0.95763408	0.0074	0	0	0	1	Ref
KF024724	Unassigned	Unassigned	Unassigned	325_35	Clustered	17	VC_325_35	17	0.0732	1	0.0732	0	0	0	0.7721	Ref
KF024725	Unassigned	Unassigned	Unassigned	889_0	Clustered	16	VC_889_0	16	0.2969	1	0.2969	0	0	0	1	Ref
KF024726	Unassigned	Unassigned	Unassigned	325_8	Clustered	2	VC_325_8	2	0.0093	0.95751268	0.0089	0	0	0	1	Ref
KF024727	Unassigned	Unassigned	Unassigned	372_1	Clustered	9	VC_372_1	9	0.194	1	0.194	0	0	0	1	Ref
KF024728	Unassigned	Unassigned	Unassigned	574_0	Clustered	7	VC_574_0	7	0.2734	1	0.2734	0	0	0	1	Ref
KF024729	Unassigned	Unassigned	Unassigned	372_3	Clustered	5	VC_372_3	5	0.1129	0.99999997	0.1129	0	0	0	1	Ref
KF024730	Unassigned	Unassigned	Unassigned	556_0	Clustered	21	VC_556_0	21	0.9912	1	0.9912	0	0	0	1	Ref
KF024731	Unassigned	Unassigned	Unassigned	888_0	Clustered	28	VC_888_0	28	0.5497	1	0.5497	0	0	0	1	Ref
KF024732	Unassigned	Unassigned	Unassigned	551_8	Clustered	19	VC_551_8	19	0.1039	1	0.1039	0	0	0	0.8713	Ref
KF024733	Unassigned	Unassigned	Unassigned	517_1	Clustered	108	VC_517_1	108	0.8483	1	0.8483	0	0	0	0.9955	Ref
KF024734	Unassigned	Unassigned	Unassigned	558_48	Clustered	2	VC_558_48	2	0.0032	0.69466126	0.0022	0	0	0	1	Ref
KF055347	Unassigned	Unassigned	Unassigned	329_16	Clustered	4	VC_329_16	4	0.0158	0.9999807	0.0158	0	0	0	1	Ref
KF056323	Unassigned	Unassigned	Unassigned	834_0	Clustered	7	VC_834_0	7	0.9845	0.94276893	0.9282	0	0	0	1	Ref
KF114874	Unassigned	Unassigned	Unassigned	520_9	Clustered/Singleton	1	VC_520_9	1	0	1	0	0	0	0	1	Ref
KF114875	Unassigned	Unassigned	Unassigned	546_4	Clustered	16	VC_546_4	16	0.2517	0.9999998	0.2517	0	0	0	0.9417	Ref
KF114876	Unassigned	Unassigned	Unassigned	687_1	Clustered	2	VC_687_1	2	0.223	0.91418477	0.2038	0	0	0	1	Ref
KF114877	Unassigned	Unassigned	Unassigned	687_1	Clustered	2	VC_687_1	2	0.223	0.91418477	0.2038	0	0	0	1	Ref
KF114879	Unassigned	Unassigned	Unassigned	687_0	Clustered	3	VC_687_0	3	0.5872	0.99252665	0.5828	0	0	0	1	Ref
KF114880	Unassigned	Unassigned	Unassigned	687_0	Clustered	3	VC_687_0	3	0.5872	0.99252665	0.5828	0	0	0	1	Ref
KF148055	Unassigned	Unassigned	Unassigned	637_2	Clustered	2	VC_637_2	2	0.0038	0.92844927	0.0035	0	0	0	1	Ref
KF148616	Unassigned	Unassigned	Unassigned	844_0	Clustered	27	VC_844_0	27	0.9385	1	0.9385	0	0	0	1	Ref
KF156338	Unassigned	Unassigned	Unassigned	433_2	Clustered/Singleton	1	VC_433_2	1	0	1	0	0	0	0	1	Ref
KF188410	Unassigned	Unassigned	Unassigned	640_0	Clustered	7	VC_640_0	7	0.9025	1	0.9025	0	0	0	1	Ref
KF188414	Unassigned	Unassigned	Unassigned	551_2	Clustered	17	VC_551_2	17	0.0973	1	0.0973	0	0	0	0.9779	Ref
KF192053	Unassigned	Unassigned	Unassigned	108_0	Clustered	33	VC_108_0	33	0.949	1	0.949	0	0	0	1	Ref
KF192075	Unassigned	Unassigned	Unassigned	779_0	Clustered	37	VC_779_0	37	0.5682	1	0.5682	0	0	0	1	Ref
KF208315	Unassigned	Unassigned	Unassigned	283_90	Clustered/Singleton	1	VC_283_90	1	0	1	0	0	0	0	1	Ref
KF208639	Unassigned	Unassigned	Unassigned	883_1	Clustered	12	VC_883_1	12	0.2441	1	0.2441	0	0	0	1	Ref
KF279411	Unassigned	Unassigned	Unassigned	372_0	Clustered	24	VC_372_0	24	0.5539	1	0.5539	0	0	0	1	Ref
KF279412	Unassigned	Unassigned	Unassigned	372_0	Clustered	24	VC_372_0	24	0.5539	1	0.5539	0	0	0	1	Ref
KF279413	Unassigned	Unassigned	Unassigned	372_0	Clustered	24	VC_372_0	24	0.5539	1	0.5539	0	0	0	1	Ref
KF279415	Unassigned	Unassigned	Unassigned	325_35	Clustered	17	VC_325_35	17	0.0732	1	0.0732	0	0	0	0.7721	Ref
KF279416	Unassigned	Unassigned	Unassigned	325_35	Clustered	17	VC_325_35	17	0.0732	1	0.0732	0	0	0	0.7721	Ref
KF279417	Unassigned	Unassigned	Unassigned	551_7	Clustered	28	VC_551_7	28	0.1661	1	0.1661	0	0	0	0.8519	Ref
KF279418	Unassigned	Unassigned	Unassigned	514_0	Clustered	75	VC_514_0	75	0.9402	1	0.9402	0	0	0	1	Ref
KF296717	Unassigned	Unassigned	Unassigned	829_0	Clustered	5	VC_829_0	5	0.8821	0.99999832	0.8821	0	0	0	1	Ref
KF302032	Unassigned	Unassigned	Unassigned	894_0	Clustered	3	VC_894_0	3	0.6358	0.99692152	0.6338	0	0	0	1	Ref
KF302033	Unassigned	Unassigned	Unassigned	894_0	Clustered	3	VC_894_0	3	0.6358	0.99692152	0.6338	0	0	0	1	Ref
KF302034	Unassigned	Unassigned	Unassigned	496_0	Clustered/Singleton	1	VC_496_0	1	0	1	0	0	0	0	1	Ref
KF302035	Unassigned	Unassigned	Unassigned	183_0	Clustered	2	VC_183_0	2	0.0667	0.95489893	0.0637	0	0	0	1	Ref
KF302036	Unassigned	Unassigned	Unassigned	895_0	Clustered	2	VC_895_0	2	0.2788	0.94251254	0.2628	0	0	0	1	Ref
KF302037	Unassigned	Unassigned	Unassigned	756_0	Clustered	2	VC_756_0	2	0.1637	0.95427014	0.1562	0	0	0	1	Ref
KF306380	Unassigned	Unassigned	Unassigned	551_3	Clustered/Singleton	1	VC_551_3	1	0	1	0	0	0	0	1	Ref
KF319020	Unassigned	Unassigned	Unassigned	112_0	Clustered	8	VC_112_0	8	0.2537	1	0.2537	0	0	0	1	Ref
KF322026	Unassigned	Unassigned	Unassigned	1027_0	Clustered	20	VC_1027_0	20	0.7114	1	0.7114	0	0	0	1	Ref
KF322032	Unassigned	Unassigned	Unassigned	228_0	Clustered	60	VC_228_0	60	0.942	1	0.942	0	0	0	1	Ref
KF356199	Unassigned	Unassigned	Unassigned	38_0	Clustered	3	VC_38_0	3	1	0	0	0	0	0	1	Ref
KF361475	Unassigned	Unassigned	Unassigned	5_0	Clustered	30	VC_5_0	30	0.9647	1	0.9647	0	0	0	1	Ref
KF381361	Unassigned	Unassigned	Unassigned	437_0	Clustered	4	VC_437_0	4	0.552	0.99998233	0.552	0	0	0	1	Ref
KF416340	Unassigned	Unassigned	Unassigned	325_49	Clustered	4	VC_325_49	4	0.014	0.99995965	0.014	0	0	0	1	Ref
KF416341	Unassigned	Unassigned	Unassigned	372_1	Clustered	9	VC_372_1	9	0.194	1	0.194	0	0	0	1	Ref
KF416342	Unassigned	Unassigned	Unassigned	568_0	Clustered	8	VC_568_0	8	0.5738	1	0.5738	0	0	0	1	Ref
KF416343	Unassigned	Unassigned	Unassigned	551_4	Clustered	2	VC_551_4	2	0.006	0.95695857	0.0058	0	0	0	1	Ref
KF416344	Unassigned	Unassigned	Unassigned	215_0	Clustered	5	VC_215_0	5	0.0724	0.99999997	0.0724	0	0	0	1	Ref
KF475786	Unassigned	Unassigned	Unassigned	179_0	Clustered	53	VC_179_0	53	0.9137	1	0.9137	0	0	0	1	Ref
KF493879	Unassigned	Unassigned	Unassigned	509_0	Clustered	37	VC_509_0	37	0.9689	1	0.9689	0	0	0	1	Ref
KF493880	Unassigned	Unassigned	Unassigned	325_3	Clustered/Singleton	1	VC_325_3	1	0	1	0	0	0	0	1	Ref
KF493881	Unassigned	Unassigned	Unassigned	372_0	Clustered	24	VC_372_0	24	0.5539	1	0.5539	0	0	0	1	Ref
KF493882	Unassigned	Unassigned	Unassigned	940_0	Clustered	34	VC_940_0	34	0.7959	1	0.7959	0	0	0	1	Ref
KF493883	Unassigned	Unassigned	Unassigned	551_8	Clustered	19	VC_551_8	19	0.1039	1	0.1039	0	0	0	0.8713	Ref
KF534715	Unassigned	Unassigned	Unassigned	792_0	Clustered	35	VC_792_0	35	0.9828	1	0.9828	0	0	0	1	Ref
KF550303	Unassigned	Unassigned	Unassigned	582_4	Clustered	3	VC_582_4	3	0.0348	0.98173503	0.0341	0	0	0	1	Ref
KF554508	Unassigned	Unassigned	Unassigned	87_1	Clustered	3	VC_87_1	3	0.1636	0.99777924	0.1632	0	0	0	1	Ref
KF560330	Unassigned	Unassigned	Unassigned	940_0	Clustered	34	VC_940_0	34	0.7959	1	0.7959	0	0	0	1	Ref
KF560331	Unassigned	Unassigned	Unassigned	325_33	Clustered	4	VC_325_33	4	0.0149	0.99998565	0.0149	0	0	0	1	Ref
KF560332	Unassigned	Unassigned	Unassigned	223_0	Clustered	38	VC_223_0	38	0.9095	1	0.9095	0	0	0	1	Ref
KF560333	Unassigned	Unassigned	Unassigned	223_0	Clustered	38	VC_223_0	38	0.9095	1	0.9095	0	0	0	1	Ref
KF560334	Unassigned	Unassigned	Unassigned	223_0	Clustered	38	VC_223_0	38	0.9095	1	0.9095	0	0	0	1	Ref
KF562099	Unassigned	Unassigned	Unassigned	551_8	Clustered	19	VC_551_8	19	0.1039	1	0.1039	0	0	0	0.8713	Ref
KF562100	Unassigned	Unassigned	Unassigned	551_8	Clustered	19	VC_551_8	19	0.1039	1	0.1039	0	0	0	0.8713	Ref
KF562101	Unassigned	Unassigned	Unassigned	551_8	Clustered	19	VC_551_8	19	0.1039	1	0.1039	0	0	0	0.8713	Ref
KF562102	Unassigned	Unassigned	Unassigned	551_8	Clustered	19	VC_551_8	19	0.1039	1	0.1039	0	0	0	0.8713	Ref
KF562340	Unassigned	Unassigned	Unassigned	779_0	Clustered	37	VC_779_0	37	0.5682	1	0.5682	0	0	0	1	Ref
KF562341	Unassigned	Unassigned	Unassigned	286_10	Clustered	9	VC_286_10	9	0.0615	0.99999878	0.0615	0	0	0	0.6667	Ref
KF562864	Unassigned	Unassigned	Unassigned	637_1	Clustered	27	VC_637_1	27	0.2578	1	0.2578	0	0	0	0.9858	Ref
KF562865	Unassigned	Unassigned	Unassigned	637_1	Clustered	27	VC_637_1	27	0.2578	1	0.2578	0	0	0	0.9858	Ref
KF582788	Unassigned	Unassigned	Unassigned	286_13	Clustered	10	VC_286_13	10	0.0698	0.99999929	0.0698	0	0	0	0.9556	Ref
KF591601	Unassigned	Unassigned	Unassigned	705_0	Clustered	11	VC_705_0	11	0.8861	1	0.8861	0	0	0	1	Ref
KF598856	Unassigned	Unassigned	Unassigned	8_0	Clustered	58	VC_8_0	58	0.8306	1	0.8306	0	0	0	0.9946	Ref
KF598865	Unassigned	Unassigned	Unassigned	777_0	Clustered	3	VC_777_0	3	0.4763	0.9961812	0.4745	0	0	0	1	Ref

KF614509	Unassigned	Unassigned	Unassigned	990_0	Clustered	3	VC_990_0	3	0.6363	0.99819562	0.6351	0	0	0	1	Ref
KF620435	Unassigned	Unassigned	Unassigned	779_0	Clustered	37	VC_779_0	37	0.5682	1	0.5682	0	0	0	1	Ref
KF623293	Unassigned	Unassigned	Unassigned	901_0	Clustered	5	VC_901_0	5	0.9265	0.99999497	0.9265	0	0	0	1	Ref
KF623294	Unassigned	Unassigned	Unassigned	488_2	Clustered	2	VC_488_2	2	0.0325	0.95511029	0.0311	0	0	0	1	Ref
KF626665	Unassigned	Unassigned	Unassigned	172_0	Clustered	5	VC_172_0	5	0.3579	0.99999997	0.3579	0	0	0	1	Ref
KF626667	Unassigned	Unassigned	Unassigned	74_0	Clustered	15	VC_74_0	15	0.5204	1	0.5204	0	0	0	1	Ref
KF626668	Unassigned	Unassigned	Unassigned	172_0	Clustered	5	VC_172_0	5	0.3579	0.99999997	0.3579	0	0	0	1	Ref
KF669647	Unassigned	Unassigned	Unassigned	883_1	Clustered	12	VC_883_1	12	0.2441	1	0.2441	0	0	0	1	Ref
KF669648	Unassigned	Unassigned	Unassigned	100_0	Clustered	15	VC_100_0	15	0.6536	1	0.6536	0	0	0	1	Ref
KF669649	Unassigned	Unassigned	Unassigned	87_0	Clustered	8	VC_87_0	8	0.5545	1	0.5545	0	0	0	1	Ref
KF669650	Unassigned	Unassigned	Unassigned	855_0	Clustered	2	VC_855_0	2	0.0285	0.89378929	0.0255	0	0	0	1	Ref
KF669651	Unassigned	Unassigned	Unassigned	100_0	Clustered	15	VC_100_0	15	0.6536	1	0.6536	0	0	0	1	Ref
KF669652	Unassigned	Unassigned	Unassigned	85_3	Clustered	19	VC_85_3	19	0.3671	1	0.3671	0	0	0	0.807	Ref
KF669653	Unassigned	Unassigned	Unassigned	133_0	Clustered	2	VC_133_0	2	0.0063	0.94038884	0.0059	0	0	0	1	Ref
KF669654	Unassigned	Unassigned	Unassigned	133_0	Clustered	2	VC_133_0	2	0.0063	0.94038884	0.0059	0	0	0	1	Ref
KF669655	Unassigned	Unassigned	Unassigned	1032_0	Clustered	7	VC_1032_0	7	0.9887	0.99999504	0.9887	0	0	0	1	Ref
KF669657	Unassigned	Unassigned	Unassigned	1032_0	Clustered	7	VC_1032_0	7	0.9887	0.99999504	0.9887	0	0	0	1	Ref
KF669659	Unassigned	Unassigned	Unassigned	100_0	Clustered	15	VC_100_0	15	0.6536	1	0.6536	0	0	0	1	Ref
KF669660	Unassigned	Unassigned	Unassigned	1032_0	Clustered	7	VC_1032_0	7	0.9887	0.99999504	0.9887	0	0	0	1	Ref
KF669662	Unassigned	Unassigned	Unassigned	883_3	Clustered	2	VC_883_3	2	0.0182	0.87900236	0.016	0	0	0	1	Ref
KF676640	Unassigned	Unassigned	Unassigned	202_0	Clustered	102	VC_202_0	102	0.4049	1	0.4049	0	0	0	0.7876	Ref
KF692088	Unassigned	Unassigned	Unassigned	1034_1	Clustered/Singleton	1	VC_1034_1	1	0	1	0	0	0	0	1	Ref
KF700371	Unassigned	Unassigned	Unassigned	386_0	Clustered	49	VC_386_0	49	0.7941	1	0.7941	0	0	0	1	Ref
KF713485	Unassigned	Unassigned	Unassigned	371_3	Clustered	22	VC_371_3	22	0.0787	1	0.0787	0	0	0	0.9048	Ref
KF713486	Unassigned	Unassigned	Unassigned	237_0	Clustered	4	VC_237_0	4	0.0429	0.99998759	0.0429	0	0	0	1	Ref
KF728385	Unassigned	Unassigned	Unassigned	695_0	Clustered	48	VC_695_0	48	0.8162	1	0.8162	0	0	0	1	Ref
KF733017	Unassigned	Unassigned	Unassigned	695_0	Clustered	48	VC_695_0	48	0.8162	1	0.8162	0	0	0	1	Ref
KF751793	Unassigned	Unassigned	Unassigned	1039_0	Clustered	5	VC_1039_0	5	1	1	1	0	0	0	1	Ref
KF751794	Unassigned	Unassigned	Unassigned	1039_0	Clustered	5	VC_1039_0	5	1	1	1	0	0	0	1	Ref
KF751795	Unassigned	Unassigned	Unassigned	1039_0	Clustered	5	VC_1039_0	5	1	1	1	0	0	0	1	Ref
KF751796	Unassigned	Unassigned	Unassigned	1039_0	Clustered	5	VC_1039_0	5	1	1	1	0	0	0	1	Ref
KF751797	Unassigned	Unassigned	Unassigned	1039_0	Clustered	5	VC_1039_0	5	1	1	1	0	0	0	1	Ref
KF765493	Unassigned	Unassigned	Unassigned	111_0	Clustered	91	VC_111_0	91	0.9587	1	0.9587	0	0	0	1	Ref
KF766114	Unassigned	Unassigned	Unassigned	149_8	Clustered	66	VC_149_8	66	0.4565	1	0.4565	0	0	0	0.8182	Ref
KF766125	Unassigned	Unassigned	Unassigned	46_0	Clustered	2	VC_46_0	2	0.0181	0.95763537	0.0173	0	0	0	1	Ref
KF767351	Unassigned	Unassigned	Unassigned	308_0	Clustered	4	VC_308_0	4	0.5014	0.99997418	0.5013	0	0	0	1	Ref
KF771236	Unassigned	Unassigned	Unassigned	455_0	Clustered	18	VC_455_0	18	0.4598	1	0.4598	0	0	0	1	Ref
KF771237	Unassigned	Unassigned	Unassigned	455_0	Clustered	18	VC_455_0	18	0.4598	1	0.4598	0	0	0	1	Ref
KF771238	Unassigned	Unassigned	Unassigned	455_0	Clustered	18	VC_455_0	18	0.4598	1	0.4598	0	0	0	1	Ref
KF771239	Unassigned	Unassigned	Unassigned	455_0	Clustered	18	VC_455_0	18	0.4598	1	0.4598	0	0	0	1	Ref
KF787094	Unassigned	Unassigned	Unassigned	779_1	Clustered	11	VC_779_1	11	0.1587	1	0.1587	0	0	0	1	Ref
KF787095	Unassigned	Unassigned	Unassigned	779_1	Clustered	11	VC_779_1	11	0.1587	1	0.1587	0	0	0	1	Ref
KF800937	Unassigned	Unassigned	Unassigned	1027_0	Clustered	20	VC_1027_0	20	0.7114	1	0.7114	0	0	0	1	Ref
KF806588	Unassigned	Unassigned	Unassigned	779_2	Clustered	5	VC_779_2	5	0.0876	0.99999997	0.0876	0	0	0	1	Ref
KF806589	Unassigned	Unassigned	Unassigned	489_1	Clustered	11	VC_489_1	11	0.4621	1	0.4621	0	0	0	1	Ref
KF831354	Unassigned	Unassigned	Unassigned	10_0	Clustered	25	VC_10_0	25	0.3467	1	0.3467	0	0	0	0.9733	Ref
KF835987	Unassigned	Unassigned	Unassigned	490_2	Clustered/Singleton	1	VC_490_2	1	0	1	0	0	0	0	1	Ref
KF841475	Unassigned	Unassigned	Unassigned	517_1	Clustered	108	VC_517_1	108	0.8483	1	0.8483	0	0	0	0.9955	Ref
KF841476	Unassigned	Unassigned	Unassigned	517_1	Clustered	108	VC_517_1	108	0.8483	1	0.8483	0	0	0	0.9955	Ref
KF841477	Unassigned	Unassigned	Unassigned	519_0	Clustered	39	VC_519_0	39	0.8156	1	0.8156	0	0	0	1	Ref
KF854250	Unassigned	Unassigned	Unassigned	498_0	Clustered	8	VC_498_0	8	0.3987	1	0.3987	0	0	0	1	Ref
KF856712	Unassigned	Unassigned	Unassigned	472_0	Clustered	47	VC_472_0	47	0.9025	1	0.9025	0	0	0	1	Ref
KF861510	Unassigned	Unassigned	Unassigned	218_0	Clustered	3	VC_218_0	3	0.0481	0.99860979	0.0481	0	0	0	1	Ref
KF925357	Unassigned	Unassigned	Unassigned	283_163	Clustered	2	VC_283_163	2	0.0029	0.93420746	0.0027	0	0	0	1	Ref
KF926093	Unassigned	Unassigned	Unassigned	474_0	Clustered	14	VC_474_0	14	0.8466	1	0.8466	0	0	0	1	Ref
KF929199	Unassigned	Unassigned	Unassigned	667_0	Clustered	7	VC_667_0	7	0.4101	1	0.4101	0	0	0	1	Ref
KF954506	Unassigned	Unassigned	Unassigned	517_1	Clustered	108	VC_517_1	108	0.8483	1	0.8483	0	0	0	0.9955	Ref
KF959563	Unassigned	Unassigned	Unassigned	517_1	Clustered	108	VC_517_1	108	0.8483	1	0.8483	0	0	0	0.9955	Ref
KF971864	Unassigned	Unassigned	Unassigned	42_0	Clustered	49	VC_42_0	49	0.4517	1	0.4517	0	0	0	0.8597	Ref
KF977490	Unassigned	Unassigned	Unassigned	124_0	Clustered	2	VC_124_0	2	0.3934	0.95337092	0.3751	0	0	0	1	Ref
KF981601	Unassigned	Unassigned	Unassigned	216_6	Clustered	42	VC_216_6	42	0.473	1	0.473	0	0	0	0.8351	Ref
KF981730	Unassigned	Unassigned	Unassigned	813_1	Clustered	24	VC_813_1	24	0.535	1	0.535	0	0	0	1	Ref
KF981875	Unassigned	Unassigned	Unassigned	80_0	Clustered	41	VC_80_0	41	0.5837	1	0.5837	0	0	0	0.9805	Ref
KF981876	Unassigned	Unassigned	Unassigned	1024_0	Clustered	2	VC_1024_0	2	1	1	1	0	0	0	1	Ref
KF986246	Unassigned	Unassigned	Unassigned	521_0	Clustered	37	VC_521_0	37	0.912	1	0.912	0	0	0	1	Ref
KF986247	Unassigned	Unassigned	Unassigned	567_0	Clustered	8	VC_567_0	8	0.4935	1	0.4935	0	0	0	1	Ref
KJ002054	Unassigned	Unassigned	Unassigned	793_0	Clustered	12	VC_793_0	12	0.8531	1	0.8531	0	0	0	1	Ref
KJ003983	Unassigned	Unassigned	Unassigned	1018_0	Clustered	2	VC_1018_0	2	0.0851	0.94401932	0.0804	0	0	0	1	Ref
KJ010547	Unassigned	Unassigned	Unassigned	85_0	Clustered	3	VC_85_0	3	0.0422	0.99823242	0.0421	0	0	0	1	Ref
KJ010548	Unassigned	Unassigned	Unassigned	85_0	Clustered	3	VC_85_0	3	0.0422	0.99823242	0.0421	0	0	0	1	Ref
KJ018213	Unassigned	Unassigned	Unassigned	827_0	Clustered	6	VC_827_0	6	0.7493	1	0.7493	0	0	0	1	Ref
KJ019028	Unassigned	Unassigned	Unassigned	656_1	Clustered	46	VC_656_1	46	0.8511	1	0.8511	0	0	0	1	Ref
KJ019029	Unassigned	Unassigned	Unassigned	656_1	Clustered	46	VC_656_1	46	0.8511	1	0.8511	0	0	0	1	Ref
KJ019031	Unassigned	Unassigned	Unassigned	656_1	Clustered	46	VC_656_1	46	0.8511	1	0.8511	0	0	0	1	Ref
KJ019032	Unassigned	Unassigned	Unassigned	656_1	Clustered	46	VC_656_1	46	0.8511	1	0.8511	0	0	0	1	Ref
KJ019034	Unassigned	Unassigned	Unassigned	656_1	Clustered	46	VC_656_1	46	0.8511	1	0.8511	0	0	0	1	Ref
KJ019035	Unassigned	Unassigned	Unassigned	656_1	Clustered	46	VC_656_1	46	0.8511	1	0.8511	0	0	0	1	Ref
KJ019036	Unassigned	Unassigned	Unassigned	656_1	Clustered	46	VC_656_1	46	0.8511	1	0.8511	0	0	0	1	Ref
KJ019037	Unassigned	Unassigned	Unassigned	657_1	Clustered	41	VC_657_1	41	0.7489	1	0.7489	0	0	0	1	Ref
KJ019045	Unassigned	Unassigned	Unassigned	657_1	Clustered	41	VC_657_1	41	0.7489	1	0.7489	0	0	0	1	Ref
KJ019046	Unassigned	Unassigned	Unassigned	656_1	Clustered	46	VC_656_1	46	0.8511	1	0.8511	0	0	0	1	Ref

KJ019047	Unassigned	Unassigned	Unassigned	656_1	Clustered	46	VC_656_1	46	0.8511	1	0.8511	0	0	0	1	Ref
KJ019048	Unassigned	Unassigned	Unassigned	656_1	Clustered	46	VC_656_1	46	0.8511	1	0.8511	0	0	0	1	Ref
KJ019050	Unassigned	Unassigned	Unassigned	656_1	Clustered	46	VC_656_1	46	0.8511	1	0.8511	0	0	0	1	Ref
KJ019052	Unassigned	Unassigned	Unassigned	657_1	Clustered	41	VC_657_1	41	0.7489	1	0.7489	0	0	0	1	Ref
KJ019053	Unassigned	Unassigned	Unassigned	657_1	Clustered	41	VC_657_1	41	0.7489	1	0.7489	0	0	0	1	Ref
KJ019054	Unassigned	Unassigned	Unassigned	433_1	Clustered	9	VC_433_1	9	0.1288	1	0.1288	0	0	0	0.8611	Ref
KJ019056	Unassigned	Unassigned	Unassigned	656_1	Clustered	46	VC_656_1	46	0.8511	1	0.8511	0	0	0	1	Ref
KJ019057	Unassigned	Unassigned	Unassigned	656_1	Clustered	46	VC_656_1	46	0.8511	1	0.8511	0	0	0	1	Ref
KJ019058	Unassigned	Unassigned	Unassigned	657_1	Clustered	41	VC_657_1	41	0.7489	1	0.7489	0	0	0	1	Ref
KJ019059	Unassigned	Unassigned	Unassigned	657_1	Clustered	41	VC_657_1	41	0.7489	1	0.7489	0	0	0	1	Ref
KJ019062	Unassigned	Unassigned	Unassigned	656_1	Clustered	46	VC_656_1	46	0.8511	1	0.8511	0	0	0	1	Ref
KJ019066	Unassigned	Unassigned	Unassigned	657_1	Clustered	41	VC_657_1	41	0.7489	1	0.7489	0	0	0	1	Ref
KJ019069	Unassigned	Unassigned	Unassigned	433_1	Clustered	9	VC_433_1	9	0.1288	1	0.1288	0	0	0	0.8611	Ref
KJ019070	Unassigned	Unassigned	Unassigned	656_1	Clustered	46	VC_656_1	46	0.8511	1	0.8511	0	0	0	1	Ref
KJ019072	Unassigned	Unassigned	Unassigned	656_1	Clustered	46	VC_656_1	46	0.8511	1	0.8511	0	0	0	1	Ref
KJ019073	Unassigned	Unassigned	Unassigned	656_1	Clustered	46	VC_656_1	46	0.8511	1	0.8511	0	0	0	1	Ref
KJ019074	Unassigned	Unassigned	Unassigned	656_1	Clustered	46	VC_656_1	46	0.8511	1	0.8511	0	0	0	1	Ref
KJ019075	Unassigned	Unassigned	Unassigned	656_1	Clustered	46	VC_656_1	46	0.8511	1	0.8511	0	0	0	1	Ref
KJ019077	Unassigned	Unassigned	Unassigned	656_1	Clustered	46	VC_656_1	46	0.8511	1	0.8511	0	0	0	1	Ref
KJ019078	Unassigned	Unassigned	Unassigned	656_1	Clustered	46	VC_656_1	46	0.8511	1	0.8511	0	0	0	1	Ref
KJ019079	Unassigned	Unassigned	Unassigned	656_1	Clustered	46	VC_656_1	46	0.8511	1	0.8511	0	0	0	1	Ref
KJ019080	Unassigned	Unassigned	Unassigned	656_1	Clustered	46	VC_656_1	46	0.8511	1	0.8511	0	0	0	1	Ref
KJ019082	Unassigned	Unassigned	Unassigned	433_1	Clustered	9	VC_433_1	9	0.1288	1	0.1288	0	0	0	0.8611	Ref
KJ019083	Unassigned	Unassigned	Unassigned	656_1	Clustered	46	VC_656_1	46	0.8511	1	0.8511	0	0	0	1	Ref
KJ019085	Unassigned	Unassigned	Unassigned	657_1	Clustered	41	VC_657_1	41	0.7489	1	0.7489	0	0	0	1	Ref
KJ019086	Unassigned	Unassigned	Unassigned	657_1	Clustered	41	VC_657_1	41	0.7489	1	0.7489	0	0	0	1	Ref
KJ019089	Unassigned	Unassigned	Unassigned	433_1	Clustered	9	VC_433_1	9	0.1288	1	0.1288	0	0	0	0.8611	Ref
KJ019090	Unassigned	Unassigned	Unassigned	657_1	Clustered	41	VC_657_1	41	0.7489	1	0.7489	0	0	0	1	Ref
KJ019091	Unassigned	Unassigned	Unassigned	657_1	Clustered	41	VC_657_1	41	0.7489	1	0.7489	0	0	0	1	Ref
KJ019092	Unassigned	Unassigned	Unassigned	657_1	Clustered	41	VC_657_1	41	0.7489	1	0.7489	0	0	0	1	Ref
KJ019093	Unassigned	Unassigned	Unassigned	657_1	Clustered	41	VC_657_1	41	0.7489	1	0.7489	0	0	0	1	Ref
KJ019094	Unassigned	Unassigned	Unassigned	433_1	Clustered	9	VC_433_1	9	0.1288	1	0.1288	0	0	0	0.8611	Ref
KJ019095	Unassigned	Unassigned	Unassigned	657_1	Clustered	41	VC_657_1	41	0.7489	1	0.7489	0	0	0	1	Ref
KJ019096	Unassigned	Unassigned	Unassigned	657_1	Clustered	41	VC_657_1	41	0.7489	1	0.7489	0	0	0	1	Ref
KJ019097	Unassigned	Unassigned	Unassigned	657_1	Clustered	41	VC_657_1	41	0.7489	1	0.7489	0	0	0	1	Ref
KJ019098	Unassigned	Unassigned	Unassigned	657_1	Clustered	41	VC_657_1	41	0.7489	1	0.7489	0	0	0	1	Ref
KJ019099	Unassigned	Unassigned	Unassigned	657_1	Clustered	41	VC_657_1	41	0.7489	1	0.7489	0	0	0	1	Ref
KJ019100	Unassigned	Unassigned	Unassigned	657_1	Clustered	41	VC_657_1	41	0.7489	1	0.7489	0	0	0	1	Ref
KJ019101	Unassigned	Unassigned	Unassigned	657_1	Clustered	41	VC_657_1	41	0.7489	1	0.7489	0	0	0	1	Ref
KJ019102	Unassigned	Unassigned	Unassigned	657_1	Clustered	41	VC_657_1	41	0.7489	1	0.7489	0	0	0	1	Ref
KJ019103	Unassigned	Unassigned	Unassigned	657_1	Clustered	41	VC_657_1	41	0.7489	1	0.7489	0	0	0	1	Ref
KJ019105	Unassigned	Unassigned	Unassigned	657_1	Clustered	41	VC_657_1	41	0.7489	1	0.7489	0	0	0	1	Ref
KJ019106	Unassigned	Unassigned	Unassigned	657_1	Clustered	41	VC_657_1	41	0.7489	1	0.7489	0	0	0	1	Ref
KJ019107	Unassigned	Unassigned	Unassigned	657_1	Clustered	41	VC_657_1	41	0.7489	1	0.7489	0	0	0	1	Ref
KJ019111	Unassigned	Unassigned	Unassigned	657_1	Clustered	41	VC_657_1	41	0.7489	1	0.7489	0	0	0	1	Ref
KJ019112	Unassigned	Unassigned	Unassigned	656_1	Clustered	46	VC_656_1	46	0.8511	1	0.8511	0	0	0	1	Ref
KJ019113	Unassigned	Unassigned	Unassigned	656_1	Clustered	46	VC_656_1	46	0.8511	1	0.8511	0	0	0	1	Ref
KJ019115	Unassigned	Unassigned	Unassigned	656_1	Clustered	46	VC_656_1	46	0.8511	1	0.8511	0	0	0	1	Ref
KJ019117	Unassigned	Unassigned	Unassigned	656_1	Clustered	46	VC_656_1	46	0.8511	1	0.8511	0	0	0	1	Ref
KJ019118	Unassigned	Unassigned	Unassigned	656_1	Clustered	46	VC_656_1	46	0.8511	1	0.8511	0	0	0	1	Ref
KJ019119	Unassigned	Unassigned	Unassigned	656_1	Clustered	46	VC_656_1	46	0.8511	1	0.8511	0	0	0	1	Ref
KJ019120	Unassigned	Unassigned	Unassigned	656_1	Clustered	46	VC_656_1	46	0.8511	1	0.8511	0	0	0	1	Ref
KJ019121	Unassigned	Unassigned	Unassigned	656_1	Clustered	46	VC_656_1	46	0.8511	1	0.8511	0	0	0	1	Ref
KJ019123	Unassigned	Unassigned	Unassigned	657_1	Clustered	41	VC_657_1	41	0.7489	1	0.7489	0	0	0	1	Ref
KJ019124	Unassigned	Unassigned	Unassigned	656_1	Clustered	46	VC_656_1	46	0.8511	1	0.8511	0	0	0	1	Ref
KJ019125	Unassigned	Unassigned	Unassigned	656_1	Clustered	46	VC_656_1	46	0.8511	1	0.8511	0	0	0	1	Ref
KJ019126	Unassigned	Unassigned	Unassigned	656_1	Clustered	46	VC_656_1	46	0.8511	1	0.8511	0	0	0	1	Ref
KJ019127	Unassigned	Unassigned	Unassigned	656_1	Clustered	46	VC_656_1	46	0.8511	1	0.8511	0	0	0	1	Ref
KJ019129	Unassigned	Unassigned	Unassigned	656_1	Clustered	46	VC_656_1	46	0.8511	1	0.8511	0	0	0	1	Ref
KJ019130	Unassigned	Unassigned	Unassigned	656_1	Clustered	46	VC_656_1	46	0.8511	1	0.8511	0	0	0	1	Ref
KJ019131	Unassigned	Unassigned	Unassigned	656_1	Clustered	46	VC_656_1	46	0.8511	1	0.8511	0	0	0	1	Ref
KJ019136	Unassigned	Unassigned	Unassigned	656_1	Clustered	46	VC_656_1	46	0.8511	1	0.8511	0	0	0	1	Ref
KJ019139	Unassigned	Unassigned	Unassigned	656_1	Clustered	46	VC_656_1	46	0.8511	1	0.8511	0	0	0	1	Ref
KJ019140	Unassigned	Unassigned	Unassigned	656_1	Clustered	46	VC_656_1	46	0.8511	1	0.8511	0	0	0	1	Ref
KJ019141	Unassigned	Unassigned	Unassigned	657_1	Clustered	41	VC_657_1	41	0.7489	1	0.7489	0	0	0	1	Ref
KJ019142	Unassigned	Unassigned	Unassigned	657_1	Clustered	41	VC_657_1	41	0.7489	1	0.7489	0	0	0	1	Ref
KJ019143	Unassigned	Unassigned	Unassigned	657_1	Clustered	41	VC_657_1	41	0.7489	1	0.7489	0	0	0	1	Ref
KJ019144	Unassigned	Unassigned	Unassigned	657_1	Clustered	41	VC_657_1	41	0.7489	1	0.7489	0	0	0	1	Ref
KJ019145	Unassigned	Unassigned	Unassigned	657_1	Clustered	41	VC_657_1	41	0.7489	1	0.7489	0	0	0	1	Ref
KJ019146	Unassigned	Unassigned	Unassigned	657_1	Clustered	41	VC_657_1	41	0.7489	1	0.7489	0	0	0	1	Ref
KJ019147	Unassigned	Unassigned	Unassigned	657_1	Clustered	41	VC_657_1	41	0.7489	1	0.7489	0	0	0	1	Ref
KJ019148	Unassigned	Unassigned	Unassigned	657_1	Clustered	41	VC_657_1	41	0.7489	1	0.7489	0	0	0	1	Ref
KJ019149	Unassigned	Unassigned	Unassigned	657_1	Clustered	41	VC_657_1	41	0.7489	1	0.7489	0	0	0	1	Ref
KJ019150	Unassigned	Unassigned	Unassigned	657_1	Clustered	41	VC_657_1	41	0.7489	1	0.7489	0	0	0	1	Ref
KJ019151	Unassigned	Unassigned	Unassigned	657_1	Clustered	41	VC_657_1	41	0.7489	1	0.7489	0	0	0	1	Ref
KJ019152	Unassigned	Unassigned	Unassigned	657_1	Clustered	41	VC_657_1	41	0.7489	1	0.7489	0	0	0	1	Ref
KJ019155	Unassigned	Unassigned	Unassigned	657_1	Clustered	41	VC_657_1	41	0.7489	1	0.7489	0	0	0	1	Ref
KJ019156	Unassigned	Unassigned	Unassigned	433_1	Clustered	9	VC_433_1	9	0.1288	1	0.1288	0	0	0	0.8611	Ref
KJ019159	Unassigned	Unassigned	Unassigned	657_1	Clustered	41	VC_657_1	41	0.7489	1	0.7489	0	0	0	1	Ref
KJ019160	Unassigned	Unassigned	Unassigned	656_1	Clustered	46	VC_656_1	46	0.8511	1	0.8511	0	0	0	1	Ref
KJ019162	Unassigned	Unassigned	Unassigned	656_1	Clustered	46	VC_656_1	46	0.8511	1	0.8511	0	0	0	1	Ref

KJ019164	Unassigned	Unassigned	Unassigned	656_1	Clustered	46	VC_656_1	46	0.8511	1	0.8511	0	0	0	1 Ref
KJ019165	Unassigned	Unassigned	Unassigned	656_1	Clustered	46	VC_656_1	46	0.8511	1	0.8511	0	0	0	1 Ref
KJ021043	Unassigned	Unassigned	Unassigned	123_0	Clustered	23	VC_123_0	23	0.817	1	0.817	0	0	0	1 Ref
KJ025956	Unassigned	Unassigned	Unassigned	520_38	Clustered	6	VC_520_38	6	0.0519	1	0.0519	0	0	0	0.8667 Ref
KJ025957	Unassigned	Unassigned	Unassigned	284_0	Clustered	3	VC_284_0	3	0.0495	0.99860966	0.0494	0	0	0	1 Ref
KJ028219	Unassigned	Unassigned	Unassigned	809_0	Clustered	2	VC_809_0	2	0.1799	0.96299906	0.1733	0	0	0	1 Ref
KJ081346	Unassigned	Unassigned	Unassigned	187_2	Clustered	2	VC_187_2	2	0.0209	0.93057595	0.0195	0	0	0	1 Ref
KJ094020	Unassigned	Unassigned	Unassigned	980_0	Clustered	10	VC_980_0	10	0.9996	1	0.9996	0	0	0	1 Ref
KJ094021	Unassigned	Unassigned	Unassigned	980_0	Clustered	10	VC_980_0	10	0.9996	1	0.9996	0	0	0	1 Ref
KJ094022	Unassigned	Unassigned	Unassigned	378_0	Clustered	9	VC_378_0	9	0.7603	1	0.7603	0	0	0	1 Ref
KJ094023	Unassigned	Unassigned	Unassigned	379_0	Clustered	5	VC_379_0	5	0.4779	0.99999992	0.4779	0	0	0	0.9 Ref
KJ094029	Unassigned	Unassigned	Unassigned	739_0	Clustered	26	VC_739_0	26	0.9571	1	0.9571	0	0	0	1 Ref
KJ094030	Unassigned	Unassigned	Unassigned	739_0	Clustered	26	VC_739_0	26	0.9571	1	0.9571	0	0	0	1 Ref
KJ094031	Unassigned	Unassigned	Unassigned	739_0	Clustered	26	VC_739_0	26	0.9571	1	0.9571	0	0	0	1 Ref
KJ094032	Unassigned	Unassigned	Unassigned	108_0	Clustered	33	VC_108_0	33	0.949	1	0.949	0	0	0	1 Ref
KJ094033	Unassigned	Unassigned	Unassigned	739_0	Clustered	26	VC_739_0	26	0.9571	1	0.9571	0	0	0	1 Ref
KJ127303	Unassigned	Unassigned	Unassigned	108_0	Clustered	33	VC_108_0	33	0.949	1	0.949	0	0	0	1 Ref
KJ127304	Unassigned	Unassigned	Unassigned	695_0	Clustered	48	VC_695_0	48	0.8162	1	0.8162	0	0	0	1 Ref
KJ133688	Unassigned	Unassigned	Unassigned	879_0	Clustered	36	VC_879_0	36	0.994	1	0.994	0	0	0	1 Ref
KJ133689	Unassigned	Unassigned	Unassigned	879_0	Clustered	36	VC_879_0	36	0.994	1	0.994	0	0	0	1 Ref
KJ133690	Unassigned	Unassigned	Unassigned	879_0	Clustered	36	VC_879_0	36	0.994	1	0.994	0	0	0	1 Ref
KJ133691	Unassigned	Unassigned	Unassigned	879_0	Clustered	36	VC_879_0	36	0.994	1	0.994	0	0	0	1 Ref
KJ133692	Unassigned	Unassigned	Unassigned	879_0	Clustered	36	VC_879_0	36	0.994	1	0.994	0	0	0	1 Ref
KJ133693	Unassigned	Unassigned	Unassigned	879_0	Clustered	36	VC_879_0	36	0.994	1	0.994	0	0	0	1 Ref
KJ133694	Unassigned	Unassigned	Unassigned	879_0	Clustered	36	VC_879_0	36	0.994	1	0.994	0	0	0	1 Ref
KJ133695	Unassigned	Unassigned	Unassigned	879_0	Clustered	36	VC_879_0	36	0.994	1	0.994	0	0	0	1 Ref
KJ133696	Unassigned	Unassigned	Unassigned	879_0	Clustered	36	VC_879_0	36	0.994	1	0.994	0	0	0	1 Ref
KJ133697	Unassigned	Unassigned	Unassigned	879_0	Clustered	36	VC_879_0	36	0.994	1	0.994	0	0	0	1 Ref
KJ133698	Unassigned	Unassigned	Unassigned	879_0	Clustered	36	VC_879_0	36	0.994	1	0.994	0	0	0	1 Ref
KJ133699	Unassigned	Unassigned	Unassigned	879_0	Clustered	36	VC_879_0	36	0.994	1	0.994	0	0	0	1 Ref
KJ133700	Unassigned	Unassigned	Unassigned	879_0	Clustered	36	VC_879_0	36	0.994	1	0.994	0	0	0	1 Ref
KJ133701	Unassigned	Unassigned	Unassigned	879_0	Clustered	36	VC_879_0	36	0.994	1	0.994	0	0	0	1 Ref
KJ133702	Unassigned	Unassigned	Unassigned	879_0	Clustered	36	VC_879_0	36	0.994	1	0.994	0	0	0	1 Ref
KJ133703	Unassigned	Unassigned	Unassigned	879_0	Clustered	36	VC_879_0	36	0.994	1	0.994	0	0	0	1 Ref
KJ133704	Unassigned	Unassigned	Unassigned	879_0	Clustered	36	VC_879_0	36	0.994	1	0.994	0	0	0	1 Ref
KJ133705	Unassigned	Unassigned	Unassigned	879_0	Clustered	36	VC_879_0	36	0.994	1	0.994	0	0	0	1 Ref
KJ133706	Unassigned	Unassigned	Unassigned	879_0	Clustered	36	VC_879_0	36	0.994	1	0.994	0	0	0	1 Ref
KJ133707	Unassigned	Unassigned	Unassigned	879_0	Clustered	36	VC_879_0	36	0.994	1	0.994	0	0	0	1 Ref
KJ135004	Unassigned	Unassigned	Unassigned	779_0	Clustered	37	VC_779_0	37	0.5682	1	0.5682	0	0	0	1 Ref
KJ140076	Unassigned	Unassigned	Unassigned	12_0	Clustered	21	VC_12_0	21	0.2274	1	0.2274	0	0	0	0.9571 Ref
KJ156985	Unassigned	Unassigned	Unassigned	515_0	Clustered	16	VC_515_0	16	0.3793	1	0.3793	0	0	0	1 Ref
KJ159566	Unassigned	Unassigned	Unassigned	102_0	Clustered	2	VC_102_0	2	0.114	0.94637553	0.1079	0	0	0	1 Ref
KJ173786	Unassigned	Unassigned	Unassigned	856_1	Clustered	13	VC_856_1	13	0.5848	1	0.5848	0	0	0	1 Ref
KJ174156	Unassigned	Unassigned	Unassigned	325_15	Clustered/Singleton	1	VC_325_15	1	0	1	0	0	0	0	1 Ref
KJ174157	Unassigned	Unassigned	Unassigned	371_3	Clustered	22	VC_371_3	22	0.0787	1	0.0787	0	0	0	0.9048 Ref
KJ174317	Unassigned	Unassigned	Unassigned	133_22	Clustered	13	VC_133_22	13	0.0804	1	0.0804	0	0	0	0.8205 Ref
KJ174318	Unassigned	Unassigned	Unassigned	133_16	Clustered/Singleton	1	VC_133_16	1	0	1	0	0	0	0	1 Ref
KJ183191	Unassigned	Unassigned	Unassigned	484_0	Clustered	3	VC_484_0	3	0.3174	0.99843638	0.3169	0	0	0	1 Ref
KJ183192	Unassigned	Unassigned	Unassigned	484_0	Clustered	3	VC_484_0	3	0.3174	0.99843638	0.3169	0	0	0	1 Ref
KJ183193	Unassigned	Unassigned	Unassigned	484_0	Clustered	3	VC_484_0	3	0.3174	0.99843638	0.3169	0	0	0	1 Ref
KJ190157	Unassigned	Unassigned	Unassigned	190_15	Clustered	3	VC_190_15	3	0.0146	0.99759465	0.0145	0	0	0	0.6667 Ref
KJ190158	Unassigned	Unassigned	Unassigned	772_16	Clustered	10	VC_772_16	10	0.0563	0.9434928	0.0531	0	0	0	0.8667 Ref
KJ194579	Unassigned	Unassigned	Unassigned	371_18	Clustered	10	VC_371_18	10	0.0302	1	0.0302	0	0	0	0.8 Ref
KJ194580	Unassigned	Unassigned	Unassigned	371_16	Clustered	42	VC_371_16	42	0.1341	1	0.1341	0	0	0	0.7282 Ref
KJ194581	Unassigned	Unassigned	Unassigned	509_0	Clustered	37	VC_509_0	37	0.9689	1	0.9689	0	0	0	1 Ref
KJ194582	Unassigned	Unassigned	Unassigned	565_0	Clustered	20	VC_565_0	20	0.8398	1	0.8398	0	0	0	1 Ref
KJ194583	Unassigned	Unassigned	Unassigned	371_16	Clustered	42	VC_371_16	42	0.1341	1	0.1341	0	0	0	0.7282 Ref
KJ194584	Unassigned	Unassigned	Unassigned	509_0	Clustered	37	VC_509_0	37	0.9689	1	0.9689	0	0	0	1 Ref
KJ194585	Unassigned	Unassigned	Unassigned	325_5	Clustered	11	VC_325_5	11	0.047	1	0.047	0	0	0	0.7636 Ref
KJ206559	Unassigned	Unassigned	Unassigned	149_8	Clustered	66	VC_149_8	66	0.4565	1	0.4565	0	0	0	0.8182 Ref
KJ206560	Unassigned	Unassigned	Unassigned	149_8	Clustered	66	VC_149_8	66	0.4565	1	0.4565	0	0	0	0.8182 Ref
KJ206561	Unassigned	Unassigned	Unassigned	149_8	Clustered	66	VC_149_8	66	0.4565	1	0.4565	0	0	0	0.8182 Ref
KJ206562	Unassigned	Unassigned	Unassigned	149_8	Clustered	66	VC_149_8	66	0.4565	1	0.4565	0	0	0	0.8182 Ref
KJ206563	Unassigned	Unassigned	Unassigned	149_8	Clustered	66	VC_149_8	66	0.4565	1	0.4565	0	0	0	0.8182 Ref
KJ210330	Unassigned	Unassigned	Unassigned	96_0	Clustered	30	VC_96_0	30	0.718	1	0.718	0	0	0	1 Ref
KJ409696	Unassigned	Unassigned	Unassigned	325_30	Clustered	4	VC_325_30	4	0.0144	0.99998616	0.0144	0	0	0	0.8333 Ref
KJ409772	Unassigned	Unassigned	Unassigned	78_3	Clustered	11	VC_78_3	11	0.2572	1	0.2572	0	0	0	1 Ref
KJ410132	Unassigned	Unassigned	Unassigned	216_6	Clustered	42	VC_216_6	42	0.473	1	0.473	0	0	0	0.8351 Ref
KJ410133	Unassigned	Unassigned	Unassigned	762_0	Clustered	9	VC_762_0	9	0.0885	1	0.0885	0	0	0	1 Ref
KJ410134	Unassigned	Unassigned	Unassigned	216_6	Clustered	42	VC_216_6	42	0.473	1	0.473	0	0	0	0.8351 Ref
KJ410740	Unassigned	Unassigned	Unassigned	927_0	Clustered	2	VC_927_0	2	0.0876	0.71936208	0.063	0	0	0	1 Ref
KJ417497	Unassigned	Unassigned	Unassigned	414_0	Clustered	37	VC_414_0	37	0.585	1	0.585	0	0	0	0.9474 Ref
KJ419279	Unassigned	Unassigned	Unassigned	722_0	Clustered	13	VC_722_0	13	0.5104	1	0.5104	0	0	0	1 Ref
KJ433973	Unassigned	Unassigned	Unassigned	372_3	Clustered	5	VC_372_3	5	0.1129	0.99999997	0.1129	0	0	0	1 Ref
KJ433974	Unassigned	Unassigned	Unassigned	372_3	Clustered	5	VC_372_3	5	0.1129	0.99999997	0.1129	0	0	0	1 Ref
KJ433975	Unassigned	Unassigned	Unassigned	372_3	Clustered	5	VC_372_3	5	0.1129	0.99999997	0.1129	0	0	0	1 Ref
KJ433976	Unassigned	Unassigned	Unassigned	372_3	Clustered	5	VC_372_3	5	0.1129	0.99999997	0.1129	0	0	0	1 Ref
KJ451625	Unassigned	Unassigned	Unassigned	187_3	Clustered	9	VC_187_3	9	0.1971	1	0.1971	0	0	0	0.8611 Ref
KJ452291	Unassigned	Unassigned	Unassigned	12_0	Clustered	21	VC_12_0	21	0.2274	1	0.2274	0	0	0	0.9571 Ref
KJ452292	Unassigned	Unassigned	Unassigned	10_0	Clustered	25	VC_10_0	25	0.3467	1	0.3467	0	0	0	0.9733 Ref
KJ473422	Unassigned	Unassigned	Unassigned	700_1	Clustered/Singleton	1	VC_700_1	1	0	1	0	0	0	0	1 Ref

KJ477077	Unassigned	Unassigned	Unassigned	179_0	Clustered	53	VC_179_0	53	0.9137	1	0.9137	0	0	0	1	Ref
KJ477684	Unassigned	Unassigned	Unassigned	283_91	Clustered	11	VC_283_91	11	0.0285	1	0.0285	0	0	0	1	Ref
KJ477685	Unassigned	Unassigned	Unassigned	283_91	Clustered	11	VC_283_91	11	0.0285	1	0.0285	0	0	0	1	Ref
KJ477686	Unassigned	Unassigned	Unassigned	283_91	Clustered	11	VC_283_91	11	0.0285	1	0.0285	0	0	0	1	Ref
KJ489010	Unassigned	Unassigned	Unassigned	801_0	Clustered	4	VC_801_0	4	0.4828	0.99998705	0.4828	0	0	0	1	Ref
KJ489011	Unassigned	Unassigned	Unassigned	801_0	Clustered	4	VC_801_0	4	0.4828	0.99998705	0.4828	0	0	0	1	Ref
KJ489012	Unassigned	Unassigned	Unassigned	801_0	Clustered	4	VC_801_0	4	0.4828	0.99998705	0.4828	0	0	0	1	Ref
KJ489013	Unassigned	Unassigned	Unassigned	801_0	Clustered	4	VC_801_0	4	0.4828	0.99998705	0.4828	0	0	0	1	Ref
KJ489397	Unassigned	Unassigned	Unassigned	883_0	Clustered	10	VC_883_0	10	0.2015	1	0.2015	0	0	0	1	Ref
KJ489398	Unassigned	Unassigned	Unassigned	883_0	Clustered	10	VC_883_0	10	0.2015	1	0.2015	0	0	0	1	Ref
KJ489399	Unassigned	Unassigned	Unassigned	884_0	Clustered	28	VC_884_0	28	0.6211	1	0.6211	0	0	0	0.9841	Ref
KJ489400	Unassigned	Unassigned	Unassigned	883_0	Clustered	10	VC_883_0	10	0.2015	1	0.2015	0	0	0	1	Ref
KJ489401	Unassigned	Unassigned	Unassigned	884_0	Clustered	28	VC_884_0	28	0.6211	1	0.6211	0	0	0	0.9841	Ref
KJ489402	Unassigned	Unassigned	Unassigned	883_1	Clustered	12	VC_883_1	12	0.2441	1	0.2441	0	0	0	1	Ref
KJ502657	Unassigned	Unassigned	Unassigned	1027_0	Clustered	20	VC_1027_0	20	0.7114	1	0.7114	0	0	0	1	Ref
KJ507099	Unassigned	Unassigned	Unassigned	129_0	Clustered	43	VC_129_0	43	0.886	1	0.886	0	0	0	1	Ref
KJ507100	Unassigned	Unassigned	Unassigned	697_0	Clustered	5	VC_697_0	5	0.8303	0.99999971	0.8303	0	0	0	1	Ref
KJ510413	Unassigned	Unassigned	Unassigned	1046_0	Clustered	7	VC_1046_0	7	0.9413	1	0.9413	0	0	0	1	Ref
KJ510414	Unassigned	Unassigned	Unassigned	517_1	Clustered	108	VC_517_1	108	0.8483	1	0.8483	0	0	0	0.9955	Ref
KJ510415	Unassigned	Unassigned	Unassigned	513_0	Clustered	32	VC_513_0	32	0.6881	1	0.6881	0	0	0	1	Ref
KJ528544	Unassigned	Unassigned	Unassigned	97_0	Clustered	5	VC_97_0	5	0.0696	0.98113835	0.0683	0	0	0	1	Ref
KJ535721	Unassigned	Unassigned	Unassigned	739_0	Clustered	26	VC_739_0	26	0.9571	1	0.9571	0	0	0	1	Ref
KJ535722	Unassigned	Unassigned	Unassigned	739_0	Clustered	26	VC_739_0	26	0.9571	1	0.9571	0	0	0	1	Ref
KJ538721	Unassigned	Unassigned	Unassigned	939_0	Clustered	15	VC_939_0	15	1	0	0	0	0	0	1	Ref
KJ538722	Unassigned	Unassigned	Unassigned	810_0	Clustered	18	VC_810_0	18	0.9121	1	0.9121	0	0	0	1	Ref
KJ538723	Unassigned	Unassigned	Unassigned	371_3	Clustered	22	VC_371_3	22	0.0787	1	0.0787	0	0	0	0.9048	Ref
KJ564036	Unassigned	Unassigned	Unassigned	640_0	Clustered	7	VC_640_0	7	0.9025	1	0.9025	0	0	0	1	Ref
KJ564037	Unassigned	Unassigned	Unassigned	640_0	Clustered	7	VC_640_0	7	0.9025	1	0.9025	0	0	0	1	Ref
KJ564038	Unassigned	Unassigned	Unassigned	640_0	Clustered	7	VC_640_0	7	0.9025	1	0.9025	0	0	0	1	Ref
KJ567042	Unassigned	Unassigned	Unassigned	238_0	Clustered	17	VC_238_0	17	0.6109	1	0.6109	0	0	0	1	Ref
KJ567043	Unassigned	Unassigned	Unassigned	563_0	Clustered	2	VC_563_0	2	0.0727	0.956426	0.0695	0	0	0	1	Ref
KJ567044	Unassigned	Unassigned	Unassigned	371_2	Clustered	8	VC_371_2	8	0.0232	1	0.0232	0	0	0	0.8571	Ref
KJ567045	Unassigned	Unassigned	Unassigned	236_0	Clustered	72	VC_236_0	72	0.8104	1	0.8104	0	0	0	1	Ref
KJ567046	Unassigned	Unassigned	Unassigned	567_0	Clustered	8	VC_567_0	8	0.4935	1	0.4935	0	0	0	1	Ref
KJ572844	Unassigned	Unassigned	Unassigned	121_0	Clustered	4	VC_121_0	4	0.1814	0.99998531	0.1814	0	0	0	1	Ref
KJ578758	Unassigned	Unassigned	Unassigned	765_0	Clustered	132	VC_765_0	132	1	1	1	0	0	0	1	Ref
KJ578759	Unassigned	Unassigned	Unassigned	765_0	Clustered	132	VC_765_0	132	1	1	1	0	0	0	1	Ref
KJ578760	Unassigned	Unassigned	Unassigned	765_0	Clustered	132	VC_765_0	132	1	1	1	0	0	0	1	Ref
KJ578761	Unassigned	Unassigned	Unassigned	765_0	Clustered	132	VC_765_0	132	1	1	1	0	0	0	1	Ref
KJ578762	Unassigned	Unassigned	Unassigned	765_0	Clustered	132	VC_765_0	132	1	1	1	0	0	0	1	Ref
KJ578763	Unassigned	Unassigned	Unassigned	765_0	Clustered	132	VC_765_0	132	1	1	1	0	0	0	1	Ref
KJ578764	Unassigned	Unassigned	Unassigned	765_0	Clustered	132	VC_765_0	132	1	1	1	0	0	0	1	Ref
KJ578765	Unassigned	Unassigned	Unassigned	765_0	Clustered	132	VC_765_0	132	1	1	1	0	0	0	1	Ref
KJ578766	Unassigned	Unassigned	Unassigned	765_0	Clustered	132	VC_765_0	132	1	1	1	0	0	0	1	Ref
KJ578767	Unassigned	Unassigned	Unassigned	765_0	Clustered	132	VC_765_0	132	1	1	1	0	0	0	1	Ref
KJ578768	Unassigned	Unassigned	Unassigned	765_0	Clustered	132	VC_765_0	132	1	1	1	0	0	0	1	Ref
KJ578769	Unassigned	Unassigned	Unassigned	765_0	Clustered	132	VC_765_0	132	1	1	1	0	0	0	1	Ref
KJ578770	Unassigned	Unassigned	Unassigned	765_0	Clustered	132	VC_765_0	132	1	1	1	0	0	0	1	Ref
KJ578771	Unassigned	Unassigned	Unassigned	765_0	Clustered	132	VC_765_0	132	1	1	1	0	0	0	1	Ref
KJ578772	Unassigned	Unassigned	Unassigned	765_0	Clustered	132	VC_765_0	132	1	1	1	0	0	0	1	Ref
KJ578773	Unassigned	Unassigned	Unassigned	765_0	Clustered	132	VC_765_0	132	1	1	1	0	0	0	1	Ref
KJ578774	Unassigned	Unassigned	Unassigned	765_0	Clustered	132	VC_765_0	132	1	1	1	0	0	0	1	Ref
KJ578775	Unassigned	Unassigned	Unassigned	765_0	Clustered	132	VC_765_0	132	1	1	1	0	0	0	1	Ref
KJ578776	Unassigned	Unassigned	Unassigned	765_0	Clustered	132	VC_765_0	132	1	1	1	0	0	0	1	Ref
KJ578777	Unassigned	Unassigned	Unassigned	765_0	Clustered	132	VC_765_0	132	1	1	1	0	0	0	1	Ref
KJ578778	Unassigned	Unassigned	Unassigned	765_0	Clustered	132	VC_765_0	132	1	1	1	0	0	0	1	Ref
KJ578779	Unassigned	Unassigned	Unassigned	765_0	Clustered	132	VC_765_0	132	1	1	1	0	0	0	1	Ref
KJ578780	Unassigned	Unassigned	Unassigned	765_0	Clustered	132	VC_765_0	132	1	1	1	0	0	0	1	Ref
KJ578781	Unassigned	Unassigned	Unassigned	765_0	Clustered	132	VC_765_0	132	1	1	1	0	0	0	1	Ref
KJ578782	Unassigned	Unassigned	Unassigned	765_0	Clustered	132	VC_765_0	132	1	1	1	0	0	0	1	Ref
KJ578783	Unassigned	Unassigned	Unassigned	765_0	Clustered	132	VC_765_0	132	1	1	1	0	0	0	1	Ref
KJ578784	Unassigned	Unassigned	Unassigned	765_0	Clustered	132	VC_765_0	132	1	1	1	0	0	0	1	Ref
KJ578785	Unassigned	Unassigned	Unassigned	765_0	Clustered	132	VC_765_0	132	1	1	1	0	0	0	1	Ref
KJ578786	Unassigned	Unassigned	Unassigned	765_0	Clustered	132	VC_765_0	132	1	1	1	0	0	0	1	Ref
KJ578787	Unassigned	Unassigned	Unassigned	765_0	Clustered	132	VC_765_0	132	1	1	1	0	0	0	1	Ref
KJ578788	Unassigned	Unassigned	Unassigned	765_0	Clustered	132	VC_765_0	132	1	1	1	0	0	0	1	Ref
KJ578789	Unassigned	Unassigned	Unassigned	765_0	Clustered	132	VC_765_0	132	1	1	1	0	0	0	1	Ref
KJ578790	Unassigned	Unassigned	Unassigned	765_0	Clustered	132	VC_765_0	132	1	1	1	0	0	0	1	Ref
KJ578791	Unassigned	Unassigned	Unassigned	765_0	Clustered	132	VC_765_0	132	1	1	1	0	0	0	1	Ref
KJ578792	Unassigned	Unassigned	Unassigned	765_0	Clustered	132	VC_765_0	132	1	1	1	0	0	0	1	Ref
KJ586794	Unassigned	Unassigned	Unassigned	739_0	Clustered	26	VC_739_0	26	0.9571	1	0.9571	0	0	0	1	Ref
KJ586795	Unassigned	Unassigned	Unassigned	739_0	Clustered	26	VC_739_0	26	0.9571	1	0.9571	0	0	0	1	Ref
KJ591604	Unassigned	Unassigned	Unassigned	739_0	Clustered	26	VC_739_0	26	0.9571	1	0.9571	0	0	0	1	Ref
KJ591605	Unassigned	Unassigned	Unassigned	739_0	Clustered	26	VC_739_0	26	0.9571	1	0.9571	0	0	0	1	Ref
KJ595575	Unassigned	Unassigned	Unassigned	558_50	Clustered	15	VC_558_50	15	0.0468	1	0.0468	0	0	0	0.9238	Ref
KJ595576	Unassigned	Unassigned	Unassigned	371_1	Clustered	11	VC_371_1	11	0.0379	1	0.0379	0	0	0	0.8545	Ref
KJ596420	Unassigned	Unassigned	Unassigned	10_0	Clustered	25	VC_10_0	25	0.3467	1	0.3467	0	0	0	0.9733	Ref
KJ603229	Unassigned	Unassigned	Unassigned	46_0	Clustered	2	VC_46_0	2	0.0181	0.95763537	0.0173	0	0	0	1	Ref
KJ608189	Unassigned	Unassigned	Unassigned	269_0	Clustered	3	VC_269_0	3	0.1948	0.99308177	0.1934	0	0	0	1	Ref
KJ617393	Unassigned	Unassigned	Unassigned	1048_0	Clustered	3	VC_1048_0	3	1	1	1	0	0	0	1	Ref
KJ619459	Unassigned	Unassigned	Unassigned	875_0	Clustered	10	VC_875_0	10	0.988	0.99948433	0.9875	0	0	0	1	Ref

KJ621082	Unassigned	Unassigned	Unassigned	833_0	Clustered	16	VC_833_0	16	0.9351	1	0.9351	0	0	0	1	Ref
KJ628499	Unassigned	Unassigned	Unassigned	700_0	Clustered	10	VC_700_0	10	0.8076	1	0.8076	0	0	0	1	Ref
KJ668713	Unassigned	Unassigned	Unassigned	455_0	Clustered	18	VC_455_0	18	0.4598	1	0.4598	0	0	0	1	Ref
KJ668714	Unassigned	Unassigned	Unassigned	283_99	Clustered/Singleton	1	VC_283_99	1	0	1	0	0	0	0	1	Ref
KJ668715	Unassigned	Unassigned	Unassigned	739_0	Clustered	26	VC_739_0	26	0.9571	1	0.9571	0	0	0	1	Ref
KJ676859	Unassigned	Unassigned	Unassigned	187_2	Clustered	2	VC_187_2	2	0.0209	0.93057595	0.0195	0	0	0	1	Ref
KJ690250	Unassigned	Unassigned	Unassigned	325_51	Clustered	2	VC_325_51	2	0.0039	0.95663826	0.0037	0	0	0	1	Ref
KJ716334	Unassigned	Unassigned	Unassigned	9_0	Clustered	38	VC_9_0	38	0.4895	1	0.4895	0	0	0	1	Ref
KJ716335	Unassigned	Unassigned	Unassigned	131_0	Clustered	17	VC_131_0	17	0.2671	1	0.2671	0	0	0	1	Ref
KJ722067	Unassigned	Unassigned	Unassigned	765_0	Clustered	132	VC_765_0	132	1	1	1	0	0	0	1	Ref
KJ725374	Unassigned	Unassigned	Unassigned	761_0	Clustered	67	VC_761_0	67	0.8947	1	0.8947	0	0	0	1	Ref
KJ746502	Unassigned	Unassigned	Unassigned	21_0	Clustered	4	VC_21_0	4	0.5696	0.87262442	0.4971	0	0	0	0.6667	Ref
KJ748011	Unassigned	Unassigned	Unassigned	451_0	Clustered	80	VC_451_0	80	0.9991	1	0.9991	0	0	0	1	Ref
KJ801817	Unassigned	Unassigned	Unassigned	91_0	Clustered	22	VC_91_0	22	0.5318	1	0.5318	0	0	0	1	Ref
KJ802832	Unassigned	Unassigned	Unassigned	1049_0	Clustered	8	VC_1049_0	8	0.6413	1	0.6413	0	0	0	1	Ref
KJ803031	Unassigned	Unassigned	Unassigned	833_0	Clustered	16	VC_833_0	16	0.9351	1	0.9351	0	0	0	1	Ref
KJ804259	Unassigned	Unassigned	Unassigned	667_0	Clustered	7	VC_667_0	7	0.4101	1	0.4101	0	0	0	1	Ref
KJ817802	Unassigned	Unassigned	Unassigned	698_0	Clustered	26	VC_698_0	26	0.9366	1	0.9366	0	0	0	1	Ref
KJ829260	Unassigned	Unassigned	Unassigned	556_0	Clustered	21	VC_556_0	21	0.9912	1	0.9912	0	0	0	1	Ref
KJ832078	Unassigned	Unassigned	Unassigned	30_1	Clustered	16	VC_30_1	16	0.1973	1	0.1973	0	0	0	0.8333	Ref
KJ858521	Unassigned	Unassigned	Unassigned	793_0	Clustered	12	VC_793_0	12	0.8531	1	0.8531	0	0	0	1	Ref
KJ888149	Unassigned	Unassigned	Unassigned	149_16	Clustered/Singleton	1	VC_149_16	1	0	1	0	0	0	0	1	Ref
KJ909655	Unassigned	Unassigned	Unassigned	39_0	Clustered	35	VC_39_0	35	0.4337	1	0.4337	0	0	0	1	Ref
KJ936628	Unassigned	Unassigned	Unassigned	530_0	Clustered	9	VC_530_0	9	0.8027	1	0.8027	0	0	0	1	Ref
KJ944841	Unassigned	Unassigned	Unassigned	234_0	Clustered	21	VC_234_0	21	0.8355	1	0.8355	0	0	0	1	Ref
KJ959591	Unassigned	Unassigned	Unassigned	747_0	Clustered	23	VC_747_0	23	0.8202	1	0.8202	0	0	0	1	Ref
KJ959632	Unassigned	Unassigned	Unassigned	216_4	Clustered	13	VC_216_4	13	0.1657	1	0.1657	0	0	0	1	Ref
KJ997912	Unassigned	Unassigned	Unassigned	303_0	Clustered	155	VC_303_0	155	0.9979	1	0.9979	0	0	0	1	Ref
KM044272	Unassigned	Unassigned	Unassigned	813_1	Clustered	24	VC_813_1	24	0.535	1	0.535	0	0	0	1	Ref
KM051843	Unassigned	Unassigned	Unassigned	85_0	Clustered	3	VC_85_0	3	0.0422	0.99823242	0.0421	0	0	0	1	Ref
KM058087	Unassigned	Unassigned	Unassigned	1051_0	Clustered	2	VC_1051_0	2	1	1	1	0	0	0	1	Ref
KM067278	Unassigned	Unassigned	Unassigned	423_0	Clustered	45	VC_423_0	45	0.9484	1	0.9484	0	0	0	1	Ref
KM091442	Unassigned	Unassigned	Unassigned	202_0	Clustered	102	VC_202_0	102	0.4049	1	0.4049	0	0	0	0.7876	Ref
KM092515	Unassigned	Unassigned	Unassigned	329_17	Clustered	4	VC_329_17	4	0.0163	0.99996247	0.0163	0	0	0	1	Ref
KM101117	Unassigned	Unassigned	Unassigned	508_0	Clustered	32	VC_508_0	32	0.906	1	0.906	0	0	0	1	Ref
KM101118	Unassigned	Unassigned	Unassigned	940_0	Clustered	34	VC_940_0	34	0.7959	1	0.7959	0	0	0	1	Ref
KM101119	Unassigned	Unassigned	Unassigned	514_0	Clustered	75	VC_514_0	75	0.9402	1	0.9402	0	0	0	1	Ref
KM101120	Unassigned	Unassigned	Unassigned	515_0	Clustered	16	VC_515_0	16	0.3793	1	0.3793	0	0	0	1	Ref
KM101122	Unassigned	Unassigned	Unassigned	520_49	Clustered/Singleton	1	VC_520_49	1	0	1	0	0	0	0	1	Ref
KM101123	Unassigned	Unassigned	Unassigned	546_7	Clustered	4	VC_546_7	4	0.041	0.9963251	0.0408	0	0	0	1	Ref
KM190144	Unassigned	Unassigned	Unassigned	582_2	Clustered/Singleton	1	VC_582_2	1	0	1	0	0	0	0	1	Ref
KM197169	Unassigned	Unassigned	Unassigned	216_4	Clustered	13	VC_216_4	13	0.1657	1	0.1657	0	0	0	1	Ref
KM199770	Unassigned	Unassigned	Unassigned	1052_3	Clustered	5	VC_1052_3	5	0.185	0.98656048	0.1825	0	0	0	1	Ref
KM199771	Unassigned	Unassigned	Unassigned	481_0	Clustered	8	VC_481_0	8	0.1348	1	0.1348	0	0	0	1	Ref
KM209228	Unassigned	Unassigned	Unassigned	131_0	Clustered	17	VC_131_0	17	0.2671	1	0.2671	0	0	0	1	Ref
KM209270	Unassigned	Unassigned	Unassigned	131_0	Clustered	17	VC_131_0	17	0.2671	1	0.2671	0	0	0	1	Ref
KM209320	Unassigned	Unassigned	Unassigned	131_0	Clustered	17	VC_131_0	17	0.2671	1	0.2671	0	0	0	1	Ref
KM216423	Unassigned	Unassigned	Unassigned	149_15	Clustered	16	VC_149_15	16	0.0788	1	0.0788	0	0	0	0.8083	Ref
KM224878	Unassigned	Unassigned	Unassigned	754_0	Clustered	8	VC_754_0	8	0.752	1	0.752	0	0	0	1	Ref
KM224879	Unassigned	Unassigned	Unassigned	754_0	Clustered	8	VC_754_0	8	0.752	1	0.752	0	0	0	1	Ref
KM233151	Unassigned	Unassigned	Unassigned	835_0	Clustered	67	VC_835_0	67	0.9624	1	0.9624	0	0	0	1	Ref
KM233261	Unassigned	Unassigned	Unassigned	907_0	Clustered	3	VC_907_0	3	0.6642	0.99633114	0.6618	0	0	0	1	Ref
KM233454	Unassigned	Unassigned	Unassigned	514_0	Clustered	75	VC_514_0	75	0.9402	1	0.9402	0	0	0	1	Ref
KM233455	Unassigned	Unassigned	Unassigned	514_0	Clustered	75	VC_514_0	75	0.9402	1	0.9402	0	0	0	1	Ref
KM233689	Unassigned	Unassigned	Unassigned	179_0	Clustered	53	VC_179_0	53	0.9137	1	0.9137	0	0	0	1	Ref
KM236237	Unassigned	Unassigned	Unassigned	669_1	Clustered	10	VC_669_1	10	0.1268	1	0.1268	0	0	0	0.9778	Ref
KM236239	Unassigned	Unassigned	Unassigned	330_0	Clustered	27	VC_330_0	27	0.5088	1	0.5088	0	0	0	0.8661	Ref
KM236241	Unassigned	Unassigned	Unassigned	584_0	Clustered	28	VC_584_0	28	0.7704	1	0.7704	0	0	0	1	Ref
KM236242	Unassigned	Unassigned	Unassigned	780_0	Clustered	8	VC_780_0	8	0.5207	1	0.5207	0	0	0	1	Ref
KM236243	Unassigned	Unassigned	Unassigned	795_0	Clustered	19	VC_795_0	19	0.6848	1	0.6848	0	0	0	1	Ref
KM236244	Unassigned	Unassigned	Unassigned	191_19	Clustered	12	VC_191_19	12	0.0655	1	0.0655	0	0	0	0.8636	Ref
KM236245	Unassigned	Unassigned	Unassigned	85_9	Clustered/Singleton	1	VC_85_9	1	0	1	0	0	0	0	1	Ref
KM236246	Unassigned	Unassigned	Unassigned	85_7	Clustered/Singleton	1	VC_85_7	1	0	1	0	0	0	0	1	Ref
KM236247	Unassigned	Unassigned	Unassigned	1032_0	Clustered	7	VC_1032_0	7	0.9887	0.99999504	0.9887	0	0	0	1	Ref
KM236248	Unassigned	Unassigned	Unassigned	1032_0	Clustered	7	VC_1032_0	7	0.9887	0.99999504	0.9887	0	0	0	1	Ref
KM236502	Unassigned	Unassigned	Unassigned	371_3	Clustered	22	VC_371_3	22	0.0787	1	0.0787	0	0	0	0.9048	Ref
KM242061	Unassigned	Unassigned	Unassigned	392_0	Clustered	126	VC_392_0	126	0.9621	1	0.9621	0	0	0	1	Ref
KM247287	Unassigned	Unassigned	Unassigned	156_0	Clustered	12	VC_156_0	12	0.9062	1	0.9062	0	0	0	1	Ref
KM253764	Unassigned	Unassigned	Unassigned	385_0	Clustered	51	VC_385_0	51	0.8754	1	0.8754	0	0	0	1	Ref
KM262191	Unassigned	Unassigned	Unassigned	862_0	Clustered	11	VC_862_0	11	0.6745	1	0.6745	0	0	0	1	Ref
KM262192	Unassigned	Unassigned	Unassigned	862_0	Clustered	11	VC_862_0	11	0.6745	1	0.6745	0	0	0	1	Ref
KM272358	Unassigned	Unassigned	Unassigned	637_2	Clustered	2	VC_637_2	2	0.0038	0.92844927	0.0035	0	0	0	1	Ref
KM279937	Unassigned	Unassigned	Unassigned	520_56	Clustered/Singleton	1	VC_520_56	1	0	1	0	0	0	0	1	Ref
KM289195	Unassigned	Unassigned	Unassigned	636_0	Clustered	9	VC_636_0	9	0.2422	1	0.2422	0	0	0	0.9722	Ref
KM347889	Unassigned	Unassigned	Unassigned	520_68	Clustered/Singleton	1	VC_520_68	1	0	1	0	0	0	0	1	Ref
KM347890	Unassigned	Unassigned	Unassigned	371_1	Clustered	11	VC_371_1	11	0.0379	1	0.0379	0	0	0	0.8545	Ref
KM359505	Unassigned	Unassigned	Unassigned	432_8	Clustered/Singleton	1	VC_432_8	1	0	1	0	0	0	0	1	Ref
KM360178	Unassigned	Unassigned	Unassigned	125_0	Clustered	17	VC_125_0	17	0.9244	1	0.9244	0	0	0	1	Ref
KM363596	Unassigned	Unassigned	Unassigned	238_0	Clustered	17	VC_238_0	17	0.6109	1	0.6109	0	0	0	1	Ref
KM363597	Unassigned	Unassigned	Unassigned	371_13	Clustered	33	VC_371_13	33	0.179	1	0.179	0	0	0	0.8769	Ref
KM366096	Unassigned	Unassigned	Unassigned	386_0	Clustered	49	VC_386_0	49	0.7941	1	0.7941	0	0	0	1	Ref

KM366097	Unassigned	Unassigned	Unassigned	452_0	Clustered	66	VC_452_0	66	0.9248	1	0.9248	0	0	0	1	Ref
KM366098	Unassigned	Unassigned	Unassigned	592_0	Clustered	50	VC_592_0	50	0.7314	1	0.7314	0	0	0	1	Ref
KM366099	Unassigned	Unassigned	Unassigned	428_0	Clustered	33	VC_428_0	33	0.9915	1	0.9915	0	0	0	1	Ref
KM366100	Unassigned	Unassigned	Unassigned	96_0	Clustered	30	VC_96_0	30	0.718	1	0.718	0	0	0	1	Ref
KM370384	Unassigned	Unassigned	Unassigned	452_0	Clustered	66	VC_452_0	66	0.9248	1	0.9248	0	0	0	1	Ref
KM373208	Unassigned	Unassigned	Unassigned	739_0	Clustered	26	VC_739_0	26	0.9571	1	0.9571	0	0	0	1	Ref
KM389210	Unassigned	Unassigned	Unassigned	297_0	Clustered	5	VC_297_0	5	0.1056	0.99999993	0.1056	0	0	0	0.9	Ref
KM400683	Unassigned	Unassigned	Unassigned	546_2	Clustered	3	VC_546_2	3	0.0275	0.85260342	0.0234	0	0	0	1	Ref
KM401838	Unassigned	Unassigned	Unassigned	223_0	Clustered	38	VC_223_0	38	0.9095	1	0.9095	0	0	0	1	Ref
KM402757	Unassigned	Unassigned	Unassigned	520_92	Clustered	5	VC_520_92	5	0.0663	0.99999997	0.0663	0	0	0	1	Ref
KM407600	Unassigned	Unassigned	Unassigned	286_10	Clustered	9	VC_286_10	9	0.0615	0.99999878	0.0615	0	0	0	0.6667	Ref
KM408320	Unassigned	Unassigned	Unassigned	371_15	Clustered	44	VC_371_15	44	0.1391	1	0.1391	0	0	0	0.7474	Ref
KM411958	Unassigned	Unassigned	Unassigned	838_0	Clustered	29	VC_838_0	29	0.8611	1	0.8611	0	0	0	1	Ref
KM411959	Unassigned	Unassigned	Unassigned	838_0	Clustered	29	VC_838_0	29	0.8611	1	0.8611	0	0	0	1	Ref
KM411960	Unassigned	Unassigned	Unassigned	838_0	Clustered	29	VC_838_0	29	0.8611	1	0.8611	0	0	0	1	Ref
KM434184	Unassigned	Unassigned	Unassigned	79_8	Clustered/Singleton	1	VC_79_8	1	0	1	0	0	0	0	1	Ref
KM434185	Unassigned	Unassigned	Unassigned	749_0	Clustered	40	VC_749_0	40	0.7517	1	0.7517	0	0	0	1	Ref
KM434186	Unassigned	Unassigned	Unassigned	77_0	Clustered	20	VC_77_0	20	0.9657	1	0.9657	0	0	0	1	Ref
KM458633	Unassigned	Unassigned	Unassigned	592_0	Clustered	50	VC_592_0	50	0.7314	1	0.7314	0	0	0	1	Ref
KM463009	Unassigned	Unassigned	Unassigned	216_6	Clustered	42	VC_216_6	42	0.473	1	0.473	0	0	0	0.8351	Ref
KM501444	Unassigned	Unassigned	Unassigned	283_145	Clustered/Singleton	1	VC_283_145	1	0	1	0	0	0	0	1	Ref
KM507819	Unassigned	Unassigned	Unassigned	164_11	Clustered	10	VC_164_11	10	0.1507	1	0.1507	0	0	0	0.9111	Ref
KM576124	Unassigned	Unassigned	Unassigned	111_0	Clustered	91	VC_111_0	91	0.9587	1	0.9587	0	0	0	1	Ref
KM581061	Unassigned	Unassigned	Unassigned	593_0	Clustered	5	VC_593_0	5	0.4608	0.99999995	0.4608	0	0	0	1	Ref
KM588359	Unassigned	Unassigned	Unassigned	521_0	Clustered	37	VC_521_0	37	0.912	1	0.912	0	0	0	1	Ref
KM589498	Unassigned	Unassigned	Unassigned	1054_0	Clustered	12	VC_1054_0	12	0.2863	0.99746644	0.2855	0	0	0	1	Ref
KM589499	Unassigned	Unassigned	Unassigned	1061_0	Clustered	2	VC_1061_0	2	0.7065	0.76024994	0.5371	0	0	0	1	Ref
KM589500	Unassigned	Unassigned	Unassigned	1062_0	Clustered	45	VC_1062_0	45	0.9734	0.50949847	0.4959	0	0	0	1	Ref
KM589502	Unassigned	Unassigned	Unassigned	995_0	Clustered	69	VC_995_0	69	0.8547	1	0.8547	0	0	0	1	Ref
KM589503	Unassigned	Unassigned	Unassigned	1062_0	Clustered	45	VC_1062_0	45	0.9734	0.50949847	0.4959	0	0	0	1	Ref
KM589504	Unassigned	Unassigned	Unassigned	1062_0	Clustered	45	VC_1062_0	45	0.9734	0.50949847	0.4959	0	0	0	1	Ref
KM589505	Unassigned	Unassigned	Unassigned	1062_0	Clustered	45	VC_1062_0	45	0.9734	0.50949847	0.4959	0	0	0	1	Ref
KM589507	Unassigned	Unassigned	Unassigned	1061_0	Clustered	2	VC_1061_0	2	0.7065	0.76024994	0.5371	0	0	0	1	Ref
KM589508	Unassigned	Unassigned	Unassigned	1066_0	Clustered	52	VC_1066_0	52	0.7682	1	0.7682	0	0	0	1	Ref
KM589509	Unassigned	Unassigned	Unassigned	1062_0	Clustered	45	VC_1062_0	45	0.9734	0.50949847	0.4959	0	0	0	1	Ref
KM589510	Unassigned	Unassigned	Unassigned	1062_0	Clustered	45	VC_1062_0	45	0.9734	0.50949847	0.4959	0	0	0	1	Ref
KM589511	Unassigned	Unassigned	Unassigned	995_0	Clustered	69	VC_995_0	69	0.8547	1	0.8547	0	0	0	1	Ref
KM589512	Unassigned	Unassigned	Unassigned	1062_0	Clustered	45	VC_1062_0	45	0.9734	0.50949847	0.4959	0	0	0	1	Ref
KM589513	Unassigned	Unassigned	Unassigned	1062_0	Clustered	45	VC_1062_0	45	0.9734	0.50949847	0.4959	0	0	0	1	Ref
KM589514	Unassigned	Unassigned	Unassigned	1068_0	Clustered	5	VC_1068_0	5	1	1	1	0	0	0	1	Ref
KM589515	Unassigned	Unassigned	Unassigned	1069_0	Clustered	4	VC_1069_0	4	1	1	1	0	0	0	1	Ref
KM589516	Unassigned	Unassigned	Unassigned	1069_0	Clustered	4	VC_1069_0	4	1	1	1	0	0	0	1	Ref
KM591905	Unassigned	Unassigned	Unassigned	1046_0	Clustered	7	VC_1046_0	7	0.9413	1	0.9413	0	0	0	1	Ref
KM591906	Unassigned	Unassigned	Unassigned	216_6	Clustered	42	VC_216_6	42	0.473	1	0.473	0	0	0	0.8351	Ref
KM592966	Unassigned	Unassigned	Unassigned	513_0	Clustered	32	VC_513_0	32	0.6881	1	0.6881	0	0	0	1	Ref
KM597530	Unassigned	Unassigned	Unassigned	520_29	Clustered/Singleton	1	VC_520_29	1	0	1	0	0	0	0	1	Ref
KM597531	Unassigned	Unassigned	Unassigned	325_35	Clustered	17	VC_325_35	17	0.0732	1	0.0732	0	0	0	0.7721	Ref
KM606994	Unassigned	Unassigned	Unassigned	283_82	Clustered	6	VC_283_82	6	0.013	1	0.013	0	0	0	1	Ref
KM606995	Unassigned	Unassigned	Unassigned	283_82	Clustered	6	VC_283_82	6	0.013	1	0.013	0	0	0	1	Ref
KM606996	Unassigned	Unassigned	Unassigned	283_82	Clustered	6	VC_283_82	6	0.013	1	0.013	0	0	0	1	Ref
KM606997	Unassigned	Unassigned	Unassigned	283_82	Clustered	6	VC_283_82	6	0.013	1	0.013	0	0	0	1	Ref
KM606998	Unassigned	Unassigned	Unassigned	283_82	Clustered	6	VC_283_82	6	0.013	1	0.013	0	0	0	1	Ref
KM606999	Unassigned	Unassigned	Unassigned	283_82	Clustered	6	VC_283_82	6	0.013	1	0.013	0	0	0	1	Ref
KM607000	Unassigned	Unassigned	Unassigned	283_83	Clustered/Singleton	1	VC_283_83	1	0	1	0	0	0	0	1	Ref
KM607001	Unassigned	Unassigned	Unassigned	283_94	Clustered	2	VC_283_94	2	0.0026	0.95293762	0.0025	0	0	0	1	Ref
KM607002	Unassigned	Unassigned	Unassigned	283_91	Clustered	11	VC_283_91	11	0.0285	1	0.0285	0	0	0	1	Ref
KM607003	Unassigned	Unassigned	Unassigned	283_91	Clustered	11	VC_283_91	11	0.0285	1	0.0285	0	0	0	1	Ref
KM607004	Unassigned	Unassigned	Unassigned	283_3	Clustered	3	VC_283_3	3	0.0055	0.9968017	0.0055	0	0	0	1	Ref
KM612259	Unassigned	Unassigned	Unassigned	607_0	Clustered	16	VC_607_0	16	0.9179	1	0.9179	0	0	0	1	Ref
KM612260	Unassigned	Unassigned	Unassigned	607_0	Clustered	16	VC_607_0	16	0.9179	1	0.9179	0	0	0	1	Ref
KM612261	Unassigned	Unassigned	Unassigned	607_0	Clustered	16	VC_607_0	16	0.9179	1	0.9179	0	0	0	1	Ref
KM612262	Unassigned	Unassigned	Unassigned	607_0	Clustered	16	VC_607_0	16	0.9179	1	0.9179	0	0	0	1	Ref
KM612263	Unassigned	Unassigned	Unassigned	607_0	Clustered	16	VC_607_0	16	0.9179	1	0.9179	0	0	0	1	Ref
KM612264	Unassigned	Unassigned	Unassigned	607_0	Clustered	16	VC_607_0	16	0.9179	1	0.9179	0	0	0	1	Ref
KM612265	Unassigned	Unassigned	Unassigned	607_0	Clustered	16	VC_607_0	16	0.9179	1	0.9179	0	0	0	1	Ref
KM652553	Unassigned	Unassigned	Unassigned	520_19	Clustered	2	VC_520_19	2	0.0098	0.95761552	0.0094	0	0	0	1	Ref
KM652554	Unassigned	Unassigned	Unassigned	1071_3	Clustered	16	VC_1071_3	16	0.2354	1	0.2354	0	0	0	0.9667	Ref
KM657822	Unassigned	Unassigned	Unassigned	329_13	Clustered/Singleton	1	VC_329_13	1	0	1	0	0	0	0	1	Ref
KM672662	Unassigned	Unassigned	Unassigned	702_0	Clustered	5	VC_702_0	5	0.7794	0.99999988	0.7794	0	0	0	1	Ref
KM677185	Unassigned	Unassigned	Unassigned	474_0	Clustered	14	VC_474_0	14	0.8466	1	0.8466	0	0	0	1	Ref
KM677210	Unassigned	Unassigned	Unassigned	216_4	Clustered	13	VC_216_4	13	0.1657	1	0.1657	0	0	0	1	Ref
KM677211	Unassigned	Unassigned	Unassigned	236_0	Clustered	72	VC_236_0	72	0.8104	1	0.8104	0	0	0	1	Ref
KM819694	Unassigned	Unassigned	Unassigned	112_0	Clustered	8	VC_112_0	8	0.2537	1	0.2537	0	0	0	1	Ref
KM819695	Unassigned	Unassigned	Unassigned	452_0	Clustered	66	VC_452_0	66	0.9248	1	0.9248	0	0	0	1	Ref
KM819696	Unassigned	Unassigned	Unassigned	452_0	Clustered	66	VC_452_0	66	0.9248	1	0.9248	0	0	0	1	Ref
KM873719	Unassigned	Unassigned	Unassigned	912_0	Clustered	76	VC_912_0	76	0.979	1	0.979	0	0	0	1	Ref
KM879463	Unassigned	Unassigned	Unassigned	1074_0	Clustered	19	VC_1074_0	19	0.9198	1	0.9198	0	0	0	1	Ref
KM881426	Unassigned	Unassigned	Unassigned	558_50	Clustered	15	VC_558_50	15	0.0468	1	0.0468	0	0	0	0.9238	Ref
KM896878	Unassigned	Unassigned	Unassigned	835_0	Clustered	67	VC_835_0	67	0.9624	1	0.9624	0	0	0	1	Ref
KM923970	Unassigned	Unassigned	Unassigned	761_0	Clustered	67	VC_761_0	67	0.8947	1	0.8947	0	0	0	1	Ref
KM923971	Unassigned	Unassigned	Unassigned	238_0	Clustered	17	VC_238_0	17	0.6109	1	0.6109	0	0	0	1	Ref

KM925136	Unassigned	Unassigned	Unassigned	546_3	Clustered	4	VC_546_3	4	0.0418	0.92194988	0.0385	0	0	0	1	Ref
KM974184	Unassigned	Unassigned	Unassigned	838_0	Clustered	29	VC_838_0	29	0.8611	1	0.8611	0	0	0	1	Ref
KM979354	Unassigned	Unassigned	Unassigned	190_22	Clustered	3	VC_190_22	3	0.0186	0.9985967	0.0185	0	0	0	1	Ref
KM979355	Unassigned	Unassigned	Unassigned	190_22	Clustered	3	VC_190_22	3	0.0186	0.9985967	0.0185	0	0	0	1	Ref
KM983328	Unassigned	Unassigned	Unassigned	649_0	Clustered	3	VC_649_0	3	0.3028	0.99838615	0.3023	0	0	0	1	Ref
KM983330	Unassigned	Unassigned	Unassigned	142_0	Clustered	2	VC_142_0	2	0.4597	0.94273135	0.4334	0	0	0	1	Ref
KM983331	Unassigned	Unassigned	Unassigned	649_0	Clustered	3	VC_649_0	3	0.3028	0.99838615	0.3023	0	0	0	1	Ref
KM983333	Unassigned	Unassigned	Unassigned	142_0	Clustered	2	VC_142_0	2	0.4597	0.94273135	0.4334	0	0	0	1	Ref
KM983334	Unassigned	Unassigned	Unassigned	649_0	Clustered	3	VC_649_0	3	0.3028	0.99838615	0.3023	0	0	0	1	Ref
KP007359	Unassigned	Unassigned	Unassigned	290_4	Clustered	2	VC_290_4	2	0.0092	0.90346068	0.0083	0	0	0	1	Ref
KP007360	Unassigned	Unassigned	Unassigned	290_1	Clustered	8	VC_290_1	8	0.0757	1	0.0757	0	0	0	0.8571	Ref
KP007361	Unassigned	Unassigned	Unassigned	290_2	Clustered	2	VC_290_2	2	0.0102	0.92371007	0.0094	0	0	0	1	Ref
KP007362	Unassigned	Unassigned	Unassigned	290_3	Clustered	2	VC_290_3	2	0.0102	0.92273843	0.0094	0	0	0	1	Ref
KP010268	Unassigned	Unassigned	Unassigned	50_0	Clustered	9	VC_50_0	9	0.5531	1	0.5531	0	0	0	1	Ref
KP010413	Unassigned	Unassigned	Unassigned	329_11	Clustered	15	VC_329_11	15	0.0791	1	0.0791	0	0	0	0.9333	Ref
KP017310	Unassigned	Unassigned	Unassigned	514_0	Clustered	75	VC_514_0	75	0.9402	1	0.9402	0	0	0	1	Ref
KP017311	Unassigned	Unassigned	Unassigned	514_0	Clustered	75	VC_514_0	75	0.9402	1	0.9402	0	0	0	1	Ref
KP025626	Unassigned	Unassigned	Unassigned	130_0	Clustered	19	VC_130_0	19	0.6068	1	0.6068	0	0	0	1	Ref
KP027195	Unassigned	Unassigned	Unassigned	764_0	Clustered	4	VC_764_0	4	0.3556	0.99997469	0.3556	0	0	0	1	Ref
KP027196	Unassigned	Unassigned	Unassigned	325_29	Clustered	3	VC_325_29	3	0.0115	0.99857442	0.0115	0	0	0	1	Ref
KP027197	Unassigned	Unassigned	Unassigned	371_18	Clustered	10	VC_371_18	10	0.0302	1	0.0302	0	0	0	0.8	Ref
KP027198	Unassigned	Unassigned	Unassigned	517_1	Clustered	108	VC_517_1	108	0.8483	1	0.8483	0	0	0	0.9955	Ref
KP027199	Unassigned	Unassigned	Unassigned	237_1	Clustered	13	VC_237_1	13	0.1773	1	0.1773	0	0	0	0.7308	Ref
KP027200	Unassigned	Unassigned	Unassigned	519_0	Clustered	39	VC_519_0	39	0.8156	1	0.8156	0	0	0	1	Ref
KP027201	Unassigned	Unassigned	Unassigned	518_1	Clustered	2	VC_518_1	2	0.0376	0.95718761	0.036	0	0	0	1	Ref
KP027202	Unassigned	Unassigned	Unassigned	514_0	Clustered	75	VC_514_0	75	0.9402	1	0.9402	0	0	0	1	Ref
KP027203	Unassigned	Unassigned	Unassigned	325_29	Clustered	3	VC_325_29	3	0.0115	0.99857442	0.0115	0	0	0	1	Ref
KP027204	Unassigned	Unassigned	Unassigned	325_8	Clustered	2	VC_325_8	2	0.0093	0.95751268	0.0089	0	0	0	1	Ref
KP027205	Unassigned	Unassigned	Unassigned	325_40	Clustered	2	VC_325_40	2	0.004	0.95574341	0.0038	0	0	0	1	Ref
KP027207	Unassigned	Unassigned	Unassigned	509_0	Clustered	37	VC_509_0	37	0.9689	1	0.9689	0	0	0	1	Ref
KP027208	Unassigned	Unassigned	Unassigned	371_15	Clustered	44	VC_371_15	44	0.1391	1	0.1391	0	0	0	0.7474	Ref
KP027209	Unassigned	Unassigned	Unassigned	371_18	Clustered	10	VC_371_18	10	0.0302	1	0.0302	0	0	0	0.8	Ref
KP027446	Unassigned	Unassigned	Unassigned	149_20	Clustered/Singleton	1	VC_149_20	1	0	1	0	0	0	0	1	Ref
KP027447	Unassigned	Unassigned	Unassigned	150_0	Clustered	13	VC_150_0	13	0.2417	1	0.2417	0	0	0	0.9872	Ref
KP057620	Unassigned	Unassigned	Unassigned	517_1	Clustered	108	VC_517_1	108	0.8483	1	0.8483	0	0	0	0.9955	Ref
KP063118	Unassigned	Unassigned	Unassigned	591_0	Clustered	14	VC_591_0	14	0.5184	1	0.5184	0	0	0	1	Ref
KP063541	Unassigned	Unassigned	Unassigned	229_0	Clustered	32	VC_229_0	32	0.9119	1	0.9119	0	0	0	1	Ref
KP064094	Unassigned	Unassigned	Unassigned	795_0	Clustered	19	VC_795_0	19	0.6848	1	0.6848	0	0	0	1	Ref
KP085586	Unassigned	Unassigned	Unassigned	575_0	Clustered	35	VC_575_0	35	0.8815	1	0.8815	0	0	0	1	Ref
KP087938	Unassigned	Unassigned	Unassigned	1077_0	Clustered	48	VC_1077_0	48	0.8525	1	0.8525	0	0	0	1	Ref
KP087939	Unassigned	Unassigned	Unassigned	398_0	Clustered	48	VC_398_0	48	0.4813	1	0.4813	0	0	0	1	Ref
KP087942	Unassigned	Unassigned	Unassigned	1077_0	Clustered	48	VC_1077_0	48	0.8525	1	0.8525	0	0	0	1	Ref
KP087943	Unassigned	Unassigned	Unassigned	1086_0	Clustered	3	VC_1086_0	3	0.8416	0.81445332	0.6855	0	0	0	1	Ref
KP087945	Unassigned	Unassigned	Unassigned	398_0	Clustered	48	VC_398_0	48	0.4813	1	0.4813	0	0	0	1	Ref
KP087948	Unassigned	Unassigned	Unassigned	1062_0	Clustered	45	VC_1062_0	45	0.9734	0.50949847	0.4959	0	0	0	1	Ref
KP087951	Unassigned	Unassigned	Unassigned	1086_0	Clustered	3	VC_1086_0	3	0.8416	0.81445332	0.6855	0	0	0	1	Ref
KP087953	Unassigned	Unassigned	Unassigned	1087_0	Clustered	5	VC_1087_0	5	0.8109	0.98759579	0.8008	0	0	0	1	Ref
KP087954	Unassigned	Unassigned	Unassigned	992_0	Clustered	8	VC_992_0	8	0.3873	1	0.3873	0	0	0	1	Ref
KP087955	Unassigned	Unassigned	Unassigned	1088_0	Clustered	3	VC_1088_0	3	0.7739	0.82711071	0.6401	0	0	0	1	Ref
KP087957	Unassigned	Unassigned	Unassigned	1086_0	Clustered	3	VC_1086_0	3	0.8416	0.81445332	0.6855	0	0	0	1	Ref
KP090453	Unassigned	Unassigned	Unassigned	386_0	Clustered	49	VC_386_0	49	0.7941	1	0.7941	0	0	0	1	Ref
KP090454	Unassigned	Unassigned	Unassigned	386_0	Clustered	49	VC_386_0	49	0.7941	1	0.7941	0	0	0	1	Ref
KP137432	Unassigned	Unassigned	Unassigned	4_0	Clustered	7	VC_4_0	7	0.7912	1	0.7912	0	0	0	1	Ref
KP137433	Unassigned	Unassigned	Unassigned	718_0	Clustered	4	VC_718_0	4	0.6393	0.99996676	0.6393	0	0	0	0.8333	Ref
KP137434	Unassigned	Unassigned	Unassigned	4_0	Clustered	7	VC_4_0	7	0.7912	1	0.7912	0	0	0	1	Ref
KP137435	Unassigned	Unassigned	Unassigned	718_0	Clustered	4	VC_718_0	4	0.6393	0.99996676	0.6393	0	0	0	0.8333	Ref
KP137436	Unassigned	Unassigned	Unassigned	4_0	Clustered	7	VC_4_0	7	0.7912	1	0.7912	0	0	0	1	Ref
KP137437	Unassigned	Unassigned	Unassigned	718_0	Clustered	4	VC_718_0	4	0.6393	0.99996676	0.6393	0	0	0	0.8333	Ref
KP137438	Unassigned	Unassigned	Unassigned	4_0	Clustered	7	VC_4_0	7	0.7912	1	0.7912	0	0	0	1	Ref
KP137439	Unassigned	Unassigned	Unassigned	718_0	Clustered	4	VC_718_0	4	0.6393	0.99996676	0.6393	0	0	0	0.8333	Ref
KP137440	Unassigned	Unassigned	Unassigned	181_0	Clustered	5	VC_181_0	5	0.1168	0.99999995	0.1168	0	0	0	1	Ref
KP143762	Unassigned	Unassigned	Unassigned	329_1	Clustered	17	VC_329_1	17	0.0867	1	0.0867	0	0	0	0.8235	Ref
KP143763	Unassigned	Unassigned	Unassigned	190_0	Clustered	2	VC_190_0	2	0.0087	0.95763473	0.0083	0	0	0	1	Ref
KP144388	Unassigned	Unassigned	Unassigned	211_1	Clustered/Singleton	1	VC_211_1	1	0	1	0	0	0	0	1	Ref
KP202158	Unassigned	Unassigned	Unassigned	490_4	Clustered/Singleton	1	VC_490_4	1	0	1	0	0	0	0	1	Ref
KP202969	Unassigned	Unassigned	Unassigned	171_0	Clustered	8	VC_171_0	8	0.5884	1	0.5884	0	0	0	1	Ref
KP202970	Unassigned	Unassigned	Unassigned	171_0	Clustered	8	VC_171_0	8	0.5884	1	0.5884	0	0	0	1	Ref
KP202971	Unassigned	Unassigned	Unassigned	1090_0	Clustered	4	VC_1090_0	4	1	1	1	0	0	0	1	Ref
KP202972	Unassigned	Unassigned	Unassigned	708_0	Clustered	47	VC_708_0	47	0.8654	1	0.8654	0	0	0	1	Ref
KP208803	Unassigned	Unassigned	Unassigned	317_3	Clustered/Singleton	1	VC_317_3	1	0	1	0	0	0	0	1	Ref
KP209285	Unassigned	Unassigned	Unassigned	12_0	Clustered	21	VC_12_0	21	0.2274	1	0.2274	0	0	0	0.9571	Ref
KP233880	Unassigned	Unassigned	Unassigned	472_0	Clustered	47	VC_472_0	47	0.9025	1	0.9025	0	0	0	1	Ref
KP238129	Unassigned	Unassigned	Unassigned	446_0	Clustered	4	VC_446_0	4	0.754	0.99987768	0.7539	0	0	0	1	Ref
KP273225	Unassigned	Unassigned	Unassigned	219_0	Clustered	5	VC_219_0	5	0.08	0.99999998	0.08	0	0	0	1	Ref
KP280062	Unassigned	Unassigned	Unassigned	784_0	Clustered	6	VC_784_0	6	0.5517	1	0.5517	0	0	0	1	Ref
KP280063	Unassigned	Unassigned	Unassigned	971_0	Clustered	3	VC_971_0	3	0.0548	0.99838914	0.0547	0	0	0	1	Ref
KP282673	Unassigned	Unassigned	Unassigned	791_0	Clustered	3	VC_791_0	3	1	1	1	0	0	0	1	Ref
KP282674	Unassigned	Unassigned	Unassigned	791_0	Clustered	3	VC_791_0	3	1	1	1	0	0	0	1	Ref
KP282675	Unassigned	Unassigned	Unassigned	348_0	Clustered	22	VC_348_0	22	0.9602	1	0.9602	0	0	0	1	Ref
KP282677	Unassigned	Unassigned	Unassigned	446_0	Clustered	4	VC_446_0	4	0.754	0.99987768	0.7539	0	0	0	1	Ref
KP282678	Unassigned	Unassigned	Unassigned	446_0	Clustered	4	VC_446_0	4	0.754	0.99987768	0.7539	0	0	0	1	Ref

KP282679	Unassigned	Unassigned	Unassigned	881_0	Clustered	2	VC_881_0	2	0.6781	0.91813282	0.6226	0	0	0	1	Ref
KP282680	Unassigned	Unassigned	Unassigned	881_0	Clustered	2	VC_881_0	2	0.6781	0.91813282	0.6226	0	0	0	1	Ref
KP296791	Unassigned	Unassigned	Unassigned	708_0	Clustered	47	VC_708_0	47	0.8654	1	0.8654	0	0	0	1	Ref
KP296793	Unassigned	Unassigned	Unassigned	708_0	Clustered	47	VC_708_0	47	0.8654	1	0.8654	0	0	0	1	Ref
KP296794	Unassigned	Unassigned	Unassigned	708_0	Clustered	47	VC_708_0	47	0.8654	1	0.8654	0	0	0	1	Ref
KP296795	Unassigned	Unassigned	Unassigned	708_0	Clustered	47	VC_708_0	47	0.8654	1	0.8654	0	0	0	1	Ref
KP296796	Unassigned	Unassigned	Unassigned	708_0	Clustered	47	VC_708_0	47	0.8654	1	0.8654	0	0	0	1	Ref
KP308307	Unassigned	Unassigned	Unassigned	813_1	Clustered	24	VC_813_1	24	0.535	1	0.535	0	0	0	1	Ref
KP313531	Unassigned	Unassigned	Unassigned	385_0	Clustered	51	VC_385_0	51	0.8754	1	0.8754	0	0	0	1	Ref
KP313532	Unassigned	Unassigned	Unassigned	751_0	Clustered	4	VC_751_0	4	0.1763	0.99998526	0.1763	0	0	0	1	Ref
KP339049	Unassigned	Unassigned	Unassigned	92_0	Clustered	22	VC_92_0	22	0.5639	1	0.5639	0	0	0	1	Ref
KP340287	Unassigned	Unassigned	Unassigned	79_0	Clustered	64	VC_79_0	64	0.4185	1	0.4185	0	0	0	0.7649	Ref
KP340288	Unassigned	Unassigned	Unassigned	79_0	Clustered	64	VC_79_0	64	0.4185	1	0.4185	0	0	0	0.7649	Ref
KP343639	Unassigned	Unassigned	Unassigned	83_0	Clustered	6	VC_83_0	6	0.6436	1	0.6436	0	0	0	1	Ref
KP399677	Unassigned	Unassigned	Unassigned	380_0	Clustered	3	VC_380_0	3	0.1631	0.99675097	0.1625	0	0	0	1	Ref
KP399678	Unassigned	Unassigned	Unassigned	378_0	Clustered	9	VC_378_0	9	0.7603	1	0.7603	0	0	0	1	Ref
KP411017	Unassigned	Unassigned	Unassigned	1032_0	Clustered	7	VC_1032_0	7	0.9887	0.99999504	0.9887	0	0	0	1	Ref
KP658157	Unassigned	Unassigned	Unassigned	938_0	Clustered	99	VC_938_0	99	0.9119	1	0.9119	0	0	0	0.9928	Ref
KP671755	Unassigned	Unassigned	Unassigned	531_0	Clustered	9	VC_531_0	9	0.26	1	0.26	0	0	0	1	Ref
KP682371	Unassigned	Unassigned	Unassigned	42_0	Clustered	49	VC_42_0	49	0.4517	1	0.4517	0	0	0	0.8597	Ref
KP682372	Unassigned	Unassigned	Unassigned	39_0	Clustered	35	VC_39_0	35	0.4337	1	0.4337	0	0	0	1	Ref
KP682373	Unassigned	Unassigned	Unassigned	39_0	Clustered	35	VC_39_0	35	0.4337	1	0.4337	0	0	0	1	Ref
KP682374	Unassigned	Unassigned	Unassigned	42_0	Clustered	49	VC_42_0	49	0.4517	1	0.4517	0	0	0	0.8597	Ref
KP682375	Unassigned	Unassigned	Unassigned	39_0	Clustered	35	VC_39_0	35	0.4337	1	0.4337	0	0	0	1	Ref
KP682376	Unassigned	Unassigned	Unassigned	39_0	Clustered	35	VC_39_0	35	0.4337	1	0.4337	0	0	0	1	Ref
KP682377	Unassigned	Unassigned	Unassigned	39_0	Clustered	35	VC_39_0	35	0.4337	1	0.4337	0	0	0	1	Ref
KP682378	Unassigned	Unassigned	Unassigned	39_0	Clustered	35	VC_39_0	35	0.4337	1	0.4337	0	0	0	1	Ref
KP682379	Unassigned	Unassigned	Unassigned	39_0	Clustered	35	VC_39_0	35	0.4337	1	0.4337	0	0	0	1	Ref
KP682380	Unassigned	Unassigned	Unassigned	39_0	Clustered	35	VC_39_0	35	0.4337	1	0.4337	0	0	0	1	Ref
KP682382	Unassigned	Unassigned	Unassigned	39_0	Clustered	35	VC_39_0	35	0.4337	1	0.4337	0	0	0	1	Ref
KP682383	Unassigned	Unassigned	Unassigned	39_0	Clustered	35	VC_39_0	35	0.4337	1	0.4337	0	0	0	1	Ref
KP682384	Unassigned	Unassigned	Unassigned	39_0	Clustered	35	VC_39_0	35	0.4337	1	0.4337	0	0	0	1	Ref
KP682385	Unassigned	Unassigned	Unassigned	39_0	Clustered	35	VC_39_0	35	0.4337	1	0.4337	0	0	0	1	Ref
KP682386	Unassigned	Unassigned	Unassigned	39_0	Clustered	35	VC_39_0	35	0.4337	1	0.4337	0	0	0	1	Ref
KP682387	Unassigned	Unassigned	Unassigned	39_0	Clustered	35	VC_39_0	35	0.4337	1	0.4337	0	0	0	1	Ref
KP682388	Unassigned	Unassigned	Unassigned	39_0	Clustered	35	VC_39_0	35	0.4337	1	0.4337	0	0	0	1	Ref
KP682389	Unassigned	Unassigned	Unassigned	39_0	Clustered	35	VC_39_0	35	0.4337	1	0.4337	0	0	0	1	Ref
KP682390	Unassigned	Unassigned	Unassigned	39_0	Clustered	35	VC_39_0	35	0.4337	1	0.4337	0	0	0	1	Ref
KP682391	Unassigned	Unassigned	Unassigned	39_0	Clustered	35	VC_39_0	35	0.4337	1	0.4337	0	0	0	1	Ref
KP682392	Unassigned	Unassigned	Unassigned	39_0	Clustered	35	VC_39_0	35	0.4337	1	0.4337	0	0	0	1	Ref
KP687431	Unassigned	Unassigned	Unassigned	149_8	Clustered	66	VC_149_8	66	0.4565	1	0.4565	0	0	0	0.8182	Ref
KP687432	Unassigned	Unassigned	Unassigned	149_8	Clustered	66	VC_149_8	66	0.4565	1	0.4565	0	0	0	0.8182	Ref
KP708985	Unassigned	Unassigned	Unassigned	111_0	Clustered	91	VC_111_0	91	0.9587	1	0.9587	0	0	0	1	Ref
KP708986	Unassigned	Unassigned	Unassigned	111_0	Clustered	91	VC_111_0	91	0.9587	1	0.9587	0	0	0	1	Ref
KP719132	Unassigned	Unassigned	Unassigned	722_0	Clustered	13	VC_722_0	13	0.5104	1	0.5104	0	0	0	1	Ref
KP719133	Unassigned	Unassigned	Unassigned	722_0	Clustered	13	VC_722_0	13	0.5104	1	0.5104	0	0	0	1	Ref
KP719134	Unassigned	Unassigned	Unassigned	722_0	Clustered	13	VC_722_0	13	0.5104	1	0.5104	0	0	0	1	Ref
KP790008	Unassigned	Unassigned	Unassigned	867_0	Clustered	6	VC_867_0	6	0.3202	1	0.3202	0	0	0	1	Ref
KP790009	Unassigned	Unassigned	Unassigned	867_0	Clustered	6	VC_867_0	6	0.3202	1	0.3202	0	0	0	1	Ref
KP790010	Unassigned	Unassigned	Unassigned	867_0	Clustered	6	VC_867_0	6	0.3202	1	0.3202	0	0	0	1	Ref
KP790011	Unassigned	Unassigned	Unassigned	406_0	Clustered	11	VC_406_0	11	0.9449	1	0.9449	0	0	0	1	Ref
KP791805	Unassigned	Unassigned	Unassigned	385_0	Clustered	51	VC_385_0	51	0.8754	1	0.8754	0	0	0	1	Ref
KP791806	Unassigned	Unassigned	Unassigned	385_0	Clustered	51	VC_385_0	51	0.8754	1	0.8754	0	0	0	1	Ref
KP791807	Unassigned	Unassigned	Unassigned	385_0	Clustered	51	VC_385_0	51	0.8754	1	0.8754	0	0	0	1	Ref
KP792623	Unassigned	Unassigned	Unassigned	806_0	Clustered	3	VC_806_0	3	0.532	0.99824026	0.5311	0	0	0	1	Ref
KP793104	Unassigned	Unassigned	Unassigned	202_0	Clustered	102	VC_202_0	102	0.4049	1	0.4049	0	0	0	0.7876	Ref
KP793105	Unassigned	Unassigned	Unassigned	202_0	Clustered	102	VC_202_0	102	0.4049	1	0.4049	0	0	0	0.7876	Ref
KP793108	Unassigned	Unassigned	Unassigned	202_0	Clustered	102	VC_202_0	102	0.4049	1	0.4049	0	0	0	0.7876	Ref
KP793109	Unassigned	Unassigned	Unassigned	202_0	Clustered	102	VC_202_0	102	0.4049	1	0.4049	0	0	0	0.7876	Ref
KP793111	Unassigned	Unassigned	Unassigned	202_0	Clustered	102	VC_202_0	102	0.4049	1	0.4049	0	0	0	0.7876	Ref
KP793113	Unassigned	Unassigned	Unassigned	202_0	Clustered	102	VC_202_0	102	0.4049	1	0.4049	0	0	0	0.7876	Ref
KP793115	Unassigned	Unassigned	Unassigned	202_0	Clustered	102	VC_202_0	102	0.4049	1	0.4049	0	0	0	0.7876	Ref
KP793116	Unassigned	Unassigned	Unassigned	202_0	Clustered	102	VC_202_0	102	0.4049	1	0.4049	0	0	0	0.7876	Ref
KP793117	Unassigned	Unassigned	Unassigned	202_0	Clustered	102	VC_202_0	102	0.4049	1	0.4049	0	0	0	0.7876	Ref
KP793118	Unassigned	Unassigned	Unassigned	202_0	Clustered	102	VC_202_0	102	0.4049	1	0.4049	0	0	0	0.7876	Ref
KP793123	Unassigned	Unassigned	Unassigned	202_0	Clustered	102	VC_202_0	102	0.4049	1	0.4049	0	0	0	0.7876	Ref
KP793124	Unassigned	Unassigned	Unassigned	202_0	Clustered	102	VC_202_0	102	0.4049	1	0.4049	0	0	0	0.7876	Ref
KP793129	Unassigned	Unassigned	Unassigned	202_0	Clustered	102	VC_202_0	102	0.4049	1	0.4049	0	0	0	0.7876	Ref
KP793134	Unassigned	Unassigned	Unassigned	202_0	Clustered	102	VC_202_0	102	0.4049	1	0.4049	0	0	0	0.7876	Ref
KP797973	Unassigned	Unassigned	Unassigned	133_9	Clustered	3	VC_133_9	3	0.0099	0.98457109	0.0098	0	0	0	1	Ref
KP836355	Unassigned	Unassigned	Unassigned	1091_0	Clustered	2	VC_1091_0	2	1	1	1	0	0	0	1	Ref
KP836356	Unassigned	Unassigned	Unassigned	1091_0	Clustered	2	VC_1091_0	2	1	1	1	0	0	0	1	Ref
KP861229	Unassigned	Unassigned	Unassigned	698_0	Clustered	26	VC_698_0	26	0.9366	1	0.9366	0	0	0	1	Ref
KP861230	Unassigned	Unassigned	Unassigned	696_1	Clustered	8	VC_696_1	8	0.5452	1	0.5452	0	0	0	0.9286	Ref
KP861231	Unassigned	Unassigned	Unassigned	698_0	Clustered	26	VC_698_0	26	0.9366	1	0.9366	0	0	0	1	Ref
KP869100	Unassigned	Unassigned	Unassigned	329_7	Clustered	18	VC_329_7	18	0.0938	1	0.0938	0	0	0	0.7778	Ref
KP869101	Unassigned	Unassigned	Unassigned	286_17	Clustered	8	VC_286_17	8	0.0535	0.99997829	0.0535	0	0	0	0.6071	Ref
KP869102	Unassigned	Unassigned	Unassigned	772_15	Clustered	5	VC_772_15	5	0.0243	0.76144861	0.0185	0	0	0	0.9	Ref
KP869103	Unassigned	Unassigned	Unassigned	772_15	Clustered	5	VC_772_15	5	0.0243	0.76144861	0.0185	0	0	0	0.9	Ref
KP869104	Unassigned	Unassigned	Unassigned	286_17	Clustered	8	VC_286_17	8	0.0535	0.99997829	0.0535	0	0	0	0.6071	Ref
KP869105	Unassigned	Unassigned	Unassigned	283_2	Clustered	2	VC_283_2	2	0.0027	0.93842619	0.0025	0	0	0	1	Ref

KP869106	Unassigned	Unassigned	Unassigned	329_7	Clustered	18	VC_329_7	18	0.0938	1	0.0938	0	0	0	0.7778	Ref
KP869107	Unassigned	Unassigned	Unassigned	476_0	Clustered	19	VC_476_0	19	0.7814	1	0.7814	0	0	0	1	Ref
KP869108	Unassigned	Unassigned	Unassigned	476_0	Clustered	19	VC_476_0	19	0.7814	1	0.7814	0	0	0	1	Ref
KP869109	Unassigned	Unassigned	Unassigned	329_7	Clustered	18	VC_329_7	18	0.0938	1	0.0938	0	0	0	0.7778	Ref
KP869110	Unassigned	Unassigned	Unassigned	329_7	Clustered	18	VC_329_7	18	0.0938	1	0.0938	0	0	0	0.7778	Ref
KP869111	Unassigned	Unassigned	Unassigned	286_17	Clustered	8	VC_286_17	8	0.0535	0.99997829	0.0535	0	0	0	0.6071	Ref
KP869112	Unassigned	Unassigned	Unassigned	772_15	Clustered	5	VC_772_15	5	0.0243	0.76144861	0.0185	0	0	0	0.9	Ref
KP869113	Unassigned	Unassigned	Unassigned	329_7	Clustered	18	VC_329_7	18	0.0938	1	0.0938	0	0	0	0.7778	Ref
KP876465	Unassigned	Unassigned	Unassigned	975_0	Clustered	11	VC_975_0	11	0.9687	1	0.9687	0	0	0	1	Ref
KP876466	Unassigned	Unassigned	Unassigned	975_0	Clustered	11	VC_975_0	11	0.9687	1	0.9687	0	0	0	1	Ref
KP881232	Unassigned	Unassigned	Unassigned	1052_1	Clustered/Singleton	1	VC_1052_1	1	0	1	0	0	0	0	1	Ref
KP881332	Unassigned	Unassigned	Unassigned	153_0	Clustered	25	VC_153_0	25	0.7729	1	0.7729	0	0	0	1	Ref
KP890822	Unassigned	Unassigned	Unassigned	452_0	Clustered	66	VC_452_0	66	0.9248	1	0.9248	0	0	0	1	Ref
KP890823	Unassigned	Unassigned	Unassigned	288_3	Clustered	3	VC_288_3	3	0.0452	0.99844025	0.0452	0	0	0	1	Ref
KP893289	Unassigned	Unassigned	Unassigned	12_0	Clustered	21	VC_12_0	21	0.2274	1	0.2274	0	0	0	0.9571	Ref
KP893290	Unassigned	Unassigned	Unassigned	12_2	Clustered	19	VC_12_2	19	0.2018	1	0.2018	0	0	0	1	Ref
KP966108	Unassigned	Unassigned	Unassigned	1_0	Clustered	54	VC_1_0	54	0.8527	1	0.8527	0	0	0	1	Ref
KP972568	Unassigned	Unassigned	Unassigned	875_0	Clustered	10	VC_875_0	10	0.988	0.99948433	0.9875	0	0	0	1	Ref
KP972569	Unassigned	Unassigned	Unassigned	875_0	Clustered	10	VC_875_0	10	0.988	0.99948433	0.9875	0	0	0	1	Ref
KP994390	Unassigned	Unassigned	Unassigned	838_0	Clustered	29	VC_838_0	29	0.8611	1	0.8611	0	0	0	1	Ref
KP994596	Unassigned	Unassigned	Unassigned	644_0	Clustered	5	VC_644_0	5	0.6649	0.99999975	0.6649	0	0	0	1	Ref
KR011062	Unassigned	Unassigned	Unassigned	866_2	Clustered	3	VC_866_2	3	0.046	0.99850411	0.046	0	0	0	1	Ref
KR011063	Unassigned	Unassigned	Unassigned	866_2	Clustered	3	VC_866_2	3	0.046	0.99850411	0.046	0	0	0	1	Ref
KR011064	Unassigned	Unassigned	Unassigned	866_2	Clustered	3	VC_866_2	3	0.046	0.99850411	0.046	0	0	0	1	Ref
KR014248	Unassigned	Unassigned	Unassigned	329_4	Clustered	2	VC_329_4	2	0.0052	0.95746454	0.005	0	0	0	1	Ref
KR029086	Unassigned	Unassigned	Unassigned	371_13	Clustered	33	VC_371_13	33	0.179	1	0.179	0	0	0	0.8769	Ref
KR029087	Unassigned	Unassigned	Unassigned	371_13	Clustered	33	VC_371_13	33	0.179	1	0.179	0	0	0	0.8769	Ref
KR049063	Unassigned	Unassigned	Unassigned	91_0	Clustered	22	VC_91_0	22	0.5318	1	0.5318	0	0	0	1	Ref
KR052142	Unassigned	Unassigned	Unassigned	838_0	Clustered	29	VC_838_0	29	0.8611	1	0.8611	0	0	0	1	Ref
KR052143	Unassigned	Unassigned	Unassigned	76_0	Clustered	37	VC_76_0	37	0.9403	1	0.9403	0	0	0	1	Ref
KR052480	Unassigned	Unassigned	Unassigned	437_0	Clustered	4	VC_437_0	4	0.552	0.99998233	0.552	0	0	0	1	Ref
KR052481	Unassigned	Unassigned	Unassigned	437_0	Clustered	4	VC_437_0	4	0.552	0.99998233	0.552	0	0	0	1	Ref
KR052482	Unassigned	Unassigned	Unassigned	437_0	Clustered	4	VC_437_0	4	0.552	0.99998233	0.552	0	0	0	1	Ref
KR053195	Unassigned	Unassigned	Unassigned	726_0	Clustered	3	VC_726_0	3	0.0381	0.88955608	0.0339	0	0	0	1	Ref
KR053197	Unassigned	Unassigned	Unassigned	949_0	Clustered	10	VC_949_0	10	0.5977	1	0.5977	0	0	0	1	Ref
KR053198	Unassigned	Unassigned	Unassigned	949_0	Clustered	10	VC_949_0	10	0.5977	1	0.5977	0	0	0	1	Ref
KR053201	Unassigned	Unassigned	Unassigned	767_0	Clustered	34	VC_767_0	34	0.9173	1	0.9173	0	0	0	1	Ref
KR054028	Unassigned	Unassigned	Unassigned	80_0	Clustered	41	VC_80_0	41	0.5837	1	0.5837	0	0	0	0.9805	Ref
KR054029	Unassigned	Unassigned	Unassigned	472_0	Clustered	47	VC_472_0	47	0.9025	1	0.9025	0	0	0	1	Ref
KR054030	Unassigned	Unassigned	Unassigned	80_0	Clustered	41	VC_80_0	41	0.5837	1	0.5837	0	0	0	0.9805	Ref
KR054031	Unassigned	Unassigned	Unassigned	423_0	Clustered	45	VC_423_0	45	0.9484	1	0.9484	0	0	0	1	Ref
KR054032	Unassigned	Unassigned	Unassigned	838_0	Clustered	29	VC_838_0	29	0.8611	1	0.8611	0	0	0	1	Ref
KR054033	Unassigned	Unassigned	Unassigned	79_7	Clustered	33	VC_79_7	33	0.216	1	0.216	0	0	0	0.8409	Ref
KR060090	Unassigned	Unassigned	Unassigned	896_0	Clustered	5	VC_896_0	5	0.4981	0.99999977	0.4981	0	0	0	1	Ref
KR063267	Unassigned	Unassigned	Unassigned	875_0	Clustered	10	VC_875_0	10	0.988	0.99948433	0.9875	0	0	0	1	Ref
KR063268	Unassigned	Unassigned	Unassigned	875_0	Clustered	10	VC_875_0	10	0.988	0.99948433	0.9875	0	0	0	1	Ref
KR063278	Unassigned	Unassigned	Unassigned	866_0	Clustered	4	VC_866_0	4	0.0795	0.99998595	0.0795	0	0	0	1	Ref
KR063279	Unassigned	Unassigned	Unassigned	867_0	Clustered	6	VC_867_0	6	0.3202	1	0.3202	0	0	0	1	Ref
KR063280	Unassigned	Unassigned	Unassigned	1096_0	Clustered	5	VC_1096_0	5	0.8566	0.99869977	0.8555	0	0	0	1	Ref
KR063281	Unassigned	Unassigned	Unassigned	1098_1	Clustered/Singleton	1	VC_1098_1	1	0	1	0	0	0	0	1	Ref
KR073660	Unassigned	Unassigned	Unassigned	228_0	Clustered	60	VC_228_0	60	0.942	1	0.942	0	0	0	1	Ref
KR073661	Unassigned	Unassigned	Unassigned	228_0	Clustered	60	VC_228_0	60	0.942	1	0.942	0	0	0	1	Ref
KR080193	Unassigned	Unassigned	Unassigned	220_0	Clustered	4	VC_220_0	4	0.0615	0.99998802	0.0615	0	0	0	1	Ref
KR080194	Unassigned	Unassigned	Unassigned	372_0	Clustered	24	VC_372_0	24	0.5539	1	0.5539	0	0	0	1	Ref
KR080195	Unassigned	Unassigned	Unassigned	519_0	Clustered	39	VC_519_0	39	0.8156	1	0.8156	0	0	0	1	Ref
KR080196	Unassigned	Unassigned	Unassigned	558_20	Clustered	17	VC_558_20	17	0.054	1	0.054	0	0	0	0.9926	Ref
KR080197	Unassigned	Unassigned	Unassigned	762_0	Clustered	9	VC_762_0	9	0.0885	1	0.0885	0	0	0	1	Ref
KR080198	Unassigned	Unassigned	Unassigned	762_0	Clustered	9	VC_762_0	9	0.0885	1	0.0885	0	0	0	1	Ref
KR080199	Unassigned	Unassigned	Unassigned	372_1	Clustered	9	VC_372_1	9	0.194	1	0.194	0	0	0	1	Ref
KR080200	Unassigned	Unassigned	Unassigned	372_0	Clustered	24	VC_372_0	24	0.5539	1	0.5539	0	0	0	1	Ref
KR080201	Unassigned	Unassigned	Unassigned	325_35	Clustered	17	VC_325_35	17	0.0732	1	0.0732	0	0	0	0.7721	Ref
KR080202	Unassigned	Unassigned	Unassigned	762_0	Clustered	9	VC_762_0	9	0.0885	1	0.0885	0	0	0	1	Ref
KR080203	Unassigned	Unassigned	Unassigned	509_0	Clustered	37	VC_509_0	37	0.9689	1	0.9689	0	0	0	1	Ref
KR080204	Unassigned	Unassigned	Unassigned	551_2	Clustered	17	VC_551_2	17	0.0973	1	0.0973	0	0	0	0.9779	Ref
KR080205	Unassigned	Unassigned	Unassigned	509_0	Clustered	37	VC_509_0	37	0.9689	1	0.9689	0	0	0	1	Ref
KR080206	Unassigned	Unassigned	Unassigned	237_1	Clustered	13	VC_237_1	13	0.1773	1	0.1773	0	0	0	0.7308	Ref
KR091952	Unassigned	Unassigned	Unassigned	129_0	Clustered	43	VC_129_0	43	0.886	1	0.886	0	0	0	1	Ref
KR093625	Unassigned	Unassigned	Unassigned	933_0	Clustered	8	VC_933_0	8	0.3648	1	0.3648	0	0	0	1	Ref
KR093626	Unassigned	Unassigned	Unassigned	933_0	Clustered	8	VC_933_0	8	0.3648	1	0.3648	0	0	0	1	Ref
KR093627	Unassigned	Unassigned	Unassigned	933_0	Clustered	8	VC_933_0	8	0.3648	1	0.3648	0	0	0	1	Ref
KR093628	Unassigned	Unassigned	Unassigned	933_0	Clustered	8	VC_933_0	8	0.3648	1	0.3648	0	0	0	1	Ref
KR093629	Unassigned	Unassigned	Unassigned	933_0	Clustered	8	VC_933_0	8	0.3648	1	0.3648	0	0	0	1	Ref
KR093630	Unassigned	Unassigned	Unassigned	933_0	Clustered	8	VC_933_0	8	0.3648	1	0.3648	0	0	0	1	Ref
KR093631	Unassigned	Unassigned	Unassigned	933_0	Clustered	8	VC_933_0	8	0.3648	1	0.3648	0	0	0	1	Ref
KR093633	Unassigned	Unassigned	Unassigned	933_0	Clustered	8	VC_933_0	8	0.3648	1	0.3648	0	0	0	1	Ref
KR093634	Unassigned	Unassigned	Unassigned	604_0	Clustered	22	VC_604_0	22	0.7846	1	0.7846	0	0	0	1	Ref
KR093635	Unassigned	Unassigned	Unassigned	604_0	Clustered	22	VC_604_0	22	0.7846	1	0.7846	0	0	0	1	Ref
KR093636	Unassigned	Unassigned	Unassigned	604_0	Clustered	22	VC_604_0	22	0.7846	1	0.7846	0	0	0	1	Ref
KR093637	Unassigned	Unassigned	Unassigned	604_0	Clustered	22	VC_604_0	22	0.7846	1	0.7846	0	0	0	1	Ref
KR093639	Unassigned	Unassigned	Unassigned	604_0	Clustered	22	VC_604_0	22	0.7846	1	0.7846	0	0	0	1	Ref
KR093640	Unassigned	Unassigned	Unassigned	604_0	Clustered	22	VC_604_0	22	0.7846	1	0.7846	0	0	0	1	Ref

KR093641	Unassigned	Unassigned	Unassigned	604_0	Clustered	22	VC_604_0	22	0.7846	1	0.7846	0	0	0	1	Ref
KR093642	Unassigned	Unassigned	Unassigned	604_0	Clustered	22	VC_604_0	22	0.7846	1	0.7846	0	0	0	1	Ref
KR093643	Unassigned	Unassigned	Unassigned	604_0	Clustered	22	VC_604_0	22	0.7846	1	0.7846	0	0	0	1	Ref
KR093644	Unassigned	Unassigned	Unassigned	604_0	Clustered	22	VC_604_0	22	0.7846	1	0.7846	0	0	0	1	Ref
KR093645	Unassigned	Unassigned	Unassigned	604_0	Clustered	22	VC_604_0	22	0.7846	1	0.7846	0	0	0	1	Ref
KR093646	Unassigned	Unassigned	Unassigned	604_0	Clustered	22	VC_604_0	22	0.7846	1	0.7846	0	0	0	1	Ref
KR093647	Unassigned	Unassigned	Unassigned	604_0	Clustered	22	VC_604_0	22	0.7846	1	0.7846	0	0	0	1	Ref
KR093648	Unassigned	Unassigned	Unassigned	604_0	Clustered	22	VC_604_0	22	0.7846	1	0.7846	0	0	0	1	Ref
KR093649	Unassigned	Unassigned	Unassigned	604_0	Clustered	22	VC_604_0	22	0.7846	1	0.7846	0	0	0	1	Ref
KR093650	Unassigned	Unassigned	Unassigned	604_0	Clustered	22	VC_604_0	22	0.7846	1	0.7846	0	0	0	1	Ref
KR093651	Unassigned	Unassigned	Unassigned	604_0	Clustered	22	VC_604_0	22	0.7846	1	0.7846	0	0	0	1	Ref
KR093652	Unassigned	Unassigned	Unassigned	604_0	Clustered	22	VC_604_0	22	0.7846	1	0.7846	0	0	0	1	Ref
KR093653	Unassigned	Unassigned	Unassigned	604_0	Clustered	22	VC_604_0	22	0.7846	1	0.7846	0	0	0	1	Ref
KR093654	Unassigned	Unassigned	Unassigned	604_0	Clustered	22	VC_604_0	22	0.7846	1	0.7846	0	0	0	1	Ref
KR093655	Unassigned	Unassigned	Unassigned	604_0	Clustered	22	VC_604_0	22	0.7846	1	0.7846	0	0	0	1	Ref
KR093656	Unassigned	Unassigned	Unassigned	604_0	Clustered	22	VC_604_0	22	0.7846	1	0.7846	0	0	0	1	Ref
KR131750	Unassigned	Unassigned	Unassigned	695_0	Clustered	48	VC_695_0	48	0.8162	1	0.8162	0	0	0	1	Ref
KR136259	Unassigned	Unassigned	Unassigned	1101_0	Clustered	2	VC_1101_0	2	0.9786	0.72985431	0.7143	0	0	0	1	Ref
KR136260	Unassigned	Unassigned	Unassigned	1101_0	Clustered	2	VC_1101_0	2	0.9786	0.72985431	0.7143	0	0	0	1	Ref
KR149290	Unassigned	Unassigned	Unassigned	699_0	Clustered	71	VC_699_0	71	0.9352	1	0.9352	0	0	0	1	Ref
KR149291	Unassigned	Unassigned	Unassigned	823_0	Clustered	124	VC_823_0	124	0.9492	1	0.9492	0	0	0	1	Ref
KR153145	Unassigned	Unassigned	Unassigned	463_0	Clustered	4	VC_463_0	4	0.7906	0.99997156	0.7906	0	0	0	1	Ref
KR233164	Unassigned	Unassigned	Unassigned	190_27	Clustered/Singleton	1	VC_190_27	1	0	1	0	0	0	0	1	Ref
KR233165	Unassigned	Unassigned	Unassigned	283_163	Clustered	2	VC_283_163	2	0.0029	0.93420746	0.0027	0	0	0	1	Ref
KR262148	Unassigned	Unassigned	Unassigned	938_0	Clustered	99	VC_938_0	99	0.9119	1	0.9119	0	0	0	0.9928	Ref
KR269718	Unassigned	Unassigned	Unassigned	283_81	Clustered/Singleton	1	VC_283_81	1	0	1	0	0	0	0	1	Ref
KR269719	Unassigned	Unassigned	Unassigned	937_0	Clustered	40	VC_937_0	40	0.6809	1	0.6809	0	0	0	0.9359	Ref
KR269720	Unassigned	Unassigned	Unassigned	491_0	Clustered	36	VC_491_0	36	0.5336	1	0.5336	0	0	0	0.9714	Ref
KR270151	Unassigned	Unassigned	Unassigned	637_0	Clustered	115	VC_637_0	115	0.731	1	0.731	0	0	0	0.8764	Ref
KR296686	Unassigned	Unassigned	Unassigned	28_1	Clustered	53	VC_28_1	53	0.7164	1	0.7164	0	0	0	0.9325	Ref
KR296687	Unassigned	Unassigned	Unassigned	28_1	Clustered	53	VC_28_1	53	0.7164	1	0.7164	0	0	0	0.9325	Ref
KR296688	Unassigned	Unassigned	Unassigned	28_1	Clustered	53	VC_28_1	53	0.7164	1	0.7164	0	0	0	0.9325	Ref
KR296689	Unassigned	Unassigned	Unassigned	592_0	Clustered	50	VC_592_0	50	0.7314	1	0.7314	0	0	0	1	Ref
KR296691	Unassigned	Unassigned	Unassigned	592_0	Clustered	50	VC_592_0	50	0.7314	1	0.7314	0	0	0	1	Ref
KR296692	Unassigned	Unassigned	Unassigned	133_18	Clustered/Singleton	1	VC_133_18	1	0	1	0	0	0	0	1	Ref
KR337643	Unassigned	Unassigned	Unassigned	765_0	Clustered	132	VC_765_0	132	1	1	1	0	0	0	1	Ref
KR337644	Unassigned	Unassigned	Unassigned	765_0	Clustered	132	VC_765_0	132	1	1	1	0	0	0	1	Ref
KR337645	Unassigned	Unassigned	Unassigned	765_0	Clustered	132	VC_765_0	132	1	1	1	0	0	0	1	Ref
KR337646	Unassigned	Unassigned	Unassigned	765_0	Clustered	132	VC_765_0	132	1	1	1	0	0	0	1	Ref
KR337647	Unassigned	Unassigned	Unassigned	765_0	Clustered	132	VC_765_0	132	1	1	1	0	0	0	1	Ref
KR337648	Unassigned	Unassigned	Unassigned	765_0	Clustered	132	VC_765_0	132	1	1	1	0	0	0	1	Ref
KR337649	Unassigned	Unassigned	Unassigned	765_0	Clustered	132	VC_765_0	132	1	1	1	0	0	0	1	Ref
KR337650	Unassigned	Unassigned	Unassigned	765_0	Clustered	132	VC_765_0	132	1	1	1	0	0	0	1	Ref
KR337651	Unassigned	Unassigned	Unassigned	765_0	Clustered	132	VC_765_0	132	1	1	1	0	0	0	1	Ref
KR337652	Unassigned	Unassigned	Unassigned	765_0	Clustered	132	VC_765_0	132	1	1	1	0	0	0	1	Ref
KR337653	Unassigned	Unassigned	Unassigned	765_0	Clustered	132	VC_765_0	132	1	1	1	0	0	0	1	Ref
KR337654	Unassigned	Unassigned	Unassigned	765_0	Clustered	132	VC_765_0	132	1	1	1	0	0	0	1	Ref
KR347352	Unassigned	Unassigned	Unassigned	765_0	Clustered	132	VC_765_0	132	1	1	1	0	0	0	1	Ref
KR347353	Unassigned	Unassigned	Unassigned	765_0	Clustered	132	VC_765_0	132	1	1	1	0	0	0	1	Ref
KR347354	Unassigned	Unassigned	Unassigned	765_0	Clustered	132	VC_765_0	132	1	1	1	0	0	0	1	Ref
KR347355	Unassigned	Unassigned	Unassigned	765_0	Clustered	132	VC_765_0	132	1	1	1	0	0	0	1	Ref
KR422352	Unassigned	Unassigned	Unassigned	286_1	Clustered	21	VC_286_1	21	0.1696	1	0.1696	0	0	0	0.9048	Ref
KR422353	Unassigned	Unassigned	Unassigned	190_10	Clustered/Singleton	1	VC_190_10	1	0	1	0	0	0	0	1	Ref
KR534323	Unassigned	Unassigned	Unassigned	496_2	Clustered/Singleton	1	VC_496_2	1	0	1	0	0	0	0	1	Ref
KR537871	Unassigned	Unassigned	Unassigned	749_0	Clustered	40	VC_749_0	40	0.7517	1	0.7517	0	0	0	1	Ref
KR537872	Unassigned	Unassigned	Unassigned	749_0	Clustered	40	VC_749_0	40	0.7517	1	0.7517	0	0	0	1	Ref
KR560069	Unassigned	Unassigned	Unassigned	506_0	Clustered	8	VC_506_0	8	0.7376	1	0.7376	0	0	0	1	Ref
KR604693	Unassigned	Unassigned	Unassigned	984_0	Clustered	38	VC_984_0	38	0.9758	1	0.9758	0	0	0	1	Ref
KR698074	Unassigned	Unassigned	Unassigned	772_8	Clustered	2	VC_772_8	2	0.006	0.58293682	0.0035	0	0	0	1	Ref
KR704913	Unassigned	Unassigned	Unassigned	1103_0	Clustered	2	VC_1103_0	2	1	1	1	0	0	0	1	Ref
KR704915	Unassigned	Unassigned	Unassigned	1103_0	Clustered	2	VC_1103_0	2	1	1	1	0	0	0	1	Ref
KR709302	Unassigned	Unassigned	Unassigned	12_2	Clustered	19	VC_12_2	19	0.2018	1	0.2018	0	0	0	1	Ref
KR709303	Unassigned	Unassigned	Unassigned	12_2	Clustered	19	VC_12_2	19	0.2018	1	0.2018	0	0	0	1	Ref
KR781349	Unassigned	Unassigned	Unassigned	371_21	Clustered	2	VC_371_21	2	0.0033	0.95770737	0.0032	0	0	0	1	Ref
KR781488	Unassigned	Unassigned	Unassigned	42_0	Clustered	49	VC_42_0	49	0.4517	1	0.4517	0	0	0	0.8597	Ref
KR816341	Unassigned	Unassigned	Unassigned	1109_0	Clustered	2	VC_1109_0	2	0.6449	0.85557782	0.5517	0	0	0	1	Ref
KR816508	Unassigned	Unassigned	Unassigned	371_18	Clustered	10	VC_371_18	10	0.0302	1	0.0302	0	0	0	0.8	Ref
KR824843	Unassigned	Unassigned	Unassigned	520_2	Clustered/Singleton	1	VC_520_2	1	0	1	0	0	0	0	1	Ref
KR869157	Unassigned	Unassigned	Unassigned	79_0	Clustered	64	VC_79_0	64	0.4185	1	0.4185	0	0	0	0.7649	Ref
KR869820	Unassigned	Unassigned	Unassigned	669_1	Clustered	10	VC_669_1	10	0.1268	1	0.1268	0	0	0	0.9778	Ref
KR902361	Unassigned	Unassigned	Unassigned	149_8	Clustered	66	VC_149_8	66	0.4565	1	0.4565	0	0	0	0.8182	Ref
KR902978	Unassigned	Unassigned	Unassigned	765_0	Clustered	132	VC_765_0	132	1	1	1	0	0	0	1	Ref
KR902979	Unassigned	Unassigned	Unassigned	765_0	Clustered	132	VC_765_0	132	1	1	1	0	0	0	1	Ref
KR902980	Unassigned	Unassigned	Unassigned	765_0	Clustered	132	VC_765_0	132	1	1	1	0	0	0	1	Ref
KR902981	Unassigned	Unassigned	Unassigned	765_0	Clustered	132	VC_765_0	132	1	1	1	0	0	0	1	Ref
KR902982	Unassigned	Unassigned	Unassigned	765_0	Clustered	132	VC_765_0	132	1	1	1	0	0	0	1	Ref
KR902983	Unassigned	Unassigned	Unassigned	765_0	Clustered	132	VC_765_0	132	1	1	1	0	0	0	1	Ref
KR902984	Unassigned	Unassigned	Unassigned	765_0	Clustered	132	VC_765_0	132	1	1	1	0	0	0	1	Ref
KR902985	Unassigned	Unassigned	Unassigned	765_0	Clustered	132	VC_765_0	132	1	1	1	0	0	0	1	Ref
KR902986	Unassigned	Unassigned	Unassigned	765_0	Clustered	132	VC_765_0	132	1	1	1	0	0	0	1	Ref
KR902987	Unassigned	Unassigned	Unassigned	765_0	Clustered	132	VC_765_0	132	1	1	1	0	0	0	1	Ref

KR905068	Unassigned	Unassigned	Unassigned	377_0	Clustered	3	VC_377_0	3	0.3906	0.99785056	0.3898	0	0	0	1	Ref
KR908644	Unassigned	Unassigned	Unassigned	149_8	Clustered	66	VC_149_8	66	0.4565	1	0.4565	0	0	0	0.8182	Ref
KR935213	Unassigned	Unassigned	Unassigned	749_0	Clustered	40	VC_749_0	40	0.7517	1	0.7517	0	0	0	1	Ref
KR935214	Unassigned	Unassigned	Unassigned	520_73	Clustered	2	VC_520_73	2	0.0107	0.95762743	0.0103	0	0	0	1	Ref
KR935215	Unassigned	Unassigned	Unassigned	749_0	Clustered	40	VC_749_0	40	0.7517	1	0.7517	0	0	0	1	Ref
KR935216	Unassigned	Unassigned	Unassigned	874_0	Clustered	3	VC_874_0	3	0.6293	0.99812483	0.6281	0	0	0	1	Ref
KR935217	Unassigned	Unassigned	Unassigned	874_0	Clustered	3	VC_874_0	3	0.6293	0.99812483	0.6281	0	0	0	1	Ref
KR997929	Unassigned	Unassigned	Unassigned	325_7	Clustered	3	VC_325_7	3	0.0085	0.99739782	0.0085	0	0	0	0.6667	Ref
KR997930	Unassigned	Unassigned	Unassigned	520_35	Clustered	3	VC_520_35	3	0.0251	0.99859857	0.0251	0	0	0	1	Ref
KR997931	Unassigned	Unassigned	Unassigned	520_35	Clustered	3	VC_520_35	3	0.0251	0.99859857	0.0251	0	0	0	1	Ref
KR997932	Unassigned	Unassigned	Unassigned	508_0	Clustered	32	VC_508_0	32	0.906	1	0.906	0	0	0	1	Ref
KR997933	Unassigned	Unassigned	Unassigned	569_0	Clustered	3	VC_569_0	3	0.2094	0.99849867	0.2091	0	0	0	1	Ref
KR997934	Unassigned	Unassigned	Unassigned	325_7	Clustered	3	VC_325_7	3	0.0085	0.99739782	0.0085	0	0	0	0.6667	Ref
KR997967	Unassigned	Unassigned	Unassigned	371_1	Clustered	11	VC_371_1	11	0.0379	1	0.0379	0	0	0	0.8545	Ref
KR997968	Unassigned	Unassigned	Unassigned	520_35	Clustered	3	VC_520_35	3	0.0251	0.99859857	0.0251	0	0	0	1	Ref
KT001911	Unassigned	Unassigned	Unassigned	1032_0	Clustered	7	VC_1032_0	7	0.9887	0.99999504	0.9887	0	0	0	1	Ref
KT001912	Unassigned	Unassigned	Unassigned	102_0	Clustered	2	VC_102_0	2	0.114	0.94637553	0.1079	0	0	0	1	Ref
KT001913	Unassigned	Unassigned	Unassigned	170_0	Clustered/Singleton	1	VC_170_0	1	0	1	0	0	0	0	1	Ref
KT001914	Unassigned	Unassigned	Unassigned	1090_0	Clustered	4	VC_1090_0	4	1	1	1	0	0	0	1	Ref
KT001916	Unassigned	Unassigned	Unassigned	330_0	Clustered	27	VC_330_0	27	0.5088	1	0.5088	0	0	0	0.8661	Ref
KT001917	Unassigned	Unassigned	Unassigned	772_19	Clustered	16	VC_772_19	16	0.0975	0.9970466	0.0972	0	0	0	0.8083	Ref
KT001918	Unassigned	Unassigned	Unassigned	668_0	Clustered	25	VC_668_0	25	0.5539	1	0.5539	0	0	0	0.96	Ref
KT001919	Unassigned	Unassigned	Unassigned	668_0	Clustered	25	VC_668_0	25	0.5539	1	0.5539	0	0	0	0.96	Ref
KT001920	Unassigned	Unassigned	Unassigned	938_0	Clustered	99	VC_938_0	99	0.9119	1	0.9119	0	0	0	0.9928	Ref
KT004677	Unassigned	Unassigned	Unassigned	940_0	Clustered	34	VC_940_0	34	0.7959	1	0.7959	0	0	0	1	Ref
KT008108	Unassigned	Unassigned	Unassigned	385_0	Clustered	51	VC_385_0	51	0.8754	1	0.8754	0	0	0	1	Ref
KT020852	Unassigned	Unassigned	Unassigned	551_7	Clustered	28	VC_551_7	28	0.1661	1	0.1661	0	0	0	0.8519	Ref
KT070866	Unassigned	Unassigned	Unassigned	1006_0	Clustered	8	VC_1006_0	8	0.7407	1	0.7407	0	0	0	1	Ref
KT070867	Unassigned	Unassigned	Unassigned	475_6	Clustered/Singleton	1	VC_475_6	1	0	1	0	0	0	0	1	Ref
KT124227	Unassigned	Unassigned	Unassigned	982_0	Clustered	62	VC_982_0	62	0.8646	1	0.8646	0	0	0	0.743	Ref
KT124228	Unassigned	Unassigned	Unassigned	982_0	Clustered	62	VC_982_0	62	0.8646	1	0.8646	0	0	0	0.743	Ref
KT124229	Unassigned	Unassigned	Unassigned	982_0	Clustered	62	VC_982_0	62	0.8646	1	0.8646	0	0	0	0.743	Ref
KT151955	Unassigned	Unassigned	Unassigned	720_0	Clustered	2	VC_720_0	2	0.5579	0.89435023	0.4989	0	0	0	1	Ref
KT151956	Unassigned	Unassigned	Unassigned	141_0	Clustered	6	VC_141_0	6	0.5999	1	0.5999	0	0	0	1	Ref
KT151957	Unassigned	Unassigned	Unassigned	720_0	Clustered	2	VC_720_0	2	0.5579	0.89435023	0.4989	0	0	0	1	Ref
KT151958	Unassigned	Unassigned	Unassigned	141_0	Clustered	6	VC_141_0	6	0.5999	1	0.5999	0	0	0	1	Ref
KT151959	Unassigned	Unassigned	Unassigned	211_2	Clustered/Singleton	1	VC_211_2	1	0	1	0	0	0	0	1	Ref
KT152029	Unassigned	Unassigned	Unassigned	982_0	Clustered	62	VC_982_0	62	0.8646	1	0.8646	0	0	0	0.743	Ref
KT160311	Unassigned	Unassigned	Unassigned	499_0	Clustered	24	VC_499_0	24	0.8569	1	0.8569	0	0	0	0.9529	Ref
KT176190	Unassigned	Unassigned	Unassigned	290_5	Clustered	14	VC_290_5	14	0.1422	1	0.1422	0	0	0	0.9231	Ref
KT184308	Unassigned	Unassigned	Unassigned	283_66	Clustered/Singleton	1	VC_283_66	1	0	1	0	0	0	0	1	Ref
KT184309	Unassigned	Unassigned	Unassigned	286_17	Clustered	8	VC_286_17	8	0.0535	0.99997829	0.0535	0	0	0	0.6071	Ref
KT184310	Unassigned	Unassigned	Unassigned	286_17	Clustered	8	VC_286_17	8	0.0535	0.99997829	0.0535	0	0	0	0.6071	Ref
KT184311	Unassigned	Unassigned	Unassigned	283_57	Clustered/Singleton	1	VC_283_57	1	0	1	0	0	0	0	1	Ref
KT184312	Unassigned	Unassigned	Unassigned	283_63	Clustered/Singleton	1	VC_283_63	1	0	1	0	0	0	0	1	Ref
KT184313	Unassigned	Unassigned	Unassigned	329_7	Clustered	18	VC_329_7	18	0.0938	1	0.0938	0	0	0	0.7778	Ref
KT184314	Unassigned	Unassigned	Unassigned	329_7	Clustered	18	VC_329_7	18	0.0938	1	0.0938	0	0	0	0.7778	Ref
KT184315	Unassigned	Unassigned	Unassigned	329_7	Clustered	18	VC_329_7	18	0.0938	1	0.0938	0	0	0	0.7778	Ref
KT184316	Unassigned	Unassigned	Unassigned	329_7	Clustered	18	VC_329_7	18	0.0938	1	0.0938	0	0	0	0.7778	Ref
KT184390	Unassigned	Unassigned	Unassigned	982_0	Clustered	62	VC_982_0	62	0.8646	1	0.8646	0	0	0	0.743	Ref
KT184391	Unassigned	Unassigned	Unassigned	982_0	Clustered	62	VC_982_0	62	0.8646	1	0.8646	0	0	0	0.743	Ref
KT184661	Unassigned	Unassigned	Unassigned	878_0	Clustered	32	VC_878_0	32	0.9965	1	0.9965	0	0	0	1	Ref
KT186228	Unassigned	Unassigned	Unassigned	982_0	Clustered	62	VC_982_0	62	0.8646	1	0.8646	0	0	0	0.743	Ref
KT186229	Unassigned	Unassigned	Unassigned	982_0	Clustered	62	VC_982_0	62	0.8646	1	0.8646	0	0	0	0.743	Ref
KT186243	Unassigned	Unassigned	Unassigned	8_0	Clustered	58	VC_8_0	58	0.8306	1	0.8306	0	0	0	0.9946	Ref
KT187252	Unassigned	Unassigned	Unassigned	187_1	Clustered	7	VC_187_1	7	0.1423	1	0.1423	0	0	0	1	Ref
KT207918	Unassigned	Unassigned	Unassigned	884_0	Clustered	28	VC_884_0	28	0.6211	1	0.6211	0	0	0	0.9841	Ref
KT221034	Unassigned	Unassigned	Unassigned	587_0	Clustered	2	VC_587_0	2	0.3252	0.91192888	0.2965	0	0	0	1	Ref
KT222940	Unassigned	Unassigned	Unassigned	810_0	Clustered	18	VC_810_0	18	0.9121	1	0.9121	0	0	0	1	Ref
KT222941	Unassigned	Unassigned	Unassigned	551_2	Clustered	17	VC_551_2	17	0.0973	1	0.0973	0	0	0	0.9779	Ref
KT222942	Unassigned	Unassigned	Unassigned	551_2	Clustered	17	VC_551_2	17	0.0973	1	0.0973	0	0	0	0.9779	Ref
KT224359	Unassigned	Unassigned	Unassigned	187_1	Clustered	7	VC_187_1	7	0.1423	1	0.1423	0	0	0	1	Ref
KT232076	Unassigned	Unassigned	Unassigned	43_0	Clustered	38	VC_43_0	38	0.5885	1	0.5885	0	0	0	0.9659	Ref
KT239446	Unassigned	Unassigned	Unassigned	491_0	Clustered	36	VC_491_0	36	0.5336	1	0.5336	0	0	0	0.9714	Ref
KT240186	Unassigned	Unassigned	Unassigned	984_0	Clustered	38	VC_984_0	38	0.9758	1	0.9758	0	0	0	1	Ref
KT246485	Unassigned	Unassigned	Unassigned	810_0	Clustered	18	VC_810_0	18	0.9121	1	0.9121	0	0	0	1	Ref
KT246486	Unassigned	Unassigned	Unassigned	940_0	Clustered	34	VC_940_0	34	0.7959	1	0.7959	0	0	0	1	Ref
KT253150	Unassigned	Unassigned	Unassigned	874_0	Clustered	3	VC_874_0	3	0.6293	0.99812483	0.6281	0	0	0	1	Ref
KT254130	Unassigned	Unassigned	Unassigned	80_0	Clustered	41	VC_80_0	41	0.5837	1	0.5837	0	0	0	0.9805	Ref
KT254131	Unassigned	Unassigned	Unassigned	80_0	Clustered	41	VC_80_0	41	0.5837	1	0.5837	0	0	0	0.9805	Ref
KT254132	Unassigned	Unassigned	Unassigned	80_0	Clustered	41	VC_80_0	41	0.5837	1	0.5837	0	0	0	0.9805	Ref
KT254133	Unassigned	Unassigned	Unassigned	80_0	Clustered	41	VC_80_0	41	0.5837	1	0.5837	0	0	0	0.9805	Ref
KT259047	Unassigned	Unassigned	Unassigned	325_23	Clustered	17	VC_325_23	17	0.0716	1	0.0716	0	0	0	0.6397	Ref
KT264749	Unassigned	Unassigned	Unassigned	1104_0	Clustered	154	VC_1104_0	154	0.8484	1	0.8484	0	0	0	1	Ref
KT264750	Unassigned	Unassigned	Unassigned	1104_0	Clustered	154	VC_1104_0	154	0.8484	1	0.8484	0	0	0	1	Ref
KT264751	Unassigned	Unassigned	Unassigned	1104_0	Clustered	154	VC_1104_0	154	0.8484	1	0.8484	0	0	0	1	Ref
KT264752	Unassigned	Unassigned	Unassigned	1104_0	Clustered	154	VC_1104_0	154	0.8484	1	0.8484	0	0	0	1	Ref
KT264753	Unassigned	Unassigned	Unassigned	1104_0	Clustered	154	VC_1104_0	154	0.8484	1	0.8484	0	0	0	1	Ref
KT264754	Unassigned	Unassigned	Unassigned	1104_0	Clustered	154	VC_1104_0	154	0.8484	1	0.8484	0	0	0	1	Ref
KT264755	Unassigned	Unassigned	Unassigned	1104_0	Clustered	154	VC_1104_0	154	0.8484	1	0.8484	0	0	0	1	Ref
KT264756	Unassigned	Unassigned	Unassigned	1104_0	Clustered	154	VC_1104_0	154	0.8484	1	0.8484	0	0	0	1	Ref

KT264757	Unassigned	Unassigned	Unassigned	1104_0	Clustered	154	VC_1104_0	154	0.8484	1	0.8484	0	0	0	1	Ref
KT264758	Unassigned	Unassigned	Unassigned	1104_0	Clustered	154	VC_1104_0	154	0.8484	1	0.8484	0	0	0	1	Ref
KT264759	Unassigned	Unassigned	Unassigned	1104_0	Clustered	154	VC_1104_0	154	0.8484	1	0.8484	0	0	0	1	Ref
KT264760	Unassigned	Unassigned	Unassigned	1104_0	Clustered	154	VC_1104_0	154	0.8484	1	0.8484	0	0	0	1	Ref
KT264761	Unassigned	Unassigned	Unassigned	1104_0	Clustered	154	VC_1104_0	154	0.8484	1	0.8484	0	0	0	1	Ref
KT264762	Unassigned	Unassigned	Unassigned	1104_0	Clustered	154	VC_1104_0	154	0.8484	1	0.8484	0	0	0	1	Ref
KT264763	Unassigned	Unassigned	Unassigned	1104_0	Clustered	154	VC_1104_0	154	0.8484	1	0.8484	0	0	0	1	Ref
KT264764	Unassigned	Unassigned	Unassigned	1104_0	Clustered	154	VC_1104_0	154	0.8484	1	0.8484	0	0	0	1	Ref
KT264765	Unassigned	Unassigned	Unassigned	1104_0	Clustered	154	VC_1104_0	154	0.8484	1	0.8484	0	0	0	1	Ref
KT264766	Unassigned	Unassigned	Unassigned	1104_0	Clustered	154	VC_1104_0	154	0.8484	1	0.8484	0	0	0	1	Ref
KT264767	Unassigned	Unassigned	Unassigned	1104_0	Clustered	154	VC_1104_0	154	0.8484	1	0.8484	0	0	0	1	Ref
KT264768	Unassigned	Unassigned	Unassigned	1104_0	Clustered	154	VC_1104_0	154	0.8484	1	0.8484	0	0	0	1	Ref
KT264769	Unassigned	Unassigned	Unassigned	1104_0	Clustered	154	VC_1104_0	154	0.8484	1	0.8484	0	0	0	1	Ref
KT264775	Unassigned	Unassigned	Unassigned	1108_0	Clustered	4	VC_1108_0	4	0.0485	0.99999021	0.0485	0	0	0	1	Ref
KT264776	Unassigned	Unassigned	Unassigned	1116_0	Clustered	14	VC_1116_0	14	0.7927	1	0.7927	0	0	0	1	Ref
KT264779	Unassigned	Unassigned	Unassigned	1104_0	Clustered	154	VC_1104_0	154	0.8484	1	0.8484	0	0	0	1	Ref
KT264781	Unassigned	Unassigned	Unassigned	1117_0	Clustered	5	VC_1117_0	5	0.4688	0.99999974	0.4688	0	0	0	1	Ref
KT264782	Unassigned	Unassigned	Unassigned	1119_0	Clustered	20	VC_1119_0	20	0.6751	0.99997892	0.6751	0	0	0	1	Ref
KT264783	Unassigned	Unassigned	Unassigned	1119_0	Clustered	20	VC_1119_0	20	0.6751	0.99997892	0.6751	0	0	0	1	Ref
KT264784	Unassigned	Unassigned	Unassigned	1123_0	Clustered	2	VC_1123_0	2	0.3187	0.93270267	0.2972	0	0	0	1	Ref
KT264785	Unassigned	Unassigned	Unassigned	1122_0	Clustered	10	VC_1122_0	10	0.9643	0.99999697	0.9643	0	0	0	1	Ref
KT264788	Unassigned	Unassigned	Unassigned	1126_0	Clustered	8	VC_1126_0	8	0.2864	1	0.2864	0	0	0	1	Ref
KT264789	Unassigned	Unassigned	Unassigned	1126_0	Clustered	8	VC_1126_0	8	0.2864	1	0.2864	0	0	0	1	Ref
KT264790	Unassigned	Unassigned	Unassigned	1126_0	Clustered	8	VC_1126_0	8	0.2864	1	0.2864	0	0	0	1	Ref
KT264791	Unassigned	Unassigned	Unassigned	1127_0	Clustered	19	VC_1127_0	19	0.7611	1	0.7611	0	0	0	1	Ref
KT264792	Unassigned	Unassigned	Unassigned	1127_0	Clustered	19	VC_1127_0	19	0.7611	1	0.7611	0	0	0	1	Ref
KT264793	Unassigned	Unassigned	Unassigned	1127_0	Clustered	19	VC_1127_0	19	0.7611	1	0.7611	0	0	0	1	Ref
KT264794	Unassigned	Unassigned	Unassigned	1128_0	Clustered	2	VC_1128_0	2	0.0904	0.95143313	0.086	0	0	0	1	Ref
KT264795	Unassigned	Unassigned	Unassigned	1132_0	Clustered	2	VC_1132_0	2	0.0978	0.83194774	0.0814	0	0	0	1	Ref
KT264796	Unassigned	Unassigned	Unassigned	1125_0	Clustered	20	VC_1125_0	20	0.5599	1	0.5599	0	0	0	1	Ref
KT264797	Unassigned	Unassigned	Unassigned	1125_0	Clustered	20	VC_1125_0	20	0.5599	1	0.5599	0	0	0	1	Ref
KT264798	Unassigned	Unassigned	Unassigned	1124_0	Clustered	10	VC_1124_0	10	0.6268	1	0.6268	0	0	0	1	Ref
KT264799	Unassigned	Unassigned	Unassigned	1124_0	Clustered	10	VC_1124_0	10	0.6268	1	0.6268	0	0	0	1	Ref
KT264800	Unassigned	Unassigned	Unassigned	1124_0	Clustered	10	VC_1124_0	10	0.6268	1	0.6268	0	0	0	1	Ref
KT264801	Unassigned	Unassigned	Unassigned	1124_0	Clustered	10	VC_1124_0	10	0.6268	1	0.6268	0	0	0	1	Ref
KT264802	Unassigned	Unassigned	Unassigned	1137_0	Clustered	3	VC_1137_0	3	0.9176	0.93067968	0.854	0	0	0	1	Ref
KT264803	Unassigned	Unassigned	Unassigned	1136_0	Clustered	2	VC_1136_0	2	0.58	0.88261817	0.5119	0	0	0	1	Ref
KT264805	Unassigned	Unassigned	Unassigned	1138_0	Clustered	4	VC_1138_0	4	0.9437	0.98964986	0.934	0	0	0	1	Ref
KT264806	Unassigned	Unassigned	Unassigned	1139_0	Clustered	4	VC_1139_0	4	1	1	1	0	0	0	1	Ref
KT264807	Unassigned	Unassigned	Unassigned	1056_0	Clustered	4	VC_1056_0	4	0.169	0.84110627	0.1421	0	0	0	1	Ref
KT264808	Unassigned	Unassigned	Unassigned	1066_0	Clustered	52	VC_1066_0	52	0.7682	1	0.7682	0	0	0	1	Ref
KT264809	Unassigned	Unassigned	Unassigned	1066_0	Clustered	52	VC_1066_0	52	0.7682	1	0.7682	0	0	0	1	Ref
KT264810	Unassigned	Unassigned	Unassigned	1066_0	Clustered	52	VC_1066_0	52	0.7682	1	0.7682	0	0	0	1	Ref
KT264811	Unassigned	Unassigned	Unassigned	398_0	Clustered	48	VC_398_0	48	0.4813	1	0.4813	0	0	0	1	Ref
KT264812	Unassigned	Unassigned	Unassigned	1085_0	Clustered	2	VC_1085_0	2	0.5907	0.9331928	0.5513	0	0	0	1	Ref
KT264813	Unassigned	Unassigned	Unassigned	995_0	Clustered	69	VC_995_0	69	0.8547	1	0.8547	0	0	0	1	Ref
KT264814	Unassigned	Unassigned	Unassigned	995_0	Clustered	69	VC_995_0	69	0.8547	1	0.8547	0	0	0	1	Ref
KT264815	Unassigned	Unassigned	Unassigned	995_0	Clustered	69	VC_995_0	69	0.8547	1	0.8547	0	0	0	1	Ref
KT264818	Unassigned	Unassigned	Unassigned	1143_0	Clustered	9	VC_1143_0	9	0.7268	1	0.7268	0	0	0	1	Ref
KT264819	Unassigned	Unassigned	Unassigned	1143_0	Clustered	9	VC_1143_0	9	0.7268	1	0.7268	0	0	0	1	Ref
KT264820	Unassigned	Unassigned	Unassigned	1140_0	Clustered	4	VC_1140_0	4	0.1325	0.66207199	0.0877	0	0	0	1	Ref
KT264821	Unassigned	Unassigned	Unassigned	1140_0	Clustered	4	VC_1140_0	4	0.1325	0.66207199	0.0877	0	0	0	1	Ref
KT264822	Unassigned	Unassigned	Unassigned	1147_0	Clustered	3	VC_1147_0	3	0.4334	0.65335909	0.2832	0	0	0	1	Ref
KT264823	Unassigned	Unassigned	Unassigned	1148_0	Clustered	16	VC_1148_0	16	0.8821	1	0.8821	0	0	0	1	Ref
KT264824	Unassigned	Unassigned	Unassigned	1148_0	Clustered	16	VC_1148_0	16	0.8821	1	0.8821	0	0	0	1	Ref
KT264825	Unassigned	Unassigned	Unassigned	1148_0	Clustered	16	VC_1148_0	16	0.8821	1	0.8821	0	0	0	1	Ref
KT264826	Unassigned	Unassigned	Unassigned	1054_0	Clustered	12	VC_1054_0	12	0.2863	0.99746644	0.2855	0	0	0	1	Ref
KT264827	Unassigned	Unassigned	Unassigned	1054_0	Clustered	12	VC_1054_0	12	0.2863	0.99746644	0.2855	0	0	0	1	Ref
KT264828	Unassigned	Unassigned	Unassigned	1151_0	Clustered	9	VC_1151_0	9	0.8548	1	0.8548	0	0	0	1	Ref
KT264829	Unassigned	Unassigned	Unassigned	1056_0	Clustered	4	VC_1056_0	4	0.169	0.84110627	0.1421	0	0	0	1	Ref
KT264830	Unassigned	Unassigned	Unassigned	1147_0	Clustered	3	VC_1147_0	3	0.4334	0.65335909	0.2832	0	0	0	1	Ref
KT264831	Unassigned	Unassigned	Unassigned	1153_0	Clustered	2	VC_1153_0	2	0.073	0.80562642	0.0588	0	0	0	1	Ref
KT264833	Unassigned	Unassigned	Unassigned	1154_0	Clustered	5	VC_1154_0	5	1	1	1	0	0	0	1	Ref
KT264834	Unassigned	Unassigned	Unassigned	1155_0	Clustered	2	VC_1155_0	2	1	1	1	0	0	0	1	Ref
KT270441	Unassigned	Unassigned	Unassigned	372_0	Clustered	24	VC_372_0	24	0.5539	1	0.5539	0	0	0	1	Ref
KT279576	Unassigned	Unassigned	Unassigned	520_60	Clustered	2	VC_520_60	2	0.0091	0.95761233	0.0087	0	0	0	1	Ref
KT281790	Unassigned	Unassigned	Unassigned	558_11	Clustered	2	VC_558_11	2	0.0032	0.78528587	0.0025	0	0	0	1	Ref
KT281791	Unassigned	Unassigned	Unassigned	889_0	Clustered	16	VC_889_0	16	0.2969	1	0.2969	0	0	0	1	Ref
KT281792	Unassigned	Unassigned	Unassigned	520_27	Clustered	2	VC_520_27	2	0.0143	0.95761635	0.0137	0	0	0	1	Ref
KT281793	Unassigned	Unassigned	Unassigned	520_13	Clustered/Singleton	1	VC_520_13	1	0	1	0	0	0	0	1	Ref
KT281794	Unassigned	Unassigned	Unassigned	889_0	Clustered	16	VC_889_0	16	0.2969	1	0.2969	0	0	0	1	Ref
KT281795	Unassigned	Unassigned	Unassigned	520_31	Clustered	11	VC_520_31	11	0.0897	1	0.0897	0	0	0	0.6	Ref
KT281796	Unassigned	Unassigned	Unassigned	556_0	Clustered	21	VC_556_0	21	0.9912	1	0.9912	0	0	0	1	Ref
KT285706	Unassigned	Unassigned	Unassigned	225_0	Clustered	32	VC_225_0	32	0.848	1	0.848	0	0	0	0.996	Ref
KT287080	Unassigned	Unassigned	Unassigned	540_0	Clustered	15	VC_540_0	15	0.2866	1	0.2866	0	0	0	1	Ref
KT307976	Unassigned	Unassigned	Unassigned	883_1	Clustered	12	VC_883_1	12	0.2441	1	0.2441	0	0	0	1	Ref
KT309034	Unassigned	Unassigned	Unassigned	520_0	Clustered/Singleton	1	VC_520_0	1	0	1	0	0	0	0	1	Ref
KT321314	Unassigned	Unassigned	Unassigned	388_0	Clustered	4	VC_388_0	4	0.0345	0.99998812	0.0345	0	0	0	1	Ref
KT321315	Unassigned	Unassigned	Unassigned	668_0	Clustered	25	VC_668_0	25	0.5539	1	0.5539	0	0	0	0.96	Ref
KT321316	Unassigned	Unassigned	Unassigned	594_0	Clustered	2	VC_594_0	2	0.0737	0.95685859	0.0705	0	0	0	1	Ref
KT321317	Unassigned	Unassigned	Unassigned	779_1	Clustered	11	VC_779_1	11	0.1587	1	0.1587	0	0	0	1	Ref

KT321476	Unassigned	Unassigned	Unassigned	558_50	Clustered	15	VC_558_50	15	0.0468	1	0.0468	0	0	0	0.9238	Ref
KT326767	Unassigned	Unassigned	Unassigned	513_0	Clustered	32	VC_513_0	32	0.6881	1	0.6881	0	0	0	1	Ref
KT326768	Unassigned	Unassigned	Unassigned	325_30	Clustered	4	VC_325_30	4	0.0144	0.99998616	0.0144	0	0	0	0.8333	Ref
KT336320	Unassigned	Unassigned	Unassigned	632_0	Clustered	37	VC_632_0	37	0.581	1	0.581	0	0	0	0.9234	Ref
KT336321	Unassigned	Unassigned	Unassigned	632_0	Clustered	37	VC_632_0	37	0.581	1	0.581	0	0	0	0.9234	Ref
KT337339	Unassigned	Unassigned	Unassigned	414_0	Clustered	37	VC_414_0	37	0.585	1	0.585	0	0	0	0.9474	Ref
KT337340	Unassigned	Unassigned	Unassigned	414_0	Clustered	37	VC_414_0	37	0.585	1	0.585	0	0	0	0.9474	Ref
KT337341	Unassigned	Unassigned	Unassigned	414_0	Clustered	37	VC_414_0	37	0.585	1	0.585	0	0	0	0.9474	Ref
KT337342	Unassigned	Unassigned	Unassigned	333_0	Clustered	25	VC_333_0	25	0.4681	1	0.4681	0	0	0	1	Ref
KT337352	Unassigned	Unassigned	Unassigned	414_0	Clustered	37	VC_414_0	37	0.585	1	0.585	0	0	0	0.9474	Ref
KT337354	Unassigned	Unassigned	Unassigned	414_0	Clustered	37	VC_414_0	37	0.585	1	0.585	0	0	0	0.9474	Ref
KT337355	Unassigned	Unassigned	Unassigned	414_0	Clustered	37	VC_414_0	37	0.585	1	0.585	0	0	0	0.9474	Ref
KT337356	Unassigned	Unassigned	Unassigned	414_0	Clustered	37	VC_414_0	37	0.585	1	0.585	0	0	0	0.9474	Ref
KT337358	Unassigned	Unassigned	Unassigned	412_0	Clustered	39	VC_412_0	39	0.845	1	0.845	0	0	0	1	Ref
KT337359	Unassigned	Unassigned	Unassigned	414_0	Clustered	37	VC_414_0	37	0.585	1	0.585	0	0	0	0.9474	Ref
KT337360	Unassigned	Unassigned	Unassigned	317_2	Clustered	20	VC_317_2	20	0.1976	1	0.1976	0	0	0	0.6842	Ref
KT337362	Unassigned	Unassigned	Unassigned	414_0	Clustered	37	VC_414_0	37	0.585	1	0.585	0	0	0	0.9474	Ref
KT337364	Unassigned	Unassigned	Unassigned	412_0	Clustered	39	VC_412_0	39	0.845	1	0.845	0	0	0	1	Ref
KT339321	Unassigned	Unassigned	Unassigned	699_0	Clustered	71	VC_699_0	71	0.9352	1	0.9352	0	0	0	1	Ref
KT344895	Unassigned	Unassigned	Unassigned	12_0	Clustered	21	VC_12_0	21	0.2274	1	0.2274	0	0	0	0.9571	Ref
KT345706	Unassigned	Unassigned	Unassigned	971_2	Clustered/Singleton	1	VC_971_2	1	0	1	0	0	0	0	1	Ref
KT347313	Unassigned	Unassigned	Unassigned	519_0	Clustered	39	VC_519_0	39	0.8156	1	0.8156	0	0	0	1	Ref
KT347314	Unassigned	Unassigned	Unassigned	761_0	Clustered	67	VC_761_0	67	0.8947	1	0.8947	0	0	0	1	Ref
KT353109	Unassigned	Unassigned	Unassigned	849_5	Clustered	3	VC_849_5	3	0.0492	0.97574727	0.048	0	0	0	1	Ref
KT355471	Unassigned	Unassigned	Unassigned	950_0	Clustered	38	VC_950_0	38	0.9443	1	0.9443	0	0	0	1	Ref
KT355472	Unassigned	Unassigned	Unassigned	761_0	Clustered	67	VC_761_0	67	0.8947	1	0.8947	0	0	0	1	Ref
KT355473	Unassigned	Unassigned	Unassigned	950_0	Clustered	38	VC_950_0	38	0.9443	1	0.9443	0	0	0	1	Ref
KT355474	Unassigned	Unassigned	Unassigned	761_0	Clustered	67	VC_761_0	67	0.8947	1	0.8947	0	0	0	1	Ref
KT355475	Unassigned	Unassigned	Unassigned	950_0	Clustered	38	VC_950_0	38	0.9443	1	0.9443	0	0	0	1	Ref
KT359365	Unassigned	Unassigned	Unassigned	514_0	Clustered	75	VC_514_0	75	0.9402	1	0.9402	0	0	0	1	Ref
KT361649	Unassigned	Unassigned	Unassigned	708_0	Clustered	47	VC_708_0	47	0.8654	1	0.8654	0	0	0	1	Ref
KT361650	Unassigned	Unassigned	Unassigned	708_0	Clustered	47	VC_708_0	47	0.8654	1	0.8654	0	0	0	1	Ref
KT361652	Unassigned	Unassigned	Unassigned	708_0	Clustered	47	VC_708_0	47	0.8654	1	0.8654	0	0	0	1	Ref
KT361654	Unassigned	Unassigned	Unassigned	620_0	Clustered	27	VC_620_0	27	0.879	1	0.879	0	0	0	1	Ref
KT361655	Unassigned	Unassigned	Unassigned	620_0	Clustered	27	VC_620_0	27	0.879	1	0.879	0	0	0	1	Ref
KT361656	Unassigned	Unassigned	Unassigned	620_0	Clustered	27	VC_620_0	27	0.879	1	0.879	0	0	0	1	Ref
KT361657	Unassigned	Unassigned	Unassigned	620_0	Clustered	27	VC_620_0	27	0.879	1	0.879	0	0	0	1	Ref
KT361920	Unassigned	Unassigned	Unassigned	234_0	Clustered	21	VC_234_0	21	0.8355	1	0.8355	0	0	0	1	Ref
KT363731	Unassigned	Unassigned	Unassigned	325_42	Clustered	4	VC_325_42	4	0.0112	0.99980442	0.0112	0	0	0	0.6667	Ref
KT363872	Unassigned	Unassigned	Unassigned	330_0	Clustered	27	VC_330_0	27	0.5088	1	0.5088	0	0	0	0.8661	Ref
KT364588	Unassigned	Unassigned	Unassigned	371_4	Clustered	5	VC_371_4	5	0.0135	0.99999971	0.0135	0	0	0	1	Ref
KT365397	Unassigned	Unassigned	Unassigned	517_1	Clustered	108	VC_517_1	108	0.8483	1	0.8483	0	0	0	0.9955	Ref
KT365398	Unassigned	Unassigned	Unassigned	558_6	Clustered	2	VC_558_6	2	0.0032	0.73149279	0.0023	0	0	0	1	Ref
KT365399	Unassigned	Unassigned	Unassigned	761_0	Clustered	67	VC_761_0	67	0.8947	1	0.8947	0	0	0	1	Ref
KT365400	Unassigned	Unassigned	Unassigned	558_37	Clustered/Singleton	1	VC_558_37	1	0	1	0	0	0	0	1	Ref
KT365401	Unassigned	Unassigned	Unassigned	1073_0	Clustered	24	VC_1073_0	24	0.8388	1	0.8388	0	0	0	1	Ref
KT365402	Unassigned	Unassigned	Unassigned	508_0	Clustered	32	VC_508_0	32	0.906	1	0.906	0	0	0	1	Ref
KT367885	Unassigned	Unassigned	Unassigned	823_0	Clustered	124	VC_823_0	124	0.9492	1	0.9492	0	0	0	1	Ref
KT367886	Unassigned	Unassigned	Unassigned	111_0	Clustered	91	VC_111_0	91	0.9587	1	0.9587	0	0	0	1	Ref
KT367887	Unassigned	Unassigned	Unassigned	836_0	Clustered	9	VC_836_0	9	0.7733	1	0.7733	0	0	0	1	Ref
KT372002	Unassigned	Unassigned	Unassigned	956_0	Clustered	39	VC_956_0	39	1	1	1	0	0	0	1	Ref
KT372003	Unassigned	Unassigned	Unassigned	889_0	Clustered	16	VC_889_0	16	0.2969	1	0.2969	0	0	0	1	Ref
KT372690	Unassigned	Unassigned	Unassigned	80_0	Clustered	41	VC_80_0	41	0.5837	1	0.5837	0	0	0	0.9805	Ref
KT372691	Unassigned	Unassigned	Unassigned	80_0	Clustered	41	VC_80_0	41	0.5837	1	0.5837	0	0	0	0.9805	Ref
KT372692	Unassigned	Unassigned	Unassigned	80_0	Clustered	41	VC_80_0	41	0.5837	1	0.5837	0	0	0	0.9805	Ref
KT372693	Unassigned	Unassigned	Unassigned	80_0	Clustered	41	VC_80_0	41	0.5837	1	0.5837	0	0	0	0.9805	Ref
KT372694	Unassigned	Unassigned	Unassigned	80_0	Clustered	41	VC_80_0	41	0.5837	1	0.5837	0	0	0	0.9805	Ref
KT372695	Unassigned	Unassigned	Unassigned	80_0	Clustered	41	VC_80_0	41	0.5837	1	0.5837	0	0	0	0.9805	Ref
KT372696	Unassigned	Unassigned	Unassigned	80_0	Clustered	41	VC_80_0	41	0.5837	1	0.5837	0	0	0	0.9805	Ref
KT372697	Unassigned	Unassigned	Unassigned	80_0	Clustered	41	VC_80_0	41	0.5837	1	0.5837	0	0	0	0.9805	Ref
KT372698	Unassigned	Unassigned	Unassigned	80_0	Clustered	41	VC_80_0	41	0.5837	1	0.5837	0	0	0	0.9805	Ref
KT372714	Unassigned	Unassigned	Unassigned	20_0	Clustered	5	VC_20_0	5	0.4387	0.99999934	0.4387	0	0	0	1	Ref
KT373978	Unassigned	Unassigned	Unassigned	551_7	Clustered	28	VC_551_7	28	0.1661	1	0.1661	0	0	0	0.8519	Ref
KT375356	Unassigned	Unassigned	Unassigned	956_0	Clustered	39	VC_956_0	39	1	1	1	0	0	0	1	Ref
KT381276	Unassigned	Unassigned	Unassigned	215_2	Clustered	5	VC_215_2	5	0.0572	0.99999997	0.0572	0	0	0	1	Ref
KT381277	Unassigned	Unassigned	Unassigned	514_0	Clustered	75	VC_514_0	75	0.9402	1	0.9402	0	0	0	1	Ref
KT381864	Unassigned	Unassigned	Unassigned	182_0	Clustered	5	VC_182_0	5	0.6184	0.99999967	0.6184	0	0	0	1	Ref
KT381865	Unassigned	Unassigned	Unassigned	182_0	Clustered	5	VC_182_0	5	0.6184	0.99999967	0.6184	0	0	0	1	Ref
KT381879	Unassigned	Unassigned	Unassigned	70_0	Clustered	27	VC_70_0	27	0.5981	1	0.5981	0	0	0	0.9886	Ref
KT381880	Unassigned	Unassigned	Unassigned	669_0	Clustered	21	VC_669_0	21	0.3623	1	0.3623	0	0	0	1	Ref
KT388014	Unassigned	Unassigned	Unassigned	325_53	Clustered	7	VC_325_53	7	0.0242	1	0.0242	0	0	0	0.9048	Ref
KT388093	Unassigned	Unassigned	Unassigned	412_0	Clustered	39	VC_412_0	39	0.845	1	0.845	0	0	0	1	Ref
KT388102	Unassigned	Unassigned	Unassigned	699_0	Clustered	71	VC_699_0	71	0.9352	1	0.9352	0	0	0	1	Ref
KT388103	Unassigned	Unassigned	Unassigned	699_0	Clustered	71	VC_699_0	71	0.9352	1	0.9352	0	0	0	1	Ref
KT429160	Unassigned	Unassigned	Unassigned	213_0	Clustered	2	VC_213_0	2	0.4293	0.95300227	0.4091	0	0	0	1	Ref
KT438500	Unassigned	Unassigned	Unassigned	325_9	Clustered	2	VC_325_9	2	0.0055	0.95750934	0.0053	0	0	0	1	Ref
KT438501	Unassigned	Unassigned	Unassigned	940_0	Clustered	34	VC_940_0	34	0.7959	1	0.7959	0	0	0	1	Ref
KT454805	Unassigned	Unassigned	Unassigned	330_0	Clustered	27	VC_330_0	27	0.5088	1	0.5088	0	0	0	0.8661	Ref
KT454806	Unassigned	Unassigned	Unassigned	330_0	Clustered	27	VC_330_0	27	0.5088	1	0.5088	0	0	0	0.8661	Ref
KT454972	Unassigned	Unassigned	Unassigned	810_0	Clustered	18	VC_810_0	18	0.9121	1	0.9121	0	0	0	1	Ref
KT588073	Unassigned	Unassigned	Unassigned	696_1	Clustered	8	VC_696_1	8	0.5452	1	0.5452	0	0	0	0.9286	Ref

KT588074	Unassigned	Unassigned	Unassigned	242_0	Clustered	2	VC_242_0	2	0.0867	0.95379254	0.0827	0	0	0	1	Ref
KT588075	Unassigned	Unassigned	Unassigned	696_0	Clustered	2	VC_696_0	2	0.1624	0.94573679	0.1536	0	0	0	1	Ref
KT588442	Unassigned	Unassigned	Unassigned	216_6	Clustered	42	VC_216_6	42	0.473	1	0.473	0	0	0	0.8351	Ref
KT591076	Unassigned	Unassigned	Unassigned	568_0	Clustered	8	VC_568_0	8	0.5738	1	0.5738	0	0	0	1	Ref
KT591489	Unassigned	Unassigned	Unassigned	888_0	Clustered	28	VC_888_0	28	0.5497	1	0.5497	0	0	0	1	Ref
KT591491	Unassigned	Unassigned	Unassigned	763_1	Clustered	9	VC_763_1	9	0.3706	1	0.3706	0	0	0	1	Ref
KT599441	Unassigned	Unassigned	Unassigned	371_15	Clustered	44	VC_371_15	44	0.1391	1	0.1391	0	0	0	0.7474	Ref
KT624200	Unassigned	Unassigned	Unassigned	88_0	Clustered/Singleton	1	VC_88_0	1	0	1	0	0	0	0	1	Ref
KT626047	Unassigned	Unassigned	Unassigned	325_5	Clustered	11	VC_325_5	11	0.047	1	0.047	0	0	0	0.7636	Ref
KT626446	Unassigned	Unassigned	Unassigned	143_0	Clustered	2	VC_143_0	2	0.1189	0.94995671	0.1129	0	0	0	1	Ref
KT630644	Unassigned	Unassigned	Unassigned	228_0	Clustered	60	VC_228_0	60	0.942	1	0.942	0	0	0	1	Ref
KT630647	Unassigned	Unassigned	Unassigned	6_0	Clustered	44	VC_6_0	44	0.908	1	0.908	0	0	0	1	Ref
KT630649	Unassigned	Unassigned	Unassigned	242_1	Clustered	20	VC_242_1	20	0.684	1	0.684	0	0	0	0.8368	Ref
KT716493	Unassigned	Unassigned	Unassigned	517_1	Clustered	108	VC_517_1	108	0.8483	1	0.8483	0	0	0	0.9955	Ref
KT717083	Unassigned	Unassigned	Unassigned	252_0	Clustered	121	VC_252_0	121	0.7376	1	0.7376	0	0	0	0.9999	Ref
KT717084	Unassigned	Unassigned	Unassigned	252_0	Clustered	121	VC_252_0	121	0.7376	1	0.7376	0	0	0	0.9999	Ref
KT717085	Unassigned	Unassigned	Unassigned	252_0	Clustered	121	VC_252_0	121	0.7376	1	0.7376	0	0	0	0.9999	Ref
KT725776	Unassigned	Unassigned	Unassigned	654_0	Clustered	4	VC_654_0	4	0.2897	0.99998263	0.2897	0	0	0	1	Ref
KT728930	Unassigned	Unassigned	Unassigned	875_0	Clustered	10	VC_875_0	10	0.988	0.99948433	0.9875	0	0	0	1	Ref
KT728931	Unassigned	Unassigned	Unassigned	875_0	Clustered	10	VC_875_0	10	0.988	0.99948433	0.9875	0	0	0	1	Ref
KT734862	Unassigned	Unassigned	Unassigned	166_0	Clustered	26	VC_166_0	26	0.7923	1	0.7923	0	0	0	1	Ref
KT736033	Unassigned	Unassigned	Unassigned	76_0	Clustered	37	VC_76_0	37	0.9403	1	0.9403	0	0	0	1	Ref
KT755656	Unassigned	Unassigned	Unassigned	1156_0	Clustered	8	VC_1156_0	8	0.8137	1	0.8137	0	0	0	1	Ref
KT783672	Unassigned	Unassigned	Unassigned	950_0	Clustered	38	VC_950_0	38	0.9443	1	0.9443	0	0	0	1	Ref
KT804908	Unassigned	Unassigned	Unassigned	699_0	Clustered	71	VC_699_0	71	0.9352	1	0.9352	0	0	0	1	Ref
KT804923	Unassigned	Unassigned	Unassigned	76_0	Clustered	37	VC_76_0	37	0.9403	1	0.9403	0	0	0	1	Ref
KT809302	Unassigned	Unassigned	Unassigned	666_0	Clustered	4	VC_666_0	4	1	1	1	0	0	0	1	Ref
KT809368	Unassigned	Unassigned	Unassigned	10_0	Clustered	25	VC_10_0	25	0.3467	1	0.3467	0	0	0	0.9733	Ref
KT809369	Unassigned	Unassigned	Unassigned	10_0	Clustered	25	VC_10_0	25	0.3467	1	0.3467	0	0	0	0.9733	Ref
KT818595	Unassigned	Unassigned	Unassigned	325_14	Clustered	3	VC_325_14	3	0.0094	0.99859159	0.0094	0	0	0	1	Ref
KT825490	Unassigned	Unassigned	Unassigned	455_0	Clustered	18	VC_455_0	18	0.4598	1	0.4598	0	0	0	1	Ref
KT852578	Unassigned	Unassigned	Unassigned	20_0	Clustered	5	VC_20_0	5	0.4387	0.99999934	0.4387	0	0	0	1	Ref
KT870145	Unassigned	Unassigned	Unassigned	977_0	Clustered	2	VC_977_0	2	0.0806	0.93920319	0.0757	0	0	0	1	Ref
KT876724	Unassigned	Unassigned	Unassigned	1157_0	Clustered	24	VC_1157_0	24	1	1	1	0	0	0	1	Ref
KT876725	Unassigned	Unassigned	Unassigned	1040_0	Clustered	23	VC_1040_0	23	0.9605	1	0.9605	0	0	0	1	Ref
KT876726	Unassigned	Unassigned	Unassigned	1157_0	Clustered	24	VC_1157_0	24	1	1	1	0	0	0	1	Ref
KT878766	Unassigned	Unassigned	Unassigned	10_0	Clustered	25	VC_10_0	25	0.3467	1	0.3467	0	0	0	0.9733	Ref
KT880194	Unassigned	Unassigned	Unassigned	508_0	Clustered	32	VC_508_0	32	0.906	1	0.906	0	0	0	1	Ref
KT881477	Unassigned	Unassigned	Unassigned	637_0	Clustered	115	VC_637_0	115	0.731	1	0.731	0	0	0	0.8764	Ref
KT887557	Unassigned	Unassigned	Unassigned	176_1	Clustered	2	VC_176_1	2	0.0363	0.95657568	0.0347	0	0	0	1	Ref
KT887558	Unassigned	Unassigned	Unassigned	173_0	Clustered	2	VC_173_0	2	0.0799	0.95489091	0.0763	0	0	0	1	Ref
KT887559	Unassigned	Unassigned	Unassigned	5_0	Clustered	30	VC_5_0	30	0.9647	1	0.9647	0	0	0	1	Ref
KT895280	Unassigned	Unassigned	Unassigned	520_85	Clustered	2	VC_520_85	2	0.0148	0.95762405	0.0142	0	0	0	1	Ref
KT895281	Unassigned	Unassigned	Unassigned	520_45	Clustered	7	VC_520_45	7	0.0635	1	0.0635	0	0	0	0.8095	Ref
KT895374	Unassigned	Unassigned	Unassigned	448_1	Clustered/Singleton	1	VC_448_1	1	0	1	0	0	0	0	1	Ref
KT919972	Unassigned	Unassigned	Unassigned	531_0	Clustered	9	VC_531_0	9	0.26	1	0.26	0	0	0	1	Ref
KT919973	Unassigned	Unassigned	Unassigned	531_0	Clustered	9	VC_531_0	9	0.26	1	0.26	0	0	0	1	Ref
KT932418	Unassigned	Unassigned	Unassigned	493_0	Clustered/Singleton	1	VC_493_0	1	0	1	0	0	0	0	1	Ref
KT932699	Unassigned	Unassigned	Unassigned	108_0	Clustered	33	VC_108_0	33	0.949	1	0.949	0	0	0	1	Ref
KT932700	Unassigned	Unassigned	Unassigned	967_0	Clustered	16	VC_967_0	16	0.8971	1	0.8971	0	0	0	1	Ref
KT932701	Unassigned	Unassigned	Unassigned	695_0	Clustered	48	VC_695_0	48	0.8162	1	0.8162	0	0	0	1	Ref
KT934381	Unassigned	Unassigned	Unassigned	765_0	Clustered	132	VC_765_0	132	1	1	1	0	0	0	1	Ref
KT934943	Unassigned	Unassigned	Unassigned	582_3	Clustered	12	VC_582_3	12	0.2357	1	0.2357	0	0	0	0.8636	Ref
KT945994	Unassigned	Unassigned	Unassigned	267_0	Clustered	2	VC_267_0	2	0.0988	0.95557934	0.0945	0	0	0	1	Ref
KT945995	Unassigned	Unassigned	Unassigned	968_0	Clustered	4	VC_968_0	4	0.2959	0.99997642	0.2959	0	0	0	1	Ref
KT949345	Unassigned	Unassigned	Unassigned	878_0	Clustered	32	VC_878_0	32	0.9965	1	0.9965	0	0	0	1	Ref
KT959213	Unassigned	Unassigned	Unassigned	956_0	Clustered	39	VC_956_0	39	1	1	1	0	0	0	1	Ref
KT962245	Unassigned	Unassigned	Unassigned	1017_0	Clustered	5	VC_1017_0	5	0.9759	0.9999961	0.9759	0	0	0	1	Ref
KT962246	Unassigned	Unassigned	Unassigned	1017_0	Clustered	5	VC_1017_0	5	0.9759	0.9999961	0.9759	0	0	0	1	Ref
KT962247	Unassigned	Unassigned	Unassigned	1017_0	Clustered	5	VC_1017_0	5	0.9759	0.9999961	0.9759	0	0	0	1	Ref
KT962832	Unassigned	Unassigned	Unassigned	637_0	Clustered	115	VC_637_0	115	0.731	1	0.731	0	0	0	0.8764	Ref
KT964103	Unassigned	Unassigned	Unassigned	111_0	Clustered	91	VC_111_0	91	0.9587	1	0.9587	0	0	0	1	Ref
KT968831	Unassigned	Unassigned	Unassigned	176_3	Clustered/Singleton	1	VC_176_3	1	0	1	0	0	0	0	1	Ref
KT968832	Unassigned	Unassigned	Unassigned	179_0	Clustered	53	VC_179_0	53	0.9137	1	0.9137	0	0	0	1	Ref
KT989433	Unassigned	Unassigned	Unassigned	975_0	Clustered	11	VC_975_0	11	0.9687	1	0.9687	0	0	0	1	Ref
KT990215	Unassigned	Unassigned	Unassigned	451_0	Clustered	80	VC_451_0	80	0.9991	1	0.9991	0	0	0	1	Ref
KT990218	Unassigned	Unassigned	Unassigned	956_0	Clustered	39	VC_956_0	39	1	1	1	0	0	0	1	Ref
KT995480	Unassigned	Unassigned	Unassigned	187_3	Clustered	9	VC_187_3	9	0.1971	1	0.1971	0	0	0	0.8611	Ref
KT997847	Unassigned	Unassigned	Unassigned	481_0	Clustered	8	VC_481_0	8	0.1348	1	0.1348	0	0	0	1	Ref
KU052037	Unassigned	Unassigned	Unassigned	37_1	Clustered	3	VC_37_1	3	0.0833	0.9484635	0.079	0	0	0	1	Ref
KU052488	Unassigned	Unassigned	Unassigned	634_0	Clustered	3	VC_634_0	3	0.2046	0.99837593	0.2043	0	0	0	1	Ref
KU055616	Unassigned	Unassigned	Unassigned	517_1	Clustered	108	VC_517_1	108	0.8483	1	0.8483	0	0	0	0.9955	Ref
KU057941	Unassigned	Unassigned	Unassigned	733_0	Clustered	5	VC_733_0	5	0.5111	0.99999994	0.5111	0	0	0	1	Ref
KU064779	Unassigned	Unassigned	Unassigned	129_0	Clustered	43	VC_129_0	43	0.886	1	0.886	0	0	0	1	Ref
KU130126	Unassigned	Unassigned	Unassigned	78_1	Clustered	6	VC_78_1	6	0.1362	1	0.1362	0	0	0	1	Ref
KU130127	Unassigned	Unassigned	Unassigned	78_1	Clustered	6	VC_78_1	6	0.1362	1	0.1362	0	0	0	1	Ref
KU130128	Unassigned	Unassigned	Unassigned	78_1	Clustered	6	VC_78_1	6	0.1362	1	0.1362	0	0	0	1	Ref
KU130129	Unassigned	Unassigned	Unassigned	78_1	Clustered	6	VC_78_1	6	0.1362	1	0.1362	0	0	0	1	Ref
KU130130	Unassigned	Unassigned	Unassigned	78_1	Clustered	6	VC_78_1	6	0.1362	1	0.1362	0	0	0	1	Ref
KU130131	Unassigned	Unassigned	Unassigned	78_1	Clustered	6	VC_78_1	6	0.1362	1	0.1362	0	0	0	1	Ref
KU160638	Unassigned	Unassigned	Unassigned	857_0	Clustered	11	VC_857_0	11	0.9712	1	0.9712	0	0	0	1	Ref

KU160639	Unassigned	Unassigned	Unassigned	857_0	Clustered	11	VC_857_0	11	0.9712	1	0.9712	0	0	0	1	Ref
KU160640	Unassigned	Unassigned	Unassigned	1160_0	Clustered	73	VC_1160_0	73	0.9835	1	0.9835	0	0	0	1	Ref
KU160641	Unassigned	Unassigned	Unassigned	1164_0	Clustered	25	VC_1164_0	25	0.7312	1	0.7312	0	0	0	1	Ref
KU160642	Unassigned	Unassigned	Unassigned	802_0	Clustered	17	VC_802_0	17	0.8025	1	0.8025	0	0	0	1	Ref
KU160643	Unassigned	Unassigned	Unassigned	1160_0	Clustered	73	VC_1160_0	73	0.9835	1	0.9835	0	0	0	1	Ref
KU160644	Unassigned	Unassigned	Unassigned	1166_0	Clustered	20	VC_1166_0	20	0.99	1	0.99	0	0	0	1	Ref
KU160645	Unassigned	Unassigned	Unassigned	1160_0	Clustered	73	VC_1160_0	73	0.9835	1	0.9835	0	0	0	1	Ref
KU160646	Unassigned	Unassigned	Unassigned	1164_0	Clustered	25	VC_1164_0	25	0.7312	1	0.7312	0	0	0	1	Ref
KU160647	Unassigned	Unassigned	Unassigned	857_0	Clustered	11	VC_857_0	11	0.9712	1	0.9712	0	0	0	1	Ref
KU160648	Unassigned	Unassigned	Unassigned	1160_0	Clustered	73	VC_1160_0	73	0.9835	1	0.9835	0	0	0	1	Ref
KU160649	Unassigned	Unassigned	Unassigned	1160_0	Clustered	73	VC_1160_0	73	0.9835	1	0.9835	0	0	0	1	Ref
KU160650	Unassigned	Unassigned	Unassigned	1167_0	Clustered	6	VC_1167_0	6	0.8642	0.99999946	0.8642	0	0	0	1	Ref
KU160651	Unassigned	Unassigned	Unassigned	1073_0	Clustered	24	VC_1073_0	24	0.8388	1	0.8388	0	0	0	1	Ref
KU160652	Unassigned	Unassigned	Unassigned	1160_0	Clustered	73	VC_1160_0	73	0.9835	1	0.9835	0	0	0	1	Ref
KU160653	Unassigned	Unassigned	Unassigned	1160_0	Clustered	73	VC_1160_0	73	0.9835	1	0.9835	0	0	0	1	Ref
KU160654	Unassigned	Unassigned	Unassigned	1169_0	Clustered	7	VC_1169_0	7	0.9811	0.99995613	0.981	0	0	0	1	Ref
KU160655	Unassigned	Unassigned	Unassigned	950_0	Clustered	38	VC_950_0	38	0.9443	1	0.9443	0	0	0	1	Ref
KU160656	Unassigned	Unassigned	Unassigned	1073_0	Clustered	24	VC_1073_0	24	0.8388	1	0.8388	0	0	0	1	Ref
KU160657	Unassigned	Unassigned	Unassigned	950_0	Clustered	38	VC_950_0	38	0.9443	1	0.9443	0	0	0	1	Ref
KU160658	Unassigned	Unassigned	Unassigned	950_0	Clustered	38	VC_950_0	38	0.9443	1	0.9443	0	0	0	1	Ref
KU160659	Unassigned	Unassigned	Unassigned	1160_0	Clustered	73	VC_1160_0	73	0.9835	1	0.9835	0	0	0	1	Ref
KU160660	Unassigned	Unassigned	Unassigned	1074_0	Clustered	19	VC_1074_0	19	0.9198	1	0.9198	0	0	0	1	Ref
KU160661	Unassigned	Unassigned	Unassigned	1160_0	Clustered	73	VC_1160_0	73	0.9835	1	0.9835	0	0	0	1	Ref
KU160662	Unassigned	Unassigned	Unassigned	1160_0	Clustered	73	VC_1160_0	73	0.9835	1	0.9835	0	0	0	1	Ref
KU160663	Unassigned	Unassigned	Unassigned	857_0	Clustered	11	VC_857_0	11	0.9712	1	0.9712	0	0	0	1	Ref
KU160664	Unassigned	Unassigned	Unassigned	1169_0	Clustered	7	VC_1169_0	7	0.9811	0.99995613	0.981	0	0	0	1	Ref
KU160665	Unassigned	Unassigned	Unassigned	1073_0	Clustered	24	VC_1073_0	24	0.8388	1	0.8388	0	0	0	1	Ref
KU160666	Unassigned	Unassigned	Unassigned	857_0	Clustered	11	VC_857_0	11	0.9712	1	0.9712	0	0	0	1	Ref
KU160667	Unassigned	Unassigned	Unassigned	950_0	Clustered	38	VC_950_0	38	0.9443	1	0.9443	0	0	0	1	Ref
KU160668	Unassigned	Unassigned	Unassigned	1073_0	Clustered	24	VC_1073_0	24	0.8388	1	0.8388	0	0	0	1	Ref
KU160669	Unassigned	Unassigned	Unassigned	1170_1	Clustered	3	VC_1170_1	3	0.308	0.99836447	0.3075	0	0	0	1	Ref
KU160670	Unassigned	Unassigned	Unassigned	950_0	Clustered	38	VC_950_0	38	0.9443	1	0.9443	0	0	0	1	Ref
KU160671	Unassigned	Unassigned	Unassigned	1160_0	Clustered	73	VC_1160_0	73	0.9835	1	0.9835	0	0	0	1	Ref
KU160672	Unassigned	Unassigned	Unassigned	1160_0	Clustered	73	VC_1160_0	73	0.9835	1	0.9835	0	0	0	1	Ref
KU160673	Unassigned	Unassigned	Unassigned	1170_1	Clustered	3	VC_1170_1	3	0.308	0.99836447	0.3075	0	0	0	1	Ref
KU160674	Unassigned	Unassigned	Unassigned	950_0	Clustered	38	VC_950_0	38	0.9443	1	0.9443	0	0	0	1	Ref
KU183006	Unassigned	Unassigned	Unassigned	823_0	Clustered	124	VC_823_0	124	0.9492	1	0.9492	0	0	0	1	Ref
KU189325	Unassigned	Unassigned	Unassigned	975_0	Clustered	11	VC_975_0	11	0.9687	1	0.9687	0	0	0	1	Ref
KU194205	Unassigned	Unassigned	Unassigned	575_0	Clustered	35	VC_575_0	35	0.8815	1	0.8815	0	0	0	1	Ref
KU194206	Unassigned	Unassigned	Unassigned	575_0	Clustered	35	VC_575_0	35	0.8815	1	0.8815	0	0	0	1	Ref
KU197013	Unassigned	Unassigned	Unassigned	74_0	Clustered	15	VC_74_0	15	0.5204	1	0.5204	0	0	0	1	Ref
KU197014	Unassigned	Unassigned	Unassigned	749_0	Clustered	40	VC_749_0	40	0.7517	1	0.7517	0	0	0	1	Ref
KU198331	Unassigned	Unassigned	Unassigned	79_7	Clustered	33	VC_79_7	33	0.216	1	0.216	0	0	0	0.8409	Ref
KU199707	Unassigned	Unassigned	Unassigned	179_0	Clustered	53	VC_179_0	53	0.9137	1	0.9137	0	0	0	1	Ref
KU199708	Unassigned	Unassigned	Unassigned	179_0	Clustered	53	VC_179_0	53	0.9137	1	0.9137	0	0	0	1	Ref
KU199709	Unassigned	Unassigned	Unassigned	179_0	Clustered	53	VC_179_0	53	0.9137	1	0.9137	0	0	0	1	Ref
KU199710	Unassigned	Unassigned	Unassigned	297_0	Clustered	5	VC_297_0	5	0.1056	0.99999993	0.1056	0	0	0	0.9	Ref
KU204984	Unassigned	Unassigned	Unassigned	169_0	Clustered	20	VC_169_0	20	0.7529	1	0.7529	0	0	0	1	Ref
KU230356	Unassigned	Unassigned	Unassigned	1173_0	Clustered	6	VC_1173_0	6	0.9951	0.99999296	0.9951	0	0	0	1	Ref
KU234099	Unassigned	Unassigned	Unassigned	888_0	Clustered	28	VC_888_0	28	0.5497	1	0.5497	0	0	0	1	Ref
KU234532	Unassigned	Unassigned	Unassigned	1174_0	Clustered	4	VC_1174_0	4	1	1	1	0	0	0	1	Ref
KU234533	Unassigned	Unassigned	Unassigned	1174_0	Clustered	4	VC_1174_0	4	1	1	1	0	0	0	1	Ref
KU238068	Unassigned	Unassigned	Unassigned	39_0	Clustered	35	VC_39_0	35	0.4337	1	0.4337	0	0	0	1	Ref
KU238069	Unassigned	Unassigned	Unassigned	39_0	Clustered	35	VC_39_0	35	0.4337	1	0.4337	0	0	0	1	Ref
KU238070	Unassigned	Unassigned	Unassigned	39_0	Clustered	35	VC_39_0	35	0.4337	1	0.4337	0	0	0	1	Ref
KU245542	Unassigned	Unassigned	Unassigned	1028_0	Clustered	8	VC_1028_0	8	0.9323	1	0.9323	0	0	0	1	Ref
KU253712	Unassigned	Unassigned	Unassigned	85_8	Clustered/Singleton	1	VC_85_8	1	0	1	0	0	0	0	1	Ref
KU255188	Unassigned	Unassigned	Unassigned	514_0	Clustered	75	VC_514_0	75	0.9402	1	0.9402	0	0	0	1	Ref
KU255730	Unassigned	Unassigned	Unassigned	286_8	Clustered/Singleton	1	VC_286_8	1	0	1	0	0	0	0	1	Ref
KU297675	Unassigned	Unassigned	Unassigned	76_0	Clustered	37	VC_76_0	37	0.9403	1	0.9403	0	0	0	1	Ref
KU297783	Unassigned	Unassigned	Unassigned	216_4	Clustered	13	VC_216_4	13	0.1657	1	0.1657	0	0	0	1	Ref
KU298437	Unassigned	Unassigned	Unassigned	42_0	Clustered	49	VC_42_0	49	0.4517	1	0.4517	0	0	0	0.8597	Ref
KU310943	Unassigned	Unassigned	Unassigned	297_1	Clustered	20	VC_297_1	20	0.5653	1	0.5653	0	0	0	0.7684	Ref
KU356689	Unassigned	Unassigned	Unassigned	464_0	Clustered	8	VC_464_0	8	0.2629	1	0.2629	0	0	0	1	Ref
KU356690	Unassigned	Unassigned	Unassigned	781_0	Clustered	2	VC_781_0	2	0.0375	0.95691208	0.0359	0	0	0	1	Ref
KU356691	Unassigned	Unassigned	Unassigned	781_0	Clustered	2	VC_781_0	2	0.0375	0.95691208	0.0359	0	0	0	1	Ref
KU497559	Unassigned	Unassigned	Unassigned	76_0	Clustered	37	VC_76_0	37	0.9403	1	0.9403	0	0	0	1	Ref
KU504502	Unassigned	Unassigned	Unassigned	5_0	Clustered	30	VC_5_0	30	0.9647	1	0.9647	0	0	0	1	Ref
KU510289	Unassigned	Unassigned	Unassigned	698_0	Clustered	26	VC_698_0	26	0.9366	1	0.9366	0	0	0	1	Ref
KU517658	Unassigned	Unassigned	Unassigned	734_0	Clustered	6	VC_734_0	6	0.1914	0.99996888	0.1914	0	0	0	1	Ref
KU521356	Unassigned	Unassigned	Unassigned	344_0	Clustered	21	VC_344_0	21	0.5436	1	0.5436	0	0	0	1	Ref
KU522583	Unassigned	Unassigned	Unassigned	848_0	Clustered	28	VC_848_0	28	0.7927	1	0.7927	0	0	0	1	Ref
KU530220	Unassigned	Unassigned	Unassigned	975_0	Clustered	11	VC_975_0	11	0.9687	1	0.9687	0	0	0	1	Ref
KU535860	Unassigned	Unassigned	Unassigned	755_0	Clustered	9	VC_755_0	9	0.4489	1	0.4489	0	0	0	1	Ref
KU568494	Unassigned	Unassigned	Unassigned	217_0	Clustered	5	VC_217_0	5	0.069	0.99999998	0.069	0	0	0	1	Ref
KU574722	Unassigned	Unassigned	Unassigned	164_5	Clustered/Singleton	1	VC_164_5	1	0	1	0	0	0	0	1	Ref
KU577463	Unassigned	Unassigned	Unassigned	187_3	Clustered	9	VC_187_3	9	0.1971	1	0.1971	0	0	0	0.8611	Ref
KU578077	Unassigned	Unassigned	Unassigned	514_0	Clustered	75	VC_514_0	75	0.9402	1	0.9402	0	0	0	1	Ref
KU594606	Unassigned	Unassigned	Unassigned	433_6	Clustered/Singleton	1	VC_433_6	1	0	1	0	0	0	0	1	Ref
KU594607	Unassigned	Unassigned	Unassigned	433_4	Clustered	9	VC_433_4	9	0.1285	1	0.1285	0	0	0	1	Ref
KU595432	Unassigned	Unassigned	Unassigned	74_0	Clustered	15	VC_74_0	15	0.5204	1	0.5204	0	0	0	1	Ref

KU595433	Unassigned	Unassigned	Unassigned	74_0	Clustered	15	VC_74_0	15	0.5204	1	0.5204	0	0	0	1 Ref
KU595434	Unassigned	Unassigned	Unassigned	637_0	Clustered	115	VC_637_0	115	0.731	1	0.731	0	0	0	0.8764 Ref
KU599877	Unassigned	Unassigned	Unassigned	1157_0	Clustered	24	VC_1157_0	24	1	1	1	0	0	0	1 Ref
KU599878	Unassigned	Unassigned	Unassigned	1157_0	Clustered	24	VC_1157_0	24	1	1	1	0	0	0	1 Ref
KU599879	Unassigned	Unassigned	Unassigned	1157_0	Clustered	24	VC_1157_0	24	1	1	1	0	0	0	1 Ref
KU599880	Unassigned	Unassigned	Unassigned	1040_0	Clustered	23	VC_1040_0	23	0.9605	1	0.9605	0	0	0	1 Ref
KU599881	Unassigned	Unassigned	Unassigned	1040_0	Clustered	23	VC_1040_0	23	0.9605	1	0.9605	0	0	0	1 Ref
KU599882	Unassigned	Unassigned	Unassigned	1040_0	Clustered	23	VC_1040_0	23	0.9605	1	0.9605	0	0	0	1 Ref
KU599883	Unassigned	Unassigned	Unassigned	1040_0	Clustered	23	VC_1040_0	23	0.9605	1	0.9605	0	0	0	1 Ref
KU599884	Unassigned	Unassigned	Unassigned	1040_0	Clustered	23	VC_1040_0	23	0.9605	1	0.9605	0	0	0	1 Ref
KU599885	Unassigned	Unassigned	Unassigned	1040_0	Clustered	23	VC_1040_0	23	0.9605	1	0.9605	0	0	0	1 Ref
KU599886	Unassigned	Unassigned	Unassigned	1157_0	Clustered	24	VC_1157_0	24	1	1	1	0	0	0	1 Ref
KU599887	Unassigned	Unassigned	Unassigned	1023_0	Clustered	8	VC_1023_0	8	0.8574	1	0.8574	0	0	0	1 Ref
KU599888	Unassigned	Unassigned	Unassigned	1023_0	Clustered	8	VC_1023_0	8	0.8574	1	0.8574	0	0	0	1 Ref
KU599889	Unassigned	Unassigned	Unassigned	1023_0	Clustered	8	VC_1023_0	8	0.8574	1	0.8574	0	0	0	1 Ref
KU613353	Unassigned	Unassigned	Unassigned	225_0	Clustered	32	VC_225_0	32	0.848	1	0.848	0	0	0	0.996 Ref
KU640380	Unassigned	Unassigned	Unassigned	85_13	Clustered/Singleton	1	VC_85_13	1	0	1	0	0	0	0	1 Ref
KU647626	Unassigned	Unassigned	Unassigned	1179_0	Clustered	3	VC_1179_0	3	0.7588	0.96819872	0.7347	0	0	0	1 Ref
KU647627	Unassigned	Unassigned	Unassigned	1179_0	Clustered	3	VC_1179_0	3	0.7588	0.96819872	0.7347	0	0	0	1 Ref
KU647628	Unassigned	Unassigned	Unassigned	802_0	Clustered	17	VC_802_0	17	0.8025	1	0.8025	0	0	0	1 Ref
KU647629	Unassigned	Unassigned	Unassigned	1073_0	Clustered	24	VC_1073_0	24	0.8388	1	0.8388	0	0	0	1 Ref
KU666550	Unassigned	Unassigned	Unassigned	111_0	Clustered	91	VC_111_0	91	0.9587	1	0.9587	0	0	0	1 Ref
KU678389	Unassigned	Unassigned	Unassigned	256_0	Clustered	14	VC_256_0	14	0.2368	1	0.2368	0	0	0	1 Ref
KU678390	Unassigned	Unassigned	Unassigned	256_0	Clustered	14	VC_256_0	14	0.2368	1	0.2368	0	0	0	1 Ref
KU678391	Unassigned	Unassigned	Unassigned	256_0	Clustered	14	VC_256_0	14	0.2368	1	0.2368	0	0	0	1 Ref
KU678392	Unassigned	Unassigned	Unassigned	256_0	Clustered	14	VC_256_0	14	0.2368	1	0.2368	0	0	0	1 Ref
KU686192	Unassigned	Unassigned	Unassigned	433_0	Clustered	6	VC_433_0	6	0.0787	1	0.0787	0	0	0	1 Ref
KU686193	Unassigned	Unassigned	Unassigned	433_0	Clustered	6	VC_433_0	6	0.0787	1	0.0787	0	0	0	1 Ref
KU686194	Unassigned	Unassigned	Unassigned	433_0	Clustered	6	VC_433_0	6	0.0787	1	0.0787	0	0	0	1 Ref
KU686195	Unassigned	Unassigned	Unassigned	433_0	Clustered	6	VC_433_0	6	0.0787	1	0.0787	0	0	0	1 Ref
KU686196	Unassigned	Unassigned	Unassigned	433_0	Clustered	6	VC_433_0	6	0.0787	1	0.0787	0	0	0	1 Ref
KU686197	Unassigned	Unassigned	Unassigned	433_3	Clustered	3	VC_433_3	3	0.0292	0.9981281	0.0292	0	0	0	1 Ref
KU686198	Unassigned	Unassigned	Unassigned	433_3	Clustered	3	VC_433_3	3	0.0292	0.9981281	0.0292	0	0	0	1 Ref
KU686199	Unassigned	Unassigned	Unassigned	433_3	Clustered	3	VC_433_3	3	0.0292	0.9981281	0.0292	0	0	0	1 Ref
KU686200	Unassigned	Unassigned	Unassigned	433_1	Clustered	9	VC_433_1	9	0.1288	1	0.1288	0	0	0	0.8611 Ref
KU686201	Unassigned	Unassigned	Unassigned	433_1	Clustered	9	VC_433_1	9	0.1288	1	0.1288	0	0	0	0.8611 Ref
KU686202	Unassigned	Unassigned	Unassigned	433_1	Clustered	9	VC_433_1	9	0.1288	1	0.1288	0	0	0	0.8611 Ref
KU686204	Unassigned	Unassigned	Unassigned	432_15	Clustered	3	VC_432_15	3	0.0418	0.99855682	0.0417	0	0	0	1 Ref
KU686205	Unassigned	Unassigned	Unassigned	432_15	Clustered	3	VC_432_15	3	0.0418	0.99855682	0.0417	0	0	0	1 Ref
KU686206	Unassigned	Unassigned	Unassigned	432_15	Clustered	3	VC_432_15	3	0.0418	0.99855682	0.0417	0	0	0	1 Ref
KU686207	Unassigned	Unassigned	Unassigned	655_5	Clustered	3	VC_655_5	3	0.0166	0.9986063	0.0165	0	0	0	1 Ref
KU686208	Unassigned	Unassigned	Unassigned	655_5	Clustered	3	VC_655_5	3	0.0166	0.9986063	0.0165	0	0	0	1 Ref
KU686209	Unassigned	Unassigned	Unassigned	655_5	Clustered	3	VC_655_5	3	0.0166	0.9986063	0.0165	0	0	0	1 Ref
KU686211	Unassigned	Unassigned	Unassigned	433_10	Clustered/Singleton	1	VC_433_10	1	0	1	0	0	0	0	1 Ref
KU686212	Unassigned	Unassigned	Unassigned	657_0	Clustered	3	VC_657_0	3	0.0217	0.92528126	0.0201	0	0	0	1 Ref
KU686213	Unassigned	Unassigned	Unassigned	657_0	Clustered	3	VC_657_0	3	0.0217	0.92528126	0.0201	0	0	0	1 Ref
KU687347	Unassigned	Unassigned	Unassigned	385_0	Clustered	51	VC_385_0	51	0.8754	1	0.8754	0	0	0	1 Ref
KU687348	Unassigned	Unassigned	Unassigned	385_0	Clustered	51	VC_385_0	51	0.8754	1	0.8754	0	0	0	1 Ref
KU687349	Unassigned	Unassigned	Unassigned	451_0	Clustered	80	VC_451_0	80	0.9991	1	0.9991	0	0	0	1 Ref
KU687350	Unassigned	Unassigned	Unassigned	451_0	Clustered	80	VC_451_0	80	0.9991	1	0.9991	0	0	0	1 Ref
KU687351	Unassigned	Unassigned	Unassigned	451_0	Clustered	80	VC_451_0	80	0.9991	1	0.9991	0	0	0	1 Ref
KU695581	Unassigned	Unassigned	Unassigned	221_0	Clustered	22	VC_221_0	22	0.7447	1	0.7447	0	0	0	1 Ref
KU695582	Unassigned	Unassigned	Unassigned	223_0	Clustered	38	VC_223_0	38	0.9095	1	0.9095	0	0	0	1 Ref
KU708004	Unassigned	Unassigned	Unassigned	298_0	Clustered/Singleton	1	VC_298_0	1	0	1	0	0	0	0	1 Ref
KU716094	Unassigned	Unassigned	Unassigned	225_0	Clustered	32	VC_225_0	32	0.848	1	0.848	0	0	0	0.996 Ref
KU716095	Unassigned	Unassigned	Unassigned	225_0	Clustered	32	VC_225_0	32	0.848	1	0.848	0	0	0	0.996 Ref
KU726251	Unassigned	Unassigned	Unassigned	959_5	Clustered	19	VC_959_5	19	0.2626	1	0.2626	0	0	0	1 Ref
KU728633	Unassigned	Unassigned	Unassigned	686_1	Clustered	2	VC_686_1	2	0.2204	0.91327915	0.2013	0	0	0	1 Ref
KU737344	Unassigned	Unassigned	Unassigned	884_0	Clustered	28	VC_884_0	28	0.6211	1	0.6211	0	0	0	0.9841 Ref
KU737345	Unassigned	Unassigned	Unassigned	883_1	Clustered	12	VC_883_1	12	0.2441	1	0.2441	0	0	0	1 Ref
KU737346	Unassigned	Unassigned	Unassigned	883_0	Clustered	10	VC_883_0	10	0.2015	1	0.2015	0	0	0	1 Ref
KU737347	Unassigned	Unassigned	Unassigned	883_1	Clustered	12	VC_883_1	12	0.2441	1	0.2441	0	0	0	1 Ref
KU737348	Unassigned	Unassigned	Unassigned	884_0	Clustered	28	VC_884_0	28	0.6211	1	0.6211	0	0	0	0.9841 Ref
KU737349	Unassigned	Unassigned	Unassigned	884_0	Clustered	28	VC_884_0	28	0.6211	1	0.6211	0	0	0	0.9841 Ref
KU737350	Unassigned	Unassigned	Unassigned	884_0	Clustered	28	VC_884_0	28	0.6211	1	0.6211	0	0	0	0.9841 Ref
KU737351	Unassigned	Unassigned	Unassigned	884_0	Clustered	28	VC_884_0	28	0.6211	1	0.6211	0	0	0	0.9841 Ref
KU737352	Unassigned	Unassigned	Unassigned	884_0	Clustered	28	VC_884_0	28	0.6211	1	0.6211	0	0	0	0.9841 Ref
KU743887	Unassigned	Unassigned	Unassigned	423_0	Clustered	45	VC_423_0	45	0.9484	1	0.9484	0	0	0	1 Ref
KU747973	Unassigned	Unassigned	Unassigned	644_0	Clustered	5	VC_644_0	5	0.6649	0.99999975	0.6649	0	0	0	1 Ref
KU760857	Unassigned	Unassigned	Unassigned	247_0	Clustered	14	VC_247_0	14	0.9119	1	0.9119	0	0	0	0.967 Ref
KU761558	Unassigned	Unassigned	Unassigned	216_3	Clustered	8	VC_216_3	8	0.0701	1	0.0701	0	0	0	0.8571 Ref
KU761559	Unassigned	Unassigned	Unassigned	216_3	Clustered	8	VC_216_3	8	0.0701	1	0.0701	0	0	0	0.8571 Ref
KU761955	Unassigned	Unassigned	Unassigned	76_0	Clustered	37	VC_76_0	37	0.9403	1	0.9403	0	0	0	1 Ref
KU831549	Unassigned	Unassigned	Unassigned	817_0	Clustered	14	VC_817_0	14	0.6342	1	0.6342	0	0	0	1 Ref
KU836751	Unassigned	Unassigned	Unassigned	100_0	Clustered	15	VC_100_0	15	0.6536	1	0.6536	0	0	0	1 Ref
KU847400	Unassigned	Unassigned	Unassigned	884_1	Clustered	8	VC_884_1	8	0.1102	0.9989323	0.1101	0	0	0	0.75 Ref
KU848187	Unassigned	Unassigned	Unassigned	377_0	Clustered	3	VC_377_0	3	0.3906	0.99785056	0.3898	0	0	0	1 Ref
KU862660	Unassigned	Unassigned	Unassigned	957_1	Clustered/Singleton	1	VC_957_1	1	0	1	0	0	0	0	1 Ref
KU865302	Unassigned	Unassigned	Unassigned	520_40	Clustered	4	VC_520_40	4	0.0396	0.99998776	0.0396	0	0	0	1 Ref
KU865303	Unassigned	Unassigned	Unassigned	551_2	Clustered	17	VC_551_2	17	0.0973	1	0.0973	0	0	0	0.9779 Ref
KU867876	Unassigned	Unassigned	Unassigned	283_25	Clustered/Singleton	1	VC_283_25	1	0	1	0	0	0	0	1 Ref

KU867906	Unassigned	Unassigned	Unassigned	517_1	Clustered	108	VC_517_1	108	0.8483	1	0.8483	0	0	0	0.9955	Ref
KU867907	Unassigned	Unassigned	Unassigned	371_15	Clustered	44	VC_371_15	44	0.1391	1	0.1391	0	0	0	0.7474	Ref
KU878088	Unassigned	Unassigned	Unassigned	448_0	Clustered	4	VC_448_0	4	0.3059	0.99978853	0.3058	0	0	0	1	Ref
KU878967	Unassigned	Unassigned	Unassigned	28_1	Clustered	53	VC_28_1	53	0.7164	1	0.7164	0	0	0	0.9325	Ref
KU878968	Unassigned	Unassigned	Unassigned	290_5	Clustered	14	VC_290_5	14	0.1422	1	0.1422	0	0	0	0.9231	Ref
KU878969	Unassigned	Unassigned	Unassigned	290_5	Clustered	14	VC_290_5	14	0.1422	1	0.1422	0	0	0	0.9231	Ref
KU884561	Unassigned	Unassigned	Unassigned	612_0	Clustered	9	VC_612_0	9	0.9545	1	0.9545	0	0	0	1	Ref
KU884562	Unassigned	Unassigned	Unassigned	612_0	Clustered	9	VC_612_0	9	0.9545	1	0.9545	0	0	0	1	Ref
KU884563	Unassigned	Unassigned	Unassigned	612_0	Clustered	9	VC_612_0	9	0.9545	1	0.9545	0	0	0	1	Ref
KU884564	Unassigned	Unassigned	Unassigned	612_0	Clustered	9	VC_612_0	9	0.9545	1	0.9545	0	0	0	1	Ref
KU884565	Unassigned	Unassigned	Unassigned	612_0	Clustered	9	VC_612_0	9	0.9545	1	0.9545	0	0	0	1	Ref
KU885988	Unassigned	Unassigned	Unassigned	833_0	Clustered	16	VC_833_0	16	0.9351	1	0.9351	0	0	0	1	Ref
KU885989	Unassigned	Unassigned	Unassigned	833_0	Clustered	16	VC_833_0	16	0.9351	1	0.9351	0	0	0	1	Ref
KU885990	Unassigned	Unassigned	Unassigned	833_0	Clustered	16	VC_833_0	16	0.9351	1	0.9351	0	0	0	1	Ref
KU886222	Unassigned	Unassigned	Unassigned	489_1	Clustered	11	VC_489_1	11	0.4621	1	0.4621	0	0	0	1	Ref
KU886223	Unassigned	Unassigned	Unassigned	489_1	Clustered	11	VC_489_1	11	0.4621	1	0.4621	0	0	0	1	Ref
KU886224	Unassigned	Unassigned	Unassigned	489_1	Clustered	11	VC_489_1	11	0.4621	1	0.4621	0	0	0	1	Ref
KU886225	Unassigned	Unassigned	Unassigned	489_1	Clustered	11	VC_489_1	11	0.4621	1	0.4621	0	0	0	1	Ref
KU925172	Unassigned	Unassigned	Unassigned	283_8	Clustered/Singleton	1	VC_283_8	1	0	1	0	0	0	0	1	Ref
KU927491	Unassigned	Unassigned	Unassigned	28_1	Clustered	53	VC_28_1	53	0.7164	1	0.7164	0	0	0	0.9325	Ref
KU927492	Unassigned	Unassigned	Unassigned	28_1	Clustered	53	VC_28_1	53	0.7164	1	0.7164	0	0	0	0.9325	Ref
KU927493	Unassigned	Unassigned	Unassigned	45_0	Clustered	12	VC_45_0	12	0.35	1	0.35	0	0	0	1	Ref
KU927494	Unassigned	Unassigned	Unassigned	28_1	Clustered	53	VC_28_1	53	0.7164	1	0.7164	0	0	0	0.9325	Ref
KU927495	Unassigned	Unassigned	Unassigned	28_1	Clustered	53	VC_28_1	53	0.7164	1	0.7164	0	0	0	0.9325	Ref
KU927496	Unassigned	Unassigned	Unassigned	28_1	Clustered	53	VC_28_1	53	0.7164	1	0.7164	0	0	0	0.9325	Ref
KU927497	Unassigned	Unassigned	Unassigned	191_19	Clustered	12	VC_191_19	12	0.0655	1	0.0655	0	0	0	0.8636	Ref
KU927498	Unassigned	Unassigned	Unassigned	28_1	Clustered	53	VC_28_1	53	0.7164	1	0.7164	0	0	0	0.9325	Ref
KU927499	Unassigned	Unassigned	Unassigned	392_0	Clustered	126	VC_392_0	126	0.9621	1	0.9621	0	0	0	1	Ref
KU927500	Unassigned	Unassigned	Unassigned	592_0	Clustered	50	VC_592_0	50	0.7314	1	0.7314	0	0	0	1	Ref
KU935726	Unassigned	Unassigned	Unassigned	521_0	Clustered	37	VC_521_0	37	0.912	1	0.912	0	0	0	1	Ref
KU935727	Unassigned	Unassigned	Unassigned	521_0	Clustered	37	VC_521_0	37	0.912	1	0.912	0	0	0	1	Ref
KU935728	Unassigned	Unassigned	Unassigned	521_0	Clustered	37	VC_521_0	37	0.912	1	0.912	0	0	0	1	Ref
KU935729	Unassigned	Unassigned	Unassigned	521_0	Clustered	37	VC_521_0	37	0.912	1	0.912	0	0	0	1	Ref
KU935730	Unassigned	Unassigned	Unassigned	521_0	Clustered	37	VC_521_0	37	0.912	1	0.912	0	0	0	1	Ref
KU935731	Unassigned	Unassigned	Unassigned	521_0	Clustered	37	VC_521_0	37	0.912	1	0.912	0	0	0	1	Ref
KU946962	Unassigned	Unassigned	Unassigned	452_0	Clustered	66	VC_452_0	66	0.9248	1	0.9248	0	0	0	1	Ref
KU948710	Unassigned	Unassigned	Unassigned	838_0	Clustered	29	VC_838_0	29	0.8611	1	0.8611	0	0	0	1	Ref
KU951146	Unassigned	Unassigned	Unassigned	637_0	Clustered	115	VC_637_0	115	0.731	1	0.731	0	0	0	0.8764	Ref
KU951147	Unassigned	Unassigned	Unassigned	637_0	Clustered	115	VC_637_0	115	0.731	1	0.731	0	0	0	0.8764	Ref
KU958700	Unassigned	Unassigned	Unassigned	588_0	Clustered	2	VC_588_0	2	0.0832	0.94395095	0.0785	0	0	0	1	Ref
KU963245	Unassigned	Unassigned	Unassigned	868_0	Clustered	25	VC_868_0	25	0.5791	1	0.5791	0	0	0	1	Ref
KU963246	Unassigned	Unassigned	Unassigned	1175_0	Clustered	22	VC_1175_0	22	0.4562	1	0.4562	0	0	0	1	Ref
KU963247	Unassigned	Unassigned	Unassigned	1175_0	Clustered	22	VC_1175_0	22	0.4562	1	0.4562	0	0	0	1	Ref
KU963249	Unassigned	Unassigned	Unassigned	525_1	Clustered	5	VC_525_1	5	0.0485	0.99999947	0.0485	0	0	0	0.9	Ref
KU963250	Unassigned	Unassigned	Unassigned	1093_0	Clustered	40	VC_1093_0	40	0.8754	1	0.8754	0	0	0	1	Ref
KU963251	Unassigned	Unassigned	Unassigned	1176_0	Clustered	41	VC_1176_0	41	0.9558	1	0.9558	0	0	0	0.9976	Ref
KU963252	Unassigned	Unassigned	Unassigned	1177_0	Clustered	14	VC_1177_0	14	0.7847	1	0.7847	0	0	0	0.989	Ref
KU963253	Unassigned	Unassigned	Unassigned	1095_0	Clustered	16	VC_1095_0	16	0.458	1	0.458	0	0	0	1	Ref
KU963254	Unassigned	Unassigned	Unassigned	1176_0	Clustered	41	VC_1176_0	41	0.9558	1	0.9558	0	0	0	0.9976	Ref
KU963255	Unassigned	Unassigned	Unassigned	525_0	Clustered	25	VC_525_0	25	0.4597	1	0.4597	0	0	0	1	Ref
KU963256	Unassigned	Unassigned	Unassigned	522_0	Clustered	3	VC_522_0	3	0.0836	0.9985266	0.0835	0	0	0	1	Ref
KU963257	Unassigned	Unassigned	Unassigned	525_0	Clustered	25	VC_525_0	25	0.4597	1	0.4597	0	0	0	1	Ref
KU963258	Unassigned	Unassigned	Unassigned	869_0	Clustered	9	VC_869_0	9	0.2624	1	0.2624	0	0	0	1	Ref
KU963259	Unassigned	Unassigned	Unassigned	1176_0	Clustered	41	VC_1176_0	41	0.9558	1	0.9558	0	0	0	0.9976	Ref
KU963260	Unassigned	Unassigned	Unassigned	919_0	Clustered	39	VC_919_0	39	0.9069	1	0.9069	0	0	0	1	Ref
KU963261	Unassigned	Unassigned	Unassigned	954_0	Clustered	13	VC_954_0	13	0.863	1	0.863	0	0	0	1	Ref
KU963262	Unassigned	Unassigned	Unassigned	869_0	Clustered	9	VC_869_0	9	0.2624	1	0.2624	0	0	0	1	Ref
KU963263	Unassigned	Unassigned	Unassigned	525_1	Clustered	5	VC_525_1	5	0.0485	0.99999947	0.0485	0	0	0	0.9	Ref
KU981050	Unassigned	Unassigned	Unassigned	269_0	Clustered	3	VC_269_0	3	0.1948	0.99308177	0.1934	0	0	0	1	Ref
KU984914	Unassigned	Unassigned	Unassigned	514_0	Clustered	75	VC_514_0	75	0.9402	1	0.9402	0	0	0	1	Ref
KU985090	Unassigned	Unassigned	Unassigned	519_0	Clustered	39	VC_519_0	39	0.8156	1	0.8156	0	0	0	1	Ref
KU985091	Unassigned	Unassigned	Unassigned	514_0	Clustered	75	VC_514_0	75	0.9402	1	0.9402	0	0	0	1	Ref
KU985092	Unassigned	Unassigned	Unassigned	517_1	Clustered	108	VC_517_1	108	0.8483	1	0.8483	0	0	0	0.9955	Ref
KU985093	Unassigned	Unassigned	Unassigned	216_6	Clustered	42	VC_216_6	42	0.473	1	0.473	0	0	0	0.8351	Ref
KU985094	Unassigned	Unassigned	Unassigned	517_1	Clustered	108	VC_517_1	108	0.8483	1	0.8483	0	0	0	0.9955	Ref
KU985095	Unassigned	Unassigned	Unassigned	216_6	Clustered	42	VC_216_6	42	0.473	1	0.473	0	0	0	0.8351	Ref
KU985096	Unassigned	Unassigned	Unassigned	223_0	Clustered	38	VC_223_0	38	0.9095	1	0.9095	0	0	0	1	Ref
KU992911	Unassigned	Unassigned	Unassigned	96_0	Clustered	30	VC_96_0	30	0.718	1	0.718	0	0	0	1	Ref
KU997639	Unassigned	Unassigned	Unassigned	888_0	Clustered	28	VC_888_0	28	0.5497	1	0.5497	0	0	0	1	Ref
KU998234	Unassigned	Unassigned	Unassigned	1178_0	Clustered	24	VC_1178_0	24	0.9524	1	0.9524	0	0	0	1	Ref
KU998235	Unassigned	Unassigned	Unassigned	1177_0	Clustered	14	VC_1177_0	14	0.7847	1	0.7847	0	0	0	0.989	Ref
KU998236	Unassigned	Unassigned	Unassigned	1176_0	Clustered	41	VC_1176_0	41	0.9558	1	0.9558	0	0	0	0.9976	Ref
KU998237	Unassigned	Unassigned	Unassigned	406_0	Clustered	11	VC_406_0	11	0.9449	1	0.9449	0	0	0	1	Ref
KU998238	Unassigned	Unassigned	Unassigned	406_0	Clustered	11	VC_406_0	11	0.9449	1	0.9449	0	0	0	1	Ref
KU998239	Unassigned	Unassigned	Unassigned	919_0	Clustered	39	VC_919_0	39	0.9069	1	0.9069	0	0	0	1	Ref
KU998240	Unassigned	Unassigned	Unassigned	866_1	Clustered	20	VC_866_1	20	0.4814	1	0.4814	0	0	0	1	Ref
KU998241	Unassigned	Unassigned	Unassigned	868_0	Clustered	25	VC_868_0	25	0.5791	1	0.5791	0	0	0	1	Ref
KU998242	Unassigned	Unassigned	Unassigned	869_0	Clustered	9	VC_869_0	9	0.2624	1	0.2624	0	0	0	1	Ref
KU998243	Unassigned	Unassigned	Unassigned	869_0	Clustered	9	VC_869_0	9	0.2624	1	0.2624	0	0	0	1	Ref
KU998244	Unassigned	Unassigned	Unassigned	1181_1	Clustered	10	VC_1181_1	10	0.5828	1	0.5828	0	0	0	1	Ref
KU998245	Unassigned	Unassigned	Unassigned	1181_0	Clustered	2	VC_1181_0	2	0.0461	0.95126432	0.0439	0	0	0	1	Ref

KU998246	Unassigned	Unassigned	Unassigned	1181_1	Clustered	10	VC_1181_1	10	0.5828	1	0.5828	0	0	0	1	Ref
KU998247	Unassigned	Unassigned	Unassigned	1181_1	Clustered	10	VC_1181_1	10	0.5828	1	0.5828	0	0	0	1	Ref
KU998248	Unassigned	Unassigned	Unassigned	1176_0	Clustered	41	VC_1176_0	41	0.9558	1	0.9558	0	0	0	0.9976	Ref
KU998249	Unassigned	Unassigned	Unassigned	224_0	Clustered	19	VC_224_0	19	0.9416	1	0.9416	0	0	0	1	Ref
KU998250	Unassigned	Unassigned	Unassigned	224_0	Clustered	19	VC_224_0	19	0.9416	1	0.9416	0	0	0	1	Ref
KU998251	Unassigned	Unassigned	Unassigned	224_0	Clustered	19	VC_224_0	19	0.9416	1	0.9416	0	0	0	1	Ref
KU998252	Unassigned	Unassigned	Unassigned	870_0	Clustered	5	VC_870_0	5	0.424	0.99999997	0.424	0	0	0	1	Ref
KU998253	Unassigned	Unassigned	Unassigned	870_0	Clustered	5	VC_870_0	5	0.424	0.99999997	0.424	0	0	0	1	Ref
KU998254	Unassigned	Unassigned	Unassigned	870_0	Clustered	5	VC_870_0	5	0.424	0.99999997	0.424	0	0	0	1	Ref
KU998255	Unassigned	Unassigned	Unassigned	949_0	Clustered	10	VC_949_0	10	0.5977	1	0.5977	0	0	0	1	Ref
KU998256	Unassigned	Unassigned	Unassigned	949_0	Clustered	10	VC_949_0	10	0.5977	1	0.5977	0	0	0	1	Ref
KX009778	Unassigned	Unassigned	Unassigned	283_139	Clustered/Singleton	1	VC_283_139	1	0	1	0	0	0	0	1	Ref
KX011028	Unassigned	Unassigned	Unassigned	149_27	Clustered/Singleton	1	VC_149_27	1	0	1	0	0	0	0	1	Ref
KX011169	Unassigned	Unassigned	Unassigned	884_1	Clustered	8	VC_884_1	8	0.1102	0.9989323	0.1101	0	0	0	0.75	Ref
KX015770	Unassigned	Unassigned	Unassigned	584_0	Clustered	28	VC_584_0	28	0.7704	1	0.7704	0	0	0	1	Ref
KX015771	Unassigned	Unassigned	Unassigned	584_0	Clustered	28	VC_584_0	28	0.7704	1	0.7704	0	0	0	1	Ref
KX017520	Unassigned	Unassigned	Unassigned	461_0	Clustered	96	VC_461_0	96	0.9307	1	0.9307	0	0	0	0.9575	Ref
KX017521	Unassigned	Unassigned	Unassigned	191_19	Clustered	12	VC_191_19	12	0.0655	1	0.0655	0	0	0	0.8636	Ref
KX058879	Unassigned	Unassigned	Unassigned	5_0	Clustered	30	VC_5_0	30	0.9647	1	0.9647	0	0	0	1	Ref
KX077179	Unassigned	Unassigned	Unassigned	182_0	Clustered	5	VC_182_0	5	0.6184	0.99999967	0.6184	0	0	0	1	Ref
KX077892	Unassigned	Unassigned	Unassigned	1187_0	Clustered	3	VC_1187_0	3	0.924	0.92554266	0.8552	0	0	0	1	Ref
KX077894	Unassigned	Unassigned	Unassigned	317_2	Clustered	20	VC_317_2	20	0.1976	1	0.1976	0	0	0	0.6842	Ref
KX077896	Unassigned	Unassigned	Unassigned	632_0	Clustered	37	VC_632_0	37	0.581	1	0.581	0	0	0	0.9234	Ref
KX078568	Unassigned	Unassigned	Unassigned	390_0	Clustered	4	VC_390_0	4	0.0468	0.9999882	0.0468	0	0	0	1	Ref
KX078569	Unassigned	Unassigned	Unassigned	288_1	Clustered/Singleton	1	VC_288_1	1	0	1	0	0	0	0	1	Ref
KX098389	Unassigned	Unassigned	Unassigned	779_2	Clustered	5	VC_779_2	5	0.0876	0.99999997	0.0876	0	0	0	1	Ref
KX098390	Unassigned	Unassigned	Unassigned	779_2	Clustered	5	VC_779_2	5	0.0876	0.99999997	0.0876	0	0	0	1	Ref
KX098391	Unassigned	Unassigned	Unassigned	779_2	Clustered	5	VC_779_2	5	0.0876	0.99999997	0.0876	0	0	0	1	Ref
KX119174	Unassigned	Unassigned	Unassigned	99_0	Clustered	32	VC_99_0	32	0.8828	1	0.8828	0	0	0	1	Ref
KX119175	Unassigned	Unassigned	Unassigned	99_0	Clustered	32	VC_99_0	32	0.8828	1	0.8828	0	0	0	1	Ref
KX119176	Unassigned	Unassigned	Unassigned	99_0	Clustered	32	VC_99_0	32	0.8828	1	0.8828	0	0	0	1	Ref
KX119177	Unassigned	Unassigned	Unassigned	99_0	Clustered	32	VC_99_0	32	0.8828	1	0.8828	0	0	0	1	Ref
KX119182	Unassigned	Unassigned	Unassigned	99_0	Clustered	32	VC_99_0	32	0.8828	1	0.8828	0	0	0	1	Ref
KX119188	Unassigned	Unassigned	Unassigned	99_0	Clustered	32	VC_99_0	32	0.8828	1	0.8828	0	0	0	1	Ref
KX119189	Unassigned	Unassigned	Unassigned	99_0	Clustered	32	VC_99_0	32	0.8828	1	0.8828	0	0	0	1	Ref
KX119190	Unassigned	Unassigned	Unassigned	99_0	Clustered	32	VC_99_0	32	0.8828	1	0.8828	0	0	0	1	Ref
KX119191	Unassigned	Unassigned	Unassigned	99_0	Clustered	32	VC_99_0	32	0.8828	1	0.8828	0	0	0	1	Ref
KX119192	Unassigned	Unassigned	Unassigned	99_0	Clustered	32	VC_99_0	32	0.8828	1	0.8828	0	0	0	1	Ref
KX119193	Unassigned	Unassigned	Unassigned	99_0	Clustered	32	VC_99_0	32	0.8828	1	0.8828	0	0	0	1	Ref
KX119194	Unassigned	Unassigned	Unassigned	99_0	Clustered	32	VC_99_0	32	0.8828	1	0.8828	0	0	0	1	Ref
KX119195	Unassigned	Unassigned	Unassigned	99_0	Clustered	32	VC_99_0	32	0.8828	1	0.8828	0	0	0	1	Ref
KX119196	Unassigned	Unassigned	Unassigned	99_0	Clustered	32	VC_99_0	32	0.8828	1	0.8828	0	0	0	1	Ref
KX119197	Unassigned	Unassigned	Unassigned	99_0	Clustered	32	VC_99_0	32	0.8828	1	0.8828	0	0	0	1	Ref
KX119198	Unassigned	Unassigned	Unassigned	99_0	Clustered	32	VC_99_0	32	0.8828	1	0.8828	0	0	0	1	Ref
KX119199	Unassigned	Unassigned	Unassigned	99_0	Clustered	32	VC_99_0	32	0.8828	1	0.8828	0	0	0	1	Ref
KX119200	Unassigned	Unassigned	Unassigned	99_0	Clustered	32	VC_99_0	32	0.8828	1	0.8828	0	0	0	1	Ref
KX119201	Unassigned	Unassigned	Unassigned	99_0	Clustered	32	VC_99_0	32	0.8828	1	0.8828	0	0	0	1	Ref
KX119202	Unassigned	Unassigned	Unassigned	99_0	Clustered	32	VC_99_0	32	0.8828	1	0.8828	0	0	0	1	Ref
KX119203	Unassigned	Unassigned	Unassigned	99_0	Clustered	32	VC_99_0	32	0.8828	1	0.8828	0	0	0	1	Ref
KX119204	Unassigned	Unassigned	Unassigned	99_0	Clustered	32	VC_99_0	32	0.8828	1	0.8828	0	0	0	1	Ref
KX119205	Unassigned	Unassigned	Unassigned	99_0	Clustered	32	VC_99_0	32	0.8828	1	0.8828	0	0	0	1	Ref
KX119206	Unassigned	Unassigned	Unassigned	99_0	Clustered	32	VC_99_0	32	0.8828	1	0.8828	0	0	0	1	Ref
KX129925	Unassigned	Unassigned	Unassigned	465_0	Clustered	7	VC_465_0	7	0.3322	1	0.3322	0	0	0	1	Ref
KX130726	Unassigned	Unassigned	Unassigned	385_0	Clustered	51	VC_385_0	51	0.8754	1	0.8754	0	0	0	1	Ref
KX130727	Unassigned	Unassigned	Unassigned	668_0	Clustered	25	VC_668_0	25	0.5539	1	0.5539	0	0	0	0.96	Ref
KX130861	Unassigned	Unassigned	Unassigned	283_114	Clustered/Singleton	1	VC_283_114	1	0	1	0	0	0	0	1	Ref
KX130862	Unassigned	Unassigned	Unassigned	283_125	Clustered/Singleton	1	VC_283_125	1	0	1	0	0	0	0	1	Ref
KX130863	Unassigned	Unassigned	Unassigned	190_0	Clustered	2	VC_190_0	2	0.0087	0.95763473	0.0083	0	0	0	1	Ref
KX130864	Unassigned	Unassigned	Unassigned	283_58	Clustered/Singleton	1	VC_283_58	1	0	1	0	0	0	0	1	Ref
KX130865	Unassigned	Unassigned	Unassigned	286_16	Clustered/Singleton	1	VC_286_16	1	0	1	0	0	0	0	1	Ref
KX130960	Unassigned	Unassigned	Unassigned	456_0	Clustered	62	VC_456_0	62	0.9026	1	0.9026	0	0	0	1	Ref
KX147096	Unassigned	Unassigned	Unassigned	132_1	Clustered	17	VC_132_1	17	0.1738	1	0.1738	0	0	0	0.9853	Ref
KX147229	Unassigned	Unassigned	Unassigned	884_0	Clustered	28	VC_884_0	28	0.6211	1	0.6211	0	0	0	0.9841	Ref
KX156152	Unassigned	Unassigned	Unassigned	884_0	Clustered	28	VC_884_0	28	0.6211	1	0.6211	0	0	0	0.9841	Ref
KX159477	Unassigned	Unassigned	Unassigned	939_0	Clustered	15	VC_939_0	15	1	0	0	0	0	0	1	Ref
KX160204	Unassigned	Unassigned	Unassigned	262_0	Clustered	5	VC_262_0	5	0.0724	0.99999989	0.0724	0	0	0	1	Ref
KX160206	Unassigned	Unassigned	Unassigned	268_0	Clustered	7	VC_268_0	7	0.3427	1	0.3427	0	0	0	1	Ref
KX160209	Unassigned	Unassigned	Unassigned	268_0	Clustered	7	VC_268_0	7	0.3427	1	0.3427	0	0	0	1	Ref
KX160210	Unassigned	Unassigned	Unassigned	268_0	Clustered	7	VC_268_0	7	0.3427	1	0.3427	0	0	0	1	Ref
KX160211	Unassigned	Unassigned	Unassigned	268_0	Clustered	7	VC_268_0	7	0.3427	1	0.3427	0	0	0	1	Ref
KX160219	Unassigned	Unassigned	Unassigned	262_0	Clustered	5	VC_262_0	5	0.0724	0.99999989	0.0724	0	0	0	1	Ref
KX160220	Unassigned	Unassigned	Unassigned	262_0	Clustered	5	VC_262_0	5	0.0724	0.99999989	0.0724	0	0	0	1	Ref
KX171208	Unassigned	Unassigned	Unassigned	79_0	Clustered	64	VC_79_0	64	0.4185	1	0.4185	0	0	0	0.7649	Ref
KX171209	Unassigned	Unassigned	Unassigned	838_0	Clustered	29	VC_838_0	29	0.8611	1	0.8611	0	0	0	1	Ref
KX171210	Unassigned	Unassigned	Unassigned	838_0	Clustered	29	VC_838_0	29	0.8611	1	0.8611	0	0	0	1	Ref
KX171211	Unassigned	Unassigned	Unassigned	133_9	Clustered	3	VC_133_9	3	0.0099	0.98457109	0.0098	0	0	0	1	Ref
KX171212	Unassigned	Unassigned	Unassigned	151_0	Clustered	6	VC_151_0	6	0.0735	1	0.0735	0	0	0	1	Ref
KX171213	Unassigned	Unassigned	Unassigned	151_0	Clustered	6	VC_151_0	6	0.0735	1	0.0735	0	0	0	1	Ref
KX179905	Unassigned	Unassigned	Unassigned	52_0	Clustered	87	VC_52_0	87	0.9858	1	0.9858	0	0	0	1	Ref
KX181651	Unassigned	Unassigned	Unassigned	177_0	Clustered	5	VC_177_0	5	0.7468	0.99885646	0.746	0	0	0	1	Ref
KX190833	Unassigned	Unassigned	Unassigned	731_0	Clustered	2	VC_731_0	2	0.3697	0.94979793	0.3512	0	0	0	1	Ref

KX190834	Unassigned	Unassigned	Unassigned	829_0	Clustered	5	VC_829_0	5	0.8821	0.99999832	0.8821	0	0	0	1	Ref
KX190835	Unassigned	Unassigned	Unassigned	20_0	Clustered	5	VC_20_0	5	0.4387	0.99999934	0.4387	0	0	0	1	Ref
KX196154	Unassigned	Unassigned	Unassigned	498_0	Clustered	8	VC_498_0	8	0.3987	1	0.3987	0	0	0	1	Ref
KX198613	Unassigned	Unassigned	Unassigned	166_0	Clustered	26	VC_166_0	26	0.7923	1	0.7923	0	0	0	1	Ref
KX198614	Unassigned	Unassigned	Unassigned	614_0	Clustered	5	VC_614_0	5	0.2395	0.99999997	0.2395	0	0	0	1	Ref
KX198615	Unassigned	Unassigned	Unassigned	904_1	Clustered	4	VC_904_1	4	0.1486	0.99997723	0.1486	0	0	0	1	Ref
KX211991	Unassigned	Unassigned	Unassigned	111_0	Clustered	91	VC_111_0	91	0.9587	1	0.9587	0	0	0	1	Ref
KX223815	Unassigned	Unassigned	Unassigned	634_1	Clustered	6	VC_634_1	6	0.4228	1	0.4228	0	0	0	1	Ref
KX227757	Unassigned	Unassigned	Unassigned	654_0	Clustered	4	VC_654_0	4	0.2897	0.99998263	0.2897	0	0	0	1	Ref
KX227758	Unassigned	Unassigned	Unassigned	652_0	Clustered	4	VC_652_0	4	0.4743	0.99998076	0.4743	0	0	0	1	Ref
KX227759	Unassigned	Unassigned	Unassigned	652_0	Clustered	4	VC_652_0	4	0.4743	0.99998076	0.4743	0	0	0	1	Ref
KX227760	Unassigned	Unassigned	Unassigned	654_0	Clustered	4	VC_654_0	4	0.2897	0.99998263	0.2897	0	0	0	1	Ref
KX229736	Unassigned	Unassigned	Unassigned	844_0	Clustered	27	VC_844_0	27	0.9385	1	0.9385	0	0	0	1	Ref
KX231828	Unassigned	Unassigned	Unassigned	125_0	Clustered	17	VC_125_0	17	0.9244	1	0.9244	0	0	0	1	Ref
KX231829	Unassigned	Unassigned	Unassigned	476_0	Clustered	19	VC_476_0	19	0.7814	1	0.7814	0	0	0	1	Ref
KX232515	Unassigned	Unassigned	Unassigned	7_0	Clustered	28	VC_7_0	28	0.3916	1	0.3916	0	0	0	0.9947	Ref
KX236333	Unassigned	Unassigned	Unassigned	844_0	Clustered	27	VC_844_0	27	0.9385	1	0.9385	0	0	0	1	Ref
KX237514	Unassigned	Unassigned	Unassigned	111_0	Clustered	91	VC_111_0	91	0.9587	1	0.9587	0	0	0	1	Ref
KX237515	Unassigned	Unassigned	Unassigned	937_0	Clustered	40	VC_937_0	40	0.6809	1	0.6809	0	0	0	0.9359	Ref
KX237516	Unassigned	Unassigned	Unassigned	123_0	Clustered	23	VC_123_0	23	0.817	1	0.817	0	0	0	1	Ref
KX241618	Unassigned	Unassigned	Unassigned	169_0	Clustered	20	VC_169_0	20	0.7529	1	0.7529	0	0	0	1	Ref
KX245012	Unassigned	Unassigned	Unassigned	133_30	Clustered	2	VC_133_30	2	0.0052	0.87738871	0.0046	0	0	0	1	Ref
KX245013	Unassigned	Unassigned	Unassigned	637_0	Clustered	115	VC_637_0	115	0.731	1	0.731	0	0	0	0.8764	Ref
KX245890	Unassigned	Unassigned	Unassigned	669_1	Clustered	10	VC_669_1	10	0.1268	1	0.1268	0	0	0	0.9778	Ref
KX257490	Unassigned	Unassigned	Unassigned	1026_0	Clustered	2	VC_1026_0	2	0.2977	0.81445332	0.2425	0	0	0	1	Ref
KX258185	Unassigned	Unassigned	Unassigned	491_0	Clustered	36	VC_491_0	36	0.5336	1	0.5336	0	0	0	0.9714	Ref
KX266300	Unassigned	Unassigned	Unassigned	360_0	Clustered	358	VC_360_0	358	0.9615	1	0.9615	0	0	0	1	Ref
KX266301	Unassigned	Unassigned	Unassigned	360_0	Clustered	358	VC_360_0	358	0.9615	1	0.9615	0	0	0	1	Ref
KX266302	Unassigned	Unassigned	Unassigned	360_0	Clustered	358	VC_360_0	358	0.9615	1	0.9615	0	0	0	1	Ref
KX266303	Unassigned	Unassigned	Unassigned	360_0	Clustered	358	VC_360_0	358	0.9615	1	0.9615	0	0	0	1	Ref
KX266304	Unassigned	Unassigned	Unassigned	360_0	Clustered	358	VC_360_0	358	0.9615	1	0.9615	0	0	0	1	Ref
KX266305	Unassigned	Unassigned	Unassigned	360_0	Clustered	358	VC_360_0	358	0.9615	1	0.9615	0	0	0	1	Ref
KX266306	Unassigned	Unassigned	Unassigned	360_0	Clustered	358	VC_360_0	358	0.9615	1	0.9615	0	0	0	1	Ref
KX266307	Unassigned	Unassigned	Unassigned	360_0	Clustered	358	VC_360_0	358	0.9615	1	0.9615	0	0	0	1	Ref
KX266308	Unassigned	Unassigned	Unassigned	360_0	Clustered	358	VC_360_0	358	0.9615	1	0.9615	0	0	0	1	Ref
KX266309	Unassigned	Unassigned	Unassigned	360_0	Clustered	358	VC_360_0	358	0.9615	1	0.9615	0	0	0	1	Ref
KX266310	Unassigned	Unassigned	Unassigned	360_0	Clustered	358	VC_360_0	358	0.9615	1	0.9615	0	0	0	1	Ref
KX266311	Unassigned	Unassigned	Unassigned	360_0	Clustered	358	VC_360_0	358	0.9615	1	0.9615	0	0	0	1	Ref
KX266312	Unassigned	Unassigned	Unassigned	360_0	Clustered	358	VC_360_0	358	0.9615	1	0.9615	0	0	0	1	Ref
KX266313	Unassigned	Unassigned	Unassigned	360_0	Clustered	358	VC_360_0	358	0.9615	1	0.9615	0	0	0	1	Ref
KX266314	Unassigned	Unassigned	Unassigned	360_0	Clustered	358	VC_360_0	358	0.9615	1	0.9615	0	0	0	1	Ref
KX266315	Unassigned	Unassigned	Unassigned	360_0	Clustered	358	VC_360_0	358	0.9615	1	0.9615	0	0	0	1	Ref
KX266316	Unassigned	Unassigned	Unassigned	360_0	Clustered	358	VC_360_0	358	0.9615	1	0.9615	0	0	0	1	Ref
KX266317	Unassigned	Unassigned	Unassigned	360_0	Clustered	358	VC_360_0	358	0.9615	1	0.9615	0	0	0	1	Ref
KX266318	Unassigned	Unassigned	Unassigned	360_0	Clustered	358	VC_360_0	358	0.9615	1	0.9615	0	0	0	1	Ref
KX266319	Unassigned	Unassigned	Unassigned	360_0	Clustered	358	VC_360_0	358	0.9615	1	0.9615	0	0	0	1	Ref
KX266320	Unassigned	Unassigned	Unassigned	360_0	Clustered	358	VC_360_0	358	0.9615	1	0.9615	0	0	0	1	Ref
KX266321	Unassigned	Unassigned	Unassigned	360_0	Clustered	358	VC_360_0	358	0.9615	1	0.9615	0	0	0	1	Ref
KX266322	Unassigned	Unassigned	Unassigned	360_0	Clustered	358	VC_360_0	358	0.9615	1	0.9615	0	0	0	1	Ref
KX266323	Unassigned	Unassigned	Unassigned	360_0	Clustered	358	VC_360_0	358	0.9615	1	0.9615	0	0	0	1	Ref
KX266324	Unassigned	Unassigned	Unassigned	360_0	Clustered	358	VC_360_0	358	0.9615	1	0.9615	0	0	0	1	Ref
KX266325	Unassigned	Unassigned	Unassigned	360_0	Clustered	358	VC_360_0	358	0.9615	1	0.9615	0	0	0	1	Ref
KX266326	Unassigned	Unassigned	Unassigned	360_0	Clustered	358	VC_360_0	358	0.9615	1	0.9615	0	0	0	1	Ref
KX266327	Unassigned	Unassigned	Unassigned	360_0	Clustered	358	VC_360_0	358	0.9615	1	0.9615	0	0	0	1	Ref
KX266328	Unassigned	Unassigned	Unassigned	360_0	Clustered	358	VC_360_0	358	0.9615	1	0.9615	0	0	0	1	Ref
KX266329	Unassigned	Unassigned	Unassigned	360_0	Clustered	358	VC_360_0	358	0.9615	1	0.9615	0	0	0	1	Ref
KX266330	Unassigned	Unassigned	Unassigned	360_0	Clustered	358	VC_360_0	358	0.9615	1	0.9615	0	0	0	1	Ref
KX266331	Unassigned	Unassigned	Unassigned	360_0	Clustered	358	VC_360_0	358	0.9615	1	0.9615	0	0	0	1	Ref
KX266332	Unassigned	Unassigned	Unassigned	360_0	Clustered	358	VC_360_0	358	0.9615	1	0.9615	0	0	0	1	Ref
KX266333	Unassigned	Unassigned	Unassigned	360_0	Clustered	358	VC_360_0	358	0.9615	1	0.9615	0	0	0	1	Ref
KX266334	Unassigned	Unassigned	Unassigned	360_0	Clustered	358	VC_360_0	358	0.9615	1	0.9615	0	0	0	1	Ref
KX266335	Unassigned	Unassigned	Unassigned	360_0	Clustered	358	VC_360_0	358	0.9615	1	0.9615	0	0	0	1	Ref
KX266336	Unassigned	Unassigned	Unassigned	360_0	Clustered	358	VC_360_0	358	0.9615	1	0.9615	0	0	0	1	Ref
KX266337	Unassigned	Unassigned	Unassigned	360_0	Clustered	358	VC_360_0	358	0.9615	1	0.9615	0	0	0	1	Ref
KX266338	Unassigned	Unassigned	Unassigned	360_0	Clustered	358	VC_360_0	358	0.9615	1	0.9615	0	0	0	1	Ref
KX266339	Unassigned	Unassigned	Unassigned	360_0	Clustered	358	VC_360_0	358	0.9615	1	0.9615	0	0	0	1	Ref
KX266340	Unassigned	Unassigned	Unassigned	360_0	Clustered	358	VC_360_0	358	0.9615	1	0.9615	0	0	0	1	Ref
KX266341	Unassigned	Unassigned	Unassigned	360_0	Clustered	358	VC_360_0	358	0.9615	1	0.9615	0	0	0	1	Ref
KX266342	Unassigned	Unassigned	Unassigned	360_0	Clustered	358	VC_360_0	358	0.9615	1	0.9615	0	0	0	1	Ref
KX266343	Unassigned	Unassigned	Unassigned	360_0	Clustered	358	VC_360_0	358	0.9615	1	0.9615	0	0	0	1	Ref
KX266344	Unassigned	Unassigned	Unassigned	360_0	Clustered	358	VC_360_0	358	0.9615	1	0.9615	0	0	0	1	Ref
KX266345	Unassigned	Unassigned	Unassigned	360_0	Clustered	358	VC_360_0	358	0.9615	1	0.9615	0	0	0	1	Ref
KX266346	Unassigned	Unassigned	Unassigned	360_0	Clustered	358	VC_360_0	358	0.9615	1	0.9615	0	0	0	1	Ref
KX266347	Unassigned	Unassigned	Unassigned	360_0	Clustered	358	VC_360_0	358	0.9615	1	0.9615	0	0	0	1	Ref
KX266348	Unassigned	Unassigned	Unassigned	360_0	Clustered	358	VC_360_0	358	0.9615	1	0.9615	0	0	0	1	Ref
KX266349	Unassigned	Unassigned	Unassigned	360_0	Clustered	358	VC_360_0	358	0.9615	1	0.9615	0	0	0	1	Ref
KX266350	Unassigned	Unassigned	Unassigned	360_0	Clustered	358	VC_360_0	358	0.9615	1	0.9615	0	0	0	1	Ref
KX266351	Unassigned	Unassigned	Unassigned	360_0	Clustered	358	VC_360_0	358	0.9615	1	0.9615	0	0	0	1	Ref
KX266352	Unassigned	Unassigned	Unassigned	360_0	Clustered	358	VC_360_0	358	0.9615	1	0.9615	0	0	0	1	Ref
KX266353	Unassigned	Unassigned	Unassigned	360_0	Clustered	358	VC_360_0	358	0.9615	1	0.9615	0	0	0	1	Ref
KX266354	Unassigned	Unassigned	Unassigned	360_0	Clustered	358	VC_360_0	358	0.9615	1	0.9615	0	0	0	1	Ref

KX266355	Unassigned	Unassigned	Unassigned	360_0	Clustered	358	VC_360_0	358	0.9615	1	0.9615	0	0	0	1	Ref
KX266356	Unassigned	Unassigned	Unassigned	360_0	Clustered	358	VC_360_0	358	0.9615	1	0.9615	0	0	0	1	Ref
KX266357	Unassigned	Unassigned	Unassigned	360_0	Clustered	358	VC_360_0	358	0.9615	1	0.9615	0	0	0	1	Ref
KX266358	Unassigned	Unassigned	Unassigned	360_0	Clustered	358	VC_360_0	358	0.9615	1	0.9615	0	0	0	1	Ref
KX266359	Unassigned	Unassigned	Unassigned	360_0	Clustered	358	VC_360_0	358	0.9615	1	0.9615	0	0	0	1	Ref
KX266360	Unassigned	Unassigned	Unassigned	360_0	Clustered	358	VC_360_0	358	0.9615	1	0.9615	0	0	0	1	Ref
KX266361	Unassigned	Unassigned	Unassigned	360_0	Clustered	358	VC_360_0	358	0.9615	1	0.9615	0	0	0	1	Ref
KX266362	Unassigned	Unassigned	Unassigned	360_0	Clustered	358	VC_360_0	358	0.9615	1	0.9615	0	0	0	1	Ref
KX266363	Unassigned	Unassigned	Unassigned	360_0	Clustered	358	VC_360_0	358	0.9615	1	0.9615	0	0	0	1	Ref
KX266364	Unassigned	Unassigned	Unassigned	360_0	Clustered	358	VC_360_0	358	0.9615	1	0.9615	0	0	0	1	Ref
KX266365	Unassigned	Unassigned	Unassigned	360_0	Clustered	358	VC_360_0	358	0.9615	1	0.9615	0	0	0	1	Ref
KX266366	Unassigned	Unassigned	Unassigned	360_0	Clustered	358	VC_360_0	358	0.9615	1	0.9615	0	0	0	1	Ref
KX266367	Unassigned	Unassigned	Unassigned	360_0	Clustered	358	VC_360_0	358	0.9615	1	0.9615	0	0	0	1	Ref
KX266368	Unassigned	Unassigned	Unassigned	360_0	Clustered	358	VC_360_0	358	0.9615	1	0.9615	0	0	0	1	Ref
KX266369	Unassigned	Unassigned	Unassigned	360_0	Clustered	358	VC_360_0	358	0.9615	1	0.9615	0	0	0	1	Ref
KX266370	Unassigned	Unassigned	Unassigned	360_0	Clustered	358	VC_360_0	358	0.9615	1	0.9615	0	0	0	1	Ref
KX266371	Unassigned	Unassigned	Unassigned	360_0	Clustered	358	VC_360_0	358	0.9615	1	0.9615	0	0	0	1	Ref
KX266372	Unassigned	Unassigned	Unassigned	360_0	Clustered	358	VC_360_0	358	0.9615	1	0.9615	0	0	0	1	Ref
KX266373	Unassigned	Unassigned	Unassigned	360_0	Clustered	358	VC_360_0	358	0.9615	1	0.9615	0	0	0	1	Ref
KX266374	Unassigned	Unassigned	Unassigned	360_0	Clustered	358	VC_360_0	358	0.9615	1	0.9615	0	0	0	1	Ref
KX266375	Unassigned	Unassigned	Unassigned	360_0	Clustered	358	VC_360_0	358	0.9615	1	0.9615	0	0	0	1	Ref
KX266376	Unassigned	Unassigned	Unassigned	360_0	Clustered	358	VC_360_0	358	0.9615	1	0.9615	0	0	0	1	Ref
KX266377	Unassigned	Unassigned	Unassigned	360_0	Clustered	358	VC_360_0	358	0.9615	1	0.9615	0	0	0	1	Ref
KX266378	Unassigned	Unassigned	Unassigned	360_0	Clustered	358	VC_360_0	358	0.9615	1	0.9615	0	0	0	1	Ref
KX266379	Unassigned	Unassigned	Unassigned	360_0	Clustered	358	VC_360_0	358	0.9615	1	0.9615	0	0	0	1	Ref
KX266380	Unassigned	Unassigned	Unassigned	360_0	Clustered	358	VC_360_0	358	0.9615	1	0.9615	0	0	0	1	Ref
KX266381	Unassigned	Unassigned	Unassigned	360_0	Clustered	358	VC_360_0	358	0.9615	1	0.9615	0	0	0	1	Ref
KX266382	Unassigned	Unassigned	Unassigned	360_0	Clustered	358	VC_360_0	358	0.9615	1	0.9615	0	0	0	1	Ref
KX266383	Unassigned	Unassigned	Unassigned	360_0	Clustered	358	VC_360_0	358	0.9615	1	0.9615	0	0	0	1	Ref
KX266384	Unassigned	Unassigned	Unassigned	360_0	Clustered	358	VC_360_0	358	0.9615	1	0.9615	0	0	0	1	Ref
KX266385	Unassigned	Unassigned	Unassigned	360_0	Clustered	358	VC_360_0	358	0.9615	1	0.9615	0	0	0	1	Ref
KX266386	Unassigned	Unassigned	Unassigned	360_0	Clustered	358	VC_360_0	358	0.9615	1	0.9615	0	0	0	1	Ref
KX266387	Unassigned	Unassigned	Unassigned	360_0	Clustered	358	VC_360_0	358	0.9615	1	0.9615	0	0	0	1	Ref
KX266388	Unassigned	Unassigned	Unassigned	360_0	Clustered	358	VC_360_0	358	0.9615	1	0.9615	0	0	0	1	Ref
KX266389	Unassigned	Unassigned	Unassigned	360_0	Clustered	358	VC_360_0	358	0.9615	1	0.9615	0	0	0	1	Ref
KX266390	Unassigned	Unassigned	Unassigned	360_0	Clustered	358	VC_360_0	358	0.9615	1	0.9615	0	0	0	1	Ref
KX266391	Unassigned	Unassigned	Unassigned	360_0	Clustered	358	VC_360_0	358	0.9615	1	0.9615	0	0	0	1	Ref
KX266392	Unassigned	Unassigned	Unassigned	360_0	Clustered	358	VC_360_0	358	0.9615	1	0.9615	0	0	0	1	Ref
KX266393	Unassigned	Unassigned	Unassigned	360_0	Clustered	358	VC_360_0	358	0.9615	1	0.9615	0	0	0	1	Ref
KX266394	Unassigned	Unassigned	Unassigned	360_0	Clustered	358	VC_360_0	358	0.9615	1	0.9615	0	0	0	1	Ref
KX266395	Unassigned	Unassigned	Unassigned	360_0	Clustered	358	VC_360_0	358	0.9615	1	0.9615	0	0	0	1	Ref
KX266396	Unassigned	Unassigned	Unassigned	360_0	Clustered	358	VC_360_0	358	0.9615	1	0.9615	0	0	0	1	Ref
KX266397	Unassigned	Unassigned	Unassigned	360_0	Clustered	358	VC_360_0	358	0.9615	1	0.9615	0	0	0	1	Ref
KX266398	Unassigned	Unassigned	Unassigned	360_0	Clustered	358	VC_360_0	358	0.9615	1	0.9615	0	0	0	1	Ref
KX266399	Unassigned	Unassigned	Unassigned	360_0	Clustered	358	VC_360_0	358	0.9615	1	0.9615	0	0	0	1	Ref
KX266400	Unassigned	Unassigned	Unassigned	360_0	Clustered	358	VC_360_0	358	0.9615	1	0.9615	0	0	0	1	Ref
KX266401	Unassigned	Unassigned	Unassigned	360_0	Clustered	358	VC_360_0	358	0.9615	1	0.9615	0	0	0	1	Ref
KX266402	Unassigned	Unassigned	Unassigned	360_0	Clustered	358	VC_360_0	358	0.9615	1	0.9615	0	0	0	1	Ref
KX266403	Unassigned	Unassigned	Unassigned	360_0	Clustered	358	VC_360_0	358	0.9615	1	0.9615	0	0	0	1	Ref
KX266404	Unassigned	Unassigned	Unassigned	360_0	Clustered	358	VC_360_0	358	0.9615	1	0.9615	0	0	0	1	Ref
KX266405	Unassigned	Unassigned	Unassigned	360_0	Clustered	358	VC_360_0	358	0.9615	1	0.9615	0	0	0	1	Ref
KX266406	Unassigned	Unassigned	Unassigned	360_0	Clustered	358	VC_360_0	358	0.9615	1	0.9615	0	0	0	1	Ref
KX266407	Unassigned	Unassigned	Unassigned	360_0	Clustered	358	VC_360_0	358	0.9615	1	0.9615	0	0	0	1	Ref
KX266408	Unassigned	Unassigned	Unassigned	360_0	Clustered	358	VC_360_0	358	0.9615	1	0.9615	0	0	0	1	Ref
KX266409	Unassigned	Unassigned	Unassigned	360_0	Clustered	358	VC_360_0	358	0.9615	1	0.9615	0	0	0	1	Ref
KX266410	Unassigned	Unassigned	Unassigned	360_0	Clustered	358	VC_360_0	358	0.9615	1	0.9615	0	0	0	1	Ref
KX266411	Unassigned	Unassigned	Unassigned	360_0	Clustered	358	VC_360_0	358	0.9615	1	0.9615	0	0	0	1	Ref
KX266412	Unassigned	Unassigned	Unassigned	360_0	Clustered	358	VC_360_0	358	0.9615	1	0.9615	0	0	0	1	Ref
KX266413	Unassigned	Unassigned	Unassigned	360_0	Clustered	358	VC_360_0	358	0.9615	1	0.9615	0	0	0	1	Ref
KX266414	Unassigned	Unassigned	Unassigned	360_0	Clustered	358	VC_360_0	358	0.9615	1	0.9615	0	0	0	1	Ref
KX266415	Unassigned	Unassigned	Unassigned	360_0	Clustered	358	VC_360_0	358	0.9615	1	0.9615	0	0	0	1	Ref
KX266416	Unassigned	Unassigned	Unassigned	360_0	Clustered	358	VC_360_0	358	0.9615	1	0.9615	0	0	0	1	Ref
KX266417	Unassigned	Unassigned	Unassigned	360_0	Clustered	358	VC_360_0	358	0.9615	1	0.9615	0	0	0	1	Ref
KX266418	Unassigned	Unassigned	Unassigned	360_0	Clustered	358	VC_360_0	358	0.9615	1	0.9615	0	0	0	1	Ref
KX266419	Unassigned	Unassigned	Unassigned	360_0	Clustered	358	VC_360_0	358	0.9615	1	0.9615	0	0	0	1	Ref
KX266420	Unassigned	Unassigned	Unassigned	360_0	Clustered	358	VC_360_0	358	0.9615	1	0.9615	0	0	0	1	Ref
KX266421	Unassigned	Unassigned	Unassigned	360_0	Clustered	358	VC_360_0	358	0.9615	1	0.9615	0	0	0	1	Ref
KX266422	Unassigned	Unassigned	Unassigned	360_0	Clustered	358	VC_360_0	358	0.9615	1	0.9615	0	0	0	1	Ref
KX266423	Unassigned	Unassigned	Unassigned	360_0	Clustered	358	VC_360_0	358	0.9615	1	0.9615	0	0	0	1	Ref
KX266424	Unassigned	Unassigned	Unassigned	360_0	Clustered	358	VC_360_0	358	0.9615	1	0.9615	0	0	0	1	Ref
KX266425	Unassigned	Unassigned	Unassigned	360_0	Clustered	358	VC_360_0	358	0.9615	1	0.9615	0	0	0	1	Ref
KX266426	Unassigned	Unassigned	Unassigned	360_0	Clustered	358	VC_360_0	358	0.9615	1	0.9615	0	0	0	1	Ref
KX266427	Unassigned	Unassigned	Unassigned	360_0	Clustered	358	VC_360_0	358	0.9615	1	0.9615	0	0	0	1	Ref
KX266428	Unassigned	Unassigned	Unassigned	360_0	Clustered	358	VC_360_0	358	0.9615	1	0.9615	0	0	0	1	Ref
KX266429	Unassigned	Unassigned	Unassigned	360_0	Clustered	358	VC_360_0	358	0.9615	1	0.9615	0	0	0	1	Ref
KX266430	Unassigned	Unassigned	Unassigned	360_0	Clustered	358	VC_360_0	358	0.9615	1	0.9615	0	0	0	1	Ref
KX266431	Unassigned	Unassigned	Unassigned	360_0	Clustered	358	VC_360_0	358	0.9615	1	0.9615	0	0	0	1	Ref
KX266432	Unassigned	Unassigned	Unassigned	360_0	Clustered	358	VC_360_0	358	0.9615	1	0.9615	0	0	0	1	Ref
KX266433	Unassigned	Unassigned	Unassigned	360_0	Clustered	358	VC_360_0	358	0.9615	1	0.9615	0	0	0	1	Ref
KX266434	Unassigned	Unassigned	Unassigned	360_0	Clustered	358	VC_360_0	358	0.9615	1	0.9615	0	0	0	1	Ref
KX266435	Unassigned	Unassigned	Unassigned	360_0	Clustered	358	VC_360_0	358	0.9615	1	0.9615	0	0	0	1	Ref

KX266436	Unassigned	Unassigned	Unassigned	360_0	Clustered	358	VC_360_0	358	0.9615	1	0.9615	0	0	0	1	Ref
KX266437	Unassigned	Unassigned	Unassigned	360_0	Clustered	358	VC_360_0	358	0.9615	1	0.9615	0	0	0	1	Ref
KX266438	Unassigned	Unassigned	Unassigned	360_0	Clustered	358	VC_360_0	358	0.9615	1	0.9615	0	0	0	1	Ref
KX266439	Unassigned	Unassigned	Unassigned	360_0	Clustered	358	VC_360_0	358	0.9615	1	0.9615	0	0	0	1	Ref
KX266440	Unassigned	Unassigned	Unassigned	360_0	Clustered	358	VC_360_0	358	0.9615	1	0.9615	0	0	0	1	Ref
KX266441	Unassigned	Unassigned	Unassigned	360_0	Clustered	358	VC_360_0	358	0.9615	1	0.9615	0	0	0	1	Ref
KX266442	Unassigned	Unassigned	Unassigned	360_0	Clustered	358	VC_360_0	358	0.9615	1	0.9615	0	0	0	1	Ref
KX266443	Unassigned	Unassigned	Unassigned	360_0	Clustered	358	VC_360_0	358	0.9615	1	0.9615	0	0	0	1	Ref
KX266444	Unassigned	Unassigned	Unassigned	360_0	Clustered	358	VC_360_0	358	0.9615	1	0.9615	0	0	0	1	Ref
KX266445	Unassigned	Unassigned	Unassigned	360_0	Clustered	358	VC_360_0	358	0.9615	1	0.9615	0	0	0	1	Ref
KX266446	Unassigned	Unassigned	Unassigned	360_0	Clustered	358	VC_360_0	358	0.9615	1	0.9615	0	0	0	1	Ref
KX266447	Unassigned	Unassigned	Unassigned	360_0	Clustered	358	VC_360_0	358	0.9615	1	0.9615	0	0	0	1	Ref
KX266448	Unassigned	Unassigned	Unassigned	360_0	Clustered	358	VC_360_0	358	0.9615	1	0.9615	0	0	0	1	Ref
KX266449	Unassigned	Unassigned	Unassigned	360_0	Clustered	358	VC_360_0	358	0.9615	1	0.9615	0	0	0	1	Ref
KX266450	Unassigned	Unassigned	Unassigned	360_0	Clustered	358	VC_360_0	358	0.9615	1	0.9615	0	0	0	1	Ref
KX266451	Unassigned	Unassigned	Unassigned	360_0	Clustered	358	VC_360_0	358	0.9615	1	0.9615	0	0	0	1	Ref
KX266452	Unassigned	Unassigned	Unassigned	360_0	Clustered	358	VC_360_0	358	0.9615	1	0.9615	0	0	0	1	Ref
KX266453	Unassigned	Unassigned	Unassigned	360_0	Clustered	358	VC_360_0	358	0.9615	1	0.9615	0	0	0	1	Ref
KX266454	Unassigned	Unassigned	Unassigned	360_0	Clustered	358	VC_360_0	358	0.9615	1	0.9615	0	0	0	1	Ref
KX266455	Unassigned	Unassigned	Unassigned	360_0	Clustered	358	VC_360_0	358	0.9615	1	0.9615	0	0	0	1	Ref
KX266456	Unassigned	Unassigned	Unassigned	360_0	Clustered	358	VC_360_0	358	0.9615	1	0.9615	0	0	0	1	Ref
KX266457	Unassigned	Unassigned	Unassigned	360_0	Clustered	358	VC_360_0	358	0.9615	1	0.9615	0	0	0	1	Ref
KX266458	Unassigned	Unassigned	Unassigned	360_0	Clustered	358	VC_360_0	358	0.9615	1	0.9615	0	0	0	1	Ref
KX266459	Unassigned	Unassigned	Unassigned	360_0	Clustered	358	VC_360_0	358	0.9615	1	0.9615	0	0	0	1	Ref
KX266460	Unassigned	Unassigned	Unassigned	360_0	Clustered	358	VC_360_0	358	0.9615	1	0.9615	0	0	0	1	Ref
KX266461	Unassigned	Unassigned	Unassigned	360_0	Clustered	358	VC_360_0	358	0.9615	1	0.9615	0	0	0	1	Ref
KX266462	Unassigned	Unassigned	Unassigned	360_0	Clustered	358	VC_360_0	358	0.9615	1	0.9615	0	0	0	1	Ref
KX266463	Unassigned	Unassigned	Unassigned	360_0	Clustered	358	VC_360_0	358	0.9615	1	0.9615	0	0	0	1	Ref
KX266464	Unassigned	Unassigned	Unassigned	360_0	Clustered	358	VC_360_0	358	0.9615	1	0.9615	0	0	0	1	Ref
KX266465	Unassigned	Unassigned	Unassigned	360_0	Clustered	358	VC_360_0	358	0.9615	1	0.9615	0	0	0	1	Ref
KX266466	Unassigned	Unassigned	Unassigned	360_0	Clustered	358	VC_360_0	358	0.9615	1	0.9615	0	0	0	1	Ref
KX266467	Unassigned	Unassigned	Unassigned	360_0	Clustered	358	VC_360_0	358	0.9615	1	0.9615	0	0	0	1	Ref
KX266468	Unassigned	Unassigned	Unassigned	360_0	Clustered	358	VC_360_0	358	0.9615	1	0.9615	0	0	0	1	Ref
KX266469	Unassigned	Unassigned	Unassigned	360_0	Clustered	358	VC_360_0	358	0.9615	1	0.9615	0	0	0	1	Ref
KX266470	Unassigned	Unassigned	Unassigned	360_0	Clustered	358	VC_360_0	358	0.9615	1	0.9615	0	0	0	1	Ref
KX266471	Unassigned	Unassigned	Unassigned	360_0	Clustered	358	VC_360_0	358	0.9615	1	0.9615	0	0	0	1	Ref
KX266472	Unassigned	Unassigned	Unassigned	360_0	Clustered	358	VC_360_0	358	0.9615	1	0.9615	0	0	0	1	Ref
KX266473	Unassigned	Unassigned	Unassigned	360_0	Clustered	358	VC_360_0	358	0.9615	1	0.9615	0	0	0	1	Ref
KX266474	Unassigned	Unassigned	Unassigned	360_0	Clustered	358	VC_360_0	358	0.9615	1	0.9615	0	0	0	1	Ref
KX266475	Unassigned	Unassigned	Unassigned	360_0	Clustered	358	VC_360_0	358	0.9615	1	0.9615	0	0	0	1	Ref
KX266476	Unassigned	Unassigned	Unassigned	360_0	Clustered	358	VC_360_0	358	0.9615	1	0.9615	0	0	0	1	Ref
KX266477	Unassigned	Unassigned	Unassigned	360_0	Clustered	358	VC_360_0	358	0.9615	1	0.9615	0	0	0	1	Ref
KX266478	Unassigned	Unassigned	Unassigned	360_0	Clustered	358	VC_360_0	358	0.9615	1	0.9615	0	0	0	1	Ref
KX266479	Unassigned	Unassigned	Unassigned	360_0	Clustered	358	VC_360_0	358	0.9615	1	0.9615	0	0	0	1	Ref
KX266480	Unassigned	Unassigned	Unassigned	360_0	Clustered	358	VC_360_0	358	0.9615	1	0.9615	0	0	0	1	Ref
KX266481	Unassigned	Unassigned	Unassigned	360_0	Clustered	358	VC_360_0	358	0.9615	1	0.9615	0	0	0	1	Ref
KX266482	Unassigned	Unassigned	Unassigned	360_0	Clustered	358	VC_360_0	358	0.9615	1	0.9615	0	0	0	1	Ref
KX266483	Unassigned	Unassigned	Unassigned	360_0	Clustered	358	VC_360_0	358	0.9615	1	0.9615	0	0	0	1	Ref
KX266484	Unassigned	Unassigned	Unassigned	360_0	Clustered	358	VC_360_0	358	0.9615	1	0.9615	0	0	0	1	Ref
KX266485	Unassigned	Unassigned	Unassigned	360_0	Clustered	358	VC_360_0	358	0.9615	1	0.9615	0	0	0	1	Ref
KX266486	Unassigned	Unassigned	Unassigned	360_0	Clustered	358	VC_360_0	358	0.9615	1	0.9615	0	0	0	1	Ref
KX266487	Unassigned	Unassigned	Unassigned	360_0	Clustered	358	VC_360_0	358	0.9615	1	0.9615	0	0	0	1	Ref
KX266488	Unassigned	Unassigned	Unassigned	360_0	Clustered	358	VC_360_0	358	0.9615	1	0.9615	0	0	0	1	Ref
KX266489	Unassigned	Unassigned	Unassigned	360_0	Clustered	358	VC_360_0	358	0.9615	1	0.9615	0	0	0	1	Ref
KX266490	Unassigned	Unassigned	Unassigned	360_0	Clustered	358	VC_360_0	358	0.9615	1	0.9615	0	0	0	1	Ref
KX266491	Unassigned	Unassigned	Unassigned	360_0	Clustered	358	VC_360_0	358	0.9615	1	0.9615	0	0	0	1	Ref
KX266492	Unassigned	Unassigned	Unassigned	360_0	Clustered	358	VC_360_0	358	0.9615	1	0.9615	0	0	0	1	Ref
KX266493	Unassigned	Unassigned	Unassigned	360_0	Clustered	358	VC_360_0	358	0.9615	1	0.9615	0	0	0	1	Ref
KX266494	Unassigned	Unassigned	Unassigned	360_0	Clustered	358	VC_360_0	358	0.9615	1	0.9615	0	0	0	1	Ref
KX266495	Unassigned	Unassigned	Unassigned	360_0	Clustered	358	VC_360_0	358	0.9615	1	0.9615	0	0	0	1	Ref
KX266496	Unassigned	Unassigned	Unassigned	360_0	Clustered	358	VC_360_0	358	0.9615	1	0.9615	0	0	0	1	Ref
KX266497	Unassigned	Unassigned	Unassigned	360_0	Clustered	358	VC_360_0	358	0.9615	1	0.9615	0	0	0	1	Ref
KX266498	Unassigned	Unassigned	Unassigned	360_0	Clustered	358	VC_360_0	358	0.9615	1	0.9615	0	0	0	1	Ref
KX266499	Unassigned	Unassigned	Unassigned	360_0	Clustered	358	VC_360_0	358	0.9615	1	0.9615	0	0	0	1	Ref
KX266500	Unassigned	Unassigned	Unassigned	360_0	Clustered	358	VC_360_0	358	0.9615	1	0.9615	0	0	0	1	Ref
KX266501	Unassigned	Unassigned	Unassigned	360_0	Clustered	358	VC_360_0	358	0.9615	1	0.9615	0	0	0	1	Ref
KX266502	Unassigned	Unassigned	Unassigned	360_0	Clustered	358	VC_360_0	358	0.9615	1	0.9615	0	0	0	1	Ref
KX266503	Unassigned	Unassigned	Unassigned	360_0	Clustered	358	VC_360_0	358	0.9615	1	0.9615	0	0	0	1	Ref
KX266504	Unassigned	Unassigned	Unassigned	360_0	Clustered	358	VC_360_0	358	0.9615	1	0.9615	0	0	0	1	Ref
KX266505	Unassigned	Unassigned	Unassigned	360_0	Clustered	358	VC_360_0	358	0.9615	1	0.9615	0	0	0	1	Ref
KX266506	Unassigned	Unassigned	Unassigned	360_0	Clustered	358	VC_360_0	358	0.9615	1	0.9615	0	0	0	1	Ref
KX266507	Unassigned	Unassigned	Unassigned	360_0	Clustered	358	VC_360_0	358	0.9615	1	0.9615	0	0	0	1	Ref
KX266508	Unassigned	Unassigned	Unassigned	360_0	Clustered	358	VC_360_0	358	0.9615	1	0.9615	0	0	0	1	Ref
KX266509	Unassigned	Unassigned	Unassigned	360_0	Clustered	358	VC_360_0	358	0.9615	1	0.9615	0	0	0	1	Ref
KX266510	Unassigned	Unassigned	Unassigned	360_0	Clustered	358	VC_360_0	358	0.9615	1	0.9615	0	0	0	1	Ref
KX266511	Unassigned	Unassigned	Unassigned	360_0	Clustered	358	VC_360_0	358	0.9615	1	0.9615	0	0	0	1	Ref
KX266512	Unassigned	Unassigned	Unassigned	360_0	Clustered	358	VC_360_0	358	0.9615	1	0.9615	0	0	0	1	Ref
KX266513	Unassigned	Unassigned	Unassigned	360_0	Clustered	358	VC_360_0	358	0.9615	1	0.9615	0	0	0	1	Ref
KX266514	Unassigned	Unassigned	Unassigned	360_0	Clustered	358	VC_360_0	358	0.9615	1	0.9615	0	0	0	1	Ref
KX266515	Unassigned	Unassigned	Unassigned	360_0	Clustered	358	VC_360_0	358	0.9615	1	0.9615	0	0	0	1	Ref
KX266516	Unassigned	Unassigned	Unassigned	360_0	Clustered	358	VC_360_0	358	0.9615	1	0.9615	0	0	0	1	Ref

KX266517	Unassigned	Unassigned	Unassigned	360_0	Clustered	358	VC_360_0	358	0.9615	1	0.9615	0	0	0	1	Ref
KX266518	Unassigned	Unassigned	Unassigned	360_0	Clustered	358	VC_360_0	358	0.9615	1	0.9615	0	0	0	1	Ref
KX266519	Unassigned	Unassigned	Unassigned	360_0	Clustered	358	VC_360_0	358	0.9615	1	0.9615	0	0	0	1	Ref
KX266520	Unassigned	Unassigned	Unassigned	360_0	Clustered	358	VC_360_0	358	0.9615	1	0.9615	0	0	0	1	Ref
KX266521	Unassigned	Unassigned	Unassigned	360_0	Clustered	358	VC_360_0	358	0.9615	1	0.9615	0	0	0	1	Ref
KX266522	Unassigned	Unassigned	Unassigned	360_0	Clustered	358	VC_360_0	358	0.9615	1	0.9615	0	0	0	1	Ref
KX266523	Unassigned	Unassigned	Unassigned	360_0	Clustered	358	VC_360_0	358	0.9615	1	0.9615	0	0	0	1	Ref
KX266524	Unassigned	Unassigned	Unassigned	360_0	Clustered	358	VC_360_0	358	0.9615	1	0.9615	0	0	0	1	Ref
KX266525	Unassigned	Unassigned	Unassigned	360_0	Clustered	358	VC_360_0	358	0.9615	1	0.9615	0	0	0	1	Ref
KX266526	Unassigned	Unassigned	Unassigned	360_0	Clustered	358	VC_360_0	358	0.9615	1	0.9615	0	0	0	1	Ref
KX266527	Unassigned	Unassigned	Unassigned	360_0	Clustered	358	VC_360_0	358	0.9615	1	0.9615	0	0	0	1	Ref
KX266528	Unassigned	Unassigned	Unassigned	360_0	Clustered	358	VC_360_0	358	0.9615	1	0.9615	0	0	0	1	Ref
KX266529	Unassigned	Unassigned	Unassigned	360_0	Clustered	358	VC_360_0	358	0.9615	1	0.9615	0	0	0	1	Ref
KX266530	Unassigned	Unassigned	Unassigned	360_0	Clustered	358	VC_360_0	358	0.9615	1	0.9615	0	0	0	1	Ref
KX266531	Unassigned	Unassigned	Unassigned	360_0	Clustered	358	VC_360_0	358	0.9615	1	0.9615	0	0	0	1	Ref
KX266532	Unassigned	Unassigned	Unassigned	360_0	Clustered	358	VC_360_0	358	0.9615	1	0.9615	0	0	0	1	Ref
KX266533	Unassigned	Unassigned	Unassigned	360_0	Clustered	358	VC_360_0	358	0.9615	1	0.9615	0	0	0	1	Ref
KX266534	Unassigned	Unassigned	Unassigned	360_0	Clustered	358	VC_360_0	358	0.9615	1	0.9615	0	0	0	1	Ref
KX266535	Unassigned	Unassigned	Unassigned	360_0	Clustered	358	VC_360_0	358	0.9615	1	0.9615	0	0	0	1	Ref
KX266536	Unassigned	Unassigned	Unassigned	360_0	Clustered	358	VC_360_0	358	0.9615	1	0.9615	0	0	0	1	Ref
KX266537	Unassigned	Unassigned	Unassigned	360_0	Clustered	358	VC_360_0	358	0.9615	1	0.9615	0	0	0	1	Ref
KX266538	Unassigned	Unassigned	Unassigned	360_0	Clustered	358	VC_360_0	358	0.9615	1	0.9615	0	0	0	1	Ref
KX266539	Unassigned	Unassigned	Unassigned	360_0	Clustered	358	VC_360_0	358	0.9615	1	0.9615	0	0	0	1	Ref
KX266540	Unassigned	Unassigned	Unassigned	360_0	Clustered	358	VC_360_0	358	0.9615	1	0.9615	0	0	0	1	Ref
KX266541	Unassigned	Unassigned	Unassigned	360_0	Clustered	358	VC_360_0	358	0.9615	1	0.9615	0	0	0	1	Ref
KX266542	Unassigned	Unassigned	Unassigned	360_0	Clustered	358	VC_360_0	358	0.9615	1	0.9615	0	0	0	1	Ref
KX266543	Unassigned	Unassigned	Unassigned	360_0	Clustered	358	VC_360_0	358	0.9615	1	0.9615	0	0	0	1	Ref
KX266544	Unassigned	Unassigned	Unassigned	360_0	Clustered	358	VC_360_0	358	0.9615	1	0.9615	0	0	0	1	Ref
KX266545	Unassigned	Unassigned	Unassigned	360_0	Clustered	358	VC_360_0	358	0.9615	1	0.9615	0	0	0	1	Ref
KX266546	Unassigned	Unassigned	Unassigned	360_0	Clustered	358	VC_360_0	358	0.9615	1	0.9615	0	0	0	1	Ref
KX266547	Unassigned	Unassigned	Unassigned	360_0	Clustered	358	VC_360_0	358	0.9615	1	0.9615	0	0	0	1	Ref
KX266548	Unassigned	Unassigned	Unassigned	360_0	Clustered	358	VC_360_0	358	0.9615	1	0.9615	0	0	0	1	Ref
KX266549	Unassigned	Unassigned	Unassigned	360_0	Clustered	358	VC_360_0	358	0.9615	1	0.9615	0	0	0	1	Ref
KX266550	Unassigned	Unassigned	Unassigned	360_0	Clustered	358	VC_360_0	358	0.9615	1	0.9615	0	0	0	1	Ref
KX266551	Unassigned	Unassigned	Unassigned	360_0	Clustered	358	VC_360_0	358	0.9615	1	0.9615	0	0	0	1	Ref
KX266552	Unassigned	Unassigned	Unassigned	360_0	Clustered	358	VC_360_0	358	0.9615	1	0.9615	0	0	0	1	Ref
KX266553	Unassigned	Unassigned	Unassigned	360_0	Clustered	358	VC_360_0	358	0.9615	1	0.9615	0	0	0	1	Ref
KX266554	Unassigned	Unassigned	Unassigned	360_0	Clustered	358	VC_360_0	358	0.9615	1	0.9615	0	0	0	1	Ref
KX266555	Unassigned	Unassigned	Unassigned	360_0	Clustered	358	VC_360_0	358	0.9615	1	0.9615	0	0	0	1	Ref
KX266556	Unassigned	Unassigned	Unassigned	360_0	Clustered	358	VC_360_0	358	0.9615	1	0.9615	0	0	0	1	Ref
KX266557	Unassigned	Unassigned	Unassigned	360_0	Clustered	358	VC_360_0	358	0.9615	1	0.9615	0	0	0	1	Ref
KX266558	Unassigned	Unassigned	Unassigned	360_0	Clustered	358	VC_360_0	358	0.9615	1	0.9615	0	0	0	1	Ref
KX266559	Unassigned	Unassigned	Unassigned	360_0	Clustered	358	VC_360_0	358	0.9615	1	0.9615	0	0	0	1	Ref
KX266560	Unassigned	Unassigned	Unassigned	360_0	Clustered	358	VC_360_0	358	0.9615	1	0.9615	0	0	0	1	Ref
KX266561	Unassigned	Unassigned	Unassigned	360_0	Clustered	358	VC_360_0	358	0.9615	1	0.9615	0	0	0	1	Ref
KX266562	Unassigned	Unassigned	Unassigned	360_0	Clustered	358	VC_360_0	358	0.9615	1	0.9615	0	0	0	1	Ref
KX266563	Unassigned	Unassigned	Unassigned	360_0	Clustered	358	VC_360_0	358	0.9615	1	0.9615	0	0	0	1	Ref
KX266564	Unassigned	Unassigned	Unassigned	360_0	Clustered	358	VC_360_0	358	0.9615	1	0.9615	0	0	0	1	Ref
KX266565	Unassigned	Unassigned	Unassigned	360_0	Clustered	358	VC_360_0	358	0.9615	1	0.9615	0	0	0	1	Ref
KX266566	Unassigned	Unassigned	Unassigned	360_0	Clustered	358	VC_360_0	358	0.9615	1	0.9615	0	0	0	1	Ref
KX266567	Unassigned	Unassigned	Unassigned	360_0	Clustered	358	VC_360_0	358	0.9615	1	0.9615	0	0	0	1	Ref
KX266568	Unassigned	Unassigned	Unassigned	360_0	Clustered	358	VC_360_0	358	0.9615	1	0.9615	0	0	0	1	Ref
KX266569	Unassigned	Unassigned	Unassigned	360_0	Clustered	358	VC_360_0	358	0.9615	1	0.9615	0	0	0	1	Ref
KX266570	Unassigned	Unassigned	Unassigned	360_0	Clustered	358	VC_360_0	358	0.9615	1	0.9615	0	0	0	1	Ref
KX266571	Unassigned	Unassigned	Unassigned	360_0	Clustered	358	VC_360_0	358	0.9615	1	0.9615	0	0	0	1	Ref
KX266572	Unassigned	Unassigned	Unassigned	360_0	Clustered	358	VC_360_0	358	0.9615	1	0.9615	0	0	0	1	Ref
KX266573	Unassigned	Unassigned	Unassigned	360_0	Clustered	358	VC_360_0	358	0.9615	1	0.9615	0	0	0	1	Ref
KX266574	Unassigned	Unassigned	Unassigned	360_0	Clustered	358	VC_360_0	358	0.9615	1	0.9615	0	0	0	1	Ref
KX266575	Unassigned	Unassigned	Unassigned	360_0	Clustered	358	VC_360_0	358	0.9615	1	0.9615	0	0	0	1	Ref
KX266576	Unassigned	Unassigned	Unassigned	360_0	Clustered	358	VC_360_0	358	0.9615	1	0.9615	0	0	0	1	Ref
KX266577	Unassigned	Unassigned	Unassigned	360_0	Clustered	358	VC_360_0	358	0.9615	1	0.9615	0	0	0	1	Ref
KX266578	Unassigned	Unassigned	Unassigned	360_0	Clustered	358	VC_360_0	358	0.9615	1	0.9615	0	0	0	1	Ref
KX266579	Unassigned	Unassigned	Unassigned	360_0	Clustered	358	VC_360_0	358	0.9615	1	0.9615	0	0	0	1	Ref
KX266580	Unassigned	Unassigned	Unassigned	360_0	Clustered	358	VC_360_0	358	0.9615	1	0.9615	0	0	0	1	Ref
KX266581	Unassigned	Unassigned	Unassigned	360_0	Clustered	358	VC_360_0	358	0.9615	1	0.9615	0	0	0	1	Ref
KX266582	Unassigned	Unassigned	Unassigned	360_0	Clustered	358	VC_360_0	358	0.9615	1	0.9615	0	0	0	1	Ref
KX266583	Unassigned	Unassigned	Unassigned	360_0	Clustered	358	VC_360_0	358	0.9615	1	0.9615	0	0	0	1	Ref
KX266584	Unassigned	Unassigned	Unassigned	360_0	Clustered	358	VC_360_0	358	0.9615	1	0.9615	0	0	0	1	Ref
KX266585	Unassigned	Unassigned	Unassigned	360_0	Clustered	358	VC_360_0	358	0.9615	1	0.9615	0	0	0	1	Ref
KX266586	Unassigned	Unassigned	Unassigned	360_0	Clustered	358	VC_360_0	358	0.9615	1	0.9615	0	0	0	1	Ref
KX266587	Unassigned	Unassigned	Unassigned	360_0	Clustered	358	VC_360_0	358	0.9615	1	0.9615	0	0	0	1	Ref
KX266588	Unassigned	Unassigned	Unassigned	360_0	Clustered	358	VC_360_0	358	0.9615	1	0.9615	0	0	0	1	Ref
KX266589	Unassigned	Unassigned	Unassigned	360_0	Clustered	358	VC_360_0	358	0.9615	1	0.9615	0	0	0	1	Ref
KX266590	Unassigned	Unassigned	Unassigned	360_0	Clustered	358	VC_360_0	358	0.9615	1	0.9615	0	0	0	1	Ref
KX266591	Unassigned	Unassigned	Unassigned	360_0	Clustered	358	VC_360_0	358	0.9615	1	0.9615	0	0	0	1	Ref
KX266592	Unassigned	Unassigned	Unassigned	360_0	Clustered	358	VC_360_0	358	0.9615	1	0.9615	0	0	0	1	Ref
KX266593	Unassigned	Unassigned	Unassigned	360_0	Clustered	358	VC_360_0	358	0.9615	1	0.9615	0	0	0	1	Ref
KX266594	Unassigned	Unassigned	Unassigned	360_0	Clustered	358	VC_360_0	358	0.9615	1	0.9615	0	0	0	1	Ref
KX266595	Unassigned	Unassigned	Unassigned	360_0	Clustered	358	VC_360_0	358	0.9615	1	0.9615	0	0	0	1	Ref
KX266596	Unassigned	Unassigned	Unassigned	360_0	Clustered	358	VC_360_0	358	0.9615	1	0.9615	0	0	0	1	Ref
KX266597	Unassigned	Unassigned	Unassigned	360_0	Clustered	358	VC_360_0	358	0.9615	1	0.9615	0	0	0	1	Ref

KX266598	Unassigned	Unassigned	Unassigned	360_0	Clustered	358	VC_360_0	358	0.9615	1	0.9615	0	0	0	1	Ref
KX266599	Unassigned	Unassigned	Unassigned	360_0	Clustered	358	VC_360_0	358	0.9615	1	0.9615	0	0	0	1	Ref
KX266600	Unassigned	Unassigned	Unassigned	360_0	Clustered	358	VC_360_0	358	0.9615	1	0.9615	0	0	0	1	Ref
KX266601	Unassigned	Unassigned	Unassigned	360_0	Clustered	358	VC_360_0	358	0.9615	1	0.9615	0	0	0	1	Ref
KX266602	Unassigned	Unassigned	Unassigned	360_0	Clustered	358	VC_360_0	358	0.9615	1	0.9615	0	0	0	1	Ref
KX266603	Unassigned	Unassigned	Unassigned	360_0	Clustered	358	VC_360_0	358	0.9615	1	0.9615	0	0	0	1	Ref
KX266604	Unassigned	Unassigned	Unassigned	360_0	Clustered	358	VC_360_0	358	0.9615	1	0.9615	0	0	0	1	Ref
KX266605	Unassigned	Unassigned	Unassigned	360_0	Clustered	358	VC_360_0	358	0.9615	1	0.9615	0	0	0	1	Ref
KX266606	Unassigned	Unassigned	Unassigned	360_0	Clustered	358	VC_360_0	358	0.9615	1	0.9615	0	0	0	1	Ref
KX266607	Unassigned	Unassigned	Unassigned	360_0	Clustered	358	VC_360_0	358	0.9615	1	0.9615	0	0	0	1	Ref
KX266608	Unassigned	Unassigned	Unassigned	360_0	Clustered	358	VC_360_0	358	0.9615	1	0.9615	0	0	0	1	Ref
KX266609	Unassigned	Unassigned	Unassigned	360_0	Clustered	358	VC_360_0	358	0.9615	1	0.9615	0	0	0	1	Ref
KX266610	Unassigned	Unassigned	Unassigned	360_0	Clustered	358	VC_360_0	358	0.9615	1	0.9615	0	0	0	1	Ref
KX266611	Unassigned	Unassigned	Unassigned	360_0	Clustered	358	VC_360_0	358	0.9615	1	0.9615	0	0	0	1	Ref
KX266612	Unassigned	Unassigned	Unassigned	360_0	Clustered	358	VC_360_0	358	0.9615	1	0.9615	0	0	0	1	Ref
KX266613	Unassigned	Unassigned	Unassigned	360_0	Clustered	358	VC_360_0	358	0.9615	1	0.9615	0	0	0	1	Ref
KX266614	Unassigned	Unassigned	Unassigned	360_0	Clustered	358	VC_360_0	358	0.9615	1	0.9615	0	0	0	1	Ref
KX266615	Unassigned	Unassigned	Unassigned	360_0	Clustered	358	VC_360_0	358	0.9615	1	0.9615	0	0	0	1	Ref
KX266616	Unassigned	Unassigned	Unassigned	360_0	Clustered	358	VC_360_0	358	0.9615	1	0.9615	0	0	0	1	Ref
KX266617	Unassigned	Unassigned	Unassigned	360_0	Clustered	358	VC_360_0	358	0.9615	1	0.9615	0	0	0	1	Ref
KX266618	Unassigned	Unassigned	Unassigned	360_0	Clustered	358	VC_360_0	358	0.9615	1	0.9615	0	0	0	1	Ref
KX266619	Unassigned	Unassigned	Unassigned	360_0	Clustered	358	VC_360_0	358	0.9615	1	0.9615	0	0	0	1	Ref
KX266620	Unassigned	Unassigned	Unassigned	360_0	Clustered	358	VC_360_0	358	0.9615	1	0.9615	0	0	0	1	Ref
KX266621	Unassigned	Unassigned	Unassigned	360_0	Clustered	358	VC_360_0	358	0.9615	1	0.9615	0	0	0	1	Ref
KX266622	Unassigned	Unassigned	Unassigned	360_0	Clustered	358	VC_360_0	358	0.9615	1	0.9615	0	0	0	1	Ref
KX266623	Unassigned	Unassigned	Unassigned	360_0	Clustered	358	VC_360_0	358	0.9615	1	0.9615	0	0	0	1	Ref
KX266624	Unassigned	Unassigned	Unassigned	360_0	Clustered	358	VC_360_0	358	0.9615	1	0.9615	0	0	0	1	Ref
KX266625	Unassigned	Unassigned	Unassigned	360_0	Clustered	358	VC_360_0	358	0.9615	1	0.9615	0	0	0	1	Ref
KX266626	Unassigned	Unassigned	Unassigned	360_0	Clustered	358	VC_360_0	358	0.9615	1	0.9615	0	0	0	1	Ref
KX266627	Unassigned	Unassigned	Unassigned	360_0	Clustered	358	VC_360_0	358	0.9615	1	0.9615	0	0	0	1	Ref
KX266628	Unassigned	Unassigned	Unassigned	360_0	Clustered	358	VC_360_0	358	0.9615	1	0.9615	0	0	0	1	Ref
KX266629	Unassigned	Unassigned	Unassigned	360_0	Clustered	358	VC_360_0	358	0.9615	1	0.9615	0	0	0	1	Ref
KX266630	Unassigned	Unassigned	Unassigned	360_0	Clustered	358	VC_360_0	358	0.9615	1	0.9615	0	0	0	1	Ref
KX266631	Unassigned	Unassigned	Unassigned	360_0	Clustered	358	VC_360_0	358	0.9615	1	0.9615	0	0	0	1	Ref
KX266632	Unassigned	Unassigned	Unassigned	360_0	Clustered	358	VC_360_0	358	0.9615	1	0.9615	0	0	0	1	Ref
KX266633	Unassigned	Unassigned	Unassigned	360_0	Clustered	358	VC_360_0	358	0.9615	1	0.9615	0	0	0	1	Ref
KX266634	Unassigned	Unassigned	Unassigned	360_0	Clustered	358	VC_360_0	358	0.9615	1	0.9615	0	0	0	1	Ref
KX266635	Unassigned	Unassigned	Unassigned	360_0	Clustered	358	VC_360_0	358	0.9615	1	0.9615	0	0	0	1	Ref
KX266636	Unassigned	Unassigned	Unassigned	360_0	Clustered	358	VC_360_0	358	0.9615	1	0.9615	0	0	0	1	Ref
KX266637	Unassigned	Unassigned	Unassigned	360_0	Clustered	358	VC_360_0	358	0.9615	1	0.9615	0	0	0	1	Ref
KX278418	Unassigned	Unassigned	Unassigned	984_0	Clustered	38	VC_984_0	38	0.9758	1	0.9758	0	0	0	1	Ref
KX278419	Unassigned	Unassigned	Unassigned	984_0	Clustered	38	VC_984_0	38	0.9758	1	0.9758	0	0	0	1	Ref
KX279892	Unassigned	Unassigned	Unassigned	452_0	Clustered	66	VC_452_0	66	0.9248	1	0.9248	0	0	0	1	Ref
KX284704	Unassigned	Unassigned	Unassigned	695_0	Clustered	48	VC_695_0	48	0.8162	1	0.8162	0	0	0	1	Ref
KX344445	Unassigned	Unassigned	Unassigned	982_0	Clustered	62	VC_982_0	62	0.8646	1	0.8646	0	0	0	0.743	Ref
KX344510	Unassigned	Unassigned	Unassigned	961_0	Clustered	3	VC_961_0	3	0.675	0.99054823	0.6686	0	0	0	1	Ref
KX346246	Unassigned	Unassigned	Unassigned	202_0	Clustered	102	VC_202_0	102	0.4049	1	0.4049	0	0	0	0.7876	Ref
KX349226	Unassigned	Unassigned	Unassigned	662_0	Clustered	62	VC_662_0	62	0.6976	1	0.6976	0	0	0	1	Ref
KX349227	Unassigned	Unassigned	Unassigned	662_0	Clustered	62	VC_662_0	62	0.6976	1	0.6976	0	0	0	1	Ref
KX349228	Unassigned	Unassigned	Unassigned	662_0	Clustered	62	VC_662_0	62	0.6976	1	0.6976	0	0	0	1	Ref
KX349229	Unassigned	Unassigned	Unassigned	662_0	Clustered	62	VC_662_0	62	0.6976	1	0.6976	0	0	0	1	Ref
KX349230	Unassigned	Unassigned	Unassigned	662_0	Clustered	62	VC_662_0	62	0.6976	1	0.6976	0	0	0	1	Ref
KX349231	Unassigned	Unassigned	Unassigned	662_0	Clustered	62	VC_662_0	62	0.6976	1	0.6976	0	0	0	1	Ref
KX349232	Unassigned	Unassigned	Unassigned	662_0	Clustered	62	VC_662_0	62	0.6976	1	0.6976	0	0	0	1	Ref
KX349233	Unassigned	Unassigned	Unassigned	662_0	Clustered	62	VC_662_0	62	0.6976	1	0.6976	0	0	0	1	Ref
KX349234	Unassigned	Unassigned	Unassigned	662_0	Clustered	62	VC_662_0	62	0.6976	1	0.6976	0	0	0	1	Ref
KX349235	Unassigned	Unassigned	Unassigned	662_0	Clustered	62	VC_662_0	62	0.6976	1	0.6976	0	0	0	1	Ref
KX349236	Unassigned	Unassigned	Unassigned	662_0	Clustered	62	VC_662_0	62	0.6976	1	0.6976	0	0	0	1	Ref
KX349237	Unassigned	Unassigned	Unassigned	662_0	Clustered	62	VC_662_0	62	0.6976	1	0.6976	0	0	0	1	Ref
KX349238	Unassigned	Unassigned	Unassigned	662_0	Clustered	62	VC_662_0	62	0.6976	1	0.6976	0	0	0	1	Ref
KX349239	Unassigned	Unassigned	Unassigned	662_0	Clustered	62	VC_662_0	62	0.6976	1	0.6976	0	0	0	1	Ref
KX349240	Unassigned	Unassigned	Unassigned	662_0	Clustered	62	VC_662_0	62	0.6976	1	0.6976	0	0	0	1	Ref
KX349241	Unassigned	Unassigned	Unassigned	662_0	Clustered	62	VC_662_0	62	0.6976	1	0.6976	0	0	0	1	Ref
KX349242	Unassigned	Unassigned	Unassigned	662_0	Clustered	62	VC_662_0	62	0.6976	1	0.6976	0	0	0	1	Ref
KX349243	Unassigned	Unassigned	Unassigned	662_0	Clustered	62	VC_662_0	62	0.6976	1	0.6976	0	0	0	1	Ref
KX349244	Unassigned	Unassigned	Unassigned	662_0	Clustered	62	VC_662_0	62	0.6976	1	0.6976	0	0	0	1	Ref
KX349245	Unassigned	Unassigned	Unassigned	662_0	Clustered	62	VC_662_0	62	0.6976	1	0.6976	0	0	0	1	Ref
KX349246	Unassigned	Unassigned	Unassigned	662_0	Clustered	62	VC_662_0	62	0.6976	1	0.6976	0	0	0	1	Ref
KX349247	Unassigned	Unassigned	Unassigned	662_0	Clustered	62	VC_662_0	62	0.6976	1	0.6976	0	0	0	1	Ref
KX349248	Unassigned	Unassigned	Unassigned	662_0	Clustered	62	VC_662_0	62	0.6976	1	0.6976	0	0	0	1	Ref
KX349249	Unassigned	Unassigned	Unassigned	662_0	Clustered	62	VC_662_0	62	0.6976	1	0.6976	0	0	0	1	Ref
KX349250	Unassigned	Unassigned	Unassigned	662_0	Clustered	62	VC_662_0	62	0.6976	1	0.6976	0	0	0	1	Ref
KX349251	Unassigned	Unassigned	Unassigned	662_0	Clustered	62	VC_662_0	62	0.6976	1	0.6976	0	0	0	1	Ref
KX349252	Unassigned	Unassigned	Unassigned	662_0	Clustered	62	VC_662_0	62	0.6976	1	0.6976	0	0	0	1	Ref
KX349253	Unassigned	Unassigned	Unassigned	662_0	Clustered	62	VC_662_0	62	0.6976	1	0.6976	0	0	0	1	Ref
KX349254	Unassigned	Unassigned	Unassigned	662_0	Clustered	62	VC_662_0	62	0.6976	1	0.6976	0	0	0	1	Ref
KX349255	Unassigned	Unassigned	Unassigned	662_0	Clustered	62	VC_662_0	62	0.6976	1	0.6976	0	0	0	1	Ref
KX349256	Unassigned	Unassigned	Unassigned	662_0	Clustered	62	VC_662_0	62	0.6976	1	0.6976	0	0	0	1	Ref
KX349257	Unassigned	Unassigned	Unassigned	662_0	Clustered	62	VC_662_0	62	0.6976	1	0.6976	0	0	0	1	Ref
KX349258	Unassigned	Unassigned	Unassigned	662_0	Clustered	62	VC_662_0	62	0.6976	1	0.6976	0	0	0	1	Ref
KX349259	Unassigned	Unassigned	Unassigned	662_0	Clustered	62	VC_662_0	62	0.6976	1	0.6976	0	0	0	1	Ref

KX349260	Unassigned	Unassigned	Unassigned	662_0	Clustered	62	VC_662_0	62	0.6976	1	0.6976	0	0	0	1	Ref
KX349261	Unassigned	Unassigned	Unassigned	662_0	Clustered	62	VC_662_0	62	0.6976	1	0.6976	0	0	0	1	Ref
KX349262	Unassigned	Unassigned	Unassigned	662_0	Clustered	62	VC_662_0	62	0.6976	1	0.6976	0	0	0	1	Ref
KX349263	Unassigned	Unassigned	Unassigned	662_0	Clustered	62	VC_662_0	62	0.6976	1	0.6976	0	0	0	1	Ref
KX349264	Unassigned	Unassigned	Unassigned	662_0	Clustered	62	VC_662_0	62	0.6976	1	0.6976	0	0	0	1	Ref
KX349265	Unassigned	Unassigned	Unassigned	662_0	Clustered	62	VC_662_0	62	0.6976	1	0.6976	0	0	0	1	Ref
KX349266	Unassigned	Unassigned	Unassigned	662_0	Clustered	62	VC_662_0	62	0.6976	1	0.6976	0	0	0	1	Ref
KX349267	Unassigned	Unassigned	Unassigned	662_0	Clustered	62	VC_662_0	62	0.6976	1	0.6976	0	0	0	1	Ref
KX349268	Unassigned	Unassigned	Unassigned	662_0	Clustered	62	VC_662_0	62	0.6976	1	0.6976	0	0	0	1	Ref
KX349269	Unassigned	Unassigned	Unassigned	662_0	Clustered	62	VC_662_0	62	0.6976	1	0.6976	0	0	0	1	Ref
KX349270	Unassigned	Unassigned	Unassigned	662_0	Clustered	62	VC_662_0	62	0.6976	1	0.6976	0	0	0	1	Ref
KX349271	Unassigned	Unassigned	Unassigned	662_0	Clustered	62	VC_662_0	62	0.6976	1	0.6976	0	0	0	1	Ref
KX349272	Unassigned	Unassigned	Unassigned	662_0	Clustered	62	VC_662_0	62	0.6976	1	0.6976	0	0	0	1	Ref
KX349273	Unassigned	Unassigned	Unassigned	662_0	Clustered	62	VC_662_0	62	0.6976	1	0.6976	0	0	0	1	Ref
KX349274	Unassigned	Unassigned	Unassigned	662_0	Clustered	62	VC_662_0	62	0.6976	1	0.6976	0	0	0	1	Ref
KX349275	Unassigned	Unassigned	Unassigned	662_0	Clustered	62	VC_662_0	62	0.6976	1	0.6976	0	0	0	1	Ref
KX349276	Unassigned	Unassigned	Unassigned	662_0	Clustered	62	VC_662_0	62	0.6976	1	0.6976	0	0	0	1	Ref
KX349277	Unassigned	Unassigned	Unassigned	662_0	Clustered	62	VC_662_0	62	0.6976	1	0.6976	0	0	0	1	Ref
KX349278	Unassigned	Unassigned	Unassigned	662_0	Clustered	62	VC_662_0	62	0.6976	1	0.6976	0	0	0	1	Ref
KX349279	Unassigned	Unassigned	Unassigned	662_0	Clustered	62	VC_662_0	62	0.6976	1	0.6976	0	0	0	1	Ref
KX349280	Unassigned	Unassigned	Unassigned	662_0	Clustered	62	VC_662_0	62	0.6976	1	0.6976	0	0	0	1	Ref
KX349281	Unassigned	Unassigned	Unassigned	662_0	Clustered	62	VC_662_0	62	0.6976	1	0.6976	0	0	0	1	Ref
KX349282	Unassigned	Unassigned	Unassigned	662_0	Clustered	62	VC_662_0	62	0.6976	1	0.6976	0	0	0	1	Ref
KX349283	Unassigned	Unassigned	Unassigned	662_0	Clustered	62	VC_662_0	62	0.6976	1	0.6976	0	0	0	1	Ref
KX349284	Unassigned	Unassigned	Unassigned	662_0	Clustered	62	VC_662_0	62	0.6976	1	0.6976	0	0	0	1	Ref
KX349291	Unassigned	Unassigned	Unassigned	433_4	Clustered	9	VC_433_4	9	0.1285	1	0.1285	0	0	0	1	Ref
KX349292	Unassigned	Unassigned	Unassigned	433_4	Clustered	9	VC_433_4	9	0.1285	1	0.1285	0	0	0	1	Ref
KX349293	Unassigned	Unassigned	Unassigned	433_4	Clustered	9	VC_433_4	9	0.1285	1	0.1285	0	0	0	1	Ref
KX349294	Unassigned	Unassigned	Unassigned	433_4	Clustered	9	VC_433_4	9	0.1285	1	0.1285	0	0	0	1	Ref
KX349295	Unassigned	Unassigned	Unassigned	433_4	Clustered	9	VC_433_4	9	0.1285	1	0.1285	0	0	0	1	Ref
KX349296	Unassigned	Unassigned	Unassigned	433_4	Clustered	9	VC_433_4	9	0.1285	1	0.1285	0	0	0	1	Ref
KX349297	Unassigned	Unassigned	Unassigned	433_4	Clustered	9	VC_433_4	9	0.1285	1	0.1285	0	0	0	1	Ref
KX349899	Unassigned	Unassigned	Unassigned	818_0	Clustered	20	VC_818_0	20	0.9601	1	0.9601	0	0	0	1	Ref
KX349900	Unassigned	Unassigned	Unassigned	818_0	Clustered	20	VC_818_0	20	0.9601	1	0.9601	0	0	0	1	Ref
KX349901	Unassigned	Unassigned	Unassigned	818_0	Clustered	20	VC_818_0	20	0.9601	1	0.9601	0	0	0	1	Ref
KX349902	Unassigned	Unassigned	Unassigned	884_0	Clustered	28	VC_884_0	28	0.6211	1	0.6211	0	0	0	0.9841	Ref
KX349903	Unassigned	Unassigned	Unassigned	884_0	Clustered	28	VC_884_0	28	0.6211	1	0.6211	0	0	0	0.9841	Ref
KX365747	Unassigned	Unassigned	Unassigned	117_0	Clustered	9	VC_117_0	9	0.9977	0.98988637	0.9876	0	0	0	1	Ref
KX365748	Unassigned	Unassigned	Unassigned	895_0	Clustered	2	VC_895_0	2	0.2788	0.94251254	0.2628	0	0	0	1	Ref
KX369583	Unassigned	Unassigned	Unassigned	558_23	Clustered/Singleton	1	VC_558_23	1	0	1	0	0	0	0	1	Ref
KX369584	Unassigned	Unassigned	Unassigned	325_28	Clustered/Singleton	1	VC_325_28	1	0	1	0	0	0	0	1	Ref
KX369585	Unassigned	Unassigned	Unassigned	371_16	Clustered	42	VC_371_16	42	0.1341	1	0.1341	0	0	0	0.7282	Ref
KX369586	Unassigned	Unassigned	Unassigned	325_4	Clustered/Singleton	1	VC_325_4	1	0	1	0	0	0	0	1	Ref
KX373685	Unassigned	Unassigned	Unassigned	690_0	Clustered	50	VC_690_0	50	0.9805	1	0.9805	0	0	0	1	Ref
KX373686	Unassigned	Unassigned	Unassigned	690_0	Clustered	50	VC_690_0	50	0.9805	1	0.9805	0	0	0	1	Ref
KX373687	Unassigned	Unassigned	Unassigned	690_0	Clustered	50	VC_690_0	50	0.9805	1	0.9805	0	0	0	1	Ref
KX373688	Unassigned	Unassigned	Unassigned	690_0	Clustered	50	VC_690_0	50	0.9805	1	0.9805	0	0	0	1	Ref
KX373689	Unassigned	Unassigned	Unassigned	690_0	Clustered	50	VC_690_0	50	0.9805	1	0.9805	0	0	0	1	Ref
KX373690	Unassigned	Unassigned	Unassigned	690_0	Clustered	50	VC_690_0	50	0.9805	1	0.9805	0	0	0	1	Ref
KX373691	Unassigned	Unassigned	Unassigned	690_0	Clustered	50	VC_690_0	50	0.9805	1	0.9805	0	0	0	1	Ref
KX373692	Unassigned	Unassigned	Unassigned	690_0	Clustered	50	VC_690_0	50	0.9805	1	0.9805	0	0	0	1	Ref
KX375815	Unassigned	Unassigned	Unassigned	223_0	Clustered	38	VC_223_0	38	0.9095	1	0.9095	0	0	0	1	Ref
KX377933	Unassigned	Unassigned	Unassigned	329_3	Clustered	24	VC_329_3	24	0.1206	1	0.1206	0	0	0	0.7283	Ref
KX379667	Unassigned	Unassigned	Unassigned	202_0	Clustered	102	VC_202_0	102	0.4049	1	0.4049	0	0	0	0.7876	Ref
KX379668	Unassigned	Unassigned	Unassigned	202_0	Clustered	102	VC_202_0	102	0.4049	1	0.4049	0	0	0	0.7876	Ref
KX379670	Unassigned	Unassigned	Unassigned	202_0	Clustered	102	VC_202_0	102	0.4049	1	0.4049	0	0	0	0.7876	Ref
KX379671	Unassigned	Unassigned	Unassigned	202_0	Clustered	102	VC_202_0	102	0.4049	1	0.4049	0	0	0	0.7876	Ref
KX397364	Unassigned	Unassigned	Unassigned	959_4	Clustered	3	VC_959_4	3	0.0237	0.77138817	0.0183	0	0	0	1	Ref
KX397365	Unassigned	Unassigned	Unassigned	959_2	Clustered	5	VC_959_2	5	0.0484	0.89346426	0.0432	0	0	0	1	Ref
KX397366	Unassigned	Unassigned	Unassigned	959_2	Clustered	5	VC_959_2	5	0.0484	0.89346426	0.0432	0	0	0	1	Ref
KX397367	Unassigned	Unassigned	Unassigned	959_0	Clustered	5	VC_959_0	5	0.0499	0.99999231	0.0499	0	0	0	1	Ref
KX397368	Unassigned	Unassigned	Unassigned	959_1	Clustered	8	VC_959_1	8	0.0878	0.98435099	0.0864	0	0	0	1	Ref
KX397369	Unassigned	Unassigned	Unassigned	959_3	Clustered	3	VC_959_3	3	0.0237	0.75911548	0.018	0	0	0	1	Ref
KX397370	Unassigned	Unassigned	Unassigned	959_1	Clustered	8	VC_959_1	8	0.0878	0.98435099	0.0864	0	0	0	1	Ref
KX397371	Unassigned	Unassigned	Unassigned	959_1	Clustered	8	VC_959_1	8	0.0878	0.98435099	0.0864	0	0	0	1	Ref
KX397372	Unassigned	Unassigned	Unassigned	959_0	Clustered	5	VC_959_0	5	0.0499	0.99999231	0.0499	0	0	0	1	Ref
KX397373	Unassigned	Unassigned	Unassigned	959_4	Clustered	3	VC_959_4	3	0.0237	0.77138817	0.0183	0	0	0	1	Ref
KX405002	Unassigned	Unassigned	Unassigned	637_0	Clustered	115	VC_637_0	115	0.731	1	0.731	0	0	0	0.8764	Ref
KX405003	Unassigned	Unassigned	Unassigned	329_16	Clustered	4	VC_329_16	4	0.0158	0.9999807	0.0158	0	0	0	1	Ref
KX431559	Unassigned	Unassigned	Unassigned	669_0	Clustered	21	VC_669_0	21	0.3623	1	0.3623	0	0	0	1	Ref
KX431560	Unassigned	Unassigned	Unassigned	669_0	Clustered	21	VC_669_0	21	0.3623	1	0.3623	0	0	0	1	Ref
KX431888	Unassigned	Unassigned	Unassigned	130_0	Clustered	19	VC_130_0	19	0.6068	1	0.6068	0	0	0	1	Ref
KX438380	Unassigned	Unassigned	Unassigned	133_14	Clustered	3	VC_133_14	3	0.0102	0.96327256	0.0098	0	0	0	1	Ref
KX443326	Unassigned	Unassigned	Unassigned	761_0	Clustered	67	VC_761_0	67	0.8947	1	0.8947	0	0	0	1	Ref
KX443552	Unassigned	Unassigned	Unassigned	669_0	Clustered	21	VC_669_0	21	0.3623	1	0.3623	0	0	0	1	Ref
KX443695	Unassigned	Unassigned	Unassigned	950_0	Clustered	38	VC_950_0	38	0.9443	1	0.9443	0	0	0	1	Ref
KX443696	Unassigned	Unassigned	Unassigned	508_0	Clustered	32	VC_508_0	32	0.906	1	0.906	0	0	0	1	Ref
KX449361	Unassigned	Unassigned	Unassigned	130_0	Clustered	19	VC_130_0	19	0.6068	1	0.6068	0	0	0	1	Ref
KX449362	Unassigned	Unassigned	Unassigned	130_0	Clustered	19	VC_130_0	19	0.6068	1	0.6068	0	0	0	1	Ref
KX449363	Unassigned	Unassigned	Unassigned	130_0	Clustered	19	VC_130_0	19	0.6068	1	0.6068	0	0	0	1	Ref
KX452695	Unassigned	Unassigned	Unassigned	582_3	Clustered	12	VC_582_3	12	0.2357	1	0.2357	0	0	0	0.8636	Ref

KX452696	Unassigned	Unassigned	Unassigned	385_0	Clustered	51	VC_385_0	51	0.8754	1	0.8754	0	0	0	1	Ref
KX452697	Unassigned	Unassigned	Unassigned	132_0	Clustered	4	VC_132_0	4	0.036	0.99998777	0.036	0	0	0	1	Ref
KX452698	Unassigned	Unassigned	Unassigned	283_143	Clustered/Singleton	1	VC_283_143	1	0	1	0	0	0	0	1	Ref
KX452699	Unassigned	Unassigned	Unassigned	592_0	Clustered	50	VC_592_0	50	0.7314	1	0.7314	0	0	0	1	Ref
KX452700	Unassigned	Unassigned	Unassigned	592_0	Clustered	50	VC_592_0	50	0.7314	1	0.7314	0	0	0	1	Ref
KX455876	Unassigned	Unassigned	Unassigned	1189_0	Clustered	9	VC_1189_0	9	0.968	1	0.968	0	0	0	1	Ref
KX456206	Unassigned	Unassigned	Unassigned	265_0	Clustered	12	VC_265_0	12	0.4401	1	0.4401	0	0	0	1	Ref
KX456208	Unassigned	Unassigned	Unassigned	265_0	Clustered	12	VC_265_0	12	0.4401	1	0.4401	0	0	0	1	Ref
KX456209	Unassigned	Unassigned	Unassigned	265_0	Clustered	12	VC_265_0	12	0.4401	1	0.4401	0	0	0	1	Ref
KX456210	Unassigned	Unassigned	Unassigned	265_0	Clustered	12	VC_265_0	12	0.4401	1	0.4401	0	0	0	1	Ref
KX456211	Unassigned	Unassigned	Unassigned	262_0	Clustered	5	VC_262_0	5	0.0724	0.99999989	0.0724	0	0	0	1	Ref
KX456213	Unassigned	Unassigned	Unassigned	265_0	Clustered	12	VC_265_0	12	0.4401	1	0.4401	0	0	0	1	Ref
KX458236	Unassigned	Unassigned	Unassigned	517_1	Clustered	108	VC_517_1	108	0.8483	1	0.8483	0	0	0	0.9955	Ref
KX458237	Unassigned	Unassigned	Unassigned	514_0	Clustered	75	VC_514_0	75	0.9402	1	0.9402	0	0	0	1	Ref
KX467643	Unassigned	Unassigned	Unassigned	349_0	Clustered	7	VC_349_0	7	0.9363	0.99999996	0.9363	0	0	0	1	Ref
KX495186	Unassigned	Unassigned	Unassigned	187_3	Clustered	9	VC_187_3	9	0.1971	1	0.1971	0	0	0	0.8611	Ref
KX507045	Unassigned	Unassigned	Unassigned	642_3	Clustered	9	VC_642_3	9	0.2131	1	0.2131	0	0	0	0.6944	Ref
KX507046	Unassigned	Unassigned	Unassigned	493_11	Clustered/Singleton	1	VC_493_11	1	0	1	0	0	0	0	1	Ref
KX507343	Unassigned	Unassigned	Unassigned	982_0	Clustered	62	VC_982_0	62	0.8646	1	0.8646	0	0	0	0.743	Ref
KX507344	Unassigned	Unassigned	Unassigned	982_0	Clustered	62	VC_982_0	62	0.8646	1	0.8646	0	0	0	0.743	Ref
KX507345	Unassigned	Unassigned	Unassigned	982_0	Clustered	62	VC_982_0	62	0.8646	1	0.8646	0	0	0	0.743	Ref
KX507361	Unassigned	Unassigned	Unassigned	888_0	Clustered	28	VC_888_0	28	0.5497	1	0.5497	0	0	0	1	Ref
KX507362	Unassigned	Unassigned	Unassigned	514_0	Clustered	75	VC_514_0	75	0.9402	1	0.9402	0	0	0	1	Ref
KX513864	Unassigned	Unassigned	Unassigned	1123_0	Clustered	2	VC_1123_0	2	0.3187	0.93270267	0.2972	0	0	0	1	Ref
KX513865	Unassigned	Unassigned	Unassigned	1104_0	Clustered	154	VC_1104_0	154	0.8484	1	0.8484	0	0	0	1	Ref
KX513866	Unassigned	Unassigned	Unassigned	1119_0	Clustered	20	VC_1119_0	20	0.6751	0.99997892	0.6751	0	0	0	1	Ref
KX513867	Unassigned	Unassigned	Unassigned	1143_0	Clustered	9	VC_1143_0	9	0.7268	1	0.7268	0	0	0	1	Ref
KX513868	Unassigned	Unassigned	Unassigned	1127_0	Clustered	19	VC_1127_0	19	0.7611	1	0.7611	0	0	0	1	Ref
KX513869	Unassigned	Unassigned	Unassigned	1056_0	Clustered	4	VC_1056_0	4	0.169	0.84110627	0.1421	0	0	0	1	Ref
KX513870	Unassigned	Unassigned	Unassigned	1119_0	Clustered	20	VC_1119_0	20	0.6751	0.99997892	0.6751	0	0	0	1	Ref
KX513871	Unassigned	Unassigned	Unassigned	1191_0	Clustered	3	VC_1191_0	3	0.1408	0.98976974	0.1394	0	0	0	1	Ref
KX513872	Unassigned	Unassigned	Unassigned	1143_0	Clustered	9	VC_1143_0	9	0.7268	1	0.7268	0	0	0	1	Ref
KX513873	Unassigned	Unassigned	Unassigned	1122_0	Clustered	10	VC_1122_0	10	0.9643	0.99999697	0.9643	0	0	0	1	Ref
KX513874	Unassigned	Unassigned	Unassigned	1143_0	Clustered	9	VC_1143_0	9	0.7268	1	0.7268	0	0	0	1	Ref
KX513875	Unassigned	Unassigned	Unassigned	1197_0	Clustered	15	VC_1197_0	15	0.8991	0.76608394	0.6888	0	0	0	1	Ref
KX513877	Unassigned	Unassigned	Unassigned	1127_0	Clustered	19	VC_1127_0	19	0.7611	1	0.7611	0	0	0	1	Ref
KX522565	Unassigned	Unassigned	Unassigned	22_0	Clustered	11	VC_22_0	11	0.5163	1	0.5163	0	0	0	1	Ref
KX522649	Unassigned	Unassigned	Unassigned	325_21	Clustered	5	VC_325_21	5	0.0209	0.99999976	0.0209	0	0	0	0.8	Ref
KX522650	Unassigned	Unassigned	Unassigned	1074_0	Clustered	19	VC_1074_0	19	0.9198	1	0.9198	0	0	0	1	Ref
KX522943	Unassigned	Unassigned	Unassigned	325_21	Clustered	5	VC_325_21	5	0.0209	0.99999976	0.0209	0	0	0	0.8	Ref
KX523125	Unassigned	Unassigned	Unassigned	514_0	Clustered	75	VC_514_0	75	0.9402	1	0.9402	0	0	0	1	Ref
KX523699	Unassigned	Unassigned	Unassigned	461_0	Clustered	96	VC_461_0	96	0.9307	1	0.9307	0	0	0	0.9575	Ref
KX532239	Unassigned	Unassigned	Unassigned	153_0	Clustered	25	VC_153_0	25	0.7729	1	0.7729	0	0	0	1	Ref
KX534004	Unassigned	Unassigned	Unassigned	761_0	Clustered	67	VC_761_0	67	0.8947	1	0.8947	0	0	0	1	Ref
KX534335	Unassigned	Unassigned	Unassigned	835_0	Clustered	67	VC_835_0	67	0.9624	1	0.9624	0	0	0	1	Ref
KX534336	Unassigned	Unassigned	Unassigned	835_0	Clustered	67	VC_835_0	67	0.9624	1	0.9624	0	0	0	1	Ref
KX534337	Unassigned	Unassigned	Unassigned	795_0	Clustered	19	VC_795_0	19	0.6848	1	0.6848	0	0	0	1	Ref
KX534338	Unassigned	Unassigned	Unassigned	835_0	Clustered	67	VC_835_0	67	0.9624	1	0.9624	0	0	0	1	Ref
KX534339	Unassigned	Unassigned	Unassigned	835_0	Clustered	67	VC_835_0	67	0.9624	1	0.9624	0	0	0	1	Ref
KX534340	Unassigned	Unassigned	Unassigned	20_0	Clustered	5	VC_20_0	5	0.4387	0.99999934	0.4387	0	0	0	1	Ref
KX534341	Unassigned	Unassigned	Unassigned	835_0	Clustered	67	VC_835_0	67	0.9624	1	0.9624	0	0	0	1	Ref
KX550065	Unassigned	Unassigned	Unassigned	412_0	Clustered	39	VC_412_0	39	0.845	1	0.845	0	0	0	1	Ref
KX550082	Unassigned	Unassigned	Unassigned	956_0	Clustered	39	VC_956_0	39	1	1	1	0	0	0	1	Ref
KX550441	Unassigned	Unassigned	Unassigned	517_1	Clustered	108	VC_517_1	108	0.8483	1	0.8483	0	0	0	0.9955	Ref
KX550442	Unassigned	Unassigned	Unassigned	517_0	Clustered	18	VC_517_0	18	0.1599	1	0.1599	0	0	0	1	Ref
KX550443	Unassigned	Unassigned	Unassigned	325_53	Clustered	7	VC_325_53	7	0.0242	1	0.0242	0	0	0	0.9048	Ref
KX552041	Unassigned	Unassigned	Unassigned	847_1	Clustered	26	VC_847_1	26	0.6323	1	0.6323	0	0	0	0.9815	Ref
KX555527	Unassigned	Unassigned	Unassigned	861_0	Clustered	9	VC_861_0	9	0.5869	1	0.5869	0	0	0	1	Ref
KX557234	Unassigned	Unassigned	Unassigned	517_1	Clustered	108	VC_517_1	108	0.8483	1	0.8483	0	0	0	0.9955	Ref
KX557272	Unassigned	Unassigned	Unassigned	1182_0	Clustered	4	VC_1182_0	4	0.2845	0.99997676	0.2845	0	0	0	1	Ref
KX557273	Unassigned	Unassigned	Unassigned	525_0	Clustered	25	VC_525_0	25	0.4597	1	0.4597	0	0	0	1	Ref
KX557274	Unassigned	Unassigned	Unassigned	1176_0	Clustered	41	VC_1176_0	41	0.9558	1	0.9558	0	0	0	0.9976	Ref
KX557275	Unassigned	Unassigned	Unassigned	1176_0	Clustered	41	VC_1176_0	41	0.9558	1	0.9558	0	0	0	0.9976	Ref
KX557276	Unassigned	Unassigned	Unassigned	1181_1	Clustered	10	VC_1181_1	10	0.5828	1	0.5828	0	0	0	1	Ref
KX557278	Unassigned	Unassigned	Unassigned	1161_0	Clustered	2	VC_1161_0	2	0.0296	0.9573719	0.0283	0	0	0	1	Ref
KX557279	Unassigned	Unassigned	Unassigned	1177_0	Clustered	14	VC_1177_0	14	0.7847	1	0.7847	0	0	0	0.989	Ref
KX557280	Unassigned	Unassigned	Unassigned	224_0	Clustered	19	VC_224_0	19	0.9416	1	0.9416	0	0	0	1	Ref
KX557281	Unassigned	Unassigned	Unassigned	867_0	Clustered	6	VC_867_0	6	0.3202	1	0.3202	0	0	0	1	Ref
KX557282	Unassigned	Unassigned	Unassigned	1184_0	Clustered	5	VC_1184_0	5	0.6591	0.99999982	0.6591	0	0	0	1	Ref
KX557283	Unassigned	Unassigned	Unassigned	224_0	Clustered	19	VC_224_0	19	0.9416	1	0.9416	0	0	0	1	Ref
KX557284	Unassigned	Unassigned	Unassigned	224_0	Clustered	19	VC_224_0	19	0.9416	1	0.9416	0	0	0	1	Ref
KX557285	Unassigned	Unassigned	Unassigned	570_0	Clustered	9	VC_570_0	9	0.499	1	0.499	0	0	0	1	Ref
KX557286	Unassigned	Unassigned	Unassigned	1178_0	Clustered	24	VC_1178_0	24	0.9524	1	0.9524	0	0	0	1	Ref
KX557287	Unassigned	Unassigned	Unassigned	525_0	Clustered	25	VC_525_0	25	0.4597	1	0.4597	0	0	0	1	Ref
KX574454	Unassigned	Unassigned	Unassigned	325_21	Clustered	5	VC_325_21	5	0.0209	0.99999976	0.0209	0	0	0	0.8	Ref
KX574455	Unassigned	Unassigned	Unassigned	215_1	Clustered	19	VC_215_1	19	0.4188	1	0.4188	0	0	0	1	Ref
KX576638	Unassigned	Unassigned	Unassigned	223_0	Clustered	38	VC_223_0	38	0.9095	1	0.9095	0	0	0	1	Ref
KX576639	Unassigned	Unassigned	Unassigned	216_6	Clustered	42	VC_216_6	42	0.473	1	0.473	0	0	0	0.8351	Ref
KX576640	Unassigned	Unassigned	Unassigned	1160_0	Clustered	73	VC_1160_0	73	0.9835	1	0.9835	0	0	0	1	Ref
KX576641	Unassigned	Unassigned	Unassigned	1160_0	Clustered	73	VC_1160_0	73	0.9835	1	0.9835	0	0	0	1	Ref
KX576642	Unassigned	Unassigned	Unassigned	950_0	Clustered	38	VC_950_0	38	0.9443	1	0.9443	0	0	0	1	Ref

KX576643	Unassigned	Unassigned	Unassigned	371_18	Clustered	10	VC_371_18	10	0.0302	1	0.0302	0	0	0	0.8	Ref
KX576644	Unassigned	Unassigned	Unassigned	520_77	Clustered	3	VC_520_77	3	0.0218	0.99859911	0.0218	0	0	0	1	Ref
KX576645	Unassigned	Unassigned	Unassigned	371_13	Clustered	33	VC_371_13	33	0.179	1	0.179	0	0	0	0.8769	Ref
KX576646	Unassigned	Unassigned	Unassigned	371_13	Clustered	33	VC_371_13	33	0.179	1	0.179	0	0	0	0.8769	Ref
KX576647	Unassigned	Unassigned	Unassigned	371_13	Clustered	33	VC_371_13	33	0.179	1	0.179	0	0	0	0.8769	Ref
KX578042	Unassigned	Unassigned	Unassigned	862_0	Clustered	11	VC_862_0	11	0.6745	1	0.6745	0	0	0	1	Ref
KX578043	Unassigned	Unassigned	Unassigned	862_0	Clustered	11	VC_862_0	11	0.6745	1	0.6745	0	0	0	1	Ref
KX578044	Unassigned	Unassigned	Unassigned	862_0	Clustered	11	VC_862_0	11	0.6745	1	0.6745	0	0	0	1	Ref
KX578071	Unassigned	Unassigned	Unassigned	371_15	Clustered	44	VC_371_15	44	0.1391	1	0.1391	0	0	0	0.7474	Ref
KX579975	Unassigned	Unassigned	Unassigned	517_0	Clustered	18	VC_517_0	18	0.1599	1	0.1599	0	0	0	1	Ref
KX580961	Unassigned	Unassigned	Unassigned	888_0	Clustered	28	VC_888_0	28	0.5497	1	0.5497	0	0	0	1	Ref
KX580962	Unassigned	Unassigned	Unassigned	888_0	Clustered	28	VC_888_0	28	0.5497	1	0.5497	0	0	0	1	Ref
KX581090	Unassigned	Unassigned	Unassigned	299_0	Clustered	24	VC_299_0	24	0.9905	1	0.9905	0	0	0	1	Ref
KX581091	Unassigned	Unassigned	Unassigned	299_0	Clustered	24	VC_299_0	24	0.9905	1	0.9905	0	0	0	1	Ref
KX581092	Unassigned	Unassigned	Unassigned	299_0	Clustered	24	VC_299_0	24	0.9905	1	0.9905	0	0	0	1	Ref
KX581093	Unassigned	Unassigned	Unassigned	299_0	Clustered	24	VC_299_0	24	0.9905	1	0.9905	0	0	0	1	Ref
KX581094	Unassigned	Unassigned	Unassigned	299_0	Clustered	24	VC_299_0	24	0.9905	1	0.9905	0	0	0	1	Ref
KX581095	Unassigned	Unassigned	Unassigned	299_0	Clustered	24	VC_299_0	24	0.9905	1	0.9905	0	0	0	1	Ref
KX581096	Unassigned	Unassigned	Unassigned	299_0	Clustered	24	VC_299_0	24	0.9905	1	0.9905	0	0	0	1	Ref
KX581097	Unassigned	Unassigned	Unassigned	299_0	Clustered	24	VC_299_0	24	0.9905	1	0.9905	0	0	0	1	Ref
KX581098	Unassigned	Unassigned	Unassigned	299_0	Clustered	24	VC_299_0	24	0.9905	1	0.9905	0	0	0	1	Ref
KX581099	Unassigned	Unassigned	Unassigned	299_0	Clustered	24	VC_299_0	24	0.9905	1	0.9905	0	0	0	1	Ref
KX581100	Unassigned	Unassigned	Unassigned	299_0	Clustered	24	VC_299_0	24	0.9905	1	0.9905	0	0	0	1	Ref
KX585251	Unassigned	Unassigned	Unassigned	238_0	Clustered	17	VC_238_0	17	0.6109	1	0.6109	0	0	0	1	Ref
KX585252	Unassigned	Unassigned	Unassigned	225_0	Clustered	32	VC_225_0	32	0.848	1	0.848	0	0	0	0.996	Ref
KX585253	Unassigned	Unassigned	Unassigned	236_0	Clustered	72	VC_236_0	72	0.8104	1	0.8104	0	0	0	1	Ref
KX587949	Unassigned	Unassigned	Unassigned	111_0	Clustered	91	VC_111_0	91	0.9587	1	0.9587	0	0	0	1	Ref
KX588251	Unassigned	Unassigned	Unassigned	761_0	Clustered	67	VC_761_0	67	0.8947	1	0.8947	0	0	0	1	Ref
KX589269	Unassigned	Unassigned	Unassigned	372_0	Clustered	24	VC_372_0	24	0.5539	1	0.5539	0	0	0	1	Ref
KX591654	Unassigned	Unassigned	Unassigned	823_0	Clustered	124	VC_823_0	124	0.9492	1	0.9492	0	0	0	1	Ref
KX592589	Unassigned	Unassigned	Unassigned	371_11	Clustered	31	VC_371_11	31	0.0927	1	0.0927	0	0	0	0.7011	Ref
KX607101	Unassigned	Unassigned	Unassigned	447_0	Clustered	3	VC_447_0	3	0.8268	0.99509929	0.8227	0	0	0	1	Ref
KX607102	Unassigned	Unassigned	Unassigned	447_0	Clustered	3	VC_447_0	3	0.8268	0.99509929	0.8227	0	0	0	1	Ref
KX610764	Unassigned	Unassigned	Unassigned	520_83	Clustered/Singleton	1	VC_520_83	1	0	1	0	0	0	0	1	Ref
KX610765	Unassigned	Unassigned	Unassigned	950_0	Clustered	38	VC_950_0	38	0.9443	1	0.9443	0	0	0	1	Ref
KX611788	Unassigned	Unassigned	Unassigned	956_0	Clustered	39	VC_956_0	39	1	1	1	0	0	0	1	Ref
KX611831	Unassigned	Unassigned	Unassigned	551_6	Clustered	22	VC_551_6	22	0.1214	1	0.1214	0	0	0	0.8095	Ref
KX619650	Unassigned	Unassigned	Unassigned	216_6	Clustered	42	VC_216_6	42	0.473	1	0.473	0	0	0	0.8351	Ref
KX619651	Unassigned	Unassigned	Unassigned	517_1	Clustered	108	VC_517_1	108	0.8483	1	0.8483	0	0	0	0.9955	Ref
KX620750	Unassigned	Unassigned	Unassigned	1186_0	Clustered	4	VC_1186_0	4	0.4582	0.99991945	0.4582	0	0	0	1	Ref
KX620751	Unassigned	Unassigned	Unassigned	1186_0	Clustered	4	VC_1186_0	4	0.4582	0.99991945	0.4582	0	0	0	1	Ref
KX620753	Unassigned	Unassigned	Unassigned	1186_0	Clustered	4	VC_1186_0	4	0.4582	0.99991945	0.4582	0	0	0	1	Ref
KX620754	Unassigned	Unassigned	Unassigned	1186_0	Clustered	4	VC_1186_0	4	0.4582	0.99991945	0.4582	0	0	0	1	Ref
KX620786	Unassigned	Unassigned	Unassigned	371_16	Clustered	42	VC_371_16	42	0.1341	1	0.1341	0	0	0	0.7282	Ref
KX621004	Unassigned	Unassigned	Unassigned	1160_0	Clustered	73	VC_1160_0	73	0.9835	1	0.9835	0	0	0	1	Ref
KX621005	Unassigned	Unassigned	Unassigned	1160_0	Clustered	73	VC_1160_0	73	0.9835	1	0.9835	0	0	0	1	Ref
KX621007	Unassigned	Unassigned	Unassigned	234_0	Clustered	21	VC_234_0	21	0.8355	1	0.8355	0	0	0	1	Ref
KX636165	Unassigned	Unassigned	Unassigned	238_0	Clustered	17	VC_238_0	17	0.6109	1	0.6109	0	0	0	1	Ref
KX640831	Unassigned	Unassigned	Unassigned	513_0	Clustered	32	VC_513_0	32	0.6881	1	0.6881	0	0	0	1	Ref
KX641260	Unassigned	Unassigned	Unassigned	517_0	Clustered	18	VC_517_0	18	0.1599	1	0.1599	0	0	0	1	Ref
KX641261	Unassigned	Unassigned	Unassigned	223_0	Clustered	38	VC_223_0	38	0.9095	1	0.9095	0	0	0	1	Ref
KX641262	Unassigned	Unassigned	Unassigned	519_0	Clustered	39	VC_519_0	39	0.8156	1	0.8156	0	0	0	1	Ref
KX641263	Unassigned	Unassigned	Unassigned	216_3	Clustered	8	VC_216_3	8	0.0701	1	0.0701	0	0	0	0.8571	Ref
KX641264	Unassigned	Unassigned	Unassigned	236_0	Clustered	72	VC_236_0	72	0.8104	1	0.8104	0	0	0	1	Ref
KX641265	Unassigned	Unassigned	Unassigned	761_0	Clustered	67	VC_761_0	67	0.8947	1	0.8947	0	0	0	1	Ref
KX641266	Unassigned	Unassigned	Unassigned	761_0	Clustered	67	VC_761_0	67	0.8947	1	0.8947	0	0	0	1	Ref
KX648373	Unassigned	Unassigned	Unassigned	517_1	Clustered	108	VC_517_1	108	0.8483	1	0.8483	0	0	0	0.9955	Ref
KX649889	Unassigned	Unassigned	Unassigned	637_0	Clustered	115	VC_637_0	115	0.731	1	0.731	0	0	0	0.8764	Ref
KX656785	Unassigned	Unassigned	Unassigned	687_0	Clustered	3	VC_687_0	3	0.5872	0.99252665	0.5828	0	0	0	1	Ref
KX657793	Unassigned	Unassigned	Unassigned	222_0	Clustered	5	VC_222_0	5	0.0565	0.99999997	0.0565	0	0	0	1	Ref
KX657794	Unassigned	Unassigned	Unassigned	236_0	Clustered	72	VC_236_0	72	0.8104	1	0.8104	0	0	0	1	Ref
KX657795	Unassigned	Unassigned	Unassigned	236_0	Clustered	72	VC_236_0	72	0.8104	1	0.8104	0	0	0	1	Ref
KX657796	Unassigned	Unassigned	Unassigned	236_0	Clustered	72	VC_236_0	72	0.8104	1	0.8104	0	0	0	1	Ref
KX660669	Unassigned	Unassigned	Unassigned	70_0	Clustered	27	VC_70_0	27	0.5981	1	0.5981	0	0	0	0.9886	Ref
KX664447	Unassigned	Unassigned	Unassigned	225_0	Clustered	32	VC_225_0	32	0.848	1	0.848	0	0	0	0.996	Ref
KX664448	Unassigned	Unassigned	Unassigned	514_0	Clustered	75	VC_514_0	75	0.9402	1	0.9402	0	0	0	1	Ref
KX664455	Unassigned	Unassigned	Unassigned	761_0	Clustered	67	VC_761_0	67	0.8947	1	0.8947	0	0	0	1	Ref
KX664695	Unassigned	Unassigned	Unassigned	847_1	Clustered	26	VC_847_1	26	0.6323	1	0.6323	0	0	0	0.9815	Ref
KX669227	Unassigned	Unassigned	Unassigned	451_0	Clustered	80	VC_451_0	80	0.9991	1	0.9991	0	0	0	1	Ref
KX670786	Unassigned	Unassigned	Unassigned	1074_0	Clustered	19	VC_1074_0	19	0.9198	1	0.9198	0	0	0	1	Ref
KX670787	Unassigned	Unassigned	Unassigned	1074_0	Clustered	19	VC_1074_0	19	0.9198	1	0.9198	0	0	0	1	Ref
KX670788	Unassigned	Unassigned	Unassigned	514_0	Clustered	75	VC_514_0	75	0.9402	1	0.9402	0	0	0	1	Ref
KX670789	Unassigned	Unassigned	Unassigned	872_0	Clustered	31	VC_872_0	31	0.8151	1	0.8151	0	0	0	0.8194	Ref
KX670790	Unassigned	Unassigned	Unassigned	872_0	Clustered	31	VC_872_0	31	0.8151	1	0.8151	0	0	0	0.8194	Ref
KX670813	Unassigned	Unassigned	Unassigned	371_15	Clustered	44	VC_371_15	44	0.1391	1	0.1391	0	0	0	0.7474	Ref
KX670828	Unassigned	Unassigned	Unassigned	513_0	Clustered	32	VC_513_0	32	0.6881	1	0.6881	0	0	0	1	Ref
KX683292	Unassigned	Unassigned	Unassigned	371_15	Clustered	44	VC_371_15	44	0.1391	1	0.1391	0	0	0	0.7474	Ref
KX683293	Unassigned	Unassigned	Unassigned	371_15	Clustered	44	VC_371_15	44	0.1391	1	0.1391	0	0	0	0.7474	Ref
KX683423	Unassigned	Unassigned	Unassigned	513_0	Clustered	32	VC_513_0	32	0.6881	1	0.6881	0	0	0	1	Ref
KX683428	Unassigned	Unassigned	Unassigned	237_1	Clustered	13	VC_237_1	13	0.1773	1	0.1773	0	0	0	0.7308	Ref
KX683875	Unassigned	Unassigned	Unassigned	216_4	Clustered	13	VC_216_4	13	0.1657	1	0.1657	0	0	0	1	Ref

KX683876	Unassigned	Unassigned	Unassigned	325_38	Clustered	6	VC_325_38	6	0.0267	1	0.0267	0	0	0	0.8	Ref
KX685355	Unassigned	Unassigned	Unassigned	225_0	Clustered	32	VC_225_0	32	0.848	1	0.848	0	0	0	0.996	Ref
KX688102	Unassigned	Unassigned	Unassigned	1160_0	Clustered	73	VC_1160_0	73	0.9835	1	0.9835	0	0	0	1	Ref
KX688103	Unassigned	Unassigned	Unassigned	1160_0	Clustered	73	VC_1160_0	73	0.9835	1	0.9835	0	0	0	1	Ref
KX689784	Unassigned	Unassigned	Unassigned	451_0	Clustered	80	VC_451_0	80	0.9991	1	0.9991	0	0	0	1	Ref
KX702319	Unassigned	Unassigned	Unassigned	371_13	Clustered	33	VC_371_13	33	0.179	1	0.179	0	0	0	0.8769	Ref
KX702320	Unassigned	Unassigned	Unassigned	325_38	Clustered	6	VC_325_38	6	0.0267	1	0.0267	0	0	0	0.8	Ref
KX711710	Unassigned	Unassigned	Unassigned	423_0	Clustered	45	VC_423_0	45	0.9484	1	0.9484	0	0	0	1	Ref
KX712070	Unassigned	Unassigned	Unassigned	823_0	Clustered	124	VC_823_0	124	0.9492	1	0.9492	0	0	0	1	Ref
KX712071	Unassigned	Unassigned	Unassigned	823_0	Clustered	124	VC_823_0	124	0.9492	1	0.9492	0	0	0	1	Ref
KX712143	Unassigned	Unassigned	Unassigned	348_0	Clustered	22	VC_348_0	22	0.9602	1	0.9602	0	0	0	1	Ref
KX712236	Unassigned	Unassigned	Unassigned	956_0	Clustered	39	VC_956_0	39	1	1	1	0	0	0	1	Ref
KX712237	Unassigned	Unassigned	Unassigned	956_0	Clustered	39	VC_956_0	39	1	1	1	0	0	0	1	Ref
KX712238	Unassigned	Unassigned	Unassigned	325_43	Clustered/Singleton	1	VC_325_43	1	0	1	0	0	0	0	1	Ref
KX721255	Unassigned	Unassigned	Unassigned	558_49	Clustered	7	VC_558_49	7	0.0194	0.99510369	0.0193	0	0	0	0.8571	Ref
KX721256	Unassigned	Unassigned	Unassigned	558_48	Clustered	2	VC_558_48	2	0.0032	0.69466126	0.0022	0	0	0	1	Ref
KX752698	Unassigned	Unassigned	Unassigned	558_81	Clustered/Singleton	1	VC_558_81	1	0	1	0	0	0	0	1	Ref
KX756439	Unassigned	Unassigned	Unassigned	521_0	Clustered	37	VC_521_0	37	0.912	1	0.912	0	0	0	1	Ref
KX756572	Unassigned	Unassigned	Unassigned	878_0	Clustered	32	VC_878_0	32	0.9965	1	0.9965	0	0	0	1	Ref
KX758538	Unassigned	Unassigned	Unassigned	215_1	Clustered	19	VC_215_1	19	0.4188	1	0.4188	0	0	0	1	Ref
KX758539	Unassigned	Unassigned	Unassigned	217_0	Clustered	5	VC_217_0	5	0.069	0.99999998	0.069	0	0	0	1	Ref
KX765862	Unassigned	Unassigned	Unassigned	592_0	Clustered	50	VC_592_0	50	0.7314	1	0.7314	0	0	0	1	Ref
KX765863	Unassigned	Unassigned	Unassigned	592_0	Clustered	50	VC_592_0	50	0.7314	1	0.7314	0	0	0	1	Ref
KX765864	Unassigned	Unassigned	Unassigned	592_0	Clustered	50	VC_592_0	50	0.7314	1	0.7314	0	0	0	1	Ref
KX765865	Unassigned	Unassigned	Unassigned	133_15	Clustered	2	VC_133_15	2	0.0051	0.84786437	0.0044	0	0	0	1	Ref
KX774321	Unassigned	Unassigned	Unassigned	1070_1	Clustered/Singleton	1	VC_1070_1	1	0	1	0	0	0	0	1	Ref
KX774374	Unassigned	Unassigned	Unassigned	5_0	Clustered	30	VC_5_0	30	0.9647	1	0.9647	0	0	0	1	Ref
KX776161	Unassigned	Unassigned	Unassigned	28_1	Clustered	53	VC_28_1	53	0.7164	1	0.7164	0	0	0	0.9325	Ref
KX778611	Unassigned	Unassigned	Unassigned	385_0	Clustered	51	VC_385_0	51	0.8754	1	0.8754	0	0	0	1	Ref
KX781992	Unassigned	Unassigned	Unassigned	558_9	Clustered	3	VC_558_9	3	0.0064	0.82911529	0.0053	0	0	0	0.6667	Ref
KX808129	Unassigned	Unassigned	Unassigned	514_0	Clustered	75	VC_514_0	75	0.9402	1	0.9402	0	0	0	1	Ref
KX808130	Unassigned	Unassigned	Unassigned	517_1	Clustered	108	VC_517_1	108	0.8483	1	0.8483	0	0	0	0.9955	Ref
KX808131	Unassigned	Unassigned	Unassigned	520_16	Clustered/Singleton	1	VC_520_16	1	0	1	0	0	0	0	1	Ref
KX815338	Unassigned	Unassigned	Unassigned	982_0	Clustered	62	VC_982_0	62	0.8646	1	0.8646	0	0	0	0.743	Ref
KX817173	Unassigned	Unassigned	Unassigned	551_2	Clustered	17	VC_551_2	17	0.0973	1	0.0973	0	0	0	0.9779	Ref
KX817174	Unassigned	Unassigned	Unassigned	517_1	Clustered	108	VC_517_1	108	0.8483	1	0.8483	0	0	0	0.9955	Ref
KX817175	Unassigned	Unassigned	Unassigned	517_1	Clustered	108	VC_517_1	108	0.8483	1	0.8483	0	0	0	0.9955	Ref
KX822733	Unassigned	Unassigned	Unassigned	496_3	Clustered/Singleton	1	VC_496_3	1	0	1	0	0	0	0	1	Ref
KX826077	Unassigned	Unassigned	Unassigned	428_0	Clustered	33	VC_428_0	33	0.9915	1	0.9915	0	0	0	1	Ref
KX827368	Unassigned	Unassigned	Unassigned	11_0	Clustered	19	VC_11_0	19	0.7562	1	0.7562	0	0	0	1	Ref
KX827369	Unassigned	Unassigned	Unassigned	11_0	Clustered	19	VC_11_0	19	0.7562	1	0.7562	0	0	0	1	Ref
KX827370	Unassigned	Unassigned	Unassigned	11_0	Clustered	19	VC_11_0	19	0.7562	1	0.7562	0	0	0	1	Ref
KX827371	Unassigned	Unassigned	Unassigned	15_0	Clustered	3	VC_15_0	3	0.0481	0.9985346	0.048	0	0	0	1	Ref
KX828710	Unassigned	Unassigned	Unassigned	575_0	Clustered	35	VC_575_0	35	0.8815	1	0.8815	0	0	0	1	Ref
KX828711	Unassigned	Unassigned	Unassigned	283_85	Clustered/Singleton	1	VC_283_85	1	0	1	0	0	0	0	1	Ref
KX831080	Unassigned	Unassigned	Unassigned	558_82	Clustered	3	VC_558_82	3	0.0064	0.88433874	0.0057	0	0	0	0.6667	Ref
KX833211	Unassigned	Unassigned	Unassigned	228_0	Clustered	60	VC_228_0	60	0.942	1	0.942	0	0	0	1	Ref
KX834009	Unassigned	Unassigned	Unassigned	551_6	Clustered	22	VC_551_6	22	0.1214	1	0.1214	0	0	0	0.8095	Ref
KX845404	Unassigned	Unassigned	Unassigned	192_0	Clustered	15	VC_192_0	15	0.5071	1	0.5071	0	0	0	1	Ref
KX855961	Unassigned	Unassigned	Unassigned	1074_0	Clustered	19	VC_1074_0	19	0.9198	1	0.9198	0	0	0	1	Ref
KX855962	Unassigned	Unassigned	Unassigned	1074_0	Clustered	19	VC_1074_0	19	0.9198	1	0.9198	0	0	0	1	Ref
KX856662	Unassigned	Unassigned	Unassigned	111_0	Clustered	91	VC_111_0	91	0.9587	1	0.9587	0	0	0	1	Ref
KX879601	Unassigned	Unassigned	Unassigned	730_0	Clustered	9	VC_730_0	9	1	1	1	0	0	0	1	Ref
KX879602	Unassigned	Unassigned	Unassigned	730_0	Clustered	9	VC_730_0	9	1	1	1	0	0	0	1	Ref
KX879627	Unassigned	Unassigned	Unassigned	844_0	Clustered	27	VC_844_0	27	0.9385	1	0.9385	0	0	0	1	Ref
KX879641	Unassigned	Unassigned	Unassigned	256_0	Clustered	14	VC_256_0	14	0.2368	1	0.2368	0	0	0	1	Ref
KX879642	Unassigned	Unassigned	Unassigned	256_0	Clustered	14	VC_256_0	14	0.2368	1	0.2368	0	0	0	1	Ref
KX879643	Unassigned	Unassigned	Unassigned	255_0	Clustered	13	VC_255_0	13	0.2048	1	0.2048	0	0	0	1	Ref
KX889068	Unassigned	Unassigned	Unassigned	930_0	Clustered	7	VC_930_0	7	0.519	1	0.519	0	0	0	1	Ref
KX897981	Unassigned	Unassigned	Unassigned	215_1	Clustered	19	VC_215_1	19	0.4188	1	0.4188	0	0	0	1	Ref
KX898399	Unassigned	Unassigned	Unassigned	465_0	Clustered	7	VC_465_0	7	0.3322	1	0.3322	0	0	0	1	Ref
KX898400	Unassigned	Unassigned	Unassigned	465_0	Clustered	7	VC_465_0	7	0.3322	1	0.3322	0	0	0	1	Ref
KX912252	Unassigned	Unassigned	Unassigned	896_0	Clustered	5	VC_896_0	5	0.4981	0.99999977	0.4981	0	0	0	1	Ref
KX925554	Unassigned	Unassigned	Unassigned	960_1	Clustered/Singleton	1	VC_960_1	1	0	1	0	0	0	0	1	Ref
KX961385	Unassigned	Unassigned	Unassigned	166_0	Clustered	26	VC_166_0	26	0.7923	1	0.7923	0	0	0	1	Ref
KX961629	Unassigned	Unassigned	Unassigned	884_0	Clustered	28	VC_884_0	28	0.6211	1	0.6211	0	0	0	0.9841	Ref
KX961630	Unassigned	Unassigned	Unassigned	187_0	Clustered	4	VC_187_0	4	0.0661	0.99994098	0.0661	0	0	0	1	Ref
KX961631	Unassigned	Unassigned	Unassigned	818_0	Clustered	20	VC_818_0	20	0.9601	1	0.9601	0	0	0	1	Ref
KX961632	Unassigned	Unassigned	Unassigned	884_0	Clustered	28	VC_884_0	28	0.6211	1	0.6211	0	0	0	0.9841	Ref
KX987127	Unassigned	Unassigned	Unassigned	805_0	Clustered	4	VC_805_0	4	0.9081	0.99622688	0.9046	0	0	0	1	Ref
KX987158	Unassigned	Unassigned	Unassigned	1049_0	Clustered	8	VC_1049_0	8	0.6413	1	0.6413	0	0	0	1	Ref
KX987999	Unassigned	Unassigned	Unassigned	187_1	Clustered	7	VC_187_1	7	0.1423	1	0.1423	0	0	0	1	Ref
KY000003	Unassigned	Unassigned	Unassigned	133_30	Clustered	2	VC_133_30	2	0.0052	0.87738871	0.0046	0	0	0	1	Ref
KY000079	Unassigned	Unassigned	Unassigned	702_0	Clustered	5	VC_702_0	5	0.7794	0.99999988	0.7794	0	0	0	1	Ref
KY000080	Unassigned	Unassigned	Unassigned	491_0	Clustered	36	VC_491_0	36	0.5336	1	0.5336	0	0	0	0.9714	Ref
KY000081	Unassigned	Unassigned	Unassigned	111_0	Clustered	91	VC_111_0	91	0.9587	1	0.9587	0	0	0	1	Ref
KY000082	Unassigned	Unassigned	Unassigned	79_11	Clustered/Singleton	1	VC_79_11	1	0	1	0	0	0	0	1	Ref
KY000083	Unassigned	Unassigned	Unassigned	76_0	Clustered	37	VC_76_0	37	0.9403	1	0.9403	0	0	0	1	Ref
KY000084	Unassigned	Unassigned	Unassigned	96_0	Clustered	30	VC_96_0	30	0.718	1	0.718	0	0	0	1	Ref
KY000085	Unassigned	Unassigned	Unassigned	96_0	Clustered	30	VC_96_0	30	0.718	1	0.718	0	0	0	1	Ref
KY000218	Unassigned	Unassigned	Unassigned	166_0	Clustered	26	VC_166_0	26	0.7923	1	0.7923	0	0	0	1	Ref

KY000219	Unassigned	Unassigned	Unassigned	166_0	Clustered	26	VC_166_0	26	0.7923	1	0.7923	0	0	0	1	Ref
KY000220	Unassigned	Unassigned	Unassigned	166_0	Clustered	26	VC_166_0	26	0.7923	1	0.7923	0	0	0	1	Ref
KY000221	Unassigned	Unassigned	Unassigned	166_0	Clustered	26	VC_166_0	26	0.7923	1	0.7923	0	0	0	1	Ref
KY002061	Unassigned	Unassigned	Unassigned	1049_0	Clustered	8	VC_1049_0	8	0.6413	1	0.6413	0	0	0	1	Ref
KY006473	Unassigned	Unassigned	Unassigned	517_1	Clustered	108	VC_517_1	108	0.8483	1	0.8483	0	0	0	0.9955	Ref
KY006474	Unassigned	Unassigned	Unassigned	371_1	Clustered	11	VC_371_1	11	0.0379	1	0.0379	0	0	0	0.8545	Ref
KY006853	Unassigned	Unassigned	Unassigned	170_1	Clustered/Singleton	1	VC_170_1	1	0	1	0	0	0	0	1	Ref
KY014601	Unassigned	Unassigned	Unassigned	592_0	Clustered	50	VC_592_0	50	0.7314	1	0.7314	0	0	0	1	Ref
KY022433	Unassigned	Unassigned	Unassigned	904_2	Clustered	7	VC_904_2	7	0.2857	1	0.2857	0	0	0	1	Ref
KY030782	Unassigned	Unassigned	Unassigned	89_2	Clustered	5	VC_89_2	5	0.2033	0.99999643	0.2033	0	0	0	1	Ref
KY045851	Unassigned	Unassigned	Unassigned	5_0	Clustered	30	VC_5_0	30	0.9647	1	0.9647	0	0	0	1	Ref
KY056619	Unassigned	Unassigned	Unassigned	879_0	Clustered	36	VC_879_0	36	0.994	1	0.994	0	0	0	1	Ref
KY056620	Unassigned	Unassigned	Unassigned	8_0	Clustered	58	VC_8_0	58	0.8306	1	0.8306	0	0	0	0.9946	Ref
KY065147	Unassigned	Unassigned	Unassigned	495_1	Clustered	41	VC_495_1	41	0.328	1	0.328	0	0	0	0.911	Ref
KY065148	Unassigned	Unassigned	Unassigned	784_0	Clustered	6	VC_784_0	6	0.5517	1	0.5517	0	0	0	1	Ref
KY065149	Unassigned	Unassigned	Unassigned	989_0	Clustered	2	VC_989_0	2	0.1205	0.95251219	0.1148	0	0	0	1	Ref
KY065449	Unassigned	Unassigned	Unassigned	415_0	Clustered/Singleton	1	VC_415_0	1	0	1	0	0	0	0	1	Ref
KY065450	Unassigned	Unassigned	Unassigned	414_0	Clustered	37	VC_414_0	37	0.585	1	0.585	0	0	0	0.9474	Ref
KY065451	Unassigned	Unassigned	Unassigned	414_0	Clustered	37	VC_414_0	37	0.585	1	0.585	0	0	0	0.9474	Ref
KY065452	Unassigned	Unassigned	Unassigned	414_0	Clustered	37	VC_414_0	37	0.585	1	0.585	0	0	0	0.9474	Ref
KY065453	Unassigned	Unassigned	Unassigned	333_0	Clustered	25	VC_333_0	25	0.4681	1	0.4681	0	0	0	1	Ref
KY065454	Unassigned	Unassigned	Unassigned	333_0	Clustered	25	VC_333_0	25	0.4681	1	0.4681	0	0	0	1	Ref
KY065455	Unassigned	Unassigned	Unassigned	412_0	Clustered	39	VC_412_0	39	0.845	1	0.845	0	0	0	1	Ref
KY065456	Unassigned	Unassigned	Unassigned	414_0	Clustered	37	VC_414_0	37	0.585	1	0.585	0	0	0	0.9474	Ref
KY065458	Unassigned	Unassigned	Unassigned	333_0	Clustered	25	VC_333_0	25	0.4681	1	0.4681	0	0	0	1	Ref
KY065459	Unassigned	Unassigned	Unassigned	333_0	Clustered	25	VC_333_0	25	0.4681	1	0.4681	0	0	0	1	Ref
KY065460	Unassigned	Unassigned	Unassigned	333_0	Clustered	25	VC_333_0	25	0.4681	1	0.4681	0	0	0	1	Ref
KY065461	Unassigned	Unassigned	Unassigned	333_0	Clustered	25	VC_333_0	25	0.4681	1	0.4681	0	0	0	1	Ref
KY065462	Unassigned	Unassigned	Unassigned	333_0	Clustered	25	VC_333_0	25	0.4681	1	0.4681	0	0	0	1	Ref
KY065463	Unassigned	Unassigned	Unassigned	333_0	Clustered	25	VC_333_0	25	0.4681	1	0.4681	0	0	0	1	Ref
KY065465	Unassigned	Unassigned	Unassigned	413_0	Clustered	3	VC_413_0	3	0.0415	0.99841754	0.0415	0	0	0	1	Ref
KY065466	Unassigned	Unassigned	Unassigned	318_0	Clustered	3	VC_318_0	3	0.0414	0.98579852	0.0408	0	0	0	1	Ref
KY065468	Unassigned	Unassigned	Unassigned	318_0	Clustered	3	VC_318_0	3	0.0414	0.98579852	0.0408	0	0	0	1	Ref
KY065469	Unassigned	Unassigned	Unassigned	333_0	Clustered	25	VC_333_0	25	0.4681	1	0.4681	0	0	0	1	Ref
KY065470	Unassigned	Unassigned	Unassigned	333_0	Clustered	25	VC_333_0	25	0.4681	1	0.4681	0	0	0	1	Ref
KY065471	Unassigned	Unassigned	Unassigned	333_0	Clustered	25	VC_333_0	25	0.4681	1	0.4681	0	0	0	1	Ref
KY065472	Unassigned	Unassigned	Unassigned	414_0	Clustered	37	VC_414_0	37	0.585	1	0.585	0	0	0	0.9474	Ref
KY065473	Unassigned	Unassigned	Unassigned	414_0	Clustered	37	VC_414_0	37	0.585	1	0.585	0	0	0	0.9474	Ref
KY065474	Unassigned	Unassigned	Unassigned	414_0	Clustered	37	VC_414_0	37	0.585	1	0.585	0	0	0	0.9474	Ref
KY065475	Unassigned	Unassigned	Unassigned	414_0	Clustered	37	VC_414_0	37	0.585	1	0.585	0	0	0	0.9474	Ref
KY065476	Unassigned	Unassigned	Unassigned	414_0	Clustered	37	VC_414_0	37	0.585	1	0.585	0	0	0	0.9474	Ref
KY065477	Unassigned	Unassigned	Unassigned	414_0	Clustered	37	VC_414_0	37	0.585	1	0.585	0	0	0	0.9474	Ref
KY065478	Unassigned	Unassigned	Unassigned	414_0	Clustered	37	VC_414_0	37	0.585	1	0.585	0	0	0	0.9474	Ref
KY065479	Unassigned	Unassigned	Unassigned	412_0	Clustered	39	VC_412_0	39	0.845	1	0.845	0	0	0	1	Ref
KY065480	Unassigned	Unassigned	Unassigned	414_0	Clustered	37	VC_414_0	37	0.585	1	0.585	0	0	0	0.9474	Ref
KY065485	Unassigned	Unassigned	Unassigned	317_2	Clustered	20	VC_317_2	20	0.1976	1	0.1976	0	0	0	0.6842	Ref
KY065486	Unassigned	Unassigned	Unassigned	414_0	Clustered	37	VC_414_0	37	0.585	1	0.585	0	0	0	0.9474	Ref
KY065487	Unassigned	Unassigned	Unassigned	412_0	Clustered	39	VC_412_0	39	0.845	1	0.845	0	0	0	1	Ref
KY065488	Unassigned	Unassigned	Unassigned	414_0	Clustered	37	VC_414_0	37	0.585	1	0.585	0	0	0	0.9474	Ref
KY065490	Unassigned	Unassigned	Unassigned	412_0	Clustered	39	VC_412_0	39	0.845	1	0.845	0	0	0	1	Ref
KY065491	Unassigned	Unassigned	Unassigned	412_0	Clustered	39	VC_412_0	39	0.845	1	0.845	0	0	0	1	Ref
KY065492	Unassigned	Unassigned	Unassigned	412_0	Clustered	39	VC_412_0	39	0.845	1	0.845	0	0	0	1	Ref
KY065493	Unassigned	Unassigned	Unassigned	412_0	Clustered	39	VC_412_0	39	0.845	1	0.845	0	0	0	1	Ref
KY065494	Unassigned	Unassigned	Unassigned	333_0	Clustered	25	VC_333_0	25	0.4681	1	0.4681	0	0	0	1	Ref
KY065495	Unassigned	Unassigned	Unassigned	333_0	Clustered	25	VC_333_0	25	0.4681	1	0.4681	0	0	0	1	Ref
KY065496	Unassigned	Unassigned	Unassigned	414_0	Clustered	37	VC_414_0	37	0.585	1	0.585	0	0	0	0.9474	Ref
KY065497	Unassigned	Unassigned	Unassigned	414_0	Clustered	37	VC_414_0	37	0.585	1	0.585	0	0	0	0.9474	Ref
KY065498	Unassigned	Unassigned	Unassigned	323_1	Clustered	9	VC_323_1	9	0.1798	1	0.1798	0	0	0	0.75	Ref
KY065499	Unassigned	Unassigned	Unassigned	333_0	Clustered	25	VC_333_0	25	0.4681	1	0.4681	0	0	0	1	Ref
KY065500	Unassigned	Unassigned	Unassigned	414_0	Clustered	37	VC_414_0	37	0.585	1	0.585	0	0	0	0.9474	Ref
KY065501	Unassigned	Unassigned	Unassigned	412_0	Clustered	39	VC_412_0	39	0.845	1	0.845	0	0	0	1	Ref
KY065502	Unassigned	Unassigned	Unassigned	412_0	Clustered	39	VC_412_0	39	0.845	1	0.845	0	0	0	1	Ref
KY065503	Unassigned	Unassigned	Unassigned	317_2	Clustered	20	VC_317_2	20	0.1976	1	0.1976	0	0	0	0.6842	Ref
KY065504	Unassigned	Unassigned	Unassigned	317_2	Clustered	20	VC_317_2	20	0.1976	1	0.1976	0	0	0	0.6842	Ref
KY065505	Unassigned	Unassigned	Unassigned	414_0	Clustered	37	VC_414_0	37	0.585	1	0.585	0	0	0	0.9474	Ref
KY073123	Unassigned	Unassigned	Unassigned	132_1	Clustered	17	VC_132_1	17	0.1738	1	0.1738	0	0	0	0.9853	Ref
KY073228	Unassigned	Unassigned	Unassigned	76_0	Clustered	37	VC_76_0	37	0.9403	1	0.9403	0	0	0	1	Ref
KY082667	Unassigned	Unassigned	Unassigned	699_0	Clustered	71	VC_699_0	71	0.9352	1	0.9352	0	0	0	1	Ref
KY083058	Unassigned	Unassigned	Unassigned	517_1	Clustered	108	VC_517_1	108	0.8483	1	0.8483	0	0	0	0.9955	Ref
KY083059	Unassigned	Unassigned	Unassigned	517_0	Clustered	18	VC_517_0	18	0.1599	1	0.1599	0	0	0	1	Ref
KY083060	Unassigned	Unassigned	Unassigned	517_1	Clustered	108	VC_517_1	108	0.8483	1	0.8483	0	0	0	0.9955	Ref
KY083061	Unassigned	Unassigned	Unassigned	517_1	Clustered	108	VC_517_1	108	0.8483	1	0.8483	0	0	0	0.9955	Ref
KY083062	Unassigned	Unassigned	Unassigned	517_1	Clustered	108	VC_517_1	108	0.8483	1	0.8483	0	0	0	0.9955	Ref
KY083063	Unassigned	Unassigned	Unassigned	517_1	Clustered	108	VC_517_1	108	0.8483	1	0.8483	0	0	0	0.9955	Ref
KY083726	Unassigned	Unassigned	Unassigned	841_0	Clustered	10	VC_841_0	10	0.5997	1	0.5997	0	0	0	1	Ref
KY084243	Unassigned	Unassigned	Unassigned	386_0	Clustered	49	VC_386_0	49	0.7941	1	0.7941	0	0	0	1	Ref
KY087898	Unassigned	Unassigned	Unassigned	792_0	Clustered	35	VC_792_0	35	0.9828	1	0.9828	0	0	0	1	Ref
KY087992	Unassigned	Unassigned	Unassigned	234_0	Clustered	21	VC_234_0	21	0.8355	1	0.8355	0	0	0	1	Ref
KY087993	Unassigned	Unassigned	Unassigned	237_1	Clustered	13	VC_237_1	13	0.1773	1	0.1773	0	0	0	0.7308	Ref
KY092479	Unassigned	Unassigned	Unassigned	727_0	Clustered	11	VC_727_0	11	0.5054	1	0.5054	0	0	0	1	Ref
KY092480	Unassigned	Unassigned	Unassigned	727_0	Clustered	11	VC_727_0	11	0.5054	1	0.5054	0	0	0	1	Ref

KY092481	Unassigned	Unassigned	Unassigned	727_0	Clustered	11	VC_727_0	11	0.5054	1	0.5054	0	0	0	1	Ref
KY092482	Unassigned	Unassigned	Unassigned	727_0	Clustered	11	VC_727_0	11	0.5054	1	0.5054	0	0	0	1	Ref
KY092483	Unassigned	Unassigned	Unassigned	727_0	Clustered	11	VC_727_0	11	0.5054	1	0.5054	0	0	0	1	Ref
KY092484	Unassigned	Unassigned	Unassigned	727_0	Clustered	11	VC_727_0	11	0.5054	1	0.5054	0	0	0	1	Ref
KY114934	Unassigned	Unassigned	Unassigned	190_21	Clustered/Singleton	1	VC_190_21	1	0	1	0	0	0	0	1	Ref
KY117485	Unassigned	Unassigned	Unassigned	154_0	Clustered	4	VC_154_0	4	0.1766	0.99995106	0.1766	0	0	0	1	Ref
KY130461	Unassigned	Unassigned	Unassigned	507_0	Clustered	8	VC_507_0	8	0.4466	1	0.4466	0	0	0	1	Ref
KY176369	Unassigned	Unassigned	Unassigned	637_1	Clustered	27	VC_637_1	27	0.2578	1	0.2578	0	0	0	0.9858	Ref
KY203335	Unassigned	Unassigned	Unassigned	409_0	Clustered	3	VC_409_0	3	0.1496	0.98294101	0.147	0	0	0	1	Ref
KY204244	Unassigned	Unassigned	Unassigned	517_1	Clustered	108	VC_517_1	108	0.8483	1	0.8483	0	0	0	0.9955	Ref
KY204245	Unassigned	Unassigned	Unassigned	517_1	Clustered	108	VC_517_1	108	0.8483	1	0.8483	0	0	0	0.9955	Ref
KY204246	Unassigned	Unassigned	Unassigned	517_1	Clustered	108	VC_517_1	108	0.8483	1	0.8483	0	0	0	0.9955	Ref
KY204247	Unassigned	Unassigned	Unassigned	517_1	Clustered	108	VC_517_1	108	0.8483	1	0.8483	0	0	0	0.9955	Ref
KY204248	Unassigned	Unassigned	Unassigned	517_1	Clustered	108	VC_517_1	108	0.8483	1	0.8483	0	0	0	0.9955	Ref
KY204249	Unassigned	Unassigned	Unassigned	517_1	Clustered	108	VC_517_1	108	0.8483	1	0.8483	0	0	0	0.9955	Ref
KY204250	Unassigned	Unassigned	Unassigned	517_1	Clustered	108	VC_517_1	108	0.8483	1	0.8483	0	0	0	0.9955	Ref
KY204251	Unassigned	Unassigned	Unassigned	517_1	Clustered	108	VC_517_1	108	0.8483	1	0.8483	0	0	0	0.9955	Ref
KY206887	Unassigned	Unassigned	Unassigned	1022_0	Clustered	4	VC_1022_0	4	0.8047	0.99996089	0.8046	0	0	0	1	Ref
KY210139	Unassigned	Unassigned	Unassigned	364_0	Clustered	16	VC_364_0	16	0.6491	1	0.6491	0	0	0	1	Ref
KY213952	Unassigned	Unassigned	Unassigned	325_48	Clustered	2	VC_325_48	2	0.0039	0.95008941	0.0037	0	0	0	1	Ref
KY223999	Unassigned	Unassigned	Unassigned	763_2	Clustered	7	VC_763_2	7	0.2612	1	0.2612	0	0	0	1	Ref
KY224000	Unassigned	Unassigned	Unassigned	372_1	Clustered	9	VC_372_1	9	0.194	1	0.194	0	0	0	1	Ref
KY224001	Unassigned	Unassigned	Unassigned	325_54	Clustered/Singleton	1	VC_325_54	1	0	1	0	0	0	0	1	Ref
KY249644	Unassigned	Unassigned	Unassigned	598_0	Clustered	4	VC_598_0	4	0.0597	0.99355358	0.0593	0	0	0	1	Ref
KY250482	Unassigned	Unassigned	Unassigned	409_0	Clustered	3	VC_409_0	3	0.1496	0.98294101	0.147	0	0	0	1	Ref
KY264020	Unassigned	Unassigned	Unassigned	961_0	Clustered	3	VC_961_0	3	0.675	0.99054823	0.6686	0	0	0	1	Ref
KY268295	Unassigned	Unassigned	Unassigned	699_0	Clustered	71	VC_699_0	71	0.9352	1	0.9352	0	0	0	1	Ref
KY268296	Unassigned	Unassigned	Unassigned	699_0	Clustered	71	VC_699_0	71	0.9352	1	0.9352	0	0	0	1	Ref
KY271395	Unassigned	Unassigned	Unassigned	31_0	Clustered	9	VC_31_0	9	0.3435	1	0.3435	0	0	0	0.6944	Ref
KY271396	Unassigned	Unassigned	Unassigned	31_0	Clustered	9	VC_31_0	9	0.3435	1	0.3435	0	0	0	0.6944	Ref
KY271397	Unassigned	Unassigned	Unassigned	229_0	Clustered	32	VC_229_0	32	0.9119	1	0.9119	0	0	0	1	Ref
KY271398	Unassigned	Unassigned	Unassigned	6_0	Clustered	44	VC_6_0	44	0.908	1	0.908	0	0	0	1	Ref
KY271399	Unassigned	Unassigned	Unassigned	35_0	Clustered	6	VC_35_0	6	0.2254	1	0.2254	0	0	0	0.9333	Ref
KY271401	Unassigned	Unassigned	Unassigned	244_0	Clustered	12	VC_244_0	12	0.4039	1	0.4039	0	0	0	1	Ref
KY271402	Unassigned	Unassigned	Unassigned	34_0	Clustered	4	VC_34_0	4	0.4349	0.99998062	0.4349	0	0	0	1	Ref
KY290756	Unassigned	Unassigned	Unassigned	1027_0	Clustered	20	VC_1027_0	20	0.7114	1	0.7114	0	0	0	1	Ref
KY290947	Unassigned	Unassigned	Unassigned	1189_0	Clustered	9	VC_1189_0	9	0.968	1	0.968	0	0	0	1	Ref
KY290948	Unassigned	Unassigned	Unassigned	600_1	Clustered	7	VC_600_1	7	0.1837	0.99999997	0.1837	0	0	0	1	Ref
KY290949	Unassigned	Unassigned	Unassigned	1189_0	Clustered	9	VC_1189_0	9	0.968	1	0.968	0	0	0	1	Ref
KY290950	Unassigned	Unassigned	Unassigned	1189_0	Clustered	9	VC_1189_0	9	0.968	1	0.968	0	0	0	1	Ref
KY290951	Unassigned	Unassigned	Unassigned	600_1	Clustered	7	VC_600_1	7	0.1837	0.99999997	0.1837	0	0	0	1	Ref
KY290952	Unassigned	Unassigned	Unassigned	1189_0	Clustered	9	VC_1189_0	9	0.968	1	0.968	0	0	0	1	Ref
KY290953	Unassigned	Unassigned	Unassigned	369_0	Clustered	6	VC_369_0	6	0.5924	1	0.5924	0	0	0	1	Ref
KY290954	Unassigned	Unassigned	Unassigned	369_0	Clustered	6	VC_369_0	6	0.5924	1	0.5924	0	0	0	1	Ref
KY290955	Unassigned	Unassigned	Unassigned	443_1	Clustered	2	VC_443_1	2	0.0287	0.89347378	0.0257	0	0	0	1	Ref
KY290956	Unassigned	Unassigned	Unassigned	600_1	Clustered	7	VC_600_1	7	0.1837	0.99999997	0.1837	0	0	0	1	Ref
KY290957	Unassigned	Unassigned	Unassigned	600_1	Clustered	7	VC_600_1	7	0.1837	0.99999997	0.1837	0	0	0	1	Ref
KY290958	Unassigned	Unassigned	Unassigned	600_1	Clustered	7	VC_600_1	7	0.1837	0.99999997	0.1837	0	0	0	1	Ref
KY290975	Unassigned	Unassigned	Unassigned	283_111	Clustered/Singleton	1	VC_283_111	1	0	1	0	0	0	0	1	Ref
KY295891	Unassigned	Unassigned	Unassigned	452_0	Clustered	66	VC_452_0	66	0.9248	1	0.9248	0	0	0	1	Ref
KY295892	Unassigned	Unassigned	Unassigned	452_0	Clustered	66	VC_452_0	66	0.9248	1	0.9248	0	0	0	1	Ref
KY295893	Unassigned	Unassigned	Unassigned	452_0	Clustered	66	VC_452_0	66	0.9248	1	0.9248	0	0	0	1	Ref
KY295894	Unassigned	Unassigned	Unassigned	451_0	Clustered	80	VC_451_0	80	0.9991	1	0.9991	0	0	0	1	Ref
KY295895	Unassigned	Unassigned	Unassigned	638_0	Clustered	45	VC_638_0	45	0.7741	1	0.7741	0	0	0	1	Ref
KY295896	Unassigned	Unassigned	Unassigned	638_0	Clustered	45	VC_638_0	45	0.7741	1	0.7741	0	0	0	1	Ref
KY295897	Unassigned	Unassigned	Unassigned	452_0	Clustered	66	VC_452_0	66	0.9248	1	0.9248	0	0	0	1	Ref
KY295898	Unassigned	Unassigned	Unassigned	638_0	Clustered	45	VC_638_0	45	0.7741	1	0.7741	0	0	0	1	Ref
KY295899	Unassigned	Unassigned	Unassigned	452_0	Clustered	66	VC_452_0	66	0.9248	1	0.9248	0	0	0	1	Ref
KY296500	Unassigned	Unassigned	Unassigned	814_0	Clustered	3	VC_814_0	3	1	1	1	0	0	0	1	Ref
KY296501	Unassigned	Unassigned	Unassigned	814_0	Clustered	3	VC_814_0	3	1	1	1	0	0	0	1	Ref
KY303907	Unassigned	Unassigned	Unassigned	108_0	Clustered	33	VC_108_0	33	0.949	1	0.949	0	0	0	1	Ref
KY316062	Unassigned	Unassigned	Unassigned	70_0	Clustered	27	VC_70_0	27	0.5981	1	0.5981	0	0	0	0.9886	Ref
KY318515	Unassigned	Unassigned	Unassigned	878_0	Clustered	32	VC_878_0	32	0.9965	1	0.9965	0	0	0	1	Ref
KY319168	Unassigned	Unassigned	Unassigned	551_2	Clustered	17	VC_551_2	17	0.0973	1	0.0973	0	0	0	0.9779	Ref
KY348865	Unassigned	Unassigned	Unassigned	520_44	Clustered/Singleton	1	VC_520_44	1	0	1	0	0	0	0	1	Ref
KY349816	Unassigned	Unassigned	Unassigned	412_0	Clustered	39	VC_412_0	39	0.845	1	0.845	0	0	0	1	Ref
KY363465	Unassigned	Unassigned	Unassigned	193_0	Clustered	2	VC_193_0	2	0.0321	0.95762656	0.0308	0	0	0	1	Ref
KY365739	Unassigned	Unassigned	Unassigned	975_0	Clustered	11	VC_975_0	11	0.9687	1	0.9687	0	0	0	1	Ref
KY368639	Unassigned	Unassigned	Unassigned	87_0	Clustered	8	VC_87_0	8	0.5545	1	0.5545	0	0	0	1	Ref
KY368640	Unassigned	Unassigned	Unassigned	85_4	Clustered	3	VC_85_4	3	0.0294	0.98125255	0.0288	0	0	0	1	Ref
KY379511	Unassigned	Unassigned	Unassigned	896_0	Clustered	5	VC_896_0	5	0.4981	0.99999977	0.4981	0	0	0	1	Ref
KY379853	Unassigned	Unassigned	Unassigned	637_0	Clustered	115	VC_637_0	115	0.731	1	0.731	0	0	0	0.8764	Ref
KY380102	Unassigned	Unassigned	Unassigned	236_0	Clustered	72	VC_236_0	72	0.8104	1	0.8104	0	0	0	1	Ref
KY381600	Unassigned	Unassigned	Unassigned	634_1	Clustered	6	VC_634_1	6	0.4228	1	0.4228	0	0	0	1	Ref
KY385380	Unassigned	Unassigned	Unassigned	371_13	Clustered	33	VC_371_13	33	0.179	1	0.179	0	0	0	0.8769	Ref
KY385381	Unassigned	Unassigned	Unassigned	764_0	Clustered	4	VC_764_0	4	0.3556	0.99997469	0.3556	0	0	0	1	Ref
KY385382	Unassigned	Unassigned	Unassigned	371_13	Clustered	33	VC_371_13	33	0.179	1	0.179	0	0	0	0.8769	Ref
KY385383	Unassigned	Unassigned	Unassigned	371_13	Clustered	33	VC_371_13	33	0.179	1	0.179	0	0	0	0.8769	Ref
KY385384	Unassigned	Unassigned	Unassigned	520_53	Clustered/Singleton	1	VC_520_53	1	0	1	0	0	0	0	1	Ref
KY385423	Unassigned	Unassigned	Unassigned	111_0	Clustered	91	VC_111_0	91	0.9587	1	0.9587	0	0	0	1	Ref
KY389063	Unassigned	Unassigned	Unassigned	7_0	Clustered	28	VC_7_0	28	0.3916	1	0.3916	0	0	0	0.9947	Ref

KY389064	Unassigned	Unassigned	Unassigned	7_0	Clustered	28	VC_7_0	28	0.3916	1	0.3916	0	0	0	0.9947	Ref
KY389315	Unassigned	Unassigned	Unassigned	823_0	Clustered	124	VC_823_0	124	0.9492	1	0.9492	0	0	0	1	Ref
KY389316	Unassigned	Unassigned	Unassigned	823_0	Clustered	124	VC_823_0	124	0.9492	1	0.9492	0	0	0	1	Ref
KY398841	Unassigned	Unassigned	Unassigned	456_0	Clustered	62	VC_456_0	62	0.9026	1	0.9026	0	0	0	1	Ref
KY434670	Unassigned	Unassigned	Unassigned	950_0	Clustered	38	VC_950_0	38	0.9443	1	0.9443	0	0	0	1	Ref
KY435490	Unassigned	Unassigned	Unassigned	452_0	Clustered	66	VC_452_0	66	0.9248	1	0.9248	0	0	0	1	Ref
KY448244	Unassigned	Unassigned	Unassigned	67_0	Clustered	2	VC_67_0	2	0.014	0.69357611	0.0097	0	0	0	1	Ref
KY450753	Unassigned	Unassigned	Unassigned	823_0	Clustered	124	VC_823_0	124	0.9492	1	0.9492	0	0	0	1	Ref
KY464836	Unassigned	Unassigned	Unassigned	70_0	Clustered	27	VC_70_0	27	0.5981	1	0.5981	0	0	0	0.9886	Ref
KY464936	Unassigned	Unassigned	Unassigned	513_0	Clustered	32	VC_513_0	32	0.6881	1	0.6881	0	0	0	1	Ref
KY471266	Unassigned	Unassigned	Unassigned	517_1	Clustered	108	VC_517_1	108	0.8483	1	0.8483	0	0	0	0.9955	Ref
KY471267	Unassigned	Unassigned	Unassigned	520_27	Clustered	2	VC_520_27	2	0.0143	0.95761635	0.0137	0	0	0	1	Ref
KY471268	Unassigned	Unassigned	Unassigned	406_0	Clustered	11	VC_406_0	11	0.9449	1	0.9449	0	0	0	1	Ref
KY471269	Unassigned	Unassigned	Unassigned	406_0	Clustered	11	VC_406_0	11	0.9449	1	0.9449	0	0	0	1	Ref
KY471460	Unassigned	Unassigned	Unassigned	221_0	Clustered	22	VC_221_0	22	0.7447	1	0.7447	0	0	0	1	Ref
KY472224	Unassigned	Unassigned	Unassigned	108_0	Clustered	33	VC_108_0	33	0.949	1	0.949	0	0	0	1	Ref
KY514263	Unassigned	Unassigned	Unassigned	849_2	Clustered	2	VC_849_2	2	0.0246	0.86513761	0.0213	0	0	0	1	Ref
KY514264	Unassigned	Unassigned	Unassigned	841_0	Clustered	10	VC_841_0	10	0.5997	1	0.5997	0	0	0	1	Ref
KY514265	Unassigned	Unassigned	Unassigned	841_0	Clustered	10	VC_841_0	10	0.5997	1	0.5997	0	0	0	1	Ref
KY549152	Unassigned	Unassigned	Unassigned	551_7	Clustered	28	VC_551_7	28	0.1661	1	0.1661	0	0	0	0.8519	Ref
KY549153	Unassigned	Unassigned	Unassigned	956_0	Clustered	39	VC_956_0	39	1	1	1	0	0	0	1	Ref
KY549154	Unassigned	Unassigned	Unassigned	956_0	Clustered	39	VC_956_0	39	1	1	1	0	0	0	1	Ref
KY549155	Unassigned	Unassigned	Unassigned	517_1	Clustered	108	VC_517_1	108	0.8483	1	0.8483	0	0	0	0.9955	Ref
KY549443	Unassigned	Unassigned	Unassigned	92_0	Clustered	22	VC_92_0	22	0.5639	1	0.5639	0	0	0	1	Ref
KY549659	Unassigned	Unassigned	Unassigned	841_0	Clustered	10	VC_841_0	10	0.5997	1	0.5997	0	0	0	1	Ref
KY554760	Unassigned	Unassigned	Unassigned	202_0	Clustered	102	VC_202_0	102	0.4049	1	0.4049	0	0	0	0.7876	Ref
KY554761	Unassigned	Unassigned	Unassigned	202_0	Clustered	102	VC_202_0	102	0.4049	1	0.4049	0	0	0	0.7876	Ref
KY554762	Unassigned	Unassigned	Unassigned	534_0	Clustered	9	VC_534_0	9	0.99	1	0.99	0	0	0	1	Ref
KY554763	Unassigned	Unassigned	Unassigned	534_0	Clustered	9	VC_534_0	9	0.99	1	0.99	0	0	0	1	Ref
KY554764	Unassigned	Unassigned	Unassigned	534_0	Clustered	9	VC_534_0	9	0.99	1	0.99	0	0	0	1	Ref
KY554765	Unassigned	Unassigned	Unassigned	534_0	Clustered	9	VC_534_0	9	0.99	1	0.99	0	0	0	1	Ref
KY554766	Unassigned	Unassigned	Unassigned	534_0	Clustered	9	VC_534_0	9	0.99	1	0.99	0	0	0	1	Ref
KY554767	Unassigned	Unassigned	Unassigned	534_0	Clustered	9	VC_534_0	9	0.99	1	0.99	0	0	0	1	Ref
KY554768	Unassigned	Unassigned	Unassigned	474_0	Clustered	14	VC_474_0	14	0.8466	1	0.8466	0	0	0	1	Ref
KY554769	Unassigned	Unassigned	Unassigned	474_0	Clustered	14	VC_474_0	14	0.8466	1	0.8466	0	0	0	1	Ref
KY554770	Unassigned	Unassigned	Unassigned	474_0	Clustered	14	VC_474_0	14	0.8466	1	0.8466	0	0	0	1	Ref
KY554771	Unassigned	Unassigned	Unassigned	474_0	Clustered	14	VC_474_0	14	0.8466	1	0.8466	0	0	0	1	Ref
KY554772	Unassigned	Unassigned	Unassigned	474_0	Clustered	14	VC_474_0	14	0.8466	1	0.8466	0	0	0	1	Ref
KY554773	Unassigned	Unassigned	Unassigned	474_0	Clustered	14	VC_474_0	14	0.8466	1	0.8466	0	0	0	1	Ref
KY554774	Unassigned	Unassigned	Unassigned	474_0	Clustered	14	VC_474_0	14	0.8466	1	0.8466	0	0	0	1	Ref
KY554775	Unassigned	Unassigned	Unassigned	474_0	Clustered	14	VC_474_0	14	0.8466	1	0.8466	0	0	0	1	Ref
KY554776	Unassigned	Unassigned	Unassigned	474_0	Clustered	14	VC_474_0	14	0.8466	1	0.8466	0	0	0	1	Ref
KY554777	Unassigned	Unassigned	Unassigned	474_0	Clustered	14	VC_474_0	14	0.8466	1	0.8466	0	0	0	1	Ref
KY555142	Unassigned	Unassigned	Unassigned	976_4	Clustered	11	VC_976_4	11	0.3364	1	0.3364	0	0	0	1	Ref
KY555143	Unassigned	Unassigned	Unassigned	976_4	Clustered	11	VC_976_4	11	0.3364	1	0.3364	0	0	0	1	Ref
KY555144	Unassigned	Unassigned	Unassigned	976_4	Clustered	11	VC_976_4	11	0.3364	1	0.3364	0	0	0	1	Ref
KY555145	Unassigned	Unassigned	Unassigned	976_4	Clustered	11	VC_976_4	11	0.3364	1	0.3364	0	0	0	1	Ref
KY555146	Unassigned	Unassigned	Unassigned	976_4	Clustered	11	VC_976_4	11	0.3364	1	0.3364	0	0	0	1	Ref
KY555147	Unassigned	Unassigned	Unassigned	976_4	Clustered	11	VC_976_4	11	0.3364	1	0.3364	0	0	0	1	Ref
KY563228	Unassigned	Unassigned	Unassigned	599_0	Clustered	50	VC_599_0	50	0.9989	1	0.9989	0	0	0	1	Ref
KY565347	Unassigned	Unassigned	Unassigned	101_0	Clustered	3	VC_101_0	3	0.3621	0.99764884	0.3612	0	0	0	1	Ref
KY566218	Unassigned	Unassigned	Unassigned	408_0	Clustered	9	VC_408_0	9	0.86	1	0.86	0	0	0	1	Ref
KY575286	Unassigned	Unassigned	Unassigned	938_0	Clustered	99	VC_938_0	99	0.9119	1	0.9119	0	0	0	0.9928	Ref
KY579375	Unassigned	Unassigned	Unassigned	599_0	Clustered	50	VC_599_0	50	0.9989	1	0.9989	0	0	0	1	Ref
KY581279	Unassigned	Unassigned	Unassigned	149_8	Clustered	66	VC_149_8	66	0.4565	1	0.4565	0	0	0	0.8182	Ref
KY593455	Unassigned	Unassigned	Unassigned	490_3	Clustered	2	VC_490_3	2	0.0135	0.95703149	0.0129	0	0	0	1	Ref
KY608966	Unassigned	Unassigned	Unassigned	428_0	Clustered	33	VC_428_0	33	0.9915	1	0.9915	0	0	0	1	Ref
KY608967	Unassigned	Unassigned	Unassigned	283_109	Clustered	2	VC_283_109	2	0.0027	0.91535723	0.0024	0	0	0	1	Ref
KY612839	Unassigned	Unassigned	Unassigned	784_0	Clustered	6	VC_784_0	6	0.5517	1	0.5517	0	0	0	1	Ref
KY613597	Unassigned	Unassigned	Unassigned	408_0	Clustered	9	VC_408_0	9	0.86	1	0.86	0	0	0	1	Ref
KY618819	Unassigned	Unassigned	Unassigned	423_0	Clustered	45	VC_423_0	45	0.9484	1	0.9484	0	0	0	1	Ref
KY620117	Unassigned	Unassigned	Unassigned	592_0	Clustered	50	VC_592_0	50	0.7314	1	0.7314	0	0	0	1	Ref
KY622015	Unassigned	Unassigned	Unassigned	498_0	Clustered	8	VC_498_0	8	0.3987	1	0.3987	0	0	0	1	Ref
KY624610	Unassigned	Unassigned	Unassigned	408_0	Clustered	9	VC_408_0	9	0.86	1	0.86	0	0	0	1	Ref
KY624611	Unassigned	Unassigned	Unassigned	408_0	Clustered	9	VC_408_0	9	0.86	1	0.86	0	0	0	1	Ref
KY624612	Unassigned	Unassigned	Unassigned	408_0	Clustered	9	VC_408_0	9	0.86	1	0.86	0	0	0	1	Ref
KY624613	Unassigned	Unassigned	Unassigned	408_0	Clustered	9	VC_408_0	9	0.86	1	0.86	0	0	0	1	Ref
KY624614	Unassigned	Unassigned	Unassigned	408_0	Clustered	9	VC_408_0	9	0.86	1	0.86	0	0	0	1	Ref
KY624615	Unassigned	Unassigned	Unassigned	408_0	Clustered	9	VC_408_0	9	0.86	1	0.86	0	0	0	1	Ref
KY624616	Unassigned	Unassigned	Unassigned	408_0	Clustered	9	VC_408_0	9	0.86	1	0.86	0	0	0	1	Ref
KY626162	Unassigned	Unassigned	Unassigned	329_1	Clustered	17	VC_329_1	17	0.0867	1	0.0867	0	0	0	0.8235	Ref
KY626163	Unassigned	Unassigned	Unassigned	637_0	Clustered	115	VC_637_0	115	0.731	1	0.731	0	0	0	0.8764	Ref
KY626176	Unassigned	Unassigned	Unassigned	1027_0	Clustered	20	VC_1027_0	20	0.7114	1	0.7114	0	0	0	1	Ref
KY626177	Unassigned	Unassigned	Unassigned	1027_0	Clustered	20	VC_1027_0	20	0.7114	1	0.7114	0	0	0	1	Ref
KY629563	Unassigned	Unassigned	Unassigned	978_0	Clustered	2	VC_978_0	2	0.0595	0.95411371	0.0568	0	0	0	1	Ref
KY629621	Unassigned	Unassigned	Unassigned	891_0	Clustered	2	VC_891_0	2	0.5746	0.92263554	0.5301	0	0	0	1	Ref
KY630163	Unassigned	Unassigned	Unassigned	133_34	Clustered	3	VC_133_34	3	0.0102	0.97369044	0.01	0	0	0	0.6667	Ref
KY630187	Unassigned	Unassigned	Unassigned	164_7	Clustered	7	VC_164_7	7	0.0956	0.99998	0.0956	0	0	0	1	Ref
KY652723	Unassigned	Unassigned	Unassigned	823_0	Clustered	124	VC_823_0	124	0.9492	1	0.9492	0	0	0	1	Ref
KY652724	Unassigned	Unassigned	Unassigned	823_0	Clustered	124	VC_823_0	124	0.9492	1	0.9492	0	0	0	1	Ref
KY652725	Unassigned	Unassigned	Unassigned	823_0	Clustered	124	VC_823_0	124	0.9492	1	0.9492	0	0	0	1	Ref

KY652726	Unassigned	Unassigned	Unassigned	582_3	Clustered	12	VC_582_3	12	0.2357	1	0.2357	0	0	0	0.8636	Ref
KY653119	Unassigned	Unassigned	Unassigned	245_0	Clustered	10	VC_245_0	10	0.1805	1	0.1805	0	0	0	0.9778	Ref
KY653123	Unassigned	Unassigned	Unassigned	10_0	Clustered	25	VC_10_0	25	0.3467	1	0.3467	0	0	0	0.9733	Ref
KY653125	Unassigned	Unassigned	Unassigned	7_0	Clustered	28	VC_7_0	28	0.3916	1	0.3916	0	0	0	0.9947	Ref
KY653237	Unassigned	Unassigned	Unassigned	303_0	Clustered	155	VC_303_0	155	0.9979	1	0.9979	0	0	0	1	Ref
KY654690	Unassigned	Unassigned	Unassigned	330_0	Clustered	27	VC_330_0	27	0.5088	1	0.5088	0	0	0	0.8661	Ref
KY657202	Unassigned	Unassigned	Unassigned	584_0	Clustered	28	VC_584_0	28	0.7704	1	0.7704	0	0	0	1	Ref
KY658673	Unassigned	Unassigned	Unassigned	299_0	Clustered	24	VC_299_0	24	0.9905	1	0.9905	0	0	0	1	Ref
KY658674	Unassigned	Unassigned	Unassigned	299_0	Clustered	24	VC_299_0	24	0.9905	1	0.9905	0	0	0	1	Ref
KY658675	Unassigned	Unassigned	Unassigned	299_0	Clustered	24	VC_299_0	24	0.9905	1	0.9905	0	0	0	1	Ref
KY658676	Unassigned	Unassigned	Unassigned	299_0	Clustered	24	VC_299_0	24	0.9905	1	0.9905	0	0	0	1	Ref
KY658677	Unassigned	Unassigned	Unassigned	299_0	Clustered	24	VC_299_0	24	0.9905	1	0.9905	0	0	0	1	Ref
KY658678	Unassigned	Unassigned	Unassigned	299_0	Clustered	24	VC_299_0	24	0.9905	1	0.9905	0	0	0	1	Ref
KY658679	Unassigned	Unassigned	Unassigned	299_0	Clustered	24	VC_299_0	24	0.9905	1	0.9905	0	0	0	1	Ref
KY658680	Unassigned	Unassigned	Unassigned	299_0	Clustered	24	VC_299_0	24	0.9905	1	0.9905	0	0	0	1	Ref
KY661963	Unassigned	Unassigned	Unassigned	730_0	Clustered	9	VC_730_0	9	1	1	1	0	0	0	1	Ref
KY670595	Unassigned	Unassigned	Unassigned	698_0	Clustered	26	VC_698_0	26	0.9366	1	0.9366	0	0	0	1	Ref
KY676783	Unassigned	Unassigned	Unassigned	371_15	Clustered	44	VC_371_15	44	0.1391	1	0.1391	0	0	0	0.7474	Ref
KY676784	Unassigned	Unassigned	Unassigned	727_0	Clustered	11	VC_727_0	11	0.5054	1	0.5054	0	0	0	1	Ref
KY677846	Unassigned	Unassigned	Unassigned	190_15	Clustered	3	VC_190_15	3	0.0146	0.99759465	0.0145	0	0	0	0.6667	Ref
KY683735	Unassigned	Unassigned	Unassigned	590_0	Clustered	27	VC_590_0	27	0.9947	1	0.9947	0	0	0	1	Ref
KY683736	Unassigned	Unassigned	Unassigned	772_9	Clustered	7	VC_772_9	7	0.0369	0.87705503	0.0324	0	0	0	1	Ref
KY684082	Unassigned	Unassigned	Unassigned	111_0	Clustered	91	VC_111_0	91	0.9587	1	0.9587	0	0	0	1	Ref
KY694971	Unassigned	Unassigned	Unassigned	584_0	Clustered	28	VC_584_0	28	0.7704	1	0.7704	0	0	0	1	Ref
KY695153	Unassigned	Unassigned	Unassigned	7_0	Clustered	28	VC_7_0	28	0.3916	1	0.3916	0	0	0	0.9947	Ref
KY703222	Unassigned	Unassigned	Unassigned	286_10	Clustered	9	VC_286_10	9	0.0615	0.99999878	0.0615	0	0	0	0.6667	Ref
KY705251	Unassigned	Unassigned	Unassigned	252_0	Clustered	121	VC_252_0	121	0.7376	1	0.7376	0	0	0	0.9999	Ref
KY705252	Unassigned	Unassigned	Unassigned	255_0	Clustered	13	VC_255_0	13	0.2048	1	0.2048	0	0	0	1	Ref
KY705253	Unassigned	Unassigned	Unassigned	255_0	Clustered	13	VC_255_0	13	0.2048	1	0.2048	0	0	0	1	Ref
KY705254	Unassigned	Unassigned	Unassigned	255_0	Clustered	13	VC_255_0	13	0.2048	1	0.2048	0	0	0	1	Ref
KY705255	Unassigned	Unassigned	Unassigned	255_0	Clustered	13	VC_255_0	13	0.2048	1	0.2048	0	0	0	1	Ref
KY705256	Unassigned	Unassigned	Unassigned	252_0	Clustered	121	VC_252_0	121	0.7376	1	0.7376	0	0	0	0.9999	Ref
KY705257	Unassigned	Unassigned	Unassigned	252_0	Clustered	121	VC_252_0	121	0.7376	1	0.7376	0	0	0	0.9999	Ref
KY705258	Unassigned	Unassigned	Unassigned	254_0	Clustered	46	VC_254_0	46	0.5159	1	0.5159	0	0	0	1	Ref
KY705259	Unassigned	Unassigned	Unassigned	252_0	Clustered	121	VC_252_0	121	0.7376	1	0.7376	0	0	0	0.9999	Ref
KY705260	Unassigned	Unassigned	Unassigned	252_0	Clustered	121	VC_252_0	121	0.7376	1	0.7376	0	0	0	0.9999	Ref
KY705261	Unassigned	Unassigned	Unassigned	252_0	Clustered	121	VC_252_0	121	0.7376	1	0.7376	0	0	0	0.9999	Ref
KY705262	Unassigned	Unassigned	Unassigned	252_0	Clustered	121	VC_252_0	121	0.7376	1	0.7376	0	0	0	0.9999	Ref
KY705263	Unassigned	Unassigned	Unassigned	252_0	Clustered	121	VC_252_0	121	0.7376	1	0.7376	0	0	0	0.9999	Ref
KY705264	Unassigned	Unassigned	Unassigned	252_0	Clustered	121	VC_252_0	121	0.7376	1	0.7376	0	0	0	0.9999	Ref
KY705265	Unassigned	Unassigned	Unassigned	252_0	Clustered	121	VC_252_0	121	0.7376	1	0.7376	0	0	0	0.9999	Ref
KY705266	Unassigned	Unassigned	Unassigned	252_0	Clustered	121	VC_252_0	121	0.7376	1	0.7376	0	0	0	0.9999	Ref
KY705267	Unassigned	Unassigned	Unassigned	252_0	Clustered	121	VC_252_0	121	0.7376	1	0.7376	0	0	0	0.9999	Ref
KY705268	Unassigned	Unassigned	Unassigned	254_0	Clustered	46	VC_254_0	46	0.5159	1	0.5159	0	0	0	1	Ref
KY705269	Unassigned	Unassigned	Unassigned	254_0	Clustered	46	VC_254_0	46	0.5159	1	0.5159	0	0	0	1	Ref
KY705270	Unassigned	Unassigned	Unassigned	252_0	Clustered	121	VC_252_0	121	0.7376	1	0.7376	0	0	0	0.9999	Ref
KY705271	Unassigned	Unassigned	Unassigned	252_0	Clustered	121	VC_252_0	121	0.7376	1	0.7376	0	0	0	0.9999	Ref
KY705272	Unassigned	Unassigned	Unassigned	252_0	Clustered	121	VC_252_0	121	0.7376	1	0.7376	0	0	0	0.9999	Ref
KY705273	Unassigned	Unassigned	Unassigned	252_0	Clustered	121	VC_252_0	121	0.7376	1	0.7376	0	0	0	0.9999	Ref
KY705274	Unassigned	Unassigned	Unassigned	252_0	Clustered	121	VC_252_0	121	0.7376	1	0.7376	0	0	0	0.9999	Ref
KY705275	Unassigned	Unassigned	Unassigned	252_0	Clustered	121	VC_252_0	121	0.7376	1	0.7376	0	0	0	0.9999	Ref
KY705276	Unassigned	Unassigned	Unassigned	252_0	Clustered	121	VC_252_0	121	0.7376	1	0.7376	0	0	0	0.9999	Ref
KY705277	Unassigned	Unassigned	Unassigned	254_0	Clustered	46	VC_254_0	46	0.5159	1	0.5159	0	0	0	1	Ref
KY705278	Unassigned	Unassigned	Unassigned	254_0	Clustered	46	VC_254_0	46	0.5159	1	0.5159	0	0	0	1	Ref
KY705279	Unassigned	Unassigned	Unassigned	254_0	Clustered	46	VC_254_0	46	0.5159	1	0.5159	0	0	0	1	Ref
KY705280	Unassigned	Unassigned	Unassigned	254_0	Clustered	46	VC_254_0	46	0.5159	1	0.5159	0	0	0	1	Ref
KY705281	Unassigned	Unassigned	Unassigned	254_0	Clustered	46	VC_254_0	46	0.5159	1	0.5159	0	0	0	1	Ref
KY705282	Unassigned	Unassigned	Unassigned	252_0	Clustered	121	VC_252_0	121	0.7376	1	0.7376	0	0	0	0.9999	Ref
KY705283	Unassigned	Unassigned	Unassigned	252_0	Clustered	121	VC_252_0	121	0.7376	1	0.7376	0	0	0	0.9999	Ref
KY705284	Unassigned	Unassigned	Unassigned	252_0	Clustered	121	VC_252_0	121	0.7376	1	0.7376	0	0	0	0.9999	Ref
KY705285	Unassigned	Unassigned	Unassigned	254_0	Clustered	46	VC_254_0	46	0.5159	1	0.5159	0	0	0	1	Ref
KY705286	Unassigned	Unassigned	Unassigned	254_0	Clustered	46	VC_254_0	46	0.5159	1	0.5159	0	0	0	1	Ref
KY705287	Unassigned	Unassigned	Unassigned	252_0	Clustered	121	VC_252_0	121	0.7376	1	0.7376	0	0	0	0.9999	Ref
KY705288	Unassigned	Unassigned	Unassigned	252_0	Clustered	121	VC_252_0	121	0.7376	1	0.7376	0	0	0	0.9999	Ref
KY705289	Unassigned	Unassigned	Unassigned	252_0	Clustered	121	VC_252_0	121	0.7376	1	0.7376	0	0	0	0.9999	Ref
KY705290	Unassigned	Unassigned	Unassigned	252_0	Clustered	121	VC_252_0	121	0.7376	1	0.7376	0	0	0	0.9999	Ref
KY705409	Unassigned	Unassigned	Unassigned	125_0	Clustered	17	VC_125_0	17	0.9244	1	0.9244	0	0	0	1	Ref
KY707339	Unassigned	Unassigned	Unassigned	747_0	Clustered	23	VC_747_0	23	0.8202	1	0.8202	0	0	0	1	Ref
KY709296	Unassigned	Unassigned	Unassigned	793_0	Clustered	12	VC_793_0	12	0.8531	1	0.8531	0	0	0	1	Ref
KY744228	Unassigned	Unassigned	Unassigned	348_0	Clustered	22	VC_348_0	22	0.9602	1	0.9602	0	0	0	1	Ref
KY744229	Unassigned	Unassigned	Unassigned	348_0	Clustered	22	VC_348_0	22	0.9602	1	0.9602	0	0	0	1	Ref
KY744230	Unassigned	Unassigned	Unassigned	348_0	Clustered	22	VC_348_0	22	0.9602	1	0.9602	0	0	0	1	Ref
KY744231	Unassigned	Unassigned	Unassigned	348_0	Clustered	22	VC_348_0	22	0.9602	1	0.9602	0	0	0	1	Ref
KY744232	Unassigned	Unassigned	Unassigned	348_0	Clustered	22	VC_348_0	22	0.9602	1	0.9602	0	0	0	1	Ref
KY744233	Unassigned	Unassigned	Unassigned	348_0	Clustered	22	VC_348_0	22	0.9602	1	0.9602	0	0	0	1	Ref
KY744234	Unassigned	Unassigned	Unassigned	348_0	Clustered	22	VC_348_0	22	0.9602	1	0.9602	0	0	0	1	Ref
KY744235	Unassigned	Unassigned	Unassigned	348_0	Clustered	22	VC_348_0	22	0.9602	1	0.9602	0	0	0	1	Ref
KY744240	Unassigned	Unassigned	Unassigned	269_0	Clustered	3	VC_269_0	3	0.1948	0.99308177	0.1934	0	0	0	1	Ref
KY744566	Unassigned	Unassigned	Unassigned	452_0	Clustered	66	VC_452_0	66	0.9248	1	0.9248	0	0	0	1	Ref
KY769270	Unassigned	Unassigned	Unassigned	841_0	Clustered	10	VC_841_0	10	0.5997	1	0.5997	0	0	0	1	Ref
KY779848	Unassigned	Unassigned	Unassigned	153_0	Clustered	25	VC_153_0	25	0.7729	1	0.7729	0	0	0	1	Ref

KY779849	Unassigned	Unassigned	Unassigned	149_15	Clustered	16	VC_149_15	16	0.0788	1	0.0788	0	0	0	0.8083	Ref
KY780482	Unassigned	Unassigned	Unassigned	937_0	Clustered	40	VC_937_0	40	0.6809	1	0.6809	0	0	0	0.9359	Ref
KY783914	Unassigned	Unassigned	Unassigned	763_2	Clustered	7	VC_763_2	7	0.2612	1	0.2612	0	0	0	1	Ref
KY787212	Unassigned	Unassigned	Unassigned	191_16	Clustered	12	VC_191_16	12	0.0643	1	0.0643	0	0	0	0.9394	Ref
KY787213	Unassigned	Unassigned	Unassigned	133_34	Clustered	3	VC_133_34	3	0.0102	0.97369044	0.01	0	0	0	0.6667	Ref
KY794641	Unassigned	Unassigned	Unassigned	149_10	Clustered/Singleton	1	VC_149_10	1	0	1	0	0	0	0	1	Ref
KY794642	Unassigned	Unassigned	Unassigned	149_29	Clustered	3	VC_149_29	3	0.012	0.99448577	0.012	0	0	0	1	Ref
KY794643	Unassigned	Unassigned	Unassigned	149_29	Clustered	3	VC_149_29	3	0.012	0.99448577	0.012	0	0	0	1	Ref
KY798120	Unassigned	Unassigned	Unassigned	129_0	Clustered	43	VC_129_0	43	0.886	1	0.886	0	0	0	1	Ref
KY798121	Unassigned	Unassigned	Unassigned	129_0	Clustered	43	VC_129_0	43	0.886	1	0.886	0	0	0	1	Ref
KY817360	Unassigned	Unassigned	Unassigned	1201_0	Clustered	2	VC_1201_0	2	0.8919	0.81445332	0.7264	0	0	0	1	Ref
KY829116	Unassigned	Unassigned	Unassigned	698_0	Clustered	26	VC_698_0	26	0.9366	1	0.9366	0	0	0	1	Ref
KY853667	Unassigned	Unassigned	Unassigned	177_0	Clustered	5	VC_177_0	5	0.7468	0.99885646	0.746	0	0	0	1	Ref
KY860935	Unassigned	Unassigned	Unassigned	461_0	Clustered	96	VC_461_0	96	0.9307	1	0.9307	0	0	0	0.9575	Ref
KY883634	Unassigned	Unassigned	Unassigned	495_1	Clustered	41	VC_495_1	41	0.328	1	0.328	0	0	0	0.911	Ref
KY883635	Unassigned	Unassigned	Unassigned	495_1	Clustered	41	VC_495_1	41	0.328	1	0.328	0	0	0	0.911	Ref
KY883636	Unassigned	Unassigned	Unassigned	495_1	Clustered	41	VC_495_1	41	0.328	1	0.328	0	0	0	0.911	Ref
KY883637	Unassigned	Unassigned	Unassigned	495_1	Clustered	41	VC_495_1	41	0.328	1	0.328	0	0	0	0.911	Ref
KY883638	Unassigned	Unassigned	Unassigned	495_0	Clustered	39	VC_495_0	39	0.308	1	0.308	0	0	0	1	Ref
KY883639	Unassigned	Unassigned	Unassigned	495_0	Clustered	39	VC_495_0	39	0.308	1	0.308	0	0	0	1	Ref
KY883640	Unassigned	Unassigned	Unassigned	495_0	Clustered	39	VC_495_0	39	0.308	1	0.308	0	0	0	1	Ref
KY883641	Unassigned	Unassigned	Unassigned	82_0	Clustered	24	VC_82_0	24	0.7351	1	0.7351	0	0	0	1	Ref
KY883642	Unassigned	Unassigned	Unassigned	607_0	Clustered	16	VC_607_0	16	0.9179	1	0.9179	0	0	0	1	Ref
KY883643	Unassigned	Unassigned	Unassigned	82_0	Clustered	24	VC_82_0	24	0.7351	1	0.7351	0	0	0	1	Ref
KY883644	Unassigned	Unassigned	Unassigned	82_0	Clustered	24	VC_82_0	24	0.7351	1	0.7351	0	0	0	1	Ref
KY883645	Unassigned	Unassigned	Unassigned	82_0	Clustered	24	VC_82_0	24	0.7351	1	0.7351	0	0	0	1	Ref
KY883646	Unassigned	Unassigned	Unassigned	82_0	Clustered	24	VC_82_0	24	0.7351	1	0.7351	0	0	0	1	Ref
KY883647	Unassigned	Unassigned	Unassigned	607_0	Clustered	16	VC_607_0	16	0.9179	1	0.9179	0	0	0	1	Ref
KY883648	Unassigned	Unassigned	Unassigned	82_0	Clustered	24	VC_82_0	24	0.7351	1	0.7351	0	0	0	1	Ref
KY883649	Unassigned	Unassigned	Unassigned	82_0	Clustered	24	VC_82_0	24	0.7351	1	0.7351	0	0	0	1	Ref
KY883650	Unassigned	Unassigned	Unassigned	82_0	Clustered	24	VC_82_0	24	0.7351	1	0.7351	0	0	0	1	Ref
KY883651	Unassigned	Unassigned	Unassigned	82_0	Clustered	24	VC_82_0	24	0.7351	1	0.7351	0	0	0	1	Ref
KY883652	Unassigned	Unassigned	Unassigned	82_0	Clustered	24	VC_82_0	24	0.7351	1	0.7351	0	0	0	1	Ref
KY883653	Unassigned	Unassigned	Unassigned	82_0	Clustered	24	VC_82_0	24	0.7351	1	0.7351	0	0	0	1	Ref
KY883654	Unassigned	Unassigned	Unassigned	971_0	Clustered	3	VC_971_0	3	0.0548	0.99838914	0.0547	0	0	0	1	Ref
KY883655	Unassigned	Unassigned	Unassigned	971_0	Clustered	3	VC_971_0	3	0.0548	0.99838914	0.0547	0	0	0	1	Ref
KY883656	Unassigned	Unassigned	Unassigned	607_0	Clustered	16	VC_607_0	16	0.9179	1	0.9179	0	0	0	1	Ref
KY883657	Unassigned	Unassigned	Unassigned	754_0	Clustered	8	VC_754_0	8	0.752	1	0.752	0	0	0	1	Ref
KY883658	Unassigned	Unassigned	Unassigned	754_0	Clustered	8	VC_754_0	8	0.752	1	0.752	0	0	0	1	Ref
KY888143	Unassigned	Unassigned	Unassigned	209_0	Clustered	4	VC_209_0	4	0.1136	0.99998779	0.1136	0	0	0	1	Ref
KY888680	Unassigned	Unassigned	Unassigned	699_0	Clustered	71	VC_699_0	71	0.9352	1	0.9352	0	0	0	1	Ref
KY888882	Unassigned	Unassigned	Unassigned	883_3	Clustered	2	VC_883_3	2	0.0182	0.87900236	0.016	0	0	0	1	Ref
KY914478	Unassigned	Unassigned	Unassigned	42_0	Clustered	49	VC_42_0	49	0.4517	1	0.4517	0	0	0	0.8597	Ref
KY926698	Unassigned	Unassigned	Unassigned	209_0	Clustered	4	VC_209_0	4	0.1136	0.99998779	0.1136	0	0	0	1	Ref
KY926699	Unassigned	Unassigned	Unassigned	209_0	Clustered	4	VC_209_0	4	0.1136	0.99998779	0.1136	0	0	0	1	Ref
KY926791	Unassigned	Unassigned	Unassigned	722_0	Clustered	13	VC_722_0	13	0.5104	1	0.5104	0	0	0	1	Ref
KY926792	Unassigned	Unassigned	Unassigned	765_0	Clustered	132	VC_765_0	132	1	1	1	0	0	0	1	Ref
KY926793	Unassigned	Unassigned	Unassigned	765_0	Clustered	132	VC_765_0	132	1	1	1	0	0	0	1	Ref
KY942056	Unassigned	Unassigned	Unassigned	131_0	Clustered	17	VC_131_0	17	0.2671	1	0.2671	0	0	0	1	Ref
KY942057	Unassigned	Unassigned	Unassigned	131_0	Clustered	17	VC_131_0	17	0.2671	1	0.2671	0	0	0	1	Ref
KY945355	Unassigned	Unassigned	Unassigned	237_2	Clustered	11	VC_237_2	11	0.1409	1	0.1409	0	0	0	0.9818	Ref
KY947509	Unassigned	Unassigned	Unassigned	818_0	Clustered	20	VC_818_0	20	0.9601	1	0.9601	0	0	0	1	Ref
KY951963	Unassigned	Unassigned	Unassigned	912_0	Clustered	76	VC_912_0	76	0.979	1	0.979	0	0	0	1	Ref
KY951964	Unassigned	Unassigned	Unassigned	912_0	Clustered	76	VC_912_0	76	0.979	1	0.979	0	0	0	1	Ref
KY953156	Unassigned	Unassigned	Unassigned	984_0	Clustered	38	VC_984_0	38	0.9758	1	0.9758	0	0	0	1	Ref
KY962008	Unassigned	Unassigned	Unassigned	451_0	Clustered	80	VC_451_0	80	0.9991	1	0.9991	0	0	0	1	Ref
KY963369	Unassigned	Unassigned	Unassigned	704_0	Clustered	16	VC_704_0	16	0.7274	1	0.7274	0	0	0	1	Ref
KY963370	Unassigned	Unassigned	Unassigned	704_0	Clustered	16	VC_704_0	16	0.7274	1	0.7274	0	0	0	1	Ref
KY963371	Unassigned	Unassigned	Unassigned	704_0	Clustered	16	VC_704_0	16	0.7274	1	0.7274	0	0	0	1	Ref
KY963424	Unassigned	Unassigned	Unassigned	190_23	Clustered	10	VC_190_23	10	0.0704	1	0.0704	0	0	0	0.8444	Ref
KY965063	Unassigned	Unassigned	Unassigned	514_0	Clustered	75	VC_514_0	75	0.9402	1	0.9402	0	0	0	1	Ref
KY965064	Unassigned	Unassigned	Unassigned	520_40	Clustered	4	VC_520_40	4	0.0396	0.99998776	0.0396	0	0	0	1	Ref
KY965065	Unassigned	Unassigned	Unassigned	216_6	Clustered	42	VC_216_6	42	0.473	1	0.473	0	0	0	0.8351	Ref
KY965066	Unassigned	Unassigned	Unassigned	371_18	Clustered	10	VC_371_18	10	0.0302	1	0.0302	0	0	0	0.8	Ref
KY965934	Unassigned	Unassigned	Unassigned	963_0	Clustered	4	VC_963_0	4	0.8505	0.99879268	0.8495	0	0	0	1	Ref
KY969628	Unassigned	Unassigned	Unassigned	568_0	Clustered	8	VC_568_0	8	0.5738	1	0.5738	0	0	0	1	Ref
KY971610	Unassigned	Unassigned	Unassigned	287_1	Clustered/Singleton	1	VC_287_1	1	0	1	0	0	0	0	1	Ref
KY971611	Unassigned	Unassigned	Unassigned	129_0	Clustered	43	VC_129_0	43	0.886	1	0.886	0	0	0	1	Ref
KY979235	Unassigned	Unassigned	Unassigned	912_0	Clustered	76	VC_912_0	76	0.979	1	0.979	0	0	0	1	Ref
KY979236	Unassigned	Unassigned	Unassigned	912_0	Clustered	76	VC_912_0	76	0.979	1	0.979	0	0	0	1	Ref
KY979237	Unassigned	Unassigned	Unassigned	912_0	Clustered	76	VC_912_0	76	0.979	1	0.979	0	0	0	1	Ref
KY979238	Unassigned	Unassigned	Unassigned	912_0	Clustered	76	VC_912_0	76	0.979	1	0.979	0	0	0	1	Ref
KY979239	Unassigned	Unassigned	Unassigned	912_0	Clustered	76	VC_912_0	76	0.979	1	0.979	0	0	0	1	Ref
KY979240	Unassigned	Unassigned	Unassigned	912_0	Clustered	76	VC_912_0	76	0.979	1	0.979	0	0	0	1	Ref
KY979241	Unassigned	Unassigned	Unassigned	912_0	Clustered	76	VC_912_0	76	0.979	1	0.979	0	0	0	1	Ref
KY979242	Unassigned	Unassigned	Unassigned	912_0	Clustered	76	VC_912_0	76	0.979	1	0.979	0	0	0	1	Ref
KY979243	Unassigned	Unassigned	Unassigned	912_0	Clustered	76	VC_912_0	76	0.979	1	0.979	0	0	0	1	Ref
KY979244	Unassigned	Unassigned	Unassigned	912_0	Clustered	76	VC_912_0	76	0.979	1	0.979	0	0	0	1	Ref
KY979245	Unassigned	Unassigned	Unassigned	912_0	Clustered	76	VC_912_0	76	0.979	1	0.979	0	0	0	1	Ref
KY979246	Unassigned	Unassigned	Unassigned	912_0	Clustered	76	VC_912_0	76	0.979	1	0.979	0	0	0	1	Ref
KY979247	Unassigned	Unassigned	Unassigned	912_0	Clustered	76	VC_912_0	76	0.979	1	0.979	0	0	0	1	Ref

KY981272	Unassigned	Unassigned	Unassigned	70_0	Clustered	27	VC_70_0	27	0.5981	1	0.5981	0	0	0	0.9886	Ref
KY984068	Unassigned	Unassigned	Unassigned	67_2	Clustered	2	VC_67_2	2	0.014	0.69357611	0.0097	0	0	0	1	Ref
KY985004	Unassigned	Unassigned	Unassigned	575_0	Clustered	35	VC_575_0	35	0.8815	1	0.8815	0	0	0	1	Ref
KY992519	Unassigned	Unassigned	Unassigned	912_0	Clustered	76	VC_912_0	76	0.979	1	0.979	0	0	0	1	Ref
KY992520	Unassigned	Unassigned	Unassigned	912_0	Clustered	76	VC_912_0	76	0.979	1	0.979	0	0	0	1	Ref
KY994101	Unassigned	Unassigned	Unassigned	77_0	Clustered	20	VC_77_0	20	0.9657	1	0.9657	0	0	0	1	Ref
KY996523	Unassigned	Unassigned	Unassigned	897_0	Clustered	6	VC_897_0	6	0.4156	1	0.4156	0	0	0	1	Ref
L67CG	Unassigned	Unassigned	Unassigned	690_0	Clustered	50	VC_690_0	50	0.9805	1	0.9805	0	0	0	1	Ref
LAMCG	Unassigned	Unassigned	Unassigned	43_0	Clustered	38	VC_43_0	38	0.5885	1	0.5885	0	0	0	0.9659	Ref
LC035386	Unassigned	Unassigned	Unassigned	1202_0	Clustered	2	VC_1202_0	2	1	1	1	0	0	0	1	Ref
LC063634	Unassigned	Unassigned	Unassigned	984_0	Clustered	38	VC_984_0	38	0.9758	1	0.9758	0	0	0	1	Ref
LC064302	Unassigned	Unassigned	Unassigned	839_0	Clustered	5	VC_839_0	5	0.165	0.99999995	0.165	0	0	0	1	Ref
LC066596	Unassigned	Unassigned	Unassigned	54_0	Clustered	6	VC_54_0	6	0.6725	0.9999999	0.6725	0	0	0	1	Ref
LC102729	Unassigned	Unassigned	Unassigned	174_0	Clustered	3	VC_174_0	3	0.9624	0.99333557	0.9559	0	0	0	1	Ref
LC102730	Unassigned	Unassigned	Unassigned	79_0	Clustered	64	VC_79_0	64	0.4185	1	0.4185	0	0	0	0.7649	Ref
LC105987	Unassigned	Unassigned	Unassigned	79_0	Clustered	64	VC_79_0	64	0.4185	1	0.4185	0	0	0	0.7649	Ref
LC105988	Unassigned	Unassigned	Unassigned	79_0	Clustered	64	VC_79_0	64	0.4185	1	0.4185	0	0	0	0.7649	Ref
LC105989	Unassigned	Unassigned	Unassigned	79_0	Clustered	64	VC_79_0	64	0.4185	1	0.4185	0	0	0	0.7649	Ref
LC105990	Unassigned	Unassigned	Unassigned	79_0	Clustered	64	VC_79_0	64	0.4185	1	0.4185	0	0	0	0.7649	Ref
LC168164	Unassigned	Unassigned	Unassigned	532_1	Clustered/Singleton	1	VC_532_1	1	0	1	0	0	0	0	1	Ref
LC210520	Unassigned	Unassigned	Unassigned	1202_0	Clustered	2	VC_1202_0	2	1	1	1	0	0	0	1	Ref
LC348379	Unassigned	Unassigned	Unassigned	283_182	Clustered/Singleton	1	VC_283_182	1	0	1	0	0	0	0	1	Ref
LC348380	Unassigned	Unassigned	Unassigned	283_92	Clustered	4	VC_283_92	4	0.0079	0.9999582	0.0079	0	0	0	1	Ref
LC373201	Unassigned	Unassigned	Unassigned	131_2	Clustered	7	VC_131_2	7	0.0685	1	0.0685	0	0	0	0.9048	Ref
LC375533	Unassigned	Unassigned	Unassigned	984_0	Clustered	38	VC_984_0	38	0.9758	1	0.9758	0	0	0	1	Ref
LC413193	Unassigned	Unassigned	Unassigned	823_0	Clustered	124	VC_823_0	124	0.9492	1	0.9492	0	0	0	1	Ref
LC413194	Unassigned	Unassigned	Unassigned	823_0	Clustered	124	VC_823_0	124	0.9492	1	0.9492	0	0	0	1	Ref
LC413195	Unassigned	Unassigned	Unassigned	823_0	Clustered	124	VC_823_0	124	0.9492	1	0.9492	0	0	0	1	Ref
LC465543	Unassigned	Unassigned	Unassigned	283_15	Clustered/Singleton	1	VC_283_15	1	0	1	0	0	0	0	1	Ref
LC472881	Unassigned	Unassigned	Unassigned	79_1	Clustered	2	VC_79_1	2	0.006	0.95748743	0.0057	0	0	0	1	Ref
LC472882	Unassigned	Unassigned	Unassigned	80_0	Clustered	41	VC_80_0	41	0.5837	1	0.5837	0	0	0	0.9805	Ref
LC472883	Unassigned	Unassigned	Unassigned	79_0	Clustered	64	VC_79_0	64	0.4185	1	0.4185	0	0	0	0.7649	Ref
LC472884	Unassigned	Unassigned	Unassigned	79_0	Clustered	64	VC_79_0	64	0.4185	1	0.4185	0	0	0	0.7649	Ref
LC473039	Unassigned	Unassigned	Unassigned	329_8	Clustered	2	VC_329_8	2	0.0048	0.95654014	0.0046	0	0	0	1	Ref
LC487410	Unassigned	Unassigned	Unassigned	971_1	Clustered	24	VC_971_1	24	0.7393	1	0.7393	0	0	0	0.8913	Ref
LC492751	Unassigned	Unassigned	Unassigned	153_0	Clustered	25	VC_153_0	25	0.7729	1	0.7729	0	0	0	1	Ref
LC492752	Unassigned	Unassigned	Unassigned	153_0	Clustered	25	VC_153_0	25	0.7729	1	0.7729	0	0	0	1	Ref
LC494302	Unassigned	Unassigned	Unassigned	164_11	Clustered	10	VC_164_11	10	0.1507	1	0.1507	0	0	0	0.9111	Ref
LC507823	Unassigned	Unassigned	Unassigned	637_0	Clustered	115	VC_637_0	115	0.731	1	0.731	0	0	0	0.8764	Ref
LC516895	Unassigned	Unassigned	Unassigned	575_0	Clustered	35	VC_575_0	35	0.8815	1	0.8815	0	0	0	1	Ref
LC519489	Unassigned	Unassigned	Unassigned	329_7	Clustered	18	VC_329_7	18	0.0938	1	0.0938	0	0	0	0.7778	Ref
LC519601	Unassigned	Unassigned	Unassigned	812_1	Clustered	4	VC_812_1	4	0.2266	0.99998426	0.2266	0	0	0	1	Ref
LC528882	Unassigned	Unassigned	Unassigned	3_0	Clustered	17	VC_3_0	17	0.5639	1	0.5639	0	0	0	1	Ref
LC541428	Unassigned	Unassigned	Unassigned	8_0	Clustered	58	VC_8_0	58	0.8306	1	0.8306	0	0	0	0.9946	Ref
LC545443	Unassigned	Unassigned	Unassigned	118_0	Clustered	3	VC_118_0	3	0.7119	0.99211494	0.7063	0	0	0	1	Ref
LC545444	Unassigned	Unassigned	Unassigned	118_0	Clustered	3	VC_118_0	3	0.7119	0.99211494	0.7063	0	0	0	1	Ref
LC545445	Unassigned	Unassigned	Unassigned	119_0	Clustered	2	VC_119_0	2	0.5516	0.91840661	0.5066	0	0	0	1	Ref
LC545446	Unassigned	Unassigned	Unassigned	119_0	Clustered	2	VC_119_0	2	0.5516	0.91840661	0.5066	0	0	0	1	Ref
LC547238	Unassigned	Unassigned	Unassigned	108_0	Clustered	33	VC_108_0	33	0.949	1	0.949	0	0	0	1	Ref
LC552830	Unassigned	Unassigned	Unassigned	749_0	Clustered	40	VC_749_0	40	0.7517	1	0.7517	0	0	0	1	Ref
LC553734	Unassigned	Unassigned	Unassigned	813_0	Clustered	12	VC_813_0	12	0.2347	1	0.2347	0	0	0	1	Ref
LC553735	Unassigned	Unassigned	Unassigned	813_1	Clustered	24	VC_813_1	24	0.535	1	0.535	0	0	0	1	Ref
LC553736	Unassigned	Unassigned	Unassigned	592_0	Clustered	50	VC_592_0	50	0.7314	1	0.7314	0	0	0	1	Ref
LC567830	Unassigned	Unassigned	Unassigned	40_0	Clustered	5	VC_40_0	5	0.0844	0.99999995	0.0844	0	0	0	1	Ref
LC567838	Unassigned	Unassigned	Unassigned	40_0	Clustered	5	VC_40_0	5	0.0844	0.99999995	0.0844	0	0	0	1	Ref
LC567842	Unassigned	Unassigned	Unassigned	40_0	Clustered	5	VC_40_0	5	0.0844	0.99999995	0.0844	0	0	0	1	Ref
LC574321	Unassigned	Unassigned	Unassigned	149_15	Clustered	16	VC_149_15	16	0.0788	1	0.0788	0	0	0	0.8083	Ref
LC576631	Unassigned	Unassigned	Unassigned	153_1	Clustered/Singleton	1	VC_153_1	1	0	1	0	0	0	0	1	Ref
LC589952	Unassigned	Unassigned	Unassigned	669_0	Clustered	21	VC_669_0	21	0.3623	1	0.3623	0	0	0	1	Ref
LC596377	Unassigned	Unassigned	Unassigned	91_0	Clustered	22	VC_91_0	22	0.5318	1	0.5318	0	0	0	1	Ref
LC596378	Unassigned	Unassigned	Unassigned	91_0	Clustered	22	VC_91_0	22	0.5318	1	0.5318	0	0	0	1	Ref
LC596379	Unassigned	Unassigned	Unassigned	91_0	Clustered	22	VC_91_0	22	0.5318	1	0.5318	0	0	0	1	Ref
LC597490	Unassigned	Unassigned	Unassigned	90_0	Clustered/Singleton	1	VC_90_0	1	0	1	0	0	0	0	1	Ref
LC616031	Unassigned	Unassigned	Unassigned	47_0	Clustered	48	VC_47_0	48	0.7783	1	0.7783	0	0	0	1	Ref
LC616032	Unassigned	Unassigned	Unassigned	47_0	Clustered	48	VC_47_0	48	0.7783	1	0.7783	0	0	0	1	Ref
LC616033	Unassigned	Unassigned	Unassigned	47_0	Clustered	48	VC_47_0	48	0.7783	1	0.7783	0	0	0	1	Ref
LC616034	Unassigned	Unassigned	Unassigned	47_0	Clustered	48	VC_47_0	48	0.7783	1	0.7783	0	0	0	1	Ref
LC616035	Unassigned	Unassigned	Unassigned	47_0	Clustered	48	VC_47_0	48	0.7783	1	0.7783	0	0	0	1	Ref
LC616036	Unassigned	Unassigned	Unassigned	47_0	Clustered	48	VC_47_0	48	0.7783	1	0.7783	0	0	0	1	Ref
LC616037	Unassigned	Unassigned	Unassigned	47_0	Clustered	48	VC_47_0	48	0.7783	1	0.7783	0	0	0	1	Ref
LC616038	Unassigned	Unassigned	Unassigned	47_0	Clustered	48	VC_47_0	48	0.7783	1	0.7783	0	0	0	1	Ref
LC616039	Unassigned	Unassigned	Unassigned	47_0	Clustered	48	VC_47_0	48	0.7783	1	0.7783	0	0	0	1	Ref
LC616040	Unassigned	Unassigned	Unassigned	47_0	Clustered	48	VC_47_0	48	0.7783	1	0.7783	0	0	0	1	Ref
LC616041	Unassigned	Unassigned	Unassigned	47_0	Clustered	48	VC_47_0	48	0.7783	1	0.7783	0	0	0	1	Ref
LC616042	Unassigned	Unassigned	Unassigned	47_0	Clustered	48	VC_47_0	48	0.7783	1	0.7783	0	0	0	1	Ref
LC616043	Unassigned	Unassigned	Unassigned	47_0	Clustered	48	VC_47_0	48	0.7783	1	0.7783	0	0	0	1	Ref
LC616044	Unassigned	Unassigned	Unassigned	47_0	Clustered	48	VC_47_0	48	0.7783	1	0.7783	0	0	0	1	Ref
LC616045	Unassigned	Unassigned	Unassigned	47_0	Clustered	48	VC_47_0	48	0.7783	1	0.7783	0	0	0	1	Ref
LC616046	Unassigned	Unassigned	Unassigned	47_0	Clustered	48	VC_47_0	48	0.7783	1	0.7783	0	0	0	1	Ref
LC616047	Unassigned	Unassigned	Unassigned	47_0	Clustered	48	VC_47_0	48	0.7783	1	0.7783	0	0	0	1	Ref
LC616048	Unassigned	Unassigned	Unassigned	47_0	Clustered	48	VC_47_0	48	0.7783	1	0.7783	0	0	0	1	Ref

LC616049	Unassigned	Unassigned	Unassigned	47_0	Clustered	48	VC_47_0	48	0.7783	1	0.7783	0	0	0	1	Ref
LC616050	Unassigned	Unassigned	Unassigned	47_0	Clustered	48	VC_47_0	48	0.7783	1	0.7783	0	0	0	1	Ref
LC616051	Unassigned	Unassigned	Unassigned	47_0	Clustered	48	VC_47_0	48	0.7783	1	0.7783	0	0	0	1	Ref
LC616052	Unassigned	Unassigned	Unassigned	47_0	Clustered	48	VC_47_0	48	0.7783	1	0.7783	0	0	0	1	Ref
LC616053	Unassigned	Unassigned	Unassigned	47_0	Clustered	48	VC_47_0	48	0.7783	1	0.7783	0	0	0	1	Ref
LC616054	Unassigned	Unassigned	Unassigned	47_0	Clustered	48	VC_47_0	48	0.7783	1	0.7783	0	0	0	1	Ref
LC616055	Unassigned	Unassigned	Unassigned	47_0	Clustered	48	VC_47_0	48	0.7783	1	0.7783	0	0	0	1	Ref
LC616056	Unassigned	Unassigned	Unassigned	47_0	Clustered	48	VC_47_0	48	0.7783	1	0.7783	0	0	0	1	Ref
LC616057	Unassigned	Unassigned	Unassigned	47_0	Clustered	48	VC_47_0	48	0.7783	1	0.7783	0	0	0	1	Ref
LC616058	Unassigned	Unassigned	Unassigned	47_0	Clustered	48	VC_47_0	48	0.7783	1	0.7783	0	0	0	1	Ref
LC616059	Unassigned	Unassigned	Unassigned	47_0	Clustered	48	VC_47_0	48	0.7783	1	0.7783	0	0	0	1	Ref
LC616060	Unassigned	Unassigned	Unassigned	47_0	Clustered	48	VC_47_0	48	0.7783	1	0.7783	0	0	0	1	Ref
LC616061	Unassigned	Unassigned	Unassigned	47_0	Clustered	48	VC_47_0	48	0.7783	1	0.7783	0	0	0	1	Ref
LC616062	Unassigned	Unassigned	Unassigned	47_0	Clustered	48	VC_47_0	48	0.7783	1	0.7783	0	0	0	1	Ref
LC616063	Unassigned	Unassigned	Unassigned	47_0	Clustered	48	VC_47_0	48	0.7783	1	0.7783	0	0	0	1	Ref
LC616064	Unassigned	Unassigned	Unassigned	47_0	Clustered	48	VC_47_0	48	0.7783	1	0.7783	0	0	0	1	Ref
LC616065	Unassigned	Unassigned	Unassigned	47_0	Clustered	48	VC_47_0	48	0.7783	1	0.7783	0	0	0	1	Ref
LC616066	Unassigned	Unassigned	Unassigned	47_0	Clustered	48	VC_47_0	48	0.7783	1	0.7783	0	0	0	1	Ref
LC616067	Unassigned	Unassigned	Unassigned	47_0	Clustered	48	VC_47_0	48	0.7783	1	0.7783	0	0	0	1	Ref
LC616068	Unassigned	Unassigned	Unassigned	47_0	Clustered	48	VC_47_0	48	0.7783	1	0.7783	0	0	0	1	Ref
LC616069	Unassigned	Unassigned	Unassigned	47_0	Clustered	48	VC_47_0	48	0.7783	1	0.7783	0	0	0	1	Ref
LC616070	Unassigned	Unassigned	Unassigned	47_0	Clustered	48	VC_47_0	48	0.7783	1	0.7783	0	0	0	1	Ref
LC616071	Unassigned	Unassigned	Unassigned	47_0	Clustered	48	VC_47_0	48	0.7783	1	0.7783	0	0	0	1	Ref
LC616072	Unassigned	Unassigned	Unassigned	47_0	Clustered	48	VC_47_0	48	0.7783	1	0.7783	0	0	0	1	Ref
LC616073	Unassigned	Unassigned	Unassigned	47_0	Clustered	48	VC_47_0	48	0.7783	1	0.7783	0	0	0	1	Ref
LC616074	Unassigned	Unassigned	Unassigned	47_0	Clustered	48	VC_47_0	48	0.7783	1	0.7783	0	0	0	1	Ref
LC616075	Unassigned	Unassigned	Unassigned	47_0	Clustered	48	VC_47_0	48	0.7783	1	0.7783	0	0	0	1	Ref
LC616076	Unassigned	Unassigned	Unassigned	47_0	Clustered	48	VC_47_0	48	0.7783	1	0.7783	0	0	0	1	Ref
LC616077	Unassigned	Unassigned	Unassigned	47_0	Clustered	48	VC_47_0	48	0.7783	1	0.7783	0	0	0	1	Ref
LC616078	Unassigned	Unassigned	Unassigned	47_0	Clustered	48	VC_47_0	48	0.7783	1	0.7783	0	0	0	1	Ref
LC616079	Unassigned	Unassigned	Unassigned	40_0	Clustered	5	VC_40_0	5	0.0844	0.99999995	0.0844	0	0	0	1	Ref
LC620505	Unassigned	Unassigned	Unassigned	283_5	Clustered/Singleton	1	VC_283_5	1	0	1	0	0	0	0	1	Ref
LC623721	Unassigned	Unassigned	Unassigned	695_0	Clustered	48	VC_695_0	48	0.8162	1	0.8162	0	0	0	1	Ref
LC625742	Unassigned	Unassigned	Unassigned	377_0	Clustered	3	VC_377_0	3	0.3906	0.99785056	0.3898	0	0	0	1	Ref
LC628082	Unassigned	Unassigned	Unassigned	97_0	Clustered	5	VC_97_0	5	0.0696	0.98113835	0.0683	0	0	0	1	Ref
LC644557	Unassigned	Unassigned	Unassigned	97_0	Clustered	5	VC_97_0	5	0.0696	0.98113835	0.0683	0	0	0	1	Ref
LC644972	Unassigned	Unassigned	Unassigned	411_0	Clustered	3	VC_411_0	3	0.1239	0.98986341	0.1227	0	0	0	1	Ref
LC644974	Unassigned	Unassigned	Unassigned	411_0	Clustered	3	VC_411_0	3	0.1239	0.98986341	0.1227	0	0	0	1	Ref
LC644975	Unassigned	Unassigned	Unassigned	1204_0	Clustered	2	VC_1204_0	2	0.883	0.9197097	0.8121	0	0	0	1	Ref
LC644976	Unassigned	Unassigned	Unassigned	1204_0	Clustered	2	VC_1204_0	2	0.883	0.9197097	0.8121	0	0	0	1	Ref
LC645408	Unassigned	Unassigned	Unassigned	41_1	Clustered	10	VC_41_1	10	0.1178	1	0.1178	0	0	0	0.8222	Ref
LC645409	Unassigned	Unassigned	Unassigned	42_0	Clustered	49	VC_42_0	49	0.4517	1	0.4517	0	0	0	0.8597	Ref
LC645410	Unassigned	Unassigned	Unassigned	42_0	Clustered	49	VC_42_0	49	0.4517	1	0.4517	0	0	0	0.8597	Ref
LC645411	Unassigned	Unassigned	Unassigned	42_0	Clustered	49	VC_42_0	49	0.4517	1	0.4517	0	0	0	0.8597	Ref
LC645412	Unassigned	Unassigned	Unassigned	42_0	Clustered	49	VC_42_0	49	0.4517	1	0.4517	0	0	0	0.8597	Ref
LC645413	Unassigned	Unassigned	Unassigned	42_0	Clustered	49	VC_42_0	49	0.4517	1	0.4517	0	0	0	0.8597	Ref
LC645414	Unassigned	Unassigned	Unassigned	42_0	Clustered	49	VC_42_0	49	0.4517	1	0.4517	0	0	0	0.8597	Ref
LC645415	Unassigned	Unassigned	Unassigned	42_0	Clustered	49	VC_42_0	49	0.4517	1	0.4517	0	0	0	0.8597	Ref
LC645416	Unassigned	Unassigned	Unassigned	42_0	Clustered	49	VC_42_0	49	0.4517	1	0.4517	0	0	0	0.8597	Ref
LC645417	Unassigned	Unassigned	Unassigned	42_0	Clustered	49	VC_42_0	49	0.4517	1	0.4517	0	0	0	0.8597	Ref
LC645418	Unassigned	Unassigned	Unassigned	42_0	Clustered	49	VC_42_0	49	0.4517	1	0.4517	0	0	0	0.8597	Ref
LC645419	Unassigned	Unassigned	Unassigned	42_0	Clustered	49	VC_42_0	49	0.4517	1	0.4517	0	0	0	0.8597	Ref
LC645420	Unassigned	Unassigned	Unassigned	42_0	Clustered	49	VC_42_0	49	0.4517	1	0.4517	0	0	0	0.8597	Ref
LC645421	Unassigned	Unassigned	Unassigned	42_0	Clustered	49	VC_42_0	49	0.4517	1	0.4517	0	0	0	0.8597	Ref
LC645422	Unassigned	Unassigned	Unassigned	42_0	Clustered	49	VC_42_0	49	0.4517	1	0.4517	0	0	0	0.8597	Ref
LC645423	Unassigned	Unassigned	Unassigned	42_0	Clustered	49	VC_42_0	49	0.4517	1	0.4517	0	0	0	0.8597	Ref
LC645424	Unassigned	Unassigned	Unassigned	42_0	Clustered	49	VC_42_0	49	0.4517	1	0.4517	0	0	0	0.8597	Ref
LC645425	Unassigned	Unassigned	Unassigned	41_1	Clustered	10	VC_41_1	10	0.1178	1	0.1178	0	0	0	0.8222	Ref
LC645426	Unassigned	Unassigned	Unassigned	41_1	Clustered	10	VC_41_1	10	0.1178	1	0.1178	0	0	0	0.8222	Ref
LC645427	Unassigned	Unassigned	Unassigned	42_0	Clustered	49	VC_42_0	49	0.4517	1	0.4517	0	0	0	0.8597	Ref
LC645428	Unassigned	Unassigned	Unassigned	48_0	Clustered	10	VC_48_0	10	0.174	1	0.174	0	0	0	1	Ref
LC645429	Unassigned	Unassigned	Unassigned	48_0	Clustered	10	VC_48_0	10	0.174	1	0.174	0	0	0	1	Ref
LC645430	Unassigned	Unassigned	Unassigned	48_0	Clustered	10	VC_48_0	10	0.174	1	0.174	0	0	0	1	Ref
LC645431	Unassigned	Unassigned	Unassigned	48_0	Clustered	10	VC_48_0	10	0.174	1	0.174	0	0	0	1	Ref
LC645432	Unassigned	Unassigned	Unassigned	48_0	Clustered	10	VC_48_0	10	0.174	1	0.174	0	0	0	1	Ref
LC645433	Unassigned	Unassigned	Unassigned	48_0	Clustered	10	VC_48_0	10	0.174	1	0.174	0	0	0	1	Ref
LC645434	Unassigned	Unassigned	Unassigned	48_0	Clustered	10	VC_48_0	10	0.174	1	0.174	0	0	0	1	Ref
LC645435	Unassigned	Unassigned	Unassigned	48_0	Clustered	10	VC_48_0	10	0.174	1	0.174	0	0	0	1	Ref
LC645436	Unassigned	Unassigned	Unassigned	48_0	Clustered	10	VC_48_0	10	0.174	1	0.174	0	0	0	1	Ref
LC645437	Unassigned	Unassigned	Unassigned	48_0	Clustered	10	VC_48_0	10	0.174	1	0.174	0	0	0	1	Ref
LC645438	Unassigned	Unassigned	Unassigned	42_0	Clustered	49	VC_42_0	49	0.4517	1	0.4517	0	0	0	0.8597	Ref
LC645442	Unassigned	Unassigned	Unassigned	42_0	Clustered	49	VC_42_0	49	0.4517	1	0.4517	0	0	0	0.8597	Ref
LC645443	Unassigned	Unassigned	Unassigned	42_0	Clustered	49	VC_42_0	49	0.4517	1	0.4517	0	0	0	0.8597	Ref
LC645444	Unassigned	Unassigned	Unassigned	42_0	Clustered	49	VC_42_0	49	0.4517	1	0.4517	0	0	0	0.8597	Ref
LC645445	Unassigned	Unassigned	Unassigned	42_0	Clustered	49	VC_42_0	49	0.4517	1	0.4517	0	0	0	0.8597	Ref
LC645446	Unassigned	Unassigned	Unassigned	42_0	Clustered	49	VC_42_0	49	0.4517	1	0.4517	0	0	0	0.8597	Ref
LC645447	Unassigned	Unassigned	Unassigned	42_0	Clustered	49	VC_42_0	49	0.4517	1	0.4517	0	0	0	0.8597	Ref
LC645448	Unassigned	Unassigned	Unassigned	42_0	Clustered	49	VC_42_0	49	0.4517	1	0.4517	0	0	0	0.8597	Ref
LC645449	Unassigned	Unassigned	Unassigned	42_0	Clustered	49	VC_42_0	49	0.4517	1	0.4517	0	0	0	0.8597	Ref
LC645450	Unassigned	Unassigned	Unassigned	42_0	Clustered	49	VC_42_0	49	0.4517	1	0.4517	0	0	0	0.8597	Ref
LC645451	Unassigned	Unassigned	Unassigned	42_0	Clustered	49	VC_42_0	49	0.4517	1	0.4517	0	0	0	0.8597	Ref

LC647030	Unassigned	Unassigned	Unassigned	152_0	Clustered/Singleton	1	VC_152_0	1	0	1	0	0	0	0	1	Ref
LC648442	Unassigned	Unassigned	Unassigned	149_7	Clustered/Singleton	1	VC_149_7	1	0	1	0	0	0	0	1	Ref
LC648443	Unassigned	Unassigned	Unassigned	472_0	Clustered	47	VC_472_0	47	0.9025	1	0.9025	0	0	0	1	Ref
LC659915	Unassigned	Unassigned	Unassigned	191_19	Clustered	12	VC_191_19	12	0.0655	1	0.0655	0	0	0	0.8636	Ref
LC667452	Unassigned	Unassigned	Unassigned	3_0	Clustered	17	VC_3_0	17	0.5639	1	0.5639	0	0	0	1	Ref
LC680884	Unassigned	Unassigned	Unassigned	448_0	Clustered	4	VC_448_0	4	0.3059	0.99978853	0.3058	0	0	0	1	Ref
LC680885	Unassigned	Unassigned	Unassigned	449_0	Clustered	7	VC_449_0	7	0.6015	1	0.6015	0	0	0	1	Ref
LC709172	Unassigned	Unassigned	Unassigned	96_0	Clustered	30	VC_96_0	30	0.718	1	0.718	0	0	0	1	Ref
LC727695	Unassigned	Unassigned	Unassigned	344_0	Clustered	21	VC_344_0	21	0.5436	1	0.5436	0	0	0	1	Ref
LC727696	Unassigned	Unassigned	Unassigned	344_0	Clustered	21	VC_344_0	21	0.5436	1	0.5436	0	0	0	1	Ref
LC727697	Unassigned	Unassigned	Unassigned	165_0	Clustered	4	VC_165_0	4	0.2247	0.99998328	0.2247	0	0	0	1	Ref
LC727698	Unassigned	Unassigned	Unassigned	165_0	Clustered	4	VC_165_0	4	0.2247	0.99998328	0.2247	0	0	0	1	Ref
LC727699	Unassigned	Unassigned	Unassigned	344_0	Clustered	21	VC_344_0	21	0.5436	1	0.5436	0	0	0	1	Ref
LC727700	Unassigned	Unassigned	Unassigned	423_0	Clustered	45	VC_423_0	45	0.9484	1	0.9484	0	0	0	1	Ref
LC727701	Unassigned	Unassigned	Unassigned	344_0	Clustered	21	VC_344_0	21	0.5436	1	0.5436	0	0	0	1	Ref
LC727840	Unassigned	Unassigned	Unassigned	49_0	Clustered	36	VC_49_0	36	0.611	1	0.611	0	0	0	1	Ref
LC727841	Unassigned	Unassigned	Unassigned	49_0	Clustered	36	VC_49_0	36	0.611	1	0.611	0	0	0	1	Ref
LC727842	Unassigned	Unassigned	Unassigned	49_0	Clustered	36	VC_49_0	36	0.611	1	0.611	0	0	0	1	Ref
LC727843	Unassigned	Unassigned	Unassigned	49_0	Clustered	36	VC_49_0	36	0.611	1	0.611	0	0	0	1	Ref
LC727844	Unassigned	Unassigned	Unassigned	49_0	Clustered	36	VC_49_0	36	0.611	1	0.611	0	0	0	1	Ref
LC727845	Unassigned	Unassigned	Unassigned	49_0	Clustered	36	VC_49_0	36	0.611	1	0.611	0	0	0	1	Ref
LC727846	Unassigned	Unassigned	Unassigned	49_0	Clustered	36	VC_49_0	36	0.611	1	0.611	0	0	0	1	Ref
LC727847	Unassigned	Unassigned	Unassigned	49_0	Clustered	36	VC_49_0	36	0.611	1	0.611	0	0	0	1	Ref
LC727848	Unassigned	Unassigned	Unassigned	49_0	Clustered	36	VC_49_0	36	0.611	1	0.611	0	0	0	1	Ref
LC727849	Unassigned	Unassigned	Unassigned	49_0	Clustered	36	VC_49_0	36	0.611	1	0.611	0	0	0	1	Ref
LC727850	Unassigned	Unassigned	Unassigned	49_0	Clustered	36	VC_49_0	36	0.611	1	0.611	0	0	0	1	Ref
LC727851	Unassigned	Unassigned	Unassigned	49_0	Clustered	36	VC_49_0	36	0.611	1	0.611	0	0	0	1	Ref
LC727852	Unassigned	Unassigned	Unassigned	49_0	Clustered	36	VC_49_0	36	0.611	1	0.611	0	0	0	1	Ref
LC727853	Unassigned	Unassigned	Unassigned	49_0	Clustered	36	VC_49_0	36	0.611	1	0.611	0	0	0	1	Ref
LC727854	Unassigned	Unassigned	Unassigned	49_0	Clustered	36	VC_49_0	36	0.611	1	0.611	0	0	0	1	Ref
LC727855	Unassigned	Unassigned	Unassigned	49_0	Clustered	36	VC_49_0	36	0.611	1	0.611	0	0	0	1	Ref
LC727856	Unassigned	Unassigned	Unassigned	49_0	Clustered	36	VC_49_0	36	0.611	1	0.611	0	0	0	1	Ref
LC727857	Unassigned	Unassigned	Unassigned	49_0	Clustered	36	VC_49_0	36	0.611	1	0.611	0	0	0	1	Ref
LC727858	Unassigned	Unassigned	Unassigned	49_0	Clustered	36	VC_49_0	36	0.611	1	0.611	0	0	0	1	Ref
LC727859	Unassigned	Unassigned	Unassigned	49_0	Clustered	36	VC_49_0	36	0.611	1	0.611	0	0	0	1	Ref
LC727860	Unassigned	Unassigned	Unassigned	49_0	Clustered	36	VC_49_0	36	0.611	1	0.611	0	0	0	1	Ref
LC727861	Unassigned	Unassigned	Unassigned	49_0	Clustered	36	VC_49_0	36	0.611	1	0.611	0	0	0	1	Ref
LC727862	Unassigned	Unassigned	Unassigned	49_0	Clustered	36	VC_49_0	36	0.611	1	0.611	0	0	0	1	Ref
LC727863	Unassigned	Unassigned	Unassigned	49_0	Clustered	36	VC_49_0	36	0.611	1	0.611	0	0	0	1	Ref
LC727864	Unassigned	Unassigned	Unassigned	49_0	Clustered	36	VC_49_0	36	0.611	1	0.611	0	0	0	1	Ref
LC727865	Unassigned	Unassigned	Unassigned	49_0	Clustered	36	VC_49_0	36	0.611	1	0.611	0	0	0	1	Ref
LC727866	Unassigned	Unassigned	Unassigned	49_0	Clustered	36	VC_49_0	36	0.611	1	0.611	0	0	0	1	Ref
LC727867	Unassigned	Unassigned	Unassigned	49_0	Clustered	36	VC_49_0	36	0.611	1	0.611	0	0	0	1	Ref
LC727868	Unassigned	Unassigned	Unassigned	49_0	Clustered	36	VC_49_0	36	0.611	1	0.611	0	0	0	1	Ref
LC727869	Unassigned	Unassigned	Unassigned	49_0	Clustered	36	VC_49_0	36	0.611	1	0.611	0	0	0	1	Ref
LC727870	Unassigned	Unassigned	Unassigned	49_0	Clustered	36	VC_49_0	36	0.611	1	0.611	0	0	0	1	Ref
LC727871	Unassigned	Unassigned	Unassigned	49_0	Clustered	36	VC_49_0	36	0.611	1	0.611	0	0	0	1	Ref
LC727872	Unassigned	Unassigned	Unassigned	49_0	Clustered	36	VC_49_0	36	0.611	1	0.611	0	0	0	1	Ref
LC727873	Unassigned	Unassigned	Unassigned	49_0	Clustered	36	VC_49_0	36	0.611	1	0.611	0	0	0	1	Ref
LC727874	Unassigned	Unassigned	Unassigned	49_0	Clustered	36	VC_49_0	36	0.611	1	0.611	0	0	0	1	Ref
LC727875	Unassigned	Unassigned	Unassigned	49_0	Clustered	36	VC_49_0	36	0.611	1	0.611	0	0	0	1	Ref
LC727891	Unassigned	Unassigned	Unassigned	41_0	Clustered	16	VC_41_0	16	0.2025	1	0.2025	0	0	0	0.9667	Ref
LC727892	Unassigned	Unassigned	Unassigned	41_0	Clustered	16	VC_41_0	16	0.2025	1	0.2025	0	0	0	0.9667	Ref
LC727893	Unassigned	Unassigned	Unassigned	41_0	Clustered	16	VC_41_0	16	0.2025	1	0.2025	0	0	0	0.9667	Ref
LC727894	Unassigned	Unassigned	Unassigned	41_0	Clustered	16	VC_41_0	16	0.2025	1	0.2025	0	0	0	0.9667	Ref
LC727895	Unassigned	Unassigned	Unassigned	41_0	Clustered	16	VC_41_0	16	0.2025	1	0.2025	0	0	0	0.9667	Ref
LC727896	Unassigned	Unassigned	Unassigned	41_0	Clustered	16	VC_41_0	16	0.2025	1	0.2025	0	0	0	0.9667	Ref
LC727897	Unassigned	Unassigned	Unassigned	41_0	Clustered	16	VC_41_0	16	0.2025	1	0.2025	0	0	0	0.9667	Ref
LC727898	Unassigned	Unassigned	Unassigned	41_0	Clustered	16	VC_41_0	16	0.2025	1	0.2025	0	0	0	0.9667	Ref
LC727899	Unassigned	Unassigned	Unassigned	41_0	Clustered	16	VC_41_0	16	0.2025	1	0.2025	0	0	0	0.9667	Ref
LC727900	Unassigned	Unassigned	Unassigned	41_0	Clustered	16	VC_41_0	16	0.2025	1	0.2025	0	0	0	0.9667	Ref
LC727901	Unassigned	Unassigned	Unassigned	41_0	Clustered	16	VC_41_0	16	0.2025	1	0.2025	0	0	0	0.9667	Ref
LC727902	Unassigned	Unassigned	Unassigned	41_0	Clustered	16	VC_41_0	16	0.2025	1	0.2025	0	0	0	0.9667	Ref
LC727903	Unassigned	Unassigned	Unassigned	42_0	Clustered	49	VC_42_0	49	0.4517	1	0.4517	0	0	0	0.8597	Ref
LC730317	Unassigned	Unassigned	Unassigned	513_0	Clustered	32	VC_513_0	32	0.6881	1	0.6881	0	0	0	1	Ref
LC730318	Unassigned	Unassigned	Unassigned	215_1	Clustered	19	VC_215_1	19	0.4188	1	0.4188	0	0	0	1	Ref
LC730319	Unassigned	Unassigned	Unassigned	129_0	Clustered	43	VC_129_0	43	0.886	1	0.886	0	0	0	1	Ref
LC730320	Unassigned	Unassigned	Unassigned	216_6	Clustered	42	VC_216_6	42	0.473	1	0.473	0	0	0	0.8351	Ref
LC730321	Unassigned	Unassigned	Unassigned	43_0	Clustered	38	VC_43_0	38	0.5885	1	0.5885	0	0	0	0.9659	Ref
LC730322	Unassigned	Unassigned	Unassigned	28_1	Clustered	53	VC_28_1	53	0.7164	1	0.7164	0	0	0	0.9325	Ref
LC730323	Unassigned	Unassigned	Unassigned	452_0	Clustered	66	VC_452_0	66	0.9248	1	0.9248	0	0	0	1	Ref
LC730324	Unassigned	Unassigned	Unassigned	385_0	Clustered	51	VC_385_0	51	0.8754	1	0.8754	0	0	0	1	Ref
LC730325	Unassigned	Unassigned	Unassigned	392_0	Clustered	126	VC_392_0	126	0.9621	1	0.9621	0	0	0	1	Ref
LC739530	Unassigned	Unassigned	Unassigned	329_20	Clustered	4	VC_329_20	4	0.0174	0.99998777	0.0174	0	0	0	1	Ref
LC739531	Unassigned	Unassigned	Unassigned	329_20	Clustered	4	VC_329_20	4	0.0174	0.99998777	0.0174	0	0	0	1	Ref
LC739532	Unassigned	Unassigned	Unassigned	329_20	Clustered	4	VC_329_20	4	0.0174	0.99998777	0.0174	0	0	0	1	Ref
LC739533	Unassigned	Unassigned	Unassigned	847_0	Clustered	5	VC_847_0	5	0.0621	0.99921937	0.0621	0	0	0	1	Ref
LC739534	Unassigned	Unassigned	Unassigned	847_0	Clustered	5	VC_847_0	5	0.0621	0.99921937	0.0621	0	0	0	1	Ref
LC739535	Unassigned	Unassigned	Unassigned	847_0	Clustered	5	VC_847_0	5	0.0621	0.99921937	0.0621	0	0	0	1	Ref
LC739536	Unassigned	Unassigned	Unassigned	848_0	Clustered	28	VC_848_0	28	0.7927	1	0.7927	0	0	0	1	Ref
LC739537	Unassigned	Unassigned	Unassigned	847_0	Clustered	5	VC_847_0	5	0.0621	0.99921937	0.0621	0	0	0	1	Ref

LC739538	Unassigned	Unassigned	Unassigned	772_16	Clustered	10	VC_772_16	10	0.0563	0.9434928	0.0531	0	0	0	0.8667	Ref
LC739539	Unassigned	Unassigned	Unassigned	847_0	Clustered	5	VC_847_0	5	0.0621	0.99921937	0.0621	0	0	0	1	Ref
LC739540	Unassigned	Unassigned	Unassigned	772_16	Clustered	10	VC_772_16	10	0.0563	0.9434928	0.0531	0	0	0	0.8667	Ref
LC739541	Unassigned	Unassigned	Unassigned	772_11	Clustered	6	VC_772_11	6	0.0305	0.84253803	0.0257	0	0	0	0.8	Ref
LC739542	Unassigned	Unassigned	Unassigned	772_11	Clustered	6	VC_772_11	6	0.0305	0.84253803	0.0257	0	0	0	0.8	Ref
LK392619	Unassigned	Unassigned	Unassigned	1048_0	Clustered	3	VC_1048_0	3	1	1	1	0	0	0	1	Ref
LK985321	Unassigned	Unassigned	Unassigned	647_0	Clustered	11	VC_647_0	11	0.598	1	0.598	0	0	0	1	Ref
LK985322	Unassigned	Unassigned	Unassigned	139_0	Clustered	2	VC_139_0	2	0.0701	0.95510185	0.067	0	0	0	1	Ref
LN610572	Unassigned	Unassigned	Unassigned	76_0	Clustered	37	VC_76_0	37	0.9403	1	0.9403	0	0	0	1	Ref
LN610573	Unassigned	Unassigned	Unassigned	77_0	Clustered	20	VC_77_0	20	0.9657	1	0.9657	0	0	0	1	Ref
LN610574	Unassigned	Unassigned	Unassigned	423_0	Clustered	45	VC_423_0	45	0.9484	1	0.9484	0	0	0	1	Ref
LN610575	Unassigned	Unassigned	Unassigned	76_0	Clustered	37	VC_76_0	37	0.9403	1	0.9403	0	0	0	1	Ref
LN610576	Unassigned	Unassigned	Unassigned	77_0	Clustered	20	VC_77_0	20	0.9657	1	0.9657	0	0	0	1	Ref
LN610577	Unassigned	Unassigned	Unassigned	464_0	Clustered	8	VC_464_0	8	0.2629	1	0.2629	0	0	0	1	Ref
LN610578	Unassigned	Unassigned	Unassigned	472_0	Clustered	47	VC_472_0	47	0.9025	1	0.9025	0	0	0	1	Ref
LN610579	Unassigned	Unassigned	Unassigned	79_0	Clustered	64	VC_79_0	64	0.4185	1	0.4185	0	0	0	0.7649	Ref
LN610580	Unassigned	Unassigned	Unassigned	423_0	Clustered	45	VC_423_0	45	0.9484	1	0.9484	0	0	0	1	Ref
LN610581	Unassigned	Unassigned	Unassigned	77_0	Clustered	20	VC_77_0	20	0.9657	1	0.9657	0	0	0	1	Ref
LN610582	Unassigned	Unassigned	Unassigned	77_0	Clustered	20	VC_77_0	20	0.9657	1	0.9657	0	0	0	1	Ref
LN610583	Unassigned	Unassigned	Unassigned	77_0	Clustered	20	VC_77_0	20	0.9657	1	0.9657	0	0	0	1	Ref
LN610584	Unassigned	Unassigned	Unassigned	464_0	Clustered	8	VC_464_0	8	0.2629	1	0.2629	0	0	0	1	Ref
LN610585	Unassigned	Unassigned	Unassigned	464_0	Clustered	8	VC_464_0	8	0.2629	1	0.2629	0	0	0	1	Ref
LN610586	Unassigned	Unassigned	Unassigned	76_0	Clustered	37	VC_76_0	37	0.9403	1	0.9403	0	0	0	1	Ref
LN610587	Unassigned	Unassigned	Unassigned	76_0	Clustered	37	VC_76_0	37	0.9403	1	0.9403	0	0	0	1	Ref
LN610588	Unassigned	Unassigned	Unassigned	79_0	Clustered	64	VC_79_0	64	0.4185	1	0.4185	0	0	0	0.7649	Ref
LN610589	Unassigned	Unassigned	Unassigned	80_0	Clustered	41	VC_80_0	41	0.5837	1	0.5837	0	0	0	0.9805	Ref
LN610590	Unassigned	Unassigned	Unassigned	179_0	Clustered	53	VC_179_0	53	0.9137	1	0.9137	0	0	0	1	Ref
LN681534	Unassigned	Unassigned	Unassigned	766_0	Clustered	2	VC_766_0	2	0.2674	0.94916107	0.2538	0	0	0	1	Ref
LN681535	Unassigned	Unassigned	Unassigned	733_0	Clustered	5	VC_733_0	5	0.5111	0.99999994	0.5111	0	0	0	1	Ref
LN681536	Unassigned	Unassigned	Unassigned	733_0	Clustered	5	VC_733_0	5	0.5111	0.99999994	0.5111	0	0	0	1	Ref
LN681537	Unassigned	Unassigned	Unassigned	473_2	Clustered	2	VC_473_2	2	0.1022	0.94602228	0.0966	0	0	0	1	Ref
LN681538	Unassigned	Unassigned	Unassigned	647_0	Clustered	11	VC_647_0	11	0.598	1	0.598	0	0	0	1	Ref
LN681540	Unassigned	Unassigned	Unassigned	647_0	Clustered	11	VC_647_0	11	0.598	1	0.598	0	0	0	1	Ref
LN681541	Unassigned	Unassigned	Unassigned	139_1	Clustered	4	VC_139_1	4	0.1775	0.99998609	0.1775	0	0	0	1	Ref
LN681542	Unassigned	Unassigned	Unassigned	139_1	Clustered	4	VC_139_1	4	0.1775	0.99998609	0.1775	0	0	0	1	Ref
LN828717	Unassigned	Unassigned	Unassigned	433_5	Clustered	2	VC_433_5	2	0.0173	0.93642158	0.0162	0	0	0	1	Ref
LN866626	Unassigned	Unassigned	Unassigned	823_0	Clustered	124	VC_823_0	124	0.9492	1	0.9492	0	0	0	1	Ref
LN878149	Unassigned	Unassigned	Unassigned	878_0	Clustered	32	VC_878_0	32	0.9965	1	0.9965	0	0	0	1	Ref
LN881725	Unassigned	Unassigned	Unassigned	795_0	Clustered	19	VC_795_0	19	0.6848	1	0.6848	0	0	0	1	Ref
LN881726	Unassigned	Unassigned	Unassigned	283_33	Clustered	4	VC_283_33	4	0.0075	0.99979471	0.0075	0	0	0	1	Ref
LN881727	Unassigned	Unassigned	Unassigned	772_9	Clustered	7	VC_772_9	7	0.0369	0.87705503	0.0324	0	0	0	1	Ref
LN881728	Unassigned	Unassigned	Unassigned	283_73	Clustered	3	VC_283_73	3	0.0052	0.9974535	0.0051	0	0	0	1	Ref
LN881729	Unassigned	Unassigned	Unassigned	283_33	Clustered	4	VC_283_33	4	0.0075	0.99979471	0.0075	0	0	0	1	Ref
LN881730	Unassigned	Unassigned	Unassigned	835_0	Clustered	67	VC_835_0	67	0.9624	1	0.9624	0	0	0	1	Ref
LN881731	Unassigned	Unassigned	Unassigned	835_0	Clustered	67	VC_835_0	67	0.9624	1	0.9624	0	0	0	1	Ref
LN881732	Unassigned	Unassigned	Unassigned	283_37	Clustered/Singleton	1	VC_283_37	1	0	1	0	0	0	0	1	Ref
LN881733	Unassigned	Unassigned	Unassigned	283_73	Clustered	3	VC_283_73	3	0.0052	0.9974535	0.0051	0	0	0	1	Ref
LN881734	Unassigned	Unassigned	Unassigned	283_33	Clustered	4	VC_283_33	4	0.0075	0.99979471	0.0075	0	0	0	1	Ref
LN881735	Unassigned	Unassigned	Unassigned	772_9	Clustered	7	VC_772_9	7	0.0369	0.87705503	0.0324	0	0	0	1	Ref
LN881736	Unassigned	Unassigned	Unassigned	283_73	Clustered	3	VC_283_73	3	0.0052	0.9974535	0.0051	0	0	0	1	Ref
LN881737	Unassigned	Unassigned	Unassigned	283_33	Clustered	4	VC_283_33	4	0.0075	0.99979471	0.0075	0	0	0	1	Ref
LN881738	Unassigned	Unassigned	Unassigned	733_0	Clustered	5	VC_733_0	5	0.5111	0.99999994	0.5111	0	0	0	1	Ref
LN887844	Unassigned	Unassigned	Unassigned	78_5	Clustered	6	VC_78_5	6	0.1087	0.99978082	0.1087	0	0	0	1	Ref
LN887948	Unassigned	Unassigned	Unassigned	190_20	Clustered	4	VC_190_20	4	0.0229	0.99997885	0.0229	0	0	0	1	Ref
LN889756	Unassigned	Unassigned	Unassigned	129_0	Clustered	43	VC_129_0	43	0.886	1	0.886	0	0	0	1	Ref
LN889995	Unassigned	Unassigned	Unassigned	129_0	Clustered	43	VC_129_0	43	0.886	1	0.886	0	0	0	1	Ref
LN898172	Unassigned	Unassigned	Unassigned	251_0	Clustered	22	VC_251_0	22	0.6508	1	0.6508	0	0	0	1	Ref
LN907801	Unassigned	Unassigned	Unassigned	179_0	Clustered	53	VC_179_0	53	0.9137	1	0.9137	0	0	0	1	Ref
LN913017	Unassigned	Unassigned	Unassigned	129_0	Clustered	43	VC_129_0	43	0.886	1	0.886	0	0	0	1	Ref
LN997803	Unassigned	Unassigned	Unassigned	245_0	Clustered	10	VC_245_0	10	0.1805	1	0.1805	0	0	0	0.9778	Ref
LR025195	Unassigned	Unassigned	Unassigned	795_0	Clustered	19	VC_795_0	19	0.6848	1	0.6848	0	0	0	1	Ref
LR025196	Unassigned	Unassigned	Unassigned	795_0	Clustered	19	VC_795_0	19	0.6848	1	0.6848	0	0	0	1	Ref
LR025197	Unassigned	Unassigned	Unassigned	795_0	Clustered	19	VC_795_0	19	0.6848	1	0.6848	0	0	0	1	Ref
LR025198	Unassigned	Unassigned	Unassigned	795_0	Clustered	19	VC_795_0	19	0.6848	1	0.6848	0	0	0	1	Ref
LR026998	Unassigned	Unassigned	Unassigned	592_0	Clustered	50	VC_592_0	50	0.7314	1	0.7314	0	0	0	1	Ref
LR027383	Unassigned	Unassigned	Unassigned	286_1	Clustered	21	VC_286_1	21	0.1696	1	0.1696	0	0	0	0.9048	Ref
LR027384	Unassigned	Unassigned	Unassigned	190_12	Clustered/Singleton	1	VC_190_12	1	0	1	0	0	0	0	1	Ref
LR027385	Unassigned	Unassigned	Unassigned	942_0	Clustered	36	VC_942_0	36	0.7848	1	0.7848	0	0	0	1	Ref
LR027386	Unassigned	Unassigned	Unassigned	286_1	Clustered	21	VC_286_1	21	0.1696	1	0.1696	0	0	0	0.9048	Ref
LR027387	Unassigned	Unassigned	Unassigned	286_1	Clustered	21	VC_286_1	21	0.1696	1	0.1696	0	0	0	0.9048	Ref
LR027388	Unassigned	Unassigned	Unassigned	942_0	Clustered	36	VC_942_0	36	0.7848	1	0.7848	0	0	0	1	Ref
LR027389	Unassigned	Unassigned	Unassigned	191_14	Clustered/Singleton	1	VC_191_14	1	0	1	0	0	0	0	1	Ref
LR027390	Unassigned	Unassigned	Unassigned	286_1	Clustered	21	VC_286_1	21	0.1696	1	0.1696	0	0	0	0.9048	Ref
LR027391	Unassigned	Unassigned	Unassigned	286_1	Clustered	21	VC_286_1	21	0.1696	1	0.1696	0	0	0	0.9048	Ref
LR031359	Unassigned	Unassigned	Unassigned	91_0	Clustered	22	VC_91_0	22	0.5318	1	0.5318	0	0	0	1	Ref
LR215718	Unassigned	Unassigned	Unassigned	149_15	Clustered	16	VC_149_15	16	0.0788	1	0.0788	0	0	0	0.8083	Ref
LR215719	Unassigned	Unassigned	Unassigned	149_13	Clustered	3	VC_149_13	3	0.0087	0.95178903	0.0083	0	0	0	1	Ref
LR215720	Unassigned	Unassigned	Unassigned	149_2	Clustered/Singleton	1	VC_149_2	1	0	1	0	0	0	0	1	Ref
LR215721	Unassigned	Unassigned	Unassigned	149_29	Clustered	3	VC_149_29	3	0.012	0.99448577	0.012	0	0	0	1	Ref
LR215722	Unassigned	Unassigned	Unassigned	283_97	Clustered/Singleton	1	VC_283_97	1	0	1	0	0	0	0	1	Ref
LR215723	Unassigned	Unassigned	Unassigned	283_71	Clustered/Singleton	1	VC_283_71	1	0	1	0	0	0	0	1	Ref

LR215724	Unassigned	Unassigned	Unassigned	283_113	Clustered/Singleton	1	VC_283_113	1	0	1	0	0	0	0	1	Ref
LR535901	Unassigned	Unassigned	Unassigned	1208_0	Clustered	11	VC_1208_0	11	1	0	0	0	0	0	1	Ref
LR535902	Unassigned	Unassigned	Unassigned	959_5	Clustered	19	VC_959_5	19	0.2626	1	0.2626	0	0	0	1	Ref
LR535903	Unassigned	Unassigned	Unassigned	1208_0	Clustered	11	VC_1208_0	11	1	0	0	0	0	0	1	Ref
LR535904	Unassigned	Unassigned	Unassigned	1208_0	Clustered	11	VC_1208_0	11	1	0	0	0	0	0	1	Ref
LR535905	Unassigned	Unassigned	Unassigned	1208_0	Clustered	11	VC_1208_0	11	1	0	0	0	0	0	1	Ref
LR535906	Unassigned	Unassigned	Unassigned	1208_0	Clustered	11	VC_1208_0	11	1	0	0	0	0	0	1	Ref
LR535907	Unassigned	Unassigned	Unassigned	1208_0	Clustered	11	VC_1208_0	11	1	0	0	0	0	0	1	Ref
LR535908	Unassigned	Unassigned	Unassigned	1208_0	Clustered	11	VC_1208_0	11	1	0	0	0	0	0	1	Ref
LR535909	Unassigned	Unassigned	Unassigned	959_5	Clustered	19	VC_959_5	19	0.2626	1	0.2626	0	0	0	1	Ref
LR535910	Unassigned	Unassigned	Unassigned	959_5	Clustered	19	VC_959_5	19	0.2626	1	0.2626	0	0	0	1	Ref
LR535911	Unassigned	Unassigned	Unassigned	1208_0	Clustered	11	VC_1208_0	11	1	0	0	0	0	0	1	Ref
LR535912	Unassigned	Unassigned	Unassigned	1208_0	Clustered	11	VC_1208_0	11	1	0	0	0	0	0	1	Ref
LR535913	Unassigned	Unassigned	Unassigned	1208_0	Clustered	11	VC_1208_0	11	1	0	0	0	0	0	1	Ref
LR535914	Unassigned	Unassigned	Unassigned	959_5	Clustered	19	VC_959_5	19	0.2626	1	0.2626	0	0	0	1	Ref
LR535915	Unassigned	Unassigned	Unassigned	959_5	Clustered	19	VC_959_5	19	0.2626	1	0.2626	0	0	0	1	Ref
LR535916	Unassigned	Unassigned	Unassigned	959_5	Clustered	19	VC_959_5	19	0.2626	1	0.2626	0	0	0	1	Ref
LR535917	Unassigned	Unassigned	Unassigned	1208_0	Clustered	11	VC_1208_0	11	1	0	0	0	0	0	1	Ref
LR535918	Unassigned	Unassigned	Unassigned	959_5	Clustered	19	VC_959_5	19	0.2626	1	0.2626	0	0	0	1	Ref
LR535919	Unassigned	Unassigned	Unassigned	959_5	Clustered	19	VC_959_5	19	0.2626	1	0.2626	0	0	0	1	Ref
LR535920	Unassigned	Unassigned	Unassigned	959_5	Clustered	19	VC_959_5	19	0.2626	1	0.2626	0	0	0	1	Ref
LR535921	Unassigned	Unassigned	Unassigned	959_5	Clustered	19	VC_959_5	19	0.2626	1	0.2626	0	0	0	1	Ref
LR588166	Unassigned	Unassigned	Unassigned	165_0	Clustered	4	VC_165_0	4	0.2247	0.99998328	0.2247	0	0	0	1	Ref
LR595850	Unassigned	Unassigned	Unassigned	43_0	Clustered	38	VC_43_0	38	0.5885	1	0.5885	0	0	0	0.9659	Ref
LR595859	Unassigned	Unassigned	Unassigned	43_0	Clustered	38	VC_43_0	38	0.5885	1	0.5885	0	0	0	0.9659	Ref
LR595860	Unassigned	Unassigned	Unassigned	43_0	Clustered	38	VC_43_0	38	0.5885	1	0.5885	0	0	0	0.9659	Ref
LR595861	Unassigned	Unassigned	Unassigned	43_0	Clustered	38	VC_43_0	38	0.5885	1	0.5885	0	0	0	0.9659	Ref
LR595863	Unassigned	Unassigned	Unassigned	43_0	Clustered	38	VC_43_0	38	0.5885	1	0.5885	0	0	0	0.9659	Ref
LR595864	Unassigned	Unassigned	Unassigned	43_0	Clustered	38	VC_43_0	38	0.5885	1	0.5885	0	0	0	0.9659	Ref
LR595865	Unassigned	Unassigned	Unassigned	43_0	Clustered	38	VC_43_0	38	0.5885	1	0.5885	0	0	0	0.9659	Ref
LR595866	Unassigned	Unassigned	Unassigned	43_0	Clustered	38	VC_43_0	38	0.5885	1	0.5885	0	0	0	0.9659	Ref
LR595867	Unassigned	Unassigned	Unassigned	50_0	Clustered	9	VC_50_0	9	0.5531	1	0.5531	0	0	0	1	Ref
LR595868	Unassigned	Unassigned	Unassigned	51_0	Clustered	10	VC_51_0	10	0.1741	1	0.1741	0	0	0	1	Ref
LR595869	Unassigned	Unassigned	Unassigned	228_0	Clustered	60	VC_228_0	60	0.942	1	0.942	0	0	0	1	Ref
LR595870	Unassigned	Unassigned	Unassigned	228_0	Clustered	60	VC_228_0	60	0.942	1	0.942	0	0	0	1	Ref
LR595873	Unassigned	Unassigned	Unassigned	228_0	Clustered	60	VC_228_0	60	0.942	1	0.942	0	0	0	1	Ref
LR595883	Unassigned	Unassigned	Unassigned	228_0	Clustered	60	VC_228_0	60	0.942	1	0.942	0	0	0	1	Ref
LR595885	Unassigned	Unassigned	Unassigned	228_0	Clustered	60	VC_228_0	60	0.942	1	0.942	0	0	0	1	Ref
LR595887	Unassigned	Unassigned	Unassigned	228_0	Clustered	60	VC_228_0	60	0.942	1	0.942	0	0	0	1	Ref
LR595891	Unassigned	Unassigned	Unassigned	229_0	Clustered	32	VC_229_0	32	0.9119	1	0.9119	0	0	0	1	Ref
LR596614	Unassigned	Unassigned	Unassigned	942_0	Clustered	36	VC_942_0	36	0.7848	1	0.7848	0	0	0	1	Ref
LR596615	Unassigned	Unassigned	Unassigned	164_8	Clustered	3	VC_164_8	3	0.0299	0.84944649	0.0254	0	0	0	1	Ref
LR596902	Unassigned	Unassigned	Unassigned	418_0	Clustered	6	VC_418_0	6	0.4575	0.9999995	0.4575	0	0	0	0.8	Ref
LR596903	Unassigned	Unassigned	Unassigned	717_0	Clustered	7	VC_717_0	7	0.5813	1	0.5813	0	0	0	0.9048	Ref
LR597635	Unassigned	Unassigned	Unassigned	43_0	Clustered	38	VC_43_0	38	0.5885	1	0.5885	0	0	0	0.9659	Ref
LR597636	Unassigned	Unassigned	Unassigned	43_0	Clustered	38	VC_43_0	38	0.5885	1	0.5885	0	0	0	0.9659	Ref
LR597637	Unassigned	Unassigned	Unassigned	5_0	Clustered	30	VC_5_0	30	0.9647	1	0.9647	0	0	0	1	Ref
LR597638	Unassigned	Unassigned	Unassigned	5_0	Clustered	30	VC_5_0	30	0.9647	1	0.9647	0	0	0	1	Ref
LR597639	Unassigned	Unassigned	Unassigned	43_0	Clustered	38	VC_43_0	38	0.5885	1	0.5885	0	0	0	0.9659	Ref
LR597640	Unassigned	Unassigned	Unassigned	5_0	Clustered	30	VC_5_0	30	0.9647	1	0.9647	0	0	0	1	Ref
LR597641	Unassigned	Unassigned	Unassigned	51_0	Clustered	10	VC_51_0	10	0.1741	1	0.1741	0	0	0	1	Ref
LR597642	Unassigned	Unassigned	Unassigned	245_0	Clustered	10	VC_245_0	10	0.1805	1	0.1805	0	0	0	0.9778	Ref
LR597643	Unassigned	Unassigned	Unassigned	43_0	Clustered	38	VC_43_0	38	0.5885	1	0.5885	0	0	0	0.9659	Ref
LR597644	Unassigned	Unassigned	Unassigned	43_0	Clustered	38	VC_43_0	38	0.5885	1	0.5885	0	0	0	0.9659	Ref
LR597646	Unassigned	Unassigned	Unassigned	835_0	Clustered	67	VC_835_0	67	0.9624	1	0.9624	0	0	0	1	Ref
LR597647	Unassigned	Unassigned	Unassigned	772_9	Clustered	7	VC_772_9	7	0.0369	0.87705503	0.0324	0	0	0	1	Ref
LR597648	Unassigned	Unassigned	Unassigned	43_0	Clustered	38	VC_43_0	38	0.5885	1	0.5885	0	0	0	0.9659	Ref
LR597649	Unassigned	Unassigned	Unassigned	5_0	Clustered	30	VC_5_0	30	0.9647	1	0.9647	0	0	0	1	Ref
LR597650	Unassigned	Unassigned	Unassigned	43_0	Clustered	38	VC_43_0	38	0.5885	1	0.5885	0	0	0	0.9659	Ref
LR597651	Unassigned	Unassigned	Unassigned	43_0	Clustered	38	VC_43_0	38	0.5885	1	0.5885	0	0	0	0.9659	Ref
LR597652	Unassigned	Unassigned	Unassigned	43_0	Clustered	38	VC_43_0	38	0.5885	1	0.5885	0	0	0	0.9659	Ref
LR597653	Unassigned	Unassigned	Unassigned	43_0	Clustered	38	VC_43_0	38	0.5885	1	0.5885	0	0	0	0.9659	Ref
LR597654	Unassigned	Unassigned	Unassigned	847_1	Clustered	26	VC_847_1	26	0.6323	1	0.6323	0	0	0	0.9815	Ref
LR597655	Unassigned	Unassigned	Unassigned	190_14	Clustered	3	VC_190_14	3	0.0144	0.99696852	0.0143	0	0	0	0.6667	Ref
LR597656	Unassigned	Unassigned	Unassigned	584_0	Clustered	28	VC_584_0	28	0.7704	1	0.7704	0	0	0	1	Ref
LR597657	Unassigned	Unassigned	Unassigned	283_137	Clustered/Singleton	1	VC_283_137	1	0	1	0	0	0	0	1	Ref
LR597658	Unassigned	Unassigned	Unassigned	329_6	Clustered	3	VC_329_6	3	0.0107	0.99833469	0.0107	0	0	0	1	Ref
LR597659	Unassigned	Unassigned	Unassigned	190_24	Clustered	5	VC_190_24	5	0.0293	0.9999996	0.0293	0	0	0	0.7	Ref
LR597660	Unassigned	Unassigned	Unassigned	283_101	Clustered/Singleton	1	VC_283_101	1	0	1	0	0	0	0	1	Ref
LR597663	Unassigned	Unassigned	Unassigned	329_9	Clustered/Singleton	1	VC_329_9	1	0	1	0	0	0	0	1	Ref
LR694165	Unassigned	Unassigned	Unassigned	772_9	Clustered	7	VC_772_9	7	0.0369	0.87705503	0.0324	0	0	0	1	Ref
LR694610	Unassigned	Unassigned	Unassigned	772_7	Clustered	2	VC_772_7	2	0.006	0.58868714	0.0035	0	0	0	1	Ref
LR694611	Unassigned	Unassigned	Unassigned	772_7	Clustered	2	VC_772_7	2	0.006	0.58868714	0.0035	0	0	0	1	Ref
LR699048	Unassigned	Unassigned	Unassigned	329_7	Clustered	18	VC_329_7	18	0.0938	1	0.0938	0	0	0	0.7778	Ref
LR699804	Unassigned	Unassigned	Unassigned	772_19	Clustered	16	VC_772_19	16	0.0975	0.9970466	0.0972	0	0	0	0.8083	Ref
LR743523	Unassigned	Unassigned	Unassigned	116_0	Clustered	13	VC_116_0	13	1	1	1	0	0	0	1	Ref
LR743524	Unassigned	Unassigned	Unassigned	172_0	Clustered	5	VC_172_0	5	0.3579	0.99999997	0.3579	0	0	0	1	Ref
LR743528	Unassigned	Unassigned	Unassigned	116_0	Clustered	13	VC_116_0	13	1	1	1	0	0	0	1	Ref
LR743529	Unassigned	Unassigned	Unassigned	116_0	Clustered	13	VC_116_0	13	1	1	1	0	0	0	1	Ref
LR743530	Unassigned	Unassigned	Unassigned	749_0	Clustered	40	VC_749_0	40	0.7517	1	0.7517	0	0	0	1	Ref
LR743531	Unassigned	Unassigned	Unassigned	116_0	Clustered	13	VC_116_0	13	1	1	1	0	0	0	1	Ref

LR743532	Unassigned	Unassigned	Unassigned	169_0	Clustered	20	VC_169_0	20	0.7529	1	0.7529	0	0	0	1	Ref
LR745206	Unassigned	Unassigned	Unassigned	533_1	Clustered/Singleton	1	VC_533_1	1	0	1	0	0	0	0	1	Ref
LR745706	Unassigned	Unassigned	Unassigned	392_0	Clustered	126	VC_392_0	126	0.9621	1	0.9621	0	0	0	1	Ref
LR745707	Unassigned	Unassigned	Unassigned	392_0	Clustered	126	VC_392_0	126	0.9621	1	0.9621	0	0	0	1	Ref
LR745708	Unassigned	Unassigned	Unassigned	392_0	Clustered	126	VC_392_0	126	0.9621	1	0.9621	0	0	0	1	Ref
LR745709	Unassigned	Unassigned	Unassigned	392_0	Clustered	126	VC_392_0	126	0.9621	1	0.9621	0	0	0	1	Ref
LR745710	Unassigned	Unassigned	Unassigned	392_0	Clustered	126	VC_392_0	126	0.9621	1	0.9621	0	0	0	1	Ref
LR746137	Unassigned	Unassigned	Unassigned	847_1	Clustered	26	VC_847_1	26	0.6323	1	0.6323	0	0	0	0.9815	Ref
LR746310	Unassigned	Unassigned	Unassigned	668_0	Clustered	25	VC_668_0	25	0.5539	1	0.5539	0	0	0	0.96	Ref
LR746311	Unassigned	Unassigned	Unassigned	283_141	Clustered/Singleton	1	VC_283_141	1	0	1	0	0	0	0	1	Ref
LR756501	Unassigned	Unassigned	Unassigned	533_0	Clustered	2	VC_533_0	2	0.2712	0.93085693	0.2525	0	0	0	1	Ref
LR756502	Unassigned	Unassigned	Unassigned	533_2	Clustered/Singleton	1	VC_533_2	1	0	1	0	0	0	0	1	Ref
LR756503	Unassigned	Unassigned	Unassigned	533_0	Clustered	2	VC_533_0	2	0.2712	0.93085693	0.2525	0	0	0	1	Ref
LR756504	Unassigned	Unassigned	Unassigned	533_3	Clustered/Singleton	1	VC_533_3	1	0	1	0	0	0	0	1	Ref
LR757892	Unassigned	Unassigned	Unassigned	938_0	Clustered	99	VC_938_0	99	0.9119	1	0.9119	0	0	0	0.9928	Ref
LR778216	Unassigned	Unassigned	Unassigned	74_0	Clustered	15	VC_74_0	15	0.5204	1	0.5204	0	0	0	1	Ref
LR794124	Unassigned	Unassigned	Unassigned	837_0	Clustered	29	VC_837_0	29	0.9955	1	0.9955	0	0	0	1	Ref
LR794146	Unassigned	Unassigned	Unassigned	29_0	Clustered	10	VC_29_0	10	0.5208	1	0.5208	0	0	0	1	Ref
LR794147	Unassigned	Unassigned	Unassigned	29_0	Clustered	10	VC_29_0	10	0.5208	1	0.5208	0	0	0	1	Ref
LR794148	Unassigned	Unassigned	Unassigned	29_0	Clustered	10	VC_29_0	10	0.5208	1	0.5208	0	0	0	1	Ref
LR794149	Unassigned	Unassigned	Unassigned	29_0	Clustered	10	VC_29_0	10	0.5208	1	0.5208	0	0	0	1	Ref
LR794150	Unassigned	Unassigned	Unassigned	29_0	Clustered	10	VC_29_0	10	0.5208	1	0.5208	0	0	0	1	Ref
LR794151	Unassigned	Unassigned	Unassigned	29_0	Clustered	10	VC_29_0	10	0.5208	1	0.5208	0	0	0	1	Ref
LR794152	Unassigned	Unassigned	Unassigned	29_0	Clustered	10	VC_29_0	10	0.5208	1	0.5208	0	0	0	1	Ref
LR797314	Unassigned	Unassigned	Unassigned	185_0	Clustered	3	VC_185_0	3	0.4487	0.97148006	0.4359	0	0	0	1	Ref
LR797923	Unassigned	Unassigned	Unassigned	29_0	Clustered	10	VC_29_0	10	0.5208	1	0.5208	0	0	0	1	Ref
LR800396	Unassigned	Unassigned	Unassigned	133_13	Clustered	2	VC_133_13	2	0.0051	0.86794768	0.0044	0	0	0	1	Ref
LR811967	Unassigned	Unassigned	Unassigned	133_28	Clustered	3	VC_133_28	3	0.0105	0.97661637	0.0103	0	0	0	0.6667	Ref
LR811968	Unassigned	Unassigned	Unassigned	133_4	Clustered/Singleton	1	VC_133_4	1	0	1	0	0	0	0	1	Ref
LR811969	Unassigned	Unassigned	Unassigned	133_28	Clustered	3	VC_133_28	3	0.0105	0.97661637	0.0103	0	0	0	0.6667	Ref
LR813619	Unassigned	Unassigned	Unassigned	793_0	Clustered	12	VC_793_0	12	0.8531	1	0.8531	0	0	0	1	Ref
LR865361	Unassigned	Unassigned	Unassigned	848_0	Clustered	28	VC_848_0	28	0.7927	1	0.7927	0	0	0	1	Ref
LR877331	Unassigned	Unassigned	Unassigned	491_0	Clustered	36	VC_491_0	36	0.5336	1	0.5336	0	0	0	0.9714	Ref
LR877332	Unassigned	Unassigned	Unassigned	283_67	Clustered	2	VC_283_67	2	0.0027	0.90726003	0.0025	0	0	0	1	Ref
LR880803	Unassigned	Unassigned	Unassigned	329_3	Clustered	24	VC_329_3	24	0.1206	1	0.1206	0	0	0	0.7283	Ref
LR881104	Unassigned	Unassigned	Unassigned	347_0	Clustered	3	VC_347_0	3	0.1545	0.9984446	0.1543	0	0	0	1	Ref
LR990702	Unassigned	Unassigned	Unassigned	835_0	Clustered	67	VC_835_0	67	0.9624	1	0.9624	0	0	0	1	Ref
LR990704	Unassigned	Unassigned	Unassigned	283_38	Clustered/Singleton	1	VC_283_38	1	0	1	0	0	0	0	1	Ref
LR990835	Unassigned	Unassigned	Unassigned	92_0	Clustered	22	VC_92_0	22	0.5639	1	0.5639	0	0	0	1	Ref
LR999870	Unassigned	Unassigned	Unassigned	835_0	Clustered	67	VC_835_0	67	0.9624	1	0.9624	0	0	0	1	Ref
LR999871	Unassigned	Unassigned	Unassigned	779_0	Clustered	37	VC_779_0	37	0.5682	1	0.5682	0	0	0	1	Ref
LT546029	Unassigned	Unassigned	Unassigned	691_1	Clustered	5	VC_691_1	5	0.4358	0.99999993	0.4358	0	0	0	1	Ref
LT594300	Unassigned	Unassigned	Unassigned	451_0	Clustered	80	VC_451_0	80	0.9991	1	0.9991	0	0	0	1	Ref
LT598654	Unassigned	Unassigned	Unassigned	67_5	Clustered/Singleton	1	VC_67_5	1	0	1	0	0	0	0	1	Ref
LT600745	Unassigned	Unassigned	Unassigned	822_0	Clustered	2	VC_822_0	2	0.038	0.89489263	0.034	0	0	0	1	Ref
LT603033	Unassigned	Unassigned	Unassigned	164_11	Clustered	10	VC_164_11	10	0.1507	1	0.1507	0	0	0	0.9111	Ref
LT603684	Unassigned	Unassigned	Unassigned	630_0	Clustered	20	VC_630_0	20	0.8513	1	0.8513	0	0	0	1	Ref
LT607758	Unassigned	Unassigned	Unassigned	668_0	Clustered	25	VC_668_0	25	0.5539	1	0.5539	0	0	0	0.96	Ref
LT608331	Unassigned	Unassigned	Unassigned	179_0	Clustered	53	VC_179_0	53	0.9137	1	0.9137	0	0	0	1	Ref
LT615366	Unassigned	Unassigned	Unassigned	691_1	Clustered	5	VC_691_1	5	0.4358	0.99999993	0.4358	0	0	0	1	Ref
LT630001	Unassigned	Unassigned	Unassigned	967_0	Clustered	16	VC_967_0	16	0.8971	1	0.8971	0	0	0	1	Ref
LT714109	Unassigned	Unassigned	Unassigned	28_1	Clustered	53	VC_28_1	53	0.7164	1	0.7164	0	0	0	0.9325	Ref
LT841304	Unassigned	Unassigned	Unassigned	942_0	Clustered	36	VC_942_0	36	0.7848	1	0.7848	0	0	0	1	Ref
LT898437	Unassigned	Unassigned	Unassigned	315_0	Clustered	12	VC_315_0	12	0.8947	1	0.8947	0	0	0	1	Ref
LT907986	Unassigned	Unassigned	Unassigned	795_0	Clustered	19	VC_795_0	19	0.6848	1	0.6848	0	0	0	1	Ref
LT960551	Unassigned	Unassigned	Unassigned	164_8	Clustered	3	VC_164_8	3	0.0299	0.84944649	0.0254	0	0	0	1	Ref
LT960552	Unassigned	Unassigned	Unassigned	164_8	Clustered	3	VC_164_8	3	0.0299	0.84944649	0.0254	0	0	0	1	Ref
LT960606	Unassigned	Unassigned	Unassigned	386_0	Clustered	49	VC_386_0	49	0.7941	1	0.7941	0	0	0	1	Ref
LT960608	Unassigned	Unassigned	Unassigned	85_3	Clustered	19	VC_85_3	19	0.3671	1	0.3671	0	0	0	0.807	Ref
LT960609	Unassigned	Unassigned	Unassigned	835_0	Clustered	67	VC_835_0	67	0.9624	1	0.9624	0	0	0	1	Ref
LT961732	Unassigned	Unassigned	Unassigned	942_0	Clustered	36	VC_942_0	36	0.7848	1	0.7848	0	0	0	1	Ref
LT961836	Unassigned	Unassigned	Unassigned	386_0	Clustered	49	VC_386_0	49	0.7941	1	0.7941	0	0	0	1	Ref
LT961837	Unassigned	Unassigned	Unassigned	386_0	Clustered	49	VC_386_0	49	0.7941	1	0.7941	0	0	0	1	Ref
LT961838	Unassigned	Unassigned	Unassigned	386_0	Clustered	49	VC_386_0	49	0.7941	1	0.7941	0	0	0	1	Ref
LT961840	Unassigned	Unassigned	Unassigned	386_0	Clustered	49	VC_386_0	49	0.7941	1	0.7941	0	0	0	1	Ref
LT961841	Unassigned	Unassigned	Unassigned	386_0	Clustered	49	VC_386_0	49	0.7941	1	0.7941	0	0	0	1	Ref
LT961842	Unassigned	Unassigned	Unassigned	386_0	Clustered	49	VC_386_0	49	0.7941	1	0.7941	0	0	0	1	Ref
LT961843	Unassigned	Unassigned	Unassigned	386_0	Clustered	49	VC_386_0	49	0.7941	1	0.7941	0	0	0	1	Ref
LT961844	Unassigned	Unassigned	Unassigned	386_0	Clustered	49	VC_386_0	49	0.7941	1	0.7941	0	0	0	1	Ref
LT961845	Unassigned	Unassigned	Unassigned	386_0	Clustered	49	VC_386_0	49	0.7941	1	0.7941	0	0	0	1	Ref
LT961846	Unassigned	Unassigned	Unassigned	386_0	Clustered	49	VC_386_0	49	0.7941	1	0.7941	0	0	0	1	Ref
LT962379	Unassigned	Unassigned	Unassigned	386_0	Clustered	49	VC_386_0	49	0.7941	1	0.7941	0	0	0	1	Ref
LT962380	Unassigned	Unassigned	Unassigned	386_0	Clustered	49	VC_386_0	49	0.7941	1	0.7941	0	0	0	1	Ref
LT962475	Unassigned	Unassigned	Unassigned	386_0	Clustered	49	VC_386_0	49	0.7941	1	0.7941	0	0	0	1	Ref
LT962906	Unassigned	Unassigned	Unassigned	386_0	Clustered	49	VC_386_0	49	0.7941	1	0.7941	0	0	0	1	Ref
LT962907	Unassigned	Unassigned	Unassigned	386_0	Clustered	49	VC_386_0	49	0.7941	1	0.7941	0	0	0	1	Ref
LT985568	Unassigned	Unassigned	Unassigned	260_0	Clustered	13	VC_260_0	13	0.3705	1	0.3705	0	0	0	1	Ref
LT985569	Unassigned	Unassigned	Unassigned	263_0	Clustered	7	VC_263_0	7	0.2364	1	0.2364	0	0	0	1	Ref
LT985571	Unassigned	Unassigned	Unassigned	260_0	Clustered	13	VC_260_0	13	0.3705	1	0.3705	0	0	0	1	Ref
LT985572	Unassigned	Unassigned	Unassigned	339_0	Clustered	9	VC_339_0	9	0.9405	0.99999771	0.9405	0	0	0	1	Ref
LT985574	Unassigned	Unassigned	Unassigned	263_0	Clustered	7	VC_263_0	7	0.2364	1	0.2364	0	0	0	1	Ref

LT985575	Unassigned	Unassigned	Unassigned	265_0	Clustered	12	VC_265_0	12	0.4401	1	0.4401	0	0	0	1	Ref
LT985576	Unassigned	Unassigned	Unassigned	265_0	Clustered	12	VC_265_0	12	0.4401	1	0.4401	0	0	0	1	Ref
LT985578	Unassigned	Unassigned	Unassigned	263_0	Clustered	7	VC_263_0	7	0.2364	1	0.2364	0	0	0	1	Ref
LT985581	Unassigned	Unassigned	Unassigned	339_0	Clustered	9	VC_339_0	9	0.9405	0.99999771	0.9405	0	0	0	1	Ref
LT985583	Unassigned	Unassigned	Unassigned	263_0	Clustered	7	VC_263_0	7	0.2364	1	0.2364	0	0	0	1	Ref
LT985584	Unassigned	Unassigned	Unassigned	339_0	Clustered	9	VC_339_0	9	0.9405	0.99999771	0.9405	0	0	0	1	Ref
LT985586	Unassigned	Unassigned	Unassigned	339_0	Clustered	9	VC_339_0	9	0.9405	0.99999771	0.9405	0	0	0	1	Ref
LT985587	Unassigned	Unassigned	Unassigned	339_0	Clustered	9	VC_339_0	9	0.9405	0.99999771	0.9405	0	0	0	1	Ref
LT985589	Unassigned	Unassigned	Unassigned	301_0	Clustered	2	VC_301_0	2	0.1292	0.94930205	0.1226	0	0	0	1	Ref
LT985593	Unassigned	Unassigned	Unassigned	339_0	Clustered	9	VC_339_0	9	0.9405	0.99999771	0.9405	0	0	0	1	Ref
LT986460	Unassigned	Unassigned	Unassigned	755_0	Clustered	9	VC_755_0	9	0.4489	1	0.4489	0	0	0	1	Ref
LT986678	Unassigned	Unassigned	Unassigned	1211_0	Clustered	25	VC_1211_0	25	1	1	1	0	0	0	1	Ref
LT993224	Unassigned	Unassigned	Unassigned	260_0	Clustered	13	VC_260_0	13	0.3705	1	0.3705	0	0	0	1	Ref
LT993227	Unassigned	Unassigned	Unassigned	339_0	Clustered	9	VC_339_0	9	0.9405	0.99999771	0.9405	0	0	0	1	Ref
LT993229	Unassigned	Unassigned	Unassigned	263_0	Clustered	7	VC_263_0	7	0.2364	1	0.2364	0	0	0	1	Ref
LT993247	Unassigned	Unassigned	Unassigned	301_0	Clustered	2	VC_301_0	2	0.1292	0.94930205	0.1226	0	0	0	1	Ref
LT996080	Unassigned	Unassigned	Unassigned	339_0	Clustered	9	VC_339_0	9	0.9405	0.99999771	0.9405	0	0	0	1	Ref
LT996081	Unassigned	Unassigned	Unassigned	265_0	Clustered	12	VC_265_0	12	0.4401	1	0.4401	0	0	0	1	Ref
LT996082	Unassigned	Unassigned	Unassigned	263_0	Clustered	7	VC_263_0	7	0.2364	1	0.2364	0	0	0	1	Ref
LT999987	Unassigned	Unassigned	Unassigned	179_0	Clustered	53	VC_179_0	53	0.9137	1	0.9137	0	0	0	1	Ref
MF001354	Unassigned	Unassigned	Unassigned	283_79	Clustered/Singleton	1	VC_283_79	1	0	1	0	0	0	0	1	Ref
MF001355	Unassigned	Unassigned	Unassigned	288_3	Clustered	3	VC_288_3	3	0.0452	0.99844025	0.0452	0	0	0	1	Ref
MF001356	Unassigned	Unassigned	Unassigned	637_0	Clustered	115	VC_637_0	115	0.731	1	0.731	0	0	0	0.8764	Ref
MF001358	Unassigned	Unassigned	Unassigned	692_0	Clustered	2	VC_692_0	2	0.4503	0.95340726	0.4294	0	0	0	1	Ref
MF001359	Unassigned	Unassigned	Unassigned	283_183	Clustered/Singleton	1	VC_283_183	1	0	1	0	0	0	0	1	Ref
MF001360	Unassigned	Unassigned	Unassigned	283_14	Clustered/Singleton	1	VC_283_14	1	0	1	0	0	0	0	1	Ref
MF001361	Unassigned	Unassigned	Unassigned	692_0	Clustered	2	VC_692_0	2	0.4503	0.95340726	0.4294	0	0	0	1	Ref
MF001362	Unassigned	Unassigned	Unassigned	133_24	Clustered/Singleton	1	VC_133_24	1	0	1	0	0	0	0	1	Ref
MF001363	Unassigned	Unassigned	Unassigned	191_16	Clustered	12	VC_191_16	12	0.0643	1	0.0643	0	0	0	0.9394	Ref
MF001365	Unassigned	Unassigned	Unassigned	149_15	Clustered	16	VC_149_15	16	0.0788	1	0.0788	0	0	0	0.8083	Ref
MF001366	Unassigned	Unassigned	Unassigned	637_0	Clustered	115	VC_637_0	115	0.731	1	0.731	0	0	0	0.8764	Ref
MF033347	Unassigned	Unassigned	Unassigned	699_0	Clustered	71	VC_699_0	71	0.9352	1	0.9352	0	0	0	1	Ref
MF033348	Unassigned	Unassigned	Unassigned	699_0	Clustered	71	VC_699_0	71	0.9352	1	0.9352	0	0	0	1	Ref
MF033349	Unassigned	Unassigned	Unassigned	699_0	Clustered	71	VC_699_0	71	0.9352	1	0.9352	0	0	0	1	Ref
MF033350	Unassigned	Unassigned	Unassigned	699_0	Clustered	71	VC_699_0	71	0.9352	1	0.9352	0	0	0	1	Ref
MF033351	Unassigned	Unassigned	Unassigned	699_0	Clustered	71	VC_699_0	71	0.9352	1	0.9352	0	0	0	1	Ref
MF034659	Unassigned	Unassigned	Unassigned	422_0	Clustered	5	VC_422_0	5	0.2592	0.99999997	0.2592	0	0	0	1	Ref
MF038790	Unassigned	Unassigned	Unassigned	1164_0	Clustered	25	VC_1164_0	25	0.7312	1	0.7312	0	0	0	1	Ref
MF038791	Unassigned	Unassigned	Unassigned	1164_0	Clustered	25	VC_1164_0	25	0.7312	1	0.7312	0	0	0	1	Ref
MF041990	Unassigned	Unassigned	Unassigned	309_0	Clustered	3	VC_309_0	3	0.1265	0.99498896	0.1258	0	0	0	1	Ref
MF042360	Unassigned	Unassigned	Unassigned	344_5	Clustered/Singleton	1	VC_344_5	1	0	1	0	0	0	0	1	Ref
MF044457	Unassigned	Unassigned	Unassigned	286_13	Clustered	10	VC_286_13	10	0.0698	0.99999929	0.0698	0	0	0	0.9556	Ref
MF044458	Unassigned	Unassigned	Unassigned	792_0	Clustered	35	VC_792_0	35	0.9828	1	0.9828	0	0	0	1	Ref
MF063068	Unassigned	Unassigned	Unassigned	344_2	Clustered/Singleton	1	VC_344_2	1	0	1	0	0	0	0	1	Ref
MF072690	Unassigned	Unassigned	Unassigned	546_3	Clustered	4	VC_546_3	4	0.0418	0.92194988	0.0385	0	0	0	1	Ref
MF074189	Unassigned	Unassigned	Unassigned	609_0	Clustered	4	VC_609_0	4	0.489	0.99992419	0.489	0	0	0	1	Ref
MF093736	Unassigned	Unassigned	Unassigned	575_0	Clustered	35	VC_575_0	35	0.8815	1	0.8815	0	0	0	1	Ref
MF098558	Unassigned	Unassigned	Unassigned	1090_0	Clustered	4	VC_1090_0	4	1	1	1	0	0	0	1	Ref
MF101922	Unassigned	Unassigned	Unassigned	1212_0	Clustered	2	VC_1212_0	2	1	1	1	0	0	0	1	Ref
MF133445	Unassigned	Unassigned	Unassigned	546_3	Clustered	4	VC_546_3	4	0.0418	0.92194988	0.0385	0	0	0	1	Ref
MF133446	Unassigned	Unassigned	Unassigned	519_0	Clustered	39	VC_519_0	39	0.8156	1	0.8156	0	0	0	1	Ref
MF140397	Unassigned	Unassigned	Unassigned	1166_0	Clustered	20	VC_1166_0	20	0.99	1	0.99	0	0	0	1	Ref
MF140398	Unassigned	Unassigned	Unassigned	237_1	Clustered	13	VC_237_1	13	0.1773	1	0.1773	0	0	0	0.7308	Ref
MF140399	Unassigned	Unassigned	Unassigned	519_0	Clustered	39	VC_519_0	39	0.8156	1	0.8156	0	0	0	1	Ref
MF140400	Unassigned	Unassigned	Unassigned	1160_0	Clustered	73	VC_1160_0	73	0.9835	1	0.9835	0	0	0	1	Ref
MF140401	Unassigned	Unassigned	Unassigned	1164_0	Clustered	25	VC_1164_0	25	0.7312	1	0.7312	0	0	0	1	Ref
MF140402	Unassigned	Unassigned	Unassigned	234_0	Clustered	21	VC_234_0	21	0.8355	1	0.8355	0	0	0	1	Ref
MF140403	Unassigned	Unassigned	Unassigned	216_3	Clustered	8	VC_216_3	8	0.0701	1	0.0701	0	0	0	0.8571	Ref
MF140404	Unassigned	Unassigned	Unassigned	1160_0	Clustered	73	VC_1160_0	73	0.9835	1	0.9835	0	0	0	1	Ref
MF140405	Unassigned	Unassigned	Unassigned	889_0	Clustered	16	VC_889_0	16	0.2969	1	0.2969	0	0	0	1	Ref
MF140406	Unassigned	Unassigned	Unassigned	237_1	Clustered	13	VC_237_1	13	0.1773	1	0.1773	0	0	0	0.7308	Ref
MF140407	Unassigned	Unassigned	Unassigned	1160_0	Clustered	73	VC_1160_0	73	0.9835	1	0.9835	0	0	0	1	Ref
MF140409	Unassigned	Unassigned	Unassigned	950_0	Clustered	38	VC_950_0	38	0.9443	1	0.9443	0	0	0	1	Ref
MF140410	Unassigned	Unassigned	Unassigned	221_0	Clustered	22	VC_221_0	22	0.7447	1	0.7447	0	0	0	1	Ref
MF140412	Unassigned	Unassigned	Unassigned	236_0	Clustered	72	VC_236_0	72	0.8104	1	0.8104	0	0	0	1	Ref
MF140413	Unassigned	Unassigned	Unassigned	889_0	Clustered	16	VC_889_0	16	0.2969	1	0.2969	0	0	0	1	Ref
MF140414	Unassigned	Unassigned	Unassigned	889_0	Clustered	16	VC_889_0	16	0.2969	1	0.2969	0	0	0	1	Ref
MF140415	Unassigned	Unassigned	Unassigned	950_0	Clustered	38	VC_950_0	38	0.9443	1	0.9443	0	0	0	1	Ref
MF140416	Unassigned	Unassigned	Unassigned	236_1	Clustered	5	VC_236_1	5	0.0358	0.99999997	0.0358	0	0	0	1	Ref
MF140417	Unassigned	Unassigned	Unassigned	950_0	Clustered	38	VC_950_0	38	0.9443	1	0.9443	0	0	0	1	Ref
MF140418	Unassigned	Unassigned	Unassigned	1169_0	Clustered	7	VC_1169_0	7	0.9811	0.99995613	0.981	0	0	0	1	Ref
MF140419	Unassigned	Unassigned	Unassigned	950_0	Clustered	38	VC_950_0	38	0.9443	1	0.9443	0	0	0	1	Ref
MF140420	Unassigned	Unassigned	Unassigned	950_0	Clustered	38	VC_950_0	38	0.9443	1	0.9443	0	0	0	1	Ref
MF140421	Unassigned	Unassigned	Unassigned	889_0	Clustered	16	VC_889_0	16	0.2969	1	0.2969	0	0	0	1	Ref
MF140422	Unassigned	Unassigned	Unassigned	888_0	Clustered	28	VC_888_0	28	0.5497	1	0.5497	0	0	0	1	Ref
MF140423	Unassigned	Unassigned	Unassigned	1164_0	Clustered	25	VC_1164_0	25	0.7312	1	0.7312	0	0	0	1	Ref
MF140424	Unassigned	Unassigned	Unassigned	1160_0	Clustered	73	VC_1160_0	73	0.9835	1	0.9835	0	0	0	1	Ref
MF140425	Unassigned	Unassigned	Unassigned	1160_0	Clustered	73	VC_1160_0	73	0.9835	1	0.9835	0	0	0	1	Ref
MF140426	Unassigned	Unassigned	Unassigned	950_0	Clustered	38	VC_950_0	38	0.9443	1	0.9443	0	0	0	1	Ref
MF140427	Unassigned	Unassigned	Unassigned	1169_0	Clustered	7	VC_1169_0	7	0.9811	0.99995613	0.981	0	0	0	1	Ref
MF140428	Unassigned	Unassigned	Unassigned	236_0	Clustered	72	VC_236_0	72	0.8104	1	0.8104	0	0	0	1	Ref

MF140429	Unassigned	Unassigned	Unassigned	950_0	Clustered	38	VC_950_0	38	0.9443	1	0.9443	0	0	0	1	Ref
MF140430	Unassigned	Unassigned	Unassigned	236_0	Clustered	72	VC_236_0	72	0.8104	1	0.8104	0	0	0	1	Ref
MF140431	Unassigned	Unassigned	Unassigned	950_0	Clustered	38	VC_950_0	38	0.9443	1	0.9443	0	0	0	1	Ref
MF140432	Unassigned	Unassigned	Unassigned	1164_0	Clustered	25	VC_1164_0	25	0.7312	1	0.7312	0	0	0	1	Ref
MF140433	Unassigned	Unassigned	Unassigned	950_0	Clustered	38	VC_950_0	38	0.9443	1	0.9443	0	0	0	1	Ref
MF140434	Unassigned	Unassigned	Unassigned	1169_0	Clustered	7	VC_1169_0	7	0.9811	0.99995613	0.981	0	0	0	1	Ref
MF140435	Unassigned	Unassigned	Unassigned	234_0	Clustered	21	VC_234_0	21	0.8355	1	0.8355	0	0	0	1	Ref
MF141540	Unassigned	Unassigned	Unassigned	762_0	Clustered	9	VC_762_0	9	0.0885	1	0.0885	0	0	0	1	Ref
MF150911	Unassigned	Unassigned	Unassigned	160_0	Clustered	2	VC_160_0	2	0.4956	0.94363853	0.4677	0	0	0	1	Ref
MF153391	Unassigned	Unassigned	Unassigned	638_0	Clustered	45	VC_638_0	45	0.7741	1	0.7741	0	0	0	1	Ref
MF155889	Unassigned	Unassigned	Unassigned	875_0	Clustered	10	VC_875_0	10	0.988	0.99948433	0.9875	0	0	0	1	Ref
MF155936	Unassigned	Unassigned	Unassigned	1046_0	Clustered	7	VC_1046_0	7	0.9413	1	0.9413	0	0	0	1	Ref
MF155946	Unassigned	Unassigned	Unassigned	1071_2	Clustered	5	VC_1071_2	5	0.0534	0.99997362	0.0534	0	0	0	0.9	Ref
MF155947	Unassigned	Unassigned	Unassigned	371_11	Clustered	31	VC_371_11	31	0.0927	1	0.0927	0	0	0	0.7011	Ref
MF156577	Unassigned	Unassigned	Unassigned	818_0	Clustered	20	VC_818_0	20	0.9601	1	0.9601	0	0	0	1	Ref
MF156578	Unassigned	Unassigned	Unassigned	818_0	Clustered	20	VC_818_0	20	0.9601	1	0.9601	0	0	0	1	Ref
MF156580	Unassigned	Unassigned	Unassigned	818_0	Clustered	20	VC_818_0	20	0.9601	1	0.9601	0	0	0	1	Ref
MF158036	Unassigned	Unassigned	Unassigned	540_0	Clustered	15	VC_540_0	15	0.2866	1	0.2866	0	0	0	1	Ref
MF158037	Unassigned	Unassigned	Unassigned	540_0	Clustered	15	VC_540_0	15	0.2866	1	0.2866	0	0	0	1	Ref
MF158038	Unassigned	Unassigned	Unassigned	461_0	Clustered	96	VC_461_0	96	0.9307	1	0.9307	0	0	0	0.9575	Ref
MF158039	Unassigned	Unassigned	Unassigned	455_0	Clustered	18	VC_455_0	18	0.4598	1	0.4598	0	0	0	1	Ref
MF158040	Unassigned	Unassigned	Unassigned	330_0	Clustered	27	VC_330_0	27	0.5088	1	0.5088	0	0	0	0.8661	Ref
MF158041	Unassigned	Unassigned	Unassigned	330_0	Clustered	27	VC_330_0	27	0.5088	1	0.5088	0	0	0	0.8661	Ref
MF158042	Unassigned	Unassigned	Unassigned	455_0	Clustered	18	VC_455_0	18	0.4598	1	0.4598	0	0	0	1	Ref
MF158043	Unassigned	Unassigned	Unassigned	330_0	Clustered	27	VC_330_0	27	0.5088	1	0.5088	0	0	0	0.8661	Ref
MF158044	Unassigned	Unassigned	Unassigned	330_0	Clustered	27	VC_330_0	27	0.5088	1	0.5088	0	0	0	0.8661	Ref
MF158045	Unassigned	Unassigned	Unassigned	283_64	Clustered/Singleton	1	VC_283_64	1	0	1	0	0	0	0	1	Ref
MF158046	Unassigned	Unassigned	Unassigned	283_189	Clustered/Singleton	1	VC_283_189	1	0	1	0	0	0	0	1	Ref
MF161328	Unassigned	Unassigned	Unassigned	252_0	Clustered	121	VC_252_0	121	0.7376	1	0.7376	0	0	0	0.9999	Ref
MF166859	Unassigned	Unassigned	Unassigned	422_0	Clustered	5	VC_422_0	5	0.2592	0.99999997	0.2592	0	0	0	1	Ref
MF172979	Unassigned	Unassigned	Unassigned	626_0	Clustered	23	VC_626_0	23	0.66	1	0.66	0	0	0	0.8379	Ref
MF176161	Unassigned	Unassigned	Unassigned	622_0	Clustered	2	VC_622_0	2	0.0507	0.6259393	0.0317	0	0	0	1	Ref
MF185717	Unassigned	Unassigned	Unassigned	238_0	Clustered	17	VC_238_0	17	0.6109	1	0.6109	0	0	0	1	Ref
MF185718	Unassigned	Unassigned	Unassigned	1074_0	Clustered	19	VC_1074_0	19	0.9198	1	0.9198	0	0	0	1	Ref
MF185719	Unassigned	Unassigned	Unassigned	238_0	Clustered	17	VC_238_0	17	0.6109	1	0.6109	0	0	0	1	Ref
MF185720	Unassigned	Unassigned	Unassigned	238_0	Clustered	17	VC_238_0	17	0.6109	1	0.6109	0	0	0	1	Ref
MF185721	Unassigned	Unassigned	Unassigned	1074_0	Clustered	19	VC_1074_0	19	0.9198	1	0.9198	0	0	0	1	Ref
MF185722	Unassigned	Unassigned	Unassigned	237_0	Clustered	4	VC_237_0	4	0.0429	0.99998759	0.0429	0	0	0	1	Ref
MF185723	Unassigned	Unassigned	Unassigned	1074_0	Clustered	19	VC_1074_0	19	0.9198	1	0.9198	0	0	0	1	Ref
MF185724	Unassigned	Unassigned	Unassigned	1074_0	Clustered	19	VC_1074_0	19	0.9198	1	0.9198	0	0	0	1	Ref
MF185725	Unassigned	Unassigned	Unassigned	1074_0	Clustered	19	VC_1074_0	19	0.9198	1	0.9198	0	0	0	1	Ref
MF185726	Unassigned	Unassigned	Unassigned	887_0	Clustered	18	VC_887_0	18	0.3663	1	0.3663	0	0	0	1	Ref
MF185727	Unassigned	Unassigned	Unassigned	216_0	Clustered	4	VC_216_0	4	0.048	0.9999878	0.048	0	0	0	1	Ref
MF185728	Unassigned	Unassigned	Unassigned	888_0	Clustered	28	VC_888_0	28	0.5497	1	0.5497	0	0	0	1	Ref
MF185729	Unassigned	Unassigned	Unassigned	514_0	Clustered	75	VC_514_0	75	0.9402	1	0.9402	0	0	0	1	Ref
MF185730	Unassigned	Unassigned	Unassigned	888_0	Clustered	28	VC_888_0	28	0.5497	1	0.5497	0	0	0	1	Ref
MF185731	Unassigned	Unassigned	Unassigned	857_0	Clustered	11	VC_857_0	11	0.9712	1	0.9712	0	0	0	1	Ref
MF185732	Unassigned	Unassigned	Unassigned	514_0	Clustered	75	VC_514_0	75	0.9402	1	0.9402	0	0	0	1	Ref
MF185733	Unassigned	Unassigned	Unassigned	514_0	Clustered	75	VC_514_0	75	0.9402	1	0.9402	0	0	0	1	Ref
MF188997	Unassigned	Unassigned	Unassigned	28_1	Clustered	53	VC_28_1	53	0.7164	1	0.7164	0	0	0	0.9325	Ref
MF189170	Unassigned	Unassigned	Unassigned	802_0	Clustered	17	VC_802_0	17	0.8025	1	0.8025	0	0	0	1	Ref
MF189171	Unassigned	Unassigned	Unassigned	802_0	Clustered	17	VC_802_0	17	0.8025	1	0.8025	0	0	0	1	Ref
MF189172	Unassigned	Unassigned	Unassigned	802_0	Clustered	17	VC_802_0	17	0.8025	1	0.8025	0	0	0	1	Ref
MF189173	Unassigned	Unassigned	Unassigned	802_0	Clustered	17	VC_802_0	17	0.8025	1	0.8025	0	0	0	1	Ref
MF189174	Unassigned	Unassigned	Unassigned	802_0	Clustered	17	VC_802_0	17	0.8025	1	0.8025	0	0	0	1	Ref
MF189175	Unassigned	Unassigned	Unassigned	802_0	Clustered	17	VC_802_0	17	0.8025	1	0.8025	0	0	0	1	Ref
MF189176	Unassigned	Unassigned	Unassigned	1073_0	Clustered	24	VC_1073_0	24	0.8388	1	0.8388	0	0	0	1	Ref
MF189177	Unassigned	Unassigned	Unassigned	1073_0	Clustered	24	VC_1073_0	24	0.8388	1	0.8388	0	0	0	1	Ref
MF189178	Unassigned	Unassigned	Unassigned	1073_0	Clustered	24	VC_1073_0	24	0.8388	1	0.8388	0	0	0	1	Ref
MF189179	Unassigned	Unassigned	Unassigned	956_0	Clustered	39	VC_956_0	39	1	1	1	0	0	0	1	Ref
MF197383	Unassigned	Unassigned	Unassigned	1200_0	Clustered	4	VC_1200_0	4	0.8691	0.9999992	0.8691	0	0	0	1	Ref
MF276773	Unassigned	Unassigned	Unassigned	228_0	Clustered	60	VC_228_0	60	0.942	1	0.942	0	0	0	1	Ref
MF278336	Unassigned	Unassigned	Unassigned	988_0	Clustered	2	VC_988_0	2	0.1586	0.95268934	0.1511	0	0	0	1	Ref
MF281061	Unassigned	Unassigned	Unassigned	519_0	Clustered	39	VC_519_0	39	0.8156	1	0.8156	0	0	0	1	Ref
MF285615	Unassigned	Unassigned	Unassigned	823_0	Clustered	124	VC_823_0	124	0.9492	1	0.9492	0	0	0	1	Ref
MF285616	Unassigned	Unassigned	Unassigned	385_0	Clustered	51	VC_385_0	51	0.8754	1	0.8754	0	0	0	1	Ref
MF285617	Unassigned	Unassigned	Unassigned	385_0	Clustered	51	VC_385_0	51	0.8754	1	0.8754	0	0	0	1	Ref
MF285618	Unassigned	Unassigned	Unassigned	488_1	Clustered	8	VC_488_1	8	0.2927	1	0.2927	0	0	0	1	Ref
MF285619	Unassigned	Unassigned	Unassigned	132_0	Clustered	4	VC_132_0	4	0.036	0.99998777	0.036	0	0	0	1	Ref
MF285620	Unassigned	Unassigned	Unassigned	385_0	Clustered	51	VC_385_0	51	0.8754	1	0.8754	0	0	0	1	Ref
MF288917	Unassigned	Unassigned	Unassigned	884_0	Clustered	28	VC_884_0	28	0.6211	1	0.6211	0	0	0	0.9841	Ref
MF288918	Unassigned	Unassigned	Unassigned	884_0	Clustered	28	VC_884_0	28	0.6211	1	0.6211	0	0	0	0.9841	Ref
MF288919	Unassigned	Unassigned	Unassigned	884_0	Clustered	28	VC_884_0	28	0.6211	1	0.6211	0	0	0	0.9841	Ref
MF288920	Unassigned	Unassigned	Unassigned	884_0	Clustered	28	VC_884_0	28	0.6211	1	0.6211	0	0	0	0.9841	Ref
MF288921	Unassigned	Unassigned	Unassigned	883_0	Clustered	10	VC_883_0	10	0.2015	1	0.2015	0	0	0	1	Ref
MF288922	Unassigned	Unassigned	Unassigned	883_0	Clustered	10	VC_883_0	10	0.2015	1	0.2015	0	0	0	1	Ref
MF319184	Unassigned	Unassigned	Unassigned	763_2	Clustered	7	VC_763_2	7	0.2612	1	0.2612	0	0	0	1	Ref
MF324898	Unassigned	Unassigned	Unassigned	956_0	Clustered	39	VC_956_0	39	1	1	1	0	0	0	1	Ref
MF324899	Unassigned	Unassigned	Unassigned	216_0	Clustered	4	VC_216_0	4	0.048	0.9999878	0.048	0	0	0	1	Ref
MF324900	Unassigned	Unassigned	Unassigned	956_0	Clustered	39	VC_956_0	39	1	1	1	0	0	0	1	Ref
MF324901	Unassigned	Unassigned	Unassigned	956_0	Clustered	39	VC_956_0	39	1	1	1	0	0	0	1	Ref

MF324902	Unassigned	Unassigned	Unassigned	956_0	Clustered	39	VC_956_0	39	1	1	1	0	0	0	1	Ref
MF324903	Unassigned	Unassigned	Unassigned	956_0	Clustered	39	VC_956_0	39	1	1	1	0	0	0	1	Ref
MF324904	Unassigned	Unassigned	Unassigned	956_0	Clustered	39	VC_956_0	39	1	1	1	0	0	0	1	Ref
MF324905	Unassigned	Unassigned	Unassigned	956_0	Clustered	39	VC_956_0	39	1	1	1	0	0	0	1	Ref
MF324906	Unassigned	Unassigned	Unassigned	802_0	Clustered	17	VC_802_0	17	0.8025	1	0.8025	0	0	0	1	Ref
MF324907	Unassigned	Unassigned	Unassigned	1073_0	Clustered	24	VC_1073_0	24	0.8388	1	0.8388	0	0	0	1	Ref
MF324908	Unassigned	Unassigned	Unassigned	237_2	Clustered	11	VC_237_2	11	0.1409	1	0.1409	0	0	0	0.9818	Ref
MF324909	Unassigned	Unassigned	Unassigned	237_2	Clustered	11	VC_237_2	11	0.1409	1	0.1409	0	0	0	0.9818	Ref
MF324910	Unassigned	Unassigned	Unassigned	888_0	Clustered	28	VC_888_0	28	0.5497	1	0.5497	0	0	0	1	Ref
MF324911	Unassigned	Unassigned	Unassigned	237_2	Clustered	11	VC_237_2	11	0.1409	1	0.1409	0	0	0	0.9818	Ref
MF324912	Unassigned	Unassigned	Unassigned	237_2	Clustered	11	VC_237_2	11	0.1409	1	0.1409	0	0	0	0.9818	Ref
MF324913	Unassigned	Unassigned	Unassigned	237_2	Clustered	11	VC_237_2	11	0.1409	1	0.1409	0	0	0	0.9818	Ref
MF324914	Unassigned	Unassigned	Unassigned	237_2	Clustered	11	VC_237_2	11	0.1409	1	0.1409	0	0	0	0.9818	Ref
MF324915	Unassigned	Unassigned	Unassigned	237_1	Clustered	13	VC_237_1	13	0.1773	1	0.1773	0	0	0	0.7308	Ref
MF327003	Unassigned	Unassigned	Unassigned	330_0	Clustered	27	VC_330_0	27	0.5088	1	0.5088	0	0	0	0.8661	Ref
MF327004	Unassigned	Unassigned	Unassigned	330_0	Clustered	27	VC_330_0	27	0.5088	1	0.5088	0	0	0	0.8661	Ref
MF327005	Unassigned	Unassigned	Unassigned	330_0	Clustered	27	VC_330_0	27	0.5088	1	0.5088	0	0	0	0.8661	Ref
MF327006	Unassigned	Unassigned	Unassigned	590_0	Clustered	27	VC_590_0	27	0.9947	1	0.9947	0	0	0	1	Ref
MF327007	Unassigned	Unassigned	Unassigned	283_158	Clustered/Singleton	1	VC_283_158	1	0	1	0	0	0	0	1	Ref
MF327008	Unassigned	Unassigned	Unassigned	283_19	Clustered/Singleton	1	VC_283_19	1	0	1	0	0	0	0	1	Ref
MF327009	Unassigned	Unassigned	Unassigned	283_20	Clustered/Singleton	1	VC_283_20	1	0	1	0	0	0	0	1	Ref
MF346584	Unassigned	Unassigned	Unassigned	698_0	Clustered	26	VC_698_0	26	0.9366	1	0.9366	0	0	0	1	Ref
MF346933	Unassigned	Unassigned	Unassigned	202_0	Clustered	102	VC_202_0	102	0.4049	1	0.4049	0	0	0	0.7876	Ref
MF347636	Unassigned	Unassigned	Unassigned	1071_3	Clustered	16	VC_1071_3	16	0.2354	1	0.2354	0	0	0	0.9667	Ref
MF347637	Unassigned	Unassigned	Unassigned	1071_3	Clustered	16	VC_1071_3	16	0.2354	1	0.2354	0	0	0	0.9667	Ref
MF347638	Unassigned	Unassigned	Unassigned	1071_3	Clustered	16	VC_1071_3	16	0.2354	1	0.2354	0	0	0	0.9667	Ref
MF347639	Unassigned	Unassigned	Unassigned	1071_3	Clustered	16	VC_1071_3	16	0.2354	1	0.2354	0	0	0	0.9667	Ref
MF351863	Unassigned	Unassigned	Unassigned	432_3	Clustered/Singleton	1	VC_432_3	1	0	1	0	0	0	0	1	Ref
MF356679	Unassigned	Unassigned	Unassigned	247_1	Clustered/Singleton	1	VC_247_1	1	0	1	0	0	0	0	1	Ref
MF358541	Unassigned	Unassigned	Unassigned	1071_3	Clustered	16	VC_1071_3	16	0.2354	1	0.2354	0	0	0	0.9667	Ref
MF358542	Unassigned	Unassigned	Unassigned	1071_3	Clustered	16	VC_1071_3	16	0.2354	1	0.2354	0	0	0	0.9667	Ref
MF360957	Unassigned	Unassigned	Unassigned	448_0	Clustered	4	VC_448_0	4	0.3059	0.99978853	0.3058	0	0	0	1	Ref
MF370225	Unassigned	Unassigned	Unassigned	329_1	Clustered	17	VC_329_1	17	0.0867	1	0.0867	0	0	0	0.8235	Ref
MF370964	Unassigned	Unassigned	Unassigned	496_1	Clustered/Singleton	1	VC_496_1	1	0	1	0	0	0	0	1	Ref
MF370965	Unassigned	Unassigned	Unassigned	896_0	Clustered	5	VC_896_0	5	0.4981	0.99999977	0.4981	0	0	0	1	Ref
MF373840	Unassigned	Unassigned	Unassigned	513_0	Clustered	32	VC_513_0	32	0.6881	1	0.6881	0	0	0	1	Ref
MF373841	Unassigned	Unassigned	Unassigned	237_1	Clustered	13	VC_237_1	13	0.1773	1	0.1773	0	0	0	0.7308	Ref
MF373842	Unassigned	Unassigned	Unassigned	956_0	Clustered	39	VC_956_0	39	1	1	1	0	0	0	1	Ref
MF373843	Unassigned	Unassigned	Unassigned	519_0	Clustered	39	VC_519_0	39	0.8156	1	0.8156	0	0	0	1	Ref
MF374379	Unassigned	Unassigned	Unassigned	43_0	Clustered	38	VC_43_0	38	0.5885	1	0.5885	0	0	0	0.9659	Ref
MF375456	Unassigned	Unassigned	Unassigned	74_0	Clustered	15	VC_74_0	15	0.5204	1	0.5204	0	0	0	1	Ref
MF377440	Unassigned	Unassigned	Unassigned	236_0	Clustered	72	VC_236_0	72	0.8104	1	0.8104	0	0	0	1	Ref
MF377441	Unassigned	Unassigned	Unassigned	1073_0	Clustered	24	VC_1073_0	24	0.8388	1	0.8388	0	0	0	1	Ref
MF377442	Unassigned	Unassigned	Unassigned	1073_0	Clustered	24	VC_1073_0	24	0.8388	1	0.8388	0	0	0	1	Ref
MF398190	Unassigned	Unassigned	Unassigned	149_8	Clustered	66	VC_149_8	66	0.4565	1	0.4565	0	0	0	0.8182	Ref
MF402939	Unassigned	Unassigned	Unassigned	190_18	Clustered/Singleton	1	VC_190_18	1	0	1	0	0	0	0	1	Ref
MF403005	Unassigned	Unassigned	Unassigned	987_0	Clustered	22	VC_987_0	22	0.8955	1	0.8955	0	0	0	1	Ref
MF403006	Unassigned	Unassigned	Unassigned	987_0	Clustered	22	VC_987_0	22	0.8955	1	0.8955	0	0	0	1	Ref
MF403007	Unassigned	Unassigned	Unassigned	1052_2	Clustered/Singleton	1	VC_1052_2	1	0	1	0	0	0	0	1	Ref
MF405094	Unassigned	Unassigned	Unassigned	149_8	Clustered	66	VC_149_8	66	0.4565	1	0.4565	0	0	0	0.8182	Ref
MF407276	Unassigned	Unassigned	Unassigned	817_0	Clustered	14	VC_817_0	14	0.6342	1	0.6342	0	0	0	1	Ref
MF415412	Unassigned	Unassigned	Unassigned	938_0	Clustered	99	VC_938_0	99	0.9119	1	0.9119	0	0	0	0.9928	Ref
MF418016	Unassigned	Unassigned	Unassigned	88_1	Clustered/Singleton	1	VC_88_1	1	0	1	0	0	0	0	1	Ref
MF422185	Unassigned	Unassigned	Unassigned	85_3	Clustered	19	VC_85_3	19	0.3671	1	0.3671	0	0	0	0.807	Ref
MF428478	Unassigned	Unassigned	Unassigned	11_0	Clustered	19	VC_11_0	19	0.7562	1	0.7562	0	0	0	1	Ref
MF428479	Unassigned	Unassigned	Unassigned	11_0	Clustered	19	VC_11_0	19	0.7562	1	0.7562	0	0	0	1	Ref
MF428480	Unassigned	Unassigned	Unassigned	11_0	Clustered	19	VC_11_0	19	0.7562	1	0.7562	0	0	0	1	Ref
MF428481	Unassigned	Unassigned	Unassigned	11_0	Clustered	19	VC_11_0	19	0.7562	1	0.7562	0	0	0	1	Ref
MF431252	Unassigned	Unassigned	Unassigned	637_0	Clustered	115	VC_637_0	115	0.731	1	0.731	0	0	0	0.8764	Ref
MF431615	Unassigned	Unassigned	Unassigned	304_0	Clustered	11	VC_304_0	11	0.7869	1	0.7869	0	0	0	1	Ref
MF431616	Unassigned	Unassigned	Unassigned	304_0	Clustered	11	VC_304_0	11	0.7869	1	0.7869	0	0	0	1	Ref
MF431617	Unassigned	Unassigned	Unassigned	304_0	Clustered	11	VC_304_0	11	0.7869	1	0.7869	0	0	0	1	Ref
MF431726	Unassigned	Unassigned	Unassigned	530_0	Clustered	9	VC_530_0	9	0.8027	1	0.8027	0	0	0	1	Ref
MF431730	Unassigned	Unassigned	Unassigned	190_7	Clustered	7	VC_190_7	7	0.0476	1	0.0476	0	0	0	1	Ref
MF431731	Unassigned	Unassigned	Unassigned	190_7	Clustered	7	VC_190_7	7	0.0476	1	0.0476	0	0	0	1	Ref
MF431732	Unassigned	Unassigned	Unassigned	190_7	Clustered	7	VC_190_7	7	0.0476	1	0.0476	0	0	0	1	Ref
MF431733	Unassigned	Unassigned	Unassigned	190_7	Clustered	7	VC_190_7	7	0.0476	1	0.0476	0	0	0	1	Ref
MF431734	Unassigned	Unassigned	Unassigned	190_7	Clustered	7	VC_190_7	7	0.0476	1	0.0476	0	0	0	1	Ref
MF431735	Unassigned	Unassigned	Unassigned	190_7	Clustered	7	VC_190_7	7	0.0476	1	0.0476	0	0	0	1	Ref
MF431736	Unassigned	Unassigned	Unassigned	191_32	Clustered	12	VC_191_32	12	0.0683	1	0.0683	0	0	0	0.7576	Ref
MF431737	Unassigned	Unassigned	Unassigned	191_32	Clustered	12	VC_191_32	12	0.0683	1	0.0683	0	0	0	0.7576	Ref
MF431738	Unassigned	Unassigned	Unassigned	191_32	Clustered	12	VC_191_32	12	0.0683	1	0.0683	0	0	0	0.7576	Ref
MF431739	Unassigned	Unassigned	Unassigned	191_32	Clustered	12	VC_191_32	12	0.0683	1	0.0683	0	0	0	0.7576	Ref
MF431740	Unassigned	Unassigned	Unassigned	191_32	Clustered	12	VC_191_32	12	0.0683	1	0.0683	0	0	0	0.7576	Ref
MF431741	Unassigned	Unassigned	Unassigned	191_32	Clustered	12	VC_191_32	12	0.0683	1	0.0683	0	0	0	0.7576	Ref
MF443119	Unassigned	Unassigned	Unassigned	690_0	Clustered	50	VC_690_0	50	0.9805	1	0.9805	0	0	0	1	Ref
MF443120	Unassigned	Unassigned	Unassigned	690_0	Clustered	50	VC_690_0	50	0.9805	1	0.9805	0	0	0	1	Ref
MF443121	Unassigned	Unassigned	Unassigned	690_0	Clustered	50	VC_690_0	50	0.9805	1	0.9805	0	0	0	1	Ref
MF443122	Unassigned	Unassigned	Unassigned	690_0	Clustered	50	VC_690_0	50	0.9805	1	0.9805	0	0	0	1	Ref
MF443123	Unassigned	Unassigned	Unassigned	690_0	Clustered	50	VC_690_0	50	0.9805	1	0.9805	0	0	0	1	Ref
MF443124	Unassigned	Unassigned	Unassigned	690_0	Clustered	50	VC_690_0	50	0.9805	1	0.9805	0	0	0	1	Ref

MF443125	Unassigned	Unassigned	Unassigned	690_0	Clustered	50	VC_690_0	50	0.9805	1	0.9805	0	0	0	1	Ref
MF443126	Unassigned	Unassigned	Unassigned	690_0	Clustered	50	VC_690_0	50	0.9805	1	0.9805	0	0	0	1	Ref
MF443783	Unassigned	Unassigned	Unassigned	1213_0	Clustered	2	VC_1213_0	2	1	1	1	0	0	0	1	Ref
MF443784	Unassigned	Unassigned	Unassigned	1213_0	Clustered	2	VC_1213_0	2	1	1	1	0	0	0	1	Ref
MF448340	Unassigned	Unassigned	Unassigned	443_2	Clustered	10	VC_443_2	10	0.3344	1	0.3344	0	0	0	0.9333	Ref
MF448554	Unassigned	Unassigned	Unassigned	202_0	Clustered	102	VC_202_0	102	0.4049	1	0.4049	0	0	0	0.7876	Ref
MF448555	Unassigned	Unassigned	Unassigned	202_0	Clustered	102	VC_202_0	102	0.4049	1	0.4049	0	0	0	0.7876	Ref
MF448556	Unassigned	Unassigned	Unassigned	202_0	Clustered	102	VC_202_0	102	0.4049	1	0.4049	0	0	0	0.7876	Ref
MF448557	Unassigned	Unassigned	Unassigned	202_0	Clustered	102	VC_202_0	102	0.4049	1	0.4049	0	0	0	0.7876	Ref
MF448558	Unassigned	Unassigned	Unassigned	202_0	Clustered	102	VC_202_0	102	0.4049	1	0.4049	0	0	0	0.7876	Ref
MF448559	Unassigned	Unassigned	Unassigned	202_0	Clustered	102	VC_202_0	102	0.4049	1	0.4049	0	0	0	0.7876	Ref
MF448560	Unassigned	Unassigned	Unassigned	202_0	Clustered	102	VC_202_0	102	0.4049	1	0.4049	0	0	0	0.7876	Ref
MF448561	Unassigned	Unassigned	Unassigned	202_0	Clustered	102	VC_202_0	102	0.4049	1	0.4049	0	0	0	0.7876	Ref
MF448562	Unassigned	Unassigned	Unassigned	202_0	Clustered	102	VC_202_0	102	0.4049	1	0.4049	0	0	0	0.7876	Ref
MF448563	Unassigned	Unassigned	Unassigned	202_0	Clustered	102	VC_202_0	102	0.4049	1	0.4049	0	0	0	0.7876	Ref
MF448564	Unassigned	Unassigned	Unassigned	202_0	Clustered	102	VC_202_0	102	0.4049	1	0.4049	0	0	0	0.7876	Ref
MF448565	Unassigned	Unassigned	Unassigned	202_0	Clustered	102	VC_202_0	102	0.4049	1	0.4049	0	0	0	0.7876	Ref
MF448566	Unassigned	Unassigned	Unassigned	202_0	Clustered	102	VC_202_0	102	0.4049	1	0.4049	0	0	0	0.7876	Ref
MF448567	Unassigned	Unassigned	Unassigned	202_0	Clustered	102	VC_202_0	102	0.4049	1	0.4049	0	0	0	0.7876	Ref
MF448568	Unassigned	Unassigned	Unassigned	202_0	Clustered	102	VC_202_0	102	0.4049	1	0.4049	0	0	0	0.7876	Ref
MF448569	Unassigned	Unassigned	Unassigned	202_0	Clustered	102	VC_202_0	102	0.4049	1	0.4049	0	0	0	0.7876	Ref
MF448570	Unassigned	Unassigned	Unassigned	202_0	Clustered	102	VC_202_0	102	0.4049	1	0.4049	0	0	0	0.7876	Ref
MF448571	Unassigned	Unassigned	Unassigned	202_0	Clustered	102	VC_202_0	102	0.4049	1	0.4049	0	0	0	0.7876	Ref
MF448572	Unassigned	Unassigned	Unassigned	202_0	Clustered	102	VC_202_0	102	0.4049	1	0.4049	0	0	0	0.7876	Ref
MF467948	Unassigned	Unassigned	Unassigned	872_0	Clustered	31	VC_872_0	31	0.8151	1	0.8151	0	0	0	0.8194	Ref
MF467949	Unassigned	Unassigned	Unassigned	872_0	Clustered	31	VC_872_0	31	0.8151	1	0.8151	0	0	0	0.8194	Ref
MF468274	Unassigned	Unassigned	Unassigned	575_0	Clustered	35	VC_575_0	35	0.8815	1	0.8815	0	0	0	1	Ref
MF472892	Unassigned	Unassigned	Unassigned	236_0	Clustered	72	VC_236_0	72	0.8104	1	0.8104	0	0	0	1	Ref
MF472894	Unassigned	Unassigned	Unassigned	519_0	Clustered	39	VC_519_0	39	0.8156	1	0.8156	0	0	0	1	Ref
MF472896	Unassigned	Unassigned	Unassigned	216_6	Clustered	42	VC_216_6	42	0.473	1	0.473	0	0	0	0.8351	Ref
MF476925	Unassigned	Unassigned	Unassigned	598_0	Clustered	4	VC_598_0	4	0.0597	0.99355358	0.0593	0	0	0	1	Ref
MF479730	Unassigned	Unassigned	Unassigned	600_2	Clustered	10	VC_600_2	10	0.3041	1	0.3041	0	0	0	1	Ref
MF481197	Unassigned	Unassigned	Unassigned	1027_0	Clustered	20	VC_1027_0	20	0.7114	1	0.7114	0	0	0	1	Ref
MF490236	Unassigned	Unassigned	Unassigned	838_0	Clustered	29	VC_838_0	29	0.8611	1	0.8611	0	0	0	1	Ref
MF490237	Unassigned	Unassigned	Unassigned	630_0	Clustered	20	VC_630_0	20	0.8513	1	0.8513	0	0	0	1	Ref
MF490238	Unassigned	Unassigned	Unassigned	838_0	Clustered	29	VC_838_0	29	0.8611	1	0.8611	0	0	0	1	Ref
MF490239	Unassigned	Unassigned	Unassigned	166_0	Clustered	26	VC_166_0	26	0.7923	1	0.7923	0	0	0	1	Ref
MF490240	Unassigned	Unassigned	Unassigned	79_0	Clustered	64	VC_79_0	64	0.4185	1	0.4185	0	0	0	0.7649	Ref
MF490241	Unassigned	Unassigned	Unassigned	79_7	Clustered	33	VC_79_7	33	0.216	1	0.216	0	0	0	0.8409	Ref
MF498773	Unassigned	Unassigned	Unassigned	443_2	Clustered	10	VC_443_2	10	0.3344	1	0.3344	0	0	0	0.9333	Ref
MF498775	Unassigned	Unassigned	Unassigned	443_2	Clustered	10	VC_443_2	10	0.3344	1	0.3344	0	0	0	0.9333	Ref
MF498901	Unassigned	Unassigned	Unassigned	883_0	Clustered	10	VC_883_0	10	0.2015	1	0.2015	0	0	0	1	Ref
MF537628	Unassigned	Unassigned	Unassigned	956_0	Clustered	39	VC_956_0	39	1	1	1	0	0	0	1	Ref
MF541403	Unassigned	Unassigned	Unassigned	982_0	Clustered	62	VC_982_0	62	0.8646	1	0.8646	0	0	0	0.743	Ref
MF541404	Unassigned	Unassigned	Unassigned	982_0	Clustered	62	VC_982_0	62	0.8646	1	0.8646	0	0	0	0.743	Ref
MF541405	Unassigned	Unassigned	Unassigned	982_0	Clustered	62	VC_982_0	62	0.8646	1	0.8646	0	0	0	0.743	Ref
MF541406	Unassigned	Unassigned	Unassigned	982_0	Clustered	62	VC_982_0	62	0.8646	1	0.8646	0	0	0	0.743	Ref
MF541407	Unassigned	Unassigned	Unassigned	982_0	Clustered	62	VC_982_0	62	0.8646	1	0.8646	0	0	0	0.743	Ref
MF541408	Unassigned	Unassigned	Unassigned	982_0	Clustered	62	VC_982_0	62	0.8646	1	0.8646	0	0	0	0.743	Ref
MF541409	Unassigned	Unassigned	Unassigned	982_0	Clustered	62	VC_982_0	62	0.8646	1	0.8646	0	0	0	0.743	Ref
MF541410	Unassigned	Unassigned	Unassigned	982_0	Clustered	62	VC_982_0	62	0.8646	1	0.8646	0	0	0	0.743	Ref
MF547662	Unassigned	Unassigned	Unassigned	473_3	Clustered	2	VC_473_3	2	0.1063	0.94203251	0.1001	0	0	0	1	Ref
MF547663	Unassigned	Unassigned	Unassigned	473_3	Clustered	2	VC_473_3	2	0.1063	0.94203251	0.1001	0	0	0	1	Ref
MF564201	Unassigned	Unassigned	Unassigned	942_0	Clustered	36	VC_942_0	36	0.7848	1	0.7848	0	0	0	1	Ref
MF574151	Unassigned	Unassigned	Unassigned	82_0	Clustered	24	VC_82_0	24	0.7351	1	0.7351	0	0	0	1	Ref
MF580410	Unassigned	Unassigned	Unassigned	8_0	Clustered	58	VC_8_0	58	0.8306	1	0.8306	0	0	0	0.9946	Ref
MF580759	Unassigned	Unassigned	Unassigned	252_0	Clustered	121	VC_252_0	121	0.7376	1	0.7376	0	0	0	0.9999	Ref
MF580760	Unassigned	Unassigned	Unassigned	252_0	Clustered	121	VC_252_0	121	0.7376	1	0.7376	0	0	0	0.9999	Ref
MF580761	Unassigned	Unassigned	Unassigned	252_0	Clustered	121	VC_252_0	121	0.7376	1	0.7376	0	0	0	0.9999	Ref
MF580762	Unassigned	Unassigned	Unassigned	252_0	Clustered	121	VC_252_0	121	0.7376	1	0.7376	0	0	0	0.9999	Ref
MF580763	Unassigned	Unassigned	Unassigned	252_0	Clustered	121	VC_252_0	121	0.7376	1	0.7376	0	0	0	0.9999	Ref
MF580764	Unassigned	Unassigned	Unassigned	252_0	Clustered	121	VC_252_0	121	0.7376	1	0.7376	0	0	0	0.9999	Ref
MF580765	Unassigned	Unassigned	Unassigned	252_0	Clustered	121	VC_252_0	121	0.7376	1	0.7376	0	0	0	0.9999	Ref
MF580766	Unassigned	Unassigned	Unassigned	252_0	Clustered	121	VC_252_0	121	0.7376	1	0.7376	0	0	0	0.9999	Ref
MF580767	Unassigned	Unassigned	Unassigned	252_0	Clustered	121	VC_252_0	121	0.7376	1	0.7376	0	0	0	0.9999	Ref
MF580768	Unassigned	Unassigned	Unassigned	252_0	Clustered	121	VC_252_0	121	0.7376	1	0.7376	0	0	0	0.9999	Ref
MF580769	Unassigned	Unassigned	Unassigned	252_0	Clustered	121	VC_252_0	121	0.7376	1	0.7376	0	0	0	0.9999	Ref
MF580770	Unassigned	Unassigned	Unassigned	252_0	Clustered	121	VC_252_0	121	0.7376	1	0.7376	0	0	0	0.9999	Ref
MF580771	Unassigned	Unassigned	Unassigned	252_0	Clustered	121	VC_252_0	121	0.7376	1	0.7376	0	0	0	0.9999	Ref
MF580772	Unassigned	Unassigned	Unassigned	252_0	Clustered	121	VC_252_0	121	0.7376	1	0.7376	0	0	0	0.9999	Ref
MF580773	Unassigned	Unassigned	Unassigned	252_0	Clustered	121	VC_252_0	121	0.7376	1	0.7376	0	0	0	0.9999	Ref
MF580774	Unassigned	Unassigned	Unassigned	252_0	Clustered	121	VC_252_0	121	0.7376	1	0.7376	0	0	0	0.9999	Ref
MF580775	Unassigned	Unassigned	Unassigned	252_0	Clustered	121	VC_252_0	121	0.7376	1	0.7376	0	0	0	0.9999	Ref
MF580955	Unassigned	Unassigned	Unassigned	1215_0	Clustered	3	VC_1215_0	3	1	1	1	0	0	0	1	Ref
MF580956	Unassigned	Unassigned	Unassigned	1216_0	Clustered	3	VC_1216_0	3	0.8134	0.99225442	0.8071	0	0	0	1	Ref
MF580957	Unassigned	Unassigned	Unassigned	1215_0	Clustered	3	VC_1215_0	3	1	1	1	0	0	0	1	Ref
MF580958	Unassigned	Unassigned	Unassigned	1215_0	Clustered	3	VC_1215_0	3	1	1	1	0	0	0	1	Ref
MF580959	Unassigned	Unassigned	Unassigned	1216_0	Clustered	3	VC_1216_0	3	0.8134	0.99225442	0.8071	0	0	0	1	Ref
MF580960	Unassigned	Unassigned	Unassigned	1216_0	Clustered	3	VC_1216_0	3	0.8134	0.99225442	0.8071	0	0	0	1	Ref
MF580961	Unassigned	Unassigned	Unassigned	1217_0	Clustered	3	VC_1217_0	3	0.7992	0.99216275	0.793	0	0	0	1	Ref
MF580962	Unassigned	Unassigned	Unassigned	1217_0	Clustered	3	VC_1217_0	3	0.7992	0.99216275	0.793	0	0	0	1	Ref

MF595878	Unassigned	Unassigned	Unassigned	734_0	Clustered	6	VC_734_0	6	0.1914	0.99996888	0.1914	0	0	0	1	Ref
MF612071	Unassigned	Unassigned	Unassigned	111_0	Clustered	91	VC_111_0	91	0.9587	1	0.9587	0	0	0	1	Ref
MF612072	Unassigned	Unassigned	Unassigned	938_0	Clustered	99	VC_938_0	99	0.9119	1	0.9119	0	0	0	0.9928	Ref
MF612073	Unassigned	Unassigned	Unassigned	359_0	Clustered	7	VC_359_0	7	0.3703	1	0.3703	0	0	0	1	Ref
MF614100	Unassigned	Unassigned	Unassigned	705_0	Clustered	11	VC_705_0	11	0.8861	1	0.8861	0	0	0	1	Ref
MF614627	Unassigned	Unassigned	Unassigned	21_0	Clustered	4	VC_21_0	4	0.5696	0.87262442	0.4971	0	0	0	0.6667	Ref
MF614628	Unassigned	Unassigned	Unassigned	21_0	Clustered	4	VC_21_0	4	0.5696	0.87262442	0.4971	0	0	0	0.6667	Ref
MF621978	Unassigned	Unassigned	Unassigned	70_0	Clustered	27	VC_70_0	27	0.5981	1	0.5981	0	0	0	0.9886	Ref
MF623055	Unassigned	Unassigned	Unassigned	79_4	Clustered	3	VC_79_4	3	0.0106	0.99632092	0.0105	0	0	0	0.6667	Ref
MF629150	Unassigned	Unassigned	Unassigned	1217_0	Clustered	3	VC_1217_0	3	0.7992	0.99216275	0.793	0	0	0	1	Ref
MF630921	Unassigned	Unassigned	Unassigned	451_0	Clustered	80	VC_451_0	80	0.9991	1	0.9991	0	0	0	1	Ref
MF663761	Unassigned	Unassigned	Unassigned	123_0	Clustered	23	VC_123_0	23	0.817	1	0.817	0	0	0	1	Ref
MF663786	Unassigned	Unassigned	Unassigned	127_0	Clustered/Singleton	1	VC_127_0	1	0	1	0	0	0	0	1	Ref
MF668266	Unassigned	Unassigned	Unassigned	1167_0	Clustered	6	VC_1167_0	6	0.8642	0.99999946	0.8642	0	0	0	1	Ref
MF668267	Unassigned	Unassigned	Unassigned	236_0	Clustered	72	VC_236_0	72	0.8104	1	0.8104	0	0	0	1	Ref
MF668268	Unassigned	Unassigned	Unassigned	761_0	Clustered	67	VC_761_0	67	0.8947	1	0.8947	0	0	0	1	Ref
MF668269	Unassigned	Unassigned	Unassigned	216_4	Clustered	13	VC_216_4	13	0.1657	1	0.1657	0	0	0	1	Ref
MF668270	Unassigned	Unassigned	Unassigned	520_15	Clustered	2	VC_520_15	2	0.0078	0.957604	0.0075	0	0	0	0	Ref
MF668272	Unassigned	Unassigned	Unassigned	761_0	Clustered	67	VC_761_0	67	0.8947	1	0.8947	0	0	0	1	Ref
MF668273	Unassigned	Unassigned	Unassigned	1167_0	Clustered	6	VC_1167_0	6	0.8642	0.99999946	0.8642	0	0	0	1	Ref
MF668274	Unassigned	Unassigned	Unassigned	1074_0	Clustered	19	VC_1074_0	19	0.9198	1	0.9198	0	0	0	1	Ref
MF668275	Unassigned	Unassigned	Unassigned	1159_0	Clustered	3	VC_1159_0	3	0.3304	0.98548359	0.3256	0	0	0	1	Ref
MF668276	Unassigned	Unassigned	Unassigned	371_11	Clustered	31	VC_371_11	31	0.0927	1	0.0927	0	0	0	0.7011	Ref
MF668277	Unassigned	Unassigned	Unassigned	551_7	Clustered	28	VC_551_7	28	0.1661	1	0.1661	0	0	0	0.8519	Ref
MF668278	Unassigned	Unassigned	Unassigned	517_0	Clustered	18	VC_517_0	18	0.1599	1	0.1599	0	0	0	1	Ref
MF668279	Unassigned	Unassigned	Unassigned	1167_0	Clustered	6	VC_1167_0	6	0.8642	0.99999946	0.8642	0	0	0	1	Ref
MF668280	Unassigned	Unassigned	Unassigned	559_0	Clustered	2	VC_559_0	2	0.0112	0.95651281	0.0107	0	0	0	1	Ref
MF668282	Unassigned	Unassigned	Unassigned	224_0	Clustered	19	VC_224_0	19	0.9416	1	0.9416	0	0	0	1	Ref
MF668283	Unassigned	Unassigned	Unassigned	325_35	Clustered	17	VC_325_35	17	0.0732	1	0.0732	0	0	0	0.7721	Ref
MF668284	Unassigned	Unassigned	Unassigned	546_3	Clustered	4	VC_546_3	4	0.0418	0.92194988	0.0385	0	0	0	1	Ref
MF668285	Unassigned	Unassigned	Unassigned	1160_0	Clustered	73	VC_1160_0	73	0.9835	1	0.9835	0	0	0	1	Ref
MF668287	Unassigned	Unassigned	Unassigned	520_3	Clustered/Singleton	1	VC_520_3	1	0	1	0	0	0	0	1	Ref
MF668288	Unassigned	Unassigned	Unassigned	223_0	Clustered	38	VC_223_0	38	0.9095	1	0.9095	0	0	0	1	Ref
MF678788	Unassigned	Unassigned	Unassigned	695_0	Clustered	48	VC_695_0	48	0.8162	1	0.8162	0	0	0	1	Ref
MF678789	Unassigned	Unassigned	Unassigned	695_0	Clustered	48	VC_695_0	48	0.8162	1	0.8162	0	0	0	1	Ref
MF678790	Unassigned	Unassigned	Unassigned	695_0	Clustered	48	VC_695_0	48	0.8162	1	0.8162	0	0	0	1	Ref
MF681663	Unassigned	Unassigned	Unassigned	191_11	Clustered/Singleton	1	VC_191_11	1	0	1	0	0	0	0	1	Ref
MF683623	Unassigned	Unassigned	Unassigned	878_0	Clustered	32	VC_878_0	32	0.9965	1	0.9965	0	0	0	1	Ref
MF695815	Unassigned	Unassigned	Unassigned	243_0	Clustered	2	VC_243_0	2	0.0637	0.95609892	0.0609	0	0	0	1	Ref
MF716957	Unassigned	Unassigned	Unassigned	52_0	Clustered	87	VC_52_0	87	0.9858	1	0.9858	0	0	0	1	Ref
MF740800	Unassigned	Unassigned	Unassigned	428_0	Clustered	33	VC_428_0	33	0.9915	1	0.9915	0	0	0	1	Ref
MF754111	Unassigned	Unassigned	Unassigned	837_0	Clustered	29	VC_837_0	29	0.9955	1	0.9955	0	0	0	1	Ref
MF754112	Unassigned	Unassigned	Unassigned	837_0	Clustered	29	VC_837_0	29	0.9955	1	0.9955	0	0	0	1	Ref
MF754113	Unassigned	Unassigned	Unassigned	882_0	Clustered	20	VC_882_0	20	0.9917	1	0.9917	0	0	0	1	Ref
MF754114	Unassigned	Unassigned	Unassigned	882_0	Clustered	20	VC_882_0	20	0.9917	1	0.9917	0	0	0	1	Ref
MF754115	Unassigned	Unassigned	Unassigned	882_0	Clustered	20	VC_882_0	20	0.9917	1	0.9917	0	0	0	1	Ref
MF754116	Unassigned	Unassigned	Unassigned	882_0	Clustered	20	VC_882_0	20	0.9917	1	0.9917	0	0	0	1	Ref
MF765814	Unassigned	Unassigned	Unassigned	883_1	Clustered	12	VC_883_1	12	0.2441	1	0.2441	0	0	0	1	Ref
MF766044	Unassigned	Unassigned	Unassigned	981_1	Clustered	26	VC_981_1	26	0.7009	1	0.7009	0	0	0	1	Ref
MF766045	Unassigned	Unassigned	Unassigned	981_1	Clustered	26	VC_981_1	26	0.7009	1	0.7009	0	0	0	1	Ref
MF766046	Unassigned	Unassigned	Unassigned	981_1	Clustered	26	VC_981_1	26	0.7009	1	0.7009	0	0	0	1	Ref
MF766047	Unassigned	Unassigned	Unassigned	981_1	Clustered	26	VC_981_1	26	0.7009	1	0.7009	0	0	0	1	Ref
MF766048	Unassigned	Unassigned	Unassigned	981_1	Clustered	26	VC_981_1	26	0.7009	1	0.7009	0	0	0	1	Ref
MF768469	Unassigned	Unassigned	Unassigned	472_0	Clustered	47	VC_472_0	47	0.9025	1	0.9025	0	0	0	1	Ref
MF768470	Unassigned	Unassigned	Unassigned	79_7	Clustered	33	VC_79_7	33	0.216	1	0.216	0	0	0	0.8409	Ref
MF773750	Unassigned	Unassigned	Unassigned	515_0	Clustered	16	VC_515_0	16	0.3793	1	0.3793	0	0	0	1	Ref
MF774688	Unassigned	Unassigned	Unassigned	190_30	Clustered/Singleton	1	VC_190_30	1	0	1	0	0	0	0	1	Ref
MF775703	Unassigned	Unassigned	Unassigned	202_0	Clustered	102	VC_202_0	102	0.4049	1	0.4049	0	0	0	0.7876	Ref
MF775704	Unassigned	Unassigned	Unassigned	202_0	Clustered	102	VC_202_0	102	0.4049	1	0.4049	0	0	0	0.7876	Ref
MF775705	Unassigned	Unassigned	Unassigned	202_0	Clustered	102	VC_202_0	102	0.4049	1	0.4049	0	0	0	0.7876	Ref
MF787246	Unassigned	Unassigned	Unassigned	526_1	Clustered/Singleton	1	VC_526_1	1	0	1	0	0	0	0	1	Ref
MF788075	Unassigned	Unassigned	Unassigned	755_0	Clustered	9	VC_755_0	9	0.4489	1	0.4489	0	0	0	1	Ref
MF805716	Unassigned	Unassigned	Unassigned	344_0	Clustered	21	VC_344_0	21	0.5436	1	0.5436	0	0	0	1	Ref
MF805809	Unassigned	Unassigned	Unassigned	848_0	Clustered	28	VC_848_0	28	0.7927	1	0.7927	0	0	0	1	Ref
MF807953	Unassigned	Unassigned	Unassigned	51_0	Clustered	10	VC_51_0	10	0.1741	1	0.1741	0	0	0	1	Ref
MF893340	Unassigned	Unassigned	Unassigned	78_6	Clustered/Singleton	1	VC_78_6	1	0	1	0	0	0	0	1	Ref
MF919490	Unassigned	Unassigned	Unassigned	216_6	Clustered	42	VC_216_6	42	0.473	1	0.473	0	0	0	0.8351	Ref
MF919491	Unassigned	Unassigned	Unassigned	765_0	Clustered	132	VC_765_0	132	1	1	1	0	0	0	1	Ref
MF919492	Unassigned	Unassigned	Unassigned	519_0	Clustered	39	VC_519_0	39	0.8156	1	0.8156	0	0	0	1	Ref
MF919493	Unassigned	Unassigned	Unassigned	558_20	Clustered	17	VC_558_20	17	0.054	1	0.054	0	0	0	0.9926	Ref
MF919494	Unassigned	Unassigned	Unassigned	558_55	Clustered/Singleton	1	VC_558_55	1	0	1	0	0	0	0	1	Ref
MF919495	Unassigned	Unassigned	Unassigned	558_74	Clustered/Singleton	1	VC_558_74	1	0	1	0	0	0	0	1	Ref
MF919496	Unassigned	Unassigned	Unassigned	517_1	Clustered	108	VC_517_1	108	0.8483	1	0.8483	0	0	0	0.9955	Ref
MF919497	Unassigned	Unassigned	Unassigned	761_0	Clustered	67	VC_761_0	67	0.8947	1	0.8947	0	0	0	1	Ref
MF919499	Unassigned	Unassigned	Unassigned	558_50	Clustered	15	VC_558_50	15	0.0468	1	0.0468	0	0	0	0.9238	Ref
MF919500	Unassigned	Unassigned	Unassigned	216_6	Clustered	42	VC_216_6	42	0.473	1	0.473	0	0	0	0.8351	Ref
MF919501	Unassigned	Unassigned	Unassigned	520_63	Clustered/Singleton	1	VC_520_63	1	0	1	0	0	0	0	1	Ref
MF919503	Unassigned	Unassigned	Unassigned	371_3	Clustered	22	VC_371_3	22	0.0787	1	0.0787	0	0	0	0.9048	Ref
MF919504	Unassigned	Unassigned	Unassigned	546_5	Clustered/Singleton	1	VC_546_5	1	0	1	0	0	0	0	1	Ref
MF919505	Unassigned	Unassigned	Unassigned	765_0	Clustered	132	VC_765_0	132	1	1	1	0	0	0	1	Ref
MF919506	Unassigned	Unassigned	Unassigned	551_8	Clustered	19	VC_551_8	19	0.1039	1	0.1039	0	0	0	0.8713	Ref

MF919507	Unassigned	Unassigned	Unassigned	371_3	Clustered	22	VC_371_3	22	0.0787	1	0.0787	0	0	0	0.9048	Ref
MF919508	Unassigned	Unassigned	Unassigned	325_56	Clustered	2	VC_325_56	2	0.0036	0.94604798	0.0034	0	0	0	0	Ref
MF919509	Unassigned	Unassigned	Unassigned	517_1	Clustered	108	VC_517_1	108	0.8483	1	0.8483	0	0	0	0.9955	Ref
MF919510	Unassigned	Unassigned	Unassigned	767_0	Clustered	34	VC_767_0	34	0.9173	1	0.9173	0	0	0	1	Ref
MF919511	Unassigned	Unassigned	Unassigned	371_13	Clustered	33	VC_371_13	33	0.179	1	0.179	0	0	0	0.8769	Ref
MF919512	Unassigned	Unassigned	Unassigned	546_6	Clustered	2	VC_546_6	2	0.0135	0.7810929	0.0105	0	0	0	1	Ref
MF919513	Unassigned	Unassigned	Unassigned	558_77	Clustered	3	VC_558_77	3	0.0065	0.90877939	0.0059	0	0	0	0.6667	Ref
MF919514	Unassigned	Unassigned	Unassigned	1093_0	Clustered	40	VC_1093_0	40	0.8754	1	0.8754	0	0	0	1	Ref
MF919515	Unassigned	Unassigned	Unassigned	765_0	Clustered	132	VC_765_0	132	1	1	1	0	0	0	1	Ref
MF919516	Unassigned	Unassigned	Unassigned	765_0	Clustered	132	VC_765_0	132	1	1	1	0	0	0	1	Ref
MF919517	Unassigned	Unassigned	Unassigned	810_0	Clustered	18	VC_810_0	18	0.9121	1	0.9121	0	0	0	1	Ref
MF919519	Unassigned	Unassigned	Unassigned	371_6	Clustered/Singleton	1	VC_371_6	1	0	1	0	0	0	0	1	Ref
MF919520	Unassigned	Unassigned	Unassigned	1181_1	Clustered	10	VC_1181_1	10	0.5828	1	0.5828	0	0	0	1	Ref
MF919521	Unassigned	Unassigned	Unassigned	1176_0	Clustered	41	VC_1176_0	41	0.9558	1	0.9558	0	0	0	0.9976	Ref
MF919522	Unassigned	Unassigned	Unassigned	765_0	Clustered	132	VC_765_0	132	1	1	1	0	0	0	1	Ref
MF919523	Unassigned	Unassigned	Unassigned	371_3	Clustered	22	VC_371_3	22	0.0787	1	0.0787	0	0	0	0.9048	Ref
MF919524	Unassigned	Unassigned	Unassigned	551_8	Clustered	19	VC_551_8	19	0.1039	1	0.1039	0	0	0	0.8713	Ref
MF919525	Unassigned	Unassigned	Unassigned	551_8	Clustered	19	VC_551_8	19	0.1039	1	0.1039	0	0	0	0.8713	Ref
MF919526	Unassigned	Unassigned	Unassigned	520_81	Clustered/Singleton	1	VC_520_81	1	0	1	0	0	0	0	1	Ref
MF919527	Unassigned	Unassigned	Unassigned	558_82	Clustered	3	VC_558_82	3	0.0064	0.88433874	0.0057	0	0	0	0.6667	Ref
MF919528	Unassigned	Unassigned	Unassigned	371_11	Clustered	31	VC_371_11	31	0.0927	1	0.0927	0	0	0	0.7011	Ref
MF919529	Unassigned	Unassigned	Unassigned	551_8	Clustered	19	VC_551_8	19	0.1039	1	0.1039	0	0	0	0.8713	Ref
MF919530	Unassigned	Unassigned	Unassigned	371_3	Clustered	22	VC_371_3	22	0.0787	1	0.0787	0	0	0	0.9048	Ref
MF919531	Unassigned	Unassigned	Unassigned	216_6	Clustered	42	VC_216_6	42	0.473	1	0.473	0	0	0	0.8351	Ref
MF919532	Unassigned	Unassigned	Unassigned	236_0	Clustered	72	VC_236_0	72	0.8104	1	0.8104	0	0	0	1	Ref
MF919533	Unassigned	Unassigned	Unassigned	765_0	Clustered	132	VC_765_0	132	1	1	1	0	0	0	1	Ref
MF919534	Unassigned	Unassigned	Unassigned	546_2	Clustered	3	VC_546_2	3	0.0275	0.85260342	0.0234	0	0	0	1	Ref
MF919535	Unassigned	Unassigned	Unassigned	551_8	Clustered	19	VC_551_8	19	0.1039	1	0.1039	0	0	0	0.8713	Ref
MF919536	Unassigned	Unassigned	Unassigned	215_2	Clustered	5	VC_215_2	5	0.0572	0.99999997	0.0572	0	0	0	1	Ref
MF919537	Unassigned	Unassigned	Unassigned	1181_1	Clustered	10	VC_1181_1	10	0.5828	1	0.5828	0	0	0	1	Ref
MF919538	Unassigned	Unassigned	Unassigned	216_4	Clustered	13	VC_216_4	13	0.1657	1	0.1657	0	0	0	1	Ref
MF919539	Unassigned	Unassigned	Unassigned	371_11	Clustered	31	VC_371_11	31	0.0927	1	0.0927	0	0	0	0.7011	Ref
MF919540	Unassigned	Unassigned	Unassigned	551_8	Clustered	19	VC_551_8	19	0.1039	1	0.1039	0	0	0	0.8713	Ref
MF919541	Unassigned	Unassigned	Unassigned	551_1	Clustered	4	VC_551_1	4	0.0159	0.99968337	0.0159	0	0	0	0.6667	Ref
MF919542	Unassigned	Unassigned	Unassigned	767_0	Clustered	34	VC_767_0	34	0.9173	1	0.9173	0	0	0	1	Ref
MF959999	Unassigned	Unassigned	Unassigned	185_0	Clustered	3	VC_185_0	3	0.4487	0.97148006	0.4359	0	0	0	1	Ref
MF974178	Unassigned	Unassigned	Unassigned	76_0	Clustered	37	VC_76_0	37	0.9403	1	0.9403	0	0	0	1	Ref
MF974396	Unassigned	Unassigned	Unassigned	688_0	Clustered	2	VC_688_0	2	0.9946	0.5	0.4973	0	0	0	1	Ref
MF974397	Unassigned	Unassigned	Unassigned	688_0	Clustered	2	VC_688_0	2	0.9946	0.5	0.4973	0	0	0	1	Ref
MF975638	Unassigned	Unassigned	Unassigned	975_0	Clustered	11	VC_975_0	11	0.9687	1	0.9687	0	0	0	1	Ref
MF975720	Unassigned	Unassigned	Unassigned	241_0	Clustered	2	VC_241_0	2	0.072	0.95433582	0.0687	0	0	0	1	Ref
MF979560	Unassigned	Unassigned	Unassigned	849_3	Clustered	2	VC_849_3	2	0.0243	0.85188229	0.0207	0	0	0	1	Ref
MF979561	Unassigned	Unassigned	Unassigned	389_0	Clustered	3	VC_389_0	3	0.0515	0.99861557	0.0514	0	0	0	1	Ref
MF979562	Unassigned	Unassigned	Unassigned	1218_0	Clustered	4	VC_1218_0	4	0.8905	0.99530591	0.8863	0	0	0	1	Ref
MF979563	Unassigned	Unassigned	Unassigned	849_3	Clustered	2	VC_849_3	2	0.0243	0.85188229	0.0207	0	0	0	1	Ref
MF996376	Unassigned	Unassigned	Unassigned	576_0	Clustered	4	VC_576_0	4	0.0606	0.99998766	0.0606	0	0	0	1	Ref
MF999224	Unassigned	Unassigned	Unassigned	272_0	Clustered	11	VC_272_0	11	0.4915	1	0.4915	0	0	0	0.8727	Ref
MG000860	Unassigned	Unassigned	Unassigned	85_3	Clustered	19	VC_85_3	19	0.3671	1	0.3671	0	0	0	0.807	Ref
MG004687	Unassigned	Unassigned	Unassigned	452_0	Clustered	66	VC_452_0	66	0.9248	1	0.9248	0	0	0	1	Ref
MG018224	Unassigned	Unassigned	Unassigned	169_0	Clustered	20	VC_169_0	20	0.7529	1	0.7529	0	0	0	1	Ref
MG018927	Unassigned	Unassigned	Unassigned	725_0	Clustered	4	VC_725_0	4	0.4268	0.99997355	0.4268	0	0	0	1	Ref
MG018928	Unassigned	Unassigned	Unassigned	779_1	Clustered	11	VC_779_1	11	0.1587	1	0.1587	0	0	0	1	Ref
MG018929	Unassigned	Unassigned	Unassigned	1027_0	Clustered	20	VC_1027_0	20	0.7114	1	0.7114	0	0	0	1	Ref
MG018930	Unassigned	Unassigned	Unassigned	78_0	Clustered	2	VC_78_0	2	0.0306	0.95617387	0.0293	0	0	0	1	Ref
MG020111	Unassigned	Unassigned	Unassigned	578_0	Clustered	2	VC_578_0	2	0.1712	0.93973606	0.1608	0	0	0	1	Ref
MG022439	Unassigned	Unassigned	Unassigned	772_9	Clustered	7	VC_772_9	7	0.0369	0.87705503	0.0324	0	0	0	1	Ref
MG022440	Unassigned	Unassigned	Unassigned	772_9	Clustered	7	VC_772_9	7	0.0369	0.87705503	0.0324	0	0	0	1	Ref
MG025518	Unassigned	Unassigned	Unassigned	1104_0	Clustered	154	VC_1104_0	154	0.8484	1	0.8484	0	0	0	1	Ref
MG029509	Unassigned	Unassigned	Unassigned	8_0	Clustered	58	VC_8_0	58	0.8306	1	0.8306	0	0	0	0.9946	Ref
MG029510	Unassigned	Unassigned	Unassigned	10_0	Clustered	25	VC_10_0	25	0.3467	1	0.3467	0	0	0	0.9733	Ref
MG029511	Unassigned	Unassigned	Unassigned	8_0	Clustered	58	VC_8_0	58	0.8306	1	0.8306	0	0	0	0.9946	Ref
MG029512	Unassigned	Unassigned	Unassigned	10_0	Clustered	25	VC_10_0	25	0.3467	1	0.3467	0	0	0	0.9733	Ref
MG029513	Unassigned	Unassigned	Unassigned	8_0	Clustered	58	VC_8_0	58	0.8306	1	0.8306	0	0	0	0.9946	Ref
MG029514	Unassigned	Unassigned	Unassigned	8_0	Clustered	58	VC_8_0	58	0.8306	1	0.8306	0	0	0	0.9946	Ref
MG029515	Unassigned	Unassigned	Unassigned	8_0	Clustered	58	VC_8_0	58	0.8306	1	0.8306	0	0	0	0.9946	Ref
MG029516	Unassigned	Unassigned	Unassigned	8_0	Clustered	58	VC_8_0	58	0.8306	1	0.8306	0	0	0	0.9946	Ref
MG029517	Unassigned	Unassigned	Unassigned	8_0	Clustered	58	VC_8_0	58	0.8306	1	0.8306	0	0	0	0.9946	Ref
MG029518	Unassigned	Unassigned	Unassigned	8_0	Clustered	58	VC_8_0	58	0.8306	1	0.8306	0	0	0	0.9946	Ref
MG030346	Unassigned	Unassigned	Unassigned	591_0	Clustered	14	VC_591_0	14	0.5184	1	0.5184	0	0	0	1	Ref
MG049919	Unassigned	Unassigned	Unassigned	575_0	Clustered	35	VC_575_0	35	0.8815	1	0.8815	0	0	0	1	Ref
MG050172	Unassigned	Unassigned	Unassigned	456_0	Clustered	62	VC_456_0	62	0.9026	1	0.9026	0	0	0	1	Ref
MG065635	Unassigned	Unassigned	Unassigned	772_19	Clustered	16	VC_772_19	16	0.0975	0.9970466	0.0972	0	0	0	0.8083	Ref
MG065636	Unassigned	Unassigned	Unassigned	941_0	Clustered	40	VC_941_0	40	0.6903	1	0.6903	0	0	0	1	Ref
MG065639	Unassigned	Unassigned	Unassigned	848_0	Clustered	28	VC_848_0	28	0.7927	1	0.7927	0	0	0	1	Ref
MG065640	Unassigned	Unassigned	Unassigned	941_0	Clustered	40	VC_941_0	40	0.6903	1	0.6903	0	0	0	1	Ref
MG065641	Unassigned	Unassigned	Unassigned	941_0	Clustered	40	VC_941_0	40	0.6903	1	0.6903	0	0	0	1	Ref
MG065642	Unassigned	Unassigned	Unassigned	191_19	Clustered	12	VC_191_19	12	0.0655	1	0.0655	0	0	0	0.8636	Ref
MG065643	Unassigned	Unassigned	Unassigned	941_0	Clustered	40	VC_941_0	40	0.6903	1	0.6903	0	0	0	1	Ref
MG065644	Unassigned	Unassigned	Unassigned	772_0	Clustered	6	VC_772_0	6	0.0305	0.8101283	0.0247	0	0	0	1	Ref
MG065645	Unassigned	Unassigned	Unassigned	848_0	Clustered	28	VC_848_0	28	0.7927	1	0.7927	0	0	0	1	Ref
MG065646	Unassigned	Unassigned	Unassigned	772_16	Clustered	10	VC_772_16	10	0.0563	0.9434928	0.0531	0	0	0	0.8667	Ref

MG065647	Unassigned	Unassigned	Unassigned	772_16	Clustered	10	VC_772_16	10	0.0563	0.9434928	0.0531	0	0	0	0.8667	Ref
MG065648	Unassigned	Unassigned	Unassigned	835_0	Clustered	67	VC_835_0	67	0.9624	1	0.9624	0	0	0	1	Ref
MG065649	Unassigned	Unassigned	Unassigned	941_0	Clustered	40	VC_941_0	40	0.6903	1	0.6903	0	0	0	1	Ref
MG065650	Unassigned	Unassigned	Unassigned	847_1	Clustered	26	VC_847_1	26	0.6323	1	0.6323	0	0	0	0.9815	Ref
MG065653	Unassigned	Unassigned	Unassigned	772_13	Clustered/Singleton	1	VC_772_13	1	0	1	0	0	0	0	1	Ref
MG065654	Unassigned	Unassigned	Unassigned	772_14	Clustered	3	VC_772_14	3	0.012	0.63979507	0.0077	0	0	0	1	Ref
MG065655	Unassigned	Unassigned	Unassigned	772_14	Clustered	3	VC_772_14	3	0.012	0.63979507	0.0077	0	0	0	1	Ref
MG065656	Unassigned	Unassigned	Unassigned	848_0	Clustered	28	VC_848_0	28	0.7927	1	0.7927	0	0	0	1	Ref
MG065657	Unassigned	Unassigned	Unassigned	941_0	Clustered	40	VC_941_0	40	0.6903	1	0.6903	0	0	0	1	Ref
MG065658	Unassigned	Unassigned	Unassigned	772_16	Clustered	10	VC_772_16	10	0.0563	0.9434928	0.0531	0	0	0	0.8667	Ref
MG065659	Unassigned	Unassigned	Unassigned	772_12	Clustered/Singleton	1	VC_772_12	1	0	1	0	0	0	0	1	Ref
MG065661	Unassigned	Unassigned	Unassigned	772_16	Clustered	10	VC_772_16	10	0.0563	0.9434928	0.0531	0	0	0	0.8667	Ref
MG065662	Unassigned	Unassigned	Unassigned	772_0	Clustered	6	VC_772_0	6	0.0305	0.8101283	0.0247	0	0	0	1	Ref
MG065663	Unassigned	Unassigned	Unassigned	941_0	Clustered	40	VC_941_0	40	0.6903	1	0.6903	0	0	0	1	Ref
MG065664	Unassigned	Unassigned	Unassigned	329_12	Clustered	2	VC_329_12	2	0.0041	0.72605466	0.003	0	0	0	1	Ref
MG065665	Unassigned	Unassigned	Unassigned	941_0	Clustered	40	VC_941_0	40	0.6903	1	0.6903	0	0	0	1	Ref
MG065666	Unassigned	Unassigned	Unassigned	772_14	Clustered	3	VC_772_14	3	0.012	0.63979507	0.0077	0	0	0	1	Ref
MG065667	Unassigned	Unassigned	Unassigned	941_0	Clustered	40	VC_941_0	40	0.6903	1	0.6903	0	0	0	1	Ref
MG065669	Unassigned	Unassigned	Unassigned	941_0	Clustered	40	VC_941_0	40	0.6903	1	0.6903	0	0	0	1	Ref
MG065670	Unassigned	Unassigned	Unassigned	941_0	Clustered	40	VC_941_0	40	0.6903	1	0.6903	0	0	0	1	Ref
MG065671	Unassigned	Unassigned	Unassigned	941_0	Clustered	40	VC_941_0	40	0.6903	1	0.6903	0	0	0	1	Ref
MG065672	Unassigned	Unassigned	Unassigned	772_0	Clustered	6	VC_772_0	6	0.0305	0.8101283	0.0247	0	0	0	1	Ref
MG065673	Unassigned	Unassigned	Unassigned	941_0	Clustered	40	VC_941_0	40	0.6903	1	0.6903	0	0	0	1	Ref
MG065674	Unassigned	Unassigned	Unassigned	772_0	Clustered	6	VC_772_0	6	0.0305	0.8101283	0.0247	0	0	0	1	Ref
MG065675	Unassigned	Unassigned	Unassigned	941_0	Clustered	40	VC_941_0	40	0.6903	1	0.6903	0	0	0	1	Ref
MG065677	Unassigned	Unassigned	Unassigned	772_0	Clustered	6	VC_772_0	6	0.0305	0.8101283	0.0247	0	0	0	1	Ref
MG065679	Unassigned	Unassigned	Unassigned	941_0	Clustered	40	VC_941_0	40	0.6903	1	0.6903	0	0	0	1	Ref
MG065680	Unassigned	Unassigned	Unassigned	941_0	Clustered	40	VC_941_0	40	0.6903	1	0.6903	0	0	0	1	Ref
MG065681	Unassigned	Unassigned	Unassigned	941_0	Clustered	40	VC_941_0	40	0.6903	1	0.6903	0	0	0	1	Ref
MG065682	Unassigned	Unassigned	Unassigned	772_16	Clustered	10	VC_772_16	10	0.0563	0.9434928	0.0531	0	0	0	0.8667	Ref
MG065687	Unassigned	Unassigned	Unassigned	941_0	Clustered	40	VC_941_0	40	0.6903	1	0.6903	0	0	0	1	Ref
MG065688	Unassigned	Unassigned	Unassigned	456_0	Clustered	62	VC_456_0	62	0.9026	1	0.9026	0	0	0	1	Ref
MG065689	Unassigned	Unassigned	Unassigned	848_0	Clustered	28	VC_848_0	28	0.7927	1	0.7927	0	0	0	1	Ref
MG065690	Unassigned	Unassigned	Unassigned	772_0	Clustered	6	VC_772_0	6	0.0305	0.8101283	0.0247	0	0	0	1	Ref
MG065691	Unassigned	Unassigned	Unassigned	1219_0	Clustered/Singleton	1	VC_1219_0	1	0	1	0	0	0	0	1	Ref
MG065692	Unassigned	Unassigned	Unassigned	190_31	Clustered/Singleton	1	VC_190_31	1	0	1	0	0	0	0	1	Ref
MG099933	Unassigned	Unassigned	Unassigned	638_0	Clustered	45	VC_638_0	45	0.7741	1	0.7741	0	0	0	1	Ref
MG099934	Unassigned	Unassigned	Unassigned	940_0	Clustered	34	VC_940_0	34	0.7959	1	0.7959	0	0	0	1	Ref
MG099935	Unassigned	Unassigned	Unassigned	866_1	Clustered	20	VC_866_1	20	0.4814	1	0.4814	0	0	0	1	Ref
MG099936	Unassigned	Unassigned	Unassigned	521_0	Clustered	37	VC_521_0	37	0.912	1	0.912	0	0	0	1	Ref
MG099937	Unassigned	Unassigned	Unassigned	940_0	Clustered	34	VC_940_0	34	0.7959	1	0.7959	0	0	0	1	Ref
MG099938	Unassigned	Unassigned	Unassigned	940_0	Clustered	34	VC_940_0	34	0.7959	1	0.7959	0	0	0	1	Ref
MG099939	Unassigned	Unassigned	Unassigned	557_1	Clustered	11	VC_557_1	11	0.4945	1	0.4945	0	0	0	0.7818	Ref
MG099940	Unassigned	Unassigned	Unassigned	868_0	Clustered	25	VC_868_0	25	0.5791	1	0.5791	0	0	0	1	Ref
MG099941	Unassigned	Unassigned	Unassigned	1175_0	Clustered	22	VC_1175_0	22	0.4562	1	0.4562	0	0	0	1	Ref
MG099942	Unassigned	Unassigned	Unassigned	517_1	Clustered	108	VC_517_1	108	0.8483	1	0.8483	0	0	0	0.9955	Ref
MG099943	Unassigned	Unassigned	Unassigned	556_0	Clustered	21	VC_556_0	21	0.9912	1	0.9912	0	0	0	1	Ref
MG099944	Unassigned	Unassigned	Unassigned	940_0	Clustered	34	VC_940_0	34	0.7959	1	0.7959	0	0	0	1	Ref
MG099945	Unassigned	Unassigned	Unassigned	223_0	Clustered	38	VC_223_0	38	0.9095	1	0.9095	0	0	0	1	Ref
MG099946	Unassigned	Unassigned	Unassigned	761_0	Clustered	67	VC_761_0	67	0.8947	1	0.8947	0	0	0	1	Ref
MG099947	Unassigned	Unassigned	Unassigned	215_0	Clustered	5	VC_215_0	5	0.0724	0.99999997	0.0724	0	0	0	1	Ref
MG099948	Unassigned	Unassigned	Unassigned	521_0	Clustered	37	VC_521_0	37	0.912	1	0.912	0	0	0	1	Ref
MG099949	Unassigned	Unassigned	Unassigned	940_0	Clustered	34	VC_940_0	34	0.7959	1	0.7959	0	0	0	1	Ref
MG099950	Unassigned	Unassigned	Unassigned	1169_0	Clustered	7	VC_1169_0	7	0.9811	0.99995613	0.981	0	0	0	1	Ref
MG099951	Unassigned	Unassigned	Unassigned	325_38	Clustered	6	VC_325_38	6	0.0267	1	0.0267	0	0	0	0.8	Ref
MG099952	Unassigned	Unassigned	Unassigned	223_0	Clustered	38	VC_223_0	38	0.9095	1	0.9095	0	0	0	1	Ref
MG099953	Unassigned	Unassigned	Unassigned	551_8	Clustered	19	VC_551_8	19	0.1039	1	0.1039	0	0	0	0.8713	Ref
MG189906	Unassigned	Unassigned	Unassigned	905_0	Clustered	3	VC_905_0	3	0.3424	0.99823775	0.3417	0	0	0	1	Ref
MG197810	Unassigned	Unassigned	Unassigned	123_0	Clustered	23	VC_123_0	23	0.817	1	0.817	0	0	0	1	Ref
MG198776	Unassigned	Unassigned	Unassigned	768_0	Clustered	8	VC_768_0	8	0.7012	1	0.7012	0	0	0	1	Ref
MG198777	Unassigned	Unassigned	Unassigned	768_0	Clustered	8	VC_768_0	8	0.7012	1	0.7012	0	0	0	1	Ref
MG198778	Unassigned	Unassigned	Unassigned	768_0	Clustered	8	VC_768_0	8	0.7012	1	0.7012	0	0	0	1	Ref
MG198779	Unassigned	Unassigned	Unassigned	1160_0	Clustered	73	VC_1160_0	73	0.9835	1	0.9835	0	0	0	1	Ref
MG198780	Unassigned	Unassigned	Unassigned	768_0	Clustered	8	VC_768_0	8	0.7012	1	0.7012	0	0	0	1	Ref
MG198781	Unassigned	Unassigned	Unassigned	1160_0	Clustered	73	VC_1160_0	73	0.9835	1	0.9835	0	0	0	1	Ref
MG198782	Unassigned	Unassigned	Unassigned	1160_0	Clustered	73	VC_1160_0	73	0.9835	1	0.9835	0	0	0	1	Ref
MG198783	Unassigned	Unassigned	Unassigned	410_0	Clustered	7	VC_410_0	7	0.7905	1	0.7905	0	0	0	1	Ref
MG198784	Unassigned	Unassigned	Unassigned	410_0	Clustered	7	VC_410_0	7	0.7905	1	0.7905	0	0	0	1	Ref
MG198785	Unassigned	Unassigned	Unassigned	519_0	Clustered	39	VC_519_0	39	0.8156	1	0.8156	0	0	0	1	Ref
MG201401	Unassigned	Unassigned	Unassigned	72_0	Clustered	2	VC_72_0	2	0.0777	0.95616461	0.0743	0	0	0	1	Ref
MG208881	Unassigned	Unassigned	Unassigned	779_0	Clustered	37	VC_779_0	37	0.5682	1	0.5682	0	0	0	1	Ref
MG209611	Unassigned	Unassigned	Unassigned	932_0	Clustered	2	VC_932_0	2	0.4438	0.89435023	0.3969	0	0	0	1	Ref
MG210949	Unassigned	Unassigned	Unassigned	1160_0	Clustered	73	VC_1160_0	73	0.9835	1	0.9835	0	0	0	1	Ref
MG214764	Unassigned	Unassigned	Unassigned	1222_0	Clustered	3	VC_1222_0	3	1	0	0	0	0	0	1	Ref
MG241338	Unassigned	Unassigned	Unassigned	584_0	Clustered	28	VC_584_0	28	0.7704	1	0.7704	0	0	0	1	Ref
MG250485	Unassigned	Unassigned	Unassigned	129_0	Clustered	43	VC_129_0	43	0.886	1	0.886	0	0	0	1	Ref
MG250486	Unassigned	Unassigned	Unassigned	582_3	Clustered	12	VC_582_3	12	0.2357	1	0.2357	0	0	0	0.8636	Ref
MG251390	Unassigned	Unassigned	Unassigned	452_0	Clustered	66	VC_452_0	66	0.9248	1	0.9248	0	0	0	1	Ref
MG251391	Unassigned	Unassigned	Unassigned	329_1	Clustered	17	VC_329_1	17	0.0867	1	0.0867	0	0	0	0.8235	Ref
MG251392	Unassigned	Unassigned	Unassigned	329_1	Clustered	17	VC_329_1	17	0.0867	1	0.0867	0	0	0	0.8235	Ref
MG252615	Unassigned	Unassigned	Unassigned	638_0	Clustered	45	VC_638_0	45	0.7741	1	0.7741	0	0	0	1	Ref

MG252693	Unassigned	Unassigned	Unassigned	634_0	Clustered	3	VC_634_0	3	0.2046	0.99837593	0.2043	0	0	0	1	Ref
MG253647	Unassigned	Unassigned	Unassigned	202_0	Clustered	102	VC_202_0	102	0.4049	1	0.4049	0	0	0	0.7876	Ref
MG253648	Unassigned	Unassigned	Unassigned	202_0	Clustered	102	VC_202_0	102	0.4049	1	0.4049	0	0	0	0.7876	Ref
MG253649	Unassigned	Unassigned	Unassigned	202_0	Clustered	102	VC_202_0	102	0.4049	1	0.4049	0	0	0	0.7876	Ref
MG253650	Unassigned	Unassigned	Unassigned	202_0	Clustered	102	VC_202_0	102	0.4049	1	0.4049	0	0	0	0.7876	Ref
MG253651	Unassigned	Unassigned	Unassigned	202_0	Clustered	102	VC_202_0	102	0.4049	1	0.4049	0	0	0	0.7876	Ref
MG253652	Unassigned	Unassigned	Unassigned	202_0	Clustered	102	VC_202_0	102	0.4049	1	0.4049	0	0	0	0.7876	Ref
MG253653	Unassigned	Unassigned	Unassigned	202_0	Clustered	102	VC_202_0	102	0.4049	1	0.4049	0	0	0	0.7876	Ref
MG264739	Unassigned	Unassigned	Unassigned	695_0	Clustered	48	VC_695_0	48	0.8162	1	0.8162	0	0	0	1	Ref
MG266157	Unassigned	Unassigned	Unassigned	131_0	Clustered	17	VC_131_0	17	0.2671	1	0.2671	0	0	0	1	Ref
MG269973	Unassigned	Unassigned	Unassigned	1223_0	Clustered	2	VC_1223_0	2	1	1	1	0	0	0	1	Ref
MG271909	Unassigned	Unassigned	Unassigned	873_0	Clustered	3	VC_873_0	3	0.9107	0.98376334	0.8959	0	0	0	1	Ref
MG280946	Unassigned	Unassigned	Unassigned	592_0	Clustered	50	VC_592_0	50	0.7314	1	0.7314	0	0	0	1	Ref
MG298964	Unassigned	Unassigned	Unassigned	982_0	Clustered	62	VC_982_0	62	0.8646	1	0.8646	0	0	0	0.743	Ref
MG309717	Unassigned	Unassigned	Unassigned	202_0	Clustered	102	VC_202_0	102	0.4049	1	0.4049	0	0	0	0.7876	Ref
MG309718	Unassigned	Unassigned	Unassigned	202_0	Clustered	102	VC_202_0	102	0.4049	1	0.4049	0	0	0	0.7876	Ref
MG324353	Unassigned	Unassigned	Unassigned	407_0	Clustered	3	VC_407_0	3	0.3352	0.99824791	0.3346	0	0	0	1	Ref
MG324354	Unassigned	Unassigned	Unassigned	407_0	Clustered	3	VC_407_0	3	0.3352	0.99824791	0.3346	0	0	0	1	Ref
MG366115	Unassigned	Unassigned	Unassigned	750_0	Clustered/Singleton	1	VC_750_0	1	0	1	0	0	0	0	1	Ref
MG383452	Unassigned	Unassigned	Unassigned	133_27	Clustered/Singleton	1	VC_133_27	1	0	1	0	0	0	0	1	Ref
MG387042	Unassigned	Unassigned	Unassigned	190_26	Clustered/Singleton	1	VC_190_26	1	0	1	0	0	0	0	1	Ref
MG407615	Unassigned	Unassigned	Unassigned	28_1	Clustered	53	VC_28_1	53	0.7164	1	0.7164	0	0	0	0.9325	Ref
MG428990	Unassigned	Unassigned	Unassigned	132_1	Clustered	17	VC_132_1	17	0.1738	1	0.1738	0	0	0	0.9853	Ref
MG428991	Unassigned	Unassigned	Unassigned	132_1	Clustered	17	VC_132_1	17	0.1738	1	0.1738	0	0	0	0.9853	Ref
MG428992	Unassigned	Unassigned	Unassigned	133_1	Clustered/Singleton	1	VC_133_1	1	0	1	0	0	0	0	1	Ref
MG432137	Unassigned	Unassigned	Unassigned	123_0	Clustered	23	VC_123_0	23	0.817	1	0.817	0	0	0	1	Ref
MG432151	Unassigned	Unassigned	Unassigned	472_0	Clustered	47	VC_472_0	47	0.9025	1	0.9025	0	0	0	1	Ref
MG459218	Unassigned	Unassigned	Unassigned	699_0	Clustered	71	VC_699_0	71	0.9352	1	0.9352	0	0	0	1	Ref
MG459987	Unassigned	Unassigned	Unassigned	192_0	Clustered	15	VC_192_0	15	0.5071	1	0.5071	0	0	0	1	Ref
MG471392	Unassigned	Unassigned	Unassigned	386_0	Clustered	49	VC_386_0	49	0.7941	1	0.7941	0	0	0	1	Ref
MG488277	Unassigned	Unassigned	Unassigned	392_0	Clustered	126	VC_392_0	126	0.9621	1	0.9621	0	0	0	1	Ref
MG515223	Unassigned	Unassigned	Unassigned	1224_0	Clustered	19	VC_1224_0	19	1	1	1	0	0	0	1	Ref
MG518519	Unassigned	Unassigned	Unassigned	1224_0	Clustered	19	VC_1224_0	19	1	1	1	0	0	0	1	Ref
MG518520	Unassigned	Unassigned	Unassigned	1224_0	Clustered	19	VC_1224_0	19	1	1	1	0	0	0	1	Ref
MG543995	Unassigned	Unassigned	Unassigned	12_1	Clustered	2	VC_12_1	2	0.0192	0.95739495	0.0184	0	0	0	1	Ref
MG545917	Unassigned	Unassigned	Unassigned	1027_0	Clustered	20	VC_1027_0	20	0.7114	1	0.7114	0	0	0	1	Ref
MG550110	Unassigned	Unassigned	Unassigned	963_0	Clustered	4	VC_963_0	4	0.8505	0.99879268	0.8495	0	0	0	1	Ref
MG550111	Unassigned	Unassigned	Unassigned	963_0	Clustered	4	VC_963_0	4	0.8505	0.99879268	0.8495	0	0	0	1	Ref
MG550112	Unassigned	Unassigned	Unassigned	459_0	Clustered	3	VC_459_0	3	0.8542	0.99054823	0.8461	0	0	0	1	Ref
MG550113	Unassigned	Unassigned	Unassigned	963_0	Clustered	4	VC_963_0	4	0.8505	0.99879268	0.8495	0	0	0	1	Ref
MG552615	Unassigned	Unassigned	Unassigned	938_0	Clustered	99	VC_938_0	99	0.9119	1	0.9119	0	0	0	0.9928	Ref
MG557618	Unassigned	Unassigned	Unassigned	149_15	Clustered	16	VC_149_15	16	0.0788	1	0.0788	0	0	0	0.8083	Ref
MG557619	Unassigned	Unassigned	Unassigned	9_0	Clustered	38	VC_9_0	38	0.4895	1	0.4895	0	0	0	1	Ref
MG557979	Unassigned	Unassigned	Unassigned	526_0	Clustered	6	VC_526_0	6	0.4386	1	0.4386	0	0	0	1	Ref
MG563770	Unassigned	Unassigned	Unassigned	360_0	Clustered	358	VC_360_0	358	0.9615	1	0.9615	0	0	0	1	Ref
MG564297	Unassigned	Unassigned	Unassigned	12_1	Clustered	2	VC_12_1	2	0.0192	0.95739495	0.0184	0	0	0	1	Ref
MG575418	Unassigned	Unassigned	Unassigned	112_0	Clustered	8	VC_112_0	8	0.2537	1	0.2537	0	0	0	1	Ref
MG575419	Unassigned	Unassigned	Unassigned	112_0	Clustered	8	VC_112_0	8	0.2537	1	0.2537	0	0	0	1	Ref
MG575421	Unassigned	Unassigned	Unassigned	426_1	Clustered	3	VC_426_1	3	0.1152	0.99804615	0.1149	0	0	0	1	Ref
MG581355	Unassigned	Unassigned	Unassigned	835_0	Clustered	67	VC_835_0	67	0.9624	1	0.9624	0	0	0	1	Ref
MG589383	Unassigned	Unassigned	Unassigned	286_12	Clustered	3	VC_286_12	3	0.0146	0.92328265	0.0135	0	0	0	1	Ref
MG589384	Unassigned	Unassigned	Unassigned	328_0	Clustered	11	VC_328_0	11	0.4707	1	0.4707	0	0	0	1	Ref
MG589385	Unassigned	Unassigned	Unassigned	472_0	Clustered	47	VC_472_0	47	0.9025	1	0.9025	0	0	0	1	Ref
MG589386	Unassigned	Unassigned	Unassigned	472_0	Clustered	47	VC_472_0	47	0.9025	1	0.9025	0	0	0	1	Ref
MG589387	Unassigned	Unassigned	Unassigned	229_0	Clustered	32	VC_229_0	32	0.9119	1	0.9119	0	0	0	1	Ref
MG592390	Unassigned	Unassigned	Unassigned	645_0	Clustered	8	VC_645_0	8	0.3138	1	0.3138	0	0	0	1	Ref
MG592391	Unassigned	Unassigned	Unassigned	1041_0	Clustered	9	VC_1041_0	9	0.6541	1	0.6541	0	0	0	1	Ref
MG592392	Unassigned	Unassigned	Unassigned	499_0	Clustered	24	VC_499_0	24	0.8569	1	0.8569	0	0	0	0.9529	Ref
MG592393	Unassigned	Unassigned	Unassigned	646_0	Clustered	29	VC_646_0	29	0.9131	1	0.9131	0	0	0	1	Ref
MG592394	Unassigned	Unassigned	Unassigned	1225_0	Clustered	4	VC_1225_0	4	0.9619	0.99374885	0.9559	0	0	0	1	Ref
MG592395	Unassigned	Unassigned	Unassigned	498_0	Clustered	8	VC_498_0	8	0.3987	1	0.3987	0	0	0	1	Ref
MG592397	Unassigned	Unassigned	Unassigned	1050_1	Clustered	29	VC_1050_1	29	0.4861	1	0.4861	0	0	0	0.9581	Ref
MG592398	Unassigned	Unassigned	Unassigned	944_0	Clustered	6	VC_944_0	6	0.8196	1	0.8196	0	0	0	1	Ref
MG592399	Unassigned	Unassigned	Unassigned	543_0	Clustered	6	VC_543_0	6	0.864	0.99999948	0.864	0	0	0	1	Ref
MG592400	Unassigned	Unassigned	Unassigned	122_0	Clustered	5	VC_122_0	5	0.2073	0.99999996	0.2073	0	0	0	1	Ref
MG592401	Unassigned	Unassigned	Unassigned	499_0	Clustered	24	VC_499_0	24	0.8569	1	0.8569	0	0	0	0.9529	Ref
MG592402	Unassigned	Unassigned	Unassigned	1225_0	Clustered	4	VC_1225_0	4	0.9619	0.99374885	0.9559	0	0	0	1	Ref
MG592403	Unassigned	Unassigned	Unassigned	296_0	Clustered	6	VC_296_0	6	0.5315	1	0.5315	0	0	0	1	Ref
MG592404	Unassigned	Unassigned	Unassigned	296_0	Clustered	6	VC_296_0	6	0.5315	1	0.5315	0	0	0	1	Ref
MG592405	Unassigned	Unassigned	Unassigned	296_0	Clustered	6	VC_296_0	6	0.5315	1	0.5315	0	0	0	1	Ref
MG592406	Unassigned	Unassigned	Unassigned	1050_1	Clustered	29	VC_1050_1	29	0.4861	1	0.4861	0	0	0	0.9581	Ref
MG592407	Unassigned	Unassigned	Unassigned	1050_1	Clustered	29	VC_1050_1	29	0.4861	1	0.4861	0	0	0	0.9581	Ref
MG592408	Unassigned	Unassigned	Unassigned	1050_1	Clustered	29	VC_1050_1	29	0.4861	1	0.4861	0	0	0	0.9581	Ref
MG592409	Unassigned	Unassigned	Unassigned	930_0	Clustered	7	VC_930_0	7	0.519	1	0.519	0	0	0	1	Ref
MG592410	Unassigned	Unassigned	Unassigned	930_0	Clustered	7	VC_930_0	7	0.519	1	0.519	0	0	0	1	Ref
MG592411	Unassigned	Unassigned	Unassigned	1050_0	Clustered	24	VC_1050_0	24	0.5274	1	0.5274	0	0	0	1	Ref
MG592412	Unassigned	Unassigned	Unassigned	248_0	Clustered	11	VC_248_0	11	0.7031	1	0.7031	0	0	0	1	Ref
MG592414	Unassigned	Unassigned	Unassigned	499_0	Clustered	24	VC_499_0	24	0.8569	1	0.8569	0	0	0	0.9529	Ref
MG592415	Unassigned	Unassigned	Unassigned	478_0	Clustered	2	VC_478_0	2	0.3505	0.94766489	0.3321	0	0	0	1	Ref
MG592416	Unassigned	Unassigned	Unassigned	1050_1	Clustered	29	VC_1050_1	29	0.4861	1	0.4861	0	0	0	0.9581	Ref
MG592417	Unassigned	Unassigned	Unassigned	1050_1	Clustered	29	VC_1050_1	29	0.4861	1	0.4861	0	0	0	0.9581	Ref

MG592418	Unassigned	Unassigned	Unassigned	543_0	Clustered	6	VC_543_0	6	0.864	0.99999948	0.864	0	0	0	1	Ref
MG592419	Unassigned	Unassigned	Unassigned	543_0	Clustered	6	VC_543_0	6	0.864	0.99999948	0.864	0	0	0	1	Ref
MG592420	Unassigned	Unassigned	Unassigned	645_0	Clustered	8	VC_645_0	8	0.3138	1	0.3138	0	0	0	1	Ref
MG592421	Unassigned	Unassigned	Unassigned	1041_0	Clustered	9	VC_1041_0	9	0.6541	1	0.6541	0	0	0	1	Ref
MG592422	Unassigned	Unassigned	Unassigned	122_0	Clustered	5	VC_122_0	5	0.2073	0.99999996	0.2073	0	0	0	1	Ref
MG592423	Unassigned	Unassigned	Unassigned	645_0	Clustered	8	VC_645_0	8	0.3138	1	0.3138	0	0	0	1	Ref
MG592425	Unassigned	Unassigned	Unassigned	122_0	Clustered	5	VC_122_0	5	0.2073	0.99999996	0.2073	0	0	0	1	Ref
MG592427	Unassigned	Unassigned	Unassigned	1225_0	Clustered	4	VC_1225_0	4	0.9619	0.99374885	0.9559	0	0	0	1	Ref
MG592428	Unassigned	Unassigned	Unassigned	1050_1	Clustered	29	VC_1050_1	29	0.4861	1	0.4861	0	0	0	0.9581	Ref
MG592429	Unassigned	Unassigned	Unassigned	948_0	Clustered	9	VC_948_0	9	0.5443	1	0.5443	0	0	0	1	Ref
MG592431	Unassigned	Unassigned	Unassigned	827_0	Clustered	6	VC_827_0	6	0.7493	1	0.7493	0	0	0	1	Ref
MG592432	Unassigned	Unassigned	Unassigned	827_0	Clustered	6	VC_827_0	6	0.7493	1	0.7493	0	0	0	1	Ref
MG592434	Unassigned	Unassigned	Unassigned	122_1	Clustered/Singleton	1	VC_122_1	1	0	1	0	0	0	0	1	Ref
MG592435	Unassigned	Unassigned	Unassigned	827_0	Clustered	6	VC_827_0	6	0.7493	1	0.7493	0	0	0	1	Ref
MG592436	Unassigned	Unassigned	Unassigned	1041_0	Clustered	9	VC_1041_0	9	0.6541	1	0.6541	0	0	0	1	Ref
MG592438	Unassigned	Unassigned	Unassigned	948_0	Clustered	9	VC_948_0	9	0.5443	1	0.5443	0	0	0	1	Ref
MG592439	Unassigned	Unassigned	Unassigned	645_0	Clustered	8	VC_645_0	8	0.3138	1	0.3138	0	0	0	1	Ref
MG592441	Unassigned	Unassigned	Unassigned	493_8	Clustered/Singleton	1	VC_493_8	1	0	1	0	0	0	0	1	Ref
MG592442	Unassigned	Unassigned	Unassigned	924_0	Clustered	34	VC_924_0	34	0.8942	1	0.8942	0	0	0	0.9768	Ref
MG592443	Unassigned	Unassigned	Unassigned	1041_0	Clustered	9	VC_1041_0	9	0.6541	1	0.6541	0	0	0	1	Ref
MG592444	Unassigned	Unassigned	Unassigned	645_0	Clustered	8	VC_645_0	8	0.3138	1	0.3138	0	0	0	1	Ref
MG592445	Unassigned	Unassigned	Unassigned	1041_0	Clustered	9	VC_1041_0	9	0.6541	1	0.6541	0	0	0	1	Ref
MG592447	Unassigned	Unassigned	Unassigned	544_0	Clustered	2	VC_544_0	2	0.3517	0.94059057	0.3308	0	0	0	1	Ref
MG592448	Unassigned	Unassigned	Unassigned	122_0	Clustered	5	VC_122_0	5	0.2073	0.99999996	0.2073	0	0	0	1	Ref
MG592449	Unassigned	Unassigned	Unassigned	1041_0	Clustered	9	VC_1041_0	9	0.6541	1	0.6541	0	0	0	1	Ref
MG592450	Unassigned	Unassigned	Unassigned	924_0	Clustered	34	VC_924_0	34	0.8942	1	0.8942	0	0	0	0.9768	Ref
MG592451	Unassigned	Unassigned	Unassigned	499_0	Clustered	24	VC_499_0	24	0.8569	1	0.8569	0	0	0	0.9529	Ref
MG592453	Unassigned	Unassigned	Unassigned	544_0	Clustered	2	VC_544_0	2	0.3517	0.94059057	0.3308	0	0	0	1	Ref
MG592454	Unassigned	Unassigned	Unassigned	645_0	Clustered	8	VC_645_0	8	0.3138	1	0.3138	0	0	0	1	Ref
MG592455	Unassigned	Unassigned	Unassigned	1225_0	Clustered	4	VC_1225_0	4	0.9619	0.99374885	0.9559	0	0	0	1	Ref
MG592456	Unassigned	Unassigned	Unassigned	531_4	Clustered/Singleton	1	VC_531_4	1	0	1	0	0	0	0	1	Ref
MG592457	Unassigned	Unassigned	Unassigned	924_0	Clustered	34	VC_924_0	34	0.8942	1	0.8942	0	0	0	0.9768	Ref
MG592458	Unassigned	Unassigned	Unassigned	827_0	Clustered	6	VC_827_0	6	0.7493	1	0.7493	0	0	0	1	Ref
MG592459	Unassigned	Unassigned	Unassigned	497_0	Clustered/Singleton	1	VC_497_0	1	0	1	0	0	0	0	1	Ref
MG592460	Unassigned	Unassigned	Unassigned	924_0	Clustered	34	VC_924_0	34	0.8942	1	0.8942	0	0	0	0.9768	Ref
MG592461	Unassigned	Unassigned	Unassigned	499_0	Clustered	24	VC_499_0	24	0.8569	1	0.8569	0	0	0	0.9529	Ref
MG592462	Unassigned	Unassigned	Unassigned	499_0	Clustered	24	VC_499_0	24	0.8569	1	0.8569	0	0	0	0.9529	Ref
MG592463	Unassigned	Unassigned	Unassigned	1050_1	Clustered	29	VC_1050_1	29	0.4861	1	0.4861	0	0	0	0.9581	Ref
MG592464	Unassigned	Unassigned	Unassigned	1050_1	Clustered	29	VC_1050_1	29	0.4861	1	0.4861	0	0	0	0.9581	Ref
MG592465	Unassigned	Unassigned	Unassigned	645_0	Clustered	8	VC_645_0	8	0.3138	1	0.3138	0	0	0	1	Ref
MG592466	Unassigned	Unassigned	Unassigned	645_0	Clustered	8	VC_645_0	8	0.3138	1	0.3138	0	0	0	1	Ref
MG592467	Unassigned	Unassigned	Unassigned	499_0	Clustered	24	VC_499_0	24	0.8569	1	0.8569	0	0	0	0.9529	Ref
MG592468	Unassigned	Unassigned	Unassigned	499_0	Clustered	24	VC_499_0	24	0.8569	1	0.8569	0	0	0	0.9529	Ref
MG592470	Unassigned	Unassigned	Unassigned	930_0	Clustered	7	VC_930_0	7	0.519	1	0.519	0	0	0	1	Ref
MG592471	Unassigned	Unassigned	Unassigned	1050_1	Clustered	29	VC_1050_1	29	0.4861	1	0.4861	0	0	0	0.9581	Ref
MG592472	Unassigned	Unassigned	Unassigned	499_0	Clustered	24	VC_499_0	24	0.8569	1	0.8569	0	0	0	0.9529	Ref
MG592473	Unassigned	Unassigned	Unassigned	497_1	Clustered/Singleton	1	VC_497_1	1	0	1	0	0	0	0	1	Ref
MG592475	Unassigned	Unassigned	Unassigned	1050_1	Clustered	29	VC_1050_1	29	0.4861	1	0.4861	0	0	0	0.9581	Ref
MG592476	Unassigned	Unassigned	Unassigned	1050_1	Clustered	29	VC_1050_1	29	0.4861	1	0.4861	0	0	0	0.9581	Ref
MG592477	Unassigned	Unassigned	Unassigned	646_0	Clustered	29	VC_646_0	29	0.9131	1	0.9131	0	0	0	1	Ref
MG592478	Unassigned	Unassigned	Unassigned	646_0	Clustered	29	VC_646_0	29	0.9131	1	0.9131	0	0	0	1	Ref
MG592482	Unassigned	Unassigned	Unassigned	924_0	Clustered	34	VC_924_0	34	0.8942	1	0.8942	0	0	0	0.9768	Ref
MG592483	Unassigned	Unassigned	Unassigned	924_0	Clustered	34	VC_924_0	34	0.8942	1	0.8942	0	0	0	0.9768	Ref
MG592484	Unassigned	Unassigned	Unassigned	1190_0	Clustered	2	VC_1190_0	2	0.5104	0.94764538	0.4837	0	0	0	1	Ref
MG592485	Unassigned	Unassigned	Unassigned	1190_0	Clustered	2	VC_1190_0	2	0.5104	0.94764538	0.4837	0	0	0	1	Ref
MG592486	Unassigned	Unassigned	Unassigned	122_0	Clustered	5	VC_122_0	5	0.2073	0.99999996	0.2073	0	0	0	1	Ref
MG592487	Unassigned	Unassigned	Unassigned	948_0	Clustered	9	VC_948_0	9	0.5443	1	0.5443	0	0	0	1	Ref
MG592488	Unassigned	Unassigned	Unassigned	924_0	Clustered	34	VC_924_0	34	0.8942	1	0.8942	0	0	0	0.9768	Ref
MG592489	Unassigned	Unassigned	Unassigned	924_0	Clustered	34	VC_924_0	34	0.8942	1	0.8942	0	0	0	0.9768	Ref
MG592490	Unassigned	Unassigned	Unassigned	924_0	Clustered	34	VC_924_0	34	0.8942	1	0.8942	0	0	0	0.9768	Ref
MG592492	Unassigned	Unassigned	Unassigned	1050_1	Clustered	29	VC_1050_1	29	0.4861	1	0.4861	0	0	0	0.9581	Ref
MG592493	Unassigned	Unassigned	Unassigned	1050_1	Clustered	29	VC_1050_1	29	0.4861	1	0.4861	0	0	0	0.9581	Ref
MG592494	Unassigned	Unassigned	Unassigned	642_3	Clustered	9	VC_642_3	9	0.2131	1	0.2131	0	0	0	0.6944	Ref
MG592495	Unassigned	Unassigned	Unassigned	493_6	Clustered/Singleton	1	VC_493_6	1	0	1	0	0	0	0	1	Ref
MG592496	Unassigned	Unassigned	Unassigned	944_0	Clustered	6	VC_944_0	6	0.8196	1	0.8196	0	0	0	1	Ref
MG592497	Unassigned	Unassigned	Unassigned	944_0	Clustered	6	VC_944_0	6	0.8196	1	0.8196	0	0	0	1	Ref
MG592499	Unassigned	Unassigned	Unassigned	499_0	Clustered	24	VC_499_0	24	0.8569	1	0.8569	0	0	0	0.9529	Ref
MG592501	Unassigned	Unassigned	Unassigned	642_3	Clustered	9	VC_642_3	9	0.2131	1	0.2131	0	0	0	0.6944	Ref
MG592502	Unassigned	Unassigned	Unassigned	924_0	Clustered	34	VC_924_0	34	0.8942	1	0.8942	0	0	0	0.9768	Ref
MG592503	Unassigned	Unassigned	Unassigned	924_0	Clustered	34	VC_924_0	34	0.8942	1	0.8942	0	0	0	0.9768	Ref
MG592504	Unassigned	Unassigned	Unassigned	924_0	Clustered	34	VC_924_0	34	0.8942	1	0.8942	0	0	0	0.9768	Ref
MG592505	Unassigned	Unassigned	Unassigned	924_0	Clustered	34	VC_924_0	34	0.8942	1	0.8942	0	0	0	0.9768	Ref
MG592506	Unassigned	Unassigned	Unassigned	642_0	Clustered	7	VC_642_0	7	0.1508	1	0.1508	0	0	0	0.7619	Ref
MG592507	Unassigned	Unassigned	Unassigned	248_0	Clustered	11	VC_248_0	11	0.7031	1	0.7031	0	0	0	1	Ref
MG592508	Unassigned	Unassigned	Unassigned	642_3	Clustered	9	VC_642_3	9	0.2131	1	0.2131	0	0	0	0.6944	Ref
MG592509	Unassigned	Unassigned	Unassigned	501_0	Clustered	16	VC_501_0	16	0.9985	1	0.9985	0	0	0	1	Ref
MG592510	Unassigned	Unassigned	Unassigned	543_0	Clustered	6	VC_543_0	6	0.864	0.99999948	0.864	0	0	0	1	Ref
MG592511	Unassigned	Unassigned	Unassigned	543_0	Clustered	6	VC_543_0	6	0.864	0.99999948	0.864	0	0	0	1	Ref
MG592513	Unassigned	Unassigned	Unassigned	248_0	Clustered	11	VC_248_0	11	0.7031	1	0.7031	0	0	0	1	Ref
MG592514	Unassigned	Unassigned	Unassigned	642_3	Clustered	9	VC_642_3	9	0.2131	1	0.2131	0	0	0	0.6944	Ref
MG592516	Unassigned	Unassigned	Unassigned	924_0	Clustered	34	VC_924_0	34	0.8942	1	0.8942	0	0	0	0.9768	Ref

MG592517	Unassigned	Unassigned	Unassigned	924_0	Clustered	34	VC_924_0	34	0.8942	1	0.8942	0	0	0	0.9768	Ref
MG592518	Unassigned	Unassigned	Unassigned	646_0	Clustered	29	VC_646_0	29	0.9131	1	0.9131	0	0	0	1	Ref
MG592519	Unassigned	Unassigned	Unassigned	930_0	Clustered	7	VC_930_0	7	0.519	1	0.519	0	0	0	1	Ref
MG592520	Unassigned	Unassigned	Unassigned	926_0	Clustered	3	VC_926_0	3	0.9445	0.99430386	0.9391	0	0	0	1	Ref
MG592521	Unassigned	Unassigned	Unassigned	930_0	Clustered	7	VC_930_0	7	0.519	1	0.519	0	0	0	1	Ref
MG592522	Unassigned	Unassigned	Unassigned	909_0	Clustered	5	VC_909_0	5	0.7406	0.9999861	0.7406	0	0	0	1	Ref
MG592523	Unassigned	Unassigned	Unassigned	115_0	Clustered	2	VC_115_0	2	0.4117	0.90744399	0.3736	0	0	0	1	Ref
MG592524	Unassigned	Unassigned	Unassigned	248_0	Clustered	11	VC_248_0	11	0.7031	1	0.7031	0	0	0	1	Ref
MG592525	Unassigned	Unassigned	Unassigned	924_0	Clustered	34	VC_924_0	34	0.8942	1	0.8942	0	0	0	0.9768	Ref
MG592526	Unassigned	Unassigned	Unassigned	499_0	Clustered	24	VC_499_0	24	0.8569	1	0.8569	0	0	0	0.9529	Ref
MG592527	Unassigned	Unassigned	Unassigned	248_0	Clustered	11	VC_248_0	11	0.7031	1	0.7031	0	0	0	1	Ref
MG592528	Unassigned	Unassigned	Unassigned	1050_1	Clustered	29	VC_1050_1	29	0.4861	1	0.4861	0	0	0	0.9581	Ref
MG592529	Unassigned	Unassigned	Unassigned	493_2	Clustered/Singleton	1	VC_493_2	1	0	1	0	0	0	0	1	Ref
MG592530	Unassigned	Unassigned	Unassigned	924_0	Clustered	34	VC_924_0	34	0.8942	1	0.8942	0	0	0	0.9768	Ref
MG592531	Unassigned	Unassigned	Unassigned	499_0	Clustered	24	VC_499_0	24	0.8569	1	0.8569	0	0	0	0.9529	Ref
MG592532	Unassigned	Unassigned	Unassigned	924_0	Clustered	34	VC_924_0	34	0.8942	1	0.8942	0	0	0	0.9768	Ref
MG592533	Unassigned	Unassigned	Unassigned	1226_0	Clustered	2	VC_1226_0	2	0.6497	0.86822376	0.564	0	0	0	1	Ref
MG592534	Unassigned	Unassigned	Unassigned	924_0	Clustered	34	VC_924_0	34	0.8942	1	0.8942	0	0	0	0.9768	Ref
MG592535	Unassigned	Unassigned	Unassigned	924_0	Clustered	34	VC_924_0	34	0.8942	1	0.8942	0	0	0	0.9768	Ref
MG592536	Unassigned	Unassigned	Unassigned	1227_0	Clustered	6	VC_1227_0	6	0.9303	0.99999969	0.9303	0	0	0	1	Ref
MG592538	Unassigned	Unassigned	Unassigned	1050_0	Clustered	24	VC_1050_0	24	0.5274	1	0.5274	0	0	0	1	Ref
MG592539	Unassigned	Unassigned	Unassigned	924_0	Clustered	34	VC_924_0	34	0.8942	1	0.8942	0	0	0	0.9768	Ref
MG592540	Unassigned	Unassigned	Unassigned	646_0	Clustered	29	VC_646_0	29	0.9131	1	0.9131	0	0	0	1	Ref
MG592541	Unassigned	Unassigned	Unassigned	499_0	Clustered	24	VC_499_0	24	0.8569	1	0.8569	0	0	0	0.9529	Ref
MG592542	Unassigned	Unassigned	Unassigned	499_0	Clustered	24	VC_499_0	24	0.8569	1	0.8569	0	0	0	0.9529	Ref
MG592543	Unassigned	Unassigned	Unassigned	924_0	Clustered	34	VC_924_0	34	0.8942	1	0.8942	0	0	0	0.9768	Ref
MG592544	Unassigned	Unassigned	Unassigned	642_5	Clustered	4	VC_642_5	4	0.0902	0.99996664	0.0902	0	0	0	1	Ref
MG592545	Unassigned	Unassigned	Unassigned	642_0	Clustered	7	VC_642_0	7	0.1508	1	0.1508	0	0	0	0.7619	Ref
MG592546	Unassigned	Unassigned	Unassigned	501_0	Clustered	16	VC_501_0	16	0.9985	1	0.9985	0	0	0	1	Ref
MG592547	Unassigned	Unassigned	Unassigned	499_0	Clustered	24	VC_499_0	24	0.8569	1	0.8569	0	0	0	0.9529	Ref
MG592549	Unassigned	Unassigned	Unassigned	807_0	Clustered	17	VC_807_0	17	0.907	1	0.907	0	0	0	1	Ref
MG592550	Unassigned	Unassigned	Unassigned	807_0	Clustered	17	VC_807_0	17	0.907	1	0.907	0	0	0	1	Ref
MG592551	Unassigned	Unassigned	Unassigned	296_0	Clustered	6	VC_296_0	6	0.5315	1	0.5315	0	0	0	1	Ref
MG592552	Unassigned	Unassigned	Unassigned	646_0	Clustered	29	VC_646_0	29	0.9131	1	0.9131	0	0	0	1	Ref
MG592553	Unassigned	Unassigned	Unassigned	493_16	Clustered/Singleton	1	VC_493_16	1	0	1	0	0	0	0	1	Ref
MG592554	Unassigned	Unassigned	Unassigned	1227_0	Clustered	6	VC_1227_0	6	0.9303	0.99999969	0.9303	0	0	0	1	Ref
MG592555	Unassigned	Unassigned	Unassigned	1227_0	Clustered	6	VC_1227_0	6	0.9303	0.99999969	0.9303	0	0	0	1	Ref
MG592556	Unassigned	Unassigned	Unassigned	1227_0	Clustered	6	VC_1227_0	6	0.9303	0.99999969	0.9303	0	0	0	1	Ref
MG592557	Unassigned	Unassigned	Unassigned	924_0	Clustered	34	VC_924_0	34	0.8942	1	0.8942	0	0	0	0.9768	Ref
MG592558	Unassigned	Unassigned	Unassigned	924_0	Clustered	34	VC_924_0	34	0.8942	1	0.8942	0	0	0	0.9768	Ref
MG592559	Unassigned	Unassigned	Unassigned	924_0	Clustered	34	VC_924_0	34	0.8942	1	0.8942	0	0	0	0.9768	Ref
MG592560	Unassigned	Unassigned	Unassigned	1226_0	Clustered	2	VC_1226_0	2	0.6497	0.86822376	0.564	0	0	0	1	Ref
MG592561	Unassigned	Unassigned	Unassigned	499_0	Clustered	24	VC_499_0	24	0.8569	1	0.8569	0	0	0	0.9529	Ref
MG592562	Unassigned	Unassigned	Unassigned	493_1	Clustered/Singleton	1	VC_493_1	1	0	1	0	0	0	0	1	Ref
MG592563	Unassigned	Unassigned	Unassigned	1050_0	Clustered	24	VC_1050_0	24	0.5274	1	0.5274	0	0	0	1	Ref
MG592564	Unassigned	Unassigned	Unassigned	1050_0	Clustered	24	VC_1050_0	24	0.5274	1	0.5274	0	0	0	1	Ref
MG592565	Unassigned	Unassigned	Unassigned	1050_0	Clustered	24	VC_1050_0	24	0.5274	1	0.5274	0	0	0	1	Ref
MG592566	Unassigned	Unassigned	Unassigned	948_0	Clustered	9	VC_948_0	9	0.5443	1	0.5443	0	0	0	1	Ref
MG592567	Unassigned	Unassigned	Unassigned	642_0	Clustered	7	VC_642_0	7	0.1508	1	0.1508	0	0	0	0.7619	Ref
MG592568	Unassigned	Unassigned	Unassigned	642_0	Clustered	7	VC_642_0	7	0.1508	1	0.1508	0	0	0	0.7619	Ref
MG592569	Unassigned	Unassigned	Unassigned	646_0	Clustered	29	VC_646_0	29	0.9131	1	0.9131	0	0	0	1	Ref
MG592570	Unassigned	Unassigned	Unassigned	646_0	Clustered	29	VC_646_0	29	0.9131	1	0.9131	0	0	0	1	Ref
MG592571	Unassigned	Unassigned	Unassigned	1050_0	Clustered	24	VC_1050_0	24	0.5274	1	0.5274	0	0	0	1	Ref
MG592572	Unassigned	Unassigned	Unassigned	499_0	Clustered	24	VC_499_0	24	0.8569	1	0.8569	0	0	0	0.9529	Ref
MG592573	Unassigned	Unassigned	Unassigned	5_0	Clustered	30	VC_5_0	30	0.9647	1	0.9647	0	0	0	1	Ref
MG592576	Unassigned	Unassigned	Unassigned	924_0	Clustered	34	VC_924_0	34	0.8942	1	0.8942	0	0	0	0.9768	Ref
MG592578	Unassigned	Unassigned	Unassigned	175_0	Clustered	4	VC_175_0	4	0.3847	0.99998259	0.3846	0	0	0	1	Ref
MG592579	Unassigned	Unassigned	Unassigned	543_0	Clustered	6	VC_543_0	6	0.864	0.99999948	0.864	0	0	0	1	Ref
MG592581	Unassigned	Unassigned	Unassigned	807_0	Clustered	17	VC_807_0	17	0.907	1	0.907	0	0	0	1	Ref
MG592582	Unassigned	Unassigned	Unassigned	807_0	Clustered	17	VC_807_0	17	0.907	1	0.907	0	0	0	1	Ref
MG592583	Unassigned	Unassigned	Unassigned	642_3	Clustered	9	VC_642_3	9	0.2131	1	0.2131	0	0	0	0.6944	Ref
MG592584	Unassigned	Unassigned	Unassigned	944_0	Clustered	6	VC_944_0	6	0.8196	1	0.8196	0	0	0	1	Ref
MG592585	Unassigned	Unassigned	Unassigned	904_0	Clustered	6	VC_904_0	6	0.233	1	0.233	0	0	0	1	Ref
MG592586	Unassigned	Unassigned	Unassigned	904_0	Clustered	6	VC_904_0	6	0.233	1	0.233	0	0	0	1	Ref
MG592587	Unassigned	Unassigned	Unassigned	642_3	Clustered	9	VC_642_3	9	0.2131	1	0.2131	0	0	0	0.6944	Ref
MG592588	Unassigned	Unassigned	Unassigned	248_0	Clustered	11	VC_248_0	11	0.7031	1	0.7031	0	0	0	1	Ref
MG592589	Unassigned	Unassigned	Unassigned	248_0	Clustered	11	VC_248_0	11	0.7031	1	0.7031	0	0	0	1	Ref
MG592590	Unassigned	Unassigned	Unassigned	499_0	Clustered	24	VC_499_0	24	0.8569	1	0.8569	0	0	0	0.9529	Ref
MG592591	Unassigned	Unassigned	Unassigned	1227_0	Clustered	6	VC_1227_0	6	0.9303	0.99999969	0.9303	0	0	0	1	Ref
MG592592	Unassigned	Unassigned	Unassigned	499_0	Clustered	24	VC_499_0	24	0.8569	1	0.8569	0	0	0	0.9529	Ref
MG592593	Unassigned	Unassigned	Unassigned	924_0	Clustered	34	VC_924_0	34	0.8942	1	0.8942	0	0	0	0.9768	Ref
MG592594	Unassigned	Unassigned	Unassigned	924_0	Clustered	34	VC_924_0	34	0.8942	1	0.8942	0	0	0	0.9768	Ref
MG592595	Unassigned	Unassigned	Unassigned	642_3	Clustered	9	VC_642_3	9	0.2131	1	0.2131	0	0	0	0.6944	Ref
MG592596	Unassigned	Unassigned	Unassigned	1229_0	Clustered	7	VC_1229_0	7	0.9924	0.99999987	0.9924	0	0	0	1	Ref
MG592597	Unassigned	Unassigned	Unassigned	924_0	Clustered	34	VC_924_0	34	0.8942	1	0.8942	0	0	0	0.9768	Ref
MG592598	Unassigned	Unassigned	Unassigned	924_0	Clustered	34	VC_924_0	34	0.8942	1	0.8942	0	0	0	0.9768	Ref
MG592599	Unassigned	Unassigned	Unassigned	757_0	Clustered	2	VC_757_0	2	0.0727	0.95527362	0.0695	0	0	0	1	Ref
MG592600	Unassigned	Unassigned	Unassigned	642_0	Clustered	7	VC_642_0	7	0.1508	1	0.1508	0	0	0	0.7619	Ref
MG592601	Unassigned	Unassigned	Unassigned	1050_1	Clustered	29	VC_1050_1	29	0.4861	1	0.4861	0	0	0	0.9581	Ref
MG592602	Unassigned	Unassigned	Unassigned	1050_1	Clustered	29	VC_1050_1	29	0.4861	1	0.4861	0	0	0	0.9581	Ref
MG592603	Unassigned	Unassigned	Unassigned	782_0	Clustered	3	VC_782_0	3	0.296	0.99856285	0.2956	0	0	0	1	Ref

MG592604	Unassigned	Unassigned	Unassigned	782_0	Clustered	3	VC_782_0	3	0.296	0.99856285	0.2956	0	0	0	1	Ref
MG592605	Unassigned	Unassigned	Unassigned	924_0	Clustered	34	VC_924_0	34	0.8942	1	0.8942	0	0	0	0.9768	Ref
MG592606	Unassigned	Unassigned	Unassigned	924_0	Clustered	34	VC_924_0	34	0.8942	1	0.8942	0	0	0	0.9768	Ref
MG592607	Unassigned	Unassigned	Unassigned	924_0	Clustered	34	VC_924_0	34	0.8942	1	0.8942	0	0	0	0.9768	Ref
MG592608	Unassigned	Unassigned	Unassigned	646_0	Clustered	29	VC_646_0	29	0.9131	1	0.9131	0	0	0	1	Ref
MG592609	Unassigned	Unassigned	Unassigned	135_0	Clustered	2	VC_135_0	2	0.0302	0.95557467	0.0288	0	0	0	1	Ref
MG592610	Unassigned	Unassigned	Unassigned	782_0	Clustered	3	VC_782_0	3	0.296	0.99856285	0.2956	0	0	0	1	Ref
MG592611	Unassigned	Unassigned	Unassigned	499_0	Clustered	24	VC_499_0	24	0.8569	1	0.8569	0	0	0	0.9529	Ref
MG592612	Unassigned	Unassigned	Unassigned	944_0	Clustered	6	VC_944_0	6	0.8196	1	0.8196	0	0	0	1	Ref
MG592613	Unassigned	Unassigned	Unassigned	944_0	Clustered	6	VC_944_0	6	0.8196	1	0.8196	0	0	0	1	Ref
MG592614	Unassigned	Unassigned	Unassigned	646_0	Clustered	29	VC_646_0	29	0.9131	1	0.9131	0	0	0	1	Ref
MG592617	Unassigned	Unassigned	Unassigned	1050_1	Clustered	29	VC_1050_1	29	0.4861	1	0.4861	0	0	0	0.9581	Ref
MG592618	Unassigned	Unassigned	Unassigned	1050_1	Clustered	29	VC_1050_1	29	0.4861	1	0.4861	0	0	0	0.9581	Ref
MG592619	Unassigned	Unassigned	Unassigned	757_0	Clustered	2	VC_757_0	2	0.0727	0.95527362	0.0695	0	0	0	1	Ref
MG592620	Unassigned	Unassigned	Unassigned	501_0	Clustered	16	VC_501_0	16	0.9985	1	0.9985	0	0	0	1	Ref
MG592621	Unassigned	Unassigned	Unassigned	135_0	Clustered	2	VC_135_0	2	0.0302	0.95557467	0.0288	0	0	0	1	Ref
MG592622	Unassigned	Unassigned	Unassigned	646_0	Clustered	29	VC_646_0	29	0.9131	1	0.9131	0	0	0	1	Ref
MG592623	Unassigned	Unassigned	Unassigned	501_0	Clustered	16	VC_501_0	16	0.9985	1	0.9985	0	0	0	1	Ref
MG592625	Unassigned	Unassigned	Unassigned	1227_0	Clustered	6	VC_1227_0	6	0.9303	0.99999969	0.9303	0	0	0	1	Ref
MG592626	Unassigned	Unassigned	Unassigned	530_0	Clustered	9	VC_530_0	9	0.8027	1	0.8027	0	0	0	1	Ref
MG592627	Unassigned	Unassigned	Unassigned	646_0	Clustered	29	VC_646_0	29	0.9131	1	0.9131	0	0	0	1	Ref
MG592628	Unassigned	Unassigned	Unassigned	646_0	Clustered	29	VC_646_0	29	0.9131	1	0.9131	0	0	0	1	Ref
MG592629	Unassigned	Unassigned	Unassigned	758_0	Clustered	18	VC_758_0	18	0.738	1	0.738	0	0	0	1	Ref
MG592630	Unassigned	Unassigned	Unassigned	642_0	Clustered	7	VC_642_0	7	0.1508	1	0.1508	0	0	0	0.7619	Ref
MG592631	Unassigned	Unassigned	Unassigned	924_0	Clustered	34	VC_924_0	34	0.8942	1	0.8942	0	0	0	0.9768	Ref
MG592632	Unassigned	Unassigned	Unassigned	1050_0	Clustered	24	VC_1050_0	24	0.5274	1	0.5274	0	0	0	1	Ref
MG592633	Unassigned	Unassigned	Unassigned	1050_0	Clustered	24	VC_1050_0	24	0.5274	1	0.5274	0	0	0	1	Ref
MG592634	Unassigned	Unassigned	Unassigned	1050_0	Clustered	24	VC_1050_0	24	0.5274	1	0.5274	0	0	0	1	Ref
MG592635	Unassigned	Unassigned	Unassigned	1050_0	Clustered	24	VC_1050_0	24	0.5274	1	0.5274	0	0	0	1	Ref
MG592636	Unassigned	Unassigned	Unassigned	1050_0	Clustered	24	VC_1050_0	24	0.5274	1	0.5274	0	0	0	1	Ref
MG592637	Unassigned	Unassigned	Unassigned	1050_0	Clustered	24	VC_1050_0	24	0.5274	1	0.5274	0	0	0	1	Ref
MG592638	Unassigned	Unassigned	Unassigned	1050_0	Clustered	24	VC_1050_0	24	0.5274	1	0.5274	0	0	0	1	Ref
MG592639	Unassigned	Unassigned	Unassigned	1050_0	Clustered	24	VC_1050_0	24	0.5274	1	0.5274	0	0	0	1	Ref
MG592640	Unassigned	Unassigned	Unassigned	1050_0	Clustered	24	VC_1050_0	24	0.5274	1	0.5274	0	0	0	1	Ref
MG592641	Unassigned	Unassigned	Unassigned	646_0	Clustered	29	VC_646_0	29	0.9131	1	0.9131	0	0	0	1	Ref
MG592642	Unassigned	Unassigned	Unassigned	1050_0	Clustered	24	VC_1050_0	24	0.5274	1	0.5274	0	0	0	1	Ref
MG592644	Unassigned	Unassigned	Unassigned	642_3	Clustered	9	VC_642_3	9	0.2131	1	0.2131	0	0	0	0.6944	Ref
MG592645	Unassigned	Unassigned	Unassigned	1050_0	Clustered	24	VC_1050_0	24	0.5274	1	0.5274	0	0	0	1	Ref
MG592646	Unassigned	Unassigned	Unassigned	1050_0	Clustered	24	VC_1050_0	24	0.5274	1	0.5274	0	0	0	1	Ref
MG592647	Unassigned	Unassigned	Unassigned	646_0	Clustered	29	VC_646_0	29	0.9131	1	0.9131	0	0	0	1	Ref
MG592648	Unassigned	Unassigned	Unassigned	1050_0	Clustered	24	VC_1050_0	24	0.5274	1	0.5274	0	0	0	1	Ref
MG592649	Unassigned	Unassigned	Unassigned	1050_0	Clustered	24	VC_1050_0	24	0.5274	1	0.5274	0	0	0	1	Ref
MG592650	Unassigned	Unassigned	Unassigned	1050_0	Clustered	24	VC_1050_0	24	0.5274	1	0.5274	0	0	0	1	Ref
MG592651	Unassigned	Unassigned	Unassigned	1050_0	Clustered	24	VC_1050_0	24	0.5274	1	0.5274	0	0	0	1	Ref
MG592652	Unassigned	Unassigned	Unassigned	1050_0	Clustered	24	VC_1050_0	24	0.5274	1	0.5274	0	0	0	1	Ref
MG592653	Unassigned	Unassigned	Unassigned	1050_0	Clustered	24	VC_1050_0	24	0.5274	1	0.5274	0	0	0	1	Ref
MG592654	Unassigned	Unassigned	Unassigned	948_0	Clustered	9	VC_948_0	9	0.5443	1	0.5443	0	0	0	1	Ref
MG592655	Unassigned	Unassigned	Unassigned	1050_1	Clustered	29	VC_1050_1	29	0.4861	1	0.4861	0	0	0	0.9581	Ref
MG592656	Unassigned	Unassigned	Unassigned	646_0	Clustered	29	VC_646_0	29	0.9131	1	0.9131	0	0	0	1	Ref
MG592657	Unassigned	Unassigned	Unassigned	948_0	Clustered	9	VC_948_0	9	0.5443	1	0.5443	0	0	0	1	Ref
MG592658	Unassigned	Unassigned	Unassigned	948_0	Clustered	9	VC_948_0	9	0.5443	1	0.5443	0	0	0	1	Ref
MG592659	Unassigned	Unassigned	Unassigned	948_0	Clustered	9	VC_948_0	9	0.5443	1	0.5443	0	0	0	1	Ref
MG592660	Unassigned	Unassigned	Unassigned	175_0	Clustered	4	VC_175_0	4	0.3847	0.99998259	0.3846	0	0	0	1	Ref
MG592661	Unassigned	Unassigned	Unassigned	1041_0	Clustered	9	VC_1041_0	9	0.6541	1	0.6541	0	0	0	1	Ref
MG592662	Unassigned	Unassigned	Unassigned	902_0	Clustered	3	VC_902_0	3	0.9978	0.87589346	0.8739	0	0	0	1	Ref
MG592664	Unassigned	Unassigned	Unassigned	642_5	Clustered	4	VC_642_5	4	0.0902	0.99996664	0.0902	0	0	0	1	Ref
MG592665	Unassigned	Unassigned	Unassigned	642_5	Clustered	4	VC_642_5	4	0.0902	0.99996664	0.0902	0	0	0	1	Ref
MG592666	Unassigned	Unassigned	Unassigned	827_0	Clustered	6	VC_827_0	6	0.7493	1	0.7493	0	0	0	1	Ref
MG592668	Unassigned	Unassigned	Unassigned	930_0	Clustered	7	VC_930_0	7	0.519	1	0.519	0	0	0	1	Ref
MG592669	Unassigned	Unassigned	Unassigned	248_0	Clustered	11	VC_248_0	11	0.7031	1	0.7031	0	0	0	1	Ref
MG592670	Unassigned	Unassigned	Unassigned	248_0	Clustered	11	VC_248_0	11	0.7031	1	0.7031	0	0	0	1	Ref
MG592671	Unassigned	Unassigned	Unassigned	164_9	Clustered/Singleton	1	VC_164_9	1	0	1	0	0	0	0	1	Ref
MG592672	Unassigned	Unassigned	Unassigned	902_0	Clustered	3	VC_902_0	3	0.9978	0.87589346	0.8739	0	0	0	1	Ref
MG593800	Unassigned	Unassigned	Unassigned	375_0	Clustered	3	VC_375_0	3	0.2479	0.99807286	0.2474	0	0	0	1	Ref
MG593802	Unassigned	Unassigned	Unassigned	981_1	Clustered	26	VC_981_1	26	0.7009	1	0.7009	0	0	0	1	Ref
MG593803	Unassigned	Unassigned	Unassigned	375_0	Clustered	3	VC_375_0	3	0.2479	0.99807286	0.2474	0	0	0	1	Ref
MG596799	Unassigned	Unassigned	Unassigned	1030_0	Clustered	3	VC_1030_0	3	0.7895	0.99542416	0.7859	0	0	0	1	Ref
MG596800	Unassigned	Unassigned	Unassigned	965_0	Clustered	2	VC_965_0	2	0.2408	0.95689931	0.2304	0	0	0	1	Ref
MG599035	Unassigned	Unassigned	Unassigned	699_0	Clustered	71	VC_699_0	71	0.9352	1	0.9352	0	0	0	1	Ref
MG602476	Unassigned	Unassigned	Unassigned	882_0	Clustered	20	VC_882_0	20	0.9917	1	0.9917	0	0	0	1	Ref
MG602477	Unassigned	Unassigned	Unassigned	187_3	Clustered	9	VC_187_3	9	0.1971	1	0.1971	0	0	0	0.8611	Ref
MG640035	Unassigned	Unassigned	Unassigned	909_0	Clustered	5	VC_909_0	5	0.7406	0.9999861	0.7406	0	0	0	1	Ref
MG641179	Unassigned	Unassigned	Unassigned	1104_0	Clustered	154	VC_1104_0	154	0.8484	1	0.8484	0	0	0	1	Ref
MG641180	Unassigned	Unassigned	Unassigned	1104_0	Clustered	154	VC_1104_0	154	0.8484	1	0.8484	0	0	0	1	Ref
MG641183	Unassigned	Unassigned	Unassigned	197_0	Clustered	5	VC_197_0	5	0.1324	0.84698401	0.1122	0	0	0	1	Ref
MG641184	Unassigned	Unassigned	Unassigned	1133_0	Clustered	4	VC_1133_0	4	0.1253	0.975067	0.1222	0	0	0	1	Ref
MG641185	Unassigned	Unassigned	Unassigned	1232_0	Clustered	2	VC_1232_0	2	0.4709	0.85557782	0.4029	0	0	0	1	Ref
MG641186	Unassigned	Unassigned	Unassigned	1104_0	Clustered	154	VC_1104_0	154	0.8484	1	0.8484	0	0	0	1	Ref
MG641188	Unassigned	Unassigned	Unassigned	1233_0	Clustered	16	VC_1233_0	16	0.8971	1	0.8971	0	0	0	1	Ref
MG646668	Unassigned	Unassigned	Unassigned	637_0	Clustered	115	VC_637_0	115	0.731	1	0.731	0	0	0	0.8764	Ref
MG646669	Unassigned	Unassigned	Unassigned	329_11	Clustered	15	VC_329_11	15	0.0791	1	0.0791	0	0	0	0.9333	Ref

MG646670	Unassigned	Unassigned	Unassigned	329_11	Clustered	15	VC_329_11	15	0.0791	1	0.0791	0	0	0	0.9333	Ref
MG646671	Unassigned	Unassigned	Unassigned	329_11	Clustered	15	VC_329_11	15	0.0791	1	0.0791	0	0	0	0.9333	Ref
MG646672	Unassigned	Unassigned	Unassigned	329_0	Clustered	2	VC_329_0	2	0.0051	0.95464582	0.0048	0	0	0	1	Ref
MG649966	Unassigned	Unassigned	Unassigned	971_1	Clustered	24	VC_971_1	24	0.7393	1	0.7393	0	0	0	0.8913	Ref
MG649967	Unassigned	Unassigned	Unassigned	971_1	Clustered	24	VC_971_1	24	0.7393	1	0.7393	0	0	0	0.8913	Ref
MG655267	Unassigned	Unassigned	Unassigned	489_1	Clustered	11	VC_489_1	11	0.4621	1	0.4621	0	0	0	1	Ref
MG655268	Unassigned	Unassigned	Unassigned	489_1	Clustered	11	VC_489_1	11	0.4621	1	0.4621	0	0	0	1	Ref
MG655269	Unassigned	Unassigned	Unassigned	489_1	Clustered	11	VC_489_1	11	0.4621	1	0.4621	0	0	0	1	Ref
MG655270	Unassigned	Unassigned	Unassigned	489_1	Clustered	11	VC_489_1	11	0.4621	1	0.4621	0	0	0	1	Ref
MG656408	Unassigned	Unassigned	Unassigned	149_8	Clustered	66	VC_149_8	66	0.4565	1	0.4565	0	0	0	0.8182	Ref
MG663582	Unassigned	Unassigned	Unassigned	1224_0	Clustered	19	VC_1224_0	19	1	1	1	0	0	0	1	Ref
MG663583	Unassigned	Unassigned	Unassigned	1224_0	Clustered	19	VC_1224_0	19	1	1	1	0	0	0	1	Ref
MG663584	Unassigned	Unassigned	Unassigned	1224_0	Clustered	19	VC_1224_0	19	1	1	1	0	0	0	1	Ref
MG663585	Unassigned	Unassigned	Unassigned	1224_0	Clustered	19	VC_1224_0	19	1	1	1	0	0	0	1	Ref
MG663586	Unassigned	Unassigned	Unassigned	1224_0	Clustered	19	VC_1224_0	19	1	1	1	0	0	0	1	Ref
MG670586	Unassigned	Unassigned	Unassigned	1234_0	Clustered	44	VC_1234_0	44	1	1	1	0	0	0	1	Ref
MG673519	Unassigned	Unassigned	Unassigned	813_1	Clustered	24	VC_813_1	24	0.535	1	0.535	0	0	0	1	Ref
MG674163	Unassigned	Unassigned	Unassigned	608_0	Clustered	5	VC_608_0	5	0.4331	0.99999996	0.4331	0	0	0	1	Ref
MG676223	Unassigned	Unassigned	Unassigned	642_2	Clustered	6	VC_642_2	6	0.1679	1	0.1679	0	0	0	1	Ref
MG676224	Unassigned	Unassigned	Unassigned	195_0	Clustered	2	VC_195_0	2	0.1201	0.95680007	0.1149	0	0	0	1	Ref
MG676225	Unassigned	Unassigned	Unassigned	195_0	Clustered	2	VC_195_0	2	0.1201	0.95680007	0.1149	0	0	0	1	Ref
MG676466	Unassigned	Unassigned	Unassigned	755_0	Clustered	9	VC_755_0	9	0.4489	1	0.4489	0	0	0	1	Ref
MG696114	Unassigned	Unassigned	Unassigned	288_2	Clustered	2	VC_288_2	2	0.0228	0.95538559	0.0218	0	0	0	1	Ref
MG707188	Unassigned	Unassigned	Unassigned	173_0	Clustered	2	VC_173_0	2	0.0799	0.95489091	0.0763	0	0	0	1	Ref
MG708273	Unassigned	Unassigned	Unassigned	252_0	Clustered	121	VC_252_0	121	0.7376	1	0.7376	0	0	0	0.9999	Ref
MG708274	Unassigned	Unassigned	Unassigned	252_0	Clustered	121	VC_252_0	121	0.7376	1	0.7376	0	0	0	0.9999	Ref
MG708275	Unassigned	Unassigned	Unassigned	252_0	Clustered	121	VC_252_0	121	0.7376	1	0.7376	0	0	0	0.9999	Ref
MG708276	Unassigned	Unassigned	Unassigned	695_0	Clustered	48	VC_695_0	48	0.8162	1	0.8162	0	0	0	1	Ref
MG710528	Unassigned	Unassigned	Unassigned	42_0	Clustered	49	VC_42_0	49	0.4517	1	0.4517	0	0	0	0.8597	Ref
MG711460	Unassigned	Unassigned	Unassigned	1235_0	Clustered	12	VC_1235_0	12	0.7997	1	0.7997	0	0	0	1	Ref
MG711461	Unassigned	Unassigned	Unassigned	717_0	Clustered	7	VC_717_0	7	0.5813	1	0.5813	0	0	0	0.9048	Ref
MG711462	Unassigned	Unassigned	Unassigned	707_1	Clustered	9	VC_707_1	9	0.2702	1	0.2702	0	0	0	0.8889	Ref
MG711463	Unassigned	Unassigned	Unassigned	804_0	Clustered	2	VC_804_0	2	0.0559	0.95690755	0.0535	0	0	0	1	Ref
MG711464	Unassigned	Unassigned	Unassigned	621_0	Clustered	7	VC_621_0	7	0.5783	1	0.5783	0	0	0	1	Ref
MG711465	Unassigned	Unassigned	Unassigned	606_0	Clustered	4	VC_606_0	4	0.4507	0.99995146	0.4507	0	0	0	1	Ref
MG711466	Unassigned	Unassigned	Unassigned	710_0	Clustered	10	VC_710_0	10	0.2446	1	0.2446	0	0	0	0.8222	Ref
MG711467	Unassigned	Unassigned	Unassigned	711_0	Clustered	3	VC_711_0	3	0.2312	0.99842495	0.2309	0	0	0	1	Ref
MG720309	Unassigned	Unassigned	Unassigned	904_0	Clustered	6	VC_904_0	6	0.233	1	0.233	0	0	0	1	Ref
MG721208	Unassigned	Unassigned	Unassigned	149_21	Clustered/Singleton	1	VC_149_21	1	0	1	0	0	0	0	1	Ref
MG727695	Unassigned	Unassigned	Unassigned	708_0	Clustered	47	VC_708_0	47	0.8654	1	0.8654	0	0	0	1	Ref
MG727696	Unassigned	Unassigned	Unassigned	708_0	Clustered	47	VC_708_0	47	0.8654	1	0.8654	0	0	0	1	Ref
MG727698	Unassigned	Unassigned	Unassigned	708_0	Clustered	47	VC_708_0	47	0.8654	1	0.8654	0	0	0	1	Ref
MG727699	Unassigned	Unassigned	Unassigned	708_0	Clustered	47	VC_708_0	47	0.8654	1	0.8654	0	0	0	1	Ref
MG727700	Unassigned	Unassigned	Unassigned	708_0	Clustered	47	VC_708_0	47	0.8654	1	0.8654	0	0	0	1	Ref
MG727701	Unassigned	Unassigned	Unassigned	708_0	Clustered	47	VC_708_0	47	0.8654	1	0.8654	0	0	0	1	Ref
MG727702	Unassigned	Unassigned	Unassigned	708_0	Clustered	47	VC_708_0	47	0.8654	1	0.8654	0	0	0	1	Ref
MG736918	Unassigned	Unassigned	Unassigned	453_0	Clustered	6	VC_453_0	6	0.114	1	0.114	0	0	0	1	Ref
MG744354	Unassigned	Unassigned	Unassigned	634_1	Clustered	6	VC_634_1	6	0.4228	1	0.4228	0	0	0	1	Ref
MG747435	Unassigned	Unassigned	Unassigned	1_0	Clustered	54	VC_1_0	54	0.8527	1	0.8527	0	0	0	1	Ref
MG748547	Unassigned	Unassigned	Unassigned	133_5	Clustered/Singleton	1	VC_133_5	1	0	1	0	0	0	0	1	Ref
MG748548	Unassigned	Unassigned	Unassigned	813_1	Clustered	24	VC_813_1	24	0.535	1	0.535	0	0	0	1	Ref
MG751100	Unassigned	Unassigned	Unassigned	491_0	Clustered	36	VC_491_0	36	0.5336	1	0.5336	0	0	0	0.9714	Ref
MG757152	Unassigned	Unassigned	Unassigned	868_0	Clustered	25	VC_868_0	25	0.5791	1	0.5791	0	0	0	1	Ref
MG757153	Unassigned	Unassigned	Unassigned	1072_0	Clustered	2	VC_1072_0	2	0.0264	0.95117401	0.0251	0	0	0	1	Ref
MG757154	Unassigned	Unassigned	Unassigned	872_0	Clustered	31	VC_872_0	31	0.8151	1	0.8151	0	0	0	0.8194	Ref
MG757155	Unassigned	Unassigned	Unassigned	868_0	Clustered	25	VC_868_0	25	0.5791	1	0.5791	0	0	0	1	Ref
MG757156	Unassigned	Unassigned	Unassigned	571_0	Clustered	4	VC_571_0	4	0.1921	0.99998591	0.1921	0	0	0	1	Ref
MG757157	Unassigned	Unassigned	Unassigned	767_0	Clustered	34	VC_767_0	34	0.9173	1	0.9173	0	0	0	1	Ref
MG757158	Unassigned	Unassigned	Unassigned	371_16	Clustered	42	VC_371_16	42	0.1341	1	0.1341	0	0	0	0.7282	Ref
MG757159	Unassigned	Unassigned	Unassigned	371_11	Clustered	31	VC_371_11	31	0.0927	1	0.0927	0	0	0	0.7011	Ref
MG757160	Unassigned	Unassigned	Unassigned	551_6	Clustered	22	VC_551_6	22	0.1214	1	0.1214	0	0	0	0.8095	Ref
MG757161	Unassigned	Unassigned	Unassigned	514_0	Clustered	75	VC_514_0	75	0.9402	1	0.9402	0	0	0	1	Ref
MG757162	Unassigned	Unassigned	Unassigned	508_0	Clustered	32	VC_508_0	32	0.906	1	0.906	0	0	0	1	Ref
MG757163	Unassigned	Unassigned	Unassigned	982_0	Clustered	62	VC_982_0	62	0.8646	1	0.8646	0	0	0	0.743	Ref
MG757164	Unassigned	Unassigned	Unassigned	371_13	Clustered	33	VC_371_13	33	0.179	1	0.179	0	0	0	0.8769	Ref
MG757165	Unassigned	Unassigned	Unassigned	371_13	Clustered	33	VC_371_13	33	0.179	1	0.179	0	0	0	0.8769	Ref
MG757166	Unassigned	Unassigned	Unassigned	767_0	Clustered	34	VC_767_0	34	0.9173	1	0.9173	0	0	0	1	Ref
MG757167	Unassigned	Unassigned	Unassigned	939_0	Clustered	15	VC_939_0	15	1	0	0	0	0	0	1	Ref
MG763894	Unassigned	Unassigned	Unassigned	884_0	Clustered	28	VC_884_0	28	0.6211	1	0.6211	0	0	0	0.9841	Ref
MG765274	Unassigned	Unassigned	Unassigned	634_1	Clustered	6	VC_634_1	6	0.4228	1	0.4228	0	0	0	1	Ref
MG765275	Unassigned	Unassigned	Unassigned	577_0	Clustered	6	VC_577_0	6	0.9267	0.99999993	0.9267	0	0	0	1	Ref
MG765276	Unassigned	Unassigned	Unassigned	634_0	Clustered	3	VC_634_0	3	0.2046	0.99837593	0.2043	0	0	0	1	Ref
MG765277	Unassigned	Unassigned	Unassigned	526_0	Clustered	6	VC_526_0	6	0.4386	1	0.4386	0	0	0	1	Ref
MG765278	Unassigned	Unassigned	Unassigned	577_0	Clustered	6	VC_577_0	6	0.9267	0.99999993	0.9267	0	0	0	1	Ref
MG765279	Unassigned	Unassigned	Unassigned	526_0	Clustered	6	VC_526_0	6	0.4386	1	0.4386	0	0	0	1	Ref
MG766218	Unassigned	Unassigned	Unassigned	96_0	Clustered	30	VC_96_0	30	0.718	1	0.718	0	0	0	1	Ref
MG766219	Unassigned	Unassigned	Unassigned	96_0	Clustered	30	VC_96_0	30	0.718	1	0.718	0	0	0	1	Ref
MG770211	Unassigned	Unassigned	Unassigned	868_0	Clustered	25	VC_868_0	25	0.5791	1	0.5791	0	0	0	1	Ref
MG770212	Unassigned	Unassigned	Unassigned	371_15	Clustered	44	VC_371_15	44	0.1391	1	0.1391	0	0	0	0.7474	Ref
MG770214	Unassigned	Unassigned	Unassigned	868_0	Clustered	25	VC_868_0	25	0.5791	1	0.5791	0	0	0	1	Ref
MG770215	Unassigned	Unassigned	Unassigned	919_0	Clustered	39	VC_919_0	39	0.9069	1	0.9069	0	0	0	1	Ref

MG770216	Unassigned	Unassigned	Unassigned	809_0	Clustered	2	VC_809_0	2	0.1799	0.96299906	0.1733	0	0	0	1	Ref
MG770228	Unassigned	Unassigned	Unassigned	779_0	Clustered	37	VC_779_0	37	0.5682	1	0.5682	0	0	0	1	Ref
MG770379	Unassigned	Unassigned	Unassigned	132_1	Clustered	17	VC_132_1	17	0.1738	1	0.1738	0	0	0	0.9853	Ref
MG770897	Unassigned	Unassigned	Unassigned	8_0	Clustered	58	VC_8_0	58	0.8306	1	0.8306	0	0	0	0.9946	Ref
MG775042	Unassigned	Unassigned	Unassigned	452_0	Clustered	66	VC_452_0	66	0.9248	1	0.9248	0	0	0	1	Ref
MG775043	Unassigned	Unassigned	Unassigned	283_24	Clustered/Singleton	1	VC_283_24	1	0	1	0	0	0	0	1	Ref
MG775258	Unassigned	Unassigned	Unassigned	129_0	Clustered	43	VC_129_0	43	0.886	1	0.886	0	0	0	1	Ref
MG775260	Unassigned	Unassigned	Unassigned	842_0	Clustered	2	VC_842_0	2	0.0559	0.95564851	0.0534	0	0	0	1	Ref
MG781190	Unassigned	Unassigned	Unassigned	283_89	Clustered	2	VC_283_89	2	0.0027	0.91264761	0.0025	0	0	0	1	Ref
MG781191	Unassigned	Unassigned	Unassigned	283_89	Clustered	2	VC_283_89	2	0.0027	0.91264761	0.0025	0	0	0	1	Ref
MG784342	Unassigned	Unassigned	Unassigned	828_0	Clustered	3	VC_828_0	3	0.4748	0.99843555	0.4741	0	0	0	1	Ref
MG788324	Unassigned	Unassigned	Unassigned	300_0	Clustered	3	VC_300_0	3	0.1218	0.99841701	0.1216	0	0	0	0.6667	Ref
MG793454	Unassigned	Unassigned	Unassigned	219_0	Clustered	5	VC_219_0	5	0.08	0.99999998	0.08	0	0	0	1	Ref
MG812486	Unassigned	Unassigned	Unassigned	325_50	Clustered	4	VC_325_50	4	0.0182	0.99998785	0.0182	0	0	0	1	Ref
MG812487	Unassigned	Unassigned	Unassigned	1093_0	Clustered	40	VC_1093_0	40	0.8754	1	0.8754	0	0	0	1	Ref
MG812488	Unassigned	Unassigned	Unassigned	520_98	Clustered/Singleton	1	VC_520_98	1	0	1	0	0	0	0	1	Ref
MG812489	Unassigned	Unassigned	Unassigned	325_50	Clustered	4	VC_325_50	4	0.0182	0.99998785	0.0182	0	0	0	1	Ref
MG812490	Unassigned	Unassigned	Unassigned	508_0	Clustered	32	VC_508_0	32	0.906	1	0.906	0	0	0	1	Ref
MG812491	Unassigned	Unassigned	Unassigned	508_0	Clustered	32	VC_508_0	32	0.906	1	0.906	0	0	0	1	Ref
MG812492	Unassigned	Unassigned	Unassigned	513_0	Clustered	32	VC_513_0	32	0.6881	1	0.6881	0	0	0	1	Ref
MG812493	Unassigned	Unassigned	Unassigned	558_72	Clustered/Singleton	1	VC_558_72	1	0	1	0	0	0	0	1	Ref
MG812494	Unassigned	Unassigned	Unassigned	558_68	Clustered/Singleton	1	VC_558_68	1	0	1	0	0	0	0	1	Ref
MG812495	Unassigned	Unassigned	Unassigned	325_50	Clustered	4	VC_325_50	4	0.0182	0.99998785	0.0182	0	0	0	1	Ref
MG812496	Unassigned	Unassigned	Unassigned	949_0	Clustered	10	VC_949_0	10	0.5977	1	0.5977	0	0	0	1	Ref
MG820632	Unassigned	Unassigned	Unassigned	765_0	Clustered	132	VC_765_0	132	1	1	1	0	0	0	1	Ref
MG820633	Unassigned	Unassigned	Unassigned	765_0	Clustered	132	VC_765_0	132	1	1	1	0	0	0	1	Ref
MG820634	Unassigned	Unassigned	Unassigned	765_0	Clustered	132	VC_765_0	132	1	1	1	0	0	0	1	Ref
MG820635	Unassigned	Unassigned	Unassigned	765_0	Clustered	132	VC_765_0	132	1	1	1	0	0	0	1	Ref
MG820636	Unassigned	Unassigned	Unassigned	765_0	Clustered	132	VC_765_0	132	1	1	1	0	0	0	1	Ref
MG820637	Unassigned	Unassigned	Unassigned	765_0	Clustered	132	VC_765_0	132	1	1	1	0	0	0	1	Ref
MG820638	Unassigned	Unassigned	Unassigned	765_0	Clustered	132	VC_765_0	132	1	1	1	0	0	0	1	Ref
MG820639	Unassigned	Unassigned	Unassigned	765_0	Clustered	132	VC_765_0	132	1	1	1	0	0	0	1	Ref
MG820640	Unassigned	Unassigned	Unassigned	765_0	Clustered	132	VC_765_0	132	1	1	1	0	0	0	1	Ref
MG820641	Unassigned	Unassigned	Unassigned	765_0	Clustered	132	VC_765_0	132	1	1	1	0	0	0	1	Ref
MG820642	Unassigned	Unassigned	Unassigned	765_0	Clustered	132	VC_765_0	132	1	1	1	0	0	0	1	Ref
MG820643	Unassigned	Unassigned	Unassigned	765_0	Clustered	132	VC_765_0	132	1	1	1	0	0	0	1	Ref
MG820644	Unassigned	Unassigned	Unassigned	765_0	Clustered	132	VC_765_0	132	1	1	1	0	0	0	1	Ref
MG820645	Unassigned	Unassigned	Unassigned	765_0	Clustered	132	VC_765_0	132	1	1	1	0	0	0	1	Ref
MG832642	Unassigned	Unassigned	Unassigned	392_0	Clustered	126	VC_392_0	126	0.9621	1	0.9621	0	0	0	1	Ref
MG833025	Unassigned	Unassigned	Unassigned	392_0	Clustered	126	VC_392_0	126	0.9621	1	0.9621	0	0	0	1	Ref
MG835568	Unassigned	Unassigned	Unassigned	823_0	Clustered	124	VC_823_0	124	0.9492	1	0.9492	0	0	0	1	Ref
MG839014	Unassigned	Unassigned	Unassigned	509_0	Clustered	37	VC_509_0	37	0.9689	1	0.9689	0	0	0	1	Ref
MG839015	Unassigned	Unassigned	Unassigned	1240_0	Clustered	82	VC_1240_0	82	0.7945	1	0.7945	0	0	0	1	Ref
MG839016	Unassigned	Unassigned	Unassigned	1240_0	Clustered	82	VC_1240_0	82	0.7945	1	0.7945	0	0	0	1	Ref
MG839017	Unassigned	Unassigned	Unassigned	1240_0	Clustered	82	VC_1240_0	82	0.7945	1	0.7945	0	0	0	1	Ref
MG839018	Unassigned	Unassigned	Unassigned	1240_0	Clustered	82	VC_1240_0	82	0.7945	1	0.7945	0	0	0	1	Ref
MG839020	Unassigned	Unassigned	Unassigned	1240_0	Clustered	82	VC_1240_0	82	0.7945	1	0.7945	0	0	0	1	Ref
MG839021	Unassigned	Unassigned	Unassigned	1240_0	Clustered	82	VC_1240_0	82	0.7945	1	0.7945	0	0	0	1	Ref
MG839022	Unassigned	Unassigned	Unassigned	1240_0	Clustered	82	VC_1240_0	82	0.7945	1	0.7945	0	0	0	1	Ref
MG839023	Unassigned	Unassigned	Unassigned	1240_0	Clustered	82	VC_1240_0	82	0.7945	1	0.7945	0	0	0	1	Ref
MG839026	Unassigned	Unassigned	Unassigned	1240_0	Clustered	82	VC_1240_0	82	0.7945	1	0.7945	0	0	0	1	Ref
MG839027	Unassigned	Unassigned	Unassigned	1163_0	Clustered	28	VC_1163_0	28	0.4923	1	0.4923	0	0	0	1	Ref
MG839028	Unassigned	Unassigned	Unassigned	1240_0	Clustered	82	VC_1240_0	82	0.7945	1	0.7945	0	0	0	1	Ref
MG839029	Unassigned	Unassigned	Unassigned	1240_0	Clustered	82	VC_1240_0	82	0.7945	1	0.7945	0	0	0	1	Ref
MG845684	Unassigned	Unassigned	Unassigned	472_0	Clustered	47	VC_472_0	47	0.9025	1	0.9025	0	0	0	1	Ref
MG845865	Unassigned	Unassigned	Unassigned	451_0	Clustered	80	VC_451_0	80	0.9991	1	0.9991	0	0	0	1	Ref
MG852086	Unassigned	Unassigned	Unassigned	638_0	Clustered	45	VC_638_0	45	0.7741	1	0.7741	0	0	0	1	Ref
MG867727	Unassigned	Unassigned	Unassigned	283_138	Clustered/Singleton	1	VC_283_138	1	0	1	0	0	0	0	1	Ref
MG872831	Unassigned	Unassigned	Unassigned	551_0	Clustered	16	VC_551_0	16	0.0915	1	0.0915	0	0	0	0.6667	Ref
MG872832	Unassigned	Unassigned	Unassigned	551_0	Clustered	16	VC_551_0	16	0.0915	1	0.0915	0	0	0	0.6667	Ref
MG872833	Unassigned	Unassigned	Unassigned	325_5	Clustered	11	VC_325_5	11	0.047	1	0.047	0	0	0	0.7636	Ref
MG872834	Unassigned	Unassigned	Unassigned	685_0	Clustered	16	VC_685_0	16	0.2972	1	0.2972	0	0	0	0.9167	Ref
MG872835	Unassigned	Unassigned	Unassigned	225_0	Clustered	32	VC_225_0	32	0.848	1	0.848	0	0	0	0.996	Ref
MG872836	Unassigned	Unassigned	Unassigned	325_50	Clustered	4	VC_325_50	4	0.0182	0.99998785	0.0182	0	0	0	1	Ref
MG872837	Unassigned	Unassigned	Unassigned	551_6	Clustered	22	VC_551_6	22	0.1214	1	0.1214	0	0	0	0.8095	Ref
MG872838	Unassigned	Unassigned	Unassigned	556_0	Clustered	21	VC_556_0	21	0.9912	1	0.9912	0	0	0	1	Ref
MG872839	Unassigned	Unassigned	Unassigned	558_14	Clustered	8	VC_558_14	8	0.0228	0.99945246	0.0228	0	0	0	0.8214	Ref
MG872840	Unassigned	Unassigned	Unassigned	225_0	Clustered	32	VC_225_0	32	0.848	1	0.848	0	0	0	0.996	Ref
MG872841	Unassigned	Unassigned	Unassigned	520_45	Clustered	7	VC_520_45	7	0.0635	1	0.0635	0	0	0	0.8095	Ref
MG872842	Unassigned	Unassigned	Unassigned	225_0	Clustered	32	VC_225_0	32	0.848	1	0.848	0	0	0	0.996	Ref
MG872843	Unassigned	Unassigned	Unassigned	551_0	Clustered	16	VC_551_0	16	0.0915	1	0.0915	0	0	0	0.6667	Ref
MG873442	Unassigned	Unassigned	Unassigned	812_0	Clustered	3	VC_812_0	3	0.1174	0.99842379	0.1172	0	0	0	1	Ref
MG878892	Unassigned	Unassigned	Unassigned	878_0	Clustered	32	VC_878_0	32	0.9965	1	0.9965	0	0	0	1	Ref
MG883709	Unassigned	Unassigned	Unassigned	1243_0	Clustered	52	VC_1243_0	52	0.9126	0.92708311	0.8461	0	0	0	1	Ref
MG883710	Unassigned	Unassigned	Unassigned	1245_0	Clustered	2	VC_1245_0	2	1	1	1	0	0	0	1	Ref
MG883712	Unassigned	Unassigned	Unassigned	1197_0	Clustered	15	VC_1197_0	15	0.8991	0.76608394	0.6888	0	0	0	1	Ref
MG883713	Unassigned	Unassigned	Unassigned	1246_0	Clustered	2	VC_1246_0	2	1	1	1	0	0	0	1	Ref
MG883714	Unassigned	Unassigned	Unassigned	1144_0	Clustered	7	VC_1144_0	7	0.7076	1	0.7076	0	0	0	1	Ref
MG883715	Unassigned	Unassigned	Unassigned	672_0	Clustered	12	VC_672_0	12	1	1	1	0	0	0	1	Ref
MG883717	Unassigned	Unassigned	Unassigned	1104_0	Clustered	154	VC_1104_0	154	0.8484	1	0.8484	0	0	0	1	Ref
MG883718	Unassigned	Unassigned	Unassigned	1108_0	Clustered	4	VC_1108_0	4	0.0485	0.99999021	0.0485	0	0	0	1	Ref

MG883719	Unassigned	Unassigned	Unassigned	1116_0	Clustered	14	VC_1116_0	14	0.7927	1	0.7927	0	0	0	1	Ref
MG883720	Unassigned	Unassigned	Unassigned	1247_0	Clustered	7	VC_1247_0	7	0.9086	0.99998959	0.9086	0	0	0	1	Ref
MG883722	Unassigned	Unassigned	Unassigned	1125_0	Clustered	20	VC_1125_0	20	0.5599	1	0.5599	0	0	0	1	Ref
MG883723	Unassigned	Unassigned	Unassigned	198_0	Clustered	28	VC_198_0	28	0.2429	0.99971175	0.2429	0	0	0	1	Ref
MG883724	Unassigned	Unassigned	Unassigned	1250_0	Clustered	5	VC_1250_0	5	0.6384	0.99918945	0.6379	0	0	0	1	Ref
MG883725	Unassigned	Unassigned	Unassigned	672_0	Clustered	12	VC_672_0	12	1	1	1	0	0	0	1	Ref
MG883726	Unassigned	Unassigned	Unassigned	1077_0	Clustered	48	VC_1077_0	48	0.8525	1	0.8525	0	0	0	1	Ref
MG893203	Unassigned	Unassigned	Unassigned	882_0	Clustered	20	VC_882_0	20	0.9917	1	0.9917	0	0	0	1	Ref
MG894052	Unassigned	Unassigned	Unassigned	938_0	Clustered	99	VC_938_0	99	0.9119	1	0.9119	0	0	0	0.9928	Ref
MG897799	Unassigned	Unassigned	Unassigned	79_0	Clustered	64	VC_79_0	64	0.4185	1	0.4185	0	0	0	0.7649	Ref
MG897800	Unassigned	Unassigned	Unassigned	749_0	Clustered	40	VC_749_0	40	0.7517	1	0.7517	0	0	0	1	Ref
MG913376	Unassigned	Unassigned	Unassigned	640_0	Clustered	7	VC_640_0	7	0.9025	1	0.9025	0	0	0	1	Ref
MG920059	Unassigned	Unassigned	Unassigned	509_0	Clustered	37	VC_509_0	37	0.9689	1	0.9689	0	0	0	1	Ref
MG920060	Unassigned	Unassigned	Unassigned	325_23	Clustered	17	VC_325_23	17	0.0716	1	0.0716	0	0	0	0.6397	Ref
MG920061	Unassigned	Unassigned	Unassigned	1240_0	Clustered	82	VC_1240_0	82	0.7945	1	0.7945	0	0	0	1	Ref
MG922974	Unassigned	Unassigned	Unassigned	779_0	Clustered	37	VC_779_0	37	0.5682	1	0.5682	0	0	0	1	Ref
MG925338	Unassigned	Unassigned	Unassigned	509_0	Clustered	37	VC_509_0	37	0.9689	1	0.9689	0	0	0	1	Ref
MG925339	Unassigned	Unassigned	Unassigned	371_3	Clustered	22	VC_371_3	22	0.0787	1	0.0787	0	0	0	0.9048	Ref
MG925340	Unassigned	Unassigned	Unassigned	325_61	Clustered	8	VC_325_61	8	0.0395	1	0.0395	0	0	0	0.7857	Ref
MG925341	Unassigned	Unassigned	Unassigned	514_0	Clustered	75	VC_514_0	75	0.9402	1	0.9402	0	0	0	1	Ref
MG925342	Unassigned	Unassigned	Unassigned	325_61	Clustered	8	VC_325_61	8	0.0395	1	0.0395	0	0	0	0.7857	Ref
MG925343	Unassigned	Unassigned	Unassigned	1163_0	Clustered	28	VC_1163_0	28	0.4923	1	0.4923	0	0	0	1	Ref
MG925344	Unassigned	Unassigned	Unassigned	325_5	Clustered	11	VC_325_5	11	0.047	1	0.047	0	0	0	0.7636	Ref
MG925345	Unassigned	Unassigned	Unassigned	1163_0	Clustered	28	VC_1163_0	28	0.4923	1	0.4923	0	0	0	1	Ref
MG925346	Unassigned	Unassigned	Unassigned	371_15	Clustered	44	VC_371_15	44	0.1391	1	0.1391	0	0	0	0.7474	Ref
MG925347	Unassigned	Unassigned	Unassigned	1163_0	Clustered	28	VC_1163_0	28	0.4923	1	0.4923	0	0	0	1	Ref
MG925348	Unassigned	Unassigned	Unassigned	371_16	Clustered	42	VC_371_16	42	0.1341	1	0.1341	0	0	0	0.7282	Ref
MG925349	Unassigned	Unassigned	Unassigned	1046_0	Clustered	7	VC_1046_0	7	0.9413	1	0.9413	0	0	0	1	Ref
MG925350	Unassigned	Unassigned	Unassigned	371_15	Clustered	44	VC_371_15	44	0.1391	1	0.1391	0	0	0	0.7474	Ref
MG925351	Unassigned	Unassigned	Unassigned	325_23	Clustered	17	VC_325_23	17	0.0716	1	0.0716	0	0	0	0.6397	Ref
MG925352	Unassigned	Unassigned	Unassigned	1240_0	Clustered	82	VC_1240_0	82	0.7945	1	0.7945	0	0	0	1	Ref
MG925353	Unassigned	Unassigned	Unassigned	509_0	Clustered	37	VC_509_0	37	0.9689	1	0.9689	0	0	0	1	Ref
MG925354	Unassigned	Unassigned	Unassigned	520_97	Clustered/Singleton	1	VC_520_97	1	0	1	0	0	0	0	1	Ref
MG925355	Unassigned	Unassigned	Unassigned	514_0	Clustered	75	VC_514_0	75	0.9402	1	0.9402	0	0	0	1	Ref
MG925356	Unassigned	Unassigned	Unassigned	371_2	Clustered	8	VC_371_2	8	0.0232	1	0.0232	0	0	0	0.8571	Ref
MG941013	Unassigned	Unassigned	Unassigned	853_0	Clustered	2	VC_853_0	2	0.27	0.72985431	0.1971	0	0	0	1	Ref
MG944216	Unassigned	Unassigned	Unassigned	1163_0	Clustered	28	VC_1163_0	28	0.4923	1	0.4923	0	0	0	1	Ref
MG944217	Unassigned	Unassigned	Unassigned	517_1	Clustered	108	VC_517_1	108	0.8483	1	0.8483	0	0	0	0.9955	Ref
MG944218	Unassigned	Unassigned	Unassigned	1163_0	Clustered	28	VC_1163_0	28	0.4923	1	0.4923	0	0	0	1	Ref
MG944219	Unassigned	Unassigned	Unassigned	1240_0	Clustered	82	VC_1240_0	82	0.7945	1	0.7945	0	0	0	1	Ref
MG944220	Unassigned	Unassigned	Unassigned	325_26	Clustered/Singleton	1	VC_325_26	1	0	1	0	0	0	0	1	Ref
MG944221	Unassigned	Unassigned	Unassigned	325_7	Clustered	3	VC_325_7	3	0.0085	0.99739782	0.0085	0	0	0	0.6667	Ref
MG944222	Unassigned	Unassigned	Unassigned	1240_0	Clustered	82	VC_1240_0	82	0.7945	1	0.7945	0	0	0	1	Ref
MG944223	Unassigned	Unassigned	Unassigned	371_13	Clustered	33	VC_371_13	33	0.179	1	0.179	0	0	0	0.8769	Ref
MG944224	Unassigned	Unassigned	Unassigned	517_1	Clustered	108	VC_517_1	108	0.8483	1	0.8483	0	0	0	0.9955	Ref
MG944225	Unassigned	Unassigned	Unassigned	371_15	Clustered	44	VC_371_15	44	0.1391	1	0.1391	0	0	0	0.7474	Ref
MG944226	Unassigned	Unassigned	Unassigned	1163_0	Clustered	28	VC_1163_0	28	0.4923	1	0.4923	0	0	0	1	Ref
MG944227	Unassigned	Unassigned	Unassigned	364_0	Clustered	16	VC_364_0	16	0.6491	1	0.6491	0	0	0	1	Ref
MG944228	Unassigned	Unassigned	Unassigned	364_0	Clustered	16	VC_364_0	16	0.6491	1	0.6491	0	0	0	1	Ref
MG944229	Unassigned	Unassigned	Unassigned	364_0	Clustered	16	VC_364_0	16	0.6491	1	0.6491	0	0	0	1	Ref
MG944230	Unassigned	Unassigned	Unassigned	364_0	Clustered	16	VC_364_0	16	0.6491	1	0.6491	0	0	0	1	Ref
MG944231	Unassigned	Unassigned	Unassigned	364_0	Clustered	16	VC_364_0	16	0.6491	1	0.6491	0	0	0	1	Ref
MG944232	Unassigned	Unassigned	Unassigned	364_0	Clustered	16	VC_364_0	16	0.6491	1	0.6491	0	0	0	1	Ref
MG944233	Unassigned	Unassigned	Unassigned	364_0	Clustered	16	VC_364_0	16	0.6491	1	0.6491	0	0	0	1	Ref
MG944234	Unassigned	Unassigned	Unassigned	364_0	Clustered	16	VC_364_0	16	0.6491	1	0.6491	0	0	0	1	Ref
MG944235	Unassigned	Unassigned	Unassigned	364_0	Clustered	16	VC_364_0	16	0.6491	1	0.6491	0	0	0	1	Ref
MG944236	Unassigned	Unassigned	Unassigned	364_0	Clustered	16	VC_364_0	16	0.6491	1	0.6491	0	0	0	1	Ref
MG945128	Unassigned	Unassigned	Unassigned	1251_0	Clustered	15	VC_1251_0	15	0.9082	1	0.9082	0	0	0	1	Ref
MG945129	Unassigned	Unassigned	Unassigned	1077_0	Clustered	48	VC_1077_0	48	0.8525	1	0.8525	0	0	0	1	Ref
MG945130	Unassigned	Unassigned	Unassigned	1233_0	Clustered	16	VC_1233_0	16	0.8971	1	0.8971	0	0	0	1	Ref
MG945131	Unassigned	Unassigned	Unassigned	1114_0	Clustered	2	VC_1114_0	2	0.0522	0.94760452	0.0495	0	0	0	1	Ref
MG945135	Unassigned	Unassigned	Unassigned	1124_0	Clustered	10	VC_1124_0	10	0.6268	1	0.6268	0	0	0	1	Ref
MG945136	Unassigned	Unassigned	Unassigned	1124_0	Clustered	10	VC_1124_0	10	0.6268	1	0.6268	0	0	0	1	Ref
MG945138	Unassigned	Unassigned	Unassigned	1126_0	Clustered	8	VC_1126_0	8	0.2864	1	0.2864	0	0	0	1	Ref
MG945139	Unassigned	Unassigned	Unassigned	1116_0	Clustered	14	VC_1116_0	14	0.7927	1	0.7927	0	0	0	1	Ref
MG945140	Unassigned	Unassigned	Unassigned	1257_0	Clustered	6	VC_1257_0	6	0.6517	0.99999969	0.6517	0	0	0	1	Ref
MG945141	Unassigned	Unassigned	Unassigned	1211_0	Clustered	25	VC_1211_0	25	1	1	1	0	0	0	1	Ref
MG945142	Unassigned	Unassigned	Unassigned	1136_0	Clustered	2	VC_1136_0	2	0.58	0.88261817	0.5119	0	0	0	1	Ref
MG945153	Unassigned	Unassigned	Unassigned	1104_0	Clustered	154	VC_1104_0	154	0.8484	1	0.8484	0	0	0	1	Ref
MG945154	Unassigned	Unassigned	Unassigned	1119_0	Clustered	20	VC_1119_0	20	0.6751	0.99997892	0.6751	0	0	0	1	Ref
MG945157	Unassigned	Unassigned	Unassigned	1124_0	Clustered	10	VC_1124_0	10	0.6268	1	0.6268	0	0	0	1	Ref
MG945158	Unassigned	Unassigned	Unassigned	1104_0	Clustered	154	VC_1104_0	154	0.8484	1	0.8484	0	0	0	1	Ref
MG945159	Unassigned	Unassigned	Unassigned	1251_0	Clustered	15	VC_1251_0	15	0.9082	1	0.9082	0	0	0	1	Ref
MG945160	Unassigned	Unassigned	Unassigned	1191_0	Clustered	3	VC_1191_0	3	0.1408	0.98976974	0.1394	0	0	0	1	Ref
MG945161	Unassigned	Unassigned	Unassigned	1000_0	Clustered	21	VC_1000_0	21	0.8437	0.99999998	0.8437	0	0	0	1	Ref
MG945162	Unassigned	Unassigned	Unassigned	1127_0	Clustered	19	VC_1127_0	19	0.7611	1	0.7611	0	0	0	1	Ref
MG945163	Unassigned	Unassigned	Unassigned	1104_0	Clustered	154	VC_1104_0	154	0.8484	1	0.8484	0	0	0	1	Ref
MG945164	Unassigned	Unassigned	Unassigned	398_0	Clustered	48	VC_398_0	48	0.4813	1	0.4813	0	0	0	1	Ref
MG945165	Unassigned	Unassigned	Unassigned	1260_0	Clustered	2	VC_1260_0	2	1	1	1	0	0	0	1	Ref
MG945166	Unassigned	Unassigned	Unassigned	1259_0	Clustered	8	VC_1259_0	8	0.8225	1	0.8225	0	0	0	1	Ref
MG945177	Unassigned	Unassigned	Unassigned	1261_0	Clustered	5	VC_1261_0	5	0.765	0.99993003	0.7649	0	0	0	1	Ref

MG945178	Unassigned	Unassigned	Unassigned	1062_0	Clustered	45	VC_1062_0	45	0.9734	0.50949847	0.4959	0	0	0	1	Ref
MG945179	Unassigned	Unassigned	Unassigned	1148_0	Clustered	16	VC_1148_0	16	0.8821	1	0.8821	0	0	0	1	Ref
MG945181	Unassigned	Unassigned	Unassigned	1110_0	Clustered	8	VC_1110_0	8	0.6915	1	0.6915	0	0	0	1	Ref
MG945183	Unassigned	Unassigned	Unassigned	1263_0	Clustered	2	VC_1263_0	2	1	1	1	0	0	0	1	Ref
MG945185	Unassigned	Unassigned	Unassigned	1191_0	Clustered	3	VC_1191_0	3	0.1408	0.98976974	0.1394	0	0	0	1	Ref
MG945186	Unassigned	Unassigned	Unassigned	1122_0	Clustered	10	VC_1122_0	10	0.9643	0.99999697	0.9643	0	0	0	1	Ref
MG945188	Unassigned	Unassigned	Unassigned	1251_0	Clustered	15	VC_1251_0	15	0.9082	1	0.9082	0	0	0	1	Ref
MG945189	Unassigned	Unassigned	Unassigned	1266_0	Clustered	5	VC_1266_0	5	1	1	1	0	0	0	1	Ref
MG945190	Unassigned	Unassigned	Unassigned	1104_0	Clustered	154	VC_1104_0	154	0.8484	1	0.8484	0	0	0	1	Ref
MG945202	Unassigned	Unassigned	Unassigned	995_0	Clustered	69	VC_995_0	69	0.8547	1	0.8547	0	0	0	1	Ref
MG945203	Unassigned	Unassigned	Unassigned	1104_0	Clustered	154	VC_1104_0	154	0.8484	1	0.8484	0	0	0	1	Ref
MG945204	Unassigned	Unassigned	Unassigned	1152_0	Clustered	4	VC_1152_0	4	0.7206	0.99971165	0.7204	0	0	0	1	Ref
MG945205	Unassigned	Unassigned	Unassigned	1104_0	Clustered	154	VC_1104_0	154	0.8484	1	0.8484	0	0	0	1	Ref
MG945206	Unassigned	Unassigned	Unassigned	1077_0	Clustered	48	VC_1077_0	48	0.8525	1	0.8525	0	0	0	1	Ref
MG945207	Unassigned	Unassigned	Unassigned	1104_0	Clustered	154	VC_1104_0	154	0.8484	1	0.8484	0	0	0	1	Ref
MG945208	Unassigned	Unassigned	Unassigned	1259_0	Clustered	8	VC_1259_0	8	0.8225	1	0.8225	0	0	0	1	Ref
MG945209	Unassigned	Unassigned	Unassigned	1267_0	Clustered	7	VC_1267_0	7	1	1	1	0	0	0	1	Ref
MG945210	Unassigned	Unassigned	Unassigned	1194_0	Clustered	5	VC_1194_0	5	0.5236	0.99999899	0.5236	0	0	0	1	Ref
MG945211	Unassigned	Unassigned	Unassigned	1110_0	Clustered	8	VC_1110_0	8	0.6915	1	0.6915	0	0	0	1	Ref
MG945212	Unassigned	Unassigned	Unassigned	1268_0	Clustered	3	VC_1268_0	3	0.0807	0.6862532	0.0554	0	0	0	1	Ref
MG945213	Unassigned	Unassigned	Unassigned	1104_0	Clustered	154	VC_1104_0	154	0.8484	1	0.8484	0	0	0	1	Ref
MG945214	Unassigned	Unassigned	Unassigned	1270_0	Clustered	3	VC_1270_0	3	0.6808	0.98688028	0.6719	0	0	0	1	Ref
MG945215	Unassigned	Unassigned	Unassigned	672_0	Clustered	12	VC_672_0	12	1	1	1	0	0	0	1	Ref
MG945227	Unassigned	Unassigned	Unassigned	1104_0	Clustered	154	VC_1104_0	154	0.8484	1	0.8484	0	0	0	1	Ref
MG945228	Unassigned	Unassigned	Unassigned	1151_0	Clustered	9	VC_1151_0	9	0.8548	1	0.8548	0	0	0	1	Ref
MG945229	Unassigned	Unassigned	Unassigned	1251_0	Clustered	15	VC_1251_0	15	0.9082	1	0.9082	0	0	0	1	Ref
MG945230	Unassigned	Unassigned	Unassigned	1271_0	Clustered	2	VC_1271_0	2	1	1	1	0	0	0	1	Ref
MG945231	Unassigned	Unassigned	Unassigned	1128_0	Clustered	2	VC_1128_0	2	0.0904	0.95143313	0.086	0	0	0	1	Ref
MG945232	Unassigned	Unassigned	Unassigned	1110_0	Clustered	8	VC_1110_0	8	0.6915	1	0.6915	0	0	0	1	Ref
MG945233	Unassigned	Unassigned	Unassigned	1127_0	Clustered	19	VC_1127_0	19	0.7611	1	0.7611	0	0	0	1	Ref
MG945234	Unassigned	Unassigned	Unassigned	1146_0	Clustered	3	VC_1146_0	3	0.3641	0.99867077	0.3636	0	0	0	1	Ref
MG945235	Unassigned	Unassigned	Unassigned	1272_0	Clustered	5	VC_1272_0	5	1	1	1	0	0	0	1	Ref
MG945236	Unassigned	Unassigned	Unassigned	1273_0	Clustered	6	VC_1273_0	6	0.7385	0.9999804	0.7385	0	0	0	1	Ref
MG945237	Unassigned	Unassigned	Unassigned	1104_0	Clustered	154	VC_1104_0	154	0.8484	1	0.8484	0	0	0	1	Ref
MG945238	Unassigned	Unassigned	Unassigned	1104_0	Clustered	154	VC_1104_0	154	0.8484	1	0.8484	0	0	0	1	Ref
MG945241	Unassigned	Unassigned	Unassigned	1256_0	Clustered	4	VC_1256_0	4	0.581	0.94751743	0.5505	0	0	0	1	Ref
MG945252	Unassigned	Unassigned	Unassigned	1275_0	Clustered	4	VC_1275_0	4	1	1	1	0	0	0	1	Ref
MG945254	Unassigned	Unassigned	Unassigned	743_0	Clustered	15	VC_743_0	15	1	1	1	0	0	0	1	Ref
MG945255	Unassigned	Unassigned	Unassigned	1251_0	Clustered	15	VC_1251_0	15	0.9082	1	0.9082	0	0	0	1	Ref
MG945256	Unassigned	Unassigned	Unassigned	1233_0	Clustered	16	VC_1233_0	16	0.8971	1	0.8971	0	0	0	1	Ref
MG945257	Unassigned	Unassigned	Unassigned	1257_0	Clustered	6	VC_1257_0	6	0.6517	0.99999969	0.6517	0	0	0	1	Ref
MG945258	Unassigned	Unassigned	Unassigned	1279_0	Clustered	4	VC_1279_0	4	0.6058	0.76972092	0.4663	0	0	0	1	Ref
MG945260	Unassigned	Unassigned	Unassigned	1243_0	Clustered	52	VC_1243_0	52	0.9126	0.92708311	0.8461	0	0	0	1	Ref
MG945261	Unassigned	Unassigned	Unassigned	1264_0	Clustered	6	VC_1264_0	6	0.7062	0.92055725	0.6501	0	0	0	1	Ref
MG945263	Unassigned	Unassigned	Unassigned	1267_0	Clustered	7	VC_1267_0	7	1	1	1	0	0	0	1	Ref
MG945275	Unassigned	Unassigned	Unassigned	1266_0	Clustered	5	VC_1266_0	5	1	1	1	0	0	0	1	Ref
MG945276	Unassigned	Unassigned	Unassigned	1256_0	Clustered	4	VC_1256_0	4	0.581	0.94751743	0.5505	0	0	0	1	Ref
MG945278	Unassigned	Unassigned	Unassigned	1281_0	Clustered	7	VC_1281_0	7	0.9212	0.99995294	0.9212	0	0	0	1	Ref
MG945279	Unassigned	Unassigned	Unassigned	1267_0	Clustered	7	VC_1267_0	7	1	1	1	0	0	0	1	Ref
MG945280	Unassigned	Unassigned	Unassigned	1282_0	Clustered	3	VC_1282_0	3	1	1	1	0	0	0	1	Ref
MG945282	Unassigned	Unassigned	Unassigned	1243_0	Clustered	52	VC_1243_0	52	0.9126	0.92708311	0.8461	0	0	0	1	Ref
MG945284	Unassigned	Unassigned	Unassigned	1197_0	Clustered	15	VC_1197_0	15	0.8991	0.76608394	0.6888	0	0	0	1	Ref
MG945285	Unassigned	Unassigned	Unassigned	1281_0	Clustered	7	VC_1281_0	7	0.9212	0.99995294	0.9212	0	0	0	1	Ref
MG945286	Unassigned	Unassigned	Unassigned	1125_0	Clustered	20	VC_1125_0	20	0.5599	1	0.5599	0	0	0	1	Ref
MG945309	Unassigned	Unassigned	Unassigned	1197_0	Clustered	15	VC_1197_0	15	0.8991	0.76608394	0.6888	0	0	0	1	Ref
MG945314	Unassigned	Unassigned	Unassigned	1263_0	Clustered	2	VC_1263_0	2	1	1	1	0	0	0	1	Ref
MG945316	Unassigned	Unassigned	Unassigned	1285_0	Clustered	25	VC_1285_0	25	1	1	1	0	0	0	1	Ref
MG945318	Unassigned	Unassigned	Unassigned	1104_0	Clustered	154	VC_1104_0	154	0.8484	1	0.8484	0	0	0	1	Ref
MG945319	Unassigned	Unassigned	Unassigned	1125_0	Clustered	20	VC_1125_0	20	0.5599	1	0.5599	0	0	0	1	Ref
MG945320	Unassigned	Unassigned	Unassigned	1139_0	Clustered	4	VC_1139_0	4	1	1	1	0	0	0	1	Ref
MG945323	Unassigned	Unassigned	Unassigned	1251_0	Clustered	15	VC_1251_0	15	0.9082	1	0.9082	0	0	0	1	Ref
MG945324	Unassigned	Unassigned	Unassigned	198_0	Clustered	28	VC_198_0	28	0.2429	0.99971175	0.2429	0	0	0	1	Ref
MG945325	Unassigned	Unassigned	Unassigned	1104_0	Clustered	154	VC_1104_0	154	0.8484	1	0.8484	0	0	0	1	Ref
MG945327	Unassigned	Unassigned	Unassigned	1194_0	Clustered	5	VC_1194_0	5	0.5236	0.99999899	0.5236	0	0	0	1	Ref
MG945328	Unassigned	Unassigned	Unassigned	1286_0	Clustered/Singleton	1	VC_1286_0	1	0	1	0	0	0	0	1	Ref
MG945329	Unassigned	Unassigned	Unassigned	1259_0	Clustered	8	VC_1259_0	8	0.8225	1	0.8225	0	0	0	1	Ref
MG945330	Unassigned	Unassigned	Unassigned	1104_0	Clustered	154	VC_1104_0	154	0.8484	1	0.8484	0	0	0	1	Ref
MG945331	Unassigned	Unassigned	Unassigned	1287_0	Clustered	7	VC_1287_0	7	1	1	1	0	0	0	1	Ref
MG945332	Unassigned	Unassigned	Unassigned	1251_0	Clustered	15	VC_1251_0	15	0.9082	1	0.9082	0	0	0	1	Ref
MG945333	Unassigned	Unassigned	Unassigned	1104_0	Clustered	154	VC_1104_0	154	0.8484	1	0.8484	0	0	0	1	Ref
MG945334	Unassigned	Unassigned	Unassigned	1127_0	Clustered	19	VC_1127_0	19	0.7611	1	0.7611	0	0	0	1	Ref
MG945335	Unassigned	Unassigned	Unassigned	1288_0	Clustered	3	VC_1288_0	3	1	1	1	0	0	0	1	Ref
MG945336	Unassigned	Unassigned	Unassigned	1289_0	Clustered	2	VC_1289_0	2	0.4661	0.85557782	0.3987	0	0	0	1	Ref
MG945337	Unassigned	Unassigned	Unassigned	1290_0	Clustered	2	VC_1290_0	2	1	1	1	0	0	0	1	Ref
MG945338	Unassigned	Unassigned	Unassigned	1125_0	Clustered	20	VC_1125_0	20	0.5599	1	0.5599	0	0	0	1	Ref
MG945339	Unassigned	Unassigned	Unassigned	1116_0	Clustered	14	VC_1116_0	14	0.7927	1	0.7927	0	0	0	1	Ref
MG945341	Unassigned	Unassigned	Unassigned	672_0	Clustered	12	VC_672_0	12	1	1	1	0	0	0	1	Ref
MG945343	Unassigned	Unassigned	Unassigned	1287_0	Clustered	7	VC_1287_0	7	1	1	1	0	0	0	1	Ref
MG945344	Unassigned	Unassigned	Unassigned	1285_0	Clustered	25	VC_1285_0	25	1	1	1	0	0	0	1	Ref
MG945345	Unassigned	Unassigned	Unassigned	1291_0	Clustered	2	VC_1291_0	2	1	1	1	0	0	0	1	Ref
MG945346	Unassigned	Unassigned	Unassigned	1104_0	Clustered	154	VC_1104_0	154	0.8484	1	0.8484	0	0	0	1	Ref

MG945347	Unassigned	Unassigned	Unassigned	1125_0	Clustered	20	VC_1125_0	20	0.5599	1	0.5599	0	0	0	1	Ref
MG945348	Unassigned	Unassigned	Unassigned	1292_0	Clustered	3	VC_1292_0	3	1	1	1	0	0	0	1	Ref
MG945349	Unassigned	Unassigned	Unassigned	1261_0	Clustered	5	VC_1261_0	5	0.765	0.99993003	0.7649	0	0	0	1	Ref
MG945350	Unassigned	Unassigned	Unassigned	1293_0	Clustered	4	VC_1293_0	4	0.7847	0.99289298	0.7791	0	0	0	1	Ref
MG945351	Unassigned	Unassigned	Unassigned	1104_0	Clustered	154	VC_1104_0	154	0.8484	1	0.8484	0	0	0	1	Ref
MG945352	Unassigned	Unassigned	Unassigned	1193_0	Clustered	3	VC_1193_0	3	0.5473	0.99523779	0.5447	0	0	0	1	Ref
MG945354	Unassigned	Unassigned	Unassigned	1294_0	Clustered	6	VC_1294_0	6	0.6626	0.99999272	0.6626	0	0	0	1	Ref
MG945355	Unassigned	Unassigned	Unassigned	1104_0	Clustered	154	VC_1104_0	154	0.8484	1	0.8484	0	0	0	1	Ref
MG945357	Unassigned	Unassigned	Unassigned	1148_0	Clustered	16	VC_1148_0	16	0.8821	1	0.8821	0	0	0	1	Ref
MG945360	Unassigned	Unassigned	Unassigned	1295_0	Clustered	3	VC_1295_0	3	1	0	0	0	0	0	1	Ref
MG945361	Unassigned	Unassigned	Unassigned	1104_0	Clustered	154	VC_1104_0	154	0.8484	1	0.8484	0	0	0	1	Ref
MG945362	Unassigned	Unassigned	Unassigned	1296_0	Clustered	4	VC_1296_0	4	0.8684	0.91151676	0.7915	0	0	0	1	Ref
MG945363	Unassigned	Unassigned	Unassigned	1104_0	Clustered	154	VC_1104_0	154	0.8484	1	0.8484	0	0	0	1	Ref
MG945364	Unassigned	Unassigned	Unassigned	1144_0	Clustered	7	VC_1144_0	7	0.7076	1	0.7076	0	0	0	1	Ref
MG945365	Unassigned	Unassigned	Unassigned	1259_0	Clustered	8	VC_1259_0	8	0.8225	1	0.8225	0	0	0	1	Ref
MG945381	Unassigned	Unassigned	Unassigned	1126_0	Clustered	8	VC_1126_0	8	0.2864	1	0.2864	0	0	0	1	Ref
MG945382	Unassigned	Unassigned	Unassigned	1126_0	Clustered	8	VC_1126_0	8	0.2864	1	0.2864	0	0	0	1	Ref
MG945383	Unassigned	Unassigned	Unassigned	1127_0	Clustered	19	VC_1127_0	19	0.7611	1	0.7611	0	0	0	1	Ref
MG945384	Unassigned	Unassigned	Unassigned	1247_0	Clustered	7	VC_1247_0	7	0.9086	0.99998959	0.9086	0	0	0	1	Ref
MG945385	Unassigned	Unassigned	Unassigned	1125_0	Clustered	20	VC_1125_0	20	0.5599	1	0.5599	0	0	0	1	Ref
MG945386	Unassigned	Unassigned	Unassigned	1108_0	Clustered	4	VC_1108_0	4	0.0485	0.99999021	0.0485	0	0	0	1	Ref
MG945387	Unassigned	Unassigned	Unassigned	1104_0	Clustered	154	VC_1104_0	154	0.8484	1	0.8484	0	0	0	1	Ref
MG945388	Unassigned	Unassigned	Unassigned	1127_0	Clustered	19	VC_1127_0	19	0.7611	1	0.7611	0	0	0	1	Ref
MG945389	Unassigned	Unassigned	Unassigned	1125_0	Clustered	20	VC_1125_0	20	0.5599	1	0.5599	0	0	0	1	Ref
MG945390	Unassigned	Unassigned	Unassigned	1243_0	Clustered	52	VC_1243_0	52	0.9126	0.92708311	0.8461	0	0	0	1	Ref
MG945391	Unassigned	Unassigned	Unassigned	1124_0	Clustered	10	VC_1124_0	10	0.6268	1	0.6268	0	0	0	1	Ref
MG945392	Unassigned	Unassigned	Unassigned	1272_0	Clustered	5	VC_1272_0	5	1	1	1	0	0	0	1	Ref
MG945393	Unassigned	Unassigned	Unassigned	1104_0	Clustered	154	VC_1104_0	154	0.8484	1	0.8484	0	0	0	1	Ref
MG945394	Unassigned	Unassigned	Unassigned	1116_0	Clustered	14	VC_1116_0	14	0.7927	1	0.7927	0	0	0	1	Ref
MG945395	Unassigned	Unassigned	Unassigned	1297_0	Clustered	7	VC_1297_0	7	1	1	1	0	0	0	1	Ref
MG945396	Unassigned	Unassigned	Unassigned	1243_0	Clustered	52	VC_1243_0	52	0.9126	0.92708311	0.8461	0	0	0	1	Ref
MG945397	Unassigned	Unassigned	Unassigned	1298_0	Clustered	3	VC_1298_0	3	0.4889	0.98167473	0.4799	0	0	0	1	Ref
MG945398	Unassigned	Unassigned	Unassigned	1104_0	Clustered	154	VC_1104_0	154	0.8484	1	0.8484	0	0	0	1	Ref
MG945401	Unassigned	Unassigned	Unassigned	1251_0	Clustered	15	VC_1251_0	15	0.9082	1	0.9082	0	0	0	1	Ref
MG945402	Unassigned	Unassigned	Unassigned	1104_0	Clustered	154	VC_1104_0	154	0.8484	1	0.8484	0	0	0	1	Ref
MG945403	Unassigned	Unassigned	Unassigned	1116_0	Clustered	14	VC_1116_0	14	0.7927	1	0.7927	0	0	0	1	Ref
MG945404	Unassigned	Unassigned	Unassigned	1267_0	Clustered	7	VC_1267_0	7	1	1	1	0	0	0	1	Ref
MG945405	Unassigned	Unassigned	Unassigned	1146_0	Clustered	3	VC_1146_0	3	0.3641	0.99867077	0.3636	0	0	0	1	Ref
MG945407	Unassigned	Unassigned	Unassigned	1104_0	Clustered	154	VC_1104_0	154	0.8484	1	0.8484	0	0	0	1	Ref
MG945408	Unassigned	Unassigned	Unassigned	1266_0	Clustered	5	VC_1266_0	5	1	1	1	0	0	0	1	Ref
MG945409	Unassigned	Unassigned	Unassigned	1299_0	Clustered	2	VC_1299_0	2	1	1	1	0	0	0	1	Ref
MG945410	Unassigned	Unassigned	Unassigned	1125_0	Clustered	20	VC_1125_0	20	0.5599	1	0.5599	0	0	0	1	Ref
MG945411	Unassigned	Unassigned	Unassigned	1261_0	Clustered	5	VC_1261_0	5	0.765	0.99993003	0.7649	0	0	0	1	Ref
MG945412	Unassigned	Unassigned	Unassigned	1300_0	Clustered	2	VC_1300_0	2	0.664	0.72985431	0.4846	0	0	0	1	Ref
MG945413	Unassigned	Unassigned	Unassigned	1148_0	Clustered	16	VC_1148_0	16	0.8821	1	0.8821	0	0	0	1	Ref
MG945414	Unassigned	Unassigned	Unassigned	1253_0	Clustered	6	VC_1253_0	6	0.6097	1	0.6097	0	0	0	1	Ref
MG945415	Unassigned	Unassigned	Unassigned	1104_0	Clustered	154	VC_1104_0	154	0.8484	1	0.8484	0	0	0	1	Ref
MG945416	Unassigned	Unassigned	Unassigned	1125_0	Clustered	20	VC_1125_0	20	0.5599	1	0.5599	0	0	0	1	Ref
MG945417	Unassigned	Unassigned	Unassigned	1301_0	Clustered	2	VC_1301_0	2	1	1	1	0	0	0	1	Ref
MG945418	Unassigned	Unassigned	Unassigned	1154_0	Clustered	5	VC_1154_0	5	1	1	1	0	0	0	1	Ref
MG945419	Unassigned	Unassigned	Unassigned	1301_0	Clustered	2	VC_1301_0	2	1	1	1	0	0	0	1	Ref
MG945420	Unassigned	Unassigned	Unassigned	1264_0	Clustered	6	VC_1264_0	6	0.7062	0.92055725	0.6501	0	0	0	1	Ref
MG945421	Unassigned	Unassigned	Unassigned	1297_0	Clustered	7	VC_1297_0	7	1	1	1	0	0	0	1	Ref
MG945424	Unassigned	Unassigned	Unassigned	1104_0	Clustered	154	VC_1104_0	154	0.8484	1	0.8484	0	0	0	1	Ref
MG945425	Unassigned	Unassigned	Unassigned	1278_0	Clustered	3	VC_1278_0	3	0.3961	0.99705286	0.395	0	0	0	1	Ref
MG945426	Unassigned	Unassigned	Unassigned	1151_0	Clustered	9	VC_1151_0	9	0.8548	1	0.8548	0	0	0	1	Ref
MG945427	Unassigned	Unassigned	Unassigned	1302_0	Clustered	3	VC_1302_0	3	1	1	1	0	0	0	1	Ref
MG945429	Unassigned	Unassigned	Unassigned	1104_0	Clustered	154	VC_1104_0	154	0.8484	1	0.8484	0	0	0	1	Ref
MG945430	Unassigned	Unassigned	Unassigned	1127_0	Clustered	19	VC_1127_0	19	0.7611	1	0.7611	0	0	0	1	Ref
MG945432	Unassigned	Unassigned	Unassigned	1293_0	Clustered	4	VC_1293_0	4	0.7847	0.99289298	0.7791	0	0	0	1	Ref
MG945433	Unassigned	Unassigned	Unassigned	1197_0	Clustered	15	VC_1197_0	15	0.8991	0.76608394	0.6888	0	0	0	1	Ref
MG945434	Unassigned	Unassigned	Unassigned	1279_0	Clustered	4	VC_1279_0	4	0.6058	0.76972092	0.4663	0	0	0	1	Ref
MG945435	Unassigned	Unassigned	Unassigned	1104_0	Clustered	154	VC_1104_0	154	0.8484	1	0.8484	0	0	0	1	Ref
MG945437	Unassigned	Unassigned	Unassigned	1125_0	Clustered	20	VC_1125_0	20	0.5599	1	0.5599	0	0	0	1	Ref
MG945438	Unassigned	Unassigned	Unassigned	1104_0	Clustered	154	VC_1104_0	154	0.8484	1	0.8484	0	0	0	1	Ref
MG945439	Unassigned	Unassigned	Unassigned	1303_0	Clustered	6	VC_1303_0	6	1	1	1	0	0	0	1	Ref
MG945441	Unassigned	Unassigned	Unassigned	1149_0	Clustered	5	VC_1149_0	5	0.4768	0.9999998	0.4768	0	0	0	1	Ref
MG945444	Unassigned	Unassigned	Unassigned	1104_0	Clustered	154	VC_1104_0	154	0.8484	1	0.8484	0	0	0	1	Ref
MG945445	Unassigned	Unassigned	Unassigned	1253_0	Clustered	6	VC_1253_0	6	0.6097	1	0.6097	0	0	0	1	Ref
MG945447	Unassigned	Unassigned	Unassigned	1127_0	Clustered	19	VC_1127_0	19	0.7611	1	0.7611	0	0	0	1	Ref
MG945448	Unassigned	Unassigned	Unassigned	1256_0	Clustered	4	VC_1256_0	4	0.581	0.94751743	0.5505	0	0	0	1	Ref
MG945449	Unassigned	Unassigned	Unassigned	1257_0	Clustered	6	VC_1257_0	6	0.6517	0.99999969	0.6517	0	0	0	1	Ref
MG945452	Unassigned	Unassigned	Unassigned	1243_0	Clustered	52	VC_1243_0	52	0.9126	0.92708311	0.8461	0	0	0	1	Ref
MG945453	Unassigned	Unassigned	Unassigned	1211_0	Clustered	25	VC_1211_0	25	1	1	1	0	0	0	1	Ref
MG945455	Unassigned	Unassigned	Unassigned	1211_0	Clustered	25	VC_1211_0	25	1	1	1	0	0	0	1	Ref
MG945456	Unassigned	Unassigned	Unassigned	1211_0	Clustered	25	VC_1211_0	25	1	1	1	0	0	0	1	Ref
MG945457	Unassigned	Unassigned	Unassigned	1233_0	Clustered	16	VC_1233_0	16	0.8971	1	0.8971	0	0	0	1	Ref
MG945458	Unassigned	Unassigned	Unassigned	1257_0	Clustered	6	VC_1257_0	6	0.6517	0.99999969	0.6517	0	0	0	1	Ref
MG945459	Unassigned	Unassigned	Unassigned	1104_0	Clustered	154	VC_1104_0	154	0.8484	1	0.8484	0	0	0	1	Ref
MG945460	Unassigned	Unassigned	Unassigned	1255_0	Clustered	4	VC_1255_0	4	0.248	0.99828153	0.2476	0	0	0	1	Ref
MG945461	Unassigned	Unassigned	Unassigned	1104_0	Clustered	154	VC_1104_0	154	0.8484	1	0.8484	0	0	0	1	Ref

MG945462	Unassigned	Unassigned	Unassigned	1261_0	Clustered	5	VC_1261_0	5	0.765	0.99993003	0.7649	0	0	0	1	Ref
MG945463	Unassigned	Unassigned	Unassigned	1062_0	Clustered	45	VC_1062_0	45	0.9734	0.50949847	0.4959	0	0	0	1	Ref
MG945464	Unassigned	Unassigned	Unassigned	1245_0	Clustered	2	VC_1245_0	2	1	1	1	0	0	0	1	Ref
MG945465	Unassigned	Unassigned	Unassigned	995_0	Clustered	69	VC_995_0	69	0.8547	1	0.8547	0	0	0	1	Ref
MG945467	Unassigned	Unassigned	Unassigned	1104_0	Clustered	154	VC_1104_0	154	0.8484	1	0.8484	0	0	0	1	Ref
MG945468	Unassigned	Unassigned	Unassigned	1285_0	Clustered	25	VC_1285_0	25	1	1	1	0	0	0	1	Ref
MG945469	Unassigned	Unassigned	Unassigned	1305_0	Clustered	5	VC_1305_0	5	0.7701	0.66985041	0.5159	0	0	0	1	Ref
MG945470	Unassigned	Unassigned	Unassigned	1264_0	Clustered	6	VC_1264_0	6	0.7062	0.92055725	0.6501	0	0	0	1	Ref
MG945472	Unassigned	Unassigned	Unassigned	1119_0	Clustered	20	VC_1119_0	20	0.6751	0.99997892	0.6751	0	0	0	1	Ref
MG945473	Unassigned	Unassigned	Unassigned	1054_0	Clustered	12	VC_1054_0	12	0.2863	0.99746644	0.2855	0	0	0	1	Ref
MG945475	Unassigned	Unassigned	Unassigned	1085_0	Clustered	2	VC_1085_0	2	0.5907	0.9331928	0.5513	0	0	0	1	Ref
MG945476	Unassigned	Unassigned	Unassigned	1104_0	Clustered	154	VC_1104_0	154	0.8484	1	0.8484	0	0	0	1	Ref
MG945477	Unassigned	Unassigned	Unassigned	995_0	Clustered	69	VC_995_0	69	0.8547	1	0.8547	0	0	0	1	Ref
MG945478	Unassigned	Unassigned	Unassigned	1295_0	Clustered	3	VC_1295_0	3	1	0	0	0	0	0	1	Ref
MG945480	Unassigned	Unassigned	Unassigned	1297_0	Clustered	7	VC_1297_0	7	1	1	1	0	0	0	1	Ref
MG945481	Unassigned	Unassigned	Unassigned	1117_0	Clustered	5	VC_1117_0	5	0.4688	0.99999974	0.4688	0	0	0	1	Ref
MG945482	Unassigned	Unassigned	Unassigned	1247_0	Clustered	7	VC_1247_0	7	0.9086	0.99998959	0.9086	0	0	0	1	Ref
MG945483	Unassigned	Unassigned	Unassigned	1306_0	Clustered	2	VC_1306_0	2	1	1	1	0	0	0	1	Ref
MG945485	Unassigned	Unassigned	Unassigned	1243_0	Clustered	52	VC_1243_0	52	0.9126	0.92708311	0.8461	0	0	0	1	Ref
MG945486	Unassigned	Unassigned	Unassigned	1104_0	Clustered	154	VC_1104_0	154	0.8484	1	0.8484	0	0	0	1	Ref
MG945487	Unassigned	Unassigned	Unassigned	1149_0	Clustered	5	VC_1149_0	5	0.4768	0.9999998	0.4768	0	0	0	1	Ref
MG945488	Unassigned	Unassigned	Unassigned	1276_0	Clustered	7	VC_1276_0	7	0.8695	0.99982476	0.8693	0	0	0	1	Ref
MG945489	Unassigned	Unassigned	Unassigned	1111_0	Clustered	2	VC_1111_0	2	0.2635	0.89847992	0.2367	0	0	0	1	Ref
MG945490	Unassigned	Unassigned	Unassigned	1287_0	Clustered	7	VC_1287_0	7	1	1	1	0	0	0	1	Ref
MG945492	Unassigned	Unassigned	Unassigned	1281_0	Clustered	7	VC_1281_0	7	0.9212	0.99995294	0.9212	0	0	0	1	Ref
MG945494	Unassigned	Unassigned	Unassigned	1277_0	Clustered	14	VC_1277_0	14	0.8218	1	0.8218	0	0	0	1	Ref
MG945495	Unassigned	Unassigned	Unassigned	1261_0	Clustered	5	VC_1261_0	5	0.765	0.99993003	0.7649	0	0	0	1	Ref
MG945496	Unassigned	Unassigned	Unassigned	1255_0	Clustered	4	VC_1255_0	4	0.248	0.99828153	0.2476	0	0	0	1	Ref
MG945498	Unassigned	Unassigned	Unassigned	1211_0	Clustered	25	VC_1211_0	25	1	1	1	0	0	0	1	Ref
MG945499	Unassigned	Unassigned	Unassigned	1104_0	Clustered	154	VC_1104_0	154	0.8484	1	0.8484	0	0	0	1	Ref
MG945500	Unassigned	Unassigned	Unassigned	1308_0	Clustered	12	VC_1308_0	12	0.8184	0.9810731	0.8029	0	0	0	1	Ref
MG945501	Unassigned	Unassigned	Unassigned	1287_0	Clustered	7	VC_1287_0	7	1	1	1	0	0	0	1	Ref
MG945502	Unassigned	Unassigned	Unassigned	1197_0	Clustered	15	VC_1197_0	15	0.8991	0.76608394	0.6888	0	0	0	1	Ref
MG945504	Unassigned	Unassigned	Unassigned	1310_0	Clustered	2	VC_1310_0	2	1	1	1	0	0	0	1	Ref
MG945505	Unassigned	Unassigned	Unassigned	1149_0	Clustered	5	VC_1149_0	5	0.4768	0.9999998	0.4768	0	0	0	1	Ref
MG945506	Unassigned	Unassigned	Unassigned	1148_0	Clustered	16	VC_1148_0	16	0.8821	1	0.8821	0	0	0	1	Ref
MG945507	Unassigned	Unassigned	Unassigned	1148_0	Clustered	16	VC_1148_0	16	0.8821	1	0.8821	0	0	0	1	Ref
MG945508	Unassigned	Unassigned	Unassigned	1104_0	Clustered	154	VC_1104_0	154	0.8484	1	0.8484	0	0	0	1	Ref
MG945509	Unassigned	Unassigned	Unassigned	1211_0	Clustered	25	VC_1211_0	25	1	1	1	0	0	0	1	Ref
MG945511	Unassigned	Unassigned	Unassigned	1243_0	Clustered	52	VC_1243_0	52	0.9126	0.92708311	0.8461	0	0	0	1	Ref
MG945512	Unassigned	Unassigned	Unassigned	1259_0	Clustered	8	VC_1259_0	8	0.8225	1	0.8225	0	0	0	1	Ref
MG945514	Unassigned	Unassigned	Unassigned	1127_0	Clustered	19	VC_1127_0	19	0.7611	1	0.7611	0	0	0	1	Ref
MG945515	Unassigned	Unassigned	Unassigned	1243_0	Clustered	52	VC_1243_0	52	0.9126	0.92708311	0.8461	0	0	0	1	Ref
MG945517	Unassigned	Unassigned	Unassigned	1104_0	Clustered	154	VC_1104_0	154	0.8484	1	0.8484	0	0	0	1	Ref
MG945518	Unassigned	Unassigned	Unassigned	1264_0	Clustered	6	VC_1264_0	6	0.7062	0.92055725	0.6501	0	0	0	1	Ref
MG945519	Unassigned	Unassigned	Unassigned	1138_0	Clustered	4	VC_1138_0	4	0.9437	0.98964986	0.934	0	0	0	1	Ref
MG945520	Unassigned	Unassigned	Unassigned	672_0	Clustered	12	VC_672_0	12	1	1	1	0	0	0	1	Ref
MG945523	Unassigned	Unassigned	Unassigned	1230_0	Clustered	3	VC_1230_0	3	0.4802	0.82711071	0.3971	0	0	0	1	Ref
MG945524	Unassigned	Unassigned	Unassigned	1285_0	Clustered	25	VC_1285_0	25	1	1	1	0	0	0	1	Ref
MG945525	Unassigned	Unassigned	Unassigned	1144_0	Clustered	7	VC_1144_0	7	0.7076	1	0.7076	0	0	0	1	Ref
MG945526	Unassigned	Unassigned	Unassigned	1104_0	Clustered	154	VC_1104_0	154	0.8484	1	0.8484	0	0	0	1	Ref
MG945529	Unassigned	Unassigned	Unassigned	1311_0	Clustered	5	VC_1311_0	5	0.8316	0.99879053	0.8306	0	0	0	1	Ref
MG945533	Unassigned	Unassigned	Unassigned	1312_0	Clustered	3	VC_1312_0	3	1	1	1	0	0	0	1	Ref
MG945534	Unassigned	Unassigned	Unassigned	1119_0	Clustered	20	VC_1119_0	20	0.6751	0.99997892	0.6751	0	0	0	1	Ref
MG945538	Unassigned	Unassigned	Unassigned	1125_0	Clustered	20	VC_1125_0	20	0.5599	1	0.5599	0	0	0	1	Ref
MG945539	Unassigned	Unassigned	Unassigned	1297_0	Clustered	7	VC_1297_0	7	1	1	1	0	0	0	1	Ref
MG945540	Unassigned	Unassigned	Unassigned	1259_0	Clustered	8	VC_1259_0	8	0.8225	1	0.8225	0	0	0	1	Ref
MG945541	Unassigned	Unassigned	Unassigned	1104_0	Clustered	154	VC_1104_0	154	0.8484	1	0.8484	0	0	0	1	Ref
MG945542	Unassigned	Unassigned	Unassigned	1116_0	Clustered	14	VC_1116_0	14	0.7927	1	0.7927	0	0	0	1	Ref
MG945544	Unassigned	Unassigned	Unassigned	1252_0	Clustered	3	VC_1252_0	3	0.3237	0.99790393	0.323	0	0	0	1	Ref
MG945546	Unassigned	Unassigned	Unassigned	198_0	Clustered	28	VC_198_0	28	0.2429	0.99971175	0.2429	0	0	0	1	Ref
MG945548	Unassigned	Unassigned	Unassigned	1104_0	Clustered	154	VC_1104_0	154	0.8484	1	0.8484	0	0	0	1	Ref
MG945549	Unassigned	Unassigned	Unassigned	1154_0	Clustered	5	VC_1154_0	5	1	1	1	0	0	0	1	Ref
MG945550	Unassigned	Unassigned	Unassigned	1116_0	Clustered	14	VC_1116_0	14	0.7927	1	0.7927	0	0	0	1	Ref
MG945551	Unassigned	Unassigned	Unassigned	672_0	Clustered	12	VC_672_0	12	1	1	1	0	0	0	1	Ref
MG945554	Unassigned	Unassigned	Unassigned	1319_0	Clustered	2	VC_1319_0	2	1	1	1	0	0	0	1	Ref
MG945556	Unassigned	Unassigned	Unassigned	1285_0	Clustered	25	VC_1285_0	25	1	1	1	0	0	0	1	Ref
MG945557	Unassigned	Unassigned	Unassigned	1233_0	Clustered	16	VC_1233_0	16	0.8971	1	0.8971	0	0	0	1	Ref
MG945559	Unassigned	Unassigned	Unassigned	1104_0	Clustered	154	VC_1104_0	154	0.8484	1	0.8484	0	0	0	1	Ref
MG945560	Unassigned	Unassigned	Unassigned	1056_0	Clustered	4	VC_1056_0	4	0.169	0.84110627	0.1421	0	0	0	1	Ref
MG945561	Unassigned	Unassigned	Unassigned	1139_0	Clustered	4	VC_1139_0	4	1	1	1	0	0	0	1	Ref
MG945562	Unassigned	Unassigned	Unassigned	1285_0	Clustered	25	VC_1285_0	25	1	1	1	0	0	0	1	Ref
MG945563	Unassigned	Unassigned	Unassigned	1277_0	Clustered	14	VC_1277_0	14	0.8218	1	0.8218	0	0	0	1	Ref
MG945564	Unassigned	Unassigned	Unassigned	1211_0	Clustered	25	VC_1211_0	25	1	1	1	0	0	0	1	Ref
MG945565	Unassigned	Unassigned	Unassigned	1104_0	Clustered	154	VC_1104_0	154	0.8484	1	0.8484	0	0	0	1	Ref
MG945566	Unassigned	Unassigned	Unassigned	1104_0	Clustered	154	VC_1104_0	154	0.8484	1	0.8484	0	0	0	1	Ref
MG945567	Unassigned	Unassigned	Unassigned	1274_0	Clustered	2	VC_1274_0	2	0.6386	0.72985431	0.4661	0	0	0	1	Ref
MG945568	Unassigned	Unassigned	Unassigned	1311_0	Clustered	5	VC_1311_0	5	0.8316	0.99879053	0.8306	0	0	0	1	Ref
MG945569	Unassigned	Unassigned	Unassigned	1122_0	Clustered	10	VC_1122_0	10	0.9643	0.99999697	0.9643	0	0	0	1	Ref
MG945570	Unassigned	Unassigned	Unassigned	1104_0	Clustered	154	VC_1104_0	154	0.8484	1	0.8484	0	0	0	1	Ref
MG945571	Unassigned	Unassigned	Unassigned	198_0	Clustered	28	VC_198_0	28	0.2429	0.99971175	0.2429	0	0	0	1	Ref

MG945572	Unassigned	Unassigned	Unassigned	1276_0	Clustered	7	VC_1276_0	7	0.8695	0.99982476	0.8693	0	0	0	1	Ref
MG945573	Unassigned	Unassigned	Unassigned	1143_0	Clustered	9	VC_1143_0	9	0.7268	1	0.7268	0	0	0	1	Ref
MG945574	Unassigned	Unassigned	Unassigned	1302_0	Clustered	3	VC_1302_0	3	1	1	1	0	0	0	1	Ref
MG945575	Unassigned	Unassigned	Unassigned	1319_0	Clustered	2	VC_1319_0	2	1	1	1	0	0	0	1	Ref
MG945576	Unassigned	Unassigned	Unassigned	1294_0	Clustered	6	VC_1294_0	6	0.6626	0.99999272	0.6626	0	0	0	1	Ref
MG945578	Unassigned	Unassigned	Unassigned	1253_0	Clustered	6	VC_1253_0	6	0.6097	1	0.6097	0	0	0	1	Ref
MG945579	Unassigned	Unassigned	Unassigned	1285_0	Clustered	25	VC_1285_0	25	1	1	1	0	0	0	1	Ref
MG945580	Unassigned	Unassigned	Unassigned	1258_0	Clustered	3	VC_1258_0	3	0.2422	0.85925819	0.2081	0	0	0	1	Ref
MG945582	Unassigned	Unassigned	Unassigned	1268_0	Clustered	3	VC_1268_0	3	0.0807	0.6862532	0.0554	0	0	0	1	Ref
MG945584	Unassigned	Unassigned	Unassigned	1275_0	Clustered	4	VC_1275_0	4	1	1	1	0	0	0	1	Ref
MG945585	Unassigned	Unassigned	Unassigned	1277_0	Clustered	14	VC_1277_0	14	0.8218	1	0.8218	0	0	0	1	Ref
MG945586	Unassigned	Unassigned	Unassigned	1104_0	Clustered	154	VC_1104_0	154	0.8484	1	0.8484	0	0	0	1	Ref
MG945587	Unassigned	Unassigned	Unassigned	1104_0	Clustered	154	VC_1104_0	154	0.8484	1	0.8484	0	0	0	1	Ref
MG945589	Unassigned	Unassigned	Unassigned	1104_0	Clustered	154	VC_1104_0	154	0.8484	1	0.8484	0	0	0	1	Ref
MG945591	Unassigned	Unassigned	Unassigned	1113_0	Clustered/Singleton	1	VC_1113_0	1	0	1	0	0	0	0	1	Ref
MG945594	Unassigned	Unassigned	Unassigned	1053_0	Clustered/Singleton	1	VC_1053_0	1	0	1	0	0	0	0	1	Ref
MG945596	Unassigned	Unassigned	Unassigned	1104_0	Clustered	154	VC_1104_0	154	0.8484	1	0.8484	0	0	0	1	Ref
MG945597	Unassigned	Unassigned	Unassigned	1105_0	Clustered	3	VC_1105_0	3	0.0755	0.99466875	0.0751	0	0	0	1	Ref
MG945602	Unassigned	Unassigned	Unassigned	1104_0	Clustered	154	VC_1104_0	154	0.8484	1	0.8484	0	0	0	1	Ref
MG945605	Unassigned	Unassigned	Unassigned	1281_0	Clustered	7	VC_1281_0	7	0.9212	0.99995294	0.9212	0	0	0	1	Ref
MG945609	Unassigned	Unassigned	Unassigned	1148_0	Clustered	16	VC_1148_0	16	0.8821	1	0.8821	0	0	0	1	Ref
MG945611	Unassigned	Unassigned	Unassigned	1253_0	Clustered	6	VC_1253_0	6	0.6097	1	0.6097	0	0	0	1	Ref
MG945612	Unassigned	Unassigned	Unassigned	1285_0	Clustered	25	VC_1285_0	25	1	1	1	0	0	0	1	Ref
MG945613	Unassigned	Unassigned	Unassigned	1119_0	Clustered	20	VC_1119_0	20	0.6751	0.99997892	0.6751	0	0	0	1	Ref
MG945614	Unassigned	Unassigned	Unassigned	1295_0	Clustered	3	VC_1295_0	3	1	0	0	0	0	0	1	Ref
MG945615	Unassigned	Unassigned	Unassigned	1322_0	Clustered	2	VC_1322_0	2	1	1	1	0	0	0	1	Ref
MG945616	Unassigned	Unassigned	Unassigned	1197_0	Clustered	15	VC_1197_0	15	0.8991	0.76608394	0.6888	0	0	0	1	Ref
MG945617	Unassigned	Unassigned	Unassigned	1310_0	Clustered	2	VC_1310_0	2	1	1	1	0	0	0	1	Ref
MG945618	Unassigned	Unassigned	Unassigned	1243_0	Clustered	52	VC_1243_0	52	0.9126	0.92708311	0.8461	0	0	0	1	Ref
MG945619	Unassigned	Unassigned	Unassigned	1324_0	Clustered	4	VC_1324_0	4	0.6008	0.54407361	0.3269	0	0	0	1	Ref
MG945620	Unassigned	Unassigned	Unassigned	1137_0	Clustered	3	VC_1137_0	3	0.9176	0.93067968	0.854	0	0	0	1	Ref
MG945621	Unassigned	Unassigned	Unassigned	1308_0	Clustered	12	VC_1308_0	12	0.8184	0.9810731	0.8029	0	0	0	1	Ref
MG945622	Unassigned	Unassigned	Unassigned	1255_0	Clustered	4	VC_1255_0	4	0.248	0.99828153	0.2476	0	0	0	1	Ref
MG945623	Unassigned	Unassigned	Unassigned	1194_0	Clustered	5	VC_1194_0	5	0.5236	0.99999899	0.5236	0	0	0	1	Ref
MG945625	Unassigned	Unassigned	Unassigned	1250_0	Clustered	5	VC_1250_0	5	0.6384	0.99918945	0.6379	0	0	0	1	Ref
MG945628	Unassigned	Unassigned	Unassigned	1303_0	Clustered	6	VC_1303_0	6	1	1	1	0	0	0	1	Ref
MG945629	Unassigned	Unassigned	Unassigned	1250_0	Clustered	5	VC_1250_0	5	0.6384	0.99918945	0.6379	0	0	0	1	Ref
MG945631	Unassigned	Unassigned	Unassigned	1274_0	Clustered	2	VC_1274_0	2	0.6386	0.72985431	0.4661	0	0	0	1	Ref
MG945632	Unassigned	Unassigned	Unassigned	1250_0	Clustered	5	VC_1250_0	5	0.6384	0.99918945	0.6379	0	0	0	1	Ref
MG945633	Unassigned	Unassigned	Unassigned	1250_0	Clustered	5	VC_1250_0	5	0.6384	0.99918945	0.6379	0	0	0	1	Ref
MG945634	Unassigned	Unassigned	Unassigned	1243_0	Clustered	52	VC_1243_0	52	0.9126	0.92708311	0.8461	0	0	0	1	Ref
MG945635	Unassigned	Unassigned	Unassigned	1125_0	Clustered	20	VC_1125_0	20	0.5599	1	0.5599	0	0	0	1	Ref
MG945637	Unassigned	Unassigned	Unassigned	1294_0	Clustered	6	VC_1294_0	6	0.6626	0.99999272	0.6626	0	0	0	1	Ref
MG945639	Unassigned	Unassigned	Unassigned	1125_0	Clustered	20	VC_1125_0	20	0.5599	1	0.5599	0	0	0	1	Ref
MG945640	Unassigned	Unassigned	Unassigned	1285_0	Clustered	25	VC_1285_0	25	1	1	1	0	0	0	1	Ref
MG945641	Unassigned	Unassigned	Unassigned	1243_0	Clustered	52	VC_1243_0	52	0.9126	0.92708311	0.8461	0	0	0	1	Ref
MG945642	Unassigned	Unassigned	Unassigned	1127_0	Clustered	19	VC_1127_0	19	0.7611	1	0.7611	0	0	0	1	Ref
MG945643	Unassigned	Unassigned	Unassigned	1256_0	Clustered	4	VC_1256_0	4	0.581	0.94751743	0.5505	0	0	0	1	Ref
MG945644	Unassigned	Unassigned	Unassigned	1140_0	Clustered	4	VC_1140_0	4	0.1325	0.66207199	0.0877	0	0	0	1	Ref
MG945645	Unassigned	Unassigned	Unassigned	1116_0	Clustered	14	VC_1116_0	14	0.7927	1	0.7927	0	0	0	1	Ref
MG945646	Unassigned	Unassigned	Unassigned	1104_0	Clustered	154	VC_1104_0	154	0.8484	1	0.8484	0	0	0	1	Ref
MG945647	Unassigned	Unassigned	Unassigned	1243_0	Clustered	52	VC_1243_0	52	0.9126	0.92708311	0.8461	0	0	0	1	Ref
MG945662	Unassigned	Unassigned	Unassigned	1257_0	Clustered	6	VC_1257_0	6	0.6517	0.99999969	0.6517	0	0	0	1	Ref
MG945663	Unassigned	Unassigned	Unassigned	995_0	Clustered	69	VC_995_0	69	0.8547	1	0.8547	0	0	0	1	Ref
MG945664	Unassigned	Unassigned	Unassigned	1211_0	Clustered	25	VC_1211_0	25	1	1	1	0	0	0	1	Ref
MG945665	Unassigned	Unassigned	Unassigned	1287_0	Clustered	7	VC_1287_0	7	1	1	1	0	0	0	1	Ref
MG945667	Unassigned	Unassigned	Unassigned	1232_0	Clustered	2	VC_1232_0	2	0.4709	0.85557782	0.4029	0	0	0	1	Ref
MG945668	Unassigned	Unassigned	Unassigned	1285_0	Clustered	25	VC_1285_0	25	1	1	1	0	0	0	1	Ref
MG945669	Unassigned	Unassigned	Unassigned	198_0	Clustered	28	VC_198_0	28	0.2429	0.99971175	0.2429	0	0	0	1	Ref
MG945670	Unassigned	Unassigned	Unassigned	1077_0	Clustered	48	VC_1077_0	48	0.8525	1	0.8525	0	0	0	1	Ref
MG945671	Unassigned	Unassigned	Unassigned	1104_0	Clustered	154	VC_1104_0	154	0.8484	1	0.8484	0	0	0	1	Ref
MG945673	Unassigned	Unassigned	Unassigned	1104_0	Clustered	154	VC_1104_0	154	0.8484	1	0.8484	0	0	0	1	Ref
MG945674	Unassigned	Unassigned	Unassigned	1104_0	Clustered	154	VC_1104_0	154	0.8484	1	0.8484	0	0	0	1	Ref
MG945676	Unassigned	Unassigned	Unassigned	1148_0	Clustered	16	VC_1148_0	16	0.8821	1	0.8821	0	0	0	1	Ref
MG945677	Unassigned	Unassigned	Unassigned	1230_0	Clustered	3	VC_1230_0	3	0.4802	0.82711071	0.3971	0	0	0	1	Ref
MG945678	Unassigned	Unassigned	Unassigned	1155_0	Clustered	2	VC_1155_0	2	1	1	1	0	0	0	1	Ref
MG945680	Unassigned	Unassigned	Unassigned	1211_0	Clustered	25	VC_1211_0	25	1	1	1	0	0	0	1	Ref
MG945681	Unassigned	Unassigned	Unassigned	1104_0	Clustered	154	VC_1104_0	154	0.8484	1	0.8484	0	0	0	1	Ref
MG945682	Unassigned	Unassigned	Unassigned	398_0	Clustered	48	VC_398_0	48	0.4813	1	0.4813	0	0	0	1	Ref
MG945683	Unassigned	Unassigned	Unassigned	1211_0	Clustered	25	VC_1211_0	25	1	1	1	0	0	0	1	Ref
MG945685	Unassigned	Unassigned	Unassigned	1251_0	Clustered	15	VC_1251_0	15	0.9082	1	0.9082	0	0	0	1	Ref
MG945686	Unassigned	Unassigned	Unassigned	672_0	Clustered	12	VC_672_0	12	1	1	1	0	0	0	1	Ref
MG945687	Unassigned	Unassigned	Unassigned	1259_0	Clustered	8	VC_1259_0	8	0.8225	1	0.8225	0	0	0	1	Ref
MG945689	Unassigned	Unassigned	Unassigned	1122_0	Clustered	10	VC_1122_0	10	0.9643	0.99999697	0.9643	0	0	0	1	Ref
MG945690	Unassigned	Unassigned	Unassigned	1326_0	Clustered	2	VC_1326_0	2	1	1	1	0	0	0	1	Ref
MG945691	Unassigned	Unassigned	Unassigned	1104_0	Clustered	154	VC_1104_0	154	0.8484	1	0.8484	0	0	0	1	Ref
MG945692	Unassigned	Unassigned	Unassigned	1247_0	Clustered	7	VC_1247_0	7	0.9086	0.99998959	0.9086	0	0	0	1	Ref
MG945693	Unassigned	Unassigned	Unassigned	1138_0	Clustered	4	VC_1138_0	4	0.9437	0.98964986	0.934	0	0	0	1	Ref
MG945694	Unassigned	Unassigned	Unassigned	1127_0	Clustered	19	VC_1127_0	19	0.7611	1	0.7611	0	0	0	1	Ref
MG945696	Unassigned	Unassigned	Unassigned	1297_0	Clustered	7	VC_1297_0	7	1	1	1	0	0	0	1	Ref
MG945697	Unassigned	Unassigned	Unassigned	1247_0	Clustered	7	VC_1247_0	7	0.9086	0.99998959	0.9086	0	0	0	1	Ref

MG945698	Unassigned	Unassigned	Unassigned	1247_0	Clustered	7	VC_1247_0	7	0.9086	0.99998959	0.9086	0	0	0	1	Ref
MG945699	Unassigned	Unassigned	Unassigned	1104_0	Clustered	154	VC_1104_0	154	0.8484	1	0.8484	0	0	0	1	Ref
MG945702	Unassigned	Unassigned	Unassigned	1292_0	Clustered	3	VC_1292_0	3	1	1	1	0	0	0	1	Ref
MG945703	Unassigned	Unassigned	Unassigned	1054_0	Clustered	12	VC_1054_0	12	0.2863	0.99746644	0.2855	0	0	0	1	Ref
MG945704	Unassigned	Unassigned	Unassigned	1120_0	Clustered	7	VC_1120_0	7	0.2688	0.9951868	0.2675	0	0	0	1	Ref
MG945705	Unassigned	Unassigned	Unassigned	1104_0	Clustered	154	VC_1104_0	154	0.8484	1	0.8484	0	0	0	1	Ref
MG945706	Unassigned	Unassigned	Unassigned	1148_0	Clustered	16	VC_1148_0	16	0.8821	1	0.8821	0	0	0	1	Ref
MG945708	Unassigned	Unassigned	Unassigned	1104_0	Clustered	154	VC_1104_0	154	0.8484	1	0.8484	0	0	0	1	Ref
MG945710	Unassigned	Unassigned	Unassigned	1104_0	Clustered	154	VC_1104_0	154	0.8484	1	0.8484	0	0	0	1	Ref
MG945712	Unassigned	Unassigned	Unassigned	1285_0	Clustered	25	VC_1285_0	25	1	1	1	0	0	0	1	Ref
MG945713	Unassigned	Unassigned	Unassigned	1211_0	Clustered	25	VC_1211_0	25	1	1	1	0	0	0	1	Ref
MG945716	Unassigned	Unassigned	Unassigned	1211_0	Clustered	25	VC_1211_0	25	1	1	1	0	0	0	1	Ref
MG945717	Unassigned	Unassigned	Unassigned	1311_0	Clustered	5	VC_1311_0	5	0.8316	0.99879053	0.8306	0	0	0	1	Ref
MG945718	Unassigned	Unassigned	Unassigned	1110_0	Clustered	8	VC_1110_0	8	0.6915	1	0.6915	0	0	0	1	Ref
MG945719	Unassigned	Unassigned	Unassigned	1144_0	Clustered	7	VC_1144_0	7	0.7076	1	0.7076	0	0	0	1	Ref
MG945721	Unassigned	Unassigned	Unassigned	1139_0	Clustered	4	VC_1139_0	4	1	1	1	0	0	0	1	Ref
MG945723	Unassigned	Unassigned	Unassigned	1252_0	Clustered	3	VC_1252_0	3	0.3237	0.99790393	0.323	0	0	0	1	Ref
MG945724	Unassigned	Unassigned	Unassigned	1327_0	Clustered	4	VC_1327_0	4	1	1	1	0	0	0	1	Ref
MG945725	Unassigned	Unassigned	Unassigned	1233_0	Clustered	16	VC_1233_0	16	0.8971	1	0.8971	0	0	0	1	Ref
MG945726	Unassigned	Unassigned	Unassigned	1151_0	Clustered	9	VC_1151_0	9	0.8548	1	0.8548	0	0	0	1	Ref
MG945727	Unassigned	Unassigned	Unassigned	1152_0	Clustered	4	VC_1152_0	4	0.7206	0.99971165	0.7204	0	0	0	1	Ref
MG945728	Unassigned	Unassigned	Unassigned	1104_0	Clustered	154	VC_1104_0	154	0.8484	1	0.8484	0	0	0	1	Ref
MG945729	Unassigned	Unassigned	Unassigned	1287_0	Clustered	7	VC_1287_0	7	1	1	1	0	0	0	1	Ref
MG945730	Unassigned	Unassigned	Unassigned	1211_0	Clustered	25	VC_1211_0	25	1	1	1	0	0	0	1	Ref
MG945731	Unassigned	Unassigned	Unassigned	1104_0	Clustered	154	VC_1104_0	154	0.8484	1	0.8484	0	0	0	1	Ref
MG945733	Unassigned	Unassigned	Unassigned	1285_0	Clustered	25	VC_1285_0	25	1	1	1	0	0	0	1	Ref
MG945734	Unassigned	Unassigned	Unassigned	1247_0	Clustered	7	VC_1247_0	7	0.9086	0.99998959	0.9086	0	0	0	1	Ref
MG945735	Unassigned	Unassigned	Unassigned	1193_0	Clustered	3	VC_1193_0	3	0.5473	0.99523779	0.5447	0	0	0	1	Ref
MG945736	Unassigned	Unassigned	Unassigned	1116_0	Clustered	14	VC_1116_0	14	0.7927	1	0.7927	0	0	0	1	Ref
MG945738	Unassigned	Unassigned	Unassigned	1132_0	Clustered	2	VC_1132_0	2	0.0978	0.83194774	0.0814	0	0	0	1	Ref
MG945739	Unassigned	Unassigned	Unassigned	1311_0	Clustered	5	VC_1311_0	5	0.8316	0.99879053	0.8306	0	0	0	1	Ref
MG945740	Unassigned	Unassigned	Unassigned	1116_0	Clustered	14	VC_1116_0	14	0.7927	1	0.7927	0	0	0	1	Ref
MG945741	Unassigned	Unassigned	Unassigned	1137_0	Clustered	3	VC_1137_0	3	0.9176	0.93067968	0.854	0	0	0	1	Ref
MG945742	Unassigned	Unassigned	Unassigned	1114_0	Clustered	2	VC_1114_0	2	0.0522	0.94760452	0.0495	0	0	0	1	Ref
MG945743	Unassigned	Unassigned	Unassigned	1243_0	Clustered	52	VC_1243_0	52	0.9126	0.92708311	0.8461	0	0	0	1	Ref
MG945744	Unassigned	Unassigned	Unassigned	1104_0	Clustered	154	VC_1104_0	154	0.8484	1	0.8484	0	0	0	1	Ref
MG945745	Unassigned	Unassigned	Unassigned	1251_0	Clustered	15	VC_1251_0	15	0.9082	1	0.9082	0	0	0	1	Ref
MG945746	Unassigned	Unassigned	Unassigned	1125_0	Clustered	20	VC_1125_0	20	0.5599	1	0.5599	0	0	0	1	Ref
MG945747	Unassigned	Unassigned	Unassigned	1154_0	Clustered	5	VC_1154_0	5	1	1	1	0	0	0	1	Ref
MG945748	Unassigned	Unassigned	Unassigned	1196_0	Clustered	3	VC_1196_0	3	0.3378	0.99743975	0.3369	0	0	0	1	Ref
MG945750	Unassigned	Unassigned	Unassigned	1267_0	Clustered	7	VC_1267_0	7	1	1	1	0	0	0	1	Ref
MG945751	Unassigned	Unassigned	Unassigned	1281_0	Clustered	7	VC_1281_0	7	0.9212	0.99995294	0.9212	0	0	0	1	Ref
MG945752	Unassigned	Unassigned	Unassigned	1193_0	Clustered	3	VC_1193_0	3	0.5473	0.99523779	0.5447	0	0	0	1	Ref
MG945753	Unassigned	Unassigned	Unassigned	1127_0	Clustered	19	VC_1127_0	19	0.7611	1	0.7611	0	0	0	1	Ref
MG945754	Unassigned	Unassigned	Unassigned	1151_0	Clustered	9	VC_1151_0	9	0.8548	1	0.8548	0	0	0	1	Ref
MG945755	Unassigned	Unassigned	Unassigned	1293_0	Clustered	4	VC_1293_0	4	0.7847	0.99289298	0.7791	0	0	0	1	Ref
MG945756	Unassigned	Unassigned	Unassigned	672_0	Clustered	12	VC_672_0	12	1	1	1	0	0	0	1	Ref
MG945757	Unassigned	Unassigned	Unassigned	1233_0	Clustered	16	VC_1233_0	16	0.8971	1	0.8971	0	0	0	1	Ref
MG945758	Unassigned	Unassigned	Unassigned	1104_0	Clustered	154	VC_1104_0	154	0.8484	1	0.8484	0	0	0	1	Ref
MG945759	Unassigned	Unassigned	Unassigned	1143_0	Clustered	9	VC_1143_0	9	0.7268	1	0.7268	0	0	0	1	Ref
MG945760	Unassigned	Unassigned	Unassigned	1143_0	Clustered	9	VC_1143_0	9	0.7268	1	0.7268	0	0	0	1	Ref
MG945761	Unassigned	Unassigned	Unassigned	1104_0	Clustered	154	VC_1104_0	154	0.8484	1	0.8484	0	0	0	1	Ref
MG945764	Unassigned	Unassigned	Unassigned	1148_0	Clustered	16	VC_1148_0	16	0.8821	1	0.8821	0	0	0	1	Ref
MG945765	Unassigned	Unassigned	Unassigned	1104_0	Clustered	154	VC_1104_0	154	0.8484	1	0.8484	0	0	0	1	Ref
MG945766	Unassigned	Unassigned	Unassigned	1328_0	Clustered	2	VC_1328_0	2	1	1	1	0	0	0	1	Ref
MG945767	Unassigned	Unassigned	Unassigned	1125_0	Clustered	20	VC_1125_0	20	0.5599	1	0.5599	0	0	0	1	Ref
MG945768	Unassigned	Unassigned	Unassigned	1104_0	Clustered	154	VC_1104_0	154	0.8484	1	0.8484	0	0	0	1	Ref
MG945769	Unassigned	Unassigned	Unassigned	1329_0	Clustered	2	VC_1329_0	2	1	1	1	0	0	0	1	Ref
MG945770	Unassigned	Unassigned	Unassigned	1104_0	Clustered	154	VC_1104_0	154	0.8484	1	0.8484	0	0	0	1	Ref
MG945771	Unassigned	Unassigned	Unassigned	1116_0	Clustered	14	VC_1116_0	14	0.7927	1	0.7927	0	0	0	1	Ref
MG945774	Unassigned	Unassigned	Unassigned	1251_0	Clustered	15	VC_1251_0	15	0.9082	1	0.9082	0	0	0	1	Ref
MG945775	Unassigned	Unassigned	Unassigned	1108_0	Clustered	4	VC_1108_0	4	0.0485	0.99999021	0.0485	0	0	0	1	Ref
MG945776	Unassigned	Unassigned	Unassigned	1267_0	Clustered	7	VC_1267_0	7	1	1	1	0	0	0	1	Ref
MG945778	Unassigned	Unassigned	Unassigned	1288_0	Clustered	3	VC_1288_0	3	1	1	1	0	0	0	1	Ref
MG945780	Unassigned	Unassigned	Unassigned	1126_0	Clustered	8	VC_1126_0	8	0.2864	1	0.2864	0	0	0	1	Ref
MG945781	Unassigned	Unassigned	Unassigned	1127_0	Clustered	19	VC_1127_0	19	0.7611	1	0.7611	0	0	0	1	Ref
MG945782	Unassigned	Unassigned	Unassigned	1287_0	Clustered	7	VC_1287_0	7	1	1	1	0	0	0	1	Ref
MG945783	Unassigned	Unassigned	Unassigned	1110_0	Clustered	8	VC_1110_0	8	0.6915	1	0.6915	0	0	0	1	Ref
MG945784	Unassigned	Unassigned	Unassigned	1211_0	Clustered	25	VC_1211_0	25	1	1	1	0	0	0	1	Ref
MG945785	Unassigned	Unassigned	Unassigned	1152_0	Clustered	4	VC_1152_0	4	0.7206	0.99971165	0.7204	0	0	0	1	Ref
MG945786	Unassigned	Unassigned	Unassigned	1104_0	Clustered	154	VC_1104_0	154	0.8484	1	0.8484	0	0	0	1	Ref
MG945787	Unassigned	Unassigned	Unassigned	1104_0	Clustered	154	VC_1104_0	154	0.8484	1	0.8484	0	0	0	1	Ref
MG945788	Unassigned	Unassigned	Unassigned	1330_0	Clustered	2	VC_1330_0	2	1	1	1	0	0	0	1	Ref
MG945789	Unassigned	Unassigned	Unassigned	1278_0	Clustered	3	VC_1278_0	3	0.3961	0.99705286	0.395	0	0	0	1	Ref
MG945790	Unassigned	Unassigned	Unassigned	1211_0	Clustered	25	VC_1211_0	25	1	1	1	0	0	0	1	Ref
MG945791	Unassigned	Unassigned	Unassigned	1267_0	Clustered	7	VC_1267_0	7	1	1	1	0	0	0	1	Ref
MG945792	Unassigned	Unassigned	Unassigned	1329_0	Clustered	2	VC_1329_0	2	1	1	1	0	0	0	1	Ref
MG945793	Unassigned	Unassigned	Unassigned	1104_0	Clustered	154	VC_1104_0	154	0.8484	1	0.8484	0	0	0	1	Ref
MG945794	Unassigned	Unassigned	Unassigned	1197_0	Clustered	15	VC_1197_0	15	0.8991	0.76608394	0.6888	0	0	0	1	Ref
MG945795	Unassigned	Unassigned	Unassigned	1330_0	Clustered	2	VC_1330_0	2	1	1	1	0	0	0	1	Ref
MG945796	Unassigned	Unassigned	Unassigned	1104_0	Clustered	154	VC_1104_0	154	0.8484	1	0.8484	0	0	0	1	Ref

MG945797	Unassigned	Unassigned	Unassigned	1122_0	Clustered	10	VC_1122_0	10	0.9643	0.99999697	0.9643	0	0	0	1	Ref
MG945798	Unassigned	Unassigned	Unassigned	1331_0	Clustered	4	VC_1331_0	4	0.7343	0.99899459	0.7335	0	0	0	1	Ref
MG945799	Unassigned	Unassigned	Unassigned	1196_0	Clustered	3	VC_1196_0	3	0.3378	0.99743975	0.3369	0	0	0	1	Ref
MG945800	Unassigned	Unassigned	Unassigned	1196_0	Clustered	3	VC_1196_0	3	0.3378	0.99743975	0.3369	0	0	0	1	Ref
MG945801	Unassigned	Unassigned	Unassigned	1104_0	Clustered	154	VC_1104_0	154	0.8484	1	0.8484	0	0	0	1	Ref
MG945802	Unassigned	Unassigned	Unassigned	1104_0	Clustered	154	VC_1104_0	154	0.8484	1	0.8484	0	0	0	1	Ref
MG945803	Unassigned	Unassigned	Unassigned	1251_0	Clustered	15	VC_1251_0	15	0.9082	1	0.9082	0	0	0	1	Ref
MG945804	Unassigned	Unassigned	Unassigned	1258_0	Clustered	3	VC_1258_0	3	0.2422	0.85925819	0.2081	0	0	0	1	Ref
MG945805	Unassigned	Unassigned	Unassigned	1144_0	Clustered	7	VC_1144_0	7	0.7076	1	0.7076	0	0	0	1	Ref
MG945806	Unassigned	Unassigned	Unassigned	1285_0	Clustered	25	VC_1285_0	25	1	1	1	0	0	0	1	Ref
MG945807	Unassigned	Unassigned	Unassigned	1117_0	Clustered	5	VC_1117_0	5	0.4688	0.99999974	0.4688	0	0	0	1	Ref
MG945808	Unassigned	Unassigned	Unassigned	1333_0	Clustered	2	VC_1333_0	2	1	1	1	0	0	0	1	Ref
MG945809	Unassigned	Unassigned	Unassigned	1138_0	Clustered	4	VC_1138_0	4	0.9437	0.98964986	0.934	0	0	0	1	Ref
MG945810	Unassigned	Unassigned	Unassigned	1152_0	Clustered	4	VC_1152_0	4	0.7206	0.99971165	0.7204	0	0	0	1	Ref
MG945811	Unassigned	Unassigned	Unassigned	1143_0	Clustered	9	VC_1143_0	9	0.7268	1	0.7268	0	0	0	1	Ref
MG945812	Unassigned	Unassigned	Unassigned	1281_0	Clustered	7	VC_1281_0	7	0.9212	0.99995294	0.9212	0	0	0	1	Ref
MG945813	Unassigned	Unassigned	Unassigned	1211_0	Clustered	25	VC_1211_0	25	1	1	1	0	0	0	1	Ref
MG945814	Unassigned	Unassigned	Unassigned	1285_0	Clustered	25	VC_1285_0	25	1	1	1	0	0	0	1	Ref
MG945815	Unassigned	Unassigned	Unassigned	1257_0	Clustered	6	VC_1257_0	6	0.6517	0.99999969	0.6517	0	0	0	1	Ref
MG945816	Unassigned	Unassigned	Unassigned	1151_0	Clustered	9	VC_1151_0	9	0.8548	1	0.8548	0	0	0	1	Ref
MG945817	Unassigned	Unassigned	Unassigned	1148_0	Clustered	16	VC_1148_0	16	0.8821	1	0.8821	0	0	0	1	Ref
MG945818	Unassigned	Unassigned	Unassigned	1291_0	Clustered	2	VC_1291_0	2	1	1	1	0	0	0	1	Ref
MG945819	Unassigned	Unassigned	Unassigned	1116_0	Clustered	14	VC_1116_0	14	0.7927	1	0.7927	0	0	0	1	Ref
MG945820	Unassigned	Unassigned	Unassigned	1104_0	Clustered	154	VC_1104_0	154	0.8484	1	0.8484	0	0	0	1	Ref
MG945821	Unassigned	Unassigned	Unassigned	1334_0	Clustered/Singleton	1	VC_1334_0	1	0	1	0	0	0	0	1	Ref
MG945822	Unassigned	Unassigned	Unassigned	1273_0	Clustered	6	VC_1273_0	6	0.7385	0.9999804	0.7385	0	0	0	1	Ref
MG945823	Unassigned	Unassigned	Unassigned	1328_0	Clustered	2	VC_1328_0	2	1	1	1	0	0	0	1	Ref
MG945824	Unassigned	Unassigned	Unassigned	1305_0	Clustered	5	VC_1305_0	5	0.7701	0.66985041	0.5159	0	0	0	1	Ref
MG945825	Unassigned	Unassigned	Unassigned	1281_0	Clustered	7	VC_1281_0	7	0.9212	0.99995294	0.9212	0	0	0	1	Ref
MG945826	Unassigned	Unassigned	Unassigned	1119_0	Clustered	20	VC_1119_0	20	0.6751	0.99997892	0.6751	0	0	0	1	Ref
MG945827	Unassigned	Unassigned	Unassigned	1146_0	Clustered	3	VC_1146_0	3	0.3641	0.99867077	0.3636	0	0	0	1	Ref
MG945828	Unassigned	Unassigned	Unassigned	1300_0	Clustered	2	VC_1300_0	2	0.664	0.72985431	0.4846	0	0	0	1	Ref
MG945829	Unassigned	Unassigned	Unassigned	1251_0	Clustered	15	VC_1251_0	15	0.9082	1	0.9082	0	0	0	1	Ref
MG945830	Unassigned	Unassigned	Unassigned	1255_0	Clustered	4	VC_1255_0	4	0.248	0.99828153	0.2476	0	0	0	1	Ref
MG945831	Unassigned	Unassigned	Unassigned	1335_0	Clustered	7	VC_1335_0	7	0.8346	0.99999844	0.8346	0	0	0	1	Ref
MG945832	Unassigned	Unassigned	Unassigned	1337_0	Clustered	2	VC_1337_0	2	1	1	1	0	0	0	1	Ref
MG945835	Unassigned	Unassigned	Unassigned	1059_0	Clustered	3	VC_1059_0	3	0.3928	0.9839946	0.3865	0	0	0	1	Ref
MG945836	Unassigned	Unassigned	Unassigned	1077_0	Clustered	48	VC_1077_0	48	0.8525	1	0.8525	0	0	0	1	Ref
MG945837	Unassigned	Unassigned	Unassigned	398_0	Clustered	48	VC_398_0	48	0.4813	1	0.4813	0	0	0	1	Ref
MG945838	Unassigned	Unassigned	Unassigned	995_0	Clustered	69	VC_995_0	69	0.8547	1	0.8547	0	0	0	1	Ref
MG945839	Unassigned	Unassigned	Unassigned	1338_0	Clustered	4	VC_1338_0	4	1	1	1	0	0	0	1	Ref
MG945840	Unassigned	Unassigned	Unassigned	1077_0	Clustered	48	VC_1077_0	48	0.8525	1	0.8525	0	0	0	1	Ref
MG945842	Unassigned	Unassigned	Unassigned	1339_0	Clustered	7	VC_1339_0	7	0.9177	0.99772305	0.9156	0	0	0	1	Ref
MG945843	Unassigned	Unassigned	Unassigned	995_0	Clustered	69	VC_995_0	69	0.8547	1	0.8547	0	0	0	1	Ref
MG945845	Unassigned	Unassigned	Unassigned	1211_0	Clustered	25	VC_1211_0	25	1	1	1	0	0	0	1	Ref
MG945846	Unassigned	Unassigned	Unassigned	1077_0	Clustered	48	VC_1077_0	48	0.8525	1	0.8525	0	0	0	1	Ref
MG945848	Unassigned	Unassigned	Unassigned	1266_0	Clustered	5	VC_1266_0	5	1	1	1	0	0	0	1	Ref
MG945849	Unassigned	Unassigned	Unassigned	1333_0	Clustered	2	VC_1333_0	2	1	1	1	0	0	0	1	Ref
MG945850	Unassigned	Unassigned	Unassigned	1340_0	Clustered	5	VC_1340_0	5	1	1	1	0	0	0	1	Ref
MG945852	Unassigned	Unassigned	Unassigned	1266_0	Clustered	5	VC_1266_0	5	1	1	1	0	0	0	1	Ref
MG945853	Unassigned	Unassigned	Unassigned	1104_0	Clustered	154	VC_1104_0	154	0.8484	1	0.8484	0	0	0	1	Ref
MG945855	Unassigned	Unassigned	Unassigned	1104_0	Clustered	154	VC_1104_0	154	0.8484	1	0.8484	0	0	0	1	Ref
MG945857	Unassigned	Unassigned	Unassigned	1342_0	Clustered	3	VC_1342_0	3	1	1	1	0	0	0	1	Ref
MG945858	Unassigned	Unassigned	Unassigned	1125_0	Clustered	20	VC_1125_0	20	0.5599	1	0.5599	0	0	0	1	Ref
MG945859	Unassigned	Unassigned	Unassigned	1342_0	Clustered	3	VC_1342_0	3	1	1	1	0	0	0	1	Ref
MG945862	Unassigned	Unassigned	Unassigned	1104_0	Clustered	154	VC_1104_0	154	0.8484	1	0.8484	0	0	0	1	Ref
MG945864	Unassigned	Unassigned	Unassigned	1104_0	Clustered	154	VC_1104_0	154	0.8484	1	0.8484	0	0	0	1	Ref
MG945866	Unassigned	Unassigned	Unassigned	1342_0	Clustered	3	VC_1342_0	3	1	1	1	0	0	0	1	Ref
MG945867	Unassigned	Unassigned	Unassigned	1124_0	Clustered	10	VC_1124_0	10	0.6268	1	0.6268	0	0	0	1	Ref
MG945869	Unassigned	Unassigned	Unassigned	1104_0	Clustered	154	VC_1104_0	154	0.8484	1	0.8484	0	0	0	1	Ref
MG945870	Unassigned	Unassigned	Unassigned	1122_0	Clustered	10	VC_1122_0	10	0.9643	0.99999697	0.9643	0	0	0	1	Ref
MG945909	Unassigned	Unassigned	Unassigned	1258_0	Clustered	3	VC_1258_0	3	0.2422	0.85925819	0.2081	0	0	0	1	Ref
MG957431	Unassigned	Unassigned	Unassigned	82_0	Clustered	24	VC_82_0	24	0.7351	1	0.7351	0	0	0	1	Ref
MG962360	Unassigned	Unassigned	Unassigned	1240_0	Clustered	82	VC_1240_0	82	0.7945	1	0.7945	0	0	0	1	Ref
MG962361	Unassigned	Unassigned	Unassigned	520_64	Clustered/Singleton	1	VC_520_64	1	0	1	0	0	0	0	1	Ref
MG962362	Unassigned	Unassigned	Unassigned	371_3	Clustered	22	VC_371_3	22	0.0787	1	0.0787	0	0	0	0.9048	Ref
MG962363	Unassigned	Unassigned	Unassigned	371_13	Clustered	33	VC_371_13	33	0.179	1	0.179	0	0	0	0.8769	Ref
MG962364	Unassigned	Unassigned	Unassigned	236_0	Clustered	72	VC_236_0	72	0.8104	1	0.8104	0	0	0	1	Ref
MG962365	Unassigned	Unassigned	Unassigned	371_16	Clustered	42	VC_371_16	42	0.1341	1	0.1341	0	0	0	0.7282	Ref
MG962367	Unassigned	Unassigned	Unassigned	1240_0	Clustered	82	VC_1240_0	82	0.7945	1	0.7945	0	0	0	1	Ref
MG962368	Unassigned	Unassigned	Unassigned	799_1	Clustered	27	VC_799_1	27	0.8611	1	0.8611	0	0	0	1	Ref
MG962369	Unassigned	Unassigned	Unassigned	799_1	Clustered	27	VC_799_1	27	0.8611	1	0.8611	0	0	0	1	Ref
MG962370	Unassigned	Unassigned	Unassigned	325_47	Clustered	2	VC_325_47	2	0.0045	0.9575092	0.0043	0	0	0	1	Ref
MG962371	Unassigned	Unassigned	Unassigned	236_0	Clustered	72	VC_236_0	72	0.8104	1	0.8104	0	0	0	1	Ref
MG962372	Unassigned	Unassigned	Unassigned	325_5	Clustered	11	VC_325_5	11	0.047	1	0.047	0	0	0	0.7636	Ref
MG962373	Unassigned	Unassigned	Unassigned	517_1	Clustered	108	VC_517_1	108	0.8483	1	0.8483	0	0	0	0.9955	Ref
MG962374	Unassigned	Unassigned	Unassigned	238_0	Clustered	17	VC_238_0	17	0.6109	1	0.6109	0	0	0	1	Ref
MG962375	Unassigned	Unassigned	Unassigned	371_2	Clustered	8	VC_371_2	8	0.0232	1	0.0232	0	0	0	0.8571	Ref
MG962376	Unassigned	Unassigned	Unassigned	567_0	Clustered	8	VC_567_0	8	0.4935	1	0.4935	0	0	0	1	Ref
MG963916	Unassigned	Unassigned	Unassigned	772_16	Clustered	10	VC_772_16	10	0.0563	0.9434928	0.0531	0	0	0	0.8667	Ref
MG966531	Unassigned	Unassigned	Unassigned	392_0	Clustered	126	VC_392_0	126	0.9621	1	0.9621	0	0	0	1	Ref

MG966533	Unassigned	Unassigned	Unassigned	277_0	Clustered	3	VC_277_0	3	1	1	1	0	0	0	1	Ref
MG967616	Unassigned	Unassigned	Unassigned	1006_0	Clustered	8	VC_1006_0	8	0.7407	1	0.7407	0	0	0	1	Ref
MG967617	Unassigned	Unassigned	Unassigned	1006_0	Clustered	8	VC_1006_0	8	0.7407	1	0.7407	0	0	0	1	Ref
MG967618	Unassigned	Unassigned	Unassigned	1006_0	Clustered	8	VC_1006_0	8	0.7407	1	0.7407	0	0	0	1	Ref
MG969404	Unassigned	Unassigned	Unassigned	329_14	Clustered/Singleton	1	VC_329_14	1	0	1	0	0	0	0	1	Ref
MG969405	Unassigned	Unassigned	Unassigned	329_1	Clustered	17	VC_329_1	17	0.0867	1	0.0867	0	0	0	0.8235	Ref
MG969406	Unassigned	Unassigned	Unassigned	329_1	Clustered	17	VC_329_1	17	0.0867	1	0.0867	0	0	0	0.8235	Ref
MG969408	Unassigned	Unassigned	Unassigned	637_0	Clustered	115	VC_637_0	115	0.731	1	0.731	0	0	0	0.8764	Ref
MG969409	Unassigned	Unassigned	Unassigned	637_0	Clustered	115	VC_637_0	115	0.731	1	0.731	0	0	0	0.8764	Ref
MG969410	Unassigned	Unassigned	Unassigned	637_0	Clustered	115	VC_637_0	115	0.731	1	0.731	0	0	0	0.8764	Ref
MG969411	Unassigned	Unassigned	Unassigned	582_6	Clustered/Singleton	1	VC_582_6	1	0	1	0	0	0	0	1	Ref
MG969412	Unassigned	Unassigned	Unassigned	190_40	Clustered	2	VC_190_40	2	0.0083	0.95556395	0.0079	0	0	0	1	Ref
MG969415	Unassigned	Unassigned	Unassigned	28_1	Clustered	53	VC_28_1	53	0.7164	1	0.7164	0	0	0	0.9325	Ref
MG972768	Unassigned	Unassigned	Unassigned	111_0	Clustered	91	VC_111_0	91	0.9587	1	0.9587	0	0	0	1	Ref
MG976803	Unassigned	Unassigned	Unassigned	813_1	Clustered	24	VC_813_1	24	0.535	1	0.535	0	0	0	1	Ref
MG983742	Unassigned	Unassigned	Unassigned	100_0	Clustered	15	VC_100_0	15	0.6536	1	0.6536	0	0	0	1	Ref
MG983743	Unassigned	Unassigned	Unassigned	779_3	Clustered	7	VC_779_3	7	0.0766	1	0.0766	0	0	0	0.7143	Ref
MG983840	Unassigned	Unassigned	Unassigned	813_1	Clustered	24	VC_813_1	24	0.535	1	0.535	0	0	0	1	Ref
MH000602	Unassigned	Unassigned	Unassigned	252_0	Clustered	121	VC_252_0	121	0.7376	1	0.7376	0	0	0	0.9999	Ref
MH000603	Unassigned	Unassigned	Unassigned	252_0	Clustered	121	VC_252_0	121	0.7376	1	0.7376	0	0	0	0.9999	Ref
MH000604	Unassigned	Unassigned	Unassigned	252_0	Clustered	121	VC_252_0	121	0.7376	1	0.7376	0	0	0	0.9999	Ref
MH000606	Unassigned	Unassigned	Unassigned	325_38	Clustered	6	VC_325_38	6	0.0267	1	0.0267	0	0	0	0.8	Ref
MH000607	Unassigned	Unassigned	Unassigned	551_7	Clustered	28	VC_551_7	28	0.1661	1	0.1661	0	0	0	0.8519	Ref
MH001446	Unassigned	Unassigned	Unassigned	507_0	Clustered	8	VC_507_0	8	0.4466	1	0.4466	0	0	0	1	Ref
MH001447	Unassigned	Unassigned	Unassigned	568_0	Clustered	8	VC_568_0	8	0.5738	1	0.5738	0	0	0	1	Ref
MH001448	Unassigned	Unassigned	Unassigned	520_59	Clustered	5	VC_520_59	5	0.0459	0.99999997	0.0459	0	0	0	1	Ref
MH001450	Unassigned	Unassigned	Unassigned	507_0	Clustered	8	VC_507_0	8	0.4466	1	0.4466	0	0	0	1	Ref
MH001451	Unassigned	Unassigned	Unassigned	1046_0	Clustered	7	VC_1046_0	7	0.9413	1	0.9413	0	0	0	1	Ref
MH001452	Unassigned	Unassigned	Unassigned	508_0	Clustered	32	VC_508_0	32	0.906	1	0.906	0	0	0	1	Ref
MH001453	Unassigned	Unassigned	Unassigned	236_0	Clustered	72	VC_236_0	72	0.8104	1	0.8104	0	0	0	1	Ref
MH001454	Unassigned	Unassigned	Unassigned	224_0	Clustered	19	VC_224_0	19	0.9416	1	0.9416	0	0	0	1	Ref
MH001455	Unassigned	Unassigned	Unassigned	761_0	Clustered	67	VC_761_0	67	0.8947	1	0.8947	0	0	0	1	Ref
MH001456	Unassigned	Unassigned	Unassigned	761_0	Clustered	67	VC_761_0	67	0.8947	1	0.8947	0	0	0	1	Ref
MH001457	Unassigned	Unassigned	Unassigned	375_0	Clustered	3	VC_375_0	3	0.2479	0.99807286	0.2474	0	0	0	1	Ref
MH001458	Unassigned	Unassigned	Unassigned	325_11	Clustered/Singleton	1	VC_325_11	1	0	1	0	0	0	0	1	Ref
MH001459	Unassigned	Unassigned	Unassigned	515_0	Clustered	16	VC_515_0	16	0.3793	1	0.3793	0	0	0	1	Ref
MH001460	Unassigned	Unassigned	Unassigned	981_1	Clustered	26	VC_981_1	26	0.7009	1	0.7009	0	0	0	1	Ref
MH015249	Unassigned	Unassigned	Unassigned	593_0	Clustered	5	VC_593_0	5	0.4608	0.99999995	0.4608	0	0	0	1	Ref
MH015250	Unassigned	Unassigned	Unassigned	833_0	Clustered	16	VC_833_0	16	0.9351	1	0.9351	0	0	0	1	Ref
MH015251	Unassigned	Unassigned	Unassigned	1212_0	Clustered	2	VC_1212_0	2	1	1	1	0	0	0	1	Ref
MH015252	Unassigned	Unassigned	Unassigned	593_0	Clustered	5	VC_593_0	5	0.4608	0.99999995	0.4608	0	0	0	1	Ref
MH015253	Unassigned	Unassigned	Unassigned	833_0	Clustered	16	VC_833_0	16	0.9351	1	0.9351	0	0	0	1	Ref
MH015254	Unassigned	Unassigned	Unassigned	593_0	Clustered	5	VC_593_0	5	0.4608	0.99999995	0.4608	0	0	0	1	Ref
MH015255	Unassigned	Unassigned	Unassigned	977_0	Clustered	2	VC_977_0	2	0.0806	0.93920319	0.0757	0	0	0	1	Ref
MH015256	Unassigned	Unassigned	Unassigned	833_0	Clustered	16	VC_833_0	16	0.9351	1	0.9351	0	0	0	1	Ref
MH015257	Unassigned	Unassigned	Unassigned	833_0	Clustered	16	VC_833_0	16	0.9351	1	0.9351	0	0	0	1	Ref
MH015258	Unassigned	Unassigned	Unassigned	593_0	Clustered	5	VC_593_0	5	0.4608	0.99999995	0.4608	0	0	0	1	Ref
MH015259	Unassigned	Unassigned	Unassigned	833_0	Clustered	16	VC_833_0	16	0.9351	1	0.9351	0	0	0	1	Ref
MH019215	Unassigned	Unassigned	Unassigned	859_0	Clustered	9	VC_859_0	9	0.9426	1	0.9426	0	0	0	1	Ref
MH019216	Unassigned	Unassigned	Unassigned	859_0	Clustered	9	VC_859_0	9	0.9426	1	0.9426	0	0	0	1	Ref
MH020235	Unassigned	Unassigned	Unassigned	520_31	Clustered	11	VC_520_31	11	0.0897	1	0.0897	0	0	0	0.6	Ref
MH020236	Unassigned	Unassigned	Unassigned	325_53	Clustered	7	VC_325_53	7	0.0242	1	0.0242	0	0	0	0.9048	Ref
MH020237	Unassigned	Unassigned	Unassigned	215_2	Clustered	5	VC_215_2	5	0.0572	0.99999997	0.0572	0	0	0	1	Ref
MH020238	Unassigned	Unassigned	Unassigned	514_0	Clustered	75	VC_514_0	75	0.9402	1	0.9402	0	0	0	1	Ref
MH020239	Unassigned	Unassigned	Unassigned	940_0	Clustered	34	VC_940_0	34	0.7959	1	0.7959	0	0	0	1	Ref
MH020240	Unassigned	Unassigned	Unassigned	223_0	Clustered	38	VC_223_0	38	0.9095	1	0.9095	0	0	0	1	Ref
MH020241	Unassigned	Unassigned	Unassigned	1176_0	Clustered	41	VC_1176_0	41	0.9558	1	0.9558	0	0	0	0.9976	Ref
MH020244	Unassigned	Unassigned	Unassigned	325_23	Clustered	17	VC_325_23	17	0.0716	1	0.0716	0	0	0	0.6397	Ref
MH020246	Unassigned	Unassigned	Unassigned	556_0	Clustered	21	VC_556_0	21	0.9912	1	0.9912	0	0	0	1	Ref
MH020247	Unassigned	Unassigned	Unassigned	551_8	Clustered	19	VC_551_8	19	0.1039	1	0.1039	0	0	0	0.8713	Ref
MH020248	Unassigned	Unassigned	Unassigned	513_0	Clustered	32	VC_513_0	32	0.6881	1	0.6881	0	0	0	1	Ref
MH020249	Unassigned	Unassigned	Unassigned	517_1	Clustered	108	VC_517_1	108	0.8483	1	0.8483	0	0	0	0.9955	Ref
MH023293	Unassigned	Unassigned	Unassigned	461_0	Clustered	96	VC_461_0	96	0.9307	1	0.9307	0	0	0	0.9575	Ref
MH025888	Unassigned	Unassigned	Unassigned	565_0	Clustered	20	VC_565_0	20	0.8398	1	0.8398	0	0	0	1	Ref
MH025889	Unassigned	Unassigned	Unassigned	866_1	Clustered	20	VC_866_1	20	0.4814	1	0.4814	0	0	0	1	Ref
MH025890	Unassigned	Unassigned	Unassigned	570_0	Clustered	9	VC_570_0	9	0.499	1	0.499	0	0	0	1	Ref
MH031343	Unassigned	Unassigned	Unassigned	386_0	Clustered	49	VC_386_0	49	0.7941	1	0.7941	0	0	0	1	Ref
MH042230	Unassigned	Unassigned	Unassigned	699_0	Clustered	71	VC_699_0	71	0.9352	1	0.9352	0	0	0	1	Ref
MH045554	Unassigned	Unassigned	Unassigned	1240_0	Clustered	82	VC_1240_0	82	0.7945	1	0.7945	0	0	0	1	Ref
MH045557	Unassigned	Unassigned	Unassigned	1344_0	Clustered	78	VC_1344_0	78	0.9732	1	0.9732	0	0	0	1	Ref
MH045558	Unassigned	Unassigned	Unassigned	1240_0	Clustered	82	VC_1240_0	82	0.7945	1	0.7945	0	0	0	1	Ref
MH045560	Unassigned	Unassigned	Unassigned	1240_0	Clustered	82	VC_1240_0	82	0.7945	1	0.7945	0	0	0	1	Ref
MH045561	Unassigned	Unassigned	Unassigned	1344_0	Clustered	78	VC_1344_0	78	0.9732	1	0.9732	0	0	0	1	Ref
MH045562	Unassigned	Unassigned	Unassigned	1240_0	Clustered	82	VC_1240_0	82	0.7945	1	0.7945	0	0	0	1	Ref
MH045563	Unassigned	Unassigned	Unassigned	1240_0	Clustered	82	VC_1240_0	82	0.7945	1	0.7945	0	0	0	1	Ref
MH045564	Unassigned	Unassigned	Unassigned	1240_0	Clustered	82	VC_1240_0	82	0.7945	1	0.7945	0	0	0	1	Ref
MH045565	Unassigned	Unassigned	Unassigned	1240_0	Clustered	82	VC_1240_0	82	0.7945	1	0.7945	0	0	0	1	Ref
MH045566	Unassigned	Unassigned	Unassigned	1234_0	Clustered	44	VC_1234_0	44	1	1	1	0	0	0	1	Ref
MH045567	Unassigned	Unassigned	Unassigned	1234_0	Clustered	44	VC_1234_0	44	1	1	1	0	0	0	1	Ref
MH045568	Unassigned	Unassigned	Unassigned	1234_0	Clustered	44	VC_1234_0	44	1	1	1	0	0	0	1	Ref
MH045569	Unassigned	Unassigned	Unassigned	761_0	Clustered	67	VC_761_0	67	0.8947	1	0.8947	0	0	0	1	Ref

MH047632	Unassigned	Unassigned	Unassigned	513_0	Clustered	32	VC_513_0	32	0.6881	1	0.6881	0	0	0	1	Ref
MH047633	Unassigned	Unassigned	Unassigned	513_0	Clustered	32	VC_513_0	32	0.6881	1	0.6881	0	0	0	1	Ref
MH051246	Unassigned	Unassigned	Unassigned	236_0	Clustered	72	VC_236_0	72	0.8104	1	0.8104	0	0	0	1	Ref
MH051247	Unassigned	Unassigned	Unassigned	517_1	Clustered	108	VC_517_1	108	0.8483	1	0.8483	0	0	0	0.9955	Ref
MH051249	Unassigned	Unassigned	Unassigned	508_0	Clustered	32	VC_508_0	32	0.906	1	0.906	0	0	0	1	Ref
MH051250	Unassigned	Unassigned	Unassigned	940_0	Clustered	34	VC_940_0	34	0.7959	1	0.7959	0	0	0	1	Ref
MH051251	Unassigned	Unassigned	Unassigned	371_13	Clustered	33	VC_371_13	33	0.179	1	0.179	0	0	0	0.8769	Ref
MH051252	Unassigned	Unassigned	Unassigned	216_6	Clustered	42	VC_216_6	42	0.473	1	0.473	0	0	0	0.8351	Ref
MH051253	Unassigned	Unassigned	Unassigned	223_0	Clustered	38	VC_223_0	38	0.9095	1	0.9095	0	0	0	1	Ref
MH051254	Unassigned	Unassigned	Unassigned	940_0	Clustered	34	VC_940_0	34	0.7959	1	0.7959	0	0	0	1	Ref
MH051255	Unassigned	Unassigned	Unassigned	238_0	Clustered	17	VC_238_0	17	0.6109	1	0.6109	0	0	0	1	Ref
MH051256	Unassigned	Unassigned	Unassigned	940_0	Clustered	34	VC_940_0	34	0.7959	1	0.7959	0	0	0	1	Ref
MH051257	Unassigned	Unassigned	Unassigned	520_51	Clustered/Singleton	1	VC_520_51	1	0	1	0	0	0	0	1	Ref
MH051258	Unassigned	Unassigned	Unassigned	234_0	Clustered	21	VC_234_0	21	0.8355	1	0.8355	0	0	0	1	Ref
MH051259	Unassigned	Unassigned	Unassigned	513_0	Clustered	32	VC_513_0	32	0.6881	1	0.6881	0	0	0	1	Ref
MH051260	Unassigned	Unassigned	Unassigned	558_61	Clustered	2	VC_558_61	2	0.0032	0.66165479	0.0021	0	0	0	1	Ref
MH051261	Unassigned	Unassigned	Unassigned	558_7	Clustered/Singleton	1	VC_558_7	1	0	1	0	0	0	0	1	Ref
MH051264	Unassigned	Unassigned	Unassigned	371_5	Clustered	2	VC_371_5	2	0.0035	0.95771071	0.0033	0	0	0	1	Ref
MH051265	Unassigned	Unassigned	Unassigned	509_0	Clustered	37	VC_509_0	37	0.9689	1	0.9689	0	0	0	1	Ref
MH051266	Unassigned	Unassigned	Unassigned	558_50	Clustered	15	VC_558_50	15	0.0468	1	0.0468	0	0	0	0.9238	Ref
MH051333	Unassigned	Unassigned	Unassigned	847_1	Clustered	26	VC_847_1	26	0.6323	1	0.6323	0	0	0	0.9815	Ref
MH051334	Unassigned	Unassigned	Unassigned	638_0	Clustered	45	VC_638_0	45	0.7741	1	0.7741	0	0	0	1	Ref
MH051335	Unassigned	Unassigned	Unassigned	618_0	Clustered	13	VC_618_0	13	0.6252	1	0.6252	0	0	0	1	Ref
MH051911	Unassigned	Unassigned	Unassigned	575_0	Clustered	35	VC_575_0	35	0.8815	1	0.8815	0	0	0	1	Ref
MH051912	Unassigned	Unassigned	Unassigned	575_0	Clustered	35	VC_575_0	35	0.8815	1	0.8815	0	0	0	1	Ref
MH051913	Unassigned	Unassigned	Unassigned	290_6	Clustered	12	VC_290_6	12	0.1149	1	0.1149	0	0	0	0.8182	Ref
MH051914	Unassigned	Unassigned	Unassigned	329_21	Clustered/Singleton	1	VC_329_21	1	0	1	0	0	0	0	1	Ref
MH051915	Unassigned	Unassigned	Unassigned	283_151	Clustered/Singleton	1	VC_283_151	1	0	1	0	0	0	0	1	Ref
MH051916	Unassigned	Unassigned	Unassigned	283_170	Clustered/Singleton	1	VC_283_170	1	0	1	0	0	0	0	1	Ref
MH051917	Unassigned	Unassigned	Unassigned	290_5	Clustered	14	VC_290_5	14	0.1422	1	0.1422	0	0	0	0.9231	Ref
MH051918	Unassigned	Unassigned	Unassigned	576_0	Clustered	4	VC_576_0	4	0.0606	0.99998766	0.0606	0	0	0	1	Ref
MH059632	Unassigned	Unassigned	Unassigned	458_0	Clustered	5	VC_458_0	5	0.1938	0.99999997	0.1938	0	0	0	1	Ref
MH059635	Unassigned	Unassigned	Unassigned	117_0	Clustered	9	VC_117_0	9	0.9977	0.98988637	0.9876	0	0	0	1	Ref
MH059636	Unassigned	Unassigned	Unassigned	490_5	Clustered/Singleton	1	VC_490_5	1	0	1	0	0	0	0	1	Ref
MH059638	Unassigned	Unassigned	Unassigned	841_0	Clustered	10	VC_841_0	10	0.5997	1	0.5997	0	0	0	1	Ref
MH059639	Unassigned	Unassigned	Unassigned	393_0	Clustered	7	VC_393_0	7	0.1775	1	0.1775	0	0	0	1	Ref
MH077576	Unassigned	Unassigned	Unassigned	216_6	Clustered	42	VC_216_6	42	0.473	1	0.473	0	0	0	0.8351	Ref
MH077577	Unassigned	Unassigned	Unassigned	236_0	Clustered	72	VC_236_0	72	0.8104	1	0.8104	0	0	0	1	Ref
MH077578	Unassigned	Unassigned	Unassigned	520_34	Clustered/Singleton	1	VC_520_34	1	0	1	0	0	0	0	1	Ref
MH077579	Unassigned	Unassigned	Unassigned	546_4	Clustered	16	VC_546_4	16	0.2517	0.9999998	0.2517	0	0	0	0.9417	Ref
MH077580	Unassigned	Unassigned	Unassigned	520_31	Clustered	11	VC_520_31	11	0.0897	1	0.0897	0	0	0	0.6	Ref
MH077581	Unassigned	Unassigned	Unassigned	517_1	Clustered	108	VC_517_1	108	0.8483	1	0.8483	0	0	0	0.9955	Ref
MH077582	Unassigned	Unassigned	Unassigned	371_2	Clustered	8	VC_371_2	8	0.0232	1	0.0232	0	0	0	0.8571	Ref
MH077583	Unassigned	Unassigned	Unassigned	514_0	Clustered	75	VC_514_0	75	0.9402	1	0.9402	0	0	0	1	Ref
MH077584	Unassigned	Unassigned	Unassigned	761_0	Clustered	67	VC_761_0	67	0.8947	1	0.8947	0	0	0	1	Ref
MH102284	Unassigned	Unassigned	Unassigned	584_0	Clustered	28	VC_584_0	28	0.7704	1	0.7704	0	0	0	1	Ref
MH102285	Unassigned	Unassigned	Unassigned	190_23	Clustered	10	VC_190_23	10	0.0704	1	0.0704	0	0	0	0.8444	Ref
MH105773	Unassigned	Unassigned	Unassigned	607_0	Clustered	16	VC_607_0	16	0.9179	1	0.9179	0	0	0	1	Ref
MH107028	Unassigned	Unassigned	Unassigned	844_0	Clustered	27	VC_844_0	27	0.9385	1	0.9385	0	0	0	1	Ref
MH107118	Unassigned	Unassigned	Unassigned	96_0	Clustered	30	VC_96_0	30	0.718	1	0.718	0	0	0	1	Ref
MH107769	Unassigned	Unassigned	Unassigned	149_12	Clustered	2	VC_149_12	2	0.0044	0.83156708	0.0037	0	0	0	1	Ref
MH107770	Unassigned	Unassigned	Unassigned	423_0	Clustered	45	VC_423_0	45	0.9484	1	0.9484	0	0	0	1	Ref
MH113812	Unassigned	Unassigned	Unassigned	1027_0	Clustered	20	VC_1027_0	20	0.7114	1	0.7114	0	0	0	1	Ref
MH113813	Unassigned	Unassigned	Unassigned	1027_0	Clustered	20	VC_1027_0	20	0.7114	1	0.7114	0	0	0	1	Ref
MH113814	Unassigned	Unassigned	Unassigned	1027_0	Clustered	20	VC_1027_0	20	0.7114	1	0.7114	0	0	0	1	Ref
MH113815	Unassigned	Unassigned	Unassigned	1027_0	Clustered	20	VC_1027_0	20	0.7114	1	0.7114	0	0	0	1	Ref
MH115576	Unassigned	Unassigned	Unassigned	696_1	Clustered	8	VC_696_1	8	0.5452	1	0.5452	0	0	0	0.9286	Ref
MH128984	Unassigned	Unassigned	Unassigned	279_0	Clustered	2	VC_279_0	2	0.1888	0.95124665	0.1796	0	0	0	1	Ref
MH133207	Unassigned	Unassigned	Unassigned	701_1	Clustered	2	VC_701_1	2	0.1304	0.95114164	0.124	0	0	0	1	Ref
MH136584	Unassigned	Unassigned	Unassigned	149_8	Clustered	66	VC_149_8	66	0.4565	1	0.4565	0	0	0	0.8182	Ref
MH142219	Unassigned	Unassigned	Unassigned	24_0	Clustered	3	VC_24_0	3	0.3455	0.99844825	0.345	0	0	0	1	Ref
MH142220	Unassigned	Unassigned	Unassigned	24_0	Clustered	3	VC_24_0	3	0.3455	0.99844825	0.345	0	0	0	1	Ref
MH153799	Unassigned	Unassigned	Unassigned	1348_0	Clustered	5	VC_1348_0	5	0.5462	0.99999985	0.5462	0	0	0	1	Ref
MH153800	Unassigned	Unassigned	Unassigned	803_0	Clustered	3	VC_803_0	3	0.0513	0.99857736	0.0512	0	0	0	0.6667	Ref
MH153801	Unassigned	Unassigned	Unassigned	803_0	Clustered	3	VC_803_0	3	0.0513	0.99857736	0.0512	0	0	0	0.6667	Ref
MH153802	Unassigned	Unassigned	Unassigned	1240_0	Clustered	82	VC_1240_0	82	0.7945	1	0.7945	0	0	0	1	Ref
MH153803	Unassigned	Unassigned	Unassigned	1171_0	Clustered	21	VC_1171_0	21	0.7125	1	0.7125	0	0	0	1	Ref
MH153804	Unassigned	Unassigned	Unassigned	726_0	Clustered	3	VC_726_0	3	0.0381	0.88955608	0.0339	0	0	0	1	Ref
MH153805	Unassigned	Unassigned	Unassigned	1240_0	Clustered	82	VC_1240_0	82	0.7945	1	0.7945	0	0	0	1	Ref
MH153806	Unassigned	Unassigned	Unassigned	1240_0	Clustered	82	VC_1240_0	82	0.7945	1	0.7945	0	0	0	1	Ref
MH153807	Unassigned	Unassigned	Unassigned	1070_0	Clustered	2	VC_1070_0	2	0.1299	0.95375267	0.1239	0	0	0	1	Ref
MH153808	Unassigned	Unassigned	Unassigned	1176_0	Clustered	41	VC_1176_0	41	0.9558	1	0.9558	0	0	0	0.9976	Ref
MH153809	Unassigned	Unassigned	Unassigned	1093_0	Clustered	40	VC_1093_0	40	0.8754	1	0.8754	0	0	0	1	Ref
MH153810	Unassigned	Unassigned	Unassigned	510_0	Clustered	12	VC_510_0	12	0.3963	1	0.3963	0	0	0	0.9697	Ref
MH153811	Unassigned	Unassigned	Unassigned	1240_0	Clustered	82	VC_1240_0	82	0.7945	1	0.7945	0	0	0	1	Ref
MH153812	Unassigned	Unassigned	Unassigned	1070_0	Clustered	2	VC_1070_0	2	0.1299	0.95375267	0.1239	0	0	0	1	Ref
MH153813	Unassigned	Unassigned	Unassigned	1171_0	Clustered	21	VC_1171_0	21	0.7125	1	0.7125	0	0	0	1	Ref
MH155864	Unassigned	Unassigned	Unassigned	514_0	Clustered	75	VC_514_0	75	0.9402	1	0.9402	0	0	0	1	Ref
MH155865	Unassigned	Unassigned	Unassigned	520_38	Clustered	6	VC_520_38	6	0.0519	1	0.0519	0	0	0	0.8667	Ref
MH155866	Unassigned	Unassigned	Unassigned	520_4	Clustered/Singleton	1	VC_520_4	1	0	1	0	0	0	0	1	Ref
MH155868	Unassigned	Unassigned	Unassigned	1224_0	Clustered	19	VC_1224_0	19	1	1	1	0	0	0	1	Ref

MH155870	Unassigned	Unassigned	Unassigned	1351_0	Clustered	2	VC_1351_0	2	1	1	1	0	0	0	1	Ref
MH155871	Unassigned	Unassigned	Unassigned	520_38	Clustered	6	VC_520_38	6	0.0519	1	0.0519	0	0	0	0.8667	Ref
MH155873	Unassigned	Unassigned	Unassigned	1352_0	Clustered	31	VC_1352_0	31	0.849	1	0.849	0	0	0	1	Ref
MH155874	Unassigned	Unassigned	Unassigned	371_13	Clustered	33	VC_371_13	33	0.179	1	0.179	0	0	0	0.8769	Ref
MH155875	Unassigned	Unassigned	Unassigned	517_1	Clustered	108	VC_517_1	108	0.8483	1	0.8483	0	0	0	0.9955	Ref
MH155876	Unassigned	Unassigned	Unassigned	223_0	Clustered	38	VC_223_0	38	0.9095	1	0.9095	0	0	0	1	Ref
MH155877	Unassigned	Unassigned	Unassigned	872_0	Clustered	31	VC_872_0	31	0.8151	1	0.8151	0	0	0	0.8194	Ref
MH155879	Unassigned	Unassigned	Unassigned	517_1	Clustered	108	VC_517_1	108	0.8483	1	0.8483	0	0	0	0.9955	Ref
MH155880	Unassigned	Unassigned	Unassigned	872_0	Clustered	31	VC_872_0	31	0.8151	1	0.8151	0	0	0	0.8194	Ref
MH159197	Unassigned	Unassigned	Unassigned	149_15	Clustered	16	VC_149_15	16	0.0788	1	0.0788	0	0	0	0.8083	Ref
MH160392	Unassigned	Unassigned	Unassigned	779_2	Clustered	5	VC_779_2	5	0.0876	0.99999997	0.0876	0	0	0	1	Ref
MH160766	Unassigned	Unassigned	Unassigned	847_1	Clustered	26	VC_847_1	26	0.6323	1	0.6323	0	0	0	0.9815	Ref
MH160767	Unassigned	Unassigned	Unassigned	247_0	Clustered	14	VC_247_0	14	0.9119	1	0.9119	0	0	0	0.967	Ref
MH165274	Unassigned	Unassigned	Unassigned	492_1	Clustered	20	VC_492_1	20	0.4176	1	0.4176	0	0	0	1	Ref
MH171093	Unassigned	Unassigned	Unassigned	982_0	Clustered	62	VC_982_0	62	0.8646	1	0.8646	0	0	0	0.743	Ref
MH171094	Unassigned	Unassigned	Unassigned	982_0	Clustered	62	VC_982_0	62	0.8646	1	0.8646	0	0	0	0.743	Ref
MH171095	Unassigned	Unassigned	Unassigned	982_0	Clustered	62	VC_982_0	62	0.8646	1	0.8646	0	0	0	0.743	Ref
MH171096	Unassigned	Unassigned	Unassigned	982_0	Clustered	62	VC_982_0	62	0.8646	1	0.8646	0	0	0	0.743	Ref
MH171097	Unassigned	Unassigned	Unassigned	982_0	Clustered	62	VC_982_0	62	0.8646	1	0.8646	0	0	0	0.743	Ref
MH171098	Unassigned	Unassigned	Unassigned	982_0	Clustered	62	VC_982_0	62	0.8646	1	0.8646	0	0	0	0.743	Ref
MH172261	Unassigned	Unassigned	Unassigned	823_0	Clustered	124	VC_823_0	124	0.9492	1	0.9492	0	0	0	1	Ref
MH172262	Unassigned	Unassigned	Unassigned	823_0	Clustered	124	VC_823_0	124	0.9492	1	0.9492	0	0	0	1	Ref
MH172263	Unassigned	Unassigned	Unassigned	823_0	Clustered	124	VC_823_0	124	0.9492	1	0.9492	0	0	0	1	Ref
MH172264	Unassigned	Unassigned	Unassigned	823_0	Clustered	124	VC_823_0	124	0.9492	1	0.9492	0	0	0	1	Ref
MH178096	Unassigned	Unassigned	Unassigned	33_0	Clustered	4	VC_33_0	4	0.06	0.99994826	0.06	0	0	0	1	Ref
MH178381	Unassigned	Unassigned	Unassigned	975_0	Clustered	11	VC_975_0	11	0.9687	1	0.9687	0	0	0	1	Ref
MH178382	Unassigned	Unassigned	Unassigned	975_0	Clustered	11	VC_975_0	11	0.9687	1	0.9687	0	0	0	1	Ref
MH179470	Unassigned	Unassigned	Unassigned	1189_0	Clustered	9	VC_1189_0	9	0.968	1	0.968	0	0	0	1	Ref
MH179471	Unassigned	Unassigned	Unassigned	1189_0	Clustered	9	VC_1189_0	9	0.968	1	0.968	0	0	0	1	Ref
MH179472	Unassigned	Unassigned	Unassigned	130_0	Clustered	19	VC_130_0	19	0.6068	1	0.6068	0	0	0	1	Ref
MH179473	Unassigned	Unassigned	Unassigned	878_0	Clustered	32	VC_878_0	32	0.9965	1	0.9965	0	0	0	1	Ref
MH179474	Unassigned	Unassigned	Unassigned	369_0	Clustered	6	VC_369_0	6	0.5924	1	0.5924	0	0	0	1	Ref
MH179475	Unassigned	Unassigned	Unassigned	130_0	Clustered	19	VC_130_0	19	0.6068	1	0.6068	0	0	0	1	Ref
MH179476	Unassigned	Unassigned	Unassigned	600_2	Clustered	10	VC_600_2	10	0.3041	1	0.3041	0	0	0	1	Ref
MH179477	Unassigned	Unassigned	Unassigned	600_2	Clustered	10	VC_600_2	10	0.3041	1	0.3041	0	0	0	1	Ref
MH179478	Unassigned	Unassigned	Unassigned	130_0	Clustered	19	VC_130_0	19	0.6068	1	0.6068	0	0	0	1	Ref
MH179479	Unassigned	Unassigned	Unassigned	1189_0	Clustered	9	VC_1189_0	9	0.968	1	0.968	0	0	0	1	Ref
MH179480	Unassigned	Unassigned	Unassigned	842_0	Clustered	2	VC_842_0	2	0.0559	0.95564851	0.0534	0	0	0	1	Ref
MH181876	Unassigned	Unassigned	Unassigned	428_0	Clustered	33	VC_428_0	33	0.9915	1	0.9915	0	0	0	1	Ref
MH183162	Unassigned	Unassigned	Unassigned	1345_1	Clustered	2	VC_1345_1	2	0.0956	0.9561938	0.0914	0	0	0	1	Ref
MH191398	Unassigned	Unassigned	Unassigned	792_0	Clustered	35	VC_792_0	35	0.9828	1	0.9828	0	0	0	1	Ref
MH193369	Unassigned	Unassigned	Unassigned	695_0	Clustered	48	VC_695_0	48	0.8162	1	0.8162	0	0	0	1	Ref
MH203051	Unassigned	Unassigned	Unassigned	190_22	Clustered	3	VC_190_22	3	0.0186	0.9985967	0.0185	0	0	0	1	Ref
MH203383	Unassigned	Unassigned	Unassigned	695_0	Clustered	48	VC_695_0	48	0.8162	1	0.8162	0	0	0	1	Ref
MH203384	Unassigned	Unassigned	Unassigned	695_0	Clustered	48	VC_695_0	48	0.8162	1	0.8162	0	0	0	1	Ref
MH206183	Unassigned	Unassigned	Unassigned	178_0	Clustered	5	VC_178_0	5	0.7741	0.99998991	0.7741	0	0	0	1	Ref
MH206184	Unassigned	Unassigned	Unassigned	178_0	Clustered	5	VC_178_0	5	0.7741	0.99998991	0.7741	0	0	0	1	Ref
MH218848	Unassigned	Unassigned	Unassigned	178_0	Clustered	5	VC_178_0	5	0.7741	0.99998991	0.7741	0	0	0	1	Ref
MH220877	Unassigned	Unassigned	Unassigned	274_0	Clustered	2	VC_274_0	2	0.0799	0.953709	0.0762	0	0	0	1	Ref
MH221128	Unassigned	Unassigned	Unassigned	959_6	Clustered/Singleton	1	VC_959_6	1	0	1	0	0	0	0	1	Ref
MH221129	Unassigned	Unassigned	Unassigned	959_5	Clustered	19	VC_959_5	19	0.2626	1	0.2626	0	0	0	1	Ref
MH229862	Unassigned	Unassigned	Unassigned	1353_0	Clustered	10	VC_1353_0	10	1	1	1	0	0	0	1	Ref
MH229863	Unassigned	Unassigned	Unassigned	1353_0	Clustered	10	VC_1353_0	10	1	1	1	0	0	0	1	Ref
MH229864	Unassigned	Unassigned	Unassigned	1353_0	Clustered	10	VC_1353_0	10	1	1	1	0	0	0	1	Ref
MH229865	Unassigned	Unassigned	Unassigned	1353_0	Clustered	10	VC_1353_0	10	1	1	1	0	0	0	1	Ref
MH230177	Unassigned	Unassigned	Unassigned	795_0	Clustered	19	VC_795_0	19	0.6848	1	0.6848	0	0	0	1	Ref
MH230874	Unassigned	Unassigned	Unassigned	371_11	Clustered	31	VC_371_11	31	0.0927	1	0.0927	0	0	0	0.7011	Ref
MH230875	Unassigned	Unassigned	Unassigned	371_3	Clustered	22	VC_371_3	22	0.0787	1	0.0787	0	0	0	0.9048	Ref
MH230876	Unassigned	Unassigned	Unassigned	325_24	Clustered	3	VC_325_24	3	0.009	0.99853949	0.009	0	0	0	1	Ref
MH230877	Unassigned	Unassigned	Unassigned	517_1	Clustered	108	VC_517_1	108	0.8483	1	0.8483	0	0	0	0.9955	Ref
MH230878	Unassigned	Unassigned	Unassigned	325_52	Clustered	5	VC_325_52	5	0.0147	0.99999073	0.0147	0	0	0	0.7	Ref
MH243438	Unassigned	Unassigned	Unassigned	286_1	Clustered	21	VC_286_1	21	0.1696	1	0.1696	0	0	0	0.9048	Ref
MH243439	Unassigned	Unassigned	Unassigned	283_181	Clustered/Singleton	1	VC_283_181	1	0	1	0	0	0	0	1	Ref
MH248069	Unassigned	Unassigned	Unassigned	898_0	Clustered	10	VC_898_0	10	0.7284	1	0.7284	0	0	0	1	Ref
MH248138	Unassigned	Unassigned	Unassigned	67_3	Clustered	20	VC_67_3	20	0.3542	1	0.3542	0	0	0	1	Ref
MH248947	Unassigned	Unassigned	Unassigned	982_0	Clustered	62	VC_982_0	62	0.8646	1	0.8646	0	0	0	0.743	Ref
MH252123	Unassigned	Unassigned	Unassigned	847_1	Clustered	26	VC_847_1	26	0.6323	1	0.6323	0	0	0	0.9815	Ref
MH252365	Unassigned	Unassigned	Unassigned	125_0	Clustered	17	VC_125_0	17	0.9244	1	0.9244	0	0	0	1	Ref
MH268168	Unassigned	Unassigned	Unassigned	130_0	Clustered	19	VC_130_0	19	0.6068	1	0.6068	0	0	0	1	Ref
MH271291	Unassigned	Unassigned	Unassigned	956_0	Clustered	39	VC_956_0	39	1	1	1	0	0	0	1	Ref
MH271292	Unassigned	Unassigned	Unassigned	1343_0	Clustered	21	VC_1343_0	21	0.9843	1	0.9843	0	0	0	1	Ref
MH271293	Unassigned	Unassigned	Unassigned	956_0	Clustered	39	VC_956_0	39	1	1	1	0	0	0	1	Ref
MH271294	Unassigned	Unassigned	Unassigned	956_0	Clustered	39	VC_956_0	39	1	1	1	0	0	0	1	Ref
MH271295	Unassigned	Unassigned	Unassigned	1175_0	Clustered	22	VC_1175_0	22	0.4562	1	0.4562	0	0	0	1	Ref
MH271296	Unassigned	Unassigned	Unassigned	949_0	Clustered	10	VC_949_0	10	0.5977	1	0.5977	0	0	0	1	Ref
MH271297	Unassigned	Unassigned	Unassigned	956_0	Clustered	39	VC_956_0	39	1	1	1	0	0	0	1	Ref
MH271298	Unassigned	Unassigned	Unassigned	1206_0	Clustered	8	VC_1206_0	8	0.1913	1	0.1913	0	0	0	1	Ref
MH271299	Unassigned	Unassigned	Unassigned	956_0	Clustered	39	VC_956_0	39	1	1	1	0	0	0	1	Ref
MH271300	Unassigned	Unassigned	Unassigned	856_1	Clustered	13	VC_856_1	13	0.5848	1	0.5848	0	0	0	1	Ref
MH271301	Unassigned	Unassigned	Unassigned	1343_0	Clustered	21	VC_1343_0	21	0.9843	1	0.9843	0	0	0	1	Ref
MH271302	Unassigned	Unassigned	Unassigned	919_0	Clustered	39	VC_919_0	39	0.9069	1	0.9069	0	0	0	1	Ref

MH271303	Unassigned	Unassigned	Unassigned	1349_0	Clustered	7	VC_1349_0	7	0.2495	1	0.2495	0	0	0	1	Ref
MH271304	Unassigned	Unassigned	Unassigned	1352_0	Clustered	31	VC_1352_0	31	0.849	1	0.849	0	0	0	1	Ref
MH271305	Unassigned	Unassigned	Unassigned	410_0	Clustered	7	VC_410_0	7	0.7905	1	0.7905	0	0	0	1	Ref
MH271306	Unassigned	Unassigned	Unassigned	956_0	Clustered	39	VC_956_0	39	1	1	1	0	0	0	1	Ref
MH271307	Unassigned	Unassigned	Unassigned	856_1	Clustered	13	VC_856_1	13	0.5848	1	0.5848	0	0	0	1	Ref
MH271308	Unassigned	Unassigned	Unassigned	1206_0	Clustered	8	VC_1206_0	8	0.1913	1	0.1913	0	0	0	1	Ref
MH271309	Unassigned	Unassigned	Unassigned	956_0	Clustered	39	VC_956_0	39	1	1	1	0	0	0	1	Ref
MH271310	Unassigned	Unassigned	Unassigned	856_1	Clustered	13	VC_856_1	13	0.5848	1	0.5848	0	0	0	1	Ref
MH271311	Unassigned	Unassigned	Unassigned	956_0	Clustered	39	VC_956_0	39	1	1	1	0	0	0	1	Ref
MH271312	Unassigned	Unassigned	Unassigned	1352_0	Clustered	31	VC_1352_0	31	0.849	1	0.849	0	0	0	1	Ref
MH271313	Unassigned	Unassigned	Unassigned	570_0	Clustered	9	VC_570_0	9	0.499	1	0.499	0	0	0	1	Ref
MH271314	Unassigned	Unassigned	Unassigned	956_0	Clustered	39	VC_956_0	39	1	1	1	0	0	0	1	Ref
MH271315	Unassigned	Unassigned	Unassigned	956_0	Clustered	39	VC_956_0	39	1	1	1	0	0	0	1	Ref
MH271316	Unassigned	Unassigned	Unassigned	856_1	Clustered	13	VC_856_1	13	0.5848	1	0.5848	0	0	0	1	Ref
MH271317	Unassigned	Unassigned	Unassigned	1200_0	Clustered	4	VC_1200_0	4	0.8691	0.9999992	0.8691	0	0	0	1	Ref
MH271318	Unassigned	Unassigned	Unassigned	410_0	Clustered	7	VC_410_0	7	0.7905	1	0.7905	0	0	0	1	Ref
MH271319	Unassigned	Unassigned	Unassigned	956_0	Clustered	39	VC_956_0	39	1	1	1	0	0	0	1	Ref
MH271320	Unassigned	Unassigned	Unassigned	1206_0	Clustered	8	VC_1206_0	8	0.1913	1	0.1913	0	0	0	1	Ref
MH271321	Unassigned	Unassigned	Unassigned	1352_0	Clustered	31	VC_1352_0	31	0.849	1	0.849	0	0	0	1	Ref
MH285980	Unassigned	Unassigned	Unassigned	575_0	Clustered	35	VC_575_0	35	0.8815	1	0.8815	0	0	0	1	Ref
MH299806	Unassigned	Unassigned	Unassigned	980_0	Clustered	10	VC_980_0	10	0.9996	1	0.9996	0	0	0	1	Ref
MH310933	Unassigned	Unassigned	Unassigned	495_0	Clustered	39	VC_495_0	39	0.308	1	0.308	0	0	0	1	Ref
MH310934	Unassigned	Unassigned	Unassigned	495_1	Clustered	41	VC_495_1	41	0.328	1	0.328	0	0	0	0.911	Ref
MH310935	Unassigned	Unassigned	Unassigned	495_0	Clustered	39	VC_495_0	39	0.308	1	0.308	0	0	0	1	Ref
MH310936	Unassigned	Unassigned	Unassigned	495_1	Clustered	41	VC_495_1	41	0.328	1	0.328	0	0	0	0.911	Ref
MH316561	Unassigned	Unassigned	Unassigned	558_14	Clustered	8	VC_558_14	8	0.0228	0.99945246	0.0228	0	0	0	0.8214	Ref
MH316562	Unassigned	Unassigned	Unassigned	565_0	Clustered	20	VC_565_0	20	0.8398	1	0.8398	0	0	0	1	Ref
MH316563	Unassigned	Unassigned	Unassigned	513_0	Clustered	32	VC_513_0	32	0.6881	1	0.6881	0	0	0	1	Ref
MH316564	Unassigned	Unassigned	Unassigned	520_45	Clustered	7	VC_520_45	7	0.0635	1	0.0635	0	0	0	0.8095	Ref
MH316565	Unassigned	Unassigned	Unassigned	509_0	Clustered	37	VC_509_0	37	0.9689	1	0.9689	0	0	0	1	Ref
MH316566	Unassigned	Unassigned	Unassigned	224_0	Clustered	19	VC_224_0	19	0.9416	1	0.9416	0	0	0	1	Ref
MH316567	Unassigned	Unassigned	Unassigned	558_50	Clustered	15	VC_558_50	15	0.0468	1	0.0468	0	0	0	0.9238	Ref
MH316568	Unassigned	Unassigned	Unassigned	371_13	Clustered	33	VC_371_13	33	0.179	1	0.179	0	0	0	0.8769	Ref
MH316569	Unassigned	Unassigned	Unassigned	956_0	Clustered	39	VC_956_0	39	1	1	1	0	0	0	1	Ref
MH316570	Unassigned	Unassigned	Unassigned	521_0	Clustered	37	VC_521_0	37	0.912	1	0.912	0	0	0	1	Ref
MH319720	Unassigned	Unassigned	Unassigned	438_0	Clustered	6	VC_438_0	6	0.0917	0.99994271	0.0917	0	0	0	1	Ref
MH319733	Unassigned	Unassigned	Unassigned	438_0	Clustered	6	VC_438_0	6	0.0917	0.99994271	0.0917	0	0	0	1	Ref
MH319737	Unassigned	Unassigned	Unassigned	659_0	Clustered	2	VC_659_0	2	0.0254	0.54598917	0.0139	0	0	0	1	Ref
MH319742	Unassigned	Unassigned	Unassigned	438_0	Clustered	6	VC_438_0	6	0.0917	0.99994271	0.0917	0	0	0	1	Ref
MH319746	Unassigned	Unassigned	Unassigned	659_0	Clustered	2	VC_659_0	2	0.0254	0.54598917	0.0139	0	0	0	1	Ref
MH319749	Unassigned	Unassigned	Unassigned	432_6	Clustered/Singleton	1	VC_432_6	1	0	1	0	0	0	0	1	Ref
MH319750	Unassigned	Unassigned	Unassigned	439_0	Clustered	2	VC_439_0	2	0.0291	0.90533848	0.0263	0	0	0	1	Ref
MH319753	Unassigned	Unassigned	Unassigned	438_0	Clustered	6	VC_438_0	6	0.0917	0.99994271	0.0917	0	0	0	1	Ref
MH319754	Unassigned	Unassigned	Unassigned	432_7	Clustered/Singleton	1	VC_432_7	1	0	1	0	0	0	0	1	Ref
MH319758	Unassigned	Unassigned	Unassigned	438_0	Clustered	6	VC_438_0	6	0.0917	0.99994271	0.0917	0	0	0	1	Ref
MH319759	Unassigned	Unassigned	Unassigned	439_0	Clustered	2	VC_439_0	2	0.0291	0.90533848	0.0263	0	0	0	1	Ref
MH321490	Unassigned	Unassigned	Unassigned	150_0	Clustered	13	VC_150_0	13	0.2417	1	0.2417	0	0	0	0.9872	Ref
MH321491	Unassigned	Unassigned	Unassigned	150_0	Clustered	13	VC_150_0	13	0.2417	1	0.2417	0	0	0	0.9872	Ref
MH321492	Unassigned	Unassigned	Unassigned	9_0	Clustered	38	VC_9_0	38	0.4895	1	0.4895	0	0	0	1	Ref
MH321493	Unassigned	Unassigned	Unassigned	461_0	Clustered	96	VC_461_0	96	0.9307	1	0.9307	0	0	0	0.9575	Ref
MH327487	Unassigned	Unassigned	Unassigned	438_0	Clustered	6	VC_438_0	6	0.0917	0.99994271	0.0917	0	0	0	1	Ref
MH333064	Unassigned	Unassigned	Unassigned	491_0	Clustered	36	VC_491_0	36	0.5336	1	0.5336	0	0	0	0.9714	Ref
MH338234	Unassigned	Unassigned	Unassigned	216_6	Clustered	42	VC_216_6	42	0.473	1	0.473	0	0	0	0.8351	Ref
MH338235	Unassigned	Unassigned	Unassigned	940_0	Clustered	34	VC_940_0	34	0.7959	1	0.7959	0	0	0	1	Ref
MH338236	Unassigned	Unassigned	Unassigned	221_0	Clustered	22	VC_221_0	22	0.7447	1	0.7447	0	0	0	1	Ref
MH338237	Unassigned	Unassigned	Unassigned	221_0	Clustered	22	VC_221_0	22	0.7447	1	0.7447	0	0	0	1	Ref
MH338238	Unassigned	Unassigned	Unassigned	325_19	Clustered	3	VC_325_19	3	0.0108	0.99859221	0.0107	0	0	0	1	Ref
MH338239	Unassigned	Unassigned	Unassigned	325_60	Clustered	2	VC_325_60	2	0.0038	0.957513	0.0036	0	0	0	1	Ref
MH338240	Unassigned	Unassigned	Unassigned	514_0	Clustered	75	VC_514_0	75	0.9402	1	0.9402	0	0	0	1	Ref
MH338241	Unassigned	Unassigned	Unassigned	940_0	Clustered	34	VC_940_0	34	0.7959	1	0.7959	0	0	0	1	Ref
MH341451	Unassigned	Unassigned	Unassigned	380_0	Clustered	3	VC_380_0	3	0.1631	0.99675097	0.1625	0	0	0	1	Ref
MH341452	Unassigned	Unassigned	Unassigned	378_0	Clustered	9	VC_378_0	9	0.7603	1	0.7603	0	0	0	1	Ref
MH341453	Unassigned	Unassigned	Unassigned	140_0	Clustered	2	VC_140_0	2	0.4111	0.94824791	0.3898	0	0	0	1	Ref
MH355583	Unassigned	Unassigned	Unassigned	695_0	Clustered	48	VC_695_0	48	0.8162	1	0.8162	0	0	0	1	Ref
MH355584	Unassigned	Unassigned	Unassigned	283_45	Clustered/Singleton	1	VC_283_45	1	0	1	0	0	0	0	1	Ref
MH356729	Unassigned	Unassigned	Unassigned	838_0	Clustered	29	VC_838_0	29	0.8611	1	0.8611	0	0	0	1	Ref
MH356730	Unassigned	Unassigned	Unassigned	667_1	Clustered/Singleton	1	VC_667_1	1	0	1	0	0	0	0	1	Ref
MH358359	Unassigned	Unassigned	Unassigned	637_0	Clustered	115	VC_637_0	115	0.731	1	0.731	0	0	0	0.8764	Ref
MH358458	Unassigned	Unassigned	Unassigned	779_0	Clustered	37	VC_779_0	37	0.5682	1	0.5682	0	0	0	1	Ref
MH359124	Unassigned	Unassigned	Unassigned	286_11	Clustered	3	VC_286_11	3	0.0146	0.93460284	0.0137	0	0	0	1	Ref
MH363700	Unassigned	Unassigned	Unassigned	134_0	Clustered	3	VC_134_0	3	0.0956	0.99858494	0.0955	0	0	0	1	Ref
MH363708	Unassigned	Unassigned	Unassigned	705_0	Clustered	11	VC_705_0	11	0.8861	1	0.8861	0	0	0	1	Ref
MH370358	Unassigned	Unassigned	Unassigned	637_0	Clustered	115	VC_637_0	115	0.731	1	0.731	0	0	0	0.8764	Ref
MH370359	Unassigned	Unassigned	Unassigned	637_0	Clustered	115	VC_637_0	115	0.731	1	0.731	0	0	0	0.8764	Ref
MH370360	Unassigned	Unassigned	Unassigned	637_0	Clustered	115	VC_637_0	115	0.731	1	0.731	0	0	0	0.8764	Ref
MH370361	Unassigned	Unassigned	Unassigned	637_0	Clustered	115	VC_637_0	115	0.731	1	0.731	0	0	0	0.8764	Ref
MH370362	Unassigned	Unassigned	Unassigned	637_0	Clustered	115	VC_637_0	115	0.731	1	0.731	0	0	0	0.8764	Ref
MH370363	Unassigned	Unassigned	Unassigned	637_0	Clustered	115	VC_637_0	115	0.731	1	0.731	0	0	0	0.8764	Ref
MH370365	Unassigned	Unassigned	Unassigned	637_0	Clustered	115	VC_637_0	115	0.731	1	0.731	0	0	0	0.8764	Ref
MH370366	Unassigned	Unassigned	Unassigned	191_4	Clustered	2	VC_191_4	2	0.0055	0.88936514	0.0049	0	0	0	1	Ref
MH370367	Unassigned	Unassigned	Unassigned	191_9	Clustered	5	VC_191_9	5	0.0228	0.99999662	0.0228	0	0	0	0.7	Ref

MH370368	Unassigned	Unassigned	Unassigned	133_3	Clustered	3	VC_133_3	3	0.0105	0.99119575	0.0104	0	0	0	1	Ref
MH370369	Unassigned	Unassigned	Unassigned	191_16	Clustered	12	VC_191_16	12	0.0643	1	0.0643	0	0	0	0.9394	Ref
MH370370	Unassigned	Unassigned	Unassigned	133_31	Clustered	6	VC_133_31	6	0.0263	0.99999694	0.0263	0	0	0	1	Ref
MH370371	Unassigned	Unassigned	Unassigned	133_3	Clustered	3	VC_133_3	3	0.0105	0.99119575	0.0104	0	0	0	1	Ref
MH370372	Unassigned	Unassigned	Unassigned	637_0	Clustered	115	VC_637_0	115	0.731	1	0.731	0	0	0	0.8764	Ref
MH370373	Unassigned	Unassigned	Unassigned	637_0	Clustered	115	VC_637_0	115	0.731	1	0.731	0	0	0	0.8764	Ref
MH370374	Unassigned	Unassigned	Unassigned	637_0	Clustered	115	VC_637_0	115	0.731	1	0.731	0	0	0	0.8764	Ref
MH370375	Unassigned	Unassigned	Unassigned	191_29	Clustered/Singleton	1	VC_191_29	1	0	1	0	0	0	0	1	Ref
MH370376	Unassigned	Unassigned	Unassigned	191_8	Clustered	2	VC_191_8	2	0.0055	0.91831352	0.0051	0	0	0	1	Ref
MH370377	Unassigned	Unassigned	Unassigned	190_13	Clustered	4	VC_190_13	4	0.0216	0.99997041	0.0216	0	0	0	0.6667	Ref
MH370378	Unassigned	Unassigned	Unassigned	190_13	Clustered	4	VC_190_13	4	0.0216	0.99997041	0.0216	0	0	0	0.6667	Ref
MH370379	Unassigned	Unassigned	Unassigned	191_23	Clustered/Singleton	1	VC_191_23	1	0	1	0	0	0	0	1	Ref
MH370380	Unassigned	Unassigned	Unassigned	191_9	Clustered	5	VC_191_9	5	0.0228	0.99999662	0.0228	0	0	0	0.7	Ref
MH370381	Unassigned	Unassigned	Unassigned	637_0	Clustered	115	VC_637_0	115	0.731	1	0.731	0	0	0	0.8764	Ref
MH370384	Unassigned	Unassigned	Unassigned	637_0	Clustered	115	VC_637_0	115	0.731	1	0.731	0	0	0	0.8764	Ref
MH370385	Unassigned	Unassigned	Unassigned	637_0	Clustered	115	VC_637_0	115	0.731	1	0.731	0	0	0	0.8764	Ref
MH370386	Unassigned	Unassigned	Unassigned	191_5	Clustered	6	VC_191_5	6	0.0285	0.99999996	0.0285	0	0	0	1	Ref
MH370387	Unassigned	Unassigned	Unassigned	28_1	Clustered	53	VC_28_1	53	0.7164	1	0.7164	0	0	0	0.9325	Ref
MH370477	Unassigned	Unassigned	Unassigned	394_0	Clustered	2	VC_394_0	2	0.0211	0.957922	0.0202	0	0	0	1	Ref
MH370478	Unassigned	Unassigned	Unassigned	76_0	Clustered	37	VC_76_0	37	0.9403	1	0.9403	0	0	0	1	Ref
MH371107	Unassigned	Unassigned	Unassigned	371_13	Clustered	33	VC_371_13	33	0.179	1	0.179	0	0	0	0.8769	Ref
MH371108	Unassigned	Unassigned	Unassigned	856_0	Clustered	7	VC_856_0	7	0.3463	1	0.3463	0	0	0	1	Ref
MH371109	Unassigned	Unassigned	Unassigned	856_0	Clustered	7	VC_856_0	7	0.3463	1	0.3463	0	0	0	1	Ref
MH371110	Unassigned	Unassigned	Unassigned	325_36	Clustered	2	VC_325_36	2	0.0043	0.95751115	0.0041	0	0	0	1	Ref
MH371111	Unassigned	Unassigned	Unassigned	1344_0	Clustered	78	VC_1344_0	78	0.9732	1	0.9732	0	0	0	1	Ref
MH371112	Unassigned	Unassigned	Unassigned	551_6	Clustered	22	VC_551_6	22	0.1214	1	0.1214	0	0	0	0.8095	Ref
MH371113	Unassigned	Unassigned	Unassigned	236_0	Clustered	72	VC_236_0	72	0.8104	1	0.8104	0	0	0	1	Ref
MH371114	Unassigned	Unassigned	Unassigned	371_16	Clustered	42	VC_371_16	42	0.1341	1	0.1341	0	0	0	0.7282	Ref
MH371115	Unassigned	Unassigned	Unassigned	520_40	Clustered	4	VC_520_40	4	0.0396	0.99998776	0.0396	0	0	0	1	Ref
MH371116	Unassigned	Unassigned	Unassigned	761_0	Clustered	67	VC_761_0	67	0.8947	1	0.8947	0	0	0	1	Ref
MH371117	Unassigned	Unassigned	Unassigned	371_16	Clustered	42	VC_371_16	42	0.1341	1	0.1341	0	0	0	0.7282	Ref
MH371118	Unassigned	Unassigned	Unassigned	1344_0	Clustered	78	VC_1344_0	78	0.9732	1	0.9732	0	0	0	1	Ref
MH371119	Unassigned	Unassigned	Unassigned	761_0	Clustered	67	VC_761_0	67	0.8947	1	0.8947	0	0	0	1	Ref
MH371120	Unassigned	Unassigned	Unassigned	520_39	Clustered/Singleton	1	VC_520_39	1	0	1	0	0	0	0	1	Ref
MH371121	Unassigned	Unassigned	Unassigned	514_0	Clustered	75	VC_514_0	75	0.9402	1	0.9402	0	0	0	1	Ref
MH371122	Unassigned	Unassigned	Unassigned	225_0	Clustered	32	VC_225_0	32	0.848	1	0.848	0	0	0	0.996	Ref
MH371123	Unassigned	Unassigned	Unassigned	520_33	Clustered	2	VC_520_33	2	0.0087	0.95761571	0.0083	0	0	0	1	Ref
MH371124	Unassigned	Unassigned	Unassigned	1344_0	Clustered	78	VC_1344_0	78	0.9732	1	0.9732	0	0	0	1	Ref
MH371125	Unassigned	Unassigned	Unassigned	371_16	Clustered	42	VC_371_16	42	0.1341	1	0.1341	0	0	0	0.7282	Ref
MH375074	Unassigned	Unassigned	Unassigned	695_0	Clustered	48	VC_695_0	48	0.8162	1	0.8162	0	0	0	1	Ref
MH375593	Unassigned	Unassigned	Unassigned	254_0	Clustered	46	VC_254_0	46	0.5159	1	0.5159	0	0	0	1	Ref
MH375594	Unassigned	Unassigned	Unassigned	252_0	Clustered	121	VC_252_0	121	0.7376	1	0.7376	0	0	0	0.9999	Ref
MH375595	Unassigned	Unassigned	Unassigned	252_0	Clustered	121	VC_252_0	121	0.7376	1	0.7376	0	0	0	0.9999	Ref
MH375596	Unassigned	Unassigned	Unassigned	252_0	Clustered	121	VC_252_0	121	0.7376	1	0.7376	0	0	0	0.9999	Ref
MH375597	Unassigned	Unassigned	Unassigned	252_0	Clustered	121	VC_252_0	121	0.7376	1	0.7376	0	0	0	0.9999	Ref
MH375598	Unassigned	Unassigned	Unassigned	252_0	Clustered	121	VC_252_0	121	0.7376	1	0.7376	0	0	0	0.9999	Ref
MH375599	Unassigned	Unassigned	Unassigned	252_0	Clustered	121	VC_252_0	121	0.7376	1	0.7376	0	0	0	0.9999	Ref
MH375600	Unassigned	Unassigned	Unassigned	252_0	Clustered	121	VC_252_0	121	0.7376	1	0.7376	0	0	0	0.9999	Ref
MH375644	Unassigned	Unassigned	Unassigned	136_0	Clustered	2	VC_136_0	2	0.0412	0.95741268	0.0394	0	0	0	1	Ref
MH378441	Unassigned	Unassigned	Unassigned	303_0	Clustered	155	VC_303_0	155	0.9979	1	0.9979	0	0	0	1	Ref
MH378442	Unassigned	Unassigned	Unassigned	303_0	Clustered	155	VC_303_0	155	0.9979	1	0.9979	0	0	0	1	Ref
MH378443	Unassigned	Unassigned	Unassigned	303_0	Clustered	155	VC_303_0	155	0.9979	1	0.9979	0	0	0	1	Ref
MH382198	Unassigned	Unassigned	Unassigned	392_0	Clustered	126	VC_392_0	126	0.9621	1	0.9621	0	0	0	1	Ref
MH383160	Unassigned	Unassigned	Unassigned	164_11	Clustered	10	VC_164_11	10	0.1507	1	0.1507	0	0	0	0.9111	Ref
MH384259	Unassigned	Unassigned	Unassigned	10_0	Clustered	25	VC_10_0	25	0.3467	1	0.3467	0	0	0	0.9733	Ref
MH384260	Unassigned	Unassigned	Unassigned	7_0	Clustered	28	VC_7_0	28	0.3916	1	0.3916	0	0	0	0.9947	Ref
MH384261	Unassigned	Unassigned	Unassigned	8_0	Clustered	58	VC_8_0	58	0.8306	1	0.8306	0	0	0	0.9946	Ref
MH389777	Unassigned	Unassigned	Unassigned	67_4	Clustered	4	VC_67_4	4	0.0431	0.90943035	0.0392	0	0	0	1	Ref
MH393889	Unassigned	Unassigned	Unassigned	899_0	Clustered	4	VC_899_0	4	0.2655	0.99997104	0.2655	0	0	0	1	Ref
MH399771	Unassigned	Unassigned	Unassigned	520_42	Clustered/Singleton	1	VC_520_42	1	0	1	0	0	0	0	1	Ref
MH399772	Unassigned	Unassigned	Unassigned	546_0	Clustered	2	VC_546_0	2	0.0136	0.63748598	0.0087	0	0	0	1	Ref
MH399773	Unassigned	Unassigned	Unassigned	371_16	Clustered	42	VC_371_16	42	0.1341	1	0.1341	0	0	0	0.7282	Ref
MH399774	Unassigned	Unassigned	Unassigned	551_0	Clustered	16	VC_551_0	16	0.0915	1	0.0915	0	0	0	0.6667	Ref
MH399775	Unassigned	Unassigned	Unassigned	371_15	Clustered	44	VC_371_15	44	0.1391	1	0.1391	0	0	0	0.7474	Ref
MH399776	Unassigned	Unassigned	Unassigned	514_0	Clustered	75	VC_514_0	75	0.9402	1	0.9402	0	0	0	1	Ref
MH399777	Unassigned	Unassigned	Unassigned	517_1	Clustered	108	VC_517_1	108	0.8483	1	0.8483	0	0	0	0.9955	Ref
MH399778	Unassigned	Unassigned	Unassigned	551_0	Clustered	16	VC_551_0	16	0.0915	1	0.0915	0	0	0	0.6667	Ref
MH399779	Unassigned	Unassigned	Unassigned	856_1	Clustered	13	VC_856_1	13	0.5848	1	0.5848	0	0	0	1	Ref
MH399780	Unassigned	Unassigned	Unassigned	371_16	Clustered	42	VC_371_16	42	0.1341	1	0.1341	0	0	0	0.7282	Ref
MH399781	Unassigned	Unassigned	Unassigned	954_0	Clustered	13	VC_954_0	13	0.863	1	0.863	0	0	0	1	Ref
MH399782	Unassigned	Unassigned	Unassigned	325_53	Clustered	7	VC_325_53	7	0.0242	1	0.0242	0	0	0	0.9048	Ref
MH399783	Unassigned	Unassigned	Unassigned	1344_0	Clustered	78	VC_1344_0	78	0.9732	1	0.9732	0	0	0	1	Ref
MH399784	Unassigned	Unassigned	Unassigned	520_45	Clustered	7	VC_520_45	7	0.0635	1	0.0635	0	0	0	0.8095	Ref
MH399785	Unassigned	Unassigned	Unassigned	517_1	Clustered	108	VC_517_1	108	0.8483	1	0.8483	0	0	0	0.9955	Ref
MH399786	Unassigned	Unassigned	Unassigned	371_15	Clustered	44	VC_371_15	44	0.1391	1	0.1391	0	0	0	0.7474	Ref
MH399787	Unassigned	Unassigned	Unassigned	521_0	Clustered	37	VC_521_0	37	0.912	1	0.912	0	0	0	1	Ref
MH399788	Unassigned	Unassigned	Unassigned	565_0	Clustered	20	VC_565_0	20	0.8398	1	0.8398	0	0	0	1	Ref
MH399789	Unassigned	Unassigned	Unassigned	1164_0	Clustered	25	VC_1164_0	25	0.7312	1	0.7312	0	0	0	1	Ref
MH400309	Unassigned	Unassigned	Unassigned	451_0	Clustered	80	VC_451_0	80	0.9991	1	0.9991	0	0	0	1	Ref
MH401414	Unassigned	Unassigned	Unassigned	7_0	Clustered	28	VC_7_0	28	0.3916	1	0.3916	0	0	0	0.9947	Ref
MH401415	Unassigned	Unassigned	Unassigned	7_0	Clustered	28	VC_7_0	28	0.3916	1	0.3916	0	0	0	0.9947	Ref

MH401416	Unassigned	Unassigned	Unassigned	10_0	Clustered	25	VC_10_0	25	0.3467	1	0.3467	0	0	0	0.9733	Ref
MH412654	Unassigned	Unassigned	Unassigned	432_1	Clustered/Singleton	1	VC_432_1	1	0	1	0	0	0	0	1	Ref
MH422554	Unassigned	Unassigned	Unassigned	247_0	Clustered	14	VC_247_0	14	0.9119	1	0.9119	0	0	0	0.967	Ref
MH424443	Unassigned	Unassigned	Unassigned	637_0	Clustered	115	VC_637_0	115	0.731	1	0.731	0	0	0	0.8764	Ref
MH424444	Unassigned	Unassigned	Unassigned	540_0	Clustered	15	VC_540_0	15	0.2866	1	0.2866	0	0	0	1	Ref
MH424445	Unassigned	Unassigned	Unassigned	637_0	Clustered	115	VC_637_0	115	0.731	1	0.731	0	0	0	0.8764	Ref
MH424446	Unassigned	Unassigned	Unassigned	461_0	Clustered	96	VC_461_0	96	0.9307	1	0.9307	0	0	0	0.9575	Ref
MH426724	Unassigned	Unassigned	Unassigned	959_3	Clustered	3	VC_959_3	3	0.0237	0.75911548	0.018	0	0	0	1	Ref
MH426725	Unassigned	Unassigned	Unassigned	328_0	Clustered	11	VC_328_0	11	0.4707	1	0.4707	0	0	0	1	Ref
MH426726	Unassigned	Unassigned	Unassigned	359_0	Clustered	7	VC_359_0	7	0.3703	1	0.3703	0	0	0	1	Ref
MH427377	Unassigned	Unassigned	Unassigned	133_22	Clustered	13	VC_133_22	13	0.0804	1	0.0804	0	0	0	0.8205	Ref
MH431931	Unassigned	Unassigned	Unassigned	708_0	Clustered	47	VC_708_0	47	0.8654	1	0.8654	0	0	0	1	Ref
MH431932	Unassigned	Unassigned	Unassigned	708_0	Clustered	47	VC_708_0	47	0.8654	1	0.8654	0	0	0	1	Ref
MH431933	Unassigned	Unassigned	Unassigned	708_0	Clustered	47	VC_708_0	47	0.8654	1	0.8654	0	0	0	1	Ref
MH431934	Unassigned	Unassigned	Unassigned	708_0	Clustered	47	VC_708_0	47	0.8654	1	0.8654	0	0	0	1	Ref
MH431935	Unassigned	Unassigned	Unassigned	708_0	Clustered	47	VC_708_0	47	0.8654	1	0.8654	0	0	0	1	Ref
MH431936	Unassigned	Unassigned	Unassigned	708_0	Clustered	47	VC_708_0	47	0.8654	1	0.8654	0	0	0	1	Ref
MH431937	Unassigned	Unassigned	Unassigned	708_0	Clustered	47	VC_708_0	47	0.8654	1	0.8654	0	0	0	1	Ref
MH431938	Unassigned	Unassigned	Unassigned	1156_0	Clustered	8	VC_1156_0	8	0.8137	1	0.8137	0	0	0	1	Ref
MH443100	Unassigned	Unassigned	Unassigned	32_0	Clustered/Singleton	1	VC_32_0	1	0	1	0	0	0	0	1	Ref
MH444512	Unassigned	Unassigned	Unassigned	851_0	Clustered	2	VC_851_0	2	0.4472	0.9410422	0.4209	0	0	0	1	Ref
MH445380	Unassigned	Unassigned	Unassigned	247_0	Clustered	14	VC_247_0	14	0.9119	1	0.9119	0	0	0	0.967	Ref
MH445381	Unassigned	Unassigned	Unassigned	247_0	Clustered	14	VC_247_0	14	0.9119	1	0.9119	0	0	0	0.967	Ref
MH445453	Unassigned	Unassigned	Unassigned	938_0	Clustered	99	VC_938_0	99	0.9119	1	0.9119	0	0	0	0.9928	Ref
MH447526	Unassigned	Unassigned	Unassigned	1354_0	Clustered	3	VC_1354_0	3	0.983	0.9595722	0.9432	0	0	0	1	Ref
MH450113	Unassigned	Unassigned	Unassigned	325_23	Clustered	17	VC_325_23	17	0.0716	1	0.0716	0	0	0	0.6397	Ref
MH450114	Unassigned	Unassigned	Unassigned	1167_0	Clustered	6	VC_1167_0	6	0.8642	0.99999946	0.8642	0	0	0	1	Ref
MH450115	Unassigned	Unassigned	Unassigned	1164_0	Clustered	25	VC_1164_0	25	0.7312	1	0.7312	0	0	0	1	Ref
MH450116	Unassigned	Unassigned	Unassigned	371_16	Clustered	42	VC_371_16	42	0.1341	1	0.1341	0	0	0	0.7282	Ref
MH450117	Unassigned	Unassigned	Unassigned	1160_0	Clustered	73	VC_1160_0	73	0.9835	1	0.9835	0	0	0	1	Ref
MH450118	Unassigned	Unassigned	Unassigned	1160_0	Clustered	73	VC_1160_0	73	0.9835	1	0.9835	0	0	0	1	Ref
MH450119	Unassigned	Unassigned	Unassigned	514_0	Clustered	75	VC_514_0	75	0.9402	1	0.9402	0	0	0	1	Ref
MH450120	Unassigned	Unassigned	Unassigned	802_0	Clustered	17	VC_802_0	17	0.8025	1	0.8025	0	0	0	1	Ref
MH450121	Unassigned	Unassigned	Unassigned	1160_0	Clustered	73	VC_1160_0	73	0.9835	1	0.9835	0	0	0	1	Ref
MH450122	Unassigned	Unassigned	Unassigned	371_16	Clustered	42	VC_371_16	42	0.1341	1	0.1341	0	0	0	0.7282	Ref
MH450124	Unassigned	Unassigned	Unassigned	514_0	Clustered	75	VC_514_0	75	0.9402	1	0.9402	0	0	0	1	Ref
MH450125	Unassigned	Unassigned	Unassigned	1164_0	Clustered	25	VC_1164_0	25	0.7312	1	0.7312	0	0	0	1	Ref
MH450126	Unassigned	Unassigned	Unassigned	514_0	Clustered	75	VC_514_0	75	0.9402	1	0.9402	0	0	0	1	Ref
MH450127	Unassigned	Unassigned	Unassigned	761_0	Clustered	67	VC_761_0	67	0.8947	1	0.8947	0	0	0	1	Ref
MH450128	Unassigned	Unassigned	Unassigned	514_0	Clustered	75	VC_514_0	75	0.9402	1	0.9402	0	0	0	1	Ref
MH450129	Unassigned	Unassigned	Unassigned	1093_0	Clustered	40	VC_1093_0	40	0.8754	1	0.8754	0	0	0	1	Ref
MH450130	Unassigned	Unassigned	Unassigned	325_23	Clustered	17	VC_325_23	17	0.0716	1	0.0716	0	0	0	0.6397	Ref
MH450131	Unassigned	Unassigned	Unassigned	1160_0	Clustered	73	VC_1160_0	73	0.9835	1	0.9835	0	0	0	1	Ref
MH450132	Unassigned	Unassigned	Unassigned	919_0	Clustered	39	VC_919_0	39	0.9069	1	0.9069	0	0	0	1	Ref
MH450133	Unassigned	Unassigned	Unassigned	325_61	Clustered	8	VC_325_61	8	0.0395	1	0.0395	0	0	0	0.7857	Ref
MH454076	Unassigned	Unassigned	Unassigned	1156_0	Clustered	8	VC_1156_0	8	0.8137	1	0.8137	0	0	0	1	Ref
MH454077	Unassigned	Unassigned	Unassigned	708_0	Clustered	47	VC_708_0	47	0.8654	1	0.8654	0	0	0	1	Ref
MH454078	Unassigned	Unassigned	Unassigned	708_0	Clustered	47	VC_708_0	47	0.8654	1	0.8654	0	0	0	1	Ref
MH454079	Unassigned	Unassigned	Unassigned	708_0	Clustered	47	VC_708_0	47	0.8654	1	0.8654	0	0	0	1	Ref
MH454080	Unassigned	Unassigned	Unassigned	1156_0	Clustered	8	VC_1156_0	8	0.8137	1	0.8137	0	0	0	1	Ref
MH454082	Unassigned	Unassigned	Unassigned	708_0	Clustered	47	VC_708_0	47	0.8654	1	0.8654	0	0	0	1	Ref
MH454083	Unassigned	Unassigned	Unassigned	708_0	Clustered	47	VC_708_0	47	0.8654	1	0.8654	0	0	0	1	Ref
MH454084	Unassigned	Unassigned	Unassigned	708_0	Clustered	47	VC_708_0	47	0.8654	1	0.8654	0	0	0	1	Ref
MH460459	Unassigned	Unassigned	Unassigned	393_0	Clustered	7	VC_393_0	7	0.1775	1	0.1775	0	0	0	1	Ref
MH460460	Unassigned	Unassigned	Unassigned	67_4	Clustered	4	VC_67_4	4	0.0431	0.90943035	0.0392	0	0	0	1	Ref
MH460461	Unassigned	Unassigned	Unassigned	67_4	Clustered	4	VC_67_4	4	0.0431	0.90943035	0.0392	0	0	0	1	Ref
MH460462	Unassigned	Unassigned	Unassigned	67_4	Clustered	4	VC_67_4	4	0.0431	0.90943035	0.0392	0	0	0	1	Ref
MH460463	Unassigned	Unassigned	Unassigned	67_3	Clustered	20	VC_67_3	20	0.3542	1	0.3542	0	0	0	1	Ref
MH460824	Unassigned	Unassigned	Unassigned	1156_0	Clustered	8	VC_1156_0	8	0.8137	1	0.8137	0	0	0	1	Ref
MH460825	Unassigned	Unassigned	Unassigned	1156_0	Clustered	8	VC_1156_0	8	0.8137	1	0.8137	0	0	0	1	Ref
MH460826	Unassigned	Unassigned	Unassigned	1156_0	Clustered	8	VC_1156_0	8	0.8137	1	0.8137	0	0	0	1	Ref
MH460827	Unassigned	Unassigned	Unassigned	1156_0	Clustered	8	VC_1156_0	8	0.8137	1	0.8137	0	0	0	1	Ref
MH460829	Unassigned	Unassigned	Unassigned	492_1	Clustered	20	VC_492_1	20	0.4176	1	0.4176	0	0	0	1	Ref
MH464253	Unassigned	Unassigned	Unassigned	451_0	Clustered	80	VC_451_0	80	0.9991	1	0.9991	0	0	0	1	Ref
MH479909	Unassigned	Unassigned	Unassigned	517_1	Clustered	108	VC_517_1	108	0.8483	1	0.8483	0	0	0	0.9955	Ref
MH479910	Unassigned	Unassigned	Unassigned	1178_0	Clustered	24	VC_1178_0	24	0.9524	1	0.9524	0	0	0	1	Ref
MH479911	Unassigned	Unassigned	Unassigned	517_1	Clustered	108	VC_517_1	108	0.8483	1	0.8483	0	0	0	0.9955	Ref
MH479912	Unassigned	Unassigned	Unassigned	525_0	Clustered	25	VC_525_0	25	0.4597	1	0.4597	0	0	0	1	Ref
MH479913	Unassigned	Unassigned	Unassigned	1183_0	Clustered	6	VC_1183_0	6	0.995	0.99996974	0.995	0	0	0	1	Ref
MH479914	Unassigned	Unassigned	Unassigned	371_16	Clustered	42	VC_371_16	42	0.1341	1	0.1341	0	0	0	0.7282	Ref
MH479915	Unassigned	Unassigned	Unassigned	1177_0	Clustered	14	VC_1177_0	14	0.7847	1	0.7847	0	0	0	0.989	Ref
MH479916	Unassigned	Unassigned	Unassigned	371_13	Clustered	33	VC_371_13	33	0.179	1	0.179	0	0	0	0.8769	Ref
MH479917	Unassigned	Unassigned	Unassigned	1178_0	Clustered	24	VC_1178_0	24	0.9524	1	0.9524	0	0	0	1	Ref
MH479918	Unassigned	Unassigned	Unassigned	371_13	Clustered	33	VC_371_13	33	0.179	1	0.179	0	0	0	0.8769	Ref
MH479919	Unassigned	Unassigned	Unassigned	325_61	Clustered	8	VC_325_61	8	0.0395	1	0.0395	0	0	0	0.7857	Ref
MH479920	Unassigned	Unassigned	Unassigned	761_0	Clustered	67	VC_761_0	67	0.8947	1	0.8947	0	0	0	1	Ref
MH479921	Unassigned	Unassigned	Unassigned	371_16	Clustered	42	VC_371_16	42	0.1341	1	0.1341	0	0	0	0.7282	Ref
MH479923	Unassigned	Unassigned	Unassigned	870_0	Clustered	5	VC_870_0	5	0.424	0.99999997	0.424	0	0	0	1	Ref
MH479924	Unassigned	Unassigned	Unassigned	1093_0	Clustered	40	VC_1093_0	40	0.8754	1	0.8754	0	0	0	1	Ref
MH479925	Unassigned	Unassigned	Unassigned	1183_0	Clustered	6	VC_1183_0	6	0.995	0.99996974	0.995	0	0	0	1	Ref
MH479926	Unassigned	Unassigned	Unassigned	1164_0	Clustered	25	VC_1164_0	25	0.7312	1	0.7312	0	0	0	1	Ref

MH479927	Unassigned	Unassigned	Unassigned	525_0	Clustered	25	VC_525_0	25	0.4597	1	0.4597	0	0	0	1	Ref
MH487649	Unassigned	Unassigned	Unassigned	187_1	Clustered	7	VC_187_1	7	0.1423	1	0.1423	0	0	0	1	Ref
MH491167	Unassigned	Unassigned	Unassigned	451_0	Clustered	80	VC_451_0	80	0.9991	1	0.9991	0	0	0	1	Ref
MH491968	Unassigned	Unassigned	Unassigned	584_0	Clustered	28	VC_584_0	28	0.7704	1	0.7704	0	0	0	1	Ref
MH491969	Unassigned	Unassigned	Unassigned	772_11	Clustered	6	VC_772_11	6	0.0305	0.84253803	0.0257	0	0	0	0.8	Ref
MH494197	Unassigned	Unassigned	Unassigned	164_2	Clustered/Singleton	1	VC_164_2	1	0	1	0	0	0	0	1	Ref
MH509443	Unassigned	Unassigned	Unassigned	517_1	Clustered	108	VC_517_1	108	0.8483	1	0.8483	0	0	0	0.9955	Ref
MH509444	Unassigned	Unassigned	Unassigned	236_0	Clustered	72	VC_236_0	72	0.8104	1	0.8104	0	0	0	1	Ref
MH509445	Unassigned	Unassigned	Unassigned	567_0	Clustered	8	VC_567_0	8	0.4935	1	0.4935	0	0	0	1	Ref
MH509446	Unassigned	Unassigned	Unassigned	954_0	Clustered	13	VC_954_0	13	0.863	1	0.863	0	0	0	1	Ref
MH509447	Unassigned	Unassigned	Unassigned	1234_0	Clustered	44	VC_1234_0	44	1	1	1	0	0	0	1	Ref
MH512890	Unassigned	Unassigned	Unassigned	655_0	Clustered	2	VC_655_0	2	0.0111	0.95704495	0.0106	0	0	0	1	Ref
MH513970	Unassigned	Unassigned	Unassigned	372_0	Clustered	24	VC_372_0	24	0.5539	1	0.5539	0	0	0	1	Ref
MH513971	Unassigned	Unassigned	Unassigned	325_0	Clustered	2	VC_325_0	2	0.0041	0.95574439	0.0039	0	0	0	1	Ref
MH513972	Unassigned	Unassigned	Unassigned	551_2	Clustered	17	VC_551_2	17	0.0973	1	0.0973	0	0	0	0.9779	Ref
MH513973	Unassigned	Unassigned	Unassigned	371_13	Clustered	33	VC_371_13	33	0.179	1	0.179	0	0	0	0.8769	Ref
MH513974	Unassigned	Unassigned	Unassigned	224_0	Clustered	19	VC_224_0	19	0.9416	1	0.9416	0	0	0	1	Ref
MH513975	Unassigned	Unassigned	Unassigned	514_0	Clustered	75	VC_514_0	75	0.9402	1	0.9402	0	0	0	1	Ref
MH513976	Unassigned	Unassigned	Unassigned	509_0	Clustered	37	VC_509_0	37	0.9689	1	0.9689	0	0	0	1	Ref
MH513977	Unassigned	Unassigned	Unassigned	236_0	Clustered	72	VC_236_0	72	0.8104	1	0.8104	0	0	0	1	Ref
MH513978	Unassigned	Unassigned	Unassigned	551_2	Clustered	17	VC_551_2	17	0.0973	1	0.0973	0	0	0	0.9779	Ref
MH513979	Unassigned	Unassigned	Unassigned	685_0	Clustered	16	VC_685_0	16	0.2972	1	0.2972	0	0	0	0.9167	Ref
MH513980	Unassigned	Unassigned	Unassigned	371_11	Clustered	31	VC_371_11	31	0.0927	1	0.0927	0	0	0	0.7011	Ref
MH513981	Unassigned	Unassigned	Unassigned	1240_0	Clustered	82	VC_1240_0	82	0.7945	1	0.7945	0	0	0	1	Ref
MH513982	Unassigned	Unassigned	Unassigned	1163_0	Clustered	28	VC_1163_0	28	0.4923	1	0.4923	0	0	0	1	Ref
MH513983	Unassigned	Unassigned	Unassigned	551_6	Clustered	22	VC_551_6	22	0.1214	1	0.1214	0	0	0	0.8095	Ref
MH513984	Unassigned	Unassigned	Unassigned	222_0	Clustered	5	VC_222_0	5	0.0565	0.99999997	0.0565	0	0	0	1	Ref
MH513985	Unassigned	Unassigned	Unassigned	215_1	Clustered	19	VC_215_1	19	0.4188	1	0.4188	0	0	0	1	Ref
MH523359	Unassigned	Unassigned	Unassigned	133_40	Clustered/Singleton	1	VC_133_40	1	0	1	0	0	0	0	1	Ref
MH533020	Unassigned	Unassigned	Unassigned	849_0	Clustered	3	VC_849_0	3	0.069	0.9984794	0.0689	0	0	0	1	Ref
MH536736	Unassigned	Unassigned	Unassigned	80_0	Clustered	41	VC_80_0	41	0.5837	1	0.5837	0	0	0	0.9805	Ref
MH536811	Unassigned	Unassigned	Unassigned	1072_4	Clustered	4	VC_1072_4	4	0.0736	0.99956559	0.0736	0	0	0	1	Ref
MH536812	Unassigned	Unassigned	Unassigned	557_2	Clustered/Singleton	1	VC_557_2	1	0	1	0	0	0	0	1	Ref
MH536813	Unassigned	Unassigned	Unassigned	514_0	Clustered	75	VC_514_0	75	0.9402	1	0.9402	0	0	0	1	Ref
MH536814	Unassigned	Unassigned	Unassigned	1072_4	Clustered	4	VC_1072_4	4	0.0736	0.99956559	0.0736	0	0	0	1	Ref
MH536815	Unassigned	Unassigned	Unassigned	1353_0	Clustered	10	VC_1353_0	10	1	1	1	0	0	0	1	Ref
MH536816	Unassigned	Unassigned	Unassigned	325_27	Clustered	2	VC_325_27	2	0.0044	0.95750955	0.0042	0	0	0	1	Ref
MH536817	Unassigned	Unassigned	Unassigned	517_1	Clustered	108	VC_517_1	108	0.8483	1	0.8483	0	0	0	0.9955	Ref
MH536818	Unassigned	Unassigned	Unassigned	1176_0	Clustered	41	VC_1176_0	41	0.9558	1	0.9558	0	0	0	0.9976	Ref
MH536819	Unassigned	Unassigned	Unassigned	767_0	Clustered	34	VC_767_0	34	0.9173	1	0.9173	0	0	0	1	Ref
MH536820	Unassigned	Unassigned	Unassigned	551_0	Clustered	16	VC_551_0	16	0.0915	1	0.0915	0	0	0	0.6667	Ref
MH536821	Unassigned	Unassigned	Unassigned	236_0	Clustered	72	VC_236_0	72	0.8104	1	0.8104	0	0	0	1	Ref
MH536822	Unassigned	Unassigned	Unassigned	236_0	Clustered	72	VC_236_0	72	0.8104	1	0.8104	0	0	0	1	Ref
MH536823	Unassigned	Unassigned	Unassigned	856_0	Clustered	7	VC_856_0	7	0.3463	1	0.3463	0	0	0	1	Ref
MH536824	Unassigned	Unassigned	Unassigned	767_0	Clustered	34	VC_767_0	34	0.9173	1	0.9173	0	0	0	1	Ref
MH536825	Unassigned	Unassigned	Unassigned	514_0	Clustered	75	VC_514_0	75	0.9402	1	0.9402	0	0	0	1	Ref
MH536827	Unassigned	Unassigned	Unassigned	551_7	Clustered	28	VC_551_7	28	0.1661	1	0.1661	0	0	0	0.8519	Ref
MH536828	Unassigned	Unassigned	Unassigned	221_0	Clustered	22	VC_221_0	22	0.7447	1	0.7447	0	0	0	1	Ref
MH536829	Unassigned	Unassigned	Unassigned	551_6	Clustered	22	VC_551_6	22	0.1214	1	0.1214	0	0	0	0.8095	Ref
MH538193	Unassigned	Unassigned	Unassigned	884_0	Clustered	28	VC_884_0	28	0.6211	1	0.6211	0	0	0	0.9841	Ref
MH540083	Unassigned	Unassigned	Unassigned	340_0	Clustered	31	VC_340_0	31	0.9549	1	0.9549	0	0	0	1	Ref
MH542234	Unassigned	Unassigned	Unassigned	150_0	Clustered	13	VC_150_0	13	0.2417	1	0.2417	0	0	0	0.9872	Ref
MH547045	Unassigned	Unassigned	Unassigned	43_0	Clustered	38	VC_43_0	38	0.5885	1	0.5885	0	0	0	0.9659	Ref
MH550421	Unassigned	Unassigned	Unassigned	283_88	Clustered	2	VC_283_88	2	0.0026	0.95596547	0.0024	0	0	0	1	Ref
MH552500	Unassigned	Unassigned	Unassigned	673_0	Clustered	15	VC_673_0	15	0.8234	1	0.8234	0	0	0	0.7619	Ref
MH553563	Unassigned	Unassigned	Unassigned	283_52	Clustered/Singleton	1	VC_283_52	1	0	1	0	0	0	0	1	Ref
MH560358	Unassigned	Unassigned	Unassigned	590_0	Clustered	27	VC_590_0	27	0.9947	1	0.9947	0	0	0	1	Ref
MH571750	Unassigned	Unassigned	Unassigned	329_7	Clustered	18	VC_329_7	18	0.0938	1	0.0938	0	0	0	0.7778	Ref
MH572269	Unassigned	Unassigned	Unassigned	398_0	Clustered	48	VC_398_0	48	0.4813	1	0.4813	0	0	0	1	Ref
MH572274	Unassigned	Unassigned	Unassigned	1355_0	Clustered	3	VC_1355_0	3	1	1	1	0	0	0	1	Ref
MH572275	Unassigned	Unassigned	Unassigned	1355_0	Clustered	3	VC_1355_0	3	1	1	1	0	0	0	1	Ref
MH572276	Unassigned	Unassigned	Unassigned	1355_0	Clustered	3	VC_1355_0	3	1	1	1	0	0	0	1	Ref
MH572277	Unassigned	Unassigned	Unassigned	198_0	Clustered	28	VC_198_0	28	0.2429	0.99971175	0.2429	0	0	0	1	Ref
MH572279	Unassigned	Unassigned	Unassigned	404_0	Clustered	25	VC_404_0	25	0.5649	1	0.5649	0	0	0	1	Ref
MH572280	Unassigned	Unassigned	Unassigned	198_0	Clustered	28	VC_198_0	28	0.2429	0.99971175	0.2429	0	0	0	1	Ref
MH572282	Unassigned	Unassigned	Unassigned	198_0	Clustered	28	VC_198_0	28	0.2429	0.99971175	0.2429	0	0	0	1	Ref
MH572283	Unassigned	Unassigned	Unassigned	995_0	Clustered	69	VC_995_0	69	0.8547	1	0.8547	0	0	0	1	Ref
MH572284	Unassigned	Unassigned	Unassigned	995_0	Clustered	69	VC_995_0	69	0.8547	1	0.8547	0	0	0	1	Ref
MH572285	Unassigned	Unassigned	Unassigned	995_0	Clustered	69	VC_995_0	69	0.8547	1	0.8547	0	0	0	1	Ref
MH572286	Unassigned	Unassigned	Unassigned	995_0	Clustered	69	VC_995_0	69	0.8547	1	0.8547	0	0	0	1	Ref
MH572287	Unassigned	Unassigned	Unassigned	1084_0	Clustered	9	VC_1084_0	9	0.6558	1	0.6558	0	0	0	1	Ref
MH572288	Unassigned	Unassigned	Unassigned	1084_0	Clustered	9	VC_1084_0	9	0.6558	1	0.6558	0	0	0	1	Ref
MH572290	Unassigned	Unassigned	Unassigned	198_0	Clustered	28	VC_198_0	28	0.2429	0.99971175	0.2429	0	0	0	1	Ref
MH572291	Unassigned	Unassigned	Unassigned	995_0	Clustered	69	VC_995_0	69	0.8547	1	0.8547	0	0	0	1	Ref
MH572292	Unassigned	Unassigned	Unassigned	995_0	Clustered	69	VC_995_0	69	0.8547	1	0.8547	0	0	0	1	Ref
MH572293	Unassigned	Unassigned	Unassigned	995_0	Clustered	69	VC_995_0	69	0.8547	1	0.8547	0	0	0	1	Ref
MH572294	Unassigned	Unassigned	Unassigned	403_0	Clustered	20	VC_403_0	20	0.6598	1	0.6598	0	0	0	1	Ref
MH572295	Unassigned	Unassigned	Unassigned	403_0	Clustered	20	VC_403_0	20	0.6598	1	0.6598	0	0	0	1	Ref
MH572296	Unassigned	Unassigned	Unassigned	403_0	Clustered	20	VC_403_0	20	0.6598	1	0.6598	0	0	0	1	Ref
MH572299	Unassigned	Unassigned	Unassigned	998_0	Clustered	2	VC_998_0	2	0.0515	0.93901809	0.0484	0	0	0	1	Ref
MH572300	Unassigned	Unassigned	Unassigned	404_0	Clustered	25	VC_404_0	25	0.5649	1	0.5649	0	0	0	1	Ref

MH572301	Unassigned	Unassigned	Unassigned	404_0	Clustered	25	VC_404_0	25	0.5649	1	0.5649	0	0	0	1	Ref
MH572302	Unassigned	Unassigned	Unassigned	404_0	Clustered	25	VC_404_0	25	0.5649	1	0.5649	0	0	0	1	Ref
MH572303	Unassigned	Unassigned	Unassigned	404_0	Clustered	25	VC_404_0	25	0.5649	1	0.5649	0	0	0	1	Ref
MH572305	Unassigned	Unassigned	Unassigned	404_0	Clustered	25	VC_404_0	25	0.5649	1	0.5649	0	0	0	1	Ref
MH572306	Unassigned	Unassigned	Unassigned	404_0	Clustered	25	VC_404_0	25	0.5649	1	0.5649	0	0	0	1	Ref
MH572307	Unassigned	Unassigned	Unassigned	404_0	Clustered	25	VC_404_0	25	0.5649	1	0.5649	0	0	0	1	Ref
MH572308	Unassigned	Unassigned	Unassigned	404_0	Clustered	25	VC_404_0	25	0.5649	1	0.5649	0	0	0	1	Ref
MH572309	Unassigned	Unassigned	Unassigned	404_0	Clustered	25	VC_404_0	25	0.5649	1	0.5649	0	0	0	1	Ref
MH572310	Unassigned	Unassigned	Unassigned	404_0	Clustered	25	VC_404_0	25	0.5649	1	0.5649	0	0	0	1	Ref
MH572311	Unassigned	Unassigned	Unassigned	404_0	Clustered	25	VC_404_0	25	0.5649	1	0.5649	0	0	0	1	Ref
MH572312	Unassigned	Unassigned	Unassigned	404_0	Clustered	25	VC_404_0	25	0.5649	1	0.5649	0	0	0	1	Ref
MH572313	Unassigned	Unassigned	Unassigned	404_0	Clustered	25	VC_404_0	25	0.5649	1	0.5649	0	0	0	1	Ref
MH572314	Unassigned	Unassigned	Unassigned	398_0	Clustered	48	VC_398_0	48	0.4813	1	0.4813	0	0	0	1	Ref
MH572315	Unassigned	Unassigned	Unassigned	398_0	Clustered	48	VC_398_0	48	0.4813	1	0.4813	0	0	0	1	Ref
MH572316	Unassigned	Unassigned	Unassigned	398_0	Clustered	48	VC_398_0	48	0.4813	1	0.4813	0	0	0	1	Ref
MH572317	Unassigned	Unassigned	Unassigned	398_0	Clustered	48	VC_398_0	48	0.4813	1	0.4813	0	0	0	1	Ref
MH572318	Unassigned	Unassigned	Unassigned	398_0	Clustered	48	VC_398_0	48	0.4813	1	0.4813	0	0	0	1	Ref
MH572319	Unassigned	Unassigned	Unassigned	398_0	Clustered	48	VC_398_0	48	0.4813	1	0.4813	0	0	0	1	Ref
MH572320	Unassigned	Unassigned	Unassigned	398_0	Clustered	48	VC_398_0	48	0.4813	1	0.4813	0	0	0	1	Ref
MH572321	Unassigned	Unassigned	Unassigned	398_0	Clustered	48	VC_398_0	48	0.4813	1	0.4813	0	0	0	1	Ref
MH572325	Unassigned	Unassigned	Unassigned	398_0	Clustered	48	VC_398_0	48	0.4813	1	0.4813	0	0	0	1	Ref
MH572326	Unassigned	Unassigned	Unassigned	398_0	Clustered	48	VC_398_0	48	0.4813	1	0.4813	0	0	0	1	Ref
MH572328	Unassigned	Unassigned	Unassigned	398_0	Clustered	48	VC_398_0	48	0.4813	1	0.4813	0	0	0	1	Ref
MH572329	Unassigned	Unassigned	Unassigned	398_0	Clustered	48	VC_398_0	48	0.4813	1	0.4813	0	0	0	1	Ref
MH572330	Unassigned	Unassigned	Unassigned	198_0	Clustered	28	VC_198_0	28	0.2429	0.99971175	0.2429	0	0	0	1	Ref
MH572334	Unassigned	Unassigned	Unassigned	198_0	Clustered	28	VC_198_0	28	0.2429	0.99971175	0.2429	0	0	0	1	Ref
MH572336	Unassigned	Unassigned	Unassigned	398_0	Clustered	48	VC_398_0	48	0.4813	1	0.4813	0	0	0	1	Ref
MH572340	Unassigned	Unassigned	Unassigned	400_0	Clustered/Singleton	1	VC_400_0	1	0	1	0	0	0	0	1	Ref
MH572341	Unassigned	Unassigned	Unassigned	199_0	Clustered	12	VC_199_0	12	0.448	1	0.448	0	0	0	1	Ref
MH572342	Unassigned	Unassigned	Unassigned	199_0	Clustered	12	VC_199_0	12	0.448	1	0.448	0	0	0	1	Ref
MH572343	Unassigned	Unassigned	Unassigned	199_0	Clustered	12	VC_199_0	12	0.448	1	0.448	0	0	0	1	Ref
MH572345	Unassigned	Unassigned	Unassigned	404_0	Clustered	25	VC_404_0	25	0.5649	1	0.5649	0	0	0	1	Ref
MH572347	Unassigned	Unassigned	Unassigned	992_0	Clustered	8	VC_992_0	8	0.3873	1	0.3873	0	0	0	1	Ref
MH572348	Unassigned	Unassigned	Unassigned	992_0	Clustered	8	VC_992_0	8	0.3873	1	0.3873	0	0	0	1	Ref
MH572349	Unassigned	Unassigned	Unassigned	992_0	Clustered	8	VC_992_0	8	0.3873	1	0.3873	0	0	0	1	Ref
MH572351	Unassigned	Unassigned	Unassigned	398_0	Clustered	48	VC_398_0	48	0.4813	1	0.4813	0	0	0	1	Ref
MH572353	Unassigned	Unassigned	Unassigned	1356_0	Clustered	8	VC_1356_0	8	0.8729	0.99998222	0.8729	0	0	0	1	Ref
MH572354	Unassigned	Unassigned	Unassigned	1356_0	Clustered	8	VC_1356_0	8	0.8729	0.99998222	0.8729	0	0	0	1	Ref
MH572355	Unassigned	Unassigned	Unassigned	1356_0	Clustered	8	VC_1356_0	8	0.8729	0.99998222	0.8729	0	0	0	1	Ref
MH572357	Unassigned	Unassigned	Unassigned	1356_0	Clustered	8	VC_1356_0	8	0.8729	0.99998222	0.8729	0	0	0	1	Ref
MH572358	Unassigned	Unassigned	Unassigned	1356_0	Clustered	8	VC_1356_0	8	0.8729	0.99998222	0.8729	0	0	0	1	Ref
MH572360	Unassigned	Unassigned	Unassigned	1079_0	Clustered	6	VC_1079_0	6	0.9301	0.89096579	0.8287	0	0	0	1	Ref
MH572361	Unassigned	Unassigned	Unassigned	1079_0	Clustered	6	VC_1079_0	6	0.9301	0.89096579	0.8287	0	0	0	1	Ref
MH572362	Unassigned	Unassigned	Unassigned	1077_0	Clustered	48	VC_1077_0	48	0.8525	1	0.8525	0	0	0	1	Ref
MH572364	Unassigned	Unassigned	Unassigned	1088_0	Clustered	3	VC_1088_0	3	0.7739	0.82711071	0.6401	0	0	0	1	Ref
MH572366	Unassigned	Unassigned	Unassigned	1082_0	Clustered	3	VC_1082_0	3	0.6925	0.61954664	0.429	0	0	0	1	Ref
MH572367	Unassigned	Unassigned	Unassigned	1062_0	Clustered	45	VC_1062_0	45	0.9734	0.50949847	0.4959	0	0	0	1	Ref
MH572368	Unassigned	Unassigned	Unassigned	1062_0	Clustered	45	VC_1062_0	45	0.9734	0.50949847	0.4959	0	0	0	1	Ref
MH572369	Unassigned	Unassigned	Unassigned	1308_0	Clustered	12	VC_1308_0	12	0.8184	0.9810731	0.8029	0	0	0	1	Ref
MH572371	Unassigned	Unassigned	Unassigned	1087_0	Clustered	5	VC_1087_0	5	0.8109	0.98759579	0.8008	0	0	0	1	Ref
MH572373	Unassigned	Unassigned	Unassigned	1087_0	Clustered	5	VC_1087_0	5	0.8109	0.98759579	0.8008	0	0	0	1	Ref
MH572374	Unassigned	Unassigned	Unassigned	1323_0	Clustered	6	VC_1323_0	6	0.598	0.99460389	0.5948	0	0	0	1	Ref
MH572375	Unassigned	Unassigned	Unassigned	1323_0	Clustered	6	VC_1323_0	6	0.598	0.99460389	0.5948	0	0	0	1	Ref
MH572376	Unassigned	Unassigned	Unassigned	1323_0	Clustered	6	VC_1323_0	6	0.598	0.99460389	0.5948	0	0	0	1	Ref
MH572377	Unassigned	Unassigned	Unassigned	1062_0	Clustered	45	VC_1062_0	45	0.9734	0.50949847	0.4959	0	0	0	1	Ref
MH572378	Unassigned	Unassigned	Unassigned	1062_0	Clustered	45	VC_1062_0	45	0.9734	0.50949847	0.4959	0	0	0	1	Ref
MH572379	Unassigned	Unassigned	Unassigned	1062_0	Clustered	45	VC_1062_0	45	0.9734	0.50949847	0.4959	0	0	0	1	Ref
MH572380	Unassigned	Unassigned	Unassigned	1087_0	Clustered	5	VC_1087_0	5	0.8109	0.98759579	0.8008	0	0	0	1	Ref
MH572382	Unassigned	Unassigned	Unassigned	995_0	Clustered	69	VC_995_0	69	0.8547	1	0.8547	0	0	0	1	Ref
MH572383	Unassigned	Unassigned	Unassigned	995_0	Clustered	69	VC_995_0	69	0.8547	1	0.8547	0	0	0	1	Ref
MH572384	Unassigned	Unassigned	Unassigned	403_0	Clustered	20	VC_403_0	20	0.6598	1	0.6598	0	0	0	1	Ref
MH572385	Unassigned	Unassigned	Unassigned	403_0	Clustered	20	VC_403_0	20	0.6598	1	0.6598	0	0	0	1	Ref
MH572386	Unassigned	Unassigned	Unassigned	403_0	Clustered	20	VC_403_0	20	0.6598	1	0.6598	0	0	0	1	Ref
MH572387	Unassigned	Unassigned	Unassigned	403_0	Clustered	20	VC_403_0	20	0.6598	1	0.6598	0	0	0	1	Ref
MH572388	Unassigned	Unassigned	Unassigned	403_0	Clustered	20	VC_403_0	20	0.6598	1	0.6598	0	0	0	1	Ref
MH572389	Unassigned	Unassigned	Unassigned	403_0	Clustered	20	VC_403_0	20	0.6598	1	0.6598	0	0	0	1	Ref
MH572390	Unassigned	Unassigned	Unassigned	403_0	Clustered	20	VC_403_0	20	0.6598	1	0.6598	0	0	0	1	Ref
MH572391	Unassigned	Unassigned	Unassigned	403_0	Clustered	20	VC_403_0	20	0.6598	1	0.6598	0	0	0	1	Ref
MH572392	Unassigned	Unassigned	Unassigned	404_0	Clustered	25	VC_404_0	25	0.5649	1	0.5649	0	0	0	1	Ref
MH572393	Unassigned	Unassigned	Unassigned	404_0	Clustered	25	VC_404_0	25	0.5649	1	0.5649	0	0	0	1	Ref
MH572394	Unassigned	Unassigned	Unassigned	398_0	Clustered	48	VC_398_0	48	0.4813	1	0.4813	0	0	0	1	Ref
MH572395	Unassigned	Unassigned	Unassigned	398_0	Clustered	48	VC_398_0	48	0.4813	1	0.4813	0	0	0	1	Ref
MH572396	Unassigned	Unassigned	Unassigned	398_0	Clustered	48	VC_398_0	48	0.4813	1	0.4813	0	0	0	1	Ref
MH572397	Unassigned	Unassigned	Unassigned	398_0	Clustered	48	VC_398_0	48	0.4813	1	0.4813	0	0	0	1	Ref
MH572399	Unassigned	Unassigned	Unassigned	199_0	Clustered	12	VC_199_0	12	0.448	1	0.448	0	0	0	1	Ref
MH572400	Unassigned	Unassigned	Unassigned	199_0	Clustered	12	VC_199_0	12	0.448	1	0.448	0	0	0	1	Ref
MH572401	Unassigned	Unassigned	Unassigned	199_0	Clustered	12	VC_199_0	12	0.448	1	0.448	0	0	0	1	Ref
MH572402	Unassigned	Unassigned	Unassigned	199_0	Clustered	12	VC_199_0	12	0.448	1	0.448	0	0	0	1	Ref
MH572403	Unassigned	Unassigned	Unassigned	199_0	Clustered	12	VC_199_0	12	0.448	1	0.448	0	0	0	1	Ref
MH572404	Unassigned	Unassigned	Unassigned	398_0	Clustered	48	VC_398_0	48	0.4813	1	0.4813	0	0	0	1	Ref
MH572405	Unassigned	Unassigned	Unassigned	992_0	Clustered	8	VC_992_0	8	0.3873	1	0.3873	0	0	0	1	Ref

MH572406	Unassigned	Unassigned	Unassigned	404_0	Clustered	25	VC_404_0	25	0.5649	1	0.5649	0	0	0	1	Ref
MH572407	Unassigned	Unassigned	Unassigned	1356_0	Clustered	8	VC_1356_0	8	0.8729	0.99998222	0.8729	0	0	0	1	Ref
MH572408	Unassigned	Unassigned	Unassigned	1356_0	Clustered	8	VC_1356_0	8	0.8729	0.99998222	0.8729	0	0	0	1	Ref
MH572409	Unassigned	Unassigned	Unassigned	1079_0	Clustered	6	VC_1079_0	6	0.9301	0.89096579	0.8287	0	0	0	1	Ref
MH572410	Unassigned	Unassigned	Unassigned	1077_0	Clustered	48	VC_1077_0	48	0.8525	1	0.8525	0	0	0	1	Ref
MH572411	Unassigned	Unassigned	Unassigned	1077_0	Clustered	48	VC_1077_0	48	0.8525	1	0.8525	0	0	0	1	Ref
MH572412	Unassigned	Unassigned	Unassigned	1077_0	Clustered	48	VC_1077_0	48	0.8525	1	0.8525	0	0	0	1	Ref
MH572414	Unassigned	Unassigned	Unassigned	1323_0	Clustered	6	VC_1323_0	6	0.598	0.99460389	0.5948	0	0	0	1	Ref
MH572418	Unassigned	Unassigned	Unassigned	992_0	Clustered	8	VC_992_0	8	0.3873	1	0.3873	0	0	0	1	Ref
MH572419	Unassigned	Unassigned	Unassigned	398_0	Clustered	48	VC_398_0	48	0.4813	1	0.4813	0	0	0	1	Ref
MH572422	Unassigned	Unassigned	Unassigned	198_0	Clustered	28	VC_198_0	28	0.2429	0.99971175	0.2429	0	0	0	1	Ref
MH572423	Unassigned	Unassigned	Unassigned	995_0	Clustered	69	VC_995_0	69	0.8547	1	0.8547	0	0	0	1	Ref
MH572424	Unassigned	Unassigned	Unassigned	995_0	Clustered	69	VC_995_0	69	0.8547	1	0.8547	0	0	0	1	Ref
MH572425	Unassigned	Unassigned	Unassigned	998_0	Clustered	2	VC_998_0	2	0.0515	0.93901809	0.0484	0	0	0	1	Ref
MH572426	Unassigned	Unassigned	Unassigned	999_0	Clustered	2	VC_999_0	2	0.0273	0.73989325	0.0202	0	0	0	1	Ref
MH572427	Unassigned	Unassigned	Unassigned	995_0	Clustered	69	VC_995_0	69	0.8547	1	0.8547	0	0	0	1	Ref
MH572428	Unassigned	Unassigned	Unassigned	995_0	Clustered	69	VC_995_0	69	0.8547	1	0.8547	0	0	0	1	Ref
MH572430	Unassigned	Unassigned	Unassigned	403_0	Clustered	20	VC_403_0	20	0.6598	1	0.6598	0	0	0	1	Ref
MH572431	Unassigned	Unassigned	Unassigned	398_0	Clustered	48	VC_398_0	48	0.4813	1	0.4813	0	0	0	1	Ref
MH572434	Unassigned	Unassigned	Unassigned	201_0	Clustered	3	VC_201_0	3	0.0998	0.69690761	0.0695	0	0	0	1	Ref
MH572435	Unassigned	Unassigned	Unassigned	404_0	Clustered	25	VC_404_0	25	0.5649	1	0.5649	0	0	0	1	Ref
MH572436	Unassigned	Unassigned	Unassigned	404_0	Clustered	25	VC_404_0	25	0.5649	1	0.5649	0	0	0	1	Ref
MH572437	Unassigned	Unassigned	Unassigned	404_0	Clustered	25	VC_404_0	25	0.5649	1	0.5649	0	0	0	1	Ref
MH572438	Unassigned	Unassigned	Unassigned	198_0	Clustered	28	VC_198_0	28	0.2429	0.99971175	0.2429	0	0	0	1	Ref
MH572439	Unassigned	Unassigned	Unassigned	398_0	Clustered	48	VC_398_0	48	0.4813	1	0.4813	0	0	0	1	Ref
MH572441	Unassigned	Unassigned	Unassigned	398_0	Clustered	48	VC_398_0	48	0.4813	1	0.4813	0	0	0	1	Ref
MH572443	Unassigned	Unassigned	Unassigned	398_0	Clustered	48	VC_398_0	48	0.4813	1	0.4813	0	0	0	1	Ref
MH572444	Unassigned	Unassigned	Unassigned	198_0	Clustered	28	VC_198_0	28	0.2429	0.99971175	0.2429	0	0	0	1	Ref
MH572445	Unassigned	Unassigned	Unassigned	398_0	Clustered	48	VC_398_0	48	0.4813	1	0.4813	0	0	0	1	Ref
MH572446	Unassigned	Unassigned	Unassigned	398_0	Clustered	48	VC_398_0	48	0.4813	1	0.4813	0	0	0	1	Ref
MH572448	Unassigned	Unassigned	Unassigned	198_0	Clustered	28	VC_198_0	28	0.2429	0.99971175	0.2429	0	0	0	1	Ref
MH572451	Unassigned	Unassigned	Unassigned	398_0	Clustered	48	VC_398_0	48	0.4813	1	0.4813	0	0	0	1	Ref
MH572452	Unassigned	Unassigned	Unassigned	198_0	Clustered	28	VC_198_0	28	0.2429	0.99971175	0.2429	0	0	0	1	Ref
MH572453	Unassigned	Unassigned	Unassigned	199_0	Clustered	12	VC_199_0	12	0.448	1	0.448	0	0	0	1	Ref
MH572454	Unassigned	Unassigned	Unassigned	198_0	Clustered	28	VC_198_0	28	0.2429	0.99971175	0.2429	0	0	0	1	Ref
MH572455	Unassigned	Unassigned	Unassigned	992_0	Clustered	8	VC_992_0	8	0.3873	1	0.3873	0	0	0	1	Ref
MH572457	Unassigned	Unassigned	Unassigned	1079_0	Clustered	6	VC_1079_0	6	0.9301	0.89096579	0.8287	0	0	0	1	Ref
MH572458	Unassigned	Unassigned	Unassigned	1088_0	Clustered	3	VC_1088_0	3	0.7739	0.82711071	0.6401	0	0	0	1	Ref
MH572459	Unassigned	Unassigned	Unassigned	1062_0	Clustered	45	VC_1062_0	45	0.9734	0.50949847	0.4959	0	0	0	1	Ref
MH572460	Unassigned	Unassigned	Unassigned	1062_0	Clustered	45	VC_1062_0	45	0.9734	0.50949847	0.4959	0	0	0	1	Ref
MH572461	Unassigned	Unassigned	Unassigned	1062_0	Clustered	45	VC_1062_0	45	0.9734	0.50949847	0.4959	0	0	0	1	Ref
MH572462	Unassigned	Unassigned	Unassigned	1062_0	Clustered	45	VC_1062_0	45	0.9734	0.50949847	0.4959	0	0	0	1	Ref
MH572463	Unassigned	Unassigned	Unassigned	1062_0	Clustered	45	VC_1062_0	45	0.9734	0.50949847	0.4959	0	0	0	1	Ref
MH572465	Unassigned	Unassigned	Unassigned	1062_0	Clustered	45	VC_1062_0	45	0.9734	0.50949847	0.4959	0	0	0	1	Ref
MH572466	Unassigned	Unassigned	Unassigned	995_0	Clustered	69	VC_995_0	69	0.8547	1	0.8547	0	0	0	1	Ref
MH572467	Unassigned	Unassigned	Unassigned	1084_0	Clustered	9	VC_1084_0	9	0.6558	1	0.6558	0	0	0	1	Ref
MH572468	Unassigned	Unassigned	Unassigned	1084_0	Clustered	9	VC_1084_0	9	0.6558	1	0.6558	0	0	0	1	Ref
MH572469	Unassigned	Unassigned	Unassigned	404_0	Clustered	25	VC_404_0	25	0.5649	1	0.5649	0	0	0	1	Ref
MH572470	Unassigned	Unassigned	Unassigned	403_0	Clustered	20	VC_403_0	20	0.6598	1	0.6598	0	0	0	1	Ref
MH572471	Unassigned	Unassigned	Unassigned	403_0	Clustered	20	VC_403_0	20	0.6598	1	0.6598	0	0	0	1	Ref
MH572472	Unassigned	Unassigned	Unassigned	403_0	Clustered	20	VC_403_0	20	0.6598	1	0.6598	0	0	0	1	Ref
MH572473	Unassigned	Unassigned	Unassigned	398_0	Clustered	48	VC_398_0	48	0.4813	1	0.4813	0	0	0	1	Ref
MH572475	Unassigned	Unassigned	Unassigned	201_0	Clustered	3	VC_201_0	3	0.0998	0.69690761	0.0695	0	0	0	1	Ref
MH572479	Unassigned	Unassigned	Unassigned	401_0	Clustered/Singleton	1	VC_401_0	1	0	1	0	0	0	0	1	Ref
MH572480	Unassigned	Unassigned	Unassigned	404_0	Clustered	25	VC_404_0	25	0.5649	1	0.5649	0	0	0	1	Ref
MH572482	Unassigned	Unassigned	Unassigned	1079_0	Clustered	6	VC_1079_0	6	0.9301	0.89096579	0.8287	0	0	0	1	Ref
MH572483	Unassigned	Unassigned	Unassigned	1079_0	Clustered	6	VC_1079_0	6	0.9301	0.89096579	0.8287	0	0	0	1	Ref
MH572484	Unassigned	Unassigned	Unassigned	1338_0	Clustered	4	VC_1338_0	4	1	1	1	0	0	0	1	Ref
MH572485	Unassigned	Unassigned	Unassigned	1323_0	Clustered	6	VC_1323_0	6	0.598	0.99460389	0.5948	0	0	0	1	Ref
MH572486	Unassigned	Unassigned	Unassigned	1077_0	Clustered	48	VC_1077_0	48	0.8525	1	0.8525	0	0	0	1	Ref
MH572487	Unassigned	Unassigned	Unassigned	398_0	Clustered	48	VC_398_0	48	0.4813	1	0.4813	0	0	0	1	Ref
MH572489	Unassigned	Unassigned	Unassigned	1089_0	Clustered	2	VC_1089_0	2	0.546	0.5	0.273	0	0	0	1	Ref
MH572490	Unassigned	Unassigned	Unassigned	1357_0	Clustered	6	VC_1357_0	6	1	1	1	0	0	0	1	Ref
MH572491	Unassigned	Unassigned	Unassigned	1357_0	Clustered	6	VC_1357_0	6	1	1	1	0	0	0	1	Ref
MH572492	Unassigned	Unassigned	Unassigned	398_0	Clustered	48	VC_398_0	48	0.4813	1	0.4813	0	0	0	1	Ref
MH572493	Unassigned	Unassigned	Unassigned	398_0	Clustered	48	VC_398_0	48	0.4813	1	0.4813	0	0	0	1	Ref
MH572494	Unassigned	Unassigned	Unassigned	398_0	Clustered	48	VC_398_0	48	0.4813	1	0.4813	0	0	0	1	Ref
MH572495	Unassigned	Unassigned	Unassigned	995_0	Clustered	69	VC_995_0	69	0.8547	1	0.8547	0	0	0	1	Ref
MH572496	Unassigned	Unassigned	Unassigned	1084_0	Clustered	9	VC_1084_0	9	0.6558	1	0.6558	0	0	0	1	Ref
MH572497	Unassigned	Unassigned	Unassigned	1084_0	Clustered	9	VC_1084_0	9	0.6558	1	0.6558	0	0	0	1	Ref
MH572498	Unassigned	Unassigned	Unassigned	1084_0	Clustered	9	VC_1084_0	9	0.6558	1	0.6558	0	0	0	1	Ref
MH572499	Unassigned	Unassigned	Unassigned	1084_0	Clustered	9	VC_1084_0	9	0.6558	1	0.6558	0	0	0	1	Ref
MH572500	Unassigned	Unassigned	Unassigned	1084_0	Clustered	9	VC_1084_0	9	0.6558	1	0.6558	0	0	0	1	Ref
MH572503	Unassigned	Unassigned	Unassigned	398_0	Clustered	48	VC_398_0	48	0.4813	1	0.4813	0	0	0	1	Ref
MH572504	Unassigned	Unassigned	Unassigned	995_0	Clustered	69	VC_995_0	69	0.8547	1	0.8547	0	0	0	1	Ref
MH572505	Unassigned	Unassigned	Unassigned	403_0	Clustered	20	VC_403_0	20	0.6598	1	0.6598	0	0	0	1	Ref
MH572506	Unassigned	Unassigned	Unassigned	403_0	Clustered	20	VC_403_0	20	0.6598	1	0.6598	0	0	0	1	Ref
MH572507	Unassigned	Unassigned	Unassigned	403_0	Clustered	20	VC_403_0	20	0.6598	1	0.6598	0	0	0	1	Ref
MH572508	Unassigned	Unassigned	Unassigned	404_0	Clustered	25	VC_404_0	25	0.5649	1	0.5649	0	0	0	1	Ref
MH572509	Unassigned	Unassigned	Unassigned	404_0	Clustered	25	VC_404_0	25	0.5649	1	0.5649	0	0	0	1	Ref
MH572510	Unassigned	Unassigned	Unassigned	398_0	Clustered	48	VC_398_0	48	0.4813	1	0.4813	0	0	0	1	Ref

MH572511	Unassigned	Unassigned	Unassigned	398_0	Clustered	48	VC_398_0	48	0.4813	1	0.4813	0	0	0	1	Ref
MH572512	Unassigned	Unassigned	Unassigned	402_0	Clustered/Singleton	1	VC_402_0	1	0	1	0	0	0	0	1	Ref
MH572513	Unassigned	Unassigned	Unassigned	199_0	Clustered	12	VC_199_0	12	0.448	1	0.448	0	0	0	1	Ref
MH572514	Unassigned	Unassigned	Unassigned	199_0	Clustered	12	VC_199_0	12	0.448	1	0.448	0	0	0	1	Ref
MH572515	Unassigned	Unassigned	Unassigned	992_0	Clustered	8	VC_992_0	8	0.3873	1	0.3873	0	0	0	1	Ref
MH572516	Unassigned	Unassigned	Unassigned	1356_0	Clustered	8	VC_1356_0	8	0.8729	0.99998222	0.8729	0	0	0	1	Ref
MH572517	Unassigned	Unassigned	Unassigned	1357_0	Clustered	6	VC_1357_0	6	1	1	1	0	0	0	1	Ref
MH572518	Unassigned	Unassigned	Unassigned	1357_0	Clustered	6	VC_1357_0	6	1	1	1	0	0	0	1	Ref
MH572519	Unassigned	Unassigned	Unassigned	1357_0	Clustered	6	VC_1357_0	6	1	1	1	0	0	0	1	Ref
MH572520	Unassigned	Unassigned	Unassigned	1357_0	Clustered	6	VC_1357_0	6	1	1	1	0	0	0	1	Ref
MH572521	Unassigned	Unassigned	Unassigned	1077_0	Clustered	48	VC_1077_0	48	0.8525	1	0.8525	0	0	0	1	Ref
MH572522	Unassigned	Unassigned	Unassigned	1323_0	Clustered	6	VC_1323_0	6	0.598	0.99460389	0.5948	0	0	0	1	Ref
MH572524	Unassigned	Unassigned	Unassigned	1324_0	Clustered	4	VC_1324_0	4	0.6008	0.54407361	0.3269	0	0	0	1	Ref
MH572525	Unassigned	Unassigned	Unassigned	1243_0	Clustered	52	VC_1243_0	52	0.9126	0.92708311	0.8461	0	0	0	1	Ref
MH572526	Unassigned	Unassigned	Unassigned	1243_0	Clustered	52	VC_1243_0	52	0.9126	0.92708311	0.8461	0	0	0	1	Ref
MH576948	Unassigned	Unassigned	Unassigned	950_0	Clustered	38	VC_950_0	38	0.9443	1	0.9443	0	0	0	1	Ref
MH576949	Unassigned	Unassigned	Unassigned	514_0	Clustered	75	VC_514_0	75	0.9402	1	0.9402	0	0	0	1	Ref
MH576950	Unassigned	Unassigned	Unassigned	950_0	Clustered	38	VC_950_0	38	0.9443	1	0.9443	0	0	0	1	Ref
MH576951	Unassigned	Unassigned	Unassigned	950_0	Clustered	38	VC_950_0	38	0.9443	1	0.9443	0	0	0	1	Ref
MH576952	Unassigned	Unassigned	Unassigned	950_0	Clustered	38	VC_950_0	38	0.9443	1	0.9443	0	0	0	1	Ref
MH576953	Unassigned	Unassigned	Unassigned	551_7	Clustered	28	VC_551_7	28	0.1661	1	0.1661	0	0	0	0.8519	Ref
MH576954	Unassigned	Unassigned	Unassigned	371_4	Clustered	5	VC_371_4	5	0.0135	0.99999971	0.0135	0	0	0	1	Ref
MH576955	Unassigned	Unassigned	Unassigned	950_0	Clustered	38	VC_950_0	38	0.9443	1	0.9443	0	0	0	1	Ref
MH576956	Unassigned	Unassigned	Unassigned	551_1	Clustered	4	VC_551_1	4	0.0159	0.99968337	0.0159	0	0	0	0.6667	Ref
MH576957	Unassigned	Unassigned	Unassigned	950_0	Clustered	38	VC_950_0	38	0.9443	1	0.9443	0	0	0	1	Ref
MH576958	Unassigned	Unassigned	Unassigned	371_13	Clustered	33	VC_371_13	33	0.179	1	0.179	0	0	0	0.8769	Ref
MH576959	Unassigned	Unassigned	Unassigned	325_23	Clustered	17	VC_325_23	17	0.0716	1	0.0716	0	0	0	0.6397	Ref
MH576960	Unassigned	Unassigned	Unassigned	767_0	Clustered	34	VC_767_0	34	0.9173	1	0.9173	0	0	0	1	Ref
MH576961	Unassigned	Unassigned	Unassigned	950_0	Clustered	38	VC_950_0	38	0.9443	1	0.9443	0	0	0	1	Ref
MH576962	Unassigned	Unassigned	Unassigned	1158_0	Clustered	3	VC_1158_0	3	0.3179	0.99335558	0.3158	0	0	0	1	Ref
MH576963	Unassigned	Unassigned	Unassigned	1344_0	Clustered	78	VC_1344_0	78	0.9732	1	0.9732	0	0	0	1	Ref
MH576964	Unassigned	Unassigned	Unassigned	1071_0	Clustered	20	VC_1071_0	20	0.3268	1	0.3268	0	0	0	0.9842	Ref
MH576965	Unassigned	Unassigned	Unassigned	1071_0	Clustered	20	VC_1071_0	20	0.3268	1	0.3268	0	0	0	0.9842	Ref
MH576966	Unassigned	Unassigned	Unassigned	238_0	Clustered	17	VC_238_0	17	0.6109	1	0.6109	0	0	0	1	Ref
MH576967	Unassigned	Unassigned	Unassigned	371_4	Clustered	5	VC_371_4	5	0.0135	0.99999971	0.0135	0	0	0	1	Ref
MH576968	Unassigned	Unassigned	Unassigned	1071_0	Clustered	20	VC_1071_0	20	0.3268	1	0.3268	0	0	0	0.9842	Ref
MH576969	Unassigned	Unassigned	Unassigned	1176_0	Clustered	41	VC_1176_0	41	0.9558	1	0.9558	0	0	0	0.9976	Ref
MH576970	Unassigned	Unassigned	Unassigned	371_15	Clustered	44	VC_371_15	44	0.1391	1	0.1391	0	0	0	0.7474	Ref
MH576971	Unassigned	Unassigned	Unassigned	325_23	Clustered	17	VC_325_23	17	0.0716	1	0.0716	0	0	0	0.6397	Ref
MH576972	Unassigned	Unassigned	Unassigned	570_0	Clustered	9	VC_570_0	9	0.499	1	0.499	0	0	0	1	Ref
MH576973	Unassigned	Unassigned	Unassigned	888_1	Clustered	3	VC_888_1	3	0.0289	0.99848448	0.0289	0	0	0	1	Ref
MH576974	Unassigned	Unassigned	Unassigned	325_23	Clustered	17	VC_325_23	17	0.0716	1	0.0716	0	0	0	0.6397	Ref
MH576975	Unassigned	Unassigned	Unassigned	325_46	Clustered	2	VC_325_46	2	0.004	0.95751617	0.0039	0	0	0	1	Ref
MH576976	Unassigned	Unassigned	Unassigned	869_0	Clustered	9	VC_869_0	9	0.2624	1	0.2624	0	0	0	1	Ref
MH576977	Unassigned	Unassigned	Unassigned	509_0	Clustered	37	VC_509_0	37	0.9689	1	0.9689	0	0	0	1	Ref
MH579717	Unassigned	Unassigned	Unassigned	480_0	Clustered	19	VC_480_0	19	0.5445	1	0.5445	0	0	0	1	Ref
MH586730	Unassigned	Unassigned	Unassigned	1049_0	Clustered	8	VC_1049_0	8	0.6413	1	0.6413	0	0	0	1	Ref
MH586731	Unassigned	Unassigned	Unassigned	329_0	Clustered	2	VC_329_0	2	0.0051	0.95464582	0.0048	0	0	0	1	Ref
MH587638	Unassigned	Unassigned	Unassigned	823_0	Clustered	124	VC_823_0	124	0.9492	1	0.9492	0	0	0	1	Ref
MH588544	Unassigned	Unassigned	Unassigned	976_3	Clustered	2	VC_976_3	2	0.0258	0.88402892	0.0228	0	0	0	1	Ref
MH588545	Unassigned	Unassigned	Unassigned	976_2	Clustered	2	VC_976_2	2	0.0278	0.95410794	0.0266	0	0	0	1	Ref
MH590587	Unassigned	Unassigned	Unassigned	551_8	Clustered	19	VC_551_8	19	0.1039	1	0.1039	0	0	0	0.8713	Ref
MH590588	Unassigned	Unassigned	Unassigned	371_11	Clustered	31	VC_371_11	31	0.0927	1	0.0927	0	0	0	0.7011	Ref
MH590589	Unassigned	Unassigned	Unassigned	1072_2	Clustered	5	VC_1072_2	5	0.1003	0.99999315	0.1003	0	0	0	1	Ref
MH590591	Unassigned	Unassigned	Unassigned	508_0	Clustered	32	VC_508_0	32	0.906	1	0.906	0	0	0	1	Ref
MH590592	Unassigned	Unassigned	Unassigned	556_0	Clustered	21	VC_556_0	21	0.9912	1	0.9912	0	0	0	1	Ref
MH590593	Unassigned	Unassigned	Unassigned	558_22	Clustered	4	VC_558_22	4	0.01	0.99312819	0.0099	0	0	0	0.8333	Ref
MH590594	Unassigned	Unassigned	Unassigned	371_10	Clustered/Singleton	1	VC_371_10	1	0	1	0	0	0	0	1	Ref
MH590595	Unassigned	Unassigned	Unassigned	325_53	Clustered	7	VC_325_53	7	0.0242	1	0.0242	0	0	0	0.9048	Ref
MH590596	Unassigned	Unassigned	Unassigned	520_78	Clustered	2	VC_520_78	2	0.008	0.95761153	0.0076	0	0	0	1	Ref
MH590597	Unassigned	Unassigned	Unassigned	1071_0	Clustered	20	VC_1071_0	20	0.3268	1	0.3268	0	0	0	0.9842	Ref
MH590598	Unassigned	Unassigned	Unassigned	520_25	Clustered/Singleton	1	VC_520_25	1	0	1	0	0	0	0	1	Ref
MH590599	Unassigned	Unassigned	Unassigned	1071_0	Clustered	20	VC_1071_0	20	0.3268	1	0.3268	0	0	0	0.9842	Ref
MH590600	Unassigned	Unassigned	Unassigned	1352_0	Clustered	31	VC_1352_0	31	0.849	1	0.849	0	0	0	1	Ref
MH590601	Unassigned	Unassigned	Unassigned	981_1	Clustered	26	VC_981_1	26	0.7009	1	0.7009	0	0	0	1	Ref
MH590602	Unassigned	Unassigned	Unassigned	520_38	Clustered	6	VC_520_38	6	0.0519	1	0.0519	0	0	0	0.8667	Ref
MH590603	Unassigned	Unassigned	Unassigned	1182_1	Clustered/Singleton	1	VC_1182_1	1	0	1	0	0	0	0	1	Ref
MH590604	Unassigned	Unassigned	Unassigned	1163_0	Clustered	28	VC_1163_0	28	0.4923	1	0.4923	0	0	0	1	Ref
MH590605	Unassigned	Unassigned	Unassigned	761_0	Clustered	67	VC_761_0	67	0.8947	1	0.8947	0	0	0	1	Ref
MH590606	Unassigned	Unassigned	Unassigned	1163_0	Clustered	28	VC_1163_0	28	0.4923	1	0.4923	0	0	0	1	Ref
MH595538	Unassigned	Unassigned	Unassigned	346_3	Clustered/Singleton	1	VC_346_3	1	0	1	0	0	0	0	1	Ref
MH598512	Unassigned	Unassigned	Unassigned	828_0	Clustered	3	VC_828_0	3	0.4748	0.99843555	0.4741	0	0	0	1	Ref
MH598798	Unassigned	Unassigned	Unassigned	480_0	Clustered	19	VC_480_0	19	0.5445	1	0.5445	0	0	0	1	Ref
MH598800	Unassigned	Unassigned	Unassigned	480_0	Clustered	19	VC_480_0	19	0.5445	1	0.5445	0	0	0	1	Ref
MH598802	Unassigned	Unassigned	Unassigned	480_0	Clustered	19	VC_480_0	19	0.5445	1	0.5445	0	0	0	1	Ref
MH598803	Unassigned	Unassigned	Unassigned	480_0	Clustered	19	VC_480_0	19	0.5445	1	0.5445	0	0	0	1	Ref
MH598804	Unassigned	Unassigned	Unassigned	480_0	Clustered	19	VC_480_0	19	0.5445	1	0.5445	0	0	0	1	Ref
MH598805	Unassigned	Unassigned	Unassigned	480_0	Clustered	19	VC_480_0	19	0.5445	1	0.5445	0	0	0	1	Ref
MH598807	Unassigned	Unassigned	Unassigned	480_0	Clustered	19	VC_480_0	19	0.5445	1	0.5445	0	0	0	1	Ref
MH599087	Unassigned	Unassigned	Unassigned	882_0	Clustered	20	VC_882_0	20	0.9917	1	0.9917	0	0	0	1	Ref
MH606185	Unassigned	Unassigned	Unassigned	85_5	Clustered/Singleton	1	VC_85_5	1	0	1	0	0	0	0	1	Ref

MH616963	Unassigned	Unassigned	Unassigned	673_0	Clustered	15	VC_673_0	15	0.8234	1	0.8234	0	0	0	0.7619	Ref
MH618488	Unassigned	Unassigned	Unassigned	108_0	Clustered	33	VC_108_0	33	0.949	1	0.949	0	0	0	1	Ref
MH629685	Unassigned	Unassigned	Unassigned	432_16	Clustered/Singleton	1	VC_432_16	1	0	1	0	0	0	0	1	Ref
MH631453	Unassigned	Unassigned	Unassigned	592_0	Clustered	50	VC_592_0	50	0.7314	1	0.7314	0	0	0	1	Ref
MH631454	Unassigned	Unassigned	Unassigned	191_19	Clustered	12	VC_191_19	12	0.0655	1	0.0655	0	0	0	0.8636	Ref
MH632117	Unassigned	Unassigned	Unassigned	325_49	Clustered	4	VC_325_49	4	0.014	0.99995965	0.014	0	0	0	1	Ref
MH632118	Unassigned	Unassigned	Unassigned	325_27	Clustered	2	VC_325_27	2	0.0044	0.95750955	0.0042	0	0	0	1	Ref
MH632119	Unassigned	Unassigned	Unassigned	520_93	Clustered	2	VC_520_93	2	0.0104	0.95762079	0.0099	0	0	0	1	Ref
MH633484	Unassigned	Unassigned	Unassigned	938_0	Clustered	99	VC_938_0	99	0.9119	1	0.9119	0	0	0	0.9928	Ref
MH633485	Unassigned	Unassigned	Unassigned	938_0	Clustered	99	VC_938_0	99	0.9119	1	0.9119	0	0	0	0.9928	Ref
MH633486	Unassigned	Unassigned	Unassigned	938_0	Clustered	99	VC_938_0	99	0.9119	1	0.9119	0	0	0	0.9928	Ref
MH633487	Unassigned	Unassigned	Unassigned	938_0	Clustered	99	VC_938_0	99	0.9119	1	0.9119	0	0	0	0.9928	Ref
MH638294	Unassigned	Unassigned	Unassigned	162_0	Clustered	18	VC_162_0	18	0.7403	1	0.7403	0	0	0	0.8627	Ref
MH638309	Unassigned	Unassigned	Unassigned	187_3	Clustered	9	VC_187_3	9	0.1971	1	0.1971	0	0	0	0.8611	Ref
MH638310	Unassigned	Unassigned	Unassigned	883_0	Clustered	10	VC_883_0	10	0.2015	1	0.2015	0	0	0	1	Ref
MH638311	Unassigned	Unassigned	Unassigned	884_0	Clustered	28	VC_884_0	28	0.6211	1	0.6211	0	0	0	0.9841	Ref
MH638312	Unassigned	Unassigned	Unassigned	187_3	Clustered	9	VC_187_3	9	0.1971	1	0.1971	0	0	0	0.8611	Ref
MH643777	Unassigned	Unassigned	Unassigned	179_0	Clustered	53	VC_179_0	53	0.9137	1	0.9137	0	0	0	1	Ref
MH643778	Unassigned	Unassigned	Unassigned	179_0	Clustered	53	VC_179_0	53	0.9137	1	0.9137	0	0	0	1	Ref
MH645904	Unassigned	Unassigned	Unassigned	972_0	Clustered	6	VC_972_0	6	0.2892	1	0.2892	0	0	0	1	Ref
MH651166	Unassigned	Unassigned	Unassigned	1093_0	Clustered	40	VC_1093_0	40	0.8754	1	0.8754	0	0	0	1	Ref
MH651167	Unassigned	Unassigned	Unassigned	685_0	Clustered	16	VC_685_0	16	0.2972	1	0.2972	0	0	0	0.9167	Ref
MH651168	Unassigned	Unassigned	Unassigned	1093_0	Clustered	40	VC_1093_0	40	0.8754	1	0.8754	0	0	0	1	Ref
MH651169	Unassigned	Unassigned	Unassigned	520_45	Clustered	7	VC_520_45	7	0.0635	1	0.0635	0	0	0	0.8095	Ref
MH651170	Unassigned	Unassigned	Unassigned	517_1	Clustered	108	VC_517_1	108	0.8483	1	0.8483	0	0	0	0.9955	Ref
MH651171	Unassigned	Unassigned	Unassigned	238_0	Clustered	17	VC_238_0	17	0.6109	1	0.6109	0	0	0	1	Ref
MH651172	Unassigned	Unassigned	Unassigned	1072_2	Clustered	5	VC_1072_2	5	0.1003	0.99999315	0.1003	0	0	0	1	Ref
MH651174	Unassigned	Unassigned	Unassigned	551_6	Clustered	22	VC_551_6	22	0.1214	1	0.1214	0	0	0	0.8095	Ref
MH651175	Unassigned	Unassigned	Unassigned	371_16	Clustered	42	VC_371_16	42	0.1341	1	0.1341	0	0	0	0.7282	Ref
MH651176	Unassigned	Unassigned	Unassigned	557_1	Clustered	11	VC_557_1	11	0.4945	1	0.4945	0	0	0	0.7818	Ref
MH651177	Unassigned	Unassigned	Unassigned	371_4	Clustered	5	VC_371_4	5	0.0135	0.99999971	0.0135	0	0	0	1	Ref
MH651178	Unassigned	Unassigned	Unassigned	520_32	Clustered/Singleton	1	VC_520_32	1	0	1	0	0	0	0	1	Ref
MH651179	Unassigned	Unassigned	Unassigned	514_0	Clustered	75	VC_514_0	75	0.9402	1	0.9402	0	0	0	1	Ref
MH651180	Unassigned	Unassigned	Unassigned	325_38	Clustered	6	VC_325_38	6	0.0267	1	0.0267	0	0	0	0.8	Ref
MH651181	Unassigned	Unassigned	Unassigned	1344_0	Clustered	78	VC_1344_0	78	0.9732	1	0.9732	0	0	0	1	Ref
MH651182	Unassigned	Unassigned	Unassigned	890_0	Clustered	3	VC_890_0	3	0.3565	0.99830951	0.3559	0	0	0	1	Ref
MH651183	Unassigned	Unassigned	Unassigned	520_41	Clustered	2	VC_520_41	2	0.0139	0.95761457	0.0134	0	0	0	1	Ref
MH651184	Unassigned	Unassigned	Unassigned	371_15	Clustered	44	VC_371_15	44	0.1391	1	0.1391	0	0	0	0.7474	Ref
MH651185	Unassigned	Unassigned	Unassigned	557_1	Clustered	11	VC_557_1	11	0.4945	1	0.4945	0	0	0	0.7818	Ref
MH651186	Unassigned	Unassigned	Unassigned	371_16	Clustered	42	VC_371_16	42	0.1341	1	0.1341	0	0	0	0.7282	Ref
MH651188	Unassigned	Unassigned	Unassigned	520_80	Clustered	3	VC_520_80	3	0.0295	0.99859904	0.0295	0	0	0	1	Ref
MH651189	Unassigned	Unassigned	Unassigned	406_0	Clustered	11	VC_406_0	11	0.9449	1	0.9449	0	0	0	1	Ref
MH651190	Unassigned	Unassigned	Unassigned	981_1	Clustered	26	VC_981_1	26	0.7009	1	0.7009	0	0	0	1	Ref
MH669000	Unassigned	Unassigned	Unassigned	1095_0	Clustered	16	VC_1095_0	16	0.458	1	0.458	0	0	0	1	Ref
MH669001	Unassigned	Unassigned	Unassigned	520_21	Clustered/Singleton	1	VC_520_21	1	0	1	0	0	0	0	1	Ref
MH669002	Unassigned	Unassigned	Unassigned	551_6	Clustered	22	VC_551_6	22	0.1214	1	0.1214	0	0	0	0.8095	Ref
MH669003	Unassigned	Unassigned	Unassigned	520_80	Clustered	3	VC_520_80	3	0.0295	0.99859904	0.0295	0	0	0	1	Ref
MH669004	Unassigned	Unassigned	Unassigned	981_1	Clustered	26	VC_981_1	26	0.7009	1	0.7009	0	0	0	1	Ref
MH669006	Unassigned	Unassigned	Unassigned	872_0	Clustered	31	VC_872_0	31	0.8151	1	0.8151	0	0	0	0.8194	Ref
MH669007	Unassigned	Unassigned	Unassigned	767_0	Clustered	34	VC_767_0	34	0.9173	1	0.9173	0	0	0	1	Ref
MH669008	Unassigned	Unassigned	Unassigned	558_50	Clustered	15	VC_558_50	15	0.0468	1	0.0468	0	0	0	0.9238	Ref
MH669011	Unassigned	Unassigned	Unassigned	325_44	Clustered	3	VC_325_44	3	0.0157	0.99859171	0.0157	0	0	0	1	Ref
MH669012	Unassigned	Unassigned	Unassigned	520_73	Clustered	2	VC_520_73	2	0.0107	0.95762743	0.0103	0	0	0	1	Ref
MH669013	Unassigned	Unassigned	Unassigned	767_0	Clustered	34	VC_767_0	34	0.9173	1	0.9173	0	0	0	1	Ref
MH669014	Unassigned	Unassigned	Unassigned	520_69	Clustered/Singleton	1	VC_520_69	1	0	1	0	0	0	0	1	Ref
MH669015	Unassigned	Unassigned	Unassigned	1178_0	Clustered	24	VC_1178_0	24	0.9524	1	0.9524	0	0	0	1	Ref
MH669016	Unassigned	Unassigned	Unassigned	981_1	Clustered	26	VC_981_1	26	0.7009	1	0.7009	0	0	0	1	Ref
MH669017	Unassigned	Unassigned	Unassigned	546_4	Clustered	16	VC_546_4	16	0.2517	0.9999998	0.2517	0	0	0	0.9417	Ref
MH669274	Unassigned	Unassigned	Unassigned	779_0	Clustered	37	VC_779_0	37	0.5682	1	0.5682	0	0	0	1	Ref
MH674343	Unassigned	Unassigned	Unassigned	233_0	Clustered	2	VC_233_0	2	0.7179	0.72985431	0.5239	0	0	0	1	Ref
MH675552	Unassigned	Unassigned	Unassigned	1018_1	Clustered	10	VC_1018_1	10	0.7518	1	0.7518	0	0	0	0.8	Ref
MH675927	Unassigned	Unassigned	Unassigned	848_0	Clustered	28	VC_848_0	28	0.7927	1	0.7927	0	0	0	1	Ref
MH684921	Unassigned	Unassigned	Unassigned	70_0	Clustered	27	VC_70_0	27	0.5981	1	0.5981	0	0	0	0.9886	Ref
MH688040	Unassigned	Unassigned	Unassigned	133_22	Clustered	13	VC_133_22	13	0.0804	1	0.0804	0	0	0	0.8205	Ref
MH688453	Unassigned	Unassigned	Unassigned	938_0	Clustered	99	VC_938_0	99	0.9119	1	0.9119	0	0	0	0.9928	Ref
MH697575	Unassigned	Unassigned	Unassigned	325_52	Clustered	5	VC_325_52	5	0.0147	0.99999073	0.0147	0	0	0	0.7	Ref
MH697576	Unassigned	Unassigned	Unassigned	521_0	Clustered	37	VC_521_0	37	0.912	1	0.912	0	0	0	1	Ref
MH697577	Unassigned	Unassigned	Unassigned	810_0	Clustered	18	VC_810_0	18	0.9121	1	0.9121	0	0	0	1	Ref
MH697578	Unassigned	Unassigned	Unassigned	236_0	Clustered	72	VC_236_0	72	0.8104	1	0.8104	0	0	0	1	Ref
MH697579	Unassigned	Unassigned	Unassigned	558_73	Clustered	2	VC_558_73	2	0.0033	0.84374839	0.0028	0	0	0	1	Ref
MH697580	Unassigned	Unassigned	Unassigned	406_0	Clustered	11	VC_406_0	11	0.9449	1	0.9449	0	0	0	1	Ref
MH697581	Unassigned	Unassigned	Unassigned	325_40	Clustered	2	VC_325_40	2	0.004	0.95574341	0.0038	0	0	0	1	Ref
MH697582	Unassigned	Unassigned	Unassigned	546_4	Clustered	16	VC_546_4	16	0.2517	0.9999998	0.2517	0	0	0	0.9417	Ref
MH697583	Unassigned	Unassigned	Unassigned	546_4	Clustered	16	VC_546_4	16	0.2517	0.9999998	0.2517	0	0	0	0.9417	Ref
MH697584	Unassigned	Unassigned	Unassigned	508_0	Clustered	32	VC_508_0	32	0.906	1	0.906	0	0	0	1	Ref
MH697585	Unassigned	Unassigned	Unassigned	521_0	Clustered	37	VC_521_0	37	0.912	1	0.912	0	0	0	1	Ref
MH697586	Unassigned	Unassigned	Unassigned	558_20	Clustered	17	VC_558_20	17	0.054	1	0.054	0	0	0	0.9926	Ref
MH697587	Unassigned	Unassigned	Unassigned	763_1	Clustered	9	VC_763_1	9	0.3706	1	0.3706	0	0	0	1	Ref
MH697588	Unassigned	Unassigned	Unassigned	517_1	Clustered	108	VC_517_1	108	0.8483	1	0.8483	0	0	0	0.9955	Ref
MH697590	Unassigned	Unassigned	Unassigned	558_20	Clustered	17	VC_558_20	17	0.054	1	0.054	0	0	0	0.9926	Ref
MH697591	Unassigned	Unassigned	Unassigned	216_6	Clustered	42	VC_216_6	42	0.473	1	0.473	0	0	0	0.8351	Ref

MH697592	Unassigned	Unassigned	Unassigned	238_0	Clustered	17	VC_238_0	17	0.6109	1	0.6109	0	0	0	1	Ref
MH697593	Unassigned	Unassigned	Unassigned	521_0	Clustered	37	VC_521_0	37	0.912	1	0.912	0	0	0	1	Ref
MH707430	Unassigned	Unassigned	Unassigned	1358_0	Clustered	5	VC_1358_0	5	1	1	1	0	0	0	1	Ref
MH709120	Unassigned	Unassigned	Unassigned	133_17	Clustered/Singleton	1	VC_133_17	1	0	1	0	0	0	0	1	Ref
MH709121	Unassigned	Unassigned	Unassigned	133_10	Clustered	10	VC_133_10	10	0.0527	1	0.0527	0	0	0	1	Ref
MH713599	Unassigned	Unassigned	Unassigned	492_1	Clustered	20	VC_492_1	20	0.4176	1	0.4176	0	0	0	1	Ref
MH717096	Unassigned	Unassigned	Unassigned	30_1	Clustered	16	VC_30_1	16	0.1973	1	0.1973	0	0	0	0.8333	Ref
MH717097	Unassigned	Unassigned	Unassigned	461_0	Clustered	96	VC_461_0	96	0.9307	1	0.9307	0	0	0	0.9575	Ref
MH717098	Unassigned	Unassigned	Unassigned	392_0	Clustered	126	VC_392_0	126	0.9621	1	0.9621	0	0	0	1	Ref
MH717099	Unassigned	Unassigned	Unassigned	392_0	Clustered	126	VC_392_0	126	0.9621	1	0.9621	0	0	0	1	Ref
MH717709	Unassigned	Unassigned	Unassigned	385_0	Clustered	51	VC_385_0	51	0.8754	1	0.8754	0	0	0	1	Ref
MH719189	Unassigned	Unassigned	Unassigned	251_0	Clustered	22	VC_251_0	22	0.6508	1	0.6508	0	0	0	1	Ref
MH719190	Unassigned	Unassigned	Unassigned	251_0	Clustered	22	VC_251_0	22	0.6508	1	0.6508	0	0	0	1	Ref
MH719191	Unassigned	Unassigned	Unassigned	251_0	Clustered	22	VC_251_0	22	0.6508	1	0.6508	0	0	0	1	Ref
MH719192	Unassigned	Unassigned	Unassigned	251_0	Clustered	22	VC_251_0	22	0.6508	1	0.6508	0	0	0	1	Ref
MH719193	Unassigned	Unassigned	Unassigned	251_0	Clustered	22	VC_251_0	22	0.6508	1	0.6508	0	0	0	1	Ref
MH719194	Unassigned	Unassigned	Unassigned	251_0	Clustered	22	VC_251_0	22	0.6508	1	0.6508	0	0	0	1	Ref
MH719195	Unassigned	Unassigned	Unassigned	251_0	Clustered	22	VC_251_0	22	0.6508	1	0.6508	0	0	0	1	Ref
MH725810	Unassigned	Unassigned	Unassigned	76_0	Clustered	37	VC_76_0	37	0.9403	1	0.9403	0	0	0	1	Ref
MH727543	Unassigned	Unassigned	Unassigned	558_2	Clustered	2	VC_558_2	2	0.0032	0.66859979	0.0021	0	0	0	1	Ref
MH727544	Unassigned	Unassigned	Unassigned	236_0	Clustered	72	VC_236_0	72	0.8104	1	0.8104	0	0	0	1	Ref
MH727546	Unassigned	Unassigned	Unassigned	508_0	Clustered	32	VC_508_0	32	0.906	1	0.906	0	0	0	1	Ref
MH727547	Unassigned	Unassigned	Unassigned	767_0	Clustered	34	VC_767_0	34	0.9173	1	0.9173	0	0	0	1	Ref
MH727548	Unassigned	Unassigned	Unassigned	520_5	Clustered/Singleton	1	VC_520_5	1	0	1	0	0	0	0	1	Ref
MH727549	Unassigned	Unassigned	Unassigned	810_0	Clustered	18	VC_810_0	18	0.9121	1	0.9121	0	0	0	1	Ref
MH727551	Unassigned	Unassigned	Unassigned	546_4	Clustered	16	VC_546_4	16	0.2517	0.9999998	0.2517	0	0	0	0.9417	Ref
MH727552	Unassigned	Unassigned	Unassigned	767_0	Clustered	34	VC_767_0	34	0.9173	1	0.9173	0	0	0	1	Ref
MH727553	Unassigned	Unassigned	Unassigned	939_0	Clustered	15	VC_939_0	15	1	0	0	0	0	0	1	Ref
MH727554	Unassigned	Unassigned	Unassigned	514_0	Clustered	75	VC_514_0	75	0.9402	1	0.9402	0	0	0	1	Ref
MH727555	Unassigned	Unassigned	Unassigned	371_16	Clustered	42	VC_371_16	42	0.1341	1	0.1341	0	0	0	0.7282	Ref
MH727557	Unassigned	Unassigned	Unassigned	551_6	Clustered	22	VC_551_6	22	0.1214	1	0.1214	0	0	0	0.8095	Ref
MH727558	Unassigned	Unassigned	Unassigned	558_56	Clustered/Singleton	1	VC_558_56	1	0	1	0	0	0	0	1	Ref
MH727559	Unassigned	Unassigned	Unassigned	889_0	Clustered	16	VC_889_0	16	0.2969	1	0.2969	0	0	0	1	Ref
MH727560	Unassigned	Unassigned	Unassigned	1199_0	Clustered	8	VC_1199_0	8	0.7928	1	0.7928	0	0	0	1	Ref
MH727561	Unassigned	Unassigned	Unassigned	372_1	Clustered	9	VC_372_1	9	0.194	1	0.194	0	0	0	1	Ref
MH727562	Unassigned	Unassigned	Unassigned	939_0	Clustered	15	VC_939_0	15	1	0	0	0	0	0	1	Ref
MH727563	Unassigned	Unassigned	Unassigned	517_1	Clustered	108	VC_517_1	108	0.8483	1	0.8483	0	0	0	0.9955	Ref
MH727564	Unassigned	Unassigned	Unassigned	1071_0	Clustered	20	VC_1071_0	20	0.3268	1	0.3268	0	0	0	0.9842	Ref
MH727593	Unassigned	Unassigned	Unassigned	184_0	Clustered	2	VC_184_0	2	0.7964	0.93668477	0.746	0	0	0	1	Ref
MH729818	Unassigned	Unassigned	Unassigned	452_0	Clustered	66	VC_452_0	66	0.9248	1	0.9248	0	0	0	1	Ref
MH729819	Unassigned	Unassigned	Unassigned	584_0	Clustered	28	VC_584_0	28	0.7704	1	0.7704	0	0	0	1	Ref
MH729874	Unassigned	Unassigned	Unassigned	491_0	Clustered	36	VC_491_0	36	0.5336	1	0.5336	0	0	0	0.9714	Ref
MH730557	Unassigned	Unassigned	Unassigned	1359_0	Clustered	11	VC_1359_0	11	1	0	0	0	0	0	1	Ref
MH733496	Unassigned	Unassigned	Unassigned	779_0	Clustered	37	VC_779_0	37	0.5682	1	0.5682	0	0	0	1	Ref
MH744414	Unassigned	Unassigned	Unassigned	325_10	Clustered	3	VC_325_10	3	0.012	0.99859169	0.012	0	0	0	1	Ref
MH744415	Unassigned	Unassigned	Unassigned	520_36	Clustered/Singleton	1	VC_520_36	1	0	1	0	0	0	0	1	Ref
MH744416	Unassigned	Unassigned	Unassigned	1344_0	Clustered	78	VC_1344_0	78	0.9732	1	0.9732	0	0	0	1	Ref
MH744417	Unassigned	Unassigned	Unassigned	371_16	Clustered	42	VC_371_16	42	0.1341	1	0.1341	0	0	0	0.7282	Ref
MH744418	Unassigned	Unassigned	Unassigned	1158_1	Clustered	2	VC_1158_1	2	0.1372	0.89820314	0.1233	0	0	0	1	Ref
MH744419	Unassigned	Unassigned	Unassigned	1074_0	Clustered	19	VC_1074_0	19	0.9198	1	0.9198	0	0	0	1	Ref
MH744420	Unassigned	Unassigned	Unassigned	588_0	Clustered	2	VC_588_0	2	0.0832	0.94395095	0.0785	0	0	0	1	Ref
MH744421	Unassigned	Unassigned	Unassigned	1160_0	Clustered	73	VC_1160_0	73	0.9835	1	0.9835	0	0	0	1	Ref
MH744422	Unassigned	Unassigned	Unassigned	558_52	Clustered/Singleton	1	VC_558_52	1	0	1	0	0	0	0	1	Ref
MH744423	Unassigned	Unassigned	Unassigned	372_2	Clustered	5	VC_372_2	5	0.072	0.99999997	0.072	0	0	0	0.9	Ref
MH744424	Unassigned	Unassigned	Unassigned	767_0	Clustered	34	VC_767_0	34	0.9173	1	0.9173	0	0	0	1	Ref
MH744425	Unassigned	Unassigned	Unassigned	887_0	Clustered	18	VC_887_0	18	0.3663	1	0.3663	0	0	0	1	Ref
MH746814	Unassigned	Unassigned	Unassigned	700_0	Clustered	10	VC_700_0	10	0.8076	1	0.8076	0	0	0	1	Ref
MH746817	Unassigned	Unassigned	Unassigned	171_0	Clustered	8	VC_171_0	8	0.5884	1	0.5884	0	0	0	1	Ref
MH751506	Unassigned	Unassigned	Unassigned	283_92	Clustered	4	VC_283_92	4	0.0079	0.9999582	0.0079	0	0	0	1	Ref
MH752385	Unassigned	Unassigned	Unassigned	104_0	Clustered	6	VC_104_0	6	0.8291	1	0.8291	0	0	0	1	Ref
MH752840	Unassigned	Unassigned	Unassigned	772_11	Clustered	6	VC_772_11	6	0.0305	0.84253803	0.0257	0	0	0	0.8	Ref
MH763831	Unassigned	Unassigned	Unassigned	699_0	Clustered	71	VC_699_0	71	0.9352	1	0.9352	0	0	0	1	Ref
MH779498	Unassigned	Unassigned	Unassigned	558_49	Clustered	7	VC_558_49	7	0.0194	0.99510369	0.0193	0	0	0	0.8571	Ref
MH779499	Unassigned	Unassigned	Unassigned	767_0	Clustered	34	VC_767_0	34	0.9173	1	0.9173	0	0	0	1	Ref
MH779500	Unassigned	Unassigned	Unassigned	371_3	Clustered	22	VC_371_3	22	0.0787	1	0.0787	0	0	0	0.9048	Ref
MH779501	Unassigned	Unassigned	Unassigned	767_0	Clustered	34	VC_767_0	34	0.9173	1	0.9173	0	0	0	1	Ref
MH779502	Unassigned	Unassigned	Unassigned	558_54	Clustered/Singleton	1	VC_558_54	1	0	1	0	0	0	0	1	Ref
MH779503	Unassigned	Unassigned	Unassigned	551_6	Clustered	22	VC_551_6	22	0.1214	1	0.1214	0	0	0	0.8095	Ref
MH779504	Unassigned	Unassigned	Unassigned	557_1	Clustered	11	VC_557_1	11	0.4945	1	0.4945	0	0	0	0.7818	Ref
MH779505	Unassigned	Unassigned	Unassigned	761_0	Clustered	67	VC_761_0	67	0.8947	1	0.8947	0	0	0	1	Ref
MH779506	Unassigned	Unassigned	Unassigned	950_0	Clustered	38	VC_950_0	38	0.9443	1	0.9443	0	0	0	1	Ref
MH779507	Unassigned	Unassigned	Unassigned	761_0	Clustered	67	VC_761_0	67	0.8947	1	0.8947	0	0	0	1	Ref
MH779508	Unassigned	Unassigned	Unassigned	767_0	Clustered	34	VC_767_0	34	0.9173	1	0.9173	0	0	0	1	Ref
MH779509	Unassigned	Unassigned	Unassigned	761_0	Clustered	67	VC_761_0	67	0.8947	1	0.8947	0	0	0	1	Ref
MH779510	Unassigned	Unassigned	Unassigned	767_0	Clustered	34	VC_767_0	34	0.9173	1	0.9173	0	0	0	1	Ref
MH779511	Unassigned	Unassigned	Unassigned	888_0	Clustered	28	VC_888_0	28	0.5497	1	0.5497	0	0	0	1	Ref
MH779512	Unassigned	Unassigned	Unassigned	1344_0	Clustered	78	VC_1344_0	78	0.9732	1	0.9732	0	0	0	1	Ref
MH779513	Unassigned	Unassigned	Unassigned	761_0	Clustered	67	VC_761_0	67	0.8947	1	0.8947	0	0	0	1	Ref
MH779514	Unassigned	Unassigned	Unassigned	761_0	Clustered	67	VC_761_0	67	0.8947	1	0.8947	0	0	0	1	Ref
MH779515	Unassigned	Unassigned	Unassigned	761_0	Clustered	67	VC_761_0	67	0.8947	1	0.8947	0	0	0	1	Ref
MH779516	Unassigned	Unassigned	Unassigned	371_16	Clustered	42	VC_371_16	42	0.1341	1	0.1341	0	0	0	0.7282	Ref

MH779517	Unassigned	Unassigned	Unassigned	223_0	Clustered	38	VC_223_0	38	0.9095	1	0.9095	0	0	0	1	Ref
MH779518	Unassigned	Unassigned	Unassigned	690_0	Clustered	50	VC_690_0	50	0.9805	1	0.9805	0	0	0	1	Ref
MH779519	Unassigned	Unassigned	Unassigned	690_0	Clustered	50	VC_690_0	50	0.9805	1	0.9805	0	0	0	1	Ref
MH779520	Unassigned	Unassigned	Unassigned	690_0	Clustered	50	VC_690_0	50	0.9805	1	0.9805	0	0	0	1	Ref
MH779521	Unassigned	Unassigned	Unassigned	690_0	Clustered	50	VC_690_0	50	0.9805	1	0.9805	0	0	0	1	Ref
MH779522	Unassigned	Unassigned	Unassigned	690_0	Clustered	50	VC_690_0	50	0.9805	1	0.9805	0	0	0	1	Ref
MH779523	Unassigned	Unassigned	Unassigned	690_0	Clustered	50	VC_690_0	50	0.9805	1	0.9805	0	0	0	1	Ref
MH779524	Unassigned	Unassigned	Unassigned	690_0	Clustered	50	VC_690_0	50	0.9805	1	0.9805	0	0	0	1	Ref
MH779525	Unassigned	Unassigned	Unassigned	690_0	Clustered	50	VC_690_0	50	0.9805	1	0.9805	0	0	0	1	Ref
MH779526	Unassigned	Unassigned	Unassigned	690_0	Clustered	50	VC_690_0	50	0.9805	1	0.9805	0	0	0	1	Ref
MH779527	Unassigned	Unassigned	Unassigned	690_0	Clustered	50	VC_690_0	50	0.9805	1	0.9805	0	0	0	1	Ref
MH779619	Unassigned	Unassigned	Unassigned	392_0	Clustered	126	VC_392_0	126	0.9621	1	0.9621	0	0	0	1	Ref
MH791395	Unassigned	Unassigned	Unassigned	592_0	Clustered	50	VC_592_0	50	0.7314	1	0.7314	0	0	0	1	Ref
MH791396	Unassigned	Unassigned	Unassigned	592_0	Clustered	50	VC_592_0	50	0.7314	1	0.7314	0	0	0	1	Ref
MH791397	Unassigned	Unassigned	Unassigned	92_0	Clustered	22	VC_92_0	22	0.5639	1	0.5639	0	0	0	1	Ref
MH791398	Unassigned	Unassigned	Unassigned	443_2	Clustered	10	VC_443_2	10	0.3344	1	0.3344	0	0	0	0.9333	Ref
MH791399	Unassigned	Unassigned	Unassigned	76_0	Clustered	37	VC_76_0	37	0.9403	1	0.9403	0	0	0	1	Ref
MH791400	Unassigned	Unassigned	Unassigned	330_0	Clustered	27	VC_330_0	27	0.5088	1	0.5088	0	0	0	0.8661	Ref
MH791402	Unassigned	Unassigned	Unassigned	461_0	Clustered	96	VC_461_0	96	0.9307	1	0.9307	0	0	0	0.9575	Ref
MH791403	Unassigned	Unassigned	Unassigned	166_0	Clustered	26	VC_166_0	26	0.7923	1	0.7923	0	0	0	1	Ref
MH791404	Unassigned	Unassigned	Unassigned	443_2	Clustered	10	VC_443_2	10	0.3344	1	0.3344	0	0	0	0.9333	Ref
MH791405	Unassigned	Unassigned	Unassigned	133_6	Clustered/Singleton	1	VC_133_6	1	0	1	0	0	0	0	1	Ref
MH791406	Unassigned	Unassigned	Unassigned	166_0	Clustered	26	VC_166_0	26	0.7923	1	0.7923	0	0	0	1	Ref
MH791408	Unassigned	Unassigned	Unassigned	76_0	Clustered	37	VC_76_0	37	0.9403	1	0.9403	0	0	0	1	Ref
MH791409	Unassigned	Unassigned	Unassigned	290_6	Clustered	12	VC_290_6	12	0.1149	1	0.1149	0	0	0	0.8182	Ref
MH791410	Unassigned	Unassigned	Unassigned	461_0	Clustered	96	VC_461_0	96	0.9307	1	0.9307	0	0	0	0.9575	Ref
MH791411	Unassigned	Unassigned	Unassigned	164_11	Clustered	10	VC_164_11	10	0.1507	1	0.1507	0	0	0	0.9111	Ref
MH791412	Unassigned	Unassigned	Unassigned	461_0	Clustered	96	VC_461_0	96	0.9307	1	0.9307	0	0	0	0.9575	Ref
MH791413	Unassigned	Unassigned	Unassigned	330_0	Clustered	27	VC_330_0	27	0.5088	1	0.5088	0	0	0	0.8661	Ref
MH791414	Unassigned	Unassigned	Unassigned	443_0	Clustered	2	VC_443_0	2	0.029	0.87409603	0.0253	0	0	0	1	Ref
MH791415	Unassigned	Unassigned	Unassigned	108_0	Clustered	33	VC_108_0	33	0.949	1	0.949	0	0	0	1	Ref
MH791416	Unassigned	Unassigned	Unassigned	76_0	Clustered	37	VC_76_0	37	0.9403	1	0.9403	0	0	0	1	Ref
MH800198	Unassigned	Unassigned	Unassigned	700_0	Clustered	10	VC_700_0	10	0.8076	1	0.8076	0	0	0	1	Ref
MH800199	Unassigned	Unassigned	Unassigned	700_0	Clustered	10	VC_700_0	10	0.8076	1	0.8076	0	0	0	1	Ref
MH800200	Unassigned	Unassigned	Unassigned	699_0	Clustered	71	VC_699_0	71	0.9352	1	0.9352	0	0	0	1	Ref
MH807810	Unassigned	Unassigned	Unassigned	458_0	Clustered	5	VC_458_0	5	0.1938	0.99999997	0.1938	0	0	0	1	Ref
MH807811	Unassigned	Unassigned	Unassigned	984_0	Clustered	38	VC_984_0	38	0.9758	1	0.9758	0	0	0	1	Ref
MH807812	Unassigned	Unassigned	Unassigned	131_0	Clustered	17	VC_131_0	17	0.2671	1	0.2671	0	0	0	1	Ref
MH807813	Unassigned	Unassigned	Unassigned	458_0	Clustered	5	VC_458_0	5	0.1938	0.99999997	0.1938	0	0	0	1	Ref
MH807814	Unassigned	Unassigned	Unassigned	984_0	Clustered	38	VC_984_0	38	0.9758	1	0.9758	0	0	0	1	Ref
MH807815	Unassigned	Unassigned	Unassigned	458_0	Clustered	5	VC_458_0	5	0.1938	0.99999997	0.1938	0	0	0	1	Ref
MH807816	Unassigned	Unassigned	Unassigned	984_0	Clustered	38	VC_984_0	38	0.9758	1	0.9758	0	0	0	1	Ref
MH807817	Unassigned	Unassigned	Unassigned	458_0	Clustered	5	VC_458_0	5	0.1938	0.99999997	0.1938	0	0	0	1	Ref
MH807818	Unassigned	Unassigned	Unassigned	984_0	Clustered	38	VC_984_0	38	0.9758	1	0.9758	0	0	0	1	Ref
MH807819	Unassigned	Unassigned	Unassigned	984_0	Clustered	38	VC_984_0	38	0.9758	1	0.9758	0	0	0	1	Ref
MH807820	Unassigned	Unassigned	Unassigned	131_0	Clustered	17	VC_131_0	17	0.2671	1	0.2671	0	0	0	1	Ref
MH809528	Unassigned	Unassigned	Unassigned	577_0	Clustered	6	VC_577_0	6	0.9267	0.99999993	0.9267	0	0	0	1	Ref
MH809529	Unassigned	Unassigned	Unassigned	526_0	Clustered	6	VC_526_0	6	0.4386	1	0.4386	0	0	0	1	Ref
MH809530	Unassigned	Unassigned	Unassigned	526_0	Clustered	6	VC_526_0	6	0.4386	1	0.4386	0	0	0	1	Ref
MH809531	Unassigned	Unassigned	Unassigned	526_0	Clustered	6	VC_526_0	6	0.4386	1	0.4386	0	0	0	1	Ref
MH809534	Unassigned	Unassigned	Unassigned	386_0	Clustered	49	VC_386_0	49	0.7941	1	0.7941	0	0	0	1	Ref
MH809535	Unassigned	Unassigned	Unassigned	283_34	Clustered	4	VC_283_34	4	0.0082	0.9997481	0.0082	0	0	0	1	Ref
MH816848	Unassigned	Unassigned	Unassigned	848_0	Clustered	28	VC_848_0	28	0.7927	1	0.7927	0	0	0	1	Ref
MH816966	Unassigned	Unassigned	Unassigned	618_0	Clustered	13	VC_618_0	13	0.6252	1	0.6252	0	0	0	1	Ref
MH817022	Unassigned	Unassigned	Unassigned	818_0	Clustered	20	VC_818_0	20	0.9601	1	0.9601	0	0	0	1	Ref
MH823906	Unassigned	Unassigned	Unassigned	669_0	Clustered	21	VC_669_0	21	0.3623	1	0.3623	0	0	0	1	Ref
MH825697	Unassigned	Unassigned	Unassigned	558_12	Clustered	3	VC_558_12	3	0.0064	0.87854253	0.0057	0	0	0	1	Ref
MH825698	Unassigned	Unassigned	Unassigned	1361_0	Clustered	6	VC_1361_0	6	0.2371	1	0.2371	0	0	0	1	Ref
MH825699	Unassigned	Unassigned	Unassigned	727_0	Clustered	11	VC_727_0	11	0.5054	1	0.5054	0	0	0	1	Ref
MH825700	Unassigned	Unassigned	Unassigned	1344_0	Clustered	78	VC_1344_0	78	0.9732	1	0.9732	0	0	0	1	Ref
MH825701	Unassigned	Unassigned	Unassigned	216_4	Clustered	13	VC_216_4	13	0.1657	1	0.1657	0	0	0	1	Ref
MH825702	Unassigned	Unassigned	Unassigned	371_15	Clustered	44	VC_371_15	44	0.1391	1	0.1391	0	0	0	0.7474	Ref
MH825703	Unassigned	Unassigned	Unassigned	1073_0	Clustered	24	VC_1073_0	24	0.8388	1	0.8388	0	0	0	1	Ref
MH825704	Unassigned	Unassigned	Unassigned	216_6	Clustered	42	VC_216_6	42	0.473	1	0.473	0	0	0	0.8351	Ref
MH825705	Unassigned	Unassigned	Unassigned	371_15	Clustered	44	VC_371_15	44	0.1391	1	0.1391	0	0	0	0.7474	Ref
MH825706	Unassigned	Unassigned	Unassigned	1353_0	Clustered	10	VC_1353_0	10	1	1	1	0	0	0	1	Ref
MH825707	Unassigned	Unassigned	Unassigned	520_30	Clustered	2	VC_520_30	2	0.0073	0.9544492	0.0069	0	0	0	1	Ref
MH825709	Unassigned	Unassigned	Unassigned	216_3	Clustered	8	VC_216_3	8	0.0701	1	0.0701	0	0	0	0.8571	Ref
MH825710	Unassigned	Unassigned	Unassigned	517_1	Clustered	108	VC_517_1	108	0.8483	1	0.8483	0	0	0	0.9955	Ref
MH825711	Unassigned	Unassigned	Unassigned	1164_0	Clustered	25	VC_1164_0	25	0.7312	1	0.7312	0	0	0	1	Ref
MH825713	Unassigned	Unassigned	Unassigned	558_50	Clustered	15	VC_558_50	15	0.0468	1	0.0468	0	0	0	0.9238	Ref
MH834594	Unassigned	Unassigned	Unassigned	951_0	Clustered	3	VC_951_0	3	0.1049	0.99921864	0.1048	0	0	0	1	Ref
MH834595	Unassigned	Unassigned	Unassigned	1166_0	Clustered	20	VC_1166_0	20	0.99	1	0.99	0	0	0	1	Ref
MH834596	Unassigned	Unassigned	Unassigned	1234_0	Clustered	44	VC_1234_0	44	1	1	1	0	0	0	1	Ref
MH834597	Unassigned	Unassigned	Unassigned	951_0	Clustered	3	VC_951_0	3	0.1049	0.99921864	0.1048	0	0	0	1	Ref
MH834598	Unassigned	Unassigned	Unassigned	1036_0	Clustered	11	VC_1036_0	11	0.656	1	0.656	0	0	0	0.8727	Ref
MH834599	Unassigned	Unassigned	Unassigned	1234_0	Clustered	44	VC_1234_0	44	1	1	1	0	0	0	1	Ref
MH834600	Unassigned	Unassigned	Unassigned	888_0	Clustered	28	VC_888_0	28	0.5497	1	0.5497	0	0	0	1	Ref
MH834602	Unassigned	Unassigned	Unassigned	1234_0	Clustered	44	VC_1234_0	44	1	1	1	0	0	0	1	Ref
MH834603	Unassigned	Unassigned	Unassigned	1035_0	Clustered	6	VC_1035_0	6	0.4854	1	0.4854	0	0	0	1	Ref
MH834604	Unassigned	Unassigned	Unassigned	1234_0	Clustered	44	VC_1234_0	44	1	1	1	0	0	0	1	Ref

MH834605	Unassigned	Unassigned	Unassigned	1035_0	Clustered	6	VC_1035_0	6	0.4854	1	0.4854	0	0	0	1	Ref
MH834606	Unassigned	Unassigned	Unassigned	1166_0	Clustered	20	VC_1166_0	20	0.99	1	0.99	0	0	0	1	Ref
MH834607	Unassigned	Unassigned	Unassigned	1362_0	Clustered	6	VC_1362_0	6	1	1	1	0	0	0	1	Ref
MH834608	Unassigned	Unassigned	Unassigned	1166_0	Clustered	20	VC_1166_0	20	0.99	1	0.99	0	0	0	1	Ref
MH834609	Unassigned	Unassigned	Unassigned	1166_0	Clustered	20	VC_1166_0	20	0.99	1	0.99	0	0	0	1	Ref
MH834610	Unassigned	Unassigned	Unassigned	1207_0	Clustered	29	VC_1207_0	29	0.8225	1	0.8225	0	0	0	1	Ref
MH834611	Unassigned	Unassigned	Unassigned	1035_0	Clustered	6	VC_1035_0	6	0.4854	1	0.4854	0	0	0	1	Ref
MH834612	Unassigned	Unassigned	Unassigned	1036_0	Clustered	11	VC_1036_0	11	0.656	1	0.656	0	0	0	0.8727	Ref
MH834613	Unassigned	Unassigned	Unassigned	571_0	Clustered	4	VC_571_0	4	0.1921	0.99998591	0.1921	0	0	0	1	Ref
MH834614	Unassigned	Unassigned	Unassigned	1035_0	Clustered	6	VC_1035_0	6	0.4854	1	0.4854	0	0	0	1	Ref
MH834615	Unassigned	Unassigned	Unassigned	565_0	Clustered	20	VC_565_0	20	0.8398	1	0.8398	0	0	0	1	Ref
MH834616	Unassigned	Unassigned	Unassigned	1166_0	Clustered	20	VC_1166_0	20	0.99	1	0.99	0	0	0	1	Ref
MH834617	Unassigned	Unassigned	Unassigned	520_45	Clustered	7	VC_520_45	7	0.0635	1	0.0635	0	0	0	0.8095	Ref
MH834618	Unassigned	Unassigned	Unassigned	1166_0	Clustered	20	VC_1166_0	20	0.99	1	0.99	0	0	0	1	Ref
MH834619	Unassigned	Unassigned	Unassigned	1207_0	Clustered	29	VC_1207_0	29	0.8225	1	0.8225	0	0	0	1	Ref
MH834620	Unassigned	Unassigned	Unassigned	1166_0	Clustered	20	VC_1166_0	20	0.99	1	0.99	0	0	0	1	Ref
MH834621	Unassigned	Unassigned	Unassigned	1037_0	Clustered	6	VC_1037_0	6	0.6798	1	0.6798	0	0	0	1	Ref
MH834622	Unassigned	Unassigned	Unassigned	1362_0	Clustered	6	VC_1362_0	6	1	1	1	0	0	0	1	Ref
MH834623	Unassigned	Unassigned	Unassigned	1035_0	Clustered	6	VC_1035_0	6	0.4854	1	0.4854	0	0	0	1	Ref
MH834624	Unassigned	Unassigned	Unassigned	1166_0	Clustered	20	VC_1166_0	20	0.99	1	0.99	0	0	0	1	Ref
MH834625	Unassigned	Unassigned	Unassigned	1036_0	Clustered	11	VC_1036_0	11	0.656	1	0.656	0	0	0	0.8727	Ref
MH834626	Unassigned	Unassigned	Unassigned	1234_0	Clustered	44	VC_1234_0	44	1	1	1	0	0	0	1	Ref
MH834627	Unassigned	Unassigned	Unassigned	1037_0	Clustered	6	VC_1037_0	6	0.6798	1	0.6798	0	0	0	1	Ref
MH834628	Unassigned	Unassigned	Unassigned	951_0	Clustered	3	VC_951_0	3	0.1049	0.99921864	0.1048	0	0	0	1	Ref
MH834629	Unassigned	Unassigned	Unassigned	1207_0	Clustered	29	VC_1207_0	29	0.8225	1	0.8225	0	0	0	1	Ref
MH837542	Unassigned	Unassigned	Unassigned	302_0	Clustered	2	VC_302_0	2	0.1917	0.94974203	0.182	0	0	0	1	Ref
MH837543	Unassigned	Unassigned	Unassigned	302_0	Clustered	2	VC_302_0	2	0.1917	0.94974203	0.182	0	0	0	1	Ref
MH837626	Unassigned	Unassigned	Unassigned	283_159	Clustered/Singleton	1	VC_283_159	1	0	1	0	0	0	0	1	Ref
MH844528	Unassigned	Unassigned	Unassigned	149_8	Clustered	66	VC_149_8	66	0.4565	1	0.4565	0	0	0	0.8182	Ref
MH844529	Unassigned	Unassigned	Unassigned	149_8	Clustered	66	VC_149_8	66	0.4565	1	0.4565	0	0	0	0.8182	Ref
MH844531	Unassigned	Unassigned	Unassigned	938_0	Clustered	99	VC_938_0	99	0.9119	1	0.9119	0	0	0	0.9928	Ref
MH853786	Unassigned	Unassigned	Unassigned	698_0	Clustered	26	VC_698_0	26	0.9366	1	0.9366	0	0	0	1	Ref
MH853787	Unassigned	Unassigned	Unassigned	698_0	Clustered	26	VC_698_0	26	0.9366	1	0.9366	0	0	0	1	Ref
MH853788	Unassigned	Unassigned	Unassigned	698_0	Clustered	26	VC_698_0	26	0.9366	1	0.9366	0	0	0	1	Ref
MH880817	Unassigned	Unassigned	Unassigned	92_0	Clustered	22	VC_92_0	22	0.5639	1	0.5639	0	0	0	1	Ref
MH884508	Unassigned	Unassigned	Unassigned	475_4	Clustered/Singleton	1	VC_475_4	1	0	1	0	0	0	0	1	Ref
MH884511	Unassigned	Unassigned	Unassigned	87_2	Clustered/Singleton	1	VC_87_2	1	0	1	0	0	0	0	1	Ref
MH884512	Unassigned	Unassigned	Unassigned	622_0	Clustered	2	VC_622_0	2	0.0507	0.6259393	0.0317	0	0	0	1	Ref
MH884513	Unassigned	Unassigned	Unassigned	100_0	Clustered	15	VC_100_0	15	0.6536	1	0.6536	0	0	0	1	Ref
MH884514	Unassigned	Unassigned	Unassigned	101_0	Clustered	3	VC_101_0	3	0.3621	0.99764884	0.3612	0	0	0	1	Ref
MH885472	Unassigned	Unassigned	Unassigned	111_0	Clustered	91	VC_111_0	91	0.9587	1	0.9587	0	0	0	1	Ref
MH892350	Unassigned	Unassigned	Unassigned	256_0	Clustered	14	VC_256_0	14	0.2368	1	0.2368	0	0	0	1	Ref
MH892351	Unassigned	Unassigned	Unassigned	252_0	Clustered	121	VC_252_0	121	0.7376	1	0.7376	0	0	0	0.9999	Ref
MH892352	Unassigned	Unassigned	Unassigned	252_0	Clustered	121	VC_252_0	121	0.7376	1	0.7376	0	0	0	0.9999	Ref
MH892353	Unassigned	Unassigned	Unassigned	252_0	Clustered	121	VC_252_0	121	0.7376	1	0.7376	0	0	0	0.9999	Ref
MH892354	Unassigned	Unassigned	Unassigned	252_0	Clustered	121	VC_252_0	121	0.7376	1	0.7376	0	0	0	0.9999	Ref
MH892355	Unassigned	Unassigned	Unassigned	255_0	Clustered	13	VC_255_0	13	0.2048	1	0.2048	0	0	0	1	Ref
MH892356	Unassigned	Unassigned	Unassigned	252_0	Clustered	121	VC_252_0	121	0.7376	1	0.7376	0	0	0	0.9999	Ref
MH892357	Unassigned	Unassigned	Unassigned	252_0	Clustered	121	VC_252_0	121	0.7376	1	0.7376	0	0	0	0.9999	Ref
MH892358	Unassigned	Unassigned	Unassigned	252_0	Clustered	121	VC_252_0	121	0.7376	1	0.7376	0	0	0	0.9999	Ref
MH892359	Unassigned	Unassigned	Unassigned	252_0	Clustered	121	VC_252_0	121	0.7376	1	0.7376	0	0	0	0.9999	Ref
MH892360	Unassigned	Unassigned	Unassigned	252_0	Clustered	121	VC_252_0	121	0.7376	1	0.7376	0	0	0	0.9999	Ref
MH892361	Unassigned	Unassigned	Unassigned	252_0	Clustered	121	VC_252_0	121	0.7376	1	0.7376	0	0	0	0.9999	Ref
MH892362	Unassigned	Unassigned	Unassigned	254_0	Clustered	46	VC_254_0	46	0.5159	1	0.5159	0	0	0	1	Ref
MH892363	Unassigned	Unassigned	Unassigned	254_0	Clustered	46	VC_254_0	46	0.5159	1	0.5159	0	0	0	1	Ref
MH892364	Unassigned	Unassigned	Unassigned	254_0	Clustered	46	VC_254_0	46	0.5159	1	0.5159	0	0	0	1	Ref
MH892365	Unassigned	Unassigned	Unassigned	256_0	Clustered	14	VC_256_0	14	0.2368	1	0.2368	0	0	0	1	Ref
MH892366	Unassigned	Unassigned	Unassigned	254_0	Clustered	46	VC_254_0	46	0.5159	1	0.5159	0	0	0	1	Ref
MH892367	Unassigned	Unassigned	Unassigned	255_0	Clustered	13	VC_255_0	13	0.2048	1	0.2048	0	0	0	1	Ref
MH892368	Unassigned	Unassigned	Unassigned	256_0	Clustered	14	VC_256_0	14	0.2368	1	0.2368	0	0	0	1	Ref
MH892369	Unassigned	Unassigned	Unassigned	256_0	Clustered	14	VC_256_0	14	0.2368	1	0.2368	0	0	0	1	Ref
MH892370	Unassigned	Unassigned	Unassigned	256_0	Clustered	14	VC_256_0	14	0.2368	1	0.2368	0	0	0	1	Ref
MH892371	Unassigned	Unassigned	Unassigned	256_0	Clustered	14	VC_256_0	14	0.2368	1	0.2368	0	0	0	1	Ref
MH892372	Unassigned	Unassigned	Unassigned	256_0	Clustered	14	VC_256_0	14	0.2368	1	0.2368	0	0	0	1	Ref
MH892373	Unassigned	Unassigned	Unassigned	255_0	Clustered	13	VC_255_0	13	0.2048	1	0.2048	0	0	0	1	Ref
MH892374	Unassigned	Unassigned	Unassigned	256_0	Clustered	14	VC_256_0	14	0.2368	1	0.2368	0	0	0	1	Ref
MH892375	Unassigned	Unassigned	Unassigned	252_0	Clustered	121	VC_252_0	121	0.7376	1	0.7376	0	0	0	0.9999	Ref
MH892376	Unassigned	Unassigned	Unassigned	254_0	Clustered	46	VC_254_0	46	0.5159	1	0.5159	0	0	0	1	Ref
MH892377	Unassigned	Unassigned	Unassigned	252_0	Clustered	121	VC_252_0	121	0.7376	1	0.7376	0	0	0	0.9999	Ref
MH892378	Unassigned	Unassigned	Unassigned	252_0	Clustered	121	VC_252_0	121	0.7376	1	0.7376	0	0	0	0.9999	Ref
MH892379	Unassigned	Unassigned	Unassigned	252_0	Clustered	121	VC_252_0	121	0.7376	1	0.7376	0	0	0	0.9999	Ref
MH892380	Unassigned	Unassigned	Unassigned	252_0	Clustered	121	VC_252_0	121	0.7376	1	0.7376	0	0	0	0.9999	Ref
MH892381	Unassigned	Unassigned	Unassigned	252_0	Clustered	121	VC_252_0	121	0.7376	1	0.7376	0	0	0	0.9999	Ref
MH892382	Unassigned	Unassigned	Unassigned	252_0	Clustered	121	VC_252_0	121	0.7376	1	0.7376	0	0	0	0.9999	Ref
MH892383	Unassigned	Unassigned	Unassigned	254_0	Clustered	46	VC_254_0	46	0.5159	1	0.5159	0	0	0	1	Ref
MH892384	Unassigned	Unassigned	Unassigned	254_0	Clustered	46	VC_254_0	46	0.5159	1	0.5159	0	0	0	1	Ref
MH892385	Unassigned	Unassigned	Unassigned	254_0	Clustered	46	VC_254_0	46	0.5159	1	0.5159	0	0	0	1	Ref
MH899585	Unassigned	Unassigned	Unassigned	780_0	Clustered	8	VC_780_0	8	0.5207	1	0.5207	0	0	0	1	Ref
MH910036	Unassigned	Unassigned	Unassigned	1036_0	Clustered	11	VC_1036_0	11	0.656	1	0.656	0	0	0	0.8727	Ref
MH910037	Unassigned	Unassigned	Unassigned	1036_0	Clustered	11	VC_1036_0	11	0.656	1	0.656	0	0	0	0.8727	Ref
MH910038	Unassigned	Unassigned	Unassigned	237_3	Clustered	8	VC_237_3	8	0.1092	1	0.1092	0	0	0	1	Ref

MH910039	Unassigned	Unassigned	Unassigned	237_3	Clustered	8	VC_237_3	8	0.1092	1	0.1092	0	0	0	1	Ref
MH910040	Unassigned	Unassigned	Unassigned	558_33	Clustered/Singleton	1	VC_558_33	1	0	1	0	0	0	0	1	Ref
MH910041	Unassigned	Unassigned	Unassigned	1036_0	Clustered	11	VC_1036_0	11	0.656	1	0.656	0	0	0	0.8727	Ref
MH910042	Unassigned	Unassigned	Unassigned	237_3	Clustered	8	VC_237_3	8	0.1092	1	0.1092	0	0	0	1	Ref
MH917278	Unassigned	Unassigned	Unassigned	575_0	Clustered	35	VC_575_0	35	0.8815	1	0.8815	0	0	0	1	Ref
MH920362	Unassigned	Unassigned	Unassigned	330_0	Clustered	27	VC_330_0	27	0.5088	1	0.5088	0	0	0	0.8661	Ref
MH920639	Unassigned	Unassigned	Unassigned	655_7	Clustered	3	VC_655_7	3	0.0166	0.99856978	0.0166	0	0	0	1	Ref
MH920640	Unassigned	Unassigned	Unassigned	655_7	Clustered	3	VC_655_7	3	0.0166	0.99856978	0.0166	0	0	0	1	Ref
MH925090	Unassigned	Unassigned	Unassigned	882_0	Clustered	20	VC_882_0	20	0.9917	1	0.9917	0	0	0	1	Ref
MH925091	Unassigned	Unassigned	Unassigned	904_2	Clustered	7	VC_904_2	7	0.2857	1	0.2857	0	0	0	1	Ref
MH925092	Unassigned	Unassigned	Unassigned	1050_1	Clustered	29	VC_1050_1	29	0.4861	1	0.4861	0	0	0	0.9581	Ref
MH925094	Unassigned	Unassigned	Unassigned	972_0	Clustered	6	VC_972_0	6	0.2892	1	0.2892	0	0	0	1	Ref
MH926055	Unassigned	Unassigned	Unassigned	521_0	Clustered	37	VC_521_0	37	0.912	1	0.912	0	0	0	1	Ref
MH926056	Unassigned	Unassigned	Unassigned	768_0	Clustered	8	VC_768_0	8	0.7012	1	0.7012	0	0	0	1	Ref
MH926057	Unassigned	Unassigned	Unassigned	768_0	Clustered	8	VC_768_0	8	0.7012	1	0.7012	0	0	0	1	Ref
MH926058	Unassigned	Unassigned	Unassigned	234_0	Clustered	21	VC_234_0	21	0.8355	1	0.8355	0	0	0	1	Ref
MH926059	Unassigned	Unassigned	Unassigned	568_0	Clustered	8	VC_568_0	8	0.5738	1	0.5738	0	0	0	1	Ref
MH926060	Unassigned	Unassigned	Unassigned	371_1	Clustered	11	VC_371_1	11	0.0379	1	0.0379	0	0	0	0.8545	Ref
MH926061	Unassigned	Unassigned	Unassigned	1199_0	Clustered	8	VC_1199_0	8	0.7928	1	0.7928	0	0	0	1	Ref
MH926062	Unassigned	Unassigned	Unassigned	556_0	Clustered	21	VC_556_0	21	0.9912	1	0.9912	0	0	0	1	Ref
MH929319	Unassigned	Unassigned	Unassigned	132_0	Clustered	4	VC_132_0	4	0.036	0.99998777	0.036	0	0	0	1	Ref
MH931003	Unassigned	Unassigned	Unassigned	1054_0	Clustered	12	VC_1054_0	12	0.2863	0.99746644	0.2855	0	0	0	1	Ref
MH931004	Unassigned	Unassigned	Unassigned	1077_0	Clustered	48	VC_1077_0	48	0.8525	1	0.8525	0	0	0	1	Ref
MH937457	Unassigned	Unassigned	Unassigned	254_0	Clustered	46	VC_254_0	46	0.5159	1	0.5159	0	0	0	1	Ref
MH937458	Unassigned	Unassigned	Unassigned	252_0	Clustered	121	VC_252_0	121	0.7376	1	0.7376	0	0	0	0.9999	Ref
MH937459	Unassigned	Unassigned	Unassigned	252_0	Clustered	121	VC_252_0	121	0.7376	1	0.7376	0	0	0	0.9999	Ref
MH937460	Unassigned	Unassigned	Unassigned	254_0	Clustered	46	VC_254_0	46	0.5159	1	0.5159	0	0	0	1	Ref
MH937461	Unassigned	Unassigned	Unassigned	252_0	Clustered	121	VC_252_0	121	0.7376	1	0.7376	0	0	0	0.9999	Ref
MH937462	Unassigned	Unassigned	Unassigned	252_0	Clustered	121	VC_252_0	121	0.7376	1	0.7376	0	0	0	0.9999	Ref
MH937463	Unassigned	Unassigned	Unassigned	254_0	Clustered	46	VC_254_0	46	0.5159	1	0.5159	0	0	0	1	Ref
MH937464	Unassigned	Unassigned	Unassigned	254_0	Clustered	46	VC_254_0	46	0.5159	1	0.5159	0	0	0	1	Ref
MH937465	Unassigned	Unassigned	Unassigned	252_0	Clustered	121	VC_252_0	121	0.7376	1	0.7376	0	0	0	0.9999	Ref
MH937466	Unassigned	Unassigned	Unassigned	252_0	Clustered	121	VC_252_0	121	0.7376	1	0.7376	0	0	0	0.9999	Ref
MH937467	Unassigned	Unassigned	Unassigned	252_0	Clustered	121	VC_252_0	121	0.7376	1	0.7376	0	0	0	0.9999	Ref
MH937468	Unassigned	Unassigned	Unassigned	252_0	Clustered	121	VC_252_0	121	0.7376	1	0.7376	0	0	0	0.9999	Ref
MH937469	Unassigned	Unassigned	Unassigned	252_0	Clustered	121	VC_252_0	121	0.7376	1	0.7376	0	0	0	0.9999	Ref
MH937470	Unassigned	Unassigned	Unassigned	252_0	Clustered	121	VC_252_0	121	0.7376	1	0.7376	0	0	0	0.9999	Ref
MH937471	Unassigned	Unassigned	Unassigned	252_0	Clustered	121	VC_252_0	121	0.7376	1	0.7376	0	0	0	0.9999	Ref
MH937472	Unassigned	Unassigned	Unassigned	252_0	Clustered	121	VC_252_0	121	0.7376	1	0.7376	0	0	0	0.9999	Ref
MH937473	Unassigned	Unassigned	Unassigned	254_0	Clustered	46	VC_254_0	46	0.5159	1	0.5159	0	0	0	1	Ref
MH937474	Unassigned	Unassigned	Unassigned	252_0	Clustered	121	VC_252_0	121	0.7376	1	0.7376	0	0	0	0.9999	Ref
MH937475	Unassigned	Unassigned	Unassigned	254_0	Clustered	46	VC_254_0	46	0.5159	1	0.5159	0	0	0	1	Ref
MH937476	Unassigned	Unassigned	Unassigned	254_0	Clustered	46	VC_254_0	46	0.5159	1	0.5159	0	0	0	1	Ref
MH937477	Unassigned	Unassigned	Unassigned	252_0	Clustered	121	VC_252_0	121	0.7376	1	0.7376	0	0	0	0.9999	Ref
MH937478	Unassigned	Unassigned	Unassigned	254_0	Clustered	46	VC_254_0	46	0.5159	1	0.5159	0	0	0	1	Ref
MH937479	Unassigned	Unassigned	Unassigned	254_0	Clustered	46	VC_254_0	46	0.5159	1	0.5159	0	0	0	1	Ref
MH937480	Unassigned	Unassigned	Unassigned	252_0	Clustered	121	VC_252_0	121	0.7376	1	0.7376	0	0	0	0.9999	Ref
MH937481	Unassigned	Unassigned	Unassigned	252_0	Clustered	121	VC_252_0	121	0.7376	1	0.7376	0	0	0	0.9999	Ref
MH937482	Unassigned	Unassigned	Unassigned	252_0	Clustered	121	VC_252_0	121	0.7376	1	0.7376	0	0	0	0.9999	Ref
MH937483	Unassigned	Unassigned	Unassigned	252_0	Clustered	121	VC_252_0	121	0.7376	1	0.7376	0	0	0	0.9999	Ref
MH937484	Unassigned	Unassigned	Unassigned	254_0	Clustered	46	VC_254_0	46	0.5159	1	0.5159	0	0	0	1	Ref
MH937485	Unassigned	Unassigned	Unassigned	252_0	Clustered	121	VC_252_0	121	0.7376	1	0.7376	0	0	0	0.9999	Ref
MH937486	Unassigned	Unassigned	Unassigned	252_0	Clustered	121	VC_252_0	121	0.7376	1	0.7376	0	0	0	0.9999	Ref
MH937487	Unassigned	Unassigned	Unassigned	252_0	Clustered	121	VC_252_0	121	0.7376	1	0.7376	0	0	0	0.9999	Ref
MH937488	Unassigned	Unassigned	Unassigned	252_0	Clustered	121	VC_252_0	121	0.7376	1	0.7376	0	0	0	0.9999	Ref
MH937489	Unassigned	Unassigned	Unassigned	252_0	Clustered	121	VC_252_0	121	0.7376	1	0.7376	0	0	0	0.9999	Ref
MH937490	Unassigned	Unassigned	Unassigned	252_0	Clustered	121	VC_252_0	121	0.7376	1	0.7376	0	0	0	0.9999	Ref
MH937491	Unassigned	Unassigned	Unassigned	252_0	Clustered	121	VC_252_0	121	0.7376	1	0.7376	0	0	0	0.9999	Ref
MH937492	Unassigned	Unassigned	Unassigned	252_0	Clustered	121	VC_252_0	121	0.7376	1	0.7376	0	0	0	0.9999	Ref
MH937493	Unassigned	Unassigned	Unassigned	252_0	Clustered	121	VC_252_0	121	0.7376	1	0.7376	0	0	0	0.9999	Ref
MH937494	Unassigned	Unassigned	Unassigned	254_0	Clustered	46	VC_254_0	46	0.5159	1	0.5159	0	0	0	1	Ref
MH937495	Unassigned	Unassigned	Unassigned	252_0	Clustered	121	VC_252_0	121	0.7376	1	0.7376	0	0	0	0.9999	Ref
MH937496	Unassigned	Unassigned	Unassigned	252_0	Clustered	121	VC_252_0	121	0.7376	1	0.7376	0	0	0	0.9999	Ref
MH937497	Unassigned	Unassigned	Unassigned	252_0	Clustered	121	VC_252_0	121	0.7376	1	0.7376	0	0	0	0.9999	Ref
MH937498	Unassigned	Unassigned	Unassigned	254_0	Clustered	46	VC_254_0	46	0.5159	1	0.5159	0	0	0	1	Ref
MH937499	Unassigned	Unassigned	Unassigned	252_0	Clustered	121	VC_252_0	121	0.7376	1	0.7376	0	0	0	0.9999	Ref
MH937500	Unassigned	Unassigned	Unassigned	252_0	Clustered	121	VC_252_0	121	0.7376	1	0.7376	0	0	0	0.9999	Ref
MH937501	Unassigned	Unassigned	Unassigned	252_0	Clustered	121	VC_252_0	121	0.7376	1	0.7376	0	0	0	0.9999	Ref
MH937502	Unassigned	Unassigned	Unassigned	254_0	Clustered	46	VC_254_0	46	0.5159	1	0.5159	0	0	0	1	Ref
MH937503	Unassigned	Unassigned	Unassigned	254_0	Clustered	46	VC_254_0	46	0.5159	1	0.5159	0	0	0	1	Ref
MH937504	Unassigned	Unassigned	Unassigned	252_0	Clustered	121	VC_252_0	121	0.7376	1	0.7376	0	0	0	0.9999	Ref
MH937505	Unassigned	Unassigned	Unassigned	254_0	Clustered	46	VC_254_0	46	0.5159	1	0.5159	0	0	0	1	Ref
MH937506	Unassigned	Unassigned	Unassigned	252_0	Clustered	121	VC_252_0	121	0.7376	1	0.7376	0	0	0	0.9999	Ref
MH937507	Unassigned	Unassigned	Unassigned	254_0	Clustered	46	VC_254_0	46	0.5159	1	0.5159	0	0	0	1	Ref
MH937508	Unassigned	Unassigned	Unassigned	252_0	Clustered	121	VC_252_0	121	0.7376	1	0.7376	0	0	0	0.9999	Ref
MH937509	Unassigned	Unassigned	Unassigned	254_0	Clustered	46	VC_254_0	46	0.5159	1	0.5159	0	0	0	1	Ref
MH937510	Unassigned	Unassigned	Unassigned	254_0	Clustered	46	VC_254_0	46	0.5159	1	0.5159	0	0	0	1	Ref
MH937511	Unassigned	Unassigned	Unassigned	254_0	Clustered	46	VC_254_0	46	0.5159	1	0.5159	0	0	0	1	Ref
MH939157	Unassigned	Unassigned	Unassigned	1363_0	Clustered	2	VC_1363_0	2	0.8326	0.72985431	0.6077	0	0	0	1	Ref
MH940212	Unassigned	Unassigned	Unassigned	133_22	Clustered	13	VC_133_22	13	0.0804	1	0.0804	0	0	0	0.8205	Ref
MH972260	Unassigned	Unassigned	Unassigned	96_0	Clustered	30	VC_96_0	30	0.718	1	0.718	0	0	0	1	Ref

MH973661	Unassigned	Unassigned	Unassigned	252_0	Clustered	121	VC_252_0	121	0.7376	1	0.7376	0	0	0	0.9999	Ref
MH973662	Unassigned	Unassigned	Unassigned	252_0	Clustered	121	VC_252_0	121	0.7376	1	0.7376	0	0	0	0.9999	Ref
MH973663	Unassigned	Unassigned	Unassigned	255_0	Clustered	13	VC_255_0	13	0.2048	1	0.2048	0	0	0	1	Ref
MH973725	Unassigned	Unassigned	Unassigned	472_0	Clustered	47	VC_472_0	47	0.9025	1	0.9025	0	0	0	1	Ref
MH976506	Unassigned	Unassigned	Unassigned	1093_0	Clustered	40	VC_1093_0	40	0.8754	1	0.8754	0	0	0	1	Ref
MH976507	Unassigned	Unassigned	Unassigned	1093_0	Clustered	40	VC_1093_0	40	0.8754	1	0.8754	0	0	0	1	Ref
MH976508	Unassigned	Unassigned	Unassigned	1093_0	Clustered	40	VC_1093_0	40	0.8754	1	0.8754	0	0	0	1	Ref
MH976509	Unassigned	Unassigned	Unassigned	1093_0	Clustered	40	VC_1093_0	40	0.8754	1	0.8754	0	0	0	1	Ref
MH976510	Unassigned	Unassigned	Unassigned	1093_0	Clustered	40	VC_1093_0	40	0.8754	1	0.8754	0	0	0	1	Ref
MH976511	Unassigned	Unassigned	Unassigned	1093_0	Clustered	40	VC_1093_0	40	0.8754	1	0.8754	0	0	0	1	Ref
MH976512	Unassigned	Unassigned	Unassigned	1093_0	Clustered	40	VC_1093_0	40	0.8754	1	0.8754	0	0	0	1	Ref
MH976513	Unassigned	Unassigned	Unassigned	1093_0	Clustered	40	VC_1093_0	40	0.8754	1	0.8754	0	0	0	1	Ref
MH976514	Unassigned	Unassigned	Unassigned	1093_0	Clustered	40	VC_1093_0	40	0.8754	1	0.8754	0	0	0	1	Ref
MH976515	Unassigned	Unassigned	Unassigned	890_1	Clustered	4	VC_890_1	4	0.4613	0.99998458	0.4613	0	0	0	1	Ref
MH976516	Unassigned	Unassigned	Unassigned	768_0	Clustered	8	VC_768_0	8	0.7012	1	0.7012	0	0	0	1	Ref
MH976517	Unassigned	Unassigned	Unassigned	513_0	Clustered	32	VC_513_0	32	0.6881	1	0.6881	0	0	0	1	Ref
MH976518	Unassigned	Unassigned	Unassigned	1093_0	Clustered	40	VC_1093_0	40	0.8754	1	0.8754	0	0	0	1	Ref
MH976519	Unassigned	Unassigned	Unassigned	1093_0	Clustered	40	VC_1093_0	40	0.8754	1	0.8754	0	0	0	1	Ref
MH979674	Unassigned	Unassigned	Unassigned	423_0	Clustered	45	VC_423_0	45	0.9484	1	0.9484	0	0	0	1	Ref
MH983004	Unassigned	Unassigned	Unassigned	272_0	Clustered	11	VC_272_0	11	0.4915	1	0.4915	0	0	0	0.8727	Ref
MH992121	Unassigned	Unassigned	Unassigned	283_188	Clustered/Singleton	1	VC_283_188	1	0	1	0	0	0	0	1	Ref
MH992122	Unassigned	Unassigned	Unassigned	286_10	Clustered	9	VC_286_10	9	0.0615	0.99999878	0.0615	0	0	0	0.6667	Ref
MH992123	Unassigned	Unassigned	Unassigned	779_0	Clustered	37	VC_779_0	37	0.5682	1	0.5682	0	0	0	1	Ref
MH992131	Unassigned	Unassigned	Unassigned	805_0	Clustered	4	VC_805_0	4	0.9081	0.99622688	0.9046	0	0	0	1	Ref
MH992154	Unassigned	Unassigned	Unassigned	1364_0	Clustered	3	VC_1364_0	3	1	1	1	0	0	0	1	Ref
MH992155	Unassigned	Unassigned	Unassigned	1365_0	Clustered	3	VC_1365_0	3	1	1	1	0	0	0	1	Ref
MH992156	Unassigned	Unassigned	Unassigned	995_0	Clustered	69	VC_995_0	69	0.8547	1	0.8547	0	0	0	1	Ref
MH992157	Unassigned	Unassigned	Unassigned	198_0	Clustered	28	VC_198_0	28	0.2429	0.99971175	0.2429	0	0	0	1	Ref
MH992158	Unassigned	Unassigned	Unassigned	327_0	Clustered	12	VC_327_0	12	0.8925	1	0.8925	0	0	0	1	Ref
MH992159	Unassigned	Unassigned	Unassigned	1339_0	Clustered	7	VC_1339_0	7	0.9177	0.99772305	0.9156	0	0	0	1	Ref
MH992160	Unassigned	Unassigned	Unassigned	327_0	Clustered	12	VC_327_0	12	0.8925	1	0.8925	0	0	0	1	Ref
MH992161	Unassigned	Unassigned	Unassigned	995_0	Clustered	69	VC_995_0	69	0.8547	1	0.8547	0	0	0	1	Ref
MH992163	Unassigned	Unassigned	Unassigned	1077_0	Clustered	48	VC_1077_0	48	0.8525	1	0.8525	0	0	0	1	Ref
MH992164	Unassigned	Unassigned	Unassigned	1077_0	Clustered	48	VC_1077_0	48	0.8525	1	0.8525	0	0	0	1	Ref
MH992165	Unassigned	Unassigned	Unassigned	1066_0	Clustered	52	VC_1066_0	52	0.7682	1	0.7682	0	0	0	1	Ref
MH992167	Unassigned	Unassigned	Unassigned	1367_0	Clustered	2	VC_1367_0	2	1	1	1	0	0	0	1	Ref
MH992169	Unassigned	Unassigned	Unassigned	997_0	Clustered/Singleton	1	VC_997_0	1	0	1	0	0	0	0	1	Ref
MH992170	Unassigned	Unassigned	Unassigned	1059_0	Clustered	3	VC_1059_0	3	0.3928	0.9839946	0.3865	0	0	0	1	Ref
MH992171	Unassigned	Unassigned	Unassigned	1368_0	Clustered	2	VC_1368_0	2	1	1	1	0	0	0	1	Ref
MH992173	Unassigned	Unassigned	Unassigned	1324_0	Clustered	4	VC_1324_0	4	0.6008	0.54407361	0.3269	0	0	0	1	Ref
MH992174	Unassigned	Unassigned	Unassigned	198_0	Clustered	28	VC_198_0	28	0.2429	0.99971175	0.2429	0	0	0	1	Ref
MH992175	Unassigned	Unassigned	Unassigned	1339_0	Clustered	7	VC_1339_0	7	0.9177	0.99772305	0.9156	0	0	0	1	Ref
MH992177	Unassigned	Unassigned	Unassigned	327_0	Clustered	12	VC_327_0	12	0.8925	1	0.8925	0	0	0	1	Ref
MH992178	Unassigned	Unassigned	Unassigned	327_0	Clustered	12	VC_327_0	12	0.8925	1	0.8925	0	0	0	1	Ref
MH992180	Unassigned	Unassigned	Unassigned	1062_0	Clustered	45	VC_1062_0	45	0.9734	0.50949847	0.4959	0	0	0	1	Ref
MH992181	Unassigned	Unassigned	Unassigned	200_0	Clustered/Singleton	1	VC_200_0	1	0	1	0	0	0	0	1	Ref
MH992182	Unassigned	Unassigned	Unassigned	198_0	Clustered	28	VC_198_0	28	0.2429	0.99971175	0.2429	0	0	0	1	Ref
MH992184	Unassigned	Unassigned	Unassigned	995_0	Clustered	69	VC_995_0	69	0.8547	1	0.8547	0	0	0	1	Ref
MH992185	Unassigned	Unassigned	Unassigned	995_0	Clustered	69	VC_995_0	69	0.8547	1	0.8547	0	0	0	1	Ref
MH992189	Unassigned	Unassigned	Unassigned	327_0	Clustered	12	VC_327_0	12	0.8925	1	0.8925	0	0	0	1	Ref
MH992191	Unassigned	Unassigned	Unassigned	327_0	Clustered	12	VC_327_0	12	0.8925	1	0.8925	0	0	0	1	Ref
MH992192	Unassigned	Unassigned	Unassigned	1077_0	Clustered	48	VC_1077_0	48	0.8525	1	0.8525	0	0	0	1	Ref
MH992193	Unassigned	Unassigned	Unassigned	1059_0	Clustered	3	VC_1059_0	3	0.3928	0.9839946	0.3865	0	0	0	1	Ref
MH992194	Unassigned	Unassigned	Unassigned	327_0	Clustered	12	VC_327_0	12	0.8925	1	0.8925	0	0	0	1	Ref
MH992195	Unassigned	Unassigned	Unassigned	398_0	Clustered	48	VC_398_0	48	0.4813	1	0.4813	0	0	0	1	Ref
MH992196	Unassigned	Unassigned	Unassigned	1365_0	Clustered	3	VC_1365_0	3	1	1	1	0	0	0	1	Ref
MH992197	Unassigned	Unassigned	Unassigned	327_0	Clustered	12	VC_327_0	12	0.8925	1	0.8925	0	0	0	1	Ref
MH992199	Unassigned	Unassigned	Unassigned	1077_0	Clustered	48	VC_1077_0	48	0.8525	1	0.8525	0	0	0	1	Ref
MH992200	Unassigned	Unassigned	Unassigned	1339_0	Clustered	7	VC_1339_0	7	0.9177	0.99772305	0.9156	0	0	0	1	Ref
MH992202	Unassigned	Unassigned	Unassigned	1077_0	Clustered	48	VC_1077_0	48	0.8525	1	0.8525	0	0	0	1	Ref
MH992205	Unassigned	Unassigned	Unassigned	1365_0	Clustered	3	VC_1365_0	3	1	1	1	0	0	0	1	Ref
MH992206	Unassigned	Unassigned	Unassigned	1077_0	Clustered	48	VC_1077_0	48	0.8525	1	0.8525	0	0	0	1	Ref
MH992207	Unassigned	Unassigned	Unassigned	327_0	Clustered	12	VC_327_0	12	0.8925	1	0.8925	0	0	0	1	Ref
MH992208	Unassigned	Unassigned	Unassigned	1077_0	Clustered	48	VC_1077_0	48	0.8525	1	0.8525	0	0	0	1	Ref
MH992210	Unassigned	Unassigned	Unassigned	198_0	Clustered	28	VC_198_0	28	0.2429	0.99971175	0.2429	0	0	0	1	Ref
MH992211	Unassigned	Unassigned	Unassigned	398_0	Clustered	48	VC_398_0	48	0.4813	1	0.4813	0	0	0	1	Ref
MH992213	Unassigned	Unassigned	Unassigned	995_0	Clustered	69	VC_995_0	69	0.8547	1	0.8547	0	0	0	1	Ref
MH992214	Unassigned	Unassigned	Unassigned	1077_0	Clustered	48	VC_1077_0	48	0.8525	1	0.8525	0	0	0	1	Ref
MH992215	Unassigned	Unassigned	Unassigned	1077_0	Clustered	48	VC_1077_0	48	0.8525	1	0.8525	0	0	0	1	Ref
MH992216	Unassigned	Unassigned	Unassigned	1077_0	Clustered	48	VC_1077_0	48	0.8525	1	0.8525	0	0	0	1	Ref
MH992217	Unassigned	Unassigned	Unassigned	1077_0	Clustered	48	VC_1077_0	48	0.8525	1	0.8525	0	0	0	1	Ref
MH992218	Unassigned	Unassigned	Unassigned	327_0	Clustered	12	VC_327_0	12	0.8925	1	0.8925	0	0	0	1	Ref
MH992219	Unassigned	Unassigned	Unassigned	1077_0	Clustered	48	VC_1077_0	48	0.8525	1	0.8525	0	0	0	1	Ref
MH992220	Unassigned	Unassigned	Unassigned	1077_0	Clustered	48	VC_1077_0	48	0.8525	1	0.8525	0	0	0	1	Ref
MH992222	Unassigned	Unassigned	Unassigned	1077_0	Clustered	48	VC_1077_0	48	0.8525	1	0.8525	0	0	0	1	Ref
MH992509	Unassigned	Unassigned	Unassigned	637_0	Clustered	115	VC_637_0	115	0.731	1	0.731	0	0	0	0.8764	Ref
MH992510	Unassigned	Unassigned	Unassigned	283_129	Clustered/Singleton	1	VC_283_129	1	0	1	0	0	0	0	1	Ref
MH992513	Unassigned	Unassigned	Unassigned	878_0	Clustered	32	VC_878_0	32	0.9965	1	0.9965	0	0	0	1	Ref
MH999279	Unassigned	Unassigned	Unassigned	897_0	Clustered	6	VC_897_0	6	0.4156	1	0.4156	0	0	0	1	Ref
MH999280	Unassigned	Unassigned	Unassigned	815_0	Clustered	8	VC_815_0	8	0.9801	1	0.9801	0	0	0	1	Ref
MK002701	Unassigned	Unassigned	Unassigned	352_0	Clustered	3	VC_352_0	3	0.7306	0.98867275	0.7223	0	0	0	1	Ref

MK005300	Unassigned	Unassigned	Unassigned	191_20	Clustered/Singleton	1	VC_191_20	1	0	1	0	0	0	0	1	Ref
MK016490	Unassigned	Unassigned	Unassigned	744_0	Clustered	10	VC_744_0	10	0.7049	1	0.7049	0	0	0	1	Ref
MK016491	Unassigned	Unassigned	Unassigned	551_5	Clustered	2	VC_551_5	2	0.0054	0.95548062	0.0052	0	0	0	1	Ref
MK016492	Unassigned	Unassigned	Unassigned	525_0	Clustered	25	VC_525_0	25	0.4597	1	0.4597	0	0	0	1	Ref
MK016494	Unassigned	Unassigned	Unassigned	520_31	Clustered	11	VC_520_31	11	0.0897	1	0.0897	0	0	0	0.6	Ref
MK016495	Unassigned	Unassigned	Unassigned	1162_0	Clustered	10	VC_1162_0	10	0.5715	1	0.5715	0	0	0	1	Ref
MK016496	Unassigned	Unassigned	Unassigned	520_77	Clustered	3	VC_520_77	3	0.0218	0.99859911	0.0218	0	0	0	1	Ref
MK016497	Unassigned	Unassigned	Unassigned	1162_0	Clustered	10	VC_1162_0	10	0.5715	1	0.5715	0	0	0	1	Ref
MK016498	Unassigned	Unassigned	Unassigned	551_5	Clustered	2	VC_551_5	2	0.0054	0.95548062	0.0052	0	0	0	1	Ref
MK016499	Unassigned	Unassigned	Unassigned	519_0	Clustered	39	VC_519_0	39	0.8156	1	0.8156	0	0	0	1	Ref
MK016500	Unassigned	Unassigned	Unassigned	744_0	Clustered	10	VC_744_0	10	0.7049	1	0.7049	0	0	0	1	Ref
MK016501	Unassigned	Unassigned	Unassigned	563_0	Clustered	2	VC_563_0	2	0.0727	0.956426	0.0695	0	0	0	1	Ref
MK016502	Unassigned	Unassigned	Unassigned	551_0	Clustered	16	VC_551_0	16	0.0915	1	0.0915	0	0	0	0.6667	Ref
MK016503	Unassigned	Unassigned	Unassigned	236_0	Clustered	72	VC_236_0	72	0.8104	1	0.8104	0	0	0	1	Ref
MK016504	Unassigned	Unassigned	Unassigned	520_90	Clustered/Singleton	1	VC_520_90	1	0	1	0	0	0	0	1	Ref
MK016662	Unassigned	Unassigned	Unassigned	340_0	Clustered	31	VC_340_0	31	0.9549	1	0.9549	0	0	0	1	Ref
MK016664	Unassigned	Unassigned	Unassigned	435_1	Clustered/Singleton	1	VC_435_1	1	0	1	0	0	0	0	1	Ref
MK017819	Unassigned	Unassigned	Unassigned	1022_0	Clustered	4	VC_1022_0	4	0.8047	0.99996089	0.8046	0	0	0	1	Ref
MK017820	Unassigned	Unassigned	Unassigned	899_0	Clustered	4	VC_899_0	4	0.2655	0.99997104	0.2655	0	0	0	1	Ref
MK024806	Unassigned	Unassigned	Unassigned	582_3	Clustered	12	VC_582_3	12	0.2357	1	0.2357	0	0	0	0.8636	Ref
MK033136	Unassigned	Unassigned	Unassigned	451_0	Clustered	80	VC_451_0	80	0.9991	1	0.9991	0	0	0	1	Ref
MK033137	Unassigned	Unassigned	Unassigned	130_0	Clustered	19	VC_130_0	19	0.6068	1	0.6068	0	0	0	1	Ref
MK034952	Unassigned	Unassigned	Unassigned	2_0	Clustered	4	VC_2_0	4	0.1311	0.99998692	0.1311	0	0	0	1	Ref
MK047717	Unassigned	Unassigned	Unassigned	286_15	Clustered	2	VC_286_15	2	0.0073	0.75626308	0.0055	0	0	0	1	Ref
MK047718	Unassigned	Unassigned	Unassigned	286_17	Clustered	8	VC_286_17	8	0.0535	0.99997829	0.0535	0	0	0	0.6071	Ref
MK050846	Unassigned	Unassigned	Unassigned	191_32	Clustered	12	VC_191_32	12	0.0683	1	0.0683	0	0	0	0.7576	Ref
MK053931	Unassigned	Unassigned	Unassigned	878_0	Clustered	32	VC_878_0	32	0.9965	1	0.9965	0	0	0	1	Ref
MK054204	Unassigned	Unassigned	Unassigned	599_0	Clustered	50	VC_599_0	50	0.9989	1	0.9989	0	0	0	1	Ref
MK054205	Unassigned	Unassigned	Unassigned	599_0	Clustered	50	VC_599_0	50	0.9989	1	0.9989	0	0	0	1	Ref
MK054206	Unassigned	Unassigned	Unassigned	599_0	Clustered	50	VC_599_0	50	0.9989	1	0.9989	0	0	0	1	Ref
MK054207	Unassigned	Unassigned	Unassigned	599_0	Clustered	50	VC_599_0	50	0.9989	1	0.9989	0	0	0	1	Ref
MK054208	Unassigned	Unassigned	Unassigned	599_0	Clustered	50	VC_599_0	50	0.9989	1	0.9989	0	0	0	1	Ref
MK054209	Unassigned	Unassigned	Unassigned	599_0	Clustered	50	VC_599_0	50	0.9989	1	0.9989	0	0	0	1	Ref
MK054210	Unassigned	Unassigned	Unassigned	599_0	Clustered	50	VC_599_0	50	0.9989	1	0.9989	0	0	0	1	Ref
MK054211	Unassigned	Unassigned	Unassigned	599_0	Clustered	50	VC_599_0	50	0.9989	1	0.9989	0	0	0	1	Ref
MK054212	Unassigned	Unassigned	Unassigned	599_0	Clustered	50	VC_599_0	50	0.9989	1	0.9989	0	0	0	1	Ref
MK054213	Unassigned	Unassigned	Unassigned	599_0	Clustered	50	VC_599_0	50	0.9989	1	0.9989	0	0	0	1	Ref
MK054214	Unassigned	Unassigned	Unassigned	599_0	Clustered	50	VC_599_0	50	0.9989	1	0.9989	0	0	0	1	Ref
MK054215	Unassigned	Unassigned	Unassigned	599_0	Clustered	50	VC_599_0	50	0.9989	1	0.9989	0	0	0	1	Ref
MK054216	Unassigned	Unassigned	Unassigned	599_0	Clustered	50	VC_599_0	50	0.9989	1	0.9989	0	0	0	1	Ref
MK054217	Unassigned	Unassigned	Unassigned	599_0	Clustered	50	VC_599_0	50	0.9989	1	0.9989	0	0	0	1	Ref
MK054218	Unassigned	Unassigned	Unassigned	599_0	Clustered	50	VC_599_0	50	0.9989	1	0.9989	0	0	0	1	Ref
MK054219	Unassigned	Unassigned	Unassigned	599_0	Clustered	50	VC_599_0	50	0.9989	1	0.9989	0	0	0	1	Ref
MK054220	Unassigned	Unassigned	Unassigned	599_0	Clustered	50	VC_599_0	50	0.9989	1	0.9989	0	0	0	1	Ref
MK054221	Unassigned	Unassigned	Unassigned	599_0	Clustered	50	VC_599_0	50	0.9989	1	0.9989	0	0	0	1	Ref
MK054222	Unassigned	Unassigned	Unassigned	599_0	Clustered	50	VC_599_0	50	0.9989	1	0.9989	0	0	0	1	Ref
MK054223	Unassigned	Unassigned	Unassigned	599_0	Clustered	50	VC_599_0	50	0.9989	1	0.9989	0	0	0	1	Ref
MK054224	Unassigned	Unassigned	Unassigned	599_0	Clustered	50	VC_599_0	50	0.9989	1	0.9989	0	0	0	1	Ref
MK054225	Unassigned	Unassigned	Unassigned	599_0	Clustered	50	VC_599_0	50	0.9989	1	0.9989	0	0	0	1	Ref
MK054226	Unassigned	Unassigned	Unassigned	599_0	Clustered	50	VC_599_0	50	0.9989	1	0.9989	0	0	0	1	Ref
MK054227	Unassigned	Unassigned	Unassigned	599_0	Clustered	50	VC_599_0	50	0.9989	1	0.9989	0	0	0	1	Ref
MK054228	Unassigned	Unassigned	Unassigned	599_0	Clustered	50	VC_599_0	50	0.9989	1	0.9989	0	0	0	1	Ref
MK054229	Unassigned	Unassigned	Unassigned	599_0	Clustered	50	VC_599_0	50	0.9989	1	0.9989	0	0	0	1	Ref
MK054230	Unassigned	Unassigned	Unassigned	599_0	Clustered	50	VC_599_0	50	0.9989	1	0.9989	0	0	0	1	Ref
MK054231	Unassigned	Unassigned	Unassigned	599_0	Clustered	50	VC_599_0	50	0.9989	1	0.9989	0	0	0	1	Ref
MK054232	Unassigned	Unassigned	Unassigned	599_0	Clustered	50	VC_599_0	50	0.9989	1	0.9989	0	0	0	1	Ref
MK054233	Unassigned	Unassigned	Unassigned	599_0	Clustered	50	VC_599_0	50	0.9989	1	0.9989	0	0	0	1	Ref
MK054234	Unassigned	Unassigned	Unassigned	599_0	Clustered	50	VC_599_0	50	0.9989	1	0.9989	0	0	0	1	Ref
MK054235	Unassigned	Unassigned	Unassigned	599_0	Clustered	50	VC_599_0	50	0.9989	1	0.9989	0	0	0	1	Ref
MK054236	Unassigned	Unassigned	Unassigned	599_0	Clustered	50	VC_599_0	50	0.9989	1	0.9989	0	0	0	1	Ref
MK054237	Unassigned	Unassigned	Unassigned	599_0	Clustered	50	VC_599_0	50	0.9989	1	0.9989	0	0	0	1	Ref
MK059749	Unassigned	Unassigned	Unassigned	372_2	Clustered	5	VC_372_2	5	0.072	0.99999997	0.072	0	0	0	0.9	Ref
MK061407	Unassigned	Unassigned	Unassigned	939_0	Clustered	15	VC_939_0	15	1	0	0	0	0	0	1	Ref
MK061408	Unassigned	Unassigned	Unassigned	216_4	Clustered	13	VC_216_4	13	0.1657	1	0.1657	0	0	0	1	Ref
MK061409	Unassigned	Unassigned	Unassigned	225_0	Clustered	32	VC_225_0	32	0.848	1	0.848	0	0	0	0.996	Ref
MK061410	Unassigned	Unassigned	Unassigned	1177_0	Clustered	14	VC_1177_0	14	0.7847	1	0.7847	0	0	0	0.989	Ref
MK061411	Unassigned	Unassigned	Unassigned	508_0	Clustered	32	VC_508_0	32	0.906	1	0.906	0	0	0	1	Ref
MK061412	Unassigned	Unassigned	Unassigned	1072_3	Clustered	6	VC_1072_3	6	0.1304	0.99999994	0.1304	0	0	0	0.8667	Ref
MK061413	Unassigned	Unassigned	Unassigned	1207_0	Clustered	29	VC_1207_0	29	0.8225	1	0.8225	0	0	0	1	Ref
MK061414	Unassigned	Unassigned	Unassigned	509_0	Clustered	37	VC_509_0	37	0.9689	1	0.9689	0	0	0	1	Ref
MK061415	Unassigned	Unassigned	Unassigned	325_37	Clustered	3	VC_325_37	3	0.008	0.99634936	0.008	0	0	0	0.6667	Ref
MK064562	Unassigned	Unassigned	Unassigned	1354_0	Clustered	3	VC_1354_0	3	0.983	0.9595722	0.9432	0	0	0	1	Ref
MK064563	Unassigned	Unassigned	Unassigned	1354_0	Clustered	3	VC_1354_0	3	0.983	0.9595722	0.9432	0	0	0	1	Ref
MK064564	Unassigned	Unassigned	Unassigned	350_0	Clustered	2	VC_350_0	2	0.324	0.93865445	0.3041	0	0	0	1	Ref
MK064565	Unassigned	Unassigned	Unassigned	348_0	Clustered	22	VC_348_0	22	0.9602	1	0.9602	0	0	0	1	Ref
MK069403	Unassigned	Unassigned	Unassigned	673_0	Clustered	15	VC_673_0	15	0.8234	1	0.8234	0	0	0	0.7619	Ref
MK071653	Unassigned	Unassigned	Unassigned	1363_0	Clustered	2	VC_1363_0	2	0.8326	0.72985431	0.6077	0	0	0	1	Ref
MK075001	Unassigned	Unassigned	Unassigned	15_1	Clustered	9	VC_15_1	9	0.182	1	0.182	0	0	0	0.8889	Ref
MK075002	Unassigned	Unassigned	Unassigned	15_0	Clustered	3	VC_15_0	3	0.0481	0.9985346	0.048	0	0	0	1	Ref
MK075003	Unassigned	Unassigned	Unassigned	11_0	Clustered	19	VC_11_0	19	0.7562	1	0.7562	0	0	0	1	Ref
MK075004	Unassigned	Unassigned	Unassigned	15_1	Clustered	9	VC_15_1	9	0.182	1	0.182	0	0	0	0.8889	Ref

MK075006	Unassigned	Unassigned	Unassigned	11_0	Clustered	19	VC_11_0	19	0.7562	1	0.7562	0	0	0	1	Ref
MK080312	Unassigned	Unassigned	Unassigned	1218_0	Clustered	4	VC_1218_0	4	0.8905	0.99530591	0.8863	0	0	0	1	Ref
MK084630	Unassigned	Unassigned	Unassigned	818_0	Clustered	20	VC_818_0	20	0.9601	1	0.9601	0	0	0	1	Ref
MK085976	Unassigned	Unassigned	Unassigned	704_0	Clustered	16	VC_704_0	16	0.7274	1	0.7274	0	0	0	1	Ref
MK088078	Unassigned	Unassigned	Unassigned	185_0	Clustered	3	VC_185_0	3	0.4487	0.97148006	0.4359	0	0	0	1	Ref
MK089780	Unassigned	Unassigned	Unassigned	699_0	Clustered	71	VC_699_0	71	0.9352	1	0.9352	0	0	0	1	Ref
MK095192	Unassigned	Unassigned	Unassigned	984_0	Clustered	38	VC_984_0	38	0.9758	1	0.9758	0	0	0	1	Ref
MK095193	Unassigned	Unassigned	Unassigned	984_0	Clustered	38	VC_984_0	38	0.9758	1	0.9758	0	0	0	1	Ref
MK095194	Unassigned	Unassigned	Unassigned	984_0	Clustered	38	VC_984_0	38	0.9758	1	0.9758	0	0	0	1	Ref
MK095195	Unassigned	Unassigned	Unassigned	984_0	Clustered	38	VC_984_0	38	0.9758	1	0.9758	0	0	0	1	Ref
MK095196	Unassigned	Unassigned	Unassigned	984_0	Clustered	38	VC_984_0	38	0.9758	1	0.9758	0	0	0	1	Ref
MK095197	Unassigned	Unassigned	Unassigned	984_0	Clustered	38	VC_984_0	38	0.9758	1	0.9758	0	0	0	1	Ref
MK095198	Unassigned	Unassigned	Unassigned	984_0	Clustered	38	VC_984_0	38	0.9758	1	0.9758	0	0	0	1	Ref
MK095199	Unassigned	Unassigned	Unassigned	984_0	Clustered	38	VC_984_0	38	0.9758	1	0.9758	0	0	0	1	Ref
MK095200	Unassigned	Unassigned	Unassigned	984_0	Clustered	38	VC_984_0	38	0.9758	1	0.9758	0	0	0	1	Ref
MK095201	Unassigned	Unassigned	Unassigned	984_0	Clustered	38	VC_984_0	38	0.9758	1	0.9758	0	0	0	1	Ref
MK095202	Unassigned	Unassigned	Unassigned	984_0	Clustered	38	VC_984_0	38	0.9758	1	0.9758	0	0	0	1	Ref
MK095203	Unassigned	Unassigned	Unassigned	984_0	Clustered	38	VC_984_0	38	0.9758	1	0.9758	0	0	0	1	Ref
MK095204	Unassigned	Unassigned	Unassigned	984_0	Clustered	38	VC_984_0	38	0.9758	1	0.9758	0	0	0	1	Ref
MK095205	Unassigned	Unassigned	Unassigned	984_0	Clustered	38	VC_984_0	38	0.9758	1	0.9758	0	0	0	1	Ref
MK095206	Unassigned	Unassigned	Unassigned	984_0	Clustered	38	VC_984_0	38	0.9758	1	0.9758	0	0	0	1	Ref
MK095207	Unassigned	Unassigned	Unassigned	984_0	Clustered	38	VC_984_0	38	0.9758	1	0.9758	0	0	0	1	Ref
MK095208	Unassigned	Unassigned	Unassigned	984_0	Clustered	38	VC_984_0	38	0.9758	1	0.9758	0	0	0	1	Ref
MK095209	Unassigned	Unassigned	Unassigned	984_0	Clustered	38	VC_984_0	38	0.9758	1	0.9758	0	0	0	1	Ref
MK095210	Unassigned	Unassigned	Unassigned	984_0	Clustered	38	VC_984_0	38	0.9758	1	0.9758	0	0	0	1	Ref
MK095211	Unassigned	Unassigned	Unassigned	984_0	Clustered	38	VC_984_0	38	0.9758	1	0.9758	0	0	0	1	Ref
MK097141	Unassigned	Unassigned	Unassigned	642_2	Clustered	6	VC_642_2	6	0.1679	1	0.1679	0	0	0	1	Ref
MK105819	Unassigned	Unassigned	Unassigned	356_0	Clustered	2	VC_356_0	2	0.1992	0.93211095	0.1857	0	0	0	1	Ref
MK105855	Unassigned	Unassigned	Unassigned	705_0	Clustered	11	VC_705_0	11	0.8861	1	0.8861	0	0	0	1	Ref
MK112526	Unassigned	Unassigned	Unassigned	1160_0	Clustered	73	VC_1160_0	73	0.9835	1	0.9835	0	0	0	1	Ref
MK112527	Unassigned	Unassigned	Unassigned	371_15	Clustered	44	VC_371_15	44	0.1391	1	0.1391	0	0	0	0.7474	Ref
MK112528	Unassigned	Unassigned	Unassigned	1160_0	Clustered	73	VC_1160_0	73	0.9835	1	0.9835	0	0	0	1	Ref
MK112529	Unassigned	Unassigned	Unassigned	1160_0	Clustered	73	VC_1160_0	73	0.9835	1	0.9835	0	0	0	1	Ref
MK112530	Unassigned	Unassigned	Unassigned	325_41	Clustered	2	VC_325_41	2	0.0036	0.94071129	0.0034	0	0	0	1	Ref
MK112531	Unassigned	Unassigned	Unassigned	1160_0	Clustered	73	VC_1160_0	73	0.9835	1	0.9835	0	0	0	1	Ref
MK112532	Unassigned	Unassigned	Unassigned	325_33	Clustered	4	VC_325_33	4	0.0149	0.99998565	0.0149	0	0	0	1	Ref
MK112533	Unassigned	Unassigned	Unassigned	558_51	Clustered/Singleton	1	VC_558_51	1	0	1	0	0	0	0	1	Ref
MK112534	Unassigned	Unassigned	Unassigned	558_10	Clustered/Singleton	1	VC_558_10	1	0	1	0	0	0	0	1	Ref
MK112535	Unassigned	Unassigned	Unassigned	1160_0	Clustered	73	VC_1160_0	73	0.9835	1	0.9835	0	0	0	1	Ref
MK112536	Unassigned	Unassigned	Unassigned	371_15	Clustered	44	VC_371_15	44	0.1391	1	0.1391	0	0	0	0.7474	Ref
MK112537	Unassigned	Unassigned	Unassigned	236_0	Clustered	72	VC_236_0	72	0.8104	1	0.8104	0	0	0	1	Ref
MK112538	Unassigned	Unassigned	Unassigned	1160_0	Clustered	73	VC_1160_0	73	0.9835	1	0.9835	0	0	0	1	Ref
MK112539	Unassigned	Unassigned	Unassigned	371_11	Clustered	31	VC_371_11	31	0.0927	1	0.0927	0	0	0	0.7011	Ref
MK112540	Unassigned	Unassigned	Unassigned	325_48	Clustered	2	VC_325_48	2	0.0039	0.95008941	0.0037	0	0	0	1	Ref
MK112541	Unassigned	Unassigned	Unassigned	558_47	Clustered	3	VC_558_47	3	0.0064	0.80043943	0.0051	0	0	0	1	Ref
MK112542	Unassigned	Unassigned	Unassigned	371_16	Clustered	42	VC_371_16	42	0.1341	1	0.1341	0	0	0	0.7282	Ref
MK112543	Unassigned	Unassigned	Unassigned	514_0	Clustered	75	VC_514_0	75	0.9402	1	0.9402	0	0	0	1	Ref
MK112544	Unassigned	Unassigned	Unassigned	371_12	Clustered	4	VC_371_12	4	0.0126	0.99998489	0.0126	0	0	0	0.8333	Ref
MK112545	Unassigned	Unassigned	Unassigned	558_47	Clustered	3	VC_558_47	3	0.0064	0.80043943	0.0051	0	0	0	1	Ref
MK112546	Unassigned	Unassigned	Unassigned	371_11	Clustered	31	VC_371_11	31	0.0927	1	0.0927	0	0	0	0.7011	Ref
MK112547	Unassigned	Unassigned	Unassigned	525_0	Clustered	25	VC_525_0	25	0.4597	1	0.4597	0	0	0	1	Ref
MK112548	Unassigned	Unassigned	Unassigned	558_47	Clustered	3	VC_558_47	3	0.0064	0.80043943	0.0051	0	0	0	1	Ref
MK112549	Unassigned	Unassigned	Unassigned	558_34	Clustered/Singleton	1	VC_558_34	1	0	1	0	0	0	0	1	Ref
MK112550	Unassigned	Unassigned	Unassigned	1224_0	Clustered	19	VC_1224_0	19	1	1	1	0	0	0	1	Ref
MK112551	Unassigned	Unassigned	Unassigned	371_11	Clustered	31	VC_371_11	31	0.0927	1	0.0927	0	0	0	0.7011	Ref
MK112552	Unassigned	Unassigned	Unassigned	371_2	Clustered	8	VC_371_2	8	0.0232	1	0.0232	0	0	0	0.8571	Ref
MK112553	Unassigned	Unassigned	Unassigned	371_16	Clustered	42	VC_371_16	42	0.1341	1	0.1341	0	0	0	0.7282	Ref
MK112554	Unassigned	Unassigned	Unassigned	558_49	Clustered	7	VC_558_49	7	0.0194	0.99510369	0.0193	0	0	0	0.8571	Ref
MK112555	Unassigned	Unassigned	Unassigned	371_15	Clustered	44	VC_371_15	44	0.1391	1	0.1391	0	0	0	0.7474	Ref
MK112556	Unassigned	Unassigned	Unassigned	520_74	Clustered	2	VC_520_74	2	0.0075	0.95760586	0.0072	0	0	0	1	Ref
MK112901	Unassigned	Unassigned	Unassigned	461_0	Clustered	96	VC_461_0	96	0.9307	1	0.9307	0	0	0	0.9575	Ref
MK125140	Unassigned	Unassigned	Unassigned	695_0	Clustered	48	VC_695_0	48	0.8162	1	0.8162	0	0	0	1	Ref
MK125141	Unassigned	Unassigned	Unassigned	705_0	Clustered	11	VC_705_0	11	0.8861	1	0.8861	0	0	0	1	Ref
MK134560	Unassigned	Unassigned	Unassigned	823_0	Clustered	124	VC_823_0	124	0.9492	1	0.9492	0	0	0	1	Ref
MK135468	Unassigned	Unassigned	Unassigned	111_0	Clustered	91	VC_111_0	91	0.9587	1	0.9587	0	0	0	1	Ref
MK138526	Unassigned	Unassigned	Unassigned	76_0	Clustered	37	VC_76_0	37	0.9403	1	0.9403	0	0	0	1	Ref
MK138527	Unassigned	Unassigned	Unassigned	835_0	Clustered	67	VC_835_0	67	0.9624	1	0.9624	0	0	0	1	Ref
MK144667	Unassigned	Unassigned	Unassigned	179_0	Clustered	53	VC_179_0	53	0.9137	1	0.9137	0	0	0	1	Ref
MK165087	Unassigned	Unassigned	Unassigned	722_0	Clustered	13	VC_722_0	13	0.5104	1	0.5104	0	0	0	1	Ref
MK165657	Unassigned	Unassigned	Unassigned	749_0	Clustered	40	VC_749_0	40	0.7517	1	0.7517	0	0	0	1	Ref
MK165658	Unassigned	Unassigned	Unassigned	749_0	Clustered	40	VC_749_0	40	0.7517	1	0.7517	0	0	0	1	Ref
MK170160	Unassigned	Unassigned	Unassigned	700_0	Clustered	10	VC_700_0	10	0.8076	1	0.8076	0	0	0	1	Ref
MK170446	Unassigned	Unassigned	Unassigned	938_0	Clustered	99	VC_938_0	99	0.9119	1	0.9119	0	0	0	0.9928	Ref
MK202159	Unassigned	Unassigned	Unassigned	255_0	Clustered	13	VC_255_0	13	0.2048	1	0.2048	0	0	0	1	Ref
MK202160	Unassigned	Unassigned	Unassigned	255_0	Clustered	13	VC_255_0	13	0.2048	1	0.2048	0	0	0	1	Ref
MK202161	Unassigned	Unassigned	Unassigned	255_0	Clustered	13	VC_255_0	13	0.2048	1	0.2048	0	0	0	1	Ref
MK203841	Unassigned	Unassigned	Unassigned	823_0	Clustered	124	VC_823_0	124	0.9492	1	0.9492	0	0	0	1	Ref
MK211557	Unassigned	Unassigned	Unassigned	12_2	Clustered	19	VC_12_2	19	0.2018	1	0.2018	0	0	0	1	Ref
MK213795	Unassigned	Unassigned	Unassigned	315_0	Clustered	12	VC_315_0	12	0.8947	1	0.8947	0	0	0	1	Ref
MK213796	Unassigned	Unassigned	Unassigned	575_0	Clustered	35	VC_575_0	35	0.8815	1	0.8815	0	0	0	1	Ref
MK214385	Unassigned	Unassigned	Unassigned	540_0	Clustered	15	VC_540_0	15	0.2866	1	0.2866	0	0	0	1	Ref

MK215646	Unassigned	Unassigned	Unassigned	883_0	Clustered	10	VC_883_0	10	0.2015	1	0.2015	0	0	0	1	Ref
MK224497	Unassigned	Unassigned	Unassigned	234_0	Clustered	21	VC_234_0	21	0.8355	1	0.8355	0	0	0	1	Ref
MK224498	Unassigned	Unassigned	Unassigned	76_0	Clustered	37	VC_76_0	37	0.9403	1	0.9403	0	0	0	1	Ref
MK224592	Unassigned	Unassigned	Unassigned	520_95	Clustered/Singleton	1	VC_520_95	1	0	1	0	0	0	0	1	Ref
MK234886	Unassigned	Unassigned	Unassigned	290_5	Clustered	14	VC_290_5	14	0.1422	1	0.1422	0	0	0	0.9231	Ref
MK238400	Unassigned	Unassigned	Unassigned	673_0	Clustered	15	VC_673_0	15	0.8234	1	0.8234	0	0	0	0.7619	Ref
MK240351	Unassigned	Unassigned	Unassigned	492_0	Clustered	2	VC_492_0	2	0.0158	0.6640397	0.0105	0	0	0	1	Ref
MK240575	Unassigned	Unassigned	Unassigned	1351_0	Clustered	2	VC_1351_0	2	1	1	1	0	0	0	1	Ref
MK249126	Unassigned	Unassigned	Unassigned	637_0	Clustered	115	VC_637_0	115	0.731	1	0.731	0	0	0	0.8764	Ref
MK249127	Unassigned	Unassigned	Unassigned	1066_0	Clustered	52	VC_1066_0	52	0.7682	1	0.7682	0	0	0	1	Ref
MK249128	Unassigned	Unassigned	Unassigned	1066_0	Clustered	52	VC_1066_0	52	0.7682	1	0.7682	0	0	0	1	Ref
MK249129	Unassigned	Unassigned	Unassigned	1066_0	Clustered	52	VC_1066_0	52	0.7682	1	0.7682	0	0	0	1	Ref
MK249131	Unassigned	Unassigned	Unassigned	1000_0	Clustered	21	VC_1000_0	21	0.8437	0.99999998	0.8437	0	0	0	1	Ref
MK249132	Unassigned	Unassigned	Unassigned	1067_0	Clustered	2	VC_1067_0	2	0.012	0.85807772	0.0103	0	0	0	1	Ref
MK249133	Unassigned	Unassigned	Unassigned	1062_0	Clustered	45	VC_1062_0	45	0.9734	0.50949847	0.4959	0	0	0	1	Ref
MK249135	Unassigned	Unassigned	Unassigned	994_0	Clustered/Singleton	1	VC_994_0	1	0	1	0	0	0	0	1	Ref
MK249138	Unassigned	Unassigned	Unassigned	1062_0	Clustered	45	VC_1062_0	45	0.9734	0.50949847	0.4959	0	0	0	1	Ref
MK249139	Unassigned	Unassigned	Unassigned	1066_0	Clustered	52	VC_1066_0	52	0.7682	1	0.7682	0	0	0	1	Ref
MK249141	Unassigned	Unassigned	Unassigned	995_0	Clustered	69	VC_995_0	69	0.8547	1	0.8547	0	0	0	1	Ref
MK249143	Unassigned	Unassigned	Unassigned	398_0	Clustered	48	VC_398_0	48	0.4813	1	0.4813	0	0	0	1	Ref
MK249144	Unassigned	Unassigned	Unassigned	1000_0	Clustered	21	VC_1000_0	21	0.8437	0.99999998	0.8437	0	0	0	1	Ref
MK249147	Unassigned	Unassigned	Unassigned	995_0	Clustered	69	VC_995_0	69	0.8547	1	0.8547	0	0	0	1	Ref
MK249148	Unassigned	Unassigned	Unassigned	1264_0	Clustered	6	VC_1264_0	6	0.7062	0.92055725	0.6501	0	0	0	1	Ref
MK249149	Unassigned	Unassigned	Unassigned	1066_0	Clustered	52	VC_1066_0	52	0.7682	1	0.7682	0	0	0	1	Ref
MK249150	Unassigned	Unassigned	Unassigned	1066_0	Clustered	52	VC_1066_0	52	0.7682	1	0.7682	0	0	0	1	Ref
MK249151	Unassigned	Unassigned	Unassigned	398_0	Clustered	48	VC_398_0	48	0.4813	1	0.4813	0	0	0	1	Ref
MK249153	Unassigned	Unassigned	Unassigned	1066_0	Clustered	52	VC_1066_0	52	0.7682	1	0.7682	0	0	0	1	Ref
MK249154	Unassigned	Unassigned	Unassigned	1066_0	Clustered	52	VC_1066_0	52	0.7682	1	0.7682	0	0	0	1	Ref
MK249155	Unassigned	Unassigned	Unassigned	1066_0	Clustered	52	VC_1066_0	52	0.7682	1	0.7682	0	0	0	1	Ref
MK249156	Unassigned	Unassigned	Unassigned	1000_0	Clustered	21	VC_1000_0	21	0.8437	0.99999998	0.8437	0	0	0	1	Ref
MK249158	Unassigned	Unassigned	Unassigned	1066_0	Clustered	52	VC_1066_0	52	0.7682	1	0.7682	0	0	0	1	Ref
MK249159	Unassigned	Unassigned	Unassigned	1000_0	Clustered	21	VC_1000_0	21	0.8437	0.99999998	0.8437	0	0	0	1	Ref
MK249160	Unassigned	Unassigned	Unassigned	1000_0	Clustered	21	VC_1000_0	21	0.8437	0.99999998	0.8437	0	0	0	1	Ref
MK249162	Unassigned	Unassigned	Unassigned	1066_0	Clustered	52	VC_1066_0	52	0.7682	1	0.7682	0	0	0	1	Ref
MK249163	Unassigned	Unassigned	Unassigned	1066_0	Clustered	52	VC_1066_0	52	0.7682	1	0.7682	0	0	0	1	Ref
MK249165	Unassigned	Unassigned	Unassigned	995_0	Clustered	69	VC_995_0	69	0.8547	1	0.8547	0	0	0	1	Ref
MK249166	Unassigned	Unassigned	Unassigned	1066_0	Clustered	52	VC_1066_0	52	0.7682	1	0.7682	0	0	0	1	Ref
MK249167	Unassigned	Unassigned	Unassigned	1000_0	Clustered	21	VC_1000_0	21	0.8437	0.99999998	0.8437	0	0	0	1	Ref
MK249168	Unassigned	Unassigned	Unassigned	1066_0	Clustered	52	VC_1066_0	52	0.7682	1	0.7682	0	0	0	1	Ref
MK249174	Unassigned	Unassigned	Unassigned	1066_0	Clustered	52	VC_1066_0	52	0.7682	1	0.7682	0	0	0	1	Ref
MK249176	Unassigned	Unassigned	Unassigned	1066_0	Clustered	52	VC_1066_0	52	0.7682	1	0.7682	0	0	0	1	Ref
MK249178	Unassigned	Unassigned	Unassigned	1066_0	Clustered	52	VC_1066_0	52	0.7682	1	0.7682	0	0	0	1	Ref
MK249179	Unassigned	Unassigned	Unassigned	1000_0	Clustered	21	VC_1000_0	21	0.8437	0.99999998	0.8437	0	0	0	1	Ref
MK249180	Unassigned	Unassigned	Unassigned	1066_0	Clustered	52	VC_1066_0	52	0.7682	1	0.7682	0	0	0	1	Ref
MK249181	Unassigned	Unassigned	Unassigned	1000_0	Clustered	21	VC_1000_0	21	0.8437	0.99999998	0.8437	0	0	0	1	Ref
MK249185	Unassigned	Unassigned	Unassigned	995_0	Clustered	69	VC_995_0	69	0.8547	1	0.8547	0	0	0	1	Ref
MK249188	Unassigned	Unassigned	Unassigned	1066_0	Clustered	52	VC_1066_0	52	0.7682	1	0.7682	0	0	0	1	Ref
MK249189	Unassigned	Unassigned	Unassigned	999_0	Clustered	2	VC_999_0	2	0.0273	0.73989325	0.0202	0	0	0	1	Ref
MK249190	Unassigned	Unassigned	Unassigned	995_0	Clustered	69	VC_995_0	69	0.8547	1	0.8547	0	0	0	1	Ref
MK249191	Unassigned	Unassigned	Unassigned	1062_0	Clustered	45	VC_1062_0	45	0.9734	0.50949847	0.4959	0	0	0	1	Ref
MK249192	Unassigned	Unassigned	Unassigned	1066_0	Clustered	52	VC_1066_0	52	0.7682	1	0.7682	0	0	0	1	Ref
MK249194	Unassigned	Unassigned	Unassigned	1066_0	Clustered	52	VC_1066_0	52	0.7682	1	0.7682	0	0	0	1	Ref
MK249195	Unassigned	Unassigned	Unassigned	1066_0	Clustered	52	VC_1066_0	52	0.7682	1	0.7682	0	0	0	1	Ref
MK249196	Unassigned	Unassigned	Unassigned	1066_0	Clustered	52	VC_1066_0	52	0.7682	1	0.7682	0	0	0	1	Ref
MK249197	Unassigned	Unassigned	Unassigned	995_0	Clustered	69	VC_995_0	69	0.8547	1	0.8547	0	0	0	1	Ref
MK249198	Unassigned	Unassigned	Unassigned	1066_0	Clustered	52	VC_1066_0	52	0.7682	1	0.7682	0	0	0	1	Ref
MK249199	Unassigned	Unassigned	Unassigned	1062_0	Clustered	45	VC_1062_0	45	0.9734	0.50949847	0.4959	0	0	0	1	Ref
MK249201	Unassigned	Unassigned	Unassigned	995_0	Clustered	69	VC_995_0	69	0.8547	1	0.8547	0	0	0	1	Ref
MK249202	Unassigned	Unassigned	Unassigned	995_0	Clustered	69	VC_995_0	69	0.8547	1	0.8547	0	0	0	1	Ref
MK249203	Unassigned	Unassigned	Unassigned	995_0	Clustered	69	VC_995_0	69	0.8547	1	0.8547	0	0	0	1	Ref
MK249204	Unassigned	Unassigned	Unassigned	1066_0	Clustered	52	VC_1066_0	52	0.7682	1	0.7682	0	0	0	1	Ref
MK249205	Unassigned	Unassigned	Unassigned	1067_0	Clustered	2	VC_1067_0	2	0.012	0.85807772	0.0103	0	0	0	1	Ref
MK249207	Unassigned	Unassigned	Unassigned	1062_0	Clustered	45	VC_1062_0	45	0.9734	0.50949847	0.4959	0	0	0	1	Ref
MK249208	Unassigned	Unassigned	Unassigned	1062_0	Clustered	45	VC_1062_0	45	0.9734	0.50949847	0.4959	0	0	0	1	Ref
MK249211	Unassigned	Unassigned	Unassigned	995_0	Clustered	69	VC_995_0	69	0.8547	1	0.8547	0	0	0	1	Ref
MK249212	Unassigned	Unassigned	Unassigned	1066_0	Clustered	52	VC_1066_0	52	0.7682	1	0.7682	0	0	0	1	Ref
MK249213	Unassigned	Unassigned	Unassigned	995_0	Clustered	69	VC_995_0	69	0.8547	1	0.8547	0	0	0	1	Ref
MK249214	Unassigned	Unassigned	Unassigned	1066_0	Clustered	52	VC_1066_0	52	0.7682	1	0.7682	0	0	0	1	Ref
MK249215	Unassigned	Unassigned	Unassigned	1087_0	Clustered	5	VC_1087_0	5	0.8109	0.98759579	0.8008	0	0	0	1	Ref
MK249216	Unassigned	Unassigned	Unassigned	1364_0	Clustered	3	VC_1364_0	3	1	1	1	0	0	0	1	Ref
MK249217	Unassigned	Unassigned	Unassigned	1364_0	Clustered	3	VC_1364_0	3	1	1	1	0	0	0	1	Ref
MK249218	Unassigned	Unassigned	Unassigned	1339_0	Clustered	7	VC_1339_0	7	0.9177	0.99772305	0.9156	0	0	0	1	Ref
MK249219	Unassigned	Unassigned	Unassigned	1066_0	Clustered	52	VC_1066_0	52	0.7682	1	0.7682	0	0	0	1	Ref
MK249221	Unassigned	Unassigned	Unassigned	995_0	Clustered	69	VC_995_0	69	0.8547	1	0.8547	0	0	0	1	Ref
MK249222	Unassigned	Unassigned	Unassigned	1068_0	Clustered	5	VC_1068_0	5	1	1	1	0	0	0	1	Ref
MK249223	Unassigned	Unassigned	Unassigned	398_0	Clustered	48	VC_398_0	48	0.4813	1	0.4813	0	0	0	1	Ref
MK249228	Unassigned	Unassigned	Unassigned	1062_0	Clustered	45	VC_1062_0	45	0.9734	0.50949847	0.4959	0	0	0	1	Ref
MK249229	Unassigned	Unassigned	Unassigned	1339_0	Clustered	7	VC_1339_0	7	0.9177	0.99772305	0.9156	0	0	0	1	Ref
MK249231	Unassigned	Unassigned	Unassigned	398_0	Clustered	48	VC_398_0	48	0.4813	1	0.4813	0	0	0	1	Ref
MK249232	Unassigned	Unassigned	Unassigned	1062_0	Clustered	45	VC_1062_0	45	0.9734	0.50949847	0.4959	0	0	0	1	Ref
MK249233	Unassigned	Unassigned	Unassigned	1339_0	Clustered	7	VC_1339_0	7	0.9177	0.99772305	0.9156	0	0	0	1	Ref

MK249308	Unassigned	Unassigned	Unassigned	592_0	Clustered	50	VC_592_0	50	0.7314	1	0.7314	0	0	0	1	Ref
MK249872	Unassigned	Unassigned	Unassigned	643_0	Clustered	2	VC_643_0	2	0.1092	0.95085347	0.1038	0	0	0	1	Ref
MK250015	Unassigned	Unassigned	Unassigned	1210_2	Clustered	4	VC_1210_2	4	0.1183	0.92062211	0.109	0	0	0	1	Ref
MK250016	Unassigned	Unassigned	Unassigned	1210_2	Clustered	4	VC_1210_2	4	0.1183	0.92062211	0.109	0	0	0	1	Ref
MK250017	Unassigned	Unassigned	Unassigned	1210_2	Clustered	4	VC_1210_2	4	0.1183	0.92062211	0.109	0	0	0	1	Ref
MK250018	Unassigned	Unassigned	Unassigned	1210_2	Clustered	4	VC_1210_2	4	0.1183	0.92062211	0.109	0	0	0	1	Ref
MK250019	Unassigned	Unassigned	Unassigned	1210_3	Clustered	2	VC_1210_3	2	0.0366	0.68120968	0.0249	0	0	0	1	Ref
MK250020	Unassigned	Unassigned	Unassigned	1210_1	Clustered	7	VC_1210_1	7	0.2693	0.99874141	0.2689	0	0	0	1	Ref
MK250021	Unassigned	Unassigned	Unassigned	1210_0	Clustered	2	VC_1210_0	2	0.0367	0.68120968	0.025	0	0	0	1	Ref
MK250022	Unassigned	Unassigned	Unassigned	1210_1	Clustered	7	VC_1210_1	7	0.2693	0.99874141	0.2689	0	0	0	1	Ref
MK250023	Unassigned	Unassigned	Unassigned	1210_0	Clustered	2	VC_1210_0	2	0.0367	0.68120968	0.025	0	0	0	1	Ref
MK250024	Unassigned	Unassigned	Unassigned	1210_1	Clustered	7	VC_1210_1	7	0.2693	0.99874141	0.2689	0	0	0	1	Ref
MK250025	Unassigned	Unassigned	Unassigned	1210_1	Clustered	7	VC_1210_1	7	0.2693	0.99874141	0.2689	0	0	0	1	Ref
MK250026	Unassigned	Unassigned	Unassigned	1210_1	Clustered	7	VC_1210_1	7	0.2693	0.99874141	0.2689	0	0	0	1	Ref
MK250027	Unassigned	Unassigned	Unassigned	1210_1	Clustered	7	VC_1210_1	7	0.2693	0.99874141	0.2689	0	0	0	1	Ref
MK250028	Unassigned	Unassigned	Unassigned	1210_1	Clustered	7	VC_1210_1	7	0.2693	0.99874141	0.2689	0	0	0	1	Ref
MK250029	Unassigned	Unassigned	Unassigned	1210_3	Clustered	2	VC_1210_3	2	0.0366	0.68120968	0.0249	0	0	0	1	Ref
MK250904	Unassigned	Unassigned	Unassigned	149_15	Clustered	16	VC_149_15	16	0.0788	1	0.0788	0	0	0	0.8083	Ref
MK257719	Unassigned	Unassigned	Unassigned	699_0	Clustered	71	VC_699_0	71	0.9352	1	0.9352	0	0	0	1	Ref
MK257720	Unassigned	Unassigned	Unassigned	699_0	Clustered	71	VC_699_0	71	0.9352	1	0.9352	0	0	0	1	Ref
MK257721	Unassigned	Unassigned	Unassigned	699_0	Clustered	71	VC_699_0	71	0.9352	1	0.9352	0	0	0	1	Ref
MK257722	Unassigned	Unassigned	Unassigned	699_0	Clustered	71	VC_699_0	71	0.9352	1	0.9352	0	0	0	1	Ref
MK257723	Unassigned	Unassigned	Unassigned	699_0	Clustered	71	VC_699_0	71	0.9352	1	0.9352	0	0	0	1	Ref
MK257744	Unassigned	Unassigned	Unassigned	477_0	Clustered	4	VC_477_0	4	0.9836	0.99748973	0.9811	0	0	0	1	Ref
MK268344	Unassigned	Unassigned	Unassigned	164_0	Clustered	3	VC_164_0	3	0.0298	0.82983726	0.0247	0	0	0	1	Ref
MK268686	Unassigned	Unassigned	Unassigned	91_0	Clustered	22	VC_91_0	22	0.5318	1	0.5318	0	0	0	1	Ref
MK278859	Unassigned	Unassigned	Unassigned	699_0	Clustered	71	VC_699_0	71	0.9352	1	0.9352	0	0	0	1	Ref
MK278860	Unassigned	Unassigned	Unassigned	492_2	Clustered	8	VC_492_2	8	0.1221	0.98786574	0.1206	0	0	0	1	Ref
MK278861	Unassigned	Unassigned	Unassigned	938_0	Clustered	99	VC_938_0	99	0.9119	1	0.9119	0	0	0	0.9928	Ref
MK279840	Unassigned	Unassigned	Unassigned	574_0	Clustered	7	VC_574_0	7	0.2734	1	0.2734	0	0	0	1	Ref
MK279841	Unassigned	Unassigned	Unassigned	728_0	Clustered	3	VC_728_0	3	0.4017	0.99850259	0.4011	0	0	0	1	Ref
MK279842	Unassigned	Unassigned	Unassigned	939_0	Clustered	15	VC_939_0	15	1	0	0	0	0	0	1	Ref
MK279843	Unassigned	Unassigned	Unassigned	371_11	Clustered	31	VC_371_11	31	0.0927	1	0.0927	0	0	0	0.7011	Ref
MK279844	Unassigned	Unassigned	Unassigned	1160_0	Clustered	73	VC_1160_0	73	0.9835	1	0.9835	0	0	0	1	Ref
MK279845	Unassigned	Unassigned	Unassigned	1160_0	Clustered	73	VC_1160_0	73	0.9835	1	0.9835	0	0	0	1	Ref
MK279846	Unassigned	Unassigned	Unassigned	371_16	Clustered	42	VC_371_16	42	0.1341	1	0.1341	0	0	0	0.7282	Ref
MK279847	Unassigned	Unassigned	Unassigned	1160_0	Clustered	73	VC_1160_0	73	0.9835	1	0.9835	0	0	0	1	Ref
MK279849	Unassigned	Unassigned	Unassigned	546_7	Clustered	4	VC_546_7	4	0.041	0.9963251	0.0408	0	0	0	1	Ref
MK279850	Unassigned	Unassigned	Unassigned	371_13	Clustered	33	VC_371_13	33	0.179	1	0.179	0	0	0	0.8769	Ref
MK279851	Unassigned	Unassigned	Unassigned	1160_0	Clustered	73	VC_1160_0	73	0.9835	1	0.9835	0	0	0	1	Ref
MK279852	Unassigned	Unassigned	Unassigned	371_16	Clustered	42	VC_371_16	42	0.1341	1	0.1341	0	0	0	0.7282	Ref
MK279853	Unassigned	Unassigned	Unassigned	1096_0	Clustered	5	VC_1096_0	5	0.8566	0.99869977	0.8555	0	0	0	1	Ref
MK279854	Unassigned	Unassigned	Unassigned	1160_0	Clustered	73	VC_1160_0	73	0.9835	1	0.9835	0	0	0	1	Ref
MK279855	Unassigned	Unassigned	Unassigned	371_15	Clustered	44	VC_371_15	44	0.1391	1	0.1391	0	0	0	0.7474	Ref
MK279856	Unassigned	Unassigned	Unassigned	1160_0	Clustered	73	VC_1160_0	73	0.9835	1	0.9835	0	0	0	1	Ref
MK279857	Unassigned	Unassigned	Unassigned	217_0	Clustered	5	VC_217_0	5	0.069	0.99999998	0.069	0	0	0	1	Ref
MK279858	Unassigned	Unassigned	Unassigned	371_13	Clustered	33	VC_371_13	33	0.179	1	0.179	0	0	0	0.8769	Ref
MK279859	Unassigned	Unassigned	Unassigned	371_15	Clustered	44	VC_371_15	44	0.1391	1	0.1391	0	0	0	0.7474	Ref
MK279860	Unassigned	Unassigned	Unassigned	1160_0	Clustered	73	VC_1160_0	73	0.9835	1	0.9835	0	0	0	1	Ref
MK279861	Unassigned	Unassigned	Unassigned	371_15	Clustered	44	VC_371_15	44	0.1391	1	0.1391	0	0	0	0.7474	Ref
MK279862	Unassigned	Unassigned	Unassigned	1160_0	Clustered	73	VC_1160_0	73	0.9835	1	0.9835	0	0	0	1	Ref
MK279863	Unassigned	Unassigned	Unassigned	1160_0	Clustered	73	VC_1160_0	73	0.9835	1	0.9835	0	0	0	1	Ref
MK279864	Unassigned	Unassigned	Unassigned	371_13	Clustered	33	VC_371_13	33	0.179	1	0.179	0	0	0	0.8769	Ref
MK279865	Unassigned	Unassigned	Unassigned	371_11	Clustered	31	VC_371_11	31	0.0927	1	0.0927	0	0	0	0.7011	Ref
MK279866	Unassigned	Unassigned	Unassigned	371_15	Clustered	44	VC_371_15	44	0.1391	1	0.1391	0	0	0	0.7474	Ref
MK279867	Unassigned	Unassigned	Unassigned	1160_0	Clustered	73	VC_1160_0	73	0.9835	1	0.9835	0	0	0	1	Ref
MK279868	Unassigned	Unassigned	Unassigned	1160_0	Clustered	73	VC_1160_0	73	0.9835	1	0.9835	0	0	0	1	Ref
MK279869	Unassigned	Unassigned	Unassigned	1160_0	Clustered	73	VC_1160_0	73	0.9835	1	0.9835	0	0	0	1	Ref
MK279870	Unassigned	Unassigned	Unassigned	371_11	Clustered	31	VC_371_11	31	0.0927	1	0.0927	0	0	0	0.7011	Ref
MK279871	Unassigned	Unassigned	Unassigned	371_16	Clustered	42	VC_371_16	42	0.1341	1	0.1341	0	0	0	0.7282	Ref
MK279872	Unassigned	Unassigned	Unassigned	1160_0	Clustered	73	VC_1160_0	73	0.9835	1	0.9835	0	0	0	1	Ref
MK279873	Unassigned	Unassigned	Unassigned	371_11	Clustered	31	VC_371_11	31	0.0927	1	0.0927	0	0	0	0.7011	Ref
MK279874	Unassigned	Unassigned	Unassigned	1160_0	Clustered	73	VC_1160_0	73	0.9835	1	0.9835	0	0	0	1	Ref
MK279875	Unassigned	Unassigned	Unassigned	1160_0	Clustered	73	VC_1160_0	73	0.9835	1	0.9835	0	0	0	1	Ref
MK279876	Unassigned	Unassigned	Unassigned	1160_0	Clustered	73	VC_1160_0	73	0.9835	1	0.9835	0	0	0	1	Ref
MK279877	Unassigned	Unassigned	Unassigned	371_3	Clustered	22	VC_371_3	22	0.0787	1	0.0787	0	0	0	0.9048	Ref
MK279878	Unassigned	Unassigned	Unassigned	371_11	Clustered	31	VC_371_11	31	0.0927	1	0.0927	0	0	0	0.7011	Ref
MK279879	Unassigned	Unassigned	Unassigned	371_15	Clustered	44	VC_371_15	44	0.1391	1	0.1391	0	0	0	0.7474	Ref
MK279880	Unassigned	Unassigned	Unassigned	1160_0	Clustered	73	VC_1160_0	73	0.9835	1	0.9835	0	0	0	1	Ref
MK279881	Unassigned	Unassigned	Unassigned	371_15	Clustered	44	VC_371_15	44	0.1391	1	0.1391	0	0	0	0.7474	Ref
MK279882	Unassigned	Unassigned	Unassigned	371_15	Clustered	44	VC_371_15	44	0.1391	1	0.1391	0	0	0	0.7474	Ref
MK279883	Unassigned	Unassigned	Unassigned	371_13	Clustered	33	VC_371_13	33	0.179	1	0.179	0	0	0	0.8769	Ref
MK279884	Unassigned	Unassigned	Unassigned	1160_0	Clustered	73	VC_1160_0	73	0.9835	1	0.9835	0	0	0	1	Ref
MK279885	Unassigned	Unassigned	Unassigned	371_15	Clustered	44	VC_371_15	44	0.1391	1	0.1391	0	0	0	0.7474	Ref
MK279886	Unassigned	Unassigned	Unassigned	1160_0	Clustered	73	VC_1160_0	73	0.9835	1	0.9835	0	0	0	1	Ref
MK279887	Unassigned	Unassigned	Unassigned	371_15	Clustered	44	VC_371_15	44	0.1391	1	0.1391	0	0	0	0.7474	Ref
MK279888	Unassigned	Unassigned	Unassigned	371_15	Clustered	44	VC_371_15	44	0.1391	1	0.1391	0	0	0	0.7474	Ref
MK279889	Unassigned	Unassigned	Unassigned	371_2	Clustered	8	VC_371_2	8	0.0232	1	0.0232	0	0	0	0.8571	Ref
MK279890	Unassigned	Unassigned	Unassigned	371_13	Clustered	33	VC_371_13	33	0.179	1	0.179	0	0	0	0.8769	Ref
MK279891	Unassigned	Unassigned	Unassigned	371_13	Clustered	33	VC_371_13	33	0.179	1	0.179	0	0	0	0.8769	Ref
MK279892	Unassigned	Unassigned	Unassigned	1160_0	Clustered	73	VC_1160_0	73	0.9835	1	0.9835	0	0	0	1	Ref

MK279893	Unassigned	Unassigned	Unassigned	954_0	Clustered	13	VC_954_0	13	0.863	1	0.863	0	0	0	1	Ref
MK279894	Unassigned	Unassigned	Unassigned	371_11	Clustered	31	VC_371_11	31	0.0927	1	0.0927	0	0	0	0.7011	Ref
MK279895	Unassigned	Unassigned	Unassigned	371_11	Clustered	31	VC_371_11	31	0.0927	1	0.0927	0	0	0	0.7011	Ref
MK279896	Unassigned	Unassigned	Unassigned	1160_0	Clustered	73	VC_1160_0	73	0.9835	1	0.9835	0	0	0	1	Ref
MK279897	Unassigned	Unassigned	Unassigned	371_11	Clustered	31	VC_371_11	31	0.0927	1	0.0927	0	0	0	0.7011	Ref
MK279898	Unassigned	Unassigned	Unassigned	517_0	Clustered	18	VC_517_0	18	0.1599	1	0.1599	0	0	0	1	Ref
MK279900	Unassigned	Unassigned	Unassigned	919_0	Clustered	39	VC_919_0	39	0.9069	1	0.9069	0	0	0	1	Ref
MK279901	Unassigned	Unassigned	Unassigned	1160_0	Clustered	73	VC_1160_0	73	0.9835	1	0.9835	0	0	0	1	Ref
MK279902	Unassigned	Unassigned	Unassigned	517_1	Clustered	108	VC_517_1	108	0.8483	1	0.8483	0	0	0	0.9955	Ref
MK279903	Unassigned	Unassigned	Unassigned	514_0	Clustered	75	VC_514_0	75	0.9402	1	0.9402	0	0	0	1	Ref
MK279904	Unassigned	Unassigned	Unassigned	371_4	Clustered	5	VC_371_4	5	0.0135	0.99999971	0.0135	0	0	0	1	Ref
MK279905	Unassigned	Unassigned	Unassigned	887_0	Clustered	18	VC_887_0	18	0.3663	1	0.3663	0	0	0	1	Ref
MK279906	Unassigned	Unassigned	Unassigned	557_1	Clustered	11	VC_557_1	11	0.4945	1	0.4945	0	0	0	0.7818	Ref
MK279907	Unassigned	Unassigned	Unassigned	1160_0	Clustered	73	VC_1160_0	73	0.9835	1	0.9835	0	0	0	1	Ref
MK279908	Unassigned	Unassigned	Unassigned	371_3	Clustered	22	VC_371_3	22	0.0787	1	0.0787	0	0	0	0.9048	Ref
MK279909	Unassigned	Unassigned	Unassigned	566_0	Clustered	2	VC_566_0	2	0.0989	0.95622526	0.0945	0	0	0	1	Ref
MK279910	Unassigned	Unassigned	Unassigned	371_1	Clustered	11	VC_371_1	11	0.0379	1	0.0379	0	0	0	0.8545	Ref
MK279911	Unassigned	Unassigned	Unassigned	1175_0	Clustered	22	VC_1175_0	22	0.4562	1	0.4562	0	0	0	1	Ref
MK279912	Unassigned	Unassigned	Unassigned	1175_0	Clustered	22	VC_1175_0	22	0.4562	1	0.4562	0	0	0	1	Ref
MK279913	Unassigned	Unassigned	Unassigned	514_0	Clustered	75	VC_514_0	75	0.9402	1	0.9402	0	0	0	1	Ref
MK284518	Unassigned	Unassigned	Unassigned	514_0	Clustered	75	VC_514_0	75	0.9402	1	0.9402	0	0	0	1	Ref
MK284519	Unassigned	Unassigned	Unassigned	513_0	Clustered	32	VC_513_0	32	0.6881	1	0.6881	0	0	0	1	Ref
MK284520	Unassigned	Unassigned	Unassigned	685_0	Clustered	16	VC_685_0	16	0.2972	1	0.2972	0	0	0	0.9167	Ref
MK284521	Unassigned	Unassigned	Unassigned	1177_0	Clustered	14	VC_1177_0	14	0.7847	1	0.7847	0	0	0	0.989	Ref
MK284522	Unassigned	Unassigned	Unassigned	216_4	Clustered	13	VC_216_4	13	0.1657	1	0.1657	0	0	0	1	Ref
MK284526	Unassigned	Unassigned	Unassigned	818_0	Clustered	20	VC_818_0	20	0.9601	1	0.9601	0	0	0	1	Ref
MK284527	Unassigned	Unassigned	Unassigned	818_0	Clustered	20	VC_818_0	20	0.9601	1	0.9601	0	0	0	1	Ref
MK284528	Unassigned	Unassigned	Unassigned	818_0	Clustered	20	VC_818_0	20	0.9601	1	0.9601	0	0	0	1	Ref
MK286578	Unassigned	Unassigned	Unassigned	592_0	Clustered	50	VC_592_0	50	0.7314	1	0.7314	0	0	0	1	Ref
MK288021	Unassigned	Unassigned	Unassigned	475_8	Clustered	2	VC_475_8	2	0.0291	0.90184699	0.0262	0	0	0	1	Ref
MK288022	Unassigned	Unassigned	Unassigned	1006_0	Clustered	8	VC_1006_0	8	0.7407	1	0.7407	0	0	0	1	Ref
MK290737	Unassigned	Unassigned	Unassigned	331_0	Clustered	4	VC_331_0	4	0.0884	0.99998775	0.0884	0	0	0	1	Ref
MK290738	Unassigned	Unassigned	Unassigned	331_0	Clustered	4	VC_331_0	4	0.0884	0.99998775	0.0884	0	0	0	1	Ref
MK290764	Unassigned	Unassigned	Unassigned	12_0	Clustered	21	VC_12_0	21	0.2274	1	0.2274	0	0	0	0.9571	Ref
MK291441	Unassigned	Unassigned	Unassigned	922_0	Clustered	2	VC_922_0	2	0.42	0.90478487	0.38	0	0	0	1	Ref
MK291443	Unassigned	Unassigned	Unassigned	922_0	Clustered	2	VC_922_0	2	0.42	0.90478487	0.38	0	0	0	1	Ref
MK295203	Unassigned	Unassigned	Unassigned	290_7	Clustered/Singleton	1	VC_290_7	1	0	1	0	0	0	0	1	Ref
MK295204	Unassigned	Unassigned	Unassigned	288_2	Clustered	2	VC_288_2	2	0.0228	0.95538559	0.0218	0	0	0	1	Ref
MK301437	Unassigned	Unassigned	Unassigned	202_0	Clustered	102	VC_202_0	102	0.4049	1	0.4049	0	0	0	0.7876	Ref
MK301442	Unassigned	Unassigned	Unassigned	690_0	Clustered	50	VC_690_0	50	0.9805	1	0.9805	0	0	0	1	Ref
MK301443	Unassigned	Unassigned	Unassigned	690_0	Clustered	50	VC_690_0	50	0.9805	1	0.9805	0	0	0	1	Ref
MK301532	Unassigned	Unassigned	Unassigned	451_0	Clustered	80	VC_451_0	80	0.9991	1	0.9991	0	0	0	1	Ref
MK305884	Unassigned	Unassigned	Unassigned	761_0	Clustered	67	VC_761_0	67	0.8947	1	0.8947	0	0	0	1	Ref
MK305885	Unassigned	Unassigned	Unassigned	954_0	Clustered	13	VC_954_0	13	0.863	1	0.863	0	0	0	1	Ref
MK305887	Unassigned	Unassigned	Unassigned	940_0	Clustered	34	VC_940_0	34	0.7959	1	0.7959	0	0	0	1	Ref
MK305888	Unassigned	Unassigned	Unassigned	727_0	Clustered	11	VC_727_0	11	0.5054	1	0.5054	0	0	0	1	Ref
MK305889	Unassigned	Unassigned	Unassigned	1178_0	Clustered	24	VC_1178_0	24	0.9524	1	0.9524	0	0	0	1	Ref
MK305890	Unassigned	Unassigned	Unassigned	1369_0	Clustered	3	VC_1369_0	3	0.9809	0.9595722	0.9412	0	0	0	1	Ref
MK305891	Unassigned	Unassigned	Unassigned	859_0	Clustered	9	VC_859_0	9	0.9426	1	0.9426	0	0	0	1	Ref
MK305892	Unassigned	Unassigned	Unassigned	939_0	Clustered	15	VC_939_0	15	1	0	0	0	0	0	1	Ref
MK305893	Unassigned	Unassigned	Unassigned	325_55	Clustered	2	VC_325_55	2	0.0061	0.95750995	0.0058	0	0	0	1	Ref
MK308637	Unassigned	Unassigned	Unassigned	1163_0	Clustered	28	VC_1163_0	28	0.4923	1	0.4923	0	0	0	1	Ref
MK308638	Unassigned	Unassigned	Unassigned	1360_0	Clustered	31	VC_1360_0	31	0.8837	1	0.8837	0	0	0	1	Ref
MK308674	Unassigned	Unassigned	Unassigned	346_0	Clustered	4	VC_346_0	4	0.4606	0.99997905	0.4606	0	0	0	1	Ref
MK308675	Unassigned	Unassigned	Unassigned	346_0	Clustered	4	VC_346_0	4	0.4606	0.99997905	0.4606	0	0	0	1	Ref
MK308676	Unassigned	Unassigned	Unassigned	346_0	Clustered	4	VC_346_0	4	0.4606	0.99997905	0.4606	0	0	0	1	Ref
MK308677	Unassigned	Unassigned	Unassigned	346_0	Clustered	4	VC_346_0	4	0.4606	0.99997905	0.4606	0	0	0	1	Ref
MK310138	Unassigned	Unassigned	Unassigned	237_0	Clustered	4	VC_237_0	4	0.0429	0.99998759	0.0429	0	0	0	1	Ref
MK310139	Unassigned	Unassigned	Unassigned	371_5	Clustered	2	VC_371_5	2	0.0035	0.95771071	0.0033	0	0	0	1	Ref
MK310140	Unassigned	Unassigned	Unassigned	514_0	Clustered	75	VC_514_0	75	0.9402	1	0.9402	0	0	0	1	Ref
MK310141	Unassigned	Unassigned	Unassigned	325_16	Clustered	2	VC_325_16	2	0.0095	0.95751393	0.0091	0	0	0	1	Ref
MK310142	Unassigned	Unassigned	Unassigned	325_61	Clustered	8	VC_325_61	8	0.0395	1	0.0395	0	0	0	0.7857	Ref
MK310143	Unassigned	Unassigned	Unassigned	558_32	Clustered/Singleton	1	VC_558_32	1	0	1	0	0	0	0	1	Ref
MK310144	Unassigned	Unassigned	Unassigned	887_0	Clustered	18	VC_887_0	18	0.3663	1	0.3663	0	0	0	1	Ref
MK310145	Unassigned	Unassigned	Unassigned	517_1	Clustered	108	VC_517_1	108	0.8483	1	0.8483	0	0	0	0.9955	Ref
MK310146	Unassigned	Unassigned	Unassigned	761_0	Clustered	67	VC_761_0	67	0.8947	1	0.8947	0	0	0	1	Ref
MK310182	Unassigned	Unassigned	Unassigned	392_0	Clustered	126	VC_392_0	126	0.9621	1	0.9621	0	0	0	1	Ref
MK310183	Unassigned	Unassigned	Unassigned	813_0	Clustered	12	VC_813_0	12	0.2347	1	0.2347	0	0	0	1	Ref
MK310184	Unassigned	Unassigned	Unassigned	290_6	Clustered	12	VC_290_6	12	0.1149	1	0.1149	0	0	0	0.8182	Ref
MK317959	Unassigned	Unassigned	Unassigned	80_0	Clustered	41	VC_80_0	41	0.5837	1	0.5837	0	0	0	0.9805	Ref
MK318076	Unassigned	Unassigned	Unassigned	79_7	Clustered	33	VC_79_7	33	0.216	1	0.216	0	0	0	0.8409	Ref
MK318083	Unassigned	Unassigned	Unassigned	195_1	Clustered/Singleton	1	VC_195_1	1	0	1	0	0	0	0	1	Ref
MK327140	Unassigned	Unassigned	Unassigned	460_0	Clustered	19	VC_460_0	19	0.3744	1	0.3744	0	0	0	1	Ref
MK327928	Unassigned	Unassigned	Unassigned	283_18	Clustered/Singleton	1	VC_283_18	1	0	1	0	0	0	0	1	Ref
MK327929	Unassigned	Unassigned	Unassigned	283_127	Clustered/Singleton	1	VC_283_127	1	0	1	0	0	0	0	1	Ref
MK327930	Unassigned	Unassigned	Unassigned	772_19	Clustered	16	VC_772_19	16	0.0975	0.9970466	0.0972	0	0	0	0.8083	Ref
MK327931	Unassigned	Unassigned	Unassigned	164_11	Clustered	10	VC_164_11	10	0.1507	1	0.1507	0	0	0	0.9111	Ref
MK327932	Unassigned	Unassigned	Unassigned	590_0	Clustered	27	VC_590_0	27	0.9947	1	0.9947	0	0	0	1	Ref
MK327933	Unassigned	Unassigned	Unassigned	286_4	Clustered/Singleton	1	VC_286_4	1	0	1	0	0	0	0	1	Ref
MK327934	Unassigned	Unassigned	Unassigned	286_1	Clustered	21	VC_286_1	21	0.1696	1	0.1696	0	0	0	0.9048	Ref
MK327935	Unassigned	Unassigned	Unassigned	590_0	Clustered	27	VC_590_0	27	0.9947	1	0.9947	0	0	0		

MK327936	Unassigned	Unassigned	Unassigned	283_133	Clustered/Singleton	1	VC_283_133	1	0	1	0	0	0	0	1	Ref
MK327937	Unassigned	Unassigned	Unassigned	283_16	Clustered/Singleton	1	VC_283_16	1	0	1	0	0	0	0	1	Ref
MK327939	Unassigned	Unassigned	Unassigned	283_119	Clustered/Singleton	1	VC_283_119	1	0	1	0	0	0	0	1	Ref
MK327940	Unassigned	Unassigned	Unassigned	283_121	Clustered	2	VC_283_121	2	0.0026	0.90700595	0.0023	0	0	0	1	Ref
MK327941	Unassigned	Unassigned	Unassigned	590_0	Clustered	27	VC_590_0	27	0.9947	1	0.9947	0	0	0	1	Ref
MK327942	Unassigned	Unassigned	Unassigned	283_156	Clustered/Singleton	1	VC_283_156	1	0	1	0	0	0	0	1	Ref
MK327943	Unassigned	Unassigned	Unassigned	286_1	Clustered	21	VC_286_1	21	0.1696	1	0.1696	0	0	0	0.9048	Ref
MK327944	Unassigned	Unassigned	Unassigned	283_134	Clustered/Singleton	1	VC_283_134	1	0	1	0	0	0	0	1	Ref
MK327945	Unassigned	Unassigned	Unassigned	283_121	Clustered	2	VC_283_121	2	0.0026	0.90700595	0.0023	0	0	0	1	Ref
MK327946	Unassigned	Unassigned	Unassigned	283_17	Clustered/Singleton	1	VC_283_17	1	0	1	0	0	0	0	1	Ref
MK327947	Unassigned	Unassigned	Unassigned	590_0	Clustered	27	VC_590_0	27	0.9947	1	0.9947	0	0	0	1	Ref
MK330684	Unassigned	Unassigned	Unassigned	878_0	Clustered	32	VC_878_0	32	0.9965	1	0.9965	0	0	0	1	Ref
MK331930	Unassigned	Unassigned	Unassigned	149_8	Clustered	66	VC_149_8	66	0.4565	1	0.4565	0	0	0	0.8182	Ref
MK331931	Unassigned	Unassigned	Unassigned	149_8	Clustered	66	VC_149_8	66	0.4565	1	0.4565	0	0	0	0.8182	Ref
MK335533	Unassigned	Unassigned	Unassigned	575_0	Clustered	35	VC_575_0	35	0.8815	1	0.8815	0	0	0	1	Ref
MK340760	Unassigned	Unassigned	Unassigned	79_9	Clustered	2	VC_79_9	2	0.0061	0.94860001	0.0058	0	0	0	1	Ref
MK340761	Unassigned	Unassigned	Unassigned	76_0	Clustered	37	VC_76_0	37	0.9403	1	0.9403	0	0	0	1	Ref
MK340941	Unassigned	Unassigned	Unassigned	242_0	Clustered	2	VC_242_0	2	0.0867	0.95379254	0.0827	0	0	0	1	Ref
MK348510	Unassigned	Unassigned	Unassigned	8_0	Clustered	58	VC_8_0	58	0.8306	1	0.8306	0	0	0	0.9946	Ref
MK359299	Unassigned	Unassigned	Unassigned	325_23	Clustered	17	VC_325_23	17	0.0716	1	0.0716	0	0	0	0.6397	Ref
MK359300	Unassigned	Unassigned	Unassigned	325_53	Clustered	7	VC_325_53	7	0.0242	1	0.0242	0	0	0	0.9048	Ref
MK359301	Unassigned	Unassigned	Unassigned	517_1	Clustered	108	VC_517_1	108	0.8483	1	0.8483	0	0	0	0.9955	Ref
MK359302	Unassigned	Unassigned	Unassigned	868_0	Clustered	25	VC_868_0	25	0.5791	1	0.5791	0	0	0	1	Ref
MK359303	Unassigned	Unassigned	Unassigned	558_14	Clustered	8	VC_558_14	8	0.0228	0.99945246	0.0228	0	0	0	0.8214	Ref
MK359304	Unassigned	Unassigned	Unassigned	325_53	Clustered	7	VC_325_53	7	0.0242	1	0.0242	0	0	0	0.9048	Ref
MK359305	Unassigned	Unassigned	Unassigned	558_62	Clustered/Singleton	1	VC_558_62	1	0	1	0	0	0	0	1	Ref
MK359306	Unassigned	Unassigned	Unassigned	558_53	Clustered/Singleton	1	VC_558_53	1	0	1	0	0	0	0	1	Ref
MK359307	Unassigned	Unassigned	Unassigned	520_92	Clustered	5	VC_520_92	5	0.0663	0.99999997	0.0663	0	0	0	1	Ref
MK359308	Unassigned	Unassigned	Unassigned	558_77	Clustered	3	VC_558_77	3	0.0065	0.90877939	0.0059	0	0	0	0.6667	Ref
MK359309	Unassigned	Unassigned	Unassigned	551_7	Clustered	28	VC_551_7	28	0.1661	1	0.1661	0	0	0	0.8519	Ref
MK359310	Unassigned	Unassigned	Unassigned	517_0	Clustered	18	VC_517_0	18	0.1599	1	0.1599	0	0	0	1	Ref
MK359311	Unassigned	Unassigned	Unassigned	558_14	Clustered	8	VC_558_14	8	0.0228	0.99945246	0.0228	0	0	0	0.8214	Ref
MK359312	Unassigned	Unassigned	Unassigned	325_35	Clustered	17	VC_325_35	17	0.0732	1	0.0732	0	0	0	0.7721	Ref
MK359313	Unassigned	Unassigned	Unassigned	1175_0	Clustered	22	VC_1175_0	22	0.4562	1	0.4562	0	0	0	1	Ref
MK359314	Unassigned	Unassigned	Unassigned	558_50	Clustered	15	VC_558_50	15	0.0468	1	0.0468	0	0	0	0.9238	Ref
MK359315	Unassigned	Unassigned	Unassigned	520_80	Clustered	3	VC_520_80	3	0.0295	0.99859904	0.0295	0	0	0	1	Ref
MK359316	Unassigned	Unassigned	Unassigned	325_33	Clustered	4	VC_325_33	4	0.0149	0.99998565	0.0149	0	0	0	1	Ref
MK359317	Unassigned	Unassigned	Unassigned	558_59	Clustered/Singleton	1	VC_558_59	1	0	1	0	0	0	0	1	Ref
MK359318	Unassigned	Unassigned	Unassigned	558_61	Clustered	2	VC_558_61	2	0.0032	0.66165479	0.0021	0	0	0	1	Ref
MK359319	Unassigned	Unassigned	Unassigned	954_0	Clustered	13	VC_954_0	13	0.863	1	0.863	0	0	0	1	Ref
MK359320	Unassigned	Unassigned	Unassigned	551_0	Clustered	16	VC_551_0	16	0.0915	1	0.0915	0	0	0	0.6667	Ref
MK359321	Unassigned	Unassigned	Unassigned	558_13	Clustered/Singleton	1	VC_558_13	1	0	1	0	0	0	0	1	Ref
MK359322	Unassigned	Unassigned	Unassigned	517_0	Clustered	18	VC_517_0	18	0.1599	1	0.1599	0	0	0	1	Ref
MK359323	Unassigned	Unassigned	Unassigned	517_1	Clustered	108	VC_517_1	108	0.8483	1	0.8483	0	0	0	0.9955	Ref
MK359324	Unassigned	Unassigned	Unassigned	558_9	Clustered	3	VC_558_9	3	0.0064	0.82911529	0.0053	0	0	0	0.6667	Ref
MK359325	Unassigned	Unassigned	Unassigned	517_1	Clustered	108	VC_517_1	108	0.8483	1	0.8483	0	0	0	0.9955	Ref
MK359326	Unassigned	Unassigned	Unassigned	558_20	Clustered	17	VC_558_20	17	0.054	1	0.054	0	0	0	0.9926	Ref
MK359327	Unassigned	Unassigned	Unassigned	558_45	Clustered/Singleton	1	VC_558_45	1	0	1	0	0	0	0	1	Ref
MK359328	Unassigned	Unassigned	Unassigned	558_17	Clustered/Singleton	1	VC_558_17	1	0	1	0	0	0	0	1	Ref
MK359329	Unassigned	Unassigned	Unassigned	558_69	Clustered/Singleton	1	VC_558_69	1	0	1	0	0	0	0	1	Ref
MK359330	Unassigned	Unassigned	Unassigned	558_66	Clustered/Singleton	1	VC_558_66	1	0	1	0	0	0	0	1	Ref
MK359331	Unassigned	Unassigned	Unassigned	325_10	Clustered	3	VC_325_10	3	0.012	0.99859169	0.012	0	0	0	1	Ref
MK359332	Unassigned	Unassigned	Unassigned	1071_0	Clustered	20	VC_1071_0	20	0.3268	1	0.3268	0	0	0	0.9842	Ref
MK359333	Unassigned	Unassigned	Unassigned	558_28	Clustered/Singleton	1	VC_558_28	1	0	1	0	0	0	0	1	Ref
MK359334	Unassigned	Unassigned	Unassigned	558_49	Clustered	7	VC_558_49	7	0.0194	0.99510369	0.0193	0	0	0	0.8571	Ref
MK359335	Unassigned	Unassigned	Unassigned	558_20	Clustered	17	VC_558_20	17	0.054	1	0.054	0	0	0	0.9926	Ref
MK359336	Unassigned	Unassigned	Unassigned	558_36	Clustered	4	VC_558_36	4	0.0096	0.85913852	0.0082	0	0	0	1	Ref
MK359337	Unassigned	Unassigned	Unassigned	558_43	Clustered/Singleton	1	VC_558_43	1	0	1	0	0	0	0	1	Ref
MK359338	Unassigned	Unassigned	Unassigned	558_20	Clustered	17	VC_558_20	17	0.054	1	0.054	0	0	0	0.9926	Ref
MK359339	Unassigned	Unassigned	Unassigned	558_20	Clustered	17	VC_558_20	17	0.054	1	0.054	0	0	0	0.9926	Ref
MK359340	Unassigned	Unassigned	Unassigned	517_1	Clustered	108	VC_517_1	108	0.8483	1	0.8483	0	0	0	0.9955	Ref
MK359341	Unassigned	Unassigned	Unassigned	514_0	Clustered	75	VC_514_0	75	0.9402	1	0.9402	0	0	0	1	Ref
MK359342	Unassigned	Unassigned	Unassigned	558_15	Clustered/Singleton	1	VC_558_15	1	0	1	0	0	0	0	1	Ref
MK359343	Unassigned	Unassigned	Unassigned	520_57	Clustered/Singleton	1	VC_520_57	1	0	1	0	0	0	0	1	Ref
MK359344	Unassigned	Unassigned	Unassigned	558_30	Clustered	3	VC_558_30	3	0.0064	0.76574053	0.0049	0	0	0	1	Ref
MK359345	Unassigned	Unassigned	Unassigned	558_44	Clustered/Singleton	1	VC_558_44	1	0	1	0	0	0	0	1	Ref
MK359346	Unassigned	Unassigned	Unassigned	558_71	Clustered/Singleton	1	VC_558_71	1	0	1	0	0	0	0	1	Ref
MK359347	Unassigned	Unassigned	Unassigned	558_20	Clustered	17	VC_558_20	17	0.054	1	0.054	0	0	0	0.9926	Ref
MK359348	Unassigned	Unassigned	Unassigned	558_12	Clustered	3	VC_558_12	3	0.0064	0.87854253	0.0057	0	0	0	1	Ref
MK359349	Unassigned	Unassigned	Unassigned	558_46	Clustered/Singleton	1	VC_558_46	1	0	1	0	0	0	0	1	Ref
MK359350	Unassigned	Unassigned	Unassigned	558_64	Clustered/Singleton	1	VC_558_64	1	0	1	0	0	0	0	1	Ref
MK359351	Unassigned	Unassigned	Unassigned	1071_0	Clustered	20	VC_1071_0	20	0.3268	1	0.3268	0	0	0	0.9842	Ref
MK359352	Unassigned	Unassigned	Unassigned	223_0	Clustered	38	VC_223_0	38	0.9095	1	0.9095	0	0	0	1	Ref
MK359353	Unassigned	Unassigned	Unassigned	558_4	Clustered/Singleton	1	VC_558_4	1	0	1	0	0	0	0	1	Ref
MK359354	Unassigned	Unassigned	Unassigned	325_5	Clustered	11	VC_325_5	11	0.047	1	0.047	0	0	0	0.7636	Ref
MK359355	Unassigned	Unassigned	Unassigned	558_3	Clustered/Singleton	1	VC_558_3	1	0	1	0	0	0	0	1	Ref
MK359356	Unassigned	Unassigned	Unassigned	517_1	Clustered	108	VC_517_1	108	0.8483	1	0.8483	0	0	0	0.9955	Ref
MK359357	Unassigned	Unassigned	Unassigned	509_0	Clustered	37	VC_509_0	37	0.9689	1	0.9689	0	0	0	1	Ref
MK359358	Unassigned	Unassigned	Unassigned	558_14	Clustered	8	VC_558_14	8	0.0228	0.99945246	0.0228	0	0	0	0.8214	Ref
MK359359	Unassigned	Unassigned	Unassigned	558_73	Clustered	2	VC_558_73	2	0.0033	0.84374839	0.0028	0	0	0	1	Ref
MK359360	Unassigned	Unassigned	Unassigned	223_0	Clustered	38	VC_223_0	38	0.9095	1	0.9095	0	0	0	1	Ref

MK359361	Unassigned	Unassigned	Unassigned	558_27	Clustered	2	VC_558_27	2	0.0032	0.64135461	0.002	0	0	0	1	Ref
MK359362	Unassigned	Unassigned	Unassigned	558_20	Clustered	17	VC_558_20	17	0.054	1	0.054	0	0	0	0.9926	Ref
MK359363	Unassigned	Unassigned	Unassigned	558_36	Clustered	4	VC_558_36	4	0.0096	0.85913852	0.0082	0	0	0	1	Ref
MK359364	Unassigned	Unassigned	Unassigned	558_39	Clustered	3	VC_558_39	3	0.0063	0.73849574	0.0047	0	0	0	0.6667	Ref
MK359365	Unassigned	Unassigned	Unassigned	558_50	Clustered	15	VC_558_50	15	0.0468	1	0.0468	0	0	0	0.9238	Ref
MK359366	Unassigned	Unassigned	Unassigned	558_36	Clustered	4	VC_558_36	4	0.0096	0.85913852	0.0082	0	0	0	1	Ref
MK359990	Unassigned	Unassigned	Unassigned	632_0	Clustered	37	VC_632_0	37	0.581	1	0.581	0	0	0	0.9234	Ref
MK360024	Unassigned	Unassigned	Unassigned	695_0	Clustered	48	VC_695_0	48	0.8162	1	0.8162	0	0	0	1	Ref
MK360025	Unassigned	Unassigned	Unassigned	968_0	Clustered	4	VC_968_0	4	0.2959	0.99997642	0.2959	0	0	0	1	Ref
MK370036	Unassigned	Unassigned	Unassigned	191_1	Clustered/Singleton	1	VC_191_1	1	0	1	0	0	0	0	1	Ref
MK372342	Unassigned	Unassigned	Unassigned	456_0	Clustered	62	VC_456_0	62	0.9026	1	0.9026	0	0	0	1	Ref
MK373770	Unassigned	Unassigned	Unassigned	813_1	Clustered	24	VC_813_1	24	0.535	1	0.535	0	0	0	1	Ref
MK373771	Unassigned	Unassigned	Unassigned	452_0	Clustered	66	VC_452_0	66	0.9248	1	0.9248	0	0	0	1	Ref
MK373772	Unassigned	Unassigned	Unassigned	1228_0	Clustered	2	VC_1228_0	2	0.9902	0.72985431	0.7227	0	0	0	1	Ref
MK373773	Unassigned	Unassigned	Unassigned	452_0	Clustered	66	VC_452_0	66	0.9248	1	0.9248	0	0	0	1	Ref
MK373774	Unassigned	Unassigned	Unassigned	286_3	Clustered	2	VC_286_3	2	0.0073	0.74885804	0.0055	0	0	0	1	Ref
MK373775	Unassigned	Unassigned	Unassigned	286_3	Clustered	2	VC_286_3	2	0.0073	0.74885804	0.0055	0	0	0	1	Ref
MK373776	Unassigned	Unassigned	Unassigned	618_0	Clustered	13	VC_618_0	13	0.6252	1	0.6252	0	0	0	1	Ref
MK373777	Unassigned	Unassigned	Unassigned	618_0	Clustered	13	VC_618_0	13	0.6252	1	0.6252	0	0	0	1	Ref
MK373778	Unassigned	Unassigned	Unassigned	286_1	Clustered	21	VC_286_1	21	0.1696	1	0.1696	0	0	0	0.9048	Ref
MK373779	Unassigned	Unassigned	Unassigned	283_124	Clustered/Singleton	1	VC_283_124	1	0	1	0	0	0	0	1	Ref
MK373780	Unassigned	Unassigned	Unassigned	772_19	Clustered	16	VC_772_19	16	0.0975	0.9970466	0.0972	0	0	0	0.8083	Ref
MK373781	Unassigned	Unassigned	Unassigned	283_53	Clustered/Singleton	1	VC_283_53	1	0	1	0	0	0	0	1	Ref
MK373782	Unassigned	Unassigned	Unassigned	286_1	Clustered	21	VC_286_1	21	0.1696	1	0.1696	0	0	0	0.9048	Ref
MK373783	Unassigned	Unassigned	Unassigned	132_1	Clustered	17	VC_132_1	17	0.1738	1	0.1738	0	0	0	0.9853	Ref
MK373784	Unassigned	Unassigned	Unassigned	286_1	Clustered	21	VC_286_1	21	0.1696	1	0.1696	0	0	0	0.9048	Ref
MK373785	Unassigned	Unassigned	Unassigned	283_12	Clustered/Singleton	1	VC_283_12	1	0	1	0	0	0	0	1	Ref
MK373786	Unassigned	Unassigned	Unassigned	283_43	Clustered/Singleton	1	VC_283_43	1	0	1	0	0	0	0	1	Ref
MK373787	Unassigned	Unassigned	Unassigned	283_76	Clustered/Singleton	1	VC_283_76	1	0	1	0	0	0	0	1	Ref
MK373788	Unassigned	Unassigned	Unassigned	847_1	Clustered	26	VC_847_1	26	0.6323	1	0.6323	0	0	0	0.9815	Ref
MK373789	Unassigned	Unassigned	Unassigned	835_0	Clustered	67	VC_835_0	67	0.9624	1	0.9624	0	0	0	1	Ref
MK373790	Unassigned	Unassigned	Unassigned	835_0	Clustered	67	VC_835_0	67	0.9624	1	0.9624	0	0	0	1	Ref
MK373791	Unassigned	Unassigned	Unassigned	835_0	Clustered	67	VC_835_0	67	0.9624	1	0.9624	0	0	0	1	Ref
MK373792	Unassigned	Unassigned	Unassigned	190_1	Clustered/Singleton	1	VC_190_1	1	0	1	0	0	0	0	1	Ref
MK373793	Unassigned	Unassigned	Unassigned	456_0	Clustered	62	VC_456_0	62	0.9026	1	0.9026	0	0	0	1	Ref
MK373794	Unassigned	Unassigned	Unassigned	722_0	Clustered	13	VC_722_0	13	0.5104	1	0.5104	0	0	0	1	Ref
MK373795	Unassigned	Unassigned	Unassigned	722_0	Clustered	13	VC_722_0	13	0.5104	1	0.5104	0	0	0	1	Ref
MK373796	Unassigned	Unassigned	Unassigned	190_9	Clustered/Singleton	1	VC_190_9	1	0	1	0	0	0	0	1	Ref
MK373797	Unassigned	Unassigned	Unassigned	190_2	Clustered	2	VC_190_2	2	0.0073	0.95531062	0.007	0	0	0	1	Ref
MK373798	Unassigned	Unassigned	Unassigned	941_0	Clustered	40	VC_941_0	40	0.6903	1	0.6903	0	0	0	1	Ref
MK373799	Unassigned	Unassigned	Unassigned	190_2	Clustered	2	VC_190_2	2	0.0073	0.95531062	0.007	0	0	0	1	Ref
MK376953	Unassigned	Unassigned	Unassigned	799_1	Clustered	27	VC_799_1	27	0.8611	1	0.8611	0	0	0	1	Ref
MK376954	Unassigned	Unassigned	Unassigned	767_0	Clustered	34	VC_767_0	34	0.9173	1	0.9173	0	0	0	1	Ref
MK376955	Unassigned	Unassigned	Unassigned	237_3	Clustered	8	VC_237_3	8	0.1092	1	0.1092	0	0	0	1	Ref
MK376956	Unassigned	Unassigned	Unassigned	1181_1	Clustered	10	VC_1181_1	10	0.5828	1	0.5828	0	0	0	1	Ref
MK376957	Unassigned	Unassigned	Unassigned	799_1	Clustered	27	VC_799_1	27	0.8611	1	0.8611	0	0	0	1	Ref
MK376958	Unassigned	Unassigned	Unassigned	954_0	Clustered	13	VC_954_0	13	0.863	1	0.863	0	0	0	1	Ref
MK376959	Unassigned	Unassigned	Unassigned	1181_1	Clustered	10	VC_1181_1	10	0.5828	1	0.5828	0	0	0	1	Ref
MK376960	Unassigned	Unassigned	Unassigned	767_0	Clustered	34	VC_767_0	34	0.9173	1	0.9173	0	0	0	1	Ref
MK376961	Unassigned	Unassigned	Unassigned	557_1	Clustered	11	VC_557_1	11	0.4945	1	0.4945	0	0	0	0.7818	Ref
MK376962	Unassigned	Unassigned	Unassigned	767_0	Clustered	34	VC_767_0	34	0.9173	1	0.9173	0	0	0	1	Ref
MK376963	Unassigned	Unassigned	Unassigned	856_1	Clustered	13	VC_856_1	13	0.5848	1	0.5848	0	0	0	1	Ref
MK380014	Unassigned	Unassigned	Unassigned	452_0	Clustered	66	VC_452_0	66	0.9248	1	0.9248	0	0	0	1	Ref
MK380015	Unassigned	Unassigned	Unassigned	823_0	Clustered	124	VC_823_0	124	0.9492	1	0.9492	0	0	0	1	Ref
MK380016	Unassigned	Unassigned	Unassigned	823_0	Clustered	124	VC_823_0	124	0.9492	1	0.9492	0	0	0	1	Ref
MK387309	Unassigned	Unassigned	Unassigned	644_0	Clustered	5	VC_644_0	5	0.6649	0.99999975	0.6649	0	0	0	1	Ref
MK387337	Unassigned	Unassigned	Unassigned	845_0	Clustered	3	VC_845_0	3	0.3348	0.99831497	0.3343	0	0	0	1	Ref
MK387869	Unassigned	Unassigned	Unassigned	112_0	Clustered	8	VC_112_0	8	0.2537	1	0.2537	0	0	0	1	Ref
MK388689	Unassigned	Unassigned	Unassigned	846_0	Clustered	2	VC_846_0	2	0.5455	0.92357512	0.5038	0	0	0	1	Ref
MK388859	Unassigned	Unassigned	Unassigned	181_0	Clustered	5	VC_181_0	5	0.1168	0.99999995	0.1168	0	0	0	1	Ref
MK392363	Unassigned	Unassigned	Unassigned	981_1	Clustered	26	VC_981_1	26	0.7009	1	0.7009	0	0	0	1	Ref
MK392364	Unassigned	Unassigned	Unassigned	981_1	Clustered	26	VC_981_1	26	0.7009	1	0.7009	0	0	0	1	Ref
MK392365	Unassigned	Unassigned	Unassigned	981_1	Clustered	26	VC_981_1	26	0.7009	1	0.7009	0	0	0	1	Ref
MK392366	Unassigned	Unassigned	Unassigned	981_1	Clustered	26	VC_981_1	26	0.7009	1	0.7009	0	0	0	1	Ref
MK392367	Unassigned	Unassigned	Unassigned	521_0	Clustered	37	VC_521_0	37	0.912	1	0.912	0	0	0	1	Ref
MK392368	Unassigned	Unassigned	Unassigned	1037_0	Clustered	6	VC_1037_0	6	0.6798	1	0.6798	0	0	0	1	Ref
MK393882	Unassigned	Unassigned	Unassigned	191_16	Clustered	12	VC_191_16	12	0.0643	1	0.0643	0	0	0	0.9394	Ref
MK408758	Unassigned	Unassigned	Unassigned	843_0	Clustered	5	VC_843_0	5	0.2843	0.99999994	0.2843	0	0	0	1	Ref
MK411746	Unassigned	Unassigned	Unassigned	1370_0	Clustered	2	VC_1370_0	2	0.8214	0.91237788	0.7494	0	0	0	1	Ref
MK416007	Unassigned	Unassigned	Unassigned	242_1	Clustered	20	VC_242_1	20	0.684	1	0.684	0	0	0	0.8368	Ref
MK416008	Unassigned	Unassigned	Unassigned	35_0	Clustered	6	VC_35_0	6	0.2254	1	0.2254	0	0	0	0.9333	Ref
MK416009	Unassigned	Unassigned	Unassigned	6_0	Clustered	44	VC_6_0	44	0.908	1	0.908	0	0	0	1	Ref
MK416010	Unassigned	Unassigned	Unassigned	476_0	Clustered	19	VC_476_0	19	0.7814	1	0.7814	0	0	0	1	Ref
MK416011	Unassigned	Unassigned	Unassigned	6_0	Clustered	44	VC_6_0	44	0.908	1	0.908	0	0	0	1	Ref
MK416012	Unassigned	Unassigned	Unassigned	6_0	Clustered	44	VC_6_0	44	0.908	1	0.908	0	0	0	1	Ref
MK416013	Unassigned	Unassigned	Unassigned	35_0	Clustered	6	VC_35_0	6	0.2254	1	0.2254	0	0	0	0.9333	Ref
MK416014	Unassigned	Unassigned	Unassigned	243_0	Clustered	2	VC_243_0	2	0.0637	0.95609892	0.0609	0	0	0	1	Ref
MK416015	Unassigned	Unassigned	Unassigned	229_0	Clustered	32	VC_229_0	32	0.9119	1	0.9119	0	0	0	1	Ref
MK416017	Unassigned	Unassigned	Unassigned	244_0	Clustered	12	VC_244_0	12	0.4039	1	0.4039	0	0	0	1	Ref
MK416018	Unassigned	Unassigned	Unassigned	228_0	Clustered	60	VC_228_0	60	0.942	1	0.942	0	0	0	1	Ref
MK416019	Unassigned	Unassigned	Unassigned	242_1	Clustered	20	VC_242_1	20	0.684	1	0.684	0	0	0	0.8368	Ref

MK416020	Unassigned	Unassigned	Unassigned	6_0	Clustered	44	VC_6_0	44	0.908	1	0.908	0	0	0	1	Ref
MK416022	Unassigned	Unassigned	Unassigned	35_0	Clustered	6	VC_35_0	6	0.2254	1	0.2254	0	0	0	0.9333	Ref
MK417514	Unassigned	Unassigned	Unassigned	149_8	Clustered	66	VC_149_8	66	0.4565	1	0.4565	0	0	0	0.8182	Ref
MK417515	Unassigned	Unassigned	Unassigned	149_8	Clustered	66	VC_149_8	66	0.4565	1	0.4565	0	0	0	0.8182	Ref
MK417516	Unassigned	Unassigned	Unassigned	149_8	Clustered	66	VC_149_8	66	0.4565	1	0.4565	0	0	0	0.8182	Ref
MK421971	Unassigned	Unassigned	Unassigned	491_0	Clustered	36	VC_491_0	36	0.5336	1	0.5336	0	0	0	0.9714	Ref
MK422448	Unassigned	Unassigned	Unassigned	476_0	Clustered	19	VC_476_0	19	0.7814	1	0.7814	0	0	0	1	Ref
MK422449	Unassigned	Unassigned	Unassigned	6_0	Clustered	44	VC_6_0	44	0.908	1	0.908	0	0	0	1	Ref
MK422451	Unassigned	Unassigned	Unassigned	723_0	Clustered	9	VC_723_0	9	0.7138	1	0.7138	0	0	0	1	Ref
MK422453	Unassigned	Unassigned	Unassigned	6_0	Clustered	44	VC_6_0	44	0.908	1	0.908	0	0	0	1	Ref
MK422454	Unassigned	Unassigned	Unassigned	6_0	Clustered	44	VC_6_0	44	0.908	1	0.908	0	0	0	1	Ref
MK422455	Unassigned	Unassigned	Unassigned	242_1	Clustered	20	VC_242_1	20	0.684	1	0.684	0	0	0	0.8368	Ref
MK424903	Unassigned	Unassigned	Unassigned	277_0	Clustered	3	VC_277_0	3	1	1	1	0	0	0	1	Ref
MK433259	Unassigned	Unassigned	Unassigned	872_0	Clustered	31	VC_872_0	31	0.8151	1	0.8151	0	0	0	0.8194	Ref
MK433260	Unassigned	Unassigned	Unassigned	872_0	Clustered	31	VC_872_0	31	0.8151	1	0.8151	0	0	0	0.8194	Ref
MK433261	Unassigned	Unassigned	Unassigned	868_0	Clustered	25	VC_868_0	25	0.5791	1	0.5791	0	0	0	1	Ref
MK433262	Unassigned	Unassigned	Unassigned	551_8	Clustered	19	VC_551_8	19	0.1039	1	0.1039	0	0	0	0.8713	Ref
MK433263	Unassigned	Unassigned	Unassigned	868_0	Clustered	25	VC_868_0	25	0.5791	1	0.5791	0	0	0	1	Ref
MK433265	Unassigned	Unassigned	Unassigned	868_0	Clustered	25	VC_868_0	25	0.5791	1	0.5791	0	0	0	1	Ref
MK433266	Unassigned	Unassigned	Unassigned	373_0	Clustered	10	VC_373_0	10	0.7471	1	0.7471	0	0	0	1	Ref
MK433267	Unassigned	Unassigned	Unassigned	868_0	Clustered	25	VC_868_0	25	0.5791	1	0.5791	0	0	0	1	Ref
MK433268	Unassigned	Unassigned	Unassigned	872_0	Clustered	31	VC_872_0	31	0.8151	1	0.8151	0	0	0	0.8194	Ref
MK433269	Unassigned	Unassigned	Unassigned	872_0	Clustered	31	VC_872_0	31	0.8151	1	0.8151	0	0	0	0.8194	Ref
MK433270	Unassigned	Unassigned	Unassigned	982_0	Clustered	62	VC_982_0	62	0.8646	1	0.8646	0	0	0	0.743	Ref
MK433271	Unassigned	Unassigned	Unassigned	982_0	Clustered	62	VC_982_0	62	0.8646	1	0.8646	0	0	0	0.743	Ref
MK433272	Unassigned	Unassigned	Unassigned	767_0	Clustered	34	VC_767_0	34	0.9173	1	0.9173	0	0	0	1	Ref
MK433273	Unassigned	Unassigned	Unassigned	514_0	Clustered	75	VC_514_0	75	0.9402	1	0.9402	0	0	0	1	Ref
MK433274	Unassigned	Unassigned	Unassigned	685_0	Clustered	16	VC_685_0	16	0.2972	1	0.2972	0	0	0	0.9167	Ref
MK433275	Unassigned	Unassigned	Unassigned	685_0	Clustered	16	VC_685_0	16	0.2972	1	0.2972	0	0	0	0.9167	Ref
MK433276	Unassigned	Unassigned	Unassigned	982_0	Clustered	62	VC_982_0	62	0.8646	1	0.8646	0	0	0	0.743	Ref
MK433277	Unassigned	Unassigned	Unassigned	761_0	Clustered	67	VC_761_0	67	0.8947	1	0.8947	0	0	0	1	Ref
MK433278	Unassigned	Unassigned	Unassigned	982_0	Clustered	62	VC_982_0	62	0.8646	1	0.8646	0	0	0	0.743	Ref
MK433279	Unassigned	Unassigned	Unassigned	761_0	Clustered	67	VC_761_0	67	0.8947	1	0.8947	0	0	0	1	Ref
MK433577	Unassigned	Unassigned	Unassigned	6_0	Clustered	44	VC_6_0	44	0.908	1	0.908	0	0	0	1	Ref
MK433578	Unassigned	Unassigned	Unassigned	6_0	Clustered	44	VC_6_0	44	0.908	1	0.908	0	0	0	1	Ref
MK433579	Unassigned	Unassigned	Unassigned	242_1	Clustered	20	VC_242_1	20	0.684	1	0.684	0	0	0	0.8368	Ref
MK433580	Unassigned	Unassigned	Unassigned	6_0	Clustered	44	VC_6_0	44	0.908	1	0.908	0	0	0	1	Ref
MK433581	Unassigned	Unassigned	Unassigned	6_0	Clustered	44	VC_6_0	44	0.908	1	0.908	0	0	0	1	Ref
MK433582	Unassigned	Unassigned	Unassigned	6_0	Clustered	44	VC_6_0	44	0.908	1	0.908	0	0	0	1	Ref
MK443264	Unassigned	Unassigned	Unassigned	76_0	Clustered	37	VC_76_0	37	0.9403	1	0.9403	0	0	0	1	Ref
MK443265	Unassigned	Unassigned	Unassigned	76_1	Clustered/Singleton	1	VC_76_1	1	0	1	0	0	0	0	1	Ref
MK443266	Unassigned	Unassigned	Unassigned	167_0	Clustered	3	VC_167_0	3	0.6952	0.99826575	0.694	0	0	0	1	Ref
MK448230	Unassigned	Unassigned	Unassigned	242_1	Clustered	20	VC_242_1	20	0.684	1	0.684	0	0	0	0.8368	Ref
MK448231	Unassigned	Unassigned	Unassigned	31_0	Clustered	9	VC_31_0	9	0.3435	1	0.3435	0	0	0	0.6944	Ref
MK448233	Unassigned	Unassigned	Unassigned	31_0	Clustered	9	VC_31_0	9	0.3435	1	0.3435	0	0	0	0.6944	Ref
MK448234	Unassigned	Unassigned	Unassigned	35_0	Clustered	6	VC_35_0	6	0.2254	1	0.2254	0	0	0	0.9333	Ref
MK448235	Unassigned	Unassigned	Unassigned	31_0	Clustered	9	VC_31_0	9	0.3435	1	0.3435	0	0	0	0.6944	Ref
MK448237	Unassigned	Unassigned	Unassigned	35_0	Clustered	6	VC_35_0	6	0.2254	1	0.2254	0	0	0	0.9333	Ref
MK448667	Unassigned	Unassigned	Unassigned	626_0	Clustered	23	VC_626_0	23	0.66	1	0.66	0	0	0	0.8379	Ref
MK448671	Unassigned	Unassigned	Unassigned	412_0	Clustered	39	VC_412_0	39	0.845	1	0.845	0	0	0	1	Ref
MK448673	Unassigned	Unassigned	Unassigned	775_1	Clustered	14	VC_775_1	14	0.2797	1	0.2797	0	0	0	0.7582	Ref
MK448674	Unassigned	Unassigned	Unassigned	632_0	Clustered	37	VC_632_0	37	0.581	1	0.581	0	0	0	0.9234	Ref
MK448675	Unassigned	Unassigned	Unassigned	293_3	Clustered	19	VC_293_3	19	0.2627	1	0.2627	0	0	0	0.8187	Ref
MK448676	Unassigned	Unassigned	Unassigned	293_3	Clustered	19	VC_293_3	19	0.2627	1	0.2627	0	0	0	0.8187	Ref
MK448678	Unassigned	Unassigned	Unassigned	773_0	Clustered	23	VC_773_0	23	0.5514	1	0.5514	0	0	0	1	Ref
MK448680	Unassigned	Unassigned	Unassigned	419_0	Clustered	5	VC_419_0	5	0.2498	0.99999997	0.2498	0	0	0	1	Ref
MK448681	Unassigned	Unassigned	Unassigned	636_0	Clustered	9	VC_636_0	9	0.2422	1	0.2422	0	0	0	0.9722	Ref
MK448683	Unassigned	Unassigned	Unassigned	636_0	Clustered	9	VC_636_0	9	0.2422	1	0.2422	0	0	0	0.9722	Ref
MK448684	Unassigned	Unassigned	Unassigned	419_0	Clustered	5	VC_419_0	5	0.2498	0.99999997	0.2498	0	0	0	1	Ref
MK448685	Unassigned	Unassigned	Unassigned	317_0	Clustered	18	VC_317_0	18	0.3304	1	0.3304	0	0	0	1	Ref
MK448686	Unassigned	Unassigned	Unassigned	416_0	Clustered	14	VC_416_0	14	0.2592	1	0.2592	0	0	0	0.989	Ref
MK448688	Unassigned	Unassigned	Unassigned	293_3	Clustered	19	VC_293_3	19	0.2627	1	0.2627	0	0	0	0.8187	Ref
MK448689	Unassigned	Unassigned	Unassigned	773_0	Clustered	23	VC_773_0	23	0.5514	1	0.5514	0	0	0	1	Ref
MK448690	Unassigned	Unassigned	Unassigned	293_3	Clustered	19	VC_293_3	19	0.2627	1	0.2627	0	0	0	0.8187	Ref
MK448692	Unassigned	Unassigned	Unassigned	431_0	Clustered	9	VC_431_0	9	0.1673	1	0.1673	0	0	0	1	Ref
MK448693	Unassigned	Unassigned	Unassigned	773_0	Clustered	23	VC_773_0	23	0.5514	1	0.5514	0	0	0	1	Ref
MK448694	Unassigned	Unassigned	Unassigned	293_3	Clustered	19	VC_293_3	19	0.2627	1	0.2627	0	0	0	0.8187	Ref
MK448696	Unassigned	Unassigned	Unassigned	773_0	Clustered	23	VC_773_0	23	0.5514	1	0.5514	0	0	0	1	Ref
MK448697	Unassigned	Unassigned	Unassigned	773_0	Clustered	23	VC_773_0	23	0.5514	1	0.5514	0	0	0	1	Ref
MK448698	Unassigned	Unassigned	Unassigned	419_0	Clustered	5	VC_419_0	5	0.2498	0.99999997	0.2498	0	0	0	1	Ref
MK448699	Unassigned	Unassigned	Unassigned	773_0	Clustered	23	VC_773_0	23	0.5514	1	0.5514	0	0	0	1	Ref
MK448700	Unassigned	Unassigned	Unassigned	626_0	Clustered	23	VC_626_0	23	0.66	1	0.66	0	0	0	0.8379	Ref
MK448702	Unassigned	Unassigned	Unassigned	431_0	Clustered	9	VC_431_0	9	0.1673	1	0.1673	0	0	0	1	Ref
MK448704	Unassigned	Unassigned	Unassigned	292_0	Clustered	4	VC_292_0	4	0.1677	0.99998671	0.1677	0	0	0	1	Ref
MK448706	Unassigned	Unassigned	Unassigned	317_1	Clustered	2	VC_317_1	2	0.0186	0.95657151	0.0178	0	0	0	1	Ref
MK448707	Unassigned	Unassigned	Unassigned	292_0	Clustered	4	VC_292_0	4	0.1677	0.99998671	0.1677	0	0	0	1	Ref
MK448708	Unassigned	Unassigned	Unassigned	412_0	Clustered	39	VC_412_0	39	0.845	1	0.845	0	0	0	1	Ref
MK448709	Unassigned	Unassigned	Unassigned	292_0	Clustered	4	VC_292_0	4	0.1677	0.99998671	0.1677	0	0	0	1	Ref
MK448710	Unassigned	Unassigned	Unassigned	292_0	Clustered	4	VC_292_0	4	0.1677	0.99998671	0.1677	0	0	0	1	Ref
MK448711	Unassigned	Unassigned	Unassigned	431_0	Clustered	9	VC_431_0	9	0.1673	1	0.1673	0	0	0	1	Ref
MK448714	Unassigned	Unassigned	Unassigned	412_0	Clustered	39	VC_412_0	39	0.845	1	0.845	0	0	0	1	Ref

MK448716	Unassigned	Unassigned	Unassigned	431_0	Clustered	9	VC_431_0	9	0.1673	1	0.1673	0	0	0	1	Ref
MK448720	Unassigned	Unassigned	Unassigned	626_0	Clustered	23	VC_626_0	23	0.66	1	0.66	0	0	0	0.8379	Ref
MK448721	Unassigned	Unassigned	Unassigned	317_1	Clustered	2	VC_317_1	2	0.0186	0.95657151	0.0178	0	0	0	1	Ref
MK448724	Unassigned	Unassigned	Unassigned	431_0	Clustered	9	VC_431_0	9	0.1673	1	0.1673	0	0	0	1	Ref
MK448730	Unassigned	Unassigned	Unassigned	431_0	Clustered	9	VC_431_0	9	0.1673	1	0.1673	0	0	0	1	Ref
MK448732	Unassigned	Unassigned	Unassigned	333_0	Clustered	25	VC_333_0	25	0.4681	1	0.4681	0	0	0	1	Ref
MK448733	Unassigned	Unassigned	Unassigned	413_0	Clustered	3	VC_413_0	3	0.0415	0.99841754	0.0415	0	0	0	1	Ref
MK448735	Unassigned	Unassigned	Unassigned	323_1	Clustered	9	VC_323_1	9	0.1798	1	0.1798	0	0	0	0.75	Ref
MK448739	Unassigned	Unassigned	Unassigned	333_0	Clustered	25	VC_333_0	25	0.4681	1	0.4681	0	0	0	1	Ref
MK448740	Unassigned	Unassigned	Unassigned	293_2	Clustered	2	VC_293_2	2	0.0311	0.95710449	0.0298	0	0	0	1	Ref
MK448741	Unassigned	Unassigned	Unassigned	632_0	Clustered	37	VC_632_0	37	0.581	1	0.581	0	0	0	0.9234	Ref
MK448744	Unassigned	Unassigned	Unassigned	431_0	Clustered	9	VC_431_0	9	0.1673	1	0.1673	0	0	0	1	Ref
MK448745	Unassigned	Unassigned	Unassigned	431_0	Clustered	9	VC_431_0	9	0.1673	1	0.1673	0	0	0	1	Ref
MK448750	Unassigned	Unassigned	Unassigned	293_0	Clustered/Singleton	1	VC_293_0	1	0	1	0	0	0	0	1	Ref
MK448752	Unassigned	Unassigned	Unassigned	632_0	Clustered	37	VC_632_0	37	0.581	1	0.581	0	0	0	0.9234	Ref
MK448757	Unassigned	Unassigned	Unassigned	323_1	Clustered	9	VC_323_1	9	0.1798	1	0.1798	0	0	0	0.75	Ref
MK448758	Unassigned	Unassigned	Unassigned	323_1	Clustered	9	VC_323_1	9	0.1798	1	0.1798	0	0	0	0.75	Ref
MK448759	Unassigned	Unassigned	Unassigned	323_1	Clustered	9	VC_323_1	9	0.1798	1	0.1798	0	0	0	0.75	Ref
MK448762	Unassigned	Unassigned	Unassigned	416_0	Clustered	14	VC_416_0	14	0.2592	1	0.2592	0	0	0	0.989	Ref
MK448763	Unassigned	Unassigned	Unassigned	317_0	Clustered	18	VC_317_0	18	0.3304	1	0.3304	0	0	0	1	Ref
MK448765	Unassigned	Unassigned	Unassigned	775_1	Clustered	14	VC_775_1	14	0.2797	1	0.2797	0	0	0	0.7582	Ref
MK448766	Unassigned	Unassigned	Unassigned	773_0	Clustered	23	VC_773_0	23	0.5514	1	0.5514	0	0	0	1	Ref
MK448767	Unassigned	Unassigned	Unassigned	317_0	Clustered	18	VC_317_0	18	0.3304	1	0.3304	0	0	0	1	Ref
MK448769	Unassigned	Unassigned	Unassigned	419_0	Clustered	5	VC_419_0	5	0.2498	0.99999997	0.2498	0	0	0	1	Ref
MK448771	Unassigned	Unassigned	Unassigned	317_0	Clustered	18	VC_317_0	18	0.3304	1	0.3304	0	0	0	1	Ref
MK448772	Unassigned	Unassigned	Unassigned	293_3	Clustered	19	VC_293_3	19	0.2627	1	0.2627	0	0	0	0.8187	Ref
MK448773	Unassigned	Unassigned	Unassigned	317_0	Clustered	18	VC_317_0	18	0.3304	1	0.3304	0	0	0	1	Ref
MK448774	Unassigned	Unassigned	Unassigned	317_0	Clustered	18	VC_317_0	18	0.3304	1	0.3304	0	0	0	1	Ref
MK448775	Unassigned	Unassigned	Unassigned	775_1	Clustered	14	VC_775_1	14	0.2797	1	0.2797	0	0	0	0.7582	Ref
MK448777	Unassigned	Unassigned	Unassigned	317_0	Clustered	18	VC_317_0	18	0.3304	1	0.3304	0	0	0	1	Ref
MK448778	Unassigned	Unassigned	Unassigned	293_3	Clustered	19	VC_293_3	19	0.2627	1	0.2627	0	0	0	0.8187	Ref
MK448779	Unassigned	Unassigned	Unassigned	773_0	Clustered	23	VC_773_0	23	0.5514	1	0.5514	0	0	0	1	Ref
MK448782	Unassigned	Unassigned	Unassigned	293_3	Clustered	19	VC_293_3	19	0.2627	1	0.2627	0	0	0	0.8187	Ref
MK448783	Unassigned	Unassigned	Unassigned	636_0	Clustered	9	VC_636_0	9	0.2422	1	0.2422	0	0	0	0.9722	Ref
MK448784	Unassigned	Unassigned	Unassigned	773_0	Clustered	23	VC_773_0	23	0.5514	1	0.5514	0	0	0	1	Ref
MK448786	Unassigned	Unassigned	Unassigned	317_0	Clustered	18	VC_317_0	18	0.3304	1	0.3304	0	0	0	1	Ref
MK448788	Unassigned	Unassigned	Unassigned	416_0	Clustered	14	VC_416_0	14	0.2592	1	0.2592	0	0	0	0.989	Ref
MK448789	Unassigned	Unassigned	Unassigned	773_0	Clustered	23	VC_773_0	23	0.5514	1	0.5514	0	0	0	1	Ref
MK448790	Unassigned	Unassigned	Unassigned	636_0	Clustered	9	VC_636_0	9	0.2422	1	0.2422	0	0	0	0.9722	Ref
MK448791	Unassigned	Unassigned	Unassigned	416_0	Clustered	14	VC_416_0	14	0.2592	1	0.2592	0	0	0	0.989	Ref
MK448793	Unassigned	Unassigned	Unassigned	416_0	Clustered	14	VC_416_0	14	0.2592	1	0.2592	0	0	0	0.989	Ref
MK448794	Unassigned	Unassigned	Unassigned	775_1	Clustered	14	VC_775_1	14	0.2797	1	0.2797	0	0	0	0.7582	Ref
MK448795	Unassigned	Unassigned	Unassigned	775_1	Clustered	14	VC_775_1	14	0.2797	1	0.2797	0	0	0	0.7582	Ref
MK448797	Unassigned	Unassigned	Unassigned	773_0	Clustered	23	VC_773_0	23	0.5514	1	0.5514	0	0	0	1	Ref
MK448798	Unassigned	Unassigned	Unassigned	636_0	Clustered	9	VC_636_0	9	0.2422	1	0.2422	0	0	0	0.9722	Ref
MK448804	Unassigned	Unassigned	Unassigned	335_0	Clustered	6	VC_335_0	6	0.3678	1	0.3678	0	0	0	1	Ref
MK448805	Unassigned	Unassigned	Unassigned	335_0	Clustered	6	VC_335_0	6	0.3678	1	0.3678	0	0	0	1	Ref
MK448807	Unassigned	Unassigned	Unassigned	335_0	Clustered	6	VC_335_0	6	0.3678	1	0.3678	0	0	0	1	Ref
MK448808	Unassigned	Unassigned	Unassigned	335_0	Clustered	6	VC_335_0	6	0.3678	1	0.3678	0	0	0	1	Ref
MK448811	Unassigned	Unassigned	Unassigned	632_0	Clustered	37	VC_632_0	37	0.581	1	0.581	0	0	0	0.9234	Ref
MK448814	Unassigned	Unassigned	Unassigned	317_2	Clustered	20	VC_317_2	20	0.1976	1	0.1976	0	0	0	0.6842	Ref
MK448815	Unassigned	Unassigned	Unassigned	335_0	Clustered	6	VC_335_0	6	0.3678	1	0.3678	0	0	0	1	Ref
MK448818	Unassigned	Unassigned	Unassigned	623_0	Clustered/Singleton	1	VC_623_0	1	0	1	0	0	0	0	1	Ref
MK448819	Unassigned	Unassigned	Unassigned	632_0	Clustered	37	VC_632_0	37	0.581	1	0.581	0	0	0	0.9234	Ref
MK448821	Unassigned	Unassigned	Unassigned	323_0	Clustered	2	VC_323_0	2	0.0281	0.95656566	0.0269	0	0	0	1	Ref
MK448822	Unassigned	Unassigned	Unassigned	323_0	Clustered	2	VC_323_0	2	0.0281	0.95656566	0.0269	0	0	0	1	Ref
MK448824	Unassigned	Unassigned	Unassigned	259_0	Clustered	3	VC_259_0	3	0.1148	0.99858602	0.1146	0	0	0	1	Ref
MK448828	Unassigned	Unassigned	Unassigned	632_0	Clustered	37	VC_632_0	37	0.581	1	0.581	0	0	0	0.9234	Ref
MK448831	Unassigned	Unassigned	Unassigned	412_0	Clustered	39	VC_412_0	39	0.845	1	0.845	0	0	0	1	Ref
MK448832	Unassigned	Unassigned	Unassigned	294_0	Clustered	2	VC_294_0	2	0.0836	0.95731776	0.0801	0	0	0	1	Ref
MK448833	Unassigned	Unassigned	Unassigned	636_0	Clustered	9	VC_636_0	9	0.2422	1	0.2422	0	0	0	0.9722	Ref
MK448834	Unassigned	Unassigned	Unassigned	412_0	Clustered	39	VC_412_0	39	0.845	1	0.845	0	0	0	1	Ref
MK448839	Unassigned	Unassigned	Unassigned	626_0	Clustered	23	VC_626_0	23	0.66	1	0.66	0	0	0	0.8379	Ref
MK448841	Unassigned	Unassigned	Unassigned	626_0	Clustered	23	VC_626_0	23	0.66	1	0.66	0	0	0	0.8379	Ref
MK448843	Unassigned	Unassigned	Unassigned	626_0	Clustered	23	VC_626_0	23	0.66	1	0.66	0	0	0	0.8379	Ref
MK448845	Unassigned	Unassigned	Unassigned	317_0	Clustered	18	VC_317_0	18	0.3304	1	0.3304	0	0	0	1	Ref
MK448850	Unassigned	Unassigned	Unassigned	293_3	Clustered	19	VC_293_3	19	0.2627	1	0.2627	0	0	0	0.8187	Ref
MK448852	Unassigned	Unassigned	Unassigned	773_0	Clustered	23	VC_773_0	23	0.5514	1	0.5514	0	0	0	1	Ref
MK448853	Unassigned	Unassigned	Unassigned	317_0	Clustered	18	VC_317_0	18	0.3304	1	0.3304	0	0	0	1	Ref
MK448854	Unassigned	Unassigned	Unassigned	775_0	Clustered	3	VC_775_0	3	0.0364	0.99850073	0.0364	0	0	0	1	Ref
MK448855	Unassigned	Unassigned	Unassigned	317_0	Clustered	18	VC_317_0	18	0.3304	1	0.3304	0	0	0	1	Ref
MK448858	Unassigned	Unassigned	Unassigned	775_1	Clustered	14	VC_775_1	14	0.2797	1	0.2797	0	0	0	0.7582	Ref
MK448859	Unassigned	Unassigned	Unassigned	293_3	Clustered	19	VC_293_3	19	0.2627	1	0.2627	0	0	0	0.8187	Ref
MK448861	Unassigned	Unassigned	Unassigned	412_0	Clustered	39	VC_412_0	39	0.845	1	0.845	0	0	0	1	Ref
MK448862	Unassigned	Unassigned	Unassigned	416_0	Clustered	14	VC_416_0	14	0.2592	1	0.2592	0	0	0	0.989	Ref
MK448864	Unassigned	Unassigned	Unassigned	775_1	Clustered	14	VC_775_1	14	0.2797	1	0.2797	0	0	0	0.7582	Ref
MK448866	Unassigned	Unassigned	Unassigned	773_0	Clustered	23	VC_773_0	23	0.5514	1	0.5514	0	0	0	1	Ref
MK448867	Unassigned	Unassigned	Unassigned	773_0	Clustered	23	VC_773_0	23	0.5514	1	0.5514	0	0	0	1	Ref
MK448868	Unassigned	Unassigned	Unassigned	773_0	Clustered	23	VC_773_0	23	0.5514	1	0.5514	0	0	0	1	Ref
MK448871	Unassigned	Unassigned	Unassigned	773_0	Clustered	23	VC_773_0	23	0.5514	1	0.5514	0	0	0	1	Ref
MK448874	Unassigned	Unassigned	Unassigned	412_0	Clustered	39	VC_412_0	39	0.845	1	0.845	0	0	0	1	Ref

MK448875	Unassigned	Unassigned	Unassigned	412_0	Clustered	39	VC_412_0	39	0.845	1	0.845	0	0	0	1	Ref
MK448877	Unassigned	Unassigned	Unassigned	293_3	Clustered	19	VC_293_3	19	0.2627	1	0.2627	0	0	0	0.8187	Ref
MK448881	Unassigned	Unassigned	Unassigned	317_2	Clustered	20	VC_317_2	20	0.1976	1	0.1976	0	0	0	0.6842	Ref
MK448882	Unassigned	Unassigned	Unassigned	317_2	Clustered	20	VC_317_2	20	0.1976	1	0.1976	0	0	0	0.6842	Ref
MK448886	Unassigned	Unassigned	Unassigned	412_0	Clustered	39	VC_412_0	39	0.845	1	0.845	0	0	0	1	Ref
MK448887	Unassigned	Unassigned	Unassigned	412_0	Clustered	39	VC_412_0	39	0.845	1	0.845	0	0	0	1	Ref
MK448890	Unassigned	Unassigned	Unassigned	625_0	Clustered	2	VC_625_0	2	0.0259	0.92480562	0.0239	0	0	0	1	Ref
MK448893	Unassigned	Unassigned	Unassigned	323_1	Clustered	9	VC_323_1	9	0.1798	1	0.1798	0	0	0	0.75	Ref
MK448895	Unassigned	Unassigned	Unassigned	419_0	Clustered	5	VC_419_0	5	0.2498	0.99999997	0.2498	0	0	0	1	Ref
MK448896	Unassigned	Unassigned	Unassigned	317_2	Clustered	20	VC_317_2	20	0.1976	1	0.1976	0	0	0	0.6842	Ref
MK448897	Unassigned	Unassigned	Unassigned	412_0	Clustered	39	VC_412_0	39	0.845	1	0.845	0	0	0	1	Ref
MK448898	Unassigned	Unassigned	Unassigned	412_0	Clustered	39	VC_412_0	39	0.845	1	0.845	0	0	0	1	Ref
MK448899	Unassigned	Unassigned	Unassigned	253_0	Clustered/Singleton	1	VC_253_0	1	0	1	0	0	0	0	1	Ref
MK448900	Unassigned	Unassigned	Unassigned	317_4	Clustered/Singleton	1	VC_317_4	1	0	1	0	0	0	0	1	Ref
MK448901	Unassigned	Unassigned	Unassigned	333_0	Clustered	25	VC_333_0	25	0.4681	1	0.4681	0	0	0	1	Ref
MK448902	Unassigned	Unassigned	Unassigned	137_1	Clustered/Singleton	1	VC_137_1	1	0	1	0	0	0	0	1	Ref
MK448904	Unassigned	Unassigned	Unassigned	413_0	Clustered	3	VC_413_0	3	0.0415	0.99841754	0.0415	0	0	0	1	Ref
MK448905	Unassigned	Unassigned	Unassigned	412_0	Clustered	39	VC_412_0	39	0.845	1	0.845	0	0	0	1	Ref
MK448909	Unassigned	Unassigned	Unassigned	317_2	Clustered	20	VC_317_2	20	0.1976	1	0.1976	0	0	0	0.6842	Ref
MK448910	Unassigned	Unassigned	Unassigned	137_0	Clustered	3	VC_137_0	3	0.1129	0.998547	0.1128	0	0	0	1	Ref
MK448912	Unassigned	Unassigned	Unassigned	333_0	Clustered	25	VC_333_0	25	0.4681	1	0.4681	0	0	0	1	Ref
MK448914	Unassigned	Unassigned	Unassigned	333_0	Clustered	25	VC_333_0	25	0.4681	1	0.4681	0	0	0	1	Ref
MK448915	Unassigned	Unassigned	Unassigned	323_1	Clustered	9	VC_323_1	9	0.1798	1	0.1798	0	0	0	0.75	Ref
MK448917	Unassigned	Unassigned	Unassigned	293_2	Clustered	2	VC_293_2	2	0.0311	0.95710449	0.0298	0	0	0	1	Ref
MK448918	Unassigned	Unassigned	Unassigned	333_0	Clustered	25	VC_333_0	25	0.4681	1	0.4681	0	0	0	1	Ref
MK448920	Unassigned	Unassigned	Unassigned	333_0	Clustered	25	VC_333_0	25	0.4681	1	0.4681	0	0	0	1	Ref
MK448923	Unassigned	Unassigned	Unassigned	431_0	Clustered	9	VC_431_0	9	0.1673	1	0.1673	0	0	0	1	Ref
MK448925	Unassigned	Unassigned	Unassigned	412_0	Clustered	39	VC_412_0	39	0.845	1	0.845	0	0	0	1	Ref
MK448927	Unassigned	Unassigned	Unassigned	412_0	Clustered	39	VC_412_0	39	0.845	1	0.845	0	0	0	1	Ref
MK448930	Unassigned	Unassigned	Unassigned	625_0	Clustered	2	VC_625_0	2	0.0259	0.92480562	0.0239	0	0	0	1	Ref
MK448931	Unassigned	Unassigned	Unassigned	293_3	Clustered	19	VC_293_3	19	0.2627	1	0.2627	0	0	0	0.8187	Ref
MK448933	Unassigned	Unassigned	Unassigned	775_0	Clustered	3	VC_775_0	3	0.0364	0.99850073	0.0364	0	0	0	1	Ref
MK448934	Unassigned	Unassigned	Unassigned	626_0	Clustered	23	VC_626_0	23	0.66	1	0.66	0	0	0	0.8379	Ref
MK448939	Unassigned	Unassigned	Unassigned	323_1	Clustered	9	VC_323_1	9	0.1798	1	0.1798	0	0	0	0.75	Ref
MK448941	Unassigned	Unassigned	Unassigned	775_1	Clustered	14	VC_775_1	14	0.2797	1	0.2797	0	0	0	0.7582	Ref
MK448942	Unassigned	Unassigned	Unassigned	317_0	Clustered	18	VC_317_0	18	0.3304	1	0.3304	0	0	0	1	Ref
MK448943	Unassigned	Unassigned	Unassigned	317_0	Clustered	18	VC_317_0	18	0.3304	1	0.3304	0	0	0	1	Ref
MK448944	Unassigned	Unassigned	Unassigned	775_1	Clustered	14	VC_775_1	14	0.2797	1	0.2797	0	0	0	0.7582	Ref
MK448945	Unassigned	Unassigned	Unassigned	775_1	Clustered	14	VC_775_1	14	0.2797	1	0.2797	0	0	0	0.7582	Ref
MK448946	Unassigned	Unassigned	Unassigned	775_1	Clustered	14	VC_775_1	14	0.2797	1	0.2797	0	0	0	0.7582	Ref
MK448947	Unassigned	Unassigned	Unassigned	773_0	Clustered	23	VC_773_0	23	0.5514	1	0.5514	0	0	0	1	Ref
MK448949	Unassigned	Unassigned	Unassigned	416_0	Clustered	14	VC_416_0	14	0.2592	1	0.2592	0	0	0	0.989	Ref
MK448950	Unassigned	Unassigned	Unassigned	775_0	Clustered	3	VC_775_0	3	0.0364	0.99850073	0.0364	0	0	0	1	Ref
MK448951	Unassigned	Unassigned	Unassigned	416_0	Clustered	14	VC_416_0	14	0.2592	1	0.2592	0	0	0	0.989	Ref
MK448952	Unassigned	Unassigned	Unassigned	773_0	Clustered	23	VC_773_0	23	0.5514	1	0.5514	0	0	0	1	Ref
MK448953	Unassigned	Unassigned	Unassigned	775_1	Clustered	14	VC_775_1	14	0.2797	1	0.2797	0	0	0	0.7582	Ref
MK448954	Unassigned	Unassigned	Unassigned	416_0	Clustered	14	VC_416_0	14	0.2592	1	0.2592	0	0	0	0.989	Ref
MK448955	Unassigned	Unassigned	Unassigned	293_3	Clustered	19	VC_293_3	19	0.2627	1	0.2627	0	0	0	0.8187	Ref
MK448957	Unassigned	Unassigned	Unassigned	775_1	Clustered	14	VC_775_1	14	0.2797	1	0.2797	0	0	0	0.7582	Ref
MK448958	Unassigned	Unassigned	Unassigned	416_0	Clustered	14	VC_416_0	14	0.2592	1	0.2592	0	0	0	0.989	Ref
MK448959	Unassigned	Unassigned	Unassigned	416_0	Clustered	14	VC_416_0	14	0.2592	1	0.2592	0	0	0	0.989	Ref
MK448960	Unassigned	Unassigned	Unassigned	416_0	Clustered	14	VC_416_0	14	0.2592	1	0.2592	0	0	0	0.989	Ref
MK448961	Unassigned	Unassigned	Unassigned	773_0	Clustered	23	VC_773_0	23	0.5514	1	0.5514	0	0	0	1	Ref
MK448962	Unassigned	Unassigned	Unassigned	293_3	Clustered	19	VC_293_3	19	0.2627	1	0.2627	0	0	0	0.8187	Ref
MK448963	Unassigned	Unassigned	Unassigned	317_0	Clustered	18	VC_317_0	18	0.3304	1	0.3304	0	0	0	1	Ref
MK448964	Unassigned	Unassigned	Unassigned	317_0	Clustered	18	VC_317_0	18	0.3304	1	0.3304	0	0	0	1	Ref
MK448965	Unassigned	Unassigned	Unassigned	416_0	Clustered	14	VC_416_0	14	0.2592	1	0.2592	0	0	0	0.989	Ref
MK448966	Unassigned	Unassigned	Unassigned	293_3	Clustered	19	VC_293_3	19	0.2627	1	0.2627	0	0	0	0.8187	Ref
MK448967	Unassigned	Unassigned	Unassigned	636_0	Clustered	9	VC_636_0	9	0.2422	1	0.2422	0	0	0	0.9722	Ref
MK448968	Unassigned	Unassigned	Unassigned	773_0	Clustered	23	VC_773_0	23	0.5514	1	0.5514	0	0	0	1	Ref
MK448969	Unassigned	Unassigned	Unassigned	317_0	Clustered	18	VC_317_0	18	0.3304	1	0.3304	0	0	0	1	Ref
MK448970	Unassigned	Unassigned	Unassigned	775_1	Clustered	14	VC_775_1	14	0.2797	1	0.2797	0	0	0	0.7582	Ref
MK448972	Unassigned	Unassigned	Unassigned	317_0	Clustered	18	VC_317_0	18	0.3304	1	0.3304	0	0	0	1	Ref
MK448973	Unassigned	Unassigned	Unassigned	293_3	Clustered	19	VC_293_3	19	0.2627	1	0.2627	0	0	0	0.8187	Ref
MK448974	Unassigned	Unassigned	Unassigned	773_0	Clustered	23	VC_773_0	23	0.5514	1	0.5514	0	0	0	1	Ref
MK448975	Unassigned	Unassigned	Unassigned	293_3	Clustered	19	VC_293_3	19	0.2627	1	0.2627	0	0	0	0.8187	Ref
MK448976	Unassigned	Unassigned	Unassigned	773_0	Clustered	23	VC_773_0	23	0.5514	1	0.5514	0	0	0	1	Ref
MK448977	Unassigned	Unassigned	Unassigned	416_0	Clustered	14	VC_416_0	14	0.2592	1	0.2592	0	0	0	0.989	Ref
MK448982	Unassigned	Unassigned	Unassigned	318_0	Clustered	3	VC_318_0	3	0.0414	0.98579852	0.0408	0	0	0	1	Ref
MK448983	Unassigned	Unassigned	Unassigned	632_0	Clustered	37	VC_632_0	37	0.581	1	0.581	0	0	0	0.9234	Ref
MK448988	Unassigned	Unassigned	Unassigned	317_2	Clustered	20	VC_317_2	20	0.1976	1	0.1976	0	0	0	0.6842	Ref
MK448989	Unassigned	Unassigned	Unassigned	317_2	Clustered	20	VC_317_2	20	0.1976	1	0.1976	0	0	0	0.6842	Ref
MK448991	Unassigned	Unassigned	Unassigned	335_0	Clustered	6	VC_335_0	6	0.3678	1	0.3678	0	0	0	1	Ref
MK448995	Unassigned	Unassigned	Unassigned	293_1	Clustered/Singleton	1	VC_293_1	1	0	1	0	0	0	0	1	Ref
MK448997	Unassigned	Unassigned	Unassigned	626_0	Clustered	23	VC_626_0	23	0.66	1	0.66	0	0	0	0.8379	Ref
MK449000	Unassigned	Unassigned	Unassigned	334_0	Clustered/Singleton	1	VC_334_0	1	0	1	0	0	0	0	1	Ref
MK449002	Unassigned	Unassigned	Unassigned	259_0	Clustered	3	VC_259_0	3	0.1148	0.99858602	0.1146	0	0	0	1	Ref
MK449003	Unassigned	Unassigned	Unassigned	259_0	Clustered	3	VC_259_0	3	0.1148	0.99858602	0.1146	0	0	0	1	Ref
MK449004	Unassigned	Unassigned	Unassigned	293_3	Clustered	19	VC_293_3	19	0.2627	1	0.2627	0	0	0	0.8187	Ref
MK449006	Unassigned	Unassigned	Unassigned	412_0	Clustered	39	VC_412_0	39	0.845	1	0.845	0	0	0	1	Ref
MK449008	Unassigned	Unassigned	Unassigned	294_0	Clustered	2	VC_294_0	2	0.0836	0.95731776	0.0801	0	0	0	1	Ref

MK449009	Unassigned	Unassigned	Unassigned	412_0	Clustered	39	VC_412_0	39	0.845	1	0.845	0	0	0	1	Ref
MK449010	Unassigned	Unassigned	Unassigned	636_0	Clustered	9	VC_636_0	9	0.2422	1	0.2422	0	0	0	0.9722	Ref
MK449012	Unassigned	Unassigned	Unassigned	317_0	Clustered	18	VC_317_0	18	0.3304	1	0.3304	0	0	0	1	Ref
MK450421	Unassigned	Unassigned	Unassigned	373_0	Clustered	10	VC_373_0	10	0.7471	1	0.7471	0	0	0	1	Ref
MK450422	Unassigned	Unassigned	Unassigned	558_65	Clustered	2	VC_558_65	2	0.0032	0.61174182	0.0019	0	0	0	1	Ref
MK450423	Unassigned	Unassigned	Unassigned	558_22	Clustered	4	VC_558_22	4	0.01	0.99312819	0.0099	0	0	0	0.8333	Ref
MK450424	Unassigned	Unassigned	Unassigned	558_41	Clustered/Singleton	1	VC_558_41	1	0	1	0	0	0	0	1	Ref
MK450425	Unassigned	Unassigned	Unassigned	558_57	Clustered/Singleton	1	VC_558_57	1	0	1	0	0	0	0	1	Ref
MK450426	Unassigned	Unassigned	Unassigned	373_0	Clustered	10	VC_373_0	10	0.7471	1	0.7471	0	0	0	1	Ref
MK450427	Unassigned	Unassigned	Unassigned	558_14	Clustered	8	VC_558_14	8	0.0228	0.99945246	0.0228	0	0	0	0.8214	Ref
MK450428	Unassigned	Unassigned	Unassigned	866_1	Clustered	20	VC_866_1	20	0.4814	1	0.4814	0	0	0	1	Ref
MK450429	Unassigned	Unassigned	Unassigned	558_50	Clustered	15	VC_558_50	15	0.0468	1	0.0468	0	0	0	0.9238	Ref
MK450430	Unassigned	Unassigned	Unassigned	866_1	Clustered	20	VC_866_1	20	0.4814	1	0.4814	0	0	0	1	Ref
MK450431	Unassigned	Unassigned	Unassigned	373_0	Clustered	10	VC_373_0	10	0.7471	1	0.7471	0	0	0	1	Ref
MK450432	Unassigned	Unassigned	Unassigned	558_20	Clustered	17	VC_558_20	17	0.054	1	0.054	0	0	0	0.9926	Ref
MK450433	Unassigned	Unassigned	Unassigned	373_0	Clustered	10	VC_373_0	10	0.7471	1	0.7471	0	0	0	1	Ref
MK450434	Unassigned	Unassigned	Unassigned	558_50	Clustered	15	VC_558_50	15	0.0468	1	0.0468	0	0	0	0.9238	Ref
MK450435	Unassigned	Unassigned	Unassigned	866_1	Clustered	20	VC_866_1	20	0.4814	1	0.4814	0	0	0	1	Ref
MK450436	Unassigned	Unassigned	Unassigned	866_1	Clustered	20	VC_866_1	20	0.4814	1	0.4814	0	0	0	1	Ref
MK450538	Unassigned	Unassigned	Unassigned	15_1	Clustered	9	VC_15_1	9	0.182	1	0.182	0	0	0	0.8889	Ref
MK450543	Unassigned	Unassigned	Unassigned	352_0	Clustered	3	VC_352_0	3	0.7306	0.98867275	0.7223	0	0	0	1	Ref
MK455769	Unassigned	Unassigned	Unassigned	1027_0	Clustered	20	VC_1027_0	20	0.7114	1	0.7114	0	0	0	1	Ref
MK455770	Unassigned	Unassigned	Unassigned	1027_0	Clustered	20	VC_1027_0	20	0.7114	1	0.7114	0	0	0	1	Ref
MK460245	Unassigned	Unassigned	Unassigned	981_1	Clustered	26	VC_981_1	26	0.7009	1	0.7009	0	0	0	1	Ref
MK460246	Unassigned	Unassigned	Unassigned	237_3	Clustered	8	VC_237_3	8	0.1092	1	0.1092	0	0	0	1	Ref
MK460247	Unassigned	Unassigned	Unassigned	236_0	Clustered	72	VC_236_0	72	0.8104	1	0.8104	0	0	0	1	Ref
MK460248	Unassigned	Unassigned	Unassigned	1071_3	Clustered	16	VC_1071_3	16	0.2354	1	0.2354	0	0	0	0.9667	Ref
MK468591	Unassigned	Unassigned	Unassigned	225_0	Clustered	32	VC_225_0	32	0.848	1	0.848	0	0	0	0.996	Ref
MK473373	Unassigned	Unassigned	Unassigned	1030_0	Clustered	3	VC_1030_0	3	0.7895	0.99542416	0.7859	0	0	0	1	Ref
MK473382	Unassigned	Unassigned	Unassigned	647_0	Clustered	11	VC_647_0	11	0.598	1	0.598	0	0	0	1	Ref
MK474470	Unassigned	Unassigned	Unassigned	837_0	Clustered	29	VC_837_0	29	0.9955	1	0.9955	0	0	0	1	Ref
MK479295	Unassigned	Unassigned	Unassigned	540_0	Clustered	15	VC_540_0	15	0.2866	1	0.2866	0	0	0	1	Ref
MK482688	Unassigned	Unassigned	Unassigned	329_7	Clustered	18	VC_329_7	18	0.0938	1	0.0938	0	0	0	0.7778	Ref
MK482689	Unassigned	Unassigned	Unassigned	329_7	Clustered	18	VC_329_7	18	0.0938	1	0.0938	0	0	0	0.7778	Ref
MK482690	Unassigned	Unassigned	Unassigned	329_7	Clustered	18	VC_329_7	18	0.0938	1	0.0938	0	0	0	0.7778	Ref
MK483107	Unassigned	Unassigned	Unassigned	530_0	Clustered	9	VC_530_0	9	0.8027	1	0.8027	0	0	0	1	Ref
MK494087	Unassigned	Unassigned	Unassigned	215_1	Clustered	19	VC_215_1	19	0.4188	1	0.4188	0	0	0	1	Ref
MK494088	Unassigned	Unassigned	Unassigned	517_1	Clustered	108	VC_517_1	108	0.8483	1	0.8483	0	0	0	0.9955	Ref
MK494089	Unassigned	Unassigned	Unassigned	1046_0	Clustered	7	VC_1046_0	7	0.9413	1	0.9413	0	0	0	1	Ref
MK494090	Unassigned	Unassigned	Unassigned	223_0	Clustered	38	VC_223_0	38	0.9095	1	0.9095	0	0	0	1	Ref
MK494091	Unassigned	Unassigned	Unassigned	940_0	Clustered	34	VC_940_0	34	0.7959	1	0.7959	0	0	0	1	Ref
MK494092	Unassigned	Unassigned	Unassigned	887_0	Clustered	18	VC_887_0	18	0.3663	1	0.3663	0	0	0	1	Ref
MK494093	Unassigned	Unassigned	Unassigned	517_1	Clustered	108	VC_517_1	108	0.8483	1	0.8483	0	0	0	0.9955	Ref
MK494094	Unassigned	Unassigned	Unassigned	517_1	Clustered	108	VC_517_1	108	0.8483	1	0.8483	0	0	0	0.9955	Ref
MK494095	Unassigned	Unassigned	Unassigned	810_0	Clustered	18	VC_810_0	18	0.9121	1	0.9121	0	0	0	1	Ref
MK494096	Unassigned	Unassigned	Unassigned	514_0	Clustered	75	VC_514_0	75	0.9402	1	0.9402	0	0	0	1	Ref
MK494097	Unassigned	Unassigned	Unassigned	517_1	Clustered	108	VC_517_1	108	0.8483	1	0.8483	0	0	0	0.9955	Ref
MK494098	Unassigned	Unassigned	Unassigned	869_0	Clustered	9	VC_869_0	9	0.2624	1	0.2624	0	0	0	1	Ref
MK494099	Unassigned	Unassigned	Unassigned	686_0	Clustered	2	VC_686_0	2	0.2147	0.9539194	0.2048	0	0	0	1	Ref
MK494100	Unassigned	Unassigned	Unassigned	887_0	Clustered	18	VC_887_0	18	0.3663	1	0.3663	0	0	0	1	Ref
MK494101	Unassigned	Unassigned	Unassigned	517_1	Clustered	108	VC_517_1	108	0.8483	1	0.8483	0	0	0	0.9955	Ref
MK494102	Unassigned	Unassigned	Unassigned	565_0	Clustered	20	VC_565_0	20	0.8398	1	0.8398	0	0	0	1	Ref
MK494103	Unassigned	Unassigned	Unassigned	950_0	Clustered	38	VC_950_0	38	0.9443	1	0.9443	0	0	0	1	Ref
MK494104	Unassigned	Unassigned	Unassigned	371_16	Clustered	42	VC_371_16	42	0.1341	1	0.1341	0	0	0	0.7282	Ref
MK494105	Unassigned	Unassigned	Unassigned	762_0	Clustered	9	VC_762_0	9	0.0885	1	0.0885	0	0	0	1	Ref
MK494106	Unassigned	Unassigned	Unassigned	866_1	Clustered	20	VC_866_1	20	0.4814	1	0.4814	0	0	0	1	Ref
MK494107	Unassigned	Unassigned	Unassigned	1074_0	Clustered	19	VC_1074_0	19	0.9198	1	0.9198	0	0	0	1	Ref
MK494108	Unassigned	Unassigned	Unassigned	225_0	Clustered	32	VC_225_0	32	0.848	1	0.848	0	0	0	0.996	Ref
MK494109	Unassigned	Unassigned	Unassigned	866_1	Clustered	20	VC_866_1	20	0.4814	1	0.4814	0	0	0	1	Ref
MK494110	Unassigned	Unassigned	Unassigned	520_45	Clustered	7	VC_520_45	7	0.0635	1	0.0635	0	0	0	0.8095	Ref
MK494111	Unassigned	Unassigned	Unassigned	950_0	Clustered	38	VC_950_0	38	0.9443	1	0.9443	0	0	0	1	Ref
MK494112	Unassigned	Unassigned	Unassigned	1071_3	Clustered	16	VC_1071_3	16	0.2354	1	0.2354	0	0	0	0.9667	Ref
MK494113	Unassigned	Unassigned	Unassigned	222_0	Clustered	5	VC_222_0	5	0.0565	0.99999997	0.0565	0	0	0	1	Ref
MK494114	Unassigned	Unassigned	Unassigned	507_0	Clustered	8	VC_507_0	8	0.4466	1	0.4466	0	0	0	1	Ref
MK494115	Unassigned	Unassigned	Unassigned	517_0	Clustered	18	VC_517_0	18	0.1599	1	0.1599	0	0	0	1	Ref
MK494116	Unassigned	Unassigned	Unassigned	325_30	Clustered	4	VC_325_30	4	0.0144	0.99998616	0.0144	0	0	0	0.8333	Ref
MK494117	Unassigned	Unassigned	Unassigned	517_0	Clustered	18	VC_517_0	18	0.1599	1	0.1599	0	0	0	1	Ref
MK494118	Unassigned	Unassigned	Unassigned	520_72	Clustered/Singleton	1	VC_520_72	1	0	1	0	0	0	0	1	Ref
MK494119	Unassigned	Unassigned	Unassigned	237_0	Clustered	4	VC_237_0	4	0.0429	0.99998759	0.0429	0	0	0	1	Ref
MK494121	Unassigned	Unassigned	Unassigned	866_1	Clustered	20	VC_866_1	20	0.4814	1	0.4814	0	0	0	1	Ref
MK494122	Unassigned	Unassigned	Unassigned	519_0	Clustered	39	VC_519_0	39	0.8156	1	0.8156	0	0	0	1	Ref
MK494123	Unassigned	Unassigned	Unassigned	939_0	Clustered	15	VC_939_0	15	1	0	0	0	0	0	1	Ref
MK494124	Unassigned	Unassigned	Unassigned	515_0	Clustered	16	VC_515_0	16	0.3793	1	0.3793	0	0	0	1	Ref
MK494125	Unassigned	Unassigned	Unassigned	517_1	Clustered	108	VC_517_1	108	0.8483	1	0.8483	0	0	0	0.9955	Ref
MK494126	Unassigned	Unassigned	Unassigned	517_1	Clustered	108	VC_517_1	108	0.8483	1	0.8483	0	0	0	0.9955	Ref
MK494127	Unassigned	Unassigned	Unassigned	513_0	Clustered	32	VC_513_0	32	0.6881	1	0.6881	0	0	0	1	Ref
MK494128	Unassigned	Unassigned	Unassigned	514_0	Clustered	75	VC_514_0	75	0.9402	1	0.9402	0	0	0	1	Ref
MK494129	Unassigned	Unassigned	Unassigned	517_0	Clustered	18	VC_517_0	18	0.1599	1	0.1599	0	0	0	1	Ref
MK494130	Unassigned	Unassigned	Unassigned	221_0	Clustered	22	VC_221_0	22	0.7447	1	0.7447	0	0	0	1	Ref
MK494131	Unassigned	Unassigned	Unassigned	507_0	Clustered	8	VC_507_0	8	0.4466	1	0.4466	0	0	0	1	Ref
MK494132	Unassigned	Unassigned	Unassigned	507_0	Clustered	8	VC_507_0	8	0.4466	1	0.4466	0	0	0	1	Ref

MK496679	Unassigned	Unassigned	Unassigned	1371_0	Clustered	5	VC_1371_0	5	0.7004	0.52188382	0.3656	0	0	0	1	Ref
MK496680	Unassigned	Unassigned	Unassigned	1371_0	Clustered	5	VC_1371_0	5	0.7004	0.52188382	0.3656	0	0	0	1	Ref
MK496681	Unassigned	Unassigned	Unassigned	1373_0	Clustered	2	VC_1373_0	2	0.6485	0.86822376	0.563	0	0	0	1	Ref
MK496682	Unassigned	Unassigned	Unassigned	1367_0	Clustered	2	VC_1367_0	2	1	1	1	0	0	0	1	Ref
MK496683	Unassigned	Unassigned	Unassigned	1374_0	Clustered	2	VC_1374_0	2	1	1	1	0	0	0	1	Ref
MK496685	Unassigned	Unassigned	Unassigned	1264_0	Clustered	6	VC_1264_0	6	0.7062	0.92055725	0.6501	0	0	0	1	Ref
MK496686	Unassigned	Unassigned	Unassigned	1375_0	Clustered	2	VC_1375_0	2	1	1	1	0	0	0	1	Ref
MK496688	Unassigned	Unassigned	Unassigned	1376_0	Clustered	3	VC_1376_0	3	1	1	1	0	0	0	1	Ref
MK496689	Unassigned	Unassigned	Unassigned	1377_0	Clustered	3	VC_1377_0	3	1	0	0	0	0	0	1	Ref
MK496690	Unassigned	Unassigned	Unassigned	1376_0	Clustered	3	VC_1376_0	3	1	1	1	0	0	0	1	Ref
MK496692	Unassigned	Unassigned	Unassigned	1326_0	Clustered	2	VC_1326_0	2	1	1	1	0	0	0	1	Ref
MK496693	Unassigned	Unassigned	Unassigned	1197_0	Clustered	15	VC_1197_0	15	0.8991	0.76608394	0.6888	0	0	0	1	Ref
MK496694	Unassigned	Unassigned	Unassigned	1243_0	Clustered	52	VC_1243_0	52	0.9126	0.92708311	0.8461	0	0	0	1	Ref
MK496696	Unassigned	Unassigned	Unassigned	1297_0	Clustered	7	VC_1297_0	7	1	1	1	0	0	0	1	Ref
MK496697	Unassigned	Unassigned	Unassigned	1378_0	Clustered	2	VC_1378_0	2	1	1	1	0	0	0	1	Ref
MK496698	Unassigned	Unassigned	Unassigned	1372_0	Clustered	2	VC_1372_0	2	0.1774	0.73645537	0.1307	0	0	0	1	Ref
MK496699	Unassigned	Unassigned	Unassigned	1379_0	Clustered	2	VC_1379_0	2	1	1	1	0	0	0	1	Ref
MK496700	Unassigned	Unassigned	Unassigned	1377_0	Clustered	3	VC_1377_0	3	1	0	0	0	0	0	1	Ref
MK496701	Unassigned	Unassigned	Unassigned	1380_0	Clustered	2	VC_1380_0	2	1	1	1	0	0	0	1	Ref
MK496702	Unassigned	Unassigned	Unassigned	1380_0	Clustered	2	VC_1380_0	2	1	1	1	0	0	0	1	Ref
MK496704	Unassigned	Unassigned	Unassigned	1296_0	Clustered	4	VC_1296_0	4	0.8684	0.91151676	0.7915	0	0	0	1	Ref
MK496710	Unassigned	Unassigned	Unassigned	1381_0	Clustered	3	VC_1381_0	3	1	1	1	0	0	0	1	Ref
MK496711	Unassigned	Unassigned	Unassigned	1297_0	Clustered	7	VC_1297_0	7	1	1	1	0	0	0	1	Ref
MK496712	Unassigned	Unassigned	Unassigned	1382_0	Clustered	2	VC_1382_0	2	1	1	1	0	0	0	1	Ref
MK496714	Unassigned	Unassigned	Unassigned	1134_0	Clustered	5	VC_1134_0	5	0.129	0.7637089	0.0985	0	0	0	1	Ref
MK496716	Unassigned	Unassigned	Unassigned	1383_0	Clustered	2	VC_1383_0	2	0.6415	0.72985431	0.4682	0	0	0	1	Ref
MK496717	Unassigned	Unassigned	Unassigned	1384_0	Clustered	2	VC_1384_0	2	1	1	1	0	0	0	1	Ref
MK496719	Unassigned	Unassigned	Unassigned	1112_0	Clustered	2	VC_1112_0	2	0.2212	0.72377355	0.1601	0	0	0	1	Ref
MK496720	Unassigned	Unassigned	Unassigned	1385_0	Clustered	2	VC_1385_0	2	1	1	1	0	0	0	1	Ref
MK496721	Unassigned	Unassigned	Unassigned	1386_0	Clustered	3	VC_1386_0	3	1	0	0	0	0	0	1	Ref
MK496723	Unassigned	Unassigned	Unassigned	1054_0	Clustered	12	VC_1054_0	12	0.2863	0.99746644	0.2855	0	0	0	1	Ref
MK496725	Unassigned	Unassigned	Unassigned	1387_0	Clustered	3	VC_1387_0	3	0.7373	0.96173875	0.709	0	0	0	1	Ref
MK496729	Unassigned	Unassigned	Unassigned	1296_0	Clustered	4	VC_1296_0	4	0.8684	0.91151676	0.7915	0	0	0	1	Ref
MK496732	Unassigned	Unassigned	Unassigned	1292_0	Clustered	3	VC_1292_0	3	1	1	1	0	0	0	1	Ref
MK496738	Unassigned	Unassigned	Unassigned	1112_0	Clustered	2	VC_1112_0	2	0.2212	0.72377355	0.1601	0	0	0	1	Ref
MK496743	Unassigned	Unassigned	Unassigned	1105_0	Clustered	3	VC_1105_0	3	0.0755	0.99466875	0.0751	0	0	0	1	Ref
MK496744	Unassigned	Unassigned	Unassigned	1105_0	Clustered	3	VC_1105_0	3	0.0755	0.99466875	0.0751	0	0	0	1	Ref
MK496745	Unassigned	Unassigned	Unassigned	1147_0	Clustered	3	VC_1147_0	3	0.4334	0.65335909	0.2832	0	0	0	1	Ref
MK496746	Unassigned	Unassigned	Unassigned	1387_0	Clustered	3	VC_1387_0	3	0.7373	0.96173875	0.709	0	0	0	1	Ref
MK496748	Unassigned	Unassigned	Unassigned	1388_0	Clustered	2	VC_1388_0	2	1	1	1	0	0	0	1	Ref
MK496750	Unassigned	Unassigned	Unassigned	1389_0	Clustered	2	VC_1389_0	2	0.6339	0.72985431	0.4627	0	0	0	1	Ref
MK496753	Unassigned	Unassigned	Unassigned	1390_0	Clustered	2	VC_1390_0	2	1	1	1	0	0	0	1	Ref
MK496754	Unassigned	Unassigned	Unassigned	1119_0	Clustered	20	VC_1119_0	20	0.6751	0.99997892	0.6751	0	0	0	1	Ref
MK496756	Unassigned	Unassigned	Unassigned	1391_0	Clustered	2	VC_1391_0	2	1	1	1	0	0	0	1	Ref
MK496757	Unassigned	Unassigned	Unassigned	1303_0	Clustered	6	VC_1303_0	6	1	1	1	0	0	0	1	Ref
MK496758	Unassigned	Unassigned	Unassigned	1392_0	Clustered	2	VC_1392_0	2	1	1	1	0	0	0	1	Ref
MK496760	Unassigned	Unassigned	Unassigned	1373_0	Clustered	2	VC_1373_0	2	0.6485	0.86822376	0.563	0	0	0	1	Ref
MK496761	Unassigned	Unassigned	Unassigned	1374_0	Clustered	2	VC_1374_0	2	1	1	1	0	0	0	1	Ref
MK496764	Unassigned	Unassigned	Unassigned	1375_0	Clustered	2	VC_1375_0	2	1	1	1	0	0	0	1	Ref
MK496765	Unassigned	Unassigned	Unassigned	1294_0	Clustered	6	VC_1294_0	6	0.6626	0.99999272	0.6626	0	0	0	1	Ref
MK496766	Unassigned	Unassigned	Unassigned	1376_0	Clustered	3	VC_1376_0	3	1	1	1	0	0	0	1	Ref
MK496767	Unassigned	Unassigned	Unassigned	1381_0	Clustered	3	VC_1381_0	3	1	1	1	0	0	0	1	Ref
MK496768	Unassigned	Unassigned	Unassigned	1381_0	Clustered	3	VC_1381_0	3	1	1	1	0	0	0	1	Ref
MK496770	Unassigned	Unassigned	Unassigned	1393_0	Clustered	2	VC_1393_0	2	1	1	1	0	0	0	1	Ref
MK496771	Unassigned	Unassigned	Unassigned	1393_0	Clustered	2	VC_1393_0	2	1	1	1	0	0	0	1	Ref
MK496772	Unassigned	Unassigned	Unassigned	1372_0	Clustered	2	VC_1372_0	2	0.1774	0.73645537	0.1307	0	0	0	1	Ref
MK496774	Unassigned	Unassigned	Unassigned	1197_0	Clustered	15	VC_1197_0	15	0.8991	0.76608394	0.6888	0	0	0	1	Ref
MK496775	Unassigned	Unassigned	Unassigned	1243_0	Clustered	52	VC_1243_0	52	0.9126	0.92708311	0.8461	0	0	0	1	Ref
MK496776	Unassigned	Unassigned	Unassigned	1371_0	Clustered	5	VC_1371_0	5	0.7004	0.52188382	0.3656	0	0	0	1	Ref
MK496779	Unassigned	Unassigned	Unassigned	1134_0	Clustered	5	VC_1134_0	5	0.129	0.7637089	0.0985	0	0	0	1	Ref
MK496780	Unassigned	Unassigned	Unassigned	1378_0	Clustered	2	VC_1378_0	2	1	1	1	0	0	0	1	Ref
MK496781	Unassigned	Unassigned	Unassigned	1386_0	Clustered	3	VC_1386_0	3	1	0	0	0	0	0	1	Ref
MK496782	Unassigned	Unassigned	Unassigned	1296_0	Clustered	4	VC_1296_0	4	0.8684	0.91151676	0.7915	0	0	0	1	Ref
MK496784	Unassigned	Unassigned	Unassigned	1377_0	Clustered	3	VC_1377_0	3	1	0	0	0	0	0	1	Ref
MK496785	Unassigned	Unassigned	Unassigned	1379_0	Clustered	2	VC_1379_0	2	1	1	1	0	0	0	1	Ref
MK496786	Unassigned	Unassigned	Unassigned	1386_0	Clustered	3	VC_1386_0	3	1	0	0	0	0	0	1	Ref
MK496787	Unassigned	Unassigned	Unassigned	1382_0	Clustered	2	VC_1382_0	2	1	1	1	0	0	0	1	Ref
MK496790	Unassigned	Unassigned	Unassigned	1383_0	Clustered	2	VC_1383_0	2	0.6415	0.72985431	0.4682	0	0	0	1	Ref
MK496792	Unassigned	Unassigned	Unassigned	1134_0	Clustered	5	VC_1134_0	5	0.129	0.7637089	0.0985	0	0	0	1	Ref
MK496796	Unassigned	Unassigned	Unassigned	1385_0	Clustered	2	VC_1385_0	2	1	1	1	0	0	0	1	Ref
MK496804	Unassigned	Unassigned	Unassigned	1384_0	Clustered	2	VC_1384_0	2	1	1	1	0	0	0	1	Ref
MK496805	Unassigned	Unassigned	Unassigned	1119_0	Clustered	20	VC_1119_0	20	0.6751	0.99997892	0.6751	0	0	0	1	Ref
MK496808	Unassigned	Unassigned	Unassigned	1392_0	Clustered	2	VC_1392_0	2	1	1	1	0	0	0	1	Ref
MK496811	Unassigned	Unassigned	Unassigned	1387_0	Clustered	3	VC_1387_0	3	0.7373	0.96173875	0.709	0	0	0	1	Ref
MK496812	Unassigned	Unassigned	Unassigned	1119_0	Clustered	20	VC_1119_0	20	0.6751	0.99997892	0.6751	0	0	0	1	Ref
MK496813	Unassigned	Unassigned	Unassigned	1119_0	Clustered	20	VC_1119_0	20	0.6751	0.99997892	0.6751	0	0	0	1	Ref
MK496815	Unassigned	Unassigned	Unassigned	1388_0	Clustered	2	VC_1388_0	2	1	1	1	0	0	0	1	Ref
MK496816	Unassigned	Unassigned	Unassigned	1389_0	Clustered	2	VC_1389_0	2	0.6339	0.72985431	0.4627	0	0	0	1	Ref
MK496818	Unassigned	Unassigned	Unassigned	1390_0	Clustered	2	VC_1390_0	2	1	1	1	0	0	0	1	Ref
MK496820	Unassigned	Unassigned	Unassigned	1394_0	Clustered	2	VC_1394_0	2	1	1	1	0	0	0	1	Ref
MK496824	Unassigned	Unassigned	Unassigned	1391_0	Clustered	2	VC_1391_0	2	1	1	1	0	0	0	1	Ref

MK496825	Unassigned	Unassigned	Unassigned	995_0	Clustered	69	VC_995_0	69	0.8547	1	0.8547	0	0	0	1	Ref
MK501729	Unassigned	Unassigned	Unassigned	1176_0	Clustered	41	VC_1176_0	41	0.9558	1	0.9558	0	0	0	0.9976	Ref
MK501730	Unassigned	Unassigned	Unassigned	1176_0	Clustered	41	VC_1176_0	41	0.9558	1	0.9558	0	0	0	0.9976	Ref
MK504442	Unassigned	Unassigned	Unassigned	578_0	Clustered	2	VC_578_0	2	0.1712	0.93973606	0.1608	0	0	0	1	Ref
MK504443	Unassigned	Unassigned	Unassigned	95_0	Clustered	3	VC_95_0	3	0.2092	0.99852577	0.2089	0	0	0	1	Ref
MK504444	Unassigned	Unassigned	Unassigned	526_3	Clustered/Singleton	1	VC_526_3	1	0	1	0	0	0	0	1	Ref
MK504446	Unassigned	Unassigned	Unassigned	95_0	Clustered	3	VC_95_0	3	0.2092	0.99852577	0.2089	0	0	0	1	Ref
MK509462	Unassigned	Unassigned	Unassigned	131_2	Clustered	7	VC_131_2	7	0.0685	1	0.0685	0	0	0	0.9048	Ref
MK510962	Unassigned	Unassigned	Unassigned	747_0	Clustered	23	VC_747_0	23	0.8202	1	0.8202	0	0	0	1	Ref
MK510963	Unassigned	Unassigned	Unassigned	747_0	Clustered	23	VC_747_0	23	0.8202	1	0.8202	0	0	0	1	Ref
MK510964	Unassigned	Unassigned	Unassigned	747_0	Clustered	23	VC_747_0	23	0.8202	1	0.8202	0	0	0	1	Ref
MK510965	Unassigned	Unassigned	Unassigned	747_0	Clustered	23	VC_747_0	23	0.8202	1	0.8202	0	0	0	1	Ref
MK510966	Unassigned	Unassigned	Unassigned	748_0	Clustered	12	VC_748_0	12	0.6879	1	0.6879	0	0	0	1	Ref
MK510967	Unassigned	Unassigned	Unassigned	748_0	Clustered	12	VC_748_0	12	0.6879	1	0.6879	0	0	0	1	Ref
MK510968	Unassigned	Unassigned	Unassigned	747_0	Clustered	23	VC_747_0	23	0.8202	1	0.8202	0	0	0	1	Ref
MK510969	Unassigned	Unassigned	Unassigned	747_0	Clustered	23	VC_747_0	23	0.8202	1	0.8202	0	0	0	1	Ref
MK510970	Unassigned	Unassigned	Unassigned	747_0	Clustered	23	VC_747_0	23	0.8202	1	0.8202	0	0	0	1	Ref
MK510971	Unassigned	Unassigned	Unassigned	747_0	Clustered	23	VC_747_0	23	0.8202	1	0.8202	0	0	0	1	Ref
MK510972	Unassigned	Unassigned	Unassigned	748_0	Clustered	12	VC_748_0	12	0.6879	1	0.6879	0	0	0	1	Ref
MK510973	Unassigned	Unassigned	Unassigned	748_0	Clustered	12	VC_748_0	12	0.6879	1	0.6879	0	0	0	1	Ref
MK510974	Unassigned	Unassigned	Unassigned	747_0	Clustered	23	VC_747_0	23	0.8202	1	0.8202	0	0	0	1	Ref
MK510975	Unassigned	Unassigned	Unassigned	747_0	Clustered	23	VC_747_0	23	0.8202	1	0.8202	0	0	0	1	Ref
MK510976	Unassigned	Unassigned	Unassigned	747_0	Clustered	23	VC_747_0	23	0.8202	1	0.8202	0	0	0	1	Ref
MK510977	Unassigned	Unassigned	Unassigned	748_0	Clustered	12	VC_748_0	12	0.6879	1	0.6879	0	0	0	1	Ref
MK510978	Unassigned	Unassigned	Unassigned	747_0	Clustered	23	VC_747_0	23	0.8202	1	0.8202	0	0	0	1	Ref
MK510979	Unassigned	Unassigned	Unassigned	748_0	Clustered	12	VC_748_0	12	0.6879	1	0.6879	0	0	0	1	Ref
MK510980	Unassigned	Unassigned	Unassigned	748_0	Clustered	12	VC_748_0	12	0.6879	1	0.6879	0	0	0	1	Ref
MK510981	Unassigned	Unassigned	Unassigned	748_0	Clustered	12	VC_748_0	12	0.6879	1	0.6879	0	0	0	1	Ref
MK510982	Unassigned	Unassigned	Unassigned	748_0	Clustered	12	VC_748_0	12	0.6879	1	0.6879	0	0	0	1	Ref
MK510983	Unassigned	Unassigned	Unassigned	748_0	Clustered	12	VC_748_0	12	0.6879	1	0.6879	0	0	0	1	Ref
MK510984	Unassigned	Unassigned	Unassigned	747_0	Clustered	23	VC_747_0	23	0.8202	1	0.8202	0	0	0	1	Ref
MK510985	Unassigned	Unassigned	Unassigned	747_0	Clustered	23	VC_747_0	23	0.8202	1	0.8202	0	0	0	1	Ref
MK510986	Unassigned	Unassigned	Unassigned	747_0	Clustered	23	VC_747_0	23	0.8202	1	0.8202	0	0	0	1	Ref
MK510987	Unassigned	Unassigned	Unassigned	747_0	Clustered	23	VC_747_0	23	0.8202	1	0.8202	0	0	0	1	Ref
MK510988	Unassigned	Unassigned	Unassigned	747_0	Clustered	23	VC_747_0	23	0.8202	1	0.8202	0	0	0	1	Ref
MK510989	Unassigned	Unassigned	Unassigned	748_0	Clustered	12	VC_748_0	12	0.6879	1	0.6879	0	0	0	1	Ref
MK510990	Unassigned	Unassigned	Unassigned	747_0	Clustered	23	VC_747_0	23	0.8202	1	0.8202	0	0	0	1	Ref
MK510991	Unassigned	Unassigned	Unassigned	748_0	Clustered	12	VC_748_0	12	0.6879	1	0.6879	0	0	0	1	Ref
MK510992	Unassigned	Unassigned	Unassigned	747_0	Clustered	23	VC_747_0	23	0.8202	1	0.8202	0	0	0	1	Ref
MK510993	Unassigned	Unassigned	Unassigned	747_0	Clustered	23	VC_747_0	23	0.8202	1	0.8202	0	0	0	1	Ref
MK511001	Unassigned	Unassigned	Unassigned	297_1	Clustered	20	VC_297_1	20	0.5653	1	0.5653	0	0	0	0.7684	Ref
MK511002	Unassigned	Unassigned	Unassigned	297_1	Clustered	20	VC_297_1	20	0.5653	1	0.5653	0	0	0	0.7684	Ref
MK511003	Unassigned	Unassigned	Unassigned	297_1	Clustered	20	VC_297_1	20	0.5653	1	0.5653	0	0	0	0.7684	Ref
MK511004	Unassigned	Unassigned	Unassigned	297_1	Clustered	20	VC_297_1	20	0.5653	1	0.5653	0	0	0	0.7684	Ref
MK511005	Unassigned	Unassigned	Unassigned	297_1	Clustered	20	VC_297_1	20	0.5653	1	0.5653	0	0	0	0.7684	Ref
MK511006	Unassigned	Unassigned	Unassigned	297_1	Clustered	20	VC_297_1	20	0.5653	1	0.5653	0	0	0	0.7684	Ref
MK511007	Unassigned	Unassigned	Unassigned	297_1	Clustered	20	VC_297_1	20	0.5653	1	0.5653	0	0	0	0.7684	Ref
MK511008	Unassigned	Unassigned	Unassigned	297_1	Clustered	20	VC_297_1	20	0.5653	1	0.5653	0	0	0	0.7684	Ref
MK511009	Unassigned	Unassigned	Unassigned	297_1	Clustered	20	VC_297_1	20	0.5653	1	0.5653	0	0	0	0.7684	Ref
MK511010	Unassigned	Unassigned	Unassigned	297_1	Clustered	20	VC_297_1	20	0.5653	1	0.5653	0	0	0	0.7684	Ref
MK511011	Unassigned	Unassigned	Unassigned	297_1	Clustered	20	VC_297_1	20	0.5653	1	0.5653	0	0	0	0.7684	Ref
MK511012	Unassigned	Unassigned	Unassigned	297_1	Clustered	20	VC_297_1	20	0.5653	1	0.5653	0	0	0	0.7684	Ref
MK511013	Unassigned	Unassigned	Unassigned	297_1	Clustered	20	VC_297_1	20	0.5653	1	0.5653	0	0	0	0.7684	Ref
MK511014	Unassigned	Unassigned	Unassigned	297_1	Clustered	20	VC_297_1	20	0.5653	1	0.5653	0	0	0	0.7684	Ref
MK511015	Unassigned	Unassigned	Unassigned	297_1	Clustered	20	VC_297_1	20	0.5653	1	0.5653	0	0	0	0.7684	Ref
MK511016	Unassigned	Unassigned	Unassigned	297_1	Clustered	20	VC_297_1	20	0.5653	1	0.5653	0	0	0	0.7684	Ref
MK511017	Unassigned	Unassigned	Unassigned	179_0	Clustered	53	VC_179_0	53	0.9137	1	0.9137	0	0	0	1	Ref
MK511018	Unassigned	Unassigned	Unassigned	179_0	Clustered	53	VC_179_0	53	0.9137	1	0.9137	0	0	0	1	Ref
MK511019	Unassigned	Unassigned	Unassigned	179_0	Clustered	53	VC_179_0	53	0.9137	1	0.9137	0	0	0	1	Ref
MK511020	Unassigned	Unassigned	Unassigned	179_0	Clustered	53	VC_179_0	53	0.9137	1	0.9137	0	0	0	1	Ref
MK511021	Unassigned	Unassigned	Unassigned	179_0	Clustered	53	VC_179_0	53	0.9137	1	0.9137	0	0	0	1	Ref
MK511022	Unassigned	Unassigned	Unassigned	179_0	Clustered	53	VC_179_0	53	0.9137	1	0.9137	0	0	0	1	Ref
MK511023	Unassigned	Unassigned	Unassigned	179_0	Clustered	53	VC_179_0	53	0.9137	1	0.9137	0	0	0	1	Ref
MK511024	Unassigned	Unassigned	Unassigned	179_0	Clustered	53	VC_179_0	53	0.9137	1	0.9137	0	0	0	1	Ref
MK511025	Unassigned	Unassigned	Unassigned	179_0	Clustered	53	VC_179_0	53	0.9137	1	0.9137	0	0	0	1	Ref
MK511026	Unassigned	Unassigned	Unassigned	179_0	Clustered	53	VC_179_0	53	0.9137	1	0.9137	0	0	0	1	Ref
MK511027	Unassigned	Unassigned	Unassigned	179_0	Clustered	53	VC_179_0	53	0.9137	1	0.9137	0	0	0	1	Ref
MK511028	Unassigned	Unassigned	Unassigned	179_0	Clustered	53	VC_179_0	53	0.9137	1	0.9137	0	0	0	1	Ref
MK511029	Unassigned	Unassigned	Unassigned	179_0	Clustered	53	VC_179_0	53	0.9137	1	0.9137	0	0	0	1	Ref
MK511030	Unassigned	Unassigned	Unassigned	179_0	Clustered	53	VC_179_0	53	0.9137	1	0.9137	0	0	0	1	Ref
MK511031	Unassigned	Unassigned	Unassigned	179_0	Clustered	53	VC_179_0	53	0.9137	1	0.9137	0	0	0	1	Ref
MK511032	Unassigned	Unassigned	Unassigned	179_0	Clustered	53	VC_179_0	53	0.9137	1	0.9137	0	0	0	1	Ref
MK511033	Unassigned	Unassigned	Unassigned	179_0	Clustered	53	VC_179_0	53	0.9137	1	0.9137	0	0	0	1	Ref
MK511034	Unassigned	Unassigned	Unassigned	179_0	Clustered	53	VC_179_0	53	0.9137	1	0.9137	0	0	0	1	Ref
MK511035	Unassigned	Unassigned	Unassigned	179_0	Clustered	53	VC_179_0	53	0.9137	1	0.9137	0	0	0	1	Ref
MK511036	Unassigned	Unassigned	Unassigned	179_0	Clustered	53	VC_179_0	53	0.9137	1	0.9137	0	0	0	1	Ref
MK511037	Unassigned	Unassigned	Unassigned	179_0	Clustered	53	VC_179_0	53	0.9137	1	0.9137	0	0	0	1	Ref
MK511038	Unassigned	Unassigned	Unassigned	179_0	Clustered	53	VC_179_0	53	0.9137	1	0.9137	0	0	0	1	Ref
MK511039	Unassigned	Unassigned	Unassigned	630_0	Clustered	20	VC_630_0	20	0.8513	1	0.8513	0	0	0	1	Ref
MK511040	Unassigned	Unassigned	Unassigned	630_0	Clustered	20	VC_630_0	20	0.8513	1	0.8513	0	0	0	1	Ref
MK511041	Unassigned	Unassigned	Unassigned	630_0	Clustered	20	VC_630_0	20	0.8513	1	0.8513	0	0	0	1	Ref

MK511042	Unassigned	Unassigned	Unassigned	630_0	Clustered	20	VC_630_0	20	0.8513	1	0.8513	0	0	0	1	Ref
MK511043	Unassigned	Unassigned	Unassigned	630_0	Clustered	20	VC_630_0	20	0.8513	1	0.8513	0	0	0	1	Ref
MK511044	Unassigned	Unassigned	Unassigned	630_0	Clustered	20	VC_630_0	20	0.8513	1	0.8513	0	0	0	1	Ref
MK511045	Unassigned	Unassigned	Unassigned	630_0	Clustered	20	VC_630_0	20	0.8513	1	0.8513	0	0	0	1	Ref
MK511046	Unassigned	Unassigned	Unassigned	630_0	Clustered	20	VC_630_0	20	0.8513	1	0.8513	0	0	0	1	Ref
MK511047	Unassigned	Unassigned	Unassigned	630_0	Clustered	20	VC_630_0	20	0.8513	1	0.8513	0	0	0	1	Ref
MK511048	Unassigned	Unassigned	Unassigned	630_0	Clustered	20	VC_630_0	20	0.8513	1	0.8513	0	0	0	1	Ref
MK511049	Unassigned	Unassigned	Unassigned	630_0	Clustered	20	VC_630_0	20	0.8513	1	0.8513	0	0	0	1	Ref
MK511050	Unassigned	Unassigned	Unassigned	630_0	Clustered	20	VC_630_0	20	0.8513	1	0.8513	0	0	0	1	Ref
MK511051	Unassigned	Unassigned	Unassigned	630_0	Clustered	20	VC_630_0	20	0.8513	1	0.8513	0	0	0	1	Ref
MK511057	Unassigned	Unassigned	Unassigned	251_0	Clustered	22	VC_251_0	22	0.6508	1	0.6508	0	0	0	1	Ref
MK511058	Unassigned	Unassigned	Unassigned	251_0	Clustered	22	VC_251_0	22	0.6508	1	0.6508	0	0	0	1	Ref
MK511059	Unassigned	Unassigned	Unassigned	251_0	Clustered	22	VC_251_0	22	0.6508	1	0.6508	0	0	0	1	Ref
MK511060	Unassigned	Unassigned	Unassigned	251_0	Clustered	22	VC_251_0	22	0.6508	1	0.6508	0	0	0	1	Ref
MK511061	Unassigned	Unassigned	Unassigned	251_0	Clustered	22	VC_251_0	22	0.6508	1	0.6508	0	0	0	1	Ref
MK511063	Unassigned	Unassigned	Unassigned	251_0	Clustered	22	VC_251_0	22	0.6508	1	0.6508	0	0	0	1	Ref
MK511064	Unassigned	Unassigned	Unassigned	251_0	Clustered	22	VC_251_0	22	0.6508	1	0.6508	0	0	0	1	Ref
MK511065	Unassigned	Unassigned	Unassigned	251_0	Clustered	22	VC_251_0	22	0.6508	1	0.6508	0	0	0	1	Ref
MK511066	Unassigned	Unassigned	Unassigned	2_0	Clustered	4	VC_2_0	4	0.1311	0.99998692	0.1311	0	0	0	1	Ref
MK512531	Unassigned	Unassigned	Unassigned	178_0	Clustered	5	VC_178_0	5	0.7741	0.99998991	0.7741	0	0	0	1	Ref
MK514281	Unassigned	Unassigned	Unassigned	489_1	Clustered	11	VC_489_1	11	0.4621	1	0.4621	0	0	0	1	Ref
MK514282	Unassigned	Unassigned	Unassigned	959_0	Clustered	5	VC_959_0	5	0.0499	0.99999231	0.0499	0	0	0	1	Ref
MK514283	Unassigned	Unassigned	Unassigned	612_0	Clustered	9	VC_612_0	9	0.9545	1	0.9545	0	0	0	1	Ref
MK521904	Unassigned	Unassigned	Unassigned	938_0	Clustered	99	VC_938_0	99	0.9119	1	0.9119	0	0	0	0.9928	Ref
MK521905	Unassigned	Unassigned	Unassigned	823_0	Clustered	124	VC_823_0	124	0.9492	1	0.9492	0	0	0	1	Ref
MK521906	Unassigned	Unassigned	Unassigned	123_0	Clustered	23	VC_123_0	23	0.817	1	0.817	0	0	0	1	Ref
MK521907	Unassigned	Unassigned	Unassigned	192_0	Clustered	15	VC_192_0	15	0.5071	1	0.5071	0	0	0	1	Ref
MK524176	Unassigned	Unassigned	Unassigned	329_18	Clustered	7	VC_329_18	7	0.0328	1	0.0328	0	0	0	0.8095	Ref
MK524177	Unassigned	Unassigned	Unassigned	230_0	Clustered	3	VC_230_0	3	0.0739	0.9934951	0.0734	0	0	0	0.6667	Ref
MK524178	Unassigned	Unassigned	Unassigned	286_11	Clustered	3	VC_286_11	3	0.0146	0.93460284	0.0137	0	0	0	1	Ref
MK524485	Unassigned	Unassigned	Unassigned	234_0	Clustered	21	VC_234_0	21	0.8355	1	0.8355	0	0	0	1	Ref
MK524486	Unassigned	Unassigned	Unassigned	372_0	Clustered	24	VC_372_0	24	0.5539	1	0.5539	0	0	0	1	Ref
MK524487	Unassigned	Unassigned	Unassigned	956_0	Clustered	39	VC_956_0	39	1	1	1	0	0	0	1	Ref
MK524488	Unassigned	Unassigned	Unassigned	234_0	Clustered	21	VC_234_0	21	0.8355	1	0.8355	0	0	0	1	Ref
MK524489	Unassigned	Unassigned	Unassigned	225_0	Clustered	32	VC_225_0	32	0.848	1	0.848	0	0	0	0.996	Ref
MK524490	Unassigned	Unassigned	Unassigned	372_1	Clustered	9	VC_372_1	9	0.194	1	0.194	0	0	0	1	Ref
MK524491	Unassigned	Unassigned	Unassigned	216_6	Clustered	42	VC_216_6	42	0.473	1	0.473	0	0	0	0.8351	Ref
MK524493	Unassigned	Unassigned	Unassigned	761_0	Clustered	67	VC_761_0	67	0.8947	1	0.8947	0	0	0	1	Ref
MK524494	Unassigned	Unassigned	Unassigned	761_0	Clustered	67	VC_761_0	67	0.8947	1	0.8947	0	0	0	1	Ref
MK524495	Unassigned	Unassigned	Unassigned	956_0	Clustered	39	VC_956_0	39	1	1	1	0	0	0	1	Ref
MK524496	Unassigned	Unassigned	Unassigned	513_0	Clustered	32	VC_513_0	32	0.6881	1	0.6881	0	0	0	1	Ref
MK524497	Unassigned	Unassigned	Unassigned	325_21	Clustered	5	VC_325_21	5	0.0209	0.99999976	0.0209	0	0	0	0.8	Ref
MK524498	Unassigned	Unassigned	Unassigned	565_0	Clustered	20	VC_565_0	20	0.8398	1	0.8398	0	0	0	1	Ref
MK524499	Unassigned	Unassigned	Unassigned	325_22	Clustered/Singleton	1	VC_325_22	1	0	1	0	0	0	0	1	Ref
MK524500	Unassigned	Unassigned	Unassigned	521_0	Clustered	37	VC_521_0	37	0.912	1	0.912	0	0	0	1	Ref
MK524501	Unassigned	Unassigned	Unassigned	1181_0	Clustered	2	VC_1181_0	2	0.0461	0.95126432	0.0439	0	0	0	1	Ref
MK524502	Unassigned	Unassigned	Unassigned	1344_0	Clustered	78	VC_1344_0	78	0.9732	1	0.9732	0	0	0	1	Ref
MK524503	Unassigned	Unassigned	Unassigned	520_87	Clustered/Singleton	1	VC_520_87	1	0	1	0	0	0	0	1	Ref
MK524504	Unassigned	Unassigned	Unassigned	546_4	Clustered	16	VC_546_4	16	0.2517	0.9999998	0.2517	0	0	0	0.9417	Ref
MK524505	Unassigned	Unassigned	Unassigned	1184_0	Clustered	5	VC_1184_0	5	0.6591	0.99999982	0.6591	0	0	0	1	Ref
MK524506	Unassigned	Unassigned	Unassigned	325_35	Clustered	17	VC_325_35	17	0.0732	1	0.0732	0	0	0	0.7721	Ref
MK524507	Unassigned	Unassigned	Unassigned	221_0	Clustered	22	VC_221_0	22	0.7447	1	0.7447	0	0	0	1	Ref
MK524508	Unassigned	Unassigned	Unassigned	519_0	Clustered	39	VC_519_0	39	0.8156	1	0.8156	0	0	0	1	Ref
MK524509	Unassigned	Unassigned	Unassigned	521_0	Clustered	37	VC_521_0	37	0.912	1	0.912	0	0	0	1	Ref
MK524510	Unassigned	Unassigned	Unassigned	1352_0	Clustered	31	VC_1352_0	31	0.849	1	0.849	0	0	0	1	Ref
MK524511	Unassigned	Unassigned	Unassigned	221_0	Clustered	22	VC_221_0	22	0.7447	1	0.7447	0	0	0	1	Ref
MK524512	Unassigned	Unassigned	Unassigned	371_1	Clustered	11	VC_371_1	11	0.0379	1	0.0379	0	0	0	0.8545	Ref
MK524513	Unassigned	Unassigned	Unassigned	221_0	Clustered	22	VC_221_0	22	0.7447	1	0.7447	0	0	0	1	Ref
MK524514	Unassigned	Unassigned	Unassigned	221_0	Clustered	22	VC_221_0	22	0.7447	1	0.7447	0	0	0	1	Ref
MK524515	Unassigned	Unassigned	Unassigned	521_0	Clustered	37	VC_521_0	37	0.912	1	0.912	0	0	0	1	Ref
MK524516	Unassigned	Unassigned	Unassigned	546_9	Clustered/Singleton	1	VC_546_9	1	0	1	0	0	0	0	1	Ref
MK524517	Unassigned	Unassigned	Unassigned	520_38	Clustered	6	VC_520_38	6	0.0519	1	0.0519	0	0	0	0.8667	Ref
MK524518	Unassigned	Unassigned	Unassigned	521_0	Clustered	37	VC_521_0	37	0.912	1	0.912	0	0	0	1	Ref
MK524519	Unassigned	Unassigned	Unassigned	221_0	Clustered	22	VC_221_0	22	0.7447	1	0.7447	0	0	0	1	Ref
MK524520	Unassigned	Unassigned	Unassigned	521_0	Clustered	37	VC_521_0	37	0.912	1	0.912	0	0	0	1	Ref
MK524521	Unassigned	Unassigned	Unassigned	546_4	Clustered	16	VC_546_4	16	0.2517	0.9999998	0.2517	0	0	0	0.9417	Ref
MK524522	Unassigned	Unassigned	Unassigned	521_0	Clustered	37	VC_521_0	37	0.912	1	0.912	0	0	0	1	Ref
MK524523	Unassigned	Unassigned	Unassigned	325_16	Clustered	2	VC_325_16	2	0.0095	0.95751393	0.0091	0	0	0	1	Ref
MK524524	Unassigned	Unassigned	Unassigned	981_1	Clustered	26	VC_981_1	26	0.7009	1	0.7009	0	0	0	1	Ref
MK524525	Unassigned	Unassigned	Unassigned	325_45	Clustered/Singleton	1	VC_325_45	1	0	1	0	0	0	0	1	Ref
MK524526	Unassigned	Unassigned	Unassigned	371_1	Clustered	11	VC_371_1	11	0.0379	1	0.0379	0	0	0	0.8545	Ref
MK524527	Unassigned	Unassigned	Unassigned	546_4	Clustered	16	VC_546_4	16	0.2517	0.9999998	0.2517	0	0	0	0.9417	Ref
MK524528	Unassigned	Unassigned	Unassigned	521_0	Clustered	37	VC_521_0	37	0.912	1	0.912	0	0	0	1	Ref
MK524529	Unassigned	Unassigned	Unassigned	546_4	Clustered	16	VC_546_4	16	0.2517	0.9999998	0.2517	0	0	0	0.9417	Ref
MK524530	Unassigned	Unassigned	Unassigned	222_0	Clustered	5	VC_222_0	5	0.0565	0.99999997	0.0565	0	0	0	1	Ref
MK524531	Unassigned	Unassigned	Unassigned	325_35	Clustered	17	VC_325_35	17	0.0732	1	0.0732	0	0	0	0.7721	Ref
MK542015	Unassigned	Unassigned	Unassigned	638_0	Clustered	45	VC_638_0	45	0.7741	1	0.7741	0	0	0	1	Ref
MK542821	Unassigned	Unassigned	Unassigned	392_0	Clustered	126	VC_392_0	126	0.9621	1	0.9621	0	0	0	1	Ref
MK552140	Unassigned	Unassigned	Unassigned	66_0	Clustered	2	VC_66_0	2	0.1995	0.95487418	0.1905	0	0	0	1	Ref
MK552141	Unassigned	Unassigned	Unassigned	66_0	Clustered	2	VC_66_0	2	0.1995	0.95487418	0.1905	0	0	0	1	Ref
MK552327	Unassigned	Unassigned	Unassigned	344_3	Clustered/Singleton	1	VC_344_3	1	0	1	0	0	0	0	1	Ref

MK559427	Unassigned	Unassigned	Unassigned	565_0	Clustered	20	VC_565_0	20	0.8398	1	0.8398	0	0	0	1	Ref
MK559428	Unassigned	Unassigned	Unassigned	225_0	Clustered	32	VC_225_0	32	0.848	1	0.848	0	0	0	0.996	Ref
MK559429	Unassigned	Unassigned	Unassigned	551_6	Clustered	22	VC_551_6	22	0.1214	1	0.1214	0	0	0	0.8095	Ref
MK559459	Unassigned	Unassigned	Unassigned	904_0	Clustered	6	VC_904_0	6	0.233	1	0.233	0	0	0	1	Ref
MK562502	Unassigned	Unassigned	Unassigned	330_0	Clustered	27	VC_330_0	27	0.5088	1	0.5088	0	0	0	0.8661	Ref
MK562503	Unassigned	Unassigned	Unassigned	111_0	Clustered	91	VC_111_0	91	0.9587	1	0.9587	0	0	0	1	Ref
MK562504	Unassigned	Unassigned	Unassigned	330_0	Clustered	27	VC_330_0	27	0.5088	1	0.5088	0	0	0	0.8661	Ref
MK562505	Unassigned	Unassigned	Unassigned	330_0	Clustered	27	VC_330_0	27	0.5088	1	0.5088	0	0	0	0.8661	Ref
MK568062	Unassigned	Unassigned	Unassigned	428_0	Clustered	33	VC_428_0	33	0.9915	1	0.9915	0	0	0	1	Ref
MK568540	Unassigned	Unassigned	Unassigned	531_0	Clustered	9	VC_531_0	9	0.26	1	0.26	0	0	0	1	Ref
MK570053	Unassigned	Unassigned	Unassigned	1395_0	Clustered	3	VC_1395_0	3	0.7761	0.9639009	0.7481	0	0	0	1	Ref
MK570054	Unassigned	Unassigned	Unassigned	1395_0	Clustered	3	VC_1395_0	3	0.7761	0.9639009	0.7481	0	0	0	1	Ref
MK570055	Unassigned	Unassigned	Unassigned	1395_0	Clustered	3	VC_1395_0	3	0.7761	0.9639009	0.7481	0	0	0	1	Ref
MK570225	Unassigned	Unassigned	Unassigned	108_0	Clustered	33	VC_108_0	33	0.949	1	0.949	0	0	0	1	Ref
MK573636	Unassigned	Unassigned	Unassigned	590_0	Clustered	27	VC_590_0	27	0.9947	1	0.9947	0	0	0	1	Ref
MK574011	Unassigned	Unassigned	Unassigned	117_0	Clustered	9	VC_117_0	9	0.9977	0.98988637	0.9876	0	0	0	1	Ref
MK574078	Unassigned	Unassigned	Unassigned	749_0	Clustered	40	VC_749_0	40	0.7517	1	0.7517	0	0	0	1	Ref
MK577502	Unassigned	Unassigned	Unassigned	600_0	Clustered	3	VC_600_0	3	0.0541	0.97110069	0.0526	0	0	0	1	Ref
MK578530	Unassigned	Unassigned	Unassigned	637_1	Clustered	27	VC_637_1	27	0.2578	1	0.2578	0	0	0	0.9858	Ref
MK580972	Unassigned	Unassigned	Unassigned	506_0	Clustered	8	VC_506_0	8	0.7376	1	0.7376	0	0	0	1	Ref
MK584893	Unassigned	Unassigned	Unassigned	149_8	Clustered	66	VC_149_8	66	0.4565	1	0.4565	0	0	0	0.8182	Ref
MK598851	Unassigned	Unassigned	Unassigned	111_0	Clustered	91	VC_111_0	91	0.9587	1	0.9587	0	0	0	1	Ref
MK599315	Unassigned	Unassigned	Unassigned	344_1	Clustered	3	VC_344_1	3	0.0359	0.93348877	0.0335	0	0	0	1	Ref
MK599416	Unassigned	Unassigned	Unassigned	461_0	Clustered	96	VC_461_0	96	0.9307	1	0.9307	0	0	0	0.9575	Ref
MK605242	Unassigned	Unassigned	Unassigned	1173_0	Clustered	6	VC_1173_0	6	0.9951	0.99999296	0.9951	0	0	0	1	Ref
MK605243	Unassigned	Unassigned	Unassigned	1173_0	Clustered	6	VC_1173_0	6	0.9951	0.99999296	0.9951	0	0	0	1	Ref
MK605244	Unassigned	Unassigned	Unassigned	1173_0	Clustered	6	VC_1173_0	6	0.9951	0.99999296	0.9951	0	0	0	1	Ref
MK605245	Unassigned	Unassigned	Unassigned	1173_0	Clustered	6	VC_1173_0	6	0.9951	0.99999296	0.9951	0	0	0	1	Ref
MK605246	Unassigned	Unassigned	Unassigned	1173_0	Clustered	6	VC_1173_0	6	0.9951	0.99999296	0.9951	0	0	0	1	Ref
MK608335	Unassigned	Unassigned	Unassigned	385_0	Clustered	51	VC_385_0	51	0.8754	1	0.8754	0	0	0	1	Ref
MK608336	Unassigned	Unassigned	Unassigned	966_0	Clustered	3	VC_966_0	3	0.3549	0.99840333	0.3543	0	0	0	1	Ref
MK610268	Unassigned	Unassigned	Unassigned	451_0	Clustered	80	VC_451_0	80	0.9991	1	0.9991	0	0	0	1	Ref
MK613343	Unassigned	Unassigned	Unassigned	305_0	Clustered	12	VC_305_0	12	0.5745	1	0.5745	0	0	0	0.8485	Ref
MK613344	Unassigned	Unassigned	Unassigned	305_0	Clustered	12	VC_305_0	12	0.5745	1	0.5745	0	0	0	0.8485	Ref
MK613345	Unassigned	Unassigned	Unassigned	305_0	Clustered	12	VC_305_0	12	0.5745	1	0.5745	0	0	0	0.8485	Ref
MK613346	Unassigned	Unassigned	Unassigned	304_0	Clustered	11	VC_304_0	11	0.7869	1	0.7869	0	0	0	1	Ref
MK618657	Unassigned	Unassigned	Unassigned	938_0	Clustered	99	VC_938_0	99	0.9119	1	0.9119	0	0	0	0.9928	Ref
MK618658	Unassigned	Unassigned	Unassigned	823_0	Clustered	124	VC_823_0	124	0.9492	1	0.9492	0	0	0	1	Ref
MK618715	Unassigned	Unassigned	Unassigned	359_0	Clustered	7	VC_359_0	7	0.3703	1	0.3703	0	0	0	1	Ref
MK618716	Unassigned	Unassigned	Unassigned	12_0	Clustered	21	VC_12_0	21	0.2274	1	0.2274	0	0	0	0.9571	Ref
MK620893	Unassigned	Unassigned	Unassigned	551_7	Clustered	28	VC_551_7	28	0.1661	1	0.1661	0	0	0	0.8519	Ref
MK620894	Unassigned	Unassigned	Unassigned	1158_0	Clustered	3	VC_1158_0	3	0.3179	0.99335558	0.3158	0	0	0	1	Ref
MK620895	Unassigned	Unassigned	Unassigned	1240_0	Clustered	82	VC_1240_0	82	0.7945	1	0.7945	0	0	0	1	Ref
MK620896	Unassigned	Unassigned	Unassigned	1072_0	Clustered	2	VC_1072_0	2	0.0264	0.95117401	0.0251	0	0	0	1	Ref
MK620897	Unassigned	Unassigned	Unassigned	1344_0	Clustered	78	VC_1344_0	78	0.9732	1	0.9732	0	0	0	1	Ref
MK620898	Unassigned	Unassigned	Unassigned	508_0	Clustered	32	VC_508_0	32	0.906	1	0.906	0	0	0	1	Ref
MK620900	Unassigned	Unassigned	Unassigned	1369_0	Clustered	3	VC_1369_0	3	0.9809	0.9595722	0.9412	0	0	0	1	Ref
MK620901	Unassigned	Unassigned	Unassigned	1163_0	Clustered	28	VC_1163_0	28	0.4923	1	0.4923	0	0	0	1	Ref
MK620902	Unassigned	Unassigned	Unassigned	1240_0	Clustered	82	VC_1240_0	82	0.7945	1	0.7945	0	0	0	1	Ref
MK620903	Unassigned	Unassigned	Unassigned	1240_0	Clustered	82	VC_1240_0	82	0.7945	1	0.7945	0	0	0	1	Ref
MK620904	Unassigned	Unassigned	Unassigned	1163_0	Clustered	28	VC_1163_0	28	0.4923	1	0.4923	0	0	0	1	Ref
MK620905	Unassigned	Unassigned	Unassigned	1240_0	Clustered	82	VC_1240_0	82	0.7945	1	0.7945	0	0	0	1	Ref
MK629527	Unassigned	Unassigned	Unassigned	743_0	Clustered	15	VC_743_0	15	1	1	1	0	0	0	1	Ref
MK629528	Unassigned	Unassigned	Unassigned	156_0	Clustered	12	VC_156_0	12	0.9062	1	0.9062	0	0	0	1	Ref
MK630230	Unassigned	Unassigned	Unassigned	192_0	Clustered	15	VC_192_0	15	0.5071	1	0.5071	0	0	0	1	Ref
MK637516	Unassigned	Unassigned	Unassigned	969_0	Clustered	3	VC_969_0	3	0.8314	0.99762851	0.8295	0	0	0	1	Ref
MK639187	Unassigned	Unassigned	Unassigned	286_13	Clustered	10	VC_286_13	10	0.0698	0.99999929	0.0698	0	0	0	0.9556	Ref
MK651787	Unassigned	Unassigned	Unassigned	705_0	Clustered	11	VC_705_0	11	0.8861	1	0.8861	0	0	0	1	Ref
MK656892	Unassigned	Unassigned	Unassigned	149_8	Clustered	66	VC_149_8	66	0.4565	1	0.4565	0	0	0	0.8182	Ref
MK658834	Unassigned	Unassigned	Unassigned	9_0	Clustered	38	VC_9_0	38	0.4895	1	0.4895	0	0	0	1	Ref
MK660712	Unassigned	Unassigned	Unassigned	949_0	Clustered	10	VC_949_0	10	0.5977	1	0.5977	0	0	0	1	Ref
MK660714	Unassigned	Unassigned	Unassigned	1160_0	Clustered	73	VC_1160_0	73	0.9835	1	0.9835	0	0	0	1	Ref
MK672798	Unassigned	Unassigned	Unassigned	795_0	Clustered	19	VC_795_0	19	0.6848	1	0.6848	0	0	0	1	Ref
MK672799	Unassigned	Unassigned	Unassigned	299_0	Clustered	24	VC_299_0	24	0.9905	1	0.9905	0	0	0	1	Ref
MK672800	Unassigned	Unassigned	Unassigned	299_0	Clustered	24	VC_299_0	24	0.9905	1	0.9905	0	0	0	1	Ref
MK672801	Unassigned	Unassigned	Unassigned	299_0	Clustered	24	VC_299_0	24	0.9905	1	0.9905	0	0	0	1	Ref
MK672803	Unassigned	Unassigned	Unassigned	299_0	Clustered	24	VC_299_0	24	0.9905	1	0.9905	0	0	0	1	Ref
MK672804	Unassigned	Unassigned	Unassigned	299_0	Clustered	24	VC_299_0	24	0.9905	1	0.9905	0	0	0	1	Ref
MK673511	Unassigned	Unassigned	Unassigned	428_0	Clustered	33	VC_428_0	33	0.9915	1	0.9915	0	0	0	1	Ref
MK685667	Unassigned	Unassigned	Unassigned	123_0	Clustered	23	VC_123_0	23	0.817	1	0.817	0	0	0	1	Ref
MK685668	Unassigned	Unassigned	Unassigned	386_0	Clustered	49	VC_386_0	49	0.7941	1	0.7941	0	0	0	1	Ref
MK686068	Unassigned	Unassigned	Unassigned	373_0	Clustered	10	VC_373_0	10	0.7471	1	0.7471	0	0	0	1	Ref
MK686069	Unassigned	Unassigned	Unassigned	373_0	Clustered	10	VC_373_0	10	0.7471	1	0.7471	0	0	0	1	Ref
MK686071	Unassigned	Unassigned	Unassigned	975_0	Clustered	11	VC_975_0	11	0.9687	1	0.9687	0	0	0	1	Ref
MK689364	Unassigned	Unassigned	Unassigned	442_2	Clustered	4	VC_442_2	4	0.0844	0.99988129	0.0844	0	0	0	0.8333	Ref
MK693005	Unassigned	Unassigned	Unassigned	111_0	Clustered	91	VC_111_0	91	0.9587	1	0.9587	0	0	0	1	Ref
MK693030	Unassigned	Unassigned	Unassigned	91_0	Clustered	22	VC_91_0	22	0.5318	1	0.5318	0	0	0	1	Ref
MK714353	Unassigned	Unassigned	Unassigned	31_0	Clustered	9	VC_31_0	9	0.3435	1	0.3435	0	0	0	0.6944	Ref
MK719701	Unassigned	Unassigned	Unassigned	1396_0	Clustered	110	VC_1396_0	110	1	1	1	0	0	0	0.9746	Ref
MK719702	Unassigned	Unassigned	Unassigned	1396_0	Clustered	110	VC_1396_0	110	1	1	1	0	0	0	0.9746	Ref
MK719703	Unassigned	Unassigned	Unassigned	1396_0	Clustered	110	VC_1396_0	110	1	1	1	0	0	0	0.9746	Ref

MK719704	Unassigned	Unassigned	Unassigned	1396_0	Clustered	110	VC_1396_0	110	1	1	1	0	0	0	0.9746	Ref
MK719705	Unassigned	Unassigned	Unassigned	1396_0	Clustered	110	VC_1396_0	110	1	1	1	0	0	0	0.9746	Ref
MK719706	Unassigned	Unassigned	Unassigned	1396_0	Clustered	110	VC_1396_0	110	1	1	1	0	0	0	0.9746	Ref
MK719707	Unassigned	Unassigned	Unassigned	1396_0	Clustered	110	VC_1396_0	110	1	1	1	0	0	0	0.9746	Ref
MK719708	Unassigned	Unassigned	Unassigned	1396_0	Clustered	110	VC_1396_0	110	1	1	1	0	0	0	0.9746	Ref
MK719709	Unassigned	Unassigned	Unassigned	1396_0	Clustered	110	VC_1396_0	110	1	1	1	0	0	0	0.9746	Ref
MK719710	Unassigned	Unassigned	Unassigned	1396_0	Clustered	110	VC_1396_0	110	1	1	1	0	0	0	0.9746	Ref
MK719711	Unassigned	Unassigned	Unassigned	1396_0	Clustered	110	VC_1396_0	110	1	1	1	0	0	0	0.9746	Ref
MK719712	Unassigned	Unassigned	Unassigned	1396_0	Clustered	110	VC_1396_0	110	1	1	1	0	0	0	0.9746	Ref
MK719713	Unassigned	Unassigned	Unassigned	1396_0	Clustered	110	VC_1396_0	110	1	1	1	0	0	0	0.9746	Ref
MK719714	Unassigned	Unassigned	Unassigned	1396_0	Clustered	110	VC_1396_0	110	1	1	1	0	0	0	0.9746	Ref
MK719715	Unassigned	Unassigned	Unassigned	1396_0	Clustered	110	VC_1396_0	110	1	1	1	0	0	0	0.9746	Ref
MK719716	Unassigned	Unassigned	Unassigned	1396_0	Clustered	110	VC_1396_0	110	1	1	1	0	0	0	0.9746	Ref
MK719717	Unassigned	Unassigned	Unassigned	1396_0	Clustered	110	VC_1396_0	110	1	1	1	0	0	0	0.9746	Ref
MK719718	Unassigned	Unassigned	Unassigned	1396_0	Clustered	110	VC_1396_0	110	1	1	1	0	0	0	0.9746	Ref
MK719719	Unassigned	Unassigned	Unassigned	1396_0	Clustered	110	VC_1396_0	110	1	1	1	0	0	0	0.9746	Ref
MK719720	Unassigned	Unassigned	Unassigned	1396_0	Clustered	110	VC_1396_0	110	1	1	1	0	0	0	0.9746	Ref
MK719721	Unassigned	Unassigned	Unassigned	1396_0	Clustered	110	VC_1396_0	110	1	1	1	0	0	0	0.9746	Ref
MK719722	Unassigned	Unassigned	Unassigned	1396_0	Clustered	110	VC_1396_0	110	1	1	1	0	0	0	0.9746	Ref
MK719723	Unassigned	Unassigned	Unassigned	1396_0	Clustered	110	VC_1396_0	110	1	1	1	0	0	0	0.9746	Ref
MK719724	Unassigned	Unassigned	Unassigned	1396_0	Clustered	110	VC_1396_0	110	1	1	1	0	0	0	0.9746	Ref
MK719725	Unassigned	Unassigned	Unassigned	1396_0	Clustered	110	VC_1396_0	110	1	1	1	0	0	0	0.9746	Ref
MK719726	Unassigned	Unassigned	Unassigned	1396_0	Clustered	110	VC_1396_0	110	1	1	1	0	0	0	0.9746	Ref
MK719727	Unassigned	Unassigned	Unassigned	1396_0	Clustered	110	VC_1396_0	110	1	1	1	0	0	0	0.9746	Ref
MK719728	Unassigned	Unassigned	Unassigned	1396_0	Clustered	110	VC_1396_0	110	1	1	1	0	0	0	0.9746	Ref
MK719729	Unassigned	Unassigned	Unassigned	1396_0	Clustered	110	VC_1396_0	110	1	1	1	0	0	0	0.9746	Ref
MK719730	Unassigned	Unassigned	Unassigned	1396_0	Clustered	110	VC_1396_0	110	1	1	1	0	0	0	0.9746	Ref
MK719731	Unassigned	Unassigned	Unassigned	1396_0	Clustered	110	VC_1396_0	110	1	1	1	0	0	0	0.9746	Ref
MK719732	Unassigned	Unassigned	Unassigned	1396_0	Clustered	110	VC_1396_0	110	1	1	1	0	0	0	0.9746	Ref
MK719733	Unassigned	Unassigned	Unassigned	1396_0	Clustered	110	VC_1396_0	110	1	1	1	0	0	0	0.9746	Ref
MK719734	Unassigned	Unassigned	Unassigned	1396_0	Clustered	110	VC_1396_0	110	1	1	1	0	0	0	0.9746	Ref
MK719735	Unassigned	Unassigned	Unassigned	1396_0	Clustered	110	VC_1396_0	110	1	1	1	0	0	0	0.9746	Ref
MK719736	Unassigned	Unassigned	Unassigned	1396_0	Clustered	110	VC_1396_0	110	1	1	1	0	0	0	0.9746	Ref
MK719737	Unassigned	Unassigned	Unassigned	1396_0	Clustered	110	VC_1396_0	110	1	1	1	0	0	0	0.9746	Ref
MK719738	Unassigned	Unassigned	Unassigned	1396_0	Clustered	110	VC_1396_0	110	1	1	1	0	0	0	0.9746	Ref
MK719739	Unassigned	Unassigned	Unassigned	1396_0	Clustered	110	VC_1396_0	110	1	1	1	0	0	0	0.9746	Ref
MK719740	Unassigned	Unassigned	Unassigned	1396_0	Clustered	110	VC_1396_0	110	1	1	1	0	0	0	0.9746	Ref
MK719741	Unassigned	Unassigned	Unassigned	1396_0	Clustered	110	VC_1396_0	110	1	1	1	0	0	0	0.9746	Ref
MK719742	Unassigned	Unassigned	Unassigned	1396_0	Clustered	110	VC_1396_0	110	1	1	1	0	0	0	0.9746	Ref
MK719743	Unassigned	Unassigned	Unassigned	1396_0	Clustered	110	VC_1396_0	110	1	1	1	0	0	0	0.9746	Ref
MK719744	Unassigned	Unassigned	Unassigned	1396_0	Clustered	110	VC_1396_0	110	1	1	1	0	0	0	0.9746	Ref
MK719745	Unassigned	Unassigned	Unassigned	1396_0	Clustered	110	VC_1396_0	110	1	1	1	0	0	0	0.9746	Ref
MK719746	Unassigned	Unassigned	Unassigned	1396_0	Clustered	110	VC_1396_0	110	1	1	1	0	0	0	0.9746	Ref
MK719747	Unassigned	Unassigned	Unassigned	1396_0	Clustered	110	VC_1396_0	110	1	1	1	0	0	0	0.9746	Ref
MK719748	Unassigned	Unassigned	Unassigned	1396_0	Clustered	110	VC_1396_0	110	1	1	1	0	0	0	0.9746	Ref
MK719749	Unassigned	Unassigned	Unassigned	1396_0	Clustered	110	VC_1396_0	110	1	1	1	0	0	0	0.9746	Ref
MK719750	Unassigned	Unassigned	Unassigned	1396_0	Clustered	110	VC_1396_0	110	1	1	1	0	0	0	0.9746	Ref
MK719751	Unassigned	Unassigned	Unassigned	1396_0	Clustered	110	VC_1396_0	110	1	1	1	0	0	0	0.9746	Ref
MK719752	Unassigned	Unassigned	Unassigned	1396_0	Clustered	110	VC_1396_0	110	1	1	1	0	0	0	0.9746	Ref
MK719753	Unassigned	Unassigned	Unassigned	1396_0	Clustered	110	VC_1396_0	110	1	1	1	0	0	0	0.9746	Ref
MK719754	Unassigned	Unassigned	Unassigned	1396_0	Clustered	110	VC_1396_0	110	1	1	1	0	0	0	0.9746	Ref
MK721183	Unassigned	Unassigned	Unassigned	967_0	Clustered	16	VC_967_0	16	0.8971	1	0.8971	0	0	0	1	Ref
MK721184	Unassigned	Unassigned	Unassigned	967_0	Clustered	16	VC_967_0	16	0.8971	1	0.8971	0	0	0	1	Ref
MK721185	Unassigned	Unassigned	Unassigned	967_0	Clustered	16	VC_967_0	16	0.8971	1	0.8971	0	0	0	1	Ref
MK721186	Unassigned	Unassigned	Unassigned	695_0	Clustered	48	VC_695_0	48	0.8162	1	0.8162	0	0	0	1	Ref
MK721187	Unassigned	Unassigned	Unassigned	695_0	Clustered	48	VC_695_0	48	0.8162	1	0.8162	0	0	0	1	Ref
MK721188	Unassigned	Unassigned	Unassigned	967_0	Clustered	16	VC_967_0	16	0.8971	1	0.8971	0	0	0	1	Ref
MK721189	Unassigned	Unassigned	Unassigned	108_0	Clustered	33	VC_108_0	33	0.949	1	0.949	0	0	0	1	Ref
MK721190	Unassigned	Unassigned	Unassigned	695_0	Clustered	48	VC_695_0	48	0.8162	1	0.8162	0	0	0	1	Ref
MK721191	Unassigned	Unassigned	Unassigned	695_0	Clustered	48	VC_695_0	48	0.8162	1	0.8162	0	0	0	1	Ref
MK721192	Unassigned	Unassigned	Unassigned	91_0	Clustered	22	VC_91_0	22	0.5318	1	0.5318	0	0	0	1	Ref
MK721193	Unassigned	Unassigned	Unassigned	967_0	Clustered	16	VC_967_0	16	0.8971	1	0.8971	0	0	0	1	Ref
MK721194	Unassigned	Unassigned	Unassigned	108_0	Clustered	33	VC_108_0	33	0.949	1	0.949	0	0	0	1	Ref
MK721195	Unassigned	Unassigned	Unassigned	967_0	Clustered	16	VC_967_0	16	0.8971	1	0.8971	0	0	0	1	Ref
MK721196	Unassigned	Unassigned	Unassigned	967_0	Clustered	16	VC_967_0	16	0.8971	1	0.8971	0	0	0	1	Ref
MK721197	Unassigned	Unassigned	Unassigned	967_0	Clustered	16	VC_967_0	16	0.8971	1	0.8971	0	0	0	1	Ref
MK721198	Unassigned	Unassigned	Unassigned	967_0	Clustered	16	VC_967_0	16	0.8971	1	0.8971	0	0	0	1	Ref
MK721199	Unassigned	Unassigned	Unassigned	695_0	Clustered	48	VC_695_0	48	0.8162	1	0.8162	0	0	0	1	Ref
MK721200	Unassigned	Unassigned	Unassigned	695_0	Clustered	48	VC_695_0	48	0.8162	1	0.8162	0	0	0	1	Ref
MK728541	Unassigned	Unassigned	Unassigned	835_0	Clustered	67	VC_835_0	67	0.9624	1	0.9624	0	0	0	1	Ref
MK728824	Unassigned	Unassigned	Unassigned	191_19	Clustered	12	VC_191_19	12	0.0655	1	0.0655	0	0	0	0.8636	Ref
MK728825	Unassigned	Unassigned	Unassigned	191_19	Clustered	12	VC_191_19	12	0.0655	1	0.0655	0	0	0	0.8636	Ref
MK733259	Unassigned	Unassigned	Unassigned	45_0	Clustered	12	VC_45_0	12	0.35	1	0.35	0	0	0	1	Ref
MK733260	Unassigned	Unassigned	Unassigned	45_0	Clustered	12	VC_45_0	12	0.35	1	0.35	0	0	0	1	Ref
MK733261	Unassigned	Unassigned	Unassigned	45_0	Clustered	12	VC_45_0	12	0.35	1	0.35	0	0	0	1	Ref
MK733262	Unassigned	Unassigned	Unassigned	281_0	Clustered	3	VC_281_0	3	0.3561	0.99828319	0.3555	0	0	0	1	Ref
MK733263	Unassigned	Unassigned	Unassigned	281_0	Clustered	3	VC_281_0	3	0.3561	0.99828319	0.3555	0	0	0	1	Ref
MK733264	Unassigned	Unassigned	Unassigned	281_0	Clustered	3	VC_281_0	3	0.3561	0.99828319	0.3555	0	0	0	1	Ref
MK737937	Unassigned	Unassigned	Unassigned	638_0	Clustered	45	VC_638_0	45	0.7741	1	0.7741	0	0	0	1	Ref
MK737941	Unassigned	Unassigned	Unassigned	1343_0	Clustered	21	VC_1343_0	21	0.9843	1	0.9843	0	0	0	1	Ref
MK737942	Unassigned	Unassigned	Unassigned	1162_0	Clustered	10	VC_1162_0	10	0.5715	1	0.5715	0	0	0	1	Ref

MK737943	Unassigned	Unassigned	Unassigned	950_0	Clustered	38	VC_950_0	38	0.9443	1	0.9443	0	0	0	1	Ref
MK737944	Unassigned	Unassigned	Unassigned	1348_0	Clustered	5	VC_1348_0	5	0.5462	0.99999985	0.5462	0	0	0	1	Ref
MK756083	Unassigned	Unassigned	Unassigned	912_0	Clustered	76	VC_912_0	76	0.979	1	0.979	0	0	0	1	Ref
MK756084	Unassigned	Unassigned	Unassigned	912_0	Clustered	76	VC_912_0	76	0.979	1	0.979	0	0	0	1	Ref
MK756085	Unassigned	Unassigned	Unassigned	912_0	Clustered	76	VC_912_0	76	0.979	1	0.979	0	0	0	1	Ref
MK756086	Unassigned	Unassigned	Unassigned	912_0	Clustered	76	VC_912_0	76	0.979	1	0.979	0	0	0	1	Ref
MK756087	Unassigned	Unassigned	Unassigned	912_0	Clustered	76	VC_912_0	76	0.979	1	0.979	0	0	0	1	Ref
MK756088	Unassigned	Unassigned	Unassigned	912_0	Clustered	76	VC_912_0	76	0.979	1	0.979	0	0	0	1	Ref
MK756089	Unassigned	Unassigned	Unassigned	912_0	Clustered	76	VC_912_0	76	0.979	1	0.979	0	0	0	1	Ref
MK756090	Unassigned	Unassigned	Unassigned	912_0	Clustered	76	VC_912_0	76	0.979	1	0.979	0	0	0	1	Ref
MK756091	Unassigned	Unassigned	Unassigned	912_0	Clustered	76	VC_912_0	76	0.979	1	0.979	0	0	0	1	Ref
MK756092	Unassigned	Unassigned	Unassigned	912_0	Clustered	76	VC_912_0	76	0.979	1	0.979	0	0	0	1	Ref
MK756093	Unassigned	Unassigned	Unassigned	912_0	Clustered	76	VC_912_0	76	0.979	1	0.979	0	0	0	1	Ref
MK756094	Unassigned	Unassigned	Unassigned	912_0	Clustered	76	VC_912_0	76	0.979	1	0.979	0	0	0	1	Ref
MK756095	Unassigned	Unassigned	Unassigned	912_0	Clustered	76	VC_912_0	76	0.979	1	0.979	0	0	0	1	Ref
MK756096	Unassigned	Unassigned	Unassigned	912_0	Clustered	76	VC_912_0	76	0.979	1	0.979	0	0	0	1	Ref
MK757445	Unassigned	Unassigned	Unassigned	551_7	Clustered	28	VC_551_7	28	0.1661	1	0.1661	0	0	0	0.8519	Ref
MK757446	Unassigned	Unassigned	Unassigned	568_0	Clustered	8	VC_568_0	8	0.5738	1	0.5738	0	0	0	1	Ref
MK757447	Unassigned	Unassigned	Unassigned	1240_0	Clustered	82	VC_1240_0	82	0.7945	1	0.7945	0	0	0	1	Ref
MK757448	Unassigned	Unassigned	Unassigned	1362_0	Clustered	6	VC_1362_0	6	1	1	1	0	0	0	1	Ref
MK757449	Unassigned	Unassigned	Unassigned	1360_0	Clustered	31	VC_1360_0	31	0.8837	1	0.8837	0	0	0	1	Ref
MK759854	Unassigned	Unassigned	Unassigned	461_0	Clustered	96	VC_461_0	96	0.9307	1	0.9307	0	0	0	0.9575	Ref
MK759883	Unassigned	Unassigned	Unassigned	637_1	Clustered	27	VC_637_1	27	0.2578	1	0.2578	0	0	0	0.9858	Ref
MK759884	Unassigned	Unassigned	Unassigned	328_0	Clustered	11	VC_328_0	11	0.4707	1	0.4707	0	0	0	1	Ref
MK759918	Unassigned	Unassigned	Unassigned	731_0	Clustered	2	VC_731_0	2	0.3697	0.94979793	0.3512	0	0	0	1	Ref
MK761195	Unassigned	Unassigned	Unassigned	637_0	Clustered	115	VC_637_0	115	0.731	1	0.731	0	0	0	0.8764	Ref
MK761196	Unassigned	Unassigned	Unassigned	637_0	Clustered	115	VC_637_0	115	0.731	1	0.731	0	0	0	0.8764	Ref
MK761197	Unassigned	Unassigned	Unassigned	637_0	Clustered	115	VC_637_0	115	0.731	1	0.731	0	0	0	0.8764	Ref
MK761198	Unassigned	Unassigned	Unassigned	637_0	Clustered	115	VC_637_0	115	0.731	1	0.731	0	0	0	0.8764	Ref
MK761199	Unassigned	Unassigned	Unassigned	637_0	Clustered	115	VC_637_0	115	0.731	1	0.731	0	0	0	0.8764	Ref
MK764384	Unassigned	Unassigned	Unassigned	96_0	Clustered	30	VC_96_0	30	0.718	1	0.718	0	0	0	1	Ref
MK764436	Unassigned	Unassigned	Unassigned	1040_0	Clustered	23	VC_1040_0	23	0.9605	1	0.9605	0	0	0	1	Ref
MK764437	Unassigned	Unassigned	Unassigned	1023_0	Clustered	8	VC_1023_0	8	0.8574	1	0.8574	0	0	0	1	Ref
MK764438	Unassigned	Unassigned	Unassigned	1040_0	Clustered	23	VC_1040_0	23	0.9605	1	0.9605	0	0	0	1	Ref
MK764439	Unassigned	Unassigned	Unassigned	1040_0	Clustered	23	VC_1040_0	23	0.9605	1	0.9605	0	0	0	1	Ref
MK764440	Unassigned	Unassigned	Unassigned	1023_0	Clustered	8	VC_1023_0	8	0.8574	1	0.8574	0	0	0	1	Ref
MK764441	Unassigned	Unassigned	Unassigned	1157_0	Clustered	24	VC_1157_0	24	1	1	1	0	0	0	1	Ref
MK764442	Unassigned	Unassigned	Unassigned	1021_0	Clustered	10	VC_1021_0	10	0.8876	1	0.8876	0	0	0	1	Ref
MK764443	Unassigned	Unassigned	Unassigned	1040_0	Clustered	23	VC_1040_0	23	0.9605	1	0.9605	0	0	0	1	Ref
MK764444	Unassigned	Unassigned	Unassigned	1040_0	Clustered	23	VC_1040_0	23	0.9605	1	0.9605	0	0	0	1	Ref
MK764445	Unassigned	Unassigned	Unassigned	1021_0	Clustered	10	VC_1021_0	10	0.8876	1	0.8876	0	0	0	1	Ref
MK764446	Unassigned	Unassigned	Unassigned	1040_0	Clustered	23	VC_1040_0	23	0.9605	1	0.9605	0	0	0	1	Ref
MK764447	Unassigned	Unassigned	Unassigned	1040_0	Clustered	23	VC_1040_0	23	0.9605	1	0.9605	0	0	0	1	Ref
MK764448	Unassigned	Unassigned	Unassigned	1021_0	Clustered	10	VC_1021_0	10	0.8876	1	0.8876	0	0	0	1	Ref
MK764449	Unassigned	Unassigned	Unassigned	1157_0	Clustered	24	VC_1157_0	24	1	1	1	0	0	0	1	Ref
MK764450	Unassigned	Unassigned	Unassigned	1023_0	Clustered	8	VC_1023_0	8	0.8574	1	0.8574	0	0	0	1	Ref
MK765551	Unassigned	Unassigned	Unassigned	1398_0	Clustered	2	VC_1398_0	2	1	1	1	0	0	0	1	Ref
MK765552	Unassigned	Unassigned	Unassigned	1283_0	Clustered	26	VC_1283_0	26	0.5446	1	0.5446	0	0	0	1	Ref
MK765553	Unassigned	Unassigned	Unassigned	1283_0	Clustered	26	VC_1283_0	26	0.5446	1	0.5446	0	0	0	1	Ref
MK765555	Unassigned	Unassigned	Unassigned	1325_0	Clustered	7	VC_1325_0	7	0.9166	0.99999981	0.9166	0	0	0	1	Ref
MK765556	Unassigned	Unassigned	Unassigned	1283_0	Clustered	26	VC_1283_0	26	0.5446	1	0.5446	0	0	0	1	Ref
MK765557	Unassigned	Unassigned	Unassigned	1211_0	Clustered	25	VC_1211_0	25	1	1	1	0	0	0	1	Ref
MK765558	Unassigned	Unassigned	Unassigned	1399_0	Clustered	9	VC_1399_0	9	1	0	0	0	0	0	1	Ref
MK765559	Unassigned	Unassigned	Unassigned	1119_0	Clustered	20	VC_1119_0	20	0.6751	0.99997892	0.6751	0	0	0	1	Ref
MK765560	Unassigned	Unassigned	Unassigned	1283_0	Clustered	26	VC_1283_0	26	0.5446	1	0.5446	0	0	0	1	Ref
MK765561	Unassigned	Unassigned	Unassigned	1243_0	Clustered	52	VC_1243_0	52	0.9126	0.92708311	0.8461	0	0	0	1	Ref
MK765562	Unassigned	Unassigned	Unassigned	1283_0	Clustered	26	VC_1283_0	26	0.5446	1	0.5446	0	0	0	1	Ref
MK765563	Unassigned	Unassigned	Unassigned	1243_0	Clustered	52	VC_1243_0	52	0.9126	0.92708311	0.8461	0	0	0	1	Ref
MK765564	Unassigned	Unassigned	Unassigned	1243_0	Clustered	52	VC_1243_0	52	0.9126	0.92708311	0.8461	0	0	0	1	Ref
MK765565	Unassigned	Unassigned	Unassigned	1283_0	Clustered	26	VC_1283_0	26	0.5446	1	0.5446	0	0	0	1	Ref
MK765567	Unassigned	Unassigned	Unassigned	1325_0	Clustered	7	VC_1325_0	7	0.9166	0.99999981	0.9166	0	0	0	1	Ref
MK765568	Unassigned	Unassigned	Unassigned	1243_0	Clustered	52	VC_1243_0	52	0.9126	0.92708311	0.8461	0	0	0	1	Ref
MK765569	Unassigned	Unassigned	Unassigned	1243_0	Clustered	52	VC_1243_0	52	0.9126	0.92708311	0.8461	0	0	0	1	Ref
MK765571	Unassigned	Unassigned	Unassigned	1285_0	Clustered	25	VC_1285_0	25	1	1	1	0	0	0	1	Ref
MK765572	Unassigned	Unassigned	Unassigned	1280_0	Clustered	2	VC_1280_0	2	0.0695	0.94944661	0.066	0	0	0	1	Ref
MK765573	Unassigned	Unassigned	Unassigned	1400_0	Clustered	2	VC_1400_0	2	1	1	1	0	0	0	1	Ref
MK765574	Unassigned	Unassigned	Unassigned	1285_0	Clustered	25	VC_1285_0	25	1	1	1	0	0	0	1	Ref
MK765575	Unassigned	Unassigned	Unassigned	1401_0	Clustered	2	VC_1401_0	2	1	1	1	0	0	0	1	Ref
MK765576	Unassigned	Unassigned	Unassigned	1285_0	Clustered	25	VC_1285_0	25	1	1	1	0	0	0	1	Ref
MK765577	Unassigned	Unassigned	Unassigned	1285_0	Clustered	25	VC_1285_0	25	1	1	1	0	0	0	1	Ref
MK765578	Unassigned	Unassigned	Unassigned	1401_0	Clustered	2	VC_1401_0	2	1	1	1	0	0	0	1	Ref
MK765579	Unassigned	Unassigned	Unassigned	1062_0	Clustered	45	VC_1062_0	45	0.9734	0.50949847	0.4959	0	0	0	1	Ref
MK765580	Unassigned	Unassigned	Unassigned	1285_0	Clustered	25	VC_1285_0	25	1	1	1	0	0	0	1	Ref
MK765581	Unassigned	Unassigned	Unassigned	1399_0	Clustered	9	VC_1399_0	9	1	0	0	0	0	0	1	Ref
MK765582	Unassigned	Unassigned	Unassigned	1062_0	Clustered	45	VC_1062_0	45	0.9734	0.50949847	0.4959	0	0	0	1	Ref
MK765583	Unassigned	Unassigned	Unassigned	1308_0	Clustered	12	VC_1308_0	12	0.8184	0.9810731	0.8029	0	0	0	1	Ref
MK765587	Unassigned	Unassigned	Unassigned	1402_0	Clustered	14	VC_1402_0	14	0.1673	0.51390354	0.086	0	0	0	1	Ref
MK765588	Unassigned	Unassigned	Unassigned	1402_0	Clustered	14	VC_1402_0	14	0.1673	0.51390354	0.086	0	0	0	1	Ref
MK765589	Unassigned	Unassigned	Unassigned	1403_0	Clustered	28	VC_1403_0	28	0.753	1	0.753	0	0	0	1	Ref
MK765590	Unassigned	Unassigned	Unassigned	1277_0	Clustered	14	VC_1277_0	14	0.8218	1	0.8218	0	0	0	1	Ref
MK765591	Unassigned	Unassigned	Unassigned	1283_0	Clustered	26	VC_1283_0	26	0.5446	1	0.5446	0	0	0	1	Ref

MK765592	Unassigned	Unassigned	Unassigned	1283_0	Clustered	26	VC_1283_0	26	0.5446	1	0.5446	0	0	0	1	Ref
MK765593	Unassigned	Unassigned	Unassigned	1283_0	Clustered	26	VC_1283_0	26	0.5446	1	0.5446	0	0	0	1	Ref
MK765594	Unassigned	Unassigned	Unassigned	1243_0	Clustered	52	VC_1243_0	52	0.9126	0.92708311	0.8461	0	0	0	1	Ref
MK765596	Unassigned	Unassigned	Unassigned	1283_0	Clustered	26	VC_1283_0	26	0.5446	1	0.5446	0	0	0	1	Ref
MK765598	Unassigned	Unassigned	Unassigned	1402_0	Clustered	14	VC_1402_0	14	0.1673	0.51390354	0.086	0	0	0	1	Ref
MK765599	Unassigned	Unassigned	Unassigned	1402_0	Clustered	14	VC_1402_0	14	0.1673	0.51390354	0.086	0	0	0	1	Ref
MK765600	Unassigned	Unassigned	Unassigned	1325_0	Clustered	7	VC_1325_0	7	0.9166	0.99999981	0.9166	0	0	0	1	Ref
MK765601	Unassigned	Unassigned	Unassigned	1303_0	Clustered	6	VC_1303_0	6	1	1	1	0	0	0	1	Ref
MK765602	Unassigned	Unassigned	Unassigned	1140_0	Clustered	4	VC_1140_0	4	0.1325	0.66207199	0.0877	0	0	0	1	Ref
MK765603	Unassigned	Unassigned	Unassigned	1119_0	Clustered	20	VC_1119_0	20	0.6751	0.99997892	0.6751	0	0	0	1	Ref
MK765604	Unassigned	Unassigned	Unassigned	1399_0	Clustered	9	VC_1399_0	9	1	0	0	0	0	0	1	Ref
MK765605	Unassigned	Unassigned	Unassigned	1406_0	Clustered	2	VC_1406_0	2	1	1	1	0	0	0	1	Ref
MK765606	Unassigned	Unassigned	Unassigned	1283_0	Clustered	26	VC_1283_0	26	0.5446	1	0.5446	0	0	0	1	Ref
MK765607	Unassigned	Unassigned	Unassigned	1283_0	Clustered	26	VC_1283_0	26	0.5446	1	0.5446	0	0	0	1	Ref
MK765609	Unassigned	Unassigned	Unassigned	1283_0	Clustered	26	VC_1283_0	26	0.5446	1	0.5446	0	0	0	1	Ref
MK765610	Unassigned	Unassigned	Unassigned	1325_0	Clustered	7	VC_1325_0	7	0.9166	0.99999981	0.9166	0	0	0	1	Ref
MK765611	Unassigned	Unassigned	Unassigned	1283_0	Clustered	26	VC_1283_0	26	0.5446	1	0.5446	0	0	0	1	Ref
MK765612	Unassigned	Unassigned	Unassigned	1283_0	Clustered	26	VC_1283_0	26	0.5446	1	0.5446	0	0	0	1	Ref
MK765613	Unassigned	Unassigned	Unassigned	1283_0	Clustered	26	VC_1283_0	26	0.5446	1	0.5446	0	0	0	1	Ref
MK765615	Unassigned	Unassigned	Unassigned	1283_0	Clustered	26	VC_1283_0	26	0.5446	1	0.5446	0	0	0	1	Ref
MK765617	Unassigned	Unassigned	Unassigned	1325_0	Clustered	7	VC_1325_0	7	0.9166	0.99999981	0.9166	0	0	0	1	Ref
MK765620	Unassigned	Unassigned	Unassigned	1104_0	Clustered	154	VC_1104_0	154	0.8484	1	0.8484	0	0	0	1	Ref
MK765621	Unassigned	Unassigned	Unassigned	1119_0	Clustered	20	VC_1119_0	20	0.6751	0.99997892	0.6751	0	0	0	1	Ref
MK765622	Unassigned	Unassigned	Unassigned	1399_0	Clustered	9	VC_1399_0	9	1	0	0	0	0	0	1	Ref
MK765623	Unassigned	Unassigned	Unassigned	1402_0	Clustered	14	VC_1402_0	14	0.1673	0.51390354	0.086	0	0	0	1	Ref
MK765625	Unassigned	Unassigned	Unassigned	1402_0	Clustered	14	VC_1402_0	14	0.1673	0.51390354	0.086	0	0	0	1	Ref
MK765626	Unassigned	Unassigned	Unassigned	1404_0	Clustered/Singleton	1	VC_1404_0	1	0	1	0	0	0	0	1	Ref
MK765628	Unassigned	Unassigned	Unassigned	1402_0	Clustered	14	VC_1402_0	14	0.1673	0.51390354	0.086	0	0	0	1	Ref
MK765630	Unassigned	Unassigned	Unassigned	1402_0	Clustered	14	VC_1402_0	14	0.1673	0.51390354	0.086	0	0	0	1	Ref
MK765631	Unassigned	Unassigned	Unassigned	1402_0	Clustered	14	VC_1402_0	14	0.1673	0.51390354	0.086	0	0	0	1	Ref
MK765633	Unassigned	Unassigned	Unassigned	1283_0	Clustered	26	VC_1283_0	26	0.5446	1	0.5446	0	0	0	1	Ref
MK765634	Unassigned	Unassigned	Unassigned	1402_0	Clustered	14	VC_1402_0	14	0.1673	0.51390354	0.086	0	0	0	1	Ref
MK765638	Unassigned	Unassigned	Unassigned	1243_0	Clustered	52	VC_1243_0	52	0.9126	0.92708311	0.8461	0	0	0	1	Ref
MK765639	Unassigned	Unassigned	Unassigned	1062_0	Clustered	45	VC_1062_0	45	0.9734	0.50949847	0.4959	0	0	0	1	Ref
MK765640	Unassigned	Unassigned	Unassigned	1399_0	Clustered	9	VC_1399_0	9	1	0	0	0	0	0	1	Ref
MK765641	Unassigned	Unassigned	Unassigned	1406_0	Clustered	2	VC_1406_0	2	1	1	1	0	0	0	1	Ref
MK765642	Unassigned	Unassigned	Unassigned	1062_0	Clustered	45	VC_1062_0	45	0.9734	0.50949847	0.4959	0	0	0	1	Ref
MK765643	Unassigned	Unassigned	Unassigned	1062_0	Clustered	45	VC_1062_0	45	0.9734	0.50949847	0.4959	0	0	0	1	Ref
MK765644	Unassigned	Unassigned	Unassigned	1062_0	Clustered	45	VC_1062_0	45	0.9734	0.50949847	0.4959	0	0	0	1	Ref
MK765645	Unassigned	Unassigned	Unassigned	1077_0	Clustered	48	VC_1077_0	48	0.8525	1	0.8525	0	0	0	1	Ref
MK765646	Unassigned	Unassigned	Unassigned	1062_0	Clustered	45	VC_1062_0	45	0.9734	0.50949847	0.4959	0	0	0	1	Ref
MK765648	Unassigned	Unassigned	Unassigned	1283_0	Clustered	26	VC_1283_0	26	0.5446	1	0.5446	0	0	0	1	Ref
MK765649	Unassigned	Unassigned	Unassigned	1282_0	Clustered	3	VC_1282_0	3	1	1	1	0	0	0	1	Ref
MK765650	Unassigned	Unassigned	Unassigned	1398_0	Clustered	2	VC_1398_0	2	1	1	1	0	0	0	1	Ref
MK765651	Unassigned	Unassigned	Unassigned	1283_0	Clustered	26	VC_1283_0	26	0.5446	1	0.5446	0	0	0	1	Ref
MK765653	Unassigned	Unassigned	Unassigned	1331_0	Clustered	4	VC_1331_0	4	0.7343	0.99899459	0.7335	0	0	0	1	Ref
MK765654	Unassigned	Unassigned	Unassigned	1399_0	Clustered	9	VC_1399_0	9	1	0	0	0	0	0	1	Ref
MK765655	Unassigned	Unassigned	Unassigned	1211_0	Clustered	25	VC_1211_0	25	1	1	1	0	0	0	1	Ref
MK765656	Unassigned	Unassigned	Unassigned	1399_0	Clustered	9	VC_1399_0	9	1	0	0	0	0	0	1	Ref
MK765657	Unassigned	Unassigned	Unassigned	1283_0	Clustered	26	VC_1283_0	26	0.5446	1	0.5446	0	0	0	1	Ref
MK765658	Unassigned	Unassigned	Unassigned	1283_0	Clustered	26	VC_1283_0	26	0.5446	1	0.5446	0	0	0	1	Ref
MK765659	Unassigned	Unassigned	Unassigned	1325_0	Clustered	7	VC_1325_0	7	0.9166	0.99999981	0.9166	0	0	0	1	Ref
MK765660	Unassigned	Unassigned	Unassigned	1283_0	Clustered	26	VC_1283_0	26	0.5446	1	0.5446	0	0	0	1	Ref
MK765661	Unassigned	Unassigned	Unassigned	1211_0	Clustered	25	VC_1211_0	25	1	1	1	0	0	0	1	Ref
MK765662	Unassigned	Unassigned	Unassigned	1400_0	Clustered	2	VC_1400_0	2	1	1	1	0	0	0	1	Ref
MK765663	Unassigned	Unassigned	Unassigned	1399_0	Clustered	9	VC_1399_0	9	1	0	0	0	0	0	1	Ref
MK765664	Unassigned	Unassigned	Unassigned	1325_0	Clustered	7	VC_1325_0	7	0.9166	0.99999981	0.9166	0	0	0	1	Ref
MK765666	Unassigned	Unassigned	Unassigned	1283_0	Clustered	26	VC_1283_0	26	0.5446	1	0.5446	0	0	0	1	Ref
MK765667	Unassigned	Unassigned	Unassigned	1211_0	Clustered	25	VC_1211_0	25	1	1	1	0	0	0	1	Ref
MK765668	Unassigned	Unassigned	Unassigned	1399_0	Clustered	9	VC_1399_0	9	1	0	0	0	0	0	1	Ref
MK765669	Unassigned	Unassigned	Unassigned	1119_0	Clustered	20	VC_1119_0	20	0.6751	0.99997892	0.6751	0	0	0	1	Ref
MK770409	Unassigned	Unassigned	Unassigned	637_0	Clustered	115	VC_637_0	115	0.731	1	0.731	0	0	0	0.8764	Ref
MK770410	Unassigned	Unassigned	Unassigned	191_31	Clustered	2	VC_191_31	2	0.0057	0.92424379	0.0052	0	0	0	1	Ref
MK770411	Unassigned	Unassigned	Unassigned	428_0	Clustered	33	VC_428_0	33	0.9915	1	0.9915	0	0	0	1	Ref
MK770412	Unassigned	Unassigned	Unassigned	328_0	Clustered	11	VC_328_0	11	0.4707	1	0.4707	0	0	0	1	Ref
MK770413	Unassigned	Unassigned	Unassigned	428_0	Clustered	33	VC_428_0	33	0.9915	1	0.9915	0	0	0	1	Ref
MK770414	Unassigned	Unassigned	Unassigned	28_1	Clustered	53	VC_28_1	53	0.7164	1	0.7164	0	0	0	0.9325	Ref
MK770415	Unassigned	Unassigned	Unassigned	28_1	Clustered	53	VC_28_1	53	0.7164	1	0.7164	0	0	0	0.9325	Ref
MK773491	Unassigned	Unassigned	Unassigned	164_0	Clustered	3	VC_164_0	3	0.0298	0.82983726	0.0247	0	0	0	1	Ref
MK774614	Unassigned	Unassigned	Unassigned	442_4	Clustered	2	VC_442_4	2	0.0254	0.91859498	0.0233	0	0	0	1	Ref
MK778457	Unassigned	Unassigned	Unassigned	585_1	Clustered	3	VC_585_1	3	0.0313	0.99857668	0.0312	0	0	0	1	Ref
MK779875	Unassigned	Unassigned	Unassigned	800_0	Clustered	3	VC_800_0	3	0.4591	0.9984737	0.4584	0	0	0	1	Ref
MK792752	Unassigned	Unassigned	Unassigned	739_0	Clustered	26	VC_739_0	26	0.9571	1	0.9571	0	0	0	1	Ref
MK795384	Unassigned	Unassigned	Unassigned	134_0	Clustered	3	VC_134_0	3	0.0956	0.99858494	0.0955	0	0	0	1	Ref
MK795385	Unassigned	Unassigned	Unassigned	823_0	Clustered	124	VC_823_0	124	0.9492	1	0.9492	0	0	0	1	Ref
MK796244	Unassigned	Unassigned	Unassigned	971_1	Clustered	24	VC_971_1	24	0.7393	1	0.7393	0	0	0	0.8913	Ref
MK796797	Unassigned	Unassigned	Unassigned	807_0	Clustered	17	VC_807_0	17	0.907	1	0.907	0	0	0	1	Ref
MK797984	Unassigned	Unassigned	Unassigned	165_0	Clustered	4	VC_165_0	4	0.2247	0.99998328	0.2247	0	0	0	1	Ref
MK798142	Unassigned	Unassigned	Unassigned	123_0	Clustered	23	VC_123_0	23	0.817	1	0.817	0	0	0	1	Ref
MK798143	Unassigned	Unassigned	Unassigned	64_0	Clustered	3	VC_64_0	3	0.5503	0.99785685	0.5491	0	0	0	1	Ref
MK798144	Unassigned	Unassigned	Unassigned	64_0	Clustered	3	VC_64_0	3	0.5503	0.99785685	0.5491	0	0	0	1	Ref

MK799650	Unassigned	Unassigned	Unassigned	77_0	Clustered	20	VC_77_0	20	0.9657	1	0.9657	0	0	0	1	Ref
MK799832	Unassigned	Unassigned	Unassigned	432_13	Clustered/Singleton	1	VC_432_13	1	0	1	0	0	0	0	1	Ref
MK800154	Unassigned	Unassigned	Unassigned	108_0	Clustered	33	VC_108_0	33	0.949	1	0.949	0	0	0	1	Ref
MK801680	Unassigned	Unassigned	Unassigned	8_0	Clustered	58	VC_8_0	58	0.8306	1	0.8306	0	0	0	0.9946	Ref
MK801681	Unassigned	Unassigned	Unassigned	8_0	Clustered	58	VC_8_0	58	0.8306	1	0.8306	0	0	0	0.9946	Ref
MK801682	Unassigned	Unassigned	Unassigned	9_0	Clustered	38	VC_9_0	38	0.4895	1	0.4895	0	0	0	1	Ref
MK801683	Unassigned	Unassigned	Unassigned	9_0	Clustered	38	VC_9_0	38	0.4895	1	0.4895	0	0	0	1	Ref
MK801721	Unassigned	Unassigned	Unassigned	1176_0	Clustered	41	VC_1176_0	41	0.9558	1	0.9558	0	0	0	0.9976	Ref
MK801722	Unassigned	Unassigned	Unassigned	1071_0	Clustered	20	VC_1071_0	20	0.3268	1	0.3268	0	0	0	0.9842	Ref
MK801723	Unassigned	Unassigned	Unassigned	949_0	Clustered	10	VC_949_0	10	0.5977	1	0.5977	0	0	0	1	Ref
MK801724	Unassigned	Unassigned	Unassigned	1176_0	Clustered	41	VC_1176_0	41	0.9558	1	0.9558	0	0	0	0.9976	Ref
MK801725	Unassigned	Unassigned	Unassigned	510_0	Clustered	12	VC_510_0	12	0.3963	1	0.3963	0	0	0	0.9697	Ref
MK801726	Unassigned	Unassigned	Unassigned	551_0	Clustered	16	VC_551_0	16	0.0915	1	0.0915	0	0	0	0.6667	Ref
MK801727	Unassigned	Unassigned	Unassigned	525_0	Clustered	25	VC_525_0	25	0.4597	1	0.4597	0	0	0	1	Ref
MK801728	Unassigned	Unassigned	Unassigned	868_0	Clustered	25	VC_868_0	25	0.5791	1	0.5791	0	0	0	1	Ref
MK801730	Unassigned	Unassigned	Unassigned	1344_0	Clustered	78	VC_1344_0	78	0.9732	1	0.9732	0	0	0	1	Ref
MK801731	Unassigned	Unassigned	Unassigned	1343_0	Clustered	21	VC_1343_0	21	0.9843	1	0.9843	0	0	0	1	Ref
MK801732	Unassigned	Unassigned	Unassigned	1343_0	Clustered	21	VC_1343_0	21	0.9843	1	0.9843	0	0	0	1	Ref
MK801733	Unassigned	Unassigned	Unassigned	868_0	Clustered	25	VC_868_0	25	0.5791	1	0.5791	0	0	0	1	Ref
MK801734	Unassigned	Unassigned	Unassigned	1175_0	Clustered	22	VC_1175_0	22	0.4562	1	0.4562	0	0	0	1	Ref
MK801735	Unassigned	Unassigned	Unassigned	216_0	Clustered	4	VC_216_0	4	0.048	0.9999878	0.048	0	0	0	1	Ref
MK803322	Unassigned	Unassigned	Unassigned	167_0	Clustered	3	VC_167_0	3	0.6952	0.99826575	0.694	0	0	0	1	Ref
MK804891	Unassigned	Unassigned	Unassigned	369_0	Clustered	6	VC_369_0	6	0.5924	1	0.5924	0	0	0	1	Ref
MK804892	Unassigned	Unassigned	Unassigned	369_0	Clustered	6	VC_369_0	6	0.5924	1	0.5924	0	0	0	1	Ref
MK804893	Unassigned	Unassigned	Unassigned	865_0	Clustered	2	VC_865_0	2	0.0766	0.9388444	0.0719	0	0	0	1	Ref
MK809530	Unassigned	Unassigned	Unassigned	191_32	Clustered	12	VC_191_32	12	0.0683	1	0.0683	0	0	0	0.7576	Ref
MK813938	Unassigned	Unassigned	Unassigned	865_1	Clustered/Singleton	1	VC_865_1	1	0	1	0	0	0	0	1	Ref
MK813939	Unassigned	Unassigned	Unassigned	865_0	Clustered	2	VC_865_0	2	0.0766	0.9388444	0.0719	0	0	0	1	Ref
MK813940	Unassigned	Unassigned	Unassigned	639_0	Clustered	2	VC_639_0	2	0.2791	0.95526917	0.2666	0	0	0	1	Ref
MK813941	Unassigned	Unassigned	Unassigned	865_2	Clustered/Singleton	1	VC_865_2	1	0	1	0	0	0	0	1	Ref
MK814748	Unassigned	Unassigned	Unassigned	216_1	Clustered/Singleton	1	VC_216_1	1	0	1	0	0	0	0	1	Ref
MK814749	Unassigned	Unassigned	Unassigned	1160_0	Clustered	73	VC_1160_0	73	0.9835	1	0.9835	0	0	0	1	Ref
MK814750	Unassigned	Unassigned	Unassigned	1184_0	Clustered	5	VC_1184_0	5	0.6591	0.99999982	0.6591	0	0	0	1	Ref
MK814751	Unassigned	Unassigned	Unassigned	508_0	Clustered	32	VC_508_0	32	0.906	1	0.906	0	0	0	1	Ref
MK814752	Unassigned	Unassigned	Unassigned	557_1	Clustered	11	VC_557_1	11	0.4945	1	0.4945	0	0	0	0.7818	Ref
MK814753	Unassigned	Unassigned	Unassigned	1234_0	Clustered	44	VC_1234_0	44	1	1	1	0	0	0	1	Ref
MK814754	Unassigned	Unassigned	Unassigned	325_31	Clustered/Singleton	1	VC_325_31	1	0	1	0	0	0	0	1	Ref
MK814755	Unassigned	Unassigned	Unassigned	525_0	Clustered	25	VC_525_0	25	0.4597	1	0.4597	0	0	0	1	Ref
MK814756	Unassigned	Unassigned	Unassigned	803_0	Clustered	3	VC_803_0	3	0.0513	0.99857736	0.0512	0	0	0	0.6667	Ref
MK814757	Unassigned	Unassigned	Unassigned	1176_0	Clustered	41	VC_1176_0	41	0.9558	1	0.9558	0	0	0	0.9976	Ref
MK814758	Unassigned	Unassigned	Unassigned	519_0	Clustered	39	VC_519_0	39	0.8156	1	0.8156	0	0	0	1	Ref
MK814759	Unassigned	Unassigned	Unassigned	1180_0	Clustered	3	VC_1180_0	3	0.5161	0.9981786	0.5151	0	0	0	1	Ref
MK814760	Unassigned	Unassigned	Unassigned	1098_0	Clustered	4	VC_1098_0	4	0.5465	0.99996462	0.5465	0	0	0	1	Ref
MK814761	Unassigned	Unassigned	Unassigned	1178_0	Clustered	24	VC_1178_0	24	0.9524	1	0.9524	0	0	0	1	Ref
MK814762	Unassigned	Unassigned	Unassigned	1163_0	Clustered	28	VC_1163_0	28	0.4923	1	0.4923	0	0	0	1	Ref
MK816297	Unassigned	Unassigned	Unassigned	796_0	Clustered	5	VC_796_0	5	0.3352	0.99999997	0.3352	0	0	0	1	Ref
MK817115	Unassigned	Unassigned	Unassigned	164_11	Clustered	10	VC_164_11	10	0.1507	1	0.1507	0	0	0	0.9111	Ref
MK819239	Unassigned	Unassigned	Unassigned	295_0	Clustered	2	VC_295_0	2	0.0336	0.94464231	0.0317	0	0	0	1	Ref
MK820013	Unassigned	Unassigned	Unassigned	1027_0	Clustered	20	VC_1027_0	20	0.7114	1	0.7114	0	0	0	1	Ref
MK820638	Unassigned	Unassigned	Unassigned	325_23	Clustered	17	VC_325_23	17	0.0716	1	0.0716	0	0	0	0.6397	Ref
MK820639	Unassigned	Unassigned	Unassigned	519_0	Clustered	39	VC_519_0	39	0.8156	1	0.8156	0	0	0	1	Ref
MK820640	Unassigned	Unassigned	Unassigned	950_0	Clustered	38	VC_950_0	38	0.9443	1	0.9443	0	0	0	1	Ref
MK820641	Unassigned	Unassigned	Unassigned	685_0	Clustered	16	VC_685_0	16	0.2972	1	0.2972	0	0	0	0.9167	Ref
MK820642	Unassigned	Unassigned	Unassigned	525_0	Clustered	25	VC_525_0	25	0.4597	1	0.4597	0	0	0	1	Ref
MK837009	Unassigned	Unassigned	Unassigned	79_5	Clustered	2	VC_79_5	2	0.0046	0.92781966	0.0043	0	0	0	1	Ref
MK837010	Unassigned	Unassigned	Unassigned	79_6	Clustered	11	VC_79_6	11	0.0528	1	0.0528	0	0	0	0.7455	Ref
MK837011	Unassigned	Unassigned	Unassigned	472_0	Clustered	47	VC_472_0	47	0.9025	1	0.9025	0	0	0	1	Ref
MK837012	Unassigned	Unassigned	Unassigned	472_0	Clustered	47	VC_472_0	47	0.9025	1	0.9025	0	0	0	1	Ref
MK838107	Unassigned	Unassigned	Unassigned	878_0	Clustered	32	VC_878_0	32	0.9965	1	0.9965	0	0	0	1	Ref
MK838108	Unassigned	Unassigned	Unassigned	878_0	Clustered	32	VC_878_0	32	0.9965	1	0.9965	0	0	0	1	Ref
MK838109	Unassigned	Unassigned	Unassigned	878_0	Clustered	32	VC_878_0	32	0.9965	1	0.9965	0	0	0	1	Ref
MK838110	Unassigned	Unassigned	Unassigned	878_0	Clustered	32	VC_878_0	32	0.9965	1	0.9965	0	0	0	1	Ref
MK838111	Unassigned	Unassigned	Unassigned	878_0	Clustered	32	VC_878_0	32	0.9965	1	0.9965	0	0	0	1	Ref
MK838113	Unassigned	Unassigned	Unassigned	597_0	Clustered	6	VC_597_0	6	0.1801	1	0.1801	0	0	0	1	Ref
MK838116	Unassigned	Unassigned	Unassigned	442_0	Clustered	4	VC_442_0	4	0.0842	0.99996321	0.0842	0	0	0	1	Ref
MK863032	Unassigned	Unassigned	Unassigned	779_3	Clustered	7	VC_779_3	7	0.0766	1	0.0766	0	0	0	0.7143	Ref
MK864263	Unassigned	Unassigned	Unassigned	1176_0	Clustered	41	VC_1176_0	41	0.9558	1	0.9558	0	0	0	0.9976	Ref
MK864264	Unassigned	Unassigned	Unassigned	1178_0	Clustered	24	VC_1178_0	24	0.9524	1	0.9524	0	0	0	1	Ref
MK864265	Unassigned	Unassigned	Unassigned	1178_0	Clustered	24	VC_1178_0	24	0.9524	1	0.9524	0	0	0	1	Ref
MK864266	Unassigned	Unassigned	Unassigned	1178_0	Clustered	24	VC_1178_0	24	0.9524	1	0.9524	0	0	0	1	Ref
MK864267	Unassigned	Unassigned	Unassigned	1178_0	Clustered	24	VC_1178_0	24	0.9524	1	0.9524	0	0	0	1	Ref
MK867354	Unassigned	Unassigned	Unassigned	432_5	Clustered/Singleton	1	VC_432_5	1	0	1	0	0	0	0	1	Ref
MK867835	Unassigned	Unassigned	Unassigned	191_27	Clustered/Singleton	1	VC_191_27	1	0	1	0	0	0	0	1	Ref
MK875791	Unassigned	Unassigned	Unassigned	919_0	Clustered	39	VC_919_0	39	0.9069	1	0.9069	0	0	0	1	Ref
MK875793	Unassigned	Unassigned	Unassigned	525_0	Clustered	25	VC_525_0	25	0.4597	1	0.4597	0	0	0	1	Ref
MK875795	Unassigned	Unassigned	Unassigned	919_0	Clustered	39	VC_919_0	39	0.9069	1	0.9069	0	0	0	1	Ref
MK875796	Unassigned	Unassigned	Unassigned	525_0	Clustered	25	VC_525_0	25	0.4597	1	0.4597	0	0	0	1	Ref
MK875797	Unassigned	Unassigned	Unassigned	1175_0	Clustered	22	VC_1175_0	22	0.4562	1	0.4562	0	0	0	1	Ref
MK875798	Unassigned	Unassigned	Unassigned	1352_0	Clustered	31	VC_1352_0	31	0.849	1	0.849	0	0	0	1	Ref
MK875799	Unassigned	Unassigned	Unassigned	517_0	Clustered	18	VC_517_0	18	0.1599	1	0.1599	0	0	0	1	Ref
MK875800	Unassigned	Unassigned	Unassigned	514_0	Clustered	75	VC_514_0	75	0.9402	1	0.9402	0	0	0	1	Ref

MK878895	Unassigned	Unassigned	Unassigned	517_1	Clustered	108	VC_517_1	108	0.8483	1	0.8483	0	0	0	0.9955	Ref
MK878896	Unassigned	Unassigned	Unassigned	1176_0	Clustered	41	VC_1176_0	41	0.9558	1	0.9558	0	0	0	0.9976	Ref
MK878897	Unassigned	Unassigned	Unassigned	558_38	Clustered	2	VC_558_38	2	0.0032	0.64328629	0.002	0	0	0	1	Ref
MK878898	Unassigned	Unassigned	Unassigned	525_0	Clustered	25	VC_525_0	25	0.4597	1	0.4597	0	0	0	1	Ref
MK878899	Unassigned	Unassigned	Unassigned	1344_0	Clustered	78	VC_1344_0	78	0.9732	1	0.9732	0	0	0	1	Ref
MK878900	Unassigned	Unassigned	Unassigned	889_0	Clustered	16	VC_889_0	16	0.2969	1	0.2969	0	0	0	1	Ref
MK878901	Unassigned	Unassigned	Unassigned	1177_0	Clustered	14	VC_1177_0	14	0.7847	1	0.7847	0	0	0	0.989	Ref
MK878903	Unassigned	Unassigned	Unassigned	866_1	Clustered	20	VC_866_1	20	0.4814	1	0.4814	0	0	0	1	Ref
MK878904	Unassigned	Unassigned	Unassigned	1360_0	Clustered	31	VC_1360_0	31	0.8837	1	0.8837	0	0	0	1	Ref
MK878905	Unassigned	Unassigned	Unassigned	517_1	Clustered	108	VC_517_1	108	0.8483	1	0.8483	0	0	0	0.9955	Ref
MK880124	Unassigned	Unassigned	Unassigned	1206_0	Clustered	8	VC_1206_0	8	0.1913	1	0.1913	0	0	0	1	Ref
MK883717	Unassigned	Unassigned	Unassigned	772_4	Clustered	4	VC_772_4	4	0.0181	0.73018303	0.0132	0	0	0	1	Ref
MK883718	Unassigned	Unassigned	Unassigned	772_4	Clustered	4	VC_772_4	4	0.0181	0.73018303	0.0132	0	0	0	1	Ref
MK886800	Unassigned	Unassigned	Unassigned	283_164	Clustered	2	VC_283_164	2	0.003	0.93475788	0.0028	0	0	0	1	Ref
MK893987	Unassigned	Unassigned	Unassigned	667_2	Clustered/Singleton	1	VC_667_2	1	0	1	0	0	0	0	1	Ref
MK894432	Unassigned	Unassigned	Unassigned	1163_0	Clustered	28	VC_1163_0	28	0.4923	1	0.4923	0	0	0	1	Ref
MK894433	Unassigned	Unassigned	Unassigned	223_0	Clustered	38	VC_223_0	38	0.9095	1	0.9095	0	0	0	1	Ref
MK894434	Unassigned	Unassigned	Unassigned	1344_0	Clustered	78	VC_1344_0	78	0.9732	1	0.9732	0	0	0	1	Ref
MK894435	Unassigned	Unassigned	Unassigned	1360_0	Clustered	31	VC_1360_0	31	0.8837	1	0.8837	0	0	0	1	Ref
MK894436	Unassigned	Unassigned	Unassigned	1098_0	Clustered	4	VC_1098_0	4	0.5465	0.99996462	0.5465	0	0	0	1	Ref
MK894438	Unassigned	Unassigned	Unassigned	1360_0	Clustered	31	VC_1360_0	31	0.8837	1	0.8837	0	0	0	1	Ref
MK895508	Unassigned	Unassigned	Unassigned	971_1	Clustered	24	VC_971_1	24	0.7393	1	0.7393	0	0	0	0.8913	Ref
MK903033	Unassigned	Unassigned	Unassigned	96_0	Clustered	30	VC_96_0	30	0.718	1	0.718	0	0	0	1	Ref
MK903277	Unassigned	Unassigned	Unassigned	451_0	Clustered	80	VC_451_0	80	0.9991	1	0.9991	0	0	0	1	Ref
MK903278	Unassigned	Unassigned	Unassigned	74_0	Clustered	15	VC_74_0	15	0.5204	1	0.5204	0	0	0	1	Ref
MK903279	Unassigned	Unassigned	Unassigned	451_0	Clustered	80	VC_451_0	80	0.9991	1	0.9991	0	0	0	1	Ref
MK903281	Unassigned	Unassigned	Unassigned	451_0	Clustered	80	VC_451_0	80	0.9991	1	0.9991	0	0	0	1	Ref
MK903282	Unassigned	Unassigned	Unassigned	618_0	Clustered	13	VC_618_0	13	0.6252	1	0.6252	0	0	0	1	Ref
MK903728	Unassigned	Unassigned	Unassigned	823_0	Clustered	124	VC_823_0	124	0.9492	1	0.9492	0	0	0	1	Ref
MK907226	Unassigned	Unassigned	Unassigned	456_0	Clustered	62	VC_456_0	62	0.9026	1	0.9026	0	0	0	1	Ref
MK907227	Unassigned	Unassigned	Unassigned	456_0	Clustered	62	VC_456_0	62	0.9026	1	0.9026	0	0	0	1	Ref
MK907228	Unassigned	Unassigned	Unassigned	456_0	Clustered	62	VC_456_0	62	0.9026	1	0.9026	0	0	0	1	Ref
MK907229	Unassigned	Unassigned	Unassigned	456_0	Clustered	62	VC_456_0	62	0.9026	1	0.9026	0	0	0	1	Ref
MK907230	Unassigned	Unassigned	Unassigned	456_0	Clustered	62	VC_456_0	62	0.9026	1	0.9026	0	0	0	1	Ref
MK907231	Unassigned	Unassigned	Unassigned	456_0	Clustered	62	VC_456_0	62	0.9026	1	0.9026	0	0	0	1	Ref
MK907232	Unassigned	Unassigned	Unassigned	456_0	Clustered	62	VC_456_0	62	0.9026	1	0.9026	0	0	0	1	Ref
MK907233	Unassigned	Unassigned	Unassigned	456_0	Clustered	62	VC_456_0	62	0.9026	1	0.9026	0	0	0	1	Ref
MK907234	Unassigned	Unassigned	Unassigned	456_0	Clustered	62	VC_456_0	62	0.9026	1	0.9026	0	0	0	1	Ref
MK907235	Unassigned	Unassigned	Unassigned	456_0	Clustered	62	VC_456_0	62	0.9026	1	0.9026	0	0	0	1	Ref
MK907236	Unassigned	Unassigned	Unassigned	228_0	Clustered	60	VC_228_0	60	0.942	1	0.942	0	0	0	1	Ref
MK907237	Unassigned	Unassigned	Unassigned	228_0	Clustered	60	VC_228_0	60	0.942	1	0.942	0	0	0	1	Ref
MK907238	Unassigned	Unassigned	Unassigned	228_0	Clustered	60	VC_228_0	60	0.942	1	0.942	0	0	0	1	Ref
MK907239	Unassigned	Unassigned	Unassigned	228_0	Clustered	60	VC_228_0	60	0.942	1	0.942	0	0	0	1	Ref
MK907240	Unassigned	Unassigned	Unassigned	456_0	Clustered	62	VC_456_0	62	0.9026	1	0.9026	0	0	0	1	Ref
MK907241	Unassigned	Unassigned	Unassigned	456_0	Clustered	62	VC_456_0	62	0.9026	1	0.9026	0	0	0	1	Ref
MK907242	Unassigned	Unassigned	Unassigned	456_0	Clustered	62	VC_456_0	62	0.9026	1	0.9026	0	0	0	1	Ref
MK907243	Unassigned	Unassigned	Unassigned	456_0	Clustered	62	VC_456_0	62	0.9026	1	0.9026	0	0	0	1	Ref
MK907244	Unassigned	Unassigned	Unassigned	456_0	Clustered	62	VC_456_0	62	0.9026	1	0.9026	0	0	0	1	Ref
MK907245	Unassigned	Unassigned	Unassigned	456_0	Clustered	62	VC_456_0	62	0.9026	1	0.9026	0	0	0	1	Ref
MK907246	Unassigned	Unassigned	Unassigned	456_0	Clustered	62	VC_456_0	62	0.9026	1	0.9026	0	0	0	1	Ref
MK907247	Unassigned	Unassigned	Unassigned	456_0	Clustered	62	VC_456_0	62	0.9026	1	0.9026	0	0	0	1	Ref
MK907248	Unassigned	Unassigned	Unassigned	456_0	Clustered	62	VC_456_0	62	0.9026	1	0.9026	0	0	0	1	Ref
MK907249	Unassigned	Unassigned	Unassigned	456_0	Clustered	62	VC_456_0	62	0.9026	1	0.9026	0	0	0	1	Ref
MK907250	Unassigned	Unassigned	Unassigned	456_0	Clustered	62	VC_456_0	62	0.9026	1	0.9026	0	0	0	1	Ref
MK907251	Unassigned	Unassigned	Unassigned	456_0	Clustered	62	VC_456_0	62	0.9026	1	0.9026	0	0	0	1	Ref
MK907252	Unassigned	Unassigned	Unassigned	456_0	Clustered	62	VC_456_0	62	0.9026	1	0.9026	0	0	0	1	Ref
MK907253	Unassigned	Unassigned	Unassigned	456_0	Clustered	62	VC_456_0	62	0.9026	1	0.9026	0	0	0	1	Ref
MK907254	Unassigned	Unassigned	Unassigned	456_0	Clustered	62	VC_456_0	62	0.9026	1	0.9026	0	0	0	1	Ref
MK907255	Unassigned	Unassigned	Unassigned	456_0	Clustered	62	VC_456_0	62	0.9026	1	0.9026	0	0	0	1	Ref
MK907256	Unassigned	Unassigned	Unassigned	456_0	Clustered	62	VC_456_0	62	0.9026	1	0.9026	0	0	0	1	Ref
MK907257	Unassigned	Unassigned	Unassigned	456_0	Clustered	62	VC_456_0	62	0.9026	1	0.9026	0	0	0	1	Ref
MK907258	Unassigned	Unassigned	Unassigned	456_0	Clustered	62	VC_456_0	62	0.9026	1	0.9026	0	0	0	1	Ref
MK907259	Unassigned	Unassigned	Unassigned	456_0	Clustered	62	VC_456_0	62	0.9026	1	0.9026	0	0	0	1	Ref
MK907260	Unassigned	Unassigned	Unassigned	456_0	Clustered	62	VC_456_0	62	0.9026	1	0.9026	0	0	0	1	Ref
MK907261	Unassigned	Unassigned	Unassigned	456_0	Clustered	62	VC_456_0	62	0.9026	1	0.9026	0	0	0	1	Ref
MK907262	Unassigned	Unassigned	Unassigned	456_0	Clustered	62	VC_456_0	62	0.9026	1	0.9026	0	0	0	1	Ref
MK907263	Unassigned	Unassigned	Unassigned	456_0	Clustered	62	VC_456_0	62	0.9026	1	0.9026	0	0	0	1	Ref
MK907264	Unassigned	Unassigned	Unassigned	456_0	Clustered	62	VC_456_0	62	0.9026	1	0.9026	0	0	0	1	Ref
MK907265	Unassigned	Unassigned	Unassigned	456_0	Clustered	62	VC_456_0	62	0.9026	1	0.9026	0	0	0	1	Ref
MK907266	Unassigned	Unassigned	Unassigned	456_0	Clustered	62	VC_456_0	62	0.9026	1	0.9026	0	0	0	1	Ref
MK907267	Unassigned	Unassigned	Unassigned	191_13	Clustered	5	VC_191_13	5	0.0233	0.99999927	0.0233	0	0	0	1	Ref
MK907268	Unassigned	Unassigned	Unassigned	191_13	Clustered	5	VC_191_13	5	0.0233	0.99999927	0.0233	0	0	0	1	Ref
MK907269	Unassigned	Unassigned	Unassigned	191_13	Clustered	5	VC_191_13	5	0.0233	0.99999927	0.0233	0	0	0	1	Ref
MK907270	Unassigned	Unassigned	Unassigned	191_13	Clustered	5	VC_191_13	5	0.0233	0.99999927	0.0233	0	0	0	1	Ref
MK907271	Unassigned	Unassigned	Unassigned	191_13	Clustered	5	VC_191_13	5	0.0233	0.99999927	0.0233	0	0	0	1	Ref
MK907272	Unassigned	Unassigned	Unassigned	456_0	Clustered	62	VC_456_0	62	0.9026	1	0.9026	0	0	0	1	Ref
MK907273	Unassigned	Unassigned	Unassigned	456_0	Clustered	62	VC_456_0	62	0.9026	1	0.9026	0	0	0	1	Ref
MK907274	Unassigned	Unassigned	Unassigned	456_0	Clustered	62	VC_456_0	62	0.9026	1	0.9026	0	0	0	1	Ref
MK907275	Unassigned	Unassigned	Unassigned	456_0	Clustered	62	VC_456_0	62	0.9026	1	0.9026	0	0	0	1	Ref
MK907276	Unassigned	Unassigned	Unassigned	456_0	Clustered	62	VC_456_0	62	0.9026	1	0.9026	0	0	0	1	Ref
MK907285	Unassigned	Unassigned	Unassigned	386_0	Clustered	49	VC_386_0	49	0.7941	1	0.7941	0	0	0	1	Ref

MK907780	Unassigned	Unassigned	Unassigned	971_1	Clustered	24	VC_971_1	24	0.7393	1	0.7393	0	0	0	0.8913	Ref
MK919469	Unassigned	Unassigned	Unassigned	525_1	Clustered	5	VC_525_1	5	0.0485	0.99999947	0.0485	0	0	0	0.9	Ref
MK919470	Unassigned	Unassigned	Unassigned	1176_0	Clustered	41	VC_1176_0	41	0.9558	1	0.9558	0	0	0	0.9976	Ref
MK919471	Unassigned	Unassigned	Unassigned	1240_0	Clustered	82	VC_1240_0	82	0.7945	1	0.7945	0	0	0	1	Ref
MK919472	Unassigned	Unassigned	Unassigned	216_6	Clustered	42	VC_216_6	42	0.473	1	0.473	0	0	0	0.8351	Ref
MK919473	Unassigned	Unassigned	Unassigned	802_0	Clustered	17	VC_802_0	17	0.8025	1	0.8025	0	0	0	1	Ref
MK919474	Unassigned	Unassigned	Unassigned	856_1	Clustered	13	VC_856_1	13	0.5848	1	0.5848	0	0	0	1	Ref
MK919475	Unassigned	Unassigned	Unassigned	761_0	Clustered	67	VC_761_0	67	0.8947	1	0.8947	0	0	0	1	Ref
MK919476	Unassigned	Unassigned	Unassigned	1160_0	Clustered	73	VC_1160_0	73	0.9835	1	0.9835	0	0	0	1	Ref
MK919477	Unassigned	Unassigned	Unassigned	866_1	Clustered	20	VC_866_1	20	0.4814	1	0.4814	0	0	0	1	Ref
MK919478	Unassigned	Unassigned	Unassigned	1183_0	Clustered	6	VC_1183_0	6	0.995	0.99996974	0.995	0	0	0	1	Ref
MK919479	Unassigned	Unassigned	Unassigned	1073_0	Clustered	24	VC_1073_0	24	0.8388	1	0.8388	0	0	0	1	Ref
MK919480	Unassigned	Unassigned	Unassigned	519_0	Clustered	39	VC_519_0	39	0.8156	1	0.8156	0	0	0	1	Ref
MK919481	Unassigned	Unassigned	Unassigned	866_1	Clustered	20	VC_866_1	20	0.4814	1	0.4814	0	0	0	1	Ref
MK919482	Unassigned	Unassigned	Unassigned	810_0	Clustered	18	VC_810_0	18	0.9121	1	0.9121	0	0	0	1	Ref
MK919483	Unassigned	Unassigned	Unassigned	525_0	Clustered	25	VC_525_0	25	0.4597	1	0.4597	0	0	0	1	Ref
MK919484	Unassigned	Unassigned	Unassigned	1160_0	Clustered	73	VC_1160_0	73	0.9835	1	0.9835	0	0	0	1	Ref
MK922546	Unassigned	Unassigned	Unassigned	96_0	Clustered	30	VC_96_0	30	0.718	1	0.718	0	0	0	1	Ref
MK922547	Unassigned	Unassigned	Unassigned	96_0	Clustered	30	VC_96_0	30	0.718	1	0.718	0	0	0	1	Ref
MK922548	Unassigned	Unassigned	Unassigned	96_0	Clustered	30	VC_96_0	30	0.718	1	0.718	0	0	0	1	Ref
MK929790	Unassigned	Unassigned	Unassigned	1407_0	Clustered	2	VC_1407_0	2	0.8176	0.81445332	0.6659	0	0	0	1	Ref
MK931297	Unassigned	Unassigned	Unassigned	779_0	Clustered	37	VC_779_0	37	0.5682	1	0.5682	0	0	0	1	Ref
MK931438	Unassigned	Unassigned	Unassigned	638_0	Clustered	45	VC_638_0	45	0.7741	1	0.7741	0	0	0	1	Ref
MK931439	Unassigned	Unassigned	Unassigned	638_0	Clustered	45	VC_638_0	45	0.7741	1	0.7741	0	0	0	1	Ref
MK931440	Unassigned	Unassigned	Unassigned	638_0	Clustered	45	VC_638_0	45	0.7741	1	0.7741	0	0	0	1	Ref
MK931441	Unassigned	Unassigned	Unassigned	638_0	Clustered	45	VC_638_0	45	0.7741	1	0.7741	0	0	0	1	Ref
MK931442	Unassigned	Unassigned	Unassigned	938_0	Clustered	99	VC_938_0	99	0.9119	1	0.9119	0	0	0	0.9928	Ref
MK931443	Unassigned	Unassigned	Unassigned	938_0	Clustered	99	VC_938_0	99	0.9119	1	0.9119	0	0	0	0.9928	Ref
MK931444	Unassigned	Unassigned	Unassigned	938_0	Clustered	99	VC_938_0	99	0.9119	1	0.9119	0	0	0	0.9928	Ref
MK931445	Unassigned	Unassigned	Unassigned	938_0	Clustered	99	VC_938_0	99	0.9119	1	0.9119	0	0	0	0.9928	Ref
MK931446	Unassigned	Unassigned	Unassigned	637_1	Clustered	27	VC_637_1	27	0.2578	1	0.2578	0	0	0	0.9858	Ref
MK932884	Unassigned	Unassigned	Unassigned	851_0	Clustered	2	VC_851_0	2	0.4472	0.9410422	0.4209	0	0	0	1	Ref
MK934841	Unassigned	Unassigned	Unassigned	251_0	Clustered	22	VC_251_0	22	0.6508	1	0.6508	0	0	0	1	Ref
MK936475	Unassigned	Unassigned	Unassigned	96_0	Clustered	30	VC_96_0	30	0.718	1	0.718	0	0	0	1	Ref
MK936476	Unassigned	Unassigned	Unassigned	96_0	Clustered	30	VC_96_0	30	0.718	1	0.718	0	0	0	1	Ref
MK937591	Unassigned	Unassigned	Unassigned	1349_0	Clustered	7	VC_1349_0	7	0.2495	1	0.2495	0	0	0	1	Ref
MK937593	Unassigned	Unassigned	Unassigned	551_0	Clustered	16	VC_551_0	16	0.0915	1	0.0915	0	0	0	0.6667	Ref
MK937594	Unassigned	Unassigned	Unassigned	1093_0	Clustered	40	VC_1093_0	40	0.8754	1	0.8754	0	0	0	1	Ref
MK937595	Unassigned	Unassigned	Unassigned	374_0	Clustered	2	VC_374_0	2	0.1258	0.95578317	0.1203	0	0	0	1	Ref
MK937596	Unassigned	Unassigned	Unassigned	856_1	Clustered	13	VC_856_1	13	0.5848	1	0.5848	0	0	0	1	Ref
MK937597	Unassigned	Unassigned	Unassigned	557_0	Clustered	3	VC_557_0	3	0.0814	0.99853279	0.0812	0	0	0	0.6667	Ref
MK937598	Unassigned	Unassigned	Unassigned	1234_0	Clustered	44	VC_1234_0	44	1	1	1	0	0	0	1	Ref
MK937599	Unassigned	Unassigned	Unassigned	1177_0	Clustered	14	VC_1177_0	14	0.7847	1	0.7847	0	0	0	0.989	Ref
MK937600	Unassigned	Unassigned	Unassigned	888_0	Clustered	28	VC_888_0	28	0.5497	1	0.5497	0	0	0	1	Ref
MK937601	Unassigned	Unassigned	Unassigned	1093_0	Clustered	40	VC_1093_0	40	0.8754	1	0.8754	0	0	0	1	Ref
MK937602	Unassigned	Unassigned	Unassigned	1163_0	Clustered	28	VC_1163_0	28	0.4923	1	0.4923	0	0	0	1	Ref
MK937603	Unassigned	Unassigned	Unassigned	1178_0	Clustered	24	VC_1178_0	24	0.9524	1	0.9524	0	0	0	1	Ref
MK937604	Unassigned	Unassigned	Unassigned	519_0	Clustered	39	VC_519_0	39	0.8156	1	0.8156	0	0	0	1	Ref
MK937606	Unassigned	Unassigned	Unassigned	1349_0	Clustered	7	VC_1349_0	7	0.2495	1	0.2495	0	0	0	1	Ref
MK937607	Unassigned	Unassigned	Unassigned	1095_0	Clustered	16	VC_1095_0	16	0.458	1	0.458	0	0	0	1	Ref
MK937608	Unassigned	Unassigned	Unassigned	1349_0	Clustered	7	VC_1349_0	7	0.2495	1	0.2495	0	0	0	1	Ref
MK937610	Unassigned	Unassigned	Unassigned	1234_0	Clustered	44	VC_1234_0	44	1	1	1	0	0	0	1	Ref
MK937611	Unassigned	Unassigned	Unassigned	1095_0	Clustered	16	VC_1095_0	16	0.458	1	0.458	0	0	0	1	Ref
MK937612	Unassigned	Unassigned	Unassigned	763_1	Clustered	9	VC_763_1	9	0.3706	1	0.3706	0	0	0	1	Ref
MK937613	Unassigned	Unassigned	Unassigned	1199_0	Clustered	8	VC_1199_0	8	0.7928	1	0.7928	0	0	0	1	Ref
MK940809	Unassigned	Unassigned	Unassigned	8_0	Clustered	58	VC_8_0	58	0.8306	1	0.8306	0	0	0	0.9946	Ref
MK947458	Unassigned	Unassigned	Unassigned	191_16	Clustered	12	VC_191_16	12	0.0643	1	0.0643	0	0	0	0.9394	Ref
MK947459	Unassigned	Unassigned	Unassigned	191_17	Clustered	4	VC_191_17	4	0.0166	0.99930063	0.0166	0	0	0	0.8333	Ref
MK947460	Unassigned	Unassigned	Unassigned	461_0	Clustered	96	VC_461_0	96	0.9307	1	0.9307	0	0	0	0.9575	Ref
MK962626	Unassigned	Unassigned	Unassigned	779_1	Clustered	11	VC_779_1	11	0.1587	1	0.1587	0	0	0	1	Ref
MK962627	Unassigned	Unassigned	Unassigned	171_0	Clustered	8	VC_171_0	8	0.5884	1	0.5884	0	0	0	1	Ref
MK962628	Unassigned	Unassigned	Unassigned	157_0	Clustered	3	VC_157_0	3	0.3732	0.99808097	0.3725	0	0	0	1	Ref
MK962629	Unassigned	Unassigned	Unassigned	779_1	Clustered	11	VC_779_1	11	0.1587	1	0.1587	0	0	0	1	Ref
MK962630	Unassigned	Unassigned	Unassigned	779_1	Clustered	11	VC_779_1	11	0.1587	1	0.1587	0	0	0	1	Ref
MK962631	Unassigned	Unassigned	Unassigned	779_1	Clustered	11	VC_779_1	11	0.1587	1	0.1587	0	0	0	1	Ref
MK962632	Unassigned	Unassigned	Unassigned	779_1	Clustered	11	VC_779_1	11	0.1587	1	0.1587	0	0	0	1	Ref
MK962633	Unassigned	Unassigned	Unassigned	171_0	Clustered	8	VC_171_0	8	0.5884	1	0.5884	0	0	0	1	Ref
MK962634	Unassigned	Unassigned	Unassigned	171_0	Clustered	8	VC_171_0	8	0.5884	1	0.5884	0	0	0	1	Ref
MK962635	Unassigned	Unassigned	Unassigned	751_0	Clustered	4	VC_751_0	4	0.1763	0.99998526	0.1763	0	0	0	1	Ref
MK962636	Unassigned	Unassigned	Unassigned	751_0	Clustered	4	VC_751_0	4	0.1763	0.99998526	0.1763	0	0	0	1	Ref
MK962637	Unassigned	Unassigned	Unassigned	751_0	Clustered	4	VC_751_0	4	0.1763	0.99998526	0.1763	0	0	0	1	Ref
MK962638	Unassigned	Unassigned	Unassigned	157_0	Clustered	3	VC_157_0	3	0.3732	0.99808097	0.3725	0	0	0	1	Ref
MK962639	Unassigned	Unassigned	Unassigned	779_1	Clustered	11	VC_779_1	11	0.1587	1	0.1587	0	0	0	1	Ref
MK962640	Unassigned	Unassigned	Unassigned	157_0	Clustered	3	VC_157_0	3	0.3732	0.99808097	0.3725	0	0	0	1	Ref
MK962641	Unassigned	Unassigned	Unassigned	779_1	Clustered	11	VC_779_1	11	0.1587	1	0.1587	0	0	0	1	Ref
MK962749	Unassigned	Unassigned	Unassigned	772_1	Clustered	2	VC_772_1	2	0.006	0.57682361	0.0034	0	0	0	1	Ref
MK962750	Unassigned	Unassigned	Unassigned	283_169	Clustered/Singleton	1	VC_283_169	1	0	1	0	0	0	0	1	Ref
MK962751	Unassigned	Unassigned	Unassigned	942_0	Clustered	36	VC_942_0	36	0.7848	1	0.7848	0	0	0	1	Ref
MK962752	Unassigned	Unassigned	Unassigned	283_120	Clustered/Singleton	1	VC_283_120	1	0	1	0	0	0	0	1	Ref
MK962753	Unassigned	Unassigned	Unassigned	590_0	Clustered	27	VC_590_0	27	0.9947	1	0.9947	0	0	0	1	Ref
MK962754	Unassigned	Unassigned	Unassigned	286_1	Clustered	21	VC_286_1	21	0.1696	1	0.1696	0	0	0	0.9048	Ref

MK962755	Unassigned	Unassigned	Unassigned	283_35	Clustered/Singleton	1	VC_283_35	1	0	1	0	0	0	0	1	Ref
MK962756	Unassigned	Unassigned	Unassigned	286_1	Clustered	21	VC_286_1	21	0.1696	1	0.1696	0	0	0	0.9048	Ref
MK962757	Unassigned	Unassigned	Unassigned	286_1	Clustered	21	VC_286_1	21	0.1696	1	0.1696	0	0	0	0.9048	Ref
MK962758	Unassigned	Unassigned	Unassigned	329_3	Clustered	24	VC_329_3	24	0.1206	1	0.1206	0	0	0	0.7283	Ref
MK965969	Unassigned	Unassigned	Unassigned	329_1	Clustered	17	VC_329_1	17	0.0867	1	0.0867	0	0	0	0.8235	Ref
MK965970	Unassigned	Unassigned	Unassigned	329_1	Clustered	17	VC_329_1	17	0.0867	1	0.0867	0	0	0	0.8235	Ref
MK967377	Unassigned	Unassigned	Unassigned	1177_0	Clustered	14	VC_1177_0	14	0.7847	1	0.7847	0	0	0	0.989	Ref
MK967378	Unassigned	Unassigned	Unassigned	890_0	Clustered	3	VC_890_0	3	0.3565	0.99830951	0.3559	0	0	0	1	Ref
MK967379	Unassigned	Unassigned	Unassigned	546_4	Clustered	16	VC_546_4	16	0.2517	0.9999998	0.2517	0	0	0	0.9417	Ref
MK967381	Unassigned	Unassigned	Unassigned	1178_0	Clustered	24	VC_1178_0	24	0.9524	1	0.9524	0	0	0	1	Ref
MK967382	Unassigned	Unassigned	Unassigned	224_0	Clustered	19	VC_224_0	19	0.9416	1	0.9416	0	0	0	1	Ref
MK967383	Unassigned	Unassigned	Unassigned	919_0	Clustered	39	VC_919_0	39	0.9069	1	0.9069	0	0	0	1	Ref
MK967384	Unassigned	Unassigned	Unassigned	525_1	Clustered	5	VC_525_1	5	0.0485	0.99999947	0.0485	0	0	0	0.9	Ref
MK967385	Unassigned	Unassigned	Unassigned	919_0	Clustered	39	VC_919_0	39	0.9069	1	0.9069	0	0	0	1	Ref
MK967386	Unassigned	Unassigned	Unassigned	872_0	Clustered	31	VC_872_0	31	0.8151	1	0.8151	0	0	0	0.8194	Ref
MK967387	Unassigned	Unassigned	Unassigned	325_19	Clustered	3	VC_325_19	3	0.0108	0.99859221	0.0107	0	0	0	1	Ref
MK967388	Unassigned	Unassigned	Unassigned	1240_0	Clustered	82	VC_1240_0	82	0.7945	1	0.7945	0	0	0	1	Ref
MK967389	Unassigned	Unassigned	Unassigned	1176_0	Clustered	41	VC_1176_0	41	0.9558	1	0.9558	0	0	0	0.9976	Ref
MK967390	Unassigned	Unassigned	Unassigned	868_0	Clustered	25	VC_868_0	25	0.5791	1	0.5791	0	0	0	1	Ref
MK967391	Unassigned	Unassigned	Unassigned	1240_0	Clustered	82	VC_1240_0	82	0.7945	1	0.7945	0	0	0	1	Ref
MK967392	Unassigned	Unassigned	Unassigned	954_0	Clustered	13	VC_954_0	13	0.863	1	0.863	0	0	0	1	Ref
MK967393	Unassigned	Unassigned	Unassigned	953_0	Clustered	2	VC_953_0	2	0.2116	0.94820857	0.2007	0	0	0	1	Ref
MK967394	Unassigned	Unassigned	Unassigned	1240_0	Clustered	82	VC_1240_0	82	0.7945	1	0.7945	0	0	0	1	Ref
MK967395	Unassigned	Unassigned	Unassigned	216_4	Clustered	13	VC_216_4	13	0.1657	1	0.1657	0	0	0	1	Ref
MK967396	Unassigned	Unassigned	Unassigned	1093_0	Clustered	40	VC_1093_0	40	0.8754	1	0.8754	0	0	0	1	Ref
MK967397	Unassigned	Unassigned	Unassigned	520_94	Clustered/Singleton	1	VC_520_94	1	0	1	0	0	0	0	1	Ref
MK967398	Unassigned	Unassigned	Unassigned	802_0	Clustered	17	VC_802_0	17	0.8025	1	0.8025	0	0	0	1	Ref
MK967399	Unassigned	Unassigned	Unassigned	514_0	Clustered	75	VC_514_0	75	0.9402	1	0.9402	0	0	0	1	Ref
MK967400	Unassigned	Unassigned	Unassigned	1240_0	Clustered	82	VC_1240_0	82	0.7945	1	0.7945	0	0	0	1	Ref
MK967401	Unassigned	Unassigned	Unassigned	325_46	Clustered	2	VC_325_46	2	0.004	0.95751617	0.0039	0	0	0	1	Ref
MK967402	Unassigned	Unassigned	Unassigned	546_4	Clustered	16	VC_546_4	16	0.2517	0.9999998	0.2517	0	0	0	0.9417	Ref
MK972685	Unassigned	Unassigned	Unassigned	28_1	Clustered	53	VC_28_1	53	0.7164	1	0.7164	0	0	0	0.9325	Ref
MK972686	Unassigned	Unassigned	Unassigned	28_1	Clustered	53	VC_28_1	53	0.7164	1	0.7164	0	0	0	0.9325	Ref
MK972687	Unassigned	Unassigned	Unassigned	28_1	Clustered	53	VC_28_1	53	0.7164	1	0.7164	0	0	0	0.9325	Ref
MK972690	Unassigned	Unassigned	Unassigned	133_21	Clustered/Singleton	1	VC_133_21	1	0	1	0	0	0	0	1	Ref
MK972691	Unassigned	Unassigned	Unassigned	637_0	Clustered	115	VC_637_0	115	0.731	1	0.731	0	0	0	0.8764	Ref
MK972692	Unassigned	Unassigned	Unassigned	28_1	Clustered	53	VC_28_1	53	0.7164	1	0.7164	0	0	0	0.9325	Ref
MK972693	Unassigned	Unassigned	Unassigned	28_1	Clustered	53	VC_28_1	53	0.7164	1	0.7164	0	0	0	0.9325	Ref
MK972694	Unassigned	Unassigned	Unassigned	28_1	Clustered	53	VC_28_1	53	0.7164	1	0.7164	0	0	0	0.9325	Ref
MK972698	Unassigned	Unassigned	Unassigned	133_48	Clustered	2	VC_133_48	2	0.0052	0.93639952	0.0048	0	0	0	1	Ref
MK972699	Unassigned	Unassigned	Unassigned	133_48	Clustered	2	VC_133_48	2	0.0052	0.93639952	0.0048	0	0	0	1	Ref
MK972700	Unassigned	Unassigned	Unassigned	637_0	Clustered	115	VC_637_0	115	0.731	1	0.731	0	0	0	0.8764	Ref
MK972701	Unassigned	Unassigned	Unassigned	637_0	Clustered	115	VC_637_0	115	0.731	1	0.731	0	0	0	0.8764	Ref
MK972702	Unassigned	Unassigned	Unassigned	637_0	Clustered	115	VC_637_0	115	0.731	1	0.731	0	0	0	0.8764	Ref
MK972703	Unassigned	Unassigned	Unassigned	637_0	Clustered	115	VC_637_0	115	0.731	1	0.731	0	0	0	0.8764	Ref
MK972704	Unassigned	Unassigned	Unassigned	191_3	Clustered	5	VC_191_3	5	0.0231	0.99999849	0.0231	0	0	0	0.8	Ref
MK972705	Unassigned	Unassigned	Unassigned	637_0	Clustered	115	VC_637_0	115	0.731	1	0.731	0	0	0	0.8764	Ref
MK972706	Unassigned	Unassigned	Unassigned	637_0	Clustered	115	VC_637_0	115	0.731	1	0.731	0	0	0	0.8764	Ref
MK972707	Unassigned	Unassigned	Unassigned	191_3	Clustered	5	VC_191_3	5	0.0231	0.99999849	0.0231	0	0	0	0.8	Ref
MK972708	Unassigned	Unassigned	Unassigned	191_31	Clustered	2	VC_191_31	2	0.0057	0.92424379	0.0052	0	0	0	1	Ref
MK972709	Unassigned	Unassigned	Unassigned	191_3	Clustered	5	VC_191_3	5	0.0231	0.99999849	0.0231	0	0	0	0.8	Ref
MK972710	Unassigned	Unassigned	Unassigned	228_0	Clustered	60	VC_228_0	60	0.942	1	0.942	0	0	0	1	Ref
MK972711	Unassigned	Unassigned	Unassigned	228_0	Clustered	60	VC_228_0	60	0.942	1	0.942	0	0	0	1	Ref
MK972712	Unassigned	Unassigned	Unassigned	5_0	Clustered	30	VC_5_0	30	0.9647	1	0.9647	0	0	0	1	Ref
MK972713	Unassigned	Unassigned	Unassigned	5_0	Clustered	30	VC_5_0	30	0.9647	1	0.9647	0	0	0	1	Ref
MK972714	Unassigned	Unassigned	Unassigned	5_0	Clustered	30	VC_5_0	30	0.9647	1	0.9647	0	0	0	1	Ref
MK972715	Unassigned	Unassigned	Unassigned	190_20	Clustered	4	VC_190_20	4	0.0229	0.99997885	0.0229	0	0	0	1	Ref
MK972716	Unassigned	Unassigned	Unassigned	190_20	Clustered	4	VC_190_20	4	0.0229	0.99997885	0.0229	0	0	0	1	Ref
MK972717	Unassigned	Unassigned	Unassigned	191_16	Clustered	12	VC_191_16	12	0.0643	1	0.0643	0	0	0	0.9394	Ref
MK972831	Unassigned	Unassigned	Unassigned	575_0	Clustered	35	VC_575_0	35	0.8815	1	0.8815	0	0	0	1	Ref
MK976036	Unassigned	Unassigned	Unassigned	22_0	Clustered	11	VC_22_0	11	0.5163	1	0.5163	0	0	0	1	Ref
MK977694	Unassigned	Unassigned	Unassigned	283_190	Clustered/Singleton	1	VC_283_190	1	0	1	0	0	0	0	1	Ref
MK977697	Unassigned	Unassigned	Unassigned	1240_0	Clustered	82	VC_1240_0	82	0.7945	1	0.7945	0	0	0	1	Ref
MK977698	Unassigned	Unassigned	Unassigned	1352_0	Clustered	31	VC_1352_0	31	0.849	1	0.849	0	0	0	1	Ref
MK977699	Unassigned	Unassigned	Unassigned	767_0	Clustered	34	VC_767_0	34	0.9173	1	0.9173	0	0	0	1	Ref
MK977700	Unassigned	Unassigned	Unassigned	768_0	Clustered	8	VC_768_0	8	0.7012	1	0.7012	0	0	0	1	Ref
MK977701	Unassigned	Unassigned	Unassigned	686_1	Clustered	2	VC_686_1	2	0.2204	0.91327915	0.2013	0	0	0	1	Ref
MK977702	Unassigned	Unassigned	Unassigned	1093_0	Clustered	40	VC_1093_0	40	0.8754	1	0.8754	0	0	0	1	Ref
MK977705	Unassigned	Unassigned	Unassigned	1182_0	Clustered	4	VC_1182_0	4	0.2845	0.99997676	0.2845	0	0	0	1	Ref
MK977706	Unassigned	Unassigned	Unassigned	1199_0	Clustered	8	VC_1199_0	8	0.7928	1	0.7928	0	0	0	1	Ref
MK977708	Unassigned	Unassigned	Unassigned	521_0	Clustered	37	VC_521_0	37	0.912	1	0.912	0	0	0	1	Ref
MK977709	Unassigned	Unassigned	Unassigned	523_0	Clustered/Singleton	1	VC_523_0	1	0	1	0	0	0	0	1	Ref
MK977710	Unassigned	Unassigned	Unassigned	1199_0	Clustered	8	VC_1199_0	8	0.7928	1	0.7928	0	0	0	1	Ref
MK977711	Unassigned	Unassigned	Unassigned	1071_3	Clustered	16	VC_1071_3	16	0.2354	1	0.2354	0	0	0	0.9667	Ref
MK977712	Unassigned	Unassigned	Unassigned	1199_0	Clustered	8	VC_1199_0	8	0.7928	1	0.7928	0	0	0	1	Ref
MK977713	Unassigned	Unassigned	Unassigned	225_0	Clustered	32	VC_225_0	32	0.848	1	0.848	0	0	0	0.996	Ref
MK977714	Unassigned	Unassigned	Unassigned	1199_0	Clustered	8	VC_1199_0	8	0.7928	1	0.7928	0	0	0	1	Ref
MK977715	Unassigned	Unassigned	Unassigned	1199_0	Clustered	8	VC_1199_0	8	0.7928	1	0.7928	0	0	0	1	Ref
MK982307	Unassigned	Unassigned	Unassigned	695_0	Clustered	48	VC_695_0	48	0.8162	1	0.8162	0	0	0	1	Ref
MK994515	Unassigned	Unassigned	Unassigned	488_1	Clustered	8	VC_488_1	8	0.2927	1	0.2927	0	0	0	1	Ref
MN010755	Unassigned	Unassigned	Unassigned	1344_0	Clustered	78	VC_1344_0	78	0.9732	1	0.9732	0	0	0	1	Ref

MN010756	Unassigned	Unassigned	Unassigned	567_0	Clustered	8	VC_567_0	8	0.4935	1	0.4935	0	0	0	1	Ref
MN010757	Unassigned	Unassigned	Unassigned	519_0	Clustered	39	VC_519_0	39	0.8156	1	0.8156	0	0	0	1	Ref
MN010758	Unassigned	Unassigned	Unassigned	406_0	Clustered	11	VC_406_0	11	0.9449	1	0.9449	0	0	0	1	Ref
MN010759	Unassigned	Unassigned	Unassigned	1344_0	Clustered	78	VC_1344_0	78	0.9732	1	0.9732	0	0	0	1	Ref
MN010760	Unassigned	Unassigned	Unassigned	1178_0	Clustered	24	VC_1178_0	24	0.9524	1	0.9524	0	0	0	1	Ref
MN010761	Unassigned	Unassigned	Unassigned	799_1	Clustered	27	VC_799_1	27	0.8611	1	0.8611	0	0	0	1	Ref
MN010762	Unassigned	Unassigned	Unassigned	1176_0	Clustered	41	VC_1176_0	41	0.9558	1	0.9558	0	0	0	0.9976	Ref
MN013074	Unassigned	Unassigned	Unassigned	385_0	Clustered	51	VC_385_0	51	0.8754	1	0.8754	0	0	0	1	Ref
MN013075	Unassigned	Unassigned	Unassigned	937_0	Clustered	40	VC_937_0	40	0.6809	1	0.6809	0	0	0	0.9359	Ref
MN013076	Unassigned	Unassigned	Unassigned	937_0	Clustered	40	VC_937_0	40	0.6809	1	0.6809	0	0	0	0.9359	Ref
MN013077	Unassigned	Unassigned	Unassigned	849_6	Clustered	7	VC_849_6	7	0.164	0.99999999	0.164	0	0	0	0.9524	Ref
MN013078	Unassigned	Unassigned	Unassigned	937_0	Clustered	40	VC_937_0	40	0.6809	1	0.6809	0	0	0	0.9359	Ref
MN013079	Unassigned	Unassigned	Unassigned	937_0	Clustered	40	VC_937_0	40	0.6809	1	0.6809	0	0	0	0.9359	Ref
MN013080	Unassigned	Unassigned	Unassigned	937_0	Clustered	40	VC_937_0	40	0.6809	1	0.6809	0	0	0	0.9359	Ref
MN013082	Unassigned	Unassigned	Unassigned	393_0	Clustered	7	VC_393_0	7	0.1775	1	0.1775	0	0	0	1	Ref
MN013083	Unassigned	Unassigned	Unassigned	937_0	Clustered	40	VC_937_0	40	0.6809	1	0.6809	0	0	0	0.9359	Ref
MN013085	Unassigned	Unassigned	Unassigned	393_0	Clustered	7	VC_393_0	7	0.1775	1	0.1775	0	0	0	1	Ref
MN013086	Unassigned	Unassigned	Unassigned	37_1	Clustered	3	VC_37_1	3	0.0833	0.9484635	0.079	0	0	0	1	Ref
MN013087	Unassigned	Unassigned	Unassigned	937_0	Clustered	40	VC_937_0	40	0.6809	1	0.6809	0	0	0	0.9359	Ref
MN013088	Unassigned	Unassigned	Unassigned	104_0	Clustered	6	VC_104_0	6	0.8291	1	0.8291	0	0	0	1	Ref
MN013089	Unassigned	Unassigned	Unassigned	187_1	Clustered	7	VC_187_1	7	0.1423	1	0.1423	0	0	0	1	Ref
MN013356	Unassigned	Unassigned	Unassigned	76_0	Clustered	37	VC_76_0	37	0.9403	1	0.9403	0	0	0	1	Ref
MN016939	Unassigned	Unassigned	Unassigned	228_0	Clustered	60	VC_228_0	60	0.942	1	0.942	0	0	0	1	Ref
MN018232	Unassigned	Unassigned	Unassigned	432_14	Clustered/Singleton	1	VC_432_14	1	0	1	0	0	0	0	1	Ref
MN019128	Unassigned	Unassigned	Unassigned	585_0	Clustered	4	VC_585_0	4	0.0533	0.99998755	0.0533	0	0	0	1	Ref
MN022785	Unassigned	Unassigned	Unassigned	283_130	Clustered/Singleton	1	VC_283_130	1	0	1	0	0	0	0	1	Ref
MN022787	Unassigned	Unassigned	Unassigned	190_14	Clustered	3	VC_190_14	3	0.0144	0.99696852	0.0143	0	0	0	0.6667	Ref
MN023176	Unassigned	Unassigned	Unassigned	860_0	Clustered	16	VC_860_0	16	0.9964	1	0.9964	0	0	0	1	Ref
MN023177	Unassigned	Unassigned	Unassigned	860_0	Clustered	16	VC_860_0	16	0.9964	1	0.9964	0	0	0	1	Ref
MN023178	Unassigned	Unassigned	Unassigned	860_0	Clustered	16	VC_860_0	16	0.9964	1	0.9964	0	0	0	1	Ref
MN023179	Unassigned	Unassigned	Unassigned	860_0	Clustered	16	VC_860_0	16	0.9964	1	0.9964	0	0	0	1	Ref
MN023180	Unassigned	Unassigned	Unassigned	860_0	Clustered	16	VC_860_0	16	0.9964	1	0.9964	0	0	0	1	Ref
MN023181	Unassigned	Unassigned	Unassigned	860_0	Clustered	16	VC_860_0	16	0.9964	1	0.9964	0	0	0	1	Ref
MN023182	Unassigned	Unassigned	Unassigned	860_0	Clustered	16	VC_860_0	16	0.9964	1	0.9964	0	0	0	1	Ref
MN023183	Unassigned	Unassigned	Unassigned	860_0	Clustered	16	VC_860_0	16	0.9964	1	0.9964	0	0	0	1	Ref
MN023184	Unassigned	Unassigned	Unassigned	860_0	Clustered	16	VC_860_0	16	0.9964	1	0.9964	0	0	0	1	Ref
MN023185	Unassigned	Unassigned	Unassigned	860_0	Clustered	16	VC_860_0	16	0.9964	1	0.9964	0	0	0	1	Ref
MN023186	Unassigned	Unassigned	Unassigned	860_0	Clustered	16	VC_860_0	16	0.9964	1	0.9964	0	0	0	1	Ref
MN023187	Unassigned	Unassigned	Unassigned	860_0	Clustered	16	VC_860_0	16	0.9964	1	0.9964	0	0	0	1	Ref
MN023188	Unassigned	Unassigned	Unassigned	860_0	Clustered	16	VC_860_0	16	0.9964	1	0.9964	0	0	0	1	Ref
MN023189	Unassigned	Unassigned	Unassigned	860_0	Clustered	16	VC_860_0	16	0.9964	1	0.9964	0	0	0	1	Ref
MN023190	Unassigned	Unassigned	Unassigned	860_0	Clustered	16	VC_860_0	16	0.9964	1	0.9964	0	0	0	1	Ref
MN023191	Unassigned	Unassigned	Unassigned	860_0	Clustered	16	VC_860_0	16	0.9964	1	0.9964	0	0	0	1	Ref
MN026740	Unassigned	Unassigned	Unassigned	392_0	Clustered	126	VC_392_0	126	0.9621	1	0.9621	0	0	0	1	Ref
MN026741	Unassigned	Unassigned	Unassigned	190_23	Clustered	10	VC_190_23	10	0.0704	1	0.0704	0	0	0	0.8444	Ref
MN027503	Unassigned	Unassigned	Unassigned	92_0	Clustered	22	VC_92_0	22	0.5639	1	0.5639	0	0	0	1	Ref
MN028536	Unassigned	Unassigned	Unassigned	11_0	Clustered	19	VC_11_0	19	0.7562	1	0.7562	0	0	0	1	Ref
MN029011	Unassigned	Unassigned	Unassigned	796_0	Clustered	5	VC_796_0	5	0.3352	0.99999997	0.3352	0	0	0	1	Ref
MN032614	Unassigned	Unassigned	Unassigned	442_3	Clustered	3	VC_442_3	3	0.0529	0.99675181	0.0527	0	0	0	1	Ref
MN038175	Unassigned	Unassigned	Unassigned	669_3	Clustered/Singleton	1	VC_669_3	1	0	1	0	0	0	0	1	Ref
MN038176	Unassigned	Unassigned	Unassigned	884_0	Clustered	28	VC_884_0	28	0.6211	1	0.6211	0	0	0	0.9841	Ref
MN038177	Unassigned	Unassigned	Unassigned	618_0	Clustered	13	VC_618_0	13	0.6252	1	0.6252	0	0	0	1	Ref
MN038178	Unassigned	Unassigned	Unassigned	883_1	Clustered	12	VC_883_1	12	0.2441	1	0.2441	0	0	0	1	Ref
MN038179	Unassigned	Unassigned	Unassigned	884_0	Clustered	28	VC_884_0	28	0.6211	1	0.6211	0	0	0	0.9841	Ref
MN043729	Unassigned	Unassigned	Unassigned	85_10	Clustered/Singleton	1	VC_85_10	1	0	1	0	0	0	0	1	Ref
MN043730	Unassigned	Unassigned	Unassigned	85_4	Clustered	3	VC_85_4	3	0.0294	0.98125255	0.0288	0	0	0	1	Ref
MN045228	Unassigned	Unassigned	Unassigned	149_12	Clustered	2	VC_149_12	2	0.0044	0.83156708	0.0037	0	0	0	1	Ref
MN045229	Unassigned	Unassigned	Unassigned	792_0	Clustered	35	VC_792_0	35	0.9828	1	0.9828	0	0	0	1	Ref
MN045230	Unassigned	Unassigned	Unassigned	132_1	Clustered	17	VC_132_1	17	0.1738	1	0.1738	0	0	0	0.9853	Ref
MN045231	Unassigned	Unassigned	Unassigned	813_1	Clustered	24	VC_813_1	24	0.535	1	0.535	0	0	0	1	Ref
MN047438	Unassigned	Unassigned	Unassigned	149_8	Clustered	66	VC_149_8	66	0.4565	1	0.4565	0	0	0	0.8182	Ref
MN047793	Unassigned	Unassigned	Unassigned	68_1	Clustered/Singleton	1	VC_68_1	1	0	1	0	0	0	0	1	Ref
MN052874	Unassigned	Unassigned	Unassigned	823_0	Clustered	124	VC_823_0	124	0.9492	1	0.9492	0	0	0	1	Ref
MN055691	Unassigned	Unassigned	Unassigned	941_0	Clustered	40	VC_941_0	40	0.6903	1	0.6903	0	0	0	1	Ref
MN061582	Unassigned	Unassigned	Unassigned	699_0	Clustered	71	VC_699_0	71	0.9352	1	0.9352	0	0	0	1	Ref
MN062185	Unassigned	Unassigned	Unassigned	530_0	Clustered	9	VC_530_0	9	0.8027	1	0.8027	0	0	0	1	Ref
MN062186	Unassigned	Unassigned	Unassigned	779_3	Clustered	7	VC_779_3	7	0.0766	1	0.0766	0	0	0	0.7143	Ref
MN062187	Unassigned	Unassigned	Unassigned	749_0	Clustered	40	VC_749_0	40	0.7517	1	0.7517	0	0	0	1	Ref
MN062188	Unassigned	Unassigned	Unassigned	591_0	Clustered	14	VC_591_0	14	0.5184	1	0.5184	0	0	0	1	Ref
MN062701	Unassigned	Unassigned	Unassigned	546_4	Clustered	16	VC_546_4	16	0.2517	0.9999998	0.2517	0	0	0	0.9417	Ref
MN062702	Unassigned	Unassigned	Unassigned	1240_0	Clustered	82	VC_1240_0	82	0.7945	1	0.7945	0	0	0	1	Ref
MN062704	Unassigned	Unassigned	Unassigned	1176_0	Clustered	41	VC_1176_0	41	0.9558	1	0.9558	0	0	0	0.9976	Ref
MN062706	Unassigned	Unassigned	Unassigned	513_0	Clustered	32	VC_513_0	32	0.6881	1	0.6881	0	0	0	1	Ref
MN062707	Unassigned	Unassigned	Unassigned	558_77	Clustered	3	VC_558_77	3	0.0065	0.90877939	0.0059	0	0	0	0.6667	Ref
MN062708	Unassigned	Unassigned	Unassigned	217_1	Clustered/Singleton	1	VC_217_1	1	0	1	0	0	0	0	1	Ref
MN062709	Unassigned	Unassigned	Unassigned	1240_0	Clustered	82	VC_1240_0	82	0.7945	1	0.7945	0	0	0	1	Ref
MN062710	Unassigned	Unassigned	Unassigned	325_52	Clustered	5	VC_325_52	5	0.0147	0.99999073	0.0147	0	0	0	0.7	Ref
MN062711	Unassigned	Unassigned	Unassigned	1361_0	Clustered	6	VC_1361_0	6	0.2371	1	0.2371	0	0	0	1	Ref
MN062712	Unassigned	Unassigned	Unassigned	513_0	Clustered	32	VC_513_0	32	0.6881	1	0.6881	0	0	0	1	Ref
MN062713	Unassigned	Unassigned	Unassigned	1240_0	Clustered	82	VC_1240_0	82	0.7945	1	0.7945	0	0	0	1	Ref
MN062714	Unassigned	Unassigned	Unassigned	1234_0	Clustered	44	VC_1234_0	44	1	1	1	0	0	0	1	Ref

MN062715	Unassigned	Unassigned	Unassigned	685_0	Clustered	16	VC_685_0	16	0.2972	1	0.2972	0	0	0	0.9167	Ref
MN062716	Unassigned	Unassigned	Unassigned	919_0	Clustered	39	VC_919_0	39	0.9069	1	0.9069	0	0	0	1	Ref
MN062717	Unassigned	Unassigned	Unassigned	1234_0	Clustered	44	VC_1234_0	44	1	1	1	0	0	0	1	Ref
MN062718	Unassigned	Unassigned	Unassigned	514_0	Clustered	75	VC_514_0	75	0.9402	1	0.9402	0	0	0	1	Ref
MN062719	Unassigned	Unassigned	Unassigned	1352_0	Clustered	31	VC_1352_0	31	0.849	1	0.849	0	0	0	1	Ref
MN065183	Unassigned	Unassigned	Unassigned	20_0	Clustered	5	VC_20_0	5	0.4387	0.99999934	0.4387	0	0	0	1	Ref
MN066127	Unassigned	Unassigned	Unassigned	133_22	Clustered	13	VC_133_22	13	0.0804	1	0.0804	0	0	0	0.8205	Ref
MN067333	Unassigned	Unassigned	Unassigned	42_0	Clustered	49	VC_42_0	49	0.4517	1	0.4517	0	0	0	0.8597	Ref
MN067430	Unassigned	Unassigned	Unassigned	451_0	Clustered	80	VC_451_0	80	0.9991	1	0.9991	0	0	0	1	Ref
MN070121	Unassigned	Unassigned	Unassigned	540_0	Clustered	15	VC_540_0	15	0.2866	1	0.2866	0	0	0	1	Ref
MN078104	Unassigned	Unassigned	Unassigned	830_0	Clustered	6	VC_830_0	6	0.6536	1	0.6536	0	0	0	1	Ref
MN080711	Unassigned	Unassigned	Unassigned	386_0	Clustered	49	VC_386_0	49	0.7941	1	0.7941	0	0	0	1	Ref
MN082624	Unassigned	Unassigned	Unassigned	818_0	Clustered	20	VC_818_0	20	0.9601	1	0.9601	0	0	0	1	Ref
MN087708	Unassigned	Unassigned	Unassigned	490_1	Clustered	5	VC_490_1	5	0.0447	0.99999996	0.0447	0	0	0	1	Ref
MN090155	Unassigned	Unassigned	Unassigned	585_1	Clustered	3	VC_585_1	3	0.0313	0.99857668	0.0312	0	0	0	1	Ref
MN091625	Unassigned	Unassigned	Unassigned	149_5	Clustered/Singleton	1	VC_149_5	1	0	1	0	0	0	0	1	Ref
MN091626	Unassigned	Unassigned	Unassigned	449_0	Clustered	7	VC_449_0	7	0.6015	1	0.6015	0	0	0	1	Ref
MN094788	Unassigned	Unassigned	Unassigned	66_1	Clustered/Singleton	1	VC_66_1	1	0	1	0	0	0	0	1	Ref
MN095770	Unassigned	Unassigned	Unassigned	196_0	Clustered	2	VC_196_0	2	0.0218	0.95762696	0.0209	0	0	0	1	Ref
MN095771	Unassigned	Unassigned	Unassigned	284_0	Clustered	3	VC_284_0	3	0.0495	0.99860966	0.0494	0	0	0	1	Ref
MN095772	Unassigned	Unassigned	Unassigned	506_0	Clustered	8	VC_506_0	8	0.7376	1	0.7376	0	0	0	1	Ref
MN096356	Unassigned	Unassigned	Unassigned	519_0	Clustered	39	VC_519_0	39	0.8156	1	0.8156	0	0	0	1	Ref
MN096357	Unassigned	Unassigned	Unassigned	1031_0	Clustered	4	VC_1031_0	4	0.7884	0.99997621	0.7884	0	0	0	1	Ref
MN096358	Unassigned	Unassigned	Unassigned	1031_0	Clustered	4	VC_1031_0	4	0.7884	0.99997621	0.7884	0	0	0	1	Ref
MN096359	Unassigned	Unassigned	Unassigned	509_0	Clustered	37	VC_509_0	37	0.9689	1	0.9689	0	0	0	1	Ref
MN096360	Unassigned	Unassigned	Unassigned	509_0	Clustered	37	VC_509_0	37	0.9689	1	0.9689	0	0	0	1	Ref
MN096361	Unassigned	Unassigned	Unassigned	551_0	Clustered	16	VC_551_0	16	0.0915	1	0.0915	0	0	0	0.6667	Ref
MN096363	Unassigned	Unassigned	Unassigned	870_0	Clustered	5	VC_870_0	5	0.424	0.99999997	0.424	0	0	0	1	Ref
MN096364	Unassigned	Unassigned	Unassigned	551_8	Clustered	19	VC_551_8	19	0.1039	1	0.1039	0	0	0	0.8713	Ref
MN096365	Unassigned	Unassigned	Unassigned	1176_0	Clustered	41	VC_1176_0	41	0.9558	1	0.9558	0	0	0	0.9976	Ref
MN096366	Unassigned	Unassigned	Unassigned	371_2	Clustered	8	VC_371_2	8	0.0232	1	0.0232	0	0	0	0.8571	Ref
MN096367	Unassigned	Unassigned	Unassigned	1163_0	Clustered	28	VC_1163_0	28	0.4923	1	0.4923	0	0	0	1	Ref
MN096368	Unassigned	Unassigned	Unassigned	514_0	Clustered	75	VC_514_0	75	0.9402	1	0.9402	0	0	0	1	Ref
MN096370	Unassigned	Unassigned	Unassigned	557_0	Clustered	3	VC_557_0	3	0.0814	0.99853279	0.0812	0	0	0	0.6667	Ref
MN096371	Unassigned	Unassigned	Unassigned	1071_3	Clustered	16	VC_1071_3	16	0.2354	1	0.2354	0	0	0	0.9667	Ref
MN096372	Unassigned	Unassigned	Unassigned	940_0	Clustered	34	VC_940_0	34	0.7959	1	0.7959	0	0	0	1	Ref
MN096373	Unassigned	Unassigned	Unassigned	982_0	Clustered	62	VC_982_0	62	0.8646	1	0.8646	0	0	0	0.743	Ref
MN096374	Unassigned	Unassigned	Unassigned	856_1	Clustered	13	VC_856_1	13	0.5848	1	0.5848	0	0	0	1	Ref
MN096375	Unassigned	Unassigned	Unassigned	982_0	Clustered	62	VC_982_0	62	0.8646	1	0.8646	0	0	0	0.743	Ref
MN096376	Unassigned	Unassigned	Unassigned	218_0	Clustered	3	VC_218_0	3	0.0481	0.99860979	0.0481	0	0	0	1	Ref
MN096377	Unassigned	Unassigned	Unassigned	889_0	Clustered	16	VC_889_0	16	0.2969	1	0.2969	0	0	0	1	Ref
MN096378	Unassigned	Unassigned	Unassigned	1163_0	Clustered	28	VC_1163_0	28	0.4923	1	0.4923	0	0	0	1	Ref
MN096379	Unassigned	Unassigned	Unassigned	982_0	Clustered	62	VC_982_0	62	0.8646	1	0.8646	0	0	0	0.743	Ref
MN096380	Unassigned	Unassigned	Unassigned	888_0	Clustered	28	VC_888_0	28	0.5497	1	0.5497	0	0	0	1	Ref
MN096381	Unassigned	Unassigned	Unassigned	982_0	Clustered	62	VC_982_0	62	0.8646	1	0.8646	0	0	0	0.743	Ref
MN096382	Unassigned	Unassigned	Unassigned	887_0	Clustered	18	VC_887_0	18	0.3663	1	0.3663	0	0	0	1	Ref
MN098325	Unassigned	Unassigned	Unassigned	96_0	Clustered	30	VC_96_0	30	0.718	1	0.718	0	0	0	1	Ref
MN098326	Unassigned	Unassigned	Unassigned	792_0	Clustered	35	VC_792_0	35	0.9828	1	0.9828	0	0	0	1	Ref
MN098327	Unassigned	Unassigned	Unassigned	242_1	Clustered	20	VC_242_1	20	0.684	1	0.684	0	0	0	0.8368	Ref
MN098328	Unassigned	Unassigned	Unassigned	506_0	Clustered	8	VC_506_0	8	0.7376	1	0.7376	0	0	0	1	Ref
MN098329	Unassigned	Unassigned	Unassigned	392_0	Clustered	126	VC_392_0	126	0.9621	1	0.9621	0	0	0	1	Ref
MN101216	Unassigned	Unassigned	Unassigned	823_0	Clustered	124	VC_823_0	124	0.9492	1	0.9492	0	0	0	1	Ref
MN101217	Unassigned	Unassigned	Unassigned	111_0	Clustered	91	VC_111_0	91	0.9587	1	0.9587	0	0	0	1	Ref
MN101218	Unassigned	Unassigned	Unassigned	823_0	Clustered	124	VC_823_0	124	0.9492	1	0.9492	0	0	0	1	Ref
MN101219	Unassigned	Unassigned	Unassigned	111_0	Clustered	91	VC_111_0	91	0.9587	1	0.9587	0	0	0	1	Ref
MN101220	Unassigned	Unassigned	Unassigned	111_0	Clustered	91	VC_111_0	91	0.9587	1	0.9587	0	0	0	1	Ref
MN101221	Unassigned	Unassigned	Unassigned	668_2	Clustered/Singleton	1	VC_668_2	1	0	1	0	0	0	0	1	Ref
MN101222	Unassigned	Unassigned	Unassigned	586_0	Clustered	3	VC_586_0	3	0.0495	0.99859933	0.0495	0	0	0	1	Ref
MN101223	Unassigned	Unassigned	Unassigned	668_0	Clustered	25	VC_668_0	25	0.5539	1	0.5539	0	0	0	0.96	Ref
MN101224	Unassigned	Unassigned	Unassigned	668_3	Clustered/Singleton	1	VC_668_3	1	0	1	0	0	0	0	1	Ref
MN101225	Unassigned	Unassigned	Unassigned	491_1	Clustered/Singleton	1	VC_491_1	1	0	1	0	0	0	0	1	Ref
MN101226	Unassigned	Unassigned	Unassigned	491_0	Clustered	36	VC_491_0	36	0.5336	1	0.5336	0	0	0	0.9714	Ref
MN101227	Unassigned	Unassigned	Unassigned	823_0	Clustered	124	VC_823_0	124	0.9492	1	0.9492	0	0	0	1	Ref
MN101228	Unassigned	Unassigned	Unassigned	192_0	Clustered	15	VC_192_0	15	0.5071	1	0.5071	0	0	0	1	Ref
MN101229	Unassigned	Unassigned	Unassigned	491_0	Clustered	36	VC_491_0	36	0.5336	1	0.5336	0	0	0	0.9714	Ref
MN101230	Unassigned	Unassigned	Unassigned	491_0	Clustered	36	VC_491_0	36	0.5336	1	0.5336	0	0	0	0.9714	Ref
MN102098	Unassigned	Unassigned	Unassigned	442_0	Clustered	4	VC_442_0	4	0.0842	0.99996321	0.0842	0	0	0	1	Ref
MN103542	Unassigned	Unassigned	Unassigned	108_0	Clustered	33	VC_108_0	33	0.949	1	0.949	0	0	0	1	Ref
MN103543	Unassigned	Unassigned	Unassigned	344_1	Clustered	3	VC_344_1	3	0.0359	0.93348877	0.0335	0	0	0	1	Ref
MN114082	Unassigned	Unassigned	Unassigned	980_0	Clustered	10	VC_980_0	10	0.9996	1	0.9996	0	0	0	1	Ref
MN114083	Unassigned	Unassigned	Unassigned	980_0	Clustered	10	VC_980_0	10	0.9996	1	0.9996	0	0	0	1	Ref
MN116494	Unassigned	Unassigned	Unassigned	111_0	Clustered	91	VC_111_0	91	0.9587	1	0.9587	0	0	0	1	Ref
MN117721	Unassigned	Unassigned	Unassigned	792_0	Clustered	35	VC_792_0	35	0.9828	1	0.9828	0	0	0	1	Ref
MN119376	Unassigned	Unassigned	Unassigned	1224_0	Clustered	19	VC_1224_0	19	1	1	1	0	0	0	1	Ref
MN119377	Unassigned	Unassigned	Unassigned	325_42	Clustered	4	VC_325_42	4	0.0112	0.99980442	0.0112	0	0	0	0.6667	Ref
MN119378	Unassigned	Unassigned	Unassigned	565_0	Clustered	20	VC_565_0	20	0.8398	1	0.8398	0	0	0	1	Ref
MN119379	Unassigned	Unassigned	Unassigned	325_10	Clustered	3	VC_325_10	3	0.012	0.99859169	0.012	0	0	0	1	Ref
MN122072	Unassigned	Unassigned	Unassigned	792_0	Clustered	35	VC_792_0	35	0.9828	1	0.9828	0	0	0	1	Ref
MN128593	Unassigned	Unassigned	Unassigned	980_0	Clustered	10	VC_980_0	10	0.9996	1	0.9996	0	0	0	1	Ref
MN128594	Unassigned	Unassigned	Unassigned	739_0	Clustered	26	VC_739_0	26	0.9571	1	0.9571	0	0	0	1	Ref
MN131137	Unassigned	Unassigned	Unassigned	442_0	Clustered	4	VC_442_0	4	0.0842	0.99996321	0.0842	0	0	0	1	Ref

MN131141	Unassigned	Unassigned	Unassigned	79_7	Clustered	33	VC_79_7	33	0.216	1	0.216	0	0	0	0.8409	Ref
MN131142	Unassigned	Unassigned	Unassigned	79_7	Clustered	33	VC_79_7	33	0.216	1	0.216	0	0	0	0.8409	Ref
MN131143	Unassigned	Unassigned	Unassigned	79_7	Clustered	33	VC_79_7	33	0.216	1	0.216	0	0	0	0.8409	Ref
MN136198	Unassigned	Unassigned	Unassigned	792_0	Clustered	35	VC_792_0	35	0.9828	1	0.9828	0	0	0	1	Ref
MN148435	Unassigned	Unassigned	Unassigned	813_1	Clustered	24	VC_813_1	24	0.535	1	0.535	0	0	0	1	Ref
MN149903	Unassigned	Unassigned	Unassigned	823_0	Clustered	124	VC_823_0	124	0.9492	1	0.9492	0	0	0	1	Ref
MN149904	Unassigned	Unassigned	Unassigned	385_0	Clustered	51	VC_385_0	51	0.8754	1	0.8754	0	0	0	1	Ref
MN150686	Unassigned	Unassigned	Unassigned	187_0	Clustered	4	VC_187_0	4	0.0661	0.99994098	0.0661	0	0	0	1	Ref
MN150710	Unassigned	Unassigned	Unassigned	96_0	Clustered	30	VC_96_0	30	0.718	1	0.718	0	0	0	1	Ref
MN153503	Unassigned	Unassigned	Unassigned	883_4	Clustered/Singleton	1	VC_883_4	1	0	1	0	0	0	0	1	Ref
MN153797	Unassigned	Unassigned	Unassigned	575_0	Clustered	35	VC_575_0	35	0.8815	1	0.8815	0	0	0	1	Ref
MN159079	Unassigned	Unassigned	Unassigned	442_0	Clustered	4	VC_442_0	4	0.0842	0.99996321	0.0842	0	0	0	1	Ref
MN161573	Unassigned	Unassigned	Unassigned	28_1	Clustered	53	VC_28_1	53	0.7164	1	0.7164	0	0	0	0.9325	Ref
MN163280	Unassigned	Unassigned	Unassigned	192_0	Clustered	15	VC_192_0	15	0.5071	1	0.5071	0	0	0	1	Ref
MN163281	Unassigned	Unassigned	Unassigned	780_0	Clustered	8	VC_780_0	8	0.5207	1	0.5207	0	0	0	1	Ref
MN164484	Unassigned	Unassigned	Unassigned	456_0	Clustered	62	VC_456_0	62	0.9026	1	0.9026	0	0	0	1	Ref
MN166083	Unassigned	Unassigned	Unassigned	698_0	Clustered	26	VC_698_0	26	0.9366	1	0.9366	0	0	0	1	Ref
MN166823	Unassigned	Unassigned	Unassigned	242_1	Clustered	20	VC_242_1	20	0.684	1	0.684	0	0	0	0.8368	Ref
MN172529	Unassigned	Unassigned	Unassigned	739_0	Clustered	26	VC_739_0	26	0.9571	1	0.9571	0	0	0	1	Ref
MN175603	Unassigned	Unassigned	Unassigned	1352_0	Clustered	31	VC_1352_0	31	0.849	1	0.849	0	0	0	1	Ref
MN175604	Unassigned	Unassigned	Unassigned	522_0	Clustered	3	VC_522_0	3	0.0836	0.9985266	0.0835	0	0	0	1	Ref
MN176217	Unassigned	Unassigned	Unassigned	618_0	Clustered	13	VC_618_0	13	0.6252	1	0.6252	0	0	0	1	Ref
MN176219	Unassigned	Unassigned	Unassigned	104_0	Clustered	6	VC_104_0	6	0.8291	1	0.8291	0	0	0	1	Ref
MN176222	Unassigned	Unassigned	Unassigned	103_0	Clustered	6	VC_103_0	6	0.7337	1	0.7337	0	0	0	1	Ref
MN176223	Unassigned	Unassigned	Unassigned	103_0	Clustered	6	VC_103_0	6	0.7337	1	0.7337	0	0	0	1	Ref
MN176224	Unassigned	Unassigned	Unassigned	103_0	Clustered	6	VC_103_0	6	0.7337	1	0.7337	0	0	0	1	Ref
MN176225	Unassigned	Unassigned	Unassigned	103_0	Clustered	6	VC_103_0	6	0.7337	1	0.7337	0	0	0	1	Ref
MN176226	Unassigned	Unassigned	Unassigned	103_0	Clustered	6	VC_103_0	6	0.7337	1	0.7337	0	0	0	1	Ref
MN176227	Unassigned	Unassigned	Unassigned	104_0	Clustered	6	VC_104_0	6	0.8291	1	0.8291	0	0	0	1	Ref
MN176228	Unassigned	Unassigned	Unassigned	104_0	Clustered	6	VC_104_0	6	0.8291	1	0.8291	0	0	0	1	Ref
MN176229	Unassigned	Unassigned	Unassigned	103_0	Clustered	6	VC_103_0	6	0.7337	1	0.7337	0	0	0	1	Ref
MN176572	Unassigned	Unassigned	Unassigned	111_0	Clustered	91	VC_111_0	91	0.9587	1	0.9587	0	0	0	1	Ref
MN176573	Unassigned	Unassigned	Unassigned	111_0	Clustered	91	VC_111_0	91	0.9587	1	0.9587	0	0	0	1	Ref
MN176574	Unassigned	Unassigned	Unassigned	823_0	Clustered	124	VC_823_0	124	0.9492	1	0.9492	0	0	0	1	Ref
MN180248	Unassigned	Unassigned	Unassigned	22_0	Clustered	11	VC_22_0	11	0.5163	1	0.5163	0	0	0	1	Ref
MN180249	Unassigned	Unassigned	Unassigned	22_0	Clustered	11	VC_22_0	11	0.5163	1	0.5163	0	0	0	1	Ref
MN180250	Unassigned	Unassigned	Unassigned	22_0	Clustered	11	VC_22_0	11	0.5163	1	0.5163	0	0	0	1	Ref
MN180251	Unassigned	Unassigned	Unassigned	22_0	Clustered	11	VC_22_0	11	0.5163	1	0.5163	0	0	0	1	Ref
MN180252	Unassigned	Unassigned	Unassigned	22_0	Clustered	11	VC_22_0	11	0.5163	1	0.5163	0	0	0	1	Ref
MN183279	Unassigned	Unassigned	Unassigned	1073_0	Clustered	24	VC_1073_0	24	0.8388	1	0.8388	0	0	0	1	Ref
MN183280	Unassigned	Unassigned	Unassigned	558_60	Clustered/Singleton	1	VC_558_60	1	0	1	0	0	0	0	1	Ref
MN183281	Unassigned	Unassigned	Unassigned	1234_0	Clustered	44	VC_1234_0	44	1	1	1	0	0	0	1	Ref
MN183282	Unassigned	Unassigned	Unassigned	1043_0	Clustered	2	VC_1043_0	2	0.0576	0.95654247	0.0551	0	0	0	1	Ref
MN183283	Unassigned	Unassigned	Unassigned	1073_0	Clustered	24	VC_1073_0	24	0.8388	1	0.8388	0	0	0	1	Ref
MN183284	Unassigned	Unassigned	Unassigned	1171_0	Clustered	21	VC_1171_0	21	0.7125	1	0.7125	0	0	0	1	Ref
MN183285	Unassigned	Unassigned	Unassigned	558_18	Clustered/Singleton	1	VC_558_18	1	0	1	0	0	0	0	1	Ref
MN183286	Unassigned	Unassigned	Unassigned	546_4	Clustered	16	VC_546_4	16	0.2517	0.9999998	0.2517	0	0	0	0.9417	Ref
MN184885	Unassigned	Unassigned	Unassigned	395_0	Clustered	5	VC_395_0	5	0.1021	0.99999998	0.1021	0	0	0	1	Ref
MN184887	Unassigned	Unassigned	Unassigned	582_0	Clustered	4	VC_582_0	4	0.0589	0.99996986	0.0589	0	0	0	1	Ref
MN187550	Unassigned	Unassigned	Unassigned	476_0	Clustered	19	VC_476_0	19	0.7814	1	0.7814	0	0	0	1	Ref
MN187969	Unassigned	Unassigned	Unassigned	191_19	Clustered	12	VC_191_19	12	0.0655	1	0.0655	0	0	0	0.8636	Ref
MN191861	Unassigned	Unassigned	Unassigned	70_0	Clustered	27	VC_70_0	27	0.5981	1	0.5981	0	0	0	0.9886	Ref
MN197465	Unassigned	Unassigned	Unassigned	191_10	Clustered/Singleton	1	VC_191_10	1	0	1	0	0	0	0	1	Ref
MN200777	Unassigned	Unassigned	Unassigned	26_0	Clustered	4	VC_26_0	4	0.7433	0.99936153	0.7428	0	0	0	1	Ref
MN200778	Unassigned	Unassigned	Unassigned	26_0	Clustered	4	VC_26_0	4	0.7433	0.99936153	0.7428	0	0	0	1	Ref
MN204492	Unassigned	Unassigned	Unassigned	872_0	Clustered	31	VC_872_0	31	0.8151	1	0.8151	0	0	0	0.8194	Ref
MN204493	Unassigned	Unassigned	Unassigned	920_0	Clustered	2	VC_920_0	2	0.1671	0.95556296	0.1597	0	0	0	1	Ref
MN204494	Unassigned	Unassigned	Unassigned	872_0	Clustered	31	VC_872_0	31	0.8151	1	0.8151	0	0	0	0.8194	Ref
MN204495	Unassigned	Unassigned	Unassigned	872_0	Clustered	31	VC_872_0	31	0.8151	1	0.8151	0	0	0	0.8194	Ref
MN204496	Unassigned	Unassigned	Unassigned	872_0	Clustered	31	VC_872_0	31	0.8151	1	0.8151	0	0	0	0.8194	Ref
MN204497	Unassigned	Unassigned	Unassigned	872_0	Clustered	31	VC_872_0	31	0.8151	1	0.8151	0	0	0	0.8194	Ref
MN204498	Unassigned	Unassigned	Unassigned	982_0	Clustered	62	VC_982_0	62	0.8646	1	0.8646	0	0	0	0.743	Ref
MN204499	Unassigned	Unassigned	Unassigned	1074_0	Clustered	19	VC_1074_0	19	0.9198	1	0.9198	0	0	0	1	Ref
MN204500	Unassigned	Unassigned	Unassigned	872_0	Clustered	31	VC_872_0	31	0.8151	1	0.8151	0	0	0	0.8194	Ref
MN204501	Unassigned	Unassigned	Unassigned	799_1	Clustered	27	VC_799_1	27	0.8611	1	0.8611	0	0	0	1	Ref
MN204503	Unassigned	Unassigned	Unassigned	1074_0	Clustered	19	VC_1074_0	19	0.9198	1	0.9198	0	0	0	1	Ref
MN204504	Unassigned	Unassigned	Unassigned	872_0	Clustered	31	VC_872_0	31	0.8151	1	0.8151	0	0	0	0.8194	Ref
MN212906	Unassigned	Unassigned	Unassigned	699_0	Clustered	71	VC_699_0	71	0.9352	1	0.9352	0	0	0	1	Ref
MN215888	Unassigned	Unassigned	Unassigned	882_0	Clustered	20	VC_882_0	20	0.9917	1	0.9917	0	0	0	1	Ref
MN218190	Unassigned	Unassigned	Unassigned	191_16	Clustered	12	VC_191_16	12	0.0643	1	0.0643	0	0	0	0.9394	Ref
MN218191	Unassigned	Unassigned	Unassigned	191_16	Clustered	12	VC_191_16	12	0.0643	1	0.0643	0	0	0	0.9394	Ref
MN218775	Unassigned	Unassigned	Unassigned	451_0	Clustered	80	VC_451_0	80	0.9991	1	0.9991	0	0	0	1	Ref
MN224564	Unassigned	Unassigned	Unassigned	1353_0	Clustered	10	VC_1353_0	10	1	1	1	0	0	0	1	Ref
MN224565	Unassigned	Unassigned	Unassigned	1353_0	Clustered	10	VC_1353_0	10	1	1	1	0	0	0	1	Ref
MN227145	Unassigned	Unassigned	Unassigned	329_11	Clustered	15	VC_329_11	15	0.0791	1	0.0791	0	0	0	0.9333	Ref
MN228696	Unassigned	Unassigned	Unassigned	783_0	Clustered	3	VC_783_0	3	0.0639	0.99854307	0.0638	0	0	0	1	Ref
MN234161	Unassigned	Unassigned	Unassigned	1176_0	Clustered	41	VC_1176_0	41	0.9558	1	0.9558	0	0	0	0.9976	Ref
MN234162	Unassigned	Unassigned	Unassigned	572_0	Clustered	16	VC_572_0	16	0.7286	1	0.7286	0	0	0	1	Ref
MN234163	Unassigned	Unassigned	Unassigned	1240_0	Clustered	82	VC_1240_0	82	0.7945	1	0.7945	0	0	0	1	Ref
MN234164	Unassigned	Unassigned	Unassigned	857_0	Clustered	11	VC_857_0	11	0.9712	1	0.9712	0	0	0	1	Ref
MN234165	Unassigned	Unassigned	Unassigned	236_1	Clustered	5	VC_236_1	5	0.0358	0.99999997	0.0358	0	0	0	1	Ref

MN234166	Unassigned	Unassigned	Unassigned	869_0	Clustered	9	VC_869_0	9	0.2624	1	0.2624	0	0	0	1	Ref
MN234167	Unassigned	Unassigned	Unassigned	225_0	Clustered	32	VC_225_0	32	0.848	1	0.848	0	0	0	0.996	Ref
MN234168	Unassigned	Unassigned	Unassigned	1164_0	Clustered	25	VC_1164_0	25	0.7312	1	0.7312	0	0	0	1	Ref
MN234169	Unassigned	Unassigned	Unassigned	215_1	Clustered	19	VC_215_1	19	0.4188	1	0.4188	0	0	0	1	Ref
MN234171	Unassigned	Unassigned	Unassigned	372_0	Clustered	24	VC_372_0	24	0.5539	1	0.5539	0	0	0	1	Ref
MN234174	Unassigned	Unassigned	Unassigned	236_1	Clustered	5	VC_236_1	5	0.0358	0.99999997	0.0358	0	0	0	1	Ref
MN234176	Unassigned	Unassigned	Unassigned	237_2	Clustered	11	VC_237_2	11	0.1409	1	0.1409	0	0	0	0.9818	Ref
MN234177	Unassigned	Unassigned	Unassigned	1240_0	Clustered	82	VC_1240_0	82	0.7945	1	0.7945	0	0	0	1	Ref
MN234179	Unassigned	Unassigned	Unassigned	1176_0	Clustered	41	VC_1176_0	41	0.9558	1	0.9558	0	0	0	0.9976	Ref
MN234181	Unassigned	Unassigned	Unassigned	1240_0	Clustered	82	VC_1240_0	82	0.7945	1	0.7945	0	0	0	1	Ref
MN234182	Unassigned	Unassigned	Unassigned	236_0	Clustered	72	VC_236_0	72	0.8104	1	0.8104	0	0	0	1	Ref
MN234183	Unassigned	Unassigned	Unassigned	762_0	Clustered	9	VC_762_0	9	0.0885	1	0.0885	0	0	0	1	Ref
MN234185	Unassigned	Unassigned	Unassigned	762_0	Clustered	9	VC_762_0	9	0.0885	1	0.0885	0	0	0	1	Ref
MN234187	Unassigned	Unassigned	Unassigned	888_1	Clustered	3	VC_888_1	3	0.0289	0.99848448	0.0289	0	0	0	1	Ref
MN234190	Unassigned	Unassigned	Unassigned	572_0	Clustered	16	VC_572_0	16	0.7286	1	0.7286	0	0	0	1	Ref
MN234192	Unassigned	Unassigned	Unassigned	981_1	Clustered	26	VC_981_1	26	0.7009	1	0.7009	0	0	0	1	Ref
MN234193	Unassigned	Unassigned	Unassigned	558_36	Clustered	4	VC_558_36	4	0.0096	0.85913852	0.0082	0	0	0	1	Ref
MN234194	Unassigned	Unassigned	Unassigned	1164_0	Clustered	25	VC_1164_0	25	0.7312	1	0.7312	0	0	0	1	Ref
MN234195	Unassigned	Unassigned	Unassigned	518_0	Clustered	6	VC_518_0	6	0.1295	1	0.1295	0	0	0	1	Ref
MN234196	Unassigned	Unassigned	Unassigned	510_0	Clustered	12	VC_510_0	12	0.3963	1	0.3963	0	0	0	0.9697	Ref
MN234197	Unassigned	Unassigned	Unassigned	236_0	Clustered	72	VC_236_0	72	0.8104	1	0.8104	0	0	0	1	Ref
MN234198	Unassigned	Unassigned	Unassigned	236_0	Clustered	72	VC_236_0	72	0.8104	1	0.8104	0	0	0	1	Ref
MN234199	Unassigned	Unassigned	Unassigned	237_1	Clustered	13	VC_237_1	13	0.1773	1	0.1773	0	0	0	0.7308	Ref
MN234200	Unassigned	Unassigned	Unassigned	225_0	Clustered	32	VC_225_0	32	0.848	1	0.848	0	0	0	0.996	Ref
MN234201	Unassigned	Unassigned	Unassigned	236_1	Clustered	5	VC_236_1	5	0.0358	0.99999997	0.0358	0	0	0	1	Ref
MN234203	Unassigned	Unassigned	Unassigned	216_6	Clustered	42	VC_216_6	42	0.473	1	0.473	0	0	0	0.8351	Ref
MN234204	Unassigned	Unassigned	Unassigned	1240_0	Clustered	82	VC_1240_0	82	0.7945	1	0.7945	0	0	0	1	Ref
MN234205	Unassigned	Unassigned	Unassigned	236_0	Clustered	72	VC_236_0	72	0.8104	1	0.8104	0	0	0	1	Ref
MN234206	Unassigned	Unassigned	Unassigned	1099_0	Clustered	2	VC_1099_0	2	0.2612	0.94673154	0.2473	0	0	0	1	Ref
MN234207	Unassigned	Unassigned	Unassigned	572_0	Clustered	16	VC_572_0	16	0.7286	1	0.7286	0	0	0	1	Ref
MN234208	Unassigned	Unassigned	Unassigned	981_1	Clustered	26	VC_981_1	26	0.7009	1	0.7009	0	0	0	1	Ref
MN234209	Unassigned	Unassigned	Unassigned	225_0	Clustered	32	VC_225_0	32	0.848	1	0.848	0	0	0	0.996	Ref
MN234211	Unassigned	Unassigned	Unassigned	1099_0	Clustered	2	VC_1099_0	2	0.2612	0.94673154	0.2473	0	0	0	1	Ref
MN234212	Unassigned	Unassigned	Unassigned	325_52	Clustered	5	VC_325_52	5	0.0147	0.99999073	0.0147	0	0	0	0.7	Ref
MN234213	Unassigned	Unassigned	Unassigned	949_0	Clustered	10	VC_949_0	10	0.5977	1	0.5977	0	0	0	1	Ref
MN234214	Unassigned	Unassigned	Unassigned	1166_0	Clustered	20	VC_1166_0	20	0.99	1	0.99	0	0	0	1	Ref
MN234215	Unassigned	Unassigned	Unassigned	919_0	Clustered	39	VC_919_0	39	0.9069	1	0.9069	0	0	0	1	Ref
MN234216	Unassigned	Unassigned	Unassigned	864_0	Clustered	2	VC_864_0	2	1	1	1	0	0	0	1	Ref
MN234217	Unassigned	Unassigned	Unassigned	857_0	Clustered	11	VC_857_0	11	0.9712	1	0.9712	0	0	0	1	Ref
MN234219	Unassigned	Unassigned	Unassigned	762_0	Clustered	9	VC_762_0	9	0.0885	1	0.0885	0	0	0	1	Ref
MN234220	Unassigned	Unassigned	Unassigned	572_0	Clustered	16	VC_572_0	16	0.7286	1	0.7286	0	0	0	1	Ref
MN234221	Unassigned	Unassigned	Unassigned	1164_0	Clustered	25	VC_1164_0	25	0.7312	1	0.7312	0	0	0	1	Ref
MN234222	Unassigned	Unassigned	Unassigned	1240_0	Clustered	82	VC_1240_0	82	0.7945	1	0.7945	0	0	0	1	Ref
MN234223	Unassigned	Unassigned	Unassigned	509_0	Clustered	37	VC_509_0	37	0.9689	1	0.9689	0	0	0	1	Ref
MN234224	Unassigned	Unassigned	Unassigned	857_0	Clustered	11	VC_857_0	11	0.9712	1	0.9712	0	0	0	1	Ref
MN234225	Unassigned	Unassigned	Unassigned	508_0	Clustered	32	VC_508_0	32	0.906	1	0.906	0	0	0	1	Ref
MN234226	Unassigned	Unassigned	Unassigned	236_1	Clustered	5	VC_236_1	5	0.0358	0.99999997	0.0358	0	0	0	1	Ref
MN234227	Unassigned	Unassigned	Unassigned	565_0	Clustered	20	VC_565_0	20	0.8398	1	0.8398	0	0	0	1	Ref
MN234228	Unassigned	Unassigned	Unassigned	888_0	Clustered	28	VC_888_0	28	0.5497	1	0.5497	0	0	0	1	Ref
MN234230	Unassigned	Unassigned	Unassigned	236_0	Clustered	72	VC_236_0	72	0.8104	1	0.8104	0	0	0	1	Ref
MN234231	Unassigned	Unassigned	Unassigned	236_0	Clustered	72	VC_236_0	72	0.8104	1	0.8104	0	0	0	1	Ref
MN234232	Unassigned	Unassigned	Unassigned	572_0	Clustered	16	VC_572_0	16	0.7286	1	0.7286	0	0	0	1	Ref
MN234233	Unassigned	Unassigned	Unassigned	1200_0	Clustered	4	VC_1200_0	4	0.8691	0.9999992	0.8691	0	0	0	1	Ref
MN234234	Unassigned	Unassigned	Unassigned	1074_0	Clustered	19	VC_1074_0	19	0.9198	1	0.9198	0	0	0	1	Ref
MN234235	Unassigned	Unassigned	Unassigned	225_0	Clustered	32	VC_225_0	32	0.848	1	0.848	0	0	0	0.996	Ref
MN234236	Unassigned	Unassigned	Unassigned	518_0	Clustered	6	VC_518_0	6	0.1295	1	0.1295	0	0	0	1	Ref
MN241318	Unassigned	Unassigned	Unassigned	92_0	Clustered	22	VC_92_0	22	0.5639	1	0.5639	0	0	0	1	Ref
MN252582	Unassigned	Unassigned	Unassigned	386_0	Clustered	49	VC_386_0	49	0.7941	1	0.7941	0	0	0	1	Ref
MN263053	Unassigned	Unassigned	Unassigned	178_0	Clustered	5	VC_178_0	5	0.7741	0.99998991	0.7741	0	0	0	1	Ref
MN270258	Unassigned	Unassigned	Unassigned	632_0	Clustered	37	VC_632_0	37	0.581	1	0.581	0	0	0	0.9234	Ref
MN270259	Unassigned	Unassigned	Unassigned	632_0	Clustered	37	VC_632_0	37	0.581	1	0.581	0	0	0	0.9234	Ref
MN270260	Unassigned	Unassigned	Unassigned	632_0	Clustered	37	VC_632_0	37	0.581	1	0.581	0	0	0	0.9234	Ref
MN270261	Unassigned	Unassigned	Unassigned	632_0	Clustered	37	VC_632_0	37	0.581	1	0.581	0	0	0	0.9234	Ref
MN270262	Unassigned	Unassigned	Unassigned	632_0	Clustered	37	VC_632_0	37	0.581	1	0.581	0	0	0	0.9234	Ref
MN270263	Unassigned	Unassigned	Unassigned	632_0	Clustered	37	VC_632_0	37	0.581	1	0.581	0	0	0	0.9234	Ref
MN270264	Unassigned	Unassigned	Unassigned	632_0	Clustered	37	VC_632_0	37	0.581	1	0.581	0	0	0	0.9234	Ref
MN270265	Unassigned	Unassigned	Unassigned	632_0	Clustered	37	VC_632_0	37	0.581	1	0.581	0	0	0	0.9234	Ref
MN270266	Unassigned	Unassigned	Unassigned	632_0	Clustered	37	VC_632_0	37	0.581	1	0.581	0	0	0	0.9234	Ref
MN270267	Unassigned	Unassigned	Unassigned	632_0	Clustered	37	VC_632_0	37	0.581	1	0.581	0	0	0	0.9234	Ref
MN270268	Unassigned	Unassigned	Unassigned	632_0	Clustered	37	VC_632_0	37	0.581	1	0.581	0	0	0	0.9234	Ref
MN270269	Unassigned	Unassigned	Unassigned	632_0	Clustered	37	VC_632_0	37	0.581	1	0.581	0	0	0	0.9234	Ref
MN270270	Unassigned	Unassigned	Unassigned	632_0	Clustered	37	VC_632_0	37	0.581	1	0.581	0	0	0	0.9234	Ref
MN270271	Unassigned	Unassigned	Unassigned	632_0	Clustered	37	VC_632_0	37	0.581	1	0.581	0	0	0	0.9234	Ref
MN270272	Unassigned	Unassigned	Unassigned	632_0	Clustered	37	VC_632_0	37	0.581	1	0.581	0	0	0	0.9234	Ref
MN270273	Unassigned	Unassigned	Unassigned	632_0	Clustered	37	VC_632_0	37	0.581	1	0.581	0	0	0	0.9234	Ref
MN270274	Unassigned	Unassigned	Unassigned	632_0	Clustered	37	VC_632_0	37	0.581	1	0.581	0	0	0	0.9234	Ref
MN270275	Unassigned	Unassigned	Unassigned	632_0	Clustered	37	VC_632_0	37	0.581	1	0.581	0	0	0	0.9234	Ref
MN270276	Unassigned	Unassigned	Unassigned	632_0	Clustered	37	VC_632_0	37	0.581	1	0.581	0	0	0	0.9234	Ref
MN270277	Unassigned	Unassigned	Unassigned	632_0	Clustered	37	VC_632_0	37	0.581	1	0.581	0	0	0	0.9234	Ref
MN270278	Unassigned	Unassigned	Unassigned	632_0	Clustered	37	VC_632_0	37	0.581	1	0.581	0	0	0	0.9234	Ref
MN270279	Unassigned	Unassigned	Unassigned	632_0	Clustered	37	VC_632_0	37	0.581	1	0.581	0	0	0	0.9234	Ref

MN270887	Unassigned	Unassigned	Unassigned	984_0	Clustered	38	VC_984_0	38	0.9758	1	0.9758	0	0	0	1	Ref
MN270888	Unassigned	Unassigned	Unassigned	984_0	Clustered	38	VC_984_0	38	0.9758	1	0.9758	0	0	0	1	Ref
MN270891	Unassigned	Unassigned	Unassigned	331_0	Clustered	4	VC_331_0	4	0.0884	0.99998775	0.0884	0	0	0	1	Ref
MN270892	Unassigned	Unassigned	Unassigned	331_0	Clustered	4	VC_331_0	4	0.0884	0.99998775	0.0884	0	0	0	1	Ref
MN271656	Unassigned	Unassigned	Unassigned	984_0	Clustered	38	VC_984_0	38	0.9758	1	0.9758	0	0	0	1	Ref
MN276049	Unassigned	Unassigned	Unassigned	701_0	Clustered	2	VC_701_0	2	0.1487	0.95249915	0.1417	0	0	0	1	Ref
MN276192	Unassigned	Unassigned	Unassigned	558_40	Clustered/Singleton	1	VC_558_40	1	0	1	0	0	0	0	1	Ref
MN284892	Unassigned	Unassigned	Unassigned	1344_0	Clustered	78	VC_1344_0	78	0.9732	1	0.9732	0	0	0	1	Ref
MN284893	Unassigned	Unassigned	Unassigned	372_1	Clustered	9	VC_372_1	9	0.194	1	0.194	0	0	0	1	Ref
MN284894	Unassigned	Unassigned	Unassigned	1224_0	Clustered	19	VC_1224_0	19	1	1	1	0	0	0	1	Ref
MN284895	Unassigned	Unassigned	Unassigned	237_3	Clustered	8	VC_237_3	8	0.1092	1	0.1092	0	0	0	1	Ref
MN284896	Unassigned	Unassigned	Unassigned	799_1	Clustered	27	VC_799_1	27	0.8611	1	0.8611	0	0	0	1	Ref
MN284897	Unassigned	Unassigned	Unassigned	802_0	Clustered	17	VC_802_0	17	0.8025	1	0.8025	0	0	0	1	Ref
MN284898	Unassigned	Unassigned	Unassigned	224_0	Clustered	19	VC_224_0	19	0.9416	1	0.9416	0	0	0	1	Ref
MN284899	Unassigned	Unassigned	Unassigned	982_0	Clustered	62	VC_982_0	62	0.8646	1	0.8646	0	0	0	0.743	Ref
MN284900	Unassigned	Unassigned	Unassigned	514_0	Clustered	75	VC_514_0	75	0.9402	1	0.9402	0	0	0	1	Ref
MN284901	Unassigned	Unassigned	Unassigned	1163_0	Clustered	28	VC_1163_0	28	0.4923	1	0.4923	0	0	0	1	Ref
MN284902	Unassigned	Unassigned	Unassigned	1164_0	Clustered	25	VC_1164_0	25	0.7312	1	0.7312	0	0	0	1	Ref
MN284904	Unassigned	Unassigned	Unassigned	799_1	Clustered	27	VC_799_1	27	0.8611	1	0.8611	0	0	0	1	Ref
MN284905	Unassigned	Unassigned	Unassigned	517_0	Clustered	18	VC_517_0	18	0.1599	1	0.1599	0	0	0	1	Ref
MN284906	Unassigned	Unassigned	Unassigned	919_0	Clustered	39	VC_919_0	39	0.9069	1	0.9069	0	0	0	1	Ref
MN284907	Unassigned	Unassigned	Unassigned	1178_0	Clustered	24	VC_1178_0	24	0.9524	1	0.9524	0	0	0	1	Ref
MN284908	Unassigned	Unassigned	Unassigned	565_0	Clustered	20	VC_565_0	20	0.8398	1	0.8398	0	0	0	1	Ref
MN294712	Unassigned	Unassigned	Unassigned	699_0	Clustered	71	VC_699_0	71	0.9352	1	0.9352	0	0	0	1	Ref
MN296515	Unassigned	Unassigned	Unassigned	575_0	Clustered	35	VC_575_0	35	0.8815	1	0.8815	0	0	0	1	Ref
MN304941	Unassigned	Unassigned	Unassigned	149_15	Clustered	16	VC_149_15	16	0.0788	1	0.0788	0	0	0	0.8083	Ref
MN310541	Unassigned	Unassigned	Unassigned	220_0	Clustered	4	VC_220_0	4	0.0615	0.99998802	0.0615	0	0	0	1	Ref
MN310542	Unassigned	Unassigned	Unassigned	225_0	Clustered	32	VC_225_0	32	0.848	1	0.848	0	0	0	0.996	Ref
MN310543	Unassigned	Unassigned	Unassigned	1163_0	Clustered	28	VC_1163_0	28	0.4923	1	0.4923	0	0	0	1	Ref
MN310544	Unassigned	Unassigned	Unassigned	520_75	Clustered/Singleton	1	VC_520_75	1	0	1	0	0	0	0	1	Ref
MN310545	Unassigned	Unassigned	Unassigned	520_82	Clustered/Singleton	1	VC_520_82	1	0	1	0	0	0	0	1	Ref
MN310546	Unassigned	Unassigned	Unassigned	1360_0	Clustered	31	VC_1360_0	31	0.8837	1	0.8837	0	0	0	1	Ref
MN310547	Unassigned	Unassigned	Unassigned	221_0	Clustered	22	VC_221_0	22	0.7447	1	0.7447	0	0	0	1	Ref
MN310548	Unassigned	Unassigned	Unassigned	520_7	Clustered/Singleton	1	VC_520_7	1	0	1	0	0	0	0	1	Ref
MN310549	Unassigned	Unassigned	Unassigned	919_0	Clustered	39	VC_919_0	39	0.9069	1	0.9069	0	0	0	1	Ref
MN311487	Unassigned	Unassigned	Unassigned	1262_0	Clustered	2	VC_1262_0	2	0.1193	0.95613564	0.1141	0	0	0	1	Ref
MN311488	Unassigned	Unassigned	Unassigned	1062_0	Clustered	45	VC_1062_0	45	0.9734	0.50949847	0.4959	0	0	0	1	Ref
MN311489	Unassigned	Unassigned	Unassigned	1062_0	Clustered	45	VC_1062_0	45	0.9734	0.50949847	0.4959	0	0	0	1	Ref
MN311490	Unassigned	Unassigned	Unassigned	1062_0	Clustered	45	VC_1062_0	45	0.9734	0.50949847	0.4959	0	0	0	1	Ref
MN311491	Unassigned	Unassigned	Unassigned	1062_0	Clustered	45	VC_1062_0	45	0.9734	0.50949847	0.4959	0	0	0	1	Ref
MN311492	Unassigned	Unassigned	Unassigned	1262_0	Clustered	2	VC_1262_0	2	0.1193	0.95613564	0.1141	0	0	0	1	Ref
MN311493	Unassigned	Unassigned	Unassigned	1243_0	Clustered	52	VC_1243_0	52	0.9126	0.92708311	0.8461	0	0	0	1	Ref
MN313256	Unassigned	Unassigned	Unassigned	644_0	Clustered	5	VC_644_0	5	0.6649	0.99999975	0.6649	0	0	0	1	Ref
MN316588	Unassigned	Unassigned	Unassigned	190_14	Clustered	3	VC_190_14	3	0.0144	0.99696852	0.0143	0	0	0	0.6667	Ref
MN317029	Unassigned	Unassigned	Unassigned	597_0	Clustered	6	VC_597_0	6	0.1801	1	0.1801	0	0	0	1	Ref
MN329673	Unassigned	Unassigned	Unassigned	1093_0	Clustered	40	VC_1093_0	40	0.8754	1	0.8754	0	0	0	1	Ref
MN329674	Unassigned	Unassigned	Unassigned	939_0	Clustered	15	VC_939_0	15	1	0	0	0	0	0	1	Ref
MN329675	Unassigned	Unassigned	Unassigned	1234_0	Clustered	44	VC_1234_0	44	1	1	1	0	0	0	1	Ref
MN329676	Unassigned	Unassigned	Unassigned	939_0	Clustered	15	VC_939_0	15	1	0	0	0	0	0	1	Ref
MN329677	Unassigned	Unassigned	Unassigned	1093_0	Clustered	40	VC_1093_0	40	0.8754	1	0.8754	0	0	0	1	Ref
MN329678	Unassigned	Unassigned	Unassigned	765_0	Clustered	132	VC_765_0	132	1	1	1	0	0	0	1	Ref
MN329679	Unassigned	Unassigned	Unassigned	1344_0	Clustered	78	VC_1344_0	78	0.9732	1	0.9732	0	0	0	1	Ref
MN334766	Unassigned	Unassigned	Unassigned	488_2	Clustered	2	VC_488_2	2	0.0325	0.95511029	0.0311	0	0	0	1	Ref
MN335248	Unassigned	Unassigned	Unassigned	177_0	Clustered	5	VC_177_0	5	0.7468	0.99885646	0.746	0	0	0	1	Ref
MN336261	Unassigned	Unassigned	Unassigned	149_8	Clustered	66	VC_149_8	66	0.4565	1	0.4565	0	0	0	0.8182	Ref
MN336262	Unassigned	Unassigned	Unassigned	149_8	Clustered	66	VC_149_8	66	0.4565	1	0.4565	0	0	0	0.8182	Ref
MN336263	Unassigned	Unassigned	Unassigned	149_8	Clustered	66	VC_149_8	66	0.4565	1	0.4565	0	0	0	0.8182	Ref
MN336264	Unassigned	Unassigned	Unassigned	637_0	Clustered	115	VC_637_0	115	0.731	1	0.731	0	0	0	0.8764	Ref
MN336265	Unassigned	Unassigned	Unassigned	637_0	Clustered	115	VC_637_0	115	0.731	1	0.731	0	0	0	0.8764	Ref
MN336266	Unassigned	Unassigned	Unassigned	637_0	Clustered	115	VC_637_0	115	0.731	1	0.731	0	0	0	0.8764	Ref
MN337573	Unassigned	Unassigned	Unassigned	575_0	Clustered	35	VC_575_0	35	0.8815	1	0.8815	0	0	0	1	Ref
MN337887	Unassigned	Unassigned	Unassigned	690_0	Clustered	50	VC_690_0	50	0.9805	1	0.9805	0	0	0	1	Ref
MN340231	Unassigned	Unassigned	Unassigned	30_1	Clustered	16	VC_30_1	16	0.1973	1	0.1973	0	0	0	0.8333	Ref
MN342150	Unassigned	Unassigned	Unassigned	133_22	Clustered	13	VC_133_22	13	0.0804	1	0.0804	0	0	0	0.8205	Ref
MN342151	Unassigned	Unassigned	Unassigned	330_0	Clustered	27	VC_330_0	27	0.5088	1	0.5088	0	0	0	0.8661	Ref
MN342247	Unassigned	Unassigned	Unassigned	575_0	Clustered	35	VC_575_0	35	0.8815	1	0.8815	0	0	0	1	Ref
MN364664	Unassigned	Unassigned	Unassigned	187_4	Clustered/Singleton	1	VC_187_4	1	0	1	0	0	0	0	1	Ref
MN369737	Unassigned	Unassigned	Unassigned	1240_0	Clustered	82	VC_1240_0	82	0.7945	1	0.7945	0	0	0	1	Ref
MN369738	Unassigned	Unassigned	Unassigned	371_14	Clustered/Singleton	1	VC_371_14	1	0	1	0	0	0	0	1	Ref
MN369739	Unassigned	Unassigned	Unassigned	520_17	Clustered/Singleton	1	VC_520_17	1	0	1	0	0	0	0	1	Ref
MN369740	Unassigned	Unassigned	Unassigned	1176_0	Clustered	41	VC_1176_0	41	0.9558	1	0.9558	0	0	0	0.9976	Ref
MN369741	Unassigned	Unassigned	Unassigned	225_0	Clustered	32	VC_225_0	32	0.848	1	0.848	0	0	0	0.996	Ref
MN369742	Unassigned	Unassigned	Unassigned	236_0	Clustered	72	VC_236_0	72	0.8104	1	0.8104	0	0	0	1	Ref
MN369743	Unassigned	Unassigned	Unassigned	1071_3	Clustered	16	VC_1071_3	16	0.2354	1	0.2354	0	0	0	0.9667	Ref
MN369744	Unassigned	Unassigned	Unassigned	371_11	Clustered	31	VC_371_11	31	0.0927	1	0.0927	0	0	0	0.7011	Ref
MN369745	Unassigned	Unassigned	Unassigned	1344_0	Clustered	78	VC_1344_0	78	0.9732	1	0.9732	0	0	0	1	Ref
MN369746	Unassigned	Unassigned	Unassigned	236_0	Clustered	72	VC_236_0	72	0.8104	1	0.8104	0	0	0	1	Ref
MN369747	Unassigned	Unassigned	Unassigned	1360_0	Clustered	31	VC_1360_0	31	0.8837	1	0.8837	0	0	0	1	Ref
MN369748	Unassigned	Unassigned	Unassigned	216_0	Clustered	4	VC_216_0	4	0.048	0.9999878	0.048	0	0	0	1	Ref
MN369749	Unassigned	Unassigned	Unassigned	520_19	Clustered	2	VC_520_19	2	0.0098	0.95761552	0.0094	0	0	0	1	Ref
MN369750	Unassigned	Unassigned	Unassigned	1071_0	Clustered	20	VC_1071_0	20	0.3268	1	0.3268	0	0	0	0.9842	Ref

MN369751	Unassigned	Unassigned	Unassigned	520_85	Clustered	2	VC_520_85	2	0.0148	0.95762405	0.0142	0	0	0	1	Ref
MN369752	Unassigned	Unassigned	Unassigned	520_10	Clustered/Singleton	1	VC_520_10	1	0	1	0	0	0	0	1	Ref
MN369753	Unassigned	Unassigned	Unassigned	767_0	Clustered	34	VC_767_0	34	0.9173	1	0.9173	0	0	0	1	Ref
MN369754	Unassigned	Unassigned	Unassigned	1072_4	Clustered	4	VC_1072_4	4	0.0736	0.99956559	0.0736	0	0	0	1	Ref
MN369755	Unassigned	Unassigned	Unassigned	520_33	Clustered	2	VC_520_33	2	0.0087	0.95761571	0.0083	0	0	0	1	Ref
MN369756	Unassigned	Unassigned	Unassigned	224_0	Clustered	19	VC_224_0	19	0.9416	1	0.9416	0	0	0	1	Ref
MN369757	Unassigned	Unassigned	Unassigned	1071_0	Clustered	20	VC_1071_0	20	0.3268	1	0.3268	0	0	0	0.9842	Ref
MN369758	Unassigned	Unassigned	Unassigned	510_0	Clustered	12	VC_510_0	12	0.3963	1	0.3963	0	0	0	0.9697	Ref
MN369759	Unassigned	Unassigned	Unassigned	372_0	Clustered	24	VC_372_0	24	0.5539	1	0.5539	0	0	0	1	Ref
MN369760	Unassigned	Unassigned	Unassigned	325_36	Clustered	2	VC_325_36	2	0.0043	0.95751115	0.0041	0	0	0	1	Ref
MN369761	Unassigned	Unassigned	Unassigned	234_0	Clustered	21	VC_234_0	21	0.8355	1	0.8355	0	0	0	1	Ref
MN369762	Unassigned	Unassigned	Unassigned	237_2	Clustered	11	VC_237_2	11	0.1409	1	0.1409	0	0	0	0.9818	Ref
MN369763	Unassigned	Unassigned	Unassigned	225_0	Clustered	32	VC_225_0	32	0.848	1	0.848	0	0	0	0.996	Ref
MN369764	Unassigned	Unassigned	Unassigned	225_0	Clustered	32	VC_225_0	32	0.848	1	0.848	0	0	0	0.996	Ref
MN369765	Unassigned	Unassigned	Unassigned	1162_0	Clustered	10	VC_1162_0	10	0.5715	1	0.5715	0	0	0	1	Ref
MN369766	Unassigned	Unassigned	Unassigned	767_0	Clustered	34	VC_767_0	34	0.9173	1	0.9173	0	0	0	1	Ref
MN379460	Unassigned	Unassigned	Unassigned	121_0	Clustered	4	VC_121_0	4	0.1814	0.99998531	0.1814	0	0	0	1	Ref
MN379629	Unassigned	Unassigned	Unassigned	1409_0	Clustered	2	VC_1409_0	2	1	1	1	0	0	0	1	Ref
MN379630	Unassigned	Unassigned	Unassigned	1122_0	Clustered	10	VC_1122_0	10	0.9643	0.99999697	0.9643	0	0	0	1	Ref
MN379632	Unassigned	Unassigned	Unassigned	1081_0	Clustered/Singleton	1	VC_1081_0	1	0	1	0	0	0	0	1	Ref
MN379633	Unassigned	Unassigned	Unassigned	1054_0	Clustered	12	VC_1054_0	12	0.2863	0.99746644	0.2855	0	0	0	1	Ref
MN379636	Unassigned	Unassigned	Unassigned	1409_0	Clustered	2	VC_1409_0	2	1	1	1	0	0	0	1	Ref
MN379637	Unassigned	Unassigned	Unassigned	1233_0	Clustered	16	VC_1233_0	16	0.8971	1	0.8971	0	0	0	1	Ref
MN379638	Unassigned	Unassigned	Unassigned	1277_0	Clustered	14	VC_1277_0	14	0.8218	1	0.8218	0	0	0	1	Ref
MN379639	Unassigned	Unassigned	Unassigned	1410_0	Clustered	2	VC_1410_0	2	1	1	1	0	0	0	1	Ref
MN379640	Unassigned	Unassigned	Unassigned	1122_0	Clustered	10	VC_1122_0	10	0.9643	0.99999697	0.9643	0	0	0	1	Ref
MN379641	Unassigned	Unassigned	Unassigned	1153_0	Clustered	2	VC_1153_0	2	0.073	0.80562642	0.0588	0	0	0	1	Ref
MN379643	Unassigned	Unassigned	Unassigned	1411_0	Clustered	2	VC_1411_0	2	1	1	1	0	0	0	1	Ref
MN379645	Unassigned	Unassigned	Unassigned	1275_0	Clustered	4	VC_1275_0	4	1	1	1	0	0	0	1	Ref
MN379646	Unassigned	Unassigned	Unassigned	1410_0	Clustered	2	VC_1410_0	2	1	1	1	0	0	0	1	Ref
MN379647	Unassigned	Unassigned	Unassigned	1233_0	Clustered	16	VC_1233_0	16	0.8971	1	0.8971	0	0	0	1	Ref
MN379648	Unassigned	Unassigned	Unassigned	1277_0	Clustered	14	VC_1277_0	14	0.8218	1	0.8218	0	0	0	1	Ref
MN379650	Unassigned	Unassigned	Unassigned	1411_0	Clustered	2	VC_1411_0	2	1	1	1	0	0	0	1	Ref
MN379651	Unassigned	Unassigned	Unassigned	1054_0	Clustered	12	VC_1054_0	12	0.2863	0.99746644	0.2855	0	0	0	1	Ref
MN379739	Unassigned	Unassigned	Unassigned	191_16	Clustered	12	VC_191_16	12	0.0643	1	0.0643	0	0	0	0.9394	Ref
MN379740	Unassigned	Unassigned	Unassigned	428_0	Clustered	33	VC_428_0	33	0.9915	1	0.9915	0	0	0	1	Ref
MN379832	Unassigned	Unassigned	Unassigned	938_0	Clustered	99	VC_938_0	99	0.9119	1	0.9119	0	0	0	0.9928	Ref
MN380459	Unassigned	Unassigned	Unassigned	111_0	Clustered	91	VC_111_0	91	0.9587	1	0.9587	0	0	0	1	Ref
MN384978	Unassigned	Unassigned	Unassigned	452_0	Clustered	66	VC_452_0	66	0.9248	1	0.9248	0	0	0	1	Ref
MN384979	Unassigned	Unassigned	Unassigned	133_9	Clustered	3	VC_133_9	3	0.0099	0.98457109	0.0098	0	0	0	1	Ref
MN385565	Unassigned	Unassigned	Unassigned	303_0	Clustered	155	VC_303_0	155	0.9979	1	0.9979	0	0	0	1	Ref
MN393078	Unassigned	Unassigned	Unassigned	637_0	Clustered	115	VC_637_0	115	0.731	1	0.731	0	0	0	0.8764	Ref
MN393079	Unassigned	Unassigned	Unassigned	540_0	Clustered	15	VC_540_0	15	0.2866	1	0.2866	0	0	0	1	Ref
MN393473	Unassigned	Unassigned	Unassigned	575_0	Clustered	35	VC_575_0	35	0.8815	1	0.8815	0	0	0	1	Ref
MN395285	Unassigned	Unassigned	Unassigned	938_0	Clustered	99	VC_938_0	99	0.9119	1	0.9119	0	0	0	0.9928	Ref
MN395286	Unassigned	Unassigned	Unassigned	938_0	Clustered	99	VC_938_0	99	0.9119	1	0.9119	0	0	0	0.9928	Ref
MN395291	Unassigned	Unassigned	Unassigned	699_0	Clustered	71	VC_699_0	71	0.9352	1	0.9352	0	0	0	1	Ref
MN402506	Unassigned	Unassigned	Unassigned	495_1	Clustered	41	VC_495_1	41	0.328	1	0.328	0	0	0	0.911	Ref
MN414250	Unassigned	Unassigned	Unassigned	451_0	Clustered	80	VC_451_0	80	0.9991	1	0.9991	0	0	0	1	Ref
MN417334	Unassigned	Unassigned	Unassigned	1022_0	Clustered	4	VC_1022_0	4	0.8047	0.99996089	0.8046	0	0	0	1	Ref
MN419153	Unassigned	Unassigned	Unassigned	495_0	Clustered	39	VC_495_0	39	0.308	1	0.308	0	0	0	1	Ref
MN428049	Unassigned	Unassigned	Unassigned	508_0	Clustered	32	VC_508_0	32	0.906	1	0.906	0	0	0	1	Ref
MN428050	Unassigned	Unassigned	Unassigned	372_0	Clustered	24	VC_372_0	24	0.5539	1	0.5539	0	0	0	1	Ref
MN428051	Unassigned	Unassigned	Unassigned	520_92	Clustered	5	VC_520_92	5	0.0663	0.99999997	0.0663	0	0	0	1	Ref
MN428052	Unassigned	Unassigned	Unassigned	556_0	Clustered	21	VC_556_0	21	0.9912	1	0.9912	0	0	0	1	Ref
MN428053	Unassigned	Unassigned	Unassigned	522_1	Clustered	5	VC_522_1	5	0.1017	0.99999997	0.1017	0	0	0	0.8	Ref
MN428054	Unassigned	Unassigned	Unassigned	325_35	Clustered	17	VC_325_35	17	0.0732	1	0.0732	0	0	0	0.7721	Ref
MN428056	Unassigned	Unassigned	Unassigned	939_0	Clustered	15	VC_939_0	15	1	0	0	0	0	0	1	Ref
MN428058	Unassigned	Unassigned	Unassigned	556_0	Clustered	21	VC_556_0	21	0.9912	1	0.9912	0	0	0	1	Ref
MN428059	Unassigned	Unassigned	Unassigned	551_6	Clustered	22	VC_551_6	22	0.1214	1	0.1214	0	0	0	0.8095	Ref
MN428060	Unassigned	Unassigned	Unassigned	1071_0	Clustered	20	VC_1071_0	20	0.3268	1	0.3268	0	0	0	0.9842	Ref
MN428061	Unassigned	Unassigned	Unassigned	939_0	Clustered	15	VC_939_0	15	1	0	0	0	0	0	1	Ref
MN428062	Unassigned	Unassigned	Unassigned	1344_0	Clustered	78	VC_1344_0	78	0.9732	1	0.9732	0	0	0	1	Ref
MN428063	Unassigned	Unassigned	Unassigned	767_0	Clustered	34	VC_767_0	34	0.9173	1	0.9173	0	0	0	1	Ref
MN428064	Unassigned	Unassigned	Unassigned	1344_0	Clustered	78	VC_1344_0	78	0.9732	1	0.9732	0	0	0	1	Ref
MN428065	Unassigned	Unassigned	Unassigned	558_38	Clustered	2	VC_558_38	2	0.0032	0.64328629	0.002	0	0	0	1	Ref
MN428066	Unassigned	Unassigned	Unassigned	558_20	Clustered	17	VC_558_20	17	0.054	1	0.054	0	0	0	0.9926	Ref
MN432485	Unassigned	Unassigned	Unassigned	941_0	Clustered	40	VC_941_0	40	0.6903	1	0.6903	0	0	0	1	Ref
MN433707	Unassigned	Unassigned	Unassigned	699_0	Clustered	71	VC_699_0	71	0.9352	1	0.9352	0	0	0	1	Ref
MN434093	Unassigned	Unassigned	Unassigned	823_0	Clustered	124	VC_823_0	124	0.9492	1	0.9492	0	0	0	1	Ref
MN434094	Unassigned	Unassigned	Unassigned	192_0	Clustered	15	VC_192_0	15	0.5071	1	0.5071	0	0	0	1	Ref
MN434095	Unassigned	Unassigned	Unassigned	491_0	Clustered	36	VC_491_0	36	0.5336	1	0.5336	0	0	0	0.9714	Ref
MN434096	Unassigned	Unassigned	Unassigned	192_0	Clustered	15	VC_192_0	15	0.5071	1	0.5071	0	0	0	1	Ref
MN444868	Unassigned	Unassigned	Unassigned	371_3	Clustered	22	VC_371_3	22	0.0787	1	0.0787	0	0	0	0.9048	Ref
MN444869	Unassigned	Unassigned	Unassigned	325_6	Clustered/Singleton	1	VC_325_6	1	0	1	0	0	0	0	1	Ref
MN444870	Unassigned	Unassigned	Unassigned	890_1	Clustered	4	VC_890_1	4	0.4613	0.99998458	0.4613	0	0	0	1	Ref
MN444871	Unassigned	Unassigned	Unassigned	919_0	Clustered	39	VC_919_0	39	0.9069	1	0.9069	0	0	0	1	Ref
MN444872	Unassigned	Unassigned	Unassigned	509_0	Clustered	37	VC_509_0	37	0.9689	1	0.9689	0	0	0	1	Ref
MN444873	Unassigned	Unassigned	Unassigned	509_0	Clustered	37	VC_509_0	37	0.9689	1	0.9689	0	0	0	1	Ref
MN444874	Unassigned	Unassigned	Unassigned	868_0	Clustered	25	VC_868_0	25	0.5791	1	0.5791	0	0	0	1	Ref
MN444875	Unassigned	Unassigned	Unassigned	761_0	Clustered	67	VC_761_0	67	0.8947	1	0.8947	0	0	0	1	Ref

MN444876	Unassigned	Unassigned	Unassigned	1071_4	Clustered/Singleton	1	VC_1071_4	1	0	1	0	0	0	0	1	Ref
MN444877	Unassigned	Unassigned	Unassigned	1352_0	Clustered	31	VC_1352_0	31	0.849	1	0.849	0	0	0	1	Ref
MN444878	Unassigned	Unassigned	Unassigned	919_0	Clustered	39	VC_919_0	39	0.9069	1	0.9069	0	0	0	1	Ref
MN445182	Unassigned	Unassigned	Unassigned	190_34	Clustered/Singleton	1	VC_190_34	1	0	1	0	0	0	0	1	Ref
MN445183	Unassigned	Unassigned	Unassigned	489_0	Clustered	3	VC_489_0	3	0.0677	0.98259158	0.0666	0	0	0	1	Ref
MN445184	Unassigned	Unassigned	Unassigned	133_42	Clustered/Singleton	1	VC_133_42	1	0	1	0	0	0	0	1	Ref
MN445185	Unassigned	Unassigned	Unassigned	959_5	Clustered	19	VC_959_5	19	0.2626	1	0.2626	0	0	0	1	Ref
MN450150	Unassigned	Unassigned	Unassigned	387_0	Clustered	3	VC_387_0	3	0.0424	0.99861589	0.0423	0	0	0	1	Ref
MN453779	Unassigned	Unassigned	Unassigned	442_3	Clustered	3	VC_442_3	3	0.0529	0.99675181	0.0527	0	0	0	1	Ref
MN461279	Unassigned	Unassigned	Unassigned	364_0	Clustered	16	VC_364_0	16	0.6491	1	0.6491	0	0	0	1	Ref
MN478375	Unassigned	Unassigned	Unassigned	74_0	Clustered	15	VC_74_0	15	0.5204	1	0.5204	0	0	0	1	Ref
MN478377	Unassigned	Unassigned	Unassigned	192_2	Clustered/Singleton	1	VC_192_2	1	0	1	0	0	0	0	1	Ref
MN478483	Unassigned	Unassigned	Unassigned	132_1	Clustered	17	VC_132_1	17	0.1738	1	0.1738	0	0	0	0.9853	Ref
MN481365	Unassigned	Unassigned	Unassigned	392_0	Clustered	126	VC_392_0	126	0.9621	1	0.9621	0	0	0	1	Ref
MN481366	Unassigned	Unassigned	Unassigned	392_0	Clustered	126	VC_392_0	126	0.9621	1	0.9621	0	0	0	1	Ref
MN481367	Unassigned	Unassigned	Unassigned	329_11	Clustered	15	VC_329_11	15	0.0791	1	0.0791	0	0	0	0.9333	Ref
MN484599	Unassigned	Unassigned	Unassigned	1071_0	Clustered	20	VC_1071_0	20	0.3268	1	0.3268	0	0	0	0.9842	Ref
MN484600	Unassigned	Unassigned	Unassigned	1166_0	Clustered	20	VC_1166_0	20	0.99	1	0.99	0	0	0	1	Ref
MN484601	Unassigned	Unassigned	Unassigned	1098_0	Clustered	4	VC_1098_0	4	0.5465	0.99996462	0.5465	0	0	0	1	Ref
MN496305	Unassigned	Unassigned	Unassigned	599_0	Clustered	50	VC_599_0	50	0.9989	1	0.9989	0	0	0	1	Ref
MN496306	Unassigned	Unassigned	Unassigned	599_0	Clustered	50	VC_599_0	50	0.9989	1	0.9989	0	0	0	1	Ref
MN496307	Unassigned	Unassigned	Unassigned	599_0	Clustered	50	VC_599_0	50	0.9989	1	0.9989	0	0	0	1	Ref
MN496308	Unassigned	Unassigned	Unassigned	599_0	Clustered	50	VC_599_0	50	0.9989	1	0.9989	0	0	0	1	Ref
MN497953	Unassigned	Unassigned	Unassigned	514_0	Clustered	75	VC_514_0	75	0.9402	1	0.9402	0	0	0	1	Ref
MN497954	Unassigned	Unassigned	Unassigned	1343_0	Clustered	21	VC_1343_0	21	0.9843	1	0.9843	0	0	0	1	Ref
MN504636	Unassigned	Unassigned	Unassigned	465_0	Clustered	7	VC_465_0	7	0.3322	1	0.3322	0	0	0	1	Ref
MN508614	Unassigned	Unassigned	Unassigned	283_0	Clustered	2	VC_283_0	2	0.0025	0.94936553	0.0024	0	0	0	1	Ref
MN508615	Unassigned	Unassigned	Unassigned	813_1	Clustered	24	VC_813_1	24	0.535	1	0.535	0	0	0	1	Ref
MN508616	Unassigned	Unassigned	Unassigned	283_110	Clustered	2	VC_283_110	2	0.0027	0.90910416	0.0024	0	0	0	1	Ref
MN508617	Unassigned	Unassigned	Unassigned	283_110	Clustered	2	VC_283_110	2	0.0027	0.90910416	0.0024	0	0	0	1	Ref
MN508618	Unassigned	Unassigned	Unassigned	283_0	Clustered	2	VC_283_0	2	0.0025	0.94936553	0.0024	0	0	0	1	Ref
MN508619	Unassigned	Unassigned	Unassigned	472_0	Clustered	47	VC_472_0	47	0.9025	1	0.9025	0	0	0	1	Ref
MN508620	Unassigned	Unassigned	Unassigned	80_0	Clustered	41	VC_80_0	41	0.5837	1	0.5837	0	0	0	0.9805	Ref
MN508621	Unassigned	Unassigned	Unassigned	669_0	Clustered	21	VC_669_0	21	0.3623	1	0.3623	0	0	0	1	Ref
MN508622	Unassigned	Unassigned	Unassigned	669_0	Clustered	21	VC_669_0	21	0.3623	1	0.3623	0	0	0	1	Ref
MN508623	Unassigned	Unassigned	Unassigned	669_0	Clustered	21	VC_669_0	21	0.3623	1	0.3623	0	0	0	1	Ref
MN508624	Unassigned	Unassigned	Unassigned	669_0	Clustered	21	VC_669_0	21	0.3623	1	0.3623	0	0	0	1	Ref
MN508817	Unassigned	Unassigned	Unassigned	669_2	Clustered/Singleton	1	VC_669_2	1	0	1	0	0	0	0	1	Ref
MN509793	Unassigned	Unassigned	Unassigned	878_0	Clustered	32	VC_878_0	32	0.9965	1	0.9965	0	0	0	1	Ref
MN510331	Unassigned	Unassigned	Unassigned	451_0	Clustered	80	VC_451_0	80	0.9991	1	0.9991	0	0	0	1	Ref
MN512538	Unassigned	Unassigned	Unassigned	354_0	Clustered	4	VC_354_0	4	0.1749	0.99998684	0.1749	0	0	0	0.8333	Ref
MN516421	Unassigned	Unassigned	Unassigned	698_0	Clustered	26	VC_698_0	26	0.9366	1	0.9366	0	0	0	1	Ref
MN516422	Unassigned	Unassigned	Unassigned	698_0	Clustered	26	VC_698_0	26	0.9366	1	0.9366	0	0	0	1	Ref
MN518893	Unassigned	Unassigned	Unassigned	451_0	Clustered	80	VC_451_0	80	0.9991	1	0.9991	0	0	0	1	Ref
MN518894	Unassigned	Unassigned	Unassigned	451_0	Clustered	80	VC_451_0	80	0.9991	1	0.9991	0	0	0	1	Ref
MN528768	Unassigned	Unassigned	Unassigned	534_0	Clustered	9	VC_534_0	9	0.99	1	0.99	0	0	0	1	Ref
MN534316	Unassigned	Unassigned	Unassigned	260_0	Clustered	13	VC_260_0	13	0.3705	1	0.3705	0	0	0	1	Ref
MN534317	Unassigned	Unassigned	Unassigned	260_0	Clustered	13	VC_260_0	13	0.3705	1	0.3705	0	0	0	1	Ref
MN534320	Unassigned	Unassigned	Unassigned	262_0	Clustered	5	VC_262_0	5	0.0724	0.99999989	0.0724	0	0	0	1	Ref
MN536023	Unassigned	Unassigned	Unassigned	16_0	Clustered	12	VC_16_0	12	0.6615	1	0.6615	0	0	0	1	Ref
MN536027	Unassigned	Unassigned	Unassigned	251_0	Clustered	22	VC_251_0	22	0.6508	1	0.6508	0	0	0	1	Ref
MN539736	Unassigned	Unassigned	Unassigned	149_8	Clustered	66	VC_149_8	66	0.4565	1	0.4565	0	0	0	0.8182	Ref
MN539737	Unassigned	Unassigned	Unassigned	149_8	Clustered	66	VC_149_8	66	0.4565	1	0.4565	0	0	0	0.8182	Ref
MN539738	Unassigned	Unassigned	Unassigned	149_8	Clustered	66	VC_149_8	66	0.4565	1	0.4565	0	0	0	0.8182	Ref
MN549360	Unassigned	Unassigned	Unassigned	1052_3	Clustered	5	VC_1052_3	5	0.185	0.98656048	0.1825	0	0	0	1	Ref
MN549361	Unassigned	Unassigned	Unassigned	1052_3	Clustered	5	VC_1052_3	5	0.185	0.98656048	0.1825	0	0	0	1	Ref
MN552143	Unassigned	Unassigned	Unassigned	800_0	Clustered	3	VC_800_0	3	0.4591	0.9984737	0.4584	0	0	0	1	Ref
MN552144	Unassigned	Unassigned	Unassigned	268_0	Clustered	7	VC_268_0	7	0.3427	1	0.3427	0	0	0	1	Ref
MN552145	Unassigned	Unassigned	Unassigned	474_0	Clustered	14	VC_474_0	14	0.8466	1	0.8466	0	0	0	1	Ref
MN552146	Unassigned	Unassigned	Unassigned	861_0	Clustered	9	VC_861_0	9	0.5869	1	0.5869	0	0	0	1	Ref
MN552147	Unassigned	Unassigned	Unassigned	862_0	Clustered	11	VC_862_0	11	0.6745	1	0.6745	0	0	0	1	Ref
MN552148	Unassigned	Unassigned	Unassigned	202_0	Clustered	102	VC_202_0	102	0.4049	1	0.4049	0	0	0	0.7876	Ref
MN552149	Unassigned	Unassigned	Unassigned	202_0	Clustered	102	VC_202_0	102	0.4049	1	0.4049	0	0	0	0.7876	Ref
MN552150	Unassigned	Unassigned	Unassigned	202_0	Clustered	102	VC_202_0	102	0.4049	1	0.4049	0	0	0	0.7876	Ref
MN553583	Unassigned	Unassigned	Unassigned	838_0	Clustered	29	VC_838_0	29	0.8611	1	0.8611	0	0	0	1	Ref
MN553584	Unassigned	Unassigned	Unassigned	838_0	Clustered	29	VC_838_0	29	0.8611	1	0.8611	0	0	0	1	Ref
MN553585	Unassigned	Unassigned	Unassigned	423_0	Clustered	45	VC_423_0	45	0.9484	1	0.9484	0	0	0	1	Ref
MN553586	Unassigned	Unassigned	Unassigned	472_0	Clustered	47	VC_472_0	47	0.9025	1	0.9025	0	0	0	1	Ref
MN553587	Unassigned	Unassigned	Unassigned	423_0	Clustered	45	VC_423_0	45	0.9484	1	0.9484	0	0	0	1	Ref
MN553588	Unassigned	Unassigned	Unassigned	423_0	Clustered	45	VC_423_0	45	0.9484	1	0.9484	0	0	0	1	Ref
MN553589	Unassigned	Unassigned	Unassigned	423_0	Clustered	45	VC_423_0	45	0.9484	1	0.9484	0	0	0	1	Ref
MN562749	Unassigned	Unassigned	Unassigned	472_0	Clustered	47	VC_472_0	47	0.9025	1	0.9025	0	0	0	1	Ref
MN563783	Unassigned	Unassigned	Unassigned	80_0	Clustered	41	VC_80_0	41	0.5837	1	0.5837	0	0	0	0.9805	Ref
MN563784	Unassigned	Unassigned	Unassigned	344_0	Clustered	21	VC_344_0	21	0.5436	1	0.5436	0	0	0	1	Ref
MN563785	Unassigned	Unassigned	Unassigned	76_0	Clustered	37	VC_76_0	37	0.9403	1	0.9403	0	0	0	1	Ref
MN563793	Unassigned	Unassigned	Unassigned	5_0	Clustered	30	VC_5_0	30	0.9647	1	0.9647	0	0	0	1	Ref
MN564818	Unassigned	Unassigned	Unassigned	80_0	Clustered	41	VC_80_0	41	0.5837	1	0.5837	0	0	0	0.9805	Ref
MN580668	Unassigned	Unassigned	Unassigned	283_179	Clustered/Singleton	1	VC_283_179	1	0	1	0	0	0	0	1	Ref
MN585130	Unassigned	Unassigned	Unassigned	823_0	Clustered	124	VC_823_0	124	0.9492	1	0.9492	0	0	0	1	Ref
MN585963	Unassigned	Unassigned	Unassigned	1176_0	Clustered	41	VC_1176_0	41	0.9558	1	0.9558	0	0	0	0.9976	Ref
MN585964	Unassigned	Unassigned	Unassigned	556_0	Clustered	21	VC_556_0	21	0.9912	1	0.9912	0	0	0	1	Ref

MN585965	Unassigned	Unassigned	Unassigned	371_13	Clustered	33	VC_371_13	33	0.179	1	0.179	0	0	0	0.8769	Ref
MN585966	Unassigned	Unassigned	Unassigned	522_1	Clustered	5	VC_522_1	5	0.1017	0.99999997	0.1017	0	0	0	0.8	Ref
MN585967	Unassigned	Unassigned	Unassigned	371_1	Clustered	11	VC_371_1	11	0.0379	1	0.0379	0	0	0	0.8545	Ref
MN585968	Unassigned	Unassigned	Unassigned	216_2	Clustered	2	VC_216_2	2	0.0139	0.95744997	0.0133	0	0	0	1	Ref
MN585969	Unassigned	Unassigned	Unassigned	514_0	Clustered	75	VC_514_0	75	0.9402	1	0.9402	0	0	0	1	Ref
MN585970	Unassigned	Unassigned	Unassigned	325_20	Clustered/Singleton	1	VC_325_20	1	0	1	0	0	0	0	1	Ref
MN585971	Unassigned	Unassigned	Unassigned	799_1	Clustered	27	VC_799_1	27	0.8611	1	0.8611	0	0	0	1	Ref
MN585972	Unassigned	Unassigned	Unassigned	1169_0	Clustered	7	VC_1169_0	7	0.9811	0.99995613	0.981	0	0	0	1	Ref
MN585973	Unassigned	Unassigned	Unassigned	520_31	Clustered	11	VC_520_31	11	0.0897	1	0.0897	0	0	0	0.6	Ref
MN585974	Unassigned	Unassigned	Unassigned	225_0	Clustered	32	VC_225_0	32	0.848	1	0.848	0	0	0	0.996	Ref
MN585975	Unassigned	Unassigned	Unassigned	520_89	Clustered/Singleton	1	VC_520_89	1	0	1	0	0	0	0	1	Ref
MN585976	Unassigned	Unassigned	Unassigned	325_24	Clustered	3	VC_325_24	3	0.009	0.99853949	0.009	0	0	0	1	Ref
MN585977	Unassigned	Unassigned	Unassigned	519_0	Clustered	39	VC_519_0	39	0.8156	1	0.8156	0	0	0	1	Ref
MN585978	Unassigned	Unassigned	Unassigned	225_0	Clustered	32	VC_225_0	32	0.848	1	0.848	0	0	0	0.996	Ref
MN585979	Unassigned	Unassigned	Unassigned	225_0	Clustered	32	VC_225_0	32	0.848	1	0.848	0	0	0	0.996	Ref
MN585980	Unassigned	Unassigned	Unassigned	1352_0	Clustered	31	VC_1352_0	31	0.849	1	0.849	0	0	0	1	Ref
MN585981	Unassigned	Unassigned	Unassigned	216_6	Clustered	42	VC_216_6	42	0.473	1	0.473	0	0	0	0.8351	Ref
MN585982	Unassigned	Unassigned	Unassigned	799_1	Clustered	27	VC_799_1	27	0.8611	1	0.8611	0	0	0	1	Ref
MN585983	Unassigned	Unassigned	Unassigned	216_6	Clustered	42	VC_216_6	42	0.473	1	0.473	0	0	0	0.8351	Ref
MN585984	Unassigned	Unassigned	Unassigned	514_0	Clustered	75	VC_514_0	75	0.9402	1	0.9402	0	0	0	1	Ref
MN585985	Unassigned	Unassigned	Unassigned	325_37	Clustered	3	VC_325_37	3	0.008	0.99634936	0.008	0	0	0	0.6667	Ref
MN585986	Unassigned	Unassigned	Unassigned	1352_0	Clustered	31	VC_1352_0	31	0.849	1	0.849	0	0	0	1	Ref
MN585987	Unassigned	Unassigned	Unassigned	520_59	Clustered	5	VC_520_59	5	0.0459	0.99999997	0.0459	0	0	0	1	Ref
MN585988	Unassigned	Unassigned	Unassigned	799_1	Clustered	27	VC_799_1	27	0.8611	1	0.8611	0	0	0	1	Ref
MN585989	Unassigned	Unassigned	Unassigned	215_0	Clustered	5	VC_215_0	5	0.0724	0.99999997	0.0724	0	0	0	1	Ref
MN585990	Unassigned	Unassigned	Unassigned	1344_0	Clustered	78	VC_1344_0	78	0.9732	1	0.9732	0	0	0	1	Ref
MN585991	Unassigned	Unassigned	Unassigned	325_51	Clustered	2	VC_325_51	2	0.0039	0.95663826	0.0037	0	0	0	1	Ref
MN585992	Unassigned	Unassigned	Unassigned	764_0	Clustered	4	VC_764_0	4	0.3556	0.99997469	0.3556	0	0	0	1	Ref
MN585993	Unassigned	Unassigned	Unassigned	571_0	Clustered	4	VC_571_0	4	0.1921	0.99998591	0.1921	0	0	0	1	Ref
MN585994	Unassigned	Unassigned	Unassigned	225_0	Clustered	32	VC_225_0	32	0.848	1	0.848	0	0	0	0.996	Ref
MN585995	Unassigned	Unassigned	Unassigned	520_11	Clustered/Singleton	1	VC_520_11	1	0	1	0	0	0	0	1	Ref
MN585996	Unassigned	Unassigned	Unassigned	799_1	Clustered	27	VC_799_1	27	0.8611	1	0.8611	0	0	0	1	Ref
MN585997	Unassigned	Unassigned	Unassigned	325_56	Clustered	2	VC_325_56	2	0.0036	0.94604798	0.0034	0	0	0	0	Ref
MN585998	Unassigned	Unassigned	Unassigned	216_6	Clustered	42	VC_216_6	42	0.473	1	0.473	0	0	0	0.8351	Ref
MN585999	Unassigned	Unassigned	Unassigned	325_5	Clustered	11	VC_325_5	11	0.047	1	0.047	0	0	0	0.7636	Ref
MN586000	Unassigned	Unassigned	Unassigned	325_35	Clustered	17	VC_325_35	17	0.0732	1	0.0732	0	0	0	0.7721	Ref
MN586001	Unassigned	Unassigned	Unassigned	520_18	Clustered/Singleton	1	VC_520_18	1	0	1	0	0	0	0	1	Ref
MN586002	Unassigned	Unassigned	Unassigned	216_2	Clustered	2	VC_216_2	2	0.0139	0.95744997	0.0133	0	0	0	1	Ref
MN586003	Unassigned	Unassigned	Unassigned	572_0	Clustered	16	VC_572_0	16	0.7286	1	0.7286	0	0	0	1	Ref
MN586004	Unassigned	Unassigned	Unassigned	520_6	Clustered	3	VC_520_6	3	0.0276	0.99859841	0.0276	0	0	0	1	Ref
MN586005	Unassigned	Unassigned	Unassigned	520_59	Clustered	5	VC_520_59	5	0.0459	0.99999997	0.0459	0	0	0	1	Ref
MN586006	Unassigned	Unassigned	Unassigned	221_0	Clustered	22	VC_221_0	22	0.7447	1	0.7447	0	0	0	1	Ref
MN586007	Unassigned	Unassigned	Unassigned	1352_0	Clustered	31	VC_1352_0	31	0.849	1	0.849	0	0	0	1	Ref
MN586008	Unassigned	Unassigned	Unassigned	1344_0	Clustered	78	VC_1344_0	78	0.9732	1	0.9732	0	0	0	1	Ref
MN586009	Unassigned	Unassigned	Unassigned	982_0	Clustered	62	VC_982_0	62	0.8646	1	0.8646	0	0	0	0.743	Ref
MN586010	Unassigned	Unassigned	Unassigned	1344_0	Clustered	78	VC_1344_0	78	0.9732	1	0.9732	0	0	0	1	Ref
MN586011	Unassigned	Unassigned	Unassigned	520_41	Clustered	2	VC_520_41	2	0.0139	0.95761457	0.0134	0	0	0	1	Ref
MN586012	Unassigned	Unassigned	Unassigned	520_66	Clustered/Singleton	1	VC_520_66	1	0	1	0	0	0	0	1	Ref
MN586013	Unassigned	Unassigned	Unassigned	551_0	Clustered	16	VC_551_0	16	0.0915	1	0.0915	0	0	0	0.6667	Ref
MN586014	Unassigned	Unassigned	Unassigned	1183_0	Clustered	6	VC_1183_0	6	0.995	0.99996974	0.995	0	0	0	1	Ref
MN586015	Unassigned	Unassigned	Unassigned	919_0	Clustered	39	VC_919_0	39	0.9069	1	0.9069	0	0	0	1	Ref
MN586016	Unassigned	Unassigned	Unassigned	940_0	Clustered	34	VC_940_0	34	0.7959	1	0.7959	0	0	0	1	Ref
MN586017	Unassigned	Unassigned	Unassigned	551_7	Clustered	28	VC_551_7	28	0.1661	1	0.1661	0	0	0	0.8519	Ref
MN586018	Unassigned	Unassigned	Unassigned	887_0	Clustered	18	VC_887_0	18	0.3663	1	0.3663	0	0	0	1	Ref
MN586019	Unassigned	Unassigned	Unassigned	551_7	Clustered	28	VC_551_7	28	0.1661	1	0.1661	0	0	0	0.8519	Ref
MN586021	Unassigned	Unassigned	Unassigned	1170_0	Clustered	3	VC_1170_0	3	0.3275	0.99834213	0.327	0	0	0	1	Ref
MN586022	Unassigned	Unassigned	Unassigned	1096_0	Clustered	5	VC_1096_0	5	0.8566	0.99869977	0.8555	0	0	0	1	Ref
MN586023	Unassigned	Unassigned	Unassigned	887_0	Clustered	18	VC_887_0	18	0.3663	1	0.3663	0	0	0	1	Ref
MN586024	Unassigned	Unassigned	Unassigned	763_1	Clustered	9	VC_763_1	9	0.3706	1	0.3706	0	0	0	1	Ref
MN586026	Unassigned	Unassigned	Unassigned	1095_0	Clustered	16	VC_1095_0	16	0.458	1	0.458	0	0	0	1	Ref
MN586027	Unassigned	Unassigned	Unassigned	1370_0	Clustered	2	VC_1370_0	2	0.8214	0.91237788	0.7494	0	0	0	1	Ref
MN586028	Unassigned	Unassigned	Unassigned	1095_0	Clustered	16	VC_1095_0	16	0.458	1	0.458	0	0	0	1	Ref
MN586029	Unassigned	Unassigned	Unassigned	546_1	Clustered	2	VC_546_1	2	0.0136	0.68405151	0.0093	0	0	0	1	Ref
MN586030	Unassigned	Unassigned	Unassigned	888_0	Clustered	28	VC_888_0	28	0.5497	1	0.5497	0	0	0	1	Ref
MN586031	Unassigned	Unassigned	Unassigned	1176_0	Clustered	41	VC_1176_0	41	0.9558	1	0.9558	0	0	0	0.9976	Ref
MN586032	Unassigned	Unassigned	Unassigned	551_2	Clustered	17	VC_551_2	17	0.0973	1	0.0973	0	0	0	0.9779	Ref
MN586034	Unassigned	Unassigned	Unassigned	551_6	Clustered	22	VC_551_6	22	0.1214	1	0.1214	0	0	0	0.8095	Ref
MN586035	Unassigned	Unassigned	Unassigned	551_7	Clustered	28	VC_551_7	28	0.1661	1	0.1661	0	0	0	0.8519	Ref
MN586036	Unassigned	Unassigned	Unassigned	919_0	Clustered	39	VC_919_0	39	0.9069	1	0.9069	0	0	0	1	Ref
MN586037	Unassigned	Unassigned	Unassigned	551_6	Clustered	22	VC_551_6	22	0.1214	1	0.1214	0	0	0	0.8095	Ref
MN586038	Unassigned	Unassigned	Unassigned	515_0	Clustered	16	VC_515_0	16	0.3793	1	0.3793	0	0	0	1	Ref
MN586039	Unassigned	Unassigned	Unassigned	223_0	Clustered	38	VC_223_0	38	0.9095	1	0.9095	0	0	0	1	Ref
MN586041	Unassigned	Unassigned	Unassigned	551_7	Clustered	28	VC_551_7	28	0.1661	1	0.1661	0	0	0	0.8519	Ref
MN586042	Unassigned	Unassigned	Unassigned	1352_0	Clustered	31	VC_1352_0	31	0.849	1	0.849	0	0	0	1	Ref
MN586043	Unassigned	Unassigned	Unassigned	551_1	Clustered	4	VC_551_1	4	0.0159	0.99968337	0.0159	0	0	0	0.6667	Ref
MN586044	Unassigned	Unassigned	Unassigned	551_1	Clustered	4	VC_551_1	4	0.0159	0.99968337	0.0159	0	0	0	0.6667	Ref
MN586045	Unassigned	Unassigned	Unassigned	508_0	Clustered	32	VC_508_0	32	0.906	1	0.906	0	0	0	1	Ref
MN586046	Unassigned	Unassigned	Unassigned	508_0	Clustered	32	VC_508_0	32	0.906	1	0.906	0	0	0	1	Ref
MN586047	Unassigned	Unassigned	Unassigned	1095_0	Clustered	16	VC_1095_0	16	0.458	1	0.458	0	0	0	1	Ref
MN586048	Unassigned	Unassigned	Unassigned	514_0	Clustered	75	VC_514_0	75	0.9402	1	0.9402	0	0	0	1	Ref
MN586049	Unassigned	Unassigned	Unassigned	919_0	Clustered	39	VC_919_0	39	0.9069	1	0.9069	0	0	0	1	Ref

MN586050	Unassigned	Unassigned	Unassigned	551_7	Clustered	28	VC_551_7	28	0.1661	1	0.1661	0	0	0	0.8519	Ref
MN586051	Unassigned	Unassigned	Unassigned	551_2	Clustered	17	VC_551_2	17	0.0973	1	0.0973	0	0	0	0.9779	Ref
MN586052	Unassigned	Unassigned	Unassigned	888_0	Clustered	28	VC_888_0	28	0.5497	1	0.5497	0	0	0	1	Ref
MN586053	Unassigned	Unassigned	Unassigned	1179_0	Clustered	3	VC_1179_0	3	0.7588	0.96819872	0.7347	0	0	0	1	Ref
MN586054	Unassigned	Unassigned	Unassigned	872_0	Clustered	31	VC_872_0	31	0.8151	1	0.8151	0	0	0	0.8194	Ref
MN586055	Unassigned	Unassigned	Unassigned	1175_0	Clustered	22	VC_1175_0	22	0.4562	1	0.4562	0	0	0	1	Ref
MN586056	Unassigned	Unassigned	Unassigned	872_0	Clustered	31	VC_872_0	31	0.8151	1	0.8151	0	0	0	0.8194	Ref
MN586057	Unassigned	Unassigned	Unassigned	919_0	Clustered	39	VC_919_0	39	0.9069	1	0.9069	0	0	0	1	Ref
MN586058	Unassigned	Unassigned	Unassigned	371_1	Clustered	11	VC_371_1	11	0.0379	1	0.0379	0	0	0	0.8545	Ref
MN586059	Unassigned	Unassigned	Unassigned	1352_0	Clustered	31	VC_1352_0	31	0.849	1	0.849	0	0	0	1	Ref
MN586060	Unassigned	Unassigned	Unassigned	940_0	Clustered	34	VC_940_0	34	0.7959	1	0.7959	0	0	0	1	Ref
MN586061	Unassigned	Unassigned	Unassigned	1160_0	Clustered	73	VC_1160_0	73	0.9835	1	0.9835	0	0	0	1	Ref
MN586062	Unassigned	Unassigned	Unassigned	940_0	Clustered	34	VC_940_0	34	0.7959	1	0.7959	0	0	0	1	Ref
MN586063	Unassigned	Unassigned	Unassigned	513_0	Clustered	32	VC_513_0	32	0.6881	1	0.6881	0	0	0	1	Ref
MN592896	Unassigned	Unassigned	Unassigned	925_0	Clustered/Singleton	1	VC_925_0	1	0	1	0	0	0	0	1	Ref
MN592897	Unassigned	Unassigned	Unassigned	296_0	Clustered	6	VC_296_0	6	0.5315	1	0.5315	0	0	0	1	Ref
MN602045	Unassigned	Unassigned	Unassigned	423_0	Clustered	45	VC_423_0	45	0.9484	1	0.9484	0	0	0	1	Ref
MN602266	Unassigned	Unassigned	Unassigned	936_0	Clustered	2	VC_936_0	2	0.9284	0.81445332	0.7561	0	0	0	1	Ref
MN602267	Unassigned	Unassigned	Unassigned	936_0	Clustered	2	VC_936_0	2	0.9284	0.81445332	0.7561	0	0	0	1	Ref
MN602268	Unassigned	Unassigned	Unassigned	977_1	Clustered	2	VC_977_1	2	0.079	0.93977186	0.0743	0	0	0	1	Ref
MN604053	Unassigned	Unassigned	Unassigned	591_0	Clustered	14	VC_591_0	14	0.5184	1	0.5184	0	0	0	1	Ref
MN604230	Unassigned	Unassigned	Unassigned	144_0	Clustered	2	VC_144_0	2	0.4237	0.94768242	0.4015	0	0	0	1	Ref
MN604238	Unassigned	Unassigned	Unassigned	699_0	Clustered	71	VC_699_0	71	0.9352	1	0.9352	0	0	0	1	Ref
MN604239	Unassigned	Unassigned	Unassigned	699_0	Clustered	71	VC_699_0	71	0.9352	1	0.9352	0	0	0	1	Ref
MN604407	Unassigned	Unassigned	Unassigned	328_0	Clustered	11	VC_328_0	11	0.4707	1	0.4707	0	0	0	1	Ref
MN604698	Unassigned	Unassigned	Unassigned	144_0	Clustered	2	VC_144_0	2	0.4237	0.94768242	0.4015	0	0	0	1	Ref
MN614471	Unassigned	Unassigned	Unassigned	699_0	Clustered	71	VC_699_0	71	0.9352	1	0.9352	0	0	0	1	Ref
MN615698	Unassigned	Unassigned	Unassigned	423_0	Clustered	45	VC_423_0	45	0.9484	1	0.9484	0	0	0	1	Ref
MN615699	Unassigned	Unassigned	Unassigned	423_0	Clustered	45	VC_423_0	45	0.9484	1	0.9484	0	0	0	1	Ref
MN615700	Unassigned	Unassigned	Unassigned	79_7	Clustered	33	VC_79_7	33	0.216	1	0.216	0	0	0	0.8409	Ref
MN615701	Unassigned	Unassigned	Unassigned	79_7	Clustered	33	VC_79_7	33	0.216	1	0.216	0	0	0	0.8409	Ref
MN615702	Unassigned	Unassigned	Unassigned	79_7	Clustered	33	VC_79_7	33	0.216	1	0.216	0	0	0	0.8409	Ref
MN617773	Unassigned	Unassigned	Unassigned	450_0	Clustered	16	VC_450_0	16	0.9564	1	0.9564	0	0	0	1	Ref
MN617840	Unassigned	Unassigned	Unassigned	1240_0	Clustered	82	VC_1240_0	82	0.7945	1	0.7945	0	0	0	1	Ref
MN617841	Unassigned	Unassigned	Unassigned	887_0	Clustered	18	VC_887_0	18	0.3663	1	0.3663	0	0	0	1	Ref
MN617842	Unassigned	Unassigned	Unassigned	950_0	Clustered	38	VC_950_0	38	0.9443	1	0.9443	0	0	0	1	Ref
MN617843	Unassigned	Unassigned	Unassigned	372_2	Clustered	5	VC_372_2	5	0.072	0.99999997	0.072	0	0	0	0.9	Ref
MN617844	Unassigned	Unassigned	Unassigned	940_0	Clustered	34	VC_940_0	34	0.7959	1	0.7959	0	0	0	1	Ref
MN617845	Unassigned	Unassigned	Unassigned	325_1	Clustered	2	VC_325_1	2	0.0043	0.95663348	0.0041	0	0	0	1	Ref
MN642089	Unassigned	Unassigned	Unassigned	442_1	Clustered	3	VC_442_1	3	0.0538	0.99419552	0.0535	0	0	0	1	Ref
MN648195	Unassigned	Unassigned	Unassigned	492_1	Clustered	20	VC_492_1	20	0.4176	1	0.4176	0	0	0	1	Ref
MN648445	Unassigned	Unassigned	Unassigned	286_17	Clustered	8	VC_286_17	8	0.0535	0.99997829	0.0535	0	0	0	0.6071	Ref
MN651570	Unassigned	Unassigned	Unassigned	699_0	Clustered	71	VC_699_0	71	0.9352	1	0.9352	0	0	0	1	Ref
MN655998	Unassigned	Unassigned	Unassigned	590_0	Clustered	27	VC_590_0	27	0.9947	1	0.9947	0	0	0	1	Ref
MN655999	Unassigned	Unassigned	Unassigned	329_11	Clustered	15	VC_329_11	15	0.0791	1	0.0791	0	0	0	0.9333	Ref
MN656993	Unassigned	Unassigned	Unassigned	836_0	Clustered	9	VC_836_0	9	0.7733	1	0.7733	0	0	0	1	Ref
MN662249	Unassigned	Unassigned	Unassigned	492_1	Clustered	20	VC_492_1	20	0.4176	1	0.4176	0	0	0	1	Ref
MN688132	Unassigned	Unassigned	Unassigned	33_0	Clustered	4	VC_33_0	4	0.06	0.99994826	0.06	0	0	0	1	Ref
MN689503	Unassigned	Unassigned	Unassigned	690_0	Clustered	50	VC_690_0	50	0.9805	1	0.9805	0	0	0	1	Ref
MN689504	Unassigned	Unassigned	Unassigned	690_0	Clustered	50	VC_690_0	50	0.9805	1	0.9805	0	0	0	1	Ref
MN689505	Unassigned	Unassigned	Unassigned	690_0	Clustered	50	VC_690_0	50	0.9805	1	0.9805	0	0	0	1	Ref
MN689506	Unassigned	Unassigned	Unassigned	690_0	Clustered	50	VC_690_0	50	0.9805	1	0.9805	0	0	0	1	Ref
MN689507	Unassigned	Unassigned	Unassigned	690_0	Clustered	50	VC_690_0	50	0.9805	1	0.9805	0	0	0	1	Ref
MN689508	Unassigned	Unassigned	Unassigned	690_0	Clustered	50	VC_690_0	50	0.9805	1	0.9805	0	0	0	1	Ref
MN689509	Unassigned	Unassigned	Unassigned	260_0	Clustered	13	VC_260_0	13	0.3705	1	0.3705	0	0	0	1	Ref
MN689510	Unassigned	Unassigned	Unassigned	690_0	Clustered	50	VC_690_0	50	0.9805	1	0.9805	0	0	0	1	Ref
MN689511	Unassigned	Unassigned	Unassigned	690_0	Clustered	50	VC_690_0	50	0.9805	1	0.9805	0	0	0	1	Ref
MN689512	Unassigned	Unassigned	Unassigned	690_0	Clustered	50	VC_690_0	50	0.9805	1	0.9805	0	0	0	1	Ref
MN689513	Unassigned	Unassigned	Unassigned	690_0	Clustered	50	VC_690_0	50	0.9805	1	0.9805	0	0	0	1	Ref
MN689514	Unassigned	Unassigned	Unassigned	202_0	Clustered	102	VC_202_0	102	0.4049	1	0.4049	0	0	0	0.7876	Ref
MN689515	Unassigned	Unassigned	Unassigned	690_0	Clustered	50	VC_690_0	50	0.9805	1	0.9805	0	0	0	1	Ref
MN689516	Unassigned	Unassigned	Unassigned	260_0	Clustered	13	VC_260_0	13	0.3705	1	0.3705	0	0	0	1	Ref
MN689517	Unassigned	Unassigned	Unassigned	202_0	Clustered	102	VC_202_0	102	0.4049	1	0.4049	0	0	0	0.7876	Ref
MN689518	Unassigned	Unassigned	Unassigned	202_0	Clustered	102	VC_202_0	102	0.4049	1	0.4049	0	0	0	0.7876	Ref
MN689519	Unassigned	Unassigned	Unassigned	202_0	Clustered	102	VC_202_0	102	0.4049	1	0.4049	0	0	0	0.7876	Ref
MN689520	Unassigned	Unassigned	Unassigned	202_0	Clustered	102	VC_202_0	102	0.4049	1	0.4049	0	0	0	0.7876	Ref
MN689521	Unassigned	Unassigned	Unassigned	260_0	Clustered	13	VC_260_0	13	0.3705	1	0.3705	0	0	0	1	Ref
MN689522	Unassigned	Unassigned	Unassigned	202_0	Clustered	102	VC_202_0	102	0.4049	1	0.4049	0	0	0	0.7876	Ref
MN689523	Unassigned	Unassigned	Unassigned	202_0	Clustered	102	VC_202_0	102	0.4049	1	0.4049	0	0	0	0.7876	Ref
MN689524	Unassigned	Unassigned	Unassigned	202_0	Clustered	102	VC_202_0	102	0.4049	1	0.4049	0	0	0	0.7876	Ref
MN689525	Unassigned	Unassigned	Unassigned	202_0	Clustered	102	VC_202_0	102	0.4049	1	0.4049	0	0	0	0.7876	Ref
MN689526	Unassigned	Unassigned	Unassigned	690_0	Clustered	50	VC_690_0	50	0.9805	1	0.9805	0	0	0	1	Ref
MN689527	Unassigned	Unassigned	Unassigned	690_0	Clustered	50	VC_690_0	50	0.9805	1	0.9805	0	0	0	1	Ref
MN689528	Unassigned	Unassigned	Unassigned	690_0	Clustered	50	VC_690_0	50	0.9805	1	0.9805	0	0	0	1	Ref
MN689529	Unassigned	Unassigned	Unassigned	690_0	Clustered	50	VC_690_0	50	0.9805	1	0.9805	0	0	0	1	Ref
MN689530	Unassigned	Unassigned	Unassigned	260_0	Clustered	13	VC_260_0	13	0.3705	1	0.3705	0	0	0	1	Ref
MN689531	Unassigned	Unassigned	Unassigned	690_0	Clustered	50	VC_690_0	50	0.9805	1	0.9805	0	0	0	1	Ref
MN689532	Unassigned	Unassigned	Unassigned	690_0	Clustered	50	VC_690_0	50	0.9805	1	0.9805	0	0	0	1	Ref
MN689533	Unassigned	Unassigned	Unassigned	690_0	Clustered	50	VC_690_0	50	0.9805	1	0.9805	0	0	0	1	Ref
MN689778	Unassigned	Unassigned	Unassigned	123_0	Clustered	23	VC_123_0	23	0.817	1	0.817	0	0	0	1	Ref
MN689779	Unassigned	Unassigned	Unassigned	938_0	Clustered	99	VC_938_0	99	0.9119	1	0.9119	0	0	0	0.9928	Ref

MN690600	Unassigned	Unassigned	Unassigned	16_0	Clustered	12	VC_16_0	12	0.6615	1	0.6615	0	0	0	1	Ref
MN692672	Unassigned	Unassigned	Unassigned	423_0	Clustered	45	VC_423_0	45	0.9484	1	0.9484	0	0	0	1	Ref
MN692673	Unassigned	Unassigned	Unassigned	461_0	Clustered	96	VC_461_0	96	0.9307	1	0.9307	0	0	0	0.9575	Ref
MN695334	Unassigned	Unassigned	Unassigned	432_0	Clustered	2	VC_432_0	2	0.0362	0.95680396	0.0346	0	0	0	1	Ref
MN695335	Unassigned	Unassigned	Unassigned	432_0	Clustered	2	VC_432_0	2	0.0362	0.95680396	0.0346	0	0	0	1	Ref
MN698239	Unassigned	Unassigned	Unassigned	470_0	Clustered	3	VC_470_0	3	0.5552	0.99823354	0.5543	0	0	0	1	Ref
MN698240	Unassigned	Unassigned	Unassigned	1003_0	Clustered	4	VC_1003_0	4	0.8718	0.99983781	0.8716	0	0	0	1	Ref
MN698241	Unassigned	Unassigned	Unassigned	470_0	Clustered	3	VC_470_0	3	0.5552	0.99823354	0.5543	0	0	0	1	Ref
MN698242	Unassigned	Unassigned	Unassigned	485_0	Clustered	3	VC_485_0	3	0.2389	0.99844364	0.2385	0	0	0	1	Ref
MN698243	Unassigned	Unassigned	Unassigned	485_0	Clustered	3	VC_485_0	3	0.2389	0.99844364	0.2385	0	0	0	1	Ref
MN698245	Unassigned	Unassigned	Unassigned	1003_0	Clustered	4	VC_1003_0	4	0.8718	0.99983781	0.8716	0	0	0	1	Ref
MN698246	Unassigned	Unassigned	Unassigned	1003_0	Clustered	4	VC_1003_0	4	0.8718	0.99983781	0.8716	0	0	0	1	Ref
MN698247	Unassigned	Unassigned	Unassigned	485_0	Clustered	3	VC_485_0	3	0.2389	0.99844364	0.2385	0	0	0	1	Ref
MN698248	Unassigned	Unassigned	Unassigned	1003_0	Clustered	4	VC_1003_0	4	0.8718	0.99983781	0.8716	0	0	0	1	Ref
MN703405	Unassigned	Unassigned	Unassigned	221_0	Clustered	22	VC_221_0	22	0.7447	1	0.7447	0	0	0	1	Ref
MN703406	Unassigned	Unassigned	Unassigned	888_0	Clustered	28	VC_888_0	28	0.5497	1	0.5497	0	0	0	1	Ref
MN703407	Unassigned	Unassigned	Unassigned	1345_0	Clustered	2	VC_1345_0	2	0.1133	0.95579173	0.1083	0	0	0	1	Ref
MN703408	Unassigned	Unassigned	Unassigned	217_0	Clustered	5	VC_217_0	5	0.069	0.99999998	0.069	0	0	0	1	Ref
MN703409	Unassigned	Unassigned	Unassigned	1166_0	Clustered	20	VC_1166_0	20	0.99	1	0.99	0	0	0	1	Ref
MN703410	Unassigned	Unassigned	Unassigned	888_0	Clustered	28	VC_888_0	28	0.5497	1	0.5497	0	0	0	1	Ref
MN703411	Unassigned	Unassigned	Unassigned	1074_0	Clustered	19	VC_1074_0	19	0.9198	1	0.9198	0	0	0	1	Ref
MN703412	Unassigned	Unassigned	Unassigned	1164_0	Clustered	25	VC_1164_0	25	0.7312	1	0.7312	0	0	0	1	Ref
MN703413	Unassigned	Unassigned	Unassigned	1207_0	Clustered	29	VC_1207_0	29	0.8225	1	0.8225	0	0	0	1	Ref
MN703414	Unassigned	Unassigned	Unassigned	1345_0	Clustered	2	VC_1345_0	2	0.1133	0.95579173	0.1083	0	0	0	1	Ref
MN703415	Unassigned	Unassigned	Unassigned	371_20	Clustered/Singleton	1	VC_371_20	1	0	1	0	0	0	0	1	Ref
MN709127	Unassigned	Unassigned	Unassigned	590_0	Clustered	27	VC_590_0	27	0.9947	1	0.9947	0	0	0	1	Ref
MN709128	Unassigned	Unassigned	Unassigned	492_1	Clustered	20	VC_492_1	20	0.4176	1	0.4176	0	0	0	1	Ref
MN710383	Unassigned	Unassigned	Unassigned	589_0	Clustered	2	VC_589_0	2	1	1	1	0	0	0	1	Ref
MN715150	Unassigned	Unassigned	Unassigned	451_0	Clustered	80	VC_451_0	80	0.9991	1	0.9991	0	0	0	1	Ref
MN715356	Unassigned	Unassigned	Unassigned	813_1	Clustered	24	VC_813_1	24	0.535	1	0.535	0	0	0	1	Ref
MN716856	Unassigned	Unassigned	Unassigned	283_131	Clustered/Singleton	1	VC_283_131	1	0	1	0	0	0	0	1	Ref
MN718463	Unassigned	Unassigned	Unassigned	766_0	Clustered	2	VC_766_0	2	0.2674	0.94916107	0.2538	0	0	0	1	Ref
MN719123	Unassigned	Unassigned	Unassigned	17_0	Clustered	9	VC_17_0	9	0.6969	1	0.6969	0	0	0	1	Ref
MN722429	Unassigned	Unassigned	Unassigned	637_0	Clustered	115	VC_637_0	115	0.731	1	0.731	0	0	0	0.8764	Ref
MN723850	Unassigned	Unassigned	Unassigned	492_1	Clustered	20	VC_492_1	20	0.4176	1	0.4176	0	0	0	1	Ref
MN727882	Unassigned	Unassigned	Unassigned	100_0	Clustered	15	VC_100_0	15	0.6536	1	0.6536	0	0	0	1	Ref
MN728683	Unassigned	Unassigned	Unassigned	223_0	Clustered	38	VC_223_0	38	0.9095	1	0.9095	0	0	0	1	Ref
MN728684	Unassigned	Unassigned	Unassigned	517_1	Clustered	108	VC_517_1	108	0.8483	1	0.8483	0	0	0	0.9955	Ref
MN729595	Unassigned	Unassigned	Unassigned	129_0	Clustered	43	VC_129_0	43	0.886	1	0.886	0	0	0	1	Ref
MN729596	Unassigned	Unassigned	Unassigned	129_0	Clustered	43	VC_129_0	43	0.886	1	0.886	0	0	0	1	Ref
MN729597	Unassigned	Unassigned	Unassigned	129_0	Clustered	43	VC_129_0	43	0.886	1	0.886	0	0	0	1	Ref
MN729598	Unassigned	Unassigned	Unassigned	129_0	Clustered	43	VC_129_0	43	0.886	1	0.886	0	0	0	1	Ref
MN729599	Unassigned	Unassigned	Unassigned	129_0	Clustered	43	VC_129_0	43	0.886	1	0.886	0	0	0	1	Ref
MN729600	Unassigned	Unassigned	Unassigned	129_0	Clustered	43	VC_129_0	43	0.886	1	0.886	0	0	0	1	Ref
MN732867	Unassigned	Unassigned	Unassigned	582_0	Clustered	4	VC_582_0	4	0.0589	0.99996986	0.0589	0	0	0	1	Ref
MN732883	Unassigned	Unassigned	Unassigned	492_1	Clustered	20	VC_492_1	20	0.4176	1	0.4176	0	0	0	1	Ref
MN732896	Unassigned	Unassigned	Unassigned	1010_0	Clustered	2	VC_1010_0	2	0.2036	0.77337265	0.1574	0	0	0	1	Ref
MN734437	Unassigned	Unassigned	Unassigned	752_0	Clustered	3	VC_752_0	3	0.0788	0.9984602	0.0787	0	0	0	1	Ref
MN734438	Unassigned	Unassigned	Unassigned	752_0	Clustered	3	VC_752_0	3	0.0788	0.9984602	0.0787	0	0	0	1	Ref
MN734439	Unassigned	Unassigned	Unassigned	752_0	Clustered	3	VC_752_0	3	0.0788	0.9984602	0.0787	0	0	0	1	Ref
MN735429	Unassigned	Unassigned	Unassigned	1160_0	Clustered	73	VC_1160_0	73	0.9835	1	0.9835	0	0	0	1	Ref
MN735430	Unassigned	Unassigned	Unassigned	1240_0	Clustered	82	VC_1240_0	82	0.7945	1	0.7945	0	0	0	1	Ref
MN735431	Unassigned	Unassigned	Unassigned	520_12	Clustered/Singleton	1	VC_520_12	1	0	1	0	0	0	0	1	Ref
MN735432	Unassigned	Unassigned	Unassigned	1046_0	Clustered	7	VC_1046_0	7	0.9413	1	0.9413	0	0	0	1	Ref
MN735434	Unassigned	Unassigned	Unassigned	558_79	Clustered/Singleton	1	VC_558_79	1	0	1	0	0	0	0	1	Ref
MN735435	Unassigned	Unassigned	Unassigned	1240_0	Clustered	82	VC_1240_0	82	0.7945	1	0.7945	0	0	0	1	Ref
MN737551	Unassigned	Unassigned	Unassigned	461_0	Clustered	96	VC_461_0	96	0.9307	1	0.9307	0	0	0	0.9575	Ref
MN737622	Unassigned	Unassigned	Unassigned	233_0	Clustered	2	VC_233_0	2	0.7179	0.72985431	0.5239	0	0	0	1	Ref
MN781580	Unassigned	Unassigned	Unassigned	283_21	Clustered/Singleton	1	VC_283_21	1	0	1	0	0	0	0	1	Ref
MN781674	Unassigned	Unassigned	Unassigned	942_0	Clustered	36	VC_942_0	36	0.7848	1	0.7848	0	0	0	1	Ref
MN782535	Unassigned	Unassigned	Unassigned	492_1	Clustered	20	VC_492_1	20	0.4176	1	0.4176	0	0	0	1	Ref
MN783016	Unassigned	Unassigned	Unassigned	938_0	Clustered	99	VC_938_0	99	0.9119	1	0.9119	0	0	0	0.9928	Ref
MN788369	Unassigned	Unassigned	Unassigned	131_0	Clustered	17	VC_131_0	17	0.2671	1	0.2671	0	0	0	1	Ref
MN794000	Unassigned	Unassigned	Unassigned	111_0	Clustered	91	VC_111_0	91	0.9587	1	0.9587	0	0	0	1	Ref
MN794001	Unassigned	Unassigned	Unassigned	111_0	Clustered	91	VC_111_0	91	0.9587	1	0.9587	0	0	0	1	Ref
MN794002	Unassigned	Unassigned	Unassigned	111_0	Clustered	91	VC_111_0	91	0.9587	1	0.9587	0	0	0	1	Ref
MN794003	Unassigned	Unassigned	Unassigned	111_0	Clustered	91	VC_111_0	91	0.9587	1	0.9587	0	0	0	1	Ref
MN794232	Unassigned	Unassigned	Unassigned	531_1	Clustered	6	VC_531_1	6	0.1481	0.99978665	0.148	0	0	0	1	Ref
MN807240	Unassigned	Unassigned	Unassigned	1027_0	Clustered	20	VC_1027_0	20	0.7114	1	0.7114	0	0	0	1	Ref
MN807246	Unassigned	Unassigned	Unassigned	1240_0	Clustered	82	VC_1240_0	82	0.7945	1	0.7945	0	0	0	1	Ref
MN807247	Unassigned	Unassigned	Unassigned	515_0	Clustered	16	VC_515_0	16	0.3793	1	0.3793	0	0	0	1	Ref
MN807248	Unassigned	Unassigned	Unassigned	519_0	Clustered	39	VC_519_0	39	0.8156	1	0.8156	0	0	0	1	Ref
MN807249	Unassigned	Unassigned	Unassigned	519_0	Clustered	39	VC_519_0	39	0.8156	1	0.8156	0	0	0	1	Ref
MN807250	Unassigned	Unassigned	Unassigned	519_0	Clustered	39	VC_519_0	39	0.8156	1	0.8156	0	0	0	1	Ref
MN807295	Unassigned	Unassigned	Unassigned	699_0	Clustered	71	VC_699_0	71	0.9352	1	0.9352	0	0	0	1	Ref
MN812203	Unassigned	Unassigned	Unassigned	1021_0	Clustered	10	VC_1021_0	10	0.8876	1	0.8876	0	0	0	1	Ref
MN812204	Unassigned	Unassigned	Unassigned	1021_0	Clustered	10	VC_1021_0	10	0.8876	1	0.8876	0	0	0	1	Ref
MN812205	Unassigned	Unassigned	Unassigned	1021_0	Clustered	10	VC_1021_0	10	0.8876	1	0.8876	0	0	0	1	Ref
MN812206	Unassigned	Unassigned	Unassigned	1013_0	Clustered	34	VC_1013_0	34	0.9939	1	0.9939	0	0	0	1	Ref
MN812207	Unassigned	Unassigned	Unassigned	1013_0	Clustered	34	VC_1013_0	34	0.9939	1	0.9939	0	0	0	1	Ref
MN812208	Unassigned	Unassigned	Unassigned	1013_0	Clustered	34	VC_1013_0	34	0.9939	1	0.9939	0	0	0	1	Ref

MN812209	Unassigned	Unassigned	Unassigned	1013_0	Clustered	34	VC_1013_0	34	0.9939	1	0.9939	0	0	0	1	Ref
MN812210	Unassigned	Unassigned	Unassigned	1013_0	Clustered	34	VC_1013_0	34	0.9939	1	0.9939	0	0	0	1	Ref
MN812212	Unassigned	Unassigned	Unassigned	1013_0	Clustered	34	VC_1013_0	34	0.9939	1	0.9939	0	0	0	1	Ref
MN812213	Unassigned	Unassigned	Unassigned	1013_0	Clustered	34	VC_1013_0	34	0.9939	1	0.9939	0	0	0	1	Ref
MN812214	Unassigned	Unassigned	Unassigned	1013_0	Clustered	34	VC_1013_0	34	0.9939	1	0.9939	0	0	0	1	Ref
MN812215	Unassigned	Unassigned	Unassigned	1013_0	Clustered	34	VC_1013_0	34	0.9939	1	0.9939	0	0	0	1	Ref
MN812216	Unassigned	Unassigned	Unassigned	1013_0	Clustered	34	VC_1013_0	34	0.9939	1	0.9939	0	0	0	1	Ref
MN812217	Unassigned	Unassigned	Unassigned	1013_0	Clustered	34	VC_1013_0	34	0.9939	1	0.9939	0	0	0	1	Ref
MN812218	Unassigned	Unassigned	Unassigned	1013_0	Clustered	34	VC_1013_0	34	0.9939	1	0.9939	0	0	0	1	Ref
MN812219	Unassigned	Unassigned	Unassigned	1013_0	Clustered	34	VC_1013_0	34	0.9939	1	0.9939	0	0	0	1	Ref
MN812220	Unassigned	Unassigned	Unassigned	1013_0	Clustered	34	VC_1013_0	34	0.9939	1	0.9939	0	0	0	1	Ref
MN812221	Unassigned	Unassigned	Unassigned	1013_0	Clustered	34	VC_1013_0	34	0.9939	1	0.9939	0	0	0	1	Ref
MN812222	Unassigned	Unassigned	Unassigned	1013_0	Clustered	34	VC_1013_0	34	0.9939	1	0.9939	0	0	0	1	Ref
MN812223	Unassigned	Unassigned	Unassigned	1013_0	Clustered	34	VC_1013_0	34	0.9939	1	0.9939	0	0	0	1	Ref
MN812224	Unassigned	Unassigned	Unassigned	1013_0	Clustered	34	VC_1013_0	34	0.9939	1	0.9939	0	0	0	1	Ref
MN812225	Unassigned	Unassigned	Unassigned	1013_0	Clustered	34	VC_1013_0	34	0.9939	1	0.9939	0	0	0	1	Ref
MN812226	Unassigned	Unassigned	Unassigned	1013_0	Clustered	34	VC_1013_0	34	0.9939	1	0.9939	0	0	0	1	Ref
MN812227	Unassigned	Unassigned	Unassigned	1013_0	Clustered	34	VC_1013_0	34	0.9939	1	0.9939	0	0	0	1	Ref
MN812228	Unassigned	Unassigned	Unassigned	1013_0	Clustered	34	VC_1013_0	34	0.9939	1	0.9939	0	0	0	1	Ref
MN812229	Unassigned	Unassigned	Unassigned	1013_0	Clustered	34	VC_1013_0	34	0.9939	1	0.9939	0	0	0	1	Ref
MN812230	Unassigned	Unassigned	Unassigned	1013_0	Clustered	34	VC_1013_0	34	0.9939	1	0.9939	0	0	0	1	Ref
MN812231	Unassigned	Unassigned	Unassigned	1013_0	Clustered	34	VC_1013_0	34	0.9939	1	0.9939	0	0	0	1	Ref
MN812232	Unassigned	Unassigned	Unassigned	1013_0	Clustered	34	VC_1013_0	34	0.9939	1	0.9939	0	0	0	1	Ref
MN812233	Unassigned	Unassigned	Unassigned	1013_0	Clustered	34	VC_1013_0	34	0.9939	1	0.9939	0	0	0	1	Ref
MN812234	Unassigned	Unassigned	Unassigned	1013_0	Clustered	34	VC_1013_0	34	0.9939	1	0.9939	0	0	0	1	Ref
MN812235	Unassigned	Unassigned	Unassigned	1013_0	Clustered	34	VC_1013_0	34	0.9939	1	0.9939	0	0	0	1	Ref
MN812236	Unassigned	Unassigned	Unassigned	1013_0	Clustered	34	VC_1013_0	34	0.9939	1	0.9939	0	0	0	1	Ref
MN812237	Unassigned	Unassigned	Unassigned	1013_0	Clustered	34	VC_1013_0	34	0.9939	1	0.9939	0	0	0	1	Ref
MN812238	Unassigned	Unassigned	Unassigned	1013_0	Clustered	34	VC_1013_0	34	0.9939	1	0.9939	0	0	0	1	Ref
MN812240	Unassigned	Unassigned	Unassigned	1013_0	Clustered	34	VC_1013_0	34	0.9939	1	0.9939	0	0	0	1	Ref
MN812241	Unassigned	Unassigned	Unassigned	1013_0	Clustered	34	VC_1013_0	34	0.9939	1	0.9939	0	0	0	1	Ref
MN812685	Unassigned	Unassigned	Unassigned	841_0	Clustered	10	VC_841_0	10	0.5997	1	0.5997	0	0	0	1	Ref
MN812686	Unassigned	Unassigned	Unassigned	451_0	Clustered	80	VC_451_0	80	0.9991	1	0.9991	0	0	0	1	Ref
MN812687	Unassigned	Unassigned	Unassigned	841_0	Clustered	10	VC_841_0	10	0.5997	1	0.5997	0	0	0	1	Ref
MN812688	Unassigned	Unassigned	Unassigned	792_0	Clustered	35	VC_792_0	35	0.9828	1	0.9828	0	0	0	1	Ref
MN812690	Unassigned	Unassigned	Unassigned	984_0	Clustered	38	VC_984_0	38	0.9758	1	0.9758	0	0	0	1	Ref
MN812691	Unassigned	Unassigned	Unassigned	841_0	Clustered	10	VC_841_0	10	0.5997	1	0.5997	0	0	0	1	Ref
MN812692	Unassigned	Unassigned	Unassigned	984_0	Clustered	38	VC_984_0	38	0.9758	1	0.9758	0	0	0	1	Ref
MN812693	Unassigned	Unassigned	Unassigned	393_0	Clustered	7	VC_393_0	7	0.1775	1	0.1775	0	0	0	1	Ref
MN812694	Unassigned	Unassigned	Unassigned	78_4	Clustered/Singleton	1	VC_78_4	1	0	1	0	0	0	0	1	Ref
MN812722	Unassigned	Unassigned	Unassigned	354_0	Clustered	4	VC_354_0	4	0.1749	0.99998684	0.1749	0	0	0	0.8333	Ref
MN813048	Unassigned	Unassigned	Unassigned	131_0	Clustered	17	VC_131_0	17	0.2671	1	0.2671	0	0	0	1	Ref
MN813049	Unassigned	Unassigned	Unassigned	131_0	Clustered	17	VC_131_0	17	0.2671	1	0.2671	0	0	0	1	Ref
MN813050	Unassigned	Unassigned	Unassigned	131_0	Clustered	17	VC_131_0	17	0.2671	1	0.2671	0	0	0	1	Ref
MN813051	Unassigned	Unassigned	Unassigned	131_0	Clustered	17	VC_131_0	17	0.2671	1	0.2671	0	0	0	1	Ref
MN813052	Unassigned	Unassigned	Unassigned	131_0	Clustered	17	VC_131_0	17	0.2671	1	0.2671	0	0	0	1	Ref
MN813053	Unassigned	Unassigned	Unassigned	131_0	Clustered	17	VC_131_0	17	0.2671	1	0.2671	0	0	0	1	Ref
MN813675	Unassigned	Unassigned	Unassigned	765_0	Clustered	132	VC_765_0	132	1	1	1	0	0	0	1	Ref
MN813676	Unassigned	Unassigned	Unassigned	1207_0	Clustered	29	VC_1207_0	29	0.8225	1	0.8225	0	0	0	1	Ref
MN813677	Unassigned	Unassigned	Unassigned	765_0	Clustered	132	VC_765_0	132	1	1	1	0	0	0	1	Ref
MN813678	Unassigned	Unassigned	Unassigned	508_0	Clustered	32	VC_508_0	32	0.906	1	0.906	0	0	0	1	Ref
MN813679	Unassigned	Unassigned	Unassigned	765_0	Clustered	132	VC_765_0	132	1	1	1	0	0	0	1	Ref
MN813680	Unassigned	Unassigned	Unassigned	1160_0	Clustered	73	VC_1160_0	73	0.9835	1	0.9835	0	0	0	1	Ref
MN813682	Unassigned	Unassigned	Unassigned	1349_0	Clustered	7	VC_1349_0	7	0.2495	1	0.2495	0	0	0	1	Ref
MN813683	Unassigned	Unassigned	Unassigned	765_0	Clustered	132	VC_765_0	132	1	1	1	0	0	0	1	Ref
MN813684	Unassigned	Unassigned	Unassigned	1349_0	Clustered	7	VC_1349_0	7	0.2495	1	0.2495	0	0	0	1	Ref
MN813687	Unassigned	Unassigned	Unassigned	573_0	Clustered	2	VC_573_0	2	0.11	0.95639318	0.1052	0	0	0	1	Ref
MN813688	Unassigned	Unassigned	Unassigned	765_0	Clustered	132	VC_765_0	132	1	1	1	0	0	0	1	Ref
MN813689	Unassigned	Unassigned	Unassigned	765_0	Clustered	132	VC_765_0	132	1	1	1	0	0	0	1	Ref
MN813690	Unassigned	Unassigned	Unassigned	765_0	Clustered	132	VC_765_0	132	1	1	1	0	0	0	1	Ref
MN813691	Unassigned	Unassigned	Unassigned	765_0	Clustered	132	VC_765_0	132	1	1	1	0	0	0	1	Ref
MN813692	Unassigned	Unassigned	Unassigned	765_0	Clustered	132	VC_765_0	132	1	1	1	0	0	0	1	Ref
MN813693	Unassigned	Unassigned	Unassigned	372_4	Clustered	3	VC_372_4	3	0.033	0.99853257	0.0329	0	0	0	1	Ref
MN813694	Unassigned	Unassigned	Unassigned	1177_0	Clustered	14	VC_1177_0	14	0.7847	1	0.7847	0	0	0	0.989	Ref
MN813695	Unassigned	Unassigned	Unassigned	950_0	Clustered	38	VC_950_0	38	0.9443	1	0.9443	0	0	0	1	Ref
MN813696	Unassigned	Unassigned	Unassigned	517_1	Clustered	108	VC_517_1	108	0.8483	1	0.8483	0	0	0	0.9955	Ref
MN813697	Unassigned	Unassigned	Unassigned	517_0	Clustered	18	VC_517_0	18	0.1599	1	0.1599	0	0	0	1	Ref
MN813698	Unassigned	Unassigned	Unassigned	1183_0	Clustered	6	VC_1183_0	6	0.995	0.99996974	0.995	0	0	0	1	Ref
MN813699	Unassigned	Unassigned	Unassigned	514_0	Clustered	75	VC_514_0	75	0.9402	1	0.9402	0	0	0	1	Ref
MN813700	Unassigned	Unassigned	Unassigned	325_12	Clustered/Singleton	1	VC_325_12	1	0	1	0	0	0	0	1	Ref
MN830254	Unassigned	Unassigned	Unassigned	877_0	Clustered	2	VC_877_0	2	0.7762	0.86822376	0.6739	0	0	0	1	Ref
MN830255	Unassigned	Unassigned	Unassigned	877_0	Clustered	2	VC_877_0	2	0.7762	0.86822376	0.6739	0	0	0	1	Ref
MN830256	Unassigned	Unassigned	Unassigned	309_0	Clustered	3	VC_309_0	3	0.1265	0.99498896	0.1258	0	0	0	1	Ref
MN830258	Unassigned	Unassigned	Unassigned	272_0	Clustered	11	VC_272_0	11	0.4915	1	0.4915	0	0	0	0.8727	Ref
MN840485	Unassigned	Unassigned	Unassigned	456_0	Clustered	62	VC_456_0	62	0.9026	1	0.9026	0	0	0	1	Ref
MN840486	Unassigned	Unassigned	Unassigned	591_0	Clustered	14	VC_591_0	14	0.5184	1	0.5184	0	0	0	1	Ref
MN844877	Unassigned	Unassigned	Unassigned	154_0	Clustered	4	VC_154_0	4	0.1766	0.99995106	0.1766	0	0	0	1	Ref
MN850564	Unassigned	Unassigned	Unassigned	329_3	Clustered	24	VC_329_3	24	0.1206	1	0.1206	0	0	0	0.7283	Ref
MN850565	Unassigned	Unassigned	Unassigned	847_1	Clustered	26	VC_847_1	26	0.6323	1	0.6323	0	0	0	0.9815	Ref
MN850566	Unassigned	Unassigned	Unassigned	329_3	Clustered	24	VC_329_3	24	0.1206	1	0.1206	0	0	0	0.7283	Ref
MN850567	Unassigned	Unassigned	Unassigned	941_0	Clustered	40	VC_941_0	40	0.6903	1	0.6903	0	0	0	1	Ref

MN850568	Unassigned	Unassigned	Unassigned	156_0	Clustered	12	VC_156_0	12	0.9062	1	0.9062	0	0	0	1	Ref
MN850569	Unassigned	Unassigned	Unassigned	941_0	Clustered	40	VC_941_0	40	0.6903	1	0.6903	0	0	0	1	Ref
MN850570	Unassigned	Unassigned	Unassigned	156_0	Clustered	12	VC_156_0	12	0.9062	1	0.9062	0	0	0	1	Ref
MN850571	Unassigned	Unassigned	Unassigned	847_1	Clustered	26	VC_847_1	26	0.6323	1	0.6323	0	0	0	0.9815	Ref
MN850572	Unassigned	Unassigned	Unassigned	941_0	Clustered	40	VC_941_0	40	0.6903	1	0.6903	0	0	0	1	Ref
MN850573	Unassigned	Unassigned	Unassigned	848_0	Clustered	28	VC_848_0	28	0.7927	1	0.7927	0	0	0	1	Ref
MN850574	Unassigned	Unassigned	Unassigned	590_0	Clustered	27	VC_590_0	27	0.9947	1	0.9947	0	0	0	1	Ref
MN850575	Unassigned	Unassigned	Unassigned	835_0	Clustered	67	VC_835_0	67	0.9624	1	0.9624	0	0	0	1	Ref
MN850576	Unassigned	Unassigned	Unassigned	772_19	Clustered	16	VC_772_19	16	0.0975	0.9970466	0.0972	0	0	0	0.8083	Ref
MN850577	Unassigned	Unassigned	Unassigned	329_3	Clustered	24	VC_329_3	24	0.1206	1	0.1206	0	0	0	0.7283	Ref
MN850578	Unassigned	Unassigned	Unassigned	772_19	Clustered	16	VC_772_19	16	0.0975	0.9970466	0.0972	0	0	0	0.8083	Ref
MN850579	Unassigned	Unassigned	Unassigned	286_1	Clustered	21	VC_286_1	21	0.1696	1	0.1696	0	0	0	0.9048	Ref
MN850580	Unassigned	Unassigned	Unassigned	329_3	Clustered	24	VC_329_3	24	0.1206	1	0.1206	0	0	0	0.7283	Ref
MN850581	Unassigned	Unassigned	Unassigned	705_0	Clustered	11	VC_705_0	11	0.8861	1	0.8861	0	0	0	1	Ref
MN850582	Unassigned	Unassigned	Unassigned	942_0	Clustered	36	VC_942_0	36	0.7848	1	0.7848	0	0	0	1	Ref
MN850583	Unassigned	Unassigned	Unassigned	156_0	Clustered	12	VC_156_0	12	0.9062	1	0.9062	0	0	0	1	Ref
MN850584	Unassigned	Unassigned	Unassigned	848_0	Clustered	28	VC_848_0	28	0.7927	1	0.7927	0	0	0	1	Ref
MN850585	Unassigned	Unassigned	Unassigned	329_3	Clustered	24	VC_329_3	24	0.1206	1	0.1206	0	0	0	0.7283	Ref
MN850586	Unassigned	Unassigned	Unassigned	942_0	Clustered	36	VC_942_0	36	0.7848	1	0.7848	0	0	0	1	Ref
MN850587	Unassigned	Unassigned	Unassigned	330_0	Clustered	27	VC_330_0	27	0.5088	1	0.5088	0	0	0	0.8661	Ref
MN850588	Unassigned	Unassigned	Unassigned	705_0	Clustered	11	VC_705_0	11	0.8861	1	0.8861	0	0	0	1	Ref
MN850589	Unassigned	Unassigned	Unassigned	835_0	Clustered	67	VC_835_0	67	0.9624	1	0.9624	0	0	0	1	Ref
MN850590	Unassigned	Unassigned	Unassigned	286_1	Clustered	21	VC_286_1	21	0.1696	1	0.1696	0	0	0	0.9048	Ref
MN850591	Unassigned	Unassigned	Unassigned	941_0	Clustered	40	VC_941_0	40	0.6903	1	0.6903	0	0	0	1	Ref
MN850592	Unassigned	Unassigned	Unassigned	156_0	Clustered	12	VC_156_0	12	0.9062	1	0.9062	0	0	0	1	Ref
MN850593	Unassigned	Unassigned	Unassigned	772_20	Clustered	2	VC_772_20	2	0.006	0.58868587	0.0035	0	0	0	1	Ref
MN850594	Unassigned	Unassigned	Unassigned	792_0	Clustered	35	VC_792_0	35	0.9828	1	0.9828	0	0	0	1	Ref
MN850595	Unassigned	Unassigned	Unassigned	772_19	Clustered	16	VC_772_19	16	0.0975	0.9970466	0.0972	0	0	0	0.8083	Ref
MN850596	Unassigned	Unassigned	Unassigned	942_0	Clustered	36	VC_942_0	36	0.7848	1	0.7848	0	0	0	1	Ref
MN850597	Unassigned	Unassigned	Unassigned	772_19	Clustered	16	VC_772_19	16	0.0975	0.9970466	0.0972	0	0	0	0.8083	Ref
MN850598	Unassigned	Unassigned	Unassigned	847_1	Clustered	26	VC_847_1	26	0.6323	1	0.6323	0	0	0	0.9815	Ref
MN850600	Unassigned	Unassigned	Unassigned	941_0	Clustered	40	VC_941_0	40	0.6903	1	0.6903	0	0	0	1	Ref
MN850601	Unassigned	Unassigned	Unassigned	848_0	Clustered	28	VC_848_0	28	0.7927	1	0.7927	0	0	0	1	Ref
MN850602	Unassigned	Unassigned	Unassigned	941_0	Clustered	40	VC_941_0	40	0.6903	1	0.6903	0	0	0	1	Ref
MN850603	Unassigned	Unassigned	Unassigned	329_3	Clustered	24	VC_329_3	24	0.1206	1	0.1206	0	0	0	0.7283	Ref
MN850604	Unassigned	Unassigned	Unassigned	942_0	Clustered	36	VC_942_0	36	0.7848	1	0.7848	0	0	0	1	Ref
MN850605	Unassigned	Unassigned	Unassigned	329_3	Clustered	24	VC_329_3	24	0.1206	1	0.1206	0	0	0	0.7283	Ref
MN850606	Unassigned	Unassigned	Unassigned	942_0	Clustered	36	VC_942_0	36	0.7848	1	0.7848	0	0	0	1	Ref
MN850607	Unassigned	Unassigned	Unassigned	941_0	Clustered	40	VC_941_0	40	0.6903	1	0.6903	0	0	0	1	Ref
MN850608	Unassigned	Unassigned	Unassigned	156_0	Clustered	12	VC_156_0	12	0.9062	1	0.9062	0	0	0	1	Ref
MN850609	Unassigned	Unassigned	Unassigned	290_6	Clustered	12	VC_290_6	12	0.1149	1	0.1149	0	0	0	0.8182	Ref
MN850610	Unassigned	Unassigned	Unassigned	329_3	Clustered	24	VC_329_3	24	0.1206	1	0.1206	0	0	0	0.7283	Ref
MN850611	Unassigned	Unassigned	Unassigned	329_3	Clustered	24	VC_329_3	24	0.1206	1	0.1206	0	0	0	0.7283	Ref
MN850612	Unassigned	Unassigned	Unassigned	772_19	Clustered	16	VC_772_19	16	0.0975	0.9970466	0.0972	0	0	0	0.8083	Ref
MN850613	Unassigned	Unassigned	Unassigned	942_0	Clustered	36	VC_942_0	36	0.7848	1	0.7848	0	0	0	1	Ref
MN850614	Unassigned	Unassigned	Unassigned	329_3	Clustered	24	VC_329_3	24	0.1206	1	0.1206	0	0	0	0.7283	Ref
MN850615	Unassigned	Unassigned	Unassigned	590_0	Clustered	27	VC_590_0	27	0.9947	1	0.9947	0	0	0	1	Ref
MN850616	Unassigned	Unassigned	Unassigned	637_0	Clustered	115	VC_637_0	115	0.731	1	0.731	0	0	0	0.8764	Ref
MN850617	Unassigned	Unassigned	Unassigned	156_0	Clustered	12	VC_156_0	12	0.9062	1	0.9062	0	0	0	1	Ref
MN850618	Unassigned	Unassigned	Unassigned	847_1	Clustered	26	VC_847_1	26	0.6323	1	0.6323	0	0	0	0.9815	Ref
MN850619	Unassigned	Unassigned	Unassigned	329_3	Clustered	24	VC_329_3	24	0.1206	1	0.1206	0	0	0	0.7283	Ref
MN850620	Unassigned	Unassigned	Unassigned	942_0	Clustered	36	VC_942_0	36	0.7848	1	0.7848	0	0	0	1	Ref
MN850621	Unassigned	Unassigned	Unassigned	290_6	Clustered	12	VC_290_6	12	0.1149	1	0.1149	0	0	0	0.8182	Ref
MN850622	Unassigned	Unassigned	Unassigned	286_17	Clustered	8	VC_286_17	8	0.0535	0.99997829	0.0535	0	0	0	0.6071	Ref
MN850623	Unassigned	Unassigned	Unassigned	705_0	Clustered	11	VC_705_0	11	0.8861	1	0.8861	0	0	0	1	Ref
MN850624	Unassigned	Unassigned	Unassigned	156_0	Clustered	12	VC_156_0	12	0.9062	1	0.9062	0	0	0	1	Ref
MN850625	Unassigned	Unassigned	Unassigned	156_0	Clustered	12	VC_156_0	12	0.9062	1	0.9062	0	0	0	1	Ref
MN850626	Unassigned	Unassigned	Unassigned	772_19	Clustered	16	VC_772_19	16	0.0975	0.9970466	0.0972	0	0	0	0.8083	Ref
MN850627	Unassigned	Unassigned	Unassigned	772_19	Clustered	16	VC_772_19	16	0.0975	0.9970466	0.0972	0	0	0	0.8083	Ref
MN850628	Unassigned	Unassigned	Unassigned	835_0	Clustered	67	VC_835_0	67	0.9624	1	0.9624	0	0	0	1	Ref
MN850629	Unassigned	Unassigned	Unassigned	835_0	Clustered	67	VC_835_0	67	0.9624	1	0.9624	0	0	0	1	Ref
MN850630	Unassigned	Unassigned	Unassigned	772_10	Clustered	8	VC_772_10	8	0.0433	0.9088183	0.0394	0	0	0	0.9286	Ref
MN850631	Unassigned	Unassigned	Unassigned	329_2	Clustered	2	VC_329_2	2	0.0049	0.95560085	0.0047	0	0	0	1	Ref
MN850632	Unassigned	Unassigned	Unassigned	848_0	Clustered	28	VC_848_0	28	0.7927	1	0.7927	0	0	0	1	Ref
MN850633	Unassigned	Unassigned	Unassigned	329_3	Clustered	24	VC_329_3	24	0.1206	1	0.1206	0	0	0	0.7283	Ref
MN850634	Unassigned	Unassigned	Unassigned	942_0	Clustered	36	VC_942_0	36	0.7848	1	0.7848	0	0	0	1	Ref
MN850635	Unassigned	Unassigned	Unassigned	329_3	Clustered	24	VC_329_3	24	0.1206	1	0.1206	0	0	0	0.7283	Ref
MN850636	Unassigned	Unassigned	Unassigned	329_3	Clustered	24	VC_329_3	24	0.1206	1	0.1206	0	0	0	0.7283	Ref
MN850637	Unassigned	Unassigned	Unassigned	329_2	Clustered	2	VC_329_2	2	0.0049	0.95560085	0.0047	0	0	0	1	Ref
MN850638	Unassigned	Unassigned	Unassigned	942_0	Clustered	36	VC_942_0	36	0.7848	1	0.7848	0	0	0	1	Ref
MN850639	Unassigned	Unassigned	Unassigned	329_3	Clustered	24	VC_329_3	24	0.1206	1	0.1206	0	0	0	0.7283	Ref
MN850640	Unassigned	Unassigned	Unassigned	941_0	Clustered	40	VC_941_0	40	0.6903	1	0.6903	0	0	0	1	Ref
MN850641	Unassigned	Unassigned	Unassigned	942_0	Clustered	36	VC_942_0	36	0.7848	1	0.7848	0	0	0	1	Ref
MN850642	Unassigned	Unassigned	Unassigned	772_16	Clustered	10	VC_772_16	10	0.0563	0.9434928	0.0531	0	0	0	0.8667	Ref
MN850643	Unassigned	Unassigned	Unassigned	942_0	Clustered	36	VC_942_0	36	0.7848	1	0.7848	0	0	0	1	Ref
MN850644	Unassigned	Unassigned	Unassigned	329_3	Clustered	24	VC_329_3	24	0.1206	1	0.1206	0	0	0	0.7283	Ref
MN850645	Unassigned	Unassigned	Unassigned	848_0	Clustered	28	VC_848_0	28	0.7927	1	0.7927	0	0	0	1	Ref
MN850646	Unassigned	Unassigned	Unassigned	772_6	Clustered	3	VC_772_6	3	0.012	0.63979507	0.0077	0	0	0	1	Ref
MN850647	Unassigned	Unassigned	Unassigned	329_3	Clustered	24	VC_329_3	24	0.1206	1	0.1206	0	0	0	0.7283	Ref
MN850648	Unassigned	Unassigned	Unassigned	847_1	Clustered	26	VC_847_1	26	0.6323	1	0.6323	0	0	0	0.9815	Ref
MN850649	Unassigned	Unassigned	Unassigned	772_6	Clustered	3	VC_772_6	3	0.012	0.63979507	0.0077	0	0	0	1	Ref

MN850650	Unassigned	Unassigned	Unassigned	835_0	Clustered	67	VC_835_0	67	0.9624	1	0.9624	0	0	0	1	Ref
MN850651	Unassigned	Unassigned	Unassigned	286_2	Clustered/Singleton	1	VC_286_2	1	0	1	0	0	0	0	1	Ref
MN854830	Unassigned	Unassigned	Unassigned	91_0	Clustered	22	VC_91_0	22	0.5318	1	0.5318	0	0	0	1	Ref
MN862068	Unassigned	Unassigned	Unassigned	467_0	Clustered	5	VC_467_0	5	0.6874	0.99999996	0.6874	0	0	0	1	Ref
MN862333	Unassigned	Unassigned	Unassigned	995_0	Clustered	69	VC_995_0	69	0.8547	1	0.8547	0	0	0	1	Ref
MN862335	Unassigned	Unassigned	Unassigned	995_0	Clustered	69	VC_995_0	69	0.8547	1	0.8547	0	0	0	1	Ref
MN862338	Unassigned	Unassigned	Unassigned	1066_0	Clustered	52	VC_1066_0	52	0.7682	1	0.7682	0	0	0	1	Ref
MN862339	Unassigned	Unassigned	Unassigned	996_0	Clustered	2	VC_996_0	2	0.0422	0.59038153	0.0249	0	0	0	1	Ref
MN862340	Unassigned	Unassigned	Unassigned	996_0	Clustered	2	VC_996_0	2	0.0422	0.59038153	0.0249	0	0	0	1	Ref
MN862341	Unassigned	Unassigned	Unassigned	995_0	Clustered	69	VC_995_0	69	0.8547	1	0.8547	0	0	0	1	Ref
MN862343	Unassigned	Unassigned	Unassigned	198_0	Clustered	28	VC_198_0	28	0.2429	0.99971175	0.2429	0	0	0	1	Ref
MN862345	Unassigned	Unassigned	Unassigned	197_0	Clustered	5	VC_197_0	5	0.1324	0.84698401	0.1122	0	0	0	1	Ref
MN862346	Unassigned	Unassigned	Unassigned	1077_0	Clustered	48	VC_1077_0	48	0.8525	1	0.8525	0	0	0	1	Ref
MN864145	Unassigned	Unassigned	Unassigned	286_6	Clustered/Singleton	1	VC_286_6	1	0	1	0	0	0	0	1	Ref
MN864865	Unassigned	Unassigned	Unassigned	492_2	Clustered	8	VC_492_2	8	0.1221	0.98786574	0.1206	0	0	0	1	Ref
MN871441	Unassigned	Unassigned	Unassigned	443_2	Clustered	10	VC_443_2	10	0.3344	1	0.3344	0	0	0	0.9333	Ref
MN871442	Unassigned	Unassigned	Unassigned	443_2	Clustered	10	VC_443_2	10	0.3344	1	0.3344	0	0	0	0.9333	Ref
MN871443	Unassigned	Unassigned	Unassigned	108_0	Clustered	33	VC_108_0	33	0.949	1	0.949	0	0	0	1	Ref
MN871444	Unassigned	Unassigned	Unassigned	347_0	Clustered	3	VC_347_0	3	0.1545	0.9984446	0.1543	0	0	0	1	Ref
MN871445	Unassigned	Unassigned	Unassigned	347_0	Clustered	3	VC_347_0	3	0.1545	0.9984446	0.1543	0	0	0	1	Ref
MN871446	Unassigned	Unassigned	Unassigned	488_0	Clustered	8	VC_488_0	8	0.2938	1	0.2938	0	0	0	1	Ref
MN871447	Unassigned	Unassigned	Unassigned	488_0	Clustered	8	VC_488_0	8	0.2938	1	0.2938	0	0	0	1	Ref
MN871448	Unassigned	Unassigned	Unassigned	488_0	Clustered	8	VC_488_0	8	0.2938	1	0.2938	0	0	0	1	Ref
MN871449	Unassigned	Unassigned	Unassigned	488_0	Clustered	8	VC_488_0	8	0.2938	1	0.2938	0	0	0	1	Ref
MN871450	Unassigned	Unassigned	Unassigned	592_0	Clustered	50	VC_592_0	50	0.7314	1	0.7314	0	0	0	1	Ref
MN871451	Unassigned	Unassigned	Unassigned	488_0	Clustered	8	VC_488_0	8	0.2938	1	0.2938	0	0	0	1	Ref
MN871452	Unassigned	Unassigned	Unassigned	488_0	Clustered	8	VC_488_0	8	0.2938	1	0.2938	0	0	0	1	Ref
MN871453	Unassigned	Unassigned	Unassigned	488_0	Clustered	8	VC_488_0	8	0.2938	1	0.2938	0	0	0	1	Ref
MN871454	Unassigned	Unassigned	Unassigned	80_0	Clustered	41	VC_80_0	41	0.5837	1	0.5837	0	0	0	0.9805	Ref
MN871455	Unassigned	Unassigned	Unassigned	423_0	Clustered	45	VC_423_0	45	0.9484	1	0.9484	0	0	0	1	Ref
MN871456	Unassigned	Unassigned	Unassigned	166_0	Clustered	26	VC_166_0	26	0.7923	1	0.7923	0	0	0	1	Ref
MN871457	Unassigned	Unassigned	Unassigned	79_6	Clustered	11	VC_79_6	11	0.0528	1	0.0528	0	0	0	0.7455	Ref
MN871458	Unassigned	Unassigned	Unassigned	79_0	Clustered	64	VC_79_0	64	0.4185	1	0.4185	0	0	0	0.7649	Ref
MN871459	Unassigned	Unassigned	Unassigned	79_0	Clustered	64	VC_79_0	64	0.4185	1	0.4185	0	0	0	0.7649	Ref
MN871460	Unassigned	Unassigned	Unassigned	79_6	Clustered	11	VC_79_6	11	0.0528	1	0.0528	0	0	0	0.7455	Ref
MN871461	Unassigned	Unassigned	Unassigned	79_0	Clustered	64	VC_79_0	64	0.4185	1	0.4185	0	0	0	0.7649	Ref
MN871462	Unassigned	Unassigned	Unassigned	79_0	Clustered	64	VC_79_0	64	0.4185	1	0.4185	0	0	0	0.7649	Ref
MN871463	Unassigned	Unassigned	Unassigned	166_0	Clustered	26	VC_166_0	26	0.7923	1	0.7923	0	0	0	1	Ref
MN871464	Unassigned	Unassigned	Unassigned	79_0	Clustered	64	VC_79_0	64	0.4185	1	0.4185	0	0	0	0.7649	Ref
MN871465	Unassigned	Unassigned	Unassigned	79_0	Clustered	64	VC_79_0	64	0.4185	1	0.4185	0	0	0	0.7649	Ref
MN871466	Unassigned	Unassigned	Unassigned	79_0	Clustered	64	VC_79_0	64	0.4185	1	0.4185	0	0	0	0.7649	Ref
MN871467	Unassigned	Unassigned	Unassigned	79_6	Clustered	11	VC_79_6	11	0.0528	1	0.0528	0	0	0	0.7455	Ref
MN871468	Unassigned	Unassigned	Unassigned	79_9	Clustered	2	VC_79_9	2	0.0061	0.94860001	0.0058	0	0	0	1	Ref
MN871469	Unassigned	Unassigned	Unassigned	166_0	Clustered	26	VC_166_0	26	0.7923	1	0.7923	0	0	0	1	Ref
MN871470	Unassigned	Unassigned	Unassigned	76_0	Clustered	37	VC_76_0	37	0.9403	1	0.9403	0	0	0	1	Ref
MN871471	Unassigned	Unassigned	Unassigned	796_0	Clustered	5	VC_796_0	5	0.3352	0.99999997	0.3352	0	0	0	1	Ref
MN871472	Unassigned	Unassigned	Unassigned	796_0	Clustered	5	VC_796_0	5	0.3352	0.99999997	0.3352	0	0	0	1	Ref
MN871473	Unassigned	Unassigned	Unassigned	79_0	Clustered	64	VC_79_0	64	0.4185	1	0.4185	0	0	0	0.7649	Ref
MN871474	Unassigned	Unassigned	Unassigned	796_0	Clustered	5	VC_796_0	5	0.3352	0.99999997	0.3352	0	0	0	1	Ref
MN871475	Unassigned	Unassigned	Unassigned	133_29	Clustered	2	VC_133_29	2	0.0052	0.8586516	0.0044	0	0	0	1	Ref
MN871476	Unassigned	Unassigned	Unassigned	472_0	Clustered	47	VC_472_0	47	0.9025	1	0.9025	0	0	0	1	Ref
MN871478	Unassigned	Unassigned	Unassigned	166_0	Clustered	26	VC_166_0	26	0.7923	1	0.7923	0	0	0	1	Ref
MN871479	Unassigned	Unassigned	Unassigned	79_0	Clustered	64	VC_79_0	64	0.4185	1	0.4185	0	0	0	0.7649	Ref
MN871480	Unassigned	Unassigned	Unassigned	344_0	Clustered	21	VC_344_0	21	0.5436	1	0.5436	0	0	0	1	Ref
MN871481	Unassigned	Unassigned	Unassigned	76_0	Clustered	37	VC_76_0	37	0.9403	1	0.9403	0	0	0	1	Ref
MN871482	Unassigned	Unassigned	Unassigned	166_0	Clustered	26	VC_166_0	26	0.7923	1	0.7923	0	0	0	1	Ref
MN871483	Unassigned	Unassigned	Unassigned	423_0	Clustered	45	VC_423_0	45	0.9484	1	0.9484	0	0	0	1	Ref
MN871484	Unassigned	Unassigned	Unassigned	472_0	Clustered	47	VC_472_0	47	0.9025	1	0.9025	0	0	0	1	Ref
MN871485	Unassigned	Unassigned	Unassigned	79_0	Clustered	64	VC_79_0	64	0.4185	1	0.4185	0	0	0	0.7649	Ref
MN871486	Unassigned	Unassigned	Unassigned	166_0	Clustered	26	VC_166_0	26	0.7923	1	0.7923	0	0	0	1	Ref
MN871487	Unassigned	Unassigned	Unassigned	79_0	Clustered	64	VC_79_0	64	0.4185	1	0.4185	0	0	0	0.7649	Ref
MN871488	Unassigned	Unassigned	Unassigned	76_0	Clustered	37	VC_76_0	37	0.9403	1	0.9403	0	0	0	1	Ref
MN871489	Unassigned	Unassigned	Unassigned	344_0	Clustered	21	VC_344_0	21	0.5436	1	0.5436	0	0	0	1	Ref
MN871490	Unassigned	Unassigned	Unassigned	461_0	Clustered	96	VC_461_0	96	0.9307	1	0.9307	0	0	0	0.9575	Ref
MN871491	Unassigned	Unassigned	Unassigned	133_36	Clustered	2	VC_133_36	2	0.0052	0.86421148	0.0045	0	0	0	1	Ref
MN871492	Unassigned	Unassigned	Unassigned	133_36	Clustered	2	VC_133_36	2	0.0052	0.86421148	0.0045	0	0	0	1	Ref
MN871493	Unassigned	Unassigned	Unassigned	133_37	Clustered/Singleton	1	VC_133_37	1	0	1	0	0	0	0	1	Ref
MN871494	Unassigned	Unassigned	Unassigned	133_12	Clustered	2	VC_133_12	2	0.0051	0.8672083	0.0044	0	0	0	1	Ref
MN871495	Unassigned	Unassigned	Unassigned	133_12	Clustered	2	VC_133_12	2	0.0051	0.8672083	0.0044	0	0	0	1	Ref
MN871496	Unassigned	Unassigned	Unassigned	133_43	Clustered	4	VC_133_43	4	0.015	0.99995682	0.015	0	0	0	1	Ref
MN871497	Unassigned	Unassigned	Unassigned	133_43	Clustered	4	VC_133_43	4	0.015	0.99995682	0.015	0	0	0	1	Ref
MN871498	Unassigned	Unassigned	Unassigned	133_43	Clustered	4	VC_133_43	4	0.015	0.99995682	0.015	0	0	0	1	Ref
MN871499	Unassigned	Unassigned	Unassigned	133_43	Clustered	4	VC_133_43	4	0.015	0.99995682	0.015	0	0	0	1	Ref
MN871500	Unassigned	Unassigned	Unassigned	133_19	Clustered	2	VC_133_19	2	0.0051	0.8696643	0.0044	0	0	0	1	Ref
MN871501	Unassigned	Unassigned	Unassigned	133_29	Clustered	2	VC_133_29	2	0.0052	0.8586516	0.0044	0	0	0	1	Ref
MN871502	Unassigned	Unassigned	Unassigned	133_38	Clustered/Singleton	1	VC_133_38	1	0	1	0	0	0	0	1	Ref
MN871503	Unassigned	Unassigned	Unassigned	461_0	Clustered	96	VC_461_0	96	0.9307	1	0.9307	0	0	0	0.9575	Ref
MN871504	Unassigned	Unassigned	Unassigned	133_10	Clustered	10	VC_133_10	10	0.0527	1	0.0527	0	0	0	1	Ref
MN871505	Unassigned	Unassigned	Unassigned	972_0	Clustered	6	VC_972_0	6	0.2892	1	0.2892	0	0	0	1	Ref
MN871506	Unassigned	Unassigned	Unassigned	904_2	Clustered	7	VC_904_2	7	0.2857	1	0.2857	0	0	0	1	Ref
MN871507	Unassigned	Unassigned	Unassigned	600_0	Clustered	3	VC_600_0	3	0.0541	0.97110069	0.0526	0	0	0	1	Ref

MN871508	Unassigned	Unassigned	Unassigned	79_0	Clustered	64	VC_79_0	64	0.4185	1	0.4185	0	0	0	0.7649	Ref
MN871509	Unassigned	Unassigned	Unassigned	79_0	Clustered	64	VC_79_0	64	0.4185	1	0.4185	0	0	0	0.7649	Ref
MN871510	Unassigned	Unassigned	Unassigned	972_0	Clustered	6	VC_972_0	6	0.2892	1	0.2892	0	0	0	1	Ref
MN871511	Unassigned	Unassigned	Unassigned	972_0	Clustered	6	VC_972_0	6	0.2892	1	0.2892	0	0	0	1	Ref
MN876841	Unassigned	Unassigned	Unassigned	348_0	Clustered	22	VC_348_0	22	0.9602	1	0.9602	0	0	0	1	Ref
MN876842	Unassigned	Unassigned	Unassigned	348_0	Clustered	22	VC_348_0	22	0.9602	1	0.9602	0	0	0	1	Ref
MN876843	Unassigned	Unassigned	Unassigned	348_0	Clustered	22	VC_348_0	22	0.9602	1	0.9602	0	0	0	1	Ref
MN876845	Unassigned	Unassigned	Unassigned	440_0	Clustered	4	VC_440_0	4	1	1	1	0	0	0	1	Ref
MN882349	Unassigned	Unassigned	Unassigned	590_0	Clustered	27	VC_590_0	27	0.9947	1	0.9947	0	0	0	1	Ref
MN882542	Unassigned	Unassigned	Unassigned	329_18	Clustered	7	VC_329_18	7	0.0328	1	0.0328	0	0	0	0.8095	Ref
MN882551	Unassigned	Unassigned	Unassigned	1413_0	Clustered	2	VC_1413_0	2	0.6796	0.89435023	0.6078	0	0	0	1	Ref
MN882552	Unassigned	Unassigned	Unassigned	1413_0	Clustered	2	VC_1413_0	2	0.6796	0.89435023	0.6078	0	0	0	1	Ref
MN882610	Unassigned	Unassigned	Unassigned	490_1	Clustered	5	VC_490_1	5	0.0447	0.99999996	0.0447	0	0	0	1	Ref
MN890136	Unassigned	Unassigned	Unassigned	721_0	Clustered	9	VC_721_0	9	1	1	1	0	0	0	1	Ref
MN890137	Unassigned	Unassigned	Unassigned	721_0	Clustered	9	VC_721_0	9	1	1	1	0	0	0	1	Ref
MN890138	Unassigned	Unassigned	Unassigned	721_0	Clustered	9	VC_721_0	9	1	1	1	0	0	0	1	Ref
MN890139	Unassigned	Unassigned	Unassigned	721_0	Clustered	9	VC_721_0	9	1	1	1	0	0	0	1	Ref
MN890140	Unassigned	Unassigned	Unassigned	721_0	Clustered	9	VC_721_0	9	1	1	1	0	0	0	1	Ref
MN890141	Unassigned	Unassigned	Unassigned	721_0	Clustered	9	VC_721_0	9	1	1	1	0	0	0	1	Ref
MN890142	Unassigned	Unassigned	Unassigned	721_0	Clustered	9	VC_721_0	9	1	1	1	0	0	0	1	Ref
MN890143	Unassigned	Unassigned	Unassigned	721_0	Clustered	9	VC_721_0	9	1	1	1	0	0	0	1	Ref
MN892483	Unassigned	Unassigned	Unassigned	1240_0	Clustered	82	VC_1240_0	82	0.7945	1	0.7945	0	0	0	1	Ref
MN892484	Unassigned	Unassigned	Unassigned	519_0	Clustered	39	VC_519_0	39	0.8156	1	0.8156	0	0	0	1	Ref
MN892486	Unassigned	Unassigned	Unassigned	519_0	Clustered	39	VC_519_0	39	0.8156	1	0.8156	0	0	0	1	Ref
MN892487	Unassigned	Unassigned	Unassigned	221_0	Clustered	22	VC_221_0	22	0.7447	1	0.7447	0	0	0	1	Ref
MN894885	Unassigned	Unassigned	Unassigned	590_0	Clustered	27	VC_590_0	27	0.9947	1	0.9947	0	0	0	1	Ref
MN895434	Unassigned	Unassigned	Unassigned	283_39	Clustered/Singleton	1	VC_283_39	1	0	1	0	0	0	0	1	Ref
MN895435	Unassigned	Unassigned	Unassigned	283_51	Clustered/Singleton	1	VC_283_51	1	0	1	0	0	0	0	1	Ref
MN895436	Unassigned	Unassigned	Unassigned	283_48	Clustered/Singleton	1	VC_283_48	1	0	1	0	0	0	0	1	Ref
MN895437	Unassigned	Unassigned	Unassigned	283_31	Clustered/Singleton	1	VC_283_31	1	0	1	0	0	0	0	1	Ref
MN895438	Unassigned	Unassigned	Unassigned	283_44	Clustered/Singleton	1	VC_283_44	1	0	1	0	0	0	0	1	Ref
MN901520	Unassigned	Unassigned	Unassigned	310_0	Clustered	33	VC_310_0	33	0.8771	1	0.8771	0	0	0	1	Ref
MN901521	Unassigned	Unassigned	Unassigned	310_0	Clustered	33	VC_310_0	33	0.8771	1	0.8771	0	0	0	1	Ref
MN901924	Unassigned	Unassigned	Unassigned	423_0	Clustered	45	VC_423_0	45	0.9484	1	0.9484	0	0	0	1	Ref
MN904502	Unassigned	Unassigned	Unassigned	980_0	Clustered	10	VC_980_0	10	0.9996	1	0.9996	0	0	0	1	Ref
MN904510	Unassigned	Unassigned	Unassigned	12_2	Clustered	19	VC_12_2	19	0.2018	1	0.2018	0	0	0	1	Ref
MN905160	Unassigned	Unassigned	Unassigned	78_3	Clustered	11	VC_78_3	11	0.2572	1	0.2572	0	0	0	1	Ref
MN906996	Unassigned	Unassigned	Unassigned	838_0	Clustered	29	VC_838_0	29	0.8611	1	0.8611	0	0	0	1	Ref
MN908683	Unassigned	Unassigned	Unassigned	519_0	Clustered	39	VC_519_0	39	0.8156	1	0.8156	0	0	0	1	Ref
MN908685	Unassigned	Unassigned	Unassigned	1100_0	Clustered	2	VC_1100_0	2	0.1285	0.94673154	0.1217	0	0	0	1	Ref
MN908689	Unassigned	Unassigned	Unassigned	1207_0	Clustered	29	VC_1207_0	29	0.8225	1	0.8225	0	0	0	1	Ref
MN908690	Unassigned	Unassigned	Unassigned	856_1	Clustered	13	VC_856_1	13	0.5848	1	0.5848	0	0	0	1	Ref
MN908691	Unassigned	Unassigned	Unassigned	1182_0	Clustered	4	VC_1182_0	4	0.2845	0.99997676	0.2845	0	0	0	1	Ref
MN908692	Unassigned	Unassigned	Unassigned	236_0	Clustered	72	VC_236_0	72	0.8104	1	0.8104	0	0	0	1	Ref
MN908693	Unassigned	Unassigned	Unassigned	510_0	Clustered	12	VC_510_0	12	0.3963	1	0.3963	0	0	0	0.9697	Ref
MN927225	Unassigned	Unassigned	Unassigned	638_0	Clustered	45	VC_638_0	45	0.7741	1	0.7741	0	0	0	1	Ref
MN927226	Unassigned	Unassigned	Unassigned	638_0	Clustered	45	VC_638_0	45	0.7741	1	0.7741	0	0	0	1	Ref
MN929097	Unassigned	Unassigned	Unassigned	831_0	Clustered	5	VC_831_0	5	0.4369	0.99999952	0.4369	0	0	0	1	Ref
MN935200	Unassigned	Unassigned	Unassigned	149_28	Clustered/Singleton	1	VC_149_28	1	0	1	0	0	0	0	1	Ref
MN937349	Unassigned	Unassigned	Unassigned	671_0	Clustered	5	VC_671_0	5	0.5289	0.99999996	0.5289	0	0	0	1	Ref
MN939408	Unassigned	Unassigned	Unassigned	108_0	Clustered	33	VC_108_0	33	0.949	1	0.949	0	0	0	1	Ref
MN939539	Unassigned	Unassigned	Unassigned	739_0	Clustered	26	VC_739_0	26	0.9571	1	0.9571	0	0	0	1	Ref
MN939540	Unassigned	Unassigned	Unassigned	739_0	Clustered	26	VC_739_0	26	0.9571	1	0.9571	0	0	0	1	Ref
MN940411	Unassigned	Unassigned	Unassigned	85_11	Clustered/Singleton	1	VC_85_11	1	0	1	0	0	0	0	1	Ref
MN945899	Unassigned	Unassigned	Unassigned	371_16	Clustered	42	VC_371_16	42	0.1341	1	0.1341	0	0	0	0.7282	Ref
MN945901	Unassigned	Unassigned	Unassigned	237_2	Clustered	11	VC_237_2	11	0.1409	1	0.1409	0	0	0	0.9818	Ref
MN945902	Unassigned	Unassigned	Unassigned	956_0	Clustered	39	VC_956_0	39	1	1	1	0	0	0	1	Ref
MN945903	Unassigned	Unassigned	Unassigned	371_13	Clustered	33	VC_371_13	33	0.179	1	0.179	0	0	0	0.8769	Ref
MN945904	Unassigned	Unassigned	Unassigned	234_0	Clustered	21	VC_234_0	21	0.8355	1	0.8355	0	0	0	1	Ref
MN945905	Unassigned	Unassigned	Unassigned	868_0	Clustered	25	VC_868_0	25	0.5791	1	0.5791	0	0	0	1	Ref
MN953776	Unassigned	Unassigned	Unassigned	133_14	Clustered	3	VC_133_14	3	0.0102	0.96327256	0.0098	0	0	0	1	Ref
MN954399	Unassigned	Unassigned	Unassigned	971_1	Clustered	24	VC_971_1	24	0.7393	1	0.7393	0	0	0	0.8913	Ref
MN956514	Unassigned	Unassigned	Unassigned	190_25	Clustered/Singleton	1	VC_190_25	1	0	1	0	0	0	0	1	Ref
MN958086	Unassigned	Unassigned	Unassigned	971_1	Clustered	24	VC_971_1	24	0.7393	1	0.7393	0	0	0	0.8913	Ref
MN966730	Unassigned	Unassigned	Unassigned	971_1	Clustered	24	VC_971_1	24	0.7393	1	0.7393	0	0	0	0.8913	Ref
MN966731	Unassigned	Unassigned	Unassigned	971_1	Clustered	24	VC_971_1	24	0.7393	1	0.7393	0	0	0	0.8913	Ref
MN966732	Unassigned	Unassigned	Unassigned	971_1	Clustered	24	VC_971_1	24	0.7393	1	0.7393	0	0	0	0.8913	Ref
MN970211	Unassigned	Unassigned	Unassigned	190_13	Clustered	4	VC_190_13	4	0.0216	0.99997041	0.0216	0	0	0	0.6667	Ref
MN974282	Unassigned	Unassigned	Unassigned	837_0	Clustered	29	VC_837_0	29	0.9955	1	0.9955	0	0	0	1	Ref
MN988459	Unassigned	Unassigned	Unassigned	886_0	Clustered	2	VC_886_0	2	0.1375	0.95352335	0.1311	0	0	0	1	Ref
MN988460	Unassigned	Unassigned	Unassigned	1403_0	Clustered	28	VC_1403_0	28	0.753	1	0.753	0	0	0	1	Ref
MN988461	Unassigned	Unassigned	Unassigned	1414_0	Clustered	4	VC_1414_0	4	1	0	0	0	0	0	1	Ref
MN988462	Unassigned	Unassigned	Unassigned	1414_0	Clustered	4	VC_1414_0	4	1	0	0	0	0	0	1	Ref
MN988463	Unassigned	Unassigned	Unassigned	1414_0	Clustered	4	VC_1414_0	4	1	0	0	0	0	0	1	Ref
MN988465	Unassigned	Unassigned	Unassigned	468_0	Clustered	4	VC_468_0	4	0.143	0.99998478	0.143	0	0	0	1	Ref
MN988466	Unassigned	Unassigned	Unassigned	987_0	Clustered	22	VC_987_0	22	0.8955	1	0.8955	0	0	0	1	Ref
MN988467	Unassigned	Unassigned	Unassigned	1403_0	Clustered	28	VC_1403_0	28	0.753	1	0.753	0	0	0	1	Ref
MN988468	Unassigned	Unassigned	Unassigned	1045_0	Clustered	2	VC_1045_0	2	0.4318	0.94398033	0.4076	0	0	0	1	Ref
MN988469	Unassigned	Unassigned	Unassigned	1403_0	Clustered	28	VC_1403_0	28	0.753	1	0.753	0	0	0	1	Ref
MN988470	Unassigned	Unassigned	Unassigned	970_0	Clustered	9	VC_970_0	9	0.9685	1	0.9685	0	0	0	1	Ref
MN988471	Unassigned	Unassigned	Unassigned	1403_0	Clustered	28	VC_1403_0	28	0.753	1	0.753	0	0	0	1	Ref

MN988472	Unassigned	Unassigned	Unassigned	1414_0	Clustered	4	VC_1414_0	4	1	0	0	0	0	0	1	Ref
MN988473	Unassigned	Unassigned	Unassigned	1403_0	Clustered	28	VC_1403_0	28	0.753	1	0.753	0	0	0	1	Ref
MN988474	Unassigned	Unassigned	Unassigned	1403_0	Clustered	28	VC_1403_0	28	0.753	1	0.753	0	0	0	1	Ref
MN988475	Unassigned	Unassigned	Unassigned	1403_0	Clustered	28	VC_1403_0	28	0.753	1	0.753	0	0	0	1	Ref
MN988478	Unassigned	Unassigned	Unassigned	1403_0	Clustered	28	VC_1403_0	28	0.753	1	0.753	0	0	0	1	Ref
MN988479	Unassigned	Unassigned	Unassigned	1403_0	Clustered	28	VC_1403_0	28	0.753	1	0.753	0	0	0	1	Ref
MN988480	Unassigned	Unassigned	Unassigned	1403_0	Clustered	28	VC_1403_0	28	0.753	1	0.753	0	0	0	1	Ref
MN988481	Unassigned	Unassigned	Unassigned	1403_0	Clustered	28	VC_1403_0	28	0.753	1	0.753	0	0	0	1	Ref
MN988482	Unassigned	Unassigned	Unassigned	946_0	Clustered	11	VC_946_0	11	0.7352	1	0.7352	0	0	0	1	Ref
MN988484	Unassigned	Unassigned	Unassigned	970_0	Clustered	9	VC_970_0	9	0.9685	1	0.9685	0	0	0	1	Ref
MN988485	Unassigned	Unassigned	Unassigned	1415_0	Clustered	2	VC_1415_0	2	1	1	1	0	0	0	1	Ref
MN988486	Unassigned	Unassigned	Unassigned	1052_4	Clustered	6	VC_1052_4	6	0.2437	0.99795925	0.2432	0	0	0	1	Ref
MN988488	Unassigned	Unassigned	Unassigned	987_0	Clustered	22	VC_987_0	22	0.8955	1	0.8955	0	0	0	1	Ref
MN988489	Unassigned	Unassigned	Unassigned	1403_0	Clustered	28	VC_1403_0	28	0.753	1	0.753	0	0	0	1	Ref
MN988490	Unassigned	Unassigned	Unassigned	771_0	Clustered	2	VC_771_0	2	0.762	0.91600907	0.698	0	0	0	1	Ref
MN988491	Unassigned	Unassigned	Unassigned	1403_0	Clustered	28	VC_1403_0	28	0.753	1	0.753	0	0	0	1	Ref
MN988492	Unassigned	Unassigned	Unassigned	987_0	Clustered	22	VC_987_0	22	0.8955	1	0.8955	0	0	0	1	Ref
MN988493	Unassigned	Unassigned	Unassigned	987_0	Clustered	22	VC_987_0	22	0.8955	1	0.8955	0	0	0	1	Ref
MN988494	Unassigned	Unassigned	Unassigned	1403_0	Clustered	28	VC_1403_0	28	0.753	1	0.753	0	0	0	1	Ref
MN988495	Unassigned	Unassigned	Unassigned	970_0	Clustered	9	VC_970_0	9	0.9685	1	0.9685	0	0	0	1	Ref
MN988496	Unassigned	Unassigned	Unassigned	770_0	Clustered	5	VC_770_0	5	0.8394	0.99999405	0.8394	0	0	0	1	Ref
MN988498	Unassigned	Unassigned	Unassigned	1403_0	Clustered	28	VC_1403_0	28	0.753	1	0.753	0	0	0	1	Ref
MN988499	Unassigned	Unassigned	Unassigned	1403_0	Clustered	28	VC_1403_0	28	0.753	1	0.753	0	0	0	1	Ref
MN988500	Unassigned	Unassigned	Unassigned	946_0	Clustered	11	VC_946_0	11	0.7352	1	0.7352	0	0	0	1	Ref
MN988501	Unassigned	Unassigned	Unassigned	946_0	Clustered	11	VC_946_0	11	0.7352	1	0.7352	0	0	0	1	Ref
MN988502	Unassigned	Unassigned	Unassigned	771_0	Clustered	2	VC_771_0	2	0.762	0.91600907	0.698	0	0	0	1	Ref
MN988503	Unassigned	Unassigned	Unassigned	1403_0	Clustered	28	VC_1403_0	28	0.753	1	0.753	0	0	0	1	Ref
MN988504	Unassigned	Unassigned	Unassigned	1403_0	Clustered	28	VC_1403_0	28	0.753	1	0.753	0	0	0	1	Ref
MN988505	Unassigned	Unassigned	Unassigned	724_1	Clustered	6	VC_724_1	6	0.4379	1	0.4379	0	0	0	1	Ref
MN988506	Unassigned	Unassigned	Unassigned	724_1	Clustered	6	VC_724_1	6	0.4379	1	0.4379	0	0	0	1	Ref
MN988507	Unassigned	Unassigned	Unassigned	759_0	Clustered	3	VC_759_0	3	0.4748	0.99813327	0.4739	0	0	0	1	Ref
MN988508	Unassigned	Unassigned	Unassigned	946_0	Clustered	11	VC_946_0	11	0.7352	1	0.7352	0	0	0	1	Ref
MN988509	Unassigned	Unassigned	Unassigned	1052_4	Clustered	6	VC_1052_4	6	0.2437	0.99795925	0.2432	0	0	0	1	Ref
MN988510	Unassigned	Unassigned	Unassigned	759_0	Clustered	3	VC_759_0	3	0.4748	0.99813327	0.4739	0	0	0	1	Ref
MN988511	Unassigned	Unassigned	Unassigned	1403_0	Clustered	28	VC_1403_0	28	0.753	1	0.753	0	0	0	1	Ref
MN988512	Unassigned	Unassigned	Unassigned	987_0	Clustered	22	VC_987_0	22	0.8955	1	0.8955	0	0	0	1	Ref
MN988513	Unassigned	Unassigned	Unassigned	987_0	Clustered	22	VC_987_0	22	0.8955	1	0.8955	0	0	0	1	Ref
MN988514	Unassigned	Unassigned	Unassigned	987_0	Clustered	22	VC_987_0	22	0.8955	1	0.8955	0	0	0	1	Ref
MN988515	Unassigned	Unassigned	Unassigned	1416_0	Clustered	5	VC_1416_0	5	0.9955	0.99988353	0.9954	0	0	0	1	Ref
MN988516	Unassigned	Unassigned	Unassigned	1045_0	Clustered	2	VC_1045_0	2	0.4318	0.94398033	0.4076	0	0	0	1	Ref
MN988518	Unassigned	Unassigned	Unassigned	1403_0	Clustered	28	VC_1403_0	28	0.753	1	0.753	0	0	0	1	Ref
MN988520	Unassigned	Unassigned	Unassigned	1052_4	Clustered	6	VC_1052_4	6	0.2437	0.99795925	0.2432	0	0	0	1	Ref
MN988521	Unassigned	Unassigned	Unassigned	1203_0	Clustered	7	VC_1203_0	7	0.6515	1	0.6515	0	0	0	1	Ref
MN988522	Unassigned	Unassigned	Unassigned	1203_0	Clustered	7	VC_1203_0	7	0.6515	1	0.6515	0	0	0	1	Ref
MN988523	Unassigned	Unassigned	Unassigned	1203_0	Clustered	7	VC_1203_0	7	0.6515	1	0.6515	0	0	0	1	Ref
MN988524	Unassigned	Unassigned	Unassigned	1203_0	Clustered	7	VC_1203_0	7	0.6515	1	0.6515	0	0	0	1	Ref
MN988525	Unassigned	Unassigned	Unassigned	1203_0	Clustered	7	VC_1203_0	7	0.6515	1	0.6515	0	0	0	1	Ref
MN988526	Unassigned	Unassigned	Unassigned	342_0	Clustered	2	VC_342_0	2	0.1817	0.93411407	0.1698	0	0	0	1	Ref
MN988527	Unassigned	Unassigned	Unassigned	759_0	Clustered	3	VC_759_0	3	0.4748	0.99813327	0.4739	0	0	0	1	Ref
MN988528	Unassigned	Unassigned	Unassigned	987_0	Clustered	22	VC_987_0	22	0.8955	1	0.8955	0	0	0	1	Ref
MN988529	Unassigned	Unassigned	Unassigned	987_0	Clustered	22	VC_987_0	22	0.8955	1	0.8955	0	0	0	1	Ref
MN988530	Unassigned	Unassigned	Unassigned	946_0	Clustered	11	VC_946_0	11	0.7352	1	0.7352	0	0	0	1	Ref
MN988531	Unassigned	Unassigned	Unassigned	970_0	Clustered	9	VC_970_0	9	0.9685	1	0.9685	0	0	0	1	Ref
MN988532	Unassigned	Unassigned	Unassigned	1052_4	Clustered	6	VC_1052_4	6	0.2437	0.99795925	0.2432	0	0	0	1	Ref
MN988533	Unassigned	Unassigned	Unassigned	908_0	Clustered	3	VC_908_0	3	0.5083	0.9972453	0.5069	0	0	0	1	Ref
MN988534	Unassigned	Unassigned	Unassigned	1052_4	Clustered	6	VC_1052_4	6	0.2437	0.99795925	0.2432	0	0	0	1	Ref
MN988535	Unassigned	Unassigned	Unassigned	1416_0	Clustered	5	VC_1416_0	5	0.9955	0.99988353	0.9954	0	0	0	1	Ref
MN988536	Unassigned	Unassigned	Unassigned	935_0	Clustered	2	VC_935_0	2	0.7079	0.72985431	0.5167	0	0	0	1	Ref
MN988537	Unassigned	Unassigned	Unassigned	970_0	Clustered	9	VC_970_0	9	0.9685	1	0.9685	0	0	0	1	Ref
MN988538	Unassigned	Unassigned	Unassigned	908_0	Clustered	3	VC_908_0	3	0.5083	0.9972453	0.5069	0	0	0	1	Ref
MN988540	Unassigned	Unassigned	Unassigned	1416_0	Clustered	5	VC_1416_0	5	0.9955	0.99988353	0.9954	0	0	0	1	Ref
MN988541	Unassigned	Unassigned	Unassigned	468_0	Clustered	4	VC_468_0	4	0.143	0.99998478	0.143	0	0	0	1	Ref
MN988543	Unassigned	Unassigned	Unassigned	724_1	Clustered	6	VC_724_1	6	0.4379	1	0.4379	0	0	0	1	Ref
MN988544	Unassigned	Unassigned	Unassigned	970_0	Clustered	9	VC_970_0	9	0.9685	1	0.9685	0	0	0	1	Ref
MN988545	Unassigned	Unassigned	Unassigned	946_0	Clustered	11	VC_946_0	11	0.7352	1	0.7352	0	0	0	1	Ref
MN988546	Unassigned	Unassigned	Unassigned	970_0	Clustered	9	VC_970_0	9	0.9685	1	0.9685	0	0	0	1	Ref
MN988547	Unassigned	Unassigned	Unassigned	724_1	Clustered	6	VC_724_1	6	0.4379	1	0.4379	0	0	0	1	Ref
MN988548	Unassigned	Unassigned	Unassigned	724_1	Clustered	6	VC_724_1	6	0.4379	1	0.4379	0	0	0	1	Ref
MN988549	Unassigned	Unassigned	Unassigned	946_0	Clustered	11	VC_946_0	11	0.7352	1	0.7352	0	0	0	1	Ref
MN988550	Unassigned	Unassigned	Unassigned	1415_0	Clustered	2	VC_1415_0	2	1	1	1	0	0	0	1	Ref
MN988551	Unassigned	Unassigned	Unassigned	744_0	Clustered	10	VC_744_0	10	0.7049	1	0.7049	0	0	0	1	Ref
MN988552	Unassigned	Unassigned	Unassigned	990_0	Clustered	3	VC_990_0	3	0.6363	0.99819562	0.6351	0	0	0	1	Ref
MN988553	Unassigned	Unassigned	Unassigned	1052_4	Clustered	6	VC_1052_4	6	0.2437	0.99795925	0.2432	0	0	0	1	Ref
MN988554	Unassigned	Unassigned	Unassigned	468_0	Clustered	4	VC_468_0	4	0.143	0.99998478	0.143	0	0	0	1	Ref
MN988556	Unassigned	Unassigned	Unassigned	342_0	Clustered	2	VC_342_0	2	0.1817	0.93411407	0.1698	0	0	0	1	Ref
MN988557	Unassigned	Unassigned	Unassigned	970_0	Clustered	9	VC_970_0	9	0.9685	1	0.9685	0	0	0	1	Ref
MN988558	Unassigned	Unassigned	Unassigned	886_0	Clustered	2	VC_886_0	2	0.1375	0.95352335	0.1311	0	0	0	1	Ref
MN988559	Unassigned	Unassigned	Unassigned	1203_0	Clustered	7	VC_1203_0	7	0.6515	1	0.6515	0	0	0	1	Ref
MN988560	Unassigned	Unassigned	Unassigned	1203_1	Clustered/Singleton	1	VC_1203_1	1	0	1	0	0	0	0	1	Ref
MN988561	Unassigned	Unassigned	Unassigned	1203_0	Clustered	7	VC_1203_0	7	0.6515	1	0.6515	0	0	0	1	Ref
MN994275	Unassigned	Unassigned	Unassigned	1224_0	Clustered	19	VC_1224_0	19	1	1	1	0	0	0	1	Ref

MN994496	Unassigned	Unassigned	Unassigned	190_15	Clustered	3	VC_190_15	3	0.0146	0.99759465	0.0145	0	0	0	0.6667	Ref
MN994497	Unassigned	Unassigned	Unassigned	283_185	Clustered/Singleton	1	VC_283_185	1	0	1	0	0	0	0	1	Ref
MN994498	Unassigned	Unassigned	Unassigned	329_10	Clustered/Singleton	1	VC_329_10	1	0	1	0	0	0	0	1	Ref
MN994499	Unassigned	Unassigned	Unassigned	329_5	Clustered/Singleton	1	VC_329_5	1	0	1	0	0	0	0	1	Ref
MN994500	Unassigned	Unassigned	Unassigned	584_0	Clustered	28	VC_584_0	28	0.7704	1	0.7704	0	0	0	1	Ref
MN994501	Unassigned	Unassigned	Unassigned	191_16	Clustered	12	VC_191_16	12	0.0643	1	0.0643	0	0	0	0.9394	Ref
MN994502	Unassigned	Unassigned	Unassigned	190_13	Clustered	4	VC_190_13	4	0.0216	0.99997041	0.0216	0	0	0	0.6667	Ref
MN994503	Unassigned	Unassigned	Unassigned	329_8	Clustered	2	VC_329_8	2	0.0048	0.95654014	0.0046	0	0	0	1	Ref
MN994504	Unassigned	Unassigned	Unassigned	190_3	Clustered/Singleton	1	VC_190_3	1	0	1	0	0	0	0	1	Ref
MN996301	Unassigned	Unassigned	Unassigned	160_0	Clustered	2	VC_160_0	2	0.4956	0.94363853	0.4677	0	0	0	1	Ref
MPU46938	Unassigned	Unassigned	Unassigned	554_0	Clustered	2	VC_554_0	2	0.2292	0.95193785	0.2181	0	0	0	1	Ref
MT001829	Unassigned	Unassigned	Unassigned	164_0	Clustered	3	VC_164_0	3	0.0298	0.82983726	0.0247	0	0	0	1	Ref
MT002875	Unassigned	Unassigned	Unassigned	896_0	Clustered	5	VC_896_0	5	0.4981	0.99999977	0.4981	0	0	0	1	Ref
MT002975	Unassigned	Unassigned	Unassigned	879_0	Clustered	36	VC_879_0	36	0.994	1	0.994	0	0	0	1	Ref
MT004791	Unassigned	Unassigned	Unassigned	191_5	Clustered	6	VC_191_5	6	0.0285	0.99999996	0.0285	0	0	0	1	Ref
MT006214	Unassigned	Unassigned	Unassigned	673_0	Clustered	15	VC_673_0	15	0.8234	1	0.8234	0	0	0	0.7619	Ref
MT006233	Unassigned	Unassigned	Unassigned	745_0	Clustered	4	VC_745_0	4	0.5585	0.99995802	0.5585	0	0	0	1	Ref
MT006234	Unassigned	Unassigned	Unassigned	1418_0	Clustered	2	VC_1418_0	2	1	1	1	0	0	0	1	Ref
MT006235	Unassigned	Unassigned	Unassigned	1418_0	Clustered	2	VC_1418_0	2	1	1	1	0	0	0	1	Ref
MT006237	Unassigned	Unassigned	Unassigned	1198_0	Clustered	3	VC_1198_0	3	0.2794	0.95148141	0.2658	0	0	0	1	Ref
MT006238	Unassigned	Unassigned	Unassigned	745_0	Clustered	4	VC_745_0	4	0.5585	0.99995802	0.5585	0	0	0	1	Ref
MT006239	Unassigned	Unassigned	Unassigned	745_0	Clustered	4	VC_745_0	4	0.5585	0.99995802	0.5585	0	0	0	1	Ref
MT006240	Unassigned	Unassigned	Unassigned	745_0	Clustered	4	VC_745_0	4	0.5585	0.99995802	0.5585	0	0	0	1	Ref
MT012302	Unassigned	Unassigned	Unassigned	125_0	Clustered	17	VC_125_0	17	0.9244	1	0.9244	0	0	0	1	Ref
MT012729	Unassigned	Unassigned	Unassigned	540_0	Clustered	15	VC_540_0	15	0.2866	1	0.2866	0	0	0	1	Ref
MT012730	Unassigned	Unassigned	Unassigned	428_0	Clustered	33	VC_428_0	33	0.9915	1	0.9915	0	0	0	1	Ref
MT013111	Unassigned	Unassigned	Unassigned	9_0	Clustered	38	VC_9_0	38	0.4895	1	0.4895	0	0	0	1	Ref
MT023084	Unassigned	Unassigned	Unassigned	576_0	Clustered	4	VC_576_0	4	0.0606	0.99998766	0.0606	0	0	0	1	Ref
MT024860	Unassigned	Unassigned	Unassigned	1234_0	Clustered	44	VC_1234_0	44	1	1	1	0	0	0	1	Ref
MT024861	Unassigned	Unassigned	Unassigned	223_0	Clustered	38	VC_223_0	38	0.9095	1	0.9095	0	0	0	1	Ref
MT024863	Unassigned	Unassigned	Unassigned	1361_0	Clustered	6	VC_1361_0	6	0.2371	1	0.2371	0	0	0	1	Ref
MT024864	Unassigned	Unassigned	Unassigned	1240_0	Clustered	82	VC_1240_0	82	0.7945	1	0.7945	0	0	0	1	Ref
MT024865	Unassigned	Unassigned	Unassigned	1072_1	Clustered	2	VC_1072_1	2	0.0259	0.95128042	0.0246	0	0	0	1	Ref
MT024866	Unassigned	Unassigned	Unassigned	519_0	Clustered	39	VC_519_0	39	0.8156	1	0.8156	0	0	0	1	Ref
MT024867	Unassigned	Unassigned	Unassigned	1362_0	Clustered	6	VC_1362_0	6	1	1	1	0	0	0	1	Ref
MT024868	Unassigned	Unassigned	Unassigned	1073_0	Clustered	24	VC_1073_0	24	0.8388	1	0.8388	0	0	0	1	Ref
MT024869	Unassigned	Unassigned	Unassigned	1207_0	Clustered	29	VC_1207_0	29	0.8225	1	0.8225	0	0	0	1	Ref
MT024870	Unassigned	Unassigned	Unassigned	1362_0	Clustered	6	VC_1362_0	6	1	1	1	0	0	0	1	Ref
MT024871	Unassigned	Unassigned	Unassigned	1207_0	Clustered	29	VC_1207_0	29	0.8225	1	0.8225	0	0	0	1	Ref
MT024872	Unassigned	Unassigned	Unassigned	1072_1	Clustered	2	VC_1072_1	2	0.0259	0.95128042	0.0246	0	0	0	1	Ref
MT028297	Unassigned	Unassigned	Unassigned	812_1	Clustered	4	VC_812_1	4	0.2266	0.99998426	0.2266	0	0	0	1	Ref
MT047590	Unassigned	Unassigned	Unassigned	440_0	Clustered	4	VC_440_0	4	1	1	1	0	0	0	1	Ref
MT066160	Unassigned	Unassigned	Unassigned	754_0	Clustered	8	VC_754_0	8	0.752	1	0.752	0	0	0	1	Ref
MT074134	Unassigned	Unassigned	Unassigned	1007_0	Clustered	19	VC_1007_0	19	0.86	1	0.86	0	0	0	1	Ref
MT074135	Unassigned	Unassigned	Unassigned	1007_0	Clustered	19	VC_1007_0	19	0.86	1	0.86	0	0	0	1	Ref
MT074136	Unassigned	Unassigned	Unassigned	1018_1	Clustered	10	VC_1018_1	10	0.7518	1	0.7518	0	0	0	0.8	Ref
MT074137	Unassigned	Unassigned	Unassigned	1419_0	Clustered	6	VC_1419_0	6	0.9543	0.99999304	0.9543	0	0	0	1	Ref
MT074138	Unassigned	Unassigned	Unassigned	1018_1	Clustered	10	VC_1018_1	10	0.7518	1	0.7518	0	0	0	0.8	Ref
MT074139	Unassigned	Unassigned	Unassigned	1419_0	Clustered	6	VC_1419_0	6	0.9543	0.99999304	0.9543	0	0	0	1	Ref
MT074140	Unassigned	Unassigned	Unassigned	1419_0	Clustered	6	VC_1419_0	6	0.9543	0.99999304	0.9543	0	0	0	1	Ref
MT074141	Unassigned	Unassigned	Unassigned	1419_0	Clustered	6	VC_1419_0	6	0.9543	0.99999304	0.9543	0	0	0	1	Ref
MT074142	Unassigned	Unassigned	Unassigned	1419_0	Clustered	6	VC_1419_0	6	0.9543	0.99999304	0.9543	0	0	0	1	Ref
MT074143	Unassigned	Unassigned	Unassigned	1007_0	Clustered	19	VC_1007_0	19	0.86	1	0.86	0	0	0	1	Ref
MT074144	Unassigned	Unassigned	Unassigned	1007_0	Clustered	19	VC_1007_0	19	0.86	1	0.86	0	0	0	1	Ref
MT074145	Unassigned	Unassigned	Unassigned	1007_0	Clustered	19	VC_1007_0	19	0.86	1	0.86	0	0	0	1	Ref
MT074146	Unassigned	Unassigned	Unassigned	1419_0	Clustered	6	VC_1419_0	6	0.9543	0.99999304	0.9543	0	0	0	1	Ref
MT074147	Unassigned	Unassigned	Unassigned	1007_0	Clustered	19	VC_1007_0	19	0.86	1	0.86	0	0	0	1	Ref
MT074148	Unassigned	Unassigned	Unassigned	1007_0	Clustered	19	VC_1007_0	19	0.86	1	0.86	0	0	0	1	Ref
MT074149	Unassigned	Unassigned	Unassigned	1007_0	Clustered	19	VC_1007_0	19	0.86	1	0.86	0	0	0	1	Ref
MT074150	Unassigned	Unassigned	Unassigned	1007_0	Clustered	19	VC_1007_0	19	0.86	1	0.86	0	0	0	1	Ref
MT074151	Unassigned	Unassigned	Unassigned	1007_0	Clustered	19	VC_1007_0	19	0.86	1	0.86	0	0	0	1	Ref
MT074152	Unassigned	Unassigned	Unassigned	1007_0	Clustered	19	VC_1007_0	19	0.86	1	0.86	0	0	0	1	Ref
MT074153	Unassigned	Unassigned	Unassigned	1007_0	Clustered	19	VC_1007_0	19	0.86	1	0.86	0	0	0	1	Ref
MT074154	Unassigned	Unassigned	Unassigned	1007_0	Clustered	19	VC_1007_0	19	0.86	1	0.86	0	0	0	1	Ref
MT074155	Unassigned	Unassigned	Unassigned	1007_0	Clustered	19	VC_1007_0	19	0.86	1	0.86	0	0	0	1	Ref
MT074156	Unassigned	Unassigned	Unassigned	1007_0	Clustered	19	VC_1007_0	19	0.86	1	0.86	0	0	0	1	Ref
MT074157	Unassigned	Unassigned	Unassigned	1007_0	Clustered	19	VC_1007_0	19	0.86	1	0.86	0	0	0	1	Ref
MT074158	Unassigned	Unassigned	Unassigned	1007_0	Clustered	19	VC_1007_0	19	0.86	1	0.86	0	0	0	1	Ref
MT074159	Unassigned	Unassigned	Unassigned	1007_0	Clustered	19	VC_1007_0	19	0.86	1	0.86	0	0	0	1	Ref
MT074160	Unassigned	Unassigned	Unassigned	1007_0	Clustered	19	VC_1007_0	19	0.86	1	0.86	0	0	0	1	Ref
MT074429	Unassigned	Unassigned	Unassigned	1218_0	Clustered	4	VC_1218_0	4	0.8905	0.99530591	0.8863	0	0	0	1	Ref
MT074430	Unassigned	Unassigned	Unassigned	637_1	Clustered	27	VC_637_1	27	0.2578	1	0.2578	0	0	0	0.9858	Ref
MT074431	Unassigned	Unassigned	Unassigned	637_0	Clustered	115	VC_637_0	115	0.731	1	0.731	0	0	0	0.8764	Ref
MT074432	Unassigned	Unassigned	Unassigned	637_0	Clustered	115	VC_637_0	115	0.731	1	0.731	0	0	0	0.8764	Ref
MT074433	Unassigned	Unassigned	Unassigned	941_0	Clustered	40	VC_941_0	40	0.6903	1	0.6903	0	0	0	1	Ref
MT074434	Unassigned	Unassigned	Unassigned	428_0	Clustered	33	VC_428_0	33	0.9915	1	0.9915	0	0	0	1	Ref
MT074435	Unassigned	Unassigned	Unassigned	428_0	Clustered	33	VC_428_0	33	0.9915	1	0.9915	0	0	0	1	Ref
MT074436	Unassigned	Unassigned	Unassigned	428_0	Clustered	33	VC_428_0	33	0.9915	1	0.9915	0	0	0	1	Ref
MT074437	Unassigned	Unassigned	Unassigned	428_0	Clustered	33	VC_428_0	33	0.9915	1	0.9915	0	0	0	1	Ref
MT074438	Unassigned	Unassigned	Unassigned	428_0	Clustered	33	VC_428_0	33	0.9915	1	0.9915	0	0	0	1	Ref
MT074439	Unassigned	Unassigned	Unassigned	428_0	Clustered	33	VC_428_0	33	0.9915	1	0.9915	0	0	0	1	Ref

MT074440	Unassigned	Unassigned	Unassigned	1218_0	Clustered	4	VC_1218_0	4	0.8905	0.99530591	0.8863	0	0	0	1	Ref
MT074441	Unassigned	Unassigned	Unassigned	428_0	Clustered	33	VC_428_0	33	0.9915	1	0.9915	0	0	0	1	Ref
MT074442	Unassigned	Unassigned	Unassigned	428_0	Clustered	33	VC_428_0	33	0.9915	1	0.9915	0	0	0	1	Ref
MT074443	Unassigned	Unassigned	Unassigned	428_0	Clustered	33	VC_428_0	33	0.9915	1	0.9915	0	0	0	1	Ref
MT074444	Unassigned	Unassigned	Unassigned	428_0	Clustered	33	VC_428_0	33	0.9915	1	0.9915	0	0	0	1	Ref
MT074445	Unassigned	Unassigned	Unassigned	428_0	Clustered	33	VC_428_0	33	0.9915	1	0.9915	0	0	0	1	Ref
MT074446	Unassigned	Unassigned	Unassigned	428_0	Clustered	33	VC_428_0	33	0.9915	1	0.9915	0	0	0	1	Ref
MT074447	Unassigned	Unassigned	Unassigned	191_30	Clustered	2	VC_191_30	2	0.0061	0.95531751	0.0058	0	0	0	1	Ref
MT074448	Unassigned	Unassigned	Unassigned	190_7	Clustered	7	VC_190_7	7	0.0476	1	0.0476	0	0	0	1	Ref
MT074449	Unassigned	Unassigned	Unassigned	191_32	Clustered	12	VC_191_32	12	0.0683	1	0.0683	0	0	0	0.7576	Ref
MT074450	Unassigned	Unassigned	Unassigned	191_16	Clustered	12	VC_191_16	12	0.0643	1	0.0643	0	0	0	0.9394	Ref
MT074451	Unassigned	Unassigned	Unassigned	637_0	Clustered	115	VC_637_0	115	0.731	1	0.731	0	0	0	0.8764	Ref
MT074452	Unassigned	Unassigned	Unassigned	191_5	Clustered	6	VC_191_5	6	0.0285	0.99999996	0.0285	0	0	0	1	Ref
MT074453	Unassigned	Unassigned	Unassigned	191_3	Clustered	5	VC_191_3	5	0.0231	0.99999849	0.0231	0	0	0	0.8	Ref
MT074454	Unassigned	Unassigned	Unassigned	191_8	Clustered	2	VC_191_8	2	0.0055	0.91831352	0.0051	0	0	0	1	Ref
MT074455	Unassigned	Unassigned	Unassigned	191_4	Clustered	2	VC_191_4	2	0.0055	0.88936514	0.0049	0	0	0	1	Ref
MT074456	Unassigned	Unassigned	Unassigned	191_5	Clustered	6	VC_191_5	6	0.0285	0.99999996	0.0285	0	0	0	1	Ref
MT074457	Unassigned	Unassigned	Unassigned	191_3	Clustered	5	VC_191_3	5	0.0231	0.99999849	0.0231	0	0	0	0.8	Ref
MT074458	Unassigned	Unassigned	Unassigned	191_16	Clustered	12	VC_191_16	12	0.0643	1	0.0643	0	0	0	0.9394	Ref
[truncated: 1,263,651 more chars]
