# Supplementary material for: Investigating the Human Intestinal DNA Virome and Predicting Disease-Associated Virus–Host Interactions in Severe Myalgic Encephalomyelitis/Chronic Fatigue Syndrome (ME/CFS)
Source: Int J Mol Sci. 2023 Dec 8;24(24):17267. doi: 10.3390/ijms242417267 (PMC10744171; doi:10.3390/ijms242417267)
Supplement: Supplementary file 1 [file ijms-24-17267-s001.zip › Table S9.pdf]

TableS9. Host predictions

Table S9-1. VLP_prediction_STD+MAGs

Virus	AAI to closest RaFAH reference	Host genus	Confidence score	List of methods
S10_vf_120738	22.73	d__Bacteria;p__Firmicutes;c__Clostridia;o__Oscillospirales;f__Oscillospiraceae;g__Dysosmobacter	95	blast;96.50 iPHoP-RF;71.90
S10_vf_120738	22.73	d__Bacteria;p__Firmicutes;c__Clostridia;o__Oscillospirales;f__Oscillospiraceae;g__Intestinimonas	93.5	blast;95.40 iPHoP-RF;64.00
S10_vf_18671	91.64	d__Bacteria;p__Bacteroidota;c__Bacteroidia;o__Bacteroidales;f__Tannerellaceae;g__Parabacteroides	91	blast;93.40 iPHoP-RF;79.00
S10_vf_24791	46.17	d__Bacteria;p__Firmicutes;c__Clostridia;o__Oscillospirales;f__Ruminococcaceae;g__Faecalibacterium	93.8	blast;95.60 CRISPR;94.10 iPHoP-RF;82.20
S10_vf_43964	91.64	d__Bacteria;p__Bacteroidota;c__Bacteroidia;o__Bacteroidales;f__Bacteroidaceae;g__Bacteroides	100	RaFAH;99.20 CRISPR;97.80 iPHoP-RF;97.10 blast;77.70
S10_vf_43964	91.64	d__Bacteria;p__Bacteroidota;c__Bacteroidia;o__Bacteroidales;f__Bacteroidaceae;g__Phocaeicola	98.2	CRISPR;98.70 iPHoP-RF;97.10 blast;92.80
S10_vf_46328	63.06	d__Bacteria;p__Firmicutes;c__Clostridia;o__Lachnospirales;f__Lachnospiraceae;g__UBA9502	91.4	CRISPR;93.70 blast;91.80 iPHoP-RF;89.50
S10_vf_62230	25.76	d__Bacteria;p__Firmicutes;c__Clostridia;o__Clostridiales;f__Clostridiaceae;g__Clostridium	90.6	blast;93.00 iPHoP-RF;61.40
S10_vf_64371	86.67	d__Bacteria;p__Firmicutes;c__Bacilli;o__Lactobacillales;f__Streptococcaceae;g__Streptococcus	98.8	RaFAH;98.90 iPHoP-RF;59.80
S10_vf_80712	98.13	d__Bacteria;p__Firmicutes;c__Clostridia;o__Lachnospirales;f__Lachnospiraceae;g__Blautia_A	100	RaFAH;99.20 blast;91.30 iPHoP-RF;86.30
S10_vf_80712	98.13	d__Bacteria;p__Firmicutes;c__Clostridia;o__Lachnospirales;f__Lachnospiraceae;g__Blautia	99	RaFAH;99.20
S10_vs_106834	44.85	d__Bacteria;p__Bacteroidota;c__Bacteroidia;o__Bacteroidales;f__Bacteroidaceae;g__Bacteroides	92.3	RaFAH;87.20 iPHoP-RF;78.70
S10_vs_120489	7.64	d__Bacteria;p__Firmicutes;c__Clostridia;o__Christensenellales;f__Borkfalkiaceae;g__QALS01	90.3	blast;92.80 iPHoP-RF;56.50
S10_vs_120541	14.85	d__Bacteria;p__Firmicutes;c__Clostridia;o__Oscillospirales;f__Oscillospiraceae;g__CAG-110	94.7	blast;96.30 iPHoP-RF;95.70 CRISPR;74.70
S10_vs_120570	98.22	d__Bacteria;p__Firmicutes;c__Clostridia;o__Oscillospirales;f__Ruminococcaceae;g__Ruminococcus_D	99.7	blast;98.90 iPHoP-RF;96.70 CRISPR;87.50 RaFAH;87.40
S10_vs_14557	99.66	d__Bacteria;p__Bacteroidota;c__Bacteroidia;o__Bacteroidales;f__Bacteroidaceae;g__Bacteroides	100	RaFAH;98.20 iPHoP-RF;97.10 blast;96.90 CRISPR;90.30
S10_vs_2538	31.92	d__Bacteria;p__Firmicutes;c__Clostridia;o__Peptostreptococcales;f__Anaerovoracaceae;g__CAG-145	94.3	blast;96.00 iPHoP-RF;76.40
S10_vs_35854	48.73	d__Bacteria;p__Bacteroidota;c__Bacteroidia;o__Bacteroidales;f__Bacteroidaceae;g__Bacteroides	99.3	iPHoP-RF;97.10 blast;96.00 RaFAH;90.40
S10_vs_35854	48.73	d__Bacteria;p__Bacteroidota;c__Bacteroidia;o__Bacteroidales;f__Bacteroidaceae;g__Phocaeicola	91.4	iPHoP-RF;93.70
S10_vs_36407	8.93	d__Bacteria;p__Firmicutes;c__Clostridia;o__Oscillospirales;f__Acutalibacteraceae;g__UBA1691	94.5	blast;96.10 CRISPR;90.70 iPHoP-RF;73.00
S10_vs_36598	93.41	d__Bacteria;p__Bacteroidota;c__Bacteroidia;o__Bacteroidales;f__Bacteroidaceae;g__Bacteroides	100	RaFAH;99.40 iPHoP-RF;96.70
S10_vs_36598	93.41	d__Bacteria;p__Bacteroidota;c__Bacteroidia;o__Bacteroidales;f__Bacteroidaceae;g__Phocaeicola	95.9	iPHoP-RF;97.10 blast;95.40
S10_vs_37379	75.26	d__Bacteria;p__Bacteroidota;c__Bacteroidia;o__Bacteroidales;f__Tannerellaceae;g__Parabacteroides	100	CRISPR;98.90 RaFAH;98.30 iPHoP-RF;97.10 blast;94.80
S10_vs_37694	99.94	d__Bacteria;p__Bacteroidota;c__Bacteroidia;o__Bacteroidales;f__Rikenellaceae;g__Alistipes	99.1	iPHoP-RF;97.10 blast;96.80 RaFAH;87.90 CRISPR;55.60
S10_vs_46819	98.03	d__Bacteria;p__Firmicutes;c__Clostridia;o__Oscillospirales;f__Ruminococcaceae;g__Faecalibacterium	99.4	blast;96.80 RaFAH;92.90 iPHoP-RF;79.00
S10_vs_58014	85.85	d__Bacteria;p__Bacteroidota;c__Bacteroidia;o__Bacteroidales;f__Bacteroidaceae;g__Bacteroides	100	RaFAH;99.50 blast;91.80 iPHoP-RF;64.00
S10_vs_58014	85.85	d__Bacteria;p__Bacteroidota;c__Bacteroidia;o__Bacteroidales;f__Bacteroidaceae;g__Phocaeicola	94.6	blast;96.20 iPHoP-RF;76.40
S10_vs_62776	44.54	d__Bacteria;p__Bacteroidota;c__Bacteroidia;o__Bacteroidales;f__Bacteroidaceae;g__Bacteroides	99	iPHoP-RF;96.70 blast;93.60 RaFAH;88.20
S10_vs_62776	44.54	d__Bacteria;p__Bacteroidota;c__Bacteroidia;o__Bacteroidales;f__Bacteroidaceae;g__Phocaeicola	92.6	iPHoP-RF;94.70 blast;84.90
S10_vs_67471	98.13	d__Bacteria;p__Bacteroidota;c__Bacteroidia;o__Bacteroidales;f__Rikenellaceae;g__Alistipes	99.9	RaFAH;97.30 blast;95.30 iPHoP-RF;52.20
S10_vs_67471	98.13	d__Bacteria;p__Bacteroidota;c__Bacteroidia;o__Bacteroidales;f__Rikenellaceae;g__Alistipes_A	96.2	RaFAH;97.30
S10_vs_71900	97	d__Bacteria;p__Bacteroidota;c__Bacteroidia;o__Bacteroidales;f__Bacteroidaceae;g__Bacteroides	100	RaFAH;99.30 iPHoP-RF;97.10 blast;90.90
S10_vs_71900	97	d__Bacteria;p__Bacteroidota;c__Bacteroidia;o__Bacteroidales;f__Bacteroidaceae;g__Phocaeicola	95.9	iPHoP-RF;97.10 blast;96.20 CRISPR;95.50
S10_vs_71900	97	d__Bacteria;p__Bacteroidota;c__Bacteroidia;o__Bacteroidales;f__Bacteroidaceae;g__OM05-12	91	iPHoP-RF;93.40
S10_vs_77004	10.42	d__Bacteria;p__Firmicutes;c__Clostridia;o__Oscillospirales;f__Acutalibacteraceae;g__Pseudoruminococcus	95.3	blast;96.70 CRISPR;89.20 iPHoP-RF;87.60
S11_vf_104033	40.07	d__Bacteria;p__Bacteroidota;c__Bacteroidia;o__Bacteroidales;f__Bacteroidaceae;g__Bacteroides	90.6	RaFAH;93.00
S11_vf_104034	21.95	d__Bacteria;p__Firmicutes;c__Clostridia;o__Oscillospirales;f__Oscillospiraceae;g__ER4	90.3	blast;92.80 iPHoP-RF;81.10
S11_vf_104035	37.57	d__Bacteria;p__Bacteroidota;c__Bacteroidia;o__Bacteroidales;f__Bacteroidaceae;g__Prevotella	91.7	RaFAH;94.00
S11_vf_104035	37.57	d__Bacteria;p__Spirochaetota;c__Spirochaetia;o__Sphaerochaetales;f__Sphaerochaetaceae;g__UBA9732	90	blast;92.50 iPHoP-RF;70.20
S11_vf_104052	38.91	d__Bacteria;p__Firmicutes;c__Clostridia;o__Oscillospirales;f__Acutalibacteraceae;g__CAG-180	91.5	blast;93.80 iPHoP-RF;55.40
S11_vf_104071	20.29	d__Bacteria;p__Bacteroidota;c__Bacteroidia;o__Bacteroidales;f__Rikenellaceae;g__Alistipes	98.5	CRISPR;98.90 iPHoP-RF;92.80
S11_vf_10907	87.86	d__Bacteria;p__Firmicutes;c__Clostridia;o__Lachnospirales;f__Lachnospiraceae;g__Bariatricus	91.6	blast;93.90 iPHoP-RF;82.80
S11_vf_27845	53.78	d__Bacteria;p__Firmicutes;c__Bacilli;o__Lactobacillales;f__Streptococcaceae;g__Streptococcus	100	CRISPR;98.80 RaFAH;97.70 iPHoP-RF;85.70
S11_vf_27845	53.78	d__Bacteria;p__Bacteroidota;c__Bacteroidia;o__Flavobacteriales;f__Flavobacteriaceae;g__Capnocytophaga	91	blast;93.40 iPHoP-RF;51.20
S11_vf_28515	95.85	d__Bacteria;p__Firmicutes;c__Clostridia;o__Lachnospirales;f__Lachnospiraceae;g__Blautia_A	100	RaFAH;98.80 blast;94.40 iPHoP-RF;90.50 CRISPR;68.00
S11_vf_28515	95.85	d__Bacteria;p__Firmicutes;c__Clostridia;o__Lachnospirales;f__Lachnospiraceae;g__Blautia	98.4	RaFAH;98.80
S11_vf_28645	6.84	d__Bacteria;p__Firmicutes;c__Clostridia;o__Lachnospirales;f__Lachnospiraceae;g__Blautia_A	92.5	blast;94.60 iPHoP-RF;51.70
S11_vf_33117	96.79	d__Bacteria;p__Firmicutes;c__Clostridia;o__Oscillospirales;f__Oscillospiraceae;g__CAG-83	97.3	RaFAH;98.10
S11_vf_33117	96.79	d__Bacteria;p__Firmicutes;c__Clostridia;o__Oscillospirales;f__Oscillospiraceae;g__Dysosmobacter	97.3	RaFAH;98.10
S11_vf_33117	96.79	d__Bacteria;p__Firmicutes;c__Clostridia;o__Oscillospirales;f__Oscillospiraceae;g__Flavonifractor	93.8	blast;95.60 iPHoP-RF;71.50
S11_vf_34008	44.72	d__Bacteria;p__Firmicutes;c__Clostridia;o__Lachnospirales;f__Lachnospiraceae;g__	93.1	iPHoP-RF;95.10 blast;93.90
S11_vf_34008	44.72	d__Bacteria;p__Firmicutes;c__Clostridia;o__Lachnospirales;f__Lachnospiraceae;g__UC5-1-2E3	91.6	blast;93.90 iPHoP-RF;93.10
S11_vf_43997	15.72	d__Bacteria;p__Bacteroidota;c__Bacteroidia;o__Bacteroidales;f__Bacteroidaceae;g__Bacteroides	91.4	blast;93.70 iPHoP-RF;78.70
S11_vf_58587	11.86	d__Bacteria;p__Actinobacteriota;c__Actinomycetia;o__Actinomycetales;f__Bifidobacteriaceae;g__Bifidobacterium	90.4	blast;92.90 iPHoP-RF;65.60
S11_vf_66522	97.65	d__Bacteria;p__Actinobacteriota;c__Actinomycetia;o__Actinomycetales;f__Actinomycetaceae;g__Pauljensenia	100	RaFAH;99.20 CRISPR;99.10 blast;93.90 iPHoP-RF;90.50
S11_vf_66522	97.65	d__Bacteria;p__Actinobacteriota;c__Actinomycetia;o__Actinomycetales;f__Actinomycetaceae;g__Actinomyces	99	RaFAH;99.20
S11_vf_68693	78.63	d__Bacteria;p__Bacteroidota;c__Bacteroidia;o__Bacteroidales;f__Tannerellaceae;g__Parabacteroides	99.3	CRISPR;97.70 blast;97.30 iPHoP-RF;91.80 RaFAH;87.20
S11_vf_70015	50.35	d__Bacteria;p__Bacteroidota;c__Bacteroidia;o__Bacteroidales;f__Rikenellaceae;g__Alistipes	97.8	blast;96.10 RaFAH;79.90
S11_vf_92391	5.33	d__Bacteria;p__Firmicutes;c__Bacilli;o__Erysipelotrichales;f__Erysipelotrichaceae;g__Clostridium_AQ	91	CRISPR;93.40
S11_vf_95067	39.9	d__Bacteria;p__Firmicutes;c__Clostridia;o__Oscillospirales;f__Oscillospiraceae;g__Flavonifractor	93.4	blast;95.30 iPHoP-RF;58.20
S11_vf_99751	8.04	d__Bacteria;p__Firmicutes;c__Clostridia;o__Oscillospirales;f__Oscillospiraceae;g__CAG-83	94.9	blast;96.40 iPHoP-RF;57.10
S11_vs_104041	16.5	d__Bacteria;p__Cyanobacteria;c__Vampirovibrionia;o__Gastranaerophilales;f__Gastranaerophilaceae;g__CAG-306	97.2	blast;98.00 iPHoP-RF;56.00
S11_vs_104042	75.46	d__Bacteria;p__Firmicutes;c__Clostridia;o__Lachnospirales;f__Lachnospiraceae;g__Eubacterium_I	98.1	blast;98.60 iPHoP-RF;84.80
S11_vs_104064	99.03	d__Bacteria;p__Firmicutes;c__Clostridia;o__Oscillospirales;f__Ruminococcaceae;g__Negativibacillus	92.1	blast;94.30
S11_vs_11994	21.97	d__Bacteria;p__Firmicutes;c__Clostridia;o__Oscillospirales;f__Ruminococcaceae;g__Faecalibacterium	91.7	RaFAH;94.00
S11_vs_13652	57.92	d__Bacteria;p__Firmicutes;c__Clostridia;o__Oscillospirales;f__Ruminococcaceae;g__Massiliocclostridium	92.9	blast;94.90 iPHoP-RF;66.50
S11_vs_14882	11.87	d__Bacteria;p__Firmicutes;c__Bacilli;o__Erysipelotrichales;f__Erysipelotrichaceae;g__Holdemania	93.1	blast;95.10 CRISPR;54.60
S11_vs_1937	99.04	d__Bacteria;p__Bacteroidota;c__Bacteroidia;o__Bacteroidales;f__Rikenellaceae;g__Alistipes	100	blast;98.60 RaFAH;96.60 iPHoP-RF;83.30
S11_vs_1937	99.04	d__Bacteria;p__Bacteroidota;c__Bacteroidia;o__Bacteroidales;f__Rikenellaceae;g__Alistipes_A	95.2	RaFAH;96.60
S11_vs_19892	45.95	d__Bacteria;p__Firmicutes;c__Clostridia;o__Oscillospirales;f__Oscillospiraceae;g__Lawsonibacter	90.6	blast;93.00 iPHoP-RF;67.50
S11_vs_23188	66.52	d__Bacteria;p__Bacteroidota;c__Bacteroidia;o__Bacteroidales;f__Bacteroidaceae;g__Bacteroides	99.9	iPHoP-RF;97.10 RaFAH;95.80 blast;93.80 CRISPR;83.30
S11_vs_26767	40.78	d__Bacteria;p__Firmicutes;c__Clostridia;o__Lachnospirales;f__Lachnospiraceae;g__Ventrimonas	90.8	blast;93.20 iPHoP-RF;72.20

S11_vs_3006	44.36	d__Bacteria;p__Firmicutes;c__Clostridia;o__Oscillospirales;f__Acutalibacteraceae;g__UMGS263	93.6	blast;95.50 iPHoP-RF;67.00
S11_vs_3277	99.63	d__Bacteria;p__Bacteroidota;c__Bacteroidia;o__Bacteroidales;f__Bacteroidaceae;g__Bacteroides	99.9	RaFAH;98.80 iPHoP-RF;83.60 blast;80.70
S11_vs_34137	17.51	d__Bacteria;p__Firmicutes;c__Clostridia;o__Lachnospirales;f__Lachnospiraceae;g__Blautia_A	91.4	blast;93.70 iPHoP-RF;83.10
S11_vs_36748	49.79	d__Bacteria;p__Bacteroidota;c__Bacteroidia;o__Bacteroidales;f__Rikenellaceae;g__Alistipes	98.5	CRISPR;98.90 blast;96.70 iPHoP-RF;91.80
S11_vs_38860	39.67	d__Bacteria;p__Firmicutes;c__Clostridia;o__Lachnospirales;f__Lachnospiraceae;g__Enterocloster	95.2	CRISPR;96.60 blast;95.90 iPHoP-RF;89.80
S11_vs_41107	98.96	d__Bacteria;p__Firmicutes;c__Clostridia;o__Clostridiales;f__Clostridiaceae;g__Clostridium	93.6	RaFAH;95.50
S11_vs_41107	98.96	d__Bacteria;p__Firmicutes;c__Clostridia;o__Clostridiales;f__Clostridiaceae;g__Clostridium_F	93.6	RaFAH;95.50
S11_vs_41107	98.96	d__Bacteria;p__Firmicutes;c__Clostridia;o__Clostridiales;f__Clostridiaceae;g__Clostridium_P	93.6	RaFAH;95.50
S11_vs_45658	40.35	d__Bacteria;p__Bacteroidota;c__Bacteroidia;o__Bacteroidales;f__Bacteroidaceae;g__Bacteroides	95	RaFAH;96.20 iPHoP-RF;53.60
S11_vs_45658	40.35	d__Bacteria;p__Bacteroidota;c__Bacteroidia;o__Bacteroidales;f__UBA11471;g__UBA11471	90	blast;92.50 iPHoP-RF;77.50
S11_vs_48109	63.66	d__Bacteria;p__Firmicutes;c__Clostridia;o__Oscillospirales;f__Acutalibacteraceae;g__An172	93.4	blast;95.30 iPHoP-RF;74.60
S11_vs_5077	84.31	d__Bacteria;p__Proteobacteria;c__Gammaproteobacteria;o__Enterobacterales;f__Enterobacteriaceae;g__Salmonella	95.3	blast;96.70
S11_vs_5077	84.31	d__Bacteria;p__Proteobacteria;c__Gammaproteobacteria;o__Enterobacterales;f__Enterobacteriaceae;g__Escherichia	94.6	blast;96.20
S11_vs_5077	84.31	d__Bacteria;p__Proteobacteria;c__Gammaproteobacteria;o__Enterobacterales;f__Enterobacteriaceae;g__Enterobacter	93.9	blast;95.70 iPHoP-RF;60.10
S11_vs_52577	78.49	d__Bacteria;p__Firmicutes;c__Clostridia;o__Oscillospirales;f__Oscillospiraceae;g__Dysosmobacter	91	blast;88.40 iPHoP-RF;73.50 RaFAH;71.60
S11_vs_55144	12.8	d__Bacteria;p__Firmicutes;c__Bacilli;o__Erysipelotrichales;f__Erysipelatoclostridiaceae;g__Massiliomicrobiota	90.3	blast;92.80
S11_vs_58930	93.75	d__Bacteria;p__Bacteroidota;c__Bacteroidia;o__Bacteroidales;f__Bacteroidaceae;g__Bacteroides	98.1	blast;92.60 RaFAH;90.60 iPHoP-RF;89.80
S11_vs_58930	93.75	d__Bacteria;p__Bacteroidota;c__Bacteroidia;o__Bacteroidales;f__Bacteroidaceae;g__Paraprevotella	90	blast;92.50
S11_vs_6458	15.38	d__Bacteria;p__Proteobacteria;c__Alphaproteobacteria;o__RF32;f__CAG-239;g__CAG-267	90.1	blast;92.60 iPHoP-RF;81.90
S11_vs_67573	11.32	d__Bacteria;p__Firmicutes;c__Clostridia;o__Lachnospirales;f__Lachnospiraceae;g__Enterocloster	92.9	CRISPR;94.90 blast;92.10 iPHoP-RF;92.10
S11_vs_68151	99.98	d__Bacteria;p__Firmicutes;c__Clostridia;o__Lachnospirales;f__Lachnospiraceae;g__Mediterraneibacter	95.3	blast;96.70
S11_vs_68151	99.98	d__Bacteria;p__Firmicutes;c__Clostridia;o__Lachnospirales;f__Lachnospiraceae;g__Hungatella	94.7	blast;87.90 RaFAH;84.60 iPHoP-RF;53.30
S11_vs_68348	49.51	d__Bacteria;p__Firmicutes;c__Bacilli;o__Erysipelotrichales;f__Erysipelatoclostridiaceae;g__Erysipelatoclostridium	95.2	blast;96.60 iPHoP-RF;64.50
S11_vs_6878	72.71	d__Bacteria;p__Firmicutes;c__Clostridia;o__Lachnospirales;f__Lachnospiraceae;g__	91	blast;93.40 iPHoP-RF;73.00
S11_vs_76784	41.57	d__Bacteria;p__Firmicutes;c__Clostridia;o__Oscillospirales;f__Ruminococcaceae;g__Ruthenibacterium	93	blast;95.00 iPHoP-RF;77.30
S11_vs_79831	17.3	d__Bacteria;p__Bacteroidota;c__Bacteroidia;o__Bacteroidales;f__UBA11471;g__UBA11471	90	blast;92.50 iPHoP-RF;73.20
S11_vs_81775	24.35	d__Bacteria;p__Firmicutes;c__Clostridia;o__Eubacteriales;f__Eubacteriaceae;g__Eubacterium	95	CRISPR;96.50 blast;93.70 iPHoP-RF;81.40
S11_vs_82331	98.94	d__Bacteria;p__Cyanobacteria;c__Vampirovibrionia;o__Gastranaerophilales;f__Gastranaerophilaceae;g__QAMI01	93.1	blast;95.10
S11_vs_82368	62.18	d__Bacteria;p__Firmicutes;c__Clostridia;o__Lachnospirales;f__Lachnospiraceae;g__Blautia_A	98.1	blast;92.60 RaFAH;90.70 iPHoP-RF;86.00
S11_vs_83666	35.28	d__Bacteria;p__Firmicutes;c__Clostridia;o__Lachnospirales;f__Lachnospiraceae;g__Dorea_A	90.9	blast;93.30 iPHoP-RF;67.70
S11_vs_84627	6.74	d__Bacteria;p__Proteobacteria;c__Alphaproteobacteria;o__RF32;f__CAG-239;g__51-20	95.6	blast;96.90 iPHoP-RF;89.50 CRISPR;76.50
S11_vs_84848	95.91	d__Bacteria;p__Bacteroidota;c__Bacteroidia;o__Bacteroidales;f__Bacteroidaceae;g__Bacteroides	95.9	blast;91.20 RaFAH;83.50 iPHoP-RF;57.90
S11_vs_84848	95.91	d__Bacteria;p__Bacteroidota;c__Bacteroidia;o__Bacteroidales;f__Tannerellaceae;g__Parabacteroides	93.2	blast;92.10 CRISPR;86.70 iPHoP-RF;83.10 RaFAH;69.90
S11_vs_98193	80.02	d__Bacteria;p__Bacteroidota;c__Bacteroidia;o__Bacteroidales;f__Rikenellaceae;g__Alistipes	96.2	iPHoP-RF;92.10 blast;92.00 RaFAH;82.70
S12_vf_105542	13.83	d__Bacteria;p__Firmicutes;c__Bacilli;o__RF39;f__UBA660;g__CAG-460	94.6	blast;96.20 iPHoP-RF;73.70
S12_vf_10887	7.67	d__Bacteria;p__Proteobacteria;c__Gammaproteobacteria;o__Enterobacterales;f__Pasteurellaceae;g__Glaesserella	94.5	iPHoP-RF;96.10 CRISPR;91.90
S12_vf_10887	7.67	d__Bacteria;p__Proteobacteria;c__Gammaproteobacteria;o__Enterobacterales;f__Pasteurellaceae;g__Bibersteinia	93.9	iPHoP-RF;95.70
S12_vf_10887	7.67	d__Bacteria;p__Proteobacteria;c__Gammaproteobacteria;o__Enterobacterales;f__Pasteurellaceae;g__Nicoletella	93.9	iPHoP-RF;95.70
S12_vf_10887	7.67	d__Bacteria;p__Proteobacteria;c__Gammaproteobacteria;o__Enterobacterales;f__Pasteurellaceae;g__Muribacter	93.5	iPHoP-RF;95.40
S12_vf_10887	7.67	d__Bacteria;p__Proteobacteria;c__Gammaproteobacteria;o__Enterobacterales;f__Pasteurellaceae;g__Actinobacillus	92.2	iPHoP-RF;94.40
S12_vf_10887	7.67	d__Bacteria;p__Proteobacteria;c__Gammaproteobacteria;o__Enterobacterales;f__Pasteurellaceae;g__Actinobacillus_C	92.2	iPHoP-RF;94.40
S12_vf_10887	7.67	d__Bacteria;p__Proteobacteria;c__Gammaproteobacteria;o__Enterobacterales;f__Pasteurellaceae;g__Mannheimia	91.8	iPHoP-RF;94.10
S12_vf_145044	32.93	d__Bacteria;p__Bacteroidota;c__Bacteroidia;o__Bacteroidales;f__Rikenellaceae;g__Alistipes_A	91.6	blast;93.90 iPHoP-RF;77.10
S12_vf_16374	100	d__Bacteria;p__Bacteroidota;c__Bacteroidia;o__Bacteroidales;f__Bacteroidaceae;g__Bacteroides	100	RaFAH;99.10 iPHoP-RF;97.10 blast;94.80
S12_vf_16374	100	d__Bacteria;p__Bacteroidota;c__Bacteroidia;o__Bacteroidales;f__Tannerellaceae;g__Parabacteroides	92.9	blast;94.90
S12_vf_170614	7.03	d__Bacteria;p__Actinobacteriota;c__Coriobacteriia;o__Coriobacteriales;f__Coriobacteriaceae;g__Collinsella	96.4	CRISPR;97.50 iPHoP-RF;95.10
S12_vf_173208	10.29	d__Bacteria;p__Firmicutes;c__Clostridia;o__Oscillospirales;f__Oscillospiraceae;g__CAG-170	93	blast;95.00 iPHoP-RF;81.40
S12_vf_173666	36.83	d__Bacteria;p__Actinobacteriota;c__Coriobacteriia;o__Coriobacteriales;f__Eggerthellaceae;g__Adlercreutzia	93.1	blast;95.10 iPHoP-RF;76.40
S12_vf_173666	36.83	d__Bacteria;p__Actinobacteriota;c__Coriobacteriia;o__Coriobacteriales;f__Coriobacteriaceae;g__Collinsella	92.6	CRISPR;94.70
S12_vf_174374	94.2	d__Bacteria;p__Bacteroidota;c__Bacteroidia;o__Bacteroidales;f__Rikenellaceae;g__Alistipes	96.2	CRISPR;97.30 iPHoP-RF;97.10 blast;96.60
S12_vf_174374	94.2	d__Bacteria;p__Bacteroidota;c__Bacteroidia;o__Bacteroidales;f__Bacteroidaceae;g__Bacteroides	91.6	RaFAH;93.90
S12_vf_176437	10.68	d__Bacteria;p__Firmicutes;c__Clostridia;o__Lachnospirales;f__Lachnospiraceae;g__OF09-33XD	95.3	CRISPR;96.70 iPHoP-RF;82.20
S12_vf_181024	98.48	d__Bacteria;p__Firmicutes;c__Clostridia;o__Oscillospirales;f__Ruminococcaceae;g__Ruminococcus_D	99.9	CRISPR;97.30 RaFAH;97.20 blast;96.90 iPHoP-RF;95.70
S12_vf_181024	98.48	d__Bacteria;p__Firmicutes;c__Clostridia;o__Oscillospirales;f__Acutalibacteraceae;g__Ruminococcus_E	96	RaFAH;97.20
S12_vf_188846		d__Bacteria;p__Bacteroidota;c__Bacteroidia;o__Bacteroidales;f__Barnesiellaceae;g__Barnesiella	94.5	blast;96.10 iPHoP-RF;68.60
S12_vf_18902	80.1	d__Bacteria;p__Firmicutes;c__Clostridia;o__Oscillospirales;f__Oscillospiraceae;g__ER4	90.6	blast;93.00 iPHoP-RF;67.00
S12_vf_60224	82.87	d__Bacteria;p__Bacteroidota;c__Bacteroidia;o__Bacteroidales;f__Bacteroidaceae;g__Bacteroides	100	RaFAH;97.10 iPHoP-RF;96.70 blast;80.90
S12_vf_60224	82.87	d__Bacteria;p__Bacteroidota;c__Bacteroidia;o__Bacteroidales;f__Bacteroidaceae;g__Paraprevotella	91.2	blast;93.60
S12_vf_61299	11.99	d__Bacteria;p__Firmicutes;c__Clostridia;o__Lachnospirales;f__Lachnospiraceae;g__Muricomes	96.9	CRISPR;97.80 iPHoP-RF;59.20
S12_vf_69136	51.25	d__Bacteria;p__Bacteroidota;c__Bacteroidia;o__Bacteroidales;f__Bacteroidaceae;g__Prevotellamassilia	98.4	CRISPR;98.80 blast;82.30 iPHoP-RF;78.30
S12_vf_69136	51.25	d__Bacteria;p__Bacteroidota;c__Bacteroidia;o__Bacteroidales;f__Bacteroidaceae;g__Paraprevotella	90.8	blast;93.20 iPHoP-RF;67.50
S12_vf_79666	99.55	d__Bacteria;p__Firmicutes;c__Clostridia;o__Oscillospirales;f__Acutalibacteraceae;g__UBA1417	91.7	blast;94.00 iPHoP-RF;58.20
S12_vs_111286	44.45	d__Bacteria;p__Bacteroidota;c__Bacteroidia;o__Bacteroidales;f__Muribaculaceae;g__UBA7173	91.6	blast;93.90 iPHoP-RF;62.50
S12_vs_121913	82.73	d__Bacteria;p__Bacteroidota;c__Bacteroidia;o__Bacteroidales;f__Rikenellaceae;g__Alistipes	98.5	blast;96.70 RaFAH;83.70
S12_vs_123620	10.35	d__Bacteria;p__Firmicutes;c__Bacilli;o__RF39;f__UBA660;g__CAG-302	95.4	blast;96.80 iPHoP-RF;64.80
S12_vs_133070	14.52	d__Bacteria;p__Firmicutes;c__Clostridia;o__Oscillospirales;f__Ruminococcaceae;g__CAG-353	91.1	blast;93.50 iPHoP-RF;79.80
S12_vs_138261	70.17	d__Bacteria;p__Bacteroidota;c__Bacteroidia;o__Bacteroidales;f__Tannerellaceae;g__Parabacteroides	98.4	blast;98.80 CRISPR;97.40 iPHoP-RF;96.10
S12_vs_141337	98.55	d__Bacteria;p__Bacteroidota;c__Bacteroidia;o__Bacteroidales;f__Bacteroidaceae;g__Bacteroides	100	RaFAH;99.40 blast;92.10 iPHoP-RF;57.90
S12_vs_142625	31.03	d__Bacteria;p__Bacteroidota;c__Bacteroidia;o__Bacteroidales;f__Rikenellaceae;g__Alistipes	95.9	iPHoP-RF;97.10 blast;96.90
S12_vs_151292	99.81	d__Bacteria;p__Bacteroidota;c__Bacteroidia;o__Bacteroidales;f__Bacteroidaceae;g__Bacteroides	100	RaFAH;98.90 iPHoP-RF;97.10 blast;95.90 CRISPR;68.40
S12_vs_151292	99.81	d__Bacteria;p__Bacteroidota;c__Bacteroidia;o__Bacteroidales;f__Bacteroidaceae;g__Phocaeicola	95.9	iPHoP-RF;97.10 blast;93.30 CRISPR;74.50
S12_vs_153780	97.5	d__Bacteria;p__Proteobacteria;c__Alphaproteobacteria;o__RF32;f__CAG-239;g__CAG-495	90.9	blast;93.30 iPHoP-RF;81.60
S12_vs_163990	99.19	d__Bacteria;p__Firmicutes;c__Clostridia;o__Lachnospirales;f__Lachnospiraceae;g__Dorea	99.3	RaFAH;99.40
S12_vs_163990	99.19	d__Bacteria;p__Firmicutes;c__Clostridia;o__Lachnospirales;f__Lachnospiraceae;g__Dorea_A	99.3	RaFAH;99.40
S12_vs_163990	99.19	d__Bacteria;p__Firmicutes;c__Clostridia;o__Oscillospirales;f__Oscillospiraceae;g__ER4	95	blast;96.50 iPHoP-RF;60.30
S12_vs_164056	11.8	d__Bacteria;p__Firmicutes;c__Clostridia;o__Oscillospirales;f__Oscillospiraceae;g__CAG-83	95.9	blast;97.10 iPHoP-RF;72.60

S12_vs_172379	18.46	d__Bacteria;p__Firmicutes;c__Bacilli;o__RF39;f__UBA660;g__CAG-877	90.8	blast;93.20
S12_vs_188820	7.83	d__Bacteria;p__Firmicutes;c__Clostridia;o__Oscillospirales;f__CAG-272;g__QALR01	93	blast;95.00 iPHoP-RF;87.00
S12_vs_188828	8.93	d__Bacteria;p__Firmicutes;c__Clostridia;o__Oscillospirales;f__UMGS1783;g__UMGS1783	92.3	blast;94.50
S12_vs_1980	94.44	d__Bacteria;p__Bacteroidota;c__Bacteroidia;o__Bacteroidales;f__Bacteroidaceae;g__Bacteroides	99.1	iPHoP-RF;97.10 blast;93.60 RaFAH;87.80 CRISPR;58.50
S12_vs_1980	94.44	d__Bacteria;p__Bacteroidota;c__Bacteroidia;o__Bacteroidales;f__Bacteroidaceae;g__Phocaeicola	94.5	iPHoP-RF;96.10 blast;70.90
S12_vs_23796	72.14	d__Bacteria;p__Bacteroidota;c__Bacteroidia;o__Bacteroidales;f__Rikenellaceae;g__Alistipes	99.1	iPHoP-RF;96.70 blast;96.60 RaFAH;89.00
S12_vs_26336	100	d__Bacteria;p__Bacteroidota;c__Bacteroidia;o__Bacteroidales;f__Bacteroidaceae;g__Bacteroides	99.6	RaFAH;99.60
S12_vs_26336	100	d__Bacteria;p__Bacteroidota;c__Bacteroidia;o__Bacteroidales;f__Rikenellaceae;g__Alistipes	95.6	blast;96.90 iPHoP-RF;96.70
S12_vs_26847	68.45	d__Bacteria;p__Firmicutes;c__Clostridia;o__Lachnospirales;f__Lachnospiraceae;g__Lachnospira	99.3	CRISPR;98.90 iPHoP-RF;95.70 blast;94.10 RaFAH;74.50
S12_vs_32669	3.93	d__Bacteria;p__Firmicutes;c__Clostridia;o__Christensenellales;f__CAG-917;g__CAG-475	94.7	blast;96.30 iPHoP-RF;68.60
S12_vs_48045	24.79	d__Bacteria;p__Firmicutes;c__Clostridia;o__Christensenellales;f__CAG-74;g__UBA11524	94.9	blast;96.40 iPHoP-RF;53.80
S12_vs_48045	24.79	d__Bacteria;p__Firmicutes;c__Clostridia;o__Christensenellales;f__CAG-74;g__UBA11524	92.7	blast;94.80 iPHoP-RF;56.00
S12_vs_6836	43.21	d__Bacteria;p__Firmicutes;c__Clostridia;o__TANB77;f__CAG-508;g__CAG-269	92.7	blast;94.80 iPHoP-RF;65.30
S12_vs_71525	30.6	d__Bacteria;p__Bacteroidota;c__Bacteroidia;o__Bacteroidales;f__Marinifilaceae;g__Butyricimonas	93.8	blast;95.60 iPHoP-RF;67.30
S12_vs_71525	30.6	d__Bacteria;p__Bacteroidota;c__Bacteroidia;o__Bacteroidales;f__Bacteroidaceae;g__Bacteroides	92.1	RaFAH;94.30
S12_vs_74280	14.1	d__Bacteria;p__Bacteroidota;c__Bacteroidia;o__Bacteroidales;f__Marinifilaceae;g__Butyricimonas	94.3	blast;96.00 iPHoP-RF;58.40
S12_vs_81729	10.77	d__Bacteria;p__Firmicutes;c__Clostridia;o__Oscillospirales;f__Ruminococcaceae;g__Faecalibacterium	97.3	CRISPR;98.10 iPHoP-RF;93.40 blast;92.10
S12_vs_86928	18.68	d__Bacteria;p__Verrucomicrobiota;c__Verrucomicrobiae;o__Verrucomicrobiales;f__Akkermansiaceae;g__Akkermansia	100	CRISPR;98.80 RaFAH;98.60 blast;96.90 iPHoP-RF;82.50
S12_vs_87088	36.55	d__Bacteria;p__Firmicutes;c__Clostridia;o__Oscillospirales;f__Ruminococcaceae;g__Faecalibacterium	97.5	blast;96.60 iPHoP-RF;92.40 RaFAH;73.80 CRISPR;53.50
S12_vs_92868	65.38	d__Bacteria;p__Firmicutes;c__Clostridia;o__Oscillospirales;f__Oscillospiraceae;g__Intestinimonas	93	blast;95.00 iPHoP-RF;58.70
S12_vs_93357	40.56	d__Bacteria;p__Proteobacteria;c__Gammaproteobacteria;o__Burkholderiales;f__Burkholderiaceae;g__Parasutterella	97	CRISPR;97.90 blast;96.90 iPHoP-RF;93.10
S12_vs_94042	10.3	d__Bacteria;p__Firmicutes;c__Clostridia;o__Christensenellales;f__CAG-74;g__UBA11524	95.6	blast;96.90 iPHoP-RF;91.80
S12_vs_94042	10.3	d__Bacteria;p__Firmicutes;c__Clostridia;o__Christensenellales;f__CAG-74;g__UBA11524	95.6	blast;96.90 CRISPR;96.60 iPHoP-RF;91.80
S12_vs_98175	95.22	d__Bacteria;p__Firmicutes;c__Clostridia;o__Lachnospirales;f__Lachnospiraceae;g__Bariatricus	90.3	iPHoP-RF;89.50 blast;81.30 RaFAH;65.60
S12_vs_99092	78.56	d__Bacteria;p__Firmicutes;c__Clostridia;o__Lachnospirales;f__Lachnospiraceae;g__Lachnospira	99.6	CRISPR;98.80 blast;98.80 iPHoP-RF;94.40 RaFAH;84.60
S12_vs_99219	99.93	d__Bacteria;p__Bacteroidota;c__Bacteroidia;o__Bacteroidales;f__Bacteroidaceae;g__Bacteroides	100	RaFAH;98.90 iPHoP-RF;96.70 blast;91.60
S12_vs_99219	99.93	d__Bacteria;p__Bacteroidota;c__Bacteroidia;o__Bacteroidales;f__Bacteroidaceae;g__Phocaeicola	92.1	blast;94.30 iPHoP-RF;94.10
S13_vf_109235	72.39	d__Bacteria;p__Firmicutes;c__Clostridia;o__Oscillospirales;f__Oscillospiraceae;g__CAG-83	90.1	blast;92.60 iPHoP-RF;68.90
S13_vf_11246	46.73	d__Bacteria;p__Bacteroidota;c__Bacteroidia;o__Bacteroidales;f__Bacteroidaceae;g__Prevotella	91.8	RaFAH;94.10
S13_vf_143422	75.96	d__Bacteria;p__Bacteroidota;c__Bacteroidia;o__Bacteroidales;f__Tannerellaceae;g__Parabacteroides	98.1	blast;98.60 iPHoP-RF;83.30
S13_vf_147589	6.46	d__Bacteria;p__Firmicutes;c__Clostridia;o__Oscillospirales;f__Oscillospiraceae;g__Flavonifractor	91.5	blast;93.80 iPHoP-RF;78.50
S13_vf_150407		d__Bacteria;p__Firmicutes;c__Clostridia;o__Oscillospirales;f__Oscillospiraceae;g__ER4	93.5	CRISPR;95.40
S13_vf_151045	98.31	d__Bacteria;p__Proteobacteria;c__Gammaproteobacteria;o__Enterobacterales;f__Enterobacteriaceae;g__Escherichia	95.7	RaFAH;97.00
S13_vf_28309	12.14	d__Bacteria;p__Firmicutes;c__Clostridia;o__Oscillospirales;f__Acutalibacteraceae;g__Ruminococcus_E	90.1	blast;92.60
S13_vf_54201	17.62	d__Bacteria;p__Firmicutes;c__Clostridia;o__Oscillospirales;f__Ruminococcaceae;g__Faecalibacterium	98.7	RaFAH;93.20 blast;89.90 iPHoP-RF;62.20
S13_vf_54201	17.62	d__Bacteria;p__Firmicutes;c__Negativicutes;o__Acidaminococcales;f__Acidaminococcaceae;g__Phascolarctobacterium	93.1	blast;95.10 iPHoP-RF;68.20
S13_vf_56074	91.56	d__Bacteria;p__Bacteroidota;c__Bacteroidia;o__Bacteroidales;f__Rikenellaceae;g__Alistipes	98.1	RaFAH;98.60
S13_vf_56074	91.56	d__Bacteria;p__Bacteroidota;c__Bacteroidia;o__Bacteroidales;f__Rikenellaceae;g__Alistipes_A	98.1	RaFAH;98.60
S13_vf_56074	91.56	d__Bacteria;p__Bacteroidota;c__Bacteroidia;o__Bacteroidales;f__Bacteroidaceae;g__UBA6398	91.6	blast;93.90 iPHoP-RF;74.30
S13_vf_66508	88.17	d__Bacteria;p__Bacteroidota;c__Bacteroidia;o__Bacteroidales;f__Bacteroidaceae;g__Bacteroides	99.4	iPHoP-RF;97.10 blast;96.60 RaFAH;91.90
S13_vf_7670	6.84	d__Bacteria;p__Verrucomicrobiota;c__Verrucomicrobiae;o__Verrucomicrobiales;f__Akkermansiaceae;g__Akkermansia	100	CRISPR;99.60 RaFAH;96.00 blast;93.60 iPHoP-RF;72.60
S13_vf_87873	97.58	d__Bacteria;p__Bacteroidota;c__Bacteroidia;o__Bacteroidales;f__Tannerellaceae;g__Marseille-P3160	98.4	CRISPR;98.80 iPHoP-RF;72.60
S13_vs_113593	40.01	d__Bacteria;p__Actinobacteriota;c__Actinomycetia;o__Actinomycetales;f__Bifidobacteriaceae;g__Bifidobacterium	91.5	blast;93.80 iPHoP-RF;88.20 CRISPR;56.60
S13_vs_121307	11.04	d__Bacteria;p__Firmicutes;c__Clostridia;o__Lachnospirales;f__Lachnospiraceae;g__Blautia_A	95.9	CRISPR;89.80 RaFAH;85.80 iPHoP-RF;84.50
S13_vs_13129	16.24	d__Bacteria;p__Firmicutes;c__Clostridia;o__Oscillospirales;f__Oscillospiraceae;g__CAG-83	98.5	CRISPR;98.90 blast;95.20 iPHoP-RF;87.90
S13_vs_148294	57.95	d__Bacteria;p__Firmicutes;c__Clostridia;o__Oscillospirales;f__Oscillospiraceae;g__	92.3	blast;94.50 iPHoP-RF;80.60
S13_vs_148294	57.95	d__Bacteria;p__Firmicutes;c__Clostridia;o__Oscillospirales;f__Oscillospiraceae;g__HGM12998	91	blast;93.40 iPHoP-RF;71.30
S13_vs_148294	57.95	d__Bacteria;p__Firmicutes;c__Clostridia;o__Oscillospirales;f__Oscillospiraceae;g__Flavonifractor	90	blast;92.50 iPHoP-RF;67.70
S13_vs_149860	61.18	d__Bacteria;p__Actinobacteriota;c__Actinomycetia;o__Actinomycetales;f__Bifidobacteriaceae;g__Bifidobacterium	99.7	CRISPR;99.70 iPHoP-RF;92.40 blast;92.00
S13_vs_15645	92	d__Bacteria;p__Bacteroidota;c__Bacteroidia;o__Bacteroidales;f__Tannerellaceae;g__Parabacteroides	99.9	CRISPR;99.20 iPHoP-RF;95.10 blast;93.30 RaFAH;90.50
S13_vs_26756	9.7	d__Bacteria;p__Firmicutes;c__Bacilli;o__RF39;f__UBA660;g__CAG-460	94	blast;95.80 iPHoP-RF;53.30
S13_vs_30101	54.46	d__Bacteria;p__Firmicutes;c__Bacilli;o__ML615J-28;f__CAG-313;g__CAG-313	95.6	blast;96.90 iPHoP-RF;81.60
S13_vs_35816	99.57	d__Bacteria;p__Firmicutes;c__Clostridia;o__Oscillospirales;f__Oscillospiraceae;g__CAG-83	99.7	RaFAH;99.00 blast;82.80
S13_vs_35816	99.57	d__Bacteria;p__Firmicutes;c__Clostridia;o__Oscillospirales;f__Oscillospiraceae;g__Dysosmobacter	98.7	RaFAH;99.00
S13_vs_45488	22.02	d__Bacteria;p__Proteobacteria;c__Alphaproteobacteria;o__RF32;f__CAG-239;g__51-20	97.9	CRISPR;98.50 blast;79.90 iPHoP-RF;74.60
S13_vs_45488	22.02	d__Bacteria;p__Proteobacteria;c__Alphaproteobacteria;o__RF32;f__CAG-239;g__CAG-267	95	blast;96.50 iPHoP-RF;72.20
S13_vs_57267	38.42	d__Bacteria;p__Bacteroidota;c__Bacteroidia;o__Bacteroidales;f__Bacteroidaceae;g__Prevotella	90.6	RaFAH;93.00
S13_vs_67429	14.26	d__Bacteria;p__Firmicutes;c__Bacilli;o__RF39;f__UBA660;g__CAG-460	94.7	blast;96.30 iPHoP-RF;53.80
S13_vs_83447	97.87	d__Bacteria;p__Bacteroidota;c__Bacteroidia;o__Bacteroidales;f__Rikenellaceae;g__Alistipes	99.9	blast;96.80 RaFAH;96.50 iPHoP-RF;82.80 CRISPR;82.60
S13_vs_83447	97.87	d__Bacteria;p__Bacteroidota;c__Bacteroidia;o__Bacteroidales;f__Rikenellaceae;g__Alistipes_A	95	RaFAH;96.50
S13_vs_90967		d__Bacteria;p__Bacteroidota;c__Bacteroidia;o__Bacteroidales;f__Barnesiellaceae;g__Barnesiella	90.3	blast;92.80 iPHoP-RF;81.90
S13_vs_9438	7.33	d__Bacteria;p__Proteobacteria;c__Alphaproteobacteria;o__RF32;f__CAG-239;g__CAG-267	91.6	blast;93.90 iPHoP-RF;83.30
S14_vf_106156	21.19	d__Bacteria;p__Firmicutes;c__Bacilli;o__Lactobacillales;f__Lactobacillaceae;g__Lactobacillus	100	RaFAH;98.90 CRISPR;96.80 iPHoP-RF;96.10 blast;95.50
S14_vf_106156	21.19	d__Bacteria;p__Firmicutes;c__Bacilli;o__Lactobacillales;f__Lactobacillaceae;g__Lacticaseibacillus	98.5	RaFAH;98.90
S14_vf_106156	21.19	d__Bacteria;p__Firmicutes;c__Bacilli;o__Lactobacillales;f__Lactobacillaceae;g__Ligilactobacillus	98.5	RaFAH;98.90
S14_vf_106156	21.19	d__Bacteria;p__Firmicutes;c__Bacilli;o__Lactobacillales;f__Lactobacillaceae;g__Limosilactobacillus	98.5	RaFAH;98.90
S14_vf_109545	12.74	d__Bacteria;p__Firmicutes;c__Clostridia;o__Oscillospirales;f__Oscillospiraceae;g__Intestinimonas	90.8	blast;93.20 iPHoP-RF;79.50
S14_vf_115736	85.01	d__Bacteria;p__Firmicutes;c__Clostridia;o__Oscillospirales;f__Oscillospiraceae;g__Flavonifractor	91.2	blast;93.60 iPHoP-RF;89.80 CRISPR;61.00
S14_vf_115736	85.01	d__Bacteria;p__Firmicutes;c__Clostridia;o__Oscillospirales;f__Oscillospiraceae;g__Intestinimonas	91.2	blast;93.60
S14_vf_117147	30.35	d__Bacteria;p__Firmicutes;c__Clostridia;o__Oscillospirales;f__Ruminococcaceae;g__Gemmiger	95.3	blast;96.70 CRISPR;94.60 iPHoP-RF;86.30
S14_vf_117150	99.65	d__Bacteria;p__Firmicutes;c__Clostridia;o__Lachnospirales;f__Lachnospiraceae;g__Lachnospira	99.9	CRISPR;98.90 RaFAH;91.00 blast;87.20 iPHoP-RF;83.10
S14_vf_117150	99.65	d__Bacteria;p__Firmicutes;c__Clostridia;o__Lachnospirales;f__Lachnospiraceae;g__Eubacterium_G	91.8	RaFAH;91.00 blast;67.20
S14_vf_117155	63.17	d__Bacteria;p__Actinobacteriota;c__Actinomycetia;o__Actinomycetales;f__Actinomycetaceae;g__Pauljensenia	98.4	RaFAH;94.80 CRISPR;83.10 iPHoP-RF;64.80
S14_vf_117155	63.17	d__Bacteria;p__Actinobacteriota;c__Actinomycetia;o__Actinomycetales;f__Actinomycetaceae;g__Actinomyces	92.7	RaFAH;94.80
S14_vf_117157	99.57	d__Bacteria;p__Firmicutes;c__Bacilli;o__Lactobacillales;f__Streptococcaceae;g__Streptococcus	100	RaFAH;99.40 CRISPR;98.80 iPHoP-RF;94.40 blast;93.60
S14_vf_117162	23.32	d__Bacteria;p__Bacteroidota;c__Bacteroidia;o__Bacteroidales;f__Bacteroidaceae;g__Bacteroides	94	RaFAH;90.00 iPHoP-RF;70.90 CRISPR;63.50

S14_vf_37500	5.58	d__Bacteria;p__Bacteroidota;c__Bacteroidia;o__Bacteroidales;f__Bacteroidaceae;g__Phocaeicola	94.3	CRISPR;96.00 iPHoP-RF;67.00
S14_vf_51134	47.7	d__Bacteria;p__Firmicutes;c__Clostridia;o__Oscillospirales;f__Ruminococcaceae;g__Ruminococcus_D	97.2	blast;97.90 iPHoP-RF;92.80 RaFAH;52.60
S14_vf_56130	100	d__Bacteria;p__Firmicutes;c__Clostridia;o__Clostridiales;f__Clostridiaceae;g__Clostridium	98.8	RaFAH;99.10
S14_vf_56130	100	d__Bacteria;p__Firmicutes;c__Clostridia;o__Clostridiales;f__Clostridiaceae;g__Clostridium_F	98.8	RaFAH;99.10
S14_vf_56130	100	d__Bacteria;p__Firmicutes;c__Clostridia;o__Clostridiales;f__Clostridiaceae;g__Clostridium_P	98.8	RaFAH;99.10
S14_vf_57689	94.04	d__Bacteria;p__Actinobacteriota;c__Actinomycetia;o__Actinomycetales;f__Actinomycetaceae;g__Actinomyces	99	blast;94.00 RaFAH;93.30 CRISPR;80.00 iPHoP-RF;75.40
S14_vf_57689	94.04	d__Bacteria;p__Actinobacteriota;c__Actinomycetia;o__Actinomycetales;f__Actinomycetaceae;g__Pauljensenia	90.9	RaFAH;93.30
S14_vf_58919	74.74	d__Bacteria;p__Firmicutes;c__Clostridia;o__Lachnospirales;f__Lachnospiraceae;g__Roseburia	95.3	blast;90.40 iPHoP-RF;87.90 RaFAH;82.70
S14_vf_58919	74.74	d__Bacteria;p__Firmicutes;c__Clostridia;o__Lachnospirales;f__Lachnospiraceae;g__Agathobacter	95.2	blast;90.20 RaFAH;82.70 iPHoP-RF;78.30
S14_vf_58919	74.74	d__Bacteria;p__Firmicutes;c__Clostridia;o__Lachnospirales;f__Lachnospiraceae;g__COE1	90.4	blast;92.90 iPHoP-RF;81.40
S14_vf_72335	11.73	d__Bacteria;p__Firmicutes;c__Bacilli;o__Erysipelotrichales;f__Erysipelotrichaceae;g__Holdemania	92.1	CRISPR;94.30
S14_vf_72953	32.12	d__Bacteria;p__Firmicutes;c__Bacilli;o__Lactobacillales;f__Lactobacillaceae;g__Lacticaseibacillus	96.7	RaFAH;97.70
S14_vf_72953	32.12	d__Bacteria;p__Firmicutes;c__Bacilli;o__Lactobacillales;f__Lactobacillaceae;g__Lactobacillus	96.7	RaFAH;97.70
S14_vf_72953	32.12	d__Bacteria;p__Firmicutes;c__Bacilli;o__Lactobacillales;f__Lactobacillaceae;g__Ligilactobacillus	96.7	RaFAH;97.70
S14_vf_72953	32.12	d__Bacteria;p__Firmicutes;c__Bacilli;o__Lactobacillales;f__Lactobacillaceae;g__Limosilactobacillus	96.7	RaFAH;97.70
S14_vf_72953	32.12	d__Bacteria;p__Firmicutes;c__Bacilli;o__Lactobacillales;f__Lactobacillaceae;g__Pediococcus	91.8	blast;94.10 iPHoP-RF;93.40 CRISPR;56.30
S14_vf_78139	74.71	d__Bacteria;p__Firmicutes;c__Bacilli;o__Lactobacillales;f__Lactobacillaceae;g__Lactobacillus	100	RaFAH;99.10 blast;96.80 CRISPR;96.50 iPHoP-RF;92.80
S14_vf_78139	74.71	d__Bacteria;p__Firmicutes;c__Bacilli;o__Lactobacillales;f__Lactobacillaceae;g__Lacticaseibacillus	98.8	RaFAH;99.10
S14_vf_78139	74.71	d__Bacteria;p__Firmicutes;c__Bacilli;o__Lactobacillales;f__Lactobacillaceae;g__Ligilactobacillus	98.8	RaFAH;99.10
S14_vf_78139	74.71	d__Bacteria;p__Firmicutes;c__Bacilli;o__Lactobacillales;f__Lactobacillaceae;g__Limosilactobacillus	98.8	RaFAH;99.10
S14_vf_85122	16.16	d__Bacteria;p__Firmicutes;c__Clostridia;o__Clostridiales;f__Clostridiaceae;g__Clostridium	95	blast;92.30 iPHoP-RF;86.00 RaFAH;77.50
S14_vf_92240	99.79	d__Bacteria;p__Firmicutes;c__Clostridia;o__Lachnospirales;f__Lachnospiraceae;g__Coprococcus	93.2	CRISPR;95.20
S14_vf_93134	12.04	d__Bacteria;p__Firmicutes;c__Clostridia;o__Lachnospirales;f__Lachnospiraceae;g__Muricomes	96.7	CRISPR;97.70
S14_vf_99344	10.5	d__Bacteria;p__Firmicutes;c__Clostridia;o__Lachnospirales;f__Lachnospiraceae;g__Eubacterium_F	96.9	CRISPR;97.80
S14_vs_100227	81.21	d__Bacteria;p__Firmicutes;c__Clostridia;o__Lachnospirales;f__Lachnospiraceae;g__Enterocloster	94.2	CRISPR;95.90 blast;94.00 iPHoP-RF;86.30
S14_vs_100227	81.21	d__Bacteria;p__Firmicutes;c__Clostridia;o__Lachnospirales;f__Lachnospiraceae;g__Hungatella_A	91	iPHoP-RF;93.40
S14_vs_101482	6.74	d__Bacteria;p__Firmicutes;c__Clostridia;o__Oscillospirales;f__Ruminococcaceae;g__QAMM01	92	blast;94.20 iPHoP-RF;69.10
S14_vs_102698	99.62	d__Bacteria;p__Firmicutes;c__Clostridia;o__Acetivibrionales;f__Acetivibrionaceae;g__Ruminiclostridium	90.1	RaFAH;92.60
S14_vs_112068	27.86	d__Bacteria;p__Firmicutes;c__Clostridia;o__Oscillospirales;f__Butyricicoccaceae;g__Agathobaculum	97.5	blast;98.20 iPHoP-RF;66.00
S14_vs_112345	71.03	d__Bacteria;p__Bacteroidota;c__Bacteroidia;o__Bacteroidales;f__Bacteroidaceae;g__Bacteroides	99.1	iPHoP-RF;96.70 RaFAH;88.90 blast;75.90
S14_vs_112345	71.03	d__Bacteria;p__Bacteroidota;c__Bacteroidia;o__Bacteroidales;f__Bacteroidaceae;g__Phocaeicola	95.6	blast;96.90 iPHoP-RF;95.70
S14_vs_11510	39.46	d__Bacteria;p__Bacteroidota;c__Bacteroidia;o__Bacteroidales;f__Bacteroidaceae;g__Bacteroides	96.9	blast;91.70 RaFAH;87.00 iPHoP-RF;81.40
S14_vs_115377	76.83	d__Bacteria;p__Firmicutes;c__Clostridia;o__Lachnospirales;f__Lachnospiraceae;g__Blautia	98.8	CRISPR;97.10 RaFAH;83.90 iPHoP-RF;60.90
S14_vs_115377	76.83	d__Bacteria;p__Firmicutes;c__Clostridia;o__Lachnospirales;f__Lachnospiraceae;g__Hespellia	94	blast;95.80 iPHoP-RF;66.80
S14_vs_115455	25.58	d__Bacteria;p__Firmicutes;c__Clostridia;o__Eubacteriales;f__Eubacteriaceae;g__Eubacterium	93	blast;95.00 CRISPR;89.70 iPHoP-RF;84.50
S14_vs_117153	96.78	d__Bacteria;p__Firmicutes;c__Clostridia;o__Lachnospirales;f__Lachnospiraceae;g__Dorea_A	94.2	blast;95.90 iPHoP-RF;94.70 CRISPR;58.40
S14_vs_117153	96.78	d__Bacteria;p__Firmicutes;c__Clostridia;o__Lachnospirales;f__Lachnospiraceae;g__Dorea	90.6	blast;93.00 iPHoP-RF;68.60
S14_vs_117166	22.09	d__Bacteria;p__Firmicutes;c__Clostridia;o__Oscillospirales;f__Oscillospiraceae;g__Evtepia	94.2	blast;95.90 iPHoP-RF;66.30
S14_vs_14737	33.52	d__Bacteria;p__Firmicutes;c__Clostridia;o__Oscillospirales;f__Ruminococcaceae;g__Ruthenibacterium	93.1	blast;95.10 iPHoP-RF;68.40
S14_vs_15228	86.47	d__Bacteria;p__Bacteroidota;c__Bacteroidia;o__Bacteroidales;f__Tannerellaceae;g__Parabacteroides	100	CRISPR;99.00 blast;98.60 RaFAH;97.90 iPHoP-RF;96.10
S14_vs_25852	26.78	d__Bacteria;p__Bacteroidota;c__Bacteroidia;o__Bacteroidales;f__Bacteroidaceae;g__Bacteroides	97.8	blast;96.60 iPHoP-RF;95.10 CRISPR;93.00 RaFAH;77.10
S14_vs_25852	26.78	d__Bacteria;p__Bacteroidota;c__Bacteroidia;o__Bacteroidales;f__Bacteroidaceae;g__Phocaeicola	90.1	blast;92.60 CRISPR;86.70
S14_vs_26320	65.93	d__Bacteria;p__Firmicutes;c__Clostridia;o__Lachnospirales;f__Lachnospiraceae;g__Ruminococcus_B	95.2	blast;96.60 iPHoP-RF;82.20
S14_vs_2870	56.13	d__Bacteria;p__Proteobacteria;c__Gammaproteobacteria;o__Burkholderiales;f__Burkholderiaceae;g__Sutterella	99	blast;96.90 RaFAH;87.10 CRISPR;78.30 iPHoP-RF;69.50
S14_vs_30108	19.83	d__Bacteria;p__Firmicutes;c__Clostridia;o__Lachnospirales;f__Lachnospiraceae;g__Enterocloster	92.3	blast;94.50 CRISPR;82.40 iPHoP-RF;78.30
S14_vs_32499	51.73	d__Bacteria;p__Bacteroidota;c__Bacteroidia;o__Bacteroidales;f__Bacteroidaceae;g__Bacteroides	99.1	blast;98.10 iPHoP-RF;96.70 RaFAH;82.60
S14_vs_3251	82.87	d__Bacteria;p__Firmicutes;c__Clostridia;o__Oscillospirales;f__Oscillospiraceae;g__Flavonifractor	95.6	blast;96.90 iPHoP-RF;65.60
S14_vs_38905	9.01	d__Bacteria;p__Firmicutes;c__Clostridia;o__Oscillospirales;f__Ruminococcaceae;g__Ruthenibacterium	93.8	blast;95.60 iPHoP-RF;81.60 CRISPR;54.30
S14_vs_39266	15.51	d__Bacteria;p__Firmicutes;c__Clostridia;o__Oscillospirales;f__Acutalibacteraceae;g__Ruminococcus_E	98.7	CRISPR;99.00 blast;96.80 iPHoP-RF;93.70
S14_vs_39797	47.42	d__Bacteria;p__Firmicutes;c__Clostridia;o__Lachnospirales;f__Lachnospiraceae;g__Anaerostipes	93.8	blast;95.60 iPHoP-RF;67.00
S14_vs_56251	20.56	d__Bacteria;p__Firmicutes;c__Clostridia;o__Lachnospirales;f__Lachnospiraceae;g__Muricomes	95.3	iPHoP-RF;96.70 blast;93.30 CRISPR;68.70
S14_vs_56410	80.58	d__Bacteria;p__Firmicutes;c__Clostridia;o__Oscillospirales;f__Oscillospiraceae;g__Flavonifractor	95.2	blast;96.60 iPHoP-RF;95.40 CRISPR;94.00
S14_vs_59347	29.43	d__Bacteria;p__Firmicutes;c__Clostridia;o__Lachnospirales;f__Lachnospiraceae;g__Muricomes	93.1	iPHoP-RF;95.10
S14_vs_59347	29.43	d__Bacteria;p__Firmicutes;c__Clostridia;o__Lachnospirales;f__Lachnospiraceae;g__Ruminococcus_B	90.7	iPHoP-RF;93.10
S14_vs_61530	22.32	d__Bacteria;p__Firmicutes;c__Clostridia;o__Oscillospirales;f__Acutalibacteraceae;g__UBA1417	92	blast;94.20 iPHoP-RF;82.80
S14_vs_632	78.66	d__Bacteria;p__Firmicutes;c__Clostridia;o__Clostridiales;f__Clostridiaceae;g__Clostridium	91	RaFAH;93.40
S14_vs_632	78.66	d__Bacteria;p__Firmicutes;c__Clostridia;o__Clostridiales;f__Clostridiaceae;g__Clostridium_F	91	RaFAH;93.40
S14_vs_632	78.66	d__Bacteria;p__Firmicutes;c__Clostridia;o__Clostridiales;f__Clostridiaceae;g__Clostridium_P	91	RaFAH;93.40
S14_vs_66139	83.75	d__Bacteria;p__Firmicutes;c__Clostridia;o__Oscillospirales;f__Acutalibacteraceae;g__Ruminococcus_E	95.6	blast;96.90 iPHoP-RF;85.70
S14_vs_66208	100	d__Bacteria;p__Bacteroidota;c__Bacteroidia;o__Bacteroidales;f__Bacteroidaceae;g__Bacteroides	100	RaFAH;98.30 iPHoP-RF;96.40 blast;93.70
S14_vs_66841	15.26	d__Bacteria;p__Firmicutes;c__Clostridia;o__Lachnospirales;f__Lachnospiraceae;g__Agathobacter	90.4	blast;92.90 iPHoP-RF;78.00
S14_vs_67094	10	d__Bacteria;p__Firmicutes;c__Clostridia;o__Oscillospirales;f__Oscillospiraceae;g__Lawsonibacter	90.7	blast;93.10
S14_vs_70985	21.93	d__Bacteria;p__Bacteroidota;c__Bacteroidia;o__Bacteroidales;f__Bacteroidaceae;g__Bacteroides	99	iPHoP-RF;96.70 blast;92.60 RaFAH;88.40
S14_vs_73301	15.25	d__Bacteria;p__Firmicutes;c__Clostridia;o__Oscillospirales;f__Ruminococcaceae;g__Massilimaliae	94.2	blast;95.90 iPHoP-RF;64.80
S14_vs_74067	100	d__Bacteria;p__Bacteroidota;c__Bacteroidia;o__Bacteroidales;f__Bacteroidaceae;g__Bacteroides	99.6	RaFAH;93.20 blast;92.00 iPHoP-RF;82.20
S14_vs_78164	61.84	d__Bacteria;p__Bacteroidota;c__Bacteroidia;o__Bacteroidales;f__Marinifilaceae;g__Odoribacter	95.4	blast;96.80 iPHoP-RF;84.80 CRISPR;56.80
S14_vs_88666	32.05	d__Bacteria;p__Firmicutes;c__Clostridia;o__Oscillospirales;f__Acutalibacteraceae;g__Fimivicinus	95.4	blast;96.80 iPHoP-RF;86.70
S14_vs_88666	32.05	d__Bacteria;p__Firmicutes;c__Clostridia;o__Oscillospirales;f__Acutalibacteraceae;g__UBA1691	95.4	blast;96.80 CRISPR;90.80 iPHoP-RF;86.70
S14_vs_90934	62.33	d__Bacteria;p__Firmicutes;c__Clostridia;o__Oscillospirales;f__Ruminococcaceae;g__Faecalibacterium	99.1	blast;96.70 RaFAH;89.00 iPHoP-RF;81.10
S14_vs_96286	99.54	d__Bacteria;p__Firmicutes;c__Clostridia;o__Lachnospirales;f__Lachnospiraceae;g__Blautia	96.3	RaFAH;97.40
S14_vs_96286	99.54	d__Bacteria;p__Firmicutes;c__Clostridia;o__Lachnospirales;f__Lachnospiraceae;g__Blautia_A	96.3	RaFAH;97.40
S14_vs_96286	99.54	d__Bacteria;p__Firmicutes;c__Clostridia;o__Lachnospirales;f__Lachnospiraceae;g__Mediterraneibacter	95.6	blast;96.90 iPHoP-RF;55.70
S14_vs_9676	24.59	d__Bacteria;p__Firmicutes;c__Clostridia;o__Oscillospirales;f__Oscillospiraceae;g__Lawsonibacter	90.7	blast;93.10 iPHoP-RF;77.50
S14_vs_97835		d__Bacteria;p__Firmicutes;c__Clostridia;o__Oscillospirales;f__Oscillospiraceae;g__CAG-83	90.6	blast;93.00 iPHoP-RF;84.20
S15_vf_2032	83.93	d__Bacteria;p__Bacteroidota;c__Bacteroidia;o__Bacteroidales;f__Bacteroidaceae;g__Bacteroides	99.3	iPHoP-RF;97.10 blast;96.20 CRISPR;91.10 RaFAH;89.30
S15_vf_2032	83.93	d__Bacteria;p__Bacteroidota;c__Bacteroidia;o__Bacteroidales;f__Bacteroidaceae;g__Phocaeicola	95.9	iPHoP-RF;97.10 blast;89.90 CRISPR;76.20

S15_vf_20644	83.74	d__Bacteria;p__Bacteroidota;c__Bacteroidia;o__Bacteroidales;f__Rikenellaceae;g__Alistipes	94.3	RaFAH;96.00
S15_vf_20644	83.74	d__Bacteria;p__Bacteroidota;c__Bacteroidia;o__Bacteroidales;f__Rikenellaceae;g__Alistipes_A	94.3	RaFAH;96.00
S15_vf_2513	25.17	d__Bacteria;p__Firmicutes;c__Clostridia;o__Lachnospirales;f__Lachnospiraceae;g__Lachnospira	94.5	CRISPR;96.10
S15_vf_75924	99.22	d__Bacteria;p__Bacteroidota;c__Bacteroidia;o__Bacteroidales;f__Bacteroidaceae;g__Bacteroides	99.6	RaFAH;93.20 blast;91.90 iPHoP-RF;82.50
S15_vs_18274	75.03	d__Bacteria;p__Bacteroidota;c__Bacteroidia;o__Bacteroidales;f__Coprobacteraceae;g__Coprobacter	98.7	CRISPR;99.00 blast;93.10 iPHoP-RF;64.00 RaFAH;50.00
S15_vs_27510	51.41	d__Bacteria;p__Bacteroidota;c__Bacteroidia;o__Bacteroidales;f__Bacteroidaceae;g__Bacteroides	99.1	iPHoP-RF;96.10 blast;93.90 RaFAH;90.50
S15_vs_27510	51.41	d__Bacteria;p__Bacteroidota;c__Bacteroidia;o__Bacteroidales;f__Bacteroidaceae;g__Phocaeicola	94.9	iPHoP-RF;96.40 blast;68.30
S15_vs_35445	27.45	d__Bacteria;p__Bacteroidota;c__Bacteroidia;o__Bacteroidales;f__Bacteroidaceae;g__Bacteroides	98.8	iPHoP-RF;96.70 blast;93.90 RaFAH;86.60
S15_vs_3868	100	d__Bacteria;p__Bacteroidota;c__Bacteroidia;o__Bacteroidales;f__Bacteroidaceae;g__Bacteroides	100	RaFAH;99.10 blast;96.50 iPHoP-RF;61.40
S15_vs_3868	100	d__Bacteria;p__Bacteroidota;c__Bacteroidia;o__Bacteroidales;f__Tannerellaceae;g__Parabacteroides	93	blast;95.00 iPHoP-RF;70.60 CRISPR;68.20
S15_vs_42043	51.36	d__Bacteria;p__Bacteroidota;c__Bacteroidia;o__Bacteroidales;f__Bacteroidaceae;g__Bacteroides	97.6	blast;96.80 iPHoP-RF;95.10 RaFAH;72.80
S15_vs_42043	51.36	d__Bacteria;p__Bacteroidota;c__Bacteroidia;o__Bacteroidales;f__Bacteroidaceae;g__Phocaeicola	94.9	iPHoP-RF;96.40 blast;88.20
S15_vs_43913	99.8	d__Bacteria;p__Bacteroidota;c__Bacteroidia;o__Bacteroidales;f__Bacteroidaceae;g__Bacteroides	100	RaFAH;99.30 iPHoP-RF;97.10 blast;96.70
S15_vs_71968	100	d__Bacteria;p__Bacteroidota;c__Bacteroidia;o__Bacteroidales;f__Rikenellaceae;g__Alistipes	100	RaFAH;98.90 blast;98.40 iPHoP-RF;95.40
S15_vs_71968	100	d__Bacteria;p__Bacteroidota;c__Bacteroidia;o__Bacteroidales;f__Rikenellaceae;g__Alistipes_A	98.5	RaFAH;98.90
S16_vf_100473	8.42	d__Bacteria;p__Actinobacteriota;c__Actinomycetia;o__Actinomycetales;f__Bifidobacteriaceae;g__Bifidobacterium	90.3	blast;92.80
S16_vf_1037	73.36	d__Bacteria;p__Firmicutes;c__Clostridia;o__Lachnospirales;f__Lachnospiraceae;g__Lachnospira	99.7	CRISPR;99.70 iPHoP-RF;94.40 blast;93.60
S16_vf_108554	18.88	d__Bacteria;p__Firmicutes;c__Clostridia;o__Oscillospirales;f__Ruminococcaceae;g__Faecalibacterium	97.9	CRISPR;98.50 blast;93.10 iPHoP-RF;92.10
S16_vf_16182	64.12	d__Bacteria;p__Actinobacteriota;c__Actinomycetia;o__Actinomycetales;f__Actinomycetaceae;g__Pauljensenia	96.9	RaFAH;95.10 CRISPR;68.30 iPHoP-RF;64.80
S16_vf_16182	64.12	d__Bacteria;p__Actinobacteriota;c__Actinomycetia;o__Actinomycetales;f__Actinomycetaceae;g__Actinomyces	93.1	RaFAH;95.10
S16_vf_169895	11.84	d__Bacteria;p__Firmicutes;c__Clostridia;o__Oscillospirales;f__Ruminococcaceae;g__Ruthenibacterium	92.7	CRISPR;94.80
S16_vf_172897	32.49	d__Bacteria;p__Actinobacteriota;c__Actinomycetia;o__Actinomycetales;f__Bifidobacteriaceae;g__Bifidobacterium	98.4	CRISPR;98.80 iPHoP-RF;97.10 blast;93.30
S16_vf_173099	55.52	d__Bacteria;p__Firmicutes;c__Clostridia;o__Oscillospirales;f__Oscillospiraceae;g__Flavonifractor	93.8	blast;92.00 iPHoP-RF;86.00 RaFAH;72.50
S16_vf_173109	7.33	d__Bacteria;p__Actinobacteriota;c__Actinomycetia;o__Actinomycetales;f__Bifidobacteriaceae;g__Bifidobacterium	97	CRISPR;97.90 iPHoP-RF;81.60
S16_vf_36146	99.74	d__Bacteria;p__Firmicutes;c__Clostridia;o__Clostridiales;f__Clostridiaceae;g__Clostridium	96.2	RaFAH;97.30
S16_vf_36146	99.74	d__Bacteria;p__Firmicutes;c__Clostridia;o__Clostridiales;f__Clostridiaceae;g__Clostridium_F	96.2	RaFAH;97.30
S16_vf_36146	99.74	d__Bacteria;p__Firmicutes;c__Clostridia;o__Clostridiales;f__Clostridiaceae;g__Clostridium_P	96.2	RaFAH;97.30
S16_vf_5809	87.43	d__Bacteria;p__Firmicutes;c__Clostridia;o__Lachnospirales;f__Lachnospiraceae;g__Lachnospira	99.9	CRISPR;99.00 iPHoP-RF;95.40 blast;93.40 RaFAH;91.70
S16_vf_6102	38.14	d__Bacteria;p__Firmicutes;c__Clostridia;o__Oscillospirales;f__Oscillospiraceae;g__ER4	91.1	blast;93.50 iPHoP-RF;66.80
S16_vf_84462	31.67	d__Bacteria;p__Firmicutes;c__Clostridia;o__Lachnospirales;f__Anaerotignaceae;g__Anaerotignum	94.2	blast;95.90 iPHoP-RF;79.50
S16_vf_87190	34.33	d__Bacteria;p__Bacteroidota;c__Bacteroidia;o__Bacteroidales;f__Bacteroidaceae;g__Bacteroides	95	RaFAH;87.80 iPHoP-RF;77.80 CRISPR;68.10
S16_vf_90328	16.6	d__Bacteria;p__Firmicutes;c__Clostridia;o__TANB77;f__CAG-508;g__CAG-269	91.4	blast;93.70 iPHoP-RF;53.00
S16_vf_9270	8.58	d__Bacteria;p__Bacteroidota;c__Bacteroidia;o__Bacteroidales;f__Rikenellaceae;g__Alistipes	98.4	CRISPR;98.80 iPHoP-RF;95.10
S16_vf_9270	8.58	d__Bacteria;p__Bacteroidota;c__Bacteroidia;o__Bacteroidales;f__Bacteroidaceae;g__Bacteroides	95.9	RaFAH;97.10
S16_vf_96483	29.87	d__Bacteria;p__Firmicutes;c__Clostridia;o__Lachnospirales;f__Lachnospiraceae;g__Blautia_A	97.6	iPHoP-RF;93.40 blast;93.30 RaFAH;87.30
S16_vf_97279	52.37	d__Bacteria;p__Firmicutes;c__Clostridia;o__Oscillospirales;f__Oscillospiraceae;g__ER4	91	blast;93.40 iPHoP-RF;60.30
S16_vs_101072	26.56	d__Bacteria;p__Firmicutes;c__Clostridia;o__Lachnospirales;f__Lachnospiraceae;g__CAG-127	95	blast;96.50 iPHoP-RF;87.30
S16_vs_103726	26.95	d__Bacteria;p__Firmicutes;c__Clostridia;o__Oscillospirales;f__Ruminococcaceae;g__Ruthenibacterium	94	blast;95.80 CRISPR;80.80 iPHoP-RF;79.80
S16_vs_107235	98.85	d__Bacteria;p__Firmicutes;c__Clostridia;o__Oscillospirales;f__Ruminococcaceae;g__Ruminococcus_D	99.7	blast;99.00 iPHoP-RF;97.10 CRISPR;94.60 RaFAH;84.50
S16_vs_116956	43.56	d__Bacteria;p__Firmicutes;c__Clostridia;o__Lachnospirales;f__Lachnospiraceae;g__Acetatifactor	91.6	blast;93.90 iPHoP-RF;75.20
S16_vs_118029	43.23	d__Bacteria;p__Firmicutes;c__Clostridia;o__Lachnospirales;f__Lachnospiraceae;g__Eubacterium_G	94.6	blast;96.20 iPHoP-RF;85.70
S16_vs_120149	12.05	d__Bacteria;p__Firmicutes;c__Clostridia;o__Oscillospirales;f__Acutalibacteraceae;g__Eubacterium_R	94.5	iPHoP-RF;96.10 blast;93.90
S16_vs_120622	63.91	d__Bacteria;p__Proteobacteria;c__Gammaproteobacteria;o__Burkholderiales;f__Burkholderiaceae;g__Parasutterella	97.2	CRISPR;98.00 blast;96.90 iPHoP-RF;93.10
S16_vs_127669	99.82	d__Bacteria;p__Bacteroidota;c__Bacteroidia;o__Bacteroidales;f__Bacteroidaceae;g__Bacteroides	100	RaFAH;99.00 CRISPR;97.60 iPHoP-RF;97.10 blast;90.90
S16_vs_127669	99.82	d__Bacteria;p__Bacteroidota;c__Bacteroidia;o__Bacteroidales;f__Bacteroidaceae;g__Phocaeicola	96.6	CRISPR;97.60 iPHoP-RF;97.10 blast;94.60
S16_vs_127905	55.65	d__Bacteria;p__Bacteroidota;c__Bacteroidia;o__Bacteroidales;f__Bacteroidaceae;g__Bacteroides	99.4	iPHoP-RF;97.10 blast;95.20 RaFAH;91.80 CRISPR;60.90
S16_vs_127905	55.65	d__Bacteria;p__Bacteroidota;c__Bacteroidia;o__Bacteroidales;f__Bacteroidaceae;g__Phocaeicola	95.9	iPHoP-RF;97.10 blast;81.50
S16_vs_132979	31.86	d__Bacteria;p__Firmicutes;c__Clostridia;o__Oscillospirales;f__Ruminococcaceae;g__Faecalibacterium	94.3	blast;96.00 iPHoP-RF;93.10
S16_vs_136935	99.58	d__Bacteria;p__Bacteroidota;c__Bacteroidia;o__Bacteroidales;f__Bacteroidaceae;g__Bacteroides	100	RaFAH;98.20 iPHoP-RF;96.70 blast;89.50
S16_vs_136935	99.58	d__Bacteria;p__Bacteroidota;c__Bacteroidia;o__Bacteroidales;f__Bacteroidaceae;g__Phocaeicola	97.6	CRISPR;98.30 blast;96.20 iPHoP-RF;95.70
S16_vs_140709	84.15	d__Bacteria;p__Firmicutes;c__Clostridia;o__Lachnospirales;f__Lachnospiraceae;g__CAG-317	94.5	iPHoP-RF;96.10
S16_vs_140709	84.15	d__Bacteria;p__Firmicutes;c__Clostridia;o__Lachnospirales;f__Lachnospiraceae;g__Bariatricus	93.5	iPHoP-RF;95.40 blast;93.80
S16_vs_140709	84.15	d__Bacteria;p__Firmicutes;c__Clostridia;o__Lachnospirales;f__Lachnospiraceae;g__Dorea_B	93.5	iPHoP-RF;95.40
S16_vs_140709	84.15	d__Bacteria;p__Firmicutes;c__Clostridia;o__Lachnospirales;f__Lachnospiraceae;g__Lachnoclostridium_B	92.6	iPHoP-RF;94.70
S16_vs_141218	10.25	d__Bacteria;p__Firmicutes;c__Bacilli;o__ML615J-28;f__CAG-313;g__CAG-313	98.7	CRISPR;99.00 blast;96.10
S16_vs_142561	99.95	d__Bacteria;p__Bacteroidota;c__Bacteroidia;o__Bacteroidales;f__Rikenellaceae;g__Alistipes_A	97.8	blast;98.20 RaFAH;56.20 iPHoP-RF;53.30
S16_vs_142561	99.95	d__Bacteria;p__Bacteroidota;c__Bacteroidia;o__Bacteroidales;f__Rikenellaceae;g__Alistipes	96	blast;96.80 RaFAH;56.20 iPHoP-RF;50.90
S16_vs_15093	19.11	d__Bacteria;p__Firmicutes;c__Clostridia;o__Christensenellales;f__CAG-74;g__UBA11524	99.6	CRISPR;99.60 blast;96.60 iPHoP-RF;85.70
S16_vs_165662	11.88	d__Bacteria;p__Firmicutes;c__Bacilli;o__RF39;f__UBA660;g__CAG-302	92.7	blast;94.80
S16_vs_173079	99.1	d__Bacteria;p__Firmicutes;c__Clostridia;o__Lachnospirales;f__Lachnospiraceae;g__Coprococcus	94	blast;95.80 iPHoP-RF;71.70
S16_vs_173096	74.56	d__Bacteria;p__Verrucomicrobiota;c__Verrucomicrobiae;o__Verrucomicrobiales;f__Akkermansiaceae;g__Akkermansia	100	RaFAH;99.70 CRISPR;99.60 blast;96.80 iPHoP-RF;81.10
S16_vs_173141		d__Bacteria;p__Firmicutes;c__Clostridia;o__Oscillospirales;f__Ruminococcaceae;g__Faecalibacterium	96	CRISPR;97.20 iPHoP-RF;82.80
S16_vs_17493	18.24	d__Bacteria;p__Firmicutes;c__Clostridia;o__Oscillospirales;f__Ruminococcaceae;g__Massilimaliae	93	blast;95.00 iPHoP-RF;58.40
S16_vs_18440	67.51	d__Bacteria;p__Firmicutes;c__Clostridia;o__Oscillospirales;f__Ruminococcaceae;g__Faecalibacterium	100	RaFAH;99.30 CRISPR;98.90 blast;93.60 iPHoP-RF;85.70
S16_vs_19968	93.01	d__Bacteria;p__Bacteroidota;c__Bacteroidia;o__Bacteroidales;f__Rikenellaceae;g__Alistipes	98.4	iPHoP-RF;97.10 blast;96.90 RaFAH;79.60
S16_vs_20060	33.58	d__Bacteria;p__Firmicutes;c__Clostridia;o__Oscillospirales;f__Oscillospiraceae;g__UMGS1766	91	blast;93.40
S16_vs_32764	17.71	d__Bacteria;p__Bacteroidota;c__Bacteroidia;o__Bacteroidales;f__Rikenellaceae;g__Alistipes	95.9	iPHoP-RF;97.10 blast;96.90
S16_vs_33281	7.09	d__Bacteria;p__Bacteroidota;c__Bacteroidia;o__Bacteroidales;f__Rikenellaceae;g__Alistipes	95.9	iPHoP-RF;97.10 blast;96.90
S16_vs_33281	7.09	d__Bacteria;p__Bacteroidota;c__Bacteroidia;o__Bacteroidales;f__Bacteroidaceae;g__Bacteroides	92.9	RaFAH;94.90
S16_vs_34021	99.81	d__Bacteria;p__Firmicutes;c__Clostridia;o__Oscillospirales;f__Ruminococcaceae;g__Ruminococcus_D	99	blast;96.90 iPHoP-RF;90.50 RaFAH;86.90
S16_vs_38863	26.24	d__Bacteria;p__Firmicutes;c__Clostridia;o__Monoglobales;f__Monoglobaceae;g__Monoglobus	95	blast;96.50 iPHoP-RF;64.50
S16_vs_4662	13.68	d__Bacteria;p__Firmicutes;c__Clostridia;o__Christensenellales;f__CAG-138;g__UBA5394	92.6	CRISPR;94.70 blast;92.60 iPHoP-RF;74.30
S16_vs_48714	5.9	d__Bacteria;p__Firmicutes;c__Clostridia;o__Oscillospirales;f__Ruminococcaceae;g__Ruminococcus_D	97	blast;97.90 iPHoP-RF;97.10 CRISPR;96.60
S16_vs_51901	16.94	d__Bacteria;p__Bacteroidota;c__Bacteroidia;o__Bacteroidales;f__Rikenellaceae;g__Alistipes	97.6	blast;93.90 iPHoP-RF;90.10 RaFAH;85.80
S16_vs_75108	32.65	d__Bacteria;p__Firmicutes;c__Clostridia;o__Oscillospirales;f__Oscillospiraceae;g__UBA5446	95	blast;96.50 CRISPR;82.40 iPHoP-RF;65.30
S16_vs_76823	20.02	d__Bacteria;p__Firmicutes;c__Clostridia;o__Oscillospirales;f__Ruminococcaceae;g__Gemmiger	98.5	CRISPR;98.90 blast;98.30 iPHoP-RF;94.40

S16_vs_78361	75.01	d__Bacteria;p__Actinobacteriota;c__Actinomycetia;o__Actinomycetales;f__Bifidobacteriaceae;g__Bifidobacterium	98.5	CRISPR;98.90 iPHoP-RF;97.10 blast;96.90
S16_vs_78429	22.27	d__Bacteria;p__Firmicutes;c__Clostridia;o__Oscillospirales;f__Oscillospiraceae;g__ER4	91.5	blast;93.80 iPHoP-RF;52.80
S16_vs_80244	99.97	d__Bacteria;p__Bacteroidota;c__Bacteroidia;o__Bacteroidales;f__Bacteroidaceae;g__Bacteroides	99.7	iPHoP-RF;97.10 RaFAH;95.20 blast;89.20
S16_vs_80244	99.97	d__Bacteria;p__Bacteroidota;c__Bacteroidia;o__Bacteroidales;f__Bacteroidaceae;g__Phocaeicola	92.2	iPHoP-RF;94.40 blast;93.80
S16_vs_81805	99.42	d__Bacteria;p__Firmicutes;c__Clostridia;o__Oscillospirales;f__Ruminococcaceae;g__Ruthenibacterium	94.7	blast;96.30 iPHoP-RF;89.50 CRISPR;57.90
S16_vs_82116	98.74	d__Bacteria;p__Firmicutes;c__Clostridia;o__Oscillospirales;f__Acutalibacteraceae;g__Ruminococcus_E	99.6	CRISPR;98.90 blast;96.90 iPHoP-RF;94.10 RaFAH;81.90
S17_vf_128147	3.97	d__Bacteria;p__Bacteroidota;c__Bacteroidia;o__Bacteroidales;f__Bacteroidaceae;g__Bacteroides	95.9	blast;97.10 iPHoP-RF;97.10 CRISPR;89.30
S17_vf_128147	3.97	d__Bacteria;p__Bacteroidota;c__Bacteroidia;o__Bacteroidales;f__Bacteroidaceae;g__Bacteroides_G	94.5	iPHoP-RF;96.10
S17_vf_128147	3.97	d__Bacteria;p__Bacteroidota;c__Bacteroidia;o__Bacteroidales;f__Bacteroidaceae;g__Phocaeicola	91	iPHoP-RF;93.40 blast;85.40
S17_vf_140653	90.58	d__Bacteria;p__Bacteroidota;c__Bacteroidia;o__Bacteroidales;f__Bacteroidaceae;g__Bacteroides	99.7	blast;96.70 RaFAH;95.30 iPHoP-RF;85.70
S17_vf_174033	68.79	d__Bacteria;p__Firmicutes;c__Clostridia;o__Oscillospirales;f__Ruminococcaceae;g__Ruminococcus_D	99.7	blast;97.00 iPHoP-RF;95.40 RaFAH;94.60 CRISPR;72.00
S17_vf_174033	68.79	d__Bacteria;p__Firmicutes;c__Clostridia;o__Oscillospirales;f__Acutalibacteraceae;g__Ruminococcus_E	92.5	RaFAH;94.60
S17_vf_35138	84.47	d__Bacteria;p__Bacteroidota;c__Bacteroidia;o__Bacteroidales;f__Barnesiellaceae;g__Barnesiella	99.1	blast;96.80 RaFAH;88.80 CRISPR;59.60
S17_vf_36452	12	d__Bacteria;p__Firmicutes;c__Clostridia;o__Lachnospirales;f__Lachnospiraceae;g__Muricomes	97.8	CRISPR;98.40 iPHoP-RF;90.80
S17_vf_46388	50.05	d__Bacteria;p__Firmicutes;c__Clostridia;o__Lachnospirales;f__Lachnospiraceae;g__Blautia_A	99.4	CRISPR;97.10 blast;94.50 iPHoP-RF;93.70 RaFAH;91.90
S17_vf_81232	50.04	d__Bacteria;p__Bacteroidota;c__Bacteroidia;o__Bacteroidales;f__Tannerellaceae;g__Parabacteroides	97.8	blast;98.40 iPHoP-RF;95.10 CRISPR;83.70
S17_vf_97671	81.31	d__Bacteria;p__Firmicutes;c__Clostridia;o__Oscillospirales;f__Oscillospiraceae;g__Lawsonibacter	90.2	blast;92.70 iPHoP-RF;73.20
S17_vs_103009	22.09	d__Bacteria;p__Firmicutes;c__Clostridia;o__Oscillospirales;f__Oscillospiraceae;g__ER4	96.7	blast;97.70 iPHoP-RF;65.10
S17_vs_110666	98.39	d__Bacteria;p__Firmicutes;c__Clostridia;o__Oscillospirales;f__Ruminococcaceae;g__Faecalibacterium	100	RaFAH;99.70 blast;96.80 iPHoP-RF;93.40 CRISPR;67.80
S17_vs_116640	83.12	d__Bacteria;p__Bacteroidota;c__Bacteroidia;o__Bacteroidales;f__Bacteroidaceae;g__Bacteroides	91	RaFAH;93.40
S17_vs_117507	40.08	d__Bacteria;p__Bacteroidota;c__Bacteroidia;o__Bacteroidales;f__Bacteroidaceae;g__Bacteroides	97.5	blast;97.40 iPHoP-RF;96.40 RaFAH;66.20
S17_vs_127519	61.22	d__Bacteria;p__Firmicutes;c__Clostridia;o__Oscillospirales;f__Oscillospiraceae;g__Dysosmobacter	91.2	blast;93.60 iPHoP-RF;86.70
S17_vs_157209	25.09	d__Bacteria;p__Bacteroidota;c__Bacteroidia;o__Bacteroidales;f__Rikenellaceae;g__Alistipes	97.6	CRISPR;98.30 blast;95.20 iPHoP-RF;92.80
S17_vs_160502	60.44	d__Bacteria;p__Bacteroidota;c__Bacteroidia;o__Bacteroidales;f__Bacteroidaceae;g__Bacteroides	99.9	iPHoP-RF;97.10 RaFAH;96.60 blast;92.60 CRISPR;56.20
S17_vs_160502	60.44	d__Bacteria;p__Bacteroidota;c__Bacteroidia;o__Bacteroidales;f__Bacteroidaceae;g__Phocaeicola	95.9	iPHoP-RF;97.10 blast;84.10
S17_vs_177196	18.1	d__Bacteria;p__Bacteroidota;c__Bacteroidia;o__Bacteroidales;f__Rikenellaceae;g__Alistipes	96.4	blast;91.90 iPHoP-RF;88.50 RaFAH;84.30
S17_vs_177203	71.95	d__Bacteria;p__Desulfobacterota;c__Desulfovibrionia;o__Desulfovibrionales;f__Desulfovibrionaceae;g__Bilophila	93.9	blast;95.70 iPHoP-RF;90.10
S17_vs_2079	67.97	d__Bacteria;p__Bacteroidota;c__Bacteroidia;o__Bacteroidales;f__Bacteroidaceae;g__Bacteroides	100	RaFAH;98.90 iPHoP-RF;94.40 blast;92.60
S17_vs_31654	36.85	d__Bacteria;p__Firmicutes;c__Bacilli;o__Erysipelotrichales;f__Erysipelatoclostridiaceae;g__Faecalibacillus	94.6	blast;96.20 iPHoP-RF;81.60 CRISPR;54.30
S17_vs_39515	82.52	d__Bacteria;p__Bacteroidota;c__Bacteroidia;o__Bacteroidales;f__Bacteroidaceae;g__Bacteroides	100	RaFAH;99.30 iPHoP-RF;97.10 blast;92.90
S17_vs_39515	82.52	d__Bacteria;p__Bacteroidota;c__Bacteroidia;o__Bacteroidales;f__Bacteroidaceae;g__Phocaeicola	95.3	iPHoP-RF;96.70
S17_vs_43307	72.14	d__Bacteria;p__Bacteroidota;c__Bacteroidia;o__Bacteroidales;f__Tannerellaceae;g__Parabacteroides	95.4	blast;96.80 CRISPR;96.70 iPHoP-RF;93.40
S17_vs_8690	6.78	d__Bacteria;p__Proteobacteria;c__Alphaproteobacteria;o__RF32;f__CAG-239;g__CAG-495	95.2	blast;96.60
S17_vs_8690	6.78	d__Bacteria;p__Proteobacteria;c__Alphaproteobacteria;o__RF32;f__CAG-239;g__Scatocola	94.9	blast;96.40 iPHoP-RF;59.50
S1_vf_136526	33.32	d__Bacteria;p__Firmicutes;c__Clostridia;o__Oscillospirales;f__Oscillospiraceae;g__CAG-170	91.6	blast;93.90 iPHoP-RF;88.50
S1_vf_158961	9.7	d__Bacteria;p__Firmicutes;c__Clostridia;o__Christensenellales;f__CAG-74;g__UBA11524	95	blast;96.50 iPHoP-RF;77.30
S1_vf_159951	22.14	d__Bacteria;p__Bacteroidota;c__Bacteroidia;o__Bacteroidales;f__Rikenellaceae;g__Alistipes	95.4	CRISPR;96.80 iPHoP-RF;72.20
S1_vf_22149	43.65	d__Bacteria;p__Firmicutes;c__Clostridia;o__Oscillospirales;f__Oscillospiraceae;g__ER4	90.8	blast;93.20 iPHoP-RF;88.90
S1_vf_28398	12.4	d__Bacteria;p__Firmicutes;c__Clostridia;o__Oscillospirales;f__Ruminococcaceae;g__Gemmiger	91.6	blast;93.90 iPHoP-RF;75.40
S1_vf_6272	25.47	d__Bacteria;p__Firmicutes;c__Clostridia;o__Oscillospirales;f__Oscillospiraceae;g__CAG-170	92	CRISPR;94.20 blast;90.80 iPHoP-RF;78.00
S1_vs_117317	21.23	d__Bacteria;p__Firmicutes;c__Clostridia;o__Oscillospirales;f__Oscillospiraceae;g__CAG-170	93.4	blast;95.30 iPHoP-RF;89.50 CRISPR;71.10
S1_vs_133577	37.09	d__Bacteria;p__Firmicutes;c__Clostridia;o__Oscillospirales;f__Ruminococcaceae;g__Gemmiger	96	blast;97.20 CRISPR;95.40 iPHoP-RF;94.40
S1_vs_143929	42.31	d__Bacteria;p__Firmicutes;c__Clostridia;o__Oscillospirales;f__Oscillospiraceae;g__ER4	91.6	blast;93.90 iPHoP-RF;75.00
S1_vs_145792	13.59	d__Bacteria;p__Firmicutes;c__Clostridia;o__Lachnospirales;f__Anaerotignaceae;g__Anaerotignum	94.6	blast;96.20 iPHoP-RF;72.60
S1_vs_155183	31.14	d__Bacteria;p__Firmicutes;c__Clostridia;o__Oscillospirales;f__Oscillospiraceae;g__ER4	95.9	CRISPR;97.10 blast;96.20 iPHoP-RF;89.20
S1_vs_23682	33.26	d__Bacteria;p__Firmicutes;c__Clostridia;o__Oscillospirales;f__Ruminococcaceae;g__Faecalibacterium	98.8	CRISPR;99.10 blast;96.20 iPHoP-RF;83.30
S2_vf_16829	81.02	d__Bacteria;p__Actinobacteriota;c__Coriobacteriia;o__Coriobacteriales;f__Eggerthellaceae;g__Eggerthella	94.9	CRISPR;96.40 iPHoP-RF;53.80 RaFAH;50.20
S2_vf_17850	11.78	d__Bacteria;p__Firmicutes;c__Clostridia;o__Lachnospirales;f__Lachnospiraceae;g__Muricomes	92.7	CRISPR;94.80 iPHoP-RF;78.70
S2_vf_20910	5.26	d__Bacteria;p__Firmicutes;c__Bacilli;o__Erysipelotrichales;f__Erysipelotrichaceae;g__Clostridium_AQ	97.2	CRISPR;98.00
S2_vs_10289	67.97	d__Bacteria;p__Bacteroidota;c__Bacteroidia;o__Bacteroidales;f__Bacteroidaceae;g__Bacteroides	100	RaFAH;98.90 blast;96.80 iPHoP-RF;87.90
S2_vs_7306	96.01	d__Bacteria;p__Bacteroidota;c__Bacteroidia;o__Bacteroidales;f__Tannerellaceae;g__Parabacteroides	93	blast;95.00 iPHoP-RF;73.70
S2_vs_7306	96.01	d__Bacteria;p__Bacteroidota;c__Bacteroidia;o__Bacteroidales;f__Marinifilaceae;g__Odoribacter	90	RaFAH;89.40 CRISPR;64.40
S3_vf_100440	98.85	d__Bacteria;p__Firmicutes;c__Clostridia;o__Clostridiales;f__Clostridiaceae;g__Clostridium	94.7	RaFAH;96.30
S3_vf_100440	98.85	d__Bacteria;p__Firmicutes;c__Clostridia;o__Clostridiales;f__Clostridiaceae;g__Clostridium_F	94.7	RaFAH;96.30
S3_vf_100440	98.85	d__Bacteria;p__Firmicutes;c__Clostridia;o__Clostridiales;f__Clostridiaceae;g__Clostridium_P	94.7	RaFAH;96.30
S3_vf_101611	23.54	d__Bacteria;p__Firmicutes;c__Clostridia;o__Monoglobales;f__Monoglobaceae;g__Monoglobus	95.2	blast;96.60
S3_vf_106401	12.56	d__Bacteria;p__Firmicutes;c__Bacilli;o__Haloplasmatales_A;f__Turicibacteraceae;g__Turicibacter	92	blast;94.20 iPHoP-RF;66.30
S3_vf_108959	42.95	d__Bacteria;p__Firmicutes;c__Clostridia;o__Lachnospirales;f__Lachnospiraceae;g__Lachnospira	99.3	CRISPR;99.30 blast;91.90 iPHoP-RF;88.90 RaFAH;55.00
S3_vf_111893	41.65	d__Bacteria;p__Firmicutes;c__Clostridia;o__Oscillospirales;f__Ruminococcaceae;g__Ruminococcus_D	95.4	blast;96.80 CRISPR;96.10 iPHoP-RF;94.10
S3_vf_11428	94.85	d__Bacteria;p__Firmicutes;c__Clostridia;o__Lachnospirales;f__Lachnospiraceae;g__Blautia_A	100	RaFAH;98.90 CRISPR;97.00 iPHoP-RF;93.40 blast;91.70
S3_vf_11428	94.85	d__Bacteria;p__Firmicutes;c__Clostridia;o__Lachnospirales;f__Lachnospiraceae;g__Blautia	98.5	RaFAH;98.90
S3_vf_115304	34.34	d__Bacteria;p__Spirochaetota;c__Spirochaetia;o__Sphaerochaetales;f__Sphaerochaetaceae;g__UBA9732	91.2	blast;93.60 iPHoP-RF;81.90
S3_vf_127392	52.43	d__Bacteria;p__Firmicutes;c__Clostridia;o__Oscillospirales;f__CAG-272;g__CAG-272	92.1	blast;94.30 iPHoP-RF;52.20
S3_vf_127399	23.09	d__Bacteria;p__Bacteroidota;c__Bacteroidia;o__Bacteroidales;f__Bacteroidaceae;g__Bacteroides	90.4	RaFAH;92.90
S3_vf_127400	54.09	d__Bacteria;p__Firmicutes;c__Clostridia;o__Oscillospirales;f__Oscillospiraceae;g__ER4	95	blast;96.50 iPHoP-RF;76.40 CRISPR;67.10
S3_vf_127405	98.09	d__Bacteria;p__Firmicutes;c__Clostridia;o__Lachnospirales;f__Lachnospiraceae;g__Lachnospira	99.9	CRISPR;98.90 RaFAH;91.60 iPHoP-RF;90.80 blast;80.40
S3_vf_127405	98.09	d__Bacteria;p__Firmicutes;c__Clostridia;o__Lachnospirales;f__Lachnospiraceae;g__Eubacterium_G	96.9	RaFAH;91.60 blast;87.20
S3_vf_127406	89.48	d__Bacteria;p__Bacteroidota;c__Bacteroidia;o__Bacteroidales;f__Rikenellaceae;g__Alistipes	97.8	RaFAH;98.40
S3_vf_127406	89.48	d__Bacteria;p__Bacteroidota;c__Bacteroidia;o__Bacteroidales;f__Rikenellaceae;g__Alistipes_A	97.8	RaFAH;98.40
S3_vf_127406	89.48	d__Bacteria;p__Bacteroidota;c__Bacteroidia;o__Bacteroidales;f__Bacteroidaceae;g__UBA6398	91.6	blast;93.90 iPHoP-RF;84.80
S3_vf_127408	6.08	d__Bacteria;p__Actinobacteriota;c__Actinomycetia;o__Actinomycetales;f__Bifidobacteriaceae;g__Bifidobacterium	98.5	blast;98.90 CRISPR;97.60 iPHoP-RF;96.70
S3_vf_127409	96.84	d__Bacteria;p__Firmicutes;c__Clostridia;o__Oscillospirales;f__Ruminococcaceae;g__Faecalibacterium	95.9	CRISPR;97.00 blast;95.70 iPHoP-RF;81.10 RaFAH;51.90
S3_vf_127450	8.34	d__Bacteria;p__Bacteroidota;c__Bacteroidia;o__Bacteroidales;f__Bacteroidaceae;g__Prevotella	92.3	RaFAH;94.50
S3_vf_18080	96.93	d__Bacteria;p__Bacteroidota;c__Bacteroidia;o__Bacteroidales;f__Bacteroidaceae;g__Bacteroides	99.7	iPHoP-RF;97.10 blast;96.10 RaFAH;94.50 CRISPR;81.40
S3_vf_18080	96.93	d__Bacteria;p__Bacteroidota;c__Bacteroidia;o__Bacteroidales;f__Bacteroidaceae;g__Phocaeicola	91.8	iPHoP-RF;94.10 CRISPR;80.00 blast;78.90
S3_vf_18829	63.94	d__Bacteria;p__Bacteroidota;c__Bacteroidia;o__Bacteroidales;f__Bacteroidaceae;g__Bacteroides	91.8	iPHoP-RF;94.10 CRISPR;73.10

S3_vf_23042	83.69	d__Bacteria;p__Firmicutes;c__Bacilli;o__Lactobacillales;f__Streptococcaceae;g__Lactococcus	99.7	blast;98.70 iPHoP-RF;90.10 RaFAH;89.50
S3_vf_44405	96.43	d__Bacteria;p__Actinobacteriota;c__Actinomycetia;o__Actinomycetales;f__Actinomycetaceae;g__Actinomyces	100	RaFAH;97.30 CRISPR;92.40 blast;92.10 iPHoP-RF;55.70
S3_vf_44405	96.43	d__Bacteria;p__Actinobacteriota;c__Actinomycetia;o__Actinomycetales;f__Actinomycetaceae;g__Pauljensenia	96.2	RaFAH;97.30
S3_vf_54353	46.9	d__Bacteria;p__Actinobacteriota;c__Actinomycetia;o__Actinomycetales;f__Actinomycetaceae;g__Actinomyces	97.8	RaFAH;97.30 iPHoP-RF;70.60
S3_vf_54353	46.9	d__Bacteria;p__Actinobacteriota;c__Actinomycetia;o__Actinomycetales;f__Actinomycetaceae;g__Pauljensenia	96.2	RaFAH;97.30
S3_vf_5525	41.37	d__Bacteria;p__Actinobacteriota;c__Actinomycetia;o__Actinomycetales;f__Actinomycetaceae;g__Actinomyces	92.6	RaFAH;85.90 iPHoP-RF;81.10
S3_vf_56363	23.06	d__Bacteria;p__Firmicutes;c__Clostridia;o__Peptostreptococcales;f__Peptostreptococcaceae;g__GCA-900066495	91.4	iPHoP-RF;93.70
S3_vf_67333	76.26	d__Bacteria;p__Firmicutes;c__Clostridia;o__Lachnospirales;f__Lachnospiraceae;g__Agathobacter	99.9	CRISPR;98.90 RaFAH;90.80 blast;85.70 iPHoP-RF;81.90
S3_vf_67333	76.26	d__Bacteria;p__Firmicutes;c__Clostridia;o__Lachnospirales;f__Lachnospiraceae;g__Roseburia	99.1	iPHoP-RF;96.10 RaFAH;90.80 blast;90.50
S3_vf_67333	76.26	d__Bacteria;p__Firmicutes;c__Clostridia;o__Lachnospirales;f__Lachnospiraceae;g__RUG115	99	RaFAH;90.80 blast;90.70 iPHoP-RF;77.50
S3_vf_67333	76.26	d__Bacteria;p__Firmicutes;c__Clostridia;o__Lachnospirales;f__Lachnospiraceae;g__Lachnospira	98.7	CRISPR;99.00 blast;90.70 iPHoP-RF;89.80
S3_vf_67333	76.26	d__Bacteria;p__Firmicutes;c__Clostridia;o__Lachnospirales;f__Lachnospiraceae;g__Eubacterium_F	90.4	blast;92.90
S3_vf_67828		d__Bacteria;p__Bacteroidota;c__Bacteroidia;o__Bacteroidales;f__Rikenellaceae;g__Alistipes	91.5	blast;93.80 iPHoP-RF;64.50
S3_vf_70746	87.64	d__Bacteria;p__Actinobacteriota;c__Actinomycetia;o__Actinomycetales;f__Bifidobacteriaceae;g__Bifidobacterium	95.3	iPHoP-RF;96.70 blast;95.90 CRISPR;78.50
S3_vf_7137	5.31	d__Bacteria;p__Actinobacteriota;c__Actinomycetia;o__Actinomycetales;f__Bifidobacteriaceae;g__Bifidobacterium	99.7	CRISPR;99.70 iPHoP-RF;77.30
S3_vf_73597	55.3	d__Bacteria;p__Bacteroidota;c__Bacteroidia;o__Bacteroidales;f__Bacteroidaceae;g__Bacteroides	99.7	CRISPR;97.20 iPHoP-RF;97.10 RaFAH;93.80 blast;93.80
S3_vf_73597	55.3	d__Bacteria;p__Bacteroidota;c__Bacteroidia;o__Bacteroidales;f__Bacteroidaceae;g__Phocaeicola	95.3	iPHoP-RF;96.70
S3_vf_80287	52.73	d__Bacteria;p__Firmicutes;c__Clostridia;o__Oscillospirales;f__Ruminococcaceae;g__D5	97.2	CRISPR;98.00 iPHoP-RF;52.20
S3_vf_8058	98.11	d__Bacteria;p__Firmicutes;c__Clostridia;o__Lachnospirales;f__Lachnospiraceae;g__Blautia_A	98.1	CRISPR;98.60 iPHoP-RF;81.60
S3_vf_8164	10.61	d__Bacteria;p__Firmicutes;c__Clostridia;o__Oscillospirales;f__Ruminococcaceae;g__Ruminococcus_D	98.2	CRISPR;98.70 blast;96.80 iPHoP-RF;95.10
S3_vf_82571	21.78	d__Bacteria;p__Firmicutes;c__Clostridia;o__Oscillospirales;f__Oscillospiraceae;g__CAG-170	93.1	blast;95.10 iPHoP-RF;73.20
S3_vf_99626	26.68	d__Bacteria;p__Actinobacteriota;c__Coriobacteriia;o__Coriobacteriales;f__Coriobacteriaceae;g__Collinsella	98.7	CRISPR;99.00 iPHoP-RF;97.10 blast;92.10
S3_vf_99734	99.18	d__Bacteria;p__Firmicutes;c__Clostridia;o__Oscillospirales;f__Ruminococcaceae;g__Faecalibacterium	95.2	CRISPR;96.60 iPHoP-RF;64.80 blast;60.50
S3_vf_99734	99.18	d__Bacteria;p__Firmicutes;c__Clostridia;o__Oscillospirales;f__Oscillospiraceae;g__CAG-83	92	RaFAH;94.20
S3_vf_99734	99.18	d__Bacteria;p__Firmicutes;c__Clostridia;o__Oscillospirales;f__Oscillospiraceae;g__Dysosmobacter	92	RaFAH;94.20
S3_vs_114271	19.01	d__Bacteria;p__Firmicutes;c__Clostridia;o__Oscillospirales;f__Acutalibacteraceae;g__Acutalibacter	92.1	blast;94.30 iPHoP-RF;56.80
S3_vs_117894	81.25	d__Bacteria;p__Bacteroidota;c__Bacteroidia;o__Bacteroidales;f__Marinifilaceae;g__Odoribacter	99.9	blast;98.40 iPHoP-RF;93.10 RaFAH;92.30 CRISPR;53.10
S3_vs_119660	91.3	d__Bacteria;p__Bacteroidota;c__Bacteroidia;o__Bacteroidales;f__Bacteroidaceae;g__Bacteroides	100	RaFAH;98.50 CRISPR;95.40 iPHoP-RF;95.40 blast;93.90
S3_vs_119660	91.3	d__Bacteria;p__Bacteroidota;c__Bacteroidia;o__Bacteroidales;f__Bacteroidaceae;g__CAG-462	96.9	CRISPR;97.80
S3_vs_119660	91.3	d__Bacteria;p__Bacteroidota;c__Bacteroidia;o__Bacteroidales;f__Bacteroidaceae;g__Phocaeicola	90.6	CRISPR;93.00 blast;88.70 iPHoP-RF;75.70
S3_vs_119771	27.39	d__Bacteria;p__Firmicutes;c__Clostridia;o__Oscillospirales;f__Ruminococcaceae;g__Gemmiger	95.9	blast;97.10 iPHoP-RF;81.40 CRISPR;55.60
S3_vs_122706	89.75	d__Bacteria;p__Firmicutes;c__Clostridia;o__Oscillospirales;f__Acutalibacteraceae;g__CAG-217	95.3	blast;96.70 iPHoP-RF;79.30
S3_vs_123834	73.11	d__Bacteria;p__Actinobacteriota;c__Actinomycetia;o__Actinomycetales;f__Bifidobacteriaceae;g__Bifidobacterium	98.4	CRISPR;98.40 blast;96.90 iPHoP-RF;96.70 RaFAH;61.70
S3_vs_127391	18.32	d__Bacteria;p__Firmicutes;c__Clostridia;o__Oscillospirales;f__Ruminococcaceae;g__Gemmiger	95.6	blast;96.90 CRISPR;96.30 iPHoP-RF;91.80
S3_vs_127401	45.54	d__Bacteria;p__Firmicutes;c__Clostridia;o__Lachnospirales;f__Lachnospiraceae;g__Lachnospira	90.4	blast;92.90 iPHoP-RF;70.60
S3_vs_127411	9.3	d__Bacteria;p__Firmicutes;c__Clostridia;o__Oscillospirales;f__Oscillospiraceae;g__ER4	95.2	blast;96.60 iPHoP-RF;73.20
S3_vs_127413	23.82	d__Bacteria;p__Bacteroidota;c__Bacteroidia;o__Bacteroidales;f__Rikenellaceae;g__Alistipes	97.9	CRISPR;98.50 iPHoP-RF;80.00
S3_vs_127421	95.6	d__Bacteria;p__Firmicutes;c__Clostridia;o__Lachnospirales;f__Lachnospiraceae;g__Agathobacter	100	CRISPR;98.90 RaFAH;94.40 iPHoP-RF;92.10
S3_vs_127421	95.6	d__Bacteria;p__Firmicutes;c__Clostridia;o__Lachnospirales;f__Lachnospiraceae;g__Roseburia	99.9	CRISPR;98.50 iPHoP-RF;96.40 blast;95.20 RaFAH;94.40
S3_vs_127421	95.6	d__Bacteria;p__Firmicutes;c__Clostridia;o__Lachnospirales;f__Lachnospiraceae;g__RUG115	98.2	RaFAH;94.40 iPHoP-RF;88.50
S3_vs_127421	95.6	d__Bacteria;p__Firmicutes;c__Clostridia;o__Lachnospirales;f__Lachnospiraceae;g__CAG-45	90.7	iPHoP-RF;93.10
S3_vs_127442		d__Bacteria;p__Bacteroidota;c__Bacteroidia;o__Bacteroidales;f__P3;g__Phil12	90.7	iPHoP-RF;93.10
S3_vs_15450	21.5	d__Bacteria;p__Firmicutes;c__Clostridia;o__Oscillospirales;f__Ruminococcaceae;g__Gemmiger	95.4	blast;96.80 iPHoP-RF;83.90 CRISPR;68.80
S3_vs_16825	80.45	d__Bacteria;p__Firmicutes;c__Clostridia;o__Lachnospirales;f__Lachnospiraceae;g__Roseburia	99.7	CRISPR;98.90 iPHoP-RF;96.10 blast;92.80 RaFAH;88.00
S3_vs_16825	80.45	d__Bacteria;p__Firmicutes;c__Clostridia;o__Lachnospirales;f__Lachnospiraceae;g__Agathobacter	99.4	CRISPR;98.10 iPHoP-RF;93.40 RaFAH;88.00
S3_vs_16825	80.45	d__Bacteria;p__Firmicutes;c__Clostridia;o__Lachnospirales;f__Lachnospiraceae;g__RUG115	97.2	iPHoP-RF;91.80 blast;90.60 RaFAH;88.00
S3_vs_17168	100	d__Bacteria;p__Bacteroidota;c__Bacteroidia;o__Bacteroidales;f__Rikenellaceae;g__Alistipes	97.5	blast;96.90 iPHoP-RF;93.10 RaFAH;71.20
S3_vs_18858	82.07	d__Bacteria;p__Firmicutes;c__Clostridia;o__Oscillospirales;f__Acutalibacteraceae;g__CAG-217	97.9	blast;98.50 iPHoP-RF;86.30 CRISPR;68.10
S3_vs_23194	42.15	d__Bacteria;p__Bacteroidota;c__Bacteroidia;o__Bacteroidales;f__Bacteroidaceae;g__Bacteroides	100	RaFAH;98.70 iPHoP-RF;97.10
S3_vs_23194	42.15	d__Bacteria;p__Bacteroidota;c__Bacteroidia;o__Bacteroidales;f__Bacteroidaceae;g__Phocaeicola	95.3	iPHoP-RF;96.70 blast;93.10 CRISPR;75.20
S3_vs_25896	11.85	d__Bacteria;p__Firmicutes;c__Clostridia;o__Oscillospirales;f__Ruminococcaceae;g__Ruthenibacterium	91.4	blast;93.70 iPHoP-RF;78.30
S3_vs_29077	34.15	d__Bacteria;p__Firmicutes;c__Clostridia;o__Oscillospirales;f__Ruminococcaceae;g__Ruminococcus_C	94.9	blast;96.40 iPHoP-RF;80.00 CRISPR;51.40
S3_vs_39774	100	d__Bacteria;p__Bacteroidota;c__Bacteroidia;o__Bacteroidales;f__Bacteroidaceae;g__Bacteroides	100	RaFAH;99.00 blast;92.00 iPHoP-RF;53.80
S3_vs_39940	5.41	d__Bacteria;p__Actinobacteriota;c__Actinomycetia;o__Actinomycetales;f__Bifidobacteriaceae;g__Bifidobacterium	95.9	iPHoP-RF;97.10 blast;96.90 CRISPR;84.00
S3_vs_40939	23.65	d__Bacteria;p__Bacteroidota;c__Bacteroidia;o__Bacteroidales;f__Bacteroidaceae;g__Bacteroides	99.3	iPHoP-RF;97.10 blast;96.60 RaFAH;89.70
S3_vs_40939	23.65	d__Bacteria;p__Bacteroidota;c__Bacteroidia;o__Bacteroidales;f__Bacteroidaceae;g__Phocaeicola	93.9	iPHoP-RF;95.70 blast;86.80
S3_vs_42569	73.75	d__Bacteria;p__Firmicutes;c__Clostridia;o__Oscillospirales;f__Ruminococcaceae;g__Faecalibacterium	99.7	CRISPR;98.30 blast;96.90 RaFAH;90.40 iPHoP-RF;73.70
S3_vs_45147	90.75	d__Bacteria;p__Bacteroidota;c__Bacteroidia;o__Bacteroidales;f__Bacteroidaceae;g__Bacteroides	99.4	iPHoP-RF;97.10 RaFAH;91.20
S3_vs_45147	90.75	d__Bacteria;p__Bacteroidota;c__Bacteroidia;o__Bacteroidales;f__Bacteroidaceae;g__Phocaeicola	95.9	iPHoP-RF;97.10 CRISPR;60.90
S3_vs_49835	13.1	d__Bacteria;p__Firmicutes;c__Clostridia;o__Oscillospirales;f__Acutalibacteraceae;g__Ruminococcus_E	99	CRISPR;99.20 blast;98.60 iPHoP-RF;93.10
S3_vs_50557	23.06	d__Bacteria;p__Actinobacteriota;c__Coriobacteriia;o__Coriobacteriales;f__Eggerthellaceae;g__Gordonibacter	93.4	CRISPR;95.30 blast;93.90 iPHoP-RF;93.10
S3_vs_50924	12.69	d__Bacteria;p__Cyanobacteria;c__Vampirovibrionia;o__Gastranaerophilales;f__Gastranaerophilaceae;g__QAMI01	90.4	blast;92.90 iPHoP-RF;57.10
S3_vs_51931	21.18	d__Bacteria;p__Proteobacteria;c__Gammaproteobacteria;o__Burkholderiales;f__Burkholderiaceae;g__Sutterella	94.6	CRISPR;96.20 iPHoP-RF;79.00
S3_vs_54303	46.83	d__Bacteria;p__Firmicutes;c__Clostridia;o__Oscillospirales;f__Oscillospiraceae;g__	91.6	blast;93.90 iPHoP-RF;80.30
S3_vs_56292	99.95	d__Bacteria;p__Bacteroidota;c__Bacteroidia;o__Bacteroidales;f__Rikenellaceae;g__Alistipes	99.9	blast;96.90 RaFAH;96.10 iPHoP-RF;83.60
S3_vs_56292	99.95	d__Bacteria;p__Bacteroidota;c__Bacteroidia;o__Bacteroidales;f__Rikenellaceae;g__Alistipes_A	94.5	RaFAH;96.10
S3_vs_64424	98.41	d__Bacteria;p__Bacteroidota;c__Bacteroidia;o__Bacteroidales;f__Bacteroidaceae;g__Bacteroides	100	RaFAH;99.60 iPHoP-RF;97.10 blast;92.20 CRISPR;69.50
S3_vs_64424	98.41	d__Bacteria;p__Bacteroidota;c__Bacteroidia;o__Bacteroidales;f__Bacteroidaceae;g__Phocaeicola	95.4	blast;96.80 iPHoP-RF;96.40 CRISPR;92.60
S3_vs_6604	75.2	d__Bacteria;p__Bacteroidota;c__Bacteroidia;o__Bacteroidales;f__Marinifilaceae;g__Butyricimonas	95.4	blast;96.80 iPHoP-RF;65.60
S3_vs_68031	27.92	d__Bacteria;p__Firmicutes;c__Clostridia;o__Oscillospirales;f__Ruminococcaceae;g__Faecalibacterium	95.6	blast;96.90 iPHoP-RF;73.20 CRISPR;56.90
S3_vs_70508	99.66	d__Bacteria;p__Bacteroidota;c__Bacteroidia;o__Bacteroidales;f__Bacteroidaceae;g__Bacteroides	100	RaFAH;99.70 iPHoP-RF;93.10 blast;92.70
S3_vs_73361	17.97	d__Bacteria;p__Firmicutes;c__Clostridia;o__Christensenellales;f__CAG-138;g__SFEL01	96.6	CRISPR;97.60 blast;96.40 iPHoP-RF;91.10
S3_vs_77156		d__Bacteria;p__Bacteroidota;c__Bacteroidia;o__Bacteroidales;f__Rikenellaceae;g__Alistipes	96.7	CRISPR;97.70 iPHoP-RF;58.40
S3_vs_80151	97.75	d__Bacteria;p__Bacteroidota;c__Bacteroidia;o__Bacteroidales;f__Tannerellaceae;g__Parabacteroides	100	RaFAH;99.60 iPHoP-RF;94.40 blast;93.80 CRISPR;92.60
S3_vs_82895	92.58	d__Bacteria;p__Bacteroidota;c__Bacteroidia;o__Bacteroidales;f__Tannerellaceae;g__Parabacteroides	100	CRISPR;99.00 RaFAH;98.20 blast;93.90 iPHoP-RF;93.70
S3_vs_87728	5.42	d__Bacteria;p__Firmicutes;c__Clostridia;o__Oscillospirales;f__Ruminococcaceae;g__Faecalibacterium	95.3	blast;96.70 iPHoP-RF;67.00

S3_vs_89485	73.42	d__Bacteria;p__Bacteroidota;c__Bacteroidia;o__Bacteroidales;f__Rikenellaceae;g__Alistipes	95 blast;96.50 iPHoP-RF;93.40
S3_vs_90331	99.17	d__Bacteria;p__Firmicutes;c__Clostridia;o__Oscillospirales;f__Ruminococcaceae;g__Faecalibacterium	100 RaFAH;98.70 CRISPR;97.70 blast;92.00 iPHoP-RF;85.70
S3_vs_90341	11.42	d__Bacteria;p__Firmicutes;c__Clostridia;o__Christensenellales;f__CAG-74;g__UBA11524	95.2 blast;96.60 CRISPR;94.50 iPHoP-RF;85.70
S3_vs_90341	11.42	d__Bacteria;p__Firmicutes;c__Clostridia;o__Christensenellales;f__CAG-74;g__UBA11524	94.9 blast;96.40 iPHoP-RF;85.10
S3_vs_9158	99.96	d__Bacteria;p__Bacteroidota;c__Bacteroidia;o__Bacteroidales;f__Bacteroidaceae;g__Bacteroides	100 RaFAH;99.60 iPHoP-RF;97.10 blast;96.90
S3_vs_9158	99.96	d__Bacteria;p__Bacteroidota;c__Bacteroidia;o__Bacteroidales;f__Bacteroidaceae;g__Phocaeicola	95.3 iPHoP-RF;96.70 blast;90.50
S3_vs_94577	45.75	d__Bacteria;p__Firmicutes;c__Bacilli;o__Erysipelotrichales;f__Erysipelatoclostridiaceae;g__Faecalibacillus	97.8 blast;98.40
S3_vs_95574	22.37	d__Bacteria;p__Firmicutes;c__Clostridia;o__Oscillospirales;f__Acutalibacteraceae;g__UBA737	90.4 blast;92.90 iPHoP-RF;70.60
S3_vs_97827	10.17	d__Bacteria;p__Bacteroidota;c__Bacteroidia;o__Bacteroidales;f__Marinifilaceae;g__Odoribacter	97.9 blast;98.50 iPHoP-RF;90.10 CRISPR;71.50
S3_vs_98675	8.18	d__Bacteria;p__Firmicutes;c__Clostridia;o__Clostridiales;f__Clostridiaceae;g__Clostridium	98.5 blast;96.40 RaFAH;85.10 iPHoP-RF;79.00
S4_vf_10506	17.1	d__Bacteria;p__Firmicutes;c__Bacilli;o__Erysipelotrichales;f__Erysipelotrichaceae;g__Holdemanella	90.7 blast;93.10
S4_vf_11308	96.94	d__Bacteria;p__Firmicutes;c__Clostridia;o__Oscillospirales;f__Acutalibacteraceae;g__Ruminococcus_E	100 CRISPR;98.90 blast;98.50 RaFAH;97.10 iPHoP-RF;93.70
S4_vf_11308	96.94	d__Bacteria;p__Firmicutes;c__Clostridia;o__Oscillospirales;f__Ruminococcaceae;g__Ruminococcus_D	95.9 RaFAH;97.10
S4_vf_12614	80.07	d__Bacteria;p__Bacteroidota;c__Bacteroidia;o__Bacteroidales;f__Barnesiellaceae;g__Barnesiella	98.7 blast;99.00 iPHoP-RF;76.10 CRISPR;67.90
S4_vf_12624	24.52	d__Bacteria;p__Actinobacteriota;c__Actinomycetia;o__Actinomycetales;f__Bifidobacteriaceae;g__Bifidobacterium	97.5 CRISPR;98.20 blast;96.50 iPHoP-RF;91.80
S4_vf_13353	93.68	d__Bacteria;p__Firmicutes;c__Clostridia;o__Lachnospirales;f__Lachnospiraceae;g__Blautia_A	99.9 CRISPR;97.90 RaFAH;95.20 blast;90.90 iPHoP-RF;89.20
S4_vf_13353	93.68	d__Bacteria;p__Firmicutes;c__Clostridia;o__Lachnospirales;f__Lachnospiraceae;g__Blautia	93.2 RaFAH;95.20
S4_vf_18803	47.59	d__Bacteria;p__Bacteroidota;c__Bacteroidia;o__Bacteroidales;f__Bacteroidaceae;g__Prevotella	98.5 CRISPR;98.90 iPHoP-RF;74.10
S4_vf_20440	44.47	d__Bacteria;p__Bacteroidota;c__Bacteroidia;o__Bacteroidales;f__Bacteroidaceae;g__Prevotella	97.8 blast;98.40 iPHoP-RF;84.20
S4_vf_30103	5.83	d__Bacteria;p__Actinobacteriota;c__Actinomycetia;o__Actinomycetales;f__Bifidobacteriaceae;g__Bifidobacterium	99.7 CRISPR;99.70 iPHoP-RF;92.10
S4_vf_31973	23.92	d__Bacteria;p__Patescibacteria;c__Paceibacteria;o__UBA9983_A;f__UBA1006;g__01-FULL-36-10b	92.2 iPHoP-RF;94.40
S4_vf_31978	62.52	d__Bacteria;p__Firmicutes;c__Clostridia;o__Lachnospirales;f__Lachnospiraceae;g__Lachnospira	99.6 CRISPR;99.60 blast;93.90 iPHoP-RF;92.40
S4_vf_31979	24.1	d__Bacteria;p__Firmicutes;c__Clostridia;o__Christensenellales;f__CAG-74;g__Ventricola	92 blast;94.20
S4_vf_31979	24.1	d__Bacteria;p__Firmicutes;c__Clostridia;o__Christensenellales;f__CAG-74;g__SFFH01	91.5 blast;93.80
S4_vf_31981	29.33	d__Bacteria;p__Bacteroidota;c__Bacteroidia;o__Bacteroidales;f__Bacteroidaceae;g__Prevotella	97.3 RaFAH;98.10
S4_vf_4838	76.93	d__Bacteria;p__Bacteroidota;c__Bacteroidia;o__Bacteroidales;f__Tannerellaceae;g__Parabacteroides	97.6 blast;98.30 iPHoP-RF;96.10 CRISPR;71.50
S4_vf_4891	28.59	d__Bacteria;p__Bacteroidota;c__Bacteroidia;o__Bacteroidales;f__Bacteroidaceae;g__Prevotella	94.6 blast;96.20 CRISPR;93.50 iPHoP-RF;92.10
S4_vs_VIRSorter_12831-c	21.81	d__Bacteria;p__Firmicutes;c__Clostridia;o__Lachnospirales;f__Lachnospiraceae;g__Blautia_A	91.6 blast;93.90 iPHoP-RF;85.10 CRISPR;58.50
S4_vs_VIRSorter_13190-c	96.31	d__Bacteria;p__Bacteroidota;c__Bacteroidia;o__Bacteroidales;f__Tannerellaceae;g__Parabacteroides	100 RaFAH;99.80 iPHoP-RF;94.40 blast;91.20 CRISPR;66.50
S4_vs_VIRSorter_15112-c	16.47	d__Bacteria;p__Firmicutes;c__Clostridia;o__Peptostreptococcales;f__Peptostreptococcaceae;g__GCA-900066495	91.6 blast;93.90 iPHoP-RF;75.70
S4_vs_VIRSorter_15837-c	96.3	d__Bacteria;p__Firmicutes;c__Clostridia;o__Oscillospirales;f__Oscillospiraceae;g__Flavonifractor	97.2 blast;98.00 CRISPR;89.70 iPHoP-RF;84.80
S4_vs_VIRSorter_16493-c	85.89	d__Bacteria;p__Actinobacteriota;c__Actinomycetia;o__Actinomycetales;f__Bifidobacteriaceae;g__Bifidobacterium	97.9 CRISPR;98.50 iPHoP-RF;96.40 blast;92.50
S4_vs_VIRSorter_19324-c	25.86	d__Bacteria;p__Firmicutes;c__Clostridia;o__Lachnospirales;f__Lachnospiraceae;g__Mediterraneibacter	91.4 blast;93.70 iPHoP-RF;76.40
S4_vs_VIRSorter_19324-c	25.86	d__Bacteria;p__Firmicutes;c__Clostridia;o__Lachnospirales;f__Lachnospiraceae;g__Faecalimonas	91 blast;93.40 iPHoP-RF;75.20
S4_vs_VIRSorter_27316-c	74.11	d__Bacteria;p__Firmicutes;c__Clostridia;o__Oscillospirales;f__Ruminococcaceae;g__Faecalibacterium	95.4 blast;96.80 iPHoP-RF;75.00 CRISPR;67.80
S4_vs_VIRSorter_2744-ca	9.58	d__Bacteria;p__Actinobacteriota;c__Actinomycetia;o__Actinomycetales;f__Bifidobacteriaceae;g__Bifidobacterium	96 CRISPR;97.20 iPHoP-RF;97.10 blast;96.70
S4_vs_VIRSorter_3006-ca	99.88	d__Bacteria;p__Firmicutes;c__Clostridia;o__Lachnospirales;f__Lachnospiraceae;g__Mediterraneibacter	91.6 blast;93.90
S4_vs_VIRSorter_30222-c	65.61	d__Bacteria;p__Firmicutes;c__Clostridia;o__Oscillospirales;f__Oscillospiraceae;g__Intestinimonas	91.5 blast;93.80
S4_vs_VIRSorter_31171-c	46.28	d__Bacteria;p__Firmicutes;c__Clostridia;o__Oscillospirales;f__Ruminococcaceae;g__QAMM01	90.2 blast;92.70 iPHoP-RF;54.90
S4_vs_VIRSorter_31196-c	93.39	d__Bacteria;p__Firmicutes;c__Clostridia;o__Oscillospirales;f__Oscillospiraceae;g__Flavonifractor	99.4 CRISPR;97.90 blast;96.20 iPHoP-RF;88.90 RaFAH;87.70
S4_vs_VIRSorter_31560-c	59.79	d__Bacteria;p__Firmicutes;c__Clostridia;o__Oscillospirales;f__Oscillospiraceae;g__Flavonifractor	90.3 blast;92.80 iPHoP-RF;86.30 CRISPR;64.80
S4_vs_VIRSorter_31916-c	39.89	d__Bacteria;p__Firmicutes;c__Clostridia;o__Lachnospirales;f__Lachnospiraceae;g__Mediterraneibacter	90.7 blast;93.10 iPHoP-RF;68.20
S4_vs_VIRSorter_31972-c	26.41	d__Bacteria;p__Firmicutes;c__Clostridia;o__Oscillospirales;f__Ruminococcaceae;g__Ruminococcus_C	97.3 blast;98.10 CRISPR;93.20 iPHoP-RF;90.50
S4_vs_VIRSorter_31975-c	88.33	d__Bacteria;p__Bacteroidota;c__Bacteroidia;o__Bacteroidales;f__Bacteroidaceae;g__Bacteroides	99.6 iPHoP-RF;97.10 blast;96.60 CRISPR;94.50 RaFAH;92.70
S4_vs_VIRSorter_31975-c	88.33	d__Bacteria;p__Bacteroidota;c__Bacteroidia;o__Bacteroidales;f__Bacteroidaceae;g__Phocaeicola	95.9 iPHoP-RF;97.10 blast;90.60 CRISPR;74.40
S4_vs_VIRSorter_31976-c	17.17	d__Bacteria;p__Firmicutes;c__Clostridia;o__Oscillospirales;f__Ruminococcaceae;g__Gemmiger	97.2 CRISPR;98.00 blast;96.20 iPHoP-RF;93.70
S4_vs_VIRSorter_31980-c	13.26	d__Bacteria;p__Proteobacteria;c__Gammaproteobacteria;o__Burkholderiales;f__Burkholderiaceae;g__Duodenibacillus	96.7 blast;97.70 CRISPR;83.90 iPHoP-RF;70.60
S4_vs_VIRSorter_4746-ca	5.45	d__Bacteria;p__Firmicutes;c__Clostridia;o__Lachnospirales;f__Lachnospiraceae;g__Roseburia	90.3 iPHoP-RF;92.80
S4_vs_VIRSorter_4839-ca	69.64	d__Bacteria;p__Firmicutes;c__Clostridia;o__Oscillospirales;f__Ruminococcaceae;g__Massilimaliae	91.4 blast;93.70 iPHoP-RF;75.70
S4_vs_VIRSorter_4959-ca	50.34	d__Bacteria;p__Firmicutes;c__Clostridia;o__Oscillospirales;f__Oscillospiraceae;g__ER4	94.2 blast;95.90 iPHoP-RF;82.50
S4_vs_VIRSorter_5698-ca	87.05	d__Bacteria;p__Actinobacteriota;c__Actinomycetia;o__Actinomycetales;f__Bifidobacteriaceae;g__Bifidobacterium	99 CRISPR;98.50 blast;96.80 iPHoP-RF;96.10 RaFAH;71.90
S4_vs_VIRSorter_6022-cir	97.71	d__Bacteria;p__Bacteroidota;c__Bacteroidia;o__Bacteroidales;f__Tannerellaceae;g__Marseille-P3160	98.4 CRISPR;98.80 iPHoP-RF;65.30
S4_vs_VIRSorter_6022-cir	97.71	d__Bacteria;p__Bacteroidota;c__Bacteroidia;o__Bacteroidales;f__Marinifilaceae;g__Odoribacter	97.3 RaFAH;98.10
S4_vs_VIRSorter_9913-ca	98.3	d__Bacteria;p__Bacteroidota;c__Bacteroidia;o__Bacteroidales;f__Marinifilaceae;g__Odoribacter	99.7 CRISPR;97.90 RaFAH;92.60
S5_vf_103206	3.75	d__Bacteria;p__Firmicutes;c__Clostridia;o__Oscillospirales;f__Oscillospiraceae;g__Lawsonibacter	90 blast;92.50
S5_vf_105728	44.2	d__Bacteria;p__Firmicutes;c__Clostridia;o__Lachnospirales;f__Lachnospiraceae;g__	94.5 iPHoP-RF;96.10 blast;93.40
S5_vf_105728	44.2	d__Bacteria;p__Firmicutes;c__Clostridia;o__Lachnospirales;f__Lachnospiraceae;g__UC5-1-2E3	93.1 iPHoP-RF;95.10 blast;91.90
S5_vf_116300	13.55	d__Bacteria;p__Firmicutes;c__Clostridia;o__Lachnospirales;f__Lachnospiraceae;g__Anaerostipes	95.2 blast;96.60 iPHoP-RF;90.50 CRISPR;69.90
S5_vf_134040	100	d__Bacteria;p__Firmicutes;c__Clostridia;o__Oscillospirales;f__Ruminococcaceae;g__Faecalibacterium	100 RaFAH;98.80 blast;96.50 iPHoP-RF;69.10
S5_vf_136076	43.14	d__Bacteria;p__Firmicutes;c__Clostridia;o__Oscillospirales;f__Oscillospiraceae;g__ER4	93 blast;95.00 iPHoP-RF;69.80
S5_vf_137260	6.74	d__Bacteria;p__Actinobacteriota;c__Coriobacteriia;o__Coriobacteriales;f__Coriobacteriaceae;g__Collinsella	92.9 CRISPR;94.90
S5_vf_145579	44.59	d__Bacteria;p__Bacteroidota;c__Bacteroidia;o__Bacteroidales;f__Bacteroidaceae;g__Prevotella	94.6 RaFAH;96.20
S5_vf_145583	10.99	d__Bacteria;p__Firmicutes;c__Clostridia;o__Clostridiales;f__Clostridiaceae;g__Clostridium	95.2 blast;93.60 iPHoP-RF;80.80 RaFAH;73.30 CRISPR;62.00
S5_vf_145585	9.85	d__Bacteria;p__Firmicutes;c__Clostridia;o__Lachnospirales;f__Lachnospiraceae;g__Enterocloster	91.2 blast;93.60 iPHoP-RF;91.40 CRISPR;53.80
S5_vf_145588	34.8	d__Bacteria;p__Firmicutes;c__Clostridia;o__Lachnospirales;f__Lachnospiraceae;g__Dorea_A	97.2 CRISPR;98.00 iPHoP-RF;64.30
S5_vf_145604	87.41	d__Bacteria;p__Firmicutes;c__Clostridia;o__Lachnospirales;f__Lachnospiraceae;g__Blautia_A	99.6 CRISPR;97.30 RaFAH;93.30 blast;92.90 iPHoP-RF;91.80
S5_vf_145604	87.41	d__Bacteria;p__Firmicutes;c__Clostridia;o__Lachnospirales;f__Lachnospiraceae;g__Blautia	90.9 RaFAH;93.30
S5_vf_18461	99.96	d__Bacteria;p__Bacteroidota;c__Bacteroidia;o__Bacteroidales;f__Rikenellaceae;g__Alistipes	92.7 RaFAH;92.00 blast;67.20
S5_vf_21869	31.69	d__Bacteria;p__Firmicutes;c__Clostridia;o__Oscillospirales;f__Oscillospiraceae;g__Flavonifractor	93.1 blast;95.10 iPHoP-RF;73.90
S5_vf_25423	81	d__Bacteria;p__Firmicutes;c__Bacilli;o__Lactobacillales;f__Streptococcaceae;g__Lactococcus	100 RaFAH;98.80 blast;98.50 iPHoP-RF;91.40
S5_vf_28061	20.02	d__Bacteria;p__Firmicutes;c__Bacilli;o__Haloplasmatales_A;f__Turicibacteraceae;g__Turicibacter	91.5 blast;93.80 iPHoP-RF;68.60
S5_vf_29332	12.73	d__Bacteria;p__Actinobacteriota;c__Coriobacteriia;o__Coriobacteriales;f__Coriobacteriaceae;g__Collinsella	97.3 CRISPR;98.10 blast;94.60 iPHoP-RF;79.50
S5_vf_55687	91.19	d__Bacteria;p__Firmicutes;c__Bacilli;o__Lactobacillales;f__Streptococcaceae;g__Lactococcus	90.2 RaFAH;92.70
S5_vf_60597	35.7	d__Bacteria;p__Firmicutes;c__Clostridia;o__Lachnospirales;f__Anaerotignaceae;g__Anaerotignum	93.9 blast;95.70 iPHoP-RF;84.20
S5_vf_6506	20.08	d__Bacteria;p__Firmicutes;c__Clostridia;o__Clostridiales;f__Clostridiaceae;g__Clostridium_J	90.3 blast;92.80 iPHoP-RF;70.90
S5_vf_74661	91.73	d__Bacteria;p__Proteobacteria;c__Gammaproteobacteria;o__Enterobacterales;f__Enterobacteriaceae;g__Escherichia	100 RaFAH;99.40 iPHoP-RF;97.10 blast;96.80 CRISPR;85.00

S5_vf_8808	97.29	d__Bacteria;p__Firmicutes;c__Negativicutes;o__Veillonellales;f__Veillonellaceae;g__Veillonella	100	CRISPR;98.80 RaFAH;98.60 blast;93.70 iPHoP-RF;78.30
S5_vf_8808	97.29	d__Bacteria;p__Firmicutes;c__Negativicutes;o__Veillonellales;f__Veillonellaceae;g__F0422	98.1	RaFAH;98.60
S5_vf_8808	97.29	d__Bacteria;p__Firmicutes;c__Negativicutes;o__Veillonellales;f__Veillonellaceae;g__Veillonella_A	98.1	RaFAH;98.60
S5_vf_8965	100	d__Bacteria;p__Bacteroidota;c__Bacteroidia;o__Bacteroidales;f__Bacteroidaceae;g__Bacteroides	100	RaFAH;99.60 iPHoP-RF;97.10 blast;96.30
S5_vf_8965	100	d__Bacteria;p__Bacteroidota;c__Bacteroidia;o__Bacteroidales;f__Bacteroidaceae;g__Phocaeicola	95.9	iPHoP-RF;97.10
S5_vs_10428	10.64	d__Bacteria;p__Firmicutes;c__Clostridia;o__Christensenellales;f__CAG-74;g__Alectryocaccomicrobium	91.4	blast;93.70
S5_vs_107105	21.85	d__Bacteria;p__Firmicutes;c__Clostridia;o__Oscillospirales;f__Oscillospiraceae;g__CAG-110	94.7	blast;96.30 iPHoP-RF;67.00
S5_vs_107105	21.85	d__Bacteria;p__Firmicutes;c__Clostridia;o__Oscillospirales;f__Oscillospiraceae;g__Faecousia	90.8	blast;93.20 iPHoP-RF;64.50
S5_vs_109527	23.09	d__Bacteria;p__Bacteroidota;c__Bacteroidia;o__Bacteroidales;f__Bacteroidaceae;g__Bacteroides	99	iPHoP-RF;96.40 blast;92.90 RaFAH;89.10
S5_vs_109527	23.09	d__Bacteria;p__Bacteroidota;c__Bacteroidia;o__Bacteroidales;f__Bacteroidaceae;g__Phocaeicola	92.6	iPHoP-RF;94.70 blast;89.10
S5_vs_117319	51.09	d__Bacteria;p__Firmicutes;c__Clostridia;o__Oscillospirales;f__Ruminococcaceae;g__Faecalibacterium	98.4	blast;95.50 RaFAH;86.90 CRISPR;78.90 iPHoP-RF;76.80
S5_vs_117417	13.57	d__Bacteria;p__Firmicutes;c__Clostridia;o__Oscillospirales;f__Oscillospiraceae;g__Enterenecus	90.7	blast;93.10 iPHoP-RF;70.90
S5_vs_118168	30.01	d__Bacteria;p__Firmicutes;c__Clostridia;o__Oscillospirales;f__Ruminococcaceae;g__Anaerotruncus	91	blast;93.40 iPHoP-RF;57.30
S5_vs_120079	88.37	d__Bacteria;p__Firmicutes;c__Clostridia;o__Lachnospirales;f__Lachnospiraceae;g__Roseburia	95.3	blast;96.70 iPHoP-RF;96.40 CRISPR;84.10
S5_vs_120079	88.37	d__Bacteria;p__Firmicutes;c__Clostridia;o__Lachnospirales;f__Lachnospiraceae;g__VUNI01	91	iPHoP-RF;93.40 blast;89.80
S5_vs_120584	98.83	d__Bacteria;p__Firmicutes;c__Clostridia;o__Lachnospirales;f__Lachnospiraceae;g__Lachnospira	97.5	CRISPR;98.20 blast;93.50 iPHoP-RF;88.90
S5_vs_121411	100	d__Bacteria;p__Firmicutes;c__Clostridia;o__Oscillospirales;f__Ruminococcaceae;g__Ruthenibacterium	95.3	blast;96.70 iPHoP-RF;87.60 CRISPR;68.40
S5_vs_122344	26.84	d__Bacteria;p__Firmicutes;c__Clostridia;o__Lachnospirales;f__Lachnospiraceae;g__Roseburia	97.6	CRISPR;98.30 blast;95.70 iPHoP-RF;93.10
S5_vs_122344	26.84	d__Bacteria;p__Firmicutes;c__Clostridia;o__Lachnospirales;f__Lachnospiraceae;g__Agathobacter	94	CRISPR;95.80 iPHoP-RF;73.90
S5_vs_122618	17.7	d__Bacteria;p__Firmicutes;c__Bacilli;o__Erysipelotrichales;f__Erysipelotrichaceae;g__Clostridium_AQ	95.4	blast;96.80 iPHoP-RF;71.10 CRISPR;66.50
S5_vs_123256	98.81	d__Bacteria;p__Bacteroidota;c__Bacteroidia;o__Bacteroidales;f__Rikenellaceae;g__Alistipes	100	RaFAH;97.10 blast;96.90 iPHoP-RF;96.10
S5_vs_123256	98.81	d__Bacteria;p__Bacteroidota;c__Bacteroidia;o__Bacteroidales;f__Rikenellaceae;g__Alistipes_A	95.9	RaFAH;97.10
S5_vs_124035	7.45	d__Bacteria;p__Firmicutes;c__Clostridia;o__Oscillospirales;f__Oscillospiraceae;g__Intestinimonas	91.2	blast;93.60 iPHoP-RF;53.30
S5_vs_124677	26.54	d__Bacteria;p__Firmicutes;c__Clostridia;o__Oscillospirales;f__Ruminococcaceae;g__Faecalibacterium	97	CRISPR;97.90 blast;96.90 iPHoP-RF;90.80
S5_vs_128398	22.85	d__Bacteria;p__Firmicutes;c__Clostridia;o__Oscillospirales;f__Ruminococcaceae;g__Negativibacillus	93.6	blast;95.50 iPHoP-RF;82.20
S5_vs_129970	93.93	d__Bacteria;p__Bacteroidota;c__Bacteroidia;o__Bacteroidales;f__Bacteroidaceae;g__Bacteroides	100	RaFAH;99.60 blast;92.90 iPHoP-RF;92.80
S5_vs_130032	98.82	d__Bacteria;p__Proteobacteria;c__Gammaproteobacteria;o__Enterobacterales;f__Enterobacteriaceae;g__Escherichia	100	RaFAH;99.70 blast;96.90 iPHoP-RF;91.40 CRISPR;72.80
S5_vs_130294	55.97	d__Bacteria;p__Firmicutes;c__Clostridia;o__Oscillospirales;f__Ruminococcaceae;g__Faecalibacterium	95.6	blast;96.90 iPHoP-RF;81.60
S5_vs_130958	18.11	d__Bacteria;p__Firmicutes;c__Clostridia;o__Oscillospirales;f__Ruminococcaceae;g__Anaerotruncus	94.2	blast;95.90 iPHoP-RF;70.40
S5_vs_131510	95	d__Bacteria;p__Bacteroidota;c__Bacteroidia;o__Bacteroidales;f__Bacteroidaceae;g__Bacteroides	100	RaFAH;97.50 blast;96.90 iPHoP-RF;96.40
S5_vs_13254	8.3	d__Bacteria;p__Firmicutes;c__Clostridia;o__Oscillospirales;f__Ruminococcaceae;g__Ruthenibacterium	95	blast;96.50 iPHoP-RF;85.40
S5_vs_1338	100	d__Bacteria;p__Bacteroidota;c__Bacteroidia;o__Bacteroidales;f__Rikenellaceae;g__Alistipes	100	RaFAH;97.80 blast;97.50 iPHoP-RF;76.10
S5_vs_1338	100	d__Bacteria;p__Bacteroidota;c__Bacteroidia;o__Bacteroidales;f__Rikenellaceae;g__Alistipes_A	99	RaFAH;97.80 blast;81.20
S5_vs_137154	10.76	d__Bacteria;p__Firmicutes;c__Clostridia;o__Clostridiales;f__Clostridiaceae;g__Clostridium	91.7	blast;94.00 iPHoP-RF;67.30
S5_vs_139401	12.88	d__Bacteria;p__Firmicutes;c__Clostridia;o__Lachnospirales;f__Lachnospiraceae;g__Anaerostipes	90.9	blast;93.30 CRISPR;68.40 iPHoP-RF;67.50
S5_vs_140946	9.68	d__Bacteria;p__Firmicutes;c__Clostridia;o__Oscillospirales;f__Acutalibacteraceae;g__UMGS856	93.9	blast;95.70 iPHoP-RF;76.80
S5_vs_141621	97.16	d__Bacteria;p__Firmicutes;c__Clostridia;o__Lachnospirales;f__Lachnospiraceae;g__Anaerostipes	96.4	blast;95.60 CRISPR;90.90 iPHoP-RF;88.90 RaFAH;72.10
S5_vs_142195	100	d__Bacteria;p__Bacteroidota;c__Bacteroidia;o__Bacteroidales;f__Bacteroidaceae;g__Bacteroides	99.9	RaFAH;99.80
S5_vs_142195	100	d__Bacteria;p__Bacteroidota;c__Bacteroidia;o__Bacteroidales;f__Rikenellaceae;g__Alistipes	95.6	blast;96.90 iPHoP-RF;94.10
S5_vs_145578	39.67	d__Bacteria;p__Firmicutes;c__Clostridia;o__Lachnospirales;f__Lachnospiraceae;g__Enterocloster	95.2	blast;96.60 iPHoP-RF;76.10 CRISPR;54.30
S5_vs_145581	9.66	d__Bacteria;p__Firmicutes;c__Clostridia;o__Christensenellales;f__Christensenellaceae;g__Christensenella	90.4	blast;92.90 iPHoP-RF;86.30
S5_vs_145590	39.74	d__Bacteria;p__Firmicutes;c__Clostridia;o__Lachnospirales;f__Lachnospiraceae;g__Clostridium_AP	91.4	iPHoP-RF;93.70
S5_vs_145590	39.74	d__Bacteria;p__Firmicutes;c__Clostridia;o__Lachnospirales;f__Lachnospiraceae;g__Lachnoclostridium_B	91	iPHoP-RF;93.40 blast;92.10
S5_vs_145597	20.03	d__Bacteria;p__Firmicutes;c__Clostridia;o__Christensenellales;f__NSJ-53;g__NSJ-53	94	blast;95.80 iPHoP-RF;60.10
S5_vs_145598	95.83	d__Bacteria;p__Firmicutes;c__Clostridia;o__Lachnospirales;f__Lachnospiraceae;g__Roseburia	92.1	blast;94.30 iPHoP-RF;91.80
S5_vs_145598	95.83	d__Bacteria;p__Firmicutes;c__Clostridia;o__Lachnospirales;f__Lachnospiraceae;g__Agathobacter	91	iPHoP-RF;93.40 blast;91.60 CRISPR;61.60
S5_vs_145602	37.74	d__Bacteria;p__Firmicutes;c__Clostridia;o__Lachnospirales;f__Lachnospiraceae;g__Anaerobutyricum	90.7	blast;93.10 iPHoP-RF;65.30
S5_vs_145605	17.51	d__Bacteria;p__Firmicutes;c__Clostridia;o__Christensenellales;f__CAG-74;g__SFTH01	90.3	blast;92.80 iPHoP-RF;68.20
S5_vs_145611	45.88	d__Bacteria;p__Firmicutes;c__Clostridia;o__Oscillospirales;f__Ruminococcaceae;g__Phocea	90.7	blast;93.10 iPHoP-RF;78.00
S5_vs_1572	97.34	d__Bacteria;p__Bacteroidota;c__Bacteroidia;o__Bacteroidales;f__Tannerellaceae;g__Parabacteroides	91.6	blast;93.90 iPHoP-RF;61.70
S5_vs_17520	39.12	d__Bacteria;p__Firmicutes;c__Clostridia;o__Lachnospirales;f__Lachnospiraceae;g__Hungatella	95.9	iPHoP-RF;97.10 CRISPR;88.40
S5_vs_17520	39.12	d__Bacteria;p__Firmicutes;c__Clostridia;o__Lachnospirales;f__Lachnospiraceae;g__Lacrimispora	91.4	iPHoP-RF;93.70
S5_vs_20268	22.4	d__Bacteria;p__Firmicutes;c__Clostridia;o__Oscillospirales;f__Acutalibacteraceae;g__Ruminococcus_E	98.5	CRISPR;98.90 blast;96.50 iPHoP-RF;89.50
S5_vs_20500	99.42	d__Bacteria;p__Bacteroidota;c__Bacteroidia;o__Bacteroidales;f__Rikenellaceae;g__Alistipes	95.6	blast;96.90 iPHoP-RF;96.70
S5_vs_21778	11.27	d__Bacteria;p__Firmicutes;c__Clostridia;o__Oscillospirales;f__Oscillospiraceae;g__Flavonifractor	90.4	blast;92.90 iPHoP-RF;59.20
S5_vs_28263	16.17	d__Bacteria;p__Firmicutes;c__Clostridia;o__Lachnospirales;f__Lachnospiraceae;g__Mediterraneibacter	91.6	blast;93.90 iPHoP-RF;86.30 CRISPR;58.00
S5_vs_30490	42.23	d__Bacteria;p__Firmicutes;c__Clostridia;o__Oscillospirales;f__Ruminococcaceae;g__Faecalibacterium	99.3	blast;97.10 RaFAH;90.30 iPHoP-RF;87.30 CRISPR;71.60
S5_vs_32885	19.8	d__Bacteria;p__Firmicutes;c__Clostridia;o__Oscillospirales;f__Ruminococcaceae;g__Anaerotruncus	92.3	blast;94.50 CRISPR;93.00 iPHoP-RF;81.90
S5_vs_3501	32.19	d__Bacteria;p__Firmicutes;c__Clostridia;o__Oscillospirales;f__Acutalibacteraceae;g__UBA1691	95.6	blast;96.90 iPHoP-RF;90.10 CRISPR;85.90
S5_vs_3501	32.19	d__Bacteria;p__Firmicutes;c__Clostridia;o__Oscillospirales;f__Acutalibacteraceae;g__Fimivicinus	94.5	blast;96.10 iPHoP-RF;85.40
S5_vs_40848	58.74	d__Bacteria;p__Firmicutes;c__Clostridia;o__Oscillospirales;f__Acutalibacteraceae;g__Ruminococcus_E	95.6	blast;96.90 iPHoP-RF;92.80
S5_vs_43207	30.09	d__Bacteria;p__Firmicutes;c__Clostridia;o__Oscillospirales;f__Butyricicoccaceae;g__Agathobaculum	91.1	blast;93.50 iPHoP-RF;70.60
S5_vs_46394	8.87	d__Bacteria;p__Firmicutes;c__Clostridia;o__Christensenellales;f__QAND01;g__UMGS1975	90.3	blast;92.80 iPHoP-RF;62.70
S5_vs_4737	20.64	d__Bacteria;p__Firmicutes;c__Clostridia;o__Christensenellales;f__CAG-74;g__Limiplasma	93.9	blast;95.70 iPHoP-RF;68.40
S5_vs_4737	20.64	d__Bacteria;p__Firmicutes;c__Clostridia;o__Christensenellales;f__CAG-74;g__Firm-11	91.5	blast;93.80 iPHoP-RF;73.00
S5_vs_51416	11.18	d__Bacteria;p__Firmicutes;c__Bacilli;o__Erysipelotrichales;f__Erysipelotrichaceae;g__Holdemania	90.3	blast;92.80 CRISPR;73.80 iPHoP-RF;66.50
S5_vs_58766	24.99	d__Bacteria;p__Firmicutes;c__Clostridia;o__Lachnospirales;f__Lachnospiraceae;g__Frisingicoccus	93.8	blast;95.60 iPHoP-RF;76.60
S5_vs_59798	40.34	d__Bacteria;p__Firmicutes;c__Clostridia;o__Lachnospirales;f__Lachnospiraceae;g__Faecalimonas	91.4	blast;93.70 iPHoP-RF;91.10 CRISPR;78.90
S5_vs_60507	96.47	d__Bacteria;p__Firmicutes;c__Bacilli;o__Lactobacillales;f__Streptococcaceae;g__Streptococcus	100	RaFAH;99.70 blast;92.80 iPHoP-RF;91.10 CRISPR;52.30
S5_vs_63924	98.89	d__Bacteria;p__Actinobacteriota;c__Actinomycetia;o__Actinomycetales;f__Bifidobacteriaceae;g__Bifidobacterium	99	CRISPR;97.70 iPHoP-RF;96.70 blast;95.40 RaFAH;82.40
S5_vs_6478	66.33	d__Bacteria;p__Firmicutes;c__Bacilli;o__Lactobacillales;f__Lactobacillaceae;g__Lactobacillus	100	RaFAH;99.60 iPHoP-RF;96.70 blast;95.40 CRISPR;68.80
S5_vs_6478	66.33	d__Bacteria;p__Firmicutes;c__Bacilli;o__Lactobacillales;f__Lactobacillaceae;g__Lacticaseibacillus	99.6	RaFAH;99.60
S5_vs_6478	66.33	d__Bacteria;p__Firmicutes;c__Bacilli;o__Lactobacillales;f__Lactobacillaceae;g__Ligilactobacillus	99.6	RaFAH;99.60
S5_vs_6478	66.33	d__Bacteria;p__Firmicutes;c__Bacilli;o__Lactobacillales;f__Lactobacillaceae;g__Limosilactobacillus	99.6	RaFAH;99.60
S5_vs_65490	3.66	d__Bacteria;p__Firmicutes;c__Clostridia;o__Oscillospirales;f__Oscillospiraceae;g__Flavonifractor	91.2	blast;93.60 iPHoP-RF;56.20
S5_vs_68254	58.14	d__Bacteria;p__Firmicutes;c__Clostridia;o__Lachnospirales;f__Lachnospiraceae;g__Blautia_A	93.8	blast;93.80 iPHoP-RF;81.90 RaFAH;64.30

S5_vs_69980	76.63	d__Bacteria;p__Firmicutes;c__Clostridia;o__Oscillospirales;f__Oscillospiraceae;g__Flavonifractor	95.3 blast;96.70 iPHoP-RF;96.40 CRISPR;95.20
S5_vs_72532	82.39	d__Bacteria;p__Bacteroidota;c__Bacteroidia;o__Bacteroidales;f__Bacteroidaceae;g__Bacteroides	99.4 blast;96.90 iPHoP-RF;96.70 RaFAH;92.20
S5_vs_74199	13.11	d__Bacteria;p__Firmicutes;c__Clostridia;o__Oscillospirales;f__Oscillospiraceae;g__CAG-83	90.4 blast;92.90 iPHoP-RF;92.10
S5_vs_76500	80.28	d__Bacteria;p__Bacteroidota;c__Bacteroidia;o__Bacteroidales;f__Rikenellaceae;g__Alistipes	99 blast;96.90 iPHoP-RF;94.40 RaFAH;87.40
S5_vs_77593	41.43	d__Bacteria;p__Firmicutes;c__Clostridia;o__Oscillospirales;f__Oscillospiraceae;g__CAG-103	90.6 blast;93.00 iPHoP-RF;71.70
S5_vs_79992	99.98	d__Bacteria;p__Bacteroidota;c__Bacteroidia;o__Bacteroidales;f__Rikenellaceae;g__Alistipes	100 RaFAH;98.20 blast;97.10 iPHoP-RF;96.70
S5_vs_79992	99.98	d__Bacteria;p__Bacteroidota;c__Bacteroidia;o__Bacteroidales;f__Rikenellaceae;g__Alistipes_A	97.5 RaFAH;98.20
S5_vs_80496	99.85	d__Bacteria;p__Bacteroidota;c__Bacteroidia;o__Bacteroidales;f__Bacteroidaceae;g__Bacteroides	99.7 blast;96.90 iPHoP-RF;96.10 RaFAH;95.90
S5_vs_80498	33.04	d__Bacteria;p__Firmicutes;c__Clostridia;o__Lachnospirales;f__Lachnospiraceae;g__Muricomes	93.2 blast;95.20 iPHoP-RF;90.50
S5_vs_8125	19.38	d__Bacteria;p__Firmicutes;c__Clostridia;o__Lachnospirales;f__Lachnospiraceae;g__Eubacterium_G	90.6 blast;93.00 iPHoP-RF;53.30
S5_vs_84574	8.38	d__Bacteria;p__Firmicutes;c__Clostridia;o__Oscillospirales;f__Butyricicoccaceae;g__Agathobaculum	91.6 blast;93.90 iPHoP-RF;81.10
S5_vs_84946	94.46	d__Bacteria;p__Firmicutes;c__Clostridia;o__Lachnospirales;f__Lachnospiraceae;g__Dorea_A	90.2 blast;92.70 iPHoP-RF;83.60
S5_vs_84946	94.46	d__Bacteria;p__Firmicutes;c__Clostridia;o__Lachnospirales;f__Lachnospiraceae;g__Dorea	90.1 blast;92.60 iPHoP-RF;75.20
S5_vs_850	18.78	d__Bacteria;p__Firmicutes;c__Clostridia;o__UBA1212;f__UBA1255;g__Egerieisoma	92.7 blast;94.80 iPHoP-RF;85.10
S5_vs_850	18.78	d__Bacteria;p__Firmicutes;c__Clostridia;o__UBA1212;f__UBA1255;g__UMGS1537	92.7 blast;94.80 CRISPR;80.00 iPHoP-RF;78.30
S5_vs_85065	67.47	d__Bacteria;p__Firmicutes;c__Clostridia;o__Oscillospirales;f__Ruminococcaceae;g__Ruthenibacterium	95.3 blast;96.70
S5_vs_86562	44.1	d__Bacteria;p__Verrucomicrobiota;c__Verrucomicrobiae;o__Verrucomicrobiales;f__Akkermansiaceae;g__Akkermansia	100 RaFAH;99.10 CRISPR;98.80 blast;96.90 iPHoP-RF;89.20
S5_vs_89540	26.84	d__Bacteria;p__Firmicutes;c__Bacilli;o__Lactobacillales;f__Lactobacillaceae;g__Limosilactobacillus	94.2 blast;93.40 iPHoP-RF;81.90 RaFAH;68.80
S5_vs_93839	98.83	d__Bacteria;p__Bacteroidota;c__Bacteroidia;o__Bacteroidales;f__Rikenellaceae;g__Alistipes	100 RaFAH;98.90 iPHoP-RF;96.70 blast;95.50
S5_vs_93839	98.83	d__Bacteria;p__Bacteroidota;c__Bacteroidia;o__Bacteroidales;f__Rikenellaceae;g__Alistipes_A	98.5 RaFAH;98.90
S5_vs_95307	57.8	d__Bacteria;p__Firmicutes;c__Clostridia;o__Lachnospirales;f__Lachnospiraceae;g__Blautia	92 blast;91.60 iPHoP-RF;73.20 RaFAH;65.20
S5_vs_9747	6.96	d__Bacteria;p__Proteobacteria;c__Alphaproteobacteria;o__RF32;f__CAG-239;g__CAG-267	93 blast;95.00 iPHoP-RF;69.80
S5_vs_97574	99.69	d__Bacteria;p__Firmicutes;c__Clostridia;o__Lachnospirales;f__Lachnospiraceae;g__Blautia_A	100 RaFAH;99.30 CRISPR;95.90 blast;93.60 iPHoP-RF;92.10
S5_vs_97574	99.69	d__Bacteria;p__Firmicutes;c__Clostridia;o__Lachnospirales;f__Lachnospiraceae;g__Blautia	99.1 RaFAH;99.30
S6_vf_25710	89.8	d__Bacteria;p__Verrucomicrobiota;c__Verrucomicrobiae;o__Verrucomicrobiales;f__Akkermansiaceae;g__Akkermansia	99.7 CRISPR;97.30 RaFAH;94.60 blast;93.60 iPHoP-RF;72.60
S6_vf_56486	65.79	d__Bacteria;p__Bacteroidota;c__Bacteroidia;o__Bacteroidales;f__Bacteroidaceae;g__Bacteroides	99.6 iPHoP-RF;96.10 RaFAH;94.70
S6_vs_1146	50.09	d__Bacteria;p__Firmicutes;c__Clostridia;o__Oscillospirales;f__Ruminococcaceae;g__Faecalibacterium	98.4 blast;96.90 iPHoP-RF;86.00 RaFAH;81.40
S6_vs_12945	99.74	d__Bacteria;p__Firmicutes;c__Clostridia;o__Lachnospirales;f__Lachnospiraceae;g__Roseburia	99.3 RaFAH;94.70 blast;88.50 iPHoP-RF;74.80
S6_vs_12945	99.74	d__Bacteria;p__Firmicutes;c__Clostridia;o__Lachnospirales;f__Lachnospiraceae;g__Agathobacter	97.9 RaFAH;94.70 blast;86.00
S6_vs_12945	99.74	d__Bacteria;p__Firmicutes;c__Clostridia;o__Lachnospirales;f__Lachnospiraceae;g__RUG115	96.4 RaFAH;94.70 blast;76.10
S6_vs_12945	99.74	d__Bacteria;p__Firmicutes;c__Clostridia;o__Lachnospirales;f__Lachnospiraceae;g__CAG-81	90.9 blast;93.30
S6_vs_15390	33.39	d__Bacteria;p__Bacteroidota;c__Bacteroidia;o__Bacteroidales;f__Tannerellaceae;g__Parabacteroides	90.8 blast;93.20 iPHoP-RF;92.10
S6_vs_15813	90.87	d__Bacteria;p__Firmicutes;c__Clostridia;o__Oscillospirales;f__Oscillospiraceae;g__Flavonifractor	99.3 blast;98.10 RaFAH;85.50 iPHoP-RF;85.10 CRISPR;56.80
S6_vs_29152	23.31	d__Bacteria;p__Firmicutes;c__Clostridia;o__Lachnospirales;f__Lachnospiraceae;g__Acetatifactor	91.2 blast;93.60
S6_vs_32886	6.93	d__Bacteria;p__Bacteroidota;c__Bacteroidia;o__Bacteroidales;f__Rikenellaceae;g__Alistipes_A	94.2 blast;95.90 iPHoP-RF;66.30
S6_vs_33711	86.98	d__Bacteria;p__Firmicutes;c__Clostridia;o__Oscillospirales;f__Ruminococcaceae;g__Ruthenibacterium	94 blast;95.80 iPHoP-RF;85.40 CRISPR;69.40
S6_vs_34453	77	d__Bacteria;p__Firmicutes;c__Clostridia;o__Oscillospirales;f__Ruminococcaceae;g__Faecalibacterium	93.6 blast;95.50 iPHoP-RF;64.30
S6_vs_38250	15.25	d__Bacteria;p__Firmicutes;c__Clostridia;o__Oscillospirales;f__Ruminococcaceae;g__UBA3818	94.3 blast;96.00 iPHoP-RF;76.10
S6_vs_39025	99.25	d__Bacteria;p__Firmicutes;c__Clostridia;o__Lachnospirales;f__Lachnospiraceae;g__Enterocloster	91.6 blast;93.90
S6_vs_39065	83.64	d__Bacteria;p__Proteobacteria;c__Alphaproteobacteria;o__RF32;f__CAG-239;g__CAG-267	94.6 blast;96.20 iPHoP-RF;75.20
S6_vs_44070	14.4	d__Bacteria;p__Proteobacteria;c__Alphaproteobacteria;o__RF32;f__CAG-239;g__51-20	94.7 blast;96.30 iPHoP-RF;72.60
S6_vs_46039	24.66	d__Bacteria;p__Bacteroidota;c__Bacteroidia;o__Bacteroidales;f__Bacteroidaceae;g__Bacteroides	93 CRISPR;93.00 blast;89.60 iPHoP-RF;82.50 RaFAH;64.10
S6_vs_46091	25.96	d__Bacteria;p__Firmicutes;c__Clostridia;o__Oscillospirales;f__Ruminococcaceae;g__Negativibacillus	96.6 blast;97.60 iPHoP-RF;83.30 CRISPR;69.80
S6_vs_53966	51.17	d__Bacteria;p__Firmicutes;c__Clostridia;o__Oscillospirales;f__Ruminococcaceae;g__Faecalibacterium	98.7 blast;96.30 iPHoP-RF;90.50 RaFAH;86.50
S6_vs_54606	99.52	d__Bacteria;p__Firmicutes;c__Clostridia;o__Lachnospirales;f__Lachnospiraceae;g__Blautia	97 RaFAH;91.10 blast;88.40
S6_vs_54606	99.52	d__Bacteria;p__Firmicutes;c__Clostridia;o__Lachnospirales;f__Lachnospiraceae;g__Ruminococcus_B	91.2 blast;93.60 iPHoP-RF;61.70
S6_vs_56485	14.92	d__Bacteria;p__Firmicutes;c__Bacilli;o__Erysipelotrichales;f__Erysipelotrichaceae;g__Holdemania	94.7 CRISPR;96.30 blast;94.30 iPHoP-RF;79.80
S6_vs_56490	26.23	d__Bacteria;p__Firmicutes;c__Clostridia;o__Oscillospirales;f__Acutalibacteraceae;g__UMGS1071	95.9 blast;97.10 iPHoP-RF;83.90 CRISPR;68.20
S6_vs_56491	92.92	d__Bacteria;p__Firmicutes;c__Clostridia;o__Lachnospirales;f__Lachnospiraceae;g__Sellimonas	97.5 blast;98.20 iPHoP-RF;75.20
S6_vs_56492	27.91	d__Bacteria;p__Firmicutes;c__Clostridia;o__Oscillospirales;f__Butyricicoccaceae;g__Agathobaculum	98.2 blast;98.70 iPHoP-RF;66.80
S6_vs_56496	61.48	d__Bacteria;p__Firmicutes;c__Clostridia;o__Lachnospirales;f__Lachnospiraceae;g__Marvinbryantia	91.2 blast;93.60 iPHoP-RF;65.10
S6_vs_56498	78.58	d__Bacteria;p__Firmicutes;c__Clostridia;o__Lachnospirales;f__Lachnospiraceae;g__Muricomes	91 blast;93.40 iPHoP-RF;69.80
S6_vs_9784	91.55	d__Bacteria;p__Bacteroidota;c__Bacteroidia;o__Bacteroidales;f__Bacteroidaceae;g__Bacteroides	100 RaFAH;97.10 iPHoP-RF;96.70 blast;93.90
S6_vs_9784	91.55	d__Bacteria;p__Bacteroidota;c__Bacteroidia;o__Bacteroidales;f__Bacteroidaceae;g__Phocaeicola	94.9 iPHoP-RF;96.40
S7_vf_114476	27.19	d__Bacteria;p__Firmicutes;c__Clostridia;o__Lachnospirales;f__Lachnospiraceae;g__Anaerobutyricum	94.3 blast;96.00 iPHoP-RF;74.30
S7_vf_117697	98.64	d__Bacteria;p__Bacteroidota;c__Bacteroidia;o__Bacteroidales;f__Rikenellaceae;g__Alistipes	96.9 CRISPR;97.80 iPHoP-RF;97.10 blast;96.80
S7_vf_117697	98.64	d__Bacteria;p__Bacteroidota;c__Bacteroidia;o__Bacteroidales;f__Bacteroidaceae;g__Bacteroides	92.6 RaFAH;94.70
S7_vf_134023	12.46	d__Bacteria;p__Bacteroidota;c__Bacteroidia;o__Bacteroidales;f__Rikenellaceae;g__Alistipes_A	95 blast;96.50 iPHoP-RF;54.40
S7_vf_13614	39.23	d__Bacteria;p__Firmicutes;c__Clostridia;o__Oscillospirales;f__Ruminococcaceae;g__Ruminococcus_D	95.4 blast;96.80 iPHoP-RF;95.40 CRISPR;50.20
S7_vf_138019	8.23	d__Bacteria;p__Firmicutes;c__Clostridia;o__Oscillospirales;f__Oscillospiraceae;g__Lawsonibacter	91.1 blast;93.50 iPHoP-RF;50.40
S7_vf_138069	78.93	d__Bacteria;p__Firmicutes;c__Bacilli;o__Lactobacillales;f__Streptococcaceae;g__Streptococcus	99.6 RaFAH;99.40 iPHoP-RF;67.30
S7_vf_27309	18.88	d__Bacteria;p__Bacteroidota;c__Bacteroidia;o__Bacteroidales;f__Bacteroidaceae;g__Bacteroides	96.3 CRISPR;97.40 iPHoP-RF;93.70
S7_vf_28429	86.01	d__Bacteria;p__Bacteroidota;c__Bacteroidia;o__Bacteroidales;f__Tannerellaceae;g__Parabacteroides	100 CRISPR;99.00 RaFAH;98.30 iPHoP-RF;94.10 blast;93.90
S7_vf_29941	79.47	d__Bacteria;p__Firmicutes;c__Bacilli;o__Lactobacillales;f__Streptococcaceae;g__Lactococcus	99.4 blast;97.20 RaFAH;91.90 iPHoP-RF;73.90
S7_vf_37279	64.97	d__Bacteria;p__Bacteroidota;c__Bacteroidia;o__Bacteroidales;f__Bacteroidaceae;g__Bacteroides	99.7 CRISPR;98.80 iPHoP-RF;97.10 blast;90.80 RaFAH;87.70
S7_vf_37279	64.97	d__Bacteria;p__Bacteroidota;c__Bacteroidia;o__Bacteroidales;f__Bacteroidaceae;g__Phocaeicola	98.2 CRISPR;98.70 iPHoP-RF;95.10 blast;91.00
S7_vf_37293	98.54	d__Bacteria;p__Firmicutes;c__Bacilli;o__Lactobacillales;f__Streptococcaceae;g__Streptococcus	100 RaFAH;99.60 CRISPR;99.00 iPHoP-RF;54.10
S7_vf_37293	98.54	d__Bacteria;p__Bacteroidota;c__Bacteroidia;o__Bacteroidales;f__Bacteroidaceae;g__Prevotella	90.1 blast;92.60
S7_vf_38697	61.28	d__Bacteria;p__Firmicutes;c__Bacilli;o__Erysipelotrichales;f__Erysipelatoclostridiaceae;g__Massiliomicrobiota	94.2 blast;95.90 iPHoP-RF;74.30
S7_vf_39724	99.39	d__Bacteria;p__Firmicutes;c__Clostridia;o__Oscillospirales;f__Ruminococcaceae;g__Faecalibacterium	99.4 RaFAH;99.50
S7_vf_49007	85	d__Bacteria;p__Firmicutes;c__Bacilli;o__Lactobacillales;f__Streptococcaceae;g__Streptococcus	100 CRISPR;98.70 RaFAH;97.80 iPHoP-RF;68.00
S7_vf_49485	64.81	d__Bacteria;p__Firmicutes;c__Clostridia;o__Lachnospirales;f__Lachnospiraceae;g__Anaerostipes	98.5 CRISPR;98.90 iPHoP-RF;56.20
S7_vf_5801	93.48	d__Bacteria;p__Bacteroidota;c__Bacteroidia;o__Bacteroidales;f__Bacteroidaceae;g__Bacteroides	100 RaFAH;96.60 iPHoP-RF;96.40 blast;91.00
S7_vf_5801	93.48	d__Bacteria;p__Bacteroidota;c__Bacteroidia;o__Bacteroidales;f__Bacteroidaceae;g__Phocaeicola	95 blast;96.50 iPHoP-RF;95.70 CRISPR;71.30
S7_vf_5843	19.99	d__Bacteria;p__Actinobacteriota;c__Actinomycetia;o__Actinomycetales;f__Bifidobacteriaceae;g__Bifidobacterium	96.3 blast;97.40 CRISPR;97.30 iPHoP-RF;93.40
S7_vf_60968	71.38	d__Bacteria;p__Firmicutes;c__Clostridia;o__Lachnospirales;f__Lachnospiraceae;g__Ruminococcus_B	97.8 blast;98.40 iPHoP-RF;81.40 CRISPR;66.90

S7_vf_63894	77.84	d__Bacteria;p__Firmicutes;c__Bacilli;o__Lactobacillales;f__Lactobacillaceae;g__Leuconostoc	96.2	iPHoP-RF;93.70 RaFAH;78.60
S7_vf_67910	11.86	d__Bacteria;p__Actinobacteriota;c__Coriobacteriia;o__Coriobacteriales;f__Eggerthellaceae;g__Eggerthella	94.5	blast;96.10
S7_vf_68239	31.22	d__Bacteria;p__Firmicutes;c__Bacilli;o__Lactobacillales;f__Streptococcaceae;g__Lactococcus	93.1	RaFAH;95.10
S7_vf_68546	29.45	d__Bacteria;p__Firmicutes;c__Clostridia;o__Lachnospirales;f__Lachnospiraceae;g__Faecalimonas	94.5	CRISPR;96.10 iPHoP-RF;76.10
S7_vf_68546	29.45	d__Bacteria;p__Firmicutes;c__Clostridia;o__Lachnospirales;f__Lachnospiraceae;g__Bariatricus	94	CRISPR;95.80 iPHoP-RF;84.80
S7_vf_71527	3.4	d__Bacteria;p__Firmicutes;c__Clostridia;o__Oscillospirales;f__Ruminococcaceae;g__Ruminococcus_D	93.1	iPHoP-RF;95.10 blast;94.30 CRISPR;92.20
S7_vf_72493	13.24	d__Bacteria;p__Firmicutes;c__Clostridia;o__Lachnospirales;f__Anaerotignaceae;g__UMGS1670	92.5	blast;94.60 iPHoP-RF;65.10
S7_vf_76463	42.93	d__Bacteria;p__Firmicutes;c__Clostridia;o__Oscillospirales;f__Oscillospiraceae;g__CAG-103	95.3	CRISPR;96.70
S7_vf_8332	10.6	d__Bacteria;p__Firmicutes;c__Clostridia;o__Oscillospirales;f__Ruminococcaceae;g__Ruminococcus_D	98.5	CRISPR;98.90 blast;96.20 iPHoP-RF;94.70
S7_vf_84974	91.7	d__Bacteria;p__Firmicutes;c__Clostridia;o__Oscillospirales;f__Ruminococcaceae;g__Faecalibacterium	99.9	RaFAH;98.40 blast;92.10 iPHoP-RF;63.30 CRISPR;56.10
S7_vf_89197	89.48	d__Bacteria;p__Firmicutes;c__Bacilli;o__Lactobacillales;f__Streptococcaceae;g__Lactococcus	96.6	RaFAH;95.80 iPHoP-RF;71.50
S7_vs_101751	99.91	d__Bacteria;p__Firmicutes;c__Clostridia;o__Lachnospirales;f__Lachnospiraceae;g__Blautia_A	99.7	RaFAH;94.40 blast;93.60 iPHoP-RF;76.80
S7_vs_101751	99.91	d__Bacteria;p__Firmicutes;c__Clostridia;o__Lachnospirales;f__Lachnospiraceae;g__Blautia	94.5	RaFAH;94.40 iPHoP-RF;64.80
S7_vs_103530	35.56	d__Bacteria;p__Firmicutes;c__Clostridia;o__Oscillospirales;f__Butyricicoccaceae;g__Agathobaculum	94.2	blast;95.90 iPHoP-RF;60.30
S7_vs_11081	95.36	d__Bacteria;p__Bacteroidota;c__Bacteroidia;o__Bacteroidales;f__Bacteroidaceae;g__Bacteroides	100	RaFAH;98.70 iPHoP-RF;94.70 blast;91.00
S7_vs_111831	38.83	d__Bacteria;p__Firmicutes;c__Clostridia;o__Oscillospirales;f__Ruminococcaceae;g__Gemmiger	97.2	blast;98.00 iPHoP-RF;88.90 CRISPR;82.40
S7_vs_118295	43.98	d__Bacteria;p__Firmicutes;c__Clostridia;o__Oscillospirales;f__Oscillospiraceae;g__Intestinimonas	91.7	blast;94.00 iPHoP-RF;75.40
S7_vs_118295	43.98	d__Bacteria;p__Firmicutes;c__Clostridia;o__Oscillospirales;f__Oscillospiraceae;g__Flavonifractor	91.4	blast;93.70 iPHoP-RF;80.80
S7_vs_119679	99.98	d__Bacteria;p__Bacteroidota;c__Bacteroidia;o__Bacteroidales;f__Rikenellaceae;g__Alistipes	100	blast;98.70 RaFAH;98.60 iPHoP-RF;97.10
S7_vs_119679	99.98	d__Bacteria;p__Bacteroidota;c__Bacteroidia;o__Bacteroidales;f__Rikenellaceae;g__Alistipes_A	98.1	RaFAH;98.60
S7_vs_129271	12.57	d__Bacteria;p__Firmicutes;c__Clostridia;o__Oscillospirales;f__Acutalibacteraceae;g__UBA1691	98.5	CRISPR;98.90 blast;97.30 iPHoP-RF;82.80
S7_vs_129271	12.57	d__Bacteria;p__Firmicutes;c__Clostridia;o__Oscillospirales;f__Acutalibacteraceae;g__Fimivicinus	93.9	blast;95.70 iPHoP-RF;80.30
S7_vs_129858	10.46	d__Bacteria;p__Firmicutes;c__Clostridia;o__Christensenellales;f__CAG-74;g__Firm-11	91.4	blast;93.70 iPHoP-RF;85.40 CRISPR;50.00
S7_vs_13041	17.74	d__Bacteria;p__Firmicutes;c__Bacilli;o__Erysipelotrichales;f__Erysipelatoclostridiaceae;g__Erysipelatoclostridium	94.5	blast;96.10 iPHoP-RF;74.60 CRISPR;54.40
S7_vs_13370	8.9	d__Bacteria;p__Firmicutes;c__Clostridia;o__Oscillospirales;f__Ruminococcaceae;g__Faecalibacterium	97	blast;97.90 iPHoP-RF;90.10 CRISPR;77.30
S7_vs_134391	17.86	d__Bacteria;p__Firmicutes;c__Clostridia;o__Oscillospirales;f__UMGS1783;g__UMGS1783	94.9	blast;96.40 iPHoP-RF;84.80 CRISPR;79.10
S7_vs_134784	77.29	d__Bacteria;p__Firmicutes;c__Negativicutes;o__Veillonellales;f__Dialisteraceae;g__Dialister	97.3	CRISPR;97.40 blast;96.60 iPHoP-RF;92.80 RaFAH;63.90
S7_vs_135322	34.72	d__Bacteria;p__Firmicutes;c__Clostridia;o__Oscillospirales;f__Ruminococcaceae;g__Faecalibacterium	99.7	CRISPR;98.30 blast;91.90 RaFAH;90.70 iPHoP-RF;90.50
S7_vs_135679	23.81	d__Bacteria;p__Firmicutes;c__Clostridia;o__Lachnospirales;f__Lachnospiraceae;g__Blautia_A	99.6	blast;96.50 RaFAH;94.00 iPHoP-RF;90.10
S7_vs_135679	23.81	d__Bacteria;p__Firmicutes;c__Clostridia;o__Lachnospirales;f__Lachnospiraceae;g__Blautia	91.7	RaFAH;94.00
S7_vs_135828	3.2	d__Bacteria;p__Bacteroidota;c__Bacteroidia;o__Bacteroidales;f__Bacteroidaceae;g__Bacteroides	93	blast;95.00 iPHoP-RF;86.00
S7_vs_137611	29.3	d__Bacteria;p__Firmicutes;c__Clostridia;o__Lachnospirales;f__Lachnospiraceae;g__Anaerobutyricum	95	blast;96.50 iPHoP-RF;87.60
S7_vs_137915	55.05	d__Bacteria;p__Bacteroidota;c__Bacteroidia;o__Bacteroidales;f__Tannerellaceae;g__Parabacteroides	98.1	CRISPR;98.30 blast;90.90 iPHoP-RF;85.40 RaFAH;59.50
S7_vs_13799	38.3	d__Bacteria;p__Firmicutes;c__Clostridia;o__Lachnospirales;f__Lachnospiraceae;g__Dorea_A	93	blast;95.00 iPHoP-RF;94.70 CRISPR;72.50
S7_vs_137998	42.1	d__Bacteria;p__Firmicutes;c__Clostridia;o__Lachnospirales;f__Lachnospiraceae;g__Blautia_A	93.4	blast;95.30 iPHoP-RF;78.00
S7_vs_138109	31.21	d__Bacteria;p__Firmicutes;c__Clostridia;o__Lachnospirales;f__Lachnospiraceae;g__Anaerobutyricum	91	blast;93.40 iPHoP-RF;75.90
S7_vs_138116	5.66	d__Bacteria;p__Firmicutes;c__Clostridia;o__Lachnospirales;f__Lachnospiraceae;g__Mediterraneibacter	91.5	blast;93.80 iPHoP-RF;89.50
S7_vs_138173	100	d__Bacteria;p__Bacteroidota;c__Bacteroidia;o__Bacteroidales;f__Rikenellaceae;g__Alistipes	100	RaFAH;98.50 blast;94.70 iPHoP-RF;74.10
S7_vs_138173	100	d__Bacteria;p__Bacteroidota;c__Bacteroidia;o__Bacteroidales;f__Rikenellaceae;g__Alistipes_A	97.9	RaFAH;98.50
S7_vs_16344	12.56	d__Bacteria;p__Firmicutes;c__Clostridia;o__Oscillospirales;f__Acutalibacteraceae;g__Ruminococcus_E	98.8	CRISPR;99.10 blast;96.90 iPHoP-RF;95.70
S7_vs_21521	35.28	d__Bacteria;p__Firmicutes;c__Clostridia;o__Oscillospirales;f__Ruminococcaceae;g__Ruminiclostridium_E	90	blast;92.50 iPHoP-RF;74.80
S7_vs_2599	89.5	d__Bacteria;p__Bacteroidota;c__Bacteroidia;o__Bacteroidales;f__Bacteroidaceae;g__Bacteroides	100	RaFAH;99.30 iPHoP-RF;94.40 blast;93.90
S7_vs_26757		d__Bacteria;p__Firmicutes;c__Clostridia;o__Oscillospirales;f__Ruminococcaceae;g__Faecalibacterium	94.6	blast;96.20 iPHoP-RF;85.10
S7_vs_28150	12.25	d__Bacteria;p__Firmicutes;c__Clostridia;o__Oscillospirales;f__Oscillospiraceae;g__Dysosmobacter	94.2	blast;95.90 iPHoP-RF;74.10
S7_vs_28733	56.54	d__Bacteria;p__Firmicutes;c__Clostridia;o__Oscillospirales;f__Acutalibacteraceae;g__Hydrogeniiclostridium	95.2	blast;96.60 iPHoP-RF;62.20
S7_vs_29595	82.91	d__Bacteria;p__Verrucomicrobiota;c__Verrucomicrobiae;o__Verrucomicrobiales;f__Akkermansiaceae;g__Akkermansia	99.4	CRISPR;99.50 iPHoP-RF;57.30
S7_vs_30640	25.04	d__Bacteria;p__Firmicutes;c__Clostridia;o__Lachnospirales;f__Lachnospiraceae;g__Eubacterium_G	94.3	blast;96.00 iPHoP-RF;75.20
S7_vs_30665	8.94	d__Bacteria;p__Firmicutes;c__Clostridia;o__Christensenellales;f__CAG-74;g__UBA11524	93.8	CRISPR;95.60 blast;92.80 iPHoP-RF;92.40
S7_vs_38113	17.89	d__Bacteria;p__Firmicutes;c__Clostridia;o__Oscillospirales;f__Acutalibacteraceae;g__Hydrogeniiclostridium	91.7	blast;94.00 iPHoP-RF;64.50
S7_vs_38834	25.72	d__Bacteria;p__Firmicutes;c__Clostridia;o__Lachnospirales;f__Lachnospiraceae;g__RUG115	91.5	blast;93.80
S7_vs_38834	25.72	d__Bacteria;p__Firmicutes;c__Clostridia;o__Lachnospirales;f__Lachnospiraceae;g__Roseburia	90.1	blast;92.60 iPHoP-RF;69.50
S7_vs_41098	97.6	d__Bacteria;p__Firmicutes;c__Clostridia;o__Lachnospirales;f__Lachnospiraceae;g__Mediterraneibacter	95.4	blast;96.80
S7_vs_42563	93.91	d__Bacteria;p__Firmicutes;c__Clostridia;o__Oscillospirales;f__Ruminococcaceae;g__Gemmiger	96.3	blast;96.80 iPHoP-RF;75.40 RaFAH;59.00
S7_vs_47050	16.97	d__Bacteria;p__Firmicutes;c__Clostridia;o__Oscillospirales;f__Acutalibacteraceae;g__CAG-180	98.7	CRISPR;99.00 blast;96.60 iPHoP-RF;92.40
S7_vs_47050	16.97	d__Bacteria;p__Firmicutes;c__Clostridia;o__Oscillospirales;f__Acutalibacteraceae;g__Fimenecus	97.5	blast;98.20 iPHoP-RF;92.10
S7_vs_48523	66.97	d__Bacteria;p__Proteobacteria;c__Gammaproteobacteria;o__Burkholderiales;f__Burkholderiaceae;g__Sutterella	91.6	blast;93.90
S7_vs_48708	42.25	d__Bacteria;p__Firmicutes;c__Clostridia;o__Lachnospirales;f__Lachnospiraceae;g__Blautia_A	90.9	blast;93.30 iPHoP-RF;69.50
S7_vs_49426	18.87	d__Bacteria;p__Firmicutes;c__Clostridia;o__Lachnospirales;f__Lachnospiraceae;g__Agathobacter	91.5	blast;93.80 iPHoP-RF;71.30
S7_vs_4956	54.17	d__Bacteria;p__Firmicutes;c__Clostridia;o__Oscillospirales;f__Oscillospiraceae;g__	91.6	blast;93.90 iPHoP-RF;83.30
S7_vs_50194	18.68	d__Bacteria;p__Bacteroidota;c__Bacteroidia;o__Bacteroidales;f__Rikenellaceae;g__Alistipes_A	96.9	blast;90.90 RaFAH;87.90 iPHoP-RF;85.70
S7_vs_50194	18.68	d__Bacteria;p__Bacteroidota;c__Bacteroidia;o__Bacteroidales;f__Rikenellaceae;g__Alistipes	96.6	blast;89.90 RaFAH;87.90
S7_vs_51332	13.21	d__Bacteria;p__Bacteroidota;c__Bacteroidia;o__Bacteroidales;f__Coprobacteraceae;g__Coprobacter	94.5	blast;96.10 iPHoP-RF;53.30
S7_vs_5236	93.85	d__Bacteria;p__Firmicutes;c__Clostridia;o__Oscillospirales;f__Oscillospiraceae;g__CAG-103	93.5	CRISPR;95.40
S7_vs_54322	65.6	d__Bacteria;p__Bacteroidota;c__Bacteroidia;o__Bacteroidales;f__Rikenellaceae;g__Alistipes	98.7	blast;96.80 CRISPR;95.60 iPHoP-RF;87.30 RaFAH;84.00
S7_vs_55031	23.56	d__Bacteria;p__Bacteroidota;c__Bacteroidia;o__Bacteroidales;f__Tannerellaceae;g__Parabacteroides	98.1	blast;98.60 CRISPR;97.70 iPHoP-RF;89.20
S7_vs_55526	22.37	d__Bacteria;p__Actinobacteriota;c__Coriobacteriia;o__Coriobacteriales;f__Coriobacteriaceae;g__Collinsella	98.7	CRISPR;99.00 blast;93.80 iPHoP-RF;84.50
S7_vs_557	66.78	d__Bacteria;p__Bacteroidota;c__Bacteroidia;o__Bacteroidales;f__Bacteroidaceae;g__Bacteroides	99.3	iPHoP-RF;97.10 blast;93.70 RaFAH;89.50
S7_vs_557	66.78	d__Bacteria;p__Bacteroidota;c__Bacteroidia;o__Bacteroidales;f__Bacteroidaceae;g__Phocaeicola	94.9	iPHoP-RF;96.40 blast;82.80
S7_vs_60775	62.59	d__Bacteria;p__Firmicutes;c__Clostridia;o__Lachnospirales;f__Lachnospiraceae;g__Eubacterium_F	91.6	blast;93.90
S7_vs_65719	47.93	d__Bacteria;p__Firmicutes;c__Clostridia;o__Lachnospirales;f__Lachnospiraceae;g__Enterocloster	95.7	CRISPR;97.00 blast;94.90 iPHoP-RF;88.90
S7_vs_69630	35.61	d__Bacteria;p__Firmicutes;c__Clostridia;o__Oscillospirales;f__Oscillospiraceae;g__Intestinimonas	94.7	blast;96.30 iPHoP-RF;65.10
S7_vs_72737	3.91	d__Bacteria;p__Firmicutes;c__Clostridia;o__Oscillospirales;f__Acutalibacteraceae;g__Ruminococcus_E	97.9	blast;98.50 iPHoP-RF;96.40
S7_vs_73280	86.44	d__Bacteria;p__Actinobacteriota;c__Actinomycetia;o__Actinomycetales;f__Bifidobacteriaceae;g__Bifidobacterium	99.1	CRISPR;99.30 iPHoP-RF;97.10 blast;96.90
S7_vs_78507	36.34	d__Bacteria;p__Firmicutes;c__Clostridia;o__Oscillospirales;f__Oscillospiraceae;g__Intestinimonas	90.8	blast;93.20 iPHoP-RF;70.90
S7_vs_83100	97.77	d__Bacteria;p__Firmicutes;c__Clostridia;o__Oscillospirales;f__Oscillospiraceae;g__Flavonifractor	97.8	blast;98.40 iPHoP-RF;85.10 CRISPR;74.00
S7_vs_83474	31.73	d__Bacteria;p__Firmicutes;c__Clostridia;o__Oscillospirales;f__Oscillospiraceae;g__Flavonifractor	93.9	blast;95.70 iPHoP-RF;90.80

S7_vs_86377	91.04	d__Bacteria;p__Firmicutes;c__Clostridia;o__Lachnospirales;f__Lachnospiraceae;g__Bariatricus	92	blast;94.20 iPHoP-RF;79.50
S7_vs_86377	91.04	d__Bacteria;p__Firmicutes;c__Clostridia;o__Lachnospirales;f__Lachnospiraceae;g__Lachnoclostridium_B	91.2	blast;93.60 iPHoP-RF;74.80
S7_vs_87279	66.77	d__Bacteria;p__Firmicutes;c__Clostridia;o__Oscillospirales;f__Ruminococcaceae;g__Faecalibacterium	95.2	blast;96.60 iPHoP-RF;62.50
S7_vs_87439	31.68	d__Bacteria;p__Verrucomicrobiota;c__Lentisphaeria;o__Victivallales;f__Victivallaceae;g__Victivallis	90.2	blast;92.70 iPHoP-RF;71.10
S7_vs_944	66.43	d__Bacteria;p__Bacteroidota;c__Bacteroidia;o__Bacteroidales;f__Bacteroidaceae;g__Bacteroides	100	RaFAH;98.90 iPHoP-RF;96.70 blast;91.80
S7_vs_94738	58.62	d__Bacteria;p__Firmicutes;c__Clostridia;o__Oscillospirales;f__Oscillospiraceae;g__Flavonifractor	91.2	blast;93.60 iPHoP-RF;71.30
S7_vs_97737	56.07	d__Bacteria;p__Firmicutes;c__Clostridia;o__Oscillospirales;f__Ruminococcaceae;g__Faecalibacterium	95.6	blast;96.90 iPHoP-RF;91.80
S8_vf_101283	99.72	d__Bacteria;p__Firmicutes;c__Clostridia;o__Oscillospirales;f__Ruminococcaceae;g__Faecalibacterium	100	RaFAH;99.60 CRISPR;98.20 blast;95.50 iPHoP-RF;84.80
S8_vf_102508	92.39	d__Bacteria;p__Actinobacteriota;c__Actinomycetia;o__Actinomycetales;f__Micrococcaceae;g__Rothia	100	RaFAH;99.40 CRISPR;98.40 iPHoP-RF;80.80
S8_vf_111790	20.45	d__Bacteria;p__Firmicutes;c__Clostridia;o__Oscillospirales;f__Oscillospiraceae;g__Flavonifractor	91.6	blast;93.90 iPHoP-RF;81.10
S8_vf_117796	71.62	d__Bacteria;p__Bacteroidota;c__Bacteroidia;o__Bacteroidales;f__Bacteroidaceae;g__Prevotella	91.1	blast;93.50 iPHoP-RF;88.50
S8_vf_117796	71.62	d__Bacteria;p__Bacteroidota;c__Bacteroidia;o__Bacteroidales;f__Bacteroidaceae;g__Bacteroides	90.9	RaFAH;93.30
S8_vf_122206	16.65	d__Bacteria;p__Bacteroidota;c__Bacteroidia;o__Bacteroidales;f__Bacteroidaceae;g__Prevotella	94.7	blast;96.30 iPHoP-RF;90.50
S8_vf_129423	87.49	d__Bacteria;p__Firmicutes;c__Bacilli;o__Lactobacillales;f__Lactobacillaceae;g__Leuconostoc	97.5	iPHoP-RF;93.70 RaFAH;86.10
S8_vf_131009	24.39	d__Bacteria;p__Firmicutes;c__Clostridia;o__Lachnospirales;f__Lachnospiraceae;g__Eubacterium_F	97.2	CRISPR;98.00 blast;96.80 iPHoP-RF;95.40
S8_vf_132697	84.84	d__Bacteria;p__Firmicutes;c__Clostridia;o__Lachnospirales;f__Lachnospiraceae;g__Agathobacter	99.9	CRISPR;98.90 RaFAH;92.10 blast;90.20 iPHoP-RF;86.30
S8_vf_132697	84.84	d__Bacteria;p__Firmicutes;c__Clostridia;o__Lachnospirales;f__Lachnospiraceae;g__Roseburia	99.3	RaFAH;92.10 blast;87.10 iPHoP-RF;85.70
S8_vf_132697	84.84	d__Bacteria;p__Firmicutes;c__Clostridia;o__Lachnospirales;f__Lachnospiraceae;g__Lachnospira	98.7	CRISPR;99.00 blast;78.00
S8_vf_132697	84.84	d__Bacteria;p__Firmicutes;c__Clostridia;o__Lachnospirales;f__Lachnospiraceae;g__RUG115	96.6	RaFAH;92.10 blast;85.00
S8_vf_134943	81.72	d__Bacteria;p__Firmicutes;c__Bacilli;o__Lactobacillales;f__Streptococcaceae;g__Streptococcus	99.7	RaFAH;99.40 iPHoP-RF;72.60
S8_vf_13709	11.95	d__Bacteria;p__Firmicutes;c__Clostridia;o__TANB77;f__CAG-508;g__CAG-354	90.2	blast;92.70 iPHoP-RF;70.60
S8_vf_13758	11.78	d__Bacteria;p__Firmicutes;c__Clostridia;o__TANB77;f__CAG-508;g__CAG-354	93.9	blast;95.70 iPHoP-RF;71.70
S8_vf_142700	5.82	d__Bacteria;p__Actinobacteriota;c__Coriobacteriia;o__Coriobacteriales;f__Coriobacteriaceae;g__Collinsella	99.4	CRISPR;99.50 iPHoP-RF;93.40 blast;91.90
S8_vf_151384	47.18	d__Bacteria;p__Firmicutes;c__Clostridia;o__Oscillospirales;f__Ruminococcaceae;g__Gemmiger	96.3	CRISPR;97.40 blast;92.00 iPHoP-RF;89.80
S8_vf_153696	11.92	d__Bacteria;p__Actinobacteriota;c__Actinomycetia;o__Actinomycetales;f__Bifidobacteriaceae;g__Bifidobacterium	91.2	blast;93.60
S8_vf_158225	14.69	d__Bacteria;p__Bacteroidota;c__Bacteroidia;o__Bacteroidales;f__Tannerellaceae;g__Parabacteroides	94	blast;95.80
S8_vf_161703	49.06	d__Bacteria;p__Firmicutes;c__Clostridia;o__Oscillospirales;f__Ruminococcaceae;g__Ruminococcus_D	99.1	CRISPR;97.90 blast;96.80 iPHoP-RF;96.40 RaFAH;83.30
S8_vf_16172	45.4	d__Bacteria;p__Bacteroidota;c__Bacteroidia;o__Bacteroidales;f__Tannerellaceae;g__Parabacteroides	95.9	iPHoP-RF;97.10 blast;96.90 CRISPR;92.40
S8_vf_16172	45.4	d__Bacteria;p__Bacteroidota;c__Bacteroidia;o__Bacteroidales;f__Bacteroidaceae;g__Bacteroides	94.9	RaFAH;96.40
S8_vf_166403	69.48	d__Bacteria;p__Firmicutes;c__Clostridia;o__Oscillospirales;f__Oscillospiraceae;g__Flavonifractor	95.2	blast;96.60 iPHoP-RF;72.20
S8_vf_166677	20.94	d__Bacteria;p__Bacteroidota;c__Bacteroidia;o__Bacteroidales;f__Bacteroidaceae;g__Prevotella	99.9	blast;98.70 CRISPR;96.60 iPHoP-RF;93.10 RaFAH;90.50
S8_vf_170878	39.33	d__Bacteria;p__Firmicutes;c__Clostridia;o__Oscillospirales;f__Oscillospiraceae;g__CAG-83	91.2	blast;93.60 iPHoP-RF;65.60
S8_vf_183045	35.16	d__Bacteria;p__Bacteroidota;c__Bacteroidia;o__Bacteroidales;f__Bacteroidaceae;g__Prevotella	98.5	CRISPR;98.90 blast;93.00 iPHoP-RF;90.50
S8_vf_187742	91.82	d__Bacteria;p__Firmicutes;c__Clostridia;o__Oscillospirales;f__Ruminococcaceae;g__Faecalibacterium	99.6	blast;96.20 RaFAH;95.10 CRISPR;86.20 iPHoP-RF;65.30
S8_vf_187742	91.82	d__Bacteria;p__Firmicutes;c__Bacilli;o__Lactobacillales;f__Streptococcaceae;g__Streptococcus	94.6	blast;96.20
S8_vf_195032	5.64	d__Bacteria;p__Actinobacteriota;c__Coriobacteriia;o__Coriobacteriales;f__Eggerthellaceae;g__Gordonibacter	94.9	CRISPR;96.40 iPHoP-RF;75.70
S8_vf_195032	5.64	d__Bacteria;p__Actinobacteriota;c__Coriobacteriia;o__Coriobacteriales;f__Eggerthellaceae;g__Eggerthella	91.6	blast;93.90 iPHoP-RF;69.30
S8_vf_197061	60.03	d__Bacteria;p__Actinobacteriota;c__Actinomycetia;o__Actinomycetales;f__Bifidobacteriaceae;g__Bifidobacterium	98.8	CRISPR;99.00 iPHoP-RF;97.10 blast;96.80 RaFAH;52.60
S8_vf_201695	30.61	d__Bacteria;p__Firmicutes;c__Clostridia;o__Lachnospirales;f__Anaerotignaceae;g__Anaerotignum	94.2	blast;95.90 iPHoP-RF;76.40
S8_vf_208110	89.6	d__Bacteria;p__Firmicutes;c__Clostridia;o__Oscillospirales;f__Oscillospiraceae;g__Intestinimonas	92	blast;94.20 iPHoP-RF;50.20
S8_vf_208111	7.17	d__Bacteria;p__Desulfobacterota;c__Desulfovibrionia;o__Desulfovibrionales;f__Desulfovibrionaceae;g__Bilophila	90.8	CRISPR;93.20 iPHoP-RF;60.30
S8_vf_208115		d__Bacteria;p__Bacteroidota;c__Bacteroidia;o__Bacteroidales;f__Barnesiellaceae;g__Barnesiella	94.2	blast;95.90 iPHoP-RF;72.80
S8_vf_208119	18.24	d__Bacteria;p__Firmicutes;c__Clostridia;o__Oscillospirales;f__Ruminococcaceae;g__Faecalibacterium	97.2	CRISPR;97.90 RaFAH;52.10
S8_vf_208120	99.62	d__Bacteria;p__Bacteroidota;c__Bacteroidia;o__Bacteroidales;f__Bacteroidaceae;g__Bacteroides	98.2	RaFAH;93.90 blast;88.30 iPHoP-RF;53.80
S8_vf_208120	99.62	d__Bacteria;p__Bacteroidota;c__Bacteroidia;o__Bacteroidales;f__Tannerellaceae;g__Parabacteroides	95.4	blast;96.80 iPHoP-RF;57.90
S8_vf_208126	48.51	d__Bacteria;p__Firmicutes;c__Clostridia;o__Oscillospirales;f__Ruminococcaceae;g__Gemmiger	91.6	blast;93.90 iPHoP-RF;60.90
S8_vf_208146	25.58	d__Bacteria;p__Firmicutes;c__Negativicutes;o__Acidaminococcales;f__Acidaminococcaceae;g__Phascolarctobacterium	93.1	CRISPR;95.10
S8_vf_28956	91.89	d__Bacteria;p__Bacteroidota;c__Bacteroidia;o__Bacteroidales;f__Bacteroidaceae;g__Bacteroides	99.6	iPHoP-RF;96.40 RaFAH;94.20 blast;93.80
S8_vf_28956	91.89	d__Bacteria;p__Bacteroidota;c__Bacteroidia;o__Bacteroidales;f__Bacteroidaceae;g__Phocaeicola	94.9	iPHoP-RF;96.40 blast;92.70
S8_vf_45776	88.6	d__Bacteria;p__Firmicutes;c__Bacilli;o__Lactobacillales;f__Streptococcaceae;g__Lactococcus	100	RaFAH;99.20 blast;98.30 iPHoP-RF;73.90
S8_vf_55476	64.72	d__Bacteria;p__Firmicutes;c__Clostridia;o__Oscillospirales;f__Oscillospiraceae;g__Intestinimonas	91.6	blast;93.90 iPHoP-RF;82.20
S8_vf_65522	12.03	d__Bacteria;p__Firmicutes;c__Clostridia;o__Christensenellales;f__CAG-74;g__UBA11524	97.2	blast;98.00 iPHoP-RF;83.10 CRISPR;55.60
S8_vf_7243	95.82	d__Bacteria;p__Actinobacteriota;c__Actinomycetia;o__Actinomycetales;f__Micrococcaceae;g__Rothia	99.4	RaFAH;99.50
S8_vf_79769	31.62	d__Bacteria;p__Bacteroidota;c__Bacteroidia;o__Bacteroidales;f__Bacteroidaceae;g__Prevotella	98.4	CRISPR;98.80 blast;96.70 iPHoP-RF;92.40
S8_vf_93976	9.53	d__Bacteria;p__Firmicutes;c__Clostridia;o__Christensenellales;f__CAG-138;g__UBA1685	95.3	blast;96.70 iPHoP-RF;66.50 CRISPR;59.10
S8_vs_102608	20	d__Bacteria;p__Firmicutes;c__Clostridia;o__Oscillospirales;f__Ruminococcaceae;g__Gemmiger	95.7	blast;97.00 CRISPR;96.10 iPHoP-RF;90.80
S8_vs_115958	68.75	d__Bacteria;p__Firmicutes;c__Clostridia;o__Oscillospirales;f__Acutalibacteraceae;g__Ruminococcus_E	91.6	blast;93.90 iPHoP-RF;67.00
S8_vs_125717	17.94	d__Bacteria;p__Firmicutes;c__Clostridia;o__Oscillospirales;f__Oscillospiraceae;g__CAG-83	95.6	blast;96.90 iPHoP-RF;64.30
S8_vs_126582	98.15	d__Bacteria;p__Bacteroidota;c__Bacteroidia;o__Bacteroidales;f__Tannerellaceae;g__Parabacteroides	100	RaFAH;99.70 iPHoP-RF;89.20 blast;86.20 CRISPR;69.40
S8_vs_134805	91.02	d__Bacteria;p__Firmicutes;c__Clostridia;o__Oscillospirales;f__Oscillospiraceae;g__Lawsonibacter	91.5	blast;93.80 iPHoP-RF;73.20
S8_vs_145173	54.47	d__Bacteria;p__Firmicutes;c__Clostridia;o__Oscillospirales;f__Oscillospiraceae;g__UBA9475	91.2	blast;93.60 iPHoP-RF;59.00
S8_vs_147670	31.23	d__Bacteria;p__Firmicutes;c__Negativicutes;o__Veillonellales;f__Megasphaeraceae;g__Caecibacter	96.7	CRISPR;97.70 blast;78.80
S8_vs_147697	41.71	d__Bacteria;p__Bacteroidota;c__Bacteroidia;o__Bacteroidales;f__Bacteroidaceae;g__Bacteroides	98.8	iPHoP-RF;95.70 blast;90.90 RaFAH;89.00
S8_vs_148389	54.68	d__Bacteria;p__Bacteroidota;c__Bacteroidia;o__Bacteroidales;f__Bacteroidaceae;g__Prevotella	99.6	blast;96.80 RaFAH;94.40 iPHoP-RF;91.80
S8_vs_173961	95.47	d__Bacteria;p__Firmicutes;c__Clostridia;o__Lachnospirales;f__Lachnospiraceae;g__Agathobacter	99.6	CRISPR;98.40 blast;90.90 RaFAH;89.00 iPHoP-RF;87.60
S8_vs_173961	95.47	d__Bacteria;p__Firmicutes;c__Clostridia;o__Lachnospirales;f__Lachnospiraceae;g__Roseburia	99.4	CRISPR;97.50 iPHoP-RF;96.40 blast;91.80 RaFAH;89.00
S8_vs_173961	95.47	d__Bacteria;p__Firmicutes;c__Clostridia;o__Lachnospirales;f__Lachnospiraceae;g__RUG115	97.9	iPHoP-RF;93.40 blast;90.90 RaFAH;89.00
S8_vs_176604	21.14	d__Bacteria;p__Firmicutes;c__Clostridia;o__Christensenellales;f__CAG-138;g__SFEL01	94.9	blast;96.40 iPHoP-RF;81.60 CRISPR;71.90
S8_vs_176604	21.14	d__Bacteria;p__Firmicutes;c__Clostridia;o__Christensenellales;f__CAG-138;g__SFEL01	94.2	blast;95.90 iPHoP-RF;83.10
S8_vs_179208	39.41	d__Bacteria;p__Bacteroidota;c__Bacteroidia;o__Bacteroidales;f__Rikenellaceae;g__Alistipes	95.4	blast;96.80 iPHoP-RF;80.80
S8_vs_179208	39.41	d__Bacteria;p__Bacteroidota;c__Bacteroidia;o__Bacteroidales;f__Bacteroidaceae;g__Bacteroides	91.7	RaFAH;85.10 blast;79.80
S8_vs_179529	98.83	d__Bacteria;p__Firmicutes;c__Clostridia;o__Oscillospirales;f__Ruminococcaceae;g__Faecalibacterium	100	CRISPR;98.50 RaFAH;98.00 blast;95.30 iPHoP-RF;86.70
S8_vs_182288	85.94	d__Bacteria;p__Bacteroidota;c__Bacteroidia;o__Bacteroidales;f__Tannerellaceae;g__Parabacteroides	100	CRISPR;99.00 RaFAH;98.40 iPHoP-RF;96.40 blast;93.80
S8_vs_206526	81.78	d__Bacteria;p__Firmicutes;c__Clostridia;o__Lachnospirales;f__Lachnospiraceae;g__Roseburia	97.9	CRISPR;98.50 blast;92.20 iPHoP-RF;87.30 RaFAH;50.70
S8_vs_206526	81.78	d__Bacteria;p__Firmicutes;c__Clostridia;o__Lachnospirales;f__Lachnospiraceae;g__RUG115	97.3	CRISPR;98.10 iPHoP-RF;56.20 RaFAH;50.70
S8_vs_206526	81.78	d__Bacteria;p__Firmicutes;c__Clostridia;o__Lachnospirales;f__Lachnospiraceae;g__UBA11774	97.3	CRISPR;98.10 iPHoP-RF;66.30

S8_vs_206526	81.78	d__Bacteria;p__Firmicutes;c__Clostridia;o__Lachnospirales;f__Lachnospiraceae;g__Lachnospira	95	blast;96.50 iPHoP-RF;50.70
S8_vs_208114	9.13	d__Bacteria;p__Desulfobacterota;c__Desulfovibrionia;o__Desulfovibrionales;f__Desulfovibrionaceae;g__Mailhella	90.1	blast;92.60 iPHoP-RF;81.40
S8_vs_28311	99.02	d__Bacteria;p__Bacteroidota;c__Bacteroidia;o__Bacteroidales;f__Bacteroidaceae;g__Bacteroides	100	RaFAH;97.40 blast;97.40 iPHoP-RF;96.70 CRISPR;54.10
S8_vs_40014	80.96	d__Bacteria;p__Bacteroidota;c__Bacteroidia;o__Bacteroidales;f__Rikenellaceae;g__Alistipes	94.6	blast;96.20 iPHoP-RF;95.70
S8_vs_4153	5.07	d__Bacteria;p__Bacteroidota;c__Bacteroidia;o__Bacteroidales;f__Barnesiellaceae;g__Barnesiella	97.9	blast;98.50 iPHoP-RF;96.40 CRISPR;53.40
S8_vs_50431	20.17	d__Bacteria;p__Firmicutes;c__Clostridia;o__Oscillospirales;f__Oscillospiraceae;g__Dysosmobacter	91.5	blast;93.80 iPHoP-RF;50.70
S8_vs_51368	31.95	d__Bacteria;p__Firmicutes;c__Clostridia;o__Oscillospirales;f__Oscillospiraceae;g__CAG-170	91.5	blast;93.80 iPHoP-RF;85.70
S8_vs_5211	70.75	d__Bacteria;p__Firmicutes;c__Clostridia;o__Lachnospirales;f__Lachnospiraceae;g__Massilistercora	90.2	blast;92.70 iPHoP-RF;58.40
S8_vs_54183	20.38	d__Bacteria;p__Firmicutes;c__Clostridia;o__Oscillospirales;f__Oscillospiraceae;g__CAG-170	91.8	CRISPR;94.10 blast;90.90 iPHoP-RF;81.90
S8_vs_59736	26.65	d__Bacteria;p__Firmicutes;c__Bacilli;o__Erysipelotrichales;f__Erysipelatoclostridiaceae;g__Catenibacterium	95.6	blast;96.90
S8_vs_63277	75.64	d__Bacteria;p__Firmicutes;c__Clostridia;o__Oscillospirales;f__Ruminococcaceae;g__Faecalibacterium	97.8	blast;98.40 iPHoP-RF;88.20
S8_vs_72117	19.43	d__Bacteria;p__Bacteroidota;c__Bacteroidia;o__Bacteroidales;f__Bacteroidaceae;g__Bacteroides	95.4	iPHoP-RF;94.40 RaFAH;71.80
S8_vs_9114	28.26	d__Bacteria;p__Bacteroidota;c__Bacteroidia;o__Bacteroidales;f__Rikenellaceae;g__Alistipes_A	91.1	blast;93.50 iPHoP-RF;82.80 CRISPR;64.80
S8_vs_99362	99.97	d__Bacteria;p__Bacteroidota;c__Bacteroidia;o__Bacteroidales;f__Bacteroidaceae;g__Bacteroides	100	RaFAH;98.30 blast;96.50 iPHoP-RF;93.40
S8_vs_99362	99.97	d__Bacteria;p__Bacteroidota;c__Bacteroidia;o__Bacteroidales;f__Bacteroidaceae;g__Paraprevotella	90.2	blast;92.70
S8_vs_9987	99.87	d__Bacteria;p__Bacteroidota;c__Bacteroidia;o__Bacteroidales;f__Rikenellaceae;g__Alistipes	100	blast;97.60 RaFAH;97.40 iPHoP-RF;93.40
S8_vs_9987	99.87	d__Bacteria;p__Bacteroidota;c__Bacteroidia;o__Bacteroidales;f__Rikenellaceae;g__Alistipes_A	96.3	RaFAH;97.40
S9_vf_13507	66.3	d__Bacteria;p__Bacteroidota;c__Bacteroidia;o__Bacteroidales;f__Bacteroidaceae;g__Prevotella	95.6	blast;96.90 iPHoP-RF;94.70
S9_vf_15012	97.67	d__Bacteria;p__Firmicutes;c__Bacilli;o__Lactobacillales;f__Streptococcaceae;g__Streptococcus	100	RaFAH;99.70 CRISPR;98.30 blast;94.40 iPHoP-RF;92.80
S9_vf_19284	15.56	d__Bacteria;p__Firmicutes;c__Bacilli;o__Lactobacillales;f__Streptococcaceae;g__Streptococcus	93	CRISPR;95.00
S9_vf_4115	96.62	d__Bacteria;p__Firmicutes;c__Bacilli;o__Lactobacillales;f__Streptococcaceae;g__Streptococcus	100	RaFAH;99.70 CRISPR;99.00 blast;96.80 iPHoP-RF;96.70
S9_vf_51656	29.36	d__Bacteria;p__Bacteroidota;c__Bacteroidia;o__Bacteroidales;f__Bacteroidaceae;g__Prevotella	99	CRISPR;99.00 blast;96.30 iPHoP-RF;96.10 RaFAH;59.50
S9_vf_64844	89.68	d__Bacteria;p__Firmicutes;c__Clostridia;o__Oscillospirales;f__Oscillospiraceae;g__CAG-83	93.4	RaFAH;95.30 blast;50.00
S9_vf_64844	89.68	d__Bacteria;p__Firmicutes;c__Clostridia;o__Oscillospirales;f__Oscillospiraceae;g__Dysosmobacter	93.4	RaFAH;95.30
S9_vf_73311	18.82	d__Bacteria;p__Firmicutes;c__Clostridia;o__Lachnospirales;f__CAG-274;g__CAG-274	97.2	CRISPR;98.00 blast;96.80 iPHoP-RF;78.70
S9_vf_76769	53.32	d__Bacteria;p__Bacteroidota;c__Bacteroidia;o__Bacteroidales;f__Bacteroidaceae;g__Prevotella	92.6	RaFAH;94.70
S9_vf_90856	94.47	d__Bacteria;p__Firmicutes;c__Bacilli;o__Lactobacillales;f__Streptococcaceae;g__Streptococcus	100	RaFAH;99.80 CRISPR;99.00 blast;95.80 iPHoP-RF;94.40
S9_vf_90866	15.47	d__Bacteria;p__Firmicutes;c__Bacilli;o__Lactobacillales;f__Streptococcaceae;g__Streptococcus	99.3	iPHoP-RF;97.10 blast;95.70 RaFAH;90.00
S9_vf_934	17.76	d__Bacteria;p__Actinobacteriota;c__Actinomycetia;o__Mycobacteriales;f__Mycobacteriaceae;g__Mycobacterium	92.2	iPHoP-RF;94.40
S9_vs_14235	12.73	d__Bacteria;p__Firmicutes;c__Clostridia;o__Oscillospirales;f__Oscillospiraceae;g__An92	91	blast;93.40 iPHoP-RF;87.00 CRISPR;73.90
S9_vs_14235	12.73	d__Bacteria;p__Firmicutes;c__Clostridia;o__Oscillospirales;f__Oscillospiraceae;g__Flavonifractor	90.4	blast;92.90 iPHoP-RF;79.00 CRISPR;60.90
S9_vs_1966	61.19	d__Bacteria;p__Firmicutes;c__Clostridia;o__Lachnospirales;f__Lachnospiraceae;g__Bariatricus	91.6	blast;93.90 iPHoP-RF;77.30
S9_vs_19689	9.42	d__Bacteria;p__Firmicutes;c__Clostridia;o__Oscillospirales;f__Butyricicoccaceae;g__Agathobaculum	91.6	blast;93.90 iPHoP-RF;68.60
S9_vs_21850	63.18	d__Bacteria;p__Bacteroidota;c__Bacteroidia;o__Bacteroidales;f__Barnesiellaceae;g__Barnesiella	97.2	blast;96.90 iPHoP-RF;85.10 CRISPR;84.10 RaFAH;68.00
S9_vs_26179	48.62	d__Bacteria;p__Firmicutes;c__Clostridia;o__Oscillospirales;f__Acutalibacteraceae;g__UMGS1071	95.7	blast;97.00 iPHoP-RF;83.30 CRISPR;81.20
S9_vs_2799	10.92	d__Bacteria;p__Bacteroidota;c__Bacteroidia;o__Bacteroidales;f__Rikenellaceae;g__Alistipes	95.9	iPHoP-RF;97.10 blast;96.90
S9_vs_28197	87.18	d__Bacteria;p__Bacteroidota;c__Bacteroidia;o__Bacteroidales;f__Bacteroidaceae;g__Bacteroides	100	RaFAH;99.40 iPHoP-RF;97.10 blast;96.30
S9_vs_28197	87.18	d__Bacteria;p__Bacteroidota;c__Bacteroidia;o__Bacteroidales;f__Bacteroidaceae;g__Phocaeicola	95.3	iPHoP-RF;96.70
S9_vs_32588	67.17	d__Bacteria;p__Firmicutes;c__Clostridia;o__Lachnospirales;f__Lachnospiraceae;g__Dorea	95	RaFAH;96.50
S9_vs_32588	67.17	d__Bacteria;p__Firmicutes;c__Clostridia;o__Lachnospirales;f__Lachnospiraceae;g__Dorea_A	95	RaFAH;96.50
S9_vs_32588	67.17	d__Bacteria;p__Firmicutes;c__Clostridia;o__Oscillospirales;f__Oscillospiraceae;g__Intestinimonas	91.6	blast;93.90 iPHoP-RF;73.70
S9_vs_34898	91.82	d__Bacteria;p__Firmicutes;c__Clostridia;o__Lachnospirales;f__Lachnospiraceae;g__Sellimonas	93.9	blast;95.70 iPHoP-RF;89.50
S9_vs_37437	14.86	d__Bacteria;p__Firmicutes;c__Bacilli;o__Erysipelotrichales;f__Erysipelotrichaceae;g__Holdemania	91.6	blast;93.90 CRISPR;91.60 iPHoP-RF;75.40
S9_vs_41371	44.35	d__Bacteria;p__Firmicutes;c__Clostridia;o__Lachnospirales;f__Lachnospiraceae;g__Enterocloster	94	blast;95.80 iPHoP-RF;85.10
S9_vs_4343	24.4	d__Bacteria;p__Firmicutes;c__Clostridia;o__Lachnospirales;f__Lachnospiraceae;g__Enterocloster	96.6	blast;97.60 CRISPR;96.60 iPHoP-RF;90.50
S9_vs_44331	98.52	d__Bacteria;p__Firmicutes;c__Clostridia;o__Lachnospirales;f__Lachnospiraceae;g__Ruminococcus_B	98.1	blast;98.60 iPHoP-RF;94.40 CRISPR;87.70
S9_vs_4558	65.18	d__Bacteria;p__Firmicutes;c__Clostridia;o__Oscillospirales;f__Oscillospiraceae;g__Flavonifractor	90.2	blast;92.70 iPHoP-RF;79.00
S9_vs_46498	26.43	d__Bacteria;p__Firmicutes;c__Bacilli;o__Erysipelotrichales;f__Erysipelatoclostridiaceae;g__Erysipelatoclostridium	96.7	blast;97.70 iPHoP-RF;74.10
S9_vs_48240	63.98	d__Bacteria;p__Firmicutes;c__Clostridia;o__Lachnospirales;f__Lachnospiraceae;g__Hungatella	92.5	blast;94.60 iPHoP-RF;94.40
S9_vs_48347	25.89	d__Bacteria;p__Firmicutes;c__Clostridia;o__Oscillospirales;f__Ruminococcaceae;g__Negativibacillus	94.2	blast;95.90 iPHoP-RF;85.40 CRISPR;59.60
S9_vs_48424	86.27	d__Bacteria;p__Bacteroidota;c__Bacteroidia;o__Bacteroidales;f__Rikenellaceae;g__Alistipes	99.3	blast;96.80 RaFAH;90.20 iPHoP-RF;54.90
S9_vs_50439	93.5	d__Bacteria;p__Firmicutes;c__Clostridia;o__Oscillospirales;f__Oscillospiraceae;g__Flavonifractor	98.1	blast;98.60 iPHoP-RF;74.30
S9_vs_55738	76	d__Bacteria;p__Firmicutes;c__Clostridia;o__Lachnospirales;f__Lachnospiraceae;g__Enterocloster	94.6	blast;96.20 iPHoP-RF;87.00 CRISPR;69.00
S9_vs_5676	19.1	d__Bacteria;p__Firmicutes;c__Clostridia;o__Oscillospirales;f__Oscillospiraceae;g__Intestinimonas	92.6	blast;94.70 iPHoP-RF;74.60
S9_vs_58086	53.1	d__Bacteria;p__Firmicutes;c__Clostridia;o__Lachnospirales;f__Lachnospiraceae;g__Dorea_A	94.3	blast;96.00 iPHoP-RF;80.60
S9_vs_58086	53.1	d__Bacteria;p__Firmicutes;c__Clostridia;o__Lachnospirales;f__Lachnospiraceae;g__Dorea	91.5	blast;93.80 iPHoP-RF;83.60
S9_vs_62803	39.71	d__Bacteria;p__Firmicutes;c__Clostridia;o__Lachnospirales;f__Lachnospiraceae;g__Sellimonas	94.9	blast;96.40 iPHoP-RF;84.50
S9_vs_62803	39.71	d__Bacteria;p__Firmicutes;c__Clostridia;o__Lachnospirales;f__Lachnospiraceae;g__Lachnoclostridium_B	92.6	iPHoP-RF;94.70
S9_vs_66877	29.56	d__Bacteria;p__Firmicutes;c__Clostridia;o__Oscillospirales;f__Ruminococcaceae;g__Ruthenibacterium	95.3	blast;96.70 CRISPR;95.50 iPHoP-RF;92.10
S9_vs_67792	30.5	d__Bacteria;p__Firmicutes;c__Negativicutes;o__Veillonellales;f__Dialisteraceae;g__Dialister	96.6	blast;97.60 iPHoP-RF;86.00 CRISPR;60.90
S9_vs_69322	20.79	d__Bacteria;p__Firmicutes;c__Clostridia;o__Oscillospirales;f__Oscillospiraceae;g__Dysosmobacter	90.7	blast;93.10 iPHoP-RF;82.20
S9_vs_75050	26.71	d__Bacteria;p__Firmicutes;c__Clostridia;o__Lachnospirales;f__Lachnospiraceae;g__CAG-127	96.4	CRISPR;97.50 blast;96.80 iPHoP-RF;86.00
S9_vs_83108	82.91	d__Bacteria;p__Bacteroidota;c__Bacteroidia;o__Bacteroidales;f__Bacteroidaceae;g__Bacteroides	99.4	iPHoP-RF;97.10 blast;93.90 RaFAH;91.80
S9_vs_83108	82.91	d__Bacteria;p__Bacteroidota;c__Bacteroidia;o__Bacteroidales;f__Bacteroidaceae;g__Phocaeicola	98.1	CRISPR;98.60 iPHoP-RF;97.10 blast;91.90
S9_vs_84046	20.91	d__Bacteria;p__Firmicutes;c__Clostridia;o__Oscillospirales;f__Oscillospiraceae;g__An92	90.7	blast;93.10 iPHoP-RF;70.60
S9_vs_88286	10.88	d__Bacteria;p__Firmicutes;c__Clostridia;o__Lachnospirales;f__Lachnospiraceae;g__Eisenbergiella	98.1	CRISPR;98.60 blast;95.70 iPHoP-RF;95.40
S9_vs_90727	57.49	d__Bacteria;p__Firmicutes;c__Clostridia;o__Oscillospirales;f__Acutalibacteraceae;g__Ruminococcus_E	95.6	blast;96.90 iPHoP-RF;88.20
S9_vs_90846	37.71	d__Bacteria;p__Firmicutes;c__Bacilli;o__Erysipelotrichales;f__Erysipelotrichaceae;g__Clostridium_AQ	97.5	blast;98.20 iPHoP-RF;62.70
S9_vs_90848	51.41	d__Bacteria;p__Firmicutes;c__Clostridia;o__Lachnospirales;f__Lachnospiraceae;g__Enterocloster	98.7	CRISPR;99.00 blast;93.90 iPHoP-RF;91.40
S9_vs_90849	70.89	d__Bacteria;p__Firmicutes;c__Clostridia;o__Lachnospirales;f__Anaerotignaceae;g__Anaerotignum	95	CRISPR;96.50 blast;93.70 iPHoP-RF;92.10
S9_vs_90851	89.56	d__Bacteria;p__Firmicutes;c__Clostridia;o__Clostridiales;f__Clostridiaceae;g__Clostridium	95.6	RaFAH;96.90
S9_vs_90851	89.56	d__Bacteria;p__Firmicutes;c__Clostridia;o__Clostridiales;f__Clostridiaceae;g__Clostridium_F	95.6	RaFAH;96.90
S9_vs_90851	89.56	d__Bacteria;p__Firmicutes;c__Clostridia;o__Clostridiales;f__Clostridiaceae;g__Clostridium_P	95.6	RaFAH;96.90
S9_vs_90859	43.06	d__Bacteria;p__Firmicutes;c__Clostridia;o__Lachnospirales;f__Lachnospiraceae;g__Blautia_A	95.3	blast;93.90 iPHoP-RF;75.90 RaFAH;73.20
S9_vs_90860	20.66	d__Bacteria;p__Firmicutes;c__Clostridia;o__Oscillospirales;f__Acutalibacteraceae;g__UMGS1591	93.4	blast;95.30 iPHoP-RF;67.00

Table S9-2. WMS_prediction_STD+MAGs

Virus	AAI to closest RaFAH reference	Host genus	Confidence score	List of methods
S1-wms-combined_100430	99.71	d__Bacteria;p__Firmicutes;c__Clostridia;o__Lachnospirales;f__Lachnospiraceae;g__Lachnospira	99.3	CRISPR;98.90 RaFAH;73.00
S1-wms-combined_100430	99.71	d__Bacteria;p__Firmicutes;c__Clostridia;o__Lachnospirales;f__Lachnospiraceae;g__Eubacterium_G	95.4	blast;94.10 RaFAH;73.00
S1-wms-combined_100430	99.71	d__Bacteria;p__Firmicutes;c__Clostridia;o__Lachnospirales;f__Lachnospiraceae;g__Roseburia	94.3	blast;96.00 iPHoP-RF;62.20
S1-wms-combined_107766	92.99	d__Bacteria;p__Bacteroidota;c__Bacteroidia;o__Bacteroidales;f__Rikenellaceae;g__Alistipes	98.8	iPHoP-RF;97.10 blast;96.90 RaFAH;83.70
S1-wms-combined_118167	33.35	d__Bacteria;p__Firmicutes;c__Clostridia;o__Oscillospirales;f__Ruminococcaceae;g__Faecalibacterium	99.1	CRISPR;96.40 blast;95.10 RaFAH;90.80 iPHoP-RF;89.50
S1-wms-combined_131270	21.6	d__Bacteria;p__Firmicutes;c__Clostridia;o__Oscillospirales;f__Ruminococcaceae;g__Faecalibacterium	99	CRISPR;99.20 blast;94.50 iPHoP-RF;84.50
S1-wms-combined_132934	48.4	d__Bacteria;p__Firmicutes;c__Clostridia;o__Oscillospirales;f__Oscillospiraceae;g__ER4	92.7	blast;94.80 iPHoP-RF;64.80
S1-wms-combined_136097	4.5	d__Bacteria;p__Firmicutes;c__Clostridia;o__Oscillospirales;f__Oscillospiraceae;g__UBA738	92	blast;94.20 iPHoP-RF;53.00
S1-wms-combined_143459	18.79	d__Bacteria;p__Desulfobacterota;c__Desulfovibrionia;o__Desulfovibrionales;f__Desulfovibrionaceae;g__Bilophila	95.2	blast;96.60 iPHoP-RF;65.80 CRISPR;58.60
S1-wms-combined_143459	18.79	d__Bacteria;p__Desulfobacterota;c__Desulfovibrionia;o__Desulfovibrionales;f__Desulfovibrionaceae;g__Bilophila	90.8	blast;93.20 iPHoP-RF;66.80
S1-wms-combined_153670		d__Bacteria;p__Bacteroidota;c__Bacteroidia;o__Bacteroidales;f__Barnesiellaceae;g__Barnesiella	94.2	blast;95.90 iPHoP-RF;72.20
S1-wms-combined_156637	11	d__Bacteria;p__Firmicutes;c__Clostridia;o__Oscillospirales;f__Oscillospiraceae;g__ER4	93	blast;95.00 iPHoP-RF;60.30
S1-wms-combined_159544	36.49	d__Bacteria;p__Desulfobacterota;c__Desulfovibrionia;o__Desulfovibrionales;f__Desulfovibrionaceae;g__Bilophila	94	blast;95.80 iPHoP-RF;79.30 CRISPR;57.40
S1-wms-combined_164485	4.67	d__Bacteria;p__Firmicutes;c__Clostridia;o__Oscillospirales;f__Oscillospiraceae;g__ER4	90.4	blast;92.90
S1-wms-combined_164894	72.02	d__Bacteria;p__Firmicutes;c__Clostridia;o__Oscillospirales;f__Ruminococcaceae;g__Faecalibacterium	91.8	blast;94.10 iPHoP-RF;70.90
S1-wms-combined_166392	37.1	d__Bacteria;p__Firmicutes;c__Clostridia;o__Oscillospirales;f__Ruminococcaceae;g__Gemmiger	95.7	blast;97.00 iPHoP-RF;95.70 CRISPR;78.40
S1-wms-combined_169788	5.96	d__Bacteria;p__Firmicutes;c__Clostridia;o__Oscillospirales;f__Oscillospiraceae;g__CAG-83	93.4	blast;95.30 iPHoP-RF;87.00
S1-wms-combined_17651	9.21	d__Bacteria;p__Firmicutes;c__Clostridia;o__Oscillospirales;f__Oscillospiraceae;g__ER4	90.6	blast;93.00 iPHoP-RF;65.10
S1-wms-combined_185336		d__Bacteria;p__Firmicutes;c__Clostridia;o__Oscillospirales;f__Oscillospiraceae;g__ER4	91.7	blast;94.00 iPHoP-RF;63.50
S1-wms-combined_186878	42.39	d__Bacteria;p__Firmicutes;c__Clostridia;o__Oscillospirales;f__Oscillospiraceae;g__ER4	91.6	blast;93.90 iPHoP-RF;76.40
S1-wms-combined_19031	37.63	d__Bacteria;p__Desulfobacterota;c__Desulfovibrionia;o__Desulfovibrionales;f__Desulfovibrionaceae;g__Bilophila	98.2	blast;98.70 iPHoP-RF;76.10
S1-wms-combined_19031	37.63	d__Bacteria;p__Desulfobacterota;c__Desulfovibrionia;o__Desulfovibrionales;f__Desulfovibrionaceae;g__Bilophila	91.4	blast;93.70 iPHoP-RF;78.30
S1-wms-combined_212892	80.18	d__Bacteria;p__Firmicutes;c__Clostridia;o__Lachnospirales;f__Lachnospiraceae;g__Anaerobutyricum	98.1	CRISPR;98.60 blast;91.50 iPHoP-RF;80.00
S1-wms-combined_220022	6.93	d__Bacteria;p__Firmicutes;c__Clostridia;o__Oscillospirales;f__Ruminococcaceae;g__Gemmiger	93.2	blast;95.20 iPHoP-RF;88.20 CRISPR;76.80
S1-wms-combined_227003	41.88	d__Bacteria;p__Firmicutes;c__Clostridia;o__Oscillospirales;f__Oscillospiraceae;g__CAG-110	94.2	blast;95.90 iPHoP-RF;87.00
S1-wms-combined_227917	27.55	d__Bacteria;p__Firmicutes;c__Clostridia;o__Oscillospirales;f__Ruminococcaceae;g__Gemmiger	95.2	blast;96.60 iPHoP-RF;95.40 CRISPR;92.50
S1-wms-combined_231411	3.46	d__Bacteria;p__Desulfobacterota;c__Desulfovibrionia;o__Desulfovibrionales;f__Desulfovibrionaceae;g__Bilophila	93	blast;95.00 iPHoP-RF;73.20 CRISPR;51.00
S1-wms-combined_231411	3.46	d__Bacteria;p__Desulfobacterota;c__Desulfovibrionia;o__Desulfovibrionales;f__Desulfovibrionaceae;g__Bilophila	90.2	blast;92.70 iPHoP-RF;76.40
S1-wms-combined_23248	61.01	d__Bacteria;p__Firmicutes;c__Clostridia;o__Oscillospirales;f__Ruminococcaceae;g__Faecalibacterium	95	blast;96.50 iPHoP-RF;68.90
S1-wms-combined_246923	43.06	d__Bacteria;p__Bacteroidota;c__Bacteroidia;o__Bacteroidales;f__UBA11471;g__UBA11471	93.9	blast;95.70 iPHoP-RF;68.60
S1-wms-combined_248622	6.16	d__Bacteria;p__Bacteroidota;c__Bacteroidia;o__Bacteroidales;f__Bacteroidaceae;g__Bacteroides	95.9	iPHoP-RF;97.10 blast;90.50
S1-wms-combined_248622	6.16	d__Bacteria;p__Bacteroidota;c__Bacteroidia;o__Bacteroidales;f__Bacteroidaceae;g__Phocaeicola	94.5	iPHoP-RF;96.10 blast;93.80
S1-wms-combined_263562	99.98	d__Bacteria;p__Bacteroidota;c__Bacteroidia;o__Bacteroidales;f__Bacteroidaceae;g__Bacteroides	100	RaFAH;99.80 blast;91.80 iPHoP-RF;82.20
S1-wms-combined_279492	44.35	d__Bacteria;p__Firmicutes;c__Clostridia;o__Oscillospirales;f__Ruminococcaceae;g__Gemmiger	97.8	blast;98.40 iPHoP-RF;96.40 CRISPR;58.00
S1-wms-combined_285012	37.37	d__Bacteria;p__Firmicutes;c__Clostridia;o__Oscillospirales;f__Ruminococcaceae;g__Faecalibacterium	95	CRISPR;96.50 iPHoP-RF;54.40
S1-wms-combined_285012	37.37	d__Bacteria;p__Firmicutes;c__Clostridia;o__Oscillospirales;f__Oscillospiraceae;g__Dysosmobacter	90	blast;92.50
S1-wms-combined_302166	9.7	d__Bacteria;p__Firmicutes;c__Clostridia;o__Christensenellales;f__CAG-74;g__UBA11524	95.6	blast;96.90 iPHoP-RF;92.40
S1-wms-combined_302166	9.7	d__Bacteria;p__Firmicutes;c__Clostridia;o__Christensenellales;f__CAG-74;g__UBA11524	95.6	blast;96.90 iPHoP-RF;84.50
S1-wms-combined_304377	48.66	d__Bacteria;p__Firmicutes;c__Clostridia;o__Oscillospirales;f__Ruminococcaceae;g__Faecalibacterium	95.6	blast;96.90 iPHoP-RF;78.50
S1-wms-combined_307323	92.27	d__Bacteria;p__Actinobacteriota;c__Actinomycetia;o__Actinomycetales;f__Bifidobacteriaceae;g__Bifidobacterium	98.7	CRISPR;99.00 iPHoP-RF;97.10 blast;96.90
S1-wms-combined_308848	5.29	d__Bacteria;p__Verrucomicrobiota;c__Verrucomicrobiae;o__Verrucomicrobiales;f__Akkermansiaceae;g__Akkermansia	95.9	CRISPR;97.10 blast;96.90 iPHoP-RF;93.10
S1-wms-combined_32863	10.89	d__Bacteria;p__Bacteroidota;c__Bacteroidia;o__Bacteroidales;f__Tannerellaceae;g__Parabacteroides	95.6	blast;96.90 iPHoP-RF;69.50
S1-wms-combined_330329	86.77	d__Bacteria;p__Bacteroidota;c__Bacteroidia;o__Bacteroidales;f__Bacteroidaceae;g__Bacteroides	98.2	RaFAH;97.70 iPHoP-RF;72.40
S1-wms-combined_331544	89.75	d__Bacteria;p__Actinobacteriota;c__Actinomycetia;o__Actinomycetales;f__Bifidobacteriaceae;g__Bifidobacterium	99.9	CRISPR;99.60 blast;96.90 iPHoP-RF;96.70 RaFAH;75.50
S1-wms-combined_331896		d__Bacteria;p__Firmicutes;c__Clostridia;o__Oscillospirales;f__Oscillospiraceae;g__ER4	91.6	blast;93.90 iPHoP-RF;58.40
S1-wms-combined_340558	85.48	d__Bacteria;p__Bacteroidota;c__Bacteroidia;o__Bacteroidales;f__Rikenellaceae;g__Alistipes	99.7	blast;96.80 iPHoP-RF;96.10 RaFAH;95.90
S1-wms-combined_340558	85.48	d__Bacteria;p__Bacteroidota;c__Bacteroidia;o__Bacteroidales;f__Rikenellaceae;g__Alistipes_A	99.1	RaFAH;95.90 blast;91.80
S1-wms-combined_351889	29.41	d__Bacteria;p__Firmicutes;c__Clostridia;o__Oscillospirales;f__Oscillospiraceae;g__ER4	96.2	CRISPR;97.30 blast;96.20 iPHoP-RF;84.50
S1-wms-combined_353757	21.95	d__Bacteria;p__Firmicutes;c__Clostridia;o__Oscillospirales;f__Oscillospiraceae;g__ER4	93.2	blast;95.20 iPHoP-RF;84.80
S1-wms-combined_357373		d__Bacteria;p__Bacteroidota;c__Bacteroidia;o__Bacteroidales;f__Barnesiellaceae;g__Barnesiella	91.8	blast;94.10 iPHoP-RF;86.30
S1-wms-combined_360285	17.11	d__Bacteria;p__Desulfobacterota;c__Desulfovibrionia;o__Desulfovibrionales;f__Desulfovibrionaceae;g__Bilophila	97.2	CRISPR;98.00 blast;96.60 iPHoP-RF;89.80
S1-wms-combined_360285	17.11	d__Bacteria;p__Desulfobacterota;c__Desulfovibrionia;o__Desulfovibrionales;f__Desulfovibrionaceae;g__Bilophila	94.2	blast;95.90 iPHoP-RF;88.20
S1-wms-combined_373308	6.25	d__Bacteria;p__Firmicutes;c__Clostridia;o__Oscillospirales;f__Oscillospiraceae;g__CAG-83	97.9	blast;98.50 iPHoP-RF;77.30
S1-wms-combined_374219	10.38	d__Bacteria;p__Firmicutes;c__Clostridia;o__Oscillospirales;f__Oscillospiraceae;g__Flavonifractor	91.5	blast;93.80 iPHoP-RF;79.80
S1-wms-combined_394574	96.28	d__Bacteria;p__Bacteroidota;c__Bacteroidia;o__Bacteroidales;f__Tannerellaceae;g__Parabacteroides	91.6	blast;93.90 iPHoP-RF;75.70
S1-wms-combined_394574	96.28	d__Bacteria;p__Bacteroidota;c__Bacteroidia;o__Bacteroidales;f__Marinifilaceae;g__Odoribacter	90	RaFAH;89.50 CRISPR;64.40
S1-wms-combined_40115	17.83	d__Bacteria;p__Firmicutes;c__Clostridia;o__Oscillospirales;f__Oscillospiraceae;g__ER4	97	blast;97.90 iPHoP-RF;71.70
S1-wms-combined_41369	37.96	d__Bacteria;p__Firmicutes;c__Clostridia;o__Oscillospirales;f__Oscillospiraceae;g__ER4	94.6	blast;96.20 iPHoP-RF;60.60
S1-wms-combined_52708	55.79	d__Bacteria;p__Firmicutes;c__Clostridia;o__Oscillospirales;f__Ruminococcaceae;g__Faecalibacterium	96.3	blast;97.10 iPHoP-RF;70.00 RaFAH;54.80
S1-wms-combined_57370	50.73	d__Bacteria;p__Firmicutes;c__Clostridia;o__Oscillospirales;f__Oscillospiraceae;g__CAG-83	91.7	blast;89.00 RaFAH;72.80
S1-wms-combined_68998	25.66	d__Bacteria;p__Firmicutes;c__Clostridia;o__Oscillospirales;f__Ruminococcaceae;g__Faecalibacterium	99.9	CRISPR;98.60 blast;92.00 iPHoP-RF;91.40 RaFAH;91.30
S1-wms-combined_72167	16.24	d__Bacteria;p__Firmicutes;c__Clostridia;o__Oscillospirales;f__Oscillospiraceae;g__CAG-83	98.5	CRISPR;98.90 blast;95.00 iPHoP-RF;90.80
S1-wms-combined_85225	64.47	d__Bacteria;p__Actinobacteriota;c__Actinomycetia;o__Actinomycetales;f__Bifidobacteriaceae;g__Bifidobacterium	99.6	CRISPR;99.60 iPHoP-RF;97.10 blast;96.90
S1-wms-combined_92523	13.27	d__Bacteria;p__Proteobacteria;c__Alphaproteobacteria;o__RF32;f__CAG-239;g__51-20	95	blast;96.50 iPHoP-RF;71.30
S1-wms-combined_94988	21.16	d__Bacteria;p__Firmicutes;c__Clostridia;o__Oscillospirales;f__Oscillospiraceae;g__CAG-83	95.6	blast;96.90 iPHoP-RF;94.10
S1-wms_102342	14.71	d__Bacteria;p__Firmicutes;c__Clostridia;o__Oscillospirales;f__Oscillospiraceae;g__Dysosmobacter	95	blast;96.50 iPHoP-RF;60.30
S1-wms_102342	14.71	d__Bacteria;p__Firmicutes;c__Clostridia;o__Oscillospirales;f__Oscillospiraceae;g__Intestinimonas	94.9	blast;96.40 iPHoP-RF;57.90
S1-wms_130214	33.32	d__Bacteria;p__Firmicutes;c__Clostridia;o__Oscillospirales;f__Oscillospiraceae;g__CAG-170	90.6	blast;93.00 iPHoP-RF;90.50 CRISPR;82.90
S1-wms_153615		d__Bacteria;p__Bacteroidota;c__Bacteroidia;o__Bacteroidales;f__Barnesiellaceae;g__Barnesiella	93.8	blast;95.60 iPHoP-RF;91.10
S1-wms_185046	36.29	d__Bacteria;p__Firmicutes;c__Clostridia;o__Oscillospirales;f__Oscillospiraceae;g__CAG-170	91.1	blast;93.50 iPHoP-RF;82.20
S1-wms_208875	95.4	d__Bacteria;p__Bacteroidota;c__Bacteroidia;o__Bacteroidales;f__Bacteroidaceae;g__Bacteroides	95.7	blast;95.00 iPHoP-RF;94.40 RaFAH;70.30
S1-wms_223620	12.07	d__Bacteria;p__Firmicutes;c__Clostridia;o__Christensenellales;f__CAG-74;g__UBA11524	97.6	blast;98.30 CRISPR;88.60 iPHoP-RF;86.00
S1-wms_236105	71.3	d__Bacteria;p__Firmicutes;c__Clostridia;o__Oscillospirales;f__Oscillospiraceae;g__ER4	92.1	blast;94.30 iPHoP-RF;89.80 CRISPR;65.50
S1-wms_244836	99.89	d__Bacteria;p__Bacteroidota;c__Bacteroidia;o__Bacteroidales;f__Bacteroidaceae;g__Bacteroides	100	RaFAH;99.60 iPHoP-RF;96.40 blast;94.20
S1-wms_244836	99.89	d__Bacteria;p__Bacteroidota;c__Bacteroidia;o__Bacteroidales;f__Bacteroidaceae;g__Phocaeicola	93.9	iPHoP-RF;95.70 blast;92.10
S1-wms_29288	41.26	d__Bacteria;p__Firmicutes;c__Clostridia;o__Oscillospirales;f__Ruminococcaceae;g__Faecalibacterium	91.6	blast;93.90 CRISPR;78.00 iPHoP-RF;75.70
S1-wms_296850	38.32	d__Bacteria;p__Firmicutes;c__Clostridia;o__Oscillospirales;f__Oscillospiraceae;g__ER4	93.1	blast;95.10 iPHoP-RF;63.80
S1-wms_394571	22.14	d__Bacteria;p__Bacteroidota;c__Bacteroidia;o__Bacteroidales;f__Rikenellaceae;g__Alistipes	95.4	CRISPR;96.80 iPHoP-RF;71.90
S1-wms_4277	93.43	d__Bacteria;p__Firmicutes;c__Clostridia;o__Oscillospirales;f__Oscillospiraceae;g__Lawsonibacter	91.4	blast;93.70
S11-wms-combined_101337	98.94	d__Bacteria;p__Proteobacteria;c__Alphaproteobacteria;o__RF32;f__CAG-239;g__CAG-495	91.6	blast;93.90 iPHoP-RF;86.30 CRISPR;51.40
S11-wms-combined_112733	59.77	d__Bacteria;p__Desulfobacterota;c__Desulfovibrionia;o__Desulfovibrionales;f__Desulfovibrionaceae;g__Desulfovibrio	97.9	blast;96.30 iPHoP-RF;88.90 RaFAH;79.80 CRISPR;67.90
S11-wms-combined_116589	25.47	d__Bacteria;p__Firmicutes;c__Clostridia;o__Oscillospirales;f__Oscillospiraceae;g__Dysosmobacter	91.6	blast;93.90 iPHoP-RF;88.90
S11-wms-combined_119215	29.67	d__Bacteria;p__Firmicutes;c__Clostridia;o__Oscillospirales;f__Oscillospiraceae;g__An92	94.9	iPHoP-RF;96.40 blast;92.70
S11-wms-combined_119215	29.67	d__Bacteria;p__Firmicutes;c__Clostridia;o__Oscillospirales;f__Oscillospiraceae;g__Flavonifractor	94.9	iPHoP-RF;96.40 blast;92.00 CRISPR;55.60
S11-wms-combined_119215	29.67	d__Bacteria;p__Firmicutes;c__Clostridia;o__Oscillospirales;f__Oscillospiraceae;g__Pseudoflavonifractor	91.4	iPHoP-RF;93.70
S11-wms-combined_120257	40.42	d__Bacteria;p__Firmicutes;c__Clostridia;o__Oscillospirales;f__Oscillospiraceae;g__Flavonifractor	91.1	blast;93.50 iPHoP-RF;66.00
S11-wms-combined_125176	94.89	d__Bacteria;p__Bacteroidota;c__Bacteroidia;o__Bacteroidales;f__Rikenellaceae;g__Alistipes	96.9	CRISPR;97.80 iPHoP-RF;97.10 blast;96.90
S11-wms-combined_140009	58.05	d__Bacteria;p__Firmicutes;c__Clostridia;o__Oscillospirales;f__Ruminococcaceae;g__Faecalibacterium	95.6	blast;96.90 iPHoP-RF;71.90
S11-wms-combined_154845	84.31	d__Bacteria;p__Proteobacteria;c__Gammaproteobacteria;o__Enterobacterales;f__Enterobacteriaceae;g__Salmonella	95.4	blast;96.80
S11-wms-combined_154845	84.31	d__Bacteria;p__Proteobacteria;c__Gammaproteobacteria;o__Enterobacterales;f__Enterobacteriaceae;g__Escherichia	95	blast;96.50
S11-wms-combined_154845	84.31	d__Bacteria;p__Proteobacteria;c__Gammaproteobacteria;o__Enterobacterales;f__Enterobacteriaceae;g__Enterobacter	93.8	blast;95.60 iPHoP-RF;66.30
S11-wms-combined_154845	84.31	d__Bacteria;p__Proteobacteria;c__Gammaproteobacteria;o__Enterobacterales;f__Enterobacteriaceae;g__Citrobacter	90	blast;92.50
S11-wms-combined_168048	95.9	d__Bacteria;p__Firmicutes;c__Clostridia;o__Oscillospirales;f__Ruminococcaceae;g__Ruminiclostridium_E	93.4	blast;95.30 iPHoP-RF;66.80
S11-wms-combined_168048	95.9	d__Bacteria;p__Firmicutes;c__Clostridia;o__Acetivibrionales;f__Acetivibrionaceae;g__Ruminiclostridium	93.1	RaFAH;95.10
S11-wms-combined_172889	100	d__Bacteria;p__Bacteroidota;c__Bacteroidia;o__Bacteroidales;f__Rikenellaceae;g__Alistipes	100	RaFAH;99.10 blast;97.90 iPHoP-RF;94.70
S11-wms-combined_172889	100	d__Bacteria;p__Bacteroidota;c__Bacteroidia;o__Bacteroidales;f__Rikenellaceae;g__Alistipes_A	98.8	RaFAH;99.10
S11-wms-combined_179605	37.98	d__Bacteria;p__Firmicutes;c__Clostridia;o__Lachnospirales;f__Lachnospiraceae;g__UBA9502	91.2	blast;93.60 iPHoP-RF;64.00
S11-wms-combined_193148	97.83	d__Bacteria;p__Proteobacteria;c__Alphaproteobacteria;o__RF32;f__CAG-239;g__CAG-267	94.9	blast;96.40 iPHoP-RF;75.00
S11-wms-combined_195425	89.45	d__Bacteria;p__Bacteroidota;c__Bacteroidia;o__Bacteroidales;f__Bacteroidaceae;g__Bacteroides	96.2	blast;90.20 RaFAH;86.00

S11-wms-combined_195425	89.45	d__Bacteria;p__Bacteroidota;c__Bacteroidia;o__Bacteroidales;f__Tannerellaceae;g__Parabacteroides	96.2	blast;96.20 iPHoP-RF;78.00 RaFAH;64.50 CRISPR;58.70
S11-wms-combined_200084	31.8	d__Bacteria;p__Firmicutes;c__Clostridia;o__Lachnospirales;f__Lachnospiraceae;g__Blautia_A	91.8	blast;94.10 iPHoP-RF;53.60
S11-wms-combined_204522	15.39	d__Bacteria;p__Proteobacteria;c__Alphaproteobacteria;o__RF32;f__CAG-239;g__CAG-267	91.7	blast;94.00 iPHoP-RF;72.60
S11-wms-combined_215723	94.77	d__Bacteria;p__Bacteroidota;c__Bacteroidia;o__Bacteroidales;f__Rikenellaceae;g__Alistipes	99.4	iPHoP-RF;97.10 blast;96.90 RaFAH;91.40
S11-wms-combined_217790	99.99	d__Bacteria;p__Bacteroidota;c__Bacteroidia;o__Bacteroidales;f__Bacteroidaceae;g__Bacteroides	99.9	RaFAH;99.80
S11-wms-combined_217790	99.99	d__Bacteria;p__Bacteroidota;c__Bacteroidia;o__Bacteroidales;f__Rikenellaceae;g__Alistipes	97.3	blast;98.10 iPHoP-RF;90.50
S11-wms-combined_226165	80.02	d__Bacteria;p__Bacteroidota;c__Bacteroidia;o__Bacteroidales;f__Rikenellaceae;g__Alistipes	98.7	iPHoP-RF;97.10 blast;93.80 RaFAH;82.70
S11-wms-combined_236214	48.8	d__Bacteria;p__Firmicutes;c__Clostridia;o__Oscillospirales;f__Ruminococcaceae;g__Faecalibacterium	94.6	blast;96.20 iPHoP-RF;57.60
S11-wms-combined_264050	47.18	d__Bacteria;p__Firmicutes;c__Clostridia;o__Oscillospirales;f__Ruminococcaceae;g__Gemmiger	97.5	blast;98.20 iPHoP-RF;93.40 CRISPR;91.60
S11-wms-combined_267993	26.2	d__Bacteria;p__Firmicutes;c__Clostridia;o__Christensenellales;f__CAG-74;g__Firm-11	91.5	blast;93.80 iPHoP-RF;75.20
S11-wms-combined_268967	35.57	d__Bacteria;p__Desulfobacterota;c__Desulfovibrionia;o__Desulfovibrionales;f__Desulfovibrionaceae;g__Desulfovibrio	93.8	blast;95.10 iPHoP-RF;81.90 RaFAH;54.80 CRISPR;51.90
S11-wms-combined_282227	60.6	d__Bacteria;p__Firmicutes;c__Clostridia;o__Lachnospirales;f__Lachnospiraceae;g__Mediterraneibacter	91.2	blast;93.60 iPHoP-RF;73.00
S11-wms-combined_283220	78.49	d__Bacteria;p__Firmicutes;c__Clostridia;o__Oscillospirales;f__Oscillospiraceae;g__Dysosmobacter	91.4	blast;93.70 iPHoP-RF;83.60
S11-wms-combined_299954	23.91	d__Bacteria;p__Firmicutes;c__Clostridia;o__Oscillospirales;f__Oscillospiraceae;g__Intestinimonas	90.2	blast;92.70 iPHoP-RF;84.20
S11-wms-combined_321938	99.59	d__Bacteria;p__Bacteroidota;c__Bacteroidia;o__Bacteroidales;f__Bacteroidaceae;g__Bacteroides	100	RaFAH;98.70 iPHoP-RF;94.10 blast;80.70
S11-wms-combined_329493	9.74	d__Bacteria;p__Firmicutes;c__Clostridia;o__Oscillospirales;f__Oscillospiraceae;g__Faecousia	96.7	blast;97.70 iPHoP-RF;80.00
S11-wms-combined_329493	9.74	d__Bacteria;p__Firmicutes;c__Clostridia;o__Oscillospirales;f__Oscillospiraceae;g__CAG-110	93.6	blast;95.50 iPHoP-RF;74.80
S11-wms-combined_3507	24.17	d__Bacteria;p__Firmicutes;c__Clostridia;o__Oscillospirales;f__Oscillospiraceae;g__CAG-83	98.1	CRISPR;98.60 blast;96.90 iPHoP-RF;92.80
S11-wms-combined_41367	40.35	d__Bacteria;p__Bacteroidota;c__Bacteroidia;o__Bacteroidales;f__Bacteroidaceae;g__Bacteroides	98.5	RaFAH;97.90 iPHoP-RF;74.10
S11-wms-combined_46816	20.69	d__Bacteria;p__Firmicutes;c__Clostridia;o__Oscillospirales;f__Ruminococcaceae;g__Gemmiger	95.4	blast;96.80 iPHoP-RF;91.40 CRISPR;69.40
S11-wms-combined_48246	99.82	d__Bacteria;p__Bacteroidota;c__Bacteroidia;o__Bacteroidales;f__Rikenellaceae;g__Alistipes	100	blast;98.50 RaFAH;96.00 iPHoP-RF;95.40
S11-wms-combined_48246	99.82	d__Bacteria;p__Bacteroidota;c__Bacteroidia;o__Bacteroidales;f__Rikenellaceae;g__Alistipes_A	94.3	RaFAH;96.00
S11-wms-combined_49632	14.66	d__Bacteria;p__Firmicutes;c__Clostridia;o__Oscillospirales;f__Oscillospiraceae;g__Dysosmobacter	91.5	blast;93.80 iPHoP-RF;84.80
S11-wms-combined_63818	50.37	d__Bacteria;p__Bacteroidota;c__Bacteroidia;o__Bacteroidales;f__Rikenellaceae;g__Alistipes	98.4	iPHoP-RF;97.10 blast;96.90 RaFAH;78.80
S11-wms-combined_64392	100	d__Bacteria;p__Bacteroidota;c__Bacteroidia;o__Bacteroidales;f__Rikenellaceae;g__Alistipes	99.6	iPHoP-RF;97.10 blast;96.80 RaFAH;92.70
S11-wms-combined_64392	100	d__Bacteria;p__Bacteroidota;c__Bacteroidia;o__Bacteroidales;f__Rikenellaceae;g__Alistipes_A	90.2	RaFAH;92.70
S11-wms-combined_73204	100	d__Bacteria;p__Bacteroidota;c__Bacteroidia;o__Bacteroidales;f__Bacteroidaceae;g__Bacteroides	99.4	RaFAH;99.30 iPHoP-RF;66.50
S11-wms-combined_83571	99.96	d__Bacteria;p__Bacteroidota;c__Bacteroidia;o__Bacteroidales;f__Rikenellaceae;g__Alistipes	100	RaFAH;98.50 blast;98.40 iPHoP-RF;96.70
S11-wms-combined_83571	99.96	d__Bacteria;p__Bacteroidota;c__Bacteroidia;o__Bacteroidales;f__Rikenellaceae;g__Alistipes_A	97.9	RaFAH;98.50
S11-wms-combined_84326	16.31	d__Bacteria;p__Desulfobacterota;c__Desulfovibrionia;o__Desulfovibrionales;f__Desulfovibrionaceae;g__Desulfovibrio	94.2	blast;95.90 iPHoP-RF;89.50 CRISPR;83.30
S11-wms-combined_92114	19.84	d__Bacteria;p__Bacteroidota;c__Bacteroidia;o__Bacteroidales;f__Rikenellaceae;g__Alistipes	97.2	CRISPR;98.00 iPHoP-RF;97.10 blast;96.90
S11-wms_14390	96.43	d__Bacteria;p__Firmicutes;c__Clostridia;o__Oscillospirales;f__Oscillospiraceae;g__CAG-83	97.3	RaFAH;98.10
S11-wms_14390	96.43	d__Bacteria;p__Firmicutes;c__Clostridia;o__Oscillospirales;f__Oscillospiraceae;g__Dysosmobacter	97.3	RaFAH;98.10
S11-wms_14390	96.43	d__Bacteria;p__Firmicutes;c__Clostridia;o__Oscillospirales;f__Oscillospiraceae;g__Flavonifractor	93.5	blast;95.40 iPHoP-RF;70.90
S11-wms_3116	99.5	d__Bacteria;p__Firmicutes;c__Clostridia;o__Oscillospirales;f__Ruminococcaceae;g__Faecalibacterium	100	RaFAH;99.60 blast;92.10
S11-wms_320555	99.4	d__Bacteria;p__Bacteroidota;c__Bacteroidia;o__Bacteroidales;f__Bacteroidaceae;g__Bacteroides	99.7	RaFAH;95.40 blast;92.70 iPHoP-RF;74.60
S11-wms_32691	12	d__Bacteria;p__Firmicutes;c__Clostridia;o__Oscillospirales;f__Oscillospiraceae;g__Pseudoflavonifractor	94.9	blast;96.40 iPHoP-RF;66.80
S11-wms_330479	20.29	d__Bacteria;p__Bacteroidota;c__Bacteroidia;o__Bacteroidales;f__Rikenellaceae;g__Alistipes	98.5	CRISPR;98.90 iPHoP-RF;78.50
S12-wms-combined_111524	100	d__Bacteria;p__Bacteroidota;c__Bacteroidia;o__Bacteroidales;f__Bacteroidaceae;g__Bacteroides	99.6	RaFAH;99.60
S12-wms-combined_111524	100	d__Bacteria;p__Bacteroidota;c__Bacteroidia;o__Bacteroidales;f__Rikenellaceae;g__Alistipes	95.6	blast;96.90 iPHoP-RF;96.70 CRISPR;80.20
S12-wms-combined_117447		d__Bacteria;p__Firmicutes;c__Clostridia;o__Christensenellales;f__CAG-917;g__	91	blast;93.40 iPHoP-RF;67.30
S12-wms-combined_117628	32.91	d__Bacteria;p__Firmicutes;c__Clostridia;o__Oscillospirales;f__Acutalibacteraceae;g__CAG-217	96.2	blast;97.30 iPHoP-RF;73.20
S12-wms-combined_120133	82.27	d__Bacteria;p__Firmicutes;c__Clostridia;o__Lachnospirales;f__Lachnospiraceae;g__Lachnospira	99.1	CRISPR;97.80 blast;97.00 iPHoP-RF;96.40 RaFAH;84.70
S12-wms-combined_129554	47.38	d__Bacteria;p__Firmicutes;c__Clostridia;o__Oscillospirales;f__Oscillospiraceae;g__F23-B02	95	blast;96.50 iPHoP-RF;75.90
S12-wms-combined_129799	52.43	d__Bacteria;p__Firmicutes;c__Clostridia;o__Oscillospirales;f__Oscillospiraceae;g__ER4	95.6	blast;96.90 iPHoP-RF;63.00
S12-wms-combined_147628	13.34	d__Bacteria;p__Firmicutes;c__Clostridia;o__Christensenellales;f__CAG-138;g__SFEL01	96	CRISPR;97.20 blast;93.30 iPHoP-RF;69.80
S12-wms-combined_148741	20.82	d__Bacteria;p__Firmicutes;c__Clostridia;o__Oscillospirales;f__Oscillospiraceae;g__CAG-83	93.9	blast;95.70 iPHoP-RF;94.70 CRISPR;93.00
S12-wms-combined_151843	24.49	d__Bacteria;p__Firmicutes;c__Clostridia;o__Christensenellales;f__CAG-74;g__UBA11524	95.6	blast;96.90 iPHoP-RF;92.80
S12-wms-combined_151843	24.49	d__Bacteria;p__Firmicutes;c__Clostridia;o__Christensenellales;f__CAG-74;g__UBA11524	95.6	blast;96.90 iPHoP-RF;90.50 CRISPR;77.70
S12-wms-combined_157167	8.42	d__Bacteria;p__Firmicutes;c__Clostridia;o__Oscillospirales;f__Oscillospiraceae;g__CAG-83	92.5	blast;94.60 iPHoP-RF;70.90
S12-wms-combined_164656	3.93	d__Bacteria;p__Firmicutes;c__Clostridia;o__Christensenellales;f__CAG-917;g__CAG-475	94.7	blast;96.30 iPHoP-RF;68.60
S12-wms-combined_168259	10.85	d__Bacteria;p__Verrucomicrobiota;c__Verrucomicrobiae;o__Verrucomicrobiales;f__Akkermansiaceae;g__Akkermansia	100	RaFAH;98.70 blast;96.90 CRISPR;95.20 iPHoP-RF;87.60
S12-wms-combined_184338	20.69	d__Bacteria;p__Firmicutes;c__Clostridia;o__Oscillospirales;f__Oscillospiraceae;g__Dysosmobacter	91.6	blast;93.90 iPHoP-RF;83.90
S12-wms-combined_189323	72.16	d__Bacteria;p__Bacteroidota;c__Bacteroidia;o__Bacteroidales;f__Rikenellaceae;g__Alistipes	99.4	blast;97.60 iPHoP-RF;96.70 RaFAH;89.00 CRISPR;53.90
S12-wms-combined_190114	93.65	d__Bacteria;p__Bacteroidota;c__Bacteroidia;o__Bacteroidales;f__Bacteroidaceae;g__Bacteroides	100	RaFAH;99.60 blast;96.70 iPHoP-RF;95.10
S12-wms-combined_201125	83.81	d__Bacteria;p__Bacteroidota;c__Bacteroidia;o__Bacteroidales;f__Bacteroidaceae;g__Bacteroides	99.4	iPHoP-RF;96.40 RaFAH;93.60 blast;77.30
S12-wms-combined_201125	83.81	d__Bacteria;p__Bacteroidota;c__Bacteroidia;o__Bacteroidales;f__Bacteroidaceae;g__Phocaeicola	95.2	blast;96.60 iPHoP-RF;80.60
S12-wms-combined_210942	9.09	d__Bacteria;p__Firmicutes;c__Clostridia;o__Oscillospirales;f__Oscillospiraceae;g__ER4	96.3	blast;97.40
S12-wms-combined_223043	99.11	d__Bacteria;p__Firmicutes;c__Clostridia;o__Lachnospirales;f__Lachnospiraceae;g__Enterocloster	91.5	blast;93.80 iPHoP-RF;64.30
S12-wms-combined_23517	28.19	d__Bacteria;p__Firmicutes;c__Clostridia;o__Oscillospirales;f__Oscillospiraceae;g__CAG-83	98.5	blast;98.90 CRISPR;98.40 iPHoP-RF;93.10
S12-wms-combined_2450	57.23	d__Bacteria;p__Bacteroidota;c__Bacteroidia;o__Bacteroidales;f__Rikenellaceae;g__Tidjanibacter	90.2	blast;92.70 iPHoP-RF;50.70
S12-wms-combined_250551	98.55	d__Bacteria;p__Bacteroidota;c__Bacteroidia;o__Bacteroidales;f__Bacteroidaceae;g__Bacteroides	100	RaFAH;99.40 blast;92.10 iPHoP-RF;83.90
S12-wms-combined_250551	98.55	d__Bacteria;p__Bacteroidota;c__Bacteroidia;o__Bacteroidales;f__Bacteroidaceae;g__Phocaeicola	92.5	blast;94.60 iPHoP-RF;75.00
S12-wms-combined_25459	9.92	d__Bacteria;p__Bacteroidota;c__Bacteroidia;o__Bacteroidales;f__Rikenellaceae;g__Alistipes	95.6	blast;96.90 iPHoP-RF;96.70
S12-wms-combined_256299	44.45	d__Bacteria;p__Bacteroidota;c__Bacteroidia;o__Bacteroidales;f__Muribaculaceae;g__UBA7173	90.1	blast;92.60 iPHoP-RF;75.70
S12-wms-combined_259783	81.56	d__Bacteria;p__Firmicutes;c__Clostridia;o__Lachnospirales;f__Lachnospiraceae;g__Roseburia	96.7	CRISPR;97.70 blast;92.20 iPHoP-RF;70.60
S12-wms-combined_259783	81.56	d__Bacteria;p__Firmicutes;c__Clostridia;o__Lachnospirales;f__Lachnospiraceae;g__RUG115	96.6	CRISPR;97.60 iPHoP-RF;50.40
S12-wms-combined_259783	81.56	d__Bacteria;p__Firmicutes;c__Clostridia;o__Lachnospirales;f__Lachnospiraceae;g__UBA11774	96.3	CRISPR;97.40 iPHoP-RF;61.70
S12-wms-combined_259783	81.56	d__Bacteria;p__Firmicutes;c__Clostridia;o__Lachnospirales;f__Lachnospiraceae;g__Lachnospira	95	blast;96.50 iPHoP-RF;52.00
S12-wms-combined_260036	44.16	d__Bacteria;p__Bacteroidota;c__Bacteroidia;o__Bacteroidales;f__Barnesiellaceae;g__Barnesiella	94.6	blast;96.20 iPHoP-RF;94.10 CRISPR;67.50
S12-wms-combined_263377	78.23	d__Bacteria;p__Firmicutes;c__Clostridia;o__Oscillospirales;f__Acutalibacteraceae;g__UBA1417	91.7	blast;94.00 iPHoP-RF;69.30
S12-wms-combined_264077	19.02	d__Bacteria;p__Firmicutes;c__Clostridia;o__Oscillospirales;f__Ruminococcaceae;g__Faecalibacterium	90.4	blast;92.90 iPHoP-RF;86.30
S12-wms-combined_264425	99.56	d__Bacteria;p__Firmicutes;c__Clostridia;o__Oscillospirales;f__Acutalibacteraceae;g__UBA1417	91.8	blast;94.10 iPHoP-RF;57.30
S12-wms-combined_264845	6.58	d__Bacteria;p__Firmicutes;c__Clostridia;o__Oscillospirales;f__Oscillospiraceae;g__CAG-110	95	blast;96.50 iPHoP-RF;90.10
S12-wms-combined_270839	20.16	d__Bacteria;p__Firmicutes;c__Clostridia;o__Oscillospirales;f__Oscillospiraceae;g__CAG-83	92.5	blast;94.60 iPHoP-RF;79.80
S12-wms-combined_282594	18.25	d__Bacteria;p__Verrucomicrobiota;c__Verrucomicrobiae;o__Verrucomicrobiales;f__Akkermansiaceae;g__Akkermansia	100	CRISPR;98.70 RaFAH;98.50 blast;96.90 iPHoP-RF;78.50
S12-wms-combined_293354	10.3	d__Bacteria;p__Firmicutes;c__Clostridia;o__Christensenellales;f__CAG-74;g__UBA11524	95.6	blast;96.90 iPHoP-RF;92.80
S12-wms-combined_293354	10.3	d__Bacteria;p__Firmicutes;c__Clostridia;o__Christensenellales;f__CAG-74;g__UBA11524	95.6	blast;96.90 CRISPR;96.60 iPHoP-RF;93.10
S12-wms-combined_297545	99.69	d__Bacteria;p__Bacteroidota;c__Bacteroidia;o__Bacteroidales;f__Bacteroidaceae;g__Bacteroides	99.9	RaFAH;98.50 blast;90.90 iPHoP-RF;65.60
S12-wms-combined_299293	62.93	d__Bacteria;p__Firmicutes;c__Clostridia;o__Christensenellales;f__CAG-74;g__SFFH01	90.4	blast;92.90 iPHoP-RF;75.40
S12-wms-combined_302876	24.68	d__Bacteria;p__Firmicutes;c__Clostridia;o__Lachnospirales;f__Lachnospiraceae;g__Lachnospira	93.9	blast;95.70 iPHoP-RF;73.50
S12-wms-combined_302876	24.68	d__Bacteria;p__Firmicutes;c__Clostridia;o__Lachnospirales;f__Lachnospiraceae;g__RUG115	93.9	blast;95.70 iPHoP-RF;70.00
S12-wms-combined_313953	97.5	d__Bacteria;p__Proteobacteria;c__Alphaproteobacteria;o__RF32;f__CAG-239;g__CAG-495	90.9	blast;93.30 iPHoP-RF;78.50
S12-wms-combined_31761	54.85	d__Bacteria;p__Firmicutes;c__Clostridia;o__Lachnospirales;f__Lachnospiraceae;g__COE1	93.6	blast;95.50 iPHoP-RF;70.20
S12-wms-combined_320262	15.45	d__Bacteria;p__Bacteroidota;c__Bacteroidia;o__Bacteroidales;f__Rikenellaceae;g__Alistipes	97.8	blast;98.40 iPHoP-RF;97.10
S12-wms-combined_320727	99.96	d__Bacteria;p__Bacteroidota;c__Bacteroidia;o__Bacteroidales;f__Bacteroidaceae;g__Bacteroides	93.8	RaFAH;93.60 blast;61.60 iPHoP-RF;55.70
S12-wms-combined_323943	18.94	d__Bacteria;p__Firmicutes;c__Clostridia;o__Oscillospirales;f__Ruminococcaceae;g__Gemmiger	94.6	CRISPR;96.20 blast;93.90 iPHoP-RF;91.80
S12-wms-combined_328554	31.03	d__Bacteria;p__Bacteroidota;c__Bacteroidia;o__Bacteroidales;f__Rikenellaceae;g__Alistipes	96.3	iPHoP-RF;96.70 blast;95.80 RaFAH;60.10
S12-wms-combined_329242	5.39	d__Bacteria;p__Firmicutes;c__Clostridia;o__Oscillospirales;f__Oscillospiraceae;g__Dysosmobacter	91.1	blast;93.50 iPHoP-RF;56.00
S12-wms-combined_334806	38.66	d__Bacteria;p__Firmicutes;c__Clostridia;o__Oscillospirales;f__Oscillospiraceae;g__CAG-83	91.6	blast;93.90 iPHoP-RF;61.90
S12-wms-combined_345948	59.64	d__Bacteria;p__Firmicutes;c__Clostridia;o__Oscillospirales;f__Ruminococcaceae;g__Faecalibacterium	98.8	blast;96.90 iPHoP-RF;92.10 RaFAH;84.90 CRISPR;63.30
S12-wms-combined_351847	11.93	d__Bacteria;p__Bacteroidota;c__Bacteroidia;o__Bacteroidales;f__Barnesiellaceae;g__Barnesiella	95	blast;96.50 iPHoP-RF;88.20 CRISPR;72.90
S12-wms-combined_358797	99.42	d__Bacteria;p__Bacteroidota;c__Bacteroidia;o__Bacteroidales;f__Rikenellaceae;g__Alistipes	100	blast;98.40 iPHoP-RF;96.10 RaFAH;95.40
S12-wms-combined_358797	99.42	d__Bacteria;p__Bacteroidota;c__Bacteroidia;o__Bacteroidales;f__Rikenellaceae;g__Alistipes_A	93.5	RaFAH;95.40
S12-wms-combined_368190	100	d__Bacteria;p__Bacteroidota;c__Bacteroidia;o__Bacteroidales;f__Bacteroidaceae;g__Bacteroides	100	RaFAH;98.90 iPHoP-RF;97.10 blast;91.30
S12-wms-combined_368190	100	d__Bacteria;p__Bacteroidota;c__Bacteroidia;o__Bacteroidales;f__Bacteroidaceae;g__Phocaeicola	93.9	blast;95.70 iPHoP-RF;95.70 CRISPR;79.60
S12-wms-combined_368562	98.38	d__Bacteria;p__Bacteroidota;c__Bacteroidia;o__Bacteroidales;f__Bacteroidaceae;g__Bacteroides	100	RaFAH;99.70 blast;93.90 iPHoP-RF;92.80
S12-wms-combined_371541	14.41	d__Bacteria;p__Bacteroidota;c__Bacteroidia;o__Bacteroidales;f__UBA11471;g__UBA11471	91.6	blast;93.90 iPHoP-RF;90.10
S12-wms-combined_371541	14.41	d__Bacteria;p__Bacteroidota;c__Bacteroidia;o__Bacteroidales;f__Bacteroidaceae;g__Bacteroides	90.7	RaFAH;93.10
S12-wms-combined_375343	20.72	d__Bacteria;p__Bacteroidota;c__Bacteroidia;o__Bacteroidales;f__UBA11471;g__UBA11471	91.6	blast;93.90 iPHoP-RF;88.50
S12-wms-combined_393703	8.02	d__Bacteria;p__Firmicutes;c__Clostridia;o__Oscillospirales;f__Oscillospiraceae;g__ER4	93.5	blast;95.40 iPHoP-RF;50.20

S12-wms-combined_48648	6.97	d__Bacteria;p__Firmicutes;c__Clostridia;o__Oscillospirales;f__Oscillospiraceae;g__ER4	90.9	blast;93.30 iPHoP-RF;72.40
S12-wms-combined_52101	35.58	d__Bacteria;p__Firmicutes;c__Clostridia;o__Oscillospirales;f__Ruminococcaceae;g__UBA3818	90.3	blast;92.80 iPHoP-RF;62.20
S12-wms-combined_63649	5.54	d__Bacteria;p__Firmicutes;c__Clostridia;o__Oscillospirales;f__Oscillospiraceae;g__CAG-83	91.7	blast;94.00 iPHoP-RF;90.10
S12-wms-combined_71235	58.55	d__Bacteria;p__Firmicutes;c__Clostridia;o__Oscillospirales;f__Ruminococcaceae;g__Faecalibacterium	95.7	blast;96.80 iPHoP-RF;84.20 RaFAH;53.60
S12-wms-combined_75733		d__Bacteria;p__Bacteroidota;c__Bacteroidia;o__Bacteroidales;f__Barnesiellaceae;g__Barnesiella	94.6	blast;96.20 iPHoP-RF;69.80
S12-wms-combined_91231	99.33	d__Bacteria;p__Bacteroidota;c__Bacteroidia;o__Bacteroidales;f__Bacteroidaceae;g__Bacteroides	100	RaFAH;99.80 iPHoP-RF;96.10 blast;94.00
S12-wms-combined_91231	99.33	d__Bacteria;p__Bacteroidota;c__Bacteroidia;o__Bacteroidales;f__Bacteroidaceae;g__Phocaeicola	91.6	blast;93.90 iPHoP-RF;82.20
S12-wms_12658	51.35	d__Bacteria;p__Bacteroidota;c__Bacteroidia;o__Bacteroidales;f__Bacteroidaceae;g__Prevotellamassilia	97.9	CRISPR;98.50 blast;60.80
S12-wms_12658	51.35	d__Bacteria;p__Bacteroidota;c__Bacteroidia;o__Bacteroidales;f__Bacteroidaceae;g__Bacteroides	92.7	blast;90.60 RaFAH;72.60
S12-wms_184276	36.83	d__Bacteria;p__Actinobacteriota;c__Coriobacteriia;o__Coriobacteriales;f__Eggerthellaceae;g__Adlercreutzia	93.1	blast;95.10 iPHoP-RF;66.80
S12-wms_184276	36.83	d__Bacteria;p__Actinobacteriota;c__Coriobacteriia;o__Coriobacteriales;f__Coriobacteriaceae;g__Collinsella	92.6	CRISPR;94.70
S12-wms_193565	96.64	d__Bacteria;p__Firmicutes;c__Clostridia;o__Oscillospirales;f__Ruminococcaceae;g__Faecalibacterium	90.2	RaFAH;92.70
S12-wms_223235		d__Bacteria;p__Firmicutes;c__Clostridia;o__Oscillospirales;f__Ruminococcaceae;g__Ruminococcus_D	95.3	blast;96.70 iPHoP-RF;74.80 CRISPR;67.70
S12-wms_235125	20.48	d__Bacteria;p__Firmicutes;c__Clostridia;o__Oscillospirales;f__Oscillospiraceae;g__ER4	90.8	blast;93.20 iPHoP-RF;64.80
S12-wms_336780	4.76	d__Bacteria;p__Firmicutes;c__Clostridia;o__Oscillospirales;f__Ruminococcaceae;g__UMGS363	95.3	blast;96.70 iPHoP-RF;93.10
S12-wms_373775	11.82	d__Bacteria;p__Firmicutes;c__Clostridia;o__Oscillospirales;f__Oscillospiraceae;g__CAG-83	91.5	blast;93.80 iPHoP-RF;82.80
S12-wms_386692	94.2	d__Bacteria;p__Bacteroidota;c__Bacteroidia;o__Bacteroidales;f__Rikenellaceae;g__Alistipes	96.2	CRISPR;97.30 iPHoP-RF;97.10 blast;96.60
S12-wms_386692	94.2	d__Bacteria;p__Bacteroidota;c__Bacteroidia;o__Bacteroidales;f__Bacteroidaceae;g__Bacteroides	91.6	RaFAH;93.90
S12-wms_401372	65.9	d__Bacteria;p__Patescibacteria;c__Paceibacteria;o__Paceibacterales;f__UBA5633;g__MWCK01	91.1	blast;93.50
S17-wms-combined_106940	77.34	d__Bacteria;p__Bacteroidota;c__Bacteroidia;o__Bacteroidales;f__Bacteroidaceae;g__Bacteroides	99.6	iPHoP-RF;97.10 blast;96.90 RaFAH;92.40
S17-wms-combined_106940	77.34	d__Bacteria;p__Bacteroidota;c__Bacteroidia;o__Bacteroidales;f__Bacteroidaceae;g__Phocaeicola	94.9	iPHoP-RF;96.40 blast;95.70
S17-wms-combined_136622	24.07	d__Bacteria;p__Firmicutes;c__Clostridia;o__Oscillospirales;f__Oscillospiraceae;g__Evtepia	90.7	blast;93.10 iPHoP-RF;81.60
S17-wms-combined_146050	6.87	d__Bacteria;p__Bacteroidota;c__Bacteroidia;o__Bacteroidales;f__Bacteroidaceae;g__Bacteroides	100	RaFAH;98.70 blast;96.80 iPHoP-RF;92.80
S17-wms-combined_146850	3.97	d__Bacteria;p__Firmicutes;c__Clostridia;o__Oscillospirales;f__Oscillospiraceae;g__Flavonifractor	91.2	blast;93.60 iPHoP-RF;74.30
S17-wms-combined_157468	3.55	d__Bacteria;p__Firmicutes;c__Clostridia;o__Oscillospirales;f__Oscillospiraceae;g__Flavonifractor	93.4	blast;95.30 iPHoP-RF;74.80
S17-wms-combined_158354	99.65	d__Bacteria;p__Bacteroidota;c__Bacteroidia;o__Bacteroidales;f__Bacteroidaceae;g__Bacteroides	100	RaFAH;99.30 iPHoP-RF;97.10 blast;92.60
S17-wms-combined_158354	99.65	d__Bacteria;p__Bacteroidota;c__Bacteroidia;o__Bacteroidales;f__Bacteroidaceae;g__Phocaeicola	95.9	iPHoP-RF;97.10 blast;96.20 CRISPR;86.70
S17-wms-combined_162786	7.07	d__Bacteria;p__Firmicutes;c__Clostridia;o__Oscillospirales;f__Oscillospiraceae;g__Marseille-P3106	94.7	blast;96.30 iPHoP-RF;71.30
S17-wms-combined_16309	27.66	d__Bacteria;p__Bacteroidota;c__Bacteroidia;o__Bacteroidales;f__Bacteroidaceae;g__Bacteroides	100	RaFAH;99.10 iPHoP-RF;97.10 blast;90.90
S17-wms-combined_16309	27.66	d__Bacteria;p__Bacteroidota;c__Bacteroidia;o__Bacteroidales;f__Bacteroidaceae;g__Phocaeicola	92.2	iPHoP-RF;94.40
S17-wms-combined_171305	56.56	d__Bacteria;p__Bacteroidota;c__Bacteroidia;o__Bacteroidales;f__Tannerellaceae;g__Parabacteroides	98.7	CRISPR;99.00 blast;98.50 iPHoP-RF;96.40
S17-wms-combined_175924	99.75	d__Bacteria;p__Firmicutes;c__Clostridia;o__Oscillospirales;f__Oscillospiraceae;g__Flavonifractor	92.1	blast;94.30 iPHoP-RF;78.00
S17-wms-combined_179771	49.28	d__Bacteria;p__Bacteroidota;c__Bacteroidia;o__Bacteroidales;f__Bacteroidaceae;g__Bacteroides	97.9	blast;98.30 iPHoP-RF;96.10 CRISPR;90.30 RaFAH;56.00
S17-wms-combined_179771	49.28	d__Bacteria;p__Bacteroidota;c__Bacteroidia;o__Bacteroidales;f__Bacteroidaceae;g__Phocaeicola	90.3	iPHoP-RF;92.80
S17-wms-combined_185239	7.9	d__Bacteria;p__Firmicutes;c__Clostridia;o__Oscillospirales;f__Oscillospiraceae;g__Flavonifractor	91.4	blast;93.70 iPHoP-RF;83.60
S17-wms-combined_185239	7.9	d__Bacteria;p__Firmicutes;c__Clostridia;o__Oscillospirales;f__Oscillospiraceae;g__Pseudoflavonifractor	91.4	blast;93.70 iPHoP-RF;52.20
S17-wms-combined_186314	29.67	d__Bacteria;p__Actinobacteriota;c__Actinomycetia;o__Actinomycetales;f__Bifidobacteriaceae;g__Bifidobacterium	99.6	CRISPR;99.60 iPHoP-RF;97.10 blast;96.90
S17-wms-combined_193958	96.31	d__Bacteria;p__Firmicutes;c__Clostridia;o__Oscillospirales;f__Ruminococcaceae;g__Faecalibacterium	94.2	blast;95.90 iPHoP-RF;53.80
S17-wms-combined_199746	69.95	d__Bacteria;p__Bacteroidota;c__Bacteroidia;o__Bacteroidales;f__Rikenellaceae;g__Alistipes	97.6	CRISPR;98.30 iPHoP-RF;97.10 blast;96.90
S17-wms-combined_199746	69.95	d__Bacteria;p__Bacteroidota;c__Bacteroidia;o__Bacteroidales;f__Bacteroidaceae;g__Bacteroides	90.8	RaFAH;93.20
S17-wms-combined_223748	4.98	d__Bacteria;p__Firmicutes;c__Clostridia;o__Lachnospirales;f__Lachnospiraceae;g__Enterocloster	91.2	blast;93.60 iPHoP-RF;75.20
S17-wms-combined_225356	10.23	d__Bacteria;p__Actinobacteriota;c__Coriobacteriia;o__Coriobacteriales;f__Eggerthellaceae;g__Eggerthella	96.3	CRISPR;97.40 blast;92.10 iPHoP-RF;87.00
S17-wms-combined_225356	10.23	d__Bacteria;p__Actinobacteriota;c__Coriobacteriia;o__Coriobacteriales;f__Eggerthellaceae;g__Arabia	90.2	CRISPR;92.70 iPHoP-RF;50.70
S17-wms-combined_233761	88	d__Bacteria;p__Bacteroidota;c__Bacteroidia;o__Bacteroidales;f__Bacteroidaceae;g__Bacteroides	100	RaFAH;99.00 blast;98.60 iPHoP-RF;96.40 CRISPR;80.00
S17-wms-combined_247494	24.14	d__Bacteria;p__Firmicutes;c__Clostridia;o__Oscillospirales;f__Oscillospiraceae;g__Flavonifractor	91.5	blast;93.80 iPHoP-RF;91.40
S17-wms-combined_247494	24.14	d__Bacteria;p__Firmicutes;c__Clostridia;o__Oscillospirales;f__Oscillospiraceae;g__Pseudoflavonifractor	90.7	iPHoP-RF;93.10 blast;89.90
S17-wms-combined_253499	25.09	d__Bacteria;p__Bacteroidota;c__Bacteroidia;o__Bacteroidales;f__Rikenellaceae;g__Alistipes	97.2	CRISPR;98.00 blast;95.10 iPHoP-RF;90.80
S17-wms-combined_258087	97.18	d__Bacteria;p__Firmicutes;c__Clostridia;o__Clostridiales;f__Clostridiaceae;g__Clostridium	98.2	RaFAH;98.70
S17-wms-combined_258087	97.18	d__Bacteria;p__Firmicutes;c__Clostridia;o__Clostridiales;f__Clostridiaceae;g__Clostridium_F	98.2	RaFAH;98.70
S17-wms-combined_258087	97.18	d__Bacteria;p__Firmicutes;c__Clostridia;o__Clostridiales;f__Clostridiaceae;g__Clostridium_P	98.2	RaFAH;98.70
S17-wms-combined_259811	66.5	d__Bacteria;p__Firmicutes;c__Clostridia;o__Oscillospirales;f__Oscillospiraceae;g__Lawsonibacter	90.9	blast;93.30
S17-wms-combined_260343	38.59	d__Bacteria;p__Firmicutes;c__Clostridia;o__Oscillospirales;f__Oscillospiraceae;g__Flavonifractor	90.8	blast;93.20
S17-wms-combined_39687	83.01	d__Bacteria;p__Firmicutes;c__Clostridia;o__Oscillospirales;f__Oscillospiraceae;g__Flavonifractor	95.2	blast;96.60 iPHoP-RF;89.20
S17-wms-combined_56006	98.39	d__Bacteria;p__Firmicutes;c__Clostridia;o__Oscillospirales;f__Ruminococcaceae;g__Faecalibacterium	100	RaFAH;98.90 blast;96.90 iPHoP-RF;87.00 CRISPR;67.80
S17-wms-combined_56061	66.23	d__Bacteria;p__Firmicutes;c__Clostridia;o__Oscillospirales;f__Ruminococcaceae;g__Faecalibacterium	98.5	blast;98.90
S17-wms-combined_66123	97.65	d__Bacteria;p__Firmicutes;c__Clostridia;o__Oscillospirales;f__Oscillospiraceae;g__Flavonifractor	90.6	blast;91.80 iPHoP-RF;85.40 CRISPR;73.10 RaFAH;57.20
S17-wms-combined_70983	7.24	d__Bacteria;p__Bacteroidota;c__Bacteroidia;o__Bacteroidales;f__Bacteroidaceae;g__Bacteroides	95.9	iPHoP-RF;97.10 blast;85.40
S17-wms-combined_70983	7.24	d__Bacteria;p__Bacteroidota;c__Bacteroidia;o__Bacteroidales;f__Bacteroidaceae;g__Phocaeicola	94.5	iPHoP-RF;96.10 blast;92.70
S17-wms-combined_72837	25.38	d__Bacteria;p__Firmicutes;c__Clostridia;o__Oscillospirales;f__Ruminococcaceae;g__Gemmiger	95.2	blast;96.60 iPHoP-RF;74.80
S17-wms-combined_75892	53.86	d__Bacteria;p__Firmicutes;c__Clostridia;o__Oscillospirales;f__Oscillospiraceae;g__HGM12998	91.4	blast;93.70 iPHoP-RF;77.50
S17-wms-combined_79709	19.94	d__Bacteria;p__Firmicutes;c__Clostridia;o__Oscillospirales;f__Oscillospiraceae;g__An92	91.4	blast;93.70 iPHoP-RF;92.10
S17-wms-combined_90378	72.41	d__Bacteria;p__Firmicutes;c__Clostridia;o__Oscillospirales;f__Ruminococcaceae;g__Faecalibacterium	98.7	blast;96.90 RaFAH;83.30 iPHoP-RF;76.80 CRISPR;69.70
S17-wms-combined_95360	17.32	d__Bacteria;p__Firmicutes;c__Negativicutes;o__Veillonellales;f__Dialisteraceae;g__Dialister	97.2	CRISPR;98.00 blast;96.90 iPHoP-RF;93.70
S17-wms_124308	51.69	d__Bacteria;p__Bacteroidota;c__Bacteroidia;o__Bacteroidales;f__Bacteroidaceae;g__Bacteroides	99.1	iPHoP-RF;96.40 RaFAH;90.70
S17-wms_254872	5.1	d__Bacteria;p__Bacteroidota;c__Bacteroidia;o__Bacteroidales;f__Bacteroidaceae;g__Bacteroides_G	95.3	iPHoP-RF;96.70
S17-wms_254872	5.1	d__Bacteria;p__Bacteroidota;c__Bacteroidia;o__Bacteroidales;f__Bacteroidaceae;g__Bacteroides	94.9	iPHoP-RF;96.40 CRISPR;89.30
S17-wms_254872	5.1	d__Bacteria;p__Proteobacteria;c__Gammaproteobacteria;o__Pseudomonadales;f__Saccharospirillaceae;g__Saccharospirillum	90	blast;92.50
S2-wms-combined_101826	26.15	d__Bacteria;p__Firmicutes;c__Clostridia;o__Oscillospirales;f__Ruminococcaceae;g__Ruminococcus_D	98.8	CRISPR;99.10 blast;96.90 iPHoP-RF;92.80
S2-wms-combined_113619	97.74	d__Bacteria;p__Bacteroidota;c__Bacteroidia;o__Bacteroidales;f__Rikenellaceae;g__Alistipes	99	blast;96.60 RaFAH;87.50 CRISPR;82.60 iPHoP-RF;77.80
S2-wms-combined_116221	80.02	d__Bacteria;p__Firmicutes;c__Clostridia;o__Lachnospirales;f__Anaerotignaceae;g__Anaerotignum	95.6	blast;96.90 iPHoP-RF;61.10
S2-wms-combined_122457	52.93	d__Bacteria;p__Firmicutes;c__Clostridia;o__Oscillospirales;f__Oscillospiraceae;g__Intestinimonas	91.6	blast;93.90 iPHoP-RF;56.50
S2-wms-combined_126134	14.26	d__Bacteria;p__Firmicutes;c__Clostridia;o__Oscillospirales;f__Oscillospiraceae;g__Lawsonibacter	90.1	blast;92.60 iPHoP-RF;75.70
S2-wms-combined_126440	24	d__Bacteria;p__Firmicutes;c__Clostridia;o__Lachnospirales;f__Lachnospiraceae;g__Acetatifactor	92.7	blast;94.80 iPHoP-RF;71.30
S2-wms-combined_127080	8.57	d__Bacteria;p__Firmicutes;c__Clostridia;o__Oscillospirales;f__Ruminococcaceae;g__Faecalibacterium	98.8	CRISPR;99.10 blast;93.50 iPHoP-RF;89.50
S2-wms-combined_13246	32.69	d__Bacteria;p__Firmicutes;c__Clostridia;o__Oscillospirales;f__Ruminococcaceae;g__Faecalibacterium	99.6	blast;96.90 RaFAH;93.60 iPHoP-RF;92.80 CRISPR;87.50
S2-wms-combined_132966		d__Bacteria;p__Bacteroidota;c__Bacteroidia;o__Bacteroidales;f__Bacteroidaceae;g__Bacteroides	93.1	iPHoP-RF;95.10
S2-wms-combined_135014	100	d__Bacteria;p__Firmicutes;c__Negativicutes;o__Veillonellales;f__Dialisteraceae;g__Dialister	100	RaFAH;97.60 blast;96.90 CRISPR;96.10 iPHoP-RF;92.40
S2-wms-combined_135139	38.63	d__Bacteria;p__Verrucomicrobiota;c__Verrucomicrobiae;o__Verrucomicrobiales;f__Akkermansiaceae;g__Akkermansia	97.8	CRISPR;98.40 blast;96.90 iPHoP-RF;88.20
S2-wms-combined_135154	34.29	d__Bacteria;p__Firmicutes;c__Negativicutes;o__Veillonellales;f__Dialisteraceae;g__Dialister	95.9	blast;97.10 iPHoP-RF;97.10 CRISPR;75.50
S2-wms-combined_137119	36.79	d__Bacteria;p__Firmicutes;c__Clostridia;o__Oscillospirales;f__Oscillospiraceae;g__An92	93.5	blast;95.40 iPHoP-RF;76.60
S2-wms-combined_140996	59.11	d__Bacteria;p__Firmicutes;c__Clostridia;o__Oscillospirales;f__Oscillospiraceae;g__ER4	95.6	blast;96.90 iPHoP-RF;70.20
S2-wms-combined_143737	99.75	d__Bacteria;p__Bacteroidota;c__Bacteroidia;o__Bacteroidales;f__Bacteroidaceae;g__Bacteroides	100	blast;97.80 RaFAH;97.50 iPHoP-RF;78.50
S2-wms-combined_147065	99.85	d__Bacteria;p__Bacteroidota;c__Bacteroidia;o__Bacteroidales;f__Bacteroidaceae;g__Bacteroides	100	RaFAH;97.00 blast;96.90 iPHoP-RF;95.10 CRISPR;58.20
S2-wms-combined_157934	99.51	d__Bacteria;p__Firmicutes;c__Clostridia;o__Lachnospirales;f__Lachnospiraceae;g__Roseburia	98.8	CRISPR;98.90 iPHoP-RF;92.80 blast;89.30 RaFAH;59.00
S2-wms-combined_157934	99.51	d__Bacteria;p__Firmicutes;c__Clostridia;o__Lachnospirales;f__Lachnospiraceae;g__Agathobacter	98.7	CRISPR;98.80 iPHoP-RF;96.70 blast;96.30 RaFAH;59.00
S2-wms-combined_157934	99.51	d__Bacteria;p__Firmicutes;c__Clostridia;o__Lachnospirales;f__Lachnospiraceae;g__UBA11774	97.9	CRISPR;98.50 iPHoP-RF;68.20
S2-wms-combined_158821	99.16	d__Bacteria;p__Firmicutes;c__Clostridia;o__Lachnospirales;f__Lachnospiraceae;g__Enterocloster	91.5	blast;93.80 iPHoP-RF;63.50
S2-wms-combined_159670	16.22	d__Bacteria;p__Firmicutes;c__Clostridia;o__Oscillospirales;f__Oscillospiraceae;g__CAG-83	91.6	blast;93.90 iPHoP-RF;88.20
S2-wms-combined_165833	74.42	d__Bacteria;p__Firmicutes;c__Clostridia;o__Lachnospirales;f__Lachnospiraceae;g__Roseburia	97.9	blast;96.20 iPHoP-RF;92.80 RaFAH;79.80
S2-wms-combined_166665	48.97	d__Bacteria;p__Firmicutes;c__Clostridia;o__Oscillospirales;f__Ruminococcaceae;g__Faecalibacterium	95.4	blast;96.80 iPHoP-RF;96.10
S2-wms-combined_176376	94.42	d__Bacteria;p__Firmicutes;c__Clostridia;o__Lachnospirales;f__Lachnospiraceae;g__Blautia_A	96.7	blast;93.20 CRISPR;89.80 iPHoP-RF;87.90 RaFAH;83.30
S2-wms-combined_181849	40.03	d__Bacteria;p__Firmicutes;c__Clostridia;o__Oscillospirales;f__Ruminococcaceae;g__Faecalibacterium	95.6	blast;96.90 CRISPR;95.80 iPHoP-RF;87.30
S2-wms-combined_185494	25.15	d__Bacteria;p__Firmicutes;c__Negativicutes;o__Veillonellales;f__Dialisteraceae;g__Dialister	96.3	CRISPR;97.40 blast;96.90 iPHoP-RF;90.10
S2-wms-combined_191613	13.4	d__Bacteria;p__Firmicutes;c__Clostridia;o__Lachnospirales;f__Anaerotignaceae;g__Anaerotignum	95.2	blast;96.60 iPHoP-RF;70.60
S2-wms-combined_196783	32.37	d__Bacteria;p__Firmicutes;c__Clostridia;o__Oscillospirales;f__Oscillospiraceae;g__Intestinimonas	95.9	CRISPR;97.10 blast;91.40 iPHoP-RF;85.10
S2-wms-combined_196783	32.37	d__Bacteria;p__Firmicutes;c__Clostridia;o__Oscillospirales;f__Oscillospiraceae;g__Flavonifractor	93.5	CRISPR;95.40 blast;91.00 iPHoP-RF;83.30
S2-wms-combined_197577	42.77	d__Bacteria;p__Firmicutes;c__Negativicutes;o__Selenomonadales;f__Selenomonadaceae;g__Megamonas	91.1	blast;93.50 iPHoP-RF;53.30
S2-wms-combined_199384	17.07	d__Bacteria;p__Firmicutes;c__Clostridia;o__Oscillospirales;f__Butyricicoccaceae;g__Agathobaculum	91.4	blast;93.70 iPHoP-RF;73.50
S2-wms-combined_199384	17.07	d__Bacteria;p__Firmicutes;c__Clostridia;o__Oscillospirales;f__Butyricicoccaceae;g__Butyricicoccus	91.1	blast;93.50 iPHoP-RF;67.70
S2-wms-combined_200390	14.49	d__Bacteria;p__Firmicutes;c__Clostridia;o__Lachnospirales;f__Lachnospiraceae;g__Eisenbergiella	92	CRISPR;94.20 iPHoP-RF;67.50
S2-wms-combined_201205	82.22	d__Bacteria;p__Verrucomicrobiota;c__Verrucomicrobiae;o__Verrucomicrobiales;f__Akkermansiaceae;g__Akkermansia	100	CRISPR;99.30 blast;96.80 RaFAH;96.70 iPHoP-RF;68.60

S2-wms-combined_204225	6.16	d__Bacteria;p__Firmicutes;c__Clostridia;o__Lachnospirales;f__Lachnospiraceae;g__Lachnoclostridium_A	91.6	blast;93.90 iPHoP-RF;79.30
S2-wms-combined_207000	92.52	d__Bacteria;p__Firmicutes;c__Clostridia;o__Lachnospirales;f__Lachnospiraceae;g__Agathobacter	93.4	blast;95.30 iPHoP-RF;93.70
S2-wms-combined_207000	92.52	d__Bacteria;p__Firmicutes;c__Clostridia;o__Lachnospirales;f__Lachnospiraceae;g__Roseburia	90.7	iPHoP-RF;93.10 blast;92.00
S2-wms-combined_208559	10.84	d__Bacteria;p__Bacteroidota;c__Bacteroidia;o__Bacteroidales;f__Rikenellaceae;g__Alistipes	95.6	blast;96.90 iPHoP-RF;96.70
S2-wms-combined_210822	37.52	d__Bacteria;p__Bacteroidota;c__Bacteroidia;o__Bacteroidales;f__Bacteroidaceae;g__Bacteroides	91.8	iPHoP-RF;89.50 blast;81.20 RaFAH;71.80
S2-wms-combined_218002	99.73	d__Bacteria;p__Firmicutes;c__Clostridia;o__Lachnospirales;f__Lachnospiraceae;g__Roseburia	99.9	blast;98.50 iPHoP-RF;92.40 RaFAH;92.30
S2-wms-combined_218002	99.73	d__Bacteria;p__Firmicutes;c__Clostridia;o__Lachnospirales;f__Lachnospiraceae;g__RUG115	99.3	RaFAH;92.30 blast;87.70 iPHoP-RF;83.10
S2-wms-combined_218002	99.73	d__Bacteria;p__Firmicutes;c__Clostridia;o__Lachnospirales;f__Lachnospiraceae;g__Agathobacter	99	RaFAH;92.30 blast;89.90 iPHoP-RF;73.00
S2-wms-combined_222535	7.99	d__Bacteria;p__Firmicutes;c__Clostridia;o__Lachnospirales;f__Lachnospiraceae;g__Agathobacter	92.1	blast;94.30 iPHoP-RF;92.10
S2-wms-combined_222535	7.99	d__Bacteria;p__Firmicutes;c__Clostridia;o__Lachnospirales;f__Lachnospiraceae;g__Roseburia	90.7	iPHoP-RF;93.10 blast;91.00
S2-wms-combined_225908	87.71	d__Bacteria;p__Firmicutes;c__Clostridia;o__Oscillospirales;f__Acutalibacteraceae;g__CAG-217	95.6	blast;96.90 CRISPR;93.30 iPHoP-RF;79.00
S2-wms-combined_229529	100	d__Bacteria;p__Bacteroidota;c__Bacteroidia;o__Bacteroidales;f__Bacteroidaceae;g__Bacteroides	99.9	blast;96.90 iPHoP-RF;96.70 RaFAH;96.10
S2-wms-combined_238929	17.21	d__Bacteria;p__Firmicutes;c__Clostridia;o__Oscillospirales;f__Oscillospiraceae;g__CAG-170	91.4	blast;93.70 iPHoP-RF;78.30
S2-wms-combined_24549	17.49	d__Bacteria;p__Firmicutes;c__Clostridia;o__Oscillospirales;f__Oscillospiraceae;g__Intestinimonas	90.9	blast;93.30
S2-wms-combined_25727	21.03	d__Bacteria;p__Firmicutes;c__Clostridia;o__Christensenellales;f__CAG-74;g__Ventricola	94.7	blast;96.30 iPHoP-RF;74.60
S2-wms-combined_25727	21.03	d__Bacteria;p__Firmicutes;c__Clostridia;o__Christensenellales;f__CAG-74;g__SFFH01	94.7	blast;96.30 iPHoP-RF;71.90
S2-wms-combined_30750	11.82	d__Bacteria;p__Firmicutes;c__Clostridia;o__Oscillospirales;f__Ruminococcaceae;g__Ruminococcus_D	95.6	blast;96.90 iPHoP-RF;82.50
S2-wms-combined_31084	22.49	d__Bacteria;p__Firmicutes;c__Clostridia;o__Lachnospirales;f__Lachnospiraceae;g__Acetivibrio_A	90	blast;92.50 iPHoP-RF;69.10
S2-wms-combined_48661	11.3	d__Bacteria;p__Firmicutes;c__Clostridia;o__Lachnospirales;f__Lachnospiraceae;g__Anaerosacchariphilus	90.8	blast;93.20 iPHoP-RF;77.30
S2-wms-combined_52568	12.66	d__Bacteria;p__Firmicutes;c__Clostridia;o__Oscillospirales;f__Oscillospiraceae;g__F23-B02	92.3	blast;94.50 iPHoP-RF;80.30
S2-wms-combined_57293	78.69	d__Bacteria;p__Firmicutes;c__Clostridia;o__Oscillospirales;f__Ruminococcaceae;g__Faecalibacterium	95.7	blast;96.90 iPHoP-RF;88.90 RaFAH;51.90
S2-wms-combined_5791	89.76	d__Bacteria;p__Firmicutes;c__Clostridia;o__Lachnospirales;f__Lachnospiraceae;g__Agathobacter	92.1	CRISPR;94.30
S2-wms-combined_5791	89.76	d__Bacteria;p__Firmicutes;c__Clostridia;o__Lachnospirales;f__Lachnospiraceae;g__Roseburia	91	CRISPR;93.40
S2-wms-combined_5791	89.76	d__Bacteria;p__Firmicutes;c__Clostridia;o__Lachnospirales;f__Lachnospiraceae;g__Eisenbergiella	90.8	blast;93.20
S2-wms-combined_58121	21.08	d__Bacteria;p__Bacteroidota;c__Bacteroidia;o__Bacteroidales;f__Bacteroidaceae;g__Bacteroides	95.9	iPHoP-RF;97.10 blast;91.00
S2-wms-combined_58121	21.08	d__Bacteria;p__Bacteroidota;c__Bacteroidia;o__Bacteroidales;f__Bacteroidaceae;g__Phocaeicola	95.2	blast;96.60 iPHoP-RF;81.90
S2-wms-combined_58563	88.99	d__Bacteria;p__Firmicutes;c__Clostridia;o__Clostridiales;f__Clostridiaceae;g__Clostridium	95.4	RaFAH;96.80
S2-wms-combined_58563	88.99	d__Bacteria;p__Firmicutes;c__Clostridia;o__Clostridiales;f__Clostridiaceae;g__Clostridium_F	95.4	RaFAH;96.80
S2-wms-combined_58563	88.99	d__Bacteria;p__Firmicutes;c__Clostridia;o__Clostridiales;f__Clostridiaceae;g__Clostridium_P	95.4	RaFAH;96.80
S2-wms-combined_58563	88.99	d__Bacteria;p__Firmicutes;c__Clostridia;o__Oscillospirales;f__Oscillospiraceae;g__F23-B02	94.6	blast;96.20 iPHoP-RF;73.70
S2-wms-combined_62839	32.39	d__Bacteria;p__Firmicutes;c__Clostridia;o__Oscillospirales;f__Ruminococcaceae;g__Gemmiger	97.6	blast;98.30 iPHoP-RF;87.30 CRISPR;73.60
S2-wms-combined_64692	24.76	d__Bacteria;p__Firmicutes;c__Clostridia;o__Oscillospirales;f__Oscillospiraceae;g__	90.8	blast;93.20 iPHoP-RF;85.40
S2-wms-combined_64692	24.76	d__Bacteria;p__Firmicutes;c__Clostridia;o__Oscillospirales;f__Oscillospiraceae;g__Flavonifractor	90.4	blast;92.90 iPHoP-RF;82.20 CRISPR;53.30
S2-wms-combined_6605	7.53	d__Bacteria;p__Firmicutes;c__Clostridia;o__Oscillospirales;f__Oscillospiraceae;g__Flavonifractor	91.4	blast;93.70 iPHoP-RF;82.50
S2-wms-combined_72725	19.31	d__Bacteria;p__Firmicutes;c__Clostridia;o__Oscillospirales;f__Oscillospiraceae;g__Intestinimonas	91.4	blast;93.70 iPHoP-RF;75.40
S2-wms-combined_73327	3.55	d__Bacteria;p__Firmicutes;c__Clostridia;o__Lachnospirales;f__Lachnospiraceae;g__Faecalimonas	91.6	blast;93.90 iPHoP-RF;73.50
S2-wms-combined_74387	38.64	d__Bacteria;p__Firmicutes;c__Clostridia;o__Lachnospirales;f__Lachnospiraceae;g__Lachnoclostridium_A	94.3	blast;96.00 iPHoP-RF;82.50
S2-wms-combined_75144	9.77	d__Bacteria;p__Bacteroidota;c__Bacteroidia;o__Bacteroidales;f__Tannerellaceae;g__Parabacteroides	95.4	blast;96.80 iPHoP-RF;95.10
S2-wms-combined_7756	9.67	d__Bacteria;p__Verrucomicrobiota;c__Verrucomicrobiae;o__Verrucomicrobiales;f__Akkermansiaceae;g__Akkermansia	96.9	blast;96.70 iPHoP-RF;91.10 CRISPR;86.00 RaFAH;66.20
S2-wms-combined_84608	21.12	d__Bacteria;p__Proteobacteria;c__Gammaproteobacteria;o__Burkholderiales;f__Burkholderiaceae;g__Sutterella	95.6	blast;96.90 iPHoP-RF;90.80 CRISPR;79.90
S2-wms-combined_85381	45.07	d__Bacteria;p__Firmicutes;c__Clostridia;o__Lachnospirales;f__Lachnospiraceae;g__CAG-81	93.8	blast;95.60 iPHoP-RF;80.60
S2-wms-combined_93845	99.44	d__Bacteria;p__Firmicutes;c__Clostridia;o__Oscillospirales;f__Oscillospiraceae;g__ER4	98.7	CRISPR;99.00 blast;91.30 iPHoP-RF;81.90
S2-wms-combined_93845	99.44	d__Bacteria;p__Firmicutes;c__Clostridia;o__Oscillospirales;f__Oscillospiraceae;g__F23-B02	95.4	blast;96.80
S2-wms-combined_93845	99.44	d__Bacteria;p__Firmicutes;c__Clostridia;o__Oscillospirales;f__Oscillospiraceae;g__Flavonifractor	90.8	RaFAH;93.20
S2-wms-combined_95987	3.24	d__Bacteria;p__Firmicutes;c__Clostridia;o__Lachnospirales;f__Lachnospiraceae;g__Lachnoclostridium_A	93.2	blast;95.20 iPHoP-RF;84.50
S2-wms_124416	99.32	d__Bacteria;p__Firmicutes;c__Clostridia;o__Oscillospirales;f__Ruminococcaceae;g__Faecalibacterium	100	RaFAH;99.60 blast;96.10 iPHoP-RF;79.30 CRISPR;55.70
S2-wms_128831	98.35	d__Bacteria;p__Proteobacteria;c__Gammaproteobacteria;o__Enterobacterales;f__Enterobacteriaceae;g__Escherichia	95.6	RaFAH;96.90
S2-wms_128831	98.35	d__Bacteria;p__Patescibacteria;c__Paceibacteria;o__Paceibacterales;f__UBA5633;g__MWCK01	92.3	blast;94.50 iPHoP-RF;66.30
S2-wms_128962	46.73	d__Bacteria;p__Bacteroidota;c__Bacteroidia;o__Bacteroidales;f__Bacteroidaceae;g__Prevotella	91.8	RaFAH;94.10
S2-wms_130687	95.89	d__Bacteria;p__Firmicutes;c__Clostridia;o__Oscillospirales;f__Acutalibacteraceae;g__Ruminococcus_E	99.7	CRISPR;98.90 blast;96.90 iPHoP-RF;92.40 RaFAH;85.50
S2-wms_140933	43.87	d__Bacteria;p__Firmicutes;c__Clostridia;o__Lachnospirales;f__Lachnospiraceae;g__Blautia_A	93.8	blast;90.60 RaFAH;76.60 iPHoP-RF;62.50
S2-wms_156439	96.43	d__Bacteria;p__Bacteroidota;c__Bacteroidia;o__Bacteroidales;f__Bacteroidaceae;g__Bacteroides	100	RaFAH;99.60 blast;96.80 iPHoP-RF;95.70
S2-wms_161986	99.14	d__Bacteria;p__Firmicutes;c__Clostridia;o__Lachnospirales;f__Lachnospiraceae;g__Lachnospira	99.9	CRISPR;98.80 RaFAH;92.10 blast;84.30 iPHoP-RF;67.70
S2-wms_161986	99.14	d__Bacteria;p__Firmicutes;c__Clostridia;o__Lachnospirales;f__Lachnospiraceae;g__Eubacterium_G	91	RaFAH;92.10 blast;58.10
S2-wms_192667	13.43	d__Bacteria;p__Firmicutes;c__Clostridia;o__Lachnospirales;f__Lachnospiraceae;g__Eubacterium_I	91.6	blast;93.90 iPHoP-RF;62.20
S2-wms_246296	98.04	d__Bacteria;p__Firmicutes;c__Clostridia;o__Lachnospirales;f__Lachnospiraceae;g__Roseburia	100	CRISPR;98.90 blast;94.40 RaFAH;93.70 iPHoP-RF;92.80
S2-wms_246296	98.04	d__Bacteria;p__Firmicutes;c__Clostridia;o__Lachnospirales;f__Lachnospiraceae;g__RUG115	99.9	CRISPR;98.20 RaFAH;93.70 blast;90.80 iPHoP-RF;84.80
S2-wms_246296	98.04	d__Bacteria;p__Firmicutes;c__Clostridia;o__Lachnospirales;f__Lachnospiraceae;g__Agathobacter	93.6	RaFAH;93.70 iPHoP-RF;64.00
S2-wms_6424	8.31	d__Bacteria;p__Bacteroidota;c__Bacteroidia;o__Bacteroidales;f__Bacteroidaceae;g__Bacteroides	93.6	RaFAH;95.50
S2-wms_79304	81.02	d__Bacteria;p__Actinobacteriota;c__Coriobacteriia;o__Coriobacteriales;f__Eggerthellaceae;g__Eggerthella	94.9	CRISPR;96.40 RaFAH;50.20
S3-wms-combined_135428	11.75	d__Bacteria;p__Actinobacteriota;c__Actinomycetia;o__Actinomycetales;f__Bifidobacteriaceae;g__Bifidobacterium	95.3	blast;96.70 iPHoP-RF;96.40 CRISPR;94.80
S3-wms-combined_45305	27.39	d__Bacteria;p__Firmicutes;c__Clostridia;o__Oscillospirales;f__Ruminococcaceae;g__Gemmiger	94.2	blast;95.90 iPHoP-RF;76.60 CRISPR;55.10
S3-wms-combined_73262	99.96	d__Bacteria;p__Bacteroidota;c__Bacteroidia;o__Bacteroidales;f__Bacteroidaceae;g__Bacteroides	100	RaFAH;99.80 blast;93.60 iPHoP-RF;79.80 CRISPR;67.80
S3-wms_22744	99.21	d__Bacteria;p__Firmicutes;c__Clostridia;o__Oscillospirales;f__Ruminococcaceae;g__Faecalibacterium	97.2	CRISPR;98.00 blast;60.70 iPHoP-RF;52.00
S3-wms_22744	99.21	d__Bacteria;p__Firmicutes;c__Clostridia;o__Oscillospirales;f__Oscillospiraceae;g__CAG-83	91.8	RaFAH;94.10
S3-wms_22744	99.21	d__Bacteria;p__Firmicutes;c__Clostridia;o__Oscillospirales;f__Oscillospiraceae;g__Dysosmobacter	91.8	RaFAH;94.10
S3-wms_23958	21.78	d__Bacteria;p__Firmicutes;c__Clostridia;o__Oscillospirales;f__Oscillospiraceae;g__CAG-170	94.2	blast;95.90 iPHoP-RF;58.70
S3-wms_94494	94.44	d__Bacteria;p__Actinobacteriota;c__Actinomycetia;o__Actinomycetales;f__Bifidobacteriaceae;g__Bifidobacterium	99.3	blast;98.30 iPHoP-RF;97.10 CRISPR;83.00 RaFAH;81.50
S4-wms-combined_10758	88.87	d__Bacteria;p__Bacteroidota;c__Bacteroidia;o__Bacteroidales;f__Tannerellaceae;g__Parabacteroides	100	RaFAH;99.50 blast;89.70 iPHoP-RF;87.00
S4-wms-combined_113721	6.49	d__Bacteria;p__Firmicutes;c__Clostridia;o__Oscillospirales;f__Ruminococcaceae;g__Gemmiger	95.6	blast;96.90 iPHoP-RF;92.80 CRISPR;57.80
S4-wms-combined_118531	66.42	d__Bacteria;p__Firmicutes;c__Negativicutes;o__Acidaminococcales;f__Acidaminococcaceae;g__Phascolarctobacterium_A	98.2	CRISPR;98.70 blast;96.80 iPHoP-RF;92.40
S4-wms-combined_118531	66.42	d__Bacteria;p__Firmicutes;c__Negativicutes;o__Acidaminococcales;f__Acidaminococcaceae;g__Phascolarctobacterium	90.7	blast;93.10 iPHoP-RF;84.20
S4-wms-combined_121978	87.01	d__Bacteria;p__Firmicutes;c__Clostridia;o__Lachnospirales;f__Lachnospiraceae;g__Mediterraneibacter	95	blast;96.50 iPHoP-RF;79.50
S4-wms-combined_14411	97.82	d__Bacteria;p__Bacteroidota;c__Bacteroidia;o__Bacteroidales;f__Tannerellaceae;g__Marseille-P3160	98.4	CRISPR;98.80 iPHoP-RF;68.90
S4-wms-combined_14411	97.82	d__Bacteria;p__Bacteroidota;c__Bacteroidia;o__Bacteroidales;f__Marinifilaceae;g__Odoribacter	97.5	RaFAH;98.20
S4-wms-combined_17284	85.97	d__Bacteria;p__Actinobacteriota;c__Actinomycetia;o__Actinomycetales;f__Bifidobacteriaceae;g__Bifidobacterium	95.3	iPHoP-RF;96.70 CRISPR;96.00 blast;95.40
S4-wms-combined_22012	25.83	d__Bacteria;p__Actinobacteriota;c__Coriobacteriia;o__Coriobacteriales;f__Coriobacteriaceae;g__Collinsella	98.7	CRISPR;99.00 blast;96.80 iPHoP-RF;89.20
S4-wms-combined_24659	6.25	d__Bacteria;p__Firmicutes;c__Clostridia;o__Lachnospirales;f__Lachnospiraceae;g__Roseburia	92.6	iPHoP-RF;94.70
S4-wms-combined_41490	99.65	d__Bacteria;p__Bacteroidota;c__Bacteroidia;o__Bacteroidales;f__Rikenellaceae;g__Alistipes	95.6	blast;96.90 iPHoP-RF;96.10
S4-wms-combined_42647	63.73	d__Bacteria;p__Firmicutes;c__Clostridia;o__Oscillospirales;f__Ruminococcaceae;g__Faecalibacterium	95.7	blast;97.00 iPHoP-RF;78.00 CRISPR;67.90
S4-wms-combined_47565	81.36	d__Bacteria;p__Bacteroidota;c__Bacteroidia;o__Bacteroidales;f__Bacteroidaceae;g__Prevotella	96.2	blast;97.30 iPHoP-RF;90.80
S4-wms-combined_51079	88.31	d__Bacteria;p__Actinobacteriota;c__Actinomycetia;o__Actinomycetales;f__Bifidobacteriaceae;g__Bifidobacterium	99.9	CRISPR;99.60 iPHoP-RF;97.10 blast;96.80 RaFAH;71.10
S4-wms-combined_57123	13.17	d__Bacteria;p__Bacteroidota;c__Bacteroidia;o__Bacteroidales;f__Bacteroidaceae;g__Prevotella	95.6	blast;96.90 iPHoP-RF;73.50
S4-wms-combined_57963	74.8	d__Bacteria;p__Firmicutes;c__Negativicutes;o__Acidaminococcales;f__Acidaminococcaceae;g__Phascolarctobacterium_A	95.6	blast;96.90 iPHoP-RF;64.80
S4-wms-combined_64632	43.29	d__Bacteria;p__Firmicutes;c__Clostridia;o__Lachnospirales;f__Lachnospiraceae;g__Dorea_A	97.6	CRISPR;98.30 iPHoP-RF;95.40 blast;92.00
S4-wms-combined_64632	43.29	d__Bacteria;p__Firmicutes;c__Clostridia;o__Lachnospirales;f__Lachnospiraceae;g__Lachnoclostridium_B	91.4	iPHoP-RF;93.70 blast;82.20
S4-wms-combined_83492	86.97	d__Bacteria;p__Bacteroidota;c__Bacteroidia;o__Bacteroidales;f__Bacteroidaceae;g__Bacteroides	95.6	blast;89.90 iPHoP-RF;86.30 RaFAH;84.50
S4-wms-combined_83492	86.97	d__Bacteria;p__Bacteroidota;c__Bacteroidia;o__Bacteroidales;f__Tannerellaceae;g__Parabacteroides	93.9	blast;95.70 iPHoP-RF;85.70
S4-wms-combined_83492	86.97	d__Bacteria;p__Bacteroidota;c__Bacteroidia;o__Bacteroidales;f__Bacteroidaceae;g__Phocaeicola	91.5	blast;93.80 iPHoP-RF;74.60
S4-wms_107182	7.59	d__Bacteria;p__Actinobacteriota;c__Coriobacteriia;o__Coriobacteriales;f__Eggerthellaceae;g__CAG-1427	96.9	blast;97.80
S4-wms_141607	37.81	d__Bacteria;p__Bacteroidota;c__Bacteroidia;o__Bacteroidales;f__Bacteroidaceae;g__Prevotella	98.4	CRISPR;98.80 blast;96.40 iPHoP-RF;79.00
S4-wms_161443	5.84	d__Bacteria;p__Actinobacteriota;c__Actinomycetia;o__Actinomycetales;f__Bifidobacteriaceae;g__Bifidobacterium	99.6	CRISPR;99.60 iPHoP-RF;78.00
S4-wms_45128	28.59	d__Bacteria;p__Bacteroidota;c__Bacteroidia;o__Bacteroidales;f__Bacteroidaceae;g__Prevotella	94.7	blast;96.30 CRISPR;93.50 iPHoP-RF;91.40
S4-wms_50475	70.49	d__Bacteria;p__Bacteroidota;c__Bacteroidia;o__Bacteroidales;f__Bacteroidaceae;g__Prevotella	97.3	blast;96.90 iPHoP-RF;94.40 RaFAH;69.20 CRISPR;50.60
S4-wms_62635	93.28	d__Bacteria;p__Firmicutes;c__Clostridia;o__Lachnospirales;f__Lachnospiraceae;g__Blautia_A	100	CRISPR;98.90 RaFAH;95.20 iPHoP-RF;93.70 blast;90.40
S4-wms_62635	93.28	d__Bacteria;p__Firmicutes;c__Clostridia;o__Lachnospirales;f__Lachnospiraceae;g__Blautia	93.2	RaFAH;95.20
S4-wms_73990	76.93	d__Bacteria;p__Bacteroidota;c__Bacteroidia;o__Bacteroidales;f__Tannerellaceae;g__Parabacteroides	97.6	blast;98.30 iPHoP-RF;82.80
S5-wms-combined_106597	25.93	d__Bacteria;p__Campylobacterota;c__Campylobacteria;o__Campylobacterales;f__Campylobacteraceae;g__Campylobacter_D	98.8	RaFAH;99.10
S5-wms-combined_106597	25.93	d__Bacteria;p__Campylobacterota;c__Campylobacteria;o__Campylobacterales;f__Campylobacteraceae;g__Campylobacter_B	91.6	blast;93.90
S5-wms-combined_108794	58.74	d__Bacteria;p__Firmicutes;c__Clostridia;o__Oscillospirales;f__Acutalibacteraceae;g__Ruminococcus_E	95.6	blast;96.90 iPHoP-RF;89.50
S5-wms-combined_117095	10.16	d__Bacteria;p__Firmicutes;c__Clostridia;o__Oscillospirales;f__Acutalibacteraceae;g__UBA1417	91	blast;93.40 iPHoP-RF;64.50

S5-wms-combined_117213	97.16	d__Bacteria;p__Actinobacteriota;c__Actinomycetia;o__Actinomycetales;f__Bifidobacteriaceae;g__Bifidobacterium	98.8	CRISPR;97.70 iPHoP-RF;97.10 blast;96.50 RaFAH;80.80
S5-wms-combined_134381	97.01	d__Bacteria;p__Firmicutes;c__Clostridia;o__Oscillospirales;f__Oscillospiraceae;g__CAG-83	98.4	RaFAH;98.30 blast;64.70
S5-wms-combined_134381	97.01	d__Bacteria;p__Firmicutes;c__Clostridia;o__Oscillospirales;f__Oscillospiraceae;g__Dysosmobacter	97.6	RaFAH;98.30
S5-wms-combined_134381	97.01	d__Bacteria;p__Firmicutes;c__Clostridia;o__Oscillospirales;f__Oscillospiraceae;g__CAG-110	90.3	blast;92.80
S5-wms-combined_145067	82.3	d__Bacteria;p__Firmicutes;c__Clostridia;o__Oscillospirales;f__Oscillospiraceae;g__Flavonifractor	95.6	blast;96.90 iPHoP-RF;95.70 CRISPR;53.80
S5-wms-combined_145067	82.3	d__Bacteria;p__Firmicutes;c__Clostridia;o__Oscillospirales;f__Oscillospiraceae;g__Intestinimonas	93.1	iPHoP-RF;95.10
S5-wms-combined_145067	82.3	d__Bacteria;p__Firmicutes;c__Clostridia;o__Oscillospirales;f__Oscillospiraceae;g__An92	92.2	iPHoP-RF;94.40
S5-wms-combined_145067	82.3	d__Bacteria;p__Firmicutes;c__Clostridia;o__Oscillospirales;f__Oscillospiraceae;g__Pseudoflavonifractor	92.2	iPHoP-RF;94.40
S5-wms-combined_156329	75.2	d__Bacteria;p__Firmicutes;c__Bacilli;o__Erysipelotrichales;f__Erysipelotrichaceae;g__Clostridium_AQ	95.9	blast;97.10 CRISPR;90.20 iPHoP-RF;75.20
S5-wms-combined_163165	3.66	d__Bacteria;p__Firmicutes;c__Clostridia;o__Oscillospirales;f__Oscillospiraceae;g__Flavonifractor	91.2	blast;93.60 iPHoP-RF;59.50
S5-wms-combined_178432	13.6	d__Bacteria;p__Bacteroidota;c__Bacteroidia;o__Bacteroidales;f__Rikenellaceae;g__Alistipes	95.9	iPHoP-RF;97.10 blast;91.90 CRISPR;84.60
S5-wms-combined_179241	13.11	d__Bacteria;p__Firmicutes;c__Clostridia;o__Oscillospirales;f__Oscillospiraceae;g__CAG-83	91	blast;93.40 iPHoP-RF;86.70
S5-wms-combined_183113	11.29	d__Bacteria;p__Bacteroidota;c__Bacteroidia;o__Bacteroidales;f__Rikenellaceae;g__Alistipes_A	97	blast;97.90 iPHoP-RF;94.70
S5-wms-combined_18356	90.68	d__Bacteria;p__Proteobacteria;c__Gammaproteobacteria;o__Enterobacterales;f__Enterobacteriaceae;g__Escherichia	98.8	iPHoP-RF;97.10 blast;96.40 RaFAH;85.20 CRISPR;70.30
S5-wms-combined_18356	90.68	d__Bacteria;p__Proteobacteria;c__Gammaproteobacteria;o__Enterobacterales;f__Enterobacteriaceae;g__Salmonella	90.8	blast;93.20
S5-wms-combined_197186	3.6	d__Bacteria;p__Firmicutes;c__Bacilli;o__Erysipelotrichales;f__Erysipelotrichaceae;g__Clostridium_AQ	94.6	blast;96.20 iPHoP-RF;73.70
S5-wms-combined_199135	16.23	d__Bacteria;p__Cyanobacteria;c__Vampirovibrionia;o__Gastranaerophilales;f__Gastranaerophilaceae;g__QAMI01	90.3	blast;92.80
S5-wms-combined_204782	6.93	d__Bacteria;p__Bacteroidota;c__Bacteroidia;o__Bacteroidales;f__Rikenellaceae;g__Alistipes_A	92.1	blast;94.30 iPHoP-RF;69.10
S5-wms-combined_205046	4.59	d__Bacteria;p__Firmicutes;c__Clostridia;o__Oscillospirales;f__Oscillospiraceae;g__Dysosmobacter	92.6	iPHoP-RF;94.70 blast;93.60
S5-wms-combined_207291	100	d__Bacteria;p__Bacteroidota;c__Bacteroidia;o__Bacteroidales;f__Bacteroidaceae;g__Bacteroides	100	RaFAH;99.80 iPHoP-RF;95.70 blast;91.90
S5-wms-combined_210959	81.3	d__Bacteria;p__Bacteroidota;c__Bacteroidia;o__Bacteroidales;f__Rikenellaceae;g__Alistipes	99.4	blast;96.90 iPHoP-RF;96.70 RaFAH;91.40 CRISPR;57.80
S5-wms-combined_26061	99.77	d__Bacteria;p__Firmicutes;c__Clostridia;o__Lachnospirales;f__Lachnospiraceae;g__Blautia_A	96.7	blast;92.60 RaFAH;84.50
S5-wms-combined_3201	23.69	d__Bacteria;p__Proteobacteria;c__Gammaproteobacteria;o__Burkholderiales;f__Burkholderiaceae;g__Oxalobacter	90.9	blast;93.30 iPHoP-RF;69.80
S5-wms-combined_39793		d__Bacteria;p__Actinobacteriota;c__Actinomycetia;o__Actinomycetales;f__Bifidobacteriaceae;g__Bifidobacterium	91.6	blast;93.90 iPHoP-RF;93.40 CRISPR;76.80
S5-wms-combined_52741	50.99	d__Bacteria;p__Verrucomicrobiota;c__Verrucomicrobiae;o__Verrucomicrobiales;f__Akkermansiaceae;g__Akkermansia	100	RaFAH;99.50 CRISPR;98.80 blast;96.90 iPHoP-RF;81.60
S5-wms-combined_59064	95	d__Bacteria;p__Bacteroidota;c__Bacteroidia;o__Bacteroidales;f__Bacteroidaceae;g__Bacteroides	100	RaFAH;97.60 blast;96.90 iPHoP-RF;96.10
S5-wms-combined_66017	9.66	d__Bacteria;p__Firmicutes;c__Clostridia;o__Christensenellales;f__Christensenellaceae;g__Christensenella	90.7	blast;93.10 iPHoP-RF;82.20
S5-wms-combined_66662	100	d__Bacteria;p__Firmicutes;c__Clostridia;o__Oscillospirales;f__Ruminococcaceae;g__Ruthenibacterium	97.2	blast;98.00 iPHoP-RF;88.50
S5-wms-combined_67650	98.81	d__Bacteria;p__Bacteroidota;c__Bacteroidia;o__Bacteroidales;f__Rikenellaceae;g__Alistipes	100	RaFAH;97.10 blast;96.90 iPHoP-RF;93.70
S5-wms-combined_67650	98.81	d__Bacteria;p__Bacteroidota;c__Bacteroidia;o__Bacteroidales;f__Rikenellaceae;g__Alistipes_A	95.9	RaFAH;97.10
S5-wms-combined_72410	60.27	d__Bacteria;p__Firmicutes;c__Clostridia;o__Oscillospirales;f__Ruminococcaceae;g__Ruthenibacterium	90.8	blast;93.20 iPHoP-RF;72.60
S5-wms-combined_7292	22.4	d__Bacteria;p__Firmicutes;c__Clostridia;o__Oscillospirales;f__Acutalibacteraceae;g__Ruminococcus_E	98.5	CRISPR;98.90 blast;96.60 iPHoP-RF;89.80
S5-wms-combined_73301	96.75	d__Bacteria;p__Firmicutes;c__Clostridia;o__Lachnospirales;f__Lachnospiraceae;g__Dorea_A	94.6	blast;96.20 iPHoP-RF;93.10 CRISPR;58.60
S5-wms-combined_73301	96.75	d__Bacteria;p__Firmicutes;c__Clostridia;o__Lachnospirales;f__Lachnospiraceae;g__Lachnoclostridium_B	90.8	blast;93.20 iPHoP-RF;77.80
S5-wms-combined_83370	82.36	d__Bacteria;p__Bacteroidota;c__Bacteroidia;o__Bacteroidales;f__Bacteroidaceae;g__Bacteroides	99.4	blast;96.90 iPHoP-RF;96.70 RaFAH;91.90
S5-wms-combined_83412	25.58	d__Bacteria;p__Firmicutes;c__Clostridia;o__Eubacteriales;f__Eubacteriaceae;g__Eubacterium	92.6	blast;94.70 iPHoP-RF;86.70 CRISPR;77.00
S5-wms-combined_90315	99.97	d__Bacteria;p__Bacteroidota;c__Bacteroidia;o__Bacteroidales;f__Rikenellaceae;g__Alistipes	98.8	RaFAH;96.10 blast;88.90
S5-wms-combined_90315	99.97	d__Bacteria;p__Bacteroidota;c__Bacteroidia;o__Bacteroidales;f__Rikenellaceae;g__Alistipes_A	94.5	RaFAH;96.10
S5-wms-combined_94482	15.25	d__Bacteria;p__Bacteroidota;c__Bacteroidia;o__Bacteroidales;f__Tannerellaceae;g__Parabacteroides	94.9	iPHoP-RF;96.40 blast;91.70
S5-wms-combined_94482	15.25	d__Bacteria;p__Bacteroidota;c__Bacteroidia;o__Bacteroidales;f__Tannerellaceae;g__Macellibacteroides	91.8	iPHoP-RF;94.10 blast;83.60
S5-wms-combined_98202	100	d__Bacteria;p__Bacteroidota;c__Bacteroidia;o__Bacteroidales;f__Bacteroidaceae;g__Bacteroides	99.4	RaFAH;99.50
S5-wms-combined_98202	100	d__Bacteria;p__Bacteroidota;c__Bacteroidia;o__Bacteroidales;f__Rikenellaceae;g__Alistipes	95.6	blast;96.90 iPHoP-RF;77.50
S5-wms_10783	3.75	d__Bacteria;p__Firmicutes;c__Clostridia;o__Oscillospirales;f__Oscillospiraceae;g__Lawsonibacter	90.8	blast;93.20
S5-wms_212596	76.97	d__Bacteria;p__Firmicutes;c__Bacilli;o__Lactobacillales;f__Lactobacillaceae;g__Lacticaseibacillus	100	blast;98.70 RaFAH;95.70 iPHoP-RF;77.10
S5-wms_212596	76.97	d__Bacteria;p__Firmicutes;c__Bacilli;o__Lactobacillales;f__Lactobacillaceae;g__Lactobacillus	93.9	RaFAH;95.70
S5-wms_212596	76.97	d__Bacteria;p__Firmicutes;c__Bacilli;o__Lactobacillales;f__Lactobacillaceae;g__Ligilactobacillus	93.9	RaFAH;95.70
S5-wms_212596	76.97	d__Bacteria;p__Firmicutes;c__Bacilli;o__Lactobacillales;f__Lactobacillaceae;g__Limosilactobacillus	93.9	RaFAH;95.70
S5-wms_49310	96.19	d__Bacteria;p__Firmicutes;c__Clostridia;o__Oscillospirales;f__Acutalibacteraceae;g__Ruminococcus_E	99.7	blast;98.00 CRISPR;97.80 RaFAH;93.50 iPHoP-RF;88.20
S5-wms_49310	96.19	d__Bacteria;p__Firmicutes;c__Clostridia;o__Oscillospirales;f__Ruminococcaceae;g__Ruminococcus_D	91.1	RaFAH;93.50
S5-wms_52566	8.51	d__Bacteria;p__Firmicutes;c__Clostridia;o__Oscillospirales;f__Oscillospiraceae;g__Intestinimonas	91.1	blast;93.50 iPHoP-RF;52.80
S5-wms_62679	11.9	d__Bacteria;p__Actinobacteriota;c__Coriobacteriia;o__Coriobacteriales;f__Eggerthellaceae;g__Eggerthella	94.6	blast;96.20
S5-wms_85650	99.85	d__Bacteria;p__Bacteroidota;c__Bacteroidia;o__Bacteroidales;f__Bacteroidaceae;g__Bacteroides	100	RaFAH;99.00 iPHoP-RF;97.10 blast;96.60
S5-wms_85650	99.85	d__Bacteria;p__Bacteroidota;c__Bacteroidia;o__Bacteroidales;f__Bacteroidaceae;g__Phocaeicola	94.9	iPHoP-RF;96.40 blast;90.30
S5-wms_94965	12.7	d__Bacteria;p__Actinobacteriota;c__Coriobacteriia;o__Coriobacteriales;f__Coriobacteriaceae;g__Collinsella	97.5	CRISPR;98.20 blast;95.40 iPHoP-RF;77.10
S6-wms-combined_110950	42.23	d__Bacteria;p__Firmicutes;c__Clostridia;o__Oscillospirales;f__Ruminococcaceae;g__Faecalibacterium	99.6	blast;96.80 RaFAH;93.40 iPHoP-RF;93.10 CRISPR;73.10
S6-wms-combined_117756	50.35	d__Bacteria;p__Firmicutes;c__Clostridia;o__Lachnospirales;f__Lachnospiraceae;g__Acetatifactor	91.6	blast;93.90 iPHoP-RF;77.50
S6-wms-combined_118427	40.24	d__Bacteria;p__Firmicutes;c__Clostridia;o__Oscillospirales;f__Oscillospiraceae;g__Dysosmobacter	97.9	iPHoP-RF;93.70 blast;91.90 RaFAH;88.40
S6-wms-combined_118427	40.24	d__Bacteria;p__Firmicutes;c__Clostridia;o__Oscillospirales;f__Oscillospiraceae;g__CAG-83	96.3	blast;88.80 RaFAH;88.40 iPHoP-RF;78.50
S6-wms-combined_119017	37.52	d__Bacteria;p__Desulfobacterota;c__Desulfovibrionia;o__Desulfovibrionales;f__Desulfovibrionaceae;g__Bilophila	94.2	blast;95.90 iPHoP-RF;92.40 CRISPR;66.40
S6-wms-combined_119017	37.52	d__Bacteria;p__Desulfobacterota;c__Desulfovibrionia;o__Desulfovibrionales;f__Desulfovibrionaceae;g__Bilophila	92.7	blast;94.80 iPHoP-RF;92.80
S6-wms-combined_119138	99.25	d__Bacteria;p__Firmicutes;c__Clostridia;o__Lachnospirales;f__Lachnospiraceae;g__Enterocloster	91.6	blast;93.90
S6-wms-combined_126236	26.53	d__Bacteria;p__Firmicutes;c__Clostridia;o__Oscillospirales;f__Ruminococcaceae;g__Faecalibacterium	97.6	blast;98.30 CRISPR;97.70 iPHoP-RF;86.70
S6-wms-combined_135223	23.09	d__Bacteria;p__Bacteroidota;c__Bacteroidia;o__Bacteroidales;f__Bacteroidaceae;g__Bacteroides	99	iPHoP-RF;96.40 blast;92.30 RaFAH;88.60
S6-wms-combined_135223	23.09	d__Bacteria;p__Bacteroidota;c__Bacteroidia;o__Bacteroidales;f__Bacteroidaceae;g__Phocaeicola	94.9	iPHoP-RF;96.40 blast;89.30
S6-wms-combined_138710	55.97	d__Bacteria;p__Firmicutes;c__Clostridia;o__Oscillospirales;f__Ruminococcaceae;g__Faecalibacterium	96.2	blast;96.90 iPHoP-RF;78.00 RaFAH;56.20
S6-wms-combined_140731	27.76	d__Bacteria;p__Firmicutes;c__Clostridia;o__Oscillospirales;f__Ruminococcaceae;g__Faecalibacterium	98.4	CRISPR;92.30 RaFAH;92.10 blast;92.10 iPHoP-RF;80.30
S6-wms-combined_32530	99.74	d__Bacteria;p__Firmicutes;c__Clostridia;o__Lachnospirales;f__Lachnospiraceae;g__Roseburia	99.3	RaFAH;94.70 blast;87.00 iPHoP-RF;77.10
S6-wms-combined_32530	99.74	d__Bacteria;p__Firmicutes;c__Clostridia;o__Lachnospirales;f__Lachnospiraceae;g__Agathobacter	97.8	RaFAH;94.70 blast;81.80 iPHoP-RF;59.80
S6-wms-combined_32530	99.74	d__Bacteria;p__Firmicutes;c__Clostridia;o__Lachnospirales;f__Lachnospiraceae;g__RUG115	96	RaFAH;94.70 blast;71.80 iPHoP-RF;53.80
S6-wms-combined_32530	99.74	d__Bacteria;p__Firmicutes;c__Clostridia;o__Lachnospirales;f__Lachnospiraceae;g__Acetatifactor	90.4	blast;92.90
S6-wms-combined_41246	21.08	d__Bacteria;p__Firmicutes;c__Clostridia;o__Oscillospirales;f__Oscillospiraceae;g__Marseille-P3106	91.2	blast;93.60
S6-wms-combined_49909	50.09	d__Bacteria;p__Firmicutes;c__Clostridia;o__Oscillospirales;f__Ruminococcaceae;g__Faecalibacterium	91	blast;93.40 iPHoP-RF;86.00
S6-wms-combined_50721	99.98	d__Bacteria;p__Bacteroidota;c__Bacteroidia;o__Bacteroidales;f__Rikenellaceae;g__Alistipes	99.6	blast;96.90 iPHoP-RF;96.70 RaFAH;94.30
S6-wms-combined_50721	99.98	d__Bacteria;p__Bacteroidota;c__Bacteroidia;o__Bacteroidales;f__Rikenellaceae;g__Alistipes_A	92.1	RaFAH;94.30
S6-wms-combined_56464	51.17	d__Bacteria;p__Firmicutes;c__Clostridia;o__Oscillospirales;f__Ruminococcaceae;g__Faecalibacterium	99	blast;96.90 iPHoP-RF;92.10 RaFAH;87.90
S6-wms-combined_65225	23.74	d__Bacteria;p__Firmicutes;c__Negativicutes;o__Veillonellales;f__Dialisteraceae;g__Dialister	94.9	blast;96.40 CRISPR;86.10 iPHoP-RF;77.30
S6-wms-combined_72228	41.84	d__Bacteria;p__Bacteroidota;c__Bacteroidia;o__Bacteroidales;f__Rikenellaceae;g__Alistipes	99	iPHoP-RF;97.10 blast;96.60 RaFAH;85.90
S6-wms-combined_745	7.61	d__Bacteria;p__Proteobacteria;c__Alphaproteobacteria;o__RF32;f__CAG-239;g__CAG-267	90.6	blast;93.00 iPHoP-RF;84.50
S6-wms-combined_82597	91.55	d__Bacteria;p__Bacteroidota;c__Bacteroidia;o__Bacteroidales;f__Bacteroidaceae;g__Bacteroides	99.7	iPHoP-RF;97.10 blast;96.90 RaFAH;95.40
S6-wms-combined_91039	100	d__Bacteria;p__Bacteroidota;c__Bacteroidia;o__Bacteroidales;f__Rikenellaceae;g__Alistipes	96.6	CRISPR;97.60 iPHoP-RF;97.10 blast;96.70
S6-wms-combined_91039	100	d__Bacteria;p__Bacteroidota;c__Bacteroidia;o__Bacteroidales;f__Bacteroidaceae;g__Bacteroides	91.5	RaFAH;93.80
S6-wms_132588	67.09	d__Bacteria;p__Firmicutes;c__Clostridia;o__Oscillospirales;f__Ruminococcaceae;g__Faecalibacterium	93.2	CRISPR;95.20 blast;94.40 iPHoP-RF;92.80
S6-wms_138342	37.57	d__Bacteria;p__Bacteroidota;c__Bacteroidia;o__Bacteroidales;f__Bacteroidaceae;g__Prevotella	91.7	RaFAH;94.00
S6-wms_138342	37.57	d__Bacteria;p__Spirochaetota;c__Spirochaetia;o__Sphaerochaetales;f__Sphaerochaetaceae;g__UBA9732	90	blast;92.50 iPHoP-RF;60.30
S6-wms_140743	9.85	d__Bacteria;p__Firmicutes;c__Clostridia;o__Lachnospirales;f__Lachnospiraceae;g__Enterocloster	91.2	blast;93.60 iPHoP-RF;92.40 CRISPR;53.80
S6-wms_54578	97	d__Bacteria;p__Bacteroidota;c__Bacteroidia;o__Bacteroidales;f__Tannerellaceae;g__Marseille-P3160	97.5	CRISPR;98.20 iPHoP-RF;69.80
S6-wms_54578	97	d__Bacteria;p__Bacteroidota;c__Bacteroidia;o__Bacteroidales;f__Tannerellaceae;g__Parabacteroides	92	blast;94.20 iPHoP-RF;68.00
S6-wms_54578	97	d__Bacteria;p__Bacteroidota;c__Bacteroidia;o__Bacteroidales;f__Marinifilaceae;g__Odoribacter	90.7	RaFAH;93.10
S7-wms-combined_104872	97.79	d__Bacteria;p__Bacteroidota;c__Bacteroidia;o__Bacteroidales;f__Bacteroidaceae;g__Bacteroides	100	RaFAH;99.70 blast;92.10 iPHoP-RF;90.50 CRISPR;53.10
S7-wms-combined_106253	71.87	d__Bacteria;p__Firmicutes;c__Clostridia;o__Lachnospirales;f__Lachnospiraceae;g__Dorea_A	95.3	blast;96.70 iPHoP-RF;94.40
S7-wms-combined_106253	71.87	d__Bacteria;p__Firmicutes;c__Clostridia;o__Lachnospirales;f__Lachnospiraceae;g__Extibacter	92.2	iPHoP-RF;94.40 blast;79.50
S7-wms-combined_107679_0	99.98	d__Bacteria;p__Bacteroidota;c__Bacteroidia;o__Bacteroidales;f__Rikenellaceae;g__Alistipes	100	RaFAH;98.50 blast;97.20 iPHoP-RF;96.40
S7-wms-combined_107679_0	99.98	d__Bacteria;p__Bacteroidota;c__Bacteroidia;o__Bacteroidales;f__Rikenellaceae;g__Alistipes_A	97.9	RaFAH;98.50
S7-wms-combined_107679_1	100	d__Bacteria;p__Bacteroidota;c__Bacteroidia;o__Bacteroidales;f__Bacteroidaceae;g__Bacteroides	99.9	RaFAH;99.80 iPHoP-RF;57.60
S7-wms-combined_107679_1	100	d__Bacteria;p__Bacteroidota;c__Bacteroidia;o__Bacteroidales;f__Rikenellaceae;g__Alistipes	98.1	blast;98.60 iPHoP-RF;64.80
S7-wms-combined_111004	34.72	d__Bacteria;p__Firmicutes;c__Clostridia;o__Oscillospirales;f__Ruminococcaceae;g__Faecalibacterium	99.7	CRISPR;98.50 blast;96.60 RaFAH;90.40 iPHoP-RF;85.70
S7-wms-combined_123085	3.26	d__Bacteria;p__Firmicutes;c__Clostridia;o__Lachnospirales;f__Lachnospiraceae;g__Fusicatenibacter	91.6	blast;93.90 iPHoP-RF;83.90
S7-wms-combined_134452	8.94	d__Bacteria;p__Firmicutes;c__Clostridia;o__Christensenellales;f__CAG-74;g__UBA11524	95.6	blast;96.90 iPHoP-RF;94.70
S7-wms-combined_134452	8.94	d__Bacteria;p__Firmicutes;c__Clostridia;o__Christensenellales;f__CAG-74;g__UBA11524	95.6	blast;96.90 CRISPR;95.90 iPHoP-RF;92.80
S7-wms-combined_134458	43.62	d__Bacteria;p__Firmicutes;c__Clostridia;o__Oscillospirales;f__Ruminococcaceae;g__Gemmiger	91.6	blast;93.90 iPHoP-RF;88.90 CRISPR;69.70
S7-wms-combined_135154	99.98	d__Bacteria;p__Bacteroidota;c__Bacteroidia;o__Bacteroidales;f__Bacteroidaceae;g__Bacteroides	100	RaFAH;99.30 iPHoP-RF;97.10 blast;92.40

S7-wms-combined_135154	99.98	d__Bacteria;p__Bacteroidota;c__Bacteroidia;o__Bacteroidales;f__Bacteroidaceae;g__Phocaeicola	93.9	iPHoP-RF;95.70 blast;83.30
S7-wms-combined_136642	81.83	d__Bacteria;p__Actinobacteriota;c__Actinomycetia;o__Actinomycetales;f__Bifidobacteriaceae;g__Bifidobacterium	99.7	CRISPR;99.40 iPHoP-RF;97.10 blast;96.80 RaFAH;72.30
S7-wms-combined_137858	55.93	d__Bacteria;p__Firmicutes;c__Clostridia;o__Oscillospirales;f__Ruminococcaceae;g__Faecalibacterium	95.6	blast;96.90 iPHoP-RF;88.20
S7-wms-combined_139683	93.85	d__Bacteria;p__Firmicutes;c__Clostridia;o__Oscillospirales;f__Oscillospiraceae;g__CAG-103	93.5	CRISPR;95.40
S7-wms-combined_14267	96.95	d__Bacteria;p__Bacteroidota;c__Bacteroidia;o__Bacteroidales;f__Rikenellaceae;g__Alistipes	97.5	CRISPR;98.20 blast;98.10 iPHoP-RF;97.10
S7-wms-combined_143218	6.41	d__Bacteria;p__Actinobacteriota;c__Actinomycetia;o__Actinomycetales;f__Bifidobacteriaceae;g__Bifidobacterium	95.2	blast;96.60 CRISPR;91.70 iPHoP-RF;81.90
S7-wms-combined_14529	25.87	d__Bacteria;p__Firmicutes;c__Clostridia;o__Christensenellales;f__CAG-74;g__UBA11524	95.6	blast;96.90 iPHoP-RF;93.40
S7-wms-combined_14529	25.87	d__Bacteria;p__Firmicutes;c__Clostridia;o__Christensenellales;f__CAG-74;g__UBA11524	95.6	blast;96.90 iPHoP-RF;92.80 CRISPR;76.00
S7-wms-combined_147158	86.44	d__Bacteria;p__Actinobacteriota;c__Actinomycetia;o__Actinomycetales;f__Bifidobacteriaceae;g__Bifidobacterium	99	CRISPR;99.20 blast;96.90 iPHoP-RF;95.70 RaFAH;50.40
S7-wms-combined_156351	22	d__Bacteria;p__Firmicutes;c__Clostridia;o__Lachnospirales;f__Lachnospiraceae;g__Blautia_A	94.5	blast;96.10 iPHoP-RF;94.10
S7-wms-combined_157260	99.48	d__Bacteria;p__Firmicutes;c__Clostridia;o__Lachnospirales;f__Lachnospiraceae;g__Lachnospira	98.7	CRISPR;99.00
S7-wms-combined_157434	16.06	d__Bacteria;p__Firmicutes;c__Clostridia;o__Lachnospirales;f__Lachnospiraceae;g__Mediterraneibacter	91.4	blast;93.70 iPHoP-RF;86.30
S7-wms-combined_163980	78.4	d__Bacteria;p__Bacteroidota;c__Bacteroidia;o__Bacteroidales;f__Rikenellaceae;g__Alistipes	95.2	blast;96.60 iPHoP-RF;93.70
S7-wms-combined_167867	10.54	d__Bacteria;p__Actinobacteriota;c__Actinomycetia;o__Actinomycetales;f__Bifidobacteriaceae;g__Bifidobacterium	97.9	CRISPR;98.50 iPHoP-RF;97.10 blast;96.70
S7-wms-combined_169375	10.64	d__Bacteria;p__Firmicutes;c__Bacilli;o__Erysipelotrichales;f__Erysipelatoclostridiaceae;g__Erysipelatoclostridium	94.7	blast;96.30
S7-wms-combined_169375	10.64	d__Bacteria;p__Firmicutes;c__Clostridia;o__Lachnospirales;f__Lachnospiraceae;g__Faecalimonas	94.5	blast;96.10 iPHoP-RF;79.00
S7-wms-combined_174935	34.84	d__Bacteria;p__Actinobacteriota;c__Actinomycetia;o__Actinomycetales;f__Bifidobacteriaceae;g__Bifidobacterium	96.3	CRISPR;97.40 blast;97.30 iPHoP-RF;97.10
S7-wms-combined_185016	95.96	d__Bacteria;p__Bacteroidota;c__Bacteroidia;o__Bacteroidales;f__Bacteroidaceae;g__Bacteroides	100	RaFAH;99.70 blast;95.30 iPHoP-RF;95.10
S7-wms-combined_189250	22.37	d__Bacteria;p__Actinobacteriota;c__Coriobacteriia;o__Coriobacteriales;f__Coriobacteriaceae;g__Collinsella	98.7	CRISPR;99.00 blast;91.90 iPHoP-RF;80.00
S7-wms-combined_190281	13.36	d__Bacteria;p__Actinobacteriota;c__Coriobacteriia;o__Coriobacteriales;f__Coriobacteriaceae;g__Collinsella	96.2	CRISPR;97.30 blast;96.90 iPHoP-RF;86.30
S7-wms-combined_20921	29.3	d__Bacteria;p__Firmicutes;c__Clostridia;o__Lachnospirales;f__Lachnospiraceae;g__Anaerobutyricum	95	blast;96.50 iPHoP-RF;77.50
S7-wms-combined_217226	97.48	d__Bacteria;p__Verrucomicrobiota;c__Verrucomicrobiae;o__Verrucomicrobiales;f__Akkermansiaceae;g__Akkermansia	100	CRISPR;99.50 RaFAH;97.70 iPHoP-RF;58.20
S7-wms-combined_218379	16.97	d__Bacteria;p__Firmicutes;c__Clostridia;o__Oscillospirales;f__Acutalibacteraceae;g__CAG-180	98.7	CRISPR;99.00 blast;96.60 iPHoP-RF;86.30
S7-wms-combined_218379	16.97	d__Bacteria;p__Firmicutes;c__Clostridia;o__Oscillospirales;f__Acutalibacteraceae;g__Fimenecus	91.7	blast;94.00 iPHoP-RF;91.80
S7-wms-combined_224523	99.68	d__Bacteria;p__Firmicutes;c__Clostridia;o__Oscillospirales;f__Ruminococcaceae;g__Ruminococcus_D	99.4	blast;96.80 iPHoP-RF;93.70 RaFAH;91.90
S7-wms-combined_235165	18.87	d__Bacteria;p__Firmicutes;c__Clostridia;o__Lachnospirales;f__Lachnospiraceae;g__Agathobacter	90.2	blast;92.70
S7-wms-combined_238231	39.27	d__Bacteria;p__Firmicutes;c__Clostridia;o__Oscillospirales;f__Ruminococcaceae;g__Negativibacillus	95.6	blast;96.90 iPHoP-RF;77.50 CRISPR;68.00
S7-wms-combined_239784	67.8	d__Bacteria;p__Bacteroidota;c__Bacteroidia;o__Bacteroidales;f__Bacteroidaceae;g__Bacteroides	99.3	iPHoP-RF;97.10 RaFAH;89.00 blast;88.60
S7-wms-combined_239784	67.8	d__Bacteria;p__Bacteroidota;c__Bacteroidia;o__Bacteroidales;f__Bacteroidaceae;g__Phocaeicola	93.5	blast;95.40 iPHoP-RF;84.80
S7-wms-combined_26217	100	d__Bacteria;p__Bacteroidota;c__Bacteroidia;o__Bacteroidales;f__Rikenellaceae;g__Alistipes	100	RaFAH;98.00 blast;96.90 iPHoP-RF;88.90
S7-wms-combined_26217	100	d__Bacteria;p__Bacteroidota;c__Bacteroidia;o__Bacteroidales;f__Rikenellaceae;g__Alistipes_A	97.2	RaFAH;98.00
S7-wms-combined_28138	12.56	d__Bacteria;p__Firmicutes;c__Clostridia;o__Oscillospirales;f__Acutalibacteraceae;g__Ruminococcus_E	98.7	CRISPR;99.00 blast;96.80 iPHoP-RF;91.10
S7-wms-combined_31068	9.44	d__Bacteria;p__Firmicutes;c__Clostridia;o__Oscillospirales;f__Oscillospiraceae;g__Lawsonibacter	91.5	blast;93.80 iPHoP-RF;81.90
S7-wms-combined_3669	8.7	d__Bacteria;p__Bacteroidota;c__Bacteroidia;o__Bacteroidales;f__Bacteroidaceae;g__Bacteroides	91.5	blast;93.80 iPHoP-RF;87.60
S7-wms-combined_3697	72.05	d__Bacteria;p__Bacteroidota;c__Bacteroidia;o__Bacteroidales;f__Rikenellaceae;g__Alistipes	98.8	iPHoP-RF;97.10 blast;96.90 CRISPR;95.90 RaFAH;84.30
S7-wms-combined_43950	99.99	d__Bacteria;p__Bacteroidota;c__Bacteroidia;o__Bacteroidales;f__Bacteroidaceae;g__Bacteroides	100	RaFAH;99.70 iPHoP-RF;95.10 blast;93.90
S7-wms-combined_43950	99.99	d__Bacteria;p__Bacteroidota;c__Bacteroidia;o__Bacteroidales;f__Bacteroidaceae;g__Phocaeicola	92.9	blast;94.90 iPHoP-RF;93.10
S7-wms-combined_5625	95.36	d__Bacteria;p__Bacteroidota;c__Bacteroidia;o__Bacteroidales;f__Bacteroidaceae;g__Bacteroides	100	RaFAH;98.60 iPHoP-RF;97.10 blast;96.90
S7-wms-combined_62119	99.99	d__Bacteria;p__Bacteroidota;c__Bacteroidia;o__Bacteroidales;f__Bacteroidaceae;g__Bacteroides	98.8	blast;96.90 iPHoP-RF;96.40 CRISPR;93.30 RaFAH;84.80
S7-wms-combined_62119	99.99	d__Bacteria;p__Bacteroidota;c__Bacteroidia;o__Bacteroidales;f__Bacteroidaceae;g__Phocaeicola	95.7	CRISPR;97.00 iPHoP-RF;96.40 blast;62.40
S7-wms-combined_69024	58.17	d__Bacteria;p__Firmicutes;c__Clostridia;o__Lachnospirales;f__Lachnospiraceae;g__Bariatricus	91.6	blast;93.90 iPHoP-RF;75.90
S7-wms-combined_71592	39.23	d__Bacteria;p__Firmicutes;c__Clostridia;o__Oscillospirales;f__Ruminococcaceae;g__Ruminococcus_D	98.1	blast;98.60 iPHoP-RF;92.40 CRISPR;61.00
S7-wms-combined_72070	35.71	d__Bacteria;p__Bacteroidota;c__Bacteroidia;o__Bacteroidales;f__Bacteroidaceae;g__Bacteroides	96.2	iPHoP-RF;97.10 blast;96.90 CRISPR;55.90 RaFAH;52.60
S7-wms-combined_74829	77.29	d__Bacteria;p__Firmicutes;c__Negativicutes;o__Veillonellales;f__Dialisteraceae;g__Dialister	97.3	CRISPR;97.40 blast;96.80 iPHoP-RF;94.70 RaFAH;63.90
S7-wms-combined_84688	29.6	d__Bacteria;p__Firmicutes;c__Clostridia;o__Oscillospirales;f__Oscillospiraceae;g__Marseille-P3106	91.6	blast;93.90 iPHoP-RF;86.70
S7-wms-combined_90393	32.36	d__Bacteria;p__Bacteroidota;c__Bacteroidia;o__Bacteroidales;f__Bacteroidaceae;g__Bacteroides	99.4	iPHoP-RF;97.10 blast;91.80 RaFAH;91.30
S7-wms-combined_90728	66.45	d__Bacteria;p__Bacteroidota;c__Bacteroidia;o__Bacteroidales;f__Bacteroidaceae;g__Bacteroides	100	RaFAH;98.90 iPHoP-RF;96.70 blast;91.80
S7-wms_113137	99.39	d__Bacteria;p__Firmicutes;c__Clostridia;o__Oscillospirales;f__Ruminococcaceae;g__Faecalibacterium	99.4	RaFAH;99.50
S7-wms_123954	100	d__Bacteria;p__Bacteroidota;c__Bacteroidia;o__Bacteroidales;f__Tannerellaceae;g__Parabacteroides	98.4	blast;96.40 RaFAH;82.90 iPHoP-RF;60.30
S7-wms_127801	10.6	d__Bacteria;p__Firmicutes;c__Clostridia;o__Oscillospirales;f__Ruminococcaceae;g__Ruminococcus_D	98.7	CRISPR;99.00 blast;97.40 iPHoP-RF;93.70
S7-wms_151112	20.08	d__Bacteria;p__Firmicutes;c__Clostridia;o__Lachnospirales;f__Lachnospiraceae;g__Anaerobutyricum	90.3	blast;92.80 iPHoP-RF;86.00
S7-wms_157264	5.31	d__Bacteria;p__Actinobacteriota;c__Actinomycetia;o__Actinomycetales;f__Bifidobacteriaceae;g__Bifidobacterium	99.7	CRISPR;99.70 iPHoP-RF;73.50
S7-wms_244866	18.88	d__Bacteria;p__Bacteroidota;c__Bacteroidia;o__Bacteroidales;f__Bacteroidaceae;g__Bacteroides	97.6	CRISPR;98.30 iPHoP-RF;92.40
S7-wms_80255	74.19	d__Bacteria;p__Firmicutes;c__Clostridia;o__Oscillospirales;f__Ruminococcaceae;g__Ruminiclostridium_E	91.5	blast;93.80 iPHoP-RF;67.00 CRISPR;57.40
S8-wms-combined_134098	81.54	d__Bacteria;p__Firmicutes;c__Clostridia;o__Oscillospirales;f__Oscillospiraceae;g__ER4	95.2	blast;96.60 iPHoP-RF;76.10
S8-wms-combined_146562	66.24	d__Bacteria;p__Firmicutes;c__Clostridia;o__Christensenellales;f__CAG-138;g__UBA1685	93	blast;95.00 iPHoP-RF;82.80 CRISPR;70.40
S8-wms-combined_164289	21.14	d__Bacteria;p__Firmicutes;c__Clostridia;o__Christensenellales;f__CAG-138;g__SFEL01	94.5	blast;96.10 iPHoP-RF;79.00 CRISPR;71.90
S8-wms-combined_169835	17.94	d__Bacteria;p__Firmicutes;c__Clostridia;o__Oscillospirales;f__Oscillospiraceae;g__CAG-83	95.6	blast;96.90 iPHoP-RF;64.30
S8-wms-combined_180957	99.92	d__Bacteria;p__Bacteroidota;c__Bacteroidia;o__Bacteroidales;f__Tannerellaceae;g__Parabacteroides	99.7	blast;96.70 RaFAH;95.30 iPHoP-RF;84.50 CRISPR;57.90
S8-wms-combined_185611	9.67	d__Bacteria;p__Firmicutes;c__Clostridia;o__Christensenellales;f__CAG-74;g__Limiplasma	94.2	blast;95.90 iPHoP-RF;76.60
S8-wms-combined_185611	9.67	d__Bacteria;p__Firmicutes;c__Clostridia;o__Christensenellales;f__CAG-74;g__Firm-11	92	blast;94.20 iPHoP-RF;85.10
S8-wms-combined_211119	22.48	d__Bacteria;p__Firmicutes;c__Clostridia;o__Christensenellales;f__CAG-138;g__UBA1685	95.9	blast;97.10 iPHoP-RF;77.50 CRISPR;68.50
S8-wms-combined_215102	12.51	d__Bacteria;p__Firmicutes;c__Clostridia;o__Christensenellales;f__CAG-74;g__UBA11524	95.6	blast;96.90 iPHoP-RF;94.70
S8-wms-combined_215102	12.51	d__Bacteria;p__Firmicutes;c__Clostridia;o__Christensenellales;f__CAG-74;g__UBA11524	95.6	blast;96.90 iPHoP-RF;93.40 CRISPR;77.10
S8-wms-combined_217866	98.95	d__Bacteria;p__Firmicutes;c__Clostridia;o__Lachnospirales;f__Lachnospiraceae;g__Roseburia	96.3	CRISPR;97.40 blast;68.80
S8-wms-combined_217866	98.95	d__Bacteria;p__Firmicutes;c__Clostridia;o__Lachnospirales;f__Lachnospiraceae;g__Agathobacter	95.7	CRISPR;97.00 blast;61.30
S8-wms-combined_217866	98.95	d__Bacteria;p__Firmicutes;c__Clostridia;o__Lachnospirales;f__Lachnospiraceae;g__Eubacterium_F	91.6	blast;93.90
S8-wms-combined_219796	14.07	d__Bacteria;p__Firmicutes;c__Clostridia;o__Christensenellales;f__CAG-138;g__UBA1685	93.5	blast;95.40 CRISPR;93.90 iPHoP-RF;88.20
S8-wms-combined_225114	99.67	d__Bacteria;p__Firmicutes;c__Clostridia;o__Oscillospirales;f__Ruminococcaceae;g__Faecalibacterium	100	CRISPR;98.40 RaFAH;98.00 blast;92.30 iPHoP-RF;88.50
S8-wms-combined_23583	28.29	d__Bacteria;p__Bacteroidota;c__Bacteroidia;o__Bacteroidales;f__Rikenellaceae;g__Alistipes_A	90.3	blast;92.80 iPHoP-RF;81.60 CRISPR;68.00
S8-wms-combined_239791	19.46	d__Bacteria;p__Bacteroidota;c__Bacteroidia;o__Bacteroidales;f__Bacteroidaceae;g__Bacteroides	96.3	iPHoP-RF;95.10 RaFAH;73.50
S8-wms-combined_246185	33.37	d__Bacteria;p__Verrucomicrobiota;c__Verrucomicrobiae;o__Verrucomicrobiales;f__Akkermansiaceae;g__Akkermansia	98.4	CRISPR;98.80 blast;96.90 iPHoP-RF;81.10
S8-wms-combined_246389	21.76	d__Bacteria;p__Firmicutes;c__Clostridia;o__Oscillospirales;f__Oscillospiraceae;g__ER4	91.6	blast;93.90 iPHoP-RF;64.50
S8-wms-combined_25607	54.47	d__Bacteria;p__Firmicutes;c__Clostridia;o__Oscillospirales;f__Oscillospiraceae;g__UBA9475	91.2	blast;93.60 iPHoP-RF;68.90
S8-wms-combined_26087	100	d__Bacteria;p__Bacteroidota;c__Bacteroidia;o__Bacteroidales;f__Rikenellaceae;g__Alistipes	98.7	blast;96.90 iPHoP-RF;91.40 RaFAH;83.40
S8-wms-combined_264711	10.73	d__Bacteria;p__Firmicutes;c__Clostridia;o__Christensenellales;f__CAG-138;g__SFEL01	90.9	blast;93.30 iPHoP-RF;74.80
S8-wms-combined_270362	99.95	d__Bacteria;p__Bacteroidota;c__Bacteroidia;o__Bacteroidales;f__Rikenellaceae;g__Alistipes	100	RaFAH;97.30 blast;96.90 iPHoP-RF;90.10
S8-wms-combined_270362	99.95	d__Bacteria;p__Bacteroidota;c__Bacteroidia;o__Bacteroidales;f__Rikenellaceae;g__Alistipes_A	96.2	RaFAH;97.30
S8-wms-combined_40666	26.57	d__Bacteria;p__Firmicutes;c__Clostridia;o__Oscillospirales;f__Oscillospiraceae;g__CAG-103	97.2	CRISPR;98.00 blast;94.30 iPHoP-RF;83.60
S8-wms-combined_49369	96.98	d__Bacteria;p__Firmicutes;c__Clostridia;o__Oscillospirales;f__Ruminococcaceae;g__Gemmiger	94	blast;95.20 iPHoP-RF;61.70 RaFAH;56.00
S8-wms-combined_68780	51.97	d__Bacteria;p__Firmicutes;c__Clostridia;o__Oscillospirales;f__Oscillospiraceae;g__F23-B02	94.6	blast;96.20 iPHoP-RF;58.40
S8-wms-combined_74275	54.68	d__Bacteria;p__Bacteroidota;c__Bacteroidia;o__Bacteroidales;f__Bacteroidaceae;g__Prevotella	99.3	blast;96.90 iPHoP-RF;91.40 RaFAH;90.60
S8-wms-combined_81597	99.93	d__Bacteria;p__Bacteroidota;c__Bacteroidia;o__Bacteroidales;f__Bacteroidaceae;g__Bacteroides	100	RaFAH;99.50 blast;95.10 iPHoP-RF;85.40
S8-wms-combined_97848	75.58	d__Bacteria;p__Bacteroidota;c__Bacteroidia;o__Bacteroidales;f__Bacteroidaceae;g__Bacteroides	98.8	RaFAH;92.20 blast;92.00 iPHoP-RF;62.20
S8-wms_152831	99.72	d__Bacteria;p__Firmicutes;c__Clostridia;o__Oscillospirales;f__Ruminococcaceae;g__Faecalibacterium	100	RaFAH;99.50 CRISPR;97.80 blast;95.40 iPHoP-RF;77.30
S8-wms_190781	93.96	d__Bacteria;p__Firmicutes;c__Clostridia;o__Oscillospirales;f__Ruminococcaceae;g__Faecalibacterium	92	CRISPR;94.20 blast;94.00 iPHoP-RF;83.30
S8-wms_20528	48.51	d__Bacteria;p__Firmicutes;c__Clostridia;o__Oscillospirales;f__Ruminococcaceae;g__Gemmiger	91.6	blast;93.90 iPHoP-RF;84.50
S8-wms_270		d__Bacteria;p__Bacteroidota;c__Bacteroidia;o__Bacteroidales;f__Barnesiellaceae;g__Barnesiella	94.6	blast;96.20 iPHoP-RF;68.20
S8-wms_30498	86.77	d__Bacteria;p__Firmicutes;c__Clostridia;o__Oscillospirales;f__Oscillospiraceae;g__CAG-83	92.7	blast;86.00 RaFAH;81.50 iPHoP-RF;63.00
S8-wms_48935	39.33	d__Bacteria;p__Firmicutes;c__Clostridia;o__Oscillospirales;f__Oscillospiraceae;g__CAG-83	90.9	blast;93.30 iPHoP-RF;77.30
S8-wms_57121	9.54	d__Bacteria;p__Firmicutes;c__Clostridia;o__Christensenellales;f__CAG-138;g__UBA1685	97.3	blast;98.10 iPHoP-RF;76.60 CRISPR;55.40
S8-wms_58515	74.23	d__Bacteria;p__Bacteroidota;c__Bacteroidia;o__Bacteroidales;f__Rikenellaceae;g__Alistipes	96.3	iPHoP-RF;97.10 blast;96.70 CRISPR;91.70 RaFAH;55.50
S8-wms_82880	30.76	d__Bacteria;p__Firmicutes;c__Clostridia;o__Oscillospirales;f__Oscillospiraceae;g__An92	93	blast;95.00 iPHoP-RF;60.10
S8-wms_82880	30.76	d__Bacteria;p__Firmicutes;c__Clostridia;o__Oscillospirales;f__Oscillospiraceae;g__Flavonifractor	91	blast;93.40 iPHoP-RF;75.90
S9-wms-combined_105462	98	d__Bacteria;p__Firmicutes;c__Clostridia;o__Lachnospirales;f__Lachnospiraceae;g__Anaerostipes	95.4	blast;96.80 CRISPR;91.30 iPHoP-RF;91.10
S9-wms-combined_105822	44.89	d__Bacteria;p__Firmicutes;c__Clostridia;o__Lachnospirales;f__Lachnospiraceae;g__Enterocloster	95	blast;96.50 iPHoP-RF;87.60 CRISPR;52.20
S9-wms-combined_10990	10.09	d__Bacteria;p__Firmicutes;c__Clostridia;o__Lachnospirales;f__Lachnospiraceae;g__Blautia_A	91.4	blast;93.70 iPHoP-RF;80.00
S9-wms-combined_122742	8.62	d__Bacteria;p__Firmicutes;c__Clostridia;o__Oscillospirales;f__Oscillospiraceae;g__Flavonifractor	90.3	blast;92.80 iPHoP-RF;72.80
S9-wms-combined_124857	79.58	d__Bacteria;p__Firmicutes;c__Clostridia;o__Oscillospirales;f__Oscillospiraceae;g__Flavonifractor	90.7	iPHoP-RF;93.10 blast;92.10 CRISPR;71.70
S9-wms-combined_2042	10.88	d__Bacteria;p__Firmicutes;c__Clostridia;o__Lachnospirales;f__Lachnospiraceae;g__Eisenbergiella	95.2	CRISPR;96.60 blast;95.10 iPHoP-RF;93.40
S9-wms-combined_20485	94.46	d__Bacteria;p__Firmicutes;c__Bacilli;o__Lactobacillales;f__Streptococcaceae;g__Streptococcus	100	RaFAH;99.70 CRISPR;99.00 iPHoP-RF;97.10 blast;96.90

S9-wms-combined_26410	99.06	d__Bacteria;p__Firmicutes;c__Clostridia;o__Lachnospirales;f__Lachnospiraceae;g__Mediterraneibacter	95.4	blast;96.80
S9-wms-combined_26410	99.06	d__Bacteria;p__Firmicutes;c__Clostridia;o__Lachnospirales;f__Lachnospiraceae;g__Hungatella	95.2	blast;88.20 RaFAH;85.60
S9-wms-combined_27311	4.98	d__Bacteria;p__Firmicutes;c__Clostridia;o__Oscillospirales;f__Acutalibacteraceae;g__Acutalibacter	91.4	blast;93.70 iPHoP-RF;76.10
S9-wms-combined_28805	94.95	d__Bacteria;p__Bacteroidota;c__Bacteroidia;o__Bacteroidales;f__Rikenellaceae;g__Alistipes	99	blast;96.10 RaFAH;89.50 iPHoP-RF;68.40
S9-wms-combined_31456	63.18	d__Bacteria;p__Bacteroidota;c__Bacteroidia;o__Bacteroidales;f__Barnesiellaceae;g__Barnesiella	97.6	CRISPR;97.40 blast;96.90 iPHoP-RF;83.60 RaFAH;68.00
S9-wms-combined_33229	87.21	d__Bacteria;p__Bacteroidota;c__Bacteroidia;o__Bacteroidales;f__Bacteroidaceae;g__Bacteroides	99.1	iPHoP-RF;97.10 blast;96.10 RaFAH;87.90
S9-wms-combined_33229	87.21	d__Bacteria;p__Bacteroidota;c__Bacteroidia;o__Bacteroidales;f__Bacteroidaceae;g__Phocaeicola	94.5	iPHoP-RF;96.10
S9-wms-combined_33230	93.11	d__Bacteria;p__Bacteroidota;c__Bacteroidia;o__Bacteroidales;f__Bacteroidaceae;g__Bacteroides	100	RaFAH;99.10 iPHoP-RF;96.40 blast;92.10
S9-wms-combined_3461	69.22	d__Bacteria;p__Firmicutes;c__Clostridia;o__Lachnospirales;f__Anaerotignaceae;g__Anaerotignum	95	CRISPR;96.50 blast;91.10 iPHoP-RF;88.90
S9-wms-combined_36459	25.89	d__Bacteria;p__Firmicutes;c__Clostridia;o__Oscillospirales;f__Ruminococcaceae;g__Negativibacillus	96.2	blast;97.30 iPHoP-RF;85.10 CRISPR;59.60
S9-wms-combined_37587	92.98	d__Bacteria;p__Bacteroidota;c__Bacteroidia;o__Bacteroidales;f__Bacteroidaceae;g__Bacteroides	99.4	blast;96.80 iPHoP-RF;96.40 RaFAH;92.80
S9-wms-combined_37587	92.98	d__Bacteria;p__Bacteroidota;c__Bacteroidia;o__Bacteroidales;f__Bacteroidaceae;g__Phocaeicola	93.1	iPHoP-RF;95.10 blast;90.70
S9-wms-combined_44402	80.74	d__Bacteria;p__Bacteroidota;c__Bacteroidia;o__Bacteroidales;f__Bacteroidaceae;g__Bacteroides	100	RaFAH;97.80 blast;96.50 iPHoP-RF;96.40
S9-wms-combined_53991	30.5	d__Bacteria;p__Firmicutes;c__Negativicutes;o__Veillonellales;f__Dialisteraceae;g__Dialister	95.4	blast;96.80 iPHoP-RF;91.80 CRISPR;60.90
S9-wms-combined_53991	30.5	d__Bacteria;p__Firmicutes;c__Negativicutes;o__Veillonellales;f__Veillonellaceae;g__F0422	90.4	RaFAH;92.90
S9-wms-combined_53991	30.5	d__Bacteria;p__Firmicutes;c__Negativicutes;o__Veillonellales;f__Veillonellaceae;g__Veillonella	90.4	RaFAH;92.90
S9-wms-combined_53991	30.5	d__Bacteria;p__Firmicutes;c__Negativicutes;o__Veillonellales;f__Veillonellaceae;g__Veillonella_A	90.4	RaFAH;92.90
S9-wms-combined_5526	86.27	d__Bacteria;p__Bacteroidota;c__Bacteroidia;o__Bacteroidales;f__Rikenellaceae;g__Alistipes	99.3	blast;96.80 iPHoP-RF;93.10 RaFAH;90.40
S9-wms-combined_64277	98.37	d__Bacteria;p__Proteobacteria;c__Gammaproteobacteria;o__Enterobacterales;f__Enterobacteriaceae;g__Escherichia	100	RaFAH;97.50 blast;96.90 iPHoP-RF;74.30
S9-wms-combined_64277	98.37	d__Bacteria;p__Proteobacteria;c__Gammaproteobacteria;o__Enterobacterales;f__Enterobacteriaceae;g__Escherichia_C	93.8	blast;95.60
S9-wms-combined_64277	98.37	d__Bacteria;p__Proteobacteria;c__Gammaproteobacteria;o__Enterobacterales;f__Enterobacteriaceae;g__Kluyvera	93.5	blast;95.40
S9-wms-combined_64277	98.37	d__Bacteria;p__Proteobacteria;c__Gammaproteobacteria;o__Enterobacterales;f__Enterobacteriaceae;g__Pseudocitrobacter	91.7	blast;94.00
S9-wms-combined_76274	91.87	d__Bacteria;p__Actinobacteriota;c__Actinomycetia;o__Actinomycetales;f__Bifidobacteriaceae;g__Bifidobacterium	99.4	CRISPR;98.60 iPHoP-RF;93.70 blast;92.70 RaFAH;81.60
S9-wms-combined_93232	57.26	d__Bacteria;p__Proteobacteria;c__Gammaproteobacteria;o__Enterobacterales;f__Enterobacteriaceae;g__Klebsiella	98.2	CRISPR;98.70 blast;96.90 iPHoP-RF;59.00
S9-wms-combined_93232	57.26	d__Bacteria;p__Proteobacteria;c__Gammaproteobacteria;o__Enterobacterales;f__Enterobacteriaceae;g__Klebsiella_A	94.7	blast;96.30
S9-wms-combined_97755	40.71	d__Bacteria;p__Firmicutes;c__Clostridia;o__Lachnospirales;f__Lachnospiraceae;g__Eisenbergiella	91	blast;93.40 iPHoP-RF;57.60
S9-wms-combined_9931	57.49	d__Bacteria;p__Firmicutes;c__Clostridia;o__Oscillospirales;f__Acutalibacteraceae;g__Ruminococcus_E	95.4	blast;96.80 iPHoP-RF;92.10
S9-wms_104169		d__Bacteria;p__Firmicutes;c__Clostridia;o__Oscillospirales;f__Ruminococcaceae;g__Ruthenibacterium	91.6	blast;93.90 CRISPR;84.10 iPHoP-RF;79.50
S9-wms_114971	60.7	d__Bacteria;p__Firmicutes;c__Negativicutes;o__Veillonellales;f__Veillonellaceae;g__Veillonella	98.4	blast;95.00 RaFAH;88.20 iPHoP-RF;73.50 CRISPR;55.70
S9-wms_19025	87.64	d__Bacteria;p__Firmicutes;c__Bacilli;o__Lactobacillales;f__Streptococcaceae;g__Streptococcus	98.4	RaFAH;98.80
S9-wms_64472	4.04	d__Bacteria;p__Firmicutes;c__Clostridia;o__Lachnospirales;f__Lachnospiraceae;g__Enterocloster	93.5	blast;95.40 iPHoP-RF;85.10

Table S9-3. VLP_STD+MAGs_tophits

Virus	Status	Realm	Kingdom	Phylum	Class	Order	Family	Subfamily	Genus	AAI to closest RaFAH reference	Host genus	Confidence score	List of methods
S10_vf_120738	Shared	n.a.	n.a.	n.a.	n.a.	n.a.	n.a.	n.a.	n.a.	22.73	d__Bacteria;p__Firmicutes;c__Clostridia;o__Oscillospirales;f__Oscillospiraceae;g__Dysosmobacter	95	blast;96.50 iPHoP-RF;71.90
S10_vf_18671	Shared	Duplodnaviria	Heunggongvirae	Uroviricota	Caudoviricetes	n.a.	Unclassified	Unclassified	Unclassified	91.64	d__Bacteria;p__Bacteroidota;c__Bacteroidia;o__Bacteroidales;f__Tannerellaceae;g__Parabacteroides	91	blast;93.40 iPHoP-RF;79.00
S10_vf_24791	SHHC	Duplodnaviria	Heunggongvirae	Uroviricota	Caudoviricetes	n.a.	Unclassified	Unclassified	Eponavirus	46.17	d__Bacteria;p__Firmicutes;c__Clostridia;o__Oscillospirales;f__Ruminococcaceae;g__Faecalibacterium	93.8	blast;95.60 CRISPR;94.10 iPHoP-RF;82.20
S10_vf_43964	SHHC	Duplodnaviria	Heunggongvirae	Uroviricota	Caudoviricetes	n.a.	Unclassified	O	O	91.64	d__Bacteria;p__Bacteroidota;c__Bacteroidia;o__Bacteroidales;f__Bacteroidaceae;g__Bacteroides	100	RaFAH;99.20 CRISPR;97.80 iPHoP-RF;97.10 blast;77.70
S10_vf_64371	Shared	n.a.	n.a.	n.a.	n.a.	n.a.	n.a.	n.a.	n.a.	86.67	d__Bacteria;p__Firmicutes;c__Bacilli;o__Lactobacillales;f__Streptococcaceae;g__Streptococcus	98.8	RaFAH;98.90 iPHoP-RF;59.80
S10_vf_80712	Shared	n.a.	n.a.	n.a.	n.a.	n.a.	n.a.	n.a.	n.a.	98.13	d__Bacteria;p__Firmicutes;c__Clostridia;o__Lachnospirales;f__Lachnospiraceae;g__Blautia_A	100	RaFAH;99.20 blast;91.30 iPHoP-RF;86.30
S10_vs_120489	SHHC	n.a.	n.a.	n.a.	n.a.	n.a.	n.a.	n.a.	n.a.	7.64	d__Bacteria;p__Firmicutes;c__Clostridia;o__Christensenellales;f__Borkfalkiaceae;g__QALS01	90.3	blast;92.80 iPHoP-RF;56.50
S10_vs_120541	SHHC	n.a.	n.a.	n.a.	n.a.	n.a.	n.a.	n.a.	n.a.	14.85	d__Bacteria;p__Firmicutes;c__Clostridia;o__Oscillospirales;f__Oscillospiraceae;g__CAG-110	94.7	blast;96.30 iPHoP-RF;95.70 CRISPR;74.70
S10_vs_14557	Shared	Duplodnaviria	Heunggongvirae	Uroviricota	Caudoviricetes	n.a.	Duneviridae	O	O	99.66	d__Bacteria;p__Bacteroidota;c__Bacteroidia;o__Bacteroidales;f__Bacteroidaceae;g__Bacteroides	100	RaFAH;98.20 iPHoP-RF;97.10 blast;96.90 CRISPR;90.30
S10_vf_46328	SHHC	Duplodnaviria	Heunggongvirae	Uroviricota	Caudoviricetes	n.a.	Unclassified	O	O	63.06	d__Bacteria;p__Firmicutes;c__Clostridia;o__Lachnospirales;f__Lachnospiraceae;g__UBA9502	91.4	CRISPR;93.70 blast;91.80 iPHoP-RF;89.50
S10_vs_2538	Shared	Duplodnaviria	Heunggongvirae	Uroviricota	Caudoviricetes	n.a.	Unclassified	O	O	31.92	d__Bacteria;p__Firmicutes;c__Clostridia;o__Peptostreptococcales;f__Anaerovoracaceae;g__CAG-145	94.3	blast;96.00 iPHoP-RF;76.40
S10_vs_35854	Shared	Duplodnaviria	Heunggongvirae	Uroviricota	Caudoviricetes	n.a.	Unclassified	O	O	48.73	d__Bacteria;p__Bacteroidota;c__Bacteroidia;o__Bacteroidales;f__Bacteroidaceae;g__Bacteroides	99.3	iPHoP-RF;97.10 blast;96.00 RaFAH;90.40
S10_vs_36407	SHHC	n.a.	n.a.	n.a.	n.a.	n.a.	n.a.	n.a.	n.a.	8.93	d__Bacteria;p__Firmicutes;c__Clostridia;o__Oscillospirales;f__Acutalibacteraceae;g__UBA1691	94.5	blast;96.10 CRISPR;90.70 iPHoP-RF;73.00
S10_vf_62230	SHHC	Duplodnaviria	Heunggongvirae	Uroviricota	Caudoviricetes	n.a.	Unclassified	O	O	25.76	d__Bacteria;p__Firmicutes;c__Clostridia;o__Clostridiales;f__Clostridiaceae;g__Clostridium	90.6	blast;93.00 iPHoP-RF;61.40
S10_vs_36598	Shared	n.a.	n.a.	n.a.	n.a.	n.a.	n.a.	n.a.	n.a.	93.41	d__Bacteria;p__Bacteroidota;c__Bacteroidia;o__Bacteroidales;f__Bacteroidaceae;g__Bacteroides	100	RaFAH;99.40 iPHoP-RF;96.70
S10_vs_37379	Shared	n.a.	n.a.	n.a.	n.a.	n.a.	n.a.	n.a.	n.a.	75.26	d__Bacteria;p__Bacteroidota;c__Bacteroidia;o__Bacteroidales;f__Tannerellaceae;g__Parabacteroides	100	CRISPR;98.90 RaFAH;98.30 iPHoP-RF;97.10 blast;94.80
S10_vs_58014	Shared	Duplodnaviria	Heunggongvirae	Uroviricota	Caudoviricetes	n.a.	Unclassified	Unclassified	Unclassified	85.85	d__Bacteria;p__Bacteroidota;c__Bacteroidia;o__Bacteroidales;f__Bacteroidaceae;g__Bacteroides	100	RaFAH;99.50 blast;91.80 iPHoP-RF;64.00
S10_vs_106834	SHHC	Duplodnaviria	Heunggongvirae	Uroviricota	Caudoviricetes	n.a.	Unclassified	O	O	44.85	d__Bacteria;p__Bacteroidota;c__Bacteroidia;o__Bacteroidales;f__Bacteroidaceae;g__Bacteroides	92.3	RaFAH;87.20 iPHoP-RF;78.70
S10_vs_67471	SHHC	n.a.	n.a.	n.a.	n.a.	n.a.	n.a.	n.a.	n.a.	98.13	d__Bacteria;p__Bacteroidota;c__Bacteroidia;o__Bacteroidales;f__Rikenellaceae;g__Alistipes	99.9	RaFAH;97.30 blast;95.30 iPHoP-RF;52.20
S10_vs_71900	Shared	n.a.	n.a.	n.a.	n.a.	n.a.	n.a.	n.a.	n.a.	97	d__Bacteria;p__Bacteroidota;c__Bacteroidia;o__Bacteroidales;f__Bacteroidaceae;g__Bacteroides	100	RaFAH;99.30 iPHoP-RF;97.10 blast;90.90
S11_vf_104035	Shared	Duplodnaviria	Heunggongvirae	Uroviricota	Caudoviricetes	Crassvirales	Suoliviridae	O	O	37.57	d__Bacteria;p__Bacteroidota;c__Bacteroidia;o__Bacteroidales;f__Bacteroidaceae;g__Prevotella	91.7	RaFAH;94.00
S10_vs_62776	Shared	Duplodnaviria	Heunggongvirae	Uroviricota	Caudoviricetes	n.a.	Unclassified	O	O	44.54	d__Bacteria;p__Bacteroidota;c__Bacteroidia;o__Bacteroidales;f__Bacteroidaceae;g__Bacteroides	99	iPHoP-RF;96.70 blast;93.60 RaFAH;88.20
S10_vs_120570	Shared	n.a.	n.a.	n.a.	n.a.	n.a.	n.a.	n.a.	n.a.	98.22	d__Bacteria;p__Firmicutes;c__Clostridia;o__Oscillospirales;f__Ruminococcaceae;g__Ruminococcus_D	99.7	blast;98.90 iPHoP-RF;96.70 CRISPR;87.50 RaFAH;87.40
S11_vf_104052	ME	Duplodnaviria	Heunggongvirae	Uroviricota	Caudoviricetes	n.a.	Unclassified	O	O	38.91	d__Bacteria;p__Firmicutes;c__Clostridia;o__Oscillospirales;f__Acutalibacteraceae;g__CAG-180	91.5	blast;93.80 iPHoP-RF;55.40
S10_vs_77004	Shared	n.a.	n.a.	n.a.	n.a.	n.a.	n.a.	n.a.	n.a.	10.42	d__Bacteria;p__Firmicutes;c__Clostridia;o__Oscillospirales;f__Acutalibacteraceae;g__Pseudoruminococcus	95.3	blast;96.70 CRISPR;89.20 iPHoP-RF;87.60
S11_vf_104033	ME	Duplodnaviria	Heunggongvirae	Uroviricota	Caudoviricetes	n.a.	Unclassified	O	O	40.07	d__Bacteria;p__Bacteroidota;c__Bacteroidia;o__Bacteroidales;f__Bacteroidaceae;g__Bacteroides	90.6	RaFAH;93.00
S11_vf_104071	ME	Duplodnaviria	Heunggongvirae	Uroviricota	Caudoviricetes	n.a.	Unclassified	O	O	20.29	d__Bacteria;p__Bacteroidota;c__Bacteroidia;o__Bacteroidales;f__Rikenellaceae;g__Alistipes	98.5	CRISPR;98.90 iPHoP-RF;92.80
S11_vf_10907	ME	Duplodnaviria	Heunggongvirae	Uroviricota	Caudoviricetes	n.a.	Unclassified	O	O	87.86	d__Bacteria;p__Firmicutes;c__Clostridia;o__Lachnospirales;f__Lachnospiraceae;g__Bariatricus	91.6	blast;93.90 iPHoP-RF;82.80
S11_vf_27845	Shared	n.a.	n.a.	n.a.	n.a.	n.a.	n.a.	n.a.	n.a.	53.78	d__Bacteria;p__Firmicutes;c__Bacilli;o__Lactobacillales;f__Streptococcaceae;g__Streptococcus	100	CRISPR;98.80 RaFAH;97.70 iPHoP-RF;85.70
S11_vf_28515	Shared	Duplodnaviria	Heunggongvirae	Uroviricota	Caudoviricetes	n.a.	Unclassified	O	O	95.85	d__Bacteria;p__Firmicutes;c__Clostridia;o__Lachnospirales;f__Lachnospiraceae;g__Blautia_A	100	RaFAH;98.80 blast;94.40 iPHoP-RF;90.50 CRISPR;68.00
S11_vf_28645	ME	n.a.	n.a.	n.a.	n.a.	n.a.	n.a.	n.a.	n.a.	6.84	d__Bacteria;p__Firmicutes;c__Clostridia;o__Lachnospirales;f__Lachnospiraceae;g__Blautia_A	92.5	blast;94.60 iPHoP-RF;51.70
S11_vf_43997	ME	Duplodnaviria	Heunggongvirae	Uroviricota	Caudoviricetes	Crassvirales	Unclassified	Unclassified	Unclassified	15.72	d__Bacteria;p__Bacteroidota;c__Bacteroidia;o__Bacteroidales;f__Bacteroidaceae;g__Bacteroides	91.4	blast;93.70 iPHoP-RF;78.70
S11_vf_66522	ME	Duplodnaviria	Heunggongvirae	Uroviricota	Caudoviricetes	n.a.	Unclassified	Unclassified	Unclassified	97.65	d__Bacteria;p__Actinobacteriota;c__Actinomycetia;o__Actinomycetales;f__Actinomycetaceae;g__Pauljensenia	100	RaFAH;99.20 CRISPR;99.10 blast;93.90 iPHoP-RF;90.50
S10_vs_37694	Shared	Duplodnaviria	Heunggongvirae	Uroviricota	Caudoviricetes	n.a.	Duneviridae	O	O	99.94	d__Bacteria;p__Bacteroidota;c__Bacteroidia;o__Bacteroidales;f__Rikenellaceae;g__Alistipes	99.1	iPHoP-RF;97.10 blast;96.80 RaFAH;87.90 CRISPR;55.60
S11_vf_68693	ME	n.a.	n.a.	n.a.	n.a.	n.a.	n.a.	n.a.	n.a.	78.63	d__Bacteria;p__Bacteroidota;c__Bacteroidia;o__Bacteroidales;f__Tannerellaceae;g__Parabacteroides	99.3	CRISPR;97.70 blast;97.30 iPHoP-RF;91.80 RaFAH;87.20
S11_vf_92391	Shared	n.a.	n.a.	n.a.	n.a.	n.a.	n.a.	n.a.	n.a.	5.33	d__Bacteria;p__Firmicutes;c__Bacilli;o__Erysipelotrichales;f__Erysipelotrichaceae;g__Clostridium_AQ	91	CRISPR;93.40
S10_vs_46819	SHHC	Duplodnaviria	Heunggongvirae	Uroviricota	Caudoviricetes	n.a.	Unclassified	O	O	98.03	d__Bacteria;p__Firmicutes;c__Clostridia;o__Oscillospirales;f__Ruminococcaceae;g__Faecalibacterium	99.4	blast;96.80 RaFAH;92.90 iPHoP-RF;79.00
S11_vf_70015	Shared	Duplodnaviria	Heunggongvirae	Uroviricota	Caudoviricetes	n.a.	Unclassified	O	O	50.35	d__Bacteria;p__Bacteroidota;c__Bacteroidia;o__Bacteroidales;f__Rikenellaceae;g__Alistipes	97.8	blast;96.10 RaFAH;79.90
S11_vf_95067	ME	n.a.	n.a.	n.a.	n.a.	n.a.	n.a.	n.a.	n.a.	39.9	d__Bacteria;p__Firmicutes;c__Clostridia;o__Oscillospirales;f__Oscillospiraceae;g__Flavonifractor	93.4	blast;95.30 iPHoP-RF;58.20
S11_vs_104041	ME	Duplodnaviria	Heunggongvirae	Uroviricota	Caudoviricetes	n.a.	Unclassified	O	O	16.5	d__Bacteria;p__Cyanobacteria;c__Vampirovibrionia;o__Gastranaerophilales;f__Gastranaerophilaceae;g__CAG-306	97.2	blast;98.00 iPHoP-RF;56.00
S11_vs_104064	Shared	Duplodnaviria	Heunggongvirae	Uroviricota	Caudoviricetes	n.a.	Unclassified	O	O	99.03	d__Bacteria;p__Firmicutes;c__Clostridia;o__Oscillospirales;f__Ruminococcaceae;g__Negativibacillus	92.1	blast;94.30
S11_vs_11994	ME	Duplodnaviria	Heunggongvirae	Uroviricota	Caudoviricetes	n.a.	Unclassified	O	O	21.97	d__Bacteria;p__Firmicutes;c__Clostridia;o__Oscillospirales;f__Ruminococcaceae;g__Faecalibacterium	91.7	RaFAH;94.00
S11_vs_14882	ME	n.a.	n.a.	n.a.	n.a.	n.a.	n.a.	n.a.	n.a.	11.87	d__Bacteria;p__Firmicutes;c__Bacilli;o__Erysipelotrichales;f__Erysipelotrichaceae;g__Holdemania	93.1	blast;95.10 CRISPR;54.60
S11_vs_1937	Shared	Duplodnaviria	Heunggongvirae	Uroviricota	Caudoviricetes	n.a.	Unclassified	O	O	99.04	d__Bacteria;p__Bacteroidota;c__Bacteroidia;o__Bacteroidales;f__Rikenellaceae;g__Alistipes	100	blast;98.60 RaFAH;96.60 iPHoP-RF;83.30
S11_vf_104034	Shared	Duplodnaviria	Heunggongvirae	Uroviricota	Caudoviricetes	n.a.	Unclassified	Unclassified	O	21.95	d__Bacteria;p__Firmicutes;c__Clostridia;o__Oscillospirales;f__Oscillospiraceae;g__ER4	90.3	blast;92.80 iPHoP-RF;81.10
S11_vs_19892	ME	Duplodnaviria	Heunggongvirae	Uroviricota	Caudoviricetes	n.a.	Unclassified	Unclassified	Eponavirus	45.95	d__Bacteria;p__Firmicutes;c__Clostridia;o__Oscillospirales;f__Oscillospiraceae;g__Lawsonibacter	90.6	blast;93.00 iPHoP-RF;67.50
S11_vs_23188	ME	n.a.	n.a.	n.a.	n.a.	n.a.	n.a.	n.a.	n.a.	66.52	d__Bacteria;p__Bacteroidota;c__Bacteroidia;o__Bacteroidales;f__Bacteroidaceae;g__Bacteroides	99.9	iPHoP-RF;97.10 RaFAH;95.80 blast;93.80 CRISPR;83.30
S11_vs_26767	ME	Duplodnaviria	Heunggongvirae	Uroviricota	Caudoviricetes	n.a.	Unclassified	O	O	40.78	d__Bacteria;p__Firmicutes;c__Clostridia;o__Lachnospirales;f__Lachnospiraceae;g__Ventrimonas	90.8	blast;93.20 iPHoP-RF;72.20
S11_vs_3277	Shared	Duplodnaviria	Heunggongvirae	Uroviricota	Caudoviricetes	n.a.	Unclassified	Unclassified	Unclassified	99.63	d__Bacteria;p__Bacteroidota;c__Bacteroidia;o__Bacteroidales;f__Bacteroidaceae;g__Bacteroides	99.9	RaFAH;98.80 iPHoP-RF;83.60 blast;80.70
S11_vs_34137	ME	Duplodnaviria	Heunggongvirae	Uroviricota	Caudoviricetes	n.a.	Unclassified	O	O	17.51	d__Bacteria;p__Firmicutes;c__Clostridia;o__Lachnospirales;f__Lachnospiraceae;g__Blautia_A	91.4	blast;93.70 iPHoP-RF;83.10
S11_vs_41107	ME	Duplodnaviria	Heunggongvirae	Uroviricota	Caudoviricetes	n.a.	Unclassified	O	O	98.96	d__Bacteria;p__Firmicutes;c__Clostridia;o__Clostridiales;f__Clostridiaceae;g__Clostridium	93.6	RaFAH;95.50
S11_vs_5077	ME	Duplodnaviria	Heunggongvirae	Uroviricota	Caudoviricetes	n.a.	Peduoviridae	Unclassified	Quadragintavirus	84.31	d__Bacteria;p__Proteobacteria;c__Gammaproteobacteria;o__Enterobacterales;f__Enterobacteriaceae;g__Salmonella	95.3	blast;96.70
S11_vs_52577	ME	Duplodnaviria	Heunggongvirae	Uroviricota	Caudoviricetes	n.a.	Unclassified	Unclassified	Mushuvirus	78.49	d__Bacteria;p__Firmicutes;c__Clostridia;o__Oscillospirales;f__Oscillospiraceae;g__Dysosmobacter	91	blast;88.40 iPHoP-RF;73.50 RaFAH;71.60
S11_vs_58930	Shared	Duplodnaviria	Heunggongvirae	Uroviricota	Caudoviricetes	n.a.	Unclassified	Unclassified	Unclassified	93.75	d__Bacteria;p__Bacteroidota;c__Bacteroidia;o__Bacteroidales;f__Bacteroidaceae;g__Bacteroides	98.1	blast;92.60 RaFAH;90.60 iPHoP-RF;89.80
S11_vs_6458	Shared	n.a.	n.a.	n.a.	n.a.	n.a.	n.a.	n.a.	n.a.	15.38	d__Bacteria;p__Proteobacteria;c__Alphaproteobacteria;o__RF32;f__CAG-239;g__CAG-267	90.1	blast;92.60 iPHoP-RF;81.90
S11_vs_68151	Shared	Duplodnaviria	Heunggongvirae	Uroviricota	Caudoviricetes	n.a.	Unclassified	O	O	99.98	d__Bacteria;p__Firmicutes;c__Clostridia;o__Lachnospirales;f__Lachnospiraceae;g__Mediterraneibacter	95.3	blast;96.70
S11_vs_76784	Shared	Duplodnaviria	Heunggongvirae	Uroviricota	Caudoviricetes	n.a.	Unclassified	O	O	41.57	d__Bacteria;p__Firmicutes;c__Clostridia;o__Oscillospirales;f__Ruminococcaceae;g__Ruthenibacterium	93	blast;95.00 iPHoP-RF;77.30
S11_vs_81775	ME	Duplodnaviria	Heunggongvirae	Uroviricota	Caudoviricetes	n.a.	Unclassified	Unclassified	Unclassified	24.35	d__Bacteria;p__Firmicutes;c__Clostridia;o__Eubacteriales;f__Eubacteriaceae;g__Eubacterium	95	CRISPR;96.50 blast;93.70 iPHoP-RF;81.40
S11_vs_84627	ME	n.a.	n.a.	n.a.	n.a.	n.a.	n.a.	n.a.	n.a.	6.74	d__Bacteria;p__Proteobacteria;c__Alphaproteobacteria;o__RF32;f__CAG-239;g__51-20	95.6	blast;96.90 iPHoP-RF;89.50 CRISPR;76.50
S11_vs_84848	ME	Duplodnaviria	Heunggongvirae	Uroviricota	Caudoviricetes	n.a.	Unclassified	Unclassified	Unclassified	95.91	d__Bacteria;p__Bacteroidota;c__Bacteroidia;o__Bacteroidales;f__Bacteroidaceae;g__Bacteroides	95.9	blast;91.20 RaFAH;83.50 iPHoP-RF;57.90
S11_vf_33117	ME	n.a.	n.a.	n.a.	n.a.	n.a.	n.a.	n.a.	n.a.	96.79	d__Bacteria;p__Firmicutes;c__Clostridia;o__Oscillospirales;f__Oscillospiraceae;g__CAG-83	97.3	RaFAH;98.10
S11_vs_98193	ME	Duplodnaviria	Heunggongvirae	Uroviricota	Caudoviricetes	n.a.	Unclassified	Unclassified	Unclassified	80.02	d__Bacteria;p__Bacteroidota;c__Bacteroidia;o__Bacteroidales;f__Rikenellaceae;g__Alistipes	96.2	iPHoP-RF;92.10 blast;92.00 RaFAH;82.70
S12_vf_10887	SHHC	n.a.	n.a.	n.a.	n.a.	n.a.	n.a.	n.a.	n.a.	7.67	d__Bacteria;p__Proteobacteria;c__Gammaproteobacteria;o__Enterobacterales;f__Pasteurellaceae;g__Glaesserella	94.5	iPHoP-RF;96.10 CRISPR;91.90
S11_vf_34008	ME	Duplodnaviria	Heunggongvirae	Uroviricota	Caudoviricetes	n.a.	Unclassified	O	O	44.72	d__Bacteria;p__Firmicutes;c__Clostridia;o__Lachnospirales;f__Lachnospiraceae;g__	93.1	iPHoP-RF;95.10 blast;93.90
S12_vf_173208	Shared	n.a.	n.a.	n.a.	n.a.	n.a.	n.a.	n.a.	n.a.	10.29	d__Bacteria;p__Firmicutes;c__Clostridia;o__Oscillospirales;f__Oscillospiraceae;g__CAG-170	93	blast;95.00 iPHoP-RF;81.40
S12_vf_174374	Shared	n.a.	n.a.	n.a.	n.a.	n.a.	n.a.	n.a.	n.a.	94.2	d__Bacteria;p__Bacteroidota;c__Bacteroidia;o__Bacteroidales;f__Rikenellaceae;g__Alistipes	96.2	CRISPR;97.30 iPHoP-RF;97.10 blast;96.60
S12_vf_176437	SHHC	Duplodnaviria	Heunggongvirae	Uroviricota	Caudoviricetes	n.a.	Salasmaviridae	O	O	10.68	d__Bacteria;p__Firmicutes;c__Clostridia;o__Lachnospirales;f__Lachnospiraceae;g__OF09-33XD	95.3	CRISPR;96.70 iPHoP-RF;82.20
S12_vf_181024	SHHC	Duplodnaviria	Heunggongvirae	Uroviricota	Caudoviricetes	n.a.	Unclassified	O	O	98.48	d__Bacteria;p__Firmicutes;c__Clostridia;o__Oscillospirales;f__Ruminococcaceae;g__Ruminococcus_D	99.9	CRISPR;97.30 RaFAH;97.20 blast;96.90 iPHoP-RF;95.70
S12_vf_60224	SHHC	Duplodnaviria	Heunggongvirae	Uroviricota	Caudoviricetes	n.a.	Unclassified	O	O	82.87	d__Bacteria;p__Bacteroidota;c__Bacteroidia;o__Bacteroidales;f__Bacteroidaceae;g__Bacteroides	100	RaFAH;97.10 iPHoP-RF;96.70 blast;80.90
S11_vf_58587	ME	n.a.	n.a.	n.a.	n.a.	n.a.	n.a.	n.a.	n.a.	11.86	d__Bacteria;p__Actinobacteriota;c__Actinomycetia;o__Actinomycetales;f__Bifidobacteriaceae;g__Bifidobacterium	90.4	blast;92.90 iPHoP-RF;65.60
S12_vf_69136	SHHC	Duplodnaviria	Heunggongvirae	Uroviricota	Caudoviricetes	n.a.	Unclassified	O	O	51.25	d__Bacteria;p__Bacteroidota;c__Bacteroidia;o__Bacteroidales;f__Bacteroidaceae;g__Prevotellamassilia	98.4	CRISPR;98.80 blast;82.30 iPHoP-RF;78.30
S12_vf_79666	Shared	n.a.	n.a.	n.a.	n.a.	n.a.	n.a.	n.a.	n.a.	99.55	d__Bacteria;p__Firmicutes;c__Clostridia;o__Oscillospirales;f__Acutalibacteraceae;g__UBA1417	91.7	blast;94.00 iPHoP-RF;58.20
S12_vs_121913	SHHC	n.a.	n.a.	n.a.	n.a.	n.a.	n.a.	n.a.	n.a.	82.73	d__Bacteria;p__Bacteroidota;c__Bacteroidia;o__Bacteroidales;f__Rikenellaceae;g__Alistipes	98.5	blast;96.70 RaFAH;83.70
S12_vs_123620	SHHC	n.a.	n.a.	n.a.	n.a.	n.a.	n.a.	n.a.	n.a.	10.35	d__Bacteria;p__Firmicutes;c__Bacilli;o__RF39;f__UBA660;g__CAG-302	95.4	blast;96.80 iPHoP-RF;64.80
S12_vs_138261	Shared	n.a.	n.a.	n.a.	n.a.	n.a.	n.a.	n.a.	n.a.	70.17	d__Bacteria;p__Bacteroidota;c__Bacteroidia;o__Bacteroidales;f__Tannerellaceae;g__Parabacteroides	98.4	blast;98.80 CRISPR;97.40 iPHoP-RF;96.10
S12_vs_141337	Shared	Duplodnaviria	Heunggongvirae	Uroviricota	Caudoviricetes	n.a.	Unclassified	Unclassified	Unclassified	98.55	d__Bacteria;p__Bacteroidota;c__Bacteroidia;o__Bacteroidales;f__Bacteroidaceae;g__Bacteroides	100	RaFAH;99.40 blast;92.10 iPHoP-RF;57.90
S12_vs_151292	Shared	Duplodnaviria	Heunggongvirae	Uroviricota	Caudoviricetes	n.a.	Unclassified	Unclassified	Unclassified	99.81	d__Bacteria;p__Bacteroidota;c__Bacteroidia;o__Bacteroidales;f__Bacteroidaceae;g__Bacteroides	100	RaFAH;98.90 iPHoP-RF;97.10 blast;95.90 CRISPR;68.40
S12_vs_163990	Shared	Duplodnaviria	Heunggongvirae	Uroviricota	Caudoviricetes	n.a.	Unclassified	O	O	99.19	d__Bacteria;p__Firmicutes;c__Clostridia;o__Lachnospirales;f__Lachnospiraceae;g__Dorea	99.3	RaFAH;99.40
S12_vs_172379	SHHC	n.a.	n.a.	n.a.	n.a.	n.a.	n.a.	n.a.	n.a.	18.46	d__Bacteria;p__Firmicutes;c__Bacilli;o__RF39;f__UBA660;g__CAG-877	90.8	blast;93.20
S11_vf_99751	ME	n.a.	n.a.	n.a.	n.a.	n.a.	n.a.	n.a.	n.a.	8.04	d__Bacteria;p__Firmicutes;c__Clostridia;o__Oscillospirales;f__Oscillospiraceae;g__CAG-83	94.9	blast;96.40 iPHoP-RF;57.10
S12_vs_188820	SHHC	n.a.	n.a.	n.a.	n.a.	n.a.	n.a.	n.a.	n.a.	7.83	d__Bacteria;p__Firmicutes;c__Clostridia;o__Oscillospirales;f__CAG-272;g__QALR01	93	blast;95.00 iPHoP-RF;87.00
S12_vs_188828	SHHC	n.a.	n.a.	n.a.	n.a.	n.a.	n.a.	n.a.	n.a.	8.93	d__Bacteria;p__Firmicutes;c__Clostridia;o__Oscillospirales;f__UMGS1783;g__UMGS1783	92.3	blast;94.50
S12_vs_23796	SHHC	Duplodnaviria	Heunggongvirae	Uroviricota	Caudoviricetes	n.a.	Unclassified	Unclassified	Unclassified	72.14	d__Bacteria;p__Bacteroidota;c__Bacteroidia;o__Bacteroidales;f__Rikenellaceae;g__Alistipes	99.1	iPHoP-RF;96.70 blast;96.60 RaFAH;89.00
S12_vs_26336	Shared	n.a.	n.a.	n.a.	n.a.	n.a.	n.a.	n.a.	n.a.	100	d__Bacteria;p__Bacteroidota;c__Bacteroidia;o__Bacteroidales;f__Bacteroidaceae;g__Bacteroides	99.6	RaFAH;99.60
S12_vs_48045	SHHC	Duplodnaviria	Heunggongvirae	Uroviricota	Caudoviricetes	n.a.	Unclassified	Unclassified	Unclassified	24.79	d__Bacteria;p__Firmicutes;c__Clostridia;o__Christensenellales;f__CAG-74;g__UBA11524	94.9	blast;96.40 iPHoP-RF;53.80
S12_vs_6836	SHHC	Duplodnaviria	Heunggongvirae	Uroviricota	Caudoviricetes	n.a.	Unclassified	O	O	43.21	d__Bacteria;p__Firmicutes;c__Clostridia;o__TANB77;f__CAG-508;g__CAG-269	92.7	blast;94.80 iPHoP-RF;65.30
S12_vs_71525	Shared	n.a.	n.a.	n.a.	n.a.	n.a.	n.a.	n.a.	n.a.	30.6	d__Bacteria;p__Bacteroidota;c__Bacteroidia;o__Bacteroidales;f__Marinifilaceae;g__Butyricimonas	93.8	blast;95.60 iPHoP-RF;67.30
S11_vs_104042	ME	n.a.	n.a.	n.a.	n.a.	n.a.	n.a.	n.a.	n.a.	75.46	d__Bacteria;p__Firmicutes;c__Clostridia;o__Lachnospirales;f__Lachnospiraceae;g__Eubacterium_I	98.1	blast;98.60 iPHoP-RF;84.80
S12_vs_86928	SHHC	Duplodnaviria	Heunggongvirae	Uroviricota	Caudoviricetes	n.a.	Unclassified	O	O	18.68	d__Bacteria;p__Verrucomicrobiota;c__Verrucomicrobiae;o__Verrucomicrobiales;f__Akkermansiaceae;g__Akkermansia	100	CRISPR;98.80 RaFAH;98.60 blast;96.90 iPHoP-RF;82.50
S12_vs_92868	Shared	Duplodnaviria	Heunggongvirae	Uroviricota	Caudoviricetes	n.a.	Unclassified	O	O	65.38	d__Bacteria;p__Firmicutes;c__Clostridia;o__Oscillospirales;f__Oscillospiraceae;g__Intestinimonas	93	blast;95.00 iPHoP-RF;58.70
S12_vs_93357	SHHC	Duplodnaviria	Heunggongvirae	Uroviricota	Caudoviricetes	n.a.	Unclassified	O	O	40.56	d__Bacteria;p__Proteobacteria;c__Gammaproteobacteria;o__Burkholderiales;f__Burkholderiaceae;g__Parasutterella	97	CRISPR;97.90 blast;96.90 iPHoP-RF;93.10
S12_vs_99092	SHHC	Duplodnaviria	Heunggongvirae	Uroviricota	Caudoviricetes	n.a.	Unclassified	O	O	78.56	d__Bacteria;p__Firmicutes;c__Clostridia;o__Lachnospirales;f__Lachnospiraceae;g__Lachnospira	99.6	CRISPR;98.80 blast;98.80 iPHoP-RF;94.40 RaFAH;84.60
S13_vf_109235	ME	n.a.	n.a.	n.a.	n.a.	n.a.	n.a.	n.a.	n.a.	72.39	d__Bacteria;p__Firmicutes;c__Clostridia;o__Oscillospirales;f__Oscillospiraceae;g__CAG-83	90.1	blast;92.60 iPHoP-RF;68.90
S13_vf_11246	Shared	Duplodnaviria	Heunggongvirae	Uroviricota	Caudoviricetes	Crassvirales	Suoliviridae	O	O	46.73	d__Bacteria;p__Bacteroidota;c__Bacteroidia;o__Bacteroidales;f__Bacteroidaceae;g__Prevotella	91.8	RaFAH;94.10
S13_vf_143422	Shared	Duplodnaviria	Heunggongvirae	Uroviricota	Caudoviricetes	Crassvirales	Suoliviridae	Boorivirinae	Canhaevirus	75.96	d__Bacteria;p__Bacteroidota;c__Bacteroidia;o__Bacteroidales;f__Tannerellaceae;g__Parabacteroides	98.1	blast;98.60 iPHoP-RF;83.30
S13_vf_150407	ME	Monodnaviria	Loebvirae	Hofneiviricota	Faserviricetes	Tubulavirales	Inoviridae	Unclassified	Inovirus		d__Bacteria;p__Firmicutes;c__Clostridia;o__Oscillospirales;f__Oscillospiraceae;g__ER4	93.5	CRISPR;95.40
S13_vf_151045	Shared	Duplodnaviria	Heunggongvirae	Uroviricota	Caudoviricetes	n.a.	Unclassified	O	O	98.31	d__Bacteria;p__Proteobacteria;c__Gammaproteobacteria;o__Enterobacterales;f__Enterobacteriaceae;g__Escherichia	95.7	RaFAH;97.00
S11_vs_13652	ME	Duplodnaviria	Heunggongvirae	Uroviricota	Caudoviricetes	n.a.	Unclassified	Unclassified	O	57.92	d__Bacteria;p__Firmicutes;c__Clostridia;o__Oscillospirales;f__Ruminococcaceae;g__Massilioclostridium	92.9	blast;94.90 iPHoP-RF;66.50
S13_vf_28309	ME	Duplodnaviria	Heunggongvirae	Uroviricota	Caudoviricetes	n.a.	Salasmaviridae	O	O	12.14	d__Bacteria;p__Firmicutes;c__Clostridia;o__Oscillospirales;f__Acutalibacteraceae;g__Ruminococcus_E	90.1	blast;92.60

S13_vf_56074	ME	Duplodnaviria	Heunggongvirae	Uroviricota	Caudoviricetes	n.a.	Unclassified	Unclassified	Unclassified	91.56	d__Bacteria;p__Bacteroidota;c__Bacteroidia;o__Bacteroidales;f__Rikenellaceae;g__Alistipes	98.1	RaFAH;98.60
S13_vf_7670	ME	n.a.	n.a.	n.a.	n.a.	n.a.	n.a.	n.a.	n.a.	6.84	d__Bacteria;p__Verrucomicrobiota;c__Verrucomicrobiae;o__Verrucomicrobiales;f__Akkermansiaceae;g__Akkermansia	100	CRISPR;99.60 RaFAH;96.00 blast;93.60 iPHoP-RF;72.60
S13_vf_87873	Shared	Duplodnaviria	Heunggongvirae	Uroviricota	Caudoviricetes	n.a.	Unclassified	Unclassified	Unclassified	97.58	d__Bacteria;p__Bacteroidota;c__Bacteroidia;o__Bacteroidales;f__Tannerellaceae;g__Marseille-P3160	98.4	CRISPR;98.80 iPHoP-RF;72.60
S13_vs_113593	ME	Duplodnaviria	Heunggongvirae	Uroviricota	Caudoviricetes	n.a.	Unclassified	Unclassified	Unclassified	40.01	d__Bacteria;p__Actinobacteriota;c__Actinomycetia;o__Actinomycetales;f__Bifidobacteriaceae;g__Bifidobacterium	91.5	blast;93.80 iPHoP-RF;88.20 CRISPR;56.60
S13_vs_121307	ME	n.a.	n.a.	n.a.	n.a.	n.a.	n.a.	n.a.	n.a.	11.04	d__Bacteria;p__Firmicutes;c__Clostridia;o__Lachnospirales;f__Lachnospiraceae;g__Blautia_A	95.9	CRISPR;89.80 RaFAH;85.80 iPHoP-RF;84.50
S13_vs_148294	ME	Duplodnaviria	Heunggongvirae	Uroviricota	Caudoviricetes	n.a.	Unclassified	O	O	57.95	d__Bacteria;p__Firmicutes;c__Clostridia;o__Oscillospirales;f__Oscillospiraceae;g__	92.3	blast;94.50 iPHoP-RF;80.60
S13_vs_15645	Shared	Duplodnaviria	Heunggongvirae	Uroviricota	Caudoviricetes	n.a.	Unclassified	O	O	92	d__Bacteria;p__Bacteroidota;c__Bacteroidia;o__Bacteroidales;f__Tannerellaceae;g__Parabacteroides	99.9	CRISPR;99.20 iPHoP-RF;95.10 blast;93.30 RaFAH;90.50
S11_vs_3006	ME	Duplodnaviria	Heunggongvirae	Uroviricota	Caudoviricetes	n.a.	Unclassified	Unclassified	O	44.36	d__Bacteria;p__Firmicutes;c__Clostridia;o__Oscillospirales;f__Acutalibacteraceae;g__UMGS263	93.6	blast;95.50 iPHoP-RF;67.00
S13_vs_30101	Shared	Duplodnaviria	Heunggongvirae	Uroviricota	Caudoviricetes	n.a.	Unclassified	O	O	54.46	d__Bacteria;p__Firmicutes;c__Bacilli;o__ML615J-28;f__CAG-313;g__CAG-313	95.6	blast;96.90 iPHoP-RF;81.60
S13_vs_26756	Shared	n.a.	n.a.	n.a.	n.a.	n.a.	n.a.	n.a.	n.a.	9.7	d__Bacteria;p__Firmicutes;c__Bacilli;o__RF39;f__UBA660;g__CAG-460	94	blast;95.80 iPHoP-RF;53.30
S13_vs_35816	Shared	Duplodnaviria	Heunggongvirae	Uroviricota	Caudoviricetes	n.a.	Unclassified	Unclassified	Lagaffevirus	99.57	d__Bacteria;p__Firmicutes;c__Clostridia;o__Oscillospirales;f__Oscillospiraceae;g__CAG-83	99.7	RaFAH;99.00 blast;82.80
S13_vs_67429	Shared	n.a.	n.a.	n.a.	n.a.	n.a.	n.a.	n.a.	n.a.	14.26	d__Bacteria;p__Firmicutes;c__Bacilli;o__RF39;f__UBA660;g__CAG-460	94.7	blast;96.30 iPHoP-RF;53.80
S11_vs_36748	ME	n.a.	n.a.	n.a.	n.a.	n.a.	n.a.	n.a.	n.a.	49.79	d__Bacteria;p__Bacteroidota;c__Bacteroidia;o__Bacteroidales;f__Rikenellaceae;g__Alistipes	98.5	CRISPR;98.90 blast;96.70 iPHoP-RF;91.80
S13_vs_83447	Shared	n.a.	n.a.	n.a.	n.a.	n.a.	n.a.	n.a.	n.a.	97.87	d__Bacteria;p__Bacteroidota;c__Bacteroidia;o__Bacteroidales;f__Rikenellaceae;g__Alistipes	99.9	blast;96.80 RaFAH;96.50 iPHoP-RF;82.80 CRISPR;82.60
S13_vs_90967	Shared	Monodnaviria	Sangervirae	Phixviricota	Malgrandaviricetes	Petitvirales	Microviridae	O	O		d__Bacteria;p__Bacteroidota;c__Bacteroidia;o__Bacteroidales;f__Barnesiellaceae;g__Barnesiella	90.3	blast;92.80 iPHoP-RF;81.90
S11_vs_38860	ME	n.a.	n.a.	n.a.	n.a.	n.a.	n.a.	n.a.	n.a.	39.67	d__Bacteria;p__Firmicutes;c__Clostridia;o__Lachnospirales;f__Lachnospiraceae;g__Enterocloster	95.2	CRISPR;96.60 blast;95.90 iPHoP-RF;89.80
S13_vs_9438	Shared	n.a.	n.a.	n.a.	n.a.	n.a.	n.a.	n.a.	n.a.	7.33	d__Bacteria;p__Proteobacteria;c__Alphaproteobacteria;o__RF32;f__CAG-239;g__CAG-267	91.6	blast;93.90 iPHoP-RF;83.30
S14_vf_106156	ME	Duplodnaviria	Heunggongvirae	Uroviricota	Caudoviricetes	n.a.	Unclassified	Unclassified	Unclassified	21.19	d__Bacteria;p__Firmicutes;c__Bacilli;o__Lactobacillales;f__Lactobacillaceae;g__Lactobacillus	100	RaFAH;98.90 CRISPR;96.80 iPHoP-RF;96.10 blast;95.50
S14_vf_109545	Shared	n.a.	n.a.	n.a.	n.a.	n.a.	n.a.	n.a.	n.a.	12.74	d__Bacteria;p__Firmicutes;c__Clostridia;o__Oscillospirales;f__Oscillospiraceae;g__Intestinimonas	90.8	blast;93.20 iPHoP-RF;79.50
S14_vf_117150	ME	Duplodnaviria	Heunggongvirae	Uroviricota	Caudoviricetes	n.a.	Unclassified	O	O	99.65	d__Bacteria;p__Firmicutes;c__Clostridia;o__Lachnospirales;f__Lachnospiraceae;g__Lachnospira	99.9	CRISPR;98.90 RaFAH;91.00 blast;87.20 iPHoP-RF;83.10
S11_vs_45658	Shared	Duplodnaviria	Heunggongvirae	Uroviricota	Caudoviricetes	n.a.	Unclassified	O	O	40.35	d__Bacteria;p__Bacteroidota;c__Bacteroidia;o__Bacteroidales;f__Bacteroidaceae;g__Bacteroides	95	RaFAH;96.20 iPHoP-RF;53.60
S14_vf_117157	Shared	n.a.	n.a.	n.a.	n.a.	n.a.	n.a.	n.a.	n.a.	99.57	d__Bacteria;p__Firmicutes;c__Bacilli;o__Lactobacillales;f__Streptococcaceae;g__Streptococcus	100	RaFAH;99.40 CRISPR;98.80 iPHoP-RF;94.40 blast;93.60
S11_vs_48109	ME	Duplodnaviria	Heunggongvirae	Uroviricota	Caudoviricetes	n.a.	Unclassified	O	O	63.66	d__Bacteria;p__Firmicutes;c__Clostridia;o__Oscillospirales;f__Acutalibacteraceae;g__An172	93.4	blast;95.30 iPHoP-RF;74.60
S14_vf_37500	ME	Monodnaviria	Sangervirae	Phixviricota	Malgrandaviricetes	Petitvirales	Microviridae	Unclassified	Unclassified	5.58	d__Bacteria;p__Bacteroidota;c__Bacteroidia;o__Bacteroidales;f__Bacteroidaceae;g__Phocaeicola	94.3	CRISPR;96.00 iPHoP-RF;67.00
S14_vf_56130	ME	Duplodnaviria	Heunggongvirae	Uroviricota	Caudoviricetes	n.a.	Unclassified	O	O	100	d__Bacteria;p__Firmicutes;c__Clostridia;o__Clostridiales;f__Clostridiaceae;g__Clostridium	98.8	RaFAH;99.10
S14_vf_58919	ME	Duplodnaviria	Heunggongvirae	Uroviricota	Caudoviricetes	n.a.	Unclassified	Unclassified	Mushuvirus	74.74	d__Bacteria;p__Firmicutes;c__Clostridia;o__Lachnospirales;f__Lachnospiraceae;g__Roseburia	95.3	blast;90.40 iPHoP-RF;87.90 RaFAH;82.70
S14_vf_72335	ME	Duplodnaviria	Heunggongvirae	Uroviricota	Caudoviricetes	n.a.	Unclassified	O	O	11.73	d__Bacteria;p__Firmicutes;c__Bacilli;o__Erysipelotrichales;f__Erysipelotrichaceae;g__Holdemania	92.1	CRISPR;94.30
S14_vf_72953	ME	Duplodnaviria	Heunggongvirae	Uroviricota	Caudoviricetes	n.a.	Unclassified	Unclassified	Unclassified	32.12	d__Bacteria;p__Firmicutes;c__Bacilli;o__Lactobacillales;f__Lactobacillaceae;g__Lacticaseibacillus	96.7	RaFAH;97.70
S11_vs_55144	ME	n.a.	n.a.	n.a.	n.a.	n.a.	n.a.	n.a.	n.a.	12.8	d__Bacteria;p__Firmicutes;c__Bacilli;o__Erysipelotrichales;f__Erysipelatoclostridiaceae;g__Massiliomicrobiota	90.3	blast;92.80
S14_vf_78139	ME	Duplodnaviria	Heunggongvirae	Uroviricota	Caudoviricetes	n.a.	Unclassified	Unclassified	Unclassified	74.71	d__Bacteria;p__Firmicutes;c__Bacilli;o__Lactobacillales;f__Lactobacillaceae;g__Lactobacillus	100	RaFAH;99.10 blast;96.80 CRISPR;96.50 iPHoP-RF;92.80
S14_vf_92240	Shared	Duplodnaviria	Heunggongvirae	Uroviricota	Caudoviricetes	n.a.	Unclassified	O	O	99.79	d__Bacteria;p__Firmicutes;c__Clostridia;o__Lachnospirales;f__Lachnospiraceae;g__Coprococcus	93.2	CRISPR;95.20
S14_vf_99344	Shared	n.a.	n.a.	n.a.	n.a.	n.a.	n.a.	n.a.	n.a.	10.5	d__Bacteria;p__Firmicutes;c__Clostridia;o__Lachnospirales;f__Lachnospiraceae;g__Eubacterium_F	96.9	CRISPR;97.80
S14_vs_102698	ME	Duplodnaviria	Heunggongvirae	Uroviricota	Caudoviricetes	n.a.	Unclassified	O	O	99.62	d__Bacteria;p__Firmicutes;c__Clostridia;o__Acetivibrionales;f__Acetivibrionaceae;g__Ruminiclostridium	90.1	RaFAH;92.60
S14_vs_11510	Shared	n.a.	n.a.	n.a.	n.a.	n.a.	n.a.	n.a.	n.a.	39.46	d__Bacteria;p__Bacteroidota;c__Bacteroidia;o__Bacteroidales;f__Bacteroidaceae;g__Bacteroides	96.9	blast;91.70 RaFAH;87.00 iPHoP-RF;81.40
S14_vs_117166	Shared	n.a.	n.a.	n.a.	n.a.	n.a.	n.a.	n.a.	n.a.	22.09	d__Bacteria;p__Firmicutes;c__Clostridia;o__Oscillospirales;f__Oscillospiraceae;g__Evtepia	94.2	blast;95.90 iPHoP-RF;66.30
S11_vs_67573	ME	n.a.	n.a.	n.a.	n.a.	n.a.	n.a.	n.a.	n.a.	11.32	d__Bacteria;p__Firmicutes;c__Clostridia;o__Lachnospirales;f__Lachnospiraceae;g__Enterocloster	92.9	CRISPR;94.90 blast;92.10 iPHoP-RF;92.10
S14_vs_14737	ME	Duplodnaviria	Heunggongvirae	Uroviricota	Caudoviricetes	n.a.	Unclassified	O	O	33.52	d__Bacteria;p__Firmicutes;c__Clostridia;o__Oscillospirales;f__Ruminococcaceae;g__Ruthenibacterium	93.1	blast;95.10 iPHoP-RF;68.40
S14_vs_15228	Shared	Duplodnaviria	Heunggongvirae	Uroviricota	Caudoviricetes	n.a.	Unclassified	O	O	86.47	d__Bacteria;p__Bacteroidota;c__Bacteroidia;o__Bacteroidales;f__Tannerellaceae;g__Parabacteroides	100	CRISPR;99.00 blast;98.60 RaFAH;97.90 iPHoP-RF;96.10
S11_vs_68348	ME	Duplodnaviria	Heunggongvirae	Uroviricota	Caudoviricetes	n.a.	Unclassified	O	O	49.51	d__Bacteria;p__Firmicutes;c__Bacilli;o__Erysipelotrichales;f__Erysipelatoclostridiaceae;g__Erysipelatoclostridium	95.2	blast;96.60 iPHoP-RF;64.50
S14_vs_25852	Shared	n.a.	n.a.	n.a.	n.a.	n.a.	n.a.	n.a.	n.a.	26.78	d__Bacteria;p__Bacteroidota;c__Bacteroidia;o__Bacteroidales;f__Bacteroidaceae;g__Bacteroides	97.8	blast;96.60 iPHoP-RF;95.10 CRISPR;93.00 RaFAH;77.10
S11_vs_6878	ME	Duplodnaviria	Heunggongvirae	Uroviricota	Caudoviricetes	n.a.	Unclassified	O	O	72.71	d__Bacteria;p__Firmicutes;c__Clostridia;o__Lachnospirales;f__Lachnospiraceae;g__	91	blast;93.40 iPHoP-RF;73.00
S14_vs_2870	ME	Duplodnaviria	Heunggongvirae	Uroviricota	Caudoviricetes	n.a.	Unclassified	O	O	56.13	d__Bacteria;p__Proteobacteria;c__Gammaproteobacteria;o__Burkholderiales;f__Burkholderiaceae;g__Sutterella	99	blast;96.90 RaFAH;87.10 CRISPR;78.30 iPHoP-RF;69.50
S14_vs_32499	Shared	Duplodnaviria	Heunggongvirae	Uroviricota	Caudoviricetes	n.a.	Unclassified	O	O	51.73	d__Bacteria;p__Bacteroidota;c__Bacteroidia;o__Bacteroidales;f__Bacteroidaceae;g__Bacteroides	99.1	blast;98.10 iPHoP-RF;96.70 RaFAH;82.60
S14_vs_3251	ME	n.a.	n.a.	n.a.	n.a.	n.a.	n.a.	n.a.	n.a.	82.87	d__Bacteria;p__Firmicutes;c__Clostridia;o__Oscillospirales;f__Oscillospiraceae;g__Flavonifractor	95.6	blast;96.90 iPHoP-RF;65.60
S14_vs_39797	ME	Duplodnaviria	Heunggongvirae	Uroviricota	Caudoviricetes	n.a.	Unclassified	O	O	47.42	d__Bacteria;p__Firmicutes;c__Clostridia;o__Lachnospirales;f__Lachnospiraceae;g__Anaerostipes	93.8	blast;95.60 iPHoP-RF;67.00
S11_vs_79831	ME	n.a.	n.a.	n.a.	n.a.	n.a.	n.a.	n.a.	n.a.	17.3	d__Bacteria;p__Bacteroidota;c__Bacteroidia;o__Bacteroidales;f__UBA11471;g__UBA11471	90	blast;92.50 iPHoP-RF;73.20
S14_vs_59347	ME	Duplodnaviria	Heunggongvirae	Uroviricota	Caudoviricetes	n.a.	Unclassified	O	O	29.43	d__Bacteria;p__Firmicutes;c__Clostridia;o__Lachnospirales;f__Lachnospiraceae;g__Muricomes	93.1	iPHoP-RF;95.10
S14_vs_61530	ME	n.a.	n.a.	n.a.	n.a.	n.a.	n.a.	n.a.	n.a.	22.32	d__Bacteria;p__Firmicutes;c__Clostridia;o__Oscillospirales;f__Acutalibacteraceae;g__UBA1417	92	blast;94.20 iPHoP-RF;82.80
S11_vs_82331	ME	Duplodnaviria	Heunggongvirae	Uroviricota	Caudoviricetes	n.a.	Unclassified	O	O	98.94	d__Bacteria;p__Cyanobacteria;c__Vampirovibrionia;o__Gastranaerophilales;f__Gastranaerophilaceae;g__QAMI01	93.1	blast;95.10
S14_vs_632	ME	Duplodnaviria	Heunggongvirae	Uroviricota	Caudoviricetes	n.a.	Unclassified	O	O	78.66	d__Bacteria;p__Firmicutes;c__Clostridia;o__Clostridiales;f__Clostridiaceae;g__Clostridium	91	RaFAH;93.40
S11_vs_82368	ME	Duplodnaviria	Heunggongvirae	Uroviricota	Caudoviricetes	n.a.	Unclassified	O	O	62.18	d__Bacteria;p__Firmicutes;c__Clostridia;o__Lachnospirales;f__Lachnospiraceae;g__Blautia_A	98.1	blast;92.60 RaFAH;90.70 iPHoP-RF;86.00
S14_vs_66139	Shared	Duplodnaviria	Heunggongvirae	Uroviricota	Caudoviricetes	n.a.	Unclassified	O	O	83.75	d__Bacteria;p__Firmicutes;c__Clostridia;o__Oscillospirales;f__Acutalibacteraceae;g__Ruminococcus_E	95.6	blast;96.90 iPHoP-RF;85.70
S11_vs_83666	ME	Duplodnaviria	Heunggongvirae	Uroviricota	Caudoviricetes	n.a.	Unclassified	O	O	35.28	d__Bacteria;p__Firmicutes;c__Clostridia;o__Lachnospirales;f__Lachnospiraceae;g__Dorea_A	90.9	blast;93.30 iPHoP-RF;67.70
S14_vs_66208	Shared	Duplodnaviria	Heunggongvirae	Uroviricota	Caudoviricetes	n.a.	Unclassified	O	O	100	d__Bacteria;p__Bacteroidota;c__Bacteroidia;o__Bacteroidales;f__Bacteroidaceae;g__Bacteroides	100	RaFAH;98.30 iPHoP-RF;96.40 blast;93.70
S14_vs_66841	Shared	Duplodnaviria	Heunggongvirae	Uroviricota	Caudoviricetes	n.a.	Unclassified	O	O	15.26	d__Bacteria;p__Firmicutes;c__Clostridia;o__Lachnospirales;f__Lachnospiraceae;g__Agathobacter	90.4	blast;92.90 iPHoP-RF;78.00
S14_vs_67094	ME	n.a.	n.a.	n.a.	n.a.	n.a.	n.a.	n.a.	n.a.	10	d__Bacteria;p__Firmicutes;c__Clostridia;o__Oscillospirales;f__Oscillospiraceae;g__Lawsonibacter	90.7	blast;93.10
S14_vs_73301	ME	Duplodnaviria	Heunggongvirae	Uroviricota	Caudoviricetes	n.a.	Unclassified	O	O	15.25	d__Bacteria;p__Firmicutes;c__Clostridia;o__Oscillospirales;f__Ruminococcaceae;g__Massilimaliae	94.2	blast;95.90 iPHoP-RF;64.80
S14_vs_78164	ME	Duplodnaviria	Heunggongvirae	Uroviricota	Caudoviricetes	n.a.	Winoviridae	O	O	61.84	d__Bacteria;p__Bacteroidota;c__Bacteroidia;o__Bacteroidales;f__Marinifilaceae;g__Odoribacter	95.4	blast;96.80 iPHoP-RF;84.80 CRISPR;56.80
S14_vs_88666	ME	Duplodnaviria	Heunggongvirae	Uroviricota	Caudoviricetes	n.a.	Unclassified	O	O	32.05	d__Bacteria;p__Firmicutes;c__Clostridia;o__Oscillospirales;f__Acutalibacteraceae;g__Fimivicinus	95.4	blast;96.80 iPHoP-RF;86.70
S14_vs_9676	ME	Duplodnaviria	Heunggongvirae	Uroviricota	Caudoviricetes	n.a.	Unclassified	O	O	24.59	d__Bacteria;p__Firmicutes;c__Clostridia;o__Oscillospirales;f__Oscillospiraceae;g__Lawsonibacter	90.7	blast;93.10 iPHoP-RF;77.50
S12_vf_105542	SHHC	Duplodnaviria	Heunggongvirae	Uroviricota	Caudoviricetes	n.a.	Unclassified	O	O	13.83	d__Bacteria;p__Firmicutes;c__Bacilli;o__RF39;f__UBA660;g__CAG-460	94.6	blast;96.20 iPHoP-RF;73.70
S15_vf_2032	Shared	Duplodnaviria	Heunggongvirae	Uroviricota	Caudoviricetes	n.a.	Unclassified	O	O	83.93	d__Bacteria;p__Bacteroidota;c__Bacteroidia;o__Bacteroidales;f__Bacteroidaceae;g__Bacteroides	99.3	iPHoP-RF;97.10 blast;96.20 CRISPR;91.10 RaFAH;89.30
S15_vf_20644	SHHC	Duplodnaviria	Heunggongvirae	Uroviricota	Caudoviricetes	n.a.	Unclassified	Unclassified	Unclassified	83.74	d__Bacteria;p__Bacteroidota;c__Bacteroidia;o__Bacteroidales;f__Rikenellaceae;g__Alistipes	94.3	RaFAH;96.00
S15_vf_2513	SHHC	n.a.	n.a.	n.a.	n.a.	n.a.	n.a.	n.a.	n.a.	25.17	d__Bacteria;p__Firmicutes;c__Clostridia;o__Lachnospirales;f__Lachnospiraceae;g__Lachnospira	94.5	CRISPR;96.10
S12_vf_145044	Shared	n.a.	n.a.	n.a.	n.a.	n.a.	n.a.	n.a.	n.a.	32.93	d__Bacteria;p__Bacteroidota;c__Bacteroidia;o__Bacteroidales;f__Rikenellaceae;g__Alistipes_A	91.6	blast;93.90 iPHoP-RF;77.10
S14_vs_70985	Shared	n.a.	n.a.	n.a.	n.a.	n.a.	n.a.	n.a.	n.a.	21.93	d__Bacteria;p__Bacteroidota;c__Bacteroidia;o__Bacteroidales;f__Bacteroidaceae;g__Bacteroides	99	iPHoP-RF;96.70 blast;92.60 RaFAH;88.40
S12_vf_16374	Shared	Duplodnaviria	Heunggongvirae	Uroviricota	Caudoviricetes	n.a.	Unclassified	O	O	100	d__Bacteria;p__Bacteroidota;c__Bacteroidia;o__Bacteroidales;f__Bacteroidaceae;g__Bacteroides	100	RaFAH;99.10 iPHoP-RF;97.10 blast;94.80
S15_vs_18274	SHHC	n.a.	n.a.	n.a.	n.a.	n.a.	n.a.	n.a.	n.a.	75.03	d__Bacteria;p__Bacteroidota;c__Bacteroidia;o__Bacteroidales;f__Coprobacteraceae;g__Coprobacter	98.7	CRISPR;99.00 blast;93.10 iPHoP-RF;64.00 RaFAH;50.00
S14_vs_74067	Shared	n.a.	n.a.	n.a.	n.a.	n.a.	n.a.	n.a.	n.a.	100	d__Bacteria;p__Bacteroidota;c__Bacteroidia;o__Bacteroidales;f__Bacteroidaceae;g__Bacteroides	99.6	RaFAH;93.20 blast;92.00 iPHoP-RF;82.20
S12_vf_170614	SHHC	Duplodnaviria	Heunggongvirae	Uroviricota	Caudoviricetes	n.a.	Salasmaviridae	O	O	7.03	d__Bacteria;p__Actinobacteriota;c__Coriobacteriia;o__Coriobacteriales;f__Coriobacteriaceae;g__Collinsella	96.4	CRISPR;97.50 iPHoP-RF;95.10
S15_vs_27510	Shared	n.a.	n.a.	n.a.	n.a.	n.a.	n.a.	n.a.	n.a.	51.41	d__Bacteria;p__Bacteroidota;c__Bacteroidia;o__Bacteroidales;f__Bacteroidaceae;g__Bacteroides	99.1	iPHoP-RF;96.10 blast;93.90 RaFAH;90.50
S15_vs_35445	Shared	n.a.	n.a.	n.a.	n.a.	n.a.	n.a.	n.a.	n.a.	27.45	d__Bacteria;p__Bacteroidota;c__Bacteroidia;o__Bacteroidales;f__Bacteroidaceae;g__Bacteroides	98.8	iPHoP-RF;96.70 blast;93.90 RaFAH;86.60
S12_vf_173666	Shared	n.a.	n.a.	n.a.	n.a.	n.a.	n.a.	n.a.	n.a.	36.83	d__Bacteria;p__Actinobacteriota;c__Coriobacteriia;o__Coriobacteriales;f__Eggerthellaceae;g__Adlercreutzia	93.1	blast;95.10 iPHoP-RF;76.40
S15_vs_71968	Shared	Duplodnaviria	Heunggongvirae	Uroviricota	Caudoviricetes	n.a.	Unclassified	O	O	100	d__Bacteria;p__Bacteroidota;c__Bacteroidia;o__Bacteroidales;f__Rikenellaceae;g__Alistipes	100	RaFAH;98.90 blast;98.40 iPHoP-RF;95.40
S16_vf_1037	SHHC	n.a.	n.a.	n.a.	n.a.	n.a.	n.a.	n.a.	n.a.	73.36	d__Bacteria;p__Firmicutes;c__Clostridia;o__Lachnospirales;f__Lachnospiraceae;g__Lachnospira	99.7	CRISPR;99.70 iPHoP-RF;94.40 blast;93.60
S15_vf_75924	Shared	Duplodnaviria	Heunggongvirae	Uroviricota	Caudoviricetes	Crassvirales	Steigviridae	O	O	99.22	d__Bacteria;p__Bacteroidota;c__Bacteroidia;o__Bacteroidales;f__Bacteroidaceae;g__Bacteroides	99.6	RaFAH;93.20 blast;91.90 iPHoP-RF;82.50
S16_vf_169895	Shared	Duplodnaviria	Heunggongvirae	Uroviricota	Caudoviricetes	n.a.	Salasmaviridae	O	O	11.84	d__Bacteria;p__Firmicutes;c__Clostridia;o__Oscillospirales;f__Ruminococcaceae;g__Ruthenibacterium	92.7	CRISPR;94.80
S16_vf_173109	SHHC	Duplodnaviria	Heunggongvirae	Uroviricota	Caudoviricetes	n.a.	Unclassified	Unclassified	Unclassified	7.33	d__Bacteria;p__Actinobacteriota;c__Actinomycetia;o__Actinomycetales;f__Bifidobacteriaceae;g__Bifidobacterium	97	CRISPR;97.90 iPHoP-RF;81.60
S16_vf_36146	Shared	Duplodnaviria	Heunggongvirae	Uroviricota	Caudoviricetes	n.a.	Unclassified	O	O	99.74	d__Bacteria;p__Firmicutes;c__Clostridia;o__Clostridiales;f__Clostridiaceae;g__Clostridium	96.2	RaFAH;97.30
S15_vs_42043	Shared	n.a.	n.a.	n.a.	n.a.	n.a.	n.a.	n.a.	n.a.	51.36	d__Bacteria;p__Bacteroidota;c__Bacteroidia;o__Bacteroidales;f__Bacteroidaceae;g__Bacteroides	97.6	blast;96.80 iPHoP-RF;95.10 RaFAH;72.80
S16_vf_6102	Shared	Duplodnaviria	Heunggongvirae	Uroviricota	Caudoviricetes	n.a.	Unclassified	Unclassified	Toutatisvirus	38.14	d__Bacteria;p__Firmicutes;c__Clostridia;o__Oscillospirales;f__Oscillospiraceae;g__ER4	91.1	blast;93.50 iPHoP-RF;66.80
S16_vf_87190	SHHC	Duplodnaviria	Heunggongvirae	Uroviricota	Caudoviricetes	n.a.	Unclassified	O	O	34.33	d__Bacteria;p__Bacteroidota;c__Bacteroidia;o__Bacteroidales;f__Bacteroidaceae;g__Bacteroides	95	RaFAH;87.80 iPHoP-RF;77.80 CRISPR;68.10
S16_vf_90328	SHHC	Duplodnaviria	Heunggongvirae	Uroviricota	Caudoviricetes	n.a.	Unclassified	O	O	16.6	d__Bacteria;p__Firmicutes;c__Clostridia;o__TANB77;f__CAG-508;g__CAG-269	91.4	blast;93.70 iPHoP-RF;53.00
S16_vf_97279	Shared	Duplodnaviria	Heunggongvirae	Uroviricota	Caudoviricetes	n.a.	Unclassified	Unclassified	Eponavirus	52.37	d__Bacteria;p__Firmicutes;c__Clostridia;o__Oscillospirales;f__Oscillospiraceae;g__ER4	91	blast;93.40 iPHoP-RF;60.30
S12_vf_188846	SHHC	Monodnaviria	Sangervirae	Phixviricota	Malgrandaviricetes	Petitvirales	Microviridae	O	O		d__Bacteria;p__Bacteroidota;c__Bacteroidia;o__Bacteroidales;f__Barnesiellaceae;g__Barnesiella	94.5	blast;96.10 iPHoP-RF;68.60
S16_vf_108554	Shared	n.a.	n.a.	n.a.	n.a.	n.a.	n.a.	n.a.	n.a.	18.88	d__Bacteria;p__Firmicutes;c__Clostridia;o__Oscillospirales;f__Ruminococcaceae;g__Faecalibacterium	97.9	CRISPR;98.50 blast;93.10 iPHoP-RF;92.10
S12_vf_18902	Shared	Duplodnaviria	Heunggongvirae	Uroviricota	Caudoviricetes	n.a.	Unclassified	O	O	80.1	d__Bacteria;p__Firmicutes;c__Clostridia;o__Oscillospirales;f__Oscillospiraceae;g__ER4	90.6	blast;93.00 iPHoP-RF;67.00
S16_vs_103726	SHHC	n.a.	n.a.	n.a.	n.a.	n.a.	n.a.	n.a.	n.a.	26.95	d__Bacteria;p__Firmicutes;c__Clostridia;o__Oscillospirales;f__Ruminococcaceae;g__Ruthenibacterium	94	blast;95.80 CRISPR;80.80 iPHoP-RF;79.80
S16_vs_116956	SHHC	n.a.	n.a.	n.a.	n.a.	n.a.	n.a.	n.a.	n.a.	43.56	d__Bacteria;p__Firmicutes;c__Clostridia;o__Lachnospirales;f__Lachnospiraceae;g__Acetatifactor	91.6	blast;93.90 iPHoP-RF;75.20
S16_vs_120622	SHHC	Duplodnaviria	Heunggongvirae	Uroviricota	Caudoviricetes	n.a.	Unclassified	O	O	63.91	d__Bacteria;p__Proteobacteria;c__Gammaproteobacteria;o__Burkholderiales;f__Burkholderiaceae;g__Parasutterella	97.2	CRISPR;98.00 blast;96.90 iPHoP-RF;93.10
S16_vf_173099	SHHC	n.a.	n.a.	n.a.	n.a.	n.a.	n.a.	n.a.	n.a.	55.52	d__Bacteria;p__Firmicutes;c__Clostridia;o__Oscillospirales;f__Oscillospiraceae;g__Flavonifractor	93.8	blast;92.00 iPHoP-RF;86.00 RaFAH;72.50
S16_vs_127669	Shared	Duplodnaviria	Heunggongvirae	Uroviricota	Caudoviricetes	n.a.	Unclassified	Unclassified	Unclassified	99.82	d__Bacteria;p__Bacteroidota;c__Bacteroidia;o__Bacteroidales;f__Bacteroidaceae;g__Bacteroides	100	RaFAH;99.00 CRISPR;97.60 iPHoP-RF;97.10 blast;90.90
S12_vf_61299	SHHC	Duplodnaviria	Heunggongvirae	Uroviricota	Caudoviricetes	n.a.	Salasmaviridae	O	O	11.99	d__Bacteria;p__Firmicutes;c__Clostridia;o__Lachnospirales;f__Lachnospiraceae;g__Muricomes	96.9	CRISPR;97.80 iPHoP-RF;59.20
S16_vs_127905	Shared	n.a.	n.a.	n.a.	n.a.	n.a.	n.a.	n.a.	n.a.	55.65	d__Bacteria;p__Bacteroidota;c__Bacteroidia;o__Bacteroidales;f__Bacteroidaceae;g__Bacteroides	99.4	iPHoP-RF;97.10 blast;95.20 RaFAH;91.80 CRISPR;60.90
S16_vs_136935	Shared	n.a.	n.a.	n.a.	n.a.	n.a.	n.a.	n.a.	n.a.	99.58	d__Bacteria;p__Bacteroidota;c__Bacteroidia;o__Bacteroidales;f__Bacteroidaceae;g__Bacteroides	100	RaFAH;98.20 iPHoP-RF;96.70 blast;89.50
S16_vs_142561	Shared	Duplodnaviria	Heunggongvirae	Uroviricota	Caudoviricetes	n.a.	Unclassified	O	O	99.95	d__Bacteria;p__Bacteroidota;c__Bacteroidia;o__Bacteroidales;f__Rikenellaceae;g__Alistipes_A	97.8	blast;98.20 RaFAH;56.20 iPHoP-RF;53.30
S16_vs_173079	SHHC	Duplodnaviria	Heunggongvirae	Uroviricota	Caudoviricetes	n.a.	Unclassified	O	O	99.1	d__Bacteria;p__Firmicutes;c__Clostridia;o__Lachnospirales;f__Lachnospiraceae;g__Coprococcus	94	blast;95.80 iPHoP-RF;71.70
S16_vs_173096	Shared	Duplodnaviria	Heunggongvirae	Uroviricota	Caudoviricetes	n.a.	Unclassified	Unclassified	Unclassified	74.56	d__Bacteria;p__Verrucomicrobiota;c__Verrucomicrobiae;o__Verrucomicrobiales;f__Akkermansiaceae;g__Akkermansia	100	RaFAH;99.70 CRISPR;99.60 blast;96.80 iPHoP-RF;81.10
S16_vs_18440	SHHC	Duplodnaviria	Heunggongvirae	Uroviricota	Caudoviricetes	n.a.	Unclassified	Unclassified	Toutatisvirus	67.51	d__Bacteria;p__Firmicutes;c__Clostridia;o__Oscillospirales;f__Ruminococcaceae;g__Faecalibacterium	100	RaFAH;99.30 CRISPR;98.90 blast;93.60 iPHoP-RF;85.70
S12_vs_111286	SHHC	n.a.	n.a.	n.a.	n.a.	n.a.	n.a.	n.a.	n.a.	44.45	d__Bacteria;p__Bacteroidota;c__Bacteroidia;o__Bacteroidales;f__Muribaculaceae;g__UBA7173	91.6	blast;93.90 iPHoP-RF;62.50
S16_vf_96483	Shared	n.a.	n.a.	n.a.	n.a.	n.a.	n.a.	n.a.	n.a.	29.87	d__Bacteria;p__Firmicutes;c__Clostridia;o__Lachnospirales;f__Lachnospiraceae;g__Blautia_A	97.6	iPHoP-RF;93.40 blast;93.30 RaFAH;87.30

S16_vs_19968	Shared	n.a.	n.a.	n.a.	n.a.	n.a.	n.a.	n.a.	n.a.	93.01	d__Bacteria;p__Bacteroidota;c__Bacteroidia;o__Bacteroidales;f__Rikenellaceae;g__Alistipes	98.4	iPHoP-RF;97.10 blast;96.90 RaFAH;79.60
S16_vs_32764	Shared	n.a.	n.a.	n.a.	n.a.	n.a.	n.a.	n.a.	n.a.	17.71	d__Bacteria;p__Bacteroidota;c__Bacteroidia;o__Bacteroidales;f__Rikenellaceae;g__Alistipes	95.9	iPHoP-RF;97.10 blast;96.90
S16_vs_33281	Shared	n.a.	n.a.	n.a.	n.a.	n.a.	n.a.	n.a.	n.a.	7.09	d__Bacteria;p__Bacteroidota;c__Bacteroidia;o__Bacteroidales;f__Rikenellaceae;g__Alistipes	95.9	iPHoP-RF;97.10 blast;96.90
S16_vs_4662	SHHC	Duplodnaviria	Heunggongvirae	Uroviricota	Caudoviricetes	n.a.	Unclassified	Unclassified	Unclassified	13.68	d__Bacteria;p__Firmicutes;c__Clostridia;o__Christensenellales;f__CAG-138;g__UBA5394	92.6	CRISPR;94.70 blast;92.60 iPHoP-RF;74.30
S12_vs_133070	Shared	Duplodnaviria	Heunggongvirae	Uroviricota	Caudoviricetes	n.a.	Unclassified	O	O	14.52	d__Bacteria;p__Firmicutes;c__Clostridia;o__Oscillospirales;f__Ruminococcaceae;g__CAG-353	91.1	blast;93.50 iPHoP-RF;79.80
S16_vs_48714	SHHC	n.a.	n.a.	n.a.	n.a.	n.a.	n.a.	n.a.	n.a.	5.9	d__Bacteria;p__Firmicutes;c__Clostridia;o__Oscillospirales;f__Ruminococcaceae;g__Ruminococcus_D	97	blast;97.90 iPHoP-RF;97.10 CRISPR;96.60
S16_vs_51901	SHHC	n.a.	n.a.	n.a.	n.a.	n.a.	n.a.	n.a.	n.a.	16.94	d__Bacteria;p__Bacteroidota;c__Bacteroidia;o__Bacteroidales;f__Rikenellaceae;g__Alistipes	97.6	blast;93.90 iPHoP-RF;90.10 RaFAH;85.80
S16_vs_75108	Shared	n.a.	n.a.	n.a.	n.a.	n.a.	n.a.	n.a.	n.a.	32.65	d__Bacteria;p__Firmicutes;c__Clostridia;o__Oscillospirales;f__Oscillospiraceae;g__UBA5446	95	blast;96.50 CRISPR;82.40 iPHoP-RF;65.30
S16_vs_78361	Shared	Duplodnaviria	Heunggongvirae	Uroviricota	Caudoviricetes	n.a.	Unclassified	O	O	75.01	d__Bacteria;p__Actinobacteriota;c__Actinomycetia;o__Actinomycetales;f__Bifidobacteriaceae;g__Bifidobacterium	98.5	CRISPR;98.90 iPHoP-RF;97.10 blast;96.90
S12_vs_142625	Shared	Duplodnaviria	Heunggongvirae	Uroviricota	Caudoviricetes	n.a.	Unclassified	O	O	31.03	d__Bacteria;p__Bacteroidota;c__Bacteroidia;o__Bacteroidales;f__Rikenellaceae;g__Alistipes	95.9	iPHoP-RF;97.10 blast;96.90
S16_vs_80244	Shared	n.a.	n.a.	n.a.	n.a.	n.a.	n.a.	n.a.	n.a.	99.97	d__Bacteria;p__Bacteroidota;c__Bacteroidia;o__Bacteroidales;f__Bacteroidaceae;g__Bacteroides	99.7	iPHoP-RF;97.10 RaFAH;95.20 blast;89.20
S16_vs_15093	SHHC	Duplodnaviria	Heunggongvirae	Uroviricota	Caudoviricetes	n.a.	Unclassified	O	O	19.11	d__Bacteria;p__Firmicutes;c__Clostridia;o__Christensenellales;f__CAG-74;g__UBA11524	99.6	CRISPR;99.60 blast;96.60 iPHoP-RF;85.70
S17_vf_174033	Shared	Duplodnaviria	Heunggongvirae	Uroviricota	Caudoviricetes	n.a.	Unclassified	O	O	68.79	d__Bacteria;p__Firmicutes;c__Clostridia;o__Oscillospirales;f__Ruminococcaceae;g__Ruminococcus_D	99.7	blast;97.00 iPHoP-RF;95.40 RaFAH;94.60 CRISPR;72.00
S17_vf_35138	ME	Duplodnaviria	Heunggongvirae	Uroviricota	Caudoviricetes	n.a.	Unclassified	O	O	84.47	d__Bacteria;p__Bacteroidota;c__Bacteroidia;o__Bacteroidales;f__Barnesiellaceae;g__Barnesiella	99.1	blast;96.80 RaFAH;88.80 CRISPR;59.60
S17_vf_81232	Shared	Duplodnaviria	Heunggongvirae	Uroviricota	Caudoviricetes	Crassvirales	Suoliviridae	Boorivirinae	Canhaevirus	50.04	d__Bacteria;p__Bacteroidota;c__Bacteroidia;o__Bacteroidales;f__Tannerellaceae;g__Parabacteroides	97.8	blast;98.40 iPHoP-RF;95.10 CRISPR;83.70
S12_vs_153780	SHHC	Duplodnaviria	Heunggongvirae	Uroviricota	Caudoviricetes	n.a.	Unclassified	O	O	97.5	d__Bacteria;p__Proteobacteria;c__Alphaproteobacteria;o__RF32;f__CAG-239;g__CAG-495	90.9	blast;93.30 iPHoP-RF;81.60
S17_vf_97671	ME	Duplodnaviria	Heunggongvirae	Uroviricota	Caudoviricetes	n.a.	Unclassified	Unclassified	Mushuvirus	81.31	d__Bacteria;p__Firmicutes;c__Clostridia;o__Oscillospirales;f__Oscillospiraceae;g__Lawsonibacter	90.2	blast;92.70 iPHoP-RF;73.20
S17_vs_103009	Shared	n.a.	n.a.	n.a.	n.a.	n.a.	n.a.	n.a.	n.a.	22.09	d__Bacteria;p__Firmicutes;c__Clostridia;o__Oscillospirales;f__Oscillospiraceae;g__ER4	96.7	blast;97.70 iPHoP-RF;65.10
S12_vs_164056	Shared	n.a.	n.a.	n.a.	n.a.	n.a.	n.a.	n.a.	n.a.	11.8	d__Bacteria;p__Firmicutes;c__Clostridia;o__Oscillospirales;f__Oscillospiraceae;g__CAG-83	95.9	blast;97.10 iPHoP-RF;72.60
S17_vs_116640	ME	Duplodnaviria	Heunggongvirae	Uroviricota	Caudoviricetes	n.a.	Unclassified	O	O	83.12	d__Bacteria;p__Bacteroidota;c__Bacteroidia;o__Bacteroidales;f__Bacteroidaceae;g__Bacteroides	91	RaFAH;93.40
S17_vs_127519	Shared	n.a.	n.a.	n.a.	n.a.	n.a.	n.a.	n.a.	n.a.	61.22	d__Bacteria;p__Firmicutes;c__Clostridia;o__Oscillospirales;f__Oscillospiraceae;g__Dysosmobacter	91.2	blast;93.60 iPHoP-RF;86.70
S17_vs_160502	ME	Duplodnaviria	Heunggongvirae	Uroviricota	Caudoviricetes	Crassvirales	Steigviridae	O	O	60.44	d__Bacteria;p__Bacteroidota;c__Bacteroidia;o__Bacteroidales;f__Bacteroidaceae;g__Bacteroides	99.9	iPHoP-RF;97.10 RaFAH;96.60 blast;92.60 CRISPR;56.20
S17_vs_177196	ME	n.a.	n.a.	n.a.	n.a.	n.a.	n.a.	n.a.	n.a.	18.1	d__Bacteria;p__Bacteroidota;c__Bacteroidia;o__Bacteroidales;f__Rikenellaceae;g__Alistipes	96.4	blast;91.90 iPHoP-RF;88.50 RaFAH;84.30
S17_vs_2079	Shared	Duplodnaviria	Heunggongvirae	Uroviricota	Caudoviricetes	n.a.	Unclassified	Unclassified	Unclassified	67.97	d__Bacteria;p__Bacteroidota;c__Bacteroidia;o__Bacteroidales;f__Bacteroidaceae;g__Bacteroides	100	RaFAH;98.90 iPHoP-RF;94.40 blast;92.60
S17_vs_39515	ME	Duplodnaviria	Heunggongvirae	Uroviricota	Caudoviricetes	Crassvirales	Intestiviridae	O	O	82.52	d__Bacteria;p__Bacteroidota;c__Bacteroidia;o__Bacteroidales;f__Bacteroidaceae;g__Bacteroides	100	RaFAH;99.30 iPHoP-RF;97.10 blast;92.90
S17_vs_43307	Shared	n.a.	n.a.	n.a.	n.a.	n.a.	n.a.	n.a.	n.a.	72.14	d__Bacteria;p__Bacteroidota;c__Bacteroidia;o__Bacteroidales;f__Tannerellaceae;g__Parabacteroides	95.4	blast;96.80 CRISPR;96.70 iPHoP-RF;93.40
S1_vf_158961	ME	Duplodnaviria	Heunggongvirae	Uroviricota	Caudoviricetes	n.a.	Unclassified	O	O	9.7	d__Bacteria;p__Firmicutes;c__Clostridia;o__Christensenellales;f__CAG-74;g__UBA11524	95	blast;96.50 iPHoP-RF;77.30
S1_vf_159951	ME	Duplodnaviria	Heunggongvirae	Uroviricota	Caudoviricetes	n.a.	Unclassified	O	O	22.14	d__Bacteria;p__Bacteroidota;c__Bacteroidia;o__Bacteroidales;f__Rikenellaceae;g__Alistipes	95.4	CRISPR;96.80 iPHoP-RF;72.20
S1_vf_22149	ME	n.a.	n.a.	n.a.	n.a.	n.a.	n.a.	n.a.	n.a.	43.65	d__Bacteria;p__Firmicutes;c__Clostridia;o__Oscillospirales;f__Oscillospiraceae;g__ER4	90.8	blast;93.20 iPHoP-RF;88.90
S1_vs_143929	ME	n.a.	n.a.	n.a.	n.a.	n.a.	n.a.	n.a.	n.a.	42.31	d__Bacteria;p__Firmicutes;c__Clostridia;o__Oscillospirales;f__Oscillospiraceae;g__ER4	91.6	blast;93.90 iPHoP-RF;75.00
S2_vf_20910	SHHC	n.a.	n.a.	n.a.	n.a.	n.a.	n.a.	n.a.	n.a.	5.26	d__Bacteria;p__Firmicutes;c__Bacilli;o__Erysipelotrichales;f__Erysipelotrichaceae;g__Clostridium_AQ	97.2	CRISPR;98.00
S12_vs_1980	Shared	Duplodnaviria	Heunggongvirae	Uroviricota	Caudoviricetes	n.a.	Unclassified	O	O	94.44	d__Bacteria;p__Bacteroidota;c__Bacteroidia;o__Bacteroidales;f__Bacteroidaceae;g__Bacteroides	99.1	iPHoP-RF;97.10 blast;93.60 RaFAH;87.80 CRISPR;58.50
S2_vs_10289	Shared	Duplodnaviria	Heunggongvirae	Uroviricota	Caudoviricetes	n.a.	Unclassified	Unclassified	Unclassified	67.97	d__Bacteria;p__Bacteroidota;c__Bacteroidia;o__Bacteroidales;f__Bacteroidaceae;g__Bacteroides	100	RaFAH;98.90 blast;96.80 iPHoP-RF;87.90
S2_vs_7306	Shared	Duplodnaviria	Heunggongvirae	Uroviricota	Caudoviricetes	n.a.	Unclassified	Unclassified	Unclassified	96.01	d__Bacteria;p__Bacteroidota;c__Bacteroidia;o__Bacteroidales;f__Tannerellaceae;g__Parabacteroides	93	blast;95.00 iPHoP-RF;73.70
S3_vf_100440	ME	Duplodnaviria	Heunggongvirae	Uroviricota	Caudoviricetes	n.a.	Unclassified	O	O	98.85	d__Bacteria;p__Firmicutes;c__Clostridia;o__Clostridiales;f__Clostridiaceae;g__Clostridium	94.7	RaFAH;96.30
S3_vf_106401	ME	Duplodnaviria	Heunggongvirae	Uroviricota	Caudoviricetes	n.a.	Unclassified	O	O	12.56	d__Bacteria;p__Firmicutes;c__Bacilli;o__Haloplasmatales_A;f__Turicibacteraceae;g__Turicibacter	92	blast;94.20 iPHoP-RF;66.30
S12_vs_26847	SHHC	n.a.	n.a.	n.a.	n.a.	n.a.	n.a.	n.a.	n.a.	68.45	d__Bacteria;p__Firmicutes;c__Clostridia;o__Lachnospirales;f__Lachnospiraceae;g__Lachnospira	99.3	CRISPR;98.90 iPHoP-RF;95.70 blast;94.10 RaFAH;74.50
S3_vf_115304	Shared	Duplodnaviria	Heunggongvirae	Uroviricota	Caudoviricetes	Crassvirales	Suoliviridae	Uncouvirinae	Aurodevirus	34.34	d__Bacteria;p__Spirochaetota;c__Spirochaetia;o__Sphaerochaetales;f__Sphaerochaetaceae;g__UBA9732	91.2	blast;93.60 iPHoP-RF;81.90
S3_vf_127399	Shared	Duplodnaviria	Heunggongvirae	Uroviricota	Caudoviricetes	n.a.	Unclassified	O	O	23.09	d__Bacteria;p__Bacteroidota;c__Bacteroidia;o__Bacteroidales;f__Bacteroidaceae;g__Bacteroides	90.4	RaFAH;92.90
S12_vs_32669	SHHC	n.a.	n.a.	n.a.	n.a.	n.a.	n.a.	n.a.	n.a.	3.93	d__Bacteria;p__Firmicutes;c__Clostridia;o__Christensenellales;f__CAG-917;g__CAG-475	94.7	blast;96.30 iPHoP-RF;68.60
S3_vf_127405	ME	Duplodnaviria	Heunggongvirae	Uroviricota	Caudoviricetes	n.a.	Unclassified	O	O	98.09	d__Bacteria;p__Firmicutes;c__Clostridia;o__Lachnospirales;f__Lachnospiraceae;g__Lachnospira	99.9	CRISPR;98.90 RaFAH;91.60 iPHoP-RF;90.80 blast;80.40
S3_vf_127406	ME	Duplodnaviria	Heunggongvirae	Uroviricota	Caudoviricetes	n.a.	Unclassified	Unclassified	Unclassified	89.48	d__Bacteria;p__Bacteroidota;c__Bacteroidia;o__Bacteroidales;f__Rikenellaceae;g__Alistipes	97.8	RaFAH;98.40
S3_vf_127409	Shared	Duplodnaviria	Heunggongvirae	Uroviricota	Caudoviricetes	n.a.	Unclassified	O	O	96.84	d__Bacteria;p__Firmicutes;c__Clostridia;o__Oscillospirales;f__Ruminococcaceae;g__Faecalibacterium	95.9	CRISPR;97.00 blast;95.70 iPHoP-RF;81.10 RaFAH;51.90
S3_vf_18080	ME	n.a.	n.a.	n.a.	n.a.	n.a.	n.a.	n.a.	n.a.	96.93	d__Bacteria;p__Bacteroidota;c__Bacteroidia;o__Bacteroidales;f__Bacteroidaceae;g__Bacteroides	99.7	iPHoP-RF;97.10 blast;96.10 RaFAH;94.50 CRISPR;81.40
S3_vf_18829	ME	Duplodnaviria	Heunggongvirae	Uroviricota	Caudoviricetes	n.a.	Unclassified	O	O	63.94	d__Bacteria;p__Bacteroidota;c__Bacteroidia;o__Bacteroidales;f__Bacteroidaceae;g__Bacteroides	91.8	iPHoP-RF;94.10 CRISPR;73.10
S3_vf_44405	Shared	Duplodnaviria	Heunggongvirae	Uroviricota	Caudoviricetes	n.a.	Unclassified	Unclassified	Unclassified	96.43	d__Bacteria;p__Actinobacteriota;c__Actinomycetia;o__Actinomycetales;f__Actinomycetaceae;g__Actinomyces	100	RaFAH;97.30 CRISPR;92.40 blast;92.10 iPHoP-RF;55.70
S3_vf_54353	ME	Duplodnaviria	Heunggongvirae	Uroviricota	Caudoviricetes	n.a.	Unclassified	Unclassified	Cimpunavirus	46.9	d__Bacteria;p__Actinobacteriota;c__Actinomycetia;o__Actinomycetales;f__Actinomycetaceae;g__Actinomyces	97.8	RaFAH;97.30 iPHoP-RF;70.60
S3_vf_67333	ME	Duplodnaviria	Heunggongvirae	Uroviricota	Caudoviricetes	n.a.	Unclassified	O	O	76.26	d__Bacteria;p__Firmicutes;c__Clostridia;o__Lachnospirales;f__Lachnospiraceae;g__Agathobacter	99.9	CRISPR;98.90 RaFAH;90.80 blast;85.70 iPHoP-RF;81.90
S12_vs_74280	Shared	n.a.	n.a.	n.a.	n.a.	n.a.	n.a.	n.a.	n.a.	14.1	d__Bacteria;p__Bacteroidota;c__Bacteroidia;o__Bacteroidales;f__Marinifilaceae;g__Butyricimonas	94.3	blast;96.00 iPHoP-RF;58.40
S3_vf_67828	Shared	Monodnaviria	Sangervirae	Phixviricota	Malgrandaviricetes	Petitvirales	Microviridae	Unclassified	Unclassified		d__Bacteria;p__Bacteroidota;c__Bacteroidia;o__Bacteroidales;f__Rikenellaceae;g__Alistipes	91.5	blast;93.80 iPHoP-RF;64.50
S3_vf_108959	ME	n.a.	n.a.	n.a.	n.a.	n.a.	n.a.	n.a.	n.a.	42.95	d__Bacteria;p__Firmicutes;c__Clostridia;o__Lachnospirales;f__Lachnospiraceae;g__Lachnospira	99.3	CRISPR;99.30 blast;91.90 iPHoP-RF;88.90 RaFAH;55.00
S3_vf_70746	Shared	Duplodnaviria	Heunggongvirae	Uroviricota	Caudoviricetes	n.a.	Unclassified	Unclassified	Unclassified	87.64	d__Bacteria;p__Actinobacteriota;c__Actinomycetia;o__Actinomycetales;f__Bifidobacteriaceae;g__Bifidobacterium	95.3	iPHoP-RF;96.70 blast;95.90 CRISPR;78.50
S12_vs_81729	SHHC	Duplodnaviria	Heunggongvirae	Uroviricota	Caudoviricetes	n.a.	Unclassified	O	O	10.77	d__Bacteria;p__Firmicutes;c__Clostridia;o__Oscillospirales;f__Ruminococcaceae;g__Faecalibacterium	97.3	CRISPR;98.10 iPHoP-RF;93.40 blast;92.10
S3_vf_73597	ME	Duplodnaviria	Heunggongvirae	Uroviricota	Caudoviricetes	n.a.	Unclassified	O	O	55.3	d__Bacteria;p__Bacteroidota;c__Bacteroidia;o__Bacteroidales;f__Bacteroidaceae;g__Bacteroides	99.7	CRISPR;97.20 iPHoP-RF;97.10 RaFAH;93.80 blast;93.80
S3_vf_80287	Shared	Duplodnaviria	Heunggongvirae	Uroviricota	Caudoviricetes	n.a.	Unclassified	O	O	52.73	d__Bacteria;p__Firmicutes;c__Clostridia;o__Oscillospirales;f__Ruminococcaceae;g__D5	97.2	CRISPR;98.00 iPHoP-RF;52.20
S3_vf_8058	ME	Duplodnaviria	Heunggongvirae	Uroviricota	Caudoviricetes	n.a.	Unclassified	Unclassified	Eponavirus	98.11	d__Bacteria;p__Firmicutes;c__Clostridia;o__Lachnospirales;f__Lachnospiraceae;g__Blautia_A	98.1	CRISPR;98.60 iPHoP-RF;81.60
S12_vs_87088	Shared	Duplodnaviria	Heunggongvirae	Uroviricota	Caudoviricetes	n.a.	Unclassified	O	O	36.55	d__Bacteria;p__Firmicutes;c__Clostridia;o__Oscillospirales;f__Ruminococcaceae;g__Faecalibacterium	97.5	blast;96.60 iPHoP-RF;92.40 RaFAH;73.80 CRISPR;53.50
S3_vf_82571	Shared	Duplodnaviria	Heunggongvirae	Uroviricota	Caudoviricetes	n.a.	Unclassified	O	O	21.78	d__Bacteria;p__Firmicutes;c__Clostridia;o__Oscillospirales;f__Oscillospiraceae;g__CAG-170	93.1	blast;95.10 iPHoP-RF;73.20
S3_vf_99734	Shared	Duplodnaviria	Heunggongvirae	Uroviricota	Caudoviricetes	n.a.	Unclassified	Unclassified	Lagaffevirus	99.18	d__Bacteria;p__Firmicutes;c__Clostridia;o__Oscillospirales;f__Ruminococcaceae;g__Faecalibacterium	95.2	CRISPR;96.60 iPHoP-RF;64.80 blast;60.50
S3_vs_114271	ME	n.a.	n.a.	n.a.	n.a.	n.a.	n.a.	n.a.	n.a.	19.01	d__Bacteria;p__Firmicutes;c__Clostridia;o__Oscillospirales;f__Acutalibacteraceae;g__Acutalibacter	92.1	blast;94.30 iPHoP-RF;56.80
S12_vs_94042	Shared	n.a.	n.a.	n.a.	n.a.	n.a.	n.a.	n.a.	n.a.	10.3	d__Bacteria;p__Firmicutes;c__Clostridia;o__Christensenellales;f__CAG-74;g__UBA11524	95.6	blast;96.90 iPHoP-RF;91.80
S3_vs_117894	Shared	n.a.	n.a.	n.a.	n.a.	n.a.	n.a.	n.a.	n.a.	81.25	d__Bacteria;p__Bacteroidota;c__Bacteroidia;o__Bacteroidales;f__Marinifilaceae;g__Odoribacter	99.9	blast;98.40 iPHoP-RF;93.10 RaFAH;92.30 CRISPR;53.10
S12_vs_98175	SHHC	Duplodnaviria	Heunggongvirae	Uroviricota	Caudoviricetes	n.a.	Unclassified	O	O	95.22	d__Bacteria;p__Firmicutes;c__Clostridia;o__Lachnospirales;f__Lachnospiraceae;g__Bariatricus	90.3	iPHoP-RF;89.50 blast;81.30 RaFAH;65.60
S3_vs_119660	Shared	Duplodnaviria	Heunggongvirae	Uroviricota	Caudoviricetes	n.a.	Unclassified	Unclassified	Unclassified	91.3	d__Bacteria;p__Bacteroidota;c__Bacteroidia;o__Bacteroidales;f__Bacteroidaceae;g__Bacteroides	100	RaFAH;98.50 CRISPR;95.40 iPHoP-RF;95.40 blast;93.90
S3_vs_123834	Shared	Duplodnaviria	Heunggongvirae	Uroviricota	Caudoviricetes	n.a.	Unclassified	O	O	73.11	d__Bacteria;p__Actinobacteriota;c__Actinomycetia;o__Actinomycetales;f__Bifidobacteriaceae;g__Bifidobacterium	98.4	CRISPR;98.40 blast;96.90 iPHoP-RF;96.70 RaFAH;61.70
S12_vs_99219	Shared	n.a.	n.a.	n.a.	n.a.	n.a.	n.a.	n.a.	n.a.	99.93	d__Bacteria;p__Bacteroidota;c__Bacteroidia;o__Bacteroidales;f__Bacteroidaceae;g__Bacteroides	100	RaFAH;98.90 iPHoP-RF;96.70 blast;91.60
S3_vs_127391	ME	n.a.	n.a.	n.a.	n.a.	n.a.	n.a.	n.a.	n.a.	18.32	d__Bacteria;p__Firmicutes;c__Clostridia;o__Oscillospirales;f__Ruminococcaceae;g__Gemmiger	95.6	blast;96.90 CRISPR;96.30 iPHoP-RF;91.80
S3_vs_127413	ME	Duplodnaviria	Heunggongvirae	Uroviricota	Caudoviricetes	n.a.	Unclassified	O	O	23.82	d__Bacteria;p__Bacteroidota;c__Bacteroidia;o__Bacteroidales;f__Rikenellaceae;g__Alistipes	97.9	CRISPR;98.50 iPHoP-RF;80.00
S3_vs_127442	ME	Monodnaviria	Sangervirae	Phixviricota	Malgrandaviricetes	Petitvirales	Microviridae	Unclassified	Unclassified		d__Bacteria;p__Bacteroidota;c__Bacteroidia;o__Bacteroidales;f__P3;g__Phil12	90.7	iPHoP-RF;93.10
S3_vs_17168	Shared	Duplodnaviria	Heunggongvirae	Uroviricota	Caudoviricetes	n.a.	Unclassified	O	O	100	d__Bacteria;p__Bacteroidota;c__Bacteroidia;o__Bacteroidales;f__Rikenellaceae;g__Alistipes	97.5	blast;96.90 iPHoP-RF;93.10 RaFAH;71.20
S3_vs_18858	ME	n.a.	n.a.	n.a.	n.a.	n.a.	n.a.	n.a.	n.a.	82.07	d__Bacteria;p__Firmicutes;c__Clostridia;o__Oscillospirales;f__Acutalibacteraceae;g__CAG-217	97.9	blast;98.50 iPHoP-RF;86.30 CRISPR;68.10
S3_vs_29077	ME	Duplodnaviria	Heunggongvirae	Uroviricota	Caudoviricetes	n.a.	Unclassified	O	O	34.15	d__Bacteria;p__Firmicutes;c__Clostridia;o__Oscillospirales;f__Ruminococcaceae;g__Ruminococcus_C	94.9	blast;96.40 iPHoP-RF;80.00 CRISPR;51.40
S13_vf_147589	ME	n.a.	n.a.	n.a.	n.a.	n.a.	n.a.	n.a.	n.a.	6.46	d__Bacteria;p__Firmicutes;c__Clostridia;o__Oscillospirales;f__Oscillospiraceae;g__Flavonifractor	91.5	blast;93.80 iPHoP-RF;78.50
S3_vs_42569	ME	Duplodnaviria	Heunggongvirae	Uroviricota	Caudoviricetes	n.a.	Unclassified	Unclassified	Toutatisvirus	73.75	d__Bacteria;p__Firmicutes;c__Clostridia;o__Oscillospirales;f__Ruminococcaceae;g__Faecalibacterium	99.7	CRISPR;98.30 blast;96.90 RaFAH;90.40 iPHoP-RF;73.70
S3_vs_45147	ME	n.a.	n.a.	n.a.	n.a.	n.a.	n.a.	n.a.	n.a.	90.75	d__Bacteria;p__Bacteroidota;c__Bacteroidia;o__Bacteroidales;f__Bacteroidaceae;g__Bacteroides	99.4	iPHoP-RF;97.10 RaFAH;91.20
S3_vs_49835	ME	n.a.	n.a.	n.a.	n.a.	n.a.	n.a.	n.a.	n.a.	13.1	d__Bacteria;p__Firmicutes;c__Clostridia;o__Oscillospirales;f__Acutalibacteraceae;g__Ruminococcus_E	99	CRISPR;99.20 blast;98.60 iPHoP-RF;93.10
S3_vs_50924	ME	n.a.	n.a.	n.a.	n.a.	n.a.	n.a.	n.a.	n.a.	12.69	d__Bacteria;p__Cyanobacteria;c__Vampirovibrionia;o__Gastranaerophilales;f__Gastranaerophilaceae;g__QAMI01	90.4	blast;92.90 iPHoP-RF;57.10
S3_vs_54303	Shared	Duplodnaviria	Heunggongvirae	Uroviricota	Caudoviricetes	n.a.	Unclassified	Unclassified	Eponavirus	46.83	d__Bacteria;p__Firmicutes;c__Clostridia;o__Oscillospirales;f__Oscillospiraceae;g__	91.6	blast;93.90 iPHoP-RF;80.30
S3_vs_64424	Shared	n.a.	n.a.	n.a.	n.a.	n.a.	n.a.	n.a.	n.a.	98.41	d__Bacteria;p__Bacteroidota;c__Bacteroidia;o__Bacteroidales;f__Bacteroidaceae;g__Bacteroides	100	RaFAH;99.60 iPHoP-RF;97.10 blast;92.20 CRISPR;69.50
S3_vs_70508	Shared	n.a.	n.a.	n.a.	n.a.	n.a.	n.a.	n.a.	n.a.	99.66	d__Bacteria;p__Bacteroidota;c__Bacteroidia;o__Bacteroidales;f__Bacteroidaceae;g__Bacteroides	100	RaFAH;99.70 iPHoP-RF;93.10 blast;92.70
S3_vs_73361	ME	n.a.	n.a.	n.a.	n.a.	n.a.	n.a.	n.a.	n.a.	17.97	d__Bacteria;p__Firmicutes;c__Clostridia;o__Christensenellales;f__CAG-138;g__SFEL01	96.6	CRISPR;97.60 blast;96.40 iPHoP-RF;91.10
S3_vs_77156	ME	Monodnaviria	Sangervirae	Phixviricota	Malgrandaviricetes	Petitvirales	Microviridae	Unclassified	Unclassified		d__Bacteria;p__Bacteroidota;c__Bacteroidia;o__Bacteroidales;f__Rikenellaceae;g__Alistipes	96.7	CRISPR;97.70 iPHoP-RF;58.40
S3_vs_80151	Shared	Duplodnaviria	Heunggongvirae	Uroviricota	Caudoviricetes	n.a.	Unclassified	O	O	97.75	d__Bacteria;p__Bacteroidota;c__Bacteroidia;o__Bacteroidales;f__Tannerellaceae;g__Parabacteroides	100	RaFAH;99.60 iPHoP-RF;94.40 blast;93.80 CRISPR;92.60
S13_vf_54201	ME	Duplodnaviria	Heunggongvirae	Uroviricota	Caudoviricetes	n.a.	Unclassified	O	O	17.62	d__Bacteria;p__Firmicutes;c__Clostridia;o__Oscillospirales;f__Ruminococcaceae;g__Faecalibacterium	98.7	RaFAH;93.20 blast;89.90 iPHoP-RF;62.20
S3_vs_82895	Shared	Duplodnaviria	Heunggongvirae	Uroviricota	Caudoviricetes	n.a.	Unclassified	O	O	92.58	d__Bacteria;p__Bacteroidota;c__Bacteroidia;o__Bacteroidales;f__Tannerellaceae;g__Parabacteroides	100	CRISPR;99.00 RaFAH;98.20 blast;93.90 iPHoP-RF;93.70
S3_vs_90341	Shared	n.a.	n.a.	n.a.	n.a.	n.a.	n.a.	n.a.	n.a.	11.42	d__Bacteria;p__Firmicutes;c__Clostridia;o__Christensenellales;f__CAG-74;g__UBA11524	95.2	blast;96.60 CRISPR;94.50 iPHoP-RF;85.70
S13_vf_66508	ME	Duplodnaviria	Heunggongvirae	Uroviricota	Caudoviricetes	n.a.	Unclassified	O	O	88.17	d__Bacteria;p__Bacteroidota;c__Bacteroidia;o__Bacteroidales;f__Bacteroidaceae;g__Bacteroides	99.4	iPHoP-RF;97.10 blast;96.60 RaFAH;91.90
S3_vs_16825	Shared	Duplodnaviria	Heunggongvirae	Uroviricota	Caudoviricetes	n.a.	Unclassified	O	O	80.45	d__Bacteria;p__Firmicutes;c__Clostridia;o__Lachnospirales;f__Lachnospiraceae;g__Roseburia	99.7	CRISPR;98.90 iPHoP-RF;96.10 blast;92.80 RaFAH;88.00
S3_vs_95574	ME	n.a.	n.a.	n.a.	n.a.	n.a.	n.a.	n.a.	n.a.	22.37	d__Bacteria;p__Firmicutes;c__Clostridia;o__Oscillospirales;f__Acutalibacteraceae;g__UBA737	90.4	blast;92.90 iPHoP-RF;70.60
S3_vs_98675	ME	Duplodnaviria	Heunggongvirae	Uroviricota	Caudoviricetes	n.a.	Unclassified	O	O	8.18	d__Bacteria;p__Firmicutes;c__Clostridia;o__Clostridiales;f__Clostridiaceae;g__Clostridium	98.5	blast;96.40 RaFAH;85.10 iPHoP-RF;79.00
S4_vf_10506	SHHC	n.a.	n.a.	n.a.	n.a.	n.a.	n.a.	n.a.	n.a.	17.1	d__Bacteria;p__Firmicutes;c__Bacilli;o__Erysipelotrichales;f__Erysipelotrichaceae;g__Holdemanella	90.7	blast;93.10
S4_vf_12614	SHHC	n.a.	n.a.	n.a.	n.a.	n.a.	n.a.	n.a.	n.a.	80.07	d__Bacteria;p__Bacteroidota;c__Bacteroidia;o__Bacteroidales;f__Barnesiellaceae;g__Barnesiella	98.7	blast;99.00 iPHoP-RF;76.10 CRISPR;67.90
S4_vf_12624	SHHC	Duplodnaviria	Heunggongvirae	Uroviricota	Caudoviricetes	n.a.	Unclassified	O	O	24.52	d__Bacteria;p__Actinobacteriota;c__Actinomycetia;o__Actinomycetales;f__Bifidobacteriaceae;g__Bifidobacterium	97.5	CRISPR;98.20 blast;96.50 iPHoP-RF;91.80
S3_vs_39774	Shared	n.a.	n.a.	n.a.	n.a.	n.a.	n.a.	n.a.	n.a.	100	d__Bacteria;p__Bacteroidota;c__Bacteroidia;o__Bacteroidales;f__Bacteroidaceae;g__Bacteroides	100	RaFAH;99.00 blast;92.00 iPHoP-RF;53.80
S4_vf_18803	SHHC	Duplodnaviria	Heunggongvirae	Uroviricota	Caudoviricetes	n.a.	Unclassified	Unclassified	Unclassified	47.59	d__Bacteria;p__Bacteroidota;c__Bacteroidia;o__Bacteroidales;f__Bacteroidaceae;g__Prevotella	98.5	CRISPR;98.90 iPHoP-RF;74.10
S4_vf_31978	SHHC	n.a.	n.a.	n.a.	n.a.	n.a.	n.a.	n.a.	n.a.	62.52	d__Bacteria;p__Firmicutes;c__Clostridia;o__Lachnospirales;f__Lachnospiraceae;g__Lachnospira	99.6	CRISPR;99.60 blast;93.90 iPHoP-RF;92.40
S13_vs_13129	ME	Duplodnaviria	Heunggongvirae	Uroviricota	Caudoviricetes	n.a.	Unclassified	O	O	16.24	d__Bacteria;p__Firmicutes;c__Clostridia;o__Oscillospirales;f__Oscillospiraceae;g__CAG-83	98.5	CRISPR;98.90 blast;95.20 iPHoP-RF;87.90
S4_vf_31981	SHHC	Duplodnaviria	Heunggongvirae	Uroviricota	Caudoviricetes	Crassvirales	Suoliviridae	Uncouvirinae	Besingivirus	29.33	d__Bacteria;p__Bacteroidota;c__Bacteroidia;o__Bacteroidales;f__Bacteroidaceae;g__Prevotella	97.3	RaFAH;98.10
S4_vf_4838	Shared	Duplodnaviria	Heunggongvirae	Uroviricota	Caudoviricetes	Crassvirales	Suoliviridae	Boorivirinae	Cohcovirus	76.93	d__Bacteria;p__Bacteroidota;c__Bacteroidia;o__Bacteroidales;f__Tannerellaceae;g__Parabacteroides	97.6	blast;98.30 iPHoP-RF;96.10 CRISPR;71.50
S4_vf_4891	SHHC	n.a.	n.a.	n.a.	n.a.	n.a.	n.a.	n.a.	n.a.	28.59	d__Bacteria;p__Bacteroidota;c__Bacteroidia;o__Bacteroidales;f__Bacteroidaceae;g__Prevotella	94.6	blast;96.20 CRISPR;93.50 iPHoP-RF;92.10
S13_vs_149860	ME	Duplodnaviria	Heunggongvirae	Uroviricota	Caudoviricetes	n.a.	Unclassified	Unclassified	O	61.18	d__Bacteria;p__Actinobacteriota;c__Actinomycetia;o__Actinomycetales;f__Bifidobacteriaceae;g__Bifidobacterium	99.7	CRISPR;99.70 iPHoP-RF;92.40 blast;92.00

S3_vs_68031	ME	Duplodnaviria	Heunggongvirae	Uroviricota	Caudoviricetes	n.a.	Unclassified	O	O	27.92	d__Bacteria;p__Firmicutes;c__Clostridia;o__Oscillospirales;f__Ruminococcaceae;g__Faecalibacterium	95.6	blast;96.90 iPHoP-RF;73.20 CRISPR;56.90
S4_vs_VIRSorter_12831-	SHHC	Duplodnaviria	Heunggongvirae	Uroviricota	Caudoviricetes	n.a.	Unclassified	O	O	21.81	d__Bacteria;p__Firmicutes;c__Clostridia;o__Lachnospirales;f__Lachnospiraceae;g__Blautia_A	91.6	blast;93.90 iPHoP-RF;85.10 CRISPR;58.50
S4_vs_VIRSorter_13190-	Shared	Duplodnaviria	Heunggongvirae	Uroviricota	Caudoviricetes	n.a.	Unclassified	O	O	96.31	d__Bacteria;p__Bacteroidota;c__Bacteroidia;o__Bacteroidales;f__Tannerellaceae;g__Parabacteroides	100	RaFAH;99.80 iPHoP-RF;94.40 blast;91.20 CRISPR;66.50
S4_vs_VIRSorter_15112-	SHHC	n.a.	n.a.	n.a.	n.a.	n.a.	n.a.	n.a.	n.a.	16.47	d__Bacteria;p__Firmicutes;c__Clostridia;o__Peptostreptococcales;f__Peptostreptococcaceae;g__GCA-900066495	91.6	blast;93.90 iPHoP-RF;75.70
S4_vs_VIRSorter_15837-	Shared	Duplodnaviria	Heunggongvirae	Uroviricota	Caudoviricetes	n.a.	Unclassified	O	O	96.3	d__Bacteria;p__Firmicutes;c__Clostridia;o__Oscillospirales;f__Oscillospiraceae;g__Flavonifractor	97.2	blast;98.00 CRISPR;89.70 iPHoP-RF;84.80
S4_vs_VIRSorter_16493-	Shared	Duplodnaviria	Heunggongvirae	Uroviricota	Caudoviricetes	n.a.	Unclassified	Unclassified	Unclassified	85.89	d__Bacteria;p__Actinobacteriota;c__Actinomycetia;o__Actinomycetales;f__Bifidobacteriaceae;g__Bifidobacterium	97.9	CRISPR;98.50 iPHoP-RF;96.40 blast;92.50
S4_vs_VIRSorter_19324-	SHHC	Duplodnaviria	Heunggongvirae	Uroviricota	Caudoviricetes	n.a.	Unclassified	O	O	25.86	d__Bacteria;p__Firmicutes;c__Clostridia;o__Lachnospirales;f__Lachnospiraceae;g__Mediterraneibacter	91.4	blast;93.70 iPHoP-RF;76.40
S4_vs_VIRSorter_3006-c	Shared	Duplodnaviria	Heunggongvirae	Uroviricota	Caudoviricetes	n.a.	Unclassified	O	O	99.88	d__Bacteria;p__Firmicutes;c__Clostridia;o__Lachnospirales;f__Lachnospiraceae;g__Mediterraneibacter	91.6	blast;93.90
S4_vs_VIRSorter_30222-	Shared	Duplodnaviria	Heunggongvirae	Uroviricota	Caudoviricetes	n.a.	Unclassified	O	O	65.61	d__Bacteria;p__Firmicutes;c__Clostridia;o__Oscillospirales;f__Oscillospiraceae;g__Intestinimonas	91.5	blast;93.80
S4_vs_VIRSorter_31171-	SHHC	Duplodnaviria	Heunggongvirae	Uroviricota	Caudoviricetes	n.a.	Unclassified	O	O	46.28	d__Bacteria;p__Firmicutes;c__Clostridia;o__Oscillospirales;f__Ruminococcaceae;g__QAMM01	90.2	blast;92.70 iPHoP-RF;54.90
S4_vs_VIRSorter_31196-	Shared	n.a.	n.a.	n.a.	n.a.	n.a.	n.a.	n.a.	n.a.	93.39	d__Bacteria;p__Firmicutes;c__Clostridia;o__Oscillospirales;f__Oscillospiraceae;g__Flavonifractor	99.4	CRISPR;97.90 blast;96.20 iPHoP-RF;88.90 RaFAH;87.70
S13_vs_45488	Shared	Duplodnaviria	Heunggongvirae	Uroviricota	Caudoviricetes	n.a.	Unclassified	O	O	22.02	d__Bacteria;p__Proteobacteria;c__Alphaproteobacteria;o__RF32;f__CAG-239;g__51-20	97.9	CRISPR;98.50 blast;79.90 iPHoP-RF;74.60
S4_vs_VIRSorter_31560-	Shared	Duplodnaviria	Heunggongvirae	Uroviricota	Caudoviricetes	n.a.	Unclassified	O	O	59.79	d__Bacteria;p__Firmicutes;c__Clostridia;o__Oscillospirales;f__Oscillospiraceae;g__Flavonifractor	90.3	blast;92.80 iPHoP-RF;86.30 CRISPR;64.80
S4_vs_VIRSorter_31976-	Shared	n.a.	n.a.	n.a.	n.a.	n.a.	n.a.	n.a.	n.a.	17.17	d__Bacteria;p__Firmicutes;c__Clostridia;o__Oscillospirales;f__Ruminococcaceae;g__Gemmiger	97.2	CRISPR;98.00 blast;96.20 iPHoP-RF;93.70
S13_vs_57267	Shared	Duplodnaviria	Heunggongvirae	Uroviricota	Caudoviricetes	n.a.	Unclassified	O	O	38.42	d__Bacteria;p__Bacteroidota;c__Bacteroidia;o__Bacteroidales;f__Bacteroidaceae;g__Prevotella	90.6	RaFAH;93.00
S4_vs_VIRSorter_31980-	SHHC	Duplodnaviria	Heunggongvirae	Uroviricota	Caudoviricetes	n.a.	Unclassified	Unclassified	Unclassified	13.26	d__Bacteria;p__Proteobacteria;c__Gammaproteobacteria;o__Burkholderiales;f__Burkholderiaceae;g__Duodenibacillus	96.7	blast;97.70 CRISPR;83.90 iPHoP-RF;70.60
S4_vs_VIRSorter_4746-c	Shared	Duplodnaviria	Heunggongvirae	Uroviricota	Caudoviricetes	n.a.	Salasmaviridae	O	O	5.45	d__Bacteria;p__Firmicutes;c__Clostridia;o__Lachnospirales;f__Lachnospiraceae;g__Roseburia	90.3	iPHoP-RF;92.80
S4_vs_VIRSorter_4839-c	SHHC	Duplodnaviria	Heunggongvirae	Uroviricota	Caudoviricetes	n.a.	Unclassified	O	O	69.64	d__Bacteria;p__Firmicutes;c__Clostridia;o__Oscillospirales;f__Ruminococcaceae;g__Massilimaliae	91.4	blast;93.70 iPHoP-RF;75.70
S4_vs_VIRSorter_4959-c	Shared	Duplodnaviria	Heunggongvirae	Uroviricota	Caudoviricetes	n.a.	Unclassified	O	O	50.34	d__Bacteria;p__Firmicutes;c__Clostridia;o__Oscillospirales;f__Oscillospiraceae;g__ER4	94.2	blast;95.90 iPHoP-RF;82.50
S4_vs_VIRSorter_6022-c	Shared	Duplodnaviria	Heunggongvirae	Uroviricota	Caudoviricetes	n.a.	Unclassified	Unclassified	Unclassified	97.71	d__Bacteria;p__Bacteroidota;c__Bacteroidia;o__Bacteroidales;f__Tannerellaceae;g__Marseille-P3160	98.4	CRISPR;98.80 iPHoP-RF;65.30
S4_vs_VIRSorter_9913-c	Shared	Duplodnaviria	Heunggongvirae	Uroviricota	Caudoviricetes	n.a.	Unclassified	Unclassified	Unclassified	98.3	d__Bacteria;p__Bacteroidota;c__Bacteroidia;o__Bacteroidales;f__Marinifilaceae;g__Odoribacter	99.7	CRISPR;97.90 RaFAH;92.60
S4_vf_31973	Shared	n.a.	n.a.	n.a.	n.a.	n.a.	n.a.	n.a.	n.a.	23.92	d__Bacteria;p__Patescibacteria;c__Paceibacteria;o__UBA9983_A;f__UBA1006;g__01-FULL-36-10b	92.2	iPHoP-RF;94.40
S5_vf_134040	Shared	Duplodnaviria	Heunggongvirae	Uroviricota	Caudoviricetes	n.a.	Unclassified	Unclassified	Mushuvirus	100	d__Bacteria;p__Firmicutes;c__Clostridia;o__Oscillospirales;f__Ruminococcaceae;g__Faecalibacterium	100	RaFAH;98.80 blast;96.50 iPHoP-RF;69.10
S5_vf_136076	Shared	n.a.	n.a.	n.a.	n.a.	n.a.	n.a.	n.a.	n.a.	43.14	d__Bacteria;p__Firmicutes;c__Clostridia;o__Oscillospirales;f__Oscillospiraceae;g__ER4	93	blast;95.00 iPHoP-RF;69.80
S5_vf_137260	Shared	Unclassified	n.a.	n.a.	n.a.	n.a.	Unclassified	O	O	6.74	d__Bacteria;p__Actinobacteriota;c__Coriobacteriia;o__Coriobacteriales;f__Coriobacteriaceae;g__Collinsella	92.9	CRISPR;94.90
S5_vf_145585	Shared	Duplodnaviria	Heunggongvirae	Uroviricota	Caudoviricetes	n.a.	Unclassified	Unclassified	Unclassified	9.85	d__Bacteria;p__Firmicutes;c__Clostridia;o__Lachnospirales;f__Lachnospiraceae;g__Enterocloster	91.2	blast;93.60 iPHoP-RF;91.40 CRISPR;53.80
S14_vf_115736	ME	Duplodnaviria	Heunggongvirae	Uroviricota	Caudoviricetes	n.a.	Unclassified	Unclassified	O	85.01	d__Bacteria;p__Firmicutes;c__Clostridia;o__Oscillospirales;f__Oscillospiraceae;g__Flavonifractor	91.2	blast;93.60 iPHoP-RF;89.80 CRISPR;61.00
S5_vf_145588	Shared	Duplodnaviria	Heunggongvirae	Uroviricota	Caudoviricetes	n.a.	Unclassified	O	O	34.8	d__Bacteria;p__Firmicutes;c__Clostridia;o__Lachnospirales;f__Lachnospiraceae;g__Dorea_A	97.2	CRISPR;98.00 iPHoP-RF;64.30
S14_vf_117147	ME	Duplodnaviria	Heunggongvirae	Uroviricota	Caudoviricetes	n.a.	Unclassified	O	O	30.35	d__Bacteria;p__Firmicutes;c__Clostridia;o__Oscillospirales;f__Ruminococcaceae;g__Gemmiger	95.3	blast;96.70 CRISPR;94.60 iPHoP-RF;86.30
S5_vf_18461	Shared	Duplodnaviria	Heunggongvirae	Uroviricota	Caudoviricetes	n.a.	Unclassified	O	O	99.96	d__Bacteria;p__Bacteroidota;c__Bacteroidia;o__Bacteroidales;f__Rikenellaceae;g__Alistipes	92.7	RaFAH;92.00 blast;67.20
S5_vf_25423	Shared	Duplodnaviria	Heunggongvirae	Uroviricota	Caudoviricetes	n.a.	Unclassified	Unclassified	Skunavirus	81	d__Bacteria;p__Firmicutes;c__Bacilli;o__Lactobacillales;f__Streptococcaceae;g__Lactococcus	100	RaFAH;98.80 blast;98.50 iPHoP-RF;91.40
S14_vf_117155	Shared	Duplodnaviria	Heunggongvirae	Uroviricota	Caudoviricetes	n.a.	Unclassified	O	O	63.17	d__Bacteria;p__Actinobacteriota;c__Actinomycetia;o__Actinomycetales;f__Actinomycetaceae;g__Pauljensenia	98.4	RaFAH;94.80 CRISPR;83.10 iPHoP-RF;64.80
S5_vf_28061	ME	n.a.	n.a.	n.a.	n.a.	n.a.	n.a.	n.a.	n.a.	20.02	d__Bacteria;p__Firmicutes;c__Bacilli;o__Haloplasmatales_A;f__Turicibacteraceae;g__Turicibacter	91.5	blast;93.80 iPHoP-RF;68.60
S5_vf_6506	ME	Duplodnaviria	Heunggongvirae	Uroviricota	Caudoviricetes	n.a.	Unclassified	O	O	20.08	d__Bacteria;p__Firmicutes;c__Clostridia;o__Clostridiales;f__Clostridiaceae;g__Clostridium_J	90.3	blast;92.80 iPHoP-RF;70.90
S14_vf_117162	ME	Duplodnaviria	Heunggongvirae	Uroviricota	Caudoviricetes	n.a.	Unclassified	Unclassified	O	23.32	d__Bacteria;p__Bacteroidota;c__Bacteroidia;o__Bacteroidales;f__Bacteroidaceae;g__Bacteroides	94	RaFAH;90.00 iPHoP-RF;70.90 CRISPR;63.50
S5_vf_74661	ME	Duplodnaviria	Heunggongvirae	Uroviricota	Caudoviricetes	n.a.	Unclassified	Unclassified	Unclassified	91.73	d__Bacteria;p__Proteobacteria;c__Gammaproteobacteria;o__Enterobacterales;f__Enterobacteriaceae;g__Escherichia	100	RaFAH;99.40 iPHoP-RF;97.10 blast;96.80 CRISPR;85.00
S5_vf_8965	ME	Duplodnaviria	Heunggongvirae	Uroviricota	Caudoviricetes	n.a.	Unclassified	O	O	100	d__Bacteria;p__Bacteroidota;c__Bacteroidia;o__Bacteroidales;f__Bacteroidaceae;g__Bacteroides	100	RaFAH;99.60 iPHoP-RF;97.10 blast;96.30
S5_vs_107105	ME	Duplodnaviria	Heunggongvirae	Uroviricota	Caudoviricetes	n.a.	Unclassified	O	O	21.85	d__Bacteria;p__Firmicutes;c__Clostridia;o__Oscillospirales;f__Oscillospiraceae;g__CAG-110	94.7	blast;96.30 iPHoP-RF;67.00
S5_vs_120079	ME	Duplodnaviria	Heunggongvirae	Uroviricota	Caudoviricetes	n.a.	Unclassified	Unclassified	Unclassified	88.37	d__Bacteria;p__Firmicutes;c__Clostridia;o__Lachnospirales;f__Lachnospiraceae;g__Roseburia	95.3	blast;96.70 iPHoP-RF;96.40 CRISPR;84.10
S4_vs_VIRSorter_31975-	Shared	Duplodnaviria	Heunggongvirae	Uroviricota	Caudoviricetes	n.a.	Unclassified	O	O	88.33	d__Bacteria;p__Bacteroidota;c__Bacteroidia;o__Bacteroidales;f__Bacteroidaceae;g__Bacteroides	99.6	iPHoP-RF;97.10 blast;96.60 CRISPR;94.50 RaFAH;92.70
S14_vf_51134	ME	Duplodnaviria	Heunggongvirae	Uroviricota	Caudoviricetes	n.a.	Unclassified	O	O	47.7	d__Bacteria;p__Firmicutes;c__Clostridia;o__Oscillospirales;f__Ruminococcaceae;g__Ruminococcus_D	97.2	blast;97.90 iPHoP-RF;92.80 RaFAH;52.60
S14_vf_57689	ME	Duplodnaviria	Heunggongvirae	Uroviricota	Caudoviricetes	n.a.	Unclassified	O	O	94.04	d__Bacteria;p__Actinobacteriota;c__Actinomycetia;o__Actinomycetales;f__Actinomycetaceae;g__Actinomyces	99	blast;94.00 RaFAH;93.30 CRISPR;80.00 iPHoP-RF;75.40
S14_vf_85122	ME	Duplodnaviria	Heunggongvirae	Uroviricota	Caudoviricetes	n.a.	Unclassified	Unclassified	O	16.16	d__Bacteria;p__Firmicutes;c__Clostridia;o__Clostridiales;f__Clostridiaceae;g__Clostridium	95	blast;92.30 iPHoP-RF;86.00 RaFAH;77.50
S14_vf_93134	ME	Duplodnaviria	Heunggongvirae	Uroviricota	Caudoviricetes	n.a.	Salasmaviridae	O	O	12.04	d__Bacteria;p__Firmicutes;c__Clostridia;o__Lachnospirales;f__Lachnospiraceae;g__Muricomes	96.7	CRISPR;97.70
S5_vf_145579	Shared	Duplodnaviria	Heunggongvirae	Uroviricota	Caudoviricetes	Crassvirales	Steigviridae	O	O	44.59	d__Bacteria;p__Bacteroidota;c__Bacteroidia;o__Bacteroidales;f__Bacteroidaceae;g__Prevotella	94.6	RaFAH;96.20
S14_vs_100227	ME	Duplodnaviria	Heunggongvirae	Uroviricota	Caudoviricetes	n.a.	Unclassified	O	O	81.21	d__Bacteria;p__Firmicutes;c__Clostridia;o__Lachnospirales;f__Lachnospiraceae;g__Enterocloster	94.2	CRISPR;95.90 blast;94.00 iPHoP-RF;86.30
S14_vs_101482	ME	n.a.	n.a.	n.a.	n.a.	n.a.	n.a.	n.a.	n.a.	6.74	d__Bacteria;p__Firmicutes;c__Clostridia;o__Oscillospirales;f__Ruminococcaceae;g__QAMM01	92	blast;94.20 iPHoP-RF;69.10
S14_vs_112068	ME	n.a.	n.a.	n.a.	n.a.	n.a.	n.a.	n.a.	n.a.	27.86	d__Bacteria;p__Firmicutes;c__Clostridia;o__Oscillospirales;f__Butyricicoccaceae;g__Agathobaculum	97.5	blast;98.20 iPHoP-RF;66.00
S14_vs_112345	Shared	n.a.	n.a.	n.a.	n.a.	n.a.	n.a.	n.a.	n.a.	71.03	d__Bacteria;p__Bacteroidota;c__Bacteroidia;o__Bacteroidales;f__Bacteroidaceae;g__Bacteroides	99.1	iPHoP-RF;96.70 RaFAH;88.90 blast;75.90
S14_vs_115377	ME	Duplodnaviria	Heunggongvirae	Uroviricota	Caudoviricetes	n.a.	Unclassified	O	O	76.83	d__Bacteria;p__Firmicutes;c__Clostridia;o__Lachnospirales;f__Lachnospiraceae;g__Blautia	98.8	CRISPR;97.10 RaFAH;83.90 iPHoP-RF;60.90
S14_vs_115455	ME	Duplodnaviria	Heunggongvirae	Uroviricota	Caudoviricetes	n.a.	Unclassified	O	O	25.58	d__Bacteria;p__Firmicutes;c__Clostridia;o__Eubacteriales;f__Eubacteriaceae;g__Eubacterium	93	blast;95.00 CRISPR;89.70 iPHoP-RF;84.50
S5_vs_109527	Shared	n.a.	n.a.	n.a.	n.a.	n.a.	n.a.	n.a.	n.a.	23.09	d__Bacteria;p__Bacteroidota;c__Bacteroidia;o__Bacteroidales;f__Bacteroidaceae;g__Bacteroides	99	iPHoP-RF;96.40 blast;92.90 RaFAH;89.10
S14_vs_117153	ME	Duplodnaviria	Heunggongvirae	Uroviricota	Caudoviricetes	n.a.	Unclassified	O	O	96.78	d__Bacteria;p__Firmicutes;c__Clostridia;o__Lachnospirales;f__Lachnospiraceae;g__Dorea_A	94.2	blast;95.90 iPHoP-RF;94.70 CRISPR;58.40
S5_vs_122344	ME	Duplodnaviria	Heunggongvirae	Uroviricota	Caudoviricetes	n.a.	Unclassified	Unclassified	O	26.84	d__Bacteria;p__Firmicutes;c__Clostridia;o__Lachnospirales;f__Lachnospiraceae;g__Roseburia	97.6	CRISPR;98.30 blast;95.70 iPHoP-RF;93.10
S14_vs_26320	ME	Duplodnaviria	Heunggongvirae	Uroviricota	Caudoviricetes	n.a.	Unclassified	O	O	65.93	d__Bacteria;p__Firmicutes;c__Clostridia;o__Lachnospirales;f__Lachnospiraceae;g__Ruminococcus_B	95.2	blast;96.60 iPHoP-RF;82.20
S14_vs_30108	ME	n.a.	n.a.	n.a.	n.a.	n.a.	n.a.	n.a.	n.a.	19.83	d__Bacteria;p__Firmicutes;c__Clostridia;o__Lachnospirales;f__Lachnospiraceae;g__Enterocloster	92.3	blast;94.50 CRISPR;82.40 iPHoP-RF;78.30
S14_vs_38905	ME	n.a.	n.a.	n.a.	n.a.	n.a.	n.a.	n.a.	n.a.	9.01	d__Bacteria;p__Firmicutes;c__Clostridia;o__Oscillospirales;f__Ruminococcaceae;g__Ruthenibacterium	93.8	blast;95.60 iPHoP-RF;81.60 CRISPR;54.30
S14_vs_39266	ME	Duplodnaviria	Heunggongvirae	Uroviricota	Caudoviricetes	n.a.	Unclassified	Unclassified	O	15.51	d__Bacteria;p__Firmicutes;c__Clostridia;o__Oscillospirales;f__Acutalibacteraceae;g__Ruminococcus_E	98.7	CRISPR;99.00 blast;96.80 iPHoP-RF;93.70
S14_vs_56251	ME	Duplodnaviria	Heunggongvirae	Uroviricota	Caudoviricetes	n.a.	Unclassified	O	O	20.56	d__Bacteria;p__Firmicutes;c__Clostridia;o__Lachnospirales;f__Lachnospiraceae;g__Muricomes	95.3	iPHoP-RF;96.70 blast;93.30 CRISPR;68.70
S14_vs_56410	ME	Duplodnaviria	Heunggongvirae	Uroviricota	Caudoviricetes	n.a.	Unclassified	O	O	80.58	d__Bacteria;p__Firmicutes;c__Clostridia;o__Oscillospirales;f__Oscillospiraceae;g__Flavonifractor	95.2	blast;96.60 iPHoP-RF;95.40 CRISPR;94.00
S14_vs_90934	ME	n.a.	n.a.	n.a.	n.a.	n.a.	n.a.	n.a.	n.a.	62.33	d__Bacteria;p__Firmicutes;c__Clostridia;o__Oscillospirales;f__Ruminococcaceae;g__Faecalibacterium	99.1	blast;96.70 RaFAH;89.00 iPHoP-RF;81.10
S14_vs_96286	Shared	Duplodnaviria	Heunggongvirae	Uroviricota	Caudoviricetes	n.a.	Unclassified	O	O	99.54	d__Bacteria;p__Firmicutes;c__Clostridia;o__Lachnospirales;f__Lachnospiraceae;g__Blautia	96.3	RaFAH;97.40
S14_vs_97835	ME	n.a.	n.a.	n.a.	n.a.	n.a.	n.a.	n.a.	n.a.		d__Bacteria;p__Firmicutes;c__Clostridia;o__Oscillospirales;f__Oscillospiraceae;g__CAG-83	90.6	blast;93.00 iPHoP-RF;84.20
S15_vs_3868	Shared	Duplodnaviria	Heunggongvirae	Uroviricota	Caudoviricetes	n.a.	Unclassified	O	O	100	d__Bacteria;p__Bacteroidota;c__Bacteroidia;o__Bacteroidales;f__Bacteroidaceae;g__Bacteroides	100	RaFAH;99.10 blast;96.50 iPHoP-RF;61.40
S15_vs_43913	Shared	Duplodnaviria	Heunggongvirae	Uroviricota	Caudoviricetes	n.a.	Unclassified	O	O	99.8	d__Bacteria;p__Bacteroidota;c__Bacteroidia;o__Bacteroidales;f__Bacteroidaceae;g__Bacteroides	100	RaFAH;99.30 iPHoP-RF;97.10 blast;96.70
S16_vf_100473	Shared	n.a.	n.a.	n.a.	n.a.	n.a.	n.a.	n.a.	n.a.	8.42	d__Bacteria;p__Actinobacteriota;c__Actinomycetia;o__Actinomycetales;f__Bifidobacteriaceae;g__Bifidobacterium	90.3	blast;92.80
S16_vf_16182	Shared	Duplodnaviria	Heunggongvirae	Uroviricota	Caudoviricetes	n.a.	Unclassified	O	O	64.12	d__Bacteria;p__Actinobacteriota;c__Actinomycetia;o__Actinomycetales;f__Actinomycetaceae;g__Pauljensenia	96.9	RaFAH;95.10 CRISPR;68.30 iPHoP-RF;64.80
S16_vf_172897	SHHC	Duplodnaviria	Heunggongvirae	Uroviricota	Caudoviricetes	n.a.	Unclassified	O	O	32.49	d__Bacteria;p__Actinobacteriota;c__Actinomycetia;o__Actinomycetales;f__Bifidobacteriaceae;g__Bifidobacterium	98.4	CRISPR;98.80 iPHoP-RF;97.10 blast;93.30
S16_vf_5809	SHHC	n.a.	n.a.	n.a.	n.a.	n.a.	n.a.	n.a.	n.a.	87.43	d__Bacteria;p__Firmicutes;c__Clostridia;o__Lachnospirales;f__Lachnospiraceae;g__Lachnospira	99.9	CRISPR;99.00 iPHoP-RF;95.40 blast;93.40 RaFAH;91.70
S16_vf_84462	Shared	n.a.	n.a.	n.a.	n.a.	n.a.	n.a.	n.a.	n.a.	31.67	d__Bacteria;p__Firmicutes;c__Clostridia;o__Lachnospirales;f__Anaerotignaceae;g__Anaerotignum	94.2	blast;95.90 iPHoP-RF;79.50
S16_vf_9270	Shared	n.a.	n.a.	n.a.	n.a.	n.a.	n.a.	n.a.	n.a.	8.58	d__Bacteria;p__Bacteroidota;c__Bacteroidia;o__Bacteroidales;f__Rikenellaceae;g__Alistipes	98.4	CRISPR;98.80 iPHoP-RF;95.10
S16_vs_101072	SHHC	Duplodnaviria	Heunggongvirae	Uroviricota	Caudoviricetes	n.a.	Unclassified	O	O	26.56	d__Bacteria;p__Firmicutes;c__Clostridia;o__Lachnospirales;f__Lachnospiraceae;g__CAG-127	95	blast;96.50 iPHoP-RF;87.30
S16_vs_107235	SHHC	n.a.	n.a.	n.a.	n.a.	n.a.	n.a.	n.a.	n.a.	98.85	d__Bacteria;p__Firmicutes;c__Clostridia;o__Oscillospirales;f__Ruminococcaceae;g__Ruminococcus_D	99.7	blast;99.00 iPHoP-RF;97.10 CRISPR;94.60 RaFAH;84.50
S16_vs_118029	SHHC	Duplodnaviria	Heunggongvirae	Uroviricota	Caudoviricetes	n.a.	Unclassified	O	O	43.23	d__Bacteria;p__Firmicutes;c__Clostridia;o__Lachnospirales;f__Lachnospiraceae;g__Eubacterium_G	94.6	blast;96.20 iPHoP-RF;85.70
S16_vs_120149	SHHC	n.a.	n.a.	n.a.	n.a.	n.a.	n.a.	n.a.	n.a.	12.05	d__Bacteria;p__Firmicutes;c__Clostridia;o__Oscillospirales;f__Acutalibacteraceae;g__Eubacterium_R	94.5	iPHoP-RF;96.10 blast;93.90
S16_vs_132979	Shared	Duplodnaviria	Heunggongvirae	Uroviricota	Caudoviricetes	n.a.	Unclassified	O	O	31.86	d__Bacteria;p__Firmicutes;c__Clostridia;o__Oscillospirales;f__Ruminococcaceae;g__Faecalibacterium	94.3	blast;96.00 iPHoP-RF;93.10
S16_vs_140709	Shared	n.a.	n.a.	n.a.	n.a.	n.a.	n.a.	n.a.	n.a.	84.15	d__Bacteria;p__Firmicutes;c__Clostridia;o__Lachnospirales;f__Lachnospiraceae;g__CAG-317	94.5	iPHoP-RF;96.10
S16_vs_141218	Shared	n.a.	n.a.	n.a.	n.a.	n.a.	n.a.	n.a.	n.a.	10.25	d__Bacteria;p__Firmicutes;c__Bacilli;o__ML615J-28;f__CAG-313;g__CAG-313	98.7	CRISPR;99.00 blast;96.10
S16_vs_165662	SHHC	Duplodnaviria	Heunggongvirae	Uroviricota	Caudoviricetes	n.a.	Unclassified	O	O	11.88	d__Bacteria;p__Firmicutes;c__Bacilli;o__RF39;f__UBA660;g__CAG-302	92.7	blast;94.80
S16_vs_173141	Shared	n.a.	n.a.	n.a.	n.a.	n.a.	n.a.	n.a.	n.a.		d__Bacteria;p__Firmicutes;c__Clostridia;o__Oscillospirales;f__Ruminococcaceae;g__Faecalibacterium	96	CRISPR;97.20 iPHoP-RF;82.80
S16_vs_17493	Shared	n.a.	n.a.	n.a.	n.a.	n.a.	n.a.	n.a.	n.a.	18.24	d__Bacteria;p__Firmicutes;c__Clostridia;o__Oscillospirales;f__Ruminococcaceae;g__Massilimaliae	93	blast;95.00 iPHoP-RF;58.40
S16_vs_20060	Shared	Duplodnaviria	Heunggongvirae	Uroviricota	Caudoviricetes	n.a.	Unclassified	Unclassified	O	33.58	d__Bacteria;p__Firmicutes;c__Clostridia;o__Oscillospirales;f__Oscillospiraceae;g__UMGS1766	91	blast;93.40
S16_vs_34021	Shared	n.a.	n.a.	n.a.	n.a.	n.a.	n.a.	n.a.	n.a.	99.81	d__Bacteria;p__Firmicutes;c__Clostridia;o__Oscillospirales;f__Ruminococcaceae;g__Ruminococcus_D	99	blast;96.90 iPHoP-RF;90.50 RaFAH;86.90
S16_vs_38863	SHHC	Duplodnaviria	Heunggongvirae	Uroviricota	Caudoviricetes	n.a.	Unclassified	O	O	26.24	d__Bacteria;p__Firmicutes;c__Clostridia;o__Monoglobales;f__Monoglobaceae;g__Monoglobus	95	blast;96.50 iPHoP-RF;64.50
S16_vs_76823	SHHC	n.a.	n.a.	n.a.	n.a.	n.a.	n.a.	n.a.	n.a.	20.02	d__Bacteria;p__Firmicutes;c__Clostridia;o__Oscillospirales;f__Ruminococcaceae;g__Gemmiger	98.5	CRISPR;98.90 blast;98.30 iPHoP-RF;94.40
S16_vs_78429	SHHC	Duplodnaviria	Heunggongvirae	Uroviricota	Caudoviricetes	n.a.	Unclassified	Unclassified	O	22.27	d__Bacteria;p__Firmicutes;c__Clostridia;o__Oscillospirales;f__Oscillospiraceae;g__ER4	91.5	blast;93.80 iPHoP-RF;52.80
S16_vs_81805	SHHC	Duplodnaviria	Heunggongvirae	Uroviricota	Caudoviricetes	n.a.	Unclassified	O	O	99.42	d__Bacteria;p__Firmicutes;c__Clostridia;o__Oscillospirales;f__Ruminococcaceae;g__Ruthenibacterium	94.7	blast;96.30 iPHoP-RF;89.50 CRISPR;57.90
S16_vs_82116	Shared	Duplodnaviria	Heunggongvirae	Uroviricota	Caudoviricetes	n.a.	Unclassified	Unclassified	O	98.74	d__Bacteria;p__Firmicutes;c__Clostridia;o__Oscillospirales;f__Acutalibacteraceae;g__Ruminococcus_E	99.6	CRISPR;98.90 blast;96.90 iPHoP-RF;94.10 RaFAH;81.90
S17_vf_128147	Shared	Monodnaviria	Sangervirae	Phixviricota	Malgrandaviricetes	Petitvirales	Microviridae	Unclassified	O	3.97	d__Bacteria;p__Bacteroidota;c__Bacteroidia;o__Bacteroidales;f__Bacteroidaceae;g__Bacteroides	95.9	blast;97.10 iPHoP-RF;97.10 CRISPR;89.30
S17_vf_140653	Shared	Duplodnaviria	Heunggongvirae	Uroviricota	Caudoviricetes	n.a.	Unclassified	O	O	90.58	d__Bacteria;p__Bacteroidota;c__Bacteroidia;o__Bacteroidales;f__Bacteroidaceae;g__Bacteroides	99.7	blast;96.70 RaFAH;95.30 iPHoP-RF;85.70
S17_vf_36452	ME	Duplodnaviria	Heunggongvirae	Uroviricota	Caudoviricetes	n.a.	Salasmaviridae	O	O	12	d__Bacteria;p__Firmicutes;c__Clostridia;o__Lachnospirales;f__Lachnospiraceae;g__Muricomes	97.8	CRISPR;98.40 iPHoP-RF;90.80
S17_vf_46388	Shared	Duplodnaviria	Heunggongvirae	Uroviricota	Caudoviricetes	n.a.	Unclassified	O	O	50.05	d__Bacteria;p__Firmicutes;c__Clostridia;o__Lachnospirales;f__Lachnospiraceae;g__Blautia_A	99.4	CRISPR;97.10 blast;94.50 iPHoP-RF;93.70 RaFAH;91.90
S17_vs_110666	ME	Duplodnaviria	Heunggongvirae	Uroviricota	Caudoviricetes	n.a.	Unclassified	O	O	98.39	d__Bacteria;p__Firmicutes;c__Clostridia;o__Oscillospirales;f__Ruminococcaceae;g__Faecalibacterium	100	RaFAH;99.70 blast;96.80 iPHoP-RF;93.40 CRISPR;67.80
S17_vs_117507	Shared	n.a.	n.a.	n.a.	n.a.	n.a.	n.a.	n.a.	n.a.	40.08	d__Bacteria;p__Bacteroidota;c__Bacteroidia;o__Bacteroidales;f__Bacteroidaceae;g__Bacteroides	97.5	blast;97.40 iPHoP-RF;96.40 RaFAH;66.20
S17_vs_157209	Shared	n.a.	n.a.	n.a.	n.a.	n.a.	n.a.	n.a.	n.a.	25.09	d__Bacteria;p__Bacteroidota;c__Bacteroidia;o__Bacteroidales;f__Rikenellaceae;g__Alistipes	97.6	CRISPR;98.30 blast;95.20 iPHoP-RF;92.80
S17_vs_177203	ME	Duplodnaviria	Heunggongvirae	Uroviricota	Caudoviricetes	n.a.	Unclassified	O	O	71.95	d__Bacteria;p__Desulfobacterota;c__Desulfovibrionia;o__Desulfovibrionales;f__Desulfovibrionaceae;g__Bilophila	93.9	blast;95.70 iPHoP-RF;90.10
S17_vs_31654	ME	Duplodnaviria	Heunggongvirae	Uroviricota	Caudoviricetes	n.a.	Unclassified	O	O	36.85	d__Bacteria;p__Firmicutes;c__Bacilli;o__Erysipelotrichales;f__Erysipelatoclostridiaceae;g__Faecalibacillus	94.6	blast;96.20 iPHoP-RF;81.60 CRISPR;54.30
S17_vs_8690	ME	n.a.	n.a.	n.a.	n.a.	n.a.	n.a.	n.a.	n.a.	6.78	d__Bacteria;p__Proteobacteria;c__Alphaproteobacteria;o__RF32;f__CAG-239;g__CAG-495	95.2	blast;96.60
S1_vf_136526	ME	Duplodnaviria	Heunggongvirae	Uroviricota	Caudoviricetes	n.a.	Unclassified	Unclassified	O	33.32	d__Bacteria;p__Firmicutes;c__Clostridia;o__Oscillospirales;f__Oscillospiraceae;g__CAG-170	91.6	blast;93.90 iPHoP-RF;88.50
S1_vf_28398	ME	n.a.	n.a.	n.a.	n.a.	n.a.	n.a.	n.a.	n.a.	12.4	d__Bacteria;p__Firmicutes;c__Clostridia;o__Oscillospirales;f__Ruminococcaceae;g__Gemmiger	91.6	blast;93.90 iPHoP-RF;75.40
S1_vf_6272	Shared	Duplodnaviria	Heunggongvirae	Uroviricota	Caudoviricetes	n.a.	Unclassified	Unclassified	O	25.47	d__Bacteria;p__Firmicutes;c__Clostridia;o__Oscillospirales;f__Oscillospiraceae;g__CAG-170	92	CRISPR;94.20 blast;90.80 iPHoP-RF;78.00

S1_vs_117317	ME	Duplodnaviria	Heunggongvirae	Uroviricota	Caudoviricetes	n.a.	Unclassified	Unclassified	O	21.23	d__Bacteria;p__Firmicutes;c__Clostridia;o__Oscillospirales;f__Oscillospiraceae;g__CAG-170	93.4	blast;95.30 iPHoP-RF;89.50 CRISPR;71.10
S1_vs_133577	ME	Duplodnaviria	Heunggongvirae	Uroviricota	Caudoviricetes	n.a.	Unclassified	O	O	37.09	d__Bacteria;p__Firmicutes;c__Clostridia;o__Oscillospirales;f__Ruminococcaceae;g__Gemmiger	96	blast;97.20 CRISPR;95.40 iPHoP-RF;94.40
S1_vs_145792	ME	n.a.	n.a.	n.a.	n.a.	n.a.	n.a.	n.a.	n.a.	13.59	d__Bacteria;p__Firmicutes;c__Clostridia;o__Lachnospirales;f__Anaerotignaceae;g__Anaerotignum	94.6	blast;96.20 iPHoP-RF;72.60
S1_vs_155183	Shared	Duplodnaviria	Heunggongvirae	Uroviricota	Caudoviricetes	n.a.	Unclassified	Unclassified	O	31.14	d__Bacteria;p__Firmicutes;c__Clostridia;o__Oscillospirales;f__Oscillospiraceae;g__ER4	95.9	CRISPR;97.10 blast;96.20 iPHoP-RF;89.20
S1_vs_23682	ME	Duplodnaviria	Heunggongvirae	Uroviricota	Caudoviricetes	n.a.	Unclassified	O	O	33.26	d__Bacteria;p__Firmicutes;c__Clostridia;o__Oscillospirales;f__Ruminococcaceae;g__Faecalibacterium	98.8	CRISPR;99.10 blast;96.20 iPHoP-RF;83.30
S2_vf_16829	Shared	Duplodnaviria	Heunggongvirae	Uroviricota	Caudoviricetes	n.a.	Unclassified	Unclassified	O	81.02	d__Bacteria;p__Actinobacteriota;c__Coriobacteriia;o__Coriobacteriales;f__Eggerthellaceae;g__Eggerthella	94.9	CRISPR;96.40 iPHoP-RF;53.80 RaFAH;50.20
S3_vf_101611	Shared	Duplodnaviria	Heunggongvirae	Uroviricota	Caudoviricetes	n.a.	Unclassified	O	O	23.54	d__Bacteria;p__Firmicutes;c__Clostridia;o__Monoglobales;f__Monoglobaceae;g__Monoglobus	95.2	blast;96.60
S2_vf_17850	Shared	Duplodnaviria	Heunggongvirae	Uroviricota	Caudoviricetes	n.a.	Salasmaviridae	O	O	11.78	d__Bacteria;p__Firmicutes;c__Clostridia;o__Lachnospirales;f__Lachnospiraceae;g__Muricomes	92.7	CRISPR;94.80 iPHoP-RF;78.70
S3_vf_111893	Shared	Duplodnaviria	Heunggongvirae	Uroviricota	Caudoviricetes	n.a.	Unclassified	O	O	41.65	d__Bacteria;p__Firmicutes;c__Clostridia;o__Oscillospirales;f__Ruminococcaceae;g__Ruminococcus_D	95.4	blast;96.80 CRISPR;96.10 iPHoP-RF;94.10
S3_vf_127392	ME	Duplodnaviria	Heunggongvirae	Uroviricota	Caudoviricetes	n.a.	Unclassified	O	O	52.43	d__Bacteria;p__Firmicutes;c__Clostridia;o__Oscillospirales;f__CAG-272;g__CAG-272	92.1	blast;94.30 iPHoP-RF;52.20
S3_vf_127408	ME	Duplodnaviria	Heunggongvirae	Uroviricota	Caudoviricetes	n.a.	Unclassified	O	O	6.08	d__Bacteria;p__Actinobacteriota;c__Actinomycetia;o__Actinomycetales;f__Bifidobacteriaceae;g__Bifidobacterium	98.5	blast;98.90 CRISPR;97.60 iPHoP-RF;96.70
S3_vf_23042	Shared	Duplodnaviria	Heunggongvirae	Uroviricota	Caudoviricetes	n.a.	Unclassified	O	O	83.69	d__Bacteria;p__Firmicutes;c__Bacilli;o__Lactobacillales;f__Streptococcaceae;g__Lactococcus	99.7	blast;98.70 iPHoP-RF;90.10 RaFAH;89.50
S3_vf_5525	ME	n.a.	n.a.	n.a.	n.a.	n.a.	n.a.	n.a.	n.a.	41.37	d__Bacteria;p__Actinobacteriota;c__Actinomycetia;o__Actinomycetales;f__Actinomycetaceae;g__Actinomyces	92.6	RaFAH;85.90 iPHoP-RF;81.10
S3_vf_56363	Shared	Duplodnaviria	Heunggongvirae	Uroviricota	Caudoviricetes	n.a.	Unclassified	O	O	23.06	d__Bacteria;p__Firmicutes;c__Clostridia;o__Peptostreptococcales;f__Peptostreptococcaceae;g__GCA-900066495	91.4	iPHoP-RF;93.70
S3_vf_99626	Shared	Duplodnaviria	Heunggongvirae	Uroviricota	Caudoviricetes	n.a.	Unclassified	O	O	26.68	d__Bacteria;p__Actinobacteriota;c__Coriobacteriia;o__Coriobacteriales;f__Coriobacteriaceae;g__Collinsella	98.7	CRISPR;99.00 iPHoP-RF;97.10 blast;92.10
S3_vs_119771	ME	Duplodnaviria	Heunggongvirae	Uroviricota	Caudoviricetes	n.a.	Unclassified	O	O	27.39	d__Bacteria;p__Firmicutes;c__Clostridia;o__Oscillospirales;f__Ruminococcaceae;g__Gemmiger	95.9	blast;97.10 iPHoP-RF;81.40 CRISPR;55.60
S3_vs_122706	ME	Duplodnaviria	Heunggongvirae	Uroviricota	Caudoviricetes	n.a.	Unclassified	O	O	89.75	d__Bacteria;p__Firmicutes;c__Clostridia;o__Oscillospirales;f__Acutalibacteraceae;g__CAG-217	95.3	blast;96.70 iPHoP-RF;79.30
S3_vs_127401	ME	Duplodnaviria	Heunggongvirae	Uroviricota	Caudoviricetes	n.a.	Unclassified	O	O	45.54	d__Bacteria;p__Firmicutes;c__Clostridia;o__Lachnospirales;f__Lachnospiraceae;g__Lachnospira	90.4	blast;92.90 iPHoP-RF;70.60
S3_vs_15450	ME	Duplodnaviria	Heunggongvirae	Uroviricota	Caudoviricetes	n.a.	Unclassified	O	O	21.5	d__Bacteria;p__Firmicutes;c__Clostridia;o__Oscillospirales;f__Ruminococcaceae;g__Gemmiger	95.4	blast;96.80 iPHoP-RF;83.90 CRISPR;68.80
S3_vs_23194	ME	Duplodnaviria	Heunggongvirae	Uroviricota	Caudoviricetes	n.a.	Duneviridae	O	O	42.15	d__Bacteria;p__Bacteroidota;c__Bacteroidia;o__Bacteroidales;f__Bacteroidaceae;g__Bacteroides	100	RaFAH;98.70 iPHoP-RF;97.10
S3_vs_40939	ME	n.a.	n.a.	n.a.	n.a.	n.a.	n.a.	n.a.	n.a.	23.65	d__Bacteria;p__Bacteroidota;c__Bacteroidia;o__Bacteroidales;f__Bacteroidaceae;g__Bacteroides	99.3	iPHoP-RF;97.10 blast;96.60 RaFAH;89.70
S3_vs_56292	Shared	Duplodnaviria	Heunggongvirae	Uroviricota	Caudoviricetes	n.a.	Unclassified	O	O	99.95	d__Bacteria;p__Bacteroidota;c__Bacteroidia;o__Bacteroidales;f__Rikenellaceae;g__Alistipes	99.9	blast;96.90 RaFAH;96.10 iPHoP-RF;83.60
S3_vs_6604	Shared	Duplodnaviria	Heunggongvirae	Uroviricota	Caudoviricetes	n.a.	Duneviridae	O	O	75.2	d__Bacteria;p__Bacteroidota;c__Bacteroidia;o__Bacteroidales;f__Marinifilaceae;g__Butyricimonas	95.4	blast;96.80 iPHoP-RF;65.60
S3_vs_94577	ME	Duplodnaviria	Heunggongvirae	Uroviricota	Caudoviricetes	n.a.	Unclassified	O	O	45.75	d__Bacteria;p__Firmicutes;c__Bacilli;o__Erysipelotrichales;f__Erysipelatoclostridiaceae;g__Faecalibacillus	97.8	blast;98.40
S3_vs_97827	ME	n.a.	n.a.	n.a.	n.a.	n.a.	n.a.	n.a.	n.a.	10.17	d__Bacteria;p__Bacteroidota;c__Bacteroidia;o__Bacteroidales;f__Marinifilaceae;g__Odoribacter	97.9	blast;98.50 iPHoP-RF;90.10 CRISPR;71.50
S4_vf_20440	SHHC	Duplodnaviria	Heunggongvirae	Uroviricota	Caudoviricetes	n.a.	Unclassified	O	O	44.47	d__Bacteria;p__Bacteroidota;c__Bacteroidia;o__Bacteroidales;f__Bacteroidaceae;g__Prevotella	97.8	blast;98.40 iPHoP-RF;84.20
S3_vf_11428	Shared	Duplodnaviria	Heunggongvirae	Uroviricota	Caudoviricetes	n.a.	Unclassified	O	O	94.85	d__Bacteria;p__Firmicutes;c__Clostridia;o__Lachnospirales;f__Lachnospiraceae;g__Blautia_A	100	RaFAH;98.90 CRISPR;97.00 iPHoP-RF;93.40 blast;91.70
S4_vs_VIRSorter_2744-c	SHHC	Duplodnaviria	Heunggongvirae	Uroviricota	Caudoviricetes	n.a.	Unclassified	O	O	9.58	d__Bacteria;p__Actinobacteriota;c__Actinomycetia;o__Actinomycetales;f__Bifidobacteriaceae;g__Bifidobacterium	96	CRISPR;97.20 iPHoP-RF;97.10 blast;96.70
S4_vs_VIRSorter_31972-	SHHC	Duplodnaviria	Heunggongvirae	Uroviricota	Caudoviricetes	n.a.	Unclassified	O	O	26.41	d__Bacteria;p__Firmicutes;c__Clostridia;o__Oscillospirales;f__Ruminococcaceae;g__Ruminococcus_C	97.3	blast;98.10 CRISPR;93.20 iPHoP-RF;90.50
S4_vs_VIRSorter_5698-c	SHHC	Duplodnaviria	Heunggongvirae	Uroviricota	Caudoviricetes	n.a.	Unclassified	O	O	87.05	d__Bacteria;p__Actinobacteriota;c__Actinomycetia;o__Actinomycetales;f__Bifidobacteriaceae;g__Bifidobacterium	99	CRISPR;98.50 blast;96.80 iPHoP-RF;96.10 RaFAH;71.90
S5_vf_103206	Shared	n.a.	n.a.	n.a.	n.a.	n.a.	n.a.	n.a.	n.a.	3.75	d__Bacteria;p__Firmicutes;c__Clostridia;o__Oscillospirales;f__Oscillospiraceae;g__Lawsonibacter	90	blast;92.50
S5_vf_116300	ME	n.a.	n.a.	n.a.	n.a.	n.a.	n.a.	n.a.	n.a.	13.55	d__Bacteria;p__Firmicutes;c__Clostridia;o__Lachnospirales;f__Lachnospiraceae;g__Anaerostipes	95.2	blast;96.60 iPHoP-RF;90.50 CRISPR;69.90
S3_vf_127400	Shared	Duplodnaviria	Heunggongvirae	Uroviricota	Caudoviricetes	n.a.	Unclassified	Unclassified	O	54.09	d__Bacteria;p__Firmicutes;c__Clostridia;o__Oscillospirales;f__Oscillospiraceae;g__ER4	95	blast;96.50 iPHoP-RF;76.40 CRISPR;67.10
S5_vf_55687	Shared	Duplodnaviria	Heunggongvirae	Uroviricota	Caudoviricetes	n.a.	Unclassified	O	O	91.19	d__Bacteria;p__Firmicutes;c__Bacilli;o__Lactobacillales;f__Streptococcaceae;g__Lactococcus	90.2	RaFAH;92.70
S5_vf_8808	ME	Duplodnaviria	Heunggongvirae	Uroviricota	Caudoviricetes	n.a.	Unclassified	O	O	97.29	d__Bacteria;p__Firmicutes;c__Negativicutes;o__Veillonellales;f__Veillonellaceae;g__Veillonella	100	CRISPR;98.80 RaFAH;98.60 blast;93.70 iPHoP-RF;78.30
S5_vs_10428	ME	Duplodnaviria	Heunggongvirae	Uroviricota	Caudoviricetes	n.a.	Unclassified	O	O	10.64	d__Bacteria;p__Firmicutes;c__Clostridia;o__Christensenellales;f__CAG-74;g__Alectryocaccomicrobium	91.4	blast;93.70
S5_vs_118168	ME	n.a.	n.a.	n.a.	n.a.	n.a.	n.a.	n.a.	n.a.	30.01	d__Bacteria;p__Firmicutes;c__Clostridia;o__Oscillospirales;f__Ruminococcaceae;g__Anaerotruncus	91	blast;93.40 iPHoP-RF;57.30
S5_vs_120584	Shared	Duplodnaviria	Heunggongvirae	Uroviricota	Caudoviricetes	n.a.	Unclassified	O	O	98.83	d__Bacteria;p__Firmicutes;c__Clostridia;o__Lachnospirales;f__Lachnospiraceae;g__Lachnospira	97.5	CRISPR;98.20 blast;93.50 iPHoP-RF;88.90
S5_vs_121411	ME	n.a.	n.a.	n.a.	n.a.	n.a.	n.a.	n.a.	n.a.	100	d__Bacteria;p__Firmicutes;c__Clostridia;o__Oscillospirales;f__Ruminococcaceae;g__Ruthenibacterium	95.3	blast;96.70 iPHoP-RF;87.60 CRISPR;68.40
S3_vf_127450	ME	n.a.	n.a.	n.a.	n.a.	n.a.	n.a.	n.a.	n.a.	8.34	d__Bacteria;p__Bacteroidota;c__Bacteroidia;o__Bacteroidales;f__Bacteroidaceae;g__Prevotella	92.3	RaFAH;94.50
S3_vf_7137	Shared	Unclassified	n.a.	n.a.	n.a.	n.a.	Unclassified	Unclassified	O	5.31	d__Bacteria;p__Actinobacteriota;c__Actinomycetia;o__Actinomycetales;f__Bifidobacteriaceae;g__Bifidobacterium	99.7	CRISPR;99.70 iPHoP-RF;77.30
S3_vf_8164	ME	Duplodnaviria	Heunggongvirae	Uroviricota	Caudoviricetes	n.a.	Unclassified	O	O	10.61	d__Bacteria;p__Firmicutes;c__Clostridia;o__Oscillospirales;f__Ruminococcaceae;g__Ruminococcus_D	98.2	CRISPR;98.70 blast;96.80 iPHoP-RF;95.10
S5_vs_117319	ME	Duplodnaviria	Heunggongvirae	Uroviricota	Caudoviricetes	n.a.	Unclassified	Unclassified	O	51.09	d__Bacteria;p__Firmicutes;c__Clostridia;o__Oscillospirales;f__Ruminococcaceae;g__Faecalibacterium	98.4	blast;95.50 RaFAH;86.90 CRISPR;78.90 iPHoP-RF;76.80
S3_vs_127411	ME	n.a.	n.a.	n.a.	n.a.	n.a.	n.a.	n.a.	n.a.	9.3	d__Bacteria;p__Firmicutes;c__Clostridia;o__Oscillospirales;f__Oscillospiraceae;g__ER4	95.2	blast;96.60 iPHoP-RF;73.20
S3_vs_51931	ME	Monodnaviria	Sangervirae	Phixviricota	Malgrandaviricetes	Petitvirales	Microviridae	Gokushovirinae	O	21.18	d__Bacteria;p__Proteobacteria;c__Gammaproteobacteria;o__Burkholderiales;f__Burkholderiaceae;g__Sutterella	94.6	CRISPR;96.20 iPHoP-RF;79.00
S3_vs_127421	Shared	Duplodnaviria	Heunggongvirae	Uroviricota	Caudoviricetes	n.a.	Unclassified	O	O	95.6	d__Bacteria;p__Firmicutes;c__Clostridia;o__Lachnospirales;f__Lachnospiraceae;g__Agathobacter	100	CRISPR;98.90 RaFAH;94.40 iPHoP-RF;92.10
S5_vf_60597	Shared	n.a.	n.a.	n.a.	n.a.	n.a.	n.a.	n.a.	n.a.	35.7	d__Bacteria;p__Firmicutes;c__Clostridia;o__Lachnospirales;f__Anaerotignaceae;g__Anaerotignum	93.9	blast;95.70 iPHoP-RF;84.20
S3_vs_25896	ME	n.a.	n.a.	n.a.	n.a.	n.a.	n.a.	n.a.	n.a.	11.85	d__Bacteria;p__Firmicutes;c__Clostridia;o__Oscillospirales;f__Ruminococcaceae;g__Ruthenibacterium	91.4	blast;93.70 iPHoP-RF;78.30
S3_vs_39940	ME	n.a.	n.a.	n.a.	n.a.	n.a.	n.a.	n.a.	n.a.	5.41	d__Bacteria;p__Actinobacteriota;c__Actinomycetia;o__Actinomycetales;f__Bifidobacteriaceae;g__Bifidobacterium	95.9	iPHoP-RF;97.10 blast;96.90 CRISPR;84.00
S3_vs_50557	ME	Duplodnaviria	Heunggongvirae	Uroviricota	Caudoviricetes	n.a.	Unclassified	O	O	23.06	d__Bacteria;p__Actinobacteriota;c__Coriobacteriia;o__Coriobacteriales;f__Eggerthellaceae;g__Gordonibacter	93.4	CRISPR;95.30 blast;93.90 iPHoP-RF;93.10
S3_vs_9158	Shared	n.a.	n.a.	n.a.	n.a.	n.a.	n.a.	n.a.	n.a.	99.96	d__Bacteria;p__Bacteroidota;c__Bacteroidia;o__Bacteroidales;f__Bacteroidaceae;g__Bacteroides	100	RaFAH;99.60 iPHoP-RF;97.10 blast;96.90
S3_vs_87728	Shared	n.a.	n.a.	n.a.	n.a.	n.a.	n.a.	n.a.	n.a.	5.42	d__Bacteria;p__Firmicutes;c__Clostridia;o__Oscillospirales;f__Ruminococcaceae;g__Faecalibacterium	95.3	blast;96.70 iPHoP-RF;67.00
S3_vs_89485	Shared	n.a.	n.a.	n.a.	n.a.	n.a.	n.a.	n.a.	n.a.	73.42	d__Bacteria;p__Bacteroidota;c__Bacteroidia;o__Bacteroidales;f__Rikenellaceae;g__Alistipes	95	blast;96.50 iPHoP-RF;93.40
S5_vs_117417	ME	n.a.	n.a.	n.a.	n.a.	n.a.	n.a.	n.a.	n.a.	13.57	d__Bacteria;p__Firmicutes;c__Clostridia;o__Oscillospirales;f__Oscillospiraceae;g__Enterenecus	90.7	blast;93.10 iPHoP-RF;70.90
S3_vs_90331	Shared	Duplodnaviria	Heunggongvirae	Uroviricota	Caudoviricetes	n.a.	Unclassified	Unclassified	O	99.17	d__Bacteria;p__Firmicutes;c__Clostridia;o__Oscillospirales;f__Ruminococcaceae;g__Faecalibacterium	100	RaFAH;98.70 CRISPR;97.70 blast;92.00 iPHoP-RF;85.70
S4_vf_30103	Shared	Unclassified	n.a.	n.a.	n.a.	n.a.	Unclassified	Unclassified	O	5.83	d__Bacteria;p__Actinobacteriota;c__Actinomycetia;o__Actinomycetales;f__Bifidobacteriaceae;g__Bifidobacterium	99.7	CRISPR;99.70 iPHoP-RF;92.10
S4_vf_11308	Shared	Duplodnaviria	Heunggongvirae	Uroviricota	Caudoviricetes	n.a.	Unclassified	Unclassified	O	96.94	d__Bacteria;p__Firmicutes;c__Clostridia;o__Oscillospirales;f__Acutalibacteraceae;g__Ruminococcus_E	100	CRISPR;98.90 blast;98.50 RaFAH;97.10 iPHoP-RF;93.70
S4_vf_13353	Shared	Duplodnaviria	Heunggongvirae	Uroviricota	Caudoviricetes	n.a.	Unclassified	O	O	93.68	d__Bacteria;p__Firmicutes;c__Clostridia;o__Lachnospirales;f__Lachnospiraceae;g__Blautia_A	99.9	CRISPR;97.90 RaFAH;95.20 blast;90.90 iPHoP-RF;89.20
S4_vs_VIRSorter_31916-	SHHC	Duplodnaviria	Heunggongvirae	Uroviricota	Caudoviricetes	n.a.	Unclassified	O	O	39.89	d__Bacteria;p__Firmicutes;c__Clostridia;o__Lachnospirales;f__Lachnospiraceae;g__Mediterraneibacter	90.7	blast;93.10 iPHoP-RF;68.20
S4_vf_31979	SHHC	Duplodnaviria	Heunggongvirae	Uroviricota	Caudoviricetes	n.a.	Unclassified	Unclassified	O	24.1	d__Bacteria;p__Firmicutes;c__Clostridia;o__Christensenellales;f__CAG-74;g__Ventricola	92	blast;94.20
S4_vs_VIRSorter_27316-	Shared	Duplodnaviria	Heunggongvirae	Uroviricota	Caudoviricetes	n.a.	Unclassified	O	O	74.11	d__Bacteria;p__Firmicutes;c__Clostridia;o__Oscillospirales;f__Ruminococcaceae;g__Faecalibacterium	95.4	blast;96.80 iPHoP-RF;75.00 CRISPR;67.80
S5_vf_145583	ME	Duplodnaviria	Heunggongvirae	Uroviricota	Caudoviricetes	n.a.	Unclassified	Unclassified	O	10.99	d__Bacteria;p__Firmicutes;c__Clostridia;o__Clostridiales;f__Clostridiaceae;g__Clostridium	95.2	blast;93.60 iPHoP-RF;80.80 RaFAH;73.30 CRISPR;62.00
S5_vf_105728	ME	Duplodnaviria	Heunggongvirae	Uroviricota	Caudoviricetes	n.a.	Unclassified	O	O	44.2	d__Bacteria;p__Firmicutes;c__Clostridia;o__Lachnospirales;f__Lachnospiraceae;g__	94.5	iPHoP-RF;96.10 blast;93.40
S5_vf_145604	Shared	Duplodnaviria	Heunggongvirae	Uroviricota	Caudoviricetes	n.a.	Unclassified	O	O	87.41	d__Bacteria;p__Firmicutes;c__Clostridia;o__Lachnospirales;f__Lachnospiraceae;g__Blautia_A	99.6	CRISPR;97.30 RaFAH;93.30 blast;92.90 iPHoP-RF;91.80
S5_vf_21869	Shared	Duplodnaviria	Heunggongvirae	Uroviricota	Caudoviricetes	n.a.	Unclassified	Unclassified	O	31.69	d__Bacteria;p__Firmicutes;c__Clostridia;o__Oscillospirales;f__Oscillospiraceae;g__Flavonifractor	93.1	blast;95.10 iPHoP-RF;73.90
S5_vf_29332	ME	Duplodnaviria	Heunggongvirae	Uroviricota	Caudoviricetes	n.a.	Unclassified	O	O	12.73	d__Bacteria;p__Actinobacteriota;c__Coriobacteriia;o__Coriobacteriales;f__Coriobacteriaceae;g__Collinsella	97.3	CRISPR;98.10 blast;94.60 iPHoP-RF;79.50
S5_vs_122618	ME	n.a.	n.a.	n.a.	n.a.	n.a.	n.a.	n.a.	n.a.	17.7	d__Bacteria;p__Firmicutes;c__Bacilli;o__Erysipelotrichales;f__Erysipelotrichaceae;g__Clostridium_AQ	95.4	blast;96.80 iPHoP-RF;71.10 CRISPR;66.50
S5_vs_123256	Shared	Duplodnaviria	Heunggongvirae	Uroviricota	Caudoviricetes	n.a.	Unclassified	Unclassified	Unclassified	98.81	d__Bacteria;p__Bacteroidota;c__Bacteroidia;o__Bacteroidales;f__Rikenellaceae;g__Alistipes	100	RaFAH;97.10 blast;96.90 iPHoP-RF;96.10
S5_vs_124035	Shared	n.a.	n.a.	n.a.	n.a.	n.a.	n.a.	n.a.	n.a.	7.45	d__Bacteria;p__Firmicutes;c__Clostridia;o__Oscillospirales;f__Oscillospiraceae;g__Intestinimonas	91.2	blast;93.60 iPHoP-RF;53.30
S5_vs_124677	ME	Duplodnaviria	Heunggongvirae	Uroviricota	Caudoviricetes	n.a.	Unclassified	Unclassified	O	26.54	d__Bacteria;p__Firmicutes;c__Clostridia;o__Oscillospirales;f__Ruminococcaceae;g__Faecalibacterium	97	CRISPR;97.90 blast;96.90 iPHoP-RF;90.80
S5_vs_128398	ME	Duplodnaviria	Heunggongvirae	Uroviricota	Caudoviricetes	n.a.	Unclassified	O	O	22.85	d__Bacteria;p__Firmicutes;c__Clostridia;o__Oscillospirales;f__Ruminococcaceae;g__Negativibacillus	93.6	blast;95.50 iPHoP-RF;82.20
S5_vs_129970	Shared	n.a.	n.a.	n.a.	n.a.	n.a.	n.a.	n.a.	n.a.	93.93	d__Bacteria;p__Bacteroidota;c__Bacteroidia;o__Bacteroidales;f__Bacteroidaceae;g__Bacteroides	100	RaFAH;99.60 blast;92.90 iPHoP-RF;92.80
S5_vs_130032	ME	Duplodnaviria	Heunggongvirae	Uroviricota	Caudoviricetes	n.a.	Unclassified	O	O	98.82	d__Bacteria;p__Proteobacteria;c__Gammaproteobacteria;o__Enterobacterales;f__Enterobacteriaceae;g__Escherichia	100	RaFAH;99.70 blast;96.90 iPHoP-RF;91.40 CRISPR;72.80
S5_vs_130294	Shared	n.a.	n.a.	n.a.	n.a.	n.a.	n.a.	n.a.	n.a.	55.97	d__Bacteria;p__Firmicutes;c__Clostridia;o__Oscillospirales;f__Ruminococcaceae;g__Faecalibacterium	95.6	blast;96.90 iPHoP-RF;81.60
S5_vs_130958	ME	Duplodnaviria	Heunggongvirae	Uroviricota	Caudoviricetes	n.a.	Unclassified	O	O	18.11	d__Bacteria;p__Firmicutes;c__Clostridia;o__Oscillospirales;f__Ruminococcaceae;g__Anaerotruncus	94.2	blast;95.90 iPHoP-RF;70.40
S5_vs_131510	Shared	Duplodnaviria	Heunggongvirae	Uroviricota	Caudoviricetes	n.a.	Unclassified	Unclassified	O	95	d__Bacteria;p__Bacteroidota;c__Bacteroidia;o__Bacteroidales;f__Bacteroidaceae;g__Bacteroides	100	RaFAH;97.50 blast;96.90 iPHoP-RF;96.40
S5_vs_13254	ME	n.a.	n.a.	n.a.	n.a.	n.a.	n.a.	n.a.	n.a.	8.3	d__Bacteria;p__Firmicutes;c__Clostridia;o__Oscillospirales;f__Ruminococcaceae;g__Ruthenibacterium	95	blast;96.50 iPHoP-RF;85.40
S5_vs_1338	ME	n.a.	n.a.	n.a.	n.a.	n.a.	n.a.	n.a.	n.a.	100	d__Bacteria;p__Bacteroidota;c__Bacteroidia;o__Bacteroidales;f__Rikenellaceae;g__Alistipes	100	RaFAH;97.80 blast;97.50 iPHoP-RF;76.10
S5_vs_137154	ME	Duplodnaviria	Heunggongvirae	Uroviricota	Caudoviricetes	n.a.	Unclassified	O	O	10.76	d__Bacteria;p__Firmicutes;c__Clostridia;o__Clostridiales;f__Clostridiaceae;g__Clostridium	91.7	blast;94.00 iPHoP-RF;67.30
S5_vs_139401	ME	n.a.	n.a.	n.a.	n.a.	n.a.	n.a.	n.a.	n.a.	12.88	d__Bacteria;p__Firmicutes;c__Clostridia;o__Lachnospirales;f__Lachnospiraceae;g__Anaerostipes	90.9	blast;93.30 CRISPR;68.40 iPHoP-RF;67.50
S5_vs_140946	ME	n.a.	n.a.	n.a.	n.a.	n.a.	n.a.	n.a.	n.a.	9.68	d__Bacteria;p__Firmicutes;c__Clostridia;o__Oscillospirales;f__Acutalibacteraceae;g__UMGS856	93.9	blast;95.70 iPHoP-RF;76.80
S5_vs_141621	ME	Duplodnaviria	Heunggongvirae	Uroviricota	Caudoviricetes	n.a.	Unclassified	O	O	97.16	d__Bacteria;p__Firmicutes;c__Clostridia;o__Lachnospirales;f__Lachnospiraceae;g__Anaerostipes	96.4	blast;95.60 CRISPR;90.90 iPHoP-RF;88.90 RaFAH;72.10
S5_vs_142195	Shared	n.a.	n.a.	n.a.	n.a.	n.a.	n.a.	n.a.	n.a.	100	d__Bacteria;p__Bacteroidota;c__Bacteroidia;o__Bacteroidales;f__Bacteroidaceae;g__Bacteroides	99.9	RaFAH;99.80
S5_vs_145578	ME	Duplodnaviria	Heunggongvirae	Uroviricota	Caudoviricetes	n.a.	Unclassified	O	O	39.67	d__Bacteria;p__Firmicutes;c__Clostridia;o__Lachnospirales;f__Lachnospiraceae;g__Enterocloster	95.2	blast;96.60 iPHoP-RF;76.10 CRISPR;54.30
S5_vs_145581	ME	n.a.	n.a.	n.a.	n.a.	n.a.	n.a.	n.a.	n.a.	9.66	d__Bacteria;p__Firmicutes;c__Clostridia;o__Christensenellales;f__Christensenellaceae;g__Christensenella	90.4	blast;92.90 iPHoP-RF;86.30
S5_vs_145590	ME	Duplodnaviria	Heunggongvirae	Uroviricota	Caudoviricetes	n.a.	Unclassified	O	O	39.74	d__Bacteria;p__Firmicutes;c__Clostridia;o__Lachnospirales;f__Lachnospiraceae;g__Clostridium_AP	91.4	iPHoP-RF;93.70
S5_vs_145597	ME	Duplodnaviria	Heunggongvirae	Uroviricota	Caudoviricetes	n.a.	Unclassified	O	O	20.03	d__Bacteria;p__Firmicutes;c__Clostridia;o__Christensenellales;f__NSJ-53;g__NSJ-53	94	blast;95.80 iPHoP-RF;60.10
S5_vs_145598	ME	Duplodnaviria	Heunggongvirae	Uroviricota	Caudoviricetes	n.a.	Unclassified	O	O	95.83	d__Bacteria;p__Firmicutes;c__Clostridia;o__Lachnospirales;f__Lachnospiraceae;g__Roseburia	92.1	blast;94.30 iPHoP-RF;91.80
S5_vs_145602	ME	Duplodnaviria	Heunggongvirae	Uroviricota	Caudoviricetes	n.a.	Unclassified	O	O	37.74	d__Bacteria;p__Firmicutes;c__Clostridia;o__Lachnospirales;f__Lachnospiraceae;g__Anaerobutyricum	90.7	blast;93.10 iPHoP-RF;65.30
S5_vs_145605	ME	Duplodnaviria	Heunggongvirae	Uroviricota	Caudoviricetes	n.a.	Unclassified	O	O	17.51	d__Bacteria;p__Firmicutes;c__Clostridia;o__Christensenellales;f__CAG-74;g__SFTH01	90.3	blast;92.80 iPHoP-RF;68.20
S5_vs_145611	ME	Duplodnaviria	Heunggongvirae	Uroviricota	Caudoviricetes	n.a.	Unclassified	O	O	45.88	d__Bacteria;p__Firmicutes;c__Clostridia;o__Oscillospirales;f__Ruminococcaceae;g__Phocea	90.7	blast;93.10 iPHoP-RF;78.00
S5_vs_1572	Shared	Duplodnaviria	Heunggongvirae	Uroviricota	Caudoviricetes	n.a.	Unclassified	Unclassified	Unclassified	97.34	d__Bacteria;p__Bacteroidota;c__Bacteroidia;o__Bacteroidales;f__Tannerellaceae;g__Parabacteroides	91.6	blast;93.90 iPHoP-RF;61.70
S5_vs_17520	ME	n.a.	n.a.	n.a.	n.a.	n.a.	n.a.	n.a.	n.a.	39.12	d__Bacteria;p__Firmicutes;c__Clostridia;o__Lachnospirales;f__Lachnospiraceae;g__Hungatella	95.9	iPHoP-RF;97.10 CRISPR;88.40
S5_vs_20268	ME	Duplodnaviria	Heunggongvirae	Uroviricota	Caudoviricetes	n.a.	Unclassified	Unclassified	Unclassified	22.4	d__Bacteria;p__Firmicutes;c__Clostridia;o__Oscillospirales;f__Acutalibacteraceae;g__Ruminococcus_E	98.5	CRISPR;98.90 blast;96.50 iPHoP-RF;89.50
S5_vs_20500	Shared	Duplodnaviria	Heunggongvirae	Uroviricota	Caudoviricetes	n.a.	Unclassified	O	O	99.42	d__Bacteria;p__Bacteroidota;c__Bacteroidia;o__Bacteroidales;f__Rikenellaceae;g__Alistipes	95.6	blast;96.90 iPHoP-RF;96.70
S5_vs_21778	ME	n.a.	n.a.	n.a.	n.a.	n.a.	n.a.	n.a.	n.a.	11.27	d__Bacteria;p__Firmicutes;c__Clostridia;o__Oscillospirales;f__Oscillospiraceae;g__Flavonifractor	90.4	blast;92.90 iPHoP-RF;59.20
S5_vs_28263	ME	n.a.	n.a.	n.a.	n.a.	n.a.	n.a.	n.a.	n.a.	16.17	d__Bacteria;p__Firmicutes;c__Clostridia;o__Lachnospirales;f__Lachnospiraceae;g__Mediterraneibacter	91.6	blast;93.90 iPHoP-RF;86.30 CRISPR;58.00
S5_vs_30490	ME	n.a.	n.a.	n.a.	n.a.	n.a.	n.a.	n.a.	n.a.	42.23	d__Bacteria;p__Firmicutes;c__Clostridia;o__Oscillospirales;f__Ruminococcaceae;g__Faecalibacterium	99.3	blast;97.10 RaFAH;90.30 iPHoP-RF;87.30 CRISPR;71.60
S5_vs_32885	ME	Duplodnaviria	Heunggongvirae	Uroviricota	Caudoviricetes	n.a.	Unclassified	O	O	19.8	d__Bacteria;p__Firmicutes;c__Clostridia;o__Oscillospirales;f__Ruminococcaceae;g__Anaerotruncus	92.3	blast;94.50 CRISPR;93.00 iPHoP-RF;81.90
S5_vs_3501	ME	Duplodnaviria	Heunggongvirae	Uroviricota	Caudoviricetes	n.a.	Unclassified	O	O	32.19	d__Bacteria;p__Firmicutes;c__Clostridia;o__Oscillospirales;f__Acutalibacteraceae;g__UBA1691	95.6	blast;96.90 iPHoP-RF;90.10 CRISPR;85.90
S5_vs_40848	ME	Duplodnaviria	Heunggongvirae	Uroviricota	Caudoviricetes	n.a.	Unclassified	O	O	58.74	d__Bacteria;p__Firmicutes;c__Clostridia;o__Oscillospirales;f__Acutalibacteraceae;g__Ruminococcus_E	95.6	blast;96.90 iPHoP-RF;92.80

S5_vs_43207	ME	Duplodnaviria	Heunggongvirae	Uroviricota	Caudoviricetes	n.a.	Unclassified	O	O	30.09	d__Bacteria;p__Firmicutes;c__Clostridia;o__Oscillospirales;f__Butyricicoccaceae;g__Agathobaculum	91.1	blast;93.50 iPHoP-RF;70.60
S5_vs_46394	ME	n.a.	n.a.	n.a.	n.a.	n.a.	n.a.	n.a.	n.a.	8.87	d__Bacteria;p__Firmicutes;c__Clostridia;o__Christensenellales;f__QAND01;g__UMGS1975	90.3	blast;92.80 iPHoP-RF;62.70
S5_vs_4737	ME	n.a.	n.a.	n.a.	n.a.	n.a.	n.a.	n.a.	n.a.	20.64	d__Bacteria;p__Firmicutes;c__Clostridia;o__Christensenellales;f__CAG-74;g__Limiplasma	93.9	blast;95.70 iPHoP-RF;68.40
S5_vs_51416	ME	Duplodnaviria	Heunggongvirae	Uroviricota	Caudoviricetes	n.a.	Unclassified	O	O	11.18	d__Bacteria;p__Firmicutes;c__Bacilli;o__Erysipelotrichales;f__Erysipelotrichaceae;g__Holdemania	90.3	blast;92.80 CRISPR;73.80 iPHoP-RF;66.50
S5_vs_58766	ME	Duplodnaviria	Heunggongvirae	Uroviricota	Caudoviricetes	n.a.	Unclassified	O	O	24.99	d__Bacteria;p__Firmicutes;c__Clostridia;o__Lachnospirales;f__Lachnospiraceae;g__Frisingicoccus	93.8	blast;95.60 iPHoP-RF;76.60
S5_vs_59798	ME	Duplodnaviria	Heunggongvirae	Uroviricota	Caudoviricetes	n.a.	Unclassified	O	O	40.34	d__Bacteria;p__Firmicutes;c__Clostridia;o__Lachnospirales;f__Lachnospiraceae;g__Faecalimonas	91.4	blast;93.70 iPHoP-RF;91.10 CRISPR;78.90
S5_vs_60507	ME	Duplodnaviria	Heunggongvirae	Uroviricota	Caudoviricetes	n.a.	Unclassified	Unclassified	O	96.47	d__Bacteria;p__Firmicutes;c__Bacilli;o__Lactobacillales;f__Streptococcaceae;g__Streptococcus	100	RaFAH;99.70 blast;92.80 iPHoP-RF;91.10 CRISPR;52.30
S5_vs_63924	Shared	Duplodnaviria	Heunggongvirae	Uroviricota	Caudoviricetes	n.a.	Unclassified	Unclassified	Unclassified	98.89	d__Bacteria;p__Actinobacteriota;c__Actinomycetia;o__Actinomycetales;f__Bifidobacteriaceae;g__Bifidobacterium	99	CRISPR;97.70 iPHoP-RF;96.70 blast;95.40 RaFAH;82.40
S5_vs_6478	ME	Duplodnaviria	Heunggongvirae	Uroviricota	Caudoviricetes	n.a.	Unclassified	Unclassified	O	66.33	d__Bacteria;p__Firmicutes;c__Bacilli;o__Lactobacillales;f__Lactobacillaceae;g__Lactobacillus	100	RaFAH;99.60 iPHoP-RF;96.70 blast;95.40 CRISPR;68.80
S5_vs_65490	ME	n.a.	n.a.	n.a.	n.a.	n.a.	n.a.	n.a.	n.a.	3.66	d__Bacteria;p__Firmicutes;c__Clostridia;o__Oscillospirales;f__Oscillospiraceae;g__Flavonifractor	91.2	blast;93.60 iPHoP-RF;56.20
S5_vs_68254	ME	Duplodnaviria	Heunggongvirae	Uroviricota	Caudoviricetes	n.a.	Unclassified	O	O	58.14	d__Bacteria;p__Firmicutes;c__Clostridia;o__Lachnospirales;f__Lachnospiraceae;g__Blautia_A	93.8	blast;93.80 iPHoP-RF;81.90 RaFAH;64.30
S5_vs_69980	ME	Duplodnaviria	Heunggongvirae	Uroviricota	Caudoviricetes	n.a.	Unclassified	O	O	76.63	d__Bacteria;p__Firmicutes;c__Clostridia;o__Oscillospirales;f__Oscillospiraceae;g__Flavonifractor	95.3	blast;96.70 iPHoP-RF;96.40 CRISPR;95.20
S5_vs_72532	ME	Duplodnaviria	Heunggongvirae	Uroviricota	Caudoviricetes	n.a.	Unclassified	O	O	82.39	d__Bacteria;p__Bacteroidota;c__Bacteroidia;o__Bacteroidales;f__Bacteroidaceae;g__Bacteroides	99.4	blast;96.90 iPHoP-RF;96.70 RaFAH;92.20
S5_vs_74199	ME	n.a.	n.a.	n.a.	n.a.	n.a.	n.a.	n.a.	n.a.	13.11	d__Bacteria;p__Firmicutes;c__Clostridia;o__Oscillospirales;f__Oscillospiraceae;g__CAG-83	90.4	blast;92.90 iPHoP-RF;92.10
S5_vs_76500	Shared	Duplodnaviria	Heunggongvirae	Uroviricota	Caudoviricetes	n.a.	Unclassified	Unclassified	Unclassified	80.28	d__Bacteria;p__Bacteroidota;c__Bacteroidia;o__Bacteroidales;f__Rikenellaceae;g__Alistipes	99	blast;96.90 iPHoP-RF;94.40 RaFAH;87.40
S5_vs_77593	ME	Duplodnaviria	Heunggongvirae	Uroviricota	Caudoviricetes	n.a.	Unclassified	O	O	41.43	d__Bacteria;p__Firmicutes;c__Clostridia;o__Oscillospirales;f__Oscillospiraceae;g__CAG-103	90.6	blast;93.00 iPHoP-RF;71.70
S5_vs_79992	Shared	Duplodnaviria	Heunggongvirae	Uroviricota	Caudoviricetes	n.a.	Winoviridae	O	O	99.98	d__Bacteria;p__Bacteroidota;c__Bacteroidia;o__Bacteroidales;f__Rikenellaceae;g__Alistipes	100	RaFAH;98.20 blast;97.10 iPHoP-RF;96.70
S5_vs_80496	Shared	n.a.	n.a.	n.a.	n.a.	n.a.	n.a.	n.a.	n.a.	99.85	d__Bacteria;p__Bacteroidota;c__Bacteroidia;o__Bacteroidales;f__Bacteroidaceae;g__Bacteroides	99.7	blast;96.90 iPHoP-RF;96.10 RaFAH;95.90
S5_vs_80498	ME	Duplodnaviria	Heunggongvirae	Uroviricota	Caudoviricetes	n.a.	Unclassified	O	O	33.04	d__Bacteria;p__Firmicutes;c__Clostridia;o__Lachnospirales;f__Lachnospiraceae;g__Muricomes	93.2	blast;95.20 iPHoP-RF;90.50
S5_vs_8125	ME	Duplodnaviria	Heunggongvirae	Uroviricota	Caudoviricetes	n.a.	Unclassified	O	O	19.38	d__Bacteria;p__Firmicutes;c__Clostridia;o__Lachnospirales;f__Lachnospiraceae;g__Eubacterium_G	90.6	blast;93.00 iPHoP-RF;53.30
S5_vs_84574	ME	n.a.	n.a.	n.a.	n.a.	n.a.	n.a.	n.a.	n.a.	8.38	d__Bacteria;p__Firmicutes;c__Clostridia;o__Oscillospirales;f__Butyricicoccaceae;g__Agathobaculum	91.6	blast;93.90 iPHoP-RF;81.10
S5_vs_84946	ME	Duplodnaviria	Heunggongvirae	Uroviricota	Caudoviricetes	n.a.	Unclassified	O	O	94.46	d__Bacteria;p__Firmicutes;c__Clostridia;o__Lachnospirales;f__Lachnospiraceae;g__Dorea_A	90.2	blast;92.70 iPHoP-RF;83.60
S5_vs_850	ME	n.a.	n.a.	n.a.	n.a.	n.a.	n.a.	n.a.	n.a.	18.78	d__Bacteria;p__Firmicutes;c__Clostridia;o__UBA1212;f__UBA1255;g__Egerieisoma	92.7	blast;94.80 iPHoP-RF;85.10
S5_vs_85065	Shared	Duplodnaviria	Heunggongvirae	Uroviricota	Caudoviricetes	n.a.	Unclassified	O	O	67.47	d__Bacteria;p__Firmicutes;c__Clostridia;o__Oscillospirales;f__Ruminococcaceae;g__Ruthenibacterium	95.3	blast;96.70
S5_vs_86562	ME	Duplodnaviria	Heunggongvirae	Uroviricota	Caudoviricetes	n.a.	Unclassified	O	O	44.1	d__Bacteria;p__Verrucomicrobiota;c__Verrucomicrobiae;o__Verrucomicrobiales;f__Akkermansiaceae;g__Akkermansia	100	RaFAH;99.10 CRISPR;98.80 blast;96.90 iPHoP-RF;89.20
S5_vs_89540	ME	Duplodnaviria	Heunggongvirae	Uroviricota	Caudoviricetes	n.a.	Unclassified	O	O	26.84	d__Bacteria;p__Firmicutes;c__Bacilli;o__Lactobacillales;f__Lactobacillaceae;g__Limosilactobacillus	94.2	blast;93.40 iPHoP-RF;81.90 RaFAH;68.80
S5_vs_93839	Shared	Duplodnaviria	Heunggongvirae	Uroviricota	Caudoviricetes	n.a.	Unclassified	O	O	98.83	d__Bacteria;p__Bacteroidota;c__Bacteroidia;o__Bacteroidales;f__Rikenellaceae;g__Alistipes	100	RaFAH;98.90 iPHoP-RF;96.70 blast;95.50
S5_vs_95307	ME	Duplodnaviria	Heunggongvirae	Uroviricota	Caudoviricetes	n.a.	Unclassified	Unclassified	O	57.8	d__Bacteria;p__Firmicutes;c__Clostridia;o__Lachnospirales;f__Lachnospiraceae;g__Blautia	92	blast;91.60 iPHoP-RF;73.20 RaFAH;65.20
S5_vs_9747	Shared	n.a.	n.a.	n.a.	n.a.	n.a.	n.a.	n.a.	n.a.	6.96	d__Bacteria;p__Proteobacteria;c__Alphaproteobacteria;o__RF32;f__CAG-239;g__CAG-267	93	blast;95.00 iPHoP-RF;69.80
S5_vs_97574	Shared	Duplodnaviria	Heunggongvirae	Uroviricota	Caudoviricetes	n.a.	Unclassified	O	O	99.69	d__Bacteria;p__Firmicutes;c__Clostridia;o__Lachnospirales;f__Lachnospiraceae;g__Blautia_A	100	RaFAH;99.30 CRISPR;95.90 blast;93.60 iPHoP-RF;92.10
S6_vf_25710	Shared	n.a.	n.a.	n.a.	n.a.	n.a.	n.a.	n.a.	n.a.	89.8	d__Bacteria;p__Verrucomicrobiota;c__Verrucomicrobiae;o__Verrucomicrobiales;f__Akkermansiaceae;g__Akkermansia	99.7	CRISPR;97.30 RaFAH;94.60 blast;93.60 iPHoP-RF;72.60
S6_vf_56486	Shared	Duplodnaviria	Heunggongvirae	Uroviricota	Caudoviricetes	n.a.	Unclassified	O	O	65.79	d__Bacteria;p__Bacteroidota;c__Bacteroidia;o__Bacteroidales;f__Bacteroidaceae;g__Bacteroides	99.6	iPHoP-RF;96.10 RaFAH;94.70
S6_vs_1146	Shared	Duplodnaviria	Heunggongvirae	Uroviricota	Caudoviricetes	n.a.	Unclassified	O	O	50.09	d__Bacteria;p__Firmicutes;c__Clostridia;o__Oscillospirales;f__Ruminococcaceae;g__Faecalibacterium	98.4	blast;96.90 iPHoP-RF;86.00 RaFAH;81.40
S6_vs_12945	Shared	Duplodnaviria	Heunggongvirae	Uroviricota	Caudoviricetes	n.a.	Unclassified	O	O	99.74	d__Bacteria;p__Firmicutes;c__Clostridia;o__Lachnospirales;f__Lachnospiraceae;g__Roseburia	99.3	RaFAH;94.70 blast;88.50 iPHoP-RF;74.80
S6_vs_15390	Shared	Duplodnaviria	Heunggongvirae	Uroviricota	Caudoviricetes	n.a.	Unclassified	O	O	33.39	d__Bacteria;p__Bacteroidota;c__Bacteroidia;o__Bacteroidales;f__Tannerellaceae;g__Parabacteroides	90.8	blast;93.20 iPHoP-RF;92.10
S6_vs_15813	Shared	n.a.	n.a.	n.a.	n.a.	n.a.	n.a.	n.a.	n.a.	90.87	d__Bacteria;p__Firmicutes;c__Clostridia;o__Oscillospirales;f__Oscillospiraceae;g__Flavonifractor	99.3	blast;98.10 RaFAH;85.50 iPHoP-RF;85.10 CRISPR;56.80
S6_vs_29152	SHHC	Duplodnaviria	Heunggongvirae	Uroviricota	Caudoviricetes	n.a.	Unclassified	O	O	23.31	d__Bacteria;p__Firmicutes;c__Clostridia;o__Lachnospirales;f__Lachnospiraceae;g__Acetatifactor	91.2	blast;93.60
S6_vs_32886	Shared	n.a.	n.a.	n.a.	n.a.	n.a.	n.a.	n.a.	n.a.	6.93	d__Bacteria;p__Bacteroidota;c__Bacteroidia;o__Bacteroidales;f__Rikenellaceae;g__Alistipes_A	94.2	blast;95.90 iPHoP-RF;66.30
S6_vs_33711	SHHC	Duplodnaviria	Heunggongvirae	Uroviricota	Caudoviricetes	n.a.	Unclassified	Unclassified	Unclassified	86.98	d__Bacteria;p__Firmicutes;c__Clostridia;o__Oscillospirales;f__Ruminococcaceae;g__Ruthenibacterium	94	blast;95.80 iPHoP-RF;85.40 CRISPR;69.40
S6_vs_34453	Shared	Duplodnaviria	Heunggongvirae	Uroviricota	Caudoviricetes	n.a.	Unclassified	Unclassified	O	77	d__Bacteria;p__Firmicutes;c__Clostridia;o__Oscillospirales;f__Ruminococcaceae;g__Faecalibacterium	93.6	blast;95.50 iPHoP-RF;64.30
S6_vs_38250	SHHC	Duplodnaviria	Heunggongvirae	Uroviricota	Caudoviricetes	n.a.	Unclassified	Unclassified	Unclassified	15.25	d__Bacteria;p__Firmicutes;c__Clostridia;o__Oscillospirales;f__Ruminococcaceae;g__UBA3818	94.3	blast;96.00 iPHoP-RF;76.10
S6_vs_39025	Shared	Duplodnaviria	Heunggongvirae	Uroviricota	Caudoviricetes	n.a.	Unclassified	O	O	99.25	d__Bacteria;p__Firmicutes;c__Clostridia;o__Lachnospirales;f__Lachnospiraceae;g__Enterocloster	91.6	blast;93.90
S6_vs_39065	Shared	n.a.	n.a.	n.a.	n.a.	n.a.	n.a.	n.a.	n.a.	83.64	d__Bacteria;p__Proteobacteria;c__Alphaproteobacteria;o__RF32;f__CAG-239;g__CAG-267	94.6	blast;96.20 iPHoP-RF;75.20
S6_vs_44070	Shared	Unclassified	n.a.	n.a.	n.a.	n.a.	Unclassified	O	O	14.4	d__Bacteria;p__Proteobacteria;c__Alphaproteobacteria;o__RF32;f__CAG-239;g__51-20	94.7	blast;96.30 iPHoP-RF;72.60
S6_vs_46039	Shared	n.a.	n.a.	n.a.	n.a.	n.a.	n.a.	n.a.	n.a.	24.66	d__Bacteria;p__Bacteroidota;c__Bacteroidia;o__Bacteroidales;f__Bacteroidaceae;g__Bacteroides	93	CRISPR;93.00 blast;89.60 iPHoP-RF;82.50 RaFAH;64.10
S6_vs_46091	Shared	Duplodnaviria	Heunggongvirae	Uroviricota	Caudoviricetes	n.a.	Unclassified	Unclassified	O	25.96	d__Bacteria;p__Firmicutes;c__Clostridia;o__Oscillospirales;f__Ruminococcaceae;g__Negativibacillus	96.6	blast;97.60 iPHoP-RF;83.30 CRISPR;69.80
S6_vs_53966	SHHC	Duplodnaviria	Heunggongvirae	Uroviricota	Caudoviricetes	n.a.	Unclassified	O	O	51.17	d__Bacteria;p__Firmicutes;c__Clostridia;o__Oscillospirales;f__Ruminococcaceae;g__Faecalibacterium	98.7	blast;96.30 iPHoP-RF;90.50 RaFAH;86.50
S6_vs_54606	Shared	Duplodnaviria	Heunggongvirae	Uroviricota	Caudoviricetes	n.a.	Unclassified	O	O	99.52	d__Bacteria;p__Firmicutes;c__Clostridia;o__Lachnospirales;f__Lachnospiraceae;g__Blautia	97	RaFAH;91.10 blast;88.40
S6_vs_56485	Shared	Duplodnaviria	Heunggongvirae	Uroviricota	Caudoviricetes	n.a.	Unclassified	O	O	14.92	d__Bacteria;p__Firmicutes;c__Bacilli;o__Erysipelotrichales;f__Erysipelotrichaceae;g__Holdemania	94.7	CRISPR;96.30 blast;94.30 iPHoP-RF;79.80
S6_vs_56490	Shared	Duplodnaviria	Heunggongvirae	Uroviricota	Caudoviricetes	n.a.	Unclassified	O	O	26.23	d__Bacteria;p__Firmicutes;c__Clostridia;o__Oscillospirales;f__Acutalibacteraceae;g__UMGS1071	95.9	blast;97.10 iPHoP-RF;83.90 CRISPR;68.20
S6_vs_56491	Shared	Duplodnaviria	Heunggongvirae	Uroviricota	Caudoviricetes	n.a.	Unclassified	O	O	92.92	d__Bacteria;p__Firmicutes;c__Clostridia;o__Lachnospirales;f__Lachnospiraceae;g__Sellimonas	97.5	blast;98.20 iPHoP-RF;75.20
S6_vs_56492	Shared	n.a.	n.a.	n.a.	n.a.	n.a.	n.a.	n.a.	n.a.	27.91	d__Bacteria;p__Firmicutes;c__Clostridia;o__Oscillospirales;f__Butyricicoccaceae;g__Agathobaculum	98.2	blast;98.70 iPHoP-RF;66.80
S6_vs_56496	Shared	Duplodnaviria	Heunggongvirae	Uroviricota	Caudoviricetes	n.a.	Unclassified	Unclassified	Eponavirus	61.48	d__Bacteria;p__Firmicutes;c__Clostridia;o__Lachnospirales;f__Lachnospiraceae;g__Marvinbryantia	91.2	blast;93.60 iPHoP-RF;65.10
S6_vs_56498	Shared	Duplodnaviria	Heunggongvirae	Uroviricota	Caudoviricetes	n.a.	Unclassified	O	O	78.58	d__Bacteria;p__Firmicutes;c__Clostridia;o__Lachnospirales;f__Lachnospiraceae;g__Muricomes	91	blast;93.40 iPHoP-RF;69.80
S6_vs_9784	Shared	n.a.	n.a.	n.a.	n.a.	n.a.	n.a.	n.a.	n.a.	91.55	d__Bacteria;p__Bacteroidota;c__Bacteroidia;o__Bacteroidales;f__Bacteroidaceae;g__Bacteroides	100	RaFAH;97.10 iPHoP-RF;96.70 blast;93.90
S7_vf_114476	ME	Duplodnaviria	Heunggongvirae	Uroviricota	Caudoviricetes	n.a.	Unclassified	O	O	27.19	d__Bacteria;p__Firmicutes;c__Clostridia;o__Lachnospirales;f__Lachnospiraceae;g__Anaerobutyricum	94.3	blast;96.00 iPHoP-RF;74.30
S7_vf_117697	Shared	n.a.	n.a.	n.a.	n.a.	n.a.	n.a.	n.a.	n.a.	98.64	d__Bacteria;p__Bacteroidota;c__Bacteroidia;o__Bacteroidales;f__Rikenellaceae;g__Alistipes	96.9	CRISPR;97.80 iPHoP-RF;97.10 blast;96.80
S7_vf_134023	ME	n.a.	n.a.	n.a.	n.a.	n.a.	n.a.	n.a.	n.a.	12.46	d__Bacteria;p__Bacteroidota;c__Bacteroidia;o__Bacteroidales;f__Rikenellaceae;g__Alistipes_A	95	blast;96.50 iPHoP-RF;54.40
S7_vf_13614	Shared	Duplodnaviria	Heunggongvirae	Uroviricota	Caudoviricetes	n.a.	Unclassified	O	O	39.23	d__Bacteria;p__Firmicutes;c__Clostridia;o__Oscillospirales;f__Ruminococcaceae;g__Ruminococcus_D	95.4	blast;96.80 iPHoP-RF;95.40 CRISPR;50.20
S7_vf_138019	ME	n.a.	n.a.	n.a.	n.a.	n.a.	n.a.	n.a.	n.a.	8.23	d__Bacteria;p__Firmicutes;c__Clostridia;o__Oscillospirales;f__Oscillospiraceae;g__Lawsonibacter	91.1	blast;93.50 iPHoP-RF;50.40
S7_vf_138069	ME	n.a.	n.a.	n.a.	n.a.	n.a.	n.a.	n.a.	n.a.	78.93	d__Bacteria;p__Firmicutes;c__Bacilli;o__Lactobacillales;f__Streptococcaceae;g__Streptococcus	99.6	RaFAH;99.40 iPHoP-RF;67.30
S7_vf_27309	ME	Duplodnaviria	Heunggongvirae	Uroviricota	Caudoviricetes	n.a.	Unclassified	Unclassified	O	18.88	d__Bacteria;p__Bacteroidota;c__Bacteroidia;o__Bacteroidales;f__Bacteroidaceae;g__Bacteroides	96.3	CRISPR;97.40 iPHoP-RF;93.70
S7_vf_28429	Shared	Duplodnaviria	Heunggongvirae	Uroviricota	Caudoviricetes	n.a.	Unclassified	O	O	86.01	d__Bacteria;p__Bacteroidota;c__Bacteroidia;o__Bacteroidales;f__Tannerellaceae;g__Parabacteroides	100	CRISPR;99.00 RaFAH;98.30 iPHoP-RF;94.10 blast;93.90
S7_vf_29941	Shared	Duplodnaviria	Heunggongvirae	Uroviricota	Caudoviricetes	n.a.	Unclassified	O	O	79.47	d__Bacteria;p__Firmicutes;c__Bacilli;o__Lactobacillales;f__Streptococcaceae;g__Lactococcus	99.4	blast;97.20 RaFAH;91.90 iPHoP-RF;73.90
S7_vf_37279	Shared	Duplodnaviria	Heunggongvirae	Uroviricota	Caudoviricetes	n.a.	Unclassified	O	O	64.97	d__Bacteria;p__Bacteroidota;c__Bacteroidia;o__Bacteroidales;f__Bacteroidaceae;g__Bacteroides	99.7	CRISPR;98.80 iPHoP-RF;97.10 blast;90.80 RaFAH;87.70
S7_vf_37293	ME	Duplodnaviria	Heunggongvirae	Uroviricota	Caudoviricetes	n.a.	Unclassified	O	O	98.54	d__Bacteria;p__Firmicutes;c__Bacilli;o__Lactobacillales;f__Streptococcaceae;g__Streptococcus	100	RaFAH;99.60 CRISPR;99.00 iPHoP-RF;54.10
S7_vf_38697	ME	Duplodnaviria	Heunggongvirae	Uroviricota	Caudoviricetes	n.a.	Unclassified	O	O	61.28	d__Bacteria;p__Firmicutes;c__Bacilli;o__Erysipelotrichales;f__Erysipelatoclostridiaceae;g__Massiliomicrobiota	94.2	blast;95.90 iPHoP-RF;74.30
S7_vf_39724	Shared	Duplodnaviria	Heunggongvirae	Uroviricota	Caudoviricetes	n.a.	Unclassified	Unclassified	Mushuvirus	99.39	d__Bacteria;p__Firmicutes;c__Clostridia;o__Oscillospirales;f__Ruminococcaceae;g__Faecalibacterium	99.4	RaFAH;99.50
S7_vf_49007	ME	Duplodnaviria	Heunggongvirae	Uroviricota	Caudoviricetes	n.a.	Unclassified	O	O	85	d__Bacteria;p__Firmicutes;c__Bacilli;o__Lactobacillales;f__Streptococcaceae;g__Streptococcus	100	CRISPR;98.70 RaFAH;97.80 iPHoP-RF;68.00
S7_vf_49485	ME	Duplodnaviria	Heunggongvirae	Uroviricota	Caudoviricetes	n.a.	Unclassified	O	O	64.81	d__Bacteria;p__Firmicutes;c__Clostridia;o__Lachnospirales;f__Lachnospiraceae;g__Anaerostipes	98.5	CRISPR;98.90 iPHoP-RF;56.20
S7_vf_5801	Shared	Duplodnaviria	Heunggongvirae	Uroviricota	Caudoviricetes	n.a.	Unclassified	O	O	93.48	d__Bacteria;p__Bacteroidota;c__Bacteroidia;o__Bacteroidales;f__Bacteroidaceae;g__Bacteroides	100	RaFAH;96.60 iPHoP-RF;96.40 blast;91.00
S7_vf_5843	ME	Duplodnaviria	Heunggongvirae	Uroviricota	Caudoviricetes	n.a.	Unclassified	O	O	19.99	d__Bacteria;p__Actinobacteriota;c__Actinomycetia;o__Actinomycetales;f__Bifidobacteriaceae;g__Bifidobacterium	96.3	blast;97.40 CRISPR;97.30 iPHoP-RF;93.40
S7_vf_60968	ME	Duplodnaviria	Heunggongvirae	Uroviricota	Caudoviricetes	n.a.	Unclassified	O	O	71.38	d__Bacteria;p__Firmicutes;c__Clostridia;o__Lachnospirales;f__Lachnospiraceae;g__Ruminococcus_B	97.8	blast;98.40 iPHoP-RF;81.40 CRISPR;66.90
S7_vf_63894	ME	Duplodnaviria	Heunggongvirae	Uroviricota	Caudoviricetes	n.a.	Unclassified	Mccleskeyvirinae	Unaquatrovirus	77.84	d__Bacteria;p__Firmicutes;c__Bacilli;o__Lactobacillales;f__Lactobacillaceae;g__Leuconostoc	96.2	iPHoP-RF;93.70 RaFAH;78.60
S7_vf_67910	ME	n.a.	n.a.	n.a.	n.a.	n.a.	n.a.	n.a.	n.a.	11.86	d__Bacteria;p__Actinobacteriota;c__Coriobacteriia;o__Coriobacteriales;f__Eggerthellaceae;g__Eggerthella	94.5	blast;96.10
S7_vf_68239	ME	Duplodnaviria	Heunggongvirae	Uroviricota	Caudoviricetes	n.a.	Unclassified	O	O	31.22	d__Bacteria;p__Firmicutes;c__Bacilli;o__Lactobacillales;f__Streptococcaceae;g__Lactococcus	93.1	RaFAH;95.10
S7_vf_68546	ME	Duplodnaviria	Heunggongvirae	Uroviricota	Caudoviricetes	n.a.	Unclassified	O	O	29.45	d__Bacteria;p__Firmicutes;c__Clostridia;o__Lachnospirales;f__Lachnospiraceae;g__Faecalimonas	94.5	CRISPR;96.10 iPHoP-RF;76.10
S7_vf_71527	ME	n.a.	n.a.	n.a.	n.a.	n.a.	n.a.	n.a.	n.a.	3.4	d__Bacteria;p__Firmicutes;c__Clostridia;o__Oscillospirales;f__Ruminococcaceae;g__Ruminococcus_D	93.1	iPHoP-RF;95.10 blast;94.30 CRISPR;92.20
S7_vf_72493	ME	n.a.	n.a.	n.a.	n.a.	n.a.	n.a.	n.a.	n.a.	13.24	d__Bacteria;p__Firmicutes;c__Clostridia;o__Lachnospirales;f__Anaerotignaceae;g__UMGS1670	92.5	blast;94.60 iPHoP-RF;65.10
S7_vf_76463	Shared	n.a.	n.a.	n.a.	n.a.	n.a.	n.a.	n.a.	n.a.	42.93	d__Bacteria;p__Firmicutes;c__Clostridia;o__Oscillospirales;f__Oscillospiraceae;g__CAG-103	95.3	CRISPR;96.70
S7_vf_8332	ME	Duplodnaviria	Heunggongvirae	Uroviricota	Caudoviricetes	n.a.	Unclassified	O	O	10.6	d__Bacteria;p__Firmicutes;c__Clostridia;o__Oscillospirales;f__Ruminococcaceae;g__Ruminococcus_D	98.5	CRISPR;98.90 blast;96.20 iPHoP-RF;94.70
S7_vf_84974	ME	Duplodnaviria	Heunggongvirae	Uroviricota	Caudoviricetes	n.a.	Unclassified	O	O	91.7	d__Bacteria;p__Firmicutes;c__Clostridia;o__Oscillospirales;f__Ruminococcaceae;g__Faecalibacterium	99.9	RaFAH;98.40 blast;92.10 iPHoP-RF;63.30 CRISPR;56.10
S7_vf_89197	Shared	Duplodnaviria	Heunggongvirae	Uroviricota	Caudoviricetes	n.a.	Unclassified	Unclassified	Ceduovirus	89.48	d__Bacteria;p__Firmicutes;c__Bacilli;o__Lactobacillales;f__Streptococcaceae;g__Lactococcus	96.6	RaFAH;95.80 iPHoP-RF;71.50
S7_vs_101751	ME	n.a.	n.a.	n.a.	n.a.	n.a.	n.a.	n.a.	n.a.	99.91	d__Bacteria;p__Firmicutes;c__Clostridia;o__Lachnospirales;f__Lachnospiraceae;g__Blautia_A	99.7	RaFAH;94.40 blast;93.60 iPHoP-RF;76.80
S7_vs_103530	ME	n.a.	n.a.	n.a.	n.a.	n.a.	n.a.	n.a.	n.a.	35.56	d__Bacteria;p__Firmicutes;c__Clostridia;o__Oscillospirales;f__Butyricicoccaceae;g__Agathobaculum	94.2	blast;95.90 iPHoP-RF;60.30
S7_vs_11081	Shared	Duplodnaviria	Heunggongvirae	Uroviricota	Caudoviricetes	n.a.	Unclassified	Unclassified	Unclassified	95.36	d__Bacteria;p__Bacteroidota;c__Bacteroidia;o__Bacteroidales;f__Bacteroidaceae;g__Bacteroides	100	RaFAH;98.70 iPHoP-RF;94.70 blast;91.00
S7_vs_111831	ME	Duplodnaviria	Heunggongvirae	Uroviricota	Caudoviricetes	n.a.	Unclassified	O	O	38.83	d__Bacteria;p__Firmicutes;c__Clostridia;o__Oscillospirales;f__Ruminococcaceae;g__Gemmiger	97.2	blast;98.00 iPHoP-RF;88.90 CRISPR;82.40
S7_vs_118295	ME	n.a.	n.a.	n.a.	n.a.	n.a.	n.a.	n.a.	n.a.	43.98	d__Bacteria;p__Firmicutes;c__Clostridia;o__Oscillospirales;f__Oscillospiraceae;g__Intestinimonas	91.7	blast;94.00 iPHoP-RF;75.40
S7_vs_119679	Shared	Duplodnaviria	Heunggongvirae	Uroviricota	Caudoviricetes	n.a.	Winoviridae	O	O	99.98	d__Bacteria;p__Bacteroidota;c__Bacteroidia;o__Bacteroidales;f__Rikenellaceae;g__Alistipes	100	blast;98.70 RaFAH;98.60 iPHoP-RF;97.10
S7_vs_129271	ME	n.a.	n.a.	n.a.	n.a.	n.a.	n.a.	n.a.	n.a.	12.57	d__Bacteria;p__Firmicutes;c__Clostridia;o__Oscillospirales;f__Acutalibacteraceae;g__UBA1691	98.5	CRISPR;98.90 blast;97.30 iPHoP-RF;82.80
S7_vs_129858	ME	n.a.	n.a.	n.a.	n.a.	n.a.	n.a.	n.a.	n.a.	10.46	d__Bacteria;p__Firmicutes;c__Clostridia;o__Christensenellales;f__CAG-74;g__Firm-11	91.4	blast;93.70 iPHoP-RF;85.40 CRISPR;50.00
S7_vs_13041	ME	Duplodnaviria	Heunggongvirae	Uroviricota	Caudoviricetes	n.a.	Unclassified	O	O	17.74	d__Bacteria;p__Firmicutes;c__Bacilli;o__Erysipelotrichales;f__Erysipelatoclostridiaceae;g__Erysipelatoclostridium	94.5	blast;96.10 iPHoP-RF;74.60 CRISPR;54.40
S7_vs_13370	Shared	n.a.	n.a.	n.a.	n.a.	n.a.	n.a.	n.a.	n.a.	8.9	d__Bacteria;p__Firmicutes;c__Clostridia;o__Oscillospirales;f__Ruminococcaceae;g__Faecalibacterium	97	blast;97.90 iPHoP-RF;90.10 CRISPR;77.30
S7_vs_134391	ME	n.a.	n.a.	n.a.	n.a.	n.a.	n.a.	n.a.	n.a.	17.86	d__Bacteria;p__Firmicutes;c__Clostridia;o__Oscillospirales;f__UMGS1783;g__UMGS1783	94.9	blast;96.40 iPHoP-RF;84.80 CRISPR;79.10
S7_vs_134784	ME	Duplodnaviria	Heunggongvirae	Uroviricota	Caudoviricetes	n.a.	Unclassified	O	O	77.29	d__Bacteria;p__Firmicutes;c__Negativicutes;o__Veillonellales;f__Dialisteraceae;g__Dialister	97.3	CRISPR;97.40 blast;96.60 iPHoP-RF;92.80 RaFAH;63.90
S7_vs_135322	Shared	Duplodnaviria	Heunggongvirae	Uroviricota	Caudoviricetes	n.a.	Unclassified	Unclassified	O	34.72	d__Bacteria;p__Firmicutes;c__Clostridia;o__Oscillospirales;f__Ruminococcaceae;g__Faecalibacterium	99.7	CRISPR;98.30 blast;91.90 RaFAH;90.70 iPHoP-RF;90.50
S7_vs_135679	ME	n.a.	n.a.	n.a.	n.a.	n.a.	n.a.	n.a.	n.a.	23.81	d__Bacteria;p__Firmicutes;c__Clostridia;o__Lachnospirales;f__Lachnospiraceae;g__Blautia_A	99.6	blast;96.50 RaFAH;94.00 iPHoP-RF;90.10
S7_vs_135828	ME	Monodnaviria	Sangervirae	Phixviricota	Malgrandaviricetes	Petitvirales	Microviridae	Unclassified	Unclassified	3.2	d__Bacteria;p__Bacteroidota;c__Bacteroidia;o__Bacteroidales;f__Bacteroidaceae;g__Bacteroides	93	blast;95.00 iPHoP-RF;86.00
S7_vs_137611	ME	Duplodnaviria	Heunggongvirae	Uroviricota	Caudoviricetes	n.a.	Unclassified	O	O	29.3	d__Bacteria;p__Firmicutes;c__Clostridia;o__Lachnospirales;f__Lachnospiraceae;g__Anaerobutyricum	95	blast;96.50 iPHoP-RF;87.60
S7_vs_137915	Shared	Duplodnaviria	Heunggongvirae	Uroviricota	Caudoviricetes	n.a.	Unclassified	O	O	55.05	d__Bacteria;p__Bacteroidota;c__Bacteroidia;o__Bacteroidales;f__Tannerellaceae;g__Parabacteroides	98.1	CRISPR;98.30 blast;90.90 iPHoP-RF;85.40 RaFAH;59.50
S7_vs_13799	ME	Duplodnaviria	Heunggongvirae	Uroviricota	Caudoviricetes	n.a.	Unclassified	O	O	38.3	d__Bacteria;p__Firmicutes;c__Clostridia;o__Lachnospirales;f__Lachnospiraceae;g__Dorea_A	93	blast;95.00 iPHoP-RF;94.70 CRISPR;72.50

S7_vs_137998	Shared	n.a.	n.a.	n.a.	n.a.	n.a.	n.a.	n.a.	n.a.	42.1	d__Bacteria;p__Firmicutes;c__Clostridia;o__Lachnospirales;f__Lachnospiraceae;g__Blautia_A	93.4	blast;95.30 iPHoP-RF;78.00
S7_vs_138109	ME	Duplodnaviria	Heunggongvirae	Uroviricota	Caudoviricetes	n.a.	Unclassified	O	O	31.21	d__Bacteria;p__Firmicutes;c__Clostridia;o__Lachnospirales;f__Lachnospiraceae;g__Anaerobutyricum	91	blast;93.40 iPHoP-RF;75.90
S7_vs_138116	ME	n.a.	n.a.	n.a.	n.a.	n.a.	n.a.	n.a.	n.a.	5.66	d__Bacteria;p__Firmicutes;c__Clostridia;o__Lachnospirales;f__Lachnospiraceae;g__Mediterraneibacter	91.5	blast;93.80 iPHoP-RF;89.50
S7_vs_138173	Shared	Duplodnaviria	Heunggongvirae	Uroviricota	Caudoviricetes	n.a.	Unclassified	O	O	100	d__Bacteria;p__Bacteroidota;c__Bacteroidia;o__Bacteroidales;f__Rikenellaceae;g__Alistipes	100	RaFAH;98.50 blast;94.70 iPHoP-RF;74.10
S7_vs_16344	Shared	Duplodnaviria	Heunggongvirae	Uroviricota	Caudoviricetes	n.a.	Unclassified	O	O	12.56	d__Bacteria;p__Firmicutes;c__Clostridia;o__Oscillospirales;f__Acutalibacteraceae;g__Ruminococcus_E	98.8	CRISPR;99.10 blast;96.90 iPHoP-RF;95.70
S7_vs_21521	Shared	n.a.	n.a.	n.a.	n.a.	n.a.	n.a.	n.a.	n.a.	35.28	d__Bacteria;p__Firmicutes;c__Clostridia;o__Oscillospirales;f__Ruminococcaceae;g__Ruminiclostridium_E	90	blast;92.50 iPHoP-RF;74.80
S7_vs_2599	ME	n.a.	n.a.	n.a.	n.a.	n.a.	n.a.	n.a.	n.a.	89.5	d__Bacteria;p__Bacteroidota;c__Bacteroidia;o__Bacteroidales;f__Bacteroidaceae;g__Bacteroides	100	RaFAH;99.30 iPHoP-RF;94.40 blast;93.90
S7_vs_26757	Shared	n.a.	n.a.	n.a.	n.a.	n.a.	n.a.	n.a.	n.a.		d__Bacteria;p__Firmicutes;c__Clostridia;o__Oscillospirales;f__Ruminococcaceae;g__Faecalibacterium	94.6	blast;96.20 iPHoP-RF;85.10
S7_vs_28150	ME	n.a.	n.a.	n.a.	n.a.	n.a.	n.a.	n.a.	n.a.	12.25	d__Bacteria;p__Firmicutes;c__Clostridia;o__Oscillospirales;f__Oscillospiraceae;g__Dysosmobacter	94.2	blast;95.90 iPHoP-RF;74.10
S7_vs_28733	ME	Duplodnaviria	Heunggongvirae	Uroviricota	Caudoviricetes	n.a.	Unclassified	O	O	56.54	d__Bacteria;p__Firmicutes;c__Clostridia;o__Oscillospirales;f__Acutalibacteraceae;g__Hydrogeniiclostridium	95.2	blast;96.60 iPHoP-RF;62.20
S7_vs_29595	ME	Duplodnaviria	Heunggongvirae	Uroviricota	Caudoviricetes	n.a.	Unclassified	O	O	82.91	d__Bacteria;p__Verrucomicrobiota;c__Verrucomicrobiae;o__Verrucomicrobiales;f__Akkermansiaceae;g__Akkermansia	99.4	CRISPR;99.50 iPHoP-RF;57.30
S7_vs_30640	ME	Duplodnaviria	Heunggongvirae	Uroviricota	Caudoviricetes	n.a.	Unclassified	O	O	25.04	d__Bacteria;p__Firmicutes;c__Clostridia;o__Lachnospirales;f__Lachnospiraceae;g__Eubacterium_G	94.3	blast;96.00 iPHoP-RF;75.20
S7_vs_30665	ME	n.a.	n.a.	n.a.	n.a.	n.a.	n.a.	n.a.	n.a.	8.94	d__Bacteria;p__Firmicutes;c__Clostridia;o__Christensenellales;f__CAG-74;g__UBA11524	93.8	CRISPR;95.60 blast;92.80 iPHoP-RF;92.40
S7_vs_38113	ME	Duplodnaviria	Heunggongvirae	Uroviricota	Caudoviricetes	n.a.	Unclassified	O	O	17.89	d__Bacteria;p__Firmicutes;c__Clostridia;o__Oscillospirales;f__Acutalibacteraceae;g__Hydrogeniiclostridium	91.7	blast;94.00 iPHoP-RF;64.50
S7_vs_38834	ME	Duplodnaviria	Heunggongvirae	Uroviricota	Caudoviricetes	n.a.	Unclassified	O	O	25.72	d__Bacteria;p__Firmicutes;c__Clostridia;o__Lachnospirales;f__Lachnospiraceae;g__RUG115	91.5	blast;93.80
S7_vs_41098	Shared	Duplodnaviria	Heunggongvirae	Uroviricota	Caudoviricetes	n.a.	Unclassified	O	O	97.6	d__Bacteria;p__Firmicutes;c__Clostridia;o__Lachnospirales;f__Lachnospiraceae;g__Mediterraneibacter	95.4	blast;96.80
S7_vs_42563	Shared	n.a.	n.a.	n.a.	n.a.	n.a.	n.a.	n.a.	n.a.	93.91	d__Bacteria;p__Firmicutes;c__Clostridia;o__Oscillospirales;f__Ruminococcaceae;g__Gemmiger	96.3	blast;96.80 iPHoP-RF;75.40 RaFAH;59.00
S7_vs_47050	ME	Duplodnaviria	Heunggongvirae	Uroviricota	Caudoviricetes	n.a.	Unclassified	O	O	16.97	d__Bacteria;p__Firmicutes;c__Clostridia;o__Oscillospirales;f__Acutalibacteraceae;g__CAG-180	98.7	CRISPR;99.00 blast;96.60 iPHoP-RF;92.40
S7_vs_48523	ME	Duplodnaviria	Heunggongvirae	Uroviricota	Caudoviricetes	n.a.	Unclassified	O	O	66.97	d__Bacteria;p__Proteobacteria;c__Gammaproteobacteria;o__Burkholderiales;f__Burkholderiaceae;g__Sutterella	91.6	blast;93.90
S7_vs_48708	ME	Duplodnaviria	Heunggongvirae	Uroviricota	Caudoviricetes	n.a.	Unclassified	O	O	42.25	d__Bacteria;p__Firmicutes;c__Clostridia;o__Lachnospirales;f__Lachnospiraceae;g__Blautia_A	90.9	blast;93.30 iPHoP-RF;69.50
S7_vs_49426	Shared	Duplodnaviria	Heunggongvirae	Uroviricota	Caudoviricetes	n.a.	Unclassified	O	O	18.87	d__Bacteria;p__Firmicutes;c__Clostridia;o__Lachnospirales;f__Lachnospiraceae;g__Agathobacter	91.5	blast;93.80 iPHoP-RF;71.30
S7_vs_4956	Shared	Duplodnaviria	Heunggongvirae	Uroviricota	Caudoviricetes	n.a.	Unclassified	Unclassified	Eponavirus	54.17	d__Bacteria;p__Firmicutes;c__Clostridia;o__Oscillospirales;f__Oscillospiraceae;g__	91.6	blast;93.90 iPHoP-RF;83.30
S7_vs_50194	Shared	n.a.	n.a.	n.a.	n.a.	n.a.	n.a.	n.a.	n.a.	18.68	d__Bacteria;p__Bacteroidota;c__Bacteroidia;o__Bacteroidales;f__Rikenellaceae;g__Alistipes_A	96.9	blast;90.90 RaFAH;87.90 iPHoP-RF;85.70
S7_vs_51332	ME	n.a.	n.a.	n.a.	n.a.	n.a.	n.a.	n.a.	n.a.	13.21	d__Bacteria;p__Bacteroidota;c__Bacteroidia;o__Bacteroidales;f__Coprobacteraceae;g__Coprobacter	94.5	blast;96.10 iPHoP-RF;53.30
S7_vs_5236	ME	Duplodnaviria	Heunggongvirae	Uroviricota	Caudoviricetes	n.a.	Unclassified	O	O	93.85	d__Bacteria;p__Firmicutes;c__Clostridia;o__Oscillospirales;f__Oscillospiraceae;g__CAG-103	93.5	CRISPR;95.40
S7_vs_54322	ME	n.a.	n.a.	n.a.	n.a.	n.a.	n.a.	n.a.	n.a.	65.6	d__Bacteria;p__Bacteroidota;c__Bacteroidia;o__Bacteroidales;f__Rikenellaceae;g__Alistipes	98.7	blast;96.80 CRISPR;95.60 iPHoP-RF;87.30 RaFAH;84.00
S7_vs_55031	ME	n.a.	n.a.	n.a.	n.a.	n.a.	n.a.	n.a.	n.a.	23.56	d__Bacteria;p__Bacteroidota;c__Bacteroidia;o__Bacteroidales;f__Tannerellaceae;g__Parabacteroides	98.1	blast;98.60 CRISPR;97.70 iPHoP-RF;89.20
S7_vs_55526	ME	n.a.	n.a.	n.a.	n.a.	n.a.	n.a.	n.a.	n.a.	22.37	d__Bacteria;p__Actinobacteriota;c__Coriobacteriia;o__Coriobacteriales;f__Coriobacteriaceae;g__Collinsella	98.7	CRISPR;99.00 blast;93.80 iPHoP-RF;84.50
S7_vs_557	ME	Duplodnaviria	Heunggongvirae	Uroviricota	Caudoviricetes	n.a.	Unclassified	O	O	66.78	d__Bacteria;p__Bacteroidota;c__Bacteroidia;o__Bacteroidales;f__Bacteroidaceae;g__Bacteroides	99.3	iPHoP-RF;97.10 blast;93.70 RaFAH;89.50
S7_vs_60775	ME	Duplodnaviria	Heunggongvirae	Uroviricota	Caudoviricetes	n.a.	Unclassified	O	O	62.59	d__Bacteria;p__Firmicutes;c__Clostridia;o__Lachnospirales;f__Lachnospiraceae;g__Eubacterium_F	91.6	blast;93.90
S7_vs_65719	Shared	Duplodnaviria	Heunggongvirae	Uroviricota	Caudoviricetes	n.a.	Unclassified	O	O	47.93	d__Bacteria;p__Firmicutes;c__Clostridia;o__Lachnospirales;f__Lachnospiraceae;g__Enterocloster	95.7	CRISPR;97.00 blast;94.90 iPHoP-RF;88.90
S7_vs_69630	ME	Duplodnaviria	Heunggongvirae	Uroviricota	Caudoviricetes	n.a.	Unclassified	O	O	35.61	d__Bacteria;p__Firmicutes;c__Clostridia;o__Oscillospirales;f__Oscillospiraceae;g__Intestinimonas	94.7	blast;96.30 iPHoP-RF;65.10
S7_vs_72737	ME	n.a.	n.a.	n.a.	n.a.	n.a.	n.a.	n.a.	n.a.	3.91	d__Bacteria;p__Firmicutes;c__Clostridia;o__Oscillospirales;f__Acutalibacteraceae;g__Ruminococcus_E	97.9	blast;98.50 iPHoP-RF;96.40
S7_vs_73280	ME	Duplodnaviria	Heunggongvirae	Uroviricota	Caudoviricetes	n.a.	Unclassified	Unclassified	Minunavirus	86.44	d__Bacteria;p__Actinobacteriota;c__Actinomycetia;o__Actinomycetales;f__Bifidobacteriaceae;g__Bifidobacterium	99.1	CRISPR;99.30 iPHoP-RF;97.10 blast;96.90
S7_vs_78507	Shared	n.a.	n.a.	n.a.	n.a.	n.a.	n.a.	n.a.	n.a.	36.34	d__Bacteria;p__Firmicutes;c__Clostridia;o__Oscillospirales;f__Oscillospiraceae;g__Intestinimonas	90.8	blast;93.20 iPHoP-RF;70.90
S7_vs_83100	ME	Duplodnaviria	Heunggongvirae	Uroviricota	Caudoviricetes	n.a.	Unclassified	O	O	97.77	d__Bacteria;p__Firmicutes;c__Clostridia;o__Oscillospirales;f__Oscillospiraceae;g__Flavonifractor	97.8	blast;98.40 iPHoP-RF;85.10 CRISPR;74.00
S7_vs_83474	ME	Duplodnaviria	Heunggongvirae	Uroviricota	Caudoviricetes	n.a.	Unclassified	Unclassified	O	31.73	d__Bacteria;p__Firmicutes;c__Clostridia;o__Oscillospirales;f__Oscillospiraceae;g__Flavonifractor	93.9	blast;95.70 iPHoP-RF;90.80
S7_vs_86377	ME	Duplodnaviria	Heunggongvirae	Uroviricota	Caudoviricetes	n.a.	Unclassified	O	O	91.04	d__Bacteria;p__Firmicutes;c__Clostridia;o__Lachnospirales;f__Lachnospiraceae;g__Bariatricus	92	blast;94.20 iPHoP-RF;79.50
S7_vs_87279	ME	Duplodnaviria	Heunggongvirae	Uroviricota	Caudoviricetes	n.a.	Unclassified	Unclassified	O	66.77	d__Bacteria;p__Firmicutes;c__Clostridia;o__Oscillospirales;f__Ruminococcaceae;g__Faecalibacterium	95.2	blast;96.60 iPHoP-RF;62.50
S7_vs_87439	ME	n.a.	n.a.	n.a.	n.a.	n.a.	n.a.	n.a.	n.a.	31.68	d__Bacteria;p__Verrucomicrobiota;c__Lentisphaeria;o__Victivallales;f__Victivallaceae;g__Victivallis	90.2	blast;92.70 iPHoP-RF;71.10
S7_vs_944	ME	n.a.	n.a.	n.a.	n.a.	n.a.	n.a.	n.a.	n.a.	66.43	d__Bacteria;p__Bacteroidota;c__Bacteroidia;o__Bacteroidales;f__Bacteroidaceae;g__Bacteroides	100	RaFAH;98.90 iPHoP-RF;96.70 blast;91.80
S7_vs_94738	ME	Duplodnaviria	Heunggongvirae	Uroviricota	Caudoviricetes	n.a.	Unclassified	Unclassified	O	58.62	d__Bacteria;p__Firmicutes;c__Clostridia;o__Oscillospirales;f__Oscillospiraceae;g__Flavonifractor	91.2	blast;93.60 iPHoP-RF;71.30
S7_vs_97737	ME	n.a.	n.a.	n.a.	n.a.	n.a.	n.a.	n.a.	n.a.	56.07	d__Bacteria;p__Firmicutes;c__Clostridia;o__Oscillospirales;f__Ruminococcaceae;g__Faecalibacterium	95.6	blast;96.90 iPHoP-RF;91.80
S8_vf_101283	SHHC	Duplodnaviria	Heunggongvirae	Uroviricota	Caudoviricetes	n.a.	Unclassified	Unclassified	Toutatisvirus	99.72	d__Bacteria;p__Firmicutes;c__Clostridia;o__Oscillospirales;f__Ruminococcaceae;g__Faecalibacterium	100	RaFAH;99.60 CRISPR;98.20 blast;95.50 iPHoP-RF;84.80
S8_vf_102508	SHHC	n.a.	n.a.	n.a.	n.a.	n.a.	n.a.	n.a.	n.a.	92.39	d__Bacteria;p__Actinobacteriota;c__Actinomycetia;o__Actinomycetales;f__Micrococcaceae;g__Rothia	100	RaFAH;99.40 CRISPR;98.40 iPHoP-RF;80.80
S8_vf_111790	Shared	Duplodnaviria	Heunggongvirae	Uroviricota	Caudoviricetes	n.a.	Unclassified	O	O	20.45	d__Bacteria;p__Firmicutes;c__Clostridia;o__Oscillospirales;f__Oscillospiraceae;g__Flavonifractor	91.6	blast;93.90 iPHoP-RF;81.10
S8_vf_117796	Shared	Duplodnaviria	Heunggongvirae	Uroviricota	Caudoviricetes	n.a.	Unclassified	Unclassified	Unclassified	71.62	d__Bacteria;p__Bacteroidota;c__Bacteroidia;o__Bacteroidales;f__Bacteroidaceae;g__Prevotella	91.1	blast;93.50 iPHoP-RF;88.50
S8_vf_122206	Shared	Duplodnaviria	Heunggongvirae	Uroviricota	Caudoviricetes	n.a.	Unclassified	O	O	16.65	d__Bacteria;p__Bacteroidota;c__Bacteroidia;o__Bacteroidales;f__Bacteroidaceae;g__Prevotella	94.7	blast;96.30 iPHoP-RF;90.50
S8_vf_129423	SHHC	Duplodnaviria	Heunggongvirae	Uroviricota	Caudoviricetes	n.a.	Unclassified	Mccleskeyvirinae	Unaquatrovirus	87.49	d__Bacteria;p__Firmicutes;c__Bacilli;o__Lactobacillales;f__Lactobacillaceae;g__Leuconostoc	97.5	iPHoP-RF;93.70 RaFAH;86.10
S8_vf_131009	SHHC	Duplodnaviria	Heunggongvirae	Uroviricota	Caudoviricetes	n.a.	Unclassified	O	O	24.39	d__Bacteria;p__Firmicutes;c__Clostridia;o__Lachnospirales;f__Lachnospiraceae;g__Eubacterium_F	97.2	CRISPR;98.00 blast;96.80 iPHoP-RF;95.40
S8_vf_132697	Shared	Duplodnaviria	Heunggongvirae	Uroviricota	Caudoviricetes	n.a.	Unclassified	O	O	84.84	d__Bacteria;p__Firmicutes;c__Clostridia;o__Lachnospirales;f__Lachnospiraceae;g__Agathobacter	99.9	CRISPR;98.90 RaFAH;92.10 blast;90.20 iPHoP-RF;86.30
S8_vf_134943	SHHC	n.a.	n.a.	n.a.	n.a.	n.a.	n.a.	n.a.	n.a.	81.72	d__Bacteria;p__Firmicutes;c__Bacilli;o__Lactobacillales;f__Streptococcaceae;g__Streptococcus	99.7	RaFAH;99.40 iPHoP-RF;72.60
S8_vf_13709	SHHC	Duplodnaviria	Heunggongvirae	Uroviricota	Caudoviricetes	n.a.	Unclassified	O	O	11.95	d__Bacteria;p__Firmicutes;c__Clostridia;o__TANB77;f__CAG-508;g__CAG-354	90.2	blast;92.70 iPHoP-RF;70.60
S8_vf_13758	SHHC	Duplodnaviria	Heunggongvirae	Uroviricota	Caudoviricetes	n.a.	Unclassified	O	O	11.78	d__Bacteria;p__Firmicutes;c__Clostridia;o__TANB77;f__CAG-508;g__CAG-354	93.9	blast;95.70 iPHoP-RF;71.70
S8_vf_142700	SHHC	Unclassified	n.a.	n.a.	n.a.	n.a.	Unclassified	O	O	5.82	d__Bacteria;p__Actinobacteriota;c__Coriobacteriia;o__Coriobacteriales;f__Coriobacteriaceae;g__Collinsella	99.4	CRISPR;99.50 iPHoP-RF;93.40 blast;91.90
S8_vf_151384	SHHC	Duplodnaviria	Heunggongvirae	Uroviricota	Caudoviricetes	n.a.	Unclassified	Unclassified	Eponavirus	47.18	d__Bacteria;p__Firmicutes;c__Clostridia;o__Oscillospirales;f__Ruminococcaceae;g__Gemmiger	96.3	CRISPR;97.40 blast;92.00 iPHoP-RF;89.80
S8_vf_153696	Shared	n.a.	n.a.	n.a.	n.a.	n.a.	n.a.	n.a.	n.a.	11.92	d__Bacteria;p__Actinobacteriota;c__Actinomycetia;o__Actinomycetales;f__Bifidobacteriaceae;g__Bifidobacterium	91.2	blast;93.60
S8_vf_158225	Shared	Duplodnaviria	Heunggongvirae	Uroviricota	Caudoviricetes	Crassvirales	Intestiviridae	Crudevirinae	Diorhovirus	14.69	d__Bacteria;p__Bacteroidota;c__Bacteroidia;o__Bacteroidales;f__Tannerellaceae;g__Parabacteroides	94	blast;95.80
S8_vf_161703	SHHC	Duplodnaviria	Heunggongvirae	Uroviricota	Caudoviricetes	n.a.	Unclassified	O	O	49.06	d__Bacteria;p__Firmicutes;c__Clostridia;o__Oscillospirales;f__Ruminococcaceae;g__Ruminococcus_D	99.1	CRISPR;97.90 blast;96.80 iPHoP-RF;96.40 RaFAH;83.30
S8_vf_16172	SHHC	n.a.	n.a.	n.a.	n.a.	n.a.	n.a.	n.a.	n.a.	45.4	d__Bacteria;p__Bacteroidota;c__Bacteroidia;o__Bacteroidales;f__Tannerellaceae;g__Parabacteroides	95.9	iPHoP-RF;97.10 blast;96.90 CRISPR;92.40
S8_vf_166403	Shared	n.a.	n.a.	n.a.	n.a.	n.a.	n.a.	n.a.	n.a.	69.48	d__Bacteria;p__Firmicutes;c__Clostridia;o__Oscillospirales;f__Oscillospiraceae;g__Flavonifractor	95.2	blast;96.60 iPHoP-RF;72.20
S8_vf_166677	Shared	Duplodnaviria	Heunggongvirae	Uroviricota	Caudoviricetes	Crassvirales	Intestiviridae	Obtuvirinae	Fohxhuevirus	20.94	d__Bacteria;p__Bacteroidota;c__Bacteroidia;o__Bacteroidales;f__Bacteroidaceae;g__Prevotella	99.9	blast;98.70 CRISPR;96.60 iPHoP-RF;93.10 RaFAH;90.50
S8_vf_170878	Shared	Duplodnaviria	Heunggongvirae	Uroviricota	Caudoviricetes	n.a.	Unclassified	Unclassified	Toutatisvirus	39.33	d__Bacteria;p__Firmicutes;c__Clostridia;o__Oscillospirales;f__Oscillospiraceae;g__CAG-83	91.2	blast;93.60 iPHoP-RF;65.60
S8_vf_183045	Shared	Duplodnaviria	Heunggongvirae	Uroviricota	Caudoviricetes	n.a.	Unclassified	Unclassified	Unclassified	35.16	d__Bacteria;p__Bacteroidota;c__Bacteroidia;o__Bacteroidales;f__Bacteroidaceae;g__Prevotella	98.5	CRISPR;98.90 blast;93.00 iPHoP-RF;90.50
S8_vf_187742	SHHC	Duplodnaviria	Heunggongvirae	Uroviricota	Caudoviricetes	n.a.	Unclassified	O	O	91.82	d__Bacteria;p__Firmicutes;c__Clostridia;o__Oscillospirales;f__Ruminococcaceae;g__Faecalibacterium	99.6	blast;96.20 RaFAH;95.10 CRISPR;86.20 iPHoP-RF;65.30
S8_vf_195032	SHHC	Duplodnaviria	Heunggongvirae	Uroviricota	Caudoviricetes	n.a.	Unclassified	O	O	5.64	d__Bacteria;p__Actinobacteriota;c__Coriobacteriia;o__Coriobacteriales;f__Eggerthellaceae;g__Gordonibacter	94.9	CRISPR;96.40 iPHoP-RF;75.70
S8_vf_197061	Shared	Duplodnaviria	Heunggongvirae	Uroviricota	Caudoviricetes	n.a.	Unclassified	Unclassified	Unclassified	60.03	d__Bacteria;p__Actinobacteriota;c__Actinomycetia;o__Actinomycetales;f__Bifidobacteriaceae;g__Bifidobacterium	98.8	CRISPR;99.00 iPHoP-RF;97.10 blast;96.80 RaFAH;52.60
S8_vf_201695	Shared	n.a.	n.a.	n.a.	n.a.	n.a.	n.a.	n.a.	n.a.	30.61	d__Bacteria;p__Firmicutes;c__Clostridia;o__Lachnospirales;f__Anaerotignaceae;g__Anaerotignum	94.2	blast;95.90 iPHoP-RF;76.40
S8_vf_208110	Shared	Duplodnaviria	Heunggongvirae	Uroviricota	Caudoviricetes	n.a.	Unclassified	O	O	89.6	d__Bacteria;p__Firmicutes;c__Clostridia;o__Oscillospirales;f__Oscillospiraceae;g__Intestinimonas	92	blast;94.20 iPHoP-RF;50.20
S8_vf_208111	Shared	n.a.	n.a.	n.a.	n.a.	n.a.	n.a.	n.a.	n.a.	7.17	d__Bacteria;p__Desulfobacterota;c__Desulfovibrionia;o__Desulfovibrionales;f__Desulfovibrionaceae;g__Bilophila	90.8	CRISPR;93.20 iPHoP-RF;60.30
S8_vf_208115	SHHC	Monodnaviria	Sangervirae	Phixviricota	Malgrandaviricetes	Petitvirales	Microviridae	O	O		d__Bacteria;p__Bacteroidota;c__Bacteroidia;o__Bacteroidales;f__Barnesiellaceae;g__Barnesiella	94.2	blast;95.90 iPHoP-RF;72.80
S8_vf_208119	SHHC	Monodnaviria	Sangervirae	Phixviricota	Malgrandaviricetes	Petitvirales	Microviridae	Gokushovirinae	Gokushovirus	18.24	d__Bacteria;p__Firmicutes;c__Clostridia;o__Oscillospirales;f__Ruminococcaceae;g__Faecalibacterium	97.2	CRISPR;97.90 RaFAH;52.10
S8_vf_208120	Shared	Duplodnaviria	Heunggongvirae	Uroviricota	Caudoviricetes	n.a.	Unclassified	O	O	99.62	d__Bacteria;p__Bacteroidota;c__Bacteroidia;o__Bacteroidales;f__Bacteroidaceae;g__Bacteroides	98.2	RaFAH;93.90 blast;88.30 iPHoP-RF;53.80
S8_vf_208126	SHHC	Duplodnaviria	Heunggongvirae	Uroviricota	Caudoviricetes	n.a.	Unclassified	O	O	48.51	d__Bacteria;p__Firmicutes;c__Clostridia;o__Oscillospirales;f__Ruminococcaceae;g__Gemmiger	91.6	blast;93.90 iPHoP-RF;60.90
S8_vf_208146	SHHC	Monodnaviria	Sangervirae	Phixviricota	Malgrandaviricetes	Petitvirales	Microviridae	Gokushovirinae	Unclassified	25.58	d__Bacteria;p__Firmicutes;c__Negativicutes;o__Acidaminococcales;f__Acidaminococcaceae;g__Phascolarctobacterium	93.1	CRISPR;95.10
S8_vf_28956	Shared	n.a.	n.a.	n.a.	n.a.	n.a.	n.a.	n.a.	n.a.	91.89	d__Bacteria;p__Bacteroidota;c__Bacteroidia;o__Bacteroidales;f__Bacteroidaceae;g__Bacteroides	99.6	iPHoP-RF;96.40 RaFAH;94.20 blast;93.80
S8_vf_45776	Shared	Duplodnaviria	Heunggongvirae	Uroviricota	Caudoviricetes	n.a.	Unclassified	Unclassified	Skunavirus	88.6	d__Bacteria;p__Firmicutes;c__Bacilli;o__Lactobacillales;f__Streptococcaceae;g__Lactococcus	100	RaFAH;99.20 blast;98.30 iPHoP-RF;73.90
S8_vf_55476	Shared	n.a.	n.a.	n.a.	n.a.	n.a.	n.a.	n.a.	n.a.	64.72	d__Bacteria;p__Firmicutes;c__Clostridia;o__Oscillospirales;f__Oscillospiraceae;g__Intestinimonas	91.6	blast;93.90 iPHoP-RF;82.20
S8_vf_65522	Shared	n.a.	n.a.	n.a.	n.a.	n.a.	n.a.	n.a.	n.a.	12.03	d__Bacteria;p__Firmicutes;c__Clostridia;o__Christensenellales;f__CAG-74;g__UBA11524	97.2	blast;98.00 iPHoP-RF;83.10 CRISPR;55.60
S8_vf_7243	SHHC	n.a.	n.a.	n.a.	n.a.	n.a.	n.a.	n.a.	n.a.	95.82	d__Bacteria;p__Actinobacteriota;c__Actinomycetia;o__Actinomycetales;f__Micrococcaceae;g__Rothia	99.4	RaFAH;99.50
S8_vf_79769	Shared	n.a.	n.a.	n.a.	n.a.	n.a.	n.a.	n.a.	n.a.	31.62	d__Bacteria;p__Bacteroidota;c__Bacteroidia;o__Bacteroidales;f__Bacteroidaceae;g__Prevotella	98.4	CRISPR;98.80 blast;96.70 iPHoP-RF;92.40
S8_vf_93976	SHHC	n.a.	n.a.	n.a.	n.a.	n.a.	n.a.	n.a.	n.a.	9.53	d__Bacteria;p__Firmicutes;c__Clostridia;o__Christensenellales;f__CAG-138;g__UBA1685	95.3	blast;96.70 iPHoP-RF;66.50 CRISPR;59.10
S8_vs_102608	SHHC	n.a.	n.a.	n.a.	n.a.	n.a.	n.a.	n.a.	n.a.	20	d__Bacteria;p__Firmicutes;c__Clostridia;o__Oscillospirales;f__Ruminococcaceae;g__Gemmiger	95.7	blast;97.00 CRISPR;96.10 iPHoP-RF;90.80
S8_vs_115958	Shared	Duplodnaviria	Heunggongvirae	Uroviricota	Caudoviricetes	n.a.	Unclassified	Unclassified	O	68.75	d__Bacteria;p__Firmicutes;c__Clostridia;o__Oscillospirales;f__Acutalibacteraceae;g__Ruminococcus_E	91.6	blast;93.90 iPHoP-RF;67.00
S8_vs_125717	Shared	n.a.	n.a.	n.a.	n.a.	n.a.	n.a.	n.a.	n.a.	17.94	d__Bacteria;p__Firmicutes;c__Clostridia;o__Oscillospirales;f__Oscillospiraceae;g__CAG-83	95.6	blast;96.90 iPHoP-RF;64.30
S8_vs_126582	SHHC	Duplodnaviria	Heunggongvirae	Uroviricota	Caudoviricetes	n.a.	Unclassified	O	O	98.15	d__Bacteria;p__Bacteroidota;c__Bacteroidia;o__Bacteroidales;f__Tannerellaceae;g__Parabacteroides	100	RaFAH;99.70 iPHoP-RF;89.20 blast;86.20 CRISPR;69.40
S8_vs_134805	Shared	Duplodnaviria	Heunggongvirae	Uroviricota	Caudoviricetes	n.a.	Unclassified	Unclassified	Mushuvirus	91.02	d__Bacteria;p__Firmicutes;c__Clostridia;o__Oscillospirales;f__Oscillospiraceae;g__Lawsonibacter	91.5	blast;93.80 iPHoP-RF;73.20
S8_vs_145173	SHHC	Duplodnaviria	Heunggongvirae	Uroviricota	Caudoviricetes	n.a.	Unclassified	O	O	54.47	d__Bacteria;p__Firmicutes;c__Clostridia;o__Oscillospirales;f__Oscillospiraceae;g__UBA9475	91.2	blast;93.60 iPHoP-RF;59.00
S8_vs_147670	SHHC	Duplodnaviria	Heunggongvirae	Uroviricota	Caudoviricetes	n.a.	Unclassified	O	O	31.23	d__Bacteria;p__Firmicutes;c__Negativicutes;o__Veillonellales;f__Megasphaeraceae;g__Caecibacter	96.7	CRISPR;97.70 blast;78.80
S8_vs_147697	Shared	n.a.	n.a.	n.a.	n.a.	n.a.	n.a.	n.a.	n.a.	41.71	d__Bacteria;p__Bacteroidota;c__Bacteroidia;o__Bacteroidales;f__Bacteroidaceae;g__Bacteroides	98.8	iPHoP-RF;95.70 blast;90.90 RaFAH;89.00
S8_vs_148389	SHHC	Duplodnaviria	Heunggongvirae	Uroviricota	Caudoviricetes	n.a.	Unclassified	O	O	54.68	d__Bacteria;p__Bacteroidota;c__Bacteroidia;o__Bacteroidales;f__Bacteroidaceae;g__Prevotella	99.6	blast;96.80 RaFAH;94.40 iPHoP-RF;91.80
S8_vs_173961	Shared	Duplodnaviria	Heunggongvirae	Uroviricota	Caudoviricetes	n.a.	Unclassified	O	O	95.47	d__Bacteria;p__Firmicutes;c__Clostridia;o__Lachnospirales;f__Lachnospiraceae;g__Agathobacter	99.6	CRISPR;98.40 blast;90.90 RaFAH;89.00 iPHoP-RF;87.60
S8_vs_176604	SHHC	n.a.	n.a.	n.a.	n.a.	n.a.	n.a.	n.a.	n.a.	21.14	d__Bacteria;p__Firmicutes;c__Clostridia;o__Christensenellales;f__CAG-138;g__SFEL01	94.9	blast;96.40 iPHoP-RF;81.60 CRISPR;71.90
S8_vs_179208	Shared	n.a.	n.a.	n.a.	n.a.	n.a.	n.a.	n.a.	n.a.	39.41	d__Bacteria;p__Bacteroidota;c__Bacteroidia;o__Bacteroidales;f__Rikenellaceae;g__Alistipes	95.4	blast;96.80 iPHoP-RF;80.80
S8_vs_179529	SHHC	Duplodnaviria	Heunggongvirae	Uroviricota	Caudoviricetes	n.a.	Unclassified	Unclassified	O	98.83	d__Bacteria;p__Firmicutes;c__Clostridia;o__Oscillospirales;f__Ruminococcaceae;g__Faecalibacterium	100	CRISPR;98.50 RaFAH;98.00 blast;95.30 iPHoP-RF;86.70
S8_vs_182288	Shared	Duplodnaviria	Heunggongvirae	Uroviricota	Caudoviricetes	n.a.	Unclassified	O	O	85.94	d__Bacteria;p__Bacteroidota;c__Bacteroidia;o__Bacteroidales;f__Tannerellaceae;g__Parabacteroides	100	CRISPR;99.00 RaFAH;98.40 iPHoP-RF;96.40 blast;93.80
S8_vs_206526	Shared	Duplodnaviria	Heunggongvirae	Uroviricota	Caudoviricetes	n.a.	Unclassified	Unclassified	Unclassified	81.78	d__Bacteria;p__Firmicutes;c__Clostridia;o__Lachnospirales;f__Lachnospiraceae;g__Roseburia	97.9	CRISPR;98.50 blast;92.20 iPHoP-RF;87.30 RaFAH;50.70
S8_vs_208114	SHHC	Duplodnaviria	Heunggongvirae	Uroviricota	Caudoviricetes	n.a.	Unclassified	O	O	9.13	d__Bacteria;p__Desulfobacterota;c__Desulfovibrionia;o__Desulfovibrionales;f__Desulfovibrionaceae;g__Mailhella	90.1	blast;92.60 iPHoP-RF;81.40
S8_vs_28311	Shared	Duplodnaviria	Heunggongvirae	Uroviricota	Caudoviricetes	n.a.	Duneviridae	O	O	99.02	d__Bacteria;p__Bacteroidota;c__Bacteroidia;o__Bacteroidales;f__Bacteroidaceae;g__Bacteroides	100	RaFAH;97.40 blast;97.40 iPHoP-RF;96.70 CRISPR;54.10
S8_vs_40014	Shared	n.a.	n.a.	n.a.	n.a.	n.a.	n.a.	n.a.	n.a.	80.96	d__Bacteria;p__Bacteroidota;c__Bacteroidia;o__Bacteroidales;f__Rikenellaceae;g__Alistipes	94.6	blast;96.20 iPHoP-RF;95.70
S8_vs_4153	Shared	n.a.	n.a.	n.a.	n.a.	n.a.	n.a.	n.a.	n.a.	5.07	d__Bacteria;p__Bacteroidota;c__Bacteroidia;o__Bacteroidales;f__Barnesiellaceae;g__Barnesiella	97.9	blast;98.50 iPHoP-RF;96.40 CRISPR;53.40

S8_vs_50431	SHHC	n.a.	n.a.	n.a.	n.a.	n.a.	n.a.	n.a.	n.a.	20.17	d__Bacteria;p__Firmicutes;c__Clostridia;o__Oscillospirales;f__Oscillospiraceae;g__Dysosmobacter	91.5	blast;93.80 iPHoP-RF;50.70
S8_vs_51368	SHHC	n.a.	n.a.	n.a.	n.a.	n.a.	n.a.	n.a.	n.a.	31.95	d__Bacteria;p__Firmicutes;c__Clostridia;o__Oscillospirales;f__Oscillospiraceae;g__CAG-170	91.5	blast;93.80 iPHoP-RF;85.70
S8_vs_5211	Shared	n.a.	n.a.	n.a.	n.a.	n.a.	n.a.	n.a.	n.a.	70.75	d__Bacteria;p__Firmicutes;c__Clostridia;o__Lachnospirales;f__Lachnospiraceae;g__Massilistercora	90.2	blast;92.70 iPHoP-RF;58.40
S8_vs_54183	SHHC	Duplodnaviria	Heunggongvirae	Uroviricota	Caudoviricetes	n.a.	Unclassified	Unclassified	O	20.38	d__Bacteria;p__Firmicutes;c__Clostridia;o__Oscillospirales;f__Oscillospiraceae;g__CAG-170	91.8	CRISPR;94.10 blast;90.90 iPHoP-RF;81.90
S8_vs_59736	Shared	Duplodnaviria	Heunggongvirae	Uroviricota	Caudoviricetes	n.a.	Unclassified	O	O	26.65	d__Bacteria;p__Firmicutes;c__Bacilli;o__Erysipelotrichales;f__Erysipelatoclostridiaceae;g__Catenibacterium	95.6	blast;96.90
S8_vs_63277	SHHC	Duplodnaviria	Heunggongvirae	Uroviricota	Caudoviricetes	n.a.	Unclassified	O	O	75.64	d__Bacteria;p__Firmicutes;c__Clostridia;o__Oscillospirales;f__Ruminococcaceae;g__Faecalibacterium	97.8	blast;98.40 iPHoP-RF;88.20
S8_vs_72117	Shared	n.a.	n.a.	n.a.	n.a.	n.a.	n.a.	n.a.	n.a.	19.43	d__Bacteria;p__Bacteroidota;c__Bacteroidia;o__Bacteroidales;f__Bacteroidaceae;g__Bacteroides	95.4	iPHoP-RF;94.40 RaFAH;71.80
S8_vs_9114	SHHC	n.a.	n.a.	n.a.	n.a.	n.a.	n.a.	n.a.	n.a.	28.26	d__Bacteria;p__Bacteroidota;c__Bacteroidia;o__Bacteroidales;f__Rikenellaceae;g__Alistipes_A	91.1	blast;93.50 iPHoP-RF;82.80 CRISPR;64.80
S8_vs_99362	Shared	Duplodnaviria	Heunggongvirae	Uroviricota	Caudoviricetes	n.a.	Unclassified	Unclassified	Unclassified	99.97	d__Bacteria;p__Bacteroidota;c__Bacteroidia;o__Bacteroidales;f__Bacteroidaceae;g__Bacteroides	100	RaFAH;98.30 blast;96.50 iPHoP-RF;93.40
S8_vs_9987	Shared	Duplodnaviria	Heunggongvirae	Uroviricota	Caudoviricetes	n.a.	Unclassified	O	O	99.87	d__Bacteria;p__Bacteroidota;c__Bacteroidia;o__Bacteroidales;f__Rikenellaceae;g__Alistipes	100	blast;97.60 RaFAH;97.40 iPHoP-RF;93.40
S9_vf_13507	Shared	n.a.	n.a.	n.a.	n.a.	n.a.	n.a.	n.a.	n.a.	66.3	d__Bacteria;p__Bacteroidota;c__Bacteroidia;o__Bacteroidales;f__Bacteroidaceae;g__Prevotella	95.6	blast;96.90 iPHoP-RF;94.70
S9_vf_15012	ME	Duplodnaviria	Heunggongvirae	Uroviricota	Caudoviricetes	n.a.	Unclassified	Unclassified	Unclassified	97.67	d__Bacteria;p__Firmicutes;c__Bacilli;o__Lactobacillales;f__Streptococcaceae;g__Streptococcus	100	RaFAH;99.70 CRISPR;98.30 blast;94.40 iPHoP-RF;92.80
S9_vf_19284	ME	n.a.	n.a.	n.a.	n.a.	n.a.	n.a.	n.a.	n.a.	15.56	d__Bacteria;p__Firmicutes;c__Bacilli;o__Lactobacillales;f__Streptococcaceae;g__Streptococcus	93	CRISPR;95.00
S9_vf_4115	ME	Duplodnaviria	Heunggongvirae	Uroviricota	Caudoviricetes	n.a.	Unclassified	Unclassified	Unclassified	96.62	d__Bacteria;p__Firmicutes;c__Bacilli;o__Lactobacillales;f__Streptococcaceae;g__Streptococcus	100	RaFAH;99.70 CRISPR;99.00 blast;96.80 iPHoP-RF;96.70
S9_vf_51656	Shared	Duplodnaviria	Heunggongvirae	Uroviricota	Caudoviricetes	Crassvirales	Intestiviridae	Obtuvirinae	Fohxhuevirus	29.36	d__Bacteria;p__Bacteroidota;c__Bacteroidia;o__Bacteroidales;f__Bacteroidaceae;g__Prevotella	99	CRISPR;99.00 blast;96.30 iPHoP-RF;96.10 RaFAH;59.50
S9_vf_64844	Shared	Duplodnaviria	Heunggongvirae	Uroviricota	Caudoviricetes	n.a.	Unclassified	Unclassified	Lagaffevirus	89.68	d__Bacteria;p__Firmicutes;c__Clostridia;o__Oscillospirales;f__Oscillospiraceae;g__CAG-83	93.4	RaFAH;95.30 blast;50.00
S9_vf_73311	ME	Duplodnaviria	Heunggongvirae	Uroviricota	Caudoviricetes	n.a.	Unclassified	Unclassified	Unclassified	18.82	d__Bacteria;p__Firmicutes;c__Clostridia;o__Lachnospirales;f__CAG-274;g__CAG-274	97.2	CRISPR;98.00 blast;96.80 iPHoP-RF;78.70
S9_vf_76769	Shared	Duplodnaviria	Heunggongvirae	Uroviricota	Caudoviricetes	Crassvirales	Suoliviridae	Loutivirinae	Buchavirus	53.32	d__Bacteria;p__Bacteroidota;c__Bacteroidia;o__Bacteroidales;f__Bacteroidaceae;g__Prevotella	92.6	RaFAH;94.70
S9_vf_90856	ME	Duplodnaviria	Heunggongvirae	Uroviricota	Caudoviricetes	n.a.	Unclassified	Unclassified	O	94.47	d__Bacteria;p__Firmicutes;c__Bacilli;o__Lactobacillales;f__Streptococcaceae;g__Streptococcus	100	RaFAH;99.80 CRISPR;99.00 blast;95.80 iPHoP-RF;94.40
S9_vf_90866	ME	Unclassified	n.a.	n.a.	n.a.	n.a.	Unclassified	Unclassified	Unclassified	15.47	d__Bacteria;p__Firmicutes;c__Bacilli;o__Lactobacillales;f__Streptococcaceae;g__Streptococcus	99.3	iPHoP-RF;97.10 blast;95.70 RaFAH;90.00
S9_vf_934	ME	Duplodnaviria	Heunggongvirae	Uroviricota	Caudoviricetes	n.a.	Unclassified	O	O	17.76	d__Bacteria;p__Actinobacteriota;c__Actinomycetia;o__Mycobacteriales;f__Mycobacteriaceae;g__Mycobacterium	92.2	iPHoP-RF;94.40
S9_vs_14235	ME	n.a.	n.a.	n.a.	n.a.	n.a.	n.a.	n.a.	n.a.	12.73	d__Bacteria;p__Firmicutes;c__Clostridia;o__Oscillospirales;f__Oscillospiraceae;g__An92	91	blast;93.40 iPHoP-RF;87.00 CRISPR;73.90
S9_vs_1966	Shared	Duplodnaviria	Heunggongvirae	Uroviricota	Caudoviricetes	n.a.	Unclassified	O	O	61.19	d__Bacteria;p__Firmicutes;c__Clostridia;o__Lachnospirales;f__Lachnospiraceae;g__Bariatricus	91.6	blast;93.90 iPHoP-RF;77.30
S9_vs_19689	ME	n.a.	n.a.	n.a.	n.a.	n.a.	n.a.	n.a.	n.a.	9.42	d__Bacteria;p__Firmicutes;c__Clostridia;o__Oscillospirales;f__Butyricicoccaceae;g__Agathobaculum	91.6	blast;93.90 iPHoP-RF;68.60
S9_vs_21850	ME	Duplodnaviria	Heunggongvirae	Uroviricota	Caudoviricetes	n.a.	Unclassified	Unclassified	Unclassified	63.18	d__Bacteria;p__Bacteroidota;c__Bacteroidia;o__Bacteroidales;f__Barnesiellaceae;g__Barnesiella	97.2	blast;96.90 iPHoP-RF;85.10 CRISPR;84.10 RaFAH;68.00
S9_vs_26179	ME	n.a.	n.a.	n.a.	n.a.	n.a.	n.a.	n.a.	n.a.	48.62	d__Bacteria;p__Firmicutes;c__Clostridia;o__Oscillospirales;f__Acutalibacteraceae;g__UMGS1071	95.7	blast;97.00 iPHoP-RF;83.30 CRISPR;81.20
S9_vs_2799	Shared	n.a.	n.a.	n.a.	n.a.	n.a.	n.a.	n.a.	n.a.	10.92	d__Bacteria;p__Bacteroidota;c__Bacteroidia;o__Bacteroidales;f__Rikenellaceae;g__Alistipes	95.9	iPHoP-RF;97.10 blast;96.90
S9_vs_28197	Shared	Duplodnaviria	Heunggongvirae	Uroviricota	Caudoviricetes	n.a.	Unclassified	O	O	87.18	d__Bacteria;p__Bacteroidota;c__Bacteroidia;o__Bacteroidales;f__Bacteroidaceae;g__Bacteroides	100	RaFAH;99.40 iPHoP-RF;97.10 blast;96.30
S9_vs_32588	ME	Duplodnaviria	Heunggongvirae	Uroviricota	Caudoviricetes	n.a.	Unclassified	O	O	67.17	d__Bacteria;p__Firmicutes;c__Clostridia;o__Lachnospirales;f__Lachnospiraceae;g__Dorea	95	RaFAH;96.50
S9_vs_34898	ME	Duplodnaviria	Heunggongvirae	Uroviricota	Caudoviricetes	n.a.	Unclassified	O	O	91.82	d__Bacteria;p__Firmicutes;c__Clostridia;o__Lachnospirales;f__Lachnospiraceae;g__Sellimonas	93.9	blast;95.70 iPHoP-RF;89.50
S9_vs_37437	ME	Duplodnaviria	Heunggongvirae	Uroviricota	Caudoviricetes	n.a.	Unclassified	O	O	14.86	d__Bacteria;p__Firmicutes;c__Bacilli;o__Erysipelotrichales;f__Erysipelotrichaceae;g__Holdemania	91.6	blast;93.90 CRISPR;91.60 iPHoP-RF;75.40
S9_vs_41371	ME	Duplodnaviria	Heunggongvirae	Uroviricota	Caudoviricetes	n.a.	Unclassified	O	O	44.35	d__Bacteria;p__Firmicutes;c__Clostridia;o__Lachnospirales;f__Lachnospiraceae;g__Enterocloster	94	blast;95.80 iPHoP-RF;85.10
S9_vs_4343	ME	n.a.	n.a.	n.a.	n.a.	n.a.	n.a.	n.a.	n.a.	24.4	d__Bacteria;p__Firmicutes;c__Clostridia;o__Lachnospirales;f__Lachnospiraceae;g__Enterocloster	96.6	blast;97.60 CRISPR;96.60 iPHoP-RF;90.50
S9_vs_44331	ME	Duplodnaviria	Heunggongvirae	Uroviricota	Caudoviricetes	n.a.	Unclassified	O	O	98.52	d__Bacteria;p__Firmicutes;c__Clostridia;o__Lachnospirales;f__Lachnospiraceae;g__Ruminococcus_B	98.1	blast;98.60 iPHoP-RF;94.40 CRISPR;87.70
S9_vs_4558	ME	Duplodnaviria	Heunggongvirae	Uroviricota	Caudoviricetes	n.a.	Unclassified	O	O	65.18	d__Bacteria;p__Firmicutes;c__Clostridia;o__Oscillospirales;f__Oscillospiraceae;g__Flavonifractor	90.2	blast;92.70 iPHoP-RF;79.00
S9_vs_46498	ME	Duplodnaviria	Heunggongvirae	Uroviricota	Caudoviricetes	n.a.	Unclassified	O	O	26.43	d__Bacteria;p__Firmicutes;c__Bacilli;o__Erysipelotrichales;f__Erysipelatoclostridiaceae;g__Erysipelatoclostridium	96.7	blast;97.70 iPHoP-RF;74.10
S9_vs_48240	ME	Duplodnaviria	Heunggongvirae	Uroviricota	Caudoviricetes	n.a.	Unclassified	Unclassified	O	63.98	d__Bacteria;p__Firmicutes;c__Clostridia;o__Lachnospirales;f__Lachnospiraceae;g__Hungatella	92.5	blast;94.60 iPHoP-RF;94.40
S9_vs_48347	ME	Duplodnaviria	Heunggongvirae	Uroviricota	Caudoviricetes	n.a.	Unclassified	Unclassified	O	25.89	d__Bacteria;p__Firmicutes;c__Clostridia;o__Oscillospirales;f__Ruminococcaceae;g__Negativibacillus	94.2	blast;95.90 iPHoP-RF;85.40 CRISPR;59.60
S9_vs_48424	Shared	n.a.	n.a.	n.a.	n.a.	n.a.	n.a.	n.a.	n.a.	86.27	d__Bacteria;p__Bacteroidota;c__Bacteroidia;o__Bacteroidales;f__Rikenellaceae;g__Alistipes	99.3	blast;96.80 RaFAH;90.20 iPHoP-RF;54.90
S9_vs_50439	ME	n.a.	n.a.	n.a.	n.a.	n.a.	n.a.	n.a.	n.a.	93.5	d__Bacteria;p__Firmicutes;c__Clostridia;o__Oscillospirales;f__Oscillospiraceae;g__Flavonifractor	98.1	blast;98.60 iPHoP-RF;74.30
S9_vs_55738	ME	Duplodnaviria	Heunggongvirae	Uroviricota	Caudoviricetes	n.a.	Unclassified	O	O	76	d__Bacteria;p__Firmicutes;c__Clostridia;o__Lachnospirales;f__Lachnospiraceae;g__Enterocloster	94.6	blast;96.20 iPHoP-RF;87.00 CRISPR;69.00
S9_vs_5676	ME	Duplodnaviria	Heunggongvirae	Uroviricota	Caudoviricetes	n.a.	Unclassified	O	O	19.1	d__Bacteria;p__Firmicutes;c__Clostridia;o__Oscillospirales;f__Oscillospiraceae;g__Intestinimonas	92.6	blast;94.70 iPHoP-RF;74.60
S9_vs_58086	ME	Duplodnaviria	Heunggongvirae	Uroviricota	Caudoviricetes	n.a.	Unclassified	O	O	53.1	d__Bacteria;p__Firmicutes;c__Clostridia;o__Lachnospirales;f__Lachnospiraceae;g__Dorea_A	94.3	blast;96.00 iPHoP-RF;80.60
S9_vs_62803	ME	Duplodnaviria	Heunggongvirae	Uroviricota	Caudoviricetes	n.a.	Unclassified	O	O	39.71	d__Bacteria;p__Firmicutes;c__Clostridia;o__Lachnospirales;f__Lachnospiraceae;g__Sellimonas	94.9	blast;96.40 iPHoP-RF;84.50
S9_vs_66877	ME	Duplodnaviria	Heunggongvirae	Uroviricota	Caudoviricetes	n.a.	Unclassified	O	O	29.56	d__Bacteria;p__Firmicutes;c__Clostridia;o__Oscillospirales;f__Ruminococcaceae;g__Ruthenibacterium	95.3	blast;96.70 CRISPR;95.50 iPHoP-RF;92.10
S9_vs_67792	ME	Duplodnaviria	Heunggongvirae	Uroviricota	Caudoviricetes	n.a.	Unclassified	O	O	30.5	d__Bacteria;p__Firmicutes;c__Negativicutes;o__Veillonellales;f__Dialisteraceae;g__Dialister	96.6	blast;97.60 iPHoP-RF;86.00 CRISPR;60.90
S9_vs_69322	ME	n.a.	n.a.	n.a.	n.a.	n.a.	n.a.	n.a.	n.a.	20.79	d__Bacteria;p__Firmicutes;c__Clostridia;o__Oscillospirales;f__Oscillospiraceae;g__Dysosmobacter	90.7	blast;93.10 iPHoP-RF;82.20
S9_vs_75050	ME	Duplodnaviria	Heunggongvirae	Uroviricota	Caudoviricetes	n.a.	Unclassified	O	O	26.71	d__Bacteria;p__Firmicutes;c__Clostridia;o__Lachnospirales;f__Lachnospiraceae;g__CAG-127	96.4	CRISPR;97.50 blast;96.80 iPHoP-RF;86.00
S9_vs_83108	ME	n.a.	n.a.	n.a.	n.a.	n.a.	n.a.	n.a.	n.a.	82.91	d__Bacteria;p__Bacteroidota;c__Bacteroidia;o__Bacteroidales;f__Bacteroidaceae;g__Bacteroides	99.4	iPHoP-RF;97.10 blast;93.90 RaFAH;91.80
S9_vs_84046	ME	Duplodnaviria	Heunggongvirae	Uroviricota	Caudoviricetes	n.a.	Unclassified	O	O	20.91	d__Bacteria;p__Firmicutes;c__Clostridia;o__Oscillospirales;f__Oscillospiraceae;g__An92	90.7	blast;93.10 iPHoP-RF;70.60
S9_vs_88286	ME	n.a.	n.a.	n.a.	n.a.	n.a.	n.a.	n.a.	n.a.	10.88	d__Bacteria;p__Firmicutes;c__Clostridia;o__Lachnospirales;f__Lachnospiraceae;g__Eisenbergiella	98.1	CRISPR;98.60 blast;95.70 iPHoP-RF;95.40
S9_vs_90727	Shared	Duplodnaviria	Heunggongvirae	Uroviricota	Caudoviricetes	n.a.	Unclassified	Unclassified	O	57.49	d__Bacteria;p__Firmicutes;c__Clostridia;o__Oscillospirales;f__Acutalibacteraceae;g__Ruminococcus_E	95.6	blast;96.90 iPHoP-RF;88.20
S9_vs_90846	ME	Duplodnaviria	Heunggongvirae	Uroviricota	Caudoviricetes	n.a.	Unclassified	O	O	37.71	d__Bacteria;p__Firmicutes;c__Bacilli;o__Erysipelotrichales;f__Erysipelotrichaceae;g__Clostridium_AQ	97.5	blast;98.20 iPHoP-RF;62.70
S9_vs_90848	Shared	Duplodnaviria	Heunggongvirae	Uroviricota	Caudoviricetes	n.a.	Unclassified	O	O	51.41	d__Bacteria;p__Firmicutes;c__Clostridia;o__Lachnospirales;f__Lachnospiraceae;g__Enterocloster	98.7	CRISPR;99.00 blast;93.90 iPHoP-RF;91.40
S9_vs_90849	ME	n.a.	n.a.	n.a.	n.a.	n.a.	n.a.	n.a.	n.a.	70.89	d__Bacteria;p__Firmicutes;c__Clostridia;o__Lachnospirales;f__Anaerotignaceae;g__Anaerotignum	95	CRISPR;96.50 blast;93.70 iPHoP-RF;92.10
S9_vs_90851	ME	Duplodnaviria	Heunggongvirae	Uroviricota	Caudoviricetes	n.a.	Unclassified	O	O	89.56	d__Bacteria;p__Firmicutes;c__Clostridia;o__Clostridiales;f__Clostridiaceae;g__Clostridium	95.6	RaFAH;96.90
S9_vs_90859	ME	Unclassified	n.a.	n.a.	n.a.	n.a.	Unclassified	O	O	43.06	d__Bacteria;p__Firmicutes;c__Clostridia;o__Lachnospirales;f__Lachnospiraceae;g__Blautia_A	95.3	blast;93.90 iPHoP-RF;75.90 RaFAH;73.20
S9_vs_90860	ME	Duplodnaviria	Heunggongvirae	Uroviricota	Caudoviricetes	n.a.	Unclassified	O	O	20.66	d__Bacteria;p__Firmicutes;c__Clostridia;o__Oscillospirales;f__Acutalibacteraceae;g__UMGS1591	93.4	blast;95.30 iPHoP-RF;67.00

Table S9-4. WMS_STD+MAGs_tophits

Virus	Status	Realm	Kingdom	Phylum	Class	Order	Family	Subfamily	Genus	AAI to closest RaFAH reference	Host genus	Confidence score	List of methods
S1-wms-combined_118167	Shared	Duplodnaviria	Heunggongvirae	Uroviricota	Caudoviricetes	n.a.	Unclassified	O	O	33.35	d__Bacteria;p__Firmicutes;c__Clostridia;o__Oscillospirales;f__Ruminococcaceae;g__Faecalibacterium	99.1	CRISPR;96.40 blast;95.10 RaFAH;90.80 iPHoP-RF;89.50
S1-wms-combined_100430	ME	Duplodnaviria	Heunggongvirae	Uroviricota	Caudoviricetes	n.a.	Unclassified	Unclassified	O	99.71	d__Bacteria;p__Firmicutes;c__Clostridia;o__Lachnospirales;f__Lachnospiraceae;g__Lachnospira	99.3	CRISPR;98.90 RaFAH;73.00
S1-wms-combined_131270	ME	Duplodnaviria	Heunggongvirae	Uroviricota	Caudoviricetes	n.a.	Unclassified	O	O	21.6	d__Bacteria;p__Firmicutes;c__Clostridia;o__Oscillospirales;f__Ruminococcaceae;g__Faecalibacterium	99	CRISPR;99.20 blast;94.50 iPHoP-RF;84.50
S1-wms-combined_132934	Shared	Duplodnaviria	Heunggongvirae	Uroviricota	Caudoviricetes	n.a.	Unclassified	O	O	48.4	d__Bacteria;p__Firmicutes;c__Clostridia;o__Oscillospirales;f__Oscillospiraceae;g__ER4	92.7	blast;94.80 iPHoP-RF;64.80
S1-wms-combined_136097	Shared	n.a.	n.a.	n.a.	n.a.	n.a.	n.a.	n.a.	n.a.	4.5	d__Bacteria;p__Firmicutes;c__Clostridia;o__Oscillospirales;f__Oscillospiraceae;g__UBA738	92	blast;94.20 iPHoP-RF;53.00
S1-wms-combined_107766	Shared	n.a.	n.a.	n.a.	n.a.	n.a.	n.a.	n.a.	n.a.	92.99	d__Bacteria;p__Bacteroidota;c__Bacteroidia;o__Bacteroidales;f__Rikenellaceae;g__Alistipes	98.8	iPHoP-RF;97.10 blast;96.90 RaFAH;83.70
S1-wms-combined_156637	Shared	n.a.	n.a.	n.a.	n.a.	n.a.	n.a.	n.a.	n.a.	11	d__Bacteria;p__Firmicutes;c__Clostridia;o__Oscillospirales;f__Oscillospiraceae;g__ER4	93	blast;95.00 iPHoP-RF;60.30
S1-wms-combined_143459	ME	Duplodnaviria	Heunggongvirae	Uroviricota	Caudoviricetes	n.a.	Unclassified	O	O	18.79	d__Bacteria;p__Desulfobacterota;c__Desulfovibrionia;o__Desulfovibrionales;f__Desulfovibrionaceae;g__Bilophila	95.2	blast;96.60 iPHoP-RF;65.80 CRISPR;58.60
S1-wms-combined_159544	ME	Duplodnaviria	Heunggongvirae	Uroviricota	Caudoviricetes	n.a.	Peduoviridae	Unclassified	Phitrevirus	36.49	d__Bacteria;p__Desulfobacterota;c__Desulfovibrionia;o__Desulfovibrionales;f__Desulfovibrionaceae;g__Bilophila	94	blast;95.80 iPHoP-RF;79.30 CRISPR;57.40
S1-wms-combined_164485	Shared	n.a.	n.a.	n.a.	n.a.	n.a.	n.a.	n.a.	n.a.	4.67	d__Bacteria;p__Firmicutes;c__Clostridia;o__Oscillospirales;f__Oscillospiraceae;g__ER4	90.4	blast;92.90
S1-wms-combined_185336	ME	n.a.	n.a.	n.a.	n.a.	n.a.	n.a.	n.a.	n.a.		d__Bacteria;p__Firmicutes;c__Clostridia;o__Oscillospirales;f__Oscillospiraceae;g__ER4	91.7	blast;94.00 iPHoP-RF;63.50
S1-wms-combined_19031	ME	Duplodnaviria	Heunggongvirae	Uroviricota	Caudoviricetes	n.a.	Unclassified	Unclassified	Unclassified	37.63	d__Bacteria;p__Desulfobacterota;c__Desulfovibrionia;o__Desulfovibrionales;f__Desulfovibrionaceae;g__Bilophila	98.2	blast;98.70 iPHoP-RF;76.10
S1-wms-combined_164894	ME	Duplodnaviria	Heunggongvirae	Uroviricota	Caudoviricetes	n.a.	Unclassified	O	O	72.02	d__Bacteria;p__Firmicutes;c__Clostridia;o__Oscillospirales;f__Ruminococcaceae;g__Faecalibacterium	91.8	blast;94.10 iPHoP-RF;70.90
S1-wms-combined_23248	Shared	Duplodnaviria	Heunggongvirae	Uroviricota	Caudoviricetes	n.a.	Unclassified	Unclassified	Lughvirus	61.01	d__Bacteria;p__Firmicutes;c__Clostridia;o__Oscillospirales;f__Ruminococcaceae;g__Faecalibacterium	95	blast;96.50 iPHoP-RF;68.90
S1-wms-combined_246923	ME	Duplodnaviria	Heunggongvirae	Uroviricota	Caudoviricetes	n.a.	Unclassified	O	O	43.06	d__Bacteria;p__Bacteroidota;c__Bacteroidia;o__Bacteroidales;f__UBA11471;g__UBA11471	93.9	blast;95.70 iPHoP-RF;68.60
S1-wms-combined_153670	ME	n.a.	n.a.	n.a.	n.a.	n.a.	n.a.	n.a.	n.a.		d__Bacteria;p__Bacteroidota;c__Bacteroidia;o__Bacteroidales;f__Barnesiellaceae;g__Barnesiella	94.2	blast;95.90 iPHoP-RF;72.20
S1-wms-combined_248622	Shared	n.a.	n.a.	n.a.	n.a.	n.a.	n.a.	n.a.	n.a.	6.16	d__Bacteria;p__Bacteroidota;c__Bacteroidia;o__Bacteroidales;f__Bacteroidaceae;g__Bacteroides	95.9	iPHoP-RF;97.10 blast;90.50
S1-wms-combined_186878	ME	n.a.	n.a.	n.a.	n.a.	n.a.	n.a.	n.a.	n.a.	42.39	d__Bacteria;p__Firmicutes;c__Clostridia;o__Oscillospirales;f__Oscillospiraceae;g__ER4	91.6	blast;93.90 iPHoP-RF;76.40
S1-wms-combined_263562	Shared	n.a.	n.a.	n.a.	n.a.	n.a.	n.a.	n.a.	n.a.	99.98	d__Bacteria;p__Bacteroidota;c__Bacteroidia;o__Bacteroidales;f__Bacteroidaceae;g__Bacteroides	100	RaFAH;99.80 blast;91.80 iPHoP-RF;82.20
S1-wms-combined_302166	Shared	Duplodnaviria	Heunggongvirae	Uroviricota	Caudoviricetes	n.a.	Unclassified	O	O	9.7	d__Bacteria;p__Firmicutes;c__Clostridia;o__Christensenellales;f__CAG-74;g__UBA11524	95.6	blast;96.90 iPHoP-RF;92.40
S1-wms-combined_212892	ME	Duplodnaviria	Heunggongvirae	Uroviricota	Caudoviricetes	n.a.	Unclassified	O	O	80.18	d__Bacteria;p__Firmicutes;c__Clostridia;o__Lachnospirales;f__Lachnospiraceae;g__Anaerobutyricum	98.1	CRISPR;98.60 blast;91.50 iPHoP-RF;80.00
S1-wms-combined_166392	ME	Duplodnaviria	Heunggongvirae	Uroviricota	Caudoviricetes	n.a.	Unclassified	O	O	37.1	d__Bacteria;p__Firmicutes;c__Clostridia;o__Oscillospirales;f__Ruminococcaceae;g__Gemmiger	95.7	blast;97.00 iPHoP-RF;95.70 CRISPR;78.40
S1-wms-combined_304377	Shared	Duplodnaviria	Heunggongvirae	Uroviricota	Caudoviricetes	n.a.	Unclassified	Unclassified	Oengusvirus	48.66	d__Bacteria;p__Firmicutes;c__Clostridia;o__Oscillospirales;f__Ruminococcaceae;g__Faecalibacterium	95.6	blast;96.90 iPHoP-RF;78.50
S1-wms-combined_169788	Shared	n.a.	n.a.	n.a.	n.a.	n.a.	n.a.	n.a.	n.a.	5.96	d__Bacteria;p__Firmicutes;c__Clostridia;o__Oscillospirales;f__Oscillospiraceae;g__CAG-83	93.4	blast;95.30 iPHoP-RF;87.00
S1-wms-combined_220022	ME	n.a.	n.a.	n.a.	n.a.	n.a.	n.a.	n.a.	n.a.	6.93	d__Bacteria;p__Firmicutes;c__Clostridia;o__Oscillospirales;f__Ruminococcaceae;g__Gemmiger	93.2	blast;95.20 iPHoP-RF;88.20 CRISPR;76.80
S1-wms-combined_307323	ME	Duplodnaviria	Heunggongvirae	Uroviricota	Caudoviricetes	n.a.	Unclassified	O	O	92.27	d__Bacteria;p__Actinobacteriota;c__Actinomycetia;o__Actinomycetales;f__Bifidobacteriaceae;g__Bifidobacterium	98.7	CRISPR;99.00 iPHoP-RF;97.10 blast;96.90
S1-wms-combined_17651	Shared	n.a.	n.a.	n.a.	n.a.	n.a.	n.a.	n.a.	n.a.	9.21	d__Bacteria;p__Firmicutes;c__Clostridia;o__Oscillospirales;f__Oscillospiraceae;g__ER4	90.6	blast;93.00 iPHoP-RF;65.10
S1-wms-combined_227003	ME	n.a.	n.a.	n.a.	n.a.	n.a.	n.a.	n.a.	n.a.	41.88	d__Bacteria;p__Firmicutes;c__Clostridia;o__Oscillospirales;f__Oscillospiraceae;g__CAG-110	94.2	blast;95.90 iPHoP-RF;87.00
S1-wms-combined_227917	ME	Duplodnaviria	Heunggongvirae	Uroviricota	Caudoviricetes	n.a.	Unclassified	O	O	27.55	d__Bacteria;p__Firmicutes;c__Clostridia;o__Oscillospirales;f__Ruminococcaceae;g__Gemmiger	95.2	blast;96.60 iPHoP-RF;95.40 CRISPR;92.50
S1-wms-combined_331544	ME	n.a.	n.a.	n.a.	n.a.	n.a.	n.a.	n.a.	n.a.	89.75	d__Bacteria;p__Actinobacteriota;c__Actinomycetia;o__Actinomycetales;f__Bifidobacteriaceae;g__Bifidobacterium	99.9	CRISPR;99.60 blast;96.90 iPHoP-RF;96.70 RaFAH;75.50
S1-wms-combined_331896	Shared	n.a.	n.a.	n.a.	n.a.	n.a.	n.a.	n.a.	n.a.		d__Bacteria;p__Firmicutes;c__Clostridia;o__Oscillospirales;f__Oscillospiraceae;g__ER4	91.6	blast;93.90 iPHoP-RF;58.40
S1-wms-combined_340558	Shared	Duplodnaviria	Heunggongvirae	Uroviricota	Caudoviricetes	n.a.	Unclassified	O	O	85.48	d__Bacteria;p__Bacteroidota;c__Bacteroidia;o__Bacteroidales;f__Rikenellaceae;g__Alistipes	99.7	blast;96.80 iPHoP-RF;96.10 RaFAH;95.90
S1-wms-combined_353757	ME	Duplodnaviria	Heunggongvirae	Uroviricota	Caudoviricetes	n.a.	Unclassified	O	O	21.95	d__Bacteria;p__Firmicutes;c__Clostridia;o__Oscillospirales;f__Oscillospiraceae;g__ER4	93.2	blast;95.20 iPHoP-RF;84.80
S1-wms-combined_279492	Shared	Duplodnaviria	Heunggongvirae	Uroviricota	Caudoviricetes	n.a.	Unclassified	O	O	44.35	d__Bacteria;p__Firmicutes;c__Clostridia;o__Oscillospirales;f__Ruminococcaceae;g__Gemmiger	97.8	blast;98.40 iPHoP-RF;96.40 CRISPR;58.00
S1-wms-combined_357373	ME	Monodnaviria	Sangervirae	Phixviricota	Malgrandaviricetes	Petitvirales	Microviridae	Unclassified	Unclassified		d__Bacteria;p__Bacteroidota;c__Bacteroidia;o__Bacteroidales;f__Barnesiellaceae;g__Barnesiella	91.8	blast;94.10 iPHoP-RF;86.30
S1-wms-combined_360285	Shared	Duplodnaviria	Heunggongvirae	Uroviricota	Caudoviricetes	n.a.	Unclassified	O	O	17.11	d__Bacteria;p__Desulfobacterota;c__Desulfovibrionia;o__Desulfovibrionales;f__Desulfovibrionaceae;g__Bilophila	97.2	CRISPR;98.00 blast;96.60 iPHoP-RF;89.80
S1-wms-combined_394574	Shared	Duplodnaviria	Heunggongvirae	Uroviricota	Caudoviricetes	n.a.	Unclassified	Unclassified	Unclassified	96.28	d__Bacteria;p__Bacteroidota;c__Bacteroidia;o__Bacteroidales;f__Tannerellaceae;g__Parabacteroides	91.6	blast;93.90 iPHoP-RF;75.70
S1-wms-combined_308848	Shared	n.a.	n.a.	n.a.	n.a.	n.a.	n.a.	n.a.	n.a.	5.29	d__Bacteria;p__Verrucomicrobiota;c__Verrucomicrobiae;o__Verrucomicrobiales;f__Akkermansiaceae;g__Akkermansia	95.9	CRISPR;97.10 blast;96.90 iPHoP-RF;93.10
S1-wms-combined_231411	ME	n.a.	n.a.	n.a.	n.a.	n.a.	n.a.	n.a.	n.a.	3.46	d__Bacteria;p__Desulfobacterota;c__Desulfovibrionia;o__Desulfovibrionales;f__Desulfovibrionaceae;g__Bilophila	93	blast;95.00 iPHoP-RF;73.20 CRISPR;51.00
S1-wms-combined_40115	ME	n.a.	n.a.	n.a.	n.a.	n.a.	n.a.	n.a.	n.a.	17.83	d__Bacteria;p__Firmicutes;c__Clostridia;o__Oscillospirales;f__Oscillospiraceae;g__ER4	97	blast;97.90 iPHoP-RF;71.70
S1-wms-combined_41369	ME	Duplodnaviria	Heunggongvirae	Uroviricota	Caudoviricetes	n.a.	Unclassified	O	O	37.96	d__Bacteria;p__Firmicutes;c__Clostridia;o__Oscillospirales;f__Oscillospiraceae;g__ER4	94.6	blast;96.20 iPHoP-RF;60.60
S1-wms-combined_68998	ME	Duplodnaviria	Heunggongvirae	Uroviricota	Caudoviricetes	n.a.	Unclassified	Unclassified	Taranisvirus	25.66	d__Bacteria;p__Firmicutes;c__Clostridia;o__Oscillospirales;f__Ruminococcaceae;g__Faecalibacterium	99.9	CRISPR;98.60 blast;92.00 iPHoP-RF;91.40 RaFAH;91.30
S1-wms-combined_72167	ME	Duplodnaviria	Heunggongvirae	Uroviricota	Caudoviricetes	n.a.	Unclassified	O	O	16.24	d__Bacteria;p__Firmicutes;c__Clostridia;o__Oscillospirales;f__Oscillospiraceae;g__CAG-83	98.5	CRISPR;98.90 blast;95.00 iPHoP-RF;90.80
S1-wms_130214	ME	Duplodnaviria	Heunggongvirae	Uroviricota	Caudoviricetes	n.a.	Unclassified	O	O	33.32	d__Bacteria;p__Firmicutes;c__Clostridia;o__Oscillospirales;f__Oscillospiraceae;g__CAG-170	90.6	blast;93.00 iPHoP-RF;90.50 CRISPR;82.90
S1-wms_185046	Shared	n.a.	n.a.	n.a.	n.a.	n.a.	n.a.	n.a.	n.a.	36.29	d__Bacteria;p__Firmicutes;c__Clostridia;o__Oscillospirales;f__Oscillospiraceae;g__CAG-170	91.1	blast;93.50 iPHoP-RF;82.20
S1-wms_244836	Shared	n.a.	n.a.	n.a.	n.a.	n.a.	n.a.	n.a.	n.a.	99.89	d__Bacteria;p__Bacteroidota;c__Bacteroidia;o__Bacteroidales;f__Bacteroidaceae;g__Bacteroides	100	RaFAH;99.60 iPHoP-RF;96.40 blast;94.20
S1-wms-combined_285012	Shared	Duplodnaviria	Heunggongvirae	Uroviricota	Caudoviricetes	n.a.	Unclassified	Unclassified	O	37.37	d__Bacteria;p__Firmicutes;c__Clostridia;o__Oscillospirales;f__Ruminococcaceae;g__Faecalibacterium	95	CRISPR;96.50 iPHoP-RF;54.40
S1-wms_296850	Shared	Duplodnaviria	Heunggongvirae	Uroviricota	Caudoviricetes	n.a.	Unclassified	Unclassified	Toutatisvirus	38.32	d__Bacteria;p__Firmicutes;c__Clostridia;o__Oscillospirales;f__Oscillospiraceae;g__ER4	93.1	blast;95.10 iPHoP-RF;63.80
S1-wms_4277	Shared	Duplodnaviria	Heunggongvirae	Uroviricota	Caudoviricetes	n.a.	Unclassified	Unclassified	Mushuvirus	93.43	d__Bacteria;p__Firmicutes;c__Clostridia;o__Oscillospirales;f__Oscillospiraceae;g__Lawsonibacter	91.4	blast;93.70
S11-wms-combined_119215	ME	Duplodnaviria	Heunggongvirae	Uroviricota	Caudoviricetes	n.a.	Unclassified	O	O	29.67	d__Bacteria;p__Firmicutes;c__Clostridia;o__Oscillospirales;f__Oscillospiraceae;g__An92	94.9	iPHoP-RF;96.40 blast;92.70
S1-wms-combined_57370	Shared	Duplodnaviria	Heunggongvirae	Uroviricota	Caudoviricetes	n.a.	Unclassified	O	O	50.73	d__Bacteria;p__Firmicutes;c__Clostridia;o__Oscillospirales;f__Oscillospiraceae;g__CAG-83	91.7	blast;89.00 RaFAH;72.80
S11-wms-combined_125176	Shared	Duplodnaviria	Heunggongvirae	Uroviricota	Caudoviricetes	n.a.	Unclassified	O	O	94.89	d__Bacteria;p__Bacteroidota;c__Bacteroidia;o__Bacteroidales;f__Rikenellaceae;g__Alistipes	96.9	CRISPR;97.80 iPHoP-RF;97.10 blast;96.90
S11-wms-combined_140009	Shared	Duplodnaviria	Heunggongvirae	Uroviricota	Caudoviricetes	n.a.	Unclassified	Unclassified	Lughvirus	58.05	d__Bacteria;p__Firmicutes;c__Clostridia;o__Oscillospirales;f__Ruminococcaceae;g__Faecalibacterium	95.6	blast;96.90 iPHoP-RF;71.90
S11-wms-combined_154845	ME	Duplodnaviria	Heunggongvirae	Uroviricota	Caudoviricetes	n.a.	Peduoviridae	Unclassified	Quadragintaviru	84.31	d__Bacteria;p__Proteobacteria;c__Gammaproteobacteria;o__Enterobacterales;f__Enterobacteriaceae;g__Salmonella	95.4	blast;96.80
S1-wms-combined_32863	Shared	n.a.	n.a.	n.a.	n.a.	n.a.	n.a.	n.a.	n.a.	10.89	d__Bacteria;p__Bacteroidota;c__Bacteroidia;o__Bacteroidales;f__Tannerellaceae;g__Parabacteroides	95.6	blast;96.90 iPHoP-RF;69.50
S1-wms-combined_85225	ME	Duplodnaviria	Heunggongvirae	Uroviricota	Caudoviricetes	n.a.	Unclassified	O	O	64.47	d__Bacteria;p__Actinobacteriota;c__Actinomycetia;o__Actinomycetales;f__Bifidobacteriaceae;g__Bifidobacterium	99.6	CRISPR;99.60 iPHoP-RF;97.10 blast;96.90
S1-wms-combined_330329	ME	Duplodnaviria	Heunggongvirae	Uroviricota	Caudoviricetes	n.a.	Unclassified	O	O	86.77	d__Bacteria;p__Bacteroidota;c__Bacteroidia;o__Bacteroidales;f__Bacteroidaceae;g__Bacteroides	98.2	RaFAH;97.70 iPHoP-RF;72.40
S1-wms-combined_92523	ME	Unclassified	n.a.	n.a.	n.a.	n.a.	Unclassified	O	O	13.27	d__Bacteria;p__Proteobacteria;c__Alphaproteobacteria;o__RF32;f__CAG-239;g__51-20	95	blast;96.50 iPHoP-RF;71.30
S11-wms-combined_172889	ME	Duplodnaviria	Heunggongvirae	Uroviricota	Caudoviricetes	n.a.	Unclassified	O	O	100	d__Bacteria;p__Bacteroidota;c__Bacteroidia;o__Bacteroidales;f__Rikenellaceae;g__Alistipes	100	RaFAH;99.10 blast;97.90 iPHoP-RF;94.70
S1-wms-combined_94988	Shared	n.a.	n.a.	n.a.	n.a.	n.a.	n.a.	n.a.	n.a.	21.16	d__Bacteria;p__Firmicutes;c__Clostridia;o__Oscillospirales;f__Oscillospiraceae;g__CAG-83	95.6	blast;96.90 iPHoP-RF;94.10
S11-wms-combined_193148	Shared	n.a.	n.a.	n.a.	n.a.	n.a.	n.a.	n.a.	n.a.	97.83	d__Bacteria;p__Proteobacteria;c__Alphaproteobacteria;o__RF32;f__CAG-239;g__CAG-267	94.9	blast;96.40 iPHoP-RF;75.00
S1-wms_102342	Shared	Duplodnaviria	Heunggongvirae	Uroviricota	Caudoviricetes	n.a.	Unclassified	O	O	14.71	d__Bacteria;p__Firmicutes;c__Clostridia;o__Oscillospirales;f__Oscillospiraceae;g__Dysosmobacter	95	blast;96.50 iPHoP-RF;60.30
S1-wms-combined_351889	Shared	Duplodnaviria	Heunggongvirae	Uroviricota	Caudoviricetes	n.a.	Unclassified	Unclassified	O	29.41	d__Bacteria;p__Firmicutes;c__Clostridia;o__Oscillospirales;f__Oscillospiraceae;g__ER4	96.2	CRISPR;97.30 blast;96.20 iPHoP-RF;84.50
S11-wms-combined_195425	ME	Duplodnaviria	Heunggongvirae	Uroviricota	Caudoviricetes	n.a.	Unclassified	Unclassified	Unclassified	89.45	d__Bacteria;p__Bacteroidota;c__Bacteroidia;o__Bacteroidales;f__Bacteroidaceae;g__Bacteroides	96.2	blast;90.20 RaFAH;86.00
S11-wms-combined_200084	ME	Duplodnaviria	Heunggongvirae	Uroviricota	Caudoviricetes	n.a.	Unclassified	O	O	31.8	d__Bacteria;p__Firmicutes;c__Clostridia;o__Lachnospirales;f__Lachnospiraceae;g__Blautia_A	91.8	blast;94.10 iPHoP-RF;53.60
S1-wms_208875	ME	Duplodnaviria	Heunggongvirae	Uroviricota	Caudoviricetes	n.a.	Unclassified	O	O	95.4	d__Bacteria;p__Bacteroidota;c__Bacteroidia;o__Bacteroidales;f__Bacteroidaceae;g__Bacteroides	95.7	blast;95.00 iPHoP-RF;94.40 RaFAH;70.30
S11-wms-combined_204522	ME	Duplodnaviria	Heunggongvirae	Uroviricota	Caudoviricetes	n.a.	Unclassified	O	O	15.39	d__Bacteria;p__Proteobacteria;c__Alphaproteobacteria;o__RF32;f__CAG-239;g__CAG-267	91.7	blast;94.00 iPHoP-RF;72.60
S11-wms-combined_217790	Shared	n.a.	n.a.	n.a.	n.a.	n.a.	n.a.	n.a.	n.a.	99.99	d__Bacteria;p__Bacteroidota;c__Bacteroidia;o__Bacteroidales;f__Bacteroidaceae;g__Bacteroides	99.9	RaFAH;99.80
S1-wms-combined_373308	Shared	n.a.	n.a.	n.a.	n.a.	n.a.	n.a.	n.a.	n.a.	6.25	d__Bacteria;p__Firmicutes;c__Clostridia;o__Oscillospirales;f__Oscillospiraceae;g__CAG-83	97.9	blast;98.50 iPHoP-RF;77.30
S1-wms_236105	Shared	Duplodnaviria	Heunggongvirae	Uroviricota	Caudoviricetes	n.a.	Unclassified	O	O	71.3	d__Bacteria;p__Firmicutes;c__Clostridia;o__Oscillospirales;f__Oscillospiraceae;g__ER4	92.1	blast;94.30 iPHoP-RF;89.80 CRISPR;65.50
S1-wms-combined_374219	ME	n.a.	n.a.	n.a.	n.a.	n.a.	n.a.	n.a.	n.a.	10.38	d__Bacteria;p__Firmicutes;c__Clostridia;o__Oscillospirales;f__Oscillospiraceae;g__Flavonifractor	91.5	blast;93.80 iPHoP-RF;79.80
S11-wms-combined_268967	ME	Duplodnaviria	Heunggongvirae	Uroviricota	Caudoviricetes	n.a.	Unclassified	Unclassified	Unclassified	35.57	d__Bacteria;p__Desulfobacterota;c__Desulfovibrionia;o__Desulfovibrionales;f__Desulfovibrionaceae;g__Desulfovibrio	93.8	blast;95.10 iPHoP-RF;81.90 RaFAH;54.80 CRISPR;51.90
S1-wms_29288	ME	Duplodnaviria	Heunggongvirae	Uroviricota	Caudoviricetes	n.a.	Unclassified	O	O	41.26	d__Bacteria;p__Firmicutes;c__Clostridia;o__Oscillospirales;f__Ruminococcaceae;g__Faecalibacterium	91.6	blast;93.90 CRISPR;78.00 iPHoP-RF;75.70
S11-wms-combined_283220	ME	Duplodnaviria	Heunggongvirae	Uroviricota	Caudoviricetes	n.a.	Unclassified	Unclassified	Mushuvirus	78.49	d__Bacteria;p__Firmicutes;c__Clostridia;o__Oscillospirales;f__Oscillospiraceae;g__Dysosmobacter	91.4	blast;93.70 iPHoP-RF;83.60
S11-wms-combined_299954	ME	n.a.	n.a.	n.a.	n.a.	n.a.	n.a.	n.a.	n.a.	23.91	d__Bacteria;p__Firmicutes;c__Clostridia;o__Oscillospirales;f__Oscillospiraceae;g__Intestinimonas	90.2	blast;92.70 iPHoP-RF;84.20
S1-wms_394571	Shared	Duplodnaviria	Heunggongvirae	Uroviricota	Caudoviricetes	n.a.	Unclassified	O	O	22.14	d__Bacteria;p__Bacteroidota;c__Bacteroidia;o__Bacteroidales;f__Rikenellaceae;g__Alistipes	95.4	CRISPR;96.80 iPHoP-RF;71.90
S11-wms-combined_41367	ME	n.a.	n.a.	n.a.	n.a.	n.a.	n.a.	n.a.	n.a.	40.35	d__Bacteria;p__Bacteroidota;c__Bacteroidia;o__Bacteroidales;f__Bacteroidaceae;g__Bacteroides	98.5	RaFAH;97.90 iPHoP-RF;74.10
S1-wms-combined_52708	Shared	n.a.	n.a.	n.a.	n.a.	n.a.	n.a.	n.a.	n.a.	55.79	d__Bacteria;p__Firmicutes;c__Clostridia;o__Oscillospirales;f__Ruminococcaceae;g__Faecalibacterium	96.3	blast;97.10 iPHoP-RF;70.00 RaFAH;54.80
S11-wms-combined_101337	Shared	Duplodnaviria	Heunggongvirae	Uroviricota	Caudoviricetes	n.a.	Unclassified	O	O	98.94	d__Bacteria;p__Proteobacteria;c__Alphaproteobacteria;o__RF32;f__CAG-239;g__CAG-495	91.6	blast;93.90 iPHoP-RF;86.30 CRISPR;51.40
S11-wms-combined_46816	Shared	n.a.	n.a.	n.a.	n.a.	n.a.	n.a.	n.a.	n.a.	20.69	d__Bacteria;p__Firmicutes;c__Clostridia;o__Oscillospirales;f__Ruminococcaceae;g__Gemmiger	95.4	blast;96.80 iPHoP-RF;91.40 CRISPR;69.40
S11-wms-combined_112733	Shared	Duplodnaviria	Heunggongvirae	Uroviricota	Caudoviricetes	n.a.	Unclassified	O	O	59.77	d__Bacteria;p__Desulfobacterota;c__Desulfovibrionia;o__Desulfovibrionales;f__Desulfovibrionaceae;g__Desulfovibrio	97.9	blast;96.30 iPHoP-RF;88.90 RaFAH;79.80 CRISPR;67.90
S11-wms-combined_48246	Shared	Duplodnaviria	Heunggongvirae	Uroviricota	Caudoviricetes	n.a.	Unclassified	O	O	99.82	d__Bacteria;p__Bacteroidota;c__Bacteroidia;o__Bacteroidales;f__Rikenellaceae;g__Alistipes	100	blast;98.50 RaFAH;96.00 iPHoP-RF;95.40
S11-wms-combined_116589	Shared	n.a.	n.a.	n.a.	n.a.	n.a.	n.a.	n.a.	n.a.	25.47	d__Bacteria;p__Firmicutes;c__Clostridia;o__Oscillospirales;f__Oscillospiraceae;g__Dysosmobacter	91.6	blast;93.90 iPHoP-RF;88.90
S11-wms-combined_49632	ME	Duplodnaviria	Heunggongvirae	Uroviricota	Caudoviricetes	n.a.	Unclassified	O	O	14.66	d__Bacteria;p__Firmicutes;c__Clostridia;o__Oscillospirales;f__Oscillospiraceae;g__Dysosmobacter	91.5	blast;93.80 iPHoP-RF;84.80
S11-wms-combined_73204	Shared	n.a.	n.a.	n.a.	n.a.	n.a.	n.a.	n.a.	n.a.	100	d__Bacteria;p__Bacteroidota;c__Bacteroidia;o__Bacteroidales;f__Bacteroidaceae;g__Bacteroides	99.4	RaFAH;99.30 iPHoP-RF;66.50
S11-wms-combined_83571	Shared	Duplodnaviria	Heunggongvirae	Uroviricota	Caudoviricetes	n.a.	Winoviridae	O	O	99.96	d__Bacteria;p__Bacteroidota;c__Bacteroidia;o__Bacteroidales;f__Rikenellaceae;g__Alistipes	100	RaFAH;98.50 blast;98.40 iPHoP-RF;96.70
S11-wms-combined_84326	ME	Duplodnaviria	Heunggongvirae	Uroviricota	Caudoviricetes	n.a.	Unclassified	O	O	16.31	d__Bacteria;p__Desulfobacterota;c__Desulfovibrionia;o__Desulfovibrionales;f__Desulfovibrionaceae;g__Desulfovibrio	94.2	blast;95.90 iPHoP-RF;89.50 CRISPR;83.30
S11-wms-combined_168048	Shared	Duplodnaviria	Heunggongvirae	Uroviricota	Caudoviricetes	n.a.	Unclassified	O	O	95.9	d__Bacteria;p__Firmicutes;c__Clostridia;o__Oscillospirales;f__Ruminococcaceae;g__Ruminiclostridium_E	93.4	blast;95.30 iPHoP-RF;66.80
S11-wms-combined_92114	Shared	n.a.	n.a.	n.a.	n.a.	n.a.	n.a.	n.a.	n.a.	19.84	d__Bacteria;p__Bacteroidota;c__Bacteroidia;o__Bacteroidales;f__Rikenellaceae;g__Alistipes	97.2	CRISPR;98.00 iPHoP-RF;97.10 blast;96.90
S1-wms_153615	ME	n.a.	n.a.	n.a.	n.a.	n.a.	n.a.	n.a.	n.a.		d__Bacteria;p__Bacteroidota;c__Bacteroidia;o__Bacteroidales;f__Barnesiellaceae;g__Barnesiella	93.8	blast;95.60 iPHoP-RF;91.10
S11-wms_14390	ME	n.a.	n.a.	n.a.	n.a.	n.a.	n.a.	n.a.	n.a.	96.43	d__Bacteria;p__Firmicutes;c__Clostridia;o__Oscillospirales;f__Oscillospiraceae;g__CAG-83	97.3	RaFAH;98.10
S11-wms_3116	Shared	Duplodnaviria	Heunggongvirae	Uroviricota	Caudoviricetes	n.a.	Unclassified	Unclassified	Mushuvirus	99.5	d__Bacteria;p__Firmicutes;c__Clostridia;o__Oscillospirales;f__Ruminococcaceae;g__Faecalibacterium	100	RaFAH;99.60 blast;92.10
S11-wms_320555	ME	Duplodnaviria	Heunggongvirae	Uroviricota	Caudoviricetes	n.a.	Unclassified	O	O	99.4	d__Bacteria;p__Bacteroidota;c__Bacteroidia;o__Bacteroidales;f__Bacteroidaceae;g__Bacteroides	99.7	RaFAH;95.40 blast;92.70 iPHoP-RF;74.60
S11-wms_330479	ME	Unclassified	n.a.	n.a.	n.a.	n.a.	Unclassified	Unclassified	Unclassified	20.29	d__Bacteria;p__Bacteroidota;c__Bacteroidia;o__Bacteroidales;f__Rikenellaceae;g__Alistipes	98.5	CRISPR;98.90 iPHoP-RF;78.50
S11-wms-combined_215723	Shared	Duplodnaviria	Heunggongvirae	Uroviricota	Caudoviricetes	n.a.	Duneviridae	O	O	94.77	d__Bacteria;p__Bacteroidota;c__Bacteroidia;o__Bacteroidales;f__Rikenellaceae;g__Alistipes	99.4	iPHoP-RF;97.10 blast;96.90 RaFAH;91.40
S1-wms_223620	Shared	Duplodnaviria	Heunggongvirae	Uroviricota	Caudoviricetes	n.a.	Unclassified	O	O	12.07	d__Bacteria;p__Firmicutes;c__Clostridia;o__Christensenellales;f__CAG-74;g__UBA11524	97.6	blast;98.30 CRISPR;88.60 iPHoP-RF;86.00
S12-wms-combined_111524	Shared	n.a.	n.a.	n.a.	n.a.	n.a.	n.a.	n.a.	n.a.	100	d__Bacteria;p__Bacteroidota;c__Bacteroidia;o__Bacteroidales;f__Bacteroidaceae;g__Bacteroides	99.6	RaFAH;99.60
S12-wms-combined_120133	Shared	Duplodnaviria	Heunggongvirae	Uroviricota	Caudoviricetes	n.a.	Unclassified	O	O	82.27	d__Bacteria;p__Firmicutes;c__Clostridia;o__Lachnospirales;f__Lachnospiraceae;g__Lachnospira	99.1	CRISPR;97.80 blast;97.00 iPHoP-RF;96.40 RaFAH;84.70
S11-wms-combined_236214	Shared	Duplodnaviria	Heunggongvirae	Uroviricota	Caudoviricetes	n.a.	Unclassified	O	O	48.8	d__Bacteria;p__Firmicutes;c__Clostridia;o__Oscillospirales;f__Ruminococcaceae;g__Faecalibacterium	94.6	blast;96.20 iPHoP-RF;57.60

S12-wms-combined_129554	SHHC	Duplodnaviria	Heunggongvirae	Uroviricota	Caudoviricetes	n.a.	Unclassified	Unclassified	Eponavirus	47.38	d__Bacteria;p__Firmicutes;c__Clostridia;o__Oscillospirales;f__Oscillospiraceae;g__F23-B02	95	blast;96.50 iPHoP-RF;75.90
S11-wms-combined_264050	Shared	Duplodnaviria	Heunggongvirae	Uroviricota	Caudoviricetes	n.a.	Unclassified	Unclassified	O	47.18	d__Bacteria;p__Firmicutes;c__Clostridia;o__Oscillospirales;f__Ruminococcaceae;g__Gemmiger	97.5	blast;98.20 iPHoP-RF;93.40 CRISPR;91.60
S12-wms-combined_147628	SHHC	Duplodnaviria	Heunggongvirae	Uroviricota	Caudoviricetes	n.a.	Unclassified	O	O	13.34	d__Bacteria;p__Firmicutes;c__Clostridia;o__Christensenellales;f__CAG-138;g__SFEL01	96	CRISPR;97.20 blast;93.30 iPHoP-RF;69.80
S11-wms-combined_267993	ME	Duplodnaviria	Heunggongvirae	Uroviricota	Caudoviricetes	n.a.	Unclassified	O	O	26.2	d__Bacteria;p__Firmicutes;c__Clostridia;o__Christensenellales;f__CAG-74;g__Firm-11	91.5	blast;93.80 iPHoP-RF;75.20
S12-wms-combined_168259	Shared	Duplodnaviria	Heunggongvirae	Uroviricota	Caudoviricetes	n.a.	Unclassified	O	O	10.85	d__Bacteria;p__Verrucomicrobiota;c__Verrucomicrobiae;o__Verrucomicrobiales;f__Akkermansiaceae;g__Akkermansia	100	RaFAH;98.70 blast;96.90 CRISPR;95.20 iPHoP-RF;87.60
S11-wms-combined_282227	ME	Duplodnaviria	Heunggongvirae	Uroviricota	Caudoviricetes	n.a.	Unclassified	O	O	60.6	d__Bacteria;p__Firmicutes;c__Clostridia;o__Lachnospirales;f__Lachnospiraceae;g__Mediterraneibacter	91.2	blast;93.60 iPHoP-RF;73.00
S12-wms-combined_210942	Shared	n.a.	n.a.	n.a.	n.a.	n.a.	n.a.	n.a.	n.a.	9.09	d__Bacteria;p__Firmicutes;c__Clostridia;o__Oscillospirales;f__Oscillospiraceae;g__ER4	96.3	blast;97.40
S11-wms-combined_329493	Shared	n.a.	n.a.	n.a.	n.a.	n.a.	n.a.	n.a.	n.a.	9.74	d__Bacteria;p__Firmicutes;c__Clostridia;o__Oscillospirales;f__Oscillospiraceae;g__Faecousia	96.7	blast;97.70 iPHoP-RF;80.00
S12-wms-combined_2450	Shared	Duplodnaviria	Heunggongvirae	Uroviricota	Caudoviricetes	n.a.	Winoviridae	O	O	57.23	d__Bacteria;p__Bacteroidota;c__Bacteroidia;o__Bacteroidales;f__Rikenellaceae;g__Tidjanibacter	90.2	blast;92.70 iPHoP-RF;50.70
S11-wms-combined_3507	Shared	Duplodnaviria	Heunggongvirae	Uroviricota	Caudoviricetes	n.a.	Unclassified	O	O	24.17	d__Bacteria;p__Firmicutes;c__Clostridia;o__Oscillospirales;f__Oscillospiraceae;g__CAG-83	98.1	CRISPR;98.60 blast;96.90 iPHoP-RF;92.80
S12-wms-combined_250551	Shared	Duplodnaviria	Heunggongvirae	Uroviricota	Caudoviricetes	n.a.	Unclassified	Unclassified	Unclassified	98.55	d__Bacteria;p__Bacteroidota;c__Bacteroidia;o__Bacteroidales;f__Bacteroidaceae;g__Bacteroides	100	RaFAH;99.40 blast;92.10 iPHoP-RF;83.90
S12-wms-combined_256299	SHHC	n.a.	n.a.	n.a.	n.a.	n.a.	n.a.	n.a.	n.a.	44.45	d__Bacteria;p__Bacteroidota;c__Bacteroidia;o__Bacteroidales;f__Muribaculaceae;g__UBA7173	90.1	blast;92.60 iPHoP-RF;75.70
S12-wms-combined_259783	Shared	Duplodnaviria	Heunggongvirae	Uroviricota	Caudoviricetes	n.a.	Unclassified	Unclassified	Unclassified	81.56	d__Bacteria;p__Firmicutes;c__Clostridia;o__Lachnospirales;f__Lachnospiraceae;g__Roseburia	96.7	CRISPR;97.70 blast;92.20 iPHoP-RF;70.60
S12-wms-combined_260036	Shared	Duplodnaviria	Heunggongvirae	Uroviricota	Caudoviricetes	Crassvirales	Steigviridae	O	O	44.16	d__Bacteria;p__Bacteroidota;c__Bacteroidia;o__Bacteroidales;f__Barnesiellaceae;g__Barnesiella	94.6	blast;96.20 iPHoP-RF;94.10 CRISPR;67.50
S11-wms-combined_63818	Shared	Duplodnaviria	Heunggongvirae	Uroviricota	Caudoviricetes	n.a.	Unclassified	O	O	50.37	d__Bacteria;p__Bacteroidota;c__Bacteroidia;o__Bacteroidales;f__Rikenellaceae;g__Alistipes	98.4	iPHoP-RF;97.10 blast;96.90 RaFAH;78.80
S11-wms-combined_120257	Shared	Duplodnaviria	Heunggongvirae	Uroviricota	Caudoviricetes	n.a.	Unclassified	Unclassified	O	40.42	d__Bacteria;p__Firmicutes;c__Clostridia;o__Oscillospirales;f__Oscillospiraceae;g__Flavonifractor	91.1	blast;93.50 iPHoP-RF;66.00
S12-wms-combined_263377	SHHC	Duplodnaviria	Heunggongvirae	Uroviricota	Caudoviricetes	n.a.	Unclassified	O	O	78.23	d__Bacteria;p__Firmicutes;c__Clostridia;o__Oscillospirales;f__Acutalibacteraceae;g__UBA1417	91.7	blast;94.00 iPHoP-RF;69.30
S12-wms-combined_264425	SHHC	n.a.	n.a.	n.a.	n.a.	n.a.	n.a.	n.a.	n.a.	99.56	d__Bacteria;p__Firmicutes;c__Clostridia;o__Oscillospirales;f__Acutalibacteraceae;g__UBA1417	91.8	blast;94.10 iPHoP-RF;57.30
S12-wms-combined_264845	Shared	n.a.	n.a.	n.a.	n.a.	n.a.	n.a.	n.a.	n.a.	6.58	d__Bacteria;p__Firmicutes;c__Clostridia;o__Oscillospirales;f__Oscillospiraceae;g__CAG-110	95	blast;96.50 iPHoP-RF;90.10
S12-wms-combined_282594	Shared	Duplodnaviria	Heunggongvirae	Uroviricota	Caudoviricetes	n.a.	Unclassified	O	O	18.25	d__Bacteria;p__Verrucomicrobiota;c__Verrucomicrobiae;o__Verrucomicrobiales;f__Akkermansiaceae;g__Akkermansia	100	CRISPR;98.70 RaFAH;98.50 blast;96.90 iPHoP-RF;78.50
S12-wms-combined_299293	Shared	n.a.	n.a.	n.a.	n.a.	n.a.	n.a.	n.a.	n.a.	62.93	d__Bacteria;p__Firmicutes;c__Clostridia;o__Christensenellales;f__CAG-74;g__SFFH01	90.4	blast;92.90 iPHoP-RF;75.40
S12-wms-combined_320727	Shared	Duplodnaviria	Heunggongvirae	Uroviricota	Caudoviricetes	n.a.	Unclassified	O	O	99.96	d__Bacteria;p__Bacteroidota;c__Bacteroidia;o__Bacteroidales;f__Bacteroidaceae;g__Bacteroides	93.8	RaFAH;93.60 blast;61.60 iPHoP-RF;55.70
S12-wms-combined_329242	Shared	n.a.	n.a.	n.a.	n.a.	n.a.	n.a.	n.a.	n.a.	5.39	d__Bacteria;p__Firmicutes;c__Clostridia;o__Oscillospirales;f__Oscillospiraceae;g__Dysosmobacter	91.1	blast;93.50 iPHoP-RF;56.00
S12-wms-combined_334806	Shared	n.a.	n.a.	n.a.	n.a.	n.a.	n.a.	n.a.	n.a.	38.66	d__Bacteria;p__Firmicutes;c__Clostridia;o__Oscillospirales;f__Oscillospiraceae;g__CAG-83	91.6	blast;93.90 iPHoP-RF;61.90
S11-wms-combined_179605	Shared	n.a.	n.a.	n.a.	n.a.	n.a.	n.a.	n.a.	n.a.	37.98	d__Bacteria;p__Firmicutes;c__Clostridia;o__Lachnospirales;f__Lachnospiraceae;g__UBA9502	91.2	blast;93.60 iPHoP-RF;64.00
S12-wms-combined_345948	SHHC	Duplodnaviria	Heunggongvirae	Uroviricota	Caudoviricetes	n.a.	Unclassified	Unclassified	Lughvirus	59.64	d__Bacteria;p__Firmicutes;c__Clostridia;o__Oscillospirales;f__Ruminococcaceae;g__Faecalibacterium	98.8	blast;96.90 iPHoP-RF;92.10 RaFAH;84.90 CRISPR;63.30
S12-wms-combined_351847	Shared	n.a.	n.a.	n.a.	n.a.	n.a.	n.a.	n.a.	n.a.	11.93	d__Bacteria;p__Bacteroidota;c__Bacteroidia;o__Bacteroidales;f__Barnesiellaceae;g__Barnesiella	95	blast;96.50 iPHoP-RF;88.20 CRISPR;72.90
S12-wms-combined_375343	Shared	n.a.	n.a.	n.a.	n.a.	n.a.	n.a.	n.a.	n.a.	20.72	d__Bacteria;p__Bacteroidota;c__Bacteroidia;o__Bacteroidales;f__UBA11471;g__UBA11471	91.6	blast;93.90 iPHoP-RF;88.50
S12-wms-combined_129799	Shared	Duplodnaviria	Heunggongvirae	Uroviricota	Caudoviricetes	n.a.	Unclassified	Unclassified	O	52.43	d__Bacteria;p__Firmicutes;c__Clostridia;o__Oscillospirales;f__Oscillospiraceae;g__ER4	95.6	blast;96.90 iPHoP-RF;63.00
S12-wms-combined_393703	Shared	n.a.	n.a.	n.a.	n.a.	n.a.	n.a.	n.a.	n.a.	8.02	d__Bacteria;p__Firmicutes;c__Clostridia;o__Oscillospirales;f__Oscillospiraceae;g__ER4	93.5	blast;95.40 iPHoP-RF;50.20
S12-wms-combined_151843	Shared	Duplodnaviria	Heunggongvirae	Uroviricota	Caudoviricetes	n.a.	Unclassified	O	O	24.49	d__Bacteria;p__Firmicutes;c__Clostridia;o__Christensenellales;f__CAG-74;g__UBA11524	95.6	blast;96.90 iPHoP-RF;92.80
S12-wms-combined_157167	SHHC	n.a.	n.a.	n.a.	n.a.	n.a.	n.a.	n.a.	n.a.	8.42	d__Bacteria;p__Firmicutes;c__Clostridia;o__Oscillospirales;f__Oscillospiraceae;g__CAG-83	92.5	blast;94.60 iPHoP-RF;70.90
S12-wms-combined_164656	SHHC	n.a.	n.a.	n.a.	n.a.	n.a.	n.a.	n.a.	n.a.	3.93	d__Bacteria;p__Firmicutes;c__Clostridia;o__Christensenellales;f__CAG-917;g__CAG-475	94.7	blast;96.30 iPHoP-RF;68.60
S12-wms-combined_71235	SHHC	Duplodnaviria	Heunggongvirae	Uroviricota	Caudoviricetes	n.a.	Unclassified	Unclassified	Lughvirus	58.55	d__Bacteria;p__Firmicutes;c__Clostridia;o__Oscillospirales;f__Ruminococcaceae;g__Faecalibacterium	95.7	blast;96.80 iPHoP-RF;84.20 RaFAH;53.60
S12-wms-combined_91231	SHHC	n.a.	n.a.	n.a.	n.a.	n.a.	n.a.	n.a.	n.a.	99.33	d__Bacteria;p__Bacteroidota;c__Bacteroidia;o__Bacteroidales;f__Bacteroidaceae;g__Bacteroides	100	RaFAH;99.80 iPHoP-RF;96.10 blast;94.00
S11-wms-combined_226165	Shared	Duplodnaviria	Heunggongvirae	Uroviricota	Caudoviricetes	n.a.	Unclassified	O	O	80.02	d__Bacteria;p__Bacteroidota;c__Bacteroidia;o__Bacteroidales;f__Rikenellaceae;g__Alistipes	98.7	iPHoP-RF;97.10 blast;93.80 RaFAH;82.70
S12-wms-combined_190114	Shared	Duplodnaviria	Heunggongvirae	Uroviricota	Caudoviricetes	n.a.	Unclassified	O	O	93.65	d__Bacteria;p__Bacteroidota;c__Bacteroidia;o__Bacteroidales;f__Bacteroidaceae;g__Bacteroides	100	RaFAH;99.60 blast;96.70 iPHoP-RF;95.10
S12-wms_12658	Shared	Duplodnaviria	Heunggongvirae	Uroviricota	Caudoviricetes	n.a.	Unclassified	O	O	51.35	d__Bacteria;p__Bacteroidota;c__Bacteroidia;o__Bacteroidales;f__Bacteroidaceae;g__Prevotellamassilia	97.9	CRISPR;98.50 blast;60.80
S12-wms-combined_201125	Shared	Duplodnaviria	Heunggongvirae	Uroviricota	Caudoviricetes	n.a.	Unclassified	O	O	83.81	d__Bacteria;p__Bacteroidota;c__Bacteroidia;o__Bacteroidales;f__Bacteroidaceae;g__Bacteroides	99.4	iPHoP-RF;96.40 RaFAH;93.60 blast;77.30
S12-wms-combined_223043	SHHC	Duplodnaviria	Heunggongvirae	Uroviricota	Caudoviricetes	n.a.	Unclassified	O	O	99.11	d__Bacteria;p__Firmicutes;c__Clostridia;o__Lachnospirales;f__Lachnospiraceae;g__Enterocloster	91.5	blast;93.80 iPHoP-RF;64.30
S12-wms_193565	SHHC	Duplodnaviria	Heunggongvirae	Uroviricota	Caudoviricetes	n.a.	Unclassified	Unclassified	Mushuvirus	96.64	d__Bacteria;p__Firmicutes;c__Clostridia;o__Oscillospirales;f__Ruminococcaceae;g__Faecalibacterium	90.2	RaFAH;92.70
S12-wms-combined_23517	Shared	Duplodnaviria	Heunggongvirae	Uroviricota	Caudoviricetes	n.a.	Unclassified	O	O	28.19	d__Bacteria;p__Firmicutes;c__Clostridia;o__Oscillospirales;f__Oscillospiraceae;g__CAG-83	98.5	blast;98.90 CRISPR;98.40 iPHoP-RF;93.10
S12-wms_373775	Shared	n.a.	n.a.	n.a.	n.a.	n.a.	n.a.	n.a.	n.a.	11.82	d__Bacteria;p__Firmicutes;c__Clostridia;o__Oscillospirales;f__Oscillospiraceae;g__CAG-83	91.5	blast;93.80 iPHoP-RF;82.80
S12-wms_386692	Shared	n.a.	n.a.	n.a.	n.a.	n.a.	n.a.	n.a.	n.a.	94.2	d__Bacteria;p__Bacteroidota;c__Bacteroidia;o__Bacteroidales;f__Rikenellaceae;g__Alistipes	96.2	CRISPR;97.30 iPHoP-RF;97.10 blast;96.60
S12-wms_401372	SHHC	Duplodnaviria	Heunggongvirae	Uroviricota	Caudoviricetes	n.a.	Unclassified	Unclassified	Unclassified	65.9	d__Bacteria;p__Patescibacteria;c__Paceibacteria;o__Paceibacterales;f__UBA5633;g__MWCK01	91.1	blast;93.50
S12-wms-combined_264077	SHHC	Duplodnaviria	Heunggongvirae	Uroviricota	Caudoviricetes	n.a.	Unclassified	O	O	19.02	d__Bacteria;p__Firmicutes;c__Clostridia;o__Oscillospirales;f__Ruminococcaceae;g__Faecalibacterium	90.4	blast;92.90 iPHoP-RF;86.30
S17-wms-combined_136622	ME	n.a.	n.a.	n.a.	n.a.	n.a.	n.a.	n.a.	n.a.	24.07	d__Bacteria;p__Firmicutes;c__Clostridia;o__Oscillospirales;f__Oscillospiraceae;g__Evtepia	90.7	blast;93.10 iPHoP-RF;81.60
S11-wms-combined_321938	Shared	Duplodnaviria	Heunggongvirae	Uroviricota	Caudoviricetes	n.a.	Unclassified	O	O	99.59	d__Bacteria;p__Bacteroidota;c__Bacteroidia;o__Bacteroidales;f__Bacteroidaceae;g__Bacteroides	100	RaFAH;98.70 iPHoP-RF;94.10 blast;80.70
S17-wms-combined_146850	ME	n.a.	n.a.	n.a.	n.a.	n.a.	n.a.	n.a.	n.a.	3.97	d__Bacteria;p__Firmicutes;c__Clostridia;o__Oscillospirales;f__Oscillospiraceae;g__Flavonifractor	91.2	blast;93.60 iPHoP-RF;74.30
S17-wms-combined_157468	ME	n.a.	n.a.	n.a.	n.a.	n.a.	n.a.	n.a.	n.a.	3.55	d__Bacteria;p__Firmicutes;c__Clostridia;o__Oscillospirales;f__Oscillospiraceae;g__Flavonifractor	93.4	blast;95.30 iPHoP-RF;74.80
S12-wms-combined_293354	Shared	Duplodnaviria	Heunggongvirae	Uroviricota	Caudoviricetes	n.a.	Unclassified	O	O	10.3	d__Bacteria;p__Firmicutes;c__Clostridia;o__Christensenellales;f__CAG-74;g__UBA11524	95.6	blast;96.90 iPHoP-RF;92.80
S17-wms-combined_158354	ME	n.a.	n.a.	n.a.	n.a.	n.a.	n.a.	n.a.	n.a.	99.65	d__Bacteria;p__Bacteroidota;c__Bacteroidia;o__Bacteroidales;f__Bacteroidaceae;g__Bacteroides	100	RaFAH;99.30 iPHoP-RF;97.10 blast;92.60
S17-wms-combined_162786	ME	n.a.	n.a.	n.a.	n.a.	n.a.	n.a.	n.a.	n.a.	7.07	d__Bacteria;p__Firmicutes;c__Clostridia;o__Oscillospirales;f__Oscillospiraceae;g__Marseille-P3106	94.7	blast;96.30 iPHoP-RF;71.30
S17-wms-combined_16309	ME	Duplodnaviria	Heunggongvirae	Uroviricota	Caudoviricetes	n.a.	Unclassified	O	O	27.66	d__Bacteria;p__Bacteroidota;c__Bacteroidia;o__Bacteroidales;f__Bacteroidaceae;g__Bacteroides	100	RaFAH;99.10 iPHoP-RF;97.10 blast;90.90
S12-wms-combined_302876	SHHC	Duplodnaviria	Heunggongvirae	Uroviricota	Caudoviricetes	n.a.	Unclassified	O	O	24.68	d__Bacteria;p__Firmicutes;c__Clostridia;o__Lachnospirales;f__Lachnospiraceae;g__Lachnospira	93.9	blast;95.70 iPHoP-RF;73.50
S17-wms-combined_175924	ME	Duplodnaviria	Heunggongvirae	Uroviricota	Caudoviricetes	n.a.	Unclassified	Unclassified	Mushuvirus	99.75	d__Bacteria;p__Firmicutes;c__Clostridia;o__Oscillospirales;f__Oscillospiraceae;g__Flavonifractor	92.1	blast;94.30 iPHoP-RF;78.00
S12-wms-combined_313953	SHHC	Duplodnaviria	Heunggongvirae	Uroviricota	Caudoviricetes	n.a.	Unclassified	O	O	97.5	d__Bacteria;p__Proteobacteria;c__Alphaproteobacteria;o__RF32;f__CAG-239;g__CAG-495	90.9	blast;93.30 iPHoP-RF;78.50
S11-wms-combined_64392	Shared	Duplodnaviria	Heunggongvirae	Uroviricota	Caudoviricetes	n.a.	Unclassified	O	O	100	d__Bacteria;p__Bacteroidota;c__Bacteroidia;o__Bacteroidales;f__Rikenellaceae;g__Alistipes	99.6	iPHoP-RF;97.10 blast;96.80 RaFAH;92.70
S17-wms-combined_179771	Shared	n.a.	n.a.	n.a.	n.a.	n.a.	n.a.	n.a.	n.a.	49.28	d__Bacteria;p__Bacteroidota;c__Bacteroidia;o__Bacteroidales;f__Bacteroidaceae;g__Bacteroides	97.9	blast;98.30 iPHoP-RF;96.10 CRISPR;90.30 RaFAH;56.00
S17-wms-combined_193958	Shared	Duplodnaviria	Heunggongvirae	Uroviricota	Caudoviricetes	n.a.	Unclassified	Unclassified	Mushuvirus	96.31	d__Bacteria;p__Firmicutes;c__Clostridia;o__Oscillospirales;f__Ruminococcaceae;g__Faecalibacterium	94.2	blast;95.90 iPHoP-RF;53.80
S17-wms-combined_199746	Shared	Duplodnaviria	Heunggongvirae	Uroviricota	Caudoviricetes	n.a.	Unclassified	O	O	69.95	d__Bacteria;p__Bacteroidota;c__Bacteroidia;o__Bacteroidales;f__Rikenellaceae;g__Alistipes	97.6	CRISPR;98.30 iPHoP-RF;97.10 blast;96.90
S12-wms-combined_323943	SHHC	Duplodnaviria	Heunggongvirae	Uroviricota	Caudoviricetes	n.a.	Unclassified	Unclassified	O	18.94	d__Bacteria;p__Firmicutes;c__Clostridia;o__Oscillospirales;f__Ruminococcaceae;g__Gemmiger	94.6	CRISPR;96.20 blast;93.90 iPHoP-RF;91.80
S12-wms-combined_328554	Shared	Duplodnaviria	Heunggongvirae	Uroviricota	Caudoviricetes	n.a.	Unclassified	O	O	31.03	d__Bacteria;p__Bacteroidota;c__Bacteroidia;o__Bacteroidales;f__Rikenellaceae;g__Alistipes	96.3	iPHoP-RF;96.70 blast;95.80 RaFAH;60.10
S17-wms-combined_223748	ME	n.a.	n.a.	n.a.	n.a.	n.a.	n.a.	n.a.	n.a.	4.98	d__Bacteria;p__Firmicutes;c__Clostridia;o__Lachnospirales;f__Lachnospiraceae;g__Enterocloster	91.2	blast;93.60 iPHoP-RF;75.20
S17-wms-combined_225356	ME	n.a.	n.a.	n.a.	n.a.	n.a.	n.a.	n.a.	n.a.	10.23	d__Bacteria;p__Actinobacteriota;c__Coriobacteriia;o__Coriobacteriales;f__Eggerthellaceae;g__Eggerthella	96.3	CRISPR;97.40 blast;92.10 iPHoP-RF;87.00
S17-wms-combined_233761	Shared	n.a.	n.a.	n.a.	n.a.	n.a.	n.a.	n.a.	n.a.	88	d__Bacteria;p__Bacteroidota;c__Bacteroidia;o__Bacteroidales;f__Bacteroidaceae;g__Bacteroides	100	RaFAH;99.00 blast;98.60 iPHoP-RF;96.40 CRISPR;80.00
S17-wms-combined_247494	Shared	Duplodnaviria	Heunggongvirae	Uroviricota	Caudoviricetes	n.a.	Unclassified	O	O	24.14	d__Bacteria;p__Firmicutes;c__Clostridia;o__Oscillospirales;f__Oscillospiraceae;g__Flavonifractor	91.5	blast;93.80 iPHoP-RF;91.40
S17-wms-combined_259811	Shared	Duplodnaviria	Heunggongvirae	Uroviricota	Caudoviricetes	n.a.	Unclassified	O	O	66.5	d__Bacteria;p__Firmicutes;c__Clostridia;o__Oscillospirales;f__Oscillospiraceae;g__Lawsonibacter	90.9	blast;93.30
S11-wms_32691	ME	n.a.	n.a.	n.a.	n.a.	n.a.	n.a.	n.a.	n.a.	12	d__Bacteria;p__Firmicutes;c__Clostridia;o__Oscillospirales;f__Oscillospiraceae;g__Pseudoflavonifractor	94.9	blast;96.40 iPHoP-RF;66.80
S12-wms-combined_358797	Shared	Duplodnaviria	Heunggongvirae	Uroviricota	Caudoviricetes	n.a.	Unclassified	O	O	99.42	d__Bacteria;p__Bacteroidota;c__Bacteroidia;o__Bacteroidales;f__Rikenellaceae;g__Alistipes	100	blast;98.40 iPHoP-RF;96.10 RaFAH;95.40
S17-wms-combined_260343	ME	Duplodnaviria	Heunggongvirae	Uroviricota	Caudoviricetes	n.a.	Unclassified	Unclassified	Toutatisvirus	38.59	d__Bacteria;p__Firmicutes;c__Clostridia;o__Oscillospirales;f__Oscillospiraceae;g__Flavonifractor	90.8	blast;93.20
S12-wms-combined_368562	Shared	Duplodnaviria	Heunggongvirae	Uroviricota	Caudoviricetes	n.a.	Unclassified	O	O	98.38	d__Bacteria;p__Bacteroidota;c__Bacteroidia;o__Bacteroidales;f__Bacteroidaceae;g__Bacteroides	100	RaFAH;99.70 blast;93.90 iPHoP-RF;92.80
S12-wms-combined_371541	Shared	n.a.	n.a.	n.a.	n.a.	n.a.	n.a.	n.a.	n.a.	14.41	d__Bacteria;p__Bacteroidota;c__Bacteroidia;o__Bacteroidales;f__UBA11471;g__UBA11471	91.6	blast;93.90 iPHoP-RF;90.10
S17-wms-combined_70983	Shared	n.a.	n.a.	n.a.	n.a.	n.a.	n.a.	n.a.	n.a.	7.24	d__Bacteria;p__Bacteroidota;c__Bacteroidia;o__Bacteroidales;f__Bacteroidaceae;g__Bacteroides	95.9	iPHoP-RF;97.10 blast;85.40
S17-wms-combined_79709	ME	Duplodnaviria	Heunggongvirae	Uroviricota	Caudoviricetes	n.a.	Unclassified	O	O	19.94	d__Bacteria;p__Firmicutes;c__Clostridia;o__Oscillospirales;f__Oscillospiraceae;g__An92	91.4	blast;93.70 iPHoP-RF;92.10
S17-wms-combined_90378	ME	Duplodnaviria	Heunggongvirae	Uroviricota	Caudoviricetes	n.a.	Unclassified	Unclassified	Lughvirus	72.41	d__Bacteria;p__Firmicutes;c__Clostridia;o__Oscillospirales;f__Ruminococcaceae;g__Faecalibacterium	98.7	blast;96.90 RaFAH;83.30 iPHoP-RF;76.80 CRISPR;69.70
S12-wms-combined_117447	SHHC	n.a.	n.a.	n.a.	n.a.	n.a.	n.a.	n.a.	n.a.		d__Bacteria;p__Firmicutes;c__Clostridia;o__Christensenellales;f__CAG-917;g__	91	blast;93.40 iPHoP-RF;67.30
S12-wms-combined_48648	Shared	n.a.	n.a.	n.a.	n.a.	n.a.	n.a.	n.a.	n.a.	6.97	d__Bacteria;p__Firmicutes;c__Clostridia;o__Oscillospirales;f__Oscillospiraceae;g__ER4	90.9	blast;93.30 iPHoP-RF;72.40
S12-wms-combined_117628	Shared	Duplodnaviria	Heunggongvirae	Uroviricota	Caudoviricetes	n.a.	Unclassified	Unclassified	O	32.91	d__Bacteria;p__Firmicutes;c__Clostridia;o__Oscillospirales;f__Acutalibacteraceae;g__CAG-217	96.2	blast;97.30 iPHoP-RF;73.20
S17-wms_124308	ME	Duplodnaviria	Heunggongvirae	Uroviricota	Caudoviricetes	n.a.	Unclassified	O	O	51.69	d__Bacteria;p__Bacteroidota;c__Bacteroidia;o__Bacteroidales;f__Bacteroidaceae;g__Bacteroides	99.1	iPHoP-RF;96.40 RaFAH;90.70
S12-wms-combined_63649	Shared	n.a.	n.a.	n.a.	n.a.	n.a.	n.a.	n.a.	n.a.	5.54	d__Bacteria;p__Firmicutes;c__Clostridia;o__Oscillospirales;f__Oscillospiraceae;g__CAG-83	91.7	blast;94.00 iPHoP-RF;90.10
S17-wms_254872	ME	Monodnaviria	Sangervirae	Phixviricota	Malgrandaviricetes	Petitvirales	Microviridae	Unclassified	Microvirus	5.1	d__Bacteria;p__Bacteroidota;c__Bacteroidia;o__Bacteroidales;f__Bacteroidaceae;g__Bacteroides_G	95.3	iPHoP-RF;96.70
S2-wms-combined_101826	Shared	Duplodnaviria	Heunggongvirae	Uroviricota	Caudoviricetes	n.a.	Unclassified	Unclassified	Unclassified	26.15	d__Bacteria;p__Firmicutes;c__Clostridia;o__Oscillospirales;f__Ruminococcaceae;g__Ruminococcus_D	98.8	CRISPR;99.10 blast;96.90 iPHoP-RF;92.80
S2-wms-combined_113619	Shared	Duplodnaviria	Heunggongvirae	Uroviricota	Caudoviricetes	n.a.	Unclassified	O	O	97.74	d__Bacteria;p__Bacteroidota;c__Bacteroidia;o__Bacteroidales;f__Rikenellaceae;g__Alistipes	99	blast;96.60 RaFAH;87.50 CRISPR;82.60 iPHoP-RF;77.80
S2-wms-combined_126134	Shared	n.a.	n.a.	n.a.	n.a.	n.a.	n.a.	n.a.	n.a.	14.26	d__Bacteria;p__Firmicutes;c__Clostridia;o__Oscillospirales;f__Oscillospiraceae;g__Lawsonibacter	90.1	blast;92.60 iPHoP-RF;75.70
S2-wms-combined_132966	SHHC	Monodnaviria	Loebvirae	Hofneiviricota	Faserviricetes	Tubulavirales	Inoviridae	Unclassified	Inovirus		d__Bacteria;p__Bacteroidota;c__Bacteroidia;o__Bacteroidales;f__Bacteroidaceae;g__Bacteroides	93.1	iPHoP-RF;95.10
S12-wms-combined_148741	Shared	n.a.	n.a.	n.a.	n.a.	n.a.	n.a.	n.a.	n.a.	20.82	d__Bacteria;p__Firmicutes;c__Clostridia;o__Oscillospirales;f__Oscillospiraceae;g__CAG-83	93.9	blast;95.70 iPHoP-RF;94.70 CRISPR;93.00
S2-wms-combined_135139	Shared	n.a.	n.a.	n.a.	n.a.	n.a.	n.a.	n.a.	n.a.	38.63	d__Bacteria;p__Verrucomicrobiota;c__Verrucomicrobiae;o__Verrucomicrobiales;f__Akkermansiaceae;g__Akkermansia	97.8	CRISPR;98.40 blast;96.90 iPHoP-RF;88.20
S2-wms-combined_135154	Shared	n.a.	n.a.	n.a.	n.a.	n.a.	n.a.	n.a.	n.a.	34.29	d__Bacteria;p__Firmicutes;c__Negativicutes;o__Veillonellales;f__Dialisteraceae;g__Dialister	95.9	blast;97.10 iPHoP-RF;97.10 CRISPR;75.50
S12-wms_235125	SHHC	n.a.	n.a.	n.a.	n.a.	n.a.	n.a.	n.a.	n.a.	20.48	d__Bacteria;p__Firmicutes;c__Clostridia;o__Oscillospirales;f__Oscillospiraceae;g__ER4	90.8	blast;93.20 iPHoP-RF;64.80
S2-wms-combined_165833	SHHC	Duplodnaviria	Heunggongvirae	Uroviricota	Caudoviricetes	n.a.	Unclassified	Unclassified	Mushuvirus	74.42	d__Bacteria;p__Firmicutes;c__Clostridia;o__Lachnospirales;f__Lachnospiraceae;g__Roseburia	97.9	blast;96.20 iPHoP-RF;92.80 RaFAH;79.80
S2-wms-combined_166665	Shared	Duplodnaviria	Heunggongvirae	Uroviricota	Caudoviricetes	n.a.	Unclassified	O	O	48.97	d__Bacteria;p__Firmicutes;c__Clostridia;o__Oscillospirales;f__Ruminococcaceae;g__Faecalibacterium	95.4	blast;96.80 iPHoP-RF;96.10
S2-wms-combined_176376	SHHC	Duplodnaviria	Heunggongvirae	Uroviricota	Caudoviricetes	n.a.	Unclassified	O	O	94.42	d__Bacteria;p__Firmicutes;c__Clostridia;o__Lachnospirales;f__Lachnospiraceae;g__Blautia_A	96.7	blast;93.20 CRISPR;89.80 iPHoP-RF;87.90 RaFAH;83.30
S17-wms-combined_106940	Shared	Duplodnaviria	Heunggongvirae	Uroviricota	Caudoviricetes	n.a.	Unclassified	O	O	77.34	d__Bacteria;p__Bacteroidota;c__Bacteroidia;o__Bacteroidales;f__Bacteroidaceae;g__Bacteroides	99.6	iPHoP-RF;97.10 blast;96.90 RaFAH;92.40
S2-wms-combined_196783	Shared	Duplodnaviria	Heunggongvirae	Uroviricota	Caudoviricetes	n.a.	Unclassified	O	O	32.37	d__Bacteria;p__Firmicutes;c__Clostridia;o__Oscillospirales;f__Oscillospiraceae;g__Intestinimonas	95.9	CRISPR;97.10 blast;91.40 iPHoP-RF;85.10
S12-wms-combined_184338	Shared	Duplodnaviria	Heunggongvirae	Uroviricota	Caudoviricetes	n.a.	Unclassified	O	O	20.69	d__Bacteria;p__Firmicutes;c__Clostridia;o__Oscillospirales;f__Oscillospiraceae;g__Dysosmobacter	91.6	blast;93.90 iPHoP-RF;83.90
S17-wms-combined_146050	Shared	n.a.	n.a.	n.a.	n.a.	n.a.	n.a.	n.a.	n.a.	6.87	d__Bacteria;p__Bacteroidota;c__Bacteroidia;o__Bacteroidales;f__Bacteroidaceae;g__Bacteroides	100	RaFAH;98.70 blast;96.80 iPHoP-RF;92.80
S12-wms-combined_189323	Shared	Duplodnaviria	Heunggongvirae	Uroviricota	Caudoviricetes	n.a.	Unclassified	O	O	72.16	d__Bacteria;p__Bacteroidota;c__Bacteroidia;o__Bacteroidales;f__Rikenellaceae;g__Alistipes	99.4	blast;97.60 iPHoP-RF;96.70 RaFAH;89.00 CRISPR;53.90
S2-wms-combined_197577	SHHC	Duplodnaviria	Heunggongvirae	Uroviricota	Caudoviricetes	n.a.	Unclassified	Unclassified	Unclassified	42.77	d__Bacteria;p__Firmicutes;c__Negativicutes;o__Selenomonadales;f__Selenomonadaceae;g__Megamonas	91.1	blast;93.50 iPHoP-RF;53.30
S2-wms-combined_210822	Shared	n.a.	n.a.	n.a.	n.a.	n.a.	n.a.	n.a.	n.a.	37.52	d__Bacteria;p__Bacteroidota;c__Bacteroidia;o__Bacteroidales;f__Bacteroidaceae;g__Bacteroides	91.8	iPHoP-RF;89.50 blast;81.20 RaFAH;71.80
S2-wms-combined_218002	Shared	Duplodnaviria	Heunggongvirae	Uroviricota	Caudoviricetes	n.a.	Unclassified	O	O	99.73	d__Bacteria;p__Firmicutes;c__Clostridia;o__Lachnospirales;f__Lachnospiraceae;g__Roseburia	99.9	blast;98.50 iPHoP-RF;92.40 RaFAH;92.30

S2-wms-combined_229529	Shared	Duplodnaviria	Heunggongvirae	Uroviricota	Caudoviricetes	n.a.	Unclassified	O	O	100	d__Bacteria;p__Bacteroidota;c__Bacteroidia;o__Bacteroidales;f__Bacteroidaceae;g__Bacteroides	99.9	blast;96.90 iPHoP-RF;96.70 RaFAH;96.10
S17-wms-combined_171305	ME	Duplodnaviria	Heunggongvirae	Uroviricota	Caudoviricetes	n.a.	Unclassified	O	O	56.56	d__Bacteria;p__Bacteroidota;c__Bacteroidia;o__Bacteroidales;f__Tannerellaceae;g__Parabacteroides	98.7	CRISPR;99.00 blast;98.50 iPHoP-RF;96.40
S2-wms-combined_238929	Shared	Duplodnaviria	Heunggongvirae	Uroviricota	Caudoviricetes	n.a.	Unclassified	O	O	17.21	d__Bacteria;p__Firmicutes;c__Clostridia;o__Oscillospirales;f__Oscillospiraceae;g__CAG-170	91.4	blast;93.70 iPHoP-RF;78.30
S17-wms-combined_185239	Shared	n.a.	n.a.	n.a.	n.a.	n.a.	n.a.	n.a.	n.a.	7.9	d__Bacteria;p__Firmicutes;c__Clostridia;o__Oscillospirales;f__Oscillospiraceae;g__Flavonifractor	91.4	blast;93.70 iPHoP-RF;83.60
S17-wms-combined_186314	ME	Duplodnaviria	Heunggongvirae	Uroviricota	Caudoviricetes	n.a.	Unclassified	O	O	29.67	d__Bacteria;p__Actinobacteriota;c__Actinomycetia;o__Actinomycetales;f__Bifidobacteriaceae;g__Bifidobacterium	99.6	CRISPR;99.60 iPHoP-RF;97.10 blast;96.90
S2-wms-combined_31084	SHHC	Duplodnaviria	Heunggongvirae	Uroviricota	Caudoviricetes	n.a.	Unclassified	O	O	22.49	d__Bacteria;p__Firmicutes;c__Clostridia;o__Lachnospirales;f__Lachnospiraceae;g__Acetivibrio_A	90	blast;92.50 iPHoP-RF;69.10
S2-wms-combined_58121	Shared	n.a.	n.a.	n.a.	n.a.	n.a.	n.a.	n.a.	n.a.	21.08	d__Bacteria;p__Bacteroidota;c__Bacteroidia;o__Bacteroidales;f__Bacteroidaceae;g__Bacteroides	95.9	iPHoP-RF;97.10 blast;91.00
S2-wms-combined_6605	Shared	n.a.	n.a.	n.a.	n.a.	n.a.	n.a.	n.a.	n.a.	7.53	d__Bacteria;p__Firmicutes;c__Clostridia;o__Oscillospirales;f__Oscillospiraceae;g__Flavonifractor	91.4	blast;93.70 iPHoP-RF;82.50
S12-wms-combined_25459	Shared	n.a.	n.a.	n.a.	n.a.	n.a.	n.a.	n.a.	n.a.	9.92	d__Bacteria;p__Bacteroidota;c__Bacteroidia;o__Bacteroidales;f__Rikenellaceae;g__Alistipes	95.6	blast;96.90 iPHoP-RF;96.70
S2-wms-combined_85381	SHHC	Duplodnaviria	Heunggongvirae	Uroviricota	Caudoviricetes	n.a.	Unclassified	Unclassified	Eponavirus	45.07	d__Bacteria;p__Firmicutes;c__Clostridia;o__Lachnospirales;f__Lachnospiraceae;g__CAG-81	93.8	blast;95.60 iPHoP-RF;80.60
S2-wms-combined_93845	Shared	Duplodnaviria	Heunggongvirae	Uroviricota	Caudoviricetes	n.a.	Unclassified	O	O	99.44	d__Bacteria;p__Firmicutes;c__Clostridia;o__Oscillospirales;f__Oscillospiraceae;g__ER4	98.7	CRISPR;99.00 blast;91.30 iPHoP-RF;81.90
S2-wms_128831	SHHC	Duplodnaviria	Heunggongvirae	Uroviricota	Caudoviricetes	n.a.	Unclassified	Unclassified	Unclassified	98.35	d__Bacteria;p__Proteobacteria;c__Gammaproteobacteria;o__Enterobacterales;f__Enterobacteriaceae;g__Escherichia	95.6	RaFAH;96.90
S2-wms_128962	Shared	Duplodnaviria	Heunggongvirae	Uroviricota	Caudoviricetes	Crassvirales	Suoliviridae	O	O	46.73	d__Bacteria;p__Bacteroidota;c__Bacteroidia;o__Bacteroidales;f__Bacteroidaceae;g__Prevotella	91.8	RaFAH;94.10
S2-wms_130687	Shared	Duplodnaviria	Heunggongvirae	Uroviricota	Caudoviricetes	n.a.	Unclassified	Unclassified	Unclassified	95.89	d__Bacteria;p__Firmicutes;c__Clostridia;o__Oscillospirales;f__Acutalibacteraceae;g__Ruminococcus_E	99.7	CRISPR;98.90 blast;96.90 iPHoP-RF;92.40 RaFAH;85.50
S17-wms-combined_72837	ME	Duplodnaviria	Heunggongvirae	Uroviricota	Caudoviricetes	n.a.	Unclassified	O	O	25.38	d__Bacteria;p__Firmicutes;c__Clostridia;o__Oscillospirales;f__Ruminococcaceae;g__Gemmiger	95.2	blast;96.60 iPHoP-RF;74.80
S2-wms_140933	Shared	Duplodnaviria	Heunggongvirae	Uroviricota	Caudoviricetes	n.a.	Unclassified	Unclassified	Unclassified	43.87	d__Bacteria;p__Firmicutes;c__Clostridia;o__Lachnospirales;f__Lachnospiraceae;g__Blautia_A	93.8	blast;90.60 RaFAH;76.60 iPHoP-RF;62.50
S2-wms_156439	Shared	Duplodnaviria	Heunggongvirae	Uroviricota	Caudoviricetes	n.a.	Unclassified	O	O	96.43	d__Bacteria;p__Bacteroidota;c__Bacteroidia;o__Bacteroidales;f__Bacteroidaceae;g__Bacteroides	100	RaFAH;99.60 blast;96.80 iPHoP-RF;95.70
S12-wms-combined_270839	Shared	n.a.	n.a.	n.a.	n.a.	n.a.	n.a.	n.a.	n.a.	20.16	d__Bacteria;p__Firmicutes;c__Clostridia;o__Oscillospirales;f__Oscillospiraceae;g__CAG-83	92.5	blast;94.60 iPHoP-RF;79.80
S17-wms-combined_95360	Shared	Duplodnaviria	Heunggongvirae	Uroviricota	Caudoviricetes	n.a.	Unclassified	O	O	17.32	d__Bacteria;p__Firmicutes;c__Negativicutes;o__Veillonellales;f__Dialisteraceae;g__Dialister	97.2	CRISPR;98.00 blast;96.90 iPHoP-RF;93.70
S2-wms_79304	SHHC	Duplodnaviria	Heunggongvirae	Uroviricota	Caudoviricetes	n.a.	Unclassified	Unclassified	Dybvigvirus	81.02	d__Bacteria;p__Actinobacteriota;c__Coriobacteriia;o__Coriobacteriales;f__Eggerthellaceae;g__Eggerthella	94.9	CRISPR;96.40 RaFAH;50.20
S3-wms-combined_73262	Shared	Duplodnaviria	Heunggongvirae	Uroviricota	Caudoviricetes	n.a.	Unclassified	Unclassified	Unclassified	99.96	d__Bacteria;p__Bacteroidota;c__Bacteroidia;o__Bacteroidales;f__Bacteroidaceae;g__Bacteroides	100	RaFAH;99.80 blast;93.60 iPHoP-RF;79.80 CRISPR;67.80
S12-wms-combined_297545	Shared	Duplodnaviria	Heunggongvirae	Uroviricota	Caudoviricetes	n.a.	Unclassified	O	O	99.69	d__Bacteria;p__Bacteroidota;c__Bacteroidia;o__Bacteroidales;f__Bacteroidaceae;g__Bacteroides	99.9	RaFAH;98.50 blast;90.90 iPHoP-RF;65.60
S3-wms_22744	Shared	Duplodnaviria	Heunggongvirae	Uroviricota	Caudoviricetes	n.a.	Unclassified	Unclassified	Lagaffevirus	99.21	d__Bacteria;p__Firmicutes;c__Clostridia;o__Oscillospirales;f__Ruminococcaceae;g__Faecalibacterium	97.2	CRISPR;98.00 blast;60.70 iPHoP-RF;52.00
S2-wms-combined_116221	Shared	Duplodnaviria	Heunggongvirae	Uroviricota	Caudoviricetes	n.a.	Unclassified	O	O	80.02	d__Bacteria;p__Firmicutes;c__Clostridia;o__Lachnospirales;f__Anaerotignaceae;g__Anaerotignum	95.6	blast;96.90 iPHoP-RF;61.10
S3-wms_23958	Shared	Duplodnaviria	Heunggongvirae	Uroviricota	Caudoviricetes	n.a.	Unclassified	O	O	21.78	d__Bacteria;p__Firmicutes;c__Clostridia;o__Oscillospirales;f__Oscillospiraceae;g__CAG-170	94.2	blast;95.90 iPHoP-RF;58.70
S4-wms-combined_113721	Shared	n.a.	n.a.	n.a.	n.a.	n.a.	n.a.	n.a.	n.a.	6.49	d__Bacteria;p__Firmicutes;c__Clostridia;o__Oscillospirales;f__Ruminococcaceae;g__Gemmiger	95.6	blast;96.90 iPHoP-RF;92.80 CRISPR;57.80
S2-wms-combined_126440	SHHC	Duplodnaviria	Heunggongvirae	Uroviricota	Caudoviricetes	n.a.	Unclassified	O	O	24	d__Bacteria;p__Firmicutes;c__Clostridia;o__Lachnospirales;f__Lachnospiraceae;g__Acetatifactor	92.7	blast;94.80 iPHoP-RF;71.30
S12-wms-combined_31761	SHHC	Duplodnaviria	Heunggongvirae	Uroviricota	Caudoviricetes	n.a.	Unclassified	Unclassified	O	54.85	d__Bacteria;p__Firmicutes;c__Clostridia;o__Lachnospirales;f__Lachnospiraceae;g__COE1	93.6	blast;95.50 iPHoP-RF;70.20
S4-wms-combined_14411	SHHC	Duplodnaviria	Heunggongvirae	Uroviricota	Caudoviricetes	n.a.	Unclassified	Unclassified	Unclassified	97.82	d__Bacteria;p__Bacteroidota;c__Bacteroidia;o__Bacteroidales;f__Tannerellaceae;g__Marseille-P3160	98.4	CRISPR;98.80 iPHoP-RF;68.90
S12-wms-combined_320262	Shared	n.a.	n.a.	n.a.	n.a.	n.a.	n.a.	n.a.	n.a.	15.45	d__Bacteria;p__Bacteroidota;c__Bacteroidia;o__Bacteroidales;f__Rikenellaceae;g__Alistipes	97.8	blast;98.40 iPHoP-RF;97.10
S2-wms-combined_127080	SHHC	n.a.	n.a.	n.a.	n.a.	n.a.	n.a.	n.a.	n.a.	8.57	d__Bacteria;p__Firmicutes;c__Clostridia;o__Oscillospirales;f__Ruminococcaceae;g__Faecalibacterium	98.8	CRISPR;99.10 blast;93.50 iPHoP-RF;89.50
S4-wms-combined_17284	Shared	Duplodnaviria	Heunggongvirae	Uroviricota	Caudoviricetes	n.a.	Unclassified	Unclassified	Unclassified	85.97	d__Bacteria;p__Actinobacteriota;c__Actinomycetia;o__Actinomycetales;f__Bifidobacteriaceae;g__Bifidobacterium	95.3	iPHoP-RF;96.70 CRISPR;96.00 blast;95.40
S2-wms-combined_13246	Shared	Duplodnaviria	Heunggongvirae	Uroviricota	Caudoviricetes	n.a.	Unclassified	Unclassified	O	32.69	d__Bacteria;p__Firmicutes;c__Clostridia;o__Oscillospirales;f__Ruminococcaceae;g__Faecalibacterium	99.6	blast;96.90 RaFAH;93.60 iPHoP-RF;92.80 CRISPR;87.50
S4-wms-combined_41490	Shared	Duplodnaviria	Heunggongvirae	Uroviricota	Caudoviricetes	n.a.	Unclassified	O	O	99.65	d__Bacteria;p__Bacteroidota;c__Bacteroidia;o__Bacteroidales;f__Rikenellaceae;g__Alistipes	95.6	blast;96.90 iPHoP-RF;96.10
S4-wms-combined_42647	Shared	Duplodnaviria	Heunggongvirae	Uroviricota	Caudoviricetes	n.a.	Unclassified	Unclassified	Lughvirus	63.73	d__Bacteria;p__Firmicutes;c__Clostridia;o__Oscillospirales;f__Ruminococcaceae;g__Faecalibacterium	95.7	blast;97.00 iPHoP-RF;78.00 CRISPR;67.90
S4-wms-combined_47565	SHHC	n.a.	n.a.	n.a.	n.a.	n.a.	n.a.	n.a.	n.a.	81.36	d__Bacteria;p__Bacteroidota;c__Bacteroidia;o__Bacteroidales;f__Bacteroidaceae;g__Prevotella	96.2	blast;97.30 iPHoP-RF;90.80
S4-wms-combined_51079	Shared	n.a.	n.a.	n.a.	n.a.	n.a.	n.a.	n.a.	n.a.	88.31	d__Bacteria;p__Actinobacteriota;c__Actinomycetia;o__Actinomycetales;f__Bifidobacteriaceae;g__Bifidobacterium	99.9	CRISPR;99.60 iPHoP-RF;97.10 blast;96.80 RaFAH;71.10
S4-wms-combined_57123	SHHC	n.a.	n.a.	n.a.	n.a.	n.a.	n.a.	n.a.	n.a.	13.17	d__Bacteria;p__Bacteroidota;c__Bacteroidia;o__Bacteroidales;f__Bacteroidaceae;g__Prevotella	95.6	blast;96.90 iPHoP-RF;73.50
S2-wms-combined_137119	Shared	Duplodnaviria	Heunggongvirae	Uroviricota	Caudoviricetes	n.a.	Unclassified	Unclassified	O	36.79	d__Bacteria;p__Firmicutes;c__Clostridia;o__Oscillospirales;f__Oscillospiraceae;g__An92	93.5	blast;95.40 iPHoP-RF;76.60
S4-wms-combined_64632	SHHC	Duplodnaviria	Heunggongvirae	Uroviricota	Caudoviricetes	n.a.	Unclassified	Unclassified	Unclassified	43.29	d__Bacteria;p__Firmicutes;c__Clostridia;o__Lachnospirales;f__Lachnospiraceae;g__Dorea_A	97.6	CRISPR;98.30 iPHoP-RF;95.40 blast;92.00
S2-wms-combined_140996	Shared	Duplodnaviria	Heunggongvirae	Uroviricota	Caudoviricetes	n.a.	Unclassified	Unclassified	O	59.11	d__Bacteria;p__Firmicutes;c__Clostridia;o__Oscillospirales;f__Oscillospiraceae;g__ER4	95.6	blast;96.90 iPHoP-RF;70.20
S4-wms_107182	SHHC	n.a.	n.a.	n.a.	n.a.	n.a.	n.a.	n.a.	n.a.	7.59	d__Bacteria;p__Actinobacteriota;c__Coriobacteriia;o__Coriobacteriales;f__Eggerthellaceae;g__CAG-1427	96.9	blast;97.80
S2-wms-combined_143737	Shared	Duplodnaviria	Heunggongvirae	Uroviricota	Caudoviricetes	n.a.	Duneviridae	O	O	99.75	d__Bacteria;p__Bacteroidota;c__Bacteroidia;o__Bacteroidales;f__Bacteroidaceae;g__Bacteroides	100	blast;97.80 RaFAH;97.50 iPHoP-RF;78.50
S12-wms-combined_368190	Shared	Duplodnaviria	Heunggongvirae	Uroviricota	Caudoviricetes	n.a.	Unclassified	O	O	100	d__Bacteria;p__Bacteroidota;c__Bacteroidia;o__Bacteroidales;f__Bacteroidaceae;g__Bacteroides	100	RaFAH;98.90 iPHoP-RF;97.10 blast;91.30
S4-wms_141607	SHHC	Duplodnaviria	Heunggongvirae	Uroviricota	Caudoviricetes	n.a.	Unclassified	Unclassified	Unclassified	37.81	d__Bacteria;p__Bacteroidota;c__Bacteroidia;o__Bacteroidales;f__Bacteroidaceae;g__Prevotella	98.4	CRISPR;98.80 blast;96.40 iPHoP-RF;79.00
S2-wms-combined_157934	Shared	Duplodnaviria	Heunggongvirae	Uroviricota	Caudoviricetes	n.a.	Unclassified	Unclassified	O	99.51	d__Bacteria;p__Firmicutes;c__Clostridia;o__Lachnospirales;f__Lachnospiraceae;g__Roseburia	98.8	CRISPR;98.90 iPHoP-RF;92.80 blast;89.30 RaFAH;59.00
S4-wms_161443	SHHC	Unclassified	n.a.	n.a.	n.a.	n.a.	Unclassified	Unclassified	Unclassified	5.84	d__Bacteria;p__Actinobacteriota;c__Actinomycetia;o__Actinomycetales;f__Bifidobacteriaceae;g__Bifidobacterium	99.6	CRISPR;99.60 iPHoP-RF;78.00
S4-wms_45128	SHHC	n.a.	n.a.	n.a.	n.a.	n.a.	n.a.	n.a.	n.a.	28.59	d__Bacteria;p__Bacteroidota;c__Bacteroidia;o__Bacteroidales;f__Bacteroidaceae;g__Prevotella	94.7	blast;96.30 CRISPR;93.50 iPHoP-RF;91.40
S2-wms-combined_159670	Shared	n.a.	n.a.	n.a.	n.a.	n.a.	n.a.	n.a.	n.a.	16.22	d__Bacteria;p__Firmicutes;c__Clostridia;o__Oscillospirales;f__Oscillospiraceae;g__CAG-83	91.6	blast;93.90 iPHoP-RF;88.20
S4-wms_62635	SHHC	Duplodnaviria	Heunggongvirae	Uroviricota	Caudoviricetes	n.a.	Unclassified	Unclassified	Unclassified	93.28	d__Bacteria;p__Firmicutes;c__Clostridia;o__Lachnospirales;f__Lachnospiraceae;g__Blautia_A	100	CRISPR;98.90 RaFAH;95.20 iPHoP-RF;93.70 blast;90.40
S4-wms_73990	SHHC	Duplodnaviria	Heunggongvirae	Uroviricota	Caudoviricetes	Crassvirales	Suoliviridae	Boorivirinae	Cohcovirus	76.93	d__Bacteria;p__Bacteroidota;c__Bacteroidia;o__Bacteroidales;f__Tannerellaceae;g__Parabacteroides	97.6	blast;98.30 iPHoP-RF;82.80
S5-wms-combined_117213	Shared	Duplodnaviria	Heunggongvirae	Uroviricota	Caudoviricetes	n.a.	Unclassified	O	O	97.16	d__Bacteria;p__Actinobacteriota;c__Actinomycetia;o__Actinomycetales;f__Bifidobacteriaceae;g__Bifidobacterium	98.8	CRISPR;97.70 iPHoP-RF;97.10 blast;96.50 RaFAH;80.80
S5-wms-combined_134381	Shared	Duplodnaviria	Heunggongvirae	Uroviricota	Caudoviricetes	n.a.	Unclassified	Unclassified	Lagaffevirus	97.01	d__Bacteria;p__Firmicutes;c__Clostridia;o__Oscillospirales;f__Oscillospiraceae;g__CAG-83	98.4	RaFAH;98.30 blast;64.70
S5-wms-combined_145067	Shared	Duplodnaviria	Heunggongvirae	Uroviricota	Caudoviricetes	n.a.	Unclassified	O	O	82.3	d__Bacteria;p__Firmicutes;c__Clostridia;o__Oscillospirales;f__Oscillospiraceae;g__Flavonifractor	95.6	blast;96.90 iPHoP-RF;95.70 CRISPR;53.80
S2-wms-combined_185494	Shared	Duplodnaviria	Heunggongvirae	Uroviricota	Caudoviricetes	n.a.	Autographiviridae	O	O	25.15	d__Bacteria;p__Firmicutes;c__Negativicutes;o__Veillonellales;f__Dialisteraceae;g__Dialister	96.3	CRISPR;97.40 blast;96.90 iPHoP-RF;90.10
S5-wms-combined_178432	Shared	n.a.	n.a.	n.a.	n.a.	n.a.	n.a.	n.a.	n.a.	13.6	d__Bacteria;p__Bacteroidota;c__Bacteroidia;o__Bacteroidales;f__Rikenellaceae;g__Alistipes	95.9	iPHoP-RF;97.10 blast;91.90 CRISPR;84.60
S2-wms-combined_191613	Shared	n.a.	n.a.	n.a.	n.a.	n.a.	n.a.	n.a.	n.a.	13.4	d__Bacteria;p__Firmicutes;c__Clostridia;o__Lachnospirales;f__Anaerotignaceae;g__Anaerotignum	95.2	blast;96.60 iPHoP-RF;70.60
S5-wms-combined_18356	ME	Duplodnaviria	Heunggongvirae	Uroviricota	Caudoviricetes	n.a.	Peduoviridae	Unclassified	Evevirus	90.68	d__Bacteria;p__Proteobacteria;c__Gammaproteobacteria;o__Enterobacterales;f__Enterobacteriaceae;g__Escherichia	98.8	iPHoP-RF;97.10 blast;96.40 RaFAH;85.20 CRISPR;70.30
S12-wms-combined_52101	SHHC	Duplodnaviria	Heunggongvirae	Uroviricota	Caudoviricetes	n.a.	Unclassified	Unclassified	O	35.58	d__Bacteria;p__Firmicutes;c__Clostridia;o__Oscillospirales;f__Ruminococcaceae;g__UBA3818	90.3	blast;92.80 iPHoP-RF;62.20
S5-wms-combined_207291	Shared	n.a.	n.a.	n.a.	n.a.	n.a.	n.a.	n.a.	n.a.	100	d__Bacteria;p__Bacteroidota;c__Bacteroidia;o__Bacteroidales;f__Bacteroidaceae;g__Bacteroides	100	RaFAH;99.80 iPHoP-RF;95.70 blast;91.90
S2-wms-combined_199384	SHHC	Duplodnaviria	Heunggongvirae	Uroviricota	Caudoviricetes	n.a.	Unclassified	O	O	17.07	d__Bacteria;p__Firmicutes;c__Clostridia;o__Oscillospirales;f__Butyricicoccaceae;g__Agathobaculum	91.4	blast;93.70 iPHoP-RF;73.50
S2-wms-combined_200390	Shared	Duplodnaviria	Heunggongvirae	Uroviricota	Caudoviricetes	n.a.	Unclassified	O	O	14.49	d__Bacteria;p__Firmicutes;c__Clostridia;o__Lachnospirales;f__Lachnospiraceae;g__Eisenbergiella	92	CRISPR;94.20 iPHoP-RF;67.50
S5-wms-combined_39793	Shared	n.a.	n.a.	n.a.	n.a.	n.a.	n.a.	n.a.	n.a.		d__Bacteria;p__Actinobacteriota;c__Actinomycetia;o__Actinomycetales;f__Bifidobacteriaceae;g__Bifidobacterium	91.6	blast;93.90 iPHoP-RF;93.40 CRISPR;76.80
S5-wms-combined_52741	Shared	Duplodnaviria	Heunggongvirae	Uroviricota	Caudoviricetes	n.a.	Unclassified	O	O	50.99	d__Bacteria;p__Verrucomicrobiota;c__Verrucomicrobiae;o__Verrucomicrobiales;f__Akkermansiaceae;g__Akkermansia	100	RaFAH;99.50 CRISPR;98.80 blast;96.90 iPHoP-RF;81.60
S12-wms-combined_75733	SHHC	Monodnaviria	Sangervirae	Phixviricota	Malgrandaviricetes	Petitvirales	Microviridae	O	O		d__Bacteria;p__Bacteroidota;c__Bacteroidia;o__Bacteroidales;f__Barnesiellaceae;g__Barnesiella	94.6	blast;96.20 iPHoP-RF;69.80
S5-wms-combined_59064	Shared	Duplodnaviria	Heunggongvirae	Uroviricota	Caudoviricetes	n.a.	Unclassified	Unclassified	Unclassified	95	d__Bacteria;p__Bacteroidota;c__Bacteroidia;o__Bacteroidales;f__Bacteroidaceae;g__Bacteroides	100	RaFAH;97.60 blast;96.90 iPHoP-RF;96.10
S5-wms-combined_66017	ME	n.a.	n.a.	n.a.	n.a.	n.a.	n.a.	n.a.	n.a.	9.66	d__Bacteria;p__Firmicutes;c__Clostridia;o__Christensenellales;f__Christensenellaceae;g__Christensenella	90.7	blast;93.10 iPHoP-RF;82.20
S5-wms-combined_7292	Shared	Duplodnaviria	Heunggongvirae	Uroviricota	Caudoviricetes	n.a.	Unclassified	Unclassified	Unclassified	22.4	d__Bacteria;p__Firmicutes;c__Clostridia;o__Oscillospirales;f__Acutalibacteraceae;g__Ruminococcus_E	98.5	CRISPR;98.90 blast;96.60 iPHoP-RF;89.80
S5-wms-combined_73301	ME	Duplodnaviria	Heunggongvirae	Uroviricota	Caudoviricetes	n.a.	Unclassified	O	O	96.75	d__Bacteria;p__Firmicutes;c__Clostridia;o__Lachnospirales;f__Lachnospiraceae;g__Dorea_A	94.6	blast;96.20 iPHoP-RF;93.10 CRISPR;58.60
S2-wms-combined_225908	Shared	Unclassified	n.a.	n.a.	n.a.	n.a.	Unclassified	O	O	87.71	d__Bacteria;p__Firmicutes;c__Clostridia;o__Oscillospirales;f__Acutalibacteraceae;g__CAG-217	95.6	blast;96.90 CRISPR;93.30 iPHoP-RF;79.00
S5-wms-combined_83370	Shared	Duplodnaviria	Heunggongvirae	Uroviricota	Caudoviricetes	n.a.	Unclassified	O	O	82.36	d__Bacteria;p__Bacteroidota;c__Bacteroidia;o__Bacteroidales;f__Bacteroidaceae;g__Bacteroides	99.4	blast;96.90 iPHoP-RF;96.70 RaFAH;91.90
S5-wms-combined_83412	ME	Duplodnaviria	Heunggongvirae	Uroviricota	Caudoviricetes	n.a.	Unclassified	Unclassified	Unclassified	25.58	d__Bacteria;p__Firmicutes;c__Clostridia;o__Eubacteriales;f__Eubacteriaceae;g__Eubacterium	92.6	blast;94.70 iPHoP-RF;86.70 CRISPR;77.00
S2-wms-combined_24549	Shared	Duplodnaviria	Heunggongvirae	Uroviricota	Caudoviricetes	n.a.	Unclassified	O	O	17.49	d__Bacteria;p__Firmicutes;c__Clostridia;o__Oscillospirales;f__Oscillospiraceae;g__Intestinimonas	90.9	blast;93.30
S5-wms-combined_98202	Shared	n.a.	n.a.	n.a.	n.a.	n.a.	n.a.	n.a.	n.a.	100	d__Bacteria;p__Bacteroidota;c__Bacteroidia;o__Bacteroidales;f__Bacteroidaceae;g__Bacteroides	99.4	RaFAH;99.50
S2-wms-combined_25727	Shared	Duplodnaviria	Heunggongvirae	Uroviricota	Caudoviricetes	n.a.	Unclassified	O	O	21.03	d__Bacteria;p__Firmicutes;c__Clostridia;o__Christensenellales;f__CAG-74;g__Ventricola	94.7	blast;96.30 iPHoP-RF;74.60
S12-wms_184276	SHHC	Duplodnaviria	Heunggongvirae	Uroviricota	Caudoviricetes	n.a.	Unclassified	O	O	36.83	d__Bacteria;p__Actinobacteriota;c__Coriobacteriia;o__Coriobacteriales;f__Eggerthellaceae;g__Adlercreutzia	93.1	blast;95.10 iPHoP-RF;66.80
S12-wms_223235	SHHC	n.a.	n.a.	n.a.	n.a.	n.a.	n.a.	n.a.	n.a.		d__Bacteria;p__Firmicutes;c__Clostridia;o__Oscillospirales;f__Ruminococcaceae;g__Ruminococcus_D	95.3	blast;96.70 iPHoP-RF;74.80 CRISPR;67.70
S2-wms-combined_52568	Shared	n.a.	n.a.	n.a.	n.a.	n.a.	n.a.	n.a.	n.a.	12.66	d__Bacteria;p__Firmicutes;c__Clostridia;o__Oscillospirales;f__Oscillospiraceae;g__F23-B02	92.3	blast;94.50 iPHoP-RF;80.30
S12-wms_336780	Shared	n.a.	n.a.	n.a.	n.a.	n.a.	n.a.	n.a.	n.a.	4.76	d__Bacteria;p__Firmicutes;c__Clostridia;o__Oscillospirales;f__Ruminococcaceae;g__UMGS363	95.3	blast;96.70 iPHoP-RF;93.10
S2-wms-combined_5791	Shared	Duplodnaviria	Heunggongvirae	Uroviricota	Caudoviricetes	n.a.	Unclassified	O	O	89.76	d__Bacteria;p__Firmicutes;c__Clostridia;o__Lachnospirales;f__Lachnospiraceae;g__Agathobacter	92.1	CRISPR;94.30
S2-wms-combined_62839	Shared	Duplodnaviria	Heunggongvirae	Uroviricota	Caudoviricetes	n.a.	Unclassified	O	O	32.39	d__Bacteria;p__Firmicutes;c__Clostridia;o__Oscillospirales;f__Ruminococcaceae;g__Gemmiger	97.6	blast;98.30 iPHoP-RF;87.30 CRISPR;73.60
S2-wms-combined_72725	Shared	n.a.	n.a.	n.a.	n.a.	n.a.	n.a.	n.a.	n.a.	19.31	d__Bacteria;p__Firmicutes;c__Clostridia;o__Oscillospirales;f__Oscillospiraceae;g__Intestinimonas	91.4	blast;93.70 iPHoP-RF;75.40
S2-wms-combined_74387	SHHC	Duplodnaviria	Heunggongvirae	Uroviricota	Caudoviricetes	n.a.	Unclassified	O	O	38.64	d__Bacteria;p__Firmicutes;c__Clostridia;o__Lachnospirales;f__Lachnospiraceae;g__Lachnoclostridium_A	94.3	blast;96.00 iPHoP-RF;82.50
S2-wms-combined_75144	Shared	n.a.	n.a.	n.a.	n.a.	n.a.	n.a.	n.a.	n.a.	9.77	d__Bacteria;p__Bacteroidota;c__Bacteroidia;o__Bacteroidales;f__Tannerellaceae;g__Parabacteroides	95.4	blast;96.80 iPHoP-RF;95.10
S2-wms-combined_7756	Shared	Duplodnaviria	Heunggongvirae	Uroviricota	Caudoviricetes	n.a.	Unclassified	O	O	9.67	d__Bacteria;p__Verrucomicrobiota;c__Verrucomicrobiae;o__Verrucomicrobiales;f__Akkermansiaceae;g__Akkermansia	96.9	blast;96.70 iPHoP-RF;91.10 CRISPR;86.00 RaFAH;66.20
S17-wms-combined_253499	ME	n.a.	n.a.	n.a.	n.a.	n.a.	n.a.	n.a.	n.a.	25.09	d__Bacteria;p__Bacteroidota;c__Bacteroidia;o__Bacteroidales;f__Rikenellaceae;g__Alistipes	97.2	CRISPR;98.00 blast;95.10 iPHoP-RF;90.80
S17-wms-combined_258087	Shared	Duplodnaviria	Heunggongvirae	Uroviricota	Caudoviricetes	n.a.	Unclassified	Unclassified	O	97.18	d__Bacteria;p__Firmicutes;c__Clostridia;o__Clostridiales;f__Clostridiaceae;g__Clostridium	98.2	RaFAH;98.70
S17-wms-combined_39687	ME	n.a.	n.a.	n.a.	n.a.	n.a.	n.a.	n.a.	n.a.	83.01	d__Bacteria;p__Firmicutes;c__Clostridia;o__Oscillospirales;f__Oscillospiraceae;g__Flavonifractor	95.2	blast;96.60 iPHoP-RF;89.20
S17-wms-combined_56006	Shared	Duplodnaviria	Heunggongvirae	Uroviricota	Caudoviricetes	n.a.	Unclassified	O	O	98.39	d__Bacteria;p__Firmicutes;c__Clostridia;o__Oscillospirales;f__Ruminococcaceae;g__Faecalibacterium	100	RaFAH;98.90 blast;96.90 iPHoP-RF;87.00 CRISPR;67.80
S17-wms-combined_56061	Shared	n.a.	n.a.	n.a.	n.a.	n.a.	n.a.	n.a.	n.a.	66.23	d__Bacteria;p__Firmicutes;c__Clostridia;o__Oscillospirales;f__Ruminococcaceae;g__Faecalibacterium	98.5	blast;98.90
S17-wms-combined_66123	ME	Duplodnaviria	Heunggongvirae	Uroviricota	Caudoviricetes	n.a.	Unclassified	Unclassified	O	97.65	d__Bacteria;p__Firmicutes;c__Clostridia;o__Oscillospirales;f__Oscillospiraceae;g__Flavonifractor	90.6	blast;91.80 iPHoP-RF;85.40 CRISPR;73.10 RaFAH;57.20
S5-wms-combined_108794	Shared	Duplodnaviria	Heunggongvirae	Uroviricota	Caudoviricetes	n.a.	Unclassified	Unclassified	O	58.74	d__Bacteria;p__Firmicutes;c__Clostridia;o__Oscillospirales;f__Acutalibacteraceae;g__Ruminococcus_E	95.6	blast;96.90 iPHoP-RF;89.50
S17-wms-combined_75892	ME	Duplodnaviria	Heunggongvirae	Uroviricota	Caudoviricetes	n.a.	Unclassified	Unclassified	O	53.86	d__Bacteria;p__Firmicutes;c__Clostridia;o__Oscillospirales;f__Oscillospiraceae;g__HGM12998	91.4	blast;93.70 iPHoP-RF;77.50
S5-wms-combined_66662	Shared	Unclassified	n.a.	n.a.	n.a.	n.a.	Unclassified	Unclassified	O	100	d__Bacteria;p__Firmicutes;c__Clostridia;o__Oscillospirales;f__Ruminococcaceae;g__Ruthenibacterium	97.2	blast;98.00 iPHoP-RF;88.50
S5-wms-combined_72410	Shared	Duplodnaviria	Heunggongvirae	Uroviricota	Caudoviricetes	n.a.	Unclassified	Unclassified	O	60.27	d__Bacteria;p__Firmicutes;c__Clostridia;o__Oscillospirales;f__Ruminococcaceae;g__Ruthenibacterium	90.8	blast;93.20 iPHoP-RF;72.60
S2-wms-combined_122457	Shared	Duplodnaviria	Heunggongvirae	Uroviricota	Caudoviricetes	n.a.	Unclassified	Unclassified	O	52.93	d__Bacteria;p__Firmicutes;c__Clostridia;o__Oscillospirales;f__Oscillospiraceae;g__Intestinimonas	91.6	blast;93.90 iPHoP-RF;56.50
S5-wms-combined_90315	Shared	Duplodnaviria	Heunggongvirae	Uroviricota	Caudoviricetes	n.a.	Unclassified	O	O	99.97	d__Bacteria;p__Bacteroidota;c__Bacteroidia;o__Bacteroidales;f__Rikenellaceae;g__Alistipes	98.8	RaFAH;96.10 blast;88.90
S2-wms-combined_135014	Shared	Duplodnaviria	Heunggongvirae	Uroviricota	Caudoviricetes	n.a.	Unclassified	Unclassified	O	100	d__Bacteria;p__Firmicutes;c__Negativicutes;o__Veillonellales;f__Dialisteraceae;g__Dialister	100	RaFAH;97.60 blast;96.90 CRISPR;96.10 iPHoP-RF;92.40
S2-wms-combined_147065	Shared	Duplodnaviria	Heunggongvirae	Uroviricota	Caudoviricetes	n.a.	Unclassified	O	O	99.85	d__Bacteria;p__Bacteroidota;c__Bacteroidia;o__Bacteroidales;f__Bacteroidaceae;g__Bacteroides	100	RaFAH;97.00 blast;96.90 iPHoP-RF;95.10 CRISPR;58.20
S2-wms-combined_158821	Shared	Duplodnaviria	Heunggongvirae	Uroviricota	Caudoviricetes	n.a.	Unclassified	Unclassified	O	99.16	d__Bacteria;p__Firmicutes;c__Clostridia;o__Lachnospirales;f__Lachnospiraceae;g__Enterocloster	91.5	blast;93.80 iPHoP-RF;63.50
S2-wms-combined_181849	Shared	n.a.	n.a.	n.a.	n.a.	n.a.	n.a.	n.a.	n.a.	40.03	d__Bacteria;p__Firmicutes;c__Clostridia;o__Oscillospirales;f__Ruminococcaceae;g__Faecalibacterium	95.6	blast;96.90 CRISPR;95.80 iPHoP-RF;87.30

S2-wms-combined_201205	Shared	Duplodnaviria	Heunggongvirae	Uroviricota	Caudoviricetes	n.a.	Unclassified	Unclassified	O	82.22	d__Bacteria;p__Verrucomicrobiota;c__Verrucomicrobiae;o__Verrucomicrobiales;f__Akkermansiaceae;g__Akkermansia	100	CRISPR;99.30 blast;96.80 RaFAH;96.70 iPHoP-RF;68.60
S2-wms-combined_204225	SHHC	n.a.	n.a.	n.a.	n.a.	n.a.	n.a.	n.a.	n.a.	6.16	d__Bacteria;p__Firmicutes;c__Clostridia;o__Lachnospirales;f__Lachnospiraceae;g__Lachnoclostridium_A	91.6	blast;93.90 iPHoP-RF;79.30
S2-wms-combined_207000	SHHC	Duplodnaviria	Heunggongvirae	Uroviricota	Caudoviricetes	n.a.	Unclassified	Unclassified	O	92.52	d__Bacteria;p__Firmicutes;c__Clostridia;o__Lachnospirales;f__Lachnospiraceae;g__Agathobacter	93.4	blast;95.30 iPHoP-RF;93.70
S2-wms-combined_84608	Shared	Duplodnaviria	Heunggongvirae	Uroviricota	Caudoviricetes	n.a.	Unclassified	O	O	21.12	d__Bacteria;p__Proteobacteria;c__Gammaproteobacteria;o__Burkholderiales;f__Burkholderiaceae;g__Sutterella	95.6	blast;96.90 iPHoP-RF;90.80 CRISPR;79.90
S2-wms_246296	Shared	n.a.	n.a.	n.a.	n.a.	n.a.	n.a.	n.a.	n.a.	98.04	d__Bacteria;p__Firmicutes;c__Clostridia;o__Lachnospirales;f__Lachnospiraceae;g__Roseburia	100	CRISPR;98.90 blast;94.40 RaFAH;93.70 iPHoP-RF;92.80
S2-wms-combined_208559	Shared	n.a.	n.a.	n.a.	n.a.	n.a.	n.a.	n.a.	n.a.	10.84	d__Bacteria;p__Bacteroidota;c__Bacteroidia;o__Bacteroidales;f__Rikenellaceae;g__Alistipes	95.6	blast;96.90 iPHoP-RF;96.70
S2-wms_6424	SHHC	Duplodnaviria	Heunggongvirae	Uroviricota	Caudoviricetes	n.a.	Pachyviridae	O	O	8.31	d__Bacteria;p__Bacteroidota;c__Bacteroidia;o__Bacteroidales;f__Bacteroidaceae;g__Bacteroides	93.6	RaFAH;95.50
S3-wms-combined_135428	ME	Duplodnaviria	Heunggongvirae	Uroviricota	Caudoviricetes	n.a.	Unclassified	O	O	11.75	d__Bacteria;p__Actinobacteriota;c__Actinomycetia;o__Actinomycetales;f__Bifidobacteriaceae;g__Bifidobacterium	95.3	blast;96.70 iPHoP-RF;96.40 CRISPR;94.80
S3-wms-combined_45305	Shared	Duplodnaviria	Heunggongvirae	Uroviricota	Caudoviricetes	n.a.	Unclassified	O	O	27.39	d__Bacteria;p__Firmicutes;c__Clostridia;o__Oscillospirales;f__Ruminococcaceae;g__Gemmiger	94.2	blast;95.90 iPHoP-RF;76.60 CRISPR;55.10
S2-wms-combined_222535	SHHC	n.a.	n.a.	n.a.	n.a.	n.a.	n.a.	n.a.	n.a.	7.99	d__Bacteria;p__Firmicutes;c__Clostridia;o__Lachnospirales;f__Lachnospiraceae;g__Agathobacter	92.1	blast;94.30 iPHoP-RF;92.10
S3-wms_94494	ME	Duplodnaviria	Heunggongvirae	Uroviricota	Caudoviricetes	n.a.	Unclassified	O	O	94.44	d__Bacteria;p__Actinobacteriota;c__Actinomycetia;o__Actinomycetales;f__Bifidobacteriaceae;g__Bifidobacterium	99.3	blast;98.30 iPHoP-RF;97.10 CRISPR;83.00 RaFAH;81.50
S4-wms-combined_10758	SHHC	Duplodnaviria	Heunggongvirae	Uroviricota	Caudoviricetes	n.a.	Unclassified	O	O	88.87	d__Bacteria;p__Bacteroidota;c__Bacteroidia;o__Bacteroidales;f__Tannerellaceae;g__Parabacteroides	100	RaFAH;99.50 blast;89.70 iPHoP-RF;87.00
S4-wms-combined_118531	SHHC	Duplodnaviria	Heunggongvirae	Uroviricota	Caudoviricetes	n.a.	Unclassified	O	O	66.42	d__Bacteria;p__Firmicutes;c__Negativicutes;o__Acidaminococcales;f__Acidaminococcaceae;g__Phascolarctobacterium_A	98.2	CRISPR;98.70 blast;96.80 iPHoP-RF;92.40
S4-wms-combined_121978	Shared	Duplodnaviria	Heunggongvirae	Uroviricota	Caudoviricetes	n.a.	Unclassified	O	O	87.01	d__Bacteria;p__Firmicutes;c__Clostridia;o__Lachnospirales;f__Lachnospiraceae;g__Mediterraneibacter	95	blast;96.50 iPHoP-RF;79.50
S4-wms-combined_22012	SHHC	Duplodnaviria	Heunggongvirae	Uroviricota	Caudoviricetes	n.a.	Unclassified	O	O	25.83	d__Bacteria;p__Actinobacteriota;c__Coriobacteriia;o__Coriobacteriales;f__Coriobacteriaceae;g__Collinsella	98.7	CRISPR;99.00 blast;96.80 iPHoP-RF;89.20
S4-wms-combined_83492	Shared	Duplodnaviria	Heunggongvirae	Uroviricota	Caudoviricetes	n.a.	Unclassified	O	O	86.97	d__Bacteria;p__Bacteroidota;c__Bacteroidia;o__Bacteroidales;f__Bacteroidaceae;g__Bacteroides	95.6	blast;89.90 iPHoP-RF;86.30 RaFAH;84.50
S4-wms_50475	SHHC	Duplodnaviria	Heunggongvirae	Uroviricota	Caudoviricetes	n.a.	Unclassified	O	O	70.49	d__Bacteria;p__Bacteroidota;c__Bacteroidia;o__Bacteroidales;f__Bacteroidaceae;g__Prevotella	97.3	blast;96.90 iPHoP-RF;94.40 RaFAH;69.20 CRISPR;50.60
S5-wms-combined_117095	Shared	n.a.	n.a.	n.a.	n.a.	n.a.	n.a.	n.a.	n.a.	10.16	d__Bacteria;p__Firmicutes;c__Clostridia;o__Oscillospirales;f__Acutalibacteraceae;g__UBA1417	91	blast;93.40 iPHoP-RF;64.50
S5-wms-combined_156329	ME	Duplodnaviria	Heunggongvirae	Uroviricota	Caudoviricetes	n.a.	Unclassified	O	O	75.2	d__Bacteria;p__Firmicutes;c__Bacilli;o__Erysipelotrichales;f__Erysipelotrichaceae;g__Clostridium_AQ	95.9	blast;97.10 CRISPR;90.20 iPHoP-RF;75.20
S5-wms-combined_179241	Shared	n.a.	n.a.	n.a.	n.a.	n.a.	n.a.	n.a.	n.a.	13.11	d__Bacteria;p__Firmicutes;c__Clostridia;o__Oscillospirales;f__Oscillospiraceae;g__CAG-83	91	blast;93.40 iPHoP-RF;86.70
S5-wms-combined_204782	ME	n.a.	n.a.	n.a.	n.a.	n.a.	n.a.	n.a.	n.a.	6.93	d__Bacteria;p__Bacteroidota;c__Bacteroidia;o__Bacteroidales;f__Rikenellaceae;g__Alistipes_A	92.1	blast;94.30 iPHoP-RF;69.10
S5-wms-combined_3201	ME	Duplodnaviria	Heunggongvirae	Uroviricota	Caudoviricetes	n.a.	Unclassified	O	O	23.69	d__Bacteria;p__Proteobacteria;c__Gammaproteobacteria;o__Burkholderiales;f__Burkholderiaceae;g__Oxalobacter	90.9	blast;93.30 iPHoP-RF;69.80
S5-wms-combined_94482	ME	n.a.	n.a.	n.a.	n.a.	n.a.	n.a.	n.a.	n.a.	15.25	d__Bacteria;p__Bacteroidota;c__Bacteroidia;o__Bacteroidales;f__Tannerellaceae;g__Parabacteroides	94.9	iPHoP-RF;96.40 blast;91.70
S2-wms-combined_30750	SHHC	n.a.	n.a.	n.a.	n.a.	n.a.	n.a.	n.a.	n.a.	11.82	d__Bacteria;p__Firmicutes;c__Clostridia;o__Oscillospirales;f__Ruminococcaceae;g__Ruminococcus_D	95.6	blast;96.90 iPHoP-RF;82.50
S2-wms-combined_48661	SHHC	n.a.	n.a.	n.a.	n.a.	n.a.	n.a.	n.a.	n.a.	11.3	d__Bacteria;p__Firmicutes;c__Clostridia;o__Lachnospirales;f__Lachnospiraceae;g__Anaerosacchariphilus	90.8	blast;93.20 iPHoP-RF;77.30
S2-wms-combined_57293	Shared	Unclassified	n.a.	n.a.	n.a.	n.a.	Unclassified	O	O	78.69	d__Bacteria;p__Firmicutes;c__Clostridia;o__Oscillospirales;f__Ruminococcaceae;g__Faecalibacterium	95.7	blast;96.90 iPHoP-RF;88.90 RaFAH;51.90
S2-wms-combined_58563	Shared	Duplodnaviria	Heunggongvirae	Uroviricota	Caudoviricetes	n.a.	Unclassified	Unclassified	O	88.99	d__Bacteria;p__Firmicutes;c__Clostridia;o__Clostridiales;f__Clostridiaceae;g__Clostridium	95.4	RaFAH;96.80
S2-wms-combined_64692	SHHC	Duplodnaviria	Heunggongvirae	Uroviricota	Caudoviricetes	n.a.	Unclassified	Unclassified	O	24.76	d__Bacteria;p__Firmicutes;c__Clostridia;o__Oscillospirales;f__Oscillospiraceae;g__	90.8	blast;93.20 iPHoP-RF;85.40
S2-wms-combined_73327	SHHC	n.a.	n.a.	n.a.	n.a.	n.a.	n.a.	n.a.	n.a.	3.55	d__Bacteria;p__Firmicutes;c__Clostridia;o__Lachnospirales;f__Lachnospiraceae;g__Faecalimonas	91.6	blast;93.90 iPHoP-RF;73.50
S2-wms-combined_95987	SHHC	n.a.	n.a.	n.a.	n.a.	n.a.	n.a.	n.a.	n.a.	3.24	d__Bacteria;p__Firmicutes;c__Clostridia;o__Lachnospirales;f__Lachnospiraceae;g__Lachnoclostridium_A	93.2	blast;95.20 iPHoP-RF;84.50
S2-wms_124416	Shared	Duplodnaviria	Heunggongvirae	Uroviricota	Caudoviricetes	n.a.	Unclassified	O	O	99.32	d__Bacteria;p__Firmicutes;c__Clostridia;o__Oscillospirales;f__Ruminococcaceae;g__Faecalibacterium	100	RaFAH;99.60 blast;96.10 iPHoP-RF;79.30 CRISPR;55.70
S2-wms_161986	Shared	Duplodnaviria	Heunggongvirae	Uroviricota	Caudoviricetes	n.a.	Unclassified	Unclassified	O	99.14	d__Bacteria;p__Firmicutes;c__Clostridia;o__Lachnospirales;f__Lachnospiraceae;g__Lachnospira	99.9	CRISPR;98.80 RaFAH;92.10 blast;84.30 iPHoP-RF;67.70
S2-wms_192667	SHHC	Duplodnaviria	Heunggongvirae	Uroviricota	Caudoviricetes	n.a.	Unclassified	Unclassified	O	13.43	d__Bacteria;p__Firmicutes;c__Clostridia;o__Lachnospirales;f__Lachnospiraceae;g__Eubacterium_I	91.6	blast;93.90 iPHoP-RF;62.20
S4-wms-combined_57963	SHHC	Duplodnaviria	Heunggongvirae	Uroviricota	Caudoviricetes	n.a.	Unclassified	Unclassified	O	74.8	d__Bacteria;p__Firmicutes;c__Negativicutes;o__Acidaminococcales;f__Acidaminococcaceae;g__Phascolarctobacterium_A	95.6	blast;96.90 iPHoP-RF;64.80
S5-wms-combined_163165	ME	n.a.	n.a.	n.a.	n.a.	n.a.	n.a.	n.a.	n.a.	3.66	d__Bacteria;p__Firmicutes;c__Clostridia;o__Oscillospirales;f__Oscillospiraceae;g__Flavonifractor	91.2	blast;93.60 iPHoP-RF;59.50
S4-wms-combined_24659	SHHC	n.a.	n.a.	n.a.	n.a.	n.a.	n.a.	n.a.	n.a.	6.25	d__Bacteria;p__Firmicutes;c__Clostridia;o__Lachnospirales;f__Lachnospiraceae;g__Roseburia	92.6	iPHoP-RF;94.70
S5-wms-combined_183113	ME	n.a.	n.a.	n.a.	n.a.	n.a.	n.a.	n.a.	n.a.	11.29	d__Bacteria;p__Bacteroidota;c__Bacteroidia;o__Bacteroidales;f__Rikenellaceae;g__Alistipes_A	97	blast;97.90 iPHoP-RF;94.70
S5-wms-combined_210959	Shared	Duplodnaviria	Heunggongvirae	Uroviricota	Caudoviricetes	n.a.	Unclassified	O	O	81.3	d__Bacteria;p__Bacteroidota;c__Bacteroidia;o__Bacteroidales;f__Rikenellaceae;g__Alistipes	99.4	blast;96.90 iPHoP-RF;96.70 RaFAH;91.40 CRISPR;57.80
S5-wms-combined_67650	Shared	Duplodnaviria	Heunggongvirae	Uroviricota	Caudoviricetes	n.a.	Unclassified	O	O	98.81	d__Bacteria;p__Bacteroidota;c__Bacteroidia;o__Bacteroidales;f__Rikenellaceae;g__Alistipes	100	RaFAH;97.10 blast;96.90 iPHoP-RF;93.70
S5-wms-combined_106597	ME	n.a.	n.a.	n.a.	n.a.	n.a.	n.a.	n.a.	n.a.	25.93	d__Bacteria;p__Campylobacterota;c__Campylobacteria;o__Campylobacterales;f__Campylobacteraceae;g__Campylobacter_D	98.8	RaFAH;99.10
S5-wms-combined_199135	ME	n.a.	n.a.	n.a.	n.a.	n.a.	n.a.	n.a.	n.a.	16.23	d__Bacteria;p__Cyanobacteria;c__Vampirovibrionia;o__Gastranaerophilales;f__Gastranaerophilaceae;g__QAMI01	90.3	blast;92.80
S5-wms-combined_26061	Shared	Duplodnaviria	Heunggongvirae	Uroviricota	Caudoviricetes	n.a.	Unclassified	Unclassified	O	99.77	d__Bacteria;p__Firmicutes;c__Clostridia;o__Lachnospirales;f__Lachnospiraceae;g__Blautia_A	96.7	blast;92.60 RaFAH;84.50
S5-wms-combined_197186	ME	n.a.	n.a.	n.a.	n.a.	n.a.	n.a.	n.a.	n.a.	3.6	d__Bacteria;p__Firmicutes;c__Bacilli;o__Erysipelotrichales;f__Erysipelotrichaceae;g__Clostridium_AQ	94.6	blast;96.20 iPHoP-RF;73.70
S5-wms-combined_205046	Shared	n.a.	n.a.	n.a.	n.a.	n.a.	n.a.	n.a.	n.a.	4.59	d__Bacteria;p__Firmicutes;c__Clostridia;o__Oscillospirales;f__Oscillospiraceae;g__Dysosmobacter	92.6	iPHoP-RF;94.70 blast;93.60
S5-wms_10783	Shared	n.a.	n.a.	n.a.	n.a.	n.a.	n.a.	n.a.	n.a.	3.75	d__Bacteria;p__Firmicutes;c__Clostridia;o__Oscillospirales;f__Oscillospiraceae;g__Lawsonibacter	90.8	blast;93.20
S5-wms_212596	ME	n.a.	n.a.	n.a.	n.a.	n.a.	n.a.	n.a.	n.a.	76.97	d__Bacteria;p__Firmicutes;c__Bacilli;o__Lactobacillales;f__Lactobacillaceae;g__Lacticaseibacillus	100	blast;98.70 RaFAH;95.70 iPHoP-RF;77.10
S5-wms_49310	Shared	Duplodnaviria	Heunggongvirae	Uroviricota	Caudoviricetes	n.a.	Unclassified	Unclassified	Unclassified	96.19	d__Bacteria;p__Firmicutes;c__Clostridia;o__Oscillospirales;f__Acutalibacteraceae;g__Ruminococcus_E	99.7	blast;98.00 CRISPR;97.80 RaFAH;93.50 iPHoP-RF;88.20
S5-wms_52566	Shared	n.a.	n.a.	n.a.	n.a.	n.a.	n.a.	n.a.	n.a.	8.51	d__Bacteria;p__Firmicutes;c__Clostridia;o__Oscillospirales;f__Oscillospiraceae;g__Intestinimonas	91.1	blast;93.50 iPHoP-RF;52.80
S5-wms_62679	ME	Duplodnaviria	Heunggongvirae	Uroviricota	Caudoviricetes	n.a.	Unclassified	O	O	11.9	d__Bacteria;p__Actinobacteriota;c__Coriobacteriia;o__Coriobacteriales;f__Eggerthellaceae;g__Eggerthella	94.6	blast;96.20
S5-wms_85650	Shared	n.a.	n.a.	n.a.	n.a.	n.a.	n.a.	n.a.	n.a.	99.85	d__Bacteria;p__Bacteroidota;c__Bacteroidia;o__Bacteroidales;f__Bacteroidaceae;g__Bacteroides	100	RaFAH;99.00 iPHoP-RF;97.10 blast;96.60
S5-wms_94965	ME	Duplodnaviria	Heunggongvirae	Uroviricota	Caudoviricetes	n.a.	Unclassified	Unclassified	Unclassified	12.7	d__Bacteria;p__Actinobacteriota;c__Coriobacteriia;o__Coriobacteriales;f__Coriobacteriaceae;g__Collinsella	97.5	CRISPR;98.20 blast;95.40 iPHoP-RF;77.10
S6-wms-combined_110950	Shared	n.a.	n.a.	n.a.	n.a.	n.a.	n.a.	n.a.	n.a.	42.23	d__Bacteria;p__Firmicutes;c__Clostridia;o__Oscillospirales;f__Ruminococcaceae;g__Faecalibacterium	99.6	blast;96.80 RaFAH;93.40 iPHoP-RF;93.10 CRISPR;73.10
S6-wms-combined_117756	SHHC	Duplodnaviria	Heunggongvirae	Uroviricota	Caudoviricetes	n.a.	Unclassified	Unclassified	Eponavirus	50.35	d__Bacteria;p__Firmicutes;c__Clostridia;o__Lachnospirales;f__Lachnospiraceae;g__Acetatifactor	91.6	blast;93.90 iPHoP-RF;77.50
S6-wms-combined_118427	Shared	Duplodnaviria	Heunggongvirae	Uroviricota	Caudoviricetes	n.a.	Unclassified	Unclassified	O	40.24	d__Bacteria;p__Firmicutes;c__Clostridia;o__Oscillospirales;f__Oscillospiraceae;g__Dysosmobacter	97.9	iPHoP-RF;93.70 blast;91.90 RaFAH;88.40
S6-wms-combined_119017	SHHC	Duplodnaviria	Heunggongvirae	Uroviricota	Caudoviricetes	n.a.	Peduoviridae	Unclassified	Phitrevirus	37.52	d__Bacteria;p__Desulfobacterota;c__Desulfovibrionia;o__Desulfovibrionales;f__Desulfovibrionaceae;g__Bilophila	94.2	blast;95.90 iPHoP-RF;92.40 CRISPR;66.40
S6-wms-combined_119138	Shared	Duplodnaviria	Heunggongvirae	Uroviricota	Caudoviricetes	n.a.	Unclassified	O	O	99.25	d__Bacteria;p__Firmicutes;c__Clostridia;o__Lachnospirales;f__Lachnospiraceae;g__Enterocloster	91.6	blast;93.90
S6-wms-combined_126236	Shared	Duplodnaviria	Heunggongvirae	Uroviricota	Caudoviricetes	n.a.	Unclassified	Unclassified	Toutatisvirus	26.53	d__Bacteria;p__Firmicutes;c__Clostridia;o__Oscillospirales;f__Ruminococcaceae;g__Faecalibacterium	97.6	blast;98.30 CRISPR;97.70 iPHoP-RF;86.70
S6-wms-combined_135223	Shared	n.a.	n.a.	n.a.	n.a.	n.a.	n.a.	n.a.	n.a.	23.09	d__Bacteria;p__Bacteroidota;c__Bacteroidia;o__Bacteroidales;f__Bacteroidaceae;g__Bacteroides	99	iPHoP-RF;96.40 blast;92.30 RaFAH;88.60
S6-wms-combined_138710	Shared	n.a.	n.a.	n.a.	n.a.	n.a.	n.a.	n.a.	n.a.	55.97	d__Bacteria;p__Firmicutes;c__Clostridia;o__Oscillospirales;f__Ruminococcaceae;g__Faecalibacterium	96.2	blast;96.90 iPHoP-RF;78.00 RaFAH;56.20
S6-wms-combined_140731	SHHC	n.a.	n.a.	n.a.	n.a.	n.a.	n.a.	n.a.	n.a.	27.76	d__Bacteria;p__Firmicutes;c__Clostridia;o__Oscillospirales;f__Ruminococcaceae;g__Faecalibacterium	98.4	CRISPR;92.30 RaFAH;92.10 blast;92.10 iPHoP-RF;80.30
S6-wms-combined_32530	Shared	Duplodnaviria	Heunggongvirae	Uroviricota	Caudoviricetes	n.a.	Unclassified	O	O	99.74	d__Bacteria;p__Firmicutes;c__Clostridia;o__Lachnospirales;f__Lachnospiraceae;g__Roseburia	99.3	RaFAH;94.70 blast;87.00 iPHoP-RF;77.10
S6-wms-combined_41246	Shared	n.a.	n.a.	n.a.	n.a.	n.a.	n.a.	n.a.	n.a.	21.08	d__Bacteria;p__Firmicutes;c__Clostridia;o__Oscillospirales;f__Oscillospiraceae;g__Marseille-P3106	91.2	blast;93.60
S6-wms-combined_49909	SHHC	Duplodnaviria	Heunggongvirae	Uroviricota	Caudoviricetes	n.a.	Unclassified	O	O	50.09	d__Bacteria;p__Firmicutes;c__Clostridia;o__Oscillospirales;f__Ruminococcaceae;g__Faecalibacterium	91	blast;93.40 iPHoP-RF;86.00
S6-wms-combined_50721	Shared	Duplodnaviria	Heunggongvirae	Uroviricota	Caudoviricetes	n.a.	Winoviridae	O	O	99.98	d__Bacteria;p__Bacteroidota;c__Bacteroidia;o__Bacteroidales;f__Rikenellaceae;g__Alistipes	99.6	blast;96.90 iPHoP-RF;96.70 RaFAH;94.30
S6-wms-combined_56464	Shared	Duplodnaviria	Heunggongvirae	Uroviricota	Caudoviricetes	n.a.	Unclassified	O	O	51.17	d__Bacteria;p__Firmicutes;c__Clostridia;o__Oscillospirales;f__Ruminococcaceae;g__Faecalibacterium	99	blast;96.90 iPHoP-RF;92.10 RaFAH;87.90
S6-wms-combined_65225	SHHC	n.a.	n.a.	n.a.	n.a.	n.a.	n.a.	n.a.	n.a.	23.74	d__Bacteria;p__Firmicutes;c__Negativicutes;o__Veillonellales;f__Dialisteraceae;g__Dialister	94.9	blast;96.40 CRISPR;86.10 iPHoP-RF;77.30
S6-wms-combined_72228	Shared	n.a.	n.a.	n.a.	n.a.	n.a.	n.a.	n.a.	n.a.	41.84	d__Bacteria;p__Bacteroidota;c__Bacteroidia;o__Bacteroidales;f__Rikenellaceae;g__Alistipes	99	iPHoP-RF;97.10 blast;96.60 RaFAH;85.90
S6-wms-combined_745	SHHC	n.a.	n.a.	n.a.	n.a.	n.a.	n.a.	n.a.	n.a.	7.61	d__Bacteria;p__Proteobacteria;c__Alphaproteobacteria;o__RF32;f__CAG-239;g__CAG-267	90.6	blast;93.00 iPHoP-RF;84.50
S6-wms-combined_82597	Shared	n.a.	n.a.	n.a.	n.a.	n.a.	n.a.	n.a.	n.a.	91.55	d__Bacteria;p__Bacteroidota;c__Bacteroidia;o__Bacteroidales;f__Bacteroidaceae;g__Bacteroides	99.7	iPHoP-RF;97.10 blast;96.90 RaFAH;95.40
S6-wms-combined_91039	Shared	n.a.	n.a.	n.a.	n.a.	n.a.	n.a.	n.a.	n.a.	100	d__Bacteria;p__Bacteroidota;c__Bacteroidia;o__Bacteroidales;f__Rikenellaceae;g__Alistipes	96.6	CRISPR;97.60 iPHoP-RF;97.10 blast;96.70
S6-wms_132588	Shared	Duplodnaviria	Heunggongvirae	Uroviricota	Caudoviricetes	n.a.	Unclassified	O	O	67.09	d__Bacteria;p__Firmicutes;c__Clostridia;o__Oscillospirales;f__Ruminococcaceae;g__Faecalibacterium	93.2	CRISPR;95.20 blast;94.40 iPHoP-RF;92.80
S6-wms_138342	SHHC	Duplodnaviria	Heunggongvirae	Uroviricota	Caudoviricetes	Crassvirales	Suoliviridae	O	O	37.57	d__Bacteria;p__Bacteroidota;c__Bacteroidia;o__Bacteroidales;f__Bacteroidaceae;g__Prevotella	91.7	RaFAH;94.00
S6-wms_140743	SHHC	Duplodnaviria	Heunggongvirae	Uroviricota	Caudoviricetes	n.a.	Unclassified	O	O	9.85	d__Bacteria;p__Firmicutes;c__Clostridia;o__Lachnospirales;f__Lachnospiraceae;g__Enterocloster	91.2	blast;93.60 iPHoP-RF;92.40 CRISPR;53.80
S6-wms_54578	SHHC	Duplodnaviria	Heunggongvirae	Uroviricota	Caudoviricetes	n.a.	Unclassified	Unclassified	Unclassified	97	d__Bacteria;p__Bacteroidota;c__Bacteroidia;o__Bacteroidales;f__Tannerellaceae;g__Marseille-P3160	97.5	CRISPR;98.20 iPHoP-RF;69.80
S7-wms-combined_104872	Shared	n.a.	n.a.	n.a.	n.a.	n.a.	n.a.	n.a.	n.a.	97.79	d__Bacteria;p__Bacteroidota;c__Bacteroidia;o__Bacteroidales;f__Bacteroidaceae;g__Bacteroides	100	RaFAH;99.70 blast;92.10 iPHoP-RF;90.50 CRISPR;53.10
S7-wms-combined_106253	Shared	Duplodnaviria	Heunggongvirae	Uroviricota	Caudoviricetes	n.a.	Unclassified	O	O	71.87	d__Bacteria;p__Firmicutes;c__Clostridia;o__Lachnospirales;f__Lachnospiraceae;g__Dorea_A	95.3	blast;96.70 iPHoP-RF;94.40
S7-wms-combined_107679_0	Shared	Duplodnaviria	Heunggongvirae	Uroviricota	Caudoviricetes	n.a.	Winoviridae	O	O	99.98	d__Bacteria;p__Bacteroidota;c__Bacteroidia;o__Bacteroidales;f__Rikenellaceae;g__Alistipes	100	RaFAH;98.50 blast;97.20 iPHoP-RF;96.40
S7-wms-combined_107679_1	Shared	Duplodnaviria	Heunggongvirae	Uroviricota	Caudoviricetes	n.a.	Unclassified	O	O	100	d__Bacteria;p__Bacteroidota;c__Bacteroidia;o__Bacteroidales;f__Bacteroidaceae;g__Bacteroides	99.9	RaFAH;99.80 iPHoP-RF;57.60
S7-wms-combined_111004	Shared	Duplodnaviria	Heunggongvirae	Uroviricota	Caudoviricetes	n.a.	Unclassified	Unclassified	Taranisvirus	34.72	d__Bacteria;p__Firmicutes;c__Clostridia;o__Oscillospirales;f__Ruminococcaceae;g__Faecalibacterium	99.7	CRISPR;98.50 blast;96.60 RaFAH;90.40 iPHoP-RF;85.70
S7-wms-combined_123085	Shared	n.a.	n.a.	n.a.	n.a.	n.a.	n.a.	n.a.	n.a.	3.26	d__Bacteria;p__Firmicutes;c__Clostridia;o__Lachnospirales;f__Lachnospiraceae;g__Fusicatenibacter	91.6	blast;93.90 iPHoP-RF;83.90
S7-wms-combined_134452	Shared	n.a.	n.a.	n.a.	n.a.	n.a.	n.a.	n.a.	n.a.	8.94	d__Bacteria;p__Firmicutes;c__Clostridia;o__Christensenellales;f__CAG-74;g__UBA11524	95.6	blast;96.90 iPHoP-RF;94.70
S7-wms-combined_134458	Shared	Duplodnaviria	Heunggongvirae	Uroviricota	Caudoviricetes	n.a.	Unclassified	Unclassified	Toutatisvirus	43.62	d__Bacteria;p__Firmicutes;c__Clostridia;o__Oscillospirales;f__Ruminococcaceae;g__Gemmiger	91.6	blast;93.90 iPHoP-RF;88.90 CRISPR;69.70
S7-wms-combined_135154	ME	n.a.	n.a.	n.a.	n.a.	n.a.	n.a.	n.a.	n.a.	99.98	d__Bacteria;p__Bacteroidota;c__Bacteroidia;o__Bacteroidales;f__Bacteroidaceae;g__Bacteroides	100	RaFAH;99.30 iPHoP-RF;97.10 blast;92.40
S7-wms-combined_136642	ME	n.a.	n.a.	n.a.	n.a.	n.a.	n.a.	n.a.	n.a.	81.83	d__Bacteria;p__Actinobacteriota;c__Actinomycetia;o__Actinomycetales;f__Bifidobacteriaceae;g__Bifidobacterium	99.7	CRISPR;99.40 iPHoP-RF;97.10 blast;96.80 RaFAH;72.30
S7-wms-combined_137858	Shared	n.a.	n.a.	n.a.	n.a.	n.a.	n.a.	n.a.	n.a.	55.93	d__Bacteria;p__Firmicutes;c__Clostridia;o__Oscillospirales;f__Ruminococcaceae;g__Faecalibacterium	95.6	blast;96.90 iPHoP-RF;88.20
S7-wms-combined_139683	Shared	Duplodnaviria	Heunggongvirae	Uroviricota	Caudoviricetes	n.a.	Unclassified	O	O	93.85	d__Bacteria;p__Firmicutes;c__Clostridia;o__Oscillospirales;f__Oscillospiraceae;g__CAG-103	93.5	CRISPR;95.40
S7-wms-combined_14267	Shared	n.a.	n.a.	n.a.	n.a.	n.a.	n.a.	n.a.	n.a.	96.95	d__Bacteria;p__Bacteroidota;c__Bacteroidia;o__Bacteroidales;f__Rikenellaceae;g__Alistipes	97.5	CRISPR;98.20 blast;98.10 iPHoP-RF;97.10
S7-wms-combined_143218	ME	n.a.	n.a.	n.a.	n.a.	n.a.	n.a.	n.a.	n.a.	6.41	d__Bacteria;p__Actinobacteriota;c__Actinomycetia;o__Actinomycetales;f__Bifidobacteriaceae;g__Bifidobacterium	95.2	blast;96.60 CRISPR;91.70 iPHoP-RF;81.90
S7-wms-combined_14529	Shared	Duplodnaviria	Heunggongvirae	Uroviricota	Caudoviricetes	n.a.	Unclassified	O	O	25.87	d__Bacteria;p__Firmicutes;c__Clostridia;o__Christensenellales;f__CAG-74;g__UBA11524	95.6	blast;96.90 iPHoP-RF;93.40
S7-wms-combined_147158	ME	Duplodnaviria	Heunggongvirae	Uroviricota	Caudoviricetes	n.a.	Unclassified	O	O	86.44	d__Bacteria;p__Actinobacteriota;c__Actinomycetia;o__Actinomycetales;f__Bifidobacteriaceae;g__Bifidobacterium	99	CRISPR;99.20 blast;96.90 iPHoP-RF;95.70 RaFAH;50.40
S7-wms-combined_156351	ME	n.a.	n.a.	n.a.	n.a.	n.a.	n.a.	n.a.	n.a.	22	d__Bacteria;p__Firmicutes;c__Clostridia;o__Lachnospirales;f__Lachnospiraceae;g__Blautia_A	94.5	blast;96.10 iPHoP-RF;94.10
S7-wms-combined_157260	Shared	Duplodnaviria	Heunggongvirae	Uroviricota	Caudoviricetes	n.a.	Unclassified	Unclassified	O	99.48	d__Bacteria;p__Firmicutes;c__Clostridia;o__Lachnospirales;f__Lachnospiraceae;g__Lachnospira	98.7	CRISPR;99.00
S7-wms-combined_157434	Shared	n.a.	n.a.	n.a.	n.a.	n.a.	n.a.	n.a.	n.a.	16.06	d__Bacteria;p__Firmicutes;c__Clostridia;o__Lachnospirales;f__Lachnospiraceae;g__Mediterraneibacter	91.4	blast;93.70 iPHoP-RF;86.30
S7-wms-combined_163980	ME	n.a.	n.a.	n.a.	n.a.	n.a.	n.a.	n.a.	n.a.	78.4	d__Bacteria;p__Bacteroidota;c__Bacteroidia;o__Bacteroidales;f__Rikenellaceae;g__Alistipes	95.2	blast;96.60 iPHoP-RF;93.70
S7-wms-combined_167867	ME	n.a.	n.a.	n.a.	n.a.	n.a.	n.a.	n.a.	n.a.	10.54	d__Bacteria;p__Actinobacteriota;c__Actinomycetia;o__Actinomycetales;f__Bifidobacteriaceae;g__Bifidobacterium	97.9	CRISPR;98.50 iPHoP-RF;97.10 blast;96.70
S7-wms-combined_169375	ME	n.a.	n.a.	n.a.	n.a.	n.a.	n.a.	n.a.	n.a.	10.64	d__Bacteria;p__Firmicutes;c__Bacilli;o__Erysipelotrichales;f__Erysipelatoclostridiaceae;g__Erysipelatoclostridium	94.7	blast;96.30
S7-wms-combined_174935	Shared	Duplodnaviria	Heunggongvirae	Uroviricota	Caudoviricetes	n.a.	Unclassified	O	O	34.84	d__Bacteria;p__Actinobacteriota;c__Actinomycetia;o__Actinomycetales;f__Bifidobacteriaceae;g__Bifidobacterium	96.3	CRISPR;97.40 blast;97.30 iPHoP-RF;97.10
S7-wms-combined_185016	Shared	Duplodnaviria	Heunggongvirae	Uroviricota	Caudoviricetes	n.a.	Unclassified	O	O	95.96	d__Bacteria;p__Bacteroidota;c__Bacteroidia;o__Bacteroidales;f__Bacteroidaceae;g__Bacteroides	100	RaFAH;99.70 blast;95.30 iPHoP-RF;95.10
S7-wms-combined_189250	Shared	Duplodnaviria	Heunggongvirae	Uroviricota	Caudoviricetes	n.a.	Unclassified	Unclassified	Unclassified	22.37	d__Bacteria;p__Actinobacteriota;c__Coriobacteriia;o__Coriobacteriales;f__Coriobacteriaceae;g__Collinsella	98.7	CRISPR;99.00 blast;91.90 iPHoP-RF;80.00
S7-wms-combined_190281	Shared	n.a.	n.a.	n.a.	n.a.	n.a.	n.a.	n.a.	n.a.	13.36	d__Bacteria;p__Actinobacteriota;c__Coriobacteriia;o__Coriobacteriales;f__Coriobacteriaceae;g__Collinsella	96.2	CRISPR;97.30 blast;96.90 iPHoP-RF;86.30
S7-wms-combined_20921	ME	Duplodnaviria	Heunggongvirae	Uroviricota	Caudoviricetes	n.a.	Unclassified	O	O	29.3	d__Bacteria;p__Firmicutes;c__Clostridia;o__Lachnospirales;f__Lachnospiraceae;g__Anaerobutyricum	95	blast;96.50 iPHoP-RF;77.50

S7-wms-combined_217226	ME	Duplodnaviria	Heunggongvirae	Uroviricota	Caudoviricetes	n.a.	Unclassified	O	O	97.48	d__Bacteria;p__Verrucomicrobiota;c__Verrucomicrobiae;o__Verrucomicrobiales;f__Akkermansiaceae;g__Akkermansia	100	CRISPR;99.50 RaFAH;97.70 iPHoP-RF;58.20
S7-wms-combined_218379	Shared	Duplodnaviria	Heunggongvirae	Uroviricota	Caudoviricetes	n.a.	Unclassified	O	O	16.97	d__Bacteria;p__Firmicutes;c__Clostridia;o__Oscillospirales;f__Acutalibacteraceae;g__CAG-180	98.7	CRISPR;99.00 blast;96.60 iPHoP-RF;86.30
S7-wms-combined_224523	Shared	Duplodnaviria	Heunggongvirae	Uroviricota	Caudoviricetes	n.a.	Unclassified	O	O	99.68	d__Bacteria;p__Firmicutes;c__Clostridia;o__Oscillospirales;f__Ruminococcaceae;g__Ruminococcus_D	99.4	blast;96.80 iPHoP-RF;93.70 RaFAH;91.90
S7-wms-combined_235165	ME	Duplodnaviria	Heunggongvirae	Uroviricota	Caudoviricetes	n.a.	Unclassified	Unclassified	Unclassified	18.87	d__Bacteria;p__Firmicutes;c__Clostridia;o__Lachnospirales;f__Lachnospiraceae;g__Agathobacter	90.2	blast;92.70
S7-wms-combined_238231	Shared	n.a.	n.a.	n.a.	n.a.	n.a.	n.a.	n.a.	n.a.	39.27	d__Bacteria;p__Firmicutes;c__Clostridia;o__Oscillospirales;f__Ruminococcaceae;g__Negativibacillus	95.6	blast;96.90 iPHoP-RF;77.50 CRISPR;68.00
S7-wms-combined_239784	Shared	n.a.	n.a.	n.a.	n.a.	n.a.	n.a.	n.a.	n.a.	67.8	d__Bacteria;p__Bacteroidota;c__Bacteroidia;o__Bacteroidales;f__Bacteroidaceae;g__Bacteroides	99.3	iPHoP-RF;97.10 RaFAH;89.00 blast;88.60
S7-wms-combined_26217	Shared	Duplodnaviria	Heunggongvirae	Uroviricota	Caudoviricetes	n.a.	Unclassified	O	O	100	d__Bacteria;p__Bacteroidota;c__Bacteroidia;o__Bacteroidales;f__Rikenellaceae;g__Alistipes	100	RaFAH;98.00 blast;96.90 iPHoP-RF;88.90
S7-wms-combined_28138	ME	Duplodnaviria	Heunggongvirae	Uroviricota	Caudoviricetes	n.a.	Unclassified	O	O	12.56	d__Bacteria;p__Firmicutes;c__Clostridia;o__Oscillospirales;f__Acutalibacteraceae;g__Ruminococcus_E	98.7	CRISPR;99.00 blast;96.80 iPHoP-RF;91.10
S7-wms-combined_31068	Shared	n.a.	n.a.	n.a.	n.a.	n.a.	n.a.	n.a.	n.a.	9.44	d__Bacteria;p__Firmicutes;c__Clostridia;o__Oscillospirales;f__Oscillospiraceae;g__Lawsonibacter	91.5	blast;93.80 iPHoP-RF;81.90
S7-wms-combined_3669	ME	Monodnaviria	Sangervirae	Phixviricota	Malgrandaviricetes	Petitvirales	Microviridae	O	O	8.7	d__Bacteria;p__Bacteroidota;c__Bacteroidia;o__Bacteroidales;f__Bacteroidaceae;g__Bacteroides	91.5	blast;93.80 iPHoP-RF;87.60
S7-wms-combined_3697	Shared	Unclassified	n.a.	n.a.	n.a.	n.a.	Unclassified	O	O	72.05	d__Bacteria;p__Bacteroidota;c__Bacteroidia;o__Bacteroidales;f__Rikenellaceae;g__Alistipes	98.8	iPHoP-RF;97.10 blast;96.90 CRISPR;95.90 RaFAH;84.30
S7-wms-combined_43950	Shared	Duplodnaviria	Heunggongvirae	Uroviricota	Caudoviricetes	n.a.	Unclassified	Unclassified	Unclassified	99.99	d__Bacteria;p__Bacteroidota;c__Bacteroidia;o__Bacteroidales;f__Bacteroidaceae;g__Bacteroides	100	RaFAH;99.70 iPHoP-RF;95.10 blast;93.90
S7-wms-combined_5625	Shared	Duplodnaviria	Heunggongvirae	Uroviricota	Caudoviricetes	n.a.	Unclassified	Unclassified	Unclassified	95.36	d__Bacteria;p__Bacteroidota;c__Bacteroidia;o__Bacteroidales;f__Bacteroidaceae;g__Bacteroides	100	RaFAH;98.60 iPHoP-RF;97.10 blast;96.90
S7-wms-combined_62119	Shared	Duplodnaviria	Heunggongvirae	Uroviricota	Caudoviricetes	n.a.	Unclassified	Unclassified	O	99.99	d__Bacteria;p__Bacteroidota;c__Bacteroidia;o__Bacteroidales;f__Bacteroidaceae;g__Bacteroides	98.8	blast;96.90 iPHoP-RF;96.40 CRISPR;93.30 RaFAH;84.80
S7-wms-combined_69024	ME	Duplodnaviria	Heunggongvirae	Uroviricota	Caudoviricetes	n.a.	Unclassified	O	O	58.17	d__Bacteria;p__Firmicutes;c__Clostridia;o__Lachnospirales;f__Lachnospiraceae;g__Bariatricus	91.6	blast;93.90 iPHoP-RF;75.90
S7-wms-combined_71592	Shared	Duplodnaviria	Heunggongvirae	Uroviricota	Caudoviricetes	n.a.	Unclassified	O	O	39.23	d__Bacteria;p__Firmicutes;c__Clostridia;o__Oscillospirales;f__Ruminococcaceae;g__Ruminococcus_D	98.1	blast;98.60 iPHoP-RF;92.40 CRISPR;61.00
S7-wms-combined_72070	Shared	n.a.	n.a.	n.a.	n.a.	n.a.	n.a.	n.a.	n.a.	35.71	d__Bacteria;p__Bacteroidota;c__Bacteroidia;o__Bacteroidales;f__Bacteroidaceae;g__Bacteroides	96.2	iPHoP-RF;97.10 blast;96.90 CRISPR;55.90 RaFAH;52.60
S7-wms-combined_74829	Shared	Duplodnaviria	Heunggongvirae	Uroviricota	Caudoviricetes	n.a.	Unclassified	O	O	77.29	d__Bacteria;p__Firmicutes;c__Negativicutes;o__Veillonellales;f__Dialisteraceae;g__Dialister	97.3	CRISPR;97.40 blast;96.80 iPHoP-RF;94.70 RaFAH;63.90
S7-wms-combined_84688	Shared	n.a.	n.a.	n.a.	n.a.	n.a.	n.a.	n.a.	n.a.	29.6	d__Bacteria;p__Firmicutes;c__Clostridia;o__Oscillospirales;f__Oscillospiraceae;g__Marseille-P3106	91.6	blast;93.90 iPHoP-RF;86.70
S7-wms-combined_90393	ME	Duplodnaviria	Heunggongvirae	Uroviricota	Caudoviricetes	n.a.	Unclassified	O	O	32.36	d__Bacteria;p__Bacteroidota;c__Bacteroidia;o__Bacteroidales;f__Bacteroidaceae;g__Bacteroides	99.4	iPHoP-RF;97.10 blast;91.80 RaFAH;91.30
S7-wms-combined_90728	ME	Duplodnaviria	Heunggongvirae	Uroviricota	Caudoviricetes	n.a.	Unclassified	O	O	66.45	d__Bacteria;p__Bacteroidota;c__Bacteroidia;o__Bacteroidales;f__Bacteroidaceae;g__Bacteroides	100	RaFAH;98.90 iPHoP-RF;96.70 blast;91.80
S7-wms_113137	Shared	Duplodnaviria	Heunggongvirae	Uroviricota	Caudoviricetes	n.a.	Unclassified	Unclassified	Mushuvirus	99.39	d__Bacteria;p__Firmicutes;c__Clostridia;o__Oscillospirales;f__Ruminococcaceae;g__Faecalibacterium	99.4	RaFAH;99.50
S7-wms_123954	ME	n.a.	n.a.	n.a.	n.a.	n.a.	n.a.	n.a.	n.a.	100	d__Bacteria;p__Bacteroidota;c__Bacteroidia;o__Bacteroidales;f__Tannerellaceae;g__Parabacteroides	98.4	blast;96.40 RaFAH;82.90 iPHoP-RF;60.30
S7-wms_127801	Shared	Duplodnaviria	Heunggongvirae	Uroviricota	Caudoviricetes	n.a.	Unclassified	O	O	10.6	d__Bacteria;p__Firmicutes;c__Clostridia;o__Oscillospirales;f__Ruminococcaceae;g__Ruminococcus_D	98.7	CRISPR;99.00 blast;97.40 iPHoP-RF;93.70
S7-wms_151112	ME	Duplodnaviria	Heunggongvirae	Uroviricota	Caudoviricetes	n.a.	Unclassified	O	O	20.08	d__Bacteria;p__Firmicutes;c__Clostridia;o__Lachnospirales;f__Lachnospiraceae;g__Anaerobutyricum	90.3	blast;92.80 iPHoP-RF;86.00
S7-wms_157264	ME	Unclassified	n.a.	n.a.	n.a.	n.a.	Unclassified	Unclassified	Unclassified	5.31	d__Bacteria;p__Actinobacteriota;c__Actinomycetia;o__Actinomycetales;f__Bifidobacteriaceae;g__Bifidobacterium	99.7	CRISPR;99.70 iPHoP-RF;73.50
S7-wms_244866	ME	Duplodnaviria	Heunggongvirae	Uroviricota	Caudoviricetes	n.a.	Unclassified	Unclassified	Unclassified	18.88	d__Bacteria;p__Bacteroidota;c__Bacteroidia;o__Bacteroidales;f__Bacteroidaceae;g__Bacteroides	97.6	CRISPR;98.30 iPHoP-RF;92.40
S7-wms_80255	ME	Duplodnaviria	Heunggongvirae	Uroviricota	Caudoviricetes	n.a.	Unclassified	O	O	74.19	d__Bacteria;p__Firmicutes;c__Clostridia;o__Oscillospirales;f__Ruminococcaceae;g__Ruminiclostridium_E	91.5	blast;93.80 iPHoP-RF;67.00 CRISPR;57.40
S8-wms-combined_134098	SHHC	n.a.	n.a.	n.a.	n.a.	n.a.	n.a.	n.a.	n.a.	81.54	d__Bacteria;p__Firmicutes;c__Clostridia;o__Oscillospirales;f__Oscillospiraceae;g__ER4	95.2	blast;96.60 iPHoP-RF;76.10
S8-wms-combined_146562	SHHC	Duplodnaviria	Heunggongvirae	Uroviricota	Caudoviricetes	n.a.	Unclassified	O	O	66.24	d__Bacteria;p__Firmicutes;c__Clostridia;o__Christensenellales;f__CAG-138;g__UBA1685	93	blast;95.00 iPHoP-RF;82.80 CRISPR;70.40
S8-wms-combined_164289	SHHC	n.a.	n.a.	n.a.	n.a.	n.a.	n.a.	n.a.	n.a.	21.14	d__Bacteria;p__Firmicutes;c__Clostridia;o__Christensenellales;f__CAG-138;g__SFEL01	94.5	blast;96.10 iPHoP-RF;79.00 CRISPR;71.90
S8-wms-combined_169835	Shared	n.a.	n.a.	n.a.	n.a.	n.a.	n.a.	n.a.	n.a.	17.94	d__Bacteria;p__Firmicutes;c__Clostridia;o__Oscillospirales;f__Oscillospiraceae;g__CAG-83	95.6	blast;96.90 iPHoP-RF;64.30
S8-wms-combined_180957	SHHC	Duplodnaviria	Heunggongvirae	Uroviricota	Caudoviricetes	n.a.	Unclassified	O	O	99.92	d__Bacteria;p__Bacteroidota;c__Bacteroidia;o__Bacteroidales;f__Tannerellaceae;g__Parabacteroides	99.7	blast;96.70 RaFAH;95.30 iPHoP-RF;84.50 CRISPR;57.90
S8-wms-combined_185611	Shared	n.a.	n.a.	n.a.	n.a.	n.a.	n.a.	n.a.	n.a.	9.67	d__Bacteria;p__Firmicutes;c__Clostridia;o__Christensenellales;f__CAG-74;g__Limiplasma	94.2	blast;95.90 iPHoP-RF;76.60
S8-wms-combined_211119	SHHC	n.a.	n.a.	n.a.	n.a.	n.a.	n.a.	n.a.	n.a.	22.48	d__Bacteria;p__Firmicutes;c__Clostridia;o__Christensenellales;f__CAG-138;g__UBA1685	95.9	blast;97.10 iPHoP-RF;77.50 CRISPR;68.50
S8-wms-combined_215102	Shared	n.a.	n.a.	n.a.	n.a.	n.a.	n.a.	n.a.	n.a.	12.51	d__Bacteria;p__Firmicutes;c__Clostridia;o__Christensenellales;f__CAG-74;g__UBA11524	95.6	blast;96.90 iPHoP-RF;94.70
S8-wms-combined_217866	SHHC	Duplodnaviria	Heunggongvirae	Uroviricota	Caudoviricetes	n.a.	Unclassified	Unclassified	Eponavirus	98.95	d__Bacteria;p__Firmicutes;c__Clostridia;o__Lachnospirales;f__Lachnospiraceae;g__Roseburia	96.3	CRISPR;97.40 blast;68.80
S8-wms-combined_219796	SHHC	Duplodnaviria	Heunggongvirae	Uroviricota	Caudoviricetes	n.a.	Unclassified	Unclassified	O	14.07	d__Bacteria;p__Firmicutes;c__Clostridia;o__Christensenellales;f__CAG-138;g__UBA1685	93.5	blast;95.40 CRISPR;93.90 iPHoP-RF;88.20
S8-wms-combined_225114	SHHC	Duplodnaviria	Heunggongvirae	Uroviricota	Caudoviricetes	n.a.	Unclassified	O	O	99.67	d__Bacteria;p__Firmicutes;c__Clostridia;o__Oscillospirales;f__Ruminococcaceae;g__Faecalibacterium	100	CRISPR;98.40 RaFAH;98.00 blast;92.30 iPHoP-RF;88.50
S8-wms-combined_23583	Shared	n.a.	n.a.	n.a.	n.a.	n.a.	n.a.	n.a.	n.a.	28.29	d__Bacteria;p__Bacteroidota;c__Bacteroidia;o__Bacteroidales;f__Rikenellaceae;g__Alistipes_A	90.3	blast;92.80 iPHoP-RF;81.60 CRISPR;68.00
S8-wms-combined_239791	SHHC	Duplodnaviria	Heunggongvirae	Uroviricota	Caudoviricetes	n.a.	Aggregaviridae	O	O	19.46	d__Bacteria;p__Bacteroidota;c__Bacteroidia;o__Bacteroidales;f__Bacteroidaceae;g__Bacteroides	96.3	iPHoP-RF;95.10 RaFAH;73.50
S8-wms-combined_246185	Shared	Duplodnaviria	Heunggongvirae	Uroviricota	Caudoviricetes	n.a.	Unclassified	O	O	33.37	d__Bacteria;p__Verrucomicrobiota;c__Verrucomicrobiae;o__Verrucomicrobiales;f__Akkermansiaceae;g__Akkermansia	98.4	CRISPR;98.80 blast;96.90 iPHoP-RF;81.10
S8-wms-combined_246389	SHHC	Duplodnaviria	Heunggongvirae	Uroviricota	Caudoviricetes	n.a.	Unclassified	Unclassified	O	21.76	d__Bacteria;p__Firmicutes;c__Clostridia;o__Oscillospirales;f__Oscillospiraceae;g__ER4	91.6	blast;93.90 iPHoP-RF;64.50
S8-wms-combined_25607	SHHC	Duplodnaviria	Heunggongvirae	Uroviricota	Caudoviricetes	n.a.	Unclassified	O	O	54.47	d__Bacteria;p__Firmicutes;c__Clostridia;o__Oscillospirales;f__Oscillospiraceae;g__UBA9475	91.2	blast;93.60 iPHoP-RF;68.90
S8-wms-combined_26087	Shared	Duplodnaviria	Heunggongvirae	Uroviricota	Caudoviricetes	n.a.	Unclassified	O	O	100	d__Bacteria;p__Bacteroidota;c__Bacteroidia;o__Bacteroidales;f__Rikenellaceae;g__Alistipes	98.7	blast;96.90 iPHoP-RF;91.40 RaFAH;83.40
S8-wms-combined_264711	SHHC	n.a.	n.a.	n.a.	n.a.	n.a.	n.a.	n.a.	n.a.	10.73	d__Bacteria;p__Firmicutes;c__Clostridia;o__Christensenellales;f__CAG-138;g__SFEL01	90.9	blast;93.30 iPHoP-RF;74.80
S8-wms-combined_270362	Shared	Duplodnaviria	Heunggongvirae	Uroviricota	Caudoviricetes	n.a.	Unclassified	O	O	99.95	d__Bacteria;p__Bacteroidota;c__Bacteroidia;o__Bacteroidales;f__Rikenellaceae;g__Alistipes	100	RaFAH;97.30 blast;96.90 iPHoP-RF;90.10
S8-wms-combined_40666	Shared	n.a.	n.a.	n.a.	n.a.	n.a.	n.a.	n.a.	n.a.	26.57	d__Bacteria;p__Firmicutes;c__Clostridia;o__Oscillospirales;f__Oscillospiraceae;g__CAG-103	97.2	CRISPR;98.00 blast;94.30 iPHoP-RF;83.60
S8-wms-combined_49369	Shared	n.a.	n.a.	n.a.	n.a.	n.a.	n.a.	n.a.	n.a.	96.98	d__Bacteria;p__Firmicutes;c__Clostridia;o__Oscillospirales;f__Ruminococcaceae;g__Gemmiger	94	blast;95.20 iPHoP-RF;61.70 RaFAH;56.00
S8-wms-combined_68780	Shared	n.a.	n.a.	n.a.	n.a.	n.a.	n.a.	n.a.	n.a.	51.97	d__Bacteria;p__Firmicutes;c__Clostridia;o__Oscillospirales;f__Oscillospiraceae;g__F23-B02	94.6	blast;96.20 iPHoP-RF;58.40
S8-wms-combined_74275	SHHC	Duplodnaviria	Heunggongvirae	Uroviricota	Caudoviricetes	n.a.	Unclassified	O	O	54.68	d__Bacteria;p__Bacteroidota;c__Bacteroidia;o__Bacteroidales;f__Bacteroidaceae;g__Prevotella	99.3	blast;96.90 iPHoP-RF;91.40 RaFAH;90.60
S8-wms-combined_81597	Shared	Duplodnaviria	Heunggongvirae	Uroviricota	Caudoviricetes	n.a.	Unclassified	O	O	99.93	d__Bacteria;p__Bacteroidota;c__Bacteroidia;o__Bacteroidales;f__Bacteroidaceae;g__Bacteroides	100	RaFAH;99.50 blast;95.10 iPHoP-RF;85.40
S8-wms-combined_97848	SHHC	Duplodnaviria	Heunggongvirae	Uroviricota	Caudoviricetes	n.a.	Unclassified	Unclassified	Unclassified	75.58	d__Bacteria;p__Bacteroidota;c__Bacteroidia;o__Bacteroidales;f__Bacteroidaceae;g__Bacteroides	98.8	RaFAH;92.20 blast;92.00 iPHoP-RF;62.20
S8-wms_152831	SHHC	Duplodnaviria	Heunggongvirae	Uroviricota	Caudoviricetes	n.a.	Unclassified	Unclassified	Toutatisvirus	99.72	d__Bacteria;p__Firmicutes;c__Clostridia;o__Oscillospirales;f__Ruminococcaceae;g__Faecalibacterium	100	RaFAH;99.50 CRISPR;97.80 blast;95.40 iPHoP-RF;77.30
S8-wms_190781	SHHC	Duplodnaviria	Heunggongvirae	Uroviricota	Caudoviricetes	n.a.	Unclassified	O	O	93.96	d__Bacteria;p__Firmicutes;c__Clostridia;o__Oscillospirales;f__Ruminococcaceae;g__Faecalibacterium	92	CRISPR;94.20 blast;94.00 iPHoP-RF;83.30
S8-wms_20528	SHHC	Duplodnaviria	Heunggongvirae	Uroviricota	Caudoviricetes	n.a.	Unclassified	Unclassified	O	48.51	d__Bacteria;p__Firmicutes;c__Clostridia;o__Oscillospirales;f__Ruminococcaceae;g__Gemmiger	91.6	blast;93.90 iPHoP-RF;84.50
S8-wms_270	SHHC	Monodnaviria	Sangervirae	Phixviricota	Malgrandaviricetes	Petitvirales	Microviridae	O	O		d__Bacteria;p__Bacteroidota;c__Bacteroidia;o__Bacteroidales;f__Barnesiellaceae;g__Barnesiella	94.6	blast;96.20 iPHoP-RF;68.20
S8-wms_30498	Shared	Duplodnaviria	Heunggongvirae	Uroviricota	Caudoviricetes	n.a.	Unclassified	Unclassified	Mushuvirus	86.77	d__Bacteria;p__Firmicutes;c__Clostridia;o__Oscillospirales;f__Oscillospiraceae;g__CAG-83	92.7	blast;86.00 RaFAH;81.50 iPHoP-RF;63.00
S8-wms_48935	Shared	Duplodnaviria	Heunggongvirae	Uroviricota	Caudoviricetes	n.a.	Unclassified	Unclassified	Toutatisvirus	39.33	d__Bacteria;p__Firmicutes;c__Clostridia;o__Oscillospirales;f__Oscillospiraceae;g__CAG-83	90.9	blast;93.30 iPHoP-RF;77.30
S8-wms_57121	SHHC	n.a.	n.a.	n.a.	n.a.	n.a.	n.a.	n.a.	n.a.	9.54	d__Bacteria;p__Firmicutes;c__Clostridia;o__Christensenellales;f__CAG-138;g__UBA1685	97.3	blast;98.10 iPHoP-RF;76.60 CRISPR;55.40
S8-wms_58515	Shared	n.a.	n.a.	n.a.	n.a.	n.a.	n.a.	n.a.	n.a.	74.23	d__Bacteria;p__Bacteroidota;c__Bacteroidia;o__Bacteroidales;f__Rikenellaceae;g__Alistipes	96.3	iPHoP-RF;97.10 blast;96.70 CRISPR;91.70 RaFAH;55.50
S8-wms_82880	Shared	Duplodnaviria	Heunggongvirae	Uroviricota	Caudoviricetes	n.a.	Unclassified	Unclassified	Brigitvirus	30.76	d__Bacteria;p__Firmicutes;c__Clostridia;o__Oscillospirales;f__Oscillospiraceae;g__An92	93	blast;95.00 iPHoP-RF;60.10
S9-wms-combined_105462	ME	Duplodnaviria	Heunggongvirae	Uroviricota	Caudoviricetes	n.a.	Unclassified	O	O	98	d__Bacteria;p__Firmicutes;c__Clostridia;o__Lachnospirales;f__Lachnospiraceae;g__Anaerostipes	95.4	blast;96.80 CRISPR;91.30 iPHoP-RF;91.10
S9-wms-combined_105822	ME	Duplodnaviria	Heunggongvirae	Uroviricota	Caudoviricetes	n.a.	Unclassified	Unclassified	O	44.89	d__Bacteria;p__Firmicutes;c__Clostridia;o__Lachnospirales;f__Lachnospiraceae;g__Enterocloster	95	blast;96.50 iPHoP-RF;87.60 CRISPR;52.20
S9-wms-combined_10990	Shared	n.a.	n.a.	n.a.	n.a.	n.a.	n.a.	n.a.	n.a.	10.09	d__Bacteria;p__Firmicutes;c__Clostridia;o__Lachnospirales;f__Lachnospiraceae;g__Blautia_A	91.4	blast;93.70 iPHoP-RF;80.00
S9-wms-combined_122742	Shared	n.a.	n.a.	n.a.	n.a.	n.a.	n.a.	n.a.	n.a.	8.62	d__Bacteria;p__Firmicutes;c__Clostridia;o__Oscillospirales;f__Oscillospiraceae;g__Flavonifractor	90.3	blast;92.80 iPHoP-RF;72.80
S9-wms-combined_124857	Shared	n.a.	n.a.	n.a.	n.a.	n.a.	n.a.	n.a.	n.a.	79.58	d__Bacteria;p__Firmicutes;c__Clostridia;o__Oscillospirales;f__Oscillospiraceae;g__Flavonifractor	90.7	iPHoP-RF;93.10 blast;92.10 CRISPR;71.70
S9-wms-combined_2042	ME	n.a.	n.a.	n.a.	n.a.	n.a.	n.a.	n.a.	n.a.	10.88	d__Bacteria;p__Firmicutes;c__Clostridia;o__Lachnospirales;f__Lachnospiraceae;g__Eisenbergiella	95.2	CRISPR;96.60 blast;95.10 iPHoP-RF;93.40
S9-wms-combined_20485	ME	Duplodnaviria	Heunggongvirae	Uroviricota	Caudoviricetes	n.a.	Unclassified	Unclassified	O	94.46	d__Bacteria;p__Firmicutes;c__Bacilli;o__Lactobacillales;f__Streptococcaceae;g__Streptococcus	100	RaFAH;99.70 CRISPR;99.00 iPHoP-RF;97.10 blast;96.90
S9-wms-combined_26410	ME	Duplodnaviria	Heunggongvirae	Uroviricota	Caudoviricetes	n.a.	Unclassified	O	O	99.06	d__Bacteria;p__Firmicutes;c__Clostridia;o__Lachnospirales;f__Lachnospiraceae;g__Mediterraneibacter	95.4	blast;96.80
S9-wms-combined_27311	ME	n.a.	n.a.	n.a.	n.a.	n.a.	n.a.	n.a.	n.a.	4.98	d__Bacteria;p__Firmicutes;c__Clostridia;o__Oscillospirales;f__Acutalibacteraceae;g__Acutalibacter	91.4	blast;93.70 iPHoP-RF;76.10
S9-wms-combined_28805	ME	Duplodnaviria	Heunggongvirae	Uroviricota	Caudoviricetes	n.a.	Winoviridae	O	O	94.95	d__Bacteria;p__Bacteroidota;c__Bacteroidia;o__Bacteroidales;f__Rikenellaceae;g__Alistipes	99	blast;96.10 RaFAH;89.50 iPHoP-RF;68.40
S9-wms-combined_31456	Shared	Duplodnaviria	Heunggongvirae	Uroviricota	Caudoviricetes	n.a.	Unclassified	Unclassified	Unclassified	63.18	d__Bacteria;p__Bacteroidota;c__Bacteroidia;o__Bacteroidales;f__Barnesiellaceae;g__Barnesiella	97.6	CRISPR;97.40 blast;96.90 iPHoP-RF;83.60 RaFAH;68.00
S9-wms-combined_33229	ME	Duplodnaviria	Heunggongvirae	Uroviricota	Caudoviricetes	n.a.	Unclassified	O	O	87.21	d__Bacteria;p__Bacteroidota;c__Bacteroidia;o__Bacteroidales;f__Bacteroidaceae;g__Bacteroides	99.1	iPHoP-RF;97.10 blast;96.10 RaFAH;87.90
S9-wms-combined_33230	Shared	Duplodnaviria	Heunggongvirae	Uroviricota	Caudoviricetes	n.a.	Unclassified	O	O	93.11	d__Bacteria;p__Bacteroidota;c__Bacteroidia;o__Bacteroidales;f__Bacteroidaceae;g__Bacteroides	100	RaFAH;99.10 iPHoP-RF;96.40 blast;92.10
S9-wms-combined_3461	ME	n.a.	n.a.	n.a.	n.a.	n.a.	n.a.	n.a.	n.a.	69.22	d__Bacteria;p__Firmicutes;c__Clostridia;o__Lachnospirales;f__Anaerotignaceae;g__Anaerotignum	95	CRISPR;96.50 blast;91.10 iPHoP-RF;88.90
S9-wms-combined_36459	ME	Duplodnaviria	Heunggongvirae	Uroviricota	Caudoviricetes	n.a.	Unclassified	O	O	25.89	d__Bacteria;p__Firmicutes;c__Clostridia;o__Oscillospirales;f__Ruminococcaceae;g__Negativibacillus	96.2	blast;97.30 iPHoP-RF;85.10 CRISPR;59.60
S9-wms-combined_37587	Shared	Duplodnaviria	Heunggongvirae	Uroviricota	Caudoviricetes	n.a.	Unclassified	O	O	92.98	d__Bacteria;p__Bacteroidota;c__Bacteroidia;o__Bacteroidales;f__Bacteroidaceae;g__Bacteroides	99.4	blast;96.80 iPHoP-RF;96.40 RaFAH;92.80
S9-wms-combined_44402	Shared	Duplodnaviria	Heunggongvirae	Uroviricota	Caudoviricetes	n.a.	Unclassified	O	O	80.74	d__Bacteria;p__Bacteroidota;c__Bacteroidia;o__Bacteroidales;f__Bacteroidaceae;g__Bacteroides	100	RaFAH;97.80 blast;96.50 iPHoP-RF;96.40
S9-wms-combined_53991	Shared	Duplodnaviria	Heunggongvirae	Uroviricota	Caudoviricetes	n.a.	Unclassified	O	O	30.5	d__Bacteria;p__Firmicutes;c__Negativicutes;o__Veillonellales;f__Dialisteraceae;g__Dialister	95.4	blast;96.80 iPHoP-RF;91.80 CRISPR;60.90
S9-wms-combined_5526	Shared	Duplodnaviria	Heunggongvirae	Uroviricota	Caudoviricetes	n.a.	Unclassified	O	O	86.27	d__Bacteria;p__Bacteroidota;c__Bacteroidia;o__Bacteroidales;f__Rikenellaceae;g__Alistipes	99.3	blast;96.80 iPHoP-RF;93.10 RaFAH;90.40
S9-wms-combined_64277	ME	Duplodnaviria	Heunggongvirae	Uroviricota	Caudoviricetes	n.a.	Unclassified	Unclassified	Bcepmuvirus	98.37	d__Bacteria;p__Proteobacteria;c__Gammaproteobacteria;o__Enterobacterales;f__Enterobacteriaceae;g__Escherichia	100	RaFAH;97.50 blast;96.90 iPHoP-RF;74.30
S9-wms-combined_76274	Shared	Duplodnaviria	Heunggongvirae	Uroviricota	Caudoviricetes	n.a.	Unclassified	O	O	91.87	d__Bacteria;p__Actinobacteriota;c__Actinomycetia;o__Actinomycetales;f__Bifidobacteriaceae;g__Bifidobacterium	99.4	CRISPR;98.60 iPHoP-RF;93.70 blast;92.70 RaFAH;81.60
S9-wms-combined_93232	ME	Duplodnaviria	Heunggongvirae	Uroviricota	Caudoviricetes	n.a.	Unclassified	O	O	57.26	d__Bacteria;p__Proteobacteria;c__Gammaproteobacteria;o__Enterobacterales;f__Enterobacteriaceae;g__Klebsiella	98.2	CRISPR;98.70 blast;96.90 iPHoP-RF;59.00
S9-wms-combined_97755	ME	Duplodnaviria	Heunggongvirae	Uroviricota	Caudoviricetes	n.a.	Unclassified	Unclassified	O	40.71	d__Bacteria;p__Firmicutes;c__Clostridia;o__Lachnospirales;f__Lachnospiraceae;g__Eisenbergiella	91	blast;93.40 iPHoP-RF;57.60
S9-wms-combined_9931	Shared	Duplodnaviria	Heunggongvirae	Uroviricota	Caudoviricetes	n.a.	Unclassified	Unclassified	O	57.49	d__Bacteria;p__Firmicutes;c__Clostridia;o__Oscillospirales;f__Acutalibacteraceae;g__Ruminococcus_E	95.4	blast;96.80 iPHoP-RF;92.10
S9-wms_104169	ME	Monodnaviria	Loebvirae	Hofneiviricota	Faserviricetes	Tubulavirales	Inoviridae	Unclassified	Inovirus		d__Bacteria;p__Firmicutes;c__Clostridia;o__Oscillospirales;f__Ruminococcaceae;g__Ruthenibacterium	91.6	blast;93.90 CRISPR;84.10 iPHoP-RF;79.50
S9-wms_114971	ME	Duplodnaviria	Heunggongvirae	Uroviricota	Caudoviricetes	n.a.	Unclassified	O	O	60.7	d__Bacteria;p__Firmicutes;c__Negativicutes;o__Veillonellales;f__Veillonellaceae;g__Veillonella	98.4	blast;95.00 RaFAH;88.20 iPHoP-RF;73.50 CRISPR;55.70
S9-wms_19025	ME	Duplodnaviria	Heunggongvirae	Uroviricota	Caudoviricetes	n.a.	Unclassified	O	O	87.64	d__Bacteria;p__Firmicutes;c__Bacilli;o__Lactobacillales;f__Streptococcaceae;g__Streptococcus	98.4	RaFAH;98.80
S9-wms_64472	Shared	n.a.	n.a.	n.a.	n.a.	n.a.	n.a.	n.a.	n.a.	4.04	d__Bacteria;p__Firmicutes;c__Clostridia;o__Lachnospirales;f__Lachnospiraceae;g__Enterocloster	93.5	blast;95.40 iPHoP-RF;85.10

Table S9-5. VLP_Hosts_pivot

Host interactions with:	# Hosts
ME-viruses-only	59
SHHC-viruses-only	26
Shared/communal viruses	90

d__Bacteria;p__Actinobacteriota;c__Actinomycetia;o__Actinomycetales;f__Actinomycetaceae;g__Actinomyces

ME
S14_vf_57689
Caudoviricetes
Unclassified
O
O
S3_vf_54353
Caudoviricetes
Unclassified
Unclassified
Cimpunavirus
S3_vf_5525
n.a.
n.a.
n.a.
n.a.
Shared
S3_vf_44405
Caudoviricetes
Unclassified
Unclassified
Unclassified

d__Bacteria;p__Actinobacteriota;c__Actinomycetia;o__Actinomycetales;f__Actinomycetaceae;g__Pauljensenia

ME
S11_vf_66522
Caudoviricetes
Unclassified
Unclassified
Unclassified
Shared
S14_vf_117155
Caudoviricetes
Unclassified
O
O
S16_vf_16182
Caudoviricetes
Unclassified
O
O

d__Bacteria;p__Actinobacteriota;c__Actinomycetia;o__Actinomycetales;f__Bifidobacteriaceae;g__Bifidobacterium

ME
S11_vf_58587
n.a.
n.a.
n.a.
n.a.
S13_vs_113593
Caudoviricetes
Unclassified
Unclassified
Unclassified
S13_vs_149860
Caudoviricetes
Unclassified
Unclassified
O
S3_vf_127408

Caudoviricetes
Unclassified
O
O
S3_vs_39940
n.a.
n.a.
n.a.
n.a.
S7_vf_5843
Caudoviricetes
Unclassified
O
O
S7_vs_73280
Caudoviricetes
Unclassified
Unclassified
Minunavirus
Shared
S16_vf_100473
n.a.
n.a.
n.a.
n.a.
S16_vs_78361
Caudoviricetes
Unclassified
O
O
S3_vf_70746
Caudoviricetes
Unclassified
Unclassified
Unclassified
S3_vf_7137
n.a.
Unclassified
Unclassified
O
S3_vs_123834
Caudoviricetes
Unclassified
O
O
S4_vf_30103
n.a.
Unclassified
Unclassified
O
S4_vs_VIRSorter_16493-cat_2
Caudoviricetes
Unclassified
Unclassified
Unclassified
S5_vs_63924
Caudoviricetes
Unclassified
Unclassified
Unclassified

S8_vf_153696
n.a.
n.a.
n.a.
n.a.
S8_vf_197061
Caudoviricetes
Unclassified
Unclassified
Unclassified
SHHC
S16_vf_172897
Caudoviricetes
Unclassified
O
O
S16_vf_173109
Caudoviricetes
Unclassified
Unclassified
Unclassified
S4_vf_12624
Caudoviricetes
Unclassified
O
O
S4_vs_VIRSorter_2744-cat_3
Caudoviricetes
Unclassified
O
O
S4_vs_VIRSorter_5698-cat_2
Caudoviricetes
Unclassified
O
O
d__Bacteria;p__Actinobacteriota;c__Actinomycetia;o__Actinomycetales;f__Micrococcaceae;g__Rothia
SHHC
S8_vf_102508
n.a.
n.a.
n.a.
n.a.
S8_vf_7243
n.a.
n.a.
n.a.
n.a.
d__Bacteria;p__Actinobacteriota;c__Actinomycetia;o__Mycobacteriales;f__Mycobacteriaceae;g__Mycobacterium
ME
S9_vf_934
Caudoviricetes
Unclassified
O
O
d__Bacteria;p__Actinobacteriota;c__Coriobacteriia;o__Coriobacteriales;f__Coriobacteriaceae;g__Collinsella
ME
S5_vf_29332
Caudoviricetes
Unclassified

O
O
S7_vs_55526
n.a.
n.a.
n.a.
n.a.
Shared
S3_vf_99626
Caudoviricetes
Unclassified
O
O
S5_vf_137260
n.a.
Unclassified
O
O
SHHC
S12_vf_170614
Caudoviricetes
Salasmaviridae
O
O
S8_vf_142700
n.a.
Unclassified
O
O

d__Bacteria;p__Actinobacteriota;c__Coriobacteriia;o__Coriobacteriales;f__Eggerthellaceae;g__Adlercreutzia

Shared
S12_vf_173666
n.a.
n.a.
n.a.
n.a.

d__Bacteria;p__Actinobacteriota;c__Coriobacteriia;o__Coriobacteriales;f__Eggerthellaceae;g__Eggerthella

ME
S7_vf_67910
n.a.
n.a.
n.a.
n.a.
Shared
S2_vf_16829
Caudoviricetes
Unclassified
Unclassified
O

d__Bacteria;p__Actinobacteriota;c__Coriobacteriia;o__Coriobacteriales;f__Eggerthellaceae;g__Gordonibacter

ME
S3_vs_50557
Caudoviricetes
Unclassified
O
O
SHHC
S8_vf_195032
Caudoviricetes
Unclassified

O
O
d__Bacteria;p__Bacteroidota;c__Bacteroidia;o__Bacteroidales;f__Bacteroidaceae;g__Bacteroides
ME
S11_vf_104033
Caudoviricetes
Unclassified
O
O
S11_vf_43997
Caudoviricetes
Unclassified
Unclassified
Unclassified
S11_vs_23188
n.a.
n.a.
n.a.
n.a.
S11_vs_84848
Caudoviricetes
Unclassified
Unclassified
Unclassified
S13_vf_66508
Caudoviricetes
Unclassified
O
O
S14_vf_117162
Caudoviricetes
Unclassified
Unclassified
O
S17_vs_116640
Caudoviricetes
Unclassified
O
O
S17_vs_160502
Caudoviricetes
Steigviridae
O
O
S17_vs_39515
Caudoviricetes
Intestiviridae
O
O
S3_vf_18080
n.a.
n.a.
n.a.
n.a.
S3_vf_18829
Caudoviricetes
Unclassified
O
O
S3_vf_73597

Caudoviricetes
Unclassified
O
O
S3_vs_23194
Caudoviricetes
Duneviridae
O
O
S3_vs_40939
n.a.
n.a.
n.a.
n.a.
S3_vs_45147
n.a.
n.a.
n.a.
n.a.
S5_vf_8965
Caudoviricetes
Unclassified
O
O
S5_vs_72532
Caudoviricetes
Unclassified
O
O
S7_vf_27309
Caudoviricetes
Unclassified
Unclassified
O
S7_vs_135828
Malgrandaviricetes
Microviridae
Unclassified
Unclassified
S7_vs_2599
n.a.
n.a.
n.a.
n.a.
S7_vs_557
Caudoviricetes
Unclassified
O
O
S7_vs_944
n.a.
n.a.
n.a.
n.a.
S9_vs_83108
n.a.
n.a.
n.a.
n.a.
Shared

S10_vs_14557
Caudoviricetes
Duneviridae
O
O
S10_vs_35854
Caudoviricetes
Unclassified
O
O
S10_vs_36598
n.a.
n.a.
n.a.
n.a.
S10_vs_58014
Caudoviricetes
Unclassified
Unclassified
Unclassified
S10_vs_62776
Caudoviricetes
Unclassified
O
O
S10_vs_71900
n.a.
n.a.
n.a.
n.a.
S11_vs_3277
Caudoviricetes
Unclassified
Unclassified
Unclassified
S11_vs_45658
Caudoviricetes
Unclassified
O
O
S11_vs_58930
Caudoviricetes
Unclassified
Unclassified
Unclassified
S12_vf_16374
Caudoviricetes
Unclassified
O
O
S12_vs_141337
Caudoviricetes
Unclassified
Unclassified
Unclassified
S12_vs_151292
Caudoviricetes
Unclassified
Unclassified
Unclassified

S12_vs_1980
Caudoviricetes
Unclassified
O
O
S12_vs_26336
n.a.
n.a.
n.a.
n.a.
S12_vs_99219
n.a.
n.a.
n.a.
n.a.
S14_vs_112345
n.a.
n.a.
n.a.
n.a.
S14_vs_11510
n.a.
n.a.
n.a.
n.a.
S14_vs_25852
n.a.
n.a.
n.a.
n.a.
S14_vs_32499
Caudoviricetes
Unclassified
O
O
S14_vs_66208
Caudoviricetes
Unclassified
O
O
S14_vs_70985
n.a.
n.a.
n.a.
n.a.
S14_vs_74067
n.a.
n.a.
n.a.
n.a.
S15_vf_2032
Caudoviricetes
Unclassified
O
O
S15_vf_75924
Caudoviricetes
Steigviridae
O
O

S15_vs_27510
n.a.
n.a.
n.a.
n.a.
S15_vs_35445
n.a.
n.a.
n.a.
n.a.
S15_vs_3868
Caudoviricetes
Unclassified
O
O
S15_vs_42043
n.a.
n.a.
n.a.
n.a.
S15_vs_43913
Caudoviricetes
Unclassified
O
O
S16_vs_127669
Caudoviricetes
Unclassified
Unclassified
Unclassified
S16_vs_127905
n.a.
n.a.
n.a.
n.a.
S16_vs_136935
n.a.
n.a.
n.a.
n.a.
S16_vs_80244
n.a.
n.a.
n.a.
n.a.
S17_vf_128147
Malgrandaviricetes
Microviridae
Unclassified
O
S17_vf_140653
Caudoviricetes
Unclassified
O
O
S17_vs_117507
n.a.
n.a.
n.a.
n.a.

S17_vs_2079
Caudoviricetes
Unclassified
Unclassified
Unclassified
S2_vs_10289
Caudoviricetes
Unclassified
Unclassified
Unclassified
S3_vf_127399
Caudoviricetes
Unclassified
O
O
S3_vs_119660
Caudoviricetes
Unclassified
Unclassified
Unclassified
S3_vs_39774
n.a.
n.a.
n.a.
n.a.
S3_vs_64424
n.a.
n.a.
n.a.
n.a.
S3_vs_70508
n.a.
n.a.
n.a.
n.a.
S3_vs_9158
n.a.
n.a.
n.a.
n.a.
S4_vs_VIRSorter_31975-circular-cat_1
Caudoviricetes
Unclassified
O
O
S5_vs_109527
n.a.
n.a.
n.a.
n.a.
S5_vs_129970
n.a.
n.a.
n.a.
n.a.
S5_vs_131510
Caudoviricetes
Unclassified
Unclassified
O

S5_vs_142195
n.a.
n.a.
n.a.
n.a.
S5_vs_80496
n.a.
n.a.
n.a.
n.a.
S6_vf_56486
Caudoviricetes
Unclassified
O
O
S6_vs_46039
n.a.
n.a.
n.a.
n.a.
S6_vs_9784
n.a.
n.a.
n.a.
n.a.
S7_vf_37279
Caudoviricetes
Unclassified
O
O
S7_vf_5801
Caudoviricetes
Unclassified
O
O
S7_vs_11081
Caudoviricetes
Unclassified
Unclassified
Unclassified
S8_vf_208120
Caudoviricetes
Unclassified
O
O
S8_vf_28956
n.a.
n.a.
n.a.
n.a.
S8_vs_147697
n.a.
n.a.
n.a.
n.a.
S8_vs_28311
Caudoviricetes
Duneviridae
O
O

S8_vs_72117
n.a.
n.a.
n.a.
n.a.
S8_vs_99362
Caudoviricetes
Unclassified
Unclassified
Unclassified
S9_vs_28197
Caudoviricetes
Unclassified
O
O
SHHC
S10_vf_43964
Caudoviricetes
Unclassified
O
O
S10_vs_106834
Caudoviricetes
Unclassified
O
O
S12_vf_60224
Caudoviricetes
Unclassified
O
O
S16_vf_87190
Caudoviricetes
Unclassified
O
O
d__Bacteria;p__Bacteroidota;c__Bacteroidia;o__Bacteroidales;f__Bacteroidaceae;g__Phocaeicola
ME
S14_vf_37500
Malgrandaviricetes
Microviridae
Unclassified
Unclassified
d__Bacteria;p__Bacteroidota;c__Bacteroidia;o__Bacteroidales;f__Bacteroidaceae;g__Prevotella
ME
S3_vf_127450
n.a.
n.a.
n.a.
n.a.
Shared
S11_vf_104035
Caudoviricetes
Suoliviridae
O
O
S13_vf_11246
Caudoviricetes
Suoliviridae
O

O
S13_vs_57267
Caudoviricetes
Unclassified
O
O
S5_vf_145579
Caudoviricetes
Steigviridae
O
O
S8_vf_117796
Caudoviricetes
Unclassified
Unclassified
Unclassified
S8_vf_122206
Caudoviricetes
Unclassified
O
O
S8_vf_166677
Caudoviricetes
Intestiviridae
Obtuvirinae
Fohxhuevirus
S8_vf_183045
Caudoviricetes
Unclassified
Unclassified
Unclassified
S8_vf_79769
n.a.
n.a.
n.a.
n.a.
S9_vf_13507
n.a.
n.a.
n.a.
n.a.
S9_vf_51656
Caudoviricetes
Intestiviridae
Obtuvirinae
Fohxhuevirus
S9_vf_76769
Caudoviricetes
Suoliviridae
Loutivirinae
Buchavirus
SHHC
S4_vf_18803
Caudoviricetes
Unclassified
Unclassified
Unclassified
S4_vf_20440
Caudoviricetes
Unclassified

O
O
S4_vf_31981
Caudoviricetes
Suoliviridae
Uncouvirinae
Besingivirus
S4_vf_4891
n.a.
n.a.
n.a.
n.a.
S8_vs_148389
Caudoviricetes
Unclassified
O
O
d__Bacteria;p__Bacteroidota;c__Bacteroidia;o__Bacteroidales;f__Bacteroidaceae;g__Prevotellamassilia
SHHC
S12_vf_69136
Caudoviricetes
Unclassified
O
O
d__Bacteria;p__Bacteroidota;c__Bacteroidia;o__Bacteroidales;f__Barnesiellaceae;g__Barnesiella
ME
S17_vf_35138
Caudoviricetes
Unclassified
O
O
S9_vs_21850
Caudoviricetes
Unclassified
Unclassified
Unclassified
Shared
S13_vs_90967
Malgrandaviricetes
Microviridae
O
O
S8_vs_4153
n.a.
n.a.
n.a.
n.a.
SHHC
S12_vf_188846
Malgrandaviricetes
Microviridae
O
O
S4_vf_12614
n.a.
n.a.
n.a.
n.a.
S8_vf_208115
Malgrandaviricetes

Microviridae
O
O
d__Bacteria;p__Bacteroidota;c__Bacteroidia;o__Bacteroidales;f__Coprobacteraceae;g__Coprobacter
ME
S7_vs_51332
n.a.
n.a.
n.a.
n.a.
SHHC
S15_vs_18274
n.a.
n.a.
n.a.
n.a.
d__Bacteria;p__Bacteroidota;c__Bacteroidia;o__Bacteroidales;f__Marinifilaceae;g__Butyricimonas
Shared
S12_vs_71525
n.a.
n.a.
n.a.
n.a.
S12_vs_74280
n.a.
n.a.
n.a.
n.a.
S3_vs_6604
Caudoviricetes
Duneviridae
O
O
d__Bacteria;p__Bacteroidota;c__Bacteroidia;o__Bacteroidales;f__Marinifilaceae;g__Odoribacter
ME
S14_vs_78164
Caudoviricetes
Winoviridae
O
O
S3_vs_97827
n.a.
n.a.
n.a.
n.a.
Shared
S3_vs_117894
n.a.
n.a.
n.a.
n.a.
S4_vs_VIRSorter_9913-cat_1
Caudoviricetes
Unclassified
Unclassified
Unclassified
d__Bacteria;p__Bacteroidota;c__Bacteroidia;o__Bacteroidales;f__Muribaculaceae;g__UBA7173
SHHC
S12_vs_111286
n.a.

n.a.
n.a.
n.a.
d__Bacteria;p__Bacteroidota;c__Bacteroidia;o__Bacteroidales;f__P3;g__Phil12
ME
S3_vs_127442
Malgrandaviricetes
Microviridae
Unclassified
Unclassified
d__Bacteria;p__Bacteroidota;c__Bacteroidia;o__Bacteroidales;f__Rikenellaceae;g__Alistipes
ME
S1_vf_159951
Caudoviricetes
Unclassified
O
O
S11_vf_104071
Caudoviricetes
Unclassified
O
O
S11_vs_36748
n.a.
n.a.
n.a.
n.a.
S11_vs_98193
Caudoviricetes
Unclassified
Unclassified
Unclassified
S13_vf_56074
Caudoviricetes
Unclassified
Unclassified
Unclassified
S17_vs_177196
n.a.
n.a.
n.a.
n.a.
S3_vf_127406
Caudoviricetes
Unclassified
Unclassified
Unclassified
S3_vs_127413
Caudoviricetes
Unclassified
O
O
S3_vs_77156
Malgrandaviricetes
Microviridae
Unclassified
Unclassified
S5_vs_1338
n.a.
n.a.

n.a.
n.a.
S7_vs_54322
n.a.
n.a.
n.a.
n.a.
Shared
S10_vs_37694
Caudoviricetes
Duneviridae
O
O
S11_vf_70015
Caudoviricetes
Unclassified
O
O
S11_vs_1937
Caudoviricetes
Unclassified
O
O
S12_vf_174374
n.a.
n.a.
n.a.
n.a.
S12_vs_142625
Caudoviricetes
Unclassified
O
O
S13_vs_83447
n.a.
n.a.
n.a.
n.a.
S15_vs_71968
Caudoviricetes
Unclassified
O
O
S16_vf_9270
n.a.
n.a.
n.a.
n.a.
S16_vs_19968
n.a.
n.a.
n.a.
n.a.
S16_vs_32764
n.a.
n.a.
n.a.
n.a.
S16_vs_33281
n.a.

n.a.
n.a.
n.a.
S17_vs_157209
n.a.
n.a.
n.a.
n.a.
S3_vf_67828
Malgrandaviricetes
Microviridae
Unclassified
Unclassified
S3_vs_17168
Caudoviricetes
Unclassified
O
O
S3_vs_56292
Caudoviricetes
Unclassified
O
O
S3_vs_89485
n.a.
n.a.
n.a.
n.a.
S5_vf_18461
Caudoviricetes
Unclassified
O
O
S5_vs_123256
Caudoviricetes
Unclassified
Unclassified
Unclassified
S5_vs_20500
Caudoviricetes
Unclassified
O
O
S5_vs_76500
Caudoviricetes
Unclassified
Unclassified
Unclassified
S5_vs_79992
Caudoviricetes
Winoviridae
O
O
S5_vs_93839
Caudoviricetes
Unclassified
O
O
S7_vf_117697
n.a.

n.a.
n.a.
n.a.
S7_vs_119679
Caudoviricetes
Winoviridae
O
O
S7_vs_138173
Caudoviricetes
Unclassified
O
O
S8_vs_179208
n.a.
n.a.
n.a.
n.a.
S8_vs_40014
n.a.
n.a.
n.a.
n.a.
S8_vs_9987
Caudoviricetes
Unclassified
O
O
S9_vs_2799
n.a.
n.a.
n.a.
n.a.
S9_vs_48424
n.a.
n.a.
n.a.
n.a.
SHHC
S10_vs_67471
n.a.
n.a.
n.a.
n.a.
S12_vs_121913
n.a.
n.a.
n.a.
n.a.
S12_vs_23796
Caudoviricetes
Unclassified
Unclassified
Unclassified
S15_vf_20644
Caudoviricetes
Unclassified
Unclassified
Unclassified
S16_vs_51901

n.a.
n.a.
n.a.
n.a.
d__Bacteria;p__Bacteroidota;c__Bacteroidia;o__Bacteroidales;f__Rikenellaceae;g__Alistipes_A
ME
S7_vf_134023
n.a.
n.a.
n.a.
n.a.
Shared
S12_vf_145044
n.a.
n.a.
n.a.
n.a.
S16_vs_142561
Caudoviricetes
Unclassified
O
O
S6_vs_32886
n.a.
n.a.
n.a.
n.a.
S7_vs_50194
n.a.
n.a.
n.a.
n.a.
SHHC
S8_vs_9114
n.a.
n.a.
n.a.
n.a.
d__Bacteria;p__Bacteroidota;c__Bacteroidia;o__Bacteroidales;f__Tannerellaceae;g__Marseille-P3160
Shared
S13_vf_87873
Caudoviricetes
Unclassified
Unclassified
Unclassified
S4_vs_VIRSorter_6022-circular-cat_1
Caudoviricetes
Unclassified
Unclassified
Unclassified
d__Bacteria;p__Bacteroidota;c__Bacteroidia;o__Bacteroidales;f__Tannerellaceae;g__Parabacteroides
ME
S11_vf_68693
n.a.
n.a.
n.a.
n.a.
S7_vs_55031
n.a.
n.a.

n.a.
n.a.
Shared
S10_vf_18671
Caudoviricetes
Unclassified
Unclassified
Unclassified
S10_vs_37379
n.a.
n.a.
n.a.
n.a.
S12_vs_138261
n.a.
n.a.
n.a.
n.a.
S13_vf_143422
Caudoviricetes
Suoliviridae
Boorivirinae
Canhaevirus
S13_vs_15645
Caudoviricetes
Unclassified
O
O
S14_vs_15228
Caudoviricetes
Unclassified
O
O
S17_vf_81232
Caudoviricetes
Suoliviridae
Boorivirinae
Canhaevirus
S17_vs_43307
n.a.
n.a.
n.a.
n.a.
S2_vs_7306
Caudoviricetes
Unclassified
Unclassified
Unclassified
S3_vs_80151
Caudoviricetes
Unclassified
O
O
S3_vs_82895
Caudoviricetes
Unclassified
O
O
S4_vf_4838
Caudoviricetes

Suoliviridae
Boorivirinae
Cohcovirus
S4_vs_VIRSorter_13190-cat_2
Caudoviricetes
Unclassified
O
O
S5_vs_1572
Caudoviricetes
Unclassified
Unclassified
Unclassified
S6_vs_15390
Caudoviricetes
Unclassified
O
O
S7_vf_28429
Caudoviricetes
Unclassified
O
O
S7_vs_137915
Caudoviricetes
Unclassified
O
O
S8_vf_158225
Caudoviricetes
Intestiviridae
Crudevirinae
Diorhovirus
S8_vs_182288
Caudoviricetes
Unclassified
O
O
SHHC
S8_vf_16172
n.a.
n.a.
n.a.
n.a.
S8_vs_126582
Caudoviricetes
Unclassified
O
O
d__Bacteria;p__Bacteroidota;c__Bacteroidia;o__Bacteroidales;f__UBA11471;g__UBA11471
ME
S11_vs_79831
n.a.
n.a.
n.a.
n.a.
d__Bacteria;p__Cyanobacteria;c__Vampirovibrionia;o__Gastranaerophilales;f__Gastranaerophilaceae;g__CAG-306
ME
S11_vs_104041
Caudoviricetes

Unclassified
O
O
d__Bacteria;p__Cyanobacteria;c__Vampirovibrionia;o__Gastranaerophilales;f__Gastranaerophilaceae;g__QAMI01
ME
S11_vs_82331
Caudoviricetes
Unclassified
O
O
S3_vs_50924
n.a.
n.a.
n.a.
n.a.
d__Bacteria;p__Desulfobacterota;c__Desulfovibrionia;o__Desulfovibrionales;f__Desulfovibrionaceae;g__Bilophila
ME
S17_vs_177203
Caudoviricetes
Unclassified
O
O
Shared
S8_vf_208111
n.a.
n.a.
n.a.
n.a.
d__Bacteria;p__Desulfobacterota;c__Desulfovibrionia;o__Desulfovibrionales;f__Desulfovibrionaceae;g__Mailhella
SHHC
S8_vs_208114
Caudoviricetes
Unclassified
O
O
d__Bacteria;p__Firmicutes;c__Bacilli;o__Erysipelotrichales;f__Erysipelatoclostridiaceae;g__Catenibacterium
Shared
S8_vs_59736
Caudoviricetes
Unclassified
O
O
d__Bacteria;p__Firmicutes;c__Bacilli;o__Erysipelotrichales;f__Erysipelatoclostridiaceae;g__Erysipelatoclostridium
ME
S11_vs_68348
Caudoviricetes
Unclassified
O
O
S7_vs_13041
Caudoviricetes
Unclassified
O
O
S9_vs_46498
Caudoviricetes
Unclassified
O
O
d__Bacteria;p__Firmicutes;c__Bacilli;o__Erysipelotrichales;f__Erysipelatoclostridiaceae;g__Faecalibacillus

ME
S17_vs_31654
Caudoviricetes
Unclassified
O
O
S3_vs_94577
Caudoviricetes
Unclassified
O
O
d__Bacteria;p__Firmicutes;c__Bacilli;o__Erysipelotrichales;f__Erysipelatoclostridiaceae;g__Massiliomicrobiota
ME
S11_vs_55144
n.a.
n.a.
n.a.
n.a.
S7_vf_38697
Caudoviricetes
Unclassified
O
O
d__Bacteria;p__Firmicutes;c__Bacilli;o__Erysipelotrichales;f__Erysipelotrichaceae;g__Clostridium_AQ
ME
S5_vs_122618
n.a.
n.a.
n.a.
n.a.
S9_vs_90846
Caudoviricetes
Unclassified
O
O
Shared
S11_vf_92391
n.a.
n.a.
n.a.
n.a.
SHHC
S2_vf_20910
n.a.
n.a.
n.a.
n.a.
d__Bacteria;p__Firmicutes;c__Bacilli;o__Erysipelotrichales;f__Erysipelotrichaceae;g__Holdemanella
SHHC
S4_vf_10506
n.a.
n.a.
n.a.
n.a.
d__Bacteria;p__Firmicutes;c__Bacilli;o__Erysipelotrichales;f__Erysipelotrichaceae;g__Holdemania
ME
S11_vs_14882
n.a.
n.a.
n.a.

n.a.
S14_vf_72335
Caudoviricetes
Unclassified
O
O
S5_vs_51416
Caudoviricetes
Unclassified
O
O
S9_vs_37437
Caudoviricetes
Unclassified
O
O
Shared
S6_vs_56485
Caudoviricetes
Unclassified
O
O
d__Bacteria;p__Firmicutes;c__Bacilli;o__Haloplasmatales_A;f__Turicibacteraceae;g__Turicibacter
ME
S3_vf_106401
Caudoviricetes
Unclassified
O
O
S5_vf_28061
n.a.
n.a.
n.a.
n.a.
d__Bacteria;p__Firmicutes;c__Bacilli;o__Lactobacillales;f__Lactobacillaceae;g__Lacticaseibacillus
ME
S14_vf_72953
Caudoviricetes
Unclassified
Unclassified
Unclassified
d__Bacteria;p__Firmicutes;c__Bacilli;o__Lactobacillales;f__Lactobacillaceae;g__Lactobacillus
ME
S14_vf_106156
Caudoviricetes
Unclassified
Unclassified
Unclassified
S14_vf_78139
Caudoviricetes
Unclassified
Unclassified
Unclassified
S5_vs_6478
Caudoviricetes
Unclassified
Unclassified
O
d__Bacteria;p__Firmicutes;c__Bacilli;o__Lactobacillales;f__Lactobacillaceae;g__Leuconostoc
ME

S7_vf_63894
Caudoviricetes
Unclassified
Mccleskeyvirinae
Unaquatrovirus
SHHC
S8_vf_129423
Caudoviricetes
Unclassified
Mccleskeyvirinae
Unaquatrovirus
d__Bacteria;p__Firmicutes;c__Bacilli;o__Lactobacillales;f__Lactobacillaceae;g__Limosilactobacillus
ME
S5_vs_89540
Caudoviricetes
Unclassified
O
O
d__Bacteria;p__Firmicutes;c__Bacilli;o__Lactobacillales;f__Streptococcaceae;g__Lactococcus
ME
S7_vf_68239
Caudoviricetes
Unclassified
O
O
Shared
S3_vf_23042
Caudoviricetes
Unclassified
O
O
S5_vf_25423
Caudoviricetes
Unclassified
Unclassified
Skunavirus
S5_vf_55687
Caudoviricetes
Unclassified
O
O
S7_vf_29941
Caudoviricetes
Unclassified
O
O
S7_vf_89197
Caudoviricetes
Unclassified
Unclassified
Ceduovirus
S8_vf_45776
Caudoviricetes
Unclassified
Unclassified
Skunavirus
d__Bacteria;p__Firmicutes;c__Bacilli;o__Lactobacillales;f__Streptococcaceae;g__Streptococcus
ME
S5_vs_60507
Caudoviricetes

Unclassified
Unclassified
O
S7_vf_138069
n.a.
n.a.
n.a.
n.a.
S7_vf_37293
Caudoviricetes
Unclassified
O
O
S7_vf_49007
Caudoviricetes
Unclassified
O
O
S9_vf_15012
Caudoviricetes
Unclassified
Unclassified
Unclassified
S9_vf_19284
n.a.
n.a.
n.a.
n.a.
S9_vf_4115
Caudoviricetes
Unclassified
Unclassified
Unclassified
S9_vf_90856
Caudoviricetes
Unclassified
Unclassified
O
S9_vf_90866
n.a.
Unclassified
Unclassified
Unclassified
Shared
S10_vf_64371
n.a.
n.a.
n.a.
n.a.
S11_vf_27845
n.a.
n.a.
n.a.
n.a.
S14_vf_117157
n.a.
n.a.
n.a.
n.a.
SHHC

S8_vf_134943
n.a.
n.a.
n.a.
n.a.
d__Bacteria;p__Firmicutes;c__Bacilli;o__ML615J-28;f__CAG-313;g__CAG-313
Shared
S13_vs_30101
Caudoviricetes
Unclassified
O
O
S16_vs_141218
n.a.
n.a.
n.a.
n.a.
d__Bacteria;p__Firmicutes;c__Bacilli;o__RF39;f__UBA660;g__CAG-302
SHHC
S12_vs_123620
n.a.
n.a.
n.a.
n.a.
S16_vs_165662
Caudoviricetes
Unclassified
O
O
d__Bacteria;p__Firmicutes;c__Bacilli;o__RF39;f__UBA660;g__CAG-460
Shared
S13_vs_26756
n.a.
n.a.
n.a.
n.a.
S13_vs_67429
n.a.
n.a.
n.a.
n.a.
SHHC
S12_vf_105542
Caudoviricetes
Unclassified
O
O
d__Bacteria;p__Firmicutes;c__Bacilli;o__RF39;f__UBA660;g__CAG-877
SHHC
S12_vs_172379
n.a.
n.a.
n.a.
n.a.
d__Bacteria;p__Firmicutes;c__Clostridia;o__Acetivibrionales;f__Acetivibrionaceae;g__Ruminiclostridium
ME
S14_vs_102698
Caudoviricetes
Unclassified
O

O
d__Bacteria;p__Firmicutes;c__Clostridia;o__Christensenellales;f__Borkfalkiaceae;g__QALS01
SHHC
S10_vs_120489
n.a.
n.a.
n.a.
n.a.
d__Bacteria;p__Firmicutes;c__Clostridia;o__Christensenellales;f__CAG-138;g__SFEL01
ME
S3_vs_73361
n.a.
n.a.
n.a.
n.a.
SHHC
S8_vs_176604
n.a.
n.a.
n.a.
n.a.
d__Bacteria;p__Firmicutes;c__Clostridia;o__Christensenellales;f__CAG-138;g__UBA1685
SHHC
S8_vf_93976
n.a.
n.a.
n.a.
n.a.
d__Bacteria;p__Firmicutes;c__Clostridia;o__Christensenellales;f__CAG-138;g__UBA5394
SHHC
S16_vs_4662
Caudoviricetes
Unclassified
Unclassified
Unclassified
d__Bacteria;p__Firmicutes;c__Clostridia;o__Christensenellales;f__CAG-74;g__Alectryocaccomicrobium
ME
S5_vs_10428
Caudoviricetes
Unclassified
O
O
d__Bacteria;p__Firmicutes;c__Clostridia;o__Christensenellales;f__CAG-74;g__Firm-11
ME
S7_vs_129858
n.a.
n.a.
n.a.
n.a.
d__Bacteria;p__Firmicutes;c__Clostridia;o__Christensenellales;f__CAG-74;g__Limiplasma
ME
S5_vs_4737
n.a.
n.a.
n.a.
n.a.
d__Bacteria;p__Firmicutes;c__Clostridia;o__Christensenellales;f__CAG-74;g__SFTH01
ME
S5_vs_145605
Caudoviricetes

Unclassified
O
O
d__Bacteria;p__Firmicutes;c__Clostridia;o__Christensenellales;f__CAG-74;g__UBA11524
ME
S1_vf_158961
Caudoviricetes
Unclassified
O
O
S7_vs_30665
n.a.
n.a.
n.a.
n.a.
Shared
S12_vs_94042
n.a.
n.a.
n.a.
n.a.
S3_vs_90341
n.a.
n.a.
n.a.
n.a.
S8_vf_65522
n.a.
n.a.
n.a.
n.a.
SHHC
S12_vs_48045
Caudoviricetes
Unclassified
Unclassified
Unclassified
S16_vs_15093
Caudoviricetes
Unclassified
O
O
d__Bacteria;p__Firmicutes;c__Clostridia;o__Christensenellales;f__CAG-74;g__Ventricola
SHHC
S4_vf_31979
Caudoviricetes
Unclassified
Unclassified
O
d__Bacteria;p__Firmicutes;c__Clostridia;o__Christensenellales;f__CAG-917;g__CAG-475
SHHC
S12_vs_32669
n.a.
n.a.
n.a.
n.a.
d__Bacteria;p__Firmicutes;c__Clostridia;o__Christensenellales;f__Christensenellaceae;g__Christensenella
ME
S5_vs_145581
n.a.

n.a.
n.a.
n.a.
d__Bacteria;p__Firmicutes;c__Clostridia;o__Christensenellales;f__NSJ-53;g__NSJ-53
ME
S5_vs_145597
Caudoviricetes
Unclassified
O
O
d__Bacteria;p__Firmicutes;c__Clostridia;o__Christensenellales;f__QAND01;g__UMGS1975
ME
S5_vs_46394
n.a.
n.a.
n.a.
n.a.
d__Bacteria;p__Firmicutes;c__Clostridia;o__Clostridiales;f__Clostridiaceae;g__Clostridium
ME
S11_vs_41107
Caudoviricetes
Unclassified
O
O
S14_vf_56130
Caudoviricetes
Unclassified
O
O
S14_vf_85122
Caudoviricetes
Unclassified
Unclassified
O
S14_vs_632
Caudoviricetes
Unclassified
O
O
S3_vf_100440
Caudoviricetes
Unclassified
O
O
S3_vs_98675
Caudoviricetes
Unclassified
O
O
S5_vf_145583
Caudoviricetes
Unclassified
Unclassified
O
S5_vs_137154
Caudoviricetes
Unclassified
O
O
S9_vs_90851

Caudoviricetes
Unclassified
O
O
Shared
S16_vf_36146
Caudoviricetes
Unclassified
O
O
SHHC
S10_vf_62230
Caudoviricetes
Unclassified
O
O
d__Bacteria;p__Firmicutes;c__Clostridia;o__Clostridiales;f__Clostridiaceae;g__Clostridium_J
ME
S5_vf_6506
Caudoviricetes
Unclassified
O
O
d__Bacteria;p__Firmicutes;c__Clostridia;o__Eubacteriales;f__Eubacteriaceae;g__Eubacterium
ME
S11_vs_81775
Caudoviricetes
Unclassified
Unclassified
Unclassified
S14_vs_115455
Caudoviricetes
Unclassified
O
O
d__Bacteria;p__Firmicutes;c__Clostridia;o__Lachnospirales;f__Anaerotignaceae;g__Anaerotignum
ME
S1_vs_145792
n.a.
n.a.
n.a.
n.a.
S9_vs_90849
n.a.
n.a.
n.a.
n.a.
Shared
S16_vf_84462
n.a.
n.a.
n.a.
n.a.
S5_vf_60597
n.a.
n.a.
n.a.
n.a.
S8_vf_201695
n.a.

n.a.
n.a.
n.a.
d__Bacteria;p__Firmicutes;c__Clostridia;o__Lachnospirales;f__Anaerotignaceae;g__UMGS1670
ME
S7_vf_72493
n.a.
n.a.
n.a.
n.a.
d__Bacteria;p__Firmicutes;c__Clostridia;o__Lachnospirales;f__CAG-274;g__CAG-274
ME
S9_vf_73311
Caudoviricetes
Unclassified
Unclassified
Unclassified
d__Bacteria;p__Firmicutes;c__Clostridia;o__Lachnospirales;f__Lachnospiraceae;g__
ME
S11_vf_34008
Caudoviricetes
Unclassified
O
O
S11_vs_6878
Caudoviricetes
Unclassified
O
O
S5_vf_105728
Caudoviricetes
Unclassified
O
O
d__Bacteria;p__Firmicutes;c__Clostridia;o__Lachnospirales;f__Lachnospiraceae;g__Acetatifactor
SHHC
S16_vs_116956
n.a.
n.a.
n.a.
n.a.
S6_vs_29152
Caudoviricetes
Unclassified
O
O
d__Bacteria;p__Firmicutes;c__Clostridia;o__Lachnospirales;f__Lachnospiraceae;g__Agathobacter
ME
S3_vf_67333
Caudoviricetes
Unclassified
O
O
Shared
S14_vs_66841
Caudoviricetes
Unclassified
O
O
S3_vs_127421

Caudoviricetes
Unclassified
O
O
S7_vs_49426
Caudoviricetes
Unclassified
O
O
S8_vf_132697
Caudoviricetes
Unclassified
O
O
S8_vs_173961
Caudoviricetes
Unclassified
O
O

d__Bacteria;p__Firmicutes;c__Clostridia;o__Lachnospirales;f__Lachnospiraceae;g__Anaerobutyricum

ME
S5_vs_145602
Caudoviricetes
Unclassified
O
O
S7_vf_114476
Caudoviricetes
Unclassified
O
O
S7_vs_137611
Caudoviricetes
Unclassified
O
O
S7_vs_138109
Caudoviricetes
Unclassified
O
O

d__Bacteria;p__Firmicutes;c__Clostridia;o__Lachnospirales;f__Lachnospiraceae;g__Anaerostipes

ME
S14_vs_39797
Caudoviricetes
Unclassified
O
O
S5_vf_116300
n.a.
n.a.
n.a.
n.a.
S5_vs_139401
n.a.
n.a.
n.a.
n.a.
S5_vs_141621
Caudoviricetes

Unclassified
O
O
S7_vf_49485
Caudoviricetes
Unclassified
O
O

d__Bacteria;p__Firmicutes;c__Clostridia;o__Lachnospirales;f__Lachnospiraceae;g__Bariatricus

ME
S11_vf_10907
Caudoviricetes
Unclassified
O
O
S7_vs_86377
Caudoviricetes
Unclassified
O
O
Shared
S9_vs_1966
Caudoviricetes
Unclassified
O
O
SHHC
S12_vs_98175
Caudoviricetes
Unclassified
O
O

d__Bacteria;p__Firmicutes;c__Clostridia;o__Lachnospirales;f__Lachnospiraceae;g__Blautia

ME
S14_vs_115377
Caudoviricetes
Unclassified
O
O
S5_vs_95307
Caudoviricetes
Unclassified
Unclassified
O
Shared
S14_vs_96286
Caudoviricetes
Unclassified
O
O
S6_vs_54606
Caudoviricetes
Unclassified
O
O

d__Bacteria;p__Firmicutes;c__Clostridia;o__Lachnospirales;f__Lachnospiraceae;g__Blautia_A

ME
S11_vf_28645
n.a.
n.a.

n.a.
n.a.
S11_vs_34137
Caudoviricetes
Unclassified
O
O
S11_vs_82368
Caudoviricetes
Unclassified
O
O
S13_vs_121307
n.a.
n.a.
n.a.
n.a.
S3_vf_8058
Caudoviricetes
Unclassified
Unclassified
Eponavirus
S5_vs_68254
Caudoviricetes
Unclassified
O
O
S7_vs_101751
n.a.
n.a.
n.a.
n.a.
S7_vs_135679
n.a.
n.a.
n.a.
n.a.
S7_vs_48708
Caudoviricetes
Unclassified
O
O
S9_vs_90859
n.a.
Unclassified
O
O
Shared
S10_vf_80712
n.a.
n.a.
n.a.
n.a.
S11_vf_28515
Caudoviricetes
Unclassified
O
O
S16_vf_96483
n.a.

n.a.
n.a.
n.a.
S17_vf_46388
Caudoviricetes
Unclassified
O
O
S3_vf_11428
Caudoviricetes
Unclassified
O
O
S4_vf_13353
Caudoviricetes
Unclassified
O
O
S5_vf_145604
Caudoviricetes
Unclassified
O
O
S5_vs_97574
Caudoviricetes
Unclassified
O
O
S7_vs_137998
n.a.
n.a.
n.a.
n.a.
SHHC
S4_vs_VIRSorter_12831-circular-cat_2
Caudoviricetes
Unclassified
O
O
d__Bacteria;p__Firmicutes;c__Clostridia;o__Lachnospirales;f__Lachnospiraceae;g__CAG-127
ME
S9_vs_75050
Caudoviricetes
Unclassified
O
O
SHHC
S16_vs_101072
Caudoviricetes
Unclassified
O
O
d__Bacteria;p__Firmicutes;c__Clostridia;o__Lachnospirales;f__Lachnospiraceae;g__CAG-317
Shared
S16_vs_140709
n.a.
n.a.
n.a.
n.a.
d__Bacteria;p__Firmicutes;c__Clostridia;o__Lachnospirales;f__Lachnospiraceae;g__Clostridium_AP

ME
S5_vs_145590
Caudoviricetes
Unclassified
O
O
d__Bacteria;p__Firmicutes;c__Clostridia;o__Lachnospirales;f__Lachnospiraceae;g__Coprococcus
Shared
S14_vf_92240
Caudoviricetes
Unclassified
O
O
SHHC
S16_vs_173079
Caudoviricetes
Unclassified
O
O
d__Bacteria;p__Firmicutes;c__Clostridia;o__Lachnospirales;f__Lachnospiraceae;g__Dorea
ME
S9_vs_32588
Caudoviricetes
Unclassified
O
O
Shared
S12_vs_163990
Caudoviricetes
Unclassified
O
O
d__Bacteria;p__Firmicutes;c__Clostridia;o__Lachnospirales;f__Lachnospiraceae;g__Dorea_A
ME
S11_vs_83666
Caudoviricetes
Unclassified
O
O
S14_vs_117153
Caudoviricetes
Unclassified
O
O
S5_vs_84946
Caudoviricetes
Unclassified
O
O
S7_vs_13799
Caudoviricetes
Unclassified
O
O
S9_vs_58086
Caudoviricetes
Unclassified
O
O
Shared

S5_vf_145588
Caudoviricetes
Unclassified
O
O
d__Bacteria;p__Firmicutes;c__Clostridia;o__Lachnospirales;f__Lachnospiraceae;g__Eisenbergiella
ME
S9_vs_88286
n.a.
n.a.
n.a.
n.a.
d__Bacteria;p__Firmicutes;c__Clostridia;o__Lachnospirales;f__Lachnospiraceae;g__Enterocloster
ME
S11_vs_38860
n.a.
n.a.
n.a.
n.a.
S11_vs_67573
n.a.
n.a.
n.a.
n.a.
S14_vs_100227
Caudoviricetes
Unclassified
O
O
S14_vs_30108
n.a.
n.a.
n.a.
n.a.
S5_vs_145578
Caudoviricetes
Unclassified
O
O
S9_vs_41371
Caudoviricetes
Unclassified
O
O
S9_vs_4343
n.a.
n.a.
n.a.
n.a.
S9_vs_55738
Caudoviricetes
Unclassified
O
O
Shared
S5_vf_145585
Caudoviricetes
Unclassified
Unclassified
Unclassified

S6_vs_39025
Caudoviricetes
Unclassified
O
O
S7_vs_65719
Caudoviricetes
Unclassified
O
O
S9_vs_90848
Caudoviricetes
Unclassified
O
O
d__Bacteria;p__Firmicutes;c__Clostridia;o__Lachnospirales;f__Lachnospiraceae;g__Eubacterium_F
ME
S7_vs_60775
Caudoviricetes
Unclassified
O
O
Shared
S14_vf_99344
n.a.
n.a.
n.a.
n.a.
SHHC
S8_vf_131009
Caudoviricetes
Unclassified
O
O
d__Bacteria;p__Firmicutes;c__Clostridia;o__Lachnospirales;f__Lachnospiraceae;g__Eubacterium_G
ME
S5_vs_8125
Caudoviricetes
Unclassified
O
O
S7_vs_30640
Caudoviricetes
Unclassified
O
O
SHHC
S16_vs_118029
Caudoviricetes
Unclassified
O
O
d__Bacteria;p__Firmicutes;c__Clostridia;o__Lachnospirales;f__Lachnospiraceae;g__Eubacterium_I
ME
S11_vs_104042
n.a.
n.a.
n.a.
n.a.
d__Bacteria;p__Firmicutes;c__Clostridia;o__Lachnospirales;f__Lachnospiraceae;g__Faecalimonas

ME
S5_vs_59798
Caudoviricetes
Unclassified
O
O
S7_vf_68546
Caudoviricetes
Unclassified
O
O
d__Bacteria;p__Firmicutes;c__Clostridia;o__Lachnospirales;f__Lachnospiraceae;g__Frisingicoccus
ME
S5_vs_58766
Caudoviricetes
Unclassified
O
O
d__Bacteria;p__Firmicutes;c__Clostridia;o__Lachnospirales;f__Lachnospiraceae;g__Hungatella
ME
S5_vs_17520
n.a.
n.a.
n.a.
n.a.
S9_vs_48240
Caudoviricetes
Unclassified
Unclassified
O
d__Bacteria;p__Firmicutes;c__Clostridia;o__Lachnospirales;f__Lachnospiraceae;g__Lachnospira
ME
S14_vf_117150
Caudoviricetes
Unclassified
O
O
S3_vf_108959
n.a.
n.a.
n.a.
n.a.
S3_vf_127405
Caudoviricetes
Unclassified
O
O
S3_vs_127401
Caudoviricetes
Unclassified
O
O
Shared
S5_vs_120584
Caudoviricetes
Unclassified
O
O
SHHC
S12_vs_26847

n.a.
n.a.
n.a.
n.a.
S12_vs_99092
Caudoviricetes
Unclassified
O
O
S15_vf_2513
n.a.
n.a.
n.a.
n.a.
S16_vf_1037
n.a.
n.a.
n.a.
n.a.
S16_vf_5809
n.a.
n.a.
n.a.
n.a.
S4_vf_31978
n.a.
n.a.
n.a.
n.a.

d__Bacteria;p__Firmicutes;c__Clostridia;o__Lachnospirales;f__Lachnospiraceae;g__Marvinbryantia

Shared
S6_vs_56496
Caudoviricetes
Unclassified
Unclassified
Eponavirus

d__Bacteria;p__Firmicutes;c__Clostridia;o__Lachnospirales;f__Lachnospiraceae;g__Massilistercora

Shared
S8_vs_5211
n.a.
n.a.
n.a.
n.a.

d__Bacteria;p__Firmicutes;c__Clostridia;o__Lachnospirales;f__Lachnospiraceae;g__Mediterraneibacter

ME
S5_vs_28263
n.a.
n.a.
n.a.
n.a.
S7_vs_138116
n.a.
n.a.
n.a.
n.a.
Shared
S11_vs_68151
Caudoviricetes
Unclassified
O

O
S4_vs_VIRSorter_3006-cat_1
Caudoviricetes
Unclassified
O
O
S7_vs_41098
Caudoviricetes
Unclassified
O
O
SHHC
S4_vs_VIRSorter_19324-cat_2
Caudoviricetes
Unclassified
O
O
S4_vs_VIRSorter_31916-circular-cat_1
Caudoviricetes
Unclassified
O
O
d__Bacteria;p__Firmicutes;c__Clostridia;o__Lachnospirales;f__Lachnospiraceae;g__Muricomes
ME
S14_vf_93134
Caudoviricetes
Salasmaviridae
O
O
S14_vs_56251
Caudoviricetes
Unclassified
O
O
S14_vs_59347
Caudoviricetes
Unclassified
O
O
S17_vf_36452
Caudoviricetes
Salasmaviridae
O
O
S5_vs_80498
Caudoviricetes
Unclassified
O
O
Shared
S2_vf_17850
Caudoviricetes
Salasmaviridae
O
O
S6_vs_56498
Caudoviricetes
Unclassified
O
O

SHHC
S12_vf_61299
Caudoviricetes
Salasmaviridae
O
O
d__Bacteria;p__Firmicutes;c__Clostridia;o__Lachnospirales;f__Lachnospiraceae;g__OF09-33XD
SHHC
S12_vf_176437
Caudoviricetes
Salasmaviridae
O
O
d__Bacteria;p__Firmicutes;c__Clostridia;o__Lachnospirales;f__Lachnospiraceae;g__Roseburia
ME
S14_vf_58919
Caudoviricetes
Unclassified
Unclassified
Mushuvirus
S5_vs_120079
Caudoviricetes
Unclassified
Unclassified
Unclassified
S5_vs_122344
Caudoviricetes
Unclassified
Unclassified
O
S5_vs_145598
Caudoviricetes
Unclassified
O
O
Shared
S3_vs_16825
Caudoviricetes
Unclassified
O
O
S4_vs_VIRSorter_4746-cat_2
Caudoviricetes
Salasmaviridae
O
O
S6_vs_12945
Caudoviricetes
Unclassified
O
O
S8_vs_206526
Caudoviricetes
Unclassified
Unclassified
Unclassified
d__Bacteria;p__Firmicutes;c__Clostridia;o__Lachnospirales;f__Lachnospiraceae;g__RUG115
ME
S7_vs_38834
Caudoviricetes

Unclassified
O
O
d__Bacteria;p__Firmicutes;c__Clostridia;o__Lachnospirales;f__Lachnospiraceae;g__Ruminococcus_B
ME
S14_vs_26320
Caudoviricetes
Unclassified
O
O
S7_vf_60968
Caudoviricetes
Unclassified
O
O
S9_vs_44331
Caudoviricetes
Unclassified
O
O
d__Bacteria;p__Firmicutes;c__Clostridia;o__Lachnospirales;f__Lachnospiraceae;g__Sellimonas
ME
S9_vs_34898
Caudoviricetes
Unclassified
O
O
S9_vs_62803
Caudoviricetes
Unclassified
O
O
Shared
S6_vs_56491
Caudoviricetes
Unclassified
O
O
d__Bacteria;p__Firmicutes;c__Clostridia;o__Lachnospirales;f__Lachnospiraceae;g__UBA9502
SHHC
S10_vf_46328
Caudoviricetes
Unclassified
O
O
d__Bacteria;p__Firmicutes;c__Clostridia;o__Lachnospirales;f__Lachnospiraceae;g__Ventrimonas
ME
S11_vs_26767
Caudoviricetes
Unclassified
O
O
d__Bacteria;p__Firmicutes;c__Clostridia;o__Monoglobales;f__Monoglobaceae;g__Monoglobus
Shared
S3_vf_101611
Caudoviricetes
Unclassified
O
O
SHHC

S16_vs_38863
Caudoviricetes
Unclassified
O
O
d__Bacteria;p__Firmicutes;c__Clostridia;o__Oscillospirales;f__Acutalibacteraceae;g__Acutalibacter
ME
S3_vs_114271
n.a.
n.a.
n.a.
n.a.
d__Bacteria;p__Firmicutes;c__Clostridia;o__Oscillospirales;f__Acutalibacteraceae;g__An172
ME
S11_vs_48109
Caudoviricetes
Unclassified
O
O
d__Bacteria;p__Firmicutes;c__Clostridia;o__Oscillospirales;f__Acutalibacteraceae;g__CAG-180
ME
S11_vf_104052
Caudoviricetes
Unclassified
O
O
S7_vs_47050
Caudoviricetes
Unclassified
O
O
d__Bacteria;p__Firmicutes;c__Clostridia;o__Oscillospirales;f__Acutalibacteraceae;g__CAG-217
ME
S3_vs_122706
Caudoviricetes
Unclassified
O
O
S3_vs_18858
n.a.
n.a.
n.a.
n.a.
d__Bacteria;p__Firmicutes;c__Clostridia;o__Oscillospirales;f__Acutalibacteraceae;g__Eubacterium_R
SHHC
S16_vs_120149
n.a.
n.a.
n.a.
n.a.
d__Bacteria;p__Firmicutes;c__Clostridia;o__Oscillospirales;f__Acutalibacteraceae;g__Fimivicinus
ME
S14_vs_88666
Caudoviricetes
Unclassified
O
O
d__Bacteria;p__Firmicutes;c__Clostridia;o__Oscillospirales;f__Acutalibacteraceae;g__Hydrogeniiclostridium
ME
S7_vs_28733

Caudoviricetes
Unclassified
O
O
S7_vs_38113
Caudoviricetes
Unclassified
O
O

d__Bacteria;p__Firmicutes;c__Clostridia;o__Oscillospirales;f__Acutalibacteraceae;g__Pseudoruminococcus

Shared
S10_vs_77004
n.a.
n.a.
n.a.
n.a.

d__Bacteria;p__Firmicutes;c__Clostridia;o__Oscillospirales;f__Acutalibacteraceae;g__Ruminococcus_E

ME
S13_vf_28309
Caudoviricetes
Salasmaviridae
O
O
S14_vs_39266
Caudoviricetes
Unclassified
Unclassified
O
S3_vs_49835
n.a.
n.a.
n.a.
n.a.
S5_vs_20268
Caudoviricetes
Unclassified
Unclassified
Unclassified
S5_vs_40848
Caudoviricetes
Unclassified
O
O
S7_vs_72737
n.a.
n.a.
n.a.
n.a.
Shared
S14_vs_66139
Caudoviricetes
Unclassified
O
O
S16_vs_82116
Caudoviricetes
Unclassified
Unclassified
O
S4_vf_11308

Caudoviricetes
Unclassified
Unclassified
O
S7_vs_16344
Caudoviricetes
Unclassified
O
O
S8_vs_115958
Caudoviricetes
Unclassified
Unclassified
O
S9_vs_90727
Caudoviricetes
Unclassified
Unclassified
O

d__Bacteria;p__Firmicutes;c__Clostridia;o__Oscillospirales;f__Acutalibacteraceae;g__UBA1417

ME
S14_vs_61530
n.a.
n.a.
n.a.
n.a.
Shared
S12_vf_79666
n.a.
n.a.
n.a.
n.a.

d__Bacteria;p__Firmicutes;c__Clostridia;o__Oscillospirales;f__Acutalibacteraceae;g__UBA1691

ME
S5_vs_3501
Caudoviricetes
Unclassified
O
O
S7_vs_129271
n.a.
n.a.
n.a.
n.a.
SHHC
S10_vs_36407
n.a.
n.a.
n.a.
n.a.

d__Bacteria;p__Firmicutes;c__Clostridia;o__Oscillospirales;f__Acutalibacteraceae;g__UBA737

ME
S3_vs_95574
n.a.
n.a.
n.a.
n.a.

d__Bacteria;p__Firmicutes;c__Clostridia;o__Oscillospirales;f__Acutalibacteraceae;g__UMGS1071

ME
S9_vs_26179

n.a.
n.a.
n.a.
n.a.
Shared
S6_vs_56490
Caudoviricetes
Unclassified
O
O
d__Bacteria;p__Firmicutes;c__Clostridia;o__Oscillospirales;f__Acutalibacteraceae;g__UMGS1591
ME
S9_vs_90860
Caudoviricetes
Unclassified
O
O
d__Bacteria;p__Firmicutes;c__Clostridia;o__Oscillospirales;f__Acutalibacteraceae;g__UMGS263
ME
S11_vs_3006
Caudoviricetes
Unclassified
Unclassified
O
d__Bacteria;p__Firmicutes;c__Clostridia;o__Oscillospirales;f__Acutalibacteraceae;g__UMGS856
ME
S5_vs_140946
n.a.
n.a.
n.a.
n.a.
d__Bacteria;p__Firmicutes;c__Clostridia;o__Oscillospirales;f__Butyricicoccaceae;g__Agathobaculum
ME
S14_vs_112068
n.a.
n.a.
n.a.
n.a.
S5_vs_43207
Caudoviricetes
Unclassified
O
O
S5_vs_84574
n.a.
n.a.
n.a.
n.a.
S7_vs_103530
n.a.
n.a.
n.a.
n.a.
S9_vs_19689
n.a.
n.a.
n.a.
n.a.
Shared
S6_vs_56492

n.a.
n.a.
n.a.
n.a.
d__Bacteria;p__Firmicutes;c__Clostridia;o__Oscillospirales;f__CAG-272;g__CAG-272
ME
S3_vf_127392
Caudoviricetes
Unclassified
O
O
d__Bacteria;p__Firmicutes;c__Clostridia;o__Oscillospirales;f__CAG-272;g__QALR01
SHHC
S12_vs_188820
n.a.
n.a.
n.a.
n.a.
d__Bacteria;p__Firmicutes;c__Clostridia;o__Oscillospirales;f__Oscillospiraceae;g__
ME
S13_vs_148294
Caudoviricetes
Unclassified
O
O
Shared
S3_vs_54303
Caudoviricetes
Unclassified
Unclassified
Eponavirus
S7_vs_4956
Caudoviricetes
Unclassified
Unclassified
Eponavirus
d__Bacteria;p__Firmicutes;c__Clostridia;o__Oscillospirales;f__Oscillospiraceae;g__An92
ME
S9_vs_14235
n.a.
n.a.
n.a.
n.a.
S9_vs_84046
Caudoviricetes
Unclassified
O
O
d__Bacteria;p__Firmicutes;c__Clostridia;o__Oscillospirales;f__Oscillospiraceae;g__CAG-103
ME
S5_vs_77593
Caudoviricetes
Unclassified
O
O
S7_vs_5236
Caudoviricetes
Unclassified
O
O

Shared
S7_vf_76463
n.a.
n.a.
n.a.
n.a.

d__Bacteria;p__Firmicutes;c__Clostridia;o__Oscillospirales;f__Oscillospiraceae;g__CAG-110

ME
S5_vs_107105
Caudoviricetes
Unclassified
O
O
SHHC
S10_vs_120541
n.a.
n.a.
n.a.
n.a.

d__Bacteria;p__Firmicutes;c__Clostridia;o__Oscillospirales;f__Oscillospiraceae;g__CAG-170

ME
S1_vf_136526
Caudoviricetes
Unclassified
Unclassified
O
S1_vs_117317
Caudoviricetes
Unclassified
Unclassified
O
Shared
S1_vf_6272
Caudoviricetes
Unclassified
Unclassified
O
S12_vf_173208
n.a.
n.a.
n.a.
n.a.
S3_vf_82571
Caudoviricetes
Unclassified
O
O
SHHC
S8_vs_51368
n.a.
n.a.
n.a.
n.a.
S8_vs_54183
Caudoviricetes
Unclassified
Unclassified
O

d__Bacteria;p__Firmicutes;c__Clostridia;o__Oscillospirales;f__Oscillospiraceae;g__CAG-83

ME

S11_vf_33117
n.a.
n.a.
n.a.
n.a.
S11_vf_99751
n.a.
n.a.
n.a.
n.a.
S13_vf_109235
n.a.
n.a.
n.a.
n.a.
S13_vs_13129
Caudoviricetes
Unclassified
O
O
S14_vs_97835
n.a.
n.a.
n.a.
n.a.
S5_vs_74199
n.a.
n.a.
n.a.
n.a.
Shared
S12_vs_164056
n.a.
n.a.
n.a.
n.a.
S13_vs_35816
Caudoviricetes
Unclassified
Unclassified
Lagaffevirus
S8_vf_170878
Caudoviricetes
Unclassified
Unclassified
Toutatisvirus
S8_vs_125717
n.a.
n.a.
n.a.
n.a.
S9_vf_64844
Caudoviricetes
Unclassified
Unclassified
Lagaffevirus
d__Bacteria;p__Firmicutes;c__Clostridia;o__Oscillospirales;f__Oscillospiraceae;g__Dysosmobacter
ME
S11_vs_52577
Caudoviricetes

Unclassified
Unclassified
Mushuvirus
S7_vs_28150
n.a.
n.a.
n.a.
n.a.
S9_vs_69322
n.a.
n.a.
n.a.
n.a.
Shared
S10_vf_120738
n.a.
n.a.
n.a.
n.a.
S17_vs_127519
n.a.
n.a.
n.a.
n.a.
SHHC
S8_vs_50431
n.a.
n.a.
n.a.
n.a.
d__Bacteria;p__Firmicutes;c__Clostridia;o__Oscillospirales;f__Oscillospiraceae;g__Enterenecus
ME
S5_vs_117417
n.a.
n.a.
n.a.
n.a.
d__Bacteria;p__Firmicutes;c__Clostridia;o__Oscillospirales;f__Oscillospiraceae;g__ER4
ME
S1_vf_22149
n.a.
n.a.
n.a.
n.a.
S1_vs_143929
n.a.
n.a.
n.a.
n.a.
S13_vf_150407
Faserviricetes
Inoviridae
Unclassified
Inovirus
S3_vs_127411
n.a.
n.a.
n.a.
n.a.
Shared

S1_vs_155183
Caudoviricetes
Unclassified
Unclassified
O
S11_vf_104034
Caudoviricetes
Unclassified
Unclassified
O
S12_vf_18902
Caudoviricetes
Unclassified
O
O
S16_vf_6102
Caudoviricetes
Unclassified
Unclassified
Toutatisvirus
S16_vf_97279
Caudoviricetes
Unclassified
Unclassified
Eponavirus
S17_vs_103009
n.a.
n.a.
n.a.
n.a.
S3_vf_127400
Caudoviricetes
Unclassified
Unclassified
O
S4_vs_VIRSorter_4959-cat_2
Caudoviricetes
Unclassified
O
O
S5_vf_136076
n.a.
n.a.
n.a.
n.a.
SHHC
S16_vs_78429
Caudoviricetes
Unclassified
Unclassified
O
d__Bacteria;p__Firmicutes;c__Clostridia;o__Oscillospirales;f__Oscillospiraceae;g__Evtepia
Shared
S14_vs_117166
n.a.
n.a.
n.a.
n.a.
d__Bacteria;p__Firmicutes;c__Clostridia;o__Oscillospirales;f__Oscillospiraceae;g__Flavonifractor
ME

S11_vf_95067
n.a.
n.a.
n.a.
n.a.
S13_vf_147589
n.a.
n.a.
n.a.
n.a.
S14_vf_115736
Caudoviricetes
Unclassified
Unclassified
O
S14_vs_3251
n.a.
n.a.
n.a.
n.a.
S14_vs_56410
Caudoviricetes
Unclassified
O
O
S5_vs_21778
n.a.
n.a.
n.a.
n.a.
S5_vs_65490
n.a.
n.a.
n.a.
n.a.
S5_vs_69980
Caudoviricetes
Unclassified
O
O
S7_vs_83100
Caudoviricetes
Unclassified
O
O
S7_vs_83474
Caudoviricetes
Unclassified
Unclassified
O
S7_vs_94738
Caudoviricetes
Unclassified
Unclassified
O
S9_vs_4558
Caudoviricetes
Unclassified
O
O

S9_vs_50439
n.a.
n.a.
n.a.
n.a.
Shared
S4_vs_VIRSorter_15837-cat_2
Caudoviricetes
Unclassified
O
O
S4_vs_VIRSorter_31196-circular-cat_2
n.a.
n.a.
n.a.
n.a.
S4_vs_VIRSorter_31560-circular-cat_2
Caudoviricetes
Unclassified
O
O
S5_vf_21869
Caudoviricetes
Unclassified
Unclassified
O
S6_vs_15813
n.a.
n.a.
n.a.
n.a.
S8_vf_111790
Caudoviricetes
Unclassified
O
O
S8_vf_166403
n.a.
n.a.
n.a.
n.a.
SHHC
S16_vf_173099
n.a.
n.a.
n.a.
n.a.
d__Bacteria;p__Firmicutes;c__Clostridia;o__Oscillospirales;f__Oscillospiraceae;g__Intestinimonas
ME
S7_vs_118295
n.a.
n.a.
n.a.
n.a.
S7_vs_69630
Caudoviricetes
Unclassified
O
O
S9_vs_5676

Caudoviricetes
Unclassified
O
O
Shared
S12_vs_92868
Caudoviricetes
Unclassified
O
O
S14_vf_109545
n.a.
n.a.
n.a.
n.a.
S4_vs_VIRSorter_30222-cat_2
Caudoviricetes
Unclassified
O
O
S5_vs_124035
n.a.
n.a.
n.a.
n.a.
S7_vs_78507
n.a.
n.a.
n.a.
n.a.
S8_vf_208110
Caudoviricetes
Unclassified
O
O
S8_vf_55476
n.a.
n.a.
n.a.
n.a.
d__Bacteria;p__Firmicutes;c__Clostridia;o__Oscillospirales;f__Oscillospiraceae;g__Lawsonibacter
ME
S11_vs_19892
Caudoviricetes
Unclassified
Unclassified
Eponavirus
S14_vs_67094
n.a.
n.a.
n.a.
n.a.
S14_vs_9676
Caudoviricetes
Unclassified
O
O
S17_vf_97671
Caudoviricetes
Unclassified

Unclassified
Mushuvirus
S7_vf_138019
n.a.
n.a.
n.a.
n.a.
Shared
S5_vf_103206
n.a.
n.a.
n.a.
n.a.
S8_vs_134805
Caudoviricetes
Unclassified
Unclassified
Mushuvirus
d__Bacteria;p__Firmicutes;c__Clostridia;o__Oscillospirales;f__Oscillospiraceae;g__UBA5446
Shared
S16_vs_75108
n.a.
n.a.
n.a.
n.a.
d__Bacteria;p__Firmicutes;c__Clostridia;o__Oscillospirales;f__Oscillospiraceae;g__UBA9475
SHHC
S8_vs_145173
Caudoviricetes
Unclassified
O
O
d__Bacteria;p__Firmicutes;c__Clostridia;o__Oscillospirales;f__Oscillospiraceae;g__UMGS1766
Shared
S16_vs_20060
Caudoviricetes
Unclassified
Unclassified
O
d__Bacteria;p__Firmicutes;c__Clostridia;o__Oscillospirales;f__Ruminococcaceae;g__Anaerotruncus
ME
S5_vs_118168
n.a.
n.a.
n.a.
n.a.
S5_vs_130958
Caudoviricetes
Unclassified
O
O
S5_vs_32885
Caudoviricetes
Unclassified
O
O
d__Bacteria;p__Firmicutes;c__Clostridia;o__Oscillospirales;f__Ruminococcaceae;g__CAG-353
Shared
S12_vs_133070
Caudoviricetes

Unclassified
O
O
d__Bacteria;p__Firmicutes;c__Clostridia;o__Oscillospirales;f__Ruminococcaceae;g__D5
Shared
S3_vf_80287
Caudoviricetes
Unclassified
O
O
d__Bacteria;p__Firmicutes;c__Clostridia;o__Oscillospirales;f__Ruminococcaceae;g__Faecalibacterium
ME
S1_vs_23682
Caudoviricetes
Unclassified
O
O
S11_vs_11994
Caudoviricetes
Unclassified
O
O
S13_vf_54201
Caudoviricetes
Unclassified
O
O
S14_vs_90934
n.a.
n.a.
n.a.
n.a.
S17_vs_110666
Caudoviricetes
Unclassified
O
O
S3_vs_42569
Caudoviricetes
Unclassified
Unclassified
Toutatisvirus
S3_vs_68031
Caudoviricetes
Unclassified
O
O
S5_vs_117319
Caudoviricetes
Unclassified
Unclassified
O
S5_vs_124677
Caudoviricetes
Unclassified
Unclassified
O
S5_vs_30490
n.a.
n.a.

n.a.
n.a.
S7_vf_84974
Caudoviricetes
Unclassified
O
O
S7_vs_87279
Caudoviricetes
Unclassified
Unclassified
O
S7_vs_97737
n.a.
n.a.
n.a.
n.a.
Shared
S12_vs_87088
Caudoviricetes
Unclassified
O
O
S16_vf_108554
n.a.
n.a.
n.a.
n.a.
S16_vs_132979
Caudoviricetes
Unclassified
O
O
S16_vs_173141
n.a.
n.a.
n.a.
n.a.
S3_vf_127409
Caudoviricetes
Unclassified
O
O
S3_vf_99734
Caudoviricetes
Unclassified
Unclassified
Lagaffevirus
S3_vs_87728
n.a.
n.a.
n.a.
n.a.
S3_vs_90331
Caudoviricetes
Unclassified
Unclassified
O
S4_vs_VIRSorter_27316-cat_2
Caudoviricetes

Unclassified
O
O
S5_vf_134040
Caudoviricetes
Unclassified
Unclassified
Mushuvirus
S5_vs_130294
n.a.
n.a.
n.a.
n.a.
S6_vs_1146
Caudoviricetes
Unclassified
O
O
S6_vs_34453
Caudoviricetes
Unclassified
Unclassified
O
S7_vf_39724
Caudoviricetes
Unclassified
Unclassified
Mushuvirus
S7_vs_13370
n.a.
n.a.
n.a.
n.a.
S7_vs_135322
Caudoviricetes
Unclassified
Unclassified
O
S7_vs_26757
n.a.
n.a.
n.a.
n.a.
SHHC
S10_vf_24791
Caudoviricetes
Unclassified
Unclassified
Eponavirus
S10_vs_46819
Caudoviricetes
Unclassified
O
O
S12_vs_81729
Caudoviricetes
Unclassified
O
O
S16_vs_18440

Caudoviricetes
Unclassified
Unclassified
Toutatisvirus
S6_vs_53966
Caudoviricetes
Unclassified
O
O
S8_vf_101283
Caudoviricetes
Unclassified
Unclassified
Toutatisvirus
S8_vf_187742
Caudoviricetes
Unclassified
O
O
S8_vf_208119
Malgrandaviricetes
Microviridae
Gokushovirinae
Gokushovirus
S8_vs_179529
Caudoviricetes
Unclassified
Unclassified
O
S8_vs_63277
Caudoviricetes
Unclassified
O
O
d__Bacteria;p__Firmicutes;c__Clostridia;o__Oscillospirales;f__Ruminococcaceae;g__Gemmiger
ME
S1_vf_28398
n.a.
n.a.
n.a.
n.a.
S1_vs_133577
Caudoviricetes
Unclassified
O
O
S14_vf_117147
Caudoviricetes
Unclassified
O
O
S3_vs_119771
Caudoviricetes
Unclassified
O
O
S3_vs_127391
n.a.
n.a.
n.a.

n.a.
S3_vs_15450
Caudoviricetes
Unclassified
O
O
S7_vs_111831
Caudoviricetes
Unclassified
O
O
Shared
S4_vs_VIRSorter_31976-circular-cat_2
n.a.
n.a.
n.a.
n.a.
S7_vs_42563
n.a.
n.a.
n.a.
n.a.
SHHC
S16_vs_76823
n.a.
n.a.
n.a.
n.a.
S8_vf_151384
Caudoviricetes
Unclassified
Unclassified
Eponavirus
S8_vf_208126
Caudoviricetes
Unclassified
O
O
S8_vs_102608
n.a.
n.a.
n.a.
n.a.
d__Bacteria;p__Firmicutes;c__Clostridia;o__Oscillospirales;f__Ruminococcaceae;g__Massilimaliae
ME
S14_vs_73301
Caudoviricetes
Unclassified
O
O
Shared
S16_vs_17493
n.a.
n.a.
n.a.
n.a.
SHHC
S4_vs_VIRSorter_4839-cat_2
Caudoviricetes
Unclassified

O
O
d__Bacteria;p__Firmicutes;c__Clostridia;o__Oscillospirales;f__Ruminococcaceae;g__Massilioclostridium
ME
S11_vs_13652
Caudoviricetes
Unclassified
Unclassified
O
d__Bacteria;p__Firmicutes;c__Clostridia;o__Oscillospirales;f__Ruminococcaceae;g__Negativibacillus
ME
S5_vs_128398
Caudoviricetes
Unclassified
O
O
S9_vs_48347
Caudoviricetes
Unclassified
Unclassified
O
Shared
S11_vs_104064
Caudoviricetes
Unclassified
O
O
S6_vs_46091
Caudoviricetes
Unclassified
Unclassified
O
d__Bacteria;p__Firmicutes;c__Clostridia;o__Oscillospirales;f__Ruminococcaceae;g__Phocea
ME
S5_vs_145611
Caudoviricetes
Unclassified
O
O
d__Bacteria;p__Firmicutes;c__Clostridia;o__Oscillospirales;f__Ruminococcaceae;g__QAMM01
ME
S14_vs_101482
n.a.
n.a.
n.a.
n.a.
SHHC
S4_vs_VIRSorter_31171-circular-cat_2
Caudoviricetes
Unclassified
O
O
d__Bacteria;p__Firmicutes;c__Clostridia;o__Oscillospirales;f__Ruminococcaceae;g__Ruminiclostridium_E
Shared
S7_vs_21521
n.a.
n.a.
n.a.
n.a.
d__Bacteria;p__Firmicutes;c__Clostridia;o__Oscillospirales;f__Ruminococcaceae;g__Ruminococcus_C

ME
S3_vs_29077
Caudoviricetes
Unclassified
O
O
SHHC
S4_vs_VIRSorter_31972-circular-cat_2
Caudoviricetes
Unclassified
O
O
d__Bacteria;p__Firmicutes;c__Clostridia;o__Oscillospirales;f__Ruminococcaceae;g__Ruminococcus_D
ME
S14_vf_51134
Caudoviricetes
Unclassified
O
O
S3_vf_8164
Caudoviricetes
Unclassified
O
O
S7_vf_71527
n.a.
n.a.
n.a.
n.a.
S7_vf_8332
Caudoviricetes
Unclassified
O
O
Shared
S10_vs_120570
n.a.
n.a.
n.a.
n.a.
S16_vs_34021
n.a.
n.a.
n.a.
n.a.
S17_vf_174033
Caudoviricetes
Unclassified
O
O
S3_vf_111893
Caudoviricetes
Unclassified
O
O
S7_vf_13614
Caudoviricetes
Unclassified
O
O

SHHC
S12_vf_181024
Caudoviricetes
Unclassified
O
O
S16_vs_107235
n.a.
n.a.
n.a.
n.a.
S16_vs_48714
n.a.
n.a.
n.a.
n.a.
S8_vf_161703
Caudoviricetes
Unclassified
O
O
d__Bacteria;p__Firmicutes;c__Clostridia;o__Oscillospirales;f__Ruminococcaceae;g__Ruthenibacterium
ME
S14_vs_14737
Caudoviricetes
Unclassified
O
O
S14_vs_38905
n.a.
n.a.
n.a.
n.a.
S3_vs_25896
n.a.
n.a.
n.a.
n.a.
S5_vs_121411
n.a.
n.a.
n.a.
n.a.
S5_vs_13254
n.a.
n.a.
n.a.
n.a.
S9_vs_66877
Caudoviricetes
Unclassified
O
O
Shared
S11_vs_76784
Caudoviricetes
Unclassified
O
O
S16_vf_169895

Caudoviricetes
Salasmaviridae
O
O
S5_vs_85065
Caudoviricetes
Unclassified
O
O
SHHC
S16_vs_103726
n.a.
n.a.
n.a.
n.a.
S16_vs_81805
Caudoviricetes
Unclassified
O
O
S6_vs_33711
Caudoviricetes
Unclassified
Unclassified
Unclassified
d__Bacteria;p__Firmicutes;c__Clostridia;o__Oscillospirales;f__Ruminococcaceae;g__UBA3818
SHHC
S6_vs_38250
Caudoviricetes
Unclassified
Unclassified
Unclassified
d__Bacteria;p__Firmicutes;c__Clostridia;o__Oscillospirales;f__UMGS1783;g__UMGS1783
ME
S7_vs_134391
n.a.
n.a.
n.a.
n.a.
SHHC
S12_vs_188828
n.a.
n.a.
n.a.
n.a.
d__Bacteria;p__Firmicutes;c__Clostridia;o__Peptostreptococcales;f__Anaerovoracaceae;g__CAG-145
Shared
S10_vs_2538
Caudoviricetes
Unclassified
O
O
d__Bacteria;p__Firmicutes;c__Clostridia;o__Peptostreptococcales;f__Peptostreptococcaceae;g__GCA-900066495
Shared
S3_vf_56363
Caudoviricetes
Unclassified
O
O
SHHC

S4_vs_VIRSorter_15112-cat_2
n.a.
n.a.
n.a.
n.a.
d__Bacteria;p__Firmicutes;c__Clostridia;o__TANB77;f__CAG-508;g__CAG-269
SHHC
S12_vs_6836
Caudoviricetes
Unclassified
O
O
S16_vf_90328
Caudoviricetes
Unclassified
O
O
d__Bacteria;p__Firmicutes;c__Clostridia;o__TANB77;f__CAG-508;g__CAG-354
SHHC
S8_vf_13709
Caudoviricetes
Unclassified
O
O
S8_vf_13758
Caudoviricetes
Unclassified
O
O
d__Bacteria;p__Firmicutes;c__Clostridia;o__UBA1212;f__UBA1255;g__Egerieisoma
ME
S5_vs_850
n.a.
n.a.
n.a.
n.a.
d__Bacteria;p__Firmicutes;c__Negativicutes;o__Acidaminococcales;f__Acidaminococcaceae;g__Phascolarctobacterium
SHHC
S8_vf_208146
Malgrandaviricetes
Microviridae
Gokushovirinae
Unclassified
d__Bacteria;p__Firmicutes;c__Negativicutes;o__Veillonellales;f__Dialisteraceae;g__Dialister
ME
S7_vs_134784
Caudoviricetes
Unclassified
O
O
S9_vs_67792
Caudoviricetes
Unclassified
O
O
d__Bacteria;p__Firmicutes;c__Negativicutes;o__Veillonellales;f__Megasphaeraceae;g__Caecibacter
SHHC
S8_vs_147670
Caudoviricetes
Unclassified

O
O
d__Bacteria;p__Firmicutes;c__Negativicutes;o__Veillonellales;f__Veillonellaceae;g__Veillonella
ME
S5_vf_8808
Caudoviricetes
Unclassified
O
O
d__Bacteria;p__Patescibacteria;c__Paceibacteria;o__UBA9983_A;f__UBA1006;g__01-FULL-36-10b
Shared
S4_vf_31973
n.a.
n.a.
n.a.
n.a.
d__Bacteria;p__Proteobacteria;c__Alphaproteobacteria;o__RF32;f__CAG-239;g__51-20
ME
S11_vs_84627
n.a.
n.a.
n.a.
n.a.
Shared
S13_vs_45488
Caudoviricetes
Unclassified
O
O
S6_vs_44070
n.a.
Unclassified
O
O
d__Bacteria;p__Proteobacteria;c__Alphaproteobacteria;o__RF32;f__CAG-239;g__CAG-267
Shared
S11_vs_6458
n.a.
n.a.
n.a.
n.a.
S13_vs_9438
n.a.
n.a.
n.a.
n.a.
S5_vs_9747
n.a.
n.a.
n.a.
n.a.
S6_vs_39065
n.a.
n.a.
n.a.
n.a.
d__Bacteria;p__Proteobacteria;c__Alphaproteobacteria;o__RF32;f__CAG-239;g__CAG-495
ME
S17_vs_8690
n.a.

n.a.
n.a.
n.a.
SHHC
S12_vs_153780
Caudoviricetes
Unclassified
O
O
d__Bacteria;p__Proteobacteria;c__Gammaproteobacteria;o__Burkholderiales;f__Burkholderiaceae;g__Duodenibacillus
SHHC
S4_vs_VIRSorter_31980-circular-cat_2
Caudoviricetes
Unclassified
Unclassified
Unclassified
d__Bacteria;p__Proteobacteria;c__Gammaproteobacteria;o__Burkholderiales;f__Burkholderiaceae;g__Parasutterella
SHHC
S12_vs_93357
Caudoviricetes
Unclassified
O
O
S16_vs_120622
Caudoviricetes
Unclassified
O
O
d__Bacteria;p__Proteobacteria;c__Gammaproteobacteria;o__Burkholderiales;f__Burkholderiaceae;g__Sutterella
ME
S14_vs_2870
Caudoviricetes
Unclassified
O
O
S3_vs_51931
Malgrandaviricetes
Microviridae
Gokushovirinae
O
S7_vs_48523
Caudoviricetes
Unclassified
O
O
d__Bacteria;p__Proteobacteria;c__Gammaproteobacteria;o__Enterobacterales;f__Enterobacteriaceae;g__Escherichia
ME
S5_vf_74661
Caudoviricetes
Unclassified
Unclassified
Unclassified
S5_vs_130032
Caudoviricetes
Unclassified
O
O
Shared
S13_vf_151045
Caudoviricetes

Unclassified
O
O
d__Bacteria;p__Proteobacteria;c__Gammaproteobacteria;o__Enterobacterales;f__Enterobacteriaceae;g__Salmonella
ME
S11_vs_5077
Caudoviricetes
Peduoviridae
Unclassified
Quadragintavirus
d__Bacteria;p__Proteobacteria;c__Gammaproteobacteria;o__Enterobacterales;f__Pasteurellaceae;g__Glaesserella
SHHC
S12_vf_10887
n.a.
n.a.
n.a.
n.a.
d__Bacteria;p__Spirochaetota;c__Spirochaetia;o__Sphaerochaetales;f__Sphaerochaetaceae;g__UBA9732
Shared
S3_vf_115304
Caudoviricetes
Suoliviridae
Uncouvirinae
Aurodevirus
d__Bacteria;p__Verrucomicrobiota;c__Lentisphaeria;o__Victivallales;f__Victivallaceae;g__Victivallis
ME
S7_vs_87439
n.a.
n.a.
n.a.
n.a.
d__Bacteria;p__Verrucomicrobiota;c__Verrucomicrobiae;o__Verrucomicrobiales;f__Akkermansiaceae;g__Akkermansia
ME
S13_vf_7670
n.a.
n.a.
n.a.
n.a.
S5_vs_86562
Caudoviricetes
Unclassified
O
O
S7_vs_29595
Caudoviricetes
Unclassified
O
O
Shared
S16_vs_173096
Caudoviricetes
Unclassified
Unclassified
Unclassified
S6_vf_25710
n.a.
n.a.
n.a.
n.a.
SHHC

S12_vs_86928
Caudoviricetes
Unclassified
O
O

Table S9-6. WMS_Hosts_pivot

Host interactions with:	# Hosts
ME-viruses-only	22
SHHC-viruses-only	23
Shared/communal viruses	61

d__Bacteria;p__Actinobacteriota;c__Actinomycetia;o__Actinomycetales;f__Bifidobacteriaceae;g__Bifidobacterium

- **ME**
  - S17-wms-combined_186314
    - **Caudoviricetes**
      - Unclassified
        - **O**
          - O
  - S1-wms-combined_307323
    - **Caudoviricetes**
      - Unclassified
        - **O**
          - O
  - S1-wms-combined_331544
    - **n.a.**
      - n.a.
        - **n.a.**
          - n.a.
  - S1-wms-combined_85225
    - **Caudoviricetes**
      - Unclassified
        - **O**
          - O
  - S3-wms_94494
    - **Caudoviricetes**
      - Unclassified
        - **O**
          - O
  - S3-wms-combined_135428
    - **Caudoviricetes**
      - Unclassified
        - **O**
          - O
  - S7-wms_157264
    - **n.a.**
      - Unclassified
        - **Unclassified**
          - Unclassified
  - S7-wms-combined_136642
    - **n.a.**
      - n.a.
        - **n.a.**
          - n.a.
  - S7-wms-combined_143218
    - **n.a.**
      - n.a.
        - **n.a.**
          - n.a.
  - S7-wms-combined_147158
    - **Caudoviricetes**
      - Unclassified
        - **O**
          - O
  - S7-wms-combined_167867
    - **n.a.**
      - n.a.
        - **n.a.**
          - n.a.
- **Shared**
  - S4-wms-combined_17284
    - **Caudoviricetes**
      - Unclassified
        - **Unclassified**
          - Unclassified
  - S4-wms-combined_51079
    - **n.a.**
      - n.a.
        - **n.a.**
          - n.a.
  - S5-wms-combined_117213
    - **Caudoviricetes**
      - Unclassified
        - **O**
          - O
  - S5-wms-combined_39793

n.a.
n.a.
n.a.
n.a.
S7-wms-combined_174935
Caudoviricetes
Unclassified
O
O
S9-wms-combined_76274
Caudoviricetes
Unclassified
O
O
SHHC
S4-wms_161443
n.a.
Unclassified
Unclassified
Unclassified
d__Bacteria;p__Actinobacteriota;c__Coriobacteriia;o__Coriobacteriales;f__Coriobacteriaceae;g__Collinsella
ME
S5-wms_94965
Caudoviricetes
Unclassified
Unclassified
Unclassified
Shared
S7-wms-combined_189250
Caudoviricetes
Unclassified
Unclassified
Unclassified
S7-wms-combined_190281
n.a.
n.a.
n.a.
n.a.
SHHC
S4-wms-combined_22012
Caudoviricetes
Unclassified
O
O
d__Bacteria;p__Actinobacteriota;c__Coriobacteriia;o__Coriobacteriales;f__Eggerthellaceae;g__Adlercreutzia
SHHC
S12-wms_184276
Caudoviricetes
Unclassified
O
O
d__Bacteria;p__Actinobacteriota;c__Coriobacteriia;o__Coriobacteriales;f__Eggerthellaceae;g__CAG-1427
SHHC
S4-wms_107182
n.a.
n.a.
n.a.
n.a.
d__Bacteria;p__Actinobacteriota;c__Coriobacteriia;o__Coriobacteriales;f__Eggerthellaceae;g__Eggerthella
ME
S17-wms-combined_225356
n.a.
n.a.
n.a.
n.a.
S5-wms_62679
Caudoviricetes
Unclassified
O
O
SHHC
S2-wms_79304
Caudoviricetes
Unclassified
Unclassified
Dybvigvirus

d__Bacteria;p__Bacteroidota;c__Bacteroidia;o__Bacteroidales;f__Bacteroidaceae;g__Bacteroides

- **ME**
  - S11-wms_320555
    - **Caudoviricetes**
      - Unclassified
        - **O**
          - O
  - S11-wms-combined_195425
    - **Caudoviricetes**
      - Unclassified
        - **Unclassified**
          - Unclassified
  - S11-wms-combined_41367
    - **n.a.**
      - n.a.
        - **n.a.**
          - n.a.
  - S17-wms_124308
    - **Caudoviricetes**
      - Unclassified
        - **O**
          - O
  - S17-wms-combined_158354
    - **n.a.**
      - n.a.
        - **n.a.**
          - n.a.
  - S17-wms-combined_16309
    - **Caudoviricetes**
      - Unclassified
        - **O**
          - O
  - S1-wms_208875
    - **Caudoviricetes**
      - Unclassified
        - **O**
          - O
  - S1-wms-combined_330329
    - **Caudoviricetes**
      - Unclassified
        - **O**
          - O
  - S7-wms_244866
    - **Caudoviricetes**
      - Unclassified
        - **Unclassified**
          - Unclassified
  - S7-wms-combined_135154
    - **n.a.**
      - n.a.
        - **n.a.**
          - n.a.
  - S7-wms-combined_3669
    - **Malgrandaviricetes**
      - Microviridae
        - **O**
          - O
  - S7-wms-combined_90393
    - **Caudoviricetes**
      - Unclassified
        - **O**
          - O
  - S7-wms-combined_90728
    - **Caudoviricetes**
      - Unclassified
        - **O**
          - O
  - S9-wms-combined_33229
    - **Caudoviricetes**
      - Unclassified
        - **O**
          - O
- **Shared**
  - S11-wms-combined_217790
    - **n.a.**
      - n.a.

n.a.
n.a.
S11-wms-combined_321938
Caudoviricetes
Unclassified
O
O
S11-wms-combined_73204
n.a.
n.a.
n.a.
n.a.
S12-wms-combined_111524
n.a.
n.a.
n.a.
n.a.
S12-wms-combined_190114
Caudoviricetes
Unclassified
O
O
S12-wms-combined_201125
Caudoviricetes
Unclassified
O
O
S12-wms-combined_250551
Caudoviricetes
Unclassified
Unclassified
Unclassified
S12-wms-combined_297545
Caudoviricetes
Unclassified
O
O
S12-wms-combined_320727
Caudoviricetes
Unclassified
O
O
S12-wms-combined_368190
Caudoviricetes
Unclassified
O
O
S12-wms-combined_368562
Caudoviricetes
Unclassified
O
O
S17-wms-combined_106940
Caudoviricetes
Unclassified
O
O
S17-wms-combined_146050
n.a.
n.a.
n.a.
n.a.
S17-wms-combined_179771
n.a.
n.a.
n.a.
n.a.
S17-wms-combined_233761
n.a.
n.a.
n.a.
n.a.
S17-wms-combined_70983
n.a.
n.a.
n.a.

n.a.
S1-wms_244836
n.a.
n.a.
n.a.
n.a.
S1-wms-combined_248622
n.a.
n.a.
n.a.
n.a.
S1-wms-combined_263562
n.a.
n.a.
n.a.
n.a.
S2-wms_156439
Caudoviricetes
Unclassified
O
O
S2-wms-combined_143737
Caudoviricetes
Duneviridae
O
O
S2-wms-combined_147065
Caudoviricetes
Unclassified
O
O
S2-wms-combined_210822
n.a.
n.a.
n.a.
n.a.
S2-wms-combined_229529
Caudoviricetes
Unclassified
O
O
S2-wms-combined_58121
n.a.
n.a.
n.a.
n.a.
S3-wms-combined_73262
Caudoviricetes
Unclassified
Unclassified
Unclassified
S4-wms-combined_83492
Caudoviricetes
Unclassified
O
O
S5-wms_85650
n.a.
n.a.
n.a.
n.a.
S5-wms-combined_207291
n.a.
n.a.
n.a.
n.a.
S5-wms-combined_59064
Caudoviricetes
Unclassified
Unclassified
Unclassified
S5-wms-combined_83370
Caudoviricetes
Unclassified
O
O

S5-wms-combined_98202
n.a.
n.a.
n.a.
n.a.
S6-wms-combined_135223
n.a.
n.a.
n.a.
n.a.
S6-wms-combined_82597
n.a.
n.a.
n.a.
n.a.
S7-wms-combined_104872
n.a.
n.a.
n.a.
n.a.
S7-wms-combined_107679_1
Caudoviricetes
Unclassified
O
O
S7-wms-combined_185016
Caudoviricetes
Unclassified
O
O
S7-wms-combined_239784
n.a.
n.a.
n.a.
n.a.
S7-wms-combined_43950
Caudoviricetes
Unclassified
Unclassified
Unclassified
S7-wms-combined_5625
Caudoviricetes
Unclassified
Unclassified
Unclassified
S7-wms-combined_62119
Caudoviricetes
Unclassified
Unclassified
O
S7-wms-combined_72070
n.a.
n.a.
n.a.
n.a.
S8-wms-combined_81597
Caudoviricetes
Unclassified
O
O
S9-wms-combined_33230
Caudoviricetes
Unclassified
O
O
S9-wms-combined_37587
Caudoviricetes
Unclassified
O
O
S9-wms-combined_44402
Caudoviricetes
Unclassified
O
O
SHHC

S12-wms-combined_91231
n.a.
n.a.
n.a.
n.a.
S2-wms_6424
Caudoviricetes
Pachyviridae
O
O
S2-wms-combined_132966
Faserviricetes
Inoviridae
Unclassified
Inovirus
S8-wms-combined_239791
Caudoviricetes
Aggregaviridae
O
O
S8-wms-combined_97848
Caudoviricetes
Unclassified
Unclassified
Unclassified
d__Bacteria;p__Bacteroidota;c__Bacteroidia;o__Bacteroidales;f__Bacteroidaceae;g__Bacteroides_G
ME
S17-wms_254872
Malgrandaviricetes
Microviridae
Unclassified
Microvirus
d__Bacteria;p__Bacteroidota;c__Bacteroidia;o__Bacteroidales;f__Bacteroidaceae;g__Prevotella
Shared
S2-wms_128962
Caudoviricetes
Suoliviridae
O
O
SHHC
S4-wms_141607
Caudoviricetes
Unclassified
Unclassified
Unclassified
S4-wms_45128
n.a.
n.a.
n.a.
n.a.
S4-wms_50475
Caudoviricetes
Unclassified
O
O
S4-wms-combined_47565
n.a.
n.a.
n.a.
n.a.
S4-wms-combined_57123
n.a.
n.a.
n.a.
n.a.
S6-wms_138342
Caudoviricetes
Suoliviridae
O
O
S8-wms-combined_74275
Caudoviricetes
Unclassified
O
O
d__Bacteria;p__Bacteroidota;c__Bacteroidia;o__Bacteroidales;f__Bacteroidaceae;g__Prevotellamassilia

Shared
S12-wms_12658
Caudoviricetes
Unclassified
O
O
d__Bacteria;p__Bacteroidota;c__Bacteroidia;o__Bacteroidales;f__Barnesiellaceae;g__Barnesiella
ME
S1-wms_153615
n.a.
n.a.
n.a.
n.a.
S1-wms-combined_153670
n.a.
n.a.
n.a.
n.a.
S1-wms-combined_357373
Malgrandaviricetes
Microviridae
Unclassified
Unclassified
Shared
S12-wms-combined_260036
Caudoviricetes
Steigviridae
O
O
S12-wms-combined_351847
n.a.
n.a.
n.a.
n.a.
S9-wms-combined_31456
Caudoviricetes
Unclassified
Unclassified
Unclassified
SHHC
S12-wms-combined_75733
Malgrandaviricetes
Microviridae
O
O
S8-wms_270
Malgrandaviricetes
Microviridae
O
O
d__Bacteria;p__Bacteroidota;c__Bacteroidia;o__Bacteroidales;f__Muribaculaceae;g__UBA7173
SHHC
S12-wms-combined_256299
n.a.
n.a.
n.a.
n.a.
d__Bacteria;p__Bacteroidota;c__Bacteroidia;o__Bacteroidales;f__Rikenellaceae;g__Alistipes
ME
S11-wms_330479
n.a.
Unclassified
Unclassified
Unclassified
S11-wms-combined_172889
Caudoviricetes
Unclassified
O
O
S17-wms-combined_253499
n.a.
n.a.
n.a.
n.a.
S7-wms-combined_163980
n.a.

n.a.
n.a.
n.a.
S9-wms-combined_28805
Caudoviricetes
Winoviridae
O
O
Shared
S11-wms-combined_125176
Caudoviricetes
Unclassified
O
O
S11-wms-combined_215723
Caudoviricetes
Duneviridae
O
O
S11-wms-combined_226165
Caudoviricetes
Unclassified
O
O
S11-wms-combined_48246
Caudoviricetes
Unclassified
O
O
S11-wms-combined_63818
Caudoviricetes
Unclassified
O
O
S11-wms-combined_64392
Caudoviricetes
Unclassified
O
O
S11-wms-combined_83571
Caudoviricetes
Winoviridae
O
O
S11-wms-combined_92114
n.a.
n.a.
n.a.
n.a.
S12-wms_386692
n.a.
n.a.
n.a.
n.a.
S12-wms-combined_189323
Caudoviricetes
Unclassified
O
O
S12-wms-combined_25459
n.a.
n.a.
n.a.
n.a.
S12-wms-combined_320262
n.a.
n.a.
n.a.
n.a.
S12-wms-combined_328554
Caudoviricetes
Unclassified
O
O
S12-wms-combined_358797
Caudoviricetes

Unclassified
O
O
S17-wms-combined_199746
Caudoviricetes
Unclassified
O
O
S1-wms_394571
Caudoviricetes
Unclassified
O
O
S1-wms-combined_107766
n.a.
n.a.
n.a.
n.a.
S1-wms-combined_340558
Caudoviricetes
Unclassified
O
O
S2-wms-combined_113619
Caudoviricetes
Unclassified
O
O
S2-wms-combined_208559
n.a.
n.a.
n.a.
n.a.
S4-wms-combined_41490
Caudoviricetes
Unclassified
O
O
S5-wms-combined_178432
n.a.
n.a.
n.a.
n.a.
S5-wms-combined_210959
Caudoviricetes
Unclassified
O
O
S5-wms-combined_67650
Caudoviricetes
Unclassified
O
O
S5-wms-combined_90315
Caudoviricetes
Unclassified
O
O
S6-wms-combined_50721
Caudoviricetes
Winoviridae
O
O
S6-wms-combined_72228
n.a.
n.a.
n.a.
n.a.
S6-wms-combined_91039
n.a.
n.a.
n.a.
n.a.
S7-wms-combined_107679_0
Caudoviricetes
Winoviridae

O
O
S7-wms-combined_14267
n.a.
n.a.
n.a.
n.a.
S7-wms-combined_26217
Caudoviricetes
Unclassified
O
O
S7-wms-combined_3697
n.a.
Unclassified
O
O
S8-wms_58515
n.a.
n.a.
n.a.
n.a.
S8-wms-combined_26087
Caudoviricetes
Unclassified
O
O
S8-wms-combined_270362
Caudoviricetes
Unclassified
O
O
S9-wms-combined_5526
Caudoviricetes
Unclassified
O
O
d__Bacteria;p__Bacteroidota;c__Bacteroidia;o__Bacteroidales;f__Rikenellaceae;g__Alistipes_A
ME
S5-wms-combined_183113
n.a.
n.a.
n.a.
n.a.
S5-wms-combined_204782
n.a.
n.a.
n.a.
n.a.
Shared
S8-wms-combined_23583
n.a.
n.a.
n.a.
n.a.
d__Bacteria;p__Bacteroidota;c__Bacteroidia;o__Bacteroidales;f__Rikenellaceae;g__Tidjanibacter
Shared
S12-wms-combined_2450
Caudoviricetes
Winoviridae
O
O
d__Bacteria;p__Bacteroidota;c__Bacteroidia;o__Bacteroidales;f__Tannerellaceae;g__Marseille-P3160
SHHC
S4-wms-combined_14411
Caudoviricetes
Unclassified
Unclassified
Unclassified
S6-wms_54578
Caudoviricetes
Unclassified
Unclassified
Unclassified
d__Bacteria;p__Bacteroidota;c__Bacteroidia;o__Bacteroidales;f__Tannerellaceae;g__Parabacteroides
ME

S17-wms-combined_171305
Caudoviricetes
Unclassified
O
O
S5-wms-combined_94482
n.a.
n.a.
n.a.
n.a.
S7-wms_123954
n.a.
n.a.
n.a.
n.a.
Shared
S1-wms-combined_32863
n.a.
n.a.
n.a.
n.a.
S1-wms-combined_394574
Caudoviricetes
Unclassified
Unclassified
Unclassified
S2-wms-combined_75144
n.a.
n.a.
n.a.
n.a.
SHHC
S4-wms_73990
Caudoviricetes
Suoliviridae
Boorivirinae
Cohcovirus
S4-wms-combined_10758
Caudoviricetes
Unclassified
O
O
S8-wms-combined_180957
Caudoviricetes
Unclassified
O
O
d__Bacteria;p__Bacteroidota;c__Bacteroidia;o__Bacteroidales;f__UBA11471;g__UBA11471
ME
S1-wms-combined_246923
Caudoviricetes
Unclassified
O
O
Shared
S12-wms-combined_371541
n.a.
n.a.
n.a.
n.a.
S12-wms-combined_375343
n.a.
n.a.
n.a.
n.a.
d__Bacteria;p__Campylobacterota;c__Campylobacteria;o__Campylobacterales;f__Campylobacteraceae;g__Campylobacter_D
ME
S5-wms-combined_106597
n.a.
n.a.
n.a.
n.a.
d__Bacteria;p__Cyanobacteria;c__Vampirovibrionia;o__Gastranaerophilales;f__Gastranaerophilaceae;g__QAMI01
ME
S5-wms-combined_199135
n.a.

n.a.
n.a.
n.a.
d__Bacteria;p__Desulfobacterota;c__Desulfovibrionia;o__Desulfovibrionales;f__Desulfovibrionaceae;g__Bilophila
ME
S1-wms-combined_143459
Caudoviricetes
Unclassified
O
O
S1-wms-combined_159544
Caudoviricetes
Peduoviridae
Unclassified
Phitrevirus
S1-wms-combined_19031
Caudoviricetes
Unclassified
Unclassified
Unclassified
S1-wms-combined_231411
n.a.
n.a.
n.a.
n.a.
Shared
S1-wms-combined_360285
Caudoviricetes
Unclassified
O
O
SHHC
S6-wms-combined_119017
Caudoviricetes
Peduoviridae
Unclassified
Phitrevirus
d__Bacteria;p__Desulfobacterota;c__Desulfovibrionia;o__Desulfovibrionales;f__Desulfovibrionaceae;g__Desulfovibrio
ME
S11-wms-combined_268967
Caudoviricetes
Unclassified
Unclassified
Unclassified
S11-wms-combined_84326
Caudoviricetes
Unclassified
O
O
Shared
S11-wms-combined_112733
Caudoviricetes
Unclassified
O
O
d__Bacteria;p__Firmicutes;c__Bacilli;o__Erysipelotrichales;f__Erysipelatoclostridiaceae;g__Erysipelatoclostridium
ME
S7-wms-combined_169375
n.a.
n.a.
n.a.
n.a.
d__Bacteria;p__Firmicutes;c__Bacilli;o__Erysipelotrichales;f__Erysipelotrichaceae;g__Clostridium_AQ
ME
S5-wms-combined_156329
Caudoviricetes
Unclassified
O
O
S5-wms-combined_197186
n.a.
n.a.
n.a.
n.a.
d__Bacteria;p__Firmicutes;c__Bacilli;o__Lactobacillales;f__Lactobacillaceae;g__Lacticaseibacillus
ME

S5-wms_212596
n.a.
n.a.
n.a.
n.a.
d__Bacteria;p__Firmicutes;c__Bacilli;o__Lactobacillales;f__Streptococcaceae;g__Streptococcus
ME
S9-wms_19025
Caudoviricetes
Unclassified
O
O
S9-wms-combined_20485
Caudoviricetes
Unclassified
Unclassified
O
d__Bacteria;p__Firmicutes;c__Clostridia;o__Christensenellales;f__CAG-138;g__SFEL01
SHHC
S12-wms-combined_147628
Caudoviricetes
Unclassified
O
O
S8-wms-combined_164289
n.a.
n.a.
n.a.
n.a.
S8-wms-combined_264711
n.a.
n.a.
n.a.
n.a.
d__Bacteria;p__Firmicutes;c__Clostridia;o__Christensenellales;f__CAG-138;g__UBA1685
SHHC
S8-wms_57121
n.a.
n.a.
n.a.
n.a.
S8-wms-combined_146562
Caudoviricetes
Unclassified
O
O
S8-wms-combined_211119
n.a.
n.a.
n.a.
n.a.
S8-wms-combined_219796
Caudoviricetes
Unclassified
Unclassified
O
d__Bacteria;p__Firmicutes;c__Clostridia;o__Christensenellales;f__CAG-74;g__Firm-11
ME
S11-wms-combined_267993
Caudoviricetes
Unclassified
O
O
d__Bacteria;p__Firmicutes;c__Clostridia;o__Christensenellales;f__CAG-74;g__Limiplasma
Shared
S8-wms-combined_185611
n.a.
n.a.
n.a.
n.a.
d__Bacteria;p__Firmicutes;c__Clostridia;o__Christensenellales;f__CAG-74;g__SFFH01
Shared
S12-wms-combined_299293
n.a.
n.a.
n.a.

n.a.
d__Bacteria;p__Firmicutes;c__Clostridia;o__Christensenellales;f__CAG-74;g__UBA11524
Shared
S12-wms-combined_151843
Caudoviricetes
Unclassified
O
O
S12-wms-combined_293354
Caudoviricetes
Unclassified
O
O
S1-wms_223620
Caudoviricetes
Unclassified
O
O
S1-wms-combined_302166
Caudoviricetes
Unclassified
O
O
S7-wms-combined_134452
n.a.
n.a.
n.a.
n.a.
S7-wms-combined_14529
Caudoviricetes
Unclassified
O
O
S8-wms-combined_215102
n.a.
n.a.
n.a.
n.a.
d__Bacteria;p__Firmicutes;c__Clostridia;o__Christensenellales;f__CAG-74;g__Ventricola
Shared
S2-wms-combined_25727
Caudoviricetes
Unclassified
O
O
d__Bacteria;p__Firmicutes;c__Clostridia;o__Christensenellales;f__CAG-917;g__
SHHC
S12-wms-combined_117447
n.a.
n.a.
n.a.
n.a.
d__Bacteria;p__Firmicutes;c__Clostridia;o__Christensenellales;f__CAG-917;g__CAG-475
SHHC
S12-wms-combined_164656
n.a.
n.a.
n.a.
n.a.
d__Bacteria;p__Firmicutes;c__Clostridia;o__Christensenellales;f__Christensenellaceae;g__Christensenella
ME
S5-wms-combined_66017
n.a.
n.a.
n.a.
n.a.
d__Bacteria;p__Firmicutes;c__Clostridia;o__Clostridiales;f__Clostridiaceae;g__Clostridium
Shared
S17-wms-combined_258087
Caudoviricetes
Unclassified
Unclassified
O
S2-wms-combined_58563
Caudoviricetes
Unclassified

Unclassified
O
d__Bacteria;p__Firmicutes;c__Clostridia;o__Eubacteriales;f__Eubacteriaceae;g__Eubacterium
ME
S5-wms-combined_83412
Caudoviricetes
Unclassified
Unclassified
Unclassified
d__Bacteria;p__Firmicutes;c__Clostridia;o__Lachnospirales;f__Anaerotignaceae;g__Anaerotignum
ME
S9-wms-combined_3461
n.a.
n.a.
n.a.
n.a.
Shared
S2-wms-combined_116221
Caudoviricetes
Unclassified
O
O
S2-wms-combined_191613
n.a.
n.a.
n.a.
n.a.
d__Bacteria;p__Firmicutes;c__Clostridia;o__Lachnospirales;f__Lachnospiraceae;g__Acetatifactor
SHHC
S2-wms-combined_126440
Caudoviricetes
Unclassified
O
O
S6-wms-combined_117756
Caudoviricetes
Unclassified
Unclassified
Eponavirus
d__Bacteria;p__Firmicutes;c__Clostridia;o__Lachnospirales;f__Lachnospiraceae;g__Acetivibrio_A
SHHC
S2-wms-combined_31084
Caudoviricetes
Unclassified
O
O
d__Bacteria;p__Firmicutes;c__Clostridia;o__Lachnospirales;f__Lachnospiraceae;g__Agathobacter
ME
S7-wms-combined_235165
Caudoviricetes
Unclassified
Unclassified
Unclassified
Shared
S2-wms-combined_5791
Caudoviricetes
Unclassified
O
O
SHHC
S2-wms-combined_207000
Caudoviricetes
Unclassified
Unclassified
O
S2-wms-combined_222535
n.a.
n.a.
n.a.
n.a.
d__Bacteria;p__Firmicutes;c__Clostridia;o__Lachnospirales;f__Lachnospiraceae;g__Anaerobutyricum
ME
S1-wms-combined_212892
Caudoviricetes
Unclassified
O

O
S7-wms_151112
Caudoviricetes
Unclassified
O
O
S7-wms-combined_20921
Caudoviricetes
Unclassified
O
O
d__Bacteria;p__Firmicutes;c__Clostridia;o__Lachnospirales;f__Lachnospiraceae;g__Anaerosacchariphilus
SHHC
S2-wms-combined_48661
n.a.
n.a.
n.a.
n.a.
d__Bacteria;p__Firmicutes;c__Clostridia;o__Lachnospirales;f__Lachnospiraceae;g__Anaerostipes
ME
S9-wms-combined_105462
Caudoviricetes
Unclassified
O
O
d__Bacteria;p__Firmicutes;c__Clostridia;o__Lachnospirales;f__Lachnospiraceae;g__Bariatricus
ME
S7-wms-combined_69024
Caudoviricetes
Unclassified
O
O
d__Bacteria;p__Firmicutes;c__Clostridia;o__Lachnospirales;f__Lachnospiraceae;g__Blautia_A
ME
S11-wms-combined_200084
Caudoviricetes
Unclassified
O
O
S7-wms-combined_156351
n.a.
n.a.
n.a.
n.a.
Shared
S2-wms_140933
Caudoviricetes
Unclassified
Unclassified
Unclassified
S5-wms-combined_26061
Caudoviricetes
Unclassified
Unclassified
O
S9-wms-combined_10990
n.a.
n.a.
n.a.
n.a.
SHHC
S2-wms-combined_176376
Caudoviricetes
Unclassified
O
O
S4-wms_62635
Caudoviricetes
Unclassified
Unclassified
Unclassified
d__Bacteria;p__Firmicutes;c__Clostridia;o__Lachnospirales;f__Lachnospiraceae;g__CAG-81
SHHC
S2-wms-combined_85381
Caudoviricetes
Unclassified

Unclassified
Eponavirus
d__Bacteria;p__Firmicutes;c__Clostridia;o__Lachnospirales;f__Lachnospiraceae;g__COE1
SHHC
S12-wms-combined_31761
Caudoviricetes
Unclassified
Unclassified
O
d__Bacteria;p__Firmicutes;c__Clostridia;o__Lachnospirales;f__Lachnospiraceae;g__Dorea_A
ME
S5-wms-combined_73301
Caudoviricetes
Unclassified
O
O
Shared
S7-wms-combined_106253
Caudoviricetes
Unclassified
O
O
SHHC
S4-wms-combined_64632
Caudoviricetes
Unclassified
Unclassified
Unclassified
d__Bacteria;p__Firmicutes;c__Clostridia;o__Lachnospirales;f__Lachnospiraceae;g__Eisenbergiella
ME
S9-wms-combined_2042
n.a.
n.a.
n.a.
n.a.
S9-wms-combined_97755
Caudoviricetes
Unclassified
Unclassified
O
Shared
S2-wms-combined_200390
Caudoviricetes
Unclassified
O
O
d__Bacteria;p__Firmicutes;c__Clostridia;o__Lachnospirales;f__Lachnospiraceae;g__Enterocloster
ME
S17-wms-combined_223748
n.a.
n.a.
n.a.
n.a.
S9-wms-combined_105822
Caudoviricetes
Unclassified
Unclassified
O
Shared
S2-wms-combined_158821
Caudoviricetes
Unclassified
Unclassified
O
S6-wms-combined_119138
Caudoviricetes
Unclassified
O
O
S9-wms_64472
n.a.
n.a.
n.a.
n.a.
SHHC
S12-wms-combined_223043

Caudoviricetes
Unclassified
O
O
S6-wms_140743
Caudoviricetes
Unclassified
O
O
d__Bacteria;p__Firmicutes;c__Clostridia;o__Lachnospirales;f__Lachnospiraceae;g__Eubacterium_I
SHHC
S2-wms_192667
Caudoviricetes
Unclassified
Unclassified
O
d__Bacteria;p__Firmicutes;c__Clostridia;o__Lachnospirales;f__Lachnospiraceae;g__Faecalimonas
SHHC
S2-wms-combined_73327
n.a.
n.a.
n.a.
n.a.
d__Bacteria;p__Firmicutes;c__Clostridia;o__Lachnospirales;f__Lachnospiraceae;g__Fusicatenibacter
Shared
S7-wms-combined_123085
n.a.
n.a.
n.a.
n.a.
d__Bacteria;p__Firmicutes;c__Clostridia;o__Lachnospirales;f__Lachnospiraceae;g__Lachnoclostridium_A
SHHC
S2-wms-combined_204225
n.a.
n.a.
n.a.
n.a.
S2-wms-combined_74387
Caudoviricetes
Unclassified
O
O
S2-wms-combined_95987
n.a.
n.a.
n.a.
n.a.
d__Bacteria;p__Firmicutes;c__Clostridia;o__Lachnospirales;f__Lachnospiraceae;g__Lachnospira
ME
S1-wms-combined_100430
Caudoviricetes
Unclassified
Unclassified
O
Shared
S12-wms-combined_120133
Caudoviricetes
Unclassified
O
O
S2-wms_161986
Caudoviricetes
Unclassified
Unclassified
O
S7-wms-combined_157260
Caudoviricetes
Unclassified
Unclassified
O
SHHC
S12-wms-combined_302876
Caudoviricetes
Unclassified
O
O

d__Bacteria;p__Firmicutes;c__Clostridia;o__Lachnospirales;f__Lachnospiraceae;g__Mediterraneibacter

ME
S11-wms-combined_282227
Caudoviricetes
Unclassified
O
O
S9-wms-combined_26410
Caudoviricetes
Unclassified
O
O
Shared
S4-wms-combined_121978
Caudoviricetes
Unclassified
O
O
S7-wms-combined_157434
n.a.
n.a.
n.a.
n.a.

d__Bacteria;p__Firmicutes;c__Clostridia;o__Lachnospirales;f__Lachnospiraceae;g__Roseburia

Shared
S12-wms-combined_259783
Caudoviricetes
Unclassified
Unclassified
Unclassified
S2-wms_246296
n.a.
n.a.
n.a.
n.a.
S2-wms-combined_157934
Caudoviricetes
Unclassified
Unclassified
O
S2-wms-combined_218002
Caudoviricetes
Unclassified
O
O
S6-wms-combined_32530
Caudoviricetes
Unclassified
O
O
SHHC
S2-wms-combined_165833
Caudoviricetes
Unclassified
Unclassified
Mushuvirus
S4-wms-combined_24659
n.a.
n.a.
n.a.
n.a.
S8-wms-combined_217866
Caudoviricetes
Unclassified
Unclassified
Eponavirus

d__Bacteria;p__Firmicutes;c__Clostridia;o__Lachnospirales;f__Lachnospiraceae;g__UBA9502

Shared
S11-wms-combined_179605
n.a.
n.a.
n.a.
n.a.

d__Bacteria;p__Firmicutes;c__Clostridia;o__Oscillospirales;f__Acutalibacteraceae;g__Acutalibacter

ME
S9-wms-combined_27311

n.a.
n.a.
n.a.
n.a.

d__Bacteria;p__Firmicutes;c__Clostridia;o__Oscillospirales;f__Acutalibacteraceae;g__CAG-180

Shared
S7-wms-combined_218379
Caudoviricetes
Unclassified
O
O

d__Bacteria;p__Firmicutes;c__Clostridia;o__Oscillospirales;f__Acutalibacteraceae;g__CAG-217

Shared
S12-wms-combined_117628
Caudoviricetes
Unclassified
Unclassified
O
S2-wms-combined_225908
n.a.
Unclassified
O
O

d__Bacteria;p__Firmicutes;c__Clostridia;o__Oscillospirales;f__Acutalibacteraceae;g__Ruminococcus_E

ME
S7-wms-combined_28138
Caudoviricetes
Unclassified
O
O
Shared
S2-wms_130687
Caudoviricetes
Unclassified
Unclassified
Unclassified
S5-wms_49310
Caudoviricetes
Unclassified
Unclassified
Unclassified
S5-wms-combined_108794
Caudoviricetes
Unclassified
Unclassified
O
S5-wms-combined_7292
Caudoviricetes
Unclassified
Unclassified
Unclassified
S9-wms-combined_9931
Caudoviricetes
Unclassified
Unclassified
O

d__Bacteria;p__Firmicutes;c__Clostridia;o__Oscillospirales;f__Acutalibacteraceae;g__UBA1417

Shared
S5-wms-combined_117095
n.a.
n.a.
n.a.
n.a.
SHHC
S12-wms-combined_263377
Caudoviricetes
Unclassified
O
O
S12-wms-combined_264425
n.a.
n.a.
n.a.
n.a.

d__Bacteria;p__Firmicutes;c__Clostridia;o__Oscillospirales;f__Butyricicoccaceae;g__Agathobaculum

SHHC

S2-wms-combined_199384
Caudoviricetes
Unclassified
O
O
d__Bacteria;p__Firmicutes;c__Clostridia;o__Oscillospirales;f__Oscillospiraceae;g__
SHHC
S2-wms-combined_64692
Caudoviricetes
Unclassified
Unclassified
O
d__Bacteria;p__Firmicutes;c__Clostridia;o__Oscillospirales;f__Oscillospiraceae;g__An92
ME
S11-wms-combined_119215
Caudoviricetes
Unclassified
O
O
S17-wms-combined_79709
Caudoviricetes
Unclassified
O
O
Shared
S2-wms-combined_137119
Caudoviricetes
Unclassified
Unclassified
O
S8-wms_82880
Caudoviricetes
Unclassified
Unclassified
Brigitvirus
d__Bacteria;p__Firmicutes;c__Clostridia;o__Oscillospirales;f__Oscillospiraceae;g__CAG-103
Shared
S7-wms-combined_139683
Caudoviricetes
Unclassified
O
O
S8-wms-combined_40666
n.a.
n.a.
n.a.
n.a.
d__Bacteria;p__Firmicutes;c__Clostridia;o__Oscillospirales;f__Oscillospiraceae;g__CAG-110
ME
S1-wms-combined_227003
n.a.
n.a.
n.a.
n.a.
Shared
S12-wms-combined_264845
n.a.
n.a.
n.a.
n.a.
d__Bacteria;p__Firmicutes;c__Clostridia;o__Oscillospirales;f__Oscillospiraceae;g__CAG-170
ME
S1-wms_130214
Caudoviricetes
Unclassified
O
O
Shared
S1-wms_185046
n.a.
n.a.
n.a.
n.a.
S2-wms-combined_238929
Caudoviricetes
Unclassified

O
O
S3-wms_23958
Caudoviricetes
Unclassified
O
O

d__Bacteria;p__Firmicutes;c__Clostridia;o__Oscillospirales;f__Oscillospiraceae;g__CAG-83

ME
S11-wms_14390
n.a.
n.a.
n.a.
n.a.
S1-wms-combined_72167
Caudoviricetes
Unclassified
O
O
Shared
S11-wms-combined_3507
Caudoviricetes
Unclassified
O
O
S12-wms_373775
n.a.
n.a.
n.a.
n.a.
S12-wms-combined_148741
n.a.
n.a.
n.a.
n.a.
S12-wms-combined_23517
Caudoviricetes
Unclassified
O
O
S12-wms-combined_270839
n.a.
n.a.
n.a.
n.a.
S12-wms-combined_334806
n.a.
n.a.
n.a.
n.a.
S12-wms-combined_63649
n.a.
n.a.
n.a.
n.a.
S1-wms-combined_169788
n.a.
n.a.
n.a.
n.a.
S1-wms-combined_373308
n.a.
n.a.
n.a.
n.a.
S1-wms-combined_57370
Caudoviricetes
Unclassified
O
O
S1-wms-combined_94988
n.a.
n.a.
n.a.
n.a.
S2-wms-combined_159670

n.a.
n.a.
n.a.
n.a.
S5-wms-combined_134381
Caudoviricetes
Unclassified
Unclassified
Lagaffevirus
S5-wms-combined_179241
n.a.
n.a.
n.a.
n.a.
S8-wms_30498
Caudoviricetes
Unclassified
Unclassified
Mushuvirus
S8-wms_48935
Caudoviricetes
Unclassified
Unclassified
Toutatisvirus
S8-wms-combined_169835
n.a.
n.a.
n.a.
n.a.
SHHC
S12-wms-combined_157167
n.a.
n.a.
n.a.
n.a.
d__Bacteria;p__Firmicutes;c__Clostridia;o__Oscillospirales;f__Oscillospiraceae;g__Dysosmobacter
ME
S11-wms-combined_283220
Caudoviricetes
Unclassified
Unclassified
Mushuvirus
S11-wms-combined_49632
Caudoviricetes
Unclassified
O
O
Shared
S11-wms-combined_116589
n.a.
n.a.
n.a.
n.a.
S12-wms-combined_184338
Caudoviricetes
Unclassified
O
O
S12-wms-combined_329242
n.a.
n.a.
n.a.
n.a.
S1-wms_102342
Caudoviricetes
Unclassified
O
O
S5-wms-combined_205046
n.a.
n.a.
n.a.
n.a.
S6-wms-combined_118427
Caudoviricetes
Unclassified

Unclassified
O
d__Bacteria;p__Firmicutes;c__Clostridia;o__Oscillospirales;f__Oscillospiraceae;g__ER4
ME
S1-wms-combined_185336
n.a.
n.a.
n.a.
n.a.
S1-wms-combined_186878
n.a.
n.a.
n.a.
n.a.
S1-wms-combined_353757
Caudoviricetes
Unclassified
O
O
S1-wms-combined_40115
n.a.
n.a.
n.a.
n.a.
S1-wms-combined_41369
Caudoviricetes
Unclassified
O
O
Shared
S12-wms-combined_129799
Caudoviricetes
Unclassified
Unclassified
O
S12-wms-combined_210942
n.a.
n.a.
n.a.
n.a.
S12-wms-combined_393703
n.a.
n.a.
n.a.
n.a.
S12-wms-combined_48648
n.a.
n.a.
n.a.
n.a.
S1-wms_236105
Caudoviricetes
Unclassified
O
O
S1-wms_296850
Caudoviricetes
Unclassified
Unclassified
Toutatisvirus
S1-wms-combined_132934
Caudoviricetes
Unclassified
O
O
S1-wms-combined_156637
n.a.
n.a.
n.a.
n.a.
S1-wms-combined_164485
n.a.
n.a.
n.a.
n.a.
S1-wms-combined_17651

n.a.
n.a.
n.a.
n.a.
S1-wms-combined_331896
n.a.
n.a.
n.a.
n.a.
S1-wms-combined_351889
Caudoviricetes
Unclassified
Unclassified
O
S2-wms-combined_140996
Caudoviricetes
Unclassified
Unclassified
O
S2-wms-combined_93845
Caudoviricetes
Unclassified
O
O
SHHC
S12-wms_235125
n.a.
n.a.
n.a.
n.a.
S8-wms-combined_134098
n.a.
n.a.
n.a.
n.a.
S8-wms-combined_246389
Caudoviricetes
Unclassified
Unclassified
O
d__Bacteria;p__Firmicutes;c__Clostridia;o__Oscillospirales;f__Oscillospiraceae;g__Evtepia
ME
S17-wms-combined_136622
n.a.
n.a.
n.a.
n.a.
d__Bacteria;p__Firmicutes;c__Clostridia;o__Oscillospirales;f__Oscillospiraceae;g__F23-B02
Shared
S2-wms-combined_52568
n.a.
n.a.
n.a.
n.a.
S8-wms-combined_68780
n.a.
n.a.
n.a.
n.a.
SHHC
S12-wms-combined_129554
Caudoviricetes
Unclassified
Unclassified
Eponavirus
d__Bacteria;p__Firmicutes;c__Clostridia;o__Oscillospirales;f__Oscillospiraceae;g__Faecousia
Shared
S11-wms-combined_329493
n.a.
n.a.
n.a.
n.a.
d__Bacteria;p__Firmicutes;c__Clostridia;o__Oscillospirales;f__Oscillospiraceae;g__Flavonifractor
ME
S17-wms-combined_146850
n.a.

n.a.
n.a.
n.a.
S17-wms-combined_157468
n.a.
n.a.
n.a.
n.a.
S17-wms-combined_175924
Caudoviricetes
Unclassified
Unclassified
Mushuvirus
S17-wms-combined_260343
Caudoviricetes
Unclassified
Unclassified
Toutatisvirus
S17-wms-combined_39687
n.a.
n.a.
n.a.
n.a.
S17-wms-combined_66123
Caudoviricetes
Unclassified
Unclassified
O
S1-wms-combined_374219
n.a.
n.a.
n.a.
n.a.
S5-wms-combined_163165
n.a.
n.a.
n.a.
n.a.
Shared
S11-wms-combined_120257
Caudoviricetes
Unclassified
Unclassified
O
S17-wms-combined_185239
n.a.
n.a.
n.a.
n.a.
S17-wms-combined_247494
Caudoviricetes
Unclassified
O
O
S2-wms-combined_6605
n.a.
n.a.
n.a.
n.a.
S5-wms-combined_145067
Caudoviricetes
Unclassified
O
O
S9-wms-combined_122742
n.a.
n.a.
n.a.
n.a.
S9-wms-combined_124857
n.a.
n.a.
n.a.
n.a.
d__Bacteria;p__Firmicutes;c__Clostridia;o__Oscillospirales;f__Oscillospiraceae;g__HGM12998
ME

S17-wms-combined_75892
Caudoviricetes
Unclassified
Unclassified
O
d__Bacteria;p__Firmicutes;c__Clostridia;o__Oscillospirales;f__Oscillospiraceae;g__Intestinimonas
ME
S11-wms-combined_299954
n.a.
n.a.
n.a.
n.a.
Shared
S2-wms-combined_122457
Caudoviricetes
Unclassified
Unclassified
O
S2-wms-combined_196783
Caudoviricetes
Unclassified
O
O
S2-wms-combined_24549
Caudoviricetes
Unclassified
O
O
S2-wms-combined_72725
n.a.
n.a.
n.a.
n.a.
S5-wms_52566
n.a.
n.a.
n.a.
n.a.
d__Bacteria;p__Firmicutes;c__Clostridia;o__Oscillospirales;f__Oscillospiraceae;g__Lawsonibacter
Shared
S17-wms-combined_259811
Caudoviricetes
Unclassified
O
O
S1-wms_4277
Caudoviricetes
Unclassified
Unclassified
Mushuvirus
S2-wms-combined_126134
n.a.
n.a.
n.a.
n.a.
S5-wms_10783
n.a.
n.a.
n.a.
n.a.
S7-wms-combined_31068
n.a.
n.a.
n.a.
n.a.
d__Bacteria;p__Firmicutes;c__Clostridia;o__Oscillospirales;f__Oscillospiraceae;g__Marseille-P3106
ME
S17-wms-combined_162786
n.a.
n.a.
n.a.
n.a.
Shared
S6-wms-combined_41246
n.a.
n.a.

n.a.
n.a.
S7-wms-combined_84688
n.a.
n.a.
n.a.
n.a.
d__Bacteria;p__Firmicutes;c__Clostridia;o__Oscillospirales;f__Oscillospiraceae;g__Pseudoflavonifractor
ME
S11-wms_32691
n.a.
n.a.
n.a.
n.a.
d__Bacteria;p__Firmicutes;c__Clostridia;o__Oscillospirales;f__Oscillospiraceae;g__UBA738
Shared
S1-wms-combined_136097
n.a.
n.a.
n.a.
n.a.
d__Bacteria;p__Firmicutes;c__Clostridia;o__Oscillospirales;f__Oscillospiraceae;g__UBA9475
SHHC
S8-wms-combined_25607
Caudoviricetes
Unclassified
O
O
d__Bacteria;p__Firmicutes;c__Clostridia;o__Oscillospirales;f__Ruminococcaceae;g__Faecalibacterium
ME
S17-wms-combined_90378
Caudoviricetes
Unclassified
Unclassified
Lughvirus
S1-wms_29288
Caudoviricetes
Unclassified
O
O
S1-wms-combined_131270
Caudoviricetes
Unclassified
O
O
S1-wms-combined_164894
Caudoviricetes
Unclassified
O
O
S1-wms-combined_68998
Caudoviricetes
Unclassified
Unclassified
Taranisvirus
Shared
S11-wms_3116
Caudoviricetes
Unclassified
Unclassified
Mushuvirus
S11-wms-combined_140009
Caudoviricetes
Unclassified
Unclassified
Lughvirus
S11-wms-combined_236214
Caudoviricetes
Unclassified
O
O
S17-wms-combined_193958
Caudoviricetes
Unclassified
Unclassified
Mushuvirus

S17-wms-combined_56006
Caudoviricetes
Unclassified
O
O
S17-wms-combined_56061
n.a.
n.a.
n.a.
n.a.
S1-wms-combined_118167
Caudoviricetes
Unclassified
O
O
S1-wms-combined_23248
Caudoviricetes
Unclassified
Unclassified
Lughvirus
S1-wms-combined_285012
Caudoviricetes
Unclassified
Unclassified
O
S1-wms-combined_304377
Caudoviricetes
Unclassified
Unclassified
Oengusvirus
S1-wms-combined_52708
n.a.
n.a.
n.a.
n.a.
S2-wms_124416
Caudoviricetes
Unclassified
O
O
S2-wms-combined_13246
Caudoviricetes
Unclassified
Unclassified
O
S2-wms-combined_166665
Caudoviricetes
Unclassified
O
O
S2-wms-combined_181849
n.a.
n.a.
n.a.
n.a.
S2-wms-combined_57293
n.a.
Unclassified
O
O
S3-wms_22744
Caudoviricetes
Unclassified
Unclassified
Lagaffevirus
S4-wms-combined_42647
Caudoviricetes
Unclassified
Unclassified
Lughvirus
S6-wms_132588
Caudoviricetes
Unclassified
O
O
S6-wms-combined_110950

n.a.
n.a.
n.a.
n.a.
S6-wms-combined_126236
Caudoviricetes
Unclassified
Unclassified
Toutatisvirus
S6-wms-combined_138710
n.a.
n.a.
n.a.
n.a.
S6-wms-combined_56464
Caudoviricetes
Unclassified
O
O
S7-wms_113137
Caudoviricetes
Unclassified
Unclassified
Mushuvirus
S7-wms-combined_111004
Caudoviricetes
Unclassified
Unclassified
Taranisvirus
S7-wms-combined_137858
n.a.
n.a.
n.a.
n.a.
SHHC
S12-wms_193565
Caudoviricetes
Unclassified
Unclassified
Mushuvirus
S12-wms-combined_264077
Caudoviricetes
Unclassified
O
O
S12-wms-combined_345948
Caudoviricetes
Unclassified
Unclassified
Lughvirus
S12-wms-combined_71235
Caudoviricetes
Unclassified
Unclassified
Lughvirus
S2-wms-combined_127080
n.a.
n.a.
n.a.
n.a.
S6-wms-combined_140731
n.a.
n.a.
n.a.
n.a.
S6-wms-combined_49909
Caudoviricetes
Unclassified
O
O
S8-wms_152831
Caudoviricetes
Unclassified
Unclassified
Toutatisvirus
S8-wms_190781

Caudoviricetes
Unclassified
O
O
S8-wms-combined_225114
Caudoviricetes
Unclassified
O
O

d__Bacteria;p__Firmicutes;c__Clostridia;o__Oscillospirales;f__Ruminococcaceae;g__Gemmiger

ME
S17-wms-combined_72837
Caudoviricetes
Unclassified
O
O
S1-wms-combined_166392
Caudoviricetes
Unclassified
O
O
S1-wms-combined_220022
n.a.
n.a.
n.a.
n.a.
S1-wms-combined_227917
Caudoviricetes
Unclassified
O
O
Shared
S11-wms-combined_264050
Caudoviricetes
Unclassified
Unclassified
O
S11-wms-combined_46816
n.a.
n.a.
n.a.
n.a.
S1-wms-combined_279492
Caudoviricetes
Unclassified
O
O
S2-wms-combined_62839
Caudoviricetes
Unclassified
O
O
S3-wms-combined_45305
Caudoviricetes
Unclassified
O
O
S4-wms-combined_113721
n.a.
n.a.
n.a.
n.a.
S7-wms-combined_134458
Caudoviricetes
Unclassified
Unclassified
Toutatisvirus
S8-wms-combined_49369
n.a.
n.a.
n.a.
n.a.
SHHC
S12-wms-combined_323943
Caudoviricetes
Unclassified

Unclassified
O
S8-wms_20528
Caudoviricetes
Unclassified
Unclassified
O
d__Bacteria;p__Firmicutes;c__Clostridia;o__Oscillospirales;f__Ruminococcaceae;g__Negativibacillus
ME
S9-wms-combined_36459
Caudoviricetes
Unclassified
O
O
Shared
S7-wms-combined_238231
n.a.
n.a.
n.a.
n.a.
d__Bacteria;p__Firmicutes;c__Clostridia;o__Oscillospirales;f__Ruminococcaceae;g__Ruminiclostridium_E
ME
S7-wms_80255
Caudoviricetes
Unclassified
O
O
Shared
S11-wms-combined_168048
Caudoviricetes
Unclassified
O
O
d__Bacteria;p__Firmicutes;c__Clostridia;o__Oscillospirales;f__Ruminococcaceae;g__Ruminococcus_D
Shared
S2-wms-combined_101826
Caudoviricetes
Unclassified
Unclassified
Unclassified
S7-wms_127801
Caudoviricetes
Unclassified
O
O
S7-wms-combined_224523
Caudoviricetes
Unclassified
O
O
S7-wms-combined_71592
Caudoviricetes
Unclassified
O
O
SHHC
S12-wms_223235
n.a.
n.a.
n.a.
n.a.
S2-wms-combined_30750
n.a.
n.a.
n.a.
n.a.
d__Bacteria;p__Firmicutes;c__Clostridia;o__Oscillospirales;f__Ruminococcaceae;g__Ruthenibacterium
ME
S9-wms_104169
Faserviricetes
Inoviridae
Unclassified
Inovirus
Shared
S5-wms-combined_66662
n.a.

Unclassified
Unclassified
O
S5-wms-combined_72410
Caudoviricetes
Unclassified
Unclassified
O
d__Bacteria;p__Firmicutes;c__Clostridia;o__Oscillospirales;f__Ruminococcaceae;g__UBA3818
SHHC
S12-wms-combined_52101
Caudoviricetes
Unclassified
Unclassified
O
d__Bacteria;p__Firmicutes;c__Clostridia;o__Oscillospirales;f__Ruminococcaceae;g__UMGS363
Shared
S12-wms_336780
n.a.
n.a.
n.a.
n.a.
d__Bacteria;p__Firmicutes;c__Negativicutes;o__Acidaminococcales;f__Acidaminococcaceae;g__Phascolarctobacterium_A
SHHC
S4-wms-combined_118531
Caudoviricetes
Unclassified
O
O
S4-wms-combined_57963
Caudoviricetes
Unclassified
Unclassified
O
d__Bacteria;p__Firmicutes;c__Negativicutes;o__Selenomonadales;f__Selenomonadaceae;g__Megamonas
SHHC
S2-wms-combined_197577
Caudoviricetes
Unclassified
Unclassified
Unclassified
d__Bacteria;p__Firmicutes;c__Negativicutes;o__Veillonellales;f__Dialisteraceae;g__Dialister
Shared
S17-wms-combined_95360
Caudoviricetes
Unclassified
O
O
S2-wms-combined_135014
Caudoviricetes
Unclassified
Unclassified
O
S2-wms-combined_135154
n.a.
n.a.
n.a.
n.a.
S2-wms-combined_185494
Caudoviricetes
Autographiviridae
O
O
S7-wms-combined_74829
Caudoviricetes
Unclassified
O
O
S9-wms-combined_53991
Caudoviricetes
Unclassified
O
O
SHHC
S6-wms-combined_65225
n.a.

n.a.
n.a.
n.a.
d__Bacteria;p__Firmicutes;c__Negativicutes;o__Veillonellales;f__Veillonellaceae;g__Veillonella
ME
S9-wms_114971
Caudoviricetes
Unclassified
O
O
d__Bacteria;p__Patescibacteria;c__Paceibacteria;o__Paceibacterales;f__UBA5633;g__MWCK01
SHHC
S12-wms_401372
Caudoviricetes
Unclassified
Unclassified
Unclassified
d__Bacteria;p__Proteobacteria;c__Alphaproteobacteria;o__RF32;f__CAG-239;g__51-20
ME
S1-wms-combined_92523
n.a.
Unclassified
O
O
d__Bacteria;p__Proteobacteria;c__Alphaproteobacteria;o__RF32;f__CAG-239;g__CAG-267
ME
S11-wms-combined_204522
Caudoviricetes
Unclassified
O
O
Shared
S11-wms-combined_193148
n.a.
n.a.
n.a.
n.a.
SHHC
S6-wms-combined_745
n.a.
n.a.
n.a.
n.a.
d__Bacteria;p__Proteobacteria;c__Alphaproteobacteria;o__RF32;f__CAG-239;g__CAG-495
Shared
S11-wms-combined_101337
Caudoviricetes
Unclassified
O
O
SHHC
S12-wms-combined_313953
Caudoviricetes
Unclassified
O
O
d__Bacteria;p__Proteobacteria;c__Gammaproteobacteria;o__Burkholderiales;f__Burkholderiaceae;g__Oxalobacter
ME
S5-wms-combined_3201
Caudoviricetes
Unclassified
O
O
d__Bacteria;p__Proteobacteria;c__Gammaproteobacteria;o__Burkholderiales;f__Burkholderiaceae;g__Sutterella
Shared
S2-wms-combined_84608
Caudoviricetes
Unclassified
O
O
d__Bacteria;p__Proteobacteria;c__Gammaproteobacteria;o__Enterobacterales;f__Enterobacteriaceae;g__Escherichia
ME
S5-wms-combined_18356
Caudoviricetes
Peduoviridae
Unclassified

Evevirus
S9-wms-combined_64277
Caudoviricetes
Unclassified
Unclassified
Bcepmuvirus
SHHC
S2-wms_128831
Caudoviricetes
Unclassified
Unclassified
Unclassified
d__Bacteria;p__Proteobacteria;c__Gammaproteobacteria;o__Enterobacterales;f__Enterobacteriaceae;g__Klebsiella
ME
S9-wms-combined_93232
Caudoviricetes
Unclassified
O
O
d__Bacteria;p__Proteobacteria;c__Gammaproteobacteria;o__Enterobacterales;f__Enterobacteriaceae;g__Salmonella
ME
S11-wms-combined_154845
Caudoviricetes
Peduoviridae
Unclassified
Quadragintavirus
d__Bacteria;p__Verrucomicrobiota;c__Verrucomicrobiae;o__Verrucomicrobiales;f__Akkermansiaceae;g__Akkermansia
ME
S7-wms-combined_217226
Caudoviricetes
Unclassified
O
O
Shared
S12-wms-combined_168259
Caudoviricetes
Unclassified
O
O
S12-wms-combined_282594
Caudoviricetes
Unclassified
O
O
S1-wms-combined_308848
n.a.
n.a.
n.a.
n.a.
S2-wms-combined_135139
n.a.
n.a.
n.a.
n.a.
S2-wms-combined_201205
Caudoviricetes
Unclassified
Unclassified
O
S2-wms-combined_7756
Caudoviricetes
Unclassified
O
O
S5-wms-combined_52741
Caudoviricetes
Unclassified
O
O
S8-wms-combined_246185
Caudoviricetes
Unclassified
O
O

Table S9-7. VLP and WMS: host_comparisons

Shared_hosts_seen_in_both_datasets	Unique_hosts_in_VLP	Unique_hosts_in_WMS
d__Bacteria;p__Actinobacteriota;c__Actinomycetia;o__Actinomycetales;f__Bifidobacteriaceae;g__Bifidobacterium	d__Bacteria;p__Actinobacteriota;c__Actinomycetia;o__Actinomycetales;f__Actinomycetaceae;g__Actinomyces	d__Bacteria;p__Actinobacteriota;c__Coriobacteriia;o__Coriobacteriales;f__Eggerthellaceae;g__CAG-1427
d__Bacteria;p__Actinobacteriota;c__Coriobacteriia;o__Coriobacteriales;f__Coriobacteriaceae;g__Collinsella	d__Bacteria;p__Actinobacteriota;c__Actinomycetia;o__Actinomycetales;f__Actinomycetaceae;g__Pauljensenia	d__Bacteria;p__Bacteroidota;c__Bacteroidia;o__Bacteroidales;f__Bacteroidaceae;g__Bacteroides_G
d__Bacteria;p__Actinobacteriota;c__Coriobacteriia;o__Coriobacteriales;f__Eggerthellaceae;g__Adlercreutzia	d__Bacteria;p__Actinobacteriota;c__Actinomycetia;o__Actinomycetales;f__Micrococcaceae;g__Rothia	d__Bacteria;p__Bacteroidota;c__Bacteroidia;o__Bacteroidales;f__Rikenellaceae;g__Tidjanibacter
d__Bacteria;p__Actinobacteriota;c__Coriobacteriia;o__Coriobacteriales;f__Eggerthellaceae;g__Eggerthella	d__Bacteria;p__Actinobacteriota;c__Actinomycetia;o__Mycobacteriales;f__Mycobacteriaceae;g__Mycobacterium	d__Bacteria;p__Campylobacterota;c__Campylobacteria;o__Campylobacterales;f__Campylobacteraceae;g__Campylobacter_D
d__Bacteria;p__Bacteroidota;c__Bacteroidia;o__Bacteroidales;f__Bacteroidaceae;g__Bacteroides	d__Bacteria;p__Actinobacteriota;c__Coriobacteriia;o__Coriobacteriales;f__Eggerthellaceae;g__Gordonibacter	d__Bacteria;p__Desulfobacterota;c__Desulfovibrionia;o__Desulfovibrionales;f__Desulfovibrionaceae;g__Desulfovibrio
d__Bacteria;p__Bacteroidota;c__Bacteroidia;o__Bacteroidales;f__Bacteroidaceae;g__Prevotella	d__Bacteria;p__Bacteroidota;c__Bacteroidia;o__Bacteroidales;f__Bacteroidaceae;g__Phocaeicola	d__Bacteria;p__Firmicutes;c__Clostridia;o__Christensenellales;f__CAG-74;g__SFFH01
d__Bacteria;p__Bacteroidota;c__Bacteroidia;o__Bacteroidales;f__Bacteroidaceae;g__Prevotellamassilia	d__Bacteria;p__Bacteroidota;c__Bacteroidia;o__Bacteroidales;f__Coprobacteraceae;g__Coprobacter	d__Bacteria;p__Firmicutes;c__Clostridia;o__Christensenellales;f__CAG-917;g__
d__Bacteria;p__Bacteroidota;c__Bacteroidia;o__Bacteroidales;f__Barnesiellaceae;g__Barnesiella	d__Bacteria;p__Bacteroidota;c__Bacteroidia;o__Bacteroidales;f__Marinifilaceae;g__Butyricimonas	d__Bacteria;p__Firmicutes;c__Clostridia;o__Lachnospirales;f__Lachnospiraceae;g__Acetivibrio_A
d__Bacteria;p__Bacteroidota;c__Bacteroidia;o__Bacteroidales;f__Muribaculaceae;g__UBA7173	d__Bacteria;p__Bacteroidota;c__Bacteroidia;o__Bacteroidales;f__Marinifilaceae;g__Odoribacter	d__Bacteria;p__Firmicutes;c__Clostridia;o__Lachnospirales;f__Lachnospiraceae;g__Anaerosacchariphilus
d__Bacteria;p__Bacteroidota;c__Bacteroidia;o__Bacteroidales;f__Rikenellaceae;g__Alistipes	d__Bacteria;p__Bacteroidota;c__Bacteroidia;o__Bacteroidales;f__P3;g__Phil12	d__Bacteria;p__Firmicutes;c__Clostridia;o__Lachnospirales;f__Lachnospiraceae;g__CAG-81
d__Bacteria;p__Bacteroidota;c__Bacteroidia;o__Bacteroidales;f__Rikenellaceae;g__Alistipes_A	d__Bacteria;p__Cyanobacteria;c__Vampirovibrionia;o__Gastranaerophilales;f__Gastranaerophilaceae;g__CAG-306	d__Bacteria;p__Firmicutes;c__Clostridia;o__Lachnospirales;f__Lachnospiraceae;g__COE1
d__Bacteria;p__Bacteroidota;c__Bacteroidia;o__Bacteroidales;f__Tannerellaceae;g__Marseille-P3160	d__Bacteria;p__Desulfobacterota;c__Desulfovibrionia;o__Desulfovibrionales;f__Desulfovibrionaceae;g__Mailhella	d__Bacteria;p__Firmicutes;c__Clostridia;o__Lachnospirales;f__Lachnospiraceae;g__Fusicatenibacter
d__Bacteria;p__Bacteroidota;c__Bacteroidia;o__Bacteroidales;f__Tannerellaceae;g__Parabacteroides	d__Bacteria;p__Firmicutes;c__Bacilli;o__Erysipelotrichales;f__Erysipelatoclostridiaceae;g__Catenibacterium	d__Bacteria;p__Firmicutes;c__Clostridia;o__Lachnospirales;f__Lachnospiraceae;g__Lachnoclostridium_A
d__Bacteria;p__Bacteroidota;c__Bacteroidia;o__Bacteroidales;f__UBA11471;g__UBA11471	d__Bacteria;p__Firmicutes;c__Bacilli;o__Erysipelotrichales;f__Erysipelatoclostridiaceae;g__Faecalibacillus	d__Bacteria;p__Firmicutes;c__Clostridia;o__Oscillospirales;f__Oscillospiraceae;g__F23-B02
d__Bacteria;p__Cyanobacteria;c__Vampirovibrionia;o__Gastranaerophilales;f__Gastranaerophilaceae;g__QAMI01	d__Bacteria;p__Firmicutes;c__Bacilli;o__Erysipelotrichales;f__Erysipelatoclostridiaceae;g__Massiliomicrobiota	d__Bacteria;p__Firmicutes;c__Clostridia;o__Oscillospirales;f__Oscillospiraceae;g__Faecousia
d__Bacteria;p__Desulfobacterota;c__Desulfovibrionia;o__Desulfovibrionales;f__Desulfovibrionaceae;g__Bilophila	d__Bacteria;p__Firmicutes;c__Bacilli;o__Erysipelotrichales;f__Erysipelotrichaceae;g__Holdemanella	d__Bacteria;p__Firmicutes;c__Clostridia;o__Oscillospirales;f__Oscillospiraceae;g__HGM12998
d__Bacteria;p__Firmicutes;c__Bacilli;o__Erysipelotrichales;f__Erysipelatoclostridiaceae;g__Erysipelatoclostridium	d__Bacteria;p__Firmicutes;c__Bacilli;o__Erysipelotrichales;f__Erysipelotrichaceae;g__Holdemania	d__Bacteria;p__Firmicutes;c__Clostridia;o__Oscillospirales;f__Oscillospiraceae;g__Marseille-P3106
d__Bacteria;p__Firmicutes;c__Bacilli;o__Erysipelotrichales;f__Erysipelotrichaceae;g__Clostridium_AQ	d__Bacteria;p__Firmicutes;c__Bacilli;o__Haloplasmatales_A;f__Turicibacteraceae;g__Turicibacter	d__Bacteria;p__Firmicutes;c__Clostridia;o__Oscillospirales;f__Oscillospiraceae;g__Pseudoflavonifractor
d__Bacteria;p__Firmicutes;c__Bacilli;o__Lactobacillales;f__Lactobacillaceae;g__Lacticaseibacillus	d__Bacteria;p__Firmicutes;c__Bacilli;o__Lactobacillales;f__Lactobacillaceae;g__Lactobacillus	d__Bacteria;p__Firmicutes;c__Clostridia;o__Oscillospirales;f__Oscillospiraceae;g__UBA738
d__Bacteria;p__Firmicutes;c__Bacilli;o__Lactobacillales;f__Streptococcaceae;g__Streptococcus	d__Bacteria;p__Firmicutes;c__Bacilli;o__Lactobacillales;f__Lactobacillaceae;g__Leuconostoc	d__Bacteria;p__Firmicutes;c__Clostridia;o__Oscillospirales;f__Ruminococcaceae;g__UMGS363
d__Bacteria;p__Firmicutes;c__Clostridia;o__Christensenellales;f__CAG-138;g__SFEL01	d__Bacteria;p__Firmicutes;c__Bacilli;o__Lactobacillales;f__Lactobacillaceae;g__Limosilactobacillus	d__Bacteria;p__Firmicutes;c__Negativicutes;o__Acidaminococcales;f__Acidaminococcaceae;g__Phascolarctobacterium_A
d__Bacteria;p__Firmicutes;c__Clostridia;o__Christensenellales;f__CAG-138;g__UBA1685	d__Bacteria;p__Firmicutes;c__Bacilli;o__Lactobacillales;f__Streptococcaceae;g__Lactococcus	d__Bacteria;p__Firmicutes;c__Negativicutes;o__Selenomonadales;f__Selenomonadaceae;g__Megamonas
d__Bacteria;p__Firmicutes;c__Clostridia;o__Christensenellales;f__CAG-74;g__Firm-11	d__Bacteria;p__Firmicutes;c__Bacilli;o__ML615J-28;f__CAG-313;g__CAG-313	d__Bacteria;p__Patescibacteria;c__Paceibacteria;o__Paceibacterales;f__UBA5633;g__MWCK01
d__Bacteria;p__Firmicutes;c__Clostridia;o__Christensenellales;f__CAG-74;g__Limiplasma	d__Bacteria;p__Firmicutes;c__Bacilli;o__RF39;f__UBA660;g__CAG-302	d__Bacteria;p__Proteobacteria;c__Gammaproteobacteria;o__Burkholderiales;f__Burkholderiaceae;g__Oxalobacter
d__Bacteria;p__Firmicutes;c__Clostridia;o__Christensenellales;f__CAG-74;g__UBA11524	d__Bacteria;p__Firmicutes;c__Bacilli;o__RF39;f__UBA660;g__CAG-460	d__Bacteria;p__Proteobacteria;c__Gammaproteobacteria;o__Enterobacterales;f__Enterobacteriaceae;g__Klebsiella
d__Bacteria;p__Firmicutes;c__Clostridia;o__Christensenellales;f__CAG-74;g__Ventricola	d__Bacteria;p__Firmicutes;c__Bacilli;o__RF39;f__UBA660;g__CAG-877	
d__Bacteria;p__Firmicutes;c__Clostridia;o__Christensenellales;f__CAG-917;g__CAG-475	d__Bacteria;p__Firmicutes;c__Clostridia;o__Acetivibrionales;f__Acetivibrionaceae;g__Ruminiclostridium	**Stats:**
d__Bacteria;p__Firmicutes;c__Clostridia;o__Christensenellales;f__Christensenellaceae;g__Christensenella	d__Bacteria;p__Firmicutes;c__Clostridia;o__Christensenellales;f__Borkfalkiaceae;g__QALS01	Hosts interact with ME-vOTUs uniquely: 6/25 (24%)
d__Bacteria;p__Firmicutes;c__Clostridia;o__Clostridiales;f__Clostridiaceae;g__Clostridium	d__Bacteria;p__Firmicutes;c__Clostridia;o__Christensenellales;f__CAG-138;g__UBA5394	Hosts interact with SHHC-vOTUs uniquely: 10/25 (40%)
d__Bacteria;p__Firmicutes;c__Clostridia;o__Eubacteriales;f__Eubacteriaceae;g__Eubacterium	d__Bacteria;p__Firmicutes;c__Clostridia;o__Christensenellales;f__CAG-74;g__Alectryocaccomicrobium	Hosts interact with common viruses (vOTUs) shared in both ME and SHHC: 9/25 (36%)
d__Bacteria;p__Firmicutes;c__Clostridia;o__Lachnospirales;f__Anaerotignaceae;g__Anaerotignum	d__Bacteria;p__Firmicutes;c__Clostridia;o__Christensenellales;f__CAG-74;g__SFTH01	
d__Bacteria;p__Firmicutes;c__Clostridia;o__Lachnospirales;f__Lachnospiraceae;g__Acetatifactor	d__Bacteria;p__Firmicutes;c__Clostridia;o__Christensenellales;f__NSJ-53;g__NSJ-53	
d__Bacteria;p__Firmicutes;c__Clostridia;o__Lachnospirales;f__Lachnospiraceae;g__Agathobacter	d__Bacteria;p__Firmicutes;c__Clostridia;o__Christensenellales;f__QAND01;g__UMGS1975	
d__Bacteria;p__Firmicutes;c__Clostridia;o__Lachnospirales;f__Lachnospiraceae;g__Anaerobutyricum	d__Bacteria;p__Firmicutes;c__Clostridia;o__Clostridiales;f__Clostridiaceae;g__Clostridium_J	
d__Bacteria;p__Firmicutes;c__Clostridia;o__Lachnospirales;f__Lachnospiraceae;g__Anaerostipes	d__Bacteria;p__Firmicutes;c__Clostridia;o__Lachnospirales;f__Anaerotignaceae;g__UMGS1670	
d__Bacteria;p__Firmicutes;c__Clostridia;o__Lachnospirales;f__Lachnospiraceae;g__Bariatricus	d__Bacteria;p__Firmicutes;c__Clostridia;o__Lachnospirales;f__CAG-274;g__CAG-274	
d__Bacteria;p__Firmicutes;c__Clostridia;o__Lachnospirales;f__Lachnospiraceae;g__Blautia_A	d__Bacteria;p__Firmicutes;c__Clostridia;o__Lachnospirales;f__Lachnospiraceae;g__	
d__Bacteria;p__Firmicutes;c__Clostridia;o__Lachnospirales;f__Lachnospiraceae;g__Dorea_A	d__Bacteria;p__Firmicutes;c__Clostridia;o__Lachnospirales;f__Lachnospiraceae;g__Blautia	
d__Bacteria;p__Firmicutes;c__Clostridia;o__Lachnospirales;f__Lachnospiraceae;g__Eisenbergiella	d__Bacteria;p__Firmicutes;c__Clostridia;o__Lachnospirales;f__Lachnospiraceae;g__CAG-127	
d__Bacteria;p__Firmicutes;c__Clostridia;o__Lachnospirales;f__Lachnospiraceae;g__Enterocloster	d__Bacteria;p__Firmicutes;c__Clostridia;o__Lachnospirales;f__Lachnospiraceae;g__CAG-317	
d__Bacteria;p__Firmicutes;c__Clostridia;o__Lachnospirales;f__Lachnospiraceae;g__Eubacterium_I	d__Bacteria;p__Firmicutes;c__Clostridia;o__Lachnospirales;f__Lachnospiraceae;g__Clostridium_AP	
d__Bacteria;p__Firmicutes;c__Clostridia;o__Lachnospirales;f__Lachnospiraceae;g__Faecalimonas	d__Bacteria;p__Firmicutes;c__Clostridia;o__Lachnospirales;f__Lachnospiraceae;g__Coprococcus	
d__Bacteria;p__Firmicutes;c__Clostridia;o__Lachnospirales;f__Anaerotignaceae;g__Anaerotignum	d__Bacteria;p__Firmicutes;c__Clostridia;o__Lachnospirales;f__Lachnospiraceae;g__Dorea	
d__Bacteria;p__Firmicutes;c__Clostridia;o__Lachnospirales;f__Lachnospiraceae;g__Mediterraneibacter	d__Bacteria;p__Firmicutes;c__Clostridia;o__Lachnospirales;f__Lachnospiraceae;g__Eubacterium_F	
d__Bacteria;p__Firmicutes;c__Clostridia;o__Lachnospirales;f__Lachnospiraceae;g__Roseburia	d__Bacteria;p__Firmicutes;c__Clostridia;o__Lachnospirales;f__Lachnospiraceae;g__Eubacterium_G	
d__Bacteria;p__Firmicutes;c__Clostridia;o__Lachnospirales;f__Lachnospiraceae;g__UBA9502	d__Bacteria;p__Firmicutes;c__Clostridia;o__Lachnospirales;f__Lachnospiraceae;g__Frisingicoccus	
d__Bacteria;p__Firmicutes;c__Clostridia;o__Oscillospirales;f__Acutalibacteraceae;g__Acutalibacter	d__Bacteria;p__Firmicutes;c__Clostridia;o__Lachnospirales;f__Lachnospiraceae;g__Hungatella	
d__Bacteria;p__Firmicutes;c__Clostridia;o__Oscillospirales;f__Acutalibacteraceae;g__CAG-180	d__Bacteria;p__Firmicutes;c__Clostridia;o__Lachnospirales;f__Lachnospiraceae;g__Marvinbryantia	
d__Bacteria;p__Firmicutes;c__Clostridia;o__Oscillospirales;f__Acutalibacteraceae;g__CAG-217	d__Bacteria;p__Firmicutes;c__Clostridia;o__Lachnospirales;f__Lachnospiraceae;g__Massilistercora	
d__Bacteria;p__Firmicutes;c__Clostridia;o__Oscillospirales;f__Acutalibacteraceae;g__Ruminococcus_E	d__Bacteria;p__Firmicutes;c__Clostridia;o__Lachnospirales;f__Lachnospiraceae;g__Muricomes	
d__Bacteria;p__Firmicutes;c__Clostridia;o__Oscillospirales;f__Acutalibacteraceae;g__UBA1417	d__Bacteria;p__Firmicutes;c__Clostridia;o__Lachnospirales;f__Lachnospiraceae;g__OF09-33XD	
d__Bacteria;p__Firmicutes;c__Clostridia;o__Oscillospirales;f__Butyricicoccaceae;g__Agathobaculum	d__Bacteria;p__Firmicutes;c__Clostridia;o__Lachnospirales;f__Lachnospiraceae;g__RUG115	
d__Bacteria;p__Firmicutes;c__Clostridia;o__Oscillospirales;f__Oscillospiraceae;g__	d__Bacteria;p__Firmicutes;c__Clostridia;o__Lachnospirales;f__Lachnospiraceae;g__Ruminococcus_B	
d__Bacteria;p__Firmicutes;c__Clostridia;o__Oscillospirales;f__Oscillospiraceae;g__An92	d__Bacteria;p__Firmicutes;c__Clostridia;o__Lachnospirales;f__Lachnospiraceae;g__Sellimonas	
d__Bacteria;p__Firmicutes;c__Clostridia;o__Oscillospirales;f__Oscillospiraceae;g__CAG-103	d__Bacteria;p__Firmicutes;c__Clostridia;o__Lachnospirales;f__Lachnospiraceae;g__Ventrimonas	
d__Bacteria;p__Firmicutes;c__Clostridia;o__Oscillospirales;f__Oscillospiraceae;g__CAG-110	d__Bacteria;p__Firmicutes;c__Clostridia;o__Monoglobales;f__Monoglobaceae;g__Monoglobus	
d__Bacteria;p__Firmicutes;c__Clostridia;o__Oscillospirales;f__Oscillospiraceae;g__CAG-170	d__Bacteria;p__Firmicutes;c__Clostridia;o__Oscillospirales;f__Acutalibacteraceae;g__An172	
d__Bacteria;p__Firmicutes;c__Clostridia;o__Oscillospirales;f__Oscillospiraceae;g__CAG-83	d__Bacteria;p__Firmicutes;c__Clostridia;o__Oscillospirales;f__Acutalibacteraceae;g__Eubacterium_R	
d__Bacteria;p__Firmicutes;c__Clostridia;o__Oscillospirales;f__Oscillospiraceae;g__Dysosmobacter	d__Bacteria;p__Firmicutes;c__Clostridia;o__Oscillospirales;f__Acutalibacteraceae;g__Fimivicinus	
d__Bacteria;p__Firmicutes;c__Clostridia;o__Oscillospirales;f__Oscillospiraceae;g__ER4	d__Bacteria;p__Firmicutes;c__Clostridia;o__Oscillospirales;f__Acutalibacteraceae;g__Hydrogeniiclostridium	
d__Bacteria;p__Firmicutes;c__Clostridia;o__Oscillospirales;f__Oscillospiraceae;g__Evtepia	d__Bacteria;p__Firmicutes;c__Clostridia;o__Oscillospirales;f__Acutalibacteraceae;g__Pseudoruminococcus	
d__Bacteria;p__Firmicutes;c__Clostridia;o__Oscillospirales;f__Oscillospiraceae;g__Flavonifractor	d__Bacteria;p__Firmicutes;c__Clostridia;o__Oscillospirales;f__Acutalibacteraceae;g__UBA1691	
d__Bacteria;p__Firmicutes;c__Clostridia;o__Oscillospirales;f__Oscillospiraceae;g__Intestinimonas	d__Bacteria;p__Firmicutes;c__Clostridia;o__Oscillospirales;f__Acutalibacteraceae;g__UBA737	
d__Bacteria;p__Firmicutes;c__Clostridia;o__Oscillospirales;f__Oscillospiraceae;g__Lawsonibacter	d__Bacteria;p__Firmicutes;c__Clostridia;o__Oscillospirales;f__Acutalibacteraceae;g__UMGS1071	
d__Bacteria;p__Firmicutes;c__Clostridia;o__Oscillospirales;f__Oscillospiraceae;g__UBA9475	d__Bacteria;p__Firmicutes;c__Clostridia;o__Oscillospirales;f__Acutalibacteraceae;g__UMGS1591	
d__Bacteria;p__Firmicutes;c__Clostridia;o__Oscillospirales;f__Ruminococcaceae;g__Faecalibacterium	d__Bacteria;p__Firmicutes;c__Clostridia;o__Oscillospirales;f__Acutalibacteraceae;g__UMGS263	
d__Bacteria;p__Firmicutes;c__Clostridia;o__Oscillospirales;f__Ruminococcaceae;g__Gemmiger	d__Bacteria;p__Firmicutes;c__Clostridia;o__Oscillospirales;f__Acutalibacteraceae;g__UMGS856	
d__Bacteria;p__Firmicutes;c__Clostridia;o__Oscillospirales;f__Ruminococcaceae;g__Negativibacillus	d__Bacteria;p__Firmicutes;c__Clostridia;o__Oscillospirales;f__CAG-272;g__CAG-272	

d__Bacteria;p__Firmicutes;c__Clostridia;o__Oscillospirales;f__Ruminococcaceae;g__Ruminiclostridium_E
d__Bacteria;p__Firmicutes;c__Clostridia;o__Oscillospirales;f__Ruminococcaceae;g__Ruminococcus_D
d__Bacteria;p__Firmicutes;c__Clostridia;o__Oscillospirales;f__Ruminococcaceae;g__Ruthenibacterium
d__Bacteria;p__Firmicutes;c__Clostridia;o__Oscillospirales;f__Ruminococcaceae;g__UBA3818
d__Bacteria;p__Firmicutes;c__Negativicutes;o__Veillonellales;f__Dialisteraceae;g__Dialister
d__Bacteria;p__Firmicutes;c__Negativicutes;o__Veillonellales;f__Veillonellaceae;g__Veillonella
d__Bacteria;p__Proteobacteria;c__Alphaproteobacteria;o__RF32;f__CAG-239;g__51-20
d__Bacteria;p__Proteobacteria;c__Alphaproteobacteria;o__RF32;f__CAG-239;g__CAG-267
d__Bacteria;p__Proteobacteria;c__Alphaproteobacteria;o__RF32;f__CAG-239;g__CAG-495
d__Bacteria;p__Proteobacteria;c__Gammaproteobacteria;o__Burkholderiales;f__Burkholderiaceae;g__Sutterella
d__Bacteria;p__Proteobacteria;c__Gammaproteobacteria;o__Enterobacterales;f__Enterobacteriaceae;g__Escherichia
d__Bacteria;p__Proteobacteria;c__Gammaproteobacteria;o__Enterobacterales;f__Enterobacteriaceae;g__Salmonella
d__Bacteria;p__Verrucomicrobiota;c__Verrucomicrobiae;o__Verrucomicrobiales;f__Akkermansiaceae;g__Akkermansia

Stats:
Hosts interact with ME-vOTUs uniquely: 11/81 (13.6%)
Hosts interact with SHHC-vOTUs uniquely: 6/81 (7.4%)
Hosts interact with communal viruses shared in both ME and SHHC: 64/81 (79%)

d__Bacteria;p__Firmicutes;c__Clostridia;o__Oscillospirales;f__CAG-272;g__QALR01
d__Bacteria;p__Firmicutes;c__Clostridia;o__Oscillospirales;f__Oscillospiraceae;g__Enterenecus
d__Bacteria;p__Firmicutes;c__Clostridia;o__Oscillospirales;f__Oscillospiraceae;g__UBA5446
d__Bacteria;p__Firmicutes;c__Clostridia;o__Oscillospirales;f__Oscillospiraceae;g__UMGS1766
d__Bacteria;p__Firmicutes;c__Clostridia;o__Oscillospirales;f__Ruminococcaceae;g__Anaerotruncus
d__Bacteria;p__Firmicutes;c__Clostridia;o__Oscillospirales;f__Ruminococcaceae;g__CAG-353
d__Bacteria;p__Firmicutes;c__Clostridia;o__Oscillospirales;f__Ruminococcaceae;g__D5
d__Bacteria;p__Firmicutes;c__Clostridia;o__Oscillospirales;f__Ruminococcaceae;g__Massilimaliae
d__Bacteria;p__Firmicutes;c__Clostridia;o__Oscillospirales;f__Ruminococcaceae;g__Massilioclostridium
d__Bacteria;p__Firmicutes;c__Clostridia;o__Oscillospirales;f__Ruminococcaceae;g__Phocea
d__Bacteria;p__Firmicutes;c__Clostridia;o__Oscillospirales;f__Ruminococcaceae;g__QAMM01
d__Bacteria;p__Firmicutes;c__Clostridia;o__Oscillospirales;f__Ruminococcaceae;g__Ruminococcus_C
d__Bacteria;p__Firmicutes;c__Clostridia;o__Oscillospirales;f__UMGS1783;g__UMGS1783
d__Bacteria;p__Firmicutes;c__Clostridia;o__Peptostreptococcales;f__Anaerovoracaceae;g__CAG-145
d__Bacteria;p__Firmicutes;c__Clostridia;o__Peptostreptococcales;f__Peptostreptococcaceae;g__GCA-900066495
d__Bacteria;p__Firmicutes;c__Clostridia;o__TANB77;f__CAG-508;g__CAG-269
d__Bacteria;p__Firmicutes;c__Clostridia;o__TANB77;f__CAG-508;g__CAG-354
d__Bacteria;p__Firmicutes;c__Clostridia;o__UBA1212;f__UBA1255;g__Egerieisoma
d__Bacteria;p__Firmicutes;c__Negativicutes;o__Acidaminococcales;f__Acidaminococcaceae;g__Phascolarctobacterium
d__Bacteria;p__Firmicutes;c__Negativicutes;o__Veillonellales;f__Megasphaeraceae;g__Caecibacter
d__Bacteria;p__Patescibacteria;c__Paceibacteria;o__UBA9983_A;f__UBA1006;g__01-FULL-36-10b
d__Bacteria;p__Proteobacteria;c__Gammaproteobacteria;o__Burkholderiales;f__Burkholderiaceae;g__Duodenibacillus
d__Bacteria;p__Proteobacteria;c__Gammaproteobacteria;o__Burkholderiales;f__Burkholderiaceae;g__Parasutterella
d__Bacteria;p__Proteobacteria;c__Gammaproteobacteria;o__Enterobacterales;f__Pasteurellaceae;g__Glaesserella
d__Bacteria;p__Spirochaetota;c__Spirochaetia;o__Sphaerochaetales;f__Sphaerochaetaceae;g__UBA9732
d__Bacteria;p__Verrucomicrobiota;c__Lentisphaeria;o__Victivallales;f__Victivallaceae;g__Victivallis
